# Supplementary material for: Genes associated with body weight gain and feed intake identified by meta-analysis of the mesenteric fat from crossbred beef steers
Source: PLoS One. 2020 Jan 7;15(1):e0227154. doi: 10.1371/journal.pone.0227154 (PMC6946124; doi:10.1371/journal.pone.0227154)
Supplement: S1 Table — Genes are ordered by adjusted meta-P-value. The individual cohort cells for DEGs identified in the meta-analysis are colored according to the sign of their log2 fold change, where green indicates up-regulation and red indicates down-regulation in high gain. Genes with all gray cell indicate those that were excluded because they were also significant for the gain by intake interaction term. (PDF) [file pone.0227154.s001.pdf]

Supplemental Table 1. Differentially expressed genes associated with the gain main effect in the individual cohort and meta analyses.
Green indicates up-regulation in animals with higher gain. Red indicates down-regulation in animals with higher gain. Genes in gray are those identfied in the interaction analysis.

Gene	Group 1 Raw P-value	Group 2 Raw P-value	Group 3 Raw P-value	Group 4 Raw P-value	Group 5 Raw P-value	Meta-P-value	Adjusted Meta-P-value
OLFML3	3.35E-06	0.27390668	0.025115229	5.75E-05	0.507691672	2.00E-08	0.000109523
GCAT	0.27956946	0.02308561	0.963199603	1.36E-07	0.016711473	2.71E-07	0.00079315
HSPH1	0.196444863	0.732021927	0.003521393	7.61E-07	0.03974559	2.90E-07	0.00079315
MRPL14	0.000141743	0.187043486	0.383077288	2.40E-06	0.585774586	2.73E-07	0.00079315
LOC100300716	0.000248838	0.002002184	7.29E-05	0.732806798	0.767393543	3.70E-07	0.000867323
AACS	0.587244494	0.262867817	0.086507126	3.67E-07	0.00938605	7.33E-07	0.001335857
EBPL	0.04217896	2.17E-06	0.006773469	0.169855825	0.422275719	7.13E-07	0.001335857
JCHAIN	0.000886369	0.011846834	1.82E-05	0.297233071	0.952863354	8.39E-07	0.001377188
ACSL3	0.043701573	0.342117358	0.005910028	0.00033889	0.015993317	5.14E-06	0.004323139
ADGRL4	0.114104269	0.824950728	0.900894345	1.21E-08	0.482656913	5.27E-06	0.004323139
FST	0.000147559	0.02770667	0.152441581	0.227705133	0.002952244	4.61E-06	0.004323139
MGC152281	0.179640238	0.011280386	1.52E-05	0.030558003	0.450392044	4.64E-06	0.004323139
MOSPD1	0.01209913	0.152622521	0.107874799	0.000470742	0.003268229	3.56E-06	0.004323139
NKAIN1	0.106073525	0.131550106	3.91E-05	0.087350546	0.008291368	4.39E-06	0.004323139
PDLIM5	0.363461538	2.37E-08	0.569603847	0.090825429	0.985006838	4.78E-06	0.004323139
PTAFR	0.246648743	0.413748955	5.36E-05	0.034687734	0.001847602	3.98E-06	0.004323139
PTGS2	0.001740884	0.040716069	0.000204794	0.256423366	0.068727448	3.06E-06	0.004323139
SFRP1	0.016583711	0.11953826	0.192187017	0.77396926	1.16E-06	3.91E-06	0.004323139
GALR3	0.000105715	0.084646107	0.073174126	0.02349653	0.049477069	7.52E-06	0.005413007
LOC100848025	0.521027674	0.995980937	8.43E-09	0.341422338	0.515085585	7.59E-06	0.005413007
PUS10	0.995206648	1.06E-05	0.000264887	0.502973392	0.502773746	7.09E-06	0.005413007
RHOH	0.777581382	3.51E-06	0.106533442	0.103072086	0.028280535	8.22E-06	0.005618886
GSTT2	0.012224038	0.518142494	0.439235788	3.93E-05	0.009883927	1.00E-05	0.006323489
H19	0.00224146	0.003907513	0.061490179	0.013366689	0.149240845	9.97E-06	0.006323489
HAS3	0.46763066	0.854057165	2.43E-08	0.5512354	0.23772525	1.14E-05	0.006945926
HPS6	0.681636999	0.379099758	0.920113156	1.02E-07	0.055046116	1.19E-05	0.006976907
LOC782367	0.000210312	0.005452975	0.273464312	0.114693929	0.044147868	1.37E-05	0.007758796
LIPG	0.290350403	0.456695811	0.775986636	1.15E-05	0.001854625	1.78E-05	0.009510624
RUFY2	0.996613211	0.413528216	7.45E-09	0.856539544	0.910580966	1.91E-05	0.009510624
SMUG1	0.316110513	0.032216535	0.538340544	0.977033729	4.31E-07	1.86E-05	0.009510624
SRPX	0.02697901	0.047512406	0.13026812	1.54E-05	0.931661801	1.91E-05	0.009510624
CCDC117	0.316902714	0.686540128	0.03116097	2.35E-06	0.204910435	2.46E-05	0.011850891
CDKN2B	4.18E-07	0.640470168	0.24035649	0.075872314	0.776344571	2.77E-05	0.012989121
ARL6IP1	0.807700252	0.351492323	1.02E-06	0.402076004	0.034938409	2.94E-05	0.013096166
CCL1	0.032999487	0.188000696	0.446196173	0.006686016	0.000224612	2.99E-05	0.013096166
GIMAP7	0.135014813	0.003102649	0.004835207	0.203882308	0.010260076	3.03E-05	0.013096166
MAP2K6	0.4722505	0.920426843	9.35E-08	0.440286036	0.291679678	3.58E-05	0.015082676

AASDH	0.069110799	0.15887845	0.000302479	0.007507493	0.24585977	4.08E-05	0.015377584
NRG2	0.00028055	0.01032675	0.163004952	0.020276093	0.641276139	4.08E-05	0.015377584
NTNG1	4.85E-06	0.781069789	0.261546223	0.748774983	0.007602292	3.81E-05	0.015377584
PLN	1.91E-05	0.288731842	0.940981689	0.599473582	0.001999223	4.12E-05	0.015377584
SLC30A2	0.001731305	0.558471934	0.000148608	0.317144664	0.12818516	3.92E-05	0.015377584
RCOR2	0.129047259	0.354423233	0.258383176	2.94E-05	0.020409002	4.58E-05	0.016340519
STT3B	0.95981012	0.287905557	4.42E-05	0.052133928	0.0110167	4.54E-05	0.016340519
DLX3	0.397915378	0.333991576	3.35E-05	0.004483092	0.39697306	5.01E-05	0.017483197
ANXA9	0.079796188	0.008270213	0.001290266	0.083269438	0.12919644	5.62E-05	0.019175109
RAB7B	0.158828637	0.438698991	0.595838018	1.34E-06	0.168639931	5.73E-05	0.019175109
LOC100848799	3.56E-06	0.443881	0.330347575	0.579780572	0.032400626	5.93E-05	0.019473611
GLCE	0.514404922	0.696497044	7.69E-07	0.041011073	0.995743148	6.62E-05	0.021303906
KCNMA1	6.48E-06	0.537848155	0.288898597	0.685441084	0.016834176	6.79E-05	0.021429024
ARHGEF26	0.00020667	0.002425741	0.407582357	0.408901814	0.15495185	7.40E-05	0.022570293
CHAC1	4.81E-05	0.20061587	0.786780829	0.014834222	0.115875691	7.44E-05	0.022570293
LOC100138922	0.780393061	0.27540503	5.58E-06	0.011324366	0.97941126	7.56E-05	0.022570293
CCDC18	0.590561877	0.30601149	0.005530697	0.814646449	1.87E-05	8.40E-05	0.024628316
ELP3	0.245554335	0.000339894	0.68151295	0.296110644	0.001029435	9.33E-05	0.02622284
MAPT	0.117018082	0.189230593	0.499467894	0.023089463	6.95E-05	9.50E-05	0.02622284
SH2B2	0.133572351	0.207033279	0.000148132	0.016444614	0.266533455	9.59E-05	0.02622284
TARDBP	0.78804219	0.91795989	0.489289253	2.93E-06	0.016397871	9.18E-05	0.02622284
ANTXRL	0.407020408	1.72E-05	0.130078334	0.582714142	0.036616864	0.000101967	0.026807218
CAB39	0.467754844	0.000617189	0.001248018	0.149146493	0.366101904	0.000103042	0.026807218
IL18BP	0.492386742	0.225739765	0.004881764	0.873213886	4.23E-05	0.000104543	0.026807218
NINL	0.65410578	0.00253947	0.000459896	0.07853033	0.317810301	0.000100525	0.026807218
AKR1B1	0.053876783	0.113066259	0.004539756	0.00150988	0.494468948	0.000107041	0.027025454
LOC101906455	0.895815089	7.67E-07	0.543803964	0.542923437	0.111765546	0.00011528	0.028024529
LOC789607	0.030482791	0.012209896	0.539772904	0.000124415	0.916010031	0.000116121	0.028024529
TMED6	0.576703584	0.613307754	0.120496782	0.001535389	0.000344225	0.00011464	0.028024529
BAG3	0.044082537	0.391170278	0.032031262	0.002697608	0.016663852	0.000123766	0.028214073
LPIN1	0.159244758	0.817443068	0.004882705	0.00465646		0.000121177	0.028214073
SLC6A16	4.49E-06	0.274628608	0.029526449	0.830100519	0.796164402	0.000120853	0.028214073
UNC5A	0.130467108	0.196489811	7.68E-05	0.024078947	0.528659785	0.000124667	0.028214073
VEGFC	0.591238245	0.987403677	1.86E-05	0.01094813	0.212608288	0.000125503	0.028214073
CD5	0.008179042	0.028327605	0.673665579	0.011122319	0.015720395	0.000133306	0.029169028
PAK5	0.006145903	0.002793834	0.102344625	0.076372688	0.202851953	0.000133054	0.029169028
CNTN4	4.47E-05	0.050236018	0.676204275	0.109115437	0.174192711	0.000139283	0.030075892
LOC783540	0.043452498	0.105825466	0.486712124	0.069594881	0.000190019	0.000142066	0.030278483
NEIL1	0.126833301	0.910125037	0.001481091	0.000209255	0.862773525	0.000146819	0.030890334
ACLY	0.269384409	0.49431706	0.185798212	0.000765705	0.001834154	0.00016108	0.033461735

ACKR4	0.000103542	0.021674309	0.042485638	0.913285954	0.416526609	0.000166576	0.033678273
CHMP1B	0.784548988	0.403985756	0.11787341	0.256468451	3.96E-06	0.000172383	0.033678273
PPTC7	0.461666645	0.860415321	0.010804172	0.000686242	0.012666258	0.000170273	0.033678273
SHROOM3	0.682816547	0.059449256	0.00249693	0.009675584	0.038493802	0.000171864	0.033678273
SLIT1	0.544143741	0.337777385	0.600064433	2.29E-05	0.014072909	0.000164197	0.033678273
ACSM3	0.020295687	0.003664858	0.132574587	0.024031029	0.162600658	0.000174634	0.033716717
TIMM13	0.014122904	0.161197586	0.066595686	0.007985408	0.033210967	0.000180536	0.034450928
DENND2D	0.297774243	0.084753246	0.000412117	0.163243054	0.02467325	0.000186408	0.035007583
PRMT7	0.890654701	0.249981963	0.724694984	1.04E-06	0.252718872	0.00018772	0.035007583
MYLK	4.68E-06	0.147071277	0.382965791	0.86724082	0.192717936	0.000193813	0.035737836
EMB	0.013686578	0.038556101	0.002775396	0.781632385	0.041326707	0.000204937	0.037369194
PDE4B	0.339121757	0.299572949	6.39E-06	0.221029781	0.34310081	0.000211363	0.038117298
GID8	0.106109635	0.428430481	0.660317743	1.30E-05	0.135189654	0.000222549	0.039698306
HSPA1A	0.471159439	0.335924108	0.166591616	0.000190075	0.011161396	0.000233445	0.040767539
PKHD1L1	0.003805645	0.013677402	0.141713703	0.491941245	0.015632431	0.000235995	0.040767539
TNNT3	0.327723253	0.050175399	0.006661634	0.033136075	0.015607972	0.00023576	0.040767539
FAM210B	0.752861637	0.179468381	7.76E-06	0.31133807	0.192747724	0.000255647	0.043702346
LDB3	1.92E-06	0.197891566	0.516323734	0.992039257	0.33422967	0.000262174	0.044355989
LOC513659	0.283966509	0.096485535	0.760994751	4.18E-06	0.772748497	0.000269807	0.045091712
RAP1A	0.436135839	0.048315129	0.001193676	0.307138015	0.008819757	0.000272018	0.045091712
CCL8	0.059268346	0.237618391	0.740695916	0.000463445	0.016160905	0.000302368	0.049621576
SGK2	0.041773343	0.542098403	0.011099605	0.000751093	0.426929279	0.000309727	0.050325967
SCG3	0.061101126	0.000484601	0.01240091	0.239266702	0.953109545	0.000318984	0.051321965
HSPB3	0.039999926	0.578881491	0.001890148	0.011368014	0.173783489	0.000326969	0.052095949
CIDEA	0.019307396	0.032520156	0.451482738	0.002415109	0.131433514	0.000337175	0.052386923
JMJD6	0.593756348	0.511363114	0.06756088	1.55E-05	0.283447809	0.000336861	0.052386923
SLF1	0.687835436	0.061853087	0.000182217	0.274284444	0.04251301	0.000338371	0.052386923
CBARP	0.341739975	0.339657156	0.000735114	0.004424095	0.26233934	0.000362986	0.054265007
CDCA4	0.069347466	0.030278651	0.03182729	0.410929249	0.003615757	0.000363729	0.054265007
LOC107132475	0.976550224	0.385870322	0.673355954	0.000631879	0.000613897	0.000361274	0.054265007
TMEM126B	0.859191365	0.193758403	0.965226735	0.120910364	5.09E-06	0.000362694	0.054265007
COL6A5	0.055236147	0.006176908	0.0627529	0.121869492	0.039838619	0.000376766	0.054506754
CYP2B6	0.00449663	0.24807484	0.042346466	0.00481989	0.465327256	0.000382309	0.054506754
DNAJA1	0.391926404	0.69048731	0.007690871	0.000734867	0.066896816	0.000372208	0.054506754
INSIG1	0.057167538	0.108715354	0.38636243	0.006988974	0.006469051	0.000389548	0.054506754
INTS6	0.335508598	0.495259629	0.909664263	2.09E-06	0.354074194	0.000398099	0.054506754
KCTD10	0.577296593	1.48E-05	0.041659713	0.755387238	0.412190376	0.00039619	0.054506754
LOC100336414	0.750826762	0.817305526	0.007355852	0.000248875	0.099561925	0.000398563	0.054506754
MGC139164	0.621277732	0.000306473	0.000799105	0.705480518	0.998275366	0.00038566	0.054506754
RIOX1	0.526783014	0.631938083	0.424749555	0.010704784	6.73E-05	0.000371034	0.054506754

SBSPON	0.02205809	0.011728086	0.099154573	0.928535843	0.004497026	0.000385534	0.054506754
LOC107133024	0.927789826	0.541741312	0.039618951	0.001345516	0.004498173	0.000422061	0.057101617
LOC618076	0.498442595	0.638346943	0.838168677	2.15E-06	0.214107921	0.000427975	0.057101617
PGF	0.022379877	0.007358536	0.075165145	0.074509684	0.132789117	0.000427285	0.057101617
CRYAB	0.117478889	0.172997614	0.039216865	0.096384665	0.001626436	0.000433876	0.057422091
ATP10D	0.779514255	0.160358755	0.13249389	2.43E-05	0.320312455	0.000444542	0.058363091
TLL2	0.049119196	0.214166882	0.00015765	0.112902298	0.747717882	0.000473351	0.061652083
LOC107131807	0.763963075	0.736343338	7.70E-07	0.954746082	0.346660986	0.000481938	0.062276278
FARSB	0.841818508	0.235687377	0.558934103	0.440417667	3.01E-06	0.000491893	0.063066009
CHI3L1	0.242893562	0.000144356	0.196146756	0.076051077	0.289956615	0.000503169	0.064011732
HOXA6	0.071698872	0.507387937	0.000678774	0.195547044	0.032864313	0.000520875	0.064840771
KPNA1	0.271388902	0.000589945	0.015913973	0.179374495	0.344349316	0.000517582	0.064840771
RNF125	0.084557213	0.18573822	0.104539698	0.033328433	0.002904895	0.000521539	0.064840771
SYS1	0.814170375	1.04E-06	0.252474838	0.842175938	0.892984864	0.000526634	0.064981832
HOGA1	0.048331004	0.149652887	0.031855052	0.00144742	0.499527676	0.000540521	0.066197685
PLEKHJ1	0.526528577	0.009028786	0.00252266	0.664723818	0.023644257	0.000593796	0.072183598
CDH2	0.048989412	0.621434559	3.28E-05	0.949094965	0.218720594	0.000638547	0.073975166
LOC100140586	5.08E-06	0.207040089	0.598676962	0.75266902	0.438790504	0.000640087	0.073975166
LOC784052	0.043506559	0.024121755	0.23391407	0.001047252	0.781140313	0.00062309	0.073975166
MIEF2	0.00219125	0.171653637	0.529153971	0.00442759	0.234587756	0.000636967	0.073975166
SOX4	0.16272633	0.333623173	2.57E-05	0.261768631	0.545452618	0.000619982	0.073975166
TCAF1	0.330288275	0.475493118	0.000651767	0.450455054	0.00442939	0.000631121	0.073975166
TUBB2A	0.521841202	0.000491499	0.004249034	0.6044013	0.313030923	0.000635703	0.073975166
CCDC188	0.843789725	0.002762442	0.015058949	0.022958609	0.263536566	0.000650131	0.074092399
LOC782706	0.099610846	0.151749988	0.00638357	0.065853822	0.033328643	0.000648758	0.074092399
B4GALT6	0.051780646	0.004359754	0.441980985	0.757794244	0.002876937	0.000662054	0.074253197
FKBP4	0.617536734	0.259356137	0.004619989	0.036045677	0.008205484	0.000665116	0.074253197
GPT	0.566868393	0.152509771	4.02E-05	0.297298263	0.210340176	0.000661192	0.074253197
TMEM165	0.085903665	0.295290606	0.469336094	0.101891231	0.000183662	0.000674166	0.074755011
WDR86	0.076776867	0.082062279	0.091386938	0.006796313	0.057669791	0.000680762	0.074979715
ACSL6	0.148331028	0.200606899	0.431179161	0.007899282	0.002345119	0.000708014	0.075477982
KCNK12	0.459125891	0.001898896	0.010025659	0.406644235	0.06653535	0.000705334	0.075477982
NCR1	0.465391756	0.699916195	3.49E-05	0.181045661	0.112979141	0.000696345	0.075477982
PAN2	0.978338712	0.000248763	0.2177002	0.951554709	0.004684496	0.000704619	0.075477982
RRAD	0.000575759	0.124232472	0.533845127	0.813000307	0.007659905	0.000708282	0.075477982
CLN5	0.588272327	0.238162436	1.28E-05	0.183696882	0.753079008	0.000729452	0.07673746
STOX2	0.650159504	0.442201703	0.004925997	0.010962063	0.01581707	0.000725706	0.07673746
CASQ1	0.005247255	0.052054134	0.35621625	0.135345175	0.019561531	0.000752461	0.077178986
EPM2AIP1	0.746804618	0.665975874	0.000150559	0.03443951	0.098190008	0.000742768	0.077178986
KBTBD8	0.767935072	0.697311185	0.001734699	0.300247585	0.000915338	0.000747358	0.077178986

ZAR1L	0.453861776	0.060843263	0.012424601	0.418725851	0.001780209	0.000748376	0.077178986
PDE4D	0.126645358	0.640062671	0.207808416	3.56E-05	0.434501619	0.000758904	0.077356327
CREB3L1	0.391304467	0.119097037	0.024344619	0.002055019	0.115225305	0.000776713	0.077807206
DEDD2	0.332040453	0.376214514	0.085931002	0.000299076	0.085836322	0.000791774	0.077807206
ITPKC	0.18161204	0.756078901	0.018799345	0.033431079	0.003162378	0.000786	0.077807206
MZF1	0.504933482	0.001731869	0.000382568	0.982466909	0.837319067	0.000791	0.077807206
PKNOX1	0.209366568	0.883831651	0.095991654	3.69E-05	0.408252915	0.000774062	0.077807206
SPRYD7	0.086296721	0.001726773	0.292417803	0.018860763	0.32957765	0.000781547	0.077807206
NOD2	0.274475052	0.446990843	0.287773305	0.065109735	0.00012355	0.000810016	0.079126056
SLC18B1	0.84671038	0.020380662	0.000428745	0.261697517	0.14809295	0.000815871	0.079226371
LOC104970711	0.915650002	0.258814558	0.000461912	0.0654156	0.043247141	0.000864601	0.08243884
SLC7A8	0.097439604	0.211008005	0.006818083	0.04772675	0.046961266	0.00087407	0.08243884
SPP1	0.724664372	0.471834121	0.023347102	0.557980128	6.98E-05	0.000867241	0.08243884
TNFAIP8L3	0.005485251	0.056610376	0.015282982	0.287427828	0.22941394	0.000871423	0.08243884
TRIP12	0.589340362	0.423952855	0.000235445	0.006824007	0.771021712	0.000864247	0.08243884
RASSF9	0.695851081	0.299355873	0.000564017	0.006174092	0.441905738	0.00088735	0.083213149
ATP6V1C2	0.024088515	0.503035719	0.009168766	0.006743338	0.444364012	0.000912973	0.083370466
FABP7	0.000820968	0.382824861	0.579864586	0.501777711	0.003601727	0.000905638	0.083370466
LAT	0.407249431	0.010680621	0.03797543	0.026452735	0.074689931	0.000899412	0.083370466
LOC530077	0.535952737	0.002142933	0.009571043	0.153699581	0.201257463	0.000927606	0.083370466
LSMEM2	0.967980941	0.041992972	0.008355329	0.012103767	0.082961151	0.000929669	0.083370466
PIK3R6	0.710030077	0.713740874	0.000732176	0.001364148	0.665008942	0.000920573	0.083370466
SH3TC1	0.607461488	0.08272742	0.005923063	0.002739196	0.413126351	0.000921049	0.083370466
ZMYND8	0.402919285	0.274561844	0.000435826	0.534568845	0.012724174	0.00090271	0.083370466
FLNC	6.05E-05	0.473476516	0.227382679	0.935347784	0.056926194	0.000941776	0.083997212
IFI6	0.154256498	0.009521733	0.092875954	0.019363483	0.133660193	0.000954186	0.084253852
RASAL2	0.495724985	0.954602948	0.460087393	0.000387199	0.00419233	0.000954921	0.084253852
AOX1	0.006933054	0.924417353	0.486257782	0.010493105	0.011043179	0.000970507	0.084689538
BTAF1	0.262487944	0.899440255	0.985419089	1.60E-06	0.986394201	0.000981469	0.084689538
KCTD12	0.009147065	0.08969925	0.011612481	0.476996488	0.080663106	0.00098152	0.084689538
LOC100297240	0.979434204	4.41E-05	0.013873176	0.897791087	0.685304214	0.000985662	0.084689538
WDR90	0.939224117	0.000319209	0.009424918	0.819646432	0.156931341	0.000975216	0.084689538
LSAMP	0.00271075	0.463119082	0.338278355	0.003106409	0.284672847	0.000999445	0.085426491
JAML	0.043867794	0.002799566	0.199727231	0.08415318	0.186577943	0.001018511	0.086605111
ADAM19	0.01531235	0.274310122	0.000704922	0.96494569	0.137918018	0.001036143	0.087650197
BBS9	0.000914605	0.551862317	0.440233995	0.095745685	0.019225691	0.001065505	0.08967177
TTC38	0.228092227	0.097496616	8.40E-05	0.943513951	0.234967202	0.00107542	0.0900445
DNAJB9	0.048302375	0.119103342	0.003775514	0.231233624	0.084149659	0.001091948	0.09096423
TNC	0.040439912	0.142810136	0.001952696	0.082039051	0.461187925	0.001099746	0.091151194
BoLA	0.970074155	0.049872309	0.00244431	0.053096157	0.071694542	0.001144697	0.092539981

LOC112445144	0.839842613	0.47895446	0.018461776	9.85E-05	0.605891913	0.00113125	0.092539981
PADI1	0.00649945	0.219694791	0.026202527	0.018022477	0.667386875	0.001144424	0.092539981
PRG4	0.027263938	0.001160177	0.196290123	0.168179979	0.424378559	0.001131318	0.092539981
RNF165	0.121626379	0.536391927	0.035150965	0.000819869	0.239428774	0.001144693	0.092539981
BCL11B	0.237816992	0.00389615	0.758261446	0.085671489	0.007717728	0.0011719	0.093359431
GIMAP4	0.366875062	0.003688364	0.118657346	0.176691429	0.016224433	0.001163882	0.093359431
RBM47	0.000207894	0.315113072	0.150464243	0.988077112	0.047394218	0.001166341	0.093359431
TFAM	0.990881143	0.001963272	0.45101974	0.008460869	0.06503538	0.001206108	0.095620491
FBXL6	0.556374868	0.100520694	8.96E-05	0.243664721	0.406589755	0.001231488	0.096698346
FCER1A	0.138150473	0.037247575	0.124469598	0.07018036	0.010981086	0.001226189	0.096698346
ALPK1	0.574825333	0.125544885	0.000178954	0.609359063	0.065358855	0.001264405	0.098810265
NIF3L1	0.507786639	0.194589017	0.806037888	0.00060715	0.010799413	0.0012788	0.098992399
PTGES3	0.195112598	0.382695825	0.018090846	0.004942389	0.077895643	0.00127485	0.098992399
LOC101906101	0.017729376	0.241063217	0.002774232	0.096024311	0.462408197	0.00128655	0.099124739
MXRA5	0.501904456	0.030432913	7.59E-05	0.615392682	0.753049093	0.001306609	0.100199813
LOC112449613	0.552396163	0.433121589	0.039550569	0.440174191	0.000131892	0.001327963	0.101363704
PTK7	0.196451308	0.024966282	0.004297922	0.044827211	0.607030038	0.001371331	0.104189428
VWA3B	0.931125055	0.408811633	0.03468079	0.007384166	0.006049189	0.001399783	0.105861015
WASF1	0.000218403	0.057831413	0.383388139	0.992493786	0.123832617	0.001409407	0.106099929
FAIM2	0.00236376	0.939457307	0.026111472	0.205310688	0.050478564	0.001419582	0.106149397
MAP3K1	0.064462403	0.000628096	0.11524256	0.346119573	0.376889882	0.001433133	0.106149397
RIMS1	0.000788316	0.028154895	0.202137839	0.381208291	0.355052351	0.001430611	0.106149397
SLC10A1	0.005989045	0.933563936	0.214415768	0.211847496	0.002402969	0.001435937	0.106149397
ANGPTL4	0.063402633	0.604098686	0.611634537	0.00055546	0.05006059	0.00150713	0.11057794
KYNU	0.115831519	0.07757695	0.035782328	0.329364989	0.006163127	0.00150932	0.11057794
KLHL25	0.121659714	0.620824733	0.239630119	0.126473632	0.000289895	0.001527961	0.11144605
FAM124B	0.278182372	0.750011672	0.000421709	0.015324916	0.495292507	0.001535204	0.11147891
EPB42	0.072892988	0.000144068	0.285784333	0.549867556	0.407478189	0.001543038	0.111554193
LOC101906565	0.937459802	5.92E-06	0.47256467	0.538679328	0.48074099	0.001554587	0.111896155
PIK3C3	0.030597145	0.059015541	0.001902465	0.290651955	0.699015122	0.001586175	0.113671266
ST6GAL2	4.11E-05	0.112413776	0.27895048	0.972704493	0.561785284	0.001596161	0.113889533
FGF10	0.417577482	0.299591354	0.964945004	0.002522275	0.002391832	0.001636874	0.116288919
LHX6	0.229042278	0.08540855	0.031099258	0.012470282	0.097938649	0.001661314	0.117012141
SMAD7	0.876992779	1.70E-05	0.876179802	0.169016762	0.334881157	0.001655026	0.117012141
SHOX2	0.03603138	0.207710806	0.052149982	0.540072288	0.003564505	0.001675077	0.117477307
ARL14EP	0.302081814	0.094630101	0.019463654	0.684967509	0.002077813	0.00174133	0.118397611
ATL2	0.296492631	0.156780133	0.650544848	0.001064578	0.024246002	0.001722913	0.118397611
FAM120B	0.628005987	0.73383188	0.010366004	0.00843513	0.019719921	0.001745816	0.118397611
GPR183	0.774075772	0.005126213	0.299149395	0.011935153	0.056056014	0.001745074	0.118397611
GSE1	0.915737336	0.194192897	2.83E-05	0.499981228	0.307776837	0.001713831	0.118397611

PDZD3	4.75E-05	0.414183504	0.807602447	0.10071896	0.479527168	0.001702559	0.118397611
TMEM266	0.015584962	0.611621496	0.035272139	0.011137014	0.205141743	0.001702652	0.118397611
TRAT1	0.580870778	0.007142372	0.141851925	0.030525407	0.044236783	0.001745916	0.118397611
LOC782951	0.374438286	0.773090553	0.064508251	0.000201963	0.212194742	0.001754926	0.118518863
EMC9	0.10420571	0.122337014	0.81019007	0.000471004	0.16913374	0.001791246	0.119496472
KCP	0.01494795	0.362925995	0.002819637	0.09770124	0.5494914	0.001788701	0.119496472
LOC112444897	0.844594786	0.37290508	0.34149775	8.79E-05	0.086407074	0.001782149	0.119496472
NDE1	0.18402993	0.807265004	0.007087631	0.819411934	0.000991805	0.001843821	0.122505827
HGH1	0.136768399	0.083170172	0.791889721	0.049346893	0.001941586	0.00185543	0.122780102
PER3	0.743006292	0.095068995	0.000174906	0.804308223	0.087828509	0.001870786	0.12329909
ALPK3	0.149499196	0.161672262	0.638379067	0.467994951	0.000122435	0.001888657	0.123494822
LENG8	0.893617188	0.018563407	0.001462927	0.392312723	0.092871799	0.001888806	0.123494822
TUBB	0.239179837	0.132281838	0.000582806	0.399031907	0.120933062	0.001897643	0.123580203
C4H7orf25	0.509202187	0.157446517	0.289581704	0.000324549	0.120156041	0.001921979	0.124179535
GSDME	0.303044542	0.774577134	0.003792932	0.249697791	0.004059967	0.001917629	0.124179535
LSM12	0.628518745	0.004516068	0.546980011	0.746642981	0.000790739	0.001939562	0.124336542
MVD	0.703442342	0.149077169	0.844748725	0.002250168	0.00459684	0.001939053	0.124336542
LOC104975559	0.02699475	0.527021113	0.058647207	0.140902418	0.007914371	0.001961008	0.124352906
LOC112442023	0.350660349	0.005123383	0.034943343	0.306015956	0.048484356	0.00196255	0.124352906
TUBB4B	0.392836099	0.020661992	0.018687265	0.264236134	0.023028224	0.001949394	0.124352906
ADCK5	0.588386746	0.000876447	0.004805829	0.478539184	0.793108921	0.001976733	0.124445023
IL7R	0.249975026	0.005220288	0.22690705	0.092969527	0.034225907	0.00197917	0.124445023
CELSR1	0.00542446	0.008287073	0.203124192	0.750668899	0.139063876	0.00199615	0.125033644
ESPN	0.000994332	0.688516628	0.651898068	0.052871399	0.041082282	0.002021023	0.125632598
IPO7	0.560778386	0.024012044	0.032841408	0.014699025	0.148554155	0.002015268	0.125632598
ATP1A2	7.58E-06	0.63500625	0.72283471	0.765786023	0.373817066	0.002062523	0.12582926
CATSPERG	0.788944195	0.230229696	0.001298503	0.044344522	0.095170645	0.002060686	0.12582926
LOC112444309	0.001484867	0.685831598	0.175014935	0.582668671	0.009492876	0.002046125	0.12582926
PPP1CB	0.563283007	0.032072439	0.053851753	0.013598157	0.074076747	0.00203722	0.12582926
TMEM176A	0.0698177	0.03949728	0.054978047	0.969514124	0.00672237	0.002049584	0.12582926
TCEA1	0.927629608	0.036744633	0.341447875	0.167144684	0.000522001	0.00209108	0.127098958
IMPA2	0.515034804	0.272827597	0.005749796	0.436592313	0.002895302	0.002099899	0.127163964
IFI27	0.404628647	0.000314036	0.514194449	0.049328091	0.321886599	0.002124206	0.127227532
LOC107131769	0.311836709	0.402106908	0.756635782	0.002359078	0.004603319	0.0021135	0.127227532
TMEM35A	0.000106455	0.703249682	0.252126828	0.732748829	0.0747457	0.002118742	0.127227532
CFB	0.06984286	0.022009308	0.035892208	0.049888739	0.388214467	0.002170766	0.129074086
ZNF182	0.90759579	0.211125833	0.000596131	0.257923922	0.036143468	0.002165258	0.129074086
PLD2	0.818363033	0.218078711	4.31E-05	0.765882611	0.182770776	0.002183952	0.129389275
CD4	0.60558435	0.002125149	0.378690313	0.36422896	0.006176981	0.002212163	0.130121153
LOC112445943	0.051675108	0.643409329	0.043050148	0.014739523	0.05184213	0.002208102	0.130121153

CABIN1	0.150863232	0.401903264	0.000929564	0.025061166	0.783141571	0.002226485	0.130495903
ARMCX4	0.364166697	0.620670542	0.002916067	0.014754422	0.11813725	0.002289054	0.131046598
CLASRP	0.937666383	0.001881337	0.001572947	0.872150317	0.480038313	0.002307749	0.131046598
COA7	0.177141023	0.484790738	0.277278038	0.174650548	0.000278261	0.002301205	0.131046598
ESRP1	0.07118865	0.299584863	0.220159686	0.000252007	0.95635181	0.002263857	0.131046598
FLT4	0.013113167	0.018859553	0.022608954	0.573763981	0.355513318	0.002276901	0.131046598
LOC112447770	0.001552552	0.180244794	0.482694718	0.272311397	0.03091739	0.002272078	0.131046598
LOC785630	0.411887037	0.240970338	9.00E-05	0.334072165	0.375666996	0.002248814	0.131046598
PLEKHA6	0.291086047	0.004654351	0.02698377	0.074338667	0.423442138	0.002291848	0.131046598
SYNDIG1	0.003517452	0.689156046	0.35202463	0.405373697	0.003287354	0.002271965	0.131046598
CA3	0.1199993	0.283236449	0.013909646	0.148243479	0.017011689	0.002351963	0.131733982
NEGR1	0.000262479	0.38402199	0.988675068	0.195663919	0.060949121	0.002346486	0.131733982
PYGM	6.34E-05	0.190432349	0.589390965	0.664649794	0.250821259	0.002343504	0.131733982
TACC1	0.163094419	0.067959016	0.14317251	0.002391727	0.309922767	0.002328889	0.131733982
BDH1	0.474835548	0.385524799	0.428844456	0.000545337	0.028534478	0.002394144	0.133187457
LOC104970537	0.984527172	0.019091732	0.02544168	0.85648557	0.00296991	0.002386712	0.133187457
SYTL3	0.332932664	0.476727463	0.001756605	0.021417027	0.208715349	0.002429368	0.134690392
DOK6	0.080571603	0.97817298	0.010422469	0.016276172	0.094351063	0.002450918	0.135427679
MAPRE1	0.823531431	0.000255386	0.012797819	0.495595368	0.96974447	0.002496352	0.136558755
SNX16	0.272599678	0.598130773	0.000398687	0.111117534	0.178874634	0.002494202	0.136558755
SPTSSA	0.345790939	0.061698469	0.6824365	0.060008299	0.001475127	0.002489623	0.136558755
CD93	0.383866443	0.907777338	0.002406231	0.003047626	0.520294477	0.002546785	0.138395001
FNDC1	0.183605267	0.017962724	0.005011956	0.214950357	0.374111582	0.002546341	0.138395001
POSTN	0.151018699	0.10800079	0.002286228	0.177061128	0.205531494	0.002584988	0.140007388
SLC32A1	0.842862937	0.023951242	0.777302456	0.001050105	0.083253839	0.002605598	0.140659411
GNAT1	0.35592786	0.244790859	0.273073074	0.003509178	0.016721788	0.002639074	0.140771413
HSP90AA1	0.48984969	0.532031779	0.01217105	0.006526327	0.067087919	0.002628992	0.140771413
RGS4	0.000307482	0.292897624	0.025379959	0.707287079	0.863983022	0.002639976	0.140771413
SIAE	0.17792693	0.542275968	0.001529667	0.125793645	0.075311909	0.002641984	0.140771413
GLUL	0.857396269	0.827005652	0.001290552	0.09587069	0.016064768	0.002657285	0.141128502
DCLRE1B	0.950045154	0.764164454	0.000115437	0.08559615	0.200870822	0.002700474	0.142959592
USPL1	0.465231601	0.699268363	0.263249342	4.41E-05	0.383826851	0.00271228	0.1431229
PI4K2A	0.497852583	0.084779571	0.000918535	0.05288466	0.712674216	0.002728038	0.143493058
TYSND1	0.68261356	0.365632913	0.551726532	2.33E-05	0.463042347	0.002764548	0.144948888
GPATCH2L	0.052839515	0.222287517	0.508579037	0.004869993	0.053005201	0.002836863	0.145243882
HK2	0.006811565	0.166727391	0.616385565	0.672551236	0.003281816	0.002840978	0.145243882
IGFBP2	0.39706005	0.002948326	0.432395404	0.048475817	0.062481302	0.002825066	0.145243882
LOC101903647	0.167727056	0.019087705	0.047218512	0.01568895	0.638468288	0.002799725	0.145243882
LOC512867	0.202223941	0.032121829	0.056173828	0.376771546	0.011134551	0.0028219	0.145243882
LOC781001	0.365260682	0.00229718	0.005031502	0.486078911	0.749362729	0.002831247	0.145243882

SFRP2	0.058409333	0.263870274	0.012268136	0.027696901	0.289680865	0.002803475	0.145243882
TATDN3	0.844298653	0.628965098	0.129388794	0.3256202	6.80E-05	0.002807857	0.145243882
SLC30A6	0.000294094	0.316409136	0.459710019	0.111564859	0.328217459	0.002869445	0.145790914
TIMM17A	0.589444601	0.158474474	0.37544066	0.107264894	0.00041551	0.002865009	0.145790914
PPP1R3B	0.004479122	0.012612021	0.47218676	0.118029607	0.504119398	0.002896953	0.146734239
ABCD4	0.00193368	0.139373675	0.263529758	0.084065936	0.268279382	0.002916317	0.147260563
SATB1	0.808751354	0.020196199	0.439882096	0.003595974	0.062354827	0.00292855	0.147424657
CNTNAP1	0.092059696	0.204916903	0.280623356	0.369648472	0.000844445	0.002982969	0.14969101
NIP7	0.62660934	0.004910592	0.139978054	0.100457604	0.038346975	0.002991813	0.14969101
SVEP1	0.039990418	0.070083107	0.194269135	0.006405859	0.496575397	0.00308628	0.153948168
LOC526966	0.871234656	0.099089496	0.506390803	0.825810097	4.84E-05	0.003106822	0.154058002
NUP210	0.643077843	0.001461798	0.229373569	0.091494827	0.088615005	0.003107257	0.154058002
DNAJC3	0.64614627	0.7846063	0.037841004	0.012520767	0.00737557	0.003137307	0.154096358
ELOVL3	0.390939357	0.329815057	0.004711863	0.031231284	0.093315581	0.003135973	0.154096358
NUDT8	0.669647294	0.576902299	0.209821008	2.66E-05	0.828529256	0.00315498	0.154096358
TMEM242	0.000242927	0.092488139	0.92663606	0.676221543	0.126136701	0.003142698	0.154096358
TTC25	0.044827716	0.981607308	0.147522274	0.003460005	0.079243135	0.003147827	0.154096358
MMRN1	0.000144592	0.341670646	0.191861518	0.61298514	0.313988037	0.003204559	0.156053461
MOB3A	0.294873613	0.07592774	0.47704473	0.434723105	0.000394816	0.003215789	0.156137023
LMO7	0.585764242	0.95406301	0.029000761	0.000388413	0.2951383	0.003247129	0.157193601
CCDC171	0.080305519	0.390406034	0.000985528	0.431572255	0.140559692	0.003267823	0.157267581
GPM6A	0.001269354	0.052175934	0.126543107	0.370254794	0.601598648	0.003258339	0.157267581
GIMAP7	0.191518304	0.005650761	0.095291007	0.208532464	0.088434192	0.003302355	0.157990336
HHIP	0.000315216	0.016187819	0.823551368	0.970163369	0.465869632	0.003299248	0.157990336
MAT2A	0.288613028	0.124983793	0.000615171	0.087755688	0.980448621	0.003311722	0.157990336
SHROOM2	0.948700796	0.041595675	0.002745131	0.117741578	0.151446366	0.003339729	0.158864627
SYNM	0.00014738	0.226496022	0.460608617	0.823297431	0.154345309	0.003367346	0.159715375
KLF15	0.092965806	0.345323165	0.002095524	0.368145214	0.080207718	0.003407854	0.161170855
MON1A	0.057067451	0.016775995	0.55308058	0.448981967	0.008501862	0.00345076	0.162731095
DNAJB4	0.032111503	0.64528605	0.221201219	0.034363782	0.013036834	0.003490325	0.163024688
NR2F1	0.000808035	0.345845944	0.027217815	0.377193324	0.717540427	0.003496721	0.163024688
TTR	0.174282304	0.008808292	0.140538735	0.773678091	0.012249004	0.003479497	0.163024688
WNT4	0.056214379	0.031549571	0.008929169	0.485607716	0.264856488	0.003469954	0.163024688
NID2	0.128806766	0.415766519	0.341748688	0.261828994	0.000433336	0.003518622	0.163581037
CAPZA2	0.442927808	0.096258869	0.003888978	0.061548881	0.204285149	0.00352873	0.163587526
SMIM33	0.040361915	0.122079267	0.041788663	0.085063106	0.120368327	0.003557299	0.164447407
GLULP	0.890349485	0.573463832	0.000326379	0.124693775	0.102414745	0.003581398	0.165096417
CCDC141	0.097235418	0.004678502	0.182288119	0.621295801	0.042572696	0.003660178	0.166403166
DNER	0.735015454	0.169771166	0.001304423	0.132630051	0.101199253	0.003649752	0.166403166
KLHL13	0.000532774	0.149153483	0.502118549	0.09112768	0.603295704	0.003660444	0.166403166

TMEM151A	0.581073569	0.32519406	0.00313504	0.026740033	0.137397284	0.003639837	0.166403166
TOP1	0.233466134	0.416817054	0.913773579	0.000163161	0.150781801	0.003653235	0.166403166
CATSPERD	0.851747884	0.052595796	0.000764587	0.704595716	0.092344519	0.003702393	0.166465688
HSPD1	0.710104155	0.478772801	0.008369275	0.243422674	0.003208521	0.003694827	0.166465688
LOC617565	0.498479761	0.063154343	0.000166551	0.898033601	0.471357349	0.003691373	0.166465688
RPGRIP1L	0.190561581	0.812763633	0.000207432	0.192281951	0.357984994	0.003681835	0.166465688
LOC782779	0.401560411	0.231869184	0.048444128	0.005720126	0.088629301	0.003771567	0.169112528
LOC107131704	0.380784739	0.007361406	0.028362445	0.949250358	0.030792818	0.003815493	0.170152339
LOC112441594	0.642642584	0.000115247	0.302953938	0.269653933	0.383244932	0.003809461	0.170152339
PSMA7	0.31017841	0.088385581	0.42660397	0.205400943	0.000972126	0.003828956	0.170289943
GPX4	0.355763228	0.296014669	0.928004542	0.000678263	0.035882094	0.003879689	0.17115477
HSP90AB1	0.630444541	0.869342723	0.052778639	0.001279575	0.064235007	0.003878618	0.17115477
KHDC4	0.469377115	0.617416944	0.528116686	5.35E-05	0.290201278	0.003875433	0.17115477
ADA	0.450278288	0.892846872	0.304311423	3.56E-05	0.560865606	0.003955153	0.173595011
LYZ	0.137912445	0.204241374	0.008020667	0.042500134	0.254542219	0.00395616	0.173595011
DNAJB1	0.043956866	0.298135524	0.059284308	0.038169715	0.083402193	0.00399009	0.174616963
AASDHPPT	0.937818512	0.11740951	0.250890011	0.133366121	0.000688601	0.004063514	0.176032369
C6H4orf19	0.012091155	0.20792184	0.21006703	0.006374303	0.749100013	0.004045974	0.176032369
EVPL	0.9528089	0.01091706	0.013742118	0.04476298	0.399663882	0.004086791	0.176032369
LOC525426	0.62615426	0.613304755	0.000115518	0.292175105	0.196940349	0.004081432	0.176032369
OGT	0.479616356	0.098817274	0.106947521	0.646893456	0.000766739	0.004037162	0.176032369
SLC25A1	0.415130969	0.364942905	0.573692106	0.001441024	0.020387327	0.004082351	0.176032369
BAHCC1	0.889682197	0.267783181	0.001610809	0.069113751	0.099552242	0.004181579	0.179174672
LOC101906178	0.471833112	0.02720985	0.768892398	0.000483623	0.552241669	0.00417702	0.179174672
CYP4V2	0.001456582	0.123739442	0.139585852	0.253740951	0.415761189	0.004197061	0.179369724
LIMK2	0.055130609	0.112460167	0.010183935	0.056085237	0.77153532	0.004285076	0.182182352
LOC101907985	0.540288999	0.856057686	7.25E-05	0.120286849	0.675943104	0.004279186	0.182182352
L3MBTL1	0.998934261	0.253998752	0.007518639	0.014598103	0.098910568	0.004310104	0.182301832
STIP1	0.597276231	0.622031576	0.016421426	0.007914965	0.056956552	0.004305452	0.182301832
LAMB3	0.097299527	0.611543418	0.016630784	0.006065706	0.469525786	0.00438126	0.183639019
NINJ2	0.256358083	0.002227621	0.208005199	0.039044695	0.608226994	0.004384136	0.183639019
NUMA1	0.801757924	0.127492244	0.041202217	0.381718501	0.001755998	0.004386478	0.183639019
PRPF40B	0.998080509	0.249689829	0.000860429	0.318623283	0.041027572	0.004364296	0.183639019
MTFP1	0.0902894	0.17498019	0.186149972	0.02212107	0.043953451	0.004426892	0.184589968
RUVBL1	0.092432475	0.384665156	0.237124751	0.39144632	0.00086774	0.004431689	0.184589968
RASD2	0.036778335	0.760655055	0.200744444	0.794190086	0.000647236	0.00445702	0.184707463
ZMYM3	0.378440289	0.631477084	0.004402884	0.204013403	0.013433214	0.004453505	0.184707463
FAM207A	0.160328889	0.553690584	0.631072863	0.000284006	0.182447473	0.004474743	0.184974846
SGPL1	0.017666755	0.655306066	0.009469013	0.051054164	0.525932585	0.004519434	0.186352825
LOC104975034	0.048009096	0.691940843	0.525184161	0.059167539	0.002869101	0.004539288	0.18670239

TXNL4A	0.465955991	0.405859954	0.015947515	0.014661093	0.067244374	0.004552011	0.186757636
GPR173	0.073208792	0.310439438	0.010661241	0.014013409	0.907318999	0.004668754	0.189424356
LOC101902293	0.887740595	0.13477456	0.192008591	0.612883993	0.000218619	0.00466592	0.189424356
LOC112441884	0.002280542	0.398505117	0.3209172	0.314421039	0.033643224	0.004673529	0.189424356
MYBL2	0.060559219	0.002077872	0.555646001	0.096141747	0.457099162	0.004660085	0.189424356
STEAP2	0.000710842	0.579797258	0.033097988	0.444567846	0.50890948	0.004674722	0.189424356
ADAMTS5	0.481484756	0.090709626	0.077256257	0.002255123	0.408760494	0.004700726	0.190008909
GALNT5	0.867688153	0.000773141	0.076712176	0.189356116	0.331002457	0.004824078	0.194515848
ELF3	0.060150632	0.174557458	0.213801126	0.008722666	0.165344503	0.004836992	0.194558536
EAF2	0.238014409	0.087201403	0.003619818	0.338648014	0.12854798	0.004872005	0.194627749
HINT3	0.040094045	0.419208313	0.294749344	0.14173958	0.004660792	0.004874292	0.194627749
LYPD6	0.004837321	0.009721989	0.498739919	0.665118093	0.208702702	0.004856328	0.194627749
FBN2	0.446758509	0.31285119	0.010614465	0.888429442	0.002515543	0.004919632	0.19596137
NAIP	0.331551134	0.000310403	0.819931642	0.250763239	0.157425619	0.004936022	0.196138148
SREBF1	0.000202474	0.254428068	0.827800463	0.404755769	0.193870095	0.004951977	0.196296866
LOC101906828	0.022979007	0.00951227	0.194875807	0.577640062	0.137328793	0.004986394	0.197184847
NR3C2	0.00330874	0.173115689	0.270592736	0.2017538	0.110036404	0.005051149	0.199265391
ERGIC2	0.881937802	0.411948839	0.417834793	0.310063968	7.42E-05	0.005106558	0.199532677
GZMB	0.170371323	0.090589995	0.001177262	0.222688406	0.860942506	0.005095551	0.199532677
SPRN	0.433261503	0.002382882	0.083737863	0.704682086	0.057311294	0.005103781	0.199532677
TMEM120A	0.232565825	0.229895946	0.009722029	0.023449796	0.285255509	0.005088766	0.199532677
CHORDC1	0.722496628	0.641545526	0.076572916	0.001077873	0.093277604	0.005183494	0.201029339
CREM	0.165953458	0.937180877	0.550808515	6.32E-05	0.68102531	0.005304107	0.201029339
HEXIM1	0.212178028	0.210926466	0.003062216	0.076078278	0.349599453	0.00526216	0.201029339
KBTBD11	3.82E-05	0.909984138	0.574169166	0.375736888	0.490868606	0.00529551	0.201029339
KCNA3	0.219670527	0.971613162	0.292734335	0.533836892	0.000109376	0.005265352	0.201029339
LMOD1	0.028879582	0.325133878	0.728620567	0.263718299	0.002024833	0.005270638	0.201029339
LOC104975686	0.157566755	0.54310539	0.00206251	0.342094313	0.059655832	0.005218013	0.201029339
MYH3	0.944114462	0.220231665	0.000156215	0.121154641	0.903677666	0.005170781	0.201029339
NPPC	0.091149566	0.134995667	0.069107069	0.042021587	0.099622667	0.005174551	0.201029339
RCAN1	0.973799154	0.612160231	0.000162759	0.297802065	0.126830867	0.005282256	0.201029339
RGS7	0.00264631	0.10239613	0.205151896	0.972205852	0.066955282	0.005235091	0.201029339
WDR19	0.332377874	0.015124522	0.016218079	0.475230417	0.094499323	0.00527893	0.201029339
ZNF341	0.869244449	0.030937797	0.02410457	0.19793676	0.028710787	0.005301886	0.201029339
ATAD3A	0.480106539	0.076359643	0.934704086	0.1005473	0.001078117	0.005333236	0.201667579
FAM136A	0.339478224	0.079603936	0.569298265	0.343511386	0.00071124	0.005378111	0.202722299
LOC533307	0.289099988	0.10891443	0.001510563	0.395075713	0.200433294	0.005385834	0.202722299
BEND6	0.310628665	0.682288686	0.030962569	0.006238989	0.09268145	0.005414316	0.202863794
HHEX	0.13732625	0.348551944	0.012472431	0.008448378	0.750157589	0.00540323	0.202863794
DDX3X	0.288243441	0.136472546	0.034921609	0.004402579	0.630339128	0.005432254	0.203072241

ASZ1	0.366914995	0.566547159	0.349490282	0.004751083	0.011198402	0.005485736	0.204605489
LOC112444164	0.062582706	0.046593185	0.048559785	0.037055458	0.73970859	0.005501696	0.204735443
ADAM12	0.690954835	0.008971991	0.005353547	0.271892151	0.436666433	0.005560866	0.205353995
C2CD2L	0.010038428	0.21720223	0.20570157	0.127327632	0.069594544	0.005594861	0.205353995
CCNJ	0.028041519	0.954671929	0.001024581	0.264082994	0.549782685	0.005602782	0.205353995
GBP5	0.081063669	0.17352581	0.157368504	0.003416719	0.526396116	0.005601838	0.205353995
KLK7	0.416058624	0.063332907	0.008204871	0.191635694	0.096134383	0.005603489	0.205353995
LOC101904573	0.701400853	0.466183509	0.411454646	7.41E-05	0.397364719	0.005581106	0.205353995
NKRF	0.31260268	0.007101193	0.060477533	0.101594392	0.292205343	0.00560591	0.205353995
HPD	0.02606425	0.772281849	0.11717504	0.002242812	0.756611395	0.005622799	0.205513929
RNF146	0.167213227	0.631360072	0.040173649	0.050068414	0.019005413	0.005655954	0.206266345
KCNH8	0.01932454	0.443617088	0.009664447	0.356455874	0.137155702	0.005670566	0.206325211
TMED10	0.319268318	0.000312423	0.174667888	0.320903834	0.726675503	0.005682713	0.206325211
LOC112441505	0.203877002	0.429115291	0.000380202	0.492314501	0.249924268	0.005712221	0.206938756
LOC516494	0.156560984	0.203697629	0.077961484	0.008707902	0.190837415	0.005750659	0.207415528
SPOCK2	0.295219154	0.001159376	0.227876421	0.288393393	0.183491878	0.005746388	0.207415528
BHLHE40	0.513568276	0.41163116	0.538342187	4.68E-05	0.782096518	0.005785219	0.20774885
TBC1D4	0.580682569	0.448200923	0.001487822	0.040298277	0.266946158	0.00578395	0.20774885
MSTO1	0.675954833	0.056081427	0.681078943	0.032641389	0.00497637	0.005811716	0.207791007
NRM	0.373672582	0.079382646	0.295040131	0.000858889	0.556966142	0.00580461	0.207791007
LIMS1	0.125543731	0.2224457	0.053342708	0.268796012	0.01071273	0.005905124	0.210671732
NUS1	0.309859461	0.072251391	0.011423842	0.149859449	0.113150711	0.005950883	0.211843674
DNAJA4	0.334796845	0.071111177	0.017986159	0.06316352	0.162691285	0.006012361	0.21274127
LOC112449258	0.930823634	0.383329821	0.000288012	0.84660899	0.050608452	0.006014987	0.21274127
SEMA3F	0.006475904	0.550408307	0.749416622	0.005635613	0.292458844	0.006014573	0.21274127
ENO2	0.012275015	0.459128033	0.542195723	0.545083773	0.002696878	0.006100395	0.214835999
JPH2	0.00012251	0.505347235	0.299296318	0.912005587	0.265643189	0.006097644	0.214835999
DTL	0.034698163	0.499644426	0.180097037	0.057603965	0.025086377	0.006119522	0.21504812
MRPL16	0.495110444	0.161704796	0.851816838	0.096547013	0.000696273	0.006188707	0.21701469
PARM1	0.618798315	0.269462803	0.504538812	0.010124615	0.00541936	0.006218699	0.21713846
PROX1	0.007196789	0.024288175	0.092841418	0.763177992	0.371889191	0.006209078	0.21713846
SLC16A14	0.273173227	0.591894011	0.058246418	0.001868162	0.263313002	0.006234553	0.217229819
CDC42EP3	0.015071982	0.06181003	0.790362825	0.030069005	0.211412427	0.006279895	0.217424818
PLD4	0.008588322	0.023070516	0.215818576	0.317083738	0.344427627	0.006269932	0.217424818
TP53INP1	0.226160533	0.227270254	0.00202837	0.53165877	0.084274929	0.006271066	0.217424818
LOC104975663	0.243261245	0.008373554	0.70119494	0.015141717	0.218266701	0.006317473	0.218198571
LOC112449100	0.221475295	0.0265093	0.771979888	0.001543686	0.676400371	0.006328836	0.218198571
TPBG	0.034673386	0.01845247	0.05157332	0.435392286	0.331985184	0.006363639	0.218938548
BAG2	0.147210542	0.134576605	0.256932588	0.637006552	0.001502058	0.006457997	0.219609182
CFI	0.006931499	0.171123819	0.010690249	0.798926579	0.482748819	0.006476805	0.219609182

MKX	0.00077673	0.548446502	0.203560188	0.819480241	0.068425258	0.006450614	0.219609182
PYCR2	0.809462473	0.615951307	0.702174143	3.17E-05	0.437680368	0.006449351	0.219609182
SIGLEC11	0.005969291	0.110695796	0.634584187	0.04930556	0.233340448	0.006414936	0.219609182
SLC4A11	0.089379661	0.008041468	0.428405077	0.416625466	0.038052711	0.006468486	0.219609182
TM9SF2	0.13084605	0.210513346	0.003616194	0.067046273	0.726734725	0.006442158	0.219609182
NAPSA	0.5759986	0.004139185	0.385773636	0.081626721	0.06539135	0.006494325	0.219749224
LOC100196897	0.003535888	0.620581119	0.045762699	0.089917204	0.545569594	0.006509912	0.219823385
GATAD2A	0.323069895	0.560537275	0.000501889	0.433925848	0.128306091	0.006634058	0.222382786
LOC101907219	0.723616581	0.177464706	0.02396123	0.101511995	0.016143361	0.006617645	0.222382786
NOA1	0.789195829	0.293287413	0.663914581	0.047350484	0.000696309	0.00663991	0.222382786
PLD5	0.561913092	0.001250633	0.016770035	0.821123066	0.51982875	0.006606524	0.222382786
LY75	0.46815923	0.016219758	0.16757447	0.156443337	0.02553342	0.006654915	0.222431387
SLC30A4	0.863461749	0.49128413	0.069155682	0.000468393	0.373256069	0.006697128	0.223387326
HSPA4L	0.509356412	0.529030441	0.183070387	0.002442631	0.042874556	0.006731411	0.224075432
DTD2	0.531856881	0.040437341	0.196798309	0.001984498	0.618447317	0.006757299	0.224481852
DNAJC16	0.155757912	0.543580634	0.416250719	0.293688597	0.000507469	0.006810084	0.22577837
CXCL10	0.04378233	0.089702229	0.016830581	0.972146037	0.083003003	0.006883774	0.225939242
DUOXA2	0.601785271	0.000871764	0.027191945	0.463629209	0.805911705	0.006880576	0.225939242
ITGA2B	0.719200421	0.006104509	0.049033255	0.276163696	0.089390173	0.00686624	0.225939242
LOC112446690	0.172676021	0.976929721	0.046473086	0.045235195	0.014957408	0.00685717	0.225939242
RRS1	0.555201231	0.021775496	0.023438623	0.211126631	0.088600808	0.006853864	0.225939242
LTB	0.535697323	0.244411139	0.070252174	0.013282762	0.043962219	0.006917678	0.226598845
TAF4B	0.647207911	0.818663456	0.000434465	0.046304572	0.510157592	0.006977852	0.227882813
UBIAD1	0.388441635	0.28372628	0.021967432	0.066933221	0.033603799	0.006984648	0.227882813
AHSA1	0.690206216	0.532452311	0.014532113	0.02730513	0.037606794	0.007019281	0.228558381
ANGPTL8	0.287393929	0.613391049	0.125838997	0.001767166	0.141883711	0.007089188	0.228935447
ARSI	0.634502326	0.048933579	0.013688022	0.022763621	0.573408153	0.007076001	0.228935447
HSPA8	0.486095407	0.418757009	0.029933813	0.010793381	0.085712551	0.007155907	0.228935447
KCNAB1	0.922705104	0.414048771	0.481193923	0.26091918	0.000117301	0.007146603	0.228935447
LOC100139916	0.030289642	0.517626095	0.089146172	0.040291552	0.099414802	0.007121717	0.228935447
MYH11	0.000258496	0.578404957	0.397445132	0.887170095	0.10723309	0.007170363	0.228935447
NTRK3	0.000172824	0.718666857	0.933193801	0.611922818	0.077920361	0.007057348	0.228935447
NUDT9	0.479457182	0.090408743	0.001694796	0.236852648	0.320300192	0.00709918	0.228935447
PDE3A	5.36E-05	0.469161102	0.716934071	0.7765757	0.403475572	0.00716713	0.228935447
PDLIM3	0.001136244	0.253922123	0.371344258	0.648911462	0.080219482	0.007102628	0.228935447
GAL	0.604651719	0.390155152	0.02628536	0.005069603	0.18196361	0.007229749	0.230383316
ARL8B	0.494801914	0.233440539	0.003842446	0.582343321	0.022392095	0.007289114	0.231376486
WAPL	0.696764017	0.046308949	0.380069408	0.000633496	0.743995176	0.007282516	0.231376486
DNAJA3	0.222412526	0.289880347	0.284123509	0.137647187	0.002373166	0.007461227	0.234571257
HEATR4	0.569612619	0.056306278	0.005651085	0.537059185	0.061164163	0.007434932	0.234571257

LOC101908206	0.138344444	0.396090081	0.000175979	0.815118435	0.759463831	0.007448804	0.234571257
LOC513508	0.411909718	0.006951732	0.059570595	0.966270117	0.036007791	0.007418606	0.234571257
PTI	0.05525109	0.080329992	0.049368651	0.51901622	0.05205758	0.00740563	0.234571257
AMIGO2	0.005148545	0.112469938	0.050670437	0.627552885	0.330550864	0.007550408	0.235339417
DAPK2	0.962118309	0.189347474	0.25901836	0.000264208	0.490176446	0.007571729	0.235339417
PTPRE	0.628178237	0.041964497	0.110926439	0.122932184	0.01704613	0.007586043	0.235339417
RRP15	0.40052752	0.051717774	0.001498025	0.770667732	0.254809376	0.007556601	0.235339417
STBD1	0.010987228	0.045616332	0.733678256	0.072399401	0.229789796	0.007577366	0.235339417
TRAPPC8	0.735951987	0.36509122	0.000568304	0.05720961	0.696646533	0.007549786	0.235339417
VWCE	0.531241833	0.096709028	0.007548367	0.287908822	0.054551665	0.007554187	0.235339417
OLFML2A	0.060501372	0.19749152	0.021329066	0.075023297	0.323636964	0.007638049	0.236270019
PCDH19	0.543465196	0.80579544	0.399039856	0.027056837	0.001310395	0.007644835	0.236270019
COX19	0.000165248	0.178771276	0.585216103	0.855538614	0.420775156	0.007668818	0.236565746
LOC783797	0.143558364	0.832233305	0.05450404	0.510301617	0.001889996	0.00771766	0.237504965
TMEM82	0.038135393	0.853890661	0.582114141	0.00398986	0.083346208	0.007737432	0.237504965
WWP1	0.870812925	7.51E-05	0.902242373	0.421495943	0.253561906	0.007742682	0.237504965
PPA1	0.550942088	0.175493661	0.892221377	0.046131476	0.001595543	0.007776863	0.238108408
LOC101902413	0.446953231	0.134593522	0.009545051	0.304464024	0.036686728	0.007831566	0.238902268
PPIF	0.873635702	0.265525221	0.635691294	0.05033934	0.000864065	0.007831907	0.238902268
LOC404103	0.078420238	0.037123671	0.13914745	0.156525963	0.101450294	0.00784774	0.238941109
COL5A2	0.38266759	0.033932437	0.008734941	0.500408319	0.114756956	0.007916157	0.239690123
TEP1	0.886824764	0.06577952	0.001116731	0.71969808	0.138401059	0.007895424	0.239690123
TFCP2	0.335110486	0.477017252	0.037715033	0.003583968	0.301331454	0.007914187	0.239690123
TRAF3	0.291529489	0.8457392	0.101943617	0.007963726	0.032783742	0.007957605	0.240501393
ZGRF1	0.816434798	0.150105488	0.022637896	0.715297425	0.003381872	0.008082981	0.243841541
ATP2B2	0.024353499	0.003796512	0.439578476	0.453946432	0.36685454	0.008130859	0.244677745
CEP131	0.884538943	0.547169368	4.58E-05	0.391855194	0.788164757	0.008192916	0.244677745
LOC100300510	0.023734043	0.01502729	0.506508853	0.371558172	0.101169767	0.008149599	0.244677745
MCMBP	0.165520426	0.518626386	0.017783872	0.014690219	0.306210447	0.00821338	0.244677745
PPID	0.976919728	0.517066744	0.017628828	0.013691922	0.056339825	0.008215065	0.244677745
RGMA	0.83031842	0.674622994	0.414316114	0.185876416	0.000157617	0.008156764	0.244677745
SRGAP3	0.000456698	0.782127788	0.851747743	0.195947623	0.114895634	0.008198658	0.244677745
GUF1	0.73947193	0.426902125	0.384789274	0.003768525	0.015050604	0.008232058	0.24473968
TMCC1	0.524800739	0.771445835	0.000214261	0.191322119	0.41984588	0.008296943	0.24622266
ECM1	0.840899787	0.048524495	0.017230112	0.513503617	0.019536111	0.008367345	0.246870483
PCBP1	0.824224854	0.115148613	0.153149005	0.009060309	0.053559755	0.008367682	0.246870483
RGS7BP	0.374876463	0.059093675	0.207344503	0.032670661	0.046883277	0.008352922	0.246870483
ZNF684	0.957401468	0.314501343	0.038221835	0.001562051	0.393112543	0.008378945	0.246870483
HOXA9	0.43345513	0.003579816	0.107501301	0.044622238	0.955448034	0.008415734	0.247510058
PUM2	0.279805202	0.302179204	0.002447461	0.045014242	0.76877453	0.008456349	0.248259655

LOC505099	0.186502253	0.000241572	0.985345085	0.319598018	0.512631436	0.008548078	0.250504491
ADAMTS17	0.423054598	0.017022214	0.003132339	0.796941145	0.411606501	0.00865068	0.250879657
CACNA1G	0.93521309	0.206603894	0.000188857	0.496599109	0.407884949	0.008644263	0.250879657
PRDM1	0.160002624	0.512398517	0.170908148	0.627717003	0.000841525	0.008652604	0.250879657
RGS12	0.461765062	0.210570612	0.000215403	0.362608974	0.969056949	0.008618489	0.250879657
SMARCA5	0.679708914	0.583032491	0.027592302	0.001157857	0.58329597	0.008639039	0.250879657
SMPD3	0.268752255	0.367043407	0.000488029	0.563944396	0.271084698	0.008618469	0.250879657
EN1	0.589171061	9.53E-05	0.65418708	0.972975153	0.209110816	0.008713225	0.252191761
APBA1	0.00043795	0.220801651	0.744265734	0.4920084	0.213564863	0.008782561	0.252926953
CX3CR1	0.712415571	0.043425852	0.001340789	0.688472289	0.26486829	0.008783982	0.252926953
TEC	0.977957634	0.049653163	0.663018257	0.00024235	0.969585138	0.008784862	0.252926953
LOC112441499	0.16001114	0.014439124	0.025936534	0.146414053	0.865889473	0.008810534	0.25322184
LOC112447832	0.001159385	0.998424108	0.960504035	0.824853401	0.008366385	0.008871362	0.254080141
TMEM203	0.830233818	0.017971931	0.122304926	0.011446767	0.366909003	0.008864619	0.254080141
NOX5	0.123960474	0.930353123	0.026644176	0.116661045	0.021607982	0.008929861	0.255310027
WDR13	0.551678433	0.000593791	0.667484955	0.055493478	0.640449392	0.008950046	0.255442106
GLIS3	0.041730007	0.156208242	0.171186412	0.04496852	0.156036918	0.008996869	0.25576994
GREB1L	0.178754775	0.274880411	0.640168778	0.032480974	0.00768092	0.009010957	0.25576994
LOC518526	0.742411261	0.306872746	0.15377049	0.947711745	0.000236511	0.009014761	0.25576994
POLR2K	0.54482437	0.00017965	0.338254346	0.874337274	0.271662806	0.009023874	0.25576994
FAM174A	0.010140061	0.331617573	0.553225801	0.448080945	0.0094601	0.009041118	0.255816879
H1F0	0.84470603	0.36572669	0.000103345	0.520993381	0.477643347	0.009088168	0.255896705
IL7	0.371968811	0.001729327	0.027097041	0.839109226	0.544346119	0.0091014	0.255896705
LOC107131817	0.307758407	0.655327406	0.041373102	0.413222713	0.002315978	0.009120374	0.255896705
PIEZO2	0.432313366	0.149066351	0.002510266	0.292561022	0.168770178	0.009121904	0.255896705
PRLR	0.000700922	0.424996065	0.072702708	0.960470192	0.383131041	0.009107753	0.255896705
MYRF	0.642234139	0.030581252	0.047734154	0.047594264	0.180668569	0.009180336	0.256221922
UBB	0.132125228	0.525764519	0.095275031	0.71400355	0.001705483	0.009178707	0.256221922
YBX3	0.309874908	0.512679668	0.025621766	0.19221744	0.010275813	0.009163308	0.256221922
LOC534967	0.212937625	0.864799948	0.842934819	0.000298505	0.175546145	0.009237435	0.257377824
CD2	0.117579136	0.013278506	0.447475861	0.426534768	0.027585292	0.00930503	0.257593401
GIMAP6	0.523168489	0.002391373	0.305559319	0.352354854	0.060792509	0.009280361	0.257593401
LOC101904177	0.541538866	0.199867532	0.803017514	0.006922179	0.013659791	0.009303588	0.257593401
PDE11A	0.083225737	0.00124123	0.202844556	0.508693348	0.771523404	0.009307957	0.257593401
C2H2orf72	0.007568301	0.175609124	0.023103203	0.411973009	0.656645619	0.009372507	0.258943116
VANGL2	0.380601624	0.34300765	0.02496412	0.087332295	0.029515462	0.009445881	0.260531685
BMPER	0.15623314	0.763184684	0.038078166	0.316532074	0.005900851	0.009507664	0.261333043
LOC104975091	0.09925191	0.373258641	0.000421553	0.599075929	0.90849289	0.009522708	0.261333043
SMTNL2	0.498679153	0.927588443	0.000355754	0.129960673	0.395655361	0.00949322	0.261333043
HBB	0.373656077	0.139820504	0.08800758	0.020926065	0.088960125	0.009568899	0.261822125

PIGZ	0.757938577	0.214509617	0.180824088	0.161018248	0.001809115	0.009572438	0.261822125
DCAF1	0.5199822	0.922783162	0.71726879	2.69E-05	0.933928936	0.009637564	0.26215719
PNPLA3	0.82846486	0.487843841	0.860614168	0.009253529	0.002691467	0.009648586	0.26215719
POLR3A	0.112354149	0.576692354	0.244445935	0.172640693	0.003166229	0.009644624	0.26215719
TSR1	0.20364229	0.105118773	0.066885881	0.555260925	0.010859482	0.009626041	0.26215719
LRRC24	0.019475648	0.347561234	0.052786862	0.054137252	0.450972082	0.009695263	0.262990007
CACNA1A	0.616141017	0.008868192	0.30058319	0.01040937	0.512456257	0.00972407	0.263336155
DARS	0.834012667	0.091758422	0.404517681	0.18476802	0.001537499	0.009749401	0.263342308
TMEM100	0.982195306	0.003478151	0.819676017	0.012173675	0.258248904	0.00975639	0.263342308
POLR1B	0.98102697	0.026728171	0.043852425	0.62920668	0.01227551	0.009815921	0.264514086
LCMT2	0.882324283	0.052297505	0.221773443	0.027939775	0.031491861	0.009909349	0.266593976
C18H19orf18	0.626027926	0.434795217	0.146761599	0.001978244	0.115381501	0.009995742	0.266731907
C5H12orf29	0.954106509	0.048464016	0.238966928	0.156053699	0.005279466	0.009984819	0.266731907
IMP3	0.445055854	0.003468974	0.86370479	0.374556025	0.018248777	0.009992958	0.266731907
RTEL1	0.374281695	0.007398161	0.019208227	0.312381009	0.545798399	0.009958038	0.266731907
ZSWIM6	0.833731033	0.800571332	0.690214783	2.51E-05	0.78176661	0.009940378	0.266731907
CACYBP	0.846771029	0.476977877	0.017688506	0.031213628	0.041079831	0.010027867	0.267154753
ZFAND2A	0.092176542	0.395047839	0.137939382	0.264867728	0.00699818	0.010140586	0.269719866
IFT20	0.033339203	0.00764047	0.624129867	0.132001372	0.445346685	0.010167233	0.269991041
LOC101903832	0.242978367	0.258557984	0.348448631	0.002975429	0.144142111	0.010199179	0.27040181
PACS2	0.14522221	0.087898424	0.224251592	0.024830866	0.132594294	0.010226087	0.270677931
ANO8	0.012532251	0.195317061	0.046750961	0.131966435	0.629347717	0.010285398	0.271388344
ARFGAP1	0.024461689	0.09157122	0.744643532	0.126271396	0.045128622	0.010286	0.271388344
DUSP16	0.740104315	0.557993058	0.800085114	0.001012258	0.028704722	0.010357188	0.272482518
PLCL2	0.959290888	0.027634448	0.084692636	0.034074841	0.125555126	0.010360678	0.272482518
C18H16orf86	0.983131496	0.005102298	0.058312727	0.443446272	0.074284691	0.01038313	0.272636068
LOC782177	0.2114052	0.036041706	0.011915664	0.840697616	0.126915481	0.010421242	0.272763961
NDUFA3	0.558311216	0.349078182	0.770129799	0.000887545	0.072654283	0.010414984	0.272763961
TBL2	0.37244968	0.006827181	0.393848412	0.023159905	0.420637597	0.010472364	0.273230457
XK	0.553624672	0.018272357	0.003359302	0.885654484	0.323793818	0.010464221	0.273230457
CHP2	0.121083799	0.204175737	0.019983614	0.09089464	0.220562579	0.010581563	0.275477445
IFIT3	0.001320773	0.614520468	0.500626941	0.037389972	0.657390763	0.010642417	0.275477445
MAX	0.419318535	0.393152151	0.033011874	0.01614256	0.113663295	0.010640904	0.275477445
PNPLA7	0.664024324	0.070042209	0.015601859	0.888056495	0.015399606	0.010595576	0.275477445
ZBTB40	0.783485192	0.025724025	0.200704593	0.253681044	0.009694727	0.010613708	0.275477445
PHAX	0.797548057	0.061611983	0.172796488	0.757635865	0.001564037	0.01069656	0.276442902
AHSA2	0.772817283	0.695148966	0.054804898	0.005249104	0.066486406	0.010852094	0.276543046
ARHGAP28	0.320583143	0.57793461	0.001819789	0.085896407	0.352657266	0.010807165	0.276543046
CCDC68	0.84858748	0.495894576	0.000179714	0.501047815	0.269584777	0.01080843	0.276543046
GPC3	0.104573647	0.014077582	0.414657226	0.082495541	0.203060694	0.010816187	0.276543046

HSD11B2	0.050752436	0.972390169	0.02810069	0.010790976	0.678095256	0.010759375	0.276543046
PCYT2	0.584470042	0.195150985	0.896912988	0.001152107	0.086809041	0.010820383	0.276543046
PROCR	0.381401906	0.001817217	0.582423672	0.217106625	0.115161745	0.010719356	0.276543046
SCN4B	0.01046475	0.81404224	0.171889667	0.500116914	0.01401414	0.01084303	0.276543046
TSC22D3	0.338130019	0.25774346	0.003159312	0.085712125	0.43008354	0.01076096	0.276543046
FCRL5	0.101942996	0.0315435	0.186161955	0.230593789	0.075051858	0.010913598	0.277394113
FKBP5	0.54635081	0.644033265	0.054579927	0.0563721	0.009595204	0.010933693	0.277394113
RASSF10	0.002136818	0.221183683	0.098630402	0.729824108	0.306128721	0.010953104	0.277394113
RNASE1	0.698387641	0.032235197	0.011625957	0.802270927	0.049515252	0.010940336	0.277394113
ERH	0.87797283	0.009086301	0.309822627	0.048183762	0.088330487	0.011028449	0.278015179
LOC100300806	0.116115152	0.282125293	0.002945252	0.141623573	0.768296467	0.011013217	0.278015179
LOC107131530	0.026565071	0.791118238	0.96481137	0.125047135	0.004134674	0.011002645	0.278015179
ABT1	0.357753768	0.003431065	0.073014249	0.524246116	0.229295496	0.011210324	0.279769977
CALM	0.956227892	0.001741564	0.021823193	0.875430464	0.334893174	0.011125619	0.279769977
COX8B	0.041035562	0.08423569	0.947544168	0.00390212	0.844080706	0.011220751	0.279769977
FERMT1	0.205519421	0.043714197	0.020904785	0.452947034	0.126797066	0.011219637	0.279769977
FUBP1	0.922751156	0.895349585	0.586735594	2.38E-05	0.933697729	0.011222421	0.279769977
KIF14	0.003384397	0.088487376	0.427517174	0.13776193	0.610155989	0.011202094	0.279769977
KMT5C	0.964070059	0.028698926	0.031923649	0.119265119	0.102430346	0.011222314	0.279769977
RBM20	0.069267597	0.167440937	0.43979659	0.65602053	0.003233812	0.01124442	0.279769977
TFRC	0.023428876	0.010058891	0.300200835	0.480548736	0.318583421	0.011251489	0.279769977
ERO1B	0.00886079	0.010234702	0.665395447	0.618214445	0.291208998	0.011274547	0.279919192
CBX2	0.863829209	0.948129411	0.020790771	0.001104602	0.582825765	0.011344831	0.280154421
CYSLTR1	0.009737952	0.192857664	0.039936544	0.629953318	0.231413506	0.011324475	0.280154421
GEMIN6	0.322405572	0.141250365	0.6940837	0.018181457	0.019093937	0.011352306	0.280154421
ZBTB26	0.678929729	0.636727929	0.015308243	0.40311016	0.004090386	0.011308764	0.280154421
CADM2	0.000668774	0.28229079	0.235344967	0.946160375	0.261946162	0.011379723	0.280188981
SHC2	0.032470776	0.95836485	0.006652243	0.140710387	0.378436055	0.011387853	0.280188981
ACTR3	0.552660551	0.001903318	0.129943858	0.118532648	0.686486996	0.01145784	0.280648666
EHF	0.048284685	0.54071304	0.964534285	0.001041272	0.423207401	0.011440069	0.280648666
LOC101905514	0.063215182	0.163751112	0.139418816	0.193404505	0.039826517	0.011453686	0.280648666
AEBP2	0.192787435	0.464371956	0.996876863	0.000539278	0.233092046	0.011525416	0.281157318
CRB3	0.035691043	0.042572858	0.102538343	0.44406982	0.162589092	0.011547135	0.281157318
MARC1	0.730686401	0.09370055	0.000682085	0.340176761	0.707972918	0.011545534	0.281157318
PGS1	0.019569359	0.059635894	0.476203165	0.323502672	0.062473028	0.011534877	0.281157318
LOC107131896	0.783481895	0.009768914	0.048296525	0.066328289	0.46256436	0.011611746	0.281622782
PKIB	0.54389481	0.017988384	0.873508007	0.08213819	0.016191575	0.011629066	0.281622782
RRAGD	0.229815618	0.132113535	0.007115947	0.543811655	0.096541614	0.011612852	0.281622782
UQCRQ	0.330225184	0.168008084	0.598124605	0.003736673	0.091729511	0.011634894	0.281622782
CEBPZ	0.156742754	0.728931666	0.019506682	0.026272819	0.196495005	0.011726689	0.283345737

SOST	0.163599442	0.273378305	0.166314937	0.068047247	0.022770967	0.011740607	0.283345737
NME2	0.216438805	0.360028007	0.62419415	0.407993919	0.000594916	0.011934948	0.287191254
SPOUT1	0.255113185	0.590057233	0.704632362	0.031570452	0.003519611	0.011921155	0.287191254
CXCR6	0.390979227	0.012301124	0.577040889	0.846309647	0.005049595	0.011972384	0.287670262
ACAP1	0.56414457	0.012070172	0.016979213	0.323808243	0.319470865	0.012041303	0.288060967
HDLBP	0.174120648	0.119932675	0.273576985	0.892657902	0.002343452	0.012035015	0.288060967
TRAF3IP3	0.609307716	0.02421324	0.185343831	0.230810156	0.018875994	0.012009001	0.288060967
SDS	0.582181294	0.126693629	0.02938955	0.031774183	0.174098579	0.012062812	0.28815548
ACTR2	0.496118715	0.023549161	0.024988422	0.431530654	0.096190381	0.012149742	0.289810192
HSF4	0.766377358	0.040993756	0.000811258	0.750174127	0.641856905	0.012254747	0.290523864
LMBR1L	0.878037896	0.001477613	0.023374292	0.46689708	0.868126441	0.012268176	0.290523864
LOC112441605	0.538043833	0.861167206	0.000173271	0.547480194	0.278745984	0.012241082	0.290523864
RELN	0.001207566	0.263306369	0.458920345	0.781897381	0.107555419	0.01225421	0.290523864
WT1	0.550749124	0.003089354	0.225430867	0.095168841	0.336424101	0.012260473	0.290523864
NDOR1	0.009433354	0.143222812	0.229350791	0.055069264	0.723041477	0.012299715	0.290851039
ABHD6	0.228958198	0.000986545	0.161127113	0.961435283	0.353622561	0.012323896	0.291003546
APH1B	0.912126483	0.805279199	0.542331452	0.539739881	5.77E-05	0.01235041	0.29121061
SLC4A8	0.49290992	0.401336277	0.001354577	0.05021818	0.930157334	0.012420947	0.292453601
BGLAP	0.839252009	0.226987397	0.006249624	0.502853447	0.020993786	0.012455767	0.292853274
EFNB2	0.224843775	0.249160922	0.000653321	0.351568854	0.987217745	0.012546618	0.292907029
LOC112441476	0.705878626	0.056812336	0.05643398	0.117478583	0.047578356	0.01251065	0.292907029
MRPS7	0.387863368	0.267145865	0.483842591	0.074453893	0.003410614	0.012565142	0.292907029
OXTR	0.204263381	0.107063204	0.022764839	0.088526863	0.288369729	0.012550736	0.292907029
RNF182	0.004123551	0.095551075	0.162455416	0.217869168	0.907179362	0.012511603	0.292907029
STK38L	0.117089981	0.352088641	0.003861675	0.347121835	0.228551078	0.012497525	0.292907029
LOC112447762	0.281977524	0.5006731	0.059934114	0.083047212	0.018168587	0.012589567	0.293060122
CCSAP	0.166993269	0.364153385	0.573415214	0.48415518	0.000757949	0.012609101	0.293099094
CEP57	0.002661023	0.859919757	0.713447393	0.328268386	0.023953974	0.012636776	0.293326927
COG2	0.777053101	0.112666824	0.229985518	0.002045912	0.313175741	0.012679402	0.293900648
ATP5MD	0.535976064	0.202385407	0.736515975	0.002473634	0.065755612	0.012742238	0.294307391
GRHL2	0.08880858	0.275325365	0.002156455	0.754829113	0.328441947	0.012793756	0.294307391
LOC101905866	0.652088169	0.896286334	0.000779897	0.128873675	0.222808849	0.01280455	0.294307391
LOC526488	0.558215634	0.028848763	0.867584194	0.089445018	0.010390377	0.012735314	0.294307391
MKS1	0.845380515	0.521549828	0.001150088	0.333753307	0.077304377	0.012800976	0.294307391
TP53BP1	0.407746409	0.456981954	0.066770658	0.093570439	0.011226772	0.012792143	0.294307391
COL5A1	0.440997768	0.020448928	0.003921344	0.587596649	0.633674402	0.012856841	0.295095974
GPX3	0.914806497	0.045484487	0.077000466	0.100225279	0.041288782	0.012917577	0.296075915
ARFGAP3	0.869100245	0.091726253	0.724004246	0.145198075	0.001597113	0.013000923	0.296153272
FZD10	0.123603242	0.013987559	0.250677553	0.280972197	0.109414645	0.012960767	0.296153272
LOC112442189	0.135255143	0.091687577	0.016086024	0.97784187	0.068402764	0.012973547	0.296153272

OSCAR	0.159266727	0.005984906	0.262183511	0.142221949	0.37729514	0.013017923	0.296153272
XPOT	0.928744624	0.205323406	0.959671702	0.743802467	9.86E-05	0.013029228	0.296153272
XRCC3	0.017014048	0.1769216	0.567742768	0.018016049	0.435590285	0.013018785	0.296153272
ABCA6	0.180615044	0.036937906	0.178657226	0.012592738	0.897366898	0.01305674	0.29636814
DNAJC2	0.84066909	0.033659368	0.2009073	0.899164691	0.00266767	0.013166775	0.296405968
FGD6	0.165633649	0.81057183	0.033734457	0.31221045	0.009615952	0.013141154	0.296405968
NDUFA4L2	0.892566144	0.108541664	0.327640349	0.001441517	0.297495354	0.013151045	0.296405968
PPP1R3D	0.749070965	0.017449602	0.756562301	0.024774011	0.055136505	0.013082354	0.296405968
SAP30L	0.000568199	0.644350702	0.616459753	0.430873639	0.139525819	0.013122215	0.296405968
SLFN11	0.02242265	0.152739544	0.129340451	0.059565973	0.513647969	0.01311204	0.296405968
IGF2BP2	0.060584062	0.099555304	0.360631761	0.11220143	0.056221618	0.013222327	0.29724877
ASAH2	0.481682266	0.535403023	0.001388788	0.069674035	0.555620817	0.013316536	0.298548738
LOC112449261	0.371333081	0.628259593	0.019085061	0.004967858	0.626429369	0.013310513	0.298548738
CCT4	0.767961962	0.464743192	0.055699421	0.0264298	0.026705644	0.013424703	0.298833335
DMPK	0.351448372	0.076963707	0.651264554	0.00297235	0.268593064	0.013445692	0.298833335
EPHB3	0.742545897	0.199581401	0.002568353	0.139611584	0.264994564	0.013457402	0.298833335
IMPA1	0.736083268	0.470394822	0.736068987	0.235115298	0.00023545	0.013474905	0.298833335
KIAA1217	0.362659814	0.270335277	0.053950854	0.004726762	0.562925154	0.01345232	0.298833335
MZT2B	0.154822239	0.038486086	0.915972807	0.003148364	0.81685085	0.013427788	0.298833335
SMU1	0.218113031	0.246822114	0.002416883	0.159793159	0.669804406	0.013356178	0.298833335
STK32C	0.503827532	0.507395107	7.04E-05	0.807686656	0.965168576	0.013417138	0.298833335
LOC101905979	0.923948507	0.000897004	0.257454216	0.312207928	0.212734382	0.013515729	0.299334178
CBL	0.919450927	0.160472351	0.032256765	0.003830099	0.77973634	0.013542873	0.299531118
THBS4	0.658751895	0.900483096	0.121196582	0.014616493	0.013564572	0.013568921	0.299703311
C1QTNF6	0.149345143	0.142801857	0.008009925	0.391720731	0.214611668	0.013637909	0.300776412
RFXANK	0.763993053	0.018255277	0.005593927	0.309156049	0.596532595	0.013655459	0.300776412
TMSB4X	0.984606499	6.43E-05	0.651393574	0.646614849	0.540604502	0.013672488	0.300776412
LARP4	0.927129301	0.144341768	0.007894539	0.483793144	0.028297355	0.013703702	0.301059502
DES	0.001036368	0.445262267	0.516107641	0.990470902	0.061909146	0.013793903	0.302231972
GPR68	0.669209207	0.05714782	0.034405477	0.027373115	0.404693526	0.013776107	0.302231972
CNOT7	0.133973823	0.034463693	0.023264897	0.209184627	0.651561481	0.013817641	0.302348401
LOC112442664	0.478786447	0.965188238	0.50852107	0.005054161	0.012353777	0.013838006	0.302390835
ACAT2	0.480251201	0.342364928	0.528031002	0.020060153	0.008514133	0.013937266	0.302946329
CSNK1B	0.959534936	0.079523189	0.227292731	0.598415649	0.001426783	0.013924424	0.302946329
TPST1	0.48486203	0.702707717	0.737350968	7.44E-05	0.788825865	0.013886291	0.302946329
ZNF605	0.789154399	0.300752154	0.025460355	0.979755023	0.002499072	0.013916398	0.302946329
ZC3H12D	0.886155288	0.004061463	0.076802437	0.429723748	0.125384389	0.01397882	0.303447647
IFFO1	0.012121375	0.222424838	0.111587575	0.098175196	0.506904956	0.014028603	0.30412604
GJA1	0.031717105	0.661783562	0.011092355	0.566928371	0.114528138	0.014120658	0.305699278
GLRB	0.043207982	0.026621562	0.607348394	0.763589726	0.028446547	0.014157056	0.305699278

IMMP1L	0.783883523	0.409114933	0.012599184	0.05359417	0.070055023	0.014154211	0.305699278
CNOT1	0.091872241	0.464071333	0.712644227	0.000501642	0.997968606	0.014179747	0.305786888
CLPX	0.361811065	0.661961523	0.006506393	0.018118148	0.541122681	0.014222066	0.306063728
LOC112441484	0.067358313	0.007015128	0.450199335	0.39597643	0.182219989	0.014267184	0.306063728
PPP1CC	0.108521879	0.907545744	0.087523213	0.012664316	0.140354322	0.014249845	0.306063728
RIPOR3	0.008339072	0.355517109	0.501600654	0.141323236	0.072984758	0.014260184	0.306063728
EIF2B2	0.156526094	0.11281676	0.430600212	0.056751003	0.035713823	0.014306082	0.306202235
SLC7A6	0.543909344	0.010336107	0.144896107	0.101698903	0.186125855	0.014310957	0.306202235
COL28A1	0.000254366	0.785505248	0.236524241	0.785391485	0.418437594	0.01438106	0.306503348
HAS1	0.644588204	0.006285992	0.004593933	0.891150804	0.933764097	0.014354852	0.306503348
PRKD2	0.062035933	0.286230089	0.134802399	0.037298322	0.173856681	0.014375162	0.306503348
ADAM11	0.592738986	0.794671435	0.67813205	0.061063193	0.00081504	0.014609509	0.307518965
ARMCX3	0.149346394	0.569654951	0.128892923	0.001900365	0.767456571	0.014668791	0.307518965
CKS2	0.543635148	0.005848996	0.032024676	0.694369924	0.225488024	0.014638272	0.307518965
CNTFR	0.003486869	0.940179059	0.864324928	0.134590556	0.042085844	0.014704141	0.307518965
HCN4	0.01421754	0.223106482	0.560879253	0.014451209	0.622562799	0.014677163	0.307518965
HSPB1	0.07440868	0.543563721	0.073048136	0.412770784	0.01326773	0.014784746	0.307518965
IDI1	0.799444588	0.340325828	0.223556093	0.033712423	0.007788794	0.014655181	0.307518965
IGFBP5	0.126288963	0.007984563	0.675955798	0.047752619	0.485755894	0.014555635	0.307518965
LNX2	0.450243754	0.065789048	0.247798376	0.015413146	0.142136003	0.014722937	0.307518965
LOC104976020	0.092746548	0.937758397	0.328800333	0.120432281	0.004674553	0.014734594	0.307518965
MICU3	0.026122033	0.632496114	0.098518933	0.102705035	0.094511271	0.014549061	0.307518965
MZB1	0.19934007	0.044918499	0.009742483	0.327319583	0.556711603	0.014608777	0.307518965
NAV1	0.183763453	0.011514179	0.025586435	0.826310176	0.361478283	0.014778733	0.307518965
OBSCN	0.650129982	0.000133263	0.845071801	0.262419831	0.824038532	0.014569181	0.307518965
PTCD2	0.280813405	0.223649799	0.493101263	0.137032261	0.003812088	0.014782892	0.307518965
SLC31A2	0.384940434	0.290087131	0.003801284	0.637086125	0.059474204	0.014724859	0.307518965
TMEM132E	0.538683731	0.033642745	0.048527777	0.285463419	0.063708924	0.014669699	0.307518965
YTHDF2	0.583103718	0.371600529	0.049877441	0.003138673	0.462593206	0.014481541	0.307518965
ZNF438	0.693224104	0.661342169	0.002146549	0.611446011	0.026149798	0.014508519	0.307518965
NUDT18	0.969068936	0.051533935	0.571131668	0.002739968	0.208493436	0.014854643	0.308191582
RPF2	0.778907147	0.046090391	0.016338275	0.548384783	0.050632777	0.014849961	0.308191582
GIMAP8	0.082162936	0.429866064	0.065910228	0.018203542	0.387473397	0.014931909	0.308618718
HSD17B12	0.455641573	0.364080717	0.919751732	0.023180125	0.00465972	0.014969258	0.308618718
NF2	0.141743208	0.314043826	0.481320149	0.001147854	0.669260054	0.014956254	0.308618718
PTPRS	0.378674445	0.050216869	0.259466407	0.051329852	0.064840439	0.014933139	0.308618718
SCYL2	0.415353524	0.002904743	0.080020013	0.537226146	0.316548925	0.01493104	0.308618718
DHX58	0.166679302	0.009656602	0.545176353	0.036377564	0.5215926	0.015072899	0.309273128
FBXO32	0.026848371	0.491894714	0.844558335	0.063917891	0.023338537	0.015066058	0.309273128
IDE	0.946550256	0.630371568	0.1385002	0.505675304	0.0003986	0.015077379	0.309273128

NDUFA1	0.88866488	0.412558554	0.37672613	0.00146538	0.082345687	0.015083028	0.309273128
RXFP3	0.715567624	0.038790523	0.061428275	0.033732791	0.290110952	0.015095227	0.309273128
RSPO2	0.174480121	0.174763339	0.509908374	0.816350226	0.001328868	0.015205471	0.310369374
SH3BGR	0.009652248	0.243878308	0.633689474	0.68157004	0.016567839	0.015191493	0.310369374
SMARCAL1	0.093129397	0.160101453	0.090000048	0.251906008	0.049813282	0.01518805	0.310369374
HIGD1B	0.015471911	0.472846544	0.683047575	0.960651305	0.003533223	0.015262352	0.310796432
TMEM30A	0.212088865	0.00112411	0.608893188	0.179404549	0.651375793	0.015264269	0.310796432
CUBN	0.406358562	0.005679939	0.112612622	0.139578989	0.468712483	0.015288784	0.310881155
CYCS	0.339318254	0.084399676	0.818537033	0.068292638	0.010640081	0.015306317	0.310881155
EXD3	0.96885776	0.067860679	0.005852267	0.253689383	0.175457829	0.015362769	0.311257295
SLC5A6	0.075114054	0.223724199	0.660595926	0.080855879	0.019070044	0.015356953	0.311257295
ACAP3	0.610630746	0.125531989	0.001245336	0.865452371	0.208208074	0.015407715	0.311294847
CROCC2	0.402097703	0.036168558	0.157488175	0.012564485	0.601851837	0.01547922	0.311294847
ENPP6	0.015014726	0.751384753	0.910589709	0.039407586	0.04255061	0.015422732	0.311294847
ERBB2	0.318508961	0.802727424	0.842717468	0.13361112	0.000600605	0.015461417	0.311294847
RWDD1	0.746317407	0.151729128	0.203008724	0.284677797	0.002632264	0.015422785	0.311294847
SELENOK	0.720291303	0.057293916	0.001536824	0.336415419	0.813177212	0.015497404	0.311294847
TPPP	0.364249536	0.290786966	0.813794768	0.001298047	0.154314241	0.015446606	0.311294847
SURF4	0.165874365	0.344474567	0.001463925	0.996824635	0.208780355	0.015532576	0.311619936
SLC2A9	0.310673596	0.001548419	0.367808522	0.340316238	0.2896863	0.01555334	0.311655506
TXK	0.62038111	0.328288748	0.043768163	0.211239648	0.009367165	0.015670268	0.313615567
PDHX	0.359448072	0.430067794	0.307262273	0.033510671	0.011135687	0.015721984	0.314267328
HERPUD1	0.941101699	0.832490361	0.019015116	0.002530177	0.473590694	0.015797349	0.315389662
C14H8orf37	0.009789616	0.818474799	0.011518756	0.637969399	0.305561248	0.01588091	0.315836263
CPM	0.452051473	0.266931291	0.009666287	0.797343435	0.01928339	0.015846541	0.315836263
GNAI3	0.249655539	0.005217489	0.035791566	0.393954848	0.982975183	0.01591779	0.315836263
LOC518495	0.804095216	0.139028376	0.137413655	0.041250475	0.028536997	0.015935191	0.315836263
SCEL	0.090725375	0.040879121	0.076267771	0.246867986	0.258383084	0.015911081	0.315836263
TMEM97	0.41045256	0.048607125	0.684569954	0.067086291	0.019714118	0.015923199	0.315836263
CCR2	0.391845328	0.162246386	0.381178461	0.041568604	0.018206614	0.016087242	0.3163122
ERICH3	0.521064868	0.003174625	0.883444187	0.492630437	0.025443404	0.016073482	0.3163122
GNB1L	0.049897026	0.011412216	0.134742284	0.630906074	0.375414381	0.015988229	0.3163122
HSPA4	0.925905155	0.381263906	0.012073644	0.04854283	0.087988072	0.016006935	0.3163122
IRF4	0.316513322	0.020003726	0.012281795	0.940374889	0.25015356	0.016058877	0.3163122
ITGAD	0.864987802	0.070912552	0.080408569	0.177372549	0.020893693	0.016050544	0.3163122
LOC782264	0.566098903	0.980727017	0.004784993	0.020742399	0.333642818	0.016113399	0.3163122
MRPS21	0.337109725	0.125711976	0.717376741	0.002192522	0.275628415	0.016105935	0.3163122
SBF1	0.371552538	0.004512483	0.37767728	0.08483367	0.343907909	0.016165957	0.31696478
LOC112448105	0.413067146	0.410376448	0.04177769	0.28756127	0.009134391	0.016240916	0.318054496
ALDH18A1	0.537252092	0.404368802	0.215169225	0.017979246	0.02218823	0.016267879	0.318202821

CASZ1	0.807911566	0.286279588	0.007510906	0.085829483	0.12560245	0.016314516	0.318356149
OTUD6B	0.981082961	0.012336596	0.013661657	0.253805771	0.44580514	0.016303668	0.318356149
RRP7A	0.043498296	0.056219217	0.26951044	0.63757202	0.044727354	0.016353763	0.318743004
BYSL	0.628951085	0.024917842	0.111423599	0.250016442	0.043146328	0.016378606	0.318745194
PTX3	0.278670065	0.02914045	0.958685994	0.071852841	0.03371842	0.016392721	0.318745194
MCRIP2	0.156756265	0.130608954	0.949581776	0.00724901	0.134797477	0.016471881	0.319905365
AK2	0.458396588	0.403215434	0.004561047	0.820695283	0.027785683	0.016603616	0.32094457
LSMEM1	0.3205085	0.383252568	0.001084832	0.99477899	0.144641956	0.016574445	0.32094457
SLC16A11	0.391762156	0.664946958	0.001298561	0.233516412	0.24255675	0.016566627	0.32094457
TMEM236	0.948397844	0.522204282	0.003185416	0.135617584	0.089758942	0.016591942	0.32094457
ITGB8	0.035900772	0.859705107	0.013771286	0.574334858	0.079255252	0.016674932	0.321665312
LOC618456	0.785753922	0.220514611	0.002144034	0.24682554	0.211094094	0.016680104	0.321665312
FAM234A	0.125260893	0.525912579	0.637070698	0.000790355	0.587063015	0.016747252	0.321859734
FLT3	0.885743187	0.015103979	0.186761165	0.212279598	0.036639277	0.016724443	0.321859734
PALB2	0.40531039	0.000494842	0.486766264	0.729391012	0.273499944	0.016749023	0.321859734
AARSD1	0.631792966	0.031055868	0.504876324	0.637581262	0.003099073	0.016805456	0.322084685
ECSIT	0.318922434	0.197590696	0.898070245	0.001697579	0.204352398	0.016839233	0.322084685
LOC104970180	0.193550724	0.73175455	0.84256779	0.476728922	0.000344298	0.016813259	0.322084685
PPP1R14B	0.102008872	0.116095218	0.570699222	0.075864122	0.03822262	0.016819615	0.322084685
OSBPL11	0.814834693	0.824624224	0.000747637	0.040105113	0.976766823	0.016866163	0.322224222
FFAR3	0.658261029	0.374855145	0.290964905	0.004079805	0.067305215	0.016886566	0.322238875
CXCL16	0.513218885	0.719203453	0.004279386	0.023611562	0.53271358	0.016974446	0.32241625
GPBP1	0.364748155	0.840001619	0.485022404	0.000750715	0.177777473	0.016954357	0.32241625
ICAM3	0.702521955	0.267496438	0.021200409	0.069465876	0.071542858	0.016935353	0.32241625
TMEM64	0.637375408	0.178526178	0.282401848	0.001246801	0.495883177	0.016974103	0.32241625
ZNF555	0.306619831	0.199662999	0.469979891	0.072727881	0.009534598	0.01702233	0.322951977
FTSJ3	0.016278689	0.149356923	0.231291572	0.143390633	0.248744602	0.017082786	0.323724704
LOC112446791	0.006204779	0.196314448	0.11575332	0.974003402	0.146506689	0.017118411	0.324002739
NOG	0.994940833	0.003073927	0.33331031	0.038119113	0.518620083	0.017136943	0.324002739
ALKBH1	0.192616496	0.688826061	0.88970546	0.000230329	0.743917931	0.01717906	0.324425264
CCT5	0.624697964	0.113424574	0.035693796	0.319009743	0.025190162	0.01723426	0.32509361
REP15	0.658977987	0.432854782	0.026840004	0.195060954	0.013634786	0.017255776	0.325125762
ABCF2	0.370998669	0.25253379	0.242548556	0.671001621	0.001349742	0.017379878	0.325299016
ARL4C	0.487036935	0.448295051	0.000726332	0.526973389	0.247620906	0.017443504	0.325299016
CDK2AP1	0.684659614	0.178700364	0.132668157	0.240727021	0.005250897	0.017344041	0.325299016
CRISPLD2	0.303114	0.515924716	0.297475924	0.003180965	0.139123795	0.017383616	0.325299016
FHL5	0.013384422	0.270511203	0.473886145	0.144011532	0.084005623	0.017479204	0.325299016
LOC101908359	0.045137031	0.870702252	0.031137932	0.063789018	0.264512588	0.017417933	0.325299016
LRRC59	0.328894524	0.076433659	0.135873802	0.844520184	0.007178651	0.017451373	0.325299016
LRRC8D	0.613737799	0.434656033	0.694699116	0.979990529	0.000112991	0.017345842	0.325299016

PARD6G	0.081039738	0.025640839	0.196236908	0.289972612	0.175605485	0.017483013	0.325299016
ST3GAL5	0.913411093	0.454354587	0.004395704	0.029392299	0.383516284	0.017370298	0.325299016
TAGLN	0.166417087	0.265791134	0.459780631	0.643095314	0.001586547	0.017475243	0.325299016
BOP1	0.172838606	0.133657963	0.659824831	0.180993603	0.007562148	0.01753874	0.325966328
MAP1LC3C	0.056152472	0.241306492	0.002723866	0.673678689	0.84822498	0.017666502	0.327969425
POMT1	0.175320561	0.036820582	0.048799177	0.196455342	0.341983655	0.017707817	0.32836495
CNN1	0.003713084	0.418553019	0.539760028	0.967074766	0.026320099	0.017812432	0.32898842
ILVBL	0.712375127	0.236599326	0.524714005	0.002805061	0.085885265	0.017787027	0.32898842
SIRPA	0.004042984	0.884861581	0.90191535	0.550787624	0.012012399	0.017810334	0.32898842
UQCR10	0.517379634	0.336746788	0.72337773	0.008835911	0.019188444	0.017821626	0.32898842
CIITA	0.395192254	0.543354246	0.111817211	0.004924387	0.181191338	0.017852375	0.329185761
C8H9orf131	0.318501282	0.18915476	0.295555167	0.050383106	0.023950701	0.017887568	0.329464515
ARHGAP20	0.114318727	0.042643724	0.084142087	0.136848999	0.386759686	0.018011725	0.329900021
CXCL13	0.134839962	0.759015786	0.005299461	0.453954187	0.088117159	0.018003559	0.329900021
EXTL1	0.144681267	0.49266249	0.011730551	0.192913849	0.133928916	0.017952267	0.329900021
LPCAT3	0.192973506	0.341322462	0.456142615	0.676426189	0.001067036	0.017997719	0.329900021
SELL	0.36229282	0.018766376	0.236180305	0.154044158	0.087378728	0.017958252	0.329900021
LOC107131975	0.674155963	0.000644761	0.12740117	0.827426341	0.476654533	0.018083772	0.330850363
DHRS1	0.126850295	0.010441585	0.657091193	0.530046044	0.047512918	0.018126711	0.331266653
TRIB2	0.214458647	0.982969274	0.001997859	0.099735162	0.524808505	0.018196157	0.332165892
ANGPTL5	0.122456953	0.823642254	0.055704538	0.587595746	0.006718861	0.018271493	0.333170529
SLC16A3	0.231526379	0.476540922	0.02305919	0.031085213	0.281100242	0.018298815	0.333298394
TMEM182	0.22491116	0.203626346	0.992991383	0.027428772	0.017924597	0.01836882	0.333832452
TYRP1	0.482106362	0.960128513	0.266976162	0.011672173	0.015482648	0.018354594	0.333832452
PLCB4	0.033068722	0.054730766	0.948613273	0.728030821	0.017922248	0.018392362	0.333890544
EIF4E	0.720958552	0.007539448	0.155236099	0.852165783	0.031629339	0.018578958	0.334390297
HSPA5	0.500571408	0.692725795	0.006226865	0.03924313	0.267452611	0.018534836	0.334390297
LOC506828	0.476690668	0.060689146	0.002235903	0.887043522	0.392357881	0.0184531	0.334390297
MPV17L2	0.246531129	0.094736159	0.102254936	0.181201248	0.052667997	0.01860529	0.334390297
PANK1	0.330295486	0.140274078	0.919947908	0.050505785	0.010596672	0.018615972	0.334390297
PEX6	0.910626679	0.005495395	0.078199831	0.120353559	0.482135048	0.018559466	0.334390297
RBM43	0.079689675	0.255305795	0.216594963	0.009365073	0.547466133	0.018497101	0.334390297
SERINC5	0.180539748	0.25349041	0.049625932	0.083809481	0.118910887	0.018519211	0.334390297
TLR7	0.354962808	0.467100527	0.00662765	0.029601058	0.701718151	0.018623651	0.334390297
VASN	0.592154279	0.616804342	0.001000118	0.169317054	0.366015495	0.018521363	0.334390297
ANKRD17	0.467292776	0.88501306	0.809449313	8.00E-05	0.859667899	0.018736751	0.334897874
CHST7	0.724635704	0.98621415	0.038509377	0.000837261	0.994687581	0.018674641	0.334897874
DIRAS2	0.769661829	0.300140264	0.042745743	0.563531435	0.004170495	0.018830448	0.334897874
ENPP1	0.38267451	0.398853613	0.435321103	0.000451007	0.773228326	0.018810859	0.334897874
GRIA1	0.986509749	0.001095324	0.07565891	0.42192687	0.671894785	0.018813696	0.334897874

LOC100847365	0.4220687	0.407783957	0.293933414	0.09983695	0.004588072	0.018812007	0.334897874
LOC112445031	0.323806228	0.016279626	0.032988189	0.319369153	0.416027874	0.018775113	0.334897874
TIMM8B	0.404510098	0.057547915	0.69300935	0.008391423	0.169949559	0.018721926	0.334897874
ZSWIM4	0.75627106	0.525042925	0.006802278	0.358455051	0.02397945	0.018835582	0.334897874
AMT	0.918179996	0.034693212	0.050969192	0.57331538	0.025242011	0.018986223	0.335744708
ARL11	0.773763044	0.006806374	0.011399499	0.522812777	0.746135385	0.018944828	0.335744708
CAP2	0.005114019	0.181486264	0.122632892	0.54485011	0.381552441	0.019075062	0.335744708
CCL21	0.004458795	0.743893058	0.01796068	0.866543998	0.45498954	0.01898172	0.335744708
FARSA	0.167178233	0.100804924	0.230647623	0.894298104	0.006787907	0.019039568	0.335744708
LOC100139881	0.28420227	0.066170364	0.066814509	0.041389995	0.452319708	0.019000925	0.335744708
LOC104973154	0.667180054	0.732111454	0.033945864	0.805058612	0.001776281	0.019101437	0.335744708
LOC104974460	0.017517867	0.126619685	0.702593682	0.865786677	0.017601102	0.01912167	0.335744708
LOC784289	0.637774263	0.457084433	0.01383139	0.022655601	0.260345538	0.019139765	0.335744708
NUAK1	0.897482529	0.28465907	0.151726166	0.002742358	0.224252536	0.01916963	0.335744708
PLEKHG2	0.787208916	0.268938328	0.00426142	0.040804223	0.646731306	0.019153579	0.335744708
RAD51D	0.925263727	0.646536419	0.043775995	0.048990673	0.018489368	0.019106813	0.335744708
SNAP23	0.926777914	0.085688047	0.27994053	0.166884594	0.006414895	0.019148928	0.335744708
ZNF771	0.85545773	0.293083876	0.0013842	0.435675722	0.154530159	0.018915599	0.335744708
PCDH1	0.634457176	0.557825941	0.003250008	0.043779311	0.475284788	0.01922064	0.33609689
PLEKHG4	0.35675861	0.060751785	0.010837208	0.226249905	0.450812687	0.019233216	0.33609689
VASP	0.582648904	0.110059562	0.520154222	0.0174592	0.041195894	0.019251178	0.33609689
GUCD1	0.131508649	0.008260985	0.073508518	0.587067593	0.512852504	0.019279472	0.33623318
CD200	0.806897889	0.000489718	0.202707621	0.736562743	0.40833803	0.019304877	0.336318834
CKAP2L	0.566619532	0.006164674	0.141672244	0.29305847	0.168027501	0.019451879	0.337929749
NDUFA5	0.403346418	0.224963668	0.548129375	0.038442706	0.012756325	0.019463553	0.337929749
PIK3R5	0.940553269	0.143516628	0.109378866	0.019988296	0.082618206	0.019459306	0.337929749
TRAM1	0.444986853	0.047945614	0.164879509	0.14851605	0.046743306	0.019479711	0.337929749
ECD	0.961026568	0.220306675	0.862294886	0.003022027	0.04450493	0.01955046	0.338798949
MADCAM1	0.620771153	0.988732854	0.000195504	0.722309531	0.284833794	0.019621008	0.339314487
PRPS2	0.135300729	0.208127969	0.067551117	0.086176042	0.150608397	0.019621562	0.339314487
PPP1R10	0.264078785	0.757071587	0.001582988	0.106092216	0.73860426	0.019679995	0.339966732
NKTR	0.732386007	0.191352783	0.005903444	0.258521307	0.117955613	0.019905429	0.343478649
ZBED5	0.407892235	0.389270792	0.009894103	0.043827493	0.366963676	0.019925152	0.343478649
TBX1	0.012901334	0.121232225	0.055224263	0.973527566	0.300979418	0.019947213	0.343498125
C2CD4B	0.978150663	0.067751119	0.00562669	0.1819461	0.378675605	0.020146763	0.344010632
FOXO6	0.851854929	0.465346392	0.331328013	0.016421408	0.011958258	0.020198907	0.344010632
GALNT10	0.006919881	0.055542796	0.739284133	0.135903976	0.661328434	0.020066991	0.344010632
HDC	0.09349196	0.096753564	0.022334175	0.568311373	0.22417992	0.020171659	0.344010632
INPP5F	0.438690159	0.030407293	0.593283176	0.109348134	0.029822905	0.020207559	0.344010632
IQGAP2	0.537924893	0.332427307	0.5471036	0.025294976	0.010346668	0.020101819	0.344010632

LOC112441452	0.536870796	0.943051168	7.82E-05	0.729829932	0.879768397	0.020003635	0.344010632
MTERF2	0.086976528	0.17437025	0.719404387	0.006013078	0.389408201	0.020071967	0.344010632
MUSTN1	0.017874033	0.159458081	0.739415382	0.712091507	0.017074025	0.020111233	0.344010632
SP100	0.210287821	0.747683052	0.427621828	0.358550344	0.001062065	0.020100903	0.344010632
TOMM70	0.313337155	0.036487131	0.427853271	0.923257509	0.005689628	0.02014885	0.344010632
SPCS1	0.513652472	0.033926941	0.948864554	0.215281123	0.007283747	0.020269962	0.344715387
OAS2	0.275540134	0.062199695	0.148969129	0.095194105	0.107577415	0.020382287	0.346266786
MEG3	0.287784642	0.076619091	0.024917625	0.173037199	0.275997271	0.020430695	0.34650191
SYCP3	0.073782272	0.13901551	0.015233711	0.731851107	0.230217935	0.020475226	0.34650191
TAS1R3	0.061707045	0.026656978	0.036637848	0.493937154	0.884722562	0.020480583	0.34650191
USP10	0.795195621	0.030747458	0.022984278	0.641547103	0.072947298	0.020461768	0.34650191
CXXC5	0.321657177	0.019286746	0.155084017	0.464892286	0.059024728	0.020513443	0.34654614
PRPF19	0.721771229	0.030962397	0.103795768	0.458560192	0.02484154	0.020525431	0.34654614
ARHGDIA	0.028148542	0.158333215	0.070731158	0.345269148	0.246158784	0.020714778	0.346887977
CRIM1	0.100572418	0.296732551	0.705284684	0.002281598	0.556499402	0.020680093	0.346887977
CYP2J2	0.204677117	0.010194609	0.722652782	0.047443181	0.371765666	0.020614004	0.346887977
LOC101902570	0.250083687	0.153910649	0.032218511	0.125052439	0.171782726	0.020636597	0.346887977
MRPL11	0.49621409	0.040836622	0.780753822	0.07674862	0.022061016	0.020712166	0.346887977
PHACTR4	0.54347332	0.320991183	0.230516697	0.77570081	0.000856136	0.020670585	0.346887977
SEMA4F	0.101162206	0.208464777	0.005555664	0.82910404	0.273095179	0.020579379	0.346887977
ZFP36L1	0.05755651	0.111647472	0.089460842	0.376537935	0.1232635	0.020658265	0.346887977
CDC25B	0.095025124	0.019818119	0.275211832	0.238516655	0.219173461	0.02086884	0.346976542
EIF3A	0.562276353	0.32950488	0.087600263	0.023399957	0.071642292	0.020926998	0.346976542
HMGCLL1	0.194690922	0.147800344	0.058825837	0.041155459	0.387060221	0.02080283	0.346976542
ITK	0.647505246	0.054419196	0.620397689	0.087196152	0.014085642	0.020744087	0.346976542
LOC514181	0.021069855	0.246926214	0.087457675	0.291890771	0.204597864	0.020909363	0.346976542
LOC789374	0.615845077	0.012083418	0.113154945	0.806972354	0.039728993	0.020818869	0.346976542
MTRF1	0.607020937	0.080160195	0.635495146	0.357037356	0.002464939	0.02092996	0.346976542
NECAB3	0.821237806	0.211121335	0.064488661	0.012681189	0.190717001	0.020842124	0.346976542
SELENOF	0.729872365	0.185707463	0.993881692	0.769676986	0.000262496	0.020931496	0.346976542
WDFY2	0.428453781	0.255491049	0.281557903	0.001364812	0.644572804	0.020878941	0.346976542
ASCL2	0.396943195	0.078151947	0.551951781	0.233014885	0.006869704	0.02102889	0.347756942
CARMIL2	0.675835731	0.001524883	0.700453639	0.136577566	0.279119346	0.021084526	0.347756942
CLDN10	0.105735773	0.180202697	0.026976499	0.068381555	0.782636337	0.021079436	0.347756942
RND2	0.737365575	0.09283003	0.521613049	0.027554762	0.027921762	0.021059916	0.347756942
TUBB2B	0.311269101	0.083753453	0.004345281	0.28966536	0.837633172	0.021067861	0.347756942
HTR2B	0.011875433	0.086072551	0.970058611	0.832866324	0.033405731	0.021119284	0.347980489
UCK2	0.260748847	0.299250633	0.756292137	0.403112513	0.00116658	0.021202364	0.34899899
BMF	0.283630503	0.114575139	0.013284023	0.158747346	0.406824486	0.021266947	0.349361229
TRADD	0.000247194	0.708882645	0.686700324	0.646109725	0.358094627	0.021247448	0.349361229

PHOSPHO2	0.43914927	0.389429642	0.489262704	0.625667443	0.00053491	0.021329207	0.35003361
PLCG2	0.000212663	0.208984048	0.817960523	0.989285759	0.78150732	0.021380679	0.350527789
GUCY1A1	0.726986356	0.006774901	0.320569466	0.216945919	0.082289313	0.021421492	0.35084641
ADM	0.756727411	0.669647525	0.204777136	0.000908095	0.301086977	0.021514342	0.352015823
CENPU	0.318467948	0.142579473	0.033950879	0.067834274	0.272951651	0.021600146	0.352715117
COL21A1	0.639390962	0.114761512	0.043360254	0.030870844	0.290810401	0.021610141	0.352715117
UGGT2	0.990615582	0.221063182	0.048713222	0.040916653	0.065492564	0.021621559	0.352715117
SLAMF1	0.343752989	0.011946246	0.877337519	0.014251982	0.558683778	0.021671745	0.353182725
AGPAT2	0.079178248	0.338934866	0.604880083	0.036998682	0.047995333	0.021740706	0.353604296
TESPA1	0.686528845	0.042242644	0.067785612	0.570496146	0.025681183	0.021728767	0.353604296
FBXO16	0.644486397	0.432214323	0.004333888	0.038798872	0.617582307	0.021791232	0.354075149
ADM5	0.110776781	0.304616404	0.010153638	0.115860389	0.732393291	0.021864127	0.354207497
CARD9	0.058758955	0.002816344	0.387686941	0.847380522	0.534641305	0.021860105	0.354207497
CCDC84	0.471785948	0.002124225	0.169127115	0.474578402	0.360701363	0.021834638	0.354207497
CCL22	0.230666293	0.175687417	0.002603747	0.297770818	0.931533844	0.021960677	0.355141554
INAFM2	0.650173353	0.007569561	0.51164582	0.046593266	0.249541936	0.021965065	0.355141554
LOC100847700	0.244547854	0.074911134	0.797631159	0.105550018	0.019052865	0.02201832	0.355652212
LOC101907302	0.868086056	0.843303362	0.000747514	0.229800809	0.235130734	0.022108609	0.355710178
SCN8A	0.396274458	0.365929713	0.130633242	0.014392577	0.108168637	0.022070406	0.355710178
SERPINE2	0.048805569	0.151928344	0.082088097	0.076364467	0.633610379	0.022050802	0.355710178
TNFRSF11A	0.394707116	0.004005615	0.058649847	0.944802846	0.337126961	0.022092473	0.355710178
DDX21	0.867818423	0.298335779	0.091239163	0.004055036	0.311228186	0.022228451	0.356589546
ICOSLG	0.009835349	0.440736378	0.551108614	0.04276969	0.291725284	0.022225985	0.356589546
TIMM10	0.977394439	0.627832833	0.089170776	0.00392053	0.138883853	0.02221981	0.356589546
CYHR1	0.014587359	0.056869169	0.766951144	0.097306231	0.482999852	0.022273002	0.35666582
NFASC	0.010501056	0.0426982	0.368149752	0.725530524	0.249846084	0.022282681	0.35666582
PSMD12	0.620832417	0.024992372	0.541476166	0.034389289	0.103675449	0.022298405	0.35666582
INHA	0.838569993	0.072113251	0.491794994	0.756155733	0.001342874	0.02241793	0.357316668
LOC112446426	0.101526039	0.54751557	0.053163868	0.023445415	0.436736535	0.022447961	0.357316668
PDGFA	0.701231306	0.031215705	0.342745698	0.030520337	0.131636242	0.02239028	0.357316668
RET	0.615481958	0.486025832	0.05603527	0.005544854	0.325118754	0.022427559	0.357316668
TMEM176B	0.180757754	0.302763415	0.009992237	0.995483416	0.055375103	0.02239165	0.357316668
ATP13A2	0.18123576	0.098709214	0.09853027	0.045788363	0.379979863	0.022646844	0.357803254
CRELD2	0.025494465	0.629590264	0.025102221	0.517437189	0.146356842	0.022571451	0.357803254
GBP6	0.831879792	0.571716532	0.544710567	0.000260153	0.457555352	0.022729084	0.357803254
LOC513779	0.002259727	0.294560767	0.139670401	0.335512133	0.974529458	0.022515128	0.357803254
LOC782598	0.566428376	0.23379329	0.030024202	0.691373552	0.011147269	0.022634588	0.357803254
LRRC51	0.617120771	0.020144775	0.008738634	0.837528952	0.339670196	0.022761964	0.357803254
RCOR1	0.94189867	0.867364429	0.219953115	0.000379676	0.45246243	0.022744663	0.357803254
RDH13	0.338335317	0.035859103	0.919633151	0.113072836	0.024226791	0.022596399	0.357803254

RYBP	0.511336025	0.108183682	0.222764877	0.004776383	0.52293588	0.022700974	0.357803254
SEPT1	0.412429277	0.020205468	0.490703416	0.179620835	0.041573306	0.022582471	0.357803254
SFI1	0.560566351	0.014876791	0.013554011	0.472315428	0.571988205	0.02258297	0.357803254
TM9SF3	0.37730846	0.410007388	0.450579226	0.080971956	0.005455519	0.02270674	0.357803254
ZNF184	0.118884427	0.188031204	0.030213009	0.67878954	0.066999538	0.02266981	0.357803254
S1PR5	0.304341265	0.070668926	0.015264108	0.209973416	0.45054895	0.022835648	0.358618004
MT1E	0.090215478	0.131013021	0.790422036	0.123647915	0.026930232	0.022860368	0.358663
LOC531747	0.828559028	0.286241175	0.017282843	0.610383558	0.012494379	0.022933417	0.359465428
CENPB	0.779933216	0.004602675	0.60774094	0.125158488	0.114761996	0.022970128	0.359512154
LOC100847509	0.351937848	0.117115098	0.973447506	0.001061095	0.73848234	0.0230203	0.359512154
PDPK1	0.276460157	0.233874251	0.004488184	0.176678067	0.611905691	0.022987805	0.359512154
TSSC4	0.141803213	0.216005625	0.988265644	0.049711464	0.02089832	0.023024025	0.359512154
CCL2	0.676058812	0.676598583	0.000243983	0.694374136	0.40852732	0.023125194	0.359838363
EFNB1	0.55039672	0.353964447	0.025083571	0.288015055	0.022477444	0.023114607	0.359838363
LOC100848703	0.762164134	0.338116783	0.349697932	0.000675001	0.520097713	0.023114974	0.359838363
MPP7	0.000554211	0.459399371	0.803033108	0.774580145	0.200002982	0.023132623	0.359838363
AMD1	0.039098599	0.537640791	0.021416174	0.326040518	0.219500535	0.023393053	0.360374055
FAP	0.792304752	0.04079635	0.25697352	0.025398749	0.152212525	0.023342243	0.360374055
GIMAP7	0.162168015	0.014702709	0.54269712	0.251963122	0.09760147	0.023203143	0.360374055
LOC100297044	0.647591508	0.048305805	0.006682931	0.178498288	0.864252597	0.023408613	0.360374055
LOC100848246	0.054743902	0.495155389	0.887309139	0.022889004	0.057895128	0.023228	0.360374055
LOC112445995	0.101043194	0.07646223	0.069230947	0.068418563	0.874053589	0.023282359	0.360374055
PFKFB4	0.00496569	0.434232505	0.288530264	0.293462892	0.175143448	0.023277924	0.360374055
PGP	0.260944998	0.133794148	0.291071026	0.063166629	0.050032118	0.023344256	0.360374055
PPARD	0.111032059	0.313649674	0.514766797	0.100935648	0.017821126	0.023406535	0.360374055
PTP4A3	0.287086362	0.609452329	0.005701213	0.066066246	0.485122421	0.023274761	0.360374055
TMEM61	0.025936178	0.085662755	0.479282009	0.374121508	0.080663962	0.023353426	0.360374055
RAB5IF	0.265192916	0.132371097	0.516547515	0.13465716	0.013257252	0.023465528	0.360911704
CLINT1	0.0020572	0.579663615	0.556247566	0.497197381	0.098426016	0.023508531	0.361234551
KCNH2	0.00246592	0.506598144	0.453688129	0.317473799	0.181050433	0.023563601	0.361742059
SERP1	0.680057286	0.050358464	0.070293109	0.612777969	0.022122321	0.023590583	0.361817813
CD79A	0.225747871	0.223372444	0.003852749	0.928563077	0.182398505	0.02371898	0.362037928
GTF2A2	0.887001786	0.197925078	0.398890699	0.539447428	0.000874005	0.023772264	0.362037928
JAK1	0.933841165	0.503620559	0.002511356	0.03722383	0.754571309	0.023846489	0.362037928
KIAA1147	0.021423995	0.229697216	0.403513595	0.03984793	0.421067856	0.023913784	0.362037928
LOC100848699	0.000536692	0.640304991	0.160769618	0.649239358	0.916076537	0.023697352	0.362037928
LOC112443499	0.769999253	0.032935571	0.169067542	0.073075808	0.104862133	0.023695746	0.362037928
LUZP1	0.109939425	0.603974414	0.813793337	0.002188633	0.280806732	0.02386308	0.362037928
NR2F2	0.023587056	0.472369338	0.009699279	0.776603334	0.39180493	0.023708495	0.362037928
PSRC1	0.954478	0.619854076	0.006637571	0.021048562	0.401288833	0.023844255	0.362037928

RASGRP3	0.921348562	0.803435078	0.020131066	0.015322996	0.145228704	0.023840424	0.362037928
RBM5	0.941387455	0.473669287	0.342599987	0.843451307	0.000258399	0.023903308	0.362037928
SLC14A1	0.166416909	0.055675841	0.505054511	0.07377861	0.095478551	0.023747021	0.362037928
TUBA1A	0.132001118	0.053149924	0.014904449	0.678089931	0.464614532	0.023737605	0.362037928
ZSWIM2	0.173782492	0.635370034	0.071387183	0.047374584	0.088993626	0.023873558	0.362037928
LOC101902444	0.323719372	0.448648844	0.269125276	0.147597123	0.005821205	0.024038602	0.363592158
LOC112441663	0.845443765	0.896467849	0.001037921	0.054560635	0.786053274	0.024111076	0.364352552
DCAF13	0.978565172	0.035318177	0.006116144	0.772826339	0.207469795	0.024183493	0.365051747
TRPC1	0.372967927	0.594261487	0.760334146	0.044092724	0.004566511	0.024201834	0.365051747
LOC104976804	0.905947475	0.170347616	0.005949856	0.854769398	0.043376888	0.024254759	0.365433126
PDLIM7	0.365139775	0.335262808	0.006020584	0.1890629	0.244582299	0.024271654	0.365433126
LIX1	0.370080132	0.900295321	0.043848253	0.006987171	0.334341185	0.024293989	0.365434148
LOC511847	0.473389531	0.406601525	0.841631933	0.00041572	0.509653265	0.024384324	0.366457086
CCL11	0.268089177	0.05433008	0.010505254	0.503544143	0.447691772	0.024463784	0.366643984
CCNG1	0.846674955	0.299394563	0.323495949	0.392714804	0.001069362	0.024437566	0.366643984
LOC112448523	0.146640654	0.113700596	0.004141249	0.896317381	0.556817682	0.024448313	0.366643984
GCHFR	0.730077999	0.225147329	0.056695417	0.015806506	0.235550944	0.024558446	0.367391659
IFRD1	0.39171415	0.208007338	0.174659006	0.013443481	0.181297719	0.024552459	0.367391659
ATP5MC1	0.266664841	0.161027603	0.974135504	0.060366256	0.013791575	0.024617323	0.367447997
P4HB	0.123429811	0.13850537	0.062962943	0.718912775	0.044953174	0.024599325	0.367447997
RAPGEFL1	0.208180614	0.049838966	0.024517143	0.414775009	0.330313849	0.024629383	0.367447997
LOC782675	0.004532296	0.838464973	0.09861005	0.802894806	0.116120873	0.024669303	0.3677093
GSC	0.020054094	0.017397953	0.591780334	0.363167281	0.466650805	0.024694116	0.367745128
CYTIP	0.918523784	0.005130449	0.198592225	0.397698195	0.094263042	0.024736662	0.367916896
FCHO1	0.97165412	0.014007424	0.158450005	0.076500319	0.212837674	0.024750488	0.367916896
DUSP12	0.158689743	0.015176113	0.749702793	0.022114551	0.884581136	0.024845377	0.368659566
HOXD3	0.518102138	0.427962525	0.00754024	0.083414548	0.252989782	0.024828048	0.368659566
ACER2	0.300879397	0.234449006	0.002994509	0.475452777	0.353307397	0.024920784	0.368778159
CCNC	0.965350236	0.062778511	0.256743763	0.618418582	0.003683678	0.024903279	0.368778159
ZNF215	0.723168449	0.444691509	0.214606734	0.022900591	0.022403936	0.024886508	0.368778159
PAK3	0.028279038	0.956289962	0.019039462	0.10458937	0.661579116	0.024986593	0.369086388
SNRPA	0.314809505	0.12021851	0.009506037	0.194440455	0.508843204	0.024971965	0.369086388
MMP28	0.188069059	0.575445694	0.286963259	0.003832238	0.300042301	0.025024415	0.369312656
EGFLAM	0.062744517	0.76122074	0.581859597	0.001565836	0.829046955	0.025192463	0.370792384
FAM118A	0.479898783	0.820209683	0.288308373	0.074997802	0.004234084	0.025173885	0.370792384
IL11RA	0.003109094	0.202860903	0.274241388	0.801508804	0.25987108	0.025169781	0.370792384
ARVCF	0.004248933	0.587910143	0.37908277	0.111298262	0.3447006	0.025307382	0.371128673
CLDN11	0.216777011	0.021351926	0.456648208	0.699305836	0.024744465	0.025418843	0.371128673
LOC101907041	0.839512238	0.475946146	0.084790258	0.010268781	0.104373828	0.025299238	0.371128673
LPP	0.001877401	0.4923586	0.089828377	0.88604808	0.496951377	0.025412957	0.371128673

MEOX2	0.566455333	0.614779091	0.008890788	0.015356829	0.761878431	0.025260104	0.371128673
MTR	0.409918732	0.47654067	0.002001246	0.216514972	0.431207162	0.025384268	0.371128673
NHSL2	0.018299858	0.041158619	0.22317894	0.243722863	0.888984324	0.025349168	0.371128673
NUP98	0.540500338	0.331823035	0.618920425	0.000333371	0.986518347	0.025388011	0.371128673
UBL3	0.936717422	0.587825477	0.045398068	0.016416376	0.089064224	0.02540729	0.371128673
C1R	0.040324202	0.509430746	0.448235469	0.005839725	0.682551234	0.025476527	0.371305055
LOC112445197	0.52877956	0.059343332	0.043820083	0.06384299	0.419201617	0.025521425	0.371305055
P2RY13	0.007032172	0.683683793	0.140733369	0.561676709	0.096443161	0.025454125	0.371305055
STK38	0.243472726	0.102962299	0.536420014	0.006592411	0.414619155	0.025501145	0.371305055
AKIRIN1	0.077816756	0.034692076	0.385718443	0.852942206	0.041897511	0.025707201	0.371921402
CCDC77	0.975848778	0.091059855	0.023427615	0.537130583	0.033235225	0.025685363	0.371921402
KLHL42	0.002496328	0.446833976	0.143283172	0.426623796	0.545553682	0.025701171	0.371921402
LOC107133071	0.62550002	0.225699476	0.016616739	0.376183767	0.042017012	0.025647287	0.371921402
MEG8	0.610845983	0.020515438	0.060056753	0.752352064	0.065423927	0.025631997	0.371921402
PARS2	0.720899432	0.636274898	0.224416367	0.000854254	0.423560015	0.02572243	0.371921402
SLA2	0.013834579	0.237328707	0.28512518	0.399892205	0.099294483	0.025689369	0.371921402
TMEM230	0.895212762	0.074643388	0.857810151	0.321539328	0.002030749	0.025804618	0.372781332
MRPL55	0.109052155	0.270478945	0.668766373	0.045618524	0.041749568	0.025868116	0.373369962
LOC100848598	0.165900919	0.130773474	0.23556322	0.284625417	0.025883271	0.025904405	0.373565193
C7H19orf70	0.294915951	0.151708766	0.965302202	0.004870391	0.179287073	0.025932286	0.373638929
MTAP	0.934611382	0.402327482	0.018600848	0.01461149	0.370661101	0.026007544	0.374394566
EOMES	0.314934493	0.035821244	0.092209032	0.308698107	0.118913691	0.026143963	0.376028552
MOK	0.700975363	0.010949937	0.060144477	0.868377949	0.095452453	0.026179529	0.376210371
KALRN	0.105281879	0.378843583	0.044394689	0.028954854	0.748256784	0.026223196	0.376508202
AK6	0.139008794	0.242311273	0.455764106	0.542107754	0.004623879	0.026275635	0.376856043
CHST13	0.355740342	0.450255685	0.004599483	0.096840197	0.540752323	0.026319323	0.376856043
LOC101908166	0.000277151	0.955159201	0.578464472	0.772720012	0.326636059	0.026350924	0.376856043
SPDYA	0.037276626	0.369041576	0.904580136	0.016629599	0.186559143	0.026331243	0.376856043
ULK3	0.894328061	0.04479741	0.033362364	0.803817777	0.035997918	0.026362241	0.376856043
RTP4	0.115909142	0.067074015	0.600289921	0.009818752	0.8459369	0.026401247	0.377085177
CD248	0.252125777	0.787422147	0.000647974	0.32818239	0.919476661	0.026425461	0.377102824
IL17RD	0.341667853	0.22491925	0.636224183	0.006718085	0.119355467	0.026595996	0.379206676
B4GALT7	0.27434858	0.085827354	0.774919584	0.135886167	0.015959001	0.026757415	0.38059558
IGFALS	0.608644202	0.313518619	0.831803035	0.102635391	0.00244395	0.0268647	0.38059558
L3MBTL2	0.98469511	0.1932152	0.462254892	0.063202198	0.007125233	0.026773048	0.38059558
LOC100196901	0.055316974	0.037445749	0.818029933	0.209239591	0.112452594	0.026889122	0.38059558
LOC527186	0.245372252	0.212580162	0.008253044	0.808936979	0.114263345	0.026854523	0.38059558
LPGAT1	0.404860739	0.096685046	0.148756445	0.009851148	0.694587001	0.026877475	0.38059558
MIS18BP1	0.255521034	0.031075828	0.098838381	0.156731893	0.321835043	0.026765421	0.38059558
TWIST2	0.000580914	0.361846839	0.403027923	0.595468062	0.787606816	0.026828617	0.38059558

WNT2	0.004711119	0.035223129	0.605200591	0.864267387	0.459691958	0.026902131	0.38059558
LOC783577	0.175855119	0.330538133	0.156323979	0.005395217	0.816713412	0.026963219	0.381131255
ADAM33	0.546447934	0.127367514	0.02711592	0.800262217	0.026817996	0.027166223	0.382345183
LOC112442284	0.270291966	0.01508506	0.473566834	0.057556003	0.364477127	0.027167522	0.382345183
NSUN6	0.885346773	0.891756276	0.015591915	0.088224372	0.037342205	0.027188887	0.382345183
PCP4L1	0.029418191	0.1643141	0.080499469	0.11883351	0.874320088	0.027133822	0.382345183
ZNF432	0.000934822	0.878858275	0.872340955	0.466807767	0.120770429	0.027123236	0.382345183
ZNF512B	0.904983972	0.352987704	0.001614709	0.654247964	0.119672202	0.027115032	0.382345183
ALDH1A2	0.62175222	0.020348257	0.068557124	0.183544996	0.259553072	0.027521138	0.383705422
ATG4B	0.609324362	0.338043312	0.004213812	0.45005966	0.104527122	0.027309087	0.383705422
CCDC78	0.055780553	0.063352987	0.081714324	0.646391557	0.219809553	0.027394826	0.383705422
CKAP4	0.503404652	0.536584788	0.001158733	0.534870999	0.246980284	0.027532849	0.383705422
FAM160A2	0.270225557	0.578208411	0.03592979	0.014749208	0.49627946	0.027422305	0.383705422
GRIK2	0.001312144	0.840093503	0.045974258	0.931477428	0.874595232	0.027506214	0.383705422
INPP5J	0.050902448	0.044111961	0.025790991	0.85004109	0.834577086	0.027418568	0.383705422
LOC112444310	0.099680242	0.040468539	0.273726295	0.043640758	0.859771751	0.027568618	0.383705422
LOC531090	0.012119957	0.03683103	0.488058288	0.629452828	0.301217352	0.027515538	0.383705422
MFSD9	0.186497728	0.198100755	0.332303487	0.076284268	0.044346933	0.027612949	0.383705422
MOG	0.11420842	0.106903697	0.355586457	0.793642051	0.011889126	0.027366985	0.383705422
PGLYRP2	0.330440394	0.341990158	0.041453709	0.20643372	0.04292898	0.027605031	0.383705422
SLC23A2	0.800076379	0.054711115	0.005505012	0.229106546	0.749320608	0.027541912	0.383705422
ZNF420	0.194003225	0.000898051	0.654336382	0.537419441	0.675694817	0.027554465	0.383705422
GP9	0.75775662	0.279612192	0.010009391	0.031018554	0.634574903	0.027704113	0.383996794
KIF4A	0.501754489	0.05780024	0.058460945	0.262213389	0.093797154	0.027684794	0.383996794
STX12	0.211280467	0.38123569	0.133540801	0.010845406	0.357546695	0.02768952	0.383996794
MRPL33	0.547667767	0.006882842	0.708178812	0.111201976	0.140882462	0.027737238	0.384131481
LTN1	0.221275212	0.138264309	0.030012174	0.061606864	0.740900579	0.027775901	0.384154025
MBNL2	0.626665089	0.954868238	0.002836411	0.151123239	0.164125018	0.027855907	0.384154025
RAB24	0.064359825	0.558336921	0.003173509	0.514183202	0.717007576	0.027832544	0.384154025
RASAL3	0.04008287	0.098755966	0.588147753	0.114726293	0.157141183	0.027802157	0.384154025
RFLNB	0.727138015	0.15840328	0.111334116	0.003553986	0.922387423	0.027830289	0.384154025
ACAN	0.188060617	0.239875923	0.024304469	0.191270877	0.201561067	0.027929755	0.384411599
MAP3K6	0.05446117	0.019307347	0.732850788	0.070233559	0.781273975	0.02793589	0.384411599
ROM1	0.203624052	0.263985401	0.014011642	0.114653289	0.489895494	0.027944856	0.384411599
DIAPH3	0.323329141	0.012610373	0.02300842	0.470730633	0.973368292	0.028235184	0.384634566
DNAH12	0.760477802	0.935317363	0.001116333	0.93157352	0.057639775	0.028086733	0.384634566
FAM189A2	0.191194455	0.598629761	0.075460172	0.006878085	0.722677484	0.028212187	0.384634566
FLNA	0.028044053	0.423954534	0.358170891	0.655356299	0.015274614	0.028083357	0.384634566
GRHPR	0.387122987	0.079325768	0.907630071	0.107107697	0.014332258	0.028150927	0.384634566
NADSYN1	0.665275838	0.046035373	0.005588485	0.280709125	0.893624302	0.028213687	0.384634566

PABPN1	0.803258303	0.034191919	0.083238589	0.041352838	0.451061356	0.028089493	0.384634566
PEX11B	0.84780497	0.654108508	0.570809009	0.000198382	0.684760486	0.028242316	0.384634566
RSPO1	0.280679607	0.883925815	0.089733035	0.007831833	0.244948972	0.028117766	0.384634566
SLC30A5	0.033490922	0.154585223	0.205572699	0.216007393	0.184862118	0.028028007	0.384634566
STAC2	0.034982756	0.044298979	0.329262291	0.141356974	0.592896333	0.02814161	0.384634566
ZBTB22	0.365575769	0.628015655	0.009936856	0.052035728	0.360484594	0.028154197	0.384634566
LOC783686	0.626302716	0.154575957	0.207435728	0.01935086	0.110887199	0.028280834	0.384839774
EPRS	0.928391901	0.064400421	0.181925542	0.612349657	0.006537688	0.028473332	0.38713823
B3GNT6	0.119557415	0.174716382	0.051154654	0.055934079	0.73763385	0.028702518	0.387411684
CACNB2	0.001063575	0.143699821	0.535279148	0.661048311	0.809127354	0.028563409	0.387411684
CCR4	0.076029622	0.342607667	0.750600446	0.007113993	0.314937527	0.028583514	0.387411684
DERL1	0.35108888	0.001466222	0.355510037	0.653667297	0.367986827	0.028674505	0.387411684
DHX57	0.873210871	0.373940457	0.038952739	0.915285924	0.003773776	0.02863753	0.387411684
IKZF1	0.240969308	0.017543946	0.462489371	0.464650445	0.048459391	0.028676101	0.387411684
LOC107131684	0.311414998	0.494139693	0.034143682	0.012545038	0.668990785	0.028705905	0.387411684
TBC1D13	0.894273254	0.483775698	0.04113956	0.175038129	0.014119023	0.028659693	0.387411684
TM7SF2	0.292466123	0.423488149	0.363530337	0.00517143	0.188545988	0.028624419	0.387411684
CCER2	0.025092263	0.375519912	0.012477722	0.768756734	0.491886035	0.028859073	0.387766204
MFSD8	0.545445465	0.827295608	0.022439419	0.021069206	0.207912626	0.028815711	0.387766204
SELENOP	0.941977226	0.211321993	0.008628661	0.066991806	0.386626695	0.02887114	0.387766204
SLC22A3	0.598009279	0.004569327	0.120787864	0.240318523	0.558951591	0.028806787	0.387766204
SOBP	0.076560591	0.383745645	0.59724961	0.003298407	0.768773303	0.028873944	0.387766204
STARD9	0.835015399	0.078834894	0.051721904	0.622922458	0.020869806	0.028776527	0.387766204
EIF2S1	0.836952411	0.006611205	0.099316401	0.673812479	0.120559104	0.028935726	0.388278168
COX7C	0.435229594	0.412147672	0.801984361	0.001388213	0.22442663	0.029010243	0.388960045
CELF1	0.494606634	0.000510615	0.652105514	0.875731953	0.312372971	0.02910757	0.389019409
CNTROB	0.686943028	0.019470294	0.007263379	0.65616549	0.715749862	0.029346538	0.389019409
CPSF1	0.401938213	0.005763407	0.054439523	0.586096509	0.612736554	0.029206581	0.389019409
DENR	0.205366228	0.021727144	0.509606299	0.304867903	0.064787805	0.029049335	0.389019409
DSTN	0.015336316	0.349766104	0.584428723	0.804416186	0.018032468	0.029283677	0.389019409
FABP1	0.025403636	0.452904881	0.900769495	0.089888751	0.048683724	0.029233116	0.389019409
GNB3	0.861810618	0.211228547	0.019523437	0.802951774	0.015813253	0.029138611	0.389019409
IFRD2	0.421316251	0.550839658	0.31985184	0.095352401	0.006431292	0.029303121	0.389019409
KCNG1	0.097949139	0.786068146	0.004907369	0.481550092	0.250602314	0.029334723	0.389019409
LOC101906664	0.694116896	0.498489733	0.292702532	0.146006855	0.003044052	0.029091308	0.389019409
NDUFS3	0.42013907	0.336852166	0.723868155	0.0039624	0.111442905	0.029185135	0.389019409
SERINC2	0.072682532	0.027010436	0.696547387	0.13282267	0.250538347	0.029296429	0.389019409
SIT1	0.517604126	0.03186268	0.652317071	0.829689315	0.005090325	0.0292677	0.389019409
UFM1	0.751963971	0.04915574	0.789240093	0.632835126	0.002462274	0.029276746	0.389019409
GABBR1	0.923957197	0.170642713	0.012550373	0.915464585	0.025252069	0.029395861	0.389358735

MANF	0.200782808	0.537988766	0.012264628	0.077884445	0.445141654	0.029473329	0.390070003
UFSP1	0.239362661	0.427372892	0.450610209	0.031106751	0.032138513	0.029536629	0.390592769
ASB3	0.601239771	0.405305487	0.220948139	0.200185844	0.004304035	0.029663668	0.390906088
DHX29	0.260746031	0.122108818	0.681205427	0.337619097	0.00632836	0.029642997	0.390906088
MCEMP1	0.138267693	0.034467013	0.16925421	0.364319953	0.157697631	0.029643479	0.390906088
SLC25A51	0.040975319	0.075975295	0.412490732	0.077359201	0.466178851	0.029630319	0.390906088
ZNF436	0.032092953	0.597950294	0.002677284	0.936568918	0.96488963	0.029679421	0.390906088
ELOF1	0.026998527	0.883441538	0.439023192	0.007333221	0.605412594	0.029704374	0.390920991
PICALM	0.882957795	0.280554385	0.00116382	0.292237348	0.554917	0.029813013	0.391826315
ZMYND15	0.712633911	0.426737926	0.004405521	0.142246559	0.245424502	0.029820917	0.391826315
MITF	0.816867566	0.085260941	0.055833865	0.312235162	0.038722322	0.029921196	0.392608023
PTHLH	0.141117485	0.911717282	0.022783597	0.477041057	0.033634297	0.029928258	0.392608023
CD96	0.00910026	0.768009896	0.058733961	0.838347225	0.137734016	0.030078697	0.394266373
BICDL1	0.256706576	0.448440289	0.002711633	0.65181402	0.233622425	0.030133976	0.394675722
CEP95	0.986444922	0.016983035	0.006054716	0.762870128	0.61818949	0.030257039	0.394712449
GMPS	0.004651164	0.172784025	0.170174302	0.40924779	0.851924328	0.030193841	0.394712449
KLHDC7A	0.003623593	0.458704339	0.493585389	0.088472708	0.657372395	0.030207691	0.394712449
MIIP	0.400667632	0.018799629	0.05538088	0.396587045	0.28863966	0.030222621	0.394712449
TLCD2	0.284723159	0.121094375	0.300412455	0.051721577	0.089232005	0.030243691	0.394712449
LOC751811	0.024733468	0.262545966	0.174689995	0.950718306	0.044505887	0.030323331	0.395263057
ATP6V0A1	0.977548208	0.110230489	0.006204767	0.86281612	0.084494318	0.030625696	0.39527679
CLASP1	0.145436089	0.00270366	0.690164173	0.244643407	0.734143527	0.03062479	0.39527679
COL3A1	0.528321783	0.048425624	0.014102514	0.482318998	0.277131692	0.030416373	0.39527679
CROCC	0.681391318	0.078290624	0.009440669	0.274272396	0.349399191	0.03043093	0.39527679
GLDN	0.612309762	0.166661114	0.013452189	0.840017538	0.042328683	0.030653452	0.39527679
HSPA13	0.87832985	0.006007166	0.048362592	0.34108029	0.561057017	0.03066159	0.39527679
LOC112446699	0.725989976	0.048017466	0.014470874	0.322280836	0.298412133	0.030533311	0.39527679
LRRC8B	0.410461333	0.211750242	0.009902428	0.161640991	0.348369905	0.030513124	0.39527679
MINK1	0.82073145	0.132144259	0.002475473	0.431729438	0.41537134	0.03038319	0.39527679
MRPL15	0.195912657	0.044500979	0.403972447	0.883312025	0.015688345	0.030651338	0.39527679
P3H1	0.623108543	0.03159764	0.010555049	0.302912	0.771301704	0.030548898	0.39527679
SAE1	0.454608474	0.027439501	0.54228761	0.48503481	0.014804796	0.03055794	0.39527679
SIGLEC1	0.755760222	0.75890378	0.935401715	0.033765246	0.002671334	0.030483111	0.39527679
TMEM167A	0.711819941	0.184868272	0.813780335	0.667528327	0.00067769	0.030504546	0.39527679
KIFC2	0.88519995	0.116430669	0.000685762	0.822068339	0.847703238	0.0308319	0.397160373
LOC101904449	0.815717133	0.002037496	0.603888699	0.293004386	0.167795547	0.030869143	0.397250634
STAP2	0.395627367	0.251320011	0.005436119	0.150256472	0.608147952	0.03088732	0.397250634
ARHGAP40	0.49167132	0.353856222	0.081508935	0.037613576	0.094008886	0.031189908	0.398642981
IL2RA	0.548888667	0.089127702	0.600908922	0.012657783	0.134505101	0.031152098	0.398642981
LOC112448070	0.22811704	0.018334838	0.652571504	0.773420438	0.023736617	0.031174976	0.398642981

PRRX1	0.01198382	0.081052648	0.237423845	0.541301783	0.401170125	0.031163774	0.398642981
SOX9	0.404522639	0.81049347	0.008968454	0.158526705	0.107490485	0.031174284	0.398642981
WDR87	0.329418752	0.180068368	0.028232941	0.621652179	0.047793199	0.031034719	0.398642981
ZBED6CL	0.070788567	0.013749735	0.24274374	0.614714797	0.344513997	0.031146742	0.398642981
ZNF451	0.467015673	0.819623688	0.129717398	0.001076321	0.936950715	0.031161364	0.398642981
MRVI1	0.050582739	0.386476973	0.54522993	0.74233239	0.006379019	0.031321327	0.400011122
APC2	0.006731856	0.136363543	0.127798252	0.559863957	0.774231342	0.031472632	0.401007274
CAPN10	0.033981258	0.046865675	0.093719244	0.419045674	0.813017486	0.031471377	0.401007274
PMS2	0.651616501	0.807396802	0.269002272	0.000558969	0.642578129	0.031465044	0.401007274
CDK3	0.656011137	0.004226031	0.275192329	0.537224898	0.124598993	0.031558489	0.401166825
CTHRC1	0.728751015	0.0374639	0.025087505	0.275073965	0.270507424	0.031517738	0.401166825
ZCCHC13	0.059477871	0.418190174	0.025885578	0.134118217	0.591019907	0.031545693	0.401166825
LOC788175	0.002833672	0.272932163	0.307215706	0.404583982	0.532557293	0.031608616	0.401493037
ATP2A2	0.395563366	0.115633738	0.136839613	0.177639378	0.046589965	0.031849129	0.402298886
C5H12orf71	0.475934239	0.965637854	0.001103858	0.128814793	0.793428748	0.031868171	0.402298886
CCNJL	0.04110632	0.096436603	0.062161173	0.27617322	0.760720496	0.031836515	0.402298886
LOC112447082	0.00290493	0.691834666	0.686812866	0.294203941	0.127370761	0.031818535	0.402298886
MYBPH	0.013864467	0.611622607	0.958565492	0.474383952	0.013419254	0.031826645	0.402298886
SEC16B	0.379115674	0.057103012	0.012908054	0.454555015	0.405255156	0.031720355	0.402298886
TAB1	0.902714465	0.294779031	0.447164913	0.000659242	0.657736396	0.031767558	0.402298886
UPK3B	0.998630593	0.069187522	0.033022355	0.096094621	0.235471675	0.031780125	0.402298886
C3	0.340711539	0.071522316	0.055491205	0.073751075	0.521091915	0.031914755	0.402320553
LOC101902537	0.332804232	0.034570643	0.751334583	0.199014555	0.030214047	0.031918918	0.402320553
GAS2L2	0.771511736	0.868771557	0.232597394	0.141846041	0.002354573	0.03195478	0.402430562
PIGO	0.681845478	0.016175898	0.011515885	0.626309056	0.655247527	0.03197669	0.402430562
CAPN8	0.116307331	0.009831811	0.0695651	0.808068672	0.812242971	0.032010788	0.402550995
FSBP	0.905159227	0.215755721	0.006957674	0.258937575	0.149027974	0.0320984	0.403343679
TAOK3	0.843589448	0.232048907	0.239304805	0.010445181	0.107296633	0.032124513	0.40336296
CFAP36	0.486539495	0.247519939	0.77544388	0.003010328	0.187533758	0.032210499	0.404133403
CCDC168	0.868173657	0.012628004	0.044670959	0.757433957	0.14341046	0.032397692	0.404934142
CD3D	0.107811732	0.009046479	0.94447528	0.629284669	0.091706785	0.032382979	0.404934142
CD83	0.081930986	0.017060133	0.758944149	0.37741518	0.132510456	0.032341133	0.404934142
LOC788801	0.470153129	0.282723066	0.015040406	0.095386585	0.278903555	0.032393351	0.404934142
RXFP1	0.225799226	0.667864459	0.157797249	0.253754363	0.008791407	0.032354146	0.404934142
C23H6orf201	0.022444807	0.281542653	0.02930459	0.897601917	0.324001003	0.032653993	0.405143858
CCNB3	0.191171304	0.410506123	0.24710648	0.173595409	0.01602231	0.032686032	0.405143858
CEBPG	0.888745142	0.069186861	0.004400684	0.531153083	0.371773551	0.03249008	0.405143858
EPOR	0.033721296	0.389260336	0.049963306	0.176664851	0.462772095	0.032561855	0.405143858
LOC615733	0.112289526	0.092228513	0.346082791	0.189873445	0.078792866	0.032562946	0.405143858
MYO1E	0.903280064	0.487839246	0.083685875	0.025027667	0.058194664	0.032597563	0.405143858

RPL22L1	0.832236155	0.13538937	0.154506578	0.328150068	0.009403729	0.032602179	0.405143858
SCRG1	0.177449666	0.54091376	0.661730034	0.002949345	0.286809964	0.032604737	0.405143858
UBE2D3	0.816729955	0.037254112	0.248962658	0.293145783	0.024278234	0.032676273	0.405143858
ZNF628	0.402974349	0.709043973	0.002743752	0.15509085	0.439492188	0.032490727	0.405143858
ZNRD1	0.324600894	0.001245845	0.539273378	0.826886306	0.298672486	0.032655817	0.405143858
DPT	0.153817251	0.944417885	0.266588554	0.003105642	0.448994512	0.032710821	0.405145124
APOC3	0.482054911	0.001513959	0.790032867	0.62679683	0.14998934	0.032790127	0.405209919
DOPEY2	0.739718964	0.324398771	0.291046381	0.031209576	0.024811101	0.032741696	0.405209919
HAGH	0.48623649	0.35947935	0.761469917	0.005274987	0.077143644	0.032773318	0.405209919
BCL7A	0.043954473	0.235079147	0.731272401	0.007487155	0.962041223	0.032875624	0.405350763
LOC615278	0.187960815	0.928886316	0.046699703	0.524100565	0.012729379	0.032863928	0.405350763
LOC781412	0.223830447	0.108276267	0.248118929	0.056520359	0.160124594	0.03287409	0.405350763
G0S2	0.054104699	0.094171198	0.73694255	0.299683152	0.048607688	0.032979979	0.406332154
STK19	0.899064935	0.070836014	0.0033841	0.794691633	0.320786935	0.033075002	0.407197197
SLC1A2	0.324360363	0.634900318	0.001173924	0.444798709	0.511912923	0.033115499	0.407390149
CDC45	0.423857918	0.058522753	0.011803343	0.917622222	0.206080776	0.03323876	0.408600218
LOC104968422	0.852174253	0.007904986	0.102303225	0.326178584	0.247335834	0.033327599	0.409385651
SDCBP	0.795382246	0.011759717	0.029843821	0.306609782	0.650688571	0.033363252	0.409517072
FFAR4	0.01893955	0.191641685	0.156703959	0.5677001	0.172777636	0.033400647	0.409669666
ARHGAP29	0.214643951	0.95511337	0.902620021	0.007254065	0.042621495	0.033943568	0.410760292
CBR4	0.044982685	0.286374495	0.321832298	0.027142555	0.507777864	0.033915846	0.410760292
COX7ALP1	0.50247308	0.647132407	0.426893811	0.008269756	0.049234935	0.033679754	0.410760292
CYP1A1	0.133717024	0.377707655	0.062431101	0.074401614	0.240897455	0.033678077	0.410760292
EBP	0.362707106	0.143705612	0.496047449	0.015593772	0.141691344	0.033911535	0.410760292
EIF2AK2	0.105274854	0.967298839	0.978656581	0.112106518	0.005129604	0.033980662	0.410760292
ELAVL1	0.48682275	0.014019566	0.02115405	0.81626067	0.486510556	0.033990158	0.410760292
HSPA6	0.227738502	0.074294862	0.212105794	0.18481331	0.084959785	0.033615424	0.410760292
IGSF10	0.001907402	0.728920261	0.14535375	0.661104942	0.422830945	0.033669518	0.410760292
LOC104969545	0.179835647	0.449953417	0.89161925	0.001022771	0.762114342	0.03357219	0.410760292
OIT3	0.144238014	0.856657213	0.001445161	0.483955577	0.661916703	0.033939731	0.410760292
PKDCC	0.473279589	0.24691744	0.004487195	0.348589268	0.313105418	0.03395159	0.410760292
RAB4A	0.645696747	0.00097923	0.761923786	0.159962259	0.735373554	0.033737293	0.410760292
RGS18	0.455665909	0.109636175	0.013533736	0.556027412	0.150640071	0.033722607	0.410760292
SCARB2	0.279083715	0.332435844	0.00188207	0.699049044	0.46566936	0.033802613	0.410760292
SZT2	0.997078497	0.59670263	0.009407278	0.466949441	0.021530397	0.033584661	0.410760292
TGM1	0.003342494	0.514530955	0.750603919	0.105087202	0.422473446	0.033981254	0.410760292
TRANK1	0.217137812	0.042846344	0.068767912	0.187194731	0.475536292	0.033844914	0.410760292
TRAPPC3	0.316967401	0.007094915	0.536162685	0.154371613	0.305764325	0.033829975	0.410760292
ZNF12	0.880349789	0.305907252	0.032711434	0.118361391	0.05449326	0.033794355	0.410760292
ENTPD2	0.257848552	0.739202611	0.000457323	0.870010648	0.757886608	0.034043237	0.410099017

UNC5C	0.013549463	0.15482529	0.102110583	0.396437042	0.677669925	0.034070678	0.411127863
CEP68	0.272455993	0.034574081	0.035328145	0.32775406	0.530220413	0.034178436	0.412125133
AKAP5	0.547613301	0.003194411	0.375863369	0.180346939	0.490436897	0.03430009	0.412265903
AVPI1	0.904776214	0.494634647	0.138624101	0.074219944	0.01268162	0.034389686	0.412265903
FN1	0.148660337	0.764097121	0.001513418	0.639951859	0.530078487	0.034360876	0.412265903
H2AFY2	0.24245033	0.196197444	0.859851968	0.005323381	0.2663694	0.034240834	0.412265903
LOC112442610	0.962789845	0.043865271	0.062103269	0.57132597	0.038777825	0.034282301	0.412265903
LOC112444461	0.301571416	0.776205382	0.602425182	0.037593089	0.010979782	0.034319524	0.412265903
LSS	0.75670181	0.331763174	0.6743377	0.007079949	0.048722193	0.03439108	0.412265903
SLC35B1	0.134249438	0.098844068	0.303750874	0.836167455	0.017240321	0.034281547	0.412265903
CAPZB	0.347931669	0.005087973	0.090725059	0.797959581	0.457903551	0.034499184	0.41325993
SMIM3	0.268939895	0.391736914	0.094613754	0.594719825	0.009917247	0.034539117	0.413436501
LOC101904103	0.002359641	0.165988617	0.500207132	0.401495999	0.750948336	0.034643791	0.414085404
PODXL2	0.717548912	0.579739425	0.001893014	0.28292885	0.265021568	0.034635195	0.414085404
ARNTL	0.832938156	0.378515695	0.105560938	0.005377774	0.333001972	0.034842146	0.414275774
BID	0.53737196	0.695842294	0.003484605	0.31342588	0.145798974	0.034820619	0.414275774
HDAC1	0.268379996	0.3734336	0.034502926	0.030833542	0.559370618	0.034857165	0.414275774
KCNH1	0.122242207	0.85696032	0.625551275	0.004778038	0.190734199	0.034886912	0.414275774
NAMPT	0.452877096	0.094804011	0.065568643	0.199639584	0.105838298	0.034798313	0.414275774
PGM3	0.366985128	0.107348263	0.019188025	0.656988904	0.119925122	0.034826414	0.414275774
RAP2B	0.868904896	0.000594221	0.461755291	0.370351374	0.674568872	0.034827961	0.414275774
RNF185	0.44325928	0.056204825	0.01499517	0.185593646	0.858868193	0.034822856	0.414275774
SNX19	0.427663883	0.208322658	0.529603132	0.043946983	0.028796816	0.034883845	0.414275774
LOC112444907	0.766817715	0.243707724	0.03593315	0.026667958	0.334478909	0.034953219	0.41476304
DISP1	0.106588086	0.258245756	0.036292838	0.400554582	0.150115995	0.035016761	0.415216805
CA11	0.050492737	0.191428427	0.019836195	0.421262899	0.747464724	0.03512939	0.415801062
KANK2	0.27773902	0.934528159	0.000802203	0.293606905	0.988104611	0.035142043	0.415801062
OLFML2B	0.021723659	0.447707818	0.229604594	0.054764148	0.493860017	0.035138203	0.415801062
C5AR2	0.687718587	0.229075804	0.53606477	0.014109206	0.050850052	0.035210149	0.41591827
DOLK	0.31195362	0.027517071	0.77730398	0.197021767	0.046145117	0.035237142	0.41591827
FAM160B2	0.961575964	0.086651892	0.261705819	0.028342063	0.098305395	0.035271345	0.41591827
GTSE1	0.138433207	0.564139502	0.184027592	0.146500495	0.028823507	0.035246072	0.41591827
HMCN2	0.013394806	0.009225889	0.750894622	0.971080787	0.674444569	0.035278669	0.41591827
KCNN1	0.302573491	0.000554813	0.440288643	0.865432297	0.952957518	0.035345868	0.416411367
KIAA1024	0.553024732	0.242353968	0.340208824	0.001698939	0.790321498	0.035444604	0.417114242
SALL2	0.003057582	0.37995767	0.064832566	0.978441961	0.831201299	0.035456363	0.417114242
LAMA2	0.211666536	0.120471697	0.706811534	0.013523412	0.251725231	0.035493121	0.417219024
NOXA1	0.576482363	0.117582485	0.004304583	0.371804272	0.566136429	0.035516116	0.417219024
APLP1	0.013304739	0.691262073	0.384805666	0.017779363	0.983005734	0.035676326	0.41785684
CALM3	0.054934281	0.671543388	0.543772895	0.005194763	0.594780488	0.035723182	0.41785684

LOC514457	0.719860683	0.00949001	0.194808127	0.372016466	0.125161403	0.035717796	0.41785684
LOC518134	0.536986572	0.029845466	0.181936991	0.027170648	0.782271476	0.035721229	0.41785684
NDUFC2	0.735708618	0.260730717	0.743793319	0.044949341	0.009625124	0.035630151	0.41785684
SLC7A4	0.691214083	0.56629918	0.007872647	0.151320024	0.13229796	0.035617025	0.41785684
LTV1	0.307287913	0.026775208	0.140850967	0.566317658	0.094716252	0.035789534	0.418334789
JSRP1	0.128107282	0.19372999	0.866482287	0.023556889	0.123028942	0.03584904	0.418732099
SLMAP	0.003702735	0.380610843	0.376844783	0.974301723	0.120937841	0.035942007	0.419519399
LOC100850659	0.446587681	0.195827915	0.287317701	0.008050578	0.311322542	0.036087684	0.420920388
ACD	0.988874854	0.006787222	0.014728652	0.685726591	0.934160576	0.036214168	0.421294423
CASTOR2	0.555226063	0.004354931	0.122645335	0.255840494	0.838447209	0.036319281	0.421294423
DFFB	0.814808262	0.006815609	0.025895194	0.94360875	0.46698481	0.036230358	0.421294423
ELL2	0.548598714	0.871440372	0.001890183	0.433910211	0.162050664	0.036292672	0.421294423
FFAR2	0.343676825	0.205844239	0.651011377	0.019766298	0.069791231	0.036290377	0.421294423
MRPL19	0.799520364	0.250040938	0.654331921	0.182060036	0.002671798	0.036325124	0.421294423
RSRP1	0.458406014	0.364911581	0.283658917	0.006583792	0.202520291	0.036193486	0.421294423
TOMM20	0.649612195	0.021800138	0.076922349	0.542854307	0.10690945	0.036176853	0.421294423
TRMT1	0.110570585	0.45657732	0.192481233	0.210602861	0.031158518	0.036374449	0.421568559
ACTG2	0.001667068	0.460014425	0.609117218	0.893595713	0.155526452	0.036791542	0.422808314
CCDC57	0.992565944	0.054593048	0.186889243	0.170494611	0.037594469	0.036788565	0.422808314
CDC20	0.613628004	0.091983029	0.04668379	0.23342732	0.10468985	0.036601631	0.422808314
DGKQ	0.054480912	0.040486353	0.152356124	0.196153689	0.984802647	0.03679088	0.422808314
ERO1A	0.720443942	0.015081204	0.050026885	0.147673667	0.807862159	0.036765119	0.422808314
FLVCR2	0.099524998	0.297835295	0.187101308	0.307648192	0.037678068	0.03656376	0.422808314
MDGA2	0.012138874	0.053271495	0.888621388	0.147814628	0.765952967	0.036842111	0.422808314
NDRG4	0.027717113	0.362292858	0.99781072	0.34137048	0.018864138	0.036649007	0.422808314
PRKAR1A	0.805672343	0.045878299	0.093921323	0.039446878	0.473658184	0.036771988	0.422808314
RGS1	0.726819585	0.064523657	0.506733792	0.003241081	0.837077211	0.03663088	0.422808314
SGF29	0.035168812	0.522394413	0.259588352	0.229191839	0.059313664	0.036760563	0.422808314
SLC31A1	0.256001249	0.343626564	0.043428965	0.96310792	0.017457286	0.036544055	0.422808314
TARBP1	0.882565278	0.033823746	0.004776587	0.720029038	0.626305959	0.036568826	0.422808314
TIGIT	0.169342722	0.012625347	0.942813424	0.692553731	0.046556391	0.036818334	0.422808314
SGIP1	0.341715663	0.111322842	0.220623508	0.04537975	0.171119691	0.036882751	0.422978912
C7H19orf25	0.497368309	0.885032937	0.128279397	0.001430221	0.809954667	0.03696893	0.423671163
EFHB	0.891748831	0.238001525	0.269230543	0.024959973	0.046020236	0.037049116	0.423702468
MAGEL2	0.31773947	0.347630952	0.00803145	0.347222961	0.212832308	0.037021488	0.423702468
TTC4	0.644685035	0.108204136	0.194945056	0.213970695	0.022552631	0.037044544	0.423702468
FCN1	0.438335199	0.201460746	0.815122103	0.012898829	0.070801353	0.037085532	0.423823586
RAMP3	0.002602363	0.171622468	0.716137045	0.819989286	0.250993109	0.037117912	0.423898433
NDST1	0.195871698	0.544938661	0.029217538	0.473369291	0.044790496	0.037223397	0.424807484
PODN	0.099772404	0.605733684	0.923241289	0.004949946	0.240393419	0.037320469	0.42561933

ZNF428	0.426590547	0.59468857	0.001017928	0.2702522	0.958663384	0.037502151	0.427394311
ATG3	0.11795412	0.133116852	0.321122437	0.064194587	0.20850862	0.037710133	0.427755813
BMP6	0.719387729	0.130772467	0.093459956	0.013002313	0.588013633	0.037615164	0.427755813
C18H16orf87	0.469072479	0.599760643	0.068028443	0.007190984	0.499720882	0.038163426	0.427755813
CCBE1	0.154008003	0.021743126	0.615005748	0.040154382	0.823688131	0.037931086	0.427755813
CRYBG2	0.236212884	0.300849083	0.092472223	0.303458306	0.034186223	0.037951775	0.427755813
DNAJC27	0.287213077	0.356971617	0.172814823	0.005445702	0.697060436	0.03762782	0.427755813
EIF5A	0.326041823	0.213427039	0.607200143	0.486230026	0.003300322	0.03782118	0.427755813
FUT1	0.884857616	0.426252458	0.004981991	0.565454117	0.064412412	0.038045866	0.427755813
GMPPB	0.064702513	0.214842685	0.191348423	0.482776928	0.053436995	0.038109494	0.427755813
GSPT1	0.83762795	0.113382318	0.045342751	0.695497075	0.022737959	0.037926156	0.427755813
LAMA3	0.018908967	0.175224483	0.2320524	0.373913446	0.239442309	0.038185502	0.427755813
LOC100849050	0.072957078	0.350294413	0.136166561	0.026870039	0.734234834	0.038121689	0.427755813
LOC100849865	0.248459817	0.038980985	0.484998003	0.01665466	0.865936038	0.037800077	0.427755813
LOC101903540	0.618506616	0.620046414	0.008927142	0.091041294	0.215360473	0.037580698	0.427755813
MDM4	0.014854774	0.949285516	0.007272533	0.843117201	0.789660653	0.037988542	0.427755813
MLYCD	0.222551951	0.256717365	0.653060466	0.00635212	0.284218576	0.037664555	0.427755813
MRPS24	0.654677393	0.24222668	0.733355655	0.00140286	0.41953639	0.038047963	0.427755813
NPHP4	0.530335268	0.315969277	0.018673534	0.880463629	0.024935534	0.038137134	0.427755813
POLD4	0.125633114	0.070452551	0.029428989	0.603642169	0.435971483	0.038084959	0.427755813
PTN	0.222076794	0.10066163	0.041878514	0.077976092	0.924551907	0.037710791	0.427755813
REC114	0.035532332	0.06858054	0.637361355	0.16432512	0.26717598	0.037957204	0.427755813
SH3BGRL	0.492123788	0.218139094	0.253851057	0.740106438	0.003382607	0.037969441	0.427755813
TONSL	0.550368759	0.001211607	0.17439097	0.741153829	0.795407408	0.03808627	0.427755813
UTP4	0.849945543	0.539515795	0.232819779	0.002763652	0.230839493	0.037929334	0.427755813
ZNF335	0.143845726	0.005972463	0.460888115	0.217181691	0.79308937	0.037961614	0.427755813
LOC100295797	0.423504673	0.248130088	0.210438642	0.376420858	0.008292702	0.038253097	0.428220723
EDA	0.063199708	0.925861418	0.040421903	0.586169061	0.049925499	0.038319572	0.428672454
LAMP3	0.702804879	0.022136313	0.767558862	0.593189722	0.009798412	0.038385465	0.429038677
LOC785408	0.079579136	0.041436285	0.067403841	0.450948595	0.69302834	0.038404595	0.429038677
CAPS	0.029908315	0.289275436	0.063111955	0.763713185	0.167386395	0.038523664	0.42949175
LMNB1	0.263480273	0.151996834	0.10271112	0.058123025	0.291382646	0.038475748	0.42949175
SMARCC1	0.876570199	0.221537789	0.026779489	0.050079436	0.267753848	0.038499316	0.42949175
AQP9	0.482964701	0.918509241	0.322751052	0.008523157	0.057333929	0.038580614	0.429834663
PPP2R1B	0.326092533	0.477579421	0.085365834	0.092053224	0.05744478	0.03869784	0.430848207
SCIN	0.42733289	0.131126205	0.007925988	0.877671992	0.180899429	0.038772274	0.431384263
LOC511617	0.585286876	0.039367509	0.687048744	0.115639937	0.038585309	0.038814413	0.431540595
POLR2F	0.09782905	0.541048885	0.49781171	0.015462248	0.173546212	0.038838916	0.431540595
ALOX15	0.88363599	0.294956484	0.007574377	0.053744004	0.670251861	0.038980011	0.43191091
CCDC158	0.014534023	0.938439665	0.035193364	0.456975518	0.323187512	0.038903663	0.43191091

CEP250	0.776048826	0.009331143	0.016109528	0.898074006	0.680883681	0.039056474	0.43191091
GLI3	0.292743091	0.092022901	0.130625683	0.104367601	0.193528639	0.038967281	0.43191091
HDAC10	0.065910047	0.152999808	0.536671928	0.013712227	0.959603911	0.03901438	0.43191091
LOC101903604	0.626151355	0.009194591	0.076204464	0.623464266	0.260398396	0.039019531	0.43191091
TPM2	0.006485537	0.526227198	0.432466767	0.859405152	0.056180325	0.039031591	0.43191091
CALR	0.635264842	0.427914802	0.006697458	0.076062036	0.515669403	0.039083199	0.431915403
PRKCQ	0.521520888	0.118000455	0.125140988	0.593309569	0.015679159	0.039162823	0.432504092
OPTC	0.775021501	0.320828948	0.629602343	0.6355895	0.000722841	0.039260661	0.433293013
PLPPR2	0.605640815	0.736396526	0.001550772	0.229905109	0.453132061	0.039304992	0.433490746
CDAN1	0.730303749	0.081088685	0.421489125	0.988064528	0.002937107	0.039437201	0.434230233
DPM1	0.6973812	0.313707777	0.296400213	0.219436922	0.005098352	0.039474993	0.434230233
LRRC46	0.623882064	0.336181904	0.337142154	0.001997787	0.513599734	0.039477881	0.434230233
PLEKHA6	0.270792852	0.038562071	0.122819409	0.091152963	0.619369817	0.03942745	0.434230233
SCN7A	0.002495998	0.850585781	0.046739779	0.977499711	0.752979011	0.039643912	0.43576439
GATA5	0.539217728	0.169086792	0.078039139	0.036350375	0.282999462	0.039697711	0.436063678
ADAP1	0.000789016	0.837135676	0.178214697	0.639929268	0.977105801	0.039837535	0.436752489
AZGP1	0.009829532	0.521904898	0.207495495	0.270075711	0.255763478	0.039811961	0.436752489
HID1	0.380206847	0.220283875	0.002955225	0.455327619	0.653173359	0.039840258	0.436752489
HOXA7	0.23839601	0.093298074	0.267627073	0.031752318	0.390574458	0.039911883	0.437245599
CCT7	0.756337962	0.254832662	0.012451967	0.815251063	0.038076184	0.040143013	0.437355808
DBF4B	0.904489065	0.836679891	0.298571033	0.001145487	0.289784552	0.040313795	0.437355808
DIP2A	0.972128202	0.363640652	0.000641331	0.799628463	0.410335207	0.040105202	0.437355808
DYNC2H1	0.002728416	0.499751029	0.522892222	0.147235162	0.711228485	0.040198082	0.437355808
GCC1	0.136290616	0.952924096	0.046889096	0.18978099	0.064998484	0.040353419	0.437355808
LCK	0.072716672	0.025952928	0.6483011	0.726657109	0.083173628	0.039954337	0.437355808
LOC100300881	0.290101234	0.20278273	0.031575744	0.973934028	0.040977831	0.040018295	0.437355808
LOC101906739	0.407837565	0.322816395	0.010663424	0.232355637	0.230495753	0.04037708	0.437355808
PCNX3	0.961165469	0.012671716	0.05257283	0.665002329	0.173944244	0.039995936	0.437355808
PGGHG	0.850767677	0.066863982	0.022654558	0.290316238	0.198353843	0.04004495	0.437355808
PRKAG2	0.000650482	0.545155494	0.735980809	0.897311971	0.318491862	0.040172778	0.437355808
SETD3	0.91803801	0.010757898	0.287149023	0.246751691	0.106986235	0.040267423	0.437355808
SHKBP1	0.153127372	0.267195259	0.653613684	0.007274459	0.386875313	0.040401646	0.437355808
SLC38A8	0.231540375	0.002260148	0.44538596	0.845589209	0.379929893	0.04027208	0.437355808
SNCG	0.666728141	0.297261547	0.242587052	0.01956893	0.07942965	0.040221899	0.437355808
TPSB2	0.534765466	0.065474173	0.057212751	0.087617174	0.426331611	0.040254527	0.437355808
ZNF311	0.529322506	0.243637683	0.005890782	0.393184359	0.251620381	0.040366769	0.437355808
ZNF618	0.670672017	0.616994781	0.092644595	0.003992288	0.487948079	0.040204513	0.437355808
RBM3	0.458962128	0.091511842	0.003392808	0.639952519	0.826785634	0.040447421	0.437562704
APEX1	0.317905746	0.198652429	0.695079766	0.890925388	0.001939245	0.040597273	0.438894496
GABARAPL1	0.913508501	0.169992874	0.007114729	0.330674289	0.209533672	0.040836915	0.440501418

NAA10	0.39980938	0.246016188	0.812836767	0.07545456	0.012685675	0.040828804	0.440501418
NDUFA6	0.45311205	0.22060918	0.850318867	0.017839156	0.05051807	0.040853279	0.440501418
SUSD3	0.237002992	0.083993584	0.274432695	0.105237913	0.132842249	0.040777004	0.440501418
ME2	0.547511286	0.563603366	0.00803457	0.102794614	0.301061803	0.040896175	0.440674413
LOC112444326	0.918936795	0.229401551	0.955080205	0.50770399	0.000753303	0.040988058	0.441374683
CDH3	0.021008945	0.830663553	0.025131552	0.388117913	0.455226546	0.041151136	0.441970745
COPS2	0.414257728	0.073151258	0.244137813	0.615837999	0.016980921	0.04111029	0.441970745
GSDMB	0.350801778	0.993761758	0.535286006	0.017760418	0.023337329	0.041103082	0.441970745
KCNMB1	0.008051464	0.128915122	0.949991818	0.308831198	0.254208167	0.04112544	0.441970745
SIRPB1	0.144295976	0.916238592	0.567880677	0.03932391	0.026289288	0.04119385	0.442140137
SNU13	0.894071568	0.019664598	0.01550721	0.393151573	0.727250762	0.041306277	0.443057065
ZNF131	0.503634653	0.946257098	0.939119917	0.000246279	0.708964122	0.041369877	0.443449409
CCDC114	0.671143144	0.061249933	0.003543399	0.592776355	0.906527935	0.041412855	0.443620347
CENPF	0.732888411	0.010786851	0.033629479	0.565160276	0.523455727	0.041538556	0.444253313
COX7A2	0.525177892	0.246130343	0.770977396	0.009344597	0.084503757	0.041553341	0.444253313
SELENOI	0.422028394	0.031026344	0.007375949	0.815027406	0.997730626	0.04150074	0.444253313
TGFBI	0.383918031	0.317188016	0.027207631	0.029541007	0.804868508	0.041580226	0.444253313
MAP3K12	0.732094993	0.10412256	0.00455841	0.550371036	0.412754614	0.041633089	0.444528709
MARCKSL1	0.56314069	0.997403058	0.050910453	0.005330237	0.521084392	0.041794781	0.445964992
CHID1	0.012814385	0.345270173	0.525073513	0.138922046	0.246952597	0.041886427	0.445970449
HJURP	0.130173684	0.061548679	0.083938347	0.734176462	0.161511171	0.041901208	0.445970449
LOC101903193	0.251104072	0.405144577	0.691422089	0.046707793	0.024274757	0.041903993	0.445970449
SLC9A1	0.680335458	0.026111729	0.23539384	0.031822561	0.598582094	0.041871325	0.445970449
LOC100335514	0.402699544	0.173564296	0.348692214	0.135485558	0.024216362	0.041972875	0.446124906
TET3	0.595006632	0.340069431	0.074436642	0.04680639	0.113319729	0.041948646	0.446124906
PLIN2	0.108217139	0.050228213	0.095434474	0.780115613	0.198825403	0.042136887	0.447578285
MED11	0.057597136	0.342601291	0.111000869	0.058375378	0.632136076	0.042257589	0.448570043
ASPSCR1	0.271228016	0.02337893	0.224868317	0.068093266	0.835151698	0.042343297	0.448881188
DUSP14	0.255650081	0.009238336	0.122371392	0.305837598	0.917231468	0.042339158	0.448881188
FANCF	0.96401223	0.849280289	0.075806476	0.012126874	0.107841774	0.042368957	0.448881188
CUL3	0.248973193	0.08348694	0.542835222	0.164488274	0.043785105	0.04240113	0.448932218
DCXR	0.378311052	0.280394999	0.715233198	0.002696536	0.400195535	0.042600588	0.449883046
LENG9	0.892217325	0.958570175	0.006754641	0.202889711	0.069689163	0.042537591	0.449883046
LOC101909003	0.720077319	0.20419328	0.072009574	0.287091056	0.026925516	0.042591289	0.449883046
ZNF624	0.036245334	0.678173757	0.130874144	0.312581524	0.081389938	0.042590454	0.449883046
TNFSF10	0.195322709	0.995745422	0.039940501	0.010879515	0.974528236	0.042759353	0.451082965
ZDHHC2	0.731779781	0.149595805	0.00806974	0.315136087	0.295951195	0.042769185	0.451082965
CDKN1A	0.498919066	0.251304376	0.015957994	0.241119572	0.171637295	0.042903841	0.452017836
FBXO36	0.071467478	0.790554089	0.067454212	0.355660037	0.061110338	0.042912911	0.452017836
LAPTM4A	0.010465353	0.089538846	0.389730941	0.943027066	0.241334888	0.043004509	0.45211211

LOC112449547	0.040442707	0.93865016	0.010615099	0.322686176	0.638248018	0.042964966	0.45211211
USP4	0.81611479	0.096035871	0.001528091	0.718486634	0.965423667	0.042991842	0.45211211
SAMD10	0.706884261	0.001239423	0.408918971	0.715122646	0.324785362	0.043036057	0.452154116
INO80B	0.057982212	0.698278874	0.253599072	0.0179393	0.454377052	0.043192443	0.453287911
SLC6A17	0.008916555	0.45021556	0.298748711	0.960449059	0.072735803	0.043220708	0.453287911
SNTA1	0.32895767	0.557505185	0.139409556	0.341211332	0.00960593	0.043226834	0.453287911
TOMM40	0.272906326	0.123059284	0.652566969	0.100926435	0.038027166	0.043327301	0.454051296
CD3E	0.116019072	0.033580996	0.849686364	0.48320468	0.052958318	0.043521316	0.454703246
HEXIM2	0.074267459	0.063150098	0.261592132	0.750628574	0.09215393	0.043570922	0.454703246
LOC512486	0.117969178	0.160320865	0.343158641	0.013879094	0.942303569	0.043575279	0.454703246
LOXL4	0.119486295	0.399488923	0.234806223	0.053192846	0.142413108	0.043583463	0.454703246
LYAR	0.653345123	0.416620487	0.364251247	0.000909007	0.936824276	0.043431134	0.454703246
NDUFB4	0.267927129	0.124366101	0.447893873	0.129090968	0.044046314	0.043568597	0.454703246
UBN1	0.659334073	0.022500939	0.900864753	0.006566297	0.967467478	0.043582647	0.454703246
COPG2	0.002876471	0.97586383	0.105377518	0.382221137	0.752962242	0.043655813	0.45516871
KIAA1143	0.689461365	0.179015819	0.230716268	0.06908119	0.043368568	0.043714395	0.45549012
TSPAN15	0.007764207	0.142572426	0.351773128	0.228355709	0.960399454	0.043742448	0.455493219
ACTA2	0.014913614	0.536854882	0.735445267	0.674500373	0.021549084	0.043801936	0.455534584
PREX1	0.105855626	0.755149852	0.945873429	0.690870776	0.00163815	0.043797482	0.455534584
ASNS	0.593422536	0.205509514	0.335386586	0.145063356	0.014499826	0.043945012	0.456733116
CADM1	0.327160551	0.06072342	0.368686733	0.592389164	0.019860871	0.043990531	0.456916836
SLC16A9	0.54508989	0.025210758	0.308709435	0.056904631	0.357334125	0.044018829	0.456921573
VDAC2	0.608774044	0.29537609	0.663629019	0.088173363	0.008222887	0.04410094	0.457294356
ZNF484	0.88636518	0.49186562	0.709499314	0.053536134	0.005226493	0.044110473	0.457294356
GCSH	0.165571788	0.374288253	0.649869891	0.02085005	0.103284696	0.044167328	0.457594705
ATG9B	0.007877842	0.858872679	0.567402267	0.044062186	0.517178161	0.044408035	0.459798269
BTBD11	0.001248281	0.480357444	0.85145468	0.934607409	0.184556061	0.044591548	0.459891896
CALM2	0.49156145	0.004947988	0.081188033	0.837433081	0.532342241	0.044581501	0.459891896
CPA3	0.481761698	0.018777573	0.530632945	0.168943495	0.108394349	0.044541354	0.459891896
DDX54	0.046493302	0.691587225	0.205252479	0.152272559	0.087621907	0.044589244	0.459891896
FAM124A	0.096499573	0.021399417	0.482257537	0.494874964	0.178594306	0.044577001	0.459891896
GMPPA	0.419638743	0.020341501	0.026523577	0.744669387	0.519706359	0.044451492	0.459891896
RAB21	0.906132707	0.001229739	0.215176749	0.539269524	0.681596979	0.044613241	0.459891896
SEMA6B	0.547498361	0.232539589	0.002226367	0.334455579	0.931233037	0.044660787	0.460093017
CHRNA1	0.029809762	0.49169754	0.542487236	0.056386693	0.19743183	0.044735877	0.460288704
LOC786139	0.966722146	0.950483459	0.228370901	0.004502928	0.093619898	0.044717222	0.460288704
MCUR1	0.045372214	0.01229412	0.75923887	0.254640899	0.822043837	0.04477771	0.460430451
SMDT1	0.315990095	0.184491997	0.539530101	0.080750593	0.034954247	0.044817915	0.460555296
ARHGAP27	0.554036501	0.004072078	0.203092702	0.515977215	0.377208956	0.044943828	0.460812327
CEP128	0.33727575	0.297905936	0.604794561	0.017826184	0.082229986	0.044911461	0.460812327

PSMF1	0.905563514	0.007136326	0.144237527	0.145374583	0.656849843	0.044889856	0.460812327
SLC25A5	0.200522377	0.232069142	0.400184606	0.813750535	0.00588716	0.044955246	0.460812327
RPP40	0.488146559	0.124070323	0.166638385	0.99091485	0.008931064	0.044987286	0.460852905
AIMP2	0.235930814	0.00648822	0.992050264	0.955844497	0.061594712	0.045015902	0.460858373
CMSS1	0.538679246	0.068874274	0.391773446	0.451976238	0.013715295	0.04523461	0.461065755
IL16	0.708095037	0.621067764	0.005000659	0.636484255	0.064228288	0.045171447	0.461065755
LIF	0.518149584	0.41076087	0.009131207	0.104166411	0.443160784	0.04511232	0.461065755
LOC104976281	0.189059839	0.783492338	0.00199835	0.709069434	0.428310812	0.045170258	0.461065755
LOC508933	0.08522516	0.009885913	0.120160058	0.929121927	0.956720464	0.045199448	0.461065755
RALGAPB	0.76607365	0.09821654	0.005020472	0.241921039	0.988780392	0.045314721	0.461065755
STAB1	0.029133828	0.252836345	0.037914119	0.841253731	0.384631418	0.045317108	0.461065755
TMED2	0.466037468	0.009175024	0.67548401	0.909007138	0.034133053	0.045081441	0.461065755
TTC19	0.737508837	0.242054514	0.092287203	0.205916845	0.026553881	0.045228021	0.461065755
TUBG2	0.437272542	0.116560874	0.083948967	0.035907482	0.587769976	0.045297748	0.461065755
ZBTB9	0.00206941	0.534576604	0.968713922	0.321628354	0.263030437	0.045408616	0.461710528
RERE	0.799688704	0.603300583	0.998065038	0.853104778	0.000221205	0.045473892	0.46180201
ZMAT5	0.003132398	0.581562544	0.917642678	0.05769567	0.941434497	0.045452248	0.46180201
NFYB	0.246386866	0.856163899	0.704639174	0.001033184	0.593271812	0.045549879	0.462287609
NDUFAF6	0.950905144	0.328785862	0.147274447	0.204648673	0.009704385	0.04565359	0.463053813
MEOX1	0.456343668	0.875391013	0.075485678	0.010382575	0.292786479	0.045723202	0.463473424
MSX1	0.178115214	0.635891119	0.012051228	0.308063754	0.218262406	0.045757376	0.463533513
RPS6KA6	0.175845256	0.224772473	0.020821359	0.922429115	0.121293955	0.045850867	0.464194063
CPZ	0.513749032	0.068371534	0.071679062	0.080007305	0.459797679	0.046019809	0.465082641
ERC2	0.001568832	0.067522765	0.989298526	0.969277276	0.910584244	0.045980635	0.465082641
PDE5A	0.250568899	0.254844284	0.372503533	0.005316123	0.732564296	0.046023655	0.465082641
XPO5	0.362007137	0.724776909	0.000792326	0.585246813	0.762647448	0.046070945	0.465274021
C15H11orf87	0.427606601	0.515864918	0.321510183	0.003127168	0.421116496	0.04625924	0.466888303
CXCL5	0.375385293	0.489854795	0.011050654	0.107465214	0.430959975	0.046479477	0.466931488
FOXK2	0.600649887	0.152620836	0.078530896	0.023263693	0.560249802	0.046392749	0.466931488
HAPLN1	0.365230837	0.225958902	0.008171642	0.257235296	0.539010881	0.04629284	0.466931488
IL6	0.053137656	0.89819491	0.024398949	0.535534291	0.151114859	0.046519589	0.466931488
LOC530973	0.989146734	0.095632646	0.203847705	0.083371962	0.05841286	0.046417064	0.466931488
NCAPD3	0.534053267	0.051915883	0.11604632	0.299337855	0.097760745	0.046493146	0.466931488
SENP1	0.001081978	0.310439419	0.729658212	0.478225124	0.80320127	0.046488434	0.466931488
STK32B	0.39008034	0.093493863	0.112911002	0.171382883	0.133426698	0.0464959	0.466931488
TYK2	0.89042942	0.212039621	0.246240059	0.056914765	0.035413335	0.046354863	0.466931488
AP5Z1	0.348878612	0.01212932	0.11801011	0.389427175	0.486985096	0.046662207	0.466993083
LOC787891	0.163444254	0.317308721	0.067426887	0.751132638	0.036003749	0.046620531	0.466993083
PFN2	0.248285017	0.040440585	0.453773549	0.035154478	0.591385663	0.046668007	0.466993083
SYT9	0.022587633	0.466853098	0.098689406	0.286705377	0.316906435	0.046616469	0.466993083

TATDN1	0.131246363	0.809858953	0.012429702	0.232317338	0.308393181	0.046646936	0.466993083
ANKRD22	0.285448053	0.029689395	0.121966118	0.467663584	0.196947003	0.046814974	0.467678144
DYNLL1	0.822969611	0.058172923	0.032220838	0.394915173	0.15684632	0.046920439	0.467678144
EIF3J	0.443401872	0.079536884	0.112820277	0.839788116	0.028643783	0.046969825	0.467678144
HMGB3	0.78134194	0.0539789	0.824939386	0.004939654	0.557330629	0.046992947	0.467678144
LOC101904526	0.676811811	0.462861545	0.000809011	0.892953783	0.422410402	0.046935082	0.467678144
LOC112448014	0.905174914	0.359542465	0.010597681	0.171494348	0.161071775	0.046835944	0.467678144
NAT14	0.665393651	0.418104254	0.594815593	0.518600888	0.00111559	0.04697858	0.467678144
RRAGC	0.710979546	0.158171617	0.004374202	0.382236595	0.508231512	0.046924385	0.467678144
STK36	0.326797791	0.112602407	0.037716435	0.22445655	0.30639348	0.04689014	0.467678144
ATF6	0.410198909	0.015170697	0.106467162	0.343670802	0.424081971	0.047230087	0.467779452
ATP5MC3	0.403879963	0.167087332	0.543063389	0.036297077	0.072563894	0.04721861	0.467779452
GPR35	0.398574058	0.014840094	0.763589957	0.064269302	0.332670693	0.047231159	0.467779452
LOC100298530	0.031420661	0.244565311	0.338567804	0.74074383	0.049931291	0.047127881	0.467779452
LOC100298890	0.143576426	0.015704454	0.445172607	0.498967041	0.192590394	0.047198468	0.467779452
LOC782456	0.218866156	0.45846685	0.040411693	0.067153066	0.353854287	0.047167565	0.467779452
LXN	0.409652691	0.288681664	0.002713571	0.702736121	0.426909784	0.04714193	0.467779452
NHP2	0.124517021	0.29928553	0.514334029	0.476569623	0.010537818	0.047137346	0.467779452
TMBIM7	0.049388073	0.322013899	0.791056129	0.014033866	0.548710996	0.047326074	0.468436794
ARID3A	0.650826287	0.309508593	0.231904775	0.013499855	0.154118867	0.047421602	0.469099402
MMP19	0.487041507	0.318045043	0.086687981	0.790616548	0.009166595	0.047459324	0.469189734
NFE2L2	0.517828433	0.566283911	0.019480296	0.063742159	0.267586488	0.047494631	0.469256109
COX5A	0.401915065	0.267870418	0.634857008	0.008670682	0.165244299	0.047645067	0.469459011
COX6B1	0.582790077	0.199639052	0.945231141	0.00718598	0.124007835	0.04766664	0.469459011
ETF1	0.679158686	0.044604943	0.048224496	0.653183791	0.102518696	0.047614083	0.469459011
HSF2	0.354499399	0.823805526	0.122004807	0.003470831	0.793004402	0.047686806	0.469459011
LOC784769	0.64310172	0.028100453	0.084650609	0.094043233	0.679159337	0.047577403	0.469459011
PET117	0.821285153	0.338970027	0.001205014	0.547093343	0.532428985	0.04758069	0.469459011
BMI1	0.775763758	0.025690236	0.278381759	0.87989773	0.020132388	0.047751006	0.469809211
TSN	0.510873504	0.062885759	0.285108303	0.995703645	0.010820019	0.047872006	0.470717493
PDGFRA	0.02279638	0.639763911	0.404179759	0.096220492	0.174453525	0.047952504	0.471226668
PIM3	0.83588351	0.25988375	0.687880464	0.001188148	0.558732928	0.048028407	0.471690122
PNPO	0.696969118	0.869199755	0.544934945	0.052749519	0.005702535	0.04805945	0.471712698
ALDH9A1	0.848370782	0.353460765	0.555168382	0.454546769	0.001319606	0.04822546	0.472494339
LPCAT2	0.426997206	0.327103153	0.074866016	0.391144838	0.024402061	0.048210567	0.472494339
LY6G5B	0.950984708	0.201907993	0.032747561	0.820986358	0.019335197	0.048212771	0.472494339
NFYC	0.391271704	0.087454328	0.379330607	0.118418855	0.065284053	0.048372678	0.473653946
COL23A1	0.830207701	0.000419044	0.598520741	0.760927198	0.636274763	0.048511732	0.473898003
MOSPD3	0.204979664	0.276088822	0.703205554	0.005169307	0.488953584	0.048444411	0.473898003
SLC5A3	0.292503686	0.559129613	0.467818446	0.002582466	0.510243093	0.04851311	0.473898003

SMIM37	0.003749537	0.254919377	0.995714724	0.214064108	0.494696524	0.048503754	0.473898003
FAM91A1	0.608976098	0.008830692	0.285276536	0.094146929	0.700407275	0.0486162	0.474058505
NKAPD1	0.642726733	0.105806524	0.031445483	0.099428145	0.474966975	0.048564242	0.474058505
RGS22	0.594629892	0.044557007	0.032867011	0.559508393	0.207490812	0.048596091	0.474058505
TNFAIP6	0.908470181	0.969002914	0.301161887	0.001823433	0.209696317	0.048678673	0.474104279
ZNF469	0.373549418	0.052291733	0.030608511	0.336398659	0.503619718	0.048655299	0.474104279
ATP5PD	0.636768732	0.147968258	0.564511557	0.017719749	0.108054085	0.048818393	0.474771894
TRA2A	0.745383482	0.46425201	0.494386948	0.008317183	0.071609218	0.048834011	0.474771894
ZNF652	0.93790608	0.030938027	0.14147433	0.212481904	0.116686888	0.048801077	0.474771894
KCTD17	0.230971832	0.502760197	0.002875486	0.621203566	0.49271936	0.04892612	0.475385767
ATP6V0E1	0.817371128	0.036601648	0.165810086	0.556468316	0.037208011	0.049076125	0.476279291
PRAG1	0.091340151	0.828859314	0.102461341	0.029576689	0.447504585	0.049065041	0.476279291
ARMCX2	0.08152225	0.662065393	0.047521136	0.102427246	0.391569199	0.049123815	0.476460362
GPR162	0.937298383	0.237360101	0.002613961	0.294122758	0.602437335	0.049175565	0.476680565
LOC100297399	0.242320759	0.704469418	0.20447509	0.010737817	0.275776803	0.049269863	0.476749839
LOC112448166	0.638047297	0.323943947	0.339953051	0.008112957	0.181153236	0.049241739	0.476749839
ROBO2	0.11823715	0.881507927	0.718520043	0.008582705	0.160588331	0.049226993	0.476749839
LONRF3	0.773293675	0.216943995	0.025450511	0.028830611	0.840748031	0.04930796	0.476837324
NRBP2	0.495085747	0.062168684	0.40287013	0.151131194	0.055328267	0.049365013	0.477107904
ATE1	0.052768905	0.1902138	0.508968081	0.062100593	0.328404133	0.049512959	0.477693813
LOC112443143	0.802591514	0.391716322	0.009985912	0.263909547	0.125686258	0.049497546	0.477693813
SLAIN2	0.634346083	0.037404495	0.302339898	0.050190617	0.289009356	0.049475166	0.477693813
MRPS30	0.826162078	0.304243223	0.300054518	0.18052645	0.007670844	0.04958758	0.478132654
PARP10	0.174481669	0.008080199	0.9032238	0.12232381	0.67207826	0.049660346	0.478272264
TRAM2	0.757015122	0.629394368	0.002302118	0.34880324	0.273520486	0.049648105	0.478272264
TIMM23	0.554386785	0.127048569	0.74453316	0.112116102	0.017868481	0.049768446	0.478653039
TMEM45A	0.185973423	0.642681111	0.020527939	0.49991429	0.085611508	0.049754047	0.478653039
TPPP3	0.237536947	0.651924239	0.331404569	0.004275475	0.479090825	0.049787383	0.478653039
SIX5	0.549636502	0.512053351	0.004525461	0.240888957	0.343150994	0.049834753	0.478827948
LOC112444603	0.567137682	0.314358727	0.891950415	0.001086357	0.610160507	0.049871426	0.478899921
ADRM1	0.349199754	0.079352064	0.546357548	0.264227237	0.026595215	0.050157993	0.479989789
CLDN15	0.189174076	0.73972439	0.040654451	0.080980144	0.230943526	0.05016041	0.479989789
GIMAP5	0.324795173	0.028177768	0.162577666	0.682187737	0.104442344	0.050048525	0.479989789
LOC112441566	0.290503898	0.154332738	0.130376256	0.075923384	0.238824391	0.050041761	0.479989789
ROGDI	0.444345094	0.015770746	0.503969883	0.058159827	0.516947021	0.050097694	0.479989789
TECPR1	0.66251649	0.007399633	0.058910633	0.429475753	0.857406706	0.05014626	0.479989789
CNTN2	0.643991482	0.410385236	0.039626115	0.076877545	0.132430745	0.050226062	0.480166772
LOC509118	0.652979025	0.144594309	0.00559204	0.202329909	0.999374222	0.050266682	0.480166772
THOP1	0.480226712	0.177443276	0.594051051	0.219944521	0.009587064	0.050260915	0.480166772
CLCN5	0.56912965	0.274253434	0.012807877	0.227210216	0.237382712	0.050576189	0.480739194

CYFIP2	0.219541622	0.005459787	0.934492253	0.488670639	0.197073399	0.05059025	0.480739194
F2RL2	0.234832863	0.303067172	0.185219869	0.18994785	0.043039252	0.05055941	0.480739194
GPR88	0.802895594	0.890552822	0.086464123	0.001818736	0.953337825	0.050393067	0.480739194
LOC112442634	0.183684791	0.628890788	0.029499595	0.047829077	0.660835804	0.050542532	0.480739194
MSL2	0.113011325	0.079981337	0.682055335	0.123135681	0.141791705	0.050522013	0.480739194
PNO1	0.81071652	0.035205665	0.043673535	0.183893237	0.469334154	0.050506388	0.480739194
TOMM5	0.464412845	0.206726389	0.848607227	0.025499467	0.051702467	0.050456149	0.480739194
TSR3	0.055530142	0.207017131	0.689274754	0.034639347	0.391956357	0.050505454	0.480739194
C2	0.156124132	0.048742522	0.256155186	0.134196629	0.413813336	0.0506995	0.481220066
VSIG10	0.717965336	0.310147406	0.152035838	0.668353571	0.004780398	0.050675007	0.481220066
NDUFB5	0.737427893	0.192035142	0.702356886	0.144420919	0.007553564	0.050772672	0.481636027
ITPKA	0.008283212	0.443228347	0.899925674	0.525132645	0.062622205	0.050815354	0.481762433
ADCY5	0.012688071	0.280895411	0.225748904	0.221264894	0.614993782	0.051055976	0.481826601
BLK	0.868676405	0.005547095	0.111326466	0.270133388	0.756688032	0.051104534	0.481826601
CEPT1	0.013334078	0.273483784	0.095912023	0.465136016	0.674017569	0.051104656	0.481826601
LOC112448373	0.413799477	0.993345904	0.000773093	0.370504236	0.924592928	0.05087583	0.481826601
LOC786474	0.095064385	0.612683807	0.60657164	0.062214565	0.04967793	0.050971958	0.481826601
MTCL1	0.745575941	0.181260146	0.00692303	0.194440994	0.603051346	0.051120201	0.481826601
SIX4	0.781492574	0.116430931	0.241586473	0.005631057	0.8804966	0.050913333	0.481826601
TENT5C	0.791332054	0.860417087	0.118049036	0.002127072	0.642892775	0.051179766	0.481826601
TMEM126A	0.522910267	0.184715249	0.774356323	0.051325216	0.028653617	0.051203802	0.481826601
TROAP	0.251763963	0.019528192	0.171548435	0.218310639	0.592048557	0.050919941	0.481826601
TRPC4	0.081624977	0.123858306	0.486000192	0.577277019	0.03856965	0.051031676	0.481826601
TUBGCP5	0.965333978	0.167114489	0.004404197	0.709156654	0.217895488	0.051143213	0.481826601
YPEL1	0.650092405	0.672596515	0.012839273	0.651979259	0.030006235	0.051155335	0.481826601
ANXA8L1	0.156770406	0.111066417	0.079359565	0.131970164	0.604972694	0.051296881	0.481873561
CDK1	0.179190511	0.558765522	0.119671235	0.030352278	0.303083292	0.051269891	0.481873561
SH2D1A	0.318005509	0.04546968	0.709351912	0.073502499	0.146311195	0.051292525	0.481873561
HAX1	0.017765209	0.060818878	0.442118628	0.790098293	0.292602053	0.051329608	0.481905143
LOC100847495	0.505966008	0.17324738	0.262207009	0.006700094	0.719125731	0.051418168	0.482460577
CCDC86	0.034649917	0.271176724	0.340226884	0.7877032	0.044045955	0.051467464	0.482594421
CENPX	0.643895385	0.462255486	0.687060806	0.001025048	0.53000685	0.051520652	0.482594421
KCNQ4	0.712423618	0.976323529	0.39255102	0.020594705	0.019754977	0.051516405	0.482594421
RASSF5	0.56071623	0.034463437	0.177105758	0.273034285	0.11935647	0.051643808	0.483472068
CD8B	0.023437541	0.23674302	0.572549415	0.065200823	0.539049937	0.051679554	0.483530875
RTCB	0.931638145	0.193083447	0.004849504	0.620044543	0.206762782	0.051731042	0.48373683
AP1G2	0.686496789	0.060936201	0.016745946	0.958283221	0.168175979	0.052033059	0.483922975
ARPC5	0.498413774	0.009271125	0.209044625	0.783154766	0.149616102	0.052114872	0.483922975
ATP1A1	0.407918464	0.291952841	0.104201233	0.016532255	0.549610947	0.051993525	0.483922975
BTG2	0.206896611	0.25523481	0.012895501	0.392029204	0.420339689	0.0518389	0.483922975

CXCR4	0.48433517	0.074787586	0.202803878	0.065093768	0.236965337	0.052151052	0.483922975
IL33	0.151818537	0.429256191	0.005309395	0.480367186	0.677058664	0.051930008	0.483922975
LOC104975162	0.319898005	0.757264285	0.135592285	0.006342593	0.545758129	0.052261153	0.483922975
LOC104976276	0.001209573	0.938856938	0.193346576	0.848349847	0.606398481	0.052049289	0.483922975
LOC112441502	0.723257171	0.006083667	0.702090728	0.267189602	0.137917531	0.052300459	0.483922975
LOC510382	0.295524151	0.262425729	0.974037374	0.001723649	0.871443543	0.052194563	0.483922975
LOC516599	0.969513537	0.31156154	0.002248664	0.606641885	0.273564642	0.051983789	0.483922975
LOC617785	0.154500233	0.141699785	0.080227988	0.08468207	0.763267524	0.052211561	0.483922975
MDM2	0.927400226	0.662204039	0.002860723	0.122424105	0.529288536	0.052300739	0.483922975
PKLR	0.119997647	0.323589516	0.036309246	0.337342441	0.237152174	0.05200358	0.483922975
PREB	0.001565403	0.21176659	0.711001258	0.660144883	0.73188378	0.052311215	0.483922975
PRR19	0.44151022	0.928846602	0.016043165	0.04660462	0.367735636	0.051993015	0.483922975
TCF15	0.678769606	0.183613784	0.337560972	0.002921536	0.922867377	0.052184722	0.483922975
TMEM70	0.917620947	0.493666783	0.465267588	0.199569338	0.002702759	0.052256713	0.483922975
TSR2	0.64771896	0.002663954	0.113315769	0.790525641	0.730441524	0.052034812	0.483922975
MRPL12	0.477968819	0.096804452	0.757250534	0.040177687	0.080980569	0.052345761	0.483969734
PAPOLA	0.427667183	0.79835692	0.099816843	0.02910116	0.115889477	0.052611024	0.486148373
ACYP2	0.426784126	0.401803064	0.202680907	0.062698801	0.053137208	0.052852776	0.486578055
ALOX12	0.056292748	0.011155508	0.45523012	0.764718938	0.536870603	0.053293179	0.486578055
ANKRA2	0.286871948	0.637535891	0.046948424	0.287174243	0.047555943	0.053264464	0.486578055
BCCIP	0.532661617	0.056941885	0.189016085	0.579105216	0.034923374	0.05289503	0.486578055
CDADC1	0.889597148	0.49494461	0.074206581	0.047755725	0.074535038	0.052994352	0.486578055
COL1A1	0.464676982	0.046633002	0.024691188	0.324428051	0.680295694	0.05349434	0.486578055
DDR1	0.832001478	0.459945326	0.164901317	0.162928248	0.011234787	0.05277211	0.486578055
DYNLRB2	0.277314043	0.412636983	0.016565918	0.482891534	0.127118331	0.053012014	0.486578055
FBF1	0.785862826	0.437076823	0.001025058	0.880290976	0.376342931	0.053090972	0.486578055
GDF10	0.304583598	0.169381038	0.090660808	0.393892199	0.063298474	0.053083344	0.486578055
GPR171	0.137557802	0.213100916	0.076088059	0.133186526	0.395629229	0.053337876	0.486578055
HSPB11	0.198151309	0.415084434	0.380522582	0.519750345	0.00719688	0.053210796	0.486578055
IKZF5	0.030928763	0.725462274	0.350469216	0.20001763	0.074948464	0.053437945	0.486578055
LIG1	0.989718621	0.005821846	0.166545043	0.329975894	0.372597326	0.053465806	0.486578055
LOC101902742	0.655328397	0.782077946	0.411533215	0.068532098	0.008172675	0.053507192	0.486578055
LOC104970249	0.034737677	0.01005128	0.347874288	0.983229136	0.988583428	0.053487513	0.486578055
LOC112442677	0.358795584	0.008765536	0.339384529	0.623813558	0.174489819	0.052961608	0.486578055
LOC112448269	0.983059357	0.696815783	0.665404877	0.003123663	0.081363808	0.052866783	0.486578055
LOC516108	0.036139237	0.401608181	0.713359851	0.057398117	0.198805607	0.053510551	0.486578055
MS4A1	0.230582737	0.039126676	0.49512199	0.952373125	0.027377032	0.053041503	0.486578055
MVB12B	0.161371641	0.873565322	0.008027224	0.630580149	0.165606736	0.05351736	0.486578055
P4HA2	0.151648319	0.547440655	0.035379492	0.151843707	0.260837305	0.053002952	0.486578055
PLEKHH2	0.926701172	0.711597902	0.001027265	0.32801326	0.524534617	0.053065336	0.486578055

RABGEF1	0.871591621	0.100383359	0.054607093	0.029215156	0.842893132	0.053373331	0.486578055
RFTN2	0.675160903	0.079616639	0.697525049	0.156974911	0.01996689	0.053336053	0.486578055
RIOK2	0.313894827	0.619242103	0.029970053	0.080465315	0.250808739	0.053349052	0.486578055
SMTN	0.003760438	0.535137731	0.58769032	0.983966984	0.10006383	0.053034336	0.486578055
STAC	0.789740107	0.741377276	0.144863607	0.00183449	0.748043658	0.053020416	0.486578055
ZFX	0.083222348	0.256774226	0.371187718	0.021458021	0.691460587	0.053383736	0.486578055
BMP1	0.392945664	0.090609882	0.00367659	0.96130111	0.941003573	0.053585475	0.486927591
MON1B	0.662600734	0.044929025	0.207579014	0.115861902	0.16589269	0.053686244	0.487127175
NHLH1	0.186978278	0.946496281	0.06481783	0.118359468	0.087588594	0.053726171	0.487127175
NMD3	0.456636587	0.087418238	0.532347177	0.213791836	0.026164906	0.053712695	0.487127175
PLIN5	0.156097695	0.908080187	0.073045773	0.06486847	0.176799828	0.053678717	0.487127175
PMM1	0.55483516	0.106564378	0.707606429	0.00452563	0.629032008	0.053776755	0.487316584
EVC2	0.739739356	0.169010284	0.103162137	0.36203151	0.025554404	0.053838078	0.487345105
NDUFS6	0.173834803	0.274503218	0.872126123	0.018504756	0.155011988	0.053852397	0.487345105
UBE4B	0.944297395	0.468970725	0.000977983	0.504782851	0.546310058	0.053868992	0.487345105
LMAN1	0.799304823	0.176292129	0.39001659	0.632019415	0.003443966	0.053921495	0.487551323
DOCK10	0.831796594	0.005349139	0.47639927	0.09663384	0.585188744	0.053988689	0.487724571
NYNRIN	0.241859896	0.360842798	0.073081325	0.094419007	0.199112599	0.054000094	0.487724571
LOC107133276	0.498575969	0.004838218	0.110799124	0.832602841	0.541434297	0.054160103	0.48877608
POLG2	0.58630834	0.22711519	0.084488262	0.444251966	0.024137313	0.054202293	0.48877608
SMAD1	0.321704764	0.199444094	0.005176032	0.699310292	0.519502863	0.054205866	0.48877608
ELAC2	0.006777306	0.32862081	0.749264211	0.348916475	0.208511081	0.054413676	0.490081625
HTR1E	0.302857888	0.00671624	0.535730541	0.209261723	0.532259577	0.054404981	0.490081625
MYO18A	0.258012718	0.497583048	0.005167138	0.506784504	0.361412135	0.054440241	0.490081625
API5	0.620737223	0.053428126	0.082040836	0.359359957	0.124489958	0.054501038	0.490091249
LOC100295712	0.972500527	0.011477387	0.328238489	0.53361134	0.062225476	0.054481293	0.490091249
NDUFAF5	0.320224343	0.09211643	0.863191908	0.048480054	0.098746563	0.054548241	0.490205595
SSBP1	0.684509136	0.067462382	0.847451946	0.018553963	0.168003547	0.054573495	0.490205595
ANP32E	0.673002938	0.000501997	0.700273304	0.652059795	0.79405524	0.054713535	0.490321477
ASIC2	0.459818412	0.01274823	0.983740634	0.676924784	0.031527432	0.054870199	0.490321477
CD22	0.04685331	0.112905679	0.067175415	0.846414457	0.407329662	0.054719114	0.490321477
LOC101903248	0.273634513	0.251031386	0.010218278	0.302217802	0.577960078	0.054742248	0.490321477
LOC101903289	0.670163977	0.677022281	0.006765443	0.47555093	0.084345428	0.054885172	0.490321477
LOC112442745	0.095685508	0.380587383	0.092718486	0.054591819	0.667432738	0.054859038	0.490321477
LRRN1	0.038383524	0.032556967	0.203496415	0.525815695	0.920300373	0.054867316	0.490321477
NDUFB9	0.650421451	0.534497528	0.994334801	0.164287282	0.002160601	0.0547699	0.490321477
SYNC	0.001266065	0.736057484	0.567595422	0.650322522	0.357836941	0.054875986	0.490321477
TBC1D22B	0.978897536	0.630965509	0.003515721	0.738206252	0.076712456	0.054843441	0.490321477
RRP12	0.134893713	0.367408629	0.723560544	0.175992534	0.019533155	0.054927367	0.49043146
PSMB6	0.344289906	0.139608243	0.469964792	0.297302471	0.018374857	0.054961584	0.490470119

DALRD3	0.07078331	0.018694122	0.802620723	0.49965395	0.232795793	0.054997917	0.490527622
SEMA4A	0.665435396	0.02251388	0.238027874	0.425402664	0.081606635	0.055069388	0.490877751
TBL1XR1	0.625072451	0.045093874	0.005421123	0.841520016	0.963524638	0.055096997	0.490877751
ABHD5	0.285395853	0.693105331	0.054016521	0.025557536	0.455803471	0.055253345	0.491736791
FAT3	0.002872134	0.578580505	0.226847044	0.79042335	0.417485604	0.055232491	0.491736791
CDC73	0.647035111	0.629711024	0.013936434	0.062150518	0.353137998	0.055295501	0.491845238
LOC783776	0.011029369	0.06185383	0.670299936	0.452815365	0.603169495	0.055368726	0.49196327
TMEM200A	0.286018199	0.543720849	0.965468373	0.000897277	0.926840479	0.055360297	0.49196327
LOC100337457	0.806682277	0.18974115	0.318990893	0.242824451	0.010594586	0.055562325	0.493149438
ZCCHC11	0.255865069	0.650485098	0.026778218	0.116280385	0.242266196	0.055547468	0.493149438
PTPRT	0.092487762	0.695336968	0.908627852	0.189579702	0.011377317	0.055678427	0.493912789
OLFML1	0.409066925	0.193376387	0.156442253	0.021326736	0.478065037	0.055715058	0.493970727
ARHGEF19	0.234049546	0.462140463	0.133578006	0.014654631	0.596671921	0.05575943	0.493990113
PPM1F	0.970032555	0.332460822	0.467075825	0.030363837	0.027636646	0.055777447	0.493990113
ADTRP	0.005537164	0.322284602	0.139861077	0.525883402	0.96929373	0.05599925	0.494353792
DHRS3	0.431893441	0.398312757	0.616880744	0.006808555	0.175557851	0.055897305	0.494353792
HENMT1	0.775883307	0.042319629	0.905449398	0.335149321	0.012721955	0.055875022	0.494353792
LURAP1	0.021527044	0.945667389	0.115264756	0.18072102	0.299768389	0.055971226	0.494353792
NGF	0.77366742	0.35820459	0.236801267	0.007792945	0.248716003	0.055991838	0.494353792
SYNCRIP	0.881581145	0.032603045	0.129296834	0.293849415	0.116243833	0.055923014	0.494353792
AKAP6	0.005455433	0.522662394	0.949693078	0.694731886	0.067722439	0.056047854	0.494516848
CSPG5	0.62669161	0.799937293	0.310235015	0.003671698	0.223506587	0.056109105	0.49452552
RAB29	0.663934459	0.129140263	0.011847713	0.239504363	0.524159975	0.056080684	0.49452552
PPARGC1A	0.227112893	0.021880173	0.245561371	0.534791539	0.195962426	0.056176578	0.494854443
LOC101907574	0.701298993	0.664006182	0.009323901	0.872572611	0.033873719	0.056297724	0.495033351
LOC107132820	0.210078286	0.904590241	0.7297672	0.123685722	0.00746708	0.05623031	0.495033351
LSP1	0.613736957	0.029140277	0.217821864	0.084620629	0.389298496	0.056297641	0.495033351
ST6GAL1	0.175395684	0.65265736	0.022919798	0.124089615	0.394399855	0.056317547	0.495033351
C15H11orf94	0.018021345	0.670049267	0.272277447	0.291881651	0.13392827	0.056349124	0.49504576
CA9	0.881812366	0.346048255	0.012339164	0.196127747	0.174551073	0.056450547	0.495141595
COL1A2	0.386584911	0.061694664	0.05363701	0.194759388	0.516544607	0.056395116	0.495141595
THAP3	0.159048447	0.01418314	0.255212426	0.54535747	0.410490698	0.056444825	0.495141595
NDUFAB1	0.632103157	0.19777045	0.835803223	0.011834817	0.104387068	0.056498433	0.495296894
DRG1	0.799411533	0.143898169	0.58433952	0.71962424	0.00267656	0.056603018	0.495419804
LOC100848208	0.542409165	0.317565517	0.336568519	0.121526691	0.018371036	0.056592284	0.495419804
PGM5	0.018181514	0.28523389	0.72256487	0.902221453	0.038267866	0.056577628	0.495419804
CHST6	0.968223599	0.044247707	0.055332826	0.16881728	0.324105961	0.056665232	0.495435867
IMP4	0.01778104	0.459552343	0.936697558	0.205433613	0.082452555	0.056650377	0.495435867
LOC101903758	0.089910946	0.206282289	0.405666604	0.155808806	0.110812061	0.056718725	0.495639504
COL8A1	0.15787123	0.143612675	0.067573761	0.098367204	0.863073134	0.056762616	0.495759067

SEMA3D	0.144819868	0.052750574	0.455668227	0.47616111	0.078721458	0.056873032	0.496459213
RAB5C	0.008584184	0.381186911	0.774901842	0.074949263	0.687979232	0.056943196	0.496807434
DAZAP2	0.658957076	0.001644062	0.391477804	0.39609403	0.781215322	0.057073682	0.496889227
LOC101902083	0.007903539	0.140089605	0.935848157	0.876992594	0.144374586	0.057063077	0.496889227
LOC112443469	0.734427185	0.281409097	0.238129815	0.203521984	0.013091957	0.057046762	0.496889227
PAQR7	0.912761298	0.430041719	0.058562668	0.014379786	0.39679348	0.057053917	0.496889227
ADAMTS2	0.262538676	0.03473641	0.13506921	0.239176699	0.44932143	0.057376536	0.497413457
COX5B	0.692818912	0.191087594	0.778445172	0.006710488	0.190612926	0.057229413	0.497413457
FADS6	0.182571317	0.907832732	0.037926129	0.275651298	0.076223083	0.057296865	0.497413457
IGF1	0.279108943	0.098998602	0.074333009	0.556391248	0.115948173	0.057410313	0.497413457
LOC100848469	0.379467472	0.261438457	0.028858626	0.364232215	0.12634874	0.057211867	0.497413457
LOC100848883	0.247603633	0.4670782	0.935848966	0.897972575	0.001364405	0.057436994	0.497413457
LOC785804	0.076593312	0.077571711	0.313692353	0.087408916	0.808755951	0.057212063	0.497413457
OSBP2	0.056167717	0.333770446	0.220126424	0.034501447	0.928781354	0.057339965	0.497413457
TARS	0.767218607	0.091056101	0.062994923	0.647165373	0.046455637	0.057358515	0.497413457
ZBP1	0.030240441	0.195112602	0.61136346	0.04933709	0.744030842	0.057386776	0.497413457
ADGRV1	0.189052107	0.922452557	0.031867065	0.939893729	0.025561277	0.057677538	0.497808539
ATP5MF	0.591090799	0.237236188	0.872469132	0.008274564	0.132093261	0.057732903	0.497808539
C11H2orf40	0.163641305	0.938600509	0.724084249	0.010501479	0.113902693	0.057549327	0.497808539
DAGLA	0.016461604	0.378507586	0.146012821	0.152451437	0.960689147	0.057606432	0.497808539
DGKB	0.190483908	0.054831467	0.769711116	0.364989409	0.045513247	0.057686064	0.497808539
DNAJC21	0.665905745	0.003810995	0.712691978	0.562371171	0.131474541	0.05773345	0.497808539
ELFN1	0.311737267	0.395773112	0.005880297	0.375702409	0.490707309	0.057740195	0.497808539
RAD50	0.393452318	0.11625805	0.008142477	0.630446469	0.569863335	0.057755619	0.497808539
SEC31A	0.441128061	0.390562821	0.042742117	0.05075237	0.357676793	0.057720539	0.497808539
DNAL4	0.211735816	0.113681483	0.196289022	0.081397975	0.348296669	0.057792381	0.497863914
DCTD	0.524819693	0.015340279	0.995058586	0.3807061	0.044085155	0.057925118	0.498325027
EGLN1	0.572600274	0.693532904	0.531469086	0.076517377	0.008328435	0.057937003	0.498325027
MTMR12	0.33109661	0.544957138	0.455452124	0.082291461	0.019855398	0.057877805	0.498325027
ZNF391	0.930919739	0.077292315	0.04683602	0.133406429	0.299475073	0.057973337	0.498376343
CUL7	0.740738609	0.014581622	0.057686423	0.989251708	0.218971898	0.058060918	0.498620354
GFI1	0.130490535	0.009997194	0.481670842	0.524502927	0.409546731	0.058062488	0.498620354
LOC787858	0.503483918	0.150573545	0.281867356	0.109287639	0.057897465	0.058123806	0.498885868
FAM167A	0.689881352	0.761188007	0.100944944	0.855574572	0.002985583	0.058175162	0.49906565
DLGAP5	0.505510921	0.019031086	0.061959937	0.417635263	0.544854468	0.058236099	0.499327388
LOC100848689	0.388765384	0.671368433	0.214338535	0.490039285	0.00495264	0.058271602	0.49937089
ATP6AP1	0.33834587	0.098836555	0.081594058	0.901114172	0.055329163	0.058341622	0.499588816
NDUFV2	0.882277115	0.149502493	0.748277035	0.023005874	0.059939766	0.058357916	0.499588816
CYC1	0.476219013	0.263103002	0.99153097	0.004925595	0.223372482	0.058510421	0.500468721
PLAA	0.654315251	0.309379416	0.407269531	0.430623293	0.003851291	0.058521691	0.500468721

ABCC5	0.944847	0.224351513	0.128982855	0.435155602	0.011504465	0.058560279	0.500506399
S100PBP	0.073809409	0.693232275	0.020132007	0.964463892	0.137878267	0.058587094	0.500506399
SMYD5	0.293019492	0.052132266	0.744066728	0.765356496	0.015853008	0.058828757	0.502309438
CCDC189	0.071807084	0.02416396	0.316441302	0.286703971	0.879487211	0.058969444	0.503123628
FAM210A	0.757732461	0.042524283	0.621002768	0.140612116	0.049228396	0.058985428	0.503123628
DYM	0.404826192	0.139888857	0.24507556	0.507515536	0.019718029	0.059083182	0.503172857
EIF4EBP1	0.864696527	0.360291059	0.000779016	0.787693367	0.725502163	0.059033009	0.503172857
GPLD1	0.046429492	0.131357115	0.56946965	0.327545566	0.121998826	0.059056728	0.503172857
MAP4K1	0.247475717	0.029659491	0.412679166	0.400867918	0.114557478	0.059138682	0.503384291
AKIP1	0.363070433	0.035738904	0.586024619	0.022384363	0.821819536	0.059341136	0.503654618
CRLF1	0.521673856	0.282728759	0.312127433	0.016976483	0.178615946	0.059266234	0.503654618
CTSB	0.433714121	0.336142501	0.003450468	0.954312803	0.291497592	0.059354581	0.503654618
NDUFB3	0.584067804	0.426661698	0.764148745	0.003690735	0.198491134	0.059242108	0.503654618
STAM	0.660305688	0.150701906	0.020415809	0.134113677	0.512342819	0.05926583	0.503654618
TMEM263	0.897171073	0.041279083	0.091076213	0.194303394	0.213452901	0.059343094	0.503654618
CDC37L1	0.698786397	0.394562146	0.104123827	0.110999223	0.043952104	0.05938614	0.503661984
PHF11	0.994344396	0.050195981	0.076292094	0.770999182	0.047789803	0.059449891	0.503682066
PRNP	0.31632322	0.334557326	0.502849488	0.106004559	0.024863154	0.059437044	0.503682066
METTL21A	0.033697399	0.077073261	0.37418658	0.305851612	0.47457944	0.059644768	0.50481191
SHANK2	0.055085034	0.578472384	0.760341367	0.018356308	0.317157381	0.059642846	0.50481191
ANKRD23	0.650544424	0.006100705	0.088999912	0.739047574	0.54282885	0.059810057	0.504946517
DNMT3A	0.674891704	0.149666342	0.031420312	0.166068827	0.268677864	0.059785484	0.504946517
FANCE	0.225217663	0.092206963	0.129233905	0.177237834	0.298252944	0.059852154	0.504946517
FASLG	0.087354158	0.328558288	0.141933435	0.192094478	0.181116847	0.059816143	0.504946517
LOC512005	0.542711228	0.019599501	0.325112454	0.361902251	0.113430517	0.059876054	0.504946517
SNCAIP	0.551722816	0.020739862	0.051146196	0.450366332	0.536218708	0.059715059	0.504946517
SPATA46	0.796090386	0.000727528	0.413261651	0.817781655	0.722813038	0.059752948	0.504946517
TEFM	0.54910413	0.033106448	0.722432683	0.602813255	0.017960488	0.059934575	0.505180434
SQSTM1	0.581363094	0.332429305	0.054003563	0.857910568	0.015907234	0.059996786	0.505445202
ZFP41	0.954240614	0.459835511	0.103861002	0.043302613	0.072258504	0.06003969	0.505547128
CCNDBP1	0.539577151	0.024894868	0.171683041	0.41562019	0.149072751	0.060112901	0.505644705
CDV3	0.303853368	0.153842389	0.587991299	0.595073009	0.008728641	0.060082769	0.505644705
NOL4L	0.696029713	0.996745668	0.160607767	0.011509563	0.111725252	0.060214315	0.505981344
SYAP1	0.748003645	0.046619999	0.232909379	0.435309032	0.040525783	0.060214586	0.505981344
GMPR	0.171363354	0.039064933	0.071400413	0.667258252	0.450634993	0.060327089	0.506201519
RNPEP	0.705107749	0.27792127	0.488561879	0.003525158	0.425331127	0.060282995	0.506201519
TMED3	0.037738034	0.132401703	0.113619326	0.544905344	0.464678193	0.060333324	0.506201519
SMARCA1	0.024181086	0.846647857	0.707144391	0.025639435	0.387746549	0.060379369	0.506328982
LOC101904976	0.220677047	0.094020742	0.506604623	0.052847731	0.260308433	0.060550525	0.507278851
PIGQ	0.961503668	0.917250708	0.825356212	0.001807128	0.10993642	0.060554462	0.507278851

CPAMD8	0.602778954	0.149477393	0.695248417	0.014359945	0.161212231	0.060657411	0.507664548
LOC112447819	0.020860973	0.7504594	0.684003684	0.456067064	0.029698455	0.060662372	0.507664548
KERA	0.038436482	0.189298158	0.947275825	0.216821365	0.097249656	0.060736619	0.507832711
NPTX1	0.61583887	0.207034221	0.013955758	0.547323388	0.149259143	0.060744355	0.507832711
AKTIP	0.346920741	0.058344435	0.075521008	0.136595708	0.701668932	0.061035687	0.507897053
EML3	0.927926327	0.176595132	0.008447461	0.34241059	0.308652945	0.06098185	0.507897053
KLK8	0.127601752	0.114127102	0.023680898	0.820945123	0.51618925	0.060941815	0.507897053
MAST4	0.221809239	0.496663325	0.529434774	0.006019299	0.417786575	0.061077356	0.507897053
MFGE8	0.320571772	0.593832542	0.097402003	0.023936043	0.328954618	0.060905911	0.507897053
NPNT	0.012482854	0.099908888	0.325976844	0.857787661	0.41992714	0.061017905	0.507897053
NPTXR	0.46294267	0.853365407	0.012032664	0.090347646	0.342227384	0.061154383	0.507897053
NUDT16L1	0.050705191	0.101112638	0.596561085	0.531993795	0.090287231	0.061136427	0.507897053
RAD23A	0.284458078	0.038750177	0.159070761	0.535674308	0.155352074	0.060885008	0.507897053
SHISA4	0.458633883	0.238608914	0.076970389	0.08818714	0.197327286	0.061052947	0.507897053
TMOD1	0.523041271	0.005358239	0.883459308	0.108299139	0.543219682	0.0608207	0.507897053
WBP2	0.265183332	0.326363008	0.062729462	0.176142891	0.153460117	0.061096561	0.507897053
ZSCAN29	0.928981092	0.435898905	0.013646506	0.596960588	0.044395078	0.061020987	0.507897053
DIRAS3	0.012990321	0.09871025	0.582916106	0.887732656	0.222130394	0.061259055	0.507995122
TSHZ3	0.699688709	0.428519115	0.019809708	0.045627449	0.543471351	0.061231443	0.507995122
VPS18	0.699419548	0.75270427	0.085609773	0.554812291	0.005891106	0.061237644	0.507995122
ACOT2	0.731846892	0.565083445	0.695000369	0.00692382	0.074215009	0.061334232	0.508091619
CHD1	0.60242427	0.982254209	0.924547236	0.000323565	0.83503508	0.061365695	0.508091619
JPT2	0.822230196	0.056396364	0.081036905	0.418088334	0.0941592	0.061394533	0.508091619
KDM7A	0.220131323	0.538577262	0.402757236	0.006664159	0.464620927	0.061373862	0.508091619
CABCOCO1	0.307774377	0.57249962	0.946530838	0.089069132	0.010057998	0.061766636	0.509238835
CLECL1	0.321909827	0.340924995	0.038398916	0.200548474	0.176538528	0.061713982	0.509238835
GNGT2	0.04812991	0.134569949	0.242222477	0.158404282	0.601461345	0.061781398	0.509238835
GPR37	0.593134772	0.009462423	0.244609951	0.289322896	0.375157505	0.06166703	0.509238835
IL17B	0.081800184	0.115906847	0.723845296	0.21999323	0.098620598	0.061637715	0.509238835
PAM	0.042407756	0.323549022	0.34026726	0.135943703	0.234380092	0.061603203	0.509238835
PRICKLE2	0.082848738	0.934268643	0.220896989	0.080095001	0.108914945	0.061702724	0.509238835
SPOPL	0.716096482	0.291264832	0.161149299	0.015018891	0.29564207	0.061724436	0.509238835
PROS1	0.038703168	0.174794984	0.108219731	0.206980029	0.988390822	0.061858	0.509358576
RORC	0.941421659	0.197756945	0.017116431	0.392480378	0.1197224	0.061847926	0.509358576
EIF5	0.827969711	0.287914926	0.422971711	0.744932735	0.001998899	0.061949569	0.50961825
LOC618169	0.699843906	0.172654267	0.040079683	0.049044004	0.632168958	0.061951643	0.50961825
ATPAF2	0.07338561	0.46192325	0.815667577	0.062615711	0.086817011	0.061991482	0.509690485
ABCB8	0.353836174	0.479881663	0.998687352	0.008439757	0.105745449	0.062249615	0.510024176
ATP6V0A2	0.557022718	0.08012228	0.84731364	0.655751695	0.006095216	0.062201081	0.510024176
BPHL	0.045886178	0.219876233	0.49984013	0.247676328	0.12071485	0.062108863	0.510024176

EIF4G2	0.885800012	0.014142907	0.033866349	0.461759018	0.772290136	0.062239278	0.510024176
KIAA1958	0.809809373	0.915718722	0.535034373	0.011183159	0.034105699	0.06224618	0.510024176
LOC104969384	0.512513326	0.212382246	0.038665919	0.308686405	0.116352847	0.062205186	0.510024176
NDUFAF4	0.576468701	0.036353137	0.99335067	0.686895809	0.010580348	0.06223688	0.510024176
CAND2	0.295149224	0.969638013	0.143187406	0.582215152	0.006368346	0.062398286	0.510477704
LOC107132469	0.187673197	0.393758273	0.192095447	0.474904748	0.022529373	0.062384211	0.510477704
LOC112446351	0.603609406	0.258548712	0.005132565	0.273020604	0.694101933	0.062362168	0.510477704
CARNS1	0.16201213	0.677558212	0.378452852	0.015964068	0.229597098	0.062481051	0.510900117
IQANK1	0.787749804	0.005998064	0.131670752	0.442879246	0.553433934	0.062535486	0.510956141
LOC101902665	0.706964411	0.328126713	0.089581186	0.358905367	0.020569329	0.062764186	0.510956141
LOC112446012	0.198184386	0.409891744	0.243702201	0.477294021	0.01616395	0.06259588	0.510956141
LOC524810	0.439602919	0.032998976	0.11331422	0.101701514	0.920609764	0.062886213	0.510956141
LOC783604	0.158498644	0.08283081	0.085111224	0.18087333	0.761300562	0.062876262	0.510956141
LOC787269	0.131010571	0.361168661	0.119205848	0.628420521	0.04334799	0.062823512	0.510956141
MTBP	0.077662682	0.545216408	0.865940968	0.216499899	0.01926701	0.062649189	0.510956141
NDUFS8	0.247687083	0.32204076	0.958679077	0.010510749	0.190901142	0.062770633	0.510956141
OMA1	0.006678747	0.793550748	0.069240009	0.480837887	0.872361317	0.062892658	0.510956141
TCP11L2	0.635382945	0.05868081	0.078009282	0.055784372	0.94376752	0.062694126	0.510956141
THBS3	0.221485894	0.235857184	0.05037762	0.070925365	0.820963554	0.062720616	0.510956141
TMEM169	0.412624539	0.121955993	0.301223866	0.188018085	0.053715699	0.062684761	0.510956141
ZNF688	0.393175841	0.00987331	0.091838071	0.766889196	0.562564603	0.062862442	0.510956141
EPHA4	0.631019754	0.997610529	0.001814227	0.367821408	0.366737777	0.062924809	0.510961514
IL6ST	0.633660525	0.466149931	0.41734197	0.701530614	0.001784924	0.062999662	0.510961514
PROKR1	0.277211698	0.205868839	0.109628033	0.537520085	0.045855973	0.062962212	0.510961514
TMEM223	0.450767268	0.138151207	0.507317323	0.027969234	0.174775509	0.06301786	0.510961514
BTG3	0.743433813	0.061006031	0.654252305	0.529284	0.009854702	0.063100929	0.511382391
APOBEC3H	0.29122329	0.473752011	0.013841199	0.737699033	0.110194732	0.063215253	0.511982576
NELL1	0.03535549	0.645176702	0.229207455	0.039350836	0.75557499	0.06326858	0.511982576
ZNF318	0.967149749	0.080179535	0.08088435	0.994867789	0.024899455	0.063249282	0.511982576
HIPK3	0.811812967	0.200456251	0.129643772	0.009092898	0.813443009	0.063415209	0.512189156
KIF27	0.394369126	0.533253298	0.094413214	0.795470944	0.00986173	0.063343658	0.512189156
KPNA2	0.593526411	0.157998894	0.222372762	0.659799046	0.011328113	0.06337	0.512189156
PSPH	0.379537286	0.199406922	0.784120482	0.015985177	0.164514455	0.063418949	0.512189156
AGBL5	0.230901782	0.167329935	0.17514751	0.185361714	0.125048339	0.063613999	0.51325926
IFI44	0.17911108	0.095054126	0.40772695	0.033122484	0.681725674	0.063586755	0.51325926
TXNL1	0.780935811	0.080268475	0.20010831	0.72383936	0.017312121	0.063695145	0.513409151
USP53	0.796723422	0.455202752	0.065225214	0.011946314	0.556135202	0.063688434	0.513409151
ATG13	0.24440986	0.053607579	0.217694972	0.463178326	0.119325114	0.063806626	0.514055248
BATF2	0.219044142	0.185897556	0.959153631	0.008418111	0.480240758	0.063868304	0.514299673
CERS4	0.216853043	0.860676999	0.066507712	0.039085811	0.326112087	0.063948095	0.514689649

ZNF554	0.013745465	0.62423573	0.038622399	0.530804139	0.902444237	0.064076275	0.515468502
IL1RAP	0.447348587	0.225244864	0.976299854	0.005049551	0.32005872	0.064135589	0.515692868
AKAP7	0.268724763	0.637329813	0.250024637	0.004026581	0.924796436	0.064249127	0.515847568
NTS	0.591887989	0.029570804	0.026952704	0.355956009	0.948133651	0.064189557	0.515847568
PERP	0.026304814	0.111902662	0.391255843	0.256676663	0.539294865	0.064241241	0.515847568
HBEGF	0.212680095	0.022512622	0.861232096	0.059521626	0.651922999	0.064383997	0.516425113
LOC112447371	0.649898053	0.086525363	0.004655719	0.615010607	0.993676114	0.064380292	0.516425113
HIST1H3G	0.445957886	0.468784658	0.1218945	0.637071225	0.009891696	0.06452442	0.517298609
HSPBP1	0.577196346	0.172777676	0.002307405	0.938086962	0.744862176	0.064573267	0.517437445
GZMA	0.746784579	0.033197695	0.03233732	0.295617895	0.684039661	0.064894222	0.518742856
LETM1	0.035337078	0.338184273	0.688036555	0.045828959	0.430069634	0.064881143	0.518742856
MFSD13A	0.051546457	0.74957766	0.22435276	0.085092671	0.219549387	0.064853808	0.518742856
PNCK	0.004523248	0.419536033	0.194109613	0.582891815	0.754366538	0.064859741	0.518742856
SHANK1	0.733426632	0.31774315	0.744239475	0.222041081	0.00420505	0.064851901	0.518742856
SLC23A1	0.108557091	0.424506443	0.056314358	0.699355584	0.089583395	0.065008706	0.519405006
WNT5A	0.288390006	0.401778216	0.017649229	0.995268823	0.079968281	0.065050587	0.519486704
PBXIP1	0.282377309	0.309593066	0.880849576	0.875641317	0.002422598	0.065193585	0.520122468
TMEM87A	0.057779142	0.295826568	0.024723878	0.409191778	0.944498481	0.065186572	0.520122468
C29H11orf24	0.268554961	0.060049914	0.267800413	0.766841109	0.049524563	0.065352525	0.520517182
MBNL3	0.08343701	0.552296065	0.167049929	0.07216532	0.295371779	0.065369929	0.520517182
PMVK	0.139554174	0.359209822	0.078988082	0.071977377	0.575681801	0.065365988	0.520517182
TRIM21	0.012807336	0.146847386	0.440715991	0.419356078	0.472039514	0.065367262	0.520517182
LOC781304	0.217181835	0.109867195	0.241209987	0.090146458	0.316873049	0.065446827	0.520624275
MAP3K13	0.042457827	0.500839532	0.062176449	0.357908038	0.34721514	0.065422429	0.520624275
ARHGEF5	0.583384938	0.016011702	0.091586916	0.298971125	0.644271904	0.065538141	0.520931289
SH3GLB1	0.960182794	0.008842858	0.346495405	0.207530255	0.269971154	0.065548907	0.520931289
GMFB	0.414259756	0.237489305	0.781998319	0.727799276	0.002952583	0.065666766	0.521615345
C5H12orf75	0.030794838	0.5510162	0.21328595	0.435424487	0.105603541	0.065927981	0.521640495
CAPS2	0.1670433	0.942018147	0.010138299	0.120624469	0.862896786	0.065841263	0.521640495
CITED2	0.307108213	0.34458331	0.066940033	0.462295545	0.051095156	0.06614566	0.521640495
FAM229A	0.491603657	0.313006421	0.706146102	0.013583202	0.113214349	0.0660902	0.521640495
FAM71E1	0.091161192	0.036644931	0.239331088	0.540525797	0.386048255	0.066027355	0.521640495
GPC4	0.188573684	0.71793635	0.012385821	0.265833619	0.375542251	0.066163057	0.521640495
ITGA3	0.325189627	0.858630605	0.170591781	0.121600262	0.028808332	0.066034021	0.521640495
LOC104971374	0.923568016	0.123166421	0.016768899	0.614315162	0.141510498	0.065786254	0.521640495
LOC616199	0.278831117	0.878225055	0.03444894	0.083318933	0.238266086	0.066178509	0.521640495
MFNG	0.018009718	0.506987255	0.323405983	0.111420985	0.507601526	0.0660696	0.521640495
MRPS34	0.302976139	0.166320703	0.931450853	0.08323736	0.042391339	0.065737356	0.521640495
SEM1	0.484358742	0.355948794	0.495312211	0.884990015	0.00221098	0.066089313	0.521640495
TENM2	0.026767568	0.240785645	0.342818244	0.138641559	0.543048722	0.065913323	0.521640495

YTHDF3	0.278395145	0.1143173	0.019198042	0.341484594	0.79479373	0.065787208	0.521640495
ZMYND19	0.089986168	0.013047119	0.439918602	0.634204176	0.509114261	0.066011415	0.521640495
ZNF862	0.206138959	0.022169141	0.563687102	0.08785942	0.737303408	0.066036572	0.521640495
CDC27	0.31923179	0.0287238	0.483442424	0.904817249	0.041812693	0.066236652	0.521848152
LOC101902656	0.468420023	0.582359684	0.002727955	0.812136695	0.278357062	0.066359305	0.522563604
CSNK2B	0.411745838	0.040790096	0.135484765	0.748331053	0.09901304	0.066448203	0.522761963
TMEM94	0.694587408	0.259840165	0.518473793	0.031243546	0.057648574	0.066434225	0.522761963
MPHOSPH9	0.68400651	0.002757794	0.693626291	0.17258077	0.749010993	0.066574352	0.52293886
SPICE1	0.269635522	0.777967011	0.042189522	0.814679888	0.023439058	0.066541294	0.52293886
TMEM119	0.629520625	0.307868882	0.004439884	0.387250705	0.508184512	0.066623558	0.52293886
TSGA10	0.712356722	0.510904778	0.003212969	0.169006383	0.856002428	0.066583021	0.52293886
TTI1	0.94380149	0.787623865	0.004268096	0.555380843	0.096118357	0.066630014	0.52293886
ARHGEF12	0.760106938	0.251318979	0.440945754	0.240163214	0.008409311	0.066807706	0.524082825
SYNE2	0.234332798	0.100878059	0.151736492	0.812269924	0.058483302	0.066872563	0.524340962
CAND1	0.329102171	0.19199745	0.275528422	0.023907021	0.41045211	0.066977231	0.524489806
DCUN1D2	0.189695014	0.077315145	0.962082927	0.508763105	0.023780113	0.066947717	0.524489806
SUN2	0.929499052	0.346279341	0.047592986	0.111805648	0.099771883	0.066987425	0.524489806
BEND3	0.867726919	0.468465864	0.009592038	0.053228321	0.827044113	0.067168936	0.525660187
NCAPD2	0.176123067	0.034787692	0.181798317	0.750104241	0.205619579	0.067203788	0.52568225
DIRC2	0.736554182	0.9198382	0.016866597	0.119211486	0.126346868	0.067279084	0.52602051
GLRX3	0.778567776	0.117484276	0.322207626	0.994456801	0.005892386	0.067415536	0.526547798
GPI	0.758897604	0.567873976	0.951443933	0.021570228	0.01956471	0.067495628	0.526547798
LOC101906226	0.62107499	0.81599258	0.016769292	0.166087264	0.122263409	0.06738648	0.526547798
LOC112445999	0.849930555	0.494180537	0.010981701	0.456921973	0.082192106	0.067539036	0.526547798
NEK4	0.030662863	0.565870906	0.453939652	0.529457422	0.041514234	0.067515233	0.526547798
TAL1	0.267600884	0.024570512	0.244552408	0.844143402	0.127355681	0.06745468	0.526547798
OGFOD2	0.20565807	0.265441118	0.40976785	0.014182402	0.548784633	0.067744363	0.527897789
SDC1	0.65796256	0.528547689	0.002609902	0.220587891	0.870669217	0.067795097	0.528042399
LOC112443141	0.969209374	0.073829905	0.026272561	0.131895968	0.704259926	0.067867604	0.528356376
ARF1	0.899022821	0.014780331	0.015593445	0.954240562	0.894356798	0.068381084	0.529629512
CBX5	0.592345378	0.172529975	0.907835377	0.235452688	0.008074617	0.06827777	0.529629512
DUSP2	0.638389671	0.026652817	0.753619166	0.04511454	0.306429763	0.068481639	0.529629512
FAM219B	0.279700009	0.249945877	0.803278893	0.008512041	0.370327702	0.068424504	0.529629512
FSCN1	0.958093363	0.689875062	0.001078745	0.94727843	0.262762487	0.068529912	0.529629512
GPX7	0.870350583	0.071945158	0.011363274	0.991006192	0.249591819	0.068186581	0.529629512
LOC101906546	0.812800265	0.920520756	0.758017416	0.010594558	0.02954932	0.068547804	0.529629512
LOC107131367	0.892491278	0.112375037	0.100550589	0.769846403	0.022689542	0.068222643	0.529629512
LOC112442593	0.889007734	0.297716922	0.105449457	0.089652675	0.070206287	0.068109912	0.529629512
LRP6	0.047587549	0.881274704	0.791199908	0.006699195	0.794383156	0.06832228	0.529629512
LRRC17	0.071165058	0.424818534	0.006773528	0.907842705	0.955285448	0.068557472	0.529629512

MPV17L	0.392157948	0.545069454	0.593600065	0.042788418	0.032590936	0.068406073	0.529629512
NKD1	0.494261741	0.743463058	0.00709619	0.172998177	0.392121417	0.068394143	0.529629512
POLA2	0.971847822	0.168929426	0.71660037	0.623919756	0.002420778	0.068579777	0.529629512
SAP130	0.677706033	0.97615536	0.003173728	0.660176467	0.12803826	0.068529122	0.529629512
SPIB	0.422921107	0.131583204	0.024421811	0.805745209	0.160789685	0.068204278	0.529629512
UNC13B	0.328976523	0.033442473	0.688685678	0.110780454	0.209947683	0.068238715	0.529629512
SLC19A2	0.834992798	0.415002504	0.001170567	0.519044284	0.846177616	0.068687104	0.53020887
PPP6R2	0.443078425	0.092080673	0.009706742	0.458268823	0.985114651	0.068832878	0.531084329
RGN	0.049254139	0.605066958	0.25306709	0.043206581	0.549163046	0.068871254	0.531130706
EDNRA	0.010807237	0.310431743	0.432220592	0.711442172	0.173747577	0.068938998	0.531352275
GOLT1B	0.644984491	0.011625633	0.083015189	0.810034419	0.35570335	0.06896474	0.531352275
RABEP2	0.532797856	0.242273059	0.004900373	0.453149472	0.626439112	0.069012605	0.531471541
KCNMB2	0.97830068	0.043590901	0.383575178	0.22762649	0.048292222	0.069069981	0.531542045
MROH6	0.143907522	0.014908144	0.176725218	0.576600135	0.824663074	0.069178649	0.531542045
MTDH	0.699552237	0.006864152	0.324821913	0.599859242	0.192637649	0.069167759	0.531542045
SEC61A1	0.155520443	0.287524764	0.43138715	0.861324442	0.010852083	0.069183707	0.531542045
ZNF704	0.325954689	0.167332028	0.319884068	0.93931197	0.010978792	0.069096187	0.531542045
AFF4	0.783940195	0.198235644	0.742875882	0.001639976	0.956718587	0.06937435	0.531614393
GTF2H1	0.481197593	0.718674078	0.418148824	0.002039004	0.61374928	0.069335353	0.531614393
POLR3K	0.347185227	0.129659806	0.817670291	0.029205494	0.168021483	0.069256774	0.531614393
SARAF	0.756274487	0.398381836	0.008239424	0.492935684	0.147737835	0.069293471	0.531614393
TEX22	0.971335563	0.008663534	0.179863541	0.225590064	0.530655067	0.069387486	0.531614393
TMEM177	0.435003373	0.343356403	0.988591718	0.002189527	0.558831854	0.069267478	0.531614393
SDHAF4	0.538884013	0.381495237	0.206468238	0.004929233	0.867072433	0.069438524	0.53175717
HECTD3	0.287743917	0.012169524	0.37065012	0.157003914	0.891429584	0.06949309	0.531926821
BLVRB	0.376354298	0.376261111	0.347853758	0.097061651	0.038048115	0.069552812	0.531935746
ENKD1	0.693492927	0.028191009	0.036233846	0.792917868	0.32391702	0.069559083	0.531935746
CYR61	0.662155452	0.162461379	0.012764046	0.787582888	0.168952015	0.069734886	0.531985104
LOC112443479	0.069239269	0.135995205	0.027434832	0.934778641	0.755378251	0.069666935	0.531985104
NEXN	0.155314295	0.426862637	0.234543755	0.713787908	0.016463202	0.069739586	0.531985104
PRMT5	0.727381528	0.57165077	0.04283239	0.037052586	0.277250413	0.069792452	0.531985104
TCTN2	0.289016208	0.182506726	0.00982945	0.666286659	0.527544644	0.069628392	0.531985104
TFDP1	0.104349209	0.135251345	0.25452658	0.273023683	0.186545376	0.06979174	0.531985104
UBR3	0.524656748	0.427991249	0.056421055	0.025304407	0.568589081	0.06963756	0.531985104
ALDOA	0.171986186	0.404383932	0.702359052	0.201004314	0.018694112	0.069927457	0.532766715
ADM2	0.919412569	0.091979073	0.270122055	0.037981459	0.211959011	0.070007195	0.532792462
F5	0.600798685	0.065742042	0.638846217	0.021727687	0.335532599	0.070020102	0.532792462
MYCN	0.319943432	0.082826222	0.015450674	0.779247586	0.576682729	0.070028234	0.532792462
CYP2S1	0.364407473	0.136946198	0.122708209	0.535235329	0.056215797	0.070087476	0.532885587
NDUFC1	0.336840262	0.239140152	0.977000772	0.043347149	0.054034065	0.070105416	0.532885587

STX18	0.526167475	0.033412655	0.079270839	0.146416804	0.904081236	0.070138995	0.532894004
AP2B1	0.667614816	0.141762152	0.162838708	0.015219732	0.788928521	0.070268883	0.53341876
LOC101907803	0.695047326	0.691838223	0.00106084	0.686537824	0.528443411	0.07027307	0.53341876
SORCS1	0.04470553	0.607737024	0.022619082	0.704460188	0.428338751	0.070356822	0.533807582
LOC112443176	0.279938265	0.229935324	0.020588466	0.522424618	0.268082756	0.070394534	0.533846904
GOT1L1	0.16100803	0.443584131	0.838131073	0.033162172	0.093623182	0.070450583	0.534025178
FAM20A	0.28834997	0.168080333	0.182730055	0.31671824	0.06645613	0.07057625	0.534730767
SPATA7	0.964752304	0.796693639	0.00611751	0.444036171	0.089416591	0.070640801	0.534940672
SSB	0.824473684	0.516205958	0.013374661	0.169682352	0.193415021	0.070669147	0.534940672
DOK5	0.035495662	0.536382553	0.242612508	0.050642901	0.800898426	0.070790928	0.535355324
EFCAB7	0.537918494	0.449778735	0.002491045	0.817916112	0.380338107	0.070821791	0.535355324
KCTD3	0.88549443	0.686673084	0.064946824	0.182847524	0.025941781	0.070783469	0.535355324
CANX	0.88100357	0.256833951	0.397076717	0.977602814	0.002151635	0.071160078	0.535988029
ENDOV	0.078232921	0.343715842	0.017149491	0.73163251	0.559705382	0.071126511	0.535988029
FKBP10	0.596002133	0.106321947	0.007345772	0.467695383	0.868819599	0.071195888	0.535988029
HYPK	0.097650131	0.077296933	0.260047719	0.568612181	0.169224921	0.071133936	0.535988029
IRAK2	0.304347635	0.909998459	0.039375111	0.038860588	0.446683239	0.071229275	0.535988029
KLRG2	0.34543875	0.000863089	0.887564718	0.993514212	0.716687051	0.07103228	0.535988029
MYH10	0.362426195	0.818030153	0.078233684	0.01037671	0.781981919	0.070984259	0.535988029
PAK2	0.671425593	0.0481802	0.096792272	0.081870871	0.735700826	0.071072175	0.535988029
SLC16A6	0.125140655	0.607971777	0.11904717	0.566883798	0.036870174	0.071232094	0.535988029
SLC43A2	0.35010262	0.034725889	0.319360935	0.272174536	0.178746957	0.071138643	0.535988029
TPM1	0.025349034	0.477522564	0.598293522	0.947419513	0.027655696	0.071332814	0.536499911
MYH9	0.529808229	0.662476602	0.809592512	0.001004818	0.665584857	0.071396652	0.536734064
NT5E	0.117177938	0.218298116	0.938939424	0.018586053	0.426078168	0.071432377	0.536756748
LLPH	0.706188512	0.009449639	0.547099123	0.768649371	0.067864454	0.071487713	0.536926706
C16H1orf159	0.031486981	0.851794299	0.085216243	0.464738305	0.180194951	0.071701162	0.537471003
CD164	0.82729403	0.374760791	0.133332857	0.633934305	0.007323237	0.071815606	0.537471003
DBR1	0.942103877	0.323606528	0.395881462	0.012060631	0.132075554	0.071892213	0.537471003
DNAJC28	0.8736876	0.330478267	0.472190052	0.013878016	0.1016787	0.071921842	0.537471003
FGD2	0.345542527	0.042881445	0.251545604	0.21110033	0.244466872	0.071914255	0.537471003
FIG4	0.310213661	0.832889301	0.054807939	0.156117406	0.087218538	0.072018691	0.537471003
KAZN	0.051067014	0.931337846	0.094820123	0.291423549	0.14617672	0.071860321	0.537471003
LOC100336476	0.295941201	0.01815992	0.176256272	0.409948308	0.494727316	0.071861098	0.537471003
MRPS18C	0.934673542	0.103425142	0.23911526	0.010860524	0.766876582	0.071951159	0.537471003
MSL1	0.996057366	0.458466033	0.186360828	0.011581127	0.195285937	0.071941098	0.537471003
NDUFA13	0.522151482	0.356857349	0.624182773	0.014707726	0.112289773	0.071854153	0.537471003
PNPT1	0.354638966	0.200556428	0.70153768	0.016640577	0.231802711	0.071940405	0.537471003
RARRES2	0.388988404	0.337375975	0.774703875	0.026734967	0.07083672	0.071956602	0.537471003
SQOR	0.696544278	0.20076371	0.09253821	0.393461639	0.037868905	0.072017539	0.537471003

PTPRCAP	0.107338268	0.322500146	0.120450968	0.41106727	0.112710487	0.072099961	0.537832934
MYL9	0.042264577	0.361743327	0.315496352	0.529140621	0.075776937	0.072150289	0.537963828
KLHL3	0.05635275	0.120541092	0.10013075	0.307089788	0.932217343	0.072440528	0.539882612
P4HA1	0.430679386	0.833509274	0.114710662	0.122020457	0.038815357	0.07251067	0.539914972
YKT6	0.431415357	0.055630934	0.491072937	0.594114291	0.027835699	0.072483329	0.539914972
MGC157082	0.979284121	0.058885079	0.10462332	0.595335621	0.054342401	0.072544416	0.539921275
C8G	0.458088286	0.008711855	0.121193078	0.471475036	0.858621883	0.072679603	0.54063484
LRRC66	0.12244463	0.653229132	0.00840135	0.923345579	0.31575175	0.072706178	0.54063484
LOC100299242	0.957090364	0.64264225	0.129362838	0.306904668	0.008051167	0.072859101	0.541526589
SCX	0.012966373	0.02897913	0.981279855	0.584006553	0.916035308	0.073002573	0.542347321
LOC101907566	0.169185552	0.131960909	0.848669666	0.138452102	0.075355468	0.073096341	0.542798213
LOC100847304	0.315136258	0.080791114	0.798207946	0.060441583	0.161128228	0.073148933	0.542943077
DEXI	0.489163235	0.057540859	0.582398639	0.08117868	0.148978067	0.073222205	0.54324123
ADO	0.39457632	0.076392753	0.146272951	0.859805639	0.05288581	0.073713318	0.543486875
ADRB3	0.088063987	0.167155146	0.466819724	0.17822548	0.164142167	0.073831758	0.543486875
ALG13	0.564303487	0.528508599	0.215797837	0.24302084	0.012761426	0.073518204	0.543486875
ANKRD26	0.926905496	0.294693428	0.053159191	0.664711011	0.02103218	0.074262603	0.543486875
BAG1	0.859782345	0.042009189	0.15903517	0.593911221	0.059020351	0.07390186	0.543486875
CCL25	0.058102759	0.306933494	0.705561911	0.075376739	0.213982351	0.074251411	0.543486875
CEMIP	0.316058885	0.503412127	0.007820622	0.295663184	0.550637489	0.074170499	0.543486875
CENPA	0.382420015	0.05308738	0.030570714	0.562073145	0.572491804	0.073542921	0.543486875
CFAP300	0.560369933	0.181300574	0.133219311	0.82131159	0.017938108	0.073474961	0.543486875
CLEC11A	0.107961508	0.346296429	0.449440655	0.049935778	0.241597443	0.074200748	0.543486875
COL26A1	0.111109587	0.066024164	0.468162284	0.438607946	0.132221467	0.073424927	0.543486875
ELP6	0.562658323	0.258899218	0.447050123	0.018031921	0.172062896	0.074055185	0.543486875
FAM98A	0.782305015	0.064560799	0.822622294	0.680245131	0.007138598	0.073990529	0.543486875
GTF3C1	0.890671382	0.020821393	0.036811944	0.669853232	0.442044665	0.074075585	0.543486875
HSP90B1	0.509694991	0.888006846	0.007981373	0.099757666	0.552844036	0.073437406	0.543486875
LAMB1	0.601779667	0.659548739	0.471341672	0.049503261	0.021784945	0.073989177	0.543486875
LOC100141253	0.00310496	0.155638203	0.860179094	0.821898655	0.58988776	0.073942344	0.543486875
LOC101903501	0.138774647	0.928270091	0.226905475	0.161297643	0.042483306	0.073671787	0.543486875
LOC509513	0.170443498	0.048645714	0.681179313	0.492877131	0.072371729	0.073926668	0.543486875
LOC768255	0.461606847	0.162432836	0.169794273	0.321712799	0.04961131	0.074305024	0.543486875
LSM14B	0.23368215	0.218876903	0.221026219	0.023799885	0.746988292	0.073821702	0.543486875
MAOB	0.963447851	0.183944742	0.330330553	0.004089512	0.848947706	0.074315066	0.543486875
MDH2	0.829397764	0.339843291	0.773464976	0.012146932	0.07517295	0.073402872	0.543486875
MRPL51	0.945936128	0.186719094	0.736245803	0.01329083	0.116460561	0.073887172	0.543486875
MTHFD2	0.224074183	0.307834735	0.050083939	0.314891351	0.185952452	0.07410694	0.543486875
RASGRP1	0.161337811	0.237340199	0.726412169	0.14044978	0.051593719	0.073948402	0.543486875
REEP1	0.642183428	0.0011352	0.516292777	0.548699062	0.965724049	0.073483932	0.543486875

SMARCC2	0.494843252	0.289038599	0.259119923	0.058329277	0.092970167	0.073821356	0.543486875
TAPT1	0.383529089	0.616385226	0.015288744	0.138166534	0.406908772	0.074305442	0.543486875
TET2	0.500936569	0.359887477	0.021976449	0.091937904	0.551940265	0.073835446	0.543486875
TMEM229A	0.057836324	0.328582505	0.276888728	0.536288345	0.071232239	0.073828528	0.543486875
TMEM245	0.018451078	0.147879517	0.177415531	0.525292405	0.791662029	0.073893048	0.543486875
LOC100848684	0.287140034	0.124359368	0.113759667	0.088767764	0.564641968	0.07439375	0.54381997
CACNA1D	0.020078466	0.043799395	0.255624423	0.929581026	0.975325376	0.074439509	0.543912191
IGSF3	0.595985617	0.371687664	0.010817505	0.166532447	0.511950714	0.074544657	0.544195893
SNRPD3	0.600174564	0.067066122	0.173967165	0.420745662	0.06931773	0.074528726	0.544195893
C17H22orf39	0.507385213	0.820896392	0.706419807	0.001284123	0.542324852	0.074676199	0.5446716
PTPRO	0.162710172	0.060377667	0.267328122	0.094523806	0.82484405	0.074644863	0.5446716
NUDT13	0.757097184	0.42202755	0.691284758	0.03711799	0.025025206	0.074733126	0.544844659
DIP2B	0.833114931	0.697400553	0.004573275	0.205678768	0.375861842	0.074786435	0.544991198
IMPG2	0.484793031	0.682904533	0.551020297	0.015304099	0.073760061	0.074897195	0.545080536
LOC104973322	0.645939826	0.264875439	0.206277584	0.608287681	0.009591813	0.074895401	0.545080536
RBFOX2	0.140882566	0.383831778	0.272410201	0.620358268	0.022535127	0.074898337	0.545080536
HIGD1A	0.51053859	0.098131639	0.462267928	0.130714625	0.068115669	0.074957682	0.545270618
ALPL	0.704073836	0.030365791	0.384856417	0.289538516	0.086796284	0.07508158	0.545688133
TIRAP	0.062103233	0.547034942	0.307161653	0.035263958	0.561562814	0.075052464	0.545688133
ABCE1	0.978318656	0.090703664	0.139317211	0.098939255	0.172067511	0.075873388	0.54630308
ARCN1	0.811851963	0.021637282	0.06227538	0.459543023	0.415934135	0.075580235	0.54630308
ATRNL1	0.563439529	0.062939785	0.085660779	0.133795472	0.512586506	0.075416687	0.54630308
CGN	0.60790372	0.411870513	0.022962811	0.1745607	0.20880237	0.075679101	0.54630308
HELB	0.277031244	0.48019728	0.003829999	0.66535196	0.620677917	0.075861705	0.54630308
KBTBD2	0.083472376	0.297486307	0.602710269	0.320542258	0.043718033	0.075716556	0.54630308
KCNIP3	0.675812409	0.014599503	0.23899793	0.643296656	0.139144843	0.076004127	0.54630308
KIF20B	0.557170911	0.095603397	0.036908412	0.638711561	0.167815032	0.075929974	0.54630308
KNTC1	0.862996576	0.037385181	0.251418799	0.032281473	0.794071984	0.075329683	0.54630308
LOC100335822	0.734481693	0.455081811	0.119657267	0.599558099	0.008810082	0.076044031	0.54630308
LOC101905510	0.939125572	0.090698305	0.01915908	0.4362344	0.296896432	0.076064989	0.54630308
LOC101905956	0.326591771	0.042582932	0.039442346	0.474956393	0.810584444	0.076026858	0.54630308
LOC104971683	0.740987138	0.186481446	0.014746577	0.194554484	0.53012255	0.075808846	0.54630308
LOC112442049	0.904258432	0.431425687	0.35131626	0.006358226	0.239245262	0.075448982	0.54630308
LOXL1	0.623638564	0.31552307	0.001215016	0.900914996	0.969205232	0.075507788	0.54630308
PCBD2	0.175322055	0.534028982	0.643209267	0.110254974	0.031821925	0.076050013	0.54630308
PLAU	0.63375407	0.949000032	0.001947377	0.210232325	0.849591103	0.075600893	0.54630308
POLR3H	0.330112921	0.179527582	0.862801909	0.121694694	0.033737067	0.075760007	0.54630308
PPIA	0.839264983	0.240378981	0.530287898	0.030984779	0.062799437	0.075380405	0.54630308
RBM39	0.819154311	0.621905671	0.712805964	0.003940273	0.147346079	0.075951125	0.54630308
RERGL	0.670136422	0.504806083	0.132991519	0.030481201	0.153090577	0.07576089	0.54630308

RSRC2	0.775619815	0.26292175	0.144727434	0.019595473	0.363595591	0.075834305	0.54630308
SNN	0.575644304	0.839595194	0.025499674	0.208994287	0.081130479	0.075552384	0.54630308
TBCA	0.392497817	0.19578424	0.308603177	0.630097052	0.01389067	0.07524999	0.54630308
TIMM44	0.331387139	0.404715651	0.840773344	0.014093543	0.131492119	0.075553136	0.54630308
USP43	0.018826719	0.885378342	0.180770102	0.231122425	0.29966392	0.07549344	0.54630308
USP54	0.242837049	0.14456446	0.465128288	0.042430891	0.303013276	0.075760968	0.54630308
PLEKHA1	0.351078928	0.124510157	0.009253698	0.697620264	0.750418442	0.076150816	0.546680248
ACTB	0.717153899	0.002502824	0.224717585	0.551448844	0.956296183	0.076351906	0.547644725
ARHGAP10	0.451293245	0.150631128	0.087482516	0.167623354	0.213350136	0.076346159	0.547644725
RAB12	0.955099674	0.007471415	0.561279494	0.169728263	0.313411754	0.076427342	0.547946315
C19H17orf113	0.096728728	0.216825361	0.580684351	0.797806788	0.022059132	0.076698361	0.548023121
CDH24	0.901491968	0.145019434	0.006795837	0.570209726	0.42197865	0.076579489	0.548023121
DTX3	0.061004698	0.903951449	0.006224874	0.740786057	0.842301544	0.076667729	0.548023121
LOC101902468	0.202861558	0.408975172	0.042751119	0.095037126	0.634670932	0.07661401	0.548023121
LOC112443006	0.424086444	0.518100767	0.564250326	0.040434975	0.042762151	0.076705204	0.548023121
LOC506495	0.607732872	0.069438963	0.030705021	0.179497053	0.921658251	0.076704725	0.548023121
NCAPG	0.6461089	0.024978485	0.074666356	0.259498385	0.683854874	0.076594057	0.548023121
VAT1	0.856059837	0.181821776	0.012186874	0.337126606	0.334820708	0.076651852	0.548023121
LOC101903026	0.591243706	0.019266745	0.024326685	0.884743897	0.876057403	0.076794366	0.548421384
LOC107131398	0.4021742	0.008108408	0.554740592	0.21067097	0.56405871	0.076832206	0.548452952
ATF4	0.187729539	0.318254536	0.067984816	0.170903034	0.310601856	0.076969128	0.549191462
ENDOG	0.940432777	0.57305128	0.734785156	0.006348049	0.086006081	0.077092996	0.549269552
FANCG	0.96643814	0.010859531	0.21514821	0.815858379	0.117411222	0.077113639	0.549269552
FZR1	0.808648745	0.902399578	0.742121737	0.147862637	0.002697715	0.077054846	0.549269552
PGBD5	0.031277021	0.20429698	0.992305373	0.248960639	0.137020906	0.077113951	0.549269552
ALG11	0.513143952	0.105930951	0.118397263	0.08944128	0.378014994	0.077388073	0.550266751
MFSD6	0.062409763	0.117959937	0.805380953	0.582781212	0.062907444	0.077340073	0.550266751
PIH1D1	0.613931068	0.907771625	0.000992277	0.660809755	0.595093873	0.077360672	0.550266751
TRPM2	0.566971443	0.00973159	0.742906096	0.22785661	0.232631649	0.07732038	0.550266751
LOC100847791	0.294636061	0.04286238	0.049465934	0.962907115	0.362140687	0.077438693	0.550388214
DDX18	0.510048976	0.097760298	0.046822581	0.118162443	0.790267775	0.077476029	0.550415201
CECR2	0.781174421	0.082096782	0.160226798	0.799021197	0.026730249	0.077781675	0.550449481
DUSP26	0.413824528	0.346279637	0.062624625	0.046086306	0.530270006	0.077748453	0.550449481
HOXD4	0.168537687	0.379991545	0.105404438	0.184610137	0.175814617	0.077704269	0.550449481
LOC112443226	0.836205911	0.799062446	0.002743246	0.381357988	0.313068743	0.07765034	0.550449481
SEC61B	0.147586645	0.044962275	0.41546195	0.730844966	0.108925278	0.077782728	0.550449481
SEMA5B	0.091223667	0.280857614	0.3177596	0.396991544	0.067672014	0.077624031	0.550449481
SLC33A1	0.828042119	0.026227975	0.08913576	0.620009751	0.181976596	0.077560586	0.550449481
SLC39A7	0.115435239	0.013488888	0.712884293	0.698326147	0.282168178	0.07762585	0.550449481
ZNF19	0.42183807	0.797436561	0.42821185	0.014737349	0.103210136	0.077704474	0.550449481

LOC112441500	0.191660137	0.820223495	0.65949521	0.010004993	0.211862989	0.077842879	0.550637711
CD52	0.350697838	0.054554597	0.214701848	0.29507217	0.181683532	0.077938072	0.5507828
LOC112441554	0.013663142	0.917028355	0.809331075	0.028216591	0.770060141	0.077964076	0.5507828
TMC4	0.011910181	0.82016622	0.262840879	0.146452635	0.585128531	0.077897454	0.5507828
IL12RB1	0.844768665	0.003581989	0.199890552	0.76715403	0.476019654	0.078078181	0.551351564
ATP5ME	0.254297237	0.290949869	0.724826094	0.018289175	0.225532025	0.078145687	0.551353771
TP53BP2	0.290231422	0.878897703	0.007173737	0.649316139	0.186100531	0.078128234	0.551353771
LOC112445176	0.220872479	0.78481866	0.022358599	0.121180731	0.471629748	0.078208998	0.55156333
LOC524576	0.584436891	0.92341416	0.122300104	0.005723609	0.586919778	0.078253704	0.551641555
NMRAL1	0.147515346	0.446640254	0.664547457	0.053038375	0.095576114	0.0783018	0.551690538
RGS16	0.073053603	0.962651793	0.008741528	0.41654102	0.86725967	0.078327887	0.551690538
CCAR1	0.836024084	0.12140403	0.761014437	0.004034154	0.714916134	0.078471769	0.551856352
MEGF8	0.645003605	0.242143036	0.023743639	0.09248338	0.648827478	0.078420688	0.551856352
PDE1B	0.414483818	0.830099406	0.809100534	0.000959184	0.834211144	0.078468112	0.551856352
SPRYD3	0.090240742	0.28846912	0.735145676	0.016160284	0.720544111	0.078485938	0.551856352
MCEE	0.116112763	0.593335265	0.334989913	0.12934799	0.075023252	0.078719555	0.553261933
ABL1	0.935095636	0.674570515	0.739757387	0.000787635	0.616216732	0.079242422	0.55384401
ACOT4	0.9007124	0.224612803	0.084870285	0.043830933	0.302972087	0.079558686	0.55384401
AKAP4	0.319073787	0.790225644	0.067775094	0.109724023	0.120970142	0.079313705	0.55384401
ATG2A	0.382187241	0.077582522	0.029731949	0.882853102	0.288710719	0.078874826	0.55384401
C18H19orf48	0.128179427	0.644427711	0.195085849	0.033150445	0.425855734	0.079451693	0.55384401
EMP1	0.134058926	0.240785558	0.011209487	0.969738373	0.646576157	0.079323835	0.55384401
FAAH	0.227572927	0.045062634	0.298520651	0.206403024	0.35986622	0.079429923	0.55384401
HHIPL1	0.058467263	0.904871353	0.258403655	0.023927146	0.696437302	0.079516666	0.55384401
LOC107131471	0.146957628	0.275828396	0.182265722	0.350092293	0.087943443	0.079446501	0.55384401
MCHR1	0.584263569	0.466658136	0.009038234	0.279269722	0.328564673	0.079167474	0.55384401
MTFR1L	0.420670337	0.045439617	0.746259311	0.096543328	0.163396702	0.078941104	0.55384401
NT5DC3	0.817387922	0.184742234	0.006144371	0.46251272	0.528375091	0.07929704	0.55384401
NUDT17	0.792309482	0.414901187	0.568332313	0.158891989	0.007640142	0.079308637	0.55384401
PHYHD1	0.447507223	0.683554219	0.004001021	0.365097016	0.508160882	0.079363473	0.55384401
PIF1	0.434661415	0.186530322	0.052128173	0.413375025	0.130193503	0.079444822	0.55384401
PRKG1	0.031162666	0.429420427	0.550251206	0.686201346	0.04494868	0.079373557	0.55384401
PTGES2	0.332108039	0.316022538	0.800093102	0.009622039	0.281994165	0.07952467	0.55384401
ROMO1	0.465566897	0.231723019	0.692240086	0.084141828	0.036264653	0.079530975	0.55384401
SAV1	0.405793161	0.717230975	0.006270591	0.261471256	0.474015978	0.079183981	0.55384401
SLC2A3	0.382830793	0.31921301	0.005086597	0.472179754	0.774453513	0.079413586	0.55384401
UBE2D2	0.991346464	0.047630449	0.151950119	0.559822375	0.056787126	0.079575098	0.55384401
USP2	0.823238321	0.611379412	0.048892804	0.026677075	0.347475357	0.079578586	0.55384401
ZNF831	0.146641111	0.479049769	0.7578251	0.23953326	0.017736725	0.079179184	0.55384401
ITGB7	0.344381633	0.060639301	0.524375412	0.27434109	0.076018945	0.079633322	0.553990014

PLEKHM2	0.067150477	0.433505781	0.272805224	0.082112684	0.350936454	0.079729337	0.554198216
PLPPR4	0.302386866	0.272371595	0.038544392	0.084483404	0.853279807	0.07973079	0.554198216
NAA38	0.427538231	0.404391308	0.052420313	0.045645058	0.554373691	0.079831003	0.554659861
SYNJ2	0.302251229	0.278719564	0.607956338	0.007525472	0.59582142	0.079894512	0.554866203
LOC617475	0.818013457	0.38259022	0.540099114	0.001431368	0.950484956	0.079960423	0.555089045
CBX6	0.649955019	0.491912315	0.033953542	0.028208004	0.753418482	0.080112726	0.555293332
LOC101906754	0.225649228	0.343450401	0.233369796	0.456549883	0.027961326	0.080147986	0.555293332
MBLAC2	0.78932448	0.248223137	0.453014197	0.048541044	0.053490288	0.080061467	0.555293332
NDUFA8	0.438470332	0.12266417	0.780094172	0.043159216	0.127619505	0.080192326	0.555293332
NOXO1	0.680992882	0.805507768	0.945770074	0.097891607	0.00454564	0.080142519	0.555293332
RPS27L	0.473309338	0.277043808	0.426337267	0.087729599	0.047120253	0.08019287	0.555293332
BRD3OS	0.11341393	0.185555178	0.282263008	0.827580141	0.047450792	0.08063483	0.556029682
BVES	0.042775512	0.064156674	0.874395391	0.801887241	0.121100306	0.080586284	0.556029682
CEP170	0.793436688	0.077072635	0.44934817	0.02253284	0.375492541	0.08047793	0.556029682
COQ7	0.09108693	0.076366674	0.515930306	0.123531349	0.526013382	0.080621472	0.556029682
EFCAB11	0.00813983	0.473035915	0.237442743	0.411129675	0.621066882	0.080671907	0.556029682
LOC101907369	0.088544754	0.791940655	0.039104915	0.743307467	0.114418691	0.080624464	0.556029682
M6PR	0.884496849	0.050511443	0.014945616	0.413783307	0.841426273	0.080475329	0.556029682
MPZL3	0.188183802	0.478813712	0.032682972	0.207115661	0.381576658	0.080527389	0.556029682
PSMD8	0.576743692	0.259735391	0.66992279	0.446722638	0.005179652	0.080419427	0.556029682
PTPRN2	0.002571414	0.323315741	0.449283265	0.743408586	0.835509309	0.080378	0.556029682
SH3GL3	0.053184986	0.10999698	0.528071879	0.568312569	0.132911317	0.08065291	0.556029682
LOC107132317	0.865190416	0.497275919	0.100719286	0.05705725	0.094666684	0.080797325	0.556660327
ADAMTS10	0.647118086	0.030714338	0.036716187	0.57330258	0.561208751	0.080946709	0.556770473
C25H16orf72	0.456182543	0.606109489	0.101478626	0.023958376	0.349457531	0.080971487	0.556770473
C3H1orf54	0.011629682	0.812368561	0.936968358	0.035251612	0.753000914	0.080982945	0.556770473
DECR1	0.663448729	0.574401431	0.534532595	0.025261146	0.045657933	0.080977011	0.556770473
EZH2	0.423185033	0.036751884	0.157862391	0.12797782	0.747772779	0.080979931	0.556770473
LOC100140431	0.587542349	0.034225982	0.024032538	0.993518214	0.490121433	0.081054842	0.557014358
LOC100336282	0.168942183	0.06717496	0.197698038	0.793469582	0.132276309	0.081086302	0.557014358
UQCRC1	0.530328279	0.379914174	0.824228339	0.010147238	0.139927038	0.081148722	0.557209906
MIC1	0.531922488	0.667321539	0.003530244	0.272005284	0.692266045	0.081183001	0.55721214
CFAP46	0.072615421	0.669092227	0.16430698	0.043021573	0.688178919	0.081262331	0.557224793
COX10	0.52511104	0.161094258	0.102906838	0.452701628	0.06044771	0.081638899	0.557224793
COX17	0.343049016	0.24735805	0.569298576	0.102742099	0.048002686	0.081646531	0.557224793
FZD2	0.590496053	0.056171155	0.020781307	0.376884796	0.917816212	0.081683209	0.557224793
GYG1	0.118927501	0.117116437	0.129471918	0.430893038	0.304475288	0.081310712	0.557224793
IFITM3	0.110746606	0.043727873	0.445775684	0.222255635	0.49542864	0.081536198	0.557224793
JMY	0.12456704	0.643356353	0.217582458	0.073051965	0.187199117	0.081688388	0.557224793
LGALS3	0.222028048	0.192411569	0.068674594	0.997203665	0.081183279	0.081497123	0.557224793

LOC100141258	0.037762719	0.455241789	0.862658247	0.055371094	0.290187938	0.081654316	0.557224793
LOC100847546	0.601542684	0.667810332	0.010250579	0.727789421	0.079586233	0.081699171	0.557224793
LOC100849652	0.211192777	0.203665415	0.066108839	0.813669353	0.102881654	0.081602867	0.557224793
MANBAP1	0.295676285	0.084310798	0.147749539	0.42577344	0.151331655	0.081458293	0.557224793
PELI2	0.468727401	0.331011325	0.729483592	0.018535513	0.113463461	0.0816026	0.557224793
SPATA21	0.206410308	0.057420916	0.751560907	0.106146525	0.252134186	0.081676082	0.557224793
TEKT3	0.551647801	0.086363991	0.373763992	0.577452754	0.023209427	0.081728114	0.557224793
ZFP1	0.693094571	0.694306492	0.279456603	0.965327314	0.001835464	0.081651203	0.557224793
EIF1	0.926936129	0.051593556	0.025339053	0.45894237	0.430564867	0.081889965	0.557740636
PIPOX	0.310865817	0.964840499	0.125221659	0.007198086	0.885102164	0.081855116	0.557740636
SLC25A15	0.824842907	0.41124372	0.929558445	0.004882851	0.155579984	0.08190573	0.557740636
RAC2	0.381031265	0.058073179	0.558353474	0.119121688	0.163062781	0.08199644	0.558126745
SLC38A4	0.297276613	0.069069203	0.153678759	0.250017455	0.304830429	0.082096121	0.558159342
SMIM26	0.944937586	0.34269783	0.192504039	0.268703402	0.014359006	0.082103263	0.558159342
TMEM109	0.971029678	0.00677512	0.156041844	0.277139667	0.845175907	0.082090414	0.558159342
F11R	0.240622914	0.817216962	0.311063923	0.028292567	0.139266831	0.08220257	0.558603056
WIPF1	0.702142358	0.017074796	0.6763925	0.051418596	0.578808761	0.082268384	0.558818891
TTN	0.879438691	0.061834144	0.270537645	0.256820028	0.063930646	0.082309051	0.558863812
SPP2	0.168399248	0.451072354	0.516010502	0.033819664	0.182355417	0.082346214	0.558884915
ZKSCAN5	0.915262373	0.505492872	0.10726669	0.865968225	0.005629019	0.08238273	0.558901607
RNF208	0.537496424	0.685470479	0.043880867	0.025597579	0.586330055	0.082530185	0.559670605
PTPRU	0.114385278	0.102610075	0.687205009	0.433742389	0.069470229	0.082608055	0.559967282
KRT7	0.210923092	0.257807616	0.070948325	0.1769882	0.356270068	0.082653772	0.560045852
SSU72	0.270997123	0.168691215	0.967743864	0.143365628	0.038383146	0.082689311	0.560055419
SLC6A1	0.409035499	0.087975614	0.128188021	0.527270006	0.100227999	0.082755351	0.560271481
PSTPIP1	0.655271291	0.196029341	0.59160103	0.023751718	0.135309016	0.082845023	0.560647286
RGS14	0.140700677	0.139299903	0.814349287	0.120154853	0.12774223	0.082995477	0.561433953
ELAVL3	0.157788827	0.01604678	0.339629699	0.723943116	0.394068948	0.083064344	0.561668297
FAM129A	0.106372075	0.213610962	0.288840109	0.688634629	0.054456881	0.083222266	0.562166932
IL18R1	0.762597474	0.140300058	0.691650285	0.059344781	0.056065158	0.083240853	0.562166932
SIGIRR	0.729580955	0.538055368	0.00853514	0.119835884	0.61284293	0.083210654	0.562166932
NSDHL	0.410072215	0.353991364	0.492128808	0.058294459	0.059357124	0.083434354	0.563241952
ESM1	0.736428628	0.013696166	0.26669113	0.159192383	0.57827416	0.083520424	0.563470195
IL10RA	0.523098105	0.058571023	0.52617227	0.044753255	0.343577691	0.083571169	0.563470195
LOC112444279	0.028088547	0.544510315	0.278609799	0.198766741	0.29252343	0.083547741	0.563470195
PCED1B	0.012744781	0.476544188	0.52489243	0.239027157	0.325827906	0.083650066	0.563770524
ADRA1D	0.391607938	0.867962708	0.067359699	0.495051201	0.022145642	0.084188775	0.564077745
CNTRL	0.806874761	0.06544457	0.142490553	0.042855667	0.778422478	0.08418859	0.564077745
COL7A1	0.822143676	0.258705812	0.229976195	0.100002884	0.051033397	0.083917628	0.564077745
DNAJC14	0.48650833	0.214237948	0.344262011	0.007846431	0.893098322	0.08427482	0.564077745

DNAJC19	0.345025681	0.218675288	0.717609727	0.037745708	0.121944785	0.083834468	0.564077745
IL17RB	0.542502671	0.154959624	0.025230556	0.355338645	0.333890956	0.084314345	0.564077745
ING1	0.172482032	0.399165427	0.032716172	0.25783783	0.432963032	0.0842759	0.564077745
LBP	0.319492486	0.122809035	0.754558159	0.199518361	0.042362394	0.084036026	0.564077745
NDUFAF8	0.183720612	0.162680486	0.690999359	0.170588255	0.070823419	0.083894178	0.564077745
OSBPL6	0.106658621	0.156040248	0.046729468	0.64221413	0.503763758	0.08430704	0.564077745
OSBPL7	0.452777982	0.063223275	0.083308071	0.406904869	0.257362517	0.083938471	0.564077745
PAK6	0.264744099	0.120608583	0.256542009	0.095859777	0.316883266	0.083758401	0.564077745
PAPPA2	0.868281902	0.034936833	0.505018448	0.113061806	0.144346726	0.083993716	0.564077745
PPP1R37	0.028483629	0.073686497	0.794569142	0.690284216	0.216692392	0.083881653	0.564077745
RAB43	0.103613977	0.375136112	0.082110601	0.118211129	0.660565405	0.083835645	0.564077745
TCAF2	0.734886321	0.139197254	0.014754202	0.517884911	0.321667206	0.084270194	0.564077745
TMEM160	0.450924275	0.221600675	0.743669677	0.057178092	0.059099842	0.084209213	0.564077745
TRPM4	0.147846943	0.648330193	0.009208045	0.790556573	0.359734078	0.084188715	0.564077745
CD6	0.024969158	0.114868599	0.908388323	0.294511568	0.329383553	0.084529462	0.564725195
CP	0.226418147	0.026937052	0.678893954	0.483305073	0.126406048	0.084571867	0.564725195
ETFBKMT	0.121312348	0.112916655	0.218225485	0.786166758	0.107738163	0.084617589	0.564725195
FEM1A	0.200987194	0.214626042	0.095894988	0.327801304	0.186427145	0.084539222	0.564725195
NACC1	0.13925244	0.524540031	0.93540237	0.029285603	0.126493736	0.084600496	0.564725195
SNAP29	0.045408933	0.077101035	0.657277973	0.912111277	0.120347518	0.084501445	0.564725195
OSGIN2	0.531898494	0.230108078	0.042494005	0.930061067	0.052562214	0.08482583	0.565884837
BLOC1S6	0.191344903	0.007870264	0.257979475	0.686544378	0.954434767	0.084886856	0.565937961
SAMD15	0.43670912	0.181477482	0.130853581	0.079604042	0.308681211	0.084937249	0.565937961
YWHAH	0.101713423	0.224519202	0.215631911	0.13901881	0.372005144	0.084905314	0.565937961
ARFGEF1	0.355382514	0.969574554	0.034414987	0.026949205	0.805061498	0.085414603	0.565973968
ATP5PB	0.676494247	0.201793819	0.716752813	0.117218928	0.022467442	0.085494453	0.565973968
DENND1A	0.57554736	0.922535605	0.857211009	0.927560807	0.000610141	0.085475022	0.565973968
DUOX2	0.610306232	0.011167008	0.068679549	0.872391513	0.630666398	0.085463379	0.565973968
GALNT1	0.015377497	0.235388922	0.508688339	0.685329156	0.203302119	0.085272399	0.565973968
GDF6	0.967683232	0.014234219	0.125950164	0.611736076	0.241267936	0.085176365	0.565973968
GEMIN4	0.359121797	0.703116379	0.009512447	0.417919759	0.256149314	0.085385513	0.565973968
IDH3A	0.495847446	0.220425266	0.586360929	0.24630603	0.016264999	0.085311402	0.565973968
LOC104975814	0.030455058	0.279757736	0.046695964	0.851001707	0.759256095	0.085373107	0.565973968
MRPS5	0.361092586	0.339669988	0.961587018	0.137438645	0.015834975	0.085298402	0.565973968
MTHFD1L	0.252061666	0.755651687	0.087060253	0.836439512	0.018470914	0.085204181	0.565973968
NLRP12	0.261206288	0.676470135	0.031322837	0.069950848	0.662441219	0.085257345	0.565973968
NT5DC2	0.021948295	0.15479235	0.604128515	0.743740702	0.168047085	0.085268671	0.565973968
PHOSPHO1	0.141941454	0.263766743	0.34880789	0.054740442	0.359883174	0.085412938	0.565973968
TMEM59L	0.26975961	0.563609937	0.10146173	0.097963314	0.169418712	0.085171008	0.565973968
TMEM62	0.215157692	0.55690835	0.366225347	0.064139251	0.090691997	0.085021646	0.565973968

DNLZ	0.15703025	0.176630108	0.444325856	0.166055293	0.126090773	0.085562839	0.566198287
APLF	0.090519543	0.148890601	0.048839256	0.799299488	0.493194027	0.08584249	0.56632464
CCT2	0.990094854	0.151541363	0.166369081	0.348033447	0.029786009	0.085704651	0.56632464
CHMP6	0.180969766	0.290714658	0.238723583	0.503333006	0.041007204	0.085793654	0.56632464
COX7A1	0.639141622	0.819073998	0.252077923	0.007721078	0.25527083	0.085961531	0.56632464
DNAJC5	0.123902398	0.217379532	0.052412079	0.380793484	0.482485993	0.085819114	0.56632464
DYNC2LI1	0.002403651	0.634239414	0.595690679	0.897224252	0.318903948	0.08591197	0.56632464
HMGN2	0.041391141	0.362484826	0.470881552	0.13661582	0.269228024	0.085914735	0.56632464
HSD17B11	0.781453409	0.170714963	0.010862635	0.312892551	0.573298795	0.085932797	0.56632464
ME1	0.840084506	0.642649854	0.461953641	0.006121185	0.169751473	0.085777581	0.56632464
PRELID3B	0.549278619	0.186615039	0.632885566	0.883790031	0.004522839	0.08580995	0.56632464
SETD7	0.906277834	0.131112175	0.040654225	0.304855188	0.175570913	0.085663332	0.56632464
GPR174	0.794174522	0.124267047	0.022818014	0.785644496	0.147560545	0.086148448	0.56732832
GRB7	0.997689157	0.734547145	0.041780747	0.066268611	0.128883519	0.086235572	0.56742774
LOC785693	0.549158845	0.203087965	0.189496177	0.500075741	0.02472789	0.086201781	0.56742774
PGD	0.332610331	0.450490308	0.917669644	0.03571475	0.053322624	0.086301849	0.56742774
RALBP1	0.670306489	0.027170808	0.15849881	0.341877052	0.265262778	0.086288081	0.56742774
PRR5L	0.937124032	0.006931511	0.084889757	0.844518492	0.563856268	0.086440423	0.567883822
TCEAL8	0.68036695	0.106758687	0.306767948	0.932018806	0.01263692	0.08641301	0.567883822
GID4	0.126988042	0.330018817	0.213151305	0.215650869	0.136943473	0.086676361	0.568735797
IRF8	0.453904116	0.998176671	0.217213606	0.20200256	0.013278346	0.08670873	0.568735797
LIFR	0.681659243	0.25858907	0.085677474	0.05182767	0.336815592	0.086643218	0.568735797
PRELID2	0.867511457	0.74581875	0.180641464	0.006343621	0.355965128	0.086698366	0.568735797
DCAF11	0.606461521	0.274167347	0.004707983	0.451758616	0.74697621	0.086744654	0.568744115
CAV3	0.020591074	0.941612701	0.679128443	0.03624846	0.554825064	0.086871062	0.568985019
CNNM3	0.846420415	0.347143208	0.681764105	0.485458228	0.002723783	0.086883274	0.568985019
MRM2	0.227229943	0.128900608	0.061533229	0.382400919	0.384346031	0.08688541	0.568985019
APBB1	0.278359724	0.908081346	0.002284422	0.985091049	0.467858301	0.087122694	0.569748831
DDX56	0.351910282	0.066000768	0.235646378	0.195038414	0.249868477	0.087237076	0.569748831
DDX6	0.533639969	0.00586131	0.0942868	0.949140198	0.951510474	0.087162523	0.569748831
DTX4	0.655359981	0.002231202	0.310142846	0.714592713	0.820880509	0.087101698	0.569748831
EXOC6	0.170200575	0.38644381	0.388324375	0.012831409	0.814175041	0.087255953	0.569748831
HEXDC	0.382043777	0.116305893	0.036317358	0.28093216	0.589621097	0.087346309	0.569748831
HPSE	0.490995609	0.108077303	0.521865197	0.0587067	0.164214533	0.087283604	0.569748831
LOC512863	0.912800517	0.998109912	0.27000388	0.01837262	0.05898228	0.087206984	0.569748831
MRPL57	0.33453232	0.457293585	0.872999387	0.025977039	0.076829082	0.087200594	0.569748831
MYOCD	0.040145415	0.68378127	0.582833016	0.275531586	0.060640065	0.087349221	0.569748831
ABLIM1	0.602386323	0.898289711	0.010657094	0.262631399	0.177165316	0.087540605	0.570723949
BCAR3	0.493494768	0.836250464	0.35854926	0.00586215	0.311189813	0.087846487	0.570723949
C2CD5	0.06068522	0.647379928	0.658113552	0.255851332	0.04074671	0.087772494	0.570723949

CD28	0.662485074	0.261886661	0.021342981	0.837674084	0.086931612	0.087793043	0.570723949
CMAS	0.083262101	0.022704503	0.431727688	0.962666545	0.341843442	0.087589674	0.570723949
FKBP1B	0.653981671	0.267688976	0.020628376	0.679211645	0.109951054	0.087801098	0.570723949
LOC112442296	0.558942209	0.037023333	0.649614935	0.075724475	0.265163382	0.087846269	0.570723949
LOC112446470	0.188130196	0.649192764	0.063596692	0.043545787	0.795206992	0.087662797	0.570723949
MSS51	0.955908321	0.594778272	0.125040852	0.383595903	0.009852047	0.087607493	0.570723949
TRIM25	0.32291446	0.02589096	0.037899904	0.874720168	0.973053071	0.087802728	0.570723949
DHODH	0.62660096	0.229608195	0.458309065	0.354937367	0.011595961	0.088123803	0.570742591
DNAJB11	0.690069729	0.613128546	0.045466626	0.063996046	0.219583962	0.087922196	0.570742591
EIF2S2	0.837052809	0.153078897	0.364951162	0.771758813	0.007538716	0.088252544	0.570742591
HNRNPD	0.623716195	0.448808954	0.002457896	0.704553186	0.561403801	0.088266693	0.570742591
LOC101906024	0.905211122	0.429340142	0.001141796	0.995468613	0.612634371	0.087978657	0.570742591
LOC781339	0.035957892	0.268714426	0.399194698	0.857794694	0.081983436	0.088098241	0.570742591
NRDE2	0.8549241	0.382768865	0.073835524	0.555409698	0.020271034	0.088245072	0.570742591
OVOS2	0.144621506	0.787361764	0.003370646	0.709543665	0.992535402	0.087916817	0.570742591
PHLPP2	0.342470427	0.070383275	0.475095563	0.494017798	0.04805068	0.08820913	0.570742591
PSD	0.110233185	0.337741559	0.815028997	0.07453943	0.120010861	0.088133417	0.570742591
WAS	0.2865588	0.09137131	0.87502499	0.08630258	0.137168193	0.088091353	0.570742591
ZBTB17	0.765708183	0.087890677	0.01191311	0.507376163	0.66581702	0.088019758	0.570742591
PTPN12	0.345523291	0.887483431	0.888337056	0.003903147	0.256160845	0.088307222	0.570779764
MRPL41	0.139386678	0.444994044	0.848923761	0.071089812	0.072950943	0.088442483	0.57114087
NUBP2	0.048913913	0.071647243	0.750415888	0.358124864	0.29008455	0.088467497	0.57114087
RAI14	0.284525815	0.444667388	0.891329493	0.148545555	0.016301409	0.088442198	0.57114087
TSPAN31	0.012238309	0.489119461	0.24993605	0.326746492	0.559540451	0.088528671	0.571311055
ACSS2	0.571152993	0.477436926	0.998118337	0.008413812	0.119626308	0.088607441	0.571537008
CLIC4	0.902208351	0.374602647	0.001721637	0.619323748	0.760589882	0.088633336	0.571537008
MMP15	0.020860126	0.732862727	0.781317743	0.049735414	0.462383121	0.088746144	0.572039657
LOC101905099	0.417488543	0.015787618	0.843450846	0.399678813	0.123833987	0.088833988	0.572325056
MKL2	0.038982098	0.760773495	0.015064018	0.859393648	0.717030987	0.08886017	0.572325056
CCDC88C	0.470997988	0.343944301	0.018163531	0.17455123	0.537362308	0.088992187	0.572444823
LOC789148	0.171738585	0.126746743	0.054433035	0.304764974	0.764984305	0.089038449	0.572444823
LRRC3	0.743890182	0.11377743	0.165843753	0.033837982	0.580236648	0.088917702	0.572444823
NEPRO	0.798304524	0.041845833	0.841292618	0.266376145	0.036928345	0.089078875	0.572444823
TBC1D10C	0.308476898	0.051835695	0.633426998	0.116985478	0.233322125	0.089080423	0.572444823
VIT	0.029577599	0.766720595	0.947036996	0.020116833	0.639990135	0.089088055	0.572444823
MSLN	0.28642484	0.147120675	0.109226107	0.208512281	0.288716603	0.089197425	0.572923263
CD79B	0.402113546	0.046519116	0.460784424	0.772051995	0.041790855	0.089388068	0.573026403
PHKG1	0.59212795	0.157635546	0.819374788	0.11818326	0.030742201	0.089344612	0.573026403
SIGMAR1	0.271682299	0.197482773	0.965452979	0.228982952	0.023444937	0.08938425	0.573026403
THAP8	0.915097465	0.060136214	0.016378622	0.315662006	0.976872819	0.089356346	0.573026403

TSC1	0.522485213	0.366585622	0.028875426	0.748091402	0.067195856	0.089372391	0.573026403
RSL24D1	0.71068837	0.219324351	0.080604052	0.76950124	0.02879869	0.089447946	0.573186348
PSMC5	0.368520847	0.180586407	0.413448026	0.336052181	0.030193017	0.089589162	0.573422874
PSMD14	0.647056067	0.219999791	0.836665091	0.606843355	0.003862719	0.089589681	0.573422874
SMAP2	0.576307304	0.714942181	0.64025331	0.069328428	0.015246769	0.089527284	0.573422874
FBXL22	0.00594422	0.545166367	0.67686574	0.360088324	0.353882358	0.089650826	0.573590526
PCF11	0.342238124	0.653330947	0.965968856	0.010232065	0.126660415	0.089727133	0.573855021
ADAMTS9	0.288466759	0.891524839	0.455974597	0.003118292	0.770643462	0.090077952	0.573914826
AGFG2	0.230615589	0.334870985	0.407969665	0.029898612	0.299048032	0.090059346	0.573914826
BZW2	0.609490826	0.75317287	0.111042837	0.130233816	0.042354637	0.089961766	0.573914826
CEP295	0.679144748	0.044846785	0.049819737	0.422818222	0.438570155	0.08999924	0.573914826
KCNJ8	0.494173294	0.307590158	0.016836041	0.371445602	0.295242347	0.089863956	0.573914826
LOC112447381	0.607419898	0.099245142	0.448975639	0.207898677	0.049870984	0.089858555	0.573914826
LOC614741	0.025795426	0.136062681	0.206876192	0.440266836	0.881246923	0.090061735	0.573914826
MEGF6	0.291449677	0.041956381	0.326488595	0.078622708	0.894155895	0.089867006	0.573914826
SPRYD4	0.505049408	0.059007141	0.787997069	0.043937415	0.273156122	0.090086198	0.573914826
WDR77	0.610463366	0.099332825	0.106401157	0.37983748	0.114999768	0.090084392	0.573914826
DGKI	0.589507786	0.378068245	0.006978946	0.241685129	0.751804803	0.090230698	0.574107653
LOC100337355	0.397997103	0.144899898	0.69033818	0.201721393	0.035215505	0.090265902	0.574107653
NDUFB1	0.408941698	0.327210444	0.99428035	0.020656323	0.102789919	0.090206079	0.574107653
PCDH17	0.46832561	0.035754786	0.061861054	0.583562539	0.468415791	0.090329118	0.574107653
SLITRK2	0.949863892	0.449602285	0.293549135	0.085743362	0.02635806	0.090361347	0.574107653
SUPT4H1	0.871331463	0.012702624	0.455886338	0.215232478	0.260555189	0.090295557	0.574107653
ZDHHC21	0.373713558	0.147230512	0.219100618	0.096913843	0.242414691	0.090342029	0.574107653
DAPK3	0.244495612	0.835646972	0.330468995	0.047924153	0.088072523	0.090668638	0.575409454
LOC107132092	0.654207896	0.633287604	0.806788275	0.58098675	0.001468317	0.09069813	0.575409454
TBXA2R	0.784206641	0.224180989	0.930129526	0.002510966	0.694186085	0.090677136	0.575409454
TNFAIP3	0.826827569	0.054496893	0.513812413	0.027137133	0.454009009	0.090717236	0.575409454
URI1	0.054233305	0.770111323	0.3907892	0.277787823	0.062942585	0.090741555	0.575409454
CILP	0.278861526	0.750744772	0.29446877	0.030871242	0.150150322	0.09081212	0.575634493
SCAPER	0.135323583	0.413034562	0.260780592	0.13668539	0.143577822	0.090866123	0.575754418
ABTB1	0.696036727	0.016189114	0.119307666	0.25027826	0.850895605	0.090912158	0.575823781
COX8A	0.788761696	0.316519336	0.75167692	0.01607903	0.094989103	0.090971549	0.575977661
CNTLN	0.515303997	0.144267209	0.172810341	0.090433431	0.247252042	0.091088368	0.576092326
DNAH10	0.461352562	0.036563959	0.310950725	0.29702736	0.184517545	0.091130076	0.576092326
LOC533597	0.65168984	0.101350485	0.083000593	0.061355675	0.854144172	0.091096187	0.576092326
UBA3	0.591023029	0.021463568	0.699228227	0.211517598	0.152979567	0.09104394	0.576092326
NSMCE4A	0.175671676	0.559425538	0.011351491	0.272739906	0.945820477	0.091184053	0.576211591
ADAMTS15	0.470341876	0.60037884	0.511251776	0.125118397	0.015959244	0.091275965	0.576469872
HABP4	0.108825268	0.649406519	0.255600795	0.023897545	0.668040476	0.09129518	0.576469872

CARD19	0.482324817	0.036124334	0.16353968	0.114167229	0.888670679	0.091427207	0.57660202
CHUK	0.173218037	0.344368434	0.146011008	0.203507111	0.163246584	0.091473791	0.57660202
CYSTM1	0.375075207	0.456230516	0.096093278	0.287216708	0.061186437	0.091404663	0.57660202
MIF	0.200861056	0.698021829	0.943707361	0.020476017	0.106793711	0.091469816	0.57660202
SLC45A3	0.548335398	0.093734225	0.049335662	0.21935817	0.520371537	0.091491784	0.57660202
UTP15	0.946184561	0.038206162	0.05394493	0.383822842	0.387153075	0.091553067	0.576766747
MDGA1	0.023070514	0.443964481	0.072744143	0.657083675	0.592710693	0.091625766	0.577003239
MAPK8IP2	0.109458233	0.07549134	0.983059705	0.042013867	0.851107438	0.091679145	0.577117931
C1H21orf91	0.225741907	0.037837046	0.300951225	0.384943663	0.294544913	0.091859722	0.577888787
LOC101906221	0.126236546	0.183888766	0.343114503	0.214182583	0.170887738	0.091872028	0.577888787
TRIM34	0.191082565	0.567417481	0.279249822	0.014474118	0.665971635	0.091932237	0.578045951
BCL6	0.013218987	0.649626049	0.329199361	0.145654712	0.710081119	0.092029188	0.578104752
LOC112441493	0.004225281	0.920791584	0.568573251	0.163625042	0.808066119	0.092047268	0.578104752
NLRC3	0.209903329	0.200043945	0.019873514	0.606762036	0.577129913	0.09199946	0.578104752
DPYSL5	0.084868614	0.16026192	0.386890384	0.106505685	0.522724164	0.092134733	0.578432712
AFTPH	0.676551868	0.366152554	0.057191784	0.024386869	0.853167111	0.092464757	0.57864368
KIF1B	0.946546662	0.602391487	0.000919217	0.740269484	0.758110788	0.092350524	0.57864368
LOC101902435	0.574543809	0.061931765	0.024169375	0.477150862	0.718518189	0.092478166	0.57864368
MARCKS	0.20556683	0.837691636	0.029964039	0.057582711	0.992578729	0.092490412	0.57864368
MORF4L1	0.697017913	0.158005731	0.03131989	0.137635654	0.621546614	0.092520932	0.57864368
MRPL43	0.169065682	0.092737878	0.780869524	0.210877875	0.113824685	0.092300813	0.57864368
PAQR8	0.460354256	0.62298587	0.175759269	0.6649925	0.008794833	0.092470437	0.57864368
PLPBP	0.305786157	0.289921245	0.300598174	0.045741055	0.242010173	0.092506393	0.57864368
PRAM1	0.329933706	0.116193655	0.164756274	0.047614146	0.978588267	0.092378347	0.57864368
TOMT	0.609811705	0.293022223	0.452060106	0.00429038	0.851222914	0.092507324	0.57864368
PTGES	0.096570158	0.236964658	0.119066358	0.529421659	0.204871427	0.092602156	0.578741857
SRSF3	0.743333014	0.03386736	0.055395983	0.632088534	0.33528815	0.092607161	0.578741857
KIAA0895	0.906176845	0.911054922	0.008220056	0.646535078	0.067572315	0.092774756	0.579113474
LOC112441545	0.018904054	0.280071519	0.140287004	0.472266615	0.84628347	0.092843066	0.579113474
RCN3	0.648980452	0.083949352	0.025954508	0.480812763	0.435874714	0.092750759	0.579113474
SOX8	0.745791685	0.954867712	0.003580711	0.138532278	0.84020248	0.092833212	0.579113474
TMEM145	0.604512963	0.181857435	0.128634139	0.025636165	0.816705615	0.09270273	0.579113474
F13A1	0.053669936	0.396657334	0.300453489	0.44713805	0.103893472	0.092893808	0.579209832
USP42	0.618280487	0.887283648	0.755139801	0.000726433	0.988508093	0.092955519	0.579374483
CNKSR3	0.079692611	0.589138344	0.204735901	0.08509229	0.364011976	0.093003184	0.579451501
ATP6AP2	0.719239131	0.472456146	0.146632648	0.690624081	0.008668545	0.093104804	0.579644516
LOC112446402	0.306018539	0.410054306	0.102624199	0.588988373	0.039319111	0.093091866	0.579644516
LOC101904794	0.977537182	0.112349611	0.015670137	0.413986104	0.41928073	0.093181236	0.579900364
PIN4	0.336789726	0.070356287	0.614480251	0.056952115	0.360979761	0.09329247	0.580372529
FCHO2	0.119365691	0.568043331	0.029059982	0.26065957	0.583988261	0.093401037	0.580827743

SMAD6	0.711414561	0.076689758	0.741328224	0.020937812	0.355017756	0.093527948	0.581396649
KLC1	0.018553634	0.432865894	0.826373281	0.066260409	0.684770163	0.093616002	0.581493182
SELENOO	0.147904498	0.582355675	0.30189025	0.082631474	0.140237363	0.093649776	0.581493182
TBC1D32	0.009245946	0.187106315	0.577284454	0.441076305	0.683825966	0.093632414	0.581493182
RANBP6	0.646032379	0.177466467	0.525007227	0.026423168	0.189672229	0.093711802	0.581557927
RTRAF	0.650191156	0.169280011	0.583368523	0.043008502	0.109301793	0.093742653	0.581557927
TBRG4	0.134645129	0.114529915	0.991817805	0.708701385	0.027858481	0.093766515	0.581557927
ENKUR	0.592701684	0.576897619	0.048091721	0.038710328	0.475159674	0.093855255	0.581668654
RAN	0.787930527	0.125033642	0.369089672	0.91177051	0.009117508	0.093822319	0.581668654
ATP7A	0.209449878	0.16620232	0.139937117	0.111977022	0.554886556	0.093894251	0.58169066
EIF4H	0.87483402	0.083105469	0.051773882	0.789094586	0.102042757	0.093968636	0.581931804
CDK15	0.672075379	0.001060579	0.767507415	0.577883381	0.964950173	0.094321419	0.581989146
GAK	0.027484907	0.061985011	0.478536049	0.385060599	0.969145239	0.094173807	0.581989146
LOC100141266	0.294418592	0.120898451	0.025634406	0.468315961	0.712356094	0.0942029	0.581989146
LOC100335990	0.031625064	0.527813768	0.334451401	0.124411215	0.437697745	0.094131825	0.581989146
LOC101907944	0.044384829	0.662638334	0.020404304	0.536202779	0.947516058	0.094291217	0.581989146
LOC785629	0.751219502	0.819981016	0.229246493	0.010678957	0.202310252	0.094325342	0.581989146
RAET1L	0.792404476	0.912386684	0.001599991	0.326858824	0.807000191	0.094332529	0.581989146
SCMH1	0.01931882	0.963308055	0.56151906	0.630270071	0.046128735	0.094097897	0.581989146
SRCIN1	0.176977744	0.11912985	0.200972874	0.148895665	0.481391479	0.094078915	0.581989146
ZNF414	0.418707095	0.320068144	0.01643219	0.220865358	0.624533634	0.094088259	0.581989146
DPH3	0.86702974	0.106935587	0.004155153	0.810602092	0.979000868	0.094440272	0.582102304
FKRP	0.99922641	0.038011883	0.942472102	0.13078011	0.065627023	0.094710124	0.582102304
IPCEF1	0.487243365	0.041947326	0.970856272	0.033760617	0.458098476	0.094646848	0.582102304
KLHL22	0.979573027	0.917975359	0.009170964	0.535884122	0.069561281	0.094741043	0.582102304
LMO3	0.021662841	0.562326218	0.643535132	0.358131759	0.108962507	0.094473226	0.582102304
LOC787122	0.481557088	0.343272862	0.720364353	0.00525057	0.490467101	0.094606695	0.582102304
MTX1	0.539017541	0.162671689	0.868100233	0.014498948	0.277908403	0.094615154	0.582102304
NUDT16	0.105285769	0.603120812	0.508339087	0.463376628	0.020544009	0.094719032	0.582102304
RMND5A	0.172683001	0.041557087	0.445490281	0.469583452	0.203926202	0.094513859	0.582102304
SFRP4	0.461242298	0.475897599	0.123836245	0.331741898	0.034088914	0.09473885	0.582102304
WDR43	0.947111654	0.438777689	0.063781617	0.022796011	0.508094083	0.094668263	0.582102304
CACNA1B	0.004321038	0.968747315	0.518906814	0.720202061	0.196798125	0.094822074	0.582164259
LOC107131567	0.05773743	0.247644618	0.375027509	0.082653097	0.694615393	0.094820773	0.582164259
GLRX5	0.263028435	0.345171168	0.950912728	0.021416008	0.166627279	0.094859856	0.58217842
MAOA	0.90988127	0.077541236	0.70779963	0.028056406	0.220112741	0.094915761	0.58230376
KCNJ2	0.010710334	0.584654825	0.18529662	0.30217077	0.881499484	0.09503456	0.582596999
OTUB2	0.977296938	0.746823285	0.264780179	0.384232884	0.004162166	0.095034458	0.582596999
ARMH4	0.298717996	0.453331815	0.825443992	0.972717615	0.002853485	0.095247684	0.582814969
BCL7B	0.541094356	0.006306642	0.244463368	0.950009805	0.391089577	0.095192146	0.582814969

LOC101903478	0.560759096	0.244230739	0.481155989	0.076016271	0.061898395	0.095212313	0.582814969
LOC101908113	0.094789296	0.324040908	0.135417719	0.926303752	0.080486709	0.095220389	0.582814969
TRIM36	0.422976477	0.559082547	0.698306222	0.02107784	0.089121768	0.095237591	0.582814969
CUL9	0.916176137	0.098512894	0.012212586	0.649718996	0.433994762	0.095344332	0.582971623
TTYH3	0.727485612	0.011402645	0.617982025	0.288832575	0.209781244	0.095309949	0.582971623
ADAM9	0.623223335	0.733441695	0.085721968	0.298916791	0.026621636	0.095521681	0.583838474
MRPL34	0.312001986	0.121835246	0.855169278	0.06980817	0.13749792	0.095559592	0.583852739
CRABP2	0.801521782	0.304935284	0.032860464	0.409275851	0.095002099	0.095605522	0.583915973
CD69	0.975551156	0.221486265	0.217979441	0.02189089	0.303472195	0.095713505	0.584358012
NLRC5	0.655083631	0.007311694	0.249279206	0.565632316	0.464299057	0.095832988	0.584869904
ACOD1	0.154073215	0.306626161	0.178350276	0.972424568	0.03851866	0.096190952	0.584990259
C11H9orf116	0.88926158	0.268574134	0.024731141	0.207205239	0.257562931	0.096125094	0.584990259
EGLN3	0.965878816	0.027895066	0.751662315	0.367397527	0.04242977	0.096209171	0.584990259
GTPBP3	0.647157579	0.076203852	0.023389792	0.40330652	0.676137493	0.096005261	0.584990259
LTBP2	0.043665305	0.294986097	0.580415645	0.725234888	0.058085699	0.096074392	0.584990259
PRCP	0.165173189	0.56975894	0.007373161	0.898492992	0.505403221	0.096101482	0.584990259
RUM1	0.615974278	0.264287086	0.059556617	0.103609558	0.313609	0.096091229	0.584990259
SLCO3A1	0.32995888	0.022523492	0.430190332	0.163852706	0.600867166	0.096044155	0.584990259
TMEM206	0.386547314	0.708364464	0.686265459	0.01149802	0.14602756	0.096174617	0.584990259
WDR89	0.921806292	0.49317984	0.137178843	0.048741716	0.103444795	0.095987135	0.584990259
ACBD4	0.23191326	0.042549596	0.291831681	0.347019698	0.31624271	0.096266523	0.585122186
NCALD	0.604701858	0.358234472	0.053844128	0.076987452	0.352247838	0.096316073	0.585206615
COLEC12	0.395971362	0.370087192	0.294665395	0.024855445	0.295280276	0.096423338	0.58520836
ECM2	0.002587542	0.70033938	0.397181829	0.576493047	0.7632153	0.09638152	0.58520836
LOC112447599	0.593798096	0.095133866	0.072495827	0.802980805	0.09636599	0.096418593	0.58520836
GNA14	0.058579979	0.560584132	0.205585457	0.129931859	0.362216452	0.096566065	0.585830721
SERPINH1	0.672827987	0.295241255	0.10327409	0.051972898	0.298523433	0.096663811	0.585830721
SYBU	0.232225159	0.954802915	0.395827771	0.006847212	0.52969107	0.096668673	0.585830721
ZNF827	0.06000685	0.115211357	0.189039762	0.730752478	0.333268483	0.096662013	0.585830721
NARF	0.634510818	0.231239382	0.702479617	0.003789703	0.816051196	0.096744639	0.586074666
C13H20orf27	0.059137278	0.19444203	0.928244583	0.045825141	0.654402747	0.096976904	0.586264635
CTC1	0.67243094	0.113678012	0.058981682	0.726044802	0.097772082	0.096971338	0.586264635
PHF7	0.151188052	0.92373716	0.011538543	0.357311801	0.554459554	0.09683166	0.586264635
PPDPF	0.941989298	0.326263184	0.004089461	0.357570302	0.712402853	0.096990341	0.586264635
SIMC1	0.573822399	0.317974143	0.364124247	0.194103814	0.024808177	0.096949223	0.586264635
SIRPB2	0.804314188	0.228001293	0.788392031	0.005744643	0.385412653	0.096980955	0.586264635
MSI2	0.549175683	0.423546516	0.730814063	0.016053366	0.117511161	0.097080184	0.586591643
TTLL11	0.614945606	0.015143106	0.123815066	0.613535501	0.454090219	0.097175914	0.586953963
AVIL	0.787355256	0.147920447	0.010086631	0.492399765	0.558469762	0.097493117	0.587014558
CCDC186	0.03508152	0.44572315	0.373778487	0.282478901	0.194751581	0.097229834	0.587014558

CCDC61	0.844683128	0.388285062	0.161692999	0.00870154	0.704553409	0.097853726	0.587014558
CDK16	0.38012237	0.296238049	0.054883975	0.054018411	0.970590689	0.097664671	0.587014558
CDT1	0.762394974	0.163383259	0.248490508	0.01530002	0.687961416	0.097971588	0.587014558
CLCN3	0.924298024	0.327977004	0.164740711	0.756685591	0.008632609	0.098044415	0.587014558
CMTM6	0.71313937	0.024539028	0.079926355	0.317416108	0.728436701	0.097555224	0.587014558
DMRT2	0.07062697	0.286160203	0.154088347	0.680781107	0.153381785	0.097864775	0.587014558
EFHD1	0.852870359	0.783220164	0.279961062	0.167777306	0.010390535	0.09800825	0.587014558
FAM120A	0.154874172	0.532551666	0.701357658	0.999083364	0.00562729	0.097871324	0.587014558
GPALPP1	0.007519125	0.218674558	0.426966629	0.520306727	0.883983767	0.097467328	0.587014558
GPR4	0.126294225	0.60507128	0.298181887	0.134912672	0.105366133	0.097643597	0.587014558
HOXB4	0.539247262	0.356194846	0.150843012	0.014914894	0.752387015	0.097855866	0.587014558
INSL3	0.267038599	0.334464088	0.920202616	0.512674645	0.00764488	0.097332432	0.587014558
KCNK1	0.190691614	0.999319046	0.469132487	0.172293436	0.021170439	0.098020497	0.587014558
LOC112445927	0.11878885	0.541735768	0.042558976	0.447212157	0.264618498	0.09767768	0.587014558
MYH7	0.892080403	0.882938532	0.267790138	0.021269025	0.072189329	0.097633826	0.587014558
MYNN	0.002049198	0.50277964	0.449032014	0.845133	0.833112536	0.097960425	0.587014558
P2RY10	0.904016025	0.117777292	0.054946778	0.400923365	0.13817145	0.09767398	0.587014558
P3H4	0.497080059	0.427027215	0.001614434	0.958581087	0.979722812	0.097282666	0.587014558
RSPRY1	0.792127563	0.577731761	0.202968814	0.030367673	0.115124417	0.097786766	0.587014558
SLC49A3	0.680740338	0.009234078	0.163717975	0.400045781	0.791685284	0.097994999	0.587014558
TRPC3	0.059396422	0.084786103	0.410933224	0.53687041	0.290490951	0.097440829	0.587014558
ZC3H6	0.028522919	0.900710354	0.062480389	0.944770573	0.212302979	0.09730476	0.587014558
CEBPZOS	0.307104319	0.254292997	0.662826592	0.155953025	0.040543384	0.098229185	0.58790633
CYREN	0.277280151	0.500079699	0.52418677	0.004692931	0.962254488	0.098391301	0.58866192
MPC2	0.551863934	0.296850253	0.570126531	0.053755696	0.065467845	0.098471529	0.588916428
STPG4	0.027829904	0.098411775	0.270225649	0.693371272	0.640919073	0.098505612	0.588916428
EPB41L3	0.840734546	0.206444949	0.037578385	0.067708437	0.74867474	0.098803985	0.589107008
LOC101906779	0.923542269	0.004712557	0.429838321	0.519316356	0.339699588	0.09870042	0.589107008
LOC101907405	0.998392136	0.217394283	0.238711931	0.057190412	0.111586251	0.098806612	0.589107008
MCM8	0.005917229	0.662235075	0.700395403	0.537185832	0.224022383	0.098745446	0.589107008
NAA50	0.561989246	0.074234943	0.099987171	0.506359387	0.156578208	0.098821473	0.589107008
SFRS18	0.723019097	0.677466152	0.071110137	0.983958859	0.009650491	0.098824666	0.589107008
SOCS6	0.885475859	0.063119262	0.021240465	0.743037543	0.374875057	0.098812195	0.589107008
ZCCHC9	0.908440603	0.7243154	0.381309018	0.313803962	0.004188539	0.098658164	0.589107008
PGK1	0.570792538	0.34440058	0.309453012	0.133848225	0.040651772	0.098868358	0.589153456
BEND5	0.290493957	0.558539951	0.14433518	0.302597843	0.046787753	0.09896433	0.589297396
LOC104975007	0.071334602	0.606429765	0.149432222	0.655527157	0.078195864	0.098929987	0.589297396
ADGRF2	0.030384522	0.776020393	0.054898345	0.314219981	0.816258985	0.099041026	0.589326425
QPRT	0.218331524	0.777376298	0.595086653	0.064893386	0.050638233	0.099022713	0.589326425
LOC112448737	0.607559252	0.344151176	0.183823851	0.186032329	0.046481877	0.099102061	0.589475869

ARRDC3	0.807611199	0.123934318	0.80072787	0.116517065	0.03575393	0.099362213	0.589561502
BAZ2B	0.291899565	0.180538659	0.037141145	0.534456946	0.318952734	0.099323531	0.589561502
FOXP3	0.055751714	0.471436115	0.643954811	0.045569978	0.432947621	0.099369901	0.589561502
GADD45A	0.411228721	0.217292446	0.026962335	0.518837385	0.2672885	0.099402322	0.589561502
HYOU1	0.444988943	0.840747351	0.007600241	0.366013975	0.320600001	0.099324224	0.589561502
LAIR1	0.262727664	0.357390888	0.044384859	0.418328529	0.191837165	0.099459863	0.589561502
LOC112441603	0.731884206	0.891182403	0.014729097	0.074194637	0.469126266	0.099448413	0.589561502
NELFB	0.473151941	0.256621451	0.110458211	0.097692138	0.255330971	0.099475705	0.589561502
PRR33	0.813451918	0.074727051	0.319272694	0.145813079	0.117629452	0.099190303	0.589561502
TAB3	0.81040662	0.056837785	0.082194426	0.685627864	0.128320237	0.099227346	0.589561502
UBAP2	0.260652664	0.98122499	0.051276889	0.07658237	0.334069699	0.09964218	0.590164388
USP15	0.219342211	0.068913181	0.210829141	0.313998771	0.33534329	0.099649352	0.590164388
TEAD2	0.108117681	0.214960823	0.335722852	0.470789679	0.091543151	0.099770322	0.590667658
CCR5	0.071753806	0.261790605	0.83854935	0.884355311	0.024219223	0.099958543	0.590929266
LOC112442053	0.757009642	0.096199841	0.027415475	0.582954991	0.289610231	0.099906445	0.590929266
MYB	0.169878123	0.777677381	0.048467784	0.063172507	0.833798975	0.099940687	0.590929266
TMOD4	0.748800187	0.220417238	0.105067351	0.742927424	0.026167937	0.099916316	0.590929266
ASGR1	0.239343191	0.969359126	0.264518528	0.031463721	0.175156464	0.100101876	0.591120219
FABP4	0.08822477	0.624947677	0.090635348	0.17592049	0.38468089	0.100095351	0.591120219
GIMAP7	0.059558459	0.260941305	0.683043168	0.277618952	0.114837366	0.10013744	0.591120219
NPC2	0.759918535	0.020552798	0.02991735	0.931654784	0.776940676	0.100102652	0.591120219
PTRH2	0.215100656	0.040144517	0.381515511	0.559221897	0.183807492	0.100171975	0.591120219
XBP1	0.724652453	0.223490228	0.089396454	0.065722278	0.356098748	0.100206962	0.591120219
HRAS	0.41594717	0.146708179	0.30699136	0.054988145	0.329817348	0.10036202	0.591609593
PDXK	0.264140766	0.994120819	0.083944039	0.095252628	0.1617565	0.100341054	0.591609593
TIMP1	0.900099625	0.217921225	0.090313759	0.033515399	0.572606372	0.100399621	0.591618738
EPB41L5	0.588761542	0.406268216	0.315355178	0.228158823	0.01977523	0.100461542	0.59161948
PHPT1	0.321128251	0.29155871	0.666573479	0.021349003	0.255480936	0.100471848	0.59161948
ZNF75D	0.92568593	0.005908152	0.242664533	0.302314108	0.848963366	0.100509076	0.591626414
WFIKKN2	0.688362239	0.083448863	0.224089482	0.109846676	0.241259503	0.100596956	0.591931392
C7H5orf63	0.780535037	0.165084556	0.98646969	0.361418899	0.00743692	0.100684233	0.592146381
INCENP	0.572102439	0.020780745	0.786563128	0.291197518	0.12551378	0.100705658	0.592146381
ARIH1	0.478641761	0.440289729	0.363334573	0.006067034	0.73710395	0.10081395	0.592358659
PAIP1	0.724628421	0.194756591	0.304384266	0.295193661	0.02699925	0.100804281	0.592358659
ERP29	0.589915207	0.161797096	0.451217047	0.876378575	0.009098984	0.10098314	0.593140412
MMP23	0.190205246	0.124435083	0.054165329	0.302188547	0.888920476	0.101142931	0.593686201
TXN2	0.788802519	0.218981021	0.761248368	0.02044762	0.128092817	0.101148414	0.593686201
CD53	0.248038588	0.373493335	0.016803178	0.511020444	0.433577907	0.101232631	0.593755793
ZNF711	0.436154822	0.084579219	0.134277548	0.270273758	0.257516798	0.101208339	0.593755793
UBR5	0.442668786	0.926551067	0.470404452	0.004776461	0.375433095	0.101414495	0.59460996

BBS12	0.023326703	0.223667133	0.092962827	0.882799843	0.813988917	0.101841956	0.59477197
DNAJB6	0.03436587	0.09075399	0.137217255	0.938257642	0.870144299	0.101985761	0.59477197
HSPB7	0.004848216	0.385584952	0.486894951	0.961698966	0.399047366	0.101970391	0.59477197
LOC107132308	0.982489603	0.215419246	0.029918537	0.162056679	0.339286188	0.10178021	0.59477197
LOC112449324	0.760367938	0.470474413	0.933423532	0.003027804	0.345360147	0.101948554	0.59477197
LOC515697	0.199158905	0.686638109	0.913930133	0.004235404	0.656069522	0.101632496	0.59477197
LOC781663	0.079202606	0.145922625	0.123254626	0.261580748	0.935962625	0.101880125	0.59477197
LOC784007	0.022427812	0.300778382	0.069086436	0.938241657	0.79574641	0.101743925	0.59477197
PDE12	0.927784615	0.319135682	0.788161432	0.498379127	0.002991597	0.101741896	0.59477197
POLR2I	0.225184207	0.008909578	0.59123359	0.339579955	0.864028576	0.101758507	0.59477197
RIPK2	0.681746321	0.255746666	0.428157416	0.252890584	0.018374073	0.101563981	0.59477197
SHOC2	0.444026967	0.345644837	0.228220923	0.026528299	0.37296513	0.101510128	0.59477197
TPI1	0.340314725	0.305278461	0.662240527	0.130621927	0.038854946	0.10195077	0.59477197
TUFM	0.531490279	0.338491916	0.755739661	0.037839833	0.06743926	0.101577856	0.59477197
YPEL3	0.964497052	0.326204062	0.29567806	0.662398193	0.005664056	0.101924008	0.59477197
MDH1	0.648307893	0.114468361	0.618498045	0.057023971	0.133677434	0.102067245	0.595035719
CGGBP1	0.540256628	0.909831087	0.547227946	0.984248504	0.001327941	0.102349238	0.595636193
FNIP1	0.226262518	0.230116976	0.132563574	0.075035498	0.678993061	0.102363375	0.595636193
ISCA1	0.744830519	0.136924057	0.16040305	0.178401904	0.120324267	0.102280398	0.595636193
LOC100848895	0.700183257	0.156928985	0.286023211	0.706604356	0.015814168	0.10228529	0.595636193
NLN	0.252221845	0.615877583	0.660790355	0.287620478	0.011916248	0.102388014	0.595636193
PDCD1	0.364786398	0.113344942	0.222223961	0.070738866	0.54110944	0.102371106	0.595636193
C22H3orf67	0.181382957	0.119280234	0.067808631	0.343613381	0.700350124	0.10259542	0.596038946
ELMO2	0.090559485	0.878602869	0.705508765	0.031439439	0.200070926	0.102602524	0.596038946
ITGA8	0.002603844	0.496791464	0.857798301	0.925014818	0.343663007	0.102544109	0.596038946
LOC101904357	0.664996348	0.174143205	0.398316664	0.012700909	0.602331789	0.102566977	0.596038946
CDR2	0.373751412	0.046404057	0.942936723	0.051606606	0.419313264	0.102734791	0.59624087
LOC107132531	0.467022971	0.113297036	0.161691845	0.302043946	0.137057667	0.102782611	0.59624087
LOC112444498	0.092757209	0.53489278	0.10858134	0.306081532	0.21467314	0.10275093	0.59624087
NCAPH2	0.364404061	0.704866281	0.008263354	0.398325661	0.418678622	0.102748349	0.59624087
NSD2	0.522888894	0.007290107	0.442145431	0.452547989	0.465126951	0.102881016	0.596600833
CYP20A1	0.018237771	0.843679451	0.068324757	0.37149761	0.90967723	0.102965911	0.596882218
ZMPSTE24	0.339852785	0.035035697	0.718260729	0.948787843	0.043829063	0.103026202	0.597020831
LOC112444896	0.084451099	0.040084941	0.400425965	0.930373918	0.282557308	0.103142687	0.59748487
C5AR1	0.245911716	0.342300582	0.685478584	0.016216404	0.381600164	0.103261074	0.597959591
ANAPC10	0.746703024	0.020284883	0.292967496	0.095026488	0.847517391	0.103314025	0.598055191
HIP1R	0.893326471	0.831278743	0.001511769	0.327876985	0.974928487	0.10355818	0.599257155
CORO1C	0.92962764	0.042882685	0.026710752	0.376722851	0.895760633	0.103635249	0.599452143
RABL6	0.058366524	0.788324155	0.412445383	0.094985766	0.199441235	0.103664931	0.599452143
MRPL18	0.164065543	0.179700113	0.648821594	0.154346744	0.121842394	0.103702915	0.59946056

CORO2B	0.029678897	0.730901648	0.06260624	0.404517336	0.655704821	0.103782506	0.599498314
DCTN4	0.026967642	0.333667045	0.260445133	0.28560433	0.538045332	0.103766983	0.599498314
CAMK2G	0.192798052	0.447668314	0.072373996	0.225542861	0.256357711	0.103939295	0.599515853
GPC1	0.034289774	0.863443943	0.198543708	0.109873463	0.558284266	0.103841697	0.599515853
LOC529196	0.581775957	0.01685746	0.582628162	0.140151109	0.451224222	0.103968199	0.599515853
PKNOX2	0.298263389	0.993990598	0.017060356	0.146102877	0.488569398	0.103917186	0.599515853
SLX4	0.22809686	0.042879959	0.3278129	0.233809211	0.481852116	0.103947239	0.599515853
FASTKD2	0.520119091	0.567761371	0.736994509	0.034931641	0.047649143	0.104115844	0.600156343
MZT1	0.524542547	0.021797748	0.412783791	0.638110973	0.12043437	0.104191837	0.600383512
FRMD4B	0.323811829	0.129574776	0.069451582	0.503844274	0.247305088	0.104255318	0.600538441
AIFM1	0.587299544	0.35885945	0.969507637	0.013534866	0.131564025	0.104379025	0.600618577
LOC788425	0.355557744	0.083710406	0.897810452	0.141655061	0.096033689	0.10432462	0.600618577
RIC1	0.688742927	0.712632838	0.443772959	0.002102704	0.794352302	0.104371973	0.600618577
SCRN2	0.017385834	0.131437222	0.621922754	0.618228199	0.414774497	0.104473061	0.600948968
CAGE1	0.035189894	0.394033765	0.210033769	0.289257644	0.434205255	0.104694329	0.602010735
ADHFE1	0.094830704	0.617034968	0.744699473	0.051611875	0.163229707	0.104910343	0.602116548
AMOTL1	0.49499867	0.680665318	0.554851949	0.014635524	0.134082041	0.10486968	0.602116548
CASK	0.166046549	0.119313125	0.218948063	0.103966267	0.815656585	0.105030397	0.602116548
CDK8	0.312250445	0.750987948	0.03860895	0.047195715	0.865610621	0.105360708	0.602116548
DMD	0.339068889	0.409398836	0.252357121	0.403027544	0.026215683	0.105401793	0.602116548
GSTM3	0.867769746	0.303692818	0.349655789	0.087584698	0.045821535	0.105350808	0.602116548
HSPB6	0.518223472	0.210384468	0.078082982	0.326363315	0.132384229	0.105025671	0.602116548
KLHL38	0.195922617	0.755438861	0.905769098	0.023659959	0.116608249	0.105360083	0.602116548
KRT19	0.333989107	0.058965403	0.384226705	0.111847403	0.434659776	0.10503502	0.602116548
LOC100296324	0.218897408	0.367923516	0.741492716	0.019157557	0.32350104	0.10539831	0.602116548
LOC101904923	0.477941454	0.553411093	0.116331768	0.01967057	0.609874088	0.105240216	0.602116548
LRRC45	0.05974532	0.106290535	0.080679251	0.945319214	0.759694544	0.105046801	0.602116548
OSR2	0.862402929	0.451875689	0.009754538	0.657537518	0.148034739	0.105384364	0.602116548
PHTF1	0.655358043	0.151185585	0.011231593	0.378271021	0.87349298	0.105007476	0.602116548
PIMREG	0.218258134	0.073465282	0.150420046	0.21638636	0.702894935	0.104867606	0.602116548
PKP1	0.814171815	0.278824111	0.094207938	0.130148696	0.132995217	0.105410614	0.602116548
RAB10	0.950221263	0.080108167	0.051251908	0.877612572	0.107019668	0.104798634	0.602116548
SGSM1	0.569632168	0.41277456	0.092864698	0.133814452	0.126238998	0.105195518	0.602116548
TFB2M	0.544263362	0.134934493	0.258948874	0.516240635	0.0376694	0.105352105	0.602116548
WDR27	0.572848329	0.346526497	0.100185133	0.53893026	0.034558681	0.105446527	0.602116548
MED12	0.389775083	0.269449961	0.379113824	0.065506885	0.142224158	0.105536992	0.60221404
PLXNA3	0.328290198	0.129728339	0.045957047	0.663110186	0.285766983	0.105525976	0.60221404
SELENOW	0.226569702	0.846222664	0.2676559	0.046127826	0.156990622	0.105644839	0.602410509
ZWILCH	0.994850906	0.573287098	0.670748464	0.075436102	0.01287483	0.105632437	0.602410509
PLAUR	0.80629512	0.33533065	0.187383349	0.031325722	0.234297066	0.105681769	0.602411778

FICD	0.097162586	0.044605175	0.192206866	0.985582901	0.453889351	0.105810893	0.602519971
HIF3A	0.763186367	0.720067921	0.005481504	0.291833755	0.423820684	0.105800488	0.602519971
LOC515578	0.189719784	0.052256294	0.121544771	0.43964822	0.702697189	0.105750444	0.602519971
GARS	0.41597971	0.206204554	0.323028305	0.855188268	0.015750577	0.105904377	0.602552567
GVQW3	0.579158747	0.453429308	0.125150193	0.270905732	0.041934677	0.105926766	0.602552567
LOC101906006	0.839365896	0.733766701	0.573316646	0.00438362	0.240950109	0.105861946	0.602552567
CENPC	0.448976926	0.327110062	0.958059493	0.005838786	0.454834613	0.105976301	0.60262546
FKBP2	0.420197451	0.009604284	0.514240434	0.391824424	0.461333066	0.106213565	0.603556376
HSPA1L	0.305370896	0.993395179	0.704276676	0.015692982	0.111885716	0.106211111	0.603556376
CPNE5	0.30232012	0.781093374	0.099763452	0.221520462	0.071930839	0.10625255	0.603568914
PRKCI	0.377593997	0.969146248	0.553885808	0.002256241	0.822664674	0.106388179	0.604130244
TNRC18	0.967319203	0.227112648	0.059377523	0.419233132	0.068876051	0.106459949	0.604328685
RHEB	0.559839852	0.021882011	0.948288648	0.144780479	0.224187131	0.10652353	0.604378823
STXBP5	0.053546165	0.573191531	0.826844083	0.01591191	0.934057339	0.106542437	0.604378823
LOC112443144	0.840450152	0.380882083	0.504484034	0.213702045	0.01093865	0.106594808	0.604466965
BHLHA15	0.506983593	0.114126997	0.026107054	0.937833362	0.267099162	0.106736912	0.6047985
CHST8	0.015177199	0.583132937	0.97382439	0.52858214	0.083190355	0.106833084	0.6047985
LOC112442713	0.005616782	0.358047569	0.535246788	0.414555035	0.850698295	0.106933237	0.6047985
PITX3	0.064983763	0.763518593	0.290738882	0.059962203	0.43857976	0.106893105	0.6047985
SEPHS2	0.821718326	0.477596063	0.34229078	0.017698545	0.159243987	0.106770666	0.6047985
SLC6A6	0.141605255	0.912431617	0.16933369	0.047547047	0.365005594	0.106948099	0.6047985
TRAF3IP1	0.971733193	0.398101309	0.965835184	0.178303045	0.005691219	0.106858638	0.6047985
TSPAN5	0.377565944	0.147600959	0.640687261	0.834604023	0.012707288	0.10678213	0.6047985
DBI	0.697163797	0.369027838	0.963935697	0.014193607	0.10802427	0.107033641	0.604821276
EIF4A2	0.048151267	0.755492392	0.779664989	0.320935823	0.041792577	0.10706269	0.604821276
SHMT1	0.572034245	0.310418932	0.047667166	0.27606346	0.162708271	0.107026905	0.604821276
C3H1orf226	0.142029486	0.555496647	0.17132	0.723185666	0.039180523	0.107473771	0.60493702
CEMIP2	0.977060766	0.096968937	0.041242201	0.101867227	0.957373324	0.107167782	0.60493702
CRYZL1	0.052153988	0.226312779	0.053218378	0.608191567	0.999298956	0.107277678	0.60493702
EDIL3	0.382819692	0.465787042	0.043760023	0.197441861	0.248982491	0.107569424	0.60493702
EMC4	0.996903188	0.30341641	0.240856844	0.357872483	0.014666527	0.107377877	0.60493702
FBXL14	0.02039094	0.64421055	0.477231853	0.614255545	0.099750627	0.107652854	0.60493702
GBE1	0.270777068	0.986802583	0.119323197	0.027603422	0.436324882	0.107636038	0.60493702
KDELR3	0.898875672	0.072567864	0.035853752	0.235348844	0.692789582	0.107207128	0.60493702
LOC112443175	0.292583452	0.928207693	0.021998936	0.993301348	0.064348021	0.107294373	0.60493702
LOC508666	0.529753454	0.247539228	0.580723008	0.034374213	0.146699825	0.107637345	0.60493702
LOC787057	0.87212987	0.053536028	0.334337188	0.045219522	0.544332057	0.107672965	0.60493702
NBEA	0.175815445	0.205266734	0.263081931	0.085265363	0.473128765	0.107478072	0.60493702
ORAI2	0.213383203	0.329122409	0.606067798	0.761925395	0.011822653	0.107541113	0.60493702
TCF19	0.398484886	0.02555583	0.119390864	0.352116128	0.89510519	0.107508248	0.60493702

TRA2B	0.852404146	0.949964533	0.270013176	0.006045902	0.288412065	0.107195955	0.60493702
TREML2	0.384681623	0.086617698	0.323944134	0.69128132	0.051249605	0.107380254	0.60493702
LOC100848263	0.998320772	0.11435089	0.038241068	0.594876276	0.148116807	0.107739085	0.605101343
GGPS1	0.075348251	0.057435972	0.277121337	0.580441264	0.554100339	0.10790894	0.605814902
STEAP4	0.82536104	0.641530719	0.006294177	0.288794227	0.401079454	0.107957756	0.605814902
SUPV3L1	0.229675833	0.070219146	0.903832181	0.204991232	0.129229204	0.107976881	0.605814902
DNM2	0.124645211	0.297937794	0.634195446	0.182283062	0.09033201	0.10823974	0.606938036
LOC107132724	0.662023209	0.533377277	0.010151262	0.115698846	0.935266128	0.108251029	0.606938036
ARHGEF1	0.694541404	0.070607784	0.043379123	0.260135136	0.703133671	0.10844616	0.607136944
BREH1	0.225282386	0.276239177	0.030227192	0.764131448	0.272678823	0.108895498	0.607136944
CD27	0.779505518	0.057646198	0.060908836	0.545989799	0.262420248	0.108927144	0.607136944
CDC42EP2	0.334922268	0.29049716	0.014933172	0.344661407	0.780463355	0.10871812	0.607136944
CHRAC1	0.283542137	0.867394741	0.098163156	0.37632483	0.043335184	0.109174403	0.607136944
DOC2A	0.068674819	0.07504566	0.329141407	0.548879614	0.419538064	0.108685553	0.607136944
F3	0.331102369	0.549685522	0.061331822	0.326267943	0.108060155	0.109148151	0.607136944
GTF2IRD1	0.228865175	0.285302836	0.209601556	0.141762255	0.200570746	0.108451851	0.607136944
GZMK	0.370958888	0.361071377	0.92760142	0.060876725	0.051893919	0.108983552	0.607136944
HOXB3	0.990416968	0.054933862	0.055862912	0.279003378	0.460387788	0.108651575	0.607136944
KDELR2	0.74359195	0.027198815	0.172869727	0.543790558	0.20522285	0.108615477	0.607136944
LCN1	0.585933964	0.929584678	0.002365018	0.429058523	0.703463357	0.108398166	0.607136944
LOC100141070	0.54556352	0.452600767	0.446874446	0.121051826	0.029432382	0.109082601	0.607136944
LOC101902854	0.272477576	0.096183064	0.877342462	0.022705061	0.751388543	0.108946194	0.607136944
LOC101904601	0.401495304	0.440337417	0.089093435	0.030065099	0.83050673	0.109107513	0.607136944
LOC112447844	0.473038798	0.281190278	0.671991105	0.382210618	0.011482051	0.108945773	0.607136944
LOC112448354	0.121324879	0.331011155	0.056353799	0.761726582	0.228206956	0.109125503	0.607136944
LOC616944	0.876995715	0.791810128	0.001169576	0.824387667	0.58669137	0.10903202	0.607136944
LRRC55	0.991226802	0.144616255	0.516926318	0.540303624	0.009809103	0.109017461	0.607136944
LYPLA1	0.548944644	0.184893316	0.434082928	0.405501844	0.02180944	0.108530099	0.607136944
MYH15	0.375659541	0.04032383	0.206112621	0.396192109	0.317244147	0.108971046	0.607136944
NDUFB7	0.423800321	0.159346303	0.778859332	0.011493976	0.644949747	0.108572505	0.607136944
TK2	0.141031684	0.777814527	0.131288676	0.031785533	0.855005387	0.108808468	0.607136944
UBR7	0.81410749	0.007345008	0.151654889	0.465054352	0.925169222	0.108614676	0.607136944
PSMA1	0.948714629	0.228686239	0.478045567	0.417090245	0.00910815	0.109219892	0.607184163
LOC101903831	0.152366301	0.097030957	0.103836199	0.970523432	0.265040702	0.109357219	0.607741728
ARHGEF2	0.748651241	0.585819916	0.407223363	0.007933145	0.279267201	0.109482333	0.607819541
LOC100139891	0.797094758	0.010080969	0.794915666	0.128924604	0.480392671	0.109471432	0.607819541
NUMB	0.97126032	0.819881495	0.56933805	0.009937713	0.087758223	0.109437927	0.607819541
ISCA2	0.528463431	0.006570963	0.232256074	0.607043966	0.809043044	0.109548312	0.607980163
SPNS2	0.385326605	0.49774112	0.06484098	0.072081094	0.442343346	0.109614188	0.60814011
CDK5RAP2	0.400747504	0.076065367	0.637185515	0.099095754	0.206175693	0.109664782	0.608215186

PARP4	0.570079111	0.932090791	0.015776536	0.489303453	0.096826379	0.109716475	0.608254512
SLC22A18	0.074831662	0.116149146	0.630432605	0.141328205	0.51311422	0.109746	0.608254512
ADAMTS6	0.767601385	0.07300813	0.025800443	0.785536354	0.353031326	0.110310695	0.608507167
CAPZA1	0.779785009	0.084478937	0.634804812	0.681251063	0.01404492	0.110177774	0.608507167
CNIH1	0.506251496	0.114427583	0.528903431	0.834811662	0.015630543	0.11012687	0.608507167
COX11	0.22279234	0.438997084	0.404661537	0.237335363	0.042421991	0.109921626	0.608507167
CTNS	0.236994566	0.64469091	0.106486885	0.321339665	0.076274241	0.109967707	0.608507167
FAM186B	0.047234163	0.180232858	0.299223259	0.641979096	0.245150667	0.110300019	0.608507167
FBXW11	0.75603684	0.171558213	0.049148033	0.080139557	0.778912176	0.109833911	0.608507167
HUNK	0.058226916	0.177872561	0.046190817	0.85968427	0.971773747	0.110106243	0.608507167
IDO1	0.354985986	0.276150508	0.304417113	0.020122815	0.667554996	0.110294603	0.608507167
KCND1	0.791061449	0.616099809	0.002285102	0.590832936	0.607792494	0.110148573	0.608507167
LY6E	0.452563912	0.516006979	0.496929805	0.025004761	0.137979756	0.110217856	0.608507167
MTG1	0.532934691	0.187032709	0.077575542	0.135129554	0.381531553	0.109948882	0.608507167
TFG	0.252781588	0.05368343	0.575299905	0.912885459	0.055999107	0.110017845	0.608507167
TREX1	0.415277542	0.243792055	0.150020566	0.035589414	0.740348865	0.110188713	0.608507167
FGF11	0.002423755	0.516842616	0.850794106	0.766973165	0.492554228	0.110568973	0.609328549
KIF22	0.314001942	0.075216933	0.147211775	0.165418579	0.699466283	0.110516453	0.609328549
OFD1	0.266720349	0.199193995	0.043310761	0.177087497	0.988105975	0.110570984	0.609328549
PANK3	0.12525158	0.314709627	0.42727418	0.342311279	0.069976938	0.1106946	0.609804996
ORM1	0.494191662	0.386462091	0.05894565	0.193719277	0.185122554	0.110739406	0.609847113
LOC101907483	0.368974069	0.14688171	0.573549952	0.301124624	0.043161343	0.110781277	0.60987304
LOC782348	0.941605846	0.0375214	0.94201352	0.604354072	0.020134354	0.110934323	0.610510788
SLC26A7	0.781212439	0.541432962	0.165630294	0.485440523	0.011918366	0.110988139	0.610602196
C15H11orf58	0.842457916	0.031304405	0.305352051	0.811597004	0.06227467	0.111248943	0.610604147
CARD6	0.799178702	0.096082819	0.443357853	0.194642259	0.061288287	0.111111563	0.610604147
CPXM1	0.040422972	0.369576304	0.195577124	0.216472492	0.643406017	0.111239061	0.610604147
GTF2E1	0.127506303	0.024738466	0.424629493	0.612450641	0.495025158	0.111104938	0.610604147
HSD11B1	0.438959713	0.565223873	0.037575257	0.161169891	0.270231653	0.11109832	0.610604147
TMBIM4	0.164077464	0.041867711	0.112297974	0.802635028	0.656825271	0.111199881	0.610604147
VAMP5	0.466018881	0.587542879	0.003231348	0.668448393	0.687946903	0.111226205	0.610604147
SET	0.604987007	0.331034353	0.167441871	0.924198697	0.013157844	0.111368925	0.610977157
ZBTB8OS	0.079328083	0.622447812	0.768385553	0.011965772	0.898553229	0.111392206	0.610977157
ZNF775	0.284037235	0.726567943	0.143140359	0.216376415	0.063858785	0.111428592	0.610977157
ACOT9	0.399526543	0.166830717	0.108459324	0.060222725	0.940904469	0.111653949	0.611395717
FMO3	0.648571718	0.209018705	0.078348692	0.317359122	0.121434717	0.111606569	0.611395717
LOC112447626	0.002939097	0.296202755	0.952652921	0.573891803	0.860425718	0.111637468	0.611395717
PDSS1	0.491297306	0.120582274	0.391873652	0.649349015	0.027132194	0.111558331	0.611395717
AZIN1	0.211574136	0.02731855	0.192717399	0.618867915	0.600809407	0.112351457	0.611702328
BMP5	0.844639723	0.998165322	0.500025461	0.001540622	0.633291165	0.111911886	0.611702328

CYP4B1	0.899696615	0.337539661	0.019568201	0.078144292	0.89693679	0.112711056	0.611702328
DLX5	0.003960233	0.397155939	0.778849432	0.579001947	0.586674809	0.112649743	0.611702328
EWSR1	0.77044413	0.472656906	0.003905278	0.693110857	0.422439032	0.112692721	0.611702328
FAM193B	0.442963171	0.175099014	0.276362056	0.412786369	0.046970714	0.112571987	0.611702328
FAM46B	0.019647759	0.628176108	0.521678385	0.081135402	0.799236151	0.112865435	0.611702328
FRRS1	0.575468317	0.123171047	0.099161352	0.277978258	0.212163527	0.11240679	0.611702328
GDF11	0.555724374	0.522280717	0.014212671	0.4943178	0.203585566	0.112500232	0.611702328
GLI4	0.83301861	0.815815037	0.0595956	0.012696658	0.799442819	0.111878625	0.611702328
GRAMD1C	0.856262343	0.766874147	0.004330289	0.216856017	0.66642995	0.111854781	0.611702328
GRID2	0.027985805	0.762341656	0.299991945	0.360000431	0.180140342	0.112488325	0.611702328
IL9R	0.29126958	0.292352809	0.060223497	0.11695676	0.695808327	0.112836146	0.611702328
KCNK17	0.284454456	0.520701602	0.738918995	0.005369657	0.706492352	0.112509035	0.611702328
LOC101903038	0.52435705	0.812910907	0.054832705	0.027526974	0.648709344	0.112841289	0.611702328
LOC101906511	0.312082987	0.288763951	0.723078719	0.022761168	0.279857622	0.112491148	0.611702328
LOC112445078	0.840794066	0.323516031	0.112436359	0.068903773	0.197698134	0.112726736	0.611702328
LOC112449614	0.704359976	0.488222253	0.139849385	0.951957754	0.009064541	0.112477377	0.611702328
MAGED1	0.881408868	0.496810018	0.00491141	0.353089475	0.547445957	0.112589243	0.611702328
MED13	0.308129245	0.678340707	0.737205531	0.002844974	0.945170869	0.112378102	0.611702328
MED29	0.903115132	0.124523228	0.753075247	0.005794133	0.844751397	0.112406087	0.611702328
MLF1	0.31225072	0.517652074	0.786519078	0.019436839	0.168158995	0.112559413	0.611702328
MRTO4	0.311149678	0.142879844	0.053372679	0.976329964	0.178745823	0.112339171	0.611702328
PET100	0.054152116	0.127429588	0.417841173	0.17810082	0.809053192	0.112551139	0.611702328
PPP1R12B	0.120357688	0.328128292	0.297624038	0.194268681	0.181808241	0.112501623	0.611702328
PRKAA2	0.871299488	0.02651845	0.760743853	0.267866696	0.087866645	0.112281408	0.611702328
SASH3	0.478479723	0.084876481	0.586507454	0.100110112	0.175015058	0.112835335	0.611702328
SERPINF1	0.767158155	0.218166426	0.09224078	0.158363442	0.168183698	0.111892793	0.611702328
TRDMT1	0.711254293	0.314055158	0.07896489	0.739493908	0.031821669	0.112489988	0.611702328
UQCRB	0.733586951	0.159862973	0.599335799	0.034694419	0.169627844	0.112271124	0.611702328
USP35	0.745396255	0.190854773	0.010503032	0.898763348	0.307311906	0.112125488	0.611702328
GDF9	0.053488847	0.788407095	0.166268892	0.341021184	0.175004304	0.113008256	0.612274177
FAM96A	0.098029379	0.394522219	0.591372768	0.752615228	0.024422848	0.113303114	0.61366911
ACSL1	0.550811847	0.136499296	0.831084973	0.192862297	0.034910761	0.113351364	0.61372789
EXOSC5	0.330815008	0.995401415	0.263994551	0.127769598	0.037945761	0.113466888	0.614099942
NDUFB11	0.507454315	0.206174444	0.472462746	0.039819505	0.214367233	0.113538365	0.614099942
SEPT6	0.024849952	0.786101624	0.518212881	0.385094206	0.108175829	0.113502363	0.614099942
TRIM31	0.017003403	0.35328316	0.972850738	0.350453794	0.206124933	0.11356976	0.614099942
AFF1	0.741881209	0.55124496	0.215902334	0.03696623	0.13030609	0.114048951	0.614103675
CENPO	0.801430994	0.005909482	0.481625336	0.469163813	0.395975219	0.113813747	0.614103675
DEPDC1	0.588754025	0.020495445	0.131393879	0.527134018	0.508968255	0.114059605	0.614103675
DPM2	0.216322461	0.103978118	0.624660179	0.069391065	0.43653925	0.114094599	0.614103675

EDF1	0.211648893	0.359559118	0.557163884	0.109835369	0.091085786	0.113879535	0.614103675
FBLIM1	0.13714614	0.892630222	0.012863936	0.673304178	0.401246571	0.114070506	0.614103675
GRPEL1	0.186679479	0.108669544	0.102520857	0.823040467	0.248772029	0.114127976	0.614103675
LOC100847995	0.886675577	0.085468118	0.294053368	0.026679555	0.714366969	0.113958417	0.614103675
LOC104969050	0.568812058	0.35534214	0.123064605	0.55469772	0.030647746	0.113678656	0.614103675
LOC112448454	0.026749922	0.32492905	0.722017243	0.816762563	0.083178301	0.11420585	0.614103675
MAML2	0.150062951	0.549776839	0.487715954	0.021967351	0.480008298	0.113893035	0.614103675
MED16	0.50121749	0.44525538	0.465523231	0.130392838	0.031338779	0.113931407	0.614103675
NDUFS5	0.418850527	0.210102856	0.720438084	0.019692066	0.339671602	0.113861202	0.614103675
TIMM8A	0.737455372	0.164845167	0.351575689	0.418382205	0.023664027	0.113721374	0.614103675
TIMM9	0.85995247	0.157634163	0.824458688	0.343616511	0.011091006	0.114142693	0.614103675
TOP3B	0.59790848	0.073372092	0.095347287	0.364740972	0.279454437	0.114206594	0.614103675
ZAP70	0.828294789	0.593275758	0.023815693	0.639054517	0.056797828	0.113970176	0.614103675
BCO2	0.429789364	0.051782046	0.805255886	0.327366215	0.072755364	0.114280512	0.614175917
CADPS2	0.073191262	0.04516871	0.912649241	0.338155366	0.41845363	0.114294879	0.614175917
HMBS	0.099847497	0.11358854	0.879867342	0.144627138	0.296008249	0.114336162	0.614196646
ZNF572	0.056512333	0.154795204	0.135845136	0.709460006	0.507070548	0.114380512	0.61423383
CAVIN3	0.51100468	0.012251261	0.640234201	0.145583562	0.734261217	0.114524127	0.614803878
MPP2	0.074550524	0.837477272	0.065298106	0.712254219	0.147853624	0.114655623	0.615308512
C16H1orf115	0.848864289	0.302589856	0.058881359	0.03033907	0.938987728	0.114885238	0.615628207
HES6	0.068972891	0.713094971	0.313210009	0.139897033	0.200011032	0.114913759	0.615628207
OVCA2	0.599509511	0.751967484	0.119096734	0.231387653	0.034661645	0.114848223	0.615628207
RIPK1	0.542871383	0.721778139	0.603557578	0.001927187	0.946146746	0.114940273	0.615628207
SCN1B	0.32803027	0.063418254	0.24218953	0.403354004	0.211856118	0.114837283	0.615628207
SMPDL3B	0.358629313	0.228210758	0.745843516	0.127454317	0.055287323	0.114777495	0.615628207
TUBA1B	0.230403989	0.092113645	0.197708176	0.54386065	0.18917267	0.115012333	0.615813179
XPO1	0.368164903	0.920959949	0.059016564	0.58103913	0.037153348	0.115053495	0.61583265
LOC112442226	0.356622304	0.32054448	0.229066096	0.062808946	0.263029712	0.115146617	0.616130136
COX7B	0.722984165	0.292146718	0.823220131	0.017134548	0.145614666	0.115331937	0.616405661
DENND6B	0.800808215	0.098295282	0.029822405	0.642452907	0.287476068	0.115290547	0.616405661
FGG	0.288750337	0.047257948	0.199742636	0.948591307	0.167657382	0.115279049	0.616405661
VCPIP1	0.803551431	0.15215308	0.004993386	0.875708456	0.81167878	0.115348351	0.616405661
SLC25A25	0.137912713	0.046653424	0.271289071	0.718139121	0.347014999	0.115504594	0.61703968
LOC112449523	0.49896303	0.125386887	0.017447348	0.895431833	0.445607326	0.115588176	0.617050458
SEC23B	0.474068957	0.006899662	0.508798556	0.690809243	0.379026699	0.115619411	0.617050458
UCKL1	0.026554883	0.223050395	0.109448173	0.683806336	0.982893685	0.115612282	0.617050458
HINT1	0.56439517	0.180509653	0.614217819	0.121638855	0.057312662	0.115692278	0.617143098
MFSD6L	0.514866564	0.642142284	0.009384319	0.159232611	0.883282526	0.115711981	0.617143098
STEAP3	0.311617732	0.916759921	0.004652088	0.815125911	0.403394706	0.115805054	0.617238305
ZNF277	0.096811036	0.651511886	0.06403265	0.352847117	0.306611844	0.115796591	0.617238305

NLRP3	0.589073549	0.23178467	0.744711706	0.034094071	0.126221351	0.115891184	0.617496827
FBXW12	0.660586864	0.021657531	0.453553627	0.623280263	0.1082678	0.115935673	0.617533376
LOC101905779	0.87985961	0.824844328	0.028495236	0.167289277	0.126685923	0.115996379	0.617585582
NEDD4	0.333935639	0.845148495	0.570400449	0.003205746	0.849593763	0.116020739	0.617585582
LOC100140403	0.270685533	0.394977928	0.298410084	0.091284108	0.150702302	0.116088469	0.61774574
KIF21B	0.610890439	0.206835049	0.295865134	0.256816167	0.045803459	0.116214215	0.618214419
STK17B	0.690741672	0.085298632	0.392353499	0.43890278	0.043399991	0.116303288	0.618487769
CENPJ	0.191081535	0.244816745	0.091105497	0.567637163	0.182858997	0.116604734	0.619288121
NUDT22	0.052487982	0.071977573	0.681725558	0.415895196	0.412423789	0.116514561	0.619288121
STOM	0.879896941	0.197345554	0.250854497	0.234363178	0.043303082	0.116559083	0.619288121
TSPAN9	0.269604287	0.33294431	0.076486211	0.067509336	0.953944495	0.116571048	0.619288121
TBC1D15	0.62696388	0.563424768	0.059072419	0.287505464	0.073808345	0.116668659	0.619427164
B3GNTL1	0.85309124	0.120589136	0.536038262	0.019287438	0.418535385	0.117015297	0.619863796
CHCHD2	0.594746943	0.216016129	0.488973648	0.078509928	0.090229952	0.116996058	0.619863796
DRAP1	0.347265891	0.111042223	0.189304083	0.375633412	0.162167132	0.116944295	0.619863796
MRPL4	0.103475333	0.406347656	0.496589396	0.07373394	0.288329765	0.116831048	0.619863796
NDUFA2	0.813794112	0.245257551	0.905929402	0.013140751	0.187242466	0.11697738	0.619863796
RPRD2	0.074029712	0.350759474	0.222637372	0.08358158	0.919730206	0.116906038	0.619863796
TH	0.797679783	0.189572959	0.068299463	0.386389271	0.111468622	0.116968921	0.619863796
P2RY6	0.822227115	0.151723533	0.411412874	0.215621284	0.040286102	0.117115806	0.620196031
ZMYND11	0.47445835	0.326183378	0.123110336	0.497953104	0.047035989	0.117177008	0.620319961
ADPRH	0.189149147	0.169940114	0.150094818	0.354618605	0.261818342	0.11742813	0.620343825
ALS2CL	0.685584934	0.538860827	0.036409775	0.490198363	0.068276385	0.117758312	0.620343825
BET1L	0.207477494	0.122220952	0.941519935	0.421120811	0.04459531	0.117491117	0.620343825
CDS1	0.17455601	0.344689092	0.03152921	0.39829327	0.595382392	0.117708999	0.620343825
COMMD5	0.3840152	0.249166027	0.034264873	0.329295733	0.415447865	0.117513452	0.620343825
DBP	0.453337516	0.545220447	0.110001834	0.088651616	0.185368038	0.117259237	0.620343825
GADD45G	0.251017109	0.339067922	0.090359771	0.133270222	0.437245037	0.117457848	0.620343825
GIGYF1	0.707870448	0.285731825	0.222496501	0.969517367	0.010255238	0.117353609	0.620343825
GRAMD1B	0.938692075	0.565804735	0.03956055	0.782360981	0.027413212	0.117822429	0.620343825
KCNK3	0.061662012	0.196606057	0.868589794	0.087548025	0.485858506	0.117421733	0.620343825
LOC100140121	0.46626128	0.010603876	0.946887429	0.5641091	0.170637646	0.117824124	0.620343825
LOC515736	0.016401155	0.659429607	0.184508639	0.951202334	0.236064356	0.117448691	0.620343825
NDUFA11	0.341027147	0.362576494	0.830652105	0.011814385	0.370348614	0.117641305	0.620343825
NFE2	0.071361643	0.074106207	0.524439734	0.524439934	0.308985172	0.117644164	0.620343825
S100A10	0.455422729	0.426230945	0.547636297	0.017871199	0.236872355	0.117731163	0.620343825
WDR12	0.90927986	0.079891166	0.112123001	0.527432449	0.104426872	0.117525791	0.620343825
ZPBP2	0.532842897	0.071157593	0.535198014	0.519233034	0.042684854	0.117693266	0.620343825
SNPH	0.565875861	0.242147465	0.032452401	0.773183425	0.13114843	0.117864243	0.620356028
ATP5IF1	0.699844861	0.265769704	0.623814286	0.020169421	0.193755865	0.11823316	0.620858733

BTG1	0.900257024	0.031677804	0.078655496	0.326649258	0.617755768	0.118116804	0.620858733
EML6	0.019606684	0.776454363	0.281697319	0.548493701	0.192675945	0.118201237	0.620858733
MRPL21	0.517418287	0.221016519	0.986883901	0.019875417	0.201856083	0.118138416	0.620858733
PLCB1	0.27640285	0.200414441	0.57786258	0.0391601	0.361265256	0.118149935	0.620858733
PMF1	0.569877408	0.242395414	0.213738037	0.114698896	0.133484948	0.118029806	0.620858733
SH3PXD2A	0.472122664	0.497208262	0.018008166	0.2958211	0.362752504	0.118262409	0.620858733
TOX2	0.687305612	0.002146037	0.609078196	0.837849574	0.602205797	0.118211819	0.620858733
ANGPTL7	0.290508962	0.346934659	0.029880059	0.694398775	0.217767739	0.118520806	0.62176264
CNBP	0.980607072	0.088379912	0.113659326	0.491690333	0.094036026	0.118528223	0.62176264
KIF17	0.49303832	0.228045903	0.986127767	0.905415537	0.004542937	0.118617265	0.62176264
PRUNE2	0.535081465	0.306901037	0.18349652	0.280786482	0.053937377	0.118661909	0.62176264
THY1	0.218286511	0.104737345	0.090569423	0.241177336	0.912974231	0.118599527	0.62176264
ZDHHC18	0.877291849	0.563609159	0.187123964	0.007273692	0.677984722	0.118648764	0.62176264
PLEKHF1	0.414541506	0.427169706	0.029968829	0.871255972	0.098854804	0.118764604	0.622102111
AREL1	0.243758075	0.596403538	0.537140249	0.022174508	0.265108917	0.11905384	0.622226931
CCDC85C	0.456716052	0.453759674	0.012538978	0.179272042	0.984104175	0.118965126	0.622226931
LOC788745	0.836764324	0.193614513	0.554612212	0.407530822	0.012512635	0.118926992	0.622226931
PAPD5	0.736465142	0.763447401	0.014480593	0.208743772	0.270052182	0.119040562	0.622226931
PFDN6	0.066763752	0.172982795	0.76348016	0.528681016	0.09840584	0.119006306	0.622226931
RSPH10B	0.791768737	0.00608891	0.670802419	0.209565641	0.67557741	0.118879077	0.622226931
SRGN	0.146224389	0.389382889	0.815589489	0.069037766	0.143187444	0.119053028	0.622226931
DHX35	0.495332867	0.036084897	0.69741468	0.070592612	0.522811601	0.119200407	0.622794613
TSPAN4	0.763463893	0.106950173	0.039394319	0.316176635	0.453034936	0.119299989	0.622958722
WFDC3	0.431735251	0.009859465	0.225677351	0.4975336	0.964126729	0.119307736	0.622958722
CAMK1G	0.178824152	0.40755594	0.765432352	0.099454272	0.08321473	0.119435391	0.62314951
GRAP2	0.474295708	0.015598364	0.888872669	0.208366837	0.337472538	0.119541943	0.62314951
NOS1	0.706078697	0.879000112	0.085217315	0.027875705	0.313744172	0.119563012	0.62314951
RNF7	0.417733389	0.034269096	0.548759865	0.179236915	0.328010751	0.119459415	0.62314951
RUNDC3B	0.59506544	0.054387718	0.084262752	0.362884594	0.466760959	0.119469368	0.62314951
SLBP	0.792698236	0.177472986	0.12289651	0.088253325	0.303193274	0.119572104	0.62314951
MUC16	0.34098218	0.141541679	0.071177586	0.400466595	0.336872185	0.119689481	0.623365302
MYOM1	0.331201064	0.493619908	0.982674878	0.466599358	0.006182067	0.119686599	0.623365302
LOC112443504	0.682220575	0.298088746	0.321946536	0.032851895	0.215635562	0.11974265	0.623375988
NMNAT1	0.298389367	0.382502619	0.131039208	0.407243383	0.076176808	0.119767503	0.623375988
PCYT1A	0.461578492	0.366114371	0.041207985	0.103314572	0.646164488	0.119899297	0.623864098
MAPK11	0.909031418	0.151871348	0.035255968	0.155901382	0.614124497	0.120061068	0.624507827
DPH5	0.221204836	0.388234377	0.690502022	0.264630506	0.029714009	0.120101478	0.624520076
FAM131A	0.56715309	0.783824837	0.930615497	0.651558633	0.001734275	0.120272949	0.624923908
KPNA4	0.396710432	0.112072501	0.06045951	0.258758491	0.672296665	0.120293378	0.624923908
SUGT1	0.445285755	0.294824821	0.199421297	0.038286981	0.466105894	0.120234024	0.624923908

ATP6V1G1	0.531725296	0.045373833	0.20806917	0.999728451	0.093356984	0.120423176	0.625225286
KIT	0.25388427	0.065480206	0.440259486	0.139509506	0.458880339	0.120427587	0.625225286
LOC785842	0.721480822	0.868078554	0.008035796	0.244273313	0.381700653	0.120529123	0.625356763
SPEN	0.246605865	0.361486692	0.237940391	0.033253449	0.664934498	0.120493058	0.625356763
ANOS1	0.549729633	0.11658251	0.189046127	0.1069639	0.362372166	0.120580518	0.62542569
N6AMT1	0.62668012	0.813478296	0.146265693	0.011825062	0.534082804	0.120767754	0.626144989
PTPN2	0.77795353	0.22024307	0.124103152	0.027288362	0.811911151	0.120795505	0.626144989
LOC104969028	0.105464604	0.087867778	0.150654418	0.694944374	0.486070847	0.120864498	0.62630479
ASB8	0.716667232	0.366132969	0.741320922	0.003141416	0.773041755	0.120977167	0.626507541
NFKBID	0.779240419	0.035024144	0.054331258	0.359690393	0.885709952	0.120979977	0.626507541
METTL8	0.245008189	0.034702426	0.175535419	0.686311177	0.461771522	0.121064829	0.626749181
PPP2CB	0.618727298	0.327558593	0.010581575	0.439087113	0.503927973	0.121283993	0.627685782
FAM13C	0.480359004	0.976138788	0.011316674	0.251684944	0.355613666	0.121342457	0.627790372
LLGL2	0.031815044	0.660337229	0.391210151	0.14042901	0.411979965	0.121422066	0.627860312
LOC104974923	0.303967543	0.459163658	0.040678356	0.098891736	0.847012738	0.121432492	0.627860312
JRKL	0.138823452	0.778008156	0.081236424	0.177662156	0.306221936	0.121686148	0.628577709
PFKFB1	0.079942575	0.435825304	0.905657743	0.054383183	0.278054142	0.121657906	0.628577709
TMCO1	0.644836655	0.306772565	0.863959296	0.524199469	0.005322605	0.121615896	0.628577709
LOC101905312	0.825830213	0.064254326	0.940778292	0.010740732	0.892959676	0.121892596	0.629050124
SLC39A2	0.983359515	0.401249294	0.020558344	0.396840956	0.148676948	0.121865731	0.629050124
SRSF5	0.983087526	0.923481083	0.066395993	0.059608103	0.133197792	0.121864087	0.629050124
STYK1	0.241384506	0.235426878	0.034238748	0.93266542	0.264450891	0.1220508	0.629668559
VIRMA	0.014848144	0.775962816	0.491262067	0.097754177	0.867903185	0.122094572	0.629696422
CDH1	0.667057638	0.424159024	0.152951543	0.020588455	0.539335541	0.122141042	0.629716947
NIPSNAP1	0.331432996	0.275422433	0.501889627	0.02065151	0.508435352	0.122213666	0.629716947
STARD7	0.37094904	0.121110021	0.072921677	0.2718923	0.53990092	0.122193407	0.629716947
TRERF1	0.77896894	0.301989477	0.059145331	0.081256524	0.425765806	0.122256214	0.629738456
LOC101903877	0.599851272	0.272343121	0.190486157	0.566584703	0.027365986	0.122419519	0.629986432
SP140L	0.451877717	0.075541663	0.151552462	0.375467779	0.248163542	0.122353691	0.629986432
TPD52L2	0.183974494	0.301532067	0.381567027	0.091001767	0.250398816	0.122395073	0.629986432
CBLN2	0.148006901	0.283293659	0.503810345	0.577891455	0.039699992	0.122722613	0.63036019
CCDC14	0.761533392	0.495927172	0.013695473	0.414853534	0.225362871	0.122571001	0.63036019
DRAM2	0.189467288	0.358765218	0.326855589	0.396151745	0.055042904	0.122697501	0.63036019
FASN	0.755226596	0.579919606	0.777810896	0.021577793	0.06591278	0.122702603	0.63036019
PIGC	0.746844494	0.046852283	0.241679286	0.132828602	0.431248768	0.122690631	0.63036019
THBD	0.064980395	0.091833918	0.338493781	0.515716002	0.464522421	0.122617086	0.63036019
ALDH5A1	0.101870089	0.853656653	0.247246205	0.08856028	0.25514393	0.122890125	0.63067924
BBS5	0.592748825	0.749053173	0.035019582	0.374726375	0.083394584	0.122900019	0.63067924
MB	0.715556229	0.417385013	0.554683935	0.094005879	0.031195458	0.122888358	0.63067924
MALT1	0.755243557	0.069298338	0.821690959	0.956178882	0.011825265	0.122950885	0.630743038

FBXL5	0.145563301	0.259112584	0.150073691	0.285310186	0.302673827	0.123309818	0.630809669
FKBP3	0.328029503	0.221148097	0.607574579	0.407330343	0.027159738	0.123140571	0.630809669
HNRNPAB	0.68822382	0.050718764	0.061289985	0.544833116	0.417977599	0.123083044	0.630809669
KBTBD7	0.965599071	0.191962496	0.120953303	0.433407319	0.050250805	0.123235879	0.630809669
MRPL37	0.436967399	0.131438138	0.875950271	0.106383753	0.091323	0.123304788	0.630809669
PHLDB2	0.542220994	0.968072698	0.137839124	0.466012282	0.0144789	0.123223139	0.630809669
SIRT4	0.82218508	0.224563241	0.129626518	0.035335571	0.575715216	0.123038381	0.630809669
SLC38A2	0.118676335	0.707387199	0.373422307	0.254762748	0.061094498	0.123186709	0.630809669
THAP5	0.240810241	0.032665483	0.299778794	0.272590477	0.7583477	0.123120945	0.630809669
C13H20orf202	0.276616548	0.767907783	0.004271442	0.953333421	0.565932108	0.123410252	0.630830863
GUCY1B1	0.421750949	0.423944309	0.363869921	0.631886464	0.011904384	0.123392199	0.630830863
TCP1	0.810775664	0.080279918	0.259857872	0.698877923	0.041423437	0.123429279	0.630830863
ANAPC15	0.213116576	0.036896546	0.415679124	0.194461779	0.771745883	0.123552795	0.63106907
SPOP	0.97716013	0.610048194	0.154620587	0.030097649	0.176781434	0.123536405	0.63106907
HBQ1	0.631196388	0.603458384	0.474051921	0.0042488	0.640781312	0.123702673	0.6312452
IDH3B	0.686570896	0.297732156	0.880940307	0.005200583	0.524688686	0.123670518	0.6312452
PHLDA2	0.063343743	0.553483454	0.389018936	0.079523313	0.452828114	0.123638063	0.6312452
A1CF	0.060802083	0.240960558	0.214848287	0.775854232	0.20294783	0.124267159	0.631292596
ANKRD13B	0.390988262	0.045821401	0.099102916	0.390200365	0.712639735	0.123998788	0.631292596
ASNA1	0.445016262	0.256896947	0.597592464	0.127396865	0.05674805	0.12402639	0.631292596
CHTF18	0.924626965	0.020188167	0.127958283	0.850787355	0.243560081	0.124171221	0.631292596
COG1	0.397467147	0.388883508	0.016836482	0.83273781	0.228763143	0.124284	0.631292596
DHX40	0.091605596	0.439403647	0.160817002	0.269444959	0.282538684	0.123869829	0.631292596
EMILIN3	0.005754153	0.58066049	0.717273285	0.480172755	0.431728986	0.124432476	0.631292596
GCFC2	0.779549437	0.400867553	0.025621048	0.628784841	0.098454407	0.124270001	0.631292596
KANK1	0.086329509	0.745639591	0.449704464	0.332008898	0.051336739	0.123953788	0.631292596
KCND3	0.630064014	0.353981025	0.120114892	0.755905026	0.024487519	0.124301076	0.631292596
LMLN	0.87438337	0.693496966	0.720751681	0.001265982	0.898552032	0.124481314	0.631292596
MAGED4B	0.205550827	0.360322254	0.017617657	0.739124719	0.514134342	0.124297768	0.631292596
NMT1	0.27518194	0.127029266	0.760038254	0.397134497	0.047043685	0.124368807	0.631292596
OCIAD2	0.147873788	0.019256464	0.998784021	0.455742534	0.383553562	0.124478434	0.631292596
OLFM2	0.027148948	0.855231805	0.42690695	0.77948426	0.064345382	0.124480014	0.631292596
POLRMT	0.085275932	0.120712013	0.340987023	0.291974336	0.482193394	0.124063187	0.631292596
RPS19BP1	0.083583472	0.302936853	0.630286391	0.211732304	0.1460257	0.123959107	0.631292596
SNURF	0.970534408	0.935894319	0.893190061	0.001507846	0.403150843	0.123923997	0.631292596
SNX22	0.265040283	0.013877742	0.569963961	0.286872366	0.823583076	0.124221463	0.631292596
TMEM128	0.235819682	0.07275114	0.234484925	0.801503621	0.154051409	0.124417933	0.631292596
ADAM1A	0.910672447	0.159681738	0.093463861	0.064114504	0.571755879	0.124629122	0.631469399
KIAA1551	0.50205583	0.204866918	0.192497009	0.147834194	0.170194136	0.124619409	0.631469399
LOC100336669	0.257945895	0.565627828	0.321035276	0.016984475	0.626295637	0.124631612	0.631469399

MED6	0.20722893	0.911629187	0.777126601	0.035228797	0.096408727	0.124684511	0.631542441
ACACA	0.718487746	0.630520673	0.827978149	0.038882131	0.034212728	0.124732618	0.631591171
CASC4	0.359075982	0.956080661	0.67480715	0.269260047	0.008015914	0.12487829	0.631698243
LOC112446456	0.102857108	0.05432726	0.188914195	0.866537846	0.547148865	0.124946225	0.631698243
MGST1	0.655441973	0.340701154	0.286512211	0.350362925	0.022317046	0.124913566	0.631698243
PQBP1	0.325538011	0.057629775	0.894710814	0.194542787	0.152935025	0.124793108	0.631698243
TOMM22	0.368651147	0.113234205	0.4007022	0.451499815	0.066249342	0.124921295	0.631698243
ETFA	0.403463461	0.491530468	0.942375768	0.010226021	0.262282685	0.125049637	0.631871529
SYT11	0.081172991	0.518312846	0.6424955	0.727425555	0.025494234	0.125057506	0.631871529
C23H6orf62	0.877667841	0.363771664	0.823162024	0.839277135	0.002275883	0.125153	0.632106537
GSTO1	0.68782174	0.129372344	0.487184125	0.319318353	0.036317848	0.125258086	0.632106537
MAP2K3	0.451406381	0.025700309	0.324536408	0.572940437	0.233045446	0.125252483	0.632106537
NIFK	0.797587889	0.08425235	0.475239472	0.121170865	0.129870143	0.125230179	0.632106537
LOC112446757	0.111979513	0.548734349	0.616353189	0.018057347	0.735645546	0.125306007	0.632153976
C1S	0.092165526	0.456513885	0.54235986	0.034024615	0.653298665	0.12587914	0.632867024
EDN1	0.887576632	0.528668148	0.029752396	0.260797019	0.139185442	0.12581344	0.632867024
EPDR1	0.217443466	0.446266313	0.766285472	0.008830314	0.771550905	0.12579159	0.632867024
IL1RL1	0.83119152	0.138599385	0.29575583	0.05745219	0.257715461	0.125497205	0.632867024
LOC112445193	0.913738846	0.239484865	0.820378675	0.010737413	0.262325696	0.125660058	0.632867024
LOC782057	0.991121435	0.005043123	0.349750243	0.435341854	0.66645732	0.125874662	0.632867024
MRPL2	0.182908223	0.138182017	0.772483496	0.087171068	0.297516868	0.125757138	0.632867024
NIN	0.629875515	0.555869824	0.037786549	0.10073642	0.378856317	0.125558853	0.632867024
QRICH2	0.581158967	0.22451328	0.074990904	0.180688132	0.286325479	0.12573737	0.632867024
TOE1	0.879600388	0.267748924	0.155759339	0.073622963	0.187257708	0.125670554	0.632867024
ZNF276	0.743990403	0.007181004	0.453352556	0.356541728	0.585953652	0.12570951	0.632867024
ZNF470	0.43103752	0.348155805	0.021909966	0.462253265	0.333884861	0.125910111	0.632867024
LOC101906966	0.980019332	0.739891686	0.007723634	0.363245946	0.249879326	0.126030843	0.633279904
RNASEK	0.394925377	0.12200553	0.730447428	0.850081832	0.017005321	0.12609113	0.633388898
POMGNT2	0.487740548	0.018508246	0.308373989	0.87341029	0.2095091	0.126175745	0.633619997
CHCHD4	0.405584168	0.063018697	0.55179608	0.497153373	0.072767536	0.126289511	0.633997296
ABCB9	0.300957131	0.008650143	0.49174041	0.821038639	0.485786052	0.126340504	0.63405933
NKD2	0.760775771	0.596272254	0.118839227	0.02399076	0.39551732	0.126469626	0.634513311
FGF7	0.127339604	0.304627776	0.047909324	0.668350032	0.413153461	0.126696036	0.634679075
PER2	0.521598153	0.437768358	0.32395124	0.038051583	0.18223229	0.126661953	0.634679075
PLSCR3	0.286964615	0.371330876	0.010413229	0.66754049	0.69228978	0.126642872	0.634679075
RTL6	0.763929725	0.587180051	0.013550164	0.930896167	0.090644874	0.12665476	0.634679075
USP12	0.748816623	0.163469207	0.018017439	0.717085723	0.324188812	0.126632051	0.634679075
CCT8	0.833706795	0.274955735	0.193550878	0.749354117	0.015466963	0.126841184	0.635212289
WDR73	0.210583239	0.70408097	0.308912616	0.089115951	0.126103879	0.126906513	0.635345571
RAD51	0.826307208	0.012762887	0.446900875	0.19178538	0.571715175	0.127187755	0.636559394

DNAJC25	0.674299769	0.167871156	0.26942367	0.025646332	0.661637237	0.127287505	0.636731036
SELENOT	0.782517157	0.604047934	0.913076686	0.881748343	0.001360089	0.127299648	0.636731036
APOL3	0.018619045	0.426581852	0.84119364	0.642437361	0.120770688	0.127407663	0.637077133
CCL19	0.695168965	0.039616251	0.193512915	0.133082562	0.731768309	0.127492441	0.63730687
LOC786586	0.821254347	0.186897717	0.003972475	0.944180259	0.903437423	0.127643641	0.637375764
PGAP3	0.996086466	0.719548058	0.28887771	0.010642064	0.236052309	0.12764514	0.637375764
TPMT	0.270120012	0.347387369	0.585581297	0.295914696	0.031994935	0.127661577	0.637375764
TVP23B	0.797676943	0.084348942	0.036752392	0.235885368	0.891043871	0.127594152	0.637375764
RBMX2	0.862195321	0.144621809	0.228841032	0.281842903	0.064811333	0.127796339	0.637854537
POLR2E	0.278945531	0.143933463	0.823883888	0.087315221	0.180590533	0.127845453	0.637905664
LOC101903574	0.776181801	0.152827186	0.007046997	0.625313918	0.999652697	0.127973452	0.638350251
CBX7	0.289849682	0.662581721	0.708078821	0.494238685	0.007796244	0.128169869	0.638608409
DLK1	0.246627954	0.305836914	0.463269072	0.078536179	0.191030653	0.128206258	0.638608409
PTPRF	0.0303914	0.199567522	0.573829075	0.278262844	0.540567439	0.128106393	0.638608409
RRBP1	0.359997999	0.301333353	0.021429712	0.29190628	0.772963151	0.128243515	0.638608409
SLC12A2	0.609226873	0.642357203	0.496022053	0.002828823	0.956469595	0.128336514	0.638608409
TECR	0.491000241	0.206291058	0.616593871	0.064549744	0.130250468	0.128320391	0.638608409
TUBB6	0.080593739	0.364909791	0.143885684	0.189907861	0.651607009	0.128123554	0.638608409
YARS	0.775844512	0.249201753	0.24943637	0.550219652	0.019775059	0.12827195	0.638608409
MTMR7	0.007700472	0.719859393	0.788649694	0.682900554	0.176185031	0.128441779	0.638938476
CORO1A	0.432968047	0.049357246	0.8190772	0.301078091	0.099919401	0.128521327	0.639059137
NAA40	0.331145283	0.247788744	0.83308851	0.098864413	0.077941177	0.128543916	0.639059137
RFC1	0.82785486	0.613365492	0.015327244	0.515074111	0.131657797	0.128684401	0.639563811
METAP1	0.966419296	0.332170805	0.010112346	0.193800693	0.839626106	0.128744579	0.639669174
H2AFY	0.17983845	0.941217924	0.274747616	0.014844834	0.765719246	0.128799038	0.639746067
GNAL	0.950732446	0.466909405	0.094224382	0.422769159	0.029912061	0.128840514	0.639758451
TIMM21	0.827800132	0.172394485	0.673220441	0.015851574	0.34826767	0.129035947	0.640535066
OXNAD1	0.070219145	0.094293548	0.466073763	0.180660724	0.952018701	0.12908694	0.64059443
PHETA1	0.025399027	0.492490545	0.825399836	0.361898748	0.142149601	0.129138115	0.640654655
GNL1	0.450610725	0.90711911	0.286779733	0.008428708	0.538061477	0.129203227	0.640746679
TMEM250	0.856449137	0.327821559	0.960708053	0.020643015	0.095520113	0.129234752	0.640746679
COQ9	0.493936492	0.417369177	0.790462125	0.008633349	0.37834194	0.129290459	0.640829272
DNAJC7	0.115315104	0.193122746	0.360836201	0.480265956	0.138573609	0.129629843	0.641307504
LOC101904265	0.058646076	0.201096491	0.29433983	0.270900127	0.567595378	0.129489172	0.641307504
LOC112448736	0.804805422	0.25740732	0.06415882	0.223692209	0.179952734	0.12966049	0.641307504
MOB4	0.554590533	0.018342336	0.33227636	0.786633876	0.201005105	0.129582619	0.641307504
REEP2	0.398391095	0.307492219	0.385988313	0.090870766	0.124148513	0.129446754	0.641307504
SNX17	0.853403379	0.264973661	0.518903585	0.015204692	0.299530756	0.129575433	0.641307504
XYLT2	0.368110814	0.758190681	0.078131786	0.06905005	0.354694172	0.129532487	0.641307504
LOC101903615	0.818717527	0.809791591	0.002007508	0.71768823	0.562066003	0.12991035	0.641383499

NDUFB6	0.74011142	0.135124382	0.749402393	0.123525123	0.057968939	0.129878702	0.641383499
PLEKHM3	0.876482547	0.236568919	0.098599001	0.758464539	0.034569808	0.12979747	0.641383499
SELENOH	0.158182221	0.465845513	0.352345482	0.297672075	0.069342611	0.129781066	0.641383499
UGP2	0.344831992	0.328894327	0.897485366	0.410454209	0.012842685	0.129864679	0.641383499
ZNF582	0.509010393	0.664664866	0.633744408	0.004972263	0.503440756	0.129886888	0.641383499
DCP1B	0.748428095	0.028696268	0.233246806	0.880507166	0.12209737	0.130132835	0.641672285
GTPBP6	0.302407137	0.450465509	0.881788104	0.014425064	0.311554431	0.130305234	0.641672285
LOC112448078	0.626597208	0.434733224	0.004127084	0.543416649	0.883006588	0.130252442	0.641672285
MED12L	0.023925283	0.057632733	0.581663447	0.830042745	0.808861674	0.13012328	0.641672285
MEG9	0.980062713	0.036731977	0.065030854	0.444508841	0.518716482	0.130298356	0.641672285
SLC2A6	0.918020348	0.949811975	0.004186498	0.78350755	0.188426925	0.130182026	0.641672285
TEAD1	0.047394006	0.68398312	0.087631922	0.383298067	0.495901202	0.130320744	0.641672285
TMF1	0.449975061	0.983837889	0.566128038	0.002426495	0.886911388	0.130241629	0.641672285
VANGL1	0.158865458	0.729305316	0.391108571	0.017620648	0.67451724	0.130136268	0.641672285
ERI3	0.983689899	0.040568086	0.455561457	0.040594796	0.732787845	0.130432915	0.642031963
C29H11orf95	0.797321271	0.117094865	0.064596776	0.266155398	0.338323593	0.13073414	0.642535613
FBXO8	0.925819666	0.170148396	0.133653558	0.095770925	0.269146646	0.130685512	0.642535613
LOC100295347	0.920928314	0.501078438	0.598363984	0.017828024	0.110186639	0.130647521	0.642535613
LOC101905262	0.657089536	0.288531635	0.051275551	0.093149805	0.599841202	0.130750281	0.642535613
LOC781022	0.20454771	0.044420907	0.137766287	0.572049151	0.758788579	0.130772353	0.642535613
PEMT	0.024669564	0.342497413	0.579791204	0.126993366	0.873276377	0.130763031	0.642535613
YIPF5	0.748996709	0.047172263	0.905684444	0.791743864	0.021457187	0.130809304	0.642535613
CXCL9	0.056248717	0.109565877	0.373940758	0.906188961	0.260465185	0.130852309	0.64255453
LOC112447032	0.577189199	0.124870788	0.105578922	0.395696147	0.180798431	0.13091122	0.642651522
AHNAK2	0.503793577	0.310052394	0.011122939	0.628281977	0.499749386	0.13106152	0.643196953
EPHA5	0.069817143	0.880282707	0.12161472	0.08091902	0.902491917	0.131103259	0.643209442
FABP5	0.06435529	0.111086246	0.90801907	0.087757936	0.961308309	0.131341105	0.644183763
FNBP1	0.7453066	0.361689987	0.700174447	0.820652056	0.003550479	0.1316481	0.644341475
LOC100848639	0.393007135	0.069261457	0.140102122	0.312277271	0.461474762	0.131598097	0.644341475
LOC112447316	0.028463482	0.134148269	0.446340691	0.533568512	0.603640715	0.131511609	0.644341475
LOC112447526	0.207212192	0.074640359	0.061536336	0.81595289	0.707600595	0.131589533	0.644341475
MATN4	0.735492315	0.856022193	0.582251841	0.004734831	0.316832801	0.131645574	0.644341475
TRIM7	0.094010956	0.178240165	0.185043683	0.25217485	0.701653476	0.131473881	0.644341475
UBE2E1	0.774533944	0.051049515	0.529079871	0.867682781	0.03029711	0.131646716	0.644341475
LOC101906916	0.457952817	0.594955247	0.003974966	0.546674133	0.930929784	0.131808953	0.64436006
LOC112448524	0.210641647	0.673882309	0.01099866	0.760704784	0.463769563	0.131759108	0.64436006
LOC112449284	0.458156128	0.093360829	0.545005391	0.300674727	0.078624231	0.131800317	0.64436006
SH3GLB2	0.025285274	0.19935532	0.850651677	0.296284609	0.433337964	0.131725034	0.64436006
CSTB	0.413955374	0.588757693	0.075356863	0.62904719	0.04781471	0.131972274	0.644966348
HPN	0.82272385	0.804704181	0.250042158	0.010296882	0.324377066	0.132039706	0.645103784

RRP8	0.476074619	0.143411105	0.739438784	0.031141357	0.351959589	0.132095268	0.645183168
ADAMTS12	0.487734395	0.790445815	0.002554912	0.7629436	0.73851516	0.13231253	0.646026207
CWF19L1	0.16204435	0.117327568	0.596396458	0.140316841	0.349095025	0.132369592	0.646026207
PRIMPOL	0.964728692	0.442106473	0.012764426	0.112228538	0.909249677	0.132385969	0.646026207
RNF219	0.340710161	0.033520495	0.428304158	0.457632085	0.249194779	0.132686301	0.647299311
AKAP8L	0.384973294	0.121308024	0.42058947	0.116400855	0.24465528	0.132886362	0.647344465
AOAH	0.992727509	0.04086036	0.659704845	0.322464127	0.064710089	0.132759159	0.647344465
C1QTNF3	0.622653623	0.044928899	0.523769004	0.085715943	0.445466867	0.13290321	0.647344465
DTNB	0.753715963	0.104028668	0.267091818	0.03122738	0.855862753	0.132932231	0.647344465
LOC107131715	0.202247458	0.320712758	0.320725566	0.11627414	0.231059255	0.132827317	0.647344465
PHLDB1	0.016259113	0.338105103	0.411598295	0.485523474	0.508963682	0.132857653	0.647344465
ANPEP	0.356423868	0.31949619	0.874773194	0.064736212	0.086848417	0.132978852	0.647376709
LOC107131209	0.169534903	0.215381996	0.039854072	0.425450647	0.90505922	0.133017748	0.647376709
DYRK1A	0.088507331	0.262848473	0.429540046	0.061850803	0.907665107	0.13310087	0.647589203
ADSL	0.893309845	0.198350004	0.739275982	0.051734248	0.082846578	0.133157381	0.647672133
GPR137B	0.517652275	0.203733355	0.024293153	0.934829924	0.235110929	0.133376638	0.648504603
THNSL2	0.821758267	0.628576544	0.013280928	0.145938868	0.562691783	0.133407564	0.648504603
LOC787074	0.951142697	0.03961939	0.73676986	0.731158802	0.027785359	0.133499174	0.648637506
ZNF800	0.506584272	0.812862964	0.822548916	0.00218151	0.763505756	0.133513954	0.648637506
GFPT2	0.80931296	0.086084598	0.517201659	0.080176058	0.195428418	0.133570563	0.64872048
ZNF566	0.389994581	0.08655612	0.318711888	0.548870839	0.095710877	0.133647168	0.648900497
ALDH3B1	0.053057019	0.292388733	0.513976516	0.62847289	0.112894766	0.133718591	0.649055248
LOC100847818	0.990177398	0.353408119	0.037789271	0.445491548	0.096092263	0.133765907	0.649092933
ACP2	0.012332996	0.340716025	0.502373088	0.545175945	0.493654408	0.134031117	0.649213857
CFAP161	0.03628274	0.145749461	0.742032481	0.227119395	0.63617369	0.133880332	0.649213857
EIF4A3	0.899464741	0.011740048	0.11125331	0.979916085	0.493066677	0.133965551	0.649213857
GPR50	0.337533685	0.388136157	0.092681116	0.236218868	0.197838476	0.133941139	0.649213857
LOC506989	0.840486696	0.194002932	0.771348434	0.613565447	0.007348073	0.133891244	0.649213857
LOC618737	0.205642863	0.014368181	0.619368908	0.783144041	0.396603165	0.134067745	0.649213857
SLC29A2	0.098920763	0.917572156	0.017801496	0.552127476	0.636697552	0.134015733	0.649213857
CCDC32	0.021962255	0.623022412	0.084050652	0.879792791	0.56440276	0.13441307	0.649239251
DNAJC24	0.599319228	0.174865783	0.720822125	0.128151663	0.058962891	0.134379496	0.649239251
GPR141	0.845676731	0.132937961	0.591154914	0.085491758	0.100512558	0.134414445	0.649239251
LARS	0.335669094	0.171524355	0.204160727	0.143687527	0.337783716	0.134340817	0.649239251
MTMR1	0.775092209	0.887071776	0.007832346	0.146590017	0.723391067	0.134411423	0.649239251
NOP10	0.309280601	0.076369036	0.458535801	0.453106264	0.116396952	0.13442904	0.649239251
RPS4Y1	0.717201362	0.1225203	0.345305193	0.933257893	0.02016887	0.134420618	0.649239251
SIVA1	0.1802404	0.598629249	0.07794408	0.069595828	0.973547633	0.134249965	0.649239251
SLC16A13	0.14930995	0.343952956	0.65589362	0.02540863	0.665104522	0.134174861	0.649239251
AARS	0.537177475	0.123453433	0.467011887	0.296049148	0.062382684	0.134529944	0.649344385

SLCO1C1	0.672524015	0.110767761	0.504772668	0.047583868	0.319550248	0.134502514	0.649344385
CNOT4	0.627064574	0.611412624	0.425853136	0.005025392	0.701423707	0.134987672	0.649643016
CRTAC1	0.052219498	0.178560774	0.083519051	0.745514538	0.990148423	0.134902833	0.649643016
JAG1	0.277834266	0.246981547	0.651273749	0.026650311	0.482569818	0.134888408	0.649643016
LOC101905499	0.402806648	0.568576425	0.060289352	0.53460597	0.077904521	0.134930501	0.649643016
LOC511386	0.723781178	0.013952132	0.600803917	0.253292055	0.373122957	0.134713789	0.649643016
MRPL54	0.244376853	0.276233551	0.635527348	0.043146871	0.310757187	0.13495079	0.649643016
POU2AF1	0.331256747	0.277701857	0.085607817	0.58434193	0.124527406	0.134668512	0.649643016
PRPS1	0.940425689	0.038404039	0.075203271	0.806932354	0.262371677	0.13492531	0.649643016
SLC35C2	0.210031658	0.200555762	0.39538637	0.088523707	0.390211547	0.134961064	0.649643016
TMEM225B	0.238150136	0.9270301	0.036335235	0.44261214	0.161655135	0.134787446	0.649643016
LOC505479	0.6772368	0.666575141	0.021261797	0.570878938	0.105104273	0.135038497	0.649697091
GLUD1	0.759705287	0.91219664	0.282176062	0.320878532	0.009192631	0.135154923	0.650066657
CACNG4	0.186755049	0.014225979	0.922794361	0.431616021	0.545652306	0.135230052	0.650084791
PRR7	0.286917528	0.383149656	0.844706633	0.033717396	0.184430809	0.135237918	0.650084791
VPS37A	0.994797631	0.025943025	0.265213922	0.435264182	0.193961259	0.135289431	0.650141978
ATP5MG	0.664360291	0.312713741	0.678936416	0.04763717	0.08606086	0.135342864	0.650208354
METTL23	0.24547305	0.284679207	0.427210111	0.963457758	0.020257144	0.135906179	0.65272353
BNIP2	0.62362226	0.062984173	0.119597315	0.126799635	0.978872236	0.135958908	0.652785734
BDP1	0.810491059	0.614399391	0.946863581	0.001523553	0.81350583	0.136128701	0.653244865
PPP4R4	0.900560082	0.397184041	0.00654801	0.457197975	0.545780786	0.136134144	0.653244865
NFE2L1	0.892881923	0.454700011	0.01496951	0.874329187	0.110116404	0.136223262	0.653481424
LOC112443444	0.287374892	0.336725939	0.540155153	0.120411942	0.093131816	0.13635395	0.65360266
MAF1	0.86834401	0.194355073	0.33571534	0.026714735	0.387363857	0.136374184	0.65360266
MCM3	0.221206986	0.293951879	0.241438566	0.232084171	0.161141303	0.136478632	0.65360266
MVK	0.529273422	0.254987251	0.482525021	0.042715696	0.21118663	0.136519752	0.65360266
NDUFA4	0.775824317	0.433137795	0.830452951	0.015203192	0.138550302	0.136567151	0.65360266
SHISA7	0.255979475	0.122201086	0.122196044	0.327200695	0.468661894	0.136353893	0.65360266
SLC1A1	0.851263703	0.614244114	0.003135933	0.360729652	0.993117524	0.136516663	0.65360266
STRIP2	0.745438222	0.943115769	0.064362471	0.022178759	0.585483752	0.13653515	0.65360266
ACO2	0.469871798	0.349416765	0.712311378	0.033181221	0.152066396	0.13685616	0.654642102
ANKRD11	0.436910018	0.077419725	0.472382998	0.052560885	0.703141089	0.136912301	0.654642102
MKI67	0.614957331	0.040707121	0.113139836	0.53134235	0.392572817	0.1369439	0.654642102
TAX1BP1	0.532800056	0.044634598	0.242565732	0.425969194	0.24038521	0.136931036	0.654642102
LOC112442386	0.621368574	0.454153231	0.030145215	0.662672696	0.10490551	0.137020412	0.654696715
TMEM88	0.702630908	0.293629179	0.271009453	0.014922509	0.708923826	0.137035112	0.654696715
SPAG5	0.826036372	0.045852575	0.041132227	0.79264385	0.479489444	0.137113068	0.65487851
DBT	0.508542464	0.391721881	0.751292254	0.067718038	0.05861151	0.137355684	0.655224443
FAHD1	0.369332982	0.123070683	0.320968435	0.136119221	0.299367425	0.13741779	0.655224443
GPR83	0.613169981	0.063443107	0.895277719	0.364440819	0.046802891	0.137359426	0.655224443

LEP	0.034140005	0.207477169	0.32136319	0.974409136	0.268058437	0.137425052	0.655224443
SMIM12	0.259041341	0.085516831	0.636780603	0.047108257	0.893066659	0.137284572	0.655224443
ZNF410	0.199295493	0.837179261	0.057574677	0.276022411	0.22402962	0.137354989	0.655224443
DLAT	0.784328686	0.207790494	0.804801135	0.067644352	0.067115283	0.137540463	0.655584242
RAB27B	0.53559755	0.377252944	0.667154404	0.075270761	0.058749524	0.137620914	0.65577724
EBD	0.216115055	0.282626279	0.970082059	0.208592439	0.04829992	0.137729208	0.656102768
MINOS1	0.247979287	0.290154991	0.806190801	0.020019632	0.514569776	0.137804485	0.656270867
CYB5R4	0.581879843	0.008033723	0.197219327	0.8549635	0.759494582	0.137941719	0.656470216
SPARCL1	0.02728285	0.268559836	0.542989635	0.781490734	0.192604808	0.13796635	0.656470216
SYT17	0.049611579	0.820191277	0.708436953	0.051039096	0.406968166	0.137957557	0.656470216
FDPS	0.363553213	0.145698229	0.913713839	0.233297423	0.05307985	0.138028865	0.656556094
HNRNPK	0.960550001	0.142783997	0.055837596	0.462971882	0.169215544	0.13810713	0.656556094
LOC107132921	0.919627736	0.08382111	0.169155562	0.39749226	0.115867155	0.138180319	0.656556094
RHBDD1	0.039163714	0.428021641	0.073020894	0.889777142	0.55142491	0.138184434	0.656556094
TMEM14A	0.279198329	0.119249431	0.023238962	0.906971387	0.85447607	0.138064988	0.656556094
FGF2	0.675920924	0.791996801	0.022685141	0.219953254	0.225274916	0.138330964	0.656659831
LOC101906607	0.66161403	0.650674714	0.253382261	0.714749274	0.007724761	0.138397247	0.656659831
LPIN3	0.712499574	0.02440348	0.082296306	0.871358585	0.482381221	0.13829605	0.656659831
SLC25A33	0.962745118	0.768612181	0.001245803	0.85068711	0.768064912	0.138406335	0.656659831
WSB2	0.563194581	0.07697743	0.106490821	0.317229331	0.411101074	0.138374962	0.656659831
ATP5F1D	0.702600042	0.314943678	0.829649443	0.016738551	0.196553117	0.138615698	0.657273514
LOC112442253	0.851928159	0.184432169	0.092401437	0.058783452	0.707718477	0.138615784	0.657273514
FBXL7	0.089491711	0.42624822	0.479952913	0.076081099	0.434667654	0.138798494	0.657759769
PRELID1	0.173009238	0.3233768	0.210592154	0.46433656	0.110613933	0.138760842	0.657759769
DLX4	0.233665851	0.028982025	0.447451504	0.302593265	0.662769224	0.139081218	0.657952355
DNAJC11	0.615813988	0.176328817	0.825523393	0.092526554	0.073257837	0.139069148	0.657952355
FAIM	0.471149625	0.017401218	0.637325361	0.312257772	0.372839995	0.13915909	0.657952355
FBXO33	0.750795325	0.64336599	0.002859037	0.835524527	0.525677184	0.138938561	0.657952355
GRAMD4	0.733826156	0.046364476	0.041288667	0.777640611	0.557645476	0.139266349	0.657952355
LOC104975460	0.576911842	0.431168672	0.095774759	0.69799108	0.036529878	0.13904865	0.657952355
LOC107131699	0.175232509	0.072271705	0.575474283	0.457597606	0.18201308	0.1389942	0.657952355
MAIP1	0.738797864	0.230288889	0.592455382	0.039278319	0.153723925	0.139196501	0.657952355
SETMAR	0.017759096	0.953514864	0.241813777	0.202966195	0.730779023	0.139036959	0.657952355
TRPT1	0.261807858	0.182325228	0.587469108	0.036786003	0.590420428	0.139250887	0.657952355
WRAP73	0.717905104	0.007105076	0.577711737	0.462589284	0.446974285	0.139280146	0.657952355
AMPD3	0.85453135	0.24434093	0.449348311	0.015236913	0.426993907	0.139420838	0.658105503
LOC101906280	0.445751481	0.08869452	0.043930826	0.647996941	0.542453535	0.13943287	0.658105503
RELT	0.565464341	0.722315213	0.186111567	0.052047952	0.15420071	0.139380314	0.658105503
TBC1D2	0.29654353	0.255249657	0.340408234	0.386867238	0.061344347	0.139555193	0.6584086
TMEM35B	0.067947325	0.106850496	0.167255437	0.777256743	0.648070661	0.139577327	0.6584086

RTCA	0.26723686	0.366105818	0.578689583	0.279532767	0.038686692	0.139652452	0.658573674
CSTF1	0.403152566	0.123939335	0.24585248	0.143490895	0.34813193	0.139824729	0.658604244
LOC786332	0.118010287	0.027220819	0.666584569	0.351774681	0.815006784	0.139856569	0.658604244
NIPA2	0.984390596	0.231435118	0.028637658	0.491400216	0.191592029	0.139899726	0.658604244
RND1	0.736749266	0.031320114	0.088680505	0.345993329	0.866712611	0.139823436	0.658604244
SEC63	0.891447783	0.039567466	0.226790233	0.212462443	0.361134408	0.139840258	0.658604244
STXBP2	0.31714813	0.687309224	0.428583269	0.014624922	0.449440525	0.139876522	0.658604244
PINX1	0.073988987	0.459340058	0.18529938	0.748260973	0.1304386	0.139950422	0.658653964
CLU	0.08682929	0.790186333	0.847675729	0.013463071	0.78674788	0.140121637	0.659117796
LARS2	0.231871802	0.51102826	0.964675675	0.029040533	0.18577492	0.140202948	0.659117796
LOC536097	0.022047643	0.937309212	0.669772697	0.608364966	0.073243148	0.140209629	0.659117796
RPAP1	0.697551955	0.03520467	0.48069384	0.99651078	0.052410261	0.140181384	0.659117796
C16H1orf105	0.349426024	0.039935585	0.924320133	0.756425586	0.063325911	0.140347713	0.659389156
LOC107132949	0.412044595	0.336720314	0.011989575	0.477096078	0.778167375	0.140314283	0.659389156
LRP11	0.039642967	0.090368739	0.734296283	0.3282467	0.716647108	0.140467231	0.659761799
LYPLA2	0.223737119	0.137107102	0.996528248	0.380220507	0.053299165	0.140552516	0.65997349
B3GALT1	0.063820303	0.097000583	0.898483746	0.240201064	0.46438606	0.140668272	0.660328091
TPK1	0.085640177	0.124786431	0.429216798	0.417260412	0.324344716	0.140710437	0.660337141
BCL11A	0.857197611	0.282298672	0.172282034	0.126454003	0.117855077	0.140776867	0.660460026
ASB10	0.390329571	0.277370258	0.796589276	0.064900895	0.111554006	0.141158406	0.660804267
CPT2	0.332990417	0.550675976	0.640352011	0.047498722	0.111932553	0.14114479	0.660804267
IL2RG	0.350140478	0.197637729	0.351806565	0.02598372	0.985302326	0.141020893	0.660804267
LOC101906317	0.871419092	0.620946548	0.033432329	0.480816155	0.071625725	0.140987015	0.660804267
LOC107132431	0.534351153	0.148146456	0.394772842	0.033367465	0.599209211	0.141212636	0.660804267
LRRC42	0.412081153	0.685011061	0.034694133	0.972018412	0.065455985	0.140998409	0.660804267
SLC25A26	0.566777754	0.290384211	0.762373806	0.006627243	0.751406071	0.141211634	0.660804267
SPIRE2	0.917860644	0.090356875	0.329526696	0.063609359	0.359158004	0.141153695	0.660804267
UQCRH	0.687956882	0.258745605	0.616961821	0.035817945	0.158818516	0.141199722	0.660804267
BBS1	0.078362746	0.559441172	0.061522577	0.28932162	0.80169947	0.141305758	0.660863152
MED9	0.390043795	0.075549764	0.075376872	0.432648022	0.650908631	0.141296564	0.660863152
CENPW	0.444152093	0.043628557	0.233639712	0.283659024	0.487785775	0.141409565	0.661160221
ADCY4	0.914202924	0.61486416	0.003014169	0.696227006	0.531620345	0.141492624	0.661360141
CLIP2	0.35599113	0.348540295	0.022140405	0.404906063	0.564528018	0.141595081	0.661462247
FBXO24	0.829166841	0.060307497	0.034239487	0.391180785	0.937214387	0.141566493	0.661462247
GORASP2	0.791157132	0.006992824	0.671253095	0.747516057	0.226928718	0.141843464	0.661680808
HS6ST1	0.91348929	0.285148042	0.919169824	0.414968216	0.006336458	0.141793442	0.661680808
LOC101901950	0.754633918	0.578448899	0.532846388	0.995986125	0.002717502	0.141792628	0.661680808
LOC104973054	0.895725944	0.077698679	0.097358659	0.168836705	0.55059992	0.141835401	0.661680808
TSEN54	0.224631477	0.394634822	0.248337741	0.08023971	0.35603329	0.141714619	0.661680808
ABCD2	0.164937332	0.101510918	0.693543453	0.780631041	0.072147928	0.144768166	0.661835557

ACSS1	0.131219978	0.872792604	0.831541508	0.125946545	0.052593895	0.141951359	0.661835557
ADCY6	0.047492678	0.363674487	0.781463039	0.078559107	0.611396814	0.144078986	0.661835557
APBB3	0.770392125	0.152764623	0.094793663	0.673866623	0.08561566	0.143515102	0.661835557
ATP6V0C	0.222935545	0.295965008	0.04927699	0.821381774	0.245168027	0.144858453	0.661835557
BLOC1S1	0.163086831	0.316598626	0.777157488	0.040318913	0.402737351	0.144476878	0.661835557
C14H8orf59	0.903653063	0.018377946	0.792796651	0.401788177	0.1250202	0.145652375	0.661835557
C26H10orf82	0.064497141	0.413824214	0.148355799	0.520471953	0.309158585	0.142724397	0.661835557
C4A	0.05261438	0.309041751	0.140185697	0.291106096	0.97828559	0.144182823	0.661835557
C7H19orf71	0.348673723	0.802834061	0.347712988	0.318064275	0.021038145	0.144444392	0.661835557
CABP7	0.480951199	0.513676489	0.045218075	0.08452885	0.691752343	0.144674476	0.661835557
CCR7	0.749915413	0.010736606	0.785047935	0.263270252	0.388573434	0.143877272	0.661835557
CCT3	0.7313404	0.278639239	0.194895479	0.41877078	0.038056823	0.142211322	0.661835557
CD164L2	0.024348516	0.252045037	0.515592483	0.580844183	0.355631157	0.144721305	0.661835557
CLSTN3	0.256362916	0.469880147	0.693586132	0.022332406	0.338322427	0.142003545	0.661835557
COX14	0.166974566	0.170129693	0.885284891	0.028024111	0.910896554	0.143312354	0.661835557
DCAF10	0.068940045	0.476742747	0.423440651	0.062727621	0.741255562	0.143935852	0.661835557
EFCAB5	0.628385336	0.378109543	0.01197022	0.422030189	0.550252615	0.145545508	0.661835557
EIF1AX	0.62964586	0.138911974	0.274187429	0.518896823	0.052519706	0.144715903	0.661835557
EME2	0.559493538	0.055278198	0.074020721	0.414264812	0.672571746	0.14281034	0.661835557
EPHA3	0.38377034	0.97522023	0.639392642	0.130891238	0.020973759	0.145122839	0.661835557
EXOSC4	0.215697215	0.115074077	0.9400079	0.229264324	0.119864294	0.143216245	0.661835557
FAM166B	0.45158989	0.711554648	0.058950802	0.918476182	0.036629842	0.142743325	0.661835557
FAM219A	0.045191782	0.321580674	0.220511536	0.735695973	0.279022325	0.14523	0.661835557
FAM43B	0.617226228	0.862194391	0.072960368	0.947627423	0.017810032	0.14492491	0.661835557
FASTKD5	0.457601692	0.040596265	0.134621981	0.763374194	0.335476729	0.143128139	0.661835557
FILIP1L	0.476508652	0.549624019	0.283835946	0.402500233	0.022081166	0.145571125	0.661835557
FOPNL	0.941244151	0.395447286	0.130483298	0.020013243	0.659239118	0.143167073	0.661835557
GCNA	0.976237259	0.176138045	0.219889834	0.832792161	0.020989015	0.145598523	0.661835557
GIPC3	0.498491323	0.03083156	0.353392203	0.11700685	0.995938155	0.142207966	0.661835557
GSTM1	0.512184506	0.717481783	0.247914073	0.250787922	0.028605627	0.144717664	0.661835557
HDAC9	0.016676796	0.289073746	0.754850457	0.337449018	0.53098843	0.144532606	0.661835557
HELLS	0.384076714	0.582428078	0.013389709	0.283067211	0.757901571	0.143387794	0.661835557
HSPA9	0.88018166	0.320776586	0.492543808	0.249002481	0.018960977	0.145078432	0.661835557
JAKMIP3	0.222404715	0.089704139	0.200907877	0.510891732	0.322794522	0.145610565	0.661835557
KCNS2	0.022866939	0.067033167	0.817012735	0.685298455	0.748419013	0.14335494	0.661835557
KCTD1	0.088186933	0.723164577	0.055817316	0.285000936	0.645374795	0.144857703	0.661835557
KLF11	0.243275337	0.546104149	0.754082342	0.238392653	0.027043515	0.143786708	0.661835557
KLF9	0.609067806	0.31994285	0.069928092	0.228312029	0.202965973	0.142027241	0.661835557
KY	0.137230384	0.397266531	0.053337644	0.333531981	0.652859638	0.142238285	0.661835557
LGALS3BP	0.077752146	0.313996639	0.486651058	0.140459915	0.389647537	0.144316114	0.661835557

LHFPL4	0.209325403	0.434791878	0.884687418	0.135703003	0.05855846	0.143053199	0.661835557
LOC101902760	0.168137632	0.024256253	0.584368417	0.344472917	0.798254463	0.144930885	0.661835557
LOC101903752	0.878756202	0.435310876	0.04538489	0.200826211	0.186706184	0.14440277	0.661835557
LOC104974057	0.134660422	0.083227844	0.9525215	0.082686279	0.74742149	0.145460085	0.661835557
LOC104974666	0.243799309	0.543356732	0.028266259	0.733159203	0.235732559	0.143941039	0.661835557
LOC112444773	0.263846314	0.668236913	0.56022933	0.54170607	0.012176864	0.14447304	0.661835557
LOC112449531	0.506922938	0.637396889	0.143878575	0.11697751	0.11676744	0.142461604	0.661835557
LOC518961	0.082584292	0.480238857	0.118640587	0.560039079	0.246729108	0.144306592	0.661835557
LRRC14	0.853178651	0.010327995	0.988654383	0.263829296	0.278368869	0.143048149	0.661835557
LRRC4	0.774180334	0.490053568	0.864599117	0.301294008	0.006462534	0.142913459	0.661835557
MGRN1	0.243505467	0.143906026	0.137386012	0.87940506	0.155133359	0.145103898	0.661835557
MLX	0.423053304	0.188990511	0.5887157	0.046397983	0.294743428	0.143522817	0.661835557
MROH1	0.819862677	0.045573453	0.053574183	0.924528568	0.346358009	0.143193657	0.661835557
MRPL53	0.942369671	0.117647611	0.503317046	0.276101815	0.04102069	0.14209446	0.661835557
NADK	0.048477306	0.616800224	0.31669648	0.157314982	0.436612464	0.144336566	0.661835557
NAXD	0.085209082	0.891624866	0.685880169	0.100095158	0.124142952	0.14398576	0.661835557
NCOR2	0.115247364	0.369004777	0.095636529	0.661393383	0.244593604	0.145243401	0.661835557
NDUFB8	0.751197413	0.390529667	0.438968186	0.033565097	0.148753207	0.143434873	0.661835557
NEO1	0.062359851	0.628424107	0.050382679	0.582664962	0.550855159	0.142304798	0.661835557
PAN3	0.421523849	0.514900634	0.32901613	0.144591865	0.062523871	0.143751391	0.661835557
PGR	0.768388119	0.038336938	0.907355978	0.624605527	0.038763004	0.143940191	0.661835557
PKN2	0.800092765	0.260746059	0.019873393	0.275204023	0.578798858	0.145538427	0.661835557
PLEKHB2	0.604395055	0.218002574	0.051922433	0.851634642	0.110223823	0.143339682	0.661835557
PPL	0.847858561	0.014754018	0.244736388	0.232343715	0.895201541	0.142678608	0.661835557
PRKX	0.476818311	0.061279059	0.353590437	0.15711435	0.403627739	0.144911019	0.661835557
PSAT1	0.133885773	0.313840946	0.700784095	0.274565644	0.081701684	0.145555353	0.661835557
REPS1	0.541765346	0.053993759	0.072771726	0.720242628	0.415885495	0.142783647	0.661835557
RGS5	0.486069102	0.01771843	0.770310406	0.239808134	0.407275031	0.144037993	0.661835557
RINL	0.446157941	0.028254116	0.649321941	0.200989113	0.389649647	0.143197804	0.661835557
RNF152	0.140859408	0.420711709	0.066436689	0.959962528	0.174751993	0.145546131	0.661835557
RPP30	0.980021591	0.105057595	0.54108216	0.055653039	0.210239591	0.144506216	0.661835557
SLC25A13	0.275885153	0.409862206	0.527773132	0.109275839	0.09749558	0.14256013	0.661835557
SLC29A3	0.863753035	0.257347027	0.770089638	0.005626387	0.686817634	0.145667542	0.661835557
SLC39A14	0.589865193	0.984277974	0.034570054	0.155118005	0.211420688	0.145277695	0.661835557
SLITRK6	0.088180674	0.79669433	0.36818505	0.031161633	0.820540071	0.145654827	0.661835557
SMC3	0.143423151	0.664405546	0.381442291	0.332819572	0.052935647	0.143118956	0.661835557
SRP19	0.427388158	0.105962243	0.808178991	0.530716056	0.034045097	0.14564511	0.661835557
STXBP6	0.303272629	0.02256899	0.656430875	0.400133267	0.352924856	0.142398823	0.661835557
SUSD2	0.960617765	0.552800218	0.194201616	0.074395785	0.084816571	0.144374247	0.661835557
SV2B	0.013684574	0.901475586	0.097325681	0.606920636	0.899051048	0.144904949	0.661835557

TAZ	0.910396421	0.034432426	0.537061419	0.207350618	0.187203794	0.144707784	0.661835557
TCP11L1	0.874461857	0.652086857	0.111755139	0.611942927	0.016533935	0.143651976	0.661835557
TCTEX1D1	0.178066787	0.255066857	0.435908927	0.826139612	0.039528694	0.143867933	0.661835557
TET1	0.268106997	0.82041796	0.047322961	0.1514305	0.412395947	0.144291435	0.661835557
THAP9	0.869071406	0.160736111	0.045946043	0.117411521	0.86291764	0.144319316	0.661835557
TLDC1	0.905249261	0.020782334	0.071057534	0.54073789	0.896789862	0.144075819	0.661835557
TMEM156	0.416775103	0.049995185	0.869651284	0.167044296	0.20980451	0.142470447	0.661835557
TMEM173	0.428478009	0.036859846	0.084028975	0.862050754	0.570196072	0.144567524	0.661835557
TOP2A	0.258678454	0.067103294	0.079658159	0.562075417	0.816767367	0.142435692	0.661835557
TRIM11	0.479620707	0.363068058	0.224141055	0.018088247	0.921495413	0.144355488	0.661835557
VARS	0.548693376	0.17668448	0.631552698	0.92077479	0.01144554	0.143710818	0.661835557
WDR74	0.446374199	0.207856152	0.154910369	0.297103638	0.153695547	0.14504709	0.661835557
ZFAND5	0.482758354	0.760314818	0.020782697	0.235789318	0.361567651	0.144326872	0.661835557
CLEC4D	0.160171106	0.185545425	0.195623531	0.22491455	0.50710341	0.14585881	0.662015874
LOC104969981	0.373559857	0.307272455	0.158167023	0.618091598	0.059076477	0.145840122	0.662015874
LOC512684	0.406390137	0.269794492	0.286286236	0.469968625	0.044954959	0.145868588	0.662015874
SLC2A1	0.27018454	0.565962905	0.010586557	0.497939519	0.821568987	0.145759233	0.662015874
BBIP1	0.268581702	0.252564332	0.97637828	0.095666921	0.104884692	0.14603609	0.662409696
RFFL	0.202055994	0.111834111	0.879479646	0.150866966	0.221586506	0.146012128	0.662409696
GPR18	0.691069114	0.076666994	0.047913215	0.539043295	0.485973727	0.146087475	0.662459672
LOC101905293	0.729921541	0.95148447	0.013281967	0.267981483	0.269534579	0.146240666	0.662971154
PPIL3	0.533941525	0.425476627	0.771952855	0.004741532	0.801809628	0.146293824	0.663028982
ZNF304	0.487513392	0.095932446	0.133162025	0.783490183	0.136768704	0.146367502	0.663179756
ROBO3	0.297446701	0.341410996	0.185944629	0.443919432	0.079653205	0.146408684	0.663183247
CHMP2B	0.55444655	0.026537697	0.286655372	0.815163467	0.194686515	0.146607601	0.663459264
KCNS3	0.462127326	0.788646565	0.92870178	0.252509488	0.007828277	0.146571071	0.663459264
KDM4A	0.960659727	0.080941845	0.828764235	0.870682599	0.01193327	0.14663133	0.663459264
MICALL1	0.044792955	0.568519259	0.266644178	0.259252328	0.38017252	0.146594187	0.663459264
FASTK	0.276357315	0.070516914	0.324825549	0.107331533	0.987321164	0.146779365	0.663763064
POGLUT1	0.036668967	0.620911318	0.951527861	0.111810079	0.276834093	0.146751858	0.663763064
AKNA	0.94416535	0.029498065	0.285556439	0.165526189	0.510705178	0.146957477	0.66386295
BUB1	0.551545006	0.102500419	0.142135363	0.313471318	0.266662136	0.146883548	0.66386295
CFDP1	0.333536187	0.019326472	0.587791857	0.497663993	0.356119082	0.146862008	0.66386295
MMADHC	0.963996344	0.125841634	0.684701903	0.160673744	0.050379731	0.146963262	0.66386295
CILP2	0.589568211	0.235124428	0.13922254	0.790497061	0.044121338	0.147052962	0.664085349
FGD3	0.2912726	0.159774113	0.899379548	0.065355213	0.246455046	0.147177355	0.664281511
KRT79	0.385262098	0.545925508	0.363912944	0.012349438	0.71319901	0.14717333	0.664281511
ABI1	0.781036267	0.238583766	0.300570876	0.019008117	0.633835237	0.147252296	0.664292802
SAMD3	0.803327281	0.352993201	0.96874401	0.255046184	0.00963239	0.147260814	0.664292802
PDZRN3	0.3302427	0.821891799	0.205793958	0.149163069	0.081218265	0.147477068	0.665085506

CENPE	0.677690731	0.068277128	0.044933538	0.60909921	0.535131607	0.147593248	0.665311038
TXNDC15	0.815537885	0.010649898	0.801158152	0.560145592	0.173899437	0.147608159	0.665311038
DAPP1	0.99695833	0.010910669	0.249809026	0.423115922	0.590832529	0.147783978	0.665354877
LOC782101	0.587949651	0.202138454	0.514183238	0.240512882	0.046238268	0.147818415	0.665354877
POLQ	0.639217208	0.062960861	0.421025782	0.077010346	0.520853954	0.147826895	0.665354877
RALA	0.658700261	0.106900031	0.085520191	0.286224449	0.39378723	0.147718008	0.665354877
RRP9	0.261438051	0.072671588	0.743496816	0.398221492	0.1205775	0.147662839	0.665354877
ZNF550	0.930056477	0.014056853	0.074051922	0.876319663	0.801460399	0.147861144	0.665354877
LOC540403	0.671373392	0.010879673	0.666739034	0.421872806	0.331366426	0.14796224	0.665627279
EPPK1	0.217221073	0.161229544	0.292300665	0.364862236	0.182557306	0.148087626	0.665748452
IFNGR1	0.627156536	0.225299201	0.432967465	0.173843342	0.064132972	0.148110877	0.665748452
LOC529399	0.632429381	0.558874332	0.056531342	0.043104606	0.791898298	0.148106863	0.665748452
AGGF1	0.860949525	0.56730818	0.007569475	0.210670106	0.876342242	0.148167976	0.665822744
ATP5PO	0.760674354	0.191720775	0.73425037	0.028640425	0.223035071	0.148339219	0.666005478
BATF3	0.040001872	0.124614673	0.832618806	0.975566131	0.168998893	0.148370972	0.666005478
SEMA4D	0.436972778	0.021424477	0.651219504	0.292973064	0.382851865	0.148318068	0.666005478
TOMM6	0.299388244	0.324272149	0.812522649	0.327242812	0.026468996	0.148250898	0.666005478
SUGP1	0.991627183	0.424151774	0.047767492	0.071584423	0.476538445	0.148497336	0.666208251
ZNF326	0.976917175	0.694702437	0.375073903	0.041057125	0.065565416	0.148482172	0.666208251
CCDC134	0.046564481	0.728055203	0.197058234	0.37599839	0.27300802	0.148544847	0.666239269
LOC112448366	0.150357959	0.267461619	0.165592592	0.110803932	0.930084244	0.148606232	0.66633248
CNTN3	0.318058378	0.084716554	0.778997147	0.037278689	0.877803436	0.148673467	0.666335155
DNASE1	0.830516236	0.404123097	0.568469787	0.006779344	0.53152307	0.148749528	0.666335155
GPRC5B	0.643853389	0.649505105	0.567939953	0.128130555	0.022602175	0.148785884	0.666335155
MRPL36	0.154090379	0.216502613	0.818678399	0.198259618	0.127062609	0.148809844	0.666335155
ZNF692	0.66908803	0.058859408	0.044586004	0.877289262	0.446296584	0.148746765	0.666335155
SERPINB5	0.11079936	0.065939354	0.900762786	0.762430466	0.137267227	0.148894387	0.666531855
LOC112446002	0.68017535	0.612640016	0.103526991	0.250337741	0.063810229	0.148938395	0.666547041
SLA	0.7231288	0.066526217	0.994817564	0.04137395	0.348348688	0.149012068	0.666694943
BLOC1S2	0.265181848	0.185850542	0.421173333	0.732930929	0.045704307	0.149662696	0.66681513
DNASE1L3	0.369900939	0.886783183	0.291394359	0.03127926	0.232465074	0.149627185	0.66681513
EFHC1	0.709071308	0.861486557	0.015616208	0.08375892	0.867707708	0.149426015	0.66681513
ELN	0.371200338	0.71179286	0.018195646	0.323244085	0.447783152	0.149725946	0.66681513
GTF2IRD2	0.642191984	0.466089327	0.053932652	0.110255565	0.390987206	0.149729877	0.66681513
IFIT5	0.094730597	0.725262738	0.35002542	0.553142007	0.052367459	0.149810943	0.66681513
INPP5B	0.770557269	0.913130203	0.005531953	0.528570484	0.337143595	0.149466126	0.66681513
KCNE4	0.230104188	0.221394628	0.284304771	0.068574507	0.695521474	0.14913397	0.66681513
LAMP2	0.576589569	0.729031249	0.016532514	0.534399788	0.18714606	0.149627295	0.66681513
LTA4H	0.085356183	0.048886533	0.380660789	0.881758631	0.495142798	0.149449111	0.66681513
PFDN4	0.965352945	0.235188298	0.618820154	0.082848683	0.059429605	0.149246625	0.66681513

PITPNM2	0.159977335	0.78298959	0.058048575	0.887651086	0.107377204	0.149396568	0.66681513
PPP1R2	0.827434111	0.016843583	0.299095598	0.431731727	0.384571592	0.149286505	0.66681513
PRRC2C	0.784958554	0.442094328	0.606221227	0.007988725	0.41421178	0.149756713	0.66681513
RDH16	0.768476186	0.56378415	0.471740293	0.947691187	0.00359292	0.14973206	0.66681513
SUOX	0.869717794	0.169923977	0.711827496	0.012887317	0.513674571	0.149787615	0.66681513
TARBP2	0.693538226	0.10787989	0.290185273	0.084162021	0.380640387	0.149686681	0.66681513
TTC14	0.789524066	0.364115111	0.015635949	0.162105302	0.951322941	0.149413909	0.66681513
VMA21	0.896758276	0.208116595	0.527204891	0.455729653	0.015492051	0.149586088	0.66681513
STX11	0.033134343	0.658102358	0.926377006	0.196321753	0.175815115	0.149885764	0.666867158
VPS4A	0.457816991	0.122132942	0.340778035	0.431228965	0.084874479	0.149903903	0.666867158
SAPCD1	0.31048691	0.016658938	0.902291873	0.259894461	0.57532918	0.149954269	0.666910437
C22H3orf22	0.361602738	0.037419893	0.35136863	0.4727551	0.31097629	0.15008686	0.666957883
HMGCS1	0.61868187	0.157778808	0.224997602	0.467628914	0.06802305	0.150047739	0.666957883
TMED4	0.805734168	0.022844293	0.227875603	0.274783569	0.605945419	0.15001788	0.666957883
CCDC153	0.375274151	0.045331732	0.999436388	0.052456158	0.784596901	0.150178177	0.667093867
TRMT10C	0.800466187	0.054050902	0.59849449	0.817155314	0.033078708	0.150198759	0.667093867
PARP6	0.695604984	0.048227242	0.11286613	0.382572765	0.483539652	0.15025593	0.667167224
C8H9orf152	0.639586618	0.078217881	0.476058781	0.186064104	0.158458138	0.150457763	0.6678827
PLA2G5	0.898928265	0.101521382	0.048553669	0.297757365	0.532920409	0.15056761	0.668182824
RBM28	0.833284356	0.191664302	0.151735043	0.072310551	0.40143624	0.150606804	0.668182824
AMPD2	0.375266313	0.148574998	0.633508645	0.43987695	0.04538292	0.150798642	0.668689398
LOC100298923	0.980465195	0.090513018	0.730194109	0.097465045	0.111703973	0.150843224	0.668689398
RNFT1	0.651161274	0.062797683	0.853943706	0.47725826	0.042318408	0.150814162	0.668689398
FAM47E	0.103113058	0.056829278	0.588001766	0.640571363	0.320419087	0.151040641	0.668890925
LAX1	0.697386174	0.981479102	0.013966805	0.178615433	0.414678709	0.151140599	0.668890925
LIME1	0.703166341	0.129948964	0.463995719	0.029603695	0.563788566	0.151088883	0.668890925
LOC100138864	0.702477874	0.190153849	0.039628813	0.413166125	0.32404327	0.151214754	0.668890925
LOC101902869	0.064555696	0.381754409	0.190059299	0.942022986	0.160554591	0.151180045	0.668890925
LOC537848	0.276391814	0.513811823	0.096114397	0.097898656	0.53016211	0.151182142	0.668890925
MEIS2	0.516464871	0.458010018	0.010811083	0.636134043	0.435345959	0.151156753	0.668890925
MPPE1	0.133848269	0.257033142	0.4672616	0.099690036	0.44091102	0.150968196	0.668890925
DEPTOR	0.089435312	0.831588068	0.123757656	0.100424077	0.768316633	0.15138238	0.668913217
EMC3	0.384119551	0.243303446	0.393884056	0.38735065	0.049786603	0.151350838	0.668913217
HEBP2	0.051333657	0.938828959	0.14801537	0.103040845	0.966012666	0.151367378	0.668913217
KIAA1324	0.004632854	0.24099203	0.839453822	0.800299844	0.948229261	0.151504168	0.668913217
LOC614732	0.873370169	0.018572533	0.34609686	0.943509112	0.134111771	0.151403598	0.668913217
TRIM66	0.524501593	0.231517875	0.68693419	0.137045536	0.062185206	0.151463534	0.668913217
ZNF503	0.003554359	0.61789123	0.779810762	0.964276436	0.430680373	0.151505114	0.668913217
CCAR2	0.634823909	0.047928923	0.14940457	0.791948045	0.197982656	0.151678396	0.66949816
BCL2L1	0.739033141	0.319054845	0.043622705	0.79253558	0.087598341	0.151832666	0.669818002

FGFR1OP	0.025057869	0.302415571	0.504656527	0.266421201	0.700829206	0.151826749	0.669818002
SPTLC1	0.549477398	0.072997346	0.063311633	0.557676839	0.504988145	0.151954934	0.669818002
TMEM136	0.299091354	0.436827113	0.586315775	0.810961799	0.011505317	0.151905809	0.669818002
TMEM254	0.280870096	0.709327977	0.024931785	0.435921922	0.330266523	0.151950217	0.669818002
PGAM5	0.332637915	0.337381048	0.41862565	0.089181824	0.170967616	0.152087973	0.669999022
SEC24A	0.255428492	0.321340998	0.152214033	0.17346683	0.330810485	0.152159305	0.669999022
SLC25A45	0.897108606	0.407589429	0.090717469	0.056873631	0.379865855	0.152124183	0.669999022
UBR2	0.407108052	0.604471265	0.055075037	0.065921731	0.802246747	0.152138671	0.669999022
CREBRF	0.1187147	0.327668783	0.068879136	0.352855422	0.759950105	0.152333925	0.670114422
GTF2H4	0.869992486	0.1986164	0.784073891	0.019233941	0.27576052	0.152348846	0.670114422
SMIM10L1	0.542372997	0.293075042	0.016917735	0.278227827	0.959902858	0.152303242	0.670114422
ZNF34	0.71101237	0.682117015	0.022382105	0.936047078	0.070678833	0.152298904	0.670114422
ACP4	0.473868014	0.056010734	0.573256454	0.083630856	0.566255066	0.152570065	0.670165785
ARMC8	0.726592188	0.953293981	0.727326995	0.052558405	0.027168161	0.152436243	0.670165785
LOC786948	0.982860057	0.131991425	0.107650069	0.175175061	0.294926856	0.15268038	0.670165785
MYCBP2	0.333019187	0.15264326	0.707615859	0.060277342	0.331813016	0.152444245	0.670165785
MYL6	0.575199905	0.126304918	0.207306496	0.097788418	0.489883505	0.152679224	0.670165785
PRSS53	0.899652337	0.276974305	0.21162707	0.609975607	0.022432438	0.152687214	0.670165785
SLC28A1	0.946240053	0.508466526	0.061631883	0.024692056	0.983727091	0.152540654	0.670165785
WDR76	0.674935329	0.517248991	0.012550969	0.938365494	0.175179943	0.152539609	0.670165785
SEC24B	0.217649215	0.656742206	0.984363337	0.005735677	0.895216211	0.152790706	0.670261503
SUSD4	0.845762257	0.020593994	0.401022972	0.438133283	0.236020446	0.152770585	0.670261503
ADRB1	0.617350042	0.108473103	0.918346829	0.080642458	0.147110502	0.153598228	0.670819749
CEP41	0.785378647	0.363843258	0.803089093	0.013761661	0.231370937	0.153725755	0.670819749
CNPPD1	0.37962692	0.891668022	0.093401527	0.045880223	0.502516953	0.153526356	0.670819749
DNAJC6	0.652013976	0.003681973	0.847421799	0.668911858	0.532454733	0.153031527	0.670819749
EIF6	0.740463218	0.047216899	0.0574083	0.760354575	0.478273025	0.153636332	0.670819749
EMC7	0.975757147	0.16182937	0.324891271	0.388984386	0.036374512	0.153179309	0.670819749
FOXM1	0.56680735	0.069788043	0.156128124	0.25310552	0.463684111	0.153056735	0.670819749
FRRS1L	0.10194634	0.240088217	0.084804471	0.885413201	0.39782768	0.153776363	0.670819749
IRF7	0.388579497	0.075470247	0.735969313	0.051293001	0.660278115	0.15375694	0.670819749
LOC112444931	0.56318035	0.300493204	0.64460828	0.069246304	0.096413977	0.153454801	0.670819749
MAGI3	0.770517283	0.093072976	0.436841979	0.21370731	0.109098187	0.153693431	0.670819749
NEK2	0.842637491	0.013897781	0.124204139	0.652487753	0.764905521	0.153186151	0.670819749
NR1H3	0.214242382	0.615019178	0.153737692	0.420013763	0.085485078	0.153343199	0.670819749
PADI2	0.383949876	0.307440089	0.169199792	0.191669981	0.190331748	0.153490132	0.670819749
PDRG1	0.161743595	0.048300272	0.348114101	0.592446176	0.452054441	0.153459236	0.670819749
PRR11	0.079081851	0.327698311	0.784899618	0.071008761	0.503042202	0.153258221	0.670819749
RRP36	0.438196707	0.029921362	0.769146074	0.962206866	0.07514074	0.153547841	0.670819749
SLC39A8	0.047673218	0.994302937	0.248950713	0.217033326	0.284977197	0.153631648	0.670819749

TELO2	0.791622582	0.029305049	0.199877205	0.291209798	0.541040857	0.153711097	0.670819749
TNFRSF21	0.005587672	0.624459318	0.677363325	0.734008148	0.417802289	0.153058057	0.670819749
WHRN	0.761838609	0.081185213	0.562848382	0.06393654	0.32698634	0.15339683	0.670819749
RBM48	0.28406539	0.424813475	0.061960416	0.14537879	0.674868641	0.154052908	0.671802962
TTC13	0.059027952	0.343543931	0.863193529	0.072255128	0.580223327	0.154083624	0.671802962
EIF3B	0.708575295	0.210619572	0.143180115	0.876741206	0.039253257	0.154256106	0.672197812
RPN1	0.929208279	0.191572184	0.1062964	0.804392957	0.0483071	0.154242485	0.672197812
FOXO3	0.640412985	0.536139603	0.218231529	0.041007812	0.23972696	0.154394672	0.672444525
NUP50	0.403516318	0.127046841	0.366799511	0.1066127	0.367254118	0.15435456	0.672444525
PNMA8B	0.65836616	0.248031249	0.176899731	0.040884907	0.624139595	0.154453391	0.672521784
LOC104975244	0.717432802	0.21363494	0.117930195	0.092967338	0.439164708	0.154547306	0.672752211
COX7B2	0.170194674	0.119453426	0.201377173	0.397074638	0.454382155	0.154626197	0.672917134
ARRB2	0.370208513	0.089394005	0.124063452	0.258450512	0.697569929	0.154801686	0.672967011
COX6A1	0.565435691	0.475946157	0.60195362	0.015817546	0.288814614	0.154782233	0.672967011
PPP1R16A	0.799021735	0.104466008	0.069543608	0.251433175	0.506651201	0.154717073	0.672967011
UQCR11	0.613249449	0.282404121	0.882107665	0.017832726	0.271488412	0.15473171	0.672967011
UBLCP1	0.576585636	0.698811914	0.371590146	0.19418818	0.025474502	0.154850209	0.672999676
RBFOX1	0.034611271	0.655542859	0.126120735	0.271811941	0.952912678	0.154909671	0.673079856
CNN2	0.68185524	0.801652318	0.045772931	0.035753692	0.829147533	0.154969719	0.673162533
POLR2D	0.379908771	0.047857662	0.514918449	0.469952597	0.168828191	0.155090407	0.673508513
ATXN2L	0.45268583	0.163253365	0.902561372	0.048270195	0.230962053	0.155184001	0.673546538
PEG3	0.208408207	0.503286185	0.614824289	0.096405234	0.119666684	0.15522229	0.673546538
RNF214	0.729273278	0.704925211	0.701287357	0.145234336	0.014202373	0.155184787	0.673546538
FBXO11	0.098946243	0.931002308	0.156673773	0.09121069	0.56548518	0.155272002	0.673584147
MAP1LC3A	0.149573585	0.536948662	0.724541806	0.175387728	0.072978819	0.155316957	0.673601103
KIAA1109	0.463369691	0.778242404	0.107632472	0.049805594	0.385508326	0.155365143	0.673602391
RAC1	0.962370114	0.061002446	0.068225414	0.621081854	0.29970302	0.155399345	0.673602391
LOC112445030	0.498399493	0.239944938	0.65504572	0.019589263	0.4867106	0.155548336	0.673892223
STX7	0.868013247	0.044323834	0.105890543	0.811850128	0.225810182	0.155546513	0.673892223
ABCC2	0.089900711	0.259179983	0.430311127	0.302504098	0.247224506	0.15587982	0.674025657
FAM107A	0.746266877	0.209747001	0.180716064	0.235214601	0.112528918	0.155753567	0.674025657
LOC101904013	0.015229052	0.966801536	0.109129261	0.596465961	0.78153226	0.155786119	0.674025657
LOC101908760	0.795761602	0.079593059	0.075988297	0.444915449	0.350293565	0.155907708	0.674025657
PTGFR	0.594053488	0.736535884	0.103731188	0.081124589	0.203344597	0.155753345	0.674025657
RRAGA	0.991211499	0.008216349	0.814158752	0.442336493	0.255571458	0.15585079	0.674025657
TBX3	0.373965516	0.122830637	0.327997242	0.099354193	0.500712574	0.155843798	0.674025657
ZFYVE21	0.201451505	0.832871959	0.036850637	0.174040413	0.696431649	0.155832076	0.674025657
SF3B1	0.694597863	0.222407164	0.161265428	0.161177729	0.186988965	0.155990664	0.674206687
FAM222A	0.628841914	0.847427188	0.015314076	0.77194471	0.119254033	0.156038808	0.674237196
AAGAB	0.104261821	0.227131237	0.472530374	0.432464021	0.155636559	0.156251173	0.674621941

MAP3K5	0.221751678	0.103243007	0.530334892	0.080282967	0.772376871	0.156219664	0.674621941
TUBG1	0.310196684	0.023596871	0.58727022	0.455947316	0.384032132	0.156196738	0.674621941
DUSP4	0.797246284	0.026580255	0.43949806	0.223841132	0.362226496	0.156470675	0.674859461
ODC1	0.712570552	0.135547238	0.095137869	0.123082822	0.667314689	0.156426447	0.674859461
RNF14	0.881184193	0.1735228	0.625122789	0.088665799	0.089011977	0.156386425	0.674859461
ZBTB11	0.532908445	0.809974261	0.756767441	0.003518821	0.656821657	0.156451739	0.674859461
BZW1	0.966390073	0.043686728	0.352923649	0.828789009	0.061201044	0.156539724	0.674979879
OAZ1	0.405525861	0.236944745	0.05056144	0.526119468	0.296087794	0.156656733	0.67504759
TIFA	0.07846657	0.296935053	0.508498613	0.583081368	0.109581934	0.15667883	0.67504759
TMEM170A	0.576787467	0.249148283	0.165283411	0.274628311	0.116019075	0.156654359	0.67504759
CISD2	0.742570505	0.117930394	0.249653742	0.850097623	0.040791449	0.156801938	0.675351608
COL6A3	0.578968458	0.243832824	0.102933341	0.258985977	0.201616412	0.156872849	0.675351608
THRSP	0.672188626	0.289427823	0.504432067	0.040992971	0.188551706	0.156847397	0.675351608
FANCA	0.212292697	0.451810931	0.164076118	0.928740583	0.051997482	0.157010286	0.675766013
EMX2	0.028862786	0.278312425	0.151495948	0.787526544	0.793855613	0.157100467	0.675799675
SLIT2	0.59291812	0.180675907	0.200719049	0.042810828	0.826454225	0.157096081	0.675799675
CCT6A	0.982094761	0.097234183	0.283053482	0.770909028	0.036585155	0.157269481	0.675992915
CYP1B1	0.236453409	0.968371917	0.642076163	0.033863038	0.153124896	0.157268926	0.675992915
LOC104976061	0.402215328	0.236234085	0.043802721	0.771570336	0.237509203	0.157310154	0.675992915
MRPL32	0.450874405	0.043671014	0.790832748	0.375781105	0.130253577	0.157251841	0.675992915
FAM214B	0.301068297	0.186381416	0.208755625	0.656375583	0.099278729	0.157379389	0.67611339
BCS1L	0.384776409	0.16466604	0.909310718	0.114894713	0.115759318	0.157703596	0.676191043
CBX4	0.964117732	0.178243616	0.272143225	0.031010829	0.528208993	0.157680221	0.676191043
CDKL1	0.034640574	0.340143641	0.622098764	0.203756769	0.512428464	0.157600667	0.676191043
RAB6A	0.96313977	0.024331907	0.152953374	0.590634953	0.362041015	0.157727092	0.676191043
SCNN1D	0.949928179	0.006086231	0.244210265	0.75343857	0.718321037	0.157467874	0.676191043
SLC35G2	0.301722142	0.212163402	0.078224863	0.210630558	0.726404853	0.157692261	0.676191043
TMEM8A	0.128949382	0.138467533	0.257515816	0.519706936	0.32064712	0.157698959	0.676191043
USP28	0.648891742	0.265420571	0.141573465	0.712977081	0.04405198	0.157654706	0.676191043
ACVR1C	0.192346615	0.831021862	0.043000131	0.337331205	0.331607865	0.157989536	0.676627158
ADGRE3	0.088405661	0.85383337	0.083387308	0.765591124	0.160824149	0.158664155	0.676627158
ALG5	0.55687918	0.137265577	0.528190152	0.299360793	0.06382921	0.158278223	0.676627158
ATP5PF	0.682406119	0.07785781	0.689176366	0.081522355	0.259675012	0.158680016	0.676627158
BANF1	0.573960581	0.28097971	0.038734081	0.180322017	0.684948551	0.158284019	0.676627158
BNC1	0.748988321	0.062854175	0.096541819	0.460602906	0.368088791	0.158176026	0.676627158
CPT1A	0.596607311	0.19974764	0.435246376	0.156132345	0.09544791	0.158441845	0.676627158
HEBP1	0.127705932	0.378997153	0.07626727	0.31066275	0.674238831	0.15846598	0.676627158
LOC100848307	0.562043885	0.502246155	0.154472837	0.410510043	0.043071657	0.158224916	0.676627158
LOC510913	0.07645597	0.025452486	0.61464789	0.716132784	0.902221953	0.158424301	0.676627158
NDUFV1	0.636600893	0.460090635	0.901899686	0.028988204	0.100598391	0.158152076	0.676627158

PCK1	0.003420088	0.328582114	0.787216953	0.983733847	0.89043018	0.158654718	0.676627158
PDK1	0.707519031	0.35287784	0.103753051	0.327128594	0.091010844	0.158248485	0.676627158
PNISR	0.992559059	0.313906827	0.035223331	0.635543172	0.110272788	0.158019344	0.676627158
POLR2L	0.162312613	0.308801229	0.365410081	0.118066899	0.356492521	0.15821243	0.676627158
RBM12	0.211830929	0.040100664	0.482671652	0.371263478	0.507609129	0.15841051	0.676627158
RBM25	0.985103217	0.626089198	0.101911447	0.024502159	0.50339842	0.158694652	0.676627158
SH3BP1	0.69859599	0.113706323	0.765289277	0.041735266	0.303112803	0.158008197	0.676627158
ST8SIA4	0.849737552	0.265530014	0.13594275	0.02669338	0.944107955	0.158444778	0.676627158
STK40	0.333190331	0.13721175	0.460530052	0.472548295	0.077808764	0.158569277	0.676627158
YBX1	0.916214744	0.068516049	0.174409994	0.860580798	0.08220031	0.158610297	0.676627158
LOC101905390	0.0944077	0.432414922	0.043409882	0.585789441	0.748081257	0.158837569	0.676884797
SLC37A3	0.570266679	0.440967468	0.117885563	0.159621579	0.16404363	0.158800186	0.676884797
KIF23	0.672121892	0.861614254	0.141149612	0.062310343	0.152633802	0.158928569	0.677096767
AHR	0.748313709	0.740928685	0.009032603	0.79896022	0.195273955	0.159358431	0.677834794
ATXN1L	0.472193216	0.757077637	0.007665935	0.838952837	0.339625421	0.159303547	0.677834794
CDCA3	0.276099455	0.144573803	0.188143561	0.210878645	0.49317451	0.159326072	0.677834794
HNRNPF	0.741057369	0.809665884	0.265784983	0.007237409	0.675610031	0.159186609	0.677834794
PAICS	0.464134335	0.065522715	0.34149088	0.276583337	0.27198923	0.159348593	0.677834794
SYNPO2	0.019758857	0.343186749	0.971810574	0.986082593	0.119990589	0.15918016	0.677834794
TTC33	0.427416118	0.899489647	0.019603823	0.597462015	0.173584036	0.159390925	0.677834794
CXHXorf36	0.684509822	0.91354583	0.018831629	0.093378272	0.711981566	0.159529382	0.678247845
GRAMD2A	0.99641113	0.148821297	0.162976227	0.162930332	0.199270799	0.159718394	0.678474041
PLK4	0.185417416	0.124870732	0.381028183	0.535435713	0.166168277	0.159747955	0.678474041
PPIE	0.772063452	0.115884783	0.622856095	0.024204989	0.581748269	0.159725004	0.678474041
ZNF608	0.828860213	0.067274317	0.170813865	0.158561035	0.519118168	0.159648137	0.678474041
LOC112444926	0.495273112	0.249107647	0.307051018	0.195386632	0.106122812	0.159811379	0.678567799
CCDC58	0.812854832	0.078915981	0.705996071	0.184994223	0.093849375	0.159894838	0.67858613
UGT8	0.165184702	0.822136218	0.671308471	0.306773864	0.02811472	0.159898395	0.67858613
DPH7	0.30265054	0.27242074	0.761448993	0.059225854	0.211675688	0.159980524	0.678759149
LPAR6	0.72283462	0.876720497	0.006661698	0.212270699	0.879345434	0.160084953	0.679026666
LOC101902968	0.373358709	0.283267869	0.074197282	0.605435705	0.16628706	0.160302624	0.679617939
PDIA6	0.302159014	0.596124707	0.012092283	0.442932229	0.819170469	0.160333115	0.679617939
ZFPM2	0.136471358	0.202561707	0.236079688	0.129519503	0.935143551	0.160348587	0.679617939
ARL14EPL	0.911788178	0.564155111	0.634303913	0.006080733	0.399850668	0.160659303	0.679729063
C10H15orf59	0.114667081	0.341883247	0.125335958	0.537655371	0.301117943	0.160894598	0.679729063
CD226	0.420540801	0.317861861	0.398221301	0.06094441	0.244106188	0.160509107	0.679729063
CFL2	0.852973195	0.045635736	0.131747891	0.498240063	0.311353216	0.160903155	0.679729063
FHDC1	0.18625429	0.205294604	0.023430123	0.926786338	0.958134063	0.160900816	0.679729063
IL6R	0.711595792	0.369831566	0.174782509	0.026349558	0.655335305	0.160763852	0.679729063
METTL14	0.016613484	0.24505073	0.616767182	0.683648564	0.462644776	0.160753232	0.679729063

PHF21A	0.980022924	0.090215064	0.062818216	0.476742702	0.300697489	0.160970208	0.679729063
PRICKLE1	0.345662198	0.123301569	0.398518551	0.09584751	0.489210272	0.160996092	0.679729063
TMEM138	0.988450471	0.18306952	0.72617814	0.566050641	0.010700245	0.160940184	0.679729063
TRAPPC6A	0.016181547	0.639502341	0.512761433	0.321743437	0.466147545	0.160929787	0.679729063
TRIP10	0.312757769	0.121969558	0.905305298	0.065438856	0.352215178	0.160947165	0.679729063
TRMT10B	0.65181553	0.561489501	0.91898228	0.056477682	0.041807403	0.160750264	0.679729063
TRMT61A	0.200235952	0.195296456	0.926564485	0.345550434	0.063422667	0.160743237	0.679729063
WDR6	0.976239603	0.84222197	0.07571243	0.038985128	0.326795416	0.160635869	0.679729063
ADGRB1	0.909063043	0.157859577	0.589697434	0.470219287	0.020036911	0.161091176	0.679955579
C1QBP	0.657799323	0.34233727	0.582678815	0.199565126	0.030494879	0.161222608	0.680335363
B3GNT7	0.776967015	0.358707123	0.033727557	0.333036962	0.257803098	0.162140818	0.680658662
BTBD3	0.074851983	0.239715366	0.717580964	0.68232245	0.091796492	0.162076638	0.680658662
CSTF2T	0.764326778	0.363221567	0.497437066	0.196998451	0.02953853	0.161769112	0.680658662
DHPS	0.454368446	0.283088365	0.365187136	0.100684774	0.169878245	0.161750558	0.680658662
EFR3A	0.442639149	0.070756818	0.120212198	0.473780815	0.450137816	0.161699347	0.680658662
FAM96B	0.179005031	0.025341027	0.715609561	0.313779817	0.7929871	0.162210294	0.680658662
FKBP7	0.7624764	0.210819613	0.013220442	0.433726647	0.872724387	0.161855849	0.680658662
LOC101907276	0.648388993	0.124674707	0.129179539	0.310697545	0.246527579	0.161365961	0.680658662
LOC112442693	0.037800274	0.250166374	0.293257132	0.821767937	0.353131752	0.161892021	0.680658662
LOC112447030	0.653815145	0.390911435	0.193802402	0.048228932	0.334895782	0.161385564	0.680658662
LOC112447492	0.435993931	0.469975427	0.12212504	0.826639349	0.038957077	0.162012303	0.680658662
LOC530348	0.512224462	0.800810899	0.511682806	0.617119221	0.006201139	0.161727584	0.680658662
MAN1A1	0.303321767	0.322166045	0.863059698	0.060960924	0.155874925	0.16153291	0.680658662
PNPLA6	0.276986313	0.151077451	0.041218397	0.891471607	0.522315446	0.161719189	0.680658662
PXMP4	0.648048973	0.20648266	0.905184585	0.156210512	0.04234393	0.16150868	0.680658662
RPS6KB2	0.779004391	0.146053047	0.4222892	0.02769374	0.607051601	0.16221249	0.680658662
SCRIB	0.185210844	0.035409657	0.230856761	0.638673876	0.835060854	0.162184646	0.680658662
SEMA3E	0.030201587	0.962623203	0.19627357	0.37686276	0.374466973	0.16194898	0.680658662
SIGLECL1	0.181124815	0.719528368	0.063921015	0.477912989	0.202156459	0.161901485	0.680658662
SLC25A39	0.400773059	0.815128186	0.622980434	0.031096006	0.127643018	0.162219025	0.680658662
TIPARP	0.755553115	0.425031381	0.32431723	0.00824319	0.940292791	0.162162017	0.680658662
ZC4H2	0.693451679	0.507610442	0.313469247	0.792096032	0.009245965	0.162253165	0.680658662
ZNHIT3	0.25769056	0.445020579	0.261899418	0.063807033	0.418475037	0.161592355	0.680658662
IER3IP1	0.811024703	0.062290472	0.51709547	0.761841554	0.040647529	0.162343576	0.680863897
ELOVL7	0.699668577	0.269663132	0.05117204	0.262691529	0.319288892	0.162433603	0.680893448
SLC25A12	0.636340311	0.876173483	0.002331861	0.703325498	0.885286858	0.162402889	0.680893448
CAVIN4	0.708504817	0.445543022	0.085410153	0.030460337	0.98730414	0.162543581	0.681180466
LOC101904595	0.802015515	0.007674191	0.515018031	0.34196627	0.749073949	0.162667028	0.681523769
HOXB5	0.186935614	0.975809738	0.264665853	0.442403799	0.038080913	0.162814007	0.681793492
NR4A2	0.20107079	0.25603116	0.371977819	0.212269443	0.200095547	0.162814496	0.681793492

ATP6V1E1	0.181195619	0.200007776	0.334928619	0.614603558	0.109357593	0.163075963	0.681883743
GNPNAT1	0.485164992	0.153347024	0.115845304	0.992683525	0.095391526	0.163110597	0.681883743
IQGAP3	0.107209372	0.11663924	0.154516337	0.618205413	0.683164325	0.163099779	0.681883743
LOC104971030	0.696857527	0.264001035	0.007236539	0.803286139	0.761876745	0.16296483	0.681883743
MAGED2	0.352622496	0.130053383	0.274007121	0.933113717	0.069443605	0.162909793	0.681883743
NOB1	0.411931344	0.420238997	0.141031545	0.357030926	0.093738275	0.163210002	0.681883743
NOV	0.070847989	0.447441712	0.766697464	0.049738295	0.675800482	0.163197417	0.681883743
SLC22A31	0.006329779	0.700216348	0.533174257	0.430907124	0.801652338	0.16312979	0.681883743
UBE2G1	0.984609374	0.059756222	0.259292106	0.820329479	0.06517813	0.163063106	0.681883743
SMIM20	0.70472611	0.243511511	0.657940372	0.114934543	0.063051192	0.16333223	0.682220725
CYP2R1	0.546882886	0.221106519	0.088377073	0.177881926	0.430984203	0.16344418	0.682320663
MACF1	0.313620521	0.899906543	0.277703449	0.056706245	0.184415599	0.163480888	0.682320663
SUSD5	0.227889758	0.476478473	0.806686331	0.047371016	0.197437397	0.16344142	0.682320663
BTBD9	0.676199563	0.336893914	0.63231193	0.822470982	0.006930458	0.163635444	0.682374805
IGF2BP3	0.363087444	0.612165223	0.358537628	0.059593896	0.172703201	0.163542092	0.682374805
KBTBD6	0.763397844	0.176555065	0.432712234	0.028842662	0.488025412	0.163620611	0.682374805
LOC100848226	0.233381527	0.613844922	0.167837817	0.141338163	0.241674469	0.163660181	0.682374805
KLHL17	0.600736845	0.033093326	0.059491542	0.899594771	0.772448625	0.163718661	0.682445249
ALS2	0.340912392	0.650530551	0.014096294	0.303667333	0.868925134	0.164040109	0.682917865
ANG	0.067028681	0.208083416	0.159439041	0.561466928	0.660276054	0.163989164	0.682917865
CDH22	0.76323307	0.046130522	0.797875808	0.602393908	0.048654608	0.163877158	0.682917865
MAGEF1	0.283070903	0.716597578	0.013382423	0.35519475	0.855448998	0.164034098	0.682917865
MT2A	0.87841385	0.389149179	0.64172339	0.117271671	0.032036555	0.163960921	0.682917865
TRIOBP	0.070467253	0.977585167	0.237788854	0.251760406	0.200145293	0.164094504	0.682971063
LOC100335828	0.007835486	0.336683481	0.467019306	0.801039297	0.837271351	0.164190369	0.683023611
MAFF	0.144163345	0.099581747	0.175602705	0.624355189	0.524741043	0.164150101	0.683023611
GXYLT2	0.455458114	0.394480586	0.049641718	0.233336304	0.39743667	0.164276643	0.683209324
ANXA7	0.294425142	0.035493682	0.169426193	0.912570502	0.51221635	0.164328047	0.683249957
EPHB6	0.496999356	0.416597643	0.015214819	0.528377606	0.49762196	0.164399969	0.683375861
POLR3D	0.298559686	0.015450496	0.967049678	0.881893367	0.211038303	0.164604875	0.684054343
COPA	0.241228856	0.15628549	0.718535506	0.043665633	0.70316781	0.164765328	0.684281255
EI24	0.585444444	0.156265485	0.136478203	0.283439592	0.23543728	0.164917372	0.684281255
LOC615989	0.065655001	0.094678617	0.300293386	0.692317492	0.64341328	0.164738262	0.684281255
NUSAP1	0.771350772	0.040639482	0.049819367	0.758152334	0.702719269	0.164793942	0.684281255
TLR6	0.045975592	0.061239831	0.939479089	0.685215496	0.459875607	0.164951352	0.684281255
TMEM106A	0.216721019	0.185012948	0.114717759	0.963961708	0.187906441	0.164914841	0.684281255
UBAC1	0.252816622	0.323173861	0.603473323	0.080331727	0.210258376	0.164875466	0.684281255
B4GALT2	0.319321159	0.888871281	0.036363655	0.765000432	0.105649637	0.165021641	0.684388711
GNS	0.635571917	0.105940717	0.060823137	0.83453877	0.244182133	0.165060662	0.684388711
SOX6	0.058506581	0.117506425	0.302603207	0.646340816	0.621186132	0.165134743	0.684522927

NINJ1	0.686906323	0.351650179	0.847731836	0.018929036	0.21592132	0.165310299	0.684559
RNF41	0.517959973	0.524854987	0.052179601	0.119664822	0.492527806	0.165217746	0.684559
SARS	0.32657498	0.107283145	0.324232074	0.653499515	0.112730626	0.165303557	0.684559
SDC2	0.196866483	0.106976438	0.470696258	0.156611332	0.538634232	0.165234796	0.684559
SNRK	0.197110625	0.748336134	0.722971448	0.056724445	0.138433119	0.165360358	0.684593549
ATP5F1E	0.864485846	0.458737235	0.797470228	0.022935937	0.115701211	0.165553315	0.684721399
CFAP100	0.421079624	0.098349521	0.101185764	0.383381746	0.522531838	0.165575475	0.684721399
EPSTI1	0.105534965	0.057963089	0.572524548	0.243312946	0.983595864	0.165438475	0.684721399
ERBIN	0.514489738	0.867814541	0.617430811	0.012125194	0.251055789	0.165545235	0.684721399
TMEM229B	0.85152321	0.029052597	0.489754823	0.681097356	0.101753884	0.165599856	0.684721399
DNAJB14	0.57669831	0.264101407	0.553254999	0.032784953	0.304398056	0.165729795	0.684741105
LOC100847454	0.219651464	0.3440704	0.309894889	0.073333464	0.489411519	0.165691468	0.684741105
VPS16	0.675011594	0.042853814	0.09599926	0.584447539	0.517679161	0.165651441	0.684741105
MDK	0.119612109	0.031774893	0.264702923	0.953601249	0.877557403	0.165831064	0.684856552
RABEP1	0.842784825	0.51110725	0.309045958	0.009235286	0.68486997	0.1658412	0.684856552
SAP25	0.048765121	0.153026613	0.857935976	0.864974379	0.152229804	0.165947534	0.685123265
AP1M1	0.687747033	0.268589729	0.304172242	0.522164358	0.028921144	0.166522238	0.685289748
CCL24	0.868922093	0.781122847	0.131128016	0.091999131	0.103768863	0.166641612	0.685289748
CCR10	0.82784386	0.036095411	0.939371289	0.062422791	0.481732873	0.16605987	0.685289748
CSF1	0.731872866	0.790210552	0.041174814	0.135303782	0.263295708	0.166502577	0.685289748
DDA1	0.147185149	0.024464734	0.585188253	0.533617506	0.754094275	0.166460224	0.685289748
LOC112448084	0.98655075	0.526583137	0.351448513	0.6750928	0.006892985	0.166636	0.685289748
MTUS2	0.651435011	0.479493449	0.32180146	0.033614027	0.251209196	0.166550232	0.685289748
MYBPHL	0.88937167	0.530171944	0.112728639	0.029980054	0.531979997	0.166441131	0.685289748
NAT1	0.886538348	0.15040391	0.439735479	0.173098606	0.083549088	0.166465851	0.685289748
ORC3	0.863160718	0.150095992	0.078485142	0.735010297	0.113245016	0.166298439	0.685289748
PDE6C	0.02597979	0.480616205	0.640076314	0.16781393	0.633033246	0.166575176	0.685289748
SLC2A10	0.503509096	0.503354762	0.009541505	0.908323875	0.386881419	0.166655986	0.685289748
STON2	0.191392759	0.217017612	0.206514722	0.813190749	0.12115374	0.166164241	0.685289748
TMEM248	0.848957368	0.22849711	0.023992308	0.412874958	0.441022335	0.16641177	0.685289748
TMEM72	0.008462946	0.808047438	0.978257828	0.218792821	0.577731158	0.166219104	0.685289748
XYLB	0.507411902	0.812636915	0.084623088	0.567630608	0.042894615	0.166635132	0.685289748
BMP2	0.317631111	0.108779964	0.157735089	0.423399946	0.368552829	0.166724015	0.685397748
FDX2	0.096563775	0.445172253	0.585579211	0.137711593	0.245502516	0.16678591	0.685415911
RALGAPA1	0.388081385	0.653271307	0.310764746	0.017081222	0.632579499	0.166811964	0.685415911
HEATR3	0.437809018	0.666767401	0.027414819	0.218266486	0.487889552	0.166907996	0.685467249
SAR1B	0.759796575	0.124998262	0.083611253	0.586048977	0.18311626	0.166902659	0.685467249
TDRD12	0.272311707	0.212010868	0.037034207	0.973814498	0.409573571	0.166965665	0.685532533
APOE	0.228117137	0.989692645	0.081771696	0.19199996	0.242275389	0.167586179	0.685613329
CLK1	0.856991041	0.286226462	0.244072616	0.196909427	0.072635618	0.167330385	0.685613329

CXXC1	0.943450657	0.05125291	0.052558575	0.838636317	0.402185001	0.167424358	0.685613329
EVA1A	0.520382729	0.623213187	0.017521666	0.23787443	0.635498946	0.167611933	0.685613329
HNF4A	0.291535783	0.950724842	0.023234253	0.135221738	0.986451967	0.167612009	0.685613329
KLHL8	0.866538669	0.412971373	0.258218815	0.051078541	0.181909002	0.167569139	0.685613329
LOC781064	0.598752128	0.268944069	0.384108822	0.07713607	0.179500682	0.16734381	0.685613329
LOC787287	0.635328736	0.908284949	0.094216736	0.031424153	0.502361196	0.167536371	0.685613329
NOSIP	0.665267479	0.747656723	0.003369695	0.544652985	0.940641822	0.167578309	0.685613329
PPP2R3A	0.973937145	0.782597283	0.020899028	0.071002271	0.755335193	0.167123199	0.685613329
SLC9B2	0.233282445	0.82262491	0.053694415	0.209676218	0.395577477	0.167161283	0.685613329
SOD2	0.877652707	0.762968553	0.589771316	0.183929916	0.011820747	0.167573604	0.685613329
THSD1	0.152550692	0.102542369	0.149643097	0.771894521	0.473231049	0.167204519	0.685613329
TTLL3	0.329877002	0.221283325	0.068260635	0.526551964	0.325575114	0.167114251	0.685613329
ZC3H4	0.291006817	0.238304902	0.017973132	0.74834323	0.920589108	0.167576941	0.685613329
PIP5K1C	0.225804524	0.490810689	0.225893097	0.232415298	0.147844021	0.167739015	0.685961867
LOC515169	0.291432644	0.60231084	0.019004086	0.982258972	0.263011142	0.167900482	0.686249911
SEC11C	0.212915361	0.130569449	0.924480881	0.315235548	0.106412393	0.1679349	0.686249911
SPG11	0.241893325	0.518857026	0.022891436	0.566167916	0.529587366	0.167863886	0.686249911
FAM171A2	0.918377821	0.393062729	0.030286988	0.085390499	0.924020046	0.167987474	0.686293861
KRTCAP3	0.485893389	0.083096842	0.180531493	0.642141252	0.184585082	0.168126036	0.686543257
PPCS	0.258391014	0.317682904	0.651031495	0.143495225	0.112674317	0.168132189	0.686543257
COX7A2L	0.592940096	0.199113206	0.655270506	0.229880722	0.048713429	0.168367754	0.68698792
CXCR1	0.847767909	0.068405761	0.73411772	0.046465045	0.437886603	0.168355556	0.68698792
GBA2	0.532931292	0.395229323	0.046940017	0.091456427	0.95942413	0.168492254	0.68698792
LYST	0.374615928	0.363016308	0.145623306	0.047899172	0.914039589	0.168440405	0.68698792
PCDH12	0.935327999	0.569614246	0.027560031	0.267393157	0.220900069	0.168468022	0.68698792
PPARGC1B	0.272210002	0.467878515	0.043775221	0.155593828	0.99847814	0.168350022	0.68698792
DEF6	0.530215173	0.29125983	0.981369845	0.076026548	0.075415235	0.168636492	0.687091207
LITAF	0.521127511	0.269524764	0.197854995	0.156473847	0.199705916	0.168581479	0.687091207
MRPS16	0.275646056	0.124220188	0.957836517	0.082753531	0.320184626	0.168643189	0.687091207
INSR	0.670188142	0.629199158	0.027903214	0.387709576	0.190864716	0.168817922	0.687465406
LOC100336941	0.458529106	0.042100106	0.308894003	0.512030611	0.285314294	0.168860706	0.687465406
SLC27A3	0.056706247	0.557718699	0.997136439	0.244627715	0.112889623	0.168836272	0.687465406
FADS3	0.934274711	0.064894036	0.166451607	0.105630348	0.817720927	0.16891883	0.68753148
ZSCAN16	0.527291068	0.282214381	0.445207081	0.513990499	0.025620674	0.168996322	0.687676329
COMMD2	0.018257773	0.246886976	0.655894757	0.528076753	0.5596033	0.169124184	0.688026026
DDT	0.447559975	0.259573575	0.91308459	0.038748454	0.212720253	0.169191939	0.688131079
LIPA	0.478127477	0.249382226	0.062673722	0.925273985	0.126752999	0.169406039	0.688831147
THEM6	0.175668086	0.777171491	0.809724181	0.016187726	0.490201938	0.16948544	0.688983293
C25H16orf45	0.424377694	0.039235851	0.188087448	0.515874755	0.544660887	0.169766211	0.689256953
KCNT1	0.832463097	0.162439194	0.099554214	0.159612358	0.409481974	0.169756047	0.689256953

LOC100299303	0.067322176	0.757632715	0.646561739	0.912192842	0.029249748	0.169759953	0.689256953
LOC509155	0.185730333	0.092946747	0.940652273	0.627829545	0.086283931	0.169735579	0.689256953
PWWP2A	0.358326406	0.81209065	0.165717444	0.266609864	0.068473804	0.169804757	0.689256953
WEE1	0.37903135	0.652622744	0.568411751	0.0181279	0.345079305	0.169725025	0.689256953
DMTN	0.020444708	0.747722892	0.599342199	0.229322142	0.419398604	0.169891092	0.68932146
FGFR3	0.226277174	0.236390566	0.174657228	0.281112691	0.335737563	0.169946659	0.68932146
H2AFZ	0.437509393	0.058968591	0.327922156	0.977250077	0.106621257	0.169924004	0.68932146
GTPBP4	0.177241649	0.035072446	0.524656381	0.571690406	0.474057546	0.170166432	0.689531188
IL1A	0.299681683	0.765575948	0.127528507	0.178250679	0.169339545	0.170093193	0.689531188
NXPE3	0.874635539	0.138863279	0.18639813	0.174746803	0.223349442	0.170135642	0.689531188
RANBP9	0.347580041	0.087145281	0.270417906	0.737716014	0.146172183	0.170102052	0.689531188
MAATS1	0.438822076	0.153822361	0.13624914	0.44100966	0.21851955	0.17041142	0.690100877
PARD3	0.612504646	0.392824258	0.98491269	0.010549813	0.354677139	0.170453602	0.690100877
PITPNM3	0.029488602	0.52093104	0.168690662	0.638959515	0.535329406	0.170419678	0.690100877
VAV1	0.499530907	0.203460908	0.566342914	0.044579645	0.345779708	0.170510798	0.690100877
ZMAT2	0.538460912	0.014231982	0.707167696	0.506172849	0.323481403	0.170517279	0.690100877
FTH1	0.378793956	0.088879838	0.289284982	0.106217734	0.858531004	0.170599314	0.690214621
LOC100847122	0.747181275	0.004990785	0.624134083	0.549623203	0.694532761	0.1706295	0.690214621
CCDC47	0.791986214	0.243182756	0.179400943	0.736841905	0.034952526	0.17077443	0.690367862
MAML3	0.858741982	0.222494638	0.09978594	0.162638969	0.287041012	0.170793585	0.690367862
THUMPD1	0.418459526	0.377797778	0.084577904	0.240708994	0.276518598	0.170786575	0.690367862
DTX1	0.363467691	0.484316209	0.022446146	0.78804011	0.286131904	0.170883212	0.690560055
LOC104968476	0.804979089	0.163849872	0.663923553	0.829628698	0.012277778	0.170987456	0.690811212
LOC783854	0.974378894	0.446387696	0.482325053	0.390269671	0.010899891	0.171032103	0.69082152
CHL1	0.130017029	0.9265891	0.03062538	0.274124367	0.884099187	0.171209216	0.691044979
LOC107131418	0.823475854	0.52325866	0.224312236	0.16184208	0.057191429	0.171256111	0.691044979
LOC112447302	0.123761424	0.132529817	0.90483493	0.188826049	0.319261765	0.171263281	0.691044979
TLR4	0.03829838	0.458202207	0.985925811	0.159880418	0.323568667	0.171297969	0.691044979
VAMP8	0.070829876	0.452490768	0.896603125	0.119349235	0.260542459	0.171148208	0.691044979
FAM160B1	0.025402933	0.503135671	0.372886721	0.228461402	0.822807738	0.171384163	0.691052949
TOMM7	0.540102791	0.022205588	0.40591009	0.220488347	0.834403585	0.171357743	0.691052949
AP1S2	0.342970217	0.461804327	0.768714556	0.135941173	0.054271136	0.171622438	0.691334274
CLPP	0.307682133	0.186042206	0.43207078	0.216348555	0.167728196	0.171545085	0.691334274
CYB5B	0.865480486	0.28286753	0.333000354	0.165879254	0.066402931	0.171594257	0.691334274
VAMP1	0.369882634	0.500858747	0.162719087	0.075498558	0.394366672	0.171550892	0.691334274
TINF2	0.489345298	0.85479726	0.00611218	0.501363122	0.701306114	0.171692423	0.691446466
INPP5D	0.890014615	0.057688963	0.442568087	0.225773154	0.17545239	0.171810797	0.691472811
LOC512672	0.089798316	0.226151769	0.373697822	0.386572733	0.306607642	0.171748429	0.691472811
OOEP	0.568281675	0.26101992	0.491350062	0.083877563	0.147356142	0.171882001	0.691472811
PCNX4	0.456211507	0.269230035	0.804372245	0.16235818	0.056176364	0.171909638	0.691472811

RHBDF1	0.880877573	0.020333934	0.302871014	0.382851199	0.433628174	0.171861068	0.691472811
GALNT9	0.016420742	0.316947277	0.995935355	0.39805883	0.437822284	0.172135772	0.69153492
GDF1	0.384422743	0.187044349	0.065773533	0.969975866	0.196621506	0.171997883	0.69153492
LOC516442	0.257958858	0.26170227	0.586240743	0.664484412	0.034349446	0.172132087	0.69153492
PLXDC1	0.026736822	0.732540447	0.478860626	0.122558344	0.785594955	0.172101466	0.69153492
RBM4	0.542388006	0.194043082	0.181171822	0.08999171	0.526045659	0.172066482	0.69153492
SNAPC5	0.725533369	0.82343554	0.369746717	0.032483759	0.125972462	0.172193758	0.691598572
ABCC3	0.472559793	0.160481489	0.017720677	0.767948844	0.876941986	0.17230511	0.691876475
GPRC5A	0.277148553	0.360411159	0.20565714	0.116590591	0.378096958	0.172358748	0.691922556
GDI1	0.290633292	0.114916126	0.083234956	0.406204572	0.802393827	0.172409678	0.691957748
DESI2	0.067125281	0.642892986	0.42287348	0.115231633	0.432650768	0.172782877	0.692269264
LSM1	0.602251396	0.281832439	0.726084533	0.588583741	0.012517935	0.172605101	0.692269264
MYCT1	0.28033319	0.965615375	0.031686807	0.113991646	0.930654558	0.172798461	0.692269264
NANOS1	0.804152782	0.637364774	0.005388794	0.846181062	0.388790541	0.172668074	0.692269264
RAPGEF1	0.955185068	0.052085277	0.451658124	0.161465259	0.25071227	0.172766629	0.692269264
STRN3	0.871269914	0.468665495	0.228124586	0.011859814	0.8233437	0.172762391	0.692269264
TNNT2	0.334251483	0.150297494	0.138028446	0.194983145	0.673214209	0.172824762	0.692269264
USP13	0.896795342	0.367232189	0.929158739	0.03347764	0.088731238	0.172701246	0.692269264
DOK7	0.542877656	0.453428199	0.510443687	0.012132292	0.59789987	0.172947716	0.692423753
NOL11	0.353996037	0.451234292	0.794391719	0.036090311	0.199002054	0.172938	0.692423753
FBXO32	0.403039423	0.695014003	0.861024865	0.073152976	0.051737661	0.173087613	0.692600548
LOC104970976	0.805449091	0.620642754	0.107649992	0.056137204	0.302298834	0.173126593	0.692600548
TPGS2	0.620775437	0.052718483	0.847005011	0.459580194	0.071667794	0.173102991	0.692600548
ZNF296	0.226396972	0.096383103	0.300382518	0.190633469	0.731135568	0.173160688	0.692600548
PEX11G	0.061497185	0.495928158	0.508210982	0.103006477	0.572669843	0.173232946	0.692720732
AGER	0.942968268	0.280563717	0.663251515	0.293762345	0.017833875	0.173730194	0.692931506
CC2D2A	0.199464963	0.333498324	0.044715285	0.55393295	0.55944023	0.173978745	0.692931506
CENPI	0.344680829	0.202739629	0.997125106	0.387694841	0.033888061	0.173349749	0.692931506
CFH	0.843461922	0.28164676	0.056752119	0.154504724	0.440233397	0.173504435	0.692931506
ENTPD6	0.262187786	0.078493083	0.903456942	0.078262251	0.631255472	0.173658564	0.692931506
KATNA1	0.631156688	0.426197391	0.135230508	0.193438648	0.130461531	0.173603696	0.692931506
LOC508131	0.191700736	0.339648963	0.524901161	0.997139882	0.027010063	0.173849237	0.692931506
MAMDC4	0.703158331	0.039639683	0.193474229	0.501653967	0.340983708	0.174045681	0.692931506
MS4A2	0.341414814	0.162533382	0.184162	0.534880237	0.168736209	0.174034174	0.692931506
NAT9	0.581404001	0.425024728	0.194103723	0.019279126	0.990817628	0.173426559	0.692931506
NDUFS7	0.293098587	0.313159017	0.571558976	0.045145948	0.388434719	0.173799308	0.692931506
NUMBL	0.300942829	0.255499881	0.638285909	0.034423849	0.543166398	0.173568711	0.692931506
POT1	0.021336049	0.859145795	0.566721062	0.115180724	0.768590389	0.173767359	0.692931506
SCML4	0.249661785	0.569844193	0.689422921	0.03360529	0.279041437	0.173776906	0.692931506
SH2D2A	0.344875702	0.2086997	0.640231477	0.672870048	0.029732981	0.173992236	0.692931506

SLC25A27	0.23132017	0.404906869	0.109827972	0.10783243	0.830364903	0.173909712	0.692931506
STAG2	0.040481965	0.357522936	0.650910071	0.152970999	0.637313235	0.173645864	0.692931506
TMEM132C	0.06613191	0.688701459	0.544335101	0.057927763	0.637892198	0.173412941	0.692931506
KDM5C	0.254470476	0.231871318	0.49997977	0.68932197	0.04540851	0.174141102	0.69314325
MRPL23	0.184249636	0.290211277	0.33618559	0.284058611	0.180980193	0.174213507	0.693263305
JPT1	0.076357406	0.573993675	0.250888961	0.817235515	0.103003235	0.174361207	0.693682854
ADAMTSL2	0.42783787	0.171435496	0.64006611	0.032783513	0.602076815	0.174461497	0.693913627
CSE1L	0.042110684	0.22643815	0.514235082	0.64770872	0.292535963	0.17470495	0.694545284
TSSK2	0.739631051	0.179858145	0.621594119	0.019963807	0.56265038	0.174677745	0.694545284
LOC101903400	0.466849797	0.793337301	0.489459923	0.053962105	0.095338508	0.175053935	0.695764137
RPS6KA3	0.134149134	0.491108638	0.033223326	0.646951957	0.658940387	0.175100026	0.695778819
RBL1	0.155538336	0.611124586	0.403430011	0.17497982	0.1391851	0.175182646	0.695938609
SMG9	0.022667646	0.416746856	0.239905032	0.656922824	0.628205233	0.175313812	0.696122664
TPC3	0.012713943	0.371106536	0.955935149	0.464646069	0.44619636	0.175297155	0.696122664
LOC101909196	0.443705137	0.010621467	0.585840672	0.494516585	0.686155569	0.175468437	0.696568097
ACIN1	0.860920652	0.067251783	0.264995482	0.451274195	0.13550635	0.175605241	0.696733941
B3GALT2	0.057053263	0.156213181	0.326674445	0.646237647	0.499366428	0.175736978	0.696733941
CCNI	0.475184182	0.437123015	0.643752976	0.078852825	0.089136219	0.175764945	0.696733941
MRPL20	0.460232613	0.030116771	0.566723619	0.514549843	0.232379567	0.175706679	0.696733941
UBA7	0.112609866	0.266846752	0.635032067	0.053927213	0.912316325	0.175665188	0.696733941
ZC3H11A	0.681279969	0.452057682	0.20228345	0.214323162	0.070283189	0.175625409	0.696733941
ABHD17B	0.705485595	0.699131468	0.431976099	0.996541769	0.004429309	0.175824915	0.696803353
TAB2	0.592064117	0.668908359	0.933249359	0.002811282	0.905769775	0.175892442	0.696902672
LOC104970821	0.249535619	0.204875211	0.045137321	0.968322402	0.422050738	0.176081379	0.697146324
LOC112443849	0.983749577	0.455449338	0.029799843	0.3586579	0.196847553	0.176040179	0.697146324
MAPK9	0.119124951	0.531796868	0.561474824	0.57025462	0.046484581	0.176063084	0.697146324
GNPTAB	0.415214359	0.118129142	0.489291249	0.054048275	0.727861834	0.17618481	0.697387584
NDUFB10	0.513575336	0.085269034	0.682421389	0.074557773	0.423922985	0.176227534	0.697388489
FHOD3	0.029715341	0.742744468	0.085049373	0.633984815	0.795501159	0.176437231	0.69771359
ICE2	0.08677177	0.597125	0.047378028	0.961550292	0.401017897	0.1764256	0.69771359
UBE2F	0.448913627	0.045470868	0.325109567	0.94105691	0.151483119	0.176372134	0.69771359
CDC42	0.99927288	0.043185982	0.043940553	0.84585469	0.59179213	0.176682065	0.697841005
LOC104973739	0.907236388	0.104894308	0.094911059	0.496643256	0.211530773	0.176650002	0.697841005
RXRA	0.111490131	0.782050895	0.347247035	0.038165164	0.820740918	0.176602129	0.697841005
ZFP30	0.129813331	0.137277668	0.147673865	0.673542919	0.535383541	0.176659791	0.697841005
ZNF521	0.158004048	0.155246569	0.133746314	0.528369499	0.546690944	0.176531322	0.697841005
DPYSL3	0.008572834	0.842221635	0.33606585	0.432240079	0.905631966	0.176745024	0.697921702
EDN3	0.065075234	0.86152361	0.044224524	0.748216816	0.513054532	0.176934299	0.698080255
HIF1A	0.929417681	0.753858477	0.173174601	0.04952471	0.158499274	0.176997864	0.698080255
HSD17B14	0.357247215	0.034636754	0.574490434	0.220628206	0.607084432	0.176968934	0.698080255

LOC615559	0.303229709	0.406767876	0.009312486	0.946965876	0.874214034	0.176848539	0.698080255
MLLT1	0.750812214	0.253908348	0.139782548	0.980951239	0.036423781	0.176967807	0.698080255
CLTB	0.425411227	0.127627514	0.401091785	0.198797397	0.220295014	0.177121633	0.698293418
ECHDC2	0.20453379	0.447051003	0.327441061	0.447629081	0.07117224	0.177137012	0.698293418
LOC104975590	0.133515916	0.251881123	0.345901776	0.932787083	0.088040841	0.177279229	0.698686222
S100G	0.127525253	0.396934814	0.164686428	0.176656812	0.649179477	0.17734785	0.698788852
FLRT1	0.505729632	0.340655961	0.839358804	0.008282825	0.799176301	0.177462498	0.698954929
POLR3B	0.322549383	0.68469969	0.063218385	0.186558646	0.367542141	0.177475181	0.698954929
CHRNB1	0.142820029	0.455376085	0.122799705	0.473780195	0.253284061	0.177577965	0.699024222
GPD1L	0.274034593	0.309530169	0.057159322	0.215766845	0.916094351	0.177573446	0.699024222
UBASH3A	0.507366736	0.386460517	0.795811105	0.708376347	0.008696127	0.177853985	0.699942863
AUP1	0.200879923	0.090771288	0.570044176	0.421770681	0.220730918	0.178479177	0.700053004
CAPG	0.250360322	0.374801142	0.247537489	0.517704702	0.080304141	0.178284147	0.700053004
IFI44L	0.040871766	0.612193013	0.91386676	0.156937209	0.269207051	0.178322802	0.700053004
MAF	0.354549634	0.207067675	0.934995645	0.091380011	0.153491629	0.17800609	0.700053004
POP4	0.615030515	0.061032751	0.176581408	0.716838487	0.203454509	0.178384428	0.700053004
REX1BD	0.048103223	0.426741076	0.683493076	0.244438382	0.281857797	0.178379902	0.700053004
RIPOR2	0.069861622	0.206153894	0.750803728	0.540629418	0.16542701	0.178420933	0.700053004
SLC1A7	0.342921945	0.974176087	0.383141435	0.709161856	0.010622962	0.178145443	0.700053004
SOWAHC	0.193454681	0.080761725	0.213200185	0.533688884	0.543550262	0.178342882	0.700053004
SP7	0.468712628	0.267850864	0.076276367	0.197166582	0.510455811	0.178102061	0.700053004
SYNE1	0.49540026	0.201904708	0.249298732	0.214644545	0.180448044	0.178298642	0.700053004
UBFD1	0.078607255	0.169660556	0.803611263	0.449840397	0.200659626	0.178452222	0.700053004
VSTM2L	0.964820939	0.070081148	0.390906669	0.054751963	0.665648368	0.178055699	0.700053004
ZMAT1	0.583420323	0.99697029	0.264560261	0.392234318	0.016031168	0.17847186	0.700053004
ATP5F1C	0.710230443	0.246355251	0.685530425	0.032675198	0.247240077	0.17860672	0.700218559
SPIRE1	0.05451155	0.170708338	0.172419885	0.74546341	0.809976155	0.178586078	0.700218559
BRI3BP	0.118777827	0.514467076	0.658080012	0.19632431	0.122971729	0.178784865	0.700316395
DDX17	0.594894749	0.648204195	0.955414753	0.075344437	0.03501326	0.178887717	0.700316395
FHOD1	0.164426623	0.48596429	0.568354133	0.07373984	0.290119745	0.17885477	0.700316395
FRS3	0.235949851	0.102878244	0.204268806	0.202376342	0.966667941	0.178705344	0.700316395
POLG	0.166313627	0.04305191	0.31125241	0.48541169	0.897829648	0.178825025	0.700316395
RPLP2	0.274202134	0.324234567	0.435730848	0.48283531	0.051881204	0.178743123	0.700316395
ANKRD39	0.509445181	0.109588022	0.3695311	0.057254298	0.826051719	0.179254779	0.70038318
ARMCX1	0.466890387	0.65686558	0.16369876	0.382573799	0.050826128	0.17930066	0.70038318
CEP72	0.128612146	0.94122814	0.04666725	0.3614533	0.477081262	0.179104957	0.70038318
COL22A1	0.690551333	0.972006356	0.844422775	0.001995642	0.863518717	0.179352651	0.70038318
DLG1	0.024427936	0.712483115	0.981127285	0.182272015	0.313746131	0.179332233	0.70038318
FCMR	0.133543088	0.245742038	0.867665206	0.837513844	0.040789286	0.178966188	0.70038318
LOC100848940	0.034151536	0.586906103	0.239366228	0.246879495	0.822667801	0.179130367	0.70038318

MEIS3	0.369176421	0.317376201	0.082500576	0.677993719	0.149000839	0.179331066	0.70038318
PLAGL1	0.195860483	0.472179713	0.352240536	0.190787643	0.157265261	0.179416909	0.70038318
SLTM	0.673984037	0.418447364	0.130707151	0.058813676	0.449280675	0.179094632	0.70038318
UBE2H	0.945488607	0.245975097	0.123489446	0.348402267	0.097553726	0.179292668	0.70038318
WDR18	0.69129412	0.456383497	0.972652446	0.829040137	0.003841249	0.179400033	0.70038318
LOC101902527	0.470075422	0.176946588	0.82825151	0.037410588	0.3799731	0.179599336	0.700428875
LOC112447010	0.780685743	0.759907396	0.681150461	0.217465672	0.011142479	0.179584858	0.700428875
NTMT1	0.485176649	0.143356483	0.38508936	0.318212631	0.114811916	0.179526542	0.700428875
SLC41A3	0.637539913	0.093715792	0.207794359	0.240352112	0.328023165	0.179553221	0.700428875
LOC104969496	0.024580122	0.140694769	0.635883823	0.766103348	0.582082414	0.179727611	0.700762609
C10H14orf93	0.218872845	0.450461068	0.059786442	0.660365435	0.252667914	0.180003951	0.701340179
COPZ1	0.651572772	0.073402694	0.185655452	0.659881982	0.167814044	0.179980291	0.701340179
MMACHC	0.539407714	0.160430087	0.930823134	0.08938635	0.136548968	0.179970001	0.701340179
LOC101906134	0.201033217	0.246583184	0.451866186	0.055501924	0.792051998	0.180116037	0.701610321
OXSM	0.915553341	0.451162892	0.731463441	0.004111448	0.793882796	0.180258887	0.701667124
PNMA1	0.162884811	0.216829513	0.388082988	0.68407525	0.105170344	0.180250184	0.701667124
RIOK3	0.666523143	0.200459883	0.359928195	0.063132388	0.324593714	0.180191587	0.701667124
ATP5MPL	0.561155969	0.340831917	0.468966907	0.033006724	0.334471688	0.18064296	0.702437649
C8H9orf40	0.326085329	0.657777998	0.208647215	0.937105609	0.023589381	0.180556057	0.702437649
NDUFB2	0.32950888	0.325203907	0.478045107	0.082828577	0.233444726	0.18067085	0.702437649
PPP4R3B	0.65737874	0.073904078	0.257559268	0.512083012	0.154560999	0.180658994	0.702437649
RLF	0.347326198	0.496856255	0.763444065	0.012699568	0.591802592	0.180639376	0.702437649
LOC112444352	0.38887597	0.316205107	0.261312765	0.059926324	0.515171354	0.180813175	0.702824493
FCHSD1	0.898499844	0.136610677	0.290345413	0.537413111	0.051830377	0.18087856	0.702912158
PTPN22	0.5955827	0.102106809	0.906716468	0.299038274	0.060269925	0.180984307	0.703156598
NAV3	0.092009158	0.183944691	0.180194997	0.659746177	0.494687902	0.181131097	0.703560341
ACKR2	0.56795612	0.31014676	0.975171703	0.116058024	0.050046056	0.18135772	0.703937592
ASTN2	0.759129569	0.209999671	0.080980551	0.14038912	0.551007517	0.181444614	0.703937592
CD40	0.07411907	0.846544494	0.167551941	0.835516688	0.113692931	0.18144714	0.703937592
CRHR2	0.18312402	0.923893891	0.187441658	0.1321602	0.238282369	0.181448957	0.703937592
LOC112444341	0.46625913	0.027599075	0.281635552	0.320709913	0.857724677	0.181283855	0.703937592
MAP4K2	0.904929399	0.469260383	0.003343822	0.860799608	0.817372677	0.181485586	0.703937592
ANP32A	0.013726095	0.880280633	0.979271462	0.155328648	0.544571044	0.181656735	0.704102193
LOC614625	0.54063006	0.453012095	0.009752223	0.770289809	0.543930116	0.181642206	0.704102193
YME1L1	0.792004178	0.147881199	0.927000862	0.221827382	0.041548817	0.181638605	0.704102193
ADAMTS7	0.368580595	0.151445336	0.048845218	0.481532356	0.764273594	0.181898864	0.704209071
CDYL2	0.220724167	0.163783022	0.160099727	0.967601322	0.178911685	0.181758846	0.704209071
HLX	0.765974767	0.576857489	0.079080243	0.074483231	0.3855076	0.181889608	0.704209071
MND1	0.946890655	0.016003197	0.614200206	0.650219712	0.165805305	0.181896753	0.704209071
UBE2A	0.580110432	0.2592164	0.786100174	0.663509203	0.012779042	0.18179203	0.704209071

DGKG	0.020815327	0.495922954	0.590333018	0.940694722	0.175833486	0.182328061	0.704209415
GART	0.894269404	0.191135973	0.448455817	0.539647633	0.024346043	0.182245734	0.704209415
GDPD1	0.187222847	0.58815207	0.061880645	0.528483778	0.279895021	0.182324847	0.704209415
LAMTOR4	0.251298648	0.238206629	0.729798278	0.095028306	0.242670966	0.182278276	0.704209415
LOC511229	0.230567276	0.503537391	0.017565249	0.757221272	0.651372972	0.18212901	0.704209415
LOC519309	0.707258623	0.118342696	0.764659859	0.204339166	0.076927463	0.182147577	0.704209415
MAB21L2	0.013151646	0.34663216	0.613699856	0.801090507	0.44882602	0.182135219	0.704209415
PRAP1	0.621449541	0.679114246	0.049755617	0.062550156	0.764964637	0.182024871	0.704209415
SACM1L	0.498510343	0.848259492	0.121437538	0.041354708	0.472808687	0.181960457	0.704209415
VDAC1	0.53736544	0.30228545	0.40817841	0.194343039	0.078198784	0.182297638	0.704209415
AGFG1	0.802841832	0.161615374	0.479009905	0.742356415	0.021878395	0.182468442	0.704382059
BIRC2	0.749601852	0.085619192	0.42310834	0.642266104	0.057898533	0.182501524	0.704382059
UBA2	0.532545997	0.532731921	0.20919951	0.034500308	0.493066196	0.182483711	0.704382059
HM13	0.422216733	0.082689393	0.287678325	0.985116058	0.102269934	0.182697231	0.704586409
PEX2	0.773699501	0.015394499	0.666806907	0.485634969	0.262290742	0.182675963	0.704586409
PRRT2	0.693655782	0.245650149	0.016200057	0.498960025	0.734879935	0.182726205	0.704586409
SYT1	0.978060496	0.898271111	0.420848245	0.004410323	0.619883297	0.182598727	0.704586409
AARS2	0.78032538	0.049670025	0.103214479	0.418851469	0.605368828	0.182930735	0.704708461
KIF18A	0.377129574	0.066871968	0.43541642	0.72117117	0.128308513	0.183093967	0.704708461
MEMO1	0.748398839	0.127643815	0.75622934	0.319201452	0.044008623	0.1829743	0.704708461
NUP153	0.335476574	0.614835779	0.611278262	0.008862782	0.909361869	0.183101388	0.704708461
PAMR1	0.226554501	0.034417625	0.731449821	0.221353524	0.804073454	0.18300315	0.704708461
RAB9A	0.598436433	0.438697684	0.062914504	0.112676115	0.54504187	0.182931844	0.704708461
SAFB	0.578437582	0.337088656	0.593701137	0.020165125	0.434680431	0.182963417	0.704708461
ZNF217	0.861926489	0.024465696	0.647212635	0.159422818	0.466942087	0.183084065	0.704708461
LMAN2L	0.020663509	0.247006334	0.526688295	0.386163119	0.979416777	0.183153537	0.704743893
LOC112449602	0.196340742	0.259718668	0.395752646	0.687444257	0.073369686	0.183260782	0.704940088
LOC614129	0.235672053	0.820906595	0.240628829	0.113676356	0.192400714	0.183290437	0.704940088
IFI30	0.466382561	0.684840347	0.376314147	0.032160407	0.263524155	0.183333987	0.704942376
P4HA3	0.64618104	0.478491923	0.036810862	0.301851907	0.296818575	0.183436526	0.705171429
BRAT1	0.626598572	0.069925123	0.142294911	0.429190283	0.38185894	0.183629338	0.70547183
CD63	0.78437377	0.093319119	0.038867019	0.510009882	0.704329994	0.183643633	0.70547183
FLCN	0.419697706	0.486449968	0.751478776	0.274730325	0.024242265	0.183630756	0.70547183
ASTE1	0.145940108	0.418637673	0.902856615	0.031838315	0.582302121	0.183709889	0.705561196
CFAP97	0.065508251	0.735808961	0.081892277	0.70403291	0.36822816	0.183772476	0.705636431
SUSD1	0.386436322	0.213327879	0.229307699	0.0598084	0.905738314	0.183837584	0.70572131
ADGRG5	0.021063243	0.55424337	0.183408464	0.558243342	0.860144433	0.184219813	0.706197464
BANP	0.370266291	0.512246307	0.115349605	0.42345218	0.110899671	0.18415434	0.706197464
FBXO41	0.397291529	0.23154096	0.533579976	0.224822434	0.093165152	0.184217549	0.706197464
MMAB	0.711735496	0.177163212	0.648326685	0.035740377	0.351182518	0.184029393	0.706197464

RAD54L2	0.450651425	0.701005289	0.754152238	0.088352369	0.048802984	0.184141501	0.706197464
TSPO	0.679035446	0.68690463	0.688760961	0.005720276	0.558944375	0.184132069	0.706197464
KRT23	0.559564339	0.908157327	0.026282404	0.086766604	0.887713635	0.184277647	0.706254195
TMEM184A	0.973390538	0.259820782	0.048971604	0.247789845	0.335461219	0.184350491	0.706368412
FNBP4	0.766151831	0.070833496	0.093469621	0.393275509	0.516874736	0.184499815	0.706729974
NFATC1	0.496128751	0.084190061	0.04267673	0.907547676	0.637574942	0.184530982	0.706729974
NUAK2	0.422560695	0.163248276	0.073729342	0.233921538	0.867423353	0.184582405	0.70676198
KIAA0754	0.746846594	0.007178503	0.51984007	0.626677379	0.59196574	0.184758728	0.70697838
LOC101905897	0.195008323	0.427335077	0.34092415	0.285939347	0.127236884	0.184734531	0.70697838
PCDHGA2	0.021457634	0.776940392	0.297221508	0.234293687	0.890651612	0.18476816	0.70697838
AGPS	0.752834862	0.245580598	0.030420502	0.723154635	0.254786059	0.184978443	0.707156665
C29H11orf80	0.336647833	0.443890446	0.080128695	0.723526929	0.119678522	0.18503247	0.707156665
LIG3	0.707037131	0.419331391	0.02707529	0.895487657	0.144555427	0.185244624	0.707156665
LOC101906837	0.667828568	0.173447691	0.050722725	0.560509867	0.314743825	0.185002744	0.707156665
LOC107131728	0.04247435	0.650457929	0.532662152	0.224343938	0.314478314	0.185163857	0.707156665
LOC112444314	0.621703846	0.177494213	0.567988628	0.959068287	0.017286794	0.185245659	0.707156665
MANEAL	0.434925695	0.026591887	0.737261954	0.258517783	0.471333721	0.185230917	0.707156665
NNAT	0.630453365	0.35326917	0.020658463	0.920945724	0.244904107	0.185115923	0.707156665
PARP12	0.523874574	0.024109249	0.911775355	0.180290469	0.498501779	0.184861252	0.707156665
PNRC1	0.940724551	0.570907089	0.351490353	0.360512745	0.015235882	0.18503667	0.707156665
ARHGEF39	0.935350644	0.168158537	0.068373646	0.589329209	0.164140845	0.18535343	0.707239046
LOC100336897	0.575176098	0.945066529	0.037437289	0.11690875	0.437174162	0.185333969	0.707239046
ABCF3	0.563942895	0.096865888	0.715709437	0.199009503	0.135646446	0.186749795	0.707266705
ACSL5	0.579767795	0.322779984	0.294233754	0.43533323	0.04356577	0.185723221	0.707266705
ADGRA2	0.984594076	0.597329064	0.97251758	0.255976617	0.007203217	0.186676104	0.707266705
AKR7A2	0.932886043	0.317018596	0.649634732	0.049375103	0.11165992	0.187099276	0.707266705
ANKRD40	0.863096202	0.190883208	0.067065891	0.177797467	0.537164998	0.186735704	0.707266705
ANO6	0.895504828	0.259974627	0.055338728	0.166695869	0.491436077	0.18674867	0.707266705
ARRDC5	0.38852096	0.111028069	0.519989781	0.780810283	0.060311353	0.186830621	0.707266705
BAALC	0.72558211	0.066525975	0.198861303	0.734253489	0.150552742	0.18727274	0.707266705
C11H9orf50	0.028870498	0.379764796	0.267645905	0.36366237	0.993529679	0.187193479	0.707266705
CFAP54	0.171266234	0.365457553	0.12011216	0.346149842	0.40626655	0.186916517	0.707266705
CHD2	0.710475351	0.175485695	0.22749714	0.314253598	0.118032384	0.186442353	0.707266705
COPE	0.564691819	0.161957616	0.45826769	0.105236769	0.240627598	0.187291164	0.707266705
CRYL1	0.815517237	0.387387257	0.012471188	0.695982476	0.381597492	0.185916772	0.707266705
CUEDC1	0.437142501	0.380224849	0.013432261	0.510523527	0.91909142	0.186027453	0.707266705
DACT3	0.17624604	0.3881768	0.113124493	0.150419047	0.896369656	0.185650973	0.707266705
EPHB2	0.134411947	0.743967685	0.166038996	0.398320152	0.157741217	0.185625165	0.707266705
GJD3	0.505715539	0.668097327	0.061074862	0.092194302	0.558078117	0.187327571	0.707266705
GRP	0.009705563	0.354569109	0.802144642	0.39228932	0.971126316	0.186399608	0.707266705

HEATR5B	0.230209243	0.554057878	0.052969865	0.762806565	0.202300638	0.185567119	0.707266705
HSPA2	0.456854573	0.658069902	0.056070883	0.135069915	0.462142369	0.186458801	0.707266705
IL17REL	0.123592794	0.106355805	0.454507254	0.404189649	0.437749868	0.186902097	0.707266705
KIFC1	0.754894798	0.074858707	0.276194616	0.37019434	0.182592952	0.186712433	0.707266705
KRBA2	0.503464882	0.136741387	0.050812466	0.908383689	0.333195349	0.187058954	0.707266705
LOC101908535	0.595887238	0.100521737	0.050100568	0.927962221	0.380612325	0.187164619	0.707266705
LOC112447005	0.049786498	0.465685142	0.253831393	0.378988528	0.473362883	0.186782104	0.707266705
LOC112447360	0.098813563	0.627226557	0.151747183	0.126115803	0.882036918	0.185900699	0.707266705
LOC787234	0.960972132	0.149532079	0.681522475	0.187605044	0.057154253	0.186257284	0.707266705
LRAT	0.302889042	0.176388641	0.122044397	0.620707191	0.262372514	0.187343146	0.707266705
LZTS1	0.900658193	0.938716866	0.260307034	0.032489538	0.148088667	0.1870675	0.707266705
MARS	0.635967429	0.057289737	0.375953068	0.990859602	0.07700902	0.185807471	0.707266705
MNF1	0.169805555	0.210249879	0.720672406	0.282791071	0.144389661	0.186303871	0.707266705
PLS3	0.197865207	0.027761278	0.950968228	0.282314089	0.715991182	0.186792287	0.707266705
PTS	0.598172602	0.095806773	0.164571143	0.414341989	0.271220586	0.187159727	0.707266705
RANGRF	0.147823293	0.29766878	0.837890725	0.217143809	0.132404191	0.187171976	0.707266705
RGS9	0.502627906	0.305633166	0.170744111	0.532291418	0.074815087	0.185747928	0.707266705
RORB	0.488928312	0.118831695	0.740127046	0.591578742	0.041138927	0.185929947	0.707266705
SEPT5	0.215889457	0.045320253	0.268640185	0.539655882	0.735694835	0.185654039	0.707266705
SOSTDC1	0.102708809	0.213763341	0.221228627	0.243965046	0.89525131	0.187248562	0.707266705
TFAP4	0.745401701	0.404340728	0.482470748	0.025705452	0.281972596	0.186619351	0.707266705
THSD4	0.017064169	0.309991057	0.448984587	0.613815787	0.720404557	0.186271074	0.707266705
TNFSF9	0.051001711	0.42861639	0.249393344	0.680251395	0.285872704	0.187187148	0.707266705
WDR17	0.696072615	0.022327761	0.446670762	0.263131394	0.576278064	0.186496784	0.707266705
ZCCHC18	0.121901242	0.065186812	0.49491064	0.704095078	0.378031152	0.185953167	0.707266705
ZDHHC13	0.008776603	0.810517389	0.515535251	0.613141524	0.465455346	0.185938101	0.707266705
ZKSCAN2	0.195854701	0.170324272	0.376788504	0.951820128	0.088652275	0.187225755	0.707266705
ZNF839	0.430241855	0.547682063	0.19939076	0.04598177	0.486333202	0.186312452	0.707266705
LOC101907084	0.494266052	0.128463389	0.705109554	0.200708061	0.118327842	0.187471175	0.707587269
LOC101906818	0.890060225	0.300899035	0.01700575	0.339477608	0.688758617	0.187620415	0.707824974
RNF122	0.58214049	0.854504877	0.339667458	0.008436975	0.74680656	0.187592833	0.707824974
LARP4B	0.170249225	0.919880867	0.036122905	0.339609441	0.554907873	0.187729408	0.707910686
RTTN	0.53464837	0.587931124	0.403709691	0.388973316	0.021597689	0.187727374	0.707910686
TIE1	0.704270268	0.12433072	0.090888004	0.613472214	0.218637108	0.1878513	0.7082076
EPN1	0.017989921	0.594751638	0.916503812	0.356969245	0.305595412	0.188060995	0.70838594
FPGT	0.650300748	0.344897054	0.058483747	0.339760813	0.239921221	0.188016977	0.70838594
FREM1	0.149588832	0.156692082	0.998864494	0.538270399	0.084819022	0.187986802	0.70838594
MAN1B1	0.473047313	0.721253946	0.020310593	0.552310148	0.279611434	0.188100666	0.70838594
MARK2	0.116823086	0.220853242	0.460111912	0.351658463	0.256614766	0.188200762	0.70838594
SLX1A	0.556099744	0.020537266	0.30402802	0.548195998	0.562390016	0.18812967	0.70838594

TGM3	0.671965838	0.295119612	0.413927005	0.061571784	0.21191884	0.188183154	0.70838594
BUD23	0.401524958	0.589662966	0.697424712	0.208340965	0.031237348	0.188507606	0.7088105
CHP1	0.436824509	0.934900215	0.012997097	0.420941144	0.481359246	0.18858568	0.7088105
DIRAS1	0.883817816	0.405597899	0.070226146	0.056935406	0.750923182	0.188659086	0.7088105
LOC101902937	0.803082634	0.273242985	0.865376148	0.022933708	0.247037568	0.188617696	0.7088105
PDE6G	0.191203484	0.095813049	0.950216598	0.272076904	0.22677617	0.188455747	0.7088105
SLC38A1	0.165608202	0.189145986	0.618459639	0.104644753	0.530791661	0.188635809	0.7088105
SMARCA2	0.240309675	0.470808869	0.030153172	0.450778044	0.697921888	0.188385152	0.7088105
STXBP3	0.208260584	0.496990404	0.101384127	0.293075574	0.34972465	0.188590548	0.7088105
HSH2D	0.430322564	0.089780945	0.195976418	0.608807891	0.234327632	0.189008658	0.709624454
LOC786512	0.03933546	0.895003096	0.038976451	0.869040514	0.90563841	0.188990618	0.709624454
MCOLN2	0.793365103	0.129479457	0.090290054	0.250098098	0.465842461	0.189048694	0.709624454
UFL1	0.078330419	0.588469762	0.402865671	0.099453574	0.584831631	0.189004196	0.709624454
LOC104974345	0.214558678	0.609265465	0.760877162	0.014494031	0.75040018	0.189158375	0.709873791
C25H7orf50	0.331791673	0.171802523	0.214356194	0.231600669	0.38358944	0.189494562	0.710344986
C26H10orf62	0.820616743	0.225362749	0.023131072	0.522254778	0.48595502	0.189507957	0.710344986
GLS	0.299713878	0.780904876	0.245751147	0.061468411	0.306656986	0.1893743	0.710344986
MLH1	0.377232149	0.666056624	0.185628851	0.05184761	0.448629791	0.189436848	0.710344986
WDR35	0.067051699	0.880171935	0.065474428	0.58845701	0.477839717	0.189586926	0.710344986
WDTC1	0.759677524	0.050694793	0.950871482	0.884841643	0.033529022	0.189576725	0.710344986
ZNF207	0.920004534	0.276450056	0.886138704	0.007969899	0.603777911	0.189404351	0.710344986
CFD	0.333308964	0.147475874	0.463177304	0.129918575	0.367766267	0.189702829	0.710617013
ADIG	0.238316575	0.361553228	0.359102536	0.113656888	0.311279297	0.190321913	0.710784303
AMZ2	0.426578307	0.170351352	0.185305176	0.429157995	0.189197208	0.190202362	0.710784303
COL15A1	0.521519471	0.060792728	0.048125119	0.723163946	0.991914873	0.190302445	0.710784303
EXD1	0.564935786	0.397348195	0.078712557	0.794385042	0.077691437	0.189942828	0.710784303
MBNL1	0.769555903	0.856102915	0.014807788	0.841005764	0.133013831	0.190018388	0.710784303
MCRS1	0.415465796	0.088145244	0.809162904	0.20907837	0.176741096	0.190350454	0.710784303
SAR1A	0.697420506	0.028251597	0.134714995	0.576972626	0.711389933	0.189851802	0.710784303
SCD	0.785734808	0.802572166	0.735351367	0.079968102	0.029526422	0.190342704	0.710784303
SDHA	0.505586917	0.538529709	0.712713603	0.031376022	0.1791705	0.189980512	0.710784303
SH3BP5	0.626637115	0.368699276	0.595460381	0.011348078	0.70140388	0.190353849	0.710784303
SNX6	0.013253524	0.869760761	0.880756371	0.57073485	0.188438687	0.190072612	0.710784303
SPTBN5	0.969097235	0.091042	0.068768427	0.615403121	0.291641744	0.189804494	0.710784303
TSTA3	0.067004564	0.357089897	0.632139353	0.116094594	0.622081825	0.190109521	0.710784303
UBE2L3	0.361030418	0.27878472	0.754074434	0.158321172	0.090816335	0.190013364	0.710784303
LOC789231	0.448472179	0.537039492	0.027487348	0.777605414	0.212869565	0.190425484	0.710890041
LOC101902106	0.329201064	0.547635352	0.472703595	0.797606635	0.016148201	0.19058601	0.711327497
STX17	0.931160029	0.138152721	0.3831104	0.469737603	0.04746631	0.190698226	0.711584492
ANKHD1	0.119871138	0.787893661	0.491515741	0.031635072	0.750575725	0.191001629	0.711737872

HAP1	0.793341995	0.16441542	0.057420643	0.327579202	0.449394056	0.191031587	0.711737872
LOC104972545	0.10138044	0.049947881	0.325905535	0.995218027	0.669678576	0.190788924	0.711737872
LOC112446716	0.066008941	0.924262689	0.025792238	0.931852867	0.75044482	0.190835686	0.711737872
NREP	0.124537559	0.556924083	0.096949959	0.49683034	0.330074825	0.191042918	0.711737872
PDE7A	0.428909769	0.377636369	0.668349589	0.351991676	0.028926546	0.190999218	0.711737872
SRP72	0.06567459	0.698283407	0.461109124	0.089449785	0.582916614	0.191032465	0.711737872
CC2D1B	0.073537396	0.349968545	0.188288326	0.31872289	0.715304592	0.191225116	0.711994916
GLYR1	0.344567954	0.028549089	0.777910892	0.891674527	0.161889439	0.191215313	0.711994916
PURG	0.268621405	0.696536933	0.267601686	0.122414271	0.180274255	0.191242069	0.711994916
BTF3L4	0.877106745	0.070084147	0.650237269	0.583135388	0.047486384	0.19141138	0.712140594
TRIM37	0.288542993	0.497572701	0.017942264	0.870468251	0.493597275	0.19140851	0.712140594
UBQLN1	0.865399126	0.118824843	0.094761572	0.554038807	0.204957528	0.191383448	0.712140594
A2M	0.951008388	0.550618921	0.779330497	0.003115726	0.871754136	0.191554493	0.712350054
TMEM265	0.802925972	0.239564588	0.214262494	0.208465905	0.128961099	0.191515194	0.712350054
ANKRD13C	0.923109874	0.022529799	0.930116524	0.511709491	0.112223887	0.191770399	0.712390889
FANCI	0.889157707	0.155776641	0.171684852	0.121542657	0.383923176	0.191663001	0.712390889
LEF1	0.631012009	0.16432043	0.6911982	0.143567942	0.107916615	0.191729488	0.712390889
RAB11FIP4	0.770418854	0.02491385	0.417374745	0.986338477	0.140626787	0.191800321	0.712390889
SLC8A1	0.026750575	0.157954728	0.490142246	0.869704173	0.617035978	0.191819034	0.712390889
SPTAN1	0.955918605	0.505517254	0.085387285	0.045498289	0.592304605	0.191869339	0.712390889
VHL	0.79002237	0.357819608	0.932595986	0.008473655	0.497629962	0.191842873	0.712390889
AOX2	0.498313605	0.079686381	0.089176874	0.347944235	0.903262112	0.19195435	0.712545315
ANLN	0.454504731	0.146787701	0.121857509	0.447241781	0.306355506	0.192042506	0.712550205
ZNF350	0.476322576	0.290433255	0.226528272	0.25953608	0.13692656	0.192021241	0.712550205
ARMC12	0.565479753	0.753665632	0.640476318	0.026302002	0.155680425	0.192379351	0.712914877
CAV2	0.305426788	0.467533398	0.186988876	0.069798368	0.60089208	0.192575203	0.712914877
CPNE9	0.542248658	0.16699266	0.479194641	0.323192834	0.079737205	0.192427152	0.712914877
KDELC2	0.024152417	0.313921512	0.512252366	0.898824513	0.320746661	0.192558124	0.712914877
PAXBP1	0.974373787	0.345878747	0.009893086	0.495611582	0.677686691	0.192569333	0.712914877
RAB2A	0.576605481	0.007540727	0.645079434	0.807408196	0.493723981	0.192416173	0.712914877
STARD3	0.494770575	0.014560636	0.270919373	0.587924734	0.973996389	0.192375163	0.712914877
STT3A	0.338283543	0.183642989	0.20039589	0.286137608	0.314147322	0.192500804	0.712914877
ZC3H15	0.844378218	0.113489143	0.706232465	0.655668873	0.025207237	0.192454219	0.712914877
ZNF609	0.874768639	0.169500401	0.689127883	0.016617611	0.657008222	0.192192145	0.712914877
KIAA0319L	0.777855142	0.072502473	0.159313746	0.552146946	0.225912911	0.192649232	0.713028088
ATP5F1B	0.805572738	0.365055722	0.356163334	0.059426318	0.180380056	0.192827953	0.71307679
LPAR3	0.031835939	0.55897585	0.686053078	0.102765411	0.895102001	0.192852584	0.71307679
MED19	0.528532759	0.80621928	0.673554109	0.015303156	0.255762169	0.192881698	0.71307679
PLCXD2	0.059786808	0.761053883	0.24078482	0.382136653	0.268338141	0.19288997	0.71307679
SIPA1L1	0.226961969	0.330627493	0.105181572	0.517227614	0.275284674	0.192923097	0.71307679

TSPYL6	0.702072715	0.927193632	0.702156828	0.017452403	0.140858005	0.192906581	0.71307679
ANKRD55	0.810096309	0.070457985	0.067962404	0.316310105	0.918100472	0.193162495	0.713319016
LOC112448395	0.941893434	0.217173474	0.388455318	0.097106533	0.145967845	0.193143301	0.713319016
SOX5	0.448983973	0.327398497	0.531488045	0.410483624	0.035116442	0.193132308	0.713319016
STX1A	0.103301742	0.983071013	0.136227489	0.123641285	0.657965542	0.193067266	0.713319016
CHMP2A	0.484281962	0.167510389	0.33122039	0.122045642	0.344105591	0.193330633	0.713779305
EPYC	0.672647142	0.327390644	0.009104455	0.892227752	0.631444472	0.1934334	0.713969126
HDAC7	0.976302534	0.09534172	0.087910859	0.641168344	0.21537315	0.193469057	0.713969126
LOC617905	0.467728974	0.097900123	0.734124283	0.195109241	0.172379988	0.193523817	0.714010649
CDK6	0.447445473	0.401393916	0.631324153	0.244839891	0.040751038	0.19358644	0.714081158
TSPAN7	0.249681323	0.029976603	0.826856981	0.419988376	0.435831571	0.1937177	0.714244255
USP47	0.721216098	0.056696479	0.285353547	0.106501738	0.911572607	0.193717218	0.714244255
ACVRL1	0.550674894	0.662108311	0.473451058	0.286984461	0.022908371	0.193901174	0.714599632
SYTL1	0.715616014	0.191400822	0.117599177	0.37695572	0.186853315	0.193870617	0.714599632
KIF2A	0.187959362	0.061172306	0.48163554	0.250749704	0.817951391	0.193982525	0.714704992
LOC112444622	0.151149957	0.39622726	0.195816611	0.782769427	0.123770908	0.194016863	0.714704992
DNAJA2	0.958521507	0.068582329	0.184085253	0.506719636	0.185460224	0.19410839	0.714881686
B3GAT3	0.161833747	0.372089752	0.717448978	0.1236699	0.213035399	0.19419413	0.71495154
COL6A1	0.122010887	0.314057147	0.119098611	0.263727801	0.946336018	0.194261369	0.71495154
ID1	0.403606575	0.180717442	0.184996532	0.371119304	0.227538219	0.194301619	0.71495154
VBP1	0.903903081	0.077453348	0.440407143	0.980732446	0.037669043	0.19426974	0.71495154
ARHGAP26	0.007456196	0.327628766	0.995280635	0.484377936	0.973247099	0.194893317	0.715136201
C7H19orf38	0.130014965	0.064401094	0.582196516	0.2479065	0.945903037	0.194624919	0.715136201
CMC2	0.465492868	0.317263466	0.968917868	0.060774226	0.131895178	0.194966396	0.715136201
CYP4A11	0.660081264	0.141520957	0.761226726	0.059018856	0.274163826	0.195282188	0.715136201
DSE	0.105085762	0.308467337	0.204976862	0.175649546	0.979873356	0.19466745	0.715136201
E2F2	0.358513171	0.066831249	0.354558686	0.539135374	0.251036407	0.195206768	0.715136201
KIF7	0.835267781	0.277580941	0.068203619	0.604876202	0.119396962	0.194530683	0.715136201
KRCC1	0.08489623	0.13240637	0.265509164	0.587736331	0.65074694	0.194481958	0.715136201
LOC101907540	0.980414199	0.058695281	0.699232736	0.032776986	0.867645915	0.194730094	0.715136201
LOC112443510	0.374571826	0.337543954	0.398048548	0.058654073	0.388809511	0.195028408	0.715136201
LOC515551	0.610156026	0.798109357	0.009311676	0.334204815	0.754447085	0.194643834	0.715136201
LOC613660	0.508411397	0.047838394	0.678476548	0.13282533	0.52376858	0.195053972	0.715136201
NFKB1	0.578947508	0.427024521	0.879341098	0.005673982	0.931216466	0.195109703	0.715136201
PARP14	0.074597275	0.153445899	0.592032113	0.242860242	0.698506517	0.195193584	0.715136201
PCOLCE	0.251332993	0.249720405	0.117590289	0.174216643	0.89362495	0.195140242	0.715136201
RHOF	0.243054166	0.084305904	0.282066302	0.79000734	0.251019701	0.194893051	0.715136201
RRAGB	0.77505584	0.736144968	0.10782856	0.162735995	0.11465414	0.195043899	0.715136201
SASS6	0.960169835	0.003441487	0.495740439	0.75806231	0.924797816	0.195089405	0.715136201
SLC25A28	0.406015466	0.672956088	0.289954366	0.960618803	0.015112734	0.195240499	0.715136201

SOD1	0.431201609	0.39375531	0.364523374	0.299443971	0.061791515	0.194806465	0.715136201
TLE4	0.399916829	0.700045988	0.751521523	0.162658161	0.033542724	0.195045831	0.715136201
YIF1B	0.189636241	0.705259444	0.136479318	0.142096845	0.44374536	0.19531049	0.715136201
LOC101907503	0.890868293	0.101937775	0.588693084	0.024054912	0.895704501	0.19539173	0.715192043
LOC112446689	0.471013652	0.605644222	0.082719583	0.069695166	0.70054403	0.195412901	0.715192043
LOC107132783	0.478158689	0.522304799	0.135644037	0.105799995	0.321859726	0.195540713	0.715364376
LY86	0.027587214	0.621926352	0.768145571	0.143622385	0.609750954	0.19559076	0.715364376
SAC3D1	0.222329159	0.288015224	0.405184172	0.22109304	0.201147234	0.195564917	0.715364376
GJA5	0.572416917	0.076377274	0.444948835	0.179088687	0.331580968	0.195678754	0.715526274
MGAT4A	0.251697004	0.355505264	0.07914619	0.866698061	0.188282492	0.195722225	0.715526274
FAM84A	0.074932243	0.308841984	0.346940791	0.576746133	0.249729478	0.195787895	0.715606938
DYNLRB1	0.511424133	0.127450876	0.589005882	0.112621862	0.267577272	0.19583446	0.71561775
LOC100848941	0.31890543	0.250696939	0.819305561	0.090203609	0.196089804	0.195978423	0.715984396
SOGA3	0.464743796	0.941812798	0.305603284	0.140165303	0.061826566	0.196028602	0.716008322
VWA5A	0.153293589	0.594122699	0.640086278	0.051246598	0.388271218	0.196095276	0.716092473
EHMT2	0.752773064	0.086788723	0.065197136	0.344573374	0.792131459	0.196326759	0.716618872
SLC22A17	0.535984347	0.635403407	0.131253449	0.061280028	0.424360471	0.196310806	0.716618872
TAOK2	0.362181878	0.137730458	0.211014728	0.273847575	0.403623604	0.196401196	0.71673116
ATG14	0.109064845	0.108763345	0.542633067	0.497021995	0.365415294	0.196885706	0.716836272
C3H1orf109	0.057649093	0.456463643	0.527807636	0.496038409	0.16920114	0.19659619	0.716836272
C6H4orf48	0.181240774	0.506735531	0.03073778	0.733479328	0.562458979	0.196502063	0.716836272
CXCL3	0.861474698	0.657740333	0.050832224	0.857322895	0.0473928	0.19699223	0.716836272
GALNT6	0.390613995	0.420076051	0.118372712	0.41342036	0.145386319	0.196747495	0.716836272
LOC101902922	0.104515759	0.29462903	0.232721594	0.218588825	0.745144385	0.196728784	0.716836272
LOC112441457	0.643941632	0.052189151	0.819235079	0.428203962	0.099208741	0.196932314	0.716836272
LOC112444726	0.346680013	0.73264048	0.777922007	0.010300662	0.574090788	0.196831074	0.716836272
LUZP6	0.573692997	0.114506593	0.312950802	0.832058241	0.068419066	0.196997842	0.716836272
TMEM200C	0.138929579	0.324117583	0.471587315	0.548174712	0.100352643	0.196809116	0.716836272
TMEM50B	0.64499864	0.211324922	0.555746383	0.66956247	0.02307483	0.196997561	0.716836272
UHRF1BP1L	0.319984072	0.55181006	0.370852417	0.020950041	0.851430987	0.196796575	0.716836272
WDR83	0.009737393	0.372651206	0.838243754	0.963014637	0.398321843	0.196686908	0.716836272
BRIX1	0.693995505	0.417218087	0.827905314	0.019727608	0.248230977	0.197304376	0.717425454
CCHCR1	0.131374337	0.191296247	0.359132623	0.841453133	0.154697003	0.197386752	0.717425454
CHDH	0.455303501	0.308732708	0.055645937	0.260286131	0.576974465	0.197372982	0.717425454
GNAZ	0.991229565	0.002799433	0.622810776	0.894856371	0.75994314	0.197422055	0.717425454
LOC101905887	0.198916678	0.595182294	0.939960876	0.019102001	0.55211295	0.197282644	0.717425454
MAGEH1	0.090215858	0.08738595	0.916633711	0.190619711	0.852504855	0.197340358	0.717425454
ARC	0.077793486	0.171128344	0.857893312	0.224608008	0.459643436	0.197751569	0.717669394
CHCHD3	0.555708649	0.204930858	0.770331933	0.216037636	0.062185227	0.197705805	0.717669394
CRLF3	0.018379807	0.45352828	0.412614605	0.994181891	0.344670242	0.197708257	0.717669394

KIAA1755	0.532257876	0.681921964	0.641931474	0.171920511	0.029386336	0.197581798	0.717669394
LOC112444936	0.931265901	0.323786737	0.382069556	0.443093071	0.023051321	0.197546135	0.717669394
LSG1	0.148164894	0.661160473	0.34012179	0.102403499	0.345507759	0.197731572	0.717669394
CCDC191	0.450423145	0.165420284	0.169464223	0.463410737	0.201613937	0.197805216	0.717705373
C7H19orf57	0.568427071	0.729041837	0.01092371	0.384884507	0.677571553	0.197877465	0.717808816
ACTG1	0.325994805	0.027847214	0.415322047	0.406995021	0.772374967	0.198278307	0.718213134
ATIC	0.470320455	0.164953312	0.760145264	0.429221166	0.046972184	0.198601621	0.718213134
CNDP2	0.174750601	0.290368659	0.043821178	0.687867319	0.777262872	0.198590284	0.718213134
LOC101906086	0.892209918	0.453923771	0.120354381	0.11835944	0.206008297	0.198560819	0.718213134
LOC112441542	0.512425735	0.756829828	0.188882059	0.230231705	0.070221749	0.198199095	0.718213134
MYOZ1	0.275008314	0.639727435	0.036537595	0.812969375	0.227420978	0.198557528	0.718213134
NDUFA10	0.517098508	0.62497332	0.778329172	0.018463053	0.255774674	0.198504432	0.718213134
NFYA	0.823344684	0.627709948	0.026533526	0.266880119	0.324254796	0.198405736	0.718213134
PSMB7	0.437097861	0.187263219	0.693676714	0.27063297	0.077344	0.19855991	0.718213134
PVR	0.581140086	0.714485468	0.764967271	0.020638407	0.180739098	0.198243311	0.718213134
SDK1	0.832364652	0.092118993	0.083914966	0.743058727	0.248659513	0.198591548	0.718213134
SLC8A3	0.020533391	0.747937443	0.93165308	0.428133906	0.193317954	0.198193316	0.718213134
SLCO4A1	0.872431447	0.845288282	0.304250417	0.079260692	0.066517064	0.19808236	0.718213134
ZBTB7C	0.899469759	0.214175019	0.229594212	0.193503286	0.138838633	0.198540994	0.718213134
MAPKBP1	0.669462578	0.322119352	0.191935306	0.329944878	0.087152995	0.198706846	0.71843535
ESRRA	0.252755205	0.177341307	0.980406882	0.352701291	0.076943072	0.198911461	0.718703676
LOC101907487	0.174593951	0.347064725	0.901475559	0.028547153	0.764709115	0.198902006	0.718703676
LOC112444920	0.499877449	0.330798975	0.030451791	0.594048582	0.398689327	0.198912443	0.718703676
ZBTB7B	0.070183407	0.695747345	0.06418383	0.394079016	0.96604664	0.198958479	0.718711774
ME3	0.951827679	0.650560206	0.014918246	0.389371974	0.332147265	0.199091502	0.718774053
PLAC8	0.679851309	0.146758562	0.449717552	0.173459137	0.153587971	0.199150912	0.718774053
TNFSF8	0.330650721	0.272362908	0.647003143	0.033003428	0.621288178	0.199095501	0.718774053
VPS53	0.948616558	0.236687534	0.214506503	0.044374281	0.559106765	0.199108824	0.718774053
IKZF3	0.109313173	0.346281297	0.337952411	0.938133739	0.099727789	0.199274744	0.718904775
IL17D	0.85775102	0.070401951	0.442501874	0.434054336	0.103177378	0.199263358	0.718904775
ADAMTS16	0.377827396	0.885548945	0.148816556	0.099015679	0.244485978	0.19999981	0.71930628
APPBP2	0.973970731	0.257458003	0.167412285	0.046842704	0.614272902	0.200218822	0.71930628
DIAPH1	0.179110933	0.258715845	0.678841607	0.968892694	0.039525349	0.199940773	0.71930628
GFM1	0.688762032	0.370701376	0.863532634	0.078763053	0.069389162	0.199969405	0.71930628
GMCL1	0.279927502	0.472509588	0.054637393	0.284868751	0.583598999	0.199667392	0.71930628
HIPK1	0.732699771	0.669890243	0.00918527	0.297979446	0.89733026	0.200010448	0.71930628
HUS1	0.10910491	0.867301775	0.208287193	0.173610334	0.352348129	0.200025637	0.71930628
IL34	0.5427775	0.16588477	0.079154843	0.215650024	0.781063343	0.199581649	0.71930628
KLHL24	0.022461925	0.598433513	0.783255844	0.258639917	0.440900354	0.199596001	0.71930628
LOC112444355	0.907464073	0.730366671	0.786680336	0.002525913	0.913880079	0.19984909	0.71930628

LRRTM2	0.225348864	0.251792399	0.406785524	0.120260002	0.433697054	0.199871468	0.71930628
NSA2	0.381563494	0.250369096	0.191028005	0.211790541	0.31163865	0.199926024	0.71930628
PLRG1	0.066813359	0.402532822	0.105008314	0.850253368	0.500705349	0.19974125	0.71930628
PSIP1	0.076424747	0.635227686	0.071537293	0.621583956	0.559070993	0.200129033	0.71930628
RUNX3	0.907063137	0.399180638	0.340431512	0.205381261	0.047400074	0.199542773	0.71930628
SCN3A	0.114602412	0.546149163	0.028244159	0.909597307	0.750195943	0.200080608	0.71930628
TMED7	0.613611076	0.158272047	0.152420752	0.626491209	0.130207166	0.20018303	0.71930628
TOMM40L	0.536168011	0.084148125	0.375225842	0.298283146	0.237464215	0.19946939	0.71930628
UNKL	0.4952413	0.197639703	0.210602441	0.530928417	0.110307889	0.200160743	0.71930628
MYL5	0.042416648	0.30708695	0.321556739	0.611985932	0.471458776	0.200265479	0.719316433
DACT1	0.019896165	0.866351165	0.107157965	0.759902663	0.864017764	0.200626657	0.719978934
EEF1E1	0.828058012	0.281369345	0.311009635	0.858117722	0.019528153	0.200757029	0.719978934
MNAT1	0.22160772	0.052873093	0.2347684	0.888055005	0.495910383	0.200516422	0.719978934
SAMSN1	0.446070626	0.199240853	0.386858226	0.091842971	0.383941668	0.200597469	0.719978934
TMEM71	0.836872133	0.515719852	0.068406789	0.766210928	0.053642918	0.200689226	0.719978934
UBE3B	0.824306489	0.743880732	0.282154879	0.046894236	0.149634441	0.200736014	0.719978934
WDR3	0.934489871	0.19862717	0.221564739	0.173152042	0.170199249	0.20056297	0.719978934
CDC37	0.466597766	0.222379654	0.360506244	0.104151789	0.31206129	0.200884322	0.720231101
GDE1	0.23549769	0.438333403	0.233572435	0.478378507	0.105462144	0.200937352	0.720231101
MUTYH	0.307106844	0.150642599	0.119406363	0.437049741	0.503975944	0.200966811	0.720231101
THUMPD3	0.861144613	0.708832806	0.391379482	0.162651677	0.031324291	0.201002891	0.720231101
TMED5	0.970903076	0.184081599	0.137662272	0.331314117	0.14946912	0.201105931	0.72044301
ACE	0.288847204	0.133750778	0.902114536	0.250434132	0.140690589	0.201911119	0.720571644
ANO1	0.218928672	0.090154957	0.690365986	0.137017778	0.655597456	0.201578752	0.720571644
CDC42EP5	0.843623944	0.241447653	0.106503632	0.905553078	0.062398312	0.2017301	0.720571644
FAM198A	0.663285869	0.130548435	0.123050426	0.360530881	0.319123681	0.201738174	0.720571644
GOLGB1	0.588954221	0.349276611	0.048728315	0.26616788	0.460550538	0.20197785	0.720571644
LOC101905743	0.908948218	0.771819301	0.058030619	0.030400033	0.990737643	0.201759002	0.720571644
LOC107131416	0.817468215	0.807491247	0.736334673	0.062368383	0.040307207	0.201399628	0.720571644
LOC783202	0.882401383	0.460428285	0.094821094	0.05239081	0.609051935	0.202019995	0.720571644
MED27	0.884068828	0.116835916	0.25066317	0.191893575	0.246903749	0.201805669	0.720571644
MYSM1	0.574506448	0.283009401	0.079750116	0.709394181	0.13301672	0.201539609	0.720571644
PABPC4	0.683520112	0.608389747	0.439819394	0.027336442	0.244643962	0.201507111	0.720571644
POLR1A	0.519118517	0.374999793	0.609708508	0.131906245	0.078288307	0.20172036	0.720571644
RIMKLB	0.464866144	0.743759789	0.040098493	0.913288363	0.097055936	0.201990527	0.720571644
RMRP	0.603417421	0.566698033	0.038262403	0.671494233	0.139079621	0.201403527	0.720571644
TDP1	0.723332813	0.008317184	0.445829395	0.760769497	0.601129796	0.201796984	0.720571644
TEPSIN	0.505390721	0.205027865	0.505800428	0.03761617	0.622153214	0.201793829	0.720571644
TMEM69	0.465055214	0.552411456	0.118219034	0.269341931	0.14911699	0.201222818	0.720571644
TRAF4	0.546163464	0.254781889	0.090017038	0.52776662	0.185917877	0.202004869	0.720571644

VDR	0.833855132	0.15687189	0.854668341	0.013505729	0.810056008	0.20150293	0.720571644
ZBTB16	0.680055982	0.929030268	0.024534692	0.198803182	0.397856336	0.201749509	0.720571644
ADGRA1	0.311910834	0.020959777	0.938415045	0.454411287	0.443670485	0.202658008	0.720793994
FBLN7	0.599834665	0.283340871	0.015965788	0.738496922	0.615150393	0.202309984	0.720793994
GPAT2	0.099979001	0.115530018	0.796769454	0.347591918	0.386727645	0.202679344	0.720793994
HDAC5	0.494625031	0.939793534	0.598863984	0.098525256	0.045002999	0.202444468	0.720793994
LDLRAP1	0.219678916	0.126231912	0.477677169	0.194317966	0.480047751	0.202554952	0.720793994
LOC112443178	0.003237643	0.912868475	0.873694619	0.980564416	0.488408045	0.202642291	0.720793994
LOC533921	0.965608495	0.309103426	0.743978219	0.036288007	0.153551301	0.202696165	0.720793994
MAT2B	0.747876065	0.049748915	0.477071989	0.490636102	0.14147991	0.20226006	0.720793994
MKRN1	0.661541193	0.688477562	0.204636343	0.173495773	0.076162356	0.202214182	0.720793994
MYL12A	0.509636866	0.138965944	0.079633228	0.400741885	0.546980589	0.202605192	0.720793994
POC5	0.890049362	0.147001177	0.021020508	0.516795468	0.866204414	0.202180502	0.720793994
PSMB10	0.872609283	0.400023891	0.024092677	0.295185333	0.498693812	0.202752887	0.720793994
RECQL4	0.220351628	0.074063692	0.151507394	0.942266208	0.529184079	0.202327773	0.720793994
SECISBP2	0.548978059	0.685992455	0.556218114	0.01520861	0.387509171	0.202459999	0.720793994
SERPINA5	0.445228708	0.337957768	0.713722481	0.077597655	0.148622783	0.202798064	0.720793994
SYNGAP1	0.3370979	0.422502762	0.044746357	0.31262558	0.621221447	0.202727765	0.720793994
TRMU	0.078434818	0.240703727	0.517954915	0.410424708	0.3086892	0.202828997	0.720793994
CERS1	0.18271869	0.25824903	0.428279117	0.43399629	0.141552498	0.203047052	0.721085975
CRYZ	0.318487918	0.416736076	0.537699786	0.113695857	0.153287818	0.203236746	0.721085975
FAM173B	0.826518338	0.611178285	0.105472392	0.214402011	0.109124108	0.203466875	0.721085975
FARS2	0.833392249	0.605597611	0.675105757	0.171317236	0.021269769	0.203051143	0.721085975
LMO2	0.387744606	0.395806619	0.089553792	0.099932514	0.906916339	0.203389886	0.721085975
LOC104972821	0.101780847	0.828538187	0.210702835	0.58284751	0.119781291	0.202961284	0.721085975
LOC112444864	0.91915301	0.52809674	0.267811599	0.029640057	0.323390236	0.203425702	0.721085975
RBBP6	0.518400593	0.444390146	0.411588979	0.067376431	0.194604162	0.203190764	0.721085975
RNF170	0.883972284	0.060155538	0.557458724	0.506626062	0.082749554	0.203149287	0.721085975
TMEM202	0.081893897	0.887897148	0.047293996	0.788989333	0.459496487	0.203482369	0.721085975
TRAPPC9	0.524711388	0.805994614	0.420185208	0.275565607	0.025419998	0.203319853	0.721085975
TRHDE	0.26742615	0.255295453	0.678343775	0.192950783	0.139304852	0.203323599	0.721085975
UTP18	0.571144019	0.417252433	0.116886	0.148225292	0.301572242	0.203350159	0.721085975
LOC101906240	0.352616065	0.50533619	0.127077402	0.824802279	0.066833926	0.203607767	0.721374583
BTBD10	0.264578855	0.567932854	0.24752498	0.045438171	0.740233395	0.203838337	0.721543459
COQ8A	0.554241389	0.790385919	0.837901757	0.141491839	0.024090729	0.203850769	0.721543459
LOC112441473	0.561419368	0.767300511	0.708928615	0.214386268	0.019105937	0.203828759	0.721543459
MPP5	0.43999015	0.777598502	0.009585718	0.636465588	0.599747923	0.203911646	0.721543459
WISP1	0.640734156	0.033519586	0.20636316	0.665060656	0.42421443	0.203789169	0.721543459
WNK2	0.046800611	0.414125474	0.601809383	0.619347559	0.173309153	0.203919235	0.721543459
POLD1	0.524157619	0.017364902	0.450942695	0.462823294	0.659731004	0.204024523	0.72176039

DCAF15	0.689344083	0.202333716	0.163633756	0.762941382	0.072047883	0.20413287	0.721988047
EIF2AK3	0.629285091	0.896494922	0.045025038	0.050068063	0.986877871	0.204176988	0.721988485
NR2F6	0.063914893	0.141635851	0.775193499	0.209814924	0.852941131	0.204241094	0.722059586
AFAP1	0.566709517	0.987176387	0.092901742	0.265261174	0.091370407	0.204557371	0.722662706
C19H17orf53	0.114987992	0.139035481	0.511144543	0.845514429	0.182365339	0.204587833	0.722662706
LOC616903	0.747288738	0.233958039	0.080613709	0.753870098	0.118564828	0.204564417	0.722662706
LOC781710	0.0475543	0.781495261	0.987639417	0.040478101	0.847580757	0.204523123	0.722662706
ATP6V1A	0.687607679	0.26015076	0.120093995	0.917239128	0.063993514	0.204664825	0.722779092
PAQR3	0.789847891	0.736795783	0.084815325	0.083364773	0.307607805	0.205059013	0.723859639
XPC	0.802745259	0.500893105	0.008450809	0.760739861	0.489566824	0.205040813	0.723859639
NPEPL1	0.675657138	0.26091772	0.479384751	0.020444365	0.733182907	0.205144524	0.724005759
IGSF9	0.323717083	0.123179373	0.27525959	0.464482983	0.24927019	0.205478424	0.724735467
LOC112448518	0.440649255	0.740076592	0.066368077	0.068658402	0.855235586	0.205483769	0.724735467
RASGRP4	0.420177776	0.171192528	0.412176889	0.247596249	0.17303149	0.205426486	0.724735467
CCM2	0.088740659	0.58856224	0.043462239	0.78805424	0.712129196	0.205733048	0.725147133
LOC616948	0.698028532	0.111718232	0.622713844	0.051278432	0.511398628	0.205693963	0.725147133
PLSCR4	0.10119594	0.430176658	0.694040083	0.135402198	0.311282167	0.205692611	0.725147133
LOC112446691	0.439892473	0.086979018	0.209488643	0.558718665	0.284613809	0.205788215	0.725185825
LOC104974348	0.759243348	0.041399208	0.251118196	0.518125904	0.312070271	0.205925386	0.72551342
TSPYL1	0.637997082	0.036136615	0.270902165	0.336707573	0.607170728	0.205974509	0.72553073
GDI2	0.954902149	0.137428269	0.494159035	0.863758859	0.02281433	0.20606136	0.725680898
LOC781256	0.067944388	0.138028959	0.663835028	0.419220377	0.490815186	0.206313312	0.726256493
NDUFAF7	0.781473683	0.611398355	0.508343674	0.020980011	0.25136667	0.206304876	0.726256493
B4GALNT3	0.502018213	0.500130108	0.028391303	0.439594019	0.409291366	0.206440974	0.726264228
GET4	0.160628878	0.182451827	0.508036771	0.201486881	0.428207514	0.206609385	0.726264228
KDM6A	0.284069961	0.640586232	0.689309278	0.040549782	0.252483221	0.206577923	0.726264228
LOC789694	0.465096786	0.296499254	0.437565777	0.025856294	0.823487835	0.206625293	0.726264228
LRRN4	0.693234815	0.073748178	0.201450363	0.349632294	0.356795325	0.206624482	0.726264228
POLR2J	0.524327327	0.177019561	0.728649878	0.043862638	0.432446514	0.206464597	0.726264228
SIX1	0.075611336	0.503388396	0.126772127	0.924629537	0.287889608	0.206594761	0.726264228
COA5	0.175704289	0.173254829	0.304866516	0.362225803	0.382419183	0.206688909	0.726330528
RENBP	0.708870046	0.155722416	0.115471297	0.125707096	0.802986664	0.206776932	0.726330528
SIGLEC15	0.701055049	0.078294109	0.174656735	0.156217198	0.858907774	0.206748347	0.726330528
LOC112442613	0.760033376	0.212107767	0.584522734	0.022954454	0.595286673	0.206855388	0.726450627
ALG14	0.637191949	0.147739503	0.375149623	0.290552084	0.125813715	0.207131681	0.726506258
ATXN7L2	0.494751005	0.348906696	0.043656509	0.982724048	0.174026254	0.20695417	0.726506258
LOC107131939	0.989479051	0.717120826	0.282827055	0.019878427	0.323139388	0.206978518	0.726506258
MLIP	0.095755817	0.110344068	0.370158295	0.419129173	0.787289243	0.207097831	0.726506258
TMEM209	0.95190029	0.402893879	0.840127852	0.2702261	0.014813472	0.207031823	0.726506258
VMAC	0.50902562	0.229195621	0.097144004	0.279991631	0.406853159	0.207136846	0.726506258

GNA15	0.056511601	0.487598555	0.57718613	0.133322293	0.609308117	0.207212282	0.726527892
ZNF622	0.869911741	0.294463705	0.164013592	0.160063783	0.192156551	0.207231556	0.726527892
PTPRZ1	0.022076117	0.849554416	0.360345207	0.631569848	0.303059659	0.207340066	0.726597868
ST6GALNAC4	0.92094111	0.030524533	0.360073455	0.460753977	0.277332966	0.207330459	0.726597868
PTP4A1	0.972869305	0.025023703	0.420979666	0.859717015	0.146967766	0.207452403	0.726828777
RAD52	0.239794342	0.628982483	0.369505334	0.770929956	0.03015124	0.207494535	0.726828777
ARID3B	0.454174101	0.963474281	0.591003893	0.008656212	0.579240695	0.207596935	0.726829475
CA4	0.372551543	0.597375407	0.97937786	0.147129627	0.040485366	0.207728766	0.726829475
CYB5R2	0.051450976	0.866533468	0.303416171	0.362293261	0.264978163	0.207754498	0.726829475
EMP2	0.234291071	0.293351061	0.447652112	0.04609379	0.916622966	0.207858863	0.726829475
GMEB2	0.15095537	0.388241854	0.465269417	0.080192507	0.594661321	0.207893337	0.726829475
LOC100335744	0.998275455	0.414778515	0.699854237	0.023569049	0.189898962	0.20762102	0.726829475
LOC101905593	0.796224287	0.690969231	0.334220397	0.050846878	0.138842439	0.207712095	0.726829475
MPHOSPH10	0.775627002	0.457669609	0.326055026	0.037016171	0.303258774	0.207806227	0.726829475
NARS2	0.32395636	0.663685787	0.29082553	0.78461877	0.026489075	0.207832076	0.726829475
SEC13	0.074522472	0.187002181	0.32144681	0.299252414	0.972114519	0.208121915	0.727473642
ENY2	0.798633481	0.124250816	0.408531429	0.036304748	0.885820816	0.208166573	0.727474793
ASAP1	0.009324588	0.550994225	0.696959486	0.491720074	0.744688304	0.208774873	0.727531689
CALM1	0.587505634	0.118704769	0.19170075	0.415212048	0.236025901	0.208691151	0.727531689
CLK4	0.36043476	0.771393093	0.625717608	0.050200351	0.149948801	0.208641604	0.727531689
DLGAP1	0.508141727	0.290129162	0.079896446	0.232790149	0.478017631	0.20873473	0.727531689
DPY19L1	0.369856032	0.299663651	0.473251805	0.411876777	0.060584392	0.208582225	0.727531689
DYNC1I2	0.562181248	0.508183779	0.377338984	0.012638549	0.960595559	0.208577232	0.727531689
GPNMB	0.706830835	0.438520108	0.099301646	0.975357462	0.0434843	0.208306921	0.727531689
LOC112443015	0.271814397	0.648618127	0.037519371	0.402598712	0.490731301	0.208422795	0.727531689
NEU3	0.887093643	0.499314015	0.564502544	0.344249642	0.015204351	0.208573296	0.727531689
SLC41A1	0.571489882	0.752765133	0.169855526	0.066262633	0.270792049	0.208768921	0.727531689
TMEM131	0.913610493	0.908826551	0.101771444	0.03918641	0.395696858	0.208699073	0.727531689
TMEM251	0.700301773	0.25516353	0.046417516	0.746958085	0.21134306	0.208626823	0.727531689
TRMT6	0.935242835	0.238029882	0.484253393	0.780325161	0.015534839	0.208417356	0.727531689
TTC22	0.57639025	0.380396	0.135570551	0.085107415	0.518452137	0.208803501	0.727531689
RNASE12	0.270426923	0.117689606	0.228350781	0.330687976	0.546024118	0.208858861	0.727570107
ACER1	0.079854071	0.108850605	0.997931585	0.576987934	0.262506948	0.208984734	0.72785409
AUTS2	0.684817409	0.567188706	0.425232753	0.01311325	0.606886834	0.209036259	0.727879069
ADA2	0.459142356	0.236901304	0.436022591	0.112780077	0.247336826	0.209720671	0.727922819
ALMS1	0.535046883	0.322010257	0.182716656	0.422102542	0.099286225	0.209427322	0.727922819
BAZ1A	0.72624485	0.143415703	0.668902823	0.026708334	0.710509161	0.209650706	0.727922819
CCRL2	0.296597753	0.540684072	0.282015365	0.32645554	0.089140664	0.209167481	0.727922819
CHD1L	0.491728337	0.285971285	0.045179741	0.565539669	0.367865137	0.209622259	0.727922819
COL6A2	0.26212636	0.268210223	0.09088881	0.236061787	0.877302432	0.209751656	0.727922819

COMMD6	0.602480165	0.494747035	0.636313863	0.092542029	0.075051665	0.209268346	0.727922819
EFNA4	0.783870809	0.231793099	0.410621145	0.994008134	0.017769598	0.209306778	0.727922819
ENPP4	0.149942568	0.084069026	0.333590523	0.491506977	0.640317123	0.209758516	0.727922819
GNPTG	0.41106133	0.142465855	0.974470645	0.162787449	0.141938505	0.209368772	0.727922819
HSD17B10	0.318632047	0.409770454	0.534546385	0.036753619	0.515262365	0.209622484	0.727922819
INSL6	0.607514763	0.360117131	0.899961557	0.040846698	0.164050368	0.20943028	0.727922819
LOC112449590	0.784852335	0.833229823	0.843552582	0.075621636	0.031567976	0.209234368	0.727922819
MCFD2	0.939098846	0.076404897	0.341234841	0.31006569	0.174083933	0.209611282	0.727922819
PHACTR1	0.279890562	0.701580323	0.011984234	0.718252866	0.778866887	0.209199849	0.727922819
SAP18	0.48710134	0.054129999	0.578207652	0.293312177	0.295146038	0.209466859	0.727922819
LOC784058	0.974257415	0.087034575	0.113980088	0.414412836	0.330638842	0.209827637	0.728008741
IRGQ	0.862145481	0.52289117	0.023126679	0.890028519	0.143060608	0.210084438	0.728745658
CWC15	0.961829491	0.056332278	0.432939175	0.958257689	0.059212777	0.210366867	0.729571143
LOC112443512	0.506503108	0.363120045	0.997945843	0.060482837	0.120218642	0.210652623	0.730407818
GIPC1	0.403320391	0.912425027	0.061259181	0.278383604	0.213021834	0.210835314	0.730886849
CARD10	0.713924861	0.013474232	0.624986193	0.706288401	0.31566651	0.211118599	0.731232904
LOXL2	0.981394977	0.188091817	0.033963454	0.306458304	0.697911343	0.211157927	0.731232904
PTPRC	0.728563955	0.090356702	0.828583914	0.109400623	0.224471402	0.211046677	0.731232904
SUGCT	0.455676028	0.65314568	0.129416526	0.049639479	0.700758333	0.211072795	0.731232904
ZNF467	0.843722131	0.265958169	0.48498148	0.024734646	0.497375919	0.210993274	0.731232904
POLA1	0.040324315	0.228069585	0.465469272	0.468936673	0.668311035	0.211212347	0.731267051
YWHAQ	0.374948465	0.458118693	0.437925733	0.023438162	0.761508612	0.211293688	0.731394373
CD1D	0.401857711	0.664157878	0.064416527	0.195261024	0.402876018	0.212078147	0.731937068
CDCP1	0.931078195	0.29898353	0.030442864	0.254022499	0.627173851	0.21189318	0.731937068
CEBPB	0.937706393	0.820960549	0.702662352	0.005616538	0.443600301	0.211700002	0.731937068
CIP2A	0.299253116	0.563956799	0.051719739	0.9946893	0.155177678	0.21166574	0.731937068
CYSLTR2	0.16699305	0.892805707	0.038914166	0.664558824	0.350967207	0.21213625	0.731937068
DLG4	0.577406695	0.148483339	0.183724383	0.218026937	0.39246424	0.211709915	0.731937068
DUSP15	0.465623982	0.287189565	0.192655175	0.074264152	0.707434101	0.212157712	0.731937068
EGR1	0.229404872	0.222305334	0.31288128	0.41822549	0.20238255	0.211926912	0.731937068
FBXO46	0.836877594	0.172089541	0.293217131	0.522627892	0.061396884	0.212280025	0.731937068
FCGRT	0.647442039	0.298117161	0.106475013	0.6760802	0.0974173	0.212162652	0.731937068
GALNT15	0.902922626	0.24768814	0.225568679	0.248480017	0.10818867	0.212369828	0.731937068
LOC100337213	0.079166164	0.647490942	0.97579106	0.048412042	0.556701231	0.211726975	0.731937068
LOC101907648	0.062566567	0.487939703	0.202901937	0.397620759	0.546566029	0.211579115	0.731937068
LOC112445980	0.95777897	0.022357796	0.511770494	0.559563204	0.220791074	0.21219486	0.731937068
LOC787497	0.943491628	0.008858628	0.872401314	0.645562014	0.288159708	0.212390664	0.731937068
MECR	0.265519272	0.60827646	0.59698962	0.15322699	0.091921944	0.212520878	0.731937068
OTUD7A	0.930170027	0.119553413	0.194142056	0.175688447	0.357547633	0.212373109	0.731937068
PRPSAP1	0.776568318	0.816811185	0.035812201	0.310567053	0.192407507	0.21246927	0.731937068

PTGDR2	0.889063569	0.413749398	0.081224436	0.171952468	0.263570312	0.21221009	0.731937068
RPGR	0.053596424	0.396063645	0.917690214	0.16542041	0.417826424	0.211597745	0.731937068
SIRT1	0.2548193	0.357308952	0.461838365	0.041265195	0.779310089	0.21206147	0.731937068
SUMF2	0.070676609	0.12660081	0.685542562	0.244328215	0.905873955	0.212488632	0.731937068
TRPM1	0.412683915	0.464085956	0.024047272	0.955517973	0.306904044	0.211928787	0.731937068
UCP2	0.706023979	0.056845332	0.275878932	0.370705064	0.329109272	0.211948092	0.731937068
PPEF1	0.262925558	0.178385147	0.983605968	0.210873353	0.139664259	0.212570708	0.731955076
CXCL14	0.436684245	0.65270505	0.442839962	0.874012218	0.012330649	0.212698239	0.732086996
MRPL44	0.543412625	0.454031537	0.247829655	0.107846448	0.206218377	0.212665427	0.732086996
UBE2V2	0.71371812	0.17130492	0.235202307	0.264351005	0.179056696	0.212766334	0.732167814
CTSO	0.145630345	0.62533827	0.246363207	0.417129605	0.145782391	0.21301642	0.732428956
GBGT1	0.495918975	0.525961612	0.084177546	0.211065765	0.294413298	0.213020742	0.732428956
SLC19A1	0.376095401	0.638116056	0.531076685	0.101988005	0.104953348	0.213011836	0.732428956
SYDE2	0.055836461	0.575165364	0.594226959	0.44450949	0.160690986	0.212921403	0.732428956
SLC6A2	0.167320891	0.256458168	0.864396795	0.066763237	0.552023706	0.213228769	0.732990644
RCBTB1	0.993263071	0.376081966	0.027658733	0.345083847	0.383920469	0.213370242	0.733323358
AQP11	0.327965672	0.045234072	0.637086756	0.547294823	0.265288457	0.213640315	0.733330101
LOC112447402	0.215445915	0.872168966	0.056406952	0.777904777	0.166348593	0.213586832	0.733330101
MTCP1	0.538238395	0.084773944	0.178391086	0.33491084	0.502925473	0.213543594	0.733330101
TMEM102	0.618977386	0.243863518	0.058078442	0.548533988	0.285341832	0.213633661	0.733330101
ZNF181	0.136166186	0.442003013	0.036002765	0.845704008	0.748784498	0.213633667	0.733330101
ZSCAN26	0.268201114	0.160457112	0.250637478	0.984471801	0.129025103	0.213469082	0.733330101
ATAT1	0.649047463	0.710243552	0.0979875	0.158800942	0.191690408	0.213857974	0.733711387
FBXO28	0.513360404	0.051281945	0.154583991	0.345931117	0.977356502	0.213929138	0.733711387
NXT2	0.253782919	0.4508735	0.070316383	0.976809312	0.17507131	0.213930229	0.733711387
PDK3	0.153762414	0.121878232	0.507930623	0.575792542	0.250852904	0.21384753	0.733711387
CCNB2	0.547952943	0.281798318	0.089852955	0.216477456	0.45883153	0.214099826	0.733832968
LOC112446481	0.546521318	0.376391636	0.501719173	0.942832906	0.014159492	0.214077482	0.733832968
RBL2	0.650701316	0.768041799	0.077385532	0.038247367	0.931011055	0.214025844	0.733832968
FTL	0.538798237	0.213130846	0.061337819	0.315177714	0.622069031	0.214327659	0.73434444
LOC112444904	0.835405349	0.487296935	0.790983554	0.490613018	0.00874261	0.214338545	0.73434444
FOXP2	0.11867877	0.59985049	0.559778822	0.180625332	0.191991316	0.214401876	0.734408098
NUDT14	0.0279961	0.653711146	0.769036295	0.127058839	0.773953649	0.214565724	0.734815963
ADGRG6	0.042001314	0.22359805	0.521773231	0.586221704	0.482563545	0.214735072	0.73497769
KPNA6	0.999362384	0.289536757	0.011429276	0.662593258	0.632421509	0.214702639	0.73497769
LOC112447459	0.059988905	0.402030892	0.30598132	0.217985577	0.86219316	0.214792091	0.73497769
RANBP1	0.270027462	0.103021049	0.665177541	0.691489058	0.108349427	0.214748626	0.73497769
HSPE1	0.508820836	0.371620208	0.485016925	0.267934765	0.056486089	0.214875467	0.735109712
REEP3	0.381781934	0.079003262	0.376970771	0.234729959	0.520788035	0.215026754	0.735341244
SHCBP1L	0.098434925	0.070576056	0.72068121	0.812648481	0.341640318	0.21503276	0.735341244

CLASP2	0.623811433	0.540888329	0.257224176	0.041010945	0.390734652	0.215090906	0.735353655
RTL5	0.315021955	0.735472796	0.033108668	0.274790848	0.659999363	0.215126007	0.735353655
FXR1	0.387473905	0.863490525	0.328929126	0.143647705	0.088101733	0.215248601	0.73561949
LOC781218	0.827856089	0.020094684	0.179310923	0.747617071	0.625179922	0.215358976	0.735843465
LOC101903678	0.349411307	0.02142605	0.607903106	0.453796373	0.676256007	0.215548726	0.736135497
NOC4L	0.310523413	0.331272493	0.452058887	0.74858078	0.040171376	0.215685012	0.736135497
OAS1X	0.520308345	0.946478242	0.521452851	0.053696146	0.101290162	0.215551542	0.736135497
PACSIN2	0.836749755	0.418700306	0.058006924	0.3785495	0.181820503	0.215713583	0.736135497
PIK3C2A	0.601749063	0.943304994	0.136238242	0.048960752	0.369217466	0.215651663	0.736135497
VPS35	0.499345581	0.279635715	0.202122074	0.94956549	0.052181236	0.215689208	0.736135497
CHKB	0.219038249	0.543485789	0.058887564	0.24144579	0.827816603	0.215899236	0.736462764
PAPD4	0.041468592	0.439625286	0.872239636	0.093100671	0.94614507	0.215864866	0.736462764
TUBB4A	0.479738178	0.74525548	0.618779541	0.054574205	0.116168541	0.216008453	0.736682196
LOC112441525	0.863717902	0.559158774	0.02814603	0.338608658	0.305045667	0.216125216	0.736774184
USP34	0.358322848	0.548273879	0.71522834	0.014681298	0.680599374	0.216121619	0.736774184
CMTM8	0.309181865	0.926981658	0.574220443	0.016708719	0.511585473	0.216335428	0.736948863
LOC531038	0.124910698	0.307476824	0.212557971	0.744310515	0.231492804	0.216324361	0.736948863
PDIA5	0.827351319	0.17855673	0.043756566	0.218966457	0.993608161	0.216304563	0.736948863
SETDB1	0.82548873	0.221631169	0.094400887	0.804087656	0.10131786	0.216356079	0.736948863
C7H19orf24	0.110419189	0.700592081	0.930064275	0.592690718	0.033141359	0.216836737	0.737653989
CCND2	0.686314576	0.672145025	0.138799271	0.122143304	0.180831072	0.216910889	0.737653989
EPS8L2	0.937503235	0.125918092	0.189652722	0.783253853	0.080847287	0.217180537	0.737653989
GJC2	0.251418983	0.557860572	0.485137537	0.028011901	0.743585967	0.217148927	0.737653989
GNA13	0.998494598	0.092016848	0.019044284	0.980420247	0.826283189	0.217163153	0.737653989
KIAA0825	0.126153127	0.31595513	0.126089959	0.681014662	0.414291957	0.2172004	0.737653989
LCTL	0.775735019	0.370478372	0.180457954	0.088923176	0.306412936	0.216824965	0.737653989
LOC104974667	0.235550107	0.756030295	0.013380232	0.825706288	0.717042243	0.216644569	0.737653989
LOC107131843	0.906198005	0.331778819	0.616875854	0.025393784	0.300829579	0.217112463	0.737653989
LOC112441887	0.803491129	0.630933057	0.24070061	0.025080195	0.461099631	0.216672398	0.737653989
NLK	0.831936712	0.063698625	0.212324402	0.4720801	0.266699926	0.217097709	0.737653989
OPA3	0.607159178	0.637866601	0.308163556	0.033857087	0.350329248	0.217017417	0.737653989
OST4	0.594177533	0.093754934	0.299059914	0.089179004	0.954121133	0.217167125	0.737653989
RPS6KL1	0.264046767	0.491058731	0.933276315	0.019227744	0.609622117	0.217237324	0.737653989
WASF2	0.127504646	0.760607384	0.104572336	0.559839995	0.248767094	0.216771142	0.737653989
RAB3A	0.661593852	0.185706288	0.118835679	0.23365346	0.416574325	0.217442835	0.738199082
ANKMY2	0.347134499	0.393732661	0.335488427	0.119157448	0.260904167	0.217782972	0.738255918
ENAH	0.637574582	0.981288157	0.200056424	0.096284835	0.118366639	0.217855697	0.738255918
HECTD1	0.454881647	0.396056753	0.025950665	0.313988677	0.97245559	0.217936653	0.738255918
KDELC1	0.616759783	0.295246528	0.021407234	0.417875286	0.877576503	0.218089373	0.738255918
LAMTOR1	0.41440565	0.316288431	0.384434876	0.064087127	0.441716371	0.217850348	0.738255918

LRPPRC	0.720538905	0.380889649	0.838190671	0.290978304	0.021328802	0.217946345	0.738255918
NDUFAF3	0.876052859	0.126767428	0.71805312	0.043639263	0.409001799	0.217611058	0.738255918
NPR2	0.842798037	0.255367062	0.440121547	0.194981782	0.077050395	0.217594105	0.738255918
NR2C2AP	0.214849919	0.310874019	0.43930677	0.895329645	0.054394016	0.21804608	0.738255918
PAX5	0.540986111	0.135388414	0.180033314	0.963336259	0.112388054	0.217944341	0.738255918
PMM2	0.161893707	0.192041096	0.708198624	0.153960146	0.421695822	0.218087798	0.738255918
PTPMT1	0.719268213	0.011353975	0.827042077	0.317470575	0.665419238	0.217880414	0.738255918
TCTEX1D2	0.332048647	0.028347431	0.997169517	0.274313946	0.554743446	0.217996317	0.738255918
YWHAB	0.787013576	0.282334897	0.062466829	0.680500732	0.15071539	0.217632551	0.738255918
STC1	0.026406133	0.123033485	0.746565859	0.947692454	0.622191179	0.218138478	0.738269864
DBN1	0.24454476	0.892897202	0.022829764	0.380426588	0.75475022	0.218226335	0.738309527
LOC107132853	0.67256736	0.769768211	0.709884981	0.161270255	0.024151945	0.218240175	0.738309527
RBPMS	0.538364034	0.304419403	0.476595059	0.405064708	0.045315597	0.218411992	0.738738501
LOC100847870	0.150665429	0.184266497	0.476928096	0.152817715	0.708865881	0.218457667	0.738740733
LIN52	0.900854826	0.053994556	0.579551067	0.684919865	0.074640693	0.218979304	0.739038593
LOC112446127	0.575497143	0.252829025	0.134653789	0.109180154	0.673822892	0.218996081	0.739038593
LOC784148	0.878849219	0.78440339	0.024460799	0.370755661	0.229893954	0.218682561	0.739038593
MANSC1	0.365120571	0.304028351	0.051155399	0.404516038	0.625569274	0.218660601	0.739038593
NDUFA9	0.709143801	0.368201465	0.752698381	0.021222299	0.345364947	0.218928538	0.739038593
PDCL3	0.574653461	0.044118425	0.094669589	0.959259889	0.625617674	0.218921023	0.739038593
PPP2R3C	0.938175208	0.524905329	0.474723839	0.432932529	0.014232527	0.218927706	0.739038593
TBC1D8	0.163846519	0.312843917	0.426882387	0.665817637	0.098827969	0.218877095	0.739038593
TRIP13	0.613623217	0.056961857	0.580610282	0.449764219	0.157357162	0.21860723	0.739038593
UPK1B	0.255748672	0.299033463	0.12509753	0.445282564	0.337828085	0.218827727	0.739038593
RAB2B	0.092739251	0.033058431	0.860407761	0.747054678	0.732867119	0.219211387	0.739322521
SLC8A2	0.107036953	0.347626138	0.747184114	0.162920986	0.318707626	0.21916462	0.739322521
SNX11	0.669502058	0.229528225	0.783127736	0.11222161	0.10694152	0.219215367	0.739322521
LRIG3	0.080060611	0.920712095	0.104532853	0.483766429	0.388076762	0.219394265	0.739621874
YPEL5	0.792055179	0.179446641	0.488045854	0.387927526	0.053742622	0.21936178	0.739621874
LOC100847708	0.655516291	0.380839069	0.111037248	0.467936396	0.111576231	0.219445834	0.739643784
CGREF1	0.02779345	0.440597666	0.166167615	0.886871894	0.803767348	0.219692075	0.73987819
GSTT1	0.018540564	0.897894742	0.860071159	0.183916108	0.551610743	0.219847767	0.73987819
LOC104972216	0.54373924	0.127733808	0.119471829	0.318325709	0.549742028	0.219811557	0.73987819
NLRC4	0.982333561	0.166079463	0.322263867	0.110866036	0.249052699	0.219781987	0.73987819
NOL6	0.754187417	0.061903855	0.626922132	0.900152941	0.055146718	0.219876055	0.73987819
PARD3B	0.42702914	0.103229288	0.662451044	0.157884054	0.314233285	0.219560665	0.73987819
PHF14	0.605001527	0.635198503	0.00728053	0.855443532	0.606874414	0.219843666	0.73987819
PRR16	0.12021205	0.304600561	0.405663192	0.342008479	0.285557718	0.219705482	0.73987819
GAPDH	0.482378891	0.237869473	0.528460072	0.124708818	0.192279005	0.219957801	0.740001533
STARD5	0.207594776	0.593912214	0.202484	0.060382123	0.965068398	0.22001567	0.74004451

RUNDC3A	0.146571827	0.419307985	0.377066016	0.089682357	0.700604894	0.220113653	0.74022237
ABHD17A	0.653541565	0.406433697	0.277151075	0.450377548	0.043957722	0.22021799	0.740341159
GTPBP10	0.103898766	0.178289305	0.777261659	0.134049104	0.755279901	0.220239202	0.740341159
DPY30	0.814722401	0.005206339	0.441127622	0.955570032	0.816952238	0.220465921	0.740951511
MYBPC2	0.237749898	0.249661181	0.129704368	0.518664472	0.366197178	0.220583572	0.741195128
DESI1	0.1930043	0.314734773	0.34318039	0.370223424	0.189767728	0.220760151	0.741205795
EPC2	0.051872594	0.42958286	0.94028739	0.138687057	0.504042828	0.220767408	0.741205795
SAMM50	0.288812919	0.34017666	0.322727844	0.828070411	0.055758671	0.220713463	0.741205795
VDAC3	0.81250685	0.187788875	0.775393473	0.196392371	0.062964241	0.220637112	0.741205795
BCKDHB	0.573189668	0.418719121	0.702533321	0.163004566	0.053345625	0.220879013	0.741330165
LPXN	0.527301182	0.24077721	0.733309545	0.053472761	0.294787253	0.220985142	0.741330165
MPI	0.740891677	0.032329607	0.565233961	0.209372458	0.517626532	0.220963893	0.741330165
TAF8	0.309533947	0.587642301	0.033062453	0.714472286	0.341509667	0.220970355	0.741330165
PPP4R2	0.873333476	0.891205532	0.183993768	0.025079371	0.40902389	0.221093229	0.741541179
ABCG2	0.835888141	0.065610749	0.23180008	0.822627219	0.141131337	0.22161349	0.742610801
GCSAML	0.857983678	0.193840158	0.03471698	0.422015503	0.60617552	0.22169742	0.742610801
GPR146	0.032244495	0.199785391	0.648090876	0.803564641	0.439837747	0.221589269	0.742610801
PLPP7	0.138439106	0.667222113	0.279343835	0.33391884	0.171165104	0.221526884	0.742610801
ROR1	0.076796305	0.81416489	0.324004521	0.54255839	0.13434131	0.221663743	0.742610801
SELENBP1	0.083511895	0.549548835	0.821287569	0.041665245	0.939565506	0.221584757	0.742610801
SPHK2	0.152964441	0.369292203	0.534326662	0.079547856	0.615344975	0.221728897	0.742610801
SUMO1	0.816513694	0.088547647	0.691340643	0.218661226	0.135581896	0.222059387	0.743565925
CD1B	0.358829504	0.013255474	0.545871345	0.711407798	0.803854854	0.222280671	0.744033929
FYB1	0.687048675	0.16693327	0.667311295	0.245382877	0.079068329	0.222289827	0.744033929
ZFC3H1	0.675959453	0.095642824	0.494913662	0.0886586	0.524069643	0.22241969	0.74431679
APOO	0.845793659	0.327684091	0.890882731	0.127843035	0.047149607	0.222543574	0.744507306
HTRA3	0.367614654	0.261786734	0.091064584	0.575301062	0.295258937	0.222567354	0.744507306
TBC1D14	0.436571135	0.983757602	0.341919267	0.1778768	0.057027736	0.222640194	0.744599191
EHD1	0.518371923	0.057093026	0.238501072	0.456294558	0.463841101	0.222962943	0.745374792
VPS29	0.893239005	0.096174004	0.39360327	0.965613562	0.045741407	0.22292893	0.745374792
ACSM5	0.709540152	0.214562803	0.085705613	0.231356678	0.496198235	0.2232581	0.745494485
BMS1	0.197157704	0.822399259	0.248098324	0.064241632	0.58069168	0.223465029	0.745494485
GINS1	0.889272408	0.537423664	0.696508919	0.011942858	0.377594573	0.223498438	0.745494485
LOC100336368	0.30736822	0.964912577	0.007209407	0.831863292	0.84311508	0.223388575	0.745494485
LOC100848324	0.610918472	0.494680956	0.214128386	0.337791698	0.068462996	0.223157748	0.745494485
LOC511936	0.014053867	0.88202467	0.700380014	0.336639504	0.513257293	0.223420791	0.745494485
METTL13	0.188977177	0.473355139	0.272903002	0.146615821	0.41897596	0.223385856	0.745494485
PLOD3	0.085395776	0.644212375	0.519117416	0.822291222	0.063894308	0.22344867	0.745494485
RADIL	0.381463684	0.077765898	0.202053681	0.424698452	0.589367912	0.223437721	0.745494485
TRMT5	0.331949563	0.143530843	0.668279908	0.43443192	0.108318427	0.223289439	0.745494485

ZNF280B	0.203922582	0.728640441	0.637469724	0.076115511	0.20784352	0.223301781	0.745494485
FAM229B	0.211548049	0.857584658	0.24018226	0.28451124	0.121409975	0.223799268	0.746346227
UPF2	0.763893026	0.456903185	0.415125199	0.022473003	0.462872018	0.223947965	0.746690382
ADNP	0.335037793	0.94642204	0.298008511	0.090802296	0.176152906	0.224266711	0.746972489
AK8	0.661888875	0.209253128	0.69681791	0.722003395	0.021755332	0.224599043	0.746972489
ASL	0.065712457	0.525398475	0.277479	0.461301758	0.342069345	0.224285273	0.746972489
C2H2orf69	0.2919892	0.213643456	0.132035756	0.245993395	0.747019389	0.224423958	0.746972489
GFER	0.138599499	0.232047975	0.581833294	0.363958916	0.221876899	0.224243106	0.746972489
GYPC	0.488516023	0.428987378	0.308826088	0.360077525	0.064985203	0.224487093	0.746972489
LOC101902768	0.718329903	0.442609214	0.012398227	0.995432488	0.386382315	0.224612405	0.746972489
LRRN3	0.746810769	0.108647685	0.357212945	0.808072675	0.064693736	0.2245447	0.746972489
NOS2	0.232448213	0.907346871	0.401026712	0.037529499	0.47767599	0.224624291	0.746972489
PRRG3	0.962113296	0.865604404	0.579411077	0.074711322	0.041925938	0.224268702	0.746972489
TMEM25	0.336511323	0.168914358	0.129660795	0.227199562	0.903340393	0.224354418	0.746972489
TMEM33	0.692066587	0.082710915	0.469443996	0.433523325	0.129943039	0.224438601	0.746972489
WWC1	0.664074372	0.04567958	0.496818827	0.189016648	0.530711013	0.224292789	0.746972489
HERC6	0.099493815	0.116645813	0.970553239	0.167234304	0.805411512	0.224688559	0.747034835
PACRGL	0.670081396	0.430221086	0.430769929	0.392740233	0.031130586	0.224773713	0.747166578
GRK3	0.586213568	0.578529198	0.08698202	0.539773817	0.095435274	0.224870405	0.747336617
ACTRT3	0.015071753	0.924384168	0.862647703	0.134194961	0.950469476	0.225852627	0.747413609
APOBR	0.015289361	0.282699863	0.823374289	0.891801374	0.485036155	0.226327819	0.747413609
APOLD1	0.943168586	0.527179438	0.015646944	0.595654276	0.329533848	0.225424263	0.747413609
ASMTL	0.404645426	0.368976592	0.590511208	0.087211169	0.199176976	0.225745955	0.747413609
CSPP1	0.77974352	0.131995838	0.34578399	0.446255229	0.096806752	0.226185304	0.747413609
DCTN6	0.804567767	0.058108665	0.125558742	0.786041042	0.332632466	0.225991758	0.747413609
EMC8	0.361116536	0.115908459	0.724568072	0.62494823	0.080951351	0.2259527	0.747413609
ERAS	0.887927193	0.525197445	0.767353265	0.095147906	0.044678275	0.224989243	0.747413609
GNG10	0.979668256	0.153483163	0.263447349	0.870852134	0.044596379	0.226255891	0.747413609
HOMEZ	0.58331871	0.013876484	0.303831676	0.666541054	0.931550979	0.225419165	0.747413609
JAM2	0.19190651	0.207456194	0.141576163	0.480167393	0.567676735	0.226105887	0.747413609
KLHL15	0.508841487	0.603745379	0.688399198	0.011891939	0.606308718	0.225256348	0.747413609
LMX1B	0.563911243	0.127821678	0.401329786	0.866171313	0.061430232	0.226314339	0.747413609
LOC100335608	0.217765056	0.266985163	0.310263629	0.203412144	0.417308902	0.225727253	0.747413609
LOC101902449	0.921213967	0.500826321	0.118202294	0.110478724	0.255559097	0.226350962	0.747413609
LOC101902851	0.68647682	0.312952741	0.937264403	0.297841573	0.025425841	0.225257444	0.747413609
LOC112442218	0.289702261	0.443985477	0.28478627	0.221142159	0.187747931	0.224962208	0.747413609
LOC530102	0.334772221	0.194507358	0.726445127	0.038489393	0.844053829	0.226131709	0.747413609
LRIG1	0.766157383	0.054497949	0.157095378	0.34200623	0.684736698	0.2260849	0.747413609
MLLT3	0.245748425	0.210329927	0.676516216	0.18809065	0.231411752	0.225049179	0.747413609
MYF6	0.067820056	0.881687526	0.995033124	0.501444485	0.051340651	0.225767158	0.747413609

PPP2CA	0.874215748	0.019746543	0.361022581	0.591668576	0.415284818	0.22573449	0.747413609
QPCT	0.357425925	0.023159643	0.237467229	0.901834033	0.863585548	0.225704658	0.747413609
RAVER2	0.26997362	0.377862838	0.470608927	0.08926621	0.35795691	0.225932896	0.747413609
S100A16	0.744407072	0.280248915	0.151022444	0.091371776	0.530061398	0.225336871	0.747413609
SEMA6A	0.462924726	0.317514021	0.15330373	0.125633981	0.540664079	0.225680565	0.747413609
SMKR1	0.82512706	0.100160376	0.889501743	0.840840554	0.024845351	0.226059768	0.747413609
SMPDL3A	0.690435358	0.007494454	0.662862798	0.959703117	0.462858804	0.225165481	0.747413609
STK24	0.446462165	0.359465818	0.056631904	0.234713171	0.714971687	0.225283936	0.747413609
TIMM22	0.551574395	0.273856055	0.279066368	0.044568009	0.817451067	0.226058878	0.747413609
TMEM258	0.171702694	0.026402075	0.590188364	0.705743007	0.814627267	0.22623867	0.747413609
TP63	0.281239415	0.419969064	0.408699958	0.084769727	0.374371616	0.225779392	0.747413609
PEX16	0.507708786	0.239567247	0.613209262	0.078672938	0.262610782	0.226440672	0.747418106
SLC25A43	0.323041213	0.198659213	0.060290412	0.598598518	0.665347857	0.226443411	0.747418106
FGF1	0.028812607	0.194276848	0.285634241	0.993063039	0.971414876	0.2265464	0.747457373
ITIH4	0.03589612	0.382823329	0.216014281	0.956337469	0.54318656	0.226518609	0.747457373
TRIM38	0.11095736	0.966570966	0.076490927	0.681165265	0.276157242	0.226601859	0.747490072
FANCC	0.409068087	0.635987955	0.200454661	0.492193407	0.060189408	0.22673395	0.747517792
HRC	0.92573891	0.510321046	0.012529889	0.608013508	0.429310777	0.226746367	0.747517792
MB21D2	0.329670277	0.846013808	0.243838025	0.083161965	0.273405373	0.226831262	0.747517792
NAP1L1	0.047341474	0.403342449	0.590007671	0.315093705	0.435486397	0.226804513	0.747517792
NAV2	0.648008368	0.976896551	0.02745391	0.531098102	0.167536044	0.226838011	0.747517792
CCDC136	0.876624706	0.270659698	0.069401866	0.659901273	0.142465622	0.226963255	0.747755236
CIT	0.460675495	0.128281532	0.044522072	0.854421298	0.690078328	0.227199669	0.747755236
CNNM4	0.144624016	0.617683483	0.110682882	0.16137721	0.975288032	0.227553476	0.747755236
EIPR1	0.254919854	0.155399962	0.940363094	0.101115541	0.411888725	0.227210846	0.747755236
HAPLN3	0.483991624	0.876497341	0.007300709	0.580372541	0.866770912	0.227684657	0.747755236
JARID2	0.995683728	0.037604196	0.549102351	0.120090168	0.6309101	0.227664116	0.747755236
LRRFIP2	0.157244112	0.133244883	0.739361103	0.676157784	0.148735152	0.227679059	0.747755236
MAP3K10	0.044756321	0.490105492	0.524831217	0.141297798	0.956282585	0.227507373	0.747755236
MGC148714	0.985420881	0.406760785	0.946393515	0.021926237	0.186925527	0.227450005	0.747755236
PSMD2	0.857998819	0.375420197	0.518838879	0.760179992	0.012232999	0.227403299	0.747755236
RPS6KA5	0.226161596	0.426273892	0.596002217	0.107101652	0.25214974	0.227227057	0.747755236
SLC7A11	0.512106791	0.171997982	0.088496681	0.987852863	0.201802386	0.227388492	0.747755236
TDRD10	0.127378432	0.31696262	0.145755127	0.94203408	0.279912711	0.227228845	0.747755236
TSPEAR	0.216563975	0.044413938	0.81308896	0.552748086	0.3590142	0.227245892	0.747755236
UBXN11	0.188684662	0.897408174	0.058193213	0.39993572	0.394812196	0.227532017	0.747755236
ZC3H14	0.804364322	0.451516067	0.310255209	0.334076744	0.041218408	0.227219859	0.747755236
ZNF446	0.640891018	0.436365377	0.276836741	0.057767482	0.347545493	0.227420792	0.747755236
SPATA24	0.40859735	0.044277506	0.152339304	0.695670316	0.814441853	0.227943066	0.748454112
ECHDC1	0.151133563	0.213171722	0.538009807	0.289336217	0.312185573	0.228241586	0.748551594

FOXN2	0.469555411	0.094835859	0.540212613	0.314714316	0.20660592	0.228133623	0.748551594
LOC112447433	0.044732025	0.40802381	0.190468505	0.917900261	0.490672958	0.228246431	0.748551594
NUCB2	0.924169238	0.542876281	0.029689205	0.219268919	0.478492496	0.228034236	0.748551594
SYT2	0.921212657	0.249103307	0.018027856	0.501704201	0.754227336	0.228225146	0.748551594
ZCCHC6	0.619715531	0.19350969	0.242202149	0.099036924	0.543735128	0.228126882	0.748551594
ATP13A4	0.262625735	0.645907789	0.050788874	0.688754306	0.264168299	0.228378931	0.748761002
HACD4	0.22737857	0.101778358	0.932098308	0.116858715	0.621985146	0.228401534	0.748761002
NAA15	0.640006585	0.125307205	0.21151235	0.524897158	0.17616876	0.228451651	0.748775722
BOLA1	0.081191178	0.149043226	0.652446352	0.360089333	0.55260624	0.228632952	0.748921232
DDX46	0.264778848	0.647235654	0.295904389	0.074542722	0.415522139	0.228607976	0.748921232
TANC1	0.675762486	0.354464251	0.946087333	0.008688147	0.797866924	0.228623376	0.748921232
WFDC1	0.210431607	0.53360733	0.148291761	0.569747543	0.165680674	0.228688512	0.748953735
KCNB1	0.302521461	0.960084095	0.047540472	0.235769943	0.483020843	0.228735439	0.748957958
HPGD	0.899687126	0.39992275	0.313803582	0.040001816	0.348418815	0.228820205	0.749086052
PEX5	0.311795492	0.154382472	0.324760134	0.923050848	0.109208514	0.228978984	0.749327491
SPSB1	0.146899685	0.103656232	0.345541973	0.757943098	0.395169093	0.228985276	0.749327491
PHF24	0.151692998	0.499338742	0.351686931	0.935476876	0.063320861	0.229132182	0.749658741
ADGRL1	0.076000979	0.832625365	0.083282446	0.613222653	0.489411999	0.229399363	0.749934617
ATAD2B	0.713004683	0.458998617	0.44719821	0.012569389	0.860833088	0.229536383	0.749934617
LOC100847345	0.087598213	0.817628982	0.857248964	0.311445112	0.082667922	0.229336872	0.749934617
LOC104971162	0.593691301	0.035862237	0.331204665	0.427220187	0.524505933	0.22928954	0.749934617
PHB	0.57764493	0.248505226	0.506300684	0.417203951	0.052221016	0.229526353	0.749934617
SMPX	0.149649561	0.203773687	0.558419074	0.681218889	0.136483588	0.229513794	0.749934617
UPF3B	0.874818794	0.194125737	0.046765294	0.696362403	0.286057957	0.22942539	0.749934617
C17H12orf65	0.22931563	0.194454698	0.893881095	0.105311602	0.378050319	0.229778309	0.750276924
IFIT2	0.024872491	0.753381819	0.859786861	0.162896297	0.604289148	0.229705651	0.750276924
LOC104969670	0.028682687	0.354456608	0.465082552	0.584330532	0.574281765	0.229762245	0.750276924
KLHL23	0.013815141	0.751943073	0.678466141	0.980612056	0.229792721	0.229868814	0.750283063
SDF2L1	0.159974299	0.289460181	0.416166053	0.279716595	0.294637968	0.229871625	0.750283063
HPCAL1	0.80957822	0.341781835	0.356987841	0.118168747	0.136165967	0.229955718	0.75040829
MRPL17	0.463356563	0.05944011	0.281974366	0.89827913	0.228205605	0.230142444	0.750605415
NRP2	0.716326749	0.019077594	0.302058779	0.700100317	0.550915275	0.230150119	0.750605415
RETREG3	0.286795637	0.863774401	0.92672784	0.467271587	0.014841887	0.230153339	0.750605415
ARHGAP30	0.453417382	0.132945622	0.842409524	0.135170629	0.23217135	0.230259384	0.750670052
ARHGEF10L	0.104721535	0.258057544	0.355321759	0.179412957	0.927131055	0.230518255	0.750670052
LOC101902366	0.808716643	0.163015639	0.040477327	0.463196269	0.645105466	0.23032437	0.750670052
LOC107131331	0.160193001	0.165515582	0.132778146	0.639537975	0.709001905	0.230453301	0.750670052
MEST	0.160741379	0.414415513	0.386987569	0.123970442	0.499884323	0.230539093	0.750670052
RPUSD2	0.560995395	0.507857202	0.152991927	0.05632511	0.650162159	0.230445505	0.750670052
SAG	0.379451478	0.979810496	0.389350594	0.011048725	0.998484431	0.230498058	0.750670052

ZFR	0.933968437	0.096708729	0.184800426	0.324164244	0.29521109	0.230525982	0.750670052
ZFYVE27	0.074687462	0.629097787	0.186446319	0.492319951	0.370671364	0.230620206	0.750785201
AFF2	0.126374717	0.095374212	0.664955419	0.577401467	0.350475451	0.232275568	0.751095797
AIF1	0.87531564	0.245515697	0.534588129	0.053106862	0.265815127	0.232268789	0.751095797
ANKRD33B	0.497663927	0.096097001	0.066118114	0.584034004	0.87796365	0.232238983	0.751095797
ANXA1	0.479745233	0.527467764	0.110992661	0.405896902	0.14177564	0.23187803	0.751095797
CAMSAP3	0.156651405	0.692142139	0.438495128	0.464339232	0.072923331	0.231422595	0.751095797
CBLL1	0.005432103	0.500559286	0.665705641	0.958854607	0.924121304	0.23099772	0.751095797
CBR1	0.196753084	0.818755158	0.143415882	0.086681336	0.80157489	0.231091446	0.751095797
COL16A1	0.449889603	0.072999838	0.136982298	0.428905008	0.830273291	0.230862365	0.751095797
CSNK1E	0.983115011	0.999702192	0.981086501	0.501883828	0.003353903	0.232359534	0.751095797
DTX3L	0.103140778	0.181285134	0.689823368	0.20774813	0.598937183	0.231066722	0.751095797
EIF3E	0.019997795	0.767715127	0.599938129	0.49381137	0.356935537	0.232387001	0.751095797
LOC100295687	0.049644317	0.218874806	0.835234533	0.406044584	0.436418439	0.231304835	0.751095797
LOC112441478	0.745851994	0.083162825	0.267619556	0.576759423	0.169260306	0.232177625	0.751095797
LOC112442967	0.990067506	0.537865623	0.137966223	0.303341932	0.072510569	0.231858688	0.751095797
LOC112443431	0.056108636	0.826435966	0.386356173	0.676965283	0.133496074	0.232074874	0.751095797
LOC509810	0.762160352	0.144536435	0.508146568	0.155020594	0.187009226	0.232340757	0.751095797
LVRN	0.347235937	0.286674361	0.640328936	0.07255401	0.347856346	0.231338503	0.751095797
MCF2L	0.714247321	0.262263505	0.100267385	0.608221771	0.141544908	0.231926782	0.751095797
PDE10A	0.626064117	0.142967203	0.165252746	0.504486181	0.215347915	0.231210615	0.751095797
PIK3CA	0.318519847	0.372512467	0.389365102	0.065597418	0.529353989	0.231018781	0.751095797
PSMB5	0.348675146	0.238085676	0.776020208	0.1740919	0.143596071	0.231462817	0.751095797
S100A2	0.059239995	0.166002788	0.361259639	0.583056196	0.779080893	0.231699316	0.751095797
SELENON	0.520969489	0.051242864	0.104283047	0.737664124	0.787669678	0.231969088	0.751095797
SFR1	0.449414738	0.419175109	0.116071145	0.808036426	0.091691001	0.232144552	0.751095797
SMIM11A	0.18160526	0.114841844	0.490471414	0.163053438	0.965744198	0.231485533	0.751095797
SRRM3	0.53716279	0.735477449	0.068435247	0.472572241	0.126307843	0.231702184	0.751095797
SSH1	0.495032476	0.059807014	0.858845212	0.502435361	0.126742628	0.232085984	0.751095797
STMN1	0.503771109	0.811174211	0.361412416	0.682270575	0.015906963	0.23092038	0.751095797
TLL1	0.069777403	0.053678384	0.848424689	0.704411039	0.716744403	0.231032169	0.751095797
TMEM51	0.138343042	0.704461238	0.572844659	0.879148487	0.032945203	0.231927663	0.751095797
TRIM24	0.224279343	0.845781531	0.193685637	0.089133162	0.491526453	0.231405381	0.751095797
TSC22D4	0.136636101	0.710093977	0.857083	0.153293067	0.127147919	0.232200513	0.751095797
TSPYL4	0.011764828	0.662536922	0.347765352	0.637400505	0.936315282	0.231983466	0.751095797
VPS9D1	0.97385118	0.294791158	0.057306738	0.291224895	0.335373905	0.231204445	0.751095797
XAF1	0.293243832	0.065122262	0.877800708	0.100403612	0.964759311	0.232409022	0.751095797
ZNF154	0.143725475	0.402691589	0.56990867	0.936921286	0.052060096	0.231349507	0.751095797
ZNF214	0.356127269	0.413191831	0.45762225	0.469691319	0.050999752	0.231646913	0.751095797
ANG2	0.767873159	0.017288674	0.871851313	0.93757667	0.150302661	0.232925016	0.751508569

APLNR	0.605296309	0.48420152	0.123167171	0.045462563	0.999201214	0.23354419	0.751508569
ATF1	0.78971665	0.773688989	0.740363627	0.004813949	0.752863875	0.233518831	0.751508569
ERAP1	0.028549044	0.215080324	0.685852097	0.404822541	0.955130688	0.232734686	0.751508569
FBXO31	0.043832169	0.433903374	0.307004593	0.329751711	0.846289052	0.232810723	0.751508569
GPR75	0.912428371	0.064475836	0.710811672	0.073360157	0.532266249	0.233050516	0.751508569
HINT2	0.384726005	0.082712444	0.28876546	0.214871865	0.824670039	0.232728635	0.751508569
L1CAM	0.050789027	0.465012335	0.159488499	0.866960889	0.499369741	0.232903346	0.751508569
LOC100295750	0.916821388	0.060017052	0.980895955	0.170355208	0.177432721	0.232952746	0.751508569
LOC101906588	0.183503424	0.773961836	0.041573134	0.455690862	0.606108131	0.232906387	0.751508569
LOC112446756	0.763372529	0.657964547	0.316670884	0.106918449	0.096018099	0.233054081	0.751508569
LOC509415	0.386191087	0.477238644	0.119232098	0.809962207	0.092094428	0.233499787	0.751508569
LOC522610	0.278592762	0.617577298	0.304874485	0.98299564	0.031572368	0.232705697	0.751508569
MCAT	0.234815566	0.558679964	0.078077464	0.161252262	0.988629916	0.2330547	0.751508569
MESD	0.369896812	0.090262556	0.072918386	0.865826032	0.77603392	0.233262383	0.751508569
MYBBP1A	0.822670451	0.338897796	0.144936156	0.504905125	0.080318123	0.233464017	0.751508569
NUFIP2	0.579527359	0.760726985	0.754585367	0.006120507	0.805092416	0.233503809	0.751508569
PLEKHF2	0.53212174	0.293443517	0.075793467	0.888880347	0.155581461	0.233324031	0.751508569
PXMP2	0.61172536	0.573386311	0.374846624	0.064611474	0.192460179	0.233201591	0.751508569
RGCC	0.432503922	0.263000876	0.022144095	0.697566234	0.931436368	0.233317485	0.751508569
SPART	0.48507076	0.547002455	0.052754677	0.70222439	0.16622115	0.2331244	0.751508569
ZNF585A	0.596108009	0.35232889	0.015263866	0.806657022	0.633578598	0.233446049	0.751508569
COPS6	0.724341623	0.067479208	0.584868084	0.269767135	0.212772352	0.233618599	0.751600634
CCSER1	0.111804294	0.130080613	0.465413926	0.305402305	0.795011523	0.233799822	0.751763516
HMGB2	0.212016476	0.454806586	0.099595975	0.541981485	0.315761531	0.233806653	0.751763516
TIPRL	0.996595862	0.042948566	0.363510309	0.7446005	0.141850932	0.233795515	0.751763516
ANKRD12	0.577343055	0.295372306	0.931425264	0.03323434	0.312024339	0.234058581	0.751984216
COL13A1	0.526986066	0.155039419	0.233311937	0.174101561	0.496274521	0.23405208	0.751984216
LOC107133095	0.140309338	0.125590796	0.515370042	0.270472317	0.67014917	0.233986776	0.751984216
RARRES1	0.695034724	0.327028403	0.091977374	0.802100569	0.098157465	0.233978054	0.751984216
ETNK2	0.571666085	0.557853961	0.065044774	0.383743775	0.207390631	0.234319555	0.752039265
HSPB8	0.08462909	0.372034788	0.820780558	0.842099859	0.075748511	0.23414942	0.752039265
LOC104969611	0.189232789	0.855056953	0.202930272	0.330402209	0.152197789	0.234341892	0.752039265
PABPC1	0.662258987	0.306704373	0.283105549	0.288993215	0.099366178	0.234350667	0.752039265
SLC9A5	0.986387342	0.13772104	0.061624309	0.729179575	0.270285733	0.234253297	0.752039265
SOX7	0.088921406	0.745105316	0.646648908	0.04061858	0.948152719	0.234263458	0.752039265
FAM92A	0.966295856	0.050401728	0.392254276	0.768644273	0.112650546	0.234553385	0.75254264
GMDS	0.084670334	0.858849714	0.765039451	0.21487834	0.13848773	0.234647994	0.752699029
UBASH3B	0.415946962	0.130075913	0.540119573	0.21526064	0.263693884	0.234875961	0.753283055
CNOT11	0.812276057	0.036605031	0.209188577	0.541735574	0.493069223	0.235059959	0.753534207
EGR2	0.088212577	0.230018715	0.625915328	0.194328352	0.673367394	0.23509202	0.753534207

WAC	0.741735982	0.528016557	0.776632787	0.011153521	0.489646446	0.235041121	0.753534207
ID2	0.500290734	0.917373348	0.034127691	0.435110007	0.2448073	0.235547422	0.754677831
SRPX2	0.371108579	0.781730815	0.038960353	0.564996804	0.261160193	0.235503272	0.754677831
UBE2M	0.185006138	0.099376524	0.491351293	0.543927547	0.33965868	0.235586773	0.754677831
ARL8A	0.189763722	0.141292337	0.087957114	0.76229944	0.930041637	0.235797126	0.754748413
COPB1	0.268582049	0.023634291	0.657693409	0.691510759	0.579190125	0.235805808	0.754748413
ETV2	0.465139993	0.527853093	0.237629384	0.170488001	0.168133314	0.235827615	0.754748413
LOC104971345	0.602835401	0.035233202	0.345431815	0.783966944	0.290787077	0.235838758	0.754748413
MERTK	0.920152113	0.160255236	0.522970198	0.120051572	0.180474724	0.235717323	0.754748413
LOC107132121	0.411338413	0.480465835	0.711934092	0.154893011	0.076802136	0.235924738	0.754876364
MRPL24	0.379046277	0.439057327	0.428050113	0.196621675	0.119594625	0.236017126	0.754877617
RPRD1B	0.524470096	0.179969267	0.166647964	0.1566396	0.679864722	0.236014988	0.754877617
RACGAP1	0.146239764	0.397278032	0.702757684	0.624393686	0.065788113	0.23615677	0.754882889
RBPJ	0.048543225	0.146440901	0.858997664	0.330532788	0.830923258	0.236153254	0.754882889
ZMYM4	0.03908959	0.818602152	0.819609461	0.068525267	0.933079999	0.236140404	0.754882889
ADAMTS3	0.516791875	0.620996654	0.465303163	0.011480053	0.979071293	0.236244706	0.755016916
GOLGA7	0.871938358	0.385232736	0.309398965	0.035308642	0.458009023	0.236401449	0.75526669
LAMA5	0.870931976	0.560541603	0.043437158	0.44402147	0.178515324	0.236414904	0.75526669
KANSL3	0.020759302	0.931011504	0.76824275	0.374939055	0.302131595	0.236492843	0.755368636
PSMA6	0.924674363	0.205345357	0.240863465	0.711468348	0.051763998	0.236655418	0.755740818
BCL6B	0.05732665	0.690328666	0.399113682	0.237851525	0.448771684	0.236766309	0.75594784
HECW1	0.22722922	0.047610483	0.653659876	0.256137373	0.93122693	0.236821868	0.755978151
ANO9	0.855509775	0.931557363	0.619028156	0.066886919	0.05146952	0.237626878	0.756195094
BTF3	0.880655812	0.646383309	0.046271461	0.586345549	0.109550984	0.237180432	0.756195094
C17H4orf46	0.276282911	0.119795326	0.325901824	0.940338184	0.166686958	0.23709614	0.756195094
DTYMK	0.181100206	0.972857614	0.14302847	0.072701705	0.926420236	0.237547894	0.756195094
FOXRED1	0.54937409	0.402611508	0.909021116	0.010656163	0.791886288	0.2375071	0.756195094
GATA6	0.174283989	0.13886436	0.736729886	0.171111296	0.557422588	0.237784265	0.756195094
LOC101904258	0.14830565	0.705879682	0.842892529	0.050833191	0.377542965	0.237286776	0.756195094
LOC101907642	0.037674265	0.832811245	0.294622468	0.884629807	0.207300349	0.237406527	0.756195094
LOC112449505	0.330244094	0.564105645	0.152486273	0.169262718	0.35363538	0.237763614	0.756195094
LOC782343	0.430407046	0.208763334	0.23674496	0.200238672	0.397494316	0.237264926	0.756195094
MCM5	0.734013952	0.803356	0.070865135	0.20966551	0.193030782	0.237131719	0.756195094
NCKAP5L	0.863263942	0.379704862	0.029727773	0.332505121	0.52175	0.237081751	0.756195094
NUPR2	0.471036922	0.259859606	0.145465868	0.51949725	0.183507247	0.237560823	0.756195094
SELP	0.623041715	0.184467538	0.059193397	0.905167924	0.276236394	0.2378114	0.756195094
SP1	0.076235781	0.292600038	0.412374135	0.507123971	0.364497	0.237760775	0.756195094
STS	0.717472323	0.624013365	0.836485361	0.134314941	0.033704552	0.237420388	0.756195094
TGFBR1	0.709643747	0.037179728	0.299815965	0.474706546	0.450885328	0.237264777	0.756195094
ZNF148	0.79025325	0.027322153	0.981772689	0.117767927	0.679062318	0.237409218	0.756195094

ZNF641	0.67496928	0.014075451	0.913652044	0.453046073	0.431768857	0.23759575	0.756195094
ZYG11B	0.482934194	0.14382118	0.541937259	0.08477253	0.532926375	0.237774108	0.756195094
C29H11orf68	0.540633293	0.151371662	0.311845824	0.14111226	0.475388455	0.238562437	0.756250281
CCDC150	0.966762506	0.465425462	0.226371657	0.335626501	0.049966743	0.238299829	0.756250281
CLEC10A	0.405859746	0.387561382	0.138185949	0.177874395	0.440888855	0.238055002	0.756250281
ELP1	0.37679612	0.024574858	0.271580363	0.806539792	0.839293929	0.237896479	0.756250281
FAM214A	0.664670805	0.58036523	0.873822083	0.021268188	0.23872336	0.238523423	0.756250281
KCNQ1	0.980985403	0.468466495	0.192502158	0.084136699	0.229617233	0.238363976	0.756250281
LOC785568	0.183357499	0.139715601	0.512393633	0.25278146	0.513428811	0.237987461	0.756250281
MGAT5B	0.132711531	0.328722198	0.996431273	0.131556935	0.298539203	0.238237921	0.756250281
NDUFS4	0.464279187	0.185969987	0.839932473	0.05159095	0.455397468	0.238003073	0.756250281
NRN1	0.083328276	0.341765588	0.923454705	0.311879628	0.208081709	0.23819931	0.756250281
PAPSS1	0.62282197	0.332800773	0.195225359	0.197175949	0.214039416	0.238273592	0.756250281
PBK	0.370964473	0.228034587	0.06915699	0.755137803	0.387120253	0.238438202	0.756250281
PIK3CD	0.683399395	0.185812165	0.416598243	0.250268108	0.129313068	0.238566066	0.756250281
PPP1R1A	0.510408081	0.972227571	0.289848213	0.024954814	0.476785966	0.238517336	0.756250281
SLC1A5	0.525374985	0.115640738	0.485423321	0.400013608	0.145108962	0.238554108	0.756250281
ZNF638	0.665790442	0.249528427	0.898724286	0.017429503	0.656510198	0.238321788	0.756250281
ATP8B4	0.269357898	0.095894004	0.735791882	0.107328244	0.839737981	0.23862554	0.756292725
LOC112442598	0.498759344	0.888786159	0.243230515	0.392383289	0.040525236	0.238736052	0.756297303
LOC786372	0.907191828	0.010280863	0.311180276	0.835621522	0.707131073	0.238765238	0.756297303
NR2C1	0.091105828	0.920301991	0.379541913	0.877468626	0.061392195	0.238719431	0.756297303
LOC100847951	0.029896317	0.742920187	0.386442921	0.469508606	0.426910441	0.239137632	0.756309053
LOC112443415	0.750187741	0.158927778	0.592120134	0.031132139	0.780918625	0.238858358	0.756309053
RBBP5	0.312042469	0.040642598	0.328778402	0.462655117	0.890402832	0.23895258	0.756309053
RBX1	0.884740622	0.121265977	0.474577686	0.046006798	0.733970832	0.239066524	0.756309053
SPEF2	0.014236998	0.983518744	0.292233181	0.522304899	0.80432739	0.23904607	0.756309053
TAGAP	0.599725339	0.098514552	0.786242645	0.473235453	0.078230505	0.23909354	0.756309053
TIMP3	0.593484875	0.10943108	0.290981166	0.22572228	0.402527568	0.238910051	0.756309053
WDR36	0.962936708	0.435731521	0.541891546	0.020730246	0.36444922	0.238960225	0.756309053
SSSCA1	0.25088111	0.62138863	0.669491338	0.326920429	0.050458275	0.239225741	0.756441935
C7	0.57189241	0.124405744	0.311895057	0.313580502	0.250266524	0.240574047	0.756768388
EIF3CL	0.564820371	0.463927535	0.215825985	0.345674575	0.088805654	0.240208789	0.756768388
GRHL1	0.022769393	0.912007593	0.353251529	0.270294828	0.877719967	0.240495833	0.756768388
KLHL4	0.110091414	0.107523974	0.273313016	0.880656847	0.611038402	0.240541177	0.756768388
LOC100847410	0.166463784	0.574290232	0.375214164	0.075369245	0.64370473	0.240491375	0.756768388
LOC101906923	0.865402871	0.450084632	0.067827108	0.465097409	0.140334513	0.239408338	0.756768388
LOC104973100	0.699119421	0.213859231	0.039689462	0.431242164	0.674381065	0.239505146	0.756768388
LOC104974891	0.700485103	0.861317771	0.030239022	0.634927752	0.14982325	0.240171082	0.756768388
LOC618541	0.439224341	0.714665195	0.748190555	0.742783126	0.009961921	0.24032682	0.756768388

LOC784087	0.491905214	0.278796026	0.741074523	0.370758483	0.046005548	0.24003455	0.756768388
LOC786352	0.858843887	0.770005313	0.767933594	0.009631932	0.352610393	0.239439772	0.756768388
LYN	0.496658417	0.416988248	0.37188488	0.050811186	0.443184308	0.240089615	0.756768388
MKKS	0.846833018	0.163888077	0.492431806	0.488277378	0.052163659	0.24052256	0.756768388
MRPS23	0.876975262	0.179433698	0.701774619	0.091752282	0.171721489	0.240469403	0.756768388
NAA25	0.302318061	0.286709271	0.561431824	0.050689278	0.703899565	0.240224385	0.756768388
NCEH1	0.909109123	0.179042369	0.070801857	0.451282812	0.332623723	0.239786654	0.756768388
NDNF	0.034639897	0.184873992	0.552713423	0.531334244	0.922820947	0.240172196	0.756768388
POLR2C	0.528008291	0.031074947	0.156719845	0.720604338	0.932941897	0.239707415	0.756768388
PSMB4	0.484558887	0.207657382	0.508273924	0.392005475	0.086308191	0.239818537	0.756768388
RAB3GAP2	0.584787098	0.675126444	0.008132761	0.642965082	0.841606912	0.240302679	0.756768388
SAPCD2	0.042669161	0.882781168	0.698352658	0.241538482	0.272678018	0.239965875	0.756768388
SURF6	0.301230284	0.832222596	0.29060913	0.27202541	0.087347948	0.239865691	0.756768388
TK1	0.188482957	0.98841021	0.784974356	0.029060393	0.408006924	0.240062428	0.756768388
TMX2	0.533467594	0.191450659	0.633647845	0.174510777	0.153878485	0.24032863	0.756768388
ZCRB1	0.980476143	0.009938813	0.709101727	0.500399359	0.501166065	0.239993064	0.756768388
ZDHHC20	0.366569061	0.091261274	0.129296672	0.837063945	0.479269378	0.240153256	0.756768388
ZNF567	0.009852172	0.430926913	0.862951138	0.624208818	0.75565093	0.239665712	0.756768388
HAS2	0.233752988	0.9052402	0.030234733	0.477142029	0.570849806	0.2406507	0.75686444
CGRRF1	0.548059627	0.188942288	0.298755544	0.823141187	0.068477306	0.240731641	0.756881352
LOC101904796	0.846634836	0.994074388	0.086620022	0.379312025	0.063070356	0.240748319	0.756881352
GFRA1	0.385144615	0.072138685	0.264561751	0.323745845	0.734576371	0.241022136	0.757379648
GPR155	0.605706035	0.47028975	0.039329214	0.278314856	0.561183185	0.24113757	0.757379648
MSRB1	0.19205003	0.106239058	0.88651168	0.220146797	0.439183239	0.241071449	0.757379648
RFX3	0.551838435	0.199086769	0.336391705	0.453555361	0.104349171	0.241092643	0.757379648
ZNF502	0.723024879	0.990174351	0.74222671	0.003386637	0.971439026	0.241029065	0.757379648
RBM18	0.554955666	0.071103451	0.519837832	0.167044147	0.511048155	0.241226528	0.757514073
EFS	0.363278891	0.475914861	0.027932956	0.697876543	0.519891875	0.241299633	0.757598675
LOC100141145	0.268141917	0.052345801	0.252769392	0.807017515	0.613248113	0.241548585	0.757614739
LOC112444633	0.01910358	0.481564392	0.461339573	0.956162794	0.432576821	0.241519836	0.757614739
PLXDC2	0.173559741	0.388285358	0.567115397	0.046183804	0.995062863	0.24158174	0.757614739
POLR1D	0.742630623	0.651131474	0.117682534	0.682935833	0.04518964	0.241571003	0.757614739
THUMPD2	0.181642265	0.221613428	0.070152424	0.98749889	0.629106367	0.241446537	0.757614739
ZFP62	0.250407911	0.771374977	0.17729621	0.595925637	0.085991462	0.241485862	0.757614739
PSMC4	0.520409185	0.181624558	0.765476722	0.29784571	0.081606584	0.241733689	0.757946422
FNDC10	0.471005046	0.553178818	0.161860953	0.053546167	0.779648191	0.241868165	0.75817103
LOC101907843	0.583739141	0.363207212	0.0208386	0.717958875	0.555171312	0.241897722	0.75817103
CDC42BPG	0.72665235	0.403895364	0.010273568	0.801087132	0.730240856	0.242087534	0.758476235
UQCRC2	0.929718887	0.251344362	0.838998492	0.06525317	0.137856779	0.242073586	0.758476235
PGM2L1	0.235193518	0.047825432	0.640396856	0.511770458	0.478839852	0.242179488	0.758619503

DHX36	0.235694801	0.625871314	0.729232948	0.058835881	0.279022612	0.242229837	0.758632414
ACYP1	0.508893445	0.2302731	0.646433156	0.164701762	0.142542846	0.243066538	0.758745161
CPLANE1	0.930187063	0.211764043	0.026769468	0.7912075	0.42603596	0.24300114	0.758745161
DHRS11	0.117262021	0.221291796	0.634307439	0.229798444	0.469744624	0.242955205	0.758745161
EPOP	0.413985813	0.592738234	0.587740501	0.507521952	0.024197322	0.24257834	0.758745161
FDFT1	0.584428226	0.199543115	0.731539698	0.297498777	0.069976049	0.242902847	0.758745161
FOSB	0.506536312	0.013595627	0.493023613	0.64326925	0.811718942	0.24269335	0.758745161
HNRNPH3	0.786593227	0.796032064	0.038117579	0.344989365	0.215160815	0.242610879	0.758745161
INPP1	0.141076019	0.376769206	0.136949685	0.610462371	0.40031669	0.243098047	0.758745161
KIAA1257	0.691860602	0.033432076	0.144034251	0.900637024	0.592488636	0.243022705	0.758745161
LOC107131792	0.813206676	0.306655508	0.111860889	0.354846203	0.179353218	0.242858678	0.758745161
MAP4K3	0.472598612	0.315160204	0.039079932	0.465499527	0.65296539	0.242449879	0.758745161
PIGA	0.73595112	0.013887283	0.768137497	0.231056096	0.977985211	0.242769779	0.758745161
PML	0.311967694	0.257302579	0.512807539	0.188367794	0.229025659	0.242891477	0.758745161
TNFRSF8	0.768775061	0.47321724	0.065995024	0.401763111	0.183666909	0.242610071	0.758745161
TP53I13	0.089667261	0.453210775	0.197992814	0.230064809	0.960094164	0.242987064	0.758745161
TRABD	0.341652356	0.0470189	0.134437404	0.999035043	0.823334317	0.242928484	0.758745161
XRCC5	0.134463308	0.439000004	0.225461243	0.214321069	0.622314805	0.242841109	0.758745161
ZFP2	0.624565232	0.336540747	0.139547534	0.639340271	0.09438083	0.242495453	0.758745161
AP3S1	0.31788403	0.121039774	0.634812555	0.7295712	0.10012364	0.24345332	0.759429479
CHRNA3	0.827725608	0.049493418	0.33161988	0.539891644	0.243650061	0.243645856	0.759429479
FAT2	0.555976103	0.923471164	0.022549241	0.731711933	0.210720525	0.243511472	0.759429479
LOC112441885	0.788580248	0.934823093	0.251687569	0.046238315	0.208460367	0.24373244	0.759429479
NFKB2	0.484680971	0.960318345	0.326566838	0.012331102	0.952088347	0.243474133	0.759429479
RABGAP1L	0.396137156	0.132526211	0.564350799	0.080583825	0.748819708	0.243693704	0.759429479
RSAD1	0.548178258	0.021833243	0.207143185	0.896511701	0.804638483	0.24373378	0.759429479
ST13	0.964003245	0.599025688	0.201857018	0.117953582	0.129858475	0.24353643	0.759429479
UBE2G2	0.833851067	0.023226416	0.138545001	0.822279586	0.809738737	0.243612459	0.759429479
LOC112446717	0.801878301	0.236241342	0.023923899	0.67309034	0.586903318	0.243862654	0.759686791
LOC112449302	0.981035361	0.358671532	0.907708302	0.720330449	0.007793734	0.244046648	0.760115683
ERCC6L	0.354498487	0.126173072	0.231556037	0.348268974	0.498565365	0.244395924	0.760193615
KIF1BP	0.061334355	0.424464082	0.378402356	0.818681132	0.22254572	0.244164257	0.760193615
MRPS36	0.504020973	0.247307601	0.802739582	0.086911248	0.20641163	0.244174794	0.760193615
PRMT3	0.430174193	0.454296754	0.835079543	0.554801571	0.019858239	0.24437284	0.760193615
S100A1	0.417628246	0.433632063	0.081655756	0.890171923	0.136431524	0.244234189	0.760193615
TXNRD3	0.269816177	0.305015627	0.499502743	0.095208893	0.459271263	0.244341033	0.760193615
WDR75	0.906257853	0.499411189	0.100765443	0.085646952	0.460264073	0.244359185	0.760193615
ELF1	0.983196113	0.54736252	0.757565898	0.006734946	0.655574412	0.244510981	0.760263301
PTGFRN	0.031757476	0.317473318	0.350579929	0.561494989	0.906852238	0.24449195	0.760263301
LOC112441506	0.173710187	0.866295483	0.410363061	0.40548682	0.071923053	0.244569591	0.760270305

POMP	0.958402574	0.104971926	0.371457334	0.859698472	0.056073793	0.244605887	0.760270305
RIN1	0.99002698	0.17762047	0.263564301	0.173563833	0.224118499	0.244696131	0.760406779
INTS6L	0.918441749	0.520267011	0.060252396	0.224362381	0.279580532	0.244901743	0.760668878
NEMF	0.440256565	0.33094268	0.030863006	0.512873601	0.782741316	0.24485106	0.760668878
VCL	0.780600201	0.179047727	0.057361842	0.484137262	0.465355226	0.244919527	0.760668878
ASB11	0.905735189	0.054821183	0.091077558	0.769460093	0.520442515	0.245235876	0.760694147
CCNQ	0.174940951	0.36917844	0.069801915	0.902559933	0.44497158	0.245203733	0.760694147
CMPK1	0.574538805	0.373759201	0.797912162	0.281843652	0.037508139	0.245258032	0.760694147
EIF2S3	0.340377785	0.782443107	0.637941708	0.28801829	0.037047143	0.245360122	0.760694147
LIMD2	0.322646588	0.251595939	0.714986755	0.135141881	0.231633949	0.245622953	0.760694147
LOC112445051	0.597294079	0.236287326	0.578990427	0.636781378	0.034900188	0.245566912	0.760694147
LYZ2	0.245874595	0.760390331	0.550550889	0.072027524	0.244179856	0.245190634	0.760694147
PTPRR	0.218332105	0.097314113	0.541645839	0.254106419	0.620982783	0.245563014	0.760694147
RPF1	0.12833331	0.430751275	0.310269803	0.40870954	0.258141672	0.245141588	0.760694147
RSU1	0.604140789	0.028945788	0.42089283	0.778900621	0.316822463	0.245587625	0.760694147
SERPINE1	0.436501513	0.209310534	0.325361618	0.324499621	0.188281608	0.245579935	0.760694147
SLC22A16	0.04245953	0.251848002	0.364008684	0.780662545	0.597796052	0.245601102	0.760694147
SLC7A2	0.218746376	0.416559336	0.342336317	0.188227177	0.308451584	0.245241442	0.760694147
TTC7A	0.358599104	0.292214049	0.10506328	0.334874534	0.49264425	0.245583123	0.760694147
ZNF697	0.552757277	0.762926552	0.251535906	0.052398786	0.326474402	0.245475547	0.760694147
C22H3orf62	0.340967186	0.290855468	0.057522199	0.618687699	0.515101519	0.245697256	0.760780691
ADH6	0.530095689	0.352754688	0.04485482	0.667844142	0.326267508	0.246332616	0.761024955
AJM1	0.008422122	0.666645423	0.493694564	0.754864651	0.871189996	0.246020139	0.761024955
KIAA1324L	0.683984563	0.043939274	0.784142755	0.612413555	0.126246352	0.245965123	0.761024955
MINPP1	0.278650975	0.108067423	0.989257549	0.568630475	0.107618956	0.24602759	0.761024955
NDUFA7	0.347986652	0.299997964	0.969172432	0.030807706	0.586314448	0.24632895	0.761024955
PDHB	0.914659377	0.225625117	0.408998336	0.235291156	0.091920444	0.246194387	0.761024955
PPM1G	0.717428196	0.012336477	0.408581608	0.99721513	0.506428583	0.246241297	0.761024955
RAB9B	0.297280398	0.898652675	0.203159798	0.173145518	0.194303682	0.246222664	0.761024955
RECQL	0.857020126	0.06965913	0.208097826	0.209916401	0.700598782	0.246295833	0.761024955
THBS1	0.441295968	0.381637181	0.564602619	0.042951535	0.44676656	0.246138114	0.761024955
TMEM132A	0.970286008	0.2780045	0.480439504	0.46433909	0.030364134	0.246305277	0.761024955
VAPA	0.525485606	0.102656934	0.151980645	0.468045226	0.47595655	0.246251558	0.761024955
PPP5C	0.40896309	0.707089868	0.053255774	0.231392018	0.513119149	0.246389941	0.761058783
LOC104971817	0.698228045	0.104708658	0.223006917	0.882178226	0.127284497	0.246539064	0.761213503
LOC112442047	0.534447452	0.295500528	0.569345879	0.200983349	0.101338679	0.246579184	0.761213503
RPP38	0.172158016	0.61195749	0.195363669	0.223082216	0.398794397	0.246560201	0.761213503
ABCG1	0.671106339	0.673724853	0.850743838	0.670382741	0.007117976	0.246850252	0.761798756
PENK	0.89016257	0.753126083	0.098946205	0.028831995	0.959802998	0.246861604	0.761798756
MID1	0.371099839	0.828369215	0.137372233	0.458794396	0.094807801	0.246940116	0.761819571

SYVN1	0.075051461	0.595278133	0.14192877	0.387234857	0.748217425	0.246961192	0.761819571
SF3B5	0.075200378	0.25482835	0.914231523	0.685619323	0.153057581	0.247047271	0.761941884
USP25	0.094362549	0.833719438	0.995890906	0.326980873	0.071825425	0.247150035	0.762115601
TERC	0.951767588	0.511876548	0.835132955	0.032015256	0.141597583	0.247436974	0.762857068
AADAT	0.420268387	0.575604019	0.641374527	0.077440928	0.153786807	0.247655895	0.762881821
ANKS6	0.418355157	0.337912056	0.039849639	0.719958232	0.45617579	0.247811943	0.762881821
DTWD1	0.167725419	0.952055096	0.018215582	0.805927653	0.789406442	0.247836433	0.762881821
DUSP19	0.594695221	0.561357559	0.053957525	0.26820437	0.383276904	0.247909862	0.762881821
GRIP1	0.347626443	0.145290375	0.39076082	0.761166606	0.123189726	0.247840369	0.762881821
IDUA	0.058694468	0.615043935	0.233794902	0.672940116	0.324981177	0.247522709	0.762881821
KCTD15	0.996399892	0.536783133	0.073582113	0.083512206	0.563365754	0.247904475	0.762881821
LOC112442559	0.654268494	0.045997071	0.596434623	0.92886119	0.11104017	0.247886321	0.762881821
MCM2	0.599496802	0.384331154	0.202735741	0.233718549	0.169416408	0.247772621	0.762881821
SLC35G1	0.413608985	0.476111017	0.185139204	0.154161715	0.329015141	0.247749686	0.762881821
BIRC3	0.523466571	0.673001855	0.040577599	0.290694041	0.447130186	0.248326891	0.763330465
CCNA2	0.68346477	0.14834132	0.094065802	0.610492847	0.318976349	0.248267644	0.763330465
IGSF1	0.414109314	0.195029453	0.395332286	0.115704915	0.50259066	0.248238275	0.763330465
LOC100848077	0.203472208	0.134414105	0.835356205	0.409537034	0.198336032	0.248174955	0.763330465
MSRA	0.227322049	0.723985975	0.368885165	0.100930523	0.302893597	0.248191414	0.763330465
TRMT2A	0.478405576	0.028162775	0.744705986	0.368993398	0.501897122	0.248334736	0.763330465
KIF24	0.652112853	0.013947988	0.950977571	0.737804765	0.291525674	0.248483342	0.763644217
LOC104972888	0.83707079	0.795785349	0.020410115	0.20876542	0.655845611	0.248550528	0.76370768
GGA1	0.700550396	0.182443691	0.023312847	0.786961475	0.79454993	0.248654982	0.763885608
NCAM1	0.180246736	0.741914631	0.034393983	0.803571476	0.504455783	0.248742382	0.7639302
PDF	0.457955885	0.243973851	0.979322133	0.327016139	0.052114933	0.248762598	0.7639302
HTR4	0.787505795	0.087034306	0.438224449	0.447513465	0.138855617	0.248870489	0.764118541
ABCB6	0.506888921	0.421657749	0.529695677	0.176956221	0.093386361	0.249160464	0.764436786
LOC782159	0.048308306	0.566434	0.953931448	0.336975417	0.212557799	0.249080825	0.764436786
RAPH1	0.031183817	0.931642011	0.891773876	0.161513966	0.447058334	0.24914894	0.764436786
SPDL1	0.170469321	0.962394013	0.114982718	0.319792356	0.309930395	0.249080938	0.764436786
GTF3C6	0.141521743	0.879159051	0.299214162	0.346037887	0.145399221	0.249302971	0.764445264
PSMB3	0.54062867	0.149470051	0.858815526	0.379707505	0.071056098	0.249259695	0.764445264
S1PR2	0.104645422	0.526931841	0.17050444	0.343122152	0.580188115	0.249210582	0.764445264
ZNF385D	0.927036663	0.389170201	0.122898882	0.049539584	0.853215976	0.249369299	0.764505804
LOC107133032	0.739737534	0.046329063	0.67531729	0.358048665	0.226322659	0.249456933	0.764631628
HEPH	0.209724986	0.405276958	0.348322506	0.113061586	0.560951029	0.249600857	0.764673993
PPP1R3F	0.099674876	0.355651712	0.880368777	0.093200743	0.645689792	0.249627406	0.764673993
SARM1	0.250228708	0.139126358	0.156649882	0.578524227	0.595424368	0.249657136	0.764673993
SLC25A22	0.196924699	0.146441839	0.199355094	0.995915985	0.327786293	0.249540021	0.764673993
LOC112443526	0.655556406	0.078696992	0.155126681	0.785255967	0.299146548	0.249748143	0.764809996

LOC101904849	0.208647198	0.461542532	0.125148736	0.720886933	0.216848931	0.250007233	0.765382404
RNASE6	0.580280422	0.22504174	0.53042397	0.139948837	0.194382472	0.250028339	0.765382404
ALKBH3	0.723893278	0.16401309	0.048312682	0.518243552	0.63487009	0.250219578	0.765646332
ATF3	0.114566346	0.265671883	0.116518367	0.726725371	0.733445757	0.250416522	0.765646332
GALNT12	0.09593262	0.304070172	0.238390541	0.469064899	0.579256821	0.250359835	0.765646332
KIAA0556	0.830630641	0.281167004	0.016362973	0.958494374	0.515409185	0.25025974	0.765646332
LOC101902059	0.590537056	0.427609464	0.292606459	0.915952954	0.02790737	0.25031419	0.765646332
LOC614785	0.082099396	0.392680102	0.629032786	0.474345311	0.196338157	0.250308865	0.765646332
OSER1	0.880386168	0.109674378	0.133119914	0.211356699	0.695962263	0.250441138	0.765646332
VAT1L	0.218283277	0.540475796	0.113853398	0.141517184	0.995681974	0.250567217	0.765889103
C20H5orf22	0.504753797	0.061592469	0.313751145	0.697416579	0.278565616	0.250718709	0.766209447
RFT1	0.630647208	0.639118396	0.30648934	0.208820218	0.073509417	0.250799115	0.766312469
FAM168B	0.058079995	0.282657503	0.36626679	0.381257079	0.827792761	0.250890518	0.76644905
DHX15	0.617068858	0.509005102	0.426690801	0.047390555	0.299331577	0.251112252	0.766960978
LOC515358	0.647319622	0.26280808	0.06374964	0.418409253	0.419099108	0.251151563	0.766960978
ADAM22	0.547073053	0.163601928	0.301484135	0.131836173	0.536907309	0.251680097	0.767141356
ANXA11	0.285443063	0.127479535	0.30655276	0.422519497	0.404639706	0.251496688	0.767141356
BAP1	0.038341813	0.227007984	0.563840015	0.73414271	0.529689099	0.251578506	0.767141356
LOC101907813	0.080454783	0.959765898	0.083496466	0.46040226	0.643770123	0.251742441	0.767141356
NFRKB	0.536950307	0.079545212	0.471076338	0.482593433	0.196244921	0.251395077	0.767141356
POR	0.532687234	0.671253816	0.964915806	0.325312774	0.016978071	0.251400332	0.767141356
RIDA	0.594468007	0.669154535	0.248833991	0.418880728	0.045930238	0.251320383	0.767141356
S100A3	0.780471195	0.643315903	0.803707889	0.652589586	0.007257449	0.25175652	0.767141356
SIRT5	0.735439833	0.490740262	0.126194056	0.283589134	0.147865963	0.251669803	0.767141356
UQCRFS1	0.723318658	0.340431053	0.782810657	0.041319737	0.23998489	0.251771577	0.767141356
VLDLR	0.875905487	0.117836708	0.688507586	0.046299915	0.580500474	0.251679322	0.767141356
ZFP57	0.141911473	0.377227776	0.107309766	0.934749982	0.355845318	0.251732193	0.767141356
COA6	0.328765758	0.251160225	0.44191455	0.160159773	0.327289424	0.251857236	0.767204848
UBL7	0.488008162	0.35339617	0.513147726	0.02200853	0.982291968	0.251885913	0.767204848
LOC787554	0.981751034	0.201345751	0.110622887	0.681799049	0.128459624	0.252012391	0.767447642
CD244	0.168516202	0.392553821	0.415499654	0.098677624	0.707105336	0.25218221	0.76753751
KHK	0.079071741	0.53890594	0.622538721	0.368173516	0.196276466	0.252127822	0.76753751
LARP1B	0.348077163	0.988317316	0.238596132	0.07072684	0.330334003	0.252170519	0.76753751
ATP6V1C1	0.805743487	0.553889963	0.017052302	0.631544304	0.399461147	0.252313998	0.767796221
ACOX3	0.076396426	0.288189925	0.311842725	0.880619416	0.318171402	0.252555286	0.768387986
LDHA	0.235742783	0.470588201	0.038597319	0.858189934	0.524076782	0.252690248	0.768656099
EPHX2	0.131314789	0.824486745	0.307879595	0.08248117	0.701199766	0.252820126	0.768667509
MYRIP	0.220996823	0.043304064	0.781163506	0.858823186	0.30030692	0.252834514	0.768667509
PABPC1L2A	0.118712788	0.656893021	0.079880593	0.676307461	0.457359074	0.252752371	0.768667509
LOC101904942	0.551292086	0.158818255	0.659675689	0.398565881	0.083803712	0.252905226	0.768669794

NUDCD1	0.61704613	0.529339241	0.086574608	0.138362105	0.493174434	0.252928943	0.768669794
GLT8D2	0.06494775	0.629949952	0.140809907	0.637323898	0.526383635	0.253128484	0.7687068
PPP1R15A	0.015999514	0.900446379	0.314029869	0.838032149	0.509507682	0.253066594	0.7687068
RHOA	0.731869606	0.130827919	0.050481495	0.729319127	0.54805988	0.253084952	0.7687068
SLC25A46	0.894885751	0.115115941	0.171569563	0.610765637	0.178923757	0.253049319	0.7687068
ABCA3	0.207719974	0.740966501	0.224019796	0.858717406	0.065330932	0.25323209	0.768879155
CHAC2	0.6614454	0.293768053	0.565145483	0.571034101	0.030867395	0.25331369	0.768910539
TXNDC17	0.126442172	0.828852424	0.774079294	0.761822527	0.031325049	0.253336133	0.768910539
TMEM144	0.166164078	0.176747625	0.249954544	0.492759655	0.537268393	0.253811172	0.770209902
TALDO1	0.193233215	0.461526364	0.453782861	0.089883905	0.534552567	0.253873653	0.770257074
APRT	0.551879346	0.708339578	0.550685704	0.222815156	0.040629697	0.254151168	0.77080525
FAM3C	0.306205273	0.883988762	0.680917108	0.123932874	0.085347928	0.254195236	0.77080525
MFAP1	0.866904342	0.056641936	0.16251756	0.448208735	0.544800841	0.254136954	0.77080525
RRAS	0.653632521	0.117750262	0.282606981	0.882371441	0.101621221	0.254246605	0.770818591
NAXE	0.388417687	0.277589429	0.89947196	0.056674924	0.355293235	0.254403547	0.77115194
HAUS5	0.759799979	0.617337674	0.009654273	0.955158995	0.453673466	0.254998091	0.771956035
LOC112443475	0.484108211	0.471418518	0.051791412	0.794099938	0.208906574	0.25490526	0.771956035
LOC112444300	0.802778987	0.545062444	0.63073029	0.049918274	0.142154534	0.254755271	0.771956035
METAP2	0.615212622	0.336526771	0.388770941	0.05352181	0.455452139	0.254984299	0.771956035
NET1	0.285694576	0.496975897	0.499786436	0.177622331	0.155576866	0.254913861	0.771956035
RSL1D1	0.88264844	0.322011074	0.265163864	0.093386469	0.27859507	0.254904175	0.771956035
YWHAG	0.271906599	0.430609171	0.068794016	0.789504107	0.308215324	0.25485644	0.771956035
ABHD16B	0.428597375	0.700037506	0.035851431	0.275368863	0.662843676	0.25506705	0.772022383
MMP14	0.188527731	0.574657578	0.655837362	0.02931075	0.943425692	0.255155742	0.772148421
LOC104968522	0.782431669	0.13900645	0.312337094	0.160832779	0.360246093	0.255372988	0.772663366
ELOVL5	0.450338286	0.320030448	0.924830977	0.106394554	0.139001993	0.255559206	0.772941787
TFB1M	0.314250163	0.681811923	0.541524039	0.103133353	0.164679468	0.255520333	0.772941787
LMTK3	0.875420534	0.730721155	0.346265303	0.136171626	0.065414082	0.255673772	0.773003365
PLAT	0.996797231	0.132549409	0.887267587	0.053837497	0.312533846	0.255641273	0.773003365
ZNF365	0.697011208	0.954960325	0.008603361	0.350141871	0.984774672	0.255770316	0.773152821
LOC615899	0.227876458	0.197287874	0.306548615	0.386954921	0.370767429	0.255937597	0.773516004
VPS41	0.564542789	0.556598738	0.031722659	0.873258244	0.227360786	0.256053278	0.773723135
AMOTL2	0.112767768	0.06735608	0.895588623	0.497139994	0.588715232	0.256791841	0.773871748
ATF5	0.239936631	0.402914819	0.741151097	0.342817162	0.081091337	0.256848778	0.773871748
C15H11orf49	0.11500469	0.8811042	0.353647849	0.190219703	0.291670569	0.25662261	0.773871748
COA3	0.654015689	0.313021524	0.701293554	0.041495922	0.333508846	0.25654066	0.773871748
EEF2KMT	0.250929919	0.716554534	0.274705763	0.061994155	0.650592902	0.256870489	0.773871748
G3BP1	0.768359611	0.167609028	0.135456661	0.172116329	0.663046139	0.256784353	0.773871748
GNG11	0.083327134	0.371423127	0.305306997	0.923304377	0.227622267	0.256476931	0.773871748
ITPR3	0.741022196	0.2552603	0.297378704	0.414032083	0.085208149	0.256387784	0.773871748

LOC101904121	0.538059985	0.396832028	0.293992605	0.069216725	0.456775111	0.25640143	0.773871748
LOC104974850	0.65664451	0.443041194	0.834848509	0.844720907	0.009685777	0.256556314	0.773871748
LOC783920	0.026070337	0.824224498	0.520126714	0.28937608	0.61581063	0.256836651	0.773871748
MTA3	0.048497021	0.747736546	0.554548302	0.709768109	0.139258705	0.25658832	0.773871748
PCGF5	0.122934443	0.132419521	0.683330165	0.776618492	0.230928775	0.257045573	0.773871748
PDPR	0.588511061	0.378794638	0.33062788	0.488535985	0.055384806	0.257000727	0.773871748
PPP3R1	0.473430702	0.141443979	0.16131703	0.729582099	0.253093556	0.257026813	0.773871748
RXYLT1	0.525964055	0.35978429	0.222066722	0.204020127	0.232497224	0.256939946	0.773871748
SLC4A2	0.822269913	0.508208276	0.29206345	0.018741361	0.868507941	0.256521611	0.773871748
SMURF2	0.252885581	0.817804348	0.547499096	0.038951289	0.449974566	0.256395548	0.773871748
SP110	0.0493067	0.161159267	0.696238769	0.476345762	0.75368518	0.25649975	0.773871748
TRIM23	0.273994702	0.83843305	0.044789841	0.476369688	0.406452545	0.256874634	0.773871748
AP3B1	0.461957454	0.124482481	0.503013143	0.14683702	0.470761349	0.257327054	0.774135521
CAPN11	0.019642693	0.826388827	0.208964135	0.6058543	0.975888788	0.257699248	0.774135521
CARF	0.161283196	0.806990075	0.203789178	0.48211468	0.156336629	0.257305196	0.774135521
GRB10	0.546879572	0.516221918	0.115300223	0.341124581	0.180014024	0.257284625	0.774135521
HPS1	0.875523189	0.674045971	0.025150876	0.665313252	0.202819412	0.257533193	0.774135521
LOC101903851	0.343876393	0.048278977	0.795145193	0.345548637	0.439115424	0.257547153	0.774135521
NDEL1	0.691928483	0.708592082	0.372211848	0.026068749	0.421277928	0.25761642	0.774135521
PEBP1	0.801141645	0.746793124	0.645786353	0.073021096	0.070922938	0.257415108	0.774135521
POU6F1	0.234290502	0.637203405	0.459751592	0.827750815	0.035295852	0.257685944	0.774135521
PRDM5	0.72826592	0.067021106	0.254478799	0.202653548	0.796416442	0.25764822	0.774135521
PUSL1	0.445872934	0.583022454	0.759026797	0.757966781	0.013387666	0.25749384	0.774135521
RUFY1	0.974580697	0.377382401	0.061782586	0.529626789	0.16657865	0.257650282	0.774135521
LYPD3	0.685972696	0.178309308	0.530032697	0.334162112	0.092647682	0.257799402	0.774142909
POLR3F	0.243424172	0.186305587	0.275709572	0.25271786	0.635405276	0.257843224	0.774142909
TMEM38B	0.975020389	0.123153872	0.246842005	0.11450055	0.591483019	0.257815283	0.774142909
ADORA1	0.12572614	0.267625949	0.634501779	0.318322109	0.295782216	0.257985794	0.774206459
DUSP23	0.189702917	0.922871493	0.319845297	0.235244799	0.152572702	0.257964741	0.774206459
PTH1R	0.477855109	0.235915558	0.099381571	0.254705991	0.705061274	0.258100271	0.774206459
TRAPPC6B	0.688799039	0.046265371	0.373486981	0.395282049	0.427423518	0.258032835	0.774206459
XIAP	0.336294736	0.105355076	0.78103045	0.239151181	0.303944504	0.258067469	0.774206459
LDLRAD3	0.584159109	0.557491059	0.079383231	0.225778723	0.344876207	0.2581632	0.774253706
FAM126A	0.939091028	0.976768882	0.640820697	0.639531795	0.005357891	0.258234347	0.774292118
PIK3CG	0.558587286	0.047568813	0.31337239	0.253398336	0.954860102	0.258270371	0.774292118
CD1A	0.967444978	0.025455942	0.172440889	0.558208087	0.850521005	0.258362003	0.774425358
STAT5A	0.891162576	0.281109597	0.86206706	0.18507278	0.050485565	0.258461492	0.774449992
TSPAN6	0.012007189	0.664241023	0.389929435	0.900334016	0.720671852	0.258464603	0.774449992
GTF3C5	0.19977012	0.252837703	0.2624107	0.408106298	0.373459377	0.258601386	0.774685346
HMGXB3	0.520763461	0.070327953	0.388526484	0.1453306	0.978332294	0.258791585	0.774685346

IRF2BPL	0.841553375	0.225532438	0.263718209	0.082490775	0.490136972	0.258826382	0.774685346
LOC112446760	0.841557818	0.946466167	0.006450899	0.747433448	0.526347609	0.258682937	0.774685346
PARP9	0.153896328	0.095707661	0.931019096	0.256222383	0.575394957	0.258700809	0.774685346
RNF168	0.080706789	0.332814115	0.653472313	0.510740428	0.225726806	0.258817394	0.774685346
ANGPT4	0.10193242	0.377765281	0.481687083	0.880801386	0.124160482	0.259115962	0.77484549
AP5M1	0.080053169	0.882297789	0.944570562	0.245596171	0.123731191	0.259049277	0.77484549
FBXO44	0.827863219	0.751293103	0.087781915	0.781881423	0.04746885	0.25898996	0.77484549
PIAS1	0.061118421	0.177798975	0.954783703	0.196134462	0.996489833	0.25907926	0.77484549
TJP3	0.390453662	0.245005078	0.096434883	0.881306353	0.249290437	0.259015444	0.77484549
CCDC62	0.83649113	0.332762892	0.402668984	0.028030634	0.646004357	0.259188464	0.774921094
SMAD2	0.410384822	0.066354426	0.110366412	0.67886158	0.995379488	0.259261911	0.774999495
AIMP1	0.709984673	0.907308711	0.189446089	0.718966652	0.023211045	0.25961464	0.775329538
ARMC6	0.215871759	0.356067812	0.479772821	0.341653909	0.161751892	0.259703033	0.775329538
CCDC157	0.498283852	0.630541675	0.018295973	0.40401864	0.875595964	0.259429792	0.775329538
LOC107132278	0.385695088	0.064921728	0.50692803	0.709475828	0.226181748	0.259638409	0.775329538
MPND	0.069188644	0.199736672	0.233170089	0.929518356	0.679656199	0.259562672	0.775329538
RCSD1	0.321889588	0.419589491	0.141589886	0.811237284	0.131286301	0.25962557	0.775329538
SCLY	0.232054723	0.163654268	0.130395339	0.893824646	0.460419999	0.259699105	0.775329538
NAPA	0.574188147	0.369129208	0.721905411	0.087501147	0.152301775	0.259769797	0.775387803
PXK	0.905740476	0.086178991	0.206714287	0.362763863	0.348661959	0.259877196	0.775567315
TACO1	0.418403933	0.454273751	0.757805235	0.066626147	0.212808974	0.259964088	0.775685572
LOC112447838	0.050999939	0.778290936	0.244310404	0.951568953	0.221403318	0.260013785	0.775692824
ASB2	0.357732967	0.387170426	0.978081344	0.592523838	0.025487103	0.26018237	0.775808749
MTPN	0.957505138	0.323529588	0.014202482	0.931695539	0.49912689	0.260194464	0.775808749
TMEM18	0.187254928	0.479435386	0.429860891	0.336338311	0.157576876	0.260152849	0.775808749
ADAMTS18	0.145710346	0.996996891	0.160107488	0.17446632	0.504665182	0.260311756	0.775929224
DNMBP	0.511454434	0.771408136	0.78996534	0.048521765	0.135439395	0.260331965	0.775929224
LOC107131542	0.814680406	0.540557029	0.029840362	0.166904626	0.934188609	0.260376713	0.775929224
GUCA1A	0.238512085	0.6551375	0.147652795	0.299134509	0.297432737	0.260608887	0.776480109
GNB1	0.345518184	0.387400923	0.045547444	0.602523918	0.559135232	0.26068022	0.776551661
LOC107132301	0.009278919	0.722718148	0.540209669	0.655688839	0.866188142	0.260897558	0.776751053
SMG5	0.074125562	0.283957075	0.922240037	0.201856709	0.525249285	0.260936479	0.776751053
TFE3	0.098219226	0.495943445	0.481729192	0.701794103	0.124940306	0.260899062	0.776751053
USP11	0.719894465	0.506086058	0.07790182	0.228071504	0.317710497	0.260841099	0.776751053
RBKS	0.431103296	0.272545442	0.136154971	0.167699052	0.767574129	0.261003386	0.776809315
ACOT7	0.649927161	0.945852531	0.049066132	0.110650545	0.617850092	0.261177497	0.776904822
FAM19A5	0.530555114	0.149846547	0.62509138	0.115556616	0.358891148	0.261111448	0.776904822
LOC112444290	0.709402418	0.597957785	0.360541458	0.271492507	0.049647683	0.261139485	0.776904822
TXNRD2	0.369214613	0.277197457	0.728594626	0.057598698	0.480514079	0.261283482	0.777079237
CCDC15	0.681734173	0.404190418	0.096318879	0.078089142	0.996580523	0.261382672	0.777118485

KATNB1	0.076079534	0.206920735	0.913675889	0.209963106	0.683972482	0.261391386	0.777118485
LOC100299503	0.15266875	0.401201805	0.183321233	0.193548541	0.951306707	0.261504491	0.777173161
TMEM161B	0.688069188	0.646802438	0.048680926	0.253184294	0.376904217	0.26150278	0.777173161
GALNT3	0.314209585	0.13008938	0.172994834	0.916859775	0.319065553	0.261574288	0.77723984
LOC112443850	0.995047459	0.916697958	0.1787171	0.052478616	0.241928059	0.261640202	0.777294958
COPS9	0.330349049	0.497041389	0.390774331	0.16719761	0.193371935	0.261933037	0.777461216
GNG5	0.887105403	0.050104912	0.927791975	0.114970565	0.437490697	0.261917516	0.777461216
LOC101906120	0.45825467	0.849966408	0.018968582	0.336502246	0.833715263	0.261826955	0.777461216
LOC515823	0.534219432	0.259905646	0.405758464	0.112906361	0.325643587	0.261744264	0.777461216
PPP2R2C	0.015213896	0.391935041	0.68154407	0.593045787	0.860231655	0.261856781	0.777461216
ASIP	0.244716719	0.009651308	0.992549071	0.970382999	0.914366179	0.262266634	0.777524388
CDC16	0.953028223	0.238436628	0.95712193	0.046863645	0.203813435	0.262106967	0.777524388
FSTL4	0.955563704	0.05343519	0.463964602	0.549943011	0.159766057	0.262356292	0.777524388
LOC101902475	0.567784477	0.304577126	0.084697593	0.956226278	0.148389009	0.262164815	0.777524388
LOC107132883	0.744594986	0.477535852	0.187817337	0.173845805	0.178958858	0.262125622	0.777524388
LOC112443837	0.116296836	0.318062726	0.914721151	0.120833175	0.509041601	0.262337368	0.777524388
METRN	0.012550583	0.812074854	0.27849314	0.760810448	0.964067168	0.262380724	0.777524388
NUTF2	0.817893068	0.215715699	0.054150974	0.387584238	0.561480444	0.262214438	0.777524388
PMPCA	0.323631026	0.538256959	0.794451097	0.049130977	0.305636328	0.2621512	0.777524388
ALAS1	0.559434556	0.151021422	0.831196914	0.095864319	0.315798256	0.265032385	0.777614506
AMMECR1	0.444470665	0.53473232	0.09994613	0.201548538	0.44455445	0.265176198	0.777614506
ANAPC11	0.211832266	0.308305864	0.924467793	0.181980515	0.190483904	0.263046341	0.777614506
ASB12	0.474002903	0.928656695	0.480368508	0.214918692	0.046639429	0.264646449	0.777614506
ATP2C2	0.119525884	0.070776282	0.378821818	0.688515523	0.9724166	0.266201834	0.777614506
ATP6V0D1	0.236506925	0.426414897	0.052639538	0.766614269	0.512921863	0.262717118	0.777614506
BPTF	0.549096706	0.461749072	0.657443083	0.030976264	0.415463223	0.266179494	0.777614506
C1QTNF9	0.14745077	0.352393184	0.690351235	0.315479318	0.184704621	0.262884795	0.777614506
CCDC80	0.148380294	0.074046402	0.25100107	0.959868976	0.79911779	0.264395403	0.777614506
CCDC89	0.157121199	0.985732892	0.981277881	0.07217911	0.191874981	0.263764602	0.777614506
CD48	0.356353463	0.162563775	0.69952737	0.146765204	0.359301129	0.265685928	0.777614506
CDC34	0.126097526	0.17386867	0.780975134	0.424200489	0.293297609	0.265291682	0.777614506
CDK10	0.197716549	0.12968832	0.336291515	0.275097216	0.891197064	0.264319863	0.777614506
CEBPA	0.855460778	0.366010789	0.868027955	0.090398061	0.087322436	0.266190644	0.777614506
CMTR2	0.305856383	0.911549695	0.656986578	0.058531878	0.196718569	0.264020073	0.777614506
CORO7	0.102255941	0.228219339	0.914213811	0.975077863	0.10304659	0.26608769	0.777614506
CRYM	0.252755415	0.522925566	0.12374762	0.646136666	0.201797083	0.265429751	0.777614506
CYTH2	0.483075083	0.761135348	0.995286688	0.718193201	0.008013587	0.263845353	0.777614506
DKKL1	0.032430704	0.976377165	0.408187601	0.525229718	0.307564416	0.262746981	0.777614506
DMBT1	0.790928013	0.872920631	0.105740035	0.47444071	0.060413862	0.263023322	0.777614506
DOHH	0.279632358	0.164828747	0.633227433	0.670180706	0.10843622	0.264736515	0.777614506

DOLPP1	0.122715617	0.187297511	0.816289391	0.365728949	0.307878507	0.264230841	0.777614506
DYRK1B	0.747552563	0.049641471	0.427356128	0.682868455	0.193543798	0.263233628	0.777614506
ELMO3	0.500848241	0.095878657	0.365029374	0.508516661	0.239191313	0.265397557	0.777614506
EMC6	0.246528865	0.159634188	0.586012462	0.21231005	0.432063121	0.264407014	0.777614506
ENTPD1	0.196887125	0.63231944	0.887456108	0.302188504	0.064081121	0.265837611	0.777614506
EPHA2	0.298623515	0.906008622	0.375331854	0.232713129	0.088656586	0.263178641	0.777614506
FBXO3	0.479120662	0.20757189	0.221122766	0.27852773	0.348952208	0.265712336	0.777614506
FOXJ3	0.461953239	0.588478711	0.332076437	0.047155404	0.491569925	0.263027364	0.777614506
GATA3	0.730652798	0.515538487	0.310505143	0.174483799	0.104540731	0.265478096	0.777614506
GEM	0.32301268	0.728476118	0.107920266	0.32590232	0.251865553	0.262536338	0.777614506
GHDC	0.323347234	0.62544374	0.219419074	0.082914419	0.581203347	0.265773478	0.777614506
GNL3	0.407754478	0.119512608	0.756849872	0.356143815	0.163150555	0.266050875	0.777614506
GPBP1L1	0.693648047	0.108074797	0.333756547	0.36643768	0.231336147	0.264733774	0.777614506
GPR137	0.272495914	0.091340876	0.596687231	0.354352201	0.399848124	0.263731437	0.777614506
GPR21	0.049026266	0.488131452	0.114608987	0.939579012	0.823926972	0.264870935	0.777614506
HIST1H1C	0.913461975	0.486998505	0.349645439	0.042803413	0.315015217	0.263310253	0.777614506
IGDCC4	0.013979133	0.825423024	0.215555384	0.864029487	0.983111256	0.264240246	0.777614506
KLK13	0.666408133	0.248691464	0.99826179	0.374765909	0.034076485	0.264244198	0.777614506
KPNA3	0.755212518	0.011033087	0.896380757	0.487917581	0.58057023	0.264419192	0.777614506
LASP1	0.945682572	0.979251657	0.972712793	0.005381405	0.441310313	0.265824518	0.777614506
LCP1	0.341292133	0.141817363	0.192661335	0.326752195	0.6983853	0.265150956	0.777614506
LGALS8	0.823093047	0.418050152	0.009124364	0.840434328	0.793924965	0.263166866	0.777614506
LOC101902128	0.605378756	0.35706656	0.240261043	0.926805044	0.043856163	0.264132815	0.777614506
LOC101903649	0.859422073	0.096243281	0.135748907	0.434375363	0.439653148	0.266124926	0.777614506
LOC112442805	0.800908132	0.058029314	0.421607044	0.433450839	0.246104637	0.262886055	0.777614506
LOC112443463	0.270478371	0.803413867	0.486868251	0.17009765	0.115964835	0.262685603	0.777614506
LOC785745	0.867357004	0.049732329	0.16540938	0.7428371	0.394875005	0.263046687	0.777614506
MGAT4B	0.517061616	0.270209558	0.033165221	0.809734312	0.564232887	0.264497109	0.777614506
MYCL	0.198593849	0.183108457	0.368306208	0.21954977	0.722387307	0.264923563	0.777614506
N4BP2L2	0.062532042	0.404126545	0.303065447	0.83237645	0.335903451	0.265949563	0.777614506
NPC1	0.74401591	0.66625133	0.038204655	0.389548171	0.288851299	0.265328941	0.777614506
OPRL1	0.128593461	0.971873505	0.469063194	0.421475512	0.0865779	0.265817273	0.777614506
PLOD2	0.068956838	0.403208952	0.408490419	0.203580235	0.91018927	0.263747347	0.777614506
POLB	0.870738265	0.038255262	0.302657525	0.814440761	0.261152211	0.266123416	0.777614506
POLDIP2	0.885466148	0.069133564	0.49364399	0.261270964	0.269140393	0.264969896	0.777614506
PPP1R36	0.092989653	0.946046348	0.030837698	0.810358368	0.970506679	0.265484846	0.777614506
PRF1	0.649427787	0.136287055	0.253985297	0.903882333	0.103089915	0.263154924	0.777614506
PSMD7	0.966505235	0.097462572	0.412694887	0.703846015	0.078175576	0.265811568	0.777614506
RABGGTA	0.733084771	0.696078516	0.799862714	0.020506741	0.251289534	0.263672235	0.777614506
RINT1	0.318187737	0.282131379	0.308665305	0.11108514	0.688387391	0.26460946	0.777614506

RMI1	0.208355705	0.772229893	0.098951717	0.448652776	0.294388354	0.263645255	0.777614506
SERF2	0.4404873	0.428806137	0.580496565	0.078254768	0.245139159	0.263677656	0.777614506
SERPIND1	0.703751943	0.464949633	0.489695863	0.388866776	0.034315559	0.265759975	0.777614506
SLC26A2	0.812265934	0.93218732	0.007879184	0.452154405	0.776552417	0.263160179	0.777614506
SNRNP48	0.963933595	0.129747292	0.926143732	0.26264221	0.06884925	0.263144862	0.777614506
SORBS2	0.137122217	0.340769701	0.69652569	0.445045528	0.146867631	0.265113693	0.777614506
STAT2	0.362706308	0.35660765	0.780248964	0.049257298	0.427716097	0.265046033	0.777614506
THBS2	0.545743497	0.16516339	0.159713127	0.343795652	0.431350525	0.265563636	0.777614506
TMEM107	0.14324428	0.887848715	0.088814187	0.333278178	0.560060577	0.263975826	0.777614506
TMEM129	0.23031312	0.080059061	0.721048372	0.990611885	0.161113256	0.264789698	0.777614506
TP53INP2	0.712969146	0.187021314	0.7234069	0.107536981	0.205347566	0.265276199	0.777614506
TSHZ2	0.430032062	0.132274896	0.176993656	0.263170145	0.805691756	0.265554213	0.777614506
USP20	0.143095805	0.747967987	0.113256604	0.261900403	0.664474001	0.264047896	0.777614506
VCAN	0.530395373	0.250953705	0.054814881	0.358342917	0.819077204	0.265956836	0.777614506
XKR5	0.493149197	0.867867344	0.009228477	0.609758557	0.882487903	0.264994336	0.777614506
YAF2	0.456551672	0.083711152	0.16780551	0.696648007	0.467585476	0.262815197	0.777614506
ZBTB14	0.682027378	0.248386206	0.051424358	0.246303368	0.9946375	0.265522536	0.777614506
ZNF584	0.701972105	0.113055846	0.997420459	0.623895071	0.043110698	0.265216465	0.777614506
ZPR1	0.914720811	0.525678932	0.087925099	0.110955768	0.455465554	0.265667255	0.777614506
HMCN1	0.068846623	0.948582081	0.1435819	0.356661081	0.642956774	0.266478892	0.778146814
LOC614923	0.502304259	0.955199822	0.314979425	0.030346804	0.468687097	0.266433232	0.778146814
LOC507696	0.885181605	0.399618102	0.023972374	0.425695605	0.596436282	0.266642484	0.778347529
PFDN2	0.38075517	0.040860359	0.31080085	0.544359093	0.817741041	0.266607139	0.778347529
CACUL1	0.984589421	0.06342728	0.153680141	0.449010506	0.500083499	0.266758898	0.778548867
XPR1	0.732550493	0.30929351	0.797403491	0.028554851	0.417926549	0.266823061	0.778597662
RAB31	0.596544453	0.308390754	0.622033827	0.530666446	0.035572071	0.267064892	0.779164789
COQ10B	0.301507612	0.419521286	0.376636207	0.118038493	0.384790154	0.267280526	0.779348387
EFR3B	0.185585612	0.398220592	0.166591945	0.296366291	0.593856811	0.267460247	0.779348387
GPC5	0.718543753	0.684948104	0.202650412	0.920843091	0.023590118	0.267443045	0.779348387
HBP1	0.406628075	0.210374369	0.336321122	0.628180817	0.119662883	0.267211823	0.779348387
LOC104969140	0.895001218	0.011800322	0.540957379	0.392904906	0.963639174	0.26723947	0.779348387
LOC112442952	0.311393707	0.804372857	0.263405604	0.129751165	0.253095323	0.26744702	0.779348387
LOC616063	0.381055152	0.919438553	0.8214371	0.096090917	0.078286322	0.267348545	0.779348387
SLC46A2	0.440969725	0.309699504	0.113888235	0.266762964	0.522463203	0.267512309	0.77936171
TAF7	0.003725974	0.663170375	0.942805206	0.996749295	0.934215304	0.267603722	0.779489649
MYH6	0.02626688	0.813716671	0.398670426	0.424888078	0.599638678	0.267703903	0.779643078
SNTB1	0.090799968	0.119620951	0.917548685	0.271624655	0.803183541	0.267894305	0.780059163
CLTC	0.648997367	0.10102379	0.05935333	0.924490905	0.606156045	0.268275534	0.780248929
INPP5E	0.950945194	0.024182883	0.808030001	0.81996387	0.143215134	0.268356956	0.780248929
LOC101903820	0.323642024	0.724924771	0.263754144	0.795367507	0.044348959	0.268396424	0.780248929

LOC101904590	0.767324593	0.563450131	0.190237586	0.113826192	0.232809949	0.26820942	0.780248929
LOC104974812	0.030925488	0.294533973	0.353052602	0.934777005	0.725717933	0.268325251	0.780248929
LOC112446454	0.295011776	0.45255689	0.30941003	0.114570828	0.46127709	0.268418398	0.780248929
SBF2	0.696333969	0.665692452	0.07294001	0.11099464	0.580875048	0.268229213	0.780248929
SETD2	0.539855232	0.352566336	0.821018788	0.016719592	0.834447691	0.268245229	0.780248929
TXN	0.3467314	0.549691151	0.776495167	0.420095978	0.035118797	0.268434919	0.780248929
UNC13C	0.901439791	0.359345779	0.214895704	0.052790235	0.592710549	0.268120512	0.780248929
LOC514507	0.064472222	0.229543719	0.597714118	0.38487042	0.642403307	0.268646049	0.780608437
TAF6L	0.838680949	0.546896583	0.145476692	0.043098985	0.76053191	0.268653736	0.780608437
IARS	0.623325399	0.225938302	0.989692011	0.808917506	0.019413364	0.268751184	0.780753352
BCAS1	0.324342277	0.594661047	0.711024002	0.130128412	0.123015826	0.269129731	0.780780632
DUSP11	0.364915796	0.201651985	0.523339558	0.291524689	0.195122216	0.2688549	0.780780632
HARBI1	0.582871312	0.631591677	0.525338386	0.019590825	0.580031849	0.26926649	0.780780632
HES2	0.689815468	0.986855135	0.004831155	0.860844839	0.773962747	0.268890782	0.780780632
IKBKE	0.160255006	0.16485091	0.387109523	0.601262856	0.357077105	0.269151248	0.780780632
LOC101906366	0.93777284	0.747736237	0.403334481	0.110266684	0.070325437	0.269004876	0.780780632
LOC112447353	0.279551675	0.639359128	0.097756842	0.264481887	0.47527582	0.269190468	0.780780632
PITPNB	0.221119511	0.510114504	0.137668111	0.311265175	0.453958586	0.269065982	0.780780632
PPP1R3C	0.129617299	0.173562476	0.246433144	0.605073264	0.654910689	0.26922365	0.780780632
SINHCAF	0.474424282	0.314572319	0.201788919	0.142427312	0.510957033	0.26891448	0.780780632
TCTA	0.233082702	0.303267735	0.674194272	0.04842129	0.952475953	0.269283918	0.780780632
SLC25A40	0.281096489	0.329384554	0.229482444	0.125031634	0.827653532	0.269332713	0.780784164
UBE2W	0.438604166	0.100471274	0.274688947	0.580492114	0.313076303	0.269399525	0.780839917
FAM76A	0.408965849	0.487124911	0.186948316	0.388129294	0.152248446	0.269451577	0.780852875
ADD2	0.013384425	0.79580948	0.552222015	0.648699537	0.577255352	0.269556926	0.780882387
LRFN4	0.668673826	0.161297571	0.206395339	0.304754587	0.324627224	0.269540089	0.780882387
LOC112443235	0.832013597	0.451013199	0.452271279	0.482429823	0.02691465	0.269618359	0.7809225
EYA3	0.955683873	0.115754514	0.651967012	0.032334628	0.94646258	0.269827952	0.781115984
KIF18B	0.775815862	0.109271334	0.183037182	0.171717782	0.828272195	0.269811192	0.781115984
TMEM183A	0.665992853	0.361610695	0.536685793	0.671304744	0.025432609	0.269796081	0.781115984
TRMT12	0.854630308	0.634478929	0.019754019	0.739705116	0.278919206	0.269987652	0.781440451
LOC104973099	0.021307776	0.331409407	0.682117773	0.668446051	0.686982702	0.27010223	0.781496421
SLC38A9	0.046307124	0.978842579	0.60144356	0.185145821	0.438090545	0.270060379	0.781496421
UBD	0.426919749	0.159017522	0.343748091	0.18069338	0.525498449	0.270330829	0.782019961
ARID1A	0.652148246	0.292270247	0.411872686	0.091876633	0.308162946	0.270726898	0.782338286
BNIP3	0.38388492	0.86145057	0.046254702	0.837614452	0.17327274	0.270573165	0.782338286
CEP126	0.51806528	0.772584133	0.142525348	0.331150473	0.117584333	0.270643093	0.782338286
KIZ	0.356170182	0.223989895	0.954194723	0.046922774	0.622193701	0.270712899	0.782338286
NCLN	0.118667909	0.46109526	0.763358229	0.339604819	0.156423392	0.270503751	0.782338286
SHC4	0.112785004	0.845469715	0.056347769	0.662326746	0.624502892	0.270713079	0.782338286

LOC107131368	0.060490551	0.168085566	0.759352937	0.817922902	0.352215416	0.27081689	0.782460559
ALDOC	0.772004482	0.112780724	0.427211102	0.74889952	0.079898846	0.270899761	0.782503244
DST	0.543998023	0.518245655	0.022314624	0.603651466	0.586266984	0.270942246	0.782503244
HEPACAM	0.486643516	0.109861215	0.614903214	0.359548728	0.18840552	0.270974708	0.782503244
CA8	0.254742159	0.289933167	0.817041609	0.623083627	0.059293838	0.271118976	0.782644418
CACNB3	0.503384987	0.846293911	0.844837068	0.040219724	0.154009378	0.271113592	0.782644418
CNGA3	0.043132594	0.231584144	0.692313921	0.548893936	0.587683886	0.271193578	0.782649064
MTMR4	0.679873842	0.683161704	0.081152901	0.410064396	0.144350074	0.271215967	0.782649064
ARMC1	0.690149665	0.199030563	0.496634879	0.677830912	0.04839492	0.27160173	0.782661281
BARX1	0.338430314	0.273966518	0.222492789	0.114302467	0.948690093	0.27155447	0.782661281
C11H2orf50	0.812844665	0.095534812	0.170783958	0.927672908	0.181802949	0.271539416	0.782661281
FCER2	0.519375364	0.162510323	0.066886687	0.486816617	0.813348083	0.271459574	0.782661281
LOC107132382	0.745933975	0.476007691	0.040566176	0.364697287	0.425340518	0.27140122	0.782661281
LOC782560	0.744816313	0.031784515	0.434526133	0.572277747	0.37996492	0.271545056	0.782661281
SCYL1	0.018779618	0.21141714	0.636073357	0.92510218	0.956415058	0.271407924	0.782661281
TXNIP	0.814846354	0.207186774	0.09808458	0.314253161	0.428926124	0.271268217	0.782661281
LYPLAL1	0.209274365	0.961685799	0.825571734	0.503905134	0.026768642	0.271797603	0.783088213
ATP6V0B	0.290113611	0.06950343	0.191417224	0.989692155	0.590383136	0.27260575	0.78316585
C3H1orf52	0.965135172	0.953866345	0.026272292	0.770281922	0.120789409	0.272326088	0.78316585
CCDC88B	0.801979316	0.081655754	0.812259444	0.176655646	0.240885554	0.273081739	0.78316585
CRACR2A	0.114491738	0.060499157	0.484608339	0.836569078	0.808275349	0.273438317	0.78316585
CUX2	0.157325582	0.85610104	0.032045138	0.888675501	0.586835734	0.272354154	0.78316585
DYRK3	0.067148224	0.476468841	0.1651948	0.618454942	0.68933813	0.272491247	0.78316585
EBF4	0.019989545	0.812195211	0.355393011	0.846696683	0.463756422	0.273203401	0.78316585
GPS1	0.464722363	0.370664618	0.604474678	0.187163658	0.115175422	0.271992746	0.78316585
LMO4	0.391934229	0.352510121	0.194140548	0.663659218	0.126786478	0.272704172	0.78316585
LOC107132465	0.572038263	0.956274314	0.031058666	0.2584041	0.51689513	0.273413401	0.78316585
LOC112442243	0.128464678	0.80715634	0.078114679	0.697496521	0.400084774	0.272898004	0.78316585
LOC112443428	0.738641169	0.175626795	0.280374556	0.2012365	0.309154518	0.273040564	0.78316585
LOC112444206	0.310686755	0.613219334	0.285196207	0.352903892	0.117732337	0.27273864	0.78316585
LOC112448744	0.930941447	0.769263079	0.978438481	0.245404325	0.013180806	0.273252723	0.78316585
LOC512869	0.811006639	0.693861879	0.186419549	0.025193232	0.851388043	0.272310618	0.78316585
LOC615112	0.030456937	0.963430904	0.767556795	0.311182475	0.323925145	0.273468717	0.78316585
LOC786614	0.674305508	0.112016254	0.594121165	0.052406916	0.964297787	0.273329752	0.78316585
MAU2	0.752458899	0.433279967	0.182962832	0.881861044	0.042852645	0.272546841	0.78316585
METTL9	0.618841473	0.384805913	0.227208254	0.190220422	0.218440551	0.272201863	0.78316585
MRPL40	0.117504683	0.416759086	0.615239153	0.297425676	0.253490196	0.273542542	0.78316585
MSMO1	0.798289234	0.263871954	0.103989635	0.676198917	0.152751369	0.273027984	0.78316585
NEMP1	0.10015584	0.098953813	0.507220224	0.878402821	0.511811915	0.27288049	0.78316585
PASK	0.918250699	0.049285352	0.265732708	0.353806938	0.53110046	0.272867849	0.78316585

PRRT4	0.062097292	0.762989709	0.625222134	0.337293844	0.2261158	0.272837335	0.78316585
PSMD10	0.654063441	0.260438744	0.565485256	0.574896907	0.040825099	0.27292664	0.78316585
PTEN	0.340360849	0.873010436	0.111867336	0.123515571	0.55115467	0.273044238	0.78316585
SDK2	0.903029933	0.034330013	0.427347935	0.322932546	0.529914608	0.273288746	0.78316585
SSR1	0.981872568	0.082094984	0.599567125	0.340429019	0.138039024	0.273516859	0.78316585
STX6	0.214139248	0.725764204	0.530945828	0.167882746	0.163742647	0.273358869	0.78316585
STX8	0.534259248	0.194803139	0.794859109	0.104140461	0.263266405	0.273341879	0.78316585
TCF25	0.657370489	0.512747249	0.012861342	0.62985932	0.831168584	0.273425453	0.78316585
TXNDC5	0.279922511	0.498454623	0.034364327	0.798105797	0.590342079	0.272828522	0.78316585
UBL5	0.69442219	0.096818129	0.500066276	0.284746105	0.23504126	0.272313426	0.78316585
VTI1B	0.087081542	0.224953722	0.293911603	0.975894397	0.403992432	0.27344926	0.78316585
ZBTB34	0.597735022	0.845192295	0.47356756	0.107015849	0.087806853	0.272197996	0.78316585
ZNF782	0.197171458	0.047470977	0.65002048	0.630640165	0.587764723	0.27260425	0.78316585
GOT2	0.831038252	0.45483938	0.730119616	0.087726954	0.093879563	0.273618366	0.783246293
CHAF1B	0.561186643	0.325487785	0.816263107	0.017684116	0.862456451	0.273682878	0.783274585
CHCHD6	0.202827492	0.0880493	0.374841989	0.685028001	0.496407831	0.273819164	0.783274585
VPS37C	0.433878599	0.473213827	0.228504644	0.829182054	0.05848307	0.273745154	0.783274585
ZNF768	0.671805249	0.267414899	0.17921358	0.096386234	0.733531038	0.273814823	0.783274585
MRPS10	0.844091211	0.24516841	0.449523242	0.504698831	0.048516332	0.27390254	0.783376539
CUEDC2	0.348324697	0.590497916	0.683019402	0.019520385	0.833584792	0.274366352	0.783593771
FRZB	0.244733532	0.971421638	0.037963911	0.584108167	0.433917506	0.274455974	0.783593771
IQCA1	0.09061125	0.909290133	0.109360291	0.483893446	0.52461756	0.274445227	0.783593771
LOC100139549	0.431235438	0.044678854	0.386470748	0.53117426	0.577879508	0.274346434	0.783593771
LOC100848331	0.400858532	0.955429793	0.236131829	0.158903952	0.159145911	0.274426572	0.783593771
LOC112442719	0.124865628	0.087632871	0.487506463	0.786927883	0.544732255	0.274406693	0.783593771
LOC504548	0.023322726	0.914201738	0.426716114	0.323698028	0.776067161	0.274344136	0.783593771
LOC513329	0.308718136	0.607502217	0.367043366	0.287023992	0.115483829	0.274124394	0.783593771
NLRX1	0.526396526	0.508275492	0.649886792	0.143191323	0.091722545	0.274237077	0.783593771
PUF60	0.725670318	0.09550919	0.164167985	0.732305751	0.274127479	0.274259656	0.783593771
FAM204A	0.479257247	0.217589593	0.389686495	0.134557912	0.419492395	0.27481095	0.784197966
LOC112441472	0.950548852	0.492350712	0.05733338	0.425111588	0.200945799	0.274715766	0.784197966
LOC510520	0.509936533	0.46367476	0.070652444	0.829271835	0.165551765	0.274790538	0.784197966
DEGS1	0.891190095	0.379648165	0.310463813	0.162280943	0.134713195	0.274956538	0.784371769
SSR3	0.789661092	0.468161696	0.046926767	0.330464256	0.400585185	0.274967448	0.784371769
DERL3	0.565083125	0.083563774	0.460317458	0.458390665	0.230932703	0.275217557	0.784401904
ELOB	0.558825286	0.382433921	0.776789157	0.079882592	0.173327757	0.275081379	0.784401904
LOC504773	0.263809386	0.865218093	0.141404086	0.209410865	0.340531013	0.275255021	0.784401904
OLA1	0.524313889	0.18456051	0.99656909	0.424410484	0.05623964	0.275264796	0.784401904
SOCS7	0.440739136	0.260041688	0.598328756	0.051394119	0.65204134	0.275050479	0.784401904
TOR2A	0.879303114	0.341764253	0.026060945	0.387584353	0.758071035	0.275224321	0.784401904

NUPR1	0.979045354	0.673959331	0.031532484	0.297828407	0.371677897	0.275343364	0.784489575
FAM92B	0.450139945	0.171121865	0.805302452	0.066080206	0.562567504	0.275502921	0.78480792
CTH	0.546526597	0.051514632	0.146843177	0.624897043	0.893215282	0.275593825	0.784930624
BASP1	0.13666809	0.502864692	0.252418368	0.318409824	0.418011518	0.27567014	0.785011743
CREB1	0.59859698	0.417992851	0.349845771	0.028966928	0.911189895	0.275753629	0.785053879
LOC112447474	0.348686743	0.654028444	0.413279043	0.576745774	0.042512937	0.275780611	0.785053879
CHRM1	0.240186341	0.166041656	0.125786612	0.647822801	0.711589881	0.275872365	0.785178872
RNF121	0.379813903	0.528288972	0.211409021	0.165013307	0.330660154	0.275987085	0.785369178
TRAPPC13	0.80508918	0.211306526	0.769921334	0.330228538	0.053538239	0.276051897	0.785417421
LOC783224	0.042840947	0.280482612	0.630199623	0.578194499	0.529241381	0.276138946	0.785528903
DUS1L	0.343703822	0.092661702	0.587451167	0.249524177	0.497002769	0.276306785	0.785597827
PPP1R9B	0.907090429	0.252902969	0.255158297	0.049661531	0.79793747	0.276269207	0.785597827
SKP1	0.935435938	0.434678653	0.274437864	0.395852393	0.052487879	0.276213613	0.785597827
CDON	0.172012224	0.160348646	0.561557384	0.453688329	0.330829528	0.276563913	0.78567918
LGI2	0.574640791	0.627720402	0.049824951	0.557775015	0.231925232	0.276574774	0.78567918
LOC101907577	0.439524639	0.871865653	0.082595309	0.464507374	0.158019493	0.276476704	0.78567918
PAPSS2	0.03574187	0.441442786	0.258739243	0.908177722	0.626194212	0.276386986	0.78567918
SLC25A30	0.378324309	0.493845515	0.298696302	0.36324936	0.114661258	0.276541638	0.78567918
TROVE2	0.534777474	0.176565963	0.193652493	0.144829789	0.879241944	0.276771743	0.786102645
ANKRD45	0.098526251	0.511424802	0.285895791	0.336556253	0.482327015	0.277336876	0.786211047
COLQ	0.08043111	0.630411205	0.085972205	0.629492893	0.850228543	0.277032294	0.786211047
ITIH1	0.126634812	0.572156596	0.476336523	0.333111213	0.20318974	0.277195512	0.786211047
LOC107131619	0.41427043	0.3577148	0.303334655	0.990903374	0.052477393	0.277278151	0.786211047
LTC4S	0.417185508	0.083492651	0.123151179	0.761980021	0.714565387	0.277173963	0.786211047
MRPL27	0.518786819	0.23324717	0.589704236	0.118836762	0.275351804	0.277136717	0.786211047
PIK3R3	0.680608414	0.861679488	0.615906708	0.009904552	0.653596517	0.277325332	0.786211047
REPIN1	0.302228206	0.828080499	0.646725326	0.120764212	0.11939851	0.277072163	0.786211047
RP2	0.426187765	0.683437958	0.033541672	0.624281523	0.383014149	0.277197673	0.786211047
SLC48A1	0.850357812	0.125734121	0.111546949	0.290209398	0.674858192	0.277184522	0.786211047
TOR1AIP2	0.594380048	0.236362035	0.066494916	0.362685158	0.690209048	0.277336893	0.786211047
AURKAIP1	0.686780977	0.215468469	0.678321277	0.094307967	0.247151911	0.277400052	0.786220459
LOC101907545	0.446187302	0.966288183	0.251069056	0.079636953	0.271479532	0.277436029	0.786220459
RNF2	0.169679591	0.930403284	0.206121733	0.225976823	0.318583477	0.277569967	0.786464213
LRP4	0.283090903	0.090813374	0.211174304	0.776901618	0.555962195	0.277695883	0.786549385
PM20D1	0.632493484	0.913222608	0.061072615	0.109235688	0.608484287	0.277685312	0.786549385
CD247	0.074163415	0.20510067	0.345690774	0.862696612	0.517417978	0.277821778	0.786674107
CNKSR1	0.924234634	0.626951354	0.041622424	0.360185679	0.270347301	0.277896752	0.786674107
DENND1B	0.172724	0.288639539	0.273543035	0.396548925	0.434171175	0.277866488	0.786674107
NT5C	0.29378578	0.365415673	0.22485479	0.39950562	0.243592708	0.27793166	0.786674107
BRB	0.270357531	0.509009564	0.694426546	0.196475424	0.125402812	0.278234158	0.786779731

DDX39A	0.555856793	0.363060077	0.146342311	0.280299869	0.284352841	0.27819949	0.786779731
HSD17B7	0.830756412	0.210045313	0.749941966	0.072250669	0.248876715	0.278153665	0.786779731
JUNB	0.89744525	0.219236261	0.069319368	0.432165474	0.399104553	0.278114658	0.786779731
LOC104974937	0.656419625	0.784951808	0.624548652	0.009179787	0.797012017	0.278228717	0.786779731
MMP16	0.316646792	0.491219073	0.248571399	0.227714543	0.267851519	0.278440237	0.786779731
MTFR1	0.717694718	0.233363511	0.217968146	0.134529036	0.480560241	0.278544284	0.786779731
PDZD11	0.484126745	0.122860189	0.485365642	0.209572579	0.389687824	0.278411211	0.786779731
PEX11A	0.214125705	0.410298846	0.096835837	0.57034356	0.486097679	0.278464167	0.786779731
SERBP1	0.906887893	0.221376635	0.293215527	0.677149811	0.059175506	0.278474409	0.786779731
SNF8	0.485232115	0.098254131	0.190143258	0.495440256	0.525055058	0.278437531	0.786779731
STK17A	0.408791744	0.056828269	0.130825351	0.969239868	0.801060585	0.278521695	0.786779731
AP4B1	0.907549548	0.476697667	0.058086908	0.32167262	0.293258444	0.279128824	0.786941195
ARFRP1	0.095410783	0.419643178	0.664151988	0.228050524	0.390344264	0.278936899	0.786941195
C5H12orf73	0.687293926	0.607873289	0.677290028	0.253860728	0.032959794	0.278963082	0.786941195
FANCM	0.514948963	0.422583212	0.032432	0.432160481	0.777397449	0.279153742	0.786941195
GLRX	0.164275593	0.192217099	0.770771681	0.401471402	0.242193609	0.278901734	0.786941195
KANSL1	0.25377535	0.607019434	0.79396148	0.021682543	0.895213156	0.279320728	0.786941195
LOC101903232	0.656015403	0.879228281	0.048693751	0.599431121	0.14082359	0.279143204	0.786941195
LOC104975324	0.736744833	0.191852682	0.893056848	0.083297619	0.225747825	0.279300102	0.786941195
LOC104976232	0.619048803	0.037802032	0.985603266	0.286370605	0.359259974	0.279257335	0.786941195
LOC107133049	0.101050869	0.638165616	0.196116807	0.306141502	0.610899854	0.278832673	0.786941195
LOC112444521	0.152996216	0.067799157	0.88662199	0.9173217	0.280188286	0.27875217	0.786941195
LOC112446423	0.089688724	0.610823257	0.773039623	0.102779269	0.542791859	0.278685107	0.786941195
MSN	0.728739831	0.306475807	0.359779161	0.036440048	0.810697228	0.279306815	0.786941195
OPHN1	0.382404696	0.141340357	0.252418336	0.551058775	0.315419369	0.279171434	0.786941195
STIM1	0.990565953	0.185430705	0.842018231	0.199658429	0.076831628	0.279237528	0.786941195
KCTD20	0.058828595	0.227834106	0.423595883	0.553783436	0.75547054	0.279390083	0.787001485
VPS13C	0.27255881	0.173658267	0.590675742	0.120327864	0.707168118	0.279595909	0.787446107
HTR1B	0.106569683	0.3796303	0.464995445	0.280182379	0.45179537	0.279727295	0.787680961
SPCS2	0.753417281	0.088408877	0.164163732	0.6162415	0.353792297	0.279873938	0.787958688
BCL2	0.697048343	0.5387429	0.008132377	0.97850201	0.799029878	0.280080458	0.787993179
IFNLR1	0.15494741	0.50210522	0.193465657	0.320052095	0.495768452	0.28010962	0.787993179
MMP25	0.310938769	0.076733812	0.531609512	0.515745707	0.365262355	0.280174285	0.787993179
NPAS3	0.524379492	0.894553469	0.165087455	0.087810162	0.351344916	0.280158941	0.787993179
PLXND1	0.152922702	0.671621799	0.15905475	0.237611107	0.614798799	0.280006336	0.787993179
RPAP2	0.609063956	0.248126293	0.023673431	0.92334054	0.722591533	0.280039364	0.787993179
CLEC5A	0.298178962	0.164140678	0.166616245	0.31667011	0.925728751	0.280237145	0.788034918
MICAL2	0.28451627	0.322808317	0.195343033	0.305094541	0.436926498	0.280295361	0.788063589
MRPS31	0.598917222	0.199086478	0.872845335	0.586221663	0.039223598	0.280375333	0.788153407
METRNL	0.532190703	0.423445019	0.243791819	0.109567314	0.397861127	0.280479157	0.788270275

PKIG	0.069613514	0.504965791	0.811527894	0.305926928	0.274492372	0.280512973	0.788270275
C5H22orf23	0.974781772	0.334346072	0.09014455	0.179289006	0.455321182	0.280667688	0.788442021
FHIT	0.123797349	0.718086588	0.088396321	0.411120074	0.742559835	0.280700572	0.788442021
IL20RA	0.292151279	0.023158306	0.682833923	0.650423024	0.798463034	0.280718221	0.788442021
HLF	0.899490316	0.189996075	0.161362401	0.168002394	0.518452794	0.280866952	0.788454935
LOC112442547	0.457070327	0.917452782	0.767860978	0.014025675	0.531711521	0.280830673	0.788454935
PALMD	0.265933084	0.064326389	0.304857981	0.677606423	0.679663505	0.280855491	0.788454935
BPI	0.217052585	0.06124011	0.424686114	0.934711647	0.456206795	0.281153804	0.788564784
C1QTNF7	0.219188836	0.909032808	0.535403392	0.227960143	0.099028611	0.281211894	0.788564784
CLBA1	0.458156808	0.101344073	0.371561467	0.384405703	0.363683776	0.281412856	0.788564784
DHX30	0.526043422	0.208286521	0.295980557	0.110367189	0.674461173	0.281530746	0.788564784
EVA1B	0.626141122	0.080556631	0.455533604	0.23191032	0.451803264	0.281170588	0.788564784
LOC614208	0.184676769	0.265298501	0.680369959	0.543126855	0.133264849	0.28145933	0.788564784
MAFK	0.300564292	0.168744857	0.133404883	0.766576404	0.465287413	0.281492112	0.788564784
ORC4	0.258881381	0.398851082	0.113656974	0.450005548	0.456333217	0.2813062	0.788564784
PIGW	0.474489033	0.05071503	0.568839166	0.457284335	0.385386812	0.281436865	0.788564784
PPFIA4	0.439201416	0.997438747	0.078246461	0.765901191	0.091698005	0.28116568	0.788564784
PRODH	0.414570262	0.395239329	0.034963749	0.502314018	0.836566381	0.281167112	0.788564784
RNF144B	0.808191464	0.700394241	0.173365566	0.718192488	0.03424388	0.281500385	0.788564784
USP24	0.606035916	0.33228836	0.307622935	0.053137735	0.730239017	0.280968252	0.788564784
DNAJC30	0.683679647	0.826626393	0.474496979	0.014953808	0.602622063	0.28166803	0.788814682
ADAMTSL1	0.469004202	0.018086149	0.482668876	0.616091321	0.958494098	0.281733575	0.788863623
ALKBH5	0.999295417	0.240917678	0.041826498	0.522879395	0.460366895	0.282073914	0.789341042
ALKBH8	0.042990415	0.618829404	0.733128475	0.165113669	0.753933121	0.282293872	0.789341042
EIF3K	0.440137639	0.244358347	0.22363205	0.548562259	0.184139793	0.282381051	0.789341042
FAM216A	0.101146937	0.376540003	0.381507451	0.466767422	0.358037915	0.282311602	0.789341042
LOC112445044	0.286304433	0.560280588	0.049438726	0.507917821	0.603391424	0.282433161	0.789341042
MRPS2	0.278341072	0.684663004	0.392644998	0.186696173	0.173447278	0.28202419	0.789341042
PHB2	0.458851102	0.106221867	0.839069755	0.340468905	0.174035778	0.282037232	0.789341042
PPAN	0.612607569	0.492688256	0.372414746	0.219511279	0.098491213	0.282415481	0.789341042
TRUB2	0.431576375	0.415677958	0.719627436	0.019373096	0.97076764	0.282293318	0.789341042
UTP6	0.559606807	0.240666482	0.154846705	0.172575708	0.674723684	0.282314585	0.789341042
ZNF644	0.269809188	0.542609053	0.944605772	0.036309582	0.48367984	0.28233522	0.789341042
ARSA	0.115304332	0.499175949	0.14331842	0.30810626	0.958671349	0.282763383	0.789591607
CSTF3	0.718837268	0.406809444	0.932140274	0.812758809	0.010985863	0.282618521	0.789591607
GHSR	0.890082022	0.432326268	0.397220252	0.332022448	0.047988924	0.282705145	0.789591607
PKD1L2	0.330266063	0.748843151	0.172311617	0.067870248	0.842397076	0.282761574	0.789591607
PPP1R14A	0.43882248	0.279054405	0.712265739	0.559177798	0.049916993	0.282655096	0.789591607
PGAM1	0.774889214	0.435481063	0.41173355	0.210678044	0.083275583	0.282821779	0.789620316
BAK1	0.421390529	0.513678548	0.647839041	0.161622552	0.107611006	0.282895301	0.789691236

LOC112443250	0.741263078	0.34460417	0.29136354	0.253209869	0.129499153	0.282979208	0.789791119
GRTP1	0.276769099	0.020533875	0.909570426	0.929922739	0.508124878	0.283091597	0.789970447
IFFO2	0.811294647	0.717218326	0.441096602	0.234320223	0.04063036	0.283147414	0.789991875
LOC539166	0.27612749	0.08868994	0.301436639	0.92816596	0.356895381	0.283246338	0.790133547
ASS1	0.307029933	0.300603216	0.867502972	0.051832847	0.590306731	0.283486187	0.79054308
FMNL3	0.851150467	0.323053265	0.176529245	0.102450252	0.492716324	0.283510444	0.79054308
POLE3	0.576520755	0.286130261	0.035754663	0.897300832	0.463069603	0.283537662	0.79054308
PRR22	0.876136896	0.424694167	0.064202087	0.385863108	0.266288709	0.283748853	0.790997525
C1QTNF2	0.276356593	0.703660595	0.048191137	0.651174769	0.403684146	0.284227166	0.791458121
CPXM2	0.721469086	0.183921442	0.169289214	0.931298106	0.117782231	0.284260503	0.791458121
KNL1	0.636407258	0.18947358	0.125635232	0.410251596	0.396579653	0.284299898	0.791458121
LOC112443243	0.394847007	0.519899761	0.038442285	0.497483398	0.627072689	0.28413941	0.791458121
LOC781494	0.140993559	0.979874591	0.110958772	0.831345972	0.193180169	0.284146056	0.791458121
MBOAT2	0.246440282	0.974649383	0.340456735	0.252604621	0.119065039	0.284014415	0.791458121
SLC46A1	0.71721208	0.160944844	0.689220804	0.560924593	0.055227428	0.284289562	0.791458121
ZNF667	0.710187349	0.832098892	0.184382392	0.21939459	0.102981988	0.284139523	0.791458121
TMEM154	0.592773386	0.138016066	0.544236883	0.106446964	0.520943393	0.284531972	0.791969843
EML4	0.221371503	0.536974928	0.480649199	0.125511291	0.344921715	0.284771255	0.792501451
COQ6	0.58466528	0.166789444	0.899696582	0.066845319	0.422019408	0.284853828	0.792532049
DNAJC4	0.565318761	0.045383951	0.276278099	0.459640696	0.760068342	0.284927127	0.792532049
EXOC3L2	0.357910216	0.998031478	0.137396651	0.082468233	0.611750821	0.284909396	0.792532049
CHCHD7	0.575954685	0.041259679	0.490300173	0.847461595	0.251519865	0.285314064	0.792777203
IFT172	0.950657726	0.054205533	0.184392386	0.839150141	0.311169602	0.285184276	0.792777203
LOC107131356	0.250429418	0.275370472	0.054611517	0.828204041	0.795947927	0.285264855	0.792777203
LOC107132032	0.491898553	0.188443797	0.756296387	0.190126163	0.185996876	0.285076177	0.792777203
MGAT3	0.362117862	0.87470836	0.164515688	0.773691762	0.061618001	0.285353418	0.792777203
NT5DC1	0.48393927	0.678074868	0.227554099	0.26458671	0.125632201	0.285238095	0.792777203
OMP	0.683359998	0.200027847	0.080146669	0.97882827	0.231425906	0.285213981	0.792777203
ARFIP1	0.178918733	0.473214185	0.459904625	0.301524137	0.211875384	0.285534759	0.793146739
ATP7B	0.049198672	0.365590281	0.483172487	0.534544775	0.536403657	0.285763818	0.793380141
IL17RA	0.447088082	0.508138793	0.105134378	0.411025008	0.253756606	0.285726696	0.793380141
RSF1	0.290381683	0.267333367	0.407819586	0.1348278	0.583537382	0.285705951	0.793380141
CRIP1	0.265762959	0.850541442	0.676210651	0.044121178	0.369736045	0.285851757	0.793396704
TMEFF2	0.228554571	0.294864392	0.65316423	0.064092284	0.883930453	0.285866474	0.793396704
HDAC2	0.816360868	0.315524783	0.583704871	0.179229907	0.092655647	0.286031071	0.793585106
SPIDR	0.667107788	0.231630171	0.575456554	0.874618642	0.032093557	0.285984395	0.793585106
NKIRAS1	0.393043131	0.401762812	0.674363543	0.042456457	0.553067065	0.286227067	0.793994657
ABCA4	0.202638602	0.889353777	0.713503335	0.102886996	0.189681453	0.286707243	0.794010325
HDAC6	0.783412464	0.366102997	0.371349124	0.726403777	0.032406764	0.286587131	0.794010325
KCNG3	0.361131685	0.866877499	0.018952231	0.537971505	0.786404884	0.286741606	0.794010325

LEPR	0.051577319	0.919769905	0.369719569	0.368849208	0.387769118	0.286662178	0.794010325
LMBR1	0.979226394	0.304054902	0.048440721	0.252495823	0.689701641	0.286825812	0.794010325
LOC100337108	0.04601308	0.922071579	0.067728434	0.986740573	0.885611671	0.286795511	0.794010325
LOC786726	0.850147345	0.185428569	0.094298273	0.68515507	0.246207405	0.286610228	0.794010325
MNS1	0.991526406	0.153323615	0.162235415	0.199747195	0.508010114	0.286346544	0.794010325
OBSL1	0.370226763	0.712493441	0.042470393	0.518305122	0.431568638	0.286519203	0.794010325
SNX5	0.298250613	0.604791847	0.063571787	0.731248255	0.299579065	0.286846497	0.794010325
STIL	0.271269389	0.741123597	0.140532397	0.841226894	0.105588506	0.286713301	0.794010325
STK16	0.352972115	0.128175495	0.64327091	0.205988197	0.419078541	0.286861691	0.794010325
XCL2	0.521773295	0.943848441	0.408457082	0.186259368	0.066968429	0.286688885	0.794010325
DNAJC1	0.280845731	0.661956278	0.776957079	0.050280016	0.346082953	0.286921354	0.794041542
OTUD1	0.699220264	0.202508547	0.072771616	0.601374735	0.405775879	0.286977405	0.794062754
GLO1	0.614629629	0.634489043	0.304820279	0.074780469	0.282994773	0.287038505	0.794097928
CD55	0.333554794	0.057399205	0.776072649	0.264131187	0.641345494	0.287112568	0.794168944
COBLL1	0.803869344	0.734829073	0.250095408	0.126707187	0.13466042	0.287308258	0.794330781
DDX24	0.553924865	0.046328325	0.625686629	0.857846208	0.18301254	0.287316283	0.794330781
WDHD1	0.607492319	0.488777721	0.187881724	0.063303812	0.71346961	0.287253511	0.794330781
CEP55	0.162212208	0.374743878	0.100495847	0.839910935	0.491676996	0.287420032	0.79439934
FBXO6	0.575347495	0.118764565	0.220356741	0.329300642	0.509932615	0.287720703	0.79439934
GARNL3	0.092200715	0.158430773	0.700071826	0.428143533	0.576925479	0.287589403	0.79439934
JAGN1	0.253239456	0.150582952	0.208257739	0.3996358	0.795838782	0.287580805	0.79439934
LOC100336208	0.509273676	0.601480503	0.367018685	0.028273539	0.794797034	0.287611681	0.79439934
LOC782021	0.329592596	0.52785238	0.355638038	0.330638019	0.123601143	0.287728333	0.79439934
PDAP1	0.068346651	0.147251993	0.712132378	0.769894077	0.458219019	0.287718973	0.79439934
SNAI2	0.204039645	0.902537554	0.77609201	0.054819669	0.32248694	0.2876254	0.79439934
ACADM	0.978417882	0.730890113	0.225962494	0.350270373	0.044733412	0.287906718	0.794537708
FCRLA	0.311216174	0.501753275	0.122117865	0.724790122	0.183134477	0.287865035	0.794537708
VPS8	0.541174766	0.246347036	0.163224647	0.345090252	0.337209377	0.287923694	0.794537708
LGR4	0.423525587	0.21939881	0.136411697	0.669425769	0.298676587	0.288037375	0.79471778
CDC40	0.058257607	0.341961215	0.455208037	0.71368318	0.392118122	0.288221426	0.794958289
MFSD10	0.094619074	0.102246523	0.764590426	0.450913286	0.760787802	0.288206169	0.794958289
COL4A6	0.012015492	0.939922987	0.725088445	0.397769623	0.77968485	0.288318894	0.794959907
PSMG2	0.804422556	0.131465746	0.636637297	0.846205148	0.044565504	0.28828406	0.794959907
ANKRD42	0.733073068	0.666718344	0.108957743	0.172226898	0.278786741	0.289235038	0.794969249
CCDC6	0.782990276	0.36199371	0.194159831	0.598818406	0.077320655	0.288764033	0.794969249
CSRP1	0.208686357	0.368761307	0.835508072	0.357418792	0.111139435	0.289083962	0.794969249
DCAF6	0.091265365	0.764587767	0.096913326	0.650018881	0.578465041	0.288488075	0.794969249
ESPL1	0.596105345	0.10434012	0.148851769	0.364916749	0.755271405	0.288956221	0.794969249
GPR45	0.064131571	0.636855404	0.823865204	0.093775119	0.809063069	0.289022724	0.794969249
LOC101905875	0.555350769	0.392844292	0.08666757	0.187167846	0.718592373	0.288499999	0.794969249

LOC107131516	0.681352802	0.117795388	0.97163468	0.054877008	0.596307695	0.288968355	0.794969249
LOC112442081	0.526603701	0.209234781	0.750723347	0.181451467	0.169841426	0.288824497	0.794969249
LOC112449072	0.09647978	0.100487315	0.320066261	0.997023059	0.822278776	0.288547695	0.794969249
LOC787714	0.595512583	0.01398018	0.901671213	0.770344564	0.441786995	0.289119528	0.794969249
MMAA	0.956454238	0.511036679	0.230632321	0.073644797	0.307107611	0.288845422	0.794969249
NUP43	0.19705607	0.791976846	0.08913236	0.562633129	0.326298199	0.289065929	0.794969249
OIP5	0.334669819	0.02956941	0.320245811	0.819816002	0.984206648	0.289242666	0.794969249
RNF19B	0.903054491	0.845069938	0.038730309	0.174803447	0.494246515	0.289057677	0.794969249
SEC31B	0.749176091	0.550538651	0.092926503	0.145963656	0.455504793	0.288776961	0.794969249
SLC19A3	0.816389351	0.082932761	0.086611579	0.624599008	0.69797967	0.28921094	0.794969249
TMEM86A	0.696332831	0.528079215	0.538364445	0.065544307	0.196682716	0.288977477	0.794969249
VMP1	0.441664845	0.036135878	0.482809515	0.856949424	0.386201459	0.288878801	0.794969249
PLEKHH3	0.398107908	0.212128334	0.176325995	0.506950509	0.339014475	0.289353003	0.79513934
GPR20	0.404168319	0.255358833	0.61731806	0.080006015	0.502779743	0.289547079	0.795473587
HSPG2	0.704966939	0.193595342	0.030312401	0.625466795	0.990633298	0.28957158	0.795473587
ANKZF1	0.888058352	0.061355743	0.761975871	0.339167632	0.182167733	0.289672141	0.795616653
SCN2B	0.070646933	0.63923102	0.724107393	0.521367666	0.150563634	0.289763962	0.795735674
LOC112446759	0.949723078	0.263230945	0.067121111	0.410785186	0.372544062	0.28981609	0.795745667
LOC782938	0.67543319	0.064555505	0.156725416	0.988963414	0.380865987	0.290134923	0.796354611
RANBP10	0.365833046	0.584883115	0.874859508	0.76963476	0.017860407	0.290090871	0.796354611
SNAPC4	0.801971722	0.046115089	0.185223559	0.722569389	0.520459225	0.290246374	0.796527297
LOC101906870	0.044312083	0.323046821	0.968442183	0.67893724	0.27383525	0.290313719	0.796578907
LOC104969409	0.011340027	0.988375087	0.704421181	0.430626762	0.759849262	0.290631185	0.796659033
LOC107131293	0.960547376	0.754323806	0.445619413	0.009778672	0.817583021	0.290523628	0.796659033
MEIOB	0.472088937	0.735117176	0.787083815	0.272625087	0.034659878	0.290505434	0.796659033
NUP160	0.192674791	0.421974615	0.395354169	0.085875932	0.935917017	0.290634186	0.796659033
PLXNA1	0.493510252	0.610721175	0.704180403	0.653726429	0.018617393	0.290612828	0.796659033
PSMC1	0.557879042	0.21722706	0.303807929	0.81967426	0.085586056	0.290600141	0.796659033
CEP112	0.255884038	0.394343061	0.346722595	0.303064109	0.243865354	0.290752252	0.796849566
CDH6	0.103702763	0.789847409	0.280494153	0.19321295	0.583452956	0.290975864	0.796904298
GRAMD1A	0.257974444	0.263729674	0.998594002	0.071031142	0.536351485	0.29088941	0.796904298
INTS12	0.352198257	0.136089912	0.735024889	0.791910308	0.092861511	0.291015741	0.796904298
LPAR2	0.385827464	0.282892813	0.118926277	0.350734152	0.568304386	0.290835956	0.796904298
PHYH	0.681962734	0.715664766	0.117499126	0.513715386	0.08789518	0.290942449	0.796904298
RAD54B	0.813807942	0.012878917	0.539326398	0.850186272	0.539278174	0.291063577	0.796904298
CX3CL1	0.091934606	0.527627505	0.074871686	0.724234687	0.987288146	0.291334986	0.797383623
EIF2A	0.856633617	0.199668466	0.915322628	0.293277685	0.056601682	0.291441367	0.797383623
IFT80	0.12423451	0.139735076	0.588100307	0.384247111	0.662055217	0.291352704	0.797383623
MYL4	0.074768423	0.555146372	0.63133949	0.985777833	0.100670695	0.291530177	0.797383623
RANBP2	0.625133369	0.39637591	0.386192736	0.027802342	0.976821765	0.291439359	0.797383623

RANGAP1	0.37700663	0.518239802	0.132934086	0.944115622	0.106018978	0.291484495	0.797383623
LOC789569	0.423252686	0.885194122	0.855487238	0.108107458	0.075134515	0.291679296	0.797525647
NMI	0.011178689	0.582813022	0.734749332	0.746026427	0.728889372	0.291656814	0.797525647
TESK2	0.524507117	0.847798059	0.278894103	0.786906508	0.026698529	0.291787907	0.797689711
LOC101903383	0.551342333	0.663931186	0.078377864	0.749294231	0.121281149	0.291878759	0.797753016
LOC101906484	0.078227062	0.888914715	0.089167642	0.610307984	0.689134971	0.291908285	0.797753016
EMILIN1	0.273977701	0.931965094	0.096992891	0.301062407	0.350707797	0.292277967	0.798231607
IL23A	0.021518878	0.715274493	0.63074509	0.277116516	0.971059657	0.292151829	0.798231607
LOC101904227	0.234019493	0.405677496	0.10108199	0.486748109	0.559756731	0.292263417	0.798231607
LOC104976082	0.483259562	0.416390477	0.082400845	0.777111899	0.202822773	0.292201579	0.798231607
MIGA2	0.198385751	0.309368784	0.965108054	0.058904108	0.750257149	0.292422896	0.798494533
CALU	0.41784113	0.300085685	0.052920524	0.432760666	0.912239924	0.292522957	0.798634878
CSGALNACT2	0.835051002	0.776937862	0.597574088	0.010061985	0.672295236	0.292679081	0.798662521
PHLDA3	0.064816942	0.761236804	0.359013955	0.273077478	0.54198401	0.292634258	0.798662521
RHPN1	0.947198932	0.420734926	0.02835889	0.266165444	0.871846018	0.29267773	0.798662521
HIST1H1E	0.068313362	0.647794285	0.827664277	0.269808436	0.265922601	0.292953295	0.798677946
LAMC2	0.596439406	0.052723571	0.561783987	0.626412219	0.237093171	0.292737178	0.798677946
NKAP	0.172656556	0.105439386	0.174496524	0.992015172	0.833293281	0.292853124	0.798677946
NTN4	0.095609842	0.452179667	0.526423436	0.426654009	0.270680947	0.292976737	0.798677946
TMEM26	0.811603736	0.216987663	0.079498905	0.203502962	0.922119057	0.292918797	0.798677946
ZNF76	0.817343437	0.066991453	0.272374973	0.654137505	0.269069268	0.292801423	0.798677946
CISD1	0.704417506	0.509134329	0.79548297	0.065180927	0.141525809	0.293155013	0.79903121
AIFM3	0.8558351	0.71343031	0.293670989	0.126215403	0.11658034	0.293498053	0.79956782
CTXN1	0.637607347	0.109817087	0.253724124	0.26849319	0.552899691	0.29344276	0.79956782
NSFL1C	0.448177312	0.145846502	0.644662302	0.273143101	0.229171935	0.293463274	0.79956782
FKTN	0.05116553	0.898143361	0.951636098	0.976592529	0.061839977	0.293635374	0.799734052
FOXJ2	0.113619947	0.112059434	0.746617261	0.627441011	0.44286369	0.293656535	0.799734052
LOC784914	0.384441809	0.617139538	0.191708145	0.374678528	0.155241746	0.293869938	0.800182437
ANXA6	0.567751785	0.232623107	0.757458329	0.420132624	0.062995162	0.293977596	0.800342788
ARF5	0.194379329	0.943996936	0.476148159	0.048624129	0.624626347	0.294283613	0.800404716
LOC101904614	0.561309276	0.315904066	0.779314508	0.188725283	0.101654885	0.294155541	0.800404716
LOC101906676	0.897987439	0.3546045	0.202159694	0.535699323	0.077064295	0.294488068	0.800404716
MAFG	0.558833204	0.470021598	0.080073468	0.821500137	0.153782904	0.294463146	0.800404716
MAGT1	0.177772173	0.119862567	0.525202681	0.772818639	0.306321179	0.294061135	0.800404716
MANBA	0.52468193	0.541728162	0.324339724	0.108331669	0.266035192	0.294453003	0.800404716
OSBPL5	0.222605221	0.486738292	0.070849046	0.916510504	0.37697523	0.294214343	0.800404716
POLR2A	0.642643809	0.544739523	0.620232753	0.014489209	0.843751528	0.29432719	0.800404716
SLC2A4	0.784718857	0.246717257	0.792684966	0.024442406	0.708169379	0.294428771	0.800404716
TACC3	0.446551732	0.118474305	0.263750297	0.486408802	0.391295775	0.294397288	0.800404716
LOC101907133	0.216471209	0.466269466	0.702880163	0.574000343	0.065325433	0.294623528	0.800566365

RAD23B	0.787348537	0.139670294	0.255411011	0.967555845	0.097902668	0.294645107	0.800566365
LOC100336564	0.228282776	0.173471508	0.1765734	0.648110369	0.588523435	0.294979032	0.801340987
RASGEF1B	0.71648803	0.15772009	0.117989632	0.40223411	0.497693862	0.295086922	0.80136877
SULT1A1	0.748141518	0.820508676	0.086135226	0.221182054	0.228210023	0.295071559	0.80136877
DUSP8	0.579459473	0.909622849	0.939225091	0.670636665	0.0080443	0.29516546	0.801449433
C18H16orf46	0.783624921	0.282382702	0.849712062	0.134871381	0.105405445	0.295282763	0.801502717
C3H1orf43	0.582880839	0.282166578	0.41498305	0.448488036	0.08729739	0.295240701	0.801502717
TEAD3	0.013383425	0.697110743	0.339575712	0.95989331	0.88148447	0.295675465	0.802038027
TICAM1	0.161153995	0.929075775	0.368713851	0.29177983	0.166360452	0.29562605	0.802038027
TMEM168	0.300037715	0.348846781	0.789092355	0.166926584	0.194393939	0.295644899	0.802038027
TNK1	0.210123926	0.397488828	0.161125212	0.468106335	0.42512854	0.295544378	0.802038027
SORT1	0.529125698	0.366158326	0.156807952	0.095495734	0.924320935	0.295725855	0.802042142
GABRE	0.764566487	0.036532212	0.630055244	0.222974773	0.685276636	0.296102058	0.802099394
GAS8	0.055478807	0.595828197	0.67198047	0.230414277	0.524815798	0.295953165	0.802099394
HECA	0.685455594	0.430515427	0.264119959	0.104045357	0.331138657	0.295915429	0.802099394
LOC618367	0.871891266	0.42292781	0.075316321	0.90566619	0.106807524	0.295974232	0.802099394
NECTIN4	0.094684009	0.931269343	0.50113627	0.12745921	0.477171229	0.296026637	0.802099394
RIMKLA	0.068607213	0.390151528	0.991235466	0.190309392	0.532771517	0.296162091	0.802099394
RPRM	0.421035026	0.239169521	0.092053876	0.325336265	0.8921969	0.296186846	0.802099394
SWT1	0.231772834	0.319604082	0.566429387	0.992494786	0.064540379	0.29603522	0.802099394
ZNF706	0.535236121	0.287247995	0.088017879	0.425910757	0.466635825	0.296126906	0.802099394
CDCA7	0.102029743	0.785785124	0.120563591	0.710763912	0.3919682	0.296302004	0.802278862
GPR17	0.13387323	0.605402227	0.345861865	0.837180784	0.114808264	0.296368476	0.80232647
BAIAP2	0.166088177	0.349186956	0.961825344	0.582495379	0.083028692	0.296552492	0.802543533
DCAF4	0.769054879	0.762687606	0.039422416	0.242309735	0.48149838	0.296552277	0.802543533
LOC514189	0.408842578	0.349300013	0.440699849	0.224589732	0.190951185	0.296614688	0.802543533
TMEM14C	0.266852344	0.436751783	0.465817176	0.236550393	0.210212394	0.296644267	0.802543533
GABRG3	0.927574335	0.753843529	0.701933112	0.021763218	0.252978854	0.296780525	0.802779824
SYT7	0.926386021	0.053486827	0.842894018	0.20847567	0.310559016	0.296868782	0.802886221
CD3EAP	0.849417799	0.514191998	0.245408845	0.537526691	0.046992541	0.297044681	0.802950605
DMAC2	0.49050873	0.334540548	0.349366771	0.070995913	0.66565172	0.297137227	0.802950605
LOC104974272	0.659186096	0.698422013	0.157792006	0.187046262	0.199105679	0.296942465	0.802950605
LOC786039	0.563739321	0.776242201	0.146279141	0.44753661	0.094549441	0.297101686	0.802950605
LOC789748	0.504419153	0.255237397	0.904995023	0.470572635	0.049406713	0.297117293	0.802950605
HR	0.426218027	0.060932471	0.256304217	0.584420402	0.698026371	0.297447077	0.803523289
LOC101908185	0.197530685	0.547313968	0.484524571	0.894805125	0.057919397	0.297417178	0.803523289
ARF2	0.747651689	0.135205292	0.052509425	0.954898625	0.538140264	0.298066622	0.803552415
BEX2	0.436130711	0.582123315	0.65052555	0.663029261	0.024839053	0.297679027	0.803552415
C1QL3	0.411301432	0.863631345	0.829784223	0.653068036	0.014152603	0.297896669	0.803552415
CDK5	0.610166819	0.432249736	0.686620007	0.046122394	0.325494846	0.29761294	0.803552415

FERMT2	0.4040707	0.043124475	0.332692885	0.535997371	0.874927899	0.297614172	0.803552415
GPATCH3	0.129118275	0.960490903	0.887338525	0.094802255	0.260774782	0.297707886	0.803552415
KIF5C	0.529465058	0.921886839	0.025348113	0.230534944	0.95677777	0.298138302	0.803552415
LOC112447066	0.859648892	0.035924454	0.28963454	0.312714273	0.974788979	0.29801476	0.803552415
MMS22L	0.382347721	0.047559795	0.728056327	0.264863213	0.778846316	0.298242799	0.803552415
NUDCD3	0.419911278	0.110081369	0.210680619	0.372897541	0.749654672	0.297800732	0.803552415
PDS5B	0.061127102	0.871434219	0.287053091	0.196146055	0.910910681	0.298290251	0.803552415
PFKFB3	0.085462258	0.104202731	0.542296761	0.571418218	0.98942538	0.298208033	0.803552415
RUSC1	0.326939473	0.475286767	0.098471361	0.193593685	0.92174378	0.298210204	0.803552415
SEC61G	0.903645511	0.012880791	0.682371049	0.923135599	0.371785941	0.29798288	0.803552415
SLC25A36	0.529033612	0.416622811	0.326472658	0.429145277	0.088073962	0.297666716	0.803552415
ZER1	0.987167059	0.736481426	0.213179962	0.833529184	0.021097615	0.297961677	0.803552415
ZNRF2	0.561450258	0.172715144	0.086974405	0.814008706	0.397327785	0.298075568	0.803552415
ASCC1	0.562055526	0.786961289	0.705230263	0.010626845	0.824800177	0.298395466	0.803583763
GYG2	0.122530394	0.237472579	0.55238621	0.707319204	0.240735896	0.298535464	0.803583763
KCNG2	0.385677787	0.38794273	0.044929817	0.479181387	0.849999613	0.298595685	0.803583763
LOC100140958	0.574411127	0.084316432	0.139757591	0.42064412	0.960915727	0.298489036	0.803583763
MED17	0.570620252	0.22841814	0.881524292	0.196945914	0.120992018	0.298586193	0.803583763
RECQL5	0.520498807	0.196450953	0.141729746	0.719409562	0.262542649	0.298552825	0.803583763
AASS	0.249596531	0.390322719	0.597450854	0.12230333	0.387898462	0.299767595	0.804004477
AHCY	0.555220114	0.571761009	0.29967396	0.589087275	0.04970051	0.30096606	0.804004477
AHI1	0.86793327	0.991041323	0.945487323	0.00384729	0.900355136	0.302548464	0.804004477
AKAP11	0.250265178	0.125363257	0.933840367	0.150372245	0.634709027	0.301515981	0.804004477
AKAP9	0.79540424	0.307387848	0.11092822	0.189217158	0.541728396	0.300706645	0.804004477
APOD	0.104971358	0.818980186	0.234910823	0.909008749	0.152172413	0.30137672	0.804004477
ASF1A	0.877726146	0.037475116	0.968554111	0.214122077	0.406430444	0.300327574	0.804004477
AVL9	0.874417796	0.246446454	0.013869421	0.950879504	0.979159412	0.300841071	0.804004477
BCL2L14	0.185354301	0.157888732	0.406913024	0.556953594	0.416603159	0.299855748	0.804004477
BGN	0.313463888	0.22925039	0.194358126	0.291202798	0.687661338	0.301543153	0.804004477
BOLA	0.528588282	0.109502815	0.533411606	0.725987385	0.125721064	0.302593406	0.804004477
CALCOCO1	0.635877866	0.738221932	0.146126447	0.892506665	0.045542988	0.3011112	0.804004477
CDK5R1	0.372243927	0.337733849	0.261104133	0.282948046	0.296812215	0.299538891	0.804004477
CEP290	0.896444077	0.312414857	0.011388584	0.953546682	0.924551619	0.30228992	0.804004477
CKAP2	0.864819843	0.09345369	0.097543163	0.476581403	0.74509629	0.301670762	0.804004477
DYNC1LI1	0.86109175	0.335014849	0.427547037	0.028277334	0.786250717	0.298803136	0.804004477
EIF2B1	0.680605243	0.169927459	0.573731313	0.519216757	0.081779984	0.302567932	0.804004477
ELAC1	0.592118541	0.553817944	0.909742347	0.077584053	0.121313053	0.302089683	0.804004477
ELF2	0.06286565	0.407484246	0.906703679	0.158197327	0.746927121	0.298921052	0.804004477
EVL	0.323169059	0.480215759	0.037719074	0.592403607	0.805805205	0.301416379	0.804004477
FAM135A	0.264417733	0.589351168	0.311541326	0.135525089	0.423907781	0.30115893	0.804004477

FAM221A	0.097426803	0.09441697	0.774670328	0.793103532	0.491900757	0.30070474	0.804004477
FAN1	0.201681123	0.120081979	0.688306828	0.271883324	0.62028168	0.302257472	0.804004477
GDF5	0.557568499	0.030567067	0.828811863	0.225547899	0.880059245	0.301892172	0.804004477
IFITM3	0.068832479	0.458058774	0.523100416	0.24392852	0.70036312	0.30257513	0.804004477
INTS13	0.201253127	0.474362916	0.299541113	0.267894644	0.359794293	0.299514536	0.804004477
IQCA1L	0.076342618	0.090897742	0.546957822	0.884427802	0.822989421	0.29983456	0.804004477
IRS2	0.725174642	0.464650864	0.563512735	0.072164756	0.202285795	0.300292421	0.804004477
KIAA0930	0.501881863	0.114200186	0.090242645	0.616144339	0.866284076	0.299736311	0.804004477
KIF3A	0.67760915	0.278805888	0.130054993	0.226993909	0.504474329	0.30237464	0.804004477
LANCL1	0.667959338	0.492253368	0.890732716	0.506115002	0.01897772	0.302348259	0.804004477
LOC100126544	0.1034175	0.742416116	0.366607233	0.109931563	0.908391019	0.302238526	0.804004477
LOC100847765	0.083406322	0.363921813	0.742996051	0.320741119	0.383515156	0.300409793	0.804004477
LOC101904087	0.660129816	0.444760453	0.028951917	0.388928691	0.853045234	0.302700268	0.804004477
LOC101906526	0.043331894	0.70281278	0.895799552	0.258290953	0.399471441	0.302436185	0.804004477
LOC104975111	0.451862084	0.983739745	0.027263206	0.678063599	0.342200615	0.302295227	0.804004477
LOC112443614	0.321888786	0.142469251	0.4197359	0.692555409	0.211457992	0.302638187	0.804004477
LOC112446753	0.138601127	0.043458098	0.748640808	0.869542648	0.700635073	0.299057065	0.804004477
LOC788599	0.366250472	0.691133534	0.941320153	0.068130116	0.171057299	0.30054652	0.804004477
LSM10	0.889763743	0.047800809	0.197978364	0.672074661	0.490366138	0.300452945	0.804004477
MAP3K8	0.10438806	0.278199607	0.113744514	0.984919216	0.849182421	0.299837365	0.804004477
METTL1	0.586159456	0.382805492	0.295315982	0.932902703	0.04507313	0.301019125	0.804004477
MFSD4A	0.820556672	0.761936449	0.03016553	0.383675387	0.387077536	0.301745099	0.804004477
MINDY1	0.131269771	0.7612798	0.547692015	0.056694928	0.888575715	0.299564314	0.804004477
NAB2	0.365310304	0.708540402	0.221321146	0.073940759	0.664241369	0.302373924	0.804004477
NDC80	0.896548226	0.058085133	0.190536825	0.785931649	0.35606617	0.300538462	0.804004477
NDUFS1	0.821790653	0.481156098	0.911307593	0.040694088	0.192013736	0.302475607	0.804004477
NEMP2	0.670620143	0.028355195	0.508318336	0.445860607	0.654475264	0.30272035	0.804004477
NLGN3	0.100688438	0.664373989	0.636285351	0.622209072	0.104521961	0.300108456	0.804004477
NLGN4X	0.280429793	0.558550539	0.369712065	0.627216988	0.076675486	0.300951772	0.804004477
PIK3R2	0.358202093	0.511773441	0.052934828	0.432412714	0.660450564	0.300267651	0.804004477
PPIP5K2	0.364799765	0.103887989	0.216812687	0.625050007	0.545106721	0.30168117	0.804004477
PPP1R14C	0.9241077	0.020447437	0.359438255	0.712483211	0.579489501	0.301907352	0.804004477
PPP3CA	0.313180284	0.067168118	0.955546114	0.179434592	0.762955853	0.299286985	0.804004477
PRKAG3	0.265387578	0.640075633	0.058836679	0.487128515	0.567286746	0.29979484	0.804004477
PRRT1	0.221403754	0.410045676	0.444848946	0.756291965	0.092054629	0.302279377	0.804004477
PTGER1	0.43357408	0.75976268	0.016920984	0.676727548	0.731856547	0.299731136	0.804004477
RAB22A	0.961236894	0.091733952	0.06882181	0.818792151	0.561583435	0.301223994	0.804004477
RB1	0.299890817	0.287855957	0.647175454	0.171763472	0.291706475	0.301660783	0.804004477
RGL3	0.768316758	0.05115598	0.368350353	0.842008982	0.228438956	0.300938998	0.804004477
RNF139	0.049976819	0.11752316	0.849387828	0.64328397	0.878602975	0.302674112	0.804004477

SIAH1	0.109745903	0.894598889	0.900828221	0.992860026	0.031544896	0.300200413	0.804004477
SKP2	0.636413429	0.732516016	0.178466322	0.277256036	0.122067323	0.302481185	0.804004477
SLAMF8	0.776843538	0.321765416	0.104798776	0.414474322	0.255100712	0.300188476	0.804004477
SLC25A37	0.545444996	0.230629086	0.169844229	0.595825118	0.221189467	0.302483087	0.804004477
SLC43A1	0.48869242	0.073586546	0.176903319	0.672894057	0.657101702	0.302338595	0.804004477
SLC9A3R1	0.232834176	0.882477939	0.209829317	0.221019803	0.294454495	0.301990838	0.804004477
SMYD3	0.139555036	0.496337939	0.279147209	0.263270151	0.54161973	0.299554192	0.804004477
SPSB4	0.191789668	0.546009688	0.522952316	0.142643327	0.359472516	0.302099541	0.804004477
TAC3	0.669272509	0.321681329	0.177164013	0.813323545	0.089891772	0.301132074	0.804004477
TDP2	0.402051478	0.124255739	0.240831911	0.66230573	0.344355015	0.298892403	0.804004477
THEMIS2	0.494738045	0.104270072	0.507373931	0.338251584	0.315624766	0.301416083	0.804004477
TNFSF13B	0.842974107	0.019705792	0.530576647	0.895431163	0.352319636	0.30072794	0.804004477
TRIM13	0.904914694	0.348664918	0.733939514	0.055370176	0.218697944	0.301904401	0.804004477
USP21	0.980941827	0.271205545	0.060647802	0.547574169	0.310900477	0.299033951	0.804004477
WDR97	0.57893806	0.440550668	0.389491141	0.400492754	0.069821301	0.30059447	0.804004477
ZNF135	0.115036634	0.649595955	0.750881257	0.355489484	0.140289206	0.301617316	0.804004477
ZNF219	0.552352806	0.202220703	0.359808001	0.203797855	0.340732282	0.301240429	0.804004477
ZNF319	0.626132743	0.969018444	0.612158775	0.444784328	0.017027904	0.302346275	0.804004477
ZNF354C	0.252356026	0.215884108	0.121405086	0.709838615	0.597129593	0.301873175	0.804004477
ZNRF1	0.53791793	0.943450964	0.043595447	0.200167953	0.626934982	0.300525823	0.804004477
ANAPC5	0.924654304	0.418593147	0.068281579	0.433624973	0.246310848	0.302828228	0.80416085
C18H19orf84	0.926976382	0.95222896	0.70557641	0.191657911	0.023709617	0.303192533	0.804998004
BAMBI	0.29730986	0.637036663	0.183372167	0.579240416	0.14073989	0.303249763	0.805019712
B4GALT5	0.646678297	0.750463474	0.923665232	0.113231019	0.056001038	0.303800708	0.805239915
BST1	0.788432897	0.367763982	0.845776877	0.297003111	0.039007096	0.303736396	0.805239915
GGA2	0.350950608	0.142836988	0.361640028	0.588734276	0.266343375	0.303810525	0.805239915
ICOS	0.852885361	0.045568091	0.520629132	0.795745979	0.176012858	0.30338354	0.805239915
KCMF1	0.759812639	0.753531039	0.154522158	0.725358673	0.044332022	0.303921518	0.805239915
KITLG	0.70207541	0.19574917	0.317786678	0.069273349	0.939068841	0.303732736	0.805239915
LOC101908014	0.710590005	0.304719865	0.195100089	0.150361271	0.446803314	0.303586707	0.805239915
LOC104972567	0.382876557	0.038208725	0.258717345	0.944218131	0.794704908	0.303681864	0.805239915
PRMT6	0.737041784	0.139552654	0.680549669	0.687461874	0.059025227	0.303698367	0.805239915
REXO2	0.133918596	0.220558367	0.832892838	0.639660588	0.1806803	0.303839385	0.805239915
TMEM140	0.122486763	0.246303999	0.411369087	0.371881518	0.614266919	0.303433507	0.805239915
VPS26B	0.669599354	0.203986661	0.030592715	0.723991638	0.940189064	0.303894051	0.805239915
ALDH1B1	0.83955344	0.029461483	0.374391405	0.431455767	0.713894298	0.304286	0.805995665
FYN	0.327990344	0.567070239	0.208136082	0.086119041	0.855683974	0.304304987	0.805995665
FAM21A	0.749842703	0.17324452	0.078199314	0.840948179	0.335066136	0.304780963	0.806552782
GLP1R	0.106784564	0.722507912	0.40794252	0.374557616	0.243006534	0.304895393	0.806552782
LOC104975593	0.123874625	0.485535226	0.050005759	0.989041649	0.964196406	0.305062305	0.806552782

LOC107132589	0.872111646	0.120132033	0.396454004	0.849245801	0.081134903	0.30475915	0.806552782
LOC112442295	0.65345794	0.04739482	0.248571009	0.920962434	0.40445751	0.305033415	0.806552782
LOC112446129	0.381595667	0.138773076	0.447304303	0.659966994	0.183502414	0.30508574	0.806552782
LOC616957	0.209209818	0.962696257	0.245195058	0.944944039	0.061365229	0.304838818	0.806552782
LOC782688	0.218515774	0.689638735	0.231019277	0.296738695	0.276869039	0.304674021	0.806552782
MAZ	0.770636451	0.186637318	0.037467869	0.757089108	0.7032044	0.305105092	0.806552782
PIK3R1	0.875216245	0.847087999	0.021880486	0.266438817	0.661866595	0.304695846	0.806552782
SESTD1	0.315124878	0.35540342	0.818642644	0.125875435	0.248564826	0.3050869	0.806552782
SPPL2A	0.38078109	0.430254397	0.272847815	0.449030389	0.142804697	0.304977053	0.806552782
ZNF275	0.767264829	0.590626115	0.573135322	0.016502177	0.670026946	0.305238603	0.806775763
EGFR	0.467309967	0.61157501	0.120297048	0.100622255	0.830779992	0.305348931	0.80693741
PMS1	0.665999499	0.63529754	0.714808867	0.148714416	0.063958495	0.305479113	0.807151461
E2F6	0.100214348	0.30066361	0.338616374	0.735366092	0.383918606	0.305663269	0.807207563
GSDMD	0.284590848	0.085761112	0.26772596	0.593849009	0.741740178	0.305555895	0.807207563
HACD2	0.41726573	0.197089365	0.491846845	0.638043928	0.111656106	0.305720672	0.807207563
ITSN1	0.45792345	0.796550263	0.765702594	0.011519596	0.895957603	0.305769887	0.807207563
NICN1	0.876084336	0.073194553	0.467029843	0.355110286	0.271043017	0.305763374	0.807207563
PPP2R5E	0.235409262	0.097131027	0.483874455	0.267956655	0.972503477	0.305795468	0.807207563
C13H10orf113	0.861626411	0.776749271	0.008011635	0.957283364	0.563279339	0.306189091	0.807280038
CYB5RL	0.337960791	0.80453028	0.02664575	0.753401934	0.529793253	0.306216455	0.807280038
LOC101903868	0.500512617	0.987427345	0.013920529	0.688905682	0.609461491	0.306058407	0.807280038
MDFI	0.053637132	0.253036521	0.668503182	0.378385689	0.841823273	0.306131589	0.807280038
MYZAP	0.049806677	0.27167656	0.570903072	0.505232657	0.739528293	0.305950357	0.807280038
PITPNM1	0.215559854	0.157441874	0.464881448	0.198050277	0.924822146	0.306117385	0.807280038
SUCLG1	0.633905277	0.314037875	0.695400983	0.100431763	0.207964193	0.306194273	0.807280038
TJAP1	0.636239607	0.337045764	0.308023843	0.192093384	0.227471331	0.30594539	0.807280038
GRIN3A	0.986876928	0.014334173	0.70540218	0.683426759	0.424364848	0.306324688	0.807343156
SLC15A4	0.876783058	0.877560873	0.171804665	0.025076704	0.873117084	0.306338787	0.807343156
EPAS1	0.684633118	0.110879264	0.503746113	0.126607096	0.598594993	0.306521546	0.807624353
LMO1	0.939270158	0.079378036	0.597647357	0.208552349	0.312099594	0.306629564	0.807624353
LOC101905367	0.174758367	0.82366753	0.322872241	0.23593252	0.264530105	0.306642334	0.807624353
SNRPD1	0.271256273	0.185076412	0.252599476	0.88974134	0.25703716	0.306622696	0.807624353
ALDH4A1	0.333590606	0.735945635	0.810203175	0.060295378	0.242397878	0.306961192	0.808283195
IFI16	0.317099532	0.029944136	0.834778059	0.381108789	0.962902876	0.307040244	0.808283195
IRAK1	0.109287677	0.162229928	0.724909426	0.757823991	0.298584557	0.307011595	0.808283195
LOC527388	0.617295234	0.175979408	0.2531195	0.826460364	0.12805237	0.307098732	0.808307506
C16H1orf74	0.645863322	0.23895229	0.826817356	0.278940561	0.08194615	0.307427279	0.808318946
CCND1	0.442234178	0.180301562	0.227829799	0.214612748	0.748511934	0.307497117	0.808318946
DBF4	0.632056649	0.068500291	0.092281939	0.864606989	0.844588184	0.307468622	0.808318946
NIPA1	0.656667752	0.40904194	0.024409057	0.453117674	0.981727708	0.307415892	0.808318946

PKP2	0.009504399	0.934874626	0.593526309	0.6580797	0.840575671	0.307450572	0.808318946
PSMA3	0.916293154	0.233753913	0.608785527	0.189723389	0.117798062	0.307302223	0.808318946
RFX2	0.407328469	0.937034229	0.436290317	0.181030573	0.096786068	0.307472422	0.808318946
VEGFD	0.04148174	0.274655108	0.818897665	0.395371183	0.789396781	0.307191143	0.808318946
FOXC2	0.0498376	0.934215257	0.741880681	0.450789861	0.18800114	0.307938029	0.808347185
LOC112441639	0.357481692	0.701634087	0.333282139	0.303531877	0.115068941	0.307569386	0.808347185
LOC787904	0.386196028	0.293694873	0.161673828	0.212373947	0.750953555	0.307803207	0.808347185
MARCH2	0.232622784	0.609333784	0.515633354	0.158983314	0.251533245	0.307717773	0.808347185
NES	0.404338899	0.458528092	0.861194532	0.023038556	0.794521578	0.307710697	0.808347185
NPEPPS	0.505226943	0.516658672	0.135476576	0.138391054	0.598047902	0.30791385	0.808347185
SENP6	0.841758515	0.947727675	0.467149514	0.012246439	0.640240462	0.307680214	0.808347185
TMEM256	0.25958088	0.28174809	0.242481857	0.215943241	0.764470282	0.307951167	0.808347185
TNFRSF25	0.77723095	0.373143844	0.104815191	0.4525004	0.21262975	0.307814714	0.808347185
CYP51A1	0.564329281	0.380989512	0.014859483	0.99783465	0.918966599	0.308046955	0.808469308
RCHY1	0.899124805	0.412193712	0.26150964	0.092315204	0.327549809	0.308096418	0.808469831
PLCH1	0.814329189	0.126860299	0.088055293	0.456766167	0.705692771	0.308171643	0.808537942
BOD1L1	0.561956012	0.193298611	0.14539061	0.312733811	0.594090447	0.308270595	0.808668276
AKT1S1	0.106044468	0.951934221	0.786999564	0.282884205	0.130699715	0.308419693	0.808671605
LOC112441456	0.904428288	0.168517827	0.362813381	0.97408401	0.054523802	0.308397891	0.808671605
MET	0.048281126	0.174718765	0.884145621	0.424629412	0.926835934	0.308322308	0.808671605
CAPN5	0.144064456	0.327215315	0.415009534	0.168567777	0.891065378	0.308478269	0.808695986
GPR65	0.549742488	0.431623136	0.713118065	0.030740045	0.565625266	0.308649629	0.809015981
PBX2	0.420977912	0.565158348	0.210217028	0.137878448	0.427053329	0.308785529	0.809242944
PLCH2	0.036056416	0.809609523	0.727061636	0.252800479	0.549470887	0.308940713	0.809520365
CAMTA1	0.688902051	0.5703261	0.906864009	0.309421897	0.02676749	0.30908079	0.809636757
PRR15L	0.109429248	0.807428133	0.104865622	0.567737572	0.561012791	0.309083803	0.809636757
HNRNPA2B1	0.357934251	0.84568147	0.073539903	0.559628535	0.236986508	0.309137863	0.809649133
LOC101907491	0.621259418	0.901617054	0.835219675	0.007245974	0.871711377	0.309271167	0.809739809
LOC790271	0.18897169	0.42354401	0.532003235	0.471326385	0.1472038	0.309233672	0.809739809
NUDT5	0.388957003	0.467345384	0.845830898	0.025327384	0.759245076	0.309346254	0.809807205
AGMO	0.685126039	0.558767934	0.019822408	0.888602095	0.440761378	0.310088773	0.809931762
CBFB	0.615538846	0.122198073	0.412550261	0.274847713	0.349409757	0.310466283	0.809931762
CDK2AP2	0.12427099	0.311107818	0.517510444	0.168537423	0.881842466	0.310160003	0.809931762
CHEK2	0.428330148	0.108552615	0.993339871	0.288991	0.222359311	0.309888544	0.809931762
CTTN	0.366755143	0.613803524	0.937630803	0.331618886	0.04247938	0.310149452	0.809931762
ENTPD7	0.702322011	0.158355532	0.44187936	0.064178855	0.944787643	0.310458289	0.809931762
GNAI1	0.102896255	0.907775998	0.110950156	0.789988633	0.364167857	0.310533271	0.809931762
KRIT1	0.056427614	0.445127319	0.844478446	0.168263013	0.829367099	0.309510911	0.809931762
LOC104970645	0.659030394	0.991976918	0.038369207	0.364152049	0.326039502	0.310374368	0.809931762
LOC104973519	0.549119042	0.10364807	0.072715241	0.861895362	0.830705173	0.309659338	0.809931762

LOC112445242	0.060272935	0.671377202	0.143889492	0.543815486	0.939748493	0.310256132	0.809931762
LOC112449102	0.214603622	0.292878277	0.426804889	0.859628117	0.129196265	0.310430409	0.809931762
MDC1	0.56581272	0.756123995	0.226370989	0.138300357	0.22128844	0.309696527	0.809931762
NLRP1	0.774325183	0.093202708	0.519710137	0.143864713	0.553096054	0.310677012	0.809931762
PDCD11	0.857309145	0.880196003	0.072715116	0.141994565	0.381292636	0.310024217	0.809931762
PDZD7	0.055242533	0.546134832	0.594278983	0.659701084	0.251432329	0.310174709	0.809931762
RAB7A	0.123472916	0.410041158	0.524623156	0.131956822	0.849985898	0.310422564	0.809931762
RCCD1	0.547903556	0.263390162	0.538307133	0.173019431	0.220630019	0.309769927	0.809931762
RNASE4	0.035507882	0.445476837	0.684828633	0.621354115	0.443352247	0.310661287	0.809931762
RPS6KB1	0.371704027	0.179678547	0.309232898	0.46254592	0.310154474	0.309645448	0.809931762
SERPINC1	0.214227379	0.735646655	0.984053343	0.360892199	0.052972938	0.309737643	0.809931762
TBL3	0.268784638	0.175244153	0.640081913	0.268547709	0.368123583	0.310490744	0.809931762
VIPR2	0.630610037	0.300663926	0.148375659	0.484580037	0.217766937	0.309923137	0.809931762
ZC3H8	0.624379724	0.046737518	0.7237845	0.253187679	0.554437136	0.309745535	0.809931762
ZNF185	0.162873987	0.279335109	0.276981573	0.652362214	0.362598518	0.310505244	0.809931762
ZNF683	0.923939965	0.779831272	0.070980353	0.625546985	0.0932852	0.310672853	0.809931762
HS3ST1	0.841809907	0.065023673	0.662719055	0.720053512	0.114396208	0.310848339	0.810249697
CBR3	0.094768032	0.551110383	0.585040706	0.565712033	0.17301013	0.310966893	0.810429053
FNTB	0.126405728	0.31732321	0.825712439	0.238335898	0.380180847	0.311466079	0.810429053
LOC101903126	0.073734977	0.246730263	0.56579878	0.319889044	0.910681151	0.311350239	0.810429053
LOC112444681	0.041126129	0.346720784	0.53345219	0.559392065	0.703766724	0.311158412	0.810429053
LOC112445007	0.77042113	0.261411517	0.020027586	0.937344479	0.792556451	0.311247754	0.810429053
NEK9	0.962445193	0.042804806	0.246272174	0.381815177	0.774356598	0.311400839	0.810429053
PISD	0.355990784	0.517481671	0.586969762	0.069256966	0.400864318	0.311509747	0.810429053
RHOT1	0.354751884	0.482821721	0.563107306	0.049502276	0.627904879	0.311316813	0.810429053
SRGAP1	0.723929271	0.137304089	0.546685631	0.060542463	0.912243125	0.311469633	0.810429053
TAF5	0.015414862	0.755981429	0.511874031	0.511183009	0.984423352	0.311497243	0.810429053
THPO	0.641908079	0.119749986	0.365579428	0.18028034	0.592202149	0.311423891	0.810429053
ZCCHC17	0.311260639	0.259550488	0.450161121	0.193882061	0.425630628	0.311468514	0.810429053
LURAP1L	0.604282304	0.456014591	0.104867613	0.530342301	0.19608723	0.311659025	0.810688899
AAMP	0.334658762	0.140287912	0.131785932	0.827059593	0.588630615	0.311988921	0.810799503
AIG1	0.864446243	0.822829915	0.368025107	0.82962726	0.013896143	0.31226194	0.810799503
C1H21orf62	0.202245105	0.492032503	0.119590302	0.77107261	0.330171999	0.312820683	0.810799503
DHRS13	0.087425972	0.868829018	0.80925472	0.191799728	0.256886263	0.312769924	0.810799503
DIDO1	0.374120625	0.310842517	0.348946827	0.371248076	0.201013018	0.312753757	0.810799503
EFEMP2	0.760563716	0.285089448	0.122489454	0.119880181	0.951683988	0.31283788	0.810799503
FIBP	0.601531562	0.348487825	0.685957343	0.163262248	0.128859835	0.312604788	0.810799503
HMG20B	0.803490204	0.57047523	0.679830444	0.114327099	0.08451779	0.311937775	0.810799503
LOC101902721	0.57617709	0.610992963	0.077661271	0.365243874	0.301774847	0.312051776	0.810799503
LOC101904447	0.886837239	0.277435623	0.366857237	0.166479009	0.201267504	0.312568607	0.810799503

LOC112442257	0.999592109	0.806709883	0.03320691	0.627584109	0.179999536	0.312593454	0.810799503
LOC616840	0.101393767	0.875793717	0.149242169	0.744319579	0.306637558	0.312586038	0.810799503
MLF2	0.314647727	0.229543744	0.718923781	0.286990541	0.203202274	0.312743678	0.810799503
PMEPA1	0.380030488	0.016214214	0.790139808	0.819300633	0.759200078	0.312759705	0.810799503
PRMT1	0.792860678	0.057607226	0.253321326	0.875149673	0.297003648	0.311765337	0.810799503
SGSM3	0.853887667	0.194242284	0.397710005	0.098524975	0.464699608	0.312369288	0.810799503
SPAG7	0.567703334	0.067373257	0.944538535	0.256673545	0.326232735	0.312601776	0.810799503
TADA3	0.720574007	0.129483477	0.122641522	0.478976045	0.549067987	0.311857416	0.810799503
TCIM	0.335119653	0.899290605	0.172289497	0.219613944	0.265661085	0.312802047	0.810799503
TIMM10B	0.770076144	0.108342347	0.204596848	0.474652489	0.373463779	0.312640484	0.810799503
TSC22D1	0.501826248	0.470868474	0.117514293	0.532955908	0.204530749	0.312685994	0.810799503
TUBD1	0.790122405	0.571771153	0.055424459	0.496945362	0.243033065	0.312554052	0.810799503
ZNF518B	0.317618465	0.698284708	0.217669267	0.431901285	0.144811959	0.312334682	0.810799503
AMHR2	0.955984787	0.105905509	0.672310595	0.151409448	0.294196541	0.31292812	0.810905318
LOC100847171	0.462498223	0.23225943	0.20662426	0.651792575	0.209702247	0.313009421	0.810987939
BCNT2	0.460262365	0.634653629	0.080105304	0.211195944	0.615718817	0.313435323	0.811067028
CCDC174	0.168328404	0.432337642	0.722561491	0.142649523	0.405251636	0.313296698	0.811067028
DNAJC10	0.061927367	0.953776163	0.483267663	0.473746428	0.224974153	0.313410265	0.811067028
G3BP2	0.669675443	0.212757372	0.10218594	0.737817873	0.28269599	0.313151444	0.811067028
LOC781977	0.052070663	0.216058362	0.482696096	0.888364515	0.629277356	0.313106149	0.811067028
MEAF6	0.555454927	0.225927895	0.173750117	0.235307776	0.592637421	0.313335789	0.811067028
SERTAD1	0.404781576	0.446841841	0.017904339	0.99014177	0.948801115	0.313413572	0.811067028
WNT9B	0.271140623	0.127439096	0.614319026	0.770023668	0.185937125	0.313267426	0.811067028
BLM	0.317332932	0.093229449	0.231728133	0.601091862	0.743939898	0.314507915	0.811099049
CRK	0.628315613	0.309322417	0.02880377	0.561474179	0.970555045	0.313803217	0.811099049
DCAKD	0.350366755	0.25350175	0.730358446	0.137491696	0.342055881	0.313811423	0.811099049
DKK2	0.10314697	0.787354018	0.506476519	0.436155014	0.170257985	0.313983038	0.811099049
DNAH2	0.054811776	0.86215962	0.437570354	0.164991147	0.895559028	0.314025923	0.811099049
DPF3	0.274021732	0.202718282	0.321107022	0.33061589	0.520177454	0.314599412	0.811099049
FAAP20	0.218128173	0.128342289	0.969385159	0.330858236	0.34013627	0.313962801	0.811099049
FYTTD1	0.4277016	0.127587032	0.196140798	0.513576281	0.555940453	0.314053706	0.811099049
GLYAT	0.74628399	0.61711691	0.582341878	0.048848289	0.233668618	0.314301267	0.811099049
GPAT4	0.755085023	0.423458063	0.018078858	0.611839287	0.867845667	0.314683301	0.811099049
LOC101906195	0.53684113	0.125599671	0.376545647	0.702557763	0.171621885	0.314303668	0.811099049
LOC112443193	0.284287055	0.795011053	0.581751316	0.035989323	0.644698618	0.31380635	0.811099049
LOC112444889	0.186426096	0.289730795	0.575014038	0.214056297	0.458597011	0.313721021	0.811099049
METTL22	0.73911987	0.243134226	0.719252244	0.080695452	0.293989621	0.314540101	0.811099049
MEX3B	0.814491239	0.151657856	0.046913837	0.591330016	0.89244301	0.314157268	0.811099049
NUP133	0.049765761	0.568031427	0.845162425	0.332840756	0.384784737	0.314234706	0.811099049
P4HTM	0.821238024	0.198439955	0.178490045	0.423863113	0.247868041	0.314056918	0.811099049

PTP4A2	0.530875393	0.413078454	0.496337662	0.988276021	0.028506251	0.31453927	0.811099049
QPCTL	0.301429497	0.083521852	0.68501872	0.272191387	0.650650975	0.313975315	0.811099049
RPA3	0.595984275	0.408744455	0.106519791	0.237673022	0.496447445	0.314324622	0.811099049
SFXN2	0.869457652	0.865574766	0.179667495	0.03213101	0.705829112	0.314547764	0.811099049
SH3GL2	0.987107942	0.232919538	0.241228726	0.474869606	0.116533032	0.314672039	0.811099049
SLC50A1	0.186337585	0.719857412	0.139855692	0.559170338	0.290788389	0.313789953	0.811099049
TPCN1	0.04512918	0.467701977	0.955736522	0.385855285	0.394095053	0.314595217	0.811099049
TUB	0.398007585	0.895778354	0.153309047	0.078017141	0.717598056	0.314245739	0.811099049
SLC10A5	0.860023517	0.610300267	0.116102291	0.141585656	0.355886298	0.314738789	0.811114676
ABCA2	0.711280217	0.117834904	0.057049318	0.703787221	0.913313079	0.314871192	0.811224325
CCDC93	0.430814896	0.472724291	0.885644394	0.108360932	0.15731582	0.314929631	0.811224325
PTPRG	0.982922065	0.105391382	0.33932107	0.869945369	0.100544373	0.314923563	0.811224325
COX20	0.252925544	0.111995222	0.712033061	0.167948278	0.909722564	0.315251349	0.811225682
DEK	0.067506546	0.226747445	0.806491502	0.347908092	0.717628009	0.31527618	0.811225682
LOC100295848	0.457204203	0.802423909	0.04783194	0.319263721	0.549235913	0.31504109	0.811225682
LOC783466	0.933800082	0.418790117	0.083813346	0.248039163	0.378728747	0.315130784	0.811225682
PRPF8	0.732497797	0.552389615	0.609866302	0.014993628	0.832641446	0.315209826	0.811225682
SAXO2	0.460298438	0.648244711	0.627229425	0.237149897	0.069417655	0.315225212	0.811225682
ZNF677	0.484843219	0.350953042	0.58557369	0.09126821	0.3383383	0.315028545	0.811225682
GFM2	0.541953478	0.588103738	0.980937946	0.120972425	0.08162331	0.315509616	0.811444649
SERTM1	0.099863838	0.552531681	0.575122694	0.649357981	0.149794293	0.315492195	0.811444649
TMEM167B	0.794821589	0.032834947	0.517637338	0.832576332	0.274439283	0.315491042	0.811444649
MTMR14	0.732467263	0.900451786	0.062078674	0.214302483	0.352261611	0.315683448	0.811554076
PALM2	0.655343035	0.249372462	0.181475917	0.113562226	0.918055493	0.315734852	0.811554076
SIPA1L2	0.770701716	0.669239188	0.118187203	0.058525172	0.866769974	0.315749971	0.811554076
VGF	0.655164387	0.707980064	0.211784747	0.137821636	0.2283158	0.315695266	0.811554076
FBXL19	0.466443512	0.125932619	0.147059064	0.833532676	0.429890095	0.315890429	0.811567943
IFT74	0.440725545	0.987106294	0.066264593	0.345805888	0.310529789	0.315903724	0.811567943
WDR53	0.842747664	0.355094476	0.027144559	0.893565307	0.42645323	0.315894668	0.811567943
LRMP	0.8144704	0.054405801	0.583054667	0.536880474	0.223694818	0.316239448	0.812049066
MOB3B	0.976609447	0.328229979	0.28084034	0.275600604	0.125057853	0.316234967	0.812049066
RER1	0.644185503	0.120620938	0.520865849	0.549079927	0.139586159	0.316197762	0.812049066
DSB	0.311519147	0.680092923	0.900407491	0.316856903	0.05137283	0.316347249	0.812071752
NT5C3B	0.714740742	0.247657036	0.215690668	0.190346361	0.427231328	0.316330511	0.812071752
FAM161B	0.683579326	0.025849256	0.663950164	0.63746131	0.415352047	0.316398229	0.812075593
APOPT1	0.172081642	0.713786262	0.226449066	0.171153612	0.653290438	0.316571541	0.812266349
CFLAR	0.996928954	0.226227247	0.088478135	0.242743716	0.641857949	0.316527252	0.812266349
RGS2	0.732394714	0.185367475	0.148836805	0.393406937	0.391791196	0.316777126	0.812666783
MICALL2	0.909450879	0.087972639	0.642432811	0.207423389	0.292517669	0.31696904	0.813032028
LHFPL2	0.413564112	0.736105126	0.083621626	0.71403343	0.171684152	0.317063805	0.813148007

FAM122A	0.26554482	0.37119079	0.683394027	0.070503793	0.659743479	0.317642723	0.814119142
LOC104974473	0.500782561	0.127209717	0.503377664	0.992201016	0.09850909	0.317691098	0.814119142
MMP2	0.844276948	0.471760548	0.174841373	0.267324068	0.16842173	0.31774012	0.814119142
S100A5	0.812887725	0.385169719	0.698128318	0.678017363	0.021134201	0.317593163	0.814119142
TOGARAM1	0.071693141	0.554355036	0.617891615	0.163645949	0.77981992	0.31767053	0.814119142
UPRT	0.874290264	0.068991384	0.979436441	0.565791639	0.093738677	0.317645484	0.814119142
DDX11	0.430509519	0.232566686	0.249484205	0.545615693	0.230161479	0.317808527	0.814167303
BANK1	0.997688472	0.177300253	0.105748859	0.489758313	0.343053453	0.318084698	0.814620472
NUDT4	0.620685492	0.918345909	0.04120214	0.235315076	0.568619937	0.318066708	0.814620472
LOC112447290	0.844483736	0.332206568	0.01729327	0.894594092	0.724416647	0.318140386	0.81463596
TMA7	0.239109064	0.59240878	0.909741838	0.024671569	0.990372956	0.318353321	0.815054033
LOC101902346	0.229305178	0.16549735	0.563048799	0.410731589	0.358998908	0.318443473	0.815094866
WWP2	0.219261797	0.388076497	0.173741287	0.358494438	0.594582238	0.318468605	0.815094866
CORO2A	0.101677821	0.601074088	0.281504211	0.804238355	0.228309623	0.318824962	0.815465003
DOCK9	0.399355283	0.187693795	0.189546244	0.368206712	0.60363016	0.318771358	0.815465003
GFRA4	0.535166493	0.780168817	0.086164755	0.43043383	0.203860657	0.318724678	0.815465003
LAG3	0.149480742	0.268820162	0.394839077	0.865631315	0.230028602	0.318836245	0.815465003
SPATA5L1	0.627617433	0.040649689	0.201905497	0.732631353	0.837283412	0.318861674	0.815465003
TOP2B	0.610423776	0.356947781	0.690829419	0.04482406	0.468735906	0.318990922	0.815668435
KCTD8	0.872275735	0.904687662	0.248343626	0.728160977	0.022175381	0.319076749	0.815760791
ZNF593	0.329224405	0.261581756	0.406423368	0.941963035	0.096021583	0.319135697	0.815784411
CD300LB	0.257566242	0.562609488	0.133217387	0.960382408	0.171408656	0.319686879	0.815903036
DENND4B	0.869860849	0.110948004	0.328721849	0.251766444	0.397211494	0.319447937	0.815903036
EMILIN2	0.2145971	0.727149565	0.278149744	0.769548006	0.095070066	0.319577432	0.815903036
LOC112448791	0.392181349	0.375358316	0.353662057	0.13245067	0.460158886	0.319469917	0.815903036
LOC112449367	0.997270968	0.186553602	0.690454352	0.587799327	0.042084859	0.319677835	0.815903036
PHLDA1	0.334793011	0.102034614	0.116853614	0.869691692	0.914509573	0.319548701	0.815903036
PRPF40A	0.5207165	0.316885739	0.905622916	0.070819008	0.300309168	0.319698894	0.815903036
SRRD	0.764850851	0.335893693	0.354706605	0.071415285	0.487917087	0.319570529	0.815903036
TRAK1	0.463762522	0.052727584	0.2541127	0.863093877	0.590925946	0.319292391	0.815903036
TRAPPC2	0.527304368	0.088397572	0.836967605	0.44937953	0.18131612	0.319728988	0.815903036
USP5	0.191953731	0.304041502	0.963993819	0.192874099	0.292199208	0.319360948	0.815903036
CTNNAL1	0.384574811	0.446361227	0.120354586	0.607037716	0.253792685	0.319917435	0.816130114
UBE2N	0.741775658	0.07203995	0.359759035	0.7155	0.231364725	0.319897327	0.816130114
RHOJ	0.282710203	0.356897966	0.059174453	0.909825855	0.58618679	0.319980658	0.816164528
SND1	0.270958311	0.277212756	0.796704171	0.826403289	0.064430524	0.320074936	0.816278132
ATP6V1B2	0.943791237	0.47707058	0.01191804	0.951499668	0.624985671	0.320290571	0.81644743
EFCAB14	0.39319384	0.026092373	0.612328262	0.52650562	0.964184234	0.320198881	0.81644743
TRNP1	0.776146354	0.185068317	0.13953169	0.635575581	0.250491457	0.320279398	0.81644743
EGR3	0.070920428	0.228587558	0.264125352	0.760658296	0.981652897	0.320571086	0.816472994

LOC100336868	0.340804355	0.128425908	0.613513132	0.124773096	0.954588427	0.320616942	0.816472994
LOC100616098	0.479574998	0.442881226	0.677104889	0.109838628	0.202228445	0.320442586	0.816472994
LOC112446645	0.15425789	0.411620632	0.25187204	0.279857789	0.713615899	0.320418961	0.816472994
MRPS33	0.825748529	0.115582389	0.371179279	0.262254312	0.344324876	0.32064886	0.816472994
RBPMS2	0.877776709	0.099795878	0.562412283	0.380831629	0.170415409	0.320575011	0.816472994
RDH10	0.352561311	0.509038693	0.787720768	0.072180785	0.313479458	0.320640233	0.816472994
TMEM267	0.928518873	0.117155446	0.536772217	0.730114924	0.075078986	0.320728644	0.816549454
ADGRF3	0.92696841	0.013753144	0.467298938	0.696275417	0.773023866	0.320990464	0.816688944
CIAO1	0.895629842	0.204695639	0.542936104	0.044940773	0.716658367	0.32095798	0.816688944
CYGB	0.690953884	0.932051583	0.227196578	0.023001387	0.955326689	0.321379054	0.816688944
DPEP3	0.11205662	0.376463829	0.099492937	0.899848251	0.85175239	0.32145906	0.816688944
EPHA1	0.917784344	0.898110549	0.121400635	0.796280969	0.040312691	0.321246079	0.816688944
LANCL2	0.502783417	0.206020944	0.04874592	0.913694441	0.697355519	0.321475856	0.816688944
LOC112442377	0.461246986	0.922901811	0.497838458	0.152327273	0.09966518	0.32148014	0.816688944
LOC112448627	0.415407641	0.110038819	0.43557607	0.707218678	0.227498315	0.320850187	0.816688944
PAWR	0.069126491	0.459444891	0.243607521	0.983118593	0.421571144	0.320993722	0.816688944
PEAK1	0.768279117	0.370777035	0.394895128	0.076462222	0.373767384	0.321367406	0.816688944
PPP1R15B	0.465668483	0.537135133	0.178061738	0.18326855	0.393229374	0.321134085	0.816688944
RBBP4	0.841861264	0.157709699	0.383516921	0.75448197	0.083551824	0.321141901	0.816688944
SKA2	0.299564215	0.987222996	0.217024645	0.356218261	0.14036539	0.321109173	0.816688944
TFIP11	0.298960997	0.520082636	0.797229254	0.608430734	0.04265149	0.321452297	0.816688944
ACTR5	0.374888123	0.226118918	0.964971045	0.095828122	0.411640407	0.321904045	0.817081815
AP1S3	0.945851388	0.275439272	0.874572057	0.147726374	0.096639566	0.323076468	0.817081815
ARRB1	0.226662939	0.113410852	0.443707482	0.549273513	0.518001811	0.322738712	0.817081815
ATP1B1	0.601804938	0.407180182	0.542911632	0.036814206	0.663081857	0.322840224	0.817081815
ATP1B3	0.212982125	0.775472658	0.087539366	0.864171824	0.260234243	0.323016063	0.817081815
CCNE2	0.281494732	0.536537908	0.711638687	0.497808604	0.060534588	0.322451514	0.817081815
CDK7	0.320846885	0.459439281	0.986142529	0.067428519	0.329411982	0.321999521	0.817081815
FAHD2A	0.339219646	0.624917791	0.699486292	0.138299952	0.157663792	0.322196839	0.817081815
GJC1	0.691372313	0.678643536	0.257494732	0.090004236	0.299083253	0.323049494	0.817081815
HOXB9	0.133072054	0.877152425	0.168569949	0.388095646	0.424037365	0.322414187	0.817081815
LIPT1	0.730944597	0.30709159	0.266379872	0.109629956	0.493491281	0.322272451	0.817081815
LOC101905818	0.28656804	0.040628831	0.907062002	0.615025891	0.500098234	0.322871682	0.817081815
LOC101905876	0.866704937	0.546473703	0.562300394	0.063758195	0.190002543	0.321884107	0.817081815
LOC112443339	0.420945425	0.226025562	0.536395638	0.645171451	0.098165078	0.322151112	0.817081815
LOC782566	0.140696967	0.183496341	0.168281263	0.875584419	0.848680513	0.321979913	0.817081815
LSM4	0.342077675	0.452461817	0.826110605	0.082078865	0.30991782	0.323064836	0.817081815
PARVA	0.923279533	0.091895813	0.138286781	0.380768455	0.726183341	0.322693394	0.817081815
PPP1CA	0.606822798	0.319511964	0.24430417	0.140775445	0.484362117	0.322042543	0.817081815
PSMD3	0.239688514	0.187219792	0.569134777	0.663773759	0.191842161	0.32304988	0.817081815

RAB37	0.602720721	0.976495904	0.091259205	0.64802573	0.092595761	0.321730697	0.817081815
RAC3	0.701823599	0.192584241	0.843200987	0.030345563	0.939466397	0.322908121	0.817081815
RAP1B	0.495069035	0.324594118	0.066060041	0.52360548	0.584728103	0.322957545	0.817081815
RUNX1	0.879154121	0.285128598	0.128841874	0.377813338	0.266209129	0.322876816	0.817081815
SRP14	0.780037753	0.165128449	0.448036698	0.410966742	0.137077918	0.322998492	0.817081815
SRRM1	0.958755727	0.246426463	0.18593143	0.103298402	0.716561231	0.323021923	0.817081815
TOB2	0.966388088	0.874432037	0.770713482	0.190215505	0.026205256	0.32279012	0.817081815
VSIG2	0.573237303	0.408538335	0.870488245	0.225238862	0.070841934	0.32307866	0.817081815
ZFAND4	0.18022455	0.549603374	0.177406508	0.238153243	0.775886326	0.32281785	0.817081815
ZKSCAN4	0.032399004	0.456499535	0.493296825	0.693416125	0.640407183	0.322496558	0.817081815
LOC112447340	0.287412768	0.058240996	0.770087394	0.26332179	0.959407998	0.323247062	0.81725582
TIMM17B	0.669345684	0.231770825	0.82529607	0.027615674	0.920891951	0.323219613	0.81725582
LOC101907247	0.44980965	0.045987611	0.879395626	0.378929687	0.472654336	0.323311331	0.817292399
RNF103	0.228051767	0.465843163	0.208128433	0.485055846	0.304052565	0.323442053	0.817496924
DNPEP	0.841973072	0.800681725	0.262177002	0.051650835	0.357847154	0.323705291	0.81759337
HSF2BP	0.624599273	0.789262956	0.236291664	0.030397185	0.921870007	0.323586547	0.81759337
NAA20	0.799618335	0.17463449	0.457090899	0.147193668	0.347592006	0.32365369	0.81759337
PPP1R18	0.157370218	0.831297407	0.494053165	0.080053706	0.630690987	0.323544867	0.81759337
TCEA2	0.503039926	0.698053414	0.379679459	0.47787085	0.051283808	0.323729311	0.81759337
ABCC9	0.414562812	0.705876377	0.180670548	0.262458532	0.23575023	0.323904353	0.817768648
PDXP	0.320631937	0.424086693	0.851759099	0.053512841	0.527974853	0.323948204	0.817768648
SCO1	0.54076578	0.681460299	0.049346602	0.554694341	0.324226468	0.323867345	0.817768648
SNX4	0.72341505	0.654350044	0.023769541	0.319792037	0.909945871	0.324033112	0.817857183
MKLN1	0.858051558	0.319971121	0.759488641	0.382676177	0.041066153	0.324154066	0.817910883
PMPCB	0.716518874	0.556183391	0.268341334	0.117574452	0.260611938	0.324147196	0.817910883
ASB13	0.925783775	0.988384517	0.653401069	0.140677676	0.039044649	0.324471106	0.818086092
HRH4	0.909620733	0.071943052	0.259198858	0.660066292	0.29328165	0.324455164	0.818086092
LOC783033	0.753149222	0.317551629	0.027758453	0.894365649	0.553436137	0.324562504	0.818086092
M1AP	0.162201914	0.845359672	0.10557256	0.338553088	0.670414128	0.324544045	0.818086092
MANBAL	0.210704906	0.133261152	0.22008847	0.585544416	0.908789314	0.324672154	0.818086092
MRC2	0.555889634	0.160475311	0.200138602	0.209244697	0.879847596	0.324601708	0.818086092
VSIG4	0.350067809	0.462908527	0.117107487	0.405742844	0.427022544	0.324650287	0.818086092
YTHDC2	0.080497384	0.967212412	0.573524824	0.260847748	0.282092456	0.324549412	0.818086092
ZDBF2	0.145525884	0.748855981	0.733527179	0.130963408	0.313584835	0.324423167	0.818086092
EIF4A1	0.611457707	0.10049617	0.397352061	0.553111067	0.243918223	0.324924864	0.818363284
SECISBP2L	0.631189359	0.260779588	0.156351117	0.324009005	0.395072788	0.324931763	0.818363284
SMIM4	0.342423927	0.256251029	0.530444167	0.195565319	0.361736171	0.324859158	0.818363284
CDC42EP1	0.369207021	0.724659237	0.386863794	0.05270504	0.604670391	0.325122123	0.818468398
LOC101904332	0.754208953	0.925835899	0.35592752	0.016798929	0.790072234	0.325123118	0.818468398
PIK3IP1	0.149885843	0.172149273	0.554834415	0.541817166	0.425178529	0.32509532	0.818468398

LOC613460	0.184526669	0.291233386	0.162631285	0.925536132	0.408035327	0.325210812	0.818563595
CDH15	0.959148767	0.107127531	0.766082344	0.132852756	0.315855294	0.325320746	0.81871473
CYP27B1	0.008705066	0.779490544	0.624877057	0.911250194	0.855657195	0.325453666	0.81892366
PCDH7	0.60517448	0.19186167	0.262349599	0.281049397	0.386607072	0.325618035	0.819211647
ANK3	0.015097967	0.778332475	0.560696499	0.95500781	0.527047189	0.325910695	0.81947381
CREB3	0.32357177	0.266115879	0.29723198	0.162777462	0.795727153	0.325852404	0.81947381
DENND1C	0.463221179	0.110521198	0.600067476	0.216004211	0.499810031	0.325921977	0.81947381
SEC24D	0.359410963	0.831693282	0.14833696	0.089161343	0.838331572	0.32581892	0.81947381
KMT2E	0.309899375	0.518177712	0.575043756	0.11721292	0.306810763	0.326106253	0.819560446
PSAP	0.824226122	0.402591829	0.012613363	0.996511549	0.795846995	0.326041258	0.819560446
TMEM11	0.314154821	0.105467639	0.648770376	0.447538748	0.345161141	0.326092765	0.819560446
RNH1	0.409708225	0.318879274	0.855483571	0.049182544	0.604519668	0.326203526	0.819679385
AGBL2	0.711509665	0.033732117	0.225815268	0.801997575	0.766237507	0.326535073	0.820261302
PSPN	0.692408948	0.284974571	0.074984556	0.801374293	0.280818738	0.326497151	0.820261302
PTCD1	0.718381644	0.078649388	0.301696533	0.912640756	0.214339247	0.326707107	0.820567849
CCDC8	0.260215734	0.90245408	0.183983441	0.308881618	0.250161209	0.32688553	0.820890348
CLGN	0.268432045	0.355497097	0.769069268	0.421416421	0.108039692	0.327014646	0.820989155
LOC104975027	0.255901424	0.697496217	0.961064385	0.170899618	0.113986361	0.327024929	0.820989155
LOC101902029	0.173985019	0.628549294	0.258358023	0.29423185	0.402154876	0.327091037	0.821029521
HMGN1	0.437877653	0.129787188	0.813580736	0.521027846	0.138844583	0.327165223	0.821090147
DNM1	0.536215812	0.155074499	0.350421529	0.216103063	0.531422944	0.327231934	0.821131997
FLOT1	0.362054793	0.219289879	0.29503592	0.86919917	0.164482814	0.327345675	0.821257524
STOML2	0.651023915	0.110527684	0.782464855	0.236382599	0.251690342	0.327382044	0.821257524
IP6K1	0.465571126	0.75464479	0.874069679	0.011550851	0.944828231	0.327458869	0.821324699
MIER1	0.213879997	0.843377267	0.586237601	0.155605386	0.203755235	0.327512306	0.821333198
GPD2	0.224898578	0.730600466	0.450663364	0.176706909	0.256340912	0.327577151	0.821369019
KLHDC4	0.079167861	0.093853267	0.784028056	0.881982084	0.653050499	0.327626689	0.821369019
ADRB2	0.108057615	0.446503436	0.161403018	0.668287085	0.645251042	0.327744448	0.821482113
CD300LF	0.685966569	0.264790133	0.969937962	0.784744193	0.024294801	0.32778059	0.821482113
CFAP44	0.864850896	0.249218187	0.309680949	0.68751341	0.073245351	0.327884595	0.821482113
COPS8	0.887208903	0.035976345	0.24176951	0.446419924	0.975876129	0.327913788	0.821482113
RBM38	0.161467195	0.215957685	0.805956373	0.144389882	0.828525567	0.327922084	0.821482113
COX4I1	0.642085996	0.417562874	0.576423665	0.130179638	0.167580789	0.328335242	0.821926403
DZIP1L	0.720349041	0.791982632	0.025000066	0.59362161	0.399522216	0.328819885	0.821926403
FAM155B	0.470513844	0.012235394	0.783296718	0.771343798	0.972297638	0.328790463	0.821926403
HDGFL3	0.289142778	0.380972945	0.439615337	0.22814664	0.305507891	0.328502254	0.821926403
KCNK7	0.492381858	0.068287076	0.345213385	0.614426308	0.474404776	0.328852727	0.821926403
KIFAP3	0.161375358	0.281427391	0.332319513	0.88357993	0.252697804	0.328261401	0.821926403
LOC101904377	0.362043107	0.727275149	0.289500995	0.770274153	0.057624762	0.328858346	0.821926403
LOC101905498	0.030198522	0.482415242	0.414788521	0.896929037	0.622461698	0.328430342	0.821926403

LOC104973105	0.143701653	0.446059885	0.260989202	0.384329072	0.525774529	0.32872804	0.821926403
MECOM	0.447048614	0.849368399	0.048382653	0.242115417	0.761154254	0.328950863	0.821926403
MRPL46	0.228657439	0.26907707	0.634051531	0.154021358	0.562225629	0.328625372	0.821926403
MTCH2	0.80245483	0.295735567	0.397637853	0.11460526	0.312952905	0.328903176	0.821926403
NSD1	0.977706122	0.332381756	0.046749649	0.758418075	0.293517473	0.32879135	0.821926403
PHC3	0.043846404	0.261616269	0.901995589	0.917591008	0.356224239	0.328794498	0.821926403
RFWD3	0.232864382	0.81597432	0.202184384	0.175701503	0.500941193	0.328765137	0.821926403
SMYD2	0.94079352	0.590340485	0.241997359	0.98981416	0.025331802	0.328268368	0.821926403
SOAT1	0.749573642	0.573073262	0.203792942	0.986652928	0.039163653	0.328823541	0.821926403
LOC112442032	0.366153717	0.485069844	0.045152496	0.470947174	0.89797204	0.329205572	0.822312427
NMT2	0.112103746	0.368719142	0.311574059	0.762091531	0.345467457	0.329173894	0.822312427
SH2D4A	0.423625097	0.629080527	0.723721647	0.088346074	0.199162751	0.329297565	0.822417037
AP1S1	0.623764381	0.322191201	0.689120768	0.118748395	0.207505002	0.330125514	0.82242597
AP3B2	0.555768807	0.589413141	0.350857217	0.249821072	0.119085147	0.330413671	0.82242597
CCDC30	0.913249588	0.142080574	0.444398169	0.070321493	0.842166925	0.330226694	0.82242597
DNAJC18	0.125204594	0.103213706	0.684108735	0.557464352	0.692638912	0.330166709	0.82242597
FAM69C	0.502387567	0.079213999	0.859472457	0.485849734	0.204802788	0.329725072	0.82242597
HIGD2A	0.320624724	0.465692611	0.515373303	0.066045613	0.671469144	0.330126334	0.82242597
KIF15	0.844932091	0.092730803	0.08147818	0.790626114	0.675979285	0.3300934	0.82242597
L2HGDH	0.663837682	0.267477441	0.853644197	0.135609389	0.16559436	0.329742318	0.82242597
LOC100337053	0.185021637	0.401976932	0.972567472	0.547947991	0.086277073	0.330429726	0.82242597
LOC100848315	0.710308078	0.098838261	0.199614154	0.404515917	0.602811138	0.330328077	0.82242597
LOC101902551	0.630679476	0.030824332	0.982373807	0.260702333	0.68403587	0.329824644	0.82242597
LOC518623	0.488800475	0.877375685	0.31876799	0.135648793	0.183484871	0.329690162	0.82242597
MIEF1	0.838434149	0.212394434	0.595304713	0.130564554	0.246098764	0.329852982	0.82242597
MRFAP1L1	0.752953723	0.182042132	0.138404445	0.507547956	0.355206833	0.330453772	0.82242597
PCBP2	0.788516765	0.393178584	0.367047948	0.190605269	0.1566875	0.329514712	0.82242597
PNPLA4	0.068978043	0.78310988	0.455475449	0.336283388	0.411053607	0.329620117	0.82242597
PPM1K	0.207017156	0.757625631	0.493355834	0.153802149	0.285531632	0.329496008	0.82242597
PPP1R7	0.535844105	0.311711846	0.124372042	0.388538083	0.423663022	0.330426503	0.82242597
PTPDC1	0.682165921	0.35540107	0.308951519	0.156672828	0.290781819	0.33011669	0.82242597
RNF13	0.080395388	0.568510157	0.463677791	0.19466724	0.824587146	0.329658483	0.82242597
ST6GALNAC3	0.817990716	0.336679514	0.444260742	0.96181439	0.028961298	0.329930034	0.82242597
TMC6	0.659368584	0.414629102	0.429897045	0.073279043	0.395534505	0.329863381	0.82242597
UBL4A	0.318710262	0.189886768	0.69461225	0.087852208	0.926005799	0.33043704	0.82242597
ACCS	0.366930569	0.257238704	0.23555341	0.240119225	0.641633229	0.330683967	0.822783559
LOC112445063	0.183721632	0.160059922	0.230087532	0.863226997	0.586719615	0.330741373	0.822783559
LRIG2	0.158523739	0.66885621	0.653258464	0.937879192	0.052753539	0.33074786	0.822783559
ALDH7A1	0.849121557	0.324728218	0.488906917	0.113036244	0.225277753	0.331000979	0.822818751
DOCK5	0.863062843	0.276297019	0.09201285	0.347747398	0.450147565	0.331080813	0.822818751

FAM177A1	0.858742029	0.098169409	0.289810923	0.857211393	0.163946655	0.331032406	0.822818751
LRP3	0.100008595	0.942738157	0.336640976	0.116986227	0.924652501	0.331020294	0.822818751
MRPS11	0.389082801	0.512597753	0.677475039	0.187412898	0.135665232	0.331112975	0.822818751
SLC39A3	0.599010203	0.253878537	0.30776785	0.239812098	0.305554279	0.330861281	0.822818751
SNAP91	0.95927618	0.129957332	0.472232465	0.062671808	0.930105549	0.330950666	0.822818751
ALKBH2	0.017584989	0.759221377	0.580023027	0.566586404	0.784151232	0.331332168	0.82323879
LOC101904768	0.947419494	0.161594601	0.874008205	0.851479416	0.030230939	0.331499183	0.823526932
LOC104968820	0.012750364	0.944566593	0.476245065	0.716895951	0.837940067	0.3315485	0.823526932
RSPO3	0.404238151	0.708361336	0.375848893	0.202666689	0.158046458	0.331622959	0.823587226
RAB14	0.815092063	0.08515278	0.329991266	0.16594491	0.907595613	0.331722694	0.823710263
ASPHD2	0.875406774	0.108076553	0.342044753	0.259105204	0.412091049	0.331972157	0.824205004
SH3PXD2B	0.642138909	0.166991921	0.199790002	0.168885196	0.95640299	0.332190152	0.824603545
ZNF385B	0.538753204	0.420534551	0.612114064	0.177795479	0.140382117	0.332233175	0.824603545
STYX	0.963997627	0.952790133	0.460577065	0.040219512	0.20357883	0.332331943	0.824723954
CDK18	0.333917421	0.313528908	0.526716694	0.117259229	0.537697874	0.332891716	0.82473825
ENO4	0.935154107	0.738400318	0.277302249	0.250954246	0.072295734	0.332774025	0.82473825
FAM169A	0.154726237	0.340457464	0.877143754	0.1966579	0.381595662	0.332492016	0.82473825
LOC100139996	0.161138008	0.336595925	0.206915495	0.330706319	0.936571708	0.332858911	0.82473825
LOC112445912	0.66384949	0.28644025	0.141123679	0.201080794	0.6424228	0.332451837	0.82473825
LOC112448038	0.149359493	0.345838789	0.418704744	0.60940617	0.263121885	0.332514553	0.82473825
LOC522763	0.506276135	0.745444337	0.510002717	0.892199025	0.020242689	0.332866359	0.82473825
PATL2	0.819672987	0.243738434	0.051301731	0.613150747	0.552449386	0.332678346	0.82473825
RPL36A	0.925279476	0.773891923	0.258415368	0.774904307	0.024255071	0.332940766	0.82473825
SLC16A5	0.606900145	0.267060821	0.8384455	0.047741455	0.535286605	0.332722154	0.82473825
TERF2IP	0.447975464	0.104889943	0.36008167	0.488592291	0.420355545	0.332814051	0.82473825
UBE2K	0.713825464	0.276001298	0.272733122	0.200029763	0.323548852	0.332924764	0.82473825
DDB2	0.145008614	0.179007697	0.690449678	0.451182837	0.431383899	0.333383752	0.824963926
FAM198B	0.955984396	0.069053196	0.845671684	0.308121916	0.202698949	0.333313918	0.824963926
LOC101906717	0.451058571	0.418309115	0.058683111	0.867250638	0.363245546	0.33337493	0.824963926
LOC112441650	0.829956034	0.087966704	0.354450883	0.611488682	0.220427936	0.333373626	0.824963926
LOC786733	0.473478109	0.841045071	0.080774957	0.649202411	0.167009629	0.333350649	0.824963926
TMEM37	0.225166955	0.225094921	0.124688944	0.628082671	0.877260858	0.333119601	0.824963926
TSPAN13	0.473572984	0.384832629	0.128111252	0.7321485	0.20390122	0.333264052	0.824963926
PIGY	0.586580412	0.071376812	0.390460365	0.345241538	0.61846082	0.333480797	0.825079657
TSLP	0.764356608	0.577959198	0.821863975	0.048519896	0.198243642	0.333554707	0.825138122
LOC101908104	0.509509467	0.037867291	0.551779334	0.591094552	0.555325746	0.333648888	0.825179936
USP32	0.484487199	0.690209278	0.261951229	0.086359561	0.46201977	0.333672175	0.825179936
COMMD3	0.922525438	0.218001404	0.602778343	0.075776133	0.381168584	0.333943521	0.825400243
GNL3L	0.605359882	0.154116376	0.532745993	0.27502037	0.256037573	0.333877021	0.825400243
LOC782293	0.34360418	0.676967429	0.407116356	0.296909096	0.124546533	0.33396244	0.825400243

SRSF6	0.845613953	0.274233711	0.17903489	0.208804915	0.403731122	0.333881287	0.825400243
ANKRD37	0.912717829	0.24311413	0.11270689	0.755707419	0.185620816	0.3342296	0.82540611
FUBP3	0.090234678	0.531785661	0.334759879	0.30230016	0.721731854	0.334085357	0.82540611
LOC613677	0.711378002	0.503044724	0.568863503	0.073977241	0.233008612	0.33426659	0.82540611
MAP10	0.092637953	0.909256415	0.299989665	0.947941801	0.146376667	0.334146303	0.82540611
PTPN23	0.746985146	0.302314708	0.118007162	0.736439854	0.178688078	0.334172871	0.82540611
THRAP3	0.993608852	0.512510606	0.128707666	0.428650738	0.124871925	0.334234253	0.82540611
CAMK4	0.432567799	0.301091782	0.124232493	0.869989224	0.249536796	0.334421794	0.825468293
KIAA1522	0.441991362	0.532916366	0.295492504	0.370196108	0.136393175	0.334492971	0.825468293
LOC618787	0.870013579	0.036611363	0.14368278	0.869796787	0.882601677	0.334453571	0.825468293
RBM33	0.105127649	0.146229969	0.768270837	0.693497872	0.428692306	0.334360522	0.825468293
USP14	0.822582185	0.268894619	0.305246795	0.688835687	0.07561175	0.334587066	0.825576357
TNNT1	0.068799013	0.540526273	0.9723511	0.46293487	0.210177464	0.334659529	0.825631018
ASPM	0.334968003	0.184677362	0.112849053	0.583157396	0.865334653	0.334851001	0.825731773
ATXN2	0.794503082	0.958961143	0.92041536	0.182249194	0.027635123	0.335237405	0.825731773
CCSER2	0.620814987	0.17185853	0.254946451	0.477291672	0.271557901	0.334969288	0.825731773
CD72	0.293539181	0.184877659	0.273505482	0.612724031	0.387547717	0.334927336	0.825731773
HERC4	0.547346361	0.73544698	0.077154838	0.391598865	0.290642129	0.335364574	0.825731773
LBH	0.067283217	0.856340586	0.699445725	0.872549772	0.100376702	0.335142027	0.825731773
LOC104970966	0.628151585	0.068653118	0.487393151	0.333540248	0.504078807	0.335321207	0.825731773
RASSF8	0.702264976	0.268830976	0.780011493	0.052946403	0.452574703	0.335099902	0.825731773
SIRT3	0.202875816	0.697914506	0.313911915	0.502430525	0.158274142	0.335347193	0.825731773
SLC4A3	0.670769676	0.727984932	0.146968032	0.808306415	0.060854349	0.335161273	0.825731773
TIGD5	0.60174401	0.449508968	0.715308314	0.063032407	0.289925375	0.33540479	0.825731773
TSTD1	0.75276278	0.040077119	0.889212014	0.301009478	0.436441957	0.334913564	0.825731773
UFSP2	0.988766665	0.02667048	0.897642691	0.489497654	0.30457775	0.335123616	0.825731773
WDR44	0.20797603	0.736353336	0.123411018	0.931297958	0.200543313	0.335148873	0.825731773
SS18L2	0.398775767	0.128551988	0.559287151	0.85674257	0.144005288	0.335465983	0.825758549
GCH1	0.306444025	0.399123521	0.132051019	0.252227039	0.870735931	0.335881975	0.826249436
LOC101902407	0.524697245	0.450233836	0.046327376	0.455427823	0.711333136	0.335812643	0.826249436
LOC101904239	0.10190166	0.130734736	0.443332345	0.710343778	0.844829856	0.335765175	0.826249436
MYH7B	0.486837096	0.818803815	0.088006047	0.242342816	0.417324777	0.335917143	0.826249436
PSMC6	0.722480031	0.218911009	0.373182586	0.547292678	0.109809589	0.335880727	0.826249436
LCA5	0.655574368	0.028420056	0.706609741	0.500542917	0.539362915	0.336182185	0.8263763
LOC783541	0.439520188	0.85126594	0.09680053	0.383062751	0.25607926	0.336119146	0.8263763
NRK	0.588597328	0.690079186	0.715734668	0.130586371	0.09364678	0.336220496	0.8263763
SFSWAP	0.843900882	0.084433429	0.241040222	0.469190815	0.441118617	0.336199903	0.8263763
ZNF385A	0.042011845	0.534161976	0.364187178	0.441189755	0.985154325	0.336096007	0.8263763
DIS3L2	0.888862867	0.232116547	0.704570464	0.644911323	0.038048872	0.336719949	0.826971427
DNTTIP2	0.901654229	0.094615059	0.507882465	0.692089575	0.118993241	0.336768647	0.826971427

HSDL1	0.935216632	0.027362352	0.350302225	0.593813372	0.670540298	0.336815369	0.826971427
LOC101907152	0.12713543	0.76746353	0.845816032	0.558380773	0.077423131	0.336752532	0.826971427
LRRC61	0.117650834	0.122923993	0.756384265	0.495272149	0.657991271	0.336627107	0.826971427
NDUFS2	0.799225954	0.402119209	0.85541778	0.06545568	0.198161681	0.336672508	0.826971427
NUF2	0.66189615	0.102575094	0.137358373	0.85172673	0.448533121	0.336538566	0.826971427
ALOX5AP	0.278829322	0.096590567	0.402913017	0.675263173	0.489734014	0.337622223	0.827217023
IER5L	0.682616374	0.689568685	0.137875984	0.069171156	0.797109483	0.337196268	0.827217023
LOC101903616	0.645326664	0.835987553	0.098890204	0.193967419	0.346029586	0.337296108	0.827217023
LOC788634	0.915437097	0.942481177	0.015519981	0.692443247	0.386905865	0.337575212	0.827217023
MEDAG	0.409505611	0.466795235	0.798788529	0.144753679	0.162220219	0.337495946	0.827217023
MEFV	0.152592877	0.068661722	0.687307616	0.657801728	0.756656996	0.337441131	0.827217023
NRIP2	0.131467875	0.645095546	0.290024367	0.461452999	0.316117255	0.337600106	0.827217023
NUCKS1	0.051255277	0.094553626	0.764113458	0.983893298	0.984599685	0.337575364	0.827217023
NUP62CL	0.134907424	0.502578226	0.22919979	0.53864564	0.428064608	0.337396884	0.827217023
PANX1	0.577188518	0.521492151	0.19975835	0.228597196	0.260997752	0.337573894	0.827217023
RAB18	0.586410795	0.149769475	0.13728372	0.576544423	0.513976957	0.336966431	0.827217023
S100B	0.251690994	0.590034609	0.704207219	0.0950402	0.360975514	0.337592105	0.827217023
SFN	0.656816171	0.269762006	0.827468008	0.2612718	0.093800982	0.337815262	0.827217023
TES	0.1159903	0.703756486	0.40669435	0.6139545	0.176258077	0.337788162	0.827217023
TGFBR3	0.40620651	0.120392806	0.893626268	0.111995066	0.733256442	0.337636173	0.827217023
TMEM147	0.733437464	0.643938938	0.716464136	0.069799125	0.152004032	0.337687785	0.827217023
TMOD2	0.214734281	0.128794521	0.992685078	0.454658548	0.287184069	0.3374625	0.827217023
USP49	0.121228457	0.95850485	0.098975708	0.566048612	0.551970715	0.337822709	0.827217023
STUB1	0.18983252	0.472597989	0.61373002	0.086155832	0.758210794	0.337967103	0.827447132
RGS20	0.419231843	0.166452207	0.253424318	0.315088761	0.645795761	0.338038314	0.827498026
LRRN2	0.54133351	0.078518087	0.383853389	0.987151503	0.223533287	0.338109566	0.827549007
LRRC4C	0.709903759	0.548780897	0.22460886	0.901601389	0.045652847	0.338172502	0.827579619
AP2S1	0.525235356	0.45413704	0.893574694	0.114230172	0.148310947	0.338559779	0.828280343
LOC514257	0.155453591	0.156927806	0.72201486	0.385072537	0.532333889	0.338541648	0.828280343
RABL3	0.724809872	0.207151994	0.399071421	0.233131861	0.258761882	0.338712274	0.828529906
CS	0.769811884	0.413053625	0.577277857	0.083207255	0.236744362	0.338764622	0.828534457
MAMLD1	0.730036502	0.140983184	0.566385464	0.08104729	0.765676316	0.33883081	0.828572853
TTC1	0.743610779	0.135182838	0.272501567	0.251331538	0.525814354	0.338937596	0.828710501
UBE3A	0.393902834	0.218549731	0.419954506	0.348989589	0.287165874	0.339066358	0.828901831
LOC615271	0.99881325	0.126755711	0.369234224	0.799889235	0.096942745	0.339139905	0.828958144
CINP	0.639273469	0.096957606	0.956093146	0.495501377	0.123504289	0.339208159	0.829001504
CENPBD1	0.214346482	0.465016638	0.324760889	0.248534763	0.451487317	0.339446087	0.82901711
PATL1	0.397348728	0.151704669	0.985956822	0.135992771	0.449466553	0.339467124	0.82901711
PIGN	0.235931035	0.539659913	0.240308753	0.538020773	0.220629118	0.339430615	0.82901711
PRKCZ	0.405097428	0.128236169	0.124802545	0.797774013	0.701860424	0.339356827	0.82901711

WRB	0.759679358	0.025521002	0.303055757	0.698991889	0.884267224	0.339420046	0.82901711
RBM10	0.657494607	0.053146776	0.197561314	0.79665185	0.660962856	0.339562517	0.829126688
CD99	0.939674946	0.187325635	0.784404623	0.638579815	0.041341684	0.339979912	0.829158916
ESPNL	0.971809291	0.056007845	0.173725804	0.490409873	0.785715669	0.339910594	0.829158916
FAM110A	0.135608298	0.114983736	0.876633472	0.381717349	0.698220825	0.339895507	0.829158916
MARCH8	0.894556521	0.106636979	0.608454433	0.273988289	0.229117225	0.339915654	0.829158916
NAT10	0.299897399	0.461074528	0.354169541	0.719094331	0.10336363	0.3397681	0.829158916
SHTN1	0.322980023	0.396396574	0.316505919	0.34103699	0.263491218	0.339818939	0.829158916
TMEM208	0.687577272	0.238536149	0.498658621	0.240217606	0.185326811	0.339807516	0.829158916
TRIM4	0.647184554	0.062621021	0.483559582	0.37418128	0.497041853	0.339964256	0.829158916
ACBD3	0.955867053	0.40878316	0.608127964	0.016972431	0.912966823	0.341499223	0.82937449
ALG9	0.614236661	0.196366691	0.624989919	0.089468438	0.544753854	0.341172531	0.82937449
BCKDK	0.402544448	0.495844688	0.962608882	0.026317464	0.727119687	0.3412814	0.82937449
BTLA	0.059523211	0.559556749	0.735720619	0.741257035	0.20239041	0.3412615	0.82937449
C18H19orf54	0.723474925	0.469457667	0.072550597	0.294221684	0.503462917	0.340183125	0.82937449
CBX8	0.80729587	0.763349347	0.739269825	0.012204966	0.660198209	0.341041336	0.82937449
CDIPT	0.498678028	0.279492103	0.824825033	0.145808593	0.219535136	0.341413594	0.82937449
CTTNBP2	0.860136908	0.35596785	0.167516035	0.38072827	0.188549902	0.341496321	0.82937449
ENO3	0.467153385	0.069977274	0.898631318	0.800926156	0.155735085	0.34076532	0.82937449
FAAP100	0.209260252	0.553337176	0.454136349	0.959838511	0.072880002	0.341354588	0.82937449
FBL	0.594613862	0.906243496	0.073939136	0.636816764	0.144918199	0.341292691	0.82937449
FES	0.51375183	0.608957977	0.599148608	0.050660058	0.386328909	0.340946705	0.82937449
FKBP14	0.651907776	0.152376827	0.474481955	0.406166938	0.192182696	0.341380451	0.82937449
FMOD	0.40405493	0.129036926	0.227633162	0.354677856	0.873166425	0.341233243	0.82937449
FUNDC2	0.808959175	0.503740246	0.412282003	0.088517039	0.245671151	0.340324132	0.82937449
GPATCH8	0.060367392	0.186757855	0.887675679	0.755501251	0.487257147	0.341584436	0.82937449
LOC101905706	0.77516564	0.647567396	0.053335233	0.902100562	0.151692877	0.340743333	0.82937449
LOC537017	0.791226395	0.903930352	0.529266761	0.068945066	0.141037038	0.341451213	0.82937449
MDFIC2	0.624477408	0.891990103	0.974234433	0.225784905	0.029820887	0.340340488	0.82937449
MTG2	0.973513704	0.747030825	0.570165391	0.011424245	0.777565464	0.341555472	0.82937449
NOC2L	0.146380007	0.437695285	0.799608461	0.704039822	0.101420767	0.340514171	0.82937449
OGFOD1	0.313302282	0.675082551	0.938533034	0.343633806	0.053878969	0.341220743	0.82937449
OGG1	0.234425637	0.135965366	0.709976139	0.644842969	0.250375983	0.340329389	0.82937449
PARP3	0.726354522	0.068165217	0.124892004	0.760527673	0.781280368	0.341179949	0.82937449
RIC8B	0.436158107	0.058460491	0.48973433	0.293243487	0.998797132	0.340485472	0.82937449
SCPEP1	0.615502786	0.204650093	0.208647885	0.389448086	0.357317951	0.34048121	0.82937449
SIRT2	0.308839343	0.423272737	0.341679773	0.464931612	0.176792013	0.341060114	0.82937449
SPON1	0.519838628	0.160982436	0.350496959	0.14187147	0.884956443	0.341521439	0.82937449
TMEM205	0.220323909	0.212947336	0.31808695	0.851009742	0.289096084	0.34107144	0.82937449
TSSK3	0.196860059	0.046350324	0.630717243	0.955943021	0.668904077	0.341414351	0.82937449

LIG4	0.261987635	0.077662395	0.269299117	0.872469334	0.770921741	0.341639489	0.829385451
DCUN1D1	0.841608557	0.300573892	0.575642748	0.263830511	0.095998165	0.341749445	0.829407002
DGKA	0.578045007	0.402357512	0.263300834	0.094179692	0.639291643	0.341707579	0.829407002
ESRP2	0.661339246	0.471000523	0.096244111	0.300339539	0.409893034	0.341854227	0.829538625
LOC112448863	0.291490573	0.255585792	0.56236058	0.116711001	0.75613616	0.342128034	0.830080302
CARS	0.446683711	0.299772636	0.754293146	0.056464107	0.649559413	0.342419687	0.830496117
CPSF3	0.928327182	0.129395229	0.855864632	0.669429777	0.053914743	0.342668771	0.830496117
HHLA2	0.291418669	0.024498832	0.727981123	0.908220404	0.785914792	0.342638252	0.830496117
IPO5	0.453441941	0.875640914	0.13060027	0.338205963	0.211408175	0.342549296	0.830496117
LOC112442280	0.025022377	0.453183768	0.471656773	0.839442324	0.826644455	0.342704266	0.830496117
MED24	0.331803284	0.575320064	0.072001755	0.458126774	0.589231025	0.342657449	0.830496117
NOTCH3	0.626270579	0.949154091	0.090798629	0.226184066	0.303770884	0.342581186	0.830496117
PCYT1B	0.533276357	0.331535652	0.3691323	0.815671281	0.069566788	0.342370167	0.830496117
ECI2	0.785818343	0.318459933	0.544331709	0.902077459	0.030285125	0.343114881	0.830877721
FNDC3A	0.359300527	0.206356048	0.309779373	0.717869004	0.225500009	0.342977014	0.830877721
KCTD2	0.436324939	0.565736785	0.100406617	0.591693674	0.253613643	0.343024893	0.830877721
KRT8	0.460047052	0.306517505	0.367410192	0.199665916	0.359686781	0.34308809	0.830877721
PDE8A	0.662604497	0.487170218	0.077104044	0.365296972	0.408942541	0.342977472	0.830877721
ACADS	0.351358604	0.726016876	0.607983036	0.058671614	0.410093766	0.343529658	0.831306414
ADCY3	0.393782496	0.424555773	0.265878648	0.360489406	0.234249633	0.344421044	0.831306414
AP4E1	0.568897283	0.340092829	0.041612558	0.560816242	0.831477562	0.344449362	0.831306414
BCAT2	0.299055583	0.394785289	0.903096029	0.188430091	0.186875149	0.344456987	0.831306414
CLCC1	0.188426605	0.466090214	0.391182989	0.468665906	0.232386207	0.343937659	0.831306414
COPG1	0.316637026	0.217425478	0.3455406	0.473161064	0.331569926	0.343549029	0.831306414
EPM2A	0.883397119	0.163866898	0.57807056	0.051284305	0.872665436	0.344075762	0.831306414
GAMT	0.715363716	0.398900068	0.695021358	0.295996571	0.063944227	0.344431957	0.831306414
LOC101902221	0.94269521	0.085580566	0.096343597	0.902340076	0.534090904	0.344108828	0.831306414
LOC112448153	0.582288715	0.498650629	0.327139346	0.844551614	0.046692269	0.344103212	0.831306414
LOC515828	0.807196871	0.033674382	0.896958907	0.46644692	0.329024405	0.3439433	0.831306414
LOC787102	0.014319477	0.995280919	0.422224442	0.686147756	0.905473544	0.343812961	0.831306414
LYSMD3	0.458804642	0.691195674	0.136905008	0.130480804	0.662413637	0.344378297	0.831306414
MARCH6	0.500809477	0.186985573	0.383514394	0.162715369	0.640083822	0.343889301	0.831306414
NDP	0.313568343	0.13269914	0.70692629	0.160140869	0.795555147	0.344176132	0.831306414
NFIC	0.813670295	0.381883739	0.076064069	0.512592029	0.308746153	0.343891	0.831306414
PCGF6	0.134254065	0.620394721	0.465166562	0.465812028	0.20675945	0.343522704	0.831306414
PDIA3	0.522942969	0.756859544	0.031743518	0.415398454	0.717079776	0.343969571	0.831306414
PNKP	0.686830449	0.131953812	0.081349402	0.516587707	0.982527255	0.343954688	0.831306414
PREP	0.966032503	0.469628425	0.078647055	0.954600155	0.110198129	0.344413424	0.831306414
SNX10	0.19577304	0.130597128	0.338605417	0.45795354	0.943350126	0.343870753	0.831306414
TMEM50A	0.782207698	0.021967581	0.891013748	0.281138693	0.866660584	0.343480137	0.831306414

TOLLIP	0.161543955	0.952271805	0.110051461	0.453736522	0.486973878	0.343899494	0.831306414
LOC112445029	0.631125728	0.059297316	0.153225857	0.768177127	0.853174257	0.344608905	0.831550764
CDKN2AIPNL	0.253095944	0.208365276	0.235893447	0.600244906	0.504890999	0.345089479	0.831719993
EIF3G	0.614156143	0.340756972	0.060307309	0.988309205	0.301974956	0.34495083	0.831719993
FDXR	0.065847119	0.838365186	0.507295426	0.653125642	0.206252622	0.345185843	0.831719993
ISOC1	0.480563919	0.363407441	0.520941495	0.056463923	0.733592318	0.345021855	0.831719993
ITGA4	0.676450133	0.048447406	0.946138993	0.299000922	0.406894237	0.345181684	0.831719993
KPNB1	0.872430383	0.35998787	0.808056539	0.05798127	0.256213863	0.345088855	0.831719993
LOC101909718	0.117127187	0.57296114	0.728952436	0.20443988	0.376327406	0.344830283	0.831719993
PCSK7	0.065468859	0.607059771	0.945999158	0.354735362	0.282398819	0.344939278	0.831719993
TOB1	0.171442818	0.674089599	0.204196561	0.661955742	0.241137539	0.344958496	0.831719993
TRAPPC1	0.320575879	0.129480336	0.696003223	0.169014785	0.771039874	0.344877241	0.831719993
TMEM104	0.238466131	0.294772545	0.943280042	0.145480949	0.391264618	0.3452574	0.831770286
LOC789337	0.437286752	0.491420334	0.777834435	0.041571021	0.544429943	0.34561134	0.832500764
TTYH2	0.048956026	0.805211518	0.343709193	0.476758435	0.585990324	0.345701534	0.832595814
BTBD6	0.124638351	0.523774112	0.336622056	0.987669087	0.174494528	0.345784236	0.832672796
PURB	0.817833579	0.32194995	0.070529071	0.386985315	0.527474053	0.345919667	0.83287671
MRPS12	0.569700657	0.372589168	0.308195346	0.464827988	0.124700155	0.345970702	0.832877392
C19H17orf80	0.818660981	0.153350204	0.197252757	0.48899159	0.313374985	0.346081392	0.833021667
CD9	0.268871159	0.364588164	0.108147657	0.932561131	0.384229657	0.346241282	0.833162124
NPW	0.784767488	0.799899012	0.111038153	0.303330868	0.179639013	0.346217927	0.833162124
SMIM17	0.280422408	0.055589913	0.803932918	0.81613922	0.371839563	0.346422824	0.833476758
LOC539009	0.04309976	0.960810852	0.320270922	0.411339423	0.698168714	0.346648878	0.833898379
VNN2	0.739348372	0.587643129	0.355777511	0.068167319	0.361616114	0.346710203	0.833923662
CDKN2AIP	0.2995633	0.405478768	0.251086453	0.132456632	0.944147997	0.346861016	0.834041924
EIF3M	0.270002734	0.97662885	0.200756891	0.30041842	0.23980439	0.346846258	0.834041924
CDKN2C	0.286030377	0.173495896	0.798971911	0.258520412	0.372951992	0.347208484	0.834514515
H2AFX	0.299075732	0.861392097	0.039337272	0.990409378	0.381003177	0.347260961	0.834514515
LOC786065	0.729110191	0.215806463	0.065682067	0.623221377	0.593661664	0.347245511	0.834514515
TAF9	0.944321098	0.046924735	0.747469564	0.76034053	0.151784935	0.347198342	0.834514515
FABP3	0.300229387	0.46987752	0.273819635	0.622605257	0.159113576	0.347364122	0.834518022
PIGF	0.640318385	0.243136916	0.554758052	0.452766553	0.097831338	0.347321968	0.834518022
JOSD2	0.147785844	0.610531063	0.976462411	0.174654302	0.24890468	0.347500133	0.834526386
LOC783045	0.370909443	0.119695294	0.119262351	0.946115038	0.764642158	0.347514945	0.834526386
SPATA6L	0.867554064	0.815097742	0.677397436	0.127780452	0.06258224	0.347520159	0.834526386
CEP89	0.56206723	0.120533261	0.332272862	0.594921179	0.286227013	0.347624053	0.834531647
LOC112449318	0.226077833	0.984250684	0.389833424	0.374942636	0.117838526	0.347601078	0.834531647
FBP2	0.5310054	0.068444596	0.37585301	0.85157412	0.330187953	0.347934223	0.834816367
HIST1H2AC	0.262556839	0.685692276	0.48419367	0.745056418	0.059144275	0.347946131	0.834816367
SDHAF2	0.280191489	0.251435416	0.557418268	0.180382995	0.541860404	0.34783047	0.834816367

TAF1	0.505212887	0.093355414	0.528935375	0.20554408	0.748767103	0.347873728	0.834816367
LOC101906756	0.583897528	0.386851586	0.828083133	0.032871924	0.625100982	0.348036668	0.834911528
ACAD10	0.268429959	0.770589524	0.086976281	0.633381727	0.339700837	0.349126606	0.834993028
ADAMTS20	0.794597371	0.943783366	0.034961552	0.842937331	0.176088849	0.349947296	0.834993028
ANKRD34A	0.111418758	0.207098869	0.260896234	0.655637862	0.983289688	0.349526958	0.834993028
ARID2	0.246271424	0.563271014	0.677718144	0.067251652	0.61216593	0.349103839	0.834993028
CDH4	0.815158512	0.667144464	0.116812392	0.110165194	0.555535707	0.349795095	0.834993028
CEP350	0.561433668	0.583488451	0.832410759	0.015711647	0.898825872	0.348331435	0.834993028
CSMD2	0.05108648	0.135987344	0.945850037	0.685706325	0.862772119	0.349778733	0.834993028
CTSZ	0.19202235	0.625553958	0.229258847	0.264517989	0.531073651	0.349033149	0.834993028
DENND2A	0.303979663	0.197407827	0.407694439	0.227714795	0.69261462	0.348635365	0.834993028
FAM173A	0.171920774	0.632799812	0.793402233	0.070376242	0.63869905	0.349477832	0.834993028
GRWD1	0.921920507	0.221663032	0.037032473	0.783055573	0.654241498	0.349368088	0.834993028
HOMER1	0.414649156	0.294183148	0.870140754	0.719238735	0.050906023	0.349732487	0.834993028
ITGA10	0.394012365	0.131799656	0.598946501	0.540166939	0.231326275	0.349744802	0.834993028
LOC101903424	0.685160575	0.152401459	0.347045816	0.129550447	0.825465087	0.34929942	0.834993028
LOC101905997	0.998410164	0.314421708	0.774048766	0.043056954	0.370979861	0.349539026	0.834993028
LOC107132486	0.874229556	0.521731773	0.116316361	0.94375868	0.077310831	0.349125833	0.834993028
LOC614531	0.022102325	0.962858043	0.47368751	0.716626226	0.534754071	0.348816331	0.834993028
LOC781989	0.925939514	0.337299654	0.062561592	0.492703133	0.401591791	0.348935562	0.834993028
LOC786015	0.550896607	0.231411092	0.725890982	0.523721624	0.079409141	0.348236503	0.834993028
MRPL50	0.811422573	0.032580343	0.815342968	0.667863512	0.269064238	0.34922194	0.834993028
NUDC	0.643406634	0.242972084	0.154967163	0.572156167	0.280585141	0.349849451	0.834993028
PA2G4	0.868728628	0.64161719	0.225340134	0.155258976	0.197507636	0.348357414	0.834993028
PDCD4	0.387991902	0.070685298	0.808815986	0.293678807	0.591217429	0.348351074	0.834993028
POU2F2	0.58113959	0.258348283	0.178636364	0.976291258	0.148297812	0.349605026	0.834993028
RAMP1	0.226728214	0.511855653	0.867907137	0.042159899	0.912269232	0.349243913	0.834993028
SELENOS	0.767239951	0.023721954	0.386573524	0.767366397	0.715338805	0.348777256	0.834993028
SORL1	0.789565012	0.736749226	0.013280256	0.656790213	0.76060821	0.348661899	0.834993028
SPATA20	0.12565075	0.266196627	0.350277052	0.401769727	0.823282642	0.349299266	0.834993028
SRP68	0.716063477	0.122248175	0.594323249	0.811201643	0.091292104	0.348406611	0.834993028
SSR4	0.901913545	0.348194797	0.212565116	0.094103895	0.615003552	0.348825318	0.834993028
TMCO4	0.878701893	0.649557919	0.211797393	0.222871387	0.144108305	0.349588514	0.834993028
TMEM204	0.786509336	0.26137545	0.324526006	0.390037695	0.148744301	0.349108238	0.834993028
TRAP1	0.398289811	0.534826191	0.797629859	0.123779051	0.184937302	0.349857976	0.834993028
USF3	0.44236152	0.330306798	0.640715479	0.108566023	0.382446296	0.349765797	0.834993028
USO1	0.657662839	0.031481117	0.869603848	0.921362439	0.232446522	0.34853009	0.834993028
VNN1	0.485777761	0.599788037	0.079089875	0.261928571	0.642615375	0.349435337	0.834993028
WDR46	0.91177458	0.283994691	0.175548749	0.54064109	0.158361458	0.349953205	0.834993028
LOC101909754	0.246958253	0.600278398	0.0398951	0.893711559	0.736689326	0.350032356	0.835060473

LOC112446761	0.946001536	0.720145682	0.165238802	0.497523248	0.069570997	0.350134426	0.835182567
SELENOV	0.062145093	0.341640505	0.885361434	0.22961887	0.903141332	0.35020321	0.835225242
ABRAXAS2	0.38513081	0.130916538	0.197199054	0.422456811	0.934392158	0.351252112	0.835269897
C2H2orf88	0.089270883	0.656822423	0.392330568	0.869377848	0.196124461	0.351156738	0.835269897
CNRIP1	0.347920567	0.188623926	0.75978304	0.212109408	0.368852544	0.350316229	0.835269897
DLK2	0.891867296	0.841984078	0.15126863	0.146098132	0.235341119	0.350499131	0.835269897
EEF1D	0.657061244	0.214858078	0.80169409	0.433873078	0.079826123	0.351058722	0.835269897
ENDOD1	0.6501739	0.061541326	0.115950035	0.88774209	0.948893646	0.350596988	0.835269897
GYS1	0.488071481	0.74728686	0.859395912	0.0347805	0.360107543	0.351292205	0.835269897
KRI1	0.615896394	0.08644731	0.461726867	0.713184985	0.223348338	0.350900995	0.835269897
LOC101903557	0.129026653	0.915045042	0.132752891	0.550449295	0.455169612	0.35133634	0.835269897
LOC101905179	0.958428066	0.597637002	0.017544236	0.895372312	0.435987238	0.35117753	0.835269897
LOC112444847	0.052566607	0.455093288	0.64551083	0.560066871	0.452529701	0.35082004	0.835269897
LUC7L3	0.88289117	0.467633124	0.203438251	0.105232536	0.441479929	0.350361167	0.835269897
NIPAL2	0.217259435	0.767414131	0.030383757	0.853582924	0.90508052	0.350813644	0.835269897
NMB	0.832476451	0.298374888	0.136997194	0.330232487	0.348422058	0.350880475	0.835269897
PLEKHA8	0.564764119	0.357956952	0.305379411	0.538495444	0.117764215	0.350866892	0.835269897
PTGDR	0.564544576	0.859782011	0.188183183	0.741243083	0.057857282	0.350956625	0.835269897
SPRED2	0.804634118	0.354952756	0.248754049	0.404916652	0.13631379	0.351119243	0.835269897
STPG1	0.712206529	0.278994718	0.122818	0.507301952	0.315901803	0.350707206	0.835269897
TCF21	0.055903652	0.741640928	0.806716874	0.189621481	0.618987807	0.351289558	0.835269897
TGDS	0.161195098	0.799407559	0.37620098	0.962692221	0.084148043	0.351341667	0.835269897
TIA1	0.78180078	0.269703939	0.278077975	0.094894721	0.704666439	0.351094781	0.835269897
TUBE1	0.664531751	0.411700077	0.764086054	0.806460804	0.023155469	0.3504213	0.835269897
ADGRD1	0.403220345	0.364256292	0.0820231	0.469044202	0.695877664	0.351540737	0.835380109
TRARG1	0.047524143	0.799883825	0.210419768	0.738614198	0.665289792	0.351478228	0.835380109
U2SURP	0.820038376	0.904226895	0.016251734	0.472040415	0.691189482	0.351524856	0.835380109
LRCH4	0.124299491	0.1748402	0.998813931	0.19713241	0.920209639	0.35175651	0.835708442
PELI1	0.600601235	0.100566127	0.393698557	0.362439271	0.45710363	0.351831676	0.835708442
TCP11	0.200490919	0.108003901	0.285962833	0.825318435	0.770740997	0.351803994	0.835708442
BRD8	0.93389267	0.128216417	0.202531483	0.879036911	0.185031446	0.352021416	0.835979282
LOC101904698	0.153287255	0.310332246	0.899179616	0.228264849	0.40496223	0.352393604	0.835979282
LOC107131239	0.784996263	0.65976828	0.13561585	0.158030541	0.355567757	0.352109209	0.835979282
LOC112443484	0.592201923	0.071899544	0.129683687	0.814282403	0.878006317	0.352152251	0.835979282
NFKBIZ	0.479675404	0.275297795	0.12404781	0.756034521	0.319287375	0.35240416	0.835979282
RHOBTB2	0.506992391	0.52350684	0.104259453	0.506121469	0.282175642	0.352315589	0.835979282
SH3TC2	0.60075369	0.419529677	0.54379643	0.739028512	0.039012438	0.352296482	0.835979282
TMUB2	0.994134829	0.232057218	0.481292307	0.403852966	0.088172826	0.352384489	0.835979282
TTC21A	0.275592783	0.062232716	0.631693171	0.592059063	0.615691388	0.352211569	0.835979282
MFSD14B	0.118903273	0.234751944	0.466562202	0.328167045	0.925824529	0.35250143	0.83608917

ADAM17	0.36907618	0.645288083	0.080412272	0.631946879	0.327824097	0.352920226	0.836499344
CD59	0.742177483	0.048691983	0.329800546	0.363298641	0.915238759	0.352742792	0.836499344
CKMT2	0.321580903	0.689932771	0.874821229	0.97819658	0.020897592	0.352929222	0.836499344
PRR12	0.53825059	0.240978109	0.0549393	0.58831683	0.945983036	0.352858451	0.836499344
TNFRSF6B	0.696997393	0.291051917	0.061075728	0.969103749	0.330339925	0.352880428	0.836499344
ARID4B	0.650627764	0.412926306	0.158625611	0.103163593	0.904350366	0.353250644	0.836722913
CCR6	0.549561343	0.451253669	0.108549986	0.412112772	0.358610098	0.353344536	0.836722913
FBXO30	0.219003092	0.163635697	0.739333998	0.275078624	0.546411887	0.353501503	0.836722913
IFT43	0.695065359	0.381709704	0.614092419	0.565188525	0.043261408	0.353551598	0.836722913
KIAA0232	0.978802867	0.942912983	0.047021404	0.288375179	0.317960757	0.353376075	0.836722913
LIPJ	0.948112184	0.838627083	0.361642319	0.61974874	0.022373048	0.353680619	0.836722913
LUC7L	0.841667012	0.095105839	0.310613843	0.312122649	0.514111488	0.353788331	0.836722913
MAP2K4	0.645772363	0.011768267	0.911892005	0.611070182	0.941825499	0.353733447	0.836722913
MSRB3	0.155876275	0.255358066	0.418389021	0.834841296	0.285869621	0.353193736	0.836722913
RORA	0.855838749	0.215231546	0.542536007	0.050610008	0.788052928	0.35363414	0.836722913
RPLP1	0.585862561	0.68949232	0.024742477	0.667622514	0.596811064	0.353498026	0.836722913
SEMA4G	0.931663529	0.413950937	0.33305342	0.523915964	0.059274966	0.353754086	0.836722913
SH2D1B	0.988139633	0.040932996	0.75472204	0.296420055	0.440459382	0.353624914	0.836722913
SLF2	0.279531724	0.683567318	0.313634184	0.280539558	0.236381563	0.353180094	0.836722913
TBC1D8B	0.300480557	0.389669458	0.145973003	0.332961716	0.700775785	0.353719825	0.836722913
TEX9	0.071975186	0.292357029	0.216209375	0.94614543	0.927641638	0.353915984	0.836904209
YIF1A	0.696085199	0.033719302	0.870864823	0.251233285	0.778076957	0.354016698	0.837021758
EMC2	0.591356598	0.148816451	0.102281294	0.724354015	0.613885104	0.354281734	0.837286511
PXYLP1	0.517427368	0.406079649	0.117500072	0.241515347	0.671205971	0.354269151	0.837286511
ZNF383	0.05216505	0.877503221	0.297135463	0.922743455	0.318782249	0.354218726	0.837286511
C11H2orf42	0.297245817	0.896565892	0.169026919	0.796660702	0.111681881	0.354487036	0.837569987
C17H12orf49	0.223145478	0.18823093	0.214122269	0.603945278	0.738028969	0.354523425	0.837569987
CXCR2	0.273441733	0.409612597	0.904457485	0.172501239	0.229446441	0.354554793	0.837569987
TSFM	0.406925531	0.252001446	0.855513605	0.238330197	0.191832479	0.354607012	0.837572781
ASB16	0.319802802	0.280414142	0.794242592	0.878839486	0.06437852	0.355335588	0.837914069
CCDC9B	0.226171195	0.74571811	0.748274935	0.043828918	0.727823977	0.355183489	0.837914069
COPS5	0.702608178	0.174897989	0.598853106	0.260246103	0.210442449	0.355353459	0.837914069
IFITM1	0.100077176	0.965031262	0.897728786	0.52962422	0.087512056	0.354896643	0.837914069
LOC112446795	0.371517515	0.205256233	0.228476442	0.398105821	0.579290813	0.354880552	0.837914069
LOC529792	0.529767567	0.137774578	0.206471352	0.870912664	0.306490632	0.355056936	0.837914069
NOL7	0.490120575	0.085774999	0.525061774	0.273613622	0.667350798	0.355364202	0.837914069
PID1	0.079814955	0.382792512	0.344645288	0.567178833	0.673518185	0.355050979	0.837914069
POLR3G	0.907898468	0.374800907	0.256306923	0.761930769	0.060624676	0.355291734	0.837914069
SCARF1	0.11274468	0.3747921	0.481039754	0.818220789	0.242276751	0.355322652	0.837914069
SRP54	0.471562603	0.074243938	0.784466511	0.328395764	0.44646556	0.355219217	0.837914069

TTC30B	0.95816156	0.786742147	0.944156389	0.075461309	0.074916291	0.3550961	0.837914069
NABP2	0.238053803	0.333372763	0.732449058	0.517747742	0.134067681	0.355528291	0.838180545
ADIPOR1	0.396164563	0.307195352	0.271597834	0.348775058	0.350126444	0.355586188	0.838196629
CCDC102B	0.964645023	0.315022891	0.702016704	0.665054111	0.028464743	0.355670022	0.838273838
HIF1AN	0.814959955	0.144014319	0.350275211	0.189174637	0.519574613	0.355756159	0.838356451
ANGPTL1	0.10421901	0.457077429	0.680437376	0.664267343	0.188149938	0.356153206	0.838617076
ATP6V0A4	0.765600612	0.854794198	0.023076562	0.620841869	0.431915306	0.356096896	0.838617076
DOCK6	0.439320077	0.18197766	0.126919614	0.711084456	0.560929165	0.356004761	0.838617076
HDHD5	0.285151714	0.298950113	0.894953395	0.062592999	0.84836296	0.356157045	0.838617076
LOC112445972	0.05662412	0.699101584	0.343974918	0.671618937	0.442835202	0.35610372	0.838617076
LOC787309	0.282597958	0.413029567	0.344700178	0.907263041	0.110994966	0.356173361	0.838617076
CCDC102A	0.988068629	0.31965427	0.016088563	0.85242787	0.935806123	0.356245039	0.838665519
FARP2	0.374318775	0.356557932	0.457838712	0.48158524	0.138227896	0.356791113	0.838868046
IGIP	0.798158678	0.632852625	0.343739899	0.314679372	0.074393605	0.356673793	0.838868046
LOC101905151	0.110855105	0.27507013	0.72541027	0.600293181	0.305951244	0.356592409	0.838868046
LOC112442298	0.11625401	0.526263692	0.433382075	0.3017757	0.508184441	0.356731932	0.838868046
PTOV1	0.303534548	0.090670616	0.956696699	0.24070033	0.641118303	0.356615826	0.838868046
SNHG12	0.297519062	0.595507396	0.061676257	0.795340957	0.46771768	0.35668506	0.838868046
SUPT16H	0.364096344	0.238856744	0.409895955	0.114631051	0.994807282	0.356689213	0.838868046
TST	0.069671307	0.419343116	0.793823871	0.179936127	0.972661591	0.356459639	0.838868046
UIMC1	0.627206426	0.19849465	0.808984822	0.104881009	0.385018959	0.356764441	0.838868046
ZNF532	0.928111591	0.644503007	0.137144479	0.587701441	0.084423218	0.356887526	0.83897453
CC2D2B	0.646782911	0.753076311	0.100924427	0.944822881	0.087717976	0.357034732	0.839080194
KIAA0895L	0.876306951	0.632288708	0.095833305	0.089062766	0.861240068	0.356990187	0.839080194
UMPS	0.941456576	0.364964068	0.513115186	0.903620842	0.025597509	0.357184025	0.839310857
CDK12	0.110277908	0.324949005	0.708172647	0.192681948	0.835680102	0.357497774	0.839327035
KIAA0040	0.703071755	0.327817061	0.094590324	0.805408296	0.232690367	0.35747928	0.839327035
KMT5B	0.059330813	0.657082892	0.807593699	0.368923292	0.351345759	0.35729697	0.839327035
LOC101907965	0.180854898	0.241187662	0.949118641	0.243691311	0.404836448	0.357426579	0.839327035
LRRC34	0.569390724	0.317308944	0.641642992	0.045338249	0.776812875	0.357369829	0.839327035
S1PR3	0.256017754	0.733806913	0.491754381	0.103300698	0.427473993	0.357242781	0.839327035
LOC112442851	0.337987988	0.077828576	0.487901207	0.967163246	0.329336358	0.357565134	0.8393651
EPHB1	0.101436682	0.794406825	0.69042658	0.131968105	0.557849377	0.357862932	0.839662926
LANCL3	0.269925183	0.947306122	0.160187072	0.233639458	0.428223505	0.357947829	0.839662926
RCN1	0.925545793	0.152839033	0.114364024	0.425550686	0.59458769	0.357772252	0.839662926
STK4	0.692878247	0.590023156	0.857300486	0.070455143	0.165873113	0.357865198	0.839662926
WDR11	0.786096819	0.628302122	0.217973258	0.823451852	0.046214425	0.35790677	0.839662926
CEP164	0.658918106	0.285532479	0.099160732	0.60689418	0.362639534	0.358248401	0.839767821
IGHMBP2	0.620277441	0.12525896	0.103701595	0.89317167	0.570521235	0.358238406	0.839767821
PDIA4	0.515743849	0.976177298	0.052752831	0.330101219	0.468315558	0.358241018	0.839767821

ZBTB1	0.636159407	0.579147867	0.07903046	0.845368825	0.166628582	0.358079886	0.839767821
ZFYVE26	0.69652721	0.024469721	0.663965629	0.828948437	0.437287738	0.358102108	0.839767821
HDGF	0.368013079	0.563657099	0.542858949	0.77939629	0.04685305	0.358480749	0.839876054
NFKBIA	0.808724231	0.548074012	0.0794698	0.15554552	0.750508483	0.358478398	0.839876054
ODF2	0.920948978	0.389318997	0.376420681	0.229306757	0.1328867	0.358499285	0.839876054
TAF10	0.730920426	0.232114858	0.644593014	0.398146059	0.094447324	0.35849127	0.839876054
AEN	0.752799209	0.542523461	0.124021148	0.215757516	0.377148248	0.358843852	0.839982234
AMDHD1	0.173582815	0.252041031	0.220552108	0.512371556	0.833336589	0.358780275	0.839982234
APCDD1	0.871962785	0.025204781	0.646531943	0.305715877	0.948624125	0.358812054	0.839982234
DCTN3	0.435954266	0.166278756	0.657562179	0.135756104	0.637719473	0.359035243	0.839982234
EXT2	0.803294274	0.22200976	0.131203532	0.48362714	0.36451799	0.358969892	0.839982234
GATM	0.491702402	0.210102185	0.902741097	0.57447051	0.077087363	0.359158816	0.839982234
LOC104973229	0.296199911	0.266250706	0.423049253	0.46998814	0.263283739	0.359097837	0.839982234
LOC614141	0.351117548	0.08436238	0.529438052	0.483965983	0.544024892	0.359123941	0.839982234
PDK4	0.491857433	0.289519	0.259586474	0.187456736	0.594630074	0.358798844	0.839982234
PRPF4B	0.362421714	0.595184384	0.131656847	0.242466339	0.598703521	0.358880176	0.839982234
SLCO2B1	0.322786747	0.580498502	0.311491224	0.12812773	0.551088672	0.35882795	0.839982234
SNX15	0.778119988	0.044499465	0.911537124	0.198793531	0.657213324	0.35892041	0.839982234
FRMPD1	0.948335555	0.878671302	0.153477289	0.052886966	0.610988079	0.359254874	0.840021666
MID2	0.565955301	0.684131369	0.353151946	0.034048454	0.888251384	0.359363761	0.840021666
TIMM50	0.750452182	0.182501828	0.650400324	0.40921185	0.11346022	0.359380423	0.840021666
ZNHIT2	0.799475902	0.273419289	0.837912361	0.08943175	0.252337881	0.359288458	0.840021666
SDHD	0.996965782	0.193801644	0.688457333	0.064498911	0.482319072	0.359466363	0.840102888
EIF3I	0.577975831	0.341885557	0.381270832	0.779550219	0.070535956	0.359638152	0.840277476
THAP4	0.075898768	0.48572277	0.925391189	0.399407164	0.304038819	0.35964347	0.840277476
BRINP1	0.316510736	0.922225666	0.20778758	0.840254118	0.0815388	0.360121458	0.840620869
LOC101902030	0.741403937	0.148243317	0.203198747	0.21556309	0.863098931	0.360109138	0.840620869
LOC790037	0.700965989	0.144773206	0.0689778	0.996948925	0.594570962	0.359887554	0.840620869
MYOM3	0.13042978	0.478520856	0.426178782	0.622879432	0.250556033	0.359962217	0.840620869
NFATC3	0.38826116	0.169586126	0.466229419	0.14389808	0.940865822	0.360149006	0.840620869
SCD5	0.429060491	0.631380308	0.694836801	0.894794593	0.024653345	0.360003027	0.840620869
UEVLD	0.472374124	0.202645871	0.245434763	0.707671561	0.249709777	0.359979841	0.840620869
ADI1	0.169288243	0.885000719	0.43737938	0.269210188	0.23576264	0.36025663	0.840640386
RCAN3	0.675558852	0.157471592	0.537235085	0.48970123	0.148608301	0.360259816	0.840640386
MOSPD2	0.674162539	0.414715583	0.090935356	0.314877732	0.51982103	0.36034616	0.840722324
ERP44	0.12300564	0.680868115	0.741253278	0.212559185	0.315680339	0.360504293	0.84082238
IQCH	0.729275806	0.30109233	0.37423941	0.253662649	0.199869292	0.360526495	0.84082238
RPH3AL	0.561020192	0.439193076	0.775037617	0.031498845	0.692687431	0.360542751	0.84082238
ABI3	0.927102646	0.228373186	0.214202261	0.20721349	0.446240726	0.361551907	0.841205288
ANKRD24	0.883650456	0.104676361	0.211535643	0.250641994	0.853968974	0.361343976	0.841205288

BRD2	0.769791325	0.398091218	0.351645195	0.086294186	0.45069646	0.361458614	0.841205288
CDH11	0.634192405	0.310262873	0.118143279	0.396574243	0.452618431	0.360769271	0.841205288
CEP152	0.407663954	0.358950231	0.138117484	0.468034858	0.44176411	0.36099924	0.841205288
COASY	0.435704384	0.493734779	0.433739504	0.079594435	0.566503616	0.362063667	0.841205288
DAZAP1	0.878161722	0.044465277	0.168612909	0.966479769	0.662437686	0.362361757	0.841205288
EFCAB2	0.569151822	0.175794162	0.752158781	0.917196863	0.061140766	0.36254682	0.841205288
ENC1	0.10528361	0.702824305	0.844136985	0.072431959	0.929373325	0.361969973	0.841205288
FASTKD1	0.792207813	0.403408725	0.611490212	0.27738387	0.077020329	0.360857276	0.841205288
FPGS	0.770689238	0.750321714	0.270376585	0.041456546	0.649970264	0.36227434	0.841205288
GPR132	0.15253877	0.362739056	0.616679715	0.177217241	0.693180386	0.361480463	0.841205288
GRIN1	0.619234224	0.920562788	0.550961625	0.682230421	0.019610582	0.361865266	0.841205288
GSKIP	0.923665427	0.219603666	0.326881025	0.842347922	0.075096076	0.361576666	0.841205288
HILPDA	0.622178749	0.906302214	0.459276684	0.73308881	0.022228609	0.362545356	0.841205288
INPP5A	0.373632575	0.543566409	0.854830328	0.130460489	0.186142841	0.362390152	0.841205288
KCNC4	0.792158611	0.266369356	0.424971052	0.125689745	0.372759932	0.361841244	0.841205288
LOC101904477	0.795393313	0.609394269	0.017893042	0.575267318	0.843690797	0.362142313	0.841205288
LOC511531	0.076127738	0.962596847	0.772035176	0.112074833	0.665781332	0.362593372	0.841205288
LOC784357	0.962471472	0.348055561	0.048396516	0.912495431	0.282338904	0.360926376	0.841205288
METTL6	0.16117178	0.2819543	0.924967132	0.487932515	0.205771268	0.362547898	0.841205288
MPG	0.527041418	0.378913323	0.529438051	0.061491414	0.647078488	0.362053252	0.841205288
NR2C2	0.973061797	0.426264028	0.880154803	0.019989292	0.57756351	0.3623441	0.841205288
OAZ2	0.485452414	0.550813787	0.356675583	0.083293477	0.527224049	0.361352083	0.841205288
PIGX	0.835583258	0.723577114	0.18070062	0.153533223	0.251026657	0.362192557	0.841205288
RNF115	0.518346106	0.293848781	0.11867871	0.337660918	0.69166345	0.36260351	0.841205288
SAFB2	0.470948596	0.272407369	0.89675688	0.037643213	0.969211394	0.36169213	0.841205288
SGCA	0.104018663	0.068311992	0.918504251	0.781408447	0.826817107	0.36241705	0.841205288
TAF4	0.731668922	0.185427926	0.97880541	0.089005283	0.354981684	0.36163271	0.841205288
TMCC3	0.75402638	0.494455387	0.304138591	0.256556434	0.144295028	0.361709186	0.841205288
TXLNA	0.433721497	0.266828048	0.736967669	0.121644483	0.404966878	0.361847735	0.841205288
USF2	0.152385163	0.672906609	0.169064227	0.805142267	0.302010421	0.362368859	0.841205288
ZDHHC4	0.623524294	0.075181574	0.812691546	0.12117196	0.909114991	0.361670165	0.841205288
ZFYVE9	0.225450581	0.078479541	0.335219245	0.880510702	0.80425638	0.361798259	0.841205288
ZNF423	0.143678504	0.703301829	0.086521611	0.484758324	0.991884507	0.361935128	0.841205288
ZNF507	0.684044747	0.576687595	0.415045714	0.029765367	0.865845659	0.362524108	0.841205288
ZSCAN30	0.9818095	0.074021724	0.419568594	0.421373869	0.327675813	0.362172658	0.841205288
LOC107131944	0.234050833	0.656533806	0.210956055	0.244984352	0.532348951	0.362821395	0.841392122
REEP4	0.118188959	0.640616185	0.29204724	0.219640869	0.870335397	0.362797386	0.841392122
ZSWIM5	0.883896403	0.563278748	0.950735069	0.277970974	0.032133334	0.362837856	0.841392122
ABCC10	0.958530694	0.137921036	0.195351174	0.979732389	0.168538557	0.36418634	0.841449958
ACHE	0.614031119	0.333080448	0.661275726	0.059655552	0.525443109	0.363257746	0.841449958

ACTL6A	0.66527536	0.01737408	0.683468242	0.713989938	0.751396348	0.363215745	0.841449958
B3GALNT2	0.173802827	0.993736379	0.168573811	0.279503841	0.522837693	0.36382842	0.841449958
C18H19orf81	0.517829694	0.317479268	0.828253193	0.689450088	0.045180275	0.363336913	0.841449958
DEFB4A	0.423330034	0.16010597	0.167241329	0.917588127	0.409808169	0.364113511	0.841449958
DVL1	0.056586134	0.582808683	0.589320827	0.26137182	0.834977367	0.363338419	0.841449958
EMC10	0.379933992	0.913885221	0.374453679	0.037585803	0.871642095	0.364005134	0.841449958
EPS15	0.398611516	0.361845023	0.584137362	0.102018835	0.493930052	0.363487732	0.841449958
GHITM	0.681824026	0.683542989	0.309635498	0.161066096	0.182915236	0.363708772	0.841449958
HAUS4	0.600196935	0.603468853	0.13726636	0.144923563	0.591060955	0.363977593	0.841449958
IFT122	0.789985189	0.185362295	0.886684148	0.071970174	0.45637707	0.364195908	0.841449958
IQCC	0.464726988	0.694701342	0.021217765	0.742721925	0.837356542	0.364031403	0.841449958
KRR1	0.321962747	0.738817548	0.766420578	0.023757128	0.979986763	0.36344818	0.841449958
LOC107131660	0.052765223	0.829780257	0.226390813	0.872429215	0.492428	0.363962571	0.841449958
LONP1	0.339111159	0.69705711	0.651074837	0.180632476	0.152889333	0.363662168	0.841449958
LRIF1	0.292556371	0.918809962	0.916275436	0.028751138	0.601090285	0.363894889	0.841449958
LRRC57	0.221474796	0.249573052	0.248045023	0.886067989	0.349151008	0.363342613	0.841449958
LSM11	0.073973564	0.831830994	0.299050696	0.308021314	0.751475904	0.364002361	0.841449958
MCL1	0.797569871	0.349932637	0.033805417	0.85677031	0.525781304	0.36366023	0.841449958
NBEAL2	0.291501081	0.131889091	0.610754357	0.908590282	0.199179352	0.363630856	0.841449958
NCOA6	0.265124211	0.635642698	0.834281305	0.147878959	0.204780118	0.363935914	0.841449958
NRARP	0.577234653	0.568017623	0.514471515	0.026099341	0.963830386	0.363405249	0.841449958
NSF	0.174251848	0.366184754	0.406180328	0.240891147	0.682383676	0.364036364	0.841449958
UBE2B	0.866877671	0.529497284	0.398505347	0.833695014	0.027924007	0.363961856	0.841449958
UNC79	0.214210239	0.628218244	0.710568846	0.172215958	0.258724915	0.364044554	0.841449958
DGCR8	0.458073491	0.033482678	0.431708301	0.84178221	0.76538996	0.364247664	0.841451072
ELP5	0.215427474	0.690780606	0.081481442	0.548710921	0.64202132	0.364452679	0.841499587
FBXO17	0.194511821	0.413891732	0.317853254	0.194506281	0.858345701	0.364475297	0.841499587
LEKR1	0.056865275	0.125959395	0.768768597	0.890031849	0.870865409	0.36432087	0.841499587
NAPEPLD	0.099470082	0.294032676	0.379657618	0.781940429	0.491839981	0.364410817	0.841499587
TNFAIP8L1	0.054422718	0.299003092	0.501809802	0.685517763	0.763446355	0.364525048	0.841499587
SLITRK5	0.163840245	0.585236407	0.331895124	0.460175088	0.292218706	0.364739929	0.841877213
ADPGK	0.857928069	0.24116243	0.22000747	0.592369594	0.159160877	0.365191691	0.842323652
CD3G	0.146458889	0.174676403	0.87653171	0.652341144	0.293538653	0.365275545	0.842323652
GUK1	0.497896233	0.737348553	0.980470738	0.047369044	0.251783611	0.365243081	0.842323652
MAP2K7	0.032706168	0.420895764	0.592223233	0.79623729	0.66143497	0.365261603	0.842323652
NFIL3	0.955267732	0.118929989	0.721537087	0.062438694	0.839026583	0.365292635	0.842323652
NIPSNAP2	0.820614631	0.213328421	0.564280249	0.113844882	0.381369871	0.365088336	0.842323652
TDRD3	0.301559208	0.384250577	0.199503761	0.48525273	0.382362454	0.36510241	0.842323652
PIAS3	0.717536225	0.026507835	0.499440285	0.77676283	0.582403934	0.365405261	0.842464983
LBR	0.343770149	0.178472522	0.27794834	0.497676251	0.507210893	0.365668878	0.842954342

ANKRD63	0.515242293	0.548301588	0.129913673	0.471409046	0.249107258	0.365862134	0.843162966
LOC104975196	0.433377211	0.072833762	0.617506476	0.37312294	0.592474574	0.365822766	0.843162966
ARMC10	0.245924483	0.068394474	0.536370741	0.663241323	0.722173083	0.366273756	0.843600777
DCAF5	0.822963497	0.662800836	0.941882879	0.069151579	0.121631786	0.366277023	0.843600777
DOCK3	0.083095668	0.56212005	0.739381957	0.175475251	0.712630812	0.36618491	0.843600777
MBP	0.491044132	0.347683138	0.393864021	0.073816312	0.870753144	0.36630913	0.843600777
TMEM17	0.132374958	0.335964183	0.458723839	0.223379955	0.94816811	0.366266084	0.843600777
ZNF654	0.643152439	0.615470111	0.574523166	0.060383957	0.314850664	0.36636623	0.84361389
AFG3L2	0.851671691	0.387735652	0.478554323	0.084249295	0.325248786	0.366609074	0.843686713
ARHGAP45	0.216722594	0.183182555	0.994364831	0.301572691	0.364348886	0.366872373	0.843686713
CFL1	0.402980061	0.23247711	0.406898784	0.515995595	0.220342563	0.366745157	0.843686713
KAT14	0.81315083	0.354735466	0.233930083	0.11013358	0.583614592	0.366859313	0.843686713
KLF7	0.730313639	0.668109208	0.822384337	0.081971622	0.131694764	0.366661036	0.843686713
LOC112446033	0.0830127	0.686502801	0.746361697	0.1372271	0.741688168	0.366563992	0.843686713
MCM6	0.408476146	0.994469521	0.040785413	0.935556214	0.279874871	0.366891814	0.843686713
MPST	0.159594832	0.435332941	0.58236917	0.148598268	0.721605113	0.366911954	0.843686713
PUDP	0.837209474	0.237382474	0.175344627	0.129030983	0.963905912	0.36674857	0.843686713
SLC44A1	0.266660532	0.3890128	0.238201371	0.313188113	0.559550659	0.366605583	0.843686713
LOC100848939	0.281612102	0.614975708	0.233781404	0.33739998	0.317813411	0.367015666	0.843688764
ZNF346	0.944116894	0.904143273	0.063123837	0.274256305	0.293706619	0.36697565	0.843688764
AATF	0.789640721	0.175702351	0.547088589	0.622485705	0.092192171	0.367545272	0.843691768
ANGPT2	0.046346528	0.684787254	0.473908958	0.575601714	0.502571665	0.36736269	0.843691768
CLSPN	0.370983868	0.302211048	0.364646198	0.970307894	0.109942602	0.367736714	0.843691768
DNM1L	0.965635605	0.392444878	0.963505282	0.485554585	0.02459383	0.367699344	0.843691768
GNE	0.043182804	0.741926459	0.88918349	0.280345336	0.544153375	0.367176592	0.843691768
KDM2B	0.279029287	0.605243386	0.087218842	0.66175297	0.447001282	0.367584195	0.843691768
LOC101902385	0.725953939	0.259385339	0.293905663	0.100967794	0.779547288	0.367545123	0.843691768
LOC104973139	0.5977804	0.050262452	0.450675979	0.509262948	0.62992218	0.367104002	0.843691768
LOC785403	0.147570462	0.220383017	0.605849786	0.977227122	0.226424019	0.367681184	0.843691768
MRPL13	0.755440731	0.190916712	0.780573372	0.117215874	0.329810181	0.36740673	0.843691768
MYADM	0.786400997	0.059474396	0.573077088	0.494814238	0.328511089	0.36757855	0.843691768
NPLOC4	0.4083088	0.165502926	0.463153791	0.900020447	0.154623067	0.367529983	0.843691768
NRAS	0.737470636	0.091414973	0.188523962	0.568972659	0.602932332	0.367691433	0.843691768
UBXN4	0.824292127	0.093205119	0.724522548	0.36523091	0.21371235	0.367137277	0.843691768
FAM89B	0.04525686	0.488297403	0.680831495	0.72860365	0.397977553	0.367789935	0.843695921
IGFLR1	0.992791439	0.042610871	0.805266867	0.237477195	0.539769862	0.367932505	0.84390501
SCAF8	0.477566827	0.418615301	0.677989264	0.034482313	0.9346495	0.367994023	0.843928159
MPPED2	0.2529872	0.558941177	0.379620141	0.25807292	0.315581476	0.368122097	0.844103916
RAB36	0.582269847	0.472564221	0.152320421	0.293652762	0.355344473	0.368180219	0.844119247
ARHGAP17	0.72625038	0.531849722	0.054760757	0.260533506	0.795157814	0.368485288	0.844190445

CMBL	0.56426259	0.412694817	0.829058768	0.058769023	0.38576846	0.368305866	0.844190445
ERCC6	0.503158699	0.538874953	0.495486072	0.156137367	0.209016899	0.368577054	0.844190445
FAM161A	0.392751191	0.509230821	0.037042512	0.736812731	0.804991904	0.368931279	0.844190445
LOC101906512	0.454686778	0.295546565	0.474379055	0.677252702	0.101565699	0.368594922	0.844190445
LOC104970450	0.616213799	0.21562057	0.304503758	0.125013103	0.867556273	0.368706776	0.844190445
PPIL6	0.442672221	0.861592074	0.279363041	0.271525598	0.151935274	0.368982881	0.844190445
PRDX1	0.480728591	0.280418432	0.440577522	0.556109397	0.132695423	0.368515758	0.844190445
PRRG1	0.544166175	0.310627738	0.355674361	0.080571	0.904007704	0.368380584	0.844190445
RHOU	0.237118484	0.428164891	0.08281928	0.556479609	0.939190487	0.368942105	0.844190445
SERF1A	0.184713937	0.052957784	0.99496464	0.545301748	0.826599327	0.368670791	0.844190445
SSNA1	0.267825119	0.071609797	0.758944581	0.335516403	0.898526317	0.368710768	0.844190445
SUZ12	0.287121854	0.344466509	0.145453752	0.349804755	0.873255089	0.368939971	0.844190445
TMEM80	0.633469817	0.194003609	0.499769603	0.080441315	0.88957588	0.368962916	0.844190445
U2AF1	0.971966742	0.271632454	0.027620633	0.926805474	0.650092321	0.368912644	0.844190445
NOP14	0.769616846	0.459814937	0.663812625	0.596780556	0.031374179	0.369080892	0.844296977
ARIH2OS	0.47289077	0.448182534	0.411647188	0.105406861	0.479273309	0.369411354	0.844739086
DDHD1	0.796465249	0.099585363	0.556489392	0.491378866	0.203303553	0.369480053	0.844739086
NCAPG2	0.503168191	0.035247617	0.547022194	0.845603537	0.537461906	0.369473348	0.844739086
PRDM15	0.703927724	0.075079664	0.671302202	0.327964272	0.378870331	0.36944524	0.844739086
ABO	0.210555874	0.529534433	0.574821616	0.490413015	0.141319785	0.370645164	0.844744534
ACAT1	0.695555056	0.69314051	0.595104661	0.039081371	0.396187825	0.370666239	0.844744534
ATP12A	0.296717921	0.16820642	0.515343599	0.391517612	0.440635408	0.370481681	0.844744534
CD68	0.478385089	0.394185879	0.127175982	0.764407097	0.242317141	0.370657283	0.844744534
CXHXorf21	0.769282036	0.10264692	0.503743526	0.463834415	0.239430609	0.369774134	0.844744534
DCBLD1	0.403106381	0.476961337	0.717357999	0.041531008	0.777298667	0.371026665	0.844744534
DEFB13	0.819913678	0.803496319	0.816288834	0.060741109	0.135507261	0.370088359	0.844744534
DSC1	0.78663717	0.734939998	0.03825474	0.331663673	0.606750253	0.370960696	0.844744534
IDNK	0.556878046	0.061544554	0.439345924	0.330745534	0.891700844	0.370612104	0.844744534
IER2	0.528549121	0.308978429	0.110347633	0.30017876	0.820056983	0.370440093	0.844744534
LATS2	0.779059668	0.207459459	0.495957572	0.864621754	0.064088245	0.370642747	0.844744534
LOC101906235	0.271253025	0.04517281	0.437863243	0.948458579	0.871730004	0.370436434	0.844744534
LOC104974144	0.147065663	0.310238128	0.697752238	0.753146971	0.185474045	0.370832644	0.844744534
LOC112443216	0.296137911	0.223899625	0.486315368	0.275803265	0.500357004	0.37093327	0.844744534
LOC112446024	0.340138689	0.15826692	0.300137138	0.762805381	0.358390919	0.369756929	0.844744534
LOC548613	0.8495615	0.988403125	0.245420939	0.034703813	0.620984849	0.370622835	0.844744534
LOC615610	0.822906671	0.194669117	0.274155249	0.138259539	0.72839191	0.369964949	0.844744534
LOC783022	0.307133051	0.156782234	0.524143302	0.331801864	0.529119112	0.370258653	0.844744534
LRRC41	0.069992105	0.459815878	0.420564156	0.59423729	0.553403415	0.370978144	0.844744534
LRRC8C	0.253992562	0.668125001	0.19125705	0.385899937	0.353130896	0.369965746	0.844744534
LUM	0.909060118	0.675878522	0.146346935	0.810425091	0.061089011	0.370997908	0.844744534

PTPN7	0.323687649	0.257381757	0.634748258	0.651953137	0.128548835	0.370289531	0.844744534
RAB1A	0.668731098	0.959877632	0.261922906	0.611935556	0.043215236	0.370800672	0.844744534
RAP1GAP2	0.470150905	0.619797685	0.040051525	0.543754084	0.699838574	0.370625749	0.844744534
ROR2	0.930286491	0.427052062	0.018992961	0.767800326	0.762749958	0.369824349	0.844744534
SEC62	0.895818427	0.43468253	0.116730553	0.697933573	0.140054668	0.370692413	0.844744534
SETDB2	0.444588002	0.375381721	0.032686927	0.836705279	0.973726166	0.370738608	0.844744534
TUBA1C	0.380260517	0.178748839	0.109510091	0.672209263	0.884485356	0.370063649	0.844744534
UNG	0.879369715	0.19127612	0.636784	0.114721105	0.361304745	0.370565112	0.844744534
WDR47	0.654344717	0.245385017	0.412898751	0.101322587	0.660111056	0.370374436	0.844744534
ARMC2	0.449192795	0.125046378	0.2764352	0.4479643	0.642018166	0.371499943	0.84484232
C3H1orf162	0.800817725	0.121243728	0.319154946	0.339419741	0.424818649	0.371590783	0.84484232
DUSP6	0.159109472	0.619529993	0.553382028	0.316486086	0.258731999	0.371536764	0.84484232
LOC104970930	0.223592654	0.68864377	0.185152884	0.333839337	0.468566229	0.371280721	0.84484232
LRWD1	0.931539338	0.059321757	0.176607188	0.831372165	0.549592807	0.371269325	0.84484232
OGFRL1	0.972029313	0.109622055	0.236574708	0.282280007	0.627950568	0.371596654	0.84484232
P2RY14	0.586301036	0.934748218	0.255199971	0.18171154	0.175586616	0.371383087	0.84484232
PDCD6	0.422986525	0.268393708	0.922675372	0.24266742	0.175767799	0.371577323	0.84484232
PI16	0.945316602	0.119122285	0.199831277	0.298090769	0.664763107	0.371265324	0.84484232
SEC22B	0.313664089	0.361428212	0.375234696	0.393975048	0.26668552	0.371635897	0.84484232
VEZF1	0.254487275	0.758770165	0.263524109	0.129471974	0.678066597	0.371557561	0.84484232
LOC112444909	0.106028638	0.266560872	0.845191697	0.43081051	0.434608283	0.37174655	0.844859803
POLL	0.823293566	0.039109688	0.527480884	0.70224399	0.374921387	0.371715291	0.844859803
CBFA2T2	0.695440706	0.139285456	0.28275979	0.421085191	0.388543705	0.372053244	0.845439737
EIF4EBP3	0.487668421	0.902634814	0.359908506	0.694333159	0.040822023	0.37238349	0.845586952
FAM107B	0.896758207	0.894817033	0.119278026	0.194679513	0.241093415	0.372451331	0.845586952
FURIN	0.026038532	0.632711606	0.727788749	0.383866648	0.976207273	0.372478708	0.845586952
KCNJ15	0.179474037	0.12682919	0.958539497	0.614175677	0.334897263	0.372289247	0.845586952
MYLIP	0.72613048	0.878299139	0.886767982	0.935445574	0.008482536	0.372280518	0.845586952
RICTOR	0.069423732	0.379130898	0.718457378	0.619031916	0.383314707	0.372264085	0.845586952
ZNF705A	0.81434357	0.639884774	0.069125703	0.310139351	0.402030308	0.372410503	0.845586952
FGL2	0.065410649	0.397886986	0.954278826	0.447273616	0.404847775	0.372625346	0.845685874
UTRN	0.660523255	0.466035015	0.441458367	0.061003953	0.542453006	0.372613568	0.845685874
SIPA1	0.35306413	0.037858687	0.407124891	0.943119676	0.87700855	0.37276114	0.845877083
BICC1	0.946245002	0.253579771	0.286903287	0.102488845	0.638376361	0.372867794	0.846002125
RNF128	0.119907727	0.85750643	0.096058928	0.614532614	0.742321782	0.372923874	0.8460124
KLHDC2	0.830325808	0.196184262	0.306359749	0.499102531	0.180954326	0.372976799	0.846015515
ABR	0.967909724	0.749005007	0.355837089	0.042631827	0.410026175	0.373055305	0.846076645
EP300	0.579640154	0.251227341	0.720675117	0.056951987	0.755022073	0.373172107	0.846107689
MRPL30	0.773095667	0.244387953	0.627762718	0.19193245	0.198184077	0.373132433	0.846107689
LOC101905925	0.237440977	0.091649533	0.650136657	0.777180073	0.410753327	0.373304703	0.846291404

ARMH3	0.955190484	0.277202584	0.383935672	0.072271394	0.615794301	0.373582763	0.846454055
LOC785087	0.200231571	0.881624471	0.214514922	0.702676492	0.169893586	0.373456812	0.846454055
SAMD4B	0.971142568	0.348663368	0.846617912	0.026384893	0.598073444	0.373560206	0.846454055
SERPING1	0.049452308	0.386586737	0.576757924	0.52674965	0.778514739	0.373490746	0.846454055
PLD3	0.464561856	0.751875536	0.040695353	0.772322711	0.412311456	0.373660438	0.846513176
GSTP1	0.448122826	0.41170498	0.733380657	0.095608576	0.350799665	0.374069843	0.846681125
LMNB2	0.238210513	0.442239167	0.202478285	0.636907143	0.334038484	0.374070045	0.846681125
MSANTD3	0.10640623	0.758453013	0.328588741	0.403580393	0.423783888	0.373979193	0.846681125
OTULIN	0.956135328	0.030573936	0.674996115	0.466206301	0.493358098	0.374085975	0.846681125
PAGR1	0.295427471	0.232447244	0.503819273	0.63556743	0.206408277	0.374095719	0.846681125
SLC25A42	0.159978747	0.576281013	0.57659665	0.106501162	0.80080669	0.373915692	0.846681125
TTC8	0.562157343	0.565476883	0.386469854	0.123209095	0.29954766	0.373932774	0.846681125
LTBP1	0.168027718	0.740048859	0.063903841	0.605802809	0.943874851	0.374271053	0.846961148
SEPT7	0.245994295	0.081426934	0.389951501	0.736033751	0.791190029	0.374443881	0.847235425
DLGAP3	0.152775855	0.551682576	0.223272323	0.652115156	0.37081305	0.374508829	0.847265563
AKNAD1	0.834400963	0.87021356	0.315103123	0.420653108	0.047456797	0.375106323	0.847332397
ANKRD2	0.394193298	0.153870218	0.429502939	0.175442928	0.997905061	0.374876949	0.847332397
ARF6	0.90203677	0.494290497	0.540952871	0.226724133	0.083407924	0.374882844	0.847332397
CDIP1	0.391528572	0.227716575	0.163043762	0.747743912	0.420162766	0.375090384	0.847332397
DDRGK1	0.515412338	0.338846314	0.214468613	0.956322675	0.127469829	0.375053595	0.847332397
DNAJC17	0.863515103	0.342661104	0.038034618	0.934581757	0.433931836	0.374986884	0.847332397
GALE	0.195458026	0.257781661	0.779058038	0.431866102	0.269170327	0.374949576	0.847332397
LOC613401	0.598065972	0.189194916	0.519069944	0.218103718	0.356057667	0.374881682	0.847332397
MEIS1	0.367943057	0.895228166	0.053595291	0.803302632	0.321804228	0.374971845	0.847332397
PER1	0.304872823	0.665624377	0.742258879	0.137324964	0.220437146	0.374833572	0.847332397
SVIL	0.099328699	0.594375657	0.350956186	0.77399107	0.284037703	0.374672181	0.847332397
LOC112443328	0.167785572	0.46349682	0.485849141	0.38831206	0.311761006	0.375339024	0.847343447
RAB3IL1	0.097272603	0.510367724	0.18286573	0.590760724	0.852497229	0.375267006	0.847343447
SNRNP70	0.682956574	0.179624826	0.059184166	0.72719004	0.866019345	0.375278038	0.847343447
TPRG1	0.013973931	0.879344866	0.578992268	0.896209002	0.717509589	0.375369378	0.847343447
UNC80	0.729780817	0.493264173	0.571480743	0.234144632	0.094879772	0.375200774	0.847343447
ADARB1	0.451058503	0.626144145	0.02822002	0.828962381	0.695701091	0.376121925	0.847503713
CAMK2N2	0.307539281	0.349742885	0.189986295	0.505162572	0.444444132	0.375825182	0.847503713
GOLGA4	0.920510629	0.699639127	0.217220551	0.042256235	0.777402621	0.376091793	0.847503713
IPPK	0.080931986	0.357574327	0.724002351	0.895057574	0.244799751	0.375924466	0.847503713
LOC100847376	0.588136709	0.555649782	0.414484362	0.339069836	0.100169672	0.376266654	0.847503713
LOC101902172	0.891915936	0.202847061	0.218190704	0.261723668	0.444131997	0.375848804	0.847503713
LOC101905757	0.550163643	0.828922464	0.210299773	0.966622941	0.049610923	0.376217053	0.847503713
LRR1	0.713026446	0.422523692	0.721990528	0.125280421	0.168764082	0.376206843	0.847503713
RETREG2	0.898351561	0.713563823	0.067133894	0.888265693	0.120119476	0.375957347	0.847503713

SHMT2	0.368527493	0.246926505	0.926878849	0.340661917	0.159442469	0.375591442	0.847503713
SLC38A3	0.306984477	0.497292701	0.064453378	0.601260097	0.775561915	0.375837371	0.847503713
SYTL2	0.389362089	0.805085324	0.682619681	0.20574988	0.104146481	0.375728435	0.847503713
TERF2	0.451034523	0.656559933	0.840027945	0.045872556	0.402994889	0.376199048	0.847503713
TLR3	0.549645611	0.501704444	0.061080457	0.494903931	0.549970235	0.375704212	0.847503713
TXLNB	0.137118339	0.426891081	0.991151106	0.095217461	0.832328249	0.376174378	0.847503713
UMAD1	0.348664172	0.664936707	0.035038685	0.872953915	0.64715945	0.375868818	0.847503713
MTMR2	0.649989381	0.851792694	0.760340148	0.013656185	0.800847263	0.376382986	0.8476494
TSPAN18	0.972834708	0.236106813	0.066962737	0.348740066	0.85870585	0.376456991	0.847699737
SNAPC2	0.130752494	0.119493141	0.967912876	0.336659417	0.905108013	0.376528603	0.847744671
ARPP19	0.372716568	0.296608096	0.161381065	0.609402468	0.424177225	0.376656552	0.84780012
ETFB	0.680234927	0.250558297	0.400307047	0.206699436	0.326990228	0.376645137	0.84780012
COLGALT1	0.730337474	0.930635763	0.033917578	0.347807999	0.577545835	0.377319386	0.848185967
DUS4L	0.967667095	0.345172958	0.06089011	0.976025485	0.232659714	0.376888499	0.848185967
FBH1	0.540099673	0.047733375	0.806091386	0.41035031	0.543109807	0.377344814	0.848185967
IP6K2	0.775484241	0.134432006	0.972642675	0.111758587	0.408240827	0.37716074	0.848185967
LOC104973252	0.67438076	0.57479405	0.266669553	0.801558493	0.05587716	0.377284615	0.848185967
RASL11A	0.400893851	0.239642096	0.491899875	0.825564914	0.118632839	0.37723454	0.848185967
RIC8A	0.533596485	0.228249846	0.311361182	0.165711344	0.736769763	0.377288895	0.848185967
SMARCAD1	0.352764826	0.510055958	0.284035426	0.977059043	0.092533479	0.376963903	0.848185967
VAPB	0.867479007	0.231087968	0.147439702	0.977492019	0.160302888	0.377338352	0.848185967
ZFAT	0.676688416	0.291938227	0.841251112	0.05243669	0.530317598	0.376994059	0.848185967
SUCLA2	0.687457814	0.220332538	0.221717899	0.551026619	0.250374161	0.377405927	0.848207158
ALDH16A1	0.097872495	0.280761937	0.734218093	0.4660854	0.493398403	0.37762838	0.848264789
C26H10orf143	0.352798681	0.595284576	0.742630488	0.994873191	0.029890102	0.377566153	0.848264789
LOC100335467	0.714776385	0.459820708	0.131807745	0.143061529	0.749139319	0.377738914	0.848264789
LOC101904435	0.264932631	0.701527261	0.324116678	0.287774336	0.267564054	0.377581634	0.848264789
PCDH18	0.086452878	0.980690259	0.41417249	0.1505767	0.877682569	0.377665708	0.848264789
SMPD4	0.403235367	0.204592477	0.16553428	0.357179026	0.951854239	0.377741702	0.848264789
LOC101905343	0.132509373	0.324126581	0.400458181	0.564808348	0.478253805	0.377847597	0.848270439
N4BP2	0.530849111	0.533857007	0.758870986	0.024243551	0.890916684	0.377817666	0.848270439
LOC101902754	0.388796129	0.551300505	0.053552852	0.419365974	0.96556102	0.377917333	0.848310951
ALG10	0.138096799	0.19771842	0.294267342	0.783034655	0.739273774	0.378026192	0.848404184
SCARA5	0.511647452	0.152173334	0.87445544	0.404125344	0.169116448	0.378096698	0.848404184
TADA1	0.667400509	0.826619406	0.865593471	0.162324592	0.060035076	0.37811396	0.848404184
CCDC124	0.564773747	0.423909701	0.457638122	0.160787131	0.264628267	0.37839699	0.848445476
CD1E	0.856969482	0.097014544	0.4107077	0.487803604	0.279932462	0.378425128	0.848445476
GINS4	0.884355053	0.522486552	0.504678361	0.097405402	0.204991124	0.378202737	0.848445476
LOC101906989	0.731464803	0.106559358	0.650561007	0.426465061	0.215706333	0.378494262	0.848445476
MED31	0.580602058	0.179541425	0.165802517	0.396651167	0.6803046	0.378467175	0.848445476

MYMK	0.41192792	0.410761391	0.036847259	0.964062278	0.775712201	0.378420828	0.848445476
RTL1	0.287632407	0.685381151	0.961717348	0.112787255	0.218093779	0.3784576	0.848445476
LOC104975222	0.967939907	0.817181704	0.085414246	0.943833824	0.073183925	0.378564545	0.848487128
EPHX3	0.46422368	0.174725237	0.281786373	0.587613854	0.347819223	0.378728037	0.848579625
RDH8	0.568153146	0.171750402	0.099403674	0.858375845	0.560994647	0.378710679	0.848579625
THTPA	0.344951045	0.318017799	0.458483753	0.103527014	0.897901554	0.378864354	0.848579625
XRRA1	0.578214106	0.524402754	0.086782829	0.41441554	0.428650415	0.378831446	0.848579625
ZXDB	0.388232229	0.061394276	0.260335706	0.762502671	0.987993107	0.378839535	0.848579625
CTXND1	0.054298275	0.877944778	0.951108189	0.362830327	0.284544995	0.379058592	0.848782993
MAN2A2	0.540093686	0.361487697	0.163103385	0.266149789	0.552180313	0.379019533	0.848782993
NAGK	0.374142659	0.387539602	0.126006698	0.461299556	0.555977106	0.379225657	0.848820803
NAPB	0.627756754	0.155827649	0.259925919	0.448718833	0.410714944	0.379230646	0.848820803
WBP11	0.897984145	0.209197603	0.141552773	0.621875596	0.283227658	0.379149796	0.848820803
TCN1	0.086136903	0.168993255	0.404317498	0.901901774	0.883272229	0.37931794	0.848900411
CREB3L4	0.234900132	0.13393108	0.382767987	0.527548165	0.738552797	0.379432874	0.849039627
TGFB1I1	0.277146039	0.572168915	0.671636184	0.805966644	0.054675932	0.379483618	0.849039627
ACSL4	0.473499433	0.33292684	0.277542999	0.524691089	0.204614775	0.379617199	0.849106973
SF1	0.937429903	0.456521034	0.074806748	0.234546042	0.62545152	0.379588505	0.849106973
APOBEC2	0.771345411	0.048654723	0.204857368	0.657112586	0.933770915	0.380312221	0.849404509
ERFE	0.224235303	0.293300089	0.965109459	0.144515786	0.513544692	0.380081504	0.849404509
IQGAP1	0.852971605	0.163515287	0.219863004	0.258552756	0.594744568	0.380244833	0.849404509
JAM3	0.674929747	0.303792103	0.186540023	0.67576947	0.182614368	0.380399773	0.849404509
LOC100336976	0.896593113	0.728794471	0.46826694	0.142902001	0.107669493	0.379984173	0.849404509
LOC104976321	0.233049854	0.832869159	0.87532795	0.030212112	0.918128685	0.380154048	0.849404509
LYZ1	0.363957448	0.909833257	0.037248974	0.665905648	0.574205858	0.380275496	0.849404509
MTMR11	0.876353977	0.593581039	0.266871926	0.132674693	0.25573907	0.380067111	0.849404509
RAF1	0.291680306	0.395596308	0.230058575	0.922422082	0.192326616	0.380037018	0.849404509
STRA6	0.696361044	0.104851771	0.27128901	0.775112487	0.306687165	0.380013156	0.849404509
TEX264	0.027715583	0.570453818	0.540555707	0.673635667	0.81949425	0.380330974	0.849404509
TOX4	0.697806715	0.072608201	0.913400338	0.103263266	0.987814238	0.380423078	0.849404509
USP6NL	0.437871291	0.394046486	0.84701588	0.03403865	0.946738247	0.380044798	0.849404509
ENPP2	0.389462541	0.483017873	0.634389657	0.943369685	0.041945022	0.380476819	0.849408934
ASPN	0.818747354	0.797145014	0.906193233	0.019462275	0.41087773	0.380725425	0.849718382
CNR1	0.767853799	0.059426108	0.424435914	0.286458109	0.852720687	0.380770762	0.849718382
TXNDC9	0.774306056	0.100028422	0.616321702	0.567849785	0.174499662	0.380746796	0.849718382
KLK12	0.523707641	0.570901168	0.541980363	0.446317413	0.065528599	0.381058762	0.850108685
LOC101905167	0.601934414	0.084607775	0.41662362	0.373706021	0.597784556	0.38108488	0.850108685
MCU	0.183863145	0.762604422	0.278575381	0.144721739	0.838860707	0.381152867	0.850108685
P2RY1	0.138668144	0.50270454	0.308585953	0.894342502	0.246466912	0.381140853	0.850108685
LOC101904275	0.794458697	0.858341355	0.335218215	0.357088821	0.058130552	0.381257081	0.850225568

ERBB4	0.380320182	0.059480121	0.371148261	0.717618651	0.787898719	0.381329134	0.850270708
CYP2D14	0.187258706	0.318068168	0.204309299	0.410333266	0.952623651	0.381656007	0.85043628
HIVEP3	0.955975965	0.547409043	0.47949755	0.122028481	0.155241347	0.381546057	0.85043628
LOC101906021	0.984232592	0.105271952	0.537181878	0.8976135	0.095165132	0.381577811	0.85043628
TTF1	0.722046909	0.408257999	0.045401245	0.490495477	0.724872663	0.381714316	0.85043628
VAV2	0.880127654	0.120922341	0.173742653	0.431354692	0.596566923	0.381709742	0.85043628
ZKSCAN8	0.388411581	0.608774238	0.051514021	0.996965379	0.391281627	0.381482401	0.85043628
IL4I1	0.226756717	0.065418162	0.779723164	0.913149702	0.450689832	0.381771849	0.850449005
LOC101906408	0.739151818	0.093940729	0.64193709	0.70517538	0.151732182	0.3820842	0.850479272
LOC112447362	0.858382739	0.422420306	0.159756426	0.141443632	0.581312576	0.381868405	0.850479272
MED10	0.844895282	0.108450589	0.155082507	0.797375148	0.420417454	0.381892247	0.850479272
SLC25A3	0.84185828	0.302352635	0.897459157	0.112038139	0.186360627	0.382096379	0.850479272
SMAD4	0.973185406	0.101986216	0.233161974	0.530298505	0.388339967	0.38196181	0.850479272
TPM4	0.313769859	0.735620591	0.56524638	0.043512341	0.840084452	0.382077995	0.850479272
CAAP1	0.902886261	0.233838414	0.0538663	0.52743484	0.795855638	0.382238996	0.850565989
NTHL1	0.774000659	0.378130177	0.104805708	0.431336357	0.360806929	0.382235028	0.850565989
CCDC96	0.178081156	0.320048712	0.994398545	0.1222318	0.689987094	0.382444775	0.850567274
FAM171A1	0.534120491	0.200703745	0.280943364	0.448901481	0.353275991	0.382317293	0.850567274
KLHL11	0.166139727	0.706571716	0.157979702	0.325534713	0.791895971	0.382473472	0.850567274
LOC101907622	0.170636279	0.934300631	0.835628215	0.126327831	0.283939995	0.382398801	0.850567274
PCMT1	0.996408664	0.110110071	0.252606021	0.653007746	0.264202811	0.382498719	0.850567274
ARFGAP2	0.887988926	0.203977622	0.172122894	0.649552122	0.236231741	0.38257984	0.850632401
CCDC162P	0.64665074	0.881639732	0.815140253	0.022906387	0.449716599	0.382694946	0.85066899
S1PR4	0.927325876	0.277382038	0.822404605	0.188916469	0.119794298	0.382699967	0.85066899
SRD5A1	0.094973814	0.961469829	0.478000327	0.138267205	0.793804699	0.382811559	0.8508018
MIB2	0.444064815	0.380340482	0.066974322	0.769706915	0.550725027	0.382956277	0.851008188
LTB4R	0.121723074	0.995331649	0.497446113	0.089625748	0.888338046	0.383072408	0.851151
FAM69A	0.026683196	0.848570698	0.978740228	0.238387833	0.908664086	0.383140866	0.851187864
ATP6V1H	0.586180933	0.6668379	0.169194937	0.691576604	0.105179843	0.383489511	0.851365224
CACHD1	0.328209503	0.263729972	0.524068349	0.207751792	0.510764501	0.383583844	0.851365224
POPDC2	0.298365567	0.610902581	0.765190899	0.426214433	0.080936198	0.383507959	0.851365224
TRAF5	0.285178621	0.070606823	0.845695512	0.415669235	0.680013015	0.383575873	0.851365224
TRMT13	0.795798595	0.214695118	0.032472645	0.884440634	0.980324656	0.383478081	0.851365224
UBE2J2	0.386937562	0.268798431	0.522385478	0.961748033	0.092065533	0.383491366	0.851365224
ZNF792	0.357419578	0.269442879	0.271686702	0.43930618	0.417990383	0.383277951	0.851365224
SAMD14	0.182804914	0.257921908	0.166952667	0.694214018	0.881247405	0.383657486	0.851413522
GEMIN5	0.328902315	0.057465437	0.940723158	0.559655483	0.484491885	0.383838055	0.85169907
ZNF202	0.328018869	0.305995653	0.290745724	0.981706762	0.168351742	0.383907018	0.851736931
SRPK3	0.150850969	0.75471957	0.376103733	0.751044475	0.15007103	0.384010834	0.851852094
ATPAF1	0.687541765	0.465936001	0.475993628	0.0908452	0.348635386	0.384121787	0.851886903

DDIT4L	0.40902959	0.714620361	0.276707902	0.334532262	0.178497891	0.384130345	0.851886903
AAED1	0.57611752	0.088737007	0.753041058	0.891275827	0.141324138	0.384782909	0.851957271
C27H4orf47	0.345714281	0.702546601	0.122161491	0.229909075	0.711011705	0.384819173	0.851957271
DOC2B	0.728251641	0.024256065	0.68345424	0.544423875	0.7374339	0.384711082	0.851957271
FAM181B	0.035607916	0.346682125	0.720857116	0.95516844	0.570638904	0.384822508	0.851957271
LOC100297097	0.374384052	0.059635214	0.427487925	0.961940623	0.527197061	0.384483672	0.851957271
LOC104968434	0.127838314	0.216964956	0.51339437	0.523437733	0.649711622	0.384566993	0.851957271
LRFN3	0.701147036	0.764488401	0.248673154	0.075219041	0.48220539	0.384297523	0.851957271
NAE1	0.918986771	0.597471291	0.467043361	0.060967883	0.309569298	0.384475153	0.851957271
PLCD3	0.69650018	0.616606508	0.032917485	0.478735648	0.715375433	0.38452888	0.851957271
RFX5	0.454803591	0.312042518	0.046859977	0.849926673	0.856601473	0.384534219	0.851957271
SCHIP1	0.301356228	0.509188968	0.458204431	0.944883362	0.072859317	0.384489866	0.851957271
SPRY4	0.665886127	0.0417076	0.445791668	0.957248072	0.40877402	0.384629818	0.851957271
TTLL5	0.732434784	0.115154812	0.3888326	0.326418286	0.453125846	0.384836954	0.851957271
CHN2	0.345897115	0.544212553	0.082313226	0.46302491	0.676408241	0.384908746	0.852001271
NEURL4	0.839948592	0.070248323	0.333676135	0.815780296	0.302625416	0.385169242	0.852119559
PITHD1	0.995484551	0.102423231	0.088129062	0.769100371	0.703009473	0.385095639	0.852119559
PRRT1B	0.382564078	0.813171649	0.614889612	0.244887502	0.103763335	0.385169879	0.852119559
RTKN2	0.143949101	0.915235413	0.071103294	0.648102147	0.79989965	0.38502587	0.852119559
AGAP2	0.609419766	0.683617962	0.033942843	0.362609999	0.954172517	0.386240931	0.852666651
CCR3	0.024788014	0.912203448	0.951839612	0.478966683	0.47283694	0.38562795	0.852666651
CHCHD5	0.098840473	0.118123177	0.998490412	0.609737237	0.686830839	0.38588812	0.852666651
DOCK2	0.625822156	0.155133508	0.851352671	0.193369108	0.306152125	0.386258052	0.852666651
E2F1	0.592201831	0.192863113	0.297142827	0.326253835	0.441803288	0.386212298	0.852666651
ESAM	0.42871759	0.518765175	0.589170008	0.19147487	0.194963778	0.386204674	0.852666651
FAT1	0.700130023	0.839898573	0.38048914	0.024802386	0.88000589	0.385933408	0.852666651
GNPDA1	0.449484678	0.13538345	0.113513515	0.797354275	0.888635749	0.386300442	0.852666651
KCNB2	0.189396349	0.666303606	0.582428733	0.549716411	0.120607412	0.385584879	0.852666651
LOC101904891	0.405724784	0.848686075	0.072624241	0.904754898	0.21571306	0.385835982	0.852666651
LOC112447802	0.024173653	0.522313681	0.921309955	0.80580281	0.52122609	0.386011408	0.852666651
PHKA1	0.154038245	0.526443051	0.165628904	0.973319312	0.374002147	0.386128375	0.852666651
PLXNA4	0.119852155	0.298021792	0.44670799	0.648281043	0.471056439	0.385567825	0.852666651
PRDX2	0.373767094	0.38084851	0.835668116	0.118515011	0.346036301	0.385766308	0.852666651
RNASE10	0.184296721	0.413806185	0.524834394	0.950851193	0.128219453	0.385811838	0.852666651
SRPRA	0.11337695	0.125737079	0.533886567	0.988130333	0.650104253	0.386124182	0.852666651
ZNF143	0.599855569	0.251624873	0.862341126	0.137250586	0.273117192	0.385789013	0.852666651
LOC101903015	0.262296297	0.348105521	0.224846353	0.958275855	0.249009229	0.386447777	0.852691208
OSR1	0.255090791	0.941226253	0.217384015	0.175857842	0.533788279	0.386467443	0.852691208
TDRD7	0.504140775	0.382952237	0.22384979	0.207518167	0.546270556	0.386456103	0.852691208
BAIAP2L1	0.045898192	0.237754947	0.734533848	0.713291195	0.857487665	0.386574467	0.852698061

SH2D5	0.274861555	0.48493582	0.372699316	0.702667649	0.140439776	0.386561393	0.852698061
ABHD1	0.088017096	0.92720109	0.265387036	0.236952904	0.959340991	0.387261023	0.852780156
ACTN1	0.712315681	0.231508483	0.351904968	0.546163701	0.155286649	0.38721058	0.852780156
ASAP2	0.748421668	0.159785178	0.2696822	0.488534387	0.312551425	0.38729719	0.852780156
BORCS7	0.352003562	0.238374644	0.482313171	0.502512845	0.242134788	0.387292991	0.852780156
HCLS1	0.808234904	0.181069495	0.821733793	0.232945558	0.175417038	0.386953994	0.852780156
INF2	0.806221747	0.587362037	0.169618237	0.379357863	0.161413471	0.387097631	0.852780156
LOC101906392	0.782129541	0.396274258	0.182270744	0.147384925	0.58960756	0.386791167	0.852780156
LOC104968435	0.443945069	0.363770302	0.758366385	0.509666184	0.078650764	0.386797449	0.852780156
LOC104975022	0.755320254	0.621865398	0.057661648	0.210302227	0.864608444	0.387307048	0.852780156
LOC112447756	0.527960522	0.342685168	0.103852555	0.617872713	0.423297749	0.386961023	0.852780156
LOC786974	0.956861384	0.686596839	0.338299698	0.345085588	0.064222022	0.38733918	0.852780156
LPAR1	0.05129932	0.958794271	0.576286119	0.26414195	0.656614042	0.387022694	0.852780156
SGPP1	0.792390502	0.881717605	0.371317667	0.864137912	0.021904815	0.386839872	0.852780156
WDSUB1	0.361759001	0.144816858	0.342666244	0.521240572	0.524959199	0.386891251	0.852780156
CAMLG	0.676841867	0.064621819	0.223430124	0.909419613	0.555331778	0.387663818	0.852984197
CRBN	0.753487391	0.821234047	0.060272678	0.257407745	0.514410589	0.387765559	0.852984197
LOC782054	0.598182457	0.385349796	0.312737263	0.612999807	0.111750616	0.38775944	0.852984197
MMP24	0.893404394	0.04351144	0.701636084	0.372523553	0.485870143	0.387706193	0.852984197
SCN2A	0.336775602	0.363955328	0.080600921	0.731702449	0.682933235	0.387707721	0.852984197
SDAD1	0.70003415	0.986552528	0.082108555	0.349418555	0.248838199	0.387500261	0.852984197
TEF	0.722850102	0.841595763	0.789878639	0.297399155	0.034563921	0.387795692	0.852984197
XG	0.999811591	0.463699765	0.448723415	0.078825352	0.301499873	0.387950969	0.853211386
KDM4B	0.701199952	0.455290997	0.226175643	0.191302311	0.358549358	0.388237207	0.853497763
LOC619159	0.057356068	0.568539106	0.379139462	0.800537381	0.500356827	0.388219938	0.853497763
RNPEPL1	0.089674943	0.811108213	0.699578019	0.300639518	0.323633609	0.388176792	0.853497763
FCF1	0.797232477	0.038239858	0.577598288	0.284566368	0.98901225	0.388337709	0.853604358
LOC112442347	0.690895771	0.796094205	0.230968283	0.723213085	0.053984577	0.388470848	0.853782654
ARHGEF18	0.289975976	0.088079117	0.781249757	0.439095806	0.566679006	0.388642893	0.853794172
CNTN1	0.137923458	0.989668037	0.207942932	0.251486498	0.695735543	0.388684191	0.853794172
DEAF1	0.122827076	0.197987594	0.871493049	0.760999728	0.307798972	0.388615122	0.853794172
FUS	0.603329626	0.134834694	0.587475516	0.640033974	0.162228796	0.388550734	0.853794172
LRP2BP	0.532256619	0.121492035	0.399935292	0.20306043	0.947696083	0.389032355	0.85444459
XPO4	0.212576675	0.97289684	0.281573533	0.355563688	0.24045413	0.389096883	0.854471959
ADD1	0.935736157	0.587534804	0.225254921	0.189068956	0.212859123	0.389266808	0.854479882
GPR34	0.367528091	0.263158652	0.653260333	0.291580168	0.270774671	0.389412896	0.854479882
LOC112448743	0.674732037	0.081828742	0.703784437	0.454131613	0.282478738	0.389293466	0.854479882
NPM1	0.70395496	0.883547828	0.618919233	0.150691982	0.085974447	0.389378121	0.854479882
RNF44	0.440954937	0.069960423	0.71735724	0.425379675	0.529310559	0.389227382	0.854479882
SSTR1	0.644197528	0.352802132	0.29587062	0.136483176	0.543394934	0.389369942	0.854479882

INO80	0.298314959	0.689559858	0.996872336	0.026321813	0.925884936	0.389714789	0.855027994
CLTA	0.4180774	0.188975811	0.098727052	0.925217284	0.692862027	0.38980181	0.855062197
RBP4	0.043860707	0.891875751	0.157289398	0.939768924	0.864919644	0.389834584	0.855062197
ALDH1A3	0.521584733	0.170926462	0.107608237	0.601953978	0.86900583	0.390397892	0.855215204
AMMECR1L	0.705947809	0.75966858	0.313558218	0.0306266	0.977525589	0.390918808	0.855215204
BTBD8	0.643458285	0.293175633	0.038649482	0.869610556	0.79560579	0.391249388	0.855215204
C15H11orf74	0.327310526	0.207006031	0.473273602	0.408440567	0.383081854	0.390363852	0.855215204
CMKLR1	0.96997753	0.167723784	0.320363289	0.101208203	0.956889488	0.391348611	0.855215204
DNAAF2	0.718720197	0.943229571	0.769295051	0.127394769	0.075893937	0.391179	0.855215204
EIF4E3	0.38567717	0.264133764	0.510912172	0.134588253	0.717916328	0.390742076	0.855215204
GATD1	0.177843751	0.89674617	0.527473122	0.092446206	0.648322813	0.391163847	0.855215204
HOMER3	0.232095567	0.343332264	0.238215578	0.316944092	0.836243083	0.390814664	0.855215204
KCNA5	0.696977425	0.205849458	0.335282899	0.467795405	0.224114466	0.391208097	0.855215204
LOC101907189	0.052051072	0.701253096	0.351711137	0.406349114	0.967973453	0.391415599	0.855215204
LOC784697	0.547780827	0.537978425	0.20511317	0.208711194	0.399599033	0.391143594	0.855215204
MKL1	0.475192674	0.050829048	0.433131508	0.510210914	0.941951245	0.390705952	0.855215204
NAA60	0.264354118	0.647080688	0.933420406	0.051546664	0.611373402	0.390838173	0.855215204
NCKAP1	0.202993926	0.251582026	0.396665025	0.377434344	0.657685667	0.3907312	0.855215204
NEDD8	0.563634591	0.161563906	0.794214909	0.103010583	0.677655841	0.391384412	0.855215204
NOP2	0.402449643	0.923612387	0.641268946	0.062764294	0.336868513	0.391097825	0.855215204
PAM16	0.295021941	0.242385806	0.920424631	0.332351481	0.230350713	0.391068411	0.855215204
PDCD1LG2	0.132270916	0.357008592	0.883964987	0.394404295	0.306311248	0.391199379	0.855215204
PSD4	0.298642774	0.64004647	0.079330918	0.665218119	0.496274896	0.389991911	0.855215204
QTRT1	0.668678995	0.095304222	0.251004931	0.4484596	0.70101827	0.390738159	0.855215204
SCML2	0.262088028	0.777209528	0.684392133	0.044917412	0.805937233	0.39132197	0.855215204
SLC26A11	0.377291273	0.483825066	0.342119846	0.544810995	0.147371327	0.390260399	0.855215204
STAR	0.382504681	0.426057242	0.141281048	0.319729197	0.680106618	0.390012913	0.855215204
SYK	0.712453888	0.268075498	0.196168465	0.166071051	0.805246279	0.390133111	0.855215204
TMEM186	0.805348027	0.851683813	0.982789537	0.050624672	0.14679055	0.390101758	0.855215204
TNFSF4	0.905568923	0.104748539	0.277101586	0.329007262	0.580135321	0.390351588	0.855215204
USP38	0.738448626	0.106043383	0.13568888	0.497471347	0.949482968	0.390412156	0.855215204
VASH1	0.737364336	0.306210155	0.080849627	0.783518255	0.35303053	0.391410966	0.855215204
ANKRD27	0.621821977	0.486913288	0.73976748	0.216753286	0.104078131	0.391523514	0.855337112
BOLA2B	0.793782171	0.131113585	0.095787839	0.752070066	0.674470654	0.391653396	0.855423765
PRDM2	0.389552475	0.519363273	0.3939828	0.174163344	0.364289426	0.391667429	0.855423765
ESD	0.311496626	0.041738563	0.833733427	0.479101917	0.97558282	0.391967851	0.855848308
GCA	0.82518241	0.312261996	0.18974129	0.744997361	0.139141715	0.392018264	0.855848308
NRBF2	0.240031136	0.394927923	0.168550031	0.689708457	0.459774887	0.391973521	0.855848308
DDX19A	0.566131424	0.218203479	0.472155063	0.123756212	0.702387807	0.392080667	0.855850519
PLEKHB1	0.246150135	0.495975561	0.159585885	0.315968567	0.823800333	0.392123579	0.855850519

LOC101907255	0.318875979	0.524717891	0.320094886	0.994015991	0.095366058	0.392310594	0.856031002
UNK	0.642018444	0.09911503	0.390882103	0.763739207	0.267223577	0.392287468	0.856031002
LOC112448833	0.087753684	0.279322952	0.371316709	0.836242721	0.668053501	0.392555931	0.856111015
MCTS1	0.966160731	0.187573174	0.580718836	0.264307439	0.182709968	0.392479102	0.856111015
NDST2	0.802470879	0.092164541	0.310978004	0.861455459	0.256463025	0.392449786	0.856111015
RAD9B	0.884292465	0.182304017	0.114948852	0.653311935	0.419922881	0.392528171	0.856111015
ARHGAP15	0.412410652	0.030461542	0.859077828	0.504931029	0.9337281	0.392672354	0.856211337
ATP6V1F	0.525034938	0.472914843	0.442609053	0.152827641	0.303252152	0.392835806	0.856211337
BEND7	0.405193498	0.892116839	0.226148437	0.110200407	0.565422792	0.392849881	0.856211337
MLC1	0.259624529	0.608402623	0.500094408	0.120723029	0.534182184	0.392862797	0.856211337
RBMS2	0.694460181	0.54312635	0.2725319	0.242914137	0.20398588	0.392843864	0.856211337
INTS5	0.610972597	0.652943955	0.785385837	0.124637671	0.130552536	0.392994592	0.856230069
LOC781726	0.727579741	0.014130724	0.741469765	0.813894602	0.82185736	0.393027915	0.856230069
PRKAA1	0.134942812	0.929844278	0.568837325	0.083072858	0.859857097	0.393001488	0.856230069
ENOSF1	0.934015594	0.533956341	0.158425135	0.882579229	0.073195891	0.393187959	0.856465038
APAF1	0.346548053	0.852465154	0.673735061	0.117042642	0.219265963	0.393309633	0.856489916
APLN	0.595243305	0.144112097	0.486843265	0.327542483	0.37349735	0.39334562	0.856489916
ARHGEF17	0.045367157	0.702428958	0.504036598	0.404593144	0.786216059	0.39335595	0.856489916
ANKLE2	0.718286664	0.08364485	0.218830196	0.405807985	0.958333441	0.393475678	0.85662975
FADS1	0.544261344	0.965991479	0.769820844	0.526367829	0.024068474	0.393942156	0.85662975
KDM3A	0.733976943	0.661630032	0.396780437	0.094334988	0.281846617	0.3937996	0.85662975
LOC112442843	0.133178746	0.729432043	0.779685395	0.721071602	0.093864472	0.393908097	0.85662975
LRRC3B	0.340653206	0.066577473	0.877392568	0.598224022	0.43039347	0.393810915	0.85662975
MGAT4C	0.634306434	0.329561515	0.540063525	0.284735573	0.159232811	0.393655582	0.85662975
PIAS2	0.282418562	0.862020488	0.348071408	0.074080785	0.81645267	0.393868912	0.85662975
PP2D1	0.081083937	0.715631138	0.390523483	0.512569105	0.440467198	0.39357288	0.85662975
RTN2	0.885496301	0.757615195	0.287425481	0.491120566	0.054098529	0.39379989	0.85662975
TRIM65	0.955234646	0.325741864	0.120866178	0.21743827	0.626929063	0.393917234	0.85662975
ARMCX6	0.482350581	0.917392672	0.141054104	0.094818643	0.867197807	0.394097115	0.856730871
DOCK7	0.139371221	0.848512863	0.568913592	0.235126309	0.3246388	0.394197478	0.856730871
IER5	0.898018853	0.400569264	0.035619406	0.577979349	0.693242357	0.394147127	0.856730871
PALM3	0.160863999	0.241235376	0.256842091	0.684303133	0.752674669	0.39413751	0.856730871
TCAP	0.282125537	0.326452842	0.89785429	0.893653112	0.069584705	0.394415148	0.85709044
LOC112444479	0.589018737	0.327306995	0.704459514	0.467770457	0.080977641	0.394486279	0.857131514
ATP9B	0.985615837	0.406759176	0.269522287	0.67650516	0.070451041	0.394661363	0.857224398
CTSC	0.548830489	0.125120897	0.672421193	0.112592035	0.990939672	0.394724613	0.857224398
MAMSTR	0.172989303	0.672158916	0.061393507	0.964794252	0.747899958	0.394697013	0.857224398
TLR10	0.361293088	0.466432028	0.909588177	0.040903499	0.821789584	0.394743484	0.857224398
TMEM150C	0.587487007	0.45981264	0.109374957	0.22656317	0.769928174	0.394790202	0.857224398
CARNMT1	0.957668442	0.08965728	0.187773205	0.92149153	0.347124648	0.394894769	0.857307628

CCDC51	0.545122357	0.273865297	0.647775866	0.089945967	0.593588527	0.395089732	0.857307628
GK	0.421834756	0.578910187	0.044860462	0.94871557	0.496715697	0.395066207	0.857307628
HAUS8	0.973215445	0.515338354	0.061059859	0.228842521	0.736296127	0.394984412	0.857307628
NBDY	0.081404536	0.341443906	0.919773527	0.537615377	0.375535787	0.395032181	0.857307628
MS4A8	0.561657254	0.515968701	0.203081864	0.457223535	0.192043405	0.395231053	0.857500901
LOC785873	0.471608115	0.871597451	0.095411558	0.793000847	0.166254888	0.395327254	0.857596242
MYL12B	0.50623245	0.226110802	0.817356622	0.056432353	0.979689311	0.395385615	0.85760948
NPAT	0.925147126	0.179043833	0.355547343	0.17586438	0.499649914	0.395466389	0.857631005
TMEM59	0.283592674	0.532816476	0.116883728	0.570618244	0.513602667	0.395500058	0.857631005
LOC107131703	0.529971194	0.506761516	0.306533837	0.064084379	0.981467417	0.395562102	0.857652219
GCNT1	0.898573588	0.487251849	0.399812419	0.778885165	0.038032914	0.395803887	0.857829845
LOC101906008	0.203881795	0.382638609	0.089746571	0.862088776	0.860077871	0.395986371	0.857829845
LOC112446659	0.536476444	0.478973991	0.657956378	0.146856647	0.209029579	0.395942949	0.857829845
LRFN1	0.699034635	0.370816466	0.678895984	0.075794019	0.389190564	0.395980005	0.857829845
SPC24	0.505504083	0.323073643	0.175911876	0.799468607	0.225963328	0.395942832	0.857829845
SREK1	0.983520424	0.390628619	0.148670948	0.158235907	0.573982483	0.395872836	0.857829845
TNFSF15	0.496818111	0.461101535	0.21085464	0.186850704	0.57526073	0.396009927	0.857829845
LOC112444505	0.65787864	0.792012107	0.769642221	0.022343909	0.579937704	0.396151235	0.858022689
ASMT	0.527635415	0.084341938	0.565452347	0.36436323	0.568407255	0.396632456	0.858498448
CPEB4	0.287281432	0.191284265	0.913492102	0.14269981	0.727302796	0.396579981	0.858498448
KIF26B	0.631355984	0.103282548	0.257533276	0.497452398	0.623331049	0.396493939	0.858498448
SREK1IP1	0.414107378	0.476388616	0.08525912	0.653830802	0.473841253	0.396611741	0.858498448
TYRO3	0.830843998	0.664736685	0.82274932	0.213173048	0.053753584	0.396483059	0.858498448
LOC101905801	0.369122226	0.643016072	0.720958334	0.187993676	0.16222672	0.39686165	0.858765286
LOC104972400	0.321423166	0.172343446	0.859573604	0.462478207	0.237005578	0.396875798	0.858765286
USP19	0.252634795	0.194943219	0.260040733	0.491506295	0.829334548	0.396912723	0.858765286
ERI1	0.341812625	0.331375268	0.19846319	0.292508545	0.794595601	0.397054592	0.858787345
GPN3	0.448738919	0.30129741	0.295852426	0.176310677	0.740962533	0.397079908	0.858787345
TRPV4	0.216234457	0.241705066	0.348886623	0.740999002	0.386668312	0.397046302	0.858787345
KATNAL1	0.479326717	0.072729277	0.942054901	0.84200012	0.189189125	0.397265745	0.85907605
MRPS18A	0.696305737	0.585099034	0.319073588	0.143982432	0.279741932	0.397404052	0.859233529
TMUB1	0.690565131	0.607697662	0.071556007	0.20735036	0.841098392	0.397443283	0.859233529
KLRK1	0.338137477	0.32712829	0.845908037	0.692417593	0.08095701	0.397698395	0.859610914
LMCD1	0.500064829	0.949312941	0.699984766	0.159238704	0.099139674	0.397722605	0.859610914
FAM241A	0.836260808	0.879431826	0.070734714	0.805044648	0.125306866	0.397780333	0.85962247
LOC788205	0.987253144	0.361654239	0.754720871	0.075492002	0.2580584	0.397840012	0.85963824
KIF16B	0.758055527	0.25957333	0.406769661	0.229295561	0.286315327	0.398001637	0.859647915
MBTPS2	0.545183751	0.214143058	0.440401451	0.133795254	0.763415805	0.397905083	0.859647915
SLAMF6	0.740327542	0.436896517	0.085365276	0.626175362	0.303924556	0.397999962	0.859647915
FLYWCH1	0.350599102	0.567439008	0.313636301	0.552650537	0.152441212	0.39806263	0.859666512

WTIP	0.502389851	0.050569709	0.244100728	0.934018173	0.90787124	0.398127937	0.859694418
DGAT2	0.493086086	0.417770514	0.917685248	0.098188499	0.28369305	0.398351305	0.860063579
AP3M2	0.855327842	0.061864877	0.67630107	0.198129441	0.7436492	0.398569668	0.860308671
FGD5	0.917248327	0.10882055	0.088080153	0.837710495	0.715742372	0.398528554	0.860308671
FOXP1	0.465632583	0.080566033	0.160051142	0.912964039	0.963883329	0.398913713	0.860683671
JDP2	0.887135366	0.44441151	0.202432962	0.201675176	0.328134991	0.398848264	0.860683671
LOC104971464	0.489002097	0.161000426	0.50345657	0.414209895	0.321986818	0.39900032	0.860683671
LOC112441611	0.956570777	0.2494903	0.047760785	0.703356673	0.659408188	0.399005628	0.860683671
TNXB	0.432447639	0.369580555	0.862090675	0.050646036	0.757173081	0.398915227	0.860683671
BBS2	0.067003544	0.452428531	0.580240887	0.474864142	0.634241299	0.399353534	0.860890345
LOC107132283	0.333918489	0.261626952	0.196974671	0.874380737	0.352060045	0.39934229	0.860890345
NAA35	0.212400536	0.267581258	0.864761713	0.767913344	0.140373982	0.399363731	0.860890345
PTPRD	0.226594001	0.500667854	0.168007979	0.503519564	0.551657922	0.39925167	0.860890345
TAF13	0.441277122	0.285990541	0.938133842	0.484160601	0.092360416	0.399247445	0.860890345
ATF7IP	0.26784442	0.718505679	0.091994919	0.500240082	0.600073334	0.399882532	0.861169554
KIAA0100	0.743165248	0.785701495	0.851371961	0.320428541	0.033351276	0.399823647	0.861169554
LOC101904308	0.901122993	0.356561743	0.083975531	0.472776821	0.416641634	0.399894406	0.861169554
LOC112443485	0.556847628	0.44533112	0.339052693	0.525720906	0.12003533	0.399610785	0.861169554
UROD	0.62920817	0.278886181	0.689183463	0.070946928	0.619190991	0.399826891	0.861169554
VGLL4	0.373788883	0.111704827	0.340841511	0.626166718	0.596458677	0.399906194	0.861169554
XCR1	0.392586743	0.031344081	0.919753313	0.619091479	0.758615444	0.399913057	0.861169554
XPO6	0.8723048	0.0728662	0.378912555	0.312156835	0.706127261	0.399702155	0.861169554
TMEM214	0.848966799	0.545295461	0.446248274	0.044116681	0.583547459	0.400004798	0.8612541
FAM110B	0.164574734	0.960510864	0.952513318	0.072195856	0.48984444	0.40020772	0.861577973
GGA3	0.063526691	0.350402096	0.579349972	0.413589657	0.999362469	0.400380323	0.861727603
ZNF527	0.938069318	0.121003454	0.358332588	0.391532681	0.334714555	0.400382242	0.861727603
BCAS2	0.682802808	0.296186394	0.965899308	0.652884166	0.041839842	0.400557631	0.861937175
LOC515042	0.969062402	0.033208445	0.436677901	0.555835862	0.683251752	0.400584659	0.861937175
DCP1A	0.547322276	0.231073273	0.691391474	0.132448225	0.461463433	0.400819505	0.862329431
ACOT8	0.35365284	0.478842104	0.63741583	0.126864047	0.390879849	0.401078407	0.862444905
ATRIP	0.750875228	0.289126338	0.631545231	0.288128004	0.135861369	0.401527061	0.862444905
CD160	0.808533518	0.326962366	0.418458223	0.820531623	0.059083195	0.401398586	0.862444905
CHTF8	0.569253375	0.619141634	0.856972153	0.241302893	0.073699751	0.401661472	0.862444905
CYB561D2	0.943203607	0.642794274	0.615279363	0.263872331	0.054539164	0.401569657	0.862444905
DNMT1	0.544609474	0.235813585	0.170211882	0.673006711	0.364495315	0.401378609	0.862444905
ERCC1	0.328961414	0.376340822	0.406270029	0.346322091	0.3072184	0.401037799	0.862444905
FAM171B	0.083664526	0.724636032	0.311457765	0.452224298	0.627888084	0.40135606	0.862444905
GRIPAP1	0.052420615	0.210055402	0.888879093	0.58145183	0.942087089	0.401351015	0.862444905
LOC101904492	0.968192614	0.887845517	0.028117468	0.252593122	0.876343641	0.401000471	0.862444905
LOC529125	0.707260595	0.977767283	0.778803327	0.022439909	0.443720753	0.40138567	0.862444905

RAB11FIP2	0.661736812	0.926976254	0.178026215	0.938629156	0.052392095	0.401625623	0.862444905
REV1	0.493866865	0.357326809	0.818786443	0.098467863	0.377137353	0.401488166	0.862444905
RHBG	0.063976265	0.314746572	0.374743497	0.716833049	0.990406643	0.401221134	0.862444905
SNX13	0.932260584	0.116356685	0.288112408	0.886843198	0.193704852	0.401579591	0.862444905
BSN	0.744732146	0.829970984	0.446116955	0.103222706	0.188884104	0.401812056	0.86265537
RYR1	0.173873687	0.628580531	0.109302459	0.896950475	0.50216974	0.401951063	0.862840927
C3AR1	0.107197317	0.411997527	0.630675581	0.540405774	0.358194619	0.402289658	0.863061273
FAM53B	0.078973445	0.75554667	0.74737874	0.199962358	0.606169715	0.402713981	0.863061273
KCTD14	0.032584543	0.99676557	0.346011981	0.835950843	0.574620833	0.402495363	0.863061273
LOC104969097	0.356703192	0.208686036	0.259651488	0.423089086	0.660799092	0.402662702	0.863061273
LOC112442191	0.838259211	0.531888067	0.036120959	0.485867664	0.688532651	0.402165265	0.863061273
LOC788648	0.873618086	0.22144242	0.038504247	0.992267149	0.731649736	0.402789976	0.863061273
NDFIP2	0.298397172	0.682523837	0.506012993	0.100086746	0.523669454	0.402591668	0.863061273
PAICSP	0.339159598	0.761826546	0.15645895	0.223624672	0.59795429	0.402722733	0.863061273
PPIL1	0.729661232	0.236792513	0.895028683	0.147109882	0.237688413	0.402771928	0.863061273
PSME2	0.470915521	0.248901773	0.811482233	0.660659365	0.085802361	0.402290916	0.863061273
SAMHD1	0.341108819	0.513839366	0.247554804	0.312077918	0.398920347	0.402604337	0.863061273
SMIM30	0.809812061	0.117929045	0.433520087	0.518712074	0.251282484	0.402435653	0.863061273
TARSL2	0.659972775	0.260029525	0.104476498	0.659074325	0.456656838	0.402431351	0.863061273
WDR62	0.858645061	0.036180727	0.697493614	0.669539328	0.372020933	0.402463315	0.863061273
SNED1	0.936332891	0.072950291	0.184151037	0.683981442	0.628783606	0.402849336	0.863075778
ATOX1	0.386071991	0.386843448	0.485820725	0.324190368	0.230572493	0.403277131	0.863119788
GTF2H5	0.882988255	0.198207119	0.741055817	0.063848685	0.655287053	0.403362499	0.863119788
LAPTM5	0.305952463	0.319969953	0.124869678	0.465947056	0.95267345	0.403359468	0.863119788
LOC107131289	0.243892185	0.282854221	0.671506484	0.55708013	0.209988621	0.403137228	0.863119788
LOC514011	0.560747264	0.081234074	0.986434796	0.33602069	0.359252861	0.403297505	0.863119788
MAGEE2	0.211592353	0.682209501	0.259883868	0.205218726	0.703525468	0.403048471	0.863119788
S100A14	0.652843717	0.504707085	0.224393487	0.080739758	0.907379725	0.403064474	0.863119788
SLC5A2	0.305895305	0.243533579	0.383286226	0.463905485	0.408959844	0.403075901	0.863119788
TTPA	0.806091145	0.825859115	0.015841675	0.555648303	0.926198045	0.403395818	0.863119788
ZSWIM1	0.313792681	0.306871724	0.790178754	0.180453519	0.394922297	0.403244315	0.863119788
SPON2	0.042699851	0.731294372	0.345905859	0.908426945	0.553443627	0.403490215	0.863209218
LOC104975635	0.658817586	0.535852989	0.67052261	0.116812653	0.196689676	0.403743121	0.863525135
MRS2	0.436334459	0.344857718	0.454890411	0.386245333	0.205670028	0.403706393	0.863525135
SLC39A6	0.454550607	0.456440262	0.432495052	0.104066436	0.582621905	0.403800632	0.863535598
S100A12	0.56399394	0.846874478	0.115039582	0.127883992	0.775423987	0.404051933	0.863960426
FAM101A	0.633524813	0.562629325	0.987202531	0.017436899	0.889846111	0.404391669	0.864123916
IRF1	0.891113697	0.821582766	0.578274591	0.047843278	0.269531936	0.404381354	0.864123916
RCL1	0.565359071	0.668993804	0.047335078	0.857012585	0.35575924	0.404351723	0.864123916
TOMM34	0.922896793	0.598958149	0.14156325	0.955481086	0.072963324	0.404256725	0.864123916

URGCP	0.06233758	0.608391797	0.94717963	0.949625503	0.159939119	0.404274428	0.864123916
ADGRB2	0.67955814	0.518215631	0.029772732	0.595471232	0.875177908	0.404521598	0.864175176
LOC781280	0.69733749	0.395097206	0.050931263	0.790706393	0.492608988	0.404574131	0.864175176
NFATC4	0.279606143	0.139006064	0.557034526	0.432537068	0.583668573	0.404575371	0.864175176
PPP1R11	0.971033206	0.052864331	0.320632709	0.334286855	0.99371107	0.404626291	0.864175176
CRAT	0.205509062	0.619023103	0.466419609	0.107297529	0.859208636	0.404710837	0.864243271
BRD9	0.03656978	0.812551928	0.681016795	0.550341991	0.492134247	0.405037095	0.864338578
CXHXorf38	0.228034329	0.436082914	0.531399715	0.838566597	0.123748662	0.405122553	0.864338578
LOC101903261	0.928535894	0.930595975	0.115463303	0.711548264	0.077249726	0.405136643	0.864338578
MTERF3	0.377645861	0.56714101	0.298320888	0.391556459	0.218894175	0.4048982	0.864338578
NRF1	0.35589261	0.679112664	0.94168877	0.035615759	0.676703943	0.405176813	0.864338578
RAB3B	0.179468665	0.639945214	0.06526391	0.951905254	0.76781503	0.404962643	0.864338578
SLC44A3	0.442322248	0.942728116	0.167658387	0.389085464	0.201502556	0.405048128	0.864338578
TNIP2	0.826881274	0.096862037	0.913303162	0.11416164	0.656744355	0.405145574	0.864338578
FAM114A2	0.422405636	0.332093611	0.147837317	0.312418166	0.847365622	0.405320718	0.864420833
RNASEL	0.240322358	0.051295054	0.654382951	0.736216108	0.924276964	0.405292761	0.864420833
CLEC4A	0.295723363	0.280869657	0.390114838	0.480678991	0.352810725	0.405474748	0.864636967
RGL2	0.846969145	0.04513027	0.178228263	0.881800988	0.915482372	0.405611171	0.864815502
BAAT	0.963683909	0.040967369	0.563583634	0.48100583	0.514161777	0.405707281	0.864908053
LOC112448335	0.2184144	0.361640973	0.632114556	0.510006637	0.216176058	0.405767975	0.864925085
LOC107132450	0.722355121	0.107544104	0.24636948	0.454718461	0.63275646	0.405831926	0.864949057
AXL	0.336444483	0.958099246	0.790563627	0.213384176	0.101323216	0.405920309	0.865025087
TMEM273	0.468418802	0.327384349	0.445219935	0.835799409	0.096608753	0.40601837	0.865121718
COPS3	0.732884301	0.122914874	0.475859662	0.622721637	0.20686599	0.406295624	0.86560009
LOC112446383	0.760793969	0.14669307	0.136413311	0.755231437	0.480726776	0.406454331	0.865772053
RAB19	0.689022682	0.347055098	0.636833415	0.796891127	0.045553955	0.406481852	0.865772053
LOC104970589	0.780617517	0.069339064	0.500638549	0.41473292	0.492671792	0.40674668	0.866010859
METTL3	0.379040054	0.658383186	0.687670754	0.044728062	0.721367803	0.406752282	0.866010859
MRM3	0.589659031	0.344688219	0.613684343	0.242388001	0.183109708	0.406718859	0.866010859
PRDM11	0.835620105	0.962256692	0.367634258	0.710396618	0.026375815	0.406807028	0.866015066
CHN1	0.854939698	0.782577848	0.023445592	0.465658367	0.758862911	0.406935794	0.866176824
AZI2	0.803659707	0.725222019	0.039853045	0.835471835	0.28591772	0.40709953	0.866188303
LOC112442214	0.218858425	0.227759641	0.258574275	0.689207669	0.624430336	0.407053759	0.866188303
WDR59	0.213741826	0.3142295	0.698336282	0.95331348	0.124038255	0.407027802	0.866188303
CEP192	0.961877764	0.179403862	0.101152426	0.323230835	0.985853177	0.407516369	0.866404528
CREG1	0.422173106	0.571826689	0.214263144	0.835906128	0.128688659	0.407573459	0.866404528
KCTD6	0.838999542	0.813383042	0.363740438	0.528888428	0.042352364	0.407453228	0.866404528
KDM2A	0.56559374	0.467747047	0.329944989	0.123461671	0.515764109	0.4073952	0.866404528
KMT5A	0.6333024	0.152207374	0.633916971	0.796292294	0.114354321	0.407575032	0.866404528
LOC112444585	0.03826558	0.662890136	0.821739497	0.54240333	0.49184071	0.407469008	0.866404528

MINDY2	0.670272536	0.116228235	0.332880725	0.388858755	0.552105225	0.4076763	0.866404528
NARS	0.77098623	0.370242391	0.208612275	0.356628777	0.262088007	0.407626161	0.866404528
RILPL1	0.496932385	0.114449658	0.774062405	0.31724185	0.39770494	0.407278442	0.866404528
LOC782799	0.094931314	0.686101922	0.636343244	0.305279525	0.44031661	0.407787553	0.866492254
RMDN2	0.402615615	0.139499694	0.493206364	0.310878512	0.647084383	0.407823178	0.866492254
CPSF2	0.400998489	0.173209491	0.608309164	0.452390387	0.291769327	0.407958024	0.866666555
CHIC1	0.131100925	0.55632542	0.84722938	0.356872654	0.253268902	0.408205974	0.866856655
RPRML	0.306323662	0.103755263	0.875641657	0.939553801	0.213524936	0.408150303	0.866856655
TLDC2	0.728785905	0.115032352	0.469368419	0.28347297	0.500670367	0.408192997	0.866856655
ABHD2	0.115576227	0.492800598	0.811590307	0.220169715	0.5516971	0.409100673	0.867024664
BUB3	0.805619052	0.251778578	0.638269367	0.370916321	0.116637694	0.408686014	0.867024664
CPOX	0.692536143	0.321587649	0.245248388	0.554836139	0.184563472	0.408449591	0.867024664
CWC25	0.665309013	0.578344116	0.740735295	0.110466544	0.177989152	0.408775217	0.867024664
GTPBP2	0.595937402	0.318636431	0.317036956	0.119552725	0.777504042	0.408530979	0.867024664
ID4	0.032512736	0.270439307	0.857000429	0.972071039	0.765559891	0.408885713	0.867024664
LCORL	0.575555431	0.538095699	0.205145502	0.151186804	0.582874824	0.40862019	0.867024664
LOC101906545	0.124814057	0.669314117	0.27201774	0.607561152	0.405034007	0.408416105	0.867024664
LOC112446369	0.406448272	0.423712139	0.274483327	0.234197249	0.506984659	0.409036135	0.867024664
LOC534578	0.251783795	0.379176477	0.259788056	0.287998675	0.784622576	0.408791748	0.867024664
RAP2A	0.440160837	0.106734823	0.202294153	0.852578926	0.691898533	0.408845438	0.867024664
RASL10B	0.721507053	0.143087781	0.487663537	0.155083472	0.719177069	0.409112286	0.867024664
RILP	0.562129518	0.207507632	0.168673188	0.578452915	0.492394277	0.408775397	0.867024664
RNF5	0.899236562	0.023863853	0.737368141	0.437187503	0.811791958	0.4091304	0.867024664
SDR16C5	0.772436385	0.999786466	0.411708019	0.126905537	0.138602676	0.408431358	0.867024664
ZNF473	0.521840505	0.018530191	0.985647546	0.669600601	0.87973051	0.409088998	0.867024664
TMEM219	0.344399408	0.330756761	0.414456907	0.290326107	0.410094943	0.409289587	0.867250021
CHD6	0.904922585	0.101782434	0.230048422	0.960936722	0.276764893	0.409712672	0.867388402
DYNLL2	0.43574102	0.081004783	0.307500518	0.549470779	0.944555281	0.409653423	0.867388402
FBXO10	0.930729151	0.422289783	0.046923606	0.736031732	0.415423146	0.409830581	0.867388402
LOC781499	0.918593725	0.568804696	0.084919616	0.249578438	0.508555508	0.40960799	0.867388402
LRRC1	0.944928588	0.297499918	0.426860509	0.107601729	0.436501615	0.409738965	0.867388402
MRPS18B	0.59051083	0.134818195	0.699374257	0.244361881	0.414380819	0.409794266	0.867388402
UBE2QL1	0.598483955	0.39425904	0.062362087	0.702601794	0.545045738	0.40970789	0.867388402
VAMP2	0.303029122	0.17243865	0.296769936	0.472840311	0.767817563	0.409557961	0.867388402
WFS1	0.185281623	0.177591243	0.525938951	0.465714699	0.698722074	0.409597704	0.867388402
CLYBL	0.436311916	0.514282598	0.109247466	0.488745002	0.472323498	0.41042186	0.867565282
FITM1	0.68161687	0.712057958	0.612515855	0.153276794	0.124156544	0.410377831	0.867565282
GALNT11	0.604808781	0.337025977	0.338995821	0.121761164	0.671979896	0.410269576	0.867565282
LOC104975974	0.543112634	0.087312577	0.329701813	0.897084401	0.402983658	0.410218171	0.867565282
LOC112448808	0.946444045	0.14878395	0.363092547	0.22346915	0.494836906	0.410272355	0.867565282

NKAIN2	0.140607281	0.37692792	0.979447687	0.4729271	0.230205278	0.410197957	0.867565282
PCBD1	0.153334973	0.478156802	0.508829304	0.248486453	0.610490419	0.41043474	0.867565282
PDIK1L	0.680438944	0.457928592	0.591542907	0.918100349	0.033421086	0.410325229	0.867565282
PLOD1	0.737542833	0.47252634	0.064773677	0.315791415	0.793914655	0.410442804	0.867565282
SEC23A	0.142477999	0.23969194	0.276312676	0.902481796	0.664167599	0.410338578	0.867565282
BICRA	0.196017109	0.348246743	0.942271783	0.195732636	0.452689438	0.411625071	0.867594031
C18H16orf74	0.554600663	0.381636666	0.477110437	0.188565316	0.298522881	0.411183868	0.867594031
CNP	0.597940909	0.704836917	0.187557537	0.711555703	0.101269968	0.411527677	0.867594031
EBAG9	0.687425698	0.037970316	0.582291229	0.988292967	0.378402776	0.411168831	0.867594031
GLYCTK	0.225090157	0.486311838	0.801459709	0.072736041	0.889966326	0.411023137	0.867594031
ISY1	0.370736894	0.044142866	0.600999564	0.969862957	0.596835041	0.411447444	0.867594031
KL	0.804321753	0.491377585	0.343988745	0.076809392	0.544420614	0.411203135	0.867594031
LOC107132262	0.084637409	0.884395965	0.398097302	0.35359896	0.539550211	0.411204271	0.867594031
LOC112446013	0.333661559	0.216963193	0.767658222	0.911961649	0.112451817	0.411618194	0.867594031
LOC112447305	0.841186122	0.901323133	0.504320329	0.693934417	0.021470001	0.411550508	0.867594031
LYRM7	0.75854714	0.192594124	0.494426232	0.546703848	0.143749957	0.410949395	0.867594031
MYO5B	0.524078782	0.403151022	0.114345056	0.4064223	0.578841252	0.411156557	0.867594031
NDUFV3	0.433384813	0.465940422	0.655722437	0.12991836	0.330633244	0.411282195	0.867594031
NHSL1	0.05921784	0.586671899	0.3143435	0.852717144	0.610062974	0.411083801	0.867594031
PECR	0.757557277	0.187878473	0.893867106	0.368478545	0.121155399	0.411040338	0.867594031
PIDD1	0.49164534	0.138968028	0.184808749	0.768462504	0.587350673	0.411620653	0.867594031
PPFIA2	0.240978675	0.371145683	0.574207271	0.121217635	0.911907814	0.410957766	0.867594031
PSMA5	0.854305864	0.108424598	0.938658401	0.177020208	0.369334972	0.411185126	0.867594031
RFC5	0.846432348	0.241074476	0.114880558	0.655530715	0.369582299	0.411030131	0.867594031
SH3D21	0.565755558	0.333373287	0.180354473	0.6236202	0.268462998	0.411496549	0.867594031
SPATS2	0.963351861	0.318372633	0.438800971	0.087947727	0.479835807	0.411034531	0.867594031
TMEM39B	0.087276633	0.63382101	0.781482201	0.295001829	0.447019383	0.411669492	0.867594031
TRIM59	0.231504378	0.889956107	0.141937694	0.327680489	0.594930102	0.411672337	0.867594031
CLEC12A	0.449328913	0.405723456	0.714638802	0.352615928	0.124207321	0.41182368	0.867595267
FARP1	0.683385567	0.135756408	0.169868739	0.39541903	0.915920287	0.411872293	0.867595267
LOC104975626	0.830504905	0.902523165	0.036775594	0.443487713	0.466744816	0.41182023	0.867595267
MFAP5	0.481668524	0.313383718	0.196772417	0.361878025	0.531049176	0.41188439	0.867595267
C29H11orf98	0.704206115	0.115788969	0.975222674	0.885245521	0.081144341	0.412004084	0.867624665
GRIK3	0.213871312	0.801039055	0.429055941	0.558371448	0.139153455	0.411982472	0.867624665
GRIA3	0.226429272	0.761870377	0.333524017	0.128266132	0.774388874	0.412089562	0.867693329
PPP1R8	0.977792939	0.250546771	0.652459459	0.396102734	0.090321083	0.412196594	0.867807352
ST3GAL6	0.603908216	0.159595739	0.679731274	0.505089968	0.173054577	0.412428818	0.86818488
PRC1	0.727060611	0.068166304	0.240164777	0.711850828	0.67655993	0.412609786	0.868454432
ABCD1	0.964329689	0.129737979	0.683743771	0.341749546	0.196210928	0.412716072	0.868505933
CCDC12	0.100706095	0.390355164	0.292722376	0.572089978	0.873067882	0.413054566	0.868505933

CLCN7	0.251215523	0.532112641	0.079699757	0.874571434	0.616681462	0.413007409	0.868505933
FAM98C	0.255523536	0.334279168	0.281068871	0.573112382	0.417470257	0.412950805	0.868505933
IL18	0.660777122	0.786388565	0.015105456	0.734365171	0.996194533	0.412897674	0.868505933
MLLT11	0.70645519	0.345045914	0.327665437	0.351725945	0.204595115	0.413057632	0.868505933
SLC17A9	0.43896845	0.338265888	0.119437454	0.648466009	0.498870475	0.412751166	0.868505933
XIRP1	0.24694895	0.550309056	0.8099795	0.073519953	0.709977057	0.412997324	0.868505933
LOC101906266	0.212140691	0.341337866	0.159926786	0.951697661	0.522159747	0.413268686	0.868838381
LOC510613	0.357444163	0.321363404	0.077642576	0.70641038	0.913955477	0.41336818	0.868936237
ICA1	0.214703414	0.097200346	0.369729764	0.815365804	0.916571754	0.413611852	0.869003213
MGAT2	0.849641012	0.350623198	0.408439525	0.956219105	0.049555032	0.413587973	0.869003213
PSMD6	0.721396065	0.191563909	0.828827243	0.507552415	0.099137083	0.413517407	0.869003213
RNF38	0.966046519	0.031935673	0.225631357	0.886132893	0.934676157	0.413581932	0.869003213
LOC101905228	0.703148649	0.427646531	0.534652567	0.222032341	0.161624556	0.41369676	0.869070344
RCC1	0.682303855	0.230982163	0.903276874	0.876607551	0.046246857	0.413750419	0.869071821
ZNF322	0.210056218	0.71315938	0.406598149	0.183875041	0.515569973	0.413841655	0.869152214
LOC104975861	0.483411623	0.584183776	0.104729646	0.948832632	0.205982539	0.414020878	0.869417355
CTSH	0.353110131	0.712216166	0.06551424	0.53654896	0.65415877	0.414096758	0.869423044
NCS1	0.778034148	0.092433333	0.191009642	0.645033852	0.652783217	0.414129543	0.869423044
YWHAE	0.919170523	0.319281876	0.160496806	0.517619894	0.237460136	0.414287735	0.869643901
ZSWIM3	0.094739634	0.644194103	0.926030502	0.704544733	0.145452176	0.414351946	0.86966745
HOXD9	0.627370603	0.487376534	0.354775583	0.089733817	0.59544799	0.414484972	0.869806457
LOC788201	0.111768597	0.816447778	0.422638793	0.165107505	0.910454569	0.414524179	0.869806457
ABHD10	0.49186155	0.358251399	0.641249409	0.127736727	0.404109603	0.415554188	0.869963987
ACO1	0.576634341	0.286466489	0.042164759	0.996512576	0.839773569	0.415435151	0.869963987
ARHGAP39	0.031292843	0.514925086	0.731288284	0.894462753	0.551997237	0.415124847	0.869963987
ARL1	0.814849134	0.131584448	0.367330791	0.686661505	0.214974141	0.4150039	0.869963987
ATP13A3	0.350284988	0.691146625	0.216415274	0.212896027	0.523068907	0.415606463	0.869963987
CARD14	0.063430989	0.495843599	0.406517019	0.598637623	0.760145407	0.41512828	0.869963987
CDK2	0.940336549	0.411241478	0.556582384	0.337008497	0.080229002	0.415166005	0.869963987
EGFL8	0.096641236	0.714439812	0.126172862	0.866757746	0.772185707	0.415491826	0.869963987
FAM43A	0.111122794	0.472002191	0.352902192	0.842558148	0.373739283	0.415435629	0.869963987
FITM2	0.118915019	0.596930275	0.322938168	0.377273064	0.672210905	0.414993333	0.869963987
GPATCH4	0.296187825	0.17037743	0.606825672	0.916570549	0.207655157	0.41542845	0.869963987
LOC100337323	0.718710573	0.723203701	0.483735945	0.031321331	0.740445455	0.415508578	0.869963987
LOC107131330	0.91806223	0.222542932	0.191584317	0.197754551	0.752485698	0.41531827	0.869963987
LOC112443151	0.975741297	0.572212057	0.101363641	0.466406288	0.220057179	0.414849541	0.869963987
SCAF11	0.2486584	0.596733319	0.96115761	0.046887781	0.870132224	0.415142909	0.869963987
SDHAF3	0.739432268	0.097441515	0.726948102	0.964110486	0.114977369	0.414775614	0.869963987
TPX2	0.868296602	0.860683747	0.347525399	0.205083043	0.109384619	0.415362795	0.869963987
TRAF6	0.246226493	0.630405833	0.266283465	0.762983483	0.184252822	0.414910096	0.869963987

ZFYVE19	0.454210287	0.998610538	0.060513553	0.322661072	0.65584455	0.414841484	0.869963987
ARL6IP4	0.170967007	0.144646098	0.758983882	0.636408847	0.488948972	0.415780984	0.870042807
NOX4	0.402616402	0.787547233	0.925085039	0.246309674	0.08079958	0.415698264	0.870042807
PGLS	0.32787287	0.6370188	0.886742097	0.193449535	0.163040528	0.415807245	0.870042807
SF3B3	0.943294749	0.769501187	0.245156763	0.054092771	0.607020456	0.415856181	0.870042807
AGPAT1	0.859153817	0.999737124	0.654290741	0.076621194	0.136707271	0.417121549	0.870450133
BEX5	0.181202684	0.594362977	0.717567057	0.211045507	0.359013624	0.416218037	0.870450133
IL15	0.858423444	0.990490456	0.168413914	0.178530642	0.230415751	0.417234152	0.870450133
KLF12	0.893923301	0.384823688	0.625281875	0.073862351	0.370351046	0.417045588	0.870450133
LOC101906206	0.942391017	0.135108809	0.83753468	0.267759864	0.206147061	0.417110626	0.870450133
LOC101907744	0.096216162	0.809264095	0.484418737	0.420982705	0.371043307	0.417270806	0.870450133
LOC112444171	0.878498648	0.497658281	0.0858145	0.707091622	0.221626297	0.416910286	0.870450133
LOC539973	0.703121303	0.242772247	0.227196782	0.742712303	0.203701386	0.416563347	0.870450133
MAP9	0.990565686	0.118219632	0.292776155	0.234634525	0.729336395	0.416556407	0.870450133
MATR3	0.142468312	0.551460372	0.671324379	0.246629142	0.450937294	0.416516055	0.870450133
MED23	0.03385099	0.64668662	0.765918242	0.439599591	0.796607391	0.416682337	0.870450133
MRPS25	0.331782523	0.615936103	0.952208168	0.092144154	0.327648321	0.416779491	0.870450133
NHLRC1	0.411217413	0.534303416	0.065984495	0.832233209	0.487742316	0.41707002	0.870450133
PI15	0.737834116	0.686151508	0.7607715	0.109414469	0.139766991	0.417217498	0.870450133
PLEKHG6	0.520527862	0.328818096	0.072714988	0.695701547	0.67866716	0.416821346	0.870450133
RAB11B	0.985950299	0.821838273	0.263031882	0.442666884	0.062235701	0.416689056	0.870450133
RPS6KA4	0.1989101	0.737505899	0.201211416	0.372258868	0.535768901	0.417133118	0.870450133
RTN4RL1	0.043188612	0.642847385	0.607751839	0.354247151	0.980001919	0.41628503	0.870450133
SCAMP2	0.831494975	0.480283786	0.024833506	0.91908409	0.646286183	0.417242378	0.870450133
TAMM41	0.106134867	0.900742626	0.735993145	0.483304288	0.17220553	0.41623153	0.870450133
TMEM199	0.936704495	0.036533195	0.637668951	0.297771783	0.902887018	0.416546239	0.870450133
TMEM200B	0.755907377	0.809974571	0.736996061	0.240100387	0.054282686	0.416961062	0.870450133
ZNF613	0.48995041	0.296097832	0.933556915	0.301988985	0.143589531	0.416718742	0.870450133
DHRS9	0.221558373	0.259717147	0.525022912	0.215740124	0.904670422	0.417404467	0.870588024
SYTL4	0.25296997	0.894425733	0.504920002	0.231785738	0.222722411	0.417443005	0.870588024
BNC2	0.287848405	0.343094835	0.467851239	0.384716218	0.332219625	0.417665046	0.870612822
DDR2	0.058639752	0.487064926	0.528757246	0.810520265	0.483238473	0.417943762	0.870612822
DGKD	0.891002212	0.573542118	0.552965348	0.023340829	0.896887823	0.417957809	0.870612822
HK3	0.586032545	0.345554627	0.124024191	0.869753238	0.270304359	0.417642405	0.870612822
IVD	0.476223953	0.704650422	0.979387173	0.038844022	0.462977884	0.417811339	0.870612822
LOC787397	0.76107389	0.537810882	0.08941968	0.724717449	0.222761097	0.417761731	0.870612822
POP1	0.594578815	0.438309198	0.744991283	0.443286169	0.068663402	0.417782607	0.870612822
RNF39	0.983610817	0.296840334	0.132538624	0.50070462	0.304840177	0.417700768	0.870612822
SNX30	0.886404705	0.246916811	0.755815964	0.07470736	0.478320509	0.417833884	0.870612822
TUBA8	0.140387524	0.338671313	0.433191851	0.359482198	0.799101633	0.417985402	0.870612822

ALG12	0.181897754	0.636444889	0.439300245	0.164043294	0.709903849	0.418158789	0.870752935
SCOC	0.448617387	0.207996394	0.506546981	0.608052813	0.206006041	0.418105824	0.870752935
ALG1	0.765757917	0.684128281	0.036040171	0.335626546	0.936012965	0.418413033	0.870840366
FAM126B	0.075200174	0.754475048	0.648834637	0.223964928	0.719134807	0.418347748	0.870840366
PAX8	0.17172391	0.911697335	0.126727575	0.855929667	0.348979632	0.418270135	0.870840366
RNF34	0.787582051	0.226376019	0.634040327	0.945509648	0.055477853	0.418363732	0.870840366
ADIRF	0.095888184	0.411764772	0.442017043	0.38708215	0.879183506	0.418641967	0.870898079
CAPN15	0.064703278	0.247510789	0.948686891	0.97427271	0.400930185	0.418506875	0.870898079
LOC100848148	0.223808675	0.418266093	0.329788867	0.322493775	0.596902095	0.418741926	0.870898079
LOC104974890	0.028756764	0.96843693	0.329236777	0.702909128	0.922178401	0.41875917	0.870898079
SH3D19	0.229555609	0.323026006	0.20385211	0.456241336	0.861736279	0.418749642	0.870898079
SLIT3	0.225794418	0.696027167	0.054718793	0.948680494	0.727981063	0.41863409	0.870898079
SHCBP1	0.995380315	0.241284344	0.148518891	0.58985568	0.282654677	0.418864644	0.871007054
ABCC1	0.597474825	0.387137781	0.971286641	0.028603971	0.933504973	0.420351325	0.871486681
ASCC2	0.062764956	0.429368174	0.38476835	0.867157	0.666052605	0.420066328	0.871486681
BLZF1	0.499724112	0.208404404	0.527381662	0.756370352	0.143965123	0.419830374	0.871486681
CHSY3	0.506277303	0.069973624	0.279220679	0.76904499	0.783495048	0.419241226	0.871486681
CRY1	0.326437559	0.832616653	0.075250435	0.401883736	0.730586901	0.420529183	0.871486681
FUT11	0.039287313	0.96710292	0.578311803	0.393538121	0.690263823	0.419489572	0.871486681
GLIS2	0.682817567	0.349511438	0.112108585	0.435672494	0.514241965	0.420216102	0.871486681
GPRIN3	0.612553337	0.433956765	0.13060891	0.2291505	0.753845263	0.420308204	0.871486681
HS1BP3	0.121942965	0.650473573	0.28394313	0.552941082	0.481672187	0.420340538	0.871486681
LOC101906632	0.641343332	0.701094356	0.157136089	0.828662156	0.102078749	0.419712882	0.871486681
LOC107131224	0.792865585	0.722357544	0.823951834	0.260449341	0.048861543	0.420536088	0.871486681
LOC112444288	0.942945386	0.12502792	0.273252558	0.652318817	0.284476736	0.419755676	0.871486681
LOC112444350	0.094405291	0.206214564	0.768431958	0.570093355	0.702743944	0.420189124	0.871486681
LOC534627	0.552779364	0.7428333	0.215128735	0.082958014	0.815005289	0.419596479	0.871486681
MGME1	0.740616114	0.068788567	0.439266622	0.460443905	0.580120073	0.419742987	0.871486681
MS4A7	0.267991577	0.147944532	0.440834277	0.497292284	0.690645323	0.420464371	0.871486681
NPR1	0.095543682	0.773926382	0.884614696	0.125812022	0.725448388	0.419527169	0.871486681
PNPLA2	0.096588324	0.598965274	0.520498053	0.35140591	0.564636256	0.419660625	0.871486681
POP5	0.575214705	0.320828084	0.749744612	0.501626189	0.086446262	0.420377578	0.871486681
RBM8A	0.515814892	0.217141342	0.546904761	0.254677128	0.384360094	0.42027277	0.871486681
RFX1	0.771003018	0.154824389	0.514199765	0.512109096	0.189997263	0.41958733	0.871486681
SHPK	0.66021076	0.548063675	0.040267907	0.428174857	0.962868816	0.420582202	0.871486681
SPTY2D1OS	0.520303933	0.121252227	0.537253961	0.896190539	0.196509319	0.419497833	0.871486681
TMEM67	0.388031986	0.885615377	0.098413033	0.265564963	0.668274872	0.420436364	0.871486681
TTC32	0.74032487	0.445346925	0.221416154	0.110082907	0.746819315	0.420426242	0.871486681
ZDHHC14	0.015479995	0.753873676	0.965427408	0.556810516	0.95748814	0.420569934	0.871486681
ZDHHC7	0.810492753	0.341210184	0.071484438	0.766434287	0.393878476	0.419462318	0.871486681

ZNF500	0.925592987	0.134848637	0.156053284	0.708598279	0.434741169	0.420388723	0.871486681
NEDD9	0.584787467	0.350484016	0.536308523	0.087892337	0.622321331	0.420734359	0.871691902
FUK	0.797093441	0.180927262	0.196134095	0.330909852	0.642856405	0.420869827	0.871726645
NXPH3	0.858449405	0.040278361	0.688417232	0.756634344	0.334023155	0.420834644	0.871726645
PDP1	0.188742534	0.552369582	0.670577296	0.19242873	0.44737813	0.420910483	0.871726645
AP3D1	0.02572338	0.611261633	0.763157875	0.79396009	0.632289164	0.421064765	0.871835881
PPCDC	0.630831142	0.528730945	0.296960787	0.149753142	0.406263317	0.421122603	0.871835881
TOR1A	0.66051154	0.680798653	0.243079843	0.190004133	0.290069808	0.421076464	0.871835881
FAM151B	0.560198728	0.383504755	0.196835623	0.611435132	0.233328732	0.421321828	0.872028316
NGLY1	0.916107769	0.982347207	0.181715001	0.074753615	0.493497545	0.421315659	0.872028316
SYNGR1	0.102126622	0.645268696	0.579203674	0.201936837	0.783082825	0.421400377	0.872080906
LOC112441839	0.72338508	0.270344796	0.156973061	0.427483123	0.460114965	0.421465096	0.872104866
LOC101906363	0.804152185	0.339590158	0.620655922	0.116577533	0.306037421	0.421716134	0.872514307
LOC104972390	0.5182451	0.265177732	0.456276797	0.223112874	0.432377105	0.421777096	0.872530433
CMIP	0.451648906	0.073115369	0.612095986	0.59283251	0.506194092	0.422247455	0.872879419
LARP1	0.61546707	0.752482897	0.082052028	0.397095352	0.401745691	0.422152021	0.872879419
MCCC1	0.675359297	0.486490785	0.433083014	0.296628936	0.143722403	0.422263417	0.872879419
NSMF	0.514857267	0.806836335	0.378403515	0.143308572	0.269079519	0.422129675	0.872879419
PLK1	0.31948132	0.487480479	0.366310525	0.714818699	0.148661784	0.422156185	0.872879419
TLCD1	0.327487138	0.054028081	0.735238106	0.47028874	0.991860578	0.422318115	0.872879419
TM4SF19	0.304496135	0.549926578	0.193997447	0.799525255	0.233631883	0.422314081	0.872879419
COX18	0.938162526	0.243802709	0.149483989	0.592321585	0.299754041	0.422388043	0.872914013
GOSR2	0.761468475	0.604203245	0.791426589	0.072345117	0.230569137	0.422475864	0.87298557
ATOH8	0.504311909	0.627294697	0.462869678	0.625573397	0.066336187	0.422555869	0.873040963
ITPA	0.268031529	0.332633729	0.882740674	0.11968428	0.645595542	0.422684233	0.873086337
LOC101902174	0.425943158	0.969408407	0.451123371	0.473584481	0.068920089	0.422650265	0.873086337
CCNY	0.765825217	0.167543354	0.171780328	0.999305957	0.276311337	0.422820508	0.87325791
ACKR1	0.194209803	0.550428437	0.07769784	0.882405074	0.83348664	0.423461627	0.873631636
AP4M1	0.924690899	0.071052213	0.902532878	0.700900434	0.147399059	0.423952937	0.873631636
CDKN1B	0.192459299	0.650935829	0.886971329	0.444215164	0.125598415	0.42600588	0.873631636
CEBPD	0.510414935	0.366680348	0.16150196	0.26866342	0.755449342	0.424196365	0.873631636
CEP170B	0.174769669	0.399408434	0.179533396	0.64132426	0.771639366	0.42606799	0.873631636
CGNL1	0.507254884	0.753105125	0.40239461	0.785250051	0.051384446	0.426089063	0.873631636
CLCN4	0.716375901	0.868855508	0.062326025	0.242930133	0.647133514	0.423178874	0.873631636
CLIC3	0.09723451	0.445517977	0.331836188	0.464976864	0.922528302	0.425075625	0.873631636
CYTH4	0.536011298	0.140900691	0.408177313	0.519314891	0.384283129	0.424678081	0.873631636
DBNDD1	0.472220856	0.449922458	0.477680465	0.072008998	0.836665948	0.423624582	0.873631636
FRMPD4	0.389286471	0.433190779	0.075003211	0.633816218	0.767331604	0.424660945	0.873631636
FUNDC1	0.124905412	0.309685905	0.865572418	0.440531092	0.416491726	0.424428741	0.873631636
GABRA1	0.314566553	0.758462039	0.084496751	0.964572599	0.316264125	0.424619891	0.873631636

GNG2	0.24300574	0.263377532	0.195354237	0.888167824	0.550716907	0.423657194	0.873631636
HIST2H2BE	0.629756423	0.660763008	0.558503847	0.323511384	0.08190529	0.42484779	0.873631636
HOOK2	0.015808682	0.775873339	0.941002	0.895451498	0.598493506	0.425614533	0.873631636
ISPD	0.902273286	0.267777613	0.6377872	0.16678876	0.240169016	0.425254736	0.873631636
L3MBTL3	0.766754662	0.341101982	0.382075618	0.110800944	0.559156679	0.425768733	0.873631636
LOC101902211	0.145571487	0.189440818	0.351084507	0.747384235	0.850277632	0.424697194	0.873631636
LOC101905141	0.805420282	0.719738679	0.285921963	0.997118173	0.037071448	0.423969906	0.873631636
LOC101905254	0.711271678	0.848477155	0.609650869	0.275852653	0.060872074	0.425405603	0.873631636
LOC101906451	0.600710826	0.292042007	0.067879509	0.648421236	0.794010799	0.424089552	0.873631636
LOC104969027	0.293338736	0.41321855	0.579327725	0.144021574	0.604564874	0.423618573	0.873631636
LOC104970812	0.356169153	0.687170467	0.850437723	0.068776429	0.432143787	0.425635439	0.873631636
LOC104972031	0.062967211	0.351072368	0.806376312	0.705876156	0.488556122	0.424548765	0.873631636
LOC107132228	0.964621368	0.052849064	0.443949068	0.297134066	0.918392018	0.425348358	0.873631636
LOC107132757	0.344769781	0.435965334	0.113925528	0.759170229	0.471497267	0.424044957	0.873631636
LOC112441654	0.549933382	0.503300106	0.026856186	0.943873167	0.873730388	0.424065549	0.873631636
LOC112447408	0.603684775	0.198558084	0.121085238	0.888507395	0.480823058	0.42603413	0.873631636
LOC112447473	0.354927188	0.633825027	0.236880084	0.269400442	0.428726603	0.424756554	0.873631636
MRAS	0.127108339	0.259483252	0.541124674	0.675713075	0.511542643	0.425156721	0.873631636
MRPL52	0.445422001	0.235064984	0.786048166	0.349611775	0.214332712	0.425100546	0.873631636
MYO1F	0.544446169	0.279169075	0.728020059	0.136659351	0.407411964	0.42492422	0.873631636
NEURL1	0.124769572	0.949499459	0.732943665	0.915304118	0.076790003	0.423301907	0.873631636
NEXMIF	0.028880475	0.868029751	0.44679432	0.674646894	0.809339429	0.423662055	0.873631636
NOC3L	0.895185747	0.951226156	0.099495227	0.202819988	0.360852136	0.426035676	0.873631636
NPRL3	0.248310981	0.696026824	0.100420405	0.616645186	0.577467794	0.425467059	0.873631636
PGM2	0.431013151	0.609646645	0.436021069	0.077663243	0.694220945	0.425380719	0.873631636
PHF6	0.201271295	0.914766181	0.109324495	0.434744676	0.707349631	0.425733341	0.873631636
PXDC1	0.835280697	0.803379446	0.104254793	0.529758421	0.165559669	0.424227637	0.873631636
RAB3GAP1	0.793346276	0.24549359	0.186737355	0.547890926	0.308259317	0.42441107	0.873631636
RTF1	0.882867869	0.298332357	0.853753737	0.057132725	0.482441339	0.425963762	0.873631636
SCAMP3	0.443835626	0.114718178	0.950315933	0.222381887	0.573494786	0.425206875	0.873631636
SCLT1	0.743634252	0.986073266	0.03459433	0.318049193	0.765772752	0.425411785	0.873631636
SEPT10	0.518741781	0.138939428	0.533716662	0.701958326	0.226938642	0.424000083	0.873631636
SIPA1L3	0.58528849	0.037633227	0.74184384	0.863595063	0.436546781	0.424906825	0.873631636
SNX24	0.147576727	0.056187458	0.861899708	0.93496342	0.912076722	0.423062831	0.873631636
SPECC1	0.493950595	0.533722245	0.123607477	0.226286896	0.834192127	0.424658663	0.873631636
TM6SF1	0.833652326	0.016438159	0.637229601	0.707385597	0.995737873	0.424645289	0.873631636
TMEFF1	0.400269199	0.672155533	0.34395075	0.147689203	0.453298912	0.425881759	0.873631636
TMEM8B	0.443845227	0.960767713	0.407775665	0.92045848	0.038573389	0.425290976	0.873631636
TMEM9B	0.916984635	0.188210528	0.36476378	0.920182856	0.106715319	0.425511351	0.873631636
TRPM6	0.861175522	0.119951697	0.890951432	0.411902197	0.162784355	0.425209883	0.873631636

ZBTB32	0.503604144	0.198156373	0.119977101	0.979744428	0.526573301	0.425371957	0.873631636
ZC3H7B	0.263882483	0.573724332	0.647649677	0.288287553	0.216547928	0.42381258	0.873631636
ZNF362	0.860962343	0.530391256	0.553346678	0.046450687	0.5220443	0.423988214	0.873631636
ZNF770	0.858539369	0.425011453	0.672744016	0.133205068	0.188373638	0.424889649	0.873631636
ZRSR2	0.189542017	0.52689889	0.604789352	0.318866303	0.316800101	0.423257085	0.873631636
CUL4A	0.113815313	0.714045738	0.390102229	0.534848995	0.366590584	0.426464089	0.873968549
OSBPL8	0.984430546	0.352576422	0.105861171	0.427345691	0.39588505	0.426466403	0.873968549
TMEM92	0.228757439	0.346161623	0.131563084	0.945886406	0.630725876	0.42644429	0.873968549
WBP4	0.580736025	0.490985507	0.643891789	0.067232359	0.503198098	0.426329005	0.873968549
NFX1	0.416547852	0.686553239	0.739832931	0.091024946	0.323165311	0.426678811	0.874294664
FAM13B	0.963205828	0.557420742	0.227986184	0.071970888	0.707025837	0.426815932	0.874357292
LOC101902361	0.553669747	0.69232237	0.601746001	0.31338321	0.086154245	0.426784873	0.874357292
EML5	0.341213027	0.494303633	0.20376553	0.517800385	0.350166381	0.426889096	0.874398023
ARFGEF3	0.47855654	0.157381512	0.303360714	0.862004009	0.318039936	0.427784244	0.874434348
ARL13B	0.159846047	0.274784079	0.37049861	0.583245268	0.661680035	0.428238916	0.874434348
ATG12	0.949063008	0.17066241	0.423732205	0.929198102	0.098151239	0.427661366	0.874434348
C16H1orf53	0.314909969	0.428031044	0.314193718	0.420714067	0.351065158	0.427544157	0.874434348
CAD	0.209829823	0.198213737	0.337437787	0.593193636	0.751206668	0.427510996	0.874434348
CATSPERE	0.965284027	0.065808496	0.126166027	0.853205502	0.916171173	0.427813114	0.874434348
CBWD2	0.908457893	0.043645146	0.514141912	0.551770186	0.556851415	0.427777902	0.874434348
CCND3	0.956847906	0.515707558	0.116230317	0.329450156	0.331929564	0.428008843	0.874434348
CDS2	0.365434411	0.161026137	0.177044874	0.976093977	0.617071117	0.42809426	0.874434348
CLN3	0.231912204	0.088197687	0.703079117	0.74229951	0.588027266	0.428152239	0.874434348
CSRNP1	0.542919985	0.667383092	0.090598649	0.685423715	0.278995116	0.428162928	0.874434348
FKBP1A	0.756173618	0.266532563	0.386058546	0.167426507	0.480090297	0.427519558	0.874434348
HDAC11	0.376695563	0.398510243	0.197628442	0.274248521	0.771723697	0.428201589	0.874434348
HNRNPA3	0.172475995	0.268682822	0.398212381	0.556646077	0.608253273	0.427350402	0.874434348
HOPX	0.729909482	0.336667806	0.064773903	0.666256575	0.588791208	0.427241635	0.874434348
IRAK3	0.291354825	0.2888607	0.445404102	0.193516019	0.861488482	0.42738396	0.874434348
KLHL30	0.219950381	0.13796524	0.886323777	0.917578699	0.252776727	0.427080059	0.874434348
LOC100297099	0.496138407	0.775094086	0.298390673	0.129316915	0.422566539	0.427965637	0.874434348
LOC101902288	0.213690506	0.254738829	0.510356516	0.764219853	0.295688572	0.42817026	0.874434348
LOC107131498	0.952635575	0.455136944	0.172352006	0.109210428	0.766429692	0.42753927	0.874434348
MAPKAPK5	0.165334231	0.423152313	0.518032252	0.248348447	0.694509797	0.427433625	0.874434348
NR3C1	0.687251022	0.853813178	0.163460555	0.098423628	0.664615912	0.428072479	0.874434348
PSMD13	0.601407851	0.301231643	0.637277623	0.164393476	0.329112508	0.427302825	0.874434348
SLC25A11	0.149515738	0.278031578	0.895279793	0.212720256	0.792198155	0.428001282	0.874434348
ZDHHC9	0.53028323	0.141842305	0.728470157	0.144207459	0.793806473	0.42801932	0.874434348
BRF1	0.480687438	0.227525141	0.191226249	0.413982714	0.727500984	0.428747277	0.874696315
CCDC66	0.928019325	0.146649024	0.178096227	0.274749946	0.944471282	0.428493019	0.874696315

HK1	0.569733558	0.520140934	0.12363881	0.192257928	0.893601906	0.428635921	0.874696315
HNRNPLL	0.666600373	0.641419761	0.300687348	0.080518994	0.607536581	0.428483669	0.874696315
LAP3	0.7558523	0.767142525	0.047393101	0.536716987	0.426993162	0.428722937	0.874696315
LOC107131948	0.904655991	0.132079636	0.247252817	0.732188092	0.291189441	0.428748629	0.874696315
MUC1	0.598411898	0.556016705	0.502083558	0.412775417	0.091367942	0.428793605	0.874696315
PREPL	0.644126198	0.557691678	0.190478023	0.095633779	0.962170628	0.428674738	0.874696315
C18H19orf12	0.098622363	0.318138613	0.507234329	0.896388488	0.443151295	0.429382394	0.874712964
COQ4	0.41048465	0.409464997	0.973076126	0.101578334	0.380341375	0.429297241	0.874712964
CPNE2	0.227265661	0.613091573	0.105999925	0.489850724	0.874572363	0.429532154	0.874712964
DHDDS	0.439944981	0.169772278	0.717170063	0.733614492	0.160688285	0.429179317	0.874712964
IZUMO4	0.722642465	0.480036462	0.096756626	0.936206193	0.200738374	0.428996308	0.874712964
LOC104970387	0.280107871	0.766449098	0.067500881	0.757271078	0.575676032	0.429262317	0.874712964
LOC104975006	0.47002317	0.349862424	0.180786606	0.419195064	0.506343234	0.429061545	0.874712964
LOC531462	0.593149275	0.747372073	0.046541727	0.776182901	0.395083618	0.429521078	0.874712964
LOC614522	0.077135306	0.95343237	0.284630493	0.466263525	0.645935789	0.428902637	0.874712964
LOC781533	0.324454379	0.144158965	0.182705858	0.863245963	0.857565659	0.429502431	0.874712964
LYRM1	0.339279415	0.437994519	0.464358455	0.649681675	0.141059961	0.429436312	0.874712964
MOB3C	0.557044739	0.786259977	0.274867761	0.086601511	0.607144405	0.429601273	0.874712964
MVP	0.549091028	0.61781898	0.298790115	0.535875265	0.116535677	0.429600044	0.874712964
UGCG	0.480851987	0.395195536	0.356152228	0.183662133	0.507423719	0.428984018	0.874712964
ZCWPW2	0.541443925	0.128911558	0.732552289	0.157459117	0.783942879	0.429098303	0.874712964
CLPB	0.847499443	0.568040178	0.068873311	0.473515934	0.403406994	0.429700979	0.874778588
ETFRF1	0.762281492	0.139438961	0.275588031	0.530749181	0.408107321	0.430010661	0.874778588
FIBIN	0.257273653	0.654914519	0.462391328	0.106742496	0.763300888	0.430090143	0.874778588
LOC101905267	0.461345135	0.122698064	0.891346693	0.894276467	0.140724865	0.43014212	0.874778588
QSER1	0.236958665	0.82499128	0.936844053	0.502119117	0.069066066	0.430185141	0.874778588
RHOV	0.597487082	0.129742755	0.196147182	0.656726439	0.635924779	0.430154494	0.874778588
SLC22A23	0.610849843	0.153396459	0.098502756	0.726389363	0.948084566	0.43032646	0.874778588
SLU7	0.925465058	0.147224689	0.321561357	0.603540255	0.239848687	0.429940637	0.874778588
SMC4	0.614921519	0.147153167	0.147581236	0.588347094	0.80873706	0.430265247	0.874778588
TGM2	0.321319532	0.361912672	0.486923552	0.644085181	0.174220844	0.430258744	0.874778588
TMEM222	0.417035993	0.543996948	0.964598074	0.209539033	0.138600848	0.430299504	0.874778588
TNFRSF4	0.560539014	0.288099685	0.200061826	0.824629106	0.238139254	0.43000225	0.874778588
UGDH	0.517986195	0.774031433	0.184649655	0.168233677	0.510114399	0.43024305	0.874778588
PIP5K1A	0.136521059	0.473077774	0.976557772	0.265213192	0.380152497	0.430393682	0.874806876
LOC618939	0.700939997	0.028818065	0.494798215	0.740181623	0.859967794	0.430478001	0.874869904
DYSF	0.403879912	0.159424855	0.686206657	0.161576398	0.89284532	0.430806732	0.874897917
LOC101905786	0.365801366	0.730674208	0.091579488	0.635518371	0.409666917	0.430770365	0.874897917
LOC107132617	0.129941451	0.457223786	0.292950824	0.388297686	0.943162211	0.430808764	0.874897917
RMND1	0.863071405	0.42027814	0.72898659	0.034202665	0.704437837	0.43072218	0.874897917

SNHG3	0.786283912	0.553578217	0.287615662	0.428731226	0.118638908	0.430633306	0.874897917
VPS52	0.754670393	0.090845795	0.345091028	0.289747316	0.929853327	0.430811655	0.874897917
EFCC1	0.282339548	0.80190454	0.157135265	0.364867302	0.491980246	0.431139678	0.875285077
RARS	0.622442605	0.247896184	0.718720139	0.184333484	0.312516455	0.431201794	0.875285077
TGFB3	0.324021704	0.64866608	0.419057685	0.076243193	0.95061198	0.431068866	0.875285077
TNFSF18	0.834115432	0.291067575	0.142857103	0.219494867	0.839256161	0.431215639	0.875285077
CYP27A1	0.102873277	0.808412323	0.25482667	0.731852061	0.412737709	0.431550265	0.875747669
LOC112447309	0.250061069	0.896631654	0.764154637	0.806810895	0.046307636	0.431545445	0.875747669
BBS7	0.277688849	0.505278595	0.468820635	0.29655877	0.328781101	0.431884527	0.875851256
CRIP2	0.544819833	0.882524981	0.800401653	0.088421342	0.188521631	0.431921529	0.875851256
CXHXorf56	0.849308199	0.338372559	0.272143401	0.096321669	0.851083536	0.43182084	0.875851256
KIF1A	0.947799548	0.11211114	0.2486231	0.701534409	0.34569891	0.431700575	0.875851256
TPD52	0.926024553	0.096070535	0.701769822	0.867786246	0.118311071	0.431777287	0.875851256
ZNF383	0.30013886	0.825168707	0.22000696	0.370095838	0.317733031	0.431710266	0.875851256
PSMB2	0.490398588	0.17450902	0.661925832	0.591962637	0.191470765	0.432068346	0.875932505
ROBO1	0.959746545	0.664722631	0.227717031	0.080941268	0.545870972	0.432020077	0.875932505
SERTAD2	0.38122883	0.511769776	0.341396513	0.143350495	0.672922712	0.432192636	0.875968055
TYW3	0.125613856	0.308355374	0.678898912	0.453641734	0.538461293	0.432143757	0.875968055
TCF3	0.682790388	0.461947214	0.858655278	0.038684328	0.613579061	0.432280809	0.876038572
BHLHE22	0.768937254	0.831110614	0.022129746	0.851261139	0.534415568	0.432426441	0.8762255
ADGB	0.500719656	0.092515259	0.275226422	0.557566322	0.908058443	0.433003153	0.876329168
ARFIP2	0.539894753	0.165503381	0.708757995	0.27967914	0.364022787	0.432800564	0.876329168
C17H5orf52	0.928517842	0.936263282	0.767016469	0.15822686	0.061051362	0.43262676	0.876329168
CACNG7	0.527461383	0.055096939	0.392799689	0.98613418	0.573451951	0.4330087	0.876329168
CLEC16A	0.069312071	0.54532017	0.819541503	0.592968166	0.351687416	0.433127774	0.876329168
FXN	0.817530822	0.275598049	0.344687163	0.211462045	0.393894948	0.433367606	0.876329168
HERPUD2	0.422880842	0.550661549	0.051911987	0.728285878	0.731697879	0.432641012	0.876329168
KIAA1211	0.433098528	0.202022259	0.687296096	0.155231349	0.692766869	0.433318773	0.876329168
LOC100847609	0.09191296	0.718213996	0.370873308	0.61704989	0.427702654	0.433167362	0.876329168
LOC101902122	0.944356802	0.935688469	0.3660625	0.592028249	0.033695099	0.432932397	0.876329168
MREG	0.340602927	0.25918626	0.22432421	0.575037104	0.567420958	0.433175102	0.876329168
PCNT	0.430864793	0.864686451	0.205578822	0.763319988	0.110425119	0.433020806	0.876329168
PODNL1	0.676244469	0.704296036	0.219591178	0.242098893	0.255502954	0.433385384	0.876329168
PTGER4	0.708290912	0.632070119	0.179246454	0.104068057	0.772143828	0.432816805	0.876329168
RNF10	0.45476713	0.598599451	0.818526349	0.373621312	0.077453059	0.432810841	0.876329168
RSBN1	0.21875933	0.215922032	0.911938936	0.190595703	0.785951651	0.432934974	0.876329168
SEMA6C	0.648096367	0.567142318	0.039927839	0.760591633	0.579069224	0.433235888	0.876329168
CHCHD10	0.401382642	0.478051286	0.745645417	0.070161532	0.645132453	0.433566037	0.876411382
EPS8	0.987801507	0.09040427	0.825933934	0.179804966	0.488362014	0.433579709	0.876411382
P2RY8	0.658046708	0.151312075	0.999164248	0.309747024	0.210179081	0.433586254	0.876411382

EXOC8	0.769056689	0.267655906	0.275863184	0.31548725	0.362411093	0.434006554	0.876802172
HPGDS	0.647632539	0.162049361	0.535623482	0.662587048	0.174259818	0.433951847	0.876802172
KDM4C	0.404483899	0.734653879	0.532592849	0.149603669	0.274007006	0.43387465	0.876802172
LOC101903545	0.48061389	0.522252914	0.90431465	0.3271955	0.087501035	0.434168992	0.876802172
LOC104974678	0.091579992	0.607118091	0.689150337	0.837621926	0.202452835	0.434145882	0.876802172
LOC112444924	0.424984824	0.187782306	0.577107224	0.89491472	0.157536109	0.434020246	0.876802172
MFHAS1	0.376463552	0.997357823	0.160738699	0.827396653	0.130086391	0.4340979	0.876802172
NQO1	0.686005261	0.432730472	0.658361588	0.213184371	0.15600815	0.43420701	0.876802172
OCLN	0.57503105	0.584822414	0.119652028	0.274217358	0.589786848	0.434412932	0.877110066
FAM50A	0.056597755	0.595151283	0.790503808	0.375134195	0.651806419	0.43449736	0.87711149
PPME1	0.40674451	0.113883776	0.947900701	0.296583695	0.500187809	0.434573976	0.87711149
PTMA	0.152718289	0.51645348	0.391913361	0.720208535	0.292529002	0.434538965	0.87711149
ARHGEF37	0.21115589	0.625311401	0.927738231	0.072128609	0.73803173	0.434766932	0.877177296
C25H16orf54	0.720510061	0.149304139	0.671199816	0.206284965	0.437731919	0.434739612	0.877177296
LOC100294792	0.651537903	0.612709315	0.773431598	0.335907693	0.062847994	0.434694635	0.877177296
PSMC2	0.84178444	0.146104082	0.855016513	0.756373394	0.082051121	0.434907788	0.877245785
ZNFX1	0.373694225	0.616515	0.742013995	0.04880095	0.782262084	0.43490545	0.877245785
C1RL	0.55565643	0.636548726	0.777136902	0.658570782	0.036146193	0.435366353	0.877303118
CCL16	0.7137876	0.642083905	0.051221858	0.34445139	0.808543926	0.435224636	0.877303118
FADS2	0.193899666	0.439601458	0.317290466	0.942143942	0.256834379	0.435391687	0.877303118
LACTB2	0.904139214	0.514193813	0.258836132	0.235615786	0.230361775	0.435045625	0.877303118
LOC615454	0.122435723	0.385145632	0.556669915	0.717646328	0.347444056	0.435416834	0.877303118
NCOA2	0.507428329	0.569476179	0.617004057	0.202604844	0.180880268	0.435116876	0.877303118
RNASEH1	0.209660967	0.631215865	0.325976786	0.42078619	0.360565225	0.435417336	0.877303118
SH3KBP1	0.947055156	0.10250676	0.808776903	0.303252753	0.27472315	0.435308512	0.877303118
TUBB1	0.731691758	0.422383142	0.800381457	0.163980527	0.160980265	0.435003397	0.877303118
MAPK14	0.876288535	0.844613804	0.606510092	0.237259424	0.061495002	0.435529468	0.877421323
BAZ1B	0.429312361	0.595601105	0.58481573	0.063091812	0.695130246	0.435762691	0.877436037
FOXS1	0.674862487	0.524351165	0.692104091	0.192374697	0.139401526	0.436018813	0.877436037
IQSEC2	0.930169021	0.445620187	0.037962523	0.610690268	0.68266791	0.435812989	0.877436037
LOC101906367	0.527279091	0.739364003	0.278152861	0.400873627	0.150742702	0.435617663	0.877436037
LOC101908154	0.463806588	0.415407368	0.565172507	0.576278601	0.104687173	0.43605647	0.877436037
OLFM1	0.678000146	0.411737992	0.067789239	0.811884108	0.427266118	0.435930216	0.877436037
PCSK1N	0.187495031	0.872310842	0.4250125	0.168126248	0.562135779	0.436065066	0.877436037
RNF215	0.663127206	0.473971483	0.053568771	0.429365643	0.908445267	0.436003693	0.877436037
VPS26A	0.536547187	0.119174596	0.367615881	0.898489853	0.311068673	0.436071435	0.877436037
ZDHHC3	0.190500555	0.752985152	0.556754344	0.24891764	0.33041102	0.436031697	0.877436037
ARHGEF3	0.424097379	0.07118143	0.886460509	0.356140381	0.690130753	0.436268004	0.87750879
FOXO4	0.221167795	0.948375235	0.243180408	0.544439733	0.236710478	0.436168975	0.87750879
NACAD	0.760220783	0.433563655	0.073515124	0.844140796	0.321488355	0.436229868	0.87750879

BRD4	0.354141504	0.554184106	0.843116288	0.043107358	0.924966118	0.436811492	0.878024257
FAM212A	0.339728743	0.91835498	0.17310413	0.235625499	0.518458668	0.436807924	0.878024257
LOC101904642	0.801006326	0.09309078	0.121381857	0.961647779	0.757325845	0.436650419	0.878024257
LOC101907916	0.307542922	0.584178936	0.159513932	0.924483708	0.24914711	0.436895888	0.878024257
LOC112443139	0.952179079	0.890573404	0.445100035	0.03273718	0.533649901	0.436710148	0.878024257
MGC126945	0.446939375	0.667553357	0.372157571	0.061555452	0.965399111	0.436827293	0.878024257
SPHK1	0.224678028	0.404409046	0.173304328	0.500700543	0.837227814	0.436898792	0.878024257
PPP2R3B	0.576470966	0.137444238	0.693241009	0.226891945	0.529832984	0.436952592	0.878024854
LOC101904062	0.875747879	0.229872884	0.504859241	0.313750044	0.207241627	0.437094101	0.878186139
SNX25	0.828842576	0.529574323	0.17614528	0.113666682	0.752157031	0.43713988	0.878186139
LOC112444348	0.56702632	0.678622586	0.132465867	0.697859872	0.185987561	0.437291205	0.878345021
SHC1	0.875094686	0.704033258	0.761438495	0.026839145	0.525560085	0.437326011	0.878345021
CPT1C	0.528526883	0.383500835	0.165360283	0.588843246	0.336107406	0.437756164	0.878384248
HTR6	0.840723987	0.468561866	0.679930256	0.034446627	0.719510064	0.437887447	0.878384248
LOC101905403	0.74085148	0.19421494	0.622845202	0.678827407	0.10915002	0.437931267	0.878384248
LOC112445939	0.55802065	0.947667288	0.863596893	0.088756035	0.164017305	0.438143872	0.878384248
LOC112448368	0.451978617	0.105500168	0.954770014	0.152791009	0.954124093	0.437848839	0.878384248
POU3F1	0.313434935	0.471590018	0.518117379	0.654128009	0.132618583	0.438024005	0.878384248
PSMC3	0.288472959	0.191729462	0.774892937	0.506383921	0.305573133	0.4377112	0.878384248
RAPGEF6	0.552108558	0.347778882	0.997681661	0.037301028	0.927992825	0.437692817	0.878384248
SARS2	0.874138108	0.319140067	0.202635379	0.175478111	0.670093645	0.438115792	0.878384248
SLC35C1	0.561848353	0.561160283	0.805353527	0.584458754	0.044611153	0.437412893	0.878384248
TMEM185A	0.915676519	0.523297106	0.814457589	0.458705968	0.03705263	0.437744053	0.878384248
TOPBP1	0.588297883	0.773490755	0.884575088	0.023096476	0.713462124	0.437740606	0.878384248
WDR55	0.621148674	0.267945831	0.924011898	0.170263379	0.253816273	0.438085868	0.878384248
XRN1	0.375510413	0.683579056	0.803992142	0.060658938	0.529604756	0.43766226	0.878384248
ZDHHC17	0.16212631	0.871927265	0.45481569	0.184341298	0.56095007	0.438148404	0.878384248
BRCA2	0.959381533	0.176147367	0.26282209	0.445619259	0.336741713	0.438581354	0.878731448
NKPD1	0.295478521	0.041700474	0.756024177	0.775661714	0.922433473	0.438589317	0.878731448
PLA2G12A	0.369367228	0.232379438	0.577150682	0.746673292	0.17999378	0.438397194	0.878731448
TAGLN2	0.426976442	0.797321625	0.206368903	0.121262468	0.782017527	0.438514579	0.878731448
VSIG10L	0.667377153	0.049997594	0.737888914	0.340102857	0.795772651	0.438549293	0.878731448
LOC615514	0.533122149	0.577217832	0.087511912	0.496002869	0.499180974	0.438656184	0.878758135
LOC101907613	0.137561082	0.822922964	0.472442731	0.629596083	0.198161611	0.438781029	0.878900947
REEP5	0.372323489	0.790205453	0.061081617	0.627633267	0.591998072	0.438906746	0.878938207
TCF20	0.780558537	0.114941691	0.137581532	0.808443515	0.66906016	0.438890947	0.878938207
IKBIP	0.158290229	0.670492568	0.450165156	0.268496237	0.521320915	0.439175413	0.879270403
TCEANC2	0.644311571	0.692764985	0.164697655	0.898046898	0.101299162	0.439179787	0.879270403
PTPA	0.320325028	0.270539875	0.510428312	0.36537829	0.413944774	0.439247528	0.879298754
LOC104971852	0.274533957	0.13231848	0.518946028	0.49574626	0.716161465	0.439314949	0.879326458

DZANK1	0.438012855	0.203364499	0.41984411	0.737407978	0.242991728	0.439532949	0.879547801
GNA12	0.754790584	0.624760744	0.350636592	0.107123369	0.378442544	0.439586318	0.879547801
IL32	0.35426103	0.442291219	0.478661154	0.119334296	0.748513071	0.439482568	0.879547801
TRIP4	0.248871327	0.640221425	0.222080116	0.413176592	0.458865505	0.439729306	0.879726642
ARMC9	0.519919247	0.274039041	0.464756871	0.292377081	0.347162797	0.440058282	0.879848497
LOC101905666	0.966376668	0.510866616	0.553250555	0.332823125	0.073929581	0.440040876	0.879848497
MOB1A	0.361592884	0.457355979	0.461517113	0.208694654	0.421511606	0.439869411	0.879848497
PCSK4	0.936909462	0.177304937	0.535654328	0.690990967	0.10922563	0.439916687	0.879848497
TNFSF12	0.335349286	0.600341647	0.574210816	0.436078571	0.133313613	0.440040438	0.879848497
BCL2L13	0.178927605	0.734542162	0.321517592	0.244325653	0.652679143	0.440509458	0.879958091
CBS	0.595553977	0.261802769	0.776911344	0.321759918	0.172860492	0.440479922	0.879958091
COMMD8	0.871905685	0.433995397	0.230133822	0.642697227	0.120276576	0.440329658	0.879958091
DKK3	0.084575626	0.641720588	0.41509182	0.410007637	0.730329876	0.440702916	0.879958091
DNAJC12	0.250364879	0.21910665	0.873077576	0.41178786	0.341526514	0.440433561	0.879958091
JADE2	0.413793417	0.458376546	0.702836643	0.149442384	0.338594547	0.440689874	0.879958091
LOC104971814	0.813373441	0.681638322	0.073798467	0.728799521	0.225716735	0.440305663	0.879958091
LOC788736	0.691668477	0.736158234	0.069030454	0.600504281	0.319438047	0.440608459	0.879958091
MAP7D1	0.149814883	0.565608195	0.539675441	0.232166219	0.634902089	0.44056662	0.879958091
SPTY2D1	0.971562712	0.538367574	0.021076405	0.773187803	0.789673886	0.440312074	0.879958091
STK26	0.938525111	0.19289975	0.678803914	0.134701893	0.407435611	0.440665819	0.879958091
FXYD1	0.270376661	0.148372903	0.521615095	0.73898658	0.436409011	0.4407667	0.879978383
CLEC6A	0.451911351	0.0528717	0.860602715	0.444847224	0.738021386	0.440829291	0.879996289
STAT6	0.246303265	0.562397779	0.861799521	0.107857729	0.52447172	0.440883571	0.879997603
DIP2C	0.056768509	0.400401233	0.737724702	0.736310031	0.547232486	0.440979151	0.880005037
UBXN7	0.545929365	0.282358138	0.478381645	0.118996186	0.770058231	0.440994542	0.880005037
H1FX	0.576019537	0.48004265	0.084521931	0.625233443	0.462574977	0.441052313	0.880013314
LOC112442392	0.810700756	0.100021562	0.581438002	0.651055726	0.220341069	0.441157618	0.880116421
C16H1orf21	0.89350753	0.270610514	0.632275544	0.373482106	0.118533221	0.441274038	0.880241673
LRRC71	0.317051594	0.90663448	0.399643562	0.245147264	0.240439382	0.441358355	0.880302864
LRRC10B	0.491160017	0.39045209	0.745274085	0.059714216	0.793790343	0.441448414	0.880375492
DHX34	0.234956995	0.807546971	0.255541124	0.238138882	0.587565159	0.441696387	0.880762989
C24H18orf25	0.432552451	0.267893106	0.335253616	0.380611042	0.45956662	0.441979771	0.881111645
CTNNBIP1	0.495266932	0.765514087	0.062533057	0.625653293	0.458524802	0.442139686	0.881111645
EARS2	0.34881717	0.671449523	0.360150609	0.528997496	0.152389026	0.442100805	0.881111645
NUDT7	0.674724562	0.817021539	0.909470385	0.635060862	0.021339768	0.441959527	0.881111645
ZBTB47	0.697678536	0.363780023	0.741822272	0.077933411	0.463289314	0.442047118	0.881111645
CHM	0.654771444	0.978273753	0.858669859	0.061386752	0.201912022	0.44255041	0.881233828
DOCK8	0.601353993	0.809602491	0.377430733	0.377585705	0.098176615	0.442408565	0.881233828
DPP3	0.29864227	0.520694528	0.799918868	0.511220633	0.107359231	0.442799427	0.881233828
EBI3	0.868127151	0.932248547	0.118583665	0.159792182	0.444729635	0.442622635	0.881233828

FAM131B	0.424294772	0.772192568	0.304782117	0.364328643	0.187259335	0.442431514	0.881233828
FOLR2	0.488728347	0.289037568	0.191063016	0.769124966	0.32790457	0.442278978	0.881233828
HIVEP1	0.679079838	0.222077415	0.508151257	0.814663427	0.109367811	0.442822913	0.881233828
HNRNPDL	0.983840756	0.885187787	0.016143513	0.514112618	0.94269561	0.442459229	0.881233828
KRTCAP2	0.995358205	0.02996614	0.780659671	0.309909931	0.945744733	0.442740035	0.881233828
LOC107131846	0.373334982	0.462504902	0.743044862	0.603092352	0.088156763	0.442654224	0.881233828
RAD21	0.68645829	0.291230523	0.532314373	0.111031228	0.577141951	0.442604613	0.881233828
TBCB	0.357620119	0.18773524	0.623237891	0.360089214	0.453220663	0.442845371	0.881233828
C25H16orf89	0.645573337	0.417202323	0.541969415	0.049857256	0.939193164	0.443011059	0.881456655
NSUN5	0.487269162	0.11220031	0.310833112	0.768829403	0.52375685	0.443213506	0.881752558
FAM160A1	0.093865591	0.247865288	0.609951297	0.624117509	0.774680944	0.443682355	0.882219329
JUP	0.896199673	0.385451308	0.676149909	0.429752839	0.068351777	0.443673975	0.882219329
LOC112449363	0.765711226	0.953362834	0.545961481	0.62854618	0.027400953	0.443755981	0.882219329
LRRC49	0.178107044	0.617704212	0.372939534	0.282686645	0.591083055	0.443539374	0.882219329
MCTP2	0.355675681	0.446086364	0.475250858	0.407632219	0.223336548	0.443770676	0.882219329
ZMYM2	0.872603892	0.647019158	0.676153376	0.053737302	0.334344907	0.443618919	0.882219329
CLIC2	0.742669229	0.418651513	0.287536149	0.683204915	0.112428675	0.443829191	0.882228786
AKAP13	0.89618909	0.11898391	0.952505542	0.125095602	0.541806913	0.444264603	0.8822394
ALKBH7	0.201394428	0.282688409	0.668488419	0.267749915	0.675226761	0.444177915	0.8822394
HASPIN	0.855033258	0.215806218	0.190481335	0.467697273	0.41826204	0.444049862	0.8822394
IQCB1	0.207055816	0.824386721	0.965221977	0.151674466	0.275436887	0.444239534	0.8822394
LMOD3	0.919738855	0.038196557	0.492982109	0.463207607	0.857037107	0.444042779	0.8822394
LOC101904344	0.285662855	0.173954197	0.584393736	0.601101416	0.393799968	0.44401132	0.8822394
LOC112442652	0.362850338	0.912553412	0.193431881	0.155879165	0.689250399	0.444198465	0.8822394
SUV39H2	0.886268487	0.622951027	0.369392051	0.483393266	0.069705932	0.443954301	0.8822394
LAMTOR2	0.285993297	0.409784214	0.74254788	0.156160375	0.506807231	0.444348679	0.882299598
CLDN5	0.31179792	0.153039178	0.917699471	0.399204275	0.394229181	0.444458689	0.882411268
CERS2	0.692455934	0.097595127	0.352982609	0.955358863	0.302671827	0.44461773	0.882620244
RIBC1	0.542015047	0.942541235	0.173083317	0.669427002	0.116571015	0.444678048	0.882633217
ALAD	0.387208082	0.173324303	0.800255088	0.520276315	0.247088523	0.444782881	0.882734534
PRKAB2	0.484904392	0.456547328	0.129552987	0.891551392	0.27011642	0.444850997	0.882762963
LOC786303	0.806693264	0.377380945	0.844791669	0.029141044	0.922252826	0.444975407	0.882903083
CENPT	0.84770508	0.175805914	0.553774956	0.149686077	0.559764767	0.445058828	0.882961851
PHLPP1	0.555630823	0.858339392	0.22213489	0.230110138	0.283783059	0.445134297	0.883004829
B4GALT3	0.805495595	0.957585081	0.775487858	0.270062354	0.042850397	0.445235866	0.883099565
LRRC8A	0.055082549	0.992892693	0.599937694	0.238173235	0.886364226	0.445354983	0.883122356
TNFRSF12A	0.455129062	0.824926518	0.530212534	0.073025786	0.476462939	0.445345848	0.883122356
NTM	0.780601717	0.710721727	0.230810027	0.879724963	0.061527609	0.445465914	0.883235606
DKK1	0.171138417	0.702614536	0.533471457	0.526002875	0.205628639	0.445647474	0.8832822
DNAJC8	0.580578379	0.022157638	0.87064919	0.904210977	0.684699443	0.445545773	0.8832822

RNF223	0.370726808	0.846174747	0.775790084	0.95241242	0.029942403	0.445697229	0.8832822
TSKU	0.259130652	0.848320505	0.332594623	0.159573417	0.594886146	0.445704704	0.8832822
EHMT1	0.597042391	0.087547371	0.678897601	0.839844812	0.233109274	0.445876534	0.883302728
LOC112445925	0.918564957	0.743005882	0.024129458	0.481301541	0.876120464	0.445803218	0.883302728
LPCAT1	0.148779983	0.49810379	0.418550354	0.630896896	0.354996724	0.445869891	0.883302728
COG6	0.100613423	0.595441923	0.994654211	0.301646774	0.387420996	0.446298618	0.883602053
DPH1	0.793427282	0.324514825	0.374484499	0.179577167	0.402201254	0.446307059	0.883602053
EXOC6B	0.591530399	0.558029656	0.761806364	0.163613384	0.169361005	0.446405129	0.883602053
GPR161	0.600102315	0.768743891	0.201516929	0.858621776	0.087314644	0.446444298	0.883602053
LOC101902839	0.478746146	0.220402721	0.966336648	0.121109414	0.563585866	0.446192644	0.883602053
LOC101907140	0.389324291	0.116187234	0.310963338	0.996462566	0.496378243	0.446137402	0.883602053
NPY5R	0.201357499	0.494230585	0.769823712	0.797618084	0.113914026	0.446222008	0.883602053
PRPSAP2	0.084352376	0.419780053	0.269952594	0.839941308	0.868133504	0.446458365	0.883602053
CWC22	0.237962627	0.337439569	0.966820507	0.143892038	0.627301985	0.447403285	0.883635262
GAPDHS	0.730650653	0.151055982	0.952372933	0.619817612	0.107422003	0.447178504	0.883635262
HTRA2	0.399819551	0.145685095	0.658240324	0.187100246	0.977235399	0.447473783	0.883635262
LOC100337044	0.908943057	0.025078313	0.869078789	0.50235714	0.704515217	0.447498182	0.883635262
LOC112444351	0.562788492	0.487941226	0.503305152	0.142887541	0.353918349	0.446946361	0.883635262
MDH1B	0.765165826	0.599074183	0.162656608	0.177892449	0.527684628	0.447189875	0.883635262
MPHOSPH8	0.902985904	0.240431685	0.185621178	0.187566931	0.926792713	0.447352406	0.883635262
OSBPL1A	0.5110831	0.975309253	0.033760203	0.594493472	0.697300342	0.446606347	0.883635262
PIGV	0.989829736	0.701886397	0.304249097	0.282647366	0.117215143	0.447289793	0.883635262
PIP4K2A	0.839833177	0.27908842	0.62393994	0.067453962	0.707091589	0.446587995	0.883635262
PLEKHG5	0.316515824	0.336242477	0.823217735	0.729036728	0.109414254	0.446923772	0.883635262
PLEKHH1	0.696739322	0.847759021	0.0788109	0.609972716	0.246419403	0.44713879	0.883635262
PPIL2	0.60546813	0.108726426	0.408512155	0.532318939	0.488682032	0.447104328	0.883635262
RABGAP1	0.234548536	0.184615442	0.909099033	0.256779961	0.692929879	0.447321173	0.883635262
RAD51AP1	0.962982946	0.185211662	0.122793733	0.521029689	0.611562341	0.446671622	0.883635262
SKA3	0.945874419	0.324849683	0.679336061	0.065482025	0.511024183	0.446833483	0.883635262
TSC2	0.510670735	0.097634471	0.161632394	0.992684599	0.872882121	0.446782737	0.883635262
UTP23	0.035268355	0.64712871	0.996143703	0.312036429	0.984418459	0.446800428	0.883635262
ZMAT3	0.803374449	0.865218175	0.114176139	0.336125865	0.262615175	0.447353088	0.883635262
CD84	0.412329392	0.249200159	0.179944094	0.534026112	0.711021963	0.447733825	0.883934064
LOC617692	0.099358004	0.405961575	0.87719053	0.220681409	0.899260878	0.447757228	0.883934064
LOC789764	0.539722988	0.496821753	0.174430089	0.727481123	0.206607286	0.447971956	0.884128007
SPTBN4	0.346548468	0.781474846	0.366539979	0.124411674	0.569389689	0.448017093	0.884128007
SYP	0.279368791	0.800081149	0.289594684	0.205703874	0.52781703	0.447918219	0.884128007
ADRA1B	0.42420642	0.206879106	0.622566279	0.244363192	0.526882682	0.448081513	0.884128755
DMAC1	0.524479396	0.35849199	0.267509023	0.43920491	0.318644598	0.44820005	0.884128755
PPT1	0.117453397	0.548824543	0.480710778	0.931364012	0.244022923	0.448286842	0.884128755

TMEM252	0.651559094	0.089762525	0.576442505	0.879476473	0.237484405	0.448258781	0.884128755
TRIM62	0.10995507	0.84766872	0.604839796	0.769682591	0.162282204	0.448258841	0.884128755
MCM4	0.524249827	0.578366313	0.348845212	0.189838316	0.350964795	0.448404403	0.884254344
IGSF6	0.668947879	0.258203958	0.632532888	0.205321223	0.314389238	0.448534163	0.884403958
CLCN2	0.645781315	0.183554788	0.509553712	0.87721397	0.133384213	0.448905754	0.8844417
CRYBG3	0.763031135	0.473594457	0.621679778	0.053500533	0.587882263	0.448870251	0.8844417
CUL1	0.340419198	0.310246792	0.233637813	0.304608181	0.942792333	0.449383424	0.8844417
FAM213B	0.58787922	0.426673511	0.90810013	0.058914282	0.527383576	0.449156026	0.8844417
JAK2	0.307159575	0.723389984	0.574567138	0.097916334	0.56612177	0.449146895	0.8844417
LLGL1	0.276655647	0.219292963	0.283881374	0.898454587	0.457328943	0.449140212	0.8844417
LOC100124497	0.14377056	0.30706959	0.802420843	0.472986072	0.423003147	0.449415595	0.8844417
LOC100849587	0.172401305	0.547442404	0.859164598	0.118587226	0.735581544	0.44905787	0.8844417
LOC112444588	0.347631103	0.795952374	0.050211178	0.802870442	0.635050612	0.44931726	0.8844417
LOC112448082	0.924077006	0.647172423	0.064984183	0.542265648	0.335555327	0.449012174	0.8844417
MBTD1	0.141480911	0.835815381	0.482526404	0.483568221	0.256637119	0.449254669	0.8844417
PDE1A	0.265912384	0.39458685	0.173551984	0.661217312	0.587054327	0.44893925	0.8844417
PPP6C	0.653707795	0.090167093	0.185564323	0.745433517	0.865436708	0.448628191	0.8844417
SERTAD4	0.820752827	0.558109266	0.488613282	0.533243777	0.059300184	0.449161741	0.8844417
UBA6	0.054326786	0.831240083	0.822440326	0.268251652	0.711101369	0.449341284	0.8844417
YJU2	0.869791512	0.49428778	0.873060038	0.04458321	0.421845231	0.448706959	0.8844417
LOC101905908	0.073255267	0.795491518	0.608840791	0.296217165	0.674813393	0.449526241	0.884553374
DENND6A	0.5588185	0.275788044	0.110106355	0.517922171	0.807222877	0.449585213	0.884560876
ENOX1	0.361869256	0.263041699	0.557927355	0.235894019	0.566467068	0.449637854	0.884560876
WWOX	0.827786175	0.800506895	0.043104118	0.885600308	0.280688508	0.449727189	0.884630576
DCTPP1	0.55018345	0.675161706	0.207128824	0.231671358	0.398678154	0.449884863	0.88483467
C10H15orf48	0.566372727	0.665945304	0.48190306	0.141166735	0.277692614	0.450353104	0.885331192
IER3	0.879633351	0.680453154	0.091432807	0.784070671	0.166034637	0.450336194	0.885331192
LOC104976274	0.878266677	0.112786278	0.135602541	0.747558779	0.709199403	0.450257908	0.885331192
NCOA3	0.720919423	0.339995118	0.102647718	0.898434073	0.315073802	0.450275474	0.885331192
CNOT10	0.252936623	0.825060997	0.583280196	0.798783557	0.073420745	0.450690409	0.885410862
LAPTM4B	0.720698789	0.195686765	0.117646005	0.748304076	0.574956707	0.450683371	0.885410862
ROCK1	0.280324577	0.643451639	0.325480789	0.57472253	0.21129152	0.450453372	0.885410862
RYK	0.884962825	0.15581269	0.872928237	0.512846358	0.115663187	0.450717345	0.885410862
TLE3	0.962458612	0.791868841	0.338390071	0.043986779	0.629267268	0.45068523	0.885410862
ZNF354A	0.638589295	0.127968224	0.099208439	0.942077597	0.934132997	0.450586166	0.885410862
KIAA2012	0.989162913	0.521431909	0.115502188	0.666225479	0.179978497	0.450802618	0.885472383
AURKB	0.071651376	0.911336196	0.541647302	0.68020468	0.297139855	0.450934528	0.885517657
BRCC3	0.957313895	0.585860453	0.700526737	0.29092412	0.062530766	0.450904478	0.885517657
PSENEN	0.868570242	0.156402963	0.451515079	0.183766767	0.634396546	0.450987543	0.885517657
AP1AR	0.6928648	0.337492669	0.080007298	0.572914553	0.668668882	0.451393699	0.885661608

CHST10	0.108574599	0.397152314	0.74673337	0.481114764	0.462757764	0.45143863	0.885661608
GIPR	0.949681248	0.345192694	0.228314152	0.285231092	0.335785341	0.451429469	0.885661608
LOC104974269	0.991723587	0.599916641	0.419841137	0.7834877	0.036618683	0.451376538	0.885661608
MFN1	0.632832134	0.4245411	0.836107728	0.095558284	0.333482305	0.451176268	0.885661608
RCBTB2	0.496270287	0.788136715	0.782931642	0.226220932	0.103387845	0.451272067	0.885661608
TRIM56	0.897009467	0.545842151	0.28992237	0.530535275	0.095062452	0.45119976	0.885661608
SPTLC3	0.74873928	0.603679092	0.602661951	0.259190552	0.101586904	0.451526201	0.885727525
MUL1	0.733156823	0.224664063	0.168699125	0.469682416	0.550191288	0.451728223	0.885934854
ULK2	0.497921993	0.731400363	0.544300498	0.361192231	0.100298899	0.451739861	0.885934854
LAGE3	0.693415331	0.19676423	0.352718169	0.196793281	0.758510134	0.451800936	0.885948759
ADIPOR2	0.249726551	0.914410106	0.074103994	0.912732652	0.467326964	0.452649264	0.886039457
CCDC71L	0.118265578	0.147498232	0.946095898	0.591886069	0.740166851	0.452950806	0.886039457
CETN2	0.176604077	0.262062814	0.698518625	0.739348346	0.302805107	0.453134464	0.886039457
CSRNP3	0.57188631	0.329541927	0.622098528	0.212817856	0.288686977	0.452282053	0.886039457
F2RL1	0.967733504	0.238304552	0.540211548	0.08400843	0.692806733	0.453458384	0.886039457
FBXO40	0.9649863	0.845365432	0.933822088	0.871128685	0.010915562	0.453282688	0.886039457
HIPK2	0.471310424	0.89354365	0.030783345	0.648099058	0.855476031	0.451902721	0.886039457
LOC101902469	0.286741752	0.647758684	0.182580143	0.635320913	0.336287439	0.453325027	0.886039457
LOC101903992	0.580837989	0.840854517	0.202511783	0.09800946	0.744327898	0.452586489	0.886039457
LOC101906018	0.560313011	0.342693919	0.234797725	0.722653399	0.221933544	0.452965515	0.886039457
LOC104976247	0.414661131	0.235162137	0.727129333	0.557301256	0.182810941	0.452793744	0.886039457
LOC107131643	0.49763987	0.171727181	0.235140328	0.868696583	0.414717001	0.453177426	0.886039457
LOC107131675	0.120417968	0.169362799	0.954629748	0.544427335	0.68146066	0.452777526	0.886039457
LOC107132852	0.507373094	0.657940943	0.151640385	0.329585459	0.432129922	0.45244448	0.886039457
LOC112441645	0.396117515	0.330783806	0.732130483	0.127068392	0.593587209	0.453086297	0.886039457
LOC112442619	0.887980452	0.02468738	0.769680459	0.552548288	0.77357082	0.452504896	0.886039457
LOC112443170	0.858008176	0.104182995	0.272027087	0.725867401	0.40951805	0.452902751	0.886039457
LOC112446375	0.341273756	0.042886218	0.724790505	0.973385959	0.698718946	0.452571042	0.886039457
LOC615663	0.35689246	0.138421343	0.292917534	0.929559269	0.535030241	0.45212915	0.886039457
LOC783641	0.422450683	0.456858641	0.447007964	0.095950381	0.869637489	0.452176977	0.886039457
NISCH	0.420004274	0.507303829	0.296801407	0.206718988	0.550482232	0.452116982	0.886039457
PCNP	0.984048745	0.295799927	0.492689796	0.363575804	0.138364804	0.452566174	0.886039457
PFKP	0.845912601	0.426600859	0.332737919	0.702712972	0.085909617	0.45341016	0.886039457
SGO2	0.340727563	0.28087902	0.430198678	0.456748014	0.382590491	0.452074011	0.886039457
SNRNP27	0.910256612	0.693501743	0.528111564	0.027079029	0.803469352	0.453520897	0.886039457
ST8SIA5	0.153527064	0.480828321	0.125471123	0.858003043	0.910925664	0.453173024	0.886039457
TNFAIP2	0.476624042	0.584201383	0.273101152	0.780026661	0.121231769	0.451984588	0.886039457
TRAF7	0.313180168	0.270945429	0.683130776	0.184569299	0.675452118	0.452863109	0.886039457
TTC37	0.980513257	0.569406306	0.685724183	0.152298319	0.124373011	0.453482778	0.886039457
VTA1	0.391021223	0.22029524	0.761836654	0.268316761	0.411913336	0.453513309	0.886039457

ZNF366	0.026302143	0.652563534	0.945823838	0.449314813	0.993138653	0.453293596	0.886039457
NEK6	0.339662131	0.198771562	0.797714988	0.554448575	0.243026076	0.453612627	0.88611318
SHANK3	0.405240332	0.286550325	0.385434386	0.222415705	0.729346987	0.453694767	0.886168153
RPN2	0.666829345	0.317015602	0.043495423	0.933036569	0.846634345	0.453764365	0.88619862
LOC515570	0.812341105	0.71165226	0.288864881	0.864473223	0.050336447	0.453847043	0.88625462
CHPF2	0.823245918	0.386524631	0.318815751	0.794078972	0.090276027	0.453989546	0.886427418
CSNK2A1	0.599480469	0.094447699	0.619919824	0.932213824	0.222375878	0.454080787	0.886500095
RMDN1	0.90803511	0.334662434	0.645102068	0.078504832	0.473068225	0.454184565	0.886597227
CCNT1	0.15682771	0.419080813	0.508951267	0.234988297	0.927267049	0.454385402	0.886778313
SMIM14	0.616081095	0.296873073	0.360862039	0.761697917	0.144963402	0.454362097	0.886778313
FXR2	0.031714153	0.673894469	0.881807201	0.862625166	0.448497585	0.454448187	0.886795385
SCUBE2	0.166632016	0.850922845	0.599677389	0.189358942	0.453317092	0.454634184	0.887052858
ETNPPL	0.102997533	0.582636086	0.417598612	0.587804869	0.495654034	0.454691983	0.887060167
ARMT1	0.943519183	0.027098225	0.901602031	0.772196904	0.410310677	0.454754463	0.887076607
DHX37	0.067946788	0.745931616	0.623128394	0.501373345	0.461922305	0.455012354	0.887171843
GLI1	0.125500655	0.869575332	0.229602406	0.387865397	0.752118138	0.454897864	0.887171843
JADE3	0.35813466	0.425787134	0.318552195	0.172073157	0.874867078	0.454970908	0.887171843
NAGA	0.657711661	0.88856353	0.809104749	0.398584753	0.038810002	0.455019524	0.887171843
ABHD3	0.853824679	0.372770369	0.037649145	0.919011916	0.668169641	0.456079169	0.887544125
ADAMTSL5	0.48770762	0.508420494	0.278852769	0.968033944	0.110023129	0.456226731	0.887544125
AGMAT	0.994659544	0.204425828	0.078277957	0.503306562	0.91927815	0.456225541	0.887544125
BTC	0.170975321	0.434908139	0.328120029	0.62398006	0.483605565	0.456182991	0.887544125
CCDC183	0.630887211	0.190550824	0.170504935	0.509317246	0.704465009	0.455986396	0.887544125
CLDN1	0.463775084	0.362277855	0.7953273	0.153341286	0.357935136	0.455498033	0.887544125
DANCR	0.750579301	0.244607358	0.398300781	0.302570069	0.332112776	0.455839092	0.887544125
HEYL	0.178686897	0.147930159	0.718375413	0.90207001	0.429408742	0.456012982	0.887544125
INTS10	0.611825085	0.488636217	0.602286275	0.298725178	0.136604615	0.455824422	0.887544125
ITGAV	0.868679916	0.893477555	0.599702328	0.270832577	0.058265015	0.455756563	0.887544125
LOC100141185	0.851524956	0.508120033	0.189221687	0.202946246	0.44162536	0.455584741	0.887544125
LOC789035	0.857288082	0.28807711	0.525668089	0.094982755	0.597047102	0.456172635	0.887544125
LOC789352	0.898257123	0.15422008	0.207018844	0.568952928	0.450708874	0.455976781	0.887544125
MACO1	0.738472775	0.156965869	0.39157719	0.993564753	0.16278031	0.455660942	0.887544125
MGC137036	0.223830952	0.631392232	0.144306028	0.672414452	0.53556709	0.455742944	0.887544125
NEURL2	0.636446003	0.555585397	0.177615181	0.689333616	0.170057322	0.45617952	0.887544125
TTC7B	0.032409274	0.966899809	0.750680351	0.594111562	0.525910637	0.455878936	0.887544125
WIF1	0.645019651	0.671466104	0.144613071	0.207507898	0.564262746	0.455482457	0.887544125
ZNF41	0.507696588	0.649585796	0.180779256	0.845424506	0.146115206	0.456238025	0.887544125
FAM196A	0.455234887	0.10262254	0.958253964	0.217237417	0.758047807	0.456415	0.887783166
LOC107133166	0.987516518	0.785927338	0.029369297	0.459746358	0.704053953	0.456560708	0.887856118
MAP1B	0.290988023	0.843301056	0.048238657	0.808122545	0.77115812	0.456531543	0.887856118

FBLN1	0.619992464	0.104768343	0.715558485	0.23679147	0.670936199	0.456710521	0.887937016
GSTA1	0.922463638	0.383396917	0.41218507	0.179067583	0.28286227	0.45669951	0.887937016
FBXO45	0.439470464	0.26532492	0.13847714	0.519911785	0.880175125	0.456823889	0.887947038
SF3B6	0.842802824	0.045671978	0.287786178	0.846775178	0.787523232	0.456779743	0.887947038
ITGA11	0.233071939	0.691834911	0.727801807	0.137923551	0.456982362	0.457012634	0.888208708
C20H5orf51	0.959779974	0.559468843	0.331507112	0.166454128	0.250248577	0.457450062	0.888231209
IPO11	0.244073247	0.084052769	0.958235095	0.465271687	0.810495419	0.457406786	0.888231209
LOC112441616	0.159498354	0.905834086	0.154776846	0.752413506	0.440268861	0.457276734	0.888231209
PIH1D2	0.285118939	0.16651157	0.37564564	0.847020443	0.490126911	0.457180401	0.888231209
RELB	0.351073341	0.802712916	0.098361757	0.32671786	0.818755013	0.457451956	0.888231209
RPL7L1	0.953103816	0.15025228	0.330062551	0.671411301	0.233330683	0.457207866	0.888231209
TGIF1	0.283959526	0.345023675	0.748161319	0.18246723	0.554419518	0.457457204	0.888231209
TMOD3	0.876773175	0.042945218	0.711257878	0.372498182	0.741739843	0.457079052	0.888231209
ARHGEF40	0.512310354	0.887157371	0.09946433	0.286560882	0.573044329	0.45765571	0.888449901
FMN1	0.130082756	0.816040575	0.661793082	0.108895791	0.970603966	0.457697111	0.888449901
TMEM192	0.370494354	0.430512859	0.260214354	0.500199689	0.357725442	0.457732248	0.888449901
YEATS2	0.877341783	0.7753145	0.811024087	0.087099968	0.15463534	0.457820759	0.888516613
ABHD13	0.389898478	0.177758562	0.262457509	0.503733813	0.81681223	0.45912141	0.888630437
C21H14orf132	0.760636337	0.038714775	0.629525824	0.936041888	0.431568974	0.459223838	0.888630437
DNAJB2	0.585468037	0.257222092	0.238527995	0.442970855	0.467296638	0.457948968	0.888630437
FCGR3A	0.53954091	0.126250011	0.342472978	0.488742301	0.655809845	0.458946459	0.888630437
HEMK1	0.537191947	0.304505647	0.510788385	0.433419553	0.205790293	0.458352818	0.888630437
IRF9	0.592305621	0.059997134	0.992221784	0.262939592	0.808026507	0.459287269	0.888630437
ITGB3	0.194028176	0.411708612	0.527229167	0.852552029	0.207786912	0.458556333	0.888630437
KLF6	0.854364879	0.510343894	0.08447107	0.206537029	0.982021567	0.458778838	0.888630437
LOC101904701	0.83432594	0.395179878	0.128897989	0.819121338	0.213893012	0.458197251	0.888630437
LOC104971021	0.207876483	0.175292283	0.80978785	0.72140759	0.350269031	0.458444523	0.888630437
LOC107131311	0.112573383	0.592274827	0.406933643	0.372911665	0.737037499	0.458467707	0.888630437
LOC112449615	0.644788729	0.771278818	0.08500407	0.83257205	0.211731139	0.458342883	0.888630437
MILR1	0.645526776	0.145287434	0.951857762	0.166728393	0.50319365	0.459242898	0.888630437
NAGS	0.10693028	0.606563927	0.227862875	0.683760739	0.739055235	0.458737075	0.888630437
NCKAP1L	0.320419538	0.217962654	0.588102172	0.275974733	0.659190944	0.458820524	0.888630437
NOL8	0.603409205	0.421296532	0.126073032	0.336260787	0.693138904	0.458772761	0.888630437
POGZ	0.317543704	0.692446068	0.465814171	0.845999086	0.086393846	0.4591587	0.888630437
PPP2R5C	0.818258332	0.121052543	0.134315911	0.599626614	0.934207849	0.458359814	0.888630437
PSMD1	0.991858644	0.303993735	0.393469173	0.893441364	0.070565507	0.459005677	0.888630437
RHOT2	0.11134547	0.13799341	0.836464536	0.704502324	0.824497736	0.458662251	0.888630437
SCARF2	0.592645194	0.856951183	0.024541892	0.666868806	0.897208243	0.458473642	0.888630437
SCRN1	0.263523386	0.217856696	0.525408546	0.942351191	0.261683006	0.458013568	0.888630437
SON	0.563575799	0.815449795	0.674150116	0.030030986	0.804936149	0.459233871	0.888630437

SRGAP2	0.26950953	0.523186249	0.103882851	0.590652631	0.864667118	0.459035251	0.888630437
STAT4	0.846320469	0.421631095	0.155639802	0.142160168	0.945125713	0.458582038	0.888630437
TCOF1	0.774805423	0.096492211	0.221873556	0.710720141	0.635160045	0.459207095	0.888630437
ATP2A3	0.992575022	0.06858179	0.469434181	0.609864765	0.384673943	0.459413637	0.888699398
LOC101902991	0.988470791	0.572523878	0.050741591	0.561593394	0.465335537	0.459593674	0.888699398
LOC112447811	0.262578206	0.095143401	0.313534878	0.988732613	0.968729645	0.459550339	0.888699398
MFAP2	0.385175518	0.213753696	0.169145776	0.713102953	0.755129678	0.459468922	0.888699398
TMEM198	0.216772646	0.514138087	0.169485501	0.887755002	0.447397944	0.459550034	0.888699398
ENO1	0.719492184	0.685932087	0.688798968	0.468928237	0.047121596	0.459765399	0.888829709
KIAA0586	0.191394201	0.220923568	0.511875167	0.622566481	0.557634469	0.459824872	0.888829709
LTB4R2	0.199675037	0.458275582	0.441475347	0.315677087	0.5891655	0.459811294	0.888829709
SLC6A4	0.769575722	0.81529791	0.423575775	0.072164256	0.391901895	0.459877708	0.888829709
HEY2	0.712923641	0.427485694	0.56573167	0.233954568	0.186453821	0.459993069	0.888940689
UCHL1	0.837330418	0.879023873	0.691998615	0.059977846	0.246267112	0.460043463	0.888940689
ITGB1BP2	0.486183151	0.646470941	0.464247359	0.203571901	0.253350802	0.460100583	0.888946394
FN3K	0.917595937	0.393884047	0.368966266	0.135809624	0.415655851	0.460155265	0.888947387
CEP76	0.948640893	0.622459898	0.249269837	0.189881538	0.269523299	0.460275371	0.88897012
MEX3A	0.632353161	0.803871052	0.097649124	0.380306399	0.398995695	0.460256499	0.88897012
NAA30	0.823502695	0.356707712	0.394684168	0.742496818	0.087559535	0.460384033	0.889075355
KHNYN	0.359369323	0.3464171	0.44959766	0.521893312	0.258226594	0.460516869	0.889227244
TLR2	0.452233018	0.722766653	0.3184074	0.267077509	0.272113295	0.461005585	0.890066195
HAUS2	0.725878764	0.022518323	0.974715209	0.802734059	0.592223581	0.461255358	0.890234233
LOC112444616	0.519108119	0.628428014	0.468985561	0.116156824	0.426050779	0.461190772	0.890234233
SDHB	0.709366833	0.365299796	0.366785439	0.318620822	0.250065039	0.46122407	0.890234233
GPSM2	0.014866478	0.779377912	0.759272645	0.98686546	0.873114643	0.461399101	0.890397429
OMD	0.664977305	0.66439313	0.310751268	0.896641977	0.061594029	0.461448427	0.890397429
NANS	0.420236183	0.422545623	0.113142411	0.855189218	0.442135584	0.461782203	0.890936719
LOC112444775	0.6803893	0.703660619	0.29310856	0.597692304	0.090709105	0.462058197	0.891364414
ABCB4	0.586127614	0.964032725	0.658560719	0.130371467	0.156948402	0.462199392	0.891485256
ACVR2A	0.2951594	0.631403631	0.147804239	0.700720161	0.394870923	0.462377686	0.891485256
CUL5	0.725975665	0.212807595	0.465133535	0.730839797	0.145224431	0.462501095	0.891485256
JAG2	0.703442245	0.966030034	0.060933386	0.194338316	0.947607877	0.462465014	0.891485256
LRMDA	0.050003335	0.89875933	0.23882956	0.833091491	0.852397566	0.462383893	0.891485256
PUS7L	0.364903115	0.239972893	0.584525887	0.353450239	0.421328179	0.462395033	0.891485256
TMEM106C	0.171604162	0.753657849	0.817972865	0.718381936	0.100253677	0.462314119	0.891485256
PROX2	0.093880871	0.964090709	0.75274804	0.46447696	0.241267341	0.462690831	0.891746239
SH2B3	0.695795586	0.550195202	0.433974367	0.064712843	0.710488136	0.462774616	0.891802985
FAM184B	0.083616065	0.168855937	0.987680842	0.780181621	0.702408203	0.462856595	0.891856239
FAM20B	0.862139552	0.30313291	0.180552625	0.284074686	0.571201679	0.463199483	0.892113491
FLI1	0.55012054	0.830936995	0.420015332	0.046667477	0.854270932	0.463143429	0.892113491

IGFBP3	0.553631276	0.094263769	0.281545774	0.616096012	0.845849562	0.463207547	0.892113491
RCN2	0.750385057	0.692210607	0.18333352	0.659332941	0.12192373	0.463166718	0.892113491
C3H1orf210	0.127011918	0.634131742	0.648234746	0.305354402	0.480525721	0.463299091	0.892185095
LIMS2	0.441194095	0.430957652	0.585297846	0.534083564	0.128937657	0.463362475	0.892202461
KCNJ10	0.270649756	0.37973518	0.32445183	0.380999963	0.604109057	0.46363111	0.892614986
EXOC5	0.714825181	0.842683984	0.599709168	0.032266775	0.659115756	0.463814807	0.892682929
MYBL1	0.328986279	0.415596532	0.458773186	0.45412794	0.269731869	0.463829586	0.892682929
RUNX2	0.595731973	0.587464188	0.089675236	0.807513837	0.303130558	0.463799068	0.892682929
PHTF2	0.939159014	0.133781488	0.679307021	0.111812356	0.805452454	0.463901144	0.892715955
CCNL2	0.821065231	0.155414438	0.215107908	0.871953783	0.321477339	0.464082703	0.892855948
CHRNA3	0.241293967	0.08358939	0.491016728	0.877849521	0.884875844	0.464052496	0.892855948
COL4A5	0.024988709	0.950873629	0.652354712	0.668664139	0.742859471	0.464205297	0.892953964
PLAG1	0.236279288	0.453103266	0.740765293	0.268230864	0.362028381	0.464242473	0.892953964
TRPA1	0.039157454	0.87105582	0.581561592	0.82737888	0.469618894	0.46438805	0.893129296
RPS23	0.458050563	0.670889562	0.070504208	0.546587953	0.651823322	0.46466298	0.893553335
KIAA0753	0.353542869	0.183698398	0.42653993	0.44932886	0.620854005	0.464866747	0.893679501
LOC101906410	0.650310165	0.925874147	0.063303392	0.291479444	0.695400174	0.464817518	0.893679501
LOC101908339	0.397710111	0.417823864	0.16428638	0.527262125	0.536949865	0.464891956	0.893679501
KREMEN1	0.588750892	0.761353149	0.134816161	0.754065255	0.169712719	0.46500029	0.893760726
SELENOM	0.706356331	0.752474787	0.035313951	0.489461322	0.84198788	0.465043132	0.893760726
CLEC2B	0.454065543	0.520654796	0.082667664	0.961403725	0.411858208	0.465114168	0.893792577
LOC614226	0.506225375	0.825290273	0.10305002	0.302492076	0.594985483	0.465347055	0.894030732
SARDH	0.255055904	0.257538866	0.745327463	0.620807018	0.254867649	0.465296251	0.894030732
TMEM63A	0.978456056	0.136338025	0.555111516	0.124023025	0.844155237	0.465448321	0.894120613
TRPV1	0.518305628	0.653720718	0.335957129	0.397375869	0.171489977	0.465547505	0.894206473
ATP8B1	0.446624122	0.215420896	0.938557768	0.197063421	0.436647015	0.46584846	0.894365826
CRTAM	0.389696087	0.076342409	0.589313532	0.909226197	0.48737003	0.465824203	0.894365826
ISLR2	0.315890519	0.513316008	0.110883243	0.90013367	0.479734646	0.465710632	0.894365826
NBR1	0.690650017	0.324246309	0.06122806	0.853277466	0.66379145	0.465756316	0.894365826
CFAP20	0.724704411	0.056472637	0.479877367	0.435231483	0.910039232	0.466048576	0.894436111
LDLRAD4	0.437319049	0.782178064	0.975046832	0.049083929	0.474991062	0.465984072	0.894436111
OSTC	0.331718432	0.310235659	0.183503817	0.739598014	0.556917793	0.466041765	0.894436111
BCORL1	0.879359566	0.449663169	0.161447957	0.232964999	0.523420354	0.466180584	0.894440732
EHHADH	0.558039078	0.646829417	0.780735078	0.115267719	0.239960415	0.466420458	0.894440732
LOC101906001	0.526908155	0.209713064	0.404526827	0.206462367	0.844667407	0.466432501	0.894440732
LOC101906656	0.174602232	0.479558766	0.458573897	0.36084917	0.561729193	0.466149867	0.894440732
LOC112444846	0.381274461	0.619779455	0.066145309	0.772287038	0.645255868	0.466287284	0.894440732
PDGFB	0.525437915	0.514657857	0.734671831	0.096691176	0.405791405	0.466427816	0.894440732
SRSF12	0.225236285	0.696405029	0.280063636	0.475711185	0.372919194	0.466382964	0.894440732
COL25A1	0.36699961	0.403238286	0.619238007	0.608062149	0.139988207	0.46655294	0.894518054

RHBDL3	0.874038067	0.062445489	0.777909013	0.327368834	0.561312411	0.466585255	0.894518054
SP140	0.234720813	0.098855388	0.8011342	0.718933904	0.58395452	0.466636345	0.894518054
AHNAK	0.997317598	0.166453242	0.180315266	0.919391921	0.284023179	0.466921933	0.894671075
ANKRD6	0.175444459	0.814698084	0.178298963	0.605898088	0.509935027	0.468246804	0.894671075
ATP5F1A	0.85027469	0.5334084	0.696489573	0.091815511	0.271238741	0.468081212	0.894671075
BDKRB2	0.09794565	0.367864237	0.601105559	0.531937215	0.682866258	0.468088697	0.894671075
BRK1	0.484926803	0.278809501	0.622520023	0.185064429	0.506789265	0.468699438	0.894671075
C23H6orf132	0.942489155	0.619026244	0.613160274	0.097743281	0.224637913	0.467801708	0.894671075
C23H6orf141	0.674184438	0.230573073	0.304624872	0.394142034	0.420998317	0.467866736	0.894671075
DCTN5	0.715657843	0.174227637	0.577420519	0.271081203	0.403889383	0.468445227	0.894671075
DDX47	0.990502215	0.187111528	0.456052261	0.567750577	0.163798714	0.467930318	0.894671075
DNHD1	0.965080047	0.410755559	0.651345434	0.308355076	0.099133351	0.468675136	0.894671075
EML1	0.291222941	0.086516965	0.5698467	0.656461492	0.838332312	0.468875461	0.894671075
FAAP24	0.763585048	0.120935909	0.832379933	0.496417792	0.207016796	0.468824202	0.894671075
FANCL	0.703545871	0.269161456	0.192700002	0.259849769	0.825038425	0.467076002	0.894671075
FCGR2B	0.114903263	0.71357321	0.56315213	0.904393025	0.188847696	0.468523863	0.894671075
GOLM1	0.610314642	0.431581613	0.11242043	0.271086399	0.984254949	0.468861701	0.894671075
GTF3A	0.16951969	0.489173857	0.147741648	0.735160234	0.87068537	0.467510755	0.894671075
IGFBP4	0.171973084	0.853469068	0.240895157	0.602596902	0.370099218	0.4685055	0.894671075
KIAA2026	0.25704627	0.581673795	0.679192639	0.179484618	0.433561483	0.468896832	0.894671075
LOC101902084	0.06310188	0.668406459	0.28882911	0.695105943	0.933034895	0.468859822	0.894671075
LOC101902561	0.719770678	0.305839748	0.12420742	0.611874139	0.472049017	0.468781582	0.894671075
LOC101907322	0.153728731	0.953687576	0.149610542	0.473243333	0.760724238	0.468760901	0.894671075
LOC107132296	0.861042217	0.354736457	0.85181677	0.144708905	0.208289478	0.467514688	0.894671075
LOC112442080	0.750455609	0.870790546	0.102412332	0.735318433	0.159785044	0.467998467	0.894671075
LOC509006	0.206717724	0.81397331	0.298505155	0.816233186	0.191490748	0.467707055	0.894671075
LSM8	0.859325987	0.454863312	0.736148482	0.349721441	0.077872528	0.46737825	0.894671075
MARK4	0.361228405	0.452773587	0.506241015	0.195023676	0.486893294	0.467973051	0.894671075
MRPL49	0.804074279	0.262445363	0.487302715	0.161247622	0.474893864	0.468256865	0.894671075
MUT	0.806147723	0.531018919	0.685420924	0.097882177	0.272809604	0.467351006	0.894671075
NFU1	0.671813167	0.273913786	0.122170227	0.566024198	0.620437671	0.468730027	0.894671075
PBRM1	0.027394196	0.712161181	0.871135988	0.530957772	0.866831912	0.467048645	0.894671075
PLPPR1	0.442429627	0.102551283	0.70970402	0.973653283	0.251321505	0.468370343	0.894671075
PRIMA1	0.649964138	0.805763457	0.375835372	0.314557786	0.127090488	0.468125551	0.894671075
RBBP9	0.666434697	0.187885729	0.620043152	0.398292854	0.252674204	0.466848825	0.894671075
RPL27	0.873110821	0.521886861	0.472539233	0.814439406	0.04463135	0.467158647	0.894671075
RWDD4	0.56587409	0.447318288	0.648735208	0.275438577	0.17342922	0.467561216	0.894671075
TMEM220	0.567422245	0.665775965	0.114995121	0.864947593	0.208087367	0.466978723	0.894671075
TSNARE1	0.882663435	0.099702273	0.811818873	0.151075092	0.729583699	0.468258342	0.894671075
URM1	0.361529982	0.31176854	0.733009985	0.399398585	0.237793952	0.467620605	0.894671075

ZBTB49	0.595199097	0.185987414	0.399213828	0.650156711	0.274418923	0.468489733	0.894671075
ZNF345	0.142843219	0.990674315	0.86958585	0.466043942	0.137390982	0.468366823	0.894671075
ADGRG2	0.757476597	0.730601183	0.736524765	0.02077248	0.934171646	0.4690593	0.894843832
IDH2	0.498051018	0.575986344	0.415967983	0.094781096	0.699688097	0.469150955	0.894843832
KYAT3	0.965970006	0.129398175	0.899837729	0.116356636	0.604659959	0.46914522	0.894843832
EHD3	0.828815778	0.378259384	0.304174831	0.088592062	0.937300063	0.469264344	0.894956089
EBF3	0.312703324	0.377458953	0.153465069	0.861079523	0.508312585	0.469489822	0.895178049
PUS7	0.586828637	0.365053853	0.314253206	0.773210331	0.152287365	0.469457995	0.895178049
PDS5A	0.879215995	0.215450993	0.766357288	0.082115889	0.665661408	0.469643958	0.895310625
THOC5	0.155634943	0.67379282	0.091651138	0.901550742	0.915910947	0.469668465	0.895310625
LOC101902531	0.172226894	0.732807938	0.140172559	0.532090477	0.84335939	0.469725755	0.895315838
BECN1	0.950273212	0.43882439	0.322673912	0.550411767	0.107291006	0.469893159	0.895423173
LOC101904378	0.030500234	0.914922231	0.621864375	0.606132443	0.755665859	0.469945755	0.895423173
SEPHS1	0.843596719	0.62529289	0.081943385	0.349550412	0.526046636	0.469939913	0.895423173
ANK1	0.158753964	0.975446327	0.171416001	0.684532468	0.438443795	0.4703654	0.895461675
AXIN2	0.108763287	0.262383672	0.851927094	0.555437473	0.589575395	0.470244021	0.895461675
LOC512464	0.495340057	0.297993267	0.108035613	0.780050072	0.641540792	0.470672364	0.895461675
NR1H2	0.670547049	0.802449774	0.05674495	0.960512059	0.271981709	0.470586163	0.895461675
NRIP3	0.162061921	0.552759542	0.602699035	0.1607738	0.918534572	0.47050764	0.895461675
NRXN2	0.274850427	0.540602375	0.115886458	0.810748568	0.570066204	0.470170024	0.895461675
PALD1	0.413068454	0.367891442	0.78627298	0.29570558	0.225690975	0.470532766	0.895461675
PIK3C2B	0.710276331	0.556515636	0.078226538	0.306059583	0.841030983	0.470194305	0.895461675
QDPR	0.281933082	0.545865472	0.880160492	0.141595679	0.416089844	0.470675303	0.895461675
RAB8B	0.857640024	0.314319927	0.647866567	0.148689205	0.306680309	0.470298941	0.895461675
UBE3C	0.773544891	0.757958367	0.120358663	0.152662841	0.740703239	0.470656609	0.895461675
ZBED8	0.423088788	0.136950783	0.231116846	0.72651231	0.818776602	0.470344035	0.895461675
ZNF672	0.362036786	0.407972776	0.305407086	0.603515677	0.292710024	0.470407855	0.895461675
HDAC3	0.284342275	0.715763957	0.141364559	0.737029922	0.376516739	0.470754763	0.895509031
KRBA1	0.495569815	0.41969033	0.150180065	0.509569544	0.502238301	0.470979202	0.895832138
LOC107131225	0.030593809	0.421674839	0.862081108	0.938688908	0.766106321	0.471067088	0.895895466
RAB26	0.367407325	0.415741296	0.193274043	0.27818253	0.974285802	0.471146609	0.895926103
TNFAIP8L2	0.413644343	0.435711004	0.946367502	0.090287292	0.519706801	0.471192383	0.895926103
HACE1	0.60171154	0.797214624	0.647742661	0.404590358	0.063698013	0.471291275	0.896010324
CETN3	0.061302935	0.943555042	0.523167751	0.929671128	0.28508425	0.471576901	0.89601125
KMT2A	0.738806014	0.445398453	0.464419318	0.144058925	0.364878376	0.471863077	0.89601125
LOC101903564	0.8345383	0.107759815	0.179697281	0.689722022	0.719166735	0.471475189	0.89601125
LOC107132225	0.566988241	0.348052014	0.159172171	0.913433534	0.280017719	0.471892342	0.89601125
LOC787257	0.774524627	0.410524628	0.227985753	0.171880395	0.643569487	0.471540138	0.89601125
NUDT21	0.798625097	0.517057865	0.742787627	0.110853474	0.236187117	0.471810723	0.89601125
STX5	0.725024518	0.290585824	0.245133657	0.377984052	0.411040507	0.471658749	0.89601125

TAF1D	0.904256474	0.098905291	0.474847986	0.370310956	0.51065231	0.471812951	0.89601125
USP7	0.869010207	0.549293459	0.096260333	0.209589072	0.833666996	0.471762987	0.89601125
ZBTB18	0.576095513	0.287935942	0.2282956	0.300110805	0.705980486	0.471647932	0.89601125
ZC3H3	0.724137768	0.240165024	0.344522507	0.773876206	0.173000601	0.471608461	0.89601125
WARS	0.069193641	0.840910964	0.938651796	0.245505656	0.59983887	0.472088145	0.896279332
FJX1	0.915203417	0.468064352	0.044161068	0.698477991	0.60935696	0.472284583	0.896341193
PRR14	0.856637169	0.061699686	0.270194789	0.899751444	0.626349969	0.472204903	0.896341193
RHBDD3	0.111750255	0.905909903	0.75620795	0.172229674	0.610623305	0.472271578	0.896341193
COTL1	0.564662936	0.161738584	0.496451938	0.194756908	0.912853302	0.472487856	0.896415978
LOC101904574	0.569312871	0.283429682	0.566824239	0.167237656	0.526910149	0.472463377	0.896415978
RBM27	0.946364236	0.030408324	0.84831228	0.399272809	0.826797691	0.47244674	0.896415978
TLNRD1	0.903048096	0.354617982	0.432814378	0.485902039	0.119729023	0.472549458	0.896429218
EOGT	0.214145895	0.244141528	0.416750653	0.517856743	0.714870909	0.472609394	0.896439293
F12	0.830637851	0.285836216	0.216325947	0.504130907	0.311790931	0.4727679	0.896558167
KDM5B	0.734982222	0.752968236	0.201488628	0.123876021	0.584500993	0.472781328	0.896558167
DDX55	0.671547844	0.107426982	0.626524943	0.435115545	0.410766324	0.47288645	0.896653903
LOC112443437	0.858649687	0.429428407	0.693447018	0.08401525	0.37623771	0.472975194	0.896693009
WFIKKN1	0.381882034	0.372657881	0.295170079	0.622416168	0.309204836	0.473016354	0.896693009
COL8A2	0.725679361	0.596103427	0.043406841	0.532113908	0.809962875	0.473206623	0.896795738
DPP9	0.424726423	0.44116491	0.210871926	0.926908666	0.221098598	0.473314313	0.896795738
FAM180B	0.343304837	0.791554183	0.720933647	0.509575472	0.081124683	0.473341674	0.896795738
PDGFC	0.623568393	0.046810089	0.572020978	0.597644544	0.811652346	0.473354688	0.896795738
PROCA1	0.345081027	0.927477527	0.266442469	0.222829204	0.426255219	0.473363557	0.896795738
TMEM158	0.740500851	0.415495725	0.251021078	0.171972811	0.609945515	0.47339842	0.896795738
SQLE	0.595564906	0.241938061	0.137910064	0.630864745	0.646737875	0.473540837	0.896858474
TBX18	0.511569338	0.217277402	0.136074153	0.65998489	0.812037128	0.473503535	0.896858474
CIB1	0.140870714	0.308197299	0.363874248	0.51721568	0.99315897	0.473707044	0.897069731
TNIP1	0.282327315	0.580954177	0.087049864	0.72630612	0.783166835	0.473851543	0.897239837
LOC100139345	0.910580338	0.283120464	0.46409878	0.543756588	0.125043212	0.474155935	0.897402036
LOC100296205	0.332691186	0.522599786	0.653831513	0.560889851	0.1275841	0.474150035	0.897402036
MRPS22	0.97735004	0.226393518	0.687088636	0.197207269	0.271147533	0.474026619	0.897402036
PDE2A	0.194116605	0.352114628	0.663198783	0.311630868	0.575556573	0.47405318	0.897402036
DNASE2	0.268595237	0.59703202	0.279390858	0.696150527	0.261133018	0.474368657	0.897651818
LOC112447103	0.764893956	0.905265622	0.244594104	0.939316782	0.051204275	0.474397308	0.897651818
ARF4	0.900337688	0.069398074	0.663852588	0.780652385	0.252171464	0.474831123	0.897868325
BAHD1	0.359249837	0.182573409	0.588435351	0.58605068	0.360599641	0.474628208	0.897868325
ELL3	0.38169706	0.411676475	0.425933175	0.54244376	0.224755017	0.474706523	0.897868325
FGL1	0.222440619	0.666603239	0.130483695	0.569415045	0.742721333	0.475213973	0.897868325
LOC107131941	0.211022185	0.196416209	0.882832275	0.781351971	0.286135392	0.475176009	0.897868325
LOC112445150	0.570155689	0.837261455	0.080216156	0.94212757	0.226210289	0.474730809	0.897868325

PALLD	0.340702132	0.725488037	0.068074653	0.644131327	0.753457239	0.474850143	0.897868325
PCDHGA8	0.405006343	0.646588003	0.725674974	0.21354087	0.201630454	0.475203988	0.897868325
PEX10	0.995993474	0.229015628	0.441312951	0.87755819	0.092530149	0.475019226	0.897868325
PHLDB3	0.746021704	0.607148157	0.040464569	0.893923463	0.498943879	0.475038328	0.897868325
SNAPC1	0.114029252	0.452700759	0.462470619	0.493657525	0.693457643	0.474989867	0.897868325
WRN	0.099588014	0.799198963	0.715729107	0.800531985	0.179163242	0.474940016	0.897868325
ZFP90	0.625082504	0.819784503	0.496674931	0.054066198	0.594674044	0.475222977	0.897868325
PLEKHO1	0.305443132	0.492470763	0.764561948	0.105510791	0.674819504	0.475346053	0.897894115
UFC1	0.673943128	0.187311856	0.676814822	0.271797482	0.35261697	0.47534564	0.897894115
ADORA3	0.238245869	0.441545202	0.470205361	0.563093969	0.294186924	0.475465147	0.898009961
AQP1	0.473978362	0.504384055	0.665279922	0.065241903	0.790599876	0.475680982	0.898009961
ATXN7L3	0.692114005	0.483257521	0.354118753	0.565294375	0.122510924	0.475658789	0.898009961
LYSMD1	0.739547474	0.085505056	0.382545256	0.543674292	0.623515861	0.475605302	0.898009961
POLR2B	0.335148586	0.44752296	0.471651658	0.133519158	0.867900947	0.475547015	0.898009961
INPP5K	0.087697304	0.530482697	0.690839629	0.540233332	0.473065381	0.475902866	0.898141516
LOC112445177	0.442317055	0.652823391	0.360432459	0.097462174	0.809690341	0.475890751	0.898141516
METTL16	0.644040556	0.468750873	0.34548616	0.116398894	0.676598519	0.475914851	0.898141516
EXTL2	0.252810758	0.47364645	0.273696158	0.546738809	0.459136187	0.476197342	0.898302077
LOC101907348	0.557193836	0.471125984	0.138683469	0.408934155	0.552377256	0.476120759	0.898302077
PFDN1	0.783539033	0.223683086	0.678847918	0.258372547	0.267657068	0.476218882	0.898302077
SYNJ1	0.669648553	0.063278325	0.507053184	0.701444774	0.545531355	0.476083626	0.898302077
C23H6orf136	0.954867504	0.341693266	0.532993088	0.177551559	0.267350661	0.476812312	0.898453526
FAM71F2	0.962803388	0.754234566	0.913476473	0.473645449	0.026290542	0.476932862	0.898453526
GAS2	0.303487473	0.063394434	0.577457338	0.949274933	0.7833093	0.476950804	0.898453526
LOC100848443	0.141783387	0.827807077	0.4098186	0.193536275	0.886686037	0.476800374	0.898453526
MAP4K4	0.732173513	0.93410906	0.603793434	0.021386849	0.935194029	0.476913652	0.898453526
MN1	0.64442861	0.186846215	0.608575988	0.19895909	0.566646342	0.476956134	0.898453526
PAFAH1B3	0.769569566	0.611815427	0.345337045	0.225235697	0.225012743	0.476497405	0.898453526
POFUT2	0.990263201	0.708210056	0.042682845	0.474955228	0.580874121	0.476893637	0.898453526
PRRX2	0.625205505	0.559161207	0.080250656	0.945605643	0.310783534	0.476589916	0.898453526
ROCK2	0.391927909	0.39996436	0.430705696	0.142935146	0.854754413	0.476678807	0.898453526
UBXN1	0.117028736	0.692882379	0.352244442	0.971936676	0.297178149	0.476705032	0.898453526
ZNF35	0.8660072	0.430183803	0.130557506	0.20909645	0.810338851	0.476512042	0.898453526
CHD4	0.924709892	0.956232634	0.092516625	0.123464051	0.818767056	0.477140655	0.898594823
CMPK2	0.209480499	0.760930339	0.937133499	0.076210396	0.726212289	0.4770895	0.898594823
ACAA1	0.50445459	0.448338359	0.144768717	0.870908861	0.291181434	0.477874713	0.898607157
AMER1	0.502688353	0.202512725	0.369342058	0.738557427	0.299265697	0.47803508	0.898607157
AP3S2	0.541302103	0.202948527	0.452350905	0.246925025	0.683604012	0.4797365	0.898607157
ATAD1	0.55915107	0.064460506	0.465568777	0.606830247	0.821941261	0.479334583	0.898607157
AVPR1A	0.125274501	0.798477267	0.879738443	0.544006833	0.174881013	0.479381283	0.898607157

BCL9L	0.076230721	0.785695845	0.522485899	0.6902976	0.388650748	0.479898024	0.898607157
CACNB1	0.7528937	0.694084795	0.121570215	0.732880804	0.178412906	0.477956106	0.898607157
CAPN7	0.712497634	0.467552209	0.613732057	0.140745332	0.289662091	0.47857967	0.898607157
CAVIN2	0.890039417	0.068645136	0.47383446	0.376161972	0.765575618	0.478617735	0.898607157
CD163	0.712552141	0.367584887	0.393003087	0.222573602	0.365067054	0.47920927	0.898607157
CLCF1	0.944577514	0.780590629	0.524757034	0.203619778	0.105962337	0.478862248	0.898607157
COQ10A	0.745749076	0.506060606	0.411955003	0.329542997	0.163470961	0.479453545	0.898607157
DDX27	0.18411462	0.784341673	0.929910477	0.162938307	0.379216362	0.477751263	0.898607157
DNMT3B	0.765508076	0.137913968	0.11168475	0.848617832	0.830546707	0.478038021	0.898607157
DOK4	0.339172138	0.452810639	0.237424482	0.405830402	0.566801352	0.479722606	0.898607157
FIGNL2	0.818112968	0.707419808	0.175154603	0.300090168	0.273138773	0.478004037	0.898607157
GOLPH3	0.891069852	0.427284558	0.165505908	0.213835294	0.615306878	0.477612194	0.898607157
KIF5B	0.560562299	0.804320074	0.381100666	0.722659517	0.067031159	0.478321365	0.898607157
KRT24	0.236574376	0.832837307	0.315797619	0.190794843	0.700495673	0.478155868	0.898607157
LMNA	0.832223642	0.966063179	0.049380906	0.241070364	0.875685394	0.479579162	0.898607157
LOC100849237	0.990443251	0.934188053	0.117517396	0.922491861	0.082866216	0.478070767	0.898607157
LOC101906200	0.722949719	0.339113406	0.571795489	0.258169301	0.231674469	0.479654834	0.898607157
LOC104972290	0.30638084	0.766770256	0.322454472	0.133353845	0.826500188	0.478884545	0.898607157
LOC104974020	0.13182883	0.581139423	0.672607225	0.303814764	0.529909135	0.477716667	0.898607157
LOC107131992	0.677573258	0.324247441	0.097134002	0.574680566	0.679533097	0.478547419	0.898607157
LOC112442271	0.294384261	0.877385745	0.286964744	0.595294191	0.190180731	0.479804226	0.898607157
LOC112442949	0.821689012	0.975608224	0.039622431	0.38659132	0.679862018	0.478864869	0.898607157
LOC527796	0.621337181	0.650785189	0.454149689	0.362338767	0.126060056	0.479730417	0.898607157
LOC617654	0.994391933	0.491289707	0.276291871	0.309933921	0.199305628	0.478635967	0.898607157
LOC781688	0.973913058	0.263904337	0.890174936	0.88841461	0.041143422	0.47918474	0.898607157
LOC781770	0.649086937	0.232248918	0.343818665	0.58477496	0.27722975	0.480049293	0.898607157
MAP6	0.227778835	0.992023024	0.580466637	0.17121263	0.369353239	0.47768626	0.898607157
MIPOL1	0.646546795	0.827802431	0.608842212	0.308039845	0.08357131	0.479746898	0.898607157
NR1D1	0.41394361	0.315792748	0.124003946	0.972742827	0.532701641	0.479984648	0.898607157
OARD1	0.129740281	0.804231187	0.823574608	0.141046113	0.686459923	0.478250839	0.898607157
OTUD5	0.461246188	0.251033489	0.501765355	0.35948279	0.400448807	0.479198208	0.898607157
PTGER3	0.709940809	0.940731319	0.043620992	0.391348058	0.729335681	0.478141221	0.898607157
RAB3IP	0.995240274	0.510814201	0.022464168	0.94705953	0.769667457	0.478346014	0.898607157
SEC16A	0.589702643	0.646492735	0.30562136	0.716771715	0.09964512	0.478285404	0.898607157
SLC25A6	0.574486192	0.756687805	0.287900896	0.687413413	0.09675132	0.478326106	0.898607157
SLC45A4	0.513560116	0.194730766	0.349949405	0.267400288	0.896431378	0.479753003	0.898607157
SSC5D	0.409299839	0.296096732	0.116023834	0.636130595	0.937201926	0.479623429	0.898607157
SYN2	0.045406872	0.361689296	0.958759793	0.66624812	0.800908112	0.480037148	0.898607157
THAP7	0.397274941	0.131975065	0.889141946	0.255839538	0.697044865	0.478102855	0.898607157
TIMP2	0.66701774	0.679069763	0.028933504	0.768008492	0.830779969	0.479162324	0.898607157

TSSK1B	0.165546446	0.458959013	0.324066778	0.809916482	0.421248667	0.480004053	0.898607157
TUBGCP6	0.973655031	0.14643385	0.262766438	0.783300455	0.282531984	0.477611724	0.898607157
TXLNG	0.547754985	0.680173175	0.135141713	0.604326358	0.271932325	0.477240453	0.898607157
VEPH1	0.958410737	0.382946356	0.082925857	0.510090064	0.534542851	0.477778124	0.898607157
WDR83OS	0.518933628	0.329247759	0.844888904	0.175757658	0.328761974	0.478710757	0.898607157
ZEB2	0.42618332	0.220303279	0.562782529	0.210896022	0.750552197	0.479205383	0.898607157
ZNF407	0.727318841	0.571688793	0.44277082	0.406407076	0.111850173	0.479312187	0.898607157
ZNF829	0.9734036	0.461189794	0.733258364	0.045151248	0.563881114	0.479575129	0.898607157
AFDN	0.428148711	0.730467814	0.036172597	0.773740973	0.960813162	0.480215575	0.898713399
ECI1	0.214188769	0.386385103	0.705031633	0.202239581	0.712669761	0.480203041	0.898713399
FH	0.802675014	0.43595485	0.631938341	0.125362354	0.303891808	0.480523581	0.898791037
LPIN2	0.262764782	0.825538216	0.317297668	0.939081465	0.130214004	0.480351123	0.898791037
PTPN9	0.949973733	0.63791877	0.251002438	0.055546299	0.996940493	0.480497153	0.898791037
USP39	0.481978884	0.787142849	0.127497323	0.188118868	0.925858236	0.480530897	0.898791037
XKRX	0.031157441	0.813151749	0.468032502	0.960716752	0.739294533	0.480473161	0.898791037
CALHM5	0.084009674	0.919791825	0.989658886	0.587301993	0.187808585	0.480750472	0.89879198
GEMIN8	0.237326106	0.223292101	0.425142316	0.771077364	0.485450375	0.480714581	0.89879198
SUFU	0.547557304	0.988476979	0.296722492	0.38608074	0.13597667	0.480669612	0.89879198
TTC39B	0.954222504	0.49029174	0.56331398	0.347931629	0.091933606	0.480641766	0.89879198
ADGRF1	0.336164878	0.114143808	0.919763505	0.717689601	0.33464816	0.48164497	0.898878282
ARSK	0.136260342	0.795130303	0.170339001	0.525550602	0.873769195	0.481614301	0.898878282
CTNND2	0.363317876	0.190060663	0.704310818	0.355470828	0.490812802	0.481837568	0.898878282
GNG7	0.872315002	0.659217708	0.088080937	0.292078193	0.572474356	0.481490553	0.898878282
IPO13	0.585502615	0.080157716	0.799721584	0.644992012	0.349311787	0.481213186	0.898878282
KIAA1549	0.962696926	0.276224702	0.182212362	0.694897536	0.251710154	0.481622009	0.898878282
KIF13A	0.403088938	0.695511341	0.111491908	0.603087432	0.449573971	0.481612928	0.898878282
LOC101905586	0.225776908	0.153457151	0.447175612	0.905656775	0.603204002	0.481378255	0.898878282
LOC104970145	0.783892596	0.084532415	0.642114918	0.294623314	0.67706901	0.481892092	0.898878282
LOC107133289	0.454670049	0.307269173	0.348516539	0.724669852	0.239475471	0.481069796	0.898878282
LOC112446734	0.600471254	0.327230645	0.52714038	0.468222186	0.174897211	0.481771913	0.898878282
LOC112447506	0.983733715	0.733866332	0.080134874	0.317671363	0.460163825	0.481224153	0.898878282
LOC784127	0.565940994	0.043952078	0.402437012	0.97855879	0.862782248	0.481110478	0.898878282
LOXL3	0.813737324	0.421566394	0.133016489	0.203604861	0.912960447	0.481767051	0.898878282
NOL10	0.409788085	0.158073022	0.557949482	0.550194774	0.425949537	0.481510462	0.898878282
POLE2	0.045991801	0.951913443	0.438041574	0.953029966	0.464321487	0.481860875	0.898878282
PVALB	0.510065754	0.918228785	0.086818254	0.83395957	0.249543959	0.481339329	0.898878282
RPS6KA1	0.308434709	0.361957351	0.665335547	0.343147138	0.332376565	0.4815465	0.898878282
XDH	0.659934181	0.459632249	0.247371791	0.198782162	0.567111892	0.481266607	0.898878282
ZNF696	0.821788095	0.388033405	0.608661907	0.516846408	0.084336228	0.481297472	0.898878282
FAM98B	0.474450061	0.160004281	0.69314928	0.39127933	0.412631775	0.48206306	0.898996321

GPR108	0.142169395	0.711301412	0.375857686	0.629723558	0.355032538	0.482106398	0.898996321
HSBP1L1	0.270273128	0.781182786	0.062406072	0.990804481	0.650999236	0.482127256	0.898996321
LOC112441886	0.045063236	0.950730673	0.898100814	0.673536657	0.328017283	0.482174494	0.898996321
LAMTOR3	0.510800821	0.144207521	0.213328026	0.912386541	0.593401135	0.48232271	0.899119557
LRRC74B	0.184672106	0.111806125	0.896184446	0.881767089	0.521508777	0.482350166	0.899119557
PIK3AP1	0.641194852	0.155202149	0.580206632	0.285211794	0.516997135	0.482453952	0.89921088
ARHGAP21	0.518533232	0.490535965	0.896139964	0.119360112	0.31327979	0.482659999	0.899242642
LOC781261	0.122689347	0.557929487	0.199706351	0.949622113	0.656685771	0.482690173	0.899242642
NCR3LG1	0.296085437	0.498234638	0.161937908	0.576668433	0.618549743	0.482611692	0.899242642
NRXN1	0.535865833	0.503036385	0.338793446	0.719250518	0.129675499	0.482539309	0.899242642
LOC112441778	0.999466017	0.72721671	0.416295252	0.160691956	0.175407022	0.48276942	0.89928819
CPEB1	0.235894271	0.673425887	0.341892447	0.634599696	0.247756708	0.483000279	0.899478061
CTGF	0.26269207	0.679307675	0.671881501	0.165759971	0.429728807	0.483024868	0.899478061
LOC112449346	0.65101681	0.48107947	0.139670835	0.947658145	0.206096061	0.483090587	0.899478061
TRAIP	0.580785998	0.592665406	0.16883064	0.226140115	0.649979462	0.483056041	0.899478061
PDE3B	0.346220171	0.569556033	0.074252152	0.745877652	0.783155852	0.483291977	0.899750951
ACTR3B	0.222351129	0.497922025	0.564249235	0.154959888	0.886555013	0.48391764	0.899791388
ADGRL3	0.207672628	0.318767107	0.647180747	0.72868496	0.276984836	0.485301616	0.899791388
B3GNT8	0.850382012	0.633846477	0.104884644	0.303602852	0.502106631	0.484683937	0.899791388
CDH20	0.536613594	0.793449279	0.383622707	0.213652961	0.245786409	0.483814373	0.899791388
COMMD4	0.136201452	0.546254573	0.29624608	0.442449798	0.884110979	0.484763485	0.899791388
CSNK2A2	0.749515879	0.817099469	0.341539262	0.078331599	0.527317308	0.485145833	0.899791388
DHRSX	0.475881823	0.355228884	0.608953718	0.115556018	0.725315392	0.484894798	0.899791388
DLD	0.668638023	0.388529851	0.652672409	0.154541629	0.328200397	0.484296949	0.899791388
DSCC1	0.567574417	0.389397798	0.787696375	0.468031321	0.106080831	0.485222537	0.899791388
EEF1AKMT3	0.666970945	0.605963207	0.406686163	0.399573512	0.130503533	0.483679182	0.899791388
FAM46A	0.38195116	0.595592614	0.304759204	0.48244382	0.257945856	0.484885562	0.899791388
FXYD5	0.322626846	0.518049228	0.440208545	0.339235125	0.344915218	0.484486033	0.899791388
GAPT	0.914538388	0.039341552	0.81075113	0.80276545	0.367390906	0.48436566	0.899791388
GGTA1	0.850125063	0.531809001	0.279558248	0.642047514	0.106068006	0.484451924	0.899791388
GTF2H3	0.576841507	0.294689946	0.841548723	0.156493182	0.386349191	0.485344868	0.899791388
HDDC2	0.35348489	0.367905847	0.956156275	0.978039425	0.070936309	0.48487378	0.899791388
LOC101902786	0.339787323	0.676026588	0.548946837	0.092866185	0.736165237	0.484735581	0.899791388
LOC101905845	0.155170767	0.29770751	0.434223309	0.780195771	0.553977391	0.485780982	0.899791388
LOC101906606	0.765775245	0.077022465	0.756197772	0.34049789	0.565292528	0.483978902	0.899791388
LOC101908205	0.889471195	0.640311382	0.097825403	0.899588952	0.172117114	0.484865987	0.899791388
LOC104968411	0.887048757	0.192663455	0.432828066	0.193541894	0.604818345	0.485550101	0.899791388
LOC112446053	0.931894183	0.327227528	0.703098794	0.139506508	0.288909244	0.485181011	0.899791388
LOC112446357	0.356941102	0.936001294	0.744465365	0.715220306	0.048725118	0.485739467	0.899791388
LOC112447080	0.11544348	0.343037298	0.714768358	0.370816782	0.82450932	0.48545241	0.899791388

METTL5	0.984070028	0.234923724	0.129476748	0.771613219	0.372025416	0.484137232	0.899791388
MFSD14A	0.679643894	0.162763368	0.365723888	0.297270914	0.716133473	0.484568214	0.899791388
MRPL22	0.713829514	0.888385101	0.644654978	0.211835724	0.100037042	0.485643482	0.899791388
MRPS35	0.872507558	0.202409948	0.584466107	0.320084028	0.260689459	0.484572458	0.899791388
MSANTD2	0.482305802	0.834449706	0.694996805	0.223235089	0.137074671	0.483423357	0.899791388
PIN1	0.482866058	0.288497378	0.544551028	0.496932378	0.229459542	0.485359249	0.899791388
RAD54L	0.747417687	0.791991222	0.104133209	0.754547399	0.186400228	0.485779922	0.899791388
RBM24	0.168398829	0.272502749	0.978085068	0.99141309	0.193765783	0.484771129	0.899791388
RBM4B	0.382366211	0.484956964	0.559868677	0.56604805	0.14724689	0.48542591	0.899791388
RFXAP	0.355670929	0.943594974	0.672622186	0.107531288	0.356688223	0.485535987	0.899791388
SEC11A	0.591576865	0.543409196	0.083364264	0.673889839	0.47977177	0.485668222	0.899791388
SFXN5	0.084655412	0.945421827	0.374362092	0.29354191	0.976374899	0.484028338	0.899791388
SIDT2	0.843363777	0.811733503	0.217927018	0.129359608	0.448346687	0.485419081	0.899791388
SMTNL1	0.675957712	0.57138961	0.858764369	0.034160802	0.760230898	0.484594248	0.899791388
SRBD1	0.499338454	0.952511857	0.097881996	0.377040794	0.493166187	0.485502628	0.899791388
TRRAP	0.485814857	0.30399868	0.955246428	0.19220476	0.316106127	0.48368907	0.899791388
TSNAX	0.457595062	0.533509069	0.707052828	0.597197371	0.08362556	0.484734642	0.899791388
TWF1	0.771699852	0.357728894	0.80888821	0.809343624	0.047506594	0.483994087	0.899791388
UHRF2	0.584885892	0.140987408	0.837738893	0.410757901	0.304968489	0.485439744	0.899791388
UNC13D	0.838945411	0.16964498	0.747410919	0.201488036	0.40252833	0.484881593	0.899791388
ZNF10	0.866142717	0.423894534	0.794169132	0.22446838	0.131201838	0.484026832	0.899791388
GTF2E2	0.95440352	0.181721614	0.626992353	0.523877151	0.152448631	0.486096092	0.900139019
KIDINS220	0.649355025	0.736933733	0.92403282	0.064687909	0.303683001	0.486133211	0.900139019
NOP16	0.483887797	0.533036722	0.26092616	0.479051163	0.269389376	0.486107904	0.900139019
PFN1	0.141135766	0.278343735	0.498057023	0.640608337	0.693543343	0.486269433	0.900289674
LOC790009	0.116955474	0.322015471	0.90676292	0.372533168	0.683643048	0.486363142	0.900361593
TMPRSS6	0.051463904	0.353018187	0.916626284	0.549523159	0.951330819	0.486542588	0.900592196
MTRF1L	0.757196344	0.049478779	0.822432758	0.570916583	0.495187651	0.486652849	0.9006947
NUBPL	0.649963295	0.58119879	0.077915398	0.94891975	0.312238867	0.486857148	0.900971206
CEP85	0.639578687	0.404985288	0.238548009	0.515970253	0.273729646	0.48698503	0.901004659
NYAP1	0.682990386	0.089421971	0.310533857	0.810613297	0.567560942	0.486959002	0.901004659
SFRP5	0.369823363	0.489617364	0.222260985	0.536491701	0.404556438	0.487154711	0.901216996
AOC1	0.347524727	0.833161206	0.687323664	0.179830399	0.244309684	0.487333871	0.901260961
SLC25A17	0.677541371	0.538446979	0.600108597	0.08622532	0.463140227	0.487323751	0.901260961
TBKBP1	0.603273688	0.23327433	0.740227579	0.322034212	0.260712899	0.48738944	0.901260961
TP53I3	0.228885394	0.476700482	0.17534216	0.716301477	0.638242667	0.487398149	0.901260961
MYO5C	0.516543195	0.39788838	0.077813973	0.834945157	0.655478357	0.487529411	0.901402113
PLA2G4B	0.804952983	0.40597324	0.090067959	0.669164876	0.444683644	0.487648261	0.901520289
AGPAT5	0.852764439	0.285348065	0.27633068	0.862874865	0.151365884	0.488150649	0.901606587
ANAPC16	0.750969597	0.352452815	0.380194442	0.206966318	0.421645752	0.488136911	0.901606587

ATF7	0.166516288	0.230230581	0.889345191	0.371055524	0.694611547	0.488261645	0.901606587
CEP104	0.300777737	0.944955469	0.071119755	0.508543394	0.856081735	0.488524101	0.901606587
DDX1	0.915131884	0.118338741	0.900928955	0.525074479	0.171496705	0.488222198	0.901606587
EVI2B	0.74179664	0.17445544	0.808042837	0.239463382	0.35104355	0.488317933	0.901606587
GRID1	0.177605398	0.31768506	0.507419886	0.310752777	0.988937917	0.488487645	0.901606587
LCP2	0.329319762	0.339715499	0.904447155	0.219231747	0.395215656	0.487830914	0.901606587
LOC100300896	0.750890935	0.658005912	0.120137926	0.183408587	0.806682168	0.488150636	0.901606587
LOC112447438	0.707749231	0.889002755	0.117042582	0.955418884	0.124739284	0.488029748	0.901606587
LOC783730	0.277435554	0.393517157	0.579519418	0.153381638	0.905414411	0.488237739	0.901606587
MAK16	0.818711367	0.218636857	0.512695138	0.906815068	0.105553445	0.488191138	0.901606587
SLC7A5	0.181265522	0.250938652	0.261895598	0.861063168	0.856143867	0.488143782	0.901606587
SNAPIN	0.824489697	0.276465545	0.320018411	0.142934787	0.843847918	0.488487157	0.901606587
TREM2	0.438196311	0.276568022	0.397512398	0.492920859	0.370688978	0.488573967	0.901606587
ZGPAT	0.295455547	0.707852275	0.303566097	0.22869891	0.605885402	0.488461439	0.901606587
LOC104973826	0.812460022	0.55791075	0.967812819	0.198434899	0.101172751	0.488672051	0.901686196
PRADC1	0.600998654	0.223211395	0.787290538	0.207372689	0.402486139	0.488836328	0.901887912
ERBB3	0.194656458	0.814801189	0.356138018	0.583635902	0.268190779	0.489386223	0.902598033
SHARPIN	0.58351446	0.119831862	0.713478132	0.188704805	0.938997889	0.489355494	0.902598033
TTC28	0.899968578	0.337583553	0.108492198	0.59642381	0.449572043	0.489317011	0.902598033
ECHDC3	0.250249898	0.807622835	0.607987873	0.21579847	0.333554023	0.489457162	0.902627428
CAMSAP2	0.827765429	0.227626428	0.432670026	0.267477787	0.406513842	0.48986338	0.902652489
CARMIL1	0.361278996	0.842386193	0.849528468	0.148046194	0.23181422	0.490041272	0.902652489
CISD3	0.928198995	0.140655511	0.508428551	0.350665241	0.381017888	0.489955011	0.902652489
CUL4B	0.416233003	0.91774134	0.083843237	0.715406012	0.387278218	0.490054546	0.902652489
LOC101904498	0.625815017	0.430116323	0.196291103	0.222538446	0.75324764	0.489704552	0.902652489
LOC104972827	0.173886278	0.682829872	0.938489611	0.259189175	0.306813126	0.489799017	0.902652489
LOC512953	0.976790167	0.446897351	0.862981461	0.040482531	0.58020946	0.489529682	0.902652489
NAP1L5	0.575528206	0.262620419	0.834009568	0.120279183	0.585495563	0.490130786	0.902652489
NELFE	0.926173636	0.081161333	0.393702727	0.545905782	0.549425802	0.49011235	0.902652489
NNT	0.870481577	0.681998929	0.224268339	0.454963564	0.146203166	0.489691526	0.902652489
SUPT3H	0.447725911	0.975277602	0.808172667	0.097318473	0.258296292	0.489994136	0.902652489
ZNF516	0.923315409	0.291080956	0.585185087	0.064804488	0.869843693	0.489886112	0.902652489
ANKRD31	0.673974896	0.356153922	0.283375438	0.189198987	0.692940307	0.490970272	0.902676043
ATG4A	0.531147828	0.248585807	0.607796045	0.717438784	0.154780623	0.490839843	0.902676043
BATF	0.667282455	0.703197675	0.118642562	0.697666107	0.22972071	0.491062459	0.902676043
BORCS6	0.651648984	0.934572882	0.873614186	0.1172714	0.142472	0.490380267	0.902676043
CPD	0.070689096	0.30568671	0.735049928	0.642444756	0.874965703	0.491188658	0.902676043
DUSP28	0.311146274	0.870806233	0.355193061	0.203958424	0.454026205	0.490849235	0.902676043
FBXL15	0.359238734	0.603330095	0.534232694	0.097464652	0.788022463	0.490459254	0.902676043
FRYL	0.684491306	0.6819759	0.037680206	0.624699597	0.810767066	0.490783176	0.902676043

GCNT3	0.202080942	0.235097507	0.450951683	0.888216248	0.468286864	0.490832641	0.902676043
GIT1	0.72738825	0.73464483	0.663747456	0.104426045	0.240945812	0.491104648	0.902676043
LOC100850276	0.691231505	0.556194849	0.098799336	0.874979448	0.268618204	0.491173866	0.902676043
LOC112442657	0.040178225	0.799381313	0.647544252	0.483920028	0.885500102	0.490850606	0.902676043
LOC782479	0.284495627	0.685698425	0.241940781	0.289403768	0.652561338	0.490879024	0.902676043
PCED1A	0.743091537	0.034698295	0.470822905	0.747734049	0.982048259	0.490897986	0.902676043
RAB11FIP1	0.235785914	0.156623276	0.668191156	0.598248122	0.604405704	0.491066433	0.902676043
RASIP1	0.310776228	0.748001325	0.871980376	0.2177474	0.201756842	0.490707013	0.902676043
SLC35A5	0.566998017	0.27712728	0.125314807	0.780918294	0.579619913	0.490865761	0.902676043
TOM1L2	0.459444381	0.780851189	0.579396313	0.052823299	0.811778949	0.49087771	0.902676043
TRPM7	0.498456311	0.668546311	0.962921912	0.058735388	0.47254138	0.490728758	0.902676043
C9H6orf203	0.852323449	0.020756344	0.940367696	0.680944206	0.788663327	0.491309054	0.902745962
EXOSC2	0.95912098	0.192943041	0.629432627	0.114499139	0.670070216	0.491359221	0.902745962
YTHDF1	0.844171991	0.415199709	0.247710199	0.116636294	0.882643378	0.49139173	0.902745962
ARL10	0.784524745	0.765054985	0.389330789	0.927992615	0.041237897	0.491480167	0.902807367
ALCAM	0.02018074	0.965268658	0.806673582	0.76724456	0.747728963	0.492968482	0.90282245
ARPC3	0.87923146	0.321528578	0.42804007	0.9526241	0.077631409	0.49161064	0.90282245
ATL3	0.12442283	0.279487112	0.532200085	0.500378896	0.970792217	0.492458693	0.90282245
CAP1	0.354230702	0.276191783	0.237849867	0.567506145	0.6811461	0.492564384	0.90282245
CD86	0.423052248	0.834795601	0.739584124	0.376420354	0.091220762	0.492019453	0.90282245
GFRA2	0.346962241	0.61946151	0.934332364	0.108651129	0.412460297	0.492650945	0.90282245
HCFC1R1	0.650384852	0.398746431	0.754883551	0.047699295	0.96413535	0.492728675	0.90282245
HECW2	0.736982382	0.917455046	0.494946462	0.071568604	0.375117691	0.492343143	0.90282245
ITGB3BP	0.468447382	0.569585762	0.047274375	0.882457644	0.807567347	0.492440111	0.90282245
LOC101907000	0.8952662	0.608319483	0.899109857	0.12746666	0.143638937	0.491950165	0.90282245
LOC101907697	0.450124205	0.849444842	0.56334214	0.080052537	0.521501368	0.492504717	0.90282245
LOC104970628	0.70293101	0.924723864	0.046741257	0.340662034	0.870535708	0.492872522	0.90282245
LOC107132490	0.757625069	0.028055504	0.613846787	0.910404565	0.754435839	0.491875246	0.90282245
LOC112442265	0.027050167	0.732764224	0.693854882	0.928205989	0.702360738	0.491968007	0.90282245
LOC112448057	0.619091604	0.591883537	0.170597003	0.163003999	0.885178371	0.493066494	0.90282245
LOC512149	0.608479461	0.025060614	0.965756716	0.690489326	0.886972006	0.493058256	0.90282245
MESP2	0.223513397	0.668174336	0.465885335	0.149808459	0.861620705	0.492272862	0.90282245
MSH6	0.050997386	0.378401769	0.994624583	0.495636265	0.945544556	0.4925621	0.90282245
PDP2	0.677164685	0.327191549	0.968026577	0.192150088	0.218618356	0.49286206	0.90282245
PHIP	0.366225037	0.39275372	0.739493643	0.105732876	0.79669619	0.491839745	0.90282245
POLH	0.630748579	0.290779497	0.605527032	0.341288937	0.236207973	0.49169734	0.90282245
SLC15A3	0.442667048	0.249196989	0.772375942	0.250281145	0.42261527	0.492909331	0.90282245
SLC7A10	0.459768553	0.365220356	0.353480946	0.192683137	0.788729839	0.493083762	0.90282245
SRRM5	0.744977766	0.369999436	0.063273848	0.926919721	0.555869129	0.492382707	0.90282245
STK32A	0.628047043	0.160477838	0.187118809	0.494271153	0.967507225	0.493046312	0.90282245

TMEM60	0.896776001	0.090111076	0.961365929	0.152689044	0.759444051	0.492838108	0.90282245
TSC22D2	0.529112395	0.656919153	0.851762482	0.048899932	0.622541077	0.492923189	0.90282245
TTLL12	0.73297942	0.895667779	0.032816507	0.730009716	0.572079179	0.492609085	0.90282245
UBE2V1	0.577298015	0.290705836	0.771054229	0.274965921	0.253026118	0.492722639	0.90282245
ARHGAP5	0.28275185	0.351417613	0.813440105	0.136925944	0.820541732	0.494321417	0.903274555
BRAP	0.723520275	0.186971372	0.46918326	0.162575407	0.878854392	0.49406565	0.903274555
CAMK2B	0.239314082	0.494761707	0.994414035	0.674590293	0.114052712	0.493868679	0.903274555
DDIAS	0.987137682	0.157202761	0.579182782	0.504226484	0.200240717	0.494189716	0.903274555
EIF1AD	0.618817269	0.140552697	0.207985425	0.569278978	0.878098104	0.493539097	0.903274555
ERLEC1	0.421842797	0.206737498	0.264101515	0.49831985	0.791145415	0.494306226	0.903274555
HAGHL	0.253109801	0.870801171	0.61113948	0.196988199	0.340923235	0.493608198	0.903274555
IBA57	0.313437216	0.544538832	0.970991055	0.055025022	0.994324503	0.494041349	0.903274555
KIAA1191	0.945719015	0.344340763	0.346010777	0.136253146	0.590841833	0.494115812	0.903274555
LOC100139764	0.692662688	0.342723474	0.60247804	0.161645573	0.392319551	0.494095668	0.903274555
MATK	0.481788758	0.233163524	0.248752631	0.796576139	0.407846619	0.494265285	0.903274555
MTPAP	0.736156298	0.991484268	0.200695759	0.092438073	0.669590279	0.494029035	0.903274555
NCAPH	0.330227061	0.301679325	0.435377563	0.24863031	0.840894609	0.494057045	0.903274555
PRKCG	0.375752661	0.091103775	0.552233578	0.621688566	0.771371483	0.494005043	0.903274555
RUFY3	0.851888005	0.116121938	0.826375972	0.14148926	0.783349112	0.493901025	0.903274555
SLAMF9	0.677906491	0.93820282	0.745435192	0.719897801	0.026575538	0.494103625	0.903274555
STK35	0.939601065	0.269715474	0.982711199	0.162574773	0.223600008	0.493750788	0.903274555
TMEM108	0.098179354	0.555314451	0.450715453	0.592881333	0.622242722	0.494001421	0.903274555
ZWINT	0.996827778	0.333211717	0.406890993	0.143472995	0.468546146	0.494406575	0.903329582
LOC616868	0.064009071	0.496817173	0.568247409	0.684198453	0.735898251	0.494676708	0.903435054
LOC790218	0.872985529	0.245879692	0.19279553	0.830867628	0.264625737	0.494683154	0.903435054
MYPOP	0.631541395	0.636313545	0.374315894	0.149163706	0.405526584	0.494684504	0.903435054
SNHG4	0.717390113	0.828221743	0.465623652	0.129224837	0.254452815	0.494640452	0.903435054
CYP3A4	0.607235133	0.537604626	0.487000528	0.060225235	0.951937704	0.495000768	0.903676751
HOMER2	0.308028525	0.310984087	0.377366749	0.98289302	0.256518673	0.494992937	0.903676751
LYPD1	0.771218024	0.94524532	0.468105446	0.158815669	0.168200827	0.495022287	0.903676751
NDUFAF1	0.959650285	0.154727833	0.682044728	0.357131455	0.252057295	0.495037109	0.903676751
METTL25	0.909382327	0.539363506	0.620098469	0.721534858	0.04155611	0.495104949	0.90370007
ECHS1	0.938424796	0.610729416	0.651725396	0.093344253	0.261840309	0.495299258	0.903733585
LIMCH1	0.721194276	0.243295584	0.350853996	0.159139821	0.932465181	0.495421694	0.903733585
LOC527744	0.950320999	0.096561701	0.824717192	0.386770933	0.311774808	0.495230275	0.903733585
LYRM4	0.845554518	0.638566614	0.505826963	0.415505721	0.08052789	0.49548506	0.903733585
MIB1	0.994995583	0.592100919	0.024575008	0.773469486	0.816402469	0.495563862	0.903733585
MRPS14	0.486073946	0.300182742	0.675976508	0.188176173	0.492495368	0.495533347	0.903733585
RPE	0.823680454	0.107230061	0.731467846	0.804430244	0.175870219	0.495518348	0.903733585
WBP1L	0.257302268	0.194407582	0.569049711	0.494813544	0.647988247	0.495247571	0.903733585

PLPPR3	0.908414636	0.771788279	0.549308243	0.126689083	0.187554933	0.495737754	0.903849826
URB2	0.950764537	0.431886976	0.218761456	0.286225468	0.355865835	0.495713187	0.903849826
MPZL2	0.95105507	0.276864357	0.309040151	0.203910747	0.551747118	0.495823724	0.903906148
NCK1	0.995162311	0.999275446	0.056958589	0.299163622	0.540522119	0.495905902	0.903955544
NDRG1	0.941860703	0.295814669	0.132770069	0.635750118	0.38979965	0.496065265	0.904145609
ETS1	0.649361634	0.303293812	0.466917919	0.139180194	0.716562715	0.496145371	0.904191193
SMG1	0.614072469	0.414116021	0.487298905	0.09635048	0.769926017	0.496579285	0.904881484
CD274	0.466513292	0.125332662	0.977625649	0.174292385	0.924162579	0.496872605	0.905075717
ELP2	0.683668697	0.227622626	0.383281889	0.439464187	0.351056835	0.496767107	0.905075717
PSMB9	0.194293316	0.479551993	0.273664907	0.742844199	0.486179529	0.496906478	0.905075717
RASSF2	0.773383415	0.055940163	0.869245783	0.260373304	0.940174958	0.496846429	0.905075717
LOC782609	0.136451702	0.363316202	0.403620588	0.506414948	0.909138825	0.496977584	0.905101929
PCDHB14	0.588831726	0.71023662	0.163703123	0.153498564	0.877670738	0.497196629	0.905101929
POLE	0.577373248	0.334284728	0.407435557	0.379125858	0.309230244	0.497115701	0.905101929
PPP1R12A	0.261164442	0.426191435	0.711467338	0.446552512	0.260791095	0.49717609	0.905101929
TMEM189	0.657781446	0.483214862	0.102376996	0.312178507	0.907273921	0.497059477	0.905101929
SH3YL1	0.95063763	0.099459446	0.124687811	0.963309575	0.812544765	0.497286683	0.905165457
IFNAR1	0.758264936	0.397951029	0.470990166	0.070663015	0.919294929	0.497376835	0.905229148
FAM162A	0.744394902	0.172681878	0.827680148	0.159001772	0.54600016	0.497461315	0.905260749
LOC101907682	0.550545103	0.292723118	0.469183614	0.938548012	0.130259886	0.497611772	0.905260749
NSUN2	0.377219364	0.251736037	0.956813182	0.619920574	0.164096105	0.497586543	0.905260749
STRIP1	0.683495547	0.596215825	0.134945685	0.222002253	0.757201695	0.497614845	0.905260749
POLDIP3	0.528625165	0.108203081	0.283413822	0.689431425	0.827911674	0.497792597	0.90548374
CHST11	0.097832329	0.924706779	0.697149333	0.151207486	0.971221111	0.497971939	0.905528572
DDX52	0.286060021	0.164691926	0.993369468	0.287494448	0.68841514	0.497978984	0.905528572
LOC107132360	0.498064309	0.747552154	0.680153014	0.073770095	0.495809856	0.497982777	0.905528572
ACTN3	0.760698085	0.327483319	0.182890376	0.228619377	0.890536807	0.498251028	0.905538252
GABARAP	0.838124811	0.082203204	0.204774999	0.732677701	0.898230702	0.498429531	0.905538252
LOC107132270	0.252027415	0.407895007	0.125715257	0.813261518	0.882104247	0.498155813	0.905538252
LOC112448893	0.536614078	0.08529155	0.825939963	0.267337702	0.91812269	0.498302362	0.905538252
LOC515227	0.159768387	0.717741166	0.848353169	0.374834759	0.254480878	0.498324964	0.905538252
MED25	0.787906934	0.112024288	0.440077726	0.689076147	0.346170023	0.498044316	0.905538252
REEP6	0.684414357	0.556659769	0.206199223	0.295858349	0.398964974	0.49819009	0.905538252
SNX3	0.777862095	0.215020875	0.51525072	0.543624043	0.198176155	0.498419558	0.905538252
LOC100848575	0.811818134	0.522168723	0.038301565	0.653625629	0.875562234	0.498569186	0.905591468
RALGDS	0.219801815	0.370062591	0.294599187	0.586885784	0.66053054	0.498518906	0.905591468
ADORA2B	0.64067969	0.732786744	0.813709726	0.129501283	0.188458494	0.499199658	0.905949441
BCL2L11	0.973457873	0.170640065	0.386589257	0.168842221	0.860079363	0.499237984	0.905949441
C17H12orf43	0.353476375	0.980411551	0.853359556	0.619333883	0.050816535	0.498880419	0.905949441
CAPN6	0.056381111	0.875347318	0.364623062	0.918707151	0.564523159	0.499387354	0.905949441

CRELD1	0.042427788	0.921643913	0.413609062	0.981939157	0.587625033	0.499375146	0.905949441
GALNS	0.460786757	0.190158639	0.544365244	0.245056699	0.798183471	0.499326971	0.905949441
LOC112443751	0.713083082	0.12934236	0.366613213	0.928598513	0.297372172	0.499472974	0.905949441
NUP62	0.15174986	0.614162655	0.733024696	0.266834297	0.511923349	0.499370202	0.905949441
OXLD1	0.980727529	0.980277521	0.256397514	0.064199648	0.588901067	0.499117758	0.905949441
PCTP	0.618724201	0.151422005	0.699400971	0.164246914	0.867413269	0.499438039	0.905949441
PNN	0.896114535	0.248710443	0.316609482	0.206596245	0.640327705	0.499425459	0.905949441
RAP1GAP	0.938743305	0.81556922	0.269094872	0.214714063	0.211090819	0.499483916	0.905949441
RBM45	0.815883694	0.226979725	0.935359321	0.31318653	0.172098814	0.49945477	0.905949441
CALHM6	0.50055501	0.226833778	0.642683656	0.719467091	0.178598926	0.500255112	0.906203292
CRCP	0.957658062	0.028231743	0.664654231	0.788732858	0.662634061	0.500554992	0.906203292
DENND5A	0.870371923	0.475953671	0.179210748	0.16329882	0.775823623	0.500825405	0.906203292
EMP3	0.216566171	0.880237244	0.088956601	0.957862484	0.578637159	0.500696346	0.906203292
FAM69B	0.877125463	0.430406617	0.173335712	0.70721418	0.202402902	0.500062586	0.906203292
FRA10AC1	0.279838259	0.517127242	0.4549541	0.184258953	0.773251832	0.500329995	0.906203292
LOC100299757	0.381521934	0.126552791	0.489567283	0.493499199	0.801493912	0.499717911	0.906203292
LOC512627	0.787433732	0.260416748	0.386540368	0.28447777	0.417047726	0.500796403	0.906203292
MRPS6	0.875900371	0.16211636	0.158979561	0.710280354	0.586237462	0.500716966	0.906203292
PACS1	0.52046931	0.493201877	0.63111033	0.344474299	0.168538531	0.500826051	0.906203292
PATJ	0.611014889	0.180924751	0.607926496	0.737077857	0.189042149	0.500009755	0.906203292
PDLIM4	0.277031302	0.432993691	0.102397021	0.870196895	0.878709431	0.500560523	0.906203292
RIPK3	0.420674307	0.19697968	0.99415555	0.196603469	0.580761455	0.500838697	0.906203292
SCFD1	0.778305354	0.032867801	0.937509804	0.774365875	0.504471854	0.500098179	0.906203292
SETD5	0.287010289	0.559770348	0.822408038	0.315892834	0.224712993	0.500304765	0.906203292
SMO	0.166615614	0.420915193	0.266162122	0.953557139	0.527147485	0.500378657	0.906203292
SS18L1	0.367860877	0.250586526	0.445194853	0.259758695	0.87894808	0.500117482	0.906203292
TESC	0.639082908	0.507292836	0.05618932	0.569633344	0.903545891	0.500241749	0.906203292
TTK	0.942947676	0.362239447	0.175516957	0.470560324	0.333169204	0.500698293	0.906203292
TWNK	0.807115457	0.536360672	0.65091243	0.096606854	0.345201373	0.500661085	0.906203292
UQCC1	0.472275903	0.417792798	0.801159724	0.12724137	0.465525522	0.499998986	0.906203292
ZC3H18	0.154624855	0.649335697	0.737502135	0.13605645	0.932357872	0.500583234	0.906203292
LOC112442079	0.614381347	0.145417642	0.675148067	0.321737707	0.485182024	0.501031306	0.906451854
LOC112441602	0.491819671	0.909910006	0.044266197	0.659464405	0.721581095	0.501241984	0.90663311
LOC531557	0.349236726	0.441883957	0.273330373	0.332908915	0.671270314	0.501235928	0.90663311
ACAD9	0.477682066	0.552618392	0.408770625	0.248532101	0.353596326	0.502347255	0.906822612
CORO6	0.280924992	0.319022635	0.226485194	0.506262559	0.92072818	0.501929533	0.906822612
DDX39B	0.9505359	0.326381999	0.157899544	0.877339915	0.219913068	0.50173066	0.906822612
KDM6B	0.853056793	0.138631423	0.42385721	0.562054697	0.334896415	0.501411164	0.906822612
LOC101906077	0.904036307	0.47473956	0.079372426	0.502326874	0.553690426	0.502188841	0.906822612
LOC522540	0.5358816	0.892628545	0.522630368	0.043415908	0.873366556	0.50228108	0.906822612

MOV10	0.314781706	0.101134423	0.49752621	0.942730568	0.635751161	0.502546481	0.906822612
MYO10	0.070103388	0.71701317	0.418053644	0.696919714	0.648269325	0.502562407	0.906822612
NFS1	0.393359329	0.430994881	0.452477332	0.183997876	0.672134492	0.502430023	0.906822612
P3H2	0.401576349	0.190447975	0.776900154	0.786033643	0.202571919	0.501917171	0.906822612
PAK4	0.980813813	0.699550276	0.065954007	0.793195837	0.264051772	0.502253875	0.906822612
PSME3	0.543193209	0.18110269	0.70319056	0.865168197	0.158182638	0.502037752	0.906822612
PVRIG	0.222099887	0.750841463	0.728121012	0.590770039	0.131574537	0.501472031	0.906822612
RNLS	0.927633633	0.770340758	0.963813043	0.225946678	0.060832207	0.502029216	0.906822612
SERINC4	0.111391399	0.588306776	0.944828919	0.299812861	0.510168007	0.502106512	0.906822612
STARD10	0.500527537	0.180530051	0.386666766	0.790588355	0.343415748	0.502411842	0.906822612
TCF24	0.618088231	0.465202216	0.147548799	0.275900136	0.806915369	0.501607906	0.906822612
TMEM121B	0.248584674	0.51779615	0.693439049	0.233029675	0.454640474	0.501827547	0.906822612
TMEM259	0.208709067	0.308050166	0.60033861	0.844248893	0.290351817	0.501927456	0.906822612
TPM3	0.881453433	0.253022758	0.49342733	0.132275566	0.650500103	0.502079526	0.906822612
USP16	0.970031638	0.276228381	0.619947426	0.47197413	0.12103851	0.502482907	0.906822612
USP46	0.896291289	0.588950046	0.738431693	0.1291187	0.187730447	0.501673839	0.906822612
PLXNB2	0.916339855	0.394229429	0.052731464	0.885068393	0.56328993	0.502625775	0.906837247
CAPRIN1	0.590985258	0.462406867	0.137723573	0.567750716	0.444700127	0.502732849	0.906907547
GDPD5	0.840446516	0.603087466	0.13430991	0.270843462	0.515638437	0.502830526	0.906907547
RGL1	0.625191787	0.830903037	0.553354659	0.12650717	0.26136719	0.502775377	0.906907547
CCDC127	0.347470647	0.319015162	0.662427099	0.376673829	0.345102455	0.503567386	0.906950748
CLCA3	0.788724776	0.712777842	0.192142843	0.122341291	0.723515802	0.503886413	0.906950748
FBXL2	0.826619994	0.63443131	0.049244763	0.618641637	0.598503987	0.503900048	0.906950748
FRMD8	0.97815354	0.601736784	0.837485157	0.299935529	0.064695994	0.503959775	0.906950748
GON4L	0.296023657	0.409651903	0.263826784	0.62371347	0.477680795	0.50331106	0.906950748
LHPP	0.246933492	0.697791982	0.572961867	0.331146025	0.291507755	0.503275978	0.906950748
LOC101902232	0.89125083	0.128952192	0.394536396	0.974085992	0.216120617	0.503579483	0.906950748
LOC101908214	0.451377569	0.74735363	0.675418977	0.068389094	0.613195258	0.503757773	0.906950748
LOC616254	0.946664973	0.978282534	0.074790803	0.149231806	0.925216704	0.503924455	0.906950748
MAP1A	0.38269364	0.788913951	0.173643921	0.980670201	0.185016038	0.502920841	0.906950748
PPP1R9A	0.418613738	0.942974226	0.631492367	0.114874164	0.332419973	0.50305716	0.906950748
PSMA2	0.955386025	0.160438114	0.644788045	0.459343035	0.210677333	0.503943625	0.906950748
RABAC1	0.358983845	0.23459418	0.86419198	0.274969292	0.477592634	0.50380888	0.906950748
SHISAL1	0.015492426	0.998307582	0.974853793	0.953862659	0.662531673	0.503239291	0.906950748
STAMBPL1	0.300083618	0.735753203	0.121782652	0.609872917	0.580265418	0.502986454	0.906950748
THAP2	0.321970272	0.077103008	0.979208116	0.529572248	0.73955	0.503084585	0.906950748
VIPR1	0.178044839	0.390567494	0.771423607	0.233630948	0.761373617	0.503509745	0.906950748
ZBTB43	0.601905414	0.151197331	0.215020324	0.741569995	0.65740867	0.503463808	0.906950748
ZNF543	0.899736179	0.535590679	0.902530258	0.027518534	0.796466912	0.503319553	0.906950748
ZSCAN2	0.711698032	0.399286741	0.071975695	0.88200648	0.529402776	0.503671789	0.906950748

ARHGEF9	0.616845991	0.413251645	0.08139302	0.52759334	0.87785264	0.504819343	0.907276667
BNIP3L	0.856713135	0.38692339	0.202932458	0.856154639	0.167426735	0.505458782	0.907276667
C17H4orf33	0.500875202	0.398158202	0.577951584	0.568836648	0.146488693	0.504720162	0.907276667
DHTKD1	0.867942113	0.66520723	0.569796811	0.075117062	0.387984974	0.504399579	0.907276667
EEPD1	0.809218254	0.64782217	0.1839032	0.153521275	0.650774645	0.505253122	0.907276667
EID1	0.630166364	0.103600252	0.563016768	0.718031731	0.364661693	0.505107667	0.907276667
FAXDC2	0.12269498	0.360526891	0.741434491	0.386499235	0.761082825	0.505557056	0.907276667
IL17RE	0.198306668	0.751743189	0.132319187	0.587457391	0.831748849	0.50537689	0.907276667
KIAA1614	0.956060026	0.246337521	0.354991435	0.539739503	0.212801653	0.50468775	0.907276667
LIN9	0.538076475	0.372316789	0.3818481	0.607518889	0.207677246	0.505633563	0.907276667
LOC104970779	0.474384897	0.516066686	0.064031083	0.964528594	0.637038225	0.505252337	0.907276667
LOC107132767	0.802975896	0.4080139	0.118131576	0.293262114	0.846189986	0.504719725	0.907276667
LOC112446044	0.818416698	0.956548083	0.753224949	0.07701234	0.212114233	0.505264351	0.907276667
LOC112448304	0.894200733	0.117730694	0.273133926	0.333397845	0.999675722	0.504314231	0.907276667
LOC112448773	0.451640649	0.168700127	0.449170496	0.49868992	0.561816921	0.504409801	0.907276667
LOC112448847	0.592335283	0.688148631	0.285712443	0.315036362	0.263002606	0.505592243	0.907276667
NODAL	0.349053753	0.718364236	0.330130627	0.258110714	0.449183325	0.504584624	0.907276667
PAAF1	0.949208981	0.10267222	0.662061524	0.193317488	0.771577377	0.505104502	0.907276667
PQLC1	0.238511801	0.951951151	0.177555508	0.638521213	0.373102855	0.504717698	0.907276667
RBM14	0.668235771	0.923210859	0.219773365	0.149329568	0.474836385	0.50490349	0.907276667
SH3RF1	0.124253971	0.693654118	0.318486632	0.581608064	0.600485918	0.504380901	0.907276667
SLC25A44	0.698441328	0.688730228	0.04696097	0.583328526	0.732374783	0.50561929	0.907276667
ST3GAL1	0.200336235	0.562063979	0.745326103	0.455016527	0.252367247	0.505356937	0.907276667
UBE2J1	0.53721139	0.067413581	0.363155328	0.877821117	0.835971559	0.505627805	0.907276667
WWC2	0.740735496	0.619640616	0.106496523	0.313367683	0.627714573	0.504928986	0.907276667
ZBTB5	0.074380032	0.876514796	0.354576825	0.874946283	0.476269074	0.505274491	0.907276667
ZNF395	0.451548302	0.06582891	0.399018392	0.824777517	0.986070471	0.505531613	0.907276667
WASF3	0.276599997	0.84420402	0.56493924	0.351696325	0.208174071	0.505762332	0.907408508
ABCB1	0.224212553	0.327296168	0.371314467	0.378704555	0.937727422	0.506116306	0.907524264
FADD	0.226022811	0.633403374	0.9860877	0.315709365	0.217075771	0.506085007	0.907524264
HIST3H2A	0.488745536	0.166263685	0.797545004	0.445531537	0.335407185	0.506273702	0.907524264
MGA	0.422327197	0.180856089	0.911885776	0.140488238	0.987754249	0.50589793	0.907524264
MRE11	0.15986562	0.431582314	0.97031994	0.491667379	0.294306252	0.506324548	0.907524264
PTBP2	0.42492871	0.744923313	0.854154555	0.734337507	0.048740224	0.506128109	0.907524264
RFC3	0.867845588	0.290952991	0.545912521	0.130942457	0.536564736	0.506275738	0.907524264
SLC1A4	0.202200586	0.444904959	0.230509919	0.983721984	0.474659841	0.506234143	0.907524264
ZNF783	0.9278518	0.443140434	0.130136207	0.309296248	0.584163336	0.505947579	0.907524264
LOC101907729	0.673421299	0.54020652	0.693366305	0.051335244	0.748984455	0.506536511	0.907575326
PDE1C	0.099942162	0.454064751	0.496228739	0.47287066	0.910217778	0.506424049	0.907575326
RETREG1	0.207884987	0.674515939	0.123943195	0.848524931	0.657783554	0.506574248	0.907575326

TRIT1	0.939665398	0.718949816	0.051735508	0.58651208	0.473034282	0.506507407	0.907575326
C1H21orf2	0.397711006	0.470060715	0.291814783	0.647955235	0.274605335	0.506701966	0.907605978
STRADB	0.908382795	0.607303313	0.32554818	0.992742353	0.054440156	0.506686023	0.907605978
EID2	0.546422205	0.477740078	0.155384386	0.562350034	0.42610066	0.506944171	0.907940718
AGT	0.875006806	0.274188762	0.995977352	0.679329402	0.060316724	0.508312843	0.907990031
AKR1C4	0.487424792	0.370364667	0.589094347	0.709171486	0.129442	0.507762091	0.907990031
ARHGAP23	0.886000853	0.76530205	0.930852857	0.196363903	0.078826422	0.507905339	0.907990031
CCNF	0.880144982	0.178503515	0.271395511	0.870857788	0.264487907	0.508884393	0.907990031
CD300A	0.527739523	0.354636942	0.964416721	0.151837033	0.358521213	0.50897229	0.907990031
CENPN	0.303115402	0.433707517	0.362398561	0.426895365	0.482893619	0.508887672	0.907990031
COIL	0.849695831	0.390935002	0.537533421	0.55717384	0.0989344	0.509295487	0.907990031
COPRS	0.446455494	0.491986907	0.296003795	0.244082827	0.614260259	0.507491202	0.907990031
COQ3	0.67166385	0.078843096	0.745542709	0.850801165	0.289785424	0.50722209	0.907990031
COQ5	0.868370849	0.372831865	0.300066042	0.23918069	0.420385463	0.507874106	0.907990031
CYB561A3	0.142272487	0.5465387	0.893925303	0.884659801	0.159891763	0.509094801	0.907990031
DPF2	0.349639323	0.441644477	0.582646787	0.405059948	0.269999671	0.509239257	0.907990031
FBP1	0.807608195	0.957507168	0.927710041	0.337334495	0.040233468	0.507269575	0.907990031
FLRT3	0.673656755	0.992718504	0.697323595	0.099812596	0.210352617	0.508314442	0.907990031
KIAA1841	0.23321857	0.98307394	0.966885692	0.737449861	0.059668435	0.507612889	0.907990031
KIAA2013	0.394952266	0.48497535	0.264656947	0.448457603	0.432719375	0.509193762	0.907990031
LOC101903713	0.616097622	0.228775139	0.658593892	0.336710048	0.3124588	0.507837966	0.907990031
LOC101906477	0.887203787	0.177717388	0.849652622	0.446853067	0.16398501	0.50880127	0.907990031
LOC107132911	0.376629988	0.588008032	0.610007195	0.114516593	0.630034274	0.507468441	0.907990031
LOC783163	0.774976151	0.128060487	0.246269917	0.899340418	0.447607592	0.509220071	0.907990031
MAN1C1	0.454420078	0.384651191	0.152473338	0.854840836	0.429463613	0.508184378	0.907990031
MARCH5	0.938593032	0.307858087	0.35161763	0.512561896	0.188274954	0.508574787	0.907990031
MASTL	0.873004121	0.176218318	0.252452333	0.622414993	0.405198242	0.508384114	0.907990031
MTERF4	0.937006784	0.601777466	0.641334154	0.227334704	0.118800446	0.507847402	0.907990031
NSD3	0.455425943	0.581118006	0.313993488	0.160910191	0.73142192	0.50810871	0.907990031
NUDT3	0.847516689	0.225289663	0.116519329	0.714632178	0.618591935	0.509150281	0.907990031
OGA	0.780052266	0.280603202	0.282877341	0.925118959	0.171694846	0.509148131	0.907990031
PARK7	0.473654528	0.291345324	0.732746695	0.451628037	0.214209578	0.508147058	0.907990031
PPP6R1	0.4460983	0.279880039	0.329299976	0.54917919	0.434473666	0.508675637	0.907990031
RNF138	0.727408982	0.944364315	0.44268349	0.039802953	0.812377161	0.509111632	0.907990031
RYR2	0.336693797	0.708432676	0.751087209	0.470931656	0.115844443	0.507980894	0.907990031
SBK1	0.222996281	0.154522772	0.811477406	0.588374603	0.59514971	0.508320582	0.907990031
SEMA4B	0.580586809	0.461146952	0.190216815	0.864066708	0.221243962	0.507257056	0.907990031
SENP8	0.727581322	0.61758438	0.11469777	0.257755915	0.734544673	0.507680876	0.907990031
SLC26A10	0.684389612	0.949188158	0.424786248	0.0466038	0.765306822	0.509283226	0.907990031
SMIM15	0.321817042	0.312438397	0.489214882	0.665352166	0.299036826	0.508235671	0.907990031

SOX15	0.707063323	0.773841713	0.827939277	0.395133683	0.054890938	0.508969168	0.907990031
SPEG	0.181801801	0.973212378	0.23573068	0.676892678	0.345331306	0.507516594	0.907990031
TPRG1L	0.433922645	0.667675032	0.087613482	0.6078431	0.635700153	0.50864141	0.907990031
VSTM1	0.284762952	0.209365798	0.326249153	0.734625587	0.684554749	0.508133918	0.907990031
ZFHX3	0.57883457	0.592806605	0.057892016	0.5746661	0.860465962	0.508918287	0.907990031
ZSCAN25	0.363585471	0.158669948	0.589139369	0.990578717	0.292349271	0.509294926	0.907990031
ASB4	0.731636152	0.929183609	0.561803962	0.088318686	0.293591337	0.510443836	0.908068539
CDPF1	0.157381897	0.33930914	0.880923595	0.546621609	0.383998551	0.509895613	0.908068539
CHMP1A	0.912376332	0.374331179	0.483951968	0.073729799	0.812266127	0.510356069	0.908068539
EMID1	0.405098892	0.491183662	0.568920961	0.105490505	0.827269377	0.509987054	0.908068539
FAM172A	0.351638007	0.214090596	0.732646659	0.616505696	0.290153332	0.509743844	0.908068539
GDPD4	0.253300808	0.591639184	0.261201422	0.37892998	0.667163749	0.510307281	0.908068539
LOC101904691	0.935814043	0.743259383	0.14817794	0.992095254	0.096617808	0.509990001	0.908068539
LOC101905668	0.420766077	0.087529779	0.70798048	0.39546449	0.959521879	0.510272707	0.908068539
LOC112445002	0.240650174	0.266355375	0.505857349	0.70981094	0.428892134	0.509835958	0.908068539
MAP3K14	0.814960994	0.243483793	0.498647013	0.586625978	0.170554959	0.510378512	0.908068539
MED18	0.657931699	0.576093476	0.989477558	0.127189681	0.206469212	0.509414053	0.908068539
PAG1	0.341912997	0.089625866	0.958923685	0.528416762	0.636777566	0.510150833	0.908068539
PPP3CC	0.979038149	0.847800065	0.296684265	0.198458297	0.202567195	0.510378598	0.908068539
SCAI	0.573098186	0.638279504	0.219338656	0.181489326	0.679604547	0.510308154	0.908068539
SCN11A	0.143756092	0.540486594	0.498939682	0.329464871	0.772973086	0.509864493	0.908068539
SPATA9	0.292564805	0.112817386	0.4834193	0.677350353	0.913492557	0.509867834	0.908068539
STEAP1	0.677469305	0.145129764	0.57966044	0.343969433	0.505176508	0.510446181	0.908068539
SYPL1	0.977502177	0.65387272	0.096883627	0.706861651	0.22537002	0.509717688	0.908068539
TEN1	0.090045384	0.818628323	0.418514579	0.465401417	0.688681514	0.510154098	0.908068539
UBN2	0.76864332	0.494140909	0.173526133	0.428095389	0.349146552	0.509457964	0.908068539
SIDT1	0.283293365	0.207639648	0.793701206	0.574496449	0.369742024	0.510707844	0.908435554
ABCA5	0.695918507	0.399170732	0.062551842	0.617565227	0.924516971	0.51077897	0.908463605
NFE2L3	0.243405998	0.089434544	0.677595957	0.980305453	0.686616208	0.510920341	0.908518118
PHF20	0.768443684	0.080916414	0.556045182	0.499334288	0.575078495	0.510917937	0.908518118
LOC112442683	0.199896247	0.678916064	0.479703497	0.177504375	0.860055503	0.511112753	0.908761797
LOC505600	0.177055039	0.665714276	0.484313139	0.453102797	0.384588216	0.511279181	0.908959229
CTF1	0.31648003	0.798381345	0.472547255	0.399318694	0.208771526	0.511398938	0.909005321
PWP1	0.905538372	0.2489243	0.574836865	0.219325841	0.350286645	0.511415888	0.909005321
B3GAT1	0.085473875	0.386488961	0.462803424	0.866747845	0.753741102	0.512041281	0.909016612
CLIP4	0.687758812	0.246023423	0.457430205	0.550499417	0.233876333	0.511609294	0.909016612
CLPTM1	0.854174791	0.520663016	0.472459618	0.915905701	0.051899536	0.51204145	0.909016612
HSPBAP1	0.945010846	0.16170506	0.58120349	0.705124914	0.159403063	0.51194176	0.909016612
IL1RN	0.05361843	0.396812042	0.805240317	0.744298997	0.782934496	0.511961593	0.909016612
IQSEC1	0.57895964	0.784720675	0.08030648	0.275846417	0.990990884	0.511769053	0.909016612

LOC101903793	0.733930359	0.413714272	0.126197103	0.790028196	0.328953987	0.511480723	0.909016612
LY6D	0.277430363	0.692885859	0.480159496	0.284598088	0.380324787	0.512086928	0.909016612
OAT	0.383214403	0.540033595	0.087316143	0.811324166	0.681403284	0.512073105	0.909016612
PAPPA	0.788925054	0.569886565	0.047807974	0.681939385	0.679715774	0.511573509	0.909016612
SNRPD2	0.411121002	0.102021721	0.459495795	0.789736462	0.65524333	0.51175942	0.909016612
SRRM2	0.853988164	0.310397041	0.392016015	0.315945174	0.303596216	0.511652734	0.909016612
ACTR1B	0.926890887	0.600301022	0.635552814	0.65280362	0.043830013	0.514468656	0.909041248
AUH	0.398306222	0.649748182	0.533599885	0.287176161	0.252320214	0.512384878	0.909041248
CASC1	0.099544494	0.547555978	0.636610602	0.519681116	0.554599184	0.512280222	0.909041248
CEP63	0.211076843	0.418067562	0.284691763	0.934396557	0.430268114	0.514135434	0.909041248
CERS5	0.896736236	0.387828072	0.418338157	0.833966631	0.08280718	0.513148821	0.909041248
CHPT1	0.646545561	0.646865343	0.226309932	0.768307895	0.138235217	0.513244901	0.909041248
CIDEB	0.597824741	0.181510878	0.2352223	0.919149949	0.426436927	0.512349442	0.909041248
DAP3	0.32930463	0.333606479	0.616122266	0.484180617	0.308137663	0.514100457	0.909041248
DUSP5	0.455915311	0.07245284	0.703622441	0.948400813	0.458855045	0.514400898	0.909041248
FAM53C	0.585483713	0.335325349	0.604099658	0.210392336	0.401952976	0.512823667	0.909041248
GABBR2	0.871994191	0.2273939	0.612736776	0.546914881	0.152142851	0.514310555	0.909041248
GALNT16	0.041108864	0.705591282	0.67959725	0.531136494	0.961501112	0.51351506	0.909041248
GGN	0.563771693	0.492777913	0.775219129	0.059771009	0.777571429	0.512441645	0.909041248
GTF3C3	0.170871798	0.53155267	0.500233394	0.493440363	0.449519275	0.513720711	0.909041248
IL5RA	0.946345362	0.349983278	0.624677282	0.470287114	0.103847095	0.514212233	0.909041248
IPO4	0.222505704	0.281843929	0.697835383	0.817257665	0.281809924	0.513740469	0.909041248
LOC100848011	0.150973496	0.469450873	0.227758974	0.92188934	0.678900727	0.514186882	0.909041248
LOC101904871	0.277729041	0.485993987	0.987617388	0.088304517	0.858849721	0.514312013	0.909041248
LOC104969159	0.964287602	0.065084805	0.524084359	0.345480638	0.883182265	0.512938707	0.909041248
LOC104969299	0.165788434	0.67259086	0.311518851	0.738891005	0.39429779	0.514507877	0.909041248
LOC112445938	0.598269826	0.780958252	0.955717069	0.381590124	0.059284618	0.514161815	0.909041248
LOC112448856	0.852329966	0.101489492	0.234312855	0.780725463	0.637821415	0.514001515	0.909041248
LOC510860	0.386594279	0.656611691	0.590488287	0.272823241	0.24514933	0.51273407	0.909041248
LOC614091	0.624204847	0.369434581	0.451083477	0.560702862	0.172364354	0.513258375	0.909041248
LOC614614	0.550508717	0.086353793	0.492825944	0.697668507	0.619446717	0.514593455	0.909041248
MANSC4	0.225386269	0.854697997	0.696182964	0.375144655	0.199106242	0.512587221	0.909041248
MPEG1	0.695863833	0.471589341	0.690845876	0.208913432	0.21368764	0.514516926	0.909041248
NOL12	0.468701755	0.696207752	0.141933983	0.933175725	0.233103423	0.513660908	0.909041248
NQO2	0.91518631	0.328535898	0.199994518	0.420695834	0.39868353	0.513865239	0.909041248
NRIP1	0.733367045	0.259915787	0.829731422	0.166986138	0.38020272	0.513036183	0.909041248
PCDH11X	0.386933603	0.328410283	0.267931451	0.754199057	0.39269675	0.513827759	0.909041248
PHF10	0.510728157	0.588034569	0.485339449	0.215931719	0.31836124	0.512642342	0.909041248
PRRC1	0.783000699	0.960459156	0.101150067	0.230510854	0.570413775	0.512303012	0.909041248
RTN4R	0.93235201	0.314520248	0.45485435	0.105701818	0.713826196	0.513463056	0.909041248

SH2B1	0.484276666	0.048310761	0.835664076	0.628510542	0.821260846	0.513975788	0.909041248
SHF	0.220403908	0.890897626	0.61711416	0.203040039	0.409157945	0.513510334	0.909041248
SLC25A34	0.49067587	0.346027874	0.423352337	0.623353255	0.225967278	0.514591415	0.909041248
SLC38A6	0.115314205	0.827161382	0.31360947	0.996923819	0.335586513	0.512406618	0.909041248
SMC2	0.16500537	0.23023872	0.34221197	0.905578702	0.854741573	0.513444423	0.909041248
STRBP	0.188251297	0.395137637	0.803455445	0.230135936	0.734179622	0.514093345	0.909041248
SUCNR1	0.275868981	0.531817619	0.51139143	0.210298099	0.638184088	0.513560034	0.909041248
TBPL1	0.066932726	0.995707104	0.614182515	0.380708082	0.644082092	0.512955735	0.909041248
TPRN	0.537226333	0.525242576	0.280614112	0.178310095	0.710569463	0.512872981	0.909041248
TUFT1	0.823287451	0.211670619	0.377098014	0.608325701	0.252934835	0.514342313	0.909041248
WDR54	0.944205265	0.282641736	0.357651943	0.286982401	0.368703747	0.514119776	0.909041248
CBLB	0.6752846	0.460346851	0.838934648	0.105526635	0.368190154	0.514741346	0.909106783
MIEN1	0.239889221	0.266385058	0.347847856	0.463826281	0.982771806	0.514734237	0.909106783
SLC41A2	0.150073909	0.946209234	0.814437845	0.121147354	0.723774764	0.514885412	0.909263369
DEF8	0.548099372	0.197809453	0.464337283	0.352439813	0.571949043	0.515021378	0.90933558
MICU1	0.632284531	0.48108594	0.332590416	0.29898746	0.33557544	0.515067159	0.90933558
PLCB3	0.205275193	0.424223587	0.247844016	0.53632799	0.877014151	0.515092533	0.90933558
PALM	0.652286058	0.218157772	0.146345743	0.670661914	0.727360773	0.515220349	0.909463391
LOC101903200	0.735537474	0.493832811	0.246365762	0.336325046	0.337711289	0.515318717	0.909539198
C26H10orf88	0.082216895	0.232378698	0.841355272	0.766625042	0.825505947	0.515478775	0.909545866
SPSB3	0.094259731	0.433633807	0.972315006	0.288542111	0.887151086	0.515488763	0.909545866
TM4SF18	0.990052087	0.508887822	0.07405048	0.510746127	0.533805154	0.515459665	0.909545866
ADD3	0.707170696	0.066304409	0.898845871	0.390346482	0.618620368	0.515559372	0.909567768
LOC112447070	0.336274173	0.39618874	0.33803557	0.293333442	0.770599097	0.515612025	0.909567768
MST1R	0.864563048	0.390505503	0.491611864	0.099789583	0.614978822	0.515717316	0.909655725
CSTF2	0.653207087	0.906836239	0.12110292	0.157345206	0.903518644	0.515948073	0.909964947
TRIM35	0.869367041	0.614192356	0.313861926	0.528438278	0.115218193	0.516050404	0.910047623
EPB41	0.751727488	0.479408228	0.296578166	0.589219334	0.162278479	0.516345259	0.91038974
NTAN1	0.976827153	0.387067466	0.588954915	0.067489053	0.680058944	0.516355353	0.91038974
LOC107131134	0.764662072	0.964050697	0.025736618	0.779144754	0.692368769	0.516619418	0.910757467
GPN1	0.394632993	0.574626797	0.094635597	0.685409511	0.696287855	0.51674763	0.910796992
MLH3	0.516057008	0.862116622	0.044842922	0.717010254	0.715981497	0.516752836	0.910796992
CHRNA7	0.674005158	0.252775555	0.370130624	0.960977834	0.169174171	0.516932896	0.910898694
HMGA1	0.369992	0.263444879	0.158588797	0.7232776	0.91674958	0.51689376	0.910898694
LOC786978	0.991819634	0.705312855	0.067874971	0.987663528	0.218660115	0.516977054	0.910898694
ARPC4	0.562763117	0.200159522	0.480049827	0.629022087	0.301922644	0.517257186	0.91106548
PRIM1	0.216414836	0.730033395	0.215362764	0.768352527	0.392672016	0.517188373	0.91106548
RPS6KC1	0.726763419	0.624838494	0.240183627	0.330891405	0.284606334	0.517293775	0.91106548
WDFY4	0.530874966	0.440810624	0.097438218	0.472236702	0.953772459	0.517269469	0.91106548
AP2A2	0.463546587	0.865871063	0.86837841	0.489752668	0.060257543	0.517557745	0.911202227

ATXN7L3B	0.173279876	0.378470195	0.691455747	0.515953723	0.439745585	0.517606502	0.911202227
DFFA	0.186231227	0.43649868	0.158839144	0.959218448	0.829924677	0.517430131	0.911202227
PPAT	0.529034349	0.187341369	0.745294689	0.979652963	0.142218211	0.517657006	0.911202227
TBC1D9	0.48883078	0.066427003	0.433069957	0.887282098	0.825011257	0.517704562	0.911202227
ZNF689	0.984345563	0.220490009	0.388282692	0.97253175	0.125526203	0.517591896	0.911202227
ARL5B	0.141129839	0.251284303	0.405554438	0.724431381	0.994111089	0.518866931	0.91127087
C8H8orf58	0.853859781	0.28466839	0.327269677	0.166146288	0.785517277	0.51930583	0.91127087
CHMP4A	0.518866336	0.322524932	0.195328121	0.42112041	0.750956048	0.518493701	0.91127087
DCAF17	0.644142293	0.63697751	0.93418764	0.718127865	0.0376505	0.518974004	0.91127087
DDX31	0.945915151	0.070492349	0.391431396	0.993444155	0.39809819	0.518224666	0.91127087
ESS2	0.363835319	0.77840029	0.866921304	0.669580476	0.063060724	0.519033264	0.91127087
FBN1	0.78351993	0.510024023	0.225243458	0.309038506	0.372229239	0.518802091	0.91127087
FKBP9	0.526628216	0.978761223	0.877641304	0.042717205	0.533193631	0.517879057	0.91127087
GDPD3	0.60227646	0.315204654	0.470601348	0.548264702	0.210989524	0.518445377	0.91127087
GUSB	0.400012245	0.788598122	0.059371427	0.780617042	0.711708605	0.519725941	0.91127087
KPTN	0.830830835	0.450757938	0.38071933	0.109223271	0.665402315	0.518951399	0.91127087
LARP7	0.30865722	0.872151347	0.351785893	0.150504504	0.724106637	0.518187951	0.91127087
LOC100847363	0.114117758	0.391101002	0.77122839	0.514289377	0.582608399	0.518062624	0.91127087
LOC101904810	0.658131295	0.065046861	0.771948573	0.398620301	0.789609065	0.519661771	0.91127087
LOC101907383	0.602653257	0.30490471	0.272343592	0.446728912	0.464944086	0.519529416	0.91127087
LOC782418	0.847221898	0.317131788	0.316765618	0.422527968	0.286554278	0.517901423	0.91127087
LOC787851	0.403566063	0.08823158	0.756379226	0.661429448	0.584262457	0.519779304	0.91127087
NME4	0.309323312	0.354297449	0.2247473	0.671967769	0.625534879	0.518784402	0.91127087
NOX1	0.867428383	0.131655138	0.111496025	0.959636262	0.845541701	0.518393228	0.91127087
NR4A3	0.289016909	0.491120488	0.544773993	0.47970731	0.279366787	0.518959364	0.91127087
PTCD3	0.823667311	0.615303762	0.488932468	0.40547171	0.103456357	0.519535672	0.91127087
RAB44	0.867121045	0.425991034	0.376064664	0.524229078	0.142243257	0.518879922	0.91127087
SIK1	0.688119115	0.54084217	0.316024861	0.338178493	0.259655867	0.518318281	0.91127087
SLIRP	0.99565156	0.130083778	0.498930802	0.689002468	0.232844917	0.519037217	0.91127087
SLITRK4	0.508494247	0.723021835	0.145563531	0.29520015	0.658880295	0.5197981	0.91127087
SNX12	0.323930022	0.155286451	0.332491896	0.719201895	0.862285385	0.519127953	0.91127087
SPAG9	0.783569264	0.278782657	0.138446412	0.403189452	0.846632685	0.518244405	0.91127087
SRPK1	0.913174399	0.315608829	0.635290532	0.208409384	0.272456565	0.519571138	0.91127087
STUM	0.231286615	0.486830623	0.232754666	0.661323261	0.600410839	0.519744309	0.91127087
TAF1C	0.821306727	0.467278025	0.078333801	0.641834866	0.538332415	0.519403744	0.91127087
TNFSF13	0.216790302	0.670441029	0.713888557	0.220652536	0.450274384	0.517979255	0.91127087
TRMT61B	0.839596837	0.415300838	0.35649611	0.147824156	0.566020096	0.5196474	0.91127087
VPS25	0.861721929	0.230402489	0.819073626	0.119311511	0.536355969	0.519754549	0.91127087
WDR92	0.540018751	0.279825859	0.673934658	0.813385189	0.125466564	0.519505631	0.91127087
ZFYVE1	0.791228602	0.303557121	0.536684594	0.493904722	0.162636495	0.518805026	0.91127087

ZNF32	0.793138314	0.843431111	0.038421896	0.496971228	0.809693821	0.518591223	0.91127087
ZNHIT1	0.056623472	0.743181154	0.552208989	0.500772306	0.890509535	0.518957089	0.91127087
LOC107133190	0.281318934	0.902923396	0.157761047	0.898107616	0.289374931	0.519896037	0.911345211
GCLM	0.627023591	0.437885602	0.129405774	0.447145057	0.656099228	0.520059668	0.911495189
RPP21	0.504188329	0.562042802	0.312052947	0.627372344	0.187921479	0.520092679	0.911495189
LOC112442082	0.999824625	0.719843264	0.135509703	0.391532558	0.273245304	0.520249071	0.911661363
SMCO4	0.42373521	0.221459867	0.492330308	0.32741318	0.689961087	0.5202986	0.911661363
CFAP69	0.512130223	0.216219523	0.230728622	0.474609957	0.861251299	0.520418612	0.911774297
XRN2	0.386652014	0.514061864	0.40924017	0.377700676	0.340080711	0.520505465	0.91182912
FMO4	0.629849307	0.450414937	0.100239075	0.816475668	0.450161167	0.520572326	0.91184891
ARV1	0.476065441	0.088790539	0.948490595	0.483214571	0.540394656	0.520884655	0.91229862
CSDC2	0.266295934	0.639233737	0.613397703	0.929122231	0.107991129	0.521019148	0.912339441
LOC784322	0.915422821	0.051756787	0.927821402	0.70335335	0.338784428	0.520985782	0.912339441
DCLK2	0.998865031	0.97930168	0.450212773	0.65337085	0.036497903	0.521471287	0.912579504
LOC101905219	0.933765123	0.094152163	0.503653577	0.306555546	0.77338477	0.52140003	0.912579504
LYRM9	0.95416074	0.533541748	0.623441243	0.05944836	0.556359476	0.521389689	0.912579504
PLA2G7	0.542776771	0.199118563	0.299626309	0.516399408	0.627925202	0.521444285	0.912579504
SNRPB	0.888618958	0.113295062	0.332512069	0.872823524	0.359156052	0.5213301	0.912579504
UQCC3	0.681111836	0.523654547	0.216891477	0.308803213	0.43966716	0.52148989	0.912579504
LOC100139548	0.247707807	0.428608889	0.98064294	0.17011868	0.593284586	0.521583338	0.912606517
LOC539893	0.962209755	0.10494354	0.547371437	0.792924638	0.239989563	0.521759219	0.912606517
NPRL2	0.581386527	0.354136058	0.35056026	0.160631181	0.907853831	0.521894593	0.912606517
PSMA4	0.900197953	0.165395867	0.934542736	0.246244038	0.30712782	0.521852528	0.912606517
SLC25A35	0.793738442	0.117863033	0.583869172	0.329665272	0.584459002	0.521874722	0.912606517
XRCC4	0.073977533	0.807087713	0.910276816	0.505941079	0.382607386	0.521809373	0.912606517
ZNF729	0.929467516	0.17130651	0.303263703	0.705276531	0.308884153	0.521781677	0.912606517
ARMC4	0.079377008	0.82531156	0.257296755	0.80186864	0.779527956	0.522084968	0.912842149
AP5B1	0.47824487	0.643882013	0.551041157	0.884377661	0.070318185	0.522373491	0.912860331
GLP2R	0.906148172	0.024211304	0.678051241	0.76634105	0.925068473	0.522256114	0.912860331
IMMP2L	0.978454993	0.293766383	0.26053979	0.240321284	0.586198208	0.522333377	0.912860331
LOC781197	0.2883324	0.562270865	0.846772196	0.191252611	0.401782984	0.522312002	0.912860331
PGGT1B	0.304015004	0.131939936	0.413017863	0.85454329	0.744712578	0.522207027	0.912860331
RTN4IP1	0.650180329	0.179708305	0.958466233	0.221280207	0.426122057	0.522507876	0.912900751
TTC39C	0.703201	0.156987744	0.464029272	0.753751924	0.273436709	0.522473327	0.912900751
ARHGEF11	0.448780664	0.331681886	0.630518238	0.482448329	0.23348479	0.522728422	0.913033068
CCL26	0.349570292	0.426697465	0.750019176	0.483323201	0.195546117	0.522750515	0.913033068
CERCAM	0.481431257	0.461805236	0.117464879	0.874102354	0.463133711	0.522731424	0.913033068
DMXL1	0.276040194	0.721226074	0.059419989	0.971562046	0.920363491	0.522834145	0.913036989
SAT2	0.339215758	0.378779241	0.193866102	0.729737587	0.582028462	0.522864032	0.913036989
PLIN3	0.12997072	0.938551277	0.519619614	0.794589019	0.210185811	0.522977034	0.913137153

BMP2K	0.65373814	0.192244544	0.841122438	0.230749922	0.4342622	0.523096834	0.913249165
HGF	0.272985506	0.397634119	0.369740346	0.823070191	0.321003186	0.523294887	0.913400594
LOC787905	0.213545141	0.440767749	0.561654899	0.62501553	0.32092352	0.523292765	0.913400594
ATP5S	0.896009486	0.588906932	0.230415542	0.699231728	0.124897906	0.523547485	0.913744313
NCF4	0.507558572	0.117748006	0.818644813	0.532571522	0.408000206	0.523776295	0.913987723
SOCS5	0.759638078	0.236791152	0.504910503	0.290337306	0.40321327	0.523798339	0.913987723
ATG101	0.984356107	0.918093617	0.048735684	0.313364124	0.770689783	0.523881348	0.914035382
LOC617875	0.834945382	0.979595037	0.101663531	0.850414424	0.150474256	0.523945494	0.914042827
MTUS1	0.589912557	0.556464122	0.516916547	0.600279792	0.1045111	0.52403192	0.914042827
SLC12A6	0.546558213	0.236869732	0.790242527	0.752268596	0.138334294	0.524052706	0.914042827
CD81	0.549644401	0.228577487	0.399335962	0.378534857	0.560773767	0.524112756	0.914050419
CLK2	0.965150956	0.250382166	0.329698673	0.357696251	0.374259624	0.524398073	0.914062324
HEATR5A	0.607050715	0.738003412	0.290301663	0.151809659	0.540159666	0.524376855	0.914062324
NF1	0.528725416	0.430322938	0.874807009	0.055806493	0.959243631	0.5242012	0.914062324
RTN1	0.993818057	0.397367413	0.283817991	0.220279777	0.431807695	0.524311448	0.914062324
ZNF518A	0.457728175	0.45350778	0.442360264	0.145414396	0.798631481	0.524362892	0.914062324
CEP85L	0.191898645	0.339773139	0.857511199	0.308433932	0.618799704	0.524489339	0.914124315
AKT2	0.371637854	0.141520194	0.715615426	0.469261399	0.604614936	0.524619313	0.914144353
FBXL3	0.070028103	0.319635052	0.863608269	0.959990919	0.575928558	0.524779223	0.914144353
JPH4	0.341933936	0.39935608	0.578228729	0.366811812	0.368759201	0.524652636	0.914144353
LOC104972830	0.374832387	0.607182302	0.479545547	0.185241815	0.528633262	0.524779352	0.914144353
TCF4	0.705858724	0.777555143	0.238129671	0.103238512	0.791880071	0.524728906	0.914144353
AGO2	0.857558089	0.501289443	0.630824016	0.08864033	0.448620173	0.526473219	0.914454845
APEX2	0.64052991	0.480313186	0.501462457	0.090482529	0.773226446	0.52664889	0.914454845
BHLHE41	0.566930631	0.216253535	0.832757996	0.152291637	0.696799895	0.527355746	0.914454845
CABLES2	0.878811848	0.451270095	0.606165135	0.433696086	0.103819475	0.527177799	0.914454845
CALY	0.769995397	0.451104329	0.086629498	0.500968716	0.714891023	0.526347293	0.914454845
CD101	0.163455088	0.783185165	0.736522153	0.144465126	0.78854987	0.525720036	0.914454845
CHRNB4	0.547068158	0.097198129	0.277052929	0.827394733	0.878694947	0.525185528	0.914454845
CPT1B	0.360050684	0.976620753	0.122033568	0.889401229	0.28113342	0.525518999	0.914454845
CYYR1	0.255354822	0.250448226	0.542498492	0.738407739	0.420543991	0.526298017	0.914454845
DMKN	0.194827657	0.67682552	0.716962293	0.602984919	0.189183678	0.526492069	0.914454845
DOCK4	0.079453087	0.832018642	0.904164104	0.400506354	0.449888578	0.526227135	0.914454845
ERGIC1	0.745502751	0.132265442	0.232061379	0.933576866	0.503101799	0.525834787	0.914454845
FAM162B	0.418012983	0.133584059	0.513119614	0.582610626	0.644830059	0.526131859	0.914454845
FBXO21	0.334803608	0.271391776	0.934168965	0.180973618	0.703222124	0.526799734	0.914454845
GADD45GIP1	0.588770913	0.429097868	0.568045777	0.427625396	0.175788733	0.5265473	0.914454845
GSAP	0.509296307	0.391760214	0.191148457	0.58397142	0.48662004	0.527419878	0.914454845
INTS4	0.06478413	0.751317294	0.841864638	0.268581927	0.9755492	0.525641388	0.914454845
IRX3	0.346265379	0.170448421	0.280103795	0.869455787	0.7503809	0.526507992	0.914454845

L3HYPDH	0.316059926	0.125534881	0.462776451	0.879111134	0.669987519	0.52701564	0.914454845
LIN54	0.81266018	0.829845914	0.948006633	0.02878747	0.582128448	0.525240873	0.914454845
LOC100849681	0.959054075	0.359662442	0.709567954	0.255419755	0.172826872	0.526833668	0.914454845
LOC101902458	0.82421818	0.293006342	0.113336759	0.655121521	0.601213419	0.526416615	0.914454845
LOC101906358	0.558328485	0.346086894	0.254365266	0.257949406	0.852943387	0.527003392	0.914454845
LOC783396	0.204048404	0.686398596	0.201899618	0.938301573	0.404972897	0.52579518	0.914454845
MAST3	0.935082122	0.147744015	0.31703648	0.903007373	0.274247374	0.527576532	0.914454845
MEF2D	0.570117811	0.582116761	0.794780934	0.359540716	0.113954887	0.526879526	0.914454845
MLXIP	0.73970554	0.309204289	0.613765446	0.253986105	0.300931949	0.525521598	0.914454845
NFAT5	0.427048134	0.328262962	0.686276942	0.291160095	0.386443181	0.527189948	0.914454845
OTOR	0.681191035	0.140826361	0.92714795	0.541207757	0.22416104	0.526584546	0.914454845
P2RY12	0.131880466	0.53397077	0.809571831	0.602695179	0.314164374	0.526663796	0.914454845
PAK1IP1	0.38501283	0.735057661	0.075167524	0.585443731	0.861333893	0.525477407	0.914454845
PHACTR3	0.526625965	0.896879194	0.575809311	0.300827899	0.13182519	0.526499758	0.914454845
PRPF18	0.888770243	0.633293653	0.079821357	0.540511156	0.442948805	0.525994796	0.914454845
PTBP3	0.586036653	0.65509769	0.471504852	0.080651291	0.735055534	0.525549205	0.914454845
R3HCC1L	0.436708383	0.626584367	0.878446853	0.082827106	0.544294202	0.527397884	0.914454845
RAD9A	0.67836251	0.880172582	0.189331245	0.262874872	0.362443158	0.526242582	0.914454845
RASGRP2	0.081036437	0.330261013	0.739295911	0.547949438	0.9904621	0.525673628	0.914454845
SNRPB2	0.980074881	0.220951363	0.16913782	0.87817956	0.333057618	0.525223131	0.914454845
SNTB2	0.837532153	0.239261452	0.739361488	0.123193778	0.588525031	0.525739134	0.914454845
TCEAL9	0.59442254	0.785438751	0.149954268	0.520834126	0.296960586	0.527254897	0.914454845
TIMM29	0.68150178	0.156340381	0.338519842	0.961828563	0.311806032	0.527054367	0.914454845
TMBIM1	0.953133438	0.338752517	0.105781273	0.471446676	0.673328198	0.527490122	0.914454845
TMEM150B	0.582592873	0.32400117	0.118922318	0.497700567	0.969728705	0.527354621	0.914454845
TMX4	0.975207808	0.305797802	0.08571108	0.630385709	0.667978078	0.526110246	0.914454845
WBP1	0.154690292	0.318442231	0.856695978	0.309108117	0.83147343	0.527566319	0.914454845
ZBTB2	0.271610629	0.67105399	0.665102736	0.099678053	0.887786548	0.525485029	0.914454845
ZW10	0.743328545	0.237320746	0.509978895	0.534640204	0.225465438	0.52753628	0.914454845
EMG1	0.86532557	0.239386249	0.406728166	0.690287144	0.186628737	0.52770234	0.914479735
VOPP1	0.154563194	0.169400085	0.747867606	0.733140088	0.755934652	0.527669369	0.914479735
SPX	0.925396042	0.532892315	0.229761254	0.173934782	0.551982456	0.528122736	0.915111626
AP1B1	0.161924625	0.706295424	0.994932576	0.548213277	0.175309371	0.529122386	0.915368998
ASRGL1	0.915265323	0.402792642	0.104306503	0.912080143	0.311559034	0.528976524	0.915368998
CTPS1	0.54554638	0.573049652	0.802409246	0.808953917	0.053882769	0.529098792	0.915368998
E2F7	0.546939339	0.200902214	0.348115032	0.618797955	0.461109033	0.528752264	0.915368998
INTS1	0.465778041	0.457506439	0.515951785	0.139815008	0.708662324	0.528394339	0.915368998
LDB2	0.949781791	0.065955781	0.408787137	0.745731459	0.571721897	0.528814227	0.915368998
LOC100847604	0.292363151	0.087023313	0.869687358	0.801741985	0.616575574	0.529163712	0.915368998
LOC101904536	0.658372442	0.412308662	0.441263243	0.195879844	0.465041186	0.528697246	0.915368998

MAPK15	0.237782137	0.403174946	0.455335523	0.886281844	0.282406171	0.528949066	0.915368998
NPY1R	0.047505707	0.891301903	0.920397049	0.807571678	0.346102794	0.528374081	0.915368998
NT5M	0.434782505	0.748505569	0.763529721	0.147769944	0.297401881	0.528849841	0.915368998
RPA1	0.654914245	0.174032363	0.715062012	0.660113542	0.202988	0.528860443	0.915368998
SLC15A2	0.532212183	0.723213256	0.602696641	0.146980055	0.320011297	0.528697532	0.915368998
SOX12	0.374518348	0.346605393	0.133880979	0.916949486	0.686047748	0.529069783	0.915368998
SUB1	0.492791346	0.045453406	0.568635011	0.999463352	0.857501341	0.528780888	0.915368998
ZBTB6	0.871379375	0.437491839	0.181129047	0.230921828	0.684692057	0.52880742	0.915368998
DEPDC7	0.552509932	0.27444485	0.450471211	0.238822212	0.670914171	0.529277827	0.915469901
LDHC	0.238441285	0.333517175	0.804160301	0.23677048	0.723909189	0.52956147	0.915596452
LOC107132870	0.077287779	0.984749384	0.309843605	0.910534951	0.510459568	0.529554106	0.915596452
SEMA3G	0.794736614	0.712299077	0.03550181	0.873009773	0.624230178	0.529407011	0.915596452
ZNF835	0.197739399	0.8073959	0.765294934	0.383798089	0.23376076	0.529574159	0.915596452
OSMR	0.245707457	0.300892643	0.985194882	0.430897645	0.349609839	0.529760428	0.915822014
LOC101910153	0.335555816	0.217460156	0.277247789	0.661393906	0.820361459	0.529833828	0.915852428
MORN2	0.631665973	0.269394804	0.138182707	0.799089298	0.584365627	0.529891606	0.915855834
CARS2	0.690384647	0.267342679	0.858681676	0.138363895	0.501010226	0.530000997	0.915948438
ACTR1A	0.569193113	0.101919191	0.628687742	0.641320294	0.469923508	0.530084707	0.915996644
ABCF1	0.638295535	0.228957736	0.849510303	0.629370039	0.140729372	0.530164959	0.916038866
LOC101906167	0.703835274	0.428088737	0.186571805	0.542381249	0.360776705	0.530233676	0.916039287
SPATA5	0.578052573	0.933451946	0.761435929	0.961579325	0.027849351	0.53027684	0.916039287
VRK2	0.439980146	0.282086732	0.606252799	0.216553955	0.675812109	0.530437821	0.916220932
BEX3	0.822458613	0.667727047	0.304030435	0.456351477	0.14484545	0.530862624	0.916613472
KIF13B	0.64616388	0.176737864	0.708799904	0.440508334	0.309324167	0.530744498	0.916613472
LOC512248	0.859259204	0.77955065	0.069370412	0.30828401	0.770548237	0.530888493	0.916613472
TFPI	0.498922659	0.564564811	0.293857642	0.383695381	0.347481646	0.530848676	0.916613472
CCNB1IP1	0.81889388	0.120549175	0.290917349	0.931051085	0.413796295	0.531337502	0.916784249
CDC26	0.699017469	0.236561111	0.378118749	0.555054732	0.319908895	0.531992956	0.916784249
FBRS	0.790265744	0.333563303	0.90856227	0.275746633	0.168096281	0.531972297	0.916784249
FBXW8	0.227016924	0.814317714	0.308775283	0.35449096	0.548564747	0.531951872	0.916784249
HIKESHI	0.71482613	0.153850944	0.462536266	0.521359949	0.41731832	0.531394245	0.916784249
IL20RB	0.714834011	0.680539137	0.213784588	0.48721364	0.218991931	0.531888311	0.916784249
ILK	0.897454499	0.256820785	0.412540509	0.550303789	0.211966099	0.531799107	0.916784249
ITPRIP	0.122072316	0.760853813	0.43657646	0.317999331	0.858307421	0.531392631	0.916784249
KIF3B	0.697295716	0.595046988	0.083381384	0.744808178	0.42891121	0.531131141	0.916784249
LOC100298868	0.721710678	0.133150272	0.269173874	0.591049921	0.72614305	0.531976667	0.916784249
LOC112446793	0.584225548	0.686102293	0.640242562	0.147721499	0.292419898	0.531705801	0.916784249
LOC781254	0.913067413	0.161221449	0.241433292	0.337854957	0.923361887	0.531732225	0.916784249
LPL	0.405256397	0.442695577	0.722578734	0.486907146	0.175169987	0.531208411	0.916784249
MAPRE3	0.502703316	0.433252238	0.453864946	0.821155608	0.136628285	0.53178476	0.916784249

PRKACA	0.812200334	0.154932494	0.200593853	0.513220808	0.855180191	0.531583722	0.916784249
STARD13	0.523382997	0.855397308	0.726634163	0.075260696	0.452190251	0.531455948	0.916784249
STRN4	0.190449751	0.414361562	0.747679227	0.499033528	0.375837827	0.53137411	0.916784249
TSKS	0.818493711	0.153110501	0.863369857	0.120216888	0.85219851	0.53168783	0.916784249
MED30	0.68247334	0.366711887	0.359360524	0.379498371	0.325460457	0.532092371	0.916859293
INTS8	0.56823993	0.517580888	0.901339388	0.193553159	0.216590686	0.532175052	0.916905488
AKAIN1	0.310608574	0.709102203	0.618270632	0.222008991	0.368206121	0.532491556	0.91691226
ASCC3	0.656873047	0.918859664	0.297035625	0.153989647	0.403258561	0.532514213	0.91691226
FAM118B	0.980430034	0.397781997	0.606848352	0.051792055	0.907785633	0.532415451	0.91691226
FKBP11	0.450529716	0.834797729	0.264759421	0.672360969	0.166149797	0.532358657	0.91691226
MFSD3	0.339301469	0.265093557	0.618407093	0.599279633	0.333536456	0.532259772	0.91691226
PI4KB	0.955872077	0.124668782	0.986823066	0.16309857	0.57981648	0.532305793	0.91691226
SMAD5	0.301691712	0.90666245	0.296807773	0.230012037	0.596396476	0.532582247	0.916933198
ATP11A	0.299807752	0.63908107	0.472015738	0.387766093	0.317936326	0.532798817	0.91701745
GRN	0.408644703	0.571809243	0.263353826	0.51043526	0.354965175	0.532797864	0.91701745
LOC511161	0.603418002	0.881817402	0.502362831	0.087872729	0.474445786	0.532706942	0.91701745
GPR39	0.426617061	0.60824092	0.23395532	0.84442104	0.217660106	0.532938467	0.917161617
C24H18orf32	0.935196031	0.045187747	0.61691979	0.655646012	0.65379729	0.533235799	0.917217965
C8H9orf72	0.300193219	0.764901817	0.634383113	0.104449103	0.734342138	0.533190649	0.917217965
LOC509972	0.534362546	0.199139184	0.245882095	0.428233789	0.997272208	0.5332138	0.917217965
SASH1	0.471871736	0.548259515	0.06508089	0.769278839	0.862881507	0.533250661	0.917217965
USP48	0.997808891	0.951982903	0.669414315	0.052867339	0.332150308	0.533073571	0.917217965
ADNP2	0.310861287	0.140882056	0.631193893	0.963010002	0.421847774	0.534157547	0.917363695
AGAP3	0.482754594	0.425334792	0.193750589	0.769575569	0.371279492	0.536469832	0.917363695
AJUBA	0.802977433	0.484463173	0.934865867	0.082599988	0.379260333	0.536898487	0.917363695
ARHGEF25	0.16396057	0.974671779	0.202986344	0.713593337	0.488849288	0.535610323	0.917363695
BACE2	0.39120576	0.639879501	0.075862996	0.728542212	0.823005168	0.536793263	0.917363695
BCL2L12	0.44508406	0.631737083	0.108834491	0.435513629	0.848866783	0.535564712	0.917363695
CBLN4	0.346181799	0.463876596	0.333411248	0.274495369	0.769802119	0.535571198	0.917363695
CCR1	0.296213861	0.38838913	0.217163529	0.478394063	0.95349356	0.536956363	0.917363695
CD34	0.450398951	0.372648884	0.47465683	0.210090861	0.681123272	0.537020758	0.917363695
CDKL2	0.533856809	0.691489062	0.164177149	0.38002153	0.490361855	0.535241312	0.917363695
CEP78	0.429676509	0.182860848	0.268230391	0.565511753	0.948096734	0.535336289	0.917363695
CRACR2B	0.951510675	0.275306331	0.095287518	0.528605759	0.853694271	0.534738939	0.917363695
CSF2RB	0.846003602	0.615631023	0.20005733	0.878942352	0.123035435	0.534799187	0.917363695
CSPG4	0.616457878	0.088043763	0.608963568	0.587403741	0.584994285	0.536307314	0.917363695
DDX41	0.691189347	0.63045664	0.402970856	0.089998852	0.713539385	0.534949352	0.917363695
DEPDC5	0.703344385	0.195623087	0.59315608	0.551782536	0.252901612	0.536830932	0.917363695
DISC1	0.535316959	0.630648078	0.097823085	0.385476306	0.884464441	0.534658998	0.917363695
EFNA2	0.333473145	0.58383141	0.294620788	0.81497498	0.241428037	0.535108211	0.917363695

EIF4E2	0.694368854	0.071871298	0.72976603	0.760807733	0.404620186	0.533842596	0.917363695
EPN3	0.761215355	0.644375463	0.07342543	0.535032398	0.587101039	0.535564585	0.917363695
FAM117A	0.207630149	0.456853601	0.481674538	0.698885706	0.356405439	0.536697264	0.917363695
FBXW7	0.745671889	0.240525523	0.63902633	0.170094386	0.58264012	0.536323266	0.917363695
FILIP1	0.147963175	0.528941784	0.697338824	0.377015766	0.545412306	0.534034218	0.917363695
GKAP1	0.714024397	0.317907633	0.300855437	0.817596606	0.204123315	0.536974195	0.917363695
H4	0.822922552	0.572201366	0.366243224	0.967866496	0.067772626	0.535547622	0.917363695
HMOX2	0.369070071	0.477752146	0.242745167	0.496753285	0.533914763	0.536216672	0.917363695
HYI	0.160306195	0.24726388	0.412102928	0.821096372	0.834703097	0.533576192	0.917363695
ING2	0.462408911	0.859591067	0.193414709	0.411473068	0.360493497	0.53708064	0.917363695
KCNMB3	0.205422645	0.295672353	0.649270191	0.936515449	0.307393571	0.536225206	0.917363695
LINGO1	0.340404282	0.33410385	0.280857739	0.454613313	0.776127008	0.534842986	0.917363695
LOC100847490	0.397411057	0.698270703	0.541139257	0.955624794	0.078116161	0.533820407	0.917363695
LOC101903301	0.607167723	0.085609693	0.810183357	0.324844361	0.830781067	0.53643665	0.917363695
LOC101903645	0.391362941	0.173634892	0.264627642	0.699288303	0.906013204	0.536903693	0.917363695
LOC104975676	0.223764842	0.298460137	0.390246824	0.531072393	0.81807809	0.53573104	0.917363695
LOC107131642	0.440207196	0.586337183	0.238002992	0.930397609	0.197508986	0.535151445	0.917363695
LOC107132524	0.074318651	0.79147262	0.333269205	0.699594107	0.822973271	0.535115691	0.917363695
LOC107133343	0.482535892	0.066823765	0.700424189	0.538818299	0.931553605	0.535952591	0.917363695
LOC112447118	0.529753416	0.794375772	0.858375748	0.661355563	0.047489278	0.53609972	0.917363695
LOC112447857	0.752461592	0.862335375	0.067471718	0.40033722	0.646451174	0.535852779	0.917363695
LOC616720	0.585927557	0.582989579	0.886712315	0.082291406	0.450036197	0.533946527	0.917363695
LOC783497	0.825848395	0.144228339	0.453355736	0.583382835	0.356033273	0.533922418	0.917363695
LOC786258	0.564987071	0.33034149	0.326966799	0.467174522	0.395219083	0.534792432	0.917363695
MBD2	0.831402435	0.929017811	0.778663478	0.751158806	0.025179024	0.53660268	0.917363695
METTL24	0.093781773	0.684189665	0.260732162	0.677647268	0.995599363	0.535123171	0.917363695
MINDY3	0.817417317	0.117935362	0.3815728	0.684080623	0.44718773	0.53454737	0.917363695
MRPL35	0.751543883	0.283350339	0.898499101	0.164254113	0.359654292	0.53539436	0.917363695
NABP1	0.25766206	0.324739636	0.467183298	0.995061233	0.291633503	0.536078926	0.917363695
NPL	0.177382689	0.404631116	0.381917097	0.515778709	0.796096383	0.534593859	0.917363695
ONECUT2	0.168271565	0.196242214	0.723808567	0.876097738	0.54233566	0.536292402	0.917363695
PAFAH1B2	0.231794424	0.282139233	0.269035101	0.719203521	0.897031559	0.536199234	0.917363695
PIGU	0.433856011	0.248465901	0.22239977	0.899163346	0.52850908	0.536901545	0.917363695
PQLC2	0.405193502	0.958622819	0.753868802	0.268953101	0.144593369	0.536810893	0.917363695
PRIM2	0.582557048	0.648124898	0.045517447	0.993525399	0.663422725	0.535809143	0.917363695
PTGER2	0.807594453	0.18932324	0.781030764	0.340051601	0.277988921	0.535149375	0.917363695
RBMS1	0.878498607	0.805162708	0.113175398	0.196155432	0.713243614	0.533651494	0.917363695
RNF187	0.96667485	0.307935671	0.946873817	0.14758833	0.273643289	0.536740081	0.917363695
SCAF4	0.840937328	0.761492608	0.57803517	0.037500347	0.809589078	0.534294005	0.917363695
SEH1L	0.675087127	0.601166669	0.158327967	0.422024127	0.418077946	0.535967925	0.917363695

SNRPF	0.697546867	0.076658021	0.356427847	0.632513911	0.942474915	0.536377789	0.917363695
SOS1	0.463531232	0.4930513	0.849574394	0.063124244	0.92173134	0.535296207	0.917363695
SPAG8	0.599387939	0.294891287	0.383842102	0.204226265	0.814348088	0.535064667	0.917363695
TBC1D2B	0.82310113	0.752054329	0.856681809	0.437755215	0.048557208	0.534873259	0.917363695
TBC1D5	0.425838401	0.108035544	0.30411639	0.92742479	0.878639827	0.537034384	0.917363695
TMEM110	0.382733743	0.482916398	0.184706749	0.716823851	0.464289545	0.536382307	0.917363695
TNFRSF18	0.085780428	0.914150579	0.669914144	0.574450144	0.373329873	0.534769598	0.917363695
TSG101	0.402417128	0.145920574	0.428774763	0.935939287	0.483698963	0.536992369	0.917363695
WNT5B	0.076800146	0.41888755	0.378692458	0.939302643	0.991887822	0.536190173	0.917363695
SLC35A3	0.370846893	0.222856678	0.257090801	0.917367769	0.585254089	0.537145654	0.917379262
RCE1	0.786961067	0.076356354	0.613425644	0.939307527	0.329635962	0.537236815	0.917439478
ADRA2B	0.817809605	0.606690305	0.345603013	0.798159505	0.083562676	0.537629629	0.917766142
CPNE1	0.553210825	0.22051952	0.977750008	0.455381992	0.210576555	0.537652688	0.917766142
CREBBP	0.591628077	0.309486236	0.910677918	0.103728258	0.661307956	0.537654595	0.917766142
CST7	0.910648701	0.690591533	0.103429954	0.773907475	0.2272843	0.537707724	0.917766142
LOC100848665	0.772254789	0.245303995	0.541645304	0.376198237	0.2963213	0.537656386	0.917766142
LOC101905525	0.361634682	0.457683786	0.302134588	0.838615299	0.273031306	0.53785432	0.917920886
PHF13	0.432280666	0.939271304	0.836509992	0.069659743	0.484256634	0.537974544	0.918030595
LOC107131429	0.473864978	0.696961743	0.970907543	0.606612947	0.058925668	0.538050042	0.918063967
PAPOLB	0.831877926	0.443984574	0.730113454	0.060872681	0.698796621	0.538196272	0.918218009
LMBRD1	0.740025302	0.365158947	0.333052159	0.521244369	0.245035571	0.53859989	0.918524659
MRPL28	0.54535683	0.247975911	0.806428392	0.248649544	0.423793735	0.538550314	0.918524659
PLXNB3	0.058757165	0.43928966	0.861310699	0.913250069	0.566076822	0.538567193	0.918524659
PTPN13	0.978812513	0.219679963	0.727279854	0.109197887	0.672985535	0.538556113	0.918524659
C14H8orf88	0.963612841	0.399613372	0.643440536	0.195210917	0.23779335	0.538706723	0.918611392
THOC1	0.642173483	0.297182828	0.31743606	0.482245631	0.393830157	0.53877463	0.918631735
LOC101907797	0.687181711	0.414821896	0.974616379	0.147546742	0.281008023	0.538996497	0.918727264
LOC613822	0.475088165	0.298111696	0.161482066	0.794913032	0.633242414	0.538887611	0.918727264
SLC3A2	0.631689387	0.415519824	0.159172914	0.336779496	0.818672077	0.538998604	0.918727264
EIF4G1	0.253755448	0.666278617	0.795131986	0.38748644	0.221410854	0.539238081	0.918944564
SERPINB8	0.877276326	0.763946934	0.260386194	0.676246221	0.097729816	0.539230883	0.918944564
DNAH5	0.15287448	0.666180771	0.651256348	0.76779928	0.226564217	0.539304863	0.918962943
SUPT6H	0.771170143	0.585268322	0.413471333	0.085532629	0.723077033	0.539370223	0.918978896
LOC101909173	0.754560261	0.339723949	0.200989796	0.785247456	0.285455365	0.53948952	0.918991334
POLR3C	0.274057667	0.539532868	0.411964632	0.352811327	0.537312048	0.539467595	0.918991334
CCDC125	0.707033443	0.206159083	0.18056226	0.475383382	0.924806653	0.539851845	0.919251101
KCNC3	0.19294734	0.840915381	0.75183441	0.258312392	0.367121978	0.539809954	0.919251101
NUP155	0.126842252	0.952165446	0.911660953	0.180175479	0.583000924	0.539768573	0.919251101
QKI	0.589279474	0.416547557	0.168020026	0.866539516	0.323788513	0.539866072	0.919251101
JMJD7	0.397341684	0.73113998	0.115377786	0.676762701	0.510485044	0.540001951	0.919259806

LOC107132487	0.946946461	0.456462584	0.212786291	0.532151204	0.23658687	0.539999161	0.919259806
POLR1E	0.770092683	0.805302728	0.389731269	0.34218918	0.140041748	0.540039229	0.919259806
HCN2	0.372034743	0.334848952	0.406913	0.326483501	0.700317327	0.540170453	0.919267435
LRBA	0.77198932	0.92811206	0.750884454	0.040765792	0.528881836	0.540323788	0.919267435
RBFA	0.359573605	0.509329428	0.512818142	0.33316081	0.370314488	0.540119724	0.919267435
RPA2	0.211555814	0.735738849	0.26228191	0.548402954	0.517988894	0.540278944	0.919267435
SGK1	0.594505413	0.644549679	0.2779418	0.145731175	0.747278207	0.540306885	0.919267435
TANGO6	0.45522358	0.631059424	0.711215281	0.35083106	0.162145259	0.540700996	0.919813832
ARHGAP22	0.30006726	0.928492775	0.3011719	0.375847666	0.369128286	0.541009484	0.919861857
EXOC1L	0.733520332	0.144957605	0.605936878	0.24558961	0.735135922	0.540859013	0.919861857
GON7	0.372542655	0.323195804	0.121608215	0.816708812	0.973115942	0.540937314	0.919861857
RUSC2	0.184176782	0.501761244	0.897233417	0.793421642	0.176871607	0.540921124	0.919861857
SLC7A7	0.613285436	0.578690023	0.294262475	0.30954265	0.36011097	0.54100931	0.919861857
CD37	0.602543771	0.284633069	0.687411481	0.197623067	0.499920307	0.541109798	0.919937108
PPIH	0.72605401	0.113877714	0.955895932	0.342178902	0.430816504	0.541168963	0.919942393
ASXL3	0.464492232	0.907280347	0.046692096	0.827213994	0.716988478	0.541489453	0.919943826
CIDEC	0.20406103	0.693905181	0.178853785	0.669476193	0.688175319	0.541445164	0.919943826
CYB5A	0.973514268	0.779045232	0.435123872	0.209605806	0.16869832	0.541462473	0.919943826
GHR	0.796683081	0.897856686	0.04438663	0.611681156	0.600739077	0.541428998	0.919943826
HAND1	0.979634478	0.131732317	0.765813911	0.54324469	0.217306646	0.541425645	0.919943826
KCTD5	0.391264833	0.512969354	0.179853472	0.992160248	0.325888092	0.541506146	0.919943826
CABP1	0.14606257	0.922626004	0.209717849	0.875761546	0.472626096	0.541933335	0.919973515
CCS	0.307249882	0.62805001	0.319117576	0.422165678	0.450096501	0.541986791	0.919973515
CHMP7	0.767520047	0.034901744	0.557472993	0.815979677	0.960260749	0.541988537	0.919973515
LOC100336104	0.171622776	0.875568352	0.851598874	0.518401511	0.17601923	0.541591845	0.919973515
LOC112444841	0.666787423	0.144126806	0.473075105	0.342351842	0.750913991	0.541767025	0.919973515
LRRC47	0.865106399	0.474712571	0.134289982	0.58641288	0.361950118	0.542061879	0.919973515
METAP1D	0.686668444	0.050771622	0.780391064	0.547963231	0.784247012	0.54183715	0.919973515
PEX13	0.835964835	0.082576243	0.383382346	0.774195917	0.571374405	0.542084205	0.919973515
RELL1	0.705360231	0.760338752	0.449312357	0.115760712	0.418851139	0.541706486	0.919973515
TF	0.666115002	0.170311751	0.597244671	0.223667281	0.771852686	0.541924281	0.919973515
ENSA	0.855015951	0.256869482	0.329455948	0.951139015	0.170171711	0.542158278	0.920004085
C1GALT1C1	0.519911138	0.369009917	0.945281647	0.203627507	0.317552574	0.542406622	0.920235199
DNASE1L1	0.443375842	0.210721136	0.544326301	0.475656384	0.484730088	0.54238565	0.920235199
SLC25A16	0.673609637	0.146448474	0.348416919	0.712213179	0.479386322	0.542541816	0.920369417
TM2D1	0.290180383	0.856181833	0.581804358	0.106767535	0.760935565	0.542678138	0.920505522
DAGLB	0.427062178	0.488835205	0.333016026	0.299229843	0.565133719	0.542887725	0.920765859
GPRC5C	0.330289089	0.489131571	0.146104653	0.528529652	0.943298895	0.543075826	0.920894543
LOC100847780	0.604334769	0.393508056	0.505663475	0.496012605	0.19728518	0.543065852	0.920894543
LRRC40	0.070111212	0.829374508	0.850370233	0.536014934	0.444365507	0.543234475	0.92097324

TBC1D31	0.764198263	0.275352752	0.382099347	0.8361982	0.175176216	0.543231261	0.92097324
LOC112442288	0.763002635	0.838949515	0.735853094	0.053058504	0.471821501	0.543464158	0.921077176
RNF167	0.614803021	0.131948098	0.335933562	0.654840805	0.660772806	0.543461827	0.921077176
SRF	0.083048773	0.361441677	0.900034362	0.577766807	0.755287083	0.543424343	0.921077176
EXT1	0.779100578	0.05753383	0.80487794	0.373533643	0.878080852	0.544135121	0.921433364
HS2ST1	0.307025806	0.189748307	0.789404059	0.309639925	0.830599717	0.544042651	0.921433364
LOC100847946	0.902734112	0.142023612	0.388570757	0.452209386	0.525198326	0.544109763	0.921433364
LOC101903997	0.123492659	0.765420598	0.557762914	0.30763428	0.729889424	0.544210643	0.921433364
LOC614423	0.858485016	0.118628277	0.439794473	0.275447565	0.957442872	0.543788229	0.921433364
NCOA5	0.2937337	0.326088891	0.955512025	0.155741931	0.829069845	0.543876247	0.921433364
RBM19	0.028460167	0.88579797	0.998456979	0.67019391	0.701924146	0.544258785	0.921433364
RBP1	0.260732756	0.177890697	0.785871459	0.324990343	0.99921253	0.544186865	0.921433364
SEMA6D	0.230615156	0.163927277	0.568390522	0.694640365	0.793194146	0.544229427	0.921433364
SLC25A21	0.875756707	0.130047117	0.326777095	0.957529555	0.332337616	0.54429194	0.921433364
WDR91	0.743761682	0.769420045	0.301923773	0.102248165	0.669277991	0.543979212	0.921433364
ARHGAP9	0.134905902	0.512511745	0.701914177	0.47749157	0.51147861	0.54444351	0.921590092
CD19	0.855017498	0.113946652	0.859749434	0.781675789	0.181076864	0.54449901	0.921590092
FNBP1L	0.116633439	0.709100322	0.302800623	0.750144255	0.631452923	0.544602507	0.921590092
LOC786616	0.526462118	0.748159947	0.478942429	0.198535416	0.316741887	0.544609147	0.921590092
MRPS9	0.747807416	0.238299743	0.800216067	0.138158239	0.602327222	0.544671419	0.921600439
ABCD3	0.785399316	0.143058742	0.413121122	0.520868227	0.491251518	0.544840503	0.921703632
C6H4orf3	0.841334607	0.842430444	0.40730192	0.501640231	0.082113304	0.545065439	0.921703632
DCPS	0.684371269	0.97032134	0.222761082	0.630466203	0.127444773	0.544979767	0.921703632
ETFDH	0.534784079	0.556518152	0.757739129	0.086389575	0.610372937	0.545069389	0.921703632
SCAMP1	0.036656425	0.800717725	0.754882263	0.545612959	0.98291093	0.544925677	0.921703632
SERPINA5	0.336761817	0.527945687	0.770920077	0.296532298	0.292251255	0.544856234	0.921703632
SLC2A13	0.873311598	0.192762104	0.319815517	0.247601543	0.892847399	0.545239685	0.921896607
DPP6	0.553189546	0.378300108	0.756538784	0.497158786	0.151439248	0.54552768	0.922288529
MRPL47	0.502884792	0.340684336	0.47074684	0.471390841	0.31376623	0.545668672	0.922320842
SCAND1	0.306747554	0.376980916	0.386007696	0.461438705	0.579018734	0.545628012	0.922320842
SLC52A2	0.252599154	0.166339946	0.561844168	0.543221139	0.930423248	0.545715396	0.922320842
TBL1X	0.383790964	0.325309425	0.983334666	0.122855424	0.791351662	0.545783669	0.92234124
ALG3	0.305866085	0.462128473	0.856952849	0.865188173	0.11405373	0.546054243	0.922418547
ERCC4	0.904389317	0.189887682	0.413978242	0.233839869	0.718987384	0.54605359	0.922418547
PJA1	0.566888858	0.264735058	0.957658318	0.434135281	0.191513637	0.545998625	0.922418547
TTL	0.794389794	0.928825326	0.061917034	0.284324	0.919991313	0.546011989	0.922418547
EEF1AKMT2	0.129378043	0.798995491	0.919084388	0.343962778	0.366440968	0.54640866	0.922506374
HDHD3	0.944413566	0.283231861	0.38291091	0.256493475	0.45630933	0.546612149	0.922506374
LOC100336589	0.13077578	0.642479651	0.632022991	0.282421223	0.798166908	0.546335921	0.922506374
LOC112444194	0.951191691	0.894730052	0.309590905	0.2084366	0.21777366	0.546167227	0.922506374

MLST8	0.210350474	0.561883166	0.783254713	0.135327836	0.95653475	0.546542853	0.922506374
PHGDH	0.656042775	0.551356415	0.119812185	0.656251979	0.420839697	0.546310499	0.922506374
RASSF3	0.841332011	0.225177566	0.147530848	0.854776886	0.501553969	0.546527609	0.922506374
VSTM4	0.391726513	0.567481548	0.150337711	0.41766343	0.858240517	0.546480167	0.922506374
ZFP3	0.416184215	0.07439618	0.589264156	0.81354465	0.807449191	0.546570358	0.922506374
MORC4	0.203225672	0.973493737	0.625159822	0.138908925	0.698677612	0.546864149	0.922565077
RXFP4	0.281108729	0.542062262	0.318265209	0.29941396	0.826483139	0.546824034	0.922565077
SMG7	0.872533086	0.137543289	0.365301404	0.99292438	0.275622006	0.546772932	0.922565077
TMEM30B	0.816747525	0.094466783	0.805053766	0.680486138	0.283998474	0.546871797	0.922565077
LOC112442997	0.430002718	0.9311595	0.991313344	0.042392988	0.713638658	0.546938545	0.922582841
ATP6V1D	0.844731836	0.409331207	0.473258165	0.272589035	0.269322169	0.547023881	0.922602869
FAM149B1	0.523405544	0.569919423	0.204378562	0.871197895	0.226385867	0.547191723	0.922602869
FAM8A1	0.92391871	0.099948655	0.552501262	0.292156744	0.806832967	0.547231511	0.922602869
PARL	0.969776849	0.12288217	0.267485374	0.931753261	0.404872736	0.547203591	0.922602869
SFXN4	0.835868834	0.324949704	0.70344382	0.314930989	0.199806329	0.547171934	0.922602869
DECR2	0.100516331	0.407934491	0.522497651	0.868250967	0.646719028	0.547288253	0.922603751
D2HGDH	0.306997637	0.280206075	0.761403585	0.22643365	0.812698298	0.547652048	0.922648368
EXOG	0.637073001	0.987993855	0.945713177	0.146625486	0.138088201	0.547639312	0.922648368
KCTD11	0.311739997	0.599138341	0.394243631	0.933835767	0.17521784	0.547579476	0.922648368
PUS3	0.756359246	0.092407866	0.627965516	0.598085119	0.458671714	0.54744932	0.922648368
RABGAP1L	0.623094606	0.282964441	0.706793836	0.116783227	0.827603485	0.547512593	0.922648368
SPRY2	0.63214008	0.418329448	0.748188012	0.41519981	0.146582202	0.547468306	0.922648368
MYLK3	0.187091972	0.68098017	0.472340421	0.3545023	0.565291381	0.547757094	0.922730617
KLHL28	0.10388599	0.200949088	0.790451247	0.894792195	0.817049318	0.547824275	0.922749069
HDGFL2	0.254720401	0.955457384	0.255149403	0.228563211	0.8507048	0.547985712	0.922780303
LOC101904705	0.609725908	0.518344035	0.532930472	0.086598942	0.828385618	0.548123965	0.922780303
LOC112446457	0.384332441	0.085794373	0.860799491	0.760114229	0.559963588	0.548096659	0.922780303
RAB38	0.075479543	0.475096682	0.832103195	0.962214485	0.420720031	0.548073308	0.922780303
TTC9C	0.84525977	0.417563904	0.707975281	0.053109089	0.909672845	0.54795508	0.922780303
FXYD3	0.332415688	0.186315109	0.477798116	0.48251425	0.847823284	0.54848595	0.922863038
KCNE3	0.38205582	0.376536641	0.452934126	0.250325944	0.741471124	0.548300646	0.922863038
NIPAL3	0.696740267	0.866101674	0.812412992	0.153880647	0.160340334	0.54833337	0.922863038
NPHP3	0.401603475	0.731463282	0.221380676	0.476502594	0.39070487	0.548509889	0.922863038
PPIL4	0.212308971	0.525008057	0.818511588	0.169166721	0.784456507	0.548509514	0.922863038
SORCS2	0.738701046	0.321820031	0.695490462	0.126341859	0.579595482	0.548510516	0.922863038
LOC616051	0.963693792	0.36767764	0.737595986	0.188317275	0.246119122	0.548605371	0.922928011
ZMYM5	0.586394273	0.729804533	0.639732455	0.071196687	0.621656859	0.548669236	0.922940839
LNPEP	0.114411093	0.937214276	0.198976688	0.755313569	0.752666815	0.548860665	0.923168225
LOC104974070	0.895803059	0.691022231	0.239690281	0.103600621	0.789889873	0.549056745	0.923308765
SLC16A12	0.36238188	0.253216668	0.557415229	0.957275886	0.247916795	0.549011217	0.923308765

DPH6	0.789368317	0.167917043	0.900377132	0.690560678	0.147418198	0.549174936	0.923412897
U2AF2	0.505487515	0.56645742	0.173786999	0.252125448	0.968711939	0.549243062	0.923432833
DDOST	0.470422896	0.453011775	0.197342583	0.361894039	0.798946532	0.549336384	0.923493154
INHBB	0.439582155	0.20709053	0.208997424	0.951325731	0.67200475	0.549391485	0.923493154
SLC2A12	0.450780405	0.497907891	0.339968356	0.242506177	0.657766816	0.5495258	0.923624325
ARPC5L	0.907016911	0.777253093	0.428370034	0.937615765	0.043004441	0.549608664	0.923669001
ALDH3A2	0.984366989	0.466912466	0.764655326	0.065026161	0.5336225	0.549893378	0.923855622
FAM78B	0.403041614	0.345058819	0.458275257	0.709273433	0.269760812	0.549885221	0.923855622
SOD3	0.536556925	0.364674234	0.932749652	0.256992404	0.260070414	0.549944889	0.923855622
SORD	0.425624281	0.467058201	0.43266697	0.307284248	0.461220149	0.549813625	0.923855622
CD209	0.161784013	0.611887901	0.916722729	0.651716667	0.206649116	0.550315831	0.924078681
FAF1	0.885359725	0.413929444	0.940649276	0.485266319	0.0731202	0.550471829	0.924078681
HMGCL	0.559856989	0.617295712	0.110824934	0.324583423	0.983654363	0.550421241	0.924078681
NEK3	0.834829237	0.940493017	0.730974804	0.066850775	0.318377491	0.550212828	0.924078681
P2RX4	0.925564628	0.495277321	0.891335507	0.350393174	0.085362495	0.550307574	0.924078681
SOGA1	0.633060072	0.530577621	0.11064462	0.890705599	0.369277414	0.550348149	0.924078681
SULT1B1	0.92852697	0.894997127	0.50637399	0.083787421	0.346716692	0.550361408	0.924078681
CNPY2	0.409291866	0.678548637	0.354192925	0.194388228	0.640648503	0.550759791	0.92416743
FBXW5	0.214774855	0.770559278	0.925058408	0.081283619	0.984255251	0.550726977	0.92416743
LOC782527	0.909584988	0.527966166	0.477452688	0.250865543	0.213023891	0.550806266	0.92416743
SLC12A9	0.894401929	0.088451175	0.598032458	0.480280391	0.538764572	0.550632328	0.92416743
STX2	0.763595869	0.54977279	0.127222526	0.504916939	0.454079717	0.550679949	0.92416743
NSUN3	0.985156817	0.261742218	0.341824255	0.337334259	0.412299225	0.550897174	0.924225467
CTPS2	0.510886771	0.219645388	0.200329403	0.726097737	0.752129104	0.551172612	0.924404062
PEX19	0.340212367	0.161569976	0.669805823	0.704224349	0.473398767	0.551136815	0.924404062
UBXN2A	0.939526086	0.140568164	0.229557964	0.879547211	0.460328992	0.551144686	0.924404062
CDC14B	0.179607345	0.813988274	0.81203542	0.84095546	0.123013252	0.55124466	0.924430422
C5H12orf45	0.462712983	0.919125698	0.756289253	0.257580275	0.148288714	0.551312262	0.924449324
AK7	0.817600697	0.885800121	0.417984739	0.487568054	0.083594743	0.552132048	0.924492353
ARL2	0.475360682	0.464801915	0.80614019	0.181785993	0.379740961	0.55146873	0.924492353
CLMN	0.598620893	0.554946925	0.774724725	0.843591053	0.05671583	0.551750188	0.924492353
DACT2	0.860402562	0.520751271	0.41903934	0.596934505	0.110465206	0.552789111	0.924492353
EAF1	0.842238414	0.325479859	0.373423746	0.125650594	0.959143413	0.552113307	0.924492353
GABPB1	0.061232561	0.674318443	0.989911634	0.389096855	0.774720536	0.551864869	0.924492353
GALNT17	0.819926699	0.370464845	0.093876071	0.497443885	0.871977352	0.552606743	0.924492353
ITGA7	0.611459731	0.70708251	0.226100724	0.356606095	0.35451624	0.552447074	0.924492353
LOC100335340	0.49049081	0.938726173	0.271239586	0.112276557	0.883252929	0.552858933	0.924492353
LOC101906230	0.410928228	0.569980504	0.767891029	0.159617919	0.429191431	0.551869984	0.924492353
LOC104975612	0.585717499	0.075673324	0.39679444	0.791522807	0.888072756	0.552510583	0.924492353
LOC112445011	0.167864037	0.55067768	0.825559974	0.290164748	0.557957629	0.552396198	0.924492353

LOC112446740	0.671614664	0.868112577	0.89222514	0.241002684	0.098651465	0.552593426	0.924492353
LOC112449106	0.069122348	0.612599423	0.754437389	0.959017014	0.402044442	0.551808327	0.924492353
NME6	0.513651671	0.138033881	0.878638512	0.402253183	0.493346724	0.552514033	0.924492353
NUB1	0.733703417	0.41645754	0.109339923	0.852659026	0.433297029	0.55221134	0.924492353
PCCA	0.493204219	0.514858533	0.645915401	0.178844106	0.422079804	0.552798178	0.924492353
PCGF1	0.918605377	0.859325119	0.477051646	0.459030808	0.071206266	0.551672884	0.924492353
PDPN	0.937878801	0.201076526	0.201794481	0.714503082	0.452707872	0.551685068	0.924492353
PLA2G6	0.396734128	0.711074948	0.814158035	0.230511433	0.232692761	0.551843812	0.924492353
POLR2M	0.730642809	0.580714705	0.148868556	0.531568834	0.367858827	0.552335225	0.924492353
RNF4	0.463307751	0.313056876	0.610812919	0.252187933	0.554284136	0.552841582	0.924492353
SC5D	0.262645322	0.738771621	0.739413219	0.203129557	0.424238127	0.552528843	0.924492353
SERPINB1	0.407119714	0.442634044	0.406703099	0.460064302	0.364738942	0.551510897	0.924492353
SYNGR3	0.611176314	0.773801528	0.108082165	0.510283055	0.474567758	0.552753983	0.924492353
VEGFA	0.474056493	0.78494191	0.100374307	0.67589742	0.489259775	0.552334728	0.924492353
WIPF3	0.345337744	0.208906796	0.434527731	0.616773369	0.640546181	0.552855895	0.924492353
GGT1	0.224656974	0.858268131	0.293748209	0.367139975	0.596183701	0.553050187	0.924562932
IRAK4	0.698874066	0.102827668	0.390642642	0.57405229	0.769369675	0.553070154	0.924562932
MCOLN3	0.450965153	0.677786271	0.189358471	0.912621411	0.234706258	0.553052803	0.924562932
TPT1	0.87169927	0.89534128	0.229825117	0.770116722	0.08979095	0.553143871	0.924591981
CTSA	0.295411172	0.929532406	0.161313254	0.874142028	0.321516982	0.553853456	0.924635615
DUSP3	0.14576835	0.15132937	0.952027168	0.934476059	0.634583403	0.553917673	0.924635615
KRAS	0.461436971	0.948936146	0.421128597	0.17434615	0.38683213	0.553655252	0.924635615
LOC101905588	0.782301983	0.08972813	0.438606362	0.735332874	0.549023282	0.553546393	0.924635615
LOC112443816	0.606684772	0.387768519	0.339239123	0.289990054	0.538065008	0.553902967	0.924635615
LOC615959	0.260449086	0.789213948	0.157905227	0.496372349	0.773149571	0.553958564	0.924635615
MIR3064	0.97315791	0.384557678	0.18600898	0.955139113	0.187179266	0.553789656	0.924635615
NAGLU	0.345978835	0.149726709	0.854518082	0.347596218	0.80808066	0.553611411	0.924635615
PEA15	0.767434658	0.666259978	0.248268409	0.98264032	0.099843146	0.553929494	0.924635615
RNF31	0.72461574	0.103507398	0.55671999	0.374893204	0.793987588	0.553539557	0.924635615
SMIM13	0.164820308	0.440896204	0.956368171	0.489643293	0.365816788	0.553839407	0.924635615
SRP9	0.758100934	0.421082868	0.728493123	0.890823948	0.060012118	0.553589786	0.924635615
SYF2	0.961720039	0.362124473	0.160292659	0.364835715	0.609339006	0.553247541	0.924635615
TMEM232	0.706505054	0.212937809	0.829954178	0.931740603	0.107070164	0.553958769	0.924635615
ARHGAP18	0.593721432	0.935467995	0.350431298	0.666154091	0.096131526	0.554077875	0.924734909
GTF2H2	0.562955994	0.085775507	0.987665738	0.270425703	0.966672702	0.554130955	0.924734909
AAMDC	0.572899248	0.203779206	0.464590711	0.335410234	0.687376691	0.554707788	0.924757163
AGPAT4	0.677224493	0.42630472	0.943986255	0.190818158	0.240339981	0.554612467	0.924757163
CDCA2	0.550502011	0.434192992	0.164721113	0.904721573	0.350800647	0.554569189	0.924757163
CLDN4	0.080136601	0.418353219	0.807530427	0.692056026	0.666680364	0.554492041	0.924757163
GMIP	0.364848761	0.340418936	0.735250354	0.287080912	0.476501664	0.554509162	0.924757163

MPP6	0.255634608	0.566241182	0.900913205	0.189076249	0.506525164	0.554470026	0.924757163
NXF1	0.72884169	0.316666962	0.53570307	0.238842003	0.423239846	0.554609441	0.924757163
PARP11	0.939398746	0.460722293	0.634955058	0.344923181	0.131687901	0.554363198	0.924757163
PEX26	0.185171855	0.706403431	0.202043446	0.6386959	0.739524907	0.554371612	0.924757163
WDR7	0.840485458	0.482647647	0.139696164	0.37109076	0.594504283	0.554663679	0.924757163
AMY2B	0.931221863	0.239691065	0.192866979	0.390464703	0.744575726	0.554873088	0.924777007
DCLK1	0.151972045	0.177328739	0.521065767	0.974452766	0.91440832	0.554821256	0.924777007
LOC107131510	0.580709043	0.370593348	0.257322221	0.800353734	0.282405706	0.554888744	0.924777007
BNIP1	0.461754837	0.052568817	0.889183692	0.830901365	0.700476419	0.555589904	0.925100032
FZD1	0.489178693	0.882215421	0.358915972	0.352957584	0.229650382	0.555479536	0.925100032
KCNJ13	0.846504821	0.683269029	0.157201939	0.287295545	0.480500531	0.555425206	0.925100032
KCNMB4	0.908440437	0.401031497	0.481232396	0.205043951	0.349406141	0.555560332	0.925100032
PIKFYVE	0.757329811	0.286580834	0.632945262	0.542667557	0.168187128	0.55521427	0.925100032
POLK	0.165995625	0.888056573	0.530233579	0.98577359	0.16302472	0.555572653	0.925100032
SBNO2	0.376796565	0.270896381	0.556638495	0.517646375	0.426671839	0.555384819	0.925100032
UTP14A	0.21361951	0.683748518	0.642840537	0.446124575	0.299548361	0.555364086	0.925100032
VIM	0.271653106	0.888030682	0.248060322	0.815465338	0.25730308	0.555490943	0.925100032
ADRA2A	0.582558209	0.257261376	0.362426136	0.962921532	0.24085822	0.556125987	0.925189964
C1GALT1	0.671545358	0.589333314	0.073766164	0.517100332	0.834257648	0.556074112	0.925189964
CCDC28A	0.943380457	0.372934426	0.407774708	0.196988859	0.445822209	0.556151301	0.925189964
FNTA	0.888442358	0.342217953	0.310512115	0.242450262	0.549846925	0.555944244	0.925189964
GPR37L1	0.95993993	0.657670029	0.28604699	0.536758602	0.129753738	0.555817408	0.925189964
IL3RA	0.626462477	0.275215097	0.249460884	0.787558138	0.371477738	0.555904485	0.925189964
PAIP2	0.717463566	0.381219694	0.352854213	0.788023699	0.165527001	0.555990066	0.925189964
SLC7A1	0.465302808	0.41016678	0.540023057	0.252575596	0.483699471	0.556032918	0.925189964
TBX21	0.797257432	0.412371733	0.192049782	0.538042077	0.37045894	0.555936524	0.925189964
TULP2	0.84706194	0.073103666	0.976554318	0.368384484	0.565884785	0.55625621	0.925270693
MAGI1	0.125385251	0.879001302	0.350911038	0.812778254	0.401404883	0.556436609	0.925328994
SOWAHD	0.878075238	0.463310154	0.776871207	0.066152516	0.603353673	0.556384909	0.925328994
TC2N	0.060199251	0.596729279	0.570959629	0.654317706	0.940327218	0.556460413	0.925328994
ERCC5	0.526087498	0.593771233	0.132685639	0.582524928	0.523028347	0.556592866	0.925370495
LOC101907998	0.439519161	0.669219891	0.244291343	0.866219911	0.202894292	0.556598145	0.925370495
ABHD17C	0.513709747	0.590338629	0.248145697	0.645376609	0.260435816	0.556902206	0.925405906
ADGRB3	0.347485617	0.505417607	0.100646967	0.836259304	0.855398866	0.556839279	0.925405906
ELOVL1	0.626427319	0.548607736	0.077162125	0.705038839	0.675747869	0.55667861	0.925405906
IMPDH1	0.087889914	0.74064158	0.611517369	0.574622814	0.552567343	0.556762476	0.925405906
RIN2	0.760216989	0.333930916	0.349924745	0.871971939	0.163340497	0.55695778	0.925405906
TCEAL4	0.766523215	0.44743442	0.824015615	0.925275733	0.048371657	0.556907782	0.925405906
ERAL1	0.6498045	0.78975172	0.490518324	0.067618519	0.745438858	0.557507171	0.925434354
FOXF1	0.809645118	0.374863932	0.224787611	0.539562972	0.343961811	0.557102913	0.925434354

GPATCH1	0.321882805	0.381914394	0.380003679	0.30455084	0.892687579	0.557686359	0.925434354
HNRNPH2	0.601187631	0.229905358	0.573209923	0.967173484	0.165365341	0.557248386	0.925434354
LOC100847320	0.267768804	0.170858083	0.550918208	0.819749771	0.615106758	0.557820768	0.925434354
LOC101905630	0.77947979	0.609394521	0.34980559	0.180549737	0.423479403	0.557752072	0.925434354
NANP	0.221581443	0.494209793	0.687881653	0.849244457	0.198287782	0.557454449	0.925434354
NRGN	0.503413795	0.337380293	0.534894092	0.154407767	0.903005045	0.557182088	0.925434354
RBM34	0.577271084	0.246094367	0.327794485	0.581800673	0.468227458	0.55746644	0.925434354
SLC22A15	0.263106808	0.298277821	0.785190766	0.702310606	0.293399479	0.557644691	0.925434354
SLC25A20	0.265770766	0.219771257	0.739016527	0.373635888	0.785718069	0.557259841	0.925434354
SWSAP1	0.718807252	0.497206412	0.088698945	0.654163246	0.612455949	0.557694207	0.925434354
TCEAL1	0.136861314	0.740429144	0.681532932	0.879080191	0.209306355	0.557797533	0.925434354
ZBTB24	0.895598725	0.030803189	0.629288308	0.807167724	0.904602863	0.557318715	0.925434354
ZUP1	0.566750507	0.538204515	0.521947526	0.246557214	0.323473665	0.557647323	0.925434354
ADSS	0.665586856	0.198501158	0.932622517	0.138889692	0.742867085	0.557882929	0.925443925
RRAS2	0.209940491	0.581559463	0.56431658	0.961501079	0.192194735	0.558170612	0.925827564
ATN1	0.891625574	0.465946456	0.124147587	0.447443889	0.552205112	0.558343969	0.925927938
DAB1	0.390104811	0.606340475	0.236995477	0.372060561	0.610951744	0.558327428	0.925927938
DLL1	0.577448118	0.353775452	0.710571027	0.421352896	0.20857654	0.558549401	0.926013419
PLK3	0.187250153	0.825414135	0.422087138	0.511577779	0.382188059	0.558516349	0.926013419
SLCO5A1	0.743665363	0.077275112	0.562116186	0.774915427	0.509679022	0.558564794	0.926013419
ADGRE5	0.449285221	0.157869043	0.279529443	0.921045391	0.699710371	0.558853474	0.9260817
ATRN	0.739685189	0.082918353	0.881200978	0.272490327	0.868180402	0.558980898	0.9260817
BMX	0.213096352	0.439211061	0.980150863	0.727715402	0.191513361	0.558967264	0.9260817
DYRK2	0.570698495	0.735082739	0.134929858	0.6669909	0.338107624	0.558667358	0.9260817
HGFAC	0.624641271	0.061987085	0.720076501	0.898022071	0.510718335	0.559000994	0.9260817
PLA2G4A	0.19146029	0.861637191	0.107228592	0.793462695	0.910870509	0.558964628	0.9260817
VRK1	0.099425877	0.43794662	0.842054815	0.480321239	0.725983301	0.558974592	0.9260817
COL12A1	0.563285508	0.566298351	0.1689159	0.276303474	0.860915047	0.55944943	0.926097889
COL4A2	0.241624969	0.6390785	0.632450828	0.695074424	0.188821831	0.559456187	0.926097889
ITFG1	0.482049551	0.462094365	0.142113228	0.521397148	0.77673282	0.559496454	0.926097889
ITGA9	0.205695151	0.618876549	0.219143168	0.900390298	0.511030841	0.55973433	0.926097889
KIF3C	0.499492444	0.895313412	0.154678597	0.937578004	0.197533421	0.559356898	0.926097889
LOC104975673	0.623659211	0.146700504	0.990853809	0.234178112	0.602652534	0.559099721	0.926097889
LZTS2	0.825511526	0.88187527	0.195363554	0.207453608	0.434981028	0.559703851	0.926097889
MAPK6	0.660938993	0.057783383	0.662393598	0.839670281	0.604167329	0.559695575	0.926097889
MBD3	0.285162916	0.257015538	0.403164086	0.812517096	0.53459179	0.559714441	0.926097889
NPAS2	0.303281131	0.254970719	0.379943567	0.954461929	0.457456754	0.559614458	0.926097889
SMOC2	0.74275133	0.545466088	0.270736542	0.732881618	0.159456924	0.559469031	0.926097889
SPATA16	0.570631317	0.233117044	0.688274407	0.519064561	0.270112057	0.559744376	0.926097889
TRPV2	0.433968843	0.430550868	0.190471894	0.916288334	0.393047935	0.559449672	0.926097889

ARID1B	0.50558306	0.174521787	0.713706479	0.21414031	0.953229757	0.560010846	0.926152299
ARL2BP	0.50138667	0.519689927	0.410530486	0.141201066	0.850904008	0.559976814	0.926152299
CNTNAP2	0.95206851	0.119545843	0.337748835	0.737804505	0.452920022	0.55987856	0.926152299
MUM1	0.416165017	0.954372171	0.370753652	0.416098632	0.209846815	0.560059437	0.926152299
SP3	0.094362761	0.466450674	0.802917504	0.39107151	0.929958905	0.559983987	0.926152299
DCN	0.277965271	0.535731953	0.835209047	0.167324456	0.618093486	0.560138965	0.926180166
LOC104975749	0.762885124	0.178405903	0.695946219	0.190537405	0.71291182	0.560189161	0.926180166
COL5A3	0.050797962	0.689073803	0.405781316	0.945963734	0.96080621	0.560833881	0.926224366
GRK6	0.639363879	0.279039791	0.302119097	0.694372795	0.344625908	0.560664807	0.926224366
HIC1	0.97362082	0.236497756	0.384345976	0.26665664	0.54623083	0.560547229	0.926224366
KMT2C	0.811812182	0.22342815	0.517554975	0.491305208	0.279910684	0.560836727	0.926224366
LOC100848439	0.564457099	0.597782608	0.297584154	0.572805926	0.224388355	0.560779553	0.926224366
LOC104974443	0.975243253	0.091163678	0.404584281	0.968316735	0.370245782	0.560628595	0.926224366
LY96	0.664008315	0.352630574	0.246914059	0.459030995	0.486185792	0.560731553	0.926224366
RNF20	0.310230328	0.556377054	0.885832205	0.122107244	0.691419483	0.560822177	0.926224366
TBRG1	0.947378413	0.053441797	0.774898707	0.537133909	0.610926971	0.560304961	0.926224366
USE1	0.2726957	0.099324136	0.716467119	0.74812279	0.887980594	0.560564023	0.926224366
ZNF784	0.988897925	0.272238958	0.140141241	0.49662829	0.687477076	0.560409597	0.926224366
HEY1	0.889086532	0.880797804	0.083743693	0.571975158	0.344618128	0.561088085	0.926469127
KIAA0513	0.357514828	0.769968039	0.160078827	0.36713197	0.799248492	0.561139981	0.926469127
SEMA4C	0.709344251	0.835094766	0.177644767	0.572058086	0.214808565	0.561154294	0.926469127
ACE2	0.109772909	0.712471027	0.290278103	0.800413578	0.712242275	0.561323652	0.92659994
LOC513767	0.576220651	0.752303635	0.035902567	0.933092021	0.891329555	0.561346451	0.92659994
AADACL3	0.73517588	0.337646099	0.17879573	0.739628492	0.395535964	0.561940565	0.926603314
ABRACL	0.819657141	0.499596492	0.527408281	0.108970834	0.554130919	0.562788633	0.926603314
ACP1	0.622244309	0.588437211	0.875748306	0.222916374	0.18243414	0.562774096	0.926603314
B3GALT4	0.755955878	0.591228879	0.829432391	0.076855216	0.458070248	0.56292944	0.926603314
EFCAB8	0.583436117	0.426258545	0.492032959	0.837616569	0.127317461	0.562909611	0.926603314
GAL3ST4	0.235360829	0.9956804	0.127949771	0.961044048	0.450804673	0.562036214	0.926603314
GCDH	0.618621035	0.573824778	0.951627056	0.045342107	0.845530883	0.561449196	0.926603314
HSPB2	0.63149492	0.13515302	0.877536447	0.514450545	0.337237921	0.562087433	0.926603314
ICA1L	0.163904703	0.536588766	0.601173425	0.769933404	0.319965606	0.562552575	0.926603314
LOC101905041	0.3512566	0.184225328	0.316382567	0.734320678	0.864097127	0.562040356	0.926603314
LOC104969833	0.304532915	0.271671936	0.556361093	0.531132189	0.533345141	0.562755584	0.926603314
LOC104973224	0.329292604	0.822290196	0.112683704	0.899546693	0.474188449	0.562398907	0.926603314
LOC112444333	0.668458408	0.466578876	0.213595627	0.225945036	0.861733404	0.56174442	0.926603314
MAFB	0.373830059	0.475323832	0.891499451	0.231686911	0.353339831	0.561705136	0.926603314
MKNK1	0.528740556	0.277798165	0.875047235	0.426193526	0.238218685	0.562906209	0.926603314
NGFR	0.162727895	0.655353343	0.672377103	0.544587746	0.333342113	0.562428015	0.926603314
NOP56	0.573253397	0.331920933	0.689242057	0.60243112	0.165128649	0.562860372	0.926603314

NUP188	0.758713245	0.118576781	0.612933703	0.962473051	0.245164224	0.562351705	0.926603314
PKMYT1	0.292482396	0.317795376	0.521058274	0.434139524	0.619445296	0.56254222	0.926603314
PRELID3A	0.816295038	0.835412872	0.759265239	0.03468029	0.726699145	0.562903122	0.926603314
PRKDC	0.648510617	0.273406107	0.338956018	0.359648217	0.600448533	0.561857441	0.926603314
SACS	0.490207836	0.744776418	0.973520818	0.148900987	0.245654992	0.562191139	0.926603314
SUCO	0.475151805	0.312941611	0.709296373	0.199426426	0.620282628	0.562866091	0.926603314
TDRKH	0.413142492	0.552912586	0.165411528	0.439840561	0.782863183	0.562336959	0.926603314
THYN1	0.432822876	0.352055642	0.397200557	0.324175976	0.662584648	0.562181814	0.926603314
TLR8	0.932183381	0.705246486	0.109475119	0.435406934	0.414638897	0.562079572	0.926603314
TUSC1	0.166627872	0.919848983	0.658307406	0.725938512	0.177870905	0.562600695	0.926603314
USP1	0.069899822	0.842951742	0.928913007	0.264597108	0.900163872	0.562717319	0.926603314
IFI27L2	0.176730936	0.910900266	0.57496921	0.215808099	0.653700786	0.563034274	0.926677991
PCM1	0.175140863	0.772844804	0.661480308	0.233078393	0.625887829	0.563087742	0.926677991
CISH	0.595724173	0.041695111	0.669531835	0.960154383	0.818318679	0.563163145	0.926709151
DACH1	0.366001643	0.994276027	0.947892936	0.076351595	0.496425326	0.563276859	0.926718314
PCIF1	0.845689108	0.912724348	0.63076978	0.616923062	0.043529119	0.563281651	0.926718314
STAP1	0.291686947	0.451630858	0.846983046	0.628300728	0.186594532	0.563374712	0.926778508
CRYBB1	0.567496242	0.321781387	0.131534799	0.800264433	0.680741493	0.563436193	0.926786746
PPIB	0.364038252	0.419825599	0.370288832	0.342913321	0.674489392	0.563496435	0.926792944
AIDA	0.466291604	0.568086914	0.332478107	0.273502425	0.544484783	0.563881008	0.926863927
FAM81A	0.076577893	0.555030262	0.83885865	0.465522668	0.790437336	0.563940843	0.926863927
LOC112443214	0.469427595	0.977826062	0.148813784	0.425170236	0.451101688	0.563671621	0.926863927
MINDY4	0.103384177	0.583746506	0.325917954	0.808527851	0.824903737	0.563926534	0.926863927
NOP9	0.374816974	0.301833199	0.46747496	0.276832029	0.896306067	0.563985302	0.926863927
PFKFB2	0.21574733	0.409212608	0.335194148	0.923827545	0.479215253	0.563672381	0.926863927
PTPN4	0.147357459	0.452284613	0.518219167	0.88397324	0.429827833	0.563991419	0.926863927
SCAP	0.471996574	0.637888642	0.961856588	0.123948369	0.365537319	0.563962791	0.926863927
SMNDC1	0.794297162	0.599774741	0.857442214	0.07827238	0.410614984	0.564075027	0.926884717
ZBTB10	0.187793922	0.996134982	0.298239976	0.401968754	0.585539377	0.564117029	0.926884717
RBM22	0.841957294	0.765633385	0.368876951	0.062770797	0.8805571	0.5642918	0.92707906
FAM57B	0.281787228	0.60656329	0.283801742	0.778215598	0.348535458	0.564492404	0.927199503
KIRREL1	0.380402781	0.617223465	0.469433964	0.219970994	0.542785686	0.564534607	0.927199503
LEPROTL1	0.703645677	0.073737862	0.601794618	0.535615855	0.786687528	0.564487594	0.927199503
RTL8C	0.494682595	0.446351963	0.65734348	0.113929016	0.796243011	0.564633188	0.927268613
ARRDC2	0.808605276	0.350024365	0.226821609	0.381927209	0.537504496	0.564813292	0.9274517
LOC112443012	0.241598008	0.461411041	0.384220639	0.449883588	0.684100325	0.564857702	0.9274517
LACTB	0.890367881	0.843534975	0.620884734	0.765798456	0.036946373	0.565029975	0.927641748
FAM206A	0.456603	0.08666279	0.720207059	0.499804044	0.926607345	0.565098573	0.927661567
ACAA2	0.553171409	0.512413826	0.279436972	0.175223586	0.955227299	0.565960126	0.927785245
ADCK2	0.896290435	0.650399197	0.314508452	0.235509823	0.306601491	0.565684958	0.927785245

AMOT	0.740843713	0.453804783	0.276116213	0.162950805	0.875081565	0.56566197	0.927785245
ANGPTL2	0.302591534	0.116969095	0.723671612	0.810949101	0.636109336	0.565307559	0.927785245
GOT1	0.948584973	0.306465094	0.564983029	0.238519585	0.338302402	0.565897065	0.927785245
HES7	0.563970621	0.972377386	0.79190715	0.052418299	0.58038534	0.565294299	0.927785245
LOC100336909	0.105920293	0.706483422	0.421973114	0.686705897	0.609956297	0.565503288	0.927785245
LOC107132192	0.03832179	0.700197195	0.823637082	0.672610173	0.891887543	0.565965394	0.927785245
PITPNC1	0.653471711	0.708962074	0.652226982	0.150417694	0.29138936	0.565763507	0.927785245
SCCPDH	0.899188071	0.569108142	0.414250515	0.284629904	0.219108863	0.565420413	0.927785245
SLAMF7	0.556401479	0.316188545	0.288124764	0.630427721	0.414103512	0.565602702	0.927785245
TACR2	0.544639497	0.102140994	0.904022887	0.740549093	0.355448578	0.56567381	0.927785245
UST	0.415759942	0.554430763	0.969730056	0.064061519	0.925231258	0.565837778	0.927785245
ZNF227	0.748088971	0.935083639	0.458087595	0.462618993	0.089249259	0.565567655	0.927785245
HMGB1	0.682232266	0.082558125	0.453104405	0.668950622	0.777350271	0.5661545	0.927884575
LOC104973058	0.943996867	0.557889537	0.737723965	0.55363999	0.06170972	0.566195609	0.927884575
LOC112444600	0.342837463	0.285108892	0.69441258	0.692478218	0.282265316	0.566100767	0.927884575
BTBD7	0.442478408	0.324979674	0.713014489	0.151524647	0.856421859	0.566650648	0.928074731
ERF	0.302017474	0.728746629	0.689345749	0.131852809	0.665094311	0.566650954	0.928074731
LOC112447342	0.202212708	0.738258761	0.292503256	0.989252775	0.307778201	0.566506316	0.928074731
PLEKHG1	0.169166347	0.574604331	0.787989265	0.467090927	0.371458485	0.56642721	0.928074731
PRKCA	0.762404566	0.928986033	0.326777244	0.257296217	0.223424069	0.566647171	0.928074731
UROC1	0.123149575	0.541306567	0.443652805	0.555712219	0.809339907	0.566598511	0.928074731
GPRASP1	0.393014691	0.878315874	0.089525552	0.563399378	0.764459557	0.566721643	0.928097882
FIS1	0.184628682	0.333785432	0.550764967	0.433372193	0.905396001	0.566835006	0.928113317
NAP1L4	0.399033093	0.412833766	0.764524825	0.127672765	0.82828371	0.566844176	0.928113317
ABCA1	0.850756041	0.093772599	0.255673789	0.668267434	0.983632284	0.568132167	0.928122339
ACVR2B	0.209811894	0.498145054	0.508974038	0.314142295	0.802704398	0.568227034	0.928122339
COL4A3BP	0.724644466	0.469230035	0.219758153	0.636657035	0.281531079	0.567926463	0.928122339
CXCL2	0.499246062	0.863010812	0.321672187	0.543712428	0.177559382	0.567735103	0.928122339
DLST	0.642347509	0.421276611	0.689697618	0.108829006	0.663592314	0.569151491	0.928122339
ENDOU	0.843785372	0.575313968	0.629075295	0.909683503	0.048514973	0.569135268	0.928122339
FGFR2	0.225399069	0.353951693	0.609872161	0.603438021	0.457550981	0.568513196	0.928122339
FHL2	0.213376145	0.807612134	0.153970743	0.656871995	0.77148833	0.568686618	0.928122339
GREM1	0.365956867	0.176885365	0.494677401	0.841487286	0.494703977	0.567014735	0.928122339
HSCB	0.391123088	0.805422667	0.836781541	0.099840819	0.506562632	0.567038667	0.928122339
HUWE1	0.759190464	0.574275792	0.519345739	0.08237153	0.718026891	0.56790779	0.928122339
KCNK13	0.813629315	0.199590549	0.862690556	0.182241095	0.526872494	0.568765092	0.928122339
KIF26A	0.990675021	0.38282327	0.113329245	0.31233922	0.996859964	0.567768419	0.928122339
LOC100139363	0.770995097	0.720620762	0.45754179	0.295477828	0.179344421	0.56904464	0.928122339
LOC100295130	0.558652143	0.864907311	0.232779522	0.83258323	0.14276133	0.567573614	0.928122339
LOC100298453	0.946303394	0.021083661	0.918494498	0.756870118	0.967817507	0.568362311	0.928122339

LOC100337495	0.474053126	0.19484448	0.272966553	0.742072629	0.714592047	0.567589217	0.928122339
LOC100847782	0.277850505	0.535115527	0.737641019	0.170411554	0.713205625	0.567006186	0.928122339
LOC101906850	0.942479001	0.033272339	0.967404674	0.517574263	0.850090374	0.567265495	0.928122339
LOC104973517	0.695552441	0.374173806	0.102989056	0.730229691	0.685167989	0.568176908	0.928122339
LOC112446042	0.385248984	0.700850354	0.800036154	0.379952013	0.164184509	0.569104879	0.928122339
LOC112446668	0.271407248	0.343769126	0.302800234	0.6312691	0.755590624	0.569108557	0.928122339
LOC112448777	0.878676506	0.347990107	0.652224637	0.964463232	0.069343943	0.567126076	0.928122339
LOC112449080	0.745722129	0.607818786	0.257209288	0.77302395	0.149195848	0.56868238	0.928122339
LOC112449280	0.615575061	0.770321825	0.1603553	0.342168641	0.512900282	0.567224919	0.928122339
LYSMD4	0.626464758	0.612443843	0.546862817	0.370404476	0.172664534	0.568296882	0.928122339
MAP4K5	0.063900732	0.767188899	0.532672017	0.82475299	0.624628678	0.568783062	0.928122339
NIPBL	0.370532034	0.415381249	0.922000645	0.097010907	0.969268123	0.567206554	0.928122339
PAQR4	0.488084066	0.356775965	0.249637429	0.867589656	0.354598673	0.567643565	0.928122339
PCDHB8	0.068613413	0.408961282	0.635613464	0.791264452	0.953759644	0.568885833	0.928122339
PDXDC1	0.761092809	0.821388209	0.403324334	0.937719596	0.056847318	0.568609438	0.928122339
PLXNC1	0.40232924	0.312988496	0.541713851	0.336514461	0.582330429	0.567555419	0.928122339
POMK	0.63720136	0.357022332	0.397132444	0.37587333	0.394131832	0.567794321	0.928122339
SERPINB6	0.622438213	0.234438153	0.928332273	0.340892881	0.290634233	0.568328904	0.928122339
SLC35E3	0.730153354	0.339254238	0.425362294	0.913530325	0.139876472	0.568939695	0.928122339
SLC6A20	0.658485189	0.903651618	0.230605067	0.667727426	0.147117764	0.569168437	0.928122339
TTC9B	0.095835129	0.95836125	0.899332327	0.229379772	0.710729485	0.56896978	0.928122339
UBP1	0.915584198	0.72938789	0.361069619	0.69675445	0.079866266	0.568283646	0.928122339
ZBED4	0.189052531	0.628571568	0.399087614	0.530885676	0.53305812	0.568323728	0.928122339
ZBTB7A	0.689076149	0.563385051	0.777085074	0.196926552	0.225600036	0.568058472	0.928122339
ZNF653	0.800553517	0.497572316	0.638939642	0.517407496	0.102354223	0.569152724	0.928122339
CSRP2	0.144692244	0.41468118	0.811000474	0.61148145	0.453807548	0.569504525	0.928301426
LOC101903913	0.701325091	0.122467672	0.378881367	0.952843845	0.435376674	0.56945794	0.928301426
NPHP1	0.969746597	0.987006597	0.591456886	0.923861664	0.025816093	0.569488147	0.928301426
VTI1A	0.279127712	0.144863697	0.714976702	0.61726313	0.756399273	0.569433414	0.928301426
PGM1	0.723401405	0.21760622	0.358314642	0.648178941	0.369834246	0.569763276	0.928450334
SPATA6	0.164255642	0.691881911	0.246195734	0.530593782	0.910809448	0.569765603	0.928450334
TMEM74	0.482584556	0.398025695	0.730437211	0.913167891	0.105476371	0.569654703	0.928450334
ARID4A	0.645626928	0.352835074	0.676779628	0.108461396	0.809421437	0.56995608	0.928454172
GMNN	0.461659874	0.761694221	0.987556159	0.52899984	0.07363982	0.569859207	0.928454172
LOC112441481	0.930741951	0.374174287	0.478349067	0.121874185	0.666859592	0.570020037	0.928454172
MRM1	0.790069154	0.703351929	0.588573643	0.502285729	0.08239701	0.569978478	0.928454172
SMARCB1	0.281196195	0.343018101	0.289540805	0.541895155	0.894771146	0.570050834	0.928454172
CABLES1	0.504111651	0.583614036	0.704784283	0.560677281	0.116579615	0.570220271	0.92851904
HHAT	0.591569906	0.390925627	0.110072558	0.945195957	0.563751298	0.570373557	0.92851904
LOC112444635	0.154070148	0.992538549	0.938875322	0.145005176	0.651423383	0.57034328	0.92851904

PTGR1	0.905428826	0.061428416	0.493996982	0.566320616	0.87162726	0.570352381	0.92851904
WDR34	0.321686576	0.761688269	0.177352779	0.317546436	0.982802102	0.570343667	0.92851904
LYNX1	0.767487156	0.940555227	0.866251776	0.030608576	0.709054355	0.57047749	0.928596121
ZNF81	0.554384501	0.56402972	0.465568418	0.224605012	0.415519034	0.570693232	0.928855165
ZNF282	0.700971677	0.281388866	0.516603535	0.749044954	0.178115398	0.570812144	0.928956574
TRPC6	0.764826167	0.794819809	0.626876089	0.101330799	0.352178159	0.570875755	0.928967974
HOXC5	0.404753414	0.319032491	0.743446715	0.438415015	0.323224017	0.570941329	0.928982565
ING4	0.138833353	0.98913566	0.57979441	0.943887534	0.18138245	0.571330796	0.929063585
LOC100847190	0.179537888	0.685734006	0.319669297	0.358138899	0.966763219	0.57126185	0.929063585
LOC112447031	0.750444965	0.97810678	0.612376855	0.099938047	0.303310388	0.571243015	0.929063585
LOC112448253	0.268583681	0.213519245	0.47645249	0.586120788	0.850537302	0.571187237	0.929063585
ORAOV1	0.093400826	0.717398931	0.27523847	0.782061888	0.945064348	0.571323616	0.929063585
PDZD4	0.380928854	0.384584675	0.20585637	0.747134613	0.604692315	0.5712392	0.929063585
AMACR	0.146430554	0.426989901	0.388648338	0.825654171	0.688355844	0.573859782	0.929079469
CALD1	0.843259377	0.71475708	0.677672748	0.911475187	0.036735333	0.571967181	0.929079469
CLDND1	0.696256511	0.532404261	0.81154372	0.128788652	0.353595834	0.572296253	0.929079469
COMMD7	0.418415176	0.464890481	0.958775186	0.717805641	0.103720781	0.574898276	0.929079469
DMTF1	0.99108551	0.148164844	0.178820728	0.945474617	0.558832436	0.57474589	0.929079469
E2F3	0.553193117	0.856096772	0.698607913	0.652572166	0.063771331	0.573267143	0.929079469
EDNRB	0.688085829	0.684861188	0.580965078	0.051422052	0.978808868	0.573425449	0.929079469
EME1	0.843884964	0.509225162	0.05968331	0.986645778	0.548731962	0.574907197	0.929079469
EPC1	0.514420666	0.521471246	0.834390755	0.134931405	0.455931564	0.573286657	0.929079469
EXOC3L1	0.938763363	0.183168775	0.236691938	0.779584864	0.43627259	0.574303201	0.929079469
FAM25A	0.132166933	0.793084771	0.817709573	0.389243516	0.41530311	0.57448847	0.929079469
G6PC3	0.445009391	0.395022769	0.968476557	0.115823409	0.702966071	0.574570054	0.929079469
HCRTR1	0.360441253	0.794655753	0.064841585	0.886030143	0.842381935	0.574576098	0.929079469
HGS	0.126181091	0.611724302	0.270487269	0.884295067	0.740524538	0.571907068	0.929079469
HYAL2	0.78549524	0.244687275	0.163676206	0.692892801	0.629558443	0.572623665	0.929079469
IFT57	0.469752771	0.656686509	0.314597915	0.449965926	0.316344364	0.573906884	0.929079469
KLHL21	0.1827264	0.771184218	0.30285175	0.969657994	0.330124637	0.571752352	0.929079469
LOC100335635	0.642627344	0.026899254	0.856209345	0.974741036	0.950557117	0.572491955	0.929079469
LOC100847284	0.698621938	0.213809293	0.904377964	0.312870626	0.327817823	0.574483143	0.929079469
LOC100848504	0.980337686	0.848409871	0.098828624	0.310244194	0.542778026	0.57429327	0.929079469
LOC101905188	0.758515235	0.073084122	0.56649861	0.548480349	0.803157633	0.574185411	0.929079469
LOC104975684	0.181130579	0.463942861	0.584140646	0.520325144	0.542971403	0.574665587	0.929079469
LOC104976344	0.189222249	0.648282913	0.48215496	0.270939957	0.855988872	0.572544384	0.929079469
LOC107132098	0.236214786	0.460902105	0.497094281	0.742068537	0.345151245	0.57456866	0.929079469
LOC112442048	0.808601928	0.830560915	0.310776913	0.381442061	0.173847341	0.57427614	0.929079469
LOC112442307	0.996036828	0.234151844	0.22227458	0.668184342	0.399333536	0.574161705	0.929079469
LOC112445989	0.438775622	0.641169719	0.397598272	0.797185727	0.153136285	0.571668452	0.929079469

LOC112448540	0.990402536	0.272672532	0.540289308	0.183038014	0.519825417	0.574867431	0.929079469
LOC112449087	0.390902027	0.954857837	0.583687121	0.567379585	0.111585306	0.573614789	0.929079469
LOC616200	0.656081539	0.515441549	0.815111071	0.094237074	0.532462277	0.574148253	0.929079469
LOC616427	0.307367375	0.805051736	0.430987727	0.54860797	0.235921775	0.573752529	0.929079469
LOC783378	0.33715583	0.155140383	0.528447184	0.584996486	0.851298755	0.573224919	0.929079469
LOC783504	0.668290019	0.690328772	0.925041849	0.123618709	0.262224155	0.574181479	0.929079469
LYL1	0.203453488	0.429955795	0.830723635	0.319729806	0.597107443	0.574734964	0.929079469
MAPK1	0.89955856	0.243798152	0.161409769	0.786719737	0.491013958	0.571938261	0.929079469
MPV17	0.817867359	0.471442755	0.613232207	0.062245368	0.938402952	0.573866864	0.929079469
MYD88	0.850033866	0.703448096	0.100324454	0.269607818	0.844190942	0.571646481	0.929079469
NECAP1	0.364201417	0.260449661	0.44800761	0.450917253	0.721605384	0.574096011	0.929079469
NTF3	0.65914021	0.924524025	0.240225904	0.521217517	0.180448278	0.573267336	0.929079469
NUP37	0.787541827	0.169875479	0.360730432	0.730957489	0.391165558	0.573691589	0.929079469
NXPE2	0.680231066	0.778684774	0.716950032	0.202352357	0.178190315	0.572203598	0.929079469
PDE9A	0.361150982	0.368226187	0.293402878	0.452707173	0.78089153	0.57361856	0.929079469
PITPNA	0.767327866	0.415214998	0.142625824	0.531279756	0.574537072	0.574695907	0.929079469
PLA2G2C	0.881225507	0.48503675	0.064250105	0.693906353	0.722283032	0.573203288	0.929079469
PNLDC1	0.665598135	0.995762872	0.531347026	0.079650293	0.48974211	0.572827894	0.929079469
PRR13	0.498664485	0.437872816	0.346953191	0.355876516	0.510255617	0.573101101	0.929079469
PRR14L	0.768198184	0.223353398	0.306784011	0.684180721	0.378996383	0.571582455	0.929079469
PTMS	0.16886726	0.610518006	0.804455433	0.441027191	0.379524562	0.574855336	0.929079469
PTPRJ	0.352642415	0.510840819	0.511399357	0.22209585	0.672463421	0.573134508	0.929079469
RHOBTB3	0.295319362	0.95975863	0.113265377	0.458772847	0.930844118	0.572438026	0.929079469
RUNDC1	0.343311448	0.275920596	0.337971968	0.697100059	0.612905168	0.571999684	0.929079469
SEPT9	0.444042264	0.786382442	0.83633087	0.239881197	0.195315449	0.572056486	0.929079469
SH2D3C	0.209859828	0.616848425	0.726931923	0.536728821	0.273822806	0.574131041	0.929079469
SHLD1	0.455779024	0.823451211	0.97829698	0.218377521	0.170866221	0.572304988	0.929079469
SKIDA1	0.561335802	0.906076386	0.588578074	0.086559845	0.527416918	0.571830774	0.929079469
SLC17A5	0.42662932	0.37571127	0.161292231	0.989870452	0.539207733	0.573697386	0.929079469
SLC20A2	0.665383126	0.243286986	0.572337605	0.157401649	0.94249896	0.572931233	0.929079469
SLC35A4	0.32207463	0.542154966	0.739502152	0.341971082	0.312002694	0.573391872	0.929079469
SNX7	0.51888515	0.199687656	0.192011502	0.796040656	0.863294863	0.571911793	0.929079469
SSX2IP	0.151442034	0.53637844	0.297429813	0.709359279	0.803675178	0.57333927	0.929079469
TEX2	0.786947487	0.362426765	0.069994146	0.893086957	0.768401971	0.572297366	0.929079469
TRIM68	0.661437313	0.502696638	0.884392179	0.089513249	0.52244661	0.573036227	0.929079469
ZC3H13	0.52471443	0.532699829	0.494105151	0.127808415	0.783703441	0.574180107	0.929079469
ATP2C1	0.900697727	0.224698338	0.115600164	0.646156625	0.920033886	0.57522435	0.929108806
DNAAF3	0.684623311	0.549219697	0.786594919	0.13233967	0.355780415	0.575465983	0.929108806
ERC1	0.342694188	0.88258077	0.794915647	0.066396106	0.872146004	0.575418297	0.929108806
LOC100847841	0.593558309	0.047874711	0.582548813	0.895938022	0.936930608	0.575049726	0.929108806

LOC101908111	0.851388754	0.072254808	0.729896075	0.746628226	0.414375036	0.574990174	0.929108806
LOC104974050	0.778967351	0.507788617	0.731827932	0.377776762	0.12739688	0.575548116	0.929108806
LOC104975607	0.351946191	0.141099362	0.796208863	0.506895917	0.693569334	0.575116471	0.929108806
LOC112448378	0.753971551	0.727626071	0.207505076	0.432704962	0.282598082	0.57539109	0.929108806
NT5C2	0.846476564	0.048687156	0.603833476	0.984597109	0.567663096	0.575231817	0.929108806
QTRT2	0.396336904	0.608422972	0.691565928	0.251111161	0.332599018	0.575497046	0.929108806
ST6GALNAC6	0.203660246	0.527477924	0.416603303	0.394836898	0.787912199	0.575424881	0.929108806
ANGEL1	0.581743575	0.378432824	0.259060742	0.596193697	0.410397347	0.575864096	0.929436043
CENPK	0.9707026	0.18578242	0.20836103	0.996403822	0.372709144	0.575863566	0.929436043
ITGB1	0.553925671	0.191415724	0.456912697	0.462115307	0.623549519	0.575939768	0.929466765
RNF180	0.197957478	0.41241527	0.902041037	0.453309379	0.418346206	0.576019964	0.929504782
CAV1	0.980861051	0.388580505	0.308897774	0.14488099	0.819699891	0.576245636	0.929507595
CLNS1A	0.581383852	0.137946295	0.50791565	0.781157482	0.439069759	0.576097215	0.929507595
PSMD9	0.456613097	0.425132781	0.417886031	0.22944226	0.750944102	0.576175301	0.929507595
ZNF548	0.356830024	0.234732593	0.789426725	0.474951633	0.445224914	0.576248265	0.929507595
DEDD	0.501814549	0.698746604	0.317652227	0.213712552	0.58787384	0.576405942	0.929559384
RNF43	0.188529263	0.941514575	0.659082373	0.401836914	0.297737807	0.576450674	0.929559384
TMEM218	0.675571043	0.123392034	0.864185764	0.374596964	0.51866506	0.576444954	0.929559384
ZNF213	0.921457006	0.456333489	0.668119128	0.05147472	0.968166786	0.576506941	0.929559384
ZNF106	0.560646178	0.636215491	0.347588345	0.765556504	0.147561811	0.576575642	0.929578825
HIBADH	0.927519826	0.516370733	0.439240435	0.112409543	0.592805246	0.57675084	0.929769945
FLAD1	0.462636665	0.339324977	0.349149108	0.460938542	0.555225951	0.576874496	0.92979326
LOC101905014	0.10564991	0.770607762	0.499921388	0.601297619	0.573353072	0.576935273	0.92979326
LOC107131710	0.584978698	0.981750035	0.351001964	0.839172436	0.082928157	0.576885268	0.92979326
MRPL39	0.829644227	0.43051147	0.580491199	0.199598033	0.339182093	0.577000209	0.929806601
RIMBP2	0.694672712	0.39209319	0.284780289	0.723812911	0.250105396	0.577074886	0.929835636
LOC100299201	0.716804953	0.576567873	0.513514483	0.144827696	0.4570439	0.577157329	0.929877177
ANAPC13	0.49533891	0.411830484	0.710074569	0.158806358	0.61427462	0.578291311	0.929884184
CCDC194	0.47589094	0.662543788	0.16470621	0.616241301	0.439560436	0.577419507	0.929884184
CCDC34	0.391043476	0.285769768	0.963795296	0.759602173	0.172563033	0.578114938	0.929884184
CCL14	0.92081235	0.102527939	0.820724511	0.26207731	0.694685213	0.577966867	0.929884184
CDK20	0.787857584	0.668098296	0.947497785	0.142405937	0.199062609	0.578393376	0.929884184
FGF9	0.547959754	0.350787788	0.107550097	0.948696407	0.720772856	0.578369073	0.929884184
GALNT2	0.852396171	0.918002312	0.461381523	0.072046038	0.542309043	0.577955342	0.929884184
HPF1	0.593871235	0.129013894	0.631524153	0.322002481	0.905360645	0.577954072	0.929884184
LOC100847118	0.624652012	0.047877055	0.80061809	0.736114138	0.801511638	0.578242498	0.929884184
LOC101904355	0.855732102	0.546302366	0.993091662	0.054792718	0.555112278	0.578161268	0.929884184
LOC112446406	0.827195316	0.358129204	0.615230392	0.17145613	0.450724109	0.577663984	0.929884184
LOC112449596	0.725142295	0.233698439	0.402433105	0.295836855	0.698178736	0.577682835	0.929884184
LPCAT4	0.530356684	0.433279742	0.248788821	0.937878983	0.263386978	0.578181112	0.929884184

PDK2	0.965285347	0.574047372	0.605070736	0.043097898	0.977714521	0.578256682	0.929884184
PRDX4	0.997133025	0.347636061	0.074244895	0.59680901	0.920132962	0.578324836	0.929884184
RABEPK	0.841069682	0.363011679	0.145129014	0.847730404	0.375394597	0.577888496	0.929884184
REXO1	0.691572728	0.065926223	0.778172331	0.462268293	0.862339789	0.57846491	0.929884184
RNF40	0.341339881	0.207284791	0.443777842	0.83712826	0.536202032	0.577793884	0.929884184
SLC40A1	0.603917303	0.507145969	0.371579685	0.524907006	0.235341151	0.577304083	0.929884184
SYPL2	0.233005618	0.508182234	0.314014616	0.455147468	0.835630203	0.578445998	0.929884184
TAPBPL	0.493509391	0.382451835	0.130509724	0.935203315	0.613443453	0.578309278	0.929884184
ZNF511	0.312975618	0.619430995	0.789425638	0.491024105	0.18793894	0.57819265	0.929884184
ZRANB1	0.863158875	0.225666452	0.370776792	0.370953093	0.525484789	0.577574774	0.929884184
B9D2	0.51379717	0.655126953	0.520833848	0.482385582	0.168217125	0.579597059	0.929913822
C8H9orf3	0.40730051	0.941652861	0.164395949	0.827064954	0.272338797	0.579269017	0.929913822
CD14	0.477031633	0.398233036	0.28598026	0.287269046	0.910037412	0.579279092	0.929913822
COG4	0.83363791	0.458430092	0.474337475	0.186824148	0.419184793	0.579193364	0.929913822
FAM3A	0.424861233	0.228567787	0.333552721	0.601734542	0.727319891	0.578916847	0.929913822
GPER1	0.716435599	0.638322806	0.512773551	0.064012357	0.945102466	0.579063612	0.929913822
HELZ	0.981597982	0.661542217	0.544350511	0.060440922	0.665333827	0.57944512	0.929913822
ISCU	0.570701462	0.763046991	0.323744688	0.174314361	0.576636708	0.57884604	0.929913822
LAYN	0.060855139	0.443314623	0.888447874	0.702028642	0.84491889	0.579476627	0.929913822
LOC112448520	0.073945431	0.806325975	0.379368931	0.982698754	0.637124206	0.578725489	0.929913822
LOC782987	0.864261751	0.750195604	0.28536715	0.972744542	0.078620282	0.578559882	0.929913822
LONRF1	0.077482113	0.599662229	0.884561325	0.46624027	0.74246991	0.579616628	0.929913822
PRDX6	0.993211096	0.409177123	0.225555478	0.343717647	0.451542764	0.579608712	0.929913822
RBP2	0.280648454	0.298523203	0.878402188	0.597708516	0.322690129	0.579163341	0.929913822
RGS3	0.654816422	0.233184976	0.247394979	0.515705351	0.728846022	0.579225454	0.929913822
RNF181	0.375392506	0.296182692	0.643869166	0.539462493	0.367753987	0.579275945	0.929913822
SYMPK	0.475000576	0.20897171	0.867483727	0.166908293	0.989509267	0.579534494	0.929913822
TRIR	0.422795279	0.271244138	0.312705542	0.562371354	0.704917158	0.579466012	0.929913822
TTLL1	0.07852335	0.906456598	0.765085975	0.637778252	0.409362719	0.57948687	0.929913822
UBA5	0.744718822	0.175234449	0.47243786	0.616695882	0.374090301	0.579562733	0.929913822
IST1	0.249024886	0.302704162	0.393889395	0.755300483	0.634970187	0.579787963	0.930097778
LOC100336602	0.464845997	0.25420718	0.241715335	0.939888556	0.531317218	0.580110684	0.930524528
CLNK	0.45374385	0.185381524	0.879299843	0.294461548	0.655293609	0.580221126	0.930610722
C23H6orf106	0.404220596	0.775969894	0.943526349	0.220047589	0.219601054	0.580618383	0.930883967
C7H1orf35	0.068925456	0.814423103	0.929075259	0.274213027	0.99998938	0.580616473	0.930883967
CDC14A	0.445531362	0.550479668	0.247013019	0.259073256	0.910725493	0.580520637	0.930883967
VPS28	0.557111887	0.379113757	0.484777892	0.158966846	0.878373095	0.580561216	0.930883967
PLEKHD1	0.618426746	0.385036868	0.347835895	0.42463294	0.407308261	0.580945946	0.931318153
ACTR10	0.850164888	0.075275231	0.676611879	0.411192853	0.806519977	0.581416555	0.931452709
CFP	0.617199921	0.538840137	0.688133762	0.109381447	0.57422178	0.581607028	0.931452709

CIART	0.581568986	0.756066747	0.178839043	0.301121074	0.609404924	0.582361488	0.931452709
DDO	0.762363642	0.584690409	0.091063103	0.465304927	0.763709335	0.582283534	0.931452709
DSN1	0.866350783	0.952489742	0.342213627	0.776327296	0.065776896	0.582226914	0.931452709
GAS1	0.822432646	0.321816216	0.377579792	0.34307715	0.421936101	0.582846132	0.931452709
GULP1	0.152939498	0.699785859	0.417582119	0.329646404	0.978417819	0.582150876	0.931452709
IFNAR2	0.295721835	0.671429044	0.12593749	0.662380192	0.871086573	0.582333065	0.931452709
ISOC2	0.690426791	0.412883948	0.802252845	0.107586511	0.584366173	0.581659648	0.931452709
KLF4	0.551608691	0.434931716	0.100710081	0.859560005	0.694482043	0.582268344	0.931452709
LCLAT1	0.100678374	0.350566283	0.508974362	0.848134896	0.949423065	0.582833382	0.931452709
LOC100140372	0.822203834	0.246670118	0.325355119	0.913397389	0.238970811	0.581998488	0.931452709
LOC104968518	0.930770769	0.487540952	0.11326602	0.402970099	0.697521736	0.582590796	0.931452709
LOC104968964	0.177017994	0.334241423	0.868324829	0.512983579	0.547092086	0.582206448	0.931452709
LOC104974883	0.703924448	0.868249628	0.868969523	0.028434111	0.956065524	0.582465008	0.931452709
LOC107131424	0.511913053	0.70045965	0.552930717	0.893452176	0.081137176	0.581588825	0.931452709
LOC112447011	0.23322587	0.877861307	0.128852695	0.844076844	0.645473318	0.581595604	0.931452709
MEF2C	0.282636335	0.380398804	0.215995865	0.646396966	0.955965987	0.581282004	0.931452709
NBAS	0.420377006	0.586515048	0.672177633	0.485634056	0.179419974	0.582500562	0.931452709
NPM3	0.882781585	0.808882611	0.786356217	0.728998782	0.035199685	0.582072361	0.931452709
PGAP2	0.364851132	0.559770711	0.201347729	0.669089239	0.524020039	0.582197844	0.931452709
RAB11A	0.951723565	0.267039175	0.171384481	0.485028155	0.684207859	0.582692785	0.931452709
RNF19A	0.624652973	0.341609586	0.288137278	0.350356815	0.669569151	0.582273692	0.931452709
RRNAD1	0.34321649	0.84367259	0.609989176	0.714063504	0.114689086	0.58283168	0.931452709
SNW1	0.559936129	0.532815211	0.926107039	0.055368083	0.938586766	0.581396295	0.931452709
SPG7	0.300133151	0.825714526	0.797760191	0.218513475	0.332408442	0.581421449	0.931452709
TLE2	0.996345273	0.616879403	0.11013993	0.40336463	0.529502063	0.582741086	0.931452709
USB1	0.032552279	0.939599149	0.952518763	0.552698643	0.897366695	0.582622903	0.931452709
UTP20	0.892873152	0.333192266	0.301405498	0.421854921	0.381992921	0.582622177	0.931452709
ZCCHC14	0.744690819	0.3167684	0.161395574	0.37907551	0.994136579	0.581249369	0.931452709
ZNF524	0.337969719	0.36376408	0.988179724	0.390857294	0.302210447	0.581285092	0.931452709
ZNF852	0.386842444	0.678182525	0.228270459	0.399463339	0.603183744	0.582355304	0.931452709
ARGLU1	0.762816935	0.258630161	0.159066856	0.544030133	0.850899755	0.583661749	0.931545345
ARIH2	0.30928799	0.610793132	0.275263066	0.784180877	0.355971635	0.583508108	0.931545345
ASF1B	0.788660027	0.956475879	0.091254793	0.22882843	0.923682232	0.583961915	0.931545345
C11H9orf16	0.514783256	0.53987259	0.323507102	0.276529247	0.585986177	0.584219321	0.931545345
CCDC166	0.218996554	0.523963648	0.964279929	0.177836902	0.743110884	0.584929759	0.931545345
CUTA	0.35344334	0.235620943	0.982680215	0.262438459	0.678164723	0.584165959	0.931545345
DNAJB5	0.742043388	0.616021225	0.431022707	0.897414112	0.082546113	0.584571008	0.931545345
EFEMP1	0.185316397	0.499161726	0.284597862	0.822041726	0.670075247	0.583314776	0.931545345
FNIP2	0.80749987	0.146264356	0.28488956	0.79480078	0.543253337	0.583678571	0.931545345
GLIPR2	0.470700083	0.89229472	0.35866306	0.151717529	0.639044292	0.584701247	0.931545345

HTT	0.461252146	0.852244597	0.552748355	0.289399282	0.231094509	0.583722822	0.931545345
IFI35	0.521268835	0.340853	0.771874757	0.655293619	0.161637089	0.583649294	0.931545345
IMPAD1	0.505707245	0.456002517	0.167179291	0.487293192	0.775095005	0.584114819	0.931545345
LDB1	0.487173092	0.852988237	0.191483894	0.670546484	0.273780918	0.584739107	0.931545345
LOC101907174	0.203115777	0.878723558	0.441303037	0.310553317	0.595861484	0.584302637	0.931545345
LOC104971510	0.367572423	0.355439131	0.197604414	0.614277013	0.918307236	0.584142935	0.931545345
LOC104973073	0.026100988	0.837432633	0.88251922	0.872198978	0.864712908	0.5839469	0.931545345
LOC112441834	0.591585435	0.44103026	0.405435038	0.486733481	0.281135804	0.582961882	0.931545345
LOC615258	0.705184336	0.100443665	0.332515046	0.633996223	0.978418043	0.584764583	0.931545345
MIER2	0.367656324	0.167248622	0.6107882	0.763251284	0.509305848	0.584627689	0.931545345
NAGPA	0.809340638	0.105603652	0.738278142	0.332626245	0.695119013	0.5844956	0.931545345
NAPG	0.192473533	0.357463754	0.39420534	0.539260435	0.999551711	0.584890541	0.931545345
NOTCH4	0.19688432	0.92422736	0.807750289	0.462885825	0.214278069	0.584349284	0.931545345
NSMAF	0.456636388	0.377417943	0.875234154	0.599056419	0.160290063	0.583085719	0.931545345
PARP16	0.651442566	0.386885488	0.55561656	0.23842124	0.438004166	0.584947583	0.931545345
PPP1R35	0.349108001	0.107391368	0.923382291	0.441604601	0.956350379	0.584904842	0.931545345
PRKCH	0.918949077	0.401058111	0.277079219	0.683492492	0.207855605	0.583401475	0.931545345
PTDSS2	0.362337628	0.499816634	0.51101566	0.241138864	0.654112562	0.584599352	0.931545345
RIT1	0.226045919	0.701029035	0.747735373	0.209289121	0.584677175	0.583287604	0.931545345
RMC1	0.346464053	0.854325528	0.292165117	0.399286462	0.419623412	0.583158375	0.931545345
RNPC3	0.780991801	0.568585712	0.466165233	0.42404006	0.166169588	0.584448549	0.931545345
SKAP1	0.940952336	0.368467538	0.657329825	0.593879101	0.107239559	0.583493123	0.931545345
SLC35A1	0.311155907	0.813012522	0.608313018	0.457497907	0.207187646	0.584455091	0.931545345
STAMBP	0.927349654	0.404938696	0.192199796	0.450260198	0.447516306	0.583875215	0.931545345
TMEM132B	0.638950008	0.910189141	0.086837538	0.48262254	0.596503638	0.583816479	0.931545345
TOPORS	0.395402041	0.508056755	0.967021328	0.114066456	0.658257782	0.584448733	0.931545345
HOXB6	0.633377285	0.344452621	0.135549836	0.890833883	0.555546924	0.58510475	0.931705226
ESF1	0.670461179	0.583147859	0.59714666	0.088162003	0.712094258	0.585392631	0.931727763
MPDU1	0.557285101	0.423898189	0.317298275	0.329618007	0.593121535	0.585351967	0.931727763
NCKAP5	0.866373848	0.712889324	0.373384244	0.729035111	0.087175678	0.585381428	0.931727763
RNF112	0.739116101	0.141084421	0.350713382	0.493335336	0.812248659	0.585358209	0.931727763
TINAGL1	0.195480101	0.495578381	0.190829969	0.995075019	0.79681526	0.585402777	0.931727763
ADAT1	0.201893236	0.762955318	0.339591434	0.702922599	0.399123137	0.585633994	0.93200538
ZNF286A	0.087778561	0.66867996	0.97473811	0.597669185	0.429398345	0.585734136	0.932074363
CHMP5	0.968965777	0.433564043	0.319025997	0.573364719	0.191162587	0.585826125	0.932130361
ATG4C	0.683347232	0.248129677	0.344774707	0.569558672	0.442226446	0.586281014	0.932552762
CDC42BPB	0.10979498	0.395935575	0.788785518	0.715010921	0.600969521	0.586411767	0.932552762
COP1	0.57755336	0.829846246	0.133652329	0.48477641	0.474652374	0.586479914	0.932552762
LOC100294994	0.605451929	0.858279645	0.567939484	0.448086597	0.111470538	0.586501911	0.932552762
LOC112442091	0.476110832	0.221257538	0.894347042	0.425181991	0.36808195	0.586546195	0.932552762

PRND	0.144159843	0.863995665	0.209833086	0.605323611	0.931638126	0.586471544	0.932552762
TMEM249	0.624896552	0.323589327	0.806829636	0.19693704	0.458530893	0.586389004	0.932552762
ZNF529	0.258606656	0.229044864	0.945287382	0.415197063	0.633581092	0.586344469	0.932552762
CNPY3	0.891067231	0.891759227	0.613943812	0.030533297	0.991184638	0.58680715	0.93260625
IL4R	0.967972881	0.835405378	0.835947837	0.055867671	0.390778835	0.586724961	0.93260625
LOC104975782	0.186618415	0.696954928	0.903046562	0.994147372	0.126441812	0.58680529	0.93260625
LOC107133459	0.479963868	0.757549166	0.123430465	0.376515547	0.873152414	0.586672835	0.93260625
C18H19orf47	0.58402043	0.262648481	0.929840299	0.763301112	0.135658257	0.586869086	0.932614367
LIPT2	0.262784068	0.449940641	0.529874015	0.501090986	0.470999796	0.587097577	0.932796818
MED1	0.571369483	0.498589394	0.774083669	0.06954095	0.963951117	0.587043635	0.932796818
ASAP3	0.865952688	0.045603253	0.73238438	0.806348492	0.634426994	0.587218056	0.932815346
C21H14orf28	0.316138988	0.47049445	0.810935774	0.645120886	0.190172553	0.587251374	0.932815346
MCRIP1	0.4216447	0.637755188	0.585583849	0.160493757	0.58563029	0.587279761	0.932815346
MLPH	0.321138387	0.552587549	0.143399892	0.722416392	0.805612247	0.587406992	0.93292714
DHX9	0.322389547	0.361154636	0.664697325	0.226477069	0.845771216	0.587594647	0.933062565
KIAA0141	0.95974835	0.257811804	0.19464804	0.809584238	0.380325415	0.587662828	0.933062565
TMEM41B	0.979066397	0.072717015	0.279831284	0.996754771	0.746776228	0.587660889	0.933062565
ABHD11	0.253508416	0.611093746	0.330510072	0.443164174	0.656506124	0.588540231	0.933359565
ADCK1	0.873869236	0.194238003	0.683438044	0.291094111	0.441149135	0.588544845	0.933359565
ANAPC1	0.48768628	0.815068091	0.657566102	0.159868402	0.356681041	0.588642341	0.933359565
C7H5orf24	0.312754148	0.070016291	0.868497376	0.868280095	0.901504416	0.588410471	0.933359565
HECTD4	0.268806361	0.549219468	0.478480695	0.842457502	0.250068543	0.588347172	0.933359565
LOC407171	0.60772364	0.471077092	0.870991981	0.354034603	0.168921778	0.58874399	0.933359565
MS4A13	0.949677835	0.067112809	0.385424467	0.915498492	0.660873157	0.588096481	0.933359565
PARVG	0.626514663	0.514154745	0.580673511	0.116375506	0.683399319	0.588273904	0.933359565
RBM23	0.777889717	0.626737276	0.207856072	0.865158223	0.169896399	0.588521274	0.933359565
SH3GL1	0.185101489	0.593176395	0.822780175	0.748794084	0.220150189	0.588482371	0.933359565
SYNE3	0.764399439	0.541775374	0.765511305	0.643586464	0.07283955	0.588082448	0.933359565
THOC3	0.914188562	0.367605294	0.049739655	0.976987354	0.913080874	0.588732745	0.933359565
TMEM47	0.056266774	0.63638422	0.680446304	0.989270681	0.61673844	0.588134379	0.933359565
TNFRSF17	0.191522327	0.379908483	0.348851922	0.996471763	0.587814126	0.588162616	0.933359565
TRIP11	0.593210676	0.733526902	0.496194731	0.087307695	0.788211606	0.588040885	0.933359565
ZNF384	0.620558101	0.470221269	0.587646762	0.565071607	0.153912735	0.588759869	0.933359565
RNF123	0.515903239	0.312808742	0.724457746	0.205500461	0.621022908	0.588850058	0.933412373
ACOT6	0.317827641	0.755620978	0.593179686	0.212270577	0.495252732	0.589574291	0.933639458
APMAP	0.346754827	0.722474589	0.9011745	0.584202848	0.113312717	0.589170393	0.933639458
CADM3	0.219048901	0.335259641	0.926239808	0.449772487	0.48931372	0.589497051	0.933639458
CD36	0.219223656	0.233033428	0.314112633	0.938592215	0.99548537	0.589800026	0.933639458
HTR2A	0.365179968	0.468988994	0.472933239	0.20083129	0.919688227	0.58936964	0.933639458
KIAA1328	0.457770137	0.565257174	0.28098616	0.372143385	0.552062227	0.589072142	0.933639458

LOC112446696	0.87439196	0.365917825	0.551474392	0.279232733	0.303849181	0.589503048	0.933639458
LOC508153	0.71659205	0.968734935	0.405229552	0.081697187	0.653449132	0.590110756	0.933639458
LOC524181	0.809208841	0.426125172	0.211063478	0.280026683	0.736270735	0.589954467	0.933639458
LRRC8E	0.861709346	0.542415455	0.681267342	0.083741611	0.562994976	0.590047769	0.933639458
NCBP1	0.203370595	0.883585786	0.406858595	0.295747009	0.694316781	0.590048727	0.933639458
NR1D2	0.264867496	0.336079031	0.787056328	0.434167969	0.493258606	0.589936781	0.933639458
OAF	0.050268514	0.636875549	0.636355406	0.91694617	0.803121115	0.589921961	0.933639458
PEF1	0.477099504	0.433494542	0.535872158	0.245699893	0.549897554	0.589548294	0.933639458
RAB34	0.168599573	0.776894632	0.302205483	0.498848261	0.760594319	0.590131138	0.933639458
RPS6KA2	0.233845776	0.954591354	0.596815636	0.2198603	0.512673809	0.590101005	0.933639458
SGMS2	0.27640763	0.536215211	0.372461373	0.519005591	0.522812546	0.589614495	0.933639458
SLCO4C1	0.402101511	0.520882367	0.963587135	0.422275498	0.175458505	0.589277976	0.933639458
SZRD1	0.370582782	0.740359874	0.771907038	0.115103754	0.615732851	0.590011356	0.933639458
ZNF512	0.076976205	0.848549218	0.615620535	0.648283765	0.57351047	0.589240586	0.933639458
ZFPL1	0.972422588	0.095271778	0.838607893	0.244909545	0.789583993	0.59019374	0.933648494
LOC100336644	0.488302534	0.726720759	0.215890522	0.810931821	0.24207017	0.590387802	0.933819564
LOC112448582	0.563019139	0.871581473	0.791055281	0.452014337	0.085721116	0.590415685	0.933819564
LOC112442384	0.394413921	0.600105188	0.695322411	0.864352896	0.1058236	0.590577526	0.933985524
B4GALT1	0.149131536	0.793489783	0.379739242	0.679362192	0.493451618	0.590713107	0.934109924
MRO	0.700978325	0.827610896	0.253856709	0.695069687	0.147242617	0.59082015	0.934189178
LOC101907893	0.63773715	0.247786422	0.685403041	0.433123347	0.321478741	0.590931108	0.934274606
C5H12orf56	0.892163574	0.168082751	0.572734669	0.185679554	0.948355916	0.59148079	0.93433355
CDKN1C	0.707027895	0.638635149	0.337785473	0.14864997	0.665912091	0.59114738	0.93433355
DYNLT3	0.750015365	0.305162567	0.447401521	0.698296739	0.211441453	0.591423678	0.93433355
FMNL2	0.986745065	0.377015633	0.895014505	0.094799398	0.478871793	0.591374506	0.93433355
IKBKB	0.62539038	0.312520336	0.14587865	0.703872393	0.753225853	0.591383971	0.93433355
ITPRIPL2	0.864921588	0.665587736	0.150217564	0.607452134	0.28767491	0.591328639	0.93433355
LOC104975004	0.624867576	0.785981631	0.255555652	0.210285387	0.572967574	0.591466667	0.93433355
PAFAH1B1	0.982764502	0.050804082	0.383486629	0.95594324	0.825838072	0.591377225	0.93433355
RAPGEF4	0.550615278	0.393644153	0.768286629	0.18070852	0.501947067	0.591236868	0.93433355
CYP8B1	0.630746765	0.225602989	0.78749112	0.377463236	0.358363282	0.591922918	0.934357161
FAM217B	0.676742999	0.953531804	0.299459339	0.471785513	0.166484542	0.592178955	0.934357161
FANCD2	0.673912651	0.639877896	0.432639039	0.124493576	0.653237077	0.592102297	0.934357161
GPR55	0.328974457	0.872982181	0.56286945	0.202864281	0.461970919	0.591811727	0.934357161
MEF2A	0.625892495	0.20635982	0.408720726	0.350101471	0.820330056	0.591963406	0.934357161
NOCT	0.262675637	0.883612427	0.911521842	0.148679346	0.482482031	0.592163341	0.934357161
NTN1	0.88829466	0.3938673	0.891419799	0.04865147	0.999416313	0.592007098	0.934357161
SARNP	0.912084587	0.045949746	0.579589608	0.984616411	0.633544022	0.591850798	0.934357161
SERGEF	0.748665183	0.414103754	0.193878337	0.380216551	0.662617171	0.591733049	0.934357161
VAMP7	0.954648939	0.333126218	0.208985273	0.643639277	0.3542688	0.591878094	0.934357161

VPS72	0.387974439	0.768066899	0.388032519	0.154712902	0.848265333	0.592140077	0.934357161
WDR1	0.764682447	0.373614591	0.151899849	0.727094311	0.480076259	0.591797309	0.934357161
PARD6A	0.241465678	0.445646131	0.996147868	0.165610758	0.855283392	0.592247314	0.934375185
SLC46A3	0.980890096	0.745577535	0.102263275	0.853678042	0.237921263	0.592333446	0.934421242
LOC107131649	0.912965951	0.214986208	0.601274884	0.459263868	0.280484697	0.59248999	0.934578356
SLC4A5	0.67192968	0.901126333	0.163550077	0.282199194	0.544378187	0.592627537	0.934705479
CCNT2	0.368479916	0.750931678	0.8707799	0.165224083	0.382306686	0.592713166	0.934750698
MAP2K1	0.969422225	0.578863949	0.417381981	0.160482191	0.405099911	0.59280468	0.934805189
LOC781379	0.988937984	0.03901187	0.731214164	0.540516589	0.999281737	0.592936942	0.934923919
EIF4B	0.290594746	0.635824542	0.701661326	0.333516523	0.35307026	0.593305971	0.934937412
EPHB4	0.643832097	0.090140949	0.739549119	0.745510905	0.477565497	0.593493096	0.934937412
INTS2	0.913247066	0.28927207	0.440538929	0.711048198	0.184692983	0.59352885	0.934937412
LAMTOR5	0.636082542	0.512550682	0.226010551	0.32718988	0.634204885	0.593609473	0.934937412
LOC112442215	0.608682316	0.250976323	0.75523629	0.539104033	0.245578249	0.593412467	0.934937412
LOC786987	0.506367166	0.444030085	0.430754256	0.332407074	0.474438707	0.593407591	0.934937412
MYL6B	0.884624017	0.930827197	0.04197794	0.565012279	0.782970765	0.593629141	0.934937412
PF4	0.459530546	0.100423807	0.779928713	0.442814312	0.957452331	0.593222506	0.934937412
RGP1	0.298986958	0.688688775	0.421603724	0.185824415	0.947732639	0.593591483	0.934937412
ULK1	0.854884992	0.195212747	0.279910724	0.762249794	0.428376895	0.593139159	0.934937412
XYLT1	0.209639005	0.952118234	0.939766927	0.53836826	0.150997519	0.593082543	0.934937412
ZSWIM8	0.304843268	0.554240122	0.873799908	0.812527647	0.127379385	0.593481958	0.934937412
ADK	0.369980865	0.257421146	0.692580327	0.48590208	0.479788739	0.594722618	0.935297416
AK1	0.529225153	0.126327318	0.931439999	0.727176229	0.338607222	0.594156189	0.935297416
AURKC	0.825145684	0.277724001	0.184580287	0.412076852	0.885818943	0.59551153	0.935297416
CAVIN1	0.853569877	0.235335063	0.313416208	0.812734619	0.301262563	0.595192629	0.935297416
COA1	0.429746648	0.270008958	0.891512131	0.243262098	0.612525396	0.595181593	0.935297416
DDAH1	0.444263289	0.631741753	0.249168607	0.228236544	0.964204718	0.594872907	0.935297416
DENND5B	0.949068959	0.532618469	0.089965011	0.392459886	0.865509579	0.595602184	0.935297416
DUS2	0.998294993	0.220667199	0.769138301	0.289775278	0.313378381	0.59482975	0.935297416
FYB2	0.280383743	0.976125059	0.664186385	0.395466882	0.214695317	0.595434612	0.935297416
GPX8	0.798779995	0.092261657	0.389877278	0.820695693	0.654262016	0.595358081	0.935297416
HMGXB4	0.486494043	0.497154139	0.360400819	0.905844859	0.194488541	0.594458088	0.935297416
HSF5	0.946947343	0.821892443	0.229083811	0.539149724	0.160257085	0.595067462	0.935297416
IGSF8	0.447237996	0.263690939	0.275293467	0.802191645	0.593850913	0.595840033	0.935297416
KLC4	0.638294975	0.961112031	0.361763319	0.071555016	0.975282931	0.596111393	0.935297416
KLHL41	0.093598741	0.865442249	0.807957133	0.492884179	0.4800017	0.596063603	0.935297416
LOC100336161	0.259837021	0.536684582	0.796912866	0.340075985	0.407413264	0.594968977	0.935297416
LOC100337507	0.308949079	0.769836406	0.238741073	0.800678646	0.338698824	0.594987942	0.935297416
LOC100847835	0.671604573	0.226509612	0.696426395	0.191605211	0.758241143	0.594900873	0.935297416
LOC101907883	0.118101037	0.657763238	0.810914526	0.958022247	0.255475255	0.595229261	0.935297416

LOC112442408	0.133766191	0.313591955	0.560976461	0.695514381	0.93664061	0.594114281	0.935297416
LOC112444967	0.901564893	0.201863258	0.12447509	0.904786915	0.747424492	0.593986723	0.935297416
LOC112447523	0.53264104	0.204364552	0.481456254	0.400356102	0.738253005	0.596137406	0.935297416
LOC404051	0.371702054	0.713820592	0.102892415	0.879339238	0.639471273	0.59438759	0.935297416
MXD4	0.564410264	0.302327922	0.30430103	0.598807521	0.497210679	0.595758854	0.935297416
PABPC5	0.035696096	0.891553102	0.658972953	0.963349529	0.76545969	0.595820477	0.935297416
PNRC2	0.668746518	0.6203464	0.356713117	0.597240277	0.174393491	0.595171626	0.935297416
PRDX5	0.324429771	0.267981244	0.683209511	0.465252116	0.557830963	0.595205573	0.935297416
PYCARD	0.471665381	0.644704988	0.450201646	0.354502558	0.317161473	0.594906137	0.935297416
RAB4B	0.491474329	0.160370653	0.321155326	0.62264563	0.982542098	0.596085527	0.935297416
RARA	0.988211983	0.979693615	0.347423531	0.151833844	0.301509216	0.594980939	0.935297416
RASD1	0.819338301	0.594745296	0.077748237	0.964469119	0.421645971	0.595094024	0.935297416
SFT2D1	0.160441034	0.289690425	0.82013587	0.614528779	0.661144326	0.596103874	0.935297416
SH3BP5L	0.614969618	0.169203465	0.766572741	0.201630205	0.962730719	0.5960603	0.935297416
SLC22A4	0.570271965	0.791395872	0.669124081	0.097978063	0.523312795	0.596058487	0.935297416
SRSF1	0.506686906	0.225327072	0.867923185	0.716731156	0.217019014	0.595169015	0.935297416
SRSF9	0.278032037	0.602660454	0.758909787	0.334889566	0.362083568	0.595249565	0.935297416
TMCO6	0.676239288	0.293993528	0.467879702	0.564428153	0.29238172	0.594381481	0.935297416
USP18	0.157947016	0.717241748	0.826837894	0.383304002	0.430146243	0.595558284	0.935297416
YBX2	0.56469443	0.383889292	0.544533264	0.486102172	0.267869509	0.594634021	0.935297416
ZBTB44	0.383104404	0.378401573	0.471885398	0.299815481	0.752845706	0.595517851	0.935297416
FGFR1	0.590565431	0.914153366	0.058704281	0.895430721	0.546013167	0.596201634	0.935308767
CCDC90B	0.928363217	0.03119679	0.930088648	0.970155826	0.59317382	0.596283983	0.935348541
APOOL	0.968775041	0.272754788	0.968935748	0.131165598	0.462102121	0.596495851	0.935582765
PRDM4	0.104803476	0.848915461	0.660431934	0.405748132	0.65108588	0.596547319	0.935582765
UGGT1	0.740355009	0.552165637	0.36069669	0.524234139	0.201473624	0.597189423	0.936500299
SOX11	0.379389635	0.581413246	0.430254607	0.651371674	0.252001336	0.597248872	0.936504036
PELO	0.992078188	0.04101341	0.563383218	0.993863107	0.684490573	0.59744712	0.936725393
CCNB1	0.729316844	0.235668464	0.162462577	0.57034397	0.983497957	0.59830444	0.936995339
DOK3	0.243145722	0.637239138	0.752734859	0.702940355	0.190687163	0.597932042	0.936995339
MFSD2B	0.576393954	0.543295335	0.450130629	0.710637597	0.156347563	0.59828286	0.936995339
MRPL42	0.762394268	0.173381138	0.65020481	0.584985937	0.311232418	0.598116308	0.936995339
MYO9A	0.30345616	0.488428116	0.857892755	0.338356892	0.363971455	0.598255372	0.936995339
NFIA	0.516647489	0.136630569	0.766464987	0.58386496	0.49535757	0.598118028	0.936995339
NKG7	0.257391498	0.806487119	0.67304153	0.242823182	0.461625377	0.598273918	0.936995339
PLCL1	0.194637742	0.218117915	0.687825404	0.60054418	0.892689625	0.598197107	0.936995339
RETN	0.524786199	0.423696698	0.660879117	0.13690105	0.776892524	0.597876519	0.936995339
SFT2D2	0.364160925	0.696267322	0.344188541	0.404441317	0.442748459	0.59785459	0.936995339
UNC119B	0.672562434	0.602895469	0.313251995	0.273525068	0.449707587	0.597817585	0.936995339
USP50	0.430988037	0.239641708	0.641400375	0.469464498	0.502669285	0.59792796	0.936995339

LOC112442227	0.535418314	0.28972647	0.394933932	0.701773966	0.364821492	0.598574515	0.937328852
CNOT3	0.564044469	0.258438649	0.327550219	0.359095394	0.916437413	0.598923409	0.937427951
CSF2RA	0.621229539	0.249941671	0.300359414	0.65890483	0.511123394	0.598842274	0.937427951
LOC519208	0.718974474	0.752459441	0.335932373	0.60595	0.142581833	0.598783488	0.937427951
MTMR6	0.739671013	0.745341631	0.079856137	0.57469045	0.620926028	0.59888518	0.937427951
PCID2	0.714606001	0.643354555	0.427907396	0.106978057	0.746358833	0.59885517	0.937427951
ATP6V1E2	0.096958361	0.784417966	0.632689644	0.396636631	0.82546127	0.599440043	0.937429335
C16H1orf116	0.305777856	0.326829384	0.228681644	0.867653231	0.793376379	0.599157807	0.937429335
CLHC1	0.694696464	0.88960619	0.191150598	0.652483345	0.204210739	0.599262178	0.937429335
LOC101905053	0.266905335	0.526280695	0.243910379	0.916989215	0.501414181	0.599418856	0.937429335
NOSTRIN	0.334774832	0.85903312	0.346256177	0.297427729	0.532042245	0.599473584	0.937429335
PSEN2	0.468869375	0.614681184	0.463457897	0.135518817	0.870868032	0.599552636	0.937429335
RALB	0.829982579	0.631456282	0.637704099	0.151926515	0.310388112	0.599510513	0.937429335
TAF1A	0.638973403	0.069397567	0.853252698	0.988345449	0.420336335	0.598990269	0.937429335
TMEM178A	0.472389966	0.969058199	0.954461807	0.091005884	0.396018857	0.599341967	0.937429335
TMEM238	0.260735178	0.312684173	0.607727594	0.321491561	0.989256937	0.599475987	0.937429335
TWISTNB	0.796367219	0.795508502	0.26087362	0.49175207	0.193941153	0.599525947	0.937429335
IRF3	0.39358971	0.723823274	0.169479401	0.33825957	0.968117246	0.600139108	0.938032277
LOC100847876	0.629129844	0.161124911	0.314707012	0.632328953	0.783599029	0.600083074	0.938032277
PLEK	0.479581964	0.172624063	0.242164651	0.804938873	0.979930676	0.600166894	0.938032277
THAP12	0.630072943	0.186074739	0.255256339	0.867294052	0.609123177	0.600118931	0.938032277
UBA1	0.582142712	0.229538917	0.511823139	0.823317182	0.280976073	0.600260687	0.938089529
ELP4	0.835208058	0.887095767	0.418771657	0.504083265	0.101220258	0.600381811	0.938100152
RERG	0.977458318	0.777747692	0.058862212	0.381494637	0.927296707	0.600367689	0.938100152
DRG2	0.758665089	0.546965991	0.224003956	0.654605216	0.260271938	0.600453931	0.938123521
AHCTF1	0.379885607	0.309969524	0.724480466	0.235846978	0.79326536	0.601968193	0.938438773
ART4	0.071930751	0.772929183	0.662021398	0.613144123	0.70676192	0.601840156	0.938438773
BPNT1	0.736471188	0.44525656	0.629325986	0.206147011	0.376139595	0.602471116	0.938438773
CHKA	0.25830005	0.885818276	0.21740068	0.784343429	0.409195	0.602021728	0.938438773
COL27A1	0.811650823	0.421337344	0.096263004	0.977970454	0.496934326	0.60243318	0.938438773
CTNNB1	0.710354073	0.651966981	0.069477294	0.507951781	0.974559914	0.601576431	0.938438773
DIABLO	0.933379449	0.076890451	0.806802898	0.364488055	0.753675042	0.601303188	0.938438773
DTNA	0.141730166	0.438152535	0.760205165	0.614157865	0.550800065	0.602078206	0.938438773
ECE1	0.526889657	0.185653996	0.920181941	0.326769408	0.543419345	0.602249533	0.938438773
ERMAP	0.84154651	0.717744067	0.084971243	0.484589316	0.639001275	0.601137034	0.938438773
FMNL1	0.522757507	0.351999178	0.8612733	0.139322214	0.725300483	0.602629739	0.938438773
GLTP	0.938703876	0.435167429	0.664723313	0.359908453	0.163651128	0.602367481	0.938438773
GSTM2	0.18088999	0.841593288	0.538855801	0.839363986	0.232253796	0.602352022	0.938438773
GTSF1	0.867457459	0.486787059	0.145655043	0.277365114	0.932025637	0.601225511	0.938438773
GXYLT1	0.313192411	0.435082336	0.320727925	0.679573196	0.53892076	0.602521729	0.938438773

HTR7	0.174457495	0.284199873	0.523680212	0.747940765	0.821909434	0.60197875	0.938438773
IPO8	0.062044819	0.276242252	0.970473724	0.988249476	0.970354342	0.601846583	0.938438773
KLC2	0.105231549	0.844706323	0.228038193	0.819430967	0.957453418	0.601268992	0.938438773
KLHL33	0.762943068	0.780912538	0.178537179	0.572008145	0.262399957	0.602031854	0.938438773
LOC112441868	0.696922637	0.31872168	0.794236504	0.179417962	0.504977805	0.602254049	0.938438773
LOC112443528	0.860655109	0.288642168	0.48633115	0.420180323	0.314629658	0.602106905	0.938438773
LOC112445968	0.831281481	0.492199655	0.949616486	0.260255179	0.158387588	0.602646319	0.938438773
LOC112446663	0.505621906	0.822332058	0.392014467	0.366649675	0.266900935	0.60184669	0.938438773
LOC509034	0.879998764	0.724579382	0.616318509	0.868979743	0.046830714	0.602357034	0.938438773
LOC510798	0.310021722	0.985706477	0.676385808	0.60143395	0.127606695	0.60077821	0.938438773
LONP2	0.958660902	0.097727796	0.550089965	0.516989845	0.60116119	0.602660507	0.938438773
LRRK1	0.566735645	0.098054356	0.953150382	0.392142732	0.765620388	0.601258753	0.938438773
MAP3K21	0.784294178	0.903527647	0.439660038	0.955511854	0.053594629	0.601899731	0.938438773
MBD6	0.87255257	0.152255059	0.230529958	0.824247688	0.630281755	0.601355986	0.938438773
MED14	0.854354629	0.385969969	0.953595571	0.275668638	0.183609041	0.601425168	0.938438773
MXI1	0.331288713	0.668577204	0.698239723	0.74166005	0.139097176	0.601895771	0.938438773
NSG1	0.127160287	0.494584637	0.710874111	0.676305778	0.529718	0.602652811	0.938438773
NUDCD2	0.714149806	0.171811562	0.676839352	0.28623147	0.668040787	0.600981054	0.938438773
PICK1	0.434783356	0.781892037	0.194285951	0.618924788	0.391931977	0.602714318	0.938438773
TIGD7	0.747309511	0.157395372	0.310559511	0.448804662	0.975244272	0.602309633	0.938438773
ZNF331	0.427388644	0.517432932	0.580238367	0.909623014	0.137241162	0.602678247	0.938438773
APBA3	0.304374919	0.297151369	0.304346677	0.721345613	0.809985007	0.603462112	0.938623516
CHMP3	0.836738267	0.145887219	0.221005119	0.648531623	0.918384719	0.603277824	0.938623516
CITED1	0.306472998	0.367155367	0.939745915	0.29460507	0.51604385	0.603374047	0.938623516
CRNKL1	0.987156599	0.14013172	0.824111385	0.249470182	0.564768895	0.603202774	0.938623516
CRY2	0.825138053	0.855331041	0.959656481	0.200749007	0.118132059	0.603202772	0.938623516
EIF1B	0.712417186	0.800737225	0.225302428	0.128764193	0.970140815	0.603123018	0.938623516
FYCO1	0.590540824	0.911677854	0.035064831	0.873542981	0.973452969	0.60309706	0.938623516
LOC788414	0.875623911	0.947496125	0.8243872	0.038025808	0.618255758	0.60341604	0.938623516
MAPK8	0.198711252	0.553731592	0.921676263	0.642043314	0.246713634	0.603229622	0.938623516
PHACTR2	0.981199024	0.658513986	0.328360234	0.089197534	0.848068101	0.603049435	0.938623516
ZMYM6	0.482115692	0.91700664	0.050067514	0.998703516	0.727509014	0.603452774	0.938623516
KMT2B	0.779149765	0.253941831	0.439799552	0.291685265	0.634637333	0.603764935	0.938797869
LOC100139325	0.822276345	0.43274252	0.491245826	0.21959377	0.420098315	0.603974645	0.938797869
LOC784522	0.759623632	0.517691561	0.111138475	0.731917738	0.503995957	0.603932494	0.938797869
MAPK3	0.628238222	0.452868969	0.442811364	0.372612831	0.343415902	0.603919283	0.938797869
SELPLG	0.288221382	0.212585129	0.965566099	0.327071216	0.833026736	0.603896148	0.938797869
TMC7	0.286938893	0.354729052	0.769257479	0.430420488	0.477845624	0.603714821	0.938797869
ZNF557	0.290213779	0.792058943	0.453291762	0.70096109	0.220539924	0.603757935	0.938797869
PKD1	0.792196977	0.351977294	0.215487417	0.301600581	0.890113557	0.604033072	0.938799767

ACOX2	0.47012079	0.223416075	0.810787603	0.354579852	0.53489376	0.604286842	0.938879038
AFAP1L1	0.528833231	0.177540717	0.516845087	0.937622974	0.354728825	0.604147327	0.938879038
PAOX	0.905349815	0.079592107	0.457531045	0.721578319	0.678713667	0.604226004	0.938879038
SNRNP35	0.855973633	0.472963544	0.495513021	0.990144905	0.081325877	0.604312917	0.938879038
C5H12orf4	0.913893928	0.240594238	0.41229441	0.395845788	0.450287881	0.60437313	0.938883703
BTD	0.539992726	0.369633438	0.174109994	0.926983167	0.502036235	0.604546004	0.939063367
CHSY1	0.689003479	0.925507776	0.323978277	0.122071309	0.641798734	0.604698692	0.939187223
MPRIP	0.34217491	0.866509791	0.247923094	0.306313141	0.718982013	0.604740198	0.939187223
BRF2	0.926376531	0.242478909	0.847409158	0.200484992	0.424656209	0.604943037	0.939190665
C7H19orf44	0.577772438	0.474527775	0.52100905	0.352446274	0.322861821	0.605526491	0.939190665
COQ8B	0.342727702	0.38707453	0.24783182	0.495080391	0.999166312	0.605634734	0.939190665
CTNNA3	0.164829654	0.944715981	0.625482937	0.479026627	0.348197999	0.605420809	0.939190665
DCAF8	0.950593236	0.619689991	0.039618985	0.834628444	0.835826439	0.605844654	0.939190665
FBXL20	0.343478537	0.275674442	0.307473133	0.904125038	0.617890181	0.605647618	0.939190665
GJC3	0.078958061	0.847353106	0.458398668	0.545822065	0.968704893	0.605065539	0.939190665
GPAT3	0.658084926	0.401872874	0.459142928	0.378017317	0.354458058	0.605715884	0.939190665
INPP4B	0.600349683	0.462811627	0.931257509	0.561800177	0.111526314	0.60501647	0.939190665
KIAA0408	0.609929827	0.825060667	0.338401249	0.482948575	0.19761045	0.605497377	0.939190665
KLKB1	0.336435786	0.461316948	0.560914398	0.341678498	0.547406658	0.60586445	0.939190665
LOC100847802	0.280412646	0.160803514	0.801816767	0.993859404	0.452249481	0.605480665	0.939190665
LOC785477	0.995010766	0.237914381	0.322915223	0.807447809	0.263907725	0.60594423	0.939190665
MRPL58	0.637501852	0.24516466	0.66115378	0.2963841	0.528777148	0.604806251	0.939190665
MRPS15	0.504131418	0.270749304	0.728155756	0.185476296	0.883393763	0.605887909	0.939190665
NEURL3	0.961743944	0.900243408	0.053346622	0.454668831	0.773782879	0.605466899	0.939190665
SFMBT2	0.674628834	0.853648816	0.035135739	0.962050633	0.834956288	0.60551821	0.939190665
SMIM7	0.901067466	0.104490553	0.751777334	0.901682057	0.254198519	0.605156772	0.939190665
TECPR2	0.866145534	0.410095916	0.784497532	0.714462927	0.081714552	0.60569429	0.939190665
TGIF2	0.428402455	0.67564274	0.541513821	0.14152599	0.733651345	0.605765034	0.939190665
ZNF200	0.603515386	0.196926442	0.36688791	0.462148653	0.805447864	0.605244291	0.939190665
RSBN1L	0.07184281	0.50889663	0.608769765	0.83941809	0.87236324	0.606051152	0.93926768
PLCE1	0.092333	0.9070632	0.391708135	0.993822851	0.500102906	0.606133697	0.939306903
EIF2B3	0.800991346	0.289009813	0.598651227	0.497086731	0.236825958	0.606246119	0.939392415
LOC104972346	0.923238513	0.723697356	0.913421754	0.050806522	0.526817718	0.606491485	0.93968389
LOC101905708	0.227352032	0.3897211	0.29696986	0.655716059	0.947782603	0.606701498	0.93992054
MTSS1	0.216886834	0.960305251	0.961282647	0.092264445	0.8855612	0.606771952	0.939940957
ANKLE1	0.927943494	0.642484492	0.902108603	0.280887695	0.108407503	0.606991367	0.940103371
TCEANC	0.73874606	0.301997463	0.353003786	0.347945915	0.597538642	0.606955665	0.940103371
PCYOX1L	0.754029641	0.842308005	0.45889732	0.213100585	0.263820796	0.607096235	0.94017706
CALCRL	0.648306366	0.660677995	0.255664086	0.736752267	0.203294689	0.607284362	0.940202232
NKAPL	0.100431051	0.250796378	0.928532065	0.890955934	0.786961212	0.607243198	0.940202232

PRPF39	0.778229874	0.390618613	0.148572367	0.42702127	0.849942922	0.607172417	0.940202232
CYB5R3	0.330148513	0.792551099	0.164734184	0.581775601	0.654332883	0.607368462	0.940243734
ARAP3	0.580646128	0.242055945	0.516657783	0.79828569	0.284112284	0.608088994	0.940272291
BRPF3	0.314478595	0.657272901	0.771338271	0.147056498	0.703631974	0.608418223	0.940272291
CDKL4	0.416788481	0.545176811	0.296195541	0.978598321	0.249701731	0.60781003	0.940272291
CEP295NL	0.604049461	0.418295697	0.319323421	0.843513647	0.241945108	0.608051714	0.940272291
EXD2	0.421549619	0.07902558	0.891722021	0.579998086	0.956840632	0.608283706	0.940272291
GM2A	0.28517081	0.602691844	0.421021464	0.836770998	0.271774751	0.607928051	0.940272291
GRIP2	0.190872216	0.71111976	0.561585187	0.40682003	0.530334921	0.607808252	0.940272291
HSD3B7	0.562727756	0.497951856	0.138725864	0.636754804	0.665012724	0.60798419	0.940272291
KIF21A	0.8874783	0.496895458	0.214005341	0.478146762	0.36549988	0.608366271	0.940272291
LOC104974749	0.891427887	0.081464003	0.558557792	0.707024467	0.574473862	0.608154659	0.940272291
LOC574091	0.07607369	0.764750262	0.749356076	0.44364864	0.851268359	0.608030315	0.940272291
LOC787812	0.066028832	0.291877296	0.990269685	0.882475393	0.976982713	0.607908532	0.940272291
MED28	0.484699583	0.242220496	0.167627449	0.981520615	0.853820524	0.608365752	0.940272291
PANX2	0.380465144	0.848018301	0.110521509	0.505690046	0.910985506	0.607587747	0.940272291
SPECC1L	0.968462694	0.060280957	0.565269437	0.570861217	0.87462585	0.608175591	0.940272291
SSPN	0.078441566	0.5890118	0.531187314	0.789821132	0.850790056	0.608354258	0.940272291
THAP11	0.991059273	0.763479298	0.077513649	0.736744516	0.380525346	0.607773177	0.940272291
WDR4	0.375797209	0.999366434	0.617979337	0.666708643	0.106257359	0.607760503	0.940272291
AES	0.931745053	0.141512451	0.477722435	0.288781016	0.908303389	0.608712698	0.940348556
ANO3	0.970837448	0.7267315	0.118744761	0.199491429	0.987922518	0.608584716	0.940348556
ARNT	0.326768768	0.575260855	0.568687273	0.192681307	0.803711185	0.609095257	0.940348556
ATRAID	0.596874376	0.739627624	0.159521914	0.537761908	0.437142844	0.609100356	0.940348556
CEP135	0.654956493	0.136578889	0.806721646	0.454309228	0.504529399	0.608933205	0.940348556
CYBRD1	0.18457147	0.919274039	0.938830992	0.147878152	0.701898452	0.608851217	0.940348556
DNAAF5	0.374286433	0.581824636	0.265089393	0.38838346	0.738998688	0.609263501	0.940348556
FXYD6	0.213416518	0.7618353	0.4073189	0.262157666	0.955497213	0.609498367	0.940348556
GAS7	0.636949615	0.86400084	0.585286032	0.730582193	0.070236934	0.608782365	0.940348556
LOC112448474	0.329909793	0.606708646	0.352780092	0.462598851	0.507383887	0.609320897	0.940348556
LOC783838	0.060480349	0.856408706	0.80331086	0.464357044	0.854487085	0.608564255	0.940348556
MLKL	0.613015575	0.476162111	0.100267564	0.729707262	0.776978613	0.609556269	0.940348556
RPUSD3	0.72320711	0.901579103	0.097018074	0.840982068	0.311870313	0.609527245	0.940348556
S100A8	0.599324812	0.733235178	0.605278294	0.131356283	0.473943426	0.609148963	0.940348556
SEMA5A	0.083813935	0.413704197	0.603529902	0.824144819	0.962038571	0.609536691	0.940348556
TMEM135	0.989268543	0.337243318	0.228532763	0.885298095	0.244730937	0.608675618	0.940348556
VAMP3	0.998774283	0.167732038	0.235238832	0.649502964	0.647134408	0.609207377	0.940348556
ZNF112	0.303711777	0.379783148	0.711324766	0.500979169	0.402612812	0.609029681	0.940348556
ZNF639	0.738176735	0.127533163	0.580993941	0.449380883	0.674783286	0.60946167	0.940348556
LOC112447027	0.928763042	0.478142807	0.524609165	0.380405089	0.187402218	0.60972473	0.940441569

LOC112449358	0.693396432	0.897346882	0.055796137	0.87308492	0.547937767	0.609731174	0.940441569
COG8	0.91812262	0.219881899	0.531131953	0.919555181	0.168775581	0.610111392	0.940462409
DCP2	0.47522103	0.647720489	0.215496541	0.491776592	0.51022908	0.610145833	0.940462409
DIO3	0.221598098	0.606818085	0.650966759	0.490405103	0.387579596	0.610075104	0.940462409
LOC112443004	0.421579382	0.878370133	0.482096523	0.275592513	0.338182785	0.610079863	0.940462409
LOC782114	0.659440063	0.292076898	0.332070625	0.59896062	0.434221937	0.610036363	0.940462409
PAXX	0.207305915	0.976136041	0.256204855	0.696162084	0.460943817	0.610060956	0.940462409
ZNF18	0.534460864	0.862843778	0.302477679	0.129777206	0.918894525	0.61003298	0.940462409
C23H6orf226	0.966207292	0.383508776	0.127661016	0.659077407	0.536164757	0.610991516	0.941075146
HBS1L	0.837136726	0.33929342	0.475633145	0.657038207	0.188135505	0.610796812	0.941075146
HDAC4	0.987417373	0.062113574	0.847141623	0.360268265	0.893050047	0.61099444	0.941075146
ICE1	0.587384983	0.079998393	0.95077386	0.412173066	0.906760515	0.61077354	0.941075146
LOC112442849	0.452601389	0.363478234	0.701814419	0.903730213	0.160098191	0.61085814	0.941075146
PDE4A	0.204260787	0.730711096	0.488046311	0.658880276	0.348308394	0.611002113	0.941075146
PSMB1	0.358691512	0.160042354	0.777747632	0.870638993	0.429992556	0.610971373	0.941075146
SPTLC2	0.423903063	0.720547511	0.72277637	0.185280179	0.408195373	0.610763033	0.941075146
CRIPT	0.997505826	0.512999596	0.792312279	0.963067656	0.043004239	0.611871598	0.941189523
ETAA1	0.226476674	0.478140857	0.295816948	0.884838086	0.591971442	0.611723303	0.941189523
GIN1	0.938658179	0.258430485	0.364092795	0.335571203	0.565840379	0.611622169	0.941189523
LOC100847819	0.298829451	0.845396542	0.258896435	0.862557449	0.296820815	0.611330232	0.941189523
LOC101902390	0.384464739	0.791331591	0.66887833	0.510163957	0.161374856	0.611427065	0.941189523
LOC101905114	0.666256787	0.722803342	0.190382951	0.355121389	0.514166398	0.61127543	0.941189523
LOC101907920	0.822291099	0.511545168	0.70836251	0.841211178	0.066777149	0.611242927	0.941189523
LOC104976574	0.710547142	0.719705382	0.078729939	0.662866425	0.627699256	0.611407925	0.941189523
LOC112442039	0.92285869	0.179544201	0.344109403	0.653468035	0.449607843	0.611406199	0.941189523
NATD1	0.082014374	0.934268728	0.488328662	0.451176131	0.994011915	0.61174342	0.941189523
NSRP1	0.107511504	0.876047836	0.46230934	0.399396367	0.965564456	0.611873347	0.941189523
PDCD5	0.632804103	0.143005009	0.867584957	0.238795935	0.895316188	0.611799241	0.941189523
RUNX1T1	0.530829262	0.188106837	0.916059182	0.459234109	0.399757778	0.611879289	0.941189523
VCPKMT	0.957061531	0.074092834	0.381897647	0.935964376	0.661204397	0.611494618	0.941189523
ADGRL2	0.674860555	0.933105735	0.300808438	0.098272813	0.90370085	0.612229388	0.941320873
GLIPR1	0.458584724	0.455480871	0.382216294	0.314293087	0.670016409	0.612106328	0.941320873
HMG20A	0.39268817	0.243976962	0.658675551	0.943550628	0.282566308	0.612256702	0.941320873
LOC101906513	0.651977313	0.287422406	0.328021978	0.358675658	0.763406005	0.612328504	0.941320873
LOC112446779	0.762228832	0.897946573	0.078567569	0.461176359	0.6784217	0.612253191	0.941320873
LYVE1	0.039174381	0.858914423	0.9275981	0.646243616	0.833606829	0.612128902	0.941320873
TM6SF2	0.467076397	0.200236228	0.945139741	0.217576808	0.875302524	0.612366196	0.941320873
AKAP17A	0.864671802	0.711701143	0.101721269	0.330291394	0.818080266	0.613290553	0.941447523
AOC2	0.571416727	0.444121304	0.32317496	0.258677038	0.79461666	0.612640557	0.941447523
C15H11orf71	0.290460393	0.472757942	0.479113318	0.326545623	0.788257641	0.61352526	0.941447523

C5H12orf66	0.372928076	0.583111155	0.3441954	0.773793957	0.292401782	0.613530109	0.941447523
CHURC1	0.680105523	0.123943142	0.531896836	0.627223981	0.602251787	0.613548144	0.941447523
CLEC4G	0.397499333	0.398828905	0.645491059	0.551546934	0.299794899	0.613365202	0.941447523
CPEB2	0.987768526	0.221317003	0.120480251	0.673939776	0.950522501	0.612803046	0.941447523
ELL	0.289868145	0.699798497	0.883467747	0.260044573	0.362975895	0.613306677	0.941447523
FBXL4	0.820799678	0.488939163	0.160045972	0.952167215	0.275967889	0.612865617	0.941447523
HPDL	0.649301556	0.547649592	0.512231869	0.732114415	0.126830469	0.613274577	0.941447523
LOC100297170	0.280546732	0.518420936	0.647379292	0.264532001	0.680329758	0.61364581	0.941447523
LOC100847861	0.261843195	0.297734222	0.247530518	0.925972285	0.947928477	0.613568549	0.941447523
LOC101904069	0.249545682	0.843588447	0.450479416	0.204990652	0.870026107	0.613276202	0.941447523
LOC104969378	0.667969186	0.489461672	0.303875625	0.470083716	0.361682678	0.613029846	0.941447523
LOC104972065	0.591675587	0.504295674	0.398711578	0.488687432	0.289918193	0.612607554	0.941447523
LOC112443502	0.913585299	0.457491608	0.483092682	0.106086875	0.789937051	0.613363789	0.941447523
MORN1	0.972304822	0.406274323	0.655331012	0.631329491	0.103451941	0.613211151	0.941447523
MTX3	0.98282668	0.528867663	0.168448932	0.490280487	0.394649552	0.613602748	0.941447523
N4BP1	0.696537613	0.587945313	0.153751017	0.288229392	0.930040949	0.612879692	0.941447523
PSMD11	0.937641151	0.31045558	0.362404204	0.823677511	0.194382239	0.613016023	0.941447523
SCN9A	0.884637801	0.178457119	0.274829437	0.397340967	0.982957889	0.61365329	0.941447523
CCDC184	0.385328874	0.847804004	0.844379741	0.566908997	0.108617859	0.614111146	0.941487618
LFNG	0.315175012	0.350839423	0.820727195	0.203829455	0.917870561	0.61403381	0.941487618
LOC101905813	0.73987397	0.132424739	0.372440905	0.807204396	0.577238227	0.614310488	0.941487618
LOC107131749	0.569526126	0.852991082	0.3099508	0.676623223	0.166743761	0.614141707	0.941487618
MTA1	0.194902185	0.143838495	0.64862506	0.956569057	0.977450239	0.614298665	0.941487618
MYEF2	0.428617148	0.304795076	0.276439793	0.669136764	0.703568352	0.614299873	0.941487618
NCK2	0.769851174	0.885985659	0.261207011	0.165716536	0.574608639	0.613875992	0.941487618
NFKBIB	0.088567234	0.893190882	0.848258388	0.899447074	0.281061851	0.613861043	0.941487618
RGS6	0.302310539	0.825175122	0.763259814	0.184196996	0.484544402	0.614204884	0.941487618
ZNF133	0.934861703	0.11484868	0.751055797	0.707858545	0.297585504	0.614122124	0.941487618
ZNF668	0.889758823	0.934311898	0.095390449	0.386758979	0.55382139	0.614111053	0.941487618
BFAR	0.999360807	0.602662775	0.730951447	0.235398815	0.164331607	0.614619659	0.941640677
DLG5	0.181214448	0.512540929	0.75298222	0.291654056	0.836716689	0.615043129	0.941640677
GEMIN7	0.363898972	0.731795805	0.586262187	0.501651265	0.217517489	0.614686254	0.941640677
LOC101905239	0.677222973	0.956844934	0.135775143	0.340829456	0.569239438	0.61507598	0.941640677
LOC107132606	0.727394647	0.224100862	0.377511368	0.282236409	0.982637484	0.615041997	0.941640677
LOC509941	0.523667609	0.776115501	0.189007349	0.228377736	0.973059468	0.615088698	0.941640677
NAPRT	0.724908499	0.267633286	0.72663104	0.269931505	0.447572776	0.614639401	0.941640677
OR51E1	0.143851176	0.7639899	0.747687244	0.306038811	0.678858234	0.6150989	0.941640677
RPTOR	0.828632899	0.613407034	0.481229707	0.281050476	0.248314527	0.615086855	0.941640677
SLC45A2	0.196449907	0.695734064	0.224651808	0.67510212	0.821994357	0.614724287	0.941640677
SLC6A14	0.225736606	0.197662559	0.834231386	0.585962203	0.782233228	0.614982998	0.941640677

TBX6	0.561605692	0.975853071	0.270488009	0.426519698	0.269660165	0.614848599	0.941640677
RN7SL1	0.605670108	0.498994945	0.207138562	0.941190116	0.289980135	0.61526176	0.941802139
DGUOK	0.817918039	0.236100336	0.771324224	0.403826872	0.284401505	0.615500767	0.942080124
ATP9A	0.449839986	0.893553774	0.835737391	0.216525116	0.235650529	0.615885089	0.942144044
C23H6orf47	0.493111868	0.612138507	0.473518211	0.407918295	0.294125542	0.615980623	0.942144044
FUT8	0.771404966	0.853288837	0.131423722	0.452224832	0.438407738	0.616001803	0.942144044
KCTD21	0.517290429	0.130462448	0.763460072	0.435247671	0.764277969	0.615871066	0.942144044
LOC100848484	0.887675181	0.746062919	0.896353221	0.049771275	0.580228799	0.615912732	0.942144044
MACROD1	0.170540234	0.62338073	0.558009494	0.615606602	0.469348904	0.615883115	0.942144044
PPT2	0.783331758	0.297473132	0.658774499	0.493799283	0.226169086	0.615924959	0.942144044
VPS33B	0.395229748	0.735666336	0.148303768	0.477541825	0.831358325	0.615640991	0.942144044
CFDP2	0.989351883	0.337033732	0.432952879	0.16933537	0.702231658	0.616185244	0.942269293
DPYD	0.918812235	0.645579103	0.141382749	0.322616715	0.635300351	0.616430621	0.942269293
FBLL1	0.615107284	0.123251606	0.571161178	0.613388304	0.646771824	0.616318263	0.942269293
LOC112442414	0.539168937	0.533066337	0.661127197	0.213478296	0.423977873	0.61654278	0.942269293
LOC782258	0.304364279	0.136034543	0.854351309	0.974234692	0.499167924	0.616588449	0.942269293
MRRF	0.382982221	0.571976158	0.598862393	0.291209672	0.450451588	0.616654934	0.942269293
PYCR1	0.764093312	0.153244509	0.471437701	0.449571203	0.692998117	0.616541929	0.942269293
SPINT2	0.719417956	0.254317864	0.539928795	0.531247871	0.327252084	0.616266796	0.942269293
TMSB10	0.110950104	0.467749514	0.371130665	0.903352839	0.988409968	0.616531253	0.942269293
ZC2HC1A	0.552474704	0.417673057	0.127753876	0.889034781	0.656602182	0.616657864	0.942269293
HLCS	0.592231322	0.288067184	0.621954745	0.454439926	0.357284432	0.616878412	0.942518538
INTS11	0.428088812	0.451577722	0.645286119	0.213317935	0.648558961	0.617220604	0.942785661
TNS2	0.508840912	0.353707459	0.72027077	0.630608632	0.211117721	0.617225589	0.942785661
ZFP64	0.139259629	0.730255896	0.768302797	0.625377298	0.35302128	0.617121758	0.942785661
AAAS	0.543781778	0.125470928	0.595081285	0.681862534	0.624146847	0.617459873	0.942871574
COL14A1	0.651647455	0.484640058	0.462048558	0.134812836	0.878192055	0.617420627	0.942871574
EPB41L4B	0.327145548	0.960887152	0.536662419	0.491612448	0.208485444	0.617588267	0.942871574
LOC112448805	0.589049841	0.357778126	0.087891105	0.989771392	0.943107942	0.617585504	0.942871574
WDR48	0.827669494	0.797264453	0.083113422	0.847211457	0.371652853	0.617339429	0.942871574
ZNF576	0.900702889	0.262576954	0.974724782	0.164011811	0.457407259	0.617626556	0.942871574
AGRN	0.526236143	0.616200383	0.781156814	0.389888597	0.175326241	0.617864868	0.943031607
CYTH1	0.641034961	0.283212074	0.494652858	0.369805142	0.521720435	0.617989193	0.943031607
DDIT3	0.501358904	0.44267451	0.727476631	0.226253495	0.474376132	0.618018703	0.943031607
IARS2	0.749884243	0.381066599	0.62151665	0.364977263	0.267226003	0.617938654	0.943031607
LOC101902301	0.470582837	0.262133814	0.389238109	0.899076935	0.401152018	0.617888253	0.943031607
BABAM2	0.731815474	0.297654166	0.221198736	0.518603542	0.698144493	0.61932578	0.943096909
C5H12orf57	0.716377978	0.841899782	0.905815801	0.137191214	0.231786522	0.618507319	0.943096909
CDC5L	0.266600748	0.512897997	0.94966311	0.136056823	0.986473022	0.619141615	0.943096909
CDO1	0.464962164	0.370167761	0.673750103	0.244651202	0.613176026	0.6187729	0.943096909

COPS7A	0.576750514	0.231517532	0.495154463	0.295805888	0.889896708	0.618867975	0.943096909
DIMT1	0.662613475	0.440851931	0.880160528	0.751420554	0.090053153	0.618795081	0.943096909
FDXACB1	0.635232251	0.449993407	0.45283282	0.548766793	0.245236858	0.619043884	0.943096909
GPR182	0.107387419	0.929966015	0.400035093	0.806275719	0.540154628	0.618805588	0.943096909
HIBCH	0.814366642	0.215115191	0.996253518	0.117730704	0.846131096	0.618656317	0.943096909
LOC100847941	0.832816929	0.955774983	0.091432579	0.319581847	0.749372011	0.619149831	0.943096909
LOC104973848	0.599022082	0.827038403	0.225328629	0.612648841	0.254379849	0.618787126	0.943096909
LOC104975610	0.939981859	0.118946602	0.319370417	0.510512877	0.951225344	0.618147262	0.943096909
LOC112442979	0.281171633	0.862707542	0.677220475	0.579426847	0.182281451	0.618259664	0.943096909
LOC112448816	0.806004769	0.173043455	0.466990205	0.605843969	0.440438352	0.61859253	0.943096909
LOC511713	0.339144009	0.269596572	0.784667693	0.534704979	0.452937074	0.618543022	0.943096909
LOC618289	0.726530778	0.549980016	0.744885121	0.786055059	0.074553269	0.619295603	0.943096909
LRRC2	0.415369139	0.729453105	0.691422933	0.270211111	0.30807171	0.61925986	0.943096909
MYO1G	0.661408368	0.357963325	0.435785937	0.287307872	0.588216029	0.61923081	0.943096909
PUM3	0.418414032	0.246857497	0.751909122	0.81371919	0.275488121	0.618929298	0.943096909
SLC25A10	0.142856066	0.845448023	0.25381152	0.695814073	0.813192133	0.618204369	0.943096909
SLC35F5	0.583265206	0.491202042	0.216247712	0.521085565	0.540166699	0.619252753	0.943096909
ZSCAN31	0.890387243	0.96717165	0.479910203	0.252404717	0.166863086	0.618886512	0.943096909
PRSS23	0.601393962	0.745142031	0.08674464	0.752055246	0.596935059	0.619389017	0.943105693
GANAB	0.892243484	0.425853618	0.380079468	0.609708474	0.19826257	0.619462543	0.943130142
FAM32A	0.368011323	0.446138254	0.753519864	0.556278099	0.254094926	0.619791835	0.943508363
LOC101903413	0.401908618	0.103813873	0.664877367	0.763526951	0.825734682	0.61982595	0.943508363
HSPA14	0.920489874	0.72922806	0.444036896	0.2069227	0.283753798	0.619943827	0.943600273
SYNJ2BP	0.219249008	0.316955845	0.97923308	0.890135855	0.289038937	0.620026514	0.943638609
APIP	0.302544021	0.292603256	0.434538908	0.943738693	0.48260349	0.620163006	0.943748666
PDLIM2	0.573423224	0.597083428	0.233043146	0.231455757	0.948936242	0.620213842	0.943748666
RBSN	0.201051376	0.783259209	0.355218452	0.416942638	0.751920975	0.620350985	0.943869832
MPZL1	0.483373523	0.777134399	0.227386375	0.476672257	0.431198651	0.620568711	0.944113574
ADGRA3	0.184126777	0.363291057	0.656351452	0.537968749	0.744508431	0.620880875	0.944238351
CALN1	0.119618484	0.667725074	0.838416204	0.45983411	0.570884641	0.62082259	0.944238351
CLIP3	0.297499251	0.81466207	0.132685662	0.711558463	0.767863889	0.620722302	0.944238351
LOC534155	0.285326824	0.607521402	0.464519996	0.4065211	0.537106458	0.620843675	0.944238351
MTMR3	0.800496963	0.828541585	0.655055154	0.557936811	0.072598211	0.621028406	0.944287703
R3HDM2	0.449263503	0.295602729	0.453914045	0.44138527	0.6613262	0.621007701	0.944287703
HERC1	0.507710424	0.856011694	0.627742411	0.074764628	0.86423785	0.62136299	0.944326404
IRF2	0.155973354	0.190631526	0.747659192	0.854544019	0.927366105	0.621240544	0.944326404
LOC100335751	0.867092165	0.073656289	0.614792547	0.787291995	0.570167213	0.621333778	0.944326404
MKRN3	0.836806665	0.698501702	0.366183452	0.991332781	0.083095259	0.621399113	0.944326404
SDHAF1	0.731106079	0.128054626	0.822747338	0.606525989	0.377103025	0.621248242	0.944326404
SLC36A4	0.273840542	0.544859061	0.527971032	0.940103333	0.237901075	0.621253834	0.944326404

FAM83G	0.290659998	0.290992416	0.422127927	0.569144654	0.869500123	0.62181124	0.944340576
HBA	0.507548448	0.987032801	0.510776296	0.111314169	0.619594167	0.621583774	0.944340576
LEMD3	0.494896602	0.782367484	0.675879386	0.072907255	0.925669949	0.621729404	0.944340576
LOC112448848	0.188495571	0.851858326	0.326398434	0.675128708	0.499297985	0.621793092	0.944340576
OGN	0.268026803	0.497840265	0.735551175	0.267403435	0.672420827	0.621580033	0.944340576
STX4	0.967503152	0.566805349	0.307570703	0.145709392	0.717787165	0.621501436	0.944340576
ZNF263	0.634039699	0.676195402	0.090117059	0.569801573	0.801962846	0.621662922	0.944340576
LOC100141168	0.726662521	0.330731435	0.674347105	0.115621099	0.944053928	0.62204616	0.944609931
TIMP4	0.732146307	0.279835623	0.488468616	0.422995779	0.418116586	0.622156511	0.94469009
LOC100297420	0.989572909	0.175861885	0.370925862	0.317181582	0.865525411	0.62239195	0.944705731
LOC104975890	0.93154536	0.843307429	0.074821523	0.684450909	0.440501133	0.622397073	0.944705731
SRSF7	0.785175468	0.344288783	0.217083429	0.31989005	0.943777748	0.622343514	0.944705731
TRIB1	0.686928795	0.773296501	0.249196385	0.209064633	0.640137112	0.622328378	0.944705731
CTSV	0.207420897	0.936447544	0.385570869	0.246324226	0.96103066	0.622477468	0.944740379
HSBP1	0.352254993	0.580988726	0.558265709	0.162543785	0.955814267	0.622713673	0.944908147
LOC100296121	0.453857706	0.276257738	0.616592268	0.734251234	0.312689083	0.622704656	0.944908147
NEK7	0.925588584	0.091625917	0.390849971	0.81115194	0.660726516	0.622875896	0.944908147
OSBPL10	0.740075792	0.090171339	0.864275573	0.381268766	0.807644561	0.622821068	0.944908147
PPRC1	0.889479304	0.745050516	0.765338428	0.044272974	0.790804457	0.622791579	0.944908147
BORCS5	0.478449238	0.583244569	0.601322851	0.273685648	0.387072112	0.622996367	0.944916208
ZNF333	0.366201921	0.240460266	0.654370387	0.659904715	0.467436467	0.622975324	0.944916208
CCM2L	0.402550872	0.150572251	0.357848491	0.829000047	0.989674879	0.623209493	0.944977454
DHH	0.16802252	0.971056571	0.635086518	0.189989665	0.903803134	0.623181034	0.944977454
LOC100299712	0.378304071	0.781078438	0.217158672	0.677251534	0.409360761	0.62314519	0.944977454
UNC93B1	0.590812895	0.171857329	0.987999503	0.378917264	0.468544764	0.623370623	0.94513445
MPLKIP	0.834554327	0.607912911	0.05477959	0.784356313	0.81737312	0.623450812	0.945168708
FUCA1	0.153296044	0.578193146	0.494110912	0.561602479	0.724981025	0.623601713	0.945310153
ALDH1A1	0.854256261	0.968827267	0.06656649	0.410970313	0.788202383	0.623761122	0.945325668
MBOAT1	0.731004113	0.350027531	0.575119644	0.169350378	0.716183852	0.623784758	0.945325668
PPIC	0.976566385	0.533763456	0.066239398	0.692112507	0.746705874	0.623740823	0.945325668
YIPF2	0.622024279	0.205854667	0.958141303	0.150080447	0.971352527	0.624193046	0.945857071
IDH3G	0.863492061	0.224959286	0.926162943	0.300199182	0.331259641	0.624251118	0.945857732
CD300E	0.424301964	0.323160769	0.39256066	0.805470584	0.413134458	0.624482256	0.94593477
LOC100300938	0.844385328	0.706882603	0.213769536	0.196523347	0.714204607	0.624450333	0.94593477
RNF11	0.921060852	0.213590982	0.232848311	0.972078387	0.40229502	0.624504171	0.94593477
SPRED3	0.60795247	0.488548941	0.171716288	0.379428072	0.925837247	0.624532523	0.94593477
PAK1	0.528016507	0.369667805	0.303524513	0.623490808	0.485426823	0.624691501	0.946088245
CCDC190	0.141295399	0.759946068	0.885080972	0.393726102	0.479527316	0.624824033	0.946201643
AR	0.816244541	0.184297218	0.688721025	0.598625857	0.289452446	0.624920183	0.946234792
LOC112442401	0.221501737	0.62866371	0.661255581	0.496941087	0.392406865	0.62496124	0.946234792

FBXW4	0.09637999	0.823940068	0.266947216	0.910890017	0.930217189	0.625029532	0.946250891
PELI3	0.603104629	0.548936518	0.424684947	0.29903679	0.427715363	0.625255763	0.946506072
ADAT2	0.133455207	0.687770729	0.985617986	0.226848935	0.887261229	0.627691626	0.94666907
ARHGAP33	0.873344712	0.511043114	0.38719919	0.2108749	0.49456707	0.625690587	0.94666907
ATP6V0D2	0.405847382	0.385012865	0.561937794	0.282795534	0.732672536	0.627526357	0.94666907
CAMK2N1	0.392215738	0.473428561	0.667449456	0.24605086	0.59661277	0.627529323	0.94666907
CCL3	0.340764252	0.339433201	0.271014189	0.62225621	0.930891747	0.627148535	0.94666907
CD7	0.328317336	0.914362846	0.132615198	0.854137254	0.531569127	0.626259783	0.94666907
CIAPIN1	0.326764922	0.298114438	0.504787966	0.679699432	0.543090597	0.627080079	0.94666907
CPPED1	0.647067986	0.81227335	0.070590456	0.904082411	0.542973604	0.627740552	0.94666907
DDX10	0.409195927	0.630543831	0.62787812	0.617885506	0.181985707	0.627777609	0.94666907
ENTR1	0.653671033	0.65160533	0.654830945	0.377828661	0.172181871	0.627007547	0.94666907
FBXW2	0.3077173	0.545530455	0.131950572	0.93671657	0.869850911	0.625963843	0.94666907
GALNT14	0.070392597	0.870068526	0.695931994	0.993657888	0.428905445	0.627228109	0.94666907
GFPT1	0.335503519	0.644741792	0.499273363	0.490851908	0.342766823	0.627284281	0.94666907
GPD1	0.383127309	0.517081623	0.599095732	0.36519516	0.416438433	0.625981403	0.94666907
HACD1	0.648068639	0.508700202	0.483886662	0.926180219	0.123058911	0.627403298	0.94666907
KCNK5	0.126318465	0.954625404	0.452040027	0.862825662	0.384818599	0.62651292	0.94666907
KDM3B	0.525238803	0.390833165	0.272911296	0.670263774	0.484434571	0.627500225	0.94666907
LOC107132971	0.814175062	0.884645854	0.23791646	0.153734301	0.688929441	0.627053372	0.94666907
LOC112441880	0.504514218	0.339308391	0.217268068	0.543525003	0.894068125	0.626242217	0.94666907
LOC112443142	0.573842422	0.06020597	0.974767412	0.684661186	0.784334385	0.62635743	0.94666907
LOC616304	0.132680198	0.308526069	0.89133213	0.656826603	0.75326751	0.626009857	0.94666907
MAGOH	0.98024047	0.365393383	0.185491455	0.359182173	0.761573057	0.627317119	0.94666907
MAN2A1	0.527732683	0.878695423	0.35171512	0.174037683	0.641799912	0.62778621	0.94666907
MFSD12	0.371394259	0.75156239	0.242494899	0.809498412	0.329535953	0.626047701	0.94666907
MOCS2	0.232890001	0.358707524	0.86884783	0.713081096	0.350058693	0.626719402	0.94666907
MSANTD4	0.97632464	0.448341173	0.592577045	0.619443112	0.113117109	0.627332353	0.94666907
MYCBPAP	0.658290974	0.454973175	0.162938867	0.375552373	0.98950018	0.626899309	0.94666907
NFATC2	0.966877976	0.197700306	0.278306433	0.989617084	0.342806003	0.625956033	0.94666907
NR5A2	0.815119605	0.9956432	0.104797537	0.268275374	0.797836953	0.627644401	0.94666907
PJVK	0.07854066	0.59016021	0.563251794	0.916525809	0.758574899	0.627077201	0.94666907
PORCN	0.564735933	0.169329412	0.532453339	0.54503702	0.650782333	0.626092458	0.94666907
R3HCC1	0.584979125	0.33645268	0.198211459	0.554055668	0.840513094	0.627248493	0.94666907
RHBDL2	0.285307932	0.731973816	0.224800683	0.586563868	0.659050403	0.627046042	0.94666907
RUBCNL	0.36535531	0.733818105	0.863903249	0.389365598	0.201049324	0.626860792	0.94666907
SESN1	0.945591335	0.735571436	0.066728462	0.411552856	0.94957363	0.626934824	0.94666907
SPATS2L	0.747629319	0.408670832	0.742369282	0.752718716	0.106482422	0.627383262	0.94666907
SPR	0.211585785	0.475837089	0.360942965	0.791712802	0.629099638	0.626519504	0.94666907
SSFA2	0.172892962	0.633078384	0.256178075	0.966678367	0.67200101	0.627760273	0.94666907

TMEM178B	0.209000114	0.613852102	0.830330806	0.473922727	0.359732666	0.627184538	0.94666907
VARS2	0.528126963	0.535143301	0.262648274	0.827833665	0.294123761	0.626243175	0.94666907
VSIR	0.990466809	0.754500504	0.465265154	0.114298454	0.456927556	0.627157035	0.94666907
ZNF664	0.928932139	0.793921074	0.523789013	0.772964454	0.060904889	0.627445694	0.94666907
ANKRD44	0.539214825	0.967571596	0.740680071	0.197870636	0.23849577	0.62798922	0.946888193
CST3	0.710641743	0.258142772	0.474353418	0.797278607	0.262945607	0.628057202	0.946903696
AGO1	0.817638287	0.856212052	0.207664931	0.255463552	0.491500877	0.628178758	0.946999962
DHRS4	0.307296772	0.755957899	0.575807823	0.278537418	0.490195352	0.628280846	0.947066866
LGALS1	0.442291537	0.244916281	0.439342535	0.392449619	0.978149505	0.62834058	0.947069917
LOC100847695	0.571051515	0.403158165	0.344788144	0.609698578	0.377621796	0.628411034	0.947089125
LOC100847453	0.520837462	0.889938633	0.05078825	0.973612455	0.797985094	0.628559985	0.947226622
CCDC137	0.97958099	0.776160572	0.200637314	0.587028972	0.204573801	0.628877921	0.947339152
FIGN	0.820312544	0.444358278	0.335108173	0.296153058	0.506900265	0.629068299	0.947339152
LOC101908149	0.393765799	0.761686381	0.414689329	0.248066269	0.594234096	0.629034981	0.947339152
MIF4GD	0.164517426	0.552058061	0.384422972	0.552984097	0.948126829	0.628730321	0.947339152
RFC4	0.133500302	0.295592953	0.983158505	0.609983762	0.774911023	0.629083409	0.947339152
RPL28	0.481403398	0.878640906	0.365522293	0.451732326	0.262321949	0.628894591	0.947339152
SRFBP1	0.622245773	0.624714263	0.641105631	0.138529268	0.531232323	0.629096464	0.947339152
ZNF174	0.104871303	0.571417718	0.96019821	0.44607668	0.713962972	0.628942377	0.947339152
ZNF33B	0.182545752	0.47607988	0.793955938	0.981986979	0.27099838	0.629331009	0.947605393
CDK9	0.78364365	0.088884481	0.409841047	0.962747059	0.668367235	0.629406125	0.947615869
PRDX3	0.953570426	0.344353484	0.934832608	0.100318185	0.596652955	0.629453451	0.947615869
HSD11B1L	0.843288159	0.996491163	0.037957134	0.954599683	0.60399375	0.629635202	0.94771561
STAG3	0.729052076	0.463896861	0.238386435	0.384468328	0.593231875	0.629612698	0.94771561
AQP4	0.221333605	0.954749452	0.406269278	0.492173202	0.435769818	0.629873293	0.947949302
PROZ	0.348483252	0.102597335	0.865979112	0.608351149	0.977729684	0.629905986	0.947949302
BLCAP	0.311536305	0.153475234	0.853840572	0.82037334	0.550184094	0.630016101	0.947968569
PI3	0.240054978	0.246364863	0.564871881	0.895903891	0.61571957	0.630034317	0.947968569
DCDC2B	0.710014606	0.31461295	0.205804808	0.983160772	0.408274734	0.630300905	0.948167482
ERMP1	0.382848652	0.986474374	0.26443035	0.541352711	0.341330063	0.630302095	0.948167482
HTATIP2	0.940928947	0.283390615	0.578973095	0.846424193	0.141245384	0.630339847	0.948167482
ZMIZ1	0.990000115	0.493561767	0.047753148	0.881663267	0.897524774	0.630414049	0.948192188
MMP11	0.587313321	0.332734257	0.108468317	0.965633077	0.903150251	0.630645472	0.948444808
TTC26	0.160723339	0.97019624	0.551924835	0.319339184	0.672802492	0.630697593	0.948444808
C10H14orf1	0.504948954	0.7264837	0.873862779	0.396461386	0.145927576	0.63128085	0.948480768
C28H1orf198	0.810305109	0.430055831	0.07076031	0.857285341	0.875682672	0.63091218	0.948480768
CSDE1	0.870927043	0.479497361	0.213498524	0.977308339	0.212474102	0.630941501	0.948480768
DDX42	0.41182674	0.637541394	0.513924019	0.832909209	0.164883796	0.631120091	0.948480768
JRK	0.631987546	0.890757029	0.442378719	0.143400987	0.518727585	0.631055314	0.948480768
LOC101903114	0.925995439	0.776865741	0.205314104	0.946224548	0.132716824	0.631298365	0.948480768

LOC112446798	0.98426619	0.2408442	0.530504106	0.606715075	0.242739431	0.63101444	0.948480768
LRRK2	0.126616283	0.66200688	0.823646215	0.283902149	0.945403837	0.63111169	0.948480768
REPS2	0.810922147	0.48260045	0.348045494	0.717640062	0.18978956	0.631337996	0.948480768
SNX2	0.554612921	0.56005017	0.241166587	0.260560281	0.950430188	0.631329268	0.948480768
UBE2Q1	0.927950238	0.585422443	0.435391161	0.368277766	0.212998008	0.631357255	0.948480768
PPP2R2A	0.385165453	0.91780195	0.856830332	0.071445424	0.857642906	0.631423539	0.948493519
KDR	0.726901012	0.365128205	0.348401493	0.218086706	0.921377854	0.631646465	0.948567911
LOC101907641	0.407637722	0.667411064	0.290033914	0.468434466	0.502563729	0.631596492	0.948567911
NEFH	0.965735824	0.332401192	0.684120873	0.094030807	0.899699487	0.631625667	0.948567911
CIRBP	0.563852666	0.786835997	0.338688854	0.917389298	0.135046255	0.632015039	0.948635485
FOXC1	0.087943817	0.381886145	0.849951909	0.775971747	0.841536984	0.632269511	0.948635485
IGFBP6	0.5440095	0.530915857	0.5405203	0.199986842	0.596266427	0.632014585	0.948635485
ITSN2	0.659191575	0.538856415	0.475713542	0.115273015	0.956794308	0.632235222	0.948635485
LOC101904520	0.222916673	0.48368555	0.944230033	0.299470222	0.611113222	0.632182296	0.948635485
LOC788334	0.651231096	0.810698875	0.93558719	0.045746646	0.823289248	0.631881747	0.948635485
NCF1	0.445143855	0.597005487	0.987034687	0.375856591	0.188922813	0.632118822	0.948635485
NUP35	0.316639168	0.771630954	0.911939123	0.212368228	0.393422201	0.632015737	0.948635485
RB1CC1	0.680019547	0.668540163	0.201569491	0.310607212	0.654252013	0.632079511	0.948635485
SF3A3	0.504126087	0.638584635	0.211681571	0.391809536	0.69747195	0.632084968	0.948635485
TMEM181	0.501380797	0.5320232	0.959416144	0.478996272	0.152126739	0.632355113	0.948677188
CALCOCO2	0.771293921	0.996236051	0.032284536	0.761390225	0.990770866	0.633036265	0.948768376
GTF2B	0.912854621	0.130121378	0.658794514	0.264258798	0.903645781	0.632753187	0.948768376
LIN7C	0.576554643	0.124671769	0.541990519	0.654799467	0.732983514	0.632876836	0.948768376
LOC104970173	0.669395215	0.520873532	0.132037889	0.450307793	0.902276248	0.632948648	0.948768376
LOC112448090	0.411331302	0.791897873	0.487968155	0.132270893	0.890620264	0.633149639	0.948768376
LOC112449406	0.638732253	0.447891591	0.11653317	0.689833553	0.812325652	0.632702748	0.948768376
LOC618071	0.502160711	0.461504897	0.619644235	0.502927681	0.259201338	0.63310239	0.948768376
LOC781646	0.938605141	0.58711559	0.353059828	0.932592095	0.103012012	0.632801102	0.948768376
MARVELD2	0.470089962	0.96588466	0.412893677	0.461574556	0.215725759	0.632555452	0.948768376
N4BP2L1	0.220050396	0.859810074	0.491752497	0.779622829	0.25765156	0.632780471	0.948768376
OSCP1	0.802376036	0.194097449	0.19159102	0.635494582	0.987561439	0.633167464	0.948768376
PRRG2	0.14373447	0.952496925	0.214486251	0.639735212	0.996490986	0.633099406	0.948768376
SLC9A8	0.259842321	0.824495299	0.429319113	0.824038396	0.246875054	0.633011847	0.948768376
KBTBD3	0.819501469	0.559459253	0.131948538	0.647706167	0.478068149	0.633231212	0.948777268
HFE	0.504200282	0.400776113	0.555298881	0.842885531	0.198161715	0.633334419	0.948803277
LOC100847182	0.740673458	0.074640458	0.610605346	0.915528495	0.606530616	0.633364201	0.948803277
PTRH1	0.523868421	0.690054262	0.719881313	0.276470954	0.26061766	0.633424226	0.948806588
LOC786173	0.59647733	0.502438725	0.738213713	0.18799844	0.450974824	0.633489892	0.948818346
LOC112447351	0.252439565	0.204344126	0.859755655	0.677975658	0.624016327	0.633552406	0.948825382
GSN	0.591225338	0.20875041	0.589051894	0.424099047	0.609019961	0.633699981	0.94895532

TNFAIP1	0.851829163	0.856843359	0.48869393	0.484472179	0.108691339	0.633754818	0.94895532
BRI3	0.718834201	0.763151308	0.08182122	0.824948316	0.507756532	0.633949555	0.948971523
CHIC2	0.392637998	0.64471963	0.596966994	0.396448997	0.315702253	0.635114964	0.948971523
CMC4	0.933786594	0.071242446	0.51572399	0.75791953	0.728047783	0.635298581	0.948971523
CXADR	0.917049806	0.751749584	0.17598938	0.666691763	0.232356259	0.633879588	0.948971523
DQX1	0.447139546	0.781206596	0.594925613	0.382517533	0.237819379	0.63502127	0.948971523
ELFN2	0.121215098	0.86085952	0.540969426	0.348765842	0.95547608	0.634052063	0.948971523
ELK4	0.386284006	0.42107471	0.941747272	0.162198164	0.760744823	0.634983019	0.948971523
FIGNL1	0.172004601	0.611910861	0.340603537	0.896598595	0.58494335	0.633950295	0.948971523
GCNT2	0.695653107	0.369804322	0.634284186	0.413775528	0.278917303	0.634264986	0.948971523
GDA	0.266792228	0.25044405	0.998637496	0.418518086	0.676911392	0.635006687	0.948971523
GLB1L	0.600991473	0.702909065	0.212350019	0.437182351	0.482680152	0.635278363	0.948971523
LOC101904963	0.556465089	0.754806464	0.183719744	0.97532833	0.250135445	0.634204977	0.948971523
LOC112442374	0.999983862	0.095808088	0.205809741	0.983575108	0.975917592	0.635251351	0.948971523
LOC512978	0.776231309	0.092631456	0.664208823	0.641640598	0.614893266	0.634380587	0.948971523
LOC783926	0.614748307	0.784695669	0.903882383	0.071373631	0.606484903	0.634705544	0.948971523
OSBPL3	0.340001131	0.450109188	0.678937039	0.416653983	0.435601832	0.634536722	0.948971523
PDGFRB	0.886302463	0.676991252	0.046519923	0.775597001	0.874859325	0.635384748	0.948971523
PTPN13	0.443544857	0.456631449	0.784381447	0.137919794	0.860643732	0.634531195	0.948971523
RBM17	0.397652363	0.18645017	0.543492356	0.655388557	0.7124035	0.634083191	0.948971523
SDC4	0.943097609	0.131246665	0.674715049	0.359605832	0.629607976	0.635062663	0.948971523
SEC14L1	0.491397495	0.719188792	0.499208119	0.138853863	0.771171822	0.6348847	0.948971523
SERPINI1	0.101285042	0.830898226	0.959745268	0.262632801	0.888321452	0.634390655	0.948971523
SLC11A1	0.97398132	0.750468011	0.09321153	0.581638476	0.474854004	0.63411962	0.948971523
SPARC	0.259635965	0.327140575	0.30000831	0.788706302	0.940393812	0.634970616	0.948971523
SSBP2	0.253703363	0.594811195	0.303808286	0.734296977	0.560196071	0.634547426	0.948971523
VCAM1	0.779548253	0.126275962	0.6006082	0.668262515	0.477452028	0.634599161	0.948971523
ZDHHC1	0.540525767	0.445557705	0.620249031	0.777103989	0.16315113	0.635374554	0.948971523
ZSCAN21	0.94040182	0.333410325	0.369089871	0.558869848	0.291735136	0.634640659	0.948971523
FUT7	0.981411742	0.443082364	0.073087791	0.724826918	0.823240402	0.635638063	0.949263469
GORASP1	0.386264749	0.358196433	0.680349086	0.24787518	0.813798655	0.635883916	0.949457824
SRPK2	0.857632082	0.636991785	0.452792837	0.173240693	0.44303223	0.635853099	0.949457824
KCNAB2	0.288327456	0.19337542	0.926566588	0.6358662	0.578396392	0.636004038	0.949464411
SCUBE3	0.155721798	0.794442538	0.350127479	0.46470449	0.943656156	0.635947151	0.949464411
ASPA	0.111696131	0.65328245	0.724809031	0.710773329	0.505826207	0.636156936	0.949510977
CCNG2	0.986796735	0.043957432	0.78657813	0.74991253	0.743437817	0.636229787	0.949510977
PYGL	0.998122662	0.069458386	0.443697762	0.633222626	0.976184765	0.636152044	0.949510977
RALGAPA2	0.805476835	0.048433504	0.94167372	0.58825459	0.880388598	0.636266664	0.949510977
KCNH4	0.256226418	0.441170383	0.229718121	0.807357499	0.908127042	0.636401085	0.949625223
HINFP	0.603401973	0.405942507	0.19542427	0.557562714	0.714280297	0.636659313	0.949803748

KCNJ12	0.179273126	0.416054503	0.593581939	0.646849645	0.666149304	0.63679823	0.949803748
LOC107133075	0.58740326	0.912746718	0.101529137	0.849886958	0.412382959	0.636806408	0.949803748
LOC112443177	0.979920355	0.475402411	0.093304041	0.658917803	0.665729583	0.636690599	0.949803748
VWA2	0.196116794	0.623046206	0.239563195	0.764272174	0.852794201	0.636810106	0.949803748
GLT8D1	0.480611686	0.50658058	0.220331358	0.568300487	0.628255273	0.637568341	0.949812458
HAUS3	0.444560297	0.814584158	0.603432661	0.115012144	0.760085639	0.63705938	0.949812458
ITGB4	0.457129312	0.122303133	0.770430556	0.912597664	0.486755093	0.637373453	0.949812458
LGI4	0.082603838	0.951539001	0.809184274	0.706664486	0.425764583	0.637399106	0.949812458
LOC100847120	0.287929686	0.676764103	0.139622525	0.760020798	0.92439309	0.637176198	0.949812458
LOC112444276	0.915915772	0.755007129	0.256676861	0.126088288	0.855322265	0.637462423	0.949812458
SAMD5	0.365814226	0.775366455	0.245229816	0.521113899	0.527767132	0.637335611	0.949812458
TCF23	0.142001842	0.704425022	0.981557115	0.391993132	0.496224471	0.637014085	0.949812458
TIGD4	0.6010104	0.913036777	0.091985333	0.884634249	0.428510202	0.637379637	0.949812458
TMEM53	0.287476454	0.398214133	0.41684999	0.470192619	0.85139276	0.637060653	0.949812458
TMX1	0.39405369	0.919786255	0.854740219	0.29117481	0.21226573	0.63751293	0.949812458
ULK4	0.178835626	0.878986633	0.245788146	0.883361406	0.560823552	0.637446265	0.949812458
XKR8	0.045065189	0.677181203	0.953566364	0.818023812	0.802771797	0.637128369	0.949812458
LOC112447087	0.801631568	0.440481394	0.161138032	0.537818936	0.626398668	0.637727548	0.949963402
ITGA2	0.683632545	0.416152874	0.151321719	0.448920888	0.992222172	0.637803231	0.94998991
LOC783963	0.390446372	0.327125437	0.886451975	0.21397074	0.79187416	0.637886811	0.950028174
CACNA1H	0.107406289	0.631553614	0.5639789	0.869709802	0.576846828	0.637975041	0.950073358
DBNL	0.728663195	0.332885135	0.871694178	0.655103585	0.139032783	0.63863833	0.950139868
FSTL1	0.617583553	0.683610966	0.151914932	0.484299222	0.620967472	0.638941993	0.950139868
GSK3B	0.727516597	0.240516463	0.698837402	0.592992982	0.265137508	0.638310943	0.950139868
LOC104970105	0.654460689	0.765885171	0.183844895	0.385564047	0.54231526	0.638743822	0.950139868
LOC107132944	0.426440738	0.530126765	0.849339036	0.57825455	0.173502922	0.638698042	0.950139868
LOC112441683	0.795767294	0.073917288	0.982486139	0.821496144	0.405723503	0.638675055	0.950139868
LOC784488	0.398292261	0.573195377	0.293724406	0.762040706	0.376414583	0.63840296	0.950139868
MARF1	0.722172195	0.308273815	0.464854569	0.49830554	0.37302784	0.638421971	0.950139868
PELP1	0.167918903	0.420184506	0.486595056	0.668377958	0.838531601	0.638474768	0.950139868
PTPN3	0.465862258	0.370666346	0.659510686	0.20252565	0.836241783	0.638935962	0.950139868
SGSH	0.524993748	0.614493383	0.106154424	0.756830246	0.744110317	0.638922843	0.950139868
SLC30A9	0.777612845	0.366758833	0.641583253	0.618765474	0.170180529	0.638737443	0.950139868
TMED1	0.216142421	0.80875475	0.55627692	0.314793385	0.628865832	0.638556897	0.950139868
TPCN2	0.933907137	0.406038623	0.470623771	0.894935794	0.120590356	0.63865487	0.950139868
VPS37D	0.86186612	0.815985183	0.537766481	0.144463604	0.353038922	0.638946048	0.950139868
WNK4	0.824344945	0.420956346	0.715559828	0.185136263	0.419406718	0.638866011	0.950139868
LOC101902895	0.671283094	0.300523691	0.188623362	0.741918026	0.683978024	0.639162409	0.950237982
LOC112444531	0.808715068	0.455444161	0.961928815	0.188098662	0.28968907	0.639123488	0.950237982
UBQLN2	0.786139182	0.598916198	0.623772165	0.848950509	0.077456163	0.639185734	0.950237982

EDC3	0.444189118	0.147169742	0.416206419	0.99892478	0.711296704	0.639387622	0.950290333
KCNN4	0.790765705	0.50218986	0.171202325	0.924319484	0.307641709	0.639393894	0.950290333
LOC112449560	0.129537383	0.23764405	0.730051933	0.963376231	0.893301045	0.639473417	0.950290333
METTL4	0.167284784	0.792370346	0.593969143	0.331497505	0.741179161	0.639510477	0.950290333
TRIO	0.684584094	0.150986314	0.281102291	0.89018196	0.747872339	0.639502845	0.950290333
ID3	0.275997099	0.690811232	0.511090637	0.688181864	0.288621012	0.639618848	0.950365316
CHST12	0.549709807	0.620606408	0.621163998	0.256390346	0.356886701	0.639976359	0.950409018
DHCR7	0.726196359	0.192137808	0.678407465	0.354895404	0.576934714	0.639884857	0.950409018
FCER1G	0.469698916	0.435048812	0.979304998	0.182474352	0.531072486	0.639995738	0.950409018
LOC101901983	0.775301892	0.477001369	0.591183385	0.260308756	0.34056025	0.63988974	0.950409018
LOC101904757	0.257640788	0.776203943	0.788417168	0.58012875	0.21198365	0.639969721	0.950409018
TNRC6B	0.799202275	0.117138215	0.373421492	0.771779262	0.718033712	0.63979852	0.950409018
FUT10	0.477328755	0.352752555	0.211789097	0.610150071	0.891739278	0.640101309	0.95042618
GPR31	0.973107454	0.27735429	0.465174632	0.995821836	0.155210495	0.640123123	0.95042618
B2M	0.301132625	0.143878804	0.711418797	0.768158361	0.820910175	0.640443243	0.950815457
CDC42SE1	0.734661608	0.47360815	0.282091802	0.300273947	0.660512991	0.640744337	0.951029221
LOC112442787	0.476265819	0.802677655	0.236307155	0.529265511	0.407427132	0.640876982	0.951029221
PCBP3	0.190237978	0.544386069	0.539333065	0.390016226	0.894108005	0.640851321	0.951029221
RAD18	0.09370719	0.523405013	0.954632759	0.637709395	0.652267747	0.640834198	0.951029221
SLC38A5	0.565716022	0.204616761	0.20933828	0.85170616	0.943674092	0.640836136	0.951029221
ETHE1	0.131036405	0.64218933	0.907299021	0.35991045	0.709648638	0.641080361	0.951190017
PRPF38B	0.710801275	0.874343061	0.485853521	0.086037588	0.750699557	0.64110126	0.951190017
C7H5orf30	0.93505627	0.420317508	0.414600823	0.252593276	0.474619497	0.641426308	0.951428287
LOC112447731	0.343211254	0.316147223	0.213627738	0.855900893	0.984893298	0.641475066	0.951428287
LOC520336	0.865971141	0.257987887	0.538375922	0.669543435	0.242658572	0.641493754	0.951428287
RPUSD1	0.64496026	0.921970706	0.301986068	0.282017727	0.385678643	0.641393143	0.951428287
ABCC11	0.994604638	0.437873728	0.082011239	0.653899209	0.837247187	0.641617168	0.951514413
BMP4	0.815808144	0.491012872	0.260436926	0.910177303	0.206034678	0.641712731	0.951514413
FLNB	0.448779471	0.725945079	0.390817463	0.547189359	0.281454887	0.642165627	0.951514413
LOC104974516	0.85849795	0.354630749	0.645041133	0.531190942	0.187843729	0.642027728	0.951514413
LOC107131566	0.577265988	0.930403833	0.112150939	0.762096286	0.427345594	0.642247587	0.951514413
LOC789587	0.094718691	0.588890087	0.418518193	0.948097813	0.884639383	0.641871838	0.951514413
MCAM	0.922865738	0.859019122	0.250636201	0.936981273	0.105211981	0.641951675	0.951514413
NXPH2	0.158339041	0.78721523	0.431626645	0.749396996	0.486482184	0.642215749	0.951514413
PAXIP1	0.794321374	0.312698013	0.76266622	0.673306113	0.153465632	0.641816036	0.951514413
PPM1L	0.673174296	0.821182738	0.282099476	0.238366167	0.527332629	0.642094469	0.951514413
SIKE1	0.970056743	0.526907115	0.97255676	0.357407297	0.110196471	0.641859957	0.951514413
TSACC	0.127628026	0.650729911	0.888971663	0.343280945	0.77341677	0.642094086	0.951514413
CACNA2D1	0.850144066	0.984587793	0.426423943	0.663518048	0.083017608	0.642684599	0.951962748
LOC101907294	0.789005971	0.17328377	0.666266934	0.420531359	0.513426387	0.642752706	0.951962748

SDHC	0.908065377	0.599021201	0.679185936	0.360602235	0.147658066	0.642784434	0.951962748
STK25	0.724405587	0.674946332	0.524690784	0.265680988	0.288697333	0.64284024	0.951962748
TYROBP	0.393430248	0.318149976	0.252052178	0.67768936	0.9195506	0.642677803	0.951962748
ADPRHL2	0.921365962	0.388979868	0.318002402	0.698834108	0.24837886	0.64388347	0.95213589
BCOR	0.189119774	0.935702824	0.911076613	0.235000931	0.52678933	0.645617489	0.95213589
CDC42EP4	0.338159457	0.663878788	0.229870702	0.570992938	0.671013174	0.643783374	0.95213589
CDC7	0.339063553	0.698823897	0.473589243	0.194121511	0.911129065	0.644524378	0.95213589
CEP120	0.57329337	0.309882908	0.641132189	0.274453974	0.639589631	0.645957579	0.95213589
CHD5	0.308977192	0.791361153	0.530757414	0.349690902	0.440624422	0.645983219	0.95213589
CREB5	0.696977463	0.430020148	0.972087261	0.321935196	0.213307821	0.646090139	0.95213589
DVL2	0.505643011	0.528362442	0.526597676	0.472610439	0.299386171	0.64510225	0.95213589
ELF4	0.8098967	0.302242545	0.837181154	0.748341939	0.128885086	0.643716703	0.95213589
FAM102B	0.289234403	0.812841698	0.256071481	0.762710075	0.432574707	0.644674276	0.95213589
FLII	0.839567502	0.814873403	0.07203942	0.953251154	0.422575318	0.644579748	0.95213589
FMR1	0.83662995	0.857826015	0.087556078	0.324074183	0.969248364	0.643442949	0.95213589
GAS2L1	0.168019385	0.966122606	0.751551464	0.268973827	0.604689128	0.644474168	0.95213589
GCNT4	0.305315672	0.84212012	0.27540235	0.667374872	0.422985995	0.645909743	0.95213589
HERC3	0.411467853	0.650101875	0.188488286	0.943197423	0.414821647	0.643338333	0.95213589
IDH1	0.852937095	0.187297232	0.373178176	0.529234197	0.627588545	0.64406736	0.95213589
IQCN	0.962996818	0.500132206	0.742490752	0.860438512	0.064349496	0.644057565	0.95213589
KLF3	0.312177475	0.961476312	0.736186301	0.101149401	0.892927377	0.645604458	0.95213589
LOC100848339	0.836301795	0.338205122	0.440881058	0.484588226	0.32903885	0.644875163	0.95213589
LOC101903326	0.98909007	0.520727931	0.390016175	0.171432757	0.580899096	0.646061293	0.95213589
LOC101906730	0.591296231	0.832862389	0.471400006	0.197282578	0.435794823	0.645618574	0.95213589
LOC104972797	0.98391781	0.476687079	0.617153332	0.168803248	0.403788035	0.643362524	0.95213589
LOC104976614	0.21297712	0.153191538	0.901372108	0.822205178	0.826001564	0.645750782	0.95213589
LOC107131458	0.174529074	0.744387299	0.814950889	0.817387577	0.230520251	0.645527136	0.95213589
LOC112442278	0.294163192	0.245629092	0.494448742	0.56082528	0.995989101	0.645588386	0.95213589
LOC112447817	0.917808365	0.816943431	0.66767638	0.254723652	0.156193216	0.645215296	0.95213589
LOC506408	0.900431386	0.085682097	0.398726424	0.941690266	0.687016155	0.645057677	0.95213589
LOC540014	0.101772908	0.686935908	0.669200846	0.832287566	0.510501345	0.644825945	0.95213589
LOC781100	0.619784545	0.368084576	0.548404636	0.277883378	0.567482368	0.643355734	0.95213589
MAP1S	0.871687544	0.583962519	0.596571165	0.197354173	0.33121436	0.64455103	0.95213589
MTX2	0.71303465	0.155779273	0.874924116	0.461131567	0.443735881	0.644900068	0.95213589
MYLK2	0.641582357	0.121094121	0.495687452	0.581230006	0.891163353	0.645506215	0.95213589
NAAA	0.409901855	0.702270769	0.301336518	0.249746634	0.922152071	0.645798202	0.95213589
NRAP	0.115926316	0.9554416	0.286080455	0.639103337	0.980834297	0.644675783	0.95213589
OTULINL	0.939698884	0.767498872	0.243869101	0.1934155	0.583617598	0.644586693	0.95213589
PARN	0.723802598	0.325692366	0.433733934	0.593178512	0.326838093	0.644283878	0.95213589
POLD3	0.347161918	0.654247542	0.585269742	0.557809034	0.267512075	0.644414226	0.95213589

PRKACB	0.428366596	0.573314742	0.942319315	0.897498316	0.095302411	0.644003002	0.95213589
PRSS42	0.314892857	0.76053577	0.105992145	0.919212261	0.854243334	0.645355564	0.95213589
PSMC3IP	0.521635232	0.260328591	0.371432247	0.951908723	0.411467908	0.643623313	0.95213589
PSMD4	0.815892819	0.260062492	0.756969013	0.502933984	0.246259315	0.644968604	0.95213589
PYM1	0.470065608	0.716662647	0.302811139	0.878184298	0.221197164	0.644212462	0.95213589
RAB28	0.547807769	0.217785054	0.350730552	0.524536924	0.904299879	0.644530737	0.95213589
RFTN1	0.776310222	0.131415487	0.925210083	0.49246971	0.42901687	0.645455592	0.95213589
SCP2D1	0.510334872	0.331742752	0.362953347	0.517147963	0.624061209	0.644364924	0.95213589
SGCD	0.181911357	0.818642181	0.178195671	0.867092096	0.865572962	0.64520565	0.95213589
SLC9A7	0.899962932	0.250042553	0.469145598	0.218939322	0.858469339	0.644475161	0.95213589
SLK	0.407146556	0.440928801	0.248226549	0.875649434	0.508818389	0.644594624	0.95213589
TBCC	0.854006584	0.512932314	0.655550774	0.354383661	0.195131717	0.644625475	0.95213589
TMEM269	0.220672019	0.334822051	0.809215309	0.505853354	0.660170333	0.645693764	0.95213589
TRAPPC5	0.593637034	0.461011471	0.813173696	0.116069757	0.768182321	0.64447726	0.95213589
UBC	0.364957837	0.922945464	0.857581916	0.741071565	0.093352075	0.645861139	0.95213589
WLS	0.215611363	0.67810793	0.38355366	0.434194323	0.815657338	0.644652137	0.95213589
ZMYM1	0.515586331	0.688074534	0.122660628	0.758831713	0.605501535	0.645961947	0.95213589
KLK10	0.271063735	0.3649007	0.619840394	0.545435031	0.598974006	0.646309877	0.952354863
YJEFN3	0.516525147	0.321277427	0.749734164	0.259526497	0.62046351	0.64635479	0.952354863
TRIM63	0.719675858	0.550067711	0.322719634	0.957273159	0.163989391	0.646558441	0.952569403
CSNK1G2	0.091780305	0.536816988	0.906985227	0.522394192	0.860157914	0.646793182	0.952692736
LOC100848995	0.753717267	0.733561019	0.073932954	0.855323568	0.574674396	0.646920159	0.952692736
LOC101907514	0.226521188	0.583913501	0.980710744	0.187789758	0.824882608	0.646932414	0.952692736
RAB23	0.196260106	0.694919905	0.771050816	0.204411843	0.933786667	0.646726234	0.952692736
TDRP	0.355322445	0.226846341	0.739454124	0.543287859	0.620448618	0.64690507	0.952692736
BLOC1S4	0.555429529	0.664462578	0.326090961	0.260414286	0.642624588	0.647380892	0.952909622
EED	0.349085415	0.170017035	0.749741158	0.794768134	0.570666558	0.647784368	0.952909622
FLYWCH2	0.600127158	0.72407036	0.350967554	0.281540918	0.469636295	0.647620741	0.952909622
LOC104975849	0.894324427	0.250798867	0.645941105	0.242496624	0.573517021	0.647473093	0.952909622
LRG1	0.899890953	0.416526303	0.342179467	0.420619892	0.373722405	0.647589743	0.952909622
MGST3	0.341192145	0.329109867	0.592222278	0.32416566	0.93643655	0.647834541	0.952909622
MMP9	0.874071128	0.290366771	0.35013759	0.801317537	0.283429314	0.647794917	0.952909622
NMUR1	0.57755222	0.723923008	0.381019191	0.16284054	0.776201629	0.647339182	0.952909622
PWWP2B	0.721407117	0.374858694	0.3265963	0.34405251	0.662676918	0.647347393	0.952909622
QSOX2	0.123949369	0.767731931	0.504618982	0.511202339	0.820289048	0.647343532	0.952909622
TBP	0.744970717	0.160286529	0.572672347	0.305571765	0.965013086	0.647618346	0.952909622
WDR20	0.821516857	0.795600569	0.947368771	0.034053516	0.957121515	0.647784953	0.952909622
ZBTB3	0.589371492	0.687268567	0.544148601	0.873148547	0.104684626	0.647445491	0.952909622
LOC107132798	0.111041949	0.735790582	0.743588829	0.698544947	0.475867528	0.647917461	0.952946178
CENPV	0.538019472	0.424569349	0.414350713	0.389044426	0.549055106	0.648131	0.953174822

ATAD5	0.226803571	0.249372942	0.443916997	0.979769945	0.82318769	0.648442854	0.953269536
BUD31	0.56371159	0.182334905	0.589892015	0.943360847	0.355326944	0.649153261	0.953269536
CCDC106	0.891457744	0.300371264	0.460308581	0.279259212	0.593877206	0.650279434	0.953269536
CCNL1	0.699508296	0.497252048	0.961690255	0.386537278	0.158049381	0.650223949	0.953269536
CHAF1A	0.211476895	0.663537028	0.325641604	0.773618705	0.578460493	0.650349619	0.953269536
COPZ2	0.227112502	0.364336518	0.647910854	0.461358744	0.819365161	0.648600673	0.953269536
CYLD	0.585818834	0.620641755	0.308720267	0.397664874	0.458523667	0.65052021	0.953269536
EPS15L1	0.492300427	0.642051354	0.52020745	0.149686056	0.822204594	0.64831428	0.953269536
GK5	0.474231774	0.80405446	0.177835381	0.598195147	0.500483372	0.648938318	0.953269536
GPCPD1	0.846236756	0.847359064	0.157406299	0.75822847	0.238455396	0.649954385	0.953269536
KCTD9	0.767408935	0.401829938	0.487575694	0.157211632	0.859784449	0.649144351	0.953269536
LOC112442867	0.316604498	0.711322495	0.332326907	0.351425112	0.774364718	0.649567527	0.953269536
LONRF2	0.669866794	0.601373497	0.446523172	0.374377467	0.300694126	0.648437197	0.953269536
MAPK8IP1	0.919055607	0.485101942	0.281984883	0.708409802	0.22974496	0.650467541	0.953269536
MED21	0.265656834	0.479234287	0.611199507	0.924852512	0.284390156	0.650515926	0.953269536
MFSD2A	0.544905394	0.424165324	0.249907621	0.456268474	0.776738942	0.650557427	0.953269536
MMS19	0.806251164	0.658409053	0.158996739	0.362713064	0.661975174	0.648593491	0.953269536
MRPS27	0.937253634	0.38379098	0.723322281	0.381354205	0.205522605	0.649812067	0.953269536
MYH14	0.158040099	0.785289764	0.370709085	0.475577937	0.93038191	0.649471367	0.953269536
N4BP3	0.593337492	0.896069275	0.339864262	0.188821516	0.59878543	0.650171277	0.953269536
PDE7B	0.029702235	0.997034412	0.923066173	0.791231869	0.946539963	0.650576979	0.953269536
PIK3R4	0.904333655	0.998089393	0.285845858	0.085140443	0.924819524	0.649071343	0.953269536
PSMD5	0.807148992	0.110632138	0.804857246	0.939245455	0.301740095	0.649584829	0.953269536
RAB5A	0.593154236	0.311761529	0.273245395	0.787064846	0.514685941	0.650541491	0.953269536
RAD51B	0.813461095	0.49215668	0.155357711	0.956261105	0.34308881	0.649939706	0.953269536
RDH11	0.611877534	0.288067611	0.395379386	0.584672754	0.497744749	0.648743202	0.953269536
RDX	0.994697611	0.179714294	0.401880555	0.859984549	0.328265459	0.648740515	0.953269536
RGS10	0.49859717	0.527160744	0.357839727	0.318588553	0.677690446	0.648990491	0.953269536
RILPL2	0.940509121	0.911458236	0.055231731	0.506413341	0.847978094	0.649230945	0.953269536
RNF130	0.853300176	0.38135025	0.512322911	0.931299935	0.130628842	0.648745805	0.953269536
SAA3	0.126631585	0.599247684	0.568586559	0.993814344	0.475049392	0.649595172	0.953269536
SIGLEC8	0.090467932	0.505726888	0.849700892	0.882276311	0.596336473	0.650396391	0.953269536
SRSF10	0.71249928	0.274116736	0.791036882	0.597183613	0.221852085	0.650538023	0.953269536
TGFBR3L	0.772788486	0.509842881	0.338497102	0.467406189	0.327695286	0.650146586	0.953269536
TMBIM6	0.251138166	0.560250656	0.510879498	0.686945352	0.41429276	0.650427979	0.953269536
TMX3	0.594395527	0.635628563	0.761759876	0.364439173	0.193897756	0.649283349	0.953269536
TRIM41	0.567751534	0.554578591	0.330636578	0.249216657	0.786083911	0.649832915	0.953269536
ZFP36L2	0.926439811	0.067401197	0.785328059	0.442115712	0.940708037	0.64983671	0.953269536
ZFPM1	0.871372493	0.62489379	0.378058766	0.154777124	0.639728456	0.649722199	0.953269536
ZNF287	0.625190077	0.260312241	0.2613613	0.962804086	0.499761142	0.65052017	0.953269536

ZNF316	0.92611699	0.061685701	0.596657106	0.696165789	0.861706568	0.650340014	0.953269536
LOC112444278	0.715485941	0.151329899	0.308970514	0.893056977	0.685977532	0.650782277	0.95348522
CASP4	0.559337291	0.507841326	0.547600179	0.827885372	0.159621553	0.651363957	0.953578
CCDC115	0.47959362	0.220970204	0.826201342	0.397997809	0.589232308	0.651154277	0.953578
CORIN	0.673431704	0.590235145	0.887851565	0.099990312	0.582596018	0.651389683	0.953578
EPB41L4A	0.453099347	0.527565419	0.637393728	0.352044809	0.382602912	0.651048072	0.953578
FBXO25	0.134866329	0.847811487	0.463600965	0.87179027	0.444946285	0.651426663	0.953578
LOC101907749	0.444452303	0.829904172	0.265740204	0.452556776	0.462844515	0.65113556	0.953578
LOC528767	0.141991339	0.714630224	0.567131977	0.481006324	0.741237956	0.651008572	0.953578
LYSMD2	0.855144026	0.786477866	0.528166316	0.146183579	0.3957012	0.651289988	0.953578
RCC1L	0.278310316	0.280041649	0.498787551	0.845372197	0.625085705	0.651241771	0.953578
TANGO2	0.147033789	0.50162698	0.54328363	0.607977407	0.842865007	0.651158713	0.953578
STK39	0.716061334	0.347732459	0.934445035	0.989022231	0.089391895	0.651510482	0.953615636
LOC112443163	0.38618463	0.951328511	0.263979702	0.579938401	0.366074548	0.651687423	0.953704504
WRAP53	0.836152477	0.436547946	0.105625931	0.747803039	0.714107839	0.65168364	0.953704504
CD8A	0.240964492	0.860700584	0.645227292	0.677427472	0.227430502	0.651948755	0.953895744
HVCN1	0.461379842	0.165887798	0.894664556	0.312791232	0.962806247	0.651992478	0.953895744
KAT6A	0.903221007	0.593461044	0.261547848	0.929509159	0.158157793	0.651882418	0.953895744
CLUAP1	0.322078732	0.717192761	0.680680981	0.827859232	0.158566774	0.652164677	0.954062623
C8H9orf85	0.988930667	0.17914919	0.520722095	0.22431759	0.997984846	0.652283098	0.954147224
LEO1	0.673694251	0.535177471	0.572699309	0.329117066	0.304444205	0.652629492	0.954147224
LOC100140533	0.957218015	0.437595817	0.509588649	0.356245617	0.272004408	0.652577321	0.954147224
LOC107131652	0.132968219	0.465150543	0.482981537	0.777203396	0.891004793	0.652603093	0.954147224
PBX1	0.343309779	0.950227917	0.155000637	0.715453456	0.571414639	0.652465495	0.954147224
TSPYL2	0.864899863	0.160814847	0.361900524	0.587429826	0.698830418	0.652388617	0.954147224
ZZEF1	0.474094985	0.168347973	0.866331903	0.309250747	0.966776358	0.652471848	0.954147224
ARAP2	0.904768621	0.901912701	0.352396573	0.19093835	0.379098179	0.653811647	0.954215012
BTBD2	0.825074216	0.749078406	0.107372038	0.400678797	0.77978619	0.653050616	0.954215012
C13H20orf96	0.713886917	0.255842713	0.168415454	0.946488527	0.713809383	0.653498951	0.954215012
CTNND1	0.778660215	0.907377678	0.440887293	0.097299348	0.684823686	0.653261164	0.954215012
EFL1	0.80271269	0.961598483	0.230366313	0.181913647	0.641213254	0.653121216	0.954215012
EZR	0.334676155	0.325087592	0.487168849	0.585401934	0.669118489	0.653310323	0.954215012
FRAT1	0.711224203	0.101892185	0.66169169	0.788165664	0.549846573	0.653490015	0.954215012
FTCDNL1	0.896480133	0.303909951	0.111987406	0.817087188	0.832212514	0.653173643	0.954215012
HMMR	0.778501161	0.046881866	0.89261461	0.692600551	0.920768642	0.653444843	0.954215012
LOC101904290	0.266084723	0.70721276	0.840164549	0.241528395	0.542970008	0.653048383	0.954215012
LOC104971220	0.2124529	0.710105751	0.294181595	0.557634055	0.839414039	0.653429225	0.954215012
LOC512323	0.497230251	0.089170204	0.661612769	0.934908244	0.756103576	0.653072199	0.954215012
MFSD4B	0.596277782	0.729976266	0.854048427	0.16372658	0.341769182	0.653682981	0.954215012
PHF8	0.787139771	0.696308996	0.704255188	0.074423105	0.724420582	0.653768511	0.954215012

PRUNE1	0.481799185	0.11539958	0.837378039	0.647567347	0.687544815	0.653003213	0.954215012
RAB40B	0.156667045	0.850788111	0.984194875	0.543286996	0.291708807	0.653578408	0.954215012
RNASEH2B	0.906121498	0.35041844	0.577098261	0.140274302	0.807316333	0.653212967	0.954215012
RTL8C	0.266878142	0.501389484	0.703472581	0.22227003	0.994492241	0.653739433	0.954215012
TRMT44	0.727367033	0.187737018	0.806524577	0.773909875	0.244121817	0.653739617	0.954215012
ZCCHC10	0.311696871	0.505986117	0.581471252	0.481323623	0.471631159	0.653838755	0.954215012
SYT3	0.120345651	0.674749326	0.938394948	0.913363891	0.299227815	0.653912987	0.954238488
FCAR	0.707916178	0.749142083	0.073842312	0.711012659	0.748351815	0.654016301	0.954304393
ABHD18	0.328272242	0.360344407	0.302073815	0.722121493	0.811348729	0.654935736	0.954444494
ABLIM2	0.686294465	0.180877527	0.862455418	0.797588911	0.244728601	0.654582385	0.954444494
ARHGAP19	0.500474767	0.969068947	0.303968065	0.454663229	0.312642634	0.655124237	0.954444494
ARPC2	0.731182711	0.236055659	0.400282022	0.781092834	0.387414233	0.654667237	0.954444494
CD320	0.475259756	0.256842056	0.206163727	0.959207731	0.864448272	0.654297591	0.954444494
CLSTN1	0.292380977	0.689529876	0.927514942	0.40661956	0.274388684	0.654260093	0.954444494
DAB2	0.828587451	0.95516991	0.820513392	0.066689661	0.482440572	0.654542615	0.954444494
GPX1	0.633485727	0.411781497	0.337090517	0.369474346	0.645476251	0.655264217	0.954444494
GTDC1	0.588162029	0.547579173	0.516437349	0.75415824	0.167332225	0.655438733	0.954444494
IQCE	0.732003472	0.858104721	0.482217811	0.737899443	0.093744954	0.655096818	0.954444494
IRS1	0.225688694	0.819057139	0.490286927	0.743150449	0.311153117	0.655134273	0.954444494
LOC101906855	0.692878899	0.195039763	0.884639618	0.703366054	0.249493194	0.655341515	0.954444494
LOC112446639	0.538373113	0.565729086	0.201178006	0.67049567	0.510808498	0.655404108	0.954444494
LOC534181	0.999804844	0.783444016	0.152078962	0.708674096	0.248627837	0.65543202	0.954444494
MCC	0.08617427	0.834835678	0.522115746	0.56816252	0.981181801	0.654973712	0.954444494
MED4	0.839905	0.249835495	0.463319236	0.528685428	0.408182386	0.655354642	0.954444494
METTL18	0.065899352	0.83421568	0.791764071	0.980368545	0.489859496	0.65463651	0.954444494
NSL1	0.921785262	0.029806092	0.882846106	0.890109821	0.969434141	0.654890335	0.954444494
PPIP5K1	0.441074427	0.864345863	0.67837914	0.10612311	0.760939774	0.654464147	0.954444494
SHPRH	0.25025668	0.528520068	0.453069154	0.798187465	0.437731415	0.654954325	0.954444494
STOML1	0.443658486	0.942954132	0.531692851	0.526471594	0.178974318	0.655152505	0.954444494
UNC45A	0.452803311	0.549048099	0.945331932	0.201181556	0.44395216	0.655449969	0.954444494
ZNF300	0.178288269	0.391532206	0.729094515	0.451950803	0.911682328	0.655261541	0.954444494
FGF18	0.34473451	0.504041628	0.318489659	0.785869587	0.482889511	0.655546953	0.954450317
TMEM134	0.629957336	0.515123039	0.266767999	0.492931075	0.492214849	0.655570287	0.954450317
BSDC1	0.759413599	0.980697808	0.38130339	0.713870562	0.104274098	0.656819252	0.954549644
C25H16orf91	0.334413333	0.18387894	0.600850659	0.736255947	0.77722942	0.65685515	0.954549644
C7H19orf53	0.764161224	0.459738877	0.35620937	0.208385439	0.812951599	0.657381612	0.954549644
CCDC146	0.504092172	0.929507217	0.163229931	0.631756582	0.438720091	0.657366444	0.954549644
COCH	0.461367108	0.110715187	0.993668124	0.424981898	0.977313632	0.656289539	0.954549644
EEA1	0.793904206	0.903324781	0.134751791	0.376514352	0.578327668	0.655931682	0.954549644
EP400	0.809177403	0.479472195	0.328378535	0.197920311	0.83956276	0.657108774	0.954549644

FGR	0.495259107	0.855880934	0.926457114	0.230261268	0.233120424	0.656277327	0.954549644
GINS3	0.085198506	0.686609752	0.698626816	0.666451473	0.775626155	0.656697223	0.954549644
HEATR1	0.494663312	0.532095808	0.731025885	0.21913111	0.501905961	0.657033949	0.954549644
IMPACT	0.224118889	0.748034485	0.292601687	0.722682727	0.596386838	0.65685177	0.954549644
ITFG2	0.811300114	0.455047502	0.209116955	0.590606942	0.464863179	0.65734464	0.954549644
JADE1	0.728615933	0.579794763	0.124771672	0.404205379	0.992363453	0.656855883	0.954549644
JOSD1	0.681927015	0.690146958	0.381385638	0.264714152	0.442933452	0.655957427	0.954549644
KTN1	0.914338673	0.527461905	0.271778508	0.445345145	0.361684021	0.656576602	0.954549644
LOC101906508	0.670395511	0.744234	0.117345481	0.784066573	0.460457677	0.656805557	0.954549644
LOC101907017	0.506887842	0.842191584	0.111599125	0.463701146	0.956886184	0.656820087	0.954549644
LOC112446381	0.423131755	0.080780992	0.944140685	0.827198646	0.791592874	0.656753321	0.954549644
LOC112448364	0.981038962	0.440493009	0.274737509	0.218388251	0.813070316	0.656290003	0.954549644
LOC614882	0.951337349	0.076254189	0.816036729	0.398315973	0.898193167	0.657189511	0.954549644
NUBP1	0.225711639	0.307518831	0.979033338	0.490957196	0.631221227	0.656087077	0.954549644
PCOLCE2	0.184265682	0.912779542	0.854458142	0.193290517	0.762630348	0.657243505	0.954549644
PLP2	0.99124188	0.549146057	0.073430089	0.960397711	0.548663396	0.656109645	0.954549644
RPS26	0.743604379	0.845873625	0.761085994	0.170373501	0.258236609	0.656110696	0.954549644
RWDD3	0.551204299	0.162360321	0.565412792	0.621801596	0.672758726	0.657083575	0.954549644
S100A13	0.558162857	0.679360851	0.185331752	0.687519819	0.43637927	0.656316928	0.954549644
SH3BGRL2	0.222926767	0.337938341	0.866154934	0.511261372	0.635446942	0.657374398	0.954549644
SMURF1	0.618420455	0.993594642	0.701959632	0.144961751	0.339061992	0.657383467	0.954549644
TMSB15B	0.141314301	0.967400189	0.258649296	0.640764217	0.935641973	0.6573717	0.954549644
WDR82	0.332681292	0.459691504	0.354771733	0.601373677	0.645380312	0.656067161	0.954549644
ZNF740	0.855391852	0.201578912	0.228809375	0.799095603	0.672916039	0.657520689	0.954664428
SPTSSB	0.34422066	0.859684632	0.838944837	0.094407343	0.905545282	0.657602249	0.954698381
LOC618256	0.808711784	0.419620086	0.83375552	0.648878501	0.115667746	0.657709448	0.954769549
CRAMP1	0.94542075	0.098455934	0.462689274	0.910343083	0.541955048	0.65782626	0.954770209
CUTC	0.945631175	0.126589843	0.52959537	0.88753816	0.37752295	0.657768121	0.954770209
ATF6B	0.949028426	0.417675154	0.417183957	0.697151342	0.184536004	0.658063471	0.954801674
GMEB1	0.687270831	0.732114057	0.694919105	0.674943681	0.09007914	0.657919498	0.954801674
LOC101902154	0.840086233	0.292450498	0.1937482	0.615154687	0.72680478	0.658136309	0.954801674
WSB1	0.920267911	0.638917603	0.465900423	0.184837961	0.420002383	0.657992383	0.954801674
ZCCHC2	0.660631157	0.855253274	0.529626808	0.995857307	0.071416868	0.658138842	0.954801674
SUPT7L	0.138691918	0.489160579	0.981242894	0.641850655	0.498242095	0.65819799	0.954803077
LOC112449282	0.946418658	0.238621007	0.485694515	0.293113493	0.662841782	0.658398629	0.95482849
MLEC	0.542640039	0.80456454	0.918052079	0.12393884	0.429105409	0.658448237	0.95482849
TAF6	0.242145696	0.173868601	0.742852415	0.759118797	0.897383511	0.658348138	0.95482849
ZC3H7A	0.395526069	0.937697804	0.218591209	0.337319826	0.779351946	0.658418966	0.95482849
LOC104976293	0.670648668	0.605374578	0.441403991	0.166564108	0.714373563	0.658515892	0.954842225
TUBGCP2	0.55615433	0.681585525	0.589006255	0.204607312	0.467052796	0.658632692	0.954927211

ABCA7	0.548463874	0.431443891	0.105407474	0.949839382	0.906705108	0.659949898	0.954960076
ALPK2	0.558127218	0.173388391	0.987069606	0.789973334	0.283993512	0.659484389	0.954960076
CARHSP1	0.415693167	0.272247348	0.963974461	0.367090304	0.536061895	0.659828803	0.954960076
EHBP1L1	0.709268512	0.779209226	0.252494063	0.493676903	0.311604015	0.659816189	0.954960076
ETV5	0.360028387	0.997445672	0.797542033	0.371418639	0.201973379	0.659984608	0.954960076
GTPBP1	0.969927154	0.724762161	0.582315681	0.176002476	0.297466637	0.659497084	0.954960076
LOC101903385	0.368738907	0.463310942	0.742225903	0.452080263	0.373475864	0.659297781	0.954960076
LOC101905770	0.558401234	0.518094036	0.308004336	0.73140069	0.328125689	0.659074305	0.954960076
LOC101908048	0.614123905	0.375889604	0.136964983	0.88870764	0.763153021	0.659607508	0.954960076
LOC112442757	0.808056671	0.245606897	0.267176903	0.690409619	0.586907467	0.659993735	0.954960076
LOC112448511	0.603514744	0.666451666	0.318731651	0.307731187	0.544028795	0.659777331	0.954960076
MACROD2	0.315745557	0.657127042	0.271680494	0.679617303	0.557179242	0.658714178	0.954960076
MYO7A	0.335115784	0.457518331	0.720577522	0.230599704	0.841150292	0.659481964	0.954960076
NACA	0.852449723	0.711344494	0.329686926	0.351456906	0.30462402	0.659244409	0.954960076
NUDT6	0.750998339	0.76907758	0.273913259	0.836807672	0.161539154	0.659082489	0.954960076
NUPL2	0.213244936	0.922665299	0.740417026	0.232409831	0.634220325	0.659875453	0.954960076
PEPD	0.197222062	0.409838258	0.931303308	0.984710682	0.288518086	0.659089301	0.954960076
PLEKHA2	0.975631238	0.378280899	0.192395994	0.386073462	0.779962701	0.659044046	0.954960076
RXRB	0.320508764	0.286782557	0.564480134	0.468204377	0.883015252	0.659674129	0.954960076
WDCP	0.650216303	0.620284147	0.198703544	0.950927566	0.281879879	0.659952441	0.954960076
WNT9A	0.843460698	0.281789201	0.237854157	0.934780138	0.405644474	0.659544091	0.954960076
YARS2	0.66505219	0.975801111	0.764212534	0.057321693	0.754832139	0.659743904	0.954960076
ZBED3	0.97032325	0.700023339	0.457207915	0.341891405	0.202262644	0.659899038	0.954960076
GLRX2	0.685070437	0.215341771	0.629022812	0.522289224	0.44352195	0.660082661	0.955004544
ABHD12	0.250087841	0.908078994	0.994912621	0.306360733	0.310774358	0.660227633	0.955050344
ACADVL	0.428054476	0.387635542	0.479436308	0.470447489	0.579905684	0.661950183	0.955050344
APBB1IP	0.405310999	0.336036313	0.51762936	0.506907785	0.606296496	0.66162992	0.955050344
AQP3	0.936538078	0.379375198	0.332730439	0.327616239	0.560683709	0.662061417	0.955050344
CCZ1	0.408755944	0.644377571	0.191269285	0.619148879	0.695823628	0.661960007	0.955050344
CDC25A	0.614068996	0.679073458	0.225401602	0.314572406	0.730303797	0.660960943	0.955050344
CLUH	0.44170829	0.294251702	0.736756113	0.563980349	0.401554872	0.661800074	0.955050344
CUX1	0.28044752	0.722052614	0.547036068	0.594707589	0.32968621	0.662092972	0.955050344
DAD1	0.68366978	0.182019659	0.803617626	0.384173014	0.561867673	0.660898151	0.955050344
EXOC1	0.984670831	0.334644554	0.473975699	0.498928887	0.277940989	0.661546836	0.955050344
FAM49A	0.726923596	0.341251236	0.252022787	0.676825948	0.512573709	0.661822028	0.955050344
GIPC2	0.656188344	0.331958354	0.234320833	0.45123022	0.935545376	0.660544443	0.955050344
ICMT	0.857316256	0.394483782	0.503183627	0.338838405	0.373572938	0.66049006	0.955050344
IQCD	0.663758246	0.155834921	0.794728526	0.609244356	0.433400624	0.661973708	0.955050344
ITM2B	0.86952164	0.966743579	0.760105202	0.583215093	0.058281897	0.662087216	0.955050344
KANSL1L	0.357387214	0.992159661	0.65640544	0.32011445	0.291144665	0.661853728	0.955050344

LHCGR	0.829203526	0.873519359	0.167135666	0.254066118	0.705644858	0.66195631	0.955050344
LOC100847612	0.79210194	0.671656487	0.803089721	0.578782673	0.087568519	0.66151713	0.955050344
LOC101905533	0.512689743	0.447918392	0.766871265	0.925656856	0.132748791	0.661382974	0.955050344
LOC112449618	0.989385851	0.722726636	0.195027602	0.176028485	0.878185882	0.66064271	0.955050344
NCAM2	0.286471376	0.217744459	0.99476994	0.41900711	0.828164532	0.660411274	0.955050344
NCBP2	0.670375143	0.208090062	0.728294571	0.759607176	0.279764755	0.66093551	0.955050344
PIM1	0.263605267	0.925627876	0.993715414	0.41831844	0.212588904	0.660685253	0.955050344
PLA2G15	0.881502507	0.110255526	0.377534921	0.883486931	0.666545043	0.661093441	0.955050344
PRKAR2B	0.542596351	0.236607709	0.246801623	0.943772622	0.724761538	0.661678668	0.955050344
RAB20	0.508816692	0.396761251	0.249472698	0.971723338	0.440314555	0.660559349	0.955050344
RALGPS2	0.093391569	0.560632415	0.786291695	0.77894461	0.67548041	0.66157675	0.955050344
RBCK1	0.68562277	0.358522524	0.574855955	0.198793102	0.766051448	0.660290643	0.955050344
SIRT7	0.434530736	0.245925148	0.361022654	0.956866621	0.585869512	0.661272532	0.955050344
TMEM184C	0.430468812	0.438846869	0.190794462	0.851336447	0.703934148	0.661023081	0.955050344
TMEM74B	0.883358937	0.931660635	0.671971995	0.040863405	0.958530799	0.661575786	0.955050344
TNNI2	0.942821895	0.135205965	0.266904704	0.719483327	0.886161865	0.661857612	0.955050344
ZDHHC6	0.978151465	0.172721302	0.635248083	0.868734571	0.232840897	0.662003817	0.955050344
ZNF358	0.362222582	0.889650975	0.197808356	0.81030025	0.419022859	0.661412969	0.955050344
SPG21	0.924319852	0.318941919	0.102706625	0.813181232	0.882996124	0.662289182	0.955165459
TERF1	0.367255738	0.678112519	0.650068888	0.584747301	0.229602197	0.662242357	0.955165459
SSBP3	0.238271913	0.790416175	0.902798621	0.134702796	0.949808234	0.662402838	0.955188713
ZADH2	0.785698897	0.817576382	0.745278338	0.12385512	0.36690808	0.662421714	0.955188713
DTX2	0.461501497	0.171517155	0.863123427	0.514525017	0.619464874	0.662602104	0.955280956
MFSD11	0.207799197	0.866181867	0.8484211	0.71891207	0.198329191	0.662579729	0.955280956
CEP162	0.964384836	0.908935769	0.102826711	0.461801043	0.525039661	0.663301008	0.955402443
CMTM3	0.171171965	0.698477687	0.585112718	0.316848813	0.986334274	0.663374661	0.955402443
LOC100297056	0.845818301	0.656346513	0.197441897	0.46742145	0.427865407	0.663896352	0.955402443
LOC100297498	0.99251883	0.970697266	0.440065996	0.058328964	0.885843397	0.663769214	0.955402443
LOC101907327	0.973775336	0.903323553	0.666805738	0.872129349	0.042769128	0.663514359	0.955402443
LOC107133150	0.338607309	0.987042458	0.154957742	0.447120136	0.941932136	0.662921386	0.955402443
LOC614617	0.531307056	0.945100663	0.791136659	0.237227563	0.231634843	0.663080413	0.955402443
MAP7	0.835135595	0.611235176	0.087249058	0.635216836	0.773800927	0.663632903	0.955402443
NDFIP1	0.964104627	0.101205871	0.690216879	0.99913295	0.325240258	0.663572736	0.955402443
NFXL1	0.642957163	0.2213177	0.850344228	0.46977248	0.383900129	0.663015259	0.955402443
NR4A1	0.579634171	0.467783504	0.337723539	0.774546327	0.308961664	0.66382773	0.955402443
OCIAD1	0.578977215	0.406134496	0.870712432	0.798571121	0.133701737	0.66335487	0.955402443
RPS2	0.897247188	0.527587148	0.381551218	0.610588551	0.198088835	0.663225413	0.955402443
SEC22C	0.85901066	0.217254717	0.658529506	0.402985901	0.441851829	0.663557722	0.955402443
SOWAHA	0.338681098	0.456838937	0.445227893	0.646799736	0.491169594	0.663570368	0.955402443
STK3	0.582884579	0.867064222	0.777056458	0.121550098	0.459252732	0.663908931	0.955402443

TRIM28	0.965944205	0.843403125	0.033095363	0.91147033	0.888678959	0.663168967	0.955402443
TYW1	0.965890346	0.051733292	0.841983909	0.786877867	0.660262852	0.663341061	0.955402443
VPS39	0.534922612	0.745396849	0.133782536	0.926209031	0.441695474	0.663020236	0.955402443
ZNF16	0.85466482	0.759354765	0.367623276	0.280257621	0.327805751	0.663874921	0.955402443
ZNF2	0.090053823	0.775856152	0.856034253	0.475916953	0.766788808	0.663052296	0.955402443
SHISA6	0.407392174	0.360162209	0.410316822	0.985519103	0.370040726	0.664200666	0.95573846
LOC107132410	0.805408175	0.611177159	0.056165094	0.911920149	0.871140885	0.664267917	0.955751428
ACTN4	0.722419765	0.437483366	0.308801009	0.406268063	0.555074832	0.664670706	0.955821392
CHMP4B	0.596742764	0.143368302	0.401474901	0.740744261	0.865854102	0.664857752	0.955821392
HACD3	0.460585215	0.465215431	0.234150464	0.982416433	0.447176578	0.664957214	0.955821392
LOC101906312	0.56086186	0.467801395	0.270452006	0.651409457	0.476697657	0.664899399	0.955821392
MAP2	0.554525623	0.523016742	0.41480989	0.951509987	0.192187102	0.664594476	0.955821392
MAP3K20	0.26189232	0.152616621	0.950503046	0.805647944	0.718949289	0.664638268	0.955821392
MCM3AP	0.875343948	0.074326992	0.827133193	0.860841706	0.475087949	0.664672479	0.955821392
MORC3	0.293138416	0.29605215	0.968049368	0.621432268	0.422110229	0.66492333	0.955821392
NR6A1	0.532781631	0.94478488	0.801825001	0.2844767	0.191688749	0.664675633	0.955821392
RBM6	0.941769461	0.333874388	0.286618804	0.787349615	0.310551178	0.664912156	0.955821392
WDR93	0.422944075	0.170731551	0.513590113	0.598210192	0.993284974	0.664915682	0.955821392
ATG5	0.310565105	0.265523372	0.499478161	0.95045256	0.564254702	0.665379864	0.956105734
SLC25A23	0.507666404	0.563293819	0.433519936	0.350536121	0.508305951	0.665381166	0.956105734
TP53I11	0.412290623	0.438266249	0.228790649	0.549053443	0.97319864	0.665388069	0.956105734
TRPC2	0.572873536	0.182377955	0.358911907	0.76156765	0.773065663	0.665275205	0.956105734
ATG2B	0.241429387	0.529183261	0.581189229	0.313301493	0.950384756	0.665558166	0.956109125
CCDC25	0.582659806	0.653566416	0.437435052	0.346445665	0.383125783	0.665566748	0.956109125
LOC100848721	0.873988245	0.159821786	0.870493774	0.271262671	0.670206699	0.665526966	0.956109125
SETX	0.497934436	0.196626964	0.583304121	0.814612108	0.475401347	0.665623469	0.956109125
CDK5RAP3	0.607638226	0.211708308	0.46426489	0.478974413	0.774910106	0.666067167	0.956495299
EZH1	0.695662695	0.828260298	0.3384904	0.948652968	0.119808596	0.666064836	0.956495299
ZNF260	0.437554902	0.471611938	0.460917526	0.278941952	0.835211968	0.66599492	0.956495299
RECK	0.230013278	0.722682371	0.85839395	0.188504107	0.825015038	0.666271881	0.95670556
KIAA0391	0.435685843	0.92511845	0.757485464	0.926906692	0.078518815	0.666533851	0.956997991
LOC101904173	0.99704793	0.902842547	0.270131182	0.949908784	0.096262242	0.666661797	0.957014237
MDM1	0.880394459	0.26254988	0.735709486	0.922277445	0.141763424	0.666653118	0.957014237
GLA	0.343257225	0.707665908	0.142699217	0.712607038	0.900500283	0.666734705	0.957035183
LOC784738	0.723894512	0.640720106	0.207697524	0.382758796	0.603995241	0.666972021	0.957292097
BET1	0.692920822	0.197617261	0.876335458	0.389310332	0.477594646	0.667330378	0.957479078
LOC100848212	0.341684521	0.326945268	0.392375321	0.636452046	0.799340829	0.667225406	0.957479078
SIN3B	0.862166881	0.509197593	0.492573648	0.957677322	0.107710759	0.667282436	0.957479078
TMEM150A	0.152437194	0.476348704	0.667081044	0.592531371	0.777392233	0.667335671	0.957479078
DAPK1	0.442024102	0.15140165	0.92517752	0.993952262	0.364605466	0.668429531	0.957673684

GPR27	0.132842498	0.342033529	0.634903596	0.910541853	0.85228663	0.667985965	0.957673684
HAT1	0.076157576	0.5833893	0.923026193	0.551017454	0.992319853	0.668300794	0.957673684
LOC100335553	0.449220482	0.373363717	0.641975537	0.658850772	0.31592644	0.668202591	0.957673684
LOC104971307	0.790044334	0.128038026	0.869793901	0.48260627	0.527785951	0.668191137	0.957673684
LOC104973551	0.520502244	0.289381574	0.389960359	0.833468815	0.457692799	0.668155249	0.957673684
LOC112444921	0.837602785	0.123886346	0.411419559	0.982596341	0.533910941	0.668071602	0.957673684
LOC513573	0.491987224	0.075985327	0.914075956	0.852623282	0.770269818	0.668463351	0.957673684
LOC780968	0.901402632	0.213994632	0.478665479	0.244453046	0.990713774	0.667761476	0.957673684
LYRM2	0.815457077	0.187975127	0.20990465	0.838243934	0.828278094	0.667570231	0.957673684
MAPK1IP1L	0.69784996	0.088231673	0.604803153	0.630349827	0.952291612	0.667696472	0.957673684
MFF	0.944121987	0.318263993	0.652717753	0.250070492	0.457480893	0.668423319	0.957673684
NRROS	0.433979434	0.742255646	0.276944501	0.269840919	0.930709867	0.668137418	0.957673684
STARD8	0.33281785	0.464735515	0.45860097	0.402197116	0.784955883	0.668045284	0.957673684
STRADA	0.395745983	0.159561013	0.457479529	0.950057217	0.817052284	0.668306138	0.957673684
STXBP4	0.331270367	0.918359221	0.838816803	0.767892206	0.114429223	0.668299398	0.957673684
SYCE2	0.758124627	0.419907942	0.942908041	0.175089212	0.42542393	0.667738147	0.957673684
ABL2	0.987249589	0.209354165	0.566554392	0.252386953	0.760522817	0.668760182	0.957830144
C27H8orf48	0.19473155	0.596806277	0.233312716	0.923109802	0.898956414	0.668970811	0.957830144
CDRT4	0.555199987	0.444725089	0.330723071	0.352846432	0.780355634	0.668829327	0.957830144
DOK2	0.510723889	0.281138929	0.578524877	0.299706698	0.90439555	0.669097847	0.957830144
LOC101905127	0.867532898	0.332351506	0.343946844	0.594756281	0.381612267	0.66903174	0.957830144
LOC107132958	0.568800859	0.351479219	0.989518398	0.345439149	0.32874067	0.668661496	0.957830144
OSBP	0.254020599	0.720989276	0.421669917	0.418882607	0.694792267	0.668754964	0.957830144
PARP8	0.704790035	0.400577	0.613151567	0.721644814	0.180197731	0.669054164	0.957830144
UCHL5	0.905812331	0.227326258	0.436086667	0.716350108	0.349643374	0.668886327	0.957830144
RGMB	0.908024655	0.998358977	0.461474461	0.845007158	0.063762567	0.669309965	0.95796667
SFXN3	0.188078922	0.708916858	0.309490176	0.567335483	0.962686544	0.669287119	0.95796667
RIOX2	0.935470944	0.719642663	0.160023942	0.364601001	0.574286387	0.669453261	0.958088207
ANKRD28	0.562070347	0.26972343	0.61355718	0.53273023	0.455779866	0.669698531	0.958112019
CCNH	0.245636325	0.212737339	0.720127146	0.971036373	0.619026818	0.669995341	0.958112019
FAM234B	0.272626494	0.715407703	0.317037962	0.731470096	0.499525123	0.66977009	0.958112019
LOC112444340	0.69647308	0.188795904	0.787479011	0.363523483	0.600587136	0.66988528	0.958112019
LOC112447359	0.751220005	0.06251441	0.989923394	0.506195719	0.96112142	0.669975275	0.958112019
MRC1	0.426581693	0.369715944	0.881993051	0.413387041	0.392718413	0.66967474	0.958112019
SPAST	0.745217564	0.976330999	0.535710925	0.060871069	0.951731392	0.669657878	0.958112019
TAF9B	0.497109235	0.801554495	0.091817621	0.827739	0.745892491	0.669722554	0.958112019
ZNF283	0.67150078	0.557146491	0.592627658	0.304468673	0.335062789	0.669984692	0.958112019
LOC101905156	0.117615126	0.301726495	0.837130309	0.997693564	0.765787785	0.670659384	0.958596822
LOC107132335	0.2853207	0.496233195	0.330111299	0.53021479	0.914788913	0.670425467	0.958596822
LSM2	0.645464675	0.611624118	0.512978888	0.199349954	0.562122432	0.670627337	0.958596822

RAD51C	0.083072278	0.387965163	0.871550316	0.925469498	0.873033195	0.670640999	0.958596822
SLC30A7	0.595539296	0.770441874	0.468252576	0.416975452	0.253391026	0.670684828	0.958596822
UPF1	0.956167583	0.570456919	0.367572324	0.811525419	0.139510584	0.670674436	0.958596822
LRRC25	0.642357874	0.365706211	0.336895425	0.344297708	0.834316632	0.670969815	0.958920634
PDZD8	0.528969644	0.187353487	0.552075246	0.599523544	0.693765024	0.671166509	0.959034705
ZBTB25	0.935579988	0.131770307	0.383479227	0.870074723	0.553124378	0.671126371	0.959034705
CAMKMT	0.925943419	0.460254017	0.447186815	0.126999814	0.942804467	0.671696867	0.959078521
GRSF1	0.343394887	0.263809073	0.632750977	0.912559092	0.436635448	0.671875955	0.959078521
HYAL3	0.799874945	0.677768947	0.667655822	0.815636016	0.077387159	0.671931981	0.959078521
IFNGR2	0.581083779	0.901276926	0.545496202	0.097289985	0.821317314	0.671773804	0.959078521
LOC104970809	0.830170829	0.536857777	0.502532506	0.952985244	0.106994471	0.671850039	0.959078521
LOC112442221	0.924323956	0.61164158	0.368943312	0.206913057	0.528526023	0.671625065	0.959078521
NCL	0.846056768	0.408488857	0.58925848	0.200461623	0.55864432	0.671587989	0.959078521
PROSER3	0.675994435	0.1344263	0.39207956	0.857686387	0.747734436	0.671956909	0.959078521
PWP2	0.364439125	0.671541042	0.49743668	0.60948912	0.307708384	0.671806347	0.959078521
RNF24	0.950679738	0.89300637	0.222466968	0.365865974	0.329893567	0.671497687	0.959078521
RXRG	0.488590973	0.153570542	0.495120729	0.824831851	0.743457243	0.67137952	0.959078521
SETD1A	0.438668426	0.195357131	0.695904719	0.417340959	0.916073818	0.671536451	0.959078521
WSCD2	0.898241554	0.61607264	0.230330441	0.502736717	0.356066983	0.671675844	0.959078521
TMED8	0.964054331	0.451639961	0.341367008	0.821482921	0.187271265	0.672094419	0.959191365
LOC104969648	0.988908857	0.954964106	0.878026567	0.193470187	0.142707424	0.672329741	0.95922304
TRMT9B	0.364382095	0.462777657	0.73574628	0.665501221	0.277125391	0.672227422	0.95922304
UBE2S	0.390024199	0.481201099	0.39771961	0.712992561	0.430173328	0.67233513	0.95922304
XPNPEP1	0.928477903	0.222786786	0.965652588	0.335981153	0.341162284	0.672350413	0.95922304
SMC6	0.313804534	0.645132702	0.632718157	0.594114166	0.301075312	0.672487079	0.959334618
LOC100847613	0.142767316	0.997752043	0.419898045	0.988734775	0.387859946	0.672704456	0.95947791
SBDS	0.768590714	0.223536961	0.46153661	0.793668455	0.364401437	0.672668621	0.95947791
DYNLT1	0.24346443	0.206805527	0.683901887	0.785880906	0.849002067	0.673020368	0.959594897
FBXO9	0.481154024	0.564926719	0.80095776	0.527665431	0.199990125	0.673018624	0.959594897
HEATR6	0.771761742	0.681922578	0.205450559	0.374850086	0.566588684	0.672928407	0.959594897
NAB1	0.051065482	0.944934841	0.743903306	0.900498214	0.710676685	0.672995237	0.959594897
TMEM106B	0.50985766	0.720734979	0.126877375	0.714832378	0.690021333	0.67320738	0.959778153
DCUN1D4	0.59613971	0.267516027	0.372306399	0.930288225	0.416542024	0.673296405	0.959821691
SLC27A1	0.591260917	0.541577162	0.881819663	0.212370237	0.383847681	0.673384499	0.959863894
PMP2	0.777954749	0.957156268	0.074217759	0.443375859	0.940741714	0.673659539	0.960089161
SP4	0.220035681	0.557020116	0.415544894	0.483800907	0.935276124	0.673615311	0.960089161
MAP3K7	0.96706925	0.546726414	0.445156343	0.974444908	0.100623058	0.67388628	0.960245527
SLC8B1	0.46052406	0.614932632	0.177052384	0.999414748	0.460520816	0.673878089	0.960245527
AGTR2	0.543818289	0.141187355	0.729524547	0.467996814	0.881684924	0.674177018	0.960326277
CLEC4E	0.833023251	0.338793415	0.398823485	0.217620272	0.943433387	0.674149129	0.960326277

LOC104974137	0.557950965	0.886515966	0.498995897	0.875331602	0.106890942	0.674019441	0.960326277
PLCG1	0.568779712	0.141073241	0.712576562	0.722999222	0.558855168	0.674092834	0.960326277
ALYREF	0.706821506	0.15073706	0.310680344	0.843567498	0.828003943	0.674244014	0.960338354
OXCT1	0.955956693	0.44770483	0.547955055	0.355333776	0.277671819	0.674399195	0.960438721
SLC2A8	0.822422132	0.247052762	0.155170629	0.906621822	0.809646479	0.674431529	0.960438721
IQCK	0.960009681	0.741642948	0.194511022	0.860447611	0.194469371	0.674688221	0.960577337
LNPK	0.734409492	0.773321321	0.549555159	0.557768334	0.133100283	0.674666803	0.960577337
RNF135	0.984144685	0.910390043	0.367627799	0.085217684	0.825661798	0.674704464	0.960577337
RAPGEF5	0.223819842	0.834630059	0.151066986	0.915024939	0.897856637	0.674782794	0.96060552
EGLN2	0.720388512	0.421696888	0.34159044	0.537720556	0.41601064	0.675020319	0.960630299
LOC101903526	0.76971622	0.495968905	0.78777844	0.34561962	0.223268433	0.674967145	0.960630299
MTRR	0.116191352	0.783285849	0.478795448	0.867201494	0.614329327	0.675034343	0.960630299
TMEM38A	0.951342663	0.635852547	0.341320741	0.616897214	0.182176781	0.674943481	0.960630299
LOC101902861	0.726415855	0.771884221	0.300365812	0.439461483	0.313889457	0.675177312	0.960667147
SPIN1	0.53781347	0.445318433	0.705189736	0.274422093	0.501211198	0.675160632	0.960667147
DERL2	0.311995386	0.319219614	0.460720949	0.969437171	0.522893466	0.675410876	0.960782104
LOC101902290	0.827850255	0.246216032	0.927389585	0.821531149	0.149843029	0.675492287	0.960782104
LY9	0.818290675	0.594437157	0.709460287	0.119934473	0.561718066	0.675319554	0.960782104
POC1A	0.958340334	0.331302814	0.889352906	0.134898466	0.610726269	0.675439475	0.960782104
AEBP1	0.397479169	0.739670073	0.308401066	0.311737194	0.823633799	0.67558166	0.960782723
LOC104975911	0.649923678	0.198981169	0.523382866	0.373844921	0.920449422	0.675668357	0.960782723
LOC112447140	0.659578058	0.909226573	0.158963568	0.483023924	0.505800987	0.675667903	0.960782723
COMMD10	0.923385544	0.451700972	0.665305194	0.521167822	0.161145854	0.675787336	0.960794926
IL17RC	0.053750337	0.862470422	0.774731955	0.792749997	0.818565838	0.67579403	0.960794926
LOC783421	0.77974668	0.454300107	0.646168206	0.195094281	0.522675126	0.676084927	0.961125238
AOC3	0.291379854	0.524373026	0.588271043	0.61435092	0.423558969	0.676481945	0.961356529
CD58	0.938029319	0.375138113	0.427241754	0.275918826	0.563679326	0.676431507	0.961356529
GAREM1	0.452728331	0.826105839	0.7965007	0.867800405	0.090433197	0.676392462	0.961356529
SLC38A7	0.207146169	0.881556506	0.51091297	0.735996647	0.340568311	0.676457988	0.961356529
BRIP1	0.858826796	0.238808241	0.813182107	0.512185039	0.273957255	0.676591154	0.961424737
REV3L	0.620717826	0.169090289	0.88147001	0.353732918	0.715289532	0.67664711	0.961424737
CIC	0.835393946	0.26905716	0.377404729	0.499633216	0.553377695	0.67702129	0.961454044
ENHO	0.348279813	0.41830259	0.756711579	0.252164608	0.844191143	0.677136423	0.961454044
IL1R1	0.235540021	0.228837745	0.776532259	0.714597031	0.784524056	0.677111961	0.961454044
LOC101903586	0.589886226	0.483505468	0.225336788	0.718272337	0.507917378	0.676962287	0.961454044
MOCS1	0.342048413	0.954758775	0.18733597	0.514162034	0.745371962	0.676958499	0.961454044
OPLAH	0.703854669	0.406154011	0.509678724	0.270219706	0.595352418	0.676907585	0.961454044
RPP25	0.541282066	0.121444859	0.941835385	0.940704654	0.402831945	0.677083234	0.961454044
TMA16	0.836663974	0.468522231	0.712909004	0.365608515	0.229338073	0.676839053	0.961454044
PPHLN1	0.846049142	0.99063944	0.051449897	0.593658239	0.917398847	0.677277547	0.961571228

LOC101904097	0.863075774	0.753555299	0.185553783	0.936738246	0.207983248	0.677496226	0.961625235
LOC112447858	0.665936568	0.47163873	0.994185883	0.588743146	0.127966553	0.677608568	0.961625235
PPOX	0.654888008	0.191532095	0.306585203	0.788406324	0.775480114	0.677496261	0.961625235
YOD1	0.396507078	0.958167504	0.590582213	0.155880073	0.671961275	0.677419083	0.961625235
ZNF74	0.874006553	0.135439968	0.330712159	0.928097722	0.647329157	0.677563199	0.961625235
PKM	0.339374384	0.835245847	0.233210652	0.400202583	0.89006014	0.677790942	0.961634577
SIL1	0.24033801	0.501814245	0.391251704	0.706080054	0.706525064	0.67772946	0.961634577
SLC35B3	0.427336352	0.517211602	0.147468112	0.837700015	0.862265628	0.677757395	0.961634577
ATG16L2	0.319045397	0.209847068	0.948321893	0.427506985	0.870758256	0.678509602	0.961943268
CLP1	0.725293532	0.550375601	0.454395728	0.141489081	0.920738399	0.678471428	0.961943268
LOC100337328	0.094773809	0.926691539	0.420237427	0.68286175	0.936451877	0.678235061	0.961943268
LOC100848527	0.4488855	0.903600473	0.228663092	0.344309757	0.739731661	0.678411316	0.961943268
LOC101907126	0.708217679	0.672677652	0.119321717	0.499239786	0.831411424	0.678182386	0.961943268
MIA3	0.73364746	0.807514337	0.813977003	0.075907596	0.645160404	0.678353818	0.961943268
NDUFAF2	0.801021673	0.19730861	0.948179769	0.310920714	0.507625562	0.678653291	0.961943268
RPUSD4	0.427163496	0.956306148	0.95374154	0.645701655	0.093744533	0.678084776	0.961943268
TEX14	0.51243154	0.977925889	0.649991716	0.873323601	0.083097087	0.678535755	0.961943268
TIMELESS	0.978150355	0.146268146	0.786229628	0.860505358	0.244294312	0.678607611	0.961943268
TXNRD1	0.678642483	0.321666774	0.405944849	0.403322386	0.66142025	0.678550689	0.961943268
ERCC6L2	0.441195475	0.754017278	0.224190531	0.59474599	0.534133261	0.678981826	0.962314624
LOC101904042	0.701323305	0.49588664	0.692127938	0.193414168	0.509197134	0.679093555	0.962314624
MUM1L1	0.875983034	0.52259555	0.819044271	0.772283431	0.081892099	0.679149837	0.962314624
TM4SF5	0.717921911	0.918215285	0.390012456	0.136331664	0.676225103	0.679060972	0.962314624
LOC100848869	0.779933199	0.187974445	0.413205891	0.970818364	0.403901199	0.67948354	0.962372001
LOC112444752	0.807240193	0.23416144	0.464324401	0.994694239	0.271892102	0.679345315	0.962372001
LOC112446733	0.951135765	0.476100871	0.220280665	0.554546041	0.429414091	0.679481721	0.962372001
SMIM1	0.772126616	0.413545625	0.673702239	0.331309034	0.333172333	0.679415007	0.962372001
SPAG1	0.216337185	0.613973853	0.743140825	0.779034976	0.308683423	0.679343788	0.962372001
ANKRD46	0.257514386	0.512673472	0.513594456	0.754831072	0.465204493	0.679938531	0.962746385
ECH1	0.704099394	0.465689623	0.563275617	0.369614315	0.348742881	0.679915918	0.962746385
LOC112444190	0.567704994	0.725387949	0.835393127	0.462434289	0.149640831	0.679906322	0.962746385
RWDD2A	0.231237693	0.601657671	0.3132515	0.60965865	0.896328187	0.679982533	0.962746385
CNTNAP4	0.63072971	0.652522054	0.137625135	0.961172332	0.43764502	0.68007368	0.962792371
SAAL1	0.215916079	0.802498236	0.326395559	0.604544335	0.697546573	0.680258762	0.962971323
ACBD5	0.173510249	0.837452876	0.307179441	0.956228029	0.566475731	0.682907879	0.963188116
ARL13A	0.861259802	0.941395116	0.175027034	0.35603349	0.477253619	0.682387198	0.963188116
BABAM1	0.579614097	0.615471296	0.788920831	0.124322141	0.685064984	0.681232917	0.963188116
BCL2L2	0.379299117	0.304037463	0.676576352	0.537319482	0.574511063	0.682166885	0.963188116
CDYL	0.661028078	0.135153679	0.568549326	0.75153455	0.633873574	0.683062441	0.963188116
CEACAM19	0.271831658	0.884697315	0.349342574	0.709368336	0.402336142	0.681298282	0.963188116

CHRM3	0.399242467	0.196839463	0.840831511	0.532931429	0.686509282	0.682887924	0.963188116
CPE	0.908075937	0.439701312	0.107350992	0.763009083	0.739668224	0.683011291	0.963188116
CWF19L2	0.143484356	0.3851091	0.772719417	0.657844874	0.852248954	0.680983238	0.963188116
DCSTAMP	0.495828239	0.545318214	0.249139657	0.559690628	0.634881629	0.680967546	0.963188116
DHDH	0.128462172	0.877583372	0.27864323	0.944430111	0.810105059	0.681751061	0.963188116
DTNBP1	0.503576037	0.938149189	0.188042411	0.9130657	0.298263015	0.683029792	0.963188116
ENGASE	0.439392579	0.834678604	0.277810395	0.328045285	0.71837934	0.68156608	0.963188116
EXO5	0.480237923	0.4760711	0.166391232	0.677628849	0.935551156	0.682416951	0.963188116
F8A1	0.72249361	0.604761625	0.64647365	0.086830321	0.987103439	0.683167355	0.963188116
FKBPL	0.227099559	0.519678905	0.570618135	0.556224046	0.639369707	0.681071758	0.963188116
GMFG	0.480748764	0.205170486	0.891788923	0.461343413	0.594016875	0.682327917	0.963188116
GSTA2	0.108624083	0.901712209	0.488771399	0.846098813	0.594831607	0.682237377	0.963188116
HNRNPL	0.056618341	0.738065098	0.627392401	0.945767556	0.975939429	0.683076164	0.963188116
KCNJ11	0.696187451	0.084447376	0.654326623	0.828191959	0.755246589	0.681975331	0.963188116
LACC1	0.12790148	0.883030472	0.663610305	0.391691208	0.817800616	0.681542695	0.963188116
LOC100296832	0.861015749	0.448327986	0.377926765	0.592010091	0.280051017	0.682979317	0.963188116
LOC101902204	0.453663502	0.258540488	0.745423866	0.303062562	0.910685796	0.682527595	0.963188116
LOC101906426	0.705257728	0.815167765	0.376833769	0.113942199	0.980800646	0.683170414	0.963188116
LOC112441770	0.460299191	0.931573089	0.556786006	0.392747267	0.256859377	0.682165694	0.963188116
LOC112443147	0.351530757	0.123100228	0.754588225	0.815563108	0.906470914	0.682606475	0.963188116
LOC617224	0.765194478	0.898858874	0.917841437	0.391591418	0.097244511	0.681798263	0.963188116
LOC785605	0.389818563	0.682381897	0.506357931	0.487942864	0.366775652	0.682327406	0.963188116
MR1	0.184920574	0.768788601	0.765438299	0.442617168	0.496406159	0.680745983	0.963188116
MT3	0.076073608	0.897203917	0.92409747	0.794295991	0.48299441	0.683062399	0.963188116
MYO1C	0.364138145	0.415205158	0.674949325	0.494283373	0.478502114	0.682569192	0.963188116
NMNAT2	0.136691928	0.466552333	0.831894429	0.579380685	0.786904644	0.68298676	0.963188116
NMRK1	0.578379569	0.115291016	0.895543443	0.848555551	0.475412145	0.682206481	0.963188116
PLAC8	0.651470221	0.163994342	0.843351366	0.384264745	0.694394436	0.68181586	0.963188116
PRPF31	0.079796736	0.877728187	0.842333952	0.588142854	0.696317083	0.682773796	0.963188116
PTPN6	0.717485654	0.458806757	0.972113704	0.13669512	0.552934747	0.682982045	0.963188116
RHPN2	0.901635364	0.837104073	0.392803733	0.273648484	0.298248609	0.683057964	0.963188116
RND3	0.228250236	0.898855076	0.967881002	0.313029547	0.385142302	0.680996295	0.963188116
SEC23IP	0.72062377	0.322604006	0.354298217	0.36966085	0.79019252	0.681956156	0.963188116
SLC39A10	0.642306319	0.60211187	0.542324724	0.182294803	0.629049961	0.681891352	0.963188116
SOS2	0.245628487	0.317935891	0.641078336	0.539096426	0.893004419	0.682297275	0.963188116
STRAP	0.861028103	0.11654265	0.691676689	0.548852638	0.629120879	0.68120386	0.963188116
TMEM143	0.800678106	0.625678936	0.645884717	0.093003518	0.801801157	0.68251156	0.963188116
TRMT11	0.862649301	0.288620717	0.231806083	0.667836613	0.628030277	0.683138089	0.963188116
TWIST1	0.595735825	0.981231293	0.363810472	0.188678742	0.596778557	0.681044004	0.963188116
ZNF236	0.669650256	0.188088582	0.891060089	0.317485064	0.67689209	0.682435687	0.963188116

ZNF317	0.157570694	0.370233503	0.72537427	0.96852941	0.590124128	0.682974257	0.963188116
DNPH1	0.308769549	0.620353935	0.88478639	0.247630988	0.577198972	0.68327415	0.963213703
TTC12	0.44292808	0.472702945	0.210723119	0.765029281	0.717798639	0.683305949	0.963213703
LOC112442248	0.486964044	0.71048667	0.35157449	0.427114588	0.46666017	0.683439936	0.963244206
RPL37A	0.598985519	0.404725345	0.1833156	0.812426187	0.671531804	0.683444978	0.963244206
LOC100297725	0.811295542	0.249876205	0.305587867	0.683884497	0.572655885	0.683574063	0.963260686
LOC788672	0.77917796	0.864209015	0.131116654	0.776207064	0.354014026	0.683571741	0.963260686
DNM3	0.51205256	0.35003542	0.705784214	0.875396022	0.219305732	0.683768363	0.963286342
LOC112444653	0.66554054	0.537212073	0.105764283	0.834468828	0.769422875	0.683715607	0.963286342
PSMG1	0.853970341	0.158105306	0.45821842	0.650535663	0.603263961	0.683718165	0.963286342
DKC1	0.155628667	0.393421295	0.802822385	0.819098975	0.60432357	0.684133303	0.963287233
FAM83H	0.368671949	0.844411631	0.205840572	0.44529048	0.853439138	0.684297274	0.963287233
LOC101905399	0.541978252	0.539274308	0.322984531	0.270057642	0.954977523	0.684244615	0.963287233
LOC112447392	0.507699074	0.232485346	0.851610842	0.428700138	0.564561387	0.684104406	0.963287233
MYC	0.417112293	0.581610816	0.291192543	0.460128091	0.747793753	0.683934528	0.963287233
ODR4	0.471043144	0.738382626	0.876863426	0.121623546	0.656498297	0.684290211	0.963287233
PLIN4	0.783708689	0.681104509	0.73870369	0.389985939	0.158102203	0.683978713	0.963287233
POLE4	0.510593981	0.62292177	0.667631457	0.574939506	0.199273288	0.684108544	0.963287233
USP8	0.624028605	0.62986605	0.270557343	0.633623051	0.360833766	0.683989666	0.963287233
LOC107133284	0.50835531	0.634009253	0.591708174	0.167473163	0.762773066	0.684372872	0.963311022
PILRA	0.710471279	0.631097602	0.318254044	0.223598239	0.763945193	0.684478114	0.963376529
ZNF329	0.505260887	0.467754371	0.861185184	0.352073247	0.340697603	0.684782785	0.96372269
ZNF292	0.233376025	0.434761281	0.475408312	0.554412841	0.913601521	0.68493042	0.963847807
KIF1C	0.190859517	0.573890131	0.685259849	0.449255937	0.726154885	0.685356214	0.96387619
LOC100296952	0.25969048	0.712436554	0.177029353	0.813190049	0.918343937	0.685144423	0.96387619
LOC533308	0.221570063	0.925749856	0.30153932	0.957944263	0.413098182	0.685276691	0.96387619
LOC786914	0.90061683	0.346748539	0.485711931	0.722135782	0.223553258	0.685361725	0.96387619
MSC	0.835495567	0.645326259	0.213903964	0.952520555	0.222649321	0.685141365	0.96387619
OXSR1	0.792483726	0.909664681	0.445383947	0.633688752	0.120226005	0.685160324	0.96387619
YIPF4	0.810189778	0.694862821	0.289566561	0.790061923	0.190097069	0.685333899	0.96387619
GLS2	0.644008487	0.337215922	0.228153969	0.558198069	0.887373804	0.685801811	0.964290529
PLA2R1	0.784960653	0.681883573	0.167320052	0.347018565	0.789828976	0.685832615	0.964290529
TMEM39A	0.646049866	0.428579009	0.395686167	0.352565592	0.635136834	0.685727262	0.964290529
ZBTB21	0.789161055	0.588107392	0.382661142	0.21037198	0.657631752	0.686017396	0.964467703
E2F8	0.980361125	0.512356883	0.190451139	0.544775128	0.471721251	0.686123086	0.964514483
LOC100848570	0.801913787	0.156462132	0.825904858	0.297977881	0.79633434	0.686168216	0.964514483
ARSB	0.835098916	0.34203303	0.209642064	0.618403355	0.664385113	0.686270375	0.964542955
FBXO38	0.627368914	0.580203553	0.177398259	0.471766157	0.808241383	0.686423568	0.964542955
LOC107132577	0.445200421	0.529055383	0.188485865	0.807171059	0.687082557	0.686418852	0.964542955
LOC112445194	0.795762255	0.363027774	0.815974033	0.781579911	0.133593279	0.686351048	0.964542955

LOC112444520	0.362381392	0.994477425	0.393489784	0.933651878	0.186056365	0.686513923	0.964587328
ACAP2	0.666644995	0.292698458	0.354823192	0.491415351	0.724958449	0.686765202	0.964617927
BCDIN3D	0.75836128	0.438485343	0.666254677	0.378024816	0.295549324	0.687446683	0.964617927
DYNC1I1	0.524309327	0.60549333	0.295553441	0.616887156	0.427561939	0.687409321	0.964617927
EIF2D	0.389278668	0.476471054	0.753487057	0.287512932	0.615781948	0.687373551	0.964617927
FGF16	0.704797465	0.958043493	0.377460683	0.513038523	0.18874974	0.686884684	0.964617927
HORMAD2	0.618556901	0.935582712	0.477255887	0.190112652	0.471480722	0.687476161	0.964617927
LOC107132327	0.988470934	0.635901809	0.504047754	0.823873788	0.094619227	0.687021671	0.964617927
LOC107132748	0.136805552	0.9845328	0.246430448	0.911646526	0.817733313	0.687377635	0.964617927
LOC107133302	0.787006629	0.09521714	0.818826491	0.806574843	0.49882733	0.686939239	0.964617927
LOC112446001	0.269980783	0.614604772	0.797054785	0.678133856	0.274944016	0.686715297	0.964617927
LOC112449552	0.409598647	0.750171754	0.383851402	0.78045761	0.268516171	0.687171221	0.964617927
MBD1	0.990971684	0.882202335	0.302656907	0.731177351	0.127809282	0.687243702	0.964617927
MTFR2	0.260684804	0.569776603	0.42502538	0.623233814	0.627889068	0.687067637	0.964617927
SDC3	0.823417747	0.602886472	0.079239076	0.707973698	0.887359905	0.687131198	0.964617927
SETD4	0.289576028	0.474888985	0.638960092	0.307241432	0.915078539	0.687066511	0.964617927
SH3BP2	0.386283895	0.218534974	0.796808631	0.496140498	0.738747261	0.686673273	0.964617927
ANKS1A	0.413692449	0.298218215	0.619294964	0.68529098	0.473307857	0.687671707	0.964644874
CLIP1	0.766012416	0.971918683	0.424561985	0.323293408	0.242406157	0.687591391	0.964644874
LOC112444763	0.945935501	0.7403509	0.709013905	0.600287699	0.083136155	0.687660696	0.964644874
TRAPPC2L	0.44838365	0.838655339	0.354372389	0.493303402	0.377216883	0.687791867	0.964730969
UHMK1	0.97715031	0.685466408	0.144792888	0.628733849	0.40689668	0.687901809	0.964802717
LOC101905319	0.707214952	0.899322544	0.818406338	0.054794802	0.871015614	0.688148644	0.964903479
MAMDC2	0.977834251	0.200224853	0.535387061	0.442886764	0.535261085	0.688196756	0.964903479
NACC2	0.601244107	0.669886507	0.261921668	0.318583823	0.739219448	0.688157262	0.964903479
RAP2C	0.892587485	0.285102848	0.390358553	0.807696824	0.309722128	0.688208837	0.964903479
TRAF2	0.618951504	0.720566851	0.839325982	0.089541179	0.741637214	0.688270707	0.964907788
CTSS	0.419001133	0.239525406	0.397136646	0.699996442	0.8913238	0.688342775	0.964926393
PPP2R5B	0.187139891	0.817004997	0.272677264	0.795712592	0.750021904	0.688445908	0.964988537
LOC104972578	0.184618513	0.79718198	0.792985067	0.979875275	0.217719135	0.688576952	0.965007374
TTC9	0.711345813	0.54895619	0.190959874	0.489471461	0.682134567	0.688573792	0.965007374
ADAMTS14	0.618604259	0.676273953	0.11715491	0.809991293	0.629775306	0.689375169	0.965030912
APPL1	0.453638523	0.349295952	0.679048025	0.27207073	0.854984357	0.689589201	0.965030912
ARSH	0.395672438	0.611333008	0.34756199	0.438666073	0.675961946	0.688815562	0.965030912
DCLRE1C	0.961192078	0.831383684	0.923912444	0.954962769	0.035374821	0.688913726	0.965030912
DSTYK	0.509937749	0.558116779	0.723740135	0.635832873	0.190494677	0.68897038	0.965030912
EMSY	0.286084065	0.833766364	0.916596616	0.161489229	0.709830776	0.68984264	0.965030912
FCHSD2	0.434922853	0.765246673	0.085241994	0.965894646	0.912530457	0.689411753	0.965030912
FRK	0.824348326	0.140832404	0.47309192	0.587154286	0.776677576	0.689725946	0.965030912
GNB2	0.739859427	0.449899723	0.350998558	0.275133212	0.779406973	0.689781761	0.965030912

LHFPL1	0.849991152	0.889883658	0.146133463	0.528922688	0.426623471	0.688918644	0.965030912
LOC112442263	0.366660178	0.664714032	0.301828745	0.503563455	0.672552903	0.688699002	0.965030912
LOC112444285	0.8735262	0.833520598	0.123032724	0.804419511	0.347486951	0.689673599	0.965030912
LOC112445035	0.972244547	0.485688798	0.440211268	0.485153783	0.247442714	0.689013972	0.965030912
LOC615183	0.056152294	0.830209855	0.987907506	0.648755665	0.838278995	0.689721297	0.965030912
MADD	0.173158522	0.467569101	0.975853274	0.366847147	0.862571802	0.689371866	0.965030912
NTRK2	0.131788778	0.616433466	0.672170775	0.779545983	0.588071791	0.68962135	0.965030912
NUP210L	0.43188289	0.177535623	0.4122104	0.96546615	0.818731659	0.689236172	0.965030912
TBC1D10B	0.947842541	0.930163344	0.240138787	0.168526313	0.701683907	0.689643826	0.965030912
TOM1	0.927763715	0.137326059	0.76064036	0.293947874	0.878049842	0.689462735	0.965030912
TRIM5	0.714978672	0.100735449	0.998825359	0.47554203	0.729413173	0.68900448	0.965030912
UBR1	0.128274621	0.747295127	0.899697435	0.335802459	0.864429152	0.689633028	0.965030912
ZCCHC3	0.157098589	0.785030029	0.976249526	0.455531993	0.457064272	0.689887433	0.965030912
LOC104974260	0.723194067	0.492274369	0.337848829	0.246523541	0.847263979	0.690307496	0.965453921
LOC521224	0.17497001	0.692397613	0.329347437	0.883528927	0.712472143	0.690263317	0.965453921
SSBP4	0.676433799	0.266094035	0.222411452	0.84784237	0.740553328	0.69040845	0.965512831
BORA	0.796309206	0.126195844	0.356750498	0.810080074	0.867231248	0.690792515	0.965551883
GRAP	0.465511403	0.496588151	0.798706238	0.190289813	0.716633538	0.690735778	0.965551883
IVNS1ABP	0.280058422	0.47860498	0.872891285	0.83350558	0.258163183	0.690719091	0.965551883
LRRC20	0.488738805	0.343628131	0.405164317	0.758818269	0.487321387	0.690613067	0.965551883
PRKCB	0.830608327	0.451675078	0.265455352	0.926416189	0.272676028	0.69057623	0.965551883
PYROXD1	0.839564607	0.452520322	0.2779093	0.581940388	0.410020874	0.690848224	0.965551883
TNPO3	0.955495807	0.099259764	0.671136801	0.716971857	0.551830266	0.690777627	0.965551883
GOLIM4	0.268480917	0.919461751	0.379680391	0.423645546	0.634939296	0.690990408	0.965586137
PHKB	0.718705275	0.371381054	0.55280477	0.45532944	0.375156877	0.690937546	0.965586137
SAYSD1	0.820417722	0.548225149	0.752789837	0.201276905	0.370075317	0.691057731	0.965597992
FCRL1	0.609666388	0.120739908	0.646123948	0.646837313	0.820347454	0.691190883	0.96570182
LOC112443419	0.110273726	0.86705561	0.848693668	0.650876929	0.478435775	0.691430804	0.965726055
LOC507581	0.693991039	0.437728694	0.148747421	0.591927521	0.944528206	0.691386119	0.965726055
MRPL38	0.941369414	0.24148547	0.956582486	0.392585497	0.296014912	0.691443614	0.965726055
TLR9	0.792129694	0.228974569	0.61378602	0.705388206	0.321692531	0.691376322	0.965726055
AQR	0.295395593	0.978550546	0.528018856	0.22155516	0.750136506	0.692170113	0.965890017
CD200R1L	0.186308113	0.561796666	0.602886544	0.42566893	0.944867951	0.692272449	0.965890017
KNOP1	0.730301912	0.786428685	0.442441934	0.601258456	0.165677131	0.691763287	0.965890017
LOC100848569	0.631681748	0.502548851	0.869189792	0.116099403	0.791158828	0.692004214	0.965890017
LOC101904468	0.430974289	0.151418919	0.854695531	0.581828533	0.779846419	0.691721229	0.965890017
LOC781224	0.933591231	0.313806242	0.387405999	0.484254344	0.461871593	0.692312314	0.965890017
LOC781612	0.293558878	0.659440926	0.282502855	0.509947739	0.910477093	0.692360745	0.965890017
LOC789715	0.889734273	0.665848974	0.207515674	0.524728673	0.393660254	0.692384995	0.965890017
OSTM1	0.79845631	0.512090043	0.121536028	0.90093332	0.56678093	0.692237328	0.965890017

PRRG4	0.300451673	0.475817893	0.450424181	0.949348319	0.415270364	0.692318593	0.965890017
RAE1	0.863755342	0.532133776	0.172391795	0.59203546	0.539923412	0.691881395	0.965890017
TCAM1	0.585622028	0.862401559	0.923797592	0.100821296	0.538484518	0.691890807	0.965890017
UBE2Z	0.813663585	0.668138604	0.155771232	0.305406316	0.981429095	0.692292879	0.965890017
ZDHHC16	0.535659265	0.41119046	0.317779628	0.75383197	0.480625454	0.692116947	0.965890017
BIN2	0.79872116	0.27727176	0.675805483	0.338703455	0.501470231	0.692581918	0.966082606
ARSJ	0.110890033	0.563729554	0.52928302	0.839615451	0.916514507	0.692885126	0.96618814
BCL9	0.739510221	0.715982919	0.123934117	0.744103083	0.521775507	0.693010822	0.96618814
BCLAF3	0.777276421	0.75517784	0.233515455	0.270481613	0.687019196	0.692963141	0.96618814
DCBLD2	0.83076413	0.094566118	0.511303119	0.732284986	0.865839711	0.692946465	0.96618814
LOC783612	0.826382771	0.236511005	0.845264691	0.478945718	0.321528391	0.692734314	0.96618814
YTHDC1	0.46919666	0.706679429	0.996971957	0.112879616	0.681986492	0.692787814	0.96618814
CCL4	0.534066169	0.703942218	0.85454428	0.865656303	0.091731232	0.693265706	0.966297223
GATAD2B	0.635868925	0.509989489	0.685007579	0.154217361	0.744373226	0.693185387	0.966297223
IFT22	0.297524631	0.63244463	0.855648321	0.19556614	0.810192314	0.693262669	0.966297223
ASGR2	0.627680036	0.894809511	0.071594817	0.70594681	0.90374542	0.694346875	0.966446445
BDKRB1	0.902810789	0.893595351	0.252063398	0.308786756	0.408339312	0.694239945	0.966446445
CD24	0.455319471	0.560205959	0.50425061	0.39249864	0.508429655	0.694440297	0.966446445
CHD7	0.56159324	0.690995113	0.924680364	0.19649054	0.36326271	0.694028845	0.966446445
CTBP1	0.348919608	0.876420445	0.958326006	0.142941316	0.611498536	0.694052511	0.966446445
DGCR6L	0.376762426	0.798766243	0.633143592	0.1581257	0.848189652	0.693600665	0.966446445
EIF3H	0.729263338	0.872720986	0.237336218	0.689660606	0.246318594	0.694385804	0.966446445
ENOX2	0.385122244	0.662193016	0.430125743	0.373236137	0.62526337	0.693928839	0.966446445
HMGN5	0.92628093	0.354461636	0.274877246	0.380619023	0.746025178	0.694136884	0.966446445
INSIG2	0.7445905	0.321746036	0.663475161	0.265315791	0.608752545	0.694475676	0.966446445
KLHDC3	0.910862198	0.737269251	0.114645157	0.847252862	0.392675074	0.694042115	0.966446445
LOC112447303	0.073099818	0.913844067	0.908853703	0.576026499	0.73016119	0.693449342	0.966446445
LOC518980	0.272135446	0.484662979	0.744824756	0.917256413	0.283472911	0.693510432	0.966446445
MAP2K2	0.19220078	0.493510351	0.743768301	0.737412517	0.493509802	0.694491678	0.966446445
MSR1	0.578784277	0.677731635	0.081709764	0.922848515	0.867438861	0.694368654	0.966446445
PIP4P2	0.544827095	0.123907554	0.545853341	0.768752733	0.90508657	0.694231953	0.966446445
PRORSD1	0.724356334	0.957559071	0.371765512	0.51534316	0.192584718	0.693875636	0.966446445
RBBP7	0.351206788	0.511193429	0.27094072	0.84332466	0.625706155	0.694443957	0.966446445
SNAI1	0.271666341	0.792474647	0.687882666	0.685384937	0.252223478	0.693942284	0.966446445
ADAR	0.509460534	0.194078333	0.585191892	0.634501382	0.700987962	0.694949646	0.966460188
BCL10	0.905602983	0.274934449	0.45667726	0.33370022	0.677941963	0.694859434	0.966460188
CUL2	0.375881348	0.272147657	0.825750556	0.943637494	0.322535182	0.694753608	0.966460188
LOC782950	0.291054356	0.509165834	0.712951407	0.724426959	0.336126333	0.694888243	0.966460188
TFEC	0.494065411	0.263135661	0.534697475	0.522465677	0.707839237	0.694743931	0.966460188
TREML1	0.916700824	0.731134116	0.271914726	0.928136143	0.151949487	0.694701234	0.966460188

ZNF140	0.474888726	0.471100417	0.215242524	0.859474906	0.62070141	0.694604219	0.966460188
ZNF568	0.330909969	0.925540608	0.29013291	0.842091878	0.343968942	0.694972682	0.966460188
CRTC2	0.384132079	0.380597569	0.966292491	0.389901029	0.468165666	0.695339496	0.966560772
LOC112449172	0.850958232	0.455223854	0.820311257	0.707975017	0.114581974	0.69526731	0.966560772
PPP1R13B	0.286664795	0.453149679	0.971081761	0.40925767	0.499301785	0.695260942	0.966560772
PSTPIP2	0.195931046	0.816435446	0.742605871	0.399257135	0.543712399	0.695337847	0.966560772
ZBTB8A	0.204095761	0.664727563	0.869570547	0.944745977	0.23129639	0.695276276	0.966560772
ANP32B	0.715243322	0.904188091	0.57039046	0.29767715	0.235626427	0.695979508	0.96668156
ATP2A1	0.911514825	0.435991902	0.239404466	0.814334372	0.334430169	0.696257678	0.96668156
BEGAIN	0.710392143	0.346310537	0.219583889	0.767518449	0.623700126	0.69587972	0.96668156
BLOC1S3	0.755143684	0.31324908	0.201383264	0.634520899	0.854205348	0.695579481	0.96668156
C15H11orf96	0.440103489	0.446050359	0.4138463	0.881632602	0.360792448	0.695744164	0.96668156
DNAI1	0.201690413	0.937976262	0.213295467	0.724191368	0.886410324	0.696197581	0.96668156
LOC112442245	0.524547231	0.488638631	0.131032617	0.997376433	0.773654285	0.696290983	0.96668156
LOC112443011	0.329780167	0.38822725	0.540865712	0.643841246	0.580243958	0.695950761	0.96668156
LOC112446882	0.333054074	0.165812538	0.944608372	0.733341306	0.677297776	0.69625196	0.96668156
LOC112447313	0.555794789	0.85030886	0.516147212	0.248362387	0.426738889	0.695828277	0.96668156
MCF2	0.449709844	0.675021486	0.697976486	0.928510783	0.13141969	0.695839509	0.96668156
MESP1	0.111957138	0.644538952	0.704011746	0.686284896	0.740962015	0.695681453	0.96668156
POMGNT1	0.487978168	0.25205666	0.893796701	0.329101906	0.713372556	0.695505575	0.96668156
TLR5	0.768426789	0.335218907	0.150705985	0.906910196	0.735994807	0.69626561	0.96668156
ZNF175	0.99142668	0.236084147	0.318826957	0.658794562	0.527191311	0.696309958	0.96668156
SMARCA4	0.568496534	0.446130719	0.208403751	0.873810462	0.561336482	0.696369846	0.966682925
LOC101907138	0.641908908	0.372736203	0.680440113	0.674329467	0.236766125	0.69686587	0.967289672
SMARCD3	0.796298742	0.449342551	0.862545651	0.321115173	0.262367971	0.696932575	0.967300448
PAFAH2	0.405044799	0.592219422	0.142088804	0.863586862	0.883956304	0.697054313	0.967387597
EXOSC7	0.767721617	0.265995684	0.305426594	0.736601565	0.566992525	0.697281234	0.967620694
ASB9	0.794592982	0.97661315	0.509759945	0.956885288	0.068962252	0.697683479	0.967657126
CYP7B1	0.952590422	0.851330827	0.228468118	0.692890803	0.20345646	0.697799449	0.967657126
FZD8	0.632875882	0.963684156	0.257014948	0.242019664	0.688281269	0.697738309	0.967657126
LOC100337293	0.526484263	0.823505635	0.451398464	0.658568981	0.202892528	0.698026339	0.967657126
LOC112441659	0.317599743	0.807780998	0.922838196	0.836631128	0.131971954	0.697953633	0.967657126
LOC784841	0.454926226	0.458902423	0.26285809	0.527438292	0.90189132	0.697685347	0.967657126
LPAR5	0.587665372	0.573499049	0.533289866	0.990255655	0.146881514	0.697964958	0.967657126
MYO19	0.619711183	0.287174534	0.494592977	0.618280991	0.480008775	0.697822372	0.967657126
PPP2R5A	0.534092566	0.34587441	0.15950982	0.985660569	0.90024828	0.69799657	0.967657126
S1PR1	0.78103643	0.520477461	0.249022256	0.492068011	0.52329579	0.697408922	0.967657126
UBALD2	0.450304167	0.724252218	0.134745984	0.885790462	0.671961722	0.698074018	0.967657126
UBE2R2	0.941221359	0.698456924	0.18727624	0.456654612	0.464575638	0.697795868	0.967657126
XPA	0.674780814	0.678524755	0.144944221	0.619685561	0.634764537	0.697687481	0.967657126

MSI1	0.890415065	0.571800005	0.164491241	0.539097953	0.580029988	0.698299763	0.967888295
GOSR1	0.369802258	0.893409997	0.147958127	0.571260352	0.938175546	0.698379289	0.967916774
LOC100462699	0.924939305	0.739526627	0.616343937	0.13143653	0.473157792	0.698527383	0.968040271
RPAP3	0.487589525	0.259761139	0.688767961	0.401330827	0.749636075	0.69872293	0.968229503
KIF9	0.372564327	0.868567306	0.677706004	0.292203241	0.410635516	0.699223904	0.968760109
NOTCH2	0.223044001	0.997436924	0.738695233	0.515338288	0.310694813	0.699215098	0.968760109
ADRA1A	0.449961889	0.17627015	0.933681906	0.672911632	0.528522267	0.69939504	0.968863645
CDK13	0.888639187	0.533691308	0.800382468	0.081943527	0.846829507	0.699416708	0.968863645
LOC112447727	0.868749409	0.77830441	0.457470322	0.456562783	0.186621406	0.699525045	0.968931931
CBLN3	0.098178593	0.650042037	0.641913857	0.873379002	0.737075603	0.699649185	0.969022093
ABRAXAS1	0.734536238	0.133410634	0.577643149	0.852802576	0.550457881	0.701100956	0.969128542
ALKAL2	0.981334436	0.432569071	0.102169436	0.87809582	0.698780548	0.701383712	0.969128542
CCDC85A	0.495904328	0.960641678	0.618488539	0.695713002	0.129821691	0.701380242	0.969128542
CLEC7A	0.683930262	0.621180446	0.149771441	0.475775242	0.876576952	0.700842411	0.969128542
DR1	0.673672306	0.977928111	0.656326748	0.714631833	0.086029503	0.701175853	0.969128542
ENG	0.912532111	0.334841912	0.521907967	0.17523615	0.944962072	0.699901172	0.969128542
EPG5	0.26738184	0.281644774	0.909457923	0.705899582	0.546031465	0.699837905	0.969128542
EXOSC3	0.901989002	0.607860853	0.385582986	0.772164101	0.162142162	0.700346939	0.969128542
FAM76B	0.265565205	0.807598824	0.852934148	0.579868755	0.250939998	0.701430398	0.969128542
FOXO1	0.727744559	0.945583911	0.195607107	0.248267381	0.792923691	0.700561587	0.969128542
IBTK	0.514966057	0.292712875	0.353289538	0.823766577	0.604683169	0.700768545	0.969128542
LOC101906067	0.611170487	0.110197461	0.931448388	0.732114682	0.577661516	0.700795954	0.969128542
LOC107132534	0.734676154	0.760035312	0.948554032	0.782894094	0.064074992	0.701077758	0.969128542
LOC112443223	0.68060558	0.667582404	0.76868358	0.172763896	0.439180732	0.700574632	0.969128542
LOC112445888	0.339801749	0.774131258	0.297976452	0.534618408	0.633426635	0.700891834	0.969128542
LOC112449086	0.689366209	0.766096712	0.525090937	0.114866286	0.834404998	0.701145863	0.969128542
MARCH9	0.662857524	0.831101729	0.181886965	0.524589308	0.504339328	0.700651478	0.969128542
MATN2	0.991466632	0.341246008	0.28817146	0.542767015	0.500760956	0.700572996	0.969128542
MRPL9	0.582164269	0.355675235	0.559138044	0.321636544	0.713026934	0.700947991	0.969128542
PMEL	0.26695627	0.289912045	0.701026672	0.698001904	0.70128175	0.700992391	0.969128542
PRKN	0.15386541	0.718127268	0.458246692	0.745156563	0.700796021	0.700149033	0.969128542
RNMT	0.726803724	0.841432349	0.396328636	0.697853948	0.157370011	0.701428793	0.969128542
SEMA3B	0.284075316	0.361332388	0.716284737	0.946637909	0.380546836	0.700474723	0.969128542
SYNPR	0.160043012	0.313605795	0.972311669	0.543745601	0.996839353	0.700222516	0.969128542
TAF11	0.985368189	0.089048851	0.543094533	0.64932861	0.855634903	0.700402814	0.969128542
TESK1	0.808327082	0.987998867	0.465713744	0.951019116	0.075257302	0.701438597	0.969128542
TPRKB	0.58346646	0.734854396	0.7889699	0.940697698	0.083429069	0.700928367	0.969128542
TRPC4AP	0.763468868	0.229322581	0.924352927	0.786137309	0.208568007	0.70082986	0.969128542
ZSCAN20	0.686409897	0.148441965	0.6805568	0.394135541	0.971128124	0.700876089	0.969128542
LOC112448075	0.889673553	0.063617709	0.673142063	0.913435422	0.765626696	0.701619544	0.969296939

CLSTN2	0.91726084	0.051751389	0.779970742	0.93027424	0.774004997	0.701724433	0.969306958
FBRSL1	0.608354636	0.802497435	0.328398882	0.940550007	0.17754198	0.702535307	0.969306958
GPR160	0.749727341	0.847634837	0.119968806	0.377459402	0.930217147	0.702513233	0.969306958
H2AFJ	0.969477173	0.839730231	0.296279077	0.124926343	0.888989567	0.702642956	0.969306958
HPS4	0.513770128	0.833370098	0.221132729	0.712539759	0.395816632	0.702040056	0.969306958
LOC100848766	0.94140322	0.849516256	0.813688137	0.324948901	0.12670537	0.70268108	0.969306958
LOC104971845	0.66224568	0.184709515	0.795764644	0.274724194	0.998028748	0.701938563	0.969306958
LOC112442292	0.370205217	0.560432465	0.666358262	0.405089352	0.477770428	0.702428783	0.969306958
LOC617648	0.298200327	0.409599223	0.496923672	0.443006644	0.994453863	0.702299963	0.969306958
MBD4	0.520992269	0.912186077	0.458874262	0.463916731	0.264095993	0.702149225	0.969306958
MYOC	0.313351933	0.902703197	0.725082743	0.559982561	0.233141677	0.702568073	0.969306958
NPR3	0.372296947	0.216262149	0.533185533	0.890198972	0.698450818	0.701955454	0.969306958
NUP205	0.553246148	0.769905014	0.513181645	0.649196878	0.18835294	0.702222148	0.969306958
PIP4K2C	0.364619362	0.974541294	0.154754159	0.926430356	0.525945193	0.702689957	0.969306958
POLI	0.390458407	0.486231674	0.427285515	0.564221498	0.584040906	0.702245805	0.969306958
SKIL	0.29387772	0.807032575	0.470374429	0.581406019	0.412961631	0.702626015	0.969306958
VCP	0.842196498	0.204897243	0.400827447	0.801320767	0.481591017	0.701964507	0.969306958
ZNF189	0.377389173	0.584011139	0.911677291	0.359039927	0.37062627	0.702290627	0.969306958
LOC112443509	0.292172843	0.633270074	0.315448967	0.538199171	0.853416706	0.702789903	0.969363346
FEZ1	0.144234548	0.94561936	0.280837178	0.758326636	0.923719542	0.702955178	0.969509827
AFF3	0.71703296	0.689947215	0.894936905	0.545987119	0.111087214	0.703112749	0.969554722
C22H3orf49	0.694338791	0.714551809	0.245443863	0.390437484	0.564809228	0.703120136	0.969554722
GATA2	0.431947494	0.177231483	0.683914384	0.742721758	0.690949756	0.703224048	0.969554722
GRO1	0.655266042	0.767575906	0.207158586	0.435517925	0.591929373	0.703167443	0.969554722
ABCG4	0.697103266	0.180829462	0.813504623	0.709229098	0.369661206	0.703343396	0.96955636
LOC100850437	0.29272208	0.732536566	0.144901762	0.98529977	0.878077271	0.703318175	0.96955636
GPS2	0.708402419	0.360114635	0.820695263	0.151427986	0.848306035	0.70340721	0.969562887
PKN3	0.880401852	0.245520914	0.18154675	0.980977001	0.698870048	0.703474135	0.969573698
HNRNPU	0.490095954	0.421842102	0.729943892	0.301796453	0.591005839	0.703568186	0.969621893
TEDC2	0.836878922	0.192746115	0.446142958	0.475154804	0.787417378	0.703628165	0.969623127
ABHD14A	0.321751304	0.39208011	0.820893554	0.452258184	0.577233715	0.704403148	0.969726187
ACBD6	0.852130397	0.818007014	0.352717363	0.128395125	0.856105634	0.704335137	0.969726187
B4GALNT1	0.483460028	0.960590291	0.388662096	0.712196548	0.209614133	0.703773232	0.969726187
CFAP126	0.328615434	0.418139536	0.389902573	0.567389531	0.887977748	0.704107156	0.969726187
DDX20	0.181233741	0.589766355	0.968623323	0.272746201	0.957200531	0.704365905	0.969726187
FBXO7	0.147136629	0.932168534	0.604051483	0.544103259	0.600175603	0.704548068	0.969726187
HDHD2	0.570546284	0.486945552	0.583649984	0.2309122	0.723171412	0.704708078	0.969726187
LGMN	0.393281264	0.487387517	0.179512066	0.832401183	0.944362425	0.704501269	0.969726187
LOC100847326	0.748940621	0.788474773	0.322294938	0.691484293	0.205807384	0.704760991	0.969726187
LOC104972724	0.823254636	0.265229617	0.89208019	0.369985853	0.375678388	0.704685837	0.969726187

LOC785503	0.882808977	0.373628583	0.368375943	0.329803771	0.674909523	0.704482128	0.969726187
PHETA2	0.341200176	0.85075892	0.20975223	0.714473285	0.621274522	0.704346628	0.969726187
PRTFDC1	0.87957595	0.828254052	0.401896291	0.209586959	0.440839309	0.70452454	0.969726187
RUBCN	0.069142999	0.511997644	0.965158392	0.887415453	0.891522376	0.704382827	0.969726187
SCO2	0.410785065	0.624425995	0.730748373	0.176373306	0.818820524	0.704652174	0.969726187
SRL	0.365550456	0.595041046	0.973583523	0.744178282	0.171815106	0.704705396	0.969726187
TCHP	0.678733221	0.925461724	0.801312768	0.358061092	0.150243547	0.704708387	0.969726187
TEX30	0.836634727	0.831615664	0.870750346	0.045187645	0.989707296	0.704825663	0.969726187
THRB	0.637024323	0.644767861	0.44047425	0.284395909	0.526557981	0.704812784	0.969726187
LOC112443859	0.808883247	0.469800501	0.23092412	0.436272745	0.708886695	0.705145078	0.969732458
LOC510193	0.607805407	0.432791378	0.923915727	0.254035447	0.439297343	0.705023853	0.969732458
MAP1LC3B	0.844366017	0.680027144	0.367445381	0.433559725	0.296674321	0.70513413	0.969732458
NTNG2	0.203718349	0.749639991	0.518601918	0.535012114	0.639880839	0.704958333	0.969732458
TMEM234	0.938167172	0.95833542	0.496139135	0.460182361	0.132066217	0.704933316	0.969732458
ZHX3	0.65490258	0.154374342	0.956569063	0.485713401	0.577890225	0.705184763	0.969732458
CDKN3	0.826884957	0.170820323	0.465163827	0.529678596	0.780686527	0.705354874	0.969885115
ATP11C	0.521269348	0.832167802	0.529579399	0.136252122	0.868478998	0.705454927	0.969928962
SHB	0.439068138	0.305404857	0.404379817	0.516642874	0.970582785	0.705504968	0.969928962
PRR15	0.739446143	0.692886261	0.557765904	0.348888361	0.27302922	0.705723335	0.970011519
TIGAR	0.702491008	0.115813521	0.844259635	0.554140452	0.715258948	0.70574234	0.970011519
TNFRSF19	0.76328556	0.699755022	0.89347047	0.926250009	0.061581963	0.705714068	0.970011519
PXN	0.839478925	0.267651457	0.514465297	0.443757452	0.531742842	0.70610508	0.970428813
REL	0.630928395	0.221386802	0.876679406	0.301346431	0.7400061	0.706321364	0.970563502
RPL22	0.594521961	0.384135273	0.408992293	0.991785564	0.294684227	0.706263209	0.970563502
AMH	0.602987403	0.662581843	0.809851912	0.254613063	0.332227862	0.706759953	0.970743218
CWC27	0.839397289	0.71191893	0.908385976	0.276276003	0.182652631	0.70692173	0.970743218
DPP7	0.192369335	0.918185737	0.224866125	0.786719328	0.875201559	0.706605079	0.970743218
HMGCR	0.992545562	0.272347374	0.154836247	0.693352793	0.942923919	0.706719049	0.970743218
LOC112442262	0.199769121	0.541364068	0.687119077	0.957320934	0.38449017	0.706637775	0.970743218
MELTF	0.607200448	0.231805158	0.78241884	0.581238763	0.427549804	0.706744828	0.970743218
TBC1D20	0.393935199	0.092274198	0.921144039	0.979514393	0.835103126	0.706897925	0.970743218
TTC30A	0.2498095	0.391617501	0.933170597	0.552587775	0.54301587	0.706925367	0.970743218
ZFAND3	0.958892301	0.673406228	0.056490968	0.875303703	0.858281178	0.706999513	0.970763806
CD82	0.589792679	0.613061596	0.133365691	0.79730803	0.71313961	0.707102496	0.970823982
DSEL	0.801982569	0.231081684	0.584695917	0.399424358	0.634017492	0.707255654	0.970871814
NLGN1	0.722597145	0.391598439	0.50221304	0.589216624	0.327687189	0.707239517	0.970871814
AMBRA1	0.56763278	0.287799574	0.809080667	0.339872724	0.611395338	0.707427974	0.97102714
AAR2	0.667270584	0.242208936	0.83689962	0.245734415	0.828929443	0.70802686	0.971217463
DGCR2	0.305919165	0.57075222	0.23645364	0.729023484	0.915535699	0.70805679	0.971217463
LOC112445033	0.563666224	0.684590095	0.858209541	0.186731781	0.445590302	0.708049238	0.971217463

LOC112446010	0.665551121	0.814330258	0.431907089	0.588544874	0.199579499	0.707639168	0.971217463
LOC505918	0.075448382	0.976384194	0.864593135	0.550836612	0.784059361	0.707721165	0.971217463
RPL36	0.246341166	0.958808849	0.77578016	0.425076256	0.353605576	0.707958492	0.971217463
SEC22A	0.307576327	0.494790311	0.732317496	0.322754683	0.76624489	0.708099259	0.971217463
TBC1D23	0.616185813	0.719266004	0.336633238	0.7908363	0.233587627	0.708090904	0.971217463
THRA	0.533246452	0.438882532	0.756988403	0.44296013	0.351036808	0.707997708	0.971217463
BSCL2	0.293442253	0.788075655	0.200005156	0.783325812	0.761762041	0.70835431	0.9712617
EVI2A	0.430308385	0.649286521	0.838262863	0.324297711	0.36334459	0.708339131	0.9712617
LOC112442383	0.491395144	0.709214419	0.534472952	0.59352874	0.249501923	0.708247118	0.9712617
SH2D3A	0.364314163	0.549661323	0.694279506	0.402732881	0.493006338	0.708390222	0.9712617
SHROOM1	0.308536888	0.215082001	0.963615628	0.796601982	0.542005126	0.70842743	0.9712617
ATXN3	0.67341461	0.295122078	0.696033307	0.876508127	0.229799301	0.7101677	0.971343226
CCDC91	0.923243513	0.211093754	0.430632186	0.837376311	0.396127279	0.710007185	0.971343226
CD80	0.526530353	0.445287064	0.849771027	0.617510607	0.224766555	0.708727228	0.971343226
CMYA5	0.444975233	0.551740186	0.597493733	0.312184899	0.604758032	0.709015576	0.971343226
EIF4G3	0.400367789	0.550940759	0.648870377	0.893135816	0.217927542	0.710138133	0.971343226
FER1L5	0.735017545	0.225268914	0.461481657	0.667742112	0.545525831	0.709973767	0.971343226
FGF14	0.308764487	0.307744172	0.809585524	0.616999675	0.587546286	0.710338162	0.971343226
GOLGA2	0.570543738	0.26097907	0.238524183	0.894938184	0.869897042	0.708703664	0.971343226
GRB2	0.7600666	0.511418184	0.147806635	0.721646475	0.667474855	0.708876559	0.971343226
IKBKG	0.465729645	0.966838474	0.786799938	0.095349411	0.818807045	0.708775679	0.971343226
KAT7	0.211559676	0.826543493	0.66809722	0.767586862	0.309334761	0.709322052	0.971343226
KCNK4	0.376784077	0.673388777	0.706073318	0.166754033	0.933366854	0.710307412	0.971343226
LAS1L	0.397209513	0.628618717	0.605326987	0.39187398	0.466467264	0.708561477	0.971343226
LOC107132924	0.370794394	0.752667979	0.299605454	0.632829438	0.524296505	0.709346413	0.971343226
LOC112442704	0.945978079	0.269080173	0.521714538	0.50285995	0.414424916	0.708879843	0.971343226
LOC788183	0.933983717	0.434997346	0.722378485	0.583642412	0.161715935	0.709056474	0.971343226
LSR	0.913754951	0.780066729	0.660178562	0.568683816	0.104172447	0.710267751	0.971343226
MAP2K5	0.711323263	0.154702599	0.599507708	0.999455116	0.421343476	0.709614081	0.971343226
MMP24OS	0.72983009	0.779447586	0.443462864	0.117377743	0.942379756	0.710457073	0.971343226
MXRA7	0.185230104	0.739124918	0.550193897	0.673330528	0.548759751	0.709964206	0.971343226
PPM1A	0.937875205	0.884727588	0.082632159	0.932383197	0.435639597	0.710082734	0.971343226
PRR36	0.139217423	0.618147972	0.998269663	0.487440516	0.665287009	0.710143833	0.971343226
RMI2	0.620056005	0.589668009	0.741845376	0.268119234	0.383234298	0.710222752	0.971343226
RNF217	0.325209997	0.575143739	0.962314104	0.425676296	0.362165784	0.709387761	0.971343226
SEPT4	0.585215249	0.556542778	0.741269886	0.606971564	0.189085543	0.709112234	0.971343226
SGCB	0.727766207	0.18615266	0.504738856	0.893397277	0.454871013	0.709658816	0.971343226
SIAH2	0.606856348	0.343304153	0.26393377	0.514759128	0.98016671	0.709352555	0.971343226
SLAIN1	0.780264166	0.511432288	0.572783303	0.510253112	0.238160473	0.709577401	0.971343226
SMIM8	0.850321391	0.353902432	0.975873221	0.442573603	0.213449374	0.709342236	0.971343226

SRC	0.851440896	0.770748186	0.190797697	0.28003398	0.79421986	0.710067845	0.971343226
TRAPPC10	0.281003113	0.951323861	0.230518086	0.463379211	0.977445868	0.710499305	0.971343226
TRAPPC4	0.214794521	0.350777813	0.703745477	0.688738832	0.764055703	0.710445162	0.971343226
TRIM45	0.52914512	0.122187795	0.832217772	0.671918437	0.769354335	0.709844177	0.971343226
VEGFB	0.453529043	0.260863157	0.510936789	0.865972696	0.529142311	0.709043873	0.971343226
ARG2	0.588378949	0.644719593	0.235887353	0.853231978	0.368328852	0.711931336	0.971353769
CLMP	0.346724556	0.477954627	0.414865255	0.60374965	0.676554599	0.711669132	0.971353769
CPLANE2	0.941301729	0.415513872	0.264134061	0.328404047	0.829887361	0.712164313	0.971353769
DPCD	0.715913115	0.365108041	0.529366503	0.260680317	0.777009361	0.711289243	0.971353769
GPR176	0.827351497	0.81394198	0.427833849	0.376966892	0.257193326	0.710652048	0.971353769
GZF1	0.722076118	0.769784231	0.661287344	0.080854647	0.945515823	0.711791107	0.971353769
INPP4A	0.248162996	0.357508147	0.515642886	0.77906791	0.787713665	0.711615415	0.971353769
KLHL7	0.106855986	0.778167437	0.740615094	0.621107549	0.731141299	0.71087648	0.971353769
LOC100335268	0.109447792	0.742835806	0.586500683	0.716706971	0.822371681	0.71181838	0.971353769
LOC101903988	0.969197599	0.253216594	0.521148729	0.679892903	0.322404281	0.71134843	0.971353769
LOC104975299	0.928241925	0.850069815	0.468137057	0.379568874	0.200714596	0.712073001	0.971353769
LOC107132532	0.495599734	0.756089302	0.817295118	0.750934895	0.122174142	0.711768574	0.971353769
LOC112443853	0.43343541	0.535471162	0.189454542	0.955815945	0.669723758	0.712106845	0.971353769
NBN	0.642241172	0.576767187	0.605925771	0.803720585	0.155191508	0.711077838	0.971353769
NCOA7	0.938441898	0.319375207	0.599146215	0.396346664	0.394834237	0.711797879	0.971353769
NOP58	0.992976502	0.379230829	0.66706865	0.253126391	0.442420398	0.711997245	0.971353769
POLN	0.88725801	0.736901936	0.578612017	0.075146891	0.989053721	0.711906085	0.971353769
PRKAG1	0.521996826	0.33352497	0.580004937	0.618608862	0.447976953	0.710993777	0.971353769
PRRC2B	0.870081821	0.575518689	0.869802248	0.620962279	0.103393518	0.710861545	0.971353769
RAMMET	0.796065065	0.214024978	0.814846485	0.743968543	0.270445256	0.710652581	0.971353769
RNASET2	0.701749923	0.386681934	0.172420247	0.632039989	0.948322985	0.711400175	0.971353769
RNF114	0.767805547	0.419902606	0.127692634	0.739721744	0.924334239	0.712118961	0.971353769
RNF145	0.120642645	0.899047115	0.97447181	0.272326182	0.974574037	0.711457884	0.971353769
RTKN	0.309947129	0.530122705	0.796531385	0.910950407	0.235490102	0.711623585	0.971353769
SEC24C	0.969391938	0.124287438	0.989988641	0.972400989	0.241134396	0.710889132	0.971353769
VPS11	0.966097725	0.493226198	0.128795279	0.817154056	0.559212782	0.711410191	0.971353769
VPS50	0.205010654	0.741520202	0.906514869	0.994667779	0.204990863	0.711778343	0.971353769
ZNF180	0.195716687	0.650423013	0.300493772	0.931855491	0.786153559	0.711264007	0.971353769
LOC104974934	0.590601457	0.485590273	0.686830154	0.898116433	0.159338033	0.712383467	0.971410475
NID1	0.146655363	0.828656756	0.996435474	0.311835891	0.746074374	0.712279965	0.971410475
VASH2	0.954687634	0.23382745	0.40036281	0.438925411	0.718525832	0.712373679	0.971410475
C24H18orf21	0.399802677	0.130496219	0.978343579	0.638232859	0.865726127	0.71248383	0.971466612
LOC522174	0.375073997	0.782837065	0.711684683	0.275645908	0.489973311	0.712617721	0.971568449
PCSK6	0.452070869	0.967444116	0.811507792	0.198326111	0.401142149	0.712706925	0.97160935
LOC100126043	0.365411806	0.648592026	0.282689608	0.866372294	0.486917488	0.712891425	0.971621076

RPSA	0.79786912	0.374085805	0.584151387	0.861398117	0.188073943	0.71277603	0.971621076
TTPAL	0.537972888	0.230577275	0.813501563	0.912912077	0.306805847	0.712893143	0.971621076
CCDC112	0.516312666	0.952645908	0.31445147	0.676390749	0.270524301	0.713144862	0.971637632
FTO	0.686528371	0.293069946	0.716032896	0.894533148	0.219461466	0.713020148	0.971637632
LOC100299281	0.273479597	0.631763337	0.426070325	0.498263435	0.771614028	0.713152716	0.971637632
NDST3	0.613727162	0.424485114	0.931484559	0.133210349	0.875748057	0.713201323	0.971637632
PCNX1	0.41250506	0.631169192	0.999289202	0.166175263	0.654475348	0.713111568	0.971637632
CLEC18C	0.495634309	0.460533534	0.488168465	0.435243703	0.585897027	0.713909549	0.97173176
E2F5	0.255560268	0.722275183	0.894124526	0.808551862	0.213219612	0.714165021	0.97173176
ESR1	0.7041921	0.432473135	0.786132119	0.213822945	0.555715729	0.714131608	0.97173176
ETNK1	0.767130475	0.359726097	0.663402858	0.344236434	0.45103495	0.713970652	0.97173176
FAM166A	0.49124259	0.675870378	0.777394637	0.454272108	0.242735053	0.714217811	0.97173176
FAM19A3	0.25052376	0.256582994	0.856973057	0.675961241	0.762190183	0.713682924	0.97173176
FUT4	0.690407061	0.150121389	0.718776251	0.592307906	0.643199502	0.713685397	0.97173176
INIP	0.596966026	0.543703595	0.92684458	0.654063947	0.144469421	0.713983505	0.97173176
JMJD4	0.949885033	0.365274102	0.090207999	0.963999087	0.939707489	0.713496692	0.97173176
LOC101905711	0.786145349	0.5672622	0.446091963	0.318482722	0.44847064	0.71390291	0.97173176
LOC101906012	0.870703417	0.880000485	0.479583992	0.152877976	0.506420547	0.714140925	0.97173176
LOC529930	0.141814458	0.505179613	0.921463431	0.816758468	0.526730218	0.713813199	0.97173176
MARCH7	0.622506949	0.780921982	0.238566735	0.563591225	0.434969675	0.714014212	0.97173176
TMC8	0.702697202	0.920707825	0.850007943	0.116255487	0.444061955	0.713744714	0.97173176
USP37	0.42133122	0.427296778	0.874633378	0.187066821	0.964444008	0.713868768	0.97173176
ZC2HC1C	0.650037248	0.496440894	0.404734218	0.386731058	0.563187327	0.714124956	0.97173176
LOC789192	0.642010679	0.885820976	0.643572064	0.810942866	0.095937901	0.714312604	0.971780166
LOC112446777	0.899786628	0.667292745	0.140335846	0.653397405	0.518039661	0.714618904	0.972116283
PRX	0.600099639	0.28334437	0.966418535	0.475835197	0.36488759	0.71468632	0.97212741
ATL1	0.592218777	0.676409749	0.90564422	0.208988536	0.37667874	0.714872209	0.972290124
SGTB	0.468577066	0.781290703	0.280624747	0.303902145	0.914974619	0.714924437	0.972290124
PSCA	0.977748736	0.304702715	0.320325403	0.918020407	0.326398724	0.715114207	0.972467621
DARS2	0.881897109	0.512167487	0.290198055	0.236351527	0.923907416	0.715296175	0.972473327
LOC112445889	0.988299355	0.262347695	0.411007879	0.704810308	0.381039302	0.715272812	0.972473327
SYT15	0.868245803	0.728841389	0.91720162	0.343370861	0.143544852	0.715199969	0.972473327
LOC100294723	0.563327597	0.583523227	0.56778582	0.688025467	0.223040345	0.715418944	0.972480268
LOC786489	0.214934872	0.604527194	0.874441985	0.760587283	0.33142924	0.715419796	0.972480268
COL4A4	0.850175808	0.071741298	0.885728963	0.990700422	0.536756697	0.715991105	0.972503566
HCK	0.807109086	0.517737185	0.968364929	0.17284347	0.410862659	0.716048728	0.972503566
HTRA1	0.365916487	0.653977763	0.33319463	0.447611092	0.804516022	0.715894744	0.972503566
LOC100848177	0.689546229	0.110491646	0.667787024	0.795265677	0.709313954	0.715808538	0.972503566
LOC107132300	0.792833018	0.818163034	0.500153586	0.413688424	0.213826595	0.715799129	0.972503566
MAPRE2	0.25020564	0.689384543	0.98272132	0.303928413	0.557132201	0.715824746	0.972503566

MOB1B	0.512584959	0.123179963	0.846723497	0.708365423	0.758956584	0.716088788	0.972503566
MUC20	0.403410195	0.331060706	0.362071826	0.93981072	0.632445178	0.716084714	0.972503566
PROSER2	0.518828745	0.132408885	0.579471193	0.773831404	0.932111664	0.7158986	0.972503566
RPL38	0.695326387	0.780707895	0.391890465	0.442532525	0.304856118	0.715808773	0.972503566
SRA1	0.801564643	0.221978377	0.845301488	0.568706069	0.3355736	0.715832595	0.972503566
BLMH	0.780800314	0.172149986	0.824198154	0.727961471	0.357224175	0.716529018	0.97269896
KEAP1	0.435286996	0.416332991	0.788608012	0.284064082	0.70923508	0.716422429	0.97269896
LOC781059	0.879625948	0.060817456	0.644505805	0.917820118	0.910158329	0.716485173	0.97269896
TBC1D19	0.545290119	0.400117511	0.211740554	0.917629291	0.679548993	0.716520018	0.97269896
TMEM42	0.647488074	0.309160347	0.257875556	0.657720265	0.847523693	0.716306282	0.97269896
AP1G1	0.678246887	0.272958025	0.635974518	0.521730648	0.470337327	0.717076421	0.972938175
ARHGAP6	0.196189452	0.622903759	0.819416407	0.880891181	0.327769423	0.717213213	0.972938175
ASXL1	0.709778634	0.167891599	0.460378822	0.729800103	0.72395576	0.717690356	0.972938175
CDC23	0.599141404	0.790150298	0.139093565	0.484897143	0.904205393	0.716938035	0.972938175
CLEC3B	0.905940733	0.223905821	0.961254781	0.910394446	0.162649715	0.716948442	0.972938175
CLEC9A	0.60808521	0.472995144	0.252024844	0.592258284	0.678176158	0.718536056	0.972938175
COLEC10	0.995854662	0.881404069	0.285213867	0.620669595	0.187676814	0.718839521	0.972938175
EXTL3	0.903602197	0.556897292	0.652851687	0.378231468	0.232933517	0.717416381	0.972938175
FEM1B	0.141158897	0.827267936	0.67793223	0.37263261	0.988196289	0.718774895	0.972938175
GNB5	0.898366704	0.548710435	0.710284424	0.973141817	0.085042809	0.717629025	0.972938175
HARS	0.373913792	0.239498282	0.825301981	0.97078901	0.404167691	0.71777344	0.972938175
KLHL6	0.48133587	0.322157441	0.598547043	0.565315583	0.553754235	0.718144755	0.972938175
LAT2	0.947282722	0.308875571	0.473655097	0.477104028	0.440267476	0.718508503	0.972938175
LOC101902428	0.485103054	0.892798408	0.929650709	0.194371984	0.371543889	0.718288237	0.972938175
LOC101904840	0.997011199	0.122089116	0.259399628	0.953310495	0.968051953	0.718693884	0.972938175
LOC101907606	0.856943241	0.701048313	0.271103893	0.221158731	0.803354656	0.717368547	0.972938175
LOC104968873	0.289197453	0.840304626	0.372332591	0.859550258	0.371502929	0.717083682	0.972938175
LOC107132697	0.930527574	0.301548058	0.688092872	0.516755536	0.292197908	0.718788121	0.972938175
LOC107133209	0.643175135	0.341266137	0.568570938	0.339506692	0.68740874	0.718602621	0.972938175
LOC112444281	0.848042861	0.526420468	0.403901349	0.758692336	0.212460618	0.718209136	0.972938175
LOC534630	0.717345656	0.357980788	0.892208435	0.633579027	0.198904279	0.71695406	0.972938175
MAP3K11	0.842257748	0.757396072	0.185346354	0.442182224	0.55285111	0.71715619	0.972938175
MME	0.455687734	0.426605134	0.295201258	0.662882306	0.764273939	0.718264676	0.972938175
NCDN	0.293351019	0.616765625	0.262429452	0.970541489	0.628235106	0.717460145	0.972938175
PCDH20	0.733569371	0.583605291	0.683011939	0.362926829	0.274535355	0.718661853	0.972938175
PHC2	0.856884846	0.534906112	0.661396889	0.264211344	0.362436319	0.71797982	0.972938175
RALY	0.656447532	0.791723488	0.47861697	0.201577744	0.578544022	0.717846625	0.972938175
RBM12B	0.354869294	0.862789251	0.618824547	0.301484966	0.508692416	0.718161758	0.972938175
RNF166	0.669288036	0.426203114	0.431051299	0.777982265	0.304455076	0.718594349	0.972938175
SLC11A2	0.429022099	0.269154914	0.930816719	0.924790885	0.291845873	0.717847768	0.972938175

SNX20	0.790016612	0.149625795	0.9007106	0.568485873	0.477776652	0.717248464	0.972938175
TGFBR2	0.806612149	0.228738143	0.727958875	0.271319985	0.800213015	0.718832902	0.972938175
TUSC2	0.585834365	0.110661886	0.657291209	0.842979208	0.809903021	0.718388573	0.972938175
WIPI1	0.1767108	0.400346313	0.563125855	0.727058126	0.997609813	0.717100499	0.972938175
ZBTB12	0.999181067	0.461842562	0.160923468	0.443290464	0.881903449	0.717989271	0.972938175
ZBTB42	0.495550079	0.64440767	0.977032146	0.619119525	0.150159477	0.717822022	0.972938175
CPSF7	0.946621937	0.836986014	0.553623931	0.140734269	0.474015564	0.719489982	0.973110684
CRABP1	0.976155214	0.57626822	0.108054516	0.843402141	0.569875079	0.719183753	0.973110684
IGF2	0.336444456	0.436917277	0.770743099	0.338698952	0.762568078	0.719495496	0.973110684
LOC101905951	0.693067017	0.118765726	0.666358173	0.554504689	0.962296978	0.719527685	0.973110684
LOC112446018	0.547382584	0.863902844	0.971713638	0.10290132	0.617733397	0.719146791	0.973110684
MPP1	0.987092444	0.540957668	0.108834688	0.719242035	0.700761797	0.719678531	0.973110684
MTHFSD	0.129288046	0.662657471	0.799306984	0.48938254	0.872960782	0.719447277	0.973110684
NUP93	0.663576919	0.622577472	0.783468717	0.983824033	0.09196739	0.719645217	0.973110684
SIK3	0.742298267	0.707957465	0.248844028	0.553120278	0.404936861	0.719672332	0.973110684
SLC37A2	0.489721367	0.480113707	0.693256784	0.395450057	0.453318578	0.719219395	0.973110684
TMEM43	0.515758956	0.525461621	0.782396629	0.553548986	0.249473486	0.719617966	0.973110684
TUBA4A	0.328337081	0.67983867	0.871375778	0.777430586	0.19324905	0.719231546	0.973110684
MLLT10	0.576288546	0.386410371	0.326913985	0.896326466	0.449093706	0.719762146	0.973143563
ABCB10	0.409980989	0.757099598	0.395561374	0.390672929	0.615613202	0.721213582	0.973157434
ASH1L	0.42638481	0.466089213	0.974784689	0.191447675	0.791307381	0.720045595	0.973157434
ATRX	0.578657582	0.933585026	0.661506928	0.083805538	0.979909009	0.720042802	0.973157434
AURKA	0.426321421	0.481887655	0.217603021	0.985707218	0.670838455	0.721415012	0.973157434
BCR	0.634373847	0.215828662	0.567429333	0.787816176	0.479792687	0.720163394	0.973157434
BOD1	0.823109706	0.671772941	0.248911462	0.296535636	0.724712529	0.721525774	0.973157434
CKS1B	0.993148146	0.432539843	0.303339542	0.284631437	0.7930308	0.720467738	0.973157434
CNKSR2	0.947230661	0.577602029	0.280198368	0.794149426	0.242877425	0.72146981	0.973157434
COLGALT2	0.513429532	0.721059268	0.55694873	0.70087934	0.203749332	0.720670353	0.973157434
COMTD1	0.959588249	0.193823853	0.70980594	0.352484304	0.632857259	0.720701259	0.973157434
CRB1	0.601074501	0.091134984	0.977135979	0.657230464	0.837335252	0.72074845	0.973157434
DCTN2	0.662875874	0.480362774	0.143678498	0.841325294	0.766886576	0.721142303	0.973157434
ESRRG	0.553054453	0.542100137	0.822596516	0.37615965	0.317749526	0.720881896	0.973157434
FAM222B	0.50551605	0.909887773	0.701781066	0.596347263	0.153634112	0.721501149	0.973157434
GPAA1	0.235238864	0.705709231	0.796513325	0.331772271	0.668849521	0.720011389	0.973157434
LMF2	0.728986713	0.222810584	0.327241914	0.984470417	0.56035586	0.719878045	0.973157434
LOC101903056	0.380356138	0.356744771	0.226911698	0.97746435	0.976562382	0.720321009	0.973157434
LOC101905357	0.437063081	0.571304061	0.376175602	0.348701547	0.899959396	0.720876424	0.973157434
LOC112442623	0.75085764	0.988029733	0.197594896	0.267341422	0.750435246	0.720441871	0.973157434
LOC112449516	0.514488549	0.36248436	0.681591808	0.796718749	0.290527276	0.720528475	0.973157434
LOC784251	0.323120809	0.433365342	0.523309563	0.573261557	0.70152545	0.720830696	0.973157434

MAPKAPK2	0.956781234	0.296959458	0.260390023	0.463647543	0.860250465	0.721080637	0.973157434
MED15	0.776937554	0.098061037	0.644204573	0.678919274	0.88127798	0.720160237	0.973157434
SLC27A5	0.374991285	0.513797942	0.765292223	0.707120341	0.283589473	0.721463197	0.973157434
SPC25	0.354771051	0.28069876	0.794609827	0.830247078	0.449173362	0.721088016	0.973157434
SPN	0.75497364	0.110702908	0.509425805	0.728155327	0.9489634	0.720512747	0.973157434
TMTC1	0.635125344	0.39600012	0.182903846	0.983119524	0.653604875	0.721408662	0.973157434
WDR45B	0.87875029	0.381485122	0.238320186	0.75519582	0.487115045	0.72031629	0.973157434
XKR6	0.068774288	0.877833434	0.835413722	0.767517512	0.764184789	0.721551377	0.973157434
ZCWPW1	0.431944649	0.973494775	0.212771403	0.749110822	0.440290315	0.721086376	0.973157434
LOC100296627	0.479527424	0.56355412	0.899494167	0.609780012	0.199732321	0.721700682	0.973278815
CNIH4	0.864153059	0.987219816	0.904638588	0.198279968	0.193595936	0.721825256	0.973366826
NOL9	0.54658975	0.169766036	0.954974454	0.932392702	0.358747048	0.721928359	0.973425872
AK3	0.882292277	0.540565017	0.381653733	0.431707575	0.378851362	0.722755081	0.973464707
ATXN7	0.153308901	0.459561425	0.958333449	0.605181527	0.72725985	0.722412813	0.973464707
CDH8	0.713693708	0.276452869	0.87124338	0.858367255	0.201245683	0.722268714	0.973464707
COL4A1	0.1852451	0.733261287	0.643774768	0.83621517	0.408627764	0.723450555	0.973464707
DAXX	0.653976853	0.652287917	0.258195273	0.572887038	0.471456586	0.722612296	0.973464707
EGFL7	0.539399587	0.54336739	0.791427316	0.345227445	0.370883597	0.722306183	0.973464707
EML2	0.520139208	0.505870337	0.673710779	0.288141404	0.58506039	0.723473769	0.973464707
ETV3	0.678405329	0.889350048	0.633329918	0.352172038	0.222141271	0.72353361	0.973464707
FAM133B	0.804551341	0.884484365	0.357846366	0.314593989	0.373189857	0.723553061	0.973464707
FAM174B	0.995739612	0.468511123	0.334541214	0.608526765	0.314802976	0.723558741	0.973464707
GNL2	0.69393127	0.379728598	0.340874259	0.505595667	0.654009807	0.72231203	0.973464707
LOC107131772	0.289077964	0.15302175	0.778210127	0.93291322	0.929904389	0.723345716	0.973464707
LOC107133268	0.542983787	0.794955952	0.11555452	0.719943327	0.828262432	0.722578504	0.973464707
LOC112441638	0.518995576	0.967627019	0.628333529	0.315109414	0.300079708	0.723178197	0.973464707
LOC112442538	0.17807845	0.409882731	0.520742181	0.871100916	0.896512098	0.722201688	0.973464707
LOC783533	0.909613044	0.501641992	0.219087409	0.617427871	0.48355774	0.723240326	0.973464707
MXRA8	0.163107485	0.869728088	0.242143247	0.997497809	0.871307417	0.723288471	0.973464707
NHEJ1	0.59476431	0.481671594	0.997228085	0.659387573	0.158120256	0.72285538	0.973464707
OSGIN1	0.427503722	0.639389022	0.170111378	0.742786663	0.861047271	0.722555147	0.973464707
OTUD3	0.595607353	0.535118017	0.202876208	0.63533587	0.725426491	0.722950697	0.973464707
PSMG3	0.292922938	0.369210929	0.811950236	0.487913239	0.697354827	0.723436798	0.973464707
RASA3	0.538371134	0.80493805	0.771283953	0.371683488	0.240164832	0.72317006	0.973464707
RMND5B	0.228861725	0.542345715	0.422853962	0.609733725	0.929652195	0.722629635	0.973464707
RTL9	0.915486884	0.858729007	0.618423378	0.678198973	0.09036141	0.722905727	0.973464707
SCNN1A	0.946507111	0.41081701	0.298335825	0.837191499	0.306687306	0.7228465	0.973464707
SGCE	0.178556121	0.766073223	0.801765588	0.399688732	0.679530683	0.722857432	0.973464707
TGOLN2	0.695057588	0.221590704	0.514434609	0.530636433	0.707226392	0.722523995	0.973464707
HCAR1	0.433546127	0.829133554	0.689064004	0.858937258	0.140597373	0.723656339	0.973516204

TBK1	0.951186903	0.758133831	0.555746043	0.277166723	0.269459807	0.72377178	0.973591695
CCDC28B	0.636772707	0.102218854	0.739851861	0.739898562	0.840710009	0.723926486	0.973690044
LOC101903397	0.422121901	0.418948596	0.54860153	0.34271904	0.901093627	0.723963556	0.973690044
SYCP2	0.590953472	0.756862704	0.519856963	0.790183349	0.163440164	0.724388774	0.974102276
TAF15	0.890167117	0.389924232	0.895221833	0.584441579	0.16530908	0.724336271	0.974102276
LOC100847831	0.703081297	0.974676331	0.79027035	0.210473261	0.26371181	0.724574655	0.974210165
LOC107132045	0.83434353	0.195374059	0.847157129	0.385279092	0.56499813	0.724587733	0.974210165
AGO3	0.554714847	0.914275371	0.964569047	0.087423726	0.704081926	0.724907192	0.974453565
CARM1	0.625888837	0.810718161	0.173506626	0.37657087	0.909909066	0.725253027	0.974453565
CROT	0.422340659	0.867495661	0.257815757	0.333774695	0.957169773	0.725320055	0.974453565
DAG1	0.782200405	0.511221541	0.997593615	0.091833764	0.822194543	0.724961635	0.974453565
FBXO34	0.778720624	0.105780347	0.959303559	0.744377391	0.512339217	0.725064131	0.974453565
KHDRBS3	0.994530713	0.739830457	0.427390848	0.114105825	0.840966076	0.725311304	0.974453565
LOC100139144	0.576743998	0.346371288	0.459233658	0.393169064	0.837191067	0.725442036	0.974453565
LOC104969340	0.909956867	0.83194886	0.062614288	0.827197945	0.769583905	0.725308866	0.974453565
LOC516849	0.342656397	0.367309797	0.375811104	0.959995706	0.664716723	0.725356932	0.974453565
MAGI2	0.454171758	0.652852023	0.347195375	0.43033291	0.681772614	0.725481302	0.974453565
SULF2	0.393783318	0.956326464	0.192488532	0.655749344	0.634849807	0.725317951	0.974453565
WSCD1	0.734710909	0.730504096	0.388886667	0.187251411	0.772490076	0.725406212	0.974453565
ELK3	0.959051892	0.721313341	0.078386556	0.673710777	0.827482496	0.725649557	0.974520039
FRG1	0.559712002	0.502572593	0.365390791	0.559390141	0.525741159	0.725635576	0.974520039
KLF14	0.771364132	0.63141203	0.619985477	0.464131317	0.215986948	0.725902619	0.974780123
SP2	0.805085166	0.673288373	0.327646889	0.910034736	0.187468503	0.726082133	0.974941407
COX4I2	0.203054088	0.552594173	0.348019249	0.880807777	0.881333474	0.726172273	0.974982669
SIK2	0.351607455	0.828787937	0.137677011	0.982991703	0.768989433	0.726255019	0.975013999
ELMSAN1	0.274732369	0.686913606	0.962830779	0.542518806	0.307949142	0.726438604	0.975021177
ITM2C	0.438745593	0.541282988	0.260563332	0.611390216	0.802382697	0.726436333	0.975021177
PNMA2	0.639472757	0.78179118	0.248477873	0.396764733	0.615752983	0.726388387	0.975021177
BAG4	0.85272404	0.163674194	0.328085172	0.726327991	0.914297437	0.726759994	0.975080348
FABP2	0.339468323	0.542130071	0.91423445	0.469600403	0.384578504	0.726620222	0.975080348
IRX5	0.752096075	0.849653928	0.155896877	0.473328898	0.644155956	0.726547703	0.975080348
LOC616094	0.771570964	0.70462428	0.158672516	0.465765599	0.756900063	0.726779771	0.975080348
LOC788724	0.922570346	0.348631188	0.703810203	0.237647022	0.565162183	0.726730092	0.975080348
URB1	0.936798344	0.680044584	0.144379362	0.881483083	0.3754078	0.72693818	0.97521315
ARPC1A	0.715829679	0.3797212	0.644605118	0.697882181	0.249042981	0.727033199	0.975260898
MFSD5	0.909274362	0.649670171	0.222686767	0.648299228	0.357263274	0.727129356	0.975310164
ABHD8	0.379841348	0.770467616	0.348590381	0.301136207	0.993046052	0.727371715	0.975330034
LOC107132952	0.580923238	0.343451737	0.5962915	0.533137078	0.480763623	0.727288875	0.975330034
PTDSS1	0.666345057	0.744316385	0.150408597	0.960963319	0.42552677	0.727353798	0.975330034
TIAL1	0.40480885	0.105428967	0.847035517	0.97705183	0.863771099	0.727381895	0.975330034

AGL	0.277010933	0.366040032	0.765064324	0.655684539	0.600221288	0.727512964	0.975359039
DCLRE1A	0.102130443	0.686657918	0.709611886	0.706133179	0.869405164	0.727641259	0.975359039
ITPKB	0.459435755	0.754546385	0.33984578	0.331022943	0.783185934	0.727593614	0.975359039
LOC101903853	0.979794752	0.275142708	0.5559752	0.267424205	0.761815369	0.727543	0.975359039
ADAM15	0.955836577	0.734653518	0.263961941	0.212211216	0.777653893	0.727874242	0.975362625
ARHGAP12	0.208945266	0.613203291	0.759805704	0.531432994	0.593508918	0.728591404	0.975362625
CAMTA2	0.626319854	0.269265797	0.301297296	0.912816919	0.660690469	0.728217933	0.975362625
ISLR	0.077229106	0.767337481	0.769703864	0.871458055	0.771775657	0.728423691	0.975362625
LGALSL	0.775626562	0.361630279	0.250183566	0.752314912	0.581414888	0.72852493	0.975362625
LOC101902809	0.882777068	0.458644758	0.154850012	0.751278215	0.650733655	0.728256308	0.975362625
LOC107132247	0.450888602	0.263498259	0.956674037	0.676710991	0.397829542	0.727938919	0.975362625
LOC112446708	0.758924752	0.681392704	0.681886993	0.165825035	0.525267322	0.728645077	0.975362625
LOC112449115	0.697170688	0.85843301	0.665856538	0.455652092	0.168672661	0.728109443	0.975362625
NFIX	0.643829579	0.634994541	0.308346336	0.451899118	0.539080094	0.728617067	0.975362625
PLEKHG3	0.776039299	0.650813149	0.468519068	0.301985009	0.428656987	0.728134752	0.975362625
RCOR3	0.557811647	0.747448616	0.513812525	0.906992771	0.158132077	0.728713738	0.975362625
RHBDD2	0.55824007	0.121267068	0.63986761	0.917910599	0.772629314	0.728682588	0.975362625
STX10	0.690804423	0.917858419	0.408581461	0.47285237	0.250417725	0.728411307	0.975362625
TNKS	0.178194271	0.736709773	0.887532911	0.295025914	0.892761381	0.728485086	0.975362625
TNRC6A	0.924107251	0.774782718	0.157560776	0.739248376	0.367929966	0.728457222	0.975362625
USHBP1	0.6302805	0.582410128	0.178361883	0.49275179	0.951860155	0.728613467	0.975362625
XPNPEP3	0.587395445	0.752929086	0.967451006	0.08723771	0.820012944	0.727994173	0.975362625
A4GALT	0.219515567	0.885006923	0.499451431	0.529765852	0.606356246	0.731408058	0.975415174
AFMID	0.334088126	0.755975793	0.521596905	0.535423112	0.439387557	0.730339517	0.975415174
ANKRD29	0.517016953	0.481195528	0.460922365	0.364474787	0.748113913	0.73200174	0.975415174
ASCL4	0.915366015	0.484610522	0.983935892	0.092530288	0.773972624	0.731947488	0.975415174
CANT1	0.44520524	0.242079186	0.681629399	0.734695018	0.57542776	0.730735796	0.975415174
CASP1	0.132013503	0.894233001	0.519066383	0.848931314	0.600715237	0.731890918	0.975415174
CD180	0.33507251	0.23604609	0.630237631	0.999931474	0.627931401	0.732189008	0.975415174
CD44	0.221749953	0.856314278	0.189370621	0.986245941	0.879996334	0.731649644	0.975415174
CNOT6L	0.549503972	0.621352558	0.80594838	0.156577436	0.715748364	0.729410995	0.975415174
COPS7B	0.901220155	0.110290177	0.841046842	0.421598745	0.884573326	0.731453057	0.975415174
CYBC1	0.204050984	0.903083325	0.789751917	0.664386655	0.319598468	0.72979109	0.975415174
DMWD	0.602313251	0.493017732	0.977059334	0.115093347	0.931400276	0.731007899	0.975415174
DNAH17	0.877903555	0.777311271	0.118002438	0.58860262	0.66024439	0.732161887	0.975415174
DTWD2	0.534770869	0.691702622	0.90897056	0.486215355	0.191123255	0.731867573	0.975415174
FAM120C	0.480864284	0.135699687	0.632791623	0.929022022	0.813388259	0.731610956	0.975415174
FSD1L	0.815795015	0.321070992	0.340877457	0.75600149	0.461230651	0.731193469	0.975415174
FZD9	0.530856352	0.189756698	0.769921163	0.74453282	0.541112737	0.731873114	0.975415174
GPIHBP1	0.265207381	0.726821583	0.584460508	0.287769283	0.955391825	0.730229395	0.975415174

HRH2	0.797651333	0.378788467	0.21386589	0.804744992	0.593606887	0.729585601	0.975415174
ISG20	0.877051228	0.203583117	0.8803837	0.228146058	0.857743417	0.728935608	0.975415174
ITIH3	0.350574033	0.914658496	0.810578038	0.385245044	0.308564243	0.729762239	0.975415174
KIAA1468	0.316033754	0.956936375	0.655314954	0.168101171	0.939194642	0.732133038	0.975415174
KRT18	0.708075933	0.579720089	0.64208916	0.300670855	0.394115884	0.731793866	0.975415174
LEPROT	0.305227943	0.31273936	0.66638725	0.491329442	0.992500721	0.730507691	0.975415174
LIX1L	0.060437009	0.803833979	0.769702073	0.910954941	0.918881855	0.732200324	0.975415174
LMF1	0.805640684	0.343779005	0.945041349	0.85519023	0.137803505	0.729449503	0.975415174
LOC104971613	0.786154206	0.859241008	0.248856464	0.594723739	0.309657795	0.730131678	0.975415174
LOC107131848	0.463054479	0.248777742	0.569547416	0.885739407	0.538549125	0.732181401	0.975415174
LOC107132796	0.487235739	0.307010688	0.334680484	0.821544989	0.752988434	0.730206927	0.975415174
LOC112443130	0.628664165	0.175690635	0.641511117	0.563047516	0.780703155	0.731271923	0.975415174
LOC112443225	0.906355374	0.88521349	0.138475181	0.555826704	0.504657699	0.731381107	0.975415174
LOC112445041	0.530455822	0.482408709	0.595920406	0.281450382	0.727974797	0.731863948	0.975415174
LOC112445952	0.595007616	0.572000558	0.345852356	0.730030989	0.362602602	0.731349576	0.975415174
LOC112446796	0.72051919	0.725202402	0.102023273	0.627847487	0.933584589	0.731881713	0.975415174
LOC112447462	0.84504036	0.778437183	0.129030792	0.372115899	0.981199082	0.730331263	0.975415174
LOC506181	0.515841863	0.475534952	0.218046688	0.75760066	0.759223764	0.728957106	0.975415174
LOC528802	0.998208405	0.612986511	0.199737412	0.403846627	0.629363297	0.730772812	0.975415174
LOC781913	0.175276724	0.419023785	0.885260616	0.486572986	0.981899116	0.730772595	0.975415174
MGMT	0.673059524	0.854447115	0.320998517	0.170543439	0.988284199	0.731079786	0.975415174
MTHFR	0.613923013	0.499564829	0.340527421	0.502944752	0.589869057	0.730289689	0.975415174
MTREX	0.283855021	0.744672763	0.984491124	0.473728406	0.314364701	0.730334429	0.975415174
OSGEPL1	0.963605969	0.087460481	0.890986227	0.453653972	0.906097369	0.729574047	0.975415174
PIGB	0.60244453	0.441783909	0.478138178	0.736854058	0.332054976	0.731215386	0.975415174
PLEC	0.853116074	0.163921988	0.472183876	0.54986025	0.853694208	0.730366519	0.975415174
PUM1	0.641427137	0.62600591	0.970998443	0.087398308	0.907760393	0.729979884	0.975415174
RNF25	0.336511168	0.314822401	0.98119535	0.396735623	0.746150548	0.72899595	0.975415174
RWDD2B	0.922285972	0.188415924	0.534315161	0.621785889	0.535046041	0.729717397	0.975415174
SMAGP	0.396431724	0.495232471	0.703663985	0.776707267	0.287873505	0.729712708	0.975415174
SMOX	0.85564499	0.564182615	0.71506561	0.159078747	0.567837788	0.731485334	0.975415174
STAT3	0.805722861	0.636650415	0.985361186	0.200810361	0.306497836	0.731052577	0.975415174
SUPT20H	0.734664287	0.260748477	0.482285204	0.859082474	0.389078654	0.729662881	0.975415174
TMEM170B	0.822585973	0.155468126	0.311120835	0.890013664	0.870781528	0.729390167	0.975415174
TMEM246	0.053402022	0.670965865	0.981138874	0.948973515	0.922663492	0.729055691	0.975415174
TNPO2	0.633959344	0.188755135	0.887569395	0.796904748	0.366540423	0.730530927	0.975415174
TPST2	0.721322737	0.213995653	0.711279734	0.692982276	0.405854383	0.729654415	0.975415174
TUSC3	0.961714245	0.373765536	0.225158735	0.63568802	0.605292214	0.731246272	0.975415174
WDR81	0.985855455	0.256581845	0.761146207	0.406239839	0.3961402	0.730291824	0.975415174
ZNF226	0.561463117	0.905626194	0.454704812	0.399270464	0.33307483	0.72884875	0.975415174

CENPQ	0.873687637	0.241060054	0.76394586	0.342722284	0.568572627	0.73251327	0.975436163
COG3	0.981139914	0.945729045	0.393309158	0.202609491	0.423975925	0.73249615	0.975436163
INTS7	0.410535377	0.319993042	0.646507036	0.373001741	0.988508201	0.732289822	0.975436163
LOC784866	0.205476595	0.961483118	0.372300341	0.814303021	0.523221027	0.732425236	0.975436163
SPPL3	0.867226893	0.276530266	0.98905144	0.559129583	0.236328015	0.732448269	0.975436163
ALG8	0.29114519	0.585413623	0.363257311	0.924248528	0.549996304	0.733232038	0.975925675
BBC3	0.536229314	0.856543928	0.163260361	0.610305084	0.687492173	0.733171695	0.975925675
EMC1	0.5871258	0.68699024	0.439285627	0.331436614	0.535579539	0.733110117	0.975925675
GFOD2	0.531843952	0.789724815	0.605829316	0.171477707	0.720670638	0.733067598	0.975925675
LOC100138933	0.980584688	0.649266255	0.095033544	0.728791114	0.713071866	0.733053098	0.975925675
NSMCE1	0.216564803	0.712777834	0.292659745	0.936573999	0.7439901	0.733266152	0.975925675
ZNF419	0.290575021	0.417498792	0.289773757	0.915027041	0.978767555	0.733297149	0.975925675
ACOT11	0.125772454	0.433056605	0.980326167	0.79671316	0.747435197	0.735149321	0.975942306
ARNT2	0.420845376	0.932284826	0.258216824	0.95595736	0.325817045	0.73372134	0.975942306
ARRDC4	0.699786898	0.918905251	0.568618783	0.110552528	0.794459023	0.737012051	0.975942306
ATG9A	0.766132131	0.829502411	0.594684356	0.739150537	0.115130518	0.737284758	0.975942306
BCL3	0.585705491	0.673978304	0.218361429	0.915346104	0.403924726	0.73558518	0.975942306
BMPR2	0.925054369	0.441697388	0.13828251	0.596389658	0.948050629	0.736030995	0.975942306
BRCA1	0.718935134	0.127578439	0.619076527	0.946329475	0.590737266	0.734835123	0.975942306
BRD1	0.938131538	0.232182186	0.813335778	0.337713026	0.527583367	0.733778947	0.975942306
BUD13	0.884147028	0.129774447	0.871301891	0.56342451	0.560887984	0.733948571	0.975942306
C1QC	0.655322058	0.589342001	0.662046669	0.318074797	0.395651803	0.73738034	0.975942306
C9H6orf120	0.819772459	0.801364519	0.358845748	0.339218245	0.396300644	0.734527497	0.975942306
CACFD1	0.79532753	0.577065377	0.157126699	0.617003274	0.721985121	0.737071834	0.975942306
CAPNS1	0.876918375	0.336606139	0.441466218	0.520245675	0.472860638	0.736677104	0.975942306
CIZ1	0.604838302	0.293940639	0.306485412	0.672574653	0.868653566	0.735373397	0.975942306
CLIC1	0.190477952	0.651622718	0.484439346	0.676112239	0.785373346	0.735925089	0.975942306
CYB561	0.76802031	0.715743329	0.228957628	0.607838056	0.417925172	0.736182098	0.975942306
DGKZ	0.872455966	0.142247854	0.685946558	0.910494563	0.41344327	0.736613551	0.975942306
DNAJC15	0.461349085	0.383674615	0.428503671	0.854533476	0.4931538	0.736133478	0.975942306
FZD6	0.72676635	0.118608716	0.782345754	0.990344417	0.475237909	0.73481761	0.975942306
GAPVD1	0.427266308	0.912485196	0.418716298	0.281135008	0.695788869	0.73595158	0.975942306
GCN1	0.854106104	0.23669813	0.752374082	0.851546326	0.24791793	0.736994295	0.975942306
GPATCH2	0.607558034	0.636449473	0.533934969	0.174380566	0.885493783	0.735644145	0.975942306
HERC5	0.136599323	0.624538505	0.75445881	0.640380237	0.768392904	0.734410677	0.975942306
HIST2H2AA4	0.627381816	0.521840454	0.574042375	0.385758767	0.440216122	0.735847524	0.975942306
HOOK3	0.875206137	0.512068923	0.774324603	0.097296858	0.948964135	0.736586686	0.975942306
HP1BP3	0.434894154	0.246482354	0.934293704	0.600312823	0.527261954	0.734580005	0.975942306
HTRA4	0.902203616	0.973343756	0.361090471	0.127988257	0.792712146	0.737345588	0.975942306
IGF2R	0.847034175	0.710313509	0.089507974	0.959534263	0.620990647	0.736865764	0.975942306

INVS	0.23986548	0.935563833	0.762855597	0.365302173	0.50515719	0.733934797	0.975942306
LGALS9	0.181213275	0.285120774	0.907207512	0.893912893	0.762316648	0.736003036	0.975942306
LOC101904442	0.921009417	0.621584778	0.198557637	0.832692791	0.33486954	0.734560414	0.975942306
LOC101904769	0.108934821	0.764287957	0.94405858	0.539443023	0.749498532	0.735046959	0.975942306
LOC101906177	0.15861144	0.363334796	0.877717739	0.748364034	0.841054029	0.735389506	0.975942306
LOC101907603	0.786203139	0.672401643	0.198281934	0.461673529	0.656595723	0.735021358	0.975942306
LOC101907653	0.982622144	0.692643747	0.125571419	0.87146068	0.432109651	0.737412991	0.975942306
LOC107131403	0.812992549	0.951478792	0.436705958	0.182729846	0.51987857	0.736877789	0.975942306
LOC107131919	0.668554056	0.204159813	0.848218657	0.347370189	0.792817827	0.735668678	0.975942306
LOC112441718	0.192723555	0.622644683	0.535261525	0.974990399	0.511152798	0.736406469	0.975942306
LOC112442585	0.513979771	0.520541164	0.573881399	0.455631212	0.451721274	0.73399739	0.975942306
LOC515333	0.414951259	0.356146105	0.390803439	0.588166971	0.947282643	0.73738494	0.975942306
LOC518775	0.919583803	0.1381527	0.453466881	0.98839503	0.562377435	0.736476494	0.975942306
LOC534913	0.981618781	0.648434157	0.174719708	0.855018067	0.336784695	0.736486652	0.975942306
LOC617141	0.410439329	0.556381245	0.587334047	0.658294316	0.363742852	0.737022076	0.975942306
LSM3	0.705410065	0.248541348	0.387682314	0.955717965	0.48914109	0.735021564	0.975942306
MARCH3	0.676590541	0.49052518	0.842158815	0.315640794	0.360687214	0.735291135	0.975942306
MDP1	0.75160089	0.638768769	0.1020335	0.704804737	0.926870337	0.736350171	0.975942306
MTURN	0.74625956	0.334913108	0.206981285	0.764676471	0.811437788	0.736920814	0.975942306
ORMDL2	0.51127954	0.893936833	0.371468339	0.390132875	0.478102537	0.734391719	0.975942306
PDLIM1	0.630923161	0.3183908	0.540164726	0.317100452	0.918458306	0.734002677	0.975942306
PGPEP1	0.708188849	0.268465825	0.743054702	0.646949433	0.346707564	0.734509073	0.975942306
PHRF1	0.256383386	0.221466269	0.842934535	0.843544348	0.787219882	0.735071221	0.975942306
POLR3GL	0.512316778	0.371134369	0.338159477	0.587262559	0.847815029	0.736420495	0.975942306
PPP2R2B	0.325199709	0.601860549	0.469259048	0.980538926	0.350785354	0.733935947	0.975942306
PTPN21	0.29195682	0.826009755	0.258442554	0.711389559	0.715129826	0.734624596	0.975942306
RNF216	0.648241627	0.519903261	0.230824908	0.803234586	0.513824551	0.73696961	0.975942306
SAT1	0.34562085	0.37222587	0.39374379	0.917565416	0.692047728	0.737311493	0.975942306
SETBP1	0.193017023	0.769076523	0.506276705	0.506525215	0.828692305	0.733669276	0.975942306
SLC35A2	0.67104213	0.128878348	0.725499607	0.886251048	0.576380884	0.736639596	0.975942306
SMYD4	0.582304291	0.323601904	0.485750308	0.369150507	0.94163116	0.735271006	0.975942306
SOX13	0.983127445	0.299652599	0.762129842	0.860648342	0.16349358	0.733943851	0.975942306
SPTA1	0.793473766	0.285695728	0.448668715	0.41706216	0.754581449	0.736396484	0.975942306
TBC1D16	0.55982602	0.895403842	0.401775649	0.517859572	0.307024094	0.736470207	0.975942306
TIMMDC1	0.934150955	0.441650323	0.302107119	0.289873028	0.87945293	0.735020181	0.975942306
TMEM98	0.73673912	0.905424872	0.314072064	0.310497149	0.484798352	0.733611711	0.975942306
TTBK2	0.419545984	0.837898991	0.35242966	0.405815799	0.631555171	0.734893747	0.975942306
USP3	0.673812502	0.424122234	0.565687738	0.221901607	0.878677732	0.733517859	0.975942306
YPEL4	0.745584101	0.534875369	0.688830159	0.121868168	0.944479092	0.734099617	0.975942306
ZBTB39	0.409340278	0.461010337	0.847295551	0.857638642	0.230506873	0.734045329	0.975942306

ZFP36	0.778281694	0.270990517	0.238959429	0.816022279	0.769434045	0.734248336	0.975942306
SMG8	0.559173078	0.788798477	0.337280349	0.227295699	0.952201754	0.737496735	0.975974431
NYX	0.829455572	0.909408545	0.10079085	0.513048836	0.826116511	0.737647281	0.976073704
SLC35E1	0.983875933	0.363638789	0.306129457	0.723253169	0.406881695	0.737690705	0.976073704
CCDC151	0.723419347	0.229636939	0.482635787	0.640328142	0.62823348	0.737820211	0.976094381
CELA1	0.837675298	0.957897016	0.462894994	0.233653036	0.371838032	0.737917985	0.976094381
FBXL21	0.406682959	0.875264217	0.317099023	0.369497236	0.774794893	0.738170026	0.976094381
HNRNPR	0.795764493	0.223523427	0.499780521	0.87610226	0.414611375	0.738038772	0.976094381
LOC100336734	0.941085066	0.176740242	0.658256457	0.667821145	0.441973843	0.738182156	0.976094381
LOC512165	0.804232082	0.262681674	0.562699324	0.621268714	0.43747238	0.738139775	0.976094381
PPARA	0.427502992	0.483302995	0.768892855	0.63214143	0.321591073	0.738064489	0.976094381
SORBS3	0.891357036	0.664636382	0.919594323	0.372709204	0.159011543	0.73801636	0.976094381
UBQLN4	0.719782091	0.952993954	0.42875971	0.46710728	0.235341064	0.738269625	0.97613139
LOC101902840	0.988548089	0.505908558	0.245553123	0.894729947	0.294474281	0.738413791	0.97621379
TAF5L	0.752495715	0.726962382	0.654536887	0.365168131	0.247512978	0.738450916	0.97621379
MGAT1	0.496371061	0.754751776	0.476234491	0.203050547	0.893976135	0.738588805	0.976317429
JCAD	0.582961699	0.601997974	0.5861685	0.477498518	0.329829209	0.738656386	0.976328121
INO80C	0.322269568	0.213886141	0.783431459	0.802558529	0.748218449	0.73882384	0.976356742
ISM1	0.91283256	0.326194129	0.714167333	0.375801721	0.406453291	0.739137079	0.976356742
LOC100847236	0.329956391	0.275637521	0.732547686	0.712503462	0.684318429	0.739153992	0.976356742
LOC782202	0.794753156	0.611838486	0.180967479	0.581959022	0.633669397	0.73895999	0.976356742
NIT1	0.51006276	0.535896683	0.524328829	0.247384519	0.916119184	0.739135474	0.976356742
ST3GAL4	0.782401221	0.211082008	0.783748801	0.793150652	0.316377757	0.7391296	0.976356742
TMEM52	0.551656166	0.109265958	0.694240506	0.943438126	0.822707922	0.739130955	0.976356742
ZFHX2	0.660036379	0.188802074	0.672295914	0.391460323	0.989174462	0.738904537	0.976356742
CNNM2	0.995197742	0.389767638	0.46153891	0.266599029	0.681385996	0.739368582	0.976397178
GFAP	0.248294154	0.921885646	0.278974447	0.794733543	0.640631781	0.739309592	0.976397178
LOC112443140	0.80226378	0.85661038	0.503159705	0.094839545	0.99209677	0.739443629	0.976397178
MTIF3	0.609810977	0.287471973	0.890807184	0.427720596	0.487331472	0.739533535	0.976397178
TLN1	0.752867426	0.469383406	0.161026553	0.580942	0.98470991	0.739544596	0.976397178
TSPAN2	0.340837199	0.751175346	0.674028788	0.412290732	0.457632135	0.739588437	0.976397178
WNT11	0.966126275	0.998790555	0.331656519	0.26359204	0.385999835	0.73960108	0.976397178
CNOT2	0.559738734	0.92034746	0.781113225	0.088108575	0.920290394	0.739978273	0.97673799
NENF	0.893038605	0.430112502	0.550423597	0.712033834	0.216725863	0.739963874	0.97673799
CYP39A1	0.93561027	0.792702031	0.374707627	0.761297072	0.154477264	0.740288353	0.976754475
EPHX1	0.496002775	0.749458632	0.350780763	0.809657538	0.309378541	0.740177932	0.976754475
LOC100847269	0.72489985	0.976712396	0.056291389	0.944479819	0.867156273	0.740056658	0.976754475
LOC104974330	0.893091532	0.33164157	0.74393319	0.232104491	0.638743165	0.740199227	0.976754475
USP31	0.953001035	0.338633137	0.682506118	0.169846269	0.873377823	0.740233097	0.976754475
CALR3	0.574903001	0.980372186	0.194490022	0.819065781	0.364378504	0.740476511	0.976767143

LOC100174924	0.960491659	0.196204611	0.658392233	0.3446392	0.76490112	0.74043489	0.976767143
YAE1D1	0.587359027	0.166532985	0.900586079	0.554763022	0.669231994	0.740415941	0.976767143
AHSP	0.262464461	0.615151993	0.888320093	0.361417416	0.632467897	0.740869546	0.97676908
C3H1orf123	0.793180417	0.355032065	0.811618607	0.150333482	0.953276662	0.740696241	0.97676908
CNIH3	0.522719114	0.855177617	0.096016358	0.871269943	0.876962534	0.740926936	0.97676908
CTSK	0.410161588	0.609136923	0.559715978	0.548498862	0.426816958	0.740605567	0.97676908
FOXRED2	0.946321815	0.41650248	0.505643388	0.219544684	0.748064233	0.740565889	0.97676908
GGACT	0.781594337	0.720027685	0.430154874	0.139829423	0.968899252	0.740939351	0.97676908
LOC107131940	0.539736451	0.984736922	0.511524493	0.241471552	0.499450004	0.740893756	0.97676908
LOC789895	0.845957776	0.85547112	0.510831755	0.100374441	0.884605486	0.741099016	0.97676908
MEA1	0.347980639	0.282061926	0.585700474	0.822268479	0.694739541	0.741187448	0.97676908
PAPLN	0.605782754	0.691468816	0.631863446	0.139531612	0.888667822	0.741064734	0.97676908
PRKAR2A	0.653155497	0.479034118	0.228809256	0.852182294	0.538163426	0.741141332	0.97676908
TAP1	0.472393255	0.676282828	0.926403309	0.219175038	0.50645092	0.741251729	0.97676908
ZNF513	0.167890373	0.439982862	0.99758757	0.604973821	0.73671449	0.741203579	0.97676908
ACADL	0.264611496	0.515305251	0.559412855	0.476559347	0.907141917	0.741954003	0.976774116
ACY1	0.251960674	0.602981717	0.484399488	0.582693684	0.77248005	0.742798446	0.976774116
ADCY7	0.830814728	0.638987965	0.936300478	0.188742317	0.351663297	0.742044252	0.976774116
AGPAT3	0.349911927	0.708856412	0.565753804	0.378223173	0.620883403	0.741827397	0.976774116
ANKRD61	0.78887234	0.41931925	0.92337383	0.742349218	0.145988235	0.742664354	0.976774116
CA5B	0.891550979	0.810240176	0.505471556	0.161273252	0.561848491	0.742571383	0.976774116
CNOT6	0.720781253	0.749545803	0.376773762	0.638146933	0.254498892	0.74242167	0.976774116
CTSL	0.286893333	0.996374158	0.346060688	0.823583956	0.404350783	0.741766785	0.976774116
EBF2	0.285180778	0.412587702	0.546971182	0.709448988	0.722797664	0.74210018	0.976774116
GSTZ1	0.609624432	0.293708817	0.683327479	0.545243587	0.496396689	0.742738254	0.976774116
LOC101904529	0.446159835	0.552806416	0.80525453	0.307522025	0.538113118	0.741331498	0.976774116
LOC101906364	0.648231381	0.778088549	0.172673227	0.756257754	0.501605903	0.742304945	0.976774116
LOC104972622	0.69448931	0.738570383	0.316797692	0.592972476	0.341568539	0.741591165	0.976774116
LOC104976078	0.527695958	0.202437478	0.559723696	0.764070541	0.723814657	0.742473232	0.976774116
LOC107131341	0.679810054	0.978106227	0.23142364	0.494302335	0.434968469	0.742568609	0.976774116
LOC508455	0.096479018	0.671559001	0.876821429	0.693482646	0.837256766	0.742007394	0.976774116
LOC784521	0.522851484	0.467163289	0.566017436	0.986360228	0.242836093	0.742737387	0.976774116
LOC789384	0.351620231	0.224829699	0.98429186	0.897274253	0.473364705	0.742371786	0.976774116
PPP1R3E	0.420725131	0.268802901	0.571891567	0.555470111	0.920308516	0.742443673	0.976774116
RIC3	0.193989405	0.426188869	0.489350066	0.944293194	0.861618722	0.741621143	0.976774116
SUMO3	0.944136128	0.261612834	0.703811869	0.261494844	0.728728155	0.742803057	0.976774116
TICAM2	0.581442495	0.730827865	0.468068177	0.198545158	0.838185821	0.742653809	0.976774116
TOR4A	0.919949616	0.91941364	0.602386785	0.098281418	0.661483882	0.742786851	0.976774116
TRMT1L	0.372448424	0.543740103	0.304487227	0.965535698	0.554749931	0.742251629	0.976774116
XRCC1	0.804434926	0.1470487	0.866327932	0.963151396	0.333086547	0.741391364	0.976774116

ZNF655	0.421471422	0.346026048	0.69395431	0.832056795	0.392004756	0.742148719	0.976774116
LOC112444778	0.842441997	0.458352187	0.756210502	0.81302658	0.139811995	0.743168136	0.977083128
LOC788467	0.507474326	0.910916043	0.220182481	0.862914872	0.377871642	0.743149396	0.977083128
ZNF132	0.208623898	0.24319637	0.953684904	0.797644754	0.860218828	0.743216665	0.977083128
CHRD	0.808580873	0.184724595	0.696072357	0.414577179	0.770959573	0.743385843	0.977148984
TTC23	0.90640607	0.414828302	0.564016354	0.195501999	0.801276833	0.743333215	0.977148984
TOP3A	0.602673525	0.391723985	0.648261093	0.930302243	0.23370146	0.743624818	0.977384823
ALDH6A1	0.782745717	0.745678288	0.228131853	0.681630683	0.368139735	0.74440463	0.977411466
BTRC	0.904642081	0.915255579	0.112721493	0.875492825	0.408425299	0.744178382	0.977411466
C28H10orf71	0.531574525	0.969075639	0.123964433	0.632152284	0.826576311	0.744149487	0.977411466
DGAT1	0.29400833	0.507152966	0.974371403	0.597289729	0.384018627	0.743908502	0.977411466
DMAP1	0.666457201	0.277671243	0.858674817	0.296989222	0.707399025	0.744240812	0.977411466
ERCC8	0.151712241	0.766548147	0.640188085	0.694301579	0.646662665	0.744478906	0.977411466
HNRNPUL2	0.991049379	0.563579721	0.401652021	0.299831833	0.496171828	0.744186648	0.977411466
HPRT1	0.382107176	0.609109221	0.571391478	0.950074742	0.263503192	0.743736141	0.977411466
LMX1A	0.788776185	0.827629538	0.076078501	0.873528619	0.76998253	0.744357475	0.977411466
LOC101905734	0.315590561	0.967756022	0.976768849	0.386411126	0.289471906	0.744155534	0.977411466
LOC104970103	0.366186953	0.570916993	0.259413296	0.7773168	0.79127863	0.744094698	0.977411466
LOC510362	0.26734134	0.540732616	0.517794568	0.604129043	0.737175938	0.743971231	0.977411466
LRSAM1	0.772978869	0.696019257	0.226307834	0.295966047	0.92753299	0.74446442	0.977411466
PBDC1	0.492675954	0.73543999	0.733116777	0.496699849	0.252924813	0.744168156	0.977411466
KLHL26	0.909558991	0.921218304	0.185428769	0.282982734	0.761472887	0.744774589	0.977601342
LOC101910094	0.994453976	0.557362767	0.602166342	0.113004419	0.887799807	0.744802241	0.977601342
SMG6	0.684405927	0.945123097	0.595753439	0.93277492	0.093114535	0.744722463	0.977601342
LOC530929	0.564553024	0.50821681	0.188474164	0.973026287	0.636616224	0.74486992	0.977611985
GATC	0.640343626	0.414715564	0.780011617	0.352366157	0.459089919	0.74493345	0.977617181
ACSS3	0.534661975	0.579889855	0.420787643	0.378204892	0.680474324	0.745305467	0.977624943
BORCS8	0.243657395	0.622111483	0.971307241	0.972105165	0.235220944	0.745804147	0.977624943
BROX	0.714420739	0.712739094	0.213272134	0.755915364	0.411848254	0.746587983	0.977624943
CASP3	0.328620974	0.638232935	0.502489897	0.579413567	0.551102153	0.745731054	0.977624943
CCDC9	0.289118332	0.936470127	0.895358567	0.194499863	0.715613159	0.746218668	0.977624943
CD38	0.448580162	0.544527024	0.645277471	0.53408776	0.401431245	0.746502881	0.977624943
CFAP70	0.83797249	0.114204693	0.808854516	0.464082709	0.942993799	0.7469547	0.977624943
ERLIN2	0.878540748	0.854430597	0.276671486	0.420449114	0.38670215	0.746359139	0.977624943
FAM104A	0.558489686	0.843202851	0.591471767	0.139238834	0.874076796	0.747083932	0.977624943
GABRB1	0.14251587	0.841180838	0.542891818	0.856495256	0.607680192	0.746945135	0.977624943
GATAD1	0.479018745	0.871725208	0.93139938	0.147270051	0.587752541	0.745797025	0.977624943
GNB4	0.903068888	0.175883459	0.834336312	0.972192104	0.263102804	0.747072573	0.977624943
KCNK6	0.475252206	0.766514623	0.441385134	0.455562904	0.45876649	0.745466017	0.977624943
KLF16	0.735236956	0.564108929	0.099409504	0.90195386	0.906974998	0.746147638	0.977624943

KMT2D	0.718920233	0.26958085	0.545906603	0.680027351	0.467491634	0.745630956	0.977624943
LOC101903438	0.277724358	0.953322084	0.598484462	0.557406458	0.383587783	0.746978134	0.977624943
LOC104970503	0.849461659	0.806438652	0.989534283	0.508585533	0.097685535	0.745866751	0.977624943
LOC107132302	0.806893068	0.995715016	0.56297779	0.514977675	0.145168228	0.746619202	0.977624943
LOC112441607	0.498232919	0.927662938	0.723851135	0.454067289	0.222725173	0.74672981	0.977624943
LOC510536	0.339740508	0.509209064	0.83996568	0.816630079	0.28429673	0.74619222	0.977624943
LOC787550	0.949393418	0.691527132	0.223914863	0.843793738	0.273261899	0.747067945	0.977624943
MPHOSPH6	0.636206756	0.433669944	0.813392329	0.450285528	0.333540632	0.746018042	0.977624943
MTM1	0.933899057	0.271585694	0.263664682	0.754556871	0.665521343	0.745342258	0.977624943
NOD1	0.434412952	0.370749958	0.669418541	0.383383328	0.814898378	0.745900098	0.977624943
PLVAP	0.34595616	0.469175135	0.891848598	0.60171705	0.387457968	0.746261343	0.977624943
PRR5	0.945282526	0.447355089	0.303302247	0.490521101	0.534457679	0.745576408	0.977624943
RN18S1	0.295159603	0.450643702	0.618568409	0.80267478	0.512055277	0.746632922	0.977624943
RPL7A	0.71846286	0.887560343	0.284938165	0.582072182	0.31938957	0.746426291	0.977624943
S100A11	0.846685373	0.365395156	0.79095156	0.161237036	0.855020523	0.746181048	0.977624943
SGMS1	0.947024224	0.527259114	0.192163012	0.474471001	0.742746209	0.746620404	0.977624943
SLC9A9	0.495787142	0.865465114	0.857424532	0.117603396	0.779579369	0.746157498	0.977624943
SMC1A	0.863111207	0.766043401	0.636192457	0.084624469	0.95028751	0.746686366	0.977624943
SMCHD1	0.145720879	0.819359203	0.810655095	0.364597894	0.95044952	0.745112588	0.977624943
UBE3D	0.19681274	0.660156891	0.373881757	0.713272442	0.976612613	0.746751582	0.977624943
WDR60	0.998221854	0.819365161	0.668471044	0.209865524	0.292740695	0.745384406	0.977624943
ZNF280D	0.266252904	0.790666808	0.299841302	0.700504907	0.764789526	0.746632071	0.977624943
DBNDD2	0.205281174	0.785583103	0.69346524	0.702446689	0.431707775	0.747159871	0.977646359
ORC1	0.885524346	0.938066871	0.345658165	0.694293069	0.170186983	0.747237134	0.977669505
CMTM4	0.876477663	0.804045157	0.081793395	0.786280696	0.750959663	0.747827166	0.977672596
CNST	0.62558816	0.474268242	0.126821799	0.997393061	0.907234585	0.747894813	0.977672596
DLL4	0.161997797	0.431573869	0.55096843	0.948846043	0.929932361	0.747573158	0.977672596
GLG1	0.92688951	0.357873532	0.353023519	0.50779109	0.571402962	0.747509472	0.977672596
GREB1	0.722114352	0.387516629	0.298225653	0.747403693	0.545216814	0.74767027	0.977672596
PXDN	0.22230464	0.441681929	0.738322259	0.50570381	0.926701232	0.747488717	0.977672596
RAI1	0.864512105	0.328833566	0.22053429	0.888144104	0.611466494	0.747889284	0.977672596
RITA1	0.577879639	0.142218582	0.721816318	0.828525851	0.692239863	0.747762932	0.977672596
TMEM88B	0.710117802	0.396706892	0.64587799	0.295080017	0.634114698	0.747880246	0.977672596
UBE2Q2	0.905191766	0.491056412	0.23277012	0.959218786	0.342341681	0.747504319	0.977672596
ZNF8	0.810286647	0.983873228	0.210851432	0.543031969	0.372569616	0.747679402	0.977672596
CSF1R	0.32332286	0.3076926	0.987391984	0.701518761	0.494488063	0.748042208	0.977787389
YBEY	0.408274115	0.689790722	0.851408783	0.419640128	0.33880244	0.748124347	0.977816873
E2F4	0.981265424	0.245410701	0.229043694	0.920080869	0.672080396	0.748215856	0.977858598
PLAC9	0.492720432	0.742890635	0.24516906	0.753143189	0.504992667	0.748347195	0.977874498
RPGRIP1	0.134811747	0.475447539	0.953653919	0.688119233	0.811376995	0.748327501	0.977874498

LOC112444871	0.730303938	0.366597405	0.850652435	0.596404932	0.251600333	0.748578939	0.977943717
RABGGTB	0.975109702	0.490047172	0.648596319	0.545928577	0.201955122	0.74856144	0.977943717
TMTC3	0.815591049	0.299043708	0.553829976	0.542175896	0.466381063	0.748479697	0.977943717
RPL23A	0.397077218	0.550153592	0.570632271	0.865399749	0.316969092	0.748686006	0.978005735
BFSP1	0.179081938	0.795819094	0.67606925	0.913821807	0.388911329	0.748950307	0.978117419
FAM129C	0.318338267	0.298626198	0.671005257	0.539381437	0.995083102	0.748919893	0.978117419
ZNF879	0.288485952	0.989028384	0.373427744	0.615013019	0.522416903	0.748895835	0.978117419
LOC104974113	0.525810152	0.762343155	0.426365587	0.321041709	0.624297138	0.749012053	0.97812022
ALG6	0.890802038	0.673218387	0.621388964	0.86624019	0.106524515	0.749724771	0.978200971
ARHGAP1	0.573309273	0.726004721	0.346493571	0.628746715	0.379383154	0.749805097	0.978200971
CD74	0.314709215	0.266756299	0.636378107	0.944260929	0.681266489	0.749622554	0.978200971
CXHXorf58	0.84890362	0.439080666	0.61940224	0.483303191	0.307347224	0.749229223	0.978200971
EXOC7	0.728112568	0.137178992	0.670236444	0.824646931	0.621311083	0.749257704	0.978200971
LOC101902757	0.288950266	0.523447152	0.34088731	0.880366201	0.75675193	0.749526896	0.978200971
LOC101903629	0.79102123	0.759472025	0.269861515	0.784226887	0.270486718	0.749741769	0.978200971
LOC104975054	0.686165921	0.875470705	0.82214747	0.487918176	0.142650662	0.749662074	0.978200971
LOC112443001	0.432838756	0.693782259	0.259458142	0.549773192	0.80299944	0.749778744	0.978200971
MCM9	0.64226307	0.672808666	0.224989857	0.69300433	0.509194456	0.749298474	0.978200971
PLCXD3	0.289029184	0.60694848	0.60533438	0.479761368	0.673773771	0.74940114	0.978200971
STK10	0.971562954	0.767190962	0.713898806	0.093876498	0.68848912	0.749757301	0.978200971
ZBTB33	0.153788912	0.746873904	0.997866813	0.454999232	0.659818452	0.749848773	0.978200971
ALKBH6	0.523404669	0.49726691	0.784726199	0.245763761	0.685993543	0.749977766	0.978235891
LOC100847999	0.949656285	0.96825719	0.231030112	0.792038054	0.20472059	0.750041648	0.978235891
LOC112444888	0.822253832	0.482381024	0.970199569	0.291065921	0.307568125	0.750065704	0.978235891
LOC781692	0.271187064	0.471422222	0.836021017	0.476445274	0.676696793	0.750113976	0.978235891
EIF2B5	0.411454756	0.390802276	0.455250507	0.567162696	0.830509103	0.750232358	0.97824248
LOC100850875	0.380813113	0.99051342	0.329850868	0.585508632	0.473478129	0.750292521	0.97824248
LOC107131531	0.605514776	0.767208606	0.233824436	0.535966425	0.592666151	0.750357464	0.97824248
MYOF	0.489473608	0.285839135	0.469430179	0.828096342	0.634412686	0.75035699	0.97824248
UBR4	0.280158156	0.480663327	0.535252727	0.483786211	0.990390146	0.75052209	0.97837938
ADPRM	0.371516317	0.601148596	0.562893883	0.637900512	0.431185497	0.750751853	0.978445732
GPR156	0.859851889	0.818493962	0.079368714	0.827452226	0.747984651	0.750718154	0.978445732
RAB11FIP5	0.673450774	0.554942252	0.256022663	0.643729004	0.561146274	0.750670171	0.978445732
HOXD8	0.627767621	0.617647476	0.513653922	0.331840914	0.523532791	0.750871156	0.978523509
LOC112447324	0.694019252	0.527548321	0.492668192	0.235947112	0.813691467	0.751032116	0.978655554
ADAM20	0.681708199	0.841851179	0.559642411	0.253762556	0.434012555	0.754939004	0.978838572
APPL2	0.85518993	0.848572836	0.365434386	0.259042991	0.51091092	0.753500468	0.978838572
B3GALNT1	0.278769929	0.961444882	0.428093049	0.914109608	0.33442662	0.753385779	0.978838572
BTBD19	0.164496097	0.631918879	0.968825058	0.844117072	0.414805644	0.754361227	0.978838572
C20H5orf34	0.701459505	0.393204152	0.425906976	0.582196472	0.516691061	0.754753463	0.978838572

C6	0.368275087	0.996889439	0.390544364	0.46945375	0.524818405	0.754692335	0.978838572
CCNI2	0.508178171	0.736820847	0.548002767	0.259295031	0.660281927	0.753672608	0.978838572
CHODL	0.552626043	0.103043451	0.753485985	0.987155588	0.823812463	0.752424311	0.978838572
CNEP1R1	0.860217092	0.180050879	0.700687652	0.521588969	0.615609448	0.75217838	0.978838572
CSRNP2	0.486437928	0.648038804	0.771848757	0.36827356	0.392695735	0.753970556	0.978838572
CST6	0.935785071	0.508525404	0.25119017	0.330412261	0.894016251	0.754608248	0.978838572
DCX	0.688351885	0.329126879	0.475608139	0.931087753	0.352969035	0.755140477	0.978838572
DDIT4	0.762024257	0.674876053	0.092870636	0.746029718	0.985977668	0.75367692	0.978838572
DHRS7B	0.632436389	0.934823407	0.677029046	0.848841275	0.103616291	0.754064138	0.978838572
DIXDC1	0.597201873	0.609732006	0.60844936	0.845619728	0.187837667	0.753993862	0.978838572
EDRF1	0.281096666	0.851904767	0.99361169	0.244463929	0.597287314	0.751626412	0.978838572
ENPP5	0.610852209	0.517238004	0.896861461	0.238973201	0.523068207	0.755186645	0.978838572
FAXC	0.997986714	0.434087536	0.155566748	0.583657787	0.889215865	0.752867028	0.978838572
FGGY	0.470730744	0.9114431	0.154785081	0.999968835	0.531841388	0.754653487	0.978838572
FLT1	0.219152132	0.301968314	0.795286938	0.797554385	0.836810588	0.753644691	0.978838572
FLT3LG	0.290134766	0.235893835	0.706300046	0.829483166	0.881842036	0.754866911	0.978838572
FNDC3B	0.810540851	0.608066671	0.246894203	0.690599064	0.415734854	0.752652829	0.978838572
GCGR	0.371958846	0.615522925	0.293201982	0.777279651	0.677186728	0.754734748	0.978838572
GCNT7	0.71920573	0.711501719	0.214953218	0.581093593	0.553786684	0.755060616	0.978838572
HCST	0.544641671	0.360308915	0.652637836	0.842080725	0.325846849	0.753732411	0.978838572
IGF1R	0.663699063	0.5906378	0.971051599	0.460562423	0.199193356	0.752575895	0.978838572
IREB2	0.93407643	0.29451659	0.343414963	0.962640755	0.383468041	0.75232412	0.978838572
ITGB2	0.484419157	0.456661115	0.287168497	0.775501647	0.715903975	0.754395109	0.978838572
JAK3	0.980208746	0.195799325	0.664902413	0.485666132	0.564251348	0.752830447	0.978838572
LCAT	0.349300647	0.772193288	0.887150701	0.243395159	0.608359615	0.755244049	0.978838572
LOC100297152	0.557362164	0.592037236	0.585293771	0.49627484	0.365887847	0.753353153	0.978838572
LOC101906283	0.505143634	0.256441028	0.919723562	0.647883619	0.451078234	0.752028576	0.978838572
LOC101908075	0.479188288	0.829684408	0.279639396	0.710046763	0.443260406	0.752942969	0.978838572
LOC104968634	0.19628983	0.751211897	0.364089632	0.688879308	0.958891504	0.75540738	0.978838572
LOC104970913	0.707284408	0.56894563	0.302633857	0.979842637	0.296078908	0.754715445	0.978838572
LOC104972045	0.584612818	0.933357674	0.280479445	0.606874225	0.380721431	0.754875174	0.978838572
LOC104974912	0.748279755	0.520791493	0.420665839	0.23622646	0.914986544	0.755249653	0.978838572
LOC112442715	0.256120798	0.661348192	0.919137851	0.235125172	0.968372253	0.755329516	0.978838572
LOC112443417	0.394506143	0.674192258	0.557511648	0.401719437	0.583623865	0.751747814	0.978838572
LOC112446676	0.973872228	0.293992899	0.14984326	0.869122502	0.929866178	0.751250863	0.978838572
LOC112447041	0.930627888	0.605145265	0.521854137	0.399822341	0.29889494	0.75362437	0.978838572
LOC112449053	0.749769205	0.722507408	0.303906957	0.518731351	0.407684718	0.752015372	0.978838572
LOC617698	0.648400975	0.397325619	0.973577137	0.249975109	0.561221197	0.753972273	0.978838572
LOC783255	0.791443647	0.532678049	0.227547787	0.47456583	0.762808883	0.751544905	0.978838572
LOC783680	0.283532669	0.231498473	0.716786828	0.855672588	0.876348672	0.754452774	0.978838572

LSM7	0.499113817	0.581236193	0.198042658	0.669645573	0.907669517	0.752569801	0.978838572
MAFA	0.556961537	0.815138822	0.262263404	0.932940385	0.312952217	0.751738912	0.978838572
MIA	0.814763513	0.255813256	0.705672819	0.959547213	0.249656405	0.754216135	0.978838572
MIS12	0.762631384	0.735555796	0.099324444	0.673569612	0.94137345	0.754708969	0.978838572
MTMR10	0.553785887	0.716284191	0.133038194	0.795333558	0.837877696	0.753862997	0.978838572
NAALAD2	0.217508899	0.898486024	0.789387938	0.388319509	0.591905435	0.755381659	0.978838572
NECTIN1	0.829911755	0.996988581	0.161237339	0.385262355	0.683899572	0.753779559	0.978838572
PCGF3	0.324266323	0.921309483	0.564760795	0.62524639	0.329300247	0.751606732	0.978838572
PHYKPL	0.293689802	0.726908047	0.42931902	0.683705819	0.564485215	0.754937322	0.978838572
PIGM	0.786905753	0.827576221	0.498179375	0.256402696	0.421789209	0.753439583	0.978838572
PPP4R3A	0.526618041	0.581977839	0.955241802	0.147575125	0.806210737	0.752100179	0.978838572
PTBP1	0.994606265	0.987484136	0.491480033	0.090361448	0.809501563	0.754606172	0.978838572
PTPN11	0.737173637	0.61896603	0.094043314	0.917335307	0.893575099	0.753901206	0.978838572
PTRHD1	0.321131329	0.672569783	0.809746769	0.216525467	0.928247704	0.753781886	0.978838572
PYGO2	0.858905265	0.430717047	0.163273029	0.888228564	0.653183592	0.753217952	0.978838572
RETSAT	0.47700008	0.964190932	0.412667704	0.525262686	0.348579859	0.751668588	0.978838572
RHOQ	0.464949045	0.534760467	0.251388082	0.943561966	0.590960144	0.752210855	0.978838572
RIN3	0.714325073	0.846971507	0.266182649	0.238856404	0.914149994	0.753848276	0.978838572
RPS17	0.773729491	0.601307576	0.299074387	0.7651343	0.33123596	0.754374172	0.978838572
SBNO1	0.708488565	0.769069837	0.463220002	0.27262141	0.512464385	0.754361203	0.978838572
SVBP	0.125171486	0.922808895	0.633963585	0.544925663	0.87756106	0.753084086	0.978838572
TCF7L1	0.605883738	0.720803504	0.533960789	0.368496268	0.410891862	0.75460114	0.978838572
TMEM185B	0.626938432	0.448393126	0.295557863	0.78795887	0.540923734	0.755147073	0.978838572
TMTC2	0.214438899	0.405474719	0.463284341	0.979818305	0.892736296	0.754223185	0.978838572
TP53	0.589685488	0.887159486	0.331555825	0.587571906	0.344113665	0.753357856	0.978838572
ZNF710	0.562021322	0.403244062	0.47554848	0.70083024	0.459996221	0.751635397	0.978838572
BCAP29	0.245572607	0.334104069	0.73507247	0.81692888	0.720528985	0.755596193	0.978851368
LOC781439	0.507270973	0.452792022	0.577383083	0.291925075	0.916674819	0.755537817	0.978851368
TRIM8	0.827444678	0.424215169	0.995484128	0.146705188	0.69217035	0.755507544	0.978851368
CENPM	0.935263686	0.142597739	0.663589758	0.658444485	0.609791996	0.7557737	0.978922
ENTPD3	0.711140551	0.479902734	0.712788127	0.459214215	0.318196337	0.755829667	0.978922
TCTN3	0.398459455	0.473292789	0.52559054	0.56369214	0.635820087	0.755727401	0.978922
C22H3orf18	0.868317724	0.082426234	0.862806648	0.677069663	0.850707195	0.755952894	0.979004336
ARL6IP5	0.917040739	0.301703344	0.364639188	0.539828592	0.653344531	0.756020188	0.979014227
AKIRIN2	0.945365931	0.310982484	0.648017177	0.257135703	0.734167623	0.757983821	0.979040806
AMFR	0.769950848	0.357087511	0.432540666	0.500762756	0.602105237	0.757430616	0.979040806
ANKRD13A	0.75933827	0.848711099	0.414681485	0.745248256	0.18004897	0.757444288	0.979040806
C10H14orf119	0.25586242	0.991776708	0.583032212	0.39438904	0.614678005	0.757479763	0.979040806
CCDC82	0.709215523	0.778898686	0.413672393	0.230858407	0.679648458	0.757420798	0.979040806
FAM192A	0.902178789	0.539967276	0.520199571	0.215234773	0.659612361	0.758047629	0.979040806

FAM57A	0.651838961	0.173303136	0.461673358	0.910729818	0.754459236	0.757319955	0.979040806
FBXL8	0.995111233	0.426745807	0.422602901	0.443026467	0.447846631	0.756147901	0.979040806
GBF1	0.331986172	0.50616802	0.884646807	0.280091693	0.861257083	0.757451136	0.979040806
GINM1	0.658831438	0.402598629	0.515129544	0.281932634	0.926312973	0.756542539	0.979040806
H3F3B	0.454215145	0.141193806	0.836996342	0.898643771	0.746510794	0.758213479	0.979040806
IMMT	0.84847215	0.552394663	0.609505435	0.300827475	0.417616192	0.757595411	0.979040806
LOC112442312	0.716554204	0.766554676	0.475330208	0.456388071	0.301440406	0.757748965	0.979040806
LOC112444215	0.287822504	0.677327503	0.54662274	0.976961026	0.344934021	0.75770722	0.979040806
LOC112445190	0.563545205	0.700648114	0.405973456	0.543006724	0.41053868	0.756804248	0.979040806
LOC112446036	0.422652327	0.495063285	0.804426617	0.309009368	0.691300159	0.757936875	0.979040806
LOC112449563	0.84033992	0.844641587	0.08471981	0.718230504	0.832284669	0.757886016	0.979040806
LOC515547	0.945045407	0.955931791	0.390275385	0.457622509	0.221756338	0.757035911	0.979040806
LOC618409	0.832102771	0.492978939	0.693632366	0.168307154	0.748866334	0.757461234	0.979040806
LOC783185	0.707413158	0.747483442	0.47950743	0.379127578	0.373962563	0.757900945	0.979040806
NUDT15	0.724808987	0.334399438	0.333780663	0.977200099	0.455154891	0.758073669	0.979040806
OCEL1	0.541285144	0.861192215	0.50594229	0.541099247	0.279618192	0.756543967	0.979040806
PIK3CB	0.928372557	0.372892294	0.455571852	0.301564512	0.756323112	0.758013429	0.979040806
PQLC3	0.271920098	0.55774418	0.811025232	0.545669154	0.534612744	0.75756163	0.979040806
PRKRIP1	0.394455268	0.746649358	0.523793318	0.405884471	0.568853565	0.756209182	0.979040806
PSKH1	0.59722444	0.794242102	0.328445319	0.327721309	0.699749298	0.756768368	0.979040806
PTK2B	0.332645718	0.353286094	0.613608078	0.66355344	0.752511291	0.758198295	0.979040806
RPL39	0.833215199	0.753402584	0.794815082	0.876701496	0.081719866	0.75686513	0.979040806
SERAC1	0.743797558	0.822419397	0.091737116	0.886395407	0.721224388	0.757524339	0.979040806
SRR	0.618925262	0.662499969	0.80107563	0.291040723	0.374572981	0.757185238	0.979040806
SYNGR2	0.976665394	0.076294783	0.6528418	0.922436296	0.79491302	0.756474271	0.979040806
TMEM201	0.478421213	0.479836526	0.285423236	0.819525757	0.669592726	0.757935893	0.979040806
TSEN15	0.951372344	0.126640548	0.513481967	0.914074382	0.633491254	0.757262443	0.979040806
UBXN8	0.844515532	0.08034577	0.604528574	0.893891503	0.981947671	0.758186557	0.979040806
USP9X	0.440349655	0.301281254	0.58270503	0.775220418	0.595778398	0.756653374	0.979040806
VPS13A	0.702664639	0.303387678	0.781171888	0.241973755	0.892562074	0.757991977	0.979040806
ZDHHC19	0.30677336	0.882372758	0.967829796	0.209527591	0.656139966	0.758248043	0.979040806
FEM1C	0.522533094	0.402727767	0.546160432	0.715809141	0.43860273	0.758588628	0.979403507
DHFR	0.854393365	0.602709311	0.785688931	0.41461512	0.215227172	0.758691679	0.979459498
HNRNPM	0.96751385	0.732472104	0.218198307	0.42837053	0.545339334	0.758787538	0.979503113
SDF2	0.913344045	0.484887517	0.853623903	0.294611847	0.324436594	0.758844835	0.979503113
PRMT9	0.57208864	0.398285525	0.745228167	0.287942383	0.739877871	0.759051628	0.979692982
LOC531152	0.775153357	0.608519052	0.316703539	0.359622362	0.674486307	0.759355713	0.979768873
LOC531679	0.548121285	0.985008508	0.128320311	0.986468365	0.530420172	0.759431818	0.979768873
POLR3E	0.412259445	0.655978168	0.310271523	0.773456951	0.558041589	0.759258862	0.979768873
TTC27	0.577690084	0.850459155	0.37271385	0.346605353	0.570671011	0.75927586	0.979768873

VPS33A	0.883759244	0.3112465	0.24977902	0.604858474	0.872478033	0.759468639	0.979768873
WDR33	0.484939083	0.929207241	0.440756838	0.381088728	0.478589535	0.759294036	0.979768873
TWSG1	0.172730693	0.927833578	0.815408982	0.559130712	0.496476088	0.759563015	0.979813602
HCFC1	0.567238607	0.237949045	0.693797969	0.455877635	0.850799064	0.759786717	0.979871942
PLCB2	0.685304198	0.383514634	0.941204046	0.238655292	0.614839666	0.759670514	0.979871942
SEPT11	0.74239084	0.998721275	0.349753211	0.242022213	0.578716362	0.759787366	0.979871942
EIF3L	0.726694408	0.748488716	0.639024452	0.764648044	0.136845578	0.760034584	0.980000471
H3F3C	0.574936096	0.528395205	0.896213613	0.164145308	0.813960835	0.760066175	0.980000471
HOXC8	0.392148362	0.547103543	0.295350068	0.655151009	0.876003229	0.760016582	0.980000471
CD46	0.373099042	0.268399931	0.425490816	0.922765413	0.929907783	0.760994104	0.980017144
CHCHD8	0.902807812	0.469805223	0.726766007	0.824599807	0.143831729	0.760984757	0.980017144
COMT	0.70494553	0.30684523	0.61549929	0.285787861	0.957357653	0.76031804	0.980017144
GIGYF2	0.933453241	0.743183152	0.403622834	0.139227335	0.936150668	0.760660432	0.980017144
GOLGA5	0.891784108	0.933295317	0.224291626	0.487607242	0.399826394	0.760155193	0.980017144
HES1	0.358848787	0.701561706	0.319405029	0.70847069	0.641212668	0.760832299	0.980017144
JKAMP	0.507355994	0.382483193	0.481632604	0.42610651	0.915567644	0.760498587	0.980017144
LDLR	0.941873438	0.341580206	0.615453147	0.459053088	0.40131901	0.76057567	0.980017144
LNX1	0.307497273	0.9141454	0.336612146	0.416380114	0.928213498	0.761034579	0.980017144
LOC100848886	0.84998123	0.902063925	0.430612213	0.114505742	0.965206106	0.760637682	0.980017144
LOC104973746	0.32098722	0.825707395	0.643980378	0.340025918	0.62762376	0.760308557	0.980017144
LOC782966	0.956857146	0.10818887	0.469696786	0.955265757	0.786529007	0.76085044	0.980017144
PES1	0.229692544	0.937354058	0.668128479	0.796979356	0.317687206	0.760291472	0.980017144
SUMO2	0.673589531	0.570027606	0.576991207	0.731146066	0.225759654	0.761028988	0.980017144
ZNF462	0.69838231	0.535447303	0.427872084	0.267309708	0.854918849	0.761009082	0.980017144
ZNF614	0.25236217	0.500142235	0.656125867	0.495010642	0.890680682	0.760747531	0.980017144
LGR6	0.314121209	0.768963943	0.373811113	0.888850217	0.456277211	0.761282002	0.980190721
UBE2E3	0.595910177	0.283626796	0.298146221	0.844465021	0.860577542	0.761288826	0.980190721
CEP57L1	0.403762245	0.634517541	0.475824254	0.922665494	0.32583765	0.761428524	0.980270958
SNRPA1	0.655734255	0.323809732	0.403918092	0.912088144	0.468612348	0.761470609	0.980270958
CAMSAP1	0.128166233	0.814618673	0.557384672	0.770584623	0.818044101	0.761604407	0.980366298
DUS3L	0.542821725	0.197634955	0.856298146	0.666279277	0.599707609	0.761714387	0.980430965
COPB2	0.783432467	0.165076921	0.716488975	0.822635064	0.481751342	0.761792408	0.980454491
EID3	0.525237486	0.636538657	0.654426545	0.288965287	0.581621462	0.762044359	0.980505214
EXOC3	0.18964748	0.534910533	0.806882663	0.778300209	0.576771478	0.761902428	0.980505214
FAM117B	0.440988594	0.440721313	0.691943743	0.405265804	0.674821436	0.762071034	0.980505214
LOC112447460	0.297520749	0.426152481	0.98303278	0.448012439	0.659307587	0.76225468	0.980505214
LOC784451	0.838798659	0.843936491	0.084043351	0.822693382	0.752481765	0.762328736	0.980505214
MIER3	0.373296468	0.364216488	0.485649464	0.596379469	0.935208168	0.76231301	0.980505214
SMCR8	0.841800426	0.426508632	0.604800448	0.170841978	0.993020879	0.76236954	0.980505214
SNX31	0.491653153	0.771357478	0.450719627	0.233435108	0.922998432	0.762321723	0.980505214

YPEL2	0.342162259	0.639232504	0.670966565	0.394990186	0.634949078	0.762207915	0.980505214
FGD4	0.485810768	0.796989078	0.174959868	0.703039572	0.774080215	0.762504543	0.980525158
LOC513580	0.917876017	0.589635719	0.570194095	0.654002003	0.182607367	0.76244755	0.980525158
GLMN	0.9428301	0.099641821	0.887385939	0.8754719	0.505465806	0.76262958	0.980609107
LOC104969024	0.942162029	0.380167228	0.270334881	0.572878761	0.665632809	0.762788257	0.980659466
PPP1R21	0.799179431	0.773612176	0.126612261	0.702631206	0.671276005	0.762776558	0.980659466
GALC	0.602903326	0.984069427	0.532349154	0.286177141	0.408665129	0.762860714	0.980675793
PSEN1	0.978461041	0.654871254	0.067228168	0.924921538	0.927596394	0.762962307	0.98072957
CYB5D2	0.5337285	0.723098704	0.791638541	0.184491113	0.657469253	0.763457605	0.980886919
IFT52	0.741318745	0.862914632	0.734939036	0.160943529	0.489125739	0.76321541	0.980886919
KHDRBS1	0.403734446	0.485822977	0.671907715	0.593048935	0.473370071	0.763155292	0.980886919
LHFPL3	0.976018611	0.400287932	0.754997992	0.351739172	0.356924476	0.763322459	0.980886919
PLPP2	0.746960588	0.533282166	0.817851893	0.330533873	0.344238123	0.763503108	0.980886919
TBC1D24	0.820943562	0.319372966	0.405792781	0.764476839	0.455583712	0.763437686	0.980886919
TSPOAP1	0.256916888	0.748900648	0.406001367	0.845461137	0.561237157	0.763494754	0.980886919
ATP8B2	0.648747183	0.297778547	0.65352382	0.596080838	0.493185408	0.763730011	0.981058817
CDKN2D	0.827917171	0.558018677	0.887486032	0.373722961	0.244172245	0.765194528	0.981058817
CSNK1A1	0.159998527	0.459076728	0.992752971	0.551478662	0.932919111	0.765684678	0.981058817
DDX50	0.776001219	0.569047242	0.473421488	0.28088254	0.635722398	0.764779292	0.981058817
DERA	0.756268358	0.307903168	0.85132678	0.998537528	0.189618896	0.765775499	0.981058817
EEF1B2	0.663119743	0.437046233	0.288411535	0.890524712	0.503949554	0.765663707	0.981058817
EEF2K	0.802560934	0.409669209	0.504669873	0.407289622	0.551534263	0.764504502	0.981058817
EFHD2	0.60060962	0.325675845	0.70212155	0.289718641	0.933218271	0.76381555	0.981058817
ELMO1	0.604277076	0.12928924	0.967766864	0.797314539	0.621395695	0.765413699	0.981058817
ELMOD3	0.195552116	0.549035518	0.710189831	0.976267293	0.502498774	0.765151824	0.981058817
FAM167B	0.656602353	0.905770581	0.094037933	0.694229684	0.958411468	0.764204796	0.981058817
FBXL12	0.429139548	0.349404263	0.656169449	0.905949739	0.419658931	0.765152728	0.981058817
FRMD6	0.562727944	0.310232893	0.768979655	0.370525392	0.748806152	0.764376365	0.981058817
HELQ	0.306102152	0.715246523	0.346297534	0.63718705	0.770598794	0.764283497	0.981058817
HIP1	0.322021339	0.606524703	0.962262078	0.912432525	0.21880802	0.765716182	0.981058817
IL15RA	0.775426431	0.300316629	0.681134511	0.668675529	0.350491778	0.764023508	0.981058817
KCNA2	0.702284323	0.817809643	0.49695542	0.158387574	0.82473475	0.764556199	0.981058817
KCTD18	0.16635812	0.727696852	0.914397187	0.473417909	0.712903869	0.764927489	0.981058817
LOC101902918	0.76824028	0.985534128	0.818917679	0.789605237	0.07655248	0.765501987	0.981058817
LOC104969425	0.301961691	0.307907924	0.767950934	0.773269663	0.679649607	0.765729444	0.981058817
LOC112443244	0.574652408	0.811985577	0.197472671	0.48735103	0.830608227	0.764631822	0.981058817
LOC614424	0.665951165	0.291305468	0.297628433	0.789126473	0.820709796	0.765093462	0.981058817
LSM6	0.971983932	0.101512326	0.946903949	0.545916874	0.735508804	0.765677581	0.981058817
MAN1A2	0.469327853	0.75406415	0.718178591	0.148610963	0.984296837	0.764043569	0.981058817
METTL26	0.288403914	0.405683385	0.749637896	0.45380781	0.939093446	0.76501773	0.981058817

NECAB2	0.820828201	0.315505702	0.785478082	0.195026825	0.938000552	0.764209863	0.981058817
PCSK5	0.544879932	0.802137654	0.837340742	0.827084383	0.123457273	0.764974947	0.981058817
PPP1R1B	0.918514373	0.744852797	0.272363117	0.652301391	0.306084673	0.764169482	0.981058817
REXO4	0.345667752	0.463734126	0.466624281	0.852674879	0.585921443	0.764974811	0.981058817
SDCCAG8	0.900413765	0.229916025	0.758448219	0.672326673	0.354902215	0.765438452	0.981058817
SEMA3A	0.421857497	0.832327016	0.15133719	0.838323236	0.839156162	0.765034067	0.981058817
SHISA5	0.391109304	0.72815454	0.932379612	0.528134454	0.265562201	0.764349849	0.981058817
ST7	0.813244201	0.200274729	0.614402319	0.611969029	0.612450253	0.765636625	0.981058817
TRAK2	0.995727508	0.582643974	0.208498995	0.480772082	0.645443457	0.765780845	0.981058817
UBXN10	0.919142027	0.767827384	0.110744103	0.570323037	0.839521538	0.765226474	0.981058817
ZNF703	0.461841697	0.510140951	0.777401957	0.702633377	0.291680215	0.76578901	0.981058817
KDSR	0.489483506	0.400428388	0.59915221	0.413920866	0.772497238	0.765851565	0.981062371
ANKRD35	0.723308144	0.989369184	0.297405386	0.259036027	0.681358064	0.765915017	0.981067074
CEND1	0.661081719	0.232164471	0.362258168	0.90043034	0.750658148	0.765996642	0.981070536
LOC112449300	0.682336027	0.466850208	0.806147279	0.25576295	0.572359938	0.766053355	0.981070536
MST1	0.189664148	0.711049713	0.62694964	0.510848503	0.870966569	0.766184729	0.981070536
PLEKHM1	0.335088499	0.919015454	0.203860096	0.844263904	0.709896498	0.766216626	0.981070536
SURF2	0.618982029	0.406313032	0.264325123	0.859243538	0.658364256	0.766121325	0.981070536
ADAMTSL3	0.520034238	0.206760478	0.910181061	0.67587007	0.576768767	0.768721725	0.981075944
ANKRD54	0.191816456	0.350712366	0.899789852	0.679977761	0.914761068	0.76633888	0.981075944
ARHGAP32	0.61025825	0.938466229	0.541456038	0.224896615	0.542709071	0.767284664	0.981075944
BARD1	0.441113436	0.663951105	0.848116064	0.303047638	0.50411321	0.767758231	0.981075944
BRAF	0.386263315	0.787755103	0.99994092	0.233115102	0.53112595	0.766437699	0.981075944
CACNB4	0.791054048	0.88032973	0.134918572	0.570918545	0.707084229	0.767669771	0.981075944
CCDC149	0.717458803	0.739790257	0.619616686	0.393807577	0.29481619	0.768876619	0.981075944
CDK5RAP1	0.355431423	0.954262728	0.820393031	0.228426579	0.596081246	0.767473402	0.981075944
DCAF16	0.205991115	0.307361305	0.680826121	0.889585077	0.991713093	0.768144662	0.981075944
EBNA1BP2	0.655867452	0.630282704	0.452077371	0.612643046	0.331277914	0.767667962	0.981075944
EIF2B4	0.635135944	0.376897452	0.458791485	0.658899925	0.527736077	0.768908858	0.981075944
EVA1C	0.460135007	0.492867864	0.571138559	0.329214069	0.887451513	0.767257443	0.981075944
EVI5	0.97338118	0.571374155	0.248270377	0.460507255	0.600058347	0.768749297	0.981075944
FAR2	0.505320228	0.58571853	0.515253716	0.91854381	0.269618171	0.766900779	0.981075944
FOCAD	0.986278304	0.428862038	0.951691198	0.684925696	0.136776869	0.766626618	0.981075944
GAREM2	0.319801824	0.191168651	0.920132012	0.694106886	0.967210665	0.76688799	0.981075944
GRID2IP	0.637487767	0.902982383	0.100714116	0.740694647	0.883908319	0.767802959	0.981075944
HEXA	0.492738063	0.299958257	0.811758626	0.852165412	0.373131636	0.76872073	0.981075944
IFT140	0.677918533	0.236054489	0.362424868	0.874588824	0.748900631	0.767948067	0.981075944
KCNJ16	0.646768839	0.907072331	0.767736643	0.195825244	0.431858759	0.768439024	0.981075944
KLHDC8B	0.556671197	0.757260982	0.315090909	0.689262553	0.41588381	0.768365353	0.981075944
KLHDC9	0.642240789	0.883108836	0.764327766	0.172358234	0.509465738	0.768324455	0.981075944

LHFPL6	0.462907221	0.854297239	0.43271784	0.263279022	0.83869299	0.766984782	0.981075944
LOC100335642	0.960388517	0.790698524	0.152808435	0.468670021	0.69266009	0.766427505	0.981075944
LOC101907132	0.688761779	0.355322657	0.192598999	0.945142677	0.852453481	0.767897257	0.981075944
LOC101910045	0.534511125	0.185303766	0.73320054	0.706560009	0.742142765	0.768391766	0.981075944
LOC104969353	0.724784223	0.26483006	0.95369931	0.330410606	0.624786822	0.767004106	0.981075944
LOC104970852	0.655374307	0.97167797	0.133781707	0.495364271	0.902013841	0.768327628	0.981075944
LOC112441644	0.803277174	0.32007372	0.268573685	0.669267796	0.816217921	0.766674938	0.981075944
LOC112445985	0.704182149	0.225152671	0.999967308	0.265311055	0.903037868	0.767936953	0.981075944
LOC112447328	0.934578291	0.61761472	0.601314522	0.308427941	0.352939328	0.766968819	0.981075944
LOC614376	0.901685052	0.341351951	0.493016125	0.498323129	0.501579669	0.767670099	0.981075944
LOC614402	0.248857491	0.562399225	0.664181369	0.616276161	0.664723665	0.768391077	0.981075944
LOC790312	0.773429503	0.53010971	0.305864184	0.702355918	0.428404276	0.766733906	0.981075944
MTHFD1	0.41131555	0.430849429	0.962614479	0.549788468	0.406789129	0.768732471	0.981075944
NEDD1	0.063327373	0.77896672	0.863591973	0.919865097	0.974548749	0.768911023	0.981075944
NEURL1B	0.703151593	0.399539971	0.285557727	0.716516879	0.66315089	0.768575783	0.981075944
RASGEF1A	0.726286376	0.666195306	0.19606914	0.692313776	0.580593851	0.768639201	0.981075944
RBM42	0.637046126	0.181539655	0.856666277	0.444355016	0.860313631	0.767407942	0.981075944
S100A9	0.411295142	0.763351252	0.699146454	0.556799255	0.309865178	0.767398193	0.981075944
SLC35F2	0.45289895	0.438056811	0.603910157	0.484148284	0.651446192	0.766998977	0.981075944
TADA2B	0.729821958	0.32043572	0.185510022	0.999163638	0.877787399	0.768246272	0.981075944
TOX	0.431407653	0.410910112	0.499487194	0.838524774	0.507880375	0.766612881	0.981075944
XRCC6	0.90436525	0.34620045	0.206508481	0.64894124	0.906586465	0.768193253	0.981075944
ZMYND12	0.245909211	0.807378786	0.567628989	0.500362678	0.676697595	0.76876506	0.981075944
CAMK2D	0.795713588	0.884822357	0.909535465	0.468160864	0.127513391	0.769092883	0.981110977
CRTAP	0.703401106	0.437183095	0.395385418	0.436834496	0.72201658	0.769662179	0.981110977
EHBP1	0.875961919	0.886418426	0.200567532	0.337356131	0.730587616	0.769826261	0.981110977
FIZ1	0.597702041	0.264343233	0.894467653	0.92706556	0.293619541	0.770228606	0.981110977
GRASP	0.551511825	0.820557913	0.392083041	0.817610088	0.264774749	0.769959112	0.981110977
INCA1	0.633236287	0.808162194	0.445050336	0.819063979	0.20552807	0.769625261	0.981110977
KANK4	0.899770498	0.878720922	0.611246551	0.130431678	0.610120614	0.770180764	0.981110977
LOC100139115	0.615810764	0.715560769	0.271677865	0.342715744	0.936501453	0.770011365	0.981110977
LOC101904579	0.790249431	0.352036614	0.861704747	0.214993528	0.744122057	0.769674213	0.981110977
LOC107131296	0.240370201	0.533412883	0.347350929	0.876681459	0.983157274	0.769839516	0.981110977
LOC107131974	0.772609956	0.184968816	0.490961307	0.680449456	0.801064739	0.769170395	0.981110977
LOC511409	0.6562946	0.672172965	0.487565172	0.53839508	0.331505647	0.76985081	0.981110977
MAEA	0.533827118	0.890245236	0.77981674	0.113711247	0.907425985	0.769148707	0.981110977
MARCH1	0.596659911	0.666498451	0.506056797	0.560005609	0.341059674	0.770076845	0.981110977
NAT8L	0.81619562	0.257038842	0.411175272	0.567017257	0.783359027	0.769506542	0.981110977
OSGEP	0.366835612	0.796431371	0.513399618	0.539344128	0.474732564	0.769928099	0.981110977
PTER	0.96072301	0.141346417	0.983944748	0.289738227	0.992614597	0.770031993	0.981110977

RTF2	0.906071273	0.203691586	0.736787204	0.294398474	0.961081697	0.770253722	0.981110977
SHBG	0.902345264	0.880404159	0.171192267	0.337575434	0.837776197	0.770197356	0.981110977
SLC35B2	0.163820155	0.722439341	0.369589764	0.893503221	0.98024243	0.769482047	0.981110977
SLC37A1	0.743065553	0.673168955	0.170958931	0.795811159	0.562026645	0.769187199	0.981110977
TKFC	0.29296972	0.534769725	0.794365156	0.342836925	0.900336124	0.76997453	0.981110977
POLM	0.344634143	0.566146236	0.598523383	0.440491064	0.748202269	0.77031641	0.981114677
EDEM3	0.355407896	0.574580761	0.907319533	0.338561024	0.614771257	0.770675841	0.981269277
ELK1	0.228478837	0.745738722	0.989229452	0.546664359	0.418618213	0.770708944	0.981269277
KCNIP2	0.666042567	0.308751595	0.890955329	0.717250743	0.293588751	0.770754216	0.981269277
LOC616400	0.825103988	0.746364594	0.161927233	0.601987104	0.64221555	0.770617117	0.981269277
NUP107	0.482437321	0.230869152	0.896662496	0.407052513	0.949344214	0.770809233	0.981269277
PDGFD	0.61628854	0.824764668	0.237183979	0.367895812	0.869183692	0.77061225	0.981269277
TMED9	0.908438611	0.166966575	0.299074072	0.928010521	0.916994166	0.770856347	0.981269277
CHEK1	0.643149625	0.437358639	0.566117285	0.87995223	0.275598193	0.770926244	0.981282136
ARRDC1	0.339010243	0.519448142	0.522648304	0.456440554	0.920772613	0.771220957	0.981465746
CDNF	0.286934617	0.504425472	0.872971103	0.584266306	0.525803393	0.77184894	0.981465746
CPSF6	0.992474282	0.718654234	0.771903132	0.159752226	0.443591624	0.772768811	0.981465746
CSNK1D	0.937593759	0.471137801	0.861593192	0.113086479	0.905632	0.772600968	0.981465746
EIF2AK4	0.934719294	0.608617379	0.232891244	0.899432811	0.32704025	0.77256834	0.981465746
FRMD5	0.238544974	0.81450746	0.469935347	0.565062238	0.752814102	0.771962386	0.981465746
GBP4	0.169339904	0.879382251	0.611566735	0.441693316	0.970102396	0.772804848	0.981465746
HNRNPUL1	0.551592536	0.663356871	0.386069329	0.882188099	0.31194872	0.77212327	0.981465746
ITGAM	0.504592235	0.550584301	0.377360408	0.598683104	0.620820608	0.772541476	0.981465746
IZUMO1	0.749903729	0.774941823	0.354170412	0.410409187	0.461011775	0.772430422	0.981465746
LGALS12	0.82747061	0.179051417	0.626807664	0.918094726	0.456481797	0.772330748	0.981465746
LOC101907943	0.956639575	0.638510003	0.767906898	0.103043454	0.803967173	0.772043638	0.981465746
LOC104973382	0.285832796	0.681164331	0.516642665	0.9910765	0.390966542	0.772590039	0.981465746
LOC104973485	0.937412961	0.283885337	0.728471464	0.291241392	0.68481656	0.771143146	0.981465746
LOC112442365	0.599121613	0.54581316	0.341706717	0.407519585	0.851996337	0.771759588	0.981465746
LOC112446034	0.835058964	0.754936407	0.471960297	0.190038622	0.684932146	0.771437368	0.981465746
LOC516742	0.339445545	0.769550049	0.889859492	0.413756387	0.40573651	0.772804002	0.981465746
MAPK13	0.85080223	0.196418066	0.352490399	0.970854077	0.678156953	0.771694352	0.981465746
MED13L	0.995549925	0.175121877	0.519860231	0.506697363	0.844600074	0.771714389	0.981465746
R3HDM1	0.878468864	0.71427248	0.22945449	0.822836356	0.328970878	0.772572123	0.981465746
SETD9	0.391314406	0.475445735	0.481738105	0.500549594	0.868630236	0.772556401	0.981465746
SMARCD2	0.335499857	0.666015651	0.515595779	0.377702198	0.889664549	0.771370746	0.981465746
SVIP	0.190217954	0.788468959	0.489382917	0.57939384	0.913373039	0.771970473	0.981465746
TCF7L2	0.905712016	0.528064064	0.811476643	0.260985305	0.381979412	0.771265637	0.981465746
THNSL1	0.231998358	0.68533432	0.907850275	0.815742724	0.329966651	0.772019386	0.981465746
TMEM86B	0.392773258	0.571836946	0.623454533	0.630464348	0.439626745	0.771827985	0.981465746

TNK2	0.197358802	0.878874684	0.579037339	0.613764205	0.630075124	0.771960907	0.981465746
UBOX5	0.597537818	0.949447265	0.367349601	0.253387482	0.738828122	0.772773819	0.981465746
ZC3H12C	0.286187999	0.739788035	0.990315091	0.998006374	0.186236415	0.772560624	0.981465746
NELFA	0.906184171	0.274608192	0.63083782	0.437392634	0.568674613	0.772914132	0.98148782
NTN3	0.339112464	0.769172779	0.307589022	0.97376999	0.499867604	0.772941843	0.98148782
TMEM175	0.665645631	0.810708631	0.300369072	0.269461029	0.894744146	0.773069473	0.981573936
CAPN1	0.516353917	0.51182138	0.546047395	0.437688129	0.620425164	0.773563331	0.981577457
CLPTM1L	0.713078391	0.403970289	0.644301317	0.36285078	0.581648685	0.773486372	0.981577457
GSTK1	0.84062291	0.524436868	0.234046441	0.380227629	0.998270959	0.773454926	0.981577457
HIC2	0.793057688	0.525151862	0.655561356	0.703005424	0.204017736	0.773430515	0.981577457
IK	0.806517048	0.297495044	0.50593692	0.339300037	0.9521107	0.773691912	0.981577457
LIN7B	0.366977283	0.380636639	0.447478734	0.65828541	0.952405264	0.773566551	0.981577457
LOC112442866	0.127000453	0.610052158	0.938531684	0.866169771	0.622111448	0.773539311	0.981577457
NCOA1	0.621386189	0.951567608	0.718570321	0.139960971	0.659106328	0.773597503	0.981577457
PREX2	0.480889173	0.830640132	0.91355271	0.195496978	0.54973289	0.773701133	0.981577457
SLC12A7	0.241870196	0.657009288	0.969961527	0.513172548	0.494430761	0.77320176	0.981577457
SMPD2	0.970840149	0.56740732	0.171316479	0.99470308	0.417847875	0.77373018	0.981577457
PLPP1	0.859982202	0.46528419	0.271905291	0.529956064	0.680509506	0.773790616	0.981578249
LOC101906469	0.65027811	0.393406793	0.210997385	0.73937465	0.983721508	0.773896276	0.981585711
PPP1R42	0.906828771	0.214823567	0.384741356	0.820717999	0.638524038	0.773975937	0.981585711
PYGO1	0.939655985	0.675440983	0.218316191	0.328178515	0.863733455	0.773969661	0.981585711
ALDH1L2	0.588168783	0.726605126	0.133296692	0.737928059	0.936708234	0.774427188	0.981775007
APP	0.983927141	0.379264923	0.5576784	0.463970895	0.407827409	0.774434808	0.981775007
ARHGEF4	0.377800397	0.979627314	0.148028285	0.817504185	0.880756836	0.774747962	0.981775007
CTDSP2	0.521338766	0.32387523	0.905556924	0.404378046	0.637187909	0.774522478	0.981775007
DCAF12	0.906150431	0.562641176	0.288684561	0.527784976	0.508248963	0.774902911	0.981775007
IL21R	0.148442601	0.45705202	0.964984233	0.809686527	0.742854664	0.774438357	0.981775007
KCNT2	0.988832592	0.387503432	0.325853558	0.409427271	0.771890616	0.774805546	0.981775007
LOC112443215	0.871827662	0.707449721	0.739157104	0.263961823	0.327637116	0.774658333	0.981775007
LOC512617	0.356074533	0.824332442	0.503061332	0.366863631	0.728323931	0.774780812	0.981775007
LOC789157	0.601118304	0.479514478	0.2006564	0.831695688	0.820490848	0.774846698	0.981775007
MAPK8IP3	0.944286416	0.876668746	0.188967334	0.681691423	0.369793592	0.77468976	0.981775007
TCEA3	0.640801169	0.598031164	0.41447212	0.358499695	0.692614467	0.774710252	0.981775007
ZNF197	0.516317486	0.487437716	0.679208191	0.319737603	0.719770432	0.774256969	0.981775007
LOC112446360	0.773789457	0.914574656	0.133062354	0.686425417	0.611274651	0.775042594	0.981876178
CASTOR1	0.261839138	0.876240692	0.586357445	0.639911045	0.459349595	0.775189867	0.981986948
ATMIN	0.470518434	0.298604463	0.551101672	0.717209683	0.713846514	0.775632721	0.982018412
BCL2L10	0.372226684	0.553564187	0.339117655	0.71031629	0.797625783	0.775393707	0.982018412
C25H7orf43	0.831941691	0.305043662	0.195523193	0.877015003	0.910843655	0.775611409	0.982018412
ENOPH1	0.587845514	0.210710212	0.758484646	0.990932419	0.42545184	0.775481748	0.982018412

GSTT4	0.390558805	0.460006785	0.39456066	0.802641504	0.6967043	0.77562401	0.982018412
RAP1GDS1	0.683004677	0.608410428	0.877625127	0.441061782	0.246007557	0.775311267	0.982018412
ZNF93	0.968382336	0.419907899	0.158277138	0.7398456	0.832525499	0.775633579	0.982018412
KCNC2	0.37896752	0.323515184	0.533011904	0.649187545	0.934905985	0.77572191	0.982032821
PTPN14	0.618195814	0.378097105	0.718690215	0.361677053	0.653172747	0.775824479	0.982032821
ZFAND1	0.71086798	0.547980373	0.122709963	0.986454871	0.841499015	0.775801554	0.982032821
MAML1	0.683145617	0.165704024	0.825040387	0.4395406	0.967829307	0.77603049	0.982035793
MEPCE	0.752067215	0.694965183	0.267748718	0.696730079	0.407435166	0.776010211	0.982035793
TMEM187	0.532123154	0.440287443	0.755186037	0.874740875	0.256661497	0.775998812	0.982035793
TRIP6	0.221018191	0.915011847	0.461288041	0.902900696	0.471779323	0.776066187	0.982035793
LOC104972584	0.688113187	0.270582794	0.975350086	0.234652347	0.933990516	0.776347608	0.982201585
OSTF1	0.622163252	0.297540829	0.239123125	0.987469222	0.910278475	0.776299095	0.982201585
TCF7	0.839845133	0.532199518	0.343047017	0.764238186	0.339705482	0.776376757	0.982201585
PDCD6IP	0.781756227	0.465093331	0.382636002	0.638304789	0.448431876	0.776444089	0.982211049
ADCY8	0.194307941	0.867188111	0.66642246	0.948928944	0.374831297	0.776980531	0.982254298
ANGPTL6	0.807058521	0.317065304	0.953018154	0.794134497	0.205767344	0.776569277	0.982254298
ARL3	0.768477463	0.152121329	0.963604378	0.628567093	0.563631733	0.776833822	0.982254298
CIPC	0.451612356	0.538645368	0.795868593	0.487485524	0.423365523	0.777048239	0.982254298
FAM212B	0.769248195	0.536795613	0.348882638	0.93219377	0.297566906	0.777072265	0.982254298
GAB3	0.900602713	0.173462886	0.367852068	0.859574483	0.808506215	0.776962756	0.982254298
RAPGEF2	0.306253284	0.843986457	0.723230063	0.286097569	0.745375606	0.776633855	0.982254298
SAMD8	0.303466551	0.610303725	0.816659266	0.313757794	0.841732064	0.776998297	0.982254298
TGFBRAP1	0.945161478	0.337725492	0.500306508	0.28266579	0.884413014	0.77690278	0.982254298
TTLL7	0.554343255	0.797052044	0.59497849	0.627954526	0.242081585	0.777076812	0.982254298
PDCD10	0.739741739	0.762796385	0.353989008	0.60391935	0.331748967	0.777328297	0.982420845
TAX1BP3	0.549239487	0.386150406	0.931230336	0.268279499	0.755149345	0.777298904	0.982420845
ABCD4	0.519387385	0.204639969	0.587317542	0.983600193	0.652294226	0.777472428	0.982481497
MSANTD1	0.804403679	0.609060852	0.179352104	0.638519741	0.713935071	0.777496021	0.982481497
CDKL5	0.561669938	0.61932822	0.587005864	0.402617399	0.488181817	0.777843688	0.98255946
CIB2	0.542269752	0.728088706	0.303297693	0.67708303	0.494793982	0.777768143	0.98255946
SOCS1	0.815866575	0.837122192	0.891363321	0.176075444	0.374108215	0.777695547	0.98255946
THSD7A	0.348520255	0.762314181	0.511989304	0.29695684	0.993653379	0.777857078	0.98255946
TTC36	0.468663787	0.329972044	0.279349946	0.972996574	0.954040974	0.777698298	0.98255946
ZBTB48	0.837406426	0.512768386	0.880251772	0.748529748	0.141974778	0.777994365	0.982657241
CIR1	0.780643259	0.842317542	0.189541863	0.604683046	0.533632276	0.778207847	0.982699983
LOC101902807	0.804778017	0.807258028	0.116036842	0.62273678	0.856442212	0.778159902	0.982699983
VGLL3	0.639546751	0.809924152	0.414591826	0.285207079	0.656571074	0.778197949	0.982699983
BBS10	0.927609062	0.342127973	0.758715991	0.447454062	0.374234987	0.778669406	0.982828665
CCDC88A	0.641979407	0.381430132	0.929157184	0.591126078	0.299777762	0.778660842	0.982828665
DHCR24	0.950994809	0.915953862	0.422004613	0.596605263	0.183581252	0.778405669	0.982828665

TRPM3	0.433003987	0.615288465	0.214273948	0.735525856	0.959861508	0.778595766	0.982828665
TUG1	0.386650636	0.772103376	0.692863338	0.196809692	0.99079149	0.77872897	0.982828665
ZBTB4	0.590944086	0.950179947	0.39309792	0.776143443	0.235264826	0.778598	0.982828665
ZNF266	0.749247835	0.388608469	0.263259448	0.750950604	0.699793045	0.778495485	0.982828665
AIF1L	0.454785743	0.385438418	0.678401007	0.397311761	0.854162006	0.778831539	0.982841856
GGNBP2	0.710475279	0.186938945	0.472536955	0.857121076	0.750344277	0.7788592	0.982841856
NRTN	0.704949255	0.169613554	0.631924391	0.691399947	0.772967738	0.778936048	0.982863254
EMCN	0.718186874	0.570517977	0.387986465	0.383497834	0.66324256	0.77917623	0.983032191
SEL1L3	0.743961343	0.249484059	0.657089726	0.797107187	0.415965173	0.779189735	0.983032191
SLC25A32	0.809567269	0.59051738	0.381701145	0.319095349	0.694955752	0.779311009	0.983082536
ST7L	0.597828142	0.83091271	0.128810411	0.721069284	0.877244327	0.779349448	0.983082536
CD2BP2	0.591286273	0.570818061	0.481812196	0.342288739	0.727562431	0.779454556	0.983139552
ACER3	0.907023587	0.601417408	0.639415941	0.193554471	0.600353554	0.779599185	0.983161603
KLB	0.684082374	0.711115004	0.111022648	0.756651579	0.991538848	0.779548745	0.983161603
LOC104975666	0.514296352	0.412819779	0.583224061	0.521924571	0.627334614	0.779651764	0.983161603
MEX3C	0.936677043	0.386150496	0.345749014	0.398216412	0.814572949	0.779750387	0.983203346
PLA2G16	0.738785627	0.341365697	0.426926069	0.629398629	0.598892851	0.779836334	0.983203346
SAP30BP	0.779661274	0.660982243	0.207934399	0.99337331	0.381329307	0.779864601	0.983203346
ABHD14B	0.322864222	0.97939434	0.597972172	0.445729234	0.483075107	0.78040195	0.98335679
ATP5MC2	0.792560736	0.579451312	0.517786362	0.470490867	0.366658853	0.781741704	0.98335679
AUNIP	0.643379692	0.246065552	0.913130482	0.330727181	0.853072718	0.780714732	0.98335679
CGAS	0.245372177	0.646674343	0.884720569	0.330062998	0.880324637	0.780735158	0.98335679
CHCHD1	0.791060944	0.433730871	0.61188756	0.293702303	0.664745395	0.781598846	0.98335679
CHTOP	0.830559072	0.641881111	0.864467854	0.281412257	0.315488944	0.781286134	0.98335679
CYSRT1	0.435015393	0.869576924	0.554170949	0.661642528	0.296100993	0.781948914	0.98335679
ESYT2	0.74763141	0.302670332	0.91204386	0.370743041	0.533531303	0.780878342	0.98335679
FBXO2	0.682806021	0.802639632	0.185103747	0.496787648	0.811798347	0.781266051	0.98335679
GABARAPL2	0.766547426	0.646024201	0.30859186	0.584227008	0.459150217	0.781616911	0.98335679
GTPBP8	0.284691538	0.829162813	0.208640832	0.859628611	0.969716816	0.781887437	0.98335679
KXD1	0.339345351	0.434258047	0.526197772	0.533206717	0.994063656	0.782083531	0.98335679
LOC101904592	0.207671253	0.812923283	0.51753466	0.93888781	0.499580223	0.781565975	0.98335679
LOC104972407	0.908554952	0.636450833	0.302785365	0.650815022	0.35997831	0.781729977	0.98335679
LOC112443864	0.578325921	0.592059935	0.482199364	0.903309339	0.274511021	0.781392696	0.98335679
LOC112447346	0.53274374	0.834246475	0.350050865	0.442589852	0.59663802	0.782004823	0.98335679
LOC507443	0.183562688	0.51358414	0.808243134	0.650078015	0.823113184	0.780655738	0.98335679
LOC509184	0.678436281	0.700570631	0.485342347	0.628469906	0.283483131	0.782072193	0.98335679
LRRC27	0.6056773	0.572241012	0.212170845	0.680877127	0.816018858	0.781029543	0.98335679
MALSU1	0.921249431	0.762513667	0.860645816	0.136181669	0.497099919	0.781331422	0.98335679
MARVELD1	0.736407415	0.320839336	0.31206595	0.666625685	0.831322307	0.781041305	0.98335679
MOSMO	0.558043347	0.434207558	0.806972938	0.69668428	0.29931728	0.780667634	0.98335679

MYO5A	0.116358809	0.935978275	0.865869321	0.681101507	0.636064772	0.781011366	0.98335679
NKAIN3	0.271257376	0.623325055	0.957204077	0.297583963	0.844428637	0.780208678	0.98335679
PDZD2	0.433716616	0.746294692	0.222377005	0.856591984	0.660884459	0.78055025	0.98335679
PGRMC1	0.918470418	0.778489226	0.56181128	0.189717718	0.53555892	0.780844463	0.98335679
PNKD	0.665970593	0.573000671	0.266774326	0.798374159	0.504544031	0.781678763	0.98335679
SNX18	0.973626708	0.627760722	0.541196008	0.219074086	0.563229619	0.780842131	0.98335679
SRSF2	0.883671092	0.321387337	0.646824043	0.36426983	0.611628626	0.781333429	0.98335679
TGFB2	0.8786466	0.785617131	0.282880152	0.352586071	0.596120682	0.781829115	0.98335679
TIPIN	0.846824021	0.181137194	0.356526897	0.84577666	0.887525351	0.781871103	0.98335679
TPR	0.917282422	0.370698993	0.479782214	0.297558704	0.84248683	0.781205535	0.98335679
TSPAN11	0.688769588	0.977951956	0.725055352	0.232718192	0.360488804	0.781525201	0.98335679
WNT2B	0.209336502	0.96814198	0.590228833	0.579843736	0.587082821	0.780430875	0.98335679
ZNF691	0.4026016	0.302767247	0.348957184	0.962039406	0.999659707	0.781245352	0.98335679
MSRB2	0.618449825	0.537844839	0.413883419	0.621248385	0.480934714	0.782223599	0.983457556
CALML4	0.75288984	0.216825512	0.267645789	0.971233489	0.970598444	0.782458638	0.983528013
LOC107131623	0.88028381	0.649743787	0.234907529	0.579480141	0.529406026	0.782591041	0.983528013
LOC112441682	0.550585724	0.42522234	0.796294473	0.525331744	0.420933524	0.782620752	0.983528013
LOC112448853	0.877498055	0.50992769	0.698818742	0.179814258	0.733269517	0.782639225	0.983528013
STXBP1	0.458064298	0.930195095	0.388378198	0.338008973	0.736607026	0.782521599	0.983528013
TFDP2	0.475854657	0.984276765	0.854319797	0.204686402	0.50321363	0.782576398	0.983528013
NCR3	0.589411266	0.767647893	0.444910744	0.238763054	0.858176737	0.78271699	0.983550423
ORC6	0.423138613	0.783778409	0.819676003	0.899656782	0.168782373	0.782849801	0.983615892
RFX7	0.543285719	0.40696903	0.290984039	0.766791055	0.836916394	0.782888964	0.983615892
ENPEP	0.14513602	0.635565813	0.807125492	0.571341683	0.974391551	0.78357871	0.983684606
FOS	0.232091534	0.824130233	0.876782091	0.381156565	0.648063262	0.783481273	0.983684606
LOC112442865	0.141461602	0.501702385	0.960152093	0.8384377	0.723914456	0.783201541	0.983684606
LOC535280	0.857041	0.388533295	0.917866995	0.154220226	0.879457344	0.783603001	0.983684606
LOC781565	0.554052937	0.341211562	0.878485161	0.279404968	0.89129993	0.783195253	0.983684606
MAST2	0.16493539	0.410302557	0.860781507	0.971387259	0.731840328	0.783420876	0.983684606
NTPCR	0.777860552	0.328824924	0.570009436	0.604320636	0.469602384	0.783268306	0.983684606
TAOK1	0.275584589	0.927152532	0.980776751	0.262124687	0.630555733	0.783456793	0.983684606
THOC7	0.746603017	0.347793009	0.517897041	0.465598631	0.661294082	0.783396617	0.983684606
TSSK4	0.516845935	0.616857767	0.864830192	0.705263776	0.212578168	0.783105279	0.983684606
ZNF853	0.163572473	0.521933278	0.993369968	0.826749135	0.591152817	0.78358015	0.983684606
HSF1	0.242516164	0.856115977	0.634627285	0.988666945	0.31845923	0.78373781	0.983778584
LOC101907570	0.40555603	0.350929729	0.503992611	0.672276502	0.860666893	0.783812244	0.983796768
RIF1	0.407391037	0.465181735	0.587645005	0.377793721	0.987126164	0.783934021	0.983874367
APTX	0.645065167	0.807925272	0.656730381	0.152006271	0.801841738	0.784723504	0.983900507
C1QB	0.68254735	0.573558835	0.755787639	0.331812734	0.424943189	0.784732639	0.983900507
GAB1	0.365318819	0.794213302	0.793002959	0.292459969	0.619658273	0.784638148	0.983900507

KCTD7	0.564816451	0.775093929	0.703555828	0.870710485	0.155237054	0.784363584	0.983900507
KLHL29	0.990152261	0.109141015	0.840354077	0.93341526	0.491757047	0.784587214	0.983900507
LOC101902838	0.497234861	0.922445629	0.290565617	0.497281704	0.629444182	0.784721146	0.983900507
LOC540707	0.328227487	0.882606073	0.733778511	0.277497044	0.706220832	0.784476283	0.983900507
MIDN	0.448021973	0.869252168	0.234587827	0.731562824	0.623359567	0.784490594	0.983900507
MOGS	0.794812223	0.70252916	0.30232982	0.495369744	0.498458478	0.784583369	0.983900507
SMIM10	0.55745831	0.391833266	0.315571967	0.870520557	0.694646469	0.784577921	0.983900507
STAT1	0.115861267	0.919993968	0.801848162	0.673658585	0.723606843	0.784497559	0.983900507
TM9SF1	0.226379349	0.6720925	0.600303378	0.679199916	0.670050818	0.784083152	0.983900507
TMEM123	0.565404306	0.413391032	0.794410311	0.757742229	0.296518561	0.784734248	0.983900507
LOC112442656	0.565124631	0.549039595	0.223580787	0.638946576	0.9416764	0.784819934	0.983923577
ZBTB45	0.592210826	0.680571841	0.355541648	0.516683244	0.563913651	0.784872582	0.983923577
ZMIZ2	0.570017482	0.618217422	0.261945115	0.463632773	0.975912741	0.784932513	0.983923577
NDN	0.569465775	0.413571129	0.882498373	0.994794591	0.202083885	0.785002614	0.983936294
CSTB	0.511341488	0.649214403	0.203761682	0.745591418	0.828860705	0.78508708	0.983967013
HNRNPH1	0.980625014	0.205094906	0.712420594	0.382089718	0.764889247	0.785394253	0.984169479
LMTK2	0.946492913	0.848077235	0.514115733	0.172109741	0.58968933	0.785428534	0.984169479
WDFY1	0.981155156	0.789358682	0.35861562	0.295408965	0.510300762	0.785366949	0.984169479
GJA10	0.93449068	0.468287615	0.668444915	0.590975321	0.242968765	0.785929531	0.984722059
CFAP53	0.942495633	0.640482962	0.941704501	0.187613174	0.394802458	0.786365505	0.984914824
CHST9	0.963252246	0.75394975	0.918922155	0.305059624	0.206844042	0.786383459	0.984914824
EIF5B	0.84098613	0.667290424	0.714571005	0.629021315	0.16693926	0.786376977	0.984914824
MICAL1	0.793255745	0.403871395	0.341303191	0.848069039	0.453975565	0.786331182	0.984914824
SLC25A19	0.750079469	0.75750682	0.746840779	0.303107247	0.326980764	0.786160354	0.984914824
ZNF581	0.397227269	0.80306197	0.163805261	0.845473801	0.954248213	0.786582114	0.985088452
FBXO15	0.585358743	0.815362906	0.372384233	0.722736573	0.329449304	0.787254814	0.985099366
GAS2L3	0.146398916	0.737313774	0.482667733	0.832609586	0.972779306	0.786751515	0.985099366
HAUS1	0.497804198	0.613326327	0.22742289	0.845836357	0.721085027	0.787386467	0.985099366
LOC100296211	0.278320704	0.433528301	0.973550324	0.484837923	0.742614183	0.787152551	0.985099366
LOC112442071	0.522546119	0.294992811	0.455829789	0.867735958	0.694067699	0.787252873	0.985099366
LOC790871	0.724604906	0.146600239	0.865950168	0.491691747	0.934384418	0.787016931	0.985099366
PSME4	0.820798119	0.957684318	0.477057022	0.205229465	0.54957051	0.787157002	0.985099366
PUS1	0.308024465	0.185835147	0.934390527	0.905409474	0.874743671	0.787431204	0.985099366
SLC2A4RG	0.262116358	0.72962163	0.991994269	0.291685076	0.763832743	0.787043791	0.985099366
TRAPPC12	0.359613125	0.399737494	0.937249814	0.752940851	0.417009873	0.787189833	0.985099366
VKORC1L1	0.95643649	0.154031682	0.45064674	0.985497856	0.64674562	0.787238015	0.985099366
VPS26C	0.746048852	0.838695382	0.697828368	0.28825592	0.335840387	0.787051034	0.985099366
ZNF445	0.74843758	0.354346796	0.531465494	0.367994179	0.816209352	0.787323332	0.985099366
ZNF774	0.333738543	0.40699583	0.92419414	0.338883541	0.991599542	0.786691409	0.985099366
KYAT1	0.981113002	0.790282841	0.48384043	0.620650683	0.182006084	0.78750004	0.985110386

AGTRAP	0.647541783	0.735714581	0.127198571	0.735931453	0.951990282	0.787821662	0.985200826
BAG6	0.851762664	0.411264685	0.355230106	0.894302844	0.381740402	0.787932536	0.985200826
CD300LG	0.207173933	0.520597186	0.526367342	0.819329043	0.91255277	0.787786329	0.985200826
ETV1	0.747287791	0.704716432	0.273820865	0.47939408	0.614282764	0.787862463	0.985200826
PRKCE	0.146610368	0.724291502	0.90085929	0.803953609	0.552370334	0.78793004	0.985200826
TMEM19	0.968915631	0.497602103	0.663346103	0.764110965	0.173608958	0.787703016	0.985200826
ACSBG1	0.724971086	0.707134018	0.441670685	0.273813187	0.686548556	0.788274913	0.985277538
ANKRD16	0.618894063	0.121210944	0.882413425	0.656343583	0.979102331	0.788170678	0.985277538
CPTP	0.809058857	0.935860069	0.394009122	0.285508522	0.499534866	0.788208236	0.985277538
F2R	0.636806332	0.477833183	0.278854311	0.664589195	0.755140237	0.788354113	0.985277538
FBLN5	0.286336247	0.601725407	0.852353838	0.467837765	0.618756117	0.788057024	0.985277538
TUBGCP4	0.961873258	0.262591521	0.984661656	0.198177381	0.863905276	0.788339458	0.985277538
CCDC120	0.580388391	0.800925772	0.237281395	0.501636659	0.7708433	0.788633058	0.985401029
OTOGL	0.43797274	0.875469452	0.392386236	0.489341151	0.57893304	0.788516273	0.985401029
RFNG	0.508595108	0.622537836	0.531435885	0.599029147	0.423013088	0.788576528	0.985401029
ARMC7	0.391290614	0.805062469	0.315179007	0.975716545	0.441747714	0.789222183	0.985545194
BACH1	0.212066955	0.561484097	0.91541353	0.565973645	0.693767862	0.789244008	0.985545194
CCDC69	0.540932931	0.717871305	0.42367558	0.283891677	0.916212262	0.789217297	0.985545194
CSNK1G3	0.216868777	0.799782091	0.30485152	0.849253003	0.953763387	0.789364108	0.985545194
E4F1	0.895036828	0.349831193	0.546866641	0.528023964	0.472829718	0.789041962	0.985545194
ESCO1	0.65508128	0.602665182	0.843998138	0.191216714	0.670533414	0.788928455	0.985545194
FAM129B	0.970323782	0.925285597	0.199148567	0.283064458	0.84568871	0.789255089	0.985545194
FNDC4	0.401654277	0.526658353	0.847648325	0.26441453	0.903881988	0.789469083	0.985545194
LOC104971266	0.702678497	0.476525069	0.806809326	0.660945032	0.23993162	0.789417281	0.985545194
LOC112447846	0.55423061	0.779104194	0.261242672	0.966985026	0.39192881	0.789049187	0.985545194
MTMR9	0.783368458	0.286261859	0.549673464	0.44720688	0.77732591	0.789449586	0.985545194
PCMTD2	0.229867273	0.547090119	0.742460843	0.869841991	0.525713183	0.788823018	0.985545194
LOC112444147	0.795663185	0.401594397	0.74742693	0.412395552	0.435327834	0.789558909	0.985582357
EIF4EBP2	0.927746268	0.641116694	0.101185464	0.872511516	0.81706899	0.789679611	0.985583093
HPS5	0.605744943	0.890849441	0.723099888	0.303990386	0.361708747	0.789678373	0.985583093
LOC101907615	0.928294351	0.601501971	0.15061603	0.873263628	0.584832385	0.789864272	0.985738598
BLOC1S5	0.730945289	0.264249823	0.684613607	0.880069375	0.369408771	0.790025445	0.985864769
AKAP2	0.528507575	0.47892119	0.546060596	0.316760598	0.98686283	0.790904412	0.98598265
ELMOD2	0.932784974	0.930489686	0.599438813	0.910215616	0.091131572	0.790705292	0.98598265
GNPDA2	0.547072439	0.757342851	0.30940488	0.6398431	0.52607472	0.790678766	0.98598265
GPR19	0.726175069	0.47775039	0.192741214	0.748542667	0.86360702	0.790987462	0.98598265
GSTP1	0.808432969	0.435491962	0.805329808	0.164393746	0.927576675	0.791021118	0.98598265
KDM1A	0.85265555	0.731925414	0.330444668	0.235239809	0.888424992	0.790470797	0.98598265
LOC101905571	0.877043357	0.577270699	0.283740098	0.552623664	0.544485909	0.790982502	0.98598265
LOC104975044	0.820109542	0.295481915	0.777136002	0.296797688	0.769953549	0.790209685	0.98598265

LOC107132251	0.441825546	0.666158769	0.832904845	0.751022613	0.234249927	0.790586234	0.98598265
LOC783803	0.635107533	0.790301043	0.634188206	0.595165974	0.228052494	0.7908991	0.98598265
OGDH	0.993072911	0.552736497	0.699813483	0.32232986	0.348800028	0.790829521	0.98598265
PPP3CB	0.643255466	0.170566213	0.933513436	0.89217665	0.471343792	0.790355574	0.98598265
PRKAR1B	0.86435388	0.452911195	0.178746246	0.926177167	0.665589183	0.790620765	0.98598265
TRIAP1	0.700435192	0.483464581	0.557429324	0.523506861	0.436167263	0.790481746	0.98598265
ZFHX4	0.809664535	0.355087688	0.66154626	0.419656068	0.540308347	0.790578029	0.98598265
WHAMM	0.860201433	0.3533645	0.219396848	0.723896178	0.895927492	0.791089585	0.985993103
ADAMTSL4	0.507575259	0.794779671	0.255565277	0.58830297	0.713652593	0.791224558	0.985999289
ICK	0.61787364	0.921577957	0.263593347	0.572516925	0.504601718	0.791533825	0.985999289
KIFC3	0.857468782	0.656660953	0.978516375	0.584069521	0.134935713	0.791782246	0.985999289
LOC112441494	0.577447104	0.65732778	0.634035217	0.238255055	0.755920903	0.791462327	0.985999289
MAGOHB	0.646231436	0.264765499	0.742213635	0.613893859	0.556012471	0.791474502	0.985999289
MRPS17	0.933964585	0.339005245	0.410972299	0.797604313	0.418234242	0.791716661	0.985999289
PPFIBP2	0.907107258	0.765344503	0.549430272	0.265127325	0.430045995	0.792055854	0.985999289
RBM11	0.353889613	0.376248364	0.410242285	0.987722801	0.802914762	0.79136601	0.985999289
RIMS3	0.406834327	0.218671968	0.984740941	0.528883571	0.937962852	0.791927165	0.985999289
RNF144A	0.839634306	0.144531389	0.509099623	0.762212115	0.92223691	0.791804191	0.985999289
SLC35F6	0.628686527	0.559199514	0.507587099	0.830122251	0.293555917	0.792034413	0.985999289
SNX8	0.528150113	0.680079687	0.293756129	0.436049695	0.944644117	0.791939227	0.985999289
TMEM164	0.851710791	0.832992161	0.529069569	0.840451601	0.137649584	0.791788053	0.985999289
TRIL	0.923096115	0.770447602	0.980985199	0.403458571	0.154278676	0.791797683	0.985999289
UBAP1	0.672980649	0.577624033	0.544257432	0.374971586	0.548002205	0.791989441	0.985999289
VKORC1	0.978384257	0.196731497	0.314357954	0.93446398	0.76701131	0.791561622	0.985999289
BFSP2	0.553713445	0.628240881	0.378504612	0.937319687	0.352531754	0.792124719	0.986010222
IKZF2	0.792500504	0.382096049	0.602243232	0.667416339	0.357726393	0.792255078	0.98602291
NUDT1	0.312299613	0.543067288	0.852626736	0.817173751	0.368392467	0.792221766	0.98602291
INPPL1	0.660990243	0.658980568	0.809575014	0.394111806	0.313563761	0.792407493	0.986094009
LOC101905265	0.907825036	0.185327441	0.422828257	0.975608107	0.628144783	0.792476382	0.986094009
PHF5A	0.944099256	0.227917866	0.469487341	0.875598698	0.492896745	0.792492467	0.986094009
RBFOX3	0.276310038	0.241086534	0.820139626	0.930555888	0.858314761	0.792639694	0.986202427
RAB33B	0.674019151	0.58685364	0.72286281	0.794831219	0.192157039	0.792777702	0.986299361
ACKR3	0.675284617	0.119817611	0.63912619	0.979136237	0.865411465	0.793369683	0.986312578
ACOX1	0.267119945	0.65221248	0.339766463	0.939622422	0.798037288	0.79561558	0.986312578
ARHGAP31	0.376794284	0.487419657	0.585319326	0.768091007	0.535014191	0.794783526	0.986312578
ATP6AP1L	0.654920734	0.817448665	0.666077822	0.333101135	0.375075052	0.79626302	0.986312578
C19H17orf49	0.234696075	0.829808901	0.907850383	0.374729168	0.671048166	0.795902886	0.986312578
C22H3orf14	0.674557139	0.555478325	0.842354711	0.563166699	0.250269136	0.796005344	0.986312578
CASP2	0.36467268	0.700868196	0.422495439	0.861197656	0.471702615	0.793559881	0.986312578
CHST3	0.876848263	0.195158311	0.612024969	0.756658151	0.552422612	0.793205481	0.986312578

CRYBA4	0.875531845	0.205682163	0.614063474	0.752819433	0.531692417	0.795126601	0.986312578
CTSD	0.371381642	0.623136779	0.356800597	0.91383269	0.580034655	0.793164522	0.986312578
CXCL12	0.702463723	0.813037178	0.392888348	0.206462035	0.958012249	0.795600441	0.986312578
EIF3F	0.917997766	0.967929504	0.414039874	0.663996775	0.181976541	0.795878648	0.986312578
ELOC	0.41781223	0.581097888	0.804026579	0.403438639	0.563701198	0.795645568	0.986312578
ERCC3	0.838738809	0.239620184	0.640177027	0.373096908	0.927152955	0.796084709	0.986312578
ETV6	0.425173133	0.380119571	0.437137577	0.854149899	0.73288116	0.794981642	0.986312578
GNG12	0.976781419	0.281529686	0.27322557	0.868561714	0.674497723	0.794159402	0.986312578
GNPAT	0.968788174	0.280950108	0.538057419	0.521143748	0.581192108	0.795499886	0.986312578
GOLPH3L	0.833261609	0.854020191	0.204492901	0.392689368	0.766756152	0.793359329	0.986312578
GRPEL2	0.953514447	0.459993109	0.623648604	0.254354772	0.630106915	0.793455289	0.986312578
GULO	0.795380874	0.91426834	0.559050144	0.151670098	0.719651352	0.795563152	0.986312578
HLTF	0.202695364	0.424408796	0.68076528	0.862735996	0.881127708	0.796132416	0.986312578
HYLS1	0.431935476	0.447578102	0.327327298	0.94198454	0.737441421	0.793925164	0.986312578
ING5	0.5247499	0.327900844	0.711128753	0.962804273	0.378091298	0.796226714	0.986312578
ISG20L2	0.705748831	0.859142201	0.346893389	0.274252663	0.77230653	0.796256337	0.986312578
JMJD8	0.675881535	0.635390667	0.571162269	0.433538971	0.411740495	0.793233991	0.986312578
LARGE1	0.566314476	0.44311753	0.38627269	0.479029602	0.954634726	0.795382115	0.986312578
LOC100139990	0.992982657	0.63013159	0.424418992	0.480321906	0.34359808	0.793406274	0.986312578
LOC101902705	0.874893874	0.44579729	0.758848146	0.514912054	0.290238689	0.795007404	0.986312578
LOC101904039	0.271650301	0.647662411	0.915293362	0.590007479	0.468180851	0.795991414	0.986312578
LOC101907581	0.668513448	0.433031332	0.786186796	0.666233803	0.29172123	0.795012972	0.986312578
LOC101909384	0.702740667	0.477051438	0.448310209	0.742005112	0.395309951	0.794424323	0.986312578
LOC112444502	0.646480346	0.57402818	0.801641471	0.388089086	0.382993959	0.794949633	0.986312578
LOC534742	0.79001527	0.594726111	0.258077978	0.440559069	0.833493788	0.796159484	0.986312578
LOC784297	0.62266111	0.688904224	0.70668524	0.726186088	0.200736449	0.794836741	0.986312578
LRRC32	0.615910685	0.305513081	0.596983475	0.872219642	0.449225602	0.794149809	0.986312578
MAP6D1	0.997177208	0.932607381	0.193391959	0.72545649	0.341487929	0.796274167	0.986312578
MFAP3L	0.68111002	0.871481961	0.25126617	0.855489162	0.347114072	0.795232218	0.986312578
MMP17	0.181660779	0.688579529	0.65765159	0.595734497	0.896082195	0.793752508	0.986312578
NCOA4	0.961385861	0.86162521	0.104546481	0.782640866	0.64568806	0.793148748	0.986312578
NDUFA12	0.373404117	0.478438544	0.872522276	0.462654445	0.612292582	0.794711002	0.986312578
NIM1K	0.87808016	0.530545087	0.663244504	0.25618408	0.553192943	0.793248575	0.986312578
NIPAL1	0.826280304	0.310700582	0.846287755	0.553472476	0.370006562	0.796032524	0.986312578
NOP53	0.414643163	0.628033081	0.556189864	0.678799775	0.452044698	0.795836283	0.986312578
OXR1	0.945526089	0.644393486	0.216685654	0.57169405	0.589210224	0.795950713	0.986312578
PLBD2	0.4487969	0.940031559	0.430360291	0.90155061	0.269424336	0.794491728	0.986312578
PPM1H	0.793453333	0.357471817	0.93098891	0.202824274	0.825475195	0.794925392	0.986312578
PRSS48	0.772851217	0.94719149	0.857681307	0.693127001	0.102131487	0.795847877	0.986312578
RING1	0.351449961	0.513684037	0.723376403	0.466847095	0.728074344	0.795624322	0.986312578

RSPH3	0.832478498	0.383375321	0.853360085	0.169319621	0.960802661	0.795302233	0.986312578
TBCD	0.487049667	0.429278021	0.73337986	0.510653298	0.56859265	0.796142788	0.986312578
TCHH	0.498746202	0.615836171	0.278727532	0.669479972	0.767186153	0.793974109	0.986312578
TOM1L1	0.766887877	0.358802412	0.388219002	0.66512539	0.617859447	0.793690378	0.986312578
TRDN	0.576052346	0.788459398	0.677714538	0.917259582	0.15651168	0.794843294	0.986312578
TWF2	0.801038833	0.847680265	0.375645121	0.186607849	0.921949714	0.793627367	0.986312578
UAP1	0.64348341	0.626614255	0.634424283	0.401448097	0.428214408	0.793992233	0.986312578
UAP1L1	0.833423694	0.637426839	0.224135268	0.543057894	0.684130565	0.795029662	0.986312578
UBE2E2	0.726789142	0.805449898	0.324607028	0.443224566	0.527807248	0.795876707	0.986312578
ZRANB3	0.269515939	0.866273259	0.681461819	0.727751842	0.384218395	0.796012245	0.986312578
INHBA	0.235910408	0.467137593	0.612378565	0.720077649	0.917684159	0.796429566	0.986348243
LOC104975925	0.510014844	0.744967769	0.55524792	0.341122413	0.619860176	0.796480309	0.986348243
SERINC1	0.906920894	0.577461468	0.317338265	0.876399092	0.306268156	0.796483269	0.986348243
HOXB2	0.48345073	0.478347594	0.964491966	0.281439209	0.713011269	0.797066123	0.986387224
IL27RA	0.713645423	0.308381765	0.470018645	0.645189669	0.669066515	0.796653101	0.986387224
IRAK1BP1	0.359396314	0.647644846	0.631755431	0.557989834	0.545649604	0.797115798	0.986387224
ISYNA1	0.856979648	0.658524594	0.292600555	0.794191757	0.341276372	0.797055656	0.986387224
LOC526769	0.943842333	0.645820355	0.799130943	0.255338723	0.359729595	0.797004758	0.986387224
MXD1	0.79739213	0.15670284	0.727336021	0.582913969	0.843518073	0.796789065	0.986387224
NEK8	0.701702587	0.250011208	0.49045333	0.53312347	0.973182042	0.796608749	0.986387224
ORAI3	0.126108789	0.67600822	0.728066608	0.965950907	0.745190436	0.79675226	0.986387224
PGA5	0.789633249	0.507040084	0.58591953	0.415695151	0.458553309	0.796904167	0.986387224
SERINC3	0.47246011	0.624678699	0.353669095	0.487797053	0.878463975	0.796948805	0.986387224
LOC100300483	0.1751667	0.690446384	0.757351603	0.851724322	0.574156231	0.797199181	0.986416026
LOC107133048	0.389019832	0.936355974	0.618719058	0.475563821	0.418397679	0.797397498	0.986587028
TMEM115	0.94280164	0.212744909	0.970432403	0.835024353	0.276048345	0.797487334	0.986623795
BOC	0.80970248	0.449820735	0.634067923	0.235185372	0.827442813	0.797774965	0.986826143
FASTKD3	0.972094297	0.372339114	0.964347843	0.864381398	0.149006032	0.797831288	0.986826143
PDHA1	0.99087778	0.646593564	0.45914073	0.197892808	0.771914328	0.797753948	0.986826143
SPRY1	0.636447761	0.699337909	0.38798801	0.280208783	0.929777476	0.797966615	0.986919143
ABI3BP	0.885895185	0.455830543	0.570422704	0.290301573	0.677043794	0.799051651	0.987001521
AHCYL1	0.957385719	0.950685879	0.212779402	0.27661833	0.843855625	0.7987941	0.987001521
ANKRD49	0.407664597	0.57967781	0.635915837	0.360204545	0.83529112	0.798823483	0.987001521
ANXA5	0.637534326	0.658568004	0.2037978	0.551306196	0.95661468	0.798487356	0.987001521
ATP13A1	0.266319814	0.766051186	0.711154484	0.382552265	0.812693232	0.798411166	0.987001521
C10H15orf65	0.812125731	0.894003828	0.627740671	0.774291337	0.127984866	0.79863588	0.987001521
CFAP298	0.507053314	0.952466075	0.636508599	0.317351373	0.461552414	0.79810233	0.987001521
ERLIN1	0.834665141	0.249093056	0.461174025	0.642258058	0.735208375	0.799055646	0.987001521
LOC100140207	0.624276473	0.996620028	0.145751484	0.785034426	0.635167501	0.798831524	0.987001521
LOC100848205	0.628492313	0.684103738	0.744551715	0.852192489	0.165569255	0.798647153	0.987001521

LOC112443007	0.144127733	0.769720729	0.587183135	0.858698023	0.806431264	0.798418943	0.987001521
MXD3	0.985952203	0.316076365	0.4249	0.620083611	0.551096765	0.798957585	0.987001521
NOM1	0.096248956	0.813529468	0.674809767	0.944805002	0.902286734	0.798169635	0.987001521
RAB32	0.353974955	0.919669323	0.537147305	0.34991861	0.73785615	0.798568247	0.987001521
RASAL1	0.692216101	0.545891037	0.406353567	0.476728851	0.618290962	0.798998779	0.987001521
TRIM2	0.294244895	0.956083144	0.643466718	0.879976837	0.284179948	0.799030383	0.987001521
ZSCAN12	0.33452404	0.944938167	0.49049913	0.463067885	0.628257848	0.798414721	0.987001521
DXO	0.750027082	0.46628781	0.707831335	0.685064572	0.267121127	0.799152012	0.98704626
KLF5	0.466309363	0.851809435	0.533870899	0.272247239	0.785368108	0.799307025	0.987163425
NGEF	0.686890494	0.72439739	0.998013098	0.232944587	0.392220618	0.79942418	0.987233818
GPR1	0.8845025	0.206492855	0.627800978	0.407472449	0.972434473	0.799663527	0.987257806
RMDN3	0.675642252	0.296828934	0.442190609	0.565124308	0.90669278	0.799684237	0.987257806
TUBA3E	0.634562776	0.919172907	0.491434188	0.578371069	0.273913888	0.799573181	0.987257806
WDR5	0.671815143	0.771467596	0.263118759	0.69103621	0.481817292	0.799551156	0.987257806
COG7	0.966656296	0.125915257	0.720511584	0.687142018	0.754613925	0.799814032	0.98734377
ALDH1L1	0.255982162	0.528387003	0.462930264	0.868042396	0.8508185	0.802709674	0.98743963
ALDH2	0.141658342	0.886249917	0.448594235	0.962036025	0.851924224	0.802388402	0.98743963
ANKRD13D	0.944134019	0.272272612	0.381756808	0.911835866	0.517210176	0.802849416	0.98743963
AP3M1	0.608887606	0.26883264	0.442944739	0.884797702	0.722326181	0.80306237	0.98743963
AQP7	0.245386633	0.617714352	0.409462607	0.79541424	0.929082935	0.801300069	0.98743963
BIVM	0.276093442	0.852680125	0.84031853	0.277486064	0.849439449	0.804135534	0.98743963
C22H3orf20	0.923108888	0.332881225	0.494774283	0.821901722	0.36886455	0.802147328	0.98743963
DOT1L	0.784967583	0.247017119	0.914074593	0.547310292	0.478306265	0.803281645	0.98743963
ELOA	0.811493819	0.262207348	0.751292352	0.578197525	0.497291801	0.80166757	0.98743963
EPHA7	0.756314603	0.988615342	0.67195646	0.231591019	0.391729232	0.800218293	0.98743963
FBXL17	0.729013073	0.926970905	0.560895003	0.824800243	0.148632908	0.803537452	0.98743963
FBXW9	0.320845616	0.408847075	0.790366456	0.825712557	0.543196404	0.803665789	0.98743963
GAB2	0.645733743	0.167983919	0.936259985	0.848290649	0.531614291	0.801045226	0.98743963
GBX1	0.421161261	0.399856691	0.492198503	0.573959424	0.960466492	0.800646878	0.98743963
GCLC	0.529996039	0.948150962	0.410101116	0.315883329	0.702550678	0.80080146	0.98743963
GLMP	0.328844769	0.551370188	0.490563768	0.610708251	0.843621509	0.801145372	0.98743963
HPCAL4	0.949225684	0.456336821	0.633424285	0.665941763	0.25079726	0.801144429	0.98743963
IFITM2	0.276815415	0.420563461	0.835205434	0.50050018	0.951040458	0.802853464	0.98743963
ITGB1BP1	0.946437117	0.777918258	0.629033156	0.277166582	0.36042756	0.802790015	0.98743963
KDELR1	0.977531152	0.921737437	0.568462738	0.239876198	0.377704527	0.803312938	0.98743963
KLF13	0.386049874	0.627342864	0.309961099	0.702071863	0.880541034	0.803315926	0.98743963
KRT10	0.64380265	0.945492429	0.520796386	0.392735074	0.374004389	0.803896614	0.98743963
LOC100847759	0.762374712	0.898060145	0.399262947	0.727768834	0.228874462	0.800037291	0.98743963
LOC100848419	0.945625708	0.607978549	0.369012704	0.241377164	0.899503362	0.802032999	0.98743963
LOC101903402	0.64044945	0.412946242	0.59015747	0.662541106	0.446887893	0.802591805	0.98743963

LOC101906347	0.932518869	0.398404378	0.708904184	0.645624869	0.272927358	0.803319826	0.98743963
LOC101907006	0.698597142	0.405406881	0.269136616	0.747152876	0.808093678	0.801879943	0.98743963
LOC112441827	0.543872353	0.917627016	0.515081681	0.823592669	0.216911331	0.801511777	0.98743963
LOC112444463	0.315412184	0.30225195	0.887221944	0.767654961	0.718606654	0.804245106	0.98743963
LOC112446004	0.909038214	0.889134481	0.64369323	0.336652638	0.264811197	0.803221027	0.98743963
LOC112447301	0.422924492	0.777568845	0.754537272	0.221270091	0.837062543	0.801642349	0.98743963
LOC112449099	0.646128501	0.856524958	0.476387512	0.228322477	0.768969849	0.802876794	0.98743963
LOC513969	0.904847292	0.559513224	0.631861616	0.295852849	0.487533733	0.802326456	0.98743963
LOC515418	0.282189691	0.247732875	0.686228917	0.968160084	0.998859114	0.803259928	0.98743963
LOC613664	0.590173591	0.509248497	0.607692926	0.580479821	0.432895742	0.801404667	0.98743963
LOC618733	0.52973384	0.735816722	0.229169952	0.734363075	0.694243931	0.80007082	0.98743963
LOC781158	0.838258254	0.440721326	0.60222188	0.572455109	0.365678422	0.80392983	0.98743963
LOC782024	0.654202673	0.351351627	0.556121182	0.571414686	0.637779815	0.803971992	0.98743963
LOC790098	0.919991527	0.946996279	0.140236457	0.627248943	0.603617805	0.802765021	0.98743963
LRP12	0.68688472	0.646408579	0.298889089	0.46682385	0.745913221	0.802585731	0.98743963
LZTFL1	0.5179315	0.796024826	0.307101549	0.710496561	0.508956605	0.80099148	0.98743963
MAD1L1	0.843222869	0.19616164	0.864682278	0.715313709	0.452600896	0.802935276	0.98743963
MAP3K9	0.24499422	0.681844699	0.989894125	0.999893665	0.28222278	0.804260428	0.98743963
METTL17	0.567845898	0.865688743	0.532822413	0.264932033	0.659950485	0.80102997	0.98743963
MFSD1	0.283358942	0.559592886	0.352454651	0.834551841	0.993532875	0.803063119	0.98743963
MMD	0.66826054	0.415456585	0.783892712	0.367299343	0.582132784	0.803784012	0.98743963
NLE1	0.709585068	0.800756845	0.265926661	0.570933982	0.529444765	0.800574163	0.98743963
NME9	0.545609195	0.638197166	0.755044648	0.384073474	0.457299896	0.802459831	0.98743963
NUDT19	0.990286023	0.288928441	0.617815448	0.463196234	0.565268846	0.802857989	0.98743963
PDCL	0.617002948	0.324634102	0.764402388	0.37329994	0.802125856	0.801221911	0.98743963
PIGP	0.626706336	0.354451779	0.723369557	0.470135345	0.618210065	0.804404388	0.98743963
PKP4	0.550974978	0.326285422	0.80219025	0.546504342	0.592144602	0.804281118	0.98743963
PLEKHA7	0.809857979	0.116383168	0.651857139	0.782664761	0.963088471	0.802962379	0.98743963
POP7	0.898427662	0.697953228	0.213691786	0.749296843	0.461391562	0.803012807	0.98743963
PRKAB1	0.98910212	0.256927265	0.48030561	0.436078907	0.870805745	0.803105644	0.98743963
PTGS1	0.425931187	0.848561149	0.412239417	0.717514405	0.429079564	0.801315899	0.98743963
RAB27A	0.48269357	0.324538881	0.857969379	0.724710675	0.478157666	0.803932443	0.98743963
RANBP17	0.744129429	0.942113392	0.273873179	0.554619606	0.431545265	0.801626368	0.98743963
RASA1	0.526297841	0.812503969	0.18032884	0.907762424	0.650872888	0.800143502	0.98743963
RNGTT	0.579076789	0.659280761	0.6076747	0.911911541	0.218167535	0.802378971	0.98743963
RNPS1	0.358955385	0.974398963	0.322649139	0.648852948	0.628640142	0.801917344	0.98743963
RPS21	0.900636579	0.974359284	0.783520176	0.269549657	0.25113382	0.80382061	0.98743963
RRP1	0.314766656	0.936407516	0.542825366	0.477089226	0.601445405	0.80146239	0.98743963
SERPINB9	0.170708688	0.607703777	0.611761855	0.794342161	0.925169483	0.804174681	0.98743963
SNX21	0.77723235	0.943556813	0.435467243	0.192179685	0.760881027	0.804387882	0.98743963

TBC1D1	0.867182853	0.342563468	0.252060653	0.971301386	0.641252369	0.804167139	0.98743963
TMCC2	0.258684858	0.640127127	0.348356755	0.844818379	0.94458716	0.80192158	0.98743963
TMEM101	0.411485681	0.353532565	0.552136648	0.569686042	0.999338802	0.800775083	0.98743963
TSPAN32	0.682634789	0.843503048	0.730976028	0.307016228	0.360908405	0.80416586	0.98743963
TTLL4	0.828254523	0.260769024	0.899323468	0.325940164	0.733966613	0.80353871	0.98743963
TXNDC16	0.57136697	0.905865173	0.1373958	0.975828769	0.669528529	0.803516928	0.98743963
ZNF169	0.359854218	0.833302277	0.308073161	0.947743396	0.528595007	0.802844952	0.98743963
ZNF569	0.264873347	0.482143278	0.715305798	0.662019897	0.770481772	0.804008998	0.98743963
ZNF597	0.618672196	0.871289538	0.380976776	0.250816656	0.899516417	0.803039034	0.98743963
ZNF830	0.177374619	0.902225229	0.420503649	0.903515494	0.767661542	0.804302163	0.98743963
TTC21B	0.277179759	0.967789323	0.282676038	0.7726019	0.797893847	0.804559075	0.987555645
LOC101908034	0.913583354	0.607311702	0.699736407	0.561884729	0.214578231	0.804793906	0.987696141
PCK2	0.707019504	0.469845418	0.677063317	0.309649941	0.672092432	0.804789365	0.987696141
CERK	0.787287077	0.118177825	0.655630808	0.788752513	0.974487992	0.805077377	0.987732445
DIS3L	0.335406065	0.684774813	0.264235566	0.973296376	0.793253526	0.804967467	0.987732445
GAN	0.298535733	0.68691359	0.423075852	0.598765213	0.903308923	0.80522085	0.987732445
LOC112443452	0.628978448	0.406061451	0.842450944	0.418019672	0.521781104	0.805239654	0.987732445
LOC614207	0.401219792	0.949401665	0.830794423	0.353281194	0.419782019	0.805244798	0.987732445
MOCS3	0.713773451	0.341309404	0.414544237	0.715005342	0.649588731	0.805148989	0.987732445
PRMT2	0.981079057	0.974729702	0.222996298	0.82172889	0.267475443	0.805019242	0.987732445
DHX32	0.538878663	0.923768109	0.396783644	0.296067471	0.803150336	0.805372927	0.987767831
LOC515150	0.340232722	0.503325062	0.849463069	0.889978916	0.362827269	0.805394025	0.987767831
PANK2	0.781337306	0.665231984	0.866063795	0.210024872	0.497119474	0.805490315	0.987812103
TXNDC11	0.396408701	0.543052235	0.539016465	0.522754014	0.775210631	0.805573406	0.987840182
TTF2	0.656177677	0.495885248	0.919156922	0.929973564	0.169126174	0.805640321	0.987848424
KIAA0355	0.917004602	0.623074224	0.200201338	0.493285412	0.83463015	0.805836068	0.988014622
LCMT1	0.467655386	0.305026702	0.373996571	0.904058925	0.977579306	0.806036728	0.988113
UBE2T	0.614123769	0.4035438	0.992376855	0.4113026	0.466093337	0.806027831	0.988113
CRLS1	0.903428878	0.352391841	0.414023541	0.455496513	0.785660039	0.806109332	0.988128193
LOC112446029	0.503386505	0.931757672	0.479201282	0.339690129	0.618468716	0.806289849	0.988139492
LOC112448169	0.857854183	0.121854931	0.774153427	0.590698387	0.987393669	0.806217653	0.988139492
SLC26A9	0.661357134	0.435084577	0.329386036	0.772998595	0.644545061	0.806299185	0.988139492
NIPSNAP3A	0.70141718	0.562679527	0.531692137	0.396263021	0.568837481	0.806583488	0.988414099
TTC17	0.553096701	0.4326475	0.835068582	0.311125542	0.761601588	0.80676102	0.988557836
MYORG	0.465156229	0.248512026	0.849521879	0.785699974	0.613982717	0.806844685	0.98858654
LOC101908204	0.250576334	0.546802289	0.923685717	0.74533775	0.503043138	0.807127388	0.988665044
LOC505199	0.406580209	0.620329861	0.281079187	0.721508726	0.927834719	0.807149733	0.988665044
ST8SIA1	0.164861579	0.855868801	0.571618922	0.674639642	0.871462298	0.807009787	0.988665044
ZNF274	0.436981681	0.26505493	0.681721599	0.988217343	0.608069379	0.807111208	0.988665044
AGO4	0.994845703	0.34167593	0.835701738	0.282906657	0.590825644	0.807233857	0.988694293

RAB8A	0.669491285	0.509352418	0.730174152	0.897150459	0.212900933	0.807512209	0.988961407
PPP1R12C	0.766860838	0.454242278	0.562519232	0.686964616	0.353532208	0.8076191	0.98896747
XPO7	0.96643317	0.654176666	0.290953226	0.651293159	0.397269853	0.807637684	0.98896747
ABI2	0.311938533	0.768640916	0.674836035	0.82618227	0.356945671	0.808075867	0.989040567
ARFGEF2	0.711546886	0.89336728	0.580010475	0.225757807	0.574331376	0.808391921	0.989040567
DND1	0.893798378	0.414182372	0.833056181	0.312814251	0.495308207	0.808310683	0.989040567
GEN1	0.806676446	0.726349547	0.606652036	0.359639342	0.373481764	0.808174914	0.989040567
ICAM1	0.78146866	0.140413199	0.898306716	0.50137927	0.968059792	0.808524405	0.989040567
LAMC1	0.465599563	0.189941398	0.781340299	0.764244845	0.906276767	0.808584401	0.989040567
LMBRD2	0.834644605	0.447468946	0.600457629	0.658847429	0.323888667	0.808569385	0.989040567
LOC100848478	0.856139187	0.833595117	0.314083717	0.21643879	0.985055174	0.808339342	0.989040567
LOC112441469	0.104873173	0.953366945	0.767331707	0.634991905	0.981408628	0.808411907	0.989040567
LOC112444626	0.599928879	0.998337314	0.944633575	0.13077836	0.647776058	0.808833374	0.989040567
LOC112447291	0.928059497	0.630435912	0.72916224	0.768249501	0.146164451	0.808748659	0.989040567
LOC514680	0.807261775	0.825050765	0.411908401	0.350165756	0.496274784	0.807927888	0.989040567
NFKBIL1	0.24716204	0.580580842	0.62384535	0.950821329	0.563024198	0.808812284	0.989040567
NME1	0.790583369	0.709371688	0.586968823	0.364891191	0.399047376	0.80884245	0.989040567
PEX14	0.654033631	0.429603349	0.324591771	0.602890771	0.86652939	0.807824129	0.989040567
PROM1	0.39809921	0.23044027	0.713791157	0.948909924	0.771088367	0.80877413	0.989040567
TBC1D7	0.516100549	0.62690003	0.369318098	0.688967768	0.579694055	0.808100256	0.989040567
THOC6	0.660328651	0.286246286	0.867362904	0.496914548	0.586182502	0.808211822	0.989040567
VPS13B	0.549606095	0.809137254	0.764035352	0.208457333	0.675183262	0.808451238	0.989040567
AOX4	0.723907787	0.291855457	0.452258447	0.950222074	0.531965347	0.810142059	0.989086032
BTK	0.667318389	0.382549747	0.684558086	0.359901298	0.768010846	0.810156076	0.989086032
CETN4	0.623147711	0.744725539	0.566432089	0.984560559	0.186182985	0.809739939	0.989086032
CHMP4C	0.556415132	0.703063087	0.950451353	0.309902506	0.419694758	0.810351286	0.989086032
CNMD	0.542316355	0.149877665	0.739821319	0.89757166	0.894668304	0.810103542	0.989086032
DOK1	0.40833168	0.477400207	0.594761516	0.736298889	0.562565047	0.809171789	0.989086032
GDPGP1	0.130982509	0.808306261	0.576883931	0.918807653	0.855395014	0.809094891	0.989086032
IRF2BP1	0.764782379	0.454424126	0.256794717	0.939233049	0.575690361	0.8099863	0.989086032
LAMP1	0.722541081	0.524282728	0.21774988	0.94367238	0.622647527	0.810729167	0.989086032
LOC100847825	0.90028172	0.303642099	0.257228013	0.854099411	0.80825511	0.810989071	0.989086032
LOC100848872	0.857615255	0.645849788	0.972431018	0.205184353	0.435839124	0.809675705	0.989086032
LOC100852077	0.407629197	0.902482827	0.645887442	0.327519291	0.62019961	0.810018265	0.989086032
LOC101905894	0.199462395	0.815421656	0.729948073	0.916913706	0.445027325	0.810651428	0.989086032
LOC101906411	0.880810398	0.294930487	0.765509789	0.32729031	0.743364249	0.81043171	0.989086032
LOC101906472	0.505874327	0.479403881	0.437293086	0.834425525	0.545041369	0.809902717	0.989086032
LOC104975290	0.461464496	0.872902441	0.823543711	0.970014792	0.15012439	0.810170997	0.989086032
LOC107132942	0.980206887	0.436951184	0.871763189	0.448319263	0.289354086	0.810618571	0.989086032
LOC112444474	0.891743571	0.620315753	0.266796239	0.65445836	0.501856396	0.810746518	0.989086032

LOC112446021	0.996799107	0.841204892	0.155538721	0.434098059	0.856803979	0.810872686	0.989086032
LOC112448260	0.406066965	0.571290919	0.481885672	0.560233116	0.76743488	0.809306389	0.989086032
LOC616295	0.736614592	0.502727018	0.602714652	0.262000359	0.822790896	0.809488875	0.989086032
MARK1	0.272721896	0.749999165	0.627310625	0.689405589	0.547487191	0.81059748	0.989086032
MASP1	0.328212087	0.286158764	0.700678161	0.868771073	0.848783109	0.810937073	0.989086032
MCCD1	0.540085917	0.715945838	0.285826621	0.454323127	0.958437182	0.809527426	0.989086032
MFN2	0.834529147	0.460560521	0.630234403	0.530809492	0.374056862	0.809422742	0.989086032
MLLT6	0.772619035	0.988262166	0.724954218	0.113514266	0.772361293	0.810951437	0.989086032
MRPL45	0.956895661	0.36931391	0.628327322	0.761546607	0.286774518	0.810820383	0.989086032
MTFMT	0.83618071	0.196815531	0.538934097	0.70070818	0.774023292	0.809453949	0.989086032
NCOR1	0.546838082	0.275380873	0.722565271	0.596272469	0.747608766	0.810861557	0.989086032
OSBPL9	0.294488293	0.762127904	0.573878303	0.879333159	0.428214621	0.810838882	0.989086032
PLEKHA4	0.571847606	0.261453782	0.633368596	0.760084777	0.666795643	0.809062605	0.989086032
PPM1D	0.791259706	0.275738521	0.866092738	0.340887625	0.749688389	0.810113415	0.989086032
SNX27	0.704627512	0.444345909	0.793982502	0.443121463	0.437360449	0.809715099	0.989086032
TAP2	0.652487242	0.512956376	0.274496402	0.802688438	0.651932068	0.809356936	0.989086032
UPP1	0.208904065	0.898150169	0.370776842	0.906135892	0.768732049	0.810700767	0.989086032
LOC101902856	0.681111952	0.884569918	0.365818595	0.713349031	0.309092443	0.811180727	0.989246259
TNIK	0.668475193	0.775977465	0.816295563	0.313046694	0.367047872	0.811378322	0.989413705
FOXK1	0.94945394	0.128387978	0.868199729	0.622409505	0.739371363	0.811551024	0.989477255
LOC112441455	0.859777722	0.405616571	0.938785759	0.201211967	0.739274426	0.811539765	0.989477255
BICRAL	0.267493336	0.224867346	0.947614039	0.897106043	0.953071914	0.811661517	0.989538455
DCHS2	0.411417675	0.743666129	0.213644936	0.838761518	0.889538769	0.81178487	0.989541818
HOXC9	0.577639036	0.332821031	0.732868056	0.546099487	0.633771777	0.811761818	0.989541818
LOC112447347	0.45063267	0.501748753	0.239771559	0.982994371	0.916194066	0.811975016	0.98963428
MCPH1	0.431604299	0.322225871	0.779207456	0.786518339	0.573070576	0.812041631	0.98963428
SLC6A3	0.112505022	0.930610775	0.92279782	0.5290412	0.955586636	0.81203769	0.98963428
ATG4D	0.531227539	0.348451135	0.457126675	0.628538541	0.918889148	0.812134223	0.989642953
LOC101902067	0.775220044	0.335869621	0.42568323	0.740103255	0.596113061	0.812231201	0.989642953
SHISAL2A	0.577821126	0.122737622	0.950601873	0.7952495	0.912563776	0.812321959	0.989642953
SLC4A7	0.846199783	0.471848134	0.865920169	0.271086699	0.521976396	0.812312488	0.989642953
TGS1	0.823206231	0.188251728	0.775042489	0.750089613	0.543153872	0.812350266	0.989642953
MON2	0.861940892	0.588426311	0.629021548	0.744204014	0.206253833	0.812474755	0.989670433
RPL12	0.816500829	0.585633539	0.48768731	0.605168771	0.347037663	0.812493434	0.989670433
FEN1	0.636768093	0.939817102	0.474224954	0.700235729	0.246739111	0.812693407	0.989688358
LIAS	0.909415289	0.425181014	0.653801826	0.285716454	0.678475004	0.812600091	0.989688358
LOC112448894	0.167633077	0.500646983	0.68625966	0.861353863	0.988082528	0.812640253	0.989688358
ZNHIT6	0.477317791	0.378221201	0.702385734	0.520149077	0.743666528	0.812749376	0.989688358
ADCYAP1R1	0.143832227	0.778096534	0.961207906	0.580973927	0.791640999	0.814207974	0.989829672
APOL3	0.239194066	0.732413798	0.896284833	0.699930578	0.453772233	0.815545335	0.989829672

AXIN1	0.481424121	0.590728817	0.829379574	0.259073421	0.811661298	0.814624632	0.989829672
B3GALT6	0.967553286	0.45420709	0.360136091	0.882617611	0.358331729	0.816166535	0.989829672
B3GNT2	0.776367266	0.68028765	0.735371272	0.536672515	0.238963634	0.815337167	0.989829672
BLNK	0.386931355	0.543862323	0.291884578	0.870705411	0.934746517	0.815952468	0.989829672
BTN3A3	0.763597508	0.365118845	0.528747042	0.5031864	0.662469595	0.813062786	0.989829672
C16H1orf174	0.647128632	0.242826538	0.608807437	0.958705209	0.537578048	0.813626611	0.989829672
C2H2orf76	0.392359967	0.772879639	0.628931891	0.463487784	0.55709601	0.813422861	0.989829672
DDAH2	0.574101804	0.989431083	0.828061997	0.447651731	0.234004442	0.813513396	0.989829672
DNAJC13	0.506523691	0.416479578	0.92277841	0.809325844	0.31797529	0.816303381	0.989829672
FZD7	0.994595017	0.805991211	0.2499348	0.262838165	0.940314491	0.814352543	0.989829672
GLDC	0.684594877	0.550007045	0.360300093	0.60552409	0.609533295	0.816221308	0.989829672
GNAO1	0.564129888	0.57996643	0.846831057	0.455964715	0.390030725	0.813516158	0.989829672
IQCG	0.908551842	0.769293499	0.559073483	0.447890886	0.282576363	0.814140812	0.989829672
KLF8	0.280981114	0.643766989	0.997782367	0.907773524	0.303058648	0.814810088	0.989829672
LOC101901960	0.929544264	0.535708289	0.450951995	0.535460276	0.412448821	0.814608765	0.989829672
LOC101902664	0.810815919	0.695827573	0.554282535	0.37940009	0.421350587	0.815951303	0.989829672
LOC101903375	0.503720484	0.984379771	0.391116524	0.583358782	0.441512036	0.815812425	0.989829672
LOC104969916	0.945989932	0.980286286	0.158453705	0.571583775	0.59529596	0.815973919	0.989829672
LOC104974669	0.911303513	0.773597084	0.400261649	0.55292455	0.315538909	0.813373206	0.989829672
LOC112448032	0.800896844	0.811201172	0.165432642	0.487394083	0.942633775	0.813881285	0.989829672
LOC112448753	0.758766327	0.19741493	0.962209518	0.430523133	0.803900584	0.815588132	0.989829672
LOC780963	0.710415783	0.329413135	0.64665941	0.345340719	0.953398079	0.815392696	0.989829672
LOC781298	0.512684596	0.410941285	0.755855336	0.370849496	0.842866295	0.815227862	0.989829672
LOC786363	0.682443903	0.221893323	0.465857747	0.886382195	0.793652151	0.814720968	0.989829672
LOC786417	0.135760875	0.955390581	0.931229543	0.698046379	0.58678699	0.814202214	0.989829672
LRCH2	0.331740483	0.717011917	0.552845602	0.638427118	0.59552102	0.815967143	0.989829672
MIOS	0.884267412	0.386040012	0.668982564	0.506619863	0.432257258	0.816012778	0.989829672
MRPL1	0.828305016	0.401963588	0.836530998	0.476239154	0.372457202	0.813963104	0.989829672
MYDGF	0.54403579	0.667931862	0.4117885	0.485250806	0.686293664	0.815415488	0.989829672
NAF1	0.533761312	0.725679595	0.477428206	0.503539914	0.537254021	0.816073971	0.989829672
NAIF1	0.363785425	0.874664423	0.277144816	0.795418791	0.705741384	0.814302182	0.989829672
OLR1	0.399831657	0.927270858	0.554872861	0.617202631	0.388115641	0.813538437	0.989829672
P2RY2	0.965312336	0.91331506	0.495491379	0.298998295	0.382506395	0.815849235	0.989829672
PLXNA2	0.908205613	0.203026758	0.684722109	0.547025729	0.721790092	0.815477517	0.989829672
PRDM8	0.355436984	0.478920555	0.652767443	0.885288916	0.506189783	0.815288944	0.989829672
PRKCSH	0.830657341	0.165096051	0.587538696	0.868388318	0.702577255	0.813125569	0.989829672
PTAR1	0.233397558	0.925130777	0.450109772	0.796331759	0.637333282	0.813698487	0.989829672
S100A4	0.790396847	0.319130345	0.47152016	0.475812041	0.88384976	0.816040669	0.989829672
SATB2	0.339740331	0.813234876	0.916617091	0.414842735	0.471553769	0.814430403	0.989829672
SFT2D3	0.135294998	0.958999232	0.938779927	0.468137863	0.871332115	0.814916739	0.989829672

SLC10A3	0.611777987	0.574945502	0.578338998	0.5014189	0.485521364	0.814370829	0.989829672
SLC10A6	0.913897865	0.967840395	0.257126622	0.410151936	0.536162854	0.816026181	0.989829672
SLC9C2	0.5641301	0.928632576	0.165269013	0.984105888	0.582070197	0.814610986	0.989829672
SMAD9	0.362934516	0.771865888	0.243487085	0.947325688	0.765962044	0.814270266	0.989829672
TENM4	0.746263531	0.524643191	0.956680411	0.29550844	0.450799994	0.815634684	0.989829672
TRAF3IP2	0.877767323	0.624357077	0.350795167	0.412933356	0.627703969	0.815411925	0.989829672
TSPYL5	0.599196643	0.769650108	0.338196534	0.541199171	0.588246601	0.814811079	0.989829672
TSTD3	0.709891482	0.318508704	0.912209999	0.739450449	0.324239903	0.814127205	0.989829672
WIPI2	0.308894655	0.897311353	0.324156717	0.569750831	0.966377195	0.814187921	0.989829672
WNT7B	0.690571116	0.777627378	0.489898955	0.413611389	0.460379121	0.81629832	0.989829672
WWC3	0.549388568	0.681075284	0.195143998	0.76728327	0.887931214	0.815127203	0.989829672
ZNF146	0.367364768	0.98371471	0.834544582	0.179837261	0.919381962	0.815524461	0.989829672
ZNF280C	0.667572087	0.658090094	0.816881757	0.93238366	0.148648785	0.815101563	0.989829672
ZNF621	0.489958547	0.278713885	0.949388354	0.441764465	0.873715899	0.816116155	0.989829672
ZNF646	0.948287117	0.184840182	0.750747831	0.570270496	0.656384703	0.813462143	0.989829672
CTCFL	0.744917487	0.57891039	0.871895465	0.546097834	0.244169019	0.816433583	0.98991441
RNF150	0.818956742	0.633432645	0.132579099	0.940608071	0.776652194	0.816791203	0.990274854
FAM114A1	0.224805178	0.60584935	0.633743208	0.742498893	0.784766528	0.816964292	0.990333969
LOC786930	0.548229118	0.525620242	0.598879203	0.466547686	0.625503806	0.817187443	0.990333969
NDC1	0.36124829	0.671442166	0.428909232	0.53455473	0.905660213	0.817202035	0.990333969
PEX7	0.960860375	0.276579157	0.662965134	0.560684866	0.509488977	0.817081302	0.990333969
RNF26	0.997615039	0.35520392	0.550282111	0.295228107	0.874298814	0.817088887	0.990333969
ZNF70	0.413762181	0.885013553	0.240114942	0.767155961	0.745762047	0.816996405	0.990333969
DCUN1D5	0.816071138	0.425410732	0.401108981	0.536504406	0.675936052	0.817641388	0.990369843
GLOD4	0.848118281	0.5603221	0.30449806	0.457205819	0.762874516	0.817551203	0.990369843
HOXC10	0.747419218	0.693197263	0.486879318	0.648895482	0.308527243	0.817654073	0.990369843
ITGA1	0.986278549	0.116684917	0.673574996	0.790936551	0.822815772	0.817474539	0.990369843
LOC112445065	0.716260528	0.716419115	0.594449448	0.562196352	0.294017951	0.817386114	0.990369843
LOC789258	0.477342885	0.487112152	0.984200677	0.256322124	0.859672831	0.817404371	0.990369843
PPIG	0.559476184	0.74490167	0.647607101	0.223492594	0.836336964	0.817471481	0.990369843
TRIM33	0.265363784	0.652661201	0.817408805	0.373857769	0.954763539	0.817754293	0.990418133
APCDD1L	0.336639692	0.779860173	0.344925349	0.748757417	0.74622391	0.817964629	0.990599773
WIZ	0.695365331	0.261583716	0.532737632	0.804315385	0.649716953	0.818107072	0.99069917
HIST1H1E	0.770143003	0.656709178	0.884844562	0.392812981	0.288383269	0.818291557	0.990719572
KANK3	0.390356341	0.433619296	0.544350561	0.77458777	0.710259138	0.818279498	0.990719572
RRM2B	0.991317771	0.36932624	0.320431215	0.543457154	0.795205151	0.818305027	0.990719572
B9D1	0.629370157	0.656303771	0.240403882	0.75917689	0.674147749	0.818707949	0.990988062
GTF3C4	0.896829187	0.291177368	0.723872388	0.417866252	0.643254537	0.818669112	0.990988062
SEC14L2	0.800401437	0.808403338	0.781330444	0.287261882	0.349840255	0.818657188	0.990988062
MAP3K2	0.622950143	0.721041114	0.544125956	0.290787847	0.715752445	0.8188621	0.991101551

ADAMTS4	0.727241015	0.527540075	0.431169448	0.588753065	0.522969564	0.819069297	0.99116073
CSK	0.771563359	0.298050211	0.657697908	0.389903941	0.863540995	0.819044984	0.99116073
LOC101903905	0.903298915	0.472579039	0.514344007	0.635328902	0.365168045	0.819092183	0.99116073
MBIP	0.592041912	0.36183433	0.3331176	0.759110355	0.941630408	0.81932025	0.991290521
NUP54	0.770152521	0.945179713	0.215786807	0.374386557	0.867265733	0.819298478	0.991290521
SRSF11	0.633923638	0.397776696	0.416351512	0.997317338	0.487537293	0.819447808	0.991371763
CELF6	0.465280235	0.662023004	0.702657097	0.777649236	0.303871675	0.819765148	0.99162188
LOC783942	0.734791632	0.333694169	0.420554082	0.915543241	0.541774165	0.819775397	0.99162188
ACADSB	0.533015595	0.826153124	0.520533306	0.318272731	0.70136045	0.819835916	0.991621995
LOC101905648	0.60478229	0.769202342	0.667819166	0.300713656	0.548576737	0.82010394	0.991873076
C7H19orf66	0.789712824	0.153414317	0.827100506	0.99966875	0.512374481	0.820352098	0.992057258
GDAP2	0.838285848	0.203197702	0.361379714	0.935406606	0.8917541	0.82042227	0.992057258
PCDHA13	0.088432587	0.890330232	0.817761836	0.996659117	0.800536618	0.820498029	0.992057258
PHC1	0.722722618	0.613189528	0.94365023	0.276346994	0.444402532	0.820456133	0.992057258
CCDC17	0.883469074	0.669861273	0.142741974	0.779603718	0.781096363	0.820723181	0.992093102
CSKMT	0.792984473	0.732284775	0.189717617	0.57469266	0.812946196	0.82081629	0.992093102
LOC104971296	0.50488062	0.261043837	0.609059784	0.763017287	0.840405798	0.82082994	0.992093102
LOC618268	0.999181054	0.679915984	0.942672729	0.834170256	0.096335044	0.820797001	0.992093102
ZNF212	0.849775464	0.275101949	0.819200886	0.951226989	0.282418686	0.820745917	0.992093102
COPS4	0.622891348	0.252693095	0.794284478	0.551305125	0.747988863	0.821091304	0.992189779
CSNK1G1	0.911016031	0.083474225	0.846091551	0.934221028	0.85711389	0.820982341	0.992189779
UBXN6	0.194702951	0.789700725	0.494860999	0.966569858	0.700821687	0.821049311	0.992189779
CRYBG1	0.751243209	0.795351169	0.317705686	0.565852726	0.480190022	0.82117239	0.992214703
LOC104968751	0.289942672	0.960444436	0.455972729	0.933772768	0.435241026	0.821253907	0.992240143
PM20D2	0.984743804	0.491051157	0.656209397	0.767732348	0.211933107	0.821332941	0.99226258
AK9	0.932698219	0.9717919	0.138829435	0.903590538	0.454612782	0.821527105	0.99230959
ARHGAP25	0.429391114	0.819325866	0.437827083	0.738175483	0.458133145	0.822806666	0.99230959
C10H15orf61	0.44893245	0.502434771	0.856648338	0.407948454	0.659919135	0.82257637	0.99230959
CDC42SE2	0.442176074	0.788728817	0.184135458	0.825222912	0.982707913	0.822765687	0.99230959
CPNE8	0.24527559	0.802441094	0.847570551	0.382207345	0.815414264	0.822485426	0.99230959
CYB561D1	0.619654487	0.855563982	0.379348649	0.677547382	0.380095748	0.821857028	0.99230959
ECSCR	0.360177239	0.387032647	0.536349968	0.942787979	0.736977029	0.822355583	0.99230959
ECT2	0.427485526	0.697930372	0.241923571	0.75722383	0.952062001	0.822630459	0.99230959
ERN1	0.969704854	0.818382201	0.889491819	0.073838386	0.994700028	0.82202446	0.99230959
GPR89A	0.425443394	0.860966079	0.298887578	0.815320363	0.581366693	0.822177865	0.99230959
LIPE	0.630031899	0.756685808	0.195285265	0.824904922	0.676579235	0.822389918	0.99230959
LOC100299025	0.450043231	0.745910791	0.815813643	0.61723913	0.308304497	0.822883505	0.99230959
LOC101902440	0.69412595	0.490549342	0.653068744	0.35772969	0.654900193	0.822824307	0.99230959
LOC101902668	0.350346743	0.330596192	0.842851654	0.864966108	0.613988449	0.822022299	0.99230959
LOC101906131	0.997982129	0.193610801	0.557250442	0.699945876	0.688434872	0.822145808	0.99230959

LOC104969719	0.909371839	0.081729551	0.89832803	0.899133331	0.865396644	0.82236121	0.99230959
LOC112443199	0.88516887	0.578766872	0.90096987	0.817676038	0.137479263	0.822156658	0.99230959
LOC786252	0.581310886	0.934698026	0.270877499	0.366229502	0.963177814	0.822253532	0.99230959
PTPN18	0.670602404	0.719593024	0.745901827	0.301398731	0.478783996	0.822330665	0.99230959
RRN3	0.712278693	0.511463575	0.514250401	0.279266109	0.990384824	0.821928859	0.99230959
SAMD11	0.934685389	0.628225115	0.182583022	0.674724602	0.719876819	0.82275417	0.99230959
SGK3	0.54547908	0.962769402	0.49231459	0.561594494	0.358080473	0.822495293	0.99230959
SMAP1	0.375225255	0.286412076	0.639779009	0.920002887	0.821737749	0.822453069	0.99230959
SYT5	0.96816265	0.279135889	0.718242229	0.385202625	0.691527746	0.821574656	0.99230959
USP22	0.899840118	0.290481808	0.439758909	0.54524398	0.831211509	0.822821024	0.99230959
ANKAR	0.456436402	0.146798303	0.888045326	0.916466842	0.957878814	0.823263607	0.992403338
LRRCC1	0.971514148	0.187167999	0.611414173	0.85259712	0.551052564	0.823259554	0.992403338
LSM14A	0.658347777	0.649207394	0.949682097	0.48648995	0.26450607	0.823249462	0.992403338
STMP1	0.994151866	0.368159047	0.489002264	0.48311172	0.603698624	0.823150592	0.992403338
TAAR1	0.826871882	0.657610336	0.916728038	0.113252481	0.925080851	0.823229743	0.992403338
LOC112448889	0.976010656	0.926244892	0.707779131	0.115639616	0.706248623	0.823331582	0.992412383
PDE6D	0.390797477	0.508837936	0.923703003	0.336180266	0.846778025	0.823431643	0.992460098
ACVR1B	0.731363958	0.650542779	0.97571194	0.188053767	0.60996091	0.826430934	0.992503333
ADAM23	0.137527341	0.961223906	0.902315129	0.50264401	0.881578041	0.825211569	0.992503333
ARHGEF6	0.781921336	0.466457856	0.82138996	0.563818675	0.313994538	0.82577712	0.992503333
ARL6IP6	0.218734888	0.940738734	0.833059462	0.335847048	0.910548013	0.823850721	0.992503333
BBOF1	0.942415285	0.800619453	0.249716642	0.774600973	0.363784382	0.825948023	0.992503333
BCAS4	0.390546397	0.901632278	0.789303906	0.900557093	0.212547905	0.826279481	0.992503333
C18H19orf33	0.177852066	0.523565039	0.925428209	0.636422592	0.97081811	0.826408509	0.992503333
CDH7	0.737719509	0.702265326	0.274367167	0.612739805	0.605188615	0.824755961	0.992503333
CERS6	0.576314804	0.856628129	0.399606275	0.652926531	0.40758547	0.824101489	0.992503333
FBXL18	0.83675467	0.346628894	0.701887153	0.650516329	0.395431597	0.823680525	0.992503333
GSC2	0.980762907	0.71457316	0.755461034	0.985870692	0.101936328	0.826301153	0.992503333
HMGN4	0.342902887	0.529551739	0.783049599	0.751276393	0.490661739	0.82382916	0.992503333
KCNQ5	0.187286506	0.73862959	0.727143616	0.785766887	0.673338317	0.826342244	0.992503333
KLHL18	0.796156207	0.241890638	0.463724762	0.928894391	0.634350155	0.824483342	0.992503333
LOC100336448	0.609512081	0.555671625	0.724731432	0.227253875	0.942766569	0.824377082	0.992503333
LOC100848007	0.630418422	0.164763345	0.85664431	0.650458739	0.907431862	0.824160896	0.992503333
LOC100848264	0.42903012	0.734954277	0.516679124	0.53530906	0.608345818	0.825829245	0.992503333
LOC101903806	0.962683571	0.239128288	0.315799713	0.977193958	0.747475478	0.825972969	0.992503333
LOC101906460	0.308355659	0.666521464	0.909583714	0.401513824	0.704735801	0.825341327	0.992503333
LOC107133473	0.779197596	0.632590065	0.794364831	0.361864834	0.371392229	0.82448188	0.992503333
LOC534520	0.81338644	0.57322782	0.277906856	0.554618221	0.738288662	0.825836678	0.992503333
LTBP4	0.487507556	0.812641329	0.959194924	0.31687193	0.434916605	0.823687032	0.992503333
MAD2L2	0.534123908	0.578799782	0.284202042	0.978338998	0.610575058	0.824047763	0.992503333

MGLL	0.519838188	0.631732702	0.313735507	0.641430765	0.801820402	0.825627851	0.992503333
MMRN2	0.564827057	0.30983808	0.823014604	0.987885995	0.368566742	0.823916596	0.992503333
MRPS28	0.852416737	0.283873341	0.994600118	0.522097525	0.418882787	0.824519858	0.992503333
NRCAM	0.146973311	0.93205475	0.874099421	0.996094599	0.442518328	0.824976346	0.992503333
PBX4	0.767659014	0.651149724	0.327574826	0.987304928	0.32657556	0.825022652	0.992503333
PHF3	0.693530354	0.432433963	0.997765747	0.185510901	0.957760545	0.826175809	0.992503333
PIGK	0.896233168	0.521025068	0.837379036	0.421042022	0.322875027	0.826146076	0.992503333
PKDREJ	0.457927304	0.451222418	0.824512323	0.377829097	0.813857516	0.823744537	0.992503333
PLBD1	0.567319475	0.365872993	0.491031529	0.873364454	0.590915515	0.824413233	0.992503333
PRR3	0.236523923	0.74547287	0.603118305	0.79079323	0.630175425	0.82564365	0.992503333
RBP7	0.392753107	0.644183809	0.315399081	0.718988245	0.91667518	0.824389996	0.992503333
SCAMP4	0.641955933	0.671415556	0.458417805	0.519197704	0.515004795	0.825139183	0.992503333
SIRT6	0.356013187	0.510217205	0.573041903	0.771678988	0.660451299	0.825814447	0.992503333
SLC10A7	0.813023063	0.512067943	0.749657255	0.681195883	0.248572035	0.825182903	0.992503333
SLC25A41	0.572713711	0.84795563	0.789272813	0.588897071	0.235678548	0.826273009	0.992503333
SNX1	0.815916131	0.614510383	0.799297383	0.57340151	0.230886393	0.825835364	0.992503333
SPI1	0.402483635	0.439537263	0.735349489	0.515891457	0.780158913	0.823649863	0.992503333
SSH2	0.512445752	0.721136109	0.921173194	0.327336763	0.472147395	0.824447581	0.992503333
STON1	0.49117649	0.465948606	0.557988992	0.640591073	0.64938015	0.826039981	0.992503333
SV2A	0.497409758	0.267475696	0.666482455	0.654300704	0.917028284	0.826291976	0.992503333
TEX12	0.46770968	0.766819476	0.921432751	0.801819451	0.200699967	0.826219971	0.992503333
TSTD2	0.971449959	0.853438589	0.860380879	0.352283582	0.210790398	0.825565218	0.992503333
TTC3	0.888051575	0.495530105	0.264436135	0.922787933	0.494909469	0.826106399	0.992503333
VAC14	0.345007473	0.865115225	0.283275105	0.704454661	0.892408641	0.826133242	0.992503333
ZFAND2B	0.294345583	0.601878555	0.769809427	0.826429617	0.469646366	0.825451516	0.992503333
ZNF268	0.957434995	0.721821181	0.991580147	0.240609587	0.322247014	0.826073064	0.992503333
CMTR1	0.323444016	0.581101654	0.987024002	0.489533306	0.587196266	0.826668751	0.992716294
AMPH	0.667997594	0.313172146	0.940340875	0.287726581	0.944831834	0.82713441	0.992884254
GPR63	0.673940862	0.677816857	0.290739929	0.460253907	0.874165958	0.827003209	0.992884254
LOC100297676	0.521248736	0.405347388	0.602637987	0.940112198	0.446683436	0.827108217	0.992884254
LOC112448770	0.90277327	0.567311479	0.145029195	0.935088875	0.769848909	0.827110351	0.992884254
LOC112449052	0.875512477	0.726409037	0.870556908	0.146610355	0.659396844	0.827275673	0.992884254
MOB2	0.759147215	0.50009833	0.955819011	0.524991125	0.281092047	0.827353128	0.992884254
NUP58	0.37851092	0.567622226	0.409431892	0.650746358	0.935402625	0.827342647	0.992884254
P2RX5	0.726609088	0.804273672	0.125241005	0.968898778	0.754463679	0.827206148	0.992884254
PRDM10	0.899016271	0.302341373	0.393476548	0.987642407	0.506916058	0.82733886	0.992884254
LOC107133294	0.620150753	0.621329782	0.899080723	0.807227182	0.191584028	0.827434446	0.992909234
CREG2	0.697684195	0.587453575	0.173770374	0.792448239	0.950340715	0.827617379	0.993056139
APOA1	0.81306805	0.298919236	0.94755394	0.539003989	0.432578317	0.827799413	0.993056738
LOC100851323	0.648388373	0.374006429	0.606459261	0.887147267	0.411524416	0.827786534	0.993056738

TANC2	0.947711286	0.886643915	0.339573451	0.212909272	0.883634004	0.827755923	0.993056738
ABHD15	0.918733175	0.355740271	0.810754718	0.638061964	0.322020876	0.830060919	0.993104817
AKAP10	0.523727228	0.303621899	0.986506093	0.69965092	0.49511526	0.829748526	0.993104817
AKT3	0.668623921	0.739215162	0.789927069	0.228094864	0.611817419	0.830180228	0.993104817
ARAF	0.550523151	0.502250848	0.433315349	0.484103545	0.939961767	0.830282029	0.993104817
ATXN10	0.51960696	0.491588176	0.618988152	0.593277257	0.572932814	0.827942079	0.993104817
B3GNT9	0.264359144	0.794287215	0.642433178	0.584098225	0.691524451	0.830186293	0.993104817
C5H12orf10	0.940460919	0.240914239	0.820983805	0.394842574	0.733334907	0.828297526	0.993104817
CCT6B	0.713589208	0.811694595	0.277500637	0.437059233	0.770595597	0.829127617	0.993104817
CLN8	0.649260846	0.519793445	0.984453962	0.163049169	0.993471812	0.828167816	0.993104817
DNAAF4	0.640430681	0.341282085	0.83021761	0.321033033	0.935111314	0.830147803	0.993104817
DOC2G	0.970657369	0.645575446	0.257178455	0.513698047	0.658151687	0.830181855	0.993104817
DUSP10	0.567773266	0.895905917	0.99195554	0.217182333	0.497391335	0.830245861	0.993104817
EAPP	0.379677267	0.486111589	0.547837321	0.739942434	0.72813981	0.83015691	0.993104817
EEF1AKMT1	0.466216471	0.370766719	0.56632645	0.860106753	0.641037119	0.828646131	0.993104817
GATD3A	0.430826147	0.623734097	0.437876313	0.577294902	0.793314446	0.828384809	0.993104817
KCNH3	0.846162872	0.879423443	0.780376513	0.935719169	0.099820527	0.829446756	0.993104817
LOC100847934	0.895862387	0.588638468	0.325438925	0.53893616	0.581565567	0.828083433	0.993104817
LOC100849067	0.856910889	0.862567989	0.280466693	0.299532406	0.874739691	0.829676195	0.993104817
LOC101904339	0.427775659	0.948214675	0.927006094	0.372984249	0.388826531	0.830320589	0.993104817
LOC101904622	0.928153538	0.12634555	0.948753521	0.821612504	0.594925572	0.829874969	0.993104817
LOC104972542	0.884726285	0.926529896	0.235986748	0.916768438	0.305317658	0.829163613	0.993104817
LOC104973285	0.986084489	0.986017694	0.208903781	0.756015372	0.352763309	0.829235583	0.993104817
LOC112444869	0.861777038	0.316952981	0.573014187	0.444432324	0.78206062	0.829926845	0.993104817
LOC112446855	0.919932455	0.499690548	0.566441896	0.342755648	0.603201581	0.828220509	0.993104817
LOC112447385	0.882242	0.986332219	0.545401184	0.129428541	0.884046275	0.829638351	0.993104817
LOC523963	0.766692893	0.265039774	0.312630282	0.899876415	0.953381346	0.830231168	0.993104817
LOC524650	0.587124046	0.979451341	0.628418294	0.273505373	0.549481297	0.829656455	0.993104817
LZIC	0.749686766	0.171240253	0.626258391	0.883926624	0.757612742	0.828236776	0.993104817
NECTIN3	0.215036961	0.883578755	0.946839771	0.507315196	0.594148131	0.829403921	0.993104817
NSUN4	0.570000092	0.635730937	0.878850697	0.206605548	0.818915595	0.828365399	0.993104817
PIP5K1B	0.437434659	0.404893157	0.864747297	0.838712163	0.423610458	0.829972314	0.993104817
PTPRK	0.336132261	0.886581445	0.643458937	0.687011711	0.412647058	0.829811103	0.993104817
RAB35	0.767830246	0.349430531	0.222760752	0.903975886	0.996862216	0.828294731	0.993104817
RAB5B	0.910019435	0.667633207	0.817989173	0.302399344	0.36114069	0.829548792	0.993104817
RAD1	0.658061069	0.669332844	0.786468406	0.975721173	0.160176581	0.829143631	0.993104817
RCC2	0.707968132	0.399448942	0.24205957	0.854208919	0.93141368	0.83011533	0.993104817
SKA1	0.767328975	0.472641413	0.691380353	0.225471681	0.95889005	0.829360273	0.993104817
SLC23A3	0.919475154	0.669666008	0.716091945	0.403210357	0.304801578	0.829294454	0.993104817
TBCE	0.545098978	0.682305403	0.679514965	0.266965453	0.800245849	0.828699139	0.993104817

TNPO1	0.787346878	0.458525867	0.257956217	0.892153177	0.650994843	0.828985289	0.993104817
UNC119	0.807946936	0.180978319	0.808148988	0.699111716	0.654498473	0.828933847	0.993104817
AIP	0.96876562	0.612653069	0.264558838	0.865196403	0.401669384	0.830428219	0.993144642
CCDC197	0.691392683	0.917533675	0.284241756	0.457212954	0.662080107	0.83047492	0.993144642
UBTD1	0.77503795	0.781655314	0.25106444	0.536581526	0.669970104	0.83075603	0.993408424
BCAP31	0.68588867	0.700774102	0.504972248	0.502006293	0.449057499	0.830866685	0.993468355
LOC112444484	0.749918543	0.411268899	0.409453804	0.477810055	0.907489757	0.830990737	0.993491249
NIPAL4	0.710144756	0.916413376	0.270850312	0.522735301	0.594340943	0.831006909	0.993491249
ABLIM3	0.408137449	0.845499016	0.689316976	0.279233294	0.826800224	0.831463444	0.993600832
CRKL	0.64879724	0.882075759	0.26291208	0.606634103	0.60288125	0.831791271	0.993600832
DCTN1	0.774588371	0.442236708	0.516541742	0.905129122	0.344744668	0.832334261	0.993600832
DNAH1	0.965419913	0.1845564	0.867386367	0.434375078	0.817408515	0.831334778	0.993600832
FGFRL1	0.132592527	0.671342163	0.778253677	0.833030891	0.955356412	0.832099802	0.993600832
ITGBL1	0.651259086	0.33793843	0.967464605	0.373047773	0.691775156	0.831556682	0.993600832
JMJD1C	0.468048955	0.652225182	0.438191747	0.427973925	0.965360709	0.8324911	0.993600832
KLK4	0.441130819	0.901283476	0.325253726	0.480386594	0.886283713	0.831877403	0.993600832
KPNA5	0.907658986	0.773009664	0.125195633	0.824704032	0.761741478	0.832245975	0.993600832
LOC100138633	0.854274881	0.436837824	0.999027133	0.820312935	0.179559234	0.831454531	0.993600832
LOC101902812	0.748219091	0.868251037	0.227493934	0.79652615	0.467688604	0.831872179	0.993600832
LOC104972595	0.562297132	0.71184505	0.243019645	0.660561244	0.859485501	0.832373444	0.993600832
LOC107131323	0.763956033	0.695409168	0.396498699	0.763782957	0.341829679	0.831696522	0.993600832
LOC510454	0.64487468	0.988966753	0.617576461	0.98796407	0.140958934	0.831267308	0.993600832
NDRG3	0.373959504	0.544868097	0.79926109	0.382824215	0.885214305	0.83226459	0.993600832
NRDC	0.61361326	0.326770609	0.611537314	0.81966113	0.548335242	0.832036559	0.993600832
PRPF4	0.951554843	0.977518529	0.222472575	0.392075238	0.681156008	0.832487873	0.993600832
RNF113A	0.825894177	0.337705612	0.643103948	0.732535318	0.419152892	0.831925985	0.993600832
RNF227	0.580530687	0.898910086	0.575092519	0.220265768	0.83561836	0.832406914	0.993600832
SLFNL1	0.281591601	0.581537839	0.760780477	0.799688363	0.553538651	0.832142735	0.993600832
SUCLG2	0.843482113	0.6216041	0.341547545	0.749462595	0.410518478	0.831992676	0.993600832
THAP6	0.479875518	0.567339593	0.477075228	0.607451417	0.696590537	0.831590973	0.993600832
TTC31	0.937574433	0.483939969	0.616462003	0.415002285	0.472294252	0.831187005	0.993600832
ERMARD	0.353850181	0.672807951	0.253352359	0.940913122	0.975050015	0.832696785	0.993701784
STYXL1	0.462742119	0.24568987	0.966806401	0.766299752	0.656895509	0.832677734	0.993701784
C1D	0.53054864	0.248778648	0.598510887	0.708187826	0.991864789	0.8331434	0.993728929
COL11A2	0.346716052	0.733784144	0.865979621	0.383635198	0.655878855	0.832987329	0.993728929
COQ2	0.858063224	0.434070675	0.482664588	0.342501303	0.900547439	0.833025293	0.993728929
GORAB	0.505240188	0.932642519	0.409354114	0.32157125	0.893837794	0.83300824	0.993728929
LOC100848991	0.201154061	0.537640267	0.821585739	0.663191306	0.941484984	0.833111545	0.993728929
RFK	0.746352083	0.772147691	0.763712834	0.12953867	0.971483328	0.832844702	0.993728929
THAP1	0.651980223	0.155050244	0.73132597	0.871382184	0.860453174	0.832973095	0.993728929

GNRH2	0.757642801	0.250338246	0.978822986	0.440997843	0.678038129	0.833208582	0.99373445
ZNF565	0.930488435	0.819619614	0.870763538	0.471716556	0.177278823	0.833273731	0.993739931
ABCA13	0.524119528	0.656191611	0.59163667	0.810193996	0.340253597	0.834887695	0.993782862
ARHGEF10	0.814266402	0.389307653	0.390403788	0.851151167	0.531673493	0.834634609	0.993782862
ARMCX5	0.917632629	0.510545929	0.201181951	0.611089104	0.976565327	0.835328752	0.993782862
ATP1B2	0.553360149	0.997821817	0.400524662	0.657133501	0.386319116	0.835029286	0.993782862
BICD2	0.919753948	0.773755379	0.143435294	0.672976388	0.812282192	0.834045633	0.993782862
DIAPH2	0.883635916	0.75541754	0.320957925	0.389833699	0.673115883	0.835247006	0.993782862
EHD4	0.690747388	0.291537769	0.995462796	0.31610173	0.879607563	0.83386615	0.993782862
ETS2	0.945967279	0.372604761	0.451794068	0.355709584	0.993594603	0.835429185	0.993782862
IGFBP7	0.70500865	0.719112074	0.206726192	0.586078011	0.906137245	0.833636109	0.993782862
INTS9	0.499006304	0.83113607	0.991366694	0.246636215	0.553065509	0.834864739	0.993782862
LOC100335936	0.557089316	0.816079394	0.137275456	0.941334329	0.951100864	0.834261896	0.993782862
LOC100848507	0.597471449	0.600829287	0.419994037	0.901245658	0.413358783	0.835100335	0.993782862
LOC101902542	0.332398969	0.902050211	0.869012042	0.812902553	0.263210041	0.833904111	0.993782862
LOC101904270	0.85079469	0.259247458	0.910674657	0.514051841	0.543031417	0.834823408	0.993782862
LOC101905046	0.801010679	0.374834907	0.258032254	0.838242906	0.862778882	0.834707488	0.993782862
LOC107132793	0.903463316	0.868752482	0.300864723	0.86736832	0.274767941	0.835420907	0.993782862
LOC112446879	0.599209162	0.179694475	0.835417162	0.852066428	0.729985643	0.834478507	0.993782862
LOC112448760	0.881819012	0.333014206	0.314535877	0.966996244	0.628937552	0.835123669	0.993782862
LOC783261	0.921140118	0.953926071	0.149902589	0.464278274	0.909676333	0.833553897	0.993782862
LOC783657	0.406944138	0.39821408	0.989109445	0.367309804	0.94603321	0.833747139	0.993782862
LRP5	0.457409245	0.955289962	0.335350683	0.637186011	0.600020747	0.834688994	0.993782862
MED7	0.966461978	0.501472167	0.786189014	0.15716072	0.937376412	0.835002114	0.993782862
MFAP3	0.761308013	0.670870799	0.707610431	0.434624614	0.35758752	0.835103468	0.993782862
POFUT1	0.504128824	0.584130104	0.539403218	0.464649004	0.754894554	0.833799646	0.993782862
PRKD1	0.48258886	0.392723443	0.705976793	0.743448369	0.563352078	0.834731045	0.993782862
RAB40C	0.748692432	0.402484134	0.848131026	0.551505619	0.398110393	0.834947276	0.993782862
RBM15	0.74022608	0.808624161	0.676124778	0.691057677	0.200572226	0.834893326	0.993782862
SLC39A13	0.953803127	0.483605416	0.792997172	0.406183531	0.3772615	0.834768518	0.993782862
ST3GAL3	0.550703728	0.700602107	0.234036675	0.750685141	0.822786297	0.833963966	0.993782862
SULT1C4	0.913123231	0.738878135	0.690420745	0.229151366	0.52677275	0.835278127	0.993782862
TMEM116	0.87824961	0.939378302	0.443988511	0.192213524	0.797568346	0.835063417	0.993782862
TUBGCP3	0.763064029	0.26718449	0.581915491	0.557609092	0.844627145	0.834264276	0.993782862
TYMS	0.578351155	0.292509766	0.521590348	0.72476458	0.879181816	0.835269015	0.993782862
UFD1	0.677881424	0.216103409	0.723296532	0.556967998	0.949887282	0.834786207	0.993782862
ZNF713	0.614031649	0.909518826	0.417106456	0.280200085	0.858240215	0.834672174	0.993782862
ABHD4	0.765066722	0.9432377	0.426377667	0.754816983	0.242443639	0.835502295	0.993797794
LOC101909140	0.569200836	0.809347901	0.229037113	0.719874513	0.741747912	0.83559569	0.993798181
ORC2	0.267026286	0.406030598	0.534708028	0.990832022	0.98099332	0.835623734	0.993798181

C14H8orf33	0.448127163	0.79745806	0.649992782	0.479953973	0.50586647	0.835756001	0.993811444
SMC5	0.67882669	0.561897686	0.570633422	0.262927957	0.985159305	0.835705496	0.993811444
ASB7	0.554398051	0.266534268	0.485465208	0.822503213	0.960231854	0.836492596	0.99383711
HDDC3	0.85116517	0.938830603	0.45324174	0.271125232	0.575358007	0.836046573	0.99383711
LOC101903165	0.460099926	0.330327573	0.899989745	0.416773403	0.992586273	0.836291803	0.99383711
LOC112444770	0.328295823	0.98153749	0.8710723	0.58361385	0.346110115	0.836610629	0.99383711
MGARP	0.883906334	0.373890026	0.98731757	0.304321092	0.570634066	0.836511117	0.99383711
NHLRC2	0.284791518	0.371074198	0.878489053	0.639803518	0.953307243	0.836402916	0.99383711
P2RX7	0.951511275	0.478015874	0.179787516	0.902164505	0.768210476	0.836542499	0.99383711
PATZ1	0.705382349	0.656881327	0.765794619	0.786680694	0.202610331	0.836210935	0.99383711
PTTG1	0.613022286	0.571512602	0.249261785	0.810076359	0.801086869	0.836535705	0.99383711
RAB1B	0.568112284	0.585660906	0.452190105	0.604976889	0.622384083	0.836474934	0.99383711
RAMP2	0.91143148	0.30136444	0.817853205	0.687000137	0.3674161	0.836625414	0.99383711
RPS10	0.875879765	0.697990344	0.545953567	0.651589323	0.260703589	0.836612198	0.99383711
SIGLEC5	0.537291525	0.576265356	0.645832327	0.796644329	0.354899289	0.836151078	0.99383711
WDR24	0.556913614	0.391166896	0.98888689	0.704159147	0.372011201	0.83585594	0.99383711
ACVR1	0.503586269	0.645196298	0.77168825	0.613195713	0.369220678	0.836804871	0.993840567
AP2M1	0.562455877	0.372166018	0.621444687	0.472245796	0.929122711	0.837680445	0.993840567
C1QA	0.541911665	0.631030833	0.81997143	0.492056608	0.413515077	0.837612484	0.993840567
CDR2L	0.999224001	0.677595003	0.603835913	0.154681036	0.898677288	0.836989302	0.993840567
CKAP5	0.718975661	0.927113865	0.697282044	0.314909776	0.388251884	0.836975523	0.993840567
CRTC1	0.684743709	0.70980999	0.226356175	0.927051127	0.558992168	0.83749749	0.993840567
FAM208B	0.886855717	0.566946863	0.791551319	0.198559126	0.720496709	0.83728544	0.993840567
FCGR1A	0.416213484	0.388835216	0.68418044	0.876818117	0.586830836	0.83738807	0.993840567
GPR62	0.973338252	0.880957749	0.270524259	0.904357196	0.272798366	0.838099129	0.993840567
IRF5	0.307976133	0.767098098	0.896961234	0.481797869	0.561342576	0.838329858	0.993840567
LAMB2	0.908132466	0.58325372	0.321408693	0.649086461	0.518553328	0.838302594	0.993840567
LIMD1	0.940006708	0.328923398	0.881253954	0.716545886	0.292088206	0.83753907	0.993840567
LOC100847567	0.861650436	0.363294713	0.496164071	0.488886113	0.751239797	0.837582136	0.993840567
LOC101902490	0.719163932	0.684618161	0.548435928	0.960660749	0.219747886	0.837469887	0.993840567
LOC101905821	0.545146297	0.415710606	0.46872821	0.983626908	0.54579563	0.837539397	0.993840567
LOC107132537	0.404869733	0.758798923	0.914012953	0.912165835	0.222662326	0.837550146	0.993840567
LOC112441543	0.439333463	0.87573435	0.800423176	0.198786117	0.933867897	0.83793398	0.993840567
LOC112442708	0.723581856	0.899276113	0.293115445	0.57368446	0.521156103	0.837530053	0.993840567
LOC789018	0.61859235	0.45287954	0.42086137	0.700495447	0.693079828	0.838138854	0.993840567
PHKA2	0.681743889	0.724724323	0.911922444	0.405106402	0.313595899	0.838130246	0.993840567
PPARG	0.855214333	0.274520497	0.977988539	0.254219862	0.976349502	0.837433549	0.993840567
PROKR2	0.778158225	0.416135004	0.651793747	0.969099302	0.280286831	0.838384548	0.993840567
PSME1	0.251301272	0.831498478	0.636695808	0.89386789	0.478724047	0.837267186	0.993840567
RHBDL1	0.551107591	0.472932447	0.414814516	0.8760548	0.603414375	0.837889302	0.993840567

TMEM159	0.747405205	0.945779403	0.79481077	0.103096263	0.9883759	0.838160875	0.993840567
TMEM9	0.98217626	0.375992066	0.285358078	0.626913835	0.863565388	0.83760407	0.993840567
YIPF1	0.294314422	0.256569839	0.800715669	0.983299224	0.963267388	0.838217212	0.993840567
ZCCHC24	0.37466316	0.87454723	0.351320723	0.524248787	0.947570032	0.837977739	0.993840567
ZYX	0.763092931	0.311183343	0.483325669	0.684412857	0.728853591	0.838167239	0.993840567
LOC100196898	0.608977774	0.32937589	0.673285889	0.651073573	0.652399199	0.838477474	0.993878933
B4GALT4	0.568121473	0.795013724	0.29470272	0.665930719	0.64799737	0.838685357	0.994053545
ABCB7	0.394168255	0.652946909	0.649195833	0.723888765	0.479454837	0.840207133	0.994100122
ACMSD	0.825621703	0.339506459	0.229907356	0.971052218	0.946170387	0.84349934	0.994100122
ACP6	0.510377255	0.624341201	0.925471787	0.41869762	0.480964906	0.843969209	0.994100122
ACRBP	0.45365685	0.636359205	0.495828841	0.814040718	0.503636099	0.842093492	0.994100122
ANGEL2	0.338824332	0.832967978	0.236301957	0.908614326	0.979981113	0.843960925	0.994100122
APELA	0.435222045	0.200451454	0.858235949	0.916012556	0.842540277	0.839644958	0.994100122
ARF3	0.649324442	0.961238202	0.564552445	0.780405881	0.216145545	0.844104475	0.994100122
ARHGEF28	0.259627964	0.835107648	0.495222415	0.630722602	0.854927895	0.839953694	0.994100122
ARL4D	0.432865925	0.820265436	0.524257882	0.670614451	0.473972604	0.84338508	0.994100122
B3GNT5	0.701117136	0.547404249	0.339200857	0.836531787	0.542405442	0.843124976	0.994100122
C25H16orf71	0.575417711	0.676110102	0.702664865	0.341563681	0.616039848	0.838915549	0.994100122
CAMK2A	0.739414229	0.807320311	0.212973553	0.804235141	0.5767141	0.842849276	0.994100122
CASP8AP2	0.975760048	0.715629749	0.677460332	0.339504576	0.368646595	0.843491073	0.994100122
CCDC126	0.980903518	0.413075663	0.539796041	0.551571093	0.491075814	0.843586983	0.994100122
CCNK	0.62471791	0.209203096	0.835881514	0.920592109	0.587538746	0.843175204	0.994100122
CCNYL1	0.996018928	0.65911336	0.789147392	0.161419992	0.695479958	0.840672473	0.994100122
CDH23	0.703075576	0.663946982	0.943145958	0.774705292	0.168777827	0.839039144	0.994100122
CENPH	0.330464811	0.541911906	0.856918896	0.535344759	0.702581285	0.839463556	0.994100122
CTSW	0.676717276	0.92438514	0.444311084	0.493469944	0.428669045	0.842386624	0.994100122
DLGAP4	0.508086162	0.94973595	0.38535933	0.483262163	0.647679941	0.840789942	0.994100122
DZIP3	0.896491412	0.832226721	0.216617895	0.998246578	0.358124106	0.839621006	0.994100122
ECE2	0.693037151	0.363911407	0.479302059	0.587640575	0.818892114	0.840698838	0.994100122
EIF4ENIF1	0.321250963	0.897511307	0.733256913	0.327649546	0.847145388	0.842087481	0.994100122
FHL3	0.5562966	0.72993387	0.520328934	0.403273546	0.677042901	0.839375617	0.994100122
FOXD2	0.339983839	0.634503949	0.924597698	0.303425109	0.957740468	0.840130309	0.994100122
FRAT2	0.4271329	0.26193816	0.779297402	0.956646912	0.711041456	0.843759522	0.994100122
FUZ	0.941920213	0.222482156	0.982218591	0.317750245	0.885421921	0.839987186	0.994100122
GALK1	0.976943205	0.662031929	0.742391112	0.183974264	0.660779077	0.841244381	0.994100122
GALNT7	0.973457229	0.473309545	0.340243154	0.49069261	0.752047519	0.83982346	0.994100122
GINS2	0.808787401	0.574452536	0.330848857	0.999980464	0.384465924	0.843199519	0.994100122
GLB1L3	0.59622803	0.268996677	0.614021749	0.995135664	0.606554669	0.844115849	0.994100122
HEXB	0.78208214	0.51873839	0.851977535	0.262348763	0.650187044	0.842828663	0.994100122
HSD17B1	0.949488694	0.535192651	0.87733402	0.54366827	0.240998967	0.841360989	0.994100122

IL12RB2	0.337488793	0.495842123	0.921778118	0.469460704	0.820053069	0.843961965	0.994100122
ITGAE	0.762685078	0.269177091	0.586844208	0.533664773	0.903892726	0.840549638	0.994100122
KRT80	0.729155616	0.958341617	0.808586417	0.161968463	0.636866644	0.841007778	0.994100122
LOC100336381	0.707972825	0.907770682	0.435615134	0.754257985	0.274364005	0.840056398	0.994100122
LOC100848407	0.886480765	0.707629385	0.122919494	0.870205867	0.874223312	0.842026424	0.994100122
LOC100848492	0.848975935	0.72254955	0.370487538	0.472538913	0.550050363	0.843130233	0.994100122
LOC101902043	0.490122053	0.647846491	0.285097111	0.997255292	0.657286646	0.843838776	0.994100122
LOC101902808	0.767915459	0.891321826	0.803769755	0.239483251	0.449844719	0.843652873	0.994100122
LOC101905595	0.367357284	0.189749588	0.945992006	0.922458851	0.949722085	0.83960159	0.994100122
LOC107132515	0.710027869	0.517568362	0.515335205	0.388865428	0.79325241	0.841371779	0.994100122
LOC112441629	0.96876151	0.856406857	0.26684876	0.555808092	0.477593808	0.842319534	0.994100122
LOC112444328	0.665797324	0.453747935	0.365026356	0.869552293	0.61237127	0.84219062	0.994100122
LOC112445982	0.84029771	0.71075051	0.537263546	0.377258498	0.488569353	0.843321249	0.994100122
LOC112446388	0.619423658	0.59540077	0.656627547	0.964934191	0.249814305	0.841257843	0.994100122
LOC112449619	0.513707405	0.566580501	0.454666727	0.455277026	0.980580947	0.843149528	0.994100122
LOC784054	0.334805786	0.746811107	0.697259988	0.483823844	0.697792708	0.842561883	0.994100122
LOC786553	0.518952848	0.514685636	0.290035449	0.762693404	0.995487203	0.842450817	0.994100122
LOC788405	0.784805352	0.663363703	0.597654122	0.195302992	0.958784186	0.840952058	0.994100122
LRRC28	0.734426995	0.217758373	0.597300883	0.941004535	0.639427842	0.838794628	0.994100122
LRRC58	0.737897289	0.832809614	0.398032781	0.265069469	0.916419915	0.844050458	0.994100122
METTL15	0.590769801	0.70344472	0.506674579	0.502792215	0.550473386	0.840991815	0.994100122
METTL27	0.531144724	0.333863293	0.436700058	0.923977848	0.811761277	0.84046305	0.994100122
NGDN	0.652712472	0.250384439	0.996434818	0.712552059	0.496244134	0.839084591	0.994100122
NOL3	0.818244484	0.787746669	0.329517384	0.398078097	0.688070557	0.84071685	0.994100122
NUP214	0.620266754	0.619536868	0.712353026	0.247303292	0.872573906	0.843128926	0.994100122
OGFOD3	0.363920339	0.670224053	0.995821201	0.295151571	0.813747782	0.841152533	0.994100122
OGFR	0.968122056	0.433914386	0.930448688	0.283612112	0.525976136	0.841070347	0.994100122
OXA1L	0.844492604	0.634053229	0.755107478	0.389122658	0.376944821	0.843753369	0.994100122
PKIA	0.350628718	0.903887094	0.655330483	0.850311126	0.332837288	0.842350861	0.994100122
PPA2	0.770255074	0.52008326	0.332645161	0.765053691	0.572616961	0.841262767	0.994100122
PRPF38A	0.460122689	0.309586332	0.908499473	0.503535414	0.886414852	0.839582045	0.994100122
RAB15	0.782779454	0.701307069	0.503958877	0.55532817	0.381050776	0.841711371	0.994100122
RALGPS1	0.390812263	0.381576834	0.454677513	0.973813461	0.884797351	0.841382567	0.994100122
RFESD	0.546275146	0.873545906	0.408957081	0.650359734	0.467728119	0.84390823	0.994100122
RHOC	0.68450059	0.800470692	0.759693339	0.537365923	0.2616084	0.841639986	0.994100122
RPIA	0.999126191	0.37803717	0.819906848	0.237992935	0.782370546	0.839306738	0.994100122
SEPT2	0.626838884	0.793887221	0.25407286	0.985152397	0.472801509	0.842650262	0.994100122
SLC35B4	0.106416708	0.857072645	0.918418179	0.78870123	0.872070328	0.839173286	0.994100122
SMPD1	0.256044833	0.50319157	0.782729658	0.837416146	0.689316766	0.840816227	0.994100122
SPATA22	0.70298971	0.279912188	0.636736076	0.489462375	0.946291114	0.840323988	0.994100122

SPATA2L	0.629160436	0.786053658	0.278754599	0.623165178	0.691174722	0.843946132	0.994100122
ST5	0.504793811	0.673490786	0.354271135	0.69761934	0.685720443	0.839178824	0.994100122
STAG1	0.145410433	0.945561345	0.979611551	0.470938904	0.921111346	0.841398112	0.994100122
TAF2	0.801308715	0.987119337	0.185806246	0.459043794	0.877654307	0.843504237	0.994100122
TBCCD1	0.89785041	0.819557846	0.669451445	0.768721751	0.154390335	0.841498774	0.994100122
TMEM121	0.46842263	0.597679786	0.528901227	0.638128798	0.616546426	0.840938112	0.994100122
TMEM255A	0.486483907	0.812779452	0.751783542	0.709439328	0.277302918	0.84153951	0.994100122
TNFRSF1B	0.855882571	0.327403321	0.819015521	0.538943659	0.478218659	0.843341673	0.994100122
TNFSF14	0.681363511	0.435024677	0.524872425	0.70180012	0.540298411	0.842918538	0.994100122
TUT1	0.853947324	0.494515077	0.462390283	0.958637602	0.308740489	0.839662712	0.994100122
UVSSA	0.961731782	0.354916384	0.9148506	0.981292643	0.193757412	0.843931788	0.994100122
VAMP4	0.580728197	0.414319148	0.362888003	0.967314602	0.691260693	0.841279313	0.994100122
WDFY3	0.746890873	0.597937799	0.86549017	0.544876465	0.27980582	0.842750132	0.994100122
WDPCP	0.931613284	0.423420786	0.311531754	0.824146232	0.578432817	0.841817776	0.994100122
WNT8B	0.350210303	0.403340339	0.4904305	0.89774704	0.945291419	0.842374822	0.994100122
ZNF382	0.382998425	0.459045714	0.89877615	0.570405761	0.639617326	0.839274721	0.994100122
RDH5	0.671563526	0.202412084	0.667166941	0.749913943	0.875780113	0.844430449	0.994399261
CEACAM1	0.502654673	0.7155144	0.647365793	0.606587943	0.423219708	0.844984605	0.994463429
GALNT4	0.33080683	0.313419648	0.763689658	0.826562043	0.914221918	0.845146103	0.994463429
HSD17B4	0.575443287	0.368376681	0.659484164	0.508271426	0.840770857	0.844903271	0.994463429
LOC101904749	0.338424722	0.557063678	0.671885392	0.892591466	0.528298263	0.844874965	0.994463429
LOC104973145	0.815551258	0.599270252	0.294806871	0.48996973	0.844371216	0.844556934	0.994463429
LOC107131273	0.27155751	0.558650308	0.67990695	0.676172485	0.85755283	0.845082923	0.994463429
PLK2	0.638719467	0.625710515	0.977019296	0.702127769	0.217986117	0.844960844	0.994463429
SLC30A1	0.594159139	0.808524499	0.382983895	0.368308552	0.883021708	0.845151511	0.994463429
SRMS	0.708385898	0.238774934	0.784630248	0.715599895	0.62980563	0.845094036	0.994463429
TM4SF1	0.454136346	0.48675365	0.756985809	0.430388721	0.828647347	0.844737563	0.994463429
ZKSCAN1	0.422382405	0.556427413	0.938059585	0.272247964	0.994529853	0.844778545	0.994463429
LOC112442633	0.985542614	0.728319221	0.933720378	0.471999954	0.189277762	0.845258338	0.994517822
GPKOW	0.576211105	0.312859386	0.436095313	0.831558439	0.917072594	0.845457955	0.994592882
GRIK5	0.186655923	0.886873386	0.463396663	0.928819789	0.841118397	0.845398845	0.994592882
ZHX2	0.710312221	0.50999196	0.393542956	0.839830469	0.500887082	0.845503949	0.994592882
LOC101904753	0.599486593	0.94966784	0.398484264	0.412020145	0.642041699	0.845614982	0.994652198
TMPPE	0.831419792	0.646951419	0.994517448	0.144721724	0.775524122	0.845683279	0.994661241
OCRL	0.687670292	0.884323683	0.584119992	0.342154022	0.494263389	0.845769877	0.994691805
B4GAT1	0.592777239	0.524594191	0.809100135	0.314282752	0.760379427	0.845912053	0.994699244
C11H2orf49	0.80181236	0.964654659	0.407692783	0.770558904	0.247805193	0.846137445	0.994699244
C14H8orf76	0.601340513	0.364178491	0.403804493	0.915389221	0.746420692	0.84667775	0.994699244
CNN3	0.837660252	0.685123171	0.335901158	0.447237278	0.697178978	0.845862384	0.994699244
ITGAX	0.678231344	0.388263399	0.420387548	0.805488513	0.675989316	0.846302862	0.994699244

LOC101903795	0.567203191	0.828174844	0.71087927	0.243495654	0.742042528	0.84645517	0.994699244
LOC112443783	0.242700722	0.776570459	0.918677888	0.446595128	0.778429984	0.846085172	0.994699244
LOC112446407	0.425704885	0.430070733	0.894731579	0.409956541	0.90135913	0.846958693	0.994699244
LOC112447461	0.684701008	0.224585561	0.9145799	0.451581951	0.953030333	0.846948194	0.994699244
LOC112447495	0.976178065	0.649246908	0.324861578	0.524568042	0.559729661	0.846757593	0.994699244
LOC112448488	0.80487101	0.537463078	0.307545104	0.97896301	0.464832119	0.846983549	0.994699244
LOC784980	0.891606331	0.892752592	0.291049822	0.793476674	0.328713068	0.846687591	0.994699244
LOC787875	0.746267499	0.600239743	0.960640356	0.317177859	0.443224707	0.846861651	0.994699244
NUP85	0.663296636	0.291088067	0.550322879	0.912031576	0.623043609	0.846563643	0.994699244
RAB30	0.919645191	0.972018711	0.568316399	0.257002268	0.462464785	0.846571792	0.994699244
ROBO4	0.431851739	0.846071505	0.688059706	0.439949521	0.544533635	0.84617401	0.994699244
TCIRG1	0.520605313	0.56226827	0.746158271	0.516937064	0.536214994	0.846988437	0.994699244
TLE6	0.343475139	0.623274575	0.492126622	0.572398963	0.998082133	0.846073633	0.994699244
TPP2	0.836317757	0.842379312	0.315888293	0.451925071	0.601680113	0.846911141	0.994699244
ZNF22	0.756385958	0.302372953	0.736851504	0.708023605	0.506230116	0.846629822	0.994699244
FBXO47	0.74162441	0.806531618	0.527664725	0.845459478	0.22719785	0.847204204	0.994728594
LOC101904855	0.790576341	0.259688611	0.828140917	0.436024894	0.818153179	0.84727112	0.994728594
PLPP4	0.820672005	0.66311135	0.218490753	0.531111742	0.959358338	0.84709482	0.994728594
POLD2	0.942176666	0.673072757	0.533027715	0.281497082	0.636971343	0.847160198	0.994728594
TSGA10IP	0.971502735	0.348901555	0.539400312	0.975616764	0.340122914	0.847316496	0.994728594
RPS15	0.966135434	0.906666676	0.336446823	0.230639164	0.893263473	0.847439043	0.994801297
WDR37	0.635198306	0.945085178	0.281194699	0.864389913	0.416570036	0.847607802	0.994928234
LOC104976573	0.756803985	0.190454669	0.749230694	0.799460212	0.70485536	0.847787925	0.995019659
LOC112446462	0.720191551	0.159504968	0.950989131	0.840875462	0.662796513	0.847867583	0.995019659
UBTF	0.869064652	0.476507079	0.550771067	0.834068136	0.319971165	0.847830038	0.995019659
ADSSL1	0.498215251	0.713907747	0.83559175	0.39834004	0.515907411	0.848358025	0.995138997
BIN1	0.59400174	0.432705036	0.723519209	0.412104482	0.797464126	0.848454381	0.995138997
CDCA5	0.87825726	0.384041505	0.312226818	0.711453428	0.814098533	0.848148426	0.995138997
FAM83F	0.33673615	0.949402879	0.334647674	0.812362998	0.702230853	0.848243167	0.995138997
LOC100848369	0.985064512	0.532817857	0.165389544	0.919093198	0.765328584	0.84831507	0.995138997
SCFD2	0.855216849	0.546835289	0.878658839	0.415405537	0.357904795	0.848398782	0.995138997
UBA52	0.677351355	0.595711716	0.413507838	0.71208808	0.513775952	0.84827297	0.995138997
WDR45	0.469217016	0.821297345	0.237360554	0.764373293	0.871952019	0.848072224	0.995138997
EPCAM	0.154034102	0.796026753	0.674719397	0.981437614	0.754159287	0.848756727	0.995318903
EXOC2	0.768223432	0.736087614	0.383642459	0.291657831	0.967984558	0.848789717	0.995318903
OTUD4	0.706908855	0.629501667	0.508945719	0.31175529	0.866911418	0.848694799	0.995318903
LDAH	0.681594622	0.520003741	0.414787896	0.740862463	0.562596524	0.848863903	0.995334775
C2CD3	0.368029636	0.330946976	0.982059412	0.515758702	0.994669374	0.849082204	0.995348279
DZIP1	0.159529147	0.789025933	0.908573488	0.997930027	0.538421828	0.849299979	0.995348279
GABPA	0.799761388	0.655070173	0.651831503	0.90548213	0.198664401	0.849253357	0.995348279

LOC112441810	0.964788019	0.689101235	0.692471624	0.150661938	0.884609067	0.849070618	0.995348279
LOC112449059	0.916775197	0.655027607	0.56246084	0.779669333	0.232923585	0.849023041	0.995348279
MCOLN1	0.39592353	0.702661051	0.437234561	0.795415786	0.6346486	0.849187539	0.995348279
NASP	0.805198981	0.917080235	0.412673107	0.441716953	0.456100928	0.849159874	0.995348279
ALKBH4	0.467545926	0.785286324	0.539220986	0.32269821	0.963186918	0.849518425	0.995421857
LOC101904057	0.895566492	0.20095662	0.736538588	0.579431114	0.801399397	0.849561325	0.995421857
LOC789867	0.881074166	0.901221119	0.563410193	0.329472263	0.417647287	0.849579364	0.995421857
MANEA	0.303069604	0.897421134	0.483070576	0.708279334	0.66163149	0.849605384	0.995421857
ANTXR1	0.110292127	0.937905608	0.777513628	0.79175939	0.97867175	0.851480816	0.995475423
ANXA4	0.857279974	0.48128214	0.59643903	0.929901249	0.270145639	0.850230198	0.995475423
AP5S1	0.763639489	0.679687687	0.825600306	0.163024196	0.897238665	0.852361622	0.995475423
ARSE	0.26101666	0.961727159	0.62881815	0.617544178	0.635377546	0.850522684	0.995475423
C1H3orf38	0.134512631	0.947149697	0.756479532	0.767896768	0.844514741	0.851922967	0.995475423
COX16	0.656676328	0.260600004	0.955286516	0.635179779	0.593798892	0.849828411	0.995475423
CTCF	0.396769005	0.943757528	0.528789262	0.374659742	0.845347662	0.852441418	0.995475423
DENND4C	0.649918756	0.389771864	0.323999514	0.77924897	0.96908768	0.850632296	0.995475423
DPP8	0.518411374	0.882832192	0.202903109	0.794336849	0.84903677	0.852235939	0.995475423
DUSP22	0.571485026	0.630318601	0.367592379	0.769662731	0.609160955	0.850884991	0.995475423
EXOSC9	0.980303531	0.106240935	0.929934919	0.711803335	0.905676811	0.851763238	0.995475423
FAM110C	0.675765625	0.320190864	0.643761542	0.952496751	0.470491908	0.851730426	0.995475423
FAM205C	0.398487827	0.905518066	0.755650216	0.943295141	0.241666569	0.851074426	0.995475423
GANC	0.998879421	0.752327678	0.372217297	0.2794705	0.798948212	0.851810378	0.995475423
GAR1	0.842441122	0.468018732	0.679251545	0.795665086	0.289992401	0.850168622	0.995475423
GATB	0.494448297	0.415592576	0.630707933	0.545785254	0.879278103	0.851169555	0.995475423
GNA11	0.83445348	0.259650601	0.326158252	0.980920159	0.890309271	0.84997058	0.995475423
GTF3C2	0.865047423	0.484133617	0.447493447	0.43914724	0.760838095	0.8522082	0.995475423
HS3ST3B1	0.879307782	0.837239266	0.119301912	0.946194636	0.754383914	0.852391043	0.995475423
HSPA12B	0.793228035	0.356126502	0.965365387	0.258643367	0.888435482	0.852323979	0.995475423
LOC101902926	0.719224798	0.31482949	0.720465396	0.640659495	0.595687726	0.85132367	0.995475423
LOC615768	0.581381026	0.548971513	0.499581239	0.918691171	0.426241323	0.851764515	0.995475423
LTBP3	0.606409899	0.484198362	0.427095485	0.494673275	0.997980508	0.850455088	0.995475423
MARK3	0.642490475	0.919279791	0.783712984	0.156897801	0.849186321	0.849862565	0.995475423
NTSR2	0.166376403	0.612945871	0.935085224	0.928585591	0.70076373	0.850811989	0.995475423
OPTN	0.757639331	0.557505454	0.226704183	0.684166449	0.949896788	0.851256312	0.995475423
PAPOLG	0.340192573	0.468137523	0.89853086	0.499227682	0.869068419	0.850892068	0.995475423
PIM2	0.977919733	0.218188828	0.91624961	0.943657866	0.335365503	0.850358071	0.995475423
PKN1	0.362353677	0.572216369	0.369341618	0.868149994	0.938711295	0.85169578	0.995475423
PYROXD2	0.536950481	0.515896206	0.750402893	0.818327997	0.368627382	0.85242475	0.995475423
QRSL1	0.666130807	0.626209304	0.501218971	0.457235479	0.650586463	0.851164749	0.995475423
RNASEH2A	0.29520704	0.839316661	0.487921772	0.723660476	0.714633091	0.851969659	0.995475423

RREB1	0.326160179	0.975805425	0.911923533	0.595715137	0.360184018	0.851365039	0.995475423
SETD6	0.62540556	0.831830887	0.343986662	0.731898857	0.477121056	0.851897839	0.995475423
SFXN1	0.582148087	0.764538598	0.439846529	0.374335564	0.843028406	0.850128382	0.995475423
SLC26A6	0.634901712	0.227280015	0.927070278	0.943391794	0.494303814	0.851631403	0.995475423
SUV39H1	0.611880563	0.678188496	0.659167488	0.679852462	0.333189421	0.850584461	0.995475423
TEX10	0.69065048	0.612740184	0.996342333	0.224082367	0.660818134	0.851761332	0.995475423
TMEM184B	0.952520674	0.726626301	0.61302435	0.150632727	0.967142592	0.850212333	0.995475423
TMEM68	0.786455389	0.643194455	0.518273872	0.627066228	0.38136838	0.852399175	0.995475423
TPRA1	0.390014621	0.66182155	0.470083031	0.572080603	0.895829172	0.851138935	0.995475423
UBE2O	0.460743405	0.634811544	0.662955931	0.479283441	0.666713158	0.850584931	0.995475423
UPF3A	0.599132995	0.769570134	0.184493658	0.867232931	0.842811325	0.851117702	0.995475423
WDR26	0.249866277	0.563819413	0.850463395	0.578723898	0.904393877	0.852434039	0.995475423
WISP2	0.616670928	0.972494939	0.198175809	0.622473744	0.847359914	0.852380287	0.995475423
YEATS4	0.649373643	0.586605292	0.652107104	0.789003798	0.317665354	0.851327008	0.995475423
ACSM1	0.692317324	0.608822559	0.586642159	0.416800634	0.6121896	0.853370812	0.995873272
CAMK1	0.53914645	0.484956615	0.52893606	0.75252382	0.605350999	0.853143779	0.995873272
CCDC43	0.426989608	0.389171022	0.753346175	0.958049181	0.52507414	0.853080391	0.995873272
CCDC85B	0.618534398	0.948155157	0.731583372	0.19612795	0.749779489	0.853369617	0.995873272
EFNA1	0.314685749	0.543918449	0.624644425	0.593863953	0.991706956	0.853063877	0.995873272
ERAP2	0.781554817	0.660174609	0.527111924	0.29152765	0.795238823	0.853270198	0.995873272
GGH	0.723799307	0.726630379	0.308898484	0.404691396	0.960141915	0.853449619	0.995873272
LOC112443476	0.717152127	0.984513663	0.268094035	0.661984249	0.502037273	0.852918956	0.995873272
LOC521580	0.451199811	0.517670946	0.915276112	0.401288819	0.735673065	0.853417815	0.995873272
RAB13	0.805370765	0.889600387	0.12029253	0.966029806	0.755366324	0.852874371	0.995873272
RPL26	0.482666785	0.943733239	0.339425864	0.789235514	0.516735498	0.853276581	0.995873272
CRTC3	0.869632901	0.564143743	0.985909867	0.829649885	0.157438086	0.853576848	0.995950917
APOM	0.321985693	0.717735263	0.401516061	0.745473675	0.916264425	0.854066429	0.995966547
ARHGAP44	0.826754862	0.596898446	0.450740205	0.3812229	0.746860711	0.853948506	0.995966547
ATXN1	0.896902417	0.504878647	0.987139371	0.173656609	0.815353168	0.85385185	0.995966547
EDEM2	0.903371947	0.970856567	0.211595379	0.936231544	0.364785564	0.854063558	0.995966547
EHD2	0.205474156	0.863695935	0.55936552	0.691024018	0.924009607	0.854075755	0.995966547
FUOM	0.90960942	0.204039523	0.91040903	0.637505324	0.588266875	0.854033714	0.995966547
LOC789996	0.506259015	0.523533907	0.411111121	0.973709727	0.597133287	0.854003278	0.995966547
RTN3	0.69161642	0.409365374	0.378210745	0.815821665	0.723605813	0.85366145	0.995966547
LOC100296900	0.682256173	0.497669242	0.681851817	0.594232803	0.461891666	0.85445993	0.996217523
MITD1	0.842587453	0.236553897	0.692228241	0.752856497	0.611323262	0.854355277	0.996217523
SPAAR	0.764711645	0.552510222	0.195123373	0.803036654	0.95990831	0.854473089	0.996217523
KCNN3	0.980025916	0.729947661	0.282575045	0.691849205	0.455495875	0.854840976	0.996221791
LOC511937	0.242850114	0.972175425	0.692187012	0.602637088	0.646330732	0.854720825	0.996221791
MRNIP	0.790826002	0.420993313	0.753986124	0.890119824	0.284792913	0.854678677	0.996221791

SCIMP	0.457199227	0.561346249	0.33674213	0.828314097	0.889721968	0.854814327	0.996221791
TPP1	0.962252876	0.757700688	0.186049124	0.961113769	0.488401254	0.854773203	0.996221791
ZXDC	0.84589732	0.32302319	0.981425534	0.74092009	0.320191364	0.854640905	0.996221791
XPNPEP2	0.518447876	0.495877209	0.568394815	0.528406331	0.825525952	0.854935512	0.996261215
PAIP2B	0.872109307	0.414159106	0.236001769	0.816988592	0.916029312	0.855058653	0.996333965
LOC533093	0.968760636	0.511279485	0.240864529	0.869016229	0.615604555	0.855128395	0.996344487
FAM213A	0.907337914	0.341331153	0.25700315	0.905963102	0.887299537	0.855509678	0.996445331
GFRA3	0.833639171	0.19626474	0.985416795	0.938797327	0.422422002	0.855402553	0.996445331
GMPR2	0.420169823	0.56159176	0.820253399	0.832465633	0.397127105	0.855518537	0.996445331
WTAP	0.833405838	0.912896154	0.599740305	0.186897225	0.749812842	0.855415933	0.996445331
ZNF575	0.746815323	0.435954838	0.719022741	0.299030377	0.914051954	0.855516459	0.996445331
ANKEF1	0.973395452	0.736135976	0.394739886	0.62596097	0.361687388	0.855641066	0.996446603
USP30	0.525092738	0.581581497	0.732793132	0.762670842	0.375135127	0.855610877	0.996446603
LOC104976062	0.396807105	0.812265911	0.640763203	0.353628451	0.877271795	0.855717328	0.996464704
LOC112447824	0.441051082	0.794047284	0.433048716	0.578867818	0.731382979	0.856046443	0.996635796
PHF19	0.332951038	0.413663789	0.593335387	0.865176674	0.907532175	0.855940948	0.996635796
PLEKHO2	0.669163496	0.349891014	0.461969902	0.61313404	0.967880753	0.855997334	0.996635796
LOC789551	0.866993378	0.894870641	0.201238365	0.565275624	0.727971261	0.856139215	0.99666343
RAVER1	0.865618111	0.423632499	0.332899469	0.679560146	0.774737396	0.856191642	0.99666343
LOC784966	0.685160132	0.407847677	0.480539859	0.605145817	0.791346735	0.856273939	0.996688531
RYR3	0.827476602	0.458643273	0.212429028	0.942296427	0.847053821	0.856377759	0.996738681
ZNF789	0.443702922	0.765995052	0.777052936	0.447152908	0.545209966	0.856463619	0.996767921
DSP	0.788285751	0.681966838	0.296798361	0.797065041	0.506531646	0.856540555	0.996786771
SEMA7A	0.919427351	0.684691546	0.576197056	0.271966245	0.65352497	0.856663515	0.996859175
FAM241B	0.46565908	0.870617717	0.41950153	0.876578879	0.432787257	0.856780196	0.996924262
FOSL2	0.656938671	0.661992934	0.338906846	0.512262449	0.856059123	0.857045629	0.99712618
SUDS3	0.761216953	0.601161033	0.215890856	0.661382734	0.989357609	0.857075248	0.99712618
ACOT13	0.720701744	0.474132281	0.485449343	0.534860058	0.750644476	0.861557788	0.99718068
ACTR6	0.450283954	0.246105157	0.921210839	0.891390619	0.731227088	0.861424601	0.99718068
AFG1L	0.706382537	0.275195718	0.847750859	0.946846262	0.419916723	0.859111441	0.99718068
ALOX5	0.606226434	0.292002442	0.693912291	0.640094111	0.833978493	0.859226599	0.99718068
ANKRD10	0.333962863	0.500063009	0.820598854	0.840288274	0.574405235	0.860532974	0.99718068
ASB1	0.847477062	0.845981568	0.576377814	0.169629805	0.94644879	0.860979745	0.99718068
ASB14	0.773980042	0.835403433	0.612670171	0.403845773	0.414930933	0.861065486	0.99718068
ASH2L	0.533971497	0.502040565	0.911907286	0.27639166	0.975507102	0.860001656	0.99718068
BIN3	0.577953661	0.408320684	0.644555472	0.921180341	0.469117175	0.859592517	0.99718068
C21H15orf40	0.93139013	0.660226984	0.460706946	0.438039981	0.527945808	0.859097656	0.99718068
CCDC142	0.719740364	0.176564793	0.95674049	0.637130394	0.847720699	0.859445151	0.99718068
CCDC160	0.697889597	0.474617781	0.411993379	0.826869522	0.575476096	0.85775352	0.99718068
CCDC36	0.755930415	0.374928487	0.446505605	0.833891746	0.627790696	0.860768092	0.99718068

CDK17	0.348395406	0.557221418	0.841813567	0.441782564	0.901742593	0.858144058	0.99718068
CHML	0.637870445	0.395975205	0.895305538	0.66901284	0.430466388	0.858192109	0.99718068
CLN6	0.949964235	0.761371897	0.871738568	0.417922571	0.250746965	0.860367673	0.99718068
CNTNAP3	0.784418326	0.425934278	0.566911927	0.766010668	0.451258481	0.858998225	0.99718068
CSF3	0.43509341	0.896817174	0.767198845	0.285093403	0.779512874	0.86139646	0.99718068
DHRS7	0.811844094	0.61912441	0.196522845	0.710764453	0.927302906	0.858145321	0.99718068
FAM45A	0.86166889	0.950689616	0.424393179	0.613893666	0.31186998	0.861468574	0.99718068
FGFR1OP2	0.981356953	0.697289938	0.999916686	0.118446481	0.811714976	0.859713434	0.99718068
FKBP8	0.551348633	0.713002036	0.585118401	0.509988534	0.558843036	0.859187237	0.99718068
GDAP1	0.597652857	0.566816555	0.308011973	0.716180095	0.870869194	0.858083877	0.99718068
GEMIN2	0.892109083	0.303603483	0.841944188	0.483047047	0.600518471	0.860541023	0.99718068
GSTCD	0.294218843	0.492339358	0.714676375	0.963953603	0.649284957	0.857422745	0.99718068
HRH1	0.983281811	0.532373638	0.3882591	0.956854537	0.337476226	0.859358548	0.99718068
IL1RAPL2	0.921896621	0.725895341	0.583528511	0.771138378	0.217787417	0.859246983	0.99718068
KLHL32	0.361488302	0.546386592	0.383686184	0.980999813	0.893490592	0.86116379	0.99718068
LOC101906836	0.520944172	0.890899464	0.248787012	0.659998132	0.871366725	0.861116205	0.99718068
LOC101907886	0.442486887	0.598309578	0.748378352	0.33586009	0.989219352	0.859806414	0.99718068
LOC104972526	0.514979519	0.282020283	0.592210121	0.909436687	0.851040093	0.861487278	0.99718068
LOC112441655	0.678927072	0.711883819	0.571733625	0.954697378	0.245633664	0.857437966	0.99718068
LOC112442544	0.780761353	0.704540155	0.342862679	0.555454957	0.630415456	0.86029792	0.99718068
LOC112445944	0.745358797	0.715145982	0.586393005	0.245876542	0.85210092	0.859029754	0.99718068
LOC112448062	0.699316909	0.497483151	0.310640926	0.72979479	0.835160734	0.859904445	0.99718068
LOC112448453	0.347728493	0.971654127	0.3993888	0.954790903	0.510902569	0.859804904	0.99718068
LOC518768	0.915452971	0.61534057	0.266622734	0.500895872	0.875381559	0.859873899	0.99718068
LOC784808	0.434786494	0.351483516	0.662126804	0.701100956	0.913975637	0.857527807	0.99718068
LRRC7	0.790812215	0.351077873	0.623332261	0.418052762	0.908874516	0.85964472	0.99718068
MAD2L1BP	0.598919445	0.371986865	0.791605646	0.862615539	0.428743774	0.858426634	0.99718068
MGST2	0.801325971	0.902605749	0.124838268	0.814676051	0.887445833	0.858552368	0.99718068
MTSS1L	0.897864542	0.947507847	0.270145866	0.41661664	0.68874299	0.86007871	0.99718068
NBEAL1	0.699483075	0.993030823	0.589396462	0.290647655	0.543689949	0.857188969	0.99718068
NCSTN	0.594297359	0.799881136	0.835395147	0.425808818	0.386571384	0.858755089	0.99718068
NFKBIE	0.587577884	0.649033682	0.383011586	0.977638283	0.459202353	0.859227388	0.99718068
NXT1	0.852616743	0.492825989	0.458512431	0.521454146	0.648871253	0.858340858	0.99718068
ORMDL3	0.548383456	0.498733936	0.454054705	0.782578694	0.685058186	0.861503907	0.99718068
P2RX3	0.840229854	0.708863723	0.417212554	0.957455652	0.273209208	0.857909153	0.99718068
PEX1	0.992318908	0.751044019	0.632949935	0.508787656	0.276823143	0.861196741	0.99718068
PJA2	0.932291812	0.302976893	0.82171135	0.595507209	0.478901947	0.860641296	0.99718068
POGK	0.853915063	0.418023954	0.31773	0.795719297	0.732838881	0.860512933	0.99718068
PRKG2	0.508378562	0.52070817	0.534315531	0.515338728	0.911180724	0.861145655	0.99718068
PRPF3	0.895957501	0.182244056	0.88884437	0.547682921	0.82664963	0.859535889	0.99718068

PRSS33	0.578828096	0.674022837	0.320308578	0.726495661	0.733132285	0.861466464	0.99718068
RARS2	0.991875026	0.396415676	0.493700526	0.363326762	0.943740368	0.861470985	0.99718068
RDM1	0.831682203	0.141582277	0.902822065	0.8502472	0.722167131	0.858541786	0.99718068
RNASE13	0.41646751	0.572927291	0.717838358	0.609797647	0.619793249	0.857284645	0.99718068
RNF6	0.343989362	0.887411808	0.966629206	0.31846453	0.691699396	0.857900926	0.99718068
SCRN3	0.526589617	0.652823442	0.264160661	0.734949117	0.978771127	0.858654054	0.99718068
SDR39U1	0.360592245	0.753808918	0.531664323	0.468453557	0.961878182	0.858176925	0.99718068
SH3BGRL3	0.38685679	0.838386585	0.869737563	0.378471659	0.612719575	0.858863511	0.99718068
SPA17	0.89750175	0.417309194	0.785665885	0.913494177	0.246062133	0.860526489	0.99718068
SPATA13	0.964583452	0.823772456	0.471095485	0.482501973	0.359818469	0.857876546	0.99718068
SPPL2B	0.732170159	0.549231744	0.759206181	0.73627078	0.296210101	0.861520392	0.99718068
TBC1D10A	0.795232813	0.43626388	0.987504551	0.756508337	0.253577059	0.859566766	0.99718068
THOC2	0.665670647	0.749786162	0.984455771	0.140062684	0.958086573	0.860056051	0.99718068
TLE1	0.993969652	0.442737228	0.437984028	0.758790599	0.45226902	0.860531496	0.99718068
TMEM231	0.627571993	0.922858059	0.258478932	0.451960841	0.972666421	0.859768263	0.99718068
UBE4A	0.793726615	0.976831631	0.322767224	0.722052052	0.36236228	0.859007059	0.99718068
UTP3	0.448952474	0.724365022	0.601348456	0.952811822	0.348944107	0.857949119	0.99718068
VPS13D	0.479310998	0.733865478	0.456623724	0.504368141	0.812881697	0.8598648	0.99718068
WASHC4	0.588041942	0.868923602	0.406661027	0.751304526	0.416391432	0.857911942	0.99718068
ZNF330	0.749910371	0.75797793	0.524962717	0.410484907	0.540991891	0.8608028	0.99718068
ATF7IP2	0.752457482	0.372206155	0.858975345	0.611065813	0.45348918	0.861704931	0.997210325
CREBL2	0.88108677	0.355269879	0.576146444	0.971108075	0.380599948	0.86168605	0.997210325
LOC783142	0.234895597	0.936744344	0.336811768	0.938320103	0.959876648	0.861893561	0.99735813
PPP6R3	0.710985763	0.841507848	0.663970598	0.171459446	0.980406184	0.861957403	0.99735813
RHEX	0.8114805	0.603760791	0.699319095	0.365892699	0.532881651	0.862014972	0.99735813
CHD3	0.511920634	0.503614566	0.618700178	0.653096086	0.64182615	0.862143043	0.997365676
SRD5A3	0.852543175	0.228379487	0.601241667	0.621561703	0.918708989	0.86211182	0.997365676
LOC112446771	0.58778613	0.609009251	0.329895338	0.943712276	0.600271669	0.862222453	0.997387233
C1H21orf58	0.324441913	0.801875798	0.68675546	0.62064021	0.606143616	0.8629273	0.997482798
CENPL	0.541605958	0.309928447	0.836047734	0.914756388	0.522988484	0.862760012	0.997482798
CPQ	0.34049454	0.745313664	0.47891384	0.604263396	0.916571574	0.863145713	0.997482798
FGD1	0.757170645	0.472909163	0.538294921	0.364944318	0.956236581	0.863037649	0.997482798
INTU	0.951263335	0.189560591	0.935118938	0.509740845	0.783184812	0.863156006	0.997482798
KIN	0.683458109	0.424299471	0.860269161	0.327624057	0.819648109	0.862433625	0.997482798
LOC788142	0.435070336	0.721433596	0.977406424	0.532788881	0.411648126	0.863081688	0.997482798
PI4K2B	0.848409405	0.471442344	0.685493109	0.314510614	0.779548776	0.862945846	0.997482798
POU2F1	0.246157147	0.618855477	0.936437046	0.572340421	0.824392708	0.863135284	0.997482798
RARB	0.580165283	0.258950327	0.716829941	0.817726148	0.760396426	0.862368522	0.997482798
SF3B4	0.926415884	0.35216745	0.258630289	0.808997064	0.981743905	0.862487767	0.997482798
SNUPN	0.712130544	0.499352751	0.262662334	0.771424426	0.933874637	0.863093504	0.997482798

TMEM120B	0.697663558	0.621593864	0.61354169	0.255280511	0.990900897	0.863127025	0.997482798
TNKS1BP1	0.832841262	0.804881195	0.459183669	0.606359034	0.359358396	0.862610701	0.997482798
GPSM1	0.803308003	0.525142798	0.641088137	0.31859625	0.783795494	0.863632003	0.997892333
MGP	0.452866242	0.874622851	0.876201721	0.221042183	0.880300856	0.863625332	0.997892333
AKAP12	0.507203547	0.732953218	0.629044218	0.35828262	0.80684575	0.863780777	0.997923712
BBX	0.979961468	0.955942575	0.176411257	0.4571074	0.894782791	0.863762668	0.997923712
TOR1B	0.935258687	0.811930871	0.284452571	0.489925121	0.639258472	0.86388699	0.997976164
SOX17	0.577066678	0.932298598	0.988604004	0.360464013	0.353325048	0.864082601	0.998061624
TTC5	0.797415672	0.55150488	0.807425318	0.945882648	0.201653061	0.864061721	0.998061624
KATNBL1	0.789030537	0.85275463	0.58019169	0.291884301	0.59525232	0.864274211	0.99814244
LOC101907518	0.852133277	0.572715352	0.442283379	0.886788765	0.354328936	0.864264842	0.99814244
AS3MT	0.893092451	0.487839218	0.280864714	0.829936824	0.668299686	0.864371545	0.998184605
FAM110D	0.616749549	0.331281263	0.68392228	0.749781453	0.648268387	0.864479086	0.998238551
STN1	0.424977317	0.615915844	0.331091309	0.824950481	0.950579	0.864563961	0.998266317
MIA2	0.590732979	0.378789387	0.735986919	0.745563051	0.553804778	0.864648875	0.998294125
LOC104975977	0.903164414	0.420089101	0.247140171	0.936235045	0.776310281	0.864979741	0.998562404
ST3GAL2	0.469459014	0.523952497	0.861249511	0.432565079	0.743823527	0.865002933	0.998562404
DDX59	0.364634999	0.455260544	0.855064589	0.570827888	0.842416479	0.865210243	0.998639149
NAA16	0.46854776	0.637141708	0.268569239	0.988746621	0.861273019	0.865251969	0.998639149
STAU1	0.526526632	0.683881143	0.377007232	0.522088399	0.962882461	0.865182576	0.998639149
HAVCR2	0.474124281	0.426502922	0.923477737	0.609463698	0.600346203	0.865360158	0.998693781
KHSRP	0.817429945	0.780675134	0.534694208	0.308942972	0.649004781	0.865551245	0.998844067
ACAD11	0.517597107	0.467129568	0.510252756	0.862736677	0.648451337	0.866845287	0.998910316
FAM84B	0.273685602	0.498779408	0.935876639	0.637165442	0.845981758	0.866514339	0.998910316
GRM8	0.998526636	0.644179712	0.415787896	0.867314453	0.297548756	0.86684632	0.998910316
LOC100847719	0.346376385	0.641376801	0.618703965	0.616875012	0.814130592	0.866867428	0.998910316
LOC782385	0.330811493	0.811575375	0.672591819	0.511197267	0.743141529	0.865947922	0.998910316
LOC782673	0.695433817	0.219348804	0.841094639	0.775234865	0.691853581	0.866409741	0.998910316
MAN2B1	0.712621283	0.519798781	0.751771437	0.266095834	0.931539712	0.866861896	0.998910316
MRGBP	0.380714468	0.571385675	0.5255532	0.643685965	0.932063845	0.865928934	0.998910316
NECAP2	0.521345323	0.883967852	0.922843158	0.963615402	0.168497468	0.86691859	0.998910316
POLR2G	0.516247628	0.388779885	0.962892702	0.663321832	0.537004995	0.866463572	0.998910316
RAB3C	0.424716156	0.462950596	0.708421532	0.497294695	0.993815102	0.866464328	0.998910316
SLC4A10	0.779335423	0.334700371	0.895038916	0.532579666	0.553522306	0.86642966	0.998910316
SPAG16	0.850332846	0.527582409	0.625171372	0.312057431	0.787609972	0.866660512	0.998910316
TBC1D22A	0.505164795	0.731950282	0.886181756	0.87400114	0.239225131	0.865756245	0.998910316
TSPAN33	0.56774851	0.543236874	0.549445383	0.481093062	0.847177385	0.866947756	0.998910316
UBAP1L	0.451628199	0.95497295	0.789915039	0.596109925	0.338185579	0.866121858	0.998910316
UNC45B	0.585768755	0.607267062	0.279551038	0.730298816	0.949287821	0.866674545	0.998910316
ZDHHC12	0.334575357	0.807955776	0.305237619	0.869590774	0.960700908	0.866660053	0.998910316

ZNF25	0.568509117	0.828379776	0.335899778	0.73317949	0.592787514	0.866275088	0.998910316
ZNF536	0.682423496	0.664990497	0.271401492	0.7212601	0.774520969	0.86638376	0.998910316
ZNF7	0.349360567	0.259598209	0.979267645	0.881594379	0.882056294	0.866937233	0.998910316
ZNF793	0.85678017	0.356355923	0.844431533	0.467838868	0.569735279	0.866207729	0.998910316
SLC35D1	0.75919571	0.765425806	0.933938438	0.933192325	0.136437975	0.867018176	0.998921321
LOC101904187	0.952551168	0.226857008	0.483593971	0.790642878	0.837345476	0.867195561	0.998985578
PHKG2	0.492620228	0.730696464	0.745487125	0.302509204	0.852273223	0.867195695	0.998985578
ARHGEF16	0.354942765	0.725332236	0.830992364	0.553111857	0.585732588	0.867465113	0.999225799
CDRT1	0.990831421	0.874428446	0.855996513	0.156861624	0.596056641	0.867531119	0.999231695
GPBAR1	0.27924209	0.756898969	0.552270061	0.727160473	0.818837485	0.867867692	0.99954921
ANXA3	0.930064889	0.707520005	0.646147436	0.170638799	0.95923308	0.868065564	0.999594065
BLVRA	0.476418182	0.950308192	0.231956833	0.907004676	0.733145902	0.868561753	0.999594065
CRISPLD1	0.737161975	0.126694108	0.802377794	0.989740992	0.93941054	0.868230462	0.999594065
IKZF4	0.660679361	0.571686497	0.345945508	0.993036828	0.536160977	0.868008768	0.999594065
LOC107132672	0.244609036	0.707625863	0.44655693	0.993672656	0.908026522	0.868371704	0.999594065
LOC112441807	0.335636495	0.794724674	0.688353567	0.504211496	0.754203662	0.868541212	0.999594065
NDRG2	0.769486702	0.662328194	0.32745249	0.971394885	0.430316775	0.868409335	0.999594065
NEU1	0.286536366	0.993162469	0.39887798	0.828809459	0.741256321	0.868361043	0.999594065
ORAI1	0.792490132	0.479826706	0.559374507	0.407148423	0.806437103	0.868576648	0.999594065
PON2	0.65811218	0.757978668	0.1648856	0.961208367	0.882619226	0.868450989	0.999594065
ZNF814	0.282882412	0.988287037	0.546608495	0.665536632	0.68619365	0.86846899	0.999594065
EEF2	0.592164893	0.968987365	0.830151368	0.665385507	0.220629957	0.868762081	0.999667263
GADD45B	0.813132978	0.677176914	0.635611591	0.307220334	0.650326532	0.868755723	0.999667263
ARHGAP24	0.709534918	0.929793949	0.624233541	0.32109877	0.533874854	0.870146465	0.999987489
BAZ2A	0.926882733	0.673275065	0.900219251	0.253727652	0.494653751	0.869962156	0.999987489
CD99L2	0.206372098	0.813133566	0.779981097	0.705456824	0.765166118	0.870259278	0.999987489
CLK3	0.802547561	0.896892658	0.559750054	0.581888415	0.301480791	0.870319987	0.999987489
COX15	0.923275508	0.465176094	0.939318168	0.269191486	0.650384266	0.870215703	0.999987489
DOCK11	0.848656899	0.703052518	0.453540427	0.977387347	0.265527857	0.869385351	0.999987489
EFNA5	0.9099206	0.474160507	0.736360816	0.694347914	0.320010283	0.870138077	0.999987489
GSTA4	0.935688186	0.489945525	0.815194118	0.424101054	0.443664181	0.869570033	0.999987489
HRASLS	0.980926638	0.47750121	0.984396446	0.536828044	0.284609321	0.869838529	0.999987489
HYKK	0.996111284	0.852508487	0.155155899	0.77177399	0.692260556	0.869727777	0.999987489
KAT2A	0.709390634	0.343407458	0.951990938	0.619720014	0.489537178	0.869652281	0.999987489
LOC101905033	0.448027774	0.966722444	0.28517227	0.868511908	0.656090867	0.869700719	0.999987489
LOC112446427	0.641882454	0.891532569	0.793539242	0.191614917	0.80867265	0.869670372	0.999987489
LOC520104	0.601793913	0.915106207	0.28424313	0.964611953	0.465332403	0.869456586	0.999987489
LRP10	0.86752832	0.597025565	0.370710197	0.940867702	0.388332501	0.869227952	0.999987489
NOLC1	0.916451319	0.756598128	0.324699744	0.530468683	0.588002892	0.869379684	0.999987489
NR1I3	0.320459312	0.5051072	0.994925828	0.595228549	0.736645228	0.870181106	0.999987489

PDSS2	0.270563454	0.647732621	0.625674431	0.88020836	0.731880953	0.870231241	0.999987489
SWI5	0.851769933	0.888469017	0.776397543	0.338312718	0.355239735	0.870180876	0.999987489
TANK	0.682756853	0.398639499	0.435643197	0.720898073	0.821758698	0.869413107	0.999987489
VPS51	0.682677422	0.908234246	0.732817918	0.189254229	0.815290555	0.869135369	0.999987489
AAK1	0.490157831	0.773696483	0.739363664	0.397768969	0.830621215	0.906742281	0.999995916
AATK	0.689863155	0.764054482	0.944121638	0.836294674	0.980691529	0.997698385	0.999995916
ABCA10	0.835421459	0.884393035	0.252168007	0.568425342	0.733669363	0.883721528	0.999995916
ABCA9	0.559859613	0.771543549	0.780459417	0.80947105	0.794178992	0.980019809	0.999995916
ABHD16A	0.974795651	0.825682832	0.422923059	0.468853762	0.695362345	0.927674074	0.999995916
ABTB2	0.60580513	0.949855364	0.676212523	0.288892168	0.99291672	0.928290772	0.999995916
ACACB	0.704868541	0.778317885	0.826318976	0.411023081	0.50606457	0.908917473	0.999995916
ACAD8	0.487789046	0.526357389	0.931400958	0.788553726	0.885806675	0.96434594	0.999995916
ACSF2	0.672986821	0.846348548	0.417222949	0.807217743	0.930627512	0.968897148	0.999995916
ACSF3	0.796455607	0.742678499	0.920554665	0.252912518	0.873732028	0.936118764	0.999995916
ACSM2B	0.510771641	0.890877064	0.930625788	0.970054717	0.807986629	0.994505754	0.999995916
ACTR8	0.618291115	0.683499187	0.974728912	0.935946844	0.595996166	0.982772364	0.999995916
ADAL	0.862313403	0.976494842	0.697600585	0.181804228	0.940200046	0.916422397	0.999995916
ADAM10	0.787973094	0.570008532	0.88251937	0.570523414	0.644214367	0.953751983	0.999995916
ADAMTS1	0.894996588	0.370002341	0.799380709	0.903350837	0.355297123	0.895733513	0.999995916
ADAP2	0.916853715	0.57209951	0.913550804	0.665110794	0.652493047	0.977912364	0.999995916
ADCY2	0.816607936	0.401723422	0.617613223	0.913571103	0.870928848	0.96175295	0.999995916
ADCY9	0.526119237	0.566981295	0.518604106	0.739264876	0.653921401	0.878392134	0.999995916
ADGRF5	0.323819053	0.840198706	0.775306037	0.55070304	0.804865319	0.907877458	0.999995916
ADGRG1	0.609019073	0.901905096	0.601175543	0.927551184	0.891836665	0.989411397	0.999995916
ADH5	0.687995284	0.247203418	0.997474115	0.593582645	0.836688025	0.89463381	0.999995916
ADIPOQ	0.982084103	0.685924596	0.385656389	0.941551788	0.353263161	0.897932957	0.999995916
ADORA2A	0.90824335	0.835169816	0.436659863	0.716280946	0.893313627	0.978896897	0.999995916
AFAP1L2	0.433647137	0.502035894	0.753518163	0.775539252	0.601117798	0.881524406	0.999995916
AGA	0.812612679	0.966822481	0.973556935	0.812471088	0.34067191	0.978841276	0.999995916
AGAP1	0.884901586	0.561386268	0.843486887	0.579819263	0.685006383	0.964082819	0.999995916
AGK	0.766423689	0.416595381	0.687861169	0.495234748	0.890167276	0.912117248	0.999995916
AGTPBP1	0.200266588	0.879272787	0.875360097	0.688185448	0.861786095	0.905090706	0.999995916
AGTR1	0.875299549	0.40182529	0.857436462	0.631788528	0.825123998	0.95985535	0.999995916
AHCYL2	0.921778075	0.551256105	0.309903876	0.892227936	0.820067583	0.931664412	0.999995916
AHDC1	0.910507864	0.681806127	0.582114748	0.781444457	0.441879926	0.939725634	0.999995916
AIFM2	0.575061266	0.389895418	0.632497817	0.91552903	0.976978043	0.941313228	0.999995916
AJAP1	0.59974993	0.528479955	0.799753764	0.561431386	0.981488639	0.950153454	0.999995916
AK4	0.674902668	0.735365356	0.331456229	0.661064421	0.826470513	0.90295782	0.999995916
AK5	0.639254366	0.914271777	0.88108247	0.924474955	0.325597451	0.958760343	0.999995916
AKAP1	0.370767354	0.984307253	0.414348749	0.927088228	0.899555402	0.94075185	0.999995916

AKAP8	0.501386824	0.506483215	0.688314515	0.542386342	0.925427667	0.899892882	0.999995916
AKR1A1	0.513742525	0.433838512	0.884787839	0.412115033	0.929618373	0.879822611	0.999995916
AKT1	0.671240931	0.948193207	0.782417179	0.547124308	0.782113033	0.979173914	0.999995916
ALG2	0.852083838	0.577707876	0.834252502	0.975167241	0.418057724	0.964507404	0.999995916
AMDHD2	0.851674539	0.973841265	0.431258538	0.715480554	0.648549343	0.963887354	0.999995916
AMIGO1	0.891066703	0.577846963	0.382671129	0.967840532	0.443253928	0.895062983	0.999995916
AMN1	0.998388761	0.435742731	0.382279166	0.925272829	0.725425261	0.928303326	0.999995916
ANAPC4	0.766786574	0.846716278	0.430795168	0.708133446	0.504957584	0.915961146	0.999995916
ANAPC7	0.962360966	0.360709711	0.821477828	0.907678189	0.491844724	0.941660665	0.999995916
ANGPT1	0.677051819	0.344604358	0.916168618	0.626600166	0.616873022	0.892050148	0.999995916
ANK2	0.878050278	0.775192924	0.564927557	0.426677798	0.736060583	0.936483742	0.999995916
ANKFY1	0.943106934	0.642415861	0.586558276	0.888622916	0.393955829	0.939432766	0.999995916
ANKH	0.522511378	0.585362197	0.58612722	0.906289926	0.548362033	0.90185204	0.999995916
ANKIB1	0.864948798	0.871103811	0.425918569	0.485145414	0.667048662	0.920312234	0.999995916
ANKRD33	0.51437289	0.750056195	0.548486326	0.851816841	0.515321155	0.907073179	0.999995916
ANKRD50	0.506672322	0.849459728	0.622070691	0.534588254	0.519906282	0.877690324	0.999995916
ANKRD52	0.402574842	0.730058947	0.854701086	0.380194347	0.779998744	0.877841281	0.999995916
ANKRD9	0.761709982	0.766547395	0.439859642	0.836516586	0.831151342	0.968913277	0.999995916
ANKS3	0.452945082	0.462829675	0.773816515	0.57526227	0.849057699	0.886404239	0.999995916
ANO10	0.851880952	0.547213219	0.31667761	0.939411336	0.872657045	0.936695632	0.999995916
ANTXR2	0.457111373	0.295051177	0.97181311	0.931383302	0.808692678	0.914433184	0.999995916
ANXA2	0.616272619	0.796237304	0.793368621	0.316216791	0.661605521	0.890132921	0.999995916
AP2A1	0.721778944	0.848754993	0.861349474	0.304608136	0.707849815	0.930335563	0.999995916
AP4S1	0.820407781	0.383038403	0.896952659	0.498619299	0.723455527	0.917882686	0.999995916
APBB2	0.699840542	0.819379125	0.454795036	0.474784291	0.95219368	0.934048521	0.999995916
APC	0.405092231	0.825616497	0.391836289	0.973383916	0.899843621	0.931267487	0.999995916
APEH	0.582388975	0.579079414	0.868320271	0.641886928	0.434951024	0.890644044	0.999995916
APH1A	0.503835886	0.922980884	0.635640999	0.796054628	0.685470935	0.961794678	0.999995916
APLP2	0.497896363	0.94651216	0.881473137	0.708751264	0.676327294	0.97554765	0.999995916
APOBEC3Z2	0.843322517	0.430114835	0.902847339	0.785933557	0.419259761	0.924611276	0.999995916
ARAP1	0.69277554	0.450065458	0.487451074	0.812988102	0.703403156	0.898682519	0.999995916
ARHGAP35	0.750773522	0.558778791	0.893461175	0.244083488	0.962507935	0.900361965	0.999995916
ARHGAP4	0.228873385	0.779501473	0.933912476	0.916954491	0.639861436	0.913268753	0.999995916
ARHGAP42	0.930477	0.919437083	0.7868146	0.593171373	0.741789658	0.991809056	0.999995916
ARHGDIB	0.445274396	0.702198297	0.924617276	0.990956763	0.743193892	0.979131506	0.999995916
ARHGEF12	0.732761844	0.696110458	0.491199259	0.753726587	0.877099986	0.963741606	0.999995916
ARHGEF15	0.92185733	0.746759322	0.602782186	0.534216156	0.532468063	0.934165202	0.999995916
ARHGEF38	0.799670147	0.96128198	0.165764404	0.814792368	0.786179477	0.890425465	0.999995916
ARHGEF7	0.766742136	0.848174177	0.45128774	0.511198136	0.513555731	0.882559422	0.999995916
ARID5A	0.548679399	0.941022849	0.89933799	0.22392932	0.783327549	0.890139616	0.999995916

ARID5B	0.652656101	0.506941552	0.482261123	0.98503385	0.705621481	0.927602108	0.999995916
ARL15	0.638526334	0.536669637	0.676594762	0.450902598	0.7766946	0.889722425	0.999995916
ARL16	0.281805658	0.934105651	0.744891207	0.882838363	0.982607673	0.965626573	0.999995916
ARL4A	0.354547873	0.45636845	0.810601328	0.755240932	0.759086745	0.87915674	0.999995916
ARL5A	0.814098455	0.584938442	0.211186969	0.798771605	0.957246918	0.88228355	0.999995916
ARL6	0.70768503	0.825181788	0.727706188	0.650418677	0.455977489	0.940692881	0.999995916
ARMC5	0.918940823	0.966010168	0.330467618	0.799341209	0.96162618	0.981915892	0.999995916
ARPC1B	0.852951954	0.945945716	0.797051989	0.513802672	0.532426605	0.967928147	0.999995916
ARPIN	0.580542306	0.823969724	0.387934651	0.721037433	0.682496677	0.904957993	0.999995916
ARSG	0.870244145	0.732702618	0.896906511	0.876109117	0.990134111	0.999209585	0.999995916
ASAH1	0.463203657	0.635365835	0.569425002	0.645391533	0.958403802	0.92009918	0.999995916
ASB5	0.925720869	0.875652418	0.402679716	0.320683055	0.860584807	0.903248041	0.999995916
ASB6	0.707800462	0.638722714	0.861643162	0.858336024	0.847852502	0.990563165	0.999995916
ASNSD1	0.905179144	0.467976969	0.781064081	0.790280365	0.352262286	0.906027516	0.999995916
ASPH	0.932443413	0.546787864	0.591451481	0.761909619	0.846101985	0.974170879	0.999995916
ASXL2	0.931235422	0.679619626	0.731070362	0.770644103	0.370453522	0.945130996	0.999995916
ATAD2	0.899710307	0.965466221	0.77393441	0.767922707	0.378468815	0.974465725	0.999995916
ATF2	0.736744228	0.698077976	0.335916324	0.972587507	0.817504467	0.948678962	0.999995916
ATG10	0.785824241	0.762106948	0.405638314	0.823581471	0.926478321	0.971301656	0.999995916
ATG16L1	0.73757323	0.29539324	0.938564282	0.715601129	0.907693276	0.945641939	0.999995916
ATG7	0.576023131	0.720191853	0.847445457	0.37916279	0.64017037	0.8963031	0.999995916
ATM	0.383159383	0.572214196	0.612213302	0.648752883	0.97525949	0.895677082	0.999995916
ATP10A	0.752915796	0.679781925	0.821205385	0.285177014	0.829973907	0.91533795	0.999995916
ATP11B	0.654490622	0.949512073	0.950736984	0.498859115	0.827084623	0.985287806	0.999995916
ATP23	0.906430979	0.441350357	0.555596472	0.876789052	0.736805501	0.952531023	0.999995916
ATP2B1	0.520126371	0.648874902	0.872402607	0.310487062	0.909649284	0.892906429	0.999995916
ATP2B4	0.389494023	0.681630221	0.739166786	0.66706458	0.956572055	0.940066927	0.999995916
ATP6V0E2	0.684344428	0.99072704	0.724251758	0.863041916	0.921295593	0.997173578	0.999995916
ATP8A1	0.866052712	0.882433274	0.320147881	0.849020339	0.404158505	0.894167107	0.999995916
ATR	0.305670168	0.997103417	0.890824515	0.580867489	0.835284144	0.944880547	0.999995916
ATXN7L1	0.757110149	0.514840979	0.474154005	0.98442216	0.620125411	0.929446629	0.999995916
AVEN	0.565853241	0.498006588	0.824103551	0.888483435	0.707323746	0.953899525	0.999995916
AVPR2	0.969422999	0.906681698	0.705950326	0.946586396	0.986878364	0.999744097	0.999995916
B3GNT3	0.772392431	0.785772802	0.32469752	0.883649738	0.476222771	0.892539848	0.999995916
BACE1	0.269043571	0.837061577	0.845510102	0.723523808	0.897107753	0.938787352	0.999995916
BACH2	0.972635105	0.664497358	0.923778413	0.758328361	0.857020707	0.997093957	0.999995916
BAD	0.42276083	0.751698024	0.626582249	0.921281555	0.78603698	0.952887109	0.999995916
BAG5	0.636772277	0.730014586	0.200841891	0.999451503	0.789554294	0.876274545	0.999995916
BAX	0.810389718	0.621723813	0.80595759	0.945273003	0.957610177	0.996327576	0.999995916
BBS4	0.681259068	0.699191055	0.33257091	0.488861322	0.951745738	0.87633515	0.999995916

BCAM	0.907811546	0.899402495	0.340757428	0.617308412	0.937109652	0.96163364	0.999995916
BCAR1	0.61294361	0.969287691	0.971999409	0.43864138	0.888416822	0.98182299	0.999995916
BCAS3	0.472711855	0.961489796	0.477561649	0.986293808	0.749254389	0.961378419	0.999995916
BCAT1	0.801592325	0.568395672	0.995933333	0.720595957	0.751686137	0.985616814	0.999995916
BCHE	0.796538458	0.809119746	0.641334188	0.800424427	0.336224395	0.927926696	0.999995916
BCKDHA	0.493274814	0.870747002	0.52795696	0.621718945	0.736362999	0.920272691	0.999995916
BCL2A1	0.784246237	0.790387434	0.898831514	0.920288868	0.643054268	0.994368998	0.999995916
BCL7C	0.792061375	0.980350618	0.648845848	0.265283555	0.975702791	0.943939966	0.999995916
BCLAF1	0.489156508	0.667788443	0.543153728	0.446706704	0.96063431	0.880902475	0.999995916
BDH2	0.392468196	0.830909718	0.616226846	0.513809047	0.946516676	0.913234973	0.999995916
BEAN1	0.873349677	0.715504881	0.263733289	0.927180863	0.946945834	0.953179166	0.999995916
BICD1	0.925883822	0.857333016	0.276120424	0.963690786	0.910469306	0.973537999	0.999995916
BIRC6	0.43557647	0.700412618	0.929512948	0.374214632	0.95749982	0.917805616	0.999995916
BMP3	0.659433444	0.678263633	0.580855471	0.895517799	0.787833954	0.970596555	0.999995916
BMP7	0.986644602	0.496441719	0.630038541	0.835320763	0.381654073	0.91402573	0.999995916
BMPR1A	0.820261947	0.532616109	0.86601717	0.7401594	0.331052342	0.906832426	0.999995916
BMT2	0.663226216	0.855326129	0.338036978	0.84978206	0.608857467	0.915021994	0.999995916
BOK	0.522515778	0.929309722	0.667723303	0.447486864	0.569291898	0.892009496	0.999995916
BOLA	0.852923758	0.662563449	0.929704519	0.758123105	0.561348956	0.981521109	0.999995916
BPGM	0.765630275	0.995160169	0.605835015	0.870923666	0.328280288	0.945049929	0.999995916
BRD3	0.883103862	0.584012942	0.316674471	0.77798868	0.796936283	0.917408247	0.999995916
BRD7	0.640566815	0.78197706	0.790128446	0.295861438	0.812125402	0.90995022	0.999995916
BRMS1	0.521191765	0.643604454	0.978775912	0.473212758	0.833008826	0.943225569	0.999995916
BRMS1L	0.733673841	0.772814105	0.822438075	0.421164162	0.897288359	0.968040549	0.999995916
BRPF1	0.359726804	0.813122336	0.970099221	0.487977756	0.672525254	0.907383136	0.999995916
BRSK1	0.784717938	0.50705638	0.601513177	0.808349548	0.635419221	0.938260018	0.999995916
BRSK2	0.587971458	0.822735289	0.841480272	0.71066978	0.493393825	0.952020479	0.999995916
BRWD1	0.577917933	0.915029491	0.523780426	0.782060902	0.938281558	0.976683447	0.999995916
BRWD3	0.691451277	0.679328307	0.490967282	0.34516	0.970455383	0.882918233	0.999995916
BTBD1	0.699436266	0.76748744	0.710912091	0.732672253	0.715125612	0.975780499	0.999995916
BTNL9	0.602806	0.571794706	0.97855739	0.770062943	0.671219335	0.9673184	0.999995916
C10H15orf41	0.683974474	0.833412876	0.437401458	0.747555765	0.582278221	0.925244058	0.999995916
C11H2orf68	0.695975145	0.958418353	0.266346191	0.570536681	0.936039219	0.909671884	0.999995916
C11H2orf92	0.800825677	0.956472005	0.393329448	0.949962837	0.408077391	0.933074376	0.999995916
C11H9orf78	0.821049646	0.63390326	0.477486522	0.708207893	0.904448933	0.960804832	0.999995916
C13H20orf194	0.616790041	0.628712486	0.741631797	0.560129491	0.487006137	0.885047479	0.999995916
C14H8orf82	0.750193096	0.980372596	0.868685679	0.776673446	0.523361956	0.987692941	0.999995916
C14H8orf89	0.509815195	0.984092423	0.348278626	0.954505578	0.966677007	0.961762041	0.999995916
C16H1orf112	0.915247987	0.405541566	0.603940227	0.594992206	0.994480608	0.945513992	0.999995916
C18H16orf70	0.964748009	0.937758088	0.326128742	0.588894983	0.496855429	0.897811776	0.999995916

C19H17orf100	0.861657424	0.318023844	0.838967426	0.666976303	0.610059203	0.907940692	0.999995916
C19H17orf75	0.394034942	0.954353569	0.440943716	0.833267887	0.522746858	0.873443625	0.999995916
C1H3orf33	0.811488473	0.920879178	0.815208075	0.465021758	0.814883653	0.982979101	0.999995916
C1H3orf58	0.472261926	0.512824654	0.821539002	0.971694091	0.569335453	0.926785966	0.999995916
C1H3orf70	0.738815596	0.52317523	0.584930722	0.777890652	0.817828733	0.952676094	0.999995916
C1QTNF1	0.739722074	0.282556367	0.760603271	0.716033766	0.692698539	0.885744275	0.999995916
C1QTNF5	0.437591044	0.89662631	0.516624122	0.508882991	0.862216298	0.901630375	0.999995916
C21H15orf39	0.620870653	0.406138308	0.979465924	0.584100123	0.574436616	0.892444911	0.999995916
C23H6orf52	0.58807344	0.528757573	0.642263503	0.597895128	0.601591397	0.872657503	0.999995916
C23H6orf89	0.797398266	0.564279032	0.985746526	0.237766693	0.73489434	0.883370493	0.999995916
C24H18orf54	0.447959281	0.530668266	0.965364074	0.810420436	0.79112792	0.954573014	0.999995916
C25H16orf58	0.784844267	0.252089201	0.841925594	0.730249154	0.720274274	0.899716975	0.999995916
C25H7orf26	0.730114603	0.486150936	0.539497318	0.918275448	0.559993736	0.914131667	0.999995916
C28H1orf131	0.970928618	0.466811343	0.959507976	0.753444995	0.574535812	0.972256349	0.999995916
C29H11orf54	0.898473157	0.503269252	0.490125343	0.862599354	0.741260413	0.951402621	0.999995916
C2CD2	0.909309904	0.767181899	0.806058518	0.939683391	0.432664865	0.982544566	0.999995916
C3H1orf216	0.419407734	0.774645861	0.758985029	0.877540539	0.905943103	0.974658367	0.999995916
C3H1orf50	0.660980852	0.439592374	0.498886046	0.998699968	0.7190632	0.920580982	0.999995916
C7H5orf15	0.894939044	0.588802282	0.678256558	0.680043947	0.694968846	0.965135862	0.999995916
C8H9orf64	0.325008587	0.893484743	0.742836279	0.475993201	0.733225109	0.879330847	0.999995916
C9H6orf163	0.82289281	0.807335616	0.855584351	0.535337137	0.929714538	0.990501827	0.999995916
CA10	0.436476981	0.924075558	0.877693255	0.960654368	0.6475037	0.980795426	0.999995916
CA2	0.917101282	0.568277964	0.926481438	0.217152168	0.88092542	0.906377358	0.999995916
CAB39L	0.862445173	0.824103192	0.367622751	0.790432261	0.491727178	0.91774459	0.999995916
CABYR	0.701616647	0.826794879	0.447337922	0.772199094	0.465437072	0.907573165	0.999995916
CACNA1C	0.459079491	0.982046963	0.44149534	0.861703019	0.772708851	0.945438529	0.999995916
CACNA1F	0.884910902	0.461358608	0.34982792	0.934018548	0.821094056	0.926249906	0.999995916
CACNG5	0.574629443	0.524336868	0.657643018	0.851923295	0.964774068	0.962508877	0.999995916
CACTIN	0.726404704	0.554919187	0.506852966	0.854499694	0.560598155	0.913406289	0.999995916
CALHM2	0.339293136	0.898323959	0.76631782	0.901414819	0.848287384	0.968926442	0.999995916
CALML6	0.901728881	0.543566085	0.279394998	0.999748715	0.910155351	0.939590286	0.999995916
CAMK1D	0.816676708	0.589549415	0.974810238	0.745171011	0.53179165	0.971511524	0.999995916
CAMKK1	0.41749502	0.64968474	0.957200203	0.801985126	0.912656231	0.972826303	0.999995916
CAMKK2	0.668694384	0.601910492	0.996947559	0.940056375	0.568812601	0.979520758	0.999995916
CAPN2	0.891591183	0.727422582	0.594100065	0.634675123	0.970615407	0.98418616	0.999995916
CAPN3	0.907033551	0.632491274	0.926270203	0.672205214	0.405111578	0.9531863	0.999995916
CAPRIN2	0.925582961	0.985431684	0.212370802	0.742194843	0.975893078	0.950538817	0.999995916
CARD11	0.475923998	0.912411044	0.799493431	0.884360469	0.820001502	0.986548971	0.999995916
CASC3	0.907159062	0.658370331	0.451283661	0.834985426	0.576491498	0.943457341	0.999995916
CASD1	0.73526479	0.965198128	0.175571065	0.982831551	0.837560989	0.91888794	0.999995916

CASKIN1	0.796241693	0.56783326	0.809229607	0.839645777	0.939932464	0.991101822	0.999995916
CASKIN2	0.73885823	0.872721026	0.83371793	0.403340576	0.861438317	0.971776981	0.999995916
CASP6	0.92708327	0.577484809	0.98700986	0.810801997	0.827012206	0.995731866	0.999995916
CASP7	0.978507759	0.534135272	0.978541899	0.975498089	0.675776126	0.994819782	0.999995916
CASP8	0.547685123	0.613635871	0.686230003	0.496784336	0.920325819	0.922033433	0.999995916
CASP9	0.78328582	0.609646929	0.870435078	0.418532346	0.676732548	0.933897137	0.999995916
CASS4	0.50383941	0.694365427	0.81286508	0.899753812	0.804306976	0.9773582	0.999995916
CAST	0.905513827	0.932802232	0.222676839	0.575268128	0.980325411	0.922700152	0.999995916
CAT	0.710145771	0.949916136	0.329622454	0.956003153	0.997317197	0.978912743	0.999995916
CATSPER2	0.902604928	0.855844406	0.607600135	0.889346661	0.567936703	0.984134528	0.999995916
CBFA2T3	0.86319875	0.75220424	0.958568794	0.949236965	0.174705129	0.919611688	0.999995916
CBX1	0.614253713	0.67815349	0.780054896	0.5321814	0.526287061	0.904532391	0.999995916
CBX3	0.850245201	0.817260476	0.412815668	0.384851264	0.753378634	0.892925591	0.999995916
CBY1	0.797148953	0.96866587	0.959458198	0.262410967	0.765354898	0.955495477	0.999995916
CC2D1A	0.913678932	0.748481903	0.419156607	0.590510583	0.523054517	0.901055263	0.999995916
CCDC107	0.661509423	0.235273988	0.850835348	0.732895165	0.800867531	0.883767032	0.999995916
CCDC121	0.931628555	0.567685198	0.735589699	0.497618147	0.836935331	0.962127912	0.999995916
CCDC130	0.90173092	0.947941233	0.374661839	0.911407214	0.683940835	0.975690423	0.999995916
CCDC138	0.603265705	0.866527424	0.855370372	0.669363461	0.734976029	0.980746571	0.999995916
CCDC167	0.465077071	0.600502889	0.8477547	0.650488255	0.881093554	0.947587789	0.999995916
CCDC170	0.707009416	0.994497369	0.627889507	0.871900471	0.326841336	0.940522246	0.999995916
CCDC22	0.9041534	0.795011427	0.702406332	0.842537134	0.335770729	0.952082204	0.999995916
CCDC3	0.798890779	0.755361999	0.3508277	0.804339303	0.8100082	0.949045452	0.999995916
CCDC50	0.487457947	0.390053788	0.932663396	0.825312078	0.492379807	0.87311351	0.999995916
CCDC59	0.829386215	0.280325045	0.653372449	0.875152417	0.542028167	0.873106912	0.999995916
CCDC71	0.821915736	0.721215951	0.792467514	0.616624304	0.518911083	0.95631904	0.999995916
CCDC92	0.958631399	0.856061158	0.902955536	0.219781742	0.711632574	0.932274628	0.999995916
CCDC97	0.993196662	0.688859428	0.50203947	0.752510903	0.482024024	0.939574962	0.999995916
CCL28	0.926193613	0.819919677	0.458553943	0.824553023	0.951223956	0.98940758	0.999995916
CCNE1	0.724328157	0.452444759	0.575087687	0.810114862	0.983961076	0.956276594	0.999995916
CCP110	0.754128013	0.790555038	0.905684332	0.52360113	0.823350876	0.983346475	0.999995916
CCPG1	0.762428098	0.721904182	0.606621123	0.758787329	0.544423971	0.949043041	0.999995916
CCR8	0.987505049	0.691709249	0.395368348	0.894850348	0.304415729	0.876067961	0.999995916
CD109	0.509089424	0.962092145	0.689679438	0.738063848	0.924701309	0.982920834	0.999995916
CD151	0.469523445	0.941454034	0.373140368	0.577787935	0.754719701	0.872841308	0.999995916
CD276	0.380164612	0.765479316	0.468293993	0.974053462	0.811280908	0.924386285	0.999995916
CD2AP	0.779029884	0.230587276	0.584265476	0.971374728	0.741637906	0.879933084	0.999995916
CD302	0.631895953	0.989840174	0.691633724	0.34754939	0.484144923	0.874556927	0.999995916
CD47	0.444180961	0.802303191	0.993352975	0.699206348	0.508106032	0.940487874	0.999995916
CDC123	0.423041599	0.506696588	0.883501825	0.943887492	0.666705558	0.935147247	0.999995916

CDC42BPA	0.881724772	0.440100123	0.564600499	0.904488653	0.835467879	0.96370789	0.999995916
CDCA7L	0.709369232	0.563459188	0.518958136	0.635391665	0.710922681	0.908141162	0.999995916
CDH13	0.787460958	0.806717814	0.227159343	0.835324356	0.891552742	0.924158211	0.999995916
CDH5	0.608909014	0.971587527	0.432858769	0.444145614	0.819504531	0.907497665	0.999995916
CDK11B	0.133607644	0.984208501	0.94739848	0.894805107	0.853440872	0.910002049	0.999995916
CDK14	0.586255153	0.29163916	0.910253295	0.594552689	0.780975416	0.873516063	0.999995916
CDK19	0.738020345	0.332755185	0.830108461	0.601605752	0.642993044	0.885755809	0.999995916
CDK4	0.418069668	0.463910733	0.581335694	0.762768623	0.890244539	0.88167942	0.999995916
CDKAL1	0.485082917	0.916602697	0.876576344	0.750388645	0.533376628	0.959255318	0.999995916
CELF2	0.980793225	0.678563326	0.325856305	0.963343311	0.598073743	0.939854517	0.999995916
CELSR2	0.548090739	0.903067717	0.97730184	0.621607128	0.578769394	0.967198107	0.999995916
CENPP	0.813932809	0.970007994	0.80654403	0.495069436	0.74467158	0.983715117	0.999995916
CENPS	0.844361489	0.621628389	0.867881142	0.489784975	0.939826153	0.978346225	0.999995916
CEP19	0.750982133	0.381205484	0.684371118	0.948580718	0.682549574	0.941315597	0.999995916
CEP44	0.701827735	0.548971199	0.661676983	0.835568961	0.917183924	0.974462168	0.999995916
CEP70	0.880293707	0.923700948	0.423987335	0.290109221	0.74440978	0.87776555	0.999995916
CEP83	0.958180795	0.858226836	0.725953641	0.308563196	0.880052345	0.962168315	0.999995916
CEP97	0.904157276	0.655156567	0.860015061	0.659077648	0.675658817	0.982193711	0.999995916
CES2	0.858873364	0.80237255	0.730840997	0.839802298	0.35950922	0.957248079	0.999995916
CH25H	0.965477447	0.77285289	0.617492721	0.999939106	0.480815392	0.981082202	0.999995916
CHAMP1	0.769584581	0.649316443	0.752709223	0.518208498	0.8256151	0.961622366	0.999995916
CHD8	0.925817007	0.566568825	0.490517533	0.520209042	0.733182852	0.913726125	0.999995916
CHD9	0.86595462	0.973887676	0.944139157	0.451964401	0.991050189	0.995842403	0.999995916
CHERP	0.861451047	0.530673883	0.513022055	0.669053497	0.467443371	0.875642745	0.999995916
CHFR	0.33667088	0.82430425	0.599940255	0.74370029	0.734729175	0.904487144	0.999995916
CHPF	0.650599616	0.856150735	0.944818596	0.92365758	0.84152114	0.997723076	0.999995916
CHRDL1	0.994528221	0.85955846	0.728442397	0.939539042	0.951528288	0.999646147	0.999995916
CHRNA5	0.929586091	0.879905599	0.994534148	0.288373109	0.981586763	0.982866993	0.999995916
CHRNE	0.490614216	0.929311754	0.774414307	0.748103965	0.575183041	0.957179706	0.999995916
CHST1	0.303193379	0.706407402	0.813351731	0.848573675	0.926202479	0.948387504	0.999995916
CHST14	0.723301343	0.388506859	0.692553853	0.963244416	0.883325309	0.963719522	0.999995916
CHST15	0.701406734	0.826969148	0.614556592	0.524168715	0.726458741	0.947615021	0.999995916
CHST2	0.530248677	0.706167943	0.683106606	0.614989904	0.70196223	0.927131038	0.999995916
CIB4	0.54728446	0.85922429	0.30498217	0.68028738	0.784547941	0.881645406	0.999995916
CITED4	0.989098398	0.759498236	0.652154357	0.878239153	0.388303656	0.964359293	0.999995916
CLCN6	0.732686398	0.87079912	0.359789246	0.500808163	0.970569148	0.928256098	0.999995916
CLDN12	0.949764894	0.881036874	0.413170724	0.907719539	0.315848059	0.914910417	0.999995916
CLDN12	0.960142162	0.988759774	0.696654438	0.990086345	0.565871827	0.996449414	0.999995916
CLDN20	0.500078186	0.690764518	0.755791495	0.880210611	0.664150005	0.9575426	0.999995916
CLEC14A	0.348974011	0.833020951	0.941241089	0.552962287	0.70758416	0.923733891	0.999995916

CLEC1A	0.527145867	0.986680194	0.266405764	0.97909839	0.636656486	0.897877569	0.999995916
CLIC5	0.881540747	0.554534129	0.220176245	0.980875794	0.951935447	0.916528965	0.999995916
CLOCK	0.942953405	0.494788304	0.6498417	0.612633709	0.923051745	0.966177224	0.999995916
CLTRN	0.735209255	0.880350385	0.964292739	0.680314376	0.66000038	0.990215511	0.999995916
CMC1	0.791890971	0.355026908	0.797832048	0.687817244	0.954987157	0.954686951	0.999995916
CMTM7	0.998016993	0.396339043	0.325416702	0.991409551	0.59076077	0.879523504	0.999995916
CNOT8	0.951810511	0.296558968	0.568610766	0.839673468	0.990667014	0.946113734	0.999995916
CNOT9	0.646459233	0.789967321	0.515974027	0.901044918	0.568887399	0.947168107	0.999995916
CNPY4	0.947131876	0.34427245	0.967872663	0.641679023	0.875452376	0.968440765	0.999995916
CNTF	0.501303695	0.541922218	0.496144513	0.915114733	0.902557708	0.928011833	0.999995916
COG5	0.833970365	0.613079936	0.841788883	0.992592914	0.604538276	0.987494148	0.999995916
COL18A1	0.961571281	0.917289883	0.355747797	0.972997236	0.873311184	0.988610809	0.999995916
COMMD1	0.665972469	0.629509847	0.801784327	0.532932469	0.981019763	0.967856192	0.999995916
COMMD9	0.639120677	0.562302227	0.421042309	0.852456439	0.677294762	0.899346743	0.999995916
COMP	0.966486703	0.768268776	0.95478213	0.570509031	0.388566532	0.95982961	0.999995916
CORO1B	0.95388295	0.852463458	0.962230994	0.475657615	0.433717532	0.961850557	0.999995916
CPEB3	0.791747221	0.881207299	0.623502034	0.997179461	0.818097262	0.995758328	0.999995916
CPED1	0.951006265	0.384876384	0.453465941	0.962691896	0.866885609	0.949418274	0.999995916
CPN2	0.614981049	0.99973866	0.707486798	0.768685975	0.649330729	0.980107427	0.999995916
CPNE3	0.847739398	0.702080323	0.549881537	0.69029373	0.863309457	0.974362629	0.999995916
CPSF4	0.889656415	0.85421654	0.733394513	0.754922025	0.947458858	0.997429695	0.999995916
CRADD	0.99638664	0.938207932	0.212904342	0.942351588	0.973000829	0.97031756	0.999995916
CREB3L2	0.992718648	0.862841106	0.445028193	0.52743155	0.808293228	0.962350176	0.999995916
CREBZF	0.612140563	0.68281435	0.575035612	0.652044124	0.56877928	0.901917035	0.999995916
CSAD	0.401729303	0.746010193	0.920479116	0.337275098	0.798583976	0.877476476	0.999995916
CSGALNACT1	0.287512611	0.66563019	0.693537264	0.881732246	0.638178069	0.878205138	0.999995916
CTBP2	0.890155697	0.578275313	0.811115323	0.250488304	0.694456628	0.8742398	0.999995916
CTDNEP1	0.579520687	0.972482608	0.408108673	0.354622345	0.879094583	0.872392317	0.999995916
CTDP1	0.72968253	0.177197121	0.917897097	0.849271531	0.842264859	0.895628721	0.999995916
CTDSP1	0.695380832	0.92634208	0.862943235	0.814523977	0.296201441	0.946523978	0.999995916
CTDSPL	0.750878064	0.676929199	0.600347506	0.941681366	0.338046016	0.912511112	0.999995916
CTDSPL2	0.710044563	0.506030743	0.980819694	0.458343146	0.752394019	0.937118704	0.999995916
CTIF	0.92258847	0.89298156	0.525187474	0.691628426	0.71121782	0.979112086	0.999995916
CTNNA1	0.831659349	0.643203893	0.559919147	0.918205449	0.900341634	0.985907772	0.999995916
CTNNBL1	0.933236073	0.732074751	0.63862049	0.43491734	0.790712387	0.956175203	0.999995916
CTR9	0.701763558	0.767654416	0.698969881	0.45630295	0.558050653	0.910946755	0.999995916
CTSF	0.832395815	0.923162178	0.332519713	0.709092464	0.570939184	0.919866372	0.999995916
CTTNBP2NL	0.665390721	0.466605694	0.947595366	0.73746624	0.971700795	0.978626792	0.999995916
CTU1	0.935046238	0.578691674	0.885577599	0.276190702	0.601774134	0.887107303	0.999995916
CTU2	0.8963409	0.503081395	0.233047488	0.841159356	0.990733496	0.899661018	0.999995916

CYB5D1	0.868592194	0.887870515	0.478556447	0.957920866	0.924244057	0.994178492	0.999995916
CYB5R1	0.900366075	0.668260935	0.66572421	0.83304923	0.330778231	0.927083413	0.999995916
CYBA	0.548818958	0.790826071	0.806011703	0.389372092	0.617300492	0.894369178	0.999995916
CYBB	0.357994071	0.712038305	0.520171718	0.644690511	0.870733726	0.877725578	0.999995916
CYFIP1	0.549788808	0.846541425	0.264228468	0.77010727	0.914571241	0.898238019	0.999995916
CYP26B1	0.934225448	0.520961889	0.992583692	0.87369274	0.557615561	0.98382371	0.999995916
CYP2U1	0.733683539	0.773473357	0.686365208	0.803896303	0.360861008	0.929602781	0.999995916
CYP3A5	0.862893626	0.950322177	0.2884372	0.458779843	0.846957796	0.905756483	0.999995916
CYS1	0.26627632	0.724758748	0.901796218	0.998821358	0.695113533	0.936539819	0.999995916
CYTH3	0.803062587	0.789076552	0.947971217	0.862199835	0.73652964	0.996866616	0.999995916
DAAM1	0.290729477	0.742947097	0.81608839	0.736446731	0.899916441	0.933101013	0.999995916
DAAM2	0.958994808	0.999571373	0.943404145	0.664785273	0.640290465	0.996988754	0.999995916
DAB2IP	0.940472101	0.876841059	0.921838352	0.842444515	0.871660578	0.999653543	0.999995916
DAP	0.861348802	0.416280394	0.89582028	0.402487568	0.98680652	0.941863004	0.999995916
DCAF7	0.875584474	0.734119332	0.374155579	0.52830937	0.878273411	0.928269174	0.999995916
DCHS1	0.896384348	0.576333677	0.853141259	0.507994314	0.994867501	0.981343212	0.999995916
DCK	0.93306458	0.741846428	0.656291519	0.898478136	0.640050921	0.987903865	0.999995916
DCLK3	0.824371897	0.723661578	0.738101484	0.490702739	0.870322791	0.97218995	0.999995916
DCUN1D3	0.960252163	0.320837626	0.723980318	0.717425226	0.581665169	0.907324564	0.999995916
DDB1	0.836755922	0.79727499	0.744894093	0.379744326	0.93229908	0.967930375	0.999995916
DDHD2	0.692078972	0.59584616	0.743460628	0.766344232	0.953883235	0.981630151	0.999995916
DDX19B	0.741637786	0.407011033	0.800208706	0.521288621	0.655693163	0.891950794	0.999995916
DDX23	0.976009657	0.982805953	0.72463646	0.859628645	0.451297701	0.988989571	0.999995916
DDX28	0.560164865	0.785943853	0.781697288	0.367778846	0.887199529	0.928940621	0.999995916
DDX49	0.567091339	0.594075558	0.894704618	0.809338232	0.434632588	0.922655386	0.999995916
DDX5	0.988255756	0.380003838	0.965290122	0.820211441	0.359437272	0.923539352	0.999995916
DDX51	0.752697712	0.603347381	0.863116214	0.651906651	0.581444625	0.955376268	0.999995916
DENND2C	0.286302099	0.8505265	0.918167711	0.927352878	0.542380515	0.929095565	0.999995916
DENND3	0.784130053	0.35803407	0.671805244	0.601455763	0.992521833	0.929222467	0.999995916
DENND4A	0.437392876	0.836326889	0.903497942	0.755491372	0.609281108	0.957286067	0.999995916
DEPDC1B	0.82686367	0.604618165	0.291231275	0.724040273	0.946978655	0.915746019	0.999995916
DEPP1	0.82611263	0.647728264	0.806672777	0.933896597	0.821131029	0.994449994	0.999995916
DET1	0.746761486	0.636151119	0.451089285	0.414579691	0.909117603	0.8890046	0.999995916
DGKE	0.652748495	0.605954747	0.62983918	0.867844458	0.336509026	0.874483067	0.999995916
DGKH	0.480027332	0.648505274	0.734803927	0.952736728	0.529567732	0.931836176	0.999995916
DHRS12	0.629416905	0.787359019	0.332455318	0.945505104	0.530398058	0.892051964	0.999995916
DHX16	0.903107307	0.975425734	0.544556816	0.448153823	0.739848659	0.960743566	0.999995916
DHX33	0.964106875	0.760027776	0.85209324	0.430635873	0.282411485	0.880530905	0.999995916
DHX38	0.98617016	0.39360784	0.952058745	0.880770055	0.922889632	0.99218479	0.999995916
DHX8	0.859925734	0.437875103	0.889977268	0.336585739	0.91821562	0.920001057	0.999995916

DIAPH2	0.702584006	0.810238461	0.629137239	0.670376395	0.943509951	0.982126442	0.999995916
DICER1	0.647626787	0.859617934	0.898610645	0.346089713	0.508675592	0.900380855	0.999995916
DIO1	0.565302431	0.634046516	0.727252815	0.813858917	0.784475183	0.964081724	0.999995916
DIO2	0.79757734	0.97752408	0.803601481	0.481207583	0.660275422	0.975531268	0.999995916
DIS3	0.847022844	0.828191006	0.672926602	0.467897132	0.833874239	0.970901253	0.999995916
DLC1	0.734058111	0.810413463	0.280438043	0.733505005	0.737721827	0.903517218	0.999995916
DLEC1	0.569691489	0.791245754	0.378762088	0.937894039	0.460235505	0.876319613	0.999995916
DLG2	0.989761518	0.513531479	0.731210737	0.547537741	0.565410205	0.931516242	0.999995916
DLG3	0.390184512	0.807182902	0.473198663	0.994306923	0.553281303	0.891020503	0.999995916
DMXL2	0.708733293	0.83818977	0.603023169	0.925752439	0.892928923	0.991803444	0.999995916
DNAJB12	0.970021026	0.612600822	0.886025042	0.746972826	0.370333506	0.953729206	0.999995916
DNAJC9	0.456326389	0.493479249	0.86752303	0.928607097	0.822717625	0.955744063	0.999995916
DNAL1	0.923555715	0.577582375	0.903496477	0.585552603	0.925370071	0.987890867	0.999995916
DNTTIP1	0.84959777	0.522766998	0.494712167	0.821224652	0.463665277	0.893709631	0.999995916
DOCK1	0.600148592	0.740203822	0.860009448	0.461667401	0.70407095	0.939253699	0.999995916
DONSON	0.940132962	0.283715775	0.827769867	0.5079527	0.89135789	0.915908424	0.999995916
DOPEY1	0.536903827	0.8453677	0.780534361	0.469174604	0.991499167	0.96337503	0.999995916
DPAGT1	0.829218008	0.799625609	0.675169684	0.591295595	0.422836237	0.928593153	0.999995916
DPF1	0.444101837	0.916418697	0.766232543	0.709829315	0.353069156	0.884525735	0.999995916
DPH2	0.977134389	0.964231217	0.838493461	0.541967397	0.928568431	0.997397589	0.999995916
DPM3	0.650505191	0.888506563	0.712467355	0.40587554	0.6309202	0.922039341	0.999995916
DPY19L3	0.838731227	0.880930225	0.517752655	0.960820539	0.697882669	0.987246623	0.999995916
DPY19L4	0.543009755	0.983485249	0.838829653	0.578599962	0.66563522	0.966608154	0.999995916
DPYSL2	0.574719381	0.57888083	0.99200199	0.66865126	0.985123045	0.980171544	0.999995916
DRAM1	0.769544661	0.811971342	0.70444846	0.679758072	0.682392489	0.97693244	0.999995916
DRD1	0.70489594	0.895308198	0.497835258	0.974964777	0.339683511	0.920529721	0.999995916
DRD2	0.890902254	0.408100483	0.996400612	0.357383861	0.918345874	0.934908122	0.999995916
DROSHA	0.860838359	0.899555826	0.305283317	0.828610265	0.593818661	0.932654581	0.999995916
DSC2	0.831066442	0.970149108	0.148698752	0.822327578	0.883889058	0.899019686	0.999995916
DTD1	0.94939756	0.856049472	0.715394369	0.353530955	0.840904265	0.966734655	0.999995916
DUSP1	0.789167606	0.886012183	0.215938882	0.708888268	0.801469219	0.896987868	0.999995916
DUSP18	0.651868981	0.8996576	0.87600003	0.406604465	0.397431706	0.892684128	0.999995916
DUSP7	0.695213119	0.989425733	0.669365843	0.838472129	0.535588509	0.977608903	0.999995916
DUT	0.523964143	0.632320486	0.868214458	0.489944861	0.980634798	0.94921957	0.999995916
DVL3	0.952028436	0.837556433	0.176999706	0.743934265	0.923836317	0.912337762	0.999995916
DYNC1H1	0.769217905	0.937776182	0.843939725	0.833131671	0.81584058	0.997845645	0.999995916
DYNC1LI2	0.690980604	0.693677042	0.306758452	0.70987077	0.979102412	0.918468822	0.999995916
EBF1	0.873082638	0.6977985	0.832522434	0.490586061	0.45436978	0.929665638	0.999995916
ECPAS	0.917435477	0.871382083	0.440565295	0.961023425	0.656466786	0.981226448	0.999995916
EDC4	0.600940679	0.626838769	0.890379215	0.731428498	0.836875838	0.977228622	0.999995916

EDEM1	0.740910049	0.507051001	0.760435751	0.695326783	0.708090205	0.950758889	0.999995916
EEF1A1	0.838546886	0.710537627	0.936839944	0.821758447	0.759853385	0.995443381	0.999995916
EEF1G	0.946099842	0.806295362	0.558689332	0.783313755	0.3543575	0.934392446	0.999995916
EEFSEC	0.88193409	0.95480338	0.721138139	0.513250739	0.276895044	0.897767525	0.999995916
EFCAB6	0.992394656	0.797098893	0.476475096	0.648719421	0.494586862	0.936622649	0.999995916
EFTUD2	0.839090113	0.546990178	0.437036439	0.981489442	0.396002605	0.884189495	0.999995916
EIF2AK1	0.877335469	0.339823638	0.797499914	0.685945775	0.971288185	0.96043617	0.999995916
EIF3D	0.81980746	0.936816136	0.863573902	0.481125331	0.44346715	0.951282194	0.999995916
EIF5A2	0.915695577	0.333020339	0.979705423	0.9928026	0.795421536	0.983928245	0.999995916
ELOVL4	0.869577649	0.34676847	0.774797561	0.377592595	0.927376067	0.890732056	0.999995916
EMD	0.839395616	0.741056968	0.283976911	0.625149724	0.952184578	0.921718474	0.999995916
ENTPD4	0.841471471	0.501602278	0.500577425	0.795121729	0.968692612	0.96245379	0.999995916
ENTPD5	0.616838688	0.453237522	0.863381341	0.858477355	0.754748734	0.959455515	0.999995916
EPB41L1	0.75528428	0.739956836	0.866521331	0.855828026	0.548694493	0.982304572	0.999995916
EPB41L2	0.906166481	0.652606653	0.907858252	0.605623579	0.251817893	0.890840325	0.999995916
EPN2	0.975483667	0.89713078	0.491007784	0.526545699	0.674332497	0.95751546	0.999995916
ERCC2	0.655482645	0.93624571	0.391994161	0.432729615	0.867796358	0.903602761	0.999995916
ERG	0.320450628	0.394427738	0.948180558	0.919458121	0.763163614	0.894386263	0.999995916
ERGIC3	0.715588842	0.9327094	0.946529366	0.645480679	0.831935037	0.994940463	0.999995916
ERI2	0.336019169	0.949340765	0.931954789	0.823797715	0.600508336	0.954536975	0.999995916
ERICH1	0.463935389	0.570210066	0.943285494	0.642157744	0.449557437	0.87306633	0.999995916
ERRFI1	0.716177331	0.834168118	0.699391045	0.275729091	0.658062955	0.880308788	0.999995916
ESCO2	0.886717233	0.619525715	0.35039439	0.786964658	0.606729813	0.905758401	0.999995916
ESYT1	0.828746796	0.942658262	0.344400716	0.599797243	0.93322883	0.956476052	0.999995916
ETV4	0.871184761	0.25276722	0.567090564	0.972246894	0.845330014	0.91896138	0.999995916
EVC	0.547666304	0.631513961	0.651970148	0.421657125	0.786847614	0.878444776	0.999995916
EVI5L	0.441809866	0.810062033	0.897915549	0.561780274	0.777354896	0.950562475	0.999995916
EXOC4	0.898861059	0.544302837	0.55308038	0.448111824	0.876511521	0.922923086	0.999995916
EXOSC1	0.792930413	0.382492171	0.627291693	0.877070994	0.612026443	0.918390512	0.999995916
EXOSC10	0.771795316	0.617489353	0.361122578	0.70635274	0.686915946	0.893457332	0.999995916
EXOSC6	0.626979608	0.534238809	0.692265836	0.549074548	0.691348482	0.900309783	0.999995916
EXOSC8	0.683240015	0.5046233	0.390586	0.843253633	0.65794221	0.878258677	0.999995916
F8	0.842186053	0.998560568	0.654123945	0.34962989	0.826106142	0.960663343	0.999995916
FA2H	0.813105004	0.895153258	0.950083906	0.619089565	0.630080124	0.989000191	0.999995916
FABP9	0.663908342	0.804928543	0.594442503	0.244526599	0.969699298	0.879403677	0.999995916
FAF2	0.952537618	0.918897644	0.781561176	0.69752504	0.443448291	0.978815198	0.999995916
FAH	0.429666749	0.890629455	0.389227604	0.841794807	0.74618183	0.907958097	0.999995916
FAM102A	0.891289955	0.556044257	0.337355371	0.841940191	0.933827925	0.944681362	0.999995916
FAM111B	0.849260213	0.393355906	0.890588617	0.868118495	0.792197975	0.977045084	0.999995916
FAM122B	0.595361391	0.843174448	0.519864874	0.993899339	0.391503201	0.917734446	0.999995916

FAM133A	0.499035712	0.724189292	0.565329947	0.359454918	0.999953222	0.875815005	0.999995916
FAM13A	0.920854125	0.414784465	0.57533036	0.379634618	0.873815415	0.874766646	0.999995916
FAM149A	0.362080086	0.372737918	0.756117282	0.954748815	0.964953502	0.908553668	0.999995916
FAM151A	0.725239326	0.738559293	0.247210478	0.887552587	0.801130565	0.908733421	0.999995916
FAM168A	0.781026975	0.60199234	0.317854761	0.864802344	0.72650354	0.908397856	0.999995916
FAM180A	0.377741113	0.893319002	0.294611158	0.855463587	0.863311104	0.875785204	0.999995916
FAM185A	0.415840726	0.746846035	0.994823277	0.705790615	0.589234836	0.942542279	0.999995916
FAM187A	0.718702948	0.687424682	0.7254581	0.484956326	0.764831509	0.945721359	0.999995916
FAM189B	0.437846046	0.68863024	0.903895788	0.600193132	0.660280358	0.924708889	0.999995916
FAM193A	0.575044423	0.483066838	0.732091138	0.409951763	0.880583744	0.875771547	0.999995916
FAM199X	0.33799429	0.645624971	0.618169987	0.678009562	0.997575145	0.904847846	0.999995916
FAM200A	0.344005372	0.533510363	0.426075619	0.999162823	0.953905788	0.877912194	0.999995916
FAM208A	0.894223598	0.534024143	0.968286175	0.356611566	0.716037255	0.934195046	0.999995916
FAM20C	0.629291985	0.941392289	0.604179106	0.978011685	0.818201429	0.990866384	0.999995916
FAM228B	0.839371554	0.384067921	0.343316868	0.938131057	0.968945222	0.916651852	0.999995916
FAM78A	0.47757059	0.412360072	0.416601855	0.925425273	0.937374317	0.871315853	0.999995916
FAM89A	0.808551057	0.459240722	0.64121355	0.92241686	0.702561043	0.958403735	0.999995916
FAR1	0.39994319	0.935095501	0.831828843	0.906959939	0.384429916	0.925177962	0.999995916
FAS	0.260659844	0.939593605	0.626021055	0.782230933	0.694722556	0.893164257	0.999995916
FAT4	0.793385627	0.91475551	0.484632112	0.977184543	0.616586977	0.978892754	0.999995916
FAU	0.854426806	0.990398369	0.831691468	0.848924995	0.407606076	0.985247369	0.999995916
FBLN2	0.800432118	0.594054965	0.542183238	0.883676905	0.696552706	0.960568626	0.999995916
FBXO22	0.801317548	0.437453074	0.88730032	0.962699087	0.461320327	0.949174727	0.999995916
FBXO4	0.918405584	0.864325582	0.69235923	0.53407732	0.480739267	0.951038035	0.999995916
FBXO42	0.924893961	0.408798231	0.988627483	0.939970084	0.414263107	0.953675553	0.999995916
FBXO48	0.362482343	0.661101201	0.97201744	0.925875867	0.71941726	0.958835888	0.999995916
FBXO5	0.726807504	0.565708391	0.562470169	0.997703553	0.705246525	0.96244772	0.999995916
FDX1	0.7473107	0.245551197	0.61386067	0.783338328	0.849728295	0.878757845	0.999995916
FECH	0.993194789	0.853646037	0.552520286	0.337173181	0.921478755	0.953670721	0.999995916
FER	0.52997925	0.786539224	0.835352794	0.452100586	0.506233494	0.887196729	0.999995916
FERMT3	0.426607814	0.420768285	0.738883433	0.806885743	0.847195128	0.904059199	0.999995916
FEZ2	0.746997767	0.336249454	0.797937764	0.978571876	0.980638423	0.97354399	0.999995916
FGF12	0.570881822	0.555483033	0.876843061	0.486917501	0.870410192	0.934000515	0.999995916
FIP1L1	0.686140808	0.95477153	0.622129084	0.253998177	0.955324934	0.914640185	0.999995916
FKBP15	0.502581878	0.776506501	0.853863808	0.908759619	0.602534344	0.970307834	0.999995916
FLOT2	0.913196957	0.779954487	0.502464493	0.948054621	0.543440156	0.970970678	0.999995916
FLRT2	0.730169466	0.267563682	0.434245138	0.906987059	0.936285602	0.873077179	0.999995916
FLVCR1	0.598645045	0.665025196	0.73153804	0.314595756	0.900925778	0.891921999	0.999995916
FMC1	0.52073787	0.449780489	0.656578543	0.567424851	0.86728116	0.880060864	0.999995916
FMO5	0.939160524	0.904784694	0.271540199	0.556360966	0.78199688	0.916399461	0.999995916

FN3KRP	0.502024345	0.481067711	0.654085443	0.976242494	0.920962311	0.951595125	0.999995916
FOLH1B	0.586392301	0.90102883	0.915836379	0.17218865	0.88191238	0.875901798	0.999995916
FOXF2	0.986212857	0.659927807	0.914565221	0.652247524	0.754223541	0.991495622	0.999995916
FOXL1	0.891817095	0.996998255	0.50644529	0.257630015	0.970241367	0.929192817	0.999995916
FOXN3	0.587782875	0.80363441	0.883711961	0.888175939	0.580759926	0.979696652	0.999995916
FOXP4	0.995469665	0.796168168	0.219015263	0.860356169	0.877519501	0.944398344	0.999995916
FOXQ1	0.286793745	0.984989595	0.554610852	0.933453552	0.854938843	0.939919023	0.999995916
FRMD3	0.995136419	0.936507452	0.602491681	0.531161865	0.656231708	0.974563858	0.999995916
FRMD4A	0.411090922	0.911891051	0.625210998	0.992049748	0.394497422	0.905510019	0.999995916
FRMPD3	0.613016643	0.630808289	0.733772038	0.449702511	0.576694615	0.876107143	0.999995916
FRS2	0.699088903	0.500827333	0.910131122	0.485762253	0.715913228	0.927517737	0.999995916
FRY	0.394456335	0.383381251	0.872949214	0.831317047	0.70527997	0.883191231	0.999995916
FSD2	0.953044501	0.955023843	0.811773457	0.948417593	0.464325649	0.994088171	0.999995916
FSTL3	0.689625641	0.925882069	0.668703277	0.998906028	0.624343939	0.988567021	0.999995916
FTSJ1	0.951684832	0.703440184	0.904782296	0.421260324	0.914494462	0.983452775	0.999995916
FUCA2	0.887054422	0.866191235	0.589805495	0.212192907	0.945421422	0.904401476	0.999995916
FXYD7	0.399353274	0.997910428	0.581774271	0.912771115	0.937692664	0.975352872	0.999995916
FZD3	0.930573931	0.716159189	0.482270492	0.297613267	0.788526367	0.879591415	0.999995916
FZD4	0.849647493	0.870276028	0.416527083	0.59461316	0.396820074	0.874322627	0.999995916
G2E3	0.459572673	0.977546708	0.472919188	0.370602153	0.980923637	0.882896533	0.999995916
G6PC	0.444310236	0.888570183	0.553947992	0.81710632	0.732864511	0.944335503	0.999995916
GAA	0.370327736	0.963439473	0.760778997	0.975398326	0.832119405	0.980819325	0.999995916
GABPB2	0.216117403	0.695806974	0.963621749	0.94359963	0.781777291	0.923562882	0.999995916
GALK2	0.61160413	0.454109417	0.820305178	0.781471903	0.617908606	0.92672741	0.999995916
GALM	0.792252243	0.895401174	0.200156067	0.972046624	0.540968979	0.878163267	0.999995916
GALNT18	0.988523466	0.613806894	0.477820293	0.394456664	0.623420178	0.871573402	0.999995916
GALT	0.376134645	0.599810399	0.791003226	0.872270915	0.619152773	0.91156913	0.999995916
GAS6	0.821018011	0.800215583	0.351505256	0.97858895	0.977772737	0.980962072	0.999995916
GAT	0.817145239	0.762327881	0.606718459	0.45178528	0.89699763	0.957821583	0.999995916
GBA	0.423089788	0.880304906	0.835093793	0.926600875	0.586777497	0.965218116	0.999995916
GCC2	0.865597275	0.62465953	0.82469688	0.611031631	0.70873541	0.973781786	0.999995916
GCK	0.922594658	0.775662203	0.444929251	0.522319088	0.492540312	0.890897409	0.999995916
GDNF	0.843457958	0.985146671	0.845397711	0.82282661	0.346465371	0.975866197	0.999995916
GFOD1	0.581895714	0.79625094	0.249142012	0.978377744	0.952710394	0.924292421	0.999995916
GGCT	0.877070477	0.310804831	0.853739182	0.941853963	0.977264742	0.979438424	0.999995916
GGCX	0.990997234	0.692013722	0.591527792	0.459397153	0.482986808	0.903144913	0.999995916
GGT5	0.587465501	0.788187278	0.434029041	0.694194034	0.605743058	0.895031178	0.999995916
GGT7	0.429895681	0.437429169	0.823818243	0.875114674	0.785029072	0.923074269	0.999995916
GIT2	0.902221707	0.755805557	0.810904103	0.162307872	0.863818866	0.88341653	0.999995916
GJA4	0.760776449	0.740375898	0.634657152	0.935187898	0.599104811	0.975873473	0.999995916

GJB2	0.256678747	0.862083545	0.770149807	0.620013987	0.958137885	0.91738147	0.999995916
GJB6	0.518482796	0.808847928	0.82400564	0.905554622	0.47224489	0.954934575	0.999995916
GLB1	0.523888588	0.742919903	0.939629569	0.984452583	0.287606256	0.919974848	0.999995916
GLCCI1	0.748013092	0.629502153	0.468009106	0.786820442	0.651323078	0.929549705	0.999995916
GLE1	0.754182267	0.340407265	0.653912218	0.897661505	0.528587045	0.887130536	0.999995916
GLI2	0.658023815	0.516430878	0.767596628	0.939840484	0.542325048	0.945730959	0.999995916
GNAI2	0.909120767	0.472301418	0.772989523	0.358813617	0.623121174	0.877300211	0.999995916
GNAQ	0.793192216	0.761021941	0.897695681	0.536813734	0.305804797	0.901654926	0.999995916
GNAS	0.699863209	0.558108835	0.705833193	0.562155438	0.531661811	0.891688782	0.999995916
GNAT2	0.936041168	0.954962394	0.911631709	0.58593194	0.504163667	0.984773835	0.999995916
GNG3	0.754089312	0.718067766	0.443009309	0.440917559	0.89638691	0.909582525	0.999995916
GOLGA1	0.792371413	0.280786919	0.965521249	0.864460668	0.636560775	0.934315545	0.999995916
GOLGA3	0.565598708	0.300817862	0.990750944	0.978828223	0.928262255	0.957821958	0.999995916
GOPC	0.338800392	0.616425182	0.693359182	0.526148382	0.995964846	0.880434518	0.999995916
GPAM	0.655129357	0.532084249	0.962985772	0.60254074	0.485898966	0.91390025	0.999995916
GPANK1	0.8093398	0.712175241	0.775110511	0.809566088	0.728662405	0.988211632	0.999995916
GPATCH11	0.821086786	0.973278061	0.412519397	0.921167471	0.829014355	0.986547573	0.999995916
GPC6	0.673997299	0.983941846	0.783573132	0.732384898	0.782130899	0.991943138	0.999995916
GPHN	0.887669742	0.831979346	0.287246041	0.61295937	0.924175251	0.935989507	0.999995916
GPM6B	0.535683011	0.971592613	0.532827795	0.593213343	0.675569404	0.927828942	0.999995916
GPN2	0.506815603	0.712112991	0.46728319	0.697248829	0.777145359	0.905046243	0.999995916
GPR107	0.985535871	0.484796851	0.577631842	0.396651202	0.784273232	0.89709416	0.999995916
GPR135	0.77153576	0.961453497	0.843648145	0.771903958	0.638381771	0.992851232	0.999995916
GPR137C	0.998039187	0.893756691	0.69106184	0.299654755	0.993291825	0.970659953	0.999995916
GPR143	0.967951761	0.910970439	0.559188279	0.366390593	0.978652438	0.968258766	0.999995916
GPR151	0.743830809	0.800502286	0.944321865	0.196228295	0.964842163	0.923105581	0.999995916
GPR153	0.319752718	0.576174758	0.6414662	0.671150638	0.913670927	0.873922026	0.999995916
GPR157	0.595725247	0.747650078	0.906256431	0.419855331	0.901786303	0.957647848	0.999995916
GPR158	0.552284553	0.623773744	0.541231021	0.936805265	0.707402644	0.938762971	0.999995916
GPR180	0.886125813	0.645098571	0.727763504	0.579280243	0.824485563	0.975425453	0.999995916
GPR52	0.633696465	0.667937264	0.720637229	0.979612359	0.85891328	0.987265662	0.999995916
GRAMD2B	0.709261862	0.400091639	0.710902182	0.395406797	0.941947989	0.8790507	0.999995916
GRB14	0.999442593	0.681959012	0.677549561	0.937039087	0.18781292	0.889844038	0.999995916
GRIN2D	0.821795023	0.361141073	0.387753283	0.621227809	0.999520155	0.871897567	0.999995916
GRINA	0.904604574	0.996705987	0.53609002	0.53807853	0.534931366	0.949804756	0.999995916
GRK2	0.895758693	0.256988774	0.90550233	0.433360852	0.898797138	0.889709613	0.999995916
GRK4	0.868538498	0.288024672	0.75097023	0.983859496	0.655687505	0.936838539	0.999995916
GRK5	0.741748505	0.71947916	0.85082801	0.872843831	0.319832736	0.941246391	0.999995916
GSK3A	0.827052173	0.94050439	0.604221283	0.753833721	0.40592356	0.952664581	0.999995916
GSPT2	0.487075982	0.949791212	0.64075361	0.62993547	0.42844908	0.887720825	0.999995916

GSR	0.655107121	0.825784676	0.49037416	0.885557741	0.333214812	0.884746318	0.999995916
GTF2A1	0.850885433	0.620927552	0.772155098	0.254292075	0.745477435	0.883075612	0.999995916
GTF2F1	0.752645086	0.567147394	0.986405858	0.692742911	0.474418784	0.949332468	0.999995916
GTF2F2	0.658344957	0.647906728	0.61021252	0.895119974	0.452916462	0.92211776	0.999995916
GTF2I	0.884370698	0.70261583	0.756435724	0.740037732	0.327890909	0.930593288	0.999995916
H2AFV	0.948928877	0.701636941	0.949226037	0.758789981	0.962115552	0.99878123	0.999995916
H2B	0.881459284	0.750906976	0.639215622	0.695599549	0.308960207	0.904421212	0.999995916
H3F3A	0.746345768	0.252197706	0.690791596	0.759202588	0.847692892	0.893735113	0.999995916
HACL1	0.795622478	0.394895405	0.72979439	0.542688635	0.904593983	0.929196527	0.999995916
HADH	0.405601037	0.826451552	0.274377773	0.81402969	0.962099362	0.87305412	0.999995916
HADHA	0.968732803	0.756128327	0.618884241	0.629900202	0.97586644	0.990041378	0.999995916
HADHB	0.908485103	0.610523555	0.227787887	0.671681843	0.981286999	0.893091385	0.999995916
HARS2	0.973159597	0.691035519	0.477897734	0.734773916	0.427388217	0.917020734	0.999995916
HAUS6	0.775022342	0.570938238	0.977410477	0.52473251	0.738824092	0.964614796	0.999995916
HAUS7	0.546296071	0.901013784	0.555508376	0.426338978	0.630892827	0.876028173	0.999995916
HBA1	0.978747496	0.616022138	0.727137766	0.786987941	0.64622461	0.981389035	0.999995916
HCFC2	0.25465905	0.880981567	0.737392878	0.502317738	0.939398023	0.884368095	0.999995916
HDAC8	0.657849605	0.493402317	0.748305792	0.360939451	0.875352123	0.882003273	0.999995916
HECTD2	0.842551974	0.716600221	0.603651613	0.472126885	0.676801302	0.932779549	0.999995916
HEG1	0.794147579	0.694186126	0.768026165	0.549406904	0.941125529	0.980514262	0.999995916
HERC2	0.617468631	0.824730503	0.879511359	0.315379137	0.86238726	0.937352181	0.999995916
HES4	0.726061256	0.747434803	0.396322193	0.349389309	0.972541502	0.875127075	0.999995916
HESX1	0.768555232	0.406464357	0.430631808	0.82240421	0.879380742	0.912695761	0.999995916
HGSNAT	0.497290999	0.585763636	0.596526887	0.842126731	0.485154808	0.870959628	0.999995916
HIRIP3	0.99607564	0.361616937	0.732472225	0.668330921	0.771395534	0.947800778	0.999995916
HIST1H1D	0.56672797	0.932268819	0.397537137	0.969468858	0.470528185	0.910855296	0.999995916
HIST1H2AK	0.593625029	0.643223934	0.769289436	0.406643839	0.635258953	0.880432703	0.999995916
HIST1H2BB	0.569895674	0.974501435	0.630482325	0.797078399	0.947546089	0.988330526	0.999995916
HIST1H2BD	0.83314912	0.706848729	0.928349766	0.416424065	0.746507764	0.965567268	0.999995916
HIST1H2BI	0.515209081	0.945778103	0.939236645	0.778204566	0.431020857	0.958001312	0.999995916
HIST1H2BL	0.655762972	0.988063317	0.879937012	0.685534823	0.313654236	0.937984039	0.999995916
HIST2H2AC	0.329303604	0.9877148	0.528368313	0.939225691	0.611096078	0.914332471	0.999995916
HIVEP2	0.857342254	0.934744318	0.151196181	0.889933169	0.934810855	0.916879598	0.999995916
HMBOX1	0.885968828	0.76798877	0.977188394	0.418006523	0.794204818	0.980911083	0.999995916
HMCES	0.718440879	0.689913988	0.968947699	0.691163828	0.80450744	0.988664341	0.999995916
HMGN3	0.585936564	0.411919589	0.930963016	0.446974729	0.89038058	0.902321166	0.999995916
HNMT	0.648757738	0.591977393	0.904623249	0.645470361	0.484402902	0.925341266	0.999995916
HNRNPA0	0.643971656	0.835863994	0.47339821	0.778699739	0.828542316	0.963199771	0.999995916
HNRNPA1	0.915971136	0.54251782	0.820096585	0.579626045	0.93862303	0.981123541	0.999995916
HNRNPC	0.878539804	0.508797104	0.567482501	0.822882844	0.541774714	0.929692408	0.999995916

HOXA2	0.789506092	0.935894054	0.772390378	0.255361769	0.996850258	0.953517605	0.999995916
HOXA3	0.240308417	0.986307596	0.920477936	0.591203009	0.888356099	0.931081233	0.999995916
HOXA4	0.8377531	0.880491948	0.824409698	0.337710349	0.857893906	0.968024845	0.999995916
HOXA5	0.874413241	0.64953987	0.309388304	0.827687359	0.927915217	0.947095261	0.999995916
HOXB7	0.879486765	0.567837874	0.624181673	0.929873577	0.979339271	0.990603865	0.999995916
HOXB8	0.991405194	0.81533181	0.828676402	0.64142447	0.768727964	0.99440469	0.999995916
HOXC4	0.865244096	0.838238881	0.826660727	0.54924803	0.9854788	0.994031677	0.999995916
HOXC6	0.765195615	0.507392044	0.925429313	0.984954201	0.234923616	0.892875232	0.999995916
HPS3	0.802995465	0.706901138	0.928140984	0.368353802	0.811252872	0.95996488	0.999995916
HRCT1	0.527500014	0.859448312	0.34554135	0.966438681	0.580950847	0.900211348	0.999995916
HS3ST2	0.825017784	0.975076004	0.887029127	0.208793263	0.965803309	0.952710473	0.999995916
HS3ST3A1	0.322010125	0.942841646	0.872521725	0.567504956	0.823753348	0.938980928	0.999995916
HS3ST4	0.861819817	0.932249969	0.331053811	0.555707377	0.776948135	0.931315234	0.999995916
HS3ST6	0.697706248	0.794961722	0.365785785	0.677686526	0.613137809	0.89470837	0.999995916
HSD17B8	0.985305479	0.630541368	0.895030199	0.343110894	0.751901174	0.952451192	0.999995916
HSDL2	0.938118419	0.887623987	0.321985627	0.695081974	0.819540586	0.9575987	0.999995916
HSPA12A	0.400913173	0.834135573	0.405506088	0.941755031	0.978404169	0.93985694	0.999995916
HYAL1	0.665764611	0.732515925	0.709855344	0.737362645	0.976824034	0.986180046	0.999995916
IAH1	0.806209262	0.442486822	0.54093619	0.899908086	0.904553962	0.95979168	0.999995916
IDS	0.669181315	0.188888757	0.997837998	0.798948555	0.754584339	0.88072459	0.999995916
IFI47	0.438063089	0.889033288	0.885139119	0.595274447	0.971169819	0.97559345	0.999995916
IFITM5	0.657103988	0.777326147	0.570031266	0.585820001	0.441823781	0.879471804	0.999995916
IFT27	0.786330179	0.8442052	0.598757306	0.339206741	0.527053147	0.871093597	0.999995916
IFT46	0.999055299	0.283112474	0.786296387	0.434913036	0.908442992	0.900087235	0.999995916
IFT81	0.684541657	0.784389725	0.803383249	0.442639683	0.668336701	0.941891563	0.999995916
IFT88	0.868385712	0.962449606	0.116982789	0.989499638	0.787391093	0.880975941	0.999995916
IGBP1	0.599049585	0.813464599	0.400539193	0.639694171	0.93619732	0.933162584	0.999995916
IL10RB	0.699380161	0.98377179	0.953571029	0.338567226	0.581558756	0.943050344	0.999995916
IL13RA1	0.722046104	0.673466542	0.849077064	0.577629037	0.300764882	0.872450664	0.999995916
IL2RB	0.897691212	0.89828562	0.415777576	0.809001742	0.290647488	0.88571557	0.999995916
ILDR2	0.551878461	0.913005331	0.410582914	0.876224758	0.454994395	0.891815558	0.999995916
ILF2	0.956793487	0.612735868	0.406913591	0.950138225	0.490277637	0.927819688	0.999995916
ILF3	0.44692684	0.843964723	0.778498829	0.976228462	0.531510599	0.95740706	0.999995916
ILKAP	0.698442642	0.834150825	0.67869816	0.792728975	0.570252226	0.968980583	0.999995916
IMPDH2	0.868033764	0.924602455	0.447653745	0.7009966	0.281753973	0.870892603	0.999995916
INAFM1	0.727258523	0.752596489	0.644888863	0.560943452	0.576677056	0.930709955	0.999995916
ING3	0.494659021	0.690252973	0.800800619	0.546468882	0.557749339	0.893192795	0.999995916
INO80D	0.451976459	0.606973422	0.694536228	0.973481031	0.593341904	0.926769716	0.999995916
INO80E	0.342591531	0.476154981	0.817445257	0.830055725	0.835663579	0.906549647	0.999995916
INTS14	0.406129463	0.565649692	0.931448816	0.649088584	0.737205913	0.918690689	0.999995916

INTS3	0.490705616	0.865969461	0.978819202	0.453980155	0.676100427	0.941929816	0.999995916
IP6K3	0.852141406	0.947378379	0.500683564	0.487336777	0.864184421	0.965680243	0.999995916
IPMK	0.672752452	0.34306806	0.79239566	0.843151439	0.587366941	0.903929024	0.999995916
IPO9	0.554479354	0.737240392	0.468705745	0.552160544	0.812853651	0.897307418	0.999995916
IPP	0.560172159	0.778109484	0.910222373	0.840306149	0.549157855	0.970523339	0.999995916
IRF2BP2	0.918349859	0.964087916	0.799645821	0.267173399	0.809002077	0.957751198	0.999995916
ISL2	0.665082311	0.594561531	0.411147153	0.927869215	0.51409735	0.883463118	0.999995916
ITCH	0.681938234	0.827470472	0.344540311	0.474497565	0.920592725	0.895676712	0.999995916
ITGA5	0.831902925	0.75889948	0.245638886	0.767914646	0.835470828	0.915351891	0.999995916
ITGA6	0.76876274	0.523237484	0.609945555	0.611686605	0.47224781	0.870712881	0.999995916
ITGAL	0.962958239	0.913638761	0.952468288	0.955668361	0.501795911	0.997523545	0.999995916
ITGB5	0.943583944	0.908533431	0.509434456	0.686659483	0.471177987	0.951153232	0.999995916
ITIH5	0.417437514	0.913289839	0.799031982	0.940695612	0.295381793	0.895241785	0.999995916
ITPK1	0.536062871	0.672653784	0.421245349	0.52727198	0.937714949	0.878986289	0.999995916
ITPR1	0.675327645	0.626744007	0.97485077	0.826661492	0.567841275	0.973957034	0.999995916
ITPR2	0.822802589	0.830143796	0.739302518	0.861329396	0.857350674	0.996543957	0.999995916
ITPRIPL1	0.853458687	0.369086615	0.544081293	0.626043057	0.982516388	0.922006931	0.999995916
IWS1	0.926160725	0.899341585	0.608495503	0.61338123	0.532530683	0.963705373	0.999995916
JAZF1	0.371509801	0.982788534	0.783821106	0.796771234	0.981067266	0.981545341	0.999995916
JPH1	0.566590237	0.957964744	0.860333826	0.958391991	0.530742781	0.984215571	0.999995916
JTB	0.629580983	0.59112468	0.714819442	0.683465047	0.967995918	0.967956355	0.999995916
JUN	0.450422025	0.966459357	0.80674363	0.889270935	0.746183469	0.983394718	0.999995916
JUND	0.77200632	0.641579123	0.719428784	0.368225464	0.539462932	0.870530314	0.999995916
KANSL2	0.741649971	0.53707773	0.941519154	0.540952622	0.604164238	0.937964773	0.999995916
KANTR	0.787405621	0.973490109	0.570038147	0.181502242	0.924243364	0.875550316	0.999995916
KARS	0.743105764	0.998575981	0.466399164	0.652648649	0.544511247	0.938305837	0.999995916
KAT2B	0.668988754	0.969977244	0.456134014	0.911091545	0.925908693	0.986233204	0.999995916
KAT5	0.892335409	0.281941346	0.932119571	0.830368492	0.697942624	0.947728029	0.999995916
KAT6B	0.758154305	0.600517656	0.978786814	0.473694387	0.626027137	0.945171573	0.999995916
KAT8	0.712832096	0.658829801	0.242363412	0.864692455	0.870891223	0.896882656	0.999995916
KAZALD1	0.703612067	0.30737159	0.858247827	0.766766615	0.568840988	0.889324607	0.999995916
KBTBD4	0.822494284	0.791169396	0.903654028	0.537766101	0.427414899	0.947229862	0.999995916
KCNA6	0.947572483	0.750702747	0.550454131	0.533541924	0.846565898	0.96828048	0.999995916
KCTD13	0.497398094	0.913290813	0.574616978	0.63851223	0.930121316	0.958770897	0.999995916
KDM1B	0.653738338	0.6376031	0.826187567	0.465104164	0.642477492	0.919266545	0.999995916
KDM5A	0.529051295	0.696982143	0.579829507	0.746535912	0.643448007	0.91904008	0.999995916
KDM8	0.317045712	0.756619964	0.586451557	0.739497611	0.856885275	0.901923458	0.999995916
KIAA1671	0.566231253	0.841063515	0.892728612	0.503189493	0.893394725	0.973169643	0.999995916
KIF25	0.560446917	0.626184654	0.806189511	0.433411599	0.739388186	0.904060314	0.999995916
KIRREL3	0.654045586	0.740294229	0.63577198	0.927289208	0.4115168	0.933668685	0.999995916

KLF10	0.787820673	0.982231324	0.680837671	0.975462055	0.570937593	0.991552352	0.999995916
KLF2	0.691525976	0.355439932	0.954889553	0.887583554	0.481968986	0.916419714	0.999995916
KLHDC1	0.857272417	0.611230635	0.763343103	0.89893747	0.613661958	0.980892899	0.999995916
KLHDC10	0.964510699	0.379203295	0.89766057	0.940799567	0.352104044	0.925473038	0.999995916
KLHL12	0.269982941	0.860370667	0.476163691	0.864989352	0.858080423	0.89119437	0.999995916
KLHL2	0.413373664	0.578546379	0.852485321	0.746297758	0.936619764	0.951886906	0.999995916
KLHL20	0.432043955	0.608259096	0.86461052	0.592271095	0.858496793	0.931944659	0.999995916
KLHL36	0.45212473	0.934143753	0.77931496	0.388953145	0.578403515	0.876992924	0.999995916
KLHL5	0.588150085	0.907364802	0.871867203	0.406420372	0.492570984	0.907414697	0.999995916
KLHL9	0.197327138	0.851951156	0.6551278	0.94113818	0.707752146	0.875668289	0.999995916
KLRD1	0.938352959	0.650025132	0.622680013	0.721922375	0.819391743	0.981745724	0.999995916
KLRF2	0.962847646	0.925027788	0.646099003	0.969312381	0.910176138	0.999318752	0.999995916
KLRG1	0.761756474	0.391887552	0.852872015	0.612645634	0.897058442	0.950305127	0.999995916
KNSTRN	0.812269348	0.30880386	0.872670443	0.999396022	0.376119481	0.891495482	0.999995916
KSR1	0.945719169	0.781859596	0.859197419	0.416642011	0.388400472	0.919157104	0.999995916
KTI12	0.940030744	0.439890157	0.685425356	0.560808293	0.8310882	0.945138624	0.999995916
LAMA4	0.513485237	0.842655176	0.501336015	0.576709551	0.639312868	0.887679014	0.999995916
LARP6	0.606741913	0.729959313	0.751854492	0.968607779	0.317679545	0.918772764	0.999995916
LATS1	0.402611953	0.577368954	0.923169833	0.656533766	0.817671445	0.931646415	0.999995916
LCN6	0.842721838	0.839521825	0.983566801	0.853775592	0.124695169	0.877058236	0.999995916
LCOR	0.806939061	0.780844486	0.656807808	0.831394924	0.550952044	0.972678337	0.999995916
LDHAL6B	0.602578578	0.52588962	0.672713856	0.485730148	0.988968174	0.918704523	0.999995916
LDHD	0.405832921	0.698501961	0.929125502	0.924904535	0.851331836	0.977767754	0.999995916
LEMD2	0.257684752	0.940227818	0.766264321	0.937676146	0.932760534	0.962289623	0.999995916
LENG1	0.581134122	0.598835266	0.963500153	0.664954639	0.920546355	0.9772135	0.999995916
LETM2	0.529126743	0.654730492	0.865642731	0.623228851	0.718554842	0.946649894	0.999995916
LETMD1	0.747885856	0.330145381	0.418263191	0.793887513	0.862992106	0.870470753	0.999995916
LGALS4	0.632513659	0.407110855	0.642294959	0.957236552	0.828928983	0.944529362	0.999995916
LGALS7	0.856928746	0.496587674	0.752078316	0.985863601	0.540321046	0.965782615	0.999995916
LIMA1	0.962406677	0.610419247	0.535252526	0.544898154	0.687780217	0.933998707	0.999995916
LIMK1	0.658161908	0.95437412	0.662249511	0.353096337	0.621595544	0.904932719	0.999995916
LIN37	0.657551806	0.622556632	0.479172793	0.797846494	0.996273494	0.95921818	0.999995916
LINS1	0.40493522	0.765431902	0.790893426	0.601486206	0.882660256	0.943750111	0.999995916
LIPC	0.970645018	0.866557585	0.385280274	0.702008723	0.548400088	0.939707455	0.999995916
LMAN2	0.363253341	0.805238166	0.502367357	0.617005426	0.924736482	0.89398973	0.999995916
LNP1	0.670374075	0.54865485	0.399060759	0.978359854	0.843390709	0.936771963	0.999995916
LOC100138078	0.785928175	0.69013579	0.447947069	0.599994707	0.786529135	0.931150929	0.999995916
LOC100138131	0.732754768	0.908176943	0.462817882	0.814858522	0.80267769	0.976194868	0.999995916
LOC100138449	0.810234079	0.957059814	0.272387256	0.995796847	0.926527218	0.974315391	0.999995916
LOC100138645	0.561938365	0.876677705	0.858146427	0.29696027	0.806624309	0.917412393	0.999995916

LOC100139360	0.529414355	0.889484903	0.727365524	0.303554372	0.850000405	0.900825305	0.999995916
LOC100139638	0.660265127	0.997037172	0.998987337	0.99272309	0.120417175	0.885333384	0.999995916
LOC100139732	0.765607357	0.887706334	0.814189703	0.956210686	0.853944445	0.998630944	0.999995916
LOC100140873	0.999286345	0.748747845	0.919370745	0.54089595	0.787988889	0.991531099	0.999995916
LOC100140915	0.91341992	0.987460248	0.401239753	0.787993085	0.907680176	0.987575812	0.999995916
LOC100297513	0.259255877	0.972059548	0.78554635	0.828124237	0.905554021	0.955310697	0.999995916
LOC100297616	0.841149561	0.900868525	0.88006077	0.593776335	0.785742627	0.993067703	0.999995916
LOC100298774	0.657535307	0.43380781	0.920401242	0.76719538	0.423698382	0.896313692	0.999995916
LOC100299705	0.426143081	0.528651497	0.547547985	0.924354794	0.850693459	0.912335762	0.999995916
LOC100299845	0.82283994	0.775989156	0.71953232	0.96680422	0.688795694	0.992654485	0.999995916
LOC100300095	0.696583392	0.857673003	0.207011657	0.955452253	0.630425455	0.877845837	0.999995916
LOC100335177	0.765728227	0.410279733	0.51713146	0.934445965	0.623535756	0.909391117	0.999995916
LOC100335190	0.466285666	0.621434124	0.88953122	0.644549586	0.960973358	0.961026998	0.999995916
LOC100335205	0.54017532	0.875325368	0.975153786	0.86176177	0.951132408	0.996736666	0.999995916
LOC100335404	0.96448288	0.730241195	0.37735339	0.847181197	0.592079704	0.945976937	0.999995916
LOC100336013	0.836876246	0.765180636	0.668824229	0.437614167	0.642606217	0.936214566	0.999995916
LOC100336369	0.917989809	0.275508366	0.912983684	0.760418497	0.405963189	0.871543957	0.999995916
LOC100336532	0.903894584	0.970180727	0.45526523	0.783873423	0.933399698	0.991428585	0.999995916
LOC100336777	0.876424626	0.796719505	0.56643227	0.854636294	0.783599767	0.98838535	0.999995916
LOC100336869	0.951934735	0.668884536	0.995130647	0.43135424	0.980938752	0.988797488	0.999995916
LOC100337081	0.916903524	0.465461231	0.803273776	0.888474802	0.529483566	0.961784855	0.999995916
LOC100337390	0.647471331	0.898907564	0.557097674	0.696600628	0.699371441	0.960220498	0.999995916
LOC100847143	0.737420454	0.727892934	0.642901513	0.925282159	0.890370234	0.990648316	0.999995916
LOC100847156	0.565765844	0.957977056	0.668675792	0.27470119	0.988400607	0.914048018	0.999995916
LOC100847180	0.737190855	0.965562172	0.870354102	0.837225491	0.29019776	0.956431473	0.999995916
LOC100847357	0.633220243	0.78057383	0.669596661	0.803208196	0.509463303	0.947411789	0.999995916
LOC100847374	0.493570345	0.995375308	0.61770415	0.877196924	0.629722496	0.964598989	0.999995916
LOC100847554	0.626411706	0.851076507	0.602135459	0.592936627	0.926499321	0.968088333	0.999995916
LOC100847573	0.218333719	0.874584394	0.814817435	0.755398673	0.954507215	0.928838178	0.999995916
LOC100847745	0.988233706	0.944839339	0.937567135	0.680017924	0.39874443	0.984188517	0.999995916
LOC100847773	0.719379746	0.544919203	0.758361383	0.958139764	0.746755014	0.979080848	0.999995916
LOC100847839	0.956245738	0.751665024	0.815910759	0.193124698	0.740040796	0.893949528	0.999995916
LOC100847890	0.277905842	0.688474977	0.837318197	0.973764258	0.929885123	0.953392937	0.999995916
LOC100847947	0.937596344	0.67837093	0.567190223	0.424350601	0.577877609	0.900951564	0.999995916
LOC100848105	0.923342181	0.869558698	0.761640412	0.665049477	0.673430846	0.989496061	0.999995916
LOC100848122	0.828318442	0.700852342	0.550259741	0.882362559	0.761727648	0.979553478	0.999995916
LOC100848138	0.95290166	0.84860188	0.998393458	0.966104779	0.97599643	0.999989963	0.999995916
LOC100848171	0.958637517	0.755469871	0.971803992	0.66847529	0.991724105	0.998856496	0.999995916
LOC100848325	0.669518349	0.887149667	0.567821355	0.58204153	0.805402914	0.96028718	0.999995916
LOC100848353	0.67953457	0.622335552	0.955013743	0.755044499	0.703881237	0.97953955	0.999995916

LOC100848357	0.984274134	0.786243489	0.960071552	0.735572741	0.786320798	0.998215206	0.999995916
LOC100848405	0.90221261	0.576543258	0.89674881	0.75279697	0.309149268	0.925270195	0.999995916
LOC100848472	0.736296114	0.50739335	0.708327887	0.976996091	0.421796626	0.925768041	0.999995916
LOC100848495	0.6675167	0.586922945	0.976872827	0.678825057	0.999908427	0.987703793	0.999995916
LOC100848538	0.733610582	0.985496935	0.726771342	0.578966013	0.443323852	0.947031028	0.999995916
LOC100848568	0.764879026	0.80823359	0.859880004	0.654190266	0.371697197	0.943107209	0.999995916
LOC100848581	0.945408897	0.59989842	0.805176518	0.744524952	0.646320765	0.980694992	0.999995916
LOC100848642	0.309479927	0.990100082	0.673179305	0.740933934	0.909183875	0.949696866	0.999995916
LOC100848815	0.530744223	0.537036715	0.719566426	0.912825971	0.936539228	0.967703058	0.999995916
LOC100848906	0.58355663	0.978416431	0.504866929	0.896608871	0.525122483	0.94760345	0.999995916
LOC100848912	0.903172113	0.678498992	0.58487491	0.383712973	0.80087863	0.926855577	0.999995916
LOC100848985	0.765839388	0.852943845	0.659385032	0.995805474	0.912841124	0.997209104	0.999995916
LOC100849023	0.688629648	0.839552917	0.815357911	0.630104954	0.984905063	0.991469708	0.999995916
LOC100849046	0.567188694	0.935110306	0.510477588	0.999246978	0.517972793	0.950436625	0.999995916
LOC100849069	0.969865941	0.629094289	0.448308781	0.987068147	0.921453003	0.986092611	0.999995916
LOC100850436	0.245952102	0.735779669	0.763628717	0.96074211	0.843956061	0.928707211	0.999995916
LOC101902036	0.88179658	0.802392679	0.972810824	0.468157092	0.435917316	0.950643417	0.999995916
LOC101902048	0.967412892	0.693377533	0.79632801	0.79396981	0.350462365	0.955407896	0.999995916
LOC101902124	0.88578247	0.744204864	0.281213792	0.684427583	0.962219761	0.937571039	0.999995916
LOC101902141	0.733149389	0.558840206	0.983660503	0.665392412	0.366801185	0.914002425	0.999995916
LOC101902207	0.916657627	0.432735945	0.759569014	0.667574922	0.964408844	0.974046126	0.999995916
LOC101902345	0.982713248	0.559130202	0.475156209	0.530558044	0.936889826	0.943483858	0.999995916
LOC101902360	0.663332437	0.783855639	0.367899101	0.882352769	0.802650206	0.947441165	0.999995916
LOC101902430	0.973578665	0.750471439	0.291070817	0.387970758	0.8819533	0.874514049	0.999995916
LOC101902644	0.902669247	0.744849523	0.545458312	0.398032743	0.869091945	0.941327622	0.999995916
LOC101902663	0.95542605	0.459450835	0.87530318	0.735986474	0.554095258	0.959600718	0.999995916
LOC101902681	0.668669501	0.806591843	0.880156962	0.564718639	0.68260316	0.970491424	0.999995916
LOC101902831	0.42268669	0.9123249	0.46217762	0.629351988	0.898289971	0.916830836	0.999995916
LOC101902841	0.812741823	0.336266932	0.865534831	0.741563596	0.534371474	0.908192561	0.999995916
LOC101902907	0.657523129	0.779614628	0.54008699	0.984595517	0.852311941	0.983262808	0.999995916
LOC101902930	0.477313119	0.38922586	0.939025249	0.958409615	0.522176068	0.899264486	0.999995916
LOC101902959	0.746793249	0.930885318	0.148278066	0.973830719	0.867160466	0.898880974	0.999995916
LOC101902983	0.923409388	0.808265779	0.242673273	0.821344823	0.791211064	0.93387517	0.999995916
LOC101902994	0.332345477	0.654605609	0.814726663	0.603817788	0.811764537	0.898631926	0.999995916
LOC101902998	0.283068403	0.871216255	0.894737525	0.377533678	0.884864822	0.87635039	0.999995916
LOC101903097	0.592964505	0.788351177	0.79304109	0.567224533	0.857470219	0.969546172	0.999995916
LOC101903098	0.910515938	0.965105367	0.6613393	0.709025616	0.675664792	0.990013469	0.999995916
LOC101903205	0.427992911	0.921553709	0.870772856	0.89265846	0.805076064	0.985782721	0.999995916
LOC101903253	0.783572145	0.793547049	0.427601821	0.478257236	0.881216411	0.928714145	0.999995916
LOC101903281	0.805505556	0.381317202	0.400778269	0.999901053	0.75035077	0.906366606	0.999995916

LOC101903356	0.844692811	0.652860632	0.283552828	0.907420506	0.97486799	0.949298312	0.999995916
LOC101903567	0.565345506	0.985541704	0.737426766	0.510428295	0.798438151	0.964520576	0.999995916
LOC101903572	0.88137169	0.952103613	0.941093337	0.834744262	0.61953552	0.997705667	0.999995916
LOC101903600	0.90150957	0.225765227	0.607676432	0.728739852	0.861506833	0.883631396	0.999995916
LOC101903682	0.454805074	0.932409157	0.392660524	0.66891705	0.692978478	0.882807056	0.999995916
LOC101903900	0.441965754	0.582464467	0.924216355	0.730396294	0.627769982	0.925809222	0.999995916
LOC101903928	0.734849393	0.787366912	0.575617916	0.856732415	0.633652198	0.969722809	0.999995916
LOC101903976	0.693943771	0.399573905	0.473852424	0.703486093	0.985210276	0.904608912	0.999995916
LOC101904084	0.583043069	0.610546471	0.799960663	0.942337623	0.963458055	0.987532513	0.999995916
LOC101904098	0.86782185	0.49345428	0.789990295	0.570484574	0.382190507	0.876441077	0.999995916
LOC101904133	0.767197407	0.627492658	0.698370673	0.839866204	0.404590859	0.93076831	0.999995916
LOC101904156	0.637067844	0.858145378	0.727231926	0.868963997	0.89708077	0.992973094	0.999995916
LOC101904248	0.896859095	0.886993646	0.118542866	0.975549521	0.864523025	0.886918239	0.999995916
LOC101904268	0.666422513	0.713487464	0.788035971	0.717045183	0.816550003	0.980616581	0.999995916
LOC101904314	0.795301837	0.56375382	0.553725053	0.83001104	0.758405738	0.959396774	0.999995916
LOC101904393	0.687179192	0.919683763	0.911261033	0.817827623	0.945226246	0.998516464	0.999995916
LOC101904396	0.727691145	0.812554754	0.784055424	0.400856737	0.665092502	0.938792216	0.999995916
LOC101904413	0.361500234	0.907007523	0.693651435	0.996409612	0.605536417	0.948590572	0.999995916
LOC101904667	0.701590349	0.646246946	0.92670552	0.85341855	0.475215563	0.965751228	0.999995916
LOC101904822	0.756705195	0.823281956	0.302145579	0.983583102	0.922569762	0.965915344	0.999995916
LOC101904902	0.720293805	0.548156756	0.445829903	0.799693734	0.996326901	0.950509241	0.999995916
LOC101904916	0.925802211	0.471628843	0.653696675	0.878514736	0.8765074	0.980704284	0.999995916
LOC101904947	0.851917091	0.991616038	0.265921176	0.833566135	0.794274397	0.955461622	0.999995916
LOC101904962	0.984102573	0.773477711	0.935899909	0.221057152	0.527671623	0.892809222	0.999995916
LOC101905010	0.955787503	0.992681701	0.787338702	0.634630236	0.436649477	0.977670318	0.999995916
LOC101905029	0.919748471	0.707263764	0.917548941	0.411118255	0.965012235	0.984085457	0.999995916
LOC101905049	0.970679116	0.984250547	0.205806172	0.478986839	0.888071836	0.893670324	0.999995916
LOC101905199	0.98589717	0.890019283	0.597737392	0.872527359	0.233877897	0.923704752	0.999995916
LOC101905203	0.712717198	0.584625027	0.769812155	0.805057913	0.960341222	0.985967707	0.999995916
LOC101905232	0.394393705	0.743847685	0.636392976	0.854717768	0.607897351	0.912344989	0.999995916
LOC101905365	0.760009108	0.747436438	0.931432338	0.19537806	0.782901223	0.889282217	0.999995916
LOC101905453	0.696091822	0.978351083	0.634778278	0.3552436	0.797578564	0.937897066	0.999995916
LOC101905493	0.743576746	0.771467597	0.846014164	0.764479049	0.720757932	0.98870934	0.999995916
LOC101905513	0.789509224	0.960783895	0.831781288	0.77825454	0.826156502	0.997631243	0.999995916
LOC101905686	0.898529114	0.928245343	0.396335121	0.696999109	0.79244975	0.970350351	0.999995916
LOC101905687	0.578176896	0.963807075	0.741428851	0.735377706	0.432903587	0.944736478	0.999995916
LOC101905723	0.611860136	0.81884893	0.834429044	0.274438405	0.994000905	0.930585868	0.999995916
LOC101905771	0.794409931	0.886357703	0.950968224	0.656237578	0.72016228	0.993467627	0.999995916
LOC101905977	0.77086668	0.890586626	0.316500231	0.440650722	0.856634971	0.891072158	0.999995916
LOC101906009	0.559214426	0.503357617	0.706452589	0.754762107	0.630102659	0.909275563	0.999995916

LOC101906110	0.669970969	0.786261197	0.578869938	0.906181707	0.727825593	0.976103531	0.999995916
LOC101906135	0.968747793	0.576570773	0.909010615	0.380135072	0.857783273	0.963706086	0.999995916
LOC101906218	0.94767991	0.711545387	0.362126606	0.809461515	0.837157703	0.963669582	0.999995916
LOC101906273	0.273562116	0.770653696	0.663753193	0.908894117	0.954564059	0.937015696	0.999995916
LOC101906276	0.905393567	0.38122412	0.678780252	0.832617025	0.847031067	0.963563643	0.999995916
LOC101906315	0.939301826	0.525718846	0.836784962	0.491581064	0.719569471	0.954024701	0.999995916
LOC101906397	0.996565112	0.78831782	0.372072851	0.709888843	0.592132849	0.938207635	0.999995916
LOC101906398	0.825553602	0.816420761	0.69403584	0.789903598	0.453993851	0.964648471	0.999995916
LOC101906457	0.419182956	0.930448911	0.655230011	0.791194597	0.641544434	0.943441074	0.999995916
LOC101906522	0.693742241	0.721074365	0.314626195	0.859587169	0.986607678	0.946091141	0.999995916
LOC101906569	0.870945196	0.530501781	0.541732717	0.720247959	0.587925439	0.922614186	0.999995916
LOC101906688	0.50630514	0.994391086	0.646383715	0.6019931	0.523688499	0.918918581	0.999995916
LOC101906914	0.983538607	0.610142511	0.85454989	0.59392077	0.875345683	0.988607473	0.999995916
LOC101907005	0.95506883	0.78637825	0.655751496	0.54407782	0.543410175	0.953708357	0.999995916
LOC101907195	0.945173932	0.361079098	0.387302262	0.958275126	0.996372253	0.940824944	0.999995916
LOC101907213	0.897874205	0.671810771	0.954412059	0.200228632	0.686557092	0.886245055	0.999995916
LOC101907250	0.859429049	0.860357698	0.54212673	0.996669033	0.588237263	0.983761818	0.999995916
LOC101907320	0.619485531	0.85234488	0.446899388	0.49452425	0.851767348	0.915234527	0.999995916
LOC101907353	0.54931829	0.680770808	0.926310101	0.929452043	0.761017512	0.985492099	0.999995916
LOC101907404	0.519398912	0.860967091	0.887209603	0.929903225	0.880153522	0.994044675	0.999995916
LOC101907523	0.442257794	0.498792492	0.850456917	0.932967215	0.719918942	0.940674095	0.999995916
LOC101907544	0.715532359	0.928078124	0.545663482	0.992071225	0.613864165	0.980898574	0.999995916
LOC101907549	0.905470987	0.932980426	0.332839992	0.695005657	0.780681058	0.95751002	0.999995916
LOC101907658	0.832797019	0.661063629	0.626150842	0.745929021	0.703092491	0.969717674	0.999995916
LOC101907661	0.474163379	0.654403883	0.469739144	0.990967686	0.543662659	0.885179794	0.999995916
LOC101907688	0.803849048	0.416388974	0.835597328	0.480358199	0.812898456	0.925930589	0.999995916
LOC101907713	0.818984594	0.817013305	0.614419813	0.626194262	0.397026419	0.918487777	0.999995916
LOC101907747	0.824639852	0.702016838	0.747166227	0.514975732	0.495391316	0.927057704	0.999995916
LOC101907800	0.999840249	0.504686097	0.521644424	0.919088629	0.529866756	0.942319629	0.999995916
LOC101907835	0.626717726	0.626908706	0.959441726	0.512692762	0.477844104	0.906354577	0.999995916
LOC101907857	0.43117899	0.912182664	0.976653456	0.668735371	0.384785033	0.914579659	0.999995916
LOC101907941	0.989307045	0.777582997	0.324804742	0.947950468	0.471058733	0.928250837	0.999995916
LOC101908046	0.766546187	0.934759757	0.890554829	0.957335863	0.593404255	0.996109545	0.999995916
LOC101908123	0.829101298	0.539160563	0.651170991	0.896489226	0.512763663	0.946317403	0.999995916
LOC101908577	0.614841309	0.795159144	0.886115388	0.807294198	0.986682096	0.995261874	0.999995916
LOC101908759	0.268324031	0.888079184	0.820729196	0.853696306	0.692707172	0.932057142	0.999995916
LOC101909083	0.813825563	0.410676571	0.456470491	0.667553549	0.778447791	0.886483294	0.999995916
LOC101909432	0.660768926	0.779439693	0.792277741	0.384696896	0.953756852	0.955998117	0.999995916
LOC104968479	0.73791397	0.460776375	0.709880561	0.975342996	0.914745997	0.979703231	0.999995916
LOC104968656	0.771538306	0.653015109	0.272092733	0.723758029	0.905139804	0.902860483	0.999995916

LOC104968671	0.918832931	0.568301984	0.924664839	0.635314372	0.789061193	0.984998806	0.999995916
LOC104968807	0.793434274	0.900501786	0.676079934	0.600526938	0.406218906	0.933992943	0.999995916
LOC104969067	0.928363689	0.923241325	0.793303182	0.299866322	0.413330772	0.894667627	0.999995916
LOC104969177	0.618880402	0.424003987	0.654558007	0.713265817	0.604614072	0.877038998	0.999995916
LOC104969192	0.620314234	0.92276834	0.754439974	0.981624976	0.676282651	0.990893899	0.999995916
LOC104969238	0.779899544	0.675801487	0.387784517	0.743080661	0.623197687	0.909373957	0.999995916
LOC104969259	0.681410837	0.939211113	0.642109122	0.833861776	0.939207239	0.993849316	0.999995916
LOC104970162	0.798147559	0.484101493	0.991437627	0.647164982	0.726847128	0.969505176	0.999995916
LOC104970698	0.408939008	0.746699438	0.936623748	0.343238052	0.89874831	0.900607916	0.999995916
LOC104970815	0.904165012	0.764844912	0.783120361	0.601037678	0.902821344	0.991594872	0.999995916
LOC104970908	0.568055359	0.556153673	0.574887228	0.758285125	0.653612885	0.903155189	0.999995916
LOC104971057	0.782376815	0.688313056	0.211406917	0.86986775	0.723306643	0.872249143	0.999995916
LOC104971501	0.960392966	0.655016247	0.917663922	0.568573797	0.991414951	0.994090279	0.999995916
LOC104971503	0.587446953	0.587292963	0.725466161	0.973553087	0.516969758	0.940644641	0.999995916
LOC104971926	0.932746149	0.194616574	0.724260832	0.7854575	0.793273794	0.890907229	0.999995916
LOC104972026	0.984588582	0.430744325	0.947041892	0.48469916	0.883108523	0.966365516	0.999995916
LOC104972409	0.624690076	0.804315936	0.531463243	0.628735802	0.434208287	0.874771478	0.999995916
LOC104972417	0.586096777	0.990853333	0.748462096	0.546063741	0.52839352	0.94021719	0.999995916
LOC104972843	0.943920574	0.50760576	0.805624152	0.766145659	0.87933674	0.98774174	0.999995916
LOC104973050	0.710142962	0.192516529	0.991707019	0.710828958	0.818107617	0.885732653	0.999995916
LOC104973390	0.624076405	0.985491365	0.583070545	0.716967381	0.96345247	0.985923526	0.999995916
LOC104973431	0.652678082	0.484619567	0.532460514	0.721788931	0.729082208	0.90118674	0.999995916
LOC104973760	0.809475523	0.519717112	0.469109834	0.767029565	0.744382444	0.929309412	0.999995916
LOC104973767	0.456887277	0.543815088	0.871953073	0.758536262	0.869461116	0.952110531	0.999995916
LOC104973803	0.349192181	0.75038553	0.604156772	0.794639413	0.799871519	0.916670318	0.999995916
LOC104974034	0.993593553	0.906911151	0.940023858	0.690881411	0.49337541	0.991099389	0.999995916
LOC104974459	0.655578921	0.905750389	0.553960602	0.318122155	0.865270665	0.90389015	0.999995916
LOC104974542	0.838170587	0.99691318	0.84890935	0.888914325	0.827048157	0.999430838	0.999995916
LOC104974758	0.975083578	0.439850934	0.767331427	0.657598896	0.909716744	0.974903771	0.999995916
LOC104974837	0.951345268	0.416746427	0.795783721	0.417590967	0.923681372	0.937254495	0.999995916
LOC104975073	0.84572315	0.830689128	0.552939109	0.735322109	0.267942139	0.881631531	0.999995916
LOC104975099	0.940747451	0.655170381	0.447313296	0.625541516	0.73230228	0.940893747	0.999995916
LOC104975283	0.81822569	0.694796714	0.608106204	0.790706044	0.801032006	0.980523252	0.999995916
LOC104975286	0.755051689	0.906156831	0.694350465	0.975997573	0.989812981	0.998744314	0.999995916
LOC104975415	0.790721946	0.674070067	0.3006187	0.898480523	0.955045341	0.948762192	0.999995916
LOC104975788	0.756356778	0.639776921	0.616370044	0.644601486	0.494853911	0.910005434	0.999995916
LOC104975811	0.722067812	0.895789687	0.670462987	0.982746012	0.201631044	0.897213237	0.999995916
LOC104975960	0.956200671	0.818841681	0.78528144	0.852179655	0.227545299	0.935189501	0.999995916
LOC104975979	0.905845718	0.670847899	0.514589494	0.736462132	0.91654695	0.978688498	0.999995916
LOC104976448	0.546324485	0.935495121	0.733057171	0.809194679	0.481786819	0.953966671	0.999995916

LOC104976575	0.926563458	0.900547477	0.909207189	0.83206269	0.180235175	0.930333536	0.999995916
LOC104976664	0.197772305	0.942107276	0.931691293	0.696879431	0.62077937	0.878981377	0.999995916
LOC107131357	0.538366767	0.834883193	0.659023744	0.97240987	0.808434145	0.983362549	0.999995916
LOC107131452	0.948688952	0.532186811	0.979864847	0.883619729	0.861307066	0.996683436	0.999995916
LOC107131455	0.783549518	0.718542967	0.852985592	0.790117568	0.806804419	0.9926701	0.999995916
LOC107131489	0.588991148	0.820806639	0.994763733	0.498807923	0.673622175	0.961930628	0.999995916
LOC107131494	0.784371904	0.808956441	0.459241848	0.881061176	0.36742973	0.908969212	0.999995916
LOC107131525	0.93886776	0.855867966	0.478267986	0.420467468	0.904999571	0.954067421	0.999995916
LOC107131573	0.493853568	0.536658298	0.888312572	0.806976041	0.914432072	0.967081066	0.999995916
LOC107131607	0.951501156	0.627317646	0.314203653	0.521959115	0.883166687	0.897998237	0.999995916
LOC107131615	0.376188797	0.925688398	0.762795821	0.921796892	0.579701578	0.951546404	0.999995916
LOC107131651	0.975180677	0.93455316	0.770569118	0.52022527	0.368167111	0.946789776	0.999995916
LOC107131834	0.844090677	0.758355459	0.314409403	0.882491977	0.955948973	0.96549837	0.999995916
LOC107131906	0.993362472	0.873273222	0.755682382	0.798167431	0.615399972	0.993860126	0.999995916
LOC107132070	0.769929691	0.769170268	0.987325599	0.609206947	0.606822514	0.979888895	0.999995916
LOC107132093	0.979403261	0.714132751	0.889879101	0.533636521	0.941513722	0.993188606	0.999995916
LOC107132175	0.978916382	0.779019033	0.728965847	0.233675041	0.578122123	0.878981368	0.999995916
LOC107132189	0.54790095	0.647143375	0.552688553	0.708003497	0.834082629	0.932120383	0.999995916
LOC107132196	0.76618335	0.247551095	0.965557118	0.979541308	0.525919673	0.908982911	0.999995916
LOC107132237	0.459754774	0.840064614	0.658913017	0.793127279	0.746012283	0.956461778	0.999995916
LOC107132243	0.912146286	0.791442789	0.723312484	0.56072201	0.949283726	0.989961522	0.999995916
LOC107132255	0.67352145	0.974436738	0.88472814	0.762242273	0.992635248	0.998408335	0.999995916
LOC107132288	0.441039356	0.619440535	0.514058504	0.864411299	0.923409707	0.928755848	0.999995916
LOC107132374	0.541036017	0.419246951	0.777124438	0.861926962	0.670944289	0.918181019	0.999995916
LOC107132386	0.861443632	0.936720835	0.559284042	0.630030942	0.821092571	0.983475598	0.999995916
LOC107132395	0.642804759	0.428685473	0.598799017	0.662813959	0.968055545	0.922491264	0.999995916
LOC107132398	0.642884315	0.531443328	0.992133355	0.99057605	0.603465609	0.976516656	0.999995916
LOC107132546	0.849141838	0.839642283	0.578103271	0.497441905	0.467644866	0.910945805	0.999995916
LOC107132556	0.971910015	0.889058342	0.518774493	0.911735688	0.493477802	0.976259622	0.999995916
LOC107132610	0.877991084	0.840012152	0.62701458	0.509447639	0.377164671	0.901511887	0.999995916
LOC107132664	0.854623623	0.779361562	0.290226784	0.8963114	0.737714469	0.942045051	0.999995916
LOC107132713	0.924548074	0.734840915	0.438303123	0.588378697	0.945833785	0.963776056	0.999995916
LOC107132735	0.907693718	0.868098378	0.400001752	0.602297954	0.693850349	0.944870015	0.999995916
LOC107132784	0.868128298	0.721404945	0.567240614	0.348187825	0.657931314	0.890024728	0.999995916
LOC107132799	0.969274353	0.772324966	0.597627894	0.806019885	0.605486095	0.980383442	0.999995916
LOC107132849	0.922378885	0.683551178	0.988346424	0.942196848	0.761501059	0.998550121	0.999995916
LOC107132851	0.953806411	0.764120352	0.567855733	0.666956413	0.916590164	0.98673566	0.999995916
LOC107132877	0.664564119	0.702243244	0.931846934	0.799040959	0.905112941	0.993324461	0.999995916
LOC107132897	0.842430886	0.92188237	0.962830751	0.518453068	0.222593191	0.897757907	0.999995916
LOC107132967	0.42248731	0.708178423	0.437661197	0.882252897	0.650756765	0.879134566	0.999995916

LOC107132987	0.697987478	0.325032275	0.859727341	0.972499048	0.419764261	0.887069716	0.999995916
LOC107132994	0.730774796	0.641353987	0.982159843	0.345358903	0.744626911	0.934461702	0.999995916
LOC107133180	0.326476236	0.807305492	0.449407181	0.880800859	0.738335363	0.882526273	0.999995916
LOC107133226	0.818326831	0.66692928	0.426725578	0.881356499	0.678728718	0.949925026	0.999995916
LOC112441491	0.843128411	0.391802708	0.929376961	0.864088108	0.401407826	0.92313701	0.999995916
LOC112441511	0.923414762	0.893786655	0.329270686	0.817768542	0.625511541	0.949732442	0.999995916
LOC112441530	0.797177749	0.795043136	0.228231083	0.982961049	0.763516956	0.925274742	0.999995916
LOC112441568	0.538807518	0.822069245	0.606634135	0.87587177	0.7789422	0.970606485	0.999995916
LOC112441619	0.831893356	0.428563054	0.92035789	0.55506588	0.548768761	0.915885567	0.999995916
LOC112441843	0.964786652	0.777502341	0.641463594	0.929635953	0.415122679	0.971412344	0.999995916
LOC112441846	0.658963569	0.951924937	0.466869554	0.838277234	0.98330212	0.984888955	0.999995916
LOC112441859	0.891341443	0.670030861	0.743000912	0.719893893	0.755041596	0.984854272	0.999995916
LOC112441879	0.661722296	0.670801464	0.652682239	0.634222998	0.583284722	0.923854329	0.999995916
LOC112441888	0.593752069	0.968573971	0.970239327	0.173070143	0.753882689	0.874577993	0.999995916
LOC112442038	0.853459977	0.961110179	0.546585647	0.846163972	0.972053866	0.996377215	0.999995916
LOC112442040	0.940532398	0.804122197	0.821169694	0.836777965	0.466579938	0.985070697	0.999995916
LOC112442083	0.678634644	0.616360586	0.864971718	0.611511454	0.530400881	0.933565536	0.999995916
LOC112442208	0.602984378	0.667634351	0.913000206	0.524251001	0.819103312	0.960156295	0.999995916
LOC112442223	0.542572539	0.996514269	0.5881461	0.329143788	0.866442866	0.904090661	0.999995916
LOC112442228	0.724693553	0.763512688	0.584050502	0.911948759	0.599268144	0.968185907	0.999995916
LOC112442244	0.763761429	0.706593536	0.985356222	0.505911536	0.863704363	0.983267784	0.999995916
LOC112442246	0.796898893	0.764830293	0.34213929	0.815571938	0.549777978	0.907883854	0.999995916
LOC112442254	0.742109038	0.711991711	0.477451636	0.914777713	0.53197646	0.938124579	0.999995916
LOC112442264	0.495010149	0.727160247	0.866190582	0.727756541	0.852444596	0.973877682	0.999995916
LOC112442323	0.500181787	0.559918465	0.803939655	0.677048647	0.62282951	0.909753701	0.999995916
LOC112442349	0.977007777	0.946411132	0.583653037	0.968152189	0.424134459	0.981099425	0.999995916
LOC112442352	0.416925825	0.964260492	0.594168724	0.627522274	0.603455085	0.903768318	0.999995916
LOC112442367	0.727066185	0.828955641	0.595337471	0.385804699	0.972018794	0.946826035	0.999995916
LOC112442382	0.792219889	0.832528945	0.386639108	0.906814639	0.448041978	0.920042448	0.999995916
LOC112442597	0.850476381	0.346472988	0.871631857	0.585855048	0.503930589	0.88033499	0.999995916
LOC112442602	0.664437011	0.981424993	0.333218547	0.881853145	0.532618539	0.918316299	0.999995916
LOC112442611	0.32563408	0.935834285	0.486582613	0.96307897	0.601332723	0.897124863	0.999995916
LOC112442625	0.665250796	0.32570218	0.765288256	0.695328727	0.934973463	0.924499069	0.999995916
LOC112442630	0.6574461	0.938600027	0.518392457	0.529608274	0.598091614	0.917482424	0.999995916
LOC112442636	0.870671333	0.786974601	0.307711476	0.771023359	0.883477157	0.952549752	0.999995916
LOC112442649	0.607435945	0.823340112	0.776724417	0.497054922	0.383899184	0.877141318	0.999995916
LOC112442676	0.805733005	0.648078844	0.931315075	0.914297055	0.585514712	0.987781085	0.999995916
LOC112442687	0.66335707	0.830754139	0.569601096	0.529896545	0.649975072	0.924818975	0.999995916
LOC112442702	0.928444173	0.830975522	0.766243519	0.824102388	0.687109233	0.994678032	0.999995916
LOC112442721	0.979844103	0.566160579	0.959194149	0.522997909	0.551022369	0.957916337	0.999995916

LOC112442740	0.40830899	0.608990409	0.573045581	0.989653485	0.747530687	0.922002305	0.999995916
LOC112442754	0.949436723	0.376352176	0.826640494	0.778488975	0.905902983	0.978001692	0.999995916
LOC112442802	0.740425903	0.937664174	0.681273442	0.618148932	0.811481834	0.98416779	0.999995916
LOC112442987	0.817418609	0.920514167	0.408716173	0.689243721	0.482543552	0.918569369	0.999995916
LOC112443159	0.896610543	0.870263698	0.630547442	0.255313281	0.620305957	0.884114153	0.999995916
LOC112443213	0.965858454	0.977280831	0.537311796	0.903397445	0.642119149	0.991626574	0.999995916
LOC112443240	0.978810333	0.221292653	0.506689703	0.792709549	0.973054229	0.895260397	0.999995916
LOC112443416	0.794845414	0.420123759	0.920243937	0.705591554	0.404152179	0.899740834	0.999995916
LOC112443422	0.862878117	0.978799797	0.802159416	0.779380612	0.377495721	0.975604709	0.999995916
LOC112443425	0.388234161	0.920447899	0.744413526	0.700959486	0.522535797	0.912874569	0.999995916
LOC112443503	0.749137609	0.811152758	0.521650987	0.952444496	0.355859802	0.924127433	0.999995916
LOC112443728	0.824186477	0.649478691	0.637763087	0.530034014	0.851820338	0.95832056	0.999995916
LOC112443767	0.925399525	0.805178631	0.642935644	0.88099434	0.635795559	0.988825672	0.999995916
LOC112443877	0.963789431	0.96472929	0.693228458	0.998796918	0.358194871	0.982931816	0.999995916
LOC112444152	0.907905156	0.757521641	0.790279923	0.562630561	0.598572318	0.970510485	0.999995916
LOC112444198	0.42328622	0.942787809	0.973092298	0.497755809	0.52092073	0.916751146	0.999995916
LOC112444207	0.971228674	0.968120313	0.775775092	0.25951206	0.865583243	0.962954485	0.999995916
LOC112444287	0.698587628	0.893311163	0.521263513	0.697298787	0.709784149	0.961657439	0.999995916
LOC112444289	0.75269382	0.47572925	0.846733134	0.637842402	0.872276848	0.965043919	0.999995916
LOC112444339	0.831798366	0.837055033	0.941734195	0.511062747	0.615095699	0.977441106	0.999995916
LOC112444346	0.887354334	0.4969326	0.777978809	0.595991712	0.50160936	0.918877213	0.999995916
LOC112444464	0.698257752	0.978477208	0.330215115	0.983363098	0.86726744	0.973568354	0.999995916
LOC112444473	0.664147188	0.967557106	0.99548641	0.799973112	0.828701435	0.998091514	0.999995916
LOC112444593	0.384638808	0.556254273	0.627056414	0.978008256	0.570594608	0.878550658	0.999995916
LOC112444613	0.761513979	0.682193005	0.667325501	0.740654635	0.691302578	0.968520758	0.999995916
LOC112444842	0.980986225	0.398149899	0.456300273	0.540992537	0.765855258	0.876596338	0.999995916
LOC112444843	0.935004721	0.681826977	0.64010221	0.5984188	0.633973746	0.95866544	0.999995916
LOC112444867	0.668170765	0.442975087	0.976334219	0.872577681	0.816999521	0.977413274	0.999995916
LOC112445001	0.932134602	0.677883368	0.352028826	0.66591295	0.776574265	0.931490754	0.999995916
LOC112445052	0.928262644	0.65420961	0.809810384	0.72985927	0.678582821	0.985252588	0.999995916
LOC112445060	0.772931987	0.980596439	0.655362827	0.250548004	0.698321319	0.89867417	0.999995916
LOC112445076	0.582610705	0.69282191	0.863155397	0.421595949	0.704726744	0.919942137	0.999995916
LOC112445088	0.413082291	0.746147976	0.831887745	0.911457669	0.338753626	0.886290787	0.999995916
LOC112445178	0.690514853	0.899262638	0.87067809	0.231550137	0.896550294	0.928887125	0.999995916
LOC112445915	0.955849526	0.832106537	0.140732583	0.845148297	0.872329833	0.89188842	0.999995916
LOC112445951	0.764644395	0.869318108	0.875215264	0.61124877	0.860516595	0.992659108	0.999995916
LOC112445965	0.70915093	0.604840594	0.503119789	0.868954987	0.567860869	0.92313529	0.999995916
LOC112445971	0.69664684	0.629846617	0.858458876	0.918997137	0.488018065	0.965145225	0.999995916
LOC112445988	0.833495133	0.758832497	0.678518811	0.828051934	0.404289224	0.952576931	0.999995916
LOC112445996	0.683249318	0.745096257	0.955448538	0.371630721	0.872028272	0.960059022	0.999995916

LOC112446007	0.798807353	0.736430721	0.267721193	0.73217159	0.900672408	0.920317502	0.999995916
LOC112446022	0.663558505	0.598568632	0.887393607	0.654701135	0.313373579	0.873612677	0.999995916
LOC112446039	0.992352737	0.832116209	0.392921378	0.58198168	0.471867924	0.901863331	0.999995916
LOC112446390	0.520275616	0.853943547	0.855904382	0.972536473	0.411344239	0.957281855	0.999995916
LOC112446417	0.971551462	0.669232653	0.961008814	0.942670521	0.974022522	0.999721402	0.999995916
LOC112446452	0.837633543	0.620103429	0.414090545	0.69804796	0.737850408	0.92748251	0.999995916
LOC112446642	0.724539248	0.663113185	0.992518488	0.709701215	0.726117615	0.985608378	0.999995916
LOC112446667	0.403060807	0.561687857	0.772690434	0.780359647	0.968715168	0.94523518	0.999995916
LOC112446701	0.916784081	0.790234606	0.720006816	0.397394061	0.556331148	0.931756502	0.999995916
LOC112446709	0.893222077	0.860342339	0.556271938	0.882528773	0.278223575	0.921519502	0.999995916
LOC112446725	0.97804641	0.924983343	0.53297623	0.704213672	0.92373379	0.993260048	0.999995916
LOC112446775	0.990794399	0.920928038	0.184990624	0.861236439	0.709606379	0.919545488	0.999995916
LOC112446799	0.518146797	0.686505408	0.614902558	0.614022061	0.802608048	0.924491428	0.999995916
LOC112446822	0.833898482	0.839068652	0.991219612	0.95555656	0.883370093	0.999764654	0.999995916
LOC112447026	0.837049433	0.751152688	0.62107658	0.629970174	0.865488776	0.979131456	0.999995916
LOC112447029	0.578103569	0.847864725	0.716010857	0.827552904	0.789065254	0.982653611	0.999995916
LOC112447085	0.465079432	0.864500157	0.621984931	0.98796424	0.357063472	0.90059488	0.999995916
LOC112447322	0.973907732	0.369423194	0.878633427	0.772464464	0.613600857	0.956063138	0.999995916
LOC112447323	0.959734541	0.98322172	0.896794412	0.721238651	0.828772726	0.999302244	0.999995916
LOC112447350	0.353184108	0.854545231	0.908560521	0.623632921	0.795417319	0.947803512	0.999995916
LOC112447370	0.797468587	0.521695797	0.754706718	0.573686972	0.694530092	0.939976791	0.999995916
LOC112447399	0.53949017	0.300669963	0.820014568	0.76702396	0.937197153	0.910610915	0.999995916
LOC112447411	0.982418221	0.586704171	0.803945612	0.794820104	0.388294497	0.952187591	0.999995916
LOC112447418	0.694908439	0.858355889	0.386483781	0.949345126	0.739375234	0.962032522	0.999995916
LOC112447420	0.752990251	0.84033218	0.666968727	0.869935464	0.665728256	0.985393172	0.999995916
LOC112447435	0.904073178	0.516970071	0.315620882	0.892083333	0.866980377	0.930627773	0.999995916
LOC112447443	0.799533087	0.499278581	0.389650475	0.922987344	0.61781882	0.90128564	0.999995916
LOC112447469	0.643773366	0.233252157	0.819801333	0.842365372	0.696801076	0.873500907	0.999995916
LOC112447499	0.512136161	0.68764647	0.970058294	0.926455718	0.936993296	0.991843179	0.999995916
LOC112447508	0.828694055	0.426474428	0.755763747	0.916970411	0.320807606	0.885259856	0.999995916
LOC112447510	0.527893737	0.738787174	0.548526755	0.917924495	0.840586979	0.963490591	0.999995916
LOC112447735	0.575534262	0.31238951	0.956064074	0.918559993	0.604653491	0.910425216	0.999995916
LOC112447769	0.993478057	0.935929603	0.357075465	0.989086268	0.255013687	0.893836847	0.999995916
LOC112447797	0.834510544	0.404925767	0.953680535	0.549392193	0.977724537	0.96683574	0.999995916
LOC112447842	0.909615647	0.759538753	0.862829439	0.565738773	0.693413091	0.983547448	0.999995916
LOC112447845	0.585800897	0.950787863	0.580849419	0.512952893	0.74102118	0.938289795	0.999995916
LOC112448021	0.955041122	0.287341055	0.865810535	0.679763502	0.515165766	0.892980636	0.999995916
LOC112448022	0.986931984	0.884244053	0.421935573	0.715388411	0.339577175	0.90235958	0.999995916
LOC112448030	0.884905632	0.724645272	0.783047812	0.289878375	0.783169667	0.930538533	0.999995916
LOC112448045	0.999380303	0.580897889	0.548233401	0.769478899	0.86668059	0.978972702	0.999995916

LOC112448056	0.709788664	0.584478771	0.62512995	0.741325138	0.750658793	0.952958647	0.999995916
LOC112448088	0.424625041	0.556909081	0.883317853	0.925542839	0.90057543	0.967229388	0.999995916
LOC112448103	0.473963231	0.61920408	0.861151226	0.769581005	0.48837229	0.909809136	0.999995916
LOC112448155	0.9142489	0.995059364	0.522923216	0.737793256	0.403656071	0.951383777	0.999995916
LOC112448271	0.862583331	0.243226313	0.591067581	0.970539054	0.714618327	0.897326375	0.999995916
LOC112448381	0.741312691	0.534798153	0.535972218	0.849883107	0.777106039	0.950562093	0.999995916
LOC112448387	0.629375643	0.939109261	0.500087723	0.991967272	0.33934977	0.915357003	0.999995916
LOC112448390	0.54306492	0.922587735	0.953761545	0.634593802	0.992366587	0.992231453	0.999995916
LOC112448430	0.97100071	0.452017172	0.683079469	0.963079118	0.559637322	0.961929783	0.999995916
LOC112448507	0.979547203	0.941238188	0.679623422	0.92343707	0.530498283	0.992736988	0.999995916
LOC112448515	0.821756013	0.575828968	0.877465821	0.970702654	0.192138859	0.883261574	0.999995916
LOC112448531	0.743861503	0.616700392	0.565658284	0.386224858	0.921234976	0.906323988	0.999995916
LOC112448579	0.450519452	0.731125724	0.93959809	0.450777036	0.555836348	0.883448759	0.999995916
LOC112448762	0.812265743	0.752763576	0.711420553	0.633735923	0.78513704	0.979954636	0.999995916
LOC112448764	0.706902303	0.986337039	0.441180728	0.973569009	0.937005514	0.990256354	0.999995916
LOC112448772	0.412081224	0.763510649	0.439059882	0.858369311	0.75073503	0.901745552	0.999995916
LOC112448776	0.836891769	0.836715778	0.217434825	0.970924585	0.708998135	0.921354637	0.999995916
LOC112448832	0.77920175	0.673227867	0.7428294	0.662792618	0.871145809	0.981812732	0.999995916
LOC112449056	0.799721069	0.864812489	0.226723008	0.816161317	0.585814872	0.878741634	0.999995916
LOC112449073	0.267887416	0.635672254	0.922923157	0.984637083	0.586922133	0.904279932	0.999995916
LOC112449075	0.499218733	0.767702596	0.675076556	0.947733314	0.463420156	0.930201356	0.999995916
LOC112449092	0.643193412	0.6202548	0.753188342	0.959104922	0.408074326	0.933788144	0.999995916
LOC112449111	0.758323599	0.852731273	0.630620456	0.552699244	0.80580473	0.970011033	0.999995916
LOC112449245	0.738260146	0.402966578	0.94317118	0.633948565	0.590890931	0.921672721	0.999995916
LOC112449247	0.566605669	0.631765831	0.815824241	0.951564244	0.76056341	0.978755164	0.999995916
LOC112449254	0.649236467	0.739780222	0.563734358	0.331707755	0.880558501	0.886148464	0.999995916
LOC112449266	0.544497893	0.810176446	0.801385615	0.88593113	0.728429303	0.982450231	0.999995916
LOC112449275	0.915720026	0.990444817	0.455053064	0.745856764	0.870663503	0.988785621	0.999995916
LOC112449338	0.823304364	0.587772749	0.588320863	0.792380696	0.909013601	0.977165816	0.999995916
LOC112449360	0.498389274	0.530547645	0.964932896	0.687985037	0.517654974	0.904338147	0.999995916
LOC112449510	0.906351213	0.475100497	0.210262123	0.930862851	0.848691176	0.872043798	0.999995916
LOC112449548	0.771932438	0.926536965	0.962225807	0.734831261	0.468649006	0.984122123	0.999995916
LOC112449558	0.941012609	0.866022702	0.795953702	0.537438133	0.225080296	0.885072063	0.999995916
LOC112449561	0.776592697	0.925172194	0.185072743	0.800625234	0.838308995	0.902069598	0.999995916
LOC112449565	0.401741765	0.706548592	0.726349384	0.961890364	0.577834441	0.93109216	0.999995916
LOC504858	0.888004549	0.824845076	0.412551271	0.776676554	0.638159757	0.956029713	0.999995916
LOC507787	0.760349507	0.610623711	0.571858885	0.800180454	0.757240736	0.961600964	0.999995916
LOC507930	0.958850105	0.683263358	0.374064389	0.358782737	0.886954495	0.884230273	0.999995916
LOC508628	0.795427968	0.92845502	0.561893496	0.945490095	0.753838323	0.991770993	0.999995916
LOC510185	0.867840522	0.668793735	0.796509276	0.448693164	0.374345488	0.883635839	0.999995916

LOC512175	0.857543114	0.761147281	0.484222701	0.577205239	0.865291778	0.960168449	0.999995916
LOC512286	0.633124456	0.565997405	0.780433035	0.584724567	0.846289014	0.949339259	0.999995916
LOC512440	0.366802592	0.586281509	0.710383507	0.983696311	0.985950166	0.955149184	0.999995916
LOC512541	0.74143663	0.731589379	0.953267029	0.544385844	0.563961821	0.960597957	0.999995916
LOC513894	0.488446912	0.734984245	0.69264783	0.791179739	0.45197008	0.901602279	0.999995916
LOC515089	0.843424878	0.677132767	0.920343006	0.830300839	0.422094189	0.970911463	0.999995916
LOC516355	0.868189293	0.884128595	0.493316255	0.368853632	0.816815312	0.93062384	0.999995916
LOC518080	0.631680827	0.697001118	0.483832525	0.623665938	0.758374394	0.916824063	0.999995916
LOC519145	0.939949501	0.641935902	0.963274279	0.338183753	0.784956761	0.958400629	0.999995916
LOC521656	0.929117673	0.288850536	0.737832889	0.595856	0.892041683	0.921827966	0.999995916
LOC523461	0.963414578	0.617422267	0.956080002	0.505420903	0.872671237	0.986409193	0.999995916
LOC532875	0.718162155	0.810227382	0.565732177	0.261091646	0.982920115	0.894986326	0.999995916
LOC534391	0.883112815	0.156803755	0.971852966	0.885539261	0.973608236	0.932393295	0.999995916
LOC539069	0.906334599	0.820097253	0.927215583	0.469801065	0.292538092	0.909463984	0.999995916
LOC541276	0.388958627	0.634970889	0.855426786	0.679852487	0.798966602	0.931242154	0.999995916
LOC613444	0.747527003	0.671869309	0.486784875	0.546666298	0.878592361	0.933631209	0.999995916
LOC613519	0.534079278	0.80216587	0.619356857	0.829253141	0.612456972	0.946964446	0.999995916
LOC613570	0.940526152	0.935186198	0.357938207	0.918141018	0.823727907	0.984318225	0.999995916
LOC614643	0.977764543	0.522823551	0.84379759	0.639146825	0.840839422	0.983164513	0.999995916
LOC614695	0.915134393	0.973387088	0.596668985	0.96919076	0.339231963	0.967476252	0.999995916
LOC614914	0.886591363	0.961360503	0.825619403	0.78593589	0.325915489	0.969525851	0.999995916
LOC614922	0.939603957	0.176274265	0.890772708	0.614487877	0.947684067	0.897189312	0.999995916
LOC615223	0.833436254	0.495010915	0.988254178	0.830015439	0.40252454	0.947931613	0.999995916
LOC615521	0.725051964	0.971931363	0.645907349	0.766456832	0.998882382	0.995440211	0.999995916
LOC615792	0.592111367	0.481261362	0.991305331	0.535872425	0.81313574	0.9383833	0.999995916
LOC616281	0.864576497	0.452194329	0.935520072	0.315592902	0.848881222	0.913543716	0.999995916
LOC616538	0.678231542	0.897178784	0.989359777	0.88398683	0.963433105	0.999361701	0.999995916
LOC616942	0.898395601	0.789663024	0.789199453	0.641115292	0.689986431	0.985917351	0.999995916
LOC618220	0.857553361	0.984704398	0.539981697	0.868409549	0.84884344	0.994759607	0.999995916
LOC618633	0.870183129	0.874921506	0.362688687	0.829450461	0.607946528	0.949877912	0.999995916
LOC619026	0.997479666	0.952924606	0.327068448	0.597741521	0.915703129	0.965653795	0.999995916
LOC619131	0.902396217	0.754521959	0.717750255	0.996647724	0.544654188	0.988437623	0.999995916
LOC619156	0.748266276	0.617508487	0.702798287	0.945046793	0.373987277	0.931255831	0.999995916
LOC781004	0.953838594	0.447888805	0.619160353	0.628276299	0.806826647	0.94650548	0.999995916
LOC781108	0.793896003	0.327822803	0.464958683	0.796264555	0.959268273	0.906461911	0.999995916
LOC781381	0.935311059	0.409963789	0.479906684	0.62640683	0.870402967	0.916333382	0.999995916
LOC781421	0.932765224	0.345730155	0.305762734	0.969630688	0.777297127	0.877505936	0.999995916
LOC781576	0.991207701	0.654312849	0.937419416	0.415168199	0.477744343	0.936337459	0.999995916
LOC781728	0.862382252	0.794116557	0.660724962	0.845258748	0.894560541	0.9951025	0.999995916
LOC781741	0.704322322	0.904237244	0.396407928	0.518154553	0.731281516	0.910668021	0.999995916

LOC781799	0.557397876	0.497010226	0.81694624	0.765010471	0.437848878	0.880299602	0.999995916
LOC781813	0.788344638	0.684820411	0.663017877	0.877653704	0.240386832	0.87976324	0.999995916
LOC781982	0.792754929	0.566139931	0.796187754	0.940426402	0.642279973	0.979816536	0.999995916
LOC782032	0.639625571	0.945610997	0.698385359	0.97075454	0.584419968	0.98458771	0.999995916
LOC782120	0.513903062	0.772882228	0.79088284	0.343682582	0.894768798	0.911843186	0.999995916
LOC782305	0.920553861	0.76379971	0.679985279	0.34884565	0.780288985	0.94374745	0.999995916
LOC782437	0.936326074	0.83095275	0.395104396	0.878464724	0.429124755	0.932263347	0.999995916
LOC782470	0.869731042	0.994019727	0.944521657	0.120593022	0.993293064	0.913334365	0.999995916
LOC782525	0.958107955	0.627400834	0.69429426	0.92260797	0.519255956	0.975777202	0.999995916
LOC782755	0.878858101	0.764352002	0.72445162	0.486071117	0.944158482	0.981468245	0.999995916
LOC782776	0.468340796	0.716152994	0.880227868	0.967182473	0.455751279	0.943743615	0.999995916
LOC782812	0.999826213	0.705237851	0.92797181	0.965922153	0.82152908	0.999413731	0.999995916
LOC782922	0.826464063	0.328732523	0.956496759	0.517992113	0.842884201	0.930041083	0.999995916
LOC782954	0.274849071	0.939663635	0.682844307	0.694077272	0.790752081	0.912081259	0.999995916
LOC783060	0.850325739	0.839606568	0.965407899	0.588506481	0.550728628	0.981478483	0.999995916
LOC783195	0.559203072	0.725343677	0.837168023	0.330052878	0.739019086	0.892374965	0.999995916
LOC783294	0.900848428	0.301608892	0.983090897	0.426303699	0.754322069	0.897155779	0.999995916
LOC783301	0.8784471	0.850754975	0.948002845	0.43377386	0.949962812	0.991412585	0.999995916
LOC783376	0.939190617	0.45503501	0.32709346	0.991581719	0.650565479	0.90337817	0.999995916
LOC783461	0.825656624	0.63252674	0.850342465	0.459539255	0.941632066	0.973492788	0.999995916
LOC783539	0.668811511	0.734945714	0.567607499	0.742568425	0.955010068	0.975186556	0.999995916
LOC783988	0.62455242	0.834673509	0.90864915	0.730004852	0.560700257	0.974016455	0.999995916
LOC784088	0.557046332	0.360818321	0.860446367	0.727476122	0.589811468	0.87729363	0.999995916
LOC784208	0.478920139	0.981295006	0.509100295	0.710954209	0.802129834	0.948079679	0.999995916
LOC784243	0.966660143	0.772315368	0.382996718	0.75260937	0.914433823	0.974875805	0.999995916
LOC784354	0.942840134	0.81477694	0.430336295	0.86310922	0.541200939	0.958466412	0.999995916
LOC784464	0.893222927	0.785382555	0.991692223	0.570859285	0.610528647	0.985069582	0.999995916
LOC784473	0.927450642	0.462794122	0.889482058	0.885093102	0.777658735	0.988110092	0.999995916
LOC784659	0.538701797	0.942258089	0.298070312	0.955102138	0.522685932	0.879787678	0.999995916
LOC784735	0.698369516	0.874775746	0.505404077	0.664777345	0.417429218	0.896830729	0.999995916
LOC785216	0.86542718	0.431939361	0.667704155	0.791329345	0.581082757	0.931254068	0.999995916
LOC785386	0.854022611	0.951027469	0.686860848	0.790988197	0.848740362	0.996607125	0.999995916
LOC785445	0.753989047	0.306630744	0.993884397	0.873546088	0.916182653	0.970805637	0.999995916
LOC785760	0.906915503	0.824572862	0.746176513	0.763954594	0.678785063	0.991161507	0.999995916
LOC785761	0.939196939	0.923841353	0.986091359	0.635358361	0.970951058	0.999476552	0.999995916
LOC785843	0.828465543	0.321041899	0.51615338	0.773697082	0.865978315	0.905855906	0.999995916
LOC786055	0.395286669	0.918225324	0.819583726	0.400172817	0.687597498	0.890800187	0.999995916
LOC786256	0.247229891	0.669144448	0.961219847	0.829747001	0.583711443	0.882502843	0.999995916
LOC786435	0.46022118	0.788723434	0.792572718	0.893030132	0.42045291	0.924726343	0.999995916
LOC786783	0.715165056	0.356359656	0.633008976	0.69797834	0.737251899	0.892679907	0.999995916

LOC787237	0.894101094	0.457913896	0.973919891	0.493565366	0.867978835	0.965922643	0.999995916
LOC787250	0.672062105	0.848287698	0.298951225	0.993303018	0.584521714	0.914710969	0.999995916
LOC787530	0.675880229	0.918454169	0.338163595	0.481312	0.891975936	0.903303928	0.999995916
LOC787679	0.430803134	0.696133083	0.421894939	0.775735843	0.91702948	0.903141061	0.999995916
LOC788293	0.901303028	0.759866133	0.970638449	0.780271491	0.772405301	0.997488465	0.999995916
LOC788541	0.887472709	0.820463623	0.488214741	0.928803491	0.661045537	0.980366909	0.999995916
LOC789388	0.534323424	0.723237803	0.59108908	0.831547985	0.616580783	0.933359866	0.999995916
LOC789494	0.815227162	0.458721692	0.395389386	0.789064456	0.997927413	0.932751945	0.999995916
LOC789626	0.817479432	0.927978613	0.797736132	0.826064327	0.887921622	0.998492764	0.999995916
LOC789733	0.487143444	0.697174712	0.785118494	0.866965436	0.721412203	0.964230661	0.999995916
LOC789960	0.471957371	0.49610881	0.610751	0.819916938	0.617131908	0.873704092	0.999995916
LOC789997	0.817954449	0.895404138	0.8019267	0.991538142	0.792152765	0.998780226	0.999995916
LOC790101	0.992034672	0.776119329	0.527890818	0.740178211	0.662201438	0.975573673	0.999995916
LOC790266	0.953845646	0.673850135	0.983659438	0.549336011	0.711409918	0.985824401	0.999995916
LOC790886	0.83107391	0.971744039	0.795944451	0.302190629	0.4007552	0.883981755	0.999995916
LOX	0.701177172	0.552758732	0.951425901	0.740038454	0.582028789	0.960637188	0.999995916
LPAR4	0.779881933	0.622540949	0.676987651	0.451440913	0.604249926	0.902653484	0.999995916
LRCH1	0.657611573	0.622682439	0.430790979	0.588328802	0.778134195	0.888987351	0.999995916
LRCH3	0.717363222	0.807147761	0.918654743	0.959562871	0.653604795	0.994609327	0.999995916
LRP1	0.985427544	0.721722266	0.632953261	0.462352464	0.909367455	0.972582841	0.999995916
LRP8	0.569327166	0.841320215	0.342517577	0.917287093	0.701913448	0.922233834	0.999995916
LRPAP1	0.329665515	0.913891955	0.858164359	0.595235675	0.620324193	0.910419026	0.999995916
LRRC69	0.648602584	0.789990235	0.562616933	0.645900526	0.668812687	0.93952955	0.999995916
LRRC72	0.658538453	0.596790421	0.952618387	0.921040562	0.640176016	0.980914713	0.999995916
LRRC75A	0.64248343	0.920337493	0.581923617	0.951925397	0.987600168	0.993961951	0.999995916
LRRFIP1	0.552197294	0.705059589	0.675416516	0.723169468	0.71759422	0.948091722	0.999995916
LRRN4CL	0.798829378	0.997396431	0.351035851	0.836365473	0.838428657	0.974684155	0.999995916
LSM5	0.828239828	0.414783428	0.728206357	0.792397694	0.617664565	0.937861608	0.999995916
LST1	0.840963008	0.417381299	0.884016607	0.658409477	0.460400795	0.908612719	0.999995916
LTBR	0.685575914	0.693077718	0.913759012	0.870208526	0.197328383	0.877959492	0.999995916
LZTR1	0.370580046	0.960400872	0.686888744	0.623749462	0.998192752	0.957327052	0.999995916
LZTS3	0.719818439	0.923778346	0.584467488	0.686725638	0.771764201	0.977404384	0.999995916
MAL	0.814854243	0.426205247	0.347625455	0.935063729	0.673439248	0.880696739	0.999995916
MAL2	0.96752238	0.535290165	0.326644565	0.933784262	0.857442991	0.947423088	0.999995916
MALL	0.964605798	0.773690528	0.366785275	0.627207397	0.925608459	0.960677414	0.999995916
MAN1B1	0.758695721	0.727469131	0.491125579	0.561755185	0.831895153	0.941183136	0.999995916
MAN2C1	0.702213846	0.53674939	0.578379798	0.724583095	0.506814565	0.88780831	0.999995916
MAP3K3	0.940960301	0.445419494	0.84731241	0.306069715	0.758804001	0.891813982	0.999995916
MAP3K4	0.58892041	0.994637266	0.375227345	0.663231744	0.86089965	0.940280726	0.999995916
MAP3K7CL	0.716284768	0.390182001	0.980959451	0.825187561	0.947341415	0.979463888	0.999995916

MAP4	0.80628478	0.91993018	0.441077652	0.495291215	0.911816166	0.95491808	0.999995916
MAPK12	0.884873363	0.395921493	0.798806181	0.908423162	0.848013168	0.979758691	0.999995916
MAPK7	0.755749093	0.896469752	0.61227408	0.972494644	0.931088548	0.9966491	0.999995916
MAPKAP1	0.753612363	0.684124818	0.5777084	0.610156383	0.71981709	0.944229458	0.999995916
MAPKAPK3	0.603868043	0.800742427	0.712457753	0.898313997	0.656537138	0.976665494	0.999995916
MARC2	0.709485394	0.56552502	0.93018483	0.916286269	0.734487855	0.986460776	0.999995916
MARS2	0.578760919	0.829041295	0.774799529	0.29112429	0.802964218	0.898668141	0.999995916
MAS1	0.472273052	0.419635105	0.971023233	0.916364026	0.700903951	0.938792988	0.999995916
MAVS	0.92036906	0.374085742	0.80594092	0.958015227	0.991844178	0.988226991	0.999995916
MBD5	0.723144397	0.704009566	0.507707987	0.333117247	0.843363583	0.874211369	0.999995916
MBLAC1	0.405756382	0.557784783	0.851755225	0.543239295	0.844957489	0.900981009	0.999995916
MBOAT7	0.647785165	0.896509987	0.793789612	0.62130578	0.415657516	0.935039459	0.999995916
MBTPS1	0.739187467	0.57044928	0.908622682	0.842195379	0.9667145	0.993129398	0.999995916
MCCC2	0.988820056	0.493385945	0.569273273	0.782858437	0.59120077	0.942580455	0.999995916
MCM10	0.896084226	0.669052917	0.824574784	0.938122525	0.633816488	0.991601803	0.999995916
MCM7	0.661341141	0.816889232	0.701905832	0.977275502	0.908239182	0.994786286	0.999995916
MCTP1	0.296047838	0.696323651	0.74128655	0.977530613	0.506599806	0.88005436	0.999995916
MCUB	0.911460401	0.419221522	0.528210195	0.613805082	0.913376734	0.929754414	0.999995916
MDFIC	0.668111718	0.663738282	0.683942583	0.867311089	0.8569676	0.981901862	0.999995916
MDN1	0.895406472	0.820991527	0.925349223	0.350110014	0.73902125	0.967957761	0.999995916
MECP2	0.737136929	0.512101009	0.966132537	0.336443503	0.605813516	0.877539802	0.999995916
MED20	0.407356555	0.740263892	0.912809141	0.502127264	0.984855647	0.947868285	0.999995916
MED22	0.57558283	0.80775071	0.999980919	0.934074642	0.79278841	0.99521928	0.999995916
MED26	0.640104307	0.790235684	0.639306172	0.728958234	0.940017692	0.981096702	0.999995916
MEGF10	0.54361442	0.996108222	0.212688372	0.955877163	0.688976256	0.880374254	0.999995916
MEGF9	0.80036955	0.848046003	0.34557234	0.950214883	0.83485964	0.971539557	0.999995916
MELK	0.920082414	0.844760944	0.598374366	0.827800593	0.337255318	0.943531571	0.999995916
MEN1	0.912646239	0.919400622	0.950885195	0.454771107	0.474266189	0.966432131	0.999995916
METTL2A	0.881144691	0.792365358	0.359593446	0.424238659	0.688836328	0.875684231	0.999995916
METTL7A	0.171374568	0.906633257	0.81558059	0.822795674	0.876707559	0.905081456	0.999995916
MEX3D	0.980734351	0.390335232	0.57254172	0.619434567	0.751859701	0.918336453	0.999995916
MFAP4	0.73697403	0.580943732	0.989974048	0.705136448	0.831462645	0.986047947	0.999995916
MGAT5	0.689557194	0.412231973	0.972752205	0.848339468	0.553874201	0.943591466	0.999995916
MGC127055	0.537405174	0.687530415	0.405221998	0.66130151	0.925622719	0.905405432	0.999995916
MICAL3	0.764983759	0.804029723	0.853096115	0.574080242	0.732635397	0.980901768	0.999995916
MICU2	0.473936577	0.477355389	0.864987252	0.574677772	0.743207935	0.893577561	0.999995916
MIGA1	0.62489387	0.75826112	0.578605799	0.970529267	0.963497329	0.987226004	0.999995916
MIPEP	0.557434653	0.436084071	0.716257255	0.846223557	0.685754944	0.917152356	0.999995916
MIS18A	0.505096727	0.725138229	0.628628191	0.74903856	0.954975125	0.963328964	0.999995916
MISP3	0.846147565	0.582830151	0.919957724	0.520183554	0.820739103	0.97395976	0.999995916

MKNK2	0.546690391	0.7069531	0.970359321	0.887830025	0.29546224	0.914019488	0.999995916
MKRN2	0.734266874	0.695398962	0.512057928	0.79692745	0.979922786	0.976933107	0.999995916
MNT	0.343505829	0.734216021	0.593787079	0.748823872	0.819472236	0.905744214	0.999995916
MOCOS	0.884069035	0.75269886	0.616184518	0.635675844	0.598329551	0.959235214	0.999995916
MOGAT1	0.565826023	0.994541715	0.600931833	0.599905287	0.874123674	0.968456819	0.999995916
MORC2	0.848872652	0.926041468	0.89697264	0.604339857	0.925597099	0.997300594	0.999995916
MORF4L2	0.841902666	0.718964999	0.681086599	0.852917577	0.284389491	0.915944787	0.999995916
MORN4	0.705645973	0.743757969	0.993022947	0.94251696	0.959517734	0.998921502	0.999995916
MPDZ	0.495692488	0.541625165	0.772991646	0.749985373	0.652976469	0.917833068	0.999995916
MRGPRF	0.73733069	0.921219426	0.860084946	0.931183309	0.980698225	0.999514718	0.999995916
MRI1	0.979886295	0.976120168	0.966795291	0.584663192	0.940281503	0.999324663	0.999995916
MRPL10	0.96655898	0.894649924	0.571748866	0.831104303	0.760529497	0.993172962	0.999995916
MRPL3	0.995902846	0.504767326	0.722888716	0.620013125	0.467920969	0.922015857	0.999995916
MRPL48	0.857204545	0.613609094	0.764678111	0.842196581	0.842348626	0.990756295	0.999995916
MRPS26	0.533916189	0.475602391	0.820821359	0.6611291	0.756295015	0.920711963	0.999995916
MSH2	0.630203855	0.603370778	0.884201953	0.918438246	0.610882405	0.972379564	0.999995916
MSH3	0.962873141	0.766605769	0.826054681	0.896928656	0.495378606	0.989143704	0.999995916
MSL3	0.779740662	0.930494166	0.914985686	0.727251046	0.995918374	0.999041655	0.999995916
MSX2	0.994031634	0.666190722	0.825765584	0.644570842	0.883618664	0.993092111	0.999995916
MT1E	0.50475011	0.724934139	0.946092713	0.924993673	0.263935335	0.895044799	0.999995916
MTA2	0.708235199	0.961791903	0.71475616	0.364442113	0.657786219	0.933006214	0.999995916
MTCH1	0.820081539	0.396711648	0.591170239	0.774437094	0.586998324	0.89944712	0.999995916
MTERF1	0.965204719	0.912875794	0.767202429	0.670711633	0.331337132	0.956274273	0.999995916
MTF1	0.984246616	0.353232939	0.571977312	0.568238599	0.967479693	0.926042479	0.999995916
MTF2	0.595762722	0.533365205	0.576812666	0.925432235	0.831735556	0.951019237	0.999995916
MTHFD2L	0.879927482	0.753299436	0.557354677	0.472882596	0.58488074	0.918452819	0.999995916
MTHFS	0.503460046	0.487402528	0.542062762	0.8623125	0.762705403	0.899522926	0.999995916
MTIF2	0.466676711	0.770751039	0.903261407	0.464854703	0.780814121	0.934069135	0.999995916
MTMR9	0.528389354	0.806858094	0.711923656	0.54687593	0.892125289	0.955102276	0.999995916
MTO1	0.396814066	0.965256923	0.764446977	0.881180059	0.536465531	0.949354413	0.999995916
MTOR	0.74979418	0.767633879	0.978013171	0.89963912	0.740954973	0.996634623	0.999995916
MUS81	0.99649352	0.538180637	0.717426954	0.943619993	0.708834676	0.987365241	0.999995916
MVB12A	0.985205065	0.620922605	0.944016816	0.483643388	0.94876599	0.988399952	0.999995916
MYCBP	0.651897954	0.468589387	0.384157237	0.778930768	0.857966172	0.884999918	0.999995916
MYO1B	0.59609366	0.801392288	0.746817428	0.63506528	0.92861624	0.978520227	0.999995916
MYO1D	0.640089026	0.742321272	0.830050509	0.654590987	0.655991093	0.965321235	0.999995916
MYO1H	0.858558225	0.988559938	0.894269753	0.638941395	0.766880351	0.996504196	0.999995916
MYO6	0.818956252	0.925948201	0.679072402	0.430149805	0.811075599	0.969311164	0.999995916
MYO9B	0.833515841	0.73338819	0.864439585	0.593458875	0.619643744	0.974147851	0.999995916
MYOZ3	0.293012529	0.958861613	0.624974764	0.601201047	0.783464273	0.892184674	0.999995916

NAALADL2	0.93979629	0.745457685	0.709719519	0.391073094	0.939245007	0.970367759	0.999995916
NADK2	0.43133435	0.95033098	0.949191997	0.939786943	0.96679541	0.995692322	0.999995916
NALCN	0.720045618	0.3279427	0.947875626	0.50371089	0.813284121	0.905466416	0.999995916
NAP1L3	0.441605287	0.877562721	0.863841687	0.355260882	0.786615934	0.907950567	0.999995916
NARFL	0.773832958	0.771420095	0.939897772	0.374564045	0.94068579	0.975139162	0.999995916
NCBP3	0.798084918	0.744627052	0.881536266	0.332553449	0.480961977	0.893910128	0.999995916
NCF2	0.522472756	0.710173708	0.427205154	0.642072377	0.798172727	0.889783755	0.999995916
NCKIPSD	0.828441082	0.4705482	0.796031644	0.746485159	0.89354925	0.977664094	0.999995916
NEBL	0.306176804	0.770990106	0.763966448	0.902828788	0.709906362	0.931994085	0.999995916
NECTIN2	0.644405026	0.896659877	0.807155525	0.763759948	0.692963221	0.985785134	0.999995916
NEDD4L	0.853165539	0.104076399	0.995525046	0.981967305	0.864256121	0.878833878	0.999995916
NEIL2	0.805249566	0.735644464	0.868090155	0.704585783	0.278530979	0.917014036	0.999995916
NEK1	0.788472315	0.954493193	0.852165974	0.362674517	0.492686971	0.931094098	0.999995916
NELFCD	0.898622673	0.589114946	0.713319142	0.92300485	0.393846744	0.948622033	0.999995916
NFAM1	0.745948396	0.225194037	0.682029686	0.80837507	0.844049802	0.884557112	0.999995916
NFATC2IP	0.469159896	0.520935823	0.885667962	0.75601174	0.735110219	0.936094726	0.999995916
NFIB	0.56198607	0.745175353	0.578404508	0.628424804	0.50532321	0.882327464	0.999995916
NGRN	0.418417908	0.387844303	0.60475062	0.909671204	0.802479535	0.872270903	0.999995916
NHLRC3	0.273157448	0.908755781	0.854903686	0.598663048	0.851841339	0.924930727	0.999995916
NHS	0.427688832	0.95090314	0.706608508	0.851319977	0.526754165	0.942820626	0.999995916
NIT2	0.733766767	0.582544666	0.751737057	0.569163923	0.435330833	0.887063614	0.999995916
NKIRAS2	0.835928008	0.32495132	0.60495447	0.654026406	0.917300886	0.914270539	0.999995916
NLGN2	0.696206891	0.751158517	0.532238055	0.838064203	0.912193865	0.979100374	0.999995916
NME3	0.792789934	0.568685824	0.800603609	0.682691491	0.794103593	0.974553319	0.999995916
NME7	0.565731842	0.966976118	0.975100215	0.401633743	0.929545379	0.975554313	0.999995916
NOBOX	0.733300731	0.674439291	0.708803427	0.973271684	0.475033251	0.962150896	0.999995916
NONO	0.62081781	0.816113553	0.623997473	0.4636179	0.945295689	0.949444049	0.999995916
NOS1AP	0.6148958	0.414588171	0.709672281	0.989625071	0.493155878	0.900704395	0.999995916
NOS3	0.789703654	0.998350954	0.787214822	0.27646799	0.860221832	0.954836471	0.999995916
NOTCH1	0.689588657	0.432458158	0.978488476	0.924201467	0.961160985	0.987625739	0.999995916
NOVA1	0.875415305	0.918028807	0.490915561	0.916601285	0.770226098	0.990027465	0.999995916
NOVA2	0.594568787	0.565063277	0.946163747	0.974602575	0.754572262	0.98353275	0.999995916
NPDC1	0.980895408	0.502609735	0.59986276	0.953311313	0.569531944	0.961456415	0.999995916
NPTN	0.655749842	0.563407648	0.860063691	0.723674667	0.877064674	0.97625899	0.999995916
NRADD	0.612062507	0.268918263	0.77253559	0.781517076	0.814443139	0.889284286	0.999995916
NRBP1	0.918881764	0.564503928	0.828886635	0.771386144	0.992062092	0.994325249	0.999995916
NRP1	0.809874683	0.633603324	0.960840531	0.512967093	0.894010656	0.982041475	0.999995916
NSMCE2	0.66407541	0.820297718	0.353811573	0.777381271	0.872454675	0.94416124	0.999995916
NSMCE3	0.983060822	0.678575187	0.651959182	0.433474755	0.809157611	0.957501083	0.999995916
NT5C3A	0.770610017	0.929621067	0.3459289	0.686220781	0.49981741	0.895786232	0.999995916

NTN5	0.775778369	0.365630782	0.78023076	0.731173681	0.9727523	0.959950618	0.999995916
NUCB1	0.882187195	0.983606743	0.890729611	0.585397553	0.797832898	0.996041911	0.999995916
NUDT12	0.846749489	0.64993728	0.590733607	0.410497653	0.824595678	0.926758922	0.999995916
NUDT2	0.659866154	0.303978704	0.982794095	0.967790022	0.853306953	0.962480626	0.999995916
NUFIP1	0.991397163	0.822254131	0.851322415	0.534068556	0.949041367	0.995605382	0.999995916
NUP88	0.940920089	0.885385625	0.991118416	0.231061542	0.68288541	0.943848699	0.999995916
NVL	0.751415175	0.949590476	0.244982246	0.589397433	0.766402461	0.885935204	0.999995916
NXN	0.429149086	0.740247821	0.610323812	0.988764799	0.462489829	0.901235623	0.999995916
NXPE4	0.876774872	0.335452986	0.556106024	0.870317147	0.673787684	0.91098457	0.999995916
OCSTAMP	0.639826019	0.370684825	0.739155903	0.972552839	0.626190903	0.923426145	0.999995916
ODF2L	0.545308036	0.864541795	0.765298641	0.915218031	0.701325265	0.983119938	0.999995916
OPA1	0.865535742	0.633822473	0.887660013	0.421207255	0.368595366	0.879922315	0.999995916
ORC5	0.335454651	0.869737729	0.638164721	0.612662532	0.782407051	0.902074636	0.999995916
ORMDL1	0.466149704	0.883057349	0.817952932	0.970564604	0.528034421	0.966618468	0.999995916
OS9	0.554144982	0.763460968	0.591810093	0.861959059	0.548531381	0.934463541	0.999995916
OSBPL2	0.728350647	0.470191327	0.453384554	0.977556796	0.554866616	0.894581156	0.999995916
OTOP1	0.505417353	0.788934713	0.918722917	0.469532245	0.558984287	0.911279711	0.999995916
OTUB1	0.51289375	0.360761464	0.960447641	0.495124841	0.876561233	0.882704098	0.999995916
OTUD7B	0.707153123	0.988476579	0.842351812	0.912259714	0.990690556	0.999505825	0.999995916
OVGP1	0.578605293	0.646261008	0.854044443	0.493172054	0.696357691	0.926390757	0.999995916
P2RX6	0.754827038	0.9260018	0.364738208	0.547510473	0.979962866	0.948304045	0.999995916
PABPC4L	0.514306254	0.92476177	0.710775318	0.869245821	0.974059925	0.990847532	0.999995916
PACSIN1	0.919649356	0.256068015	0.985139413	0.756910895	0.851170047	0.955861707	0.999995916
PACSIN3	0.499005329	0.977515326	0.748600605	0.566813482	0.693490259	0.95249853	0.999995916
PAF1	0.410529459	0.844110209	0.868455045	0.601133275	0.904613304	0.962865491	0.999995916
PANK4	0.541272707	0.967886053	0.824831432	0.694257949	0.541423243	0.962313412	0.999995916
PAPD7	0.948709952	0.926389191	0.194118269	0.570392711	0.928731833	0.903658515	0.999995916
PAQR5	0.703220738	0.769885825	0.698103019	0.901058716	0.897810401	0.992638588	0.999995916
PAQR6	0.340151113	0.706976199	0.965033805	0.629876181	0.662561336	0.912154128	0.999995916
PARG	0.990543545	0.679537736	0.808635901	0.820655517	0.29781437	0.945782963	0.999995916
PARP1	0.988745256	0.859091762	0.78541379	0.912592888	0.744891174	0.998658505	0.999995916
PARP2	0.969065651	0.534994011	0.474168651	0.794932054	0.943760575	0.97098551	0.999995916
PARPBP	0.934875579	0.907741132	0.363229221	0.577075479	0.623850896	0.927667423	0.999995916
PARVB	0.945022893	0.511983929	0.595182975	0.821064984	0.968534871	0.982620591	0.999995916
PBX3	0.202026323	0.831197943	0.96488604	0.770240381	0.662562954	0.892153542	0.999995916
PC	0.872202743	0.762975868	0.661426314	0.522482735	0.934310677	0.979592068	0.999995916
PCBP4	0.835466351	0.471705822	0.366877392	0.742903134	0.702007199	0.879551129	0.999995916
PCCB	0.923519268	0.768629672	0.987604209	0.507448483	0.984207285	0.995524197	0.999995916
PCDH9	0.547679914	0.870255102	0.867507408	0.672092261	0.74381052	0.977590927	0.999995916
PCDHB11	0.817176726	0.819978308	0.483825878	0.782531846	0.629481762	0.96104723	0.999995916

PCDHGB4	0.814776983	0.651548483	0.803592383	0.532362416	0.940940864	0.979316306	0.999995916
PCDHGC3	0.97439551	0.652634849	0.832571701	0.690868342	0.391594682	0.952322315	0.999995916
PCGF2	0.71383771	0.901450158	0.909009584	0.725753599	0.441568358	0.971995834	0.999995916
PCMTD1	0.929444336	0.603415376	0.779186419	0.528466549	0.398671196	0.905976193	0.999995916
PCNA	0.268349422	0.941877464	0.710119836	0.722292544	0.94734399	0.93816276	0.999995916
PCYOX1	0.822529849	0.84511058	0.50467327	0.855961219	0.747564222	0.981706561	0.999995916
PDCD2	0.244451217	0.917057154	0.725994019	0.614169688	0.93731311	0.908130907	0.999995916
PDCD2L	0.866799093	0.98069397	0.527014725	0.573148959	0.814312986	0.978197473	0.999995916
PDCD7	0.891368749	0.272661547	0.680351819	0.604536707	0.796554272	0.887077043	0.999995916
PDE4C	0.672350479	0.525543687	0.479388144	0.922821518	0.893158642	0.950113442	0.999995916
PDE4DIP	0.897739064	0.945252658	0.936490817	0.86679292	0.764763949	0.999469398	0.999995916
PDE6A	0.76379489	0.953980143	0.713839058	0.858868325	0.916533206	0.99773315	0.999995916
PDE8B	0.80024035	0.835973999	0.352559482	0.585287485	0.640791623	0.900939562	0.999995916
PDGFRL	0.800987	0.629815819	0.882312295	0.814933058	0.449969825	0.962670053	0.999995916
PDZD9	0.866062138	0.861845035	0.558722466	0.839080773	0.656270812	0.982746749	0.999995916
PEAR1	0.816603634	0.599326655	0.330824762	0.590739323	0.804749585	0.882420494	0.999995916
PECAM1	0.626557831	0.439812	0.542673399	0.919780145	0.692268466	0.910106101	0.999995916
PEG10	0.957269956	0.946255588	0.985474751	0.808674204	0.707994494	0.999348008	0.999995916
PEX12	0.82235008	0.527330493	0.900704144	0.790905963	0.674603013	0.978023909	0.999995916
PEX3	0.916255573	0.228394388	0.879671391	0.477656333	0.944488013	0.89273245	0.999995916
PFAS	0.914983151	0.244311699	0.747987047	0.873391102	0.519585929	0.880428977	0.999995916
PFDN5	0.746164302	0.35292895	0.433235221	0.659939597	0.968357519	0.874789438	0.999995916
PFKL	0.616754931	0.487969559	0.649479322	0.866264458	0.605349373	0.918813445	0.999995916
PFKM	0.983296128	0.936063086	0.323927739	0.665566167	0.42852229	0.895846315	0.999995916
PGAM2	0.811513925	0.769776357	0.805040453	0.619994595	0.410313354	0.942129174	0.999995916
PGAP1	0.239328443	0.864617977	0.996469021	0.94885449	0.82708135	0.962035075	0.999995916
PGBD1	0.664538481	0.971109484	0.424360639	0.823235041	0.350748582	0.886132499	0.999995916
PGBD2	0.571358617	0.769747707	0.956094157	0.373842113	0.547808872	0.897487494	0.999995916
PGRMC2	0.827035192	0.51881162	0.327700354	0.83876361	0.845758256	0.915648884	0.999995916
PHF1	0.494198841	0.973097301	0.498165949	0.852761341	0.496584396	0.917624102	0.999995916
PHF12	0.848763727	0.930670196	0.698296124	0.379021972	0.796287441	0.964104963	0.999995916
PHF2	0.819346151	0.501874888	0.889168021	0.4540176	0.831844223	0.949146537	0.999995916
PHF20L1	0.946547879	0.756550998	0.976901012	0.742888404	0.501196546	0.987799283	0.999995916
PHF23	0.998409174	0.509147627	0.94409157	0.222618284	0.90667162	0.912175316	0.999995916
PI4KA	0.673353078	0.944871308	0.953049126	0.430544041	0.522711154	0.94809159	0.999995916
PIAS4	0.764425942	0.99056668	0.585928383	0.889541061	0.259302253	0.918630298	0.999995916
PIBF1	0.810344728	0.947241604	0.852984299	0.257918052	0.861020184	0.953587422	0.999995916
PIEZO1	0.375909319	0.750800532	0.830602668	0.917675647	0.510668889	0.926573426	0.999995916
PIGG	0.691562053	0.180991803	0.930263843	0.951967238	0.906073702	0.916453465	0.999995916
PIGH	0.569778579	0.524588102	0.687086825	0.531194574	0.760733502	0.892637071	0.999995916

PIGL	0.816710436	0.371238994	0.899214341	0.59881408	0.911639909	0.955517266	0.999995916
PIGS	0.997017235	0.992981892	0.438971386	0.939981979	0.466319761	0.972972065	0.999995916
PIGT	0.443833149	0.850932671	0.595685484	0.728890109	0.538435567	0.900703387	0.999995916
PINK1	0.427349882	0.816118317	0.505773466	0.66753421	0.830085054	0.913251409	0.999995916
PIP4K2B	0.726224579	0.790627405	0.833803075	0.923082522	0.788432287	0.995437494	0.999995916
PIP4P1	0.721491643	0.576208912	0.609663207	0.9548039	0.959466754	0.983235906	0.999995916
PITRM1	0.464516123	0.684590424	0.732358466	0.752208655	0.949353743	0.964033163	0.999995916
PKD2	0.545257236	0.692845245	0.836278315	0.908565787	0.929422179	0.98863009	0.999995916
PLA1A	0.599786549	0.949305311	0.37853466	0.684824763	0.638976275	0.908942073	0.999995916
PLA2G2D4	0.801854724	0.562772698	0.578273246	0.838815015	0.938965538	0.977288265	0.999995916
PLAGL2	0.795517348	0.506433576	0.958026506	0.442000978	0.959272931	0.962863895	0.999995916
PLB1	0.42382982	0.712729594	0.640026008	0.726998268	0.807174143	0.93003457	0.999995916
PLCD1	0.719730598	0.650036767	0.726433367	0.586175681	0.670970659	0.946223213	0.999995916
PLCD4	0.652653339	0.858704877	0.93811378	0.995297078	0.650701288	0.995011217	0.999995916
PLD1	0.666880532	0.604127166	0.547038675	0.990955089	0.816196889	0.968799339	0.999995916
PLEKHA3	0.263907376	0.480522398	0.918191222	0.946499947	0.941262034	0.920184544	0.999995916
PLEKHA5	0.886490565	0.493189666	0.610455403	0.396935841	0.868424056	0.905884877	0.999995916
PLEKHG7	0.956827254	0.571500569	0.415056269	0.883524053	0.860195103	0.966593724	0.999995916
PLGRKT	0.666311626	0.243510143	0.981696033	0.607834022	0.858494601	0.89284225	0.999995916
PLIN1	0.740842879	0.830268874	0.249720621	0.929037868	0.725887172	0.920019788	0.999995916
PLPP3	0.900366254	0.703070292	0.814112856	0.687260314	0.936459508	0.994491266	0.999995916
PLPP5	0.677094325	0.996864495	0.924925438	0.390285165	0.584156062	0.951780368	0.999995916
PLPP6	0.746647495	0.829957007	0.779510364	0.262394979	0.827432981	0.921426069	0.999995916
PLPPR5	0.920073671	0.38498121	0.295761003	0.913623842	0.972025561	0.90726811	0.999995916
PLSCR2	0.384106536	0.67603419	0.950291028	0.667500741	0.568159001	0.907990696	0.999995916
PLTP	0.579169526	0.807493006	0.631654643	0.831032627	0.478805051	0.933736093	0.999995916
PLXNB1	0.212265598	0.972829523	0.590008376	0.712242818	0.887024789	0.882423309	0.999995916
PMAIP1	0.738406252	0.799994044	0.799111098	0.377367707	0.797751525	0.95164585	0.999995916
PMP22	0.411300961	0.984081767	0.959414958	0.80051845	0.755771173	0.983748495	0.999995916
PNMA8A	0.927143827	0.523077793	0.858631649	0.30688147	0.758780041	0.912293837	0.999995916
PNP	0.97650723	0.733090054	0.275126286	0.542973667	0.819363504	0.899728746	0.999995916
PNPLA8	0.391742617	0.764243336	0.760483263	0.725920844	0.61092298	0.917074881	0.999995916
POC1B	0.55268965	0.850701807	0.96888996	0.484670123	0.342101833	0.879789983	0.999995916
PODXL	0.485023785	0.818853499	0.431626977	0.931040997	0.962036354	0.958019157	0.999995916
POLR1C	0.960556482	0.427058814	0.947655297	0.593350696	0.34793853	0.888146396	0.999995916
POLR2H	0.924530383	0.932316095	0.654807846	0.452532448	0.86246391	0.980814018	0.999995916
POMC	0.868854909	0.976920252	0.606837587	0.867733249	0.595967217	0.988577699	0.999995916
POMT2	0.396404124	0.471065565	0.787849348	0.927899403	0.811924274	0.92753531	0.999995916
PON3	0.981906937	0.866561611	0.389047998	0.924380505	0.644942636	0.975041514	0.999995916
POPDC3	0.920638767	0.543183196	0.608121784	0.775136426	0.548365364	0.94311013	0.999995916

POU2F3	0.948201935	0.315856501	0.680289256	0.563368604	0.66788434	0.881860615	0.999995916
PPFIA1	0.26662167	0.864419533	0.779664939	0.852877516	0.755795377	0.932214338	0.999995916
PPFIA3	0.876534747	0.804642561	0.932439292	0.496650889	0.308324657	0.916767801	0.999995916
PPFIBP1	0.840269298	0.409409504	0.577915451	0.673636172	0.785217597	0.92173136	0.999995916
PPM1B	0.786589897	0.52877151	0.64645392	0.707760248	0.641477915	0.937562764	0.999995916
PPM1J	0.530040116	0.759867763	0.768216437	0.478750939	0.86528097	0.942307924	0.999995916
PPM1M	0.941344222	0.456061799	0.763190157	0.48225734	0.852502274	0.94692391	0.999995916
PPM1N	0.695345315	0.486407254	0.987592303	0.696547167	0.620793732	0.953027965	0.999995916
PPP1R13L	0.697732691	0.770542646	0.957594135	0.966374429	0.167916077	0.89351622	0.999995916
PPP1R16B	0.992328178	0.496369643	0.537088149	0.650448724	0.784939572	0.947169051	0.999995916
PPP1R26	0.81093572	0.280917035	0.728039423	0.494021392	0.939854617	0.882483163	0.999995916
PPP1R3G	0.362217264	0.991922585	0.900703175	0.896738811	0.621667297	0.969581088	0.999995916
PPP2R1A	0.843307648	0.705956448	0.954208384	0.519537609	0.551591463	0.962479563	0.999995916
PPP2R5D	0.990144535	0.748887667	0.256051721	0.706400077	0.937789169	0.940496158	0.999995916
PPP4C	0.585052563	0.736389466	0.98639147	0.94669139	0.527321597	0.978947291	0.999995916
PPP4R1	0.849036938	0.404735088	0.856228095	0.995419465	0.869373184	0.98697379	0.999995916
PPWD1	0.914274847	0.675624074	0.926047577	0.409702681	0.884433912	0.977739019	0.999995916
PPYR1	0.774770788	0.562214258	0.994427808	0.925558777	0.34643957	0.949657678	0.999995916
PRAF2	0.813960283	0.924460269	0.490111987	0.696647512	0.791521687	0.976713398	0.999995916
PRCC	0.77944455	0.441352856	0.725571794	0.648644414	0.834509792	0.947196998	0.999995916
PRDM16	0.572803027	0.854764731	0.776270556	0.925783507	0.211054622	0.877402312	0.999995916
PRDM6	0.737621831	0.887949197	0.62868614	0.73861076	0.973509453	0.991799743	0.999995916
PRELP	0.81582691	0.830222193	0.755932128	0.290833268	0.92141865	0.948577729	0.999995916
PRICKLE3	0.719890081	0.757348563	0.71599648	0.75759193	0.777968245	0.982830107	0.999995916
PRKCD	0.663212953	0.912611908	0.775242184	0.30188771	0.555270235	0.88540282	0.999995916
PRKD3	0.565646913	0.966593708	0.370025722	0.427756564	0.922049448	0.887364795	0.999995916
PRKRA	0.667087734	0.864685592	0.436885693	0.707293582	0.96873007	0.966662973	0.999995916
PRL	0.94686844	0.884975056	0.781483521	0.975654581	0.805320987	0.99937653	0.999995916
PROB1	0.48280664	0.790378124	0.633897762	0.972300269	0.930449936	0.980494792	0.999995916
PROSER1	0.663613831	0.778807932	0.999281046	0.740859179	0.573737104	0.980646733	0.999995916
PRPF6	0.688280349	0.980309765	0.782999305	0.821364355	0.984248478	0.998158516	0.999995916
PRR18	0.596374874	0.906910875	0.213433385	0.949828995	0.844145612	0.906631352	0.999995916
PRR29	0.891466829	0.790284676	0.605622855	0.873062261	0.494310098	0.970885533	0.999995916
PRRC2A	0.851380556	0.623816773	0.494176214	0.322720056	0.875050906	0.877126191	0.999995916
PRSS2	0.904238034	0.864419669	0.376000433	0.687709248	0.756415493	0.95767746	0.999995916
PSD3	0.532192682	0.762235893	0.960879015	0.648923586	0.749071714	0.972647611	0.999995916
PSMB8	0.481052328	0.593916133	0.747363022	0.631758427	0.637347407	0.897278659	0.999995916
PSMG4	0.929122781	0.653475587	0.220957802	0.674739878	0.847215977	0.881913148	0.999995916
PSPC1	0.759049997	0.514450101	0.629513471	0.967425791	0.401430815	0.910419171	0.999995916
PSTK	0.540111418	0.551956548	0.582625953	0.869384644	0.715795298	0.924793672	0.999995916

PTCH1	0.943901403	0.831830183	0.647670455	0.24945485	0.887145775	0.929173976	0.999995916
PTGDS	0.940321374	0.881447579	0.240291026	0.988913915	0.730500054	0.952699602	0.999995916
PTGIS	0.523809734	0.636521921	0.84729549	0.495584044	0.998478768	0.950222027	0.999995916
PTGR2	0.951893818	0.478367875	0.651001777	0.490830056	0.625690056	0.904572655	0.999995916
PTK2	0.724982211	0.72046971	0.554425691	0.852654865	0.940011739	0.983220467	0.999995916
PTMA	0.951825121	0.7689254	0.859222353	0.756045044	0.424112976	0.976247498	0.999995916
PTPN1	0.646584031	0.768471454	0.936414873	0.738008271	0.54286226	0.971651859	0.999995916
PTPRA	0.835774978	0.526152658	0.931984224	0.685712798	0.473702037	0.945848552	0.999995916
PTPRB	0.414783605	0.44186183	0.892069054	0.853148953	0.671114616	0.908027299	0.999995916
PTPRH	0.43942051	0.768037113	0.988107065	0.558634678	0.440591888	0.891167906	0.999995916
PTPRM	0.405085306	0.729203829	0.878069717	0.402858478	0.825322263	0.897674198	0.999995916
PTTG1IP	0.670431166	0.425498132	0.503530264	0.920075325	0.855748669	0.929700184	0.999995916
PURA	0.976126493	0.817033456	0.345265529	0.803663875	0.714866393	0.960332853	0.999995916
PWWP2B	0.723938409	0.449256378	0.571925118	0.645510019	0.847526744	0.917980923	0.999995916
PYCR3	0.586199185	0.542868926	0.984157964	0.743470054	0.555871561	0.943233606	0.999995916
PYGB	0.95251109	0.732880377	0.623175514	0.978095339	0.762444237	0.994024345	0.999995916
PYURF	0.414677164	0.834300692	0.975888915	0.433938387	0.513910968	0.879342453	0.999995916
QARS	0.97971919	0.335776442	0.842842656	0.865042056	0.67001075	0.96151828	0.999995916
QRICH1	0.857818582	0.999734818	0.442013742	0.809429801	0.24495856	0.87909628	0.999995916
QSOX1	0.619711288	0.818874554	0.595778941	0.782100303	0.783608863	0.971277481	0.999995916
R3HDM4	0.801750137	0.807673013	0.419052626	0.767529208	0.466342289	0.912496711	0.999995916
RAB11FIP3	0.951268275	0.539390712	0.875876628	0.65629123	0.779941993	0.98282375	0.999995916
RAB33A	0.670413614	0.896330225	0.355185762	0.906683223	0.861317674	0.964192177	0.999995916
RAB39A	0.668425963	0.909868192	0.861893254	0.737752709	0.267344277	0.919800356	0.999995916
RAB3D	0.96344744	0.981715019	0.555942739	0.715548343	0.646166087	0.985179337	0.999995916
RAB42	0.665334116	0.948186074	0.311512882	0.770282919	0.787060405	0.935117958	0.999995916
RAB6B	0.630751585	0.424612922	0.875192148	0.470377711	0.951568063	0.921467718	0.999995916
RABIF	0.629208042	0.605637598	0.946597098	0.89634041	0.507234563	0.963021929	0.999995916
RABL2B	0.767816218	0.752414905	0.826134086	0.45249074	0.327983797	0.870628141	0.999995916
RACK1	0.833932807	0.780694805	0.846971969	0.549201161	0.700791068	0.978966769	0.999995916
RAD17	0.833359781	0.79152196	0.413074831	0.821257285	0.968372397	0.980012562	0.999995916
RAI2	0.269795135	0.766711853	0.926645336	0.740884287	0.744394374	0.922321347	0.999995916
RANBP3	0.660072793	0.694109669	0.831881989	0.583970545	0.324911236	0.873620068	0.999995916
RAPGEF3	0.674392103	0.599221502	0.372431445	0.961200001	0.885718536	0.942276937	0.999995916
RARG	0.872356156	0.787530516	0.852609306	0.776765401	0.601586252	0.989476871	0.999995916
RASA2	0.475249748	0.998023463	0.974556265	0.455531736	0.667569634	0.950704457	0.999995916
RASEF	0.499895484	0.748538256	0.826883486	0.949152687	0.381702572	0.928754445	0.999995916
RASGRF2	0.69926328	0.702422929	0.498891795	0.967172707	0.492104479	0.932929252	0.999995916
RASL11B	0.599447081	0.34518258	0.657249725	0.845937775	0.88297935	0.917775182	0.999995916
RASSF1	0.741546991	0.724201859	0.601800932	0.790502165	0.302366397	0.882917557	0.999995916

RASSF4	0.237902875	0.500956175	0.901097456	0.769109028	0.934237052	0.882767232	0.999995916
RASSF7	0.436591333	0.673912717	0.81517915	0.718775775	0.78984043	0.947896573	0.999995916
RBAK	0.762067835	0.819763651	0.969377482	0.620538511	0.839964914	0.99340859	0.999995916
RBBP8	0.937529539	0.697266043	0.891931191	0.715219024	0.432341316	0.96954058	0.999995916
RBM15B	0.898471365	0.52383058	0.621034713	0.420492875	0.988193858	0.937055189	0.999995916
RBM26	0.712258086	0.746763767	0.937194248	0.353407526	0.954047985	0.964784165	0.999995916
RBM41	0.836062517	0.539908651	0.995534032	0.657791028	0.463818839	0.948511161	0.999995916
RBM7	0.347100415	0.726617263	0.346881347	0.886680915	0.981963019	0.880971287	0.999995916
RBMS3	0.585318391	0.993211302	0.761088321	0.568542331	0.39234865	0.914403168	0.999995916
RC3H1	0.684978127	0.529352112	0.854003538	0.604566019	0.956643012	0.969105658	0.999995916
RC3H2	0.775948776	0.641172616	0.929903249	0.795284791	0.815559888	0.992156578	0.999995916
RCAN2	0.405964762	0.982883516	0.919100945	0.854263674	0.419646328	0.94469523	0.999995916
RDH14	0.934808468	0.665552114	0.766736036	0.917117648	0.715544844	0.993214256	0.999995916
RELA	0.938572235	0.369662499	0.83781274	0.714203293	0.828081604	0.966363161	0.999995916
REM1	0.777106882	0.687694412	0.319097702	0.984870795	0.637194065	0.923689699	0.999995916
RESP18	0.412488712	0.874672432	0.458629751	0.838583935	0.612437231	0.895763691	0.999995916
REST	0.595560923	0.990759667	0.994422076	0.556142549	0.872657354	0.990697589	0.999995916
REXO5	0.364142289	0.964405142	0.378602294	0.88209792	0.70841027	0.892787734	0.999995916
RFC2	0.987734233	0.765848731	0.367422425	0.34022108	0.77683077	0.875856468	0.999995916
RGS11	0.647621574	0.943702626	0.866280754	0.327715615	0.46576233	0.889080321	0.999995916
RGS13	0.885315967	0.716747161	0.86829592	0.931941396	0.266001318	0.948172785	0.999995916
RGS19	0.60125958	0.534860623	0.576140771	0.848724013	0.683577145	0.924184192	0.999995916
RGS2	0.901313459	0.451016741	0.418499095	0.952983734	0.888249963	0.952776536	0.999995916
RHBDF2	0.561123279	0.747840636	0.829172413	0.553412996	0.497527063	0.910845626	0.999995916
RHNO1	0.839234591	0.308861662	0.824406377	0.493000205	0.762019473	0.888185962	0.999995916
RHOB	0.9101736	0.854290378	0.546250475	0.618514835	0.744968106	0.974561249	0.999995916
RHOBTB1	0.868029343	0.991902402	0.599366173	0.461584179	0.732559988	0.967379871	0.999995916
RHOG	0.841480827	0.688304037	0.498299093	0.636931558	0.783847234	0.952826228	0.999995916
RIOK1	0.999776567	0.509993901	0.318710005	0.804773517	0.749764133	0.913627501	0.999995916
RIPOR1	0.912702919	0.460865517	0.55397668	0.694125097	0.453988892	0.875804613	0.999995916
RIPPLY3	0.589052952	0.755343886	0.706607404	0.418963199	0.946948124	0.939686034	0.999995916
RLIM	0.731272897	0.9975705	0.874727985	0.59521159	0.275055164	0.920984645	0.999995916
RNASEH2C	0.641830966	0.446369604	0.649840903	0.635736265	0.716139567	0.895422823	0.999995916
RNF111	0.715762461	0.621439133	0.883616388	0.389909959	0.621736817	0.910183422	0.999995916
RNF126	0.556455656	0.851301294	0.794963108	0.874725649	0.292374007	0.911482317	0.999995916
RNF141	0.62746377	0.61277868	0.533416114	0.789812036	0.876741532	0.951592895	0.999995916
RNF149	0.955011559	0.918423419	0.593964761	0.787477795	0.727452418	0.992012468	0.999995916
RNF157	0.8139169	0.868221269	0.295631549	0.661070411	0.740305762	0.918520025	0.999995916
RNF169	0.593368901	0.593553638	0.955267507	0.455640709	0.918329631	0.950833492	0.999995916
RNF220	0.663409051	0.719279773	0.812008324	0.443927142	0.567093017	0.913009059	0.999995916

RNF8	0.844931083	0.995704886	0.671950865	0.857810839	0.726082068	0.995622922	0.999995916
RNFT2	0.702465486	0.651214487	0.434622699	0.913577533	0.90564638	0.963241956	0.999995916
RP9	0.925739031	0.789651477	0.761838476	0.779452394	0.524220236	0.982333642	0.999995916
RPA4	0.663247221	0.820750025	0.810517263	0.597306325	0.904583839	0.98436897	0.999995916
RPAIN	0.579627744	0.771234147	0.77054736	0.83779839	0.442825971	0.942025013	0.999995916
RPL10	0.966100386	0.960118162	0.948444544	0.957359081	0.718784834	0.999824882	0.999995916
RPL10A	0.906624076	0.960356108	0.859655406	0.790439791	0.913397881	0.999557678	0.999995916
RPL11	0.869729552	0.963064516	0.583752026	0.96511498	0.808116161	0.996862162	0.999995916
RPL13	0.783469633	0.988836832	0.876423237	0.700926517	0.239165081	0.930379987	0.999995916
RPL13A	0.864161278	0.897259228	0.844664461	0.979813706	0.429429467	0.989692427	0.999995916
RPL14	0.766036818	0.877944862	0.992537946	0.920475783	0.840155071	0.999390183	0.999995916
RPL15	0.619928209	0.775252523	0.566105973	0.752297695	0.691316206	0.951274925	0.999995916
RPL17	0.749635585	0.825187622	0.522566303	0.984470993	0.782185249	0.986113326	0.999995916
RPL18	0.902905802	0.897237323	0.698899373	0.829171116	0.6363992	0.992041999	0.999995916
RPL18A	0.885057228	0.951355464	0.528124654	0.451231797	0.556921534	0.928419946	0.999995916
RPL19	0.68327902	0.935455906	0.743923686	0.980533645	0.410871102	0.973307047	0.999995916
RPL21	0.926870956	0.603034691	0.675967272	0.61775371	0.446691605	0.920755645	0.999995916
RPL23	0.82163189	0.83090201	0.419061185	0.941103407	0.710562422	0.973228331	0.999995916
RPL24	0.789468132	0.934094605	0.697920328	0.986418277	0.232096502	0.933986988	0.999995916
RPL26L1	0.595069726	0.704483156	0.644184086	0.684627545	0.579347268	0.923789764	0.999995916
RPL27A	0.879715314	0.861644173	0.766094767	0.875972519	0.792196144	0.997555592	0.999995916
RPL29	0.877155303	0.985043642	0.933404518	0.793758935	0.632722963	0.997613869	0.999995916
RPL3	0.743634943	0.94581924	0.483188493	0.985268547	0.446635451	0.95590959	0.999995916
RPL30	0.680329993	0.693621179	0.447587847	0.960953979	0.685189653	0.949769667	0.999995916
RPL31	0.780032945	0.715492027	0.379135472	0.921404157	0.673452547	0.944575524	0.999995916
RPL32	0.989446667	0.906275057	0.820841182	0.970613384	0.944381892	0.999943271	0.999995916
RPL34	0.859697255	0.743205733	0.777260369	0.918449896	0.628438363	0.99088963	0.999995916
RPL35	0.766734063	0.790755014	0.669889692	0.906909533	0.806542605	0.991891007	0.999995916
RPL35A	0.91488777	0.710783374	0.524987996	0.957195196	0.57489681	0.972129593	0.999995916
RPL36A	0.866599674	0.881874182	0.465751854	0.964924937	0.513164547	0.968050558	0.999995916
RPL36AL	0.961449694	0.144411864	0.902554805	0.99191812	0.801677585	0.915535947	0.999995916
RPL37	0.801741824	0.938442856	0.842548689	0.991520456	0.705281863	0.998482345	0.999995916
RPL4	0.825253486	0.854835402	0.661077397	0.952962718	0.299127977	0.945721324	0.999995916
RPL5	0.741694187	0.753954817	0.80824699	0.959338595	0.644184338	0.990114264	0.999995916
RPL6	0.788919698	0.881256778	0.640317249	0.901584577	0.872262874	0.995522558	0.999995916
RPL7	0.910275531	0.940644183	0.526796389	0.82195923	0.525410379	0.974291934	0.999995916
RPL8	0.980561936	0.92179082	0.668933689	0.48815159	0.330107225	0.912870182	0.999995916
RPL9	0.532800456	0.624541652	0.527430009	0.954732677	0.685086537	0.93127472	0.999995916
RPLP0	0.988676846	0.497542331	0.574676648	0.706526927	0.458247457	0.905237992	0.999995916
RPP14	0.777089125	0.500075544	0.421607324	0.489002718	0.955930761	0.881724284	0.999995916

RPP25L	0.542464472	0.999504594	0.60838034	0.469529708	0.889677939	0.948956151	0.999995916
RPRD1A	0.597065029	0.838023312	0.706981504	0.790226224	0.755279908	0.978700448	0.999995916
RPS11	0.829066162	0.827352831	0.538897119	0.99299966	0.825911182	0.992422007	0.999995916
RPS12	0.956403294	0.94880561	0.728915846	0.649935711	0.726311332	0.993152666	0.999995916
RPS13	0.860952249	0.69687303	0.413952368	0.929563493	0.81586896	0.972289573	0.999995916
RPS14	0.599755642	0.95125814	0.944056766	0.994240146	0.977227226	0.999444345	0.999995916
RPS15A	0.811316133	0.82811816	0.692413019	0.969052629	0.552179562	0.986114977	0.999995916
RPS16	0.746524267	0.799533946	0.91670189	0.731350582	0.994142105	0.997404782	0.999995916
RPS18	0.764815032	0.915911354	0.816285622	0.549098171	0.609859328	0.973281516	0.999995916
RPS19	0.686661439	0.864150745	0.994760271	0.625090677	0.598702355	0.980948285	0.999995916
RPS20	0.959706324	0.953385349	0.822933559	0.611700423	0.236750942	0.925761621	0.999995916
RPS24	0.91476383	0.890076418	0.949869579	0.898433371	0.498559913	0.995333071	0.999995916
RPS25	0.890118081	0.564747925	0.368737495	0.995365673	0.751453835	0.949500643	0.999995916
RPS27	0.956959838	0.776444892	0.811029914	0.791151183	0.453617499	0.979915145	0.999995916
RPS27A	0.968247381	0.851731904	0.924936778	0.933263125	0.156977486	0.928419563	0.999995916
RPS27P	0.984378555	0.97741404	0.654354088	0.715385502	0.79958982	0.996003327	0.999995916
RPS28	0.71843715	0.910564512	0.57729123	0.690729526	0.488436244	0.941739723	0.999995916
RPS29	0.833454046	0.750356862	0.624355018	0.79986689	0.322645441	0.916840814	0.999995916
RPS3	0.697545542	0.984858234	0.739404668	0.985197167	0.433326735	0.980049067	0.999995916
RPS3A	0.958284514	0.657042596	0.672887955	0.900512326	0.68685476	0.988012628	0.999995916
RPS4X	0.450899441	0.911548329	0.544444601	0.67233676	0.73418199	0.92716746	0.999995916
RPS5	0.786282442	0.951138256	0.791474227	0.928757843	0.874307171	0.999039522	0.999995916
RPS6	0.85711115	0.960482566	0.72492414	0.683285961	0.235587592	0.911177942	0.999995916
RPS7	0.85750216	0.878670134	0.622027365	0.929725911	0.247911168	0.924728291	0.999995916
RPS8	0.990635454	0.986543925	0.772850876	0.957291357	0.335224295	0.985055748	0.999995916
RPS9	0.998279955	0.915817754	0.589330793	0.824042458	0.272506072	0.936671168	0.999995916
RRH	0.699038653	0.87286732	0.891967968	0.430567486	0.536138338	0.940388607	0.999995916
RRM1	0.680689475	0.689538013	0.920591966	0.65847963	0.737660484	0.978394206	0.999995916
RRP1B	0.963390151	0.798560937	0.93856962	0.456728487	0.947537559	0.993171474	0.999995916
RSRC1	0.697497376	0.905281372	0.731247284	0.419469756	0.452321033	0.899704405	0.999995916
RTN4	0.760012973	0.539607655	0.50585198	0.945017518	0.486722759	0.910362565	0.999995916
RTN4RL2	0.948252064	0.89561317	0.562723649	0.529656127	0.881958452	0.981448252	0.999995916
RUVBL2	0.678077945	0.990101509	0.523686298	0.660097756	0.6805781	0.960213134	0.999995916
SAMD1	0.91324118	0.971657615	0.385148534	0.429181004	0.501554139	0.876068943	0.999995916
SAMD12	0.862630829	0.867901312	0.526546299	0.911809068	0.547709557	0.974902256	0.999995916
SAMD13	0.679513945	0.978417732	0.323641104	0.777768984	0.663729731	0.927771401	0.999995916
SAMD4A	0.973120323	0.915201082	0.493307251	0.536786175	0.707386888	0.9642537	0.999995916
SAO	0.960367941	0.98974566	0.524639157	0.369070303	0.431820065	0.886820384	0.999995916
SAP30	0.30352361	0.705767807	0.551037813	0.993173342	0.785752412	0.9060428	0.999995916
SART1	0.564874697	0.857550481	0.86118701	0.957804648	0.94408345	0.996710293	0.999995916

SART3	0.982034818	0.353560919	0.940280774	0.459572378	0.807280554	0.936782165	0.999995916
SCAF1	0.510022122	0.967895765	0.723402559	0.763542305	0.842347583	0.98275303	0.999995916
SCAMP5	0.204488053	0.505841627	0.932097683	0.90485171	0.920701917	0.888260294	0.999995916
SCARA3	0.801633053	0.629761229	0.783396178	0.93150867	0.909168505	0.994689377	0.999995916
SCARB1	0.553007527	0.766066969	0.813609248	0.788098069	0.787165497	0.979347564	0.999995916
SCG5	0.492843748	0.850106427	0.866389038	0.610709273	0.753489199	0.964343863	0.999995916
SCML1	0.958953859	0.314111076	0.832742208	0.879403226	0.52489401	0.932174089	0.999995916
SCN3B	0.380617493	0.655712111	0.883939905	0.392888168	0.920717576	0.887379771	0.999995916
SCNM1	0.952863299	0.206168579	0.962946792	0.773210313	0.668562996	0.913308015	0.999995916
SCYL3	0.916242396	0.741909742	0.497825117	0.71106414	0.316858467	0.881102315	0.999995916
SDE2	0.257042352	0.86341958	0.575658281	0.740560513	0.971188496	0.905730696	0.999995916
SDF4	0.608554407	0.690648861	0.474349925	0.833086833	0.869936007	0.953058454	0.999995916
SDR42E1	0.974939632	0.866175719	0.519201805	0.77478594	0.705008195	0.984561636	0.999995916
SDSL	0.821278282	0.676174303	0.916235382	0.69410367	0.753168345	0.988529499	0.999995916
SEC61A2	0.856109531	0.461476646	0.849553189	0.947305522	0.71668323	0.982396124	0.999995916
SEL1L	0.973173657	0.428145341	0.294497601	0.774802663	0.790371166	0.87906317	0.999995916
SEMA3C	0.60564746	0.864684463	0.41804261	0.929578044	0.995401147	0.976501744	0.999995916
SENP2	0.960892012	0.836233402	0.837824003	0.498178205	0.263047216	0.900599176	0.999995916
SENP3	0.82324393	0.623350769	0.427280938	0.434468617	0.991931114	0.90917871	0.999995916
SENP5	0.855334611	0.919235265	0.497781849	0.389037887	0.878682215	0.946305309	0.999995916
SENP7	0.618139175	0.630230575	0.973432356	0.368134585	0.88978388	0.939281601	0.999995916
SEPSECS	0.829219717	0.598024838	0.617088981	0.63391767	0.518079233	0.916529779	0.999995916
SEPT3	0.837517409	0.523424385	0.731355337	0.577132999	0.658575957	0.9373876	0.999995916
SEPT8	0.960272007	0.253138137	0.707665811	0.6494998	0.966852292	0.924727684	0.999995916
SERP2	0.904127145	0.827064816	0.907367746	0.459483396	0.91821729	0.990851256	0.999995916
SERTAD3	0.421673754	0.897093096	0.665053195	0.996745507	0.320097967	0.888165942	0.999995916
SESN2	0.48637093	0.639585616	0.866729735	0.334885279	0.954715563	0.897620343	0.999995916
SESN3	0.726982837	0.832948735	0.601527181	0.999484716	0.210347044	0.88171082	0.999995916
SETD1B	0.94261117	0.203582338	0.860168554	0.652836399	0.985308931	0.922811702	0.999995916
SF3A1	0.943277178	0.808749896	0.867191565	0.588374367	0.787338694	0.992696769	0.999995916
SF3A2	0.752867394	0.794576497	0.585879414	0.655744952	0.874632684	0.976075004	0.999995916
SF3B2	0.883151451	0.872276097	0.590105332	0.715471392	0.931982639	0.992419088	0.999995916
SFMBT1	0.82297329	0.607998592	0.857880238	0.883626819	0.671090744	0.986962309	0.999995916
SFPQ	0.755858709	0.691707799	0.974693787	0.40748582	0.612456047	0.941565239	0.999995916
SGO1	0.678982827	0.455338251	0.500036156	0.87774646	0.975523205	0.94532844	0.999995916
SGSM2	0.940400556	0.718508662	0.574774559	0.936409159	0.459344892	0.964350424	0.999995916
SGTA	0.832173963	0.795453432	0.383233852	0.899878175	0.47256702	0.924580343	0.999995916
SH2D7	0.692624837	0.869049923	0.730793423	0.913875064	0.847172919	0.995014733	0.999995916
SH3BP4	0.778222071	0.518798414	0.812798554	0.750821156	0.342204856	0.894731123	0.999995916
SH3RF3	0.994968371	0.901741122	0.61140665	0.823874987	0.822105755	0.996489843	0.999995916

SHE	0.41502953	0.595011487	0.875287682	0.600935369	0.963581696	0.94002076	0.999995916
SHISA3	0.69159163	0.492305173	0.722506535	0.920813842	0.866205946	0.974708446	0.999995916
SHLD2	0.764794666	0.983461211	0.275568815	0.589455713	0.625869173	0.881504711	0.999995916
SHQ1	0.523545696	0.804986902	0.428125958	0.640111584	0.628636971	0.874191934	0.999995916
SHROOM4	0.744522035	0.97002842	0.493228049	0.635551706	0.327076931	0.87699207	0.999995916
SIGLEC10	0.736089104	0.76175397	0.984399097	0.241094576	0.55908909	0.877667743	0.999995916
SIN3A	0.405452264	0.818950527	0.739862315	0.635783641	0.90123032	0.950826023	0.999995916
SKAP2	0.851058522	0.780630852	0.402573987	0.443167995	0.845460007	0.916192639	0.999995916
SKI	0.959081477	0.666011575	0.81360293	0.922228604	0.422223387	0.976444678	0.999995916
SLC12A4	0.833280389	0.931474277	0.647971848	0.253417534	0.644083675	0.891188269	0.999995916
SLC13A5	0.702718458	0.476765399	0.764971774	0.928339056	0.948555104	0.981954628	0.999995916
SLC16A10	0.689897263	0.883038906	0.983133858	0.278837488	0.926645751	0.958634492	0.999995916
SLC16A2	0.572277047	0.931978491	0.356485848	0.858743955	0.578598964	0.909145391	0.999995916
SLC18A2	0.833817755	0.492182209	0.988602385	0.736000169	0.256200338	0.88157182	0.999995916
SLC1A3	0.81832826	0.605204764	0.616883597	0.902665296	0.320845207	0.900975886	0.999995916
SLC20A1	0.96483583	0.444789003	0.583653913	0.517136614	0.611857037	0.886438875	0.999995916
SLC22A5	0.954825316	0.87173835	0.233056117	0.746434686	0.591812883	0.896850806	0.999995916
SLC24A3	0.637453436	0.803503508	0.442894551	0.997583663	0.33671841	0.881018973	0.999995916
SLC25A14	0.541485926	0.583240363	0.891544612	0.788096686	0.426961437	0.909496203	0.999995916
SLC25A29	0.457705421	0.897809473	0.571619725	0.884928269	0.564197307	0.933502617	0.999995916
SLC25A37	0.850959959	0.486677896	0.343239742	0.672241303	0.74822213	0.871985869	0.999995916
SLC25A38	0.830216578	0.236145351	0.597850234	0.773530477	0.900293519	0.890426436	0.999995916
SLC25A53	0.759772326	0.902811277	0.425122136	0.549934768	0.515881794	0.892218974	0.999995916
SLC26A1	0.781763372	0.381110482	0.733212448	0.605482538	0.702043805	0.907033389	0.999995916
SLC27A4	0.460968125	0.800701589	0.818534434	0.596553624	0.961097114	0.966880686	0.999995916
SLC27A6	0.959647381	0.604026647	0.730020303	0.859104886	0.74483751	0.989125955	0.999995916
SLC29A1	0.966769053	0.656204012	0.704170385	0.461675554	0.51345106	0.922513074	0.999995916
SLC35D2	0.692507943	0.724700618	0.797797224	0.791603873	0.854590262	0.98913589	0.999995916
SLC35E2	0.984440859	0.884433039	0.899863713	0.74970808	0.67217672	0.997313375	0.999995916
SLC35E4	0.843366004	0.918531015	0.978455213	0.584318159	0.946169265	0.997974857	0.999995916
SLC36A1	0.422027021	0.917847966	0.815323939	0.68765728	0.93122548	0.976411448	0.999995916
SLC37A4	0.47745773	0.533662768	0.915171095	0.938870169	0.817657444	0.969076546	0.999995916
SLC38A10	0.728842126	0.531398746	0.428639492	0.678500983	0.965047284	0.92541888	0.999995916
SLC39A1	0.405267594	0.975168999	0.412682256	0.76266239	0.726241591	0.903598687	0.999995916
SLC39A11	0.738579692	0.866899279	0.578770934	0.707335996	0.524301023	0.948721637	0.999995916
SLC39A9	0.495206029	0.421537835	0.851766567	0.963611052	0.578414932	0.914887263	0.999995916
SLC43A3	0.982913066	0.799882322	0.462335405	0.839521275	0.560164333	0.965970251	0.999995916
SLC44A2	0.627618351	0.533440608	0.386965129	0.699416067	0.83670891	0.880314996	0.999995916
SLC4A1AP	0.299233475	0.88297477	0.762116742	0.908573985	0.623814801	0.930663385	0.999995916
SLC4A4	0.992268724	0.950304848	0.94643203	0.971513621	0.524774139	0.998682341	0.999995916

SLC6A9	0.245176194	0.760190542	0.978793186	0.906061997	0.654614773	0.924909615	0.999995916
SLC7A6OS	0.552483875	0.992923544	0.762735787	0.674791787	0.711904057	0.976072154	0.999995916
SLC9A3R2	0.939925052	0.617808127	0.694428532	0.261310209	0.84182249	0.901297315	0.999995916
SLCO2A1	0.939800903	0.857461711	0.942554337	0.625953916	0.605890113	0.991033239	0.999995916
SLX4IP	0.876817254	0.904182036	0.688141143	0.863856493	0.889472021	0.997978244	0.999995916
SMAD3	0.853523406	0.868147807	0.337666625	0.638582719	0.564347071	0.903369686	0.999995916
SMARCD1	0.702170841	0.359582386	0.685670017	0.975477954	0.444277315	0.878850302	0.999995916
SMARCE1	0.889093008	0.514749827	0.787389401	0.892856378	0.611115886	0.974829985	0.999995916
SMCO3	0.359033419	0.925889058	0.879344566	0.701616359	0.522500521	0.923840309	0.999995916
SMIM15	0.773745741	0.944674767	0.886462744	0.838927411	0.915410464	0.999224568	0.999995916
SMIM19	0.970238816	0.385646741	0.667510505	0.778718624	0.908594103	0.968226346	0.999995916
SMIM5	0.388646491	0.997623632	0.776344722	0.494787545	0.650856834	0.912258935	0.999995916
SMN2	0.94033811	0.813472551	0.62897569	0.530567888	0.438107974	0.928503653	0.999995916
SMOC1	0.594688164	0.715019294	0.789959392	0.745882027	0.962647293	0.984852524	0.999995916
SMPD5	0.624861836	0.622764919	0.894074319	0.845396372	0.830188147	0.985355149	0.999995916
SMS	0.650330301	0.623584635	0.855589256	0.473872348	0.876066196	0.952797826	0.999995916
SNAP47	0.75060499	0.58446402	0.63807378	0.535206894	0.628446217	0.908733128	0.999995916
SNAPC3	0.820611393	0.704943026	0.236012581	0.977006681	0.823927452	0.926619467	0.999995916
SNIP1	0.837149464	0.347796393	0.837899609	0.982276038	0.562956916	0.947059364	0.999995916
SNRNP200	0.911150747	0.78336445	0.708645778	0.825615118	0.68806775	0.990960024	0.999995916
SNRNP40	0.864895721	0.723192672	0.623615358	0.688831795	0.830262324	0.981413745	0.999995916
SNRPC	0.907726436	0.428415245	0.664304783	0.754726973	0.441069849	0.897311009	0.999995916
SNRPE	0.590705017	0.310908564	0.741004135	0.814956885	0.792763822	0.900166101	0.999995916
SNRPG	0.853505736	0.208456791	0.53511949	0.997446805	0.836801218	0.886805154	0.999995916
SNRPN	0.699998574	0.563955918	0.870667994	0.350203275	0.703607031	0.895316698	0.999995916
SNX14	0.535889033	0.805669299	0.788315036	0.611361955	0.664272791	0.949230965	0.999995916
SNX29	0.989845832	0.872615677	0.865254593	0.885214927	0.396511523	0.988049281	0.999995916
SNX33	0.854829048	0.721409492	0.316008593	0.902963888	0.866512997	0.957466275	0.999995916
SNX9	0.8253417	0.782124622	0.439690971	0.673758867	0.665319325	0.941603406	0.999995916
SOCS2	0.312249296	0.963014852	0.884786673	0.638523633	0.934047566	0.96056413	0.999995916
SOCS4	0.815971661	0.977731223	0.22428136	0.730593987	0.735776158	0.911326527	0.999995916
SORBS1	0.637735333	0.939328677	0.365103612	0.691857837	0.790092969	0.935469009	0.999995916
SOX18	0.824391169	0.857830411	0.514149761	0.518630274	0.576533521	0.925434456	0.999995916
SPAM1	0.559362948	0.734343164	0.859088453	0.353984133	0.936180338	0.933207265	0.999995916
SPATA2	0.654452505	0.370431885	0.85904072	0.519369151	0.66876642	0.873657564	0.999995916
SPEF1	0.746609191	0.36397888	0.955523076	0.922296969	0.455275694	0.925749976	0.999995916
SPESP1	0.848860108	0.742188235	0.609367326	0.511377993	0.433519813	0.895960113	0.999995916
SPIN2	0.91236248	0.904381542	0.550296311	0.865508916	0.8636238	0.994949134	0.999995916
SPIN2B	0.460493285	0.91260498	0.751674509	0.877410663	0.755648727	0.978284631	0.999995916
SPIN4	0.592682249	0.924103826	0.353909696	0.796069942	0.804807561	0.939258087	0.999995916

SPINDOC	0.585914643	0.62763551	0.651196412	0.76301756	0.46428093	0.89553509	0.999995916
SPNS1	0.596969179	0.71369475	0.73911818	0.535584519	0.913859015	0.958317666	0.999995916
SPRED1	0.66962441	0.663452628	0.876223158	0.538779991	0.664535349	0.949961809	0.999995916
SPRTN	0.856523161	0.69034011	0.887258301	0.423272345	0.323504933	0.872665806	0.999995916
SPSB2	0.703232944	0.975648242	0.4329657	0.427375678	0.987626275	0.940194623	0.999995916
SPTBN1	0.486409025	0.695349986	0.860800943	0.943637326	0.297738037	0.890710993	0.999995916
SREBF2	0.665890201	0.989267244	0.936095551	0.834197033	0.944200573	0.999098491	0.999995916
SRI	0.765679858	0.948096845	0.512902481	0.913813666	0.9764819	0.994526293	0.999995916
SRM	0.836392154	0.972892144	0.807080121	0.641247943	0.724779851	0.992595015	0.999995916
SRRT	0.835773802	0.685584333	0.490704171	0.294571133	0.990979065	0.891165792	0.999995916
SRSF4	0.95461929	0.393348724	0.755656356	0.647432271	0.38932968	0.872032409	0.999995916
SS18	0.717463556	0.823711846	0.732531082	0.512847282	0.550923913	0.937759423	0.999995916
SSH3	0.714519181	0.795988787	0.337296287	0.819739704	0.601571823	0.909313854	0.999995916
SSR2	0.815358701	0.788771746	0.779731906	0.729496609	0.939228162	0.995181771	0.999995916
SSRP1	0.589938646	0.930490277	0.801382522	0.901124689	0.616859158	0.985411607	0.999995916
SSUH2	0.89806438	0.775086737	0.457587431	0.69667289	0.864257632	0.97337362	0.999995916
ST6GALNAC2	0.993531274	0.270250325	0.63521189	0.648509754	0.85695247	0.909550485	0.999995916
ST6GALNAC5	0.535591024	0.920453296	0.793597046	0.967275038	0.689610387	0.987866748	0.999995916
STAM2	0.377954577	0.882113504	0.799444549	0.475514818	0.891557576	0.929606821	0.999995916
STARD3NL	0.629583002	0.390443105	0.973996227	0.649382455	0.497039727	0.882972338	0.999995916
STAT5B	0.945808132	0.28101077	0.735340164	0.750140064	0.665898986	0.913107696	0.999995916
STAU2	0.948875717	0.643606181	0.589245836	0.4591229	0.638492081	0.922082558	0.999995916
STIM2	0.58580852	0.472908872	0.901245583	0.636705941	0.960489296	0.957576419	0.999995916
STK11	0.351104858	0.757661676	0.929369087	0.599210152	0.752761739	0.928195398	0.999995916
STK11IP	0.759118256	0.615248976	0.782755935	0.913810239	0.507845714	0.965445456	0.999995916
STMN3	0.52837163	0.869332068	0.738000126	0.562561787	0.509590414	0.912560113	0.999995916
STRN	0.395072063	0.581464912	0.914907044	0.397609101	0.968044086	0.889220509	0.999995916
STX16	0.506149301	0.858721923	0.520918753	0.926958797	0.955622442	0.97595019	0.999995916
STX3	0.940784516	0.741411435	0.732429372	0.223541657	0.708706215	0.889286175	0.999995916
SUGP2	0.730814075	0.219458773	0.49288488	0.993693736	0.911758426	0.872229501	0.999995916
SULF1	0.68033993	0.572301161	0.970175704	0.799900109	0.693105848	0.978281928	0.999995916
SUMF1	0.633922563	0.790049187	0.363398069	0.991282658	0.620707467	0.928645557	0.999995916
SUN1	0.87343908	0.642448123	0.90720108	0.967706145	0.894068833	0.998429278	0.999995916
SUPT5H	0.705100859	0.968668374	0.524430647	0.893532401	0.978601795	0.993226174	0.999995916
SUSD6	0.959571315	0.520903953	0.874269054	0.843278977	0.524592853	0.973845724	0.999995916
SWAP70	0.797067728	0.937639928	0.791115394	0.573564164	0.807338288	0.989484984	0.999995916
SYCE1L	0.573030563	0.83820449	0.731095642	0.848272232	0.696139895	0.977761498	0.999995916
SYDE1	0.919834061	0.961394745	0.932574335	0.655906182	0.517870506	0.990203419	0.999995916
SYN1	0.695212643	0.989594291	0.914772271	0.828035224	0.433327041	0.981981382	0.999995916
SYN3	0.901089759	0.970694494	0.455034165	0.213174299	0.884938808	0.878952216	0.999995916

SYNPO	0.904413067	0.693304667	0.869933531	0.582485609	0.716940577	0.982381313	0.999995916
SYNRG	0.777158927	0.552783558	0.641060628	0.967772175	0.573878571	0.957713248	0.999995916
SYT12	0.916343269	0.999389546	0.790112932	0.764860473	0.966362311	0.999523173	0.999995916
SYT4	0.99081979	0.286752844	0.833795412	0.992874254	0.887407807	0.978106599	0.999995916
TACC2	0.98915115	0.755150334	0.781056893	0.2916404	0.64039503	0.92567892	0.999995916
TADA2A	0.841706443	0.998545124	0.625361936	0.443013095	0.826023571	0.97354634	0.999995916
TAF12	0.871682366	0.470110087	0.977694994	0.958779956	0.461570001	0.968446315	0.999995916
TAF1B	0.762805903	0.472538448	0.54078256	0.854919878	0.5728178	0.910410458	0.999995916
TAF3	0.876144003	0.856756819	0.995993885	0.268559111	0.767649723	0.95831897	0.999995916
TAPBP	0.637139482	0.942972404	0.980376784	0.92460134	0.960389265	0.999442317	0.999995916
TARS2	0.776468491	0.703251013	0.961253772	0.252206696	0.990421222	0.944442621	0.999995916
TASP1	0.706518796	0.534191004	0.759477958	0.894483774	0.929174249	0.984340971	0.999995916
TATDN2	0.933383205	0.741832644	0.860072213	0.765664849	0.645233069	0.991626729	0.999995916
TBC1D12	0.734522278	0.424162739	0.81250219	0.447948099	0.768841145	0.89907961	0.999995916
TBC1D17	0.434602176	0.919493195	0.547386978	0.41999463	0.958450131	0.900356332	0.999995916
TBC1D25	0.696672836	0.72790913	0.996936809	0.981463949	0.628180707	0.993110989	0.999995916
TBC1D30	0.808306311	0.646932961	0.282014736	0.893603429	0.655496329	0.897889435	0.999995916
TBC1D9B	0.558141986	0.913218432	0.302441015	0.824316047	0.567807328	0.873295366	0.999995916
TBCEL	0.673885143	0.502786628	0.270388963	0.854555179	0.932483945	0.874971771	0.999995916
TBCK	0.524473074	0.846962599	0.890831638	0.951493691	0.728846175	0.989559963	0.999995916
TBX2	0.98120104	0.841127241	0.643335081	0.396426681	0.955898725	0.976127177	0.999995916
TBXAS1	0.931189129	0.395238468	0.89541409	0.549683045	0.954027669	0.966723294	0.999995916
TCAIM	0.992566992	0.625586284	0.319655998	0.736226653	0.887718196	0.943444664	0.999995916
TCERG1	0.939037772	0.425469385	0.449782637	0.72833972	0.743457715	0.91271733	0.999995916
TCF12	0.976583154	0.85564544	0.560386863	0.855615612	0.864066305	0.995321025	0.999995916
TCFL5	0.879418115	0.588851285	0.423876121	0.448855176	0.751708371	0.877019802	0.999995916
TCN2	0.761054725	0.786640774	0.775741614	0.971690075	0.610407382	0.989679628	0.999995916
TCTN1	0.636263504	0.905069205	0.847856167	0.819453399	0.80361614	0.993827728	0.999995916
TDG	0.69736257	0.955244209	0.259559467	0.966321188	0.51624202	0.897699346	0.999995916
TDRD6	0.888279882	0.568422764	0.616725206	0.888572713	0.845487639	0.983564688	0.999995916
TDRD9	0.97883819	0.606198375	0.413969814	0.95307602	0.896936557	0.978419357	0.999995916
TEAD4	0.417247529	0.449539147	0.965755125	0.75554879	0.611903956	0.893841391	0.999995916
TEDC1	0.999810401	0.891596389	0.360001809	0.53729768	0.986512782	0.965627925	0.999995916
TEK	0.714084401	0.646419151	0.90390353	0.799571272	0.834558065	0.990014879	0.999995916
TENM1	0.283987556	0.601484151	0.636123432	0.94510075	0.980978969	0.91680818	0.999995916
TENM3	0.814686233	0.670159909	0.636933094	0.258073171	0.96332175	0.89799515	0.999995916
TEX11	0.869676669	0.591371758	0.325125122	0.732425412	0.712345632	0.899167537	0.999995916
TEX261	0.709514817	0.595719577	0.52858763	0.674225615	0.983270949	0.955121085	0.999995916
TEX35	0.80218269	0.772050219	0.346258435	0.452691225	0.847513129	0.891486638	0.999995916
TFEB	0.750955985	0.942969854	0.982446048	0.52877652	0.412951663	0.957170411	0.999995916

TFPI2	0.486190781	0.659809641	0.569378689	0.642828712	0.63435156	0.877819657	0.999995916
TFPT	0.52444016	0.940133102	0.604854254	0.848184949	0.99412625	0.98650356	0.999995916
TG	0.708157957	0.996242316	0.912066394	0.934994805	0.79874731	0.999038387	0.999995916
TGFB1	0.923336608	0.423174528	0.858622414	0.283652971	0.867921826	0.892002761	0.999995916
THADA	0.628805987	0.858990187	0.798689854	0.791874555	0.293442344	0.916232999	0.999995916
THEM4	0.852400547	0.521521115	0.982570026	0.617969985	0.618511591	0.964308895	0.999995916
THG1L	0.78780103	0.934861803	0.334240385	0.544398259	0.613129838	0.891309727	0.999995916
TIAM1	0.583026991	0.330738786	0.886134499	0.526981101	0.887425196	0.887561173	0.999995916
TIAM2	0.738289149	0.701251047	0.80883494	0.436111303	0.746378874	0.947989847	0.999995916
TICRR	0.910489983	0.197396717	0.965882736	0.912700092	0.690671757	0.926148876	0.999995916
TIGD2	0.917521864	0.992684529	0.749225752	0.231857856	0.665251049	0.921832994	0.999995916
TJP1	0.501551789	0.835343702	0.567719038	0.744130404	0.534014188	0.909207301	0.999995916
TJP2	0.267681641	0.919623164	0.515631021	0.974792416	0.800873175	0.914876151	0.999995916
TLK1	0.60865123	0.805685661	0.706865096	0.314334313	0.911149927	0.915095618	0.999995916
TLK2	0.842927297	0.646423473	0.492129364	0.485275681	0.681822945	0.901325258	0.999995916
TLN2	0.927995928	0.562940056	0.727867424	0.983287725	0.707574861	0.988343336	0.999995916
TM2D2	0.593994724	0.527079054	0.800586573	0.869374807	0.816301815	0.968660151	0.999995916
TM2D3	0.832421007	0.570300716	0.76617927	0.344531341	0.596770543	0.8783918	0.999995916
TM7SF3	0.747333347	0.773772448	0.614770407	0.820109793	0.958316077	0.990123124	0.999995916
TM9SF4	0.860136488	0.799205843	0.760612049	0.954250273	0.787080944	0.997246787	0.999995916
TMCO3	0.654185795	0.330395651	0.937781628	0.979173314	0.871886308	0.966811758	0.999995916
TMEM117	0.590031615	0.839558602	0.921630882	0.503018919	0.654961763	0.956374055	0.999995916
TMEM127	0.805141727	0.367637798	0.4147399	0.846186019	0.819401351	0.895975305	0.999995916
TMEM131L	0.667281593	0.896001667	0.430693874	0.64702888	0.762310804	0.94143795	0.999995916
TMEM141	0.662815081	0.927784206	0.800290367	0.415033164	0.727994917	0.955441612	0.999995916
TMEM161A	0.724636595	0.795836085	0.744744101	0.51949567	0.938928972	0.978297448	0.999995916
TMEM163	0.987866914	0.751245789	0.596840046	0.632953293	0.802289582	0.981799012	0.999995916
TMEM179B	0.662238196	0.516988962	0.929154179	0.712069811	0.702022362	0.96072788	0.999995916
TMEM233	0.797346319	0.577231152	0.425782991	0.485239084	0.812765214	0.882987078	0.999995916
TMEM237	0.843605331	0.72024176	0.978102745	0.668814457	0.241695132	0.911178371	0.999995916
TMEM240	0.986547348	0.151569066	0.862440842	0.865346618	0.710132814	0.886429263	0.999995916
TMEM241	0.938001287	0.608862068	0.761805083	0.938727976	0.548914857	0.981645648	0.999995916
TMEM243	0.927725695	0.869939513	0.236720777	0.836909688	0.447900895	0.872218833	0.999995916
TMEM260	0.855209569	0.294323918	0.443339847	0.703772654	0.916787476	0.872991355	0.999995916
TMEM268	0.94459373	0.745761236	0.679519463	0.454807591	0.557088609	0.936913994	0.999995916
TMEM41A	0.939334746	0.329915586	0.865757988	0.897510596	0.446352864	0.924172037	0.999995916
TMEM44	0.891221108	0.356988915	0.666893048	0.872355056	0.456474729	0.89500292	0.999995916
TMEM63B	0.329641914	0.852283113	0.839984688	0.718765488	0.929582381	0.960082225	0.999995916
TMEM65	0.773644536	0.9477938	0.552525308	0.799593673	0.304409556	0.914302738	0.999995916
TMEM81	0.726489199	0.899237144	0.371497386	0.866201678	0.657798384	0.949270534	0.999995916

TMEM87B	0.889235543	0.276829971	0.971896795	0.879363896	0.615809429	0.943325162	0.999995916
TMLHE	0.762275524	0.956520532	0.634717346	0.794287508	0.991165794	0.996189941	0.999995916
TMPO	0.379671145	0.857388825	0.578394743	0.895001657	0.796947906	0.946648865	0.999995916
TMTC4	0.837579863	0.906267849	0.126448364	0.898436248	0.868657853	0.878624822	0.999995916
TNFAIP8	0.556990777	0.887753987	0.975374965	0.628432244	0.527401774	0.961120011	0.999995916
TNFRSF1A	0.658679749	0.895501048	0.738024478	0.719011023	0.643096948	0.976151602	0.999995916
TNKS2	0.963451816	0.37652584	0.322294384	0.854984969	0.785221371	0.885119322	0.999995916
TNNI3	0.718718373	0.942206495	0.911917845	0.638523525	0.966540556	0.996853833	0.999995916
TNRC6C	0.816646995	0.306186467	0.599149825	0.840886014	0.82618276	0.920561766	0.999995916
TNS1	0.916855461	0.595320625	0.59984288	0.865289128	0.53209461	0.956551475	0.999995916
TNS3	0.962019257	0.678108218	0.710805252	0.227363601	0.805819598	0.895722821	0.999995916
TOR1AIP1	0.84491787	0.763891311	0.291507717	0.629366933	0.691348232	0.890818758	0.999995916
TOR3A	0.743780574	0.505427534	0.313524949	0.929627051	0.929916352	0.918123819	0.999995916
TP53RK	0.935570198	0.466846035	0.466306298	0.793992188	0.63292139	0.918643917	0.999995916
TPBGL	0.628276599	0.564396585	0.831769229	0.550186396	0.475559628	0.882778951	0.999995916
TPGS1	0.8344225	0.947715608	0.899693154	0.49160705	0.739227157	0.987534766	0.999995916
TPO	0.825810994	0.932448838	0.98532243	0.894541907	0.261785229	0.968585072	0.999995916
TRABD2B	0.33573087	0.837553041	0.992507271	0.377226672	0.893242727	0.908586438	0.999995916
TRAF1	0.777792682	0.710187486	0.67787753	0.292116209	0.975407778	0.923351018	0.999995916
TRAFD1	0.844176504	0.891532287	0.222675393	0.927549281	0.868148199	0.947089506	0.999995916
TRAM1L1	0.435799863	0.914345768	0.914087817	0.294416139	0.899945896	0.911729341	0.999995916
TRAPPC11	0.967107159	0.702839233	0.806380705	0.684358812	0.828316903	0.993034403	0.999995916
TRIM14	0.586119122	0.59197033	0.628075724	0.956437269	0.408083367	0.895876713	0.999995916
TRIM16	0.615743961	0.673295809	0.604021245	0.646308707	0.77797163	0.940599362	0.999995916
TRIM17	0.478785019	0.638952676	0.881620722	0.379508985	0.894047391	0.90521988	0.999995916
TRIM27	0.689879149	0.95615588	0.636998944	0.313863051	0.963059417	0.941436029	0.999995916
TRIM3	0.786248459	0.605445339	0.98049253	0.683898066	0.376501988	0.935997327	0.999995916
TRIM32	0.515383096	0.716935586	0.585393047	0.450368747	0.978342216	0.910215451	0.999995916
TRIM47	0.932346242	0.250127609	0.977924558	0.932617872	0.645540408	0.948638178	0.999995916
TRIM52	0.832331311	0.919854473	0.920961963	0.653845821	0.564079667	0.98774271	0.999995916
TRIM9	0.775322901	0.891912333	0.392133419	0.780196803	0.834217559	0.968141398	0.999995916
TRIQK	0.927685969	0.893638664	0.887397209	0.965015273	0.76215846	0.999561739	0.999995916
TRMO	0.541251698	0.863474309	0.72048796	0.862332399	0.605428257	0.967878765	0.999995916
TRMT10A	0.796244767	0.276024798	0.967901884	0.453220106	0.840689331	0.88948179	0.999995916
TRMT112	0.674249549	0.688550836	0.773358339	0.525909558	0.893813981	0.965076894	0.999995916
TRMT2B	0.484457259	0.869907979	0.85272488	0.656954421	0.7385082	0.967323631	0.999995916
TRNAU1AP	0.590627391	0.775247302	0.925954541	0.896395739	0.302096561	0.931291892	0.999995916
TRNT1	0.973314696	0.649220925	0.806339441	0.533654234	0.864677126	0.983780078	0.999995916
TRPS1	0.940176641	0.579722345	0.443006995	0.474519758	0.671337643	0.882326542	0.999995916
TRUB1	0.785577219	0.69250027	0.249524478	0.615073512	0.970430884	0.889433086	0.999995916

TSEN2	0.840218977	0.995774272	0.77300522	0.521907154	0.828741906	0.990160422	0.999995916
TSEN34	0.657763834	0.831384822	0.436064437	0.951549404	0.777846047	0.968145323	0.999995916
TSHZ1	0.800152181	0.222952452	0.694286179	0.944396572	0.942193228	0.926921085	0.999995916
TSPAN12	0.667347498	0.970427307	0.872492205	0.667897337	0.912775101	0.995229279	0.999995916
TSPAN14	0.680569823	0.489507782	0.890126641	0.367087031	0.888668705	0.912013994	0.999995916
TSPAN17	0.896585926	0.989598189	0.474677421	0.939318637	0.98123795	0.997099247	0.999995916
TSPAN3	0.183043079	0.477025651	0.876735091	0.978718823	0.991122744	0.877395636	0.999995916
TTC39A	0.581112354	0.792041188	0.67227449	0.710299944	0.730801334	0.96148008	0.999995916
TTI2	0.838676118	0.617087303	0.740755071	0.98283718	0.715772308	0.988993838	0.999995916
TTLL9	0.479784076	0.452833961	0.696459583	0.738910638	0.936070247	0.921191644	0.999995916
TUBB3	0.553185762	0.721823019	0.580885018	0.609254547	0.820708569	0.932348811	0.999995916
TULP3	0.993135818	0.475693836	0.797252301	0.536105038	0.74046684	0.955889973	0.999995916
TULP4	0.959814087	0.924778238	0.976408942	0.543153463	0.949543125	0.998548174	0.999995916
TXNDC12	0.78372691	0.465139305	0.601375592	0.964952576	0.866908781	0.970629545	0.999995916
TXNL4B	0.794679152	0.571280855	0.65564779	0.862944579	0.47257935	0.936999466	0.999995916
TYW5	0.994272666	0.613311231	0.413723405	0.972859281	0.404063884	0.914972005	0.999995916
U2AF1L4	0.893759799	0.418376612	0.804427617	0.372665	0.717184229	0.888379964	0.999995916
UACA	0.939832177	0.889824694	0.423096696	0.286721443	0.710303182	0.873111296	0.999995916
UBAC2	0.5497438	0.821949567	0.729835827	0.9873019	0.753071561	0.985520949	0.999995916
UBALD1	0.857548825	0.50950597	0.684318577	0.71406598	0.804619405	0.96631245	0.999995916
UBAP2L	0.953949207	0.466130532	0.861950833	0.364962789	0.965988878	0.947206639	0.999995916
UBE2D1	0.915565051	0.769711212	0.861909511	0.791918101	0.84690205	0.99767774	0.999995916
UBE2D4	0.255667528	0.967563373	0.887309537	0.761583479	0.481293709	0.888483753	0.999995916
UBE2I	0.766811096	0.351607565	0.742029033	0.752920052	0.57487785	0.898208096	0.999995916
UBE2L6	0.857585943	0.700605405	0.890138307	0.817227823	0.851661853	0.996517464	0.999995916
UBTD2	0.567775869	0.6383993	0.667326627	0.561456771	0.923276497	0.940196297	0.999995916
UBXN2B	0.974749588	0.75121448	0.810368736	0.798922153	0.529158361	0.986412381	0.999995916
UCK1	0.586725202	0.629143824	0.737194068	0.797566322	0.675339725	0.954257383	0.999995916
UHRF1BP1	0.923758976	0.693514725	0.731991169	0.649277291	0.954605526	0.991288051	0.999995916
ULBP21	0.949933092	0.385111775	0.919139435	0.935294175	0.854486791	0.988874572	0.999995916
UNC50	0.95109869	0.581523509	0.843514705	0.612981276	0.940028418	0.988886783	0.999995916
UNC5B	0.481263821	0.669469985	0.980513913	0.592233572	0.701016484	0.944472735	0.999995916
UROS	0.439250134	0.753810955	0.939890509	0.404441097	0.929525857	0.933253526	0.999995916
USF1	0.842428647	0.716929584	0.800184398	0.447027245	0.460637282	0.915377475	0.999995916
USP27X	0.631410325	0.699014193	0.905621685	0.710822824	0.412169443	0.933351802	0.999995916
USP33	0.510084262	0.96987217	0.698813655	0.829393964	0.568102061	0.962524206	0.999995916
USP36	0.845821083	0.351022366	0.585321405	0.565192017	0.908860558	0.902101482	0.999995916
USP40	0.775678502	0.66849136	0.480967409	0.781256409	0.55792622	0.925423513	0.999995916
USP45	0.765190216	0.742088786	0.495106749	0.800930726	0.445868675	0.916411605	0.999995916
UTP11	0.633906035	0.255306755	0.894986593	0.976838055	0.65728895	0.907221112	0.999995916

UTP25	0.805511971	0.442214177	0.391511194	0.668940933	0.834895133	0.88405602	0.999995916
UVRAG	0.918949748	0.736409508	0.244917381	0.43779079	0.982309777	0.871534256	0.999995916
UXS1	0.981012526	0.984792123	0.192789618	0.514640664	0.837529938	0.888187616	0.999995916
UXT	0.822295444	0.815136647	0.990477777	0.46802009	0.603050227	0.971970988	0.999995916
VAV3	0.799025243	0.884256396	0.584004948	0.582523052	0.442977685	0.923124998	0.999995916
VIPAS39	0.829595085	0.477917575	0.726641731	0.975870093	0.360738431	0.917589765	0.999995916
VN1R1	0.406678276	0.704777129	0.791630906	0.783052912	0.584630748	0.920333712	0.999995916
VPS35L	0.618172774	0.81158514	0.602973603	0.494165086	0.780055736	0.932913231	0.999995916
VPS36	0.389437178	0.583668038	0.670241437	0.948410521	0.816315284	0.934088105	0.999995916
VPS37B	0.862851273	0.836781715	0.82899649	0.586359813	0.595504477	0.978175381	0.999995916
VPS45	0.671210865	0.733768744	0.857656659	0.427824271	0.440733258	0.887114624	0.999995916
VPS4B	0.994019718	0.763048094	0.339256443	0.969966924	0.872920567	0.980279823	0.999995916
VPS54	0.923225929	0.752245594	0.901580357	0.763162404	0.987449371	0.998928491	0.999995916
VRK3	0.608793674	0.906586668	0.412851973	0.457425415	0.759640391	0.886308208	0.999995916
VWA1	0.480297596	0.961490963	0.812118474	0.837814595	0.989475894	0.993049808	0.999995916
VWA5B2	0.452582917	0.762285545	0.895881895	0.833795509	0.445630819	0.931319038	0.999995916
VWA8	0.851398149	0.820576298	0.385366425	0.964790966	0.87566796	0.982313496	0.999995916
VWF	0.268792074	0.725636133	0.92653181	0.644757289	0.624097014	0.874413819	0.999995916
WARS2	0.968089224	0.429214983	0.674510659	0.348376887	0.745878283	0.874628557	0.999995916
WASHC1	0.647551163	0.83644978	0.671996795	0.565470819	0.498702964	0.918973814	0.999995916
WASHC3	0.834738977	0.476249525	0.411076888	0.713308458	0.74186126	0.898034332	0.999995916
WASHC5	0.630486899	0.870971051	0.64621793	0.876689922	0.773149646	0.984740469	0.999995916
WASL	0.388248253	0.70129968	0.880972484	0.665286109	0.476583006	0.88075222	0.999995916
WDR25	0.875314484	0.684202626	0.818033986	0.969618626	0.842902101	0.997481514	0.999995916
WDR31	0.467183889	0.494581815	0.473199759	0.871070018	0.847003407	0.888841763	0.999995916
WDR41	0.970685672	0.648983624	0.717274571	0.634401755	0.961721311	0.98970549	0.999995916
WDR5B	0.81751453	0.740134749	0.458423312	0.592927874	0.966954295	0.960732062	0.999995916
WDR61	0.989460004	0.354317202	0.637886228	0.68908185	0.487096017	0.878912196	0.999995916
WDR70	0.910920527	0.947534247	0.804646437	0.880805001	0.485609258	0.9918889	0.999995916
WDR78	0.909917433	0.990531672	0.204978697	0.824769626	0.890256557	0.947557783	0.999995916
WDYHV1	0.953607383	0.708230949	0.996340757	0.681445337	0.707741497	0.994032104	0.999995916
WIPF2	0.995764253	0.421514847	0.583583233	0.461800604	0.841579339	0.910077164	0.999995916
WNK1	0.561875702	0.818208886	0.390131307	0.598778766	0.994462301	0.923463919	0.999995916
WNT10B	0.373094629	0.869378523	0.599449536	0.480685166	0.800366589	0.878429674	0.999995916
WNT16	0.779715213	0.626539909	0.558945927	0.856955832	0.918083187	0.979583081	0.999995916
WRNIP1	0.708013034	0.835507659	0.990146875	0.687429693	0.962152765	0.997073092	0.999995916
WWTR1	0.978612522	0.886112403	0.292183437	0.540162669	0.992396537	0.947669234	0.999995916
XAB2	0.927188632	0.511183535	0.70435126	0.825290987	0.826131732	0.982344161	0.999995916
XKR4	0.642715252	0.812243111	0.778017923	0.702878521	0.888350983	0.986824348	0.999995916
XRCC2	0.905044309	0.97293128	0.588805918	0.862940651	0.855131884	0.996906723	0.999995916

XXYLT1	0.725952796	0.910713279	0.223039816	0.616204516	0.94306519	0.896848478	0.999995916
YAP1	0.759746871	0.508328824	0.876051258	0.999923316	0.575208432	0.974231025	0.999995916
YES1	0.868038368	0.697933637	0.605605983	0.96121479	0.717160268	0.986722424	0.999995916
YIPF3	0.97167115	0.661990067	0.993720744	0.732003763	0.894498086	0.997962377	0.999995916
YIPF6	0.895328271	0.4921786	0.450522812	0.713542114	0.538886101	0.881271125	0.999995916
YLPM1	0.767660239	0.622032539	0.436704522	0.423622526	0.999907037	0.900756515	0.999995916
YRDC	0.884345236	0.612288641	0.543885134	0.71205024	0.854313676	0.969126484	0.999995916
YWHAZ	0.903307733	0.6180191	0.335760781	0.721253048	0.690473775	0.907680487	0.999995916
YY1	0.922185427	0.978035373	0.24254351	0.349131415	0.955164583	0.874869733	0.999995916
ZBED1	0.445065255	0.773611586	0.700419865	0.327506374	0.928441283	0.875608356	0.999995916
ZBTB38	0.247628252	0.804049154	0.784117118	0.692876991	0.703483495	0.880832981	0.999995916
ZBTB41	0.948495624	0.861395413	0.903502033	0.653433506	0.711977956	0.995172972	0.999995916
ZBTB46	0.411259359	0.732704755	0.479632385	0.914746273	0.921401326	0.93735197	0.999995916
ZC3H10	0.842750723	0.960692384	0.57809027	0.742462562	0.666498809	0.983125044	0.999995916
ZC3H12A	0.617305699	0.917624642	0.741154074	0.730653411	0.702655082	0.979747803	0.999995916
ZC3HAV1	0.703955597	0.998154452	0.284563482	0.7254411	0.667944471	0.912199447	0.999995916
ZC3HAV1L	0.499898698	0.965951273	0.760500002	0.917991749	0.715326237	0.984845917	0.999995916
ZC3HC1	0.924716356	0.644628301	0.418360474	0.981554549	0.527729985	0.943049372	0.999995916
ZCCHC4	0.610380483	0.837917179	0.407730018	0.665470469	0.631461895	0.899737637	0.999995916
ZCCHC7	0.698442445	0.94412862	0.570007077	0.372845721	0.91722831	0.942581675	0.999995916
ZCCHC8	0.833788827	0.572034083	0.702808507	0.682124405	0.357830463	0.890744483	0.999995916
ZDHHC15	0.948466921	0.621608188	0.331434289	0.743097951	0.79982194	0.932491602	0.999995916
ZDHHC24	0.271148005	0.928887388	0.686036182	0.767202389	0.720052953	0.910403511	0.999995916
ZDHHC5	0.664358204	0.559295453	0.47100451	0.682703846	0.763118272	0.904765506	0.999995916
ZDHHC8	0.700639675	0.898728617	0.422400285	0.69080503	0.74723097	0.948635409	0.999995916
ZEB1	0.810199693	0.831592781	0.410253834	0.861720492	0.764936395	0.970216225	0.999995916
ZFAND6	0.900294333	0.792821361	0.522800386	0.866668367	0.335773373	0.925304947	0.999995916
ZFP28	0.76332246	0.426411102	0.758390738	0.413212468	0.761439756	0.883665552	0.999995916
ZFP37	0.504124091	0.877352293	0.659490506	0.911410664	0.970425659	0.987455034	0.999995916
ZFP69	0.692335615	0.854174249	0.779329468	0.417039127	0.911664658	0.96766008	0.999995916
ZFP91	0.778868486	0.405275559	0.839503241	0.979482969	0.70622036	0.970600568	0.999995916
ZFYVE16	0.391472258	0.858773705	0.928951711	0.363754118	0.907855388	0.919518768	0.999995916
ZHX1	0.922529792	0.344375373	0.756981793	0.917243993	0.47442381	0.921183351	0.999995916
ZKSCAN7	0.705189898	0.316531282	0.583661348	0.819852572	0.811358626	0.898309368	0.999995916
ZNF142	0.933107334	0.49728836	0.446577043	0.939250331	0.48341229	0.908650158	0.999995916
ZNF165	0.85140618	0.607915568	0.746465962	0.450253766	0.709776099	0.938690455	0.999995916
ZNF177	0.611482017	0.816603831	0.439606361	0.525098195	0.677609388	0.884439672	0.999995916
ZNF205	0.770152832	0.544105152	0.494454916	0.815635162	0.85774472	0.953331228	0.999995916
ZNF23	0.95615963	0.649768266	0.605385947	0.450712738	0.832517801	0.951049479	0.999995916
ZNF235	0.817821642	0.357609233	0.700970199	0.610735189	0.745768578	0.907714885	0.999995916

ZNF239	0.257776658	0.880080578	0.947151748	0.498780258	0.954414114	0.918576142	0.999995916
ZNF24	0.992706051	0.997921847	0.369572053	0.732424787	0.628711178	0.965001373	0.999995916
ZNF248	0.932758285	0.636508279	0.672754607	0.813173878	0.624395585	0.976563707	0.999995916
ZNF281	0.704528966	0.987448507	0.87936177	0.552448232	0.887853804	0.992155951	0.999995916
ZNF3	0.891371928	0.784002117	0.567410859	0.686623608	0.277209783	0.879682291	0.999995916
ZNF367	0.713130854	0.768283923	0.392149284	0.865864795	0.706665238	0.944690365	0.999995916
ZNF389	0.903683579	0.673366425	0.355896885	0.753111656	0.772786206	0.940699256	0.999995916
ZNF394	0.529012665	0.806203398	0.788592616	0.799080456	0.569063457	0.957705479	0.999995916
ZNF397	0.895489113	0.539745951	0.994380925	0.727633564	0.441468161	0.958449367	0.999995916
ZNF398	0.805158035	0.337801905	0.953085536	0.624403126	0.560365534	0.904107829	0.999995916
ZNF404	0.503238035	0.915942601	0.561853982	0.412493513	0.733958438	0.884969477	0.999995916
ZNF408	0.758023521	0.758757472	0.931564536	0.92116088	0.335174263	0.963649055	0.999995916
ZNF444	0.42157136	0.634209051	0.794809076	0.601567293	0.740155095	0.909335585	0.999995916
ZNF449	0.950254195	0.633167016	0.806054993	0.81859986	0.623284534	0.98588197	0.999995916
ZNF45	0.670234258	0.985968138	0.819449329	0.931797526	0.220508796	0.927952902	0.999995916
ZNF461	0.963551579	0.987849225	0.926250153	0.917842385	0.985661606	0.999995916	0.999995916
ZNF48	0.954051916	0.499793925	0.546671961	0.58601219	0.875959996	0.946316905	0.999995916
ZNF496	0.566623137	0.540501833	0.770895042	0.951177089	0.563213378	0.941034779	0.999995916
ZNF514	0.54897172	0.919365354	0.673298779	0.702224192	0.676572791	0.961865053	0.999995916
ZNF526	0.381453594	0.906758429	0.492750157	0.928122332	0.851845999	0.946954604	0.999995916
ZNF570	0.603883746	0.876363227	0.950062089	0.553828025	0.671200324	0.971814562	0.999995916
ZNF574	0.798339379	0.582119491	0.783758101	0.43434627	0.640147848	0.917422658	0.999995916
ZNF579	0.896281963	0.850777839	0.879303428	0.536351157	0.68985853	0.985983598	0.999995916
ZNF580	0.474008859	0.939322455	0.613979107	0.384593038	0.746190266	0.88504909	0.999995916
ZNF583	0.509727673	0.217537316	0.808685933	0.953424948	0.98025064	0.893932978	0.999995916
ZNF592	0.827904136	0.317518268	0.943684507	0.555940369	0.564362005	0.883958631	0.999995916
ZNF596	0.708496648	0.750566828	0.932433688	0.198676782	0.873675684	0.897417993	0.999995916
ZNF598	0.749817984	0.420880778	0.553154286	0.779955095	0.995630386	0.947496008	0.999995916
ZNF599	0.904495872	0.799293447	0.310292412	0.728209129	0.456051108	0.877852744	0.999995916
ZNF606	0.373536759	0.901821708	0.822647357	0.804899294	0.524487381	0.933247807	0.999995916
ZNF623	0.921890612	0.320815693	0.623164786	0.677621528	0.910744041	0.930304587	0.999995916
ZNF629	0.668519892	0.837359551	0.542649339	0.776226687	0.324694187	0.881678288	0.999995916
ZNF674	0.528479679	0.566369913	0.50466314	0.75995868	0.976256655	0.92872616	0.999995916
ZNF687	0.721088828	0.60831699	0.586279142	0.91188714	0.340426922	0.887432338	0.999995916
ZNF699	0.577967467	0.571461155	0.639937245	0.840994157	0.813488809	0.953124234	0.999995916
ZNF746	0.647126714	0.863887547	0.268261431	0.928218536	0.932745169	0.94353156	0.999995916
ZNF75A	0.298549347	0.547626753	0.964345632	0.801186503	0.735174842	0.907043589	0.999995916
ZNF75D	0.689131223	0.322178849	0.650139501	0.612797946	0.877925827	0.883647156	0.999995916
ZNF777	0.8744382	0.4785405	0.891315227	0.433413152	0.951151244	0.958126703	0.999995916
ZNF787	0.856139112	0.815269107	0.818132648	0.918041161	0.759267261	0.997411466	0.999995916

ZNF821	0.633132832	0.789328613	0.895379877	0.633595381	0.917087911	0.98773533	0.999995916
ZNF845	0.987722108	0.741060966	0.291659413	0.563279585	0.952607906	0.931053243	0.999995916
ZNF865	0.989265968	0.20047959	0.939154467	0.957614786	0.659805515	0.933860673	0.999995916
ZNF891	0.927966839	0.767459138	0.884623679	0.732425044	0.429816211	0.975322451	0.999995916
ZNRF3	0.870639959	0.79825798	0.904262561	0.605101383	0.270390545	0.919175417	0.999995916
ZPBP	0.949546369	0.336286066	0.862510697	0.862486827	0.690114186	0.962989798	0.999995916
ZRANB2	0.904978311	0.56288341	0.47331362	0.863145597	0.438278251	0.904807732	0.999995916
ZSCAN23	0.310301411	0.827365442	0.965284222	0.702259224	0.573436639	0.915709074	0.999995916
ZSCAN4	0.958292335	0.43339926	0.439872153	0.823916596	0.946939085	0.951901601	0.999995916
ZSWIM7	0.416175672	0.94182089	0.381900223	0.779833303	0.620252961	0.873794476	0.999995916
ZSWIM9	0.147109245	0.880728365	0.88142305	0.957896171	0.840145371	0.905754689	0.999995916
ZZZ3	0.30380048	0.81770875	0.60828527	0.560718208	0.956298374	0.889438933	0.999995916
A2ML1	0.146405151	NaN	0.80644757	0.113026604	0.959274464	NaN	NaN
ABAT	0.785622257	0.705805317	NaN	0.054090127	0.186870991	NaN	NaN
ACBD7	0.10815266	0.216952312	0.308741991	NaN	0.055806046	NaN	NaN
ACOT2	0.490746044	0.648307378	NaN	0.039378559	0.985424526	NaN	NaN
ACP5	0.591070488	0.709488395	0.29588307	0.362019225	NaN	NaN	NaN
ACTA1	0.837783196	NaN	0.197238786	0.264274828	0.408623461	NaN	NaN
ACTN2	0.803245402	NaN	0.693581767	NaN	0.867648862	NaN	NaN
ADAM8	0.018993846	0.054728106	0.506893292	NaN	0.032555209	NaN	NaN
ADAMTS19	7.39E-07	0.057495986	0.378142107	NaN	0.543382388	NaN	NaN
ADRA2C	0.682820493	NaN	0.242015797	0.416452425	0.797078186	NaN	NaN
ALB	0.185045521	0.646713316	NaN	0.921889669	0.196333173	NaN	NaN
ALDOB	NaN	0.987010954	0.18604224	0.375494317	0.549953085	NaN	NaN
AMZ1	0.579371788	0.046603734	0.046368079	0.595520383	NaN	NaN	NaN
APOA4	NaN	0.321393468	0.77594946	0.024289895	0.600752343	NaN	NaN
ARHGAP11A	0.478526804	0.014297549	NaN	0.680490439	0.246152896	NaN	NaN
ART3	0.028659346	0.613847074	0.099538231	NaN	0.572126554	NaN	NaN
ATP10B	0.353099946	0.950729714	NaN	0.807678391	0.784578469	NaN	NaN
BAIAP2L2	NaN	0.389368186	0.923300777	0.481003518	0.255776407	NaN	NaN
BCL2L15	NaN	0.230360679	0.66210143	0.244583855	0.128970051	NaN	NaN
BIRC5	0.547756464	0.04203456	NaN	0.351445548	0.48044538	NaN	NaN
BOLA	0.133443628	0.852243458	0.935345495	NaN	0.728994098	NaN	NaN
BOLA3	0.623259106	0.312160079	NaN	0.02917769	0.08023382	NaN	NaN
BSG	0.244268337	0.507829594	NaN	0.143510578	0.202141412	NaN	NaN
BUB1B	0.459744575	0.183618343	NaN	0.753102505	0.747953423	NaN	NaN
C10H15orf62	0.599633565	NaN	0.248688173	0.559289216	0.569841304	NaN	NaN
C19H17orf58	0.220976558	0.10782092	0.128110392	NaN	0.062096182	NaN	NaN
C4BPA	NaN	0.344221278	NaN	0.443351023	0.700585136	NaN	NaN
CA12	NaN	0.289748066	0.173429629	0.069068508	0.574064288	NaN	NaN

CA13	NaN	0.402399334	0.182503018	0.238381503	NaN	NaN	NaN
CADM4	0.100915254	0.865282488	0.091932212	NaN	0.353770027	NaN	NaN
CATHL5	0.210345696	0.633846392	NaN	0.186360801	0.910008372	NaN	NaN
CCDC73	0.274794017	0.688906331	NaN	0.610885351	0.294381723	NaN	NaN
CCL17	0.288589647	0.803020079	0.092104879	NaN	0.877302039	NaN	NaN
CCL5	NaN	0.329737964	0.079847795	0.997759083	0.541003742	NaN	NaN
CCR9	0.912004562	0.398134756	0.859454097	0.877212466	NaN	NaN	NaN
CD5L	0.201255545	0.168660818	0.209324932	0.112631138	NaN	NaN	NaN
CDC6	0.311268369	0.069513215	NaN	0.422965492	0.884600972	NaN	NaN
CDCA8	0.843092319	0.079251468	NaN	0.327938699	0.258105185	NaN	NaN
CDH17	NaN	0.030481436	0.835581281	0.049982157	0.496828274	NaN	NaN
CDH19	0.06497098	0.97652163	0.074966553	NaN	0.82207708	NaN	NaN
CDKN2A	0.632050541	0.508831393	NaN	0.4683104	0.850669821	NaN	NaN
CERS3	0.321960436	0.220640428	NaN	0.481750245	0.389068708	NaN	NaN
CFHR5	0.369333362	NaN	NaN	0.839186851	0.91294548	NaN	NaN
CHAD	NaN	0.250663358	0.16492836	0.308951014	0.303622433	NaN	NaN
CHGA	NaN	0.003740994	0.873673026	0.379189834	0.913006242	NaN	NaN
CKB	0.385639481	0.556010191	NaN	0.247344715	0.000191206	NaN	NaN
CKMT1A	NaN	0.471078717	0.640256282	0.016130778	0.988982929	NaN	NaN
CLDN3	NaN	0.225328457	0.026857636	0.795930172	0.249294734	NaN	NaN
COBL	0.80138493	0.744679105	NaN	0.788401234	0.47857972	NaN	NaN
COL24A1	0.300913409	NaN	0.019342984	0.543694685	0.974171864	NaN	NaN
COL2A1	0.058611169	0.918814863	0.143094677	NaN	0.227368467	NaN	NaN
CR2	0.175144797	0.457668779	0.996196832	0.496682978	NaN	NaN	NaN
CRB2	NaN	0.184916518	0.215117486	0.317477577	0.235712785	NaN	NaN
CXCL11	0.220496334	0.001217399	0.436903522	0.348127954	NaN	NaN	NaN
CXCL8	0.529949407	0.014738569	0.567784161	NaN	0.008945121	NaN	NaN
CXHXorf57	0.542410545	0.40757799	0.11701332	NaN	0.460252671	NaN	NaN
CYP21	0.831444627	0.605105879	NaN	0.911323429	0.698482041	NaN	NaN
CYP46A1	0.774475447	0.907861646	NaN	0.793049186	0.035083097	NaN	NaN
DDX58	0.046663716	0.450631487	0.662725394	NaN	0.943557728	NaN	NaN
DEFB7	0.219455877	0.912426262	0.282310389	NaN	0.39100063	NaN	NaN
DMRT3	0.606324979	NaN	0.473424516	0.137874151	0.420130819	NaN	NaN
DNAH9	0.601921234	0.103108821	0.783296528	NaN	0.894821056	NaN	NaN
DNAJB13	NaN	0.936264029	0.089720194	5.20E-07	0.071801713	NaN	NaN
DPP10	0.000340544	0.756595144	0.414072932	NaN	0.773629194	NaN	NaN
DPP4	0.123588241	0.124735838	NaN	0.237689544	0.520281915	NaN	NaN
DSG2	NaN	0.417761103	0.830048829	0.3440877	0.945543127	NaN	NaN
EEF1A2	0.745493053	NaN	0.304212044	0.457569999	0.006389885	NaN	NaN
ELOVL6	0.312334156	0.376059023	NaN	0.082545356	0.033299373	NaN	NaN

FAM3B	NaN	0.943482364	0.969839435	0.020852917	0.900640738	NaN	NaN
FAM49B	0.341509347	0.278323641	NaN	0.453808	0.166118777	NaN	NaN
FAM83D	0.015759921	NaN	0.433539953	0.029624376	0.03493939	NaN	NaN
FCGBP	NaN	0.708116195	0.333903296	NaN	0.191057067	NaN	NaN
FCGR2A	0.747413808	0.055313174	NaN	0.88222503	0.032212399	NaN	NaN
FGB	0.789745144	0.596265501	NaN	0.976179762	0.018345283	NaN	NaN
FOSL1	0.333102404	0.777278947	0.36948428	0.39205219	NaN	NaN	NaN
FOXD1	0.771085134	0.333009987	0.01003778	NaN	0.89318343	NaN	NaN
FOXD3	0.144178914	0.768194406	0.343257289	NaN	0.460553021	NaN	NaN
FREM2	0.441898543	0.563301448	0.494438242	NaN	0.460964052	NaN	NaN
FZD5	NaN	0.18930365	0.083943538	0.032768221	0.945858237	NaN	NaN
G6PD	0.101408862	0.288373043	NaN	0.000624371	0.015307112	NaN	NaN
GABRB3	0.8272372	0.871521052	0.434042083	NaN	0.679562866	NaN	NaN
GOLGA7B	0.23554794	0.331520449	0.853407075	0.207978652	NaN	NaN	NaN
GPT2	0.348881836	0.943688616	NaN	0.372507988	0.04774296	NaN	NaN
GRIA2	NaN	0.174688797	0.310813336	0.506584196	0.218291397	NaN	NaN
GRIK4	0.477895049	0.149562745	0.578730473	NaN	0.014731426	NaN	NaN
GSS	0.437256373	0.482311525	NaN	0.073191991	0.198450563	NaN	NaN
GSTA3	NaN	0.233327561	NaN	0.73589	0.472270317	NaN	NaN
GSTM1	0.138204329	0.813933493	NaN	0.493607064	0.077032102	NaN	NaN
GUCY2C	NaN	0.597678153	0.268588762	0.545780995	0.471295789	NaN	NaN
GYS2	0.573335843	0.958860763	0.392454491	NaN	0.567934878	NaN	NaN
HAND2	0.344710691	NaN	0.862865612	0.066503151	0.9000809	NaN	NaN
HMOX1	0.834579552	0.636578148	NaN	0.018608289	0.090113345	NaN	NaN
HNF4G	NaN	0.874119793	0.161356847	0.342100566	0.470549062	NaN	NaN
HP	0.014539446	NaN	0.010327882	0.07846458	0.099399992	NaN	NaN
HPSE2	0.022696268	NaN	0.847783704	0.816782411	0.004102026	NaN	NaN
IFIH1	0.110355485	0.027449759	0.846708926	NaN	0.564813932	NaN	NaN
IGLL1	0.09139849	NaN	NaN	0.216555392	0.848891958	NaN	NaN
IGSF11	0.018000598	0.375382998	0.639796097	NaN	0.28877464	NaN	NaN
IGSF5	NaN	0.150142899	NaN	0.667473478	0.781207342	NaN	NaN
IGSF9B	0.100330256	0.066511306	0.374228942	NaN	0.352684441	NaN	NaN
IL1B	0.309642127	0.401661678	0.000303356	NaN	0.269468632	NaN	NaN
IRX1	0.916183732	0.633690752	NaN	0.707971503	0.438215339	NaN	NaN
ISG15	0.143794258	0.705415751	0.178333844	NaN	0.918914467	NaN	NaN
JAKMIP2	0.357265968	0.62976064	0.772695699	NaN	0.145569553	NaN	NaN
KCNA4	0.492565499	0.811224994	NaN	0.435902654	0.129566215	NaN	NaN
KCNK2	0.44063998	0.221533684	NaN	0.107185193	0.829840389	NaN	NaN
KCNN2	0.314269257	0.082151722	NaN	0.272579219	0.9162886	NaN	NaN
KIAA1211L	NaN	0.241381314	0.766498632	0.439075476	0.231453202	NaN	NaN

KIF11	0.507000903	0.129184056	NaN	0.682653312	0.759690891	NaN	NaN
KIF20A	0.250521231	0.099073301	NaN	0.349823085	0.488764588	NaN	NaN
KIF2C	0.910815212	0.023451229	NaN	0.176237012	0.240901629	NaN	NaN
KLHL31	0.593360515	0.344058971	NaN	0.249293872	0.151912885	NaN	NaN
LAMC3	0.078584829	0.74024857	NaN	0.081250616	0.429793698	NaN	NaN
LCN2	0.736715839	0.338971831	0.014382022	0.272185688	NaN	NaN	NaN
LDHB	0.752452578	0.65091235	NaN	0.278978488	0.368384702	NaN	NaN
LGI1	0.264861107	0.918586176	0.252902878	NaN	0.964084508	NaN	NaN
LGR5	0.301302992	0.568556139	0.390245182	0.878367091	NaN	NaN	NaN
LIN7A	0.203961995	0.408799538	NaN	0.210079174	0.289673367	NaN	NaN
LOC100138641	0.941498597	0.846187141	NaN	0.353403679	0.012051277	NaN	NaN
LOC100139670	NaN	0.720597742	0.765683203	NaN	0.987707679	NaN	NaN
LOC100139885	NaN	0.59100704	0.735519394	0.288377579	0.539847747	NaN	NaN
LOC100140226	NaN	0.886791539	0.141966505	0.33942269	0.885372568	NaN	NaN
LOC100297192	0.014596654	NaN	0.001440701	0.315068067	0.925863425	NaN	NaN
LOC100297779	NaN	0.579315747	0.140680319	0.437016098	NaN	NaN	NaN
LOC100298356	0.123544592	0.253417997	0.330479992	NaN	0.380616401	NaN	NaN
LOC100300115	0.041004758	0.958087351	0.669555583	0.196973846	NaN	NaN	NaN
LOC100301224	0.312889295	0.349785879	NaN	0.743619932	0.828110268	NaN	NaN
LOC100847119	0.076262801	NaN	1.64E-05	0.424983681	0.511106796	NaN	NaN
LOC100847415	0.713229319	NaN	0.693129573	0.275931376	0.609212566	NaN	NaN
LOC100847724	0.79689379	NaN	0.01252191	0.156830477	0.457675822	NaN	NaN
LOC100847981	0.33717551	0.337365603	0.88731316	NaN	0.875775495	NaN	NaN
LOC100848536	0.847305539	0.005232686	0.168820303	NaN	0.89341992	NaN	NaN
LOC100851369	0.637088938	0.401706225	NaN	0.514233007	0.554304064	NaN	NaN
LOC101901948	0.285024915	0.145797215	0.043549121	0.283008744	NaN	NaN	NaN
LOC101902787	0.502334245	0.087082073	0.765572306	NaN	0.055275358	NaN	NaN
LOC101903284	0.951061218	0.323570934	NaN	NaN	NaN	NaN	NaN
LOC101903734	NaN	0.330732254	0.179314545	0.901462908	0.72815874	NaN	NaN
LOC101905242	0.025007609	0.182849281	NaN	0.051539181	9.21E-05	NaN	NaN
LOC101905509	0.028067173	0.156215684	0.020307533	NaN	0.132902289	NaN	NaN
LOC101906743	NaN	0.850621014	0.058183282	0.158296461	0.009990516	NaN	NaN
LOC101907335	0.414054803	0.840000286	NaN	0.555216724	0.010700013	NaN	NaN
LOC104968484	0.086582863	0.02169009	NaN	0.386063857	0.861005303	NaN	NaN
LOC104973965	0.195859417	0.615215438	NaN	0.063628424	0.03374531	NaN	NaN
LOC104974214	NaN	0.924998353	0.090986854	0.169677655	0.272472389	NaN	NaN
LOC104974444	0.727112517	0.795475767	NaN	0.38244534	0.797137522	NaN	NaN
LOC104974455	0.220797358	NaN	0.454073195	0.179699054	0.818300284	NaN	NaN
LOC104976942	0.290036598	NaN	0.47377159	0.294344109	0.108836355	NaN	NaN
LOC107131864	0.828199698	0.999472394	NaN	NaN	0.587972769	NaN	NaN

LOC107131942	0.000318512	NaN	NaN	0.979275885	NaN	NaN	NaN
LOC112441507	0.107962297	0.199634536	0.395397215	NaN	0.649245949	NaN	NaN
LOC112441557	0.020182247	0.685324591	0.986043739	NaN	0.930386082	NaN	NaN
LOC112441777	0.724299027	NaN	0.776982559	0.286353516	0.346084929	NaN	NaN
LOC112442062	0.073401797	NaN	0.003535422	0.214979761	0.212670238	NaN	NaN
LOC112443013	0.260719096	0.645682508	NaN	0.342984897	0.708274489	NaN	NaN
LOC112443862	0.343018165	0.995467643	0.104202372	0.863799682	NaN	NaN	NaN
LOC112444652	0.714981482	0.266508074	0.25870537	0.26551838	NaN	NaN	NaN
LOC112445090	0.773574863	0.380819412	NaN	0.773861971	0.4160636	NaN	NaN
LOC112446680	0.899121714	0.940539524	0.951376268	NaN	NaN	NaN	NaN
LOC112446726	0.499892547	NaN	0.956080775	0.888141168	0.385260378	NaN	NaN
LOC112447079	NaN	0.864261497	0.022357116	0.731922432	0.047406785	NaN	NaN
LOC112447816	0.766364872	NaN	0.336311554	0.115626771	0.492069259	NaN	NaN
LOC112448034	0.175751817	0.139965926	NaN	0.857204853	0.624789429	NaN	NaN
LOC505033	0.795613148	0.093487092	0.012392669	NaN	0.266406455	NaN	NaN
LOC507055	0.363268137	0.414125846	0.974266918	NaN	0.666350172	NaN	NaN
LOC508459	0.16736908	0.048449715	0.135511129	0.032814656	NaN	NaN	NaN
LOC509283	0.131096794	0.158067991	0.723927116	NaN	0.893257689	NaN	NaN
LOC509911	0.154860276	NaN	0.375037036	0.873378984	0.408410713	NaN	NaN
LOC511683	0.811489644	NaN	0.518346996	0.135893877	0.683613848	NaN	NaN
LOC513210	0.839670526	0.228991158	NaN	0.898780603	0.705191391	NaN	NaN
LOC514978	NaN	0.803208586	0.596055293	0.153159099	1.07E-07	NaN	NaN
LOC515676	0.006948493	0.252979652	0.121802347	NaN	NaN	NaN	NaN
LOC516421	0.568045703	NaN	0.305850303	0.750535284	0.883503329	NaN	NaN
LOC519274	0.056730938	NaN	0.088324855	0.020351292	NaN	NaN	NaN
LOC530653	NaN	0.404897413	0.91847814	0.92430275	0.702470596	NaN	NaN
LOC615051	0.350156515	0.990449441	NaN	NaN	NaN	NaN	NaN
LOC616782	NaN	0.414490035	0.97480636	0.134613265	0.370150855	NaN	NaN
LOC616830	0.006954986	0.179384315	NaN	0.18808781	0.120484304	NaN	NaN
LOC618297	0.999544349	0.96012468	NaN	0.014493938	0.958578377	NaN	NaN
LOC781736	NaN	0.437254466	0.942355433	0.901155924	0.938447098	NaN	NaN
LOC781796	NaN	0.257356749	0.130751144	0.077132081	0.006570703	NaN	NaN
LOC783106	0.181824811	0.980521547	NaN	0.053130597	0.571656343	NaN	NaN
LOC784266	0.57277126	0.887960232	0.249265782	0.034914709	NaN	NaN	NaN
LOC785161	0.907179308	NaN	0.524721675	0.247382169	0.498568967	NaN	NaN
LOC789829	NaN	0.373193387	0.507296586	0.018738755	0.042867941	NaN	NaN
LTF	NaN	0.590097461	0.949031122	0.024003581	0.168396648	NaN	NaN
LUZP2	0.76379201	0.895993196	0.094390223	NaN	0.906050435	NaN	NaN
MAPK4	0.221166148	NaN	0.813005661	0.024395333	0.773990982	NaN	NaN
MARCO	0.091484381	0.9848485	NaN	0.12113358	0.744789214	NaN	NaN

MEF2B	NaN	0.69477054	0.721826773	NaN	0.063877802	NaN	NaN
MEGF11	NaN	0.906661372	NaN	0.33456536	0.892071735	NaN	NaN
MID1IP1	0.413401166	0.198372809	NaN	0.163852848	0.002451574	NaN	NaN
MISP	NaN	0.378802035	0.044031765	0.209559895	0.366778733	NaN	NaN
MLXIPL	0.15125532	0.34991402	NaN	0.129169394	0.214075683	NaN	NaN
MMP12	0.757092564	0.836563676	0.413808006	NaN	0.162779266	NaN	NaN
MOXD1	0.938594417	NaN	0.258727106	NaN	0.908935701	NaN	NaN
MPC1	0.268006845	0.510468083	NaN	0.09948597	0.162755528	NaN	NaN
MPZ	0.180626556	0.854653025	0.031939926	NaN	0.306651954	NaN	NaN
MT1A	0.75028708	0.777383754	0.474199978	0.152239101	NaN	NaN	NaN
MUSK	0.092481179	NaN	0.202684682	0.030828789	0.443462754	NaN	NaN
MX1	0.200388398	0.040183154	0.326741839	NaN	0.692392088	NaN	NaN
MX2	NaN	0.277660043	0.895846708	NaN	0.786233707	NaN	NaN
MYO1A	NaN	0.55856356	0.550166711	0.301830546	0.100979001	NaN	NaN
NAALADL1	NaN	NaN	0.499940607	0.928347521	0.801708542	NaN	NaN
NPFFR2	0.318438399	NaN	0.428813847	0.582733941	0.290047455	NaN	NaN
OAS1Y	0.151174665	0.036195694	0.611577635	NaN	0.897026305	NaN	NaN
OAS1Z	NaN	0.123780787	0.590736936	0.075363149	0.588278015	NaN	NaN
OPCML	0.647740559	NaN	0.860684957	0.323645065	0.122676425	NaN	NaN
OSM	0.014910164	0.001578225	0.139999289	NaN	0.08694731	NaN	NaN
P2RX1	0.180199232	NaN	0.315956044	0.330940554	0.168572819	NaN	NaN
PARD6B	0.192521773	0.080969716	NaN	0.596350986	0.660033952	NaN	NaN
PBLD	0.60432869	0.695261325	NaN	0.682833795	0.220758601	NaN	NaN
PCDH10	0.021499288	0.714389557	0.111754413	NaN	0.358190629	NaN	NaN
PCLAF	0.714007103	0.215533325	NaN	0.073661063	0.83043785	NaN	NaN
PDZK1	NaN	0.126920189	0.118036997	0.173501825	0.349656373	NaN	NaN
PDZRN4	0.039643751	0.34786161	0.999737122	NaN	0.019946177	NaN	NaN
PEBP4	0.01337025	NaN	0.836147451	NaN	0.614905555	NaN	NaN
PHF21B	0.718003518	NaN	0.506235303	0.842082254	0.290084493	NaN	NaN
PIR	0.66376221	0.753550234	NaN	0.128241855	0.070528014	NaN	NaN
PKD2L1	0.128515222	0.755042196	NaN	NaN	0.034500585	NaN	NaN
PLA2G2D1	NaN	0.752043575	0.101808955	0.073223081	0.006403638	NaN	NaN
PLEK2	NaN	0.294788877	0.301034335	0.300732564	0.310600738	NaN	NaN
PLP1	0.078174239	0.922034204	0.016810562	NaN	0.794304183	NaN	NaN
PPDPFL	0.922183681	NaN	0.856147737	0.267331413	0.697121028	NaN	NaN
PPM1E	0.004674513	0.194281526	NaN	0.492990713	0.071083442	NaN	NaN
PRSS35	0.308961408	NaN	0.000559004	0.508673922	0.905220299	NaN	NaN
PTCH2	0.779393565	0.29237999	NaN	0.747975248	0.948959919	NaN	NaN
PTGIR	0.794071474	NaN	0.00101481	0.613346253	0.496376797	NaN	NaN
PTPRQ	0.825542271	0.501098219	NaN	0.174723827	0.761005267	NaN	NaN

RASL12	0.23303219	NaN	0.593682952	0.682204556	0.056950375	NaN	NaN
REC8	0.601568619	0.017237403	0.241708807	0.000146896	NaN	NaN	NaN
RHOD	0.803323078	0.431117513	0.005505369	0.575774417	NaN	NaN	NaN
RIPK4	NaN	0.644982885	0.976679573	0.005318617	0.005970963	NaN	NaN
RRM2	0.318853414	0.340511807	NaN	0.557495641	0.525986609	NaN	NaN
RSAD2	0.077673088	0.595449141	0.761399092	NaN	0.643711438	NaN	NaN
SAMD9	0.267033393	0.126335787	0.517048873	NaN	0.002399473	NaN	NaN
SCP2	0.7947544	0.675441077	NaN	0.349569086	0.099846726	NaN	NaN
SCUBE1	0.80582216	0.93132643	0.126369972	NaN	0.618638748	NaN	NaN
SECTM1A	0.449076907	0.624319144	0.856969347	0.642930173	NaN	NaN	NaN
SERHL2	0.248324626	0.302150325	NaN	0.448271496	0.144611155	NaN	NaN
SGCG	0.376436857	0.81671918	0.120396497	NaN	0.892541412	NaN	NaN
SH3RF2	0.347263161	0.297737902	NaN	0.886324157	0.894662031	NaN	NaN
SIX2	0.521672557	NaN	0.057085122	0.248328595	0.4306771	NaN	NaN
SLC13A3	0.13028325	0.760245726	NaN	0.57486981	0.318980715	NaN	NaN
SLC15A1	NaN	0.2306441	0.991533476	0.823669221	0.28585079	NaN	NaN
SLC16A1	0.179197186	0.161865841	NaN	0.083930098	0.134546874	NaN	NaN
SLC16A7	0.528444928	0.445179721	NaN	0.460515062	0.030254604	NaN	NaN
SLC25A4	0.804635494	0.750610501	NaN	0.066530332	0.078522595	NaN	NaN
SLC35F1	0.001328115	0.803856577	0.057708332	NaN	0.836843591	NaN	NaN
SLC51B	NaN	0.479065418	0.95332944	0.731638415	0.280078636	NaN	NaN
SLC5A9	0.654799904	0.795160674	NaN	0.611894826	0.491137827	NaN	NaN
SLC6A8	NaN	0.553706968	0.459696056	0.224040055	0.049869083	NaN	NaN
SLC9A2	0.245505443	0.855820311	0.555569306	0.285934981	NaN	NaN	NaN
SLC9A3	NaN	0.880149771	0.175197015	0.463319167	0.660274792	NaN	NaN
SNAP25	0.961011949	0.956776219	0.138856835	NaN	0.378716085	NaN	NaN
SNCA	0.01246963	0.74857869	0.547140668	NaN	0.785130854	NaN	NaN
SNRNP25	0.308062069	0.275320225	NaN	0.061889585	0.028839308	NaN	NaN
SOCS3	0.522390616	0.410375625	0.907331898	0.060593884	NaN	NaN	NaN
SOX10	0.019449554	0.744440632	0.156672079	NaN	0.60759809	NaN	NaN
SPCS3	0.940559972	0.014162906	0.552078602	0.087845776	NaN	NaN	NaN
SPHAR	0.013683432	0.015192233	0.02530901	NaN	NaN	NaN	NaN
SPOCK1	0.329886726	0.997395957	0.445266474	NaN	0.301702959	NaN	NaN
SPRY3	0.628192447	0.065777826	NaN	0.058442029	0.064108269	NaN	NaN
SPTB	0.954575448	0.563126338	0.052924025	0.711417399	NaN	NaN	NaN
SRXN1	0.062589985	0.294612959	NaN	0.505939894	0.357624468	NaN	NaN
ST14	NaN	0.68226166	0.133799127	0.258597408	0.505085381	NaN	NaN
ST8SIA2	0.134679057	0.172558514	0.091724427	0.975421704	NaN	NaN	NaN
SVOP	0.965542562	0.898583471	0.002004542	0.255786601	NaN	NaN	NaN
TDH	0.01259964	0.257211021	NaN	0.612044258	0.060669	NaN	NaN

TECTB	0.401515317	0.341574312	NaN	0.299983759	0.078329719	NaN	NaN
TGM5	0.203757266	0.125489832	NaN	0.001386706	0.13541289	NaN	NaN
TIMD4	0.212982766	0.952938434	0.278431397	0.562929151	NaN	NaN	NaN
TKT	0.534102054	0.27530783	NaN	0.334275053	0.004462251	NaN	NaN
TMEM179	0.124355394	0.20646127	NaN	0.314598113	0.994585999	NaN	NaN
TMEM216	0.052141092	0.452515584	0.195808978	0.09729347	NaN	NaN	NaN
TMEM45B	NaN	0.937745322	0.971517588	0.69408336	0.477105479	NaN	NaN
TMPRSS2	NaN	0.649701896	0.799581781	0.013643983	0.030297385	NaN	NaN
TMPRSS5	0.638900841	0.649486513	0.429677747	NaN	0.853063062	NaN	NaN
TNFRSF11B	0.840939409	0.066727788	0.130460216	NaN	0.258618801	NaN	NaN
TRIB3	0.963685895	0.278951347	0.029384566	NaN	0.022217346	NaN	NaN
TRIM44	0.105693899	0.438604404	NaN	0.215393322	0.631613975	NaN	NaN
TTYH1	0.247410584	0.49651121	0.195459767	NaN	0.650066894	NaN	NaN
TUBA1D	NaN	0.980707814	0.360520553	0.906127888	0.538787507	NaN	NaN
UBE2C	0.39771076	0.067457754	NaN	0.381385809	0.579303943	NaN	NaN
UCHL3	0.525064791	0.207730721	NaN	0.345440793	0.064369482	NaN	NaN
UCMA	0.325020704	0.375478199	NaN	0.848545285	0.482958983	NaN	NaN
UCP1	NaN	0.301076707	0.497066507	0.502859076	0.557680729	NaN	NaN
UHRF1	0.737757913	0.171281086	NaN	0.624513813	0.694656681	NaN	NaN
VIL1	NaN	0.725660343	0.632070538	0.00328602	0.899987942	NaN	NaN
WNT6	0.605568407	0.873577773	0.459854363	NaN	0.193355112	NaN	NaN
YDJC	0.290474928	0.374474188	NaN	0.0113094	0.168155571	NaN	NaN
ZBTB37	NaN	0.000237069	NaN	0.001811751	0.413532142	NaN	NaN
ZDHHC23	0.221187065	0.525657962	0.675970588	NaN	NaN	NaN	NaN
