# Supplementary material for: Genes associated with body weight gain and feed intake identified by meta-analysis of the mesenteric fat from crossbred beef steers
Source: PLoS One. 2020 Jan 7;15(1):e0227154. doi: 10.1371/journal.pone.0227154 (PMC6946124; doi:10.1371/journal.pone.0227154)
Supplement: S2 Table — Genes are ordered by adjusted meta-P-value. The individual cohort cells for DEGs identified in the meta-analysis are colored according to the sign of their log2 fold change, where green indicates up-regulation and red indicates down-regulation in high intake. Genes with all gray cell indicate those that were excluded because they were also significant for the gain by intake interaction term. (PDF) [file pone.0227154.s002.pdf]

Supplemental Table 2. Differentially expressed genes associated with the intake main effect in the individual cohort and meta analyses. Green indicates up-regulation in animals with higher intake. Red indicates down-regulation in animals with higher intake. Genes in gray are those identfied in the interaction analysis.

Gene	Group 1 Raw P-value	Group 2 Raw P-value	Group 3 Raw P-value	Group 4 Raw P-value	Group 5 Raw P-value	Meta-P-value	Adjusted Meta-P-value
IL18BP	0.493792941	0.319586355	2.73E-08	0.425172679	1.99E-05	1.59E-09	8.71E-06
LOC100300716	0.000261462	0.001371626	7.35E-05	0.181076394	0.409472491	5.02E-08	0.000205934
LOC112445144	2.14E-05	0.180940343	0.014945839	7.50E-05	0.699864163	7.31E-08	0.000239889
NF2	0.509016946	0.884231579	0.005641444	1.67E-08	0.703793175	5.09E-07	0.001391992
ECE2	0.692798004	0.364925856	5.35E-06	7.16E-05	0.652010674	9.56E-07	0.001743624
FST	5.92E-06	0.08283651	0.308542628	0.169468313	0.001941268	7.83E-07	0.001743624
PRMT7	0.919824541	0.72554778	0.848481147	4.91E-10	0.205564953	8.79E-07	0.001743624
JCHAIN	0.000287482	0.032603648	0.002521078	0.014108338	0.328366166	1.51E-06	0.002484096
SPP1	0.366828513	0.53611369	1.05E-05	0.9290687	6.98E-05	1.78E-06	0.002662926
SOBP	0.173390831	0.418539713	0.353288782	7.59E-09	0.811858373	2.05E-06	0.002809374
RIPK1	0.132152506	0.589577128	0.646799846	1.86E-08	0.48798269	4.95E-06	0.006251633
AACS	0.548278217	0.970050537	0.085546772	1.02E-06	0.012415112	5.97E-06	0.006579026
LY86	0.013843767	0.165706606	4.68E-07	0.892473164	0.654385265	6.41E-06	0.006579026
MRPL14	0.000195666	0.274745708	0.26156024	0.000197151	0.224463966	6.38E-06	0.006579026
PTAFR	0.240903286	0.8991105	3.73E-05	0.034398216	0.003616911	9.45E-06	0.009117906
LOC782367	4.75E-05	0.049463635	0.236681375	0.124852355	0.016121394	1.03E-05	0.009403144
PTRH1	0.777272877	0.88886631	0.091226678	6.23E-07	0.041982857	1.41E-05	0.012202208
GCAT	0.355425972	0.327768005	0.095222436	7.59E-07	0.400679231	2.52E-05	0.01975543
PDLIM5	0.138845707	1.93E-06	0.526271344	0.027017664	0.887664116	2.53E-05	0.01975543
CCDC117	0.295100098	0.417792653	0.966709872	4.44E-08	0.701452296	2.72E-05	0.019841436
CPM	0.702953598	0.262314807	2.34E-06	0.575508514	0.015344621	2.78E-05	0.019841436
H19	0.003848877	0.001394606	0.068911759	0.024158279	0.471073413	3.02E-05	0.020627354
CD5	0.001619567	0.143113552	0.452358993	0.003357449	0.017930335	4.18E-05	0.025659453
HPS6	0.475514726	0.380610536	0.920389737	6.22E-07	0.058926371	4.06E-05	0.025659453
LOC101907219	0.76136343	0.325743824	8.54E-05	0.016512626	0.018307369	4.22E-05	0.025659453
DHRS9	0.435752181	0.594931555	3.96E-06	0.096260975	0.072958102	4.65E-05	0.027237602
LOC112441663	0.37338351	0.780418107	1.50E-06	0.026480758	0.772836243	5.52E-05	0.031216637
B3GAT1	0.066279133	0.777270426	6.02E-07	0.35278908	0.902119641	5.97E-05	0.032213091
GLCE	0.793815497	0.664584359	1.94E-07	0.10009287	0.988835005	6.08E-05	0.032213091
LOC100138922	0.808695721	0.985263139	1.64E-06	0.00931894	0.970396137	6.90E-05	0.033305234
LYZ	0.449461837	0.170641236	5.51E-06	0.047390964	0.551169767	6.52E-05	0.033305234
RCOR2	0.220473615	0.621403573	0.299133171	1.60E-05	0.018045563	6.88E-05	0.033305234
CD53	0.212431293	0.385562373	1.18E-06	0.28692883	0.483527399	7.61E-05	0.035678354
SRPX	0.026822949	0.046763396	0.144187389	0.000142117	0.629808138	8.83E-05	0.040269792
NOG	0.649289748	1.15E-05	0.057192767	0.105288869	0.497903025	0.000114419	0.050749425
HOXA9	0.960479071	0.000186386	0.073405314	0.002473569	0.737934795	0.000120453	0.052019736
ITGB8	0.32834272	0.00021645	0.035317247	0.314191296	0.031532512	0.000123915	0.052142675
EPM2AIP1	0.588673386	0.615216366	6.31E-05	0.005772687	0.210592911	0.000135118	0.052816256
GSTT2	0.151988618	0.157567367	0.478344305	6.35E-06	0.381774904	0.00013517	0.052816256
PYCR2	0.077073842	0.617863882	0.632826458	9.64E-07	0.920558947	0.000131255	0.052816256
DLK1	0.372622732	0.000976251	0.747957675	0.000149636	0.902909739	0.000168334	0.062885086
RGS12	0.785158982	0.128071063	1.88E-06	0.340709095	0.571230458	0.000168603	0.062885086

ADGRL4	0.166562451	0.902064937	0.84728248	7.76E-07	0.465686285	0.000200547	0.067443872
BTAF1	0.254610888	0.980310664	0.971125459	2.41E-07	0.746926942	0.000192621	0.067443872
CLEC5A	0.359666847	0.326080809	1.78E-06	0.245551169	0.832502461	0.000189124	0.067443872
LOC107131807	0.091864638	0.849215639	1.65E-06	0.704497825	0.510846735	0.000201374	0.067443872
OLFML3	0.278713488	0.263254718	0.030239442	4.11E-05	0.502511418	0.000199883	0.067443872
HSPH1	0.197523891	0.152561686	0.914095713	2.60E-06	0.679235525	0.000209565	0.068783516
FADS6	0.436307879	0.906558541	5.33E-06	0.287526287	0.09964642	0.000247946	0.079652178
MZF1	0.15921536	0.001752902	0.000630419	0.366788382	0.958502444	0.000252386	0.079652178
SCIN	0.454004938	0.222087293	5.95E-06	0.620969205	0.172478034	0.000259827	0.080453228
KBTBD8	0.765134927	0.10655614	0.000255832	0.933331485	0.003862105	0.000293532	0.088908499
PDE4B	0.774912285	0.723238394	1.60E-06	0.432159858	0.198221359	0.000297969	0.088908499
MXRA5	0.487201656	0.009539507	0.000108385	0.16991072	0.917905509	0.000303669	0.088991259
CYP1B1	0.259277617	0.961104576	0.624541197	3.90E-06	0.151811827	0.000343347	0.097149509
PLPPR2	0.787632118	0.856431836	1.15E-06	0.184741495	0.639946716	0.000342928	0.097149509
ENTPD2	0.464744512	0.507825088	0.000703075	0.009607692	0.060763761	0.000356876	0.097642973
SBF1	0.800861	0.004376484	0.650266769	4.86E-05	0.875036435	0.000356991	0.097642973
LIPA	0.579242165	0.244077609	1.32E-05	0.553564832	0.102612841	0.000383176	0.09981439
SLC6A16	5.49E-06	0.971833756	0.043420478	0.531640515	0.848571198	0.000378333	0.09981439
TENM2	0.049624239	0.001181366	0.217058863	0.012031382	0.6691201	0.000372554	0.09981439
BOP1	0.359045426	0.998755692	0.174448517	0.002344534	0.000761663	0.000398185	0.100532435
CAMK1G	0.390108043	0.209896926	0.003158008	0.004898798	0.087098816	0.00039441	0.100532435
BYSL	0.03363412	0.116670514	0.033827053	0.050104368	0.019844677	0.000452473	0.103791728
CAB39	0.876861527	0.000918525	0.002463608	0.138640058	0.490403308	0.000460119	0.103791728
ELN	0.932469008	0.394629676	1.70E-06	0.277738315	0.858292859	0.000497438	0.103791728
ITGAD	0.908888005	0.097933787	0.000267929	0.273348077	0.020826977	0.000462368	0.103791728
KCNA3	0.174776616	0.982109795	0.114121626	0.107390451	6.27E-05	0.000452016	0.103791728
KCNK12	0.443589264	0.00149368	0.009398203	0.066451567	0.363139017	0.000499637	0.103791728
LGALS3	0.203255861	0.253772249	2.37E-05	0.783051334	0.15036293	0.000484123	0.103791728
LOC516494	0.975996208	0.12512423	0.009616549	0.024791694	0.004595097	0.000457189	0.103791728
NUMB	0.964890894	0.829682931	0.583677741	3.54E-06	0.084551659	0.000472889	0.103791728
PTK7	0.476286358	0.014523411	0.001415429	0.020954929	0.729632695	0.000498193	0.103791728
RAB7B	0.462276484	0.534325849	0.030286934	0.000491876	0.039512355	0.000487221	0.103791728
SLC7A8	0.950722371	0.01405862	0.008016099	0.001796482	0.668392111	0.000443699	0.103791728
STAP2	0.075698574	0.406027834	0.020872883	0.00037413	0.623196708	0.000497894	0.103791728
SYS1	0.268827611	6.36E-06	0.206682603	0.516515349	0.743897675	0.000462239	0.103791728
LOC513659	0.970504903	0.168204152	0.3882981	9.29E-06	0.26465738	0.000513776	0.105394749
VWA3B	0.918643198	0.539895895	0.010576553	0.000310978	0.09875066	0.000526871	0.106746651
IL1RN	0.048650622	0.680959183	6.20E-05	0.193643017	0.412353496	0.000534383	0.106948279
KYNU	0.097159434	0.288037953	0.005030391	0.664335619	0.001947286	0.000578479	0.111767696
LOC789607	0.114248583	0.018985454	0.718724494	0.000122565	0.954033297	0.000578896	0.111767696
NCR1	0.42175088	0.926993046	0.004237857	0.000513825	0.210992593	0.000572437	0.111767696
CNTFR	2.28E-05	0.548036677	0.867179394	0.037883331	0.466949457	0.00060222	0.112243805
FHL5	0.014074378	0.223568062	0.211403133	0.132173692	0.002166683	0.000598611	0.112243805
LOC104969545	0.360359246	0.774595425	0.920969269	9.27E-07	0.803076832	0.000600509	0.112243805
LRRC8B	0.467336216	0.266845386	0.000253143	0.022299273	0.276639939	0.00060872	0.112243805

KCTD10	0.107688314	1.64E-05	0.443990281	0.259152111	0.99457816	0.000625803	0.114111679
LOC782951	0.39544304	0.403074513	0.902922763	1.69E-06	0.922531036	0.000678956	0.12244345
TNNT3	0.291029483	0.033435346	0.010469772	0.03513215	0.064468008	0.000692317	0.123495787
CCNJ	0.042084691	0.661314163	0.013399983	0.001042449	0.609430757	0.000706322	0.124639201
MAP2K6	0.253502989	0.634131797	0.000144486	0.045851907	0.233068364	0.000731643	0.125655673
TBL1X	0.026756533	0.955391075	0.07571353	0.000177359	0.744849676	0.00074823	0.125655673
TIMM13	0.10272037	0.368836132	0.107028386	0.001089259	0.05786411	0.000747999	0.125655673
TREML1	0.896056164	0.736500345	1.35E-06	0.949831427	0.302354656	0.000750366	0.125655673
USP50	5.31E-06	0.166576959	0.860298275	0.759495806	0.424934216	0.000725529	0.125655673
ARHGEF26	0.000291062	0.215700946	0.408471823	0.889329357	0.011812459	0.000778363	0.126870503
FBLN7	0.64702915	0.29199072	1.48E-05	0.179391408	0.540007524	0.000780813	0.126870503
UNC5A	0.597911812	0.064601286	0.000785021	0.051708657	0.168510434	0.000767005	0.126870503
DRAM1	0.873810627	0.448777496	0.625685141	2.02E-06	0.563705321	0.000798607	0.128489644
ASAH2	0.23208213	0.424160473	0.000853686	0.475427206	0.007273784	0.00082417	0.128813854
CDS1	0.219114712	0.404546167	0.000404795	0.016443216	0.490010986	0.000820961	0.128813854
PDE4D	0.126832185	0.325816759	0.546669987	1.94E-05	0.650634978	0.000813147	0.128813854
SRGN	0.074507231	0.56658167	0.000249856	0.178239148	0.157747261	0.000836858	0.12956304
ADAM12	0.633747315	0.001653008	0.00752143	0.085257581	0.456311471	0.000857984	0.131592282
VEGFC	0.61188175	0.268379499	0.000393313	0.04078366	0.118552575	0.00087007	0.132210343
CDCA4	0.229896086	0.015393979	0.044094166	0.520032826	0.003983583	0.000893009	0.133447348
ECM1	0.656080809	0.068911502	0.00923218	0.692729593	0.0011217	0.000895217	0.133447348
SHTN1	0.465536221	0.57914838	1.03E-05	0.345706374	0.339952782	0.000902605	0.133447348
HK3	0.726956554	0.387737957	6.69E-06	0.670454408	0.269041827	0.000928096	0.135990858
NDST1	0.342675459	0.819504258	0.001355537	0.012998308	0.071246198	0.000953107	0.137882527
PKNOX1	0.162329067	0.086283423	0.892034155	3.03E-05	0.938608244	0.000957809	0.137882527
MMP19	0.520179824	0.379302447	0.000100816	0.773508343	0.024139375	0.000991187	0.141446686
SNAP23	0.886943737	0.000102376	0.567890679	0.163758721	0.045409791	0.00101519	0.143623094
CA3	0.03489106	0.085876448	0.031131112	0.005863819	0.751931688	0.001069909	0.148151146
IGFBP2	0.249570186	0.002269908	0.026606018	0.093647865	0.297758768	0.001087388	0.148151146
KLK7	0.244414471	0.074195497	0.01058486	0.014889283	0.146248379	0.00108292	0.148151146
PLIN5	0.088366144	0.889998169	0.004781138	0.012809306	0.086302753	0.001078454	0.148151146
PRLR	0.000709591	0.005517446	0.538494604	0.974503792	0.20580965	0.001092334	0.148151146
LOC100139916	0.042393179	0.734155235	0.036338752	0.012181656	0.031082279	0.001102726	0.148334691
LOC101906455	0.918207221	9.18E-06	0.389735416	0.753877771	0.178749951	0.00113018	0.150791704
LOC512486	0.885136015	0.086174643	0.00067461	0.019845366	0.442679442	0.001148288	0.151972255
GSE1	0.836621499	0.476002619	1.25E-05	0.420927582	0.223424861	0.001176892	0.152078591
PERP	0.001096906	0.030913268	0.834661291	0.102730441	0.158531762	0.001165101	0.152078591
SYNJ2	0.006170389	0.032940393	0.206342581	0.017922685	0.616254327	0.001169423	0.152078591
ATAD3A	0.559513685	0.549519722	0.201656549	0.004102317	0.001922276	0.001217536	0.152485938
CD83	0.355255296	0.542879981	9.99E-06	0.565750905	0.46875365	0.001257939	0.152485938
FOSB	0.177350398	0.010421711	0.117201071	0.017633383	0.134547469	0.00126367	0.152485938
KPNA1	0.253954635	0.001018805	0.01869906	0.130472385	0.780124701	0.001224032	0.152485938
LOC107132475	0.854239571	0.340737253	0.637366511	0.001209628	0.002138742	0.001200807	0.152485938
LOC783797	0.069239793	0.953933303	0.074543621	0.103366348	0.000996994	0.001251674	0.152485938
NTNG1	3.70E-05	0.771252722	0.244266436	0.780626544	0.091940062	0.001238257	0.152485938

RASSF9	0.782559722	0.463365134	0.000562144	0.006950455	0.355054519	0.001243623	0.152485938
TRIP12	0.65303864	0.478703898	0.000817486	0.002291711	0.87307097	0.001258868	0.152485938
MAPRE1	0.417274044	0.000126751	0.068019575	0.249892925	0.606847438	0.001321117	0.158254401
ACSL6	0.608440537	0.416519038	0.127743721	0.006765199	0.002696681	0.001401422	0.158270366
ADGRB2	0.692048368	0.492999161	1.98E-06	0.935214078	0.941101112	0.001408048	0.158270366
BHLHA15	0.235896008	0.132703844	0.071263177	0.002745182	0.094416814	0.001379502	0.158270366
CHI3L1	0.373722568	0.000107445	0.183850863	0.170895419	0.459669098	0.001382541	0.158270366
KIFC2	0.124293938	0.157707211	0.000153359	0.216146306	0.912700039	0.001405705	0.158270366
LOC101906101	0.033890284	0.331858737	0.003577375	0.028885697	0.476580531	0.00133608	0.158270366
LOC112441499	0.164413975	0.019075992	0.008900076	0.045779817	0.443667032	0.001359483	0.158270366
PCDH19	0.92150769	0.007686487	0.407142009	0.07950911	0.002536238	0.001385393	0.158270366
SLC31A2	0.38430473	0.355295094	0.000202277	0.424101769	0.049743213	0.001387378	0.158270366
FAM210B	0.950281607	0.548036114	2.46E-05	0.1640669	0.291269897	0.001438445	0.160210802
TSPAN9	0.098374484	0.866092978	0.105921692	7.18E-05	0.950200432	0.001444836	0.160210802
B3GNT6	0.040310059	0.011133176	0.117472948	0.022176543	0.544208748	0.001481042	0.160962807
C16H1orf115	0.462207845	0.888414014	0.118563951	4.52E-05	0.287084591	0.001474034	0.160962807
LOC112442745	0.084464447	0.848140074	0.000103534	0.172241718	0.4901361	0.001463558	0.160962807
LOC100850659	0.408761883	0.068034732	0.023847417	0.013985942	0.069396819	0.001493855	0.161287167
CCDC188	0.02402205	0.002765475	0.778581467	0.02312651	0.572267101	0.001563539	0.161674787
ETNK2	0.128283821	0.661400117	0.000167637	0.405819118	0.119196998	0.001569448	0.161674787
GALNT5	0.628616063	0.001126897	0.02343023	0.043372674	0.961350846	0.001576258	0.161674787
GIMAP8	0.201498132	0.091046626	0.00081753	0.110207242	0.401055041	0.001526798	0.161674787
GTPBP4	0.238898487	0.193072227	0.288483355	0.002584531	0.019983731	0.001568131	0.161674787
IMPA2	0.50372835	0.316505639	0.001416042	0.407295793	0.007525813	0.001576185	0.161674787
NR3C2	0.004928129	0.146603095	0.402684785	0.020036941	0.112397437	0.001513633	0.161674787
TSR1	0.193039327	0.40435477	0.03775861	0.025348321	0.008886421	0.001528487	0.161674787
SVEP1	0.0545457	0.07987618	0.03452489	0.006299361	0.738762675	0.00158966	0.162036736
ACTR3	0.851446637	0.001887911	0.020330246	0.056824758	0.382940491	0.001608294	0.162924197
CREB3L1	0.222311337	0.124880019	0.034721922	0.003891484	0.197720637	0.001659133	0.164024289
SLAMF7	0.55116168	0.281569742	2.65E-05	0.533537218	0.338430423	0.00165906	0.164024289
SLC25A51	0.079617193	0.103588237	0.000587753	0.292005465	0.515182956	0.001638471	0.164024289
ZNF775	0.431504052	0.453451926	0.009194743	0.166112417	0.002465594	0.001651114	0.164024289
LOC617565	0.638559434	0.473735324	1.33E-05	0.736338294	0.254847854	0.001683854	0.16492686
MYLK	2.65E-05	0.280168676	0.202138519	0.916435344	0.551838697	0.001688362	0.16492686
LOC101906664	0.692719054	0.005764374	0.308036858	0.006318351	0.09858782	0.001699457	0.165028376
PLAC8	0.991540274	0.216086595	0.00133768	0.070965118	0.038231237	0.001718098	0.16585706
FAM124B	0.61702542	0.700226109	0.000220715	0.01456067	0.569036477	0.001738492	0.166844427
CASQ1	0.001894842	0.587379015	0.061296904	0.080872552	0.15287381	0.001824307	0.170106297
CLINT1	0.003607194	0.070066628	0.457784172	0.07441221	0.097578715	0.001818979	0.170106297
CNOT6	0.000128226	0.196825966	0.291692167	0.21341291	0.531650922	0.001811242	0.170106297
IL7R	0.243222134	0.152944893	0.105541547	0.006555793	0.032755383	0.001823704	0.170106297
SCG3	0.007439926	0.178490314	0.002582109	0.335606491	0.713877653	0.001789182	0.170106297
LOC107131896	0.744952981	0.029890864	0.146896685	0.033394649	0.007972511	0.001867797	0.170896857
NID2	0.068102295	0.291996061	0.155572602	0.266979268	0.001038884	0.001847522	0.170896857
NLRC5	0.888034054	0.000173123	0.017847464	0.378806798	0.841908922	0.00187444	0.170896857

SUSD5	0.100253785	0.11273449	0.863422227	0.035024464	0.002548056	0.001867829	0.170896857
REEP2	0.310765156	0.226796116	0.320159438	0.000368955	0.10757142	0.001906682	0.172875971
STYK1	0.056293963	0.026590148	0.048846386	0.575228422	0.021947506	0.001949588	0.175794981
NDOR1	0.012841751	0.06640136	0.278402037	0.006464526	0.627147804	0.00201038	0.179306265
SNX16	0.292028645	0.100326525	0.001248485	0.158398097	0.16594558	0.002008884	0.179306265
HSPB3	0.731545342	0.19638142	0.000460361	0.016422305	0.914176082	0.002056895	0.182463282
ITGB2	0.544097864	0.506758832	1.12E-05	0.588354953	0.562025507	0.002095463	0.18467923
RRS1	0.496654194	0.083950352	0.016544372	0.019771708	0.075099759	0.002104382	0.18467923
CD93	0.585870413	0.42476647	0.008896829	0.000588441	0.808698236	0.002148434	0.187542314
FCER1A	0.133017951	0.121442391	0.111500235	0.074172224	0.008013309	0.002173724	0.18874595
LOC101903647	0.67314975	0.018487003	0.079136017	0.001134319	0.975017227	0.002201371	0.190140526
MARC1	0.093706334	0.278595904	0.000656553	0.08565984	0.749277116	0.00221752	0.190532573
HHIPL1	0.139922909	0.770151917	0.016179413	0.00093126	0.682771865	0.002230051	0.190586142
LSAMP	0.002145025	0.350129307	0.011003206	0.180906641	0.746694175	0.00224137	0.190586142
PLAU	0.795977654	0.424498542	0.002227569	0.003976966	0.388458737	0.002309338	0.195353331
TXNDC5	0.019097502	0.342455766	0.00628216	0.038339609	0.755691688	0.002349254	0.197710791
NOD2	0.917464612	0.863015336	0.483457233	0.081673643	3.89E-05	0.002388094	0.199954156
IFRD1	0.440867683	0.047396631	0.241886044	0.001448761	0.169501902	0.002422122	0.201773842
TARDBP	0.918362292	0.417953562	0.722527538	0.000927759	0.005032222	0.002498004	0.206274468
WNT4	0.670133529	0.012900503	0.000363974	0.81455691	0.50607319	0.002501287	0.206274468
DUSP16	0.446578498	0.987289265	0.785221796	3.00E-05	0.127421767	0.002537682	0.206552107
EN1	0.624458312	0.000286825	0.061088095	0.166926839	0.725616436	0.002540814	0.206552107
MRPL16	0.213279758	0.362330646	0.852460518	0.071058486	0.00028336	0.002542412	0.206552107
CCDC84	0.028178558	0.002256113	0.507193885	0.076459729	0.552078683	0.00259067	0.20881881
NIP7	0.542480532	0.004474164	0.541002459	0.01387226	0.075462335	0.002609361	0.20881881
RXFP1	0.086335254	0.797263403	0.049059804	0.272597149	0.001502577	0.00262121	0.20881881
SMAD7	0.175061396	6.78E-05	0.355999914	0.456015506	0.714987955	0.002615014	0.20881881
IKZF1	0.170949082	0.531981532	0.000777049	0.405029169	0.048931244	0.0026451	0.209704067
HOXA7	0.017146455	0.495761417	0.23250781	0.034120548	0.020948209	0.00266184	0.210016596
CFH	0.002622099	0.268932977	0.065529524	0.151675645	0.203131627	0.002676945	0.210197786
ACAN	0.097172622	0.124987525	0.005537342	0.05473451	0.400436598	0.002745447	0.21053069
CBX6	0.668965341	0.570556177	0.000488881	0.010144099	0.7999773	0.002799695	0.21053069
CLECL1	0.271207297	0.828871613	0.008369889	0.004102408	0.187831488	0.002712579	0.21053069
COL5A1	0.393512505	0.012723269	0.001033709	0.379176255	0.766849173	0.002787123	0.21053069
DHODH	0.559970074	0.813734616	0.216341918	0.001395086	0.010665961	0.002735729	0.21053069
FARSB	0.842113071	0.734619361	0.294549224	0.087438132	9.55E-05	0.002809471	0.21053069
PHF13	0.380149915	0.502535294	0.343647762	4.90E-05	0.46934617	0.002795038	0.21053069
PLEK	0.699347362	0.420289418	1.21E-05	0.436033077	0.968933801	0.002784515	0.21053069
PTGES3	0.126860599	0.306590308	0.389859553	0.000128895	0.776068111	0.002803134	0.21053069
WT1	0.6526678	0.000281472	0.181047081	0.089830697	0.500758965	0.002775324	0.21053069
LOC101903868	0.011927242	0.062048322	0.058267341	0.668780112	0.053252137	0.002828507	0.210993745
CABIN1	0.907799553	0.330279562	0.001590558	0.003376009	0.964035309	0.002850361	0.211124928
ELOVL3	0.419440888	0.540903101	0.008039913	0.041004395	0.021004184	0.002875542	0.211124928
PTCD2	0.257561456	0.262137379	0.505733581	0.002168079	0.021085975	0.00286222	0.211124928
SV2B	0.612360227	0.011114897	0.052490691	0.023565317	0.187152059	0.002881725	0.211124928

ELOF1	0.038344058	0.589382753	0.030260862	0.003113205	0.754285744	0.002921771	0.213107497
PSMB8	0.0732548	0.952921844	0.02704825	0.001488843	0.577025272	0.00294311	0.213714086
ELP3	0.219659752	0.024563518	0.883319794	0.218093588	0.001584681	0.002976066	0.214157315
LOC101907041	0.616286028	0.299653339	0.087872765	0.000533795	0.190619921	0.00298131	0.214157315
WDR13	0.626752781	0.000542041	0.700697205	0.009795703	0.710426095	0.002988363	0.214157315
STK17B	0.682639524	0.989720849	0.061763135	0.000554081	0.072284934	0.003007588	0.214597913
RNF165	0.886308438	0.615057502	0.041564961	0.000421485	0.176240802	0.003022994	0.214763451
CHAC1	0.001299392	0.471029	0.762300272	0.017203365	0.213132575	0.003058819	0.214868048
GK5	0.194704627	0.766413665	0.000113852	0.994665531	0.101924049	0.00307395	0.214868048
LRRC3	0.508825922	0.173539802	0.000237267	0.141348098	0.582375425	0.003076838	0.214868048
PTN	0.347662785	0.059212766	0.00552276	0.016822559	0.900391421	0.003073529	0.214868048
DRAM2	0.275377009	0.049943644	0.024041017	0.153019834	0.034334697	0.003093009	0.215082092
ZNF684	0.852355331	0.263545061	0.023372398	0.00128829	0.264482235	0.003159433	0.218774097
CACNA1G	0.541296192	0.237442025	5.07E-05	0.451599348	0.640857859	0.003281913	0.223911813
CLDN10	0.041642124	0.099630355	0.557059844	0.001658551	0.493217035	0.003288267	0.223911813
FEM1A	0.103804825	0.497557349	0.006270395	0.023069292	0.254496111	0.00330185	0.223911813
HSPA1A	0.494290958	0.18694705	0.762421341	0.0001092	0.246965628	0.003300113	0.223911813
LARP7	0.057219643	0.00402061	0.691034165	0.153316775	0.077405746	0.00328338	0.223911813
ERAP2	0.323053974	0.012444892	0.010766921	0.050680974	0.878162477	0.003333339	0.225117007
THOP1	0.643634242	0.571708943	0.150357394	0.035921942	0.00097699	0.003352302	0.225469788
ACSM3	0.442232566	0.005322526	0.697134435	0.005746868	0.21091171	0.003410914	0.225573547
CCDC158	0.022333595	0.727666918	0.033356016	0.005071925	0.73436515	0.003448137	0.225573547
CRYM	0.169112329	0.02100076	0.053503286	0.458142864	0.023117478	0.003440024	0.225573547
H1F0	0.685726023	0.139202489	5.95E-05	0.596888452	0.585316415	0.003405826	0.225573547
HOGA1	0.097612312	0.582572608	0.19523247	0.000611935	0.297422984	0.003450062	0.225573547
LGALS9	0.149022931	0.984196662	0.105562202	0.000186954	0.688478389	0.003415738	0.225573547
NFAM1	0.893998856	0.758827504	8.66E-05	0.384967467	0.087524774	0.003400145	0.225573547
TRIM36	0.979547375	0.123607003	0.5900558	0.000336355	0.084602473	0.003465315	0.225671772
ACKR3	0.428974629	0.742438972	0.580290914	0.000197031	0.057691549	0.003548211	0.22823402
ADCK5	0.212946978	0.000970463	0.056236942	0.185692305	0.969570959	0.003537992	0.22823402
ATG9B	0.034710028	0.863557601	0.585562953	0.000170371	0.708718077	0.003570678	0.22823402
EMB	0.07567783	0.042138271	0.002427021	0.553410783	0.500994766	0.003602868	0.22823402
EMID1	0.978061112	0.158771899	0.027886148	0.001005692	0.488344914	0.00357976	0.22823402
GALNT6	0.478085218	0.441186772	0.000169676	0.4147878	0.144430302	0.003600681	0.22823402
LOC112447770	0.012933009	0.100809986	0.466665268	0.091796173	0.038614199	0.003615919	0.22823402
TUBB4B	0.057788521	0.336288656	0.140171731	0.17339751	0.004447738	0.003548284	0.22823402
ANXA9	0.109118871	0.76542628	0.001510479	0.326900692	0.054628606	0.003731414	0.23204055
CYP2B6	0.005815325	0.332678202	0.041841586	0.028766066	0.993622898	0.00380348	0.23204055
ERI3	0.245228939	0.075417147	0.171248758	0.004392045	0.162056643	0.003732953	0.23204055
ITGAL	0.91445854	0.496825075	1.43E-05	0.586073781	0.599021427	0.003771302	0.23204055
LOC107131944	0.023361155	0.101487199	0.003642212	0.792661756	0.337455599	0.003798868	0.23204055
PUS10	0.323914749	3.25E-05	0.594030046	0.477297694	0.769278858	0.003786269	0.23204055
SIGLEC11	0.010071804	0.853703474	0.098509198	0.012697952	0.208473596	0.003718594	0.23204055
SYNM	0.000108736	0.420884052	0.262748311	0.900624052	0.211465499	0.003775566	0.23204055
TYSND1	0.919436276	0.623221741	0.799832185	1.14E-05	0.437286548	0.003779778	0.23204055

LOC112448523	0.090531046	0.010695872	0.013463661	0.648866295	0.276250822	0.003830812	0.232842407
WISP1	0.754689065	0.024046244	0.000746448	0.188361883	0.937701401	0.003896223	0.235944337
ATP2A2	0.96954125	0.041844662	0.003899103	0.172709722	0.088477916	0.003924996	0.236579692
CASP1	0.023096595	0.581403788	0.000333343	0.916403016	0.591483598	0.003935547	0.236579692
NIF3L1	0.740456967	0.734324498	0.809699578	0.000635353	0.009186076	0.00410083	0.245615791
ZBTB11	0.318641078	0.791912027	0.776824645	1.75E-05	0.762046625	0.00415035	0.247677822
COL6A5	0.034828188	0.429075053	0.032224884	0.09945775	0.055660609	0.00421039	0.247850488
HSD11B2	0.014433828	0.659712592	0.030473984	0.014713547	0.634480962	0.004258963	0.247850488
KCP	0.00653817	0.690836198	0.007509921	0.163716725	0.486112724	0.004248415	0.247850488
LOC101906565	0.840049428	2.53E-05	0.35883954	0.702253131	0.502271228	0.004240568	0.247850488
MVD	0.858183509	0.606007268	0.400929186	0.001216553	0.01039701	0.004178047	0.247850488
SOX4	0.47621089	0.738118966	1.21E-05	0.858925537	0.73303862	0.004213883	0.247850488
TEC	0.971588165	0.617521525	0.046847736	0.000126446	0.745546424	0.004192118	0.247850488
ADAMTS17	0.470987818	0.017452257	0.002954431	0.255171908	0.443547807	0.004303447	0.248112524
DDX54	0.228954836	0.813046917	0.07996064	0.002817843	0.065825143	0.004317232	0.248112524
HHEX	0.4846014	0.399002102	0.013616186	0.001396215	0.744313841	0.004289401	0.248112524
TMEM100	0.59664333	0.001597866	0.459859824	0.076897265	0.082073189	0.00432394	0.248112524
GPR37	0.014972927	0.004858978	0.203756999	0.927648186	0.204092187	0.00436817	0.248954942
SDR16C5	0.778759341	0.235676453	0.271840656	0.000283828	0.198241827	0.004368961	0.248954942
CENPB	0.251488232	0.000904922	0.396147514	0.119059592	0.26654772	0.00442862	0.250601629
HSPA6	0.387535064	0.058360535	0.664676401	0.000288136	0.668779305	0.004468107	0.250601629
INTS6	0.727925142	0.6600796	0.789398964	7.97E-06	0.960352366	0.004474211	0.250601629
LOC100848883	0.004041738	0.229790141	0.827566964	0.201890602	0.018504887	0.00444015	0.250601629
LOC104976082	0.594262848	0.488956913	5.36E-05	0.936331214	0.198783971	0.004472296	0.250601629
HACD4	0.927405869	0.821558485	6.76E-05	0.063222555	0.898437534	0.004501961	0.251298241
CBARP	0.416950784	0.746699366	0.002925434	0.008922789	0.373608111	0.004620595	0.2533422
CD300E	0.42383606	0.580060756	3.24E-05	0.799367082	0.471792535	0.004581645	0.2533422
LTBP2	0.087789847	0.470624169	0.001170828	0.187695085	0.332945486	0.004606195	0.2533422
PADI2	0.890947333	0.110659882	0.001233723	0.141725749	0.176453083	0.00462667	0.2533422
PTPRZ1	0.881771205	0.113990208	0.665875852	0.046471173	0.000958818	0.004561748	0.2533422
TTC38	0.50434776	0.09978706	0.000277097	0.650745342	0.335649669	0.004631202	0.2533422
ATG4B	0.916031312	0.929110755	0.001105972	0.034680905	0.095078965	0.004693694	0.253593496
LOC513508	0.003227127	0.002930158	0.754898458	0.927050998	0.463294803	0.004652721	0.253593496
OIT3	0.268545029	0.006301041	0.00362483	0.766121719	0.661286405	0.004697607	0.253593496
PCDH1	0.809566448	0.953061541	0.007214734	0.029711032	0.01876849	0.004694033	0.253593496
NEIL1	0.338912363	0.20592903	0.219479618	0.000266261	0.773209904	0.004747201	0.255430567
NCLN	0.255934628	0.65288291	0.348324493	0.002832815	0.019437792	0.004802107	0.257540458
LAPTM5	0.380692022	0.406879691	0.000129131	0.181736323	0.890077962	0.004834709	0.258444309
LOC100847719	0.060799379	0.155968325	0.025449628	0.223773185	0.060210711	0.004851854	0.258518752
LCP1	0.40902112	0.430759809	0.000122496	0.203344277	0.752582011	0.004906155	0.258955918
MYBBP1A	0.300097272	0.690824822	0.024713929	0.036802802	0.017490853	0.004901128	0.258955918
NAT14	0.325208318	0.236490173	0.005873907	0.816090378	0.008961972	0.004907397	0.258955918
PNO1	0.22937988	0.140649535	0.029978713	0.055658314	0.062031906	0.004944588	0.260082149
HMGCLL1	0.009930133	0.022070873	0.349692488	0.139666424	0.315305007	0.004982244	0.261225564
NRM	0.248946717	0.687347757	0.199504736	0.000139533	0.71523876	0.005015634	0.261659632

PAK5	0.010343618	0.788914001	0.094077114	0.084022015	0.053155076	0.005038355	0.261659632
PGLYRP2	0.854379997	0.249340255	0.045514122	0.003252064	0.108507693	0.005030817	0.261659632
ITGAX	0.609313458	0.328507987	6.40E-05	0.627549691	0.432125194	0.005087281	0.262538873
TMEM156	0.354123431	0.279103414	0.01629403	0.01292976	0.166482679	0.005077958	0.262538873
CD68	0.532304895	0.353577776	0.000144075	0.802878177	0.161281234	0.005124351	0.263622985
GDF11	0.806047276	0.835880373	0.007295185	0.064487104	0.011318624	0.005203225	0.266844122
CD200R1L	0.266163547	0.653855927	9.76E-05	0.514829937	0.415124462	0.005248423	0.268305966
SLAMF6	0.752551726	0.473092314	5.24E-05	0.640474742	0.305371871	0.005264428	0.268305966
EMILIN3	0.001753051	0.440332537	0.152604981	0.061847017	0.511279668	0.005343781	0.271507112
CTGF	0.378265293	0.00114556	0.142368547	0.17301863	0.350534259	0.00536065	0.271523536
MGC157082	0.999316398	0.204476749	0.000278299	0.640883712	0.103497933	0.00539152	0.272246859
TRPC1	0.730496974	0.471449161	0.675043387	0.003327184	0.004907749	0.005415999	0.272644042
ALPK3	0.555147309	0.973041272	0.814408993	0.302703456	2.88E-05	0.005454641	0.273175616
NOC4L	0.373389134	0.610738379	0.093455683	0.019977896	0.009056899	0.005476496	0.273175616
TAB1	0.488794223	0.213149018	0.261674824	0.00016542	0.853923523	0.005471375	0.273175616
SH2B2	0.363004747	0.996514906	0.000493555	0.061708948	0.360789525	0.005595485	0.277424468
TUBB	0.022168912	0.568648572	0.007083497	0.523265199	0.084783326	0.005582135	0.277424468
GZMB	0.057261695	0.66164619	0.001160283	0.207538819	0.442332367	0.005655694	0.278725486
LOC788801	0.230970183	0.655505864	0.004329396	0.018791802	0.326751125	0.005645046	0.278725486
ARL11	0.352198903	0.014654831	0.002121151	0.543605156	0.69115723	0.005732657	0.280831727
GTF2E1	0.001304957	0.022502507	0.517720788	0.640285239	0.420892997	0.005716503	0.280831727
KIF14	0.000869771	0.562495148	0.116619238	0.304147754	0.241739985	0.005812773	0.28390898
SLC30A4	0.885231359	0.530414247	0.147811897	0.000132068	0.463573084	0.005865687	0.285643294
NKD1	0.220145319	0.781750095	0.000363786	0.135309049	0.509360545	0.005929747	0.287908537
ATP6V1C2	0.005753915	0.867272901	0.003537626	0.382232785	0.64967593	0.005996432	0.288714659
BMP6	0.343099024	0.187497506	0.055779287	0.016593179	0.075285439	0.006091405	0.288714659
FARSA	0.223494367	0.691687289	0.23378	0.037702882	0.003326985	0.006139871	0.288714659
HMGA1	0.133416446	0.357155587	0.000177481	0.547487366	0.954850228	0.006032311	0.288714659
JMJD6	0.626447017	0.335711558	0.768284922	2.75E-05	0.991702835	0.00602522	0.288714659
KCNJ10	0.69904001	0.029102659	0.032934794	0.019085303	0.347312431	0.006051766	0.288714659
LOC107131398	0.354714839	0.000179169	0.238530597	0.453007204	0.651806855	0.006085284	0.288714659
MYO1G	0.874031469	0.817286118	1.37E-05	0.526988759	0.873374405	0.006121051	0.288714659
PPL	0.608183685	0.020436859	0.456334688	0.004887117	0.162594378	0.006114816	0.288714659
TEPSIN	0.741053353	0.754568739	0.279565679	4.35E-05	0.665156047	0.00612884	0.288714659
WAPL	0.60714628	0.044348016	0.57118022	0.000393286	0.746640158	0.006123458	0.288714659
MAT2A	0.323868827	0.203829938	0.000819316	0.105022372	0.805116796	0.006178062	0.289324648
MERTK	0.8564821	0.294282534	0.002923355	0.041249294	0.150816471	0.006188103	0.289324648
CTSB	0.341282144	0.197481721	0.000152529	0.636384821	0.709807992	0.006244822	0.289502188
SLC41A3	0.773616457	0.040928239	0.833790406	0.000194154	0.905657869	0.006243429	0.289502188
TUBA1A	0.002023066	0.339858717	0.038547203	0.576191724	0.303035019	0.006229728	0.289502188
MEOX2	0.705330254	0.003930103	0.043946542	0.052253711	0.739716157	0.006306411	0.291533838
CREM	0.164715569	0.918216359	0.65151058	5.27E-05	0.922910858	0.006389357	0.294538587
ATF3	0.122816699	0.000861573	0.337226338	0.158475096	0.88190832	0.006566587	0.297702705
CD48	0.269801599	0.923811993	0.000408649	0.134145659	0.361346859	0.006520228	0.297702705
KHDC4	0.370677729	0.636138575	0.814168987	7.96E-05	0.32631806	0.006566838	0.297702705

PIEZO2	0.386242603	0.074196691	0.001070059	0.161710029	0.995484239	0.006519642	0.297702705
TP53BP2	0.236763181	0.386236898	0.000703474	0.363220888	0.212365781	0.006543411	0.297702705
ZMYND8	0.221045646	0.137935742	0.007237774	0.30808495	0.072032631	0.006483207	0.297702705
AOX1	0.190381011	0.414772765	0.283732442	0.016129837	0.01431724	0.006738534	0.304644856
CXHXorf21	0.666948546	0.840246644	7.88E-05	0.559808846	0.210873636	0.006776404	0.30551531
RRAGD	0.62902982	0.127389809	0.007201585	0.060754254	0.150052742	0.006817671	0.306533684
L3MBTL2	0.412958032	0.319171691	0.375902443	0.010785554	0.010113432	0.006947663	0.311524864
NDRG4	0.17650898	0.239258161	0.904435439	0.328223475	0.000433158	0.006971046	0.311721628
ZSWIM6	0.885633614	0.997332846	0.323169929	2.18E-05	0.877822314	0.007006912	0.312473989
ARMCX3	0.352540862	0.020124973	0.554018138	0.001820451	0.796283908	0.007209972	0.312515869
C4H7orf25	0.955540925	0.225854543	0.328496981	0.000742554	0.107113678	0.007157464	0.312515869
DNASE1L3	0.057058915	0.064036007	0.421768777	0.028743545	0.128911078	0.007220871	0.312515869
DUSP26	0.479914241	0.220643405	0.016271281	0.02866722	0.11174955	0.007051172	0.312515869
INSIG1	0.077724089	0.722318616	0.835215856	0.010062665	0.012080375	0.007211842	0.312515869
JAML	0.011907107	0.414994946	0.062945116	0.100213574	0.182065872	0.007189664	0.312515869
LOC506828	0.499675969	0.064575493	0.0030373	0.142663255	0.399609164	0.00711154	0.312515869
LOC788175	0.001778392	0.080668861	0.720180071	0.240308027	0.226628507	0.007146793	0.312515869
MMP2	0.098601165	0.400841367	0.001131758	0.30159556	0.410298398	0.007065126	0.312515869
PRMT1	0.581491611	0.439066302	0.000791647	0.284955695	0.099448988	0.007236368	0.312515869
RIMS1	0.015888628	0.164304372	0.227203922	0.279805458	0.034036628	0.007166375	0.312515869
TBRG4	0.095531733	0.657454186	0.321644224	0.038125943	0.007151182	0.007040707	0.312515869
GRP	0.00499762	0.273144716	0.887603402	0.010116362	0.469509097	0.007260441	0.312732535
SLC25A27	0.329166955	0.31094759	0.355452552	0.000319266	0.499283259	0.007299643	0.313597993
ARHGAP4	0.442303202	0.369721241	0.004920289	0.014536829	0.50270164	0.0073703	0.315710725
HGH1	0.563179774	0.732579604	0.580782574	0.007884631	0.003157418	0.007445009	0.315710725
PEX6	0.041167975	0.058853318	0.066166184	0.05222097	0.708951653	0.007418679	0.315710725
RUVBL1	0.273948851	0.725450755	0.266105039	0.068771825	0.001633696	0.007424463	0.315710725
SCN4B	0.333147079	0.050572713	0.136373757	0.041594538	0.06220782	0.007427478	0.315710725
LOC112441594	0.589451113	0.000133874	0.304585373	0.650055934	0.385370974	0.007493746	0.316958424
CSPG5	0.473632141	0.742657478	0.328448939	0.000358327	0.148685951	0.007609936	0.31697123
EHF	0.020943252	0.889029414	0.853801062	0.001170879	0.327911196	0.007565251	0.31697123
GMPR	0.271580969	0.009811094	0.004618608	0.579230067	0.850339497	0.00752866	0.31697123
TAS1R3	0.020445605	0.022013078	0.020158777	0.787455736	0.856013755	0.007575806	0.31697123
TRIM25	0.338992389	0.007791269	0.00452282	0.72976303	0.70406909	0.00759475	0.31697123
ZBTB8OS	0.07030512	0.13824725	0.84228205	0.012509315	0.059436239	0.00755067	0.31697123
CTSS	0.464569741	0.412438969	0.000576969	0.104084197	0.540397494	0.007664148	0.317617015
MZB1	0.145143179	0.054250712	0.009975725	0.21843541	0.361227201	0.007646777	0.317617015
LOC618076	0.978161173	0.732324548	0.844150751	1.08E-05	0.963173092	0.007708757	0.318660996
TFRC	0.042190502	0.008641581	0.112180553	0.520713272	0.296540169	0.007747702	0.31894088
TIRAP	0.493789387	0.215031878	0.079685588	0.020056978	0.037261068	0.007754397	0.31894088
TM4SF19	0.356647741	0.488022924	0.000206896	0.786986447	0.224266066	0.007782067	0.319278746
CTHRC1	0.711423246	0.01613452	0.010402319	0.074220013	0.721267583	0.007813	0.319748503
LOC112447408	0.646905967	0.26319676	7.63E-05	0.905835597	0.549797949	0.007882618	0.321795123
HOXA6	0.297924939	0.4057463	0.040109308	0.090505499	0.01493115	0.007948938	0.323697313
GRK3	0.409944402	0.793311675	0.000318275	0.856000923	0.074515188	0.007991345	0.324618715

EPHA5	0.120335485	0.002443816	0.198175364	0.375405692	0.303583614	0.008024759	0.325085747
LOC784052	0.258231874	0.062479009	0.460744416	0.001046241	0.859712504	0.008062152	0.325085747
SMPD3	0.953238591	0.266540339	0.000369526	0.471336293	0.151097508	0.008062269	0.325085747
SOST	0.747898952	0.26225706	0.406834027	0.000554102	0.152011947	0.008091501	0.325464759
DDX56	0.10546058	0.52955315	0.024020213	0.051505318	0.098378099	0.008154958	0.327215188
GIMAP7	0.390717527	0.408688363	0.100353047	0.041718122	0.010271539	0.008212922	0.32873721
SLC2A3	0.164097968	0.053622695	0.007560693	0.896489567	0.115569123	0.008234725	0.328807961
TMEM177	0.983188745	0.881377017	0.747966156	1.95E-05	0.546515115	0.008256296	0.328869102
MFN1	0.085662273	0.875612041	0.046038026	0.013677359	0.148143936	0.008320967	0.329131565
MPHOSPH9	0.401951616	0.000270728	0.486358126	0.141064385	0.935287382	0.008309273	0.329131565
OXTR	0.211156773	0.090411432	0.029825292	0.28690508	0.042845894	0.008323052	0.329131565
LPAR2	0.406218537	0.411101845	0.000204525	0.373300832	0.553622781	0.008372069	0.32948208
MTFP1	0.136554586	0.551739064	0.185362026	0.009097721	0.055423619	0.00835815	0.32948208
EFNB2	0.809791272	0.058101265	0.000546358	0.393634879	0.708663115	0.00846426	0.329495788
IL2RG	0.098080072	0.847823673	0.004784515	0.024318091	0.73655491	0.008427623	0.329495788
LOC101904923	0.025470178	0.625201255	0.094738985	0.008380992	0.563156089	0.008422847	0.329495788
PRPSAP2	0.066135652	0.576926126	0.433949458	0.005190953	0.083064314	0.00843839	0.329495788
SCYL2	0.187478053	0.003176704	0.072794712	0.290229813	0.570730152	0.008472806	0.329495788
LOC100336414	0.710700373	0.662515907	0.91851163	1.99E-05	0.842610987	0.008518982	0.330508303
LY9	0.789335297	0.928922309	9.65E-05	0.155411532	0.669819654	0.008622743	0.333744905
LOC101905979	0.630326031	0.000936308	0.154786742	0.341045522	0.239937234	0.008712264	0.335693048
POU3F1	0.315513799	0.600829953	0.001329057	0.400479601	0.074106734	0.008713987	0.335693048
CASTOR2	0.954725009	0.014517613	0.011855312	0.123036309	0.377650659	0.008841007	0.33793374
COL5A2	0.318461968	0.031875494	0.004348595	0.588302664	0.293617603	0.008833051	0.33793374
H2AFJ	0.161223365	0.681177094	0.208841043	0.000361876	0.918247788	0.008829906	0.33793374
PMF1	0.521096979	0.66856519	0.958432344	0.000213282	0.10744541	0.008854519	0.33793374
LOC100848703	0.171335593	0.753616424	0.809053312	0.000128196	0.574834248	0.008891731	0.338566585
ACSL3	0.801281851	0.024747545	0.02606435	0.04879152	0.308858087	0.008964051	0.339273332
DAPK3	0.095521222	0.432119227	0.346199711	0.031332744	0.017418723	0.008972313	0.339273332
THBS4	0.769548621	0.913465936	0.020137716	0.022869413	0.023933495	0.008931663	0.339273332
MYH10	0.260806965	0.168700806	0.004925272	0.042198963	0.85807332	0.009010302	0.339926599
C18H16orf86	0.179111562	0.003734839	0.073475658	0.491047212	0.335297843	0.009204921	0.342027897
ENDOV	0.415682838	0.582109632	0.152649873	0.000340428	0.63498239	0.009119579	0.342027897
HAS3	0.868003399	0.400359714	4.14E-05	0.794775876	0.710907053	0.009226286	0.342027897
LOC101902413	0.516695401	0.056668104	0.009568394	0.781399193	0.036896934	0.009192936	0.342027897
LOC101904121	0.048454112	0.877697741	0.012365058	0.055153316	0.277299548	0.009165383	0.342027897
SEPT1	0.052003013	0.041520789	0.628780876	0.084166751	0.071129822	0.009232732	0.342027897
SURF2	0.283059006	0.348511507	0.054580837	0.129520536	0.011481774	0.009137506	0.342027897
TRAF3	0.521959739	0.710078013	0.044692004	0.020583848	0.023559719	0.009157744	0.342027897
TYROBP	0.401767382	0.459504682	9.07E-05	0.558856146	0.872373512	0.009258805	0.342221298
DNAJB1	0.394466455	0.281190887	0.676171715	0.000132981	0.823680466	0.009301125	0.34285922
PHC3	0.000703476	0.201906143	0.247972232	0.593770688	0.395036299	0.009337352	0.34285922
XK	0.504681527	0.032878184	0.004888941	0.212548682	0.479239753	0.009338741	0.34285922
TF	0.055752088	0.737439376	0.842381833	0.000291797	0.820997989	0.009365081	0.343058803
LOC514457	0.047475794	0.029023868	0.381959182	0.231955957	0.068445266	0.00941103	0.343974208

BHLHE40	0.198990991	0.616519056	0.366919936	0.000776812	0.242683032	0.009512138	0.344272311
CLASRP	0.123782637	0.00260649	0.082328304	0.48581049	0.662872536	0.009564576	0.344272311
DCAF1	0.460977504	0.983387783	0.737435604	2.66E-05	0.948988253	0.009470467	0.344272311
GALNT10	0.004166691	0.062480248	0.834795731	0.058906253	0.668316253	0.009566033	0.344272311
LOC112442851	0.000789743	0.175901225	0.678940855	0.780418686	0.11616063	0.009561708	0.344272311
TRPV2	0.606982749	0.460303903	9.93E-05	0.651411646	0.471363568	0.00953825	0.344272311
VASP	0.239679142	0.123093018	0.355673769	0.011479928	0.0701245	0.009482175	0.344272311
MAOB	0.898741267	0.177849948	0.728198833	8.20E-05	0.907515345	0.009651647	0.346593399
AHCY	0.708543138	0.35829126	0.00054453	0.311518039	0.202734634	0.009700517	0.34725505
DNAJB4	0.060108561	0.495856255	0.67590652	0.002993335	0.145034457	0.009712392	0.34725505
AFF4	0.808506193	0.07999792	0.755314713	0.000192958	0.949825528	0.009870975	0.347623547
CDK16	0.715699564	0.126440858	0.018270682	0.005791832	0.934152421	0.009864854	0.347623547
IFI27	0.509741938	0.002327953	0.05190926	0.504299331	0.288123968	0.009868466	0.347623547
LYAR	0.647669015	0.993463233	0.593460113	0.001211035	0.019070669	0.009768345	0.347623547
POSTN	0.164892451	0.107464707	0.001059343	0.920684126	0.510488826	0.009771154	0.347623547
TPST1	0.703472148	0.454794707	0.608473174	5.59E-05	0.816501604	0.009821511	0.347623547
ZNF391	0.857805988	0.067190729	0.000973541	0.58711533	0.268759139	0.009795081	0.347623547
SMYD5	0.495950291	0.482113863	0.153748202	0.030310272	0.008114426	0.009937802	0.349227542
CXCL13	0.093136772	0.332674429	0.007089987	0.563125951	0.075181626	0.01013304	0.349355727
DEFB4A	0.442578118	0.167060448	0.000333491	0.919276274	0.405174987	0.010045527	0.349355727
DKK2	0.184927831	0.442167332	0.385824049	0.003455293	0.084616379	0.010075554	0.349355727
GK	0.424992007	0.57674084	6.81E-05	0.952967552	0.581374663	0.01009112	0.349355727
LOC100847376	0.001313335	0.832267597	0.435616676	0.239497746	0.081408002	0.01012032	0.349355727
LOC784289	0.301299185	0.644967376	0.008338087	0.008540821	0.667437938	0.010085073	0.349355727
PKDCC	0.692292753	0.074263617	0.004405083	0.117425953	0.348060951	0.010099914	0.349355727
PPP2R2C	0.925861512	0.422739379	0.683919007	4.15E-05	0.832954788	0.010096708	0.349355727
PTPRT	0.074600482	0.133141955	0.53319979	0.168639487	0.010310185	0.010063645	0.349355727
WDR86	0.073789673	0.073842047	0.022673545	0.130146932	0.582386337	0.010180739	0.350264368
GIMAP4	0.093254091	0.279361966	0.104239167	0.123263271	0.028115454	0.010216014	0.350742701
AQP9	0.78211188	0.886377178	0.04022879	0.006258473	0.054240121	0.010257707	0.351111897
POMT1	0.468971575	0.016265955	0.195885996	0.582422054	0.010896185	0.010269558	0.351111897
LOC529792	0.57548009	0.180075457	0.000357682	0.837288636	0.30769452	0.010319093	0.352072001
SMU1	0.453101146	0.100640197	0.002340234	0.098404388	0.917223012	0.010380402	0.353429009
CYP20A1	0.02046871	0.803266651	0.055354187	0.011498882	0.928913488	0.010446662	0.354948582
DNAH2	0.112150803	0.828229708	0.323320584	0.000397603	0.831633058	0.01060057	0.355032545
FAM160A2	0.068507652	0.39925851	0.044482265	0.014696911	0.554524254	0.010589815	0.355032545
GLULP	0.949793188	0.052901842	0.001094212	0.432100083	0.414181796	0.010533796	0.355032545
LAG3	0.543694033	0.18131064	0.097600391	0.004105041	0.247762506	0.010493938	0.355032545
SCIMP	0.700818901	0.673526538	8.71E-05	0.529892938	0.449920718	0.010503649	0.355032545
SLC14A1	0.140499822	0.063489823	0.63042136	0.012716242	0.138432959	0.010577791	0.355032545
SYCP3	0.820411227	0.007813952	0.222575443	0.486852199	0.014239365	0.01057207	0.355032545
ADAMTSL2	0.833453146	0.077738999	0.02462053	0.010114703	0.636884876	0.010852772	0.355374399
BCL11B	0.059015104	0.322107843	0.573890822	0.081179073	0.011317136	0.010667995	0.355374399
CLEC12A	0.679310013	0.323057334	0.001406406	0.341289586	0.095311699	0.010680719	0.355374399
CLGN	0.651417667	0.25833743	0.36965883	0.011703608	0.01395279	0.010767256	0.355374399

CYSLTR1	0.08713526	0.246588239	0.036408037	0.015415035	0.838451103	0.010732519	0.355374399
ERBB3	0.42587266	0.038476393	0.209910452	0.117481203	0.025430812	0.010852861	0.355374399
FLVCR2	0.066889218	0.535403162	0.026025753	0.473898144	0.023592168	0.010957251	0.355374399
GBP5	0.631442574	0.038654248	0.04509593	0.012617756	0.736055795	0.010813885	0.355374399
LOC112441505	0.568752766	0.912932194	0.000374328	0.685414576	0.078157802	0.010951144	0.355374399
NLK	0.027849516	0.148342467	0.221542537	0.027088126	0.416269678	0.010884772	0.355374399
SDS	0.590969135	0.192931075	0.012237041	0.028459922	0.258715506	0.010850507	0.355374399
SFRP1	0.01558813	0.727249082	0.053770677	0.786896677	0.021365925	0.010832724	0.355374399
TPPP	0.299004399	0.256780003	0.959162045	0.000384084	0.36800192	0.010948831	0.355374399
UCK2	0.59897614	0.9246841	0.569026118	0.012625619	0.002553594	0.010769144	0.355374399
WDR18	0.3779114	0.944614494	0.206864762	0.039622761	0.00353505	0.010901601	0.355374399
ZNF207	0.17702155	0.363146731	0.732397372	0.000250128	0.85809463	0.010728556	0.355374399
HK2	0.169767739	0.367129313	0.553588891	0.081828645	0.003716801	0.011010059	0.356382813
PTPN22	0.534584447	0.94874149	0.008826152	0.199219597	0.01189295	0.011090716	0.357582993
TNFSF10	0.315196083	0.400274714	0.039932582	0.003640479	0.57803841	0.01108764	0.357582993
ENC1	0.067961475	0.528060021	0.037026435	0.016811065	0.481108671	0.011191669	0.359639851
OSBPL8	0.658102915	0.499532429	0.00278288	0.013265162	0.886358588	0.01119834	0.359639851
CAPG	0.576944373	0.673644182	0.0006195	0.561831613	0.08024441	0.011268423	0.36118378
LOC107131817	0.282009756	0.758281918	0.155047654	0.652184259	0.000503883	0.011297492	0.361409624
CAPZB	0.127062159	0.007827767	0.033920273	0.578620303	0.565001709	0.011392282	0.362555356
KLRG2	0.340236578	0.000494846	0.189914814	0.995371716	0.347853228	0.011421676	0.362555356
RNF182	0.100025444	0.418407162	0.028412636	0.155926225	0.059449191	0.01138742	0.362555356
SEPT5	0.154132524	0.026215553	0.023573344	0.143767704	0.807034333	0.011407939	0.362555356
CYP3A4	0.390307662	0.006831307	0.265305276	0.021374254	0.736991246	0.011472554	0.363467354
NOA1	0.817361961	0.157194724	0.869800178	0.018289317	0.005535562	0.011592898	0.366572349
CEP72	0.090123351	0.655659604	0.000279553	0.726730392	0.965746902	0.011787788	0.366643018
GPBP1	0.289830281	0.878779857	0.831055938	0.000121938	0.44344258	0.011684555	0.366643018
LOC100297044	0.761786836	0.028343652	0.007087664	0.312598549	0.239038417	0.011677505	0.366643018
LOC512867	0.135655923	0.357279061	0.037069003	0.298529059	0.021693229	0.011816614	0.366643018
LOC514181	0.01763735	0.41148012	0.118931673	0.075796693	0.177886072	0.011818546	0.366643018
LOC618541	0.42755631	0.650481911	0.940163692	0.023979137	0.001841017	0.011752709	0.366643018
POLRMT	0.628998225	0.093557275	0.634302789	0.000819513	0.374028848	0.011681878	0.366643018
RPLP1	0.163102411	0.555769822	0.036625729	0.004919035	0.709566831	0.011784046	0.366643018
SLC7A7	0.583948942	0.82836871	0.000156065	0.361023547	0.423781424	0.011757462	0.366643018
TPBG	0.030322035	0.056884384	0.038937496	0.271833173	0.636653037	0.01180837	0.366643018
CLN5	0.655606575	0.417139423	6.36E-05	0.689000267	0.981709945	0.011907566	0.367401252
GNL3	0.326331352	0.389659689	0.628326686	0.002586048	0.057244855	0.01195003	0.367401252
LOC784148	0.021028718	0.777234386	0.019205517	0.146933789	0.256596486	0.011954925	0.367401252
MATK	0.564064672	0.311996573	0.000190749	0.701156244	0.499912954	0.011907757	0.367401252
MED9	0.266976352	0.143043879	0.008135524	0.089489148	0.425500311	0.011951816	0.367401252
PPP1R1A	0.699961779	0.227814609	0.264543589	0.000564408	0.500090179	0.01200451	0.368235535
CYP4V2	0.006432893	0.124321985	0.197279365	0.081118754	0.93621552	0.012056316	0.36844731
ELAC2	0.949976109	0.721062036	0.182603918	0.001634711	0.058561565	0.012050935	0.36844731
FTH1	0.325880311	0.082380017	0.013514176	0.039608715	0.841414527	0.012131177	0.369359454
LOC101902527	0.125218148	0.166429011	0.605702725	0.003557653	0.269225327	0.01213051	0.369359454

BBS9	0.310147023	0.924618157	0.143516638	0.021573482	0.013846859	0.012269857	0.369851701
C26H10orf82	0.039287785	0.121823203	0.363537813	0.020792024	0.338049878	0.012225809	0.369851701
CCL2	0.749724067	0.494876203	0.000234899	0.622471667	0.228189633	0.012327639	0.369851701
CDK8	0.232574794	0.382872546	0.019580027	0.029464195	0.240700908	0.012318109	0.369851701
GPT	0.718467521	0.723332147	0.000160611	0.291661777	0.504776219	0.012265911	0.369851701
LOC101907985	0.92273472	0.900987846	0.000142029	0.12066501	0.865133563	0.012291725	0.369851701
LOC112444603	0.386554596	0.511406228	0.138039051	0.000605506	0.737711113	0.012198352	0.369851701
PPP1R26	0.356980483	0.613933681	0.000681378	0.14267707	0.576369395	0.012260319	0.369851701
ABI1	0.892905202	0.336528166	0.271940862	0.000198461	0.782746542	0.012540468	0.369977525
AKAP7	0.383940252	0.038970914	0.288042763	0.00537677	0.540034328	0.012419134	0.369977525
BCAT2	0.274785118	0.399939825	0.562135303	0.001082377	0.188966934	0.012501068	0.369977525
BGLAP	0.344058369	0.481514367	0.00134353	0.16687962	0.342034118	0.012547614	0.369977525
HMGB3	0.686486227	0.197522549	0.550066454	0.000487815	0.350246621	0.012573841	0.369977525
LOC100140431	0.664843206	0.064801813	0.000768558	0.804023624	0.473092074	0.012473709	0.369977525
LOC101904344	0.002308705	0.186612361	0.435948685	0.339196915	0.200170159	0.012579822	0.369977525
NIPAL2	0.133435302	0.768899051	0.002770031	0.857563474	0.051812221	0.012495771	0.369977525
POMGNT2	0.133131998	0.197564675	0.028510301	0.094360631	0.178114981	0.01247934	0.369977525
RAB7A	0.00817092	0.464406713	0.036413447	0.108770059	0.848358774	0.012578336	0.369977525
SLC16A3	0.16088167	0.895754058	0.015213335	0.016957319	0.34113014	0.012532571	0.369977525
PAQR8	0.39444246	0.680604501	0.049864817	0.481122979	0.00198553	0.012603722	0.370017321
LOC782706	0.221473424	0.936253609	0.005014515	0.786725443	0.015747834	0.012666799	0.371205057
FAM136A	0.518278073	0.459329825	0.508430984	0.063594578	0.001692415	0.012763661	0.373376898
CEBPZ	0.15956953	0.819453265	0.014176885	0.030841431	0.232060442	0.012923556	0.376723348
FGG	0.291388268	0.113272036	0.009056626	0.251136453	0.178591188	0.013015791	0.376723348
GID8	0.012950077	0.423719296	0.677755582	0.025224474	0.142366682	0.012981655	0.376723348
KCNG1	0.112280275	0.522262762	0.003890642	0.276433033	0.210558181	0.012931359	0.376723348
NFIC	0.332990899	0.404256116	0.002913761	0.112077367	0.30455946	0.013003606	0.376723348
VASN	0.860728222	0.334563622	0.000475284	0.174176716	0.559900786	0.012976465	0.376723348
LOC515418	0.575971055	0.315348376	0.00013426	0.829935134	0.665734445	0.013059727	0.37732954
CARD9	0.122721146	0.010920788	0.039639211	0.559751231	0.455316951	0.01310323	0.377921117
IFI44	0.91669727	0.157404062	0.008134587	0.045675976	0.254829461	0.013183617	0.379572516
CKAP4	0.257821148	0.4310067	0.00123992	0.525476504	0.190130436	0.013251585	0.380861238
MAGEL2	0.246816117	0.19264071	0.003834576	0.535912552	0.141918048	0.013317445	0.382084949
GNA15	0.053268479	0.892397723	0.007010052	0.069552833	0.600224728	0.013346807	0.382189289
SLF1	0.205788579	0.060770088	0.302614816	0.053437616	0.068949092	0.013367659	0.382189289
PRAG1	0.027927063	0.855241765	0.103259262	0.06025284	0.094829855	0.013463909	0.384271676
MAFG	0.497990839	0.004908791	0.054968619	0.957540431	0.110447518	0.013541104	0.385803917
CACNB2	0.002130126	0.601932061	0.589264095	0.687402919	0.027756779	0.013673315	0.386218202
FBXO40	0.9396679	0.466181484	0.032498377	0.126719134	0.007957417	0.013634121	0.386218202
KNTC1	0.668203764	0.006066751	0.151644053	0.04479021	0.522382045	0.013652371	0.386218202
NUAK1	0.964721817	0.027610815	0.192187883	0.005590637	0.50339394	0.013667621	0.386218202
VARS	0.655603032	0.800056949	0.083616145	0.071017176	0.004619587	0.013655714	0.386218202
LOC104970249	0.025557485	0.002243398	0.753195998	0.395996032	0.85327743	0.013786303	0.388372518
LRFN4	0.689417503	0.059362279	0.022483944	0.218353825	0.072891718	0.013820581	0.388372518
PLEKHG4	0.471164327	0.034927376	0.004104368	0.227327437	0.951422462	0.01379699	0.388372518

CAP2	0.002201086	0.481330479	0.026058862	0.591525553	0.907298348	0.013930041	0.389785792
RGS10	0.195954637	0.967174384	0.000362803	0.327445885	0.657339672	0.013919198	0.389785792
SCNN1D	0.031141315	0.002732496	0.854654331	0.594324933	0.343245197	0.013942128	0.389785792
LOC782348	0.18345197	0.034463076	0.242026214	0.454424424	0.021403928	0.013972145	0.389960677
COL21A1	0.691978217	0.118289349	0.054252944	0.030574759	0.112385999	0.014210372	0.393265444
INPP5F	0.706465613	0.188028043	0.06735633	0.038834956	0.043640866	0.014150067	0.393265444
NSUN2	0.361589935	0.474974628	0.125865475	0.004822588	0.146036954	0.014188234	0.393265444
NT5C3A	0.642117463	0.989575418	0.007612858	0.011989927	0.262782784	0.014199035	0.393265444
SLCO2B1	0.399429081	0.994196996	0.028527286	0.004141823	0.322533658	0.014130909	0.393265444
LOC782456	0.216311851	0.028394231	0.09390688	0.057642835	0.461051307	0.014253971	0.393807947
GEMIN6	0.420932384	0.300133621	0.488081135	0.002544832	0.097952269	0.014280429	0.39387583
ACOD1	0.358449542	0.427325209	0.001315686	0.633968891	0.124714716	0.01463227	0.394165022
ASS1	0.092787584	0.233720739	0.039265701	0.075492917	0.24879324	0.014669216	0.394165022
CALM3	0.037569228	0.544319411	0.798720479	0.004520845	0.215418164	0.014615395	0.394165022
DNAJA1	0.442293928	0.38983969	0.971232973	0.000111127	0.853531601	0.014601206	0.394165022
KLHL25	0.139875349	0.845322457	0.389054463	0.040306256	0.008631003	0.014675207	0.394165022
LOC100848307	0.190623797	0.695428598	0.219478973	0.005177062	0.102933484	0.014364547	0.394165022
LOC101906024	0.877858157	0.473480588	0.002149379	0.991770098	0.01766779	0.014458136	0.394165022
LOC615278	0.090753784	0.963608265	0.047837174	0.448284838	0.008326433	0.014433642	0.394165022
LPAR3	0.516444286	0.254959692	0.089349583	0.002312242	0.5760316	0.014467915	0.394165022
LRRC46	0.498272961	0.228679098	0.333008145	0.001939334	0.214272475	0.014528871	0.394165022
MSL2	0.098456505	0.459673595	0.619148461	0.032985692	0.017284253	0.01465826	0.394165022
PPTC7	0.390916895	0.112794875	0.133092612	0.003491802	0.765172996	0.014473998	0.394165022
SAMSN1	0.64559182	0.929095629	0.00054857	0.097046072	0.497859451	0.014609767	0.394165022
SPN	0.412779814	0.75110337	0.000283704	0.186031653	0.951256419	0.014402738	0.394165022
THBS2	0.437395328	0.140963764	0.007437086	0.164687543	0.204339246	0.0143184	0.394165022
ZNF335	0.663704354	0.010283313	0.405274502	0.006511568	0.881023488	0.014591471	0.394165022
HAS1	0.934854091	0.024528827	0.001267011	0.887206657	0.626934859	0.014772319	0.396125048
MBLAC2	0.659784095	0.762840192	0.361868318	0.000671944	0.132495465	0.014806171	0.396385111
ACER2	0.039491215	0.858681891	0.005721603	0.525003429	0.159605634	0.014832458	0.396442119
ACLY	0.830902787	0.650594434	0.556808481	0.00045115	0.124029188	0.015190477	0.396617602
AKIP1	0.938946767	0.238127965	0.441327482	0.000183436	0.937248166	0.015264686	0.396617602
APLP1	0.004433263	0.549884639	0.438435694	0.022386941	0.708461343	0.015256667	0.396617602
APOC3	0.101176608	0.003247983	0.124514857	0.606861477	0.691308536	0.015386605	0.396617602
B4GALT7	0.225689842	0.965096447	0.044356899	0.118523764	0.014575725	0.015098005	0.396617602
C2CD2L	0.004901782	0.222276143	0.570807241	0.245293578	0.113876727	0.015510761	0.396617602
CCR1	0.338998137	0.554354082	0.000305868	0.298704271	0.950906901	0.01487489	0.396617602
CCSAP	0.038404945	0.906789132	0.541109122	0.933483205	0.000954545	0.015159133	0.396617602
CD1B	0.411491532	0.013184582	0.004574476	0.944487517	0.716730887	0.015164778	0.396617602
CIITA	0.776390926	0.666524327	0.123348424	0.002914175	0.093230001	0.015492585	0.396617602
DDX3X	0.304771676	0.119872214	0.129979638	0.004574389	0.780737342	0.015261275	0.396617602
DEDD2	0.412505211	0.694559978	0.974164403	0.000138871	0.445533708	0.015448492	0.396617602
GATA5	0.383984023	0.070586626	0.007530884	0.40921724	0.202421131	0.015230149	0.396617602
HBQ1	0.534050438	0.022617739	0.857790355	0.002758654	0.607257579	0.015501752	0.396617602
HTR2B	0.009106705	0.249782781	0.98147937	0.04467063	0.167545532	0.015109274	0.396617602

KLHL6	0.408127794	0.30523156	0.013021186	0.027370391	0.381196276	0.015239952	0.396617602
LOC112442226	0.052355345	0.197667891	0.259770297	0.019661676	0.313839501	0.015035637	0.396617602
LOC112448378	0.00442337	0.898444888	0.243573414	0.073859635	0.240081155	0.015385728	0.396617602
NUP98	0.359856538	0.498909826	0.888236551	0.000107721	0.997835945	0.0153714	0.396617602
P3H1	0.339554964	0.015336195	0.0068637	0.484854392	0.95899378	0.015054297	0.396617602
PENK	0.826598043	0.026779334	0.123643113	0.006273111	0.999287148	0.015380896	0.396617602
RPTOR	0.387091789	0.958789853	0.052481924	0.002162823	0.390994369	0.01496394	0.396617602
SAP30L	0.000441144	0.387662949	0.382232106	0.56429571	0.458998932	0.015244083	0.396617602
SLC5A3	0.238797542	0.436187019	0.341040889	0.000653888	0.734698498	0.01532573	0.396617602
SLC7A11	0.486417798	0.682436655	0.016229549	0.240243035	0.013325764	0.01543549	0.396617602
SPRN	0.03313959	0.043280234	0.047735679	0.48480184	0.523624015	0.015515721	0.396617602
TMED6	0.697912017	0.583670095	0.18955911	0.003619787	0.060340444	0.015204468	0.396617602
TRA2A	0.522980011	0.427481658	0.495519353	0.000690437	0.223819444	0.015358186	0.396617602
ADAMTS2	0.153030403	0.014329635	0.070364834	0.125145673	0.904519749	0.015567265	0.397316312
LOC616840	0.000995021	0.539382439	0.148720174	0.29483518	0.747968919	0.015648557	0.397536322
MCAT	0.334720546	0.334971535	0.003110124	0.068762259	0.732740477	0.015629315	0.397536322
SLFN11	0.569984403	0.405489751	0.002801327	0.098811654	0.274113814	0.015609398	0.397536322
TRAT1	0.368670916	0.640421553	0.203236566	0.002528123	0.146727889	0.015766755	0.39991997
CCL22	0.167134456	0.463257088	0.001357667	0.180596834	0.944277296	0.015842004	0.399974038
ESRP1	0.442072359	0.113777688	0.257691096	0.001430325	0.965560102	0.015826623	0.399974038
LOC107131975	0.373914108	0.00056282	0.689856275	0.360370294	0.342248348	0.015829722	0.399974038
TRIM7	0.191354981	0.624016883	0.030603665	0.182196588	0.02712082	0.015919766	0.401319932
KIF5C	0.380819894	0.092663219	0.007466842	0.119118678	0.577367006	0.015957853	0.401663067
EIF4EBP1	0.981001965	0.365395132	0.000365925	0.190769131	0.733030236	0.016088291	0.404326096
MKX	0.003829563	0.134814911	0.147107608	0.743845296	0.326802744	0.016159043	0.404913665
NPY1R	0.017837805	0.356195104	0.878164575	0.185547112	0.017836517	0.016161017	0.404913665
PCDHGA2	0.018034923	0.179964631	0.10245137	0.08569596	0.653304314	0.016249398	0.40650743
AVPI1	0.852914529	0.574737405	0.229998988	0.147330943	0.001126193	0.016302576	0.406758664
LRRC66	0.134762061	0.387339193	0.000845295	0.953911991	0.445199143	0.016320795	0.406758664
UGT8	0.124346157	0.617624393	0.385478297	0.095253512	0.006652843	0.016333798	0.406758664
CACNA1A	0.693038598	0.005158244	0.595218771	0.019944797	0.446177773	0.01643584	0.407258129
FN1	0.026242445	0.739203588	0.005122344	0.590530767	0.322835755	0.01644061	0.407258129
FNDC1	0.82542997	0.06633137	0.003071641	0.167065752	0.671008969	0.016387941	0.407258129
MED13	0.493122602	0.683856245	0.953824783	6.20E-05	0.954927938	0.016502752	0.407258129
OLFML1	0.627566351	0.149677492	0.04940277	0.004647078	0.881105194	0.01647403	0.407258129
WDR90	0.369323777	0.000666226	0.263933478	0.871276726	0.336394537	0.016493226	0.407258129
LOC112445031	0.249841579	0.004045008	0.023290425	0.963368223	0.844690075	0.016562757	0.408125234
ABRACL	0.829369863	0.173137321	0.000483319	0.319938672	0.871214102	0.016673473	0.408400541
DAPK2	0.47556785	0.512008721	0.312299853	0.000255894	0.993105303	0.016661976	0.408400541
SLX1A	0.485626736	0.058616648	0.109364236	0.085650093	0.07254348	0.016672462	0.408400541
TLL2	0.171130217	0.168023934	0.003993132	0.422941331	0.397699918	0.016655014	0.408400541
IL6	0.075865151	0.60393542	0.373575757	0.080960583	0.014113785	0.01679658	0.409786761
NLN	0.204229472	0.81547323	0.548281972	0.007009925	0.030527234	0.016786322	0.409786761
PPP1CB	0.642027998	0.032250268	0.07456417	0.013490415	0.939744128	0.016804978	0.409786761
ABCF2	0.67022755	0.825762597	0.165412069	0.232984672	0.000938638	0.017061492	0.410291917

AFAP1	0.580556442	0.982623391	0.0034376	0.222373489	0.047798883	0.017528347	0.410291917
ANTXRL	0.648657974	0.001220905	0.738293165	0.575023802	0.059293856	0.017012918	0.410291917
APC2	0.002897185	0.140403833	0.095417022	0.589093137	0.880875879	0.017130288	0.410291917
ARMCX4	0.527936905	0.506536312	0.004155571	0.090209172	0.201963021	0.017190242	0.410291917
ATP7A	0.260596665	0.182485719	0.131385366	0.006458504	0.519734179	0.01760056	0.410291917
BLNK	0.372746597	0.880290221	0.000103291	0.717375829	0.876406228	0.017788382	0.410291917
BMPER	0.022541685	0.776914105	0.055252665	0.412338039	0.051934689	0.017459268	0.410291917
C1QL3	0.238646935	0.431659963	0.790781968	0.509003008	0.000509714	0.017691379	0.410291917
CARD6	0.803983352	0.080676598	0.10705732	0.485139743	0.006068769	0.017302754	0.410291917
CCDC68	0.938832086	0.013689901	0.078645151	0.022083194	0.91582439	0.017301575	0.410291917
CEP170B	0.032561301	0.946208224	0.006392584	0.157980394	0.686461433	0.01781656	0.410291917
CPZ	0.206700971	0.089808817	0.005127135	0.462410005	0.485685813	0.017825735	0.410291917
DBF4B	0.820409694	0.187099088	0.213895501	0.001093697	0.592129773	0.017762751	0.410291917
DEXI	0.59009207	0.995024702	0.017342234	0.019915737	0.104222108	0.017691738	0.410291917
DHX58	0.886516317	0.062712846	0.03444989	0.083623048	0.132118486	0.017705514	0.410291917
DIP2B	0.913984477	0.575445459	0.008514958	0.086191781	0.054323337	0.017598387	0.410291917
DLX5	0.000624836	0.244965857	0.829380164	0.244749842	0.663548677	0.017400119	0.410291917
EAPP	0.16160587	0.590983095	0.567903615	0.013435236	0.027887859	0.017233262	0.410291917
EIF4G1	0.529451755	0.747786279	0.141931452	0.002997347	0.120303182	0.01719954	0.410291917
FGF10	0.472333061	0.063123487	0.748575693	0.051200379	0.017451544	0.017017123	0.410291917
FGFR1	0.368692985	0.955683055	0.000557285	0.18130162	0.561249775	0.017038983	0.410291917
ILVBL	0.416227333	0.494911748	0.245123269	0.006821669	0.059097357	0.017252778	0.410291917
LOC101905956	0.100422769	0.173313956	0.006375921	0.381423298	0.486829583	0.017394019	0.410291917
LOC518134	0.661716063	0.771059759	0.000387963	0.132523428	0.789354958	0.017450976	0.410291917
MCOLN3	0.54650406	0.520676059	0.000924198	0.659074655	0.116185184	0.017128478	0.410291917
MREG	0.313973972	0.493460612	0.000710418	0.509869978	0.373801076	0.017603321	0.410291917
MRPL15	0.472627104	0.165008532	0.07262006	0.039750249	0.094567793	0.017777695	0.410291917
NOL6	0.651007511	0.125642892	0.271107618	0.079947689	0.01143513	0.017205269	0.410291917
NTN1	0.377448117	0.261353474	0.032461082	0.006407471	0.997355349	0.0173136	0.410291917
POLR1B	0.974467365	0.100865933	0.163299926	0.263391809	0.004830459	0.017289693	0.410291917
RHEX	0.96050208	0.598223468	0.000439712	0.218851469	0.358604261	0.016951955	0.410291917
RPL22L1	0.316422616	0.054519792	0.23622972	0.382693098	0.01346011	0.017611334	0.410291917
RRBP1	0.131670654	0.330385001	0.006536211	0.097023107	0.736349389	0.017228672	0.410291917
RRP9	0.062210865	0.454855664	0.253100971	0.113924202	0.025858777	0.017671131	0.410291917
SERPINE1	0.120592705	0.005013795	0.33501916	0.28692067	0.365408107	0.01774852	0.410291917
SNTA1	0.832051023	0.201129049	0.128141414	0.288495393	0.003252313	0.017118822	0.410291917
SNX10	0.708846696	0.403197055	0.010537325	0.030490673	0.229204105	0.017642102	0.410291917
ST3GAL5	0.33584739	0.726766119	0.022305655	0.004702709	0.829126727	0.017743699	0.410291917
TOP1	0.583907605	0.406191632	0.913189331	0.000259797	0.375511231	0.0176884	0.410291917
TBL1XR1	0.567339873	0.04528745	0.005761067	0.538551139	0.269714618	0.01789542	0.41131895
SLC23A2	0.953348889	0.094065179	0.001186207	0.237508639	0.856877957	0.017977729	0.412632889
LIG3	0.13737924	0.964638475	0.001126617	0.928909378	0.156769585	0.018029142	0.413234975
ELP1	0.54768022	0.001368296	0.20892946	0.373763898	0.373177178	0.018082485	0.413303143
P2RX7	0.979597223	0.083116216	0.027821865	0.01463635	0.657499956	0.018061061	0.413303143
GPR162	0.972270613	0.272393794	0.00022727	0.414703176	0.877069227	0.018112472	0.413412758

ALKBH1	0.158869185	0.845006608	0.898159757	0.0002032	0.897851666	0.018170556	0.413748131
C18H19orf48	0.039395751	0.765536828	0.279276891	0.010745754	0.24323382	0.01817978	0.413748131
KY	0.083510743	0.539886402	0.022562476	0.641692025	0.033811891	0.018210985	0.413748131
PQBP1	0.264259746	0.424205031	0.501570097	0.00237365	0.165607819	0.018228012	0.413748131
LOC783577	0.231813273	0.377379327	0.22020745	0.008404676	0.137038252	0.01827498	0.414241303
WDFY4	0.566951951	0.769234432	0.000136034	0.401619361	0.940014004	0.018390158	0.416277069
ANKRD17	0.465570186	0.898324261	0.809277659	6.88E-05	0.969468071	0.018479463	0.416574814
CD226	0.191792725	0.358758281	0.196440234	0.008541869	0.194677599	0.018433436	0.416574814
PLEKHM2	0.783043035	0.998306613	0.445952263	0.000119731	0.54039626	0.018476564	0.416574814
GAL	0.622104523	0.491934027	0.019365716	0.015778601	0.241903012	0.018512269	0.416741901
CNOT1	0.321445719	0.767356579	0.773332441	0.000120636	0.9911649	0.01861421	0.418462742
LOC782177	0.078716458	0.274311193	0.013375068	0.794732194	0.099749105	0.018661179	0.418472138
PTPN6	0.697844075	0.77643215	0.00109954	0.05442011	0.707860564	0.018691127	0.418472138
SIX4	0.80670566	0.08849847	0.039639377	0.008349114	0.969652925	0.018669754	0.418472138
LOC107132537	0.038068097	0.041136465	0.038224358	0.887064357	0.434482292	0.018756467	0.419362905
LNX2	0.41871122	0.98847555	0.659758636	0.000300823	0.281511013	0.01878574	0.419445952
BRI3BP	0.551710357	0.367496673	0.712563254	0.001288557	0.12491801	0.018856275	0.419878337
LOC112442023	0.144051243	0.006166049	0.453358764	0.186696198	0.309267092	0.018853915	0.419878337
NPTX1	0.652825961	0.182135154	0.000739586	0.666553495	0.39997097	0.018958486	0.421527099
PBXIP1	0.070456773	0.287284753	0.335963787	0.877821987	0.003950676	0.019033062	0.421527099
STPG4	0.017755361	0.125234581	0.051700242	0.31896324	0.642030237	0.019010819	0.421527099
TOB2	0.472348865	0.697488209	0.617798221	0.001963319	0.058916589	0.01901176	0.421527099
ADM5	0.42946043	0.124986212	0.011358798	0.082433857	0.471320581	0.019089541	0.422208168
DAZAP2	0.283272962	0.001754476	0.380765854	0.126793019	0.991941621	0.019149644	0.422398945
ICAM3	0.629190657	0.722020506	0.014463367	0.088513347	0.040918257	0.019147744	0.422398945
C7H19orf25	0.290876387	0.79518565	0.127737273	0.00098977	0.821795727	0.019273227	0.424554269
ZNF404	0.000340403	0.949247197	0.543883885	0.248271577	0.552054969	0.019302613	0.424631619
ZFX	0.128985894	0.025938867	0.410206244	0.021032391	0.841645925	0.019412999	0.426488245
RAPGEFL1	0.141396219	0.033727033	0.109835097	0.460982918	0.100870349	0.019445461	0.426630292
IGF2BP3	0.401708783	0.558363404	0.000714088	0.989612456	0.154925722	0.019551758	0.427248867
LAMB3	0.083919053	0.51917796	0.018703286	0.03995662	0.753955639	0.019547587	0.427248867
ZMAT1	0.079405139	0.559301231	0.888555378	0.013813196	0.044986973	0.019533555	0.427248867
LOC101906870	0.455135185	0.312139163	0.107683487	0.006591066	0.245610982	0.019661881	0.428514125
WAS	0.229426188	0.4921607	0.006677295	0.187486832	0.175129972	0.019657087	0.428514125
ALKBH8	0.000267404	0.906810971	0.746122134	0.283185295	0.487328849	0.019768786	0.429044245
KLHL17	0.344793588	0.02281567	0.105651935	0.035488155	0.844912396	0.019743931	0.429044245
LOC100336868	0.025616963	0.046335938	0.202222604	0.115354053	0.901100958	0.019759531	0.429044245
RASSF4	0.376486376	0.518407576	0.000441193	0.313382925	0.926810716	0.01979078	0.429044245
ALG5	0.419390715	0.156762402	0.78336555	0.002303208	0.216585761	0.02014685	0.429209733
CHORDC1	0.203168067	0.07388526	0.953994583	0.002510382	0.704446803	0.019955731	0.429209733
CLK4	0.11124245	0.566369494	0.629241385	0.002823441	0.228746127	0.020101743	0.429209733
FABP1	0.006517124	0.254961292	0.890456168	0.086010283	0.202911532	0.020214991	0.429209733
FCGR1A	0.513253295	0.841460768	0.001011704	0.097376707	0.608165999	0.020242675	0.429209733
GRIK2	0.392593821	0.01072356	0.060870645	0.183041936	0.542184481	0.020011927	0.429209733
ITIH4	0.040891764	0.28111599	0.005181717	0.812548561	0.535578696	0.020266518	0.429209733

LOC112445943	0.024980158	0.693841863	0.833925179	0.033145064	0.054224701	0.020295336	0.429209733
LPAR6	0.075753438	0.919245547	0.01136873	0.037112272	0.878421607	0.020207787	0.429209733
NUDT9	0.669957799	0.143207382	0.002944776	0.16176163	0.549967971	0.019856303	0.429209733
OAS2	0.777074289	0.186183511	0.068456121	0.162794713	0.015774153	0.020012366	0.429209733
POT1	0.028939807	0.833574376	0.048032572	0.031577414	0.693876744	0.019988914	0.429209733
RYBP	0.705839529	0.100420817	0.288588946	0.001798173	0.686887577	0.019924638	0.429209733
SELL	0.498701689	0.215079053	0.016279398	0.224783102	0.064176589	0.01988483	0.429209733
SPRY2	0.341949297	0.00175418	0.75464433	0.084566187	0.672792852	0.020179734	0.429209733
TAOK3	0.824684824	0.94787969	0.030320959	0.010388181	0.104137873	0.020120542	0.429209733
TMBIM7	0.455452618	0.120253376	0.401243464	0.001514326	0.779143916	0.020270159	0.429209733
TMEM203	0.452420533	0.072443078	0.089569471	0.010613551	0.829773304	0.020231027	0.429209733
USPL1	0.46351857	0.761212429	0.994683775	7.62E-05	0.969955012	0.02028224	0.429209733
SLC29A2	0.689109054	0.950892652	0.016545082	0.005250598	0.458960027	0.020372073	0.430278115
GDF6	0.787500171	0.031624178	0.006790062	0.614780553	0.252024545	0.02041048	0.430535193
AKR1B1	0.056375073	0.107856007	0.019942295	0.307393504	0.710064899	0.020547687	0.430987541
DFFB	0.023597445	0.008315281	0.275166342	0.819107235	0.60738272	0.020750438	0.430987541
DGKI	0.202330601	0.386710079	0.006473713	0.057377814	0.90569015	0.020473391	0.430987541
FAIM2	0.602367376	0.974158973	0.023936402	0.213628165	0.008827068	0.020557899	0.430987541
FRRS1L	0.74180377	0.174999634	0.001985887	0.79418571	0.131283403	0.020759047	0.430987541
GPR137B	0.535136311	0.239563724	0.000948493	0.588668614	0.371359407	0.02060685	0.430987541
MYH3	0.304772007	0.162972205	0.001597066	0.348356959	0.965901458	0.020662968	0.430987541
NEU3	0.972113056	0.08659498	0.336501781	0.047573231	0.019966617	0.020773332	0.430987541
PSAP	0.220892318	0.558831258	0.000409532	0.632927611	0.826711342	0.020540088	0.430987541
RAC2	0.210046711	0.588612407	0.017737322	0.082884217	0.147446945	0.020718855	0.430987541
TIMM44	0.639554134	0.662284394	0.399178262	0.000553702	0.284219036	0.020620466	0.430987541
TMEM236	0.741203571	0.596841545	0.004061857	0.175907339	0.08494627	0.020744401	0.430987541
TREM2	0.352747825	0.437565151	0.000831272	0.559041676	0.37021504	0.020593146	0.430987541
BAALC	0.40841516	0.049907083	0.728276714	0.195423052	0.009467649	0.021057324	0.435228889
RGS4	0.07768235	0.76982075	0.001542817	0.332786401	0.892850904	0.021031444	0.435228889
ZNF19	0.435949809	0.689617418	0.177096212	0.003241972	0.159027871	0.02104957	0.435228889
C25H16orf54	0.627636202	0.248595922	0.012420378	0.033030626	0.433563898	0.021202972	0.435336314
CFI	0.011244558	0.100151183	0.094714967	0.665141675	0.395304002	0.021350455	0.435336314
LOC781001	0.341098988	0.001873531	0.224186195	0.220407902	0.873117686	0.021110985	0.435336314
PGF	0.090834937	0.300656218	0.068404543	0.01694828	0.883211851	0.021309444	0.435336314
PTGS2	0.349431448	0.032877711	0.06983882	0.256910151	0.136094226	0.021354319	0.435336314
RABGEF1	0.694966612	0.044602675	0.074688429	0.04560263	0.263787615	0.021251935	0.435336314
SCML4	0.377542665	0.040000743	0.683703261	0.011064065	0.243682037	0.021246035	0.435336314
SIT1	0.050129538	0.311472463	0.518297213	0.719575014	0.004798338	0.021298443	0.435336314
STXBP3	0.057527118	0.621974731	0.076402053	0.042282766	0.242081471	0.021318418	0.435336314
UBD	0.007933635	0.74153807	0.356158017	0.033792281	0.392573519	0.021224931	0.435336314
UNC5C	0.003070839	0.210480276	0.153823852	0.727693283	0.381248894	0.021117378	0.435336314
ABCF3	0.420456855	0.714954229	0.30987781	0.001939267	0.155826904	0.021402732	0.435781927
LOC101908206	0.720708487	0.784170451	0.000134637	0.833393864	0.445205409	0.021444282	0.436086878
EVI2B	0.787206315	0.926582626	0.00104995	0.10203679	0.362422713	0.021488935	0.436454099
LMOD1	0.104420367	0.277712941	0.481980029	0.269115501	0.007563794	0.021553575	0.437225852

CYB5D2	0.024398674	0.344821909	0.315556705	0.073566573	0.146244723	0.021609688	0.437283104
XRCC5	0.043360368	0.19409991	0.513170404	0.011899819	0.555635747	0.021606772	0.437283104
ARHGDIA	0.009318142	0.422745285	0.466739383	0.248116027	0.063534438	0.021819123	0.439920108
CAPN8	0.275919514	0.007716666	0.041891324	0.562226616	0.578652245	0.021836322	0.439920108
NFE2L2	0.749100649	0.642856663	0.013474473	0.006025985	0.7426768	0.02184723	0.439920108
SURF6	0.440898212	0.200474229	0.082219887	0.182102212	0.021863087	0.021794407	0.439920108
CRABP1	0.962724341	0.340821749	0.076872535	0.022501922	0.051436564	0.021923612	0.440377479
LOC618737	0.338566983	0.356749782	0.009402438	0.089954242	0.285684233	0.021919108	0.440377479
AQP4	0.802207747	0.018424322	0.105760867	0.08074905	0.235684456	0.02219753	0.440488102
C2CD4B	0.964507188	0.051429471	0.006168789	0.140345179	0.68391319	0.022011134	0.440488102
CDC16	0.697541594	0.97154887	0.834512373	0.00077031	0.068064049	0.022149723	0.440488102
CTNND2	0.102435416	0.437785628	0.005497023	0.329855826	0.363423397	0.022100202	0.440488102
GJC3	0.634515967	0.012016115	0.213413228	0.098104376	0.185699351	0.022145548	0.440488102
LOC789035	0.006567004	0.177293792	0.988814915	0.120802041	0.211803453	0.022053518	0.440488102
PAK2	0.521147873	0.052338533	0.133679651	0.008612637	0.93700602	0.022038231	0.440488102
SLC32A1	0.990150019	0.020886473	0.928273124	0.003832672	0.398321815	0.021979799	0.440488102
TENT5C	0.91127493	0.849677725	0.813645259	0.000112084	0.420718812	0.022177459	0.440488102
TNNT2	0.324923798	0.139765274	0.102341777	0.007805868	0.818221658	0.022165694	0.440488102
CHST13	0.542427854	0.824829171	0.008114529	0.016823745	0.48955894	0.022272599	0.441443991
HCN4	0.014046239	0.779682126	0.723565695	0.014467036	0.261541289	0.02231245	0.441700392
CSTB	0.287800437	0.469354008	0.001305335	0.187675417	0.909590197	0.022369769	0.442301549
PSRC1	0.918982003	0.712107168	0.014910681	0.004336733	0.713928224	0.022424116	0.442842566
CD4	0.385771853	0.089917058	0.382135372	0.358512893	0.006468048	0.022680532	0.445660955
CKS2	0.930081194	0.004228895	0.053832007	0.666508402	0.217012685	0.02262599	0.445660955
EDIL3	0.356616496	0.183972402	0.222337095	0.066803893	0.031544456	0.022681321	0.445660955
LOC617141	0.614359059	0.00626544	0.01529392	0.607735974	0.859988421	0.022695393	0.445660955
POPDC2	0.022102975	0.279174981	0.976609159	0.062799848	0.081339867	0.022702612	0.445660955
ACTB	0.075990906	0.001676473	0.529913205	0.654973541	0.704840985	0.022887957	0.446762905
HDAC10	0.273730879	0.126261184	0.681023535	0.001452637	0.912740611	0.02290815	0.446762905
LOC781022	0.325526766	0.049533214	0.004861357	0.503584345	0.789267746	0.022883085	0.446762905
MEGF8	0.746543889	0.265126031	0.088773231	0.002688263	0.663693038	0.022976534	0.446762905
PLEKHJ1	0.446086432	0.010058355	0.212861858	0.513547452	0.063743467	0.022935912	0.446762905
SH3TC1	0.473692329	0.323765315	0.008709551	0.030274134	0.774249553	0.022957281	0.446762905
SMUG1	0.392569874	0.014157719	0.725711919	0.994847845	0.007807049	0.022965727	0.446762905
ZNF428	0.375157229	0.643551856	0.000244897	0.595694108	0.888510563	0.022950022	0.446762905
LIMK2	0.586243209	0.462939088	0.013721913	0.010029592	0.845537022	0.023088219	0.447596127
LOC107132724	0.007913399	0.202148404	0.108685697	0.377721032	0.480814593	0.023085507	0.447596127
SMTNL2	0.777862106	0.921548655	0.000417014	0.167981711	0.630594235	0.023128482	0.447596127
WASF1	0.00468913	0.544917349	0.393796185	0.485456467	0.064758257	0.02311296	0.447596127
LOC782675	0.004637095	0.350019043	0.515774764	0.34081337	0.112041914	0.023272802	0.449858612
LOC781977	0.062287902	0.348043197	0.002115829	0.8032211	0.869654391	0.023308183	0.450012465
ANPEP	0.086082171	0.39325556	0.320993771	0.016868297	0.176236328	0.023433728	0.451904707
BID	0.369010849	0.380908675	0.002612978	0.208226539	0.423502797	0.023474036	0.452150716
ALG11	0.628803915	0.183906629	0.003439022	0.200370623	0.412908736	0.023718227	0.456086468
ZNF311	0.010079378	0.278374138	0.697243359	0.02369635	0.710470169	0.023733949	0.456086468

PMP2	0.062835113	0.844559782	0.016104031	0.06842998	0.565229376	0.023790097	0.456097296
TNNI2	0.962209906	0.227110509	0.035534976	0.009179307	0.463439993	0.023780323	0.456097296
BMP1	0.412628373	0.024519356	0.005437268	0.673151997	0.897324012	0.0238719	0.456598782
PRPF19	0.370684535	0.301184804	0.037636459	0.390303478	0.020246518	0.023860645	0.456598782
NCF2	0.546904702	0.858581794	0.000154354	0.467736993	0.98339889	0.023923549	0.457053982
RBM39	0.227809669	0.620962025	0.708154172	0.002247593	0.149244503	0.024048057	0.458898454
ADAM19	0.857563902	0.471449831	0.000681665	0.992922021	0.124608358	0.024279737	0.459578738
LOC100300806	0.636015275	0.282056069	0.001478811	0.178267539	0.720444469	0.02426704	0.459578738
M6PR	0.287601412	0.049778287	0.007116292	0.408238518	0.818962567	0.024262437	0.459578738
NFYB	0.181420601	0.948190361	0.741781427	0.000394179	0.676506007	0.024246352	0.459578738
PLEKHA6	0.618889238	0.003570196	0.4052879	0.09115474	0.416831208	0.024245803	0.459578738
VSTM2L	0.574955724	0.05054717	0.030280419	0.061843642	0.624028894	0.024216002	0.459578738
VWCE	0.52754861	0.718333155	0.003418865	0.367456076	0.071405967	0.024231255	0.459578738
ABT1	0.378765269	0.011674802	0.183671135	0.485039982	0.087079508	0.024375827	0.460526418
PDZD3	0.035618735	0.392259734	0.880844705	0.023953757	0.116531113	0.024398371	0.460526418
TMED10	0.697397692	0.000895624	0.345733184	0.2100394	0.758129968	0.02441399	0.460526418
JAK1	0.974245862	0.610187437	0.001820218	0.032045824	0.996353041	0.024489511	0.461420621
GRN	0.480292503	0.743788736	0.000429675	0.383109375	0.59242918	0.0246233	0.463409384
RENBP	0.683927384	0.549858248	0.005388287	0.021217881	0.812119186	0.024659639	0.463561659
MCM8	0.00369441	0.3738444	0.058369881	0.568000792	0.765374634	0.024719702	0.463867585
POLG2	0.036906553	0.257693552	0.188023516	0.231415064	0.084757678	0.024732444	0.463867585
LOC112445011	0.075061859	0.015124755	0.989114872	0.149598532	0.210407774	0.024857697	0.465684558
CNDP2	0.171473279	0.285530994	0.000978933	0.886826001	0.838792864	0.024998539	0.467117001
LOC101907405	0.991031351	0.004636149	0.488094889	0.054994318	0.289143857	0.025001741	0.467117001
TARBP1	0.450573813	0.043737319	0.006772269	0.431142104	0.620413243	0.025019551	0.467117001
NAAA	0.6873665	0.485067487	0.007195047	0.019825611	0.756357445	0.025145038	0.46892638
FBXO28	0.469727495	0.0021966	0.125519115	0.287532314	0.970573483	0.025222495	0.469304274
PPP1R14B	0.076414567	0.589076959	0.447237994	0.052315005	0.034279259	0.025204389	0.469304274
CBL	0.928846086	0.219939648	0.034400413	0.007445382	0.692378932	0.025261226	0.469492616
BEND6	0.321622611	0.944821617	0.035926618	0.008726663	0.382314704	0.025350305	0.470391156
INHA	0.787499787	0.003765182	0.34897319	0.126263231	0.27906902	0.025366899	0.470391156
LOC107131704	0.389921679	0.007807721	0.507089256	0.989493228	0.024000959	0.025459017	0.471049398
MED21	0.402130581	0.422741264	0.009274445	0.092204648	0.252647454	0.025488506	0.471049398
ZNF469	0.97305436	0.040891708	0.02082893	0.124740689	0.354765595	0.025465198	0.471049398
ARC	0.03283351	0.079076287	0.915793742	0.247403064	0.062632353	0.025540965	0.471487942
DNAJC2	0.462289233	0.871487613	0.312829122	0.459881817	0.000637294	0.025583363	0.471739957
TXLNA	0.606070467	0.911830182	0.276733577	0.001096744	0.221935568	0.025712974	0.473597783
LARS2	0.761720217	0.779260689	0.360792813	0.000575903	0.303332775	0.025797079	0.474614188
LOC783680	0.267386257	0.416940863	0.015777471	0.024563616	0.867782817	0.025833904	0.47475947
MITF	0.931947276	0.956041184	0.000458363	0.788380137	0.116744679	0.025876463	0.475009664
CA4	0.123574904	0.869579445	0.901493954	0.014975485	0.025961445	0.025909982	0.475093533
HBB	0.251917872	0.231105279	0.223897373	0.028628972	0.101530074	0.026011577	0.47536301
HSD11B1	0.05276423	0.511564707	0.006206037	0.440712052	0.512601747	0.025990935	0.47536301
SIRPA	0.009027148	0.822382242	0.749723696	0.330612322	0.020522696	0.025955423	0.47536301
CADM1	0.612524166	0.310966949	0.023133791	0.422427474	0.020525743	0.02615319	0.475831486

CD84	0.503403281	0.283697863	0.000857939	0.41543074	0.7496041	0.026130589	0.475831486
FBXL7	0.12211497	0.524673793	0.634103127	0.002498779	0.375794386	0.026128122	0.475831486
PHACTR4	0.089887276	0.537223602	0.307208014	0.487552314	0.005275272	0.026130303	0.475831486
GPATCH4	0.241888436	0.885738374	0.028954253	0.068947226	0.090029759	0.026286982	0.477736057
CX3CL1	0.123180965	0.306430543	0.03573788	0.504585969	0.057214746	0.026481358	0.479146151
GNA12	0.233711777	0.49924241	0.021877589	0.073395724	0.207387461	0.026441565	0.479146151
INO80B	0.017626519	0.308306715	0.498880012	0.016098675	0.890226995	0.026441237	0.479146151
NOP14	0.825819077	0.149469672	0.096402371	0.021919326	0.149135438	0.026460961	0.479146151
TBC1D32	0.000936547	0.152653018	0.963665937	0.285203821	0.99610866	0.026567892	0.480182459
SRGAP3	0.000592092	0.660137065	0.961604243	0.201603071	0.518093749	0.026619795	0.480591262
USP4	0.807044312	0.114334174	0.004672706	0.864848327	0.105484445	0.026653526	0.480671441
KCNH1	0.174864142	0.8067454	0.232144028	0.011432785	0.106574396	0.026903474	0.481153383
LOC101902211	0.044328409	0.356949342	0.457536185	0.026733637	0.206650002	0.02694412	0.481153383
METTL1	0.540800738	0.52776989	0.101465037	0.232280376	0.005915485	0.026855216	0.481153383
MTPAP	0.061069323	0.533691789	0.018075703	0.089451929	0.7538854	0.026827177	0.481153383
RDH13	0.342268737	0.383466847	0.541340517	0.021263411	0.026347327	0.026860604	0.481153383
RGS14	0.131750279	0.412203528	0.026083876	0.169283491	0.166785244	0.026944119	0.481153383
SEC16B	0.371572411	0.083674842	0.008245808	0.411496741	0.377833064	0.026884872	0.481153383
STEAP2	0.111045794	0.39502353	0.006343877	0.445711692	0.322172547	0.026928578	0.481153383
TNPO2	0.875460271	0.76331357	0.036052076	0.004970368	0.330871647	0.026779545	0.481153383
IPO7	0.602323258	0.021630597	0.187442409	0.051192263	0.321350976	0.027022608	0.482030449
AP2B1	0.47615904	0.143393709	0.17845425	0.007971677	0.417898428	0.027204432	0.482137083
KIAA1191	0.191647351	0.30623315	0.101561527	0.008600973	0.793102439	0.027234238	0.482137083
LOC104969384	0.405471782	0.095088941	0.048264969	0.081322816	0.267912327	0.027184013	0.482137083
SCARB2	0.880384599	0.226562424	0.0003172	0.665355073	0.959793399	0.027122938	0.482137083
SH2D1B	0.865254768	0.483478021	0.000831898	0.296075501	0.394239942	0.027217644	0.482137083
SLC38A4	0.08349209	0.025897306	0.160570733	0.565583734	0.205112821	0.027067399	0.482137083
SLC6A6	0.113565758	0.320079533	0.003048346	0.380794263	0.958565225	0.027141479	0.482137083
HECTD3	0.121284184	0.014385907	0.917692186	0.032090755	0.795249024	0.027322426	0.482248839
LOC781710	0.247864427	0.327938343	0.089404414	0.076093375	0.073920169	0.027328708	0.482248839
VSTM1	0.298500235	0.242205142	0.001851877	0.425248764	0.716616093	0.027295794	0.482248839
SLC37A2	0.597703574	0.879034712	0.000215998	0.38061135	0.952611024	0.027446022	0.483798785
SFN	0.227612692	0.002263873	0.81556972	0.449432214	0.218222944	0.027476239	0.483811764
ACTR2	0.660554444	0.01592303	0.034174958	0.271760381	0.423672773	0.02754968	0.484274758
ARHGAP20	0.342940212	0.002747669	0.946176988	0.058968722	0.787716991	0.027561552	0.484274758
PIP4K2A	0.829778517	0.921106246	0.036762873	0.002042546	0.724562084	0.027635068	0.48504717
CD63	0.259547255	0.084615628	0.088911955	0.027281956	0.784659519	0.027728391	0.485758491
GCHFR	0.714977713	0.628799289	0.043924725	0.026679081	0.079369558	0.027734794	0.485758491
SIRPB2	0.818278934	0.68006302	0.005507714	0.031968461	0.428992267	0.027828406	0.486878441
MYCT1	0.610469211	0.41584401	0.030021151	0.005817839	0.952298919	0.027910168	0.487788882
LRRC47	0.757609669	0.181304198	0.012438367	0.18775693	0.132373094	0.028012748	0.489060858
OAZ2	0.787459897	0.563433232	0.210946315	0.000863123	0.529308073	0.02813902	0.490743314
LOC101905029	0.14150135	0.000951909	0.877750021	0.37716321	0.965886896	0.028272005	0.492539136
SGPL1	0.694158481	0.204161821	0.028946461	0.01535761	0.688998164	0.028415316	0.494510869
MBNL1	0.091315203	0.962421399	0.039754903	0.033566547	0.374626394	0.028638026	0.494714369

SERPINE2	0.285245756	0.198704043	0.06178483	0.091577839	0.136714793	0.028600022	0.494714369
SGK2	0.420103273	0.490140313	0.068077615	0.003777398	0.825547559	0.028544707	0.494714369
SLITRK5	0.708069958	0.067033103	0.946113948	0.147326308	0.006580088	0.028468387	0.494714369
TP53INP1	0.416680145	0.63855519	0.00154492	0.493214754	0.216535411	0.028623855	0.494714369
TSSC4	0.274739003	0.710970823	0.469233943	0.01534579	0.031179205	0.028604379	0.494714369
ZNF182	0.606542631	0.140747374	0.00586869	0.713817941	0.122315355	0.028557295	0.494714369
PALB2	0.313093942	0.002453978	0.373437964	0.746878546	0.205965435	0.028723731	0.494721127
RAB43	0.468106705	0.996491891	0.003572078	0.043618753	0.60617678	0.028689657	0.494721127
WARS	0.083901865	0.805029253	0.080975903	0.095756653	0.084298474	0.028728855	0.494721127
KCNE4	0.012135097	0.285165846	0.388012547	0.040902496	0.806438088	0.028788005	0.495220073
PRRT4	0.712645908	0.241768463	0.430796457	0.006280256	0.095762401	0.028934713	0.497222587
DNAJC16	0.330216498	0.132762704	0.898166639	0.082623546	0.013793138	0.029033209	0.497257184
MARCKS	0.179881816	0.870394365	0.004514237	0.071412391	0.8902432	0.029057927	0.497257184
PIPOX	0.370059842	0.847457508	0.042531094	0.005450915	0.617020593	0.029027449	0.497257184
PKIB	0.530624417	0.167452029	0.056847755	0.112810109	0.078590483	0.028994748	0.497257184
NUDT18	0.919657664	0.120737825	0.590811845	0.002624921	0.261512569	0.029099409	0.497448329
EIF2S1	0.343149281	0.016692397	0.117604741	0.340105904	0.197905263	0.029228434	0.497840797
MRPS21	0.884150214	0.355443615	0.639087129	0.000458583	0.491444439	0.029195872	0.497840797
PRG4	0.361159593	0.002324615	0.437835631	0.535060377	0.229804171	0.029168596	0.497840797
SERPINI1	0.117029724	0.253084982	0.918839206	0.001851985	0.900346199	0.02924371	0.497840797
C2H2orf72	0.308786838	0.083812834	0.033041729	0.703270226	0.076673223	0.029547759	0.498544928
CDH2	0.302805022	0.597186688	0.00127218	0.520812269	0.387217861	0.029664939	0.498544928
CORO1A	0.076663786	0.994463811	0.019210307	0.312229036	0.101435035	0.029661248	0.498544928
CRIP1	0.046987109	0.659402412	0.536215196	0.008676913	0.321199891	0.029627478	0.498544928
KIAA1217	0.450518436	0.27188623	0.077782189	0.033439554	0.144217305	0.029480115	0.498544928
LOC101906178	0.722059303	0.130595157	0.613564234	0.000837557	0.943027748	0.029376787	0.498544928
LOC112441884	0.017495602	0.399127384	0.232605228	0.300103692	0.093897033	0.029406729	0.498544928
LOC112449547	0.673513831	0.769120264	0.010063031	0.724970431	0.012260979	0.029640967	0.498544928
MMP28	0.535563249	0.904824077	0.499019458	0.000324524	0.58506189	0.029466242	0.498544928
PPDPF	0.428775778	0.117732178	0.005288572	0.234928171	0.732450368	0.029476579	0.498544928
SHISA4	0.119329312	0.231761591	0.025498639	0.074100322	0.885840932	0.029621856	0.498544928
TMEM126B	0.36972313	0.534106152	0.85819545	0.418762931	0.000645445	0.029421382	0.498544928
YJU2	0.542421682	0.053334627	0.746494776	0.002521967	0.852485702	0.029679995	0.498544928
EMILIN1	0.025191412	0.848200272	0.040043171	0.205430936	0.265164318	0.029753395	0.499266834
OSBPL11	0.977856583	0.122077926	0.014986042	0.047326801	0.553413161	0.029855131	0.500462264
DIAPH3	0.695043889	0.015440125	0.021152164	0.215130418	0.964096535	0.029947785	0.501503159
CCAR1	0.332842117	0.115100143	0.599655368	0.00241283	0.851932751	0.030006051	0.50169508
LOC782264	0.580918978	0.991803755	0.003665404	0.027535777	0.813942409	0.030050957	0.50169508
PPIF	0.758911966	0.402597497	0.722657339	0.008846936	0.024195072	0.030022413	0.50169508
GTPBP6	0.848446538	0.901962516	0.145473126	0.00165877	0.256756678	0.03008445	0.501743812
LENG9	0.4316469	0.188936824	0.011252691	0.082007654	0.631655321	0.030135115	0.502078546
TNFRSF18	0.067883881	0.905049483	0.003396923	0.720708131	0.316691687	0.030174744	0.502228928
AP4E1	0.007906458	0.388425171	0.043175762	0.492367617	0.738837942	0.030419859	0.504459611
CCR2	0.289320985	0.726811966	0.245182577	0.046961156	0.020060917	0.030556234	0.504459611
CYTH4	0.545163462	0.915967879	0.005413696	0.050607893	0.35564247	0.030590336	0.504459611

ECD	0.959070392	0.487810312	0.780294706	0.00551996	0.024177152	0.030616158	0.504459611
EIF4A3	0.317026129	0.017560601	0.041923671	0.705027516	0.292385848	0.030369678	0.504459611
GAS2L3	0.449060961	0.056689764	0.068839904	0.341428097	0.081261691	0.030576838	0.504459611
PLA2G7	0.66989528	0.583330821	0.002726798	0.046196231	0.981935617	0.03046037	0.504459611
SDK2	0.871932975	0.071107811	0.008651643	0.123351453	0.731428312	0.030485281	0.504459611
SERPINB6	0.14620425	0.713963834	0.028029144	0.028343166	0.582692115	0.030454453	0.504459611
TELO2	0.748671529	0.014377379	0.25404726	0.021284788	0.832208458	0.030502118	0.504459611
DNAJC11	0.69101663	0.680750204	0.489197643	0.002446661	0.087192994	0.030767118	0.505813991
DOLK	0.48494755	0.366325581	0.260824312	0.065020128	0.016322394	0.030800257	0.505813991
PEG3	0.318900482	0.075465326	0.691509816	0.046645881	0.0634155	0.030821643	0.505813991
VAT1	0.159697938	0.39041097	0.018219777	0.257681453	0.16786377	0.030784851	0.505813991
LOC101906312	0.640851423	0.58812469	0.000608986	0.3883856	0.55433173	0.030897503	0.506552375
LOC112447626	0.001777139	0.135985357	0.80825613	0.272421663	0.934763676	0.031027849	0.507675011
VSIG4	0.296850952	0.432539033	0.002374758	0.369852645	0.440562852	0.031005485	0.507675011
ICOSLG	0.027158269	0.611358321	0.559735937	0.008714273	0.61528079	0.031063729	0.507755829
APH1B	0.902284603	0.26970876	0.640359863	0.32278644	0.001007622	0.031405777	0.507783449
C5AR1	0.2830934	0.536282003	0.0598716	0.013395632	0.414491296	0.031319679	0.507783449
CLEC4A	0.286611942	0.165026819	0.004627649	0.519100033	0.442215697	0.031230332	0.507783449
HEBP2	0.851239029	0.678453422	0.273114221	0.000499123	0.636005993	0.031160542	0.507783449
HEY2	0.147233945	0.261768663	0.474805766	0.222649937	0.01227904	0.031144089	0.507783449
RGL3	0.005774853	0.068466971	0.572028446	0.948525167	0.233879195	0.031201912	0.507783449
SCRG1	0.654471189	0.560065779	0.704685715	0.00019811	0.988882907	0.031373289	0.507783449
SLC25A15	0.959822246	0.554722848	0.183750807	0.00069584	0.741990045	0.031337376	0.507783449
SLC2A10	0.394219222	0.946966683	0.006364305	0.06126096	0.346425091	0.031300851	0.507783449
TMEM17	0.001215968	0.139318316	0.451959763	0.976951287	0.677497843	0.031402769	0.507783449
VPS18	0.106927415	0.748686115	0.288788826	0.423490667	0.005139272	0.03125905	0.507783449
BST1	0.88986361	0.103201275	0.002021261	0.731202811	0.375130314	0.031497793	0.50829293
CD3EAP	0.144383324	0.064378462	0.119631659	0.629103994	0.072992512	0.031556322	0.50829293
FMO3	0.162537746	0.480423797	0.111887963	0.020467225	0.286055162	0.031592151	0.50829293
MSR1	0.673043833	0.599210711	0.00017653	0.871791769	0.821752394	0.031532505	0.50829293
TOMM40	0.443695048	0.690374311	0.720808838	0.003198033	0.072349129	0.03156572	0.50829293
LPIN1	0.986411252	0.060911591	0.012369069	0.556475776	0.123909546	0.031628428	0.508378193
EPCAM	0.3383907	0.079118881	0.646309566	0.015188322	0.196445839	0.031780751	0.509195541
FICD	0.032094283	0.064901323	0.087394799	0.592143997	0.480945717	0.031865445	0.509195541
GNAZ	0.007147943	0.192689333	0.168970331	0.3605092	0.617344626	0.031846733	0.509195541
MPV17L2	0.240780805	0.341757263	0.041241035	0.14217085	0.107238025	0.031824841	0.509195541
SLC11A1	0.972684826	0.705048181	0.000262874	0.734302631	0.391512711	0.031859265	0.509195541
ZNF711	0.038799966	0.083469605	0.107367693	0.228538869	0.648120658	0.031731753	0.509195541
MMP9	0.957548727	0.228897429	0.000725771	0.636056115	0.513923512	0.03192703	0.509683355
DOK7	0.087941133	0.398433021	0.801386915	0.003590908	0.517041663	0.031980176	0.510035638
HDLBP	0.190923246	0.40882618	0.12378007	0.137919954	0.039242379	0.032042261	0.510164297
SLC1A2	0.507737154	0.781889491	0.001435418	0.394891499	0.232467549	0.032050417	0.510164297
LOC101904976	0.153924285	0.100025355	0.271997851	0.033157937	0.378473085	0.032145473	0.510450274
OTUD1	0.842191349	0.003433874	0.208721257	0.126282201	0.691028872	0.0321928	0.510450274
PAN2	0.329963898	0.000452642	0.635730121	0.991200307	0.5583164	0.032142159	0.510450274

SLIT1	0.241678933	0.297418236	0.638674636	0.006653573	0.172349656	0.032180959	0.510450274
CD1E	0.858991601	0.083057259	0.000912046	0.909564378	0.895425004	0.032318965	0.510477892
LOC101905876	0.009033582	0.596014383	0.549444249	0.114712094	0.155470067	0.032225951	0.510477892
LOC101908014	0.007698985	0.196010379	0.300066824	0.230715667	0.506967141	0.032306614	0.510477892
SMIM15	0.118517176	0.007295463	0.267756979	0.626781788	0.364915871	0.032301679	0.510477892
AMPD3	0.841132371	0.271622476	0.031544411	0.011278029	0.657880621	0.032505133	0.510500558
EFCAB11	0.005517023	0.444356384	0.091958139	0.54809343	0.432154198	0.032475704	0.510500558
GOLGA2	0.620251732	0.69762764	0.032149972	0.003856578	0.997514435	0.032522465	0.510500558
LOC101907813	0.174058559	0.914142916	0.04427377	0.017852147	0.423586569	0.032426261	0.510500558
PUM2	0.248716222	0.448789169	0.023104708	0.027739454	0.747271358	0.032499872	0.510500558
TAF4B	0.778249231	0.793582465	0.019260305	0.044464062	0.101410683	0.032569257	0.510500558
TBL3	0.261284525	0.190402566	0.061669193	0.088546885	0.197246511	0.032548604	0.510500558
USP13	0.667550183	0.869759174	0.050992855	0.024420785	0.073694019	0.032430838	0.510500558
GNAI3	0.363607541	0.004338288	0.104475722	0.378788444	0.862973012	0.032660493	0.511442134
CLTB	0.059017938	0.156582768	0.122554784	0.221220165	0.216406917	0.032795503	0.513066729
DMBT1	0.401253077	0.415745052	0.005362711	0.709930362	0.085871472	0.032918729	0.513525918
LOC782779	0.452637353	0.250500015	0.820784436	0.000840357	0.69546101	0.032861883	0.513525918
TUBB2A	0.041877477	0.007240197	0.844706181	0.731359548	0.290748896	0.032889152	0.513525918
FAM118A	0.42306001	0.885432331	0.283956147	0.095377523	0.005410305	0.033054158	0.515148889
PACS2	0.952617858	0.961045211	0.377454084	0.00279741	0.057700613	0.033396568	0.519991539
CD3E	0.023055252	0.276150287	0.515668971	0.217474248	0.078318314	0.033450491	0.520337447
TBC1D22B	0.351028815	0.283181125	0.003484089	0.513581324	0.315131862	0.03350183	0.520642548
ASGR1	0.192642023	0.686613044	0.148598058	0.019133493	0.15148979	0.033851968	0.522278728
DLX3	0.460281092	0.335127824	0.000629901	0.90920655	0.641655926	0.033743277	0.522278728
EPHB3	0.792795258	0.561810074	0.000316569	0.926119192	0.437762117	0.033925362	0.522278728
FYB1	0.582704678	0.423700203	0.040622975	0.084128626	0.067486702	0.033841352	0.522278728
GUCD1	0.721032016	0.008573621	0.032013609	0.563344619	0.51200288	0.033894185	0.522278728
HDC	0.035623428	0.179516555	0.026757623	0.644884558	0.51724941	0.033892672	0.522278728
LOC100849652	0.017587464	0.113558776	0.288652881	0.635537885	0.155446162	0.033845359	0.522278728
RFT1	0.297853811	0.545878402	0.905862866	0.001057803	0.363973319	0.033751481	0.522278728
STOX2	0.645542109	0.605099447	0.005650013	0.043708849	0.585018779	0.033647419	0.522278728
TRIM34	0.931902967	0.874732763	0.051652197	0.001737933	0.776979546	0.0338083	0.522278728
KCNMA1	0.000464894	0.60375538	0.317205324	0.648232393	0.996870181	0.034065532	0.523945115
AARSD1	0.555862677	0.617173791	0.263643276	0.631520807	0.001029749	0.034549442	0.524021159
AIF1	0.412755197	0.901498165	0.014679154	0.037147216	0.285321215	0.034200897	0.524021159
CA8	0.504077212	0.968846312	0.002261362	0.074179679	0.707682441	0.034232453	0.524021159
CENPA	0.395981657	0.026313594	0.020693102	0.588601721	0.454877205	0.034139428	0.524021159
CENPC	0.144428252	0.382456143	0.877361477	0.00362275	0.33263224	0.034392483	0.524021159
CHD1	0.615936579	0.950414875	0.957337604	0.000136475	0.761932639	0.034345471	0.524021159
FGD2	0.276342792	0.268645069	0.003901143	0.514189641	0.394943965	0.034547761	0.524021159
FNDC10	0.388303453	0.54112929	0.127013098	0.003883354	0.559210941	0.034225302	0.524021159
IL10RA	0.469172086	0.91046358	0.014414072	0.027304856	0.348709432	0.03447707	0.524021159
LOC617475	0.752303035	0.455337231	0.419363877	0.000429863	0.947380347	0.03443078	0.524021159
NKG7	0.315555616	0.926535918	0.002247456	0.271459325	0.327924528	0.034427558	0.524021159
PIGZ	0.887940049	0.338209218	0.974773693	0.381057206	0.000520011	0.034244066	0.524021159

POLR2K	0.102250397	0.004132627	0.415894926	0.876895981	0.380997177	0.034510536	0.524021159
TMEM242	0.001115963	0.083223485	0.894379881	0.712033539	0.985236221	0.034344122	0.524021159
ZNF451	0.466256556	0.585227801	0.58976386	0.000380504	0.958158601	0.034494278	0.524021159
DPT	0.005161644	0.623684018	0.10697434	0.185459835	0.922957284	0.034597891	0.524271452
ZNF148	0.660721557	0.029826206	0.744399645	0.008587552	0.469563134	0.034675417	0.524961496
KIF1BP	0.035453406	0.084159927	0.211413118	0.675267139	0.139174394	0.034723582	0.525206177
TNFAIP8L3	0.037222911	0.777940736	0.00440689	0.881954792	0.527542398	0.034757418	0.525233881
LOC618268	0.725271223	0.810314667	0.001122556	0.910333652	0.099011292	0.034790882	0.525255909
CBX2	0.902131573	0.861878251	0.009956375	0.010872952	0.709386428	0.034883037	0.526163158
GIMAP6	0.232532434	0.451687577	0.147752369	0.04255996	0.090801816	0.03498079	0.526513205
GPR20	0.161058729	0.13615587	0.279382557	0.06641869	0.147491293	0.034997483	0.526513205
TRMT1	0.20863422	0.720165414	0.240222714	0.046857139	0.035494936	0.035002493	0.526513205
COL3A1	0.594578181	0.054533465	0.009238145	0.385676911	0.525958448	0.035273835	0.528629039
LOC100298890	0.196575566	0.013448622	0.277726747	0.47976328	0.172011104	0.035210526	0.528629039
SPIB	0.014749123	0.930332532	0.024808183	0.952905911	0.187572353	0.035304212	0.528629039
TROAP	0.292765114	0.016436145	0.102111377	0.166660818	0.741429303	0.035256477	0.528629039
WDR12	0.811342803	0.559821114	0.129788825	0.020764585	0.049615891	0.035263639	0.528629039
LOC112443504	0.23759227	0.359026341	0.311503429	0.010313836	0.223802181	0.035485418	0.530857975
HTR6	0.838911235	0.194200713	0.672375179	0.001385458	0.404982089	0.035532398	0.531076673
CXCL3	0.052150273	0.451313226	0.715315774	0.912220721	0.0040518	0.035813558	0.531285939
F5	0.486300263	0.883087919	0.022149563	0.021438238	0.304511525	0.035765589	0.531285939
LOC616063	0.603909095	0.924218589	0.103109599	0.009272573	0.117003858	0.035890603	0.531285939
MEG8	0.602355554	0.003785529	0.097499845	0.419096965	0.670461851	0.035902511	0.531285939
NKD2	0.778150112	0.346606758	0.04383836	0.017626108	0.299097033	0.035852596	0.531285939
REPS1	0.894309015	0.13201152	0.001374292	0.622168164	0.612864818	0.035680939	0.531285939
RGS7	0.017566436	0.720518471	0.193888306	0.936395761	0.027037782	0.035778414	0.531285939
RHOH	0.467130802	0.760585353	0.029618811	0.213690945	0.027492182	0.03566516	0.531285939
SLC2A9	0.563498295	0.016496846	0.471107136	0.023878494	0.596368823	0.035863735	0.531285939
TMC4	0.018095549	0.516750822	0.036996335	0.200627074	0.89107399	0.035673822	0.531285939
ZNF395	0.325091306	0.022574277	0.909997411	0.094540356	0.097664075	0.035605667	0.531285939
TIMP1	0.244150216	0.168839375	0.089131185	0.037997423	0.448166021	0.035938405	0.531337985
ATRIP	0.945405945	0.407544451	0.874722061	0.002145692	0.086838368	0.036022211	0.531644432
FCHO2	0.092175378	0.184982345	0.020693676	0.302119846	0.601391772	0.036499113	0.531644432
HTR7	0.043364205	0.857482251	0.009083456	0.786540146	0.240967126	0.036465283	0.531644432
IFNGR2	0.183445044	0.262648044	0.816141666	0.001863844	0.86782808	0.036316182	0.531644432
LCMT2	0.476012966	0.046972687	0.250964081	0.361605736	0.031620412	0.036518306	0.531644432
LCN1	0.626299135	0.842058761	0.000899713	0.252763651	0.529924133	0.036298498	0.531644432
LOC100847365	0.422202452	0.353940949	0.281334719	0.077928096	0.019388734	0.036285841	0.531644432
LOC112441494	0.573433295	0.709397738	0.001859274	0.19833772	0.425916811	0.036420446	0.531644432
LOC112443444	0.017601133	0.528885946	0.600685588	0.956873322	0.01200389	0.036542253	0.531644432
LOC518526	0.775436408	0.182265579	0.985036505	0.323917372	0.001408385	0.036282667	0.531644432
LOC783540	0.133697196	0.573875418	0.747417188	0.137927014	0.008021045	0.036257405	0.531644432
PCYT2	0.71832813	0.770557666	0.671024229	0.000988975	0.174036254	0.036433514	0.531644432
RGS1	0.737167486	0.617584736	0.050202153	0.006477869	0.431596358	0.036423047	0.531644432
SAC3D1	0.039516139	0.118154648	0.257859504	0.170735006	0.307274208	0.036155219	0.531644432

SEMA6A	0.190864161	0.529191139	0.311261996	0.003735945	0.542261204	0.036347214	0.531644432
SLC4A11	0.021870816	0.291881224	0.459590285	0.210680836	0.10281127	0.036295907	0.531644432
TMCC1	0.862938992	0.441528195	0.001971181	0.190347698	0.43979285	0.036049596	0.531644432
ZNF131	0.339888404	0.968069234	0.896656634	0.000245969	0.879028866	0.036383473	0.531644432
CEMIP	0.061111561	0.533785308	0.011332712	0.304669012	0.574443347	0.036712389	0.533646604
FAP	0.324778716	0.555729665	0.005594648	0.431138221	0.148833226	0.036746846	0.533674764
ITGAM	0.572077031	0.678395732	0.000643705	0.677787421	0.383726416	0.036811396	0.534139535
C11H9orf116	0.842367706	0.31261601	0.038516318	0.045457816	0.142858313	0.037132228	0.534284515
CAGE1	0.011240581	0.246593728	0.144838039	0.208922999	0.785356167	0.037134104	0.534284515
CCL3	0.902647672	0.339103433	0.000842119	0.274194974	0.926647473	0.036998114	0.534284515
IRF1	0.146254934	0.089971028	0.250703197	0.314568776	0.063413229	0.03711034	0.534284515
MAP3K6	0.859003228	0.013172537	0.678712474	0.012821183	0.668931321	0.037131571	0.534284515
MILR1	0.867477406	0.598287379	0.001302384	0.133876577	0.728335306	0.037146952	0.534284515
MON1B	0.304070628	0.045209869	0.203668208	0.148435987	0.15853424	0.037138824	0.534284515
RPP40	0.975689675	0.623655185	0.187303615	0.04024063	0.014335677	0.037089551	0.534284515
ZBP1	0.890105297	0.157195496	0.085537397	0.016440885	0.334924711	0.03714516	0.534284515
ZNF432	0.001698736	0.962180841	0.530329612	0.637871962	0.118060996	0.036920836	0.534284515
LAIR1	0.282861486	0.293717218	0.068256033	0.05469897	0.212809497	0.037183446	0.534341096
C25H16orf72	0.16947732	0.522988287	0.30604869	0.007029305	0.348718083	0.03735597	0.534489398
CD180	0.64572598	0.729594808	0.000560426	0.563126724	0.445975137	0.037289625	0.534489398
EARS2	0.49944392	0.668293481	0.439088112	0.001907167	0.237733777	0.037339832	0.534489398
GRIK3	0.373438872	0.023967967	0.816397696	0.156178572	0.058575006	0.03748204	0.534489398
LOC112449613	0.48783762	0.832172832	0.568150721	0.165663245	0.001761827	0.037649732	0.534489398
MED30	0.79037618	0.224772388	0.234764456	0.21638506	0.00745426	0.037633194	0.534489398
NDUFAF4	0.688672883	0.185030618	0.603962129	0.111557057	0.007725299	0.037295828	0.534489398
PDCD1	0.050399046	0.172734618	0.195445349	0.071996264	0.546295839	0.037508262	0.534489398
PGGHG	0.835069938	0.043330561	0.033963523	0.077460627	0.698646095	0.037360733	0.534489398
PGP	0.48934977	0.716318279	0.109480796	0.015611653	0.111837788	0.037537645	0.534489398
PIGO	0.292676925	0.002775738	0.133262959	0.677576694	0.91316834	0.037531332	0.534489398
PRICKLE2	0.849724298	0.106427291	0.179028246	0.131749671	0.031456873	0.037571491	0.534489398
TMEM64	0.498921428	0.11563344	0.27466065	0.004668917	0.905600369	0.03753567	0.534489398
ZNF75D	0.937850807	0.006004766	0.092900735	0.346159749	0.371603469	0.037642228	0.534489398
EIF6	0.124751751	0.121990806	0.047342143	0.728285848	0.128668901	0.037718831	0.534545538
LOC101904573	0.056998987	0.474973256	0.43428989	0.006438032	0.890947925	0.037692636	0.534545538
FABP3	0.310556026	0.458160199	0.001964439	0.695151371	0.349926377	0.037887461	0.5355271
FOLR2	0.448133935	0.324948351	0.000954436	0.614252613	0.796714852	0.037896691	0.5355271
POU2AF1	0.031249151	0.336586231	0.091647675	0.71997105	0.097756549	0.037835773	0.5355271
PREX1	0.14182814	0.504974817	0.448318148	0.97170928	0.002182028	0.037918621	0.5355271
LOC618169	0.896107727	0.640297339	0.003727538	0.053948902	0.591633899	0.037984247	0.535992679
CNTN2	0.687261594	0.458290516	0.000314413	0.947213235	0.732666279	0.038146317	0.537050271
ERO1A	0.305140017	0.016551203	0.153414605	0.092617644	0.955604121	0.038093482	0.537050271
HS6ST1	0.327987415	0.845944396	0.101347191	0.392812549	0.006224715	0.038157371	0.537050271
CLASP1	0.80631706	0.002757877	0.698898671	0.109350992	0.406378265	0.038265115	0.538105226
MUSTN1	0.043032074	0.110348468	0.307495438	0.730218422	0.064936714	0.038326126	0.538501753
LOC101902665	0.045588347	0.289844352	0.102808106	0.754802162	0.067692659	0.038386568	0.538874852

UBASH3A	0.028353484	0.587370817	0.788916035	0.640036357	0.008264832	0.038418352	0.538874852
C15H11orf87	0.953252187	0.763750116	0.362146708	0.000277543	0.956339017	0.03858663	0.540773004
ARNTL	0.487922417	0.472093013	0.054029096	0.013002962	0.433561197	0.038649285	0.541188922
C3H1orf52	0.93944934	0.147254037	0.00525275	0.341847183	0.283868917	0.038772188	0.542224647
LAX1	0.010422651	0.981539135	0.046280474	0.291367531	0.511511925	0.038789333	0.542224647
DCTN6	0.870548252	0.033778163	0.116754796	0.534306021	0.039248537	0.039286062	0.543053189
DLGAP5	0.712194957	0.018279178	0.03073846	0.270354782	0.661351288	0.039131176	0.543053189
EIF3B	0.287470667	0.639707914	0.090941569	0.053824879	0.078932572	0.038959047	0.543053189
ELF3	0.070333525	0.279925226	0.749289758	0.007333647	0.663653309	0.039217394	0.543053189
IL34	0.243621498	0.083965638	0.81847618	0.010915178	0.394100124	0.039294269	0.543053189
KDM7A	0.506596297	0.326142294	0.21346213	0.004085862	0.500809705	0.039344966	0.543053189
LOC104970180	0.16648279	0.926942001	0.804248592	0.593689079	0.000979216	0.039339434	0.543053189
MLYCD	0.581261774	0.777105902	0.604972456	0.000641197	0.404637427	0.038906205	0.543053189
SLC25A30	0.498761835	0.223144461	0.489379377	0.00406523	0.323337204	0.039146099	0.543053189
SPDYA	0.114381867	0.113713703	0.313359896	0.131271095	0.133967982	0.039175623	0.543053189
SPRED2	0.805752754	0.727161731	0.449887237	0.27677487	0.000973798	0.038956483	0.543053189
STBD1	0.058551064	0.358171348	0.638015596	0.015668359	0.342637079	0.039228778	0.543053189
TNFAIP8L2	0.360944373	0.789453778	0.006052884	0.073708073	0.563180918	0.039147614	0.543053189
TTR	0.174020146	0.296514703	0.402579208	0.236593704	0.014575788	0.039161297	0.543053189
ZSWIM7	0.002796454	0.228097978	0.574982131	0.21108042	0.922350215	0.039081251	0.543053189
ABCC5	0.082460778	0.236997635	0.207002745	0.936043742	0.01922063	0.03955651	0.544349148
CCL11	0.038227108	0.349951042	0.031704921	0.365124543	0.470669649	0.039592858	0.544349148
CHRNA1	0.019001191	0.546313486	0.843380315	0.055080383	0.150661059	0.039511233	0.544349148
DOC2A	0.979338611	0.003396854	0.186581077	0.262080446	0.448287221	0.039604709	0.544349148
ZFPM2	0.530473247	0.315981064	0.007124542	0.105430811	0.578471953	0.039573782	0.544349148
SCN1B	0.157457555	0.282928826	0.007090552	0.312938002	0.738741097	0.039639463	0.544370897
LOC785629	0.625857507	0.966262003	0.03031702	0.019526086	0.204672115	0.039723698	0.54474481
RASGRP1	0.257052488	0.555879065	0.173144562	0.182104123	0.016269055	0.039733078	0.54474481
CCDC168	0.1345389	0.041796178	0.116875806	0.742191475	0.15160496	0.039955856	0.545343496
FASLG	0.016978321	0.98512144	0.507673438	0.05925464	0.146145835	0.039813166	0.545343496
HOXD3	0.135364536	0.456953158	0.031561797	0.063551595	0.597302352	0.040009358	0.545343496
LOC100139881	0.320487138	0.340813359	0.078196776	0.039357303	0.220381593	0.040001084	0.545343496
LOC112447082	0.00457641	0.771319134	0.315195635	0.483545977	0.137584141	0.0399795	0.545343496
LOC789374	0.094063819	0.020808076	0.830187319	0.936687138	0.048433403	0.039876455	0.545343496
TMEM263	0.504705906	0.249158646	0.221118358	0.008559631	0.30934473	0.039845549	0.545343496
TRMT6	0.935581816	0.62562041	0.286239511	0.244750859	0.001811863	0.040074126	0.545773009
P2RY13	0.051878154	0.854202384	0.014617152	0.634626153	0.181507337	0.040181906	0.546787121
CLIP2	0.900870797	0.092111538	0.001562444	0.671546844	0.863367341	0.04037106	0.547226733
GPX7	0.889439799	0.075345457	0.002796378	0.786593253	0.509284359	0.040337478	0.547226733
KLHL24	0.001193676	0.87396979	0.768301609	0.722951986	0.129423047	0.040311443	0.547226733
LOC112444310	0.425145819	0.029317749	0.149688655	0.043348885	0.927217111	0.040310342	0.547226733
SMTN	0.002463043	0.480325817	0.276617124	0.953441209	0.241013054	0.040380938	0.547226733
S100A12	0.655273943	0.084434137	0.006537546	0.277566355	0.752644078	0.040503734	0.547534416
SNX19	0.811870233	0.753986705	0.087397064	0.016198544	0.087178639	0.040499089	0.547534416
TLR6	0.085994063	0.685375819	0.003886162	0.681684945	0.483122491	0.040459463	0.547534416

ANKRD13B	0.324707435	0.049950588	0.037288196	0.307108965	0.407584882	0.040550961	0.547706637
PIM3	0.226422913	0.455971856	0.660334674	0.002233189	0.498517943	0.040616597	0.547706637
PTPRC	0.628008904	0.494500618	0.007199013	0.131270245	0.258548788	0.040609857	0.547706637
HSPA4L	0.029539026	0.239440913	0.836758686	0.0663109	0.193681664	0.040654317	0.547765188
ESS2	0.076287418	0.564086278	0.36039109	0.089740972	0.054982285	0.040826848	0.549188041
TMEM165	0.843566479	0.279195757	0.548351031	0.16033202	0.003692746	0.040807326	0.549188041
SLAIN2	0.738668433	0.036389797	0.162240422	0.021895991	0.80310869	0.040882639	0.549488121
LAMP3	0.726910985	0.57586448	0.1928252	0.102338748	0.009324184	0.040994837	0.550094678
LOC100847509	0.319132383	0.607702293	0.952097019	0.000563718	0.740845445	0.041025536	0.550094678
SH3BGR	0.016245843	0.221389883	0.820233547	0.708519977	0.036896873	0.041028328	0.550094678
PSTPIP1	0.523828846	0.452933261	0.270065827	0.009565819	0.126569991	0.041181352	0.551245655
TRAPPC8	0.528592331	0.376482444	0.004007308	0.134237877	0.724294089	0.041167153	0.551245655
LOC104976276	0.019669032	0.115056042	0.410446887	0.286996223	0.292167589	0.041283669	0.551849154
STEAP1	0.327379359	0.439832994	0.288625179	0.006948204	0.269857614	0.041297015	0.551849154
XAF1	0.704713833	0.21707685	0.01721791	0.052992683	0.558938303	0.041327318	0.551849154
BATF3	0.005584688	0.786784315	0.105671995	0.735602421	0.22933143	0.041431362	0.551891298
FSCN1	0.95833204	0.576205621	0.000963548	0.654464242	0.224727511	0.041406628	0.551891298
PHOSPHO1	0.271012827	0.005430192	0.30839146	0.33648107	0.512659235	0.041417889	0.551891298
ANKRD63	0.673275762	0.567456153	0.003189799	0.263610374	0.244600724	0.041514766	0.552553797
CYP4B1	0.809082421	0.345580114	0.015633734	0.094073536	0.192171332	0.04166248	0.553272543
SCN8A	0.514606073	0.798762263	0.051148379	0.014344421	0.261564925	0.041615823	0.553272543
SEMA4F	0.027737702	0.234611857	0.053828212	0.235514118	0.958155566	0.041669908	0.553272543
LPP	0.08997766	0.561645251	0.048781922	0.537494549	0.060094999	0.041862559	0.555381132
BANK1	0.882506668	0.536286761	0.00398543	0.169585928	0.252114099	0.042197746	0.556230687
FSTL4	0.84802548	0.186618796	0.228083948	0.346086613	0.006449636	0.042173439	0.556230687
FTL	0.27215604	0.166057715	0.021232072	0.14485055	0.575763093	0.041994009	0.556230687
LENG8	0.266489164	0.019452372	0.089974531	0.889734199	0.194225104	0.042183103	0.556230687
LOC786586	0.07768156	0.170554043	0.008549295	0.779330068	0.911022478	0.042123526	0.556230687
PLA2G2C	0.517497807	0.13542464	0.056402698	0.403172102	0.050528808	0.042158585	0.556230687
RASAL2	0.625010502	0.446532608	0.474154129	0.016257596	0.037280837	0.042052935	0.556230687
TMEM129	0.55951294	0.45096921	0.086039406	0.022073712	0.167600169	0.042089609	0.556230687
LIPG	0.22234686	0.936666171	0.1711308	0.023266576	0.097871427	0.042366301	0.558004304
DIRAS2	0.850633241	0.645649862	0.028566708	0.915551623	0.005720944	0.042699539	0.558335416
FAM167A	0.36828399	0.610661024	0.091007923	0.746891138	0.005345705	0.042550046	0.558335416
FASTKD2	0.523493566	0.970709132	0.802292636	0.00691904	0.029190381	0.042753195	0.558335416
GCC1	0.195875133	0.895767756	0.028606688	0.087790676	0.186952045	0.042765683	0.558335416
GET4	0.166589713	0.847970984	0.177701625	0.008398841	0.390155555	0.042725939	0.558335416
GIMAP7	0.069919187	0.777590239	0.115076863	0.072955032	0.179565454	0.042629409	0.558335416
LXN	0.479010692	0.85785277	0.001534204	0.227245576	0.574705146	0.042750882	0.558335416
PDE1B	0.628761349	0.480971389	0.673076131	0.000963229	0.415799151	0.042485974	0.558335416
PI4K2A	0.83553911	0.059709925	0.087123934	0.057379803	0.326866914	0.042485868	0.558335416
RAB21	0.698857359	0.001159643	0.514106734	0.217701525	0.901684228	0.042572041	0.558335416
RAD23A	0.087564257	0.221780573	0.051587527	0.368598958	0.221365581	0.042558337	0.558335416
FILIP1L	0.667153465	0.410092452	0.283074425	0.068638715	0.015531462	0.042825604	0.558673282
F11R	0.201235976	0.966243872	0.737322627	0.002240495	0.257614432	0.042885961	0.559016288

DOK2	0.586869165	0.60400188	0.000618126	0.396220919	0.955429162	0.04294974	0.559316572
MMACHC	0.795472066	0.570879663	0.225492035	0.002854409	0.284064741	0.042977161	0.559316572
GRAMD4	0.929801955	0.048763012	0.02443116	0.931234009	0.080631218	0.043023934	0.559481605
CRHR2	0.370364132	0.159968372	0.051977679	0.186581314	0.145201257	0.043106762	0.559671728
RRP7A	0.121407391	0.920199974	0.232028796	0.077965252	0.041248898	0.043085857	0.559671728
SLC7A5	0.107282896	0.017092679	0.911195938	0.076002546	0.658388047	0.04316558	0.559992359
XCR1	0.002201203	0.258649909	0.647988562	0.428617051	0.5297471	0.043216443	0.560209357
C22H3orf67	0.334027884	0.0351019	0.027304387	0.28560136	0.922705832	0.043409785	0.560943291
CARNS1	0.184618518	0.358663855	0.451054026	0.010884781	0.259265531	0.043383886	0.560943291
CHST6	0.967925296	0.104319281	0.052788287	0.101153569	0.156202234	0.043362377	0.560943291
VSIG10	0.291884093	0.83482973	0.321287445	0.445165621	0.002419119	0.043391714	0.560943291
CENPF	0.88283351	0.011813711	0.020888611	0.442693894	0.879563169	0.043558917	0.561103132
LIPJ	0.061424803	0.734260046	0.223743807	0.338784147	0.02479092	0.043534728	0.561103132
MIEF1	0.993708211	0.450327403	0.102517019	0.00698829	0.26425526	0.043523254	0.561103132
TSR2	0.368479903	0.004327024	0.108092254	0.867216257	0.566554914	0.043510019	0.561103132
UTP4	0.907840312	0.74644707	0.45542717	0.002800988	0.098349914	0.043619766	0.561446264
BIN2	0.786751099	0.771135755	0.000999855	0.258042198	0.548886165	0.04390801	0.562615248
CLIC4	0.740639279	0.293243641	0.000706795	0.654727001	0.856504057	0.043961261	0.562615248
DTX4	0.607615317	0.002562203	0.293634159	0.598588519	0.31392865	0.043903579	0.562615248
GPNMB	0.838008129	0.297871503	0.006200406	0.974936895	0.056809784	0.043845921	0.562615248
KIF4A	0.701793358	0.060942425	0.036388161	0.227385599	0.243466072	0.043984849	0.562615248
PVR	0.053388406	0.541684166	0.70800577	0.04732639	0.088801361	0.043950763	0.562615248
SMARCA5	0.730295868	0.594654962	0.099208007	0.002354294	0.845405576	0.04385474	0.562615248
TMEM106A	0.500134904	0.346924491	0.005634662	0.206385871	0.423786698	0.043777687	0.562615248
CD28	0.693675979	0.182919834	0.007587012	0.476817734	0.188826769	0.044150916	0.563072157
GABARAP	0.325991669	0.121229084	0.204677443	0.01232183	0.867400665	0.044079307	0.563072157
PIK3R6	0.649743417	0.717994087	0.013484855	0.017563308	0.784849199	0.044163478	0.563072157
RFXANK	0.42032353	0.017596005	0.051048987	0.248958237	0.920485158	0.04410155	0.563072157
TFEC	0.501241361	0.610241893	0.001075465	0.374215719	0.705154576	0.044192123	0.563072157
TIGD5	0.723374978	0.857602214	0.006654292	0.028961916	0.727022427	0.044228762	0.563101798
LOC104969719	0.019360985	0.071392148	0.692783619	0.915890987	0.099922055	0.044455877	0.565554571
CUL3	0.084950156	0.043679369	0.197936935	0.161695148	0.73975185	0.044524442	0.565988084
CRLF1	0.442382112	0.22203173	0.028029549	0.040669999	0.787416338	0.044624399	0.566258258
FTSJ3	0.097802925	0.334700402	0.210123799	0.091727976	0.139864699	0.04464921	0.566258258
POMK	0.560921945	0.866982445	0.013639455	0.019813158	0.670880266	0.044624476	0.566258258
PEX11B	0.691002866	0.652714911	0.431455203	0.000839399	0.541258726	0.044701906	0.566488789
IGSF10	0.033021983	0.855555382	0.148834877	0.059646841	0.353467411	0.044777957	0.567014695
TGFBI	0.741117849	0.248849062	0.011774827	0.051541354	0.795270313	0.044891274	0.568011337
LOC784697	0.090323485	0.645292818	0.481027455	0.003330111	0.957085789	0.045000512	0.568162402
NR2C2AP	0.150894367	0.49506679	0.13039529	0.414269631	0.022169973	0.045033553	0.568162402
RGS5	0.069223875	0.141959178	0.821131768	0.014176586	0.78229173	0.045041697	0.568162402
SCAPER	0.118662268	0.169517901	0.882866173	0.019058845	0.263588465	0.044955785	0.568162402
C1QBP	0.727985907	0.493594141	0.718677294	0.010557726	0.032962194	0.045161155	0.568358682
CD248	0.553499014	0.72977813	0.000638353	0.440251448	0.79012827	0.045106207	0.568358682
RRP12	0.791449293	0.919724231	0.235180736	0.025723452	0.020397649	0.045146762	0.568358682

C13H20orf202	0.656058521	0.360639522	0.000880142	0.781155858	0.556546204	0.045369151	0.569675471
DDX21	0.773327028	0.333734523	0.49435062	0.003637139	0.195804072	0.045471907	0.569675471
EIF1	0.423516156	0.133514692	0.019545518	0.349608869	0.233953989	0.045326843	0.569675471
LOC768255	0.257397527	0.399874805	0.357244665	0.032829868	0.075267991	0.045471424	0.569675471
PSMF1	0.152612162	0.023047708	0.157196752	0.268730403	0.611556735	0.045474064	0.569675471
SLC16A14	0.486304353	0.943803936	0.39063273	0.002958069	0.170846715	0.045393017	0.569675471
COQ10B	0.257455307	0.041466469	0.313202334	0.062798929	0.435241572	0.045637307	0.571284397
ANP32E	0.764167099	0.000538463	0.607283848	0.45151293	0.814014042	0.04577735	0.571848668
CD86	0.555058863	0.91472232	0.018244668	0.096861156	0.102517789	0.045821766	0.571848668
MRPS26	0.336485595	0.188435069	0.007895774	0.210399023	0.872493354	0.045796462	0.571848668
TUBA1B	0.006052518	0.469016736	0.453462402	0.615368874	0.116082475	0.0458122	0.571848668
SURF4	0.046382984	0.352382922	0.041924256	0.323518007	0.415532924	0.04586324	0.571931325
B2M	0.37181896	0.351393405	0.030781756	0.023942981	0.958666324	0.045923996	0.572254136
BRIX1	0.322872938	0.299155909	0.430815039	0.016552491	0.134345272	0.045992765	0.572676225
NEDD4	0.404120921	0.749184011	0.538472569	0.00081462	0.69768962	0.046030774	0.572714963
IFIT3	0.895104266	0.241699847	0.034855538	0.034877747	0.354894027	0.046241994	0.573740043
PCF11	0.214287358	0.770307114	0.717863101	0.006141648	0.127926195	0.046167528	0.573740043
STX18	0.533436232	0.111538115	0.085421817	0.116931426	0.156898217	0.046212455	0.573740043
TCAF1	0.575883141	0.519801431	0.003713496	0.49311264	0.170346734	0.046253006	0.573740043
LOC100298530	0.605518429	0.257081961	0.019366583	0.496478666	0.062537662	0.046323412	0.574179388
NAT10	0.236063826	0.816924321	0.121120625	0.053359354	0.075396113	0.046436234	0.575143419
ADRA2B	0.059963677	0.117282545	0.414039746	0.416138905	0.077852225	0.046548602	0.57610038
FADS1	0.464191706	0.892939331	0.609255017	0.523111671	0.000715968	0.046624383	0.576169242
UBR5	0.611694328	0.767601359	0.854496547	0.000319404	0.737651995	0.046608668	0.576169242
LOC530929	0.897179147	0.00979988	0.068924231	0.69608658	0.224595802	0.046673241	0.576339026
NEK4	0.376100301	0.240771827	0.502564086	0.084226131	0.024931403	0.046925464	0.578161119
ROBO2	0.511463131	0.316865109	0.860777392	0.003541303	0.193326671	0.046907977	0.578161119
ZNF354A	0.065321431	0.234841102	0.007029461	0.957447178	0.92564181	0.046926489	0.578161119
LOC112446717	0.756392608	0.236376248	0.000919639	0.810623356	0.723685883	0.047198244	0.580202537
LOC787309	0.103805871	0.177170608	0.030115744	0.255596868	0.680321538	0.047153575	0.580202537
SLIT2	0.486605974	0.180729852	0.025636983	0.044586686	0.958987573	0.047181663	0.580202537
ZDHHC20	0.932384924	0.013641607	0.06746099	0.17683808	0.63671209	0.047245265	0.580345841
OSR1	0.128921248	0.720528186	0.188436221	0.007300559	0.759067137	0.047363464	0.581362607
ALDH1A2	0.321105768	0.010636575	0.238701739	0.490674792	0.243527055	0.047490539	0.582486718
ARL4C	0.52648923	0.363591918	0.018051089	0.172521438	0.165203474	0.047812813	0.582953992
C6H4orf19	0.613273983	0.189967734	0.720058451	0.001746415	0.670583837	0.047739851	0.582953992
CCNJL	0.041053563	0.041703566	0.193881587	0.595961731	0.496350571	0.047723782	0.582953992
FAM174A	0.560699329	0.175397569	0.556358808	0.059123219	0.030190302	0.04756458	0.582953992
FBXW12	0.090455009	0.021261327	0.328417303	0.579881826	0.268673625	0.047788465	0.582953992
KLF15	0.900675388	0.171033361	0.001793032	0.646613416	0.548348403	0.04764669	0.582953992
MAFF	0.501181135	0.070987887	0.067656763	0.110514491	0.369985579	0.047793983	0.582953992
PARD3	0.500916351	0.390019114	0.095266317	0.014165635	0.372981468	0.04776796	0.582953992
GOT1L1	0.054832656	0.425002637	0.807004338	0.015831375	0.332065467	0.047927915	0.583490366
KCNA5	0.661248113	0.030368665	0.166880387	0.118470184	0.248752414	0.047895182	0.583490366
NOX1	0.007481521	0.187183295	0.093967344	0.76675814	0.985990538	0.048114552	0.584894746

TBXA2R	0.480227824	0.952252149	0.116446153	0.002050014	0.910871766	0.048098948	0.584894746
DMRT2	0.036840968	0.180763444	0.353427521	0.308539377	0.137731191	0.048274489	0.586404622
ARRB2	0.078437585	0.722246652	0.010115947	0.364554551	0.480957845	0.048412832	0.587331947
CACFD1	0.354305313	0.950627326	0.00175095	0.203156438	0.838941716	0.048422407	0.587331947
FGF1	0.103642442	0.019813273	0.271435898	0.365800302	0.497694291	0.048710098	0.589797816
MED19	0.673703228	0.836420819	0.910963934	0.000571693	0.346056447	0.048733523	0.589797816
PLN	0.007646303	0.243095287	0.802685392	0.665984765	0.102117387	0.048707979	0.589797816
UBN1	0.95183773	0.034693992	0.857873361	0.004550848	0.789198466	0.04879029	0.589919733
ZNF536	0.800151046	0.109168585	0.290112507	0.353188908	0.011377245	0.048815489	0.589919733
BDH1	0.944131825	0.652439624	0.42676985	0.000941116	0.415942485	0.049134715	0.591599266
FAM91A1	0.989973793	0.009867737	0.27263639	0.056422143	0.683476836	0.049075473	0.591599266
GPRC5A	0.181042214	0.465138862	0.289021197	0.100080486	0.042163482	0.049074041	0.591599266
SLC18B1	0.378579203	0.035868929	0.057290317	0.437408339	0.302148472	0.049108101	0.591599266
TRABD2B	0.823040838	0.8456812	0.471464555	0.00036179	0.863791445	0.049029546	0.591599266
CYBB	0.416924354	0.618747777	0.001956238	0.476991085	0.433517774	0.049562076	0.592278132
FAM47E	0.03388943	0.102325546	0.914592915	0.090793232	0.358844086	0.049260617	0.592278132
IFT43	0.209750847	0.174108984	0.246155633	0.210833548	0.055070339	0.049567899	0.592278132
IL2RA	0.204471696	0.660167201	0.577346771	0.009896971	0.134605754	0.049405295	0.592278132
LOC101903383	0.570442011	0.712596049	0.003719686	0.510091053	0.134365207	0.049349326	0.592278132
LOXL4	0.09575974	0.295246378	0.239594236	0.032082929	0.476533528	0.049329299	0.592278132
RNF185	0.809195531	0.044173213	0.011502147	0.353573808	0.714384777	0.049413327	0.592278132
RRAGB	0.53810237	0.013238233	0.644883966	0.16594367	0.136782262	0.049537835	0.592278132
TMEM120A	0.459476157	0.634386149	0.101251977	0.012643171	0.279899016	0.04958809	0.592278132
TMEM144	0.188130549	0.260457379	0.010173789	0.488828038	0.425199942	0.049344557	0.592278132
WDR46	0.310118176	0.963428021	0.094530244	0.091691178	0.040218178	0.049502518	0.592278132
ADAMTS7	0.317171919	0.153986257	0.007532081	0.35189102	0.814266888	0.049871074	0.592619983
ATG13	0.89489384	0.200649418	0.038614416	0.391564213	0.038893327	0.049925893	0.592619983
CCDC125	0.20901473	0.421919832	0.073108707	0.087483303	0.187054229	0.049899674	0.592619983
CD14	0.444211211	0.730770281	0.002082906	0.181864197	0.860158918	0.049977823	0.592619983
DNAJA3	0.777084052	0.589368674	0.275142557	0.027386693	0.030357299	0.049682842	0.592619983
ME2	0.97885792	0.412467807	0.008202801	0.09836877	0.32263891	0.049784319	0.592619983
NAT9	0.55667933	0.550922912	0.261696295	0.001340677	0.980061717	0.049885612	0.592619983
SPATA7	0.292822763	0.579426285	0.412930553	0.009397183	0.160072933	0.049866025	0.592619983
TMEM132A	0.99420683	0.127316894	0.647863216	0.025288949	0.050774242	0.049839341	0.592619983
TMEM62	0.01945249	0.291413952	0.908815885	0.021004973	0.9765269	0.049949449	0.592619983
PTPRS	0.684035106	0.175255607	0.019174565	0.081981529	0.563747119	0.050114111	0.593806981
ATP7B	0.084048789	0.053945377	0.464745747	0.343404167	0.147441582	0.050246466	0.594744041
OBSL1	0.490311667	0.482290702	0.000601991	0.759574476	0.987310157	0.050265675	0.594744041
BTG2	0.535933758	0.001150388	0.728193646	0.412958367	0.578391503	0.050404499	0.595956941
IL16	0.552205363	0.939330382	0.665587907	0.012680546	0.024549963	0.050475127	0.596346476
LOC112443250	0.869830303	0.132054915	0.097866685	0.083970128	0.113985845	0.050510121	0.596346476
BAHCC1	0.988414297	0.248457387	0.008783102	0.080719462	0.620592552	0.050641511	0.597037859
CAPN11	0.024684722	0.058233448	0.154040809	0.556926782	0.879260766	0.050751872	0.597037859
CDK3	0.165056779	0.006724958	0.358802266	0.564673681	0.480208951	0.050625809	0.597037859
CNKSR1	0.218904037	0.634472017	0.001387978	0.600999349	0.935684406	0.050744628	0.597037859

RND2	0.504848438	0.390227283	0.261507556	0.161904598	0.013014147	0.050786963	0.597037859
TOMM70	0.122189532	0.23052339	0.372063894	0.942756612	0.010980835	0.050769878	0.597037859
DDX49	0.190965342	0.792055041	0.110132291	0.052263301	0.124860513	0.050831	0.597127808
BTK	0.700104061	0.922109579	0.000544924	0.388684791	0.798178353	0.050956491	0.597288935
LOC107131542	0.344820372	0.208826649	0.047294958	0.071066725	0.451424052	0.050990299	0.597288935
MOB3A	0.265418382	0.212167838	0.458668601	0.506723843	0.008334558	0.050940883	0.597288935
SMCO4	0.110815644	0.467677976	0.347765683	0.742802966	0.00813775	0.050900963	0.597288935
ASTE1	0.089199711	0.509342978	0.412060666	0.010611827	0.551306766	0.051068279	0.597349621
CSMD2	0.096606783	0.089512321	0.126456655	0.200077267	0.500086252	0.051036047	0.597349621
DLX4	0.165524219	0.014068391	0.289639605	0.196357912	0.830374574	0.051196909	0.597914933
LOC100850436	0.00030426	0.946582993	0.656803909	0.729774122	0.799201715	0.051298777	0.597914933
LRIF1	0.304152609	0.984203916	0.490841297	0.001005308	0.744003726	0.051175479	0.597914933
PIKFYVE	0.772060715	0.264848796	0.000820507	0.774471232	0.847820953	0.051251902	0.597914933
ZNF550	0.034611175	0.01827624	0.786563306	0.254182848	0.87172981	0.051275532	0.597914933
CEP128	0.292632117	0.892389073	0.900566793	0.002335468	0.201828021	0.051449588	0.598549357
SEC24B	0.260688256	0.802677159	0.830408944	0.001054031	0.604959469	0.051433967	0.598549357
SLBP	0.457834961	0.34013846	0.074881152	0.025479621	0.373674786	0.051499098	0.598549357
VEGFD	0.03775158	0.196483484	0.192418236	0.102324047	0.760080855	0.051493309	0.598549357
ARIH1	0.546227424	0.560591446	0.532520464	0.000787775	0.866530276	0.051581061	0.599077702
MROH6	0.043084572	0.01667153	0.317891755	0.617035463	0.791244911	0.051629128	0.599211898
CD74	0.082134685	0.932774742	0.015977627	0.152274666	0.598773699	0.051666459	0.599221383
STX12	0.446746926	0.366010588	0.137868106	0.039106595	0.126921104	0.051747077	0.599732539
CLPX	0.661434245	0.204847181	0.013384323	0.065994377	0.942372329	0.052000627	0.60134027
EXOC6	0.19810173	0.10653068	0.046399465	0.161844801	0.710645292	0.051955214	0.60134027
FCN1	0.258228362	0.353581207	0.215348629	0.086588812	0.066298873	0.052027274	0.60134027
LOC781304	0.210616744	0.55769549	0.627341488	0.003496768	0.438241059	0.052039902	0.60134027
PRMT3	0.195975748	0.337554442	0.196495535	0.24293691	0.035791118	0.05206901	0.60134027
DDX41	0.864446278	0.932415392	0.725805235	0.000344723	0.56367625	0.052254231	0.601363384
GMPPB	0.07026494	0.944648085	0.344717836	0.080013812	0.061984881	0.052199011	0.601363384
LOC112447011	0.048906649	0.856562831	0.123604613	0.035731406	0.61232667	0.052145059	0.601363384
MSTO1	0.494477155	0.914142781	0.354471814	0.060266381	0.011764612	0.052233975	0.601363384
ZNF446	0.585899797	0.830036723	0.066864669	0.004523089	0.770059689	0.052136307	0.601363384
DCAF13	0.650575652	0.250676764	0.01063176	0.624461535	0.105666018	0.052461659	0.602904963
RDH10	0.17833151	0.062219	0.634732401	0.122495252	0.132500042	0.05243329	0.602904963
INAFM2	0.532369678	0.015214831	0.348833495	0.046313012	0.877078277	0.052564666	0.603666008
GPR183	0.419507933	0.778113357	0.314088951	0.022716455	0.049400662	0.052644474	0.604159761
TMEM35B	0.11138884	0.186953136	0.022120843	0.302220808	0.827672711	0.05269306	0.604294763
RAB3A	0.767784225	0.377217946	0.006777635	0.205024918	0.286714167	0.052738415	0.604392551
B3GALT4	0.103167308	0.138512932	0.306071627	0.033675165	0.785179997	0.052810966	0.604790538
LOC112445925	0.35268067	0.78457976	0.000484226	0.97705599	0.884735559	0.052860803	0.604790538
LOC618733	0.018159549	0.797105233	0.215947863	0.039401826	0.941063025	0.052883701	0.604790538
CDKN1A	0.571211267	0.134594062	0.018406807	0.225346785	0.364181153	0.052948647	0.605111592
ADAMTS9	0.247939145	0.078008068	0.950431453	0.007387373	0.856623359	0.053002399	0.605304358
PTCD3	0.520737787	0.924510937	0.732972874	0.006981481	0.047292686	0.053053172	0.605462873
C3AR1	0.434401818	0.923533434	0.009374646	0.097578524	0.318939387	0.053203903	0.606709901

LRRC24	0.153448859	0.324546255	0.052651429	0.088019249	0.508222204	0.053273351	0.606709901
SPATA46	0.517050674	0.000735654	0.671765773	0.603306025	0.760280233	0.053247233	0.606709901
SLC25A22	0.297618754	0.260935301	0.008776595	0.70553052	0.244402889	0.0533383	0.607028319
ARHGEF5	0.270182321	0.002507364	0.354811565	0.952119314	0.51432412	0.053388146	0.607174546
ACAT2	0.431979277	0.490110853	0.610787314	0.044842833	0.021138287	0.054735715	0.609318618
ANOS1	0.186451538	0.302149476	0.146837061	0.106680262	0.135487441	0.053905383	0.609318618
ARHGAP30	0.467189207	0.842253573	0.007269998	0.187717326	0.229709098	0.054948328	0.609318618
BLOC1S6	0.111390537	0.012528482	0.251075718	0.567801671	0.602058609	0.05396497	0.609318618
CDC42BPB	0.775631829	0.562433739	0.058194619	0.018812963	0.251774841	0.05409442	0.609318618
CHPF2	0.160308492	0.90085041	0.028903308	0.365613114	0.078207782	0.053846021	0.609318618
COTL1	0.332684102	0.903503081	0.002484851	0.174455313	0.925241286	0.054180383	0.609318618
ELP5	0.233973164	0.672189048	0.040470406	0.420006843	0.045881672	0.054757433	0.609318618
ERP29	0.910229225	0.122168771	0.409809804	0.690974564	0.003903249	0.054826452	0.609318618
FBN2	0.216669098	0.358585773	0.041857182	0.851772339	0.044352017	0.054812136	0.609318618
GCNA	0.143024514	0.20361447	0.395620692	0.377802928	0.027974514	0.054512924	0.609318618
GLI3	0.401378652	0.050400921	0.335384396	0.098475782	0.184591493	0.054942442	0.609318618
GSTP1	0.143619591	0.372628461	0.064180163	0.038207913	0.932346394	0.054674689	0.609318618
IGF1	0.756146457	0.096428309	0.015268286	0.712930877	0.155771128	0.05502469	0.609318618
KDELC2	0.002490302	0.216804158	0.900799318	0.908390599	0.277332652	0.054721107	0.609318618
LOC101905743	0.98765499	0.296545416	0.057533945	0.008547473	0.842052803	0.054379811	0.609318618
LOC101906916	0.737630673	0.681583285	0.001882205	0.269709915	0.479536833	0.05468417	0.609318618
LOC104976020	0.26655533	0.701177954	0.431408473	0.266796466	0.005538067	0.053786122	0.609318618
LOC112441484	0.435903356	0.007017543	0.103169366	0.482800001	0.799819883	0.054540654	0.609318618
LOC530973	0.072117217	0.097264139	0.648267611	0.402487003	0.06593839	0.054213747	0.609318618
LOC784007	0.110309571	0.135965507	0.0662282	0.404690832	0.30687949	0.054950162	0.609318618
MIS18BP1	0.70488152	0.025250774	0.067654412	0.308826852	0.328658103	0.054637992	0.609318618
ODC1	0.300319954	0.122213976	0.067253007	0.124443264	0.398235899	0.054667533	0.609318618
PDGFA	0.613169595	0.473958362	0.084650152	0.07985228	0.062477969	0.054778504	0.609318618
PIGN	0.225060295	0.25850534	0.094924178	0.078538422	0.281661371	0.054623188	0.609318618
PIK3AP1	0.743822765	0.612254633	0.000982823	0.521801438	0.51632004	0.054188053	0.609318618
PLD4	0.034211959	0.33263056	0.194749511	0.138422789	0.394413809	0.054301271	0.609318618
PLXNC1	0.390267352	0.685778571	0.001243739	0.418458268	0.858090403	0.053894247	0.609318618
PRKCQ	0.604581161	0.674226621	0.097221857	0.075310849	0.041291198	0.054916257	0.609318618
SIAE	0.210356506	0.763272915	0.056219233	0.241063628	0.054625956	0.053710675	0.609318618
SLA	0.409436238	0.476642632	0.042910826	0.049586908	0.294744622	0.054685345	0.609318618
SMIM10L1	0.542783725	0.34280106	0.055595749	0.105855085	0.109184122	0.053903487	0.609318618
SPI1	0.463013384	0.72958052	0.001159605	0.375482844	0.807057006	0.053666545	0.609318618
TFDP1	0.134716007	0.12706198	0.176584531	0.080053477	0.500837147	0.054354159	0.609318618
TMEM176B	0.425387549	0.115071669	0.01001092	0.786875866	0.308948123	0.053784325	0.609318618
TTYH2	0.210733689	0.817886458	0.008827121	0.137369396	0.591235348	0.055006133	0.609318618
USP15	0.018063718	0.26190452	0.105396617	0.310993374	0.795525538	0.054950625	0.609318618
ZNF436	0.124305921	0.399661691	0.003149609	0.918430279	0.842124246	0.054307965	0.609318618
ZNF831	0.058617749	0.872324527	0.973237158	0.153424778	0.01574902	0.054094081	0.609318618
LOC514507	0.103889212	0.193782791	0.388202002	0.120980302	0.131010336	0.05508915	0.60962107
SLC28A1	0.299936898	0.653448127	0.138236487	0.008675475	0.529633866	0.055258233	0.611080092

GEMIN4	0.57494378	0.572474143	0.039278681	0.065642613	0.146862912	0.055297541	0.611102996
FGF2	0.174062333	0.385305061	0.030873253	0.298652288	0.202461239	0.055451102	0.611313317
LOC112444600	0.009717199	0.070758646	0.631707234	0.857385694	0.335443601	0.055375393	0.611313317
PRKX	0.461552589	0.167765363	0.004451098	0.974275949	0.373002543	0.055465574	0.611313317
ZNF12	0.972905212	0.909761104	0.204650992	0.091261046	0.00756248	0.055401145	0.611313317
ESM1	0.514709741	0.038900267	0.024442041	0.348638221	0.73529825	0.055520938	0.611325418
GPR34	0.380352603	0.828558391	0.004488764	0.402347847	0.220579805	0.05554546	0.611325418
MBP	0.367988196	0.899193834	0.015020304	0.086511755	0.292269085	0.055578424	0.611325418
LOC104973519	0.532287536	0.068449798	0.046050407	0.315268441	0.238170958	0.05566459	0.611863084
ALOX5AP	0.080482865	0.405094372	0.112821942	0.068144675	0.503566723	0.05572913	0.611924437
ARL14EP	0.137083621	0.098630665	0.025070707	0.910794511	0.410452083	0.055863378	0.611924437
CCDC114	0.387223243	0.095749291	0.006163099	0.683174184	0.813352979	0.055931184	0.611924437
ITGA2B	0.189884995	0.00681255	0.286220358	0.531339005	0.64496095	0.055907461	0.611924437
LOC112441545	0.029562927	0.879503172	0.8294183	0.007443255	0.78701752	0.055757738	0.611924437
SERTM1	0.004496234	0.90958119	0.74049238	0.350159252	0.119669339	0.05591168	0.611924437
SLC5A6	0.972073869	0.39317342	0.748581852	0.014750456	0.030043063	0.05588115	0.611924437
LOC509118	0.608628941	0.60286329	0.00400541	0.091943178	0.942337119	0.056028928	0.612402536
VNN2	0.462885264	0.77743489	0.01149117	0.063948077	0.481809653	0.056049516	0.612402536
HPD	0.498945063	0.993195334	0.065658771	0.004342818	0.904128294	0.056142667	0.612720451
TET2	0.76081872	0.840652446	0.015178099	0.033960846	0.387634422	0.056153285	0.612720451
PARP14	0.916018137	0.160693644	0.023524946	0.115406573	0.321401529	0.056327298	0.613395679
SPATA5L1	0.051477794	0.035852639	0.118221339	0.748224632	0.786506576	0.056316291	0.613395679
TNC	0.063350645	0.374104953	0.023203913	0.879440831	0.265258917	0.056285412	0.613395679
CILP2	0.030533371	0.184062272	0.685980329	0.770999301	0.043390177	0.056469367	0.613572432
GPC3	0.118668204	0.081884403	0.438187839	0.030606244	0.990349579	0.056493081	0.613572432
MANBA	0.198482248	0.828300869	0.063082879	0.033031183	0.376241159	0.056446694	0.613572432
PXDC1	0.244966337	0.831886956	0.055712745	0.509434071	0.022270785	0.056425774	0.613572432
FGF11	0.00156207	0.561637229	0.595298008	0.745294503	0.332076972	0.056545901	0.613739939
ABCA6	0.041439261	0.533379842	0.687277685	0.02084592	0.411741513	0.056847033	0.613975188
ASPHD2	0.17901918	0.094988692	0.271746469	0.080617411	0.35036905	0.056884156	0.613975188
DNAAF5	0.24223232	0.389906255	0.143320566	0.012766876	0.750379565	0.056658303	0.613975188
EVI2A	0.554500486	0.994175309	0.003399218	0.261587315	0.265144866	0.056736389	0.613975188
GPX3	0.668567328	0.490805559	0.102823436	0.166632688	0.023212675	0.056879521	0.613975188
LOC533307	0.098385008	0.595096781	0.01438846	0.590341867	0.262429235	0.056880685	0.613975188
PSKH1	0.136873775	0.546825374	0.017383655	0.176461192	0.568829288	0.056904287	0.613975188
PTPRN2	0.66521811	0.419204873	0.019464206	0.049257403	0.485534553	0.056694427	0.613975188
TLR7	0.320381861	0.876722176	0.042479691	0.014708168	0.743917352	0.056892535	0.613975188
TMUB1	0.504353026	0.634759219	0.013752998	0.110631197	0.268525666	0.056957479	0.614145331
ACAP1	0.240897424	0.019668821	0.072592103	0.426292772	0.895015119	0.05707261	0.614176128
DHRS1	0.086271804	0.110671633	0.671514952	0.436353333	0.046866148	0.057042094	0.614176128
SNCG	0.72991657	0.275890398	0.438271271	0.097338604	0.015259037	0.057034592	0.614176128
TCEA1	0.710267782	0.470069985	0.19786118	0.367008181	0.00541938	0.057115412	0.614233958
RSRP1	0.148299935	0.253037355	0.828622915	0.009230227	0.459211828	0.057223066	0.614988698
LYN	0.674211542	0.479220789	0.047791621	0.016710999	0.512086477	0.057313471	0.615557178
LOC100848940	0.449977188	0.008838538	0.212403246	0.177039543	0.885238938	0.057380759	0.615876802

HECA	0.493932262	0.822636763	0.241624	0.002857166	0.47357443	0.057500107	0.616351574
RAD50	0.027371273	0.078951516	0.176349987	0.626898526	0.555529856	0.057467453	0.616351574
ACAP3	0.315423467	0.142838572	0.007074414	0.600497441	0.709789772	0.058292444	0.616410679
BCL7A	0.693542796	0.18880331	0.426866855	0.008671449	0.279857413	0.058237821	0.616410679
CD300LB	0.282016939	0.628641845	0.235680998	0.951021635	0.003391328	0.058005725	0.616410679
ELAVL3	0.170642156	0.094915555	0.119877516	0.480339735	0.142835486	0.057597838	0.616410679
FLNC	0.004285059	0.381811306	0.375848684	0.830157378	0.265842506	0.058254167	0.616410679
GCN1	0.379771839	0.462592674	0.138969321	0.021064971	0.26088933	0.057850498	0.616410679
LOC100300510	0.015128656	0.415692745	0.778644311	0.186587363	0.146270881	0.057711877	0.616410679
LOC101908359	0.933565519	0.352900101	0.031479473	0.157574544	0.082893916	0.058191095	0.616410679
LOC104972821	0.613869315	0.092827341	0.194866701	0.166158994	0.072268869	0.057631762	0.616410679
LOC515551	0.343620335	0.064025282	0.010783701	0.584777398	0.980163399	0.058326566	0.616410679
LOC534967	0.423575287	0.660814549	0.855070069	0.001141025	0.49689264	0.058251402	0.616410679
LSMEM1	0.317533157	0.307527333	0.053662311	0.181018557	0.14244808	0.058100296	0.616410679
MZT2B	0.388076678	0.920655943	0.999263778	0.000470324	0.810428136	0.058352738	0.616410679
NCAPD2	0.117513385	0.146899451	0.066742386	0.720623996	0.163980869	0.05836952	0.616410679
PARVG	0.661509283	0.982087066	0.000485969	0.579018087	0.730867187	0.057701555	0.616410679
PCNX3	0.976316284	0.013453514	0.182522727	0.062339079	0.907967393	0.058251741	0.616410679
PINX1	0.201453021	0.91491722	0.33803763	0.349319473	0.006164407	0.057848638	0.616410679
POLR1A	0.721221282	0.93234347	0.18298703	0.01756561	0.062695088	0.058201628	0.616410679
SIRT1	0.16184805	0.542499754	0.472710236	0.004567346	0.710092592	0.057966405	0.616410679
SLC22A23	0.813576176	0.445050193	0.001783174	0.645824483	0.323292556	0.058017787	0.616410679
SLC9B2	0.012074808	0.732543477	0.103749046	0.376167819	0.390749767	0.058039574	0.616410679
SOX5	0.123817804	0.192096817	0.531389127	0.228908877	0.046648629	0.058059108	0.616410679
SRGAP2	0.147060123	0.465734566	0.139763715	0.016346028	0.859096054	0.05791759	0.616410679
LOC101906818	0.298342048	0.07555813	0.132880324	0.109645141	0.416727377	0.058557342	0.61799649
TRMT61A	0.355014362	0.951583709	0.31027109	0.026757065	0.048875966	0.058612502	0.618181086
ITIH1	0.02369383	0.572524834	0.217573209	0.367707277	0.126599727	0.058695044	0.618654055
RNF112	0.728609372	0.231634536	0.135233624	0.007434245	0.811727387	0.058782466	0.619177828
B3GNT5	0.022752194	0.766029771	0.114447111	0.800176666	0.086446014	0.058846451	0.619454209
DCXR	0.418782992	0.422316718	0.537767151	0.002339504	0.62243929	0.058981912	0.619939597
HSPA1L	0.687743943	0.297593627	0.668723377	0.002091157	0.484734934	0.059043665	0.619939597
THAP9	0.64541282	0.029526967	0.756323211	0.014105093	0.681856389	0.059014239	0.619939597
ZNF688	0.34298199	0.008651859	0.354419145	0.180951153	0.727826003	0.058985591	0.619939597
ZDHHC13	0.009157436	0.71639496	0.563244798	0.041089705	0.915377268	0.059107414	0.620212125
FREM1	0.200090833	0.136417623	0.264562898	0.268442617	0.072019538	0.059271177	0.620343937
LY75	0.486257901	0.830608122	0.083933635	0.147814696	0.027832046	0.059232371	0.620343937
PLEKHB2	0.538013709	0.121562322	0.007300241	0.898971284	0.325092671	0.059250733	0.620343937
RIPOR3	0.047306306	0.145888852	0.837214455	0.267808587	0.090122277	0.059230117	0.620343937
SPTBN5	0.173524741	0.177362678	0.322749819	0.053353652	0.264044379	0.059354586	0.620820975
SP140	0.856103253	0.278954232	0.027060998	0.029719814	0.72943241	0.05939651	0.620863774
CHCHD6	0.027862121	0.123682031	0.426794671	0.543311768	0.176330432	0.059603645	0.622236268
LOC530077	0.692820257	0.796946984	0.025670569	0.070140904	0.141585912	0.059566941	0.622236268
TYRP1	0.516888253	0.938518247	0.388319268	0.127068757	0.005896807	0.059667856	0.622510608
CUL1	0.428418991	0.31634055	0.006110539	0.176081037	0.980786343	0.060146975	0.624729115

GTF2H4	0.683902459	0.313309308	0.783913899	0.003678181	0.231238593	0.060108148	0.624729115
IFI6	0.609933198	0.276074942	0.003816809	0.274268676	0.809736791	0.060074517	0.624729115
LRRN3	0.409960645	0.006370622	0.35604897	0.829715579	0.184311315	0.059938724	0.624729115
PMM1	0.413387091	0.529562377	0.708231089	0.001368007	0.67378779	0.060119206	0.624729115
RCC1	0.380308861	0.896895723	0.872588842	0.257345327	0.001858566	0.059977995	0.624729115
SPINT2	0.088240547	0.258909146	0.343073682	0.038920639	0.467137426	0.060015433	0.624729115
MCRIP2	0.586308307	0.128515209	0.965964047	0.003410297	0.576979541	0.060197891	0.624862482
PRR11	0.160969047	0.364156084	0.567998743	0.009980461	0.432441809	0.060321256	0.625435126
TOB1	0.534244884	0.098751818	0.387986418	0.030281384	0.231885097	0.060329279	0.625435126
SCN7A	0.50993404	0.046229361	0.106741958	0.335039007	0.170672646	0.060369496	0.625456945
C3	0.266974469	0.009429468	0.390640114	0.318078315	0.462264144	0.060549972	0.625565545
DLK2	0.520609912	0.825459096	0.103391834	0.077062731	0.042218608	0.06054018	0.625565545
LOC513894	0.131153436	0.437668002	0.686245399	0.076430729	0.048053948	0.060570572	0.625565545
LPXN	0.275736818	0.797470209	0.002980294	0.559357675	0.394126151	0.060519049	0.625565545
PIK3CG	0.986873717	0.034684702	0.238835288	0.101354244	0.174081358	0.060459677	0.625565545
LOC104970537	0.110020012	0.028674706	0.39607947	0.982523805	0.117979791	0.060613216	0.625612252
IMPG2	0.707161689	0.673308253	0.631320813	0.002905396	0.16624346	0.060700585	0.625939071
MRTO4	0.262587189	0.812406193	0.033614911	0.123195929	0.16443706	0.060721163	0.625939071
SYPL2	0.265954212	0.545211167	0.297267148	0.081366134	0.041525933	0.060815301	0.626515952
SIRPB1	0.148190695	0.822954679	0.180610274	0.513393953	0.012908861	0.060900156	0.626996522
FANCI	0.149518614	0.630042038	0.076260591	0.025802372	0.790845385	0.061056986	0.627633074
LOC112447302	0.021408111	0.137103235	0.938785754	0.143526928	0.37059215	0.061048956	0.627633074
NHP2	0.322061535	0.626072835	0.561358935	0.096938816	0.013367343	0.061076718	0.627633074
ARMC8	0.197533675	0.085061882	0.376344928	0.04024317	0.578202354	0.061195336	0.627672909
LOC784769	0.085919013	0.201907195	0.084854933	0.109201941	0.914868481	0.061175905	0.627672909
ZDHHC2	0.324209276	0.119382441	0.009001831	0.701706566	0.600675868	0.061123325	0.627672909
S1PR5	0.35381544	0.02053581	0.261000849	0.389080331	0.199846685	0.061275196	0.628099468
HMGN5	0.895354495	0.237526875	0.002351491	0.343293766	0.860946416	0.061363198	0.628216746
MAP6	0.316203123	0.537945383	0.454895027	0.224133586	0.008521744	0.061359586	0.628216746
P4HB	0.11956949	0.560417843	0.184569023	0.297669663	0.040412769	0.06160828	0.629547623
PSPH	0.585025255	0.383011761	0.872304466	0.001772047	0.428930598	0.061554287	0.629547623
RAP1A	0.599030871	0.149040002	0.004852901	0.385742531	0.890030709	0.061600675	0.629547623
C5H12orf29	0.985979911	0.622022988	0.203527763	0.079302893	0.015060743	0.061684896	0.629938292
SH3BGRL3	0.093071428	0.867954665	0.054600343	0.046050599	0.735729688	0.06177364	0.630452246
HIPK3	0.810579725	0.390056621	0.159033921	0.003464998	0.859421465	0.061847912	0.630817954
LOC104973154	0.114607204	0.795833413	0.979894237	0.706679663	0.002378996	0.061978285	0.630971242
MYO1E	0.706219849	0.851059274	0.125808253	0.017289485	0.114858453	0.061954413	0.630971242
UCKL1	0.26786601	0.334353788	0.175550471	0.024212467	0.394133208	0.061924479	0.630971242
YIPF2	0.110187391	0.205559372	0.043827353	0.157239299	0.964037935	0.062033714	0.631144001
FXN	0.852835047	0.325845923	0.540840714	0.001793996	0.561194517	0.062243006	0.632249945
LOC112442218	0.022214295	0.492231301	0.40996337	0.083113042	0.406480204	0.062275507	0.632249945
LPCAT2	0.009615273	0.707944119	0.099693334	0.330760989	0.675081182	0.062296518	0.632249945
NOC2L	0.273802357	0.953371231	0.408747593	0.064583231	0.021979435	0.062278577	0.632249945
DNAJC3	0.892697989	0.310460713	0.31155012	0.007469101	0.237096674	0.062639689	0.633709702
HSH2D	0.027922505	0.409076794	0.060865116	0.703932957	0.312329743	0.062625652	0.633709702

INSL6	0.048688456	0.445559574	0.740415658	0.032955521	0.28770262	0.062486587	0.633709702
KCNH3	0.749080671	0.476246249	0.24696154	0.010957014	0.158012329	0.062547078	0.633709702
LTV1	0.483324376	0.459773452	0.534995514	0.033715602	0.038164533	0.062656357	0.633709702
SPAG5	0.96111765	0.041827364	0.029585417	0.719273701	0.178893291	0.06267204	0.633709702
C1QTNF6	0.603689222	0.15723565	0.008378708	0.666828895	0.290477238	0.062923024	0.635464456
LOC107132524	0.41710298	0.529784543	0.459289778	0.018414072	0.082357487	0.062889182	0.635464456
ABL1	0.463377416	0.946073899	0.574315487	0.000894425	0.687078518	0.063089351	0.6355797
CYTIP	0.993114544	0.247054287	0.022341988	0.329840555	0.085553743	0.063080171	0.6355797
MAP3K12	0.231110011	0.121900816	0.016186767	0.666645452	0.508924282	0.063086939	0.6355797
SRSF6	0.125460884	0.318027415	0.575337665	0.008553111	0.786411158	0.06301097	0.6355797
ADGRB1	0.127595633	0.008628383	0.591396238	0.646747465	0.369937388	0.063348964	0.636049161
ALG3	0.347352634	0.836378978	0.892163062	0.00528464	0.113533797	0.063282933	0.636049161
GIMAP7	0.163862848	0.394111565	0.121710616	0.140863012	0.140353954	0.0632557	0.636049161
GPIHBP1	0.028155503	0.733905067	0.891277268	0.009369808	0.902718652	0.063348288	0.636049161
KLK13	0.704898262	0.08469675	0.065833502	0.639053969	0.061944546	0.063302537	0.636049161
PF4	0.21913579	0.637485222	0.003937053	0.509024939	0.556716615	0.063368495	0.636049161
FKBP10	0.676329319	0.098243805	0.003822675	0.794764945	0.778420335	0.063683234	0.637419598
HSPA2	0.672043202	0.816105483	0.082675007	0.004266099	0.813380845	0.063732832	0.637419598
LARS	0.557441969	0.142178698	0.754086188	0.039011195	0.067492763	0.063738076	0.637419598
MRPS24	0.652333707	0.705623102	0.950999457	0.000865508	0.414461146	0.063656101	0.637419598
PUSL1	0.654884009	0.575821434	0.372877212	0.089759227	0.012463931	0.063724809	0.637419598
PYGM	0.005034103	0.545355763	0.219302411	0.76323606	0.341574854	0.06363947	0.637419598
DERL1	0.352713491	0.013933743	0.145234621	0.634768076	0.34895457	0.063919629	0.638457113
TNFSF8	0.450294419	0.801841538	0.012270735	0.068931646	0.51767692	0.063918661	0.638457113
ADGRE3	0.094596799	0.762343948	0.00354213	0.738710485	0.84468248	0.06423324	0.640303369
H2AFY2	0.649697884	0.131534946	0.64038904	0.004187169	0.69558647	0.064233997	0.640303369
INPP5J	0.219840924	0.029127429	0.096213203	0.550444289	0.470333807	0.064260535	0.640303369
YPEL1	0.085560087	0.720932739	0.011719888	0.310070036	0.710699949	0.064213203	0.640303369
NINL	0.231303708	0.054441079	0.636188322	0.193709177	0.102979956	0.064335071	0.640657065
EMC9	0.54660142	0.330888768	0.926428492	0.000961669	0.993848482	0.064416814	0.640747012
PTPN12	0.367124592	0.720226878	0.75318299	0.002671306	0.301369287	0.064461234	0.640747012
SUSD4	0.9560823	0.023349471	0.058660024	0.48924374	0.250150539	0.064446472	0.640747012
ATP6AP1	0.146879275	0.121823109	0.031720124	0.543013312	0.525545401	0.06486053	0.640768672
CAPZA2	0.36039169	0.119160491	0.01039297	0.475045789	0.75903674	0.064608389	0.640768672
CD6	0.022736121	0.060408157	0.867964551	0.386683139	0.352371376	0.064970999	0.640768672
CLK1	0.04832333	0.287082878	0.454264657	0.198620961	0.128521783	0.064593103	0.640768672
FBXL3	0.0380567	0.209473874	0.207389004	0.737257669	0.13282067	0.064841223	0.640768672
FFAR3	0.594896181	0.237415838	0.352138722	0.011157003	0.291763975	0.064842322	0.640768672
FUBP1	0.638178604	0.903439815	0.937867521	0.000354888	0.841670531	0.064749691	0.640768672
FZD2	0.818131818	0.117953465	0.011455439	0.210854619	0.695615678	0.064901341	0.640768672
GPR4	0.049595601	0.4516781	0.406160017	0.301995739	0.059090554	0.064954601	0.640768672
LOC783604	0.511958223	0.056943429	0.347193718	0.148505659	0.107640065	0.06481736	0.640768672
LRP4	0.247117795	0.562493517	0.014770163	0.448237441	0.176141003	0.064890262	0.640768672
MAGED1	0.502817303	0.284744926	0.003603867	0.348173008	0.90087591	0.064828903	0.640768672
STK38L	0.810830437	0.693244462	0.004092263	0.145651245	0.479453474	0.064536137	0.640768672

SAT2	0.184601261	0.331410148	0.194017115	0.023532428	0.582950643	0.065067637	0.64133633
AEBP2	0.222244219	0.944236389	0.802572593	0.003086629	0.317151326	0.065558348	0.641437856
ANGPTL5	0.099401674	0.402457002	0.138678226	0.609703779	0.049100741	0.065848639	0.641437856
ARHGEF2	0.352681901	0.013539258	0.435227702	0.112311785	0.702133122	0.065321345	0.641437856
ATL2	0.247321479	0.275813598	0.975739561	0.009872868	0.254055616	0.066055084	0.641437856
ATP6V0A1	0.786819361	0.13243387	0.005239765	0.821782042	0.36445613	0.065235037	0.641437856
C23H6orf62	0.073813865	0.569373755	0.43685818	0.835242248	0.010828047	0.065840418	0.641437856
CDKN2B	0.094258806	0.337387777	0.439912916	0.015111514	0.788881232	0.066014212	0.641437856
COL1A2	0.930255337	0.058788943	0.026025948	0.138930628	0.837654445	0.065742575	0.641437856
EME2	0.172019267	0.064773687	0.190778644	0.080085602	0.959580663	0.06519458	0.641437856
FKBP1B	0.573855734	0.129647679	0.017216343	0.636908667	0.202990598	0.065732915	0.641437856
GALNT12	0.006166974	0.353374233	0.655372768	0.355188969	0.327554535	0.065867846	0.641437856
GIMAP7	0.107168915	0.277117668	0.201648022	0.327506985	0.083989305	0.065524215	0.641437856
KIF20B	0.316077206	0.174992758	0.026402608	0.230588018	0.493387624	0.065862964	0.641437856
LSS	0.956987174	0.95431126	0.52822449	0.003932136	0.087951974	0.066028072	0.641437856
MINPP1	0.037295265	0.705884827	0.354886593	0.14110751	0.12377081	0.065149665	0.641437856
RABEP1	0.901058535	0.699302287	0.302070388	0.002169384	0.40281531	0.065907182	0.641437856
RASGRP3	0.949795851	0.811577742	0.024743303	0.093785507	0.092098168	0.065527239	0.641437856
REEP4	0.069121047	0.02664549	0.489414051	0.347766858	0.531880493	0.06600277	0.641437856
SAMD3	0.344840294	0.349864232	0.903612845	0.215279224	0.00708669	0.065905072	0.641437856
SLC39A14	0.791939796	0.67252419	0.014454427	0.123177579	0.173533945	0.065482862	0.641437856
SRRM3	0.546379657	0.764947989	0.029873598	0.251506264	0.052464922	0.065529697	0.641437856
TMED4	0.455278481	0.021767213	0.068918779	0.282594973	0.851491472	0.065432416	0.641437856
TMEM88	0.394662359	0.917199089	0.338642193	0.002025036	0.663156421	0.065497608	0.641437856
WDR6	0.976152059	0.935288957	0.022017713	0.017715885	0.463757806	0.065626036	0.641437856
ZC3H7B	0.926573123	0.497900471	0.043135013	0.010534332	0.789847378	0.065727522	0.641437856
ATP13A2	0.337613081	0.053476553	0.290560328	0.054221154	0.588606888	0.06616788	0.641773686
SLC15A2	0.3674909	0.965717761	0.350922898	0.006968111	0.192792345	0.06614037	0.641773686
GJA1	0.086362286	0.767390209	0.007276684	0.496803963	0.700781646	0.066280764	0.642488852
USP42	0.55179544	0.902906635	0.842840238	0.000577699	0.69287539	0.066325061	0.642538713
CLEC10A	0.57269521	0.603409774	0.006998591	0.206327312	0.338376901	0.066507517	0.643546496
P3H4	0.528151209	0.304445697	0.001489477	0.82635805	0.852974807	0.066498515	0.643546496
PICALM	0.662456269	0.360961757	0.001955093	0.510780719	0.708152121	0.066566837	0.643740932
MAP4K1	0.641501592	0.156308305	0.029296666	0.395800767	0.146049209	0.066735674	0.644993609
HSPB11	0.405738284	0.813294404	0.19872561	0.539095571	0.004817553	0.066852894	0.645746227
CPSF1	0.660456497	0.012966445	0.060176896	0.346377457	0.959363333	0.06707448	0.647124802
LOC508153	0.927293652	0.943449043	0.025535936	0.019450675	0.393963325	0.067060573	0.647124802
CHRD	0.670710067	0.186837882	0.021464518	0.074321585	0.859164479	0.067193955	0.647614401
DPH1	0.701294546	0.992948638	0.030706165	0.010437571	0.771380834	0.06728793	0.647614401
EPOR	0.835499457	0.392430999	0.043985486	0.022173141	0.538613251	0.067306761	0.647614401
LOC112447526	0.284974435	0.054011398	0.013648173	0.825107728	0.994067415	0.067322538	0.647614401
MFSD9	0.149673819	0.867619665	0.64819725	0.045242371	0.045179857	0.067264775	0.647614401
ADAMTS10	0.147897699	0.0305485	0.123032659	0.534020668	0.581460876	0.067393244	0.647651011
AFTPH	0.666434785	0.343926412	0.038817807	0.029995591	0.659524363	0.068190021	0.647651011
ANKMY2	0.343639721	0.333940168	0.241636918	0.031746895	0.199948261	0.068191129	0.647651011

ARFGEF1	0.252193139	0.982515737	0.030146389	0.026694542	0.87964324	0.068048172	0.647651011
ARID4A	0.363729778	0.47835117	0.284353831	0.005427614	0.655028735	0.068162824	0.647651011
ARRDC5	0.177807917	0.198625588	0.562545434	0.530182143	0.016568429	0.067842577	0.647651011
AXIN2	0.102125548	0.485792776	0.063402021	0.297476828	0.185063421	0.067525145	0.647651011
FLT3	0.355239309	0.331419938	0.156343363	0.263221144	0.03579834	0.067590665	0.647651011
HHIP	0.127110328	0.056119526	0.503919251	0.796918414	0.060925609	0.06784438	0.647651011
ITGB7	0.049103787	0.399088863	0.389777654	0.235658772	0.097061199	0.067887702	0.647651011
KCNK17	0.715658388	0.19958212	0.628224742	0.002670929	0.7223082	0.067512522	0.647651011
LIF	0.668150072	0.644478871	0.008329232	0.069207881	0.705523252	0.067983941	0.647651011
LOC101905630	0.057452509	0.258124047	0.123607947	0.179965081	0.527453809	0.067721203	0.647651011
LOC112444931	0.591656796	0.132741283	0.066548907	0.079285247	0.420585174	0.067787468	0.647651011
MYOM3	0.017477481	0.251416746	0.4524805	0.207805861	0.418797517	0.067494533	0.647651011
PLEKHA1	0.457616651	0.130912976	0.008462297	0.512664362	0.672014068	0.067873937	0.647651011
TCF21	0.043292445	0.924504912	0.345096431	0.018053603	0.705935319	0.068194561	0.647651011
TMEM245	0.021888081	0.148459192	0.175416018	0.388181868	0.793421432	0.068084527	0.647651011
TMSB10	0.005021188	0.419289864	0.365429317	0.7100677	0.317559096	0.067598697	0.647651011
TOMM34	0.734198765	0.836198085	0.111305164	0.066395238	0.038442316	0.067817634	0.647651011
TRABD	0.158448323	0.011301113	0.351849927	0.489710845	0.569213751	0.068099434	0.647651011
ZNF213	0.542870344	0.825409439	0.1166299	0.006764488	0.497183391	0.068131996	0.647651011
CCR4	0.039495483	0.979619668	0.916121784	0.01351341	0.378732348	0.069436903	0.648827454
CD52	0.014048246	0.834370788	0.523018967	0.155889019	0.185629434	0.068513137	0.648827454
CNTLN	0.273370879	0.148017901	0.51011015	0.032667091	0.266116515	0.068983597	0.648827454
COL1A1	0.865190243	0.043977203	0.018344817	0.296978918	0.886150886	0.069959204	0.648827454
CXCL16	0.151912449	0.503627722	0.005749772	0.545161183	0.759657431	0.069613564	0.648827454
CXCR6	0.141236351	0.906274072	0.676175445	0.472268644	0.004353486	0.068639284	0.648827454
ECSIT	0.640173394	0.581876341	0.890918786	0.001173204	0.472566536	0.070028419	0.648827454
ENKD1	0.621154223	0.027180662	0.125440304	0.390445094	0.22455718	0.07041385	0.648827454
FAM160B2	0.878172552	0.950062451	0.064859438	0.006988923	0.477661964	0.069262696	0.648827454
FAM180B	0.336256827	0.416002614	0.002391373	0.610945391	0.904865131	0.070240994	0.648827454
FAM222A	0.524326793	0.046006428	0.036867394	0.688222161	0.297512991	0.069594473	0.648827454
GCFC2	0.023319376	0.434360236	0.351228475	0.161527837	0.312534782	0.069020742	0.648827454
GPR155	0.420180634	0.280920607	0.013966389	0.74882136	0.148991736	0.070013098	0.648827454
GPR173	0.195091461	0.737209566	0.010417134	0.172010064	0.718337658	0.07028549	0.648827454
GTF2H1	0.345984791	0.570488312	0.409696657	0.002679781	0.839339341	0.069546984	0.648827454
HMCN1	0.099640686	0.693064982	0.145131945	0.019057118	0.962272052	0.069982646	0.648827454
ITGA3	0.881169531	0.347883043	0.643165889	0.236108987	0.003866779	0.069113777	0.648827454
ITPKA	0.01118551	0.842060822	0.981160163	0.984507575	0.020283452	0.07015382	0.648827454
KCNS3	0.450371357	0.774605216	0.576349382	0.109903983	0.00803778	0.068562932	0.648827454
LAMA2	0.130533732	0.025095187	0.821762681	0.073803176	0.929938056	0.070201103	0.648827454
LCK	0.079959284	0.592727468	0.097117916	0.56807393	0.068160503	0.068702803	0.648827454
LDB3	0.003894503	0.540676156	0.225248615	0.988740453	0.394189648	0.07022533	0.648827454
LOC100297240	0.834814774	0.000696514	0.357229898	0.90156527	0.979913762	0.069917392	0.648827454
LOC101906397	0.006142842	0.866051581	0.221902496	0.467755669	0.332449746	0.069933581	0.648827454
LOC112445030	0.265508201	0.28549501	0.594452846	0.008207993	0.494582022	0.069784083	0.648827454
LOC519309	0.740517877	0.042218371	0.100803826	0.72881069	0.080557456	0.070263646	0.648827454

LOC613822	0.4673708	0.297300684	0.003076033	0.764851909	0.566867513	0.070328865	0.648827454
LOC615733	0.104083116	0.13946777	0.490457293	0.268911473	0.096906819	0.07037848	0.648827454
LOC789748	0.069487078	0.450601923	0.159060119	0.885293792	0.040570017	0.068853931	0.648827454
LRRC8D	0.833309895	0.986606446	0.968704448	0.099487304	0.002335452	0.070268095	0.648827454
MEG9	0.939717608	0.008351803	0.06085603	0.455902594	0.842209016	0.069890456	0.648827454
MN1	0.268365226	0.230921607	0.132636091	0.043077233	0.516132583	0.069744847	0.648827454
NCF1	0.424060986	0.740950391	0.007107763	0.319183821	0.253937444	0.069347089	0.648827454
NPC2	0.696360045	0.020274355	0.023359533	0.540698793	0.997360853	0.068616616	0.648827454
PGM5	0.004649847	0.297849809	0.406688302	0.741365823	0.441814492	0.070141258	0.648827454
PIK3C3	0.003416634	0.682081334	0.359477429	0.311044641	0.692809157	0.069234622	0.648827454
PSMB9	0.046048385	0.882661908	0.04912507	0.159463433	0.560694847	0.068772976	0.648827454
PTOV1	0.332294414	0.624715612	0.212772969	0.006597107	0.627608571	0.069773977	0.648827454
PTP4A3	0.873805285	0.361990945	0.01357753	0.05005445	0.841307745	0.069310017	0.648827454
RALGAPB	0.799027195	0.098510038	0.00849797	0.271856404	0.996262295	0.06938085	0.648827454
RNASE6	0.656825075	0.804655804	0.016695929	0.074113696	0.278360694	0.069582878	0.648827454
RPS26	0.40647055	0.19382569	0.812168884	0.036140928	0.078456258	0.06944248	0.648827454
SDK1	0.740240641	0.062494145	0.014533744	0.743059215	0.362612124	0.069379411	0.648827454
SEPHS2	0.151520779	0.615976596	0.412173008	0.028523119	0.16497652	0.069348727	0.648827454
SMURF2	0.794892324	0.896720285	0.818106931	0.000740893	0.426439635	0.070084981	0.648827454
TAB2	0.577652019	0.853336709	0.982654904	0.001712376	0.215343268	0.068793759	0.648827454
TMEM109	0.379619889	0.009108268	0.126884609	0.691957445	0.587341997	0.068722101	0.648827454
TRANK1	0.987042887	0.064145131	0.070591261	0.124168023	0.327327257	0.069493204	0.648827454
TWIST2	0.011313099	0.913104258	0.18161841	0.134709977	0.733808354	0.070361526	0.648827454
UBIAD1	0.664055061	0.08979132	0.099976607	0.073280008	0.424111555	0.070318685	0.648827454
WFS1	0.238530394	0.25492157	0.555889932	0.046140824	0.114094913	0.068639192	0.648827454
ZNF385B	0.282609362	0.176459242	0.672979551	0.046285833	0.117223024	0.069594113	0.648827454
ZNF800	0.151876114	0.702877884	0.901529922	0.002437382	0.769547463	0.069231367	0.648827454
SAA3	0.104414393	0.37717469	0.017439691	0.606939446	0.448496417	0.070701928	0.651116355
SFMBT2	0.661708856	0.310476142	0.001421221	0.934020609	0.688945291	0.070911853	0.652683349
ZBTB40	0.430654383	0.104375909	0.212675786	0.666089038	0.029606555	0.071055492	0.653638835
LYNX1	0.843170806	0.916787262	0.862334482	0.000371104	0.763000245	0.071105678	0.653734055
CHPF	0.078962528	0.843495767	0.014355274	0.222607022	0.891488488	0.071330212	0.654988697
KCTD15	0.992844376	0.566000153	0.026844842	0.0891215	0.141237951	0.071361879	0.654988697
ZNF470	0.152725803	0.336818866	0.021905532	0.535664267	0.314475414	0.0713471	0.654988697
KCNMB1	0.039685899	0.075581259	0.903993211	0.308349198	0.227474375	0.071430504	0.655252098
CAD	0.33878724	0.18380428	0.153870241	0.033822048	0.58902213	0.071585791	0.65594328
TUBG2	0.430870938	0.682344797	0.054152449	0.022904826	0.523150663	0.071561578	0.65594328
ABHD16B	0.457172182	0.705817783	0.019804896	0.055372215	0.54701692	0.072185816	0.657039062
AMT	0.183470092	0.042273017	0.196666418	0.821089722	0.154373496	0.072134671	0.657039062
CD37	0.560533155	0.767909624	0.008345972	0.103894618	0.516246421	0.071987662	0.657039062
EPPK1	0.348674518	0.185573562	0.873011566	0.611765424	0.005588777	0.072088662	0.657039062
FBLIM1	0.76796725	0.888559706	0.00174328	0.840765855	0.193382292	0.072151143	0.657039062
ITPKC	0.815815344	0.249393167	0.136885856	0.027892605	0.24745211	0.071886433	0.657039062
KIF1A	0.91991651	0.090470626	0.004031347	0.778513018	0.737066886	0.07195209	0.657039062
METTL3	0.087087079	0.171919503	0.943901646	0.014863348	0.916380214	0.071944069	0.657039062

PFN1	0.009454561	0.208427175	0.451980187	0.772643951	0.280892134	0.072125947	0.657039062
PPP1R3B	0.027731771	0.328706266	0.416674476	0.107748983	0.469678383	0.071884555	0.657039062
RAI1	0.943293983	0.540134376	0.102184193	0.006766192	0.548392908	0.072099635	0.657039062
RANBP2	0.569885743	0.417344547	0.368997794	0.002231946	0.978823507	0.07177568	0.657039062
RASGEF1A	0.755031933	0.853279839	0.000549453	0.981316222	0.55943686	0.072355641	0.658219744
LOC112445197	0.546145838	0.033553023	0.323791884	0.034658784	0.947211056	0.072457062	0.658777201
CACNB3	0.228179752	0.919316581	0.09879941	0.055932372	0.168445633	0.072562	0.658868903
GLIS3	0.169203688	0.524279682	0.054886588	0.04978285	0.806328767	0.072603031	0.658868903
LOC100848799	0.120496417	0.299465497	0.26086318	0.986304366	0.021093023	0.072688112	0.658868903
MYO1F	0.545730888	0.999886466	0.005424262	0.124299528	0.531208132	0.072600346	0.658868903
UCP2	0.191691753	0.301125255	0.21128277	0.437150823	0.03674828	0.072708036	0.658868903
ZBTB7B	0.153275279	0.69304396	0.018999984	0.100296502	0.966746645	0.072658272	0.658868903
MRPL23	0.295048539	0.384810198	0.211022543	0.037409506	0.218917072	0.072772732	0.659091232
FCGR2B	0.118934617	0.769045089	0.145171141	0.096147613	0.15443922	0.072983512	0.659544277
LOC104970976	0.738625695	0.563021129	0.003344274	0.158358772	0.893871725	0.072916575	0.659544277
SLC45A3	0.199053235	0.371006435	0.114793875	0.039181561	0.592990419	0.072939663	0.659544277
TRAM2	0.828990976	0.656786532	0.001432949	0.413970311	0.610371735	0.072976688	0.659544277
CFAP46	0.150342029	0.6851735	0.278260344	0.012956454	0.53176861	0.073054161	0.659571327
CYSLTR2	0.026712637	0.626194398	0.036009298	0.632996906	0.51817052	0.07307159	0.659571327
MAP1LC3C	0.05623247	0.870482814	0.011854698	0.582970797	0.584499181	0.073107077	0.659571327
TCF19	0.644249906	0.0213573	0.079663834	0.186496029	0.968199057	0.073150006	0.659596019
WIPF1	0.69007598	0.468921288	0.012209432	0.069416786	0.722671824	0.073210458	0.659778602
DTNBP1	0.093843502	0.224110593	0.057074422	0.25388038	0.65159985	0.073292932	0.659797206
SUPT3H	0.614513536	0.989395719	0.806438853	0.000681213	0.594047497	0.073258635	0.659797206
ABLIM1	0.495745869	0.704194614	0.172510072	0.019287468	0.171646764	0.073470053	0.661029082
C17H22orf39	0.701280279	0.961154818	0.441084248	0.000776225	0.865769878	0.07356282	0.661336752
DGKQ	0.237856427	0.043416218	0.51419025	0.039590741	0.951447892	0.073610526	0.661336752
MLLT1	0.82343203	0.850412316	0.012124793	0.173950858	0.135473984	0.073625144	0.661336752
TTC39C	0.82853465	0.287630703	0.390589765	0.746274663	0.002883491	0.073672618	0.66140117
CFB	0.155348685	0.024414338	0.268472921	0.421276104	0.470691631	0.074024264	0.661806223
DNER	0.099324266	0.304989241	0.056923922	0.794222634	0.147121828	0.073932749	0.661806223
EBPL	0.824981315	0.420329751	0.0200849	0.112935108	0.255592323	0.073834131	0.661806223
FABP4	0.066924366	0.500820068	0.090410142	0.149581979	0.444990136	0.073979265	0.661806223
GNGT2	0.023453055	0.537306733	0.246157867	0.098830046	0.655955642	0.0738461	0.661806223
MFSD8	0.563262527	0.575183923	0.12721599	0.021183146	0.231347634	0.074040353	0.661806223
RRP36	0.545149302	0.480330642	0.129675839	0.088591156	0.066906314	0.073883923	0.661806223
TRUB2	0.543532963	0.437113769	0.693517717	0.001356721	0.899299863	0.073833354	0.661806223
LOC104975814	0.073035796	0.479753372	0.00994483	0.692058176	0.839250911	0.074128787	0.662235997
NRIP1	0.703962625	0.046353732	0.16751946	0.344303257	0.107684857	0.074190769	0.662429112
ARHGAP25	0.434305765	0.783625715	0.01208761	0.194885616	0.253221492	0.074265007	0.662731396
RIOX1	0.565954598	0.789692585	0.212775549	0.005014452	0.426841169	0.074379702	0.663394179
DUSP2	0.708366007	0.143302925	0.061581402	0.228496654	0.142803073	0.074474394	0.663871513
NT5C3B	0.305629817	0.167803389	0.117244309	0.062215682	0.545733932	0.074514126	0.663871513
SH2D2A	0.074946357	0.557272349	0.641955897	0.287823416	0.026487284	0.074566994	0.663982063
WNK4	0.626366411	0.371107418	0.281364836	0.195267403	0.016026402	0.074625947	0.664146651

WDR19	0.233106498	0.022117139	0.119917304	0.494312658	0.673588353	0.07488166	0.666061205
LOC112448166	0.561438969	0.488351299	0.362589977	0.009484593	0.218882851	0.074996538	0.666530973
SHOC2	0.425412954	0.382634585	0.331362158	0.004087757	0.936450546	0.075015703	0.666530973
ABLIM2	0.545979055	0.013167684	0.12348018	0.667182839	0.351646634	0.075403357	0.66663841
AGPS	0.428694567	0.298299505	0.016801596	0.363151978	0.266202639	0.075281488	0.66663841
COG6	0.381462208	0.033521844	0.978295901	0.018459986	0.900906907	0.07535457	0.66663841
IGSF6	0.666427282	0.765578588	0.007422042	0.169088445	0.325028109	0.075368993	0.66663841
LOC101902043	0.678273128	0.387238247	0.00098395	0.971579708	0.824027135	0.075108993	0.66663841
LOC112447819	0.019718069	0.030697648	0.786221842	0.474076068	0.921336793	0.075315408	0.66663841
LOC781339	0.035975798	0.396056723	0.226641301	0.580595026	0.110944343	0.075346777	0.66663841
PARM1	0.933774679	0.289251536	0.205629375	0.007591606	0.494304394	0.075434009	0.66663841
SUSD1	0.424204656	0.888370033	0.0014961	0.411625635	0.89249159	0.07515639	0.66663841
TM9SF2	0.616199018	0.260757968	0.041839631	0.036214966	0.851974874	0.075220675	0.66663841
LOC112446690	0.392352785	0.991520955	0.278746551	0.01250683	0.154232635	0.075597291	0.66736264
NDUFAF5	0.272604514	0.496280744	0.231674636	0.012700729	0.525215416	0.075575932	0.66736264
GSDME	0.104270304	0.621125447	0.011434026	0.39864399	0.710039558	0.075689913	0.667542495
RABGGTA	0.393672087	0.686873957	0.63381335	0.00152313	0.803127885	0.075699018	0.667542495
CAMK2N2	0.26869993	0.74010185	0.196075558	0.014625427	0.367971657	0.075741534	0.667558706
ESCO1	0.049356825	0.512277881	0.562768855	0.020368356	0.724862659	0.075792212	0.667646801
XPR1	0.753109093	0.703018193	0.10015484	0.006781429	0.585327132	0.075877412	0.668038742
CDC20	0.74676964	0.080496743	0.034147339	0.189188755	0.543811198	0.076027872	0.669004507
HOMER1	0.970161519	0.533223537	0.331241082	0.005478159	0.225697728	0.076172872	0.669203966
PSD4	0.508289613	0.735158682	0.001840602	0.598229245	0.514744648	0.076157494	0.669203966
TTLL11	0.830836163	0.009935895	0.126474715	0.614543812	0.329761213	0.076112428	0.669203966
DUSP4	0.087319187	0.014810623	0.880158215	0.516809288	0.361686285	0.076365337	0.669487889
GSAP	0.518967202	0.54479563	0.007382951	0.315807377	0.322543621	0.07633489	0.669487889
PUS7	0.111416797	0.346299074	0.404189774	0.081966472	0.166460663	0.076368371	0.669487889
VWF	0.043828123	0.970204761	0.989827583	0.008951913	0.563711436	0.076286334	0.669487889
JPH2	0.009531203	0.591063049	0.154944489	0.913332855	0.267529599	0.076475218	0.669995817
PPP1R9A	0.659987715	0.422282352	0.192686235	0.004979464	0.798178246	0.076507962	0.669995817
POR	0.72319053	0.62153039	0.199477123	0.104371314	0.022872061	0.076635834	0.670495455
SLC7A6	0.533347679	0.265223623	0.195606106	0.023217741	0.333252751	0.076646729	0.670495455
TBC1D14	0.40751442	0.382390606	0.394498919	0.063678902	0.05474776	0.076694811	0.67055863
LOC112442677	0.075241171	0.014348053	0.777557578	0.258701197	0.990178636	0.076845249	0.67151618
LOC101902128	0.018033977	0.356750532	0.642348107	0.508138254	0.102894013	0.077066434	0.673090606
MLLT3	0.256336491	0.494171942	0.473307277	0.009758482	0.370119283	0.077167163	0.673107825
NSL1	0.000555165	0.745897983	0.801746227	0.693023653	0.94133639	0.077175117	0.673107825
SELENOK	0.38525302	0.082273531	0.023266433	0.377741015	0.777778888	0.077191453	0.673107825
CCDC171	0.088216963	0.289375666	0.345878916	0.128768117	0.191271594	0.077361287	0.673872651
GLUL	0.998601953	0.240419863	0.004086872	0.58030292	0.381709857	0.077334365	0.673872651
CCL19	0.099956069	0.566595259	0.049425899	0.087435802	0.890013683	0.077437906	0.674182215
IFI44L	0.722711294	0.822720062	0.015957124	0.049087044	0.468862246	0.077550059	0.67480065
C23H6orf141	0.921347958	0.696844759	0.006603348	0.775427088	0.066538693	0.077629996	0.675138244
MTMR2	0.582212826	0.779154328	0.58900865	0.001118965	0.732609045	0.077691053	0.675311373
LOC104970387	0.133597907	0.061228269	0.061694145	0.743473262	0.585263246	0.077807298	0.675963775

SAMHD1	0.626092679	0.64959599	0.001696514	0.608037582	0.525071723	0.077953413	0.676874846
AASDH	0.031472578	0.444770063	0.418427023	0.059007581	0.645133558	0.078513747	0.676877264
AASDHPPT	0.33061163	0.305269111	0.234695785	0.137112046	0.068708415	0.078550837	0.676877264
AK5	0.85034103	0.713639928	0.698617693	0.001438154	0.363975613	0.078294216	0.676877264
ANK3	0.653540101	0.164843945	0.394026684	0.132644824	0.039360887	0.078234129	0.676877264
COL6A2	0.071596305	0.301498653	0.055612001	0.250169799	0.742480719	0.078515835	0.676877264
ITGB3	0.392634191	0.63741667	0.016230669	0.318095295	0.172834197	0.078587544	0.676877264
LOC112447316	0.116624127	0.27589012	0.133008573	0.147636733	0.353655026	0.078613617	0.676877264
MAP3K1	0.343939617	0.044723865	0.182424244	0.62140713	0.128134436	0.078610162	0.676877264
PLXDC1	0.037741371	0.6408011	0.495136483	0.036170209	0.510069405	0.078087157	0.676877264
POLR3B	0.328504099	0.888005383	0.02621998	0.105247162	0.274881379	0.078161503	0.676877264
RASSF5	0.242295683	0.997163812	0.01611085	0.345497503	0.166079405	0.078593587	0.676877264
SMIM3	0.369718334	0.376991116	0.182323135	0.319441786	0.027435636	0.078460895	0.676877264
SORBS2	0.299449857	0.341115282	0.59619663	0.080096898	0.045467154	0.07826635	0.676877264
TMEM119	0.95613104	0.338200728	0.002051737	0.917111729	0.364522737	0.07826978	0.676877264
TMEM206	0.457762388	0.928682328	0.554450323	0.006691529	0.140533848	0.07823948	0.676877264
TRIM5	0.432877664	0.089316588	0.676129887	0.085588798	0.09884355	0.078134691	0.676877264
RAMP1	0.241043213	0.235595249	0.956168122	0.018907792	0.219315617	0.078970573	0.679360092
TIMM17A	0.889725951	0.55581032	0.157787984	0.018111359	0.159378709	0.07898477	0.679360092
PTPRO	0.2234319	0.217399217	0.228262619	0.097674545	0.208292049	0.079055093	0.679608765
EVPL	0.970361146	0.002756987	0.267917333	0.656935437	0.482654485	0.079403918	0.682246709
RCN3	0.228000423	0.237357095	0.010637198	0.605797997	0.652253113	0.079445095	0.682246709
HENMT1	0.822005435	0.012244207	0.894577559	0.422109929	0.059971487	0.079540737	0.682556796
PTI	0.327154135	0.372202538	0.054484212	0.672001963	0.051149263	0.079564387	0.682556796
MEG3	0.292377957	0.031306446	0.035243265	0.750438397	0.943510378	0.079640584	0.682853515
ABCE1	0.659523626	0.434920836	0.50359833	0.011342005	0.139861656	0.079792008	0.68341753
CASP8	0.616923013	0.428810553	0.168089405	0.010416974	0.502568338	0.080539242	0.68341753
COL4A4	0.544435738	0.002140477	0.633329141	0.661659076	0.47666983	0.080535418	0.68341753
DOK3	0.789734861	0.577872295	0.132122464	0.017429881	0.22099543	0.080429289	0.68341753
FLT4	0.157281525	0.692931078	0.004209712	0.601087762	0.838058133	0.080196311	0.68341753
GADD45G	0.745159421	0.341087392	0.094510497	0.027195654	0.35366328	0.080180212	0.68341753
GDF10	0.304437536	0.330907185	0.059478497	0.68582785	0.056110736	0.080086687	0.68341753
GFRA1	0.096829559	0.081675109	0.146094662	0.277402626	0.716562188	0.079898717	0.68341753
IL11RA	0.001259251	0.922047664	0.492313894	0.465918238	0.864269073	0.080004113	0.68341753
MKNK1	0.247997692	0.297497905	0.022636155	0.498483968	0.279404309	0.080500428	0.68341753
NFE2L1	0.778245905	0.587591976	0.015502643	0.086075979	0.379274061	0.080261864	0.68341753
PAQR7	0.672478448	0.450364051	0.079840186	0.024325035	0.3916433	0.080041243	0.68341753
PDE11A	0.381867317	0.030666314	0.032607603	0.95310461	0.6391248	0.080500501	0.68341753
PIGA	0.00758359	0.143780406	0.84565507	0.254372705	0.978604541	0.079871466	0.68341753
PSME2	0.189639504	0.331667739	0.236155794	0.090221108	0.173307025	0.080427912	0.68341753
RPF1	0.045102345	0.24893642	0.438179865	0.271933981	0.173059256	0.080279829	0.68341753
SOS2	0.338345138	0.295405595	0.291577075	0.009324631	0.846635549	0.079981697	0.68341753
TACO1	0.426730546	0.453970646	0.08916179	0.063325122	0.211699712	0.080286187	0.68341753
TRIM66	0.353700288	0.209283195	0.393071551	0.147916342	0.053814822	0.080297407	0.68341753
USP10	0.481354614	0.170610997	0.097358981	0.363360984	0.080078273	0.080509465	0.68341753

CNOT7	0.215335161	0.05543364	0.139399995	0.182942074	0.766191017	0.080629881	0.683833063
LOC618289	0.742703582	0.575718272	0.02500873	0.455774129	0.047924094	0.080697676	0.684054521
UGGT2	0.993628966	0.214800872	0.014382594	0.096380029	0.790405827	0.080754002	0.684178592
C3H1orf162	0.802679935	0.124317162	0.016738861	0.305157926	0.460321276	0.080913152	0.685023947
CSNK1B	0.294621956	0.087300298	0.242307459	0.579757609	0.065192224	0.081100567	0.685023947
HSPA13	0.880665873	0.022449023	0.085143278	0.198762428	0.704740301	0.081148176	0.685023947
KDM6A	0.362983598	0.485095928	0.930753413	0.003177977	0.452394062	0.081114554	0.685023947
ST8SIA4	0.532751021	0.250042424	0.236747406	0.013850149	0.540109896	0.081174322	0.685023947
TIMM10	0.894887902	0.840421345	0.158505668	0.004676266	0.423322823	0.081187714	0.685023947
TMEM132E	0.988475202	0.722600181	0.079952826	0.152497826	0.026995087	0.081007753	0.685023947
ZNF599	0.005139218	0.692071464	0.217555943	0.746753785	0.408206666	0.081165142	0.685023947
KCNN4	0.814945399	0.419424638	0.002502075	0.559177951	0.494433706	0.081282446	0.685470827
ADAMTS5	0.940958686	0.53392647	0.275827252	0.04091942	0.041876208	0.081486118	0.686210391
C3H1orf210	0.076172673	0.621490064	0.880813302	0.015857077	0.359579944	0.081546901	0.686210391
DNAH10	0.493270611	0.015712265	0.324365904	0.385508318	0.245291284	0.081540186	0.686210391
RHOF	0.081674149	0.144358451	0.29226905	0.317303591	0.217054363	0.081460391	0.686210391
TRAF5	0.913596834	0.254594344	0.755704895	0.515683196	0.002624759	0.081579214	0.686210391
ADNP	0.244361345	0.795354435	0.223072094	0.015858156	0.34972089	0.082087784	0.686662198
ANKRD23	0.617020056	0.004224441	0.898331662	0.19702715	0.522538878	0.082214366	0.686662198
BRIP1	0.436771255	0.046931288	0.403633586	0.098637664	0.294633654	0.082089589	0.686662198
CATSPERD	0.074870794	0.05677603	0.19556899	0.9066042	0.318728206	0.08204363	0.686662198
CREBRF	0.017167033	0.841158173	0.065461423	0.867188231	0.294369469	0.08226055	0.686662198
DTX1	0.717479436	0.393559581	0.019586017	0.381083548	0.114404105	0.082223047	0.686662198
FARS2	0.302608078	0.733469516	0.699853807	0.08388887	0.018493988	0.082198202	0.686662198
LMNB1	0.484061112	0.06687067	0.072524399	0.252996795	0.405373329	0.08215204	0.686662198
LOC112446791	0.011389202	0.711185879	0.4325788	0.381546683	0.179222036	0.081917536	0.686662198
MAST4	0.372939819	0.973054632	0.831433788	0.001961243	0.403414696	0.081740729	0.686662198
MTX1	0.690656608	0.70255475	0.752016313	0.001726743	0.378758919	0.08172669	0.686662198
PLAG1	0.245074719	0.401309681	0.808946069	0.008315108	0.363013786	0.082029519	0.686662198
PPP1R14C	0.705836823	0.016314588	0.204043189	0.445072299	0.229859994	0.082074854	0.686662198
QTRT1	0.211898203	0.210821818	0.334523654	0.04925832	0.327534948	0.082220709	0.686662198
XKR5	0.405797661	0.462195946	0.004135035	0.905092569	0.34089997	0.081856941	0.686662198
LOC781494	0.166310909	0.120981851	0.070148759	0.852400179	0.200823299	0.082322318	0.686828447
AHSA2	0.565192534	0.593863997	0.833975195	0.003249263	0.266216917	0.082427032	0.687003565
PIK3CD	0.598156291	0.987009829	0.007145506	0.121902183	0.470838892	0.082426566	0.687003565
LOC100848419	0.900541555	0.267930476	0.031251835	0.038984238	0.82502517	0.082505271	0.687306604
UBA7	0.767046975	0.241521855	0.107927717	0.01839304	0.660973077	0.082616067	0.687880403
PMS2	0.995434717	0.825653922	0.534316104	0.000561064	0.990285664	0.082798988	0.689053849
ATP10D	0.979665706	0.389212503	0.128030746	0.009225726	0.544315776	0.083028829	0.68984339
BOLA	0.496918965	0.075059233	0.231116655	0.066741556	0.426326564	0.083054589	0.68984339
LOC511617	0.324670344	0.811744007	0.030919326	0.529317751	0.056923469	0.083104039	0.68984339
NLRP3	0.606234878	0.401423114	0.316217144	0.024269871	0.131353169	0.08306325	0.68984339
TSPYL1	0.332060562	0.030213704	0.216976471	0.256655669	0.438178	0.082961991	0.68984339
TAPBP	0.131089282	0.425856661	0.698481227	0.006620228	0.952633702	0.083181066	0.690133709
SOX8	0.925736094	0.079162405	0.014357001	0.293052402	0.798627095	0.083245794	0.690321738

HSD17B11	0.638713599	0.519823017	0.006931156	0.157401321	0.680393167	0.083288195	0.690324531
TLR2	0.829523575	0.925185799	0.001235179	0.643345748	0.40467608	0.083356259	0.690539912
ACKR4	0.017263742	0.909791538	0.043169933	0.472719506	0.771029382	0.083423153	0.690745394
HRAS	0.170312582	0.573007262	0.127823376	0.036824398	0.538969648	0.083511703	0.690863088
RIC1	0.791630223	0.760980269	0.935130502	0.000690118	0.636961505	0.083521563	0.690863088
LOC112446470	0.29816759	0.303736611	0.254140164	0.043584173	0.247082145	0.083566373	0.690885515
ACTG1	0.182589304	0.006924973	0.535107197	0.639820736	0.577474609	0.083988158	0.691238548
CCL1	0.041595872	0.292990837	0.31779743	0.214770599	0.299097942	0.083752083	0.691238548
ELFN1	0.65156032	0.275631496	0.008149154	0.369104151	0.461644291	0.083866444	0.691238548
GPATCH8	0.319763606	0.023331844	0.98196904	0.108359571	0.314578038	0.08393666	0.691238548
KCTD17	0.971729185	0.526040993	0.001278428	0.796858808	0.477911025	0.083766415	0.691238548
KIF18B	0.852291853	0.097978564	0.083002545	0.213882863	0.168339094	0.083902658	0.691238548
LOC100847861	0.162025211	0.480868927	0.188221899	0.049591761	0.343584927	0.083965197	0.691238548
UHRF1BP1L	0.132568289	0.557805192	0.591192097	0.011555474	0.4928299	0.083785233	0.691238548
ZNF365	0.89624984	0.56448179	0.008646467	0.125470627	0.45358883	0.083783702	0.691238548
ALOX15	0.653726139	0.397446459	0.097466023	0.220666536	0.044806759	0.084065634	0.691445037
EFR3B	0.225558868	0.057380752	0.188851684	0.115914863	0.884305243	0.084097513	0.691445037
WFIKKN2	0.694350104	0.091763279	0.112761371	0.103657452	0.33730791	0.084227882	0.692170139
VHL	0.704874925	0.132052083	0.70936029	0.006425424	0.593134776	0.084312642	0.692519906
LOC100847171	0.037599846	0.250748177	0.061477758	0.573406351	0.759360334	0.084457719	0.693364498
WNT2	0.011431084	0.657704863	0.567123451	0.378755413	0.157297266	0.084780351	0.695665171
CTSC	0.493401118	0.229035728	0.117935694	0.023674764	0.806808375	0.08488668	0.695841811
LOC112442610	0.645522234	0.079577131	0.095644966	0.457262688	0.113259318	0.084863382	0.695841811
TTC33	0.303766464	0.633752605	0.013996667	0.487326464	0.194848314	0.08513863	0.697558692
NLRC3	0.208790539	0.089797234	0.068747844	0.33891848	0.586232668	0.085184275	0.697584399
CX3CR1	0.808970079	0.341079344	0.001883617	0.745742997	0.663789792	0.085414633	0.697596336
LOC104975607	0.333170815	0.584886754	0.003101932	0.701029932	0.607127546	0.085413172	0.697596336
OPTC	0.776784614	0.392053019	0.582645352	0.708209706	0.002046828	0.085402287	0.697596336
PQLC2	0.529469952	0.960591494	0.278308492	0.007991041	0.22695373	0.085304918	0.697596336
UTP15	0.729859943	0.032990741	0.043104128	0.573523682	0.432439402	0.08544078	0.697596336
VIPR1	0.022480774	0.171644232	0.300942762	0.713221291	0.310582929	0.085406123	0.697596336
DMD	0.516389932	0.163340943	0.173636487	0.21291507	0.082884735	0.085644686	0.697621857
FAM129A	0.15100623	0.218348424	0.803538905	0.753659179	0.012916007	0.085536242	0.697621857
KCNB1	0.281907843	0.267066213	0.283913033	0.022341163	0.541351628	0.085656452	0.697621857
SPOUT1	0.73098511	0.70565061	0.54285175	0.027339004	0.033678308	0.085519786	0.697621857
SUPT4H1	0.387623693	0.025116657	0.457249626	0.149664143	0.387472083	0.085585689	0.697621857
ADAM33	0.091400873	0.130584703	0.270018373	0.326674742	0.246277192	0.085804279	0.698180721
APBA3	0.942545346	0.153111079	0.137597069	0.016605919	0.789069957	0.085980332	0.698180721
CHP2	0.535969319	0.36242451	0.041617895	0.084622084	0.379097868	0.085815492	0.698180721
EML6	0.074589349	0.052928046	0.631520578	0.369500106	0.282047516	0.085909741	0.698180721
NPTXR	0.464234368	0.815274841	0.019301898	0.040434455	0.880740775	0.085973909	0.698180721
TUBB6	0.025210498	0.367440278	0.553080294	0.221861416	0.228363588	0.085860888	0.698180721
PDF	0.511972115	0.580752842	0.318344203	0.028306785	0.097302974	0.086079428	0.698639712
AMIGO2	0.059796689	0.497877631	0.049478104	0.547011669	0.324104535	0.086165674	0.698648652
XPOT	0.343444946	0.948034148	0.477023263	0.704654436	0.00238561	0.086154279	0.698648652

ADAMTS15	0.081054001	0.529909195	0.054134214	0.250356943	0.450421	0.086366895	0.699243767
CAMK2G	0.314409808	0.417342726	0.068699084	0.208833593	0.139149321	0.086319829	0.699243767
DHX35	0.512132998	0.01875316	0.773639913	0.078135786	0.451567439	0.086360226	0.699243767
AATK	0.830255771	0.003131936	0.414128556	0.264284731	0.927848965	0.086726239	0.699374181
ATF6	0.524933525	0.013815686	0.225342821	0.350309861	0.462216223	0.086832387	0.699374181
CALM	0.951012358	0.001022055	0.812969604	0.822223731	0.407774443	0.08689398	0.699374181
CLDN11	0.001508267	0.922471096	0.367887659	0.541623822	0.955717391	0.086897318	0.699374181
DNAJB14	0.194932543	0.276819077	0.5307865	0.010256457	0.900658021	0.086825312	0.699374181
EZH2	0.387252136	0.040266626	0.114701689	0.196689727	0.74840264	0.086576495	0.699374181
JPT2	0.871179197	0.140660764	0.261643021	0.098026207	0.083840478	0.086618437	0.699374181
LCP2	0.244701811	0.589159884	0.007206167	0.632951975	0.402294035	0.086817306	0.699374181
LOC100849050	0.299031166	0.275728196	0.271529615	0.01329723	0.883256567	0.086510887	0.699374181
LOC112443484	0.049244358	0.071308817	0.149487043	0.89797384	0.557811173	0.086509868	0.699374181
LOC510520	0.471414189	0.465798092	0.011295016	0.303846383	0.350679444	0.086766202	0.699374181
USP2	0.654888954	0.42503063	0.044640249	0.064171792	0.329954668	0.086540022	0.699374181
ZNF608	0.981214537	0.035935835	0.197816966	0.216230947	0.175808397	0.086937014	0.699374181
CDC42EP3	0.024472272	0.108862847	0.947428133	0.570087841	0.184759713	0.087070091	0.699564604
DUOXA2	0.988115358	0.018201796	0.032752876	0.468334923	0.964464165	0.087112825	0.699564604
EBP	0.595321109	0.50550301	0.506956071	0.006764376	0.258180553	0.087181145	0.699564604
KMT5C	0.582094671	0.053837283	0.042127935	0.968573753	0.208350456	0.0871785	0.699564604
POLR2F	0.175194531	0.439057639	0.391933469	0.012202864	0.723494975	0.087128974	0.699564604
TPP1	0.910267193	0.211237082	0.004745167	0.544031401	0.537134499	0.087216451	0.699564604
TMEM37	0.073695754	0.177590627	0.34699398	0.181754791	0.323482843	0.087289694	0.699810045
PTGER2	0.51734488	0.236452885	0.107806407	0.07266082	0.279276399	0.087405107	0.700393167
SPOPL	0.22059522	0.42661781	0.205689927	0.09403644	0.14725498	0.087488656	0.700720515
CISH	0.93152169	0.008874643	0.209035303	0.231960042	0.67278854	0.087799878	0.702870148
LOC112448084	0.875915912	0.710584901	0.188930441	0.735813526	0.003123013	0.08790221	0.703016872
NOP16	0.723721441	0.671945278	0.119652596	0.0457408	0.101532432	0.087903883	0.703016872
TSPAN18	0.653048299	0.332477603	0.007599385	0.206718848	0.79300011	0.08795156	0.703055552
ARMC10	0.112588241	0.03939048	0.561690572	0.16165875	0.673867128	0.088119016	0.703170595
CBX7	0.431233169	0.35425507	0.678300623	0.488895173	0.005350996	0.088065267	0.703170595
CP	0.209000751	0.085811666	0.686385465	0.257657613	0.085728999	0.088223037	0.703170595
GDI1	0.095931665	0.16174785	0.28520921	0.214292408	0.28566177	0.088032386	0.703170595
LOC522763	0.332961098	0.170381452	0.253796046	0.916380367	0.020597694	0.088194837	0.703170595
SYP	0.297828365	0.039496485	0.217204449	0.172227688	0.617651176	0.088200102	0.703170595
BARX1	0.838356678	0.026633558	0.549941528	0.082702364	0.268890672	0.088440792	0.70347819
CENPX	0.534431537	0.815996696	0.758549568	0.000901727	0.91675384	0.08851496	0.70347819
HJURP	0.331839229	0.058172888	0.039439268	0.898223132	0.401098357	0.088671986	0.70347819
HTR1B	0.194613056	0.136666132	0.963918551	0.474694681	0.022573881	0.088753155	0.70347819
IDH3A	0.569102271	0.475041306	0.326836182	0.086675518	0.035615489	0.088383672	0.70347819
KANK2	0.691679244	0.558859682	0.003912414	0.478111776	0.380407214	0.08881712	0.70347819
LMAN1	0.76283363	0.995920325	0.177988668	0.30242943	0.006705635	0.088659895	0.70347819
MAP2K4	0.621022083	0.012383915	0.466527407	0.07687005	0.989081683	0.088388931	0.70347819
PGM2L1	0.116399972	0.065343788	0.255792701	0.169187146	0.834132155	0.088723818	0.70347819
RPS20	0.271480572	0.655207561	0.921450184	0.006777948	0.245962927	0.088475154	0.70347819

SCRIB	0.270138849	0.024172739	0.138219497	0.312188952	0.974804765	0.08874405	0.70347819
STX1A	0.113466821	0.955646217	0.20214799	0.297446299	0.042189201	0.08881889	0.70347819
TTC25	0.136206473	0.623470855	0.242060312	0.014730206	0.90378031	0.08855263	0.70347819
SFI1	0.16639713	0.01099494	0.217088561	0.70350918	0.985366482	0.088866338	0.70351446
CENPW	0.448364115	0.014559032	0.156871769	0.271734545	0.994199431	0.089115154	0.703591007
LOC100336476	0.079794406	0.05206032	0.7162636	0.319406308	0.290316573	0.088976653	0.703591007
LOC101909196	0.305374199	0.01161538	0.565054195	0.343852717	0.401890623	0.089176119	0.703591007
MTAP	0.806819339	0.412556901	0.145393445	0.01344898	0.425409134	0.08915996	0.703591007
SELENBP1	0.14878019	0.567879195	0.709468687	0.009798988	0.469837227	0.088988054	0.703591007
TMEM200A	0.633845285	0.459281845	0.87506217	0.001195637	0.907157846	0.089050243	0.703591007
TMEM30B	0.76661788	0.281958072	0.011717317	0.24554952	0.444336224	0.08905729	0.703591007
FERMT2	0.171788263	0.090449623	0.329126906	0.156059815	0.347490161	0.08924327	0.703782465
FRRS1	0.53250518	0.948668048	0.470935596	0.007298405	0.160658099	0.089546995	0.705499636
TNFRSF21	0.010051266	0.820617691	0.56186374	0.191669688	0.313923242	0.089527706	0.705499636
FADS2	0.085577148	0.477057182	0.175776867	0.932277714	0.041934966	0.089845409	0.707482088
GPR65	0.257790343	0.573245916	0.261295179	0.014196284	0.513707252	0.090040443	0.707482088
KBTBD2	0.185311642	0.288338535	0.421263597	0.085492027	0.146032998	0.089932689	0.707482088
LOC104972031	0.240575705	0.009246948	0.438802005	0.865460121	0.332403832	0.08989604	0.707482088
MGC152281	0.52649219	0.076728262	0.454279593	0.02656388	0.577026868	0.089983587	0.707482088
PRKAR1A	0.478312642	0.076341508	0.228561178	0.038864814	0.868606078	0.090067613	0.707482088
REP15	0.44132161	0.897074232	0.025762579	0.512249355	0.054004346	0.090143504	0.707482088
TMEM26	0.694080252	0.029018451	0.262202224	0.059741811	0.893871909	0.09011825	0.707482088
RAET1L	0.804179298	0.55537506	0.002051256	0.603950355	0.510488984	0.09019952	0.707583332
CD151	0.073103003	0.7836416	0.096487587	0.130337891	0.392618954	0.090274408	0.707832446
LOC100140586	0.003339753	0.23649564	0.556766632	0.647822592	0.994038165	0.090335342	0.707971964
MRPL12	0.509086957	0.427065272	0.939144993	0.003470422	0.401409714	0.090567868	0.709117023
ZFC3H1	0.052566793	0.139608694	0.649802456	0.10857414	0.548984169	0.090531783	0.709117023
INPP5A	0.106816475	0.552726353	0.154366021	0.133025307	0.234898641	0.090631662	0.709243819
LOC512627	0.750044418	0.290194048	0.002418162	0.665680286	0.813407571	0.090670497	0.709243819
KIAA1024	0.451209694	0.954389003	0.446999461	0.001891971	0.78716506	0.090981317	0.711336061
EIF4EBP3	0.160560949	0.255574449	0.293327284	0.583193287	0.041040596	0.091242835	0.711664495
FGD6	0.193501374	0.315945761	0.407113043	0.11314272	0.102635005	0.09141361	0.711664495
LOC100141145	0.17704388	0.126201022	0.068844241	0.305980574	0.61199343	0.091232584	0.711664495
MYRF	0.793151251	0.019672783	0.153700164	0.356384038	0.336537669	0.091158887	0.711664495
PABPC1	0.731971497	0.788377927	0.04845434	0.056800237	0.181513089	0.091277439	0.711664495
RAB12	0.947058277	0.00597528	0.31889601	0.635041902	0.251976012	0.091366162	0.711664495
RGS18	0.672856154	0.402687883	0.013227797	0.537235389	0.149849019	0.091323543	0.711664495
STK32C	0.643392748	0.566934924	0.00121519	0.841221365	0.772244223	0.091216273	0.711664495
USP35	0.794488247	0.193322604	0.013299826	0.461542316	0.306359365	0.091379991	0.711664495
UFSP1	0.278956504	0.549337496	0.241285272	0.045741296	0.171059589	0.091466046	0.711735079
A1CF	0.007085539	0.466996987	0.768209397	0.736492752	0.155364453	0.091750787	0.712603002
ABCB8	0.749381064	0.827092816	0.468462709	0.005844652	0.173543869	0.092416962	0.712603002
ABHD17B	0.044820012	0.569584039	0.306673786	0.96067526	0.038746566	0.091852603	0.712603002
AFF3	0.095677746	0.428825419	0.745121834	0.722885156	0.013441947	0.092880977	0.712603002
C6H4orf48	0.151653498	0.479211954	0.006402254	0.887596257	0.707211566	0.09197074	0.712603002

CKAP2L	0.95233033	0.009219562	0.104142688	0.369565351	0.867027518	0.092139664	0.712603002
CNTNAP1	0.129535565	0.101182929	0.118135898	0.370721164	0.510471239	0.09214533	0.712603002
CPAMD8	0.887186068	0.191053143	0.617213564	0.014593837	0.19472453	0.092923675	0.712603002
DALRD3	0.316929043	0.313416252	0.724912839	0.02289412	0.176842367	0.091872663	0.712603002
DHX30	0.703260864	0.38636455	0.241879831	0.005973999	0.739579743	0.091662112	0.712603002
EPB42	0.016580745	0.82336014	0.450066195	0.394281797	0.120800209	0.092077364	0.712603002
IFFO1	0.010307739	0.251908411	0.508140097	0.263829037	0.853725867	0.092903339	0.712603002
KIF21B	0.725046624	0.113461455	0.270609448	0.341107795	0.039058666	0.092796202	0.712603002
LOC101904794	0.800350311	0.053071255	0.023988056	0.393314539	0.740906837	0.09285468	0.712603002
LOC104968422	0.857725253	0.076385876	0.041433528	0.40165	0.266759423	0.091750052	0.712603002
LOC104976232	0.038761072	0.064471899	0.816337771	0.335517241	0.4305044	0.092445196	0.712603002
LOC404051	0.18324893	0.69142648	0.003973214	0.805095816	0.730841135	0.092726052	0.712603002
LOC788425	0.743021948	0.030808704	0.514298709	0.103527499	0.242144962	0.092530821	0.712603002
LRR1	0.005766817	0.355433616	0.209230277	0.737645526	0.92938039	0.092325711	0.712603002
LRRC71	0.266561856	0.89251905	0.009247642	0.925274914	0.143394374	0.091942668	0.712603002
MMP23	0.383687179	0.117800828	0.02429726	0.288967221	0.925981131	0.092297741	0.712603002
NAV2	0.699714889	0.312970178	0.01280526	0.696305654	0.151480978	0.092648147	0.712603002
NKTR	0.373899047	0.183875583	0.062302119	0.474067596	0.145416104	0.092557067	0.712603002
PARP9	0.744957792	0.104979725	0.112910469	0.126584938	0.261301826	0.091972953	0.712603002
PDE12	0.914097495	0.800462704	0.404147738	0.179989388	0.005516296	0.092252692	0.712603002
RLF	0.254014068	0.350379433	0.842484518	0.006268133	0.621929377	0.092014977	0.712603002
RRP15	0.548391681	0.17629399	0.045265282	0.432919	0.15626247	0.092695723	0.712603002
SIPA1L2	0.794404739	0.374090258	0.319916515	0.016421208	0.189850535	0.092759724	0.712603002
SMAD1	0.543975479	0.429409779	0.003506919	0.669393367	0.539463087	0.092654293	0.712603002
TSHZ3	0.698591869	0.527195408	0.025897691	0.056829724	0.543495894	0.092432777	0.712603002
ZC3H12D	0.893961926	0.068004964	0.045585329	0.674158216	0.158334136	0.092654413	0.712603002
LAMP2	0.209064101	0.419253266	0.00913472	0.495730625	0.751926671	0.093132018	0.713200907
LOC787234	0.954382787	0.247644689	0.101762276	0.186875974	0.066353226	0.093092442	0.713200907
PROKR1	0.784082426	0.111018928	0.135193394	0.557494223	0.045432855	0.093063954	0.713200907
GSDMD	0.286872446	0.118942099	0.021106045	0.619976347	0.669933225	0.09325209	0.713344645
VLDLR	0.982850956	0.160382656	0.959163069	0.002401173	0.823042497	0.093195521	0.713344645
VPS37A	0.524253277	0.025267478	0.124752519	0.225993256	0.801353089	0.09328119	0.713344645
GTSE1	0.130854567	0.469051951	0.199594749	0.079031645	0.309398803	0.093331839	0.713399537
ADAMTS16	0.658494496	0.891385832	0.570584394	0.002582737	0.346944063	0.093431351	0.71350732
NUS1	0.27328594	0.028380814	0.719466457	0.121900659	0.441195431	0.093432894	0.71350732
MPPE1	0.07587134	0.100773135	0.326484727	0.24977199	0.481811125	0.093484784	0.713571533
CDH15	0.313367679	0.256239618	0.197258887	0.071797269	0.269821147	0.094640116	0.714426866
CNGA3	0.019864422	0.457068932	0.642329694	0.126825401	0.413989571	0.094525511	0.714426866
COL12A1	0.58096307	0.683417649	0.007213736	0.215011709	0.498679286	0.094685177	0.714426866
FMOD	0.350699379	0.308965298	0.021578068	0.198265541	0.661936711	0.094640115	0.714426866
HBEGF	0.34298439	0.073607533	0.759920624	0.047776385	0.331305153	0.094072542	0.714426866
IRF4	0.369313898	0.926225388	0.100531626	0.0360044	0.247475511	0.09456223	0.714426866
JMY	0.046532415	0.492726792	0.248635879	0.109003632	0.493218913	0.094575257	0.714426866
LOC101907857	0.346529755	0.757918937	0.096683973	0.329445137	0.036407914	0.094234458	0.714426866
LOC112446481	0.194944435	0.201137119	0.443909951	0.958831539	0.018246987	0.094226265	0.714426866

LOC524810	0.481916468	0.02863829	0.317520574	0.894708325	0.077480609	0.094092469	0.714426866
MED6	0.253758751	0.793875033	0.722329319	0.019135296	0.108850628	0.093968298	0.714426866
MFHAS1	0.820447218	0.994080022	0.167602115	0.932247323	0.002409844	0.09468431	0.714426866
MRPL57	0.52529109	0.722707128	0.161765129	0.021726424	0.228126075	0.094198524	0.714426866
NDUFA1	0.993863028	0.792035728	0.337242967	0.001330571	0.858942611	0.094023664	0.714426866
NDUFS3	0.652882759	0.482761533	0.603832599	0.002223957	0.721872965	0.094407257	0.714426866
OSCAR	0.191374069	0.167443519	0.062726504	0.557990239	0.272594757	0.094441852	0.714426866
PTPRK	0.349469459	0.413356604	0.757026502	0.043664296	0.063991404	0.094409984	0.714426866
RANBP6	0.312571282	0.476761848	0.557884841	0.018504301	0.198259219	0.094310401	0.714426866
RGS16	0.156718808	0.993922602	0.007362863	0.312342447	0.854265457	0.094491892	0.714426866
SLC49A3	0.544323199	0.026723735	0.072513978	0.380712828	0.752990618	0.093841636	0.714426866
SNPH	0.744495782	0.345863049	0.018841012	0.525987751	0.118115829	0.093665558	0.714426866
TMEM59L	0.402869807	0.391584431	0.02049098	0.098452597	0.963370063	0.094596287	0.714426866
TMSB4X	0.776852561	0.001497718	0.601962768	0.873099198	0.498322964	0.094261591	0.714426866
TTC19	0.782625322	0.627172786	0.660210783	0.021554472	0.043342141	0.093905298	0.714426866
ZNF654	0.153286029	0.287926674	0.288036638	0.027065325	0.886736681	0.09432786	0.714426866
CREB1	0.400870651	0.495053641	0.708016019	0.002207534	0.991869449	0.094783985	0.714515381
LOC112442284	0.539268739	0.12323753	0.476308103	0.019495694	0.498159445	0.094743816	0.714515381
DYNC1I2	0.950785833	0.660012295	0.294299949	0.002118726	0.787828356	0.094893813	0.714601281
FAM78A	0.41715105	0.5664193	0.003692415	0.52421527	0.67537918	0.095003527	0.714601281
LOC782021	0.194852304	0.593367116	0.236910147	0.073929974	0.152112834	0.09485185	0.714601281
TSPO	0.75668477	0.634084242	0.746048093	0.002219702	0.388822501	0.095013101	0.714601281
TTC4	0.72526994	0.288854185	0.186798796	0.14580529	0.054067939	0.094936047	0.714601281
TUBB2B	0.011389859	0.09319174	0.681968827	0.786362551	0.543702144	0.095110438	0.715005678
AREL1	0.311401488	0.518544146	0.669506674	0.005071952	0.567415796	0.095401207	0.716207321
IDI1	0.90601999	0.819349302	0.091276676	0.040115597	0.114284584	0.095316154	0.716207321
LPGAT1	0.901262289	0.381844877	0.067392451	0.017784532	0.754231004	0.095395919	0.716207321
FERMT1	0.328467907	0.347678856	0.162756193	0.442880817	0.037850308	0.09548084	0.716477395
ARF1	0.310949795	0.03214186	0.094916755	0.733392185	0.450319265	0.095785611	0.718116739
ARHGAP45	0.201681803	0.817965655	0.016623844	0.287700921	0.397270548	0.095811077	0.718116739
CHRNB1	0.157929944	0.488450601	0.084656353	0.25435897	0.188765309	0.095830581	0.718116739
ADA2	0.605160658	0.828821223	0.036611585	0.088279485	0.193884485	0.095963064	0.718781309
EPSTI1	0.653275136	0.151590637	0.068952048	0.082729134	0.55707028	0.096030861	0.718960974
LOC614625	0.52870475	0.55259497	0.009631072	0.171948719	0.650988425	0.096079747	0.718998961
CD79A	0.123894486	0.589600647	0.027772765	0.925767404	0.16817722	0.0962371	0.719491149
FCF1	0.309700012	0.014537712	0.619953822	0.38515901	0.294249832	0.096320886	0.719491149
PDLIM7	0.763580545	0.970696488	0.004558474	0.117141547	0.798831063	0.096291551	0.719491149
PDSS1	0.724130166	0.560016683	0.346963412	0.087395094	0.025673322	0.096207981	0.719491149
MFSD11	0.197802599	0.725753939	0.388335754	0.027329029	0.207944237	0.096403727	0.71978233
CHMP6	0.205736949	0.627772515	0.122804119	0.44908773	0.044519206	0.096455781	0.719843487
MYBL2	0.354269146	0.002048051	0.606804534	0.840973631	0.858922011	0.096616476	0.720714993
CYB5R2	0.103848374	0.335588342	0.400523004	0.110006872	0.20772027	0.096780061	0.721607257
GNL3L	0.404628244	0.689556678	0.553297649	0.009229521	0.224900729	0.0970403	0.72199873
LOC527796	0.06196396	0.647592923	0.111738237	0.404497394	0.176723683	0.097052995	0.72199873
PLPBP	0.388972179	0.659685862	0.484252005	0.004704186	0.547267808	0.096944549	0.72199873

RPP38	0.163747272	0.738877264	0.319941551	0.017287379	0.479654555	0.097133065	0.72199873
RSPRY1	0.467123005	0.658335579	0.19324472	0.016559546	0.325648675	0.097044029	0.72199873
SERPINF1	0.376965646	0.199009105	0.073966396	0.058724946	0.985146508	0.097140527	0.72199873
SYNGR1	0.04399001	0.206945692	0.922684153	0.246033131	0.155300954	0.097127453	0.72199873
MAN2A2	0.325215578	0.309958069	0.229460882	0.031093554	0.447219243	0.097249049	0.722478111
FBXO32	0.177448033	0.364323573	0.876363527	0.298203583	0.019056722	0.097304568	0.722563471
BCORL1	0.454629802	0.17580423	0.210559496	0.037390369	0.513453909	0.097500966	0.723292939
CBFB	0.544771406	0.225250413	0.088908634	0.033428069	0.887834977	0.097623171	0.723292939
EIF5A	0.320061723	0.548208936	0.842181317	0.198097232	0.011056082	0.097596979	0.723292939
MCMBP	0.513634033	0.800049071	0.091201594	0.011788262	0.73228669	0.09757609	0.723292939
MYB	0.200855994	0.809023031	0.033560651	0.06965956	0.851643246	0.097577479	0.723292939
ATP1A1	0.714928518	0.084142034	0.200954032	0.041336094	0.649216619	0.097729945	0.723757274
AGMO	0.593216944	0.696466513	0.002445252	0.479677361	0.671951676	0.097941871	0.724346124
LOC504548	0.010751747	0.957668077	0.65785291	0.066701761	0.720340227	0.097912406	0.724346124
PLD2	0.201072935	0.256585441	0.008075076	0.946511694	0.825448064	0.097918925	0.724346124
MYL9	0.033916457	0.269206137	0.51009125	0.505999849	0.138339616	0.098008852	0.724514983
OLFM1	0.980309436	0.36614545	0.002943507	0.533816472	0.580723754	0.098269481	0.726114564
KCNK1	0.045573869	0.988443591	0.335602435	0.115677399	0.187654124	0.098381043	0.726611744
NUDT8	0.591504076	0.465920001	0.80722215	0.001860842	0.793657935	0.098446972	0.726771593
PET100	0.012602925	0.325913937	0.505343286	0.18581793	0.853573326	0.098562388	0.727296466
NT5DC3	0.145605999	0.144281169	0.10903702	0.224066659	0.641931395	0.098606735	0.72729669
CD79B	0.174838402	0.126285423	0.420482738	0.709601388	0.050064606	0.09866616	0.727408064
ADH6	0.260965133	0.302658142	0.030893383	0.578695342	0.234081508	0.098788303	0.727695765
MYBPC2	0.233969026	0.248910802	0.069256101	0.157467984	0.520484161	0.098793868	0.727695765
ADRA1D	0.644828916	0.309101772	0.036355235	0.3835343	0.119955763	0.099276246	0.728306869
ATE1	0.052935954	0.223345664	0.619711764	0.115580096	0.391648211	0.098982109	0.728306869
HAPLN1	0.35637585	0.208780164	0.123403385	0.063243534	0.57388755	0.099253805	0.728306869
MYBPH	0.050547456	0.383075274	0.65541955	0.051365705	0.510492287	0.099174702	0.728306869
NR4A2	0.468302211	0.0856046	0.743924606	0.025110409	0.443155126	0.099016796	0.728306869
RASD2	0.502144238	0.241374281	0.121831507	0.223767457	0.100768578	0.09920568	0.728306869
SMAP2	0.964820656	0.597881824	0.255879009	0.065231242	0.034518042	0.099100298	0.728306869
TCAF2	0.183562452	0.211128889	0.034953715	0.692347567	0.355348704	0.099258569	0.728306869
THSD4	0.023089093	0.766813368	0.122421592	0.63119829	0.2430666	0.099132748	0.728306869
FAM107B	0.038027653	0.338862396	0.850621492	0.101051893	0.301336669	0.099344263	0.728480207
PRKCI	0.593120283	0.802269672	0.522600373	0.00271524	0.495058346	0.099428695	0.728773703
C7H19orf57	0.299186955	0.192306597	0.021694536	0.37569837	0.71614452	0.099696096	0.729861836
DENR	0.21310945	0.345113475	0.444924376	0.120605576	0.085052562	0.099666875	0.729861836
LOC508666	0.781325041	0.949389792	0.053304268	0.052388896	0.162259021	0.099743485	0.729861836
ZNHIT2	0.50665761	0.339021262	0.048196021	0.061323549	0.662207417	0.099755048	0.729861836
DHRS11	0.097536452	0.390732574	0.26842215	0.143262009	0.230068907	0.099924078	0.730579761
LOC112441839	0.827739241	0.616623382	0.008657776	0.118292533	0.645614174	0.099976474	0.730579761
LOC782598	0.219901207	0.251702456	0.052206985	0.515540499	0.226579861	0.099986725	0.730579761
TAF15	0.176236116	0.574157204	0.868035477	0.282642615	0.013635545	0.100151553	0.731458447
ARPC5	0.440016331	0.008930147	0.11662369	0.829659055	0.891873074	0.100250058	0.731852178
LOC101903248	0.081874387	0.197432749	0.107525946	0.252256396	0.774368196	0.100323421	0.732062096

ASB1	0.81152517	0.125270008	0.09665987	0.038030524	0.917343083	0.100880343	0.733058352
ATIC	0.454631096	0.34791996	0.454921689	0.13417228	0.035629936	0.10107955	0.733058352
BCR	0.284420291	0.614031446	0.029553171	0.142323108	0.468095356	0.101054464	0.733058352
CYB5RL	0.099615587	0.934045937	0.04182601	0.17538273	0.503659689	0.101041248	0.733058352
GABARAPL1	0.621570491	0.967991548	0.003751626	0.573671654	0.264882033	0.10091206	0.733058352
ITK	0.609685026	0.569860024	0.695015819	0.079362242	0.01795546	0.101096359	0.733058352
LOC101903501	0.34643596	0.972146609	0.21248539	0.081341347	0.059066473	0.101050619	0.733058352
LOC112441683	0.284606102	0.014690191	0.461527	0.370043329	0.48152971	0.101051717	0.733058352
MYL12A	0.435944099	0.136470015	0.099862901	0.169810146	0.341267398	0.10112998	0.733058352
OXLD1	0.760887079	0.739558031	0.236932068	0.007540822	0.340417986	0.100785511	0.733058352
SUSD2	0.102636692	0.219065707	0.103495085	0.464213819	0.317021455	0.100819603	0.733058352
TEAD2	0.273036959	0.913651785	0.267201868	0.057499831	0.088868107	0.100507105	0.733058352
WDR43	0.589297433	0.486361752	0.232047771	0.021814986	0.235793231	0.100760233	0.733058352
ZNF770	0.165787867	0.830347946	0.528343644	0.028557654	0.164644111	0.100738993	0.733058352
ZNF827	0.051945359	0.315612556	0.178202485	0.161833926	0.726184978	0.100969958	0.733058352
AGAP1	0.169146129	0.968052452	0.006995449	0.496005411	0.607324906	0.101257373	0.733377773
CD2	0.11602439	0.644252623	0.490740285	0.353030255	0.026728071	0.101438364	0.733377773
CORO2B	0.208897931	0.464665207	0.117827047	0.038080437	0.795581117	0.101501174	0.733377773
F2RL1	0.185131354	0.253444969	0.554195268	0.014687774	0.907913683	0.101544232	0.733377773
FKBP14	0.180289618	0.57708547	0.661027664	0.01208781	0.415851107	0.101368653	0.733377773
LOC101902537	0.262576425	0.026601955	0.965765697	0.440189503	0.116412553	0.101363913	0.733377773
SH3GL1	0.915148037	0.831184782	0.392332242	0.014027507	0.082878591	0.10157624	0.733377773
SYT2	0.882359571	0.100730043	0.017407218	0.799790631	0.279335978	0.101358108	0.733377773
ZNF644	0.146457809	0.976864229	0.695878099	0.007193426	0.48437094	0.10156658	0.733377773
FABP7	0.391839579	0.808034425	0.896047948	0.68660523	0.001785368	0.101715532	0.733941529
RTP4	0.739563984	0.192934429	0.060420439	0.07521934	0.536556742	0.101743768	0.733941529
PDXK	0.544719049	0.2174263	0.107339068	0.46500246	0.059078802	0.101960768	0.735183728
KRI1	0.784975755	0.059090307	0.722071633	0.044674776	0.234056234	0.102123087	0.736030732
CCDC18	0.270820101	0.249282337	0.243967083	0.32918706	0.064835316	0.102341842	0.737283566
LGI2	0.350338251	0.095968277	0.022628998	0.49976923	0.927040253	0.102502618	0.738117798
AP4B1	0.329963762	0.556451873	0.01328404	0.473219542	0.308705952	0.103137094	0.738942339
AVL9	0.177400941	0.177575631	0.024829364	0.473804655	0.961498714	0.103139976	0.738942339
C1H21orf62	0.009746685	0.992771723	0.737722763	0.517337657	0.096577179	0.103193681	0.738942339
CAND2	0.648618866	0.736316446	0.114603412	0.662690185	0.009798473	0.102986247	0.738942339
CORO1C	0.498572406	0.061583962	0.213665335	0.056772027	0.955943118	0.103091552	0.738942339
FMNL1	0.441886646	0.745846051	0.00874046	0.152646089	0.808508251	0.103005778	0.738942339
GDA	0.997342144	0.346789916	0.013059961	0.093229263	0.841724072	0.102830855	0.738942339
KCNJ8	0.181568159	0.927883867	0.015811199	0.663499893	0.201510468	0.103110593	0.738942339
MAGEF1	0.437469036	0.902666996	0.006334122	0.349386746	0.40594717	0.102880017	0.738942339
MEGF10	0.528456348	0.050113534	0.18805399	0.271675087	0.263643055	0.103202476	0.738942339
PPP1CC	0.105955672	0.863406234	0.756015511	0.005173072	0.9947262	0.103067903	0.738942339
RRAGC	0.730790917	0.156217995	0.007826039	0.404622416	0.984189441	0.103050609	0.738942339
SPIDR	0.688113614	0.430334935	0.189316499	0.09305305	0.068138782	0.102994336	0.738942339
MLF1	0.18350583	0.596381461	0.600691518	0.026098898	0.208307722	0.103316732	0.739364048
RIMKLA	0.145094376	0.346156402	0.780569506	0.029144716	0.31297817	0.103351479	0.739364048

CALCOCO1	0.397549822	0.560165223	0.119017699	0.790194818	0.017150833	0.103614461	0.740424824
FOXK2	0.315402199	0.113929315	0.846105068	0.029536574	0.399666478	0.103565873	0.740424824
TRMT9B	0.035278229	0.084382862	0.680471801	0.420604421	0.421740243	0.103635112	0.740424824
DOHH	0.245188422	0.491842499	0.119701205	0.077667719	0.320830334	0.10369658	0.740541587
COL8A1	0.088070719	0.372380241	0.023298406	0.574614461	0.821409838	0.10385232	0.741331196
FAM120B	0.969208152	0.134530581	0.291506811	0.016815925	0.565777587	0.104012659	0.741788767
LDLRAP1	0.039533791	0.199458177	0.957842723	0.068431446	0.700117047	0.104052023	0.741788767
ZSWIM2	0.036309817	0.791566584	0.476462155	0.214554312	0.123121274	0.104034144	0.741788767
ADRA1B	0.867790825	0.560334792	0.640701604	0.001682154	0.697777147	0.104677868	0.742303601
BNIP2	0.880955005	0.05731845	0.155309398	0.0575385	0.807988233	0.104500302	0.742303601
C8H9orf72	0.580954414	0.020393	0.563781007	0.073906283	0.742972017	0.104855041	0.742303601
CCL8	0.0696309	0.271244122	0.917712032	0.044707207	0.474889371	0.10505521	0.742303601
CDC27	0.146819377	0.092214768	0.496355566	0.918915078	0.059503399	0.10496623	0.742303601
CEMIP2	0.925546466	0.407133767	0.024458791	0.039982138	0.998100062	0.105023698	0.742303601
CERS4	0.399847628	0.263070716	0.245187676	0.020764122	0.687091494	0.105049006	0.742303601
CFAP69	0.537282636	0.167318179	0.505530437	0.018044231	0.444426899	0.104475912	0.742303601
DPYSL5	0.191956549	0.302140463	0.127888616	0.103582083	0.476572533	0.104754475	0.742303601
ENDOG	0.873474856	0.507825172	0.426222233	0.005983831	0.321066188	0.104275876	0.742303601
EPC2	0.024404851	0.595507585	0.946101445	0.051414241	0.51505706	0.104422073	0.742303601
FXYD3	0.701488808	0.02889742	0.164409525	0.127191181	0.859636337	0.104468577	0.742303601
HSPBP1	0.167698626	0.668916561	0.012286876	0.410725687	0.643718454	0.104470252	0.742303601
LOC100848246	0.24067458	0.475277564	0.635250047	0.07747997	0.064868067	0.104601083	0.742303601
NPPC	0.148475291	0.286025863	0.395761785	0.220604793	0.099281286	0.105074113	0.742303601
PER3	0.13566188	0.101981941	0.212013123	0.731235842	0.171618471	0.105072685	0.742303601
SEMA6B	0.634950664	0.411050235	0.003546357	0.416328948	0.951259153	0.104822716	0.742303601
SFRP2	0.269759007	0.210013442	0.023026571	0.318538539	0.873377806	0.104226767	0.742303601
TMEM35A	0.038785856	0.736064653	0.210733722	0.747778978	0.081259907	0.104660274	0.742303601
ZMAT5	0.046059184	0.839445701	0.322747259	0.0343676	0.849702883	0.104469836	0.742303601
ZNF219	0.183375133	0.372729599	0.050030253	0.226704525	0.474183578	0.104991035	0.742303601
CLHC1	0.148502521	0.726036178	0.493878635	0.639168923	0.010848855	0.105258721	0.743287809
ARHGAP9	0.149969329	0.684552551	0.006325886	0.942973256	0.605015305	0.105464148	0.743400839
GPR50	0.091758913	0.169263359	0.117709876	0.347029479	0.585230833	0.10559182	0.743400839
LOC101907540	0.330981984	0.045070197	0.678996799	0.045458812	0.806229942	0.105580848	0.743400839
LRRC4	0.932858542	0.081669773	0.135388988	0.391177181	0.091970903	0.105559487	0.743400839
NEK2	0.868788596	0.008329857	0.066794879	0.845722964	0.907879178	0.105568716	0.743400839
NYNRIN	0.88801323	0.231117001	0.040900738	0.623143851	0.070740039	0.105386575	0.743400839
PARP10	0.762043087	0.050046577	0.371742976	0.059884046	0.437176049	0.105570538	0.743400839
LOC104975663	0.986090238	0.28123787	0.532295794	0.002982723	0.844496165	0.105680094	0.743703267
LOC618367	0.575258047	0.434825229	0.098480625	0.287778422	0.052682414	0.105943715	0.745238877
CCDC28B	0.63695869	0.02507336	0.917731921	0.58815955	0.043398368	0.106048888	0.745504837
PCOLCE	0.125590402	0.16129281	0.100459762	0.184762101	0.995406529	0.106072378	0.745504837
RBM12	0.123109324	0.036275712	0.48788621	0.342456013	0.502102232	0.10613439	0.74562135
PNMA1	0.316942041	0.194661562	0.656448198	0.344749745	0.026954236	0.106408917	0.747230094
CD300LG	0.093905269	0.558528315	0.795365501	0.009595641	0.944002223	0.106654448	0.748628507
DNAJC5	0.390059035	0.336759112	0.110554776	0.064085904	0.406330832	0.106699292	0.748628507

ABCF1	0.643225016	0.775791306	0.610743227	0.041080802	0.030253701	0.106799281	0.74900983
PDCD1LG2	0.524459863	0.518976629	0.083642404	0.069442145	0.23992551	0.106883628	0.749281172
PHGDH	0.332586694	0.440329672	0.126846366	0.1065903	0.191983471	0.107017683	0.749589743
WDR77	0.329207288	0.662007087	0.127734451	0.268560003	0.05084758	0.107018997	0.749589743
ATG14	0.006444269	0.802166224	0.846300738	0.986187445	0.088231356	0.107102264	0.749852921
DDX51	0.277176221	0.562061296	0.205365115	0.032175306	0.372893539	0.107612629	0.749964572
EIF3CL	0.784437527	0.946266806	0.09442166	0.139843684	0.039257102	0.107757995	0.749964572
FBXO11	0.331214702	0.981654737	0.224313091	0.007676732	0.683161052	0.107394172	0.749964572
IDUA	0.033751651	0.762052814	0.080385899	0.217202883	0.856034318	0.107703264	0.749964572
LCORL	0.224329141	0.685981994	0.017948838	0.258324822	0.535896396	0.107374372	0.749964572
NEXN	0.267286964	0.253029807	0.157597085	0.609522853	0.05904742	0.107572426	0.749964572
OSBPL1A	0.773198671	0.959284414	0.003015296	0.241176903	0.708418698	0.107333919	0.749964572
RASSF10	0.075392698	0.65476	0.025369066	0.883715901	0.346626833	0.107572735	0.749964572
RERE	0.905501757	0.756458268	0.888317387	0.897423722	0.000704057	0.107707628	0.749964572
RMRP	0.649882603	0.247327966	0.046494768	0.695522728	0.073743868	0.107524659	0.749964572
S100A1	0.596053604	0.460793643	0.010677058	0.806483569	0.16132648	0.107242617	0.749964572
TERF2IP	0.285702274	0.209779041	0.032592657	0.434721749	0.452854687	0.107724292	0.749964572
TMEM176A	0.213533028	0.129341171	0.057775849	0.89802573	0.267757911	0.107585207	0.749964572
TSLP	0.931291985	0.068276009	0.383906576	0.047147987	0.334134788	0.107724092	0.749964572
ID2	0.570294237	0.171060166	0.014831653	0.275473279	0.967048129	0.107865322	0.750393299
ELL2	0.675543231	0.880679777	0.001817577	0.480275025	0.743204697	0.107949706	0.750662129
MOSPD1	0.478440595	0.050387519	0.243928816	0.160808469	0.409851783	0.108202571	0.752101816
COX10	0.657570892	0.552650206	0.563983266	0.083761764	0.022625754	0.108338324	0.752726606
UTP18	0.506881876	0.148904284	0.097262572	0.312861724	0.169254284	0.108387068	0.752746585
FAM20A	0.284781902	0.773686691	0.008478513	0.433776893	0.481289348	0.108588329	0.753825325
NDEL1	0.563101455	0.594739088	0.454289306	0.009410904	0.272710535	0.108660651	0.754008436
ZNF362	0.772589314	0.51897542	0.361644445	0.008310844	0.325377651	0.108921175	0.755496792
TAP1	0.184513359	0.732500116	0.156763582	0.037563943	0.493073062	0.108971461	0.755526252
TIA1	0.136287142	0.194983067	0.288566938	0.080728326	0.636722455	0.109244066	0.757096439
ADCY5	0.351751258	0.138771563	0.144026942	0.074452845	0.754634684	0.109375988	0.757690727
PCDH7	0.622097944	0.677299816	0.455539725	0.007199052	0.286089832	0.109425416	0.757713293
FCER1G	0.483464927	0.654550137	0.006986755	0.231619006	0.773627678	0.109560738	0.758330355
LMTK3	0.751877214	0.823663325	0.105241598	0.349178267	0.017459004	0.109741381	0.758580412
NOM1	0.103724333	0.890198139	0.061758753	0.121017935	0.576058086	0.109774514	0.758580412
PLEKHG2	0.585982053	0.269801883	0.009818846	0.366460832	0.698783182	0.109771343	0.758580412
PRRT2	0.932599294	0.906815425	0.045382134	0.823416957	0.012590298	0.109827985	0.758580412
TMEM248	0.907422745	0.416960011	0.001873809	0.940405109	0.596623405	0.109812194	0.758580412
ISCA2	0.88843093	0.00717604	0.156956384	0.436172883	0.913000749	0.109923062	0.758917698
MTR	0.740446479	0.480447149	0.004468378	0.708498723	0.355260441	0.110175635	0.760341608
PABPC4	0.399835657	0.550266161	0.962634552	0.01831808	0.103289118	0.110272781	0.760372523
RASAL3	0.086374784	0.599986519	0.656141581	0.082884963	0.142105194	0.110238446	0.760372523
ANGPTL8	0.790113304	0.82501876	0.518773425	0.002229982	0.533310014	0.110497767	0.760937467
C14H8orf37	0.064192584	0.396229264	0.554983714	0.158421479	0.180559777	0.110748142	0.760937467
C2	0.417111111	0.047063586	0.257796755	0.128764176	0.623965977	0.111185694	0.760937467
CCNDBP1	0.821630843	0.048938142	0.120921252	0.461339372	0.180257688	0.110834053	0.760937467

CRABP2	0.856639466	0.400928735	0.03116743	0.463383276	0.082036744	0.111235694	0.760937467
GPR88	0.860459908	0.872893987	0.101991444	0.005378091	0.977286721	0.110569019	0.760937467
HHLA2	0.091655185	0.931504145	0.018736483	0.294925887	0.860774956	0.11110776	0.760937467
L1CAM	0.416683174	0.028535784	0.769995779	0.208434614	0.212440274	0.111000364	0.760937467
LOC100847495	0.673153913	0.232187945	0.269027809	0.097597621	0.099005043	0.111138964	0.760937467
LOC101905514	0.532521162	0.269332049	0.344642272	0.191547706	0.042706636	0.110837609	0.760937467
LOC112441650	0.322677254	0.014683624	0.497281028	0.635836429	0.268966723	0.11061829	0.760937467
LOC112443428	0.900311141	0.034096047	0.112372859	0.161845034	0.724489896	0.110855542	0.760937467
LOC784087	0.011155291	0.323761124	0.316618392	0.670139214	0.530817641	0.11121207	0.760937467
MCUR1	0.840532393	0.019343113	0.495228982	0.629804082	0.080116895	0.111134426	0.760937467
PAMR1	0.307036747	0.046913924	0.080722528	0.769544279	0.449679916	0.110528336	0.760937467
PILRA	0.8893544	0.844479116	0.001294606	0.491903244	0.84624106	0.11089683	0.760937467
POC1A	0.905140062	0.352123173	0.397193534	0.003415501	0.940532538	0.111195934	0.760937467
SLC26A2	0.761787761	0.776775199	0.008236063	0.085726292	0.96715001	0.110792964	0.760937467
TTC22	0.338102522	0.068293289	0.123204411	0.286214915	0.499170706	0.111160164	0.760937467
FERMT3	0.46089194	0.760089066	0.002077734	0.756291507	0.741145453	0.111399834	0.761742778
GPN3	0.065413733	0.08768659	0.335038552	0.327158064	0.649536304	0.111459644	0.76183433
GPX4	0.782227043	0.554094846	0.922035655	0.002098076	0.487544825	0.111523934	0.761956402
BEND3	0.05052656	0.530499171	0.212842809	0.082912972	0.86675408	0.111710312	0.762912167
OPA3	0.139051552	0.399677133	0.229333194	0.03318899	0.970291358	0.111778226	0.763058432
CDR2	0.245489261	0.199931096	0.911023124	0.040910585	0.225660095	0.112140537	0.763309148
CFL1	0.036987326	0.258377763	0.440822336	0.616064103	0.158754947	0.112022613	0.763309148
LOC112444346	0.543827822	0.870504865	0.01197748	0.146797613	0.495360277	0.112068351	0.763309148
LOC112447381	0.090396016	0.088383457	0.534408404	0.601055304	0.160741345	0.112097155	0.763309148
MANSC1	0.835118319	0.769215847	0.046200652	0.015886531	0.873718402	0.11201095	0.763309148
SAMD15	0.196179459	0.131558744	0.144650954	0.448074372	0.246512354	0.112074932	0.763309148
TM7SF2	0.534063434	0.442957576	0.573988988	0.013106503	0.231397391	0.111990372	0.763309148
LOC112448736	0.06636609	0.251730078	0.7232161	0.427245571	0.080048271	0.112205722	0.763436194
FAM43B	0.75868744	0.983773854	0.108865679	0.408561626	0.012472473	0.112334413	0.763995045
LOC112448893	0.060010388	0.089959517	0.881983972	0.159356656	0.546942454	0.112478812	0.764660224
LOC101907682	0.674386816	0.018219612	0.341160898	0.723817445	0.137146997	0.112651211	0.764881679
RETN	0.257875555	0.166550598	0.094017542	0.229705314	0.448252202	0.11259785	0.764881679
TEX11	0.339453473	0.714737495	0.003120317	0.761719373	0.721530281	0.11264422	0.764881679
EPDR1	0.239575814	0.431857418	0.824930281	0.007480788	0.653085431	0.112782512	0.764999815
PRDM5	0.882411021	0.034563016	0.225738094	0.093933445	0.644704787	0.112775266	0.764999815
RSRC2	0.533500588	0.301161245	0.470767188	0.009471036	0.582313732	0.112808455	0.764999815
STK40	0.416246632	0.376666253	0.261724966	0.130994241	0.077830538	0.112993553	0.765938535
AIMP2	0.223591924	0.394552639	0.136046487	0.693779394	0.0505918	0.113434542	0.767287186
GMPPA	0.418767192	0.08465196	0.021596407	0.811950823	0.679084194	0.113566545	0.767287186
LOC101906195	0.885026574	0.149099074	0.007817099	0.487695864	0.838299115	0.113504618	0.767287186
LOC509513	0.057483993	0.325266872	0.703304946	0.434432815	0.073549242	0.11326966	0.767287186
OIP5	0.286789155	0.012176433	0.500182796	0.819233154	0.293998352	0.113348154	0.767287186
SAE1	0.378645863	0.141121768	0.636606414	0.488234283	0.025412042	0.113554966	0.767287186
TRPA1	0.012608617	0.27175454	0.312475336	0.820840783	0.479583223	0.11346891	0.767287186
ZAR1L	0.469332946	0.045499701	0.347925468	0.54570135	0.103715107	0.113319986	0.767287186

TSKU	0.056514942	0.356357165	0.14007727	0.479337973	0.313302613	0.113799682	0.768545916
BRD3OS	0.07834365	0.311425508	0.166188491	0.830413615	0.126435454	0.114110611	0.768944584
GRWD1	0.356298584	0.485872672	0.016733638	0.513715569	0.285208069	0.113916444	0.768944584
KATNAL1	0.421223944	0.006817224	0.981339493	0.846571283	0.178661923	0.114186701	0.768944584
LOC101903289	0.616289362	0.658200641	0.006593028	0.872121242	0.182409743	0.114070667	0.768944584
LOC112448791	0.449945742	0.617142095	0.154301555	0.086578146	0.114557634	0.113995876	0.768944584
LOC539973	0.284343036	0.099305462	0.236978579	0.243578051	0.261211946	0.114115614	0.768944584
P2RX5	0.662476801	0.761887544	0.070737565	0.08770696	0.136091609	0.114178048	0.768944584
HNMT	0.740979784	0.917822344	0.004228187	0.206448179	0.720316257	0.114397202	0.769213245
IL33	0.167520271	0.647463454	0.012111861	0.481921196	0.67541768	0.114395487	0.769213245
NCOR2	0.932551444	0.369097278	0.039518222	0.066803981	0.471394435	0.114507828	0.769213245
NUCB2	0.694544734	0.226265424	0.026025306	0.289270449	0.361201359	0.114354753	0.769213245
PLS3	0.659621024	0.033113635	0.835565445	0.043783432	0.535913609	0.114491262	0.769213245
STT3B	0.63818137	0.135143485	0.021113429	0.278273984	0.844877905	0.114473148	0.769213245
SSTR1	0.62434119	0.337563988	0.264580001	0.010504704	0.732654044	0.114629811	0.769717605
POLD4	0.25458466	0.147904522	0.058494226	0.321182827	0.608426774	0.114818857	0.770671684
NRG2	0.066625841	0.273327898	0.662098665	0.060149055	0.594149939	0.114891443	0.770843612
ARG2	0.505049959	0.561945603	0.003866044	0.625846034	0.629631459	0.115111577	0.770892705
CKAP2	0.746288566	0.08011954	0.04916138	0.357288685	0.410760878	0.114966525	0.770892705
FAM212A	0.983431756	0.953352614	0.201297024	0.143898209	0.015924139	0.115126086	0.770892705
IFT57	0.553436496	0.20234505	0.28613109	0.042389961	0.317899242	0.11502626	0.770892705
PSMA7	0.362125778	0.573237872	0.653467536	0.201074178	0.015857049	0.115133631	0.770892705
BRD8	0.065253511	0.277487409	0.248596232	0.922413633	0.10430898	0.115222109	0.771170484
ABCA3	0.431250661	0.367523816	0.294989336	0.235041936	0.039479693	0.1153345	0.771335001
CACNB1	0.025535073	0.505804442	0.330641466	0.849313594	0.119799157	0.115433004	0.771335001
OGFOD2	0.280072488	0.810631868	0.790889272	0.00441689	0.547877969	0.115434694	0.771335001
SKA3	0.159128831	0.122584851	0.448300573	0.055042581	0.901524607	0.115348062	0.771335001
COL6A1	0.067840844	0.35226955	0.090549021	0.25967359	0.776564892	0.11571127	0.77161221
IRX3	0.90000709	0.050729755	0.092976672	0.275000931	0.373620006	0.115679969	0.77161221
TNFSF13	0.659040778	0.9939975	0.008315488	0.282902395	0.283048519	0.115685351	0.77161221
TRIM24	0.153380949	0.827675101	0.052980593	0.087864237	0.737503733	0.11563161	0.77161221
ZFPL1	0.862017136	0.9830668	0.391300057	0.00254202	0.517306766	0.115663322	0.77161221
KCNE3	0.393853943	0.378579339	0.015815118	0.387640034	0.478251602	0.115830432	0.771825922
LOC101905312	0.26643161	0.125770698	0.502543508	0.034880729	0.74434012	0.11583738	0.771825922
ABCD4	0.378429643	0.168074426	0.276170208	0.048524945	0.515225241	0.116127493	0.772715522
LOC100336669	0.867638836	0.680493568	0.463576875	0.023616035	0.068065201	0.116246592	0.772715522
LOC781726	0.80092131	0.013299469	0.103180895	0.762048711	0.523892659	0.116070726	0.772715522
PABPN1	0.24795582	0.032594716	0.220732142	0.424868479	0.579757418	0.116166944	0.772715522
SLITRK6	0.880020318	0.886354524	0.411759085	0.007963983	0.172023762	0.116253405	0.772715522
WBP2	0.116972056	0.439436674	0.060524568	0.211764095	0.666812775	0.116148648	0.772715522
DPM1	0.071706252	0.754672777	0.392077166	0.15542281	0.133623548	0.116347262	0.772747166
HMGN1	0.433507895	0.314435075	0.751769721	0.165766412	0.025998363	0.116493601	0.772747166
LOC107133024	0.759475197	0.722602326	0.301434073	0.003152346	0.845716825	0.116404415	0.772747166
LOC540403	0.504350966	0.024049795	0.562518507	0.251248002	0.257573534	0.116482969	0.772747166
LOC783641	0.824692274	0.241440246	0.513405371	0.006778898	0.637043157	0.116468648	0.772747166

LOC101907152	0.182715666	0.335102786	0.164916497	0.571464092	0.076845276	0.116760742	0.773195546
NACC1	0.60664229	0.84387092	0.538369269	0.003495413	0.45993133	0.116708804	0.773195546
NPM3	0.897740325	0.719859779	0.830588171	0.142137679	0.005805623	0.116687948	0.773195546
RBM47	0.814672276	0.275654581	0.147768722	0.209377664	0.063855938	0.116796768	0.773195546
RTEL1	0.433181751	0.009654643	0.282726388	0.386390853	0.96865004	0.116631146	0.773195546
MCRS1	0.160935518	0.57199774	0.176381684	0.088302991	0.309908037	0.116894443	0.77353012
KRBA2	0.223823616	0.125054457	0.643030526	0.909412278	0.027177108	0.116969037	0.773711756
AARS2	0.641664506	0.042335919	0.38905509	0.048538808	0.894362046	0.11901777	0.773738878
ADORA3	0.355226872	0.85567093	0.005773321	0.652863927	0.399810669	0.118907888	0.773738878
ARFGAP2	0.549349286	0.18058638	0.102112926	0.282883876	0.161781283	0.119714194	0.773738878
ARID4B	0.614024835	0.438840385	0.205506963	0.009496041	0.855223809	0.117688757	0.773738878
ARPC3	0.884275532	0.394446104	0.014180057	0.173717639	0.532942098	0.11888668	0.773738878
ARSI	0.628197471	0.257919355	0.035163887	0.225099151	0.34820207	0.117223881	0.773738878
ASPA	0.113655502	0.464668209	0.013865517	0.700761273	0.912819362	0.120406884	0.773738878
CCT5	0.233865215	0.364455645	0.206057628	0.189745475	0.13569208	0.118052047	0.773738878
CEP95	0.280184551	0.019857014	0.140530213	0.749621495	0.801680522	0.12061608	0.773738878
CMSS1	0.865010553	0.395864935	0.376770963	0.062097336	0.055859765	0.117365908	0.773738878
CTBP1	0.423387921	0.896526281	0.038983278	0.047565555	0.6459502	0.118410633	0.773738878
CXCR4	0.546448585	0.797255804	0.116121839	0.033487263	0.266350783	0.117910124	0.773738878
CYB561A3	0.124755866	0.899010879	0.221170014	0.614572971	0.030229051	0.119313094	0.773738878
CYP27A1	0.126893114	0.972612921	0.010372349	0.741051394	0.489886771	0.119876233	0.773738878
DENND6A	0.234128857	0.260574092	0.055727538	0.238080423	0.575952531	0.120087802	0.773738878
DGCR8	0.690008412	0.021115237	0.108185112	0.32750588	0.891311801	0.11920786	0.773738878
DHX40	0.227249479	0.169015547	0.180870546	0.171346902	0.37686766	0.117524631	0.773738878
DIP2C	0.019001799	0.519751112	0.125451563	0.534467265	0.67829945	0.117607706	0.773738878
DMPK	0.52625856	0.317376978	0.929564923	0.003370472	0.868431297	0.118381428	0.773738878
ELAVL1	0.536727504	0.02293678	0.197126975	0.287963729	0.640180513	0.117343838	0.773738878
EPB41L5	0.150407383	0.424127652	0.5090094	0.373612635	0.03776335	0.118918376	0.773738878
EXD3	0.691020201	0.064853602	0.088949374	0.128274137	0.915643203	0.120377191	0.773738878
FAM131A	0.375397047	0.421500474	0.718352542	0.029655517	0.137438038	0.119666052	0.773738878
FAN1	0.492398196	0.650766889	0.234713728	0.010164066	0.602180618	0.119239879	0.773738878
FCMR	0.085221274	0.152592143	0.939787474	0.907988231	0.040208059	0.117166395	0.773738878
GRB7	0.785279592	0.51369336	0.5639989	0.061266555	0.032851418	0.118887739	0.773738878
GZMK	0.345927196	0.676337326	0.929011287	0.059166113	0.035082052	0.117899839	0.773738878
HMGCR	0.191583464	0.325898515	0.154930658	0.143453103	0.32766305	0.118417327	0.773738878
HSF2	0.270556312	0.833054095	0.65196896	0.003170903	0.967940924	0.117878874	0.773738878
IFI27L2	0.007035009	0.914951379	0.351199683	0.303206026	0.666759904	0.118755756	0.773738878
KCTD14	0.210398555	0.250359576	0.087779924	0.141349928	0.719254575	0.120648603	0.773738878
KLHDC7A	0.702460739	0.015797852	0.655248197	0.169657292	0.363016841	0.117412581	0.773738878
LOC100141266	0.006615277	0.524546239	0.273220924	0.806768735	0.603754459	0.119452546	0.773738878
LOC100337213	0.078716941	0.728096304	0.502268406	0.028089401	0.578119186	0.120271648	0.773738878
LOC100848684	0.352642441	0.262803651	0.2517858	0.09903332	0.193728884	0.117389446	0.773738878
LOC101902293	0.238465393	0.102650464	0.17085281	0.568267897	0.195920361	0.120006473	0.773738878
LOC101905267	0.414280918	0.185133167	0.045794491	0.988594179	0.135483327	0.120697731	0.773738878
LOC101908760	0.353258033	0.034848596	0.28995268	0.434267128	0.302611552	0.120503537	0.773738878

LOC112448805	0.636130575	0.394195772	0.002126596	0.996096338	0.850796953	0.118013215	0.773738878
LOC506495	0.227322554	0.062872828	0.059715031	0.653266684	0.818161527	0.118632271	0.773738878
LOC511713	0.476596607	0.889267879	0.461251893	0.586015566	0.003894232	0.11715874	0.773738878
LOC788599	0.345144238	0.496080869	0.951441969	0.017759768	0.160985901	0.12002496	0.773738878
LRIG3	0.218620528	0.211815204	0.187841515	0.455339167	0.113554195	0.117694302	0.773738878
LUZP1	0.091538274	0.603480352	0.956663931	0.038698679	0.228152726	0.120146764	0.773738878
MGC139164	0.677749134	0.017894534	0.210060548	0.283831423	0.626664792	0.118190191	0.773738878
MOG	0.700873908	0.105767413	0.984749352	0.762145508	0.008330915	0.11969834	0.773738878
MSX1	0.473221158	0.447835508	0.04404492	0.311044603	0.155690457	0.118027361	0.773738878
MYADM	0.06585243	0.060466505	0.87834521	0.431947479	0.3086396	0.120098347	0.773738878
MYH11	0.004191509	0.594233289	0.501250172	0.906483478	0.408134563	0.119466429	0.773738878
NAV1	0.202508635	0.027791712	0.198330927	0.558721062	0.74176679	0.119569231	0.773738878
NRCAM	0.675754965	0.957800976	0.003908913	0.371112234	0.484672834	0.118472315	0.773738878
NTRK3	0.082995538	0.830022655	0.647178029	0.616735135	0.016488372	0.118223083	0.773738878
PKLR	0.031622351	0.335183489	0.423125002	0.422496022	0.237453371	0.117719966	0.773738878
PLCB4	0.073963564	0.570229002	0.838062666	0.816409305	0.016075623	0.119755691	0.773738878
PLCXD2	0.014815853	0.938742183	0.356118038	0.432609549	0.215422183	0.119421458	0.773738878
PLIN2	0.913873682	0.048746604	0.102892442	0.378149034	0.270923758	0.120577674	0.773738878
PNMA8A	0.143792026	0.359219956	0.502806188	0.440495666	0.04015029	0.11909396	0.773738878
PTPRG	0.435383837	0.074020583	0.190330761	0.799508086	0.095488834	0.120389004	0.773738878
RAB9A	0.598359813	0.459555362	0.284680383	0.009032074	0.663279069	0.120487176	0.773738878
SENP6	0.214238584	0.690145039	0.458978833	0.010135357	0.659106004	0.118220046	0.773738878
SFR1	0.433834383	0.205085992	0.112713311	0.197075045	0.232765938	0.119195492	0.773738878
SH2D4A	0.042037192	0.837663798	0.775359742	0.855379655	0.01992459	0.1199622	0.773738878
SLC25A33	0.762066425	0.417584609	0.0036296	0.541901064	0.71267103	0.117151533	0.773738878
SLC39A8	0.034929182	0.525456004	0.498848357	0.213507826	0.235412385	0.119218605	0.773738878
SLCO4A1	0.68985249	0.908043072	0.404631016	0.004682246	0.383551518	0.118491505	0.773738878
SLITRK2	0.519028532	0.355795715	0.022502883	0.20519326	0.54400013	0.119751494	0.773738878
SLMAP	0.016594733	0.309920245	0.608556732	0.829960823	0.17664272	0.119024541	0.773738878
SMARCC1	0.941641988	0.248231404	0.223939187	0.055656794	0.161475225	0.120697796	0.773738878
SNAP47	0.567434598	0.264436041	0.075799867	0.113707758	0.356267819	0.119300997	0.773738878
SPSB1	0.006689421	0.414980646	0.377700239	0.743775152	0.575358221	0.117536957	0.773738878
TH	0.193841937	0.346416	0.457096998	0.133555284	0.112121018	0.119136402	0.773738878
TMEM108	0.08441464	0.052185904	0.583721571	0.350228792	0.512888246	0.119467373	0.773738878
TPPP3	0.287239195	0.798928253	0.37261001	0.007021844	0.774571059	0.119925599	0.773738878
UTRN	0.634516525	0.88603673	0.844135571	0.002625074	0.376014895	0.120411382	0.773738878
YTHDF2	0.789149475	0.35159742	0.342237996	0.008281448	0.573530319	0.117879835	0.773738878
ZBTB14	0.467544279	0.037652186	0.128132004	0.207963925	0.998584545	0.120409829	0.773738878
ZDHHC18	0.909413847	0.851079241	0.01362427	0.061016153	0.706719774	0.118420645	0.773738878
ZNF341	0.99047659	0.022868327	0.067557246	0.308373121	0.970601791	0.118900646	0.773738878
ZNF638	0.107441561	0.391067495	0.827504274	0.016827676	0.792235739	0.119702164	0.773738878
FKRP	0.297422251	0.053563326	0.157090264	0.280396536	0.670891144	0.120747304	0.773754008
TMEM128	0.039069365	0.076826357	0.239661389	0.790430221	0.829366303	0.120862431	0.774189446
CORO2A	0.032900923	0.788467568	0.073858675	0.303517884	0.813529484	0.12108005	0.775063245
LOC112446423	0.862029893	0.472108386	0.76354014	0.007359804	0.20690634	0.121093301	0.775063245

LRRC59	0.28464047	0.733623948	0.297971108	0.813283951	0.009378728	0.121295344	0.775147155
LRRC61	0.061485301	0.831113078	0.102184678	0.437313197	0.20755094	0.121202292	0.775147155
SLC9A1	0.52396421	0.099164094	0.249610037	0.052231299	0.700514077	0.121285252	0.775147155
SPATA5	0.140076817	0.777913344	0.048274677	0.663728488	0.135821341	0.121239891	0.775147155
ZKSCAN5	0.754296735	0.502672633	0.935231352	0.906470527	0.00147794	0.12136201	0.775271292
CFAP44	0.186432432	0.26229027	0.671383551	0.909405469	0.015948338	0.12151683	0.775798607
RPS12	0.612630154	0.416553708	0.705239448	0.004292612	0.616541229	0.121539103	0.775798607
MVK	0.186666882	0.315078657	0.653156097	0.043940655	0.283739448	0.12191508	0.776838435
NELL1	0.875279602	0.31941668	0.260935128	0.014238767	0.460887171	0.121885976	0.776838435
SDHAF4	0.805747766	0.67499225	0.136350144	0.006703766	0.963757161	0.121938688	0.776838435
SYK	0.756734711	0.938884141	0.004103429	0.196035958	0.837036866	0.121835634	0.776838435
TRAF3IP3	0.577127003	0.683258193	0.118398065	0.297496413	0.034459703	0.121868749	0.776838435
CPNE9	0.267613434	0.169751788	0.536829215	0.223186688	0.088302931	0.122152525	0.777295496
NRP2	0.679446978	0.078257193	0.032610811	0.361586477	0.765960505	0.122098823	0.777295496
RBP7	0.054778867	0.095768774	0.477970439	0.24691313	0.776141928	0.122140194	0.777295496
STK38	0.413894041	0.882062384	0.407571962	0.010223934	0.316283561	0.122228853	0.777479729
ANO1	0.226791903	0.125288381	0.334495742	0.064380029	0.787776887	0.122354041	0.777974495
CD3D	0.120985505	0.163238388	0.872851998	0.308116084	0.091209344	0.122695379	0.778335475
COG2	0.663036723	0.528693569	0.747136274	0.003076969	0.599534137	0.122510717	0.778335475
PIK3R5	0.838520488	0.422058649	0.088679999	0.162217908	0.095138456	0.122682306	0.778335475
PPP1R3F	0.308647236	0.742433502	0.711302786	0.025828093	0.114735526	0.1224928	0.778335475
PPP6R2	0.345857867	0.268848352	0.045822428	0.116034975	0.978812358	0.122619931	0.778335475
TAP2	0.037958305	0.572339428	0.610957919	0.069841173	0.522334056	0.12266095	0.778335475
DRG1	0.956943791	0.446009921	0.255149891	0.550755928	0.008094303	0.122839781	0.778348895
LOC100847120	0.054969679	0.067570759	0.274595015	0.767144886	0.619855415	0.122773083	0.778348895
RERGL	0.348777157	0.343797068	0.625774384	0.027580585	0.23446189	0.122804851	0.778348895
LOC101905925	0.249350916	0.031857051	0.43027597	0.305689408	0.465303365	0.122936956	0.778363571
MAP4K4	0.499069723	0.897604732	0.390036598	0.005790436	0.480368217	0.122914365	0.778363571
C2CD5	0.633596471	0.838421422	0.492894643	0.204589514	0.009112163	0.123214325	0.779011151
CCBE1	0.913147847	0.041517148	0.346531131	0.041785	0.890141239	0.123286546	0.779011151
LOC101906754	0.994740156	0.192211553	0.196645283	0.889677823	0.014575593	0.123134815	0.779011151
MDM4	0.01044675	0.879204435	0.075009235	0.895213786	0.790177238	0.12310403	0.779011151
PRAP1	0.905238719	0.6162591	0.249898054	0.008701703	0.402676015	0.123264243	0.779011151
UBE3B	0.831691261	0.768660681	0.317376974	0.002860096	0.842508137	0.123324049	0.779011151
SOCS6	0.573671797	0.110527598	0.012343592	0.753160803	0.830197499	0.123390281	0.77912963
FLCN	0.370446922	0.763713689	0.555346737	0.054909306	0.056856099	0.123548682	0.779829775
COL7A1	0.826308692	0.179412725	0.09296613	0.289927099	0.123582103	0.124012873	0.780546379
HERPUD1	0.476591138	0.931952559	0.640150147	0.001922406	0.902626934	0.123950861	0.780546379
IL18R1	0.619267053	0.273896393	0.730112128	0.059781958	0.066689531	0.123999652	0.780546379
IRF5	0.840109376	0.99648035	0.001835739	0.437854549	0.734187123	0.124042713	0.780546379
LOC101902059	0.671966236	0.665438084	0.291521163	0.090517009	0.041671305	0.123715169	0.780546379
PDHX	0.797754231	0.791246415	0.302293883	0.005587234	0.461822431	0.123808858	0.780546379
SNRK	0.127095672	0.38408009	0.816349627	0.097163341	0.127517689	0.124002832	0.780546379
WDR76	0.884387697	0.638664819	0.027645579	0.227542443	0.13853824	0.123791591	0.780546379
CHTF18	0.931052088	0.015375439	0.166247124	0.321931549	0.645293148	0.124094318	0.780571812

S100A5	0.933755868	0.1174505	0.510827962	0.303490701	0.029115158	0.124182126	0.780824851
PM20D1	0.831414769	0.451380111	0.06208034	0.266874985	0.079804019	0.124344676	0.781248267
RXRA	0.56773452	0.701407827	0.509472718	0.003031135	0.806386214	0.124302944	0.781248267
ACSL4	0.099888293	0.683108071	0.286392288	0.282289209	0.091521014	0.125551507	0.78134379
ADAT2	0.091739687	0.066364721	0.98575231	0.161675522	0.516595278	0.125049954	0.78134379
AKAP8L	0.245755501	0.145000208	0.434573519	0.154803022	0.210253476	0.125435307	0.78134379
ALDH18A1	0.723564924	0.683387463	0.053716829	0.083035102	0.227999112	0.125273386	0.78134379
ANGPTL4	0.125638323	0.951151078	0.430202711	0.108961836	0.090122156	0.125546735	0.78134379
C7H5orf63	0.768836938	0.792771338	0.437607197	0.269433412	0.007030496	0.12560357	0.78134379
CBX4	0.420049827	0.107344808	0.276846425	0.052777256	0.757464389	0.124741331	0.78134379
CENPE	0.484359832	0.070532639	0.024459001	0.720579969	0.832898406	0.125084287	0.78134379
CXHXorf38	0.68003821	0.298175931	0.066799242	0.743358705	0.050141288	0.125550046	0.78134379
DES	0.00857014	0.36296259	0.465795661	0.788041995	0.436588928	0.124667364	0.78134379
DMWD	0.403338801	0.240766879	0.085504851	0.066040776	0.912437235	0.124924269	0.78134379
FAM135A	0.505491791	0.430928922	0.303718242	0.008556776	0.879516603	0.124583848	0.78134379
FAM198A	0.867022604	0.118302633	0.043661683	0.381886556	0.295599804	0.125645376	0.78134379
FAM234A	0.236229623	0.597263103	0.519429464	0.00695487	0.975600862	0.12449487	0.78134379
GPR151	0.010126604	0.223406855	0.312284131	0.714435122	0.994241067	0.125131362	0.78134379
LOC101903038	0.706361514	0.740912066	0.093936519	0.025287581	0.404730053	0.125313711	0.78134379
LOC404103	0.172766173	0.365856407	0.12584386	0.564080024	0.110899269	0.12454007	0.78134379
MESD	0.122136591	0.177319993	0.352845099	0.215913486	0.305681321	0.125480078	0.78134379
PABPC1L2A	0.110407005	0.723673338	0.017440034	0.783511195	0.462402835	0.125546968	0.78134379
PLAUR	0.841498185	0.768883834	0.046181375	0.105189191	0.160291109	0.12540374	0.78134379
PTX3	0.590488107	0.327977085	0.831555527	0.081303182	0.038075093	0.1246717	0.78134379
RBM34	0.417047433	0.044146328	0.34844639	0.379109856	0.205383063	0.124807879	0.78134379
RGS2	0.7942952	0.089433358	0.099749193	0.158838526	0.44708536	0.125319535	0.78134379
ST3GAL6	0.520416381	0.581899693	0.015206651	0.453308412	0.240584599	0.125184228	0.78134379
TNFAIP8L1	0.086279408	0.185074081	0.058741573	0.737041456	0.721854592	0.124742959	0.78134379
ZNF512B	0.822069057	0.357838768	0.002957314	0.697157528	0.828000069	0.125177842	0.78134379
ZNF704	0.405955509	0.468985928	0.919352267	0.31691098	0.00896002	0.12445952	0.78134379
AFG3L2	0.455384856	0.417785652	0.13497572	0.061171084	0.323642182	0.126037859	0.782750706
ARHGAP19	0.836898989	0.347437789	0.033195223	0.087694067	0.601392252	0.1261274	0.782750706
GALR3	0.399086518	0.045121283	0.649719482	0.102500785	0.424659035	0.126157798	0.782750706
LOC614207	0.672994309	0.469460453	0.216277369	0.029701085	0.250893349	0.126148269	0.782750706
OTUD6B	0.815254124	0.359266811	0.012155032	0.429382394	0.332091536	0.125936096	0.782750706
PTGES2	0.400875837	0.694150769	0.525236119	0.005547476	0.627534686	0.126094734	0.782750706
ANKRD33B	0.989985399	0.101414891	0.03575616	0.156476366	0.918588972	0.12708281	0.78297171
CD164L2	0.018742786	0.836394636	0.176087511	0.47898096	0.386613472	0.126420849	0.78297171
DAPP1	0.33865874	0.37444041	0.036761279	0.274947111	0.40052273	0.126719305	0.78297171
DNASE2	0.253685692	0.587994607	0.013488569	0.436803709	0.587443435	0.127121202	0.78297171
DPH7	0.37172113	0.554809965	0.225066084	0.027013059	0.411477504	0.127073067	0.78297171
DTX3L	0.715377907	0.907373663	0.042600636	0.072007284	0.256738132	0.126426578	0.78297171
IFIT2	0.912002954	0.683903453	0.017492442	0.354384656	0.132261811	0.126450247	0.78297171
IL12RB1	0.953081763	0.03078079	0.039448448	0.917844066	0.484055218	0.126831488	0.78297171
LOC100336869	0.224811542	0.828926728	0.027571879	0.249377465	0.400755815	0.126739767	0.78297171

LOC101903026	0.910472075	0.014141182	0.066211596	0.915287094	0.660859095	0.127034261	0.78297171
LOC107131418	0.058617238	0.611728918	0.480204676	0.441660769	0.067532633	0.126751946	0.78297171
LOC786352	0.789872163	0.556310835	0.43023617	0.007264618	0.372533788	0.126484046	0.78297171
MYL12B	0.216729473	0.109542224	0.52948841	0.044974704	0.912964632	0.127103432	0.78297171
NOL11	0.245571052	0.248108898	0.997986441	0.045313076	0.187448677	0.127147621	0.78297171
PCMTD2	0.252508012	0.34091822	0.410514713	0.125258715	0.116012829	0.126744619	0.78297171
PLXNB3	0.095573087	0.013712694	0.910885418	0.596328962	0.724144242	0.127014856	0.78297171
PSME1	0.122679585	0.795905845	0.144779604	0.079005486	0.460140051	0.126796612	0.78297171
RBFOX2	0.351818291	0.272716017	0.15685046	0.63324181	0.053735866	0.126547026	0.78297171
SWI5	0.894023514	0.835816512	0.009251548	0.118472188	0.622734957	0.126263145	0.78297171
TIGIT	0.130242616	0.307237709	0.57091182	0.708914569	0.031731842	0.126796155	0.78297171
ARMCX1	0.748736453	0.124013277	0.209156083	0.227910479	0.117181558	0.127447269	0.78364074
CD244	0.633198159	0.582608543	0.010596034	0.187158428	0.708693895	0.127420666	0.78364074
IPCEF1	0.333601822	0.981385462	0.080563968	0.040927509	0.479924522	0.127366798	0.78364074
LOC104975676	0.049758152	0.228189175	0.736393154	0.700020737	0.08860253	0.127436653	0.78364074
KRT79	0.606957212	0.686099938	0.082729221	0.017392218	0.866285191	0.127500881	0.783676762
CAPN7	0.216992284	0.469313802	0.46410987	0.142181088	0.077812863	0.128023526	0.78375428
CD3G	0.135752217	0.330888215	0.716848324	0.076431786	0.211989485	0.1278637	0.78375428
DCTPP1	0.502215354	0.875573867	0.279405327	0.037933615	0.112236589	0.128048796	0.78375428
ITGA9	0.009257643	0.809986242	0.108311416	0.679336941	0.949041299	0.128121847	0.78375428
LOC101904275	0.510649522	0.663553165	0.53022195	0.281329915	0.010331178	0.127925512	0.78375428
LOC112446734	0.041144608	0.192719597	0.505393882	0.176581547	0.738738019	0.128004378	0.78375428
LOC112449523	0.255543234	0.056209034	0.090549743	0.62546563	0.642244542	0.127964657	0.78375428
MGC126945	0.437242669	0.518094328	0.061049248	0.063882591	0.590806461	0.127895662	0.78375428
OSCP1	0.326172491	0.79582887	0.22624618	0.40665393	0.021881455	0.127979017	0.78375428
PLAT	0.196732939	0.960056533	0.605597774	0.022826615	0.20066208	0.128161506	0.78375428
PRF1	0.341123374	0.35522126	0.181442355	0.272488469	0.086743403	0.127584581	0.78375428
SAV1	0.387320835	0.619153172	0.005253996	0.521460938	0.792080419	0.127685073	0.78375428
SOX11	0.448365054	0.244323692	0.152769171	0.270411262	0.115806507	0.128182103	0.78375428
TNFRSF25	0.167578172	0.351743563	0.351210708	0.116138909	0.217591806	0.128057871	0.78375428
FAM219B	0.270526029	0.580007606	0.693147574	0.006514928	0.740237427	0.128240776	0.783820997
GAPT	0.742053393	0.245198444	0.020173751	0.328795475	0.436131885	0.128492226	0.784520621
NEFH	0.696641853	0.862957756	0.565172059	0.06104131	0.025353686	0.128420316	0.784520621
SEPT9	0.236769311	0.627903288	0.294715781	0.061455492	0.195628211	0.12854646	0.784520621
SNAP29	0.276046678	0.348266433	0.103051033	0.472099694	0.112590407	0.128524776	0.784520621
CAP1	0.402059029	0.249631422	0.490081467	0.742355747	0.01455842	0.129199345	0.786162385
EVA1B	0.160886806	0.489654758	0.038328591	0.205986126	0.852355541	0.129003075	0.786162385
FBXL18	0.557752216	0.432308735	0.022019457	0.234621924	0.427150417	0.12926655	0.786162385
GSTO1	0.856534168	0.532423648	0.794202814	0.395771078	0.003718469	0.129390324	0.786162385
HEATR4	0.508910856	0.173950657	0.327809549	0.607352383	0.030194074	0.129276526	0.786162385
HELLS	0.487043785	0.52870722	0.010161039	0.285107317	0.714187207	0.129357335	0.786162385
HMGN2	0.331668539	0.296546265	0.467759453	0.014541697	0.795471568	0.129277714	0.786162385
LIG1	0.674488069	0.011205308	0.164644233	0.60986142	0.702197727	0.129372741	0.786162385
NCKAP1L	0.476901453	0.708037494	0.007673526	0.28995306	0.708439315	0.129286151	0.786162385
RGS20	0.493050521	0.320681099	0.03500723	0.258024305	0.372339571	0.129222203	0.786162385

SASH3	0.291289317	0.978664458	0.037410969	0.261868324	0.190052275	0.129088913	0.786162385
TK1	0.036220715	0.838665897	0.840987266	0.031417384	0.66239055	0.129205263	0.786162385
SLC16A9	0.530058553	0.274883112	0.1059877	0.05346506	0.646584491	0.129502743	0.786554223
BAZ1A	0.775108576	0.320100736	0.45471615	0.012780255	0.372055459	0.129852256	0.787049415
FBXO33	0.731118714	0.853444375	0.007917464	0.217700408	0.499177969	0.129906406	0.787049415
LOC112441476	0.537405421	0.132155392	0.981867877	0.383913831	0.020058178	0.129923186	0.787049415
LOC781663	0.460230083	0.221085329	0.081938433	0.067115593	0.959555558	0.129915057	0.787049415
LSM10	0.384614947	0.246834926	0.064171101	0.238456211	0.368752138	0.129749449	0.787049415
MTCL1	0.713922121	0.238420028	0.006284116	0.912664396	0.550410327	0.129967943	0.787049415
SIRT4	0.869369173	0.41625266	0.451324631	0.003932471	0.834683606	0.129802769	0.787049415
UBL4A	0.364920413	0.47764203	0.064374024	0.052794291	0.90515348	0.129816468	0.787049415
INSR	0.881624874	0.604329897	0.013503695	0.249842542	0.299876771	0.130197407	0.787857537
NFASC	0.025028197	0.753998952	0.159730503	0.637134159	0.280618069	0.130183299	0.787857537
LHX6	0.423509078	0.138718858	0.031199853	0.87228343	0.337414316	0.130255838	0.787920591
COMP	0.513779848	0.465398921	0.011145535	0.644268239	0.316150845	0.130703316	0.788013269
CYP2J2	0.031339545	0.452581816	0.238909086	0.164585358	0.970679313	0.130507881	0.788013269
GFM2	0.272104349	0.76362562	0.921400713	0.025795041	0.109421111	0.130377022	0.788013269
MKI67	0.64610898	0.049559131	0.054873589	0.5277709	0.582629208	0.130365176	0.788013269
PPAT	0.097100347	0.762216033	0.141052985	0.880917502	0.058921687	0.130574737	0.788013269
SH2D5	0.087187876	0.95548955	0.458100333	0.111746215	0.127211844	0.130659815	0.788013269
SRRD	0.711562268	0.317460261	0.284381129	0.010185683	0.828334037	0.130592561	0.788013269
TMOD3	0.87640821	0.89598114	0.39981669	0.002136431	0.809069083	0.130682711	0.788013269
UIMC1	0.706903809	0.088596705	0.340356545	0.063398218	0.401427447	0.130658666	0.788013269
LSM5	0.080501463	0.165634925	0.247922862	0.248109728	0.663627607	0.13089918	0.7886147
NDUFAF6	0.619433746	0.491478748	0.182583408	0.157277315	0.06225312	0.130890451	0.7886147
NAIP	0.745302007	0.457133287	0.036215764	0.297511111	0.148466518	0.130993197	0.788891504
CDAN1	0.968080558	0.083451275	0.753882204	0.992994224	0.00903523	0.131181639	0.789435415
EMG1	0.581805485	0.532971006	0.272201133	0.06059164	0.106982932	0.131275928	0.789435415
GPD1L	0.363918851	0.106327959	0.305904457	0.05143482	0.898447527	0.131257428	0.789435415
TRIOBP	0.772184091	0.056849252	0.019211808	0.841682252	0.769606583	0.131164419	0.789435415
KCNH8	0.418646556	0.479904827	0.076368073	0.273104457	0.131197789	0.131622318	0.79122852
LOC101906739	0.431460173	0.355376584	0.009755946	0.411169127	0.895503039	0.131759111	0.791471002
SF3B1	0.127567442	0.240087546	0.554993783	0.129697643	0.249765428	0.131738256	0.791471002
STARD5	0.161360034	0.540638388	0.130318207	0.05798494	0.837364915	0.131918629	0.792139267
BDP1	0.602145985	0.86273953	0.935233648	0.001510163	0.755339852	0.132209073	0.793378366
LOC112441807	0.099326314	0.099280157	0.407413245	0.342556279	0.402817937	0.132231737	0.793378366
SPP2	0.046683275	0.38301887	0.279133798	0.21799762	0.510119839	0.132318359	0.793378366
TMCC2	0.275192701	0.570863231	0.122182299	0.810221587	0.035680929	0.132301435	0.793378366
AOX4	0.711023932	0.016152588	0.148226428	0.838320819	0.39282596	0.133050695	0.793781328
ARFGAP1	0.024250253	0.866696524	0.800884039	0.125985782	0.262898813	0.132645836	0.793781328
BROX	0.004961267	0.612956432	0.226895507	0.93441243	0.87103303	0.133178972	0.793781328
DDR1	0.878074727	0.278884393	0.168264775	0.064031413	0.211327937	0.132651864	0.793781328
KIF22	0.298060917	0.071552159	0.071861666	0.513529014	0.708530388	0.132660229	0.793781328
LOC104974516	0.793083899	0.482460676	0.003660685	0.831116525	0.478712098	0.132614929	0.793781328
LOC104976247	0.120888537	0.165322444	0.914068927	0.20130049	0.15274054	0.133191434	0.793781328

LOC107131134	0.490096004	0.782711615	0.005907971	0.498088989	0.496364394	0.133011234	0.793781328
LOC618456	0.322176509	0.254921024	0.014895594	0.645335963	0.708255671	0.132860033	0.793781328
LOC790009	0.022804832	0.669136957	0.909432191	0.323200345	0.124475672	0.132747893	0.793781328
RASL10B	0.198475669	0.715624163	0.031272613	0.205871822	0.614382258	0.133207835	0.793781328
RNF138	0.207430119	0.661244729	0.82784471	0.006553803	0.754923061	0.133205638	0.793781328
SDC4	0.176972436	0.031396204	0.857298062	0.204628611	0.574122981	0.13291955	0.793781328
SSC5D	0.254584723	0.221344535	0.03693752	0.270976024	0.99251754	0.132945218	0.793781328
TANC1	0.600569594	0.578914496	0.947135247	0.001708887	0.988486474	0.13247919	0.793781328
TMEM250	0.412897707	0.580923487	0.478745334	0.022000257	0.221991215	0.133078926	0.793781328
TPD52L2	0.238793106	0.969385725	0.386660907	0.037549408	0.166796723	0.133046784	0.793781328
HSPG2	0.575053019	0.561229354	0.003186276	0.556999305	0.984197318	0.133457745	0.794981868
DNAJC27	0.357021486	0.461296314	0.695534971	0.010451976	0.472656512	0.133740694	0.795224104
GPR83	0.581778683	0.858843688	0.486220204	0.058384097	0.039802052	0.133565594	0.795224104
PSAT1	0.273132946	0.470387149	0.559873607	0.369873356	0.021254391	0.133687058	0.795224104
STK19	0.125883629	0.041136667	0.30315482	0.727654897	0.495122496	0.13370058	0.795224104
ZFP2	0.130865757	0.236867413	0.515540396	0.353316576	0.100087517	0.13363901	0.795224104
PRKD2	0.046494893	0.909635907	0.869989456	0.043924743	0.350591762	0.133835822	0.79550151
UBFD1	0.122226701	0.379135684	0.385990786	0.13886408	0.228564341	0.13397823	0.796059641
FYB2	0.38460262	0.697998511	0.782958846	0.022686287	0.119199911	0.134064746	0.796133947
LSMEM2	0.965114397	0.211422388	0.063888707	0.054452792	0.801477861	0.134136272	0.796133947
SNU13	0.286135665	0.136782308	0.089354363	0.436180779	0.37292768	0.134126623	0.796133947
ELF1	0.993943224	0.973005736	0.550985234	0.001457636	0.733990998	0.134287961	0.796746106
MACF1	0.260877743	0.744506267	0.700998283	0.01688454	0.248192643	0.134346499	0.796805348
GPR171	0.175465837	0.776710204	0.079522531	0.101224683	0.521139344	0.134496743	0.796832508
MAML3	0.629074376	0.197895175	0.545303574	0.041389414	0.203325099	0.134441125	0.796832508
STUB1	0.378189908	0.944473176	0.356167153	0.005834602	0.769816198	0.134457476	0.796832508
CTSV	0.091905724	0.78525657	0.051385056	0.224739714	0.688010773	0.134715818	0.797554577
RBBP6	0.306104329	0.516876525	0.355742896	0.090309762	0.112754993	0.134681154	0.797554577
LOC112446756	0.887866505	0.898300013	0.138542717	0.083557731	0.062298402	0.134946533	0.798388987
NIPA2	0.195676527	0.405244351	0.012034566	0.905494571	0.66571504	0.134954058	0.798388987
TERF2	0.329345253	0.629629884	0.443643831	0.052497548	0.11922296	0.135024142	0.798515748
GAS2	0.719834234	0.045569488	0.407819094	0.045608611	0.945046699	0.135127536	0.798839337
LOC101904177	0.352401442	0.113986245	0.696315382	0.344233925	0.06014075	0.135443556	0.80041923
CFAP36	0.753763329	0.22523432	0.417716922	0.009604927	0.85122613	0.135541703	0.800710903
CRYBG2	0.103581944	0.196585548	0.524137276	0.301079013	0.180750408	0.135670434	0.801182978
MAT2B	0.928580949	0.038500287	0.159534032	0.486616253	0.209664283	0.135809221	0.80142579
PPP1R16A	0.787194454	0.083922842	0.077405596	0.158281689	0.718832107	0.135799691	0.80142579
COL16A1	0.436520157	0.070770404	0.056411576	0.402921364	0.829408659	0.135871966	0.801507849
CCNA2	0.893861503	0.179489686	0.053976168	0.478684641	0.1412122	0.136254274	0.803402317
YKT6	0.621433988	0.661507013	0.486614532	0.053269885	0.054960464	0.136291027	0.803402317
ABCC2	0.188620853	0.509648609	0.248067277	0.304739136	0.081199345	0.136854805	0.803646245
ADSL	0.867113234	0.611495218	0.960369341	0.029187383	0.039759587	0.136964436	0.803646245
ANKRD44	0.339823622	0.884591388	0.048051308	0.088438892	0.465428538	0.13742452	0.803646245
AP3B2	0.513414513	0.499573227	0.317317496	0.145754233	0.04998693	0.13722344	0.803646245
ATXN1L	0.555487392	0.764512903	0.007666764	0.781735381	0.231692432	0.136808433	0.803646245

CCDC61	0.755718764	0.422040123	0.694251549	0.003978616	0.674458291	0.137375938	0.803646245
CDC25A	0.476824948	0.565356472	0.488639054	0.004523139	0.998355578	0.137458717	0.803646245
CROCC2	0.166389324	0.023256496	0.372195841	0.616486214	0.66245902	0.136614979	0.803646245
DCAF11	0.443893568	0.671151232	0.003879445	0.620434428	0.828950495	0.137406375	0.803646245
DDX24	0.696843436	0.485901521	0.243186782	0.026772681	0.268872247	0.137192632	0.803646245
GLI4	0.995691563	0.865866451	0.120943427	0.0071941	0.78899102	0.137079149	0.803646245
GPR35	0.253240111	0.076429434	0.743349089	0.092799564	0.442842777	0.137005519	0.803646245
GTF3C1	0.600483123	0.143431251	0.028470952	0.502642523	0.478856481	0.136872176	0.803646245
LMAN2	0.15404982	0.808978748	0.048709211	0.099312722	0.975290177	0.13658466	0.803646245
LOC107131209	0.440793305	0.237357654	0.020217235	0.311292457	0.899088849	0.137100751	0.803646245
NAA10	0.614895282	0.644827301	0.105619863	0.057758952	0.243738487	0.136789556	0.803646245
NANOS1	0.914217931	0.277413576	0.041976079	0.149041863	0.374584924	0.137396757	0.803646245
PES1	0.366069276	0.996537173	0.359047081	0.031247895	0.144317844	0.136930641	0.803646245
PIK3CA	0.143173503	0.420196649	0.472260896	0.025303029	0.820149764	0.136794626	0.803646245
RSL1D1	0.382813378	0.336605056	0.946010919	0.041719511	0.116030268	0.13685543	0.803646245
TEAD1	0.106657267	0.663876603	0.018603725	0.512460726	0.878128565	0.137198725	0.803646245
TIMM9	0.39004149	0.488760511	0.808134245	0.050448318	0.075751077	0.136684494	0.803646245
YBX1	0.140442525	0.360733213	0.213294978	0.14101655	0.387639786	0.136933208	0.803646245
LOC112444505	0.879553812	0.117098405	0.731500699	0.011670254	0.678130264	0.137637141	0.804116457
NFKB1	0.288688678	0.780381954	0.755381962	0.005478481	0.639257094	0.137605452	0.804116457
FLNA	0.070057487	0.320970633	0.534179019	0.592488734	0.083929273	0.137772568	0.804334974
ZBTB16	0.742401466	0.645570087	0.082007122	0.214265857	0.070882048	0.137724341	0.804334974
CDC45	0.284672882	0.047982045	0.116910262	0.48449098	0.772727569	0.137840328	0.804344648
MCEMP1	0.245612624	0.470226944	0.269456079	0.130542534	0.147231221	0.137876633	0.804344648
TRAM1	0.517537253	0.03825518	0.798987794	0.062788212	0.602565563	0.137921263	0.804344648
MYCL	0.090446568	0.489465699	0.245881859	0.146616446	0.375433966	0.138008826	0.804569396
PTPN7	0.329475066	0.890184576	0.065146672	0.128842566	0.243726842	0.138113492	0.80489365
LOC783686	0.959511145	0.886708714	0.274147994	0.029132583	0.088407063	0.13820496	0.805126175
MOK	0.929983473	0.045708926	0.454868429	0.969238368	0.032074227	0.138251512	0.805126175
ARMCX2	0.40151487	0.974561723	0.072222585	0.043676987	0.495699503	0.139601807	0.805247801
CCDC77	0.473142664	0.202069018	0.12610984	0.737333218	0.067782211	0.138438602	0.805247801
CD300A	0.617987768	0.593838079	0.013832278	0.313315307	0.379379219	0.13853934	0.805247801
CPEB4	0.226812485	0.044839391	0.937743831	0.067438607	0.959419639	0.140249257	0.805247801
DEPDC1	0.582229785	0.019318354	0.116219131	0.678710521	0.693914907	0.140073592	0.805247801
EIPR1	0.157494506	0.419568721	0.834052941	0.021246065	0.522151388	0.139545852	0.805247801
FAHD2A	0.341752664	0.625692278	0.205748881	0.081006927	0.171356544	0.139457229	0.805247801
GALNT1	0.011150416	0.435001766	0.618921661	0.836997222	0.246309939	0.140478251	0.805247801
HSBP1L1	0.145957402	0.685090787	0.027388153	0.852982168	0.259360663	0.138851541	0.805247801
IER3	0.456876701	0.117027795	0.049469058	0.780186143	0.293735048	0.13888563	0.805247801
ITGB1BP2	0.076467031	0.661127938	0.45482154	0.198722267	0.133240375	0.139220112	0.805247801
LOC100336282	0.051220902	0.605329069	0.149799394	0.880382542	0.149126755	0.139341738	0.805247801
LOC100847995	0.862724506	0.106194915	0.495283478	0.026494003	0.503969566	0.138851634	0.805247801
LOC104969670	0.04895749	0.16012529	0.152006841	0.575121875	0.903096174	0.140480437	0.805247801
LOC107132382	0.47978033	0.222161685	0.030751876	0.408934994	0.455662596	0.139465639	0.805247801
LOC112446351	0.534422079	0.691818538	0.004887141	0.379705859	0.894400137	0.139822823	0.805247801

LTN1	0.156104279	0.137976143	0.320269265	0.096089524	0.915517917	0.138973468	0.805247801
MRM3	0.62007484	0.442015302	0.650179416	0.026849262	0.127139128	0.139157471	0.805247801
MRPS18C	0.932208494	0.934679405	0.293432289	0.00342223	0.703670306	0.14007872	0.805247801
MTBP	0.02948641	0.429872623	0.87011268	0.917273242	0.060872324	0.140095266	0.805247801
NDUFA3	0.945015629	0.281693055	0.720127974	0.003703713	0.864469585	0.139840677	0.805247801
NFKBID	0.15911034	0.062416578	0.13512188	0.524497533	0.877771353	0.140341268	0.805247801
P2RY6	0.498940112	0.330401981	0.42415056	0.217270598	0.039873476	0.138836674	0.805247801
PANK1	0.900916184	0.525004207	0.642748612	0.067775248	0.029910682	0.140153499	0.805247801
PDE4A	0.140371449	0.294753728	0.353180598	0.07840126	0.527295817	0.138629479	0.805247801
RANBP1	0.214097931	0.434334913	0.278919421	0.352830062	0.066113587	0.138744952	0.805247801
RAPGEF6	0.402411069	0.405900374	0.625404818	0.006569264	0.913878196	0.139777922	0.805247801
RBM28	0.805222329	0.581555105	0.106079418	0.044658712	0.277230971	0.139994708	0.805247801
RPS6KA1	0.249469505	0.940786923	0.005983508	0.64897521	0.660195923	0.138324251	0.805247801
SERPINB5	0.111737607	0.061333934	0.411478525	0.789014483	0.273927532	0.139305245	0.805247801
SH3YL1	0.290269218	0.013407737	0.247169961	0.821987325	0.763538024	0.138583251	0.805247801
SLC30A2	0.48210904	0.076281931	0.043682358	0.631527569	0.607826777	0.140199297	0.805247801
STXBP5	0.121428747	0.727991703	0.999798336	0.007042323	0.985843323	0.139818174	0.805247801
SYT17	0.310361634	0.725670361	0.020111312	0.628447589	0.216069258	0.139998959	0.805247801
TCHH	0.13459664	0.01762715	0.524945499	0.633955543	0.772219573	0.139333718	0.805247801
TLR4	0.478965521	0.949064169	0.022950178	0.150645471	0.38678342	0.139101965	0.805247801
TMEM138	0.929634116	0.163399757	0.695896433	0.582807364	0.00995405	0.139773916	0.805247801
TNFRSF1B	0.835618536	0.70945501	0.005646498	0.357157069	0.51449755	0.140006864	0.805247801
TPRN	0.123575739	0.722953442	0.143537729	0.069209199	0.697312198	0.140474178	0.805247801
TUFM	0.805806737	0.456853005	0.472031258	0.019768612	0.180006962	0.140411037	0.805247801
UMPS	0.916502261	0.563817216	0.647549324	0.137321424	0.013316977	0.139607704	0.805247801
UPF1	0.941163445	0.461182551	0.029529233	0.061064903	0.776215397	0.139058854	0.805247801
WRB	0.224310208	0.027043354	0.154046119	0.692550172	0.943457908	0.139439843	0.805247801
ZNF385D	0.809653154	0.795420458	0.046734273	0.031350065	0.654682962	0.14033276	0.805247801
ZNF605	0.065876963	0.344496366	0.372060149	0.945155231	0.076471084	0.139404279	0.805247801
CAVIN3	0.503703282	0.048701661	0.225722635	0.133134007	0.840470653	0.140563429	0.80532699
NLGN4X	0.912299752	0.061870315	0.457306374	0.118236237	0.203091232	0.140592397	0.80532699
CDC25B	0.288397962	0.564023177	0.237825729	0.03474687	0.464146911	0.141097592	0.806022756
DTL	0.058039362	0.726346261	0.11457888	0.269722155	0.477832635	0.14092801	0.806022756
FAM217B	0.023666947	0.598307538	0.530484529	0.588849625	0.140454539	0.140771367	0.806022756
LOC112445999	0.128541507	0.576347097	0.099006001	0.238725904	0.355176057	0.14085158	0.806022756
OLFM2	0.169330918	0.918090158	0.481438227	0.186718018	0.044650106	0.141106781	0.806022756
PISD	0.163068115	0.522719316	0.47172141	0.146193714	0.105928891	0.140946273	0.806022756
RNF167	0.613106012	0.707515182	0.474425069	0.007246285	0.417985495	0.141025974	0.806022756
TIMM8B	0.800440087	0.306598713	0.342875642	0.010024448	0.739683232	0.141101655	0.806022756
SP4	0.210569941	0.224747375	0.649472568	0.020668082	0.983382366	0.141196191	0.806252849
LIME1	0.366053291	0.095934942	0.942560554	0.047312197	0.399642819	0.141338544	0.80678499
NRF1	0.22943636	0.366454704	0.702921855	0.090061749	0.117696235	0.141412692	0.80692757
RND1	0.136062716	0.046654728	0.915239411	0.167102974	0.646630819	0.141575953	0.807578371
BORA	0.293823717	0.186725075	0.069083841	0.20268289	0.819920229	0.14183331	0.808206512
CRISPLD2	0.921191845	0.192522213	0.443348169	0.01648764	0.485738237	0.141812272	0.808206512

LOC107132921	0.988923622	0.063180632	0.164377976	0.184223127	0.333118829	0.141883064	0.808206512
TRHDE	0.173138182	0.423623964	0.112070329	0.213932105	0.358242481	0.141844438	0.808206512
CEP57L1	0.007038093	0.626830661	0.664434381	0.793755974	0.271064731	0.141933662	0.808214199
CA11	0.511777888	0.337038428	0.005453055	0.843651186	0.795692483	0.142021391	0.808433244
HNRNPF	0.698076084	0.803823381	0.491510659	0.002845382	0.805274036	0.142087748	0.808530522
LOC507581	0.884243715	0.491806816	0.02786562	0.056629763	0.922032731	0.142185202	0.808609078
LOC515358	0.293765243	0.176625818	0.048143827	0.519647519	0.487534802	0.142200097	0.808609078
ESPN	0.12113824	0.775876923	0.152021833	0.053033057	0.836442547	0.14231684	0.808682337
FAM207A	0.192755628	0.289649177	0.281038361	0.249586752	0.162655585	0.142705749	0.808682337
FANCC	0.820666643	0.486512268	0.541919594	0.027247492	0.107824404	0.142545174	0.808682337
IZUMO4	0.951001195	0.15091202	0.117501402	0.860035169	0.04391964	0.1427035	0.808682337
LOC100849865	0.244316719	0.236548377	0.740410677	0.015356759	0.969104257	0.142684322	0.808682337
MADCAM1	0.651518494	0.377516313	0.005951349	0.626485079	0.692581976	0.142477293	0.808682337
PLRG1	0.082851309	0.306681024	0.091324666	0.557792277	0.490332456	0.142419621	0.808682337
REPS2	0.810401894	0.657395091	0.014587905	0.398813046	0.205016717	0.142516427	0.808682337
SSSCA1	0.699896953	0.436051472	0.161911653	0.065128437	0.197803716	0.142655506	0.808682337
TMBIM1	0.718493956	0.378796421	0.049994598	0.355537778	0.131066867	0.142346863	0.808682337
ARHGAP26	0.032262774	0.561105689	0.318615741	0.527649823	0.209797969	0.142889572	0.809444519
TK2	0.104207598	0.87918936	0.842085958	0.008453701	0.980364388	0.142999391	0.809787094
IL17B	0.032260685	0.31776687	0.868954713	0.264628928	0.271945697	0.143201687	0.81037341
LOC537848	0.37289027	0.893960905	0.727500326	0.006942729	0.380545082	0.143160397	0.81037341
NOC3L	0.836091152	0.950665513	0.057418934	0.045422673	0.309526694	0.143275109	0.810509415
CD99	0.52087479	0.122668181	0.784540075	0.725234015	0.017676714	0.143392529	0.810550146
HMMR	0.457986625	0.031717842	0.422756059	0.277631928	0.377018419	0.143413789	0.810550146
TSPYL4	0.05914709	0.868129144	0.401193933	0.295213661	0.105721297	0.143430481	0.810550146
LOC101904239	0.545671479	0.511103734	0.260636804	0.077317402	0.114473589	0.143482247	0.810563566
DACT3	0.568686487	0.339772005	0.030252657	0.128204319	0.864070962	0.143990209	0.810587024
HDAC2	0.93916869	0.454350772	0.177761897	0.144806264	0.058676406	0.143620165	0.810587024
HERC6	0.963397315	0.263567697	0.103801555	0.225054464	0.108784333	0.14371587	0.810587024
IFITM5	0.724206982	0.161861603	0.354029367	0.020525346	0.760603944	0.144029722	0.810587024
NSDHL	0.400913811	0.608630287	0.463968285	0.055740001	0.102520435	0.143916954	0.810587024
PARP6	0.148958203	0.047469049	0.421744388	0.339118412	0.638913224	0.143817221	0.810587024
PIGQ	0.15124202	0.917970353	0.611347576	0.013200844	0.577813959	0.143972686	0.810587024
RBM3	0.411176206	0.675925964	0.006195935	0.545382347	0.688729969	0.143901384	0.810587024
SNX17	0.558438141	0.698172256	0.415513702	0.01366317	0.291763741	0.143779034	0.810587024
SPATA9	0.328355302	0.020518923	0.351284689	0.638824232	0.427955233	0.143929095	0.810587024
TMEM252	0.509385143	0.125073163	0.488571316	0.753166288	0.027553431	0.143796915	0.810587024
BUB1	0.497520349	0.092526574	0.076388721	0.247696619	0.745711729	0.144228985	0.811082592
CRELD2	0.076567296	0.706698419	0.156965616	0.411957252	0.185841078	0.144315469	0.811082592
FGFR3	0.015589117	0.350082421	0.295680428	0.692343009	0.581141021	0.144196375	0.811082592
PKD1	0.868375898	0.339443494	0.050429219	0.047857472	0.913684633	0.14428431	0.811082592
DENND1A	0.743617188	0.913855412	0.933852532	0.499498328	0.002059498	0.144627761	0.811170944
FAM19A3	0.288658357	0.146070233	0.366240965	0.049387741	0.854399345	0.144481434	0.811170944
FBLL1	0.665934845	0.141413274	0.017697979	0.57656934	0.678985982	0.144583788	0.811170944
NKAIN1	0.781872023	0.701062719	0.002749389	0.475250712	0.911123554	0.144596626	0.811170944

PALLD	0.179150146	0.60281798	0.010851222	0.890979115	0.624149594	0.144489652	0.811170944
UCHL1	0.782928914	0.746580895	0.608948505	0.099879018	0.018349772	0.144570519	0.811170944
AKAP6	0.068023161	0.458114586	0.88666483	0.707935026	0.033680756	0.145347809	0.811281264
ALKBH5	0.292211005	0.246792678	0.009725844	0.97224243	0.972555258	0.145872568	0.811281264
ASPSCR1	0.412292048	0.094094579	0.516292454	0.040043031	0.835146611	0.146661028	0.811281264
ASTN2	0.786798116	0.029126877	0.452601104	0.54471882	0.117509079	0.145958681	0.811281264
BAG3	0.063153501	0.49161308	0.706241735	0.044212488	0.680509577	0.145454804	0.811281264
CAPS	0.003668919	0.370543822	0.812086526	0.856789941	0.697651949	0.145480251	0.811281264
CAV2	0.084092718	0.807401553	0.237821152	0.077982589	0.534262971	0.147009027	0.811281264
CCDC12	0.122413755	0.214920761	0.056059473	0.529098461	0.859667046	0.146783796	0.811281264
CCDC186	0.04337056	0.612482498	0.600582969	0.362365892	0.115887766	0.146677826	0.811281264
CCR5	0.22291032	0.978442663	0.394644899	0.328413245	0.023581523	0.146278166	0.811281264
CD96	0.229788413	0.717864254	0.135680721	0.306225804	0.097428946	0.146416131	0.811281264
CLEC7A	0.550116947	0.731296596	0.009241752	0.454251439	0.397256068	0.146792122	0.811281264
DANCR	0.05598522	0.348368701	0.1214653	0.784064694	0.354291553	0.145258989	0.811281264
DBF4	0.296065348	0.057598943	0.06020546	0.741354072	0.873290301	0.146051041	0.811281264
EPN1	0.078665014	0.70068316	0.12326273	0.171092199	0.56255579	0.144760153	0.811281264
FFAR2	0.31385858	0.646565807	0.950508669	0.01776073	0.193175318	0.145702957	0.811281264
GATAD2A	0.656314399	0.817429404	0.010236577	0.400589228	0.30162753	0.14591678	0.811281264
GCA	0.345271416	0.228515773	0.098613592	0.52663143	0.159679298	0.144803338	0.811281264
GNAT1	0.352249695	0.25753949	0.511537222	0.51456143	0.027438466	0.144912176	0.811281264
GYG1	0.109431921	0.089831223	0.50098277	0.491289774	0.271248044	0.145044684	0.811281264
KBTBD7	0.776750366	0.266956458	0.136411654	0.468748522	0.050835492	0.147161414	0.811281264
KIAA0040	0.352100276	0.271442368	0.156112927	0.17449592	0.258769738	0.147124886	0.811281264
KMT5B	0.014201757	0.880940975	0.867574121	0.162266266	0.382987654	0.147221765	0.811281264
LOC100335514	0.85472366	0.166784295	0.706914253	0.11386648	0.057523304	0.145497401	0.811281264
LOC101906511	0.604949745	0.39573521	0.92352115	0.011966699	0.253459143	0.146752385	0.811281264
LOC101907276	0.462643704	0.123917244	0.074331586	0.247549051	0.637756824	0.147012152	0.811281264
LOC107131471	0.830987352	0.038067808	0.616542926	0.563315704	0.060385592	0.14590101	0.811281264
LOC112441478	0.496249457	0.073623404	0.101848405	0.374409499	0.470158518	0.144892678	0.811281264
LONRF3	0.715464655	0.100251041	0.080602208	0.131155465	0.889187914	0.14718415	0.811281264
MAP2	0.374603411	0.477174369	0.420034405	0.037009431	0.243010732	0.14730696	0.811281264
MARCKSL1	0.4488769	0.997542072	0.062830149	0.02598607	0.916609101	0.146697028	0.811281264
MYOM1	0.288215527	0.614144317	0.855339262	0.496209115	0.008988224	0.147305724	0.811281264
NACC2	0.732418481	0.831494969	0.016076369	0.093801178	0.735189265	0.147296427	0.811281264
NAT8L	0.22361328	0.770592085	0.069663349	0.063494625	0.87073694	0.145928326	0.811281264
NLE1	0.668587352	0.253893163	0.082006719	0.16482244	0.291915311	0.146656773	0.811281264
PCYT1A	0.801012476	0.268753901	0.154627472	0.025571039	0.786893089	0.146659159	0.811281264
PKN2	0.704744507	0.145047384	0.036300683	0.206359317	0.880898794	0.147221399	0.811281264
PTGFR	0.398193937	0.576644961	0.088657347	0.098378047	0.332911451	0.146292847	0.811281264
RAVER2	0.330770029	0.837112277	0.426603708	0.060782795	0.091449007	0.145080236	0.811281264
RITA1	0.178724897	0.221033676	0.200384861	0.139372854	0.609837369	0.147018114	0.811281264
RPL8	0.34162311	0.700338369	0.759149465	0.006375307	0.577231289	0.146492136	0.811281264
RSPO3	0.554907768	0.46056005	0.295075069	0.009285734	0.95510204	0.146542002	0.811281264
SCARF2	0.580871052	0.447886165	0.004250858	0.68030299	0.872189273	0.145033545	0.811281264

SELENOO	0.298755248	0.426145286	0.695965639	0.023527312	0.323918361	0.147306473	0.811281264
SEMA3E	0.16306797	0.772050857	0.042395034	0.33495131	0.377758725	0.147316932	0.811281264
SHARPIN	0.605285012	0.121676004	0.223720776	0.042928869	0.929469981	0.14518235	0.811281264
SMAD5	0.098527816	0.743526911	0.389721913	0.029149389	0.797151568	0.145897349	0.811281264
SMOC2	0.263147864	0.938930701	0.014198787	0.772416394	0.247159096	0.146653271	0.811281264
SPARCL1	0.019681384	0.43151866	0.5126304	0.632575306	0.242479793	0.146421434	0.811281264
SPON1	0.720243875	0.184832488	0.034431051	0.168115729	0.871728254	0.146889006	0.811281264
THBS1	0.345909951	0.512535844	0.812640731	0.035366137	0.130263492	0.145936691	0.811281264
XRCC3	0.276483215	0.155790673	0.984848781	0.023570627	0.664419508	0.146009207	0.811281264
ZNF181	0.075493949	0.956207219	0.058705902	0.326507364	0.476769258	0.14545366	0.811281264
ZNF276	0.768969687	0.010512184	0.421914844	0.237171289	0.816705977	0.145564347	0.811281264
PSMD12	0.851312815	0.108781493	0.561551422	0.03390799	0.383606563	0.14744507	0.811714539
LOC112443528	0.727096347	0.053238252	0.242482818	0.239223767	0.301639718	0.147549668	0.811745758
TRPT1	0.653758359	0.947508581	0.312794669	0.00838798	0.416507511	0.14750368	0.811745758
KIAA1257	0.366530525	0.027683789	0.157196857	0.537749193	0.793326468	0.147922598	0.812316228
LOC616903	0.018962651	0.167553567	0.536719667	0.887573417	0.449692771	0.147942763	0.812316228
MPRIP	0.971935282	0.845254835	0.040490408	0.032079388	0.637908352	0.147950351	0.812316228
RABEP2	0.750338887	0.218394432	0.013943928	0.362074001	0.82051047	0.147729615	0.812316228
SHISA3	0.954566232	0.088075025	0.03284331	0.879661293	0.279842559	0.147834678	0.812316228
YBX3	0.572176466	0.27364697	0.025358424	0.355417897	0.481561019	0.147815944	0.812316228
AHR	0.727483895	0.745971232	0.015439954	0.648509238	0.12757315	0.149416401	0.81252289
AKT1	0.112527569	0.530777127	0.041481362	0.332809014	0.84315283	0.149650923	0.81252289
ANG2	0.361056936	0.113713829	0.70674788	0.036359165	0.657555911	0.149478453	0.81252289
AP5Z1	0.141509427	0.100085998	0.369057753	0.136258463	0.972864976	0.149379569	0.81252289
ATP6V1B2	0.988463009	0.412894317	0.003510319	0.880010749	0.546867924	0.148979242	0.81252289
BACE2	0.270833039	0.231361182	0.023812843	0.649254238	0.718700298	0.149770382	0.81252289
CYB5R3	0.130897697	0.749590191	0.047816242	0.311508779	0.467448122	0.148241202	0.81252289
DHRSX	0.227305573	0.153813902	0.451461772	0.383262708	0.112793471	0.148143577	0.81252289
E2F2	0.239418181	0.034900979	0.247081062	0.576382344	0.583534589	0.149555101	0.81252289
ESRRG	0.183710463	0.940129843	0.023203986	0.535945132	0.320331495	0.148809893	0.81252289
FAM206A	0.436518218	0.242851427	0.222371313	0.03100198	0.945833866	0.149185495	0.81252289
FASTK	0.43509599	0.310935837	0.144268514	0.03544468	0.993439628	0.148720031	0.81252289
FOXP3	0.36507002	0.865816706	0.682464983	0.007228824	0.440160096	0.148616343	0.81252289
GALC	0.855874926	0.092339243	0.487121945	0.092760603	0.191081691	0.148145383	0.81252289
HSF2BP	0.964099667	0.682610631	0.184880773	0.007949229	0.704907066	0.148076372	0.81252289
LOC100295797	0.379538393	0.221136158	0.425445389	0.376163356	0.051482126	0.149216401	0.81252289
LOC101904013	0.024120154	0.916878066	0.447557697	0.070951857	0.984941799	0.149239238	0.81252289
LOC101905499	0.02550903	0.571877704	0.334491385	0.543634815	0.261961555	0.149613888	0.81252289
LOC104973322	0.277567952	0.958149649	0.701914522	0.451906276	0.008097158	0.148228781	0.81252289
LOC112446426	0.590697744	0.796578141	0.035764624	0.044742289	0.911377488	0.148598371	0.81252289
LOC112448070	0.317574107	0.061384224	0.943180106	0.9089973	0.041496587	0.149454495	0.81252289
MEGF6	0.294193019	0.095466372	0.158205204	0.164971942	0.949278266	0.149722097	0.81252289
PADI1	0.279222908	0.861619446	0.197516115	0.018576911	0.786817791	0.149574722	0.81252289
PHYHD1	0.712813073	0.897802132	0.003791879	0.354675184	0.794698043	0.148335319	0.81252289
PLEKHH1	0.783392206	0.777524815	0.192074734	0.905525217	0.006541016	0.149387097	0.81252289

PTPMT1	0.34838541	0.132414021	0.230381175	0.26298119	0.24896525	0.149721569	0.81252289
PYCARD	0.378899957	0.760542079	0.018728944	0.35187194	0.360848798	0.148488512	0.81252289
RNF146	0.213066599	0.287972786	0.086271787	0.165358999	0.785655613	0.14877118	0.81252289
SH2B1	0.674526161	0.056523298	0.194935665	0.175646961	0.53153411	0.149495088	0.81252289
SHANK2	0.057608678	0.877107413	0.46088293	0.063246639	0.467258916	0.148832548	0.81252289
SHKBP1	0.729151462	0.382086375	0.910870513	0.00579566	0.470993369	0.149358081	0.81252289
TAGAP	0.671372053	0.720564366	0.157544381	0.127481957	0.070738923	0.148724884	0.81252289
TMEM189	0.140756186	0.307392646	0.092736375	0.332862788	0.51782693	0.14922918	0.81252289
TOMM5	0.477519118	0.416550045	0.656019345	0.015668757	0.339458791	0.149514988	0.81252289
TRA2B	0.756825598	0.986922542	0.714991177	0.001797838	0.723933768	0.149632908	0.81252289
USP19	0.34078768	0.176614775	0.237497119	0.055797421	0.870712688	0.149563821	0.81252289
EXT2	0.155934189	0.226438639	0.044931636	0.542378562	0.810946739	0.149952006	0.813055135
KERA	0.477584629	0.270479697	0.513651872	0.484371623	0.021716496	0.149967576	0.813055135
TATDN1	0.197544418	0.774209864	0.011285765	0.569896555	0.710060686	0.150028034	0.813114291
LAT	0.432688124	0.242653201	0.24894218	0.168280732	0.159098873	0.150180383	0.813671268
RIOK3	0.409857603	0.201575925	0.355430173	0.024740669	0.965210678	0.150348486	0.814313205
BRAF	0.046569733	0.846930922	0.972972814	0.033997958	0.537966729	0.150423595	0.814439909
TTLL12	0.157520903	0.739401959	0.020618103	0.742734087	0.393743757	0.150471135	0.814439909
CEPT1	0.074644952	0.237485157	0.11849295	0.502883224	0.665468147	0.150547816	0.814586286
CLDND1	0.806280851	0.614492659	0.529326743	0.006116282	0.440185063	0.150909042	0.814651393
LOC112444864	0.875997397	0.838484957	0.126698358	0.026784142	0.282754146	0.150760049	0.814651393
LOC786987	0.240918093	0.282741724	0.130426843	0.141342556	0.561539063	0.150801967	0.814651393
PCSK1N	0.659349458	0.467443231	0.705519372	0.127593244	0.025364049	0.150637013	0.814651393
PDZRN3	0.995869515	0.588642494	0.009937985	0.189800935	0.638437651	0.150893939	0.814651393
PPIL3	0.557128894	0.36980563	0.700118993	0.005084606	0.96140697	0.150799412	0.814651393
PPM1F	0.686470882	0.371825924	0.355778006	0.122097344	0.063689289	0.150921026	0.814651393
ZNF318	0.920729811	0.197619475	0.184128223	0.929040639	0.022698016	0.150956973	0.814651393
AGMAT	0.939057606	0.264471506	0.008075273	0.572703149	0.626641998	0.152479365	0.814875519
ARHGEF19	0.168302464	0.625878718	0.659218931	0.016654892	0.622224516	0.152464563	0.814875519
C7H19orf70	0.533379469	0.369389762	0.96517624	0.004571405	0.820743537	0.151762555	0.814875519
CARF	0.024870008	0.13836441	0.773593736	0.359971807	0.742193665	0.151501309	0.814875519
CCAR2	0.512646365	0.013796274	0.338257714	0.319518894	0.942502371	0.152560619	0.814875519
CIDEA	0.511442188	0.446010522	0.719886476	0.00561191	0.777983419	0.15215946	0.814875519
CNOT4	0.501346094	0.57377249	0.636985781	0.00407624	0.963586958	0.15247563	0.814875519
CNPPD1	0.159518393	0.175991272	0.701855951	0.070153171	0.518894577	0.152195311	0.814875519
COMMD5	0.378335073	0.592617528	0.015971439	0.326344047	0.607246132	0.151319927	0.814875519
CSRNP1	0.100864192	0.16600401	0.09915805	0.893879628	0.480359201	0.151695378	0.814875519
EPHA4	0.997544538	0.985725215	0.025479547	0.137291155	0.208762213	0.152289496	0.814875519
FBXO3	0.479530243	0.342438141	0.318787421	0.023295751	0.586296028	0.151933932	0.814875519
FOXS1	0.851531696	0.224339975	0.73962963	0.17360185	0.029381286	0.152587248	0.814875519
HSP90AA1	0.550839746	0.309882954	0.59358949	0.008865303	0.792985277	0.151627348	0.814875519
IFRD2	0.552716553	0.774014563	0.493175574	0.02260449	0.149426626	0.151666556	0.814875519
ITSN2	0.526319255	0.412739501	0.272859371	0.013494048	0.889130922	0.151496249	0.814875519
LGMN	0.412151273	0.594301365	0.00706692	0.652871562	0.628320358	0.151370143	0.814875519
LOC101907574	0.793987361	0.928983782	0.046752757	0.958538505	0.021773379	0.152476886	0.814875519

LOC112443175	0.912829165	0.82038071	0.027045406	0.849968728	0.041531215	0.151930734	0.814875519
LOC112449261	0.330604198	0.852874715	0.026801756	0.140898073	0.665899593	0.151251263	0.814875519
LOC536097	0.025907256	0.90716126	0.559305674	0.660070267	0.082140358	0.151671465	0.814875519
LRRC57	0.204027693	0.018911288	0.419228795	0.957843368	0.462980523	0.152203294	0.814875519
NT5E	0.454668979	0.206935629	0.400028315	0.019152972	0.993366246	0.152061222	0.814875519
PMS1	0.086582369	0.656226975	0.559486824	0.418967673	0.053114985	0.151063313	0.814875519
RGCC	0.573565153	0.307459851	0.013401377	0.316729186	0.962807106	0.15258744	0.814875519
RTCB	0.597822926	0.408216471	0.048914265	0.62896665	0.094461823	0.151272315	0.814875519
RUFY3	0.35271668	0.815274791	0.028597329	0.116654631	0.747439156	0.152168273	0.814875519
TIMM8A	0.503727196	0.264486926	0.566193031	0.088320521	0.106463158	0.151280317	0.814875519
TIMMDC1	0.230988289	0.321074884	0.257103366	0.118946172	0.312382577	0.151189325	0.814875519
TMEM82	0.943906765	0.82401448	0.656717118	0.009567704	0.147026735	0.152340965	0.814875519
TOP2A	0.668024569	0.086304415	0.058814933	0.237892353	0.891275658	0.15239003	0.814875519
TUBA4A	0.038458112	0.247340889	0.957771583	0.226623754	0.345892499	0.151839439	0.814875519
CFAP100	0.564034361	0.097611951	0.080038404	0.350090615	0.468018367	0.152739545	0.81515729
CNOT11	0.500312318	0.016085619	0.179177928	0.939327095	0.532909316	0.152717415	0.81515729
GUF1	0.488420614	0.446568512	0.582622198	0.249399196	0.022828914	0.152910125	0.81580236
ARMC9	0.346034292	0.840557663	0.086591908	0.067083335	0.429458303	0.153147591	0.816008156
BMP2K	0.879521984	0.400102752	0.280813833	0.016135266	0.454834853	0.15310331	0.816008156
LOC100848895	0.242135674	0.052267988	0.32160303	0.818344334	0.217642155	0.153070476	0.816008156
STRIP2	0.709638731	0.838239581	0.301566437	0.01970632	0.204997413	0.153041716	0.816008156
SRF	0.013754153	0.37971078	0.757720717	0.527771634	0.348807293	0.153476296	0.817228909
ZMYM4	0.06882296	0.807694742	0.825860861	0.018451964	0.859538904	0.153432146	0.817228909
BNIP3	0.384254047	0.997820899	0.030430227	0.488362426	0.128243356	0.153728909	0.817513001
IL23A	0.006664743	0.858914145	0.996492835	0.130030765	0.984709326	0.153692339	0.817513001
LOC100196897	0.425211191	0.325026624	0.040771215	0.192943125	0.670949968	0.153584703	0.817513001
ZNRD1	0.954683776	0.009036076	0.512779421	0.553890184	0.298011999	0.153666772	0.817513001
PRELID1	0.189530897	0.492106474	0.755278385	0.297319827	0.034952122	0.153879631	0.817823022
TMED3	0.12993302	0.132924932	0.130068859	0.574881221	0.566896914	0.153886874	0.817823022
APLF	0.147537362	0.283031788	0.913268489	0.039977274	0.480987115	0.15402065	0.817960955
AQP3	0.740906091	0.040058654	0.342933883	0.275515804	0.261336043	0.153969752	0.817960955
LOC524576	0.237682796	0.89348729	0.093319176	0.072101312	0.514004041	0.15415139	0.817960955
SLC16A6	0.099165104	0.618803412	0.418437744	0.835575086	0.034207118	0.154089805	0.817960955
TLCD2	0.428025267	0.041991261	0.277389914	0.535434437	0.275167517	0.15416204	0.817960955
PRPF8	0.32665359	0.653556669	0.285378351	0.018323046	0.659683112	0.154372814	0.818814562
OGFOD1	0.18078203	0.670079308	0.541074584	0.284697868	0.039589405	0.154636458	0.819947952
DARS	0.557584775	0.379179061	0.412919273	0.462966869	0.018300257	0.15473721	0.819950096
DHX29	0.188557385	0.586931816	0.691851544	0.406607249	0.023955649	0.155428523	0.819950096
F13A1	0.583360367	0.761852426	0.157853734	0.092513588	0.114616513	0.155214725	0.819950096
FAM71E1	0.730631926	0.053438201	0.228289004	0.502850105	0.166565586	0.155511681	0.819950096
FHDC1	0.234273852	0.681790701	0.008351715	0.810585113	0.689857586	0.155444198	0.819950096
GRIN1	0.14487027	0.219987483	0.612405854	0.525406349	0.072848925	0.155564767	0.819950096
KANK4	0.912131719	0.025104614	0.84850392	0.081163186	0.474395896	0.155686095	0.819950096
LOC104969050	0.263287632	0.251099823	0.989918564	0.238484423	0.047707557	0.155293187	0.819950096
LOC112449300	0.817699937	0.791168478	0.003890956	0.361508988	0.82190047	0.155665416	0.819950096

MRPL19	0.766008808	0.561740321	0.554356868	0.106360822	0.029477724	0.155660945	0.819950096
NUDT17	0.518008325	0.368616073	0.052433717	0.38645538	0.192284688	0.155224404	0.819950096
PSMD8	0.343979372	0.463270215	0.492092616	0.444890274	0.021400082	0.155516049	0.819950096
RALGAPA1	0.321032944	0.920888279	0.930199217	0.004146793	0.65387042	0.155411088	0.819950096
SH3BP5L	0.708404584	0.220842949	0.252734367	0.029617277	0.631912932	0.154776332	0.819950096
SIX5	0.935246059	0.303871197	0.008787947	0.307166081	0.966033352	0.154898436	0.819950096
SLC35B1	0.2333699	0.456716239	0.624428825	0.549263315	0.02034884	0.155210714	0.819950096
SLC9A5	0.212212209	0.170708425	0.045746807	0.745317838	0.602494997	0.155246576	0.819950096
TIAM1	0.598845121	0.616864139	0.007261468	0.348247671	0.797452704	0.155331279	0.819950096
UHRF2	0.021640487	0.488102088	0.700828579	0.373991711	0.269218858	0.155376539	0.819950096
XPO1	0.328624639	0.270108912	0.969335895	0.344263195	0.025209335	0.155532091	0.819950096
ZNF879	0.240539657	0.209767468	0.292698901	0.656256637	0.076598248	0.155046276	0.819950096
BBS5	0.764800156	0.925574488	0.071970768	0.166042127	0.088761323	0.15599318	0.819988495
BCCIP	0.776400911	0.466636407	0.353020653	0.281529239	0.020847744	0.155971916	0.819988495
LOC101905151	0.003402878	0.78307965	0.596011287	0.481236959	0.98060879	0.155839644	0.819988495
NKAPD1	0.503117668	0.035896921	0.350648801	0.134455074	0.8813322	0.155946566	0.819988495
SP100	0.992632489	0.561776236	0.570366744	0.912040754	0.002583493	0.155833081	0.819988495
WSB1	0.645270648	0.423649486	0.495739739	0.011181141	0.494880029	0.155883372	0.819988495
ASZ1	0.427984214	0.366781568	0.361592928	0.016552676	0.810085118	0.157134528	0.820923805
C3H1orf54	0.051805303	0.539177183	0.864780661	0.050777132	0.617169539	0.156675015	0.820923805
CCDC3	0.775499877	0.361775635	0.891750413	0.004906405	0.619917069	0.157116527	0.820923805
CUEDC1	0.034538715	0.609495787	0.178445229	0.505677952	0.400149858	0.157022824	0.820923805
DNAJB9	0.03481117	0.406844515	0.973280249	0.078558462	0.700609255	0.156862879	0.820923805
ERLIN1	0.590480616	0.388279277	0.010090641	0.869510237	0.377843902	0.157018404	0.820923805
ESAM	0.270629454	0.503819757	0.62887158	0.045416357	0.19420297	0.156596794	0.820923805
FAXC	0.653006322	0.268166068	0.037276638	0.128161226	0.90622245	0.156803869	0.820923805
GRHL2	0.932767404	0.237531083	0.019557991	0.638912816	0.274649879	0.157053977	0.820923805
LOC100139549	0.193459253	0.364707825	0.379680743	0.287843953	0.09834272	0.156823845	0.820923805
LOC101904042	0.224054561	0.121512084	0.996331982	0.035472368	0.791059591	0.157138928	0.820923805
LOC101905403	0.794407078	0.019218641	0.320436104	0.653561899	0.237748258	0.157029183	0.820923805
LOC112444633	0.020527502	0.340094321	0.976451675	0.846090782	0.131528799	0.156857104	0.820923805
LOC785842	0.401911057	0.635880932	0.006210599	0.562620813	0.845178845	0.15642714	0.820923805
LRRC41	0.128310729	0.516091386	0.021411495	0.721306632	0.736688	0.156279505	0.820923805
METTL16	0.655910022	0.382793148	0.089098214	0.066840861	0.504903896	0.156451224	0.820923805
RAB5C	0.027124781	0.596255865	0.491792986	0.134060812	0.712287417	0.156956832	0.820923805
TMEM150C	0.120245776	0.801891411	0.033801845	0.462476828	0.503100654	0.156827368	0.820923805
TYK2	0.893388724	0.715582363	0.053112089	0.077193661	0.290515755	0.157171568	0.820923805
ZSCAN29	0.35888884	0.726812001	0.097833378	0.519720324	0.057406487	0.157162823	0.820923805
GRAMD1C	0.364024347	0.668543942	0.035406031	0.209399248	0.422956438	0.157358775	0.821640106
TSHZ2	0.706518384	0.145817423	0.044788296	0.266875844	0.621583501	0.157611197	0.822696358
SDC1	0.746891723	0.708196517	0.00495305	0.63852454	0.457978736	0.157689231	0.822841962
COL6A3	0.4922387	0.314421615	0.020768554	0.289365887	0.824253792	0.157746811	0.822880772
GNS	0.866702253	0.174216945	0.024557102	0.607829616	0.341102168	0.15798089	0.82353484
LOC100848025	0.550712656	0.648218286	0.013383239	0.213923511	0.752069256	0.15796532	0.82353484
ZNF862	0.172172347	0.174173837	0.650276403	0.056036231	0.703890545	0.158022741	0.82353484

IL1A	0.41120275	0.235256257	0.133904952	0.063455595	0.937034652	0.15814001	0.823622885
LOC107131948	0.052049194	0.1558737	0.190046124	0.652540397	0.765301707	0.158113968	0.823622885
LOC614732	0.018322487	0.203616052	0.261236216	0.946540813	0.835929873	0.15824145	0.823889735
EPHB1	0.040844018	0.647152834	0.679658174	0.101960735	0.421530982	0.158348745	0.823992871
FUT4	0.009916183	0.285036649	0.729021057	0.728883516	0.514267992	0.158377005	0.823992871
LOC112444290	0.136554771	0.509136026	0.582985688	0.367201582	0.051916846	0.158411889	0.823992871
CLDN4	0.327234853	0.007688912	0.683264937	0.685697524	0.658832652	0.158844276	0.82415582
GRAMD1A	0.327209654	0.499013267	0.906671995	0.032127725	0.163288812	0.158844973	0.82415582
KIAA1147	0.613274859	0.894781189	0.265608843	0.052411232	0.101515413	0.158716921	0.82415582
LOXL1	0.32295343	0.720688624	0.004275087	0.845866905	0.920446515	0.158631248	0.82415582
NFE2L3	0.287578048	0.605715121	0.073820226	0.473225033	0.127457139	0.158728765	0.82415582
POLR3F	0.011148045	0.758567101	0.221957645	0.585665981	0.705770275	0.158757114	0.82415582
S1PR4	0.670374236	0.205171791	0.984366996	0.103437497	0.055347244	0.158676818	0.82415582
ZNF624	0.168438774	0.183934248	0.383882058	0.254193154	0.256584394	0.158741527	0.82415582
GALNT17	0.371536779	0.175139636	0.037364888	0.346896462	0.922041038	0.158957691	0.824219486
USP12	0.955260689	0.151040301	0.055623613	0.203081295	0.477035868	0.15893708	0.824219486
CACNA1D	0.022117485	0.832354755	0.130926828	0.882068348	0.366307623	0.159079445	0.824423573
GSPT1	0.907047433	0.359651538	0.115208309	0.527431945	0.039296246	0.159097523	0.824423573
KBTBD6	0.752348649	0.545048244	0.54409885	0.048367145	0.072234683	0.159159299	0.824483352
COX7C	0.60985869	0.588231416	0.784569832	0.002868207	0.96942926	0.159494608	0.82476214
CUL7	0.757385451	0.014590866	0.127412621	0.721079688	0.771798472	0.159605112	0.82476214
LOC101904357	0.358316364	0.82835851	0.202927447	0.0135393	0.960886085	0.159604374	0.82476214
LOC104970930	0.285192948	0.818856418	0.184070177	0.032016974	0.568010705	0.159402486	0.82476214
MCM6	0.709597353	0.359241674	0.040927255	0.225842327	0.332765721	0.159655774	0.82476214
MRPS27	0.332161344	0.811620068	0.398295747	0.065987347	0.110671577	0.159665427	0.82476214
SEC14L1	0.18622446	0.163947491	0.500439296	0.078772808	0.651318703	0.159637588	0.82476214
SPOP	0.964806002	0.594182357	0.742740062	0.002905945	0.633689754	0.159656852	0.82476214
WWP1	0.888422825	0.009750997	0.875009711	0.312989764	0.330448313	0.159647913	0.82476214
FAM53B	0.528085823	0.76392436	0.534046437	0.005968557	0.610769572	0.159798522	0.824785828
MTMR12	0.398246773	0.490836887	0.566109504	0.056646519	0.125315311	0.159815576	0.824785828
STARD7	0.717673954	0.2381672	0.031227282	0.166108861	0.886569644	0.159871045	0.824785828
TMEM269	0.019464099	0.357345243	0.340216517	0.549633937	0.6043343	0.159867008	0.824785828
ZNF354C	0.051365856	0.447129957	0.063864401	0.75568736	0.710741797	0.16006259	0.825514506
CAND1	0.515981442	0.208539186	0.388821932	0.048169141	0.391177178	0.160120507	0.825553765
ATP13A4	0.237180283	0.71689945	0.047183675	0.195268454	0.506329669	0.160649504	0.8258629
AVIL	0.771613599	0.22814864	0.016468996	0.722233509	0.379760377	0.16086278	0.8258629
C18H16orf46	0.437320108	0.21797248	0.11445711	0.611729334	0.119347814	0.161010398	0.8258629
GPBP1L1	0.704424037	0.120577328	0.363780226	0.217537911	0.117785934	0.160486107	0.8258629
HIGD2A	0.608721042	0.847636315	0.301818407	0.008606364	0.596366821	0.161305589	0.8258629
HMGCS1	0.320833805	0.626368103	0.176063221	0.453945621	0.049723094	0.161232915	0.8258629
HNRNPK	0.526459905	0.20296789	0.073336226	0.454428806	0.222165195	0.160424041	0.8258629
IKZF5	0.23840258	0.483341152	0.455738198	0.166276289	0.091731776	0.161488882	0.8258629
KPNA6	0.271872954	0.449475727	0.078299172	0.281113486	0.295213007	0.160738999	0.8258629
LOC104975054	0.715452636	0.026296079	0.738640122	0.300646549	0.189694676	0.160574798	0.8258629
LOC781989	0.534294464	0.079622837	0.054749851	0.505453347	0.6794595	0.161372881	0.8258629

LOC786039	0.237898922	0.752205111	0.23747975	0.204362273	0.09125206	0.160571358	0.8258629
MIEF2	0.321444092	0.543785988	0.693120022	0.021884323	0.297739091	0.160238056	0.8258629
NOXO1	0.419503857	0.510238336	0.41415456	0.14001753	0.064307761	0.161188682	0.8258629
OXSM	0.981570203	0.621642271	0.550389879	0.00266504	0.890100641	0.161021805	0.8258629
PHLPP1	0.978701664	0.747700588	0.192468051	0.273521094	0.020665476	0.160961927	0.8258629
PLEKHF1	0.219905884	0.421872514	0.125002239	0.570148508	0.121058001	0.161426757	0.8258629
PPARD	0.354699288	0.692393165	0.541655896	0.062838871	0.095755861	0.161429326	0.8258629
PPP1R2	0.857692567	0.024314248	0.193106478	0.85044176	0.231647927	0.160663004	0.8258629
PRKG2	0.132004196	0.370905566	0.55929147	0.033750263	0.866280122	0.161448082	0.8258629
RBL2	0.701170717	0.246645204	0.04152997	0.303860896	0.366239025	0.161303748	0.8258629
STAM	0.759213466	0.151935062	0.073741983	0.117862014	0.790029311	0.160522952	0.8258629
SYTL3	0.33370188	0.371469726	0.65229096	0.02439	0.406010128	0.161457476	0.8258629
TMEM107	0.110954858	0.883132617	0.027354765	0.59017294	0.50262937	0.160854909	0.8258629
UBE2H	0.860730325	0.601641357	0.312574954	0.072796736	0.067923606	0.161421644	0.8258629
ZBTB32	0.016829533	0.199811559	0.425210818	0.733833495	0.754232213	0.160452978	0.8258629
ADGRV1	0.305728149	0.925350897	0.049728387	0.09568575	0.598164927	0.161942971	0.826003165
AMD1	0.016364673	0.494344386	0.661752433	0.388291073	0.387074266	0.16187725	0.826003165
CACNB4	0.121588528	0.378309096	0.334853102	0.072721968	0.719492884	0.162016898	0.826003165
KCND1	0.810556746	0.566082289	0.006097725	0.803330088	0.359197077	0.162170629	0.826003165
KCNJ2	0.017350419	0.887403686	0.228322343	0.258642826	0.88578919	0.16196207	0.826003165
LOC112441452	0.930271471	0.456830137	0.005863697	0.371918561	0.86752173	0.161813595	0.826003165
LOC514257	0.082879278	0.733755579	0.659578392	0.130837947	0.153282407	0.161858475	0.826003165
LSP1	0.581017169	0.035139132	0.114481417	0.363438129	0.948596955	0.162005225	0.826003165
MFSD6L	0.417439561	0.373749493	0.009736821	0.617444787	0.857393904	0.161834622	0.826003165
OVOS2	0.325790575	0.940671649	0.003898008	0.878856476	0.768249019	0.16208729	0.826003165
SNRPA	0.311827231	0.719334457	0.02138728	0.310301106	0.5421009	0.162132026	0.826003165
YTHDF3	0.427356534	0.505585774	0.049264736	0.082348452	0.919954792	0.162068163	0.826003165
ZMAT2	0.359695191	0.0390778	0.74377832	0.54161871	0.142119439	0.161891416	0.826003165
SIVA1	0.185297869	0.900285399	0.038200078	0.166384719	0.763589758	0.162416017	0.826996358
BCL2A1	0.749052782	0.836176917	0.031704417	0.229516075	0.178576967	0.162871511	0.827139486
CHUK	0.172752442	0.37320911	0.269577504	0.124189785	0.376962729	0.162846607	0.827139486
EIF3M	0.320252221	0.984928697	0.063880786	0.069017514	0.582823811	0.162510879	0.827139486
EIF4A1	0.218915193	0.288792728	0.977317188	0.447846115	0.029356744	0.162704704	0.827139486
IMPA1	0.640296909	0.76771012	0.739329006	0.497083261	0.004503722	0.162841159	0.827139486
LOC101906508	0.628875122	0.744013568	0.263823223	0.008098353	0.814490155	0.162905892	0.827139486
LOC526488	0.531005686	0.183983614	0.450461239	0.191914547	0.096451378	0.162948142	0.827139486
METTL13	0.241433479	0.538375262	0.208817402	0.107865819	0.277194026	0.162621328	0.827139486
MICU3	0.036976868	0.588004166	0.10689644	0.466330593	0.750166681	0.162782852	0.827139486
PTPN2	0.763514627	0.36990214	0.171797453	0.035107006	0.477610863	0.162835518	0.827139486
GTSF1	0.777853803	0.420981616	0.037985223	0.171535417	0.38202146	0.163001216	0.827153048
SSR3	0.777545476	0.377610317	0.014405447	0.207999425	0.927726942	0.163113569	0.82746732
LOC100847818	0.264501951	0.14804409	0.122299888	0.395631425	0.431453211	0.163252121	0.827914263
PLD3	0.39319201	0.507194874	0.010156418	0.903759093	0.446960701	0.163326589	0.828036035
CNN2	0.358670673	0.869001201	0.043102249	0.089081407	0.684352367	0.163414972	0.828119176
WNK2	0.096837785	0.705112615	0.473900882	0.731177717	0.034626893	0.16344391	0.828119176

CPA3	0.767084523	0.643284454	0.861614341	0.153868278	0.012532035	0.163504596	0.828170964
C26H10orf62	0.135863975	0.453651331	0.03719039	0.53056665	0.677716002	0.163969536	0.828990777
FAM187A	0.698445003	0.056550388	0.645792198	0.334247964	0.096674281	0.163968609	0.828990777
PLIN3	0.226598187	0.634433737	0.20729659	0.034466424	0.80229666	0.16395368	0.828990777
PLXNA1	0.501196768	0.69380509	0.252378871	0.148784295	0.063022896	0.163830697	0.828990777
RNASET2	0.359303569	0.530942057	0.005541335	0.844490885	0.922785625	0.163924092	0.828990777
ZCCHC13	0.465908535	0.615372676	0.033679684	0.214166706	0.397537747	0.163746933	0.828990777
CCDC189	0.435698156	0.017616576	0.806712817	0.154849476	0.863520068	0.164364709	0.829487128
CD1D	0.323668406	0.806672172	0.029962649	0.149038047	0.710994692	0.164472068	0.829487128
COPS3	0.951551502	0.617951546	0.132513689	0.524994181	0.020229233	0.164318984	0.829487128
FAM19A5	0.766250055	0.688708275	0.52051845	0.008408584	0.357982497	0.164247545	0.829487128
PLEKHA6	0.986059615	0.031461881	0.430920284	0.095524815	0.648176271	0.164341697	0.829487128
SUN2	0.814687251	0.331476718	0.010638223	0.551798634	0.522544634	0.164406755	0.829487128
SVIL	0.0725769	0.590119643	0.031169248	0.743887894	0.834539829	0.164447531	0.829487128
ZNF215	0.930990395	0.926150663	0.087174908	0.060808053	0.181040963	0.164313933	0.829487128
CYCS	0.409125817	0.441730453	0.74792366	0.021029009	0.292603557	0.164760286	0.830430297
TSR3	0.378966082	0.777547527	0.47350377	0.011000852	0.541667497	0.164727969	0.830430297
TUBGCP5	0.092945006	0.160872094	0.086907042	0.90192462	0.710561154	0.164874074	0.830748675
TNRC18	0.649780568	0.893565162	0.070832979	0.605987679	0.033446621	0.164957115	0.830911975
BAK1	0.229633445	0.535516563	0.368936841	0.163942097	0.112219294	0.165073049	0.831240811
P4HA2	0.119298903	0.50693259	0.031553335	0.445065632	0.983615456	0.165146501	0.831355593
ATPAF2	0.338205523	0.678511069	0.76418372	0.024775913	0.192786639	0.165381385	0.832027563
DRG2	0.744573253	0.487103886	0.145018623	0.441695721	0.036044895	0.165356633	0.832027563
TESPA1	0.900595	0.407421921	0.07720558	0.96120802	0.030821061	0.165552677	0.832634073
DDX18	0.711254428	0.153870593	0.146136435	0.122716982	0.42827482	0.165690274	0.833042501
LOC104969027	0.521628895	0.027172149	0.707744391	0.139224075	0.602854935	0.16583693	0.833042501
LOC112442634	0.194065298	0.414726107	0.029890705	0.525299703	0.665512419	0.165739439	0.833042501
PRCP	0.768541979	0.949631013	0.007659353	0.2958227	0.508896549	0.165793572	0.833042501
FXR1	0.068501953	0.750413188	0.398552325	0.050053114	0.822231629	0.165963769	0.833256334
GLT8D2	0.062143097	0.638810135	0.138480985	0.499279204	0.307256302	0.165981047	0.833256334
ADGRB3	0.337543661	0.137210983	0.057821563	0.348991587	0.906526699	0.166388667	0.833268612
AMOTL1	0.424412228	0.372090308	0.147575796	0.156213428	0.232263734	0.166215145	0.833268612
ARSA	0.104624519	0.593279199	0.079136373	0.299718577	0.575108898	0.166333378	0.833268612
IL7	0.874623917	0.38765523	0.032358577	0.230418645	0.333804883	0.166035401	0.833268612
MAPT	0.136879613	0.919315728	0.551266336	0.959631756	0.012725639	0.166377462	0.833268612
SMARCAD1	0.16234657	0.290414248	0.288449175	0.711944623	0.087504968	0.166389692	0.833268612
TMEM223	0.70457223	0.326588996	0.259997776	0.026290821	0.538610115	0.166382794	0.833268612
UBE2V2	0.09903894	0.382485346	0.241514408	0.251261678	0.368039273	0.166262545	0.833268612
PSD	0.066445574	0.406934226	0.746015252	0.078648808	0.535911463	0.166698001	0.834303414
UTP6	0.188895766	0.272287682	0.140385938	0.170537598	0.690185075	0.166664653	0.834303414
ANKRD13C	0.88096583	0.019302438	0.526015129	0.943763459	0.100893752	0.166855748	0.834838316
ERAP1	0.040784717	0.233666868	0.935358827	0.268982235	0.35551475	0.166928611	0.834866139
LOC512869	0.752475638	0.955658498	0.031691315	0.04552177	0.822465198	0.167013926	0.834866139
VPS13C	0.684119779	0.752990063	0.356150021	0.009877054	0.470742982	0.166992196	0.834866139
PAX8	0.126505598	0.980842508	0.10559045	0.413910618	0.157448997	0.167076809	0.83492616

MRPS12	0.482085677	0.54497356	0.430868058	0.039541233	0.191072487	0.167222279	0.835398728
HLX	0.648189446	0.324360019	0.093032667	0.226605333	0.193268491	0.167366094	0.835862743
CUBN	0.214769764	0.395841527	0.135871111	0.179732589	0.413211512	0.167493916	0.836126544
PELI1	0.671582556	0.185802888	0.486498904	0.039624409	0.356743268	0.167520814	0.836126544
DUSP11	0.476398234	0.515498715	0.298661358	0.059185708	0.197923441	0.16763104	0.836422317
AMDHD1	0.032053187	0.072733548	0.510320492	0.78709298	0.919770437	0.16784892	0.83644565
C17H12orf65	0.226623901	0.50314986	0.844872414	0.017406817	0.51385912	0.167890559	0.83644565
CHID1	0.292855513	0.539385458	0.499967881	0.054246057	0.20079101	0.16773678	0.83644565
TBX3	0.474627845	0.038856829	0.200214183	0.25111491	0.928842458	0.167843897	0.83644565
UFM1	0.395583463	0.319593432	0.840862344	0.411607445	0.019682655	0.16784345	0.83644565
HSF4	0.192986955	0.030559336	0.268603856	0.740425099	0.737014769	0.168174431	0.837605643
SRPK3	0.806401126	0.447962185	0.480793185	0.450717298	0.01104995	0.168230664	0.837631502
APOBEC3H	0.71935356	0.72868164	0.066751658	0.204009354	0.121265565	0.168295257	0.837698956
LOC104975559	0.354735307	0.504041344	0.072882506	0.543276612	0.122410142	0.168398481	0.837958601
LOC112443510	0.250052165	0.564764078	0.621792128	0.021236234	0.465047621	0.168457171	0.837996552
MIER1	0.238988918	0.859002615	0.708870829	0.021082469	0.282914859	0.168539152	0.83815031
KIF18A	0.317239282	0.097206069	0.19923069	0.917798217	0.154348521	0.168779828	0.838838813
NEMF	0.244039413	0.271992817	0.087684638	0.186810811	0.800443215	0.168777191	0.838838813
GPC5	0.653635999	0.349645226	0.196373706	0.815522567	0.023807776	0.168885976	0.839112249
LOC101907302	0.161896245	0.676419043	0.010881199	0.955927516	0.765970609	0.16900233	0.839436211
ABHD5	0.970940469	0.253343943	0.142224004	0.055996649	0.446715227	0.169270936	0.840316094
PLXNB2	0.842769978	0.595469195	0.003710334	0.479965198	0.979333971	0.169281885	0.840316094
HIST1H3G	0.571808708	0.197128889	0.25170838	0.913729016	0.033789569	0.169359684	0.840448072
P2RY12	0.839367069	0.852794423	0.006910864	0.209453559	0.846834387	0.169507581	0.840673592
TMEM186	0.936026393	0.54725709	0.68919316	0.016359546	0.151895199	0.169491233	0.840673592
FAM102B	0.850749992	0.694987761	0.139960088	0.501384412	0.02120264	0.169741011	0.841019489
LOC101903540	0.1373507	0.748587338	0.561261872	0.081684683	0.186778648	0.169816039	0.841019489
NRROS	0.669334097	0.374054924	0.313603109	0.013065434	0.857766466	0.169763249	0.841019489
RAC1	0.741669928	0.065240619	0.124294528	0.193287402	0.75695216	0.169763487	0.841019489
TSC22D3	0.603954857	0.652989705	0.006544702	0.585969326	0.582258523	0.169833562	0.841019489
MTURN	0.778081107	0.060135654	0.173657345	0.259830211	0.417714149	0.169963443	0.841408769
C29H11orf24	0.267389329	0.624579313	0.201463416	0.283415244	0.092898849	0.170366042	0.843147501
MORF4L1	0.50219641	0.229607953	0.216936246	0.04659691	0.760774502	0.17045824	0.843349466
ACOT4	0.469395416	0.762569575	0.159666935	0.040978375	0.381627563	0.171169691	0.843938867
C18H16orf87	0.473831656	0.373370149	0.083149442	0.080476245	0.754161819	0.17107131	0.843938867
GM2A	0.250742156	0.471466855	0.009549288	0.813157962	0.970696289	0.170894979	0.843938867
GPR37L1	0.810114412	0.102239506	0.285609117	0.437053772	0.086187402	0.170896652	0.843938867
KEAP1	0.083896883	0.94517009	0.132156379	0.0986221	0.862774979	0.170959032	0.843938867
LOC104970711	0.112793662	0.243988008	0.058551707	0.962294351	0.57465829	0.170895512	0.843938867
LOC112444461	0.636203131	0.843941941	0.960479263	0.049234641	0.035069298	0.170829772	0.843938867
LOC507787	0.322705321	0.571190909	0.115608484	0.87339055	0.047806294	0.170762514	0.843938867
LOC789388	0.360477608	0.56837712	0.394118996	0.011396493	0.966591961	0.170738813	0.843938867
PRR12	0.629220725	0.303852949	0.041653961	0.422828571	0.265320865	0.171134445	0.843938867
TMED1	0.209682833	0.685892774	0.239134564	0.046652623	0.557192113	0.171193581	0.843938867
ZFP36L1	0.489670241	0.362284116	0.087814143	0.078448388	0.731552096	0.171194472	0.843938867

TAGLN	0.424861933	0.16591618	0.833924154	0.530928789	0.028710371	0.171401071	0.844703596
LOC100126544	0.139475804	0.759689466	0.30143016	0.054968447	0.511201801	0.171544834	0.845158291
NCAPG	0.936551942	0.034638685	0.084899594	0.341612993	0.955844112	0.171731179	0.845315049
NUP210	0.804695743	0.129538222	0.161681598	0.295970729	0.180227643	0.171697784	0.845315049
TIAL1	0.025992801	0.337925748	0.125044103	0.937556744	0.873182432	0.171714433	0.845315049
DCTD	0.879177621	0.104708905	0.81089474	0.255923144	0.047128394	0.171834898	0.845318499
ZPBP2	0.110609001	0.880406074	0.955367069	0.246787086	0.039194901	0.1717887	0.845318499
LLPH	0.698759172	0.126387085	0.397331236	0.674378578	0.038158953	0.172099592	0.846366919
LMO7	0.410672415	0.952400165	0.624094009	0.004272508	0.868284298	0.172356341	0.847375648
USP7	0.776223054	0.780028585	0.009607109	0.248721564	0.626360598	0.172422907	0.847449035
DUSP3	0.022633205	0.115265369	0.621306725	0.901157302	0.620981943	0.17250733	0.847610116
TMEM94	0.544971104	0.124475855	0.923065143	0.022330193	0.650770479	0.172796898	0.848778779
CDK1	0.410882727	0.82917087	0.111072633	0.034264053	0.703771434	0.173054787	0.849791174
CCDC82	0.159561626	0.853532606	0.125179344	0.082585454	0.649395401	0.173234441	0.849998454
SYT1	0.279531445	0.730658548	0.382782004	0.023447265	0.498858087	0.17324955	0.849998454
TMEM30A	0.369331325	0.070796983	0.615242528	0.059901176	0.949001116	0.173252381	0.849998454
ADGRL1	0.240463507	0.978260311	0.04430424	0.752011207	0.117320651	0.173751063	0.850284439
AK2	0.436861456	0.207699772	0.397516005	0.840410692	0.030250701	0.173501131	0.850284439
C5H12orf57	0.759472297	0.701436553	0.93868928	0.005229704	0.35137769	0.173693272	0.850284439
CYB5R4	0.492310091	0.079316974	0.19199017	0.178033228	0.690117284	0.173911367	0.850284439
GP9	0.748336779	0.497046014	0.086692343	0.056750215	0.501925367	0.173652723	0.850284439
IFT22	0.774926603	0.673850166	0.131507218	0.025647733	0.52026037	0.173434151	0.850284439
LOC112443417	0.810445717	0.094100113	0.922536694	0.025586278	0.510489301	0.173697062	0.850284439
LSM14B	0.230716902	0.223778692	0.354725439	0.067264735	0.747279239	0.173859264	0.850284439
POLR3A	0.920340019	0.498390022	0.204820266	0.188122968	0.051953181	0.173623955	0.850284439
RNF168	0.228378639	0.753583672	0.280591967	0.111239145	0.170751009	0.173526764	0.850284439
TMEM97	0.466097899	0.510999851	0.490707933	0.117752682	0.06689124	0.173859311	0.850284439
TRAK2	0.794850886	0.623499908	0.390683557	0.008772373	0.542429546	0.173932415	0.850284439
ADD2	0.005709902	0.80024579	0.550357707	0.664610478	0.553524167	0.174311092	0.850757996
GFRA2	0.864013727	0.697225369	0.059035452	0.215755601	0.120440903	0.174214304	0.850757996
LAMA3	0.070937441	0.153781303	0.369307072	0.655592176	0.350755367	0.174438774	0.850757996
MUL1	0.529066854	0.574461223	0.085320674	0.06919842	0.516217828	0.174428193	0.850757996
OAS1X	0.278962068	0.721165909	0.0312188	0.363693582	0.405599439	0.174444011	0.850757996
PHLDB1	0.226649455	0.232286432	0.083560906	0.403876507	0.520411657	0.174263845	0.850757996
SMAD6	0.379836208	0.12982536	0.227601162	0.08925639	0.923634401	0.174325666	0.850757996
STARD13	0.788449033	0.823660683	0.442914909	0.00576054	0.558046101	0.174263479	0.850757996
ENAH	0.556378079	0.793248078	0.058755228	0.376066177	0.095057162	0.174495863	0.850758051
NAP1L4	0.462861457	0.996912335	0.517902489	0.015281536	0.254362606	0.174686667	0.850929916
RPRD1B	0.603191374	0.234606826	0.046492171	0.157349825	0.896831001	0.17463929	0.850929916
TMEM70	0.893262178	0.727110064	0.377567369	0.06638042	0.05705784	0.174676635	0.850929916
CDCA3	0.240239892	0.163002996	0.130802448	0.197754321	0.918387835	0.17482061	0.851023646
ID1	0.054144668	0.536764751	0.087246106	0.381942208	0.961722815	0.174931784	0.851023646
LOC107132820	0.155181598	0.667736677	0.810974076	0.413071694	0.026819342	0.174887841	0.851023646
RPS6KL1	0.727947429	0.168798246	0.509597544	0.015344246	0.969728363	0.174965193	0.851023646
VPS8	0.502435954	0.182945786	0.272047243	0.106547326	0.349373875	0.174878188	0.851023646

IL32	0.070879527	0.983800357	0.786136037	0.018162081	0.937268511	0.175106053	0.851204219
TMF1	0.477862455	0.915917524	0.959314298	0.002666187	0.833412505	0.175088172	0.851204219
RPS6KB2	0.44009499	0.368313608	0.932950177	0.010505443	0.587875373	0.175183952	0.85133072
RPL36A	0.837566856	0.473020287	0.48587992	0.01649858	0.294801555	0.175413749	0.851392829
SLC43A1	0.131797169	0.079084664	0.3370353	0.386661798	0.689155568	0.175396273	0.851392829
VDR	0.05792016	0.102277123	0.693810602	0.283202678	0.804743506	0.17545613	0.851392829
VMA21	0.174055575	0.306130104	0.538519753	0.525951441	0.062003372	0.175361075	0.851392829
ZBTB47	0.472255138	0.655630629	0.212127099	0.027329567	0.521550254	0.175404497	0.851392829
TMEM266	0.901189324	0.67304127	0.780925661	0.003793323	0.521661961	0.175514005	0.851421915
PPP2R1A	0.189895357	0.839123894	0.29991977	0.139310038	0.140952054	0.175625188	0.851709502
HOXB3	0.377080346	0.341888523	0.05311746	0.679725378	0.201961001	0.1757863	0.851735745
KIAA1958	0.82040627	0.994846336	0.17575947	0.01043055	0.627939669	0.175737816	0.851735745
LOC112447362	0.894408353	0.464095877	0.032159265	0.779414122	0.090277472	0.175709833	0.851735745
ZBTB7C	0.941461353	0.227894836	0.634919268	0.146788502	0.047048369	0.175857683	0.851830116
ABTB1	0.862820724	0.030130235	0.180577836	0.253727947	0.803019103	0.177393582	0.851890519
CCT8	0.872494791	0.584700443	0.539443972	0.071739316	0.048163947	0.176845499	0.851890519
CD22	0.080632016	0.530339331	0.101364809	0.961812809	0.22835865	0.176959865	0.851890519
CD47	0.534757501	0.483939919	0.564024541	0.006916037	0.939932277	0.176648489	0.851890519
CDH20	0.507259496	0.415497844	0.79219239	0.0136232	0.414132528	0.175976078	0.851890519
CLU	0.499248037	0.600159857	0.935499863	0.014496068	0.235010783	0.177239996	0.851890519
DNAJC28	0.899875941	0.553154634	0.400233341	0.014149417	0.335736718	0.176409252	0.851890519
DNLZ	0.258173881	0.4048501	0.319140696	0.092643765	0.309470945	0.177381386	0.851890519
FIP1L1	0.829046197	0.933813005	0.038264422	0.211437765	0.151138226	0.176432909	0.851890519
FLOT1	0.10350145	0.678056207	0.275024236	0.284809203	0.174413559	0.177615717	0.851890519
FOXRED1	0.488549994	0.492625835	0.833248496	0.005360393	0.879626082	0.176327231	0.851890519
FOXRED2	0.892496999	0.151293675	0.376615514	0.276621677	0.066997393	0.176023311	0.851890519
GNG11	0.280239063	0.871539201	0.110429473	0.361431751	0.098428881	0.177686993	0.851890519
H1FX	0.201290527	0.598197847	0.107999735	0.157152869	0.469387582	0.177664527	0.851890519
HABP4	0.35465303	0.84700589	0.302094439	0.016950361	0.618576204	0.176906044	0.851890519
ILK	0.229183131	0.375115757	0.304910279	0.583265809	0.062271728	0.17696433	0.851890519
KCNG3	0.906382784	0.860635689	0.007816995	0.550535747	0.281764157	0.176358516	0.851890519
LGALS8	0.733487516	0.503055686	0.004115394	0.991439979	0.636601972	0.17758079	0.851890519
LOC101903758	0.523243137	0.379274258	0.319209013	0.018888284	0.795043583	0.176886901	0.851890519
LOC112442253	0.346894148	0.340681218	0.221308402	0.043339217	0.841303218	0.177113987	0.851890519
LOC516108	0.171096343	0.674886801	0.582789312	0.026043488	0.546937423	0.17759483	0.851890519
NSMCE4A	0.531231258	0.739317934	0.011987238	0.330110807	0.613855384	0.177153197	0.851890519
PIMREG	0.983150459	0.073209117	0.079819332	0.299489445	0.553267317	0.176949852	0.851890519
PRKCSH	0.348295188	0.077889972	0.061076356	0.851381074	0.673520124	0.176771691	0.851890519
RAB30	0.140553325	0.44084876	0.653532197	0.025397617	0.928753484	0.177267015	0.851890519
RHEB	0.820078822	0.029299881	0.620430419	0.356169717	0.17944065	0.177031718	0.851890519
RIOK2	0.146369818	0.581092615	0.117065253	0.13158057	0.724369587	0.176664866	0.851890519
RSPH10B	0.745866193	0.013431109	0.537657701	0.246085258	0.713673196	0.176363117	0.851890519
SHROOM3	0.297583383	0.109703472	0.260243798	0.853790817	0.131948454	0.17745437	0.851890519
SSBP1	0.677132693	0.472038053	0.902435131	0.006746448	0.489509905	0.177012688	0.851890519
TNFRSF19	0.253776429	0.609569422	0.800790149	0.453113309	0.016838871	0.176288305	0.851890519

TPSB2	0.787978283	0.35964606	0.060502603	0.264084839	0.210279808	0.176970151	0.851890519
UPF3B	0.029504626	0.171568995	0.987575832	0.704544488	0.270533799	0.177039629	0.851890519
WFDC3	0.682493954	0.010834482	0.307037347	0.451699644	0.930851912	0.17721075	0.851890519
ZNF414	0.889319647	0.355187713	0.052524595	0.081855334	0.703699057	0.177314442	0.851890519
CLEC4D	0.163952722	0.432685275	0.049892237	0.294941268	0.921319546	0.177906086	0.852305651
F3	0.902655843	0.210089851	0.046585697	0.658688551	0.166052536	0.178344007	0.852305651
FRA10AC1	0.002609827	0.983554173	0.828604874	0.650343209	0.698214869	0.178297434	0.852305651
LARP4B	0.813211361	0.763434986	0.01213948	0.154720415	0.828350606	0.178307817	0.852305651
MAPK13	0.035531912	0.449683967	0.328911591	0.249274481	0.737042911	0.17827302	0.852305651
PAOX	0.837649946	0.073041202	0.148667085	0.22891387	0.46303529	0.178134396	0.852305651
PIAS2	0.416237299	0.566080214	0.160749504	0.029790418	0.85869625	0.178598062	0.852305651
RCOR1	0.871068618	0.872204736	0.185805037	0.008377889	0.818682438	0.178532025	0.852305651
RPL36A	0.753545009	0.509346035	0.641253298	0.036971236	0.105981593	0.178159061	0.852305651
RRP8	0.582350179	0.728628963	0.651145295	0.020137675	0.173790928	0.178408499	0.852305651
S100B	0.496708575	0.570445523	0.785674853	0.008940618	0.485820229	0.178407812	0.852305651
SETMAR	0.010671885	0.846243102	0.733480738	0.204379439	0.709996085	0.177852067	0.852305651
SLC19A2	0.734197639	0.177926655	0.048333584	0.283636864	0.540927476	0.178580804	0.852305651
SLTM	0.514441171	0.409228649	0.42956631	0.03212793	0.332807724	0.178409171	0.852305651
USHBP1	0.788277391	0.610230591	0.022172314	0.097111233	0.932135006	0.178263679	0.852305651
ZMYM6	0.009803048	0.847568032	0.334895095	0.405375881	0.85903815	0.178604542	0.852305651
PPA1	0.896595008	0.272091081	0.875552468	0.293590618	0.01547009	0.178715149	0.852585556
DDX19A	0.678129581	0.190337528	0.323182411	0.057695451	0.403838922	0.178889212	0.853167931
IQGAP3	0.260994853	0.118487658	0.063458795	0.619482939	0.801425783	0.179117032	0.853828374
LOC785408	0.778191476	0.15974096	0.15168908	0.07433883	0.695508577	0.17917878	0.853828374
NUPR1	0.960446149	0.723955939	0.065495796	0.057946606	0.369668559	0.179235802	0.853828374
SEC13	0.015910912	0.62438736	0.214473464	0.584127445	0.78375594	0.179229771	0.853828374
APBB3	0.356696736	0.035797135	0.450897964	0.48353712	0.353265292	0.179997484	0.854252947
BATF2	0.27348664	0.222304724	0.111183318	0.233008114	0.624769785	0.18005368	0.854252947
C19H17orf53	0.22232966	0.093871894	0.159225865	0.300366802	0.985196124	0.179989839	0.854252947
CTSO	0.665747453	0.835412865	0.472108841	0.023778357	0.156613266	0.179457301	0.854252947
HCLS1	0.620733438	0.506961933	0.10119968	0.170151622	0.181253089	0.179872719	0.854252947
KCNK4	0.069644111	0.602721564	0.669444165	0.037306429	0.937890879	0.179974713	0.854252947
KCNT2	0.668578999	0.08302352	0.440069901	0.230239065	0.173750574	0.1793962	0.854252947
LOC781412	0.09402728	0.241361008	0.770215058	0.353949977	0.158179242	0.179535468	0.854252947
SEC22A	0.050333995	0.497765148	0.350716407	0.174141591	0.642155822	0.179916898	0.854252947
SFRP5	0.284195428	0.536916296	0.088877928	0.276357806	0.261398497	0.17963681	0.854252947
SLC31A1	0.260420607	0.441591186	0.238025103	0.849597519	0.042060818	0.179488952	0.854252947
SNX20	0.688275713	0.758271845	0.008084298	0.468921132	0.497327508	0.180044235	0.854252947
UQCRQ	0.615802636	0.50231902	0.981625904	0.004068524	0.796464041	0.180043859	0.854252947
ZC2HC1A	0.108790952	0.350823842	0.148047663	0.286864306	0.604787461	0.179695359	0.854252947
DNAJC14	0.33311049	0.147374103	0.80475622	0.02974324	0.838299574	0.180150437	0.854464976
AKAP4	0.401922552	0.394383267	0.07486253	0.380777573	0.21972664	0.180893628	0.855532765
ARHGDIB	0.114244123	0.824606793	0.227013445	0.087764051	0.53296444	0.181606455	0.855532765
EMP3	0.029657992	0.958601289	0.110785364	0.435042679	0.729158835	0.181490448	0.855532765
F8	0.601490251	0.35858912	0.665995751	0.007265801	0.954641696	0.181229631	0.855532765

GPR75	0.957627053	0.512016701	0.047399137	0.065771168	0.65578437	0.181803247	0.855532765
IMP4	0.421136462	0.598114117	0.916682643	0.117559365	0.03658487	0.180916546	0.855532765
IQCD	0.863855705	0.002856094	0.544598596	0.833779418	0.890889419	0.181391983	0.855532765
KNL1	0.878286066	0.250267278	0.066270504	0.098089635	0.70040039	0.181646349	0.855532765
LOC101902851	0.483190553	0.702763594	0.930671447	0.234834396	0.013431227	0.181268988	0.855532765
LOC104972843	0.912780153	0.351036627	0.007751407	0.873404865	0.45901222	0.181168126	0.855532765
LOC112449100	0.047012693	0.067215044	0.863375801	0.42163718	0.87141131	0.181802371	0.855532765
LOC531557	0.418947356	0.453991941	0.249185094	0.215329606	0.097267237	0.180876325	0.855532765
LOC784914	0.008108952	0.587677047	0.491907669	0.762625811	0.555896053	0.180983527	0.855532765
LRBA	0.434623612	0.994132087	0.766917368	0.00688166	0.437684187	0.181390874	0.855532765
LST1	0.714423915	0.593792648	0.007639853	0.959296695	0.321424961	0.181510583	0.855532765
NEPRO	0.488871099	0.705725585	0.924804679	0.226647434	0.013775053	0.181208532	0.855532765
NUFIP2	0.332148917	0.599447803	0.837420131	0.007843656	0.758439781	0.180803881	0.855532765
PUS1	0.149960704	0.1962771	0.771842182	0.095492549	0.459278547	0.181229673	0.855532765
RAB3GAP2	0.673502516	0.847525234	0.008884743	0.373935653	0.527068689	0.181530783	0.855532765
RNF2	0.372853945	0.87605759	0.409206611	0.011930615	0.626665719	0.181511561	0.855532765
SLC9A3R1	0.107832589	0.613027606	0.629413241	0.252026551	0.095550927	0.18175942	0.855532765
TACC1	0.981505303	0.13268213	0.077490398	0.106354562	0.935322646	0.181939513	0.855532765
TBC1D15	0.185968172	0.209984372	0.087621506	0.316579664	0.920303511	0.181279659	0.855532765
THUMPD1	0.181608561	0.247505802	0.242411243	0.232370279	0.39301409	0.181107976	0.855532765
TMEM182	0.975182607	0.138658719	0.78275728	0.323375294	0.029317223	0.181899991	0.855532765
TMEM231	0.832933778	0.711739208	0.135940618	0.013033546	0.952216714	0.181591543	0.855532765
TXNRD3	0.572636625	0.348667182	0.512762939	0.009743761	0.997054716	0.181062045	0.855532765
USP53	0.801153796	0.416842634	0.100317388	0.041823068	0.715819388	0.181854861	0.855532765
VAV1	0.602667949	0.8513737	0.038851141	0.139222297	0.356915408	0.180675238	0.855532765
ZNF184	0.227935716	0.70693859	0.062922178	0.480012062	0.205786088	0.181719817	0.855532765
MBTPS2	0.029903833	0.598723085	0.675810332	0.145541028	0.57043094	0.182004214	0.85559185
AKT1S1	0.756041318	0.544797633	0.692266168	0.005136075	0.688010864	0.18229206	0.855814396
C4A	0.370946997	0.350635717	0.075973979	0.10673125	0.95501891	0.182260485	0.855814396
KCNA2	0.129603484	0.455142511	0.529501807	0.067601354	0.477293995	0.182312298	0.855814396
PLCL2	0.960942603	0.203861663	0.422881741	0.049388591	0.24593998	0.182166563	0.855814396
TET1	0.886333313	0.726443637	0.02361419	0.318908367	0.20762115	0.182210776	0.855814396
WDR3	0.937868498	0.682232123	0.465535606	0.059660122	0.05678167	0.182432087	0.856131822
AGBL2	0.660456305	0.112346971	0.196986398	0.086452688	0.799096295	0.182497882	0.856195751
LOC511386	0.883178964	0.05486001	0.259930769	0.254995789	0.314788275	0.182606381	0.856459934
COL25A1	0.367873301	0.733979679	0.548499091	0.339976116	0.02010263	0.182726157	0.856532125
PPP2CB	0.584716134	0.373990534	0.018744954	0.483152394	0.510978067	0.182707924	0.856532125
NR4A3	0.447857193	0.01524759	0.897071168	0.22734507	0.728331115	0.182928808	0.8572372
RFXAP	0.019870854	0.874624225	0.727120144	0.096108085	0.836010732	0.183024186	0.857439314
RNF19B	0.544661133	0.86558136	0.030219608	0.142530149	0.501162821	0.183242542	0.857972426
SLC4A2	0.286544687	0.517066027	0.06250144	0.134929074	0.814332187	0.183226442	0.857972426
C25H16orf45	0.458853248	0.017432714	0.20773482	0.959061325	0.639695697	0.183409684	0.858020619
IRF8	0.51421277	0.985261642	0.292054431	0.450458444	0.015282748	0.183331843	0.858020619
LOC511229	0.591386922	0.634912503	0.044201479	0.106925586	0.57425482	0.183374617	0.858020619
ATP1B1	0.254346681	0.872651649	0.394151193	0.060423555	0.193152038	0.183556726	0.858463789

ADM	0.879415188	0.740935884	0.247155343	0.105523474	0.060402876	0.184067644	0.859139392
GSTA4	0.46293187	0.734031693	0.169185312	0.017873381	0.997532515	0.183930096	0.859139392
LOC616944	0.861049877	0.97506655	0.001447573	0.914438361	0.923467189	0.184051697	0.859139392
LOC785745	0.871445918	0.010421684	0.133276434	0.945708699	0.895604024	0.183947046	0.859139392
OLFML2B	0.240238902	0.569994093	0.149637227	0.076961808	0.650742955	0.184042641	0.859139392
PHKG1	0.667937498	0.619840945	0.137854891	0.03182439	0.565008797	0.18404606	0.859139392
PLEKHF2	0.416546471	0.379501443	0.073656006	0.094860089	0.927502296	0.18387608	0.859139392
CBX3	0.034460148	0.82110132	0.34249387	0.136831734	0.775668429	0.184261718	0.859312034
LOC614522	0.012963864	0.894973069	0.818364325	0.888945444	0.121788538	0.18420492	0.859312034
TRIM21	0.651063801	0.060637218	0.275476435	0.116567556	0.811287323	0.18425509	0.859312034
DOCK2	0.63132921	0.856277252	0.033867888	0.161326407	0.348458901	0.184324899	0.859362477
LOC112441616	0.054670523	0.261195088	0.251559395	0.630028202	0.455219861	0.184418788	0.85939628
SLC35A5	0.01906568	0.454977145	0.143559369	0.978079855	0.846007132	0.184436884	0.85939628
BTG3	0.343075173	0.110917925	0.375032809	0.764904553	0.094574082	0.184617888	0.85949631
CNTRL	0.69597728	0.136324528	0.145111839	0.076333456	0.983900942	0.18477259	0.85949631
ESPL1	0.630207313	0.094457922	0.089575198	0.197326997	0.980213789	0.184524313	0.85949631
LOC783045	0.675898659	0.01406409	0.148486844	0.857435388	0.853613429	0.184685239	0.85949631
SHCBP1	0.932353142	0.284929405	0.099619454	0.52357389	0.074608811	0.184748611	0.85949631
STK32A	0.257859888	0.158299554	0.249183668	0.116800952	0.870352723	0.184769604	0.85949631
PSMB6	0.288858314	0.526978344	0.418028861	0.266476086	0.061089714	0.184943689	0.860048421
MFGE8	0.882688415	0.465530901	0.326306139	0.018831712	0.410675144	0.185045508	0.860278141
FOPNL	0.320123774	0.34248419	0.43853463	0.02417821	0.892859475	0.185134295	0.860447157
ASF1B	0.755773807	0.482757927	0.044669079	0.190509792	0.335164524	0.185386049	0.860589231
NSA2	0.959690716	0.316469902	0.141487634	0.09821396	0.246567787	0.185383418	0.860589231
PLA2R1	0.736994151	0.475475396	0.155203217	0.134508123	0.14220355	0.185352324	0.860589231
SYT9	0.339117773	0.241760776	0.141209865	0.166343778	0.540383693	0.185387521	0.860589231
XYLB	0.588400535	0.675529883	0.132570501	0.326847342	0.060447555	0.185427062	0.860589231
OSGIN1	0.475934575	0.761541163	0.044874133	0.085324617	0.751186515	0.185553873	0.860934295
LOC107131403	0.817531746	0.459884999	0.418971651	0.012340081	0.53665607	0.185619173	0.860993852
MTSS1	0.280434426	0.994087849	0.02631403	0.479633856	0.2966588	0.185676539	0.861016582
FAM169A	0.228016692	0.144098877	0.877833643	0.091565954	0.395608098	0.185771778	0.861214874
NAP1L1	0.042785153	0.895148504	0.574823173	0.062457119	0.760302438	0.185827882	0.861231678
ALG13	0.168753294	0.502820882	0.578275633	0.174715498	0.122080483	0.185936371	0.861491186
HAPLN3	0.737087252	0.964269551	0.007379528	0.240492228	0.830393325	0.186014917	0.861497137
SMC4	0.441278541	0.140804614	0.135317847	0.126988043	0.981313367	0.186042645	0.861497137
ATP6V0C	0.149473177	0.225666089	0.047902425	0.815301999	0.797478708	0.186303278	0.861637134
CNTN4	0.023093211	0.671068815	0.687303567	0.109017583	0.904331907	0.186258615	0.861637134
DNAJC25	0.580548844	0.224227944	0.739714766	0.018732201	0.582622266	0.186335396	0.861637134
PNPLA7	0.537811819	0.023535929	0.229975556	0.954616965	0.377960807	0.186279776	0.861637134
POP1	0.622769947	0.254246692	0.410218042	0.313972558	0.051422811	0.186129516	0.861637134
AMOTL2	0.068973817	0.05798164	0.462136961	0.616255445	0.923695532	0.186439442	0.861875406
C7H19orf24	0.924374398	0.546976046	0.180672135	0.062638692	0.184111196	0.186572295	0.862003924
SLAMF8	0.441781491	0.55376201	0.045046027	0.527747996	0.181081803	0.18654066	0.862003924
HSP90AB1	0.528542062	0.408431201	0.803991385	0.006098339	0.996771092	0.186712242	0.86216923
MIIP	0.376849031	0.018849743	0.849783445	0.369653415	0.472808313	0.186713146	0.86216923

LOC112447010	0.537156175	0.804890166	0.758884428	0.254588642	0.012640916	0.186795705	0.862307822
DIRAS3	0.006587462	0.63224713	0.594007424	0.819112779	0.522003771	0.186970935	0.862631434
GPR141	0.861420853	0.739186551	0.703649849	0.021608012	0.10923988	0.186950327	0.862631434
ANO9	0.177890363	0.945229307	0.64526266	0.057851043	0.168659975	0.187045903	0.86273477
KIAA1551	0.246701219	0.351079812	0.369077126	0.131917787	0.251189945	0.187101959	0.862750845
LOC100294723	0.506977798	0.883462619	0.07277435	0.147802515	0.22006186	0.187187495	0.862902802
CBR4	0.344352286	0.501383588	0.31900803	0.028727244	0.671059146	0.187332467	0.862965445
IRGQ	0.778963776	0.419289233	0.009810306	0.92029626	0.360168727	0.187358837	0.862965445
NSUN5	0.446020665	0.068419686	0.268697129	0.219670469	0.589292624	0.187303866	0.862965445
ANP32B	0.472743694	0.89441174	0.846658773	0.011200548	0.265763534	0.187685943	0.863326354
ARFIP2	0.564884797	0.819028771	0.183254479	0.034327683	0.365457281	0.187503953	0.863326354
KIAA0895L	0.460396789	0.99773712	0.081237222	0.030272921	0.943515753	0.187708524	0.863326354
SLC19A1	0.214938378	0.512860583	0.178253899	0.413567117	0.131087678	0.187653168	0.863326354
SNRPB	0.147249284	0.251166083	0.507641731	0.406027175	0.139732985	0.187645394	0.863326354
SOAT1	0.822679455	0.444908683	0.004919764	0.835033842	0.709176876	0.187752834	0.863326354
ADAM23	0.707023004	0.208959225	0.496393261	0.162192274	0.089881565	0.188003695	0.863402446
ARSH	0.503382903	0.538799909	0.960079366	0.393559323	0.010415938	0.187850976	0.863402446
CYC1	0.754679615	0.608139317	0.789581802	0.003290216	0.896938168	0.188032438	0.863402446
LOC101902435	0.233526959	0.049628794	0.466207855	0.267388802	0.739539108	0.187943709	0.863402446
SLC4A8	0.482708802	0.9604035	0.050646934	0.056735974	0.802212864	0.187962925	0.863402446
EEF2K	0.802052977	0.330970719	0.644610709	0.775250483	0.00806714	0.18810031	0.863472498
CASZ1	0.900753316	0.44358644	0.016205241	0.250709841	0.661454567	0.188428486	0.86364539
CTSZ	0.130305861	0.163671675	0.150044328	0.659815091	0.508767587	0.188470731	0.86364539
EDNRB	0.528135743	0.946304489	0.547659748	0.004423419	0.885997509	0.188331018	0.86364539
ETAA1	0.046656605	0.486720719	0.233982653	0.302049296	0.667483772	0.188199979	0.86364539
HPGDS	0.463966136	0.495418057	0.524842879	0.106014847	0.083949139	0.188419333	0.86364539
ITGBL1	0.651209388	0.294114505	0.024871352	0.226455697	0.996178698	0.188506355	0.86364539
NMD3	0.51537531	0.348421326	0.5490877	0.415217018	0.026247685	0.188501676	0.86364539
CYP3A5	0.742552302	0.13489746	0.107163572	0.205081344	0.489790878	0.188834776	0.864463022
IPO13	0.3918155	0.40699749	0.161930622	0.121745694	0.343067754	0.188862052	0.864463022
LOC101902742	0.365178861	0.836265389	0.127682365	0.615644866	0.044909891	0.188820818	0.864463022
SIGLEC1	0.760817614	0.940786187	0.461563757	0.018749224	0.174179794	0.188895521	0.864463022
C10H15orf65	0.507421532	0.646171922	0.153444889	0.190955318	0.112594083	0.189151019	0.865390958
ADGRF2	0.017617258	0.797815348	0.242472482	0.326718273	0.973304626	0.189333626	0.865502545
FAM166B	0.314824058	0.948558899	0.105079541	0.848584757	0.040691478	0.189316394	0.865502545
HTR4	0.009748091	0.927840276	0.361376633	0.337306258	0.982400343	0.189274988	0.865502545
CDC37L1	0.073869472	0.424282362	0.111167653	0.333783749	0.935746386	0.189739797	0.866140398
DLG1	0.838159537	0.256607022	0.994093375	0.055033701	0.092503236	0.189759328	0.866140398
EIF4A2	0.078503528	0.41534263	0.400090314	0.178615448	0.466864783	0.189704068	0.866140398
LOC107131530	0.292506063	0.759338467	0.844959595	0.104152469	0.055610035	0.189628303	0.866140398
MRPS7	0.828846404	0.836796756	0.41298586	0.034245783	0.110756247	0.189577555	0.866140398
RPUSD1	0.999961818	0.916537227	0.071584413	0.043059208	0.385411607	0.189789828	0.866140398
CBX8	0.664595842	0.836905832	0.283436624	0.012035861	0.574150357	0.18984646	0.866157979
LOC512672	0.595321271	0.293489533	0.56856596	0.021655218	0.506743731	0.189910499	0.86620934
CRYBB1	0.400609117	0.486997282	0.209665811	0.125398186	0.213028399	0.190143397	0.866792387

ROR2	0.949457366	0.334597766	0.012513069	0.359473214	0.765086149	0.190196782	0.866792387
SBSPON	0.077583493	0.529307312	0.16185414	0.607476715	0.270666747	0.190157414	0.866792387
CFP	0.591734626	0.987553608	0.008305517	0.39313161	0.573513485	0.190286748	0.866961636
SNX4	0.110171131	0.589094679	0.041691825	0.497091644	0.814389016	0.190385717	0.867095989
SOX9	0.630717921	0.277096611	0.056158901	0.119757734	0.932273022	0.190421909	0.867095989
EXD1	0.671391741	0.374377592	0.045065958	0.780902979	0.124147875	0.190635139	0.867550836
SLC16A13	0.294463327	0.617757004	0.987657254	0.009469152	0.645391215	0.190616619	0.867550836
ZBTB18	0.862840743	0.207788924	0.222863321	0.317308831	0.086655515	0.190680389	0.867550836
ADA	0.631512631	0.342897888	0.370732449	0.015321179	0.903279851	0.19178526	0.867742781
APOLD1	0.856598656	0.33273019	0.019637496	0.691049487	0.287638101	0.191919802	0.867742781
BICC1	0.896545297	0.237201694	0.109070509	0.06271672	0.766238114	0.192110152	0.867742781
C16H1orf105	0.19051491	0.161944358	0.999336592	0.645115131	0.056110031	0.19223357	0.867742781
CACNA1F	0.878781069	0.45880882	0.003593095	0.934163572	0.813727705	0.190910673	0.867742781
CASS4	0.642209187	0.854380648	0.002770254	0.871307716	0.837628829	0.191636668	0.867742781
CCDC66	0.2055123	0.179100965	0.576630115	0.061978925	0.84585064	0.191933396	0.867742781
CNTN3	0.533252113	0.066087542	0.812016492	0.057053022	0.676637508	0.191222118	0.867742781
DPM2	0.309807327	0.36150452	0.518008053	0.03844178	0.498198944	0.191791255	0.867742781
DYRK1A	0.509460695	0.346174984	0.92583026	0.007531606	0.900877553	0.19150449	0.867742781
EIF2B1	0.751294668	0.745706254	0.373679537	0.096277072	0.055218928	0.191960225	0.867742781
FBLN1	0.852521909	0.101581349	0.081900025	0.158215815	0.988919339	0.191669424	0.867742781
FBXO7	0.284209829	0.22817944	0.409242185	0.091138132	0.459290696	0.191776544	0.867742781
FLI1	0.518611883	0.99597002	0.511807549	0.005405513	0.780096381	0.192118413	0.867742781
FRZB	0.99186663	0.027247388	0.151814145	0.52295218	0.522455214	0.19267235	0.867742781
GNB1L	0.384573587	0.031722916	0.551595149	0.479555266	0.347407322	0.192683617	0.867742781
LOC100848263	0.492869055	0.139008205	0.085655068	0.588795572	0.324066355	0.192562894	0.867742781
LOC101906230	0.779778595	0.922490545	0.199076913	0.01788435	0.437632522	0.192657562	0.867742781
LOC112444164	0.63516711	0.093629244	0.448226934	0.064939055	0.647838322	0.192710982	0.867742781
LOC112448743	0.673513531	0.04891412	0.770855359	0.206460813	0.210092078	0.19093743	0.867742781
LOC515570	0.491289221	0.393131751	0.068525475	0.60519807	0.13769321	0.191059525	0.867742781
LOC533921	0.868331511	0.3737583	0.286858808	0.014155811	0.848302816	0.192404204	0.867742781
METAP2	0.482261122	0.735274156	0.335120397	0.066237844	0.141783958	0.192229312	0.867742781
MTMR1	0.955507181	0.617411644	0.009172074	0.327327808	0.625484346	0.191500977	0.867742781
NDUFB3	0.730588631	0.593914431	0.689969765	0.003852962	0.959407967	0.19139824	0.867742781
NLRC4	0.989409439	0.835142664	0.046157896	0.14060002	0.207059602	0.191726272	0.867742781
PELI2	0.672155299	0.622848179	0.55973824	0.032863194	0.143864653	0.191506674	0.867742781
PNPT1	0.626511424	0.679239358	0.371138186	0.017039073	0.411421768	0.191442854	0.867742781
PPFIA2	0.74763176	0.133404947	0.127360858	0.109419697	0.806708041	0.192696658	0.867742781
RGS9	0.130221328	0.307760954	0.27303283	0.813739658	0.124451818	0.191528689	0.867742781
STRN3	0.585736548	0.285762443	0.306309772	0.022933126	0.951111143	0.192434546	0.867742781
SYNCRIP	0.392201466	0.078385072	0.53756493	0.299605586	0.223171293	0.191247162	0.867742781
SYNPR	0.046989149	0.67639555	0.983492169	0.086617328	0.41258434	0.19232561	0.867742781
TAPT1	0.225375613	0.520167867	0.130220077	0.130399567	0.558059546	0.191776842	0.867742781
TPGS2	0.404330011	0.098494218	0.849295739	0.441452426	0.074676478	0.192139624	0.867742781
VCL	0.507393076	0.204142817	0.067663217	0.415842396	0.384858525	0.192731853	0.867742781
VMP1	0.624799732	0.432591305	0.458038173	0.864159254	0.01048151	0.192703621	0.867742781

ZBED6CL	0.020574161	0.128608008	0.942578679	0.725787082	0.619044212	0.192635062	0.867742781
ATP2B2	0.049399186	0.205776031	0.400920721	0.684801046	0.402999269	0.193002524	0.86855038
IFITM3	0.456996785	0.084197047	0.181224388	0.161813337	0.996928999	0.193017076	0.86855038
ANXA1	0.339258442	0.495590802	0.06078271	0.294347638	0.375067606	0.193315235	0.868701073
CD101	0.179063586	0.707014308	0.524590216	0.147282286	0.115310081	0.193285236	0.868701073
LOC107133284	0.182238659	0.272333714	0.410258911	0.127243411	0.435048624	0.193216252	0.868701073
SLA2	0.321642971	0.360909856	0.217408299	0.42791265	0.104386315	0.193233374	0.868701073
THAP3	0.501294346	0.013422695	0.826499082	0.452676599	0.447267715	0.193114668	0.868701073
SUPV3L1	0.29836808	0.421100503	0.660580332	0.101072783	0.134654248	0.193433317	0.868993747
RAD17	0.00805207	0.860664856	0.446452421	0.393656082	0.927954855	0.193489068	0.869006318
EIF4E	0.878334703	0.009874131	0.737755158	0.711725401	0.248459801	0.193599231	0.869260235
SRPRA	0.252502031	0.458403144	0.085483685	0.30704593	0.372621732	0.19365154	0.869260235
SPTSSB	0.681306411	0.982635656	0.960346457	0.003282994	0.537280121	0.193827907	0.869683998
STOML2	0.631135516	0.836387968	0.275133704	0.106300356	0.073473241	0.193851932	0.869683998
LMX1B	0.360785093	0.176276724	0.43766818	0.744458966	0.054787404	0.193937559	0.869830359
EVL	0.278924842	0.405184865	0.022684273	0.595333457	0.744433508	0.194016205	0.869945339
EOMES	0.498876217	0.275432467	0.242581459	0.267858172	0.127596039	0.194282672	0.870133282
EPB41L4B	0.657109756	0.933082088	0.02144798	0.121351633	0.713085659	0.194172896	0.870133282
GSTT1	0.512240509	0.917273831	0.031423496	0.090605854	0.85179947	0.194308829	0.870133282
MED11	0.258393728	0.678490289	0.174157903	0.064376313	0.580120815	0.194376248	0.870133282
RBFOX1	0.208537415	0.727062855	0.070041185	0.242272815	0.442270103	0.19416603	0.870133282
WWC1	0.460471528	0.00718702	0.545515592	0.970518201	0.650783159	0.194372892	0.870133282
ASPM	0.404142889	0.150383949	0.080913829	0.335029526	0.692533545	0.194438483	0.870174515
PLA2G4B	0.187258382	0.434334322	0.382448272	0.050835338	0.722705917	0.194595992	0.870454906
SELP	0.487826784	0.552623672	0.078121372	0.793550755	0.068387128	0.194607218	0.870454906
CXCL10	0.140824062	0.346494074	0.575194116	0.414415412	0.098412239	0.194759218	0.87067529
SLC25A1	0.867622512	0.71683591	0.553858818	0.007800362	0.426237778	0.194815652	0.87067529
TACC3	0.293152503	0.107952046	0.11674101	0.496584054	0.624186694	0.194801522	0.87067529
ERBB4	0.224749467	0.754152095	0.235404527	0.6742893	0.042635229	0.194970762	0.871131276
IER5	0.192802954	0.135630068	0.336615182	0.205062619	0.636177357	0.195081967	0.871153787
NUSAP1	0.987020045	0.0586628	0.034206297	0.713728189	0.812129582	0.195055349	0.871153787
DPP9	0.121250936	0.445124972	0.72774333	0.131663242	0.222320176	0.195202453	0.871230529
MTHFD2	0.06001717	0.710835252	0.354019295	0.414058365	0.183851922	0.195205329	0.871230529
BSN	0.746150955	0.030953155	0.413143142	0.26011953	0.463957632	0.195363319	0.871461653
DUSP14	0.168714468	0.085227981	0.490120026	0.170864161	0.956280311	0.195360936	0.871461653
ARHGAP40	0.861996867	0.256413774	0.961884408	0.036292371	0.14997079	0.195852178	0.871510328
DDX27	0.941314632	0.679670664	0.291428922	0.012232749	0.505707762	0.195526951	0.871510328
KCTD5	0.168624612	0.366207392	0.042689187	0.462713727	0.948272154	0.195810264	0.871510328
LYPLA2	0.262275217	0.750673944	0.96342383	0.50464772	0.012063916	0.195645861	0.871510328
MAPKAPK3	0.543303948	0.766226688	0.005478212	0.80906309	0.62626558	0.195710176	0.871510328
RPSA	0.283277098	0.293383873	0.40676383	0.222868589	0.153015752	0.195477205	0.871510328
TMEM92	0.168700383	0.43076103	0.030555567	0.924253514	0.56369408	0.195825597	0.871510328
TUBA1C	0.293093534	0.087767885	0.401787652	0.425225873	0.263238288	0.195832509	0.871510328
WAC	0.733172175	0.824786929	0.71205688	0.010155936	0.264537032	0.195823618	0.871510328
C19H17orf113	0.841396506	0.018087357	0.63298028	0.811187287	0.148617899	0.196215954	0.872183107

CDKN1B	0.030780496	0.528664047	0.759677154	0.133155517	0.705328668	0.196187348	0.872183107
PSMB10	0.47759401	0.089948093	0.175295027	0.263572048	0.584511781	0.196112503	0.872183107
SRPX2	0.342126582	0.362540173	0.091691856	0.383769962	0.265666869	0.196058853	0.872183107
KIF1B	0.89093069	0.602928367	0.006796604	0.418866913	0.760237004	0.196324849	0.87243084
DNAH17	0.793958485	0.615761067	0.005065576	0.602018231	0.782002302	0.196610989	0.872553472
LRRC34	0.193059323	0.260907583	0.510175313	0.04726219	0.96001296	0.196618289	0.872553472
RET	0.815596781	0.523559987	0.023335555	0.157748442	0.741127867	0.196532272	0.872553472
SLCO1C1	0.07588795	0.68826599	0.600231829	0.043945174	0.84469162	0.196425061	0.872553472
ZNF410	0.167755546	0.865228028	0.124345873	0.276624576	0.233408857	0.196562207	0.872553472
GID4	0.624947111	0.607084019	0.284045177	0.03579702	0.302697714	0.196769106	0.872750757
SLC22A18	0.133275235	0.227525556	0.570473054	0.079547416	0.848259796	0.196730496	0.872750757
C1R	0.661449843	0.452120938	0.788001006	0.005645002	0.88116186	0.197156706	0.873570867
CD8A	0.518753741	0.260327886	0.260959773	0.293543578	0.113266102	0.197117029	0.873570867
FANCF	0.787403879	0.83823645	0.587705857	0.019610162	0.154112065	0.19716693	0.873570867
N4BP1	0.380625416	0.649022325	0.173649318	0.034797544	0.78523185	0.197151781	0.873570867
BCL7B	0.333451692	0.020463735	0.275627896	0.933594199	0.670852762	0.197653738	0.873793289
BMS1	0.465878589	0.594107206	0.171949966	0.063771806	0.388264214	0.197693339	0.873793289
BoLA	0.491765796	0.088064786	0.567367177	0.434033118	0.110669732	0.197852567	0.873793289
CD69	0.964902885	0.666158482	0.390036572	0.015597869	0.300698594	0.197475388	0.873793289
FUT1	0.821662302	0.540132704	0.012561119	0.298878888	0.707448357	0.197720557	0.873793289
LOC100847454	0.262651316	0.547041012	0.365498606	0.0615634	0.364203223	0.197612881	0.873793289
LOC107131429	0.909359753	0.025221247	0.696482921	0.273761528	0.269099148	0.197553503	0.873793289
LOC107131843	0.82753103	0.830610393	0.44095155	0.012115873	0.32078125	0.197656038	0.873793289
NAMPT	0.422853814	0.160137117	0.02523684	0.972626593	0.708181021	0.197579244	0.873793289
PNRC2	0.162835736	0.55421662	0.258987819	0.062440589	0.809176827	0.197909308	0.873793289
POLR3K	0.661484043	0.476969234	0.54819638	0.008428814	0.809382612	0.197826762	0.873793289
RPF2	0.790043912	0.15464219	0.277994274	0.213803494	0.162623564	0.197907976	0.873793289
WDR5B	0.004483628	0.659491577	0.590948515	0.773280809	0.873045671	0.197802525	0.873793289
KLF5	0.187673599	0.255641308	0.556907204	0.279595241	0.158361122	0.198091053	0.874125374
POLR2A	0.441330091	0.963425076	0.661865462	0.005500804	0.763826932	0.198038823	0.874125374
ATG3	0.426073531	0.467827364	0.276063191	0.211082007	0.10213752	0.198376875	0.874446117
LRP6	0.38488709	0.817060121	0.560921215	0.006725871	0.999729453	0.198354611	0.874446117
MRPL43	0.453157146	0.342441567	0.48954064	0.08588906	0.181774366	0.198348589	0.874446117
POC1B	0.05052786	0.63527746	0.852220633	0.312708238	0.138500473	0.198241346	0.874446117
TSHZ1	0.669243862	0.546367862	0.059616401	0.135311511	0.402577314	0.198471643	0.874628931
SQOR	0.729587762	0.20309001	0.174251939	0.277809543	0.165825913	0.198641493	0.874907551
TAX1BP1	0.457644194	0.027411848	0.238494249	0.77687086	0.511616591	0.198616701	0.874907551
TRMT2A	0.787064988	0.564710928	0.221399973	0.036382978	0.332662096	0.198775924	0.875264742
CCDC88B	0.790849893	0.546645962	0.022866543	0.243498234	0.495794764	0.198983254	0.875303059
FKBP7	0.807904026	0.254894854	0.014608954	0.450112102	0.881455574	0.198997972	0.875303059
LOC100295712	0.996478243	0.012295768	0.501439958	0.240534517	0.807309305	0.198950661	0.875303059
NFE2	0.094248732	0.150524075	0.695588381	0.453746489	0.266158392	0.198839648	0.875303059
ATF1	0.827265102	0.76505611	0.805258018	0.002650664	0.884427517	0.199098793	0.875511868
NIFK	0.720481767	0.484218091	0.742166185	0.075703995	0.061034236	0.199232267	0.875864112
JUND	0.259671479	0.083237268	0.517309417	0.138942895	0.77128115	0.199393237	0.876102385

PLXNA3	0.464771133	0.130423065	0.063731063	0.494612622	0.627007582	0.199379392	0.876102385
KIF23	0.483305829	0.897860835	0.0977425	0.054770685	0.517167945	0.199664744	0.877060522
METTL24	0.946576238	0.38857514	0.020279401	0.223214968	0.72352008	0.199939513	0.878032471
CNTROB	0.689301498	0.020875836	0.109496951	0.852809688	0.897655051	0.200070974	0.87824739
TIMM23	0.651947837	0.520135225	0.593841337	0.032809542	0.182608449	0.200095484	0.87824739
ACTA2	0.200712217	0.395032025	0.734717302	0.605112469	0.034591229	0.201185543	0.878370556
APBB1IP	0.760461135	0.99133717	0.004013341	0.527075331	0.767589686	0.20158361	0.878370556
BLM	0.705287457	0.469475201	0.014668375	0.424463788	0.593127352	0.201475154	0.878370556
CALM1	0.201935578	0.132320512	0.262523758	0.311039794	0.553798687	0.200250368	0.878370556
FAM13C	0.567056813	0.422235469	0.111781919	0.154697978	0.294057209	0.201029323	0.878370556
FNTB	0.303741599	0.973085969	0.446403964	0.145006892	0.063936297	0.201515563	0.878370556
GTF2F1	0.444438876	0.528280738	0.029154513	0.531548926	0.333217675	0.200599566	0.878370556
HERC5	0.962956314	0.817711757	0.111620373	0.022494207	0.619148701	0.201585905	0.878370556
HM13	0.36729571	0.978376629	0.188291472	0.34326854	0.052720825	0.201622198	0.878370556
HOXD4	0.171754442	0.405126123	0.170578838	0.210958506	0.488660199	0.201541137	0.878370556
INTS13	0.015217605	0.819225386	0.502473939	0.239172975	0.813987914	0.201200916	0.878370556
KLF4	0.02521723	0.496010062	0.284052538	0.911846127	0.376349405	0.201178635	0.878370556
LOC104974923	0.342939997	0.592772901	0.08090475	0.10275726	0.718610359	0.200772903	0.878370556
LOC107131516	0.359581853	0.188960312	0.794391804	0.039376303	0.571178625	0.200731135	0.878370556
MAX	0.833744207	0.132580038	0.099468895	0.12262589	0.908059174	0.201604578	0.878370556
PDZD7	0.22418863	0.529160768	0.561255296	0.335227281	0.054725396	0.201366127	0.878370556
PLCB2	0.735312518	0.942759934	0.002372198	0.942484547	0.781654593	0.200518998	0.878370556
PLSCR3	0.563214177	0.452694041	0.008769002	0.961473587	0.565197305	0.200815385	0.878370556
PPM1G	0.553614619	0.039218316	0.239662226	0.88224291	0.264438651	0.200730941	0.878370556
PSMD6	0.771384031	0.698334832	0.456424636	0.153569143	0.032098923	0.20056266	0.878370556
PTPRF	0.718668599	0.173192883	0.469781048	0.058022376	0.3598155	0.201304368	0.878370556
SH3TC2	0.225940776	0.179182214	0.422300285	0.664192121	0.107555145	0.201353282	0.878370556
SMIM5	0.808947677	0.114126636	0.593425823	0.075851142	0.292079186	0.20071377	0.878370556
ST6GALNAC4	0.195774659	0.046230792	0.277041265	0.628461623	0.769855883	0.200662625	0.878370556
TIMM50	0.464762961	0.801098697	0.487690903	0.042785056	0.155917949	0.200504151	0.878370556
TNFRSF6B	0.452182963	0.261088678	0.092071968	0.335610382	0.334620432	0.201301502	0.878370556
TXK	0.580381784	0.69112941	0.204274578	0.494789181	0.029856821	0.200433424	0.878370556
UBR3	0.5130392	0.316360601	0.051435735	0.163861677	0.888620822	0.200868847	0.878370556
AHI1	0.480240613	0.745067932	0.976675074	0.003895941	0.900782809	0.201780999	0.87882908
CLEC4E	0.683913916	0.766792102	0.016492238	0.208389821	0.682275916	0.202056297	0.879117559
GTF3C6	0.094519115	0.874481524	0.186151061	0.666048869	0.119998335	0.20206151	0.879117559
STK32B	0.441474737	0.108218531	0.168782258	0.381548734	0.399519472	0.202015159	0.879117559
TP63	0.465341864	0.904480232	0.239531001	0.024332389	0.501223545	0.202045342	0.879117559
UBLCP1	0.735466348	0.66392336	0.520713003	0.175131886	0.027683642	0.202311533	0.879972054
COL22A1	0.858352832	0.972426255	0.991622482	0.001717701	0.872504498	0.202959169	0.880688241
CUX2	0.247858236	0.771123491	0.025423254	0.306059383	0.832831869	0.202801049	0.880688241
FBXO36	0.088323852	0.918267513	0.214920193	0.230874037	0.308188802	0.202943021	0.880688241
IL1RAPL2	0.558883725	0.190920219	0.26358823	0.251607963	0.174611207	0.202556495	0.880688241
MMP15	0.125549787	0.952631579	0.801522142	0.026133699	0.494054595	0.202732074	0.880688241
PA2G4	0.374394641	0.567130863	0.270998954	0.146911291	0.14651864	0.202802745	0.880688241

SDCBP	0.800284625	0.278270585	0.024289226	0.293579092	0.778942226	0.202666139	0.880688241
TSSK3	0.245498292	0.025094	0.639468986	0.977795248	0.321299491	0.202723371	0.880688241
ZNF326	0.135485155	0.75999354	0.452970297	0.041410669	0.64160747	0.202856291	0.880688241
ACP4	0.114971829	0.478246984	0.47403583	0.080016987	0.59838486	0.203588619	0.880935278
ARMC12	0.721769146	0.492456171	0.750950227	0.6831529	0.006845382	0.203606575	0.880935278
ATP5MD	0.655379414	0.364902221	0.886170502	0.006059623	0.968845412	0.203270324	0.880935278
BTBD11	0.018669796	0.714573123	0.908665081	0.949060378	0.108107089	0.203234807	0.880935278
CYREN	0.491860879	0.863920899	0.809395607	0.004597102	0.787280656	0.203319116	0.880935278
FAM189A2	0.18678185	0.387634589	0.106786344	0.185692634	0.869034848	0.203561613	0.880935278
NME9	0.01809296	0.585962761	0.755185567	0.288825701	0.539141064	0.203481899	0.880935278
P4HTM	0.107229305	0.563758764	0.212964421	0.304409421	0.317942547	0.20342187	0.880935278
PTHLH	0.140947925	0.499886287	0.039561598	0.576591347	0.774450475	0.203313062	0.880935278
QRICH2	0.533940182	0.071219004	0.734527709	0.213613725	0.208695612	0.203354575	0.880935278
TEDC2	0.239376757	0.167737748	0.170202231	0.27210207	0.668353912	0.203157938	0.880935278
DENND1C	0.460141464	0.294775288	0.048138083	0.306588832	0.624241742	0.203723315	0.881204837
LOC101905156	0.120617334	0.339741291	0.97957708	0.039478811	0.789422628	0.203840646	0.881204837
PI16	0.388435825	0.109227416	0.120238798	0.246101741	0.996872527	0.203883661	0.881204837
SLC25A13	0.587441263	0.819814898	0.529450079	0.042269831	0.11608919	0.203854359	0.881204837
LOC107132897	0.046505734	0.415658325	0.727075977	0.412107289	0.216608374	0.204137357	0.881892458
PALD1	0.364573224	0.380505157	0.131200932	0.200862751	0.343226141	0.204150231	0.881892458
GPR45	0.044045447	0.484211146	0.47725875	0.160090957	0.770531853	0.204218611	0.881940714
LMCD1	0.525777033	0.078526649	0.712770215	0.345181975	0.123686998	0.204288973	0.881940714
LOC101904942	0.543619462	0.165476584	0.626762154	0.338066327	0.065973315	0.204376366	0.881940714
TGM1	0.012692481	0.353431971	0.874593504	0.345453141	0.927577864	0.204350828	0.881940714
ACSM5	0.642053912	0.225738886	0.072738398	0.20214953	0.590379713	0.204434275	0.881958699
CDC42EP2	0.651423556	0.384920012	0.017586699	0.349921376	0.817112441	0.204656402	0.882119063
EXTL1	0.424535542	0.573042438	0.055200432	0.306295289	0.306786089	0.204740205	0.882119063
MNS1	0.582619505	0.144528062	0.186519883	0.242494943	0.330761457	0.204562545	0.882119063
NUDT4	0.61638966	0.208519488	0.082528759	0.59633015	0.199484616	0.204736405	0.882119063
PCED1B	0.048828758	0.499287131	0.599898649	0.273856016	0.315034143	0.204731684	0.882119063
ANG	0.229875928	0.874529282	0.115231933	0.056326273	0.969621208	0.205013046	0.882394824
LOC112449072	0.086534125	0.144452408	0.123087507	0.861671531	0.954387987	0.205022443	0.882394824
LOC783224	0.025160763	0.53512253	0.657561052	0.651024809	0.219240015	0.204887155	0.882394824
LOXL2	0.788726247	0.340324224	0.038192305	0.630805702	0.195626299	0.205004397	0.882394824
YWHAQ	0.918763985	0.457995038	0.698021152	0.004362623	0.987920823	0.205073052	0.882394824
WDR74	0.632120242	0.333029658	0.047333878	0.521943003	0.24388848	0.205281529	0.883060332
C1H21orf91	0.367885399	0.028542775	0.517945107	0.896284159	0.260692206	0.205473609	0.883423473
LOC787122	0.575551488	0.837571265	0.613358651	0.008676489	0.495084717	0.205420038	0.883423473
TRPM1	0.492235412	0.554092115	0.012982494	0.89797092	0.399869543	0.205528402	0.883427606
NHLRC1	0.273380129	0.44635999	0.071918633	0.2418382	0.599447169	0.205594928	0.883482159
NUP153	0.337690978	0.653743598	0.985483334	0.008972863	0.652652675	0.205744461	0.883893287
ACTN1	0.061248281	0.352331577	0.170254091	0.562365513	0.619468668	0.206225479	0.885146449
ANKRA2	0.778377166	0.220859762	0.125218644	0.209315027	0.28443331	0.20636463	0.885146449
BEND7	0.34518272	0.983217961	0.10296094	0.091840436	0.399413219	0.206381844	0.885146449
CDC73	0.620811064	0.452710921	0.123185769	0.060803671	0.609388175	0.206463384	0.885146449

FAM198B	0.116678488	0.293936584	0.540955759	0.317111034	0.217704797	0.206299092	0.885146449
H2AFY	0.179618904	0.550650874	0.787246407	0.016543719	0.997573571	0.20664548	0.885146449
LOC100299712	0.377677419	0.763420555	0.132710787	0.045716661	0.73253695	0.206349777	0.885146449
MAP4K5	0.011571839	0.809096924	0.417308968	0.7486352	0.439055444	0.20658087	0.885146449
NMT2	0.138961141	0.081473578	0.659652639	0.839374678	0.204559855	0.206423938	0.885146449
SPHK1	0.18926012	0.62885295	0.199625982	0.260853752	0.207349221	0.206647329	0.885146449
TUBG1	0.14990357	0.429633382	0.14634404	0.352631457	0.38621969	0.206530102	0.885146449
WEE1	0.57732445	0.955133128	0.576645605	0.020258445	0.199560584	0.206683395	0.885146449
ENG	0.509111877	0.532939945	0.183029923	0.036624091	0.707619632	0.206806098	0.885440875
BTBD10	0.36582224	0.430377084	0.21021699	0.048658192	0.800120716	0.206931673	0.885454816
TFAM	0.931627481	0.072770365	0.049417341	0.566496293	0.679185484	0.206971219	0.885454816
VGF	0.612603032	0.015543529	0.24458426	0.97738077	0.566170024	0.206948491	0.885454816
CORO7	0.11414388	0.789407073	0.031399814	0.923931829	0.493444114	0.207043213	0.885531968
CRYL1	0.446493699	0.380854565	0.013252437	0.939211173	0.609902412	0.207124516	0.885648886
MYOC	0.319925764	0.500787501	0.353305284	0.045789723	0.498419535	0.20720264	0.885752155
C1H21orf2	0.025551577	0.776591422	0.53933987	0.411987416	0.294164518	0.207623003	0.885792655
CEP68	0.122820467	0.274274153	0.140760409	0.480708042	0.569426476	0.207697894	0.885792655
HBP1	0.04609552	0.806514328	0.507002692	0.868332577	0.079263706	0.207645964	0.885792655
LOC101906569	0.890933029	0.485531195	0.016188986	0.393395117	0.469564778	0.207345934	0.885792655
LOC112444775	0.149733726	0.360984717	0.313928829	0.505398108	0.151305928	0.20766727	0.885792655
MDM2	0.677369055	0.910070215	0.010469995	0.290051816	0.692405097	0.207558733	0.885792655
MICALL1	0.143461286	0.561154291	0.150538503	0.283819048	0.376230958	0.207383403	0.885792655
PNPO	0.81766921	0.966108912	0.443100379	0.029297587	0.126085607	0.207296632	0.885792655
USP32	0.471813969	0.462221288	0.461825914	0.02801663	0.459834672	0.207664217	0.885792655
COL26A1	0.176920867	0.245693718	0.530864948	0.325493179	0.173103451	0.207880169	0.885963817
GNA13	0.711843726	0.171822479	0.019421084	0.711145275	0.769991522	0.207923376	0.885963817
HNRNPAB	0.292289201	0.066770738	0.442200934	0.452553694	0.332755581	0.207833783	0.885963817
ZNF705A	0.362135037	0.375838552	0.071551063	0.426250965	0.313440274	0.207953971	0.885963817
CDKL1	0.07070594	0.339957769	0.626984873	0.856792417	0.100827964	0.20802367	0.886030743
EIF3A	0.901654482	0.324932765	0.399804059	0.040234529	0.276708519	0.208195981	0.88626882
ELOB	0.436319793	0.392358042	0.12086604	0.070345599	0.89564142	0.208162026	0.88626882
GPALPP1	0.03896817	0.142361011	0.319900303	0.736650878	0.997957598	0.20824158	0.88626882
RAP2B	0.981695609	0.505860388	0.022764562	0.202684366	0.569711497	0.2083031	0.886300795
TJP3	0.202792547	0.537064408	0.232234604	0.830054603	0.062217207	0.208371428	0.886361715
PRUNE2	0.418173038	0.291725953	0.045540186	0.490390939	0.480149157	0.20852306	0.88677687
PNCK	0.021989271	0.935472569	0.099867204	0.787723475	0.809195495	0.208632208	0.887011181
LOC101902841	0.01418885	0.530974811	0.995994318	0.369668799	0.472367835	0.208700288	0.887070819
RFNG	0.243362285	0.612410407	0.166649017	0.173026494	0.305236577	0.208816629	0.887335498
IQSEC1	0.150068236	0.95671114	0.139752614	0.110738656	0.590716112	0.208880065	0.887375291
MRPL11	0.478861124	0.117953754	0.783269852	0.079251065	0.374703734	0.208982213	0.887579477
CCDC78	0.395877074	0.063328162	0.463395626	0.678493294	0.166935374	0.20914768	0.887822704
LOC618071	0.151163808	0.953666613	0.055918977	0.365627504	0.446249506	0.209102987	0.887822704
ADAMTS12	0.471914134	0.494238453	0.008311776	0.714735188	0.954873847	0.209730352	0.888000702
C2H2orf69	0.39881531	0.132657297	0.126992061	0.253598122	0.775475754	0.209585703	0.888000702
CELF1	0.585245643	0.012083757	0.934892746	0.780129699	0.256251895	0.209620487	0.888000702

CTNS	0.238682848	0.721127839	0.051645861	0.184037578	0.808797656	0.209737919	0.888000702
FBXO41	0.656193265	0.243553649	0.780079247	0.465239983	0.022833885	0.209837531	0.888000702
HDGFL2	0.576681151	0.94684556	0.026849308	0.090485129	0.99496695	0.209473131	0.888000702
LMBR1L	0.733922805	0.018317674	0.252149085	0.422326865	0.925596461	0.209893043	0.888000702
LOC104975590	0.255456354	0.225686315	0.680130775	0.797768339	0.042303429	0.209750497	0.888000702
LOC104976274	0.021262247	0.112034409	0.946089937	0.7261132	0.808515133	0.209730598	0.888000702
NAA40	0.406380658	0.270313936	0.300087666	0.117966633	0.340032621	0.209667871	0.888000702
PAPD5	0.359386072	0.71532783	0.722342179	0.013716442	0.519946772	0.209834527	0.888000702
SUZ12	0.042771211	0.405187356	0.172834208	0.509056413	0.869019416	0.209889131	0.888000702
TMEM204	0.129040059	0.157535159	0.317818256	0.519910397	0.394264598	0.209831454	0.888000702
SIGLEC15	0.703457091	0.48751068	0.051606094	0.15578186	0.48112034	0.210002094	0.888233083
PAPOLA	0.063334893	0.632629066	0.533073718	0.105595289	0.588578503	0.21008426	0.888351661
PSIP1	0.480919117	0.634973664	0.197993918	0.08028562	0.274019572	0.210297863	0.889025819
AASS	0.123304643	0.25478827	0.80171871	0.567996256	0.093152426	0.210499602	0.889153534
CACNA1B	0.018630026	0.792271256	0.494509018	0.757165258	0.240975726	0.210427152	0.889153534
MCFD2	0.932203875	0.094583028	0.127382506	0.463747544	0.255889424	0.210511231	0.889153534
NRBP2	0.424888362	0.027896905	0.612776544	0.279840658	0.655934806	0.210544795	0.889153534
ARHGEF16	0.127627348	0.040421306	0.794033702	0.3444697	0.946566428	0.21073912	0.889197361
LOC613660	0.364946357	0.064710943	0.949616163	0.330411762	0.180399482	0.210825309	0.889197361
MVB12B	0.390198135	0.469189875	0.136650299	0.271784794	0.196598071	0.210826088	0.889197361
SORT1	0.406603953	0.489350315	0.052754799	0.140237972	0.907033416	0.210701555	0.889197361
UBA6	0.033468941	0.844340795	0.231339021	0.287453814	0.710815196	0.210748529	0.889197361
MINDY2	0.411155272	0.11339577	0.332702127	0.108554087	0.794688171	0.210937377	0.889209681
MPEG1	0.906828398	0.972832846	0.016456827	0.220199046	0.418529324	0.210924963	0.889209681
AMPD2	0.332154855	0.991786122	0.540680073	0.382415542	0.019954959	0.212611735	0.889919388
B3GNT8	0.248007029	0.641170593	0.078948035	0.693732258	0.155578348	0.212273915	0.889919388
BRF1	0.472702721	0.131306398	0.142026944	0.173635373	0.889467154	0.212791894	0.889919388
CEP131	0.613841224	0.355713183	0.029010239	0.268629779	0.795220695	0.212132815	0.889919388
CIT	0.462499792	0.168031081	0.032145676	0.671334211	0.803882219	0.211739119	0.889919388
CMTM6	0.922375417	0.022332836	0.37700989	0.177152715	0.976110299	0.211318971	0.889919388
COL14A1	0.219374672	0.544048758	0.09475431	0.150102787	0.797649259	0.212200747	0.889919388
CYR61	0.28070704	0.167903992	0.068472087	0.913078564	0.457623239	0.211761126	0.889919388
DKC1	0.598193229	0.450050029	0.256137064	0.091110878	0.216338143	0.212609922	0.889919388
EI24	0.404392897	0.233768771	0.162660851	0.312092985	0.283970237	0.212895224	0.889919388
GALNT3	0.093477053	0.977750857	0.157023828	0.200128956	0.474259139	0.21284431	0.889919388
HCN2	0.347151118	0.289682022	0.081925472	0.69922366	0.233977004	0.211712819	0.889919388
LOC107131239	0.896297426	0.284275793	0.084992869	0.166211461	0.376905285	0.212409386	0.889919388
LOC107132851	0.169688988	0.388312825	0.479795103	0.369485261	0.116139863	0.212409861	0.889919388
LOC112442740	0.484312255	0.637980852	0.012027974	0.904475509	0.401834765	0.211940304	0.889919388
LOC534181	0.489497854	0.949029046	0.133082675	0.067190682	0.325542932	0.212063624	0.889919388
LUC7L	0.288597501	0.10589692	0.23845765	0.359086457	0.518004765	0.212323366	0.889919388
MDFIC2	0.580394449	0.995233247	0.454099408	0.114375531	0.045367036	0.21275715	0.889919388
MRPL4	0.235434483	0.735921813	0.602899926	0.037151289	0.350301657	0.212630969	0.889919388
MUC16	0.326286219	0.053024124	0.346250265	0.717827319	0.313550983	0.211747895	0.889919388
NDUFB7	0.453376607	0.478100166	0.799260172	0.008422681	0.929606684	0.212396533	0.889919388

NELFE	0.100322065	0.586097058	0.216945483	0.552264793	0.192464899	0.212347003	0.889919388
PCDH20	0.609675961	0.834516382	0.501240019	0.014532343	0.363084321	0.211533853	0.889919388
PPIE	0.989053865	0.213332627	0.862147157	0.045378773	0.165011298	0.212844468	0.889919388
PPIG	0.026538554	0.505762489	0.752207033	0.304791874	0.439312537	0.212031285	0.889919388
SLAMF1	0.714449129	0.402360243	0.651807852	0.01397073	0.518269688	0.212412638	0.889919388
SLC1A4	0.0470087	0.427495776	0.64189252	0.345228425	0.304476556	0.212351276	0.889919388
SLC39A7	0.134183662	0.777460056	0.292428837	0.206309349	0.216015269	0.212640432	0.889919388
SPRYD4	0.779039322	0.413655618	0.661161013	0.025220456	0.253445099	0.212823983	0.889919388
SRSF11	0.641604201	0.430801865	0.481652075	0.022881717	0.446255184	0.212627758	0.889919388
TMEM202	0.131476226	0.940218968	0.030346753	0.776391639	0.465818252	0.212413865	0.889919388
TWNK	0.652687807	0.142824643	0.329653675	0.10780508	0.408974578	0.212269501	0.889919388
ZNRF2	0.627457685	0.147274461	0.023725443	0.853014184	0.724404552	0.212259991	0.889919388
GPS1	0.661033075	0.804916514	0.368335971	0.068336249	0.101966496	0.213117588	0.88996466
GZMA	0.403009493	0.057291873	0.364335248	0.237799272	0.682407578	0.213077178	0.88996466
KAZN	0.88423981	0.989324173	0.120467094	0.053374219	0.242737613	0.213097484	0.88996466
LOC112442713	0.767950367	0.361412325	0.368715819	0.350999959	0.038020126	0.213122973	0.88996466
ICE2	0.115644076	0.264778765	0.175570479	0.978929364	0.259957296	0.213312261	0.890075644
RBM5	0.712715467	0.478025521	0.677062187	0.62793434	0.00944301	0.213289703	0.890075644
STAT4	0.251153315	0.937764358	0.274298451	0.041127356	0.514587386	0.213246375	0.890075644
LOC112448354	0.04840024	0.23518838	0.353565825	0.467750563	0.727223109	0.213387785	0.890164447
APELA	0.440216473	0.066208732	0.550953734	0.11220805	0.767477106	0.214474991	0.890593638
ATP6V1C1	0.991804225	0.260538089	0.013737243	0.690081721	0.570703129	0.215654328	0.890593638
BCL2	0.668580824	0.673625098	0.008163125	0.423101252	0.889630122	0.214548845	0.890593638
C11H9orf16	0.237024476	0.339198206	0.035423952	0.571936865	0.845248432	0.213999348	0.890593638
CCNB2	0.535176059	0.28910079	0.043543777	0.227981355	0.900273996	0.21446501	0.890593638
CEP295	0.181490577	0.063980188	0.349032908	0.411402866	0.840733513	0.215950884	0.890593638
CH25H	0.043199923	0.30223452	0.961136474	0.535079844	0.204679664	0.213807615	0.890593638
CHMP2B	0.040618688	0.140288812	0.311691354	0.852493304	0.920112389	0.215278603	0.890593638
CIART	0.511229613	0.448501003	0.172626515	0.365307296	0.095017507	0.213768709	0.890593638
CLPTM1L	0.38862335	0.763912629	0.274453489	0.039845727	0.425368324	0.214326003	0.890593638
CNN1	0.028330193	0.343274781	0.779535064	0.96776449	0.189331732	0.214957478	0.890593638
CNP	0.112430634	0.728587917	0.320737355	0.643177118	0.082672261	0.215578998	0.890593638
CSTB	0.525438872	0.563393646	0.109260287	0.880839869	0.049147159	0.215825675	0.890593638
DACH1	0.556402627	0.652415288	0.771320473	0.009257139	0.537070941	0.215191396	0.890593638
DHDDS	0.31663976	0.594086327	0.496167627	0.147735774	0.100387226	0.214580052	0.890593638
DYNLT3	0.016381111	0.511423669	0.354267253	0.904042041	0.521386548	0.215728452	0.890593638
FAM101A	0.213690432	0.981573883	0.691767141	0.010190012	0.94885136	0.216038415	0.890593638
FAM114A2	0.174331384	0.479267692	0.228539714	0.138591537	0.529735352	0.215955755	0.890593638
FKBP9	0.209223687	0.992985319	0.564080072	0.029446529	0.401929157	0.214797196	0.890593638
G2E3	0.089138032	0.344936963	0.449824168	0.101365078	0.987032082	0.214544555	0.890593638
GORASP2	0.779006858	0.160750389	0.189578832	0.203571865	0.287757491	0.215084192	0.890593638
HR	0.164606174	0.058896005	0.348808103	0.666794467	0.620488588	0.215738638	0.890593638
IFNLR1	0.144189954	0.392029847	0.089543803	0.382872303	0.712161975	0.214259787	0.890593638
IMMP2L	0.528304449	0.287059182	0.270988181	0.167321337	0.202222728	0.21507415	0.890593638
KCNK3	0.128137895	0.072644063	0.585243654	0.361390047	0.707330488	0.215230475	0.890593638

LOC100297099	0.116906014	0.406638366	0.335383585	0.125192036	0.698549211	0.215367405	0.890593638
LOC100335608	0.414820753	0.271908209	0.302415458	0.113868721	0.35999403	0.215674097	0.890593638
LOC112446002	0.946102758	0.916370284	0.06349288	0.216897348	0.115810841	0.214462941	0.890593638
LOC112449280	0.067638406	0.553859413	0.069375054	0.748142005	0.719704654	0.215761244	0.890593638
MICALL2	0.865188964	0.027711496	0.065161827	0.968110348	0.90885327	0.213826872	0.890593638
NUBP2	0.145665276	0.92537345	0.519324938	0.06411774	0.311799257	0.215768993	0.890593638
PALM3	0.168841619	0.176926808	0.256349297	0.787270088	0.232877868	0.216117725	0.890593638
PARP12	0.896188582	0.056856196	0.140547351	0.257574941	0.756150232	0.21540513	0.890593638
POFUT2	0.2016255	0.601247449	0.077381912	0.253011732	0.583791769	0.2146871	0.890593638
PRMT5	0.847679458	0.648773794	0.486566632	0.011316304	0.460307578	0.21533222	0.890593638
RALBP1	0.470139546	0.061929151	0.238595353	0.426436664	0.463392439	0.213678843	0.890593638
RASD1	0.337730926	0.369826446	0.016161486	0.947944135	0.720971906	0.214217143	0.890593638
RMND5A	0.216654575	0.658201371	0.424177852	0.084084431	0.276118155	0.216149806	0.890593638
RSPO2	0.015502662	0.816434265	0.201870209	0.93574826	0.584692905	0.215649077	0.890593638
SCO2	0.43769471	0.341418272	0.543896413	0.023373491	0.732599884	0.21516842	0.890593638
SLC43A2	0.338814548	0.34660567	0.715756124	0.119372714	0.139841724	0.21605439	0.890593638
SLC6A17	0.050482825	0.356115095	0.262395371	0.972607274	0.301998454	0.214686014	0.890593638
TAGLN2	0.1225617	0.80507362	0.302270586	0.082106837	0.569525493	0.215396922	0.890593638
TBC1D24	0.081837487	0.161165202	0.267150334	0.958942621	0.409187706	0.214452147	0.890593638
TIMP2	0.249826246	0.589905373	0.013666408	0.742680362	0.919794202	0.213922524	0.890593638
TRPV1	0.627682645	0.704093282	0.066328855	0.097769685	0.482134741	0.214390092	0.890593638
USP34	0.517583706	0.96054563	0.713321322	0.007296422	0.535982125	0.21478871	0.890593638
VCPIP1	0.773024564	0.116528503	0.024070315	0.774659571	0.835033735	0.216008651	0.890593638
ZSCAN21	0.470427033	0.05445837	0.525875319	0.400468846	0.256743861	0.214655101	0.890593638
BCNT2	0.060276657	0.416128803	0.661277934	0.176552275	0.479815733	0.216205878	0.89060107
KIAA1143	0.299371309	0.356399451	0.386199049	0.071134098	0.479786012	0.216299959	0.890765025
RFC5	0.026034036	0.394953573	0.351197353	0.743000844	0.524449559	0.216362191	0.890797771
ADRA2A	0.519914363	0.231149217	0.286763118	0.219288646	0.186309769	0.216429327	0.890850684
PACRGL	0.804117778	0.512528513	0.495426636	0.383686652	0.01798854	0.216527328	0.891002364
TET3	0.623325207	0.425051899	0.084377398	0.315171765	0.200100635	0.216574763	0.891002364
APAF1	0.754248134	0.802804117	0.621015831	0.004944007	0.75957214	0.216749626	0.891308437
SPRYD7	0.679340482	0.067432243	0.313547608	0.162124101	0.606458206	0.216757783	0.891308437
MYO5C	0.604340905	0.487343557	0.217838806	0.949225314	0.023204929	0.216831921	0.891389942
ALDH1B1	0.781559051	0.053955485	0.443895102	0.086427483	0.884065281	0.218145151	0.891568091
ALPK1	0.799799212	0.166890816	0.090423179	0.380457489	0.310115478	0.217667926	0.891568091
APEX1	0.538650945	0.803922287	0.457544817	0.263133869	0.027374336	0.217908234	0.891568091
ATAD2B	0.45202172	0.79142634	0.302824976	0.013965825	0.94615766	0.218240545	0.891568091
CBR3	0.228721213	0.737259082	0.109818801	0.459664463	0.166794347	0.217341049	0.891568091
CCDC146	0.115290977	0.366126495	0.06874793	0.585449736	0.846533773	0.218753472	0.891568091
CCDC190	0.149966493	0.505437033	0.891727505	0.656368707	0.032161319	0.217882927	0.891568091
CCL21	0.614377979	0.717770955	0.022787958	0.344125437	0.413526853	0.218128352	0.891568091
CDNF	0.296300153	0.317251092	0.277024104	0.137119344	0.401860183	0.218502066	0.891568091
CHMP2A	0.314636177	0.57115231	0.224918945	0.165127074	0.215527709	0.218775751	0.891568091
CTPS2	0.248133364	0.566770998	0.101845075	0.223816387	0.446060398	0.218121012	0.891568091
DDX6	0.897524565	0.006666847	0.56580357	0.490500544	0.854183307	0.217239712	0.891568091

DEFB13	0.807147545	0.777571934	0.680371283	0.069645053	0.048394578	0.218831044	0.891568091
ELOVL5	0.375712344	0.822129566	0.916436367	0.007904422	0.63852517	0.218027044	0.891568091
HPN	0.265615717	0.517416303	0.460780039	0.069088392	0.3255035	0.21767436	0.891568091
KNOP1	0.501896491	0.088194047	0.479746675	0.407693665	0.166154209	0.21877643	0.891568091
LARP4	0.481998254	0.260610925	0.06922252	0.381012905	0.430262605	0.217777879	0.891568091
LHFPL4	0.222941402	0.515790582	0.811810952	0.288551747	0.052953805	0.2178488	0.891568091
LOC100847700	0.30493039	0.489515202	0.715043614	0.041678326	0.322542278	0.218494498	0.891568091
LOC107132469	0.138785131	0.448681024	0.830702774	0.708829041	0.038594684	0.216981097	0.891568091
LOC515169	0.488287734	0.655731505	0.12743492	0.830109317	0.042297584	0.218328873	0.891568091
LOC789867	0.46666292	0.949882424	0.248048524	0.038150372	0.338565097	0.217372585	0.891568091
LUC7L3	0.090427016	0.398570899	0.174386272	0.256038412	0.893191896	0.218689183	0.891568091
MPP7	0.095086838	0.595838409	0.280274677	0.985625606	0.091443762	0.218217107	0.891568091
NLRX1	0.302759539	0.953252343	0.618429672	0.071654083	0.1123188	0.218619215	0.891568091
NOP10	0.487387441	0.428881608	0.429273383	0.054021994	0.293540538	0.217582631	0.891568091
OBSCN	0.17381651	0.258044405	0.838670116	0.130117461	0.292377423	0.218206303	0.891568091
PNISR	0.187489546	0.310530876	0.185902687	0.52493578	0.250599063	0.21765037	0.891568091
RAB31	0.378321763	0.294398949	0.601439494	0.266829604	0.080333811	0.218576835	0.891568091
RUNX1	0.731123858	0.096861513	0.27960591	0.382608686	0.188842294	0.218177657	0.891568091
SETD7	0.906546167	0.371623725	0.012626236	0.935670586	0.358917712	0.218011629	0.891568091
TMEM181	0.509077839	0.832337548	0.042043084	0.370022212	0.215004144	0.217146533	0.891568091
TREML2	0.532304051	0.446604661	0.181639537	0.659659313	0.050156171	0.218024843	0.891568091
VAT1L	0.153354825	0.610415941	0.117304325	0.139155526	0.928897065	0.217310793	0.891568091
ZNF296	0.180793533	0.161343214	0.191701537	0.407591788	0.631386229	0.218819213	0.891568091
ZNF484	0.803479206	0.910400683	0.896038954	0.059796282	0.036567386	0.218369575	0.891568091
PRKCZ	0.09494693	0.719970182	0.050938516	0.825826407	0.501499939	0.219052635	0.891806697
SELPLG	0.293317486	0.634157515	0.047712515	0.266659538	0.608884254	0.218965969	0.891806697
SPON2	0.014180172	0.734033391	0.250114638	0.96496801	0.573786673	0.219001562	0.891806697
ARL14EPL	0.195113709	0.364537042	0.60544599	0.152992393	0.219145055	0.219180613	0.891900547
CLN3	0.231887018	0.101947178	0.454639804	0.298258135	0.450635216	0.219238959	0.891900547
DIRC2	0.98187561	0.527962885	0.037804779	0.097438956	0.756360953	0.219221662	0.891900547
ETF1	0.654751849	0.228162684	0.12851426	0.57250503	0.131555707	0.219346769	0.891900547
TCN1	0.050681248	0.120362323	0.396885592	0.636017173	0.939057577	0.219347426	0.891900547
IDO1	0.980810125	0.544717063	0.01548048	0.535183853	0.327113348	0.219493899	0.892132139
PIGM	0.028005197	0.98048636	0.57326867	0.48327076	0.190459468	0.219567467	0.892132139
THPO	0.35537813	0.116968431	0.469852402	0.140664489	0.527271758	0.219544196	0.892132139
MRPL21	0.661513841	0.481525473	0.486208631	0.01364603	0.686535392	0.219724539	0.892549359
HNRNPH3	0.891794382	0.794654101	0.039701786	0.174637539	0.295559195	0.219822299	0.892659921
MRPS18B	0.802097114	0.434617347	0.538106072	0.02485032	0.312099929	0.220023727	0.892659921
SETDB2	0.177236042	0.805459713	0.013026996	0.836904768	0.933476822	0.219869234	0.892659921
SH3GL3	0.100427847	0.18285832	0.458420425	0.573571122	0.301232737	0.219996965	0.892659921
SP110	0.631224716	0.240583967	0.225315179	0.129205274	0.328906116	0.219964183	0.892659921
ATP5MC1	0.921369907	0.307743255	0.923242895	0.007634495	0.730802007	0.22045397	0.893903289
CNTNAP2	0.609560417	0.301027436	0.02740054	0.454025794	0.640046805	0.220493603	0.893903289
SECISBP2	0.470167478	0.692148677	0.485421676	0.022662008	0.4079382	0.220440052	0.893903289
LAMB2	0.916332202	0.486950806	0.145601648	0.696654784	0.032338546	0.220689304	0.894254857

LMO3	0.214179567	0.452466365	0.641845047	0.415968041	0.056558228	0.220665822	0.894254857
GANC	0.278310462	0.790872743	0.142554922	0.223284874	0.209105346	0.220791344	0.89435747
TTC9C	0.400422007	0.658027935	0.841968025	0.058537238	0.112844219	0.220823623	0.89435747
EPRS	0.795476445	0.647756279	0.372619395	0.509011535	0.015010303	0.220938901	0.894382906
HARBI1	0.600204618	0.62339507	0.492845612	0.024841379	0.320221789	0.220933464	0.894382906
LOC101905099	0.983320689	0.01887412	0.768375046	0.919066576	0.111996424	0.221006441	0.894435687
CCDC15	0.253509653	0.267324421	0.256014377	0.085643375	0.988516081	0.221080113	0.894466223
LOC112445044	0.836685284	0.501313388	0.019153275	0.499365411	0.366324479	0.221137246	0.894466223
NCOA7	0.935174744	0.157427956	0.871485568	0.017280279	0.663087741	0.221177499	0.894466223
CENPI	0.147895805	0.599584656	0.390055786	0.471958918	0.090188688	0.22133907	0.894899109
LOC112444479	0.612654873	0.819845223	0.096520156	0.372261556	0.081665482	0.221457385	0.894932259
LOC514189	0.588267378	0.849497681	0.343091053	0.270268404	0.031819944	0.221505565	0.894932259
TFCP2	0.795266939	0.53613892	0.049789962	0.195941518	0.354663604	0.221565399	0.894932259
WWOX	0.247491056	0.774471696	0.013175626	0.90492138	0.645375523	0.221535877	0.894932259
AEBP1	0.411842344	0.739144666	0.018293692	0.347786415	0.765812369	0.22215945	0.894981166
ARHGAP23	0.725925121	0.834418586	0.034961743	0.194266862	0.361307512	0.222402319	0.894981166
C17H12orf43	0.384936273	0.616063872	0.638984064	0.199929074	0.048930471	0.222098792	0.894981166
CDT1	0.762444814	0.661459077	0.20279725	0.040725988	0.356649206	0.222336124	0.894981166
CKMT2	0.267150862	0.949070931	0.944169336	0.908906336	0.00682441	0.222285643	0.894981166
CLIC5	0.352489499	0.191401506	0.350428613	0.468224007	0.134274154	0.222400422	0.894981166
CWF19L1	0.05834831	0.191551804	0.417789287	0.422375923	0.749813963	0.221833751	0.894981166
DYRK3	0.097964296	0.104870856	0.743557622	0.242383693	0.803978497	0.222566561	0.894981166
GNPDA1	0.359512901	0.186213082	0.043652382	0.805753077	0.632344207	0.222592992	0.894981166
GXYLT2	0.676658094	0.398530239	0.023980729	0.441620501	0.521597952	0.222641712	0.894981166
IL1RAP	0.346661178	0.890381927	0.784269098	0.006151037	0.998831381	0.222464513	0.894981166
LOC100297399	0.177501563	0.88046073	0.117086682	0.141097109	0.576653005	0.222583811	0.894981166
MAFK	0.3555785	0.262475383	0.059601631	0.550192683	0.483901851	0.22199493	0.894981166
PAPPA2	0.670907462	0.058331625	0.451680785	0.424143118	0.197993479	0.222252911	0.894981166
PRPF40A	0.217747587	0.290271599	0.867001204	0.079274234	0.341271877	0.222111648	0.894981166
RAB5IF	0.507891037	0.441993524	0.322434424	0.100102793	0.203833308	0.221686598	0.894981166
SLC18A2	0.264734126	0.079142834	0.988600138	0.196771577	0.363451863	0.222019802	0.894981166
STAC	0.802856012	0.380536341	0.176701104	0.041434757	0.666106206	0.222668217	0.894981166
TBC1D23	0.030406686	0.546165786	0.261679226	0.491283794	0.694990616	0.222204894	0.894981166
VCPKMT	0.180163748	0.069930857	0.531877486	0.365479709	0.604031749	0.221871208	0.894981166
GPRC5C	0.31195751	0.708240519	0.583561837	0.125774019	0.091966418	0.222771375	0.89514792
TLR10	0.39186729	0.778110812	0.234158458	0.023416167	0.892404823	0.222818795	0.89514792
NRAS	0.747038078	0.077558907	0.145597101	0.182560746	0.969287792	0.222875089	0.895154941
ANKRD22	0.748357782	0.1059106	0.107210171	0.944162747	0.186261488	0.222995113	0.895417863
DENND6B	0.237478065	0.111479773	0.568029295	0.152448054	0.653557025	0.223288377	0.895525471
HACE1	0.217855698	0.296408953	0.80012934	0.426369852	0.068116137	0.223458461	0.895525471
HEXIM1	0.980102278	0.221573356	0.088255766	0.137294671	0.569898256	0.223387996	0.895525471
LOC100848598	0.248769698	0.94408419	0.335301908	0.187804818	0.101332554	0.223315189	0.895525471
LOC104974460	0.171166974	0.370963819	0.832065802	0.790709619	0.035908393	0.223423795	0.895525471
LOC112445888	0.376582621	0.187526811	0.305610815	0.119536546	0.579951147	0.223131253	0.895525471
LOC515697	0.171872558	0.686323467	0.965169468	0.035861772	0.367474004	0.223443127	0.895525471

NMT1	0.772613637	0.702140924	0.658939097	0.182488381	0.022934834	0.22312567	0.895525471
LOC101906001	0.178343236	0.390372342	0.560976371	0.047226826	0.813966786	0.22351475	0.895532366
ANKRD12	0.229228558	0.748182211	0.853724555	0.03229257	0.318174854	0.223743012	0.895725093
CCDC121	0.039965406	0.43128792	0.638462611	0.512698582	0.266983936	0.223890338	0.895725093
CCDC86	0.201269083	0.880417148	0.101538078	0.362286121	0.231020248	0.223854781	0.895725093
GADD45A	0.982427697	0.22857101	0.02620577	0.466743288	0.548037957	0.22380613	0.895725093
KLK8	0.802681227	0.148633525	0.059795564	0.724504118	0.290934123	0.223692031	0.895725093
LOC104971345	0.42252224	0.03690039	0.44475614	0.977283706	0.222065355	0.223779483	0.895725093
FDXACB1	0.694807032	0.583327241	0.21562404	0.131417545	0.131344668	0.224046324	0.89613069
LIMS1	0.34878614	0.29666787	0.121944434	0.260959484	0.458974747	0.224256297	0.896193974
LOC534913	0.946085071	0.256977699	0.467321353	0.015240803	0.87220647	0.224182726	0.896193974
SOS1	0.279240278	0.488948093	0.979804806	0.014019865	0.805372237	0.224196258	0.896193974
TBL2	0.863354688	0.469226341	0.429965197	0.023440739	0.370229894	0.224280583	0.896193974
INCENP	0.881642869	0.022591185	0.342279896	0.483037716	0.459411505	0.224370462	0.896334871
LAS1L	0.081099516	0.658698487	0.136277803	0.344377451	0.604030084	0.224480774	0.896450177
LOC617785	0.25889691	0.913137208	0.015669077	0.677916818	0.603181864	0.224508575	0.896450177
BMP2	0.343797601	0.091169766	0.181242428	0.294982855	0.904631091	0.224599182	0.896593815
PPAN	0.404910354	0.964574002	0.458998505	0.262253911	0.032263427	0.224665401	0.896640052
ATG16L1	0.613648037	0.309619354	0.357477423	0.025764625	0.867734128	0.224786444	0.896824611
FGL1	0.362223396	0.69281403	0.149951566	0.360379314	0.112004638	0.22482094	0.896824611
LOC104973739	0.895941983	0.231157821	0.160228393	0.60848182	0.075283636	0.224907837	0.896953225
MYCN	0.601670969	0.235823816	0.057193971	0.530966502	0.353080737	0.225001447	0.897108541
HPSE	0.803818604	0.294072129	0.659060505	0.063050275	0.155036091	0.225109813	0.897216095
TTN	0.328986637	0.093162656	0.062315803	0.809625311	0.985059882	0.225137766	0.897216095
ESYT2	0.340430402	0.257967354	0.212514125	0.243665996	0.335823475	0.225428217	0.897581274
LOC787904	0.568945961	0.425506013	0.194552022	0.150028524	0.215967893	0.225347572	0.897581274
OSER1	0.827236552	0.072452429	0.154305408	0.26095995	0.632887098	0.225448176	0.897581274
PTBP3	0.545000808	0.642229962	0.033900105	0.266034181	0.483539367	0.225368947	0.897581274
CCDC141	0.644964892	0.690976135	0.197608998	0.32326867	0.053700342	0.225547303	0.897758136
GLP1R	0.140883086	0.475951821	0.132254024	0.503079431	0.343736476	0.225896418	0.89784115
JUNB	0.726069002	0.018357848	0.935342125	0.550148104	0.223210986	0.225707712	0.89784115
LOC615610	0.997592241	0.732359295	0.186531455	0.106608909	0.105452067	0.225788769	0.89784115
PLAC8	0.310028248	0.924062619	0.120164052	0.152763959	0.29148336	0.225849456	0.89784115
SIAH1	0.273814148	0.308454513	0.900298196	0.217160317	0.092745459	0.225744315	0.89784115
WDR44	0.44577227	0.574417027	0.07809724	0.63037628	0.121423643	0.225685365	0.89784115
LOC786065	0.117726396	0.390285043	0.109501447	0.541969778	0.56310705	0.226038816	0.898189589
AURKB	0.015116697	0.888375018	0.358510541	0.895423608	0.356873924	0.226261324	0.89839377
FANCG	0.812662434	0.019940318	0.322447324	0.929492344	0.316818742	0.2262767	0.89839377
KIAA1841	0.11864684	0.821017952	0.899225676	0.660771192	0.026596336	0.226326766	0.89839377
MAP4K2	0.522226777	0.061770124	0.06332663	0.867418835	0.868409116	0.226281759	0.89839377
SEPHS1	0.852948348	0.624794252	0.012257163	0.480789502	0.490330302	0.226363917	0.89839377
TEX22	0.994771673	0.008098786	0.666672191	0.80078872	0.358232632	0.226427686	0.898429583
POP4	0.391617498	0.134296981	0.25621889	0.543073971	0.210802159	0.226566704	0.898763881
ALDH5A1	0.100705824	0.945096065	0.320826222	0.169747781	0.300977077	0.22783433	0.899160055
CDK2AP2	0.161403934	0.444949589	0.366522027	0.067141886	0.879899057	0.227472458	0.899160055

CDK9	0.678760739	0.084021323	0.143992595	0.288249361	0.65354513	0.226883285	0.899160055
CHST7	0.525960795	0.377928591	0.072137307	0.130872227	0.828471374	0.227445504	0.899160055
DDX31	0.680284706	0.251677104	0.042592527	0.532636879	0.401903208	0.227908141	0.899160055
DTYMK	0.205857141	0.991657098	0.176356119	0.078186254	0.552463239	0.227472561	0.899160055
EMP1	0.636550013	0.240606008	0.013160664	0.865813571	0.89398517	0.227843102	0.899160055
FAM126B	0.024015863	0.756946553	0.796142995	0.115432543	0.928035721	0.227132542	0.899160055
KCNH2	0.091489646	0.419762094	0.300237773	0.324756651	0.413463872	0.226973312	0.899160055
LAMTOR3	0.327131755	0.296266821	0.021966266	0.991093255	0.732905143	0.226840058	0.899160055
LOC101906240	0.4029747	0.036591809	0.257791903	0.591892315	0.691247558	0.227487025	0.899160055
LOC107131416	0.561218594	0.810730429	0.844825135	0.102835928	0.039234234	0.227168487	0.899160055
LOC112444585	0.014639149	0.301652354	0.940143583	0.789107156	0.473790086	0.227260551	0.899160055
LOC112447005	0.066084579	0.550438116	0.643276074	0.482976755	0.137915461	0.227731791	0.899160055
MRPL55	0.250217193	0.754958968	0.689054409	0.054109125	0.221502182	0.227835534	0.899160055
PFN2	0.324328762	0.120787568	0.600887883	0.083262525	0.796601323	0.227926746	0.899160055
RNF122	0.545804989	0.260509213	0.860983385	0.078767896	0.16036151	0.226834978	0.899160055
SEC61A1	0.215417686	0.77671907	0.422075356	0.285484529	0.07715982	0.227513597	0.899160055
TMEM200C	0.557755896	0.282033421	0.370557653	0.112312658	0.23794502	0.227669311	0.899160055
TMEM209	0.981466903	0.589764687	0.877870631	0.229139448	0.013299483	0.226995045	0.899160055
TREX1	0.698779438	0.671049832	0.074573476	0.078963116	0.564879152	0.227813746	0.899160055
ZGRF1	0.33901354	0.141006736	0.153119594	0.881394602	0.24137718	0.227629749	0.899160055
ZNF593	0.368302575	0.458910585	0.340431913	0.459995928	0.0589329	0.227817869	0.899160055
ASCC1	0.451277335	0.305276401	0.674398363	0.0236974	0.711627657	0.228322781	0.899581967
BTNL9	0.415454686	0.183069636	0.955896302	0.060765655	0.354294038	0.22820928	0.899581967
GAK	0.604614616	0.041702511	0.321438216	0.204818151	0.951680073	0.229263585	0.899581967
GALK2	0.711156235	0.365286601	0.041209029	0.24748462	0.592984177	0.228630473	0.899581967
GMIP	0.388230458	0.709697164	0.01575853	0.569853057	0.634028106	0.228464848	0.899581967
IMP3	0.489218228	0.165612254	0.543737302	0.359359136	0.098797532	0.228127246	0.899581967
KIAA0586	0.035676029	0.283575426	0.470449708	0.620291969	0.534033854	0.229034197	0.899581967
LOC101904378	0.10900763	0.482468718	0.198449064	0.170262654	0.883970555	0.22861734	0.899581967
LOC104971162	0.294748511	0.061545109	0.430628009	0.32767021	0.614976701	0.228855904	0.899581967
MTHFD1L	0.238625081	0.659874822	0.543239138	0.506708924	0.036425802	0.229195002	0.899581967
NDUFS8	0.583748645	0.626567105	0.994533121	0.007474884	0.580148617	0.229095351	0.899581967
PCTP	0.635078208	0.211848914	0.420873214	0.030185256	0.918159483	0.228508807	0.899581967
PLOD1	0.614678523	0.227049748	0.051533486	0.39946099	0.546959717	0.228658186	0.899581967
RAB44	0.116041849	0.60427612	0.576209263	0.272277708	0.142570449	0.228444474	0.899581967
ROBO3	0.430488924	0.643012112	0.082008943	0.74053633	0.093550871	0.228749399	0.899581967
S100A8	0.655899526	0.064150302	0.59574355	0.140517368	0.444234415	0.228173928	0.899581967
SHC4	0.250781205	0.24824675	0.0653945	0.756611412	0.512797998	0.229248161	0.899581967
SLC30A5	0.117575007	0.252095857	0.317878645	0.235297435	0.71278107	0.229294459	0.899581967
SLC7A4	0.411957025	0.524720302	0.198637085	0.585057098	0.062849411	0.229195842	0.899581967
SREK1	0.193195104	0.411535037	0.224352598	0.158223235	0.558138233	0.228935315	0.899581967
TRIB2	0.501675302	0.933821108	0.015586846	0.426632532	0.505762255	0.228961253	0.899581967
USP21	0.536769923	0.111895208	0.098138718	0.754528436	0.355156814	0.229247324	0.899581967
VANGL1	0.700520903	0.360237505	0.475363645	0.015023667	0.869295843	0.228315653	0.899581967
DUSP19	0.026775315	0.798901497	0.408721672	0.388601052	0.46535241	0.229353773	0.89959961

PRDM1	0.907174393	0.515529485	0.333737362	0.960736942	0.010562412	0.22955734	0.900182918
FMO4	0.112682806	0.426873245	0.095944277	0.81685052	0.422103231	0.230088436	0.900544081
H4	0.984783303	0.039242926	0.112504247	0.975761652	0.374604702	0.229944022	0.900544081
IFI16	0.733276769	0.485018148	0.03086762	0.219426954	0.659524381	0.229907715	0.900544081
P2RY10	0.858100269	0.438837883	0.058501178	0.405797199	0.177498716	0.229766046	0.900544081
SGTB	0.484168903	0.86565154	0.662131406	0.024320628	0.235526103	0.229972972	0.900544081
SLC16A11	0.652025765	0.32363345	0.091612802	0.119651593	0.686821128	0.229904182	0.900544081
SLC16A5	0.091089954	0.620017108	0.825302987	0.058400283	0.58432321	0.230040744	0.900544081
USP28	0.303406917	0.258400871	0.521018708	0.636870671	0.061112205	0.229987161	0.900544081
ANLN	0.958000691	0.176651863	0.059137427	0.500315042	0.318383234	0.230299874	0.901156707
ARHGEF10L	0.335361161	0.22887039	0.402846063	0.052396885	0.98676698	0.230621637	0.901770715
DIS3	0.005064235	0.832301454	0.841418593	0.48083324	0.937391874	0.23061127	0.901770715
STRBP	0.032464519	0.797639124	0.353962174	0.235629888	0.739881442	0.230569772	0.901770715
LOC518080	0.696999568	0.734203992	0.009543369	0.437174325	0.749375165	0.230712148	0.901909735
ANGPTL7	0.774257086	0.690415955	0.212215953	0.883785139	0.016395019	0.233820198	0.902213739
ARFIP1	0.0856986	0.467668315	0.355907284	0.465682159	0.246524102	0.233384962	0.902213739
BCL11A	0.958061414	0.752717188	0.172313548	0.12793871	0.10304127	0.233427587	0.902213739
BET1	0.022085073	0.707672275	0.842776937	0.208088152	0.591890012	0.232305478	0.902213739
BRCA2	0.378612574	0.079055151	0.346080428	0.635691109	0.24959076	0.233805341	0.902213739
CACUL1	0.325791553	0.049937839	0.165718146	0.896668917	0.679622471	0.233768742	0.902213739
CENPBD1	0.107989558	0.576423798	0.531170161	0.277557286	0.177101699	0.232517773	0.902213739
COPG1	0.211057002	0.959326001	0.18210382	0.202064471	0.218024737	0.232450959	0.902213739
DYNLRB2	0.651156472	0.137488914	0.034176543	0.84782124	0.632447856	0.233601197	0.902213739
FAT3	0.064734782	0.578807311	0.302043873	0.888923147	0.160047507	0.23143809	0.902213739
FDPS	0.290339656	0.577502009	0.56424235	0.182792344	0.095089102	0.233868579	0.902213739
FNIP1	0.320237021	0.54977357	0.215759531	0.07257122	0.581283693	0.230889659	0.902213739
GFPT2	0.782313898	0.079974384	0.515206913	0.088850228	0.574150146	0.233864149	0.902213739
ITGA10	0.108835339	0.128018458	0.697083791	0.386120513	0.428283423	0.231154573	0.902213739
KANSL1L	0.150282618	0.628435934	0.872041699	0.181089921	0.10993126	0.233524628	0.902213739
KIT	0.385608681	0.27732276	0.554246315	0.065495686	0.418689101	0.232519573	0.902213739
LOC100848941	0.746381846	0.610996236	0.09007724	0.053493173	0.747308159	0.233708367	0.902213739
LOC101903752	0.437732311	0.468518748	0.053575256	0.167519615	0.872940122	0.231199018	0.902213739
LOC101905757	0.571141574	0.732405213	0.683981712	0.90067093	0.006315085	0.232664313	0.902213739
LOC101907369	0.344647437	0.81626725	0.084819536	0.505416129	0.135900862	0.23348528	0.902213739
LOC101907642	0.060816931	0.367436883	0.242413934	0.506560244	0.598324608	0.233687077	0.902213739
LOC107132301	0.009814761	0.488103093	0.521160662	0.83478432	0.782108524	0.232855219	0.902213739
LOC112443006	0.081278596	0.556456532	0.524748281	0.625114224	0.108391054	0.231294457	0.902213739
LOC112443526	0.493013845	0.066614846	0.084554412	0.648949513	0.912094949	0.233816125	0.902213739
LOC112444896	0.68659873	0.024152618	0.528355022	0.798911978	0.231230857	0.232042064	0.902213739
LOC112446013	0.200445334	0.204658635	0.401543021	0.880939508	0.112322741	0.23284663	0.902213739
LOC112447359	0.483996408	0.061340617	0.663631455	0.082705544	0.991317531	0.231810927	0.902213739
LOC112448808	0.076664758	0.149017779	0.373317469	0.822793262	0.462621143	0.232384037	0.902213739
LOC615559	0.164415122	0.426357213	0.041836088	0.70323409	0.793514908	0.233311534	0.902213739
LOC783497	0.533301913	0.039665179	0.42875348	0.407802192	0.438817651	0.232356331	0.902213739
MED12	0.381596868	0.654912858	0.513713334	0.089337585	0.142628326	0.233265719	0.902213739

MRPL46	0.438220626	0.483181847	0.616517806	0.131815467	0.093618067	0.231496302	0.902213739
NRXN1	0.534229346	0.912457839	0.404917295	0.022775794	0.35957985	0.231893263	0.902213739
PDGFB	0.19761374	0.834151173	0.441590592	0.024419436	0.920534733	0.233294534	0.902213739
RBM48	0.072337079	0.902558855	0.056773072	0.672745169	0.654008105	0.232910555	0.902213739
RUNX3	0.658163295	0.904754574	0.41763231	0.177732781	0.036714303	0.232339762	0.902213739
SERTAD1	0.054200721	0.228537801	0.442548448	0.362997634	0.819124685	0.232848184	0.902213739
SKP2	0.520437938	0.386284099	0.15519333	0.465104456	0.112236385	0.232756662	0.902213739
SLC15A3	0.532023545	0.797184526	0.066767355	0.112655289	0.504570478	0.231404379	0.902213739
SLC38A8	0.672475477	0.014349283	0.664988955	0.735323189	0.345052404	0.232717221	0.902213739
SLX4	0.342017163	0.050427874	0.488389728	0.334646072	0.572176377	0.231635288	0.902213739
STAT5A	0.936999398	0.784544432	0.597696749	0.090696924	0.040571485	0.231913237	0.902213739
SYPL1	0.515808469	0.079266001	0.096072813	0.425981359	0.968517561	0.232184107	0.902213739
TDRD3	0.226510208	0.55980232	0.070954672	0.290162019	0.624647974	0.232901728	0.902213739
TEFM	0.708260162	0.194732743	0.720366304	0.984533822	0.016743582	0.233402693	0.902213739
TIMM21	0.902061322	0.825030114	0.40007846	0.014491158	0.371968729	0.2310698	0.902213739
TMX3	0.070111154	0.241169369	0.471012506	0.292586778	0.705278667	0.233800083	0.902213739
TULP2	0.173951233	0.069686693	0.452553943	0.51551142	0.57307745	0.232190813	0.902213739
TWF2	0.472519775	0.958408641	0.223369531	0.119266545	0.136282212	0.233852108	0.902213739
UNC79	0.219148984	0.200005035	0.694078509	0.061527239	0.872293532	0.233045089	0.902213739
UNC93B1	0.63302557	0.9548919	0.007913297	0.617690704	0.542848003	0.230996339	0.902213739
XYLT2	0.578033177	0.661099336	0.057009557	0.10095992	0.732752755	0.231548786	0.902213739
ZBTB1	0.449210063	0.905093327	0.081916534	0.567382644	0.086929502	0.233746951	0.902213739
ZNF383	0.302348458	0.076309765	0.406295109	0.479163401	0.364729937	0.233434701	0.902213739
ZNF385A	0.01598737	0.778570696	0.348054079	0.458851201	0.820474784	0.232922854	0.902213739
ZNF652	0.831808679	0.029071519	0.172503056	0.707234459	0.553768246	0.233114272	0.902213739
GGA3	0.219725701	0.357293253	0.990063293	0.023219643	0.911813095	0.233952699	0.902326146
ASB13	0.730364682	0.658177444	0.729959417	0.175079825	0.026837817	0.234175232	0.902548082
CNOT6L	0.289756974	0.967972068	0.554317608	0.013010504	0.815016771	0.234162244	0.902548082
EMC10	0.313280774	0.761661144	0.219823135	0.047083578	0.667217654	0.234106213	0.902548082
IL1RL1	0.609375462	0.547188592	0.546851972	0.023330329	0.388150868	0.234348201	0.902806879
MAP4K3	0.347761487	0.274755034	0.062249071	0.409963422	0.677210732	0.234352404	0.902806879
DOK5	0.465205663	0.72395929	0.264447295	0.048567452	0.382381563	0.234542332	0.903326499
ADRM1	0.613750957	0.523963353	0.292739458	0.08306401	0.212741271	0.235210125	0.903470463
BRD9	0.080206624	0.863359033	0.660131145	0.061384574	0.590561813	0.234760266	0.903470463
CAPN10	0.217253957	0.065076207	0.227495117	0.719221502	0.722206608	0.235704752	0.903470463
CIDEB	0.080681342	0.182457999	0.214453281	0.863042293	0.611595168	0.235404398	0.903470463
CREB5	0.544795975	0.14357418	0.658885609	0.082545103	0.390775006	0.23512969	0.903470463
CTSH	0.693148402	0.28164	0.021512102	0.739072032	0.53840844	0.235735819	0.903470463
EXTL2	0.280072631	0.667516379	0.054165084	0.394625808	0.417158848	0.235451019	0.903470463
FCHO1	0.968066269	0.239200123	0.946462079	0.062040793	0.122620922	0.235470476	0.903470463
GABRE	0.814418878	0.0242071	0.398442854	0.592581383	0.358150978	0.235459539	0.903470463
GALNT4	0.229512093	0.571831937	0.334090741	0.566800577	0.067203207	0.235670605	0.903470463
GRPEL2	0.138791738	0.835981354	0.851659005	0.029118655	0.580368416	0.23565515	0.903470463
LOC509415	0.380856786	0.203188251	0.114553753	0.561475864	0.333598471	0.234992334	0.903470463
MAN1C1	0.232223448	0.876351941	0.784634034	0.037873399	0.274569587	0.234996002	0.903470463

NFATC1	0.545646609	0.543858575	0.008547769	0.843778581	0.776812709	0.235144858	0.903470463
PHF11	0.335778877	0.112204958	0.225618185	0.89728944	0.217785812	0.235039639	0.903470463
PPME1	0.541927387	0.672655518	0.797687536	0.011270757	0.506383841	0.234932117	0.903470463
PPP2R5E	0.186825254	0.149030806	0.72217184	0.15467929	0.533089765	0.234821241	0.903470463
PRR15L	0.129676154	0.292332067	0.747041054	0.472624684	0.12467592	0.235569149	0.903470463
TERC	0.960245082	0.352853567	0.96025935	0.025287514	0.202411108	0.235335004	0.903470463
TMEM126A	0.841664475	0.555349879	0.66919519	0.023722535	0.223831252	0.235023232	0.903470463
TSSK2	0.454533795	0.772218882	0.413917637	0.017452996	0.654007754	0.234844947	0.903470463
LOC524650	0.023360397	0.847962852	0.814008283	0.311637461	0.333245412	0.235976507	0.904181755
CDK15	0.641504265	0.011469246	0.924173776	0.465834468	0.529113055	0.236075349	0.904349336
ZPR1	0.520849174	0.406142773	0.053764102	0.290832465	0.507221416	0.236197571	0.904606379
CAPS2	0.22944984	0.815023811	0.020082141	0.450739057	0.992923483	0.236408478	0.90511736
SMIM10	0.416682331	0.187516544	0.547430625	0.049786251	0.789492546	0.236441297	0.90511736
SAPCD1	0.746122342	0.02083354	0.897248554	0.401888437	0.300146314	0.236520068	0.905207749
NTF3	0.241818977	0.675666365	0.041150688	0.940640076	0.26626566	0.236631329	0.905422415
LOC104975593	0.192672865	0.5837563	0.037707258	0.587447981	0.67671233	0.236769028	0.905738115
NCK1	0.105457137	0.808378045	0.07725542	0.41767642	0.613420352	0.236867732	0.905904533
LOC100849587	0.990297993	0.004425878	0.813963191	0.665007906	0.712772348	0.237118039	0.906650545
CDIPT	0.311896294	0.58896421	0.64138428	0.135404425	0.106272018	0.237420104	0.90683849
GLO1	0.166094415	0.672227937	0.373891611	0.080130674	0.507753554	0.237635718	0.90683849
LOC786726	0.725693717	0.124845662	0.083058973	0.731478192	0.308553118	0.237629144	0.90683849
LSG1	0.230733632	0.869387456	0.142660927	0.082784466	0.716388347	0.2375434	0.90683849
PGAM5	0.712124373	0.70425086	0.307999572	0.03850169	0.284643117	0.237243544	0.90683849
RXYLT1	0.597013411	0.177472406	0.156408815	0.149425696	0.686079472	0.237664515	0.90683849
SERGEF	0.2780502	0.392177438	0.606626834	0.031230405	0.819920275	0.237314344	0.90683849
TMSB15B	0.151452605	0.740148061	0.439179473	0.815544298	0.042292406	0.237602891	0.90683849
ZMPSTE24	0.327146467	0.121376868	0.46337217	0.957601519	0.096272522	0.237481028	0.90683849
IL17D	0.074806332	0.071343033	0.979119503	0.817769794	0.398082865	0.237814039	0.907198092
SPAG8	0.281486828	0.306886826	0.120728354	0.214030189	0.763334113	0.238004348	0.907713073
BCL2L1	0.310647154	0.266986936	0.055287344	0.632893205	0.589549266	0.238490229	0.907739746
C11H2orf50	0.420004533	0.095744645	0.133794846	0.934422279	0.33999962	0.238380961	0.907739746
CEP57	0.166635615	0.892027463	0.894347775	0.250093593	0.051434086	0.238428701	0.907739746
DRAP1	0.278608765	0.452327067	0.526752385	0.37218445	0.069191261	0.238390113	0.907739746
LOC112443012	0.015614442	0.394657859	0.866111293	0.442197412	0.722405948	0.238080791	0.907739746
LOC787554	0.01006285	0.728680939	0.686079681	0.800513139	0.424155445	0.238299074	0.907739746
NARFL	0.660745153	0.163783375	0.627792903	0.031625843	0.796418521	0.238509158	0.907739746
RECQL	0.874413403	0.096004602	0.056477954	0.403110851	0.892472643	0.238131776	0.907739746
STEAP3	0.702305739	0.926361779	0.186871971	0.030887946	0.455342198	0.238421091	0.907739746
ESPNL	0.513802238	0.045007987	0.166393495	0.488366265	0.91260737	0.238765578	0.907769504
HELB	0.346215509	0.880730253	0.008538072	0.913500451	0.721268365	0.23879355	0.907769504
MDGA1	0.512746619	0.57006306	0.191654426	0.068658029	0.445677533	0.238712831	0.907769504
RAP1GAP2	0.121329321	0.341125824	0.057087989	0.795196925	0.912946292	0.238790877	0.907769504
RNPC3	0.025560478	0.527131751	0.953080836	0.780600788	0.170971353	0.238689448	0.907769504
ADM2	0.592582533	0.114037361	0.361587173	0.232217595	0.306778931	0.240523591	0.907930539
AGRN	0.352856348	0.434380589	0.747230562	0.389238498	0.038977466	0.240312131	0.907930539

ANAPC10	0.796071712	0.045339459	0.888607901	0.070088895	0.76544046	0.239157626	0.907930539
ARHGAP28	0.953008688	0.447608475	0.011320617	0.556960843	0.644289574	0.239991033	0.907930539
C5H12orf71	0.378663042	0.938734688	0.023557926	0.248799381	0.836475459	0.240661225	0.907930539
CRLS1	0.043751399	0.219836985	0.621130797	0.449254396	0.642841611	0.239475558	0.907930539
CTSA	0.366490323	0.589430179	0.041279743	0.576886537	0.337484036	0.240209662	0.907930539
ENY2	0.357238558	0.363927961	0.349049524	0.058520091	0.652344343	0.239956171	0.907930539
FBLN5	0.034996085	0.760741045	0.366500252	0.294276722	0.604603664	0.240204708	0.907930539
FBXO16	0.876604615	0.566992973	0.173899861	0.034405485	0.585844742	0.240621904	0.907930539
HIF1AN	0.569238973	0.516575789	0.262469913	0.033484437	0.66698359	0.239365529	0.907930539
IQCB1	0.069209398	0.782267047	0.98193069	0.102721984	0.314520985	0.238944896	0.907930539
KPNA2	0.606824493	0.59302844	0.459870297	0.601492073	0.017510303	0.240678167	0.907930539
LBP	0.978783679	0.19781788	0.721033638	0.491553184	0.025104274	0.239300357	0.907930539
LOC100140403	0.777380372	0.726652683	0.163872743	0.152727674	0.123118676	0.240517924	0.907930539
LOC101902869	0.067294706	0.321020263	0.54743057	0.800635988	0.183809096	0.240500414	0.907930539
LOC112443226	0.89818255	0.316482043	0.092826292	0.484198251	0.135120697	0.239545461	0.907930539
LOC788334	0.038340164	0.978326112	0.474465903	0.28695143	0.338157269	0.239583434	0.907930539
MAGI3	0.812924764	0.493098801	0.570510173	0.083325249	0.090678762	0.23965403	0.907930539
MANBAP1	0.915732248	0.162328516	0.052970736	0.589084183	0.370700435	0.239076473	0.907930539
MED27	0.526071373	0.571695	0.215775907	0.126455511	0.210510582	0.239625211	0.907930539
MPND	0.264713194	0.316917264	0.71298183	0.095094597	0.304757965	0.240029023	0.907930539
MS4A1	0.183307692	0.928483774	0.378267034	0.975378325	0.027378795	0.23906053	0.907930539
NAPSA	0.594643193	0.467005155	0.446403018	0.212905333	0.065661639	0.239999857	0.907930539
OSBPL7	0.275612352	0.054082483	0.590639672	0.322131676	0.611471796	0.240077097	0.907930539
PRPF40B	0.438569848	0.24365237	0.098121844	0.745050048	0.220679302	0.23938066	0.907930539
SLC25A5	0.350726286	0.516415557	0.514867046	0.824590529	0.022674562	0.240716944	0.907930539
SNAPC5	0.786249016	0.863278675	0.360769472	0.012170366	0.58349705	0.240402115	0.907930539
TOP3B	0.334825098	0.058981927	0.522065227	0.192195876	0.876385412	0.240243764	0.907930539
TOR1A	0.119157626	0.465987996	0.909341967	0.145684204	0.235502056	0.239952986	0.907930539
UBE2D3	0.150669381	0.196787919	0.380473858	0.215511043	0.709176837	0.239394265	0.907930539
UEVLD	0.567350913	0.22214689	0.104615147	0.423883468	0.309618742	0.239825153	0.907930539
USP54	0.267991919	0.344944738	0.320464449	0.161802577	0.363124693	0.24051342	0.907930539
ZSWIM4	0.74375703	0.430939202	0.012367262	0.541157039	0.801931924	0.239125691	0.907930539
CYTH2	0.750272627	0.700628196	0.97477357	0.452458867	0.007537526	0.240983471	0.908138957
GABBR1	0.47847711	0.128538093	0.577358258	0.970470301	0.050710712	0.240984007	0.908138957
KIF15	0.786007082	0.117397335	0.07031198	0.517593192	0.520019033	0.240902659	0.908138957
SMIM12	0.453515808	0.260910899	0.533084041	0.035417601	0.78263441	0.241048888	0.908138957
ZCCHC18	0.028072043	0.306000324	0.779034751	0.584419941	0.446977379	0.241022965	0.908138957
LOC107131330	0.907905997	0.227358975	0.098501593	0.206966067	0.415959838	0.241182035	0.908292345
RNF40	0.376724601	0.455264448	0.347715484	0.1080209	0.27176543	0.241200295	0.908292345
MYH15	0.052381187	0.538492715	0.240650485	0.761634621	0.338913116	0.241299359	0.908456935
LOC511936	0.943259306	0.075359527	0.361495036	0.276076125	0.247715741	0.241647927	0.909413053
SOWAHD	0.457784628	0.934274558	0.027447263	0.251627272	0.595005482	0.241664147	0.909413053
EPHB6	0.57920305	0.601291027	0.466765494	0.583280776	0.018560547	0.241819659	0.909581119
SPIRE2	0.332192557	0.621103001	0.042660992	0.649557683	0.307711399	0.241782239	0.909581119
FIBP	0.570676528	0.58424407	0.712381308	0.165594819	0.044821173	0.242023025	0.909720538

SEC31A	0.813032464	0.6273029	0.125607018	0.062904574	0.437443084	0.24201648	0.909720538
SSR4	0.557424618	0.226965138	0.214878316	0.273182035	0.237336647	0.242003347	0.909720538
ZFP90	0.12977732	0.845317732	0.569215875	0.049928574	0.565834627	0.242107153	0.909828369
ELK1	0.192140368	0.275356154	0.986673577	0.035952125	0.943527524	0.24255366	0.910259088
KLHL13	0.847040791	0.5145262	0.152194646	0.070288461	0.379530588	0.242464934	0.910259088
NCOA2	0.547910516	0.913211115	0.499641213	0.034956485	0.202629429	0.242554567	0.910259088
TP53BP1	0.550869864	0.635632202	0.05573485	0.159041521	0.570348818	0.242517176	0.910259088
ZFYVE21	0.691929301	0.899756876	0.028215878	0.128797839	0.780921941	0.242288299	0.910259088
ZNF502	0.386931	0.705880728	0.72650831	0.011868942	0.751052122	0.242422638	0.910259088
GNPTAB	0.936322397	0.516483665	0.099118881	0.050566308	0.731576644	0.242716214	0.910350756
LOC112447857	0.807407865	0.388021537	0.064691354	0.130718078	0.669690554	0.24278346	0.910350756
MGST2	0.77869191	0.842435325	0.059279292	0.048645662	0.938035079	0.242800881	0.910350756
TMED7	0.668877403	0.318368922	0.149321626	0.329138105	0.169489455	0.242760406	0.910350756
CATSPERG	0.376777556	0.181436894	0.123379386	0.282353173	0.746176309	0.24297055	0.91049814
FDX2	0.181101195	0.482543639	0.659815027	0.065528467	0.470935676	0.243130825	0.91049814
HEMK1	0.053260971	0.241119783	0.758001486	0.776665146	0.235679061	0.243294125	0.91049814
HMGXB3	0.295652684	0.073095362	0.760535084	0.111828692	0.96995755	0.243357284	0.91049814
INPP5B	0.950161486	0.246596787	0.077130368	0.286449205	0.344596727	0.243432004	0.91049814
LOC107131715	0.726046379	0.09219326	0.382342126	0.081947466	0.849384576	0.243263601	0.91049814
MRPL1	0.199283209	0.800458494	0.880827904	0.434254103	0.029113834	0.242931219	0.91049814
RHOT2	0.095083833	0.1337768	0.891486792	0.190054303	0.827013455	0.24332868	0.91049814
TBXAS1	0.931929194	0.291996098	0.012688593	0.529573163	0.975746225	0.243450481	0.91049814
TIGD4	0.316913012	0.18931209	0.091665591	0.799379709	0.405408465	0.243322646	0.91049814
TIMM17B	0.449755626	0.61955866	0.826360264	0.011747691	0.657571761	0.243090338	0.91049814
ELP6	0.832470023	0.977852284	0.041753995	0.256221088	0.205054898	0.243557221	0.910689803
APLP2	0.280768486	0.806968456	0.59957903	0.019984486	0.661265771	0.244188415	0.911185991
B3GNT7	0.324877454	0.84853567	0.390345736	0.030756612	0.542686346	0.244245151	0.911185991
CEP350	0.677096117	0.998966229	0.781242915	0.004050278	0.837972237	0.24407318	0.911185991
GREB1L	0.294041912	0.275079068	0.964232336	0.115003644	0.199536892	0.243820596	0.911185991
MDK	0.082768486	0.380708195	0.183725407	0.60101523	0.51449571	0.243850954	0.911185991
MTMR14	0.654240523	0.89716345	0.035201305	0.181626094	0.478572223	0.244236069	0.911185991
POLR2L	0.186709113	0.875969438	0.583989046	0.029609933	0.635048678	0.244238848	0.911185991
TBX1	0.192029615	0.566984389	0.018799326	0.981844818	0.89303609	0.244153482	0.911185991
VANGL2	0.606407116	0.406072505	0.01688434	0.700873959	0.614671129	0.243917224	0.911185991
ZNF516	0.465165099	0.38216492	0.913976749	0.064911014	0.170296741	0.244242763	0.911185991
CEP55	0.348790973	0.538800445	0.067441675	0.225847562	0.627790153	0.244307079	0.911209881
LOC539893	0.450077001	0.107173502	0.533354481	0.362358103	0.193132423	0.244536223	0.911857297
MANEAL	0.130978694	0.287863598	0.39342889	0.252206079	0.481562302	0.24461292	0.911936082
HCK	0.917414275	0.835013406	0.008747602	0.55026149	0.489063207	0.244728799	0.912160871
C23H6orf201	0.006416154	0.804825622	0.413556893	0.915340343	0.923798923	0.244891458	0.912240224
CARMIL2	0.936989553	0.064242323	0.893395009	0.118548494	0.284361352	0.245359115	0.912240224
CTCFL	0.223330414	0.602582246	0.309697369	0.354478334	0.122467917	0.245123749	0.912240224
FAT2	0.724973201	0.252236385	0.197311332	0.509950699	0.098362471	0.245158417	0.912240224
GCSAML	0.936339561	0.434918032	0.101235172	0.231539719	0.189857312	0.245320148	0.912240224
GMFG	0.472339048	0.979941803	0.013908039	0.453282082	0.621459403	0.245396468	0.912240224

INSIG2	0.038842297	0.329814405	0.64913187	0.943001401	0.231449358	0.245499927	0.912240224
IRS2	0.496353805	0.072719628	0.427158586	0.581460036	0.202437513	0.245490872	0.912240224
KDELR3	0.44911599	0.244209074	0.063432943	0.317743075	0.816735445	0.244869602	0.912240224
MARCH6	0.279596026	0.203728611	0.571654403	0.076303026	0.73066808	0.245528308	0.912240224
NUMBL	0.685698601	0.274562505	0.703304151	0.043429754	0.315657586	0.245512502	0.912240224
RBPMS	0.49071982	0.318669364	0.426240788	0.185087873	0.146552227	0.245036964	0.912240224
ST6GAL1	0.845730233	0.635126399	0.052445603	0.254593711	0.252692825	0.245324744	0.912240224
UNC13B	0.223267682	0.276847337	0.684277998	0.197721229	0.216198938	0.245038715	0.912240224
BRI3	0.418260641	0.332088703	0.122433302	0.120746143	0.885226103	0.245680259	0.912429049
LOC529196	0.435520032	0.478020222	0.368703225	0.027445452	0.862904694	0.245690328	0.912429049
RBBP5	0.469579058	0.051472115	0.448890829	0.203993588	0.821798388	0.24575634	0.912467715
LOC112442262	0.525395512	0.011637287	0.532423176	0.972905602	0.575904431	0.246091962	0.9133985
RCHY1	0.060865021	0.406938842	0.275828997	0.602761575	0.443025865	0.246118345	0.9133985
FAAH	0.798833439	0.04294165	0.919567147	0.111076478	0.521062553	0.246206651	0.913480125
LOC112446456	0.106941911	0.063934784	0.386134648	0.960334521	0.72035479	0.246251665	0.913480125
MTG1	0.470724231	0.237131841	0.441179655	0.05790614	0.640991137	0.246350186	0.913639073
EXOC3	0.374419399	0.855700994	0.153262829	0.060610416	0.614549318	0.246425744	0.913712806
ACSL1	0.736619872	0.622861622	0.997148272	0.11102039	0.036114682	0.246775129	0.914044568
CNKSR3	0.093853413	0.55811533	0.817888376	0.090070652	0.474796832	0.246630315	0.914044568
ELP4	0.443993788	0.987134281	0.413968037	0.100908552	0.10021163	0.246798642	0.914044568
LOC101903564	0.043006716	0.106825698	0.589395836	0.924617275	0.732080257	0.246680403	0.914044568
ST13	0.892653519	0.530956639	0.276073131	0.015586223	0.899483483	0.246780373	0.914044568
TSPYL6	0.338141446	0.994349601	0.757863652	0.009815982	0.733815126	0.246849401	0.914044568
BRF2	0.258927867	0.185699276	0.57550549	0.152655515	0.435011445	0.246988916	0.914084304
ENPP5	0.756366492	0.632024866	0.258130199	0.031531798	0.472054078	0.246931762	0.914084304
FAM98A	0.49236927	0.546140301	0.54272285	0.25024781	0.050370545	0.247119013	0.914084304
HMGB2	0.588377801	0.79686956	0.138165567	0.03518668	0.808296544	0.247305728	0.914084304
KCTD3	0.436903275	0.846515056	0.448645883	0.572287424	0.019399499	0.247287955	0.914084304
LOC513580	0.691645714	0.224621588	0.345931505	0.732879533	0.046756162	0.247251379	0.914084304
RPS4Y1	0.545857927	0.230362604	0.339846219	0.64598828	0.066694947	0.247222172	0.914084304
UBAP2	0.963324431	0.941953285	0.062315839	0.071451058	0.455511846	0.247171371	0.914084304
ITGA4	0.8621091	0.56365737	0.039694175	0.26082629	0.367003696	0.247565261	0.914694834
LOC112447492	0.006524662	0.499037058	0.712267942	0.957033142	0.832005804	0.247582381	0.914694834
APOBEC2	0.56607543	0.121099486	0.300600657	0.104425109	0.861586712	0.248061867	0.914734303
CC2D1B	0.126199359	0.296375235	0.201071798	0.31524109	0.782592098	0.248150455	0.914734303
CISD2	0.366819142	0.111503836	0.903133656	0.288255263	0.174029975	0.248001903	0.914734303
GPR68	0.747221392	0.066504895	0.049050131	0.887000759	0.857774058	0.24809794	0.914734303
GPX1	0.271869797	0.506223241	0.044521396	0.693501307	0.436390719	0.248085303	0.914734303
LOC104974669	0.361823299	0.912120426	0.284351966	0.861583354	0.022862769	0.247705745	0.914734303
MRPL54	0.329976777	0.753850542	0.523515286	0.023716204	0.600354606	0.248073161	0.914734303
PPP1R12B	0.493983308	0.392630291	0.421590801	0.194369931	0.116662482	0.248072182	0.914734303
RPL12	0.702468259	0.633553144	0.376626018	0.519633472	0.021289163	0.248080059	0.914734303
ZNF674	0.175776066	0.450089167	0.569831248	0.087783636	0.468532029	0.248074944	0.914734303
RNASEH2C	0.629241813	0.416121899	0.015591301	0.647766622	0.702593354	0.248321715	0.915117112
SEMA4B	0.144451411	0.573525594	0.756223457	0.415276845	0.071472495	0.248421591	0.915117112

ZBTB22	0.571861656	0.279354302	0.362685499	0.038288942	0.838125103	0.248409701	0.915117112
AP1M1	0.164105658	0.533960896	0.921099146	0.565735966	0.040930511	0.249035233	0.915491485
ARHGEF9	0.923189081	0.650236339	0.00827008	0.474186319	0.792166066	0.24876622	0.915491485
ATP1A2	0.061960444	0.609184674	0.676693787	0.795970751	0.091902199	0.249001256	0.915491485
BFSP2	0.772523946	0.551874581	0.013374167	0.965881785	0.340174946	0.249327257	0.915491485
ELMOD2	0.007172933	0.749952326	0.598118032	0.652460547	0.891731535	0.249232318	0.915491485
GBGT1	0.328344749	0.906610902	0.188025858	0.11233041	0.29803509	0.249351849	0.915491485
IDH2	0.118811352	0.47500869	0.412500582	0.106213402	0.757877668	0.249359999	0.915491485
LOC112448395	0.926330843	0.211474264	0.379587935	0.062029748	0.405948398	0.249260594	0.915491485
LRRC10B	0.58805954	0.219694431	0.751944789	0.031578282	0.60711597	0.248612644	0.915491485
MGAT4A	0.269765353	0.28990847	0.079131363	0.621775	0.486739111	0.249293668	0.915491485
PCBP2	0.692179717	0.312146581	0.317322217	0.054223398	0.501419899	0.248718603	0.915491485
RNF214	0.236444085	0.845337229	0.745738406	0.473007387	0.026529681	0.249131491	0.915491485
SAR1B	0.770552376	0.608079233	0.064896426	0.289136799	0.212917015	0.249229837	0.915491485
SOX7	0.236960286	0.785456092	0.357555543	0.039189447	0.716820795	0.249068859	0.915491485
STAP1	0.231168777	0.796952298	0.088580646	0.349490433	0.326813482	0.248710058	0.915491485
ATP5MC3	0.505023832	0.583372165	0.97096938	0.017271634	0.379700301	0.24949283	0.915646963
CXXC5	0.330324599	0.491870491	0.32196603	0.523632983	0.068674881	0.249825605	0.915646963
KLHL21	0.348126147	0.765394499	0.037990252	0.858271378	0.216221049	0.249654891	0.915646963
LOC786974	0.966139308	0.709573218	0.411685942	0.037935744	0.175480872	0.249672981	0.915646963
LOC787891	0.181537748	0.392993335	0.726712467	0.173381801	0.209386708	0.249893006	0.915646963
LRRN4	0.552753028	0.039731191	0.327079781	0.720455366	0.363734843	0.2499045	0.915646963
LYSMD3	0.229911226	0.55337988	0.775793045	0.019133089	0.99582738	0.249789749	0.915646963
POLR2C	0.394832271	0.086899215	0.122703214	0.611532807	0.729526657	0.249634914	0.915646963
RECQL4	0.203019611	0.11649213	0.145975211	0.668697874	0.814902455	0.249831936	0.915646963
LOC101903604	0.693124288	0.015673658	0.312660849	0.929635594	0.597741713	0.250233127	0.915906841
MSI2	0.797950111	0.922921075	0.299124182	0.019361651	0.442060435	0.250102679	0.915906841
PHETA1	0.057982634	0.560490212	0.518287035	0.235758081	0.474614521	0.250054505	0.915906841
PRRC2C	0.808408846	0.870890552	0.837151806	0.007674485	0.417358617	0.25025448	0.915906841
TGFB2	0.830723392	0.906814516	0.012536052	0.442469755	0.451399417	0.250148747	0.915906841
SPRYD3	0.102961246	0.494367746	0.842439019	0.045703081	0.963860638	0.250328675	0.915974111
ATRNL1	0.460860579	0.125338264	0.734417745	0.135407307	0.329349648	0.250517601	0.915994069
ERBB2	0.2513938	0.859196474	0.394242295	0.126488904	0.175781732	0.250613209	0.915994069
LOC101907483	0.771767872	0.272296031	0.721119216	0.058441191	0.213445321	0.250418039	0.915994069
MVP	0.284484137	0.585497578	0.31621145	0.325252128	0.110390672	0.250466424	0.915994069
PDPK1	0.96939177	0.235130533	0.039847242	0.263528665	0.790784879	0.250572914	0.915994069
CFAP20	0.871436661	0.022540057	0.40190439	0.254241677	0.947402313	0.251135129	0.916065481
EIF3K	0.263062181	0.665646898	0.256848953	0.2418698	0.174655304	0.251036427	0.916065481
LOC100848484	0.986598472	0.67909995	0.772847186	0.00469487	0.781910226	0.251093936	0.916065481
LOC112448269	0.980120355	0.856492122	0.73174949	0.004784806	0.645158015	0.250798262	0.916065481
NOB1	0.39765101	0.76652485	0.717920308	0.088497664	0.098111787	0.251040362	0.916065481
RHOU	0.189325336	0.179443437	0.16520515	0.414841877	0.816407413	0.251094281	0.916065481
RHPN1	0.978834866	0.416062889	0.0170152	0.310025265	0.884521584	0.251055378	0.916065481
STAG2	0.295857725	0.715946353	0.544766426	0.018201735	0.904067006	0.250964382	0.916065481
USP6NL	0.289709399	0.973122343	0.688508587	0.011022544	0.887944532	0.251026176	0.916065481

ANKS6	0.485798123	0.344833678	0.057337196	0.271820016	0.730316368	0.251472486	0.916164437
ANKZF1	0.658490078	0.057612039	0.627653612	0.172520688	0.473017105	0.253789174	0.916164437
ARMC1	0.664447192	0.235373476	0.232760013	0.204387509	0.258005813	0.25229384	0.916164437
ARVCF	0.201718417	0.939933526	0.704885455	0.040858808	0.351158023	0.252163938	0.916164437
CAPN6	0.033803025	0.909483794	0.170733665	0.690629008	0.526986386	0.251703128	0.916164437
CCDC124	0.22369823	0.409581138	0.08345147	0.324516342	0.787716145	0.254510086	0.916164437
CCDC71L	0.05867722	0.170990213	0.441957047	0.619521264	0.70038806	0.252577569	0.916164437
CD164	0.162553682	0.399472036	0.115563335	0.861163666	0.299828482	0.253438462	0.916164437
CDS2	0.498112415	0.162321988	0.167095581	0.226900694	0.626193875	0.252295001	0.916164437
COLEC12	0.378156507	0.697402162	0.270775537	0.033802166	0.797810871	0.252688512	0.916164437
CRB3	0.128512647	0.209909857	0.354582138	0.460966226	0.436023673	0.252481311	0.916164437
CRYAB	0.469734806	0.620721741	0.053899371	0.122285663	0.999438176	0.252365672	0.916164437
CSRP2	0.282329208	0.174464764	0.996985958	0.101050749	0.390585027	0.253479742	0.916164437
CUEDC2	0.174483672	0.987487057	0.446296665	0.027701298	0.895558102	0.251531381	0.916164437
CXCR1	0.767138277	0.096554373	0.719173587	0.048341683	0.749610099	0.252978833	0.916164437
DCLRE1B	0.953032565	0.199942641	0.034791023	0.661262529	0.44462153	0.254170166	0.916164437
DHX37	0.69753849	0.785315066	0.531484626	0.021309425	0.310996649	0.252920548	0.916164437
DNAJC30	0.564063296	0.914957747	0.436808648	0.0140938	0.610054054	0.253482241	0.916164437
EPYC	0.850913527	0.499034305	0.006003863	0.768877052	0.984121599	0.252898521	0.916164437
FILIP1	0.265237505	0.283092503	0.83567953	0.041275576	0.754064661	0.254414149	0.916164437
FNDC4	0.365235838	0.326564399	0.022329061	0.969088131	0.756685807	0.254410387	0.916164437
GIMAP5	0.502909211	0.833382979	0.084680024	0.440831194	0.12179811	0.25139755	0.916164437
IK	0.596882307	0.101024094	0.328053101	0.149771757	0.647356431	0.252186966	0.916164437
IPO4	0.463377939	0.537035407	0.236461119	0.283451206	0.117123691	0.254447148	0.916164437
LOC101907886	0.833801492	0.630694576	0.018025112	0.401972117	0.501302778	0.251686763	0.916164437
LOC112449619	0.02541673	0.196225992	0.941781382	0.466664731	0.869717045	0.251448527	0.916164437
LOC534578	0.973640949	0.128661998	0.161969186	0.120362321	0.799568507	0.254392286	0.916164437
LOC781256	0.034578618	0.664161216	0.416379444	0.345190772	0.585159206	0.253054329	0.916164437
MTFR1L	0.790204725	0.34171227	0.62088801	0.027227636	0.428034715	0.254470229	0.916164437
MYH7B	0.17726823	0.324967434	0.667450083	0.387015639	0.129294293	0.252572379	0.916164437
NDUFAB1	0.855822371	0.573888354	0.929376912	0.004837545	0.872206555	0.252699382	0.916164437
NFKBIZ	0.085028593	0.195679748	0.285132979	0.917813647	0.441393263	0.25244288	0.916164437
NIN	0.771806468	0.758405798	0.06117573	0.120245127	0.448239719	0.252959199	0.916164437
NPAS3	0.262817992	0.916113727	0.142460145	0.058360083	0.972606284	0.254030124	0.916164437
NUDT19	0.637777603	0.433371139	0.202719194	0.050204578	0.680211493	0.251899559	0.916164437
OST4	0.211914772	0.223817817	0.929815428	0.055417817	0.790951048	0.253152715	0.916164437
PDCL3	0.522000202	0.286577974	0.097731022	0.82110183	0.160397273	0.252669072	0.916164437
PTPRJ	0.574154935	0.611507242	0.142966392	0.044598982	0.857406709	0.25228433	0.916164437
RP2	0.341521187	0.56867175	0.017735262	0.783143406	0.716054444	0.2530545	0.916164437
S1PR3	0.72112755	0.512945872	0.589109842	0.012234651	0.728398377	0.253715502	0.916164437
SCMH1	0.033904228	0.809929176	0.526593457	0.609959425	0.221372229	0.254384538	0.916164437
SEC23B	0.176121592	0.088992726	0.743169248	0.437468337	0.383283667	0.254418969	0.916164437
SLC35G2	0.399006988	0.226271882	0.118173686	0.193117831	0.925766161	0.251517821	0.916164437
SPTLC1	0.901589779	0.114217623	0.067883025	0.521164499	0.528701069	0.252710967	0.916164437
SRP19	0.189625965	0.498213502	0.530986946	0.234347969	0.16506744	0.25362469	0.916164437

SSFA2	0.563165898	0.113330704	0.337604243	0.134820718	0.67128242	0.254229435	0.916164437
STRA6	0.603885552	0.149521132	0.088913895	0.670452525	0.360770775	0.253711477	0.916164437
SYNC	0.048359856	0.81969616	0.315625025	0.351250728	0.442483464	0.2538807	0.916164437
TCF15	0.792981372	0.730897057	0.452902887	0.008348769	0.883592002	0.253363956	0.916164437
TCP11	0.081198409	0.506917273	0.771914764	0.141502628	0.425189608	0.251783994	0.916164437
TINAGL1	0.123135082	0.215064006	0.277148658	0.825399584	0.32136132	0.254021429	0.916164437
TM2D1	0.283572312	0.373301832	0.55070027	0.14752978	0.221530184	0.251376111	0.916164437
TMEM52	0.526259284	0.287819059	0.065441859	0.907797699	0.215202132	0.253367369	0.916164437
TMEM87A	0.414166283	0.291099388	0.023507546	0.749402114	0.90876174	0.252965201	0.916164437
TUG1	0.078336503	0.222377721	0.287024519	0.615875253	0.634719368	0.254511832	0.916164437
UBL3	0.899447369	0.796683104	0.08104908	0.05079253	0.658174942	0.25369007	0.916164437
UBL5	0.441070868	0.278502166	0.394532424	0.178319008	0.226023299	0.254432796	0.916164437
USP5	0.399690599	0.930948452	0.620270139	0.055057785	0.153636101	0.254368081	0.916164437
ZCCHC17	0.219864241	0.520124693	0.359208398	0.128925937	0.369041038	0.25450461	0.916164437
ZNF185	0.196133883	0.224769566	0.950697727	0.241584572	0.191282665	0.253386271	0.916164437
ADCY6	0.790516171	0.814828901	0.50463282	0.007522261	0.80189568	0.254900945	0.91640285
AP1G2	0.275200228	0.083388331	0.134279181	0.964768513	0.659567783	0.254908445	0.91640285
E2F6	0.081574439	0.109488738	0.322276048	0.723142638	0.94078533	0.254743869	0.91640285
GALNT9	0.313179449	0.30267513	0.887645108	0.038667335	0.601783466	0.254723351	0.91640285
LOC100847410	0.34421912	0.732513442	0.375249797	0.033467219	0.62038188	0.255137149	0.91640285
LOC112447070	0.043303673	0.831058726	0.924206121	0.066270096	0.890183556	0.254987219	0.91640285
NSFL1C	0.424514555	0.788411158	0.241162569	0.170305485	0.142610526	0.254877228	0.91640285
SAP18	0.210428961	0.308818183	0.523900436	0.220517161	0.26190355	0.2552484	0.91640285
SCEL	0.348693735	0.040705236	0.698763897	0.245957234	0.806402448	0.255303993	0.91640285
STK36	0.266147646	0.219035104	0.405906827	0.141271091	0.587762929	0.255156607	0.91640285
TBK1	0.303312777	0.917633548	0.044070659	0.390142568	0.410820116	0.255232372	0.91640285
TOX4	0.556959084	0.051912416	0.815774597	0.167447746	0.496635155	0.254947146	0.91640285
VSIG10L	0.246107289	0.046453187	0.428348061	0.665827153	0.602822808	0.255204895	0.91640285
RARRES2	0.417923916	0.451425641	0.239244466	0.125351382	0.347890815	0.255378297	0.916469107
NAT1	0.200669292	0.362673086	0.218488533	0.229004891	0.541021596	0.255488397	0.916549954
TRAPPC9	0.563968082	0.892422617	0.412788206	0.262149592	0.036179907	0.255512525	0.916549954
ANXA7	0.750410989	0.032572481	0.180442155	0.694792589	0.643623396	0.255628136	0.91676428
FAIM	0.882605016	0.010798912	0.528320348	0.660653031	0.593864293	0.255835912	0.91730897
CD8B	0.04623653	0.798612859	0.472287473	0.168136627	0.674154332	0.255905803	0.917341891
PRR36	0.284624305	0.102446893	0.954784231	0.394782025	0.179926636	0.25595689	0.917341891
ERBIN	0.910465493	0.895647858	0.854999074	0.010723051	0.264648111	0.256020961	0.917370431
RPS6	0.514505574	0.523500302	0.864768881	0.012718517	0.668198255	0.256076652	0.917370431
ALDH3A2	0.464381047	0.34769389	0.295481157	0.072006494	0.57697421	0.25624223	0.917763255
DVL1	0.577908417	0.873177961	0.397393267	0.010649429	0.928742827	0.256320602	0.917826457
FAM69A	0.192283046	0.486050592	0.594520851	0.046178785	0.774440146	0.25655349	0.917826457
GBA2	0.721847027	0.473513443	0.070789849	0.090624856	0.907244691	0.256696386	0.917826457
LAMB1	0.349327886	0.29418327	0.610520093	0.037242478	0.852420758	0.256845978	0.917826457
LOC101905897	0.289398835	0.251761423	0.376697712	0.121033308	0.599470144	0.256819582	0.917826457
LOC107131567	0.055234674	0.246322206	0.81109475	0.568265941	0.317179314	0.256674448	0.917826457
NPY5R	0.075314679	0.398542961	0.547617046	0.399082161	0.303662953	0.256858006	0.917826457

PTMS	0.030315005	0.645262099	0.867091119	0.558186779	0.209914923	0.256571048	0.917826457
PURG	0.262264933	0.777211338	0.379035201	0.086137925	0.29848359	0.256511757	0.917826457
SUGT1	0.195199643	0.451606302	0.447985315	0.050997558	0.989221221	0.256875079	0.917826457
VTI1B	0.044330742	0.360738743	0.292821372	0.966877913	0.438624796	0.256480813	0.917826457
CGREF1	0.199291167	0.466969686	0.156022063	0.229435353	0.598440974	0.256960497	0.917844814
FBXW11	0.745608902	0.381242988	0.225263176	0.125956924	0.247246218	0.256992074	0.917844814
DUSP8	0.468735102	0.212427158	0.786286755	0.871163393	0.029261694	0.257095791	0.917928288
LOC112449565	0.463057098	0.005818241	0.934635879	0.884784666	0.896027763	0.257127313	0.917928288
CNIH1	0.11007683	0.068594727	0.852427337	0.853640108	0.363671461	0.257241821	0.918137349
RSAD1	0.935421846	0.023431324	0.140094912	0.713263473	0.913046029	0.25733949	0.918286231
C13H20orf27	0.229410601	0.677786722	0.901848814	0.020617513	0.695647631	0.25805472	0.918409193
CNTNAP4	0.389823218	0.461339305	0.712043036	0.26274766	0.059718098	0.257932386	0.918409193
DCAF10	0.130582762	0.643410305	0.928685121	0.027532169	0.93669092	0.258115874	0.918409193
DDX47	0.454463464	0.286396833	0.166624151	0.913691063	0.101993837	0.258661099	0.918409193
EIF4E2	0.549771009	0.118440508	0.548593049	0.211233129	0.266902401	0.258221392	0.918409193
FCRL5	0.290077597	0.759718321	0.296807564	0.469274831	0.06566121	0.258316374	0.918409193
HECTD1	0.631278038	0.404354208	0.024087401	0.333530246	0.985454111	0.258651268	0.918409193
HK1	0.1378973	0.495939522	0.150540071	0.491498357	0.399396159	0.258656533	0.918409193
KLHL3	0.257966858	0.60481425	0.111792957	0.121307616	0.947783696	0.257691209	0.918409193
LOC100336208	0.725324488	0.356911216	0.366438515	0.0232304	0.910073115	0.257699503	0.918409193
LOC104970812	0.428311356	0.473265278	0.961732506	0.024644089	0.420208241	0.258522487	0.918409193
LOC107132531	0.575080099	0.097822692	0.758817131	0.157888291	0.297579633	0.25770855	0.918409193
LOC112447371	0.199564991	0.03575483	0.331654436	0.8518102	0.995140817	0.257729768	0.918409193
LOC782966	0.056805165	0.399691734	0.686457354	0.164306509	0.787963476	0.258462633	0.918409193
LOC786474	0.08648939	0.928877881	0.780279101	0.116354471	0.276944268	0.258594583	0.918409193
MON2	0.8305434	0.089653865	0.791815612	0.383011873	0.088934439	0.257874593	0.918409193
RNF7	0.388158288	0.101082865	0.175020126	0.361167711	0.809491421	0.257833877	0.918409193
RTL6	0.335144236	0.592634435	0.011024572	0.930722131	0.986804268	0.258044842	0.918409193
SAP130	0.765759032	0.854670801	0.005631118	0.68636294	0.798232555	0.258543468	0.918409193
VOPP1	0.098725494	0.141964178	0.56620113	0.471626792	0.534935705	0.257485343	0.918409193
WNT9B	0.325457673	0.675877349	0.562550105	0.049242278	0.330516109	0.258223536	0.918409193
YEATS2	0.852479242	0.633159789	0.256973657	0.076501037	0.190166321	0.258462421	0.918409193
ZBED5	0.237331622	0.352743096	0.261190592	0.096785652	0.949117319	0.257893201	0.918409193
BAZ2B	0.539001273	0.183013249	0.291293248	0.134739253	0.523589095	0.259037358	0.91895705
CEP85L	0.196444657	0.892412281	0.944600755	0.022590366	0.541620967	0.258975622	0.91895705
FGF14	0.345024073	0.30032425	0.063619414	0.377395797	0.816040265	0.259224526	0.91895705
GEMIN2	0.236290531	0.65993909	0.940595072	0.021417195	0.646555436	0.259277222	0.91895705
KRT19	0.211217227	0.061213299	0.900498621	0.515173763	0.338306634	0.25916247	0.91895705
METTL4	0.053049361	0.889612654	0.127392024	0.338650295	0.997913406	0.259319365	0.91895705
MTCP1	0.059338563	0.078382405	0.976092817	0.700329828	0.63885055	0.259285105	0.91895705
NINJ2	0.241896197	0.047520894	0.874021059	0.311129845	0.649017731	0.259136966	0.91895705
SNAPC2	0.284080786	0.536941715	0.40633225	0.0360799	0.906833926	0.259082201	0.91895705
PXMP4	0.577278822	0.7155531	0.905265914	0.122289142	0.044493201	0.259497152	0.919388549
MRPS16	0.2839287	0.482067309	0.510715037	0.038585733	0.75513756	0.259630572	0.919662708
ZNF135	0.082514676	0.397234736	0.656682284	0.105128237	0.900829811	0.259730665	0.919818719

INPP4B	0.684449836	0.417605967	0.369432243	0.544049506	0.035510749	0.259830241	0.919956147
PHPT1	0.333004851	0.414017345	0.75751137	0.020784007	0.940225341	0.259881585	0.919956147
NCF4	0.403606156	0.154169461	0.101720674	0.877950733	0.368048156	0.260147095	0.92069743
TBC1D4	0.377625209	0.198282591	0.05825367	0.746865986	0.628267467	0.260238256	0.920821479
GRPEL1	0.147261433	0.430425424	0.166087676	0.810996494	0.240066011	0.260416607	0.921055374
OSR2	0.645902628	0.77596034	0.033439588	0.328768247	0.371889857	0.260387071	0.921055374
PHLDB2	0.554562563	0.750234283	0.773532782	0.261707436	0.024349558	0.260491484	0.921121687
CSF1	0.880244045	0.148287823	0.040761637	0.512897297	0.752268423	0.260615715	0.921143424
FBH1	0.782789035	0.140276448	0.571636983	0.086040955	0.380144128	0.260627235	0.921143424
SELENOP	0.929085191	0.894678489	0.015914061	0.189490432	0.819307141	0.26066602	0.921143424
ACTR1A	0.270873411	0.30296759	0.563332483	0.185480587	0.240052191	0.260953295	0.921227317
LOC112443751	0.190739699	0.08772722	0.366780993	0.944358693	0.355234317	0.260982991	0.921227317
LOC112444841	0.232921251	0.095353365	0.694123981	0.289825125	0.460002498	0.260764508	0.921227317
NCAM2	0.225933247	0.048489656	0.727172794	0.361282046	0.714935239	0.260909484	0.921227317
PLK4	0.645180687	0.144783129	0.324773891	0.125408162	0.540681187	0.260871187	0.921227317
PLPP1	0.804566801	0.371850956	0.029462783	0.393205048	0.594238806	0.261026569	0.921227317
HEXIM2	0.871116276	0.069663961	0.405442984	0.099767688	0.839809752	0.261142283	0.921437541
PTPRE	0.338064011	0.543790319	0.273459164	0.821655886	0.049980859	0.261325078	0.921488152
SAFB	0.856235939	0.57200657	0.598384372	0.016651082	0.422826828	0.261256518	0.921488152
SPECC1	0.939323601	0.177432759	0.047701733	0.63059425	0.411734662	0.26130599	0.921488152
PCBP1	0.382235128	0.320476807	0.651526226	0.025917789	0.998549619	0.261385904	0.921504635
HCRTR1	0.566505151	0.188824966	0.076786561	0.386164134	0.652492075	0.261637563	0.921549356
KLHDC2	0.07871707	0.550382678	0.939485312	0.347616623	0.146520037	0.261847825	0.921549356
LOC100848405	0.754559011	0.967307149	0.09637949	0.224726467	0.131124641	0.261836096	0.921549356
PHLDA2	0.280658428	0.433808508	0.403844434	0.102755848	0.410284662	0.261836477	0.921549356
SRSF12	0.205806717	0.535795489	0.172412194	0.678743509	0.160538491	0.261758034	0.921549356
TBRG1	0.865449871	0.044739001	0.825473565	0.12268275	0.528602561	0.261825477	0.921549356
YTHDF1	0.345976385	0.573281375	0.236073718	0.116048546	0.380555562	0.261528659	0.921549356
ZNF566	0.32099524	0.211822233	0.177621018	0.550155246	0.311706159	0.261724328	0.921549356
CTSK	0.47791694	0.29859748	0.13702188	0.317090198	0.334806845	0.26201727	0.92155281
GAS8	0.129609324	0.250641245	0.403545872	0.222272318	0.712048549	0.261950876	0.92155281
TMEM45A	0.606411125	0.646434411	0.010983424	0.504499478	0.955503328	0.261993098	0.92155281
TERF1	0.064124801	0.189059717	0.660439513	0.388765025	0.667549247	0.262139953	0.92178675
FOXO6	0.983317997	0.277885542	0.559023427	0.05301376	0.256721654	0.262201601	0.921806013
LOC107132697	0.351412736	0.76742496	0.105663247	0.967996436	0.075424686	0.262295507	0.921938651
LOC101907566	0.28598979	0.538312729	0.647607013	0.02132486	0.981001821	0.262611455	0.922851519
NCS1	0.755223469	0.254328707	0.032950466	0.488385483	0.676512061	0.262936376	0.923795517
MAFB	0.735246907	0.961161698	0.023948792	0.289256956	0.427719325	0.263106582	0.924195659
DCAF5	0.747845152	0.674795797	0.438439584	0.077841188	0.121656366	0.263188666	0.924207592
ENPP2	0.017421464	0.403313948	0.542909099	0.847967858	0.649363148	0.263504194	0.924207592
LOC112447756	0.381532664	0.299539067	0.0502138	0.659854423	0.554580271	0.263474722	0.924207592
LOC787851	0.363462223	0.137325796	0.727064888	0.187814561	0.308163083	0.263496158	0.924207592
MGAT3	0.150064564	0.208450497	0.244361494	0.409974838	0.670080589	0.263468101	0.924207592
PRKRIP1	0.397127442	0.039066154	0.817767671	0.181857337	0.909561854	0.263388167	0.924207592
ZNF277	0.350026427	0.857960972	0.16181925	0.110927583	0.388840073	0.263238429	0.924207592

BCL3	0.05199077	0.774815343	0.231155103	0.770431957	0.292937538	0.263566876	0.924229915
CCNG2	0.071879893	0.077277839	0.883853756	0.678684278	0.635260673	0.264478337	0.924753052
CYB561D2	0.62365319	0.899961404	0.467550927	0.116832679	0.069283725	0.26492508	0.924753052
DESI1	0.372412602	0.613273051	0.43863147	0.099391939	0.213027546	0.264741942	0.924753052
ERGIC1	0.148237904	0.743518323	0.137712115	0.291317775	0.480514338	0.264955752	0.924753052
GJA5	0.973983391	0.065040444	0.525517355	0.128257467	0.493814169	0.263983943	0.924753052
GOLGA3	0.750658173	0.729807625	0.027907995	0.138584907	0.999441567	0.26453383	0.924753052
GTPBP3	0.12719816	0.079520936	0.803604778	0.295577981	0.878729468	0.264148382	0.924753052
KIFAP3	0.409466508	0.602834312	0.945174841	0.821648254	0.011076211	0.264870748	0.924753052
KLC2	0.381121804	0.696294354	0.032634571	0.245026191	0.996338041	0.264329803	0.924753052
LOC100337053	0.035246204	0.351125861	0.658003957	0.644226419	0.403935187	0.264621837	0.924753052
MAMDC4	0.237284317	0.059184387	0.360592401	0.628923021	0.662536052	0.264080609	0.924753052
MTERF2	0.356244189	0.880133964	0.630308733	0.019661486	0.545545105	0.264663613	0.924753052
NFATC3	0.568754769	0.684026925	0.477970486	0.019507173	0.584970897	0.264789568	0.924753052
NR2F2	0.403669271	0.79189166	0.008175636	0.850242068	0.95477996	0.264769385	0.924753052
PIK3R4	0.992709238	0.933733876	0.038739101	0.060145888	0.976442429	0.264006975	0.924753052
PRC1	0.687141874	0.091347396	0.187265603	0.392768516	0.457537304	0.26421598	0.924753052
SAG	0.413243396	0.842423867	0.362538631	0.01898995	0.883895888	0.264581503	0.924753052
SEC63	0.773761211	0.124341533	0.170395556	0.160196169	0.808671413	0.264899976	0.924753052
SERINC3	0.815784891	0.50037156	0.483473954	0.01504279	0.70934216	0.263825691	0.924753052
SERPINA5	0.405950332	0.138615997	0.619043389	0.280664484	0.216229229	0.264316788	0.924753052
SNX11	0.52638728	0.289657304	0.817745059	0.126689592	0.13377063	0.264258507	0.924753052
UPK3B	0.863726373	0.016771834	0.878772626	0.736809271	0.225947771	0.264634257	0.924753052
AEN	0.494685224	0.542765988	0.313956655	0.066571225	0.379560827	0.265272484	0.925015057
LOC513329	0.877488479	0.597707261	0.276282959	0.235318289	0.062479281	0.265300746	0.925015057
PAGR1	0.336311525	0.029191038	0.579712155	0.768563461	0.487027539	0.265289388	0.925015057
PTRH2	0.636690878	0.55918074	0.164810976	0.333485193	0.108866365	0.265289473	0.925015057
REC114	0.111759564	0.654064219	0.586455756	0.146364866	0.339577764	0.265312648	0.925015057
FAM84A	0.102379501	0.331320401	0.38294336	0.432024656	0.380017983	0.265427392	0.92521855
LOC100141258	0.190323652	0.810008888	0.619962338	0.158110839	0.141258958	0.265549525	0.925259987
PSMB2	0.342655893	0.464145011	0.547454491	0.471669392	0.051980025	0.265552041	0.925259987
COQ8A	0.098761517	0.945813698	0.875941195	0.259562671	0.101125496	0.266325199	0.926061705
FOXO3	0.922468801	0.558433019	0.289129977	0.399783894	0.036088496	0.266395285	0.926061705
ITGB3BP	0.437329005	0.557272864	0.105932122	0.162814236	0.509447972	0.265951146	0.926061705
KCTD11	0.268142378	0.981467882	0.047950047	0.927429417	0.183118115	0.26605281	0.926061705
LOC101903193	0.618205477	0.905942855	0.277183589	0.043349504	0.31926513	0.266373801	0.926061705
MLIP	0.017845993	0.751611214	0.591843584	0.376153989	0.719663026	0.266402858	0.926061705
MPP5	0.663572552	0.739592062	0.021647609	0.447172439	0.452094642	0.266332177	0.926061705
SELENOT	0.139274312	0.661164657	0.903589025	0.887374718	0.029070953	0.266250871	0.926061705
SNAPC4	0.274742533	0.044747184	0.357444561	0.556385624	0.878572003	0.266349372	0.926061705
TAF1D	0.065644877	0.167289953	0.394845481	0.584237432	0.846523362	0.266135943	0.926061705
VPS37C	0.076255232	0.47325012	0.463442262	0.233730741	0.549131911	0.266259854	0.926061705
AK6	0.131426655	0.938118037	0.487850494	0.144234558	0.247904752	0.266504472	0.92607217
LOC101903126	0.456111138	0.526898788	0.318635731	0.622594159	0.045121764	0.266534304	0.92607217
ZCCHC6	0.227860548	0.294818235	0.471117929	0.109962615	0.618335817	0.266575159	0.92607217

LOC104973099	0.056314744	0.990043762	0.402985652	0.132124867	0.725219903	0.266633255	0.926077958
CPT1C	0.085931974	0.382707583	0.325689541	0.224857187	0.8948069	0.266762935	0.926332316
PPP4R2	0.857843778	0.858152259	0.539698951	0.01719858	0.315743744	0.266906864	0.926636038
JDP2	0.276235888	0.71280347	0.885404265	0.038944319	0.318397702	0.267158013	0.92690022
LOC104974348	0.954553018	0.054352869	0.260341433	0.512257643	0.312218201	0.267071814	0.92690022
NAA15	0.477162486	0.306320646	0.205312574	0.533770274	0.134973084	0.267173932	0.92690022
SLC25A26	0.808959596	0.869278144	0.606983233	0.005094258	0.994569763	0.267208881	0.92690022
FCGR3A	0.611907652	0.874772547	0.007558123	0.809728255	0.660727082	0.267320087	0.927090016
CANT1	0.510933067	0.160379105	0.179656822	0.245933968	0.598417959	0.267444374	0.927325085
LOC783033	0.870239355	0.311801441	0.032789213	0.556553722	0.438239742	0.267646981	0.927799541
UBE2W	0.117648784	0.121601965	0.271354252	0.59782656	0.935378475	0.26769428	0.927799541
FBXL14	0.09018289	0.906136923	0.507805753	0.076524483	0.683954983	0.267757997	0.927824429
COX11	0.155259472	0.846180589	0.381433332	0.080531192	0.539854607	0.268152215	0.927891837
DCLK2	0.86281071	0.466008236	0.481308269	0.548514781	0.020514278	0.268092125	0.927891837
EEF1E1	0.626442304	0.585268607	0.270613722	0.290648648	0.07556098	0.268173236	0.927891837
EIF3E	0.061057983	0.897254131	0.50024906	0.162962081	0.487673388	0.268116549	0.927891837
LIX1	0.500284459	0.673815053	0.591822288	0.086282392	0.126580552	0.268169162	0.927891837
MYPOP	0.725654009	0.433146374	0.365493182	0.020745673	0.913466189	0.268058675	0.927891837
RTL1	0.713655945	0.125060123	0.477523781	0.060490932	0.843459711	0.26790943	0.927891837
ABHD13	0.423293796	0.17807822	0.245599756	0.142520828	0.832783054	0.269248171	0.927894371
BBC3	0.360469286	0.445014315	0.126853292	0.782823163	0.138148734	0.269444572	0.927894371
CACHD1	0.244389099	0.46448747	0.74198511	0.120955864	0.214375495	0.26846865	0.927894371
CCR6	0.796180057	0.489470542	0.140006672	0.344412825	0.116764728	0.269066786	0.927894371
COQ9	0.972886455	0.80961941	0.559389771	0.0054361	0.915155434	0.268937378	0.927894371
EIF2B2	0.418491648	0.394279062	0.429764663	0.173770448	0.178631581	0.269474412	0.927894371
FAM46B	0.164791725	0.354668432	0.521393842	0.089829829	0.800385604	0.268879395	0.927894371
GNG7	0.878936441	0.807250158	0.086056326	0.060672814	0.590630333	0.268706432	0.927894371
LOC100196901	0.049328231	0.92932795	0.7854608	0.078693636	0.775330847	0.269225948	0.927894371
LOC112448520	0.038294908	0.127582836	0.852615003	0.992609906	0.53207994	0.269411178	0.927894371
NME2	0.211529673	0.641141546	0.798228022	0.396513636	0.051094255	0.269009075	0.927894371
PLEKHH3	0.311023103	0.398609609	0.178875533	0.354157993	0.279588844	0.269164572	0.927894371
PSMA1	0.748159884	0.481543363	0.362203068	0.218990933	0.076504407	0.268598991	0.927894371
REXO4	0.139594715	0.939036905	0.301775666	0.113045461	0.490607068	0.269050585	0.927894371
RNF103	0.194122537	0.727316145	0.81073514	0.074959973	0.254223675	0.268312371	0.927894371
SEPT6	0.745243913	0.387141824	0.950747218	0.039912799	0.199391353	0.268409318	0.927894371
SLC46A2	0.457650737	0.846936638	0.057188575	0.241103909	0.411339167	0.269309658	0.927894371
THEM6	0.529867519	0.949123432	0.925457405	0.006190951	0.757338128	0.268362538	0.927894371
TMEM59	0.364870289	0.707629958	0.051934556	0.22383243	0.729090222	0.268720352	0.927894371
TNFRSF12A	0.111217606	0.616294616	0.770751895	0.473475468	0.087985615	0.26945378	0.927894371
TRPM3	0.214004916	0.794312585	0.21471277	0.651527516	0.091898653	0.268545969	0.927894371
WDR97	0.104756924	0.317794412	0.422687367	0.621442598	0.251365633	0.269296788	0.927894371
ZNF768	0.830556824	0.413364841	0.092765261	0.087387394	0.78871366	0.269119668	0.927894371
TCEA2	0.621254426	0.476335838	0.019804256	0.894131108	0.42038228	0.269572561	0.928037611
SUGP2	0.305956662	0.168812571	0.581775006	0.092693998	0.791607732	0.269689388	0.928163157
WSCD1	0.808985138	0.052302561	0.812953261	0.115587234	0.554693453	0.269722144	0.928163157

SLC8A2	0.089072543	0.448131293	0.86711624	0.177479658	0.359343801	0.269838633	0.92836935
DOCK8	0.476665954	0.704297734	0.209170016	0.03563456	0.885017253	0.270256007	0.929610424
AGTR1	0.513930319	0.439585974	0.028256599	0.425239071	0.817767857	0.270564094	0.929695843
C1QTNF3	0.830290756	0.06284835	0.458506147	0.099686465	0.929738437	0.270426089	0.929695843
LRRC45	0.060709464	0.155374731	0.355889886	0.93145599	0.709809386	0.270541933	0.929695843
RPRD2	0.492047336	0.393231448	0.221826033	0.072268748	0.715183915	0.270476449	0.929695843
TMEM106C	0.37842027	0.577985436	0.612460477	0.184830913	0.089538508	0.270392731	0.929695843
ERCC6L	0.543134074	0.096868949	0.11878006	0.356885184	0.997221778	0.270808893	0.929702032
PIK3R1	0.987695234	0.153220801	0.077792597	0.955446597	0.19751874	0.270671736	0.929702032
POLR3G	0.604041959	0.54770822	0.172903586	0.313655014	0.12399905	0.270849151	0.929702032
PPM1B	0.008609509	0.938077051	0.775497496	0.600705777	0.591271475	0.270835799	0.929702032
SLF2	0.260752207	0.990391538	0.181653054	0.182490966	0.259855293	0.270837147	0.929702032
BNC1	0.725953307	0.029039367	0.718803053	0.807501562	0.182046269	0.271009606	0.929706381
IRF7	0.687466482	0.119889674	0.218902545	0.250539996	0.492497187	0.270930563	0.929706381
LAMC2	0.38339483	0.321176902	0.288188801	0.29472773	0.212999439	0.271020372	0.929706381
LIMS2	0.21635576	0.322445428	0.356642414	0.537054923	0.166881378	0.271144386	0.929937411
IKBKE	0.160766599	0.372452891	0.11383557	0.723389509	0.452897333	0.271332615	0.93019418
TNFSF14	0.914832404	0.48546205	0.016261622	0.400959798	0.771123074	0.271323121	0.93019418
LIMD2	0.141515146	0.615674851	0.74289587	0.249317998	0.138576538	0.27151368	0.930534065
LOC101908113	0.06462892	0.931684731	0.872748121	0.166991557	0.255023089	0.271612865	0.930534065
LTBP3	0.285986049	0.438482678	0.056262315	0.32691711	0.970643547	0.271658565	0.930534065
THY1	0.951481413	0.099772763	0.092666371	0.278840741	0.912449433	0.271624975	0.930534065
NT5DC2	0.029270618	0.181129689	0.787459147	0.671635407	0.798918953	0.271740027	0.93059834
TCAP	0.520670389	0.122774371	0.79259717	0.515426437	0.085815619	0.271790741	0.93059834
PRKAG2	0.067045751	0.74321919	0.20236568	0.945864958	0.235157413	0.271897003	0.930767983
ANKRD45	0.12571141	0.50994276	0.244406065	0.244950473	0.585197566	0.272068672	0.930967259
CALR	0.06705635	0.251092211	0.331666428	0.466770101	0.861549799	0.272059485	0.930967259
TMED5	0.935801973	0.15720777	0.144093899	0.165617733	0.640091147	0.272147168	0.931041727
AAGAB	0.166159778	0.542110254	0.479104248	0.327190711	0.160481039	0.273226792	0.931434333
ANKRD55	0.811436753	0.267783536	0.042164733	0.357142222	0.6892338	0.272607613	0.931434333
B4GALT2	0.240029709	0.807800279	0.022385315	0.638215279	0.812958771	0.272420278	0.931434333
CHCHD4	0.427504528	0.367336226	0.308359316	0.291887751	0.16013568	0.273076515	0.931434333
CRIM1	0.387974305	0.330426794	0.70920124	0.034404835	0.723992742	0.273147016	0.931434333
DENND5B	0.409350938	0.474513368	0.218862328	0.055194137	0.965461736	0.273189428	0.931434333
HOXD9	0.301383865	0.849021906	0.194571462	0.073983536	0.612935152	0.272748528	0.931434333
LOC112445078	0.807955175	0.336739671	0.092493409	0.143784669	0.622282517	0.272397779	0.931434333
MED17	0.626106884	0.420288574	0.586226117	0.502190985	0.02910686	0.272586667	0.931434333
OSBPL9	0.563712511	0.224909816	0.809844134	0.072596622	0.303261484	0.272907887	0.931434333
PAK3	0.20333034	0.80472783	0.298198099	0.111256738	0.416208992	0.272845952	0.931434333
RIT1	0.314909466	0.723700818	0.694529113	0.023076688	0.617192796	0.272558033	0.931434333
SKIL	0.344293834	0.073884656	0.443985845	0.592437214	0.337159441	0.272647798	0.931434333
SYNGAP1	0.185121528	0.28624596	0.089043893	0.481759205	0.992813198	0.27269787	0.931434333
TMEM173	0.949589725	0.260908035	0.025648939	0.5261239	0.675540095	0.272797618	0.931434333
TNFAIP8	0.347989201	0.538282438	0.212130299	0.424255973	0.134396966	0.273205226	0.931434333
TTC21A	0.099615143	0.071823796	0.625945427	0.8150123	0.620713129	0.273201989	0.931434333

ADAMTS6	0.827347334	0.076863609	0.083851141	0.603239149	0.704791372	0.273286438	0.931444183
AMMECR1	0.19561286	0.519319875	0.103232655	0.481438958	0.449432527	0.273402323	0.931527533
GNB3	0.269030317	0.210355258	0.058475811	0.807476013	0.849314539	0.273424418	0.931527533
ENTPD1	0.285653604	0.738921792	0.448015673	0.351094583	0.068438926	0.273582901	0.931874011
LOC101904595	0.591476841	0.043045939	0.278021438	0.652062978	0.493013314	0.273773798	0.932330734
EFHB	0.982073332	0.534672599	0.191889829	0.0282171	0.80153622	0.273960362	0.932506405
GFM1	0.987600666	0.668260566	0.5058403	0.075421287	0.090469685	0.273905939	0.932506405
PFKFB3	0.461955793	0.095185839	0.532512591	0.595780083	0.163398913	0.273995849	0.932506405
DCAKD	0.371466995	0.970609229	0.449311594	0.080489855	0.174986689	0.274121897	0.932741955
CSF1R	0.356838721	0.908905991	0.030556826	0.480995448	0.480534229	0.274633541	0.932911148
CSPG4	0.172532597	0.183112282	0.30105555	0.300235359	0.799812254	0.27424981	0.932911148
KAT14	0.348018914	0.681723681	0.087314373	0.150254405	0.735050726	0.274476624	0.932911148
LOC112441542	0.52138854	0.768549942	0.368770487	0.224903991	0.068803151	0.274402757	0.932911148
MRPL2	0.434421552	0.601055011	0.49347893	0.02651023	0.66976944	0.274474085	0.932911148
NREP	0.912180352	0.885896308	0.077733487	0.072212228	0.505402	0.274740088	0.932911148
RASGRP4	0.650822514	0.634812307	0.072689496	0.180909378	0.420556041	0.274303585	0.932911148
SGCD	0.02313323	0.851760417	0.160129047	0.866250599	0.838207455	0.274649879	0.932911148
SZRD1	0.263998791	0.300298613	0.652913007	0.045883168	0.96506416	0.274710284	0.932911148
UNC13D	0.64041311	0.963987823	0.024492151	0.300618742	0.504315838	0.27472745	0.932911148
CCL14	0.153866832	0.500583042	0.527024162	0.082465433	0.685358275	0.274836706	0.933046169
GCSH	0.385689562	0.721494493	0.703400638	0.021093812	0.556350735	0.274997719	0.933399704
CSPP1	0.425120324	0.133178018	0.882937384	0.383841332	0.11994249	0.275244635	0.934044603
DUOX2	0.770931747	0.074264652	0.068527488	0.636024524	0.923990257	0.275486691	0.934286623
FUCA1	0.3097451	0.582963517	0.381066019	0.045660808	0.733283273	0.275383619	0.934286623
GRB10	0.473438379	0.302407959	0.256767164	0.435735688	0.143941173	0.275486745	0.934286623
CUL4A	0.593574282	0.83223917	0.120984283	0.179246792	0.215564152	0.275690057	0.934647096
LOC101906006	0.928697635	0.765991302	0.831259329	0.004416151	0.884418075	0.27570694	0.934647096
MPHOSPH10	0.859865725	0.672670607	0.125152102	0.104029301	0.306913002	0.275799038	0.934766214
SLC36A4	0.056653431	0.576601387	0.578109396	0.192603467	0.636070318	0.275931194	0.935021026
ANKLE2	0.631592795	0.091756721	0.112047129	0.421887545	0.863105771	0.278788991	0.935484754
API5	0.542846107	0.068679826	0.328011329	0.514951824	0.371971418	0.277557615	0.935484754
ATP8B4	0.141554444	0.191196907	0.457943747	0.196633839	0.969219125	0.278655605	0.935484754
CDH8	0.345976902	0.176962245	0.419999569	0.298865473	0.307781473	0.27883701	0.935484754
CHODL	0.163003258	0.038055538	0.812157561	0.625920013	0.736120996	0.276365457	0.935484754
CHRM1	0.63559011	0.126436802	0.112158423	0.355508916	0.733098536	0.277928062	0.935484754
CLEC2B	0.295271953	0.833825503	0.056737328	0.201076921	0.839159951	0.278375646	0.935484754
CRISPLD1	0.046696286	0.635143675	0.360247291	0.969149536	0.225529347	0.277158042	0.935484754
DOCK4	0.146804046	0.268038512	0.489698904	0.22737548	0.53178233	0.27685396	0.935484754
ERH	0.838797066	0.149227104	0.507527872	0.143595758	0.255037748	0.27666345	0.935484754
GARS	0.261900245	0.682347785	0.791876272	0.545904868	0.030660554	0.279019453	0.935484754
GFAP	0.683712573	0.089686476	0.576622617	0.336979708	0.197420637	0.278107634	0.935484754
HIF1A	0.920268209	0.557412728	0.279299292	0.070873428	0.231084347	0.277782717	0.935484754
IDNK	0.700558177	0.422208961	0.542469834	0.016438467	0.885350061	0.277150394	0.935484754
IFIT5	0.728528783	0.955485074	0.172136185	0.054246825	0.35956054	0.277260838	0.935484754
IFT122	0.833472139	0.475657411	0.159167603	0.191974811	0.194106785	0.278058124	0.935484754

ING2	0.499956289	0.733906417	0.064004863	0.583363385	0.172059814	0.278385066	0.935484754
KRR1	0.079535726	0.462666823	0.664375964	0.133627915	0.720571983	0.278207758	0.935484754
LOC100335635	0.039139849	0.644682187	0.213478897	0.562087006	0.77901458	0.278465255	0.935484754
LOC101903261	0.678665314	0.784938252	0.180374088	0.336471248	0.072906595	0.278377721	0.935484754
LOC107132853	0.83935579	0.901841165	0.664503168	0.146216042	0.03154115	0.27628204	0.935484754
LOC107133276	0.460015721	0.082126044	0.086588604	0.894864937	0.802228286	0.277890046	0.935484754
LOC112442189	0.081305043	0.066434887	0.98377146	0.981907299	0.44435871	0.276211622	0.935484754
LOC515828	0.3502843	0.28765628	0.492420046	0.1457457	0.323152039	0.277243847	0.935484754
LOC789337	0.119704648	0.744129848	0.801210522	0.044023065	0.751492741	0.27859942	0.935484754
LTA4H	0.015088004	0.861329468	0.357113742	0.629532784	0.811147508	0.27908923	0.935484754
MLX	0.93176174	0.716307859	0.483163517	0.017412364	0.415251465	0.276952297	0.935484754
MRPS30	0.98237254	0.461153103	0.712553931	0.301858556	0.023843546	0.276483329	0.935484754
MS4A2	0.137733533	0.571740496	0.308515409	0.549188808	0.177507458	0.279006644	0.935484754
MSI1	0.586077609	0.689884533	0.013619155	0.704709245	0.59941443	0.276635775	0.935484754
NLGN3	0.544071249	0.618104328	0.551009855	0.571866776	0.02196441	0.276717984	0.935484754
PHF14	0.270385475	0.679483482	0.017930173	0.885490001	0.809111656	0.278547093	0.935484754
PLA2G2D4	0.431635028	0.430876802	0.018339371	0.740049532	0.935247884	0.278578289	0.935484754
PPP2R2A	0.50157469	0.468326038	0.871740676	0.016219014	0.707118565	0.277895537	0.935484754
PRMT2	0.994826082	0.860887932	0.083192492	0.066965542	0.495559762	0.278785602	0.935484754
RANGRF	0.095671392	0.878242075	0.759863749	0.225729894	0.162867545	0.277825256	0.935484754
RUM1	0.98786218	0.182414918	0.86833287	0.024468947	0.609565212	0.27707634	0.935484754
SESN1	0.606655805	0.721054664	0.039140595	0.901068335	0.15345036	0.278948925	0.935484754
SGSH	0.372848316	0.862533864	0.040532332	0.317058654	0.56919841	0.278115122	0.935484754
SOCS2	0.192961369	0.369923503	0.955315757	0.046626569	0.729398763	0.276246468	0.935484754
SOWAHA	0.060709932	0.863321111	0.370680079	0.791305319	0.153103925	0.278190395	0.935484754
SPARC	0.294773802	0.149496394	0.277498545	0.258065832	0.736952457	0.276615091	0.935484754
SSH2	0.864974574	0.519056553	0.765127356	0.106142135	0.064332108	0.277738015	0.935484754
SUFU	0.515866517	0.379162058	0.505524793	0.149896342	0.157472241	0.277082776	0.935484754
SYVN1	0.159290952	0.927239801	0.265612035	0.124254885	0.481324638	0.277772849	0.935484754
TAB3	0.97133941	0.236477274	0.073504838	0.720357938	0.192609802	0.277567336	0.935484754
TSPAN7	0.250796186	0.109938679	0.770846725	0.363307619	0.303513527	0.277624481	0.935484754
UBR7	0.986151566	0.027088456	0.201314641	0.550679226	0.792775515	0.277854464	0.935484754
UQCRC1	0.970210185	0.634561937	0.54159173	0.010775832	0.659511262	0.279076797	0.935484754
WNK1	0.571686056	0.903876323	0.110887374	0.050098712	0.816782233	0.277681807	0.935484754
ZBTB10	0.533722718	0.453204064	0.489729361	0.031816539	0.628741389	0.279078536	0.935484754
ZNF217	0.614857631	0.204542024	0.847275247	0.127515445	0.172204087	0.27741157	0.935484754
ZNF461	0.054723689	0.793882221	0.137610126	0.409624317	0.962747535	0.278408	0.935484754
ACTR3B	0.342765804	0.491642062	0.749257683	0.020298843	0.93427045	0.280456509	0.935547743
ADARB1	0.991795493	0.525272928	0.008426954	0.807411818	0.686170229	0.282528463	0.935547743
AGGF1	0.104387477	0.693516044	0.072654019	0.506287	0.892693107	0.279495549	0.935547743
ASMTL	0.542278402	0.38570477	0.496043589	0.087614111	0.265427486	0.281461782	0.935547743
BCL6B	0.049391085	0.248498826	0.443136874	0.448662472	0.983537394	0.280761219	0.935547743
C28H1orf198	0.492016551	0.330316003	0.062114985	0.30024933	0.794597986	0.281223038	0.935547743
CBLL1	0.004606783	0.76884358	0.894814459	0.914684383	0.836873018	0.282190149	0.935547743
CDCA7	0.920029972	0.519657077	0.112580331	0.135578689	0.328365707	0.28055064	0.935547743

CYBC1	0.266401023	0.849437151	0.137540797	0.288924644	0.26912995	0.281867666	0.935547743
DEF6	0.845578646	0.884191861	0.056832626	0.280530607	0.203856972	0.282405775	0.935547743
DPH3	0.994255483	0.490209881	0.007143119	0.919591591	0.758675202	0.282349063	0.935547743
DYNC1LI1	0.881869033	0.382207173	0.600843081	0.018510442	0.64338043	0.281410328	0.935547743
EBF4	0.036749719	0.671716944	0.324531132	0.770557709	0.387260994	0.280238434	0.935547743
ENHO	0.535073074	0.190026503	0.603959743	0.264606276	0.14834042	0.281332848	0.935547743
ENPP4	0.219787367	0.067175884	0.476347398	0.639415338	0.540456707	0.282430211	0.935547743
EXOSC4	0.266655893	0.979678949	0.415412704	0.21957289	0.101807161	0.282181639	0.935547743
FBXL5	0.60683552	0.653608667	0.154103583	0.173283705	0.227846734	0.281487732	0.935547743
FGD3	0.748243864	0.507114798	0.183989991	0.10927022	0.312083442	0.279693954	0.935547743
FGF7	0.566284405	0.304269399	0.033435187	0.690786581	0.602113089	0.280546991	0.935547743
GIPC1	0.363964472	0.281539549	0.157754076	0.661841314	0.226567102	0.282076983	0.935547743
GSTT4	0.705336944	0.409755006	0.227630342	0.368942959	0.099560416	0.281669494	0.935547743
IL20RA	0.125941812	0.243263916	0.75118129	0.117534835	0.876914299	0.279207742	0.935547743
IL9R	0.333905199	0.277887344	0.087030237	0.552787663	0.540082731	0.281359058	0.935547743
IQCN	0.885051871	0.318439634	0.721448076	0.92293067	0.012859866	0.281489065	0.935547743
KHDRBS3	0.578453042	0.628906586	0.075638812	0.158243495	0.556237547	0.281971581	0.935547743
LOC104970821	0.540111438	0.171439596	0.087880503	0.906788505	0.328190191	0.281951252	0.935547743
LOC107132796	0.407861891	0.804788703	0.010561305	0.753991802	0.923438145	0.281513685	0.935547743
LOC112441502	0.609045077	0.034925934	0.863371722	0.779724122	0.168940726	0.281814259	0.935547743
LOC112442032	0.341302352	0.556457224	0.07535059	0.42364762	0.393630302	0.280008735	0.935547743
LOC112442271	0.73367907	0.710076977	0.371066378	0.531649502	0.023105222	0.279354319	0.935547743
LOC112443479	0.128374232	0.604554979	0.117216811	0.847367602	0.313461808	0.281658209	0.935547743
LOC112448515	0.022762424	0.500037127	0.970323565	0.763008936	0.283405559	0.280107437	0.935547743
LOC506989	0.863210007	0.649105036	0.938830092	0.608488714	0.007460421	0.280094955	0.935547743
LOC512286	0.819579414	0.776546224	0.120492799	0.03568344	0.869700451	0.279645207	0.935547743
LOC574091	0.063231208	0.817149586	0.580132724	0.542288939	0.148913782	0.281892357	0.935547743
LOC781381	0.942538787	0.849543412	0.881970287	0.010037223	0.340941843	0.281680394	0.935547743
LRP3	0.665783713	0.747916525	0.012488322	0.641085459	0.604357665	0.281273448	0.935547743
LRRC17	0.949250235	0.382838487	0.007015602	0.960584219	0.978503658	0.280559344	0.935547743
MAPK9	0.119830385	0.963052616	0.743457246	0.300609008	0.09418992	0.282367605	0.935547743
MND1	0.86352018	0.117731097	0.620406785	0.289280657	0.131994523	0.281217026	0.935547743
MRVI1	0.078942385	0.55025719	0.359656916	0.81910217	0.188713244	0.281580226	0.935547743
NAGA	0.335917168	0.952259193	0.509840406	0.040584845	0.362519048	0.280729186	0.935547743
PDCD11	0.198819207	0.906303758	0.384057245	0.158908392	0.219130647	0.281297164	0.935547743
PEX5	0.413247774	0.950182124	0.278422537	0.107965395	0.202400263	0.280150688	0.935547743
PIP5K1C	0.33589009	0.675459341	0.281984071	0.554804808	0.067544355	0.280617592	0.935547743
PWP2	0.340983061	0.647574917	0.521692071	0.080233947	0.260579434	0.281223078	0.935547743
ROCK2	0.502054872	0.397186956	0.272487612	0.051902546	0.84275258	0.27947068	0.935547743
SAP30BP	0.402660954	0.732236346	0.077999726	0.450914093	0.229695843	0.279759221	0.935547743
SEMA6C	0.790116859	0.515402836	0.012626892	0.772818789	0.597519178	0.279343807	0.935547743
SLAMF9	0.741518152	0.995927671	0.303823689	0.703858709	0.015355008	0.282131141	0.935547743
SLC38A2	0.080838373	0.75834317	0.597566539	0.232731202	0.28242165	0.281188544	0.935547743
SLC6A4	0.657283237	0.413394092	0.588329244	0.020558573	0.732237683	0.281115322	0.935547743
SON	0.60117942	0.749496152	0.663680119	0.00996319	0.807221679	0.281036368	0.935547743

SYNE2	0.119373964	0.428061846	0.628463949	0.645866685	0.117242921	0.282504359	0.935547743
TMEM170A	0.317593876	0.433942458	0.167148115	0.135292661	0.769050871	0.280582482	0.935547743
TMEM222	0.348166891	0.907744853	0.657572691	0.059035816	0.194943639	0.280303615	0.935547743
TMEM71	0.941088152	0.996629264	0.161528744	0.709267489	0.022513523	0.281813241	0.935547743
UPF2	0.323578427	0.452758037	0.92692743	0.019886199	0.88584092	0.280327766	0.935547743
ZC3H14	0.635849482	0.710139031	0.361366029	0.288242566	0.050969712	0.280606945	0.935547743
ZWILCH	0.543021354	0.250516016	0.720592817	0.657535287	0.037227432	0.280731664	0.935547743
ARHGAP27	0.234607197	0.167695524	0.371911111	0.475446369	0.350726759	0.282947547	0.936100923
CEP120	0.027447648	0.530627993	0.911575336	0.262812614	0.69914262	0.282925306	0.936100923
HSD17B14	0.352339847	0.076963651	0.181206608	0.960505837	0.517081672	0.282980725	0.936100923
LOC104969409	0.095595783	0.834974904	0.975387159	0.154696267	0.202422713	0.282842056	0.936100923
MRPL36	0.180403337	0.550977807	0.501238522	0.100964149	0.485147931	0.282975781	0.936100923
GGA2	0.363097846	0.11564023	0.236114429	0.879932578	0.280118559	0.283154249	0.93615201
PDP1	0.060682941	0.465717028	0.76787245	0.193269006	0.582705261	0.283167301	0.93615201
PIGW	0.877029515	0.106303876	0.254382116	0.366685707	0.280989158	0.283150262	0.93615201
C8H9orf131	0.821520107	0.289126104	0.497860139	0.156358306	0.132446476	0.283438945	0.936283748
DNAJC8	0.631446644	0.008959181	0.572850183	0.897782342	0.84198137	0.283483559	0.936283748
IL17RB	0.524353885	0.607739168	0.145270539	0.32728378	0.161462012	0.283297733	0.936283748
LOC112442047	0.145316753	0.415581455	0.516752842	0.646125403	0.121588976	0.283589851	0.936283748
REEP1	0.640411242	0.023560395	0.264533854	0.916820921	0.670573507	0.283708999	0.936283748
SUOX	0.924921226	0.700640583	0.288087122	0.022735579	0.577457899	0.28355438	0.936283748
TIMM29	0.963884471	0.192953595	0.060172185	0.429526483	0.510105878	0.283607716	0.936283748
TMUB2	0.027934396	0.756249648	0.744547274	0.499955528	0.312078165	0.283720619	0.936283748
YARS	0.651167277	0.541664034	0.637954849	0.445416016	0.02441429	0.283330976	0.936283748
LOC112445176	0.622849974	0.754108686	0.117968433	0.053954625	0.821553154	0.283830001	0.936456402
PHB2	0.466765229	0.659450854	0.293285686	0.134444742	0.202582933	0.283974726	0.936745575
CAVIN1	0.291980234	0.207245546	0.357537906	0.994349145	0.114396172	0.284095443	0.936767192
LRRC25	0.880610582	0.456887173	0.015332258	0.432568908	0.922045578	0.284063319	0.936767192
TROVE2	0.499013773	0.204967645	0.222049399	0.109385031	0.991302135	0.284187766	0.936883371
ABCA2	0.715199467	0.127401171	0.041910556	0.690222379	0.937220194	0.284601103	0.936966053
ATP5ME	0.466708478	0.306303021	0.940184328	0.018695133	0.984167976	0.284740607	0.936966053
KCTD1	0.419505887	0.709606552	0.031566447	0.280947212	0.93740487	0.284840877	0.936966053
LOC112444355	0.641356948	0.808666961	0.429132583	0.019976384	0.554560087	0.284346208	0.936966053
MYH7	0.406626141	0.319292227	0.521930764	0.214422397	0.170035867	0.284617273	0.936966053
NCEH1	0.92840562	0.178174686	0.073485163	0.565704329	0.359854301	0.2848305	0.936966053
NUMA1	0.892893988	0.264922702	0.471464684	0.819564781	0.027037327	0.284650541	0.936966053
PGA5	0.514601789	0.604492379	0.048938712	0.344079522	0.470884432	0.28439585	0.936966053
PIF1	0.418590563	0.278626842	0.210423259	0.757126101	0.132900433	0.284553709	0.936966053
SNX22	0.281275928	0.013244912	0.774576198	0.878973791	0.973886076	0.284593458	0.936966053
WRAP53	0.618085489	0.095213738	0.122129147	0.479018863	0.71841321	0.284767291	0.936966053
ATP11C	0.51502398	0.60577791	0.171653031	0.056955819	0.813451157	0.285188692	0.936986704
C18H16orf74	0.676603672	0.623340351	0.016847954	0.742884831	0.470730042	0.285385511	0.936986704
LEKR1	0.512643365	0.342687242	0.043400824	0.950715662	0.342305673	0.285193025	0.936986704
LOC101902561	0.348249647	0.925925314	0.081777261	0.170033818	0.553164752	0.285135378	0.936986704
LOC101906410	0.634692137	0.898495172	0.026889112	0.290925502	0.556710541	0.285313459	0.936986704

LOC783776	0.028151459	0.350707668	0.442849373	0.789112075	0.720029872	0.285351964	0.936986704
LRPPRC	0.65012721	0.778689943	0.818503779	0.367242506	0.016278828	0.284972232	0.936986704
NUDT16	0.026651806	0.86982418	0.581480815	0.46557011	0.39502707	0.285078798	0.936986704
PRPS1	0.953085278	0.060369915	0.094242489	0.915910363	0.50044204	0.285418106	0.936986704
RAB13	0.823156537	0.981690504	0.105930967	0.061450748	0.471504345	0.285136556	0.936986704
IL21R	0.17422962	0.473088272	0.282364817	0.305074807	0.350495964	0.285590364	0.937177259
LOC107133268	0.520247567	0.109005957	0.105298861	0.713900744	0.583749927	0.285585586	0.937177259
ZFP41	0.511719377	0.605414007	0.771401755	0.081086458	0.12875954	0.285938813	0.93813312
ACER3	0.085419098	0.793343633	0.700919957	0.0863332	0.609382588	0.286142706	0.938284771
FAM241A	0.093003996	0.524774547	0.062049643	0.94894721	0.870014966	0.286213732	0.938284771
PSME3	0.544076678	0.622205831	0.707617128	0.194935562	0.05351593	0.286147047	0.938284771
SERPIND1	0.402989084	0.20078638	0.484810741	0.211514747	0.30122067	0.286165131	0.938284771
B4GALT5	0.615006299	0.277649088	0.891535354	0.02045873	0.804173939	0.286448904	0.938411188
FXYD5	0.23707384	0.930019888	0.072831125	0.53775687	0.289865915	0.28636632	0.938411188
LURAP1	0.606341228	0.937012768	0.05551944	0.176173795	0.450964682	0.286525756	0.938411188
PRSS53	0.154793554	0.26426838	0.54345634	0.607322225	0.185437394	0.286398238	0.938411188
QDPR	0.147663829	0.809916532	0.415309404	0.166352442	0.303418297	0.286576977	0.938411188
SCX	0.954077317	0.409169691	0.960375829	0.619360547	0.010798037	0.286595386	0.938411188
LUM	0.049953159	0.912878249	0.080286231	0.782325128	0.876094589	0.286700146	0.938566944
ASAP3	0.35132892	0.031400321	0.698188953	0.500248922	0.652691442	0.286997306	0.938790669
IRAK2	0.799248589	0.178358903	0.376843404	0.047911729	0.976126628	0.286864328	0.938790669
LOC526966	0.814150754	0.759149677	0.636212733	0.779094573	0.008202368	0.286888611	0.938790669
RCL1	0.944516083	0.62949411	0.083304519	0.964543176	0.052636859	0.286986831	0.938790669
CLEC6A	0.457129037	0.372820202	0.045937427	0.511550787	0.628700751	0.287159184	0.939132996
MPZL3	0.356400946	0.616813562	0.176746947	0.104083232	0.622936063	0.287230844	0.939180192
TMA16	0.592374005	0.03588407	0.407235273	0.365204109	0.798035898	0.287426801	0.93963371
LOC107132534	0.196968484	0.571013088	0.772092534	0.482237762	0.060333552	0.287622507	0.939811788
NENF	0.408367334	0.453953683	0.044697185	0.889284144	0.34309449	0.287704628	0.939811788
NFXL1	0.552706977	0.397120068	0.854901	0.398892263	0.033735715	0.287543692	0.939811788
PTPN13	0.182099603	0.414336127	0.641035899	0.148474028	0.352063319	0.287710342	0.939811788
ACTG2	0.04159726	0.398381687	0.582124438	0.65874503	0.399821261	0.288377195	0.939875426
ARID5A	0.303824247	0.161394035	0.901626912	0.254931589	0.224677683	0.2879291	0.939875426
ARIH2OS	0.450597156	0.431008027	0.409766874	0.093180232	0.342381647	0.288278315	0.939875426
ELL3	0.586506815	0.368526228	0.504610143	0.127121142	0.183187365	0.288329836	0.939875426
FOXJ3	0.372486861	0.475817427	0.855318495	0.017726546	0.942839562	0.287997621	0.939875426
KRIT1	0.087154431	0.353596238	0.446941918	0.21592426	0.854558619	0.288417077	0.939875426
LOC112441655	0.716647622	0.353777448	0.569673355	0.132502049	0.132465433	0.288073932	0.939875426
MSL1	0.928472842	0.474403976	0.459566218	0.071962629	0.173914843	0.287987749	0.939875426
PROSER2	0.4616762	0.227967457	0.278017136	0.635338652	0.136662139	0.288368568	0.939875426
SLC23A1	0.065950783	0.601804811	0.493142708	0.882240141	0.146967556	0.288219787	0.939875426
SYDE2	0.056030557	0.788565454	0.559541622	0.513730173	0.199895797	0.288274868	0.939875426
TMEM72	0.041917333	0.684351391	0.881543518	0.10084169	0.992013774	0.287790686	0.939875426
DNMT3A	0.5127258	0.153661761	0.055048337	0.838714762	0.700971715	0.288858141	0.940026481
FBXL6	0.941403984	0.099283554	0.232832276	0.147162561	0.794425203	0.288558624	0.940026481
GEMIN5	0.527589783	0.098913645	0.143577322	0.535294895	0.636594723	0.289040057	0.940026481

GMCL1	0.267605022	0.904131156	0.065771724	0.256479931	0.62437424	0.288781402	0.940026481
KITLG	0.934205535	0.154513027	0.40562136	0.046635749	0.934648509	0.288978141	0.940026481
LOC104969097	0.036409283	0.382431536	0.412548976	0.692202804	0.64190057	0.288992713	0.940026481
LOC112443235	0.38717821	0.919246176	0.717977672	0.117221587	0.085251375	0.289061254	0.940026481
LOC112448737	0.328174574	0.492198028	0.746837489	0.608747289	0.034782305	0.289093513	0.940026481
MRPL48	0.716121173	0.996932587	0.727551955	0.922098795	0.005321729	0.288807309	0.940026481
SERPINC1	0.13123307	0.650846328	0.733546959	0.665735466	0.061220034	0.289055733	0.940026481
SLC17A5	0.32937189	0.695802353	0.35095879	0.300716782	0.105397793	0.288829447	0.940026481
MBTD1	0.117396678	0.451628638	0.69952084	0.323016278	0.213320468	0.289164671	0.940071594
ARL8B	0.980219476	0.234717211	0.025456599	0.770034996	0.568238092	0.289542999	0.940624005
AZIN1	0.575283477	0.042216704	0.29423644	0.596950543	0.601256638	0.289652541	0.940624005
LOC112442377	0.643365799	0.960165893	0.584877684	0.153121881	0.046285166	0.289430493	0.940624005
LOC112447402	0.686482095	0.97583055	0.021085706	0.468208193	0.387894298	0.289678491	0.940624005
SLC25A45	0.971352761	0.441232655	0.259858747	0.032111683	0.71705128	0.289632072	0.940624005
WIZ	0.429337204	0.861031781	0.045581879	0.211565254	0.718548092	0.289480883	0.940624005
GABRA1	0.373291108	0.885810842	0.105457694	0.435480318	0.169459155	0.290101515	0.940823617
GRID2	0.135295178	0.303144311	0.815958008	0.357479185	0.215083764	0.290088242	0.940823617
LOC101902468	0.404055659	0.180225069	0.813529935	0.159703221	0.272231234	0.290220453	0.940823617
LOC101903795	0.78794175	0.336516031	0.904663723	0.011335549	0.948370091	0.290384646	0.940823617
LOC112442719	0.634668915	0.084079271	0.318754123	0.9273367	0.163537212	0.290427911	0.940823617
PCDH12	0.945176599	0.953240732	0.020620428	0.172872482	0.801605215	0.290163668	0.940823617
PPP1CA	0.184803168	0.424113619	0.889169638	0.139733611	0.263675427	0.289804023	0.940823617
RRAD	0.034584112	0.848776688	0.495053808	0.892848569	0.198706788	0.290355144	0.940823617
TLE3	0.544133692	0.876481474	0.265847327	0.054749251	0.371534901	0.290400567	0.940823617
TMEM168	0.581840052	0.316144254	0.689695674	0.113466377	0.178971509	0.290256643	0.940823617
TMEM38B	0.653524088	0.192191926	0.167734069	0.130797493	0.933116717	0.289993227	0.940823617
ZNF438	0.73470276	0.566247157	0.051204293	0.794293854	0.152096365	0.290108892	0.940823617
AADAT	0.93517907	0.820483203	0.563752692	0.01191556	0.503852113	0.291342295	0.940939658
AP3M2	0.221104743	0.199800362	0.959706979	0.145390277	0.421020454	0.291248684	0.940939658
ARHGAP33	0.115875487	0.510890361	0.388107353	0.219122959	0.51375567	0.290793356	0.940939658
CTXND1	0.329668804	0.067178957	0.830829136	0.982100732	0.143541958	0.291181783	0.940939658
DHX57	0.281732948	0.297402763	0.471162873	0.931900265	0.070197917	0.290584511	0.940939658
GRTP1	0.114438049	0.470253796	0.098627334	0.730770764	0.669095946	0.291248079	0.940939658
GSTM3	0.162367903	0.366587227	0.276337813	0.800133435	0.197468382	0.291438443	0.940939658
MAIP1	0.668442595	0.588175651	0.538649382	0.093867585	0.130023514	0.290699344	0.940939658
MMP11	0.669367064	0.321912175	0.019559773	0.953452275	0.646675817	0.291430093	0.940939658
NAF1	0.010621887	0.812801981	0.444409418	0.927721825	0.728780238	0.291190015	0.940939658
NFKB2	0.430460052	0.614469522	0.292380245	0.067933592	0.4920109	0.290707363	0.940939658
RAD51AP1	0.813800959	0.352434506	0.070537007	0.647118128	0.198415907	0.291374547	0.940939658
RIC8B	0.231627567	0.065978211	0.470528327	0.361005333	0.999492593	0.291216832	0.940939658
SPNS2	0.576907153	0.558824288	0.057979382	0.15011646	0.925668503	0.291363555	0.940939658
TM9SF3	0.281550966	0.825184559	0.548504111	0.077762296	0.262192647	0.291407066	0.940939658
USP22	0.303899098	0.150810148	0.128169073	0.468360098	0.944556704	0.291430094	0.940939658
VPS26B	0.600069746	0.963566662	0.01251078	0.36280232	0.987582097	0.291073357	0.940939658
SIPA1L3	0.330818087	0.065592393	0.757255642	0.655701615	0.241585169	0.29165262	0.941075726

TMEM25	0.298509367	0.106572314	0.439384457	0.204021413	0.912123913	0.291563323	0.941075726
TOLLIP	0.535107861	0.94651335	0.02362145	0.290669035	0.748452332	0.291645035	0.941075726
LOC101902754	0.504290377	0.514746779	0.032623102	0.679054907	0.453118794	0.291795095	0.941211526
MEOX1	0.237200224	0.377232619	0.089494645	0.347085661	0.937571022	0.291809411	0.941211526
RDH16	0.866259772	0.627634714	0.34518242	0.550786041	0.025235632	0.291946882	0.941469894
ABHD10	0.051601457	0.702315012	0.685757346	0.207596294	0.5061577	0.292093901	0.941758941
CCDC102A	0.917463135	0.357257251	0.011884303	0.968865511	0.692355654	0.292177407	0.941786163
LY6E	0.943132302	0.562306725	0.873475059	0.008091313	0.697601377	0.2922673	0.941786163
U2AF2	0.648811785	0.548023948	0.01979418	0.371698793	0.99953673	0.292274507	0.941786163
POLE	0.948192785	0.210685128	0.274878992	0.130425456	0.365856851	0.292556912	0.942511088
CSGALNACT2	0.885746582	0.772193491	0.95286687	0.006548896	0.614259385	0.292632925	0.942570938
HDHD3	0.706603143	0.314696976	0.435186295	0.029860263	0.908070286	0.292748695	0.942642723
WDTC1	0.142672665	0.898708264	0.253649256	0.981365315	0.082224409	0.292770091	0.942642723
MARVELD1	0.100384601	0.306268443	0.231176804	0.757497238	0.487891981	0.292893782	0.942671082
POLR3H	0.527559991	0.30016409	0.710120756	0.065484157	0.356689453	0.29288466	0.942671082
LOC101904590	0.486588423	0.803114636	0.457360304	0.068213304	0.215553701	0.292957881	0.942692506
LOC100847708	0.353087731	0.448220774	0.449904652	0.447525074	0.082533077	0.293057859	0.942829351
MTERF1	0.112198792	0.242984486	0.24157202	0.977807447	0.40936205	0.293382887	0.943331219
RPL39	0.853232624	0.33834235	0.646784199	0.026493304	0.532528106	0.293284638	0.943331219
ZUP1	0.141257495	0.538701263	0.428438448	0.237066634	0.341088246	0.293386298	0.943331219
MAPK3	0.085076224	0.573343469	0.53417376	0.253766759	0.398871879	0.293445642	0.943337204
ARL5B	0.452430737	0.039388204	0.632652228	0.249644337	0.938653842	0.293677463	0.943345469
GNAL	0.120627568	0.738243749	0.266587684	0.24326079	0.457485664	0.2936855	0.943345469
LOC100299242	0.330647687	0.729442089	0.090722337	0.557451715	0.21645721	0.29359543	0.943345469
MCTP2	0.535206251	0.219967783	0.104740427	0.701916424	0.305312612	0.293713423	0.943345469
TMEM63A	0.58116028	0.176586503	0.475339172	0.116325817	0.465759933	0.293735625	0.943345469
AKR1C4	0.781487405	0.53016851	0.108164772	0.867965916	0.068236961	0.294318033	0.943470926
C29H11orf95	0.660968818	0.632640557	0.051170809	0.357210475	0.347436919	0.294386234	0.943470926
CEP126	0.270677509	0.675802362	0.581412326	0.093105132	0.267620865	0.294099193	0.943470926
CPE	0.912786711	0.566087424	0.00867981	0.875172624	0.676511623	0.294377276	0.943470926
GALE	0.357786805	0.3073973	0.762274051	0.087259431	0.362932514	0.294358908	0.943470926
KCNG2	0.681909333	0.317147725	0.031770533	0.428288259	0.901822676	0.294294691	0.943470926
KIF3A	0.245714118	0.968481544	0.097171102	0.220094558	0.521511056	0.294314033	0.943470926
MYCBP2	0.424530921	0.234326445	0.88449605	0.064135965	0.4701887	0.294272721	0.943470926
PDE7A	0.27736864	0.798000446	0.06641108	0.647583033	0.27901661	0.294407081	0.943470926
SGMS1	0.943893751	0.261103388	0.047640273	0.438220082	0.515333086	0.29417496	0.943470926
TRMT5	0.558977725	0.890921793	0.65073791	0.113711613	0.071905979	0.29408669	0.943470926
AAMP	0.300948841	0.655208308	0.062232522	0.347915704	0.649778044	0.300409092	0.943842536
ABHD3	0.474537469	0.373414418	0.023496666	0.829783061	0.784930329	0.297266087	0.943842536
ABHD6	0.334874016	0.946579405	0.045662129	0.563838619	0.326233551	0.294738766	0.943842536
AP2M1	0.057963944	0.215951017	0.797815483	0.542459718	0.510791708	0.300056993	0.943842536
ARFRP1	0.481972984	0.717788558	0.198545766	0.118329298	0.32792072	0.294884859	0.943842536
ATP6V0E2	0.004363466	0.961429133	0.782281016	0.907145608	0.920016658	0.298639883	0.943842536
ATP8B2	0.106409458	0.235375727	0.544414541	0.462104949	0.435450232	0.298884021	0.943842536
BAG2	0.153097715	0.272610758	0.211577072	0.748650437	0.403902737	0.295135938	0.943842536

BTBD9	0.957818952	0.195292188	0.641558143	0.920700754	0.024916541	0.299349418	0.943842536
C1QTNF2	0.649109215	0.702477095	0.066191689	0.992557365	0.092141893	0.299718188	0.943842536
CD300LF	0.490115169	0.229056219	0.052723832	0.849143602	0.551951657	0.30040936	0.943842536
CD82	0.191125634	0.540504135	0.1323113	0.310886027	0.640724903	0.297813758	0.943842536
CHST10	0.077437046	0.61224469	0.139330018	0.510010651	0.792590414	0.295139216	0.943842536
CLBA1	0.824626004	0.44278456	0.13395503	0.11355923	0.488171621	0.297247034	0.943842536
COX6B1	0.816793656	0.379565626	0.896752777	0.011262843	0.861417949	0.296527226	0.943842536
CSNK1G2	0.072601574	0.555933907	0.421702491	0.335348104	0.473240394	0.296723465	0.943842536
DDIAS	0.503253003	0.103723232	0.167339625	0.420767866	0.748408095	0.299231948	0.943842536
DENND2D	0.571377988	0.296094851	0.705688688	0.307104884	0.073504181	0.29641075	0.943842536
DHPS	0.97840723	0.618809439	0.164163109	0.076266432	0.352067322	0.295064915	0.943842536
DYNC2LI1	0.236788155	0.566770533	0.128334708	0.479651447	0.330164733	0.29806178	0.943842536
EED	0.099293252	0.453561426	0.321544047	0.518555678	0.355135204	0.294962024	0.943842536
EIF4H	0.216882908	0.12738451	0.216962915	0.751978547	0.614083971	0.30009971	0.943842536
FHL2	0.125041189	0.600984641	0.057175094	0.841696145	0.749778928	0.297249711	0.943842536
FOXO4	0.208901787	0.792325421	0.158768852	0.480225245	0.217435094	0.298895144	0.943842536
GAS2L2	0.296721698	0.54075198	0.473872324	0.579729792	0.061320923	0.296815427	0.943842536
GATD1	0.885654037	0.939355118	0.192289641	0.025996272	0.649989874	0.29682316	0.943842536
GNA11	0.097672829	0.299572617	0.106542121	0.929841941	0.947884682	0.299078779	0.943842536
GUCY1A1	0.737750972	0.236491555	0.478493335	0.237290915	0.136987622	0.297361406	0.943842536
HDAC11	0.861719948	0.17450884	0.211189145	0.191640386	0.454881095	0.300125092	0.943842536
HSPB8	0.075645037	0.263074633	0.431534422	0.587473452	0.549510217	0.300316477	0.943842536
IKBKG	0.921763843	0.999209064	0.903858733	0.004567586	0.724659866	0.299472864	0.943842536
INPP1	0.213806415	0.267096656	0.346831124	0.327873813	0.425592881	0.299891315	0.943842536
KLF11	0.422523497	0.244451854	0.937201749	0.309234385	0.091928936	0.299287776	0.943842536
KLF7	0.692717976	0.902130781	0.639032278	0.043453287	0.157926173	0.298716684	0.943842536
LOC100139548	0.32272643	0.548257635	0.377265648	0.082706198	0.497429894	0.299006922	0.943842536
LOC100337328	0.489282661	0.525058086	0.096399079	0.11609658	0.954735294	0.298945107	0.943842536
LOC100849237	0.96398501	0.987542156	0.129612617	0.867007501	0.025774417	0.299564577	0.943842536
LOC101902721	0.256527088	0.092738042	0.692411987	0.26117814	0.640738296	0.299528938	0.943842536
LOC101904087	0.412629089	0.363783325	0.096251574	0.318279054	0.60333141	0.300423776	0.943842536
LOC101905041	0.220435421	0.077829944	0.208123685	0.875739353	0.858829522	0.295924791	0.943842536
LOC101907603	0.33539111	0.742514493	0.213651701	0.069436238	0.74527825	0.299367569	0.943842536
LOC104972797	0.267024028	0.395873117	0.708767813	0.17998591	0.204866609	0.299831178	0.943842536
LOC104972827	0.026495354	0.696158221	0.786510816	0.964623336	0.191197926	0.29541623	0.943842536
LOC104975626	0.950917064	0.87328571	0.05375338	0.294754978	0.209982972	0.299840749	0.943842536
LOC107131675	0.128133873	0.088749945	0.893231138	0.520950625	0.510947299	0.296855225	0.943842536
LOC112443485	0.203557352	0.337021933	0.336584238	0.256673087	0.456475944	0.296940348	0.943842536
LOC112444152	0.470197042	0.711370572	0.699537216	0.04654633	0.253793049	0.299905192	0.943842536
LOC112446759	0.547707706	0.341895467	0.143274839	0.265881293	0.387902276	0.300055478	0.943842536
LOC112446796	0.271354929	0.314763029	0.101363445	0.474436426	0.673406983	0.300003146	0.943842536
LOC112448105	0.157686756	0.562961783	0.242514326	0.36223539	0.344939989	0.29615095	0.943842536
LOC112448153	0.567554985	0.069059882	0.607311007	0.472864443	0.241624366	0.297665862	0.943842536
LOC531747	0.663103971	0.217598766	0.838868588	0.591927182	0.038127301	0.298274442	0.943842536
LOC781197	0.194543982	0.954655073	0.536891956	0.205284814	0.135073506	0.299945565	0.943842536

LOC785568	0.178976237	0.169123179	0.218997767	0.476678921	0.848417717	0.295685138	0.943842536
LOC789148	0.300685943	0.123872206	0.17437153	0.474629915	0.881990858	0.297621209	0.943842536
LRRC72	0.036132182	0.621445727	0.998393854	0.698127447	0.173610553	0.297534919	0.943842536
LRRN2	0.722352384	0.133933614	0.221810238	0.752046043	0.171703664	0.300254269	0.943842536
LRRTM2	0.243306074	0.231714401	0.555015343	0.131697042	0.652602508	0.296116052	0.943842536
LYRM4	0.278044892	0.633293696	0.312230941	0.061866664	0.795778513	0.297006319	0.943842536
MAGED4B	0.786304848	0.400121124	0.030162267	0.950314792	0.294905487	0.294586904	0.943842536
MAP3K10	0.548730076	0.776073335	0.299487508	0.034604092	0.604709292	0.295065877	0.943842536
MED29	0.893527112	0.670697767	0.823101364	0.005698607	0.956281799	0.296054889	0.943842536
MEF2A	0.1295019	0.326528562	0.407848391	0.205014855	0.781671184	0.299890412	0.943842536
MRO	0.683894908	0.500573875	0.293700738	0.121574715	0.224052478	0.298630725	0.943842536
MTA1	0.968318494	0.150132075	0.079134955	0.240553736	0.978110316	0.297011106	0.943842536
NDUFC2	0.675607563	0.475700904	0.490867252	0.022007232	0.783472785	0.297684791	0.943842536
NEMP2	0.51239814	0.026719126	0.988211456	0.44453229	0.448220723	0.296445169	0.943842536
NGF	0.694621873	0.331817244	0.198083548	0.174642495	0.346159228	0.299703981	0.943842536
NLRP1	0.74758095	0.400508679	0.020177253	0.54859375	0.816886871	0.297038058	0.943842536
NR1H3	0.380374589	0.072769971	0.116251299	0.93403746	0.906679741	0.297938376	0.943842536
NR2F6	0.065052411	0.727975959	0.530615203	0.123693173	0.892772469	0.300446859	0.943842536
NUDCD3	0.28071493	0.293781624	0.1015624	0.454085051	0.728277121	0.300194399	0.943842536
PCNX4	0.738347684	0.825305353	0.876235631	0.04771028	0.106029908	0.29671833	0.943842536
PDLIM3	0.037656444	0.598594065	0.418887839	0.997214271	0.2916697	0.299011016	0.943842536
PGBD5	0.030067904	0.232224031	0.980077079	0.942042543	0.416030593	0.295746008	0.943842536
PPP1R3C	0.169378195	0.074541963	0.429629093	0.650515463	0.770044568	0.297539798	0.943842536
PTGR1	0.699266206	0.103301648	0.208144421	0.178522436	0.992491881	0.294819627	0.943842536
PURB	0.839765521	0.206703503	0.114149057	0.147214579	0.939455913	0.298710291	0.943842536
RGMA	0.835658524	0.65532532	0.329139601	0.098148915	0.15361099	0.297555119	0.943842536
RHBDF1	0.709061926	0.019964001	0.350871105	0.576544517	0.939193254	0.296126006	0.943842536
RNASE1	0.71757812	0.097294477	0.358555611	0.846234955	0.129632173	0.299000942	0.943842536
RPS6KA3	0.570374738	0.480723425	0.015808252	0.854761359	0.728598707	0.296634596	0.943842536
RUNDC3B	0.060353683	0.233432249	0.75134809	0.496014242	0.511119284	0.295826959	0.943842536
SELENOS	0.903071999	0.034738236	0.234439363	0.473086249	0.774783453	0.2964472	0.943842536
SLC13A5	0.681672041	0.686654405	0.011056626	0.892586491	0.587997426	0.297487889	0.943842536
SLC8A1	0.01417031	0.461445298	0.491194658	0.928446889	0.8961222	0.29524376	0.943842536
SNX5	0.275574448	0.698724977	0.078563732	0.761424566	0.240555611	0.300243214	0.943842536
SPATA20	0.048372216	0.240079678	0.326693225	0.821169421	0.859977338	0.295602102	0.943842536
SPSB4	0.065283563	0.338297227	0.930305683	0.287087006	0.455084082	0.295862047	0.943842536
STX7	0.688539989	0.070821417	0.093618642	0.741214605	0.801377133	0.297257682	0.943842536
SYTL1	0.737186836	0.063506405	0.342183402	0.890595579	0.187817145	0.295620607	0.943842536
TEX264	0.071901192	0.842495563	0.325740072	0.322367759	0.434764627	0.299978316	0.943842536
TFG	0.252266131	0.412340057	0.812510046	0.194123266	0.166442162	0.298226024	0.943842536
THBS3	0.759193459	0.189673464	0.059860673	0.32213849	0.968095145	0.296060833	0.943842536
TOM1L2	0.630481231	0.939570083	0.522277474	0.01071923	0.811526239	0.296221507	0.943842536
TPI1	0.420682541	0.561072363	0.746988011	0.092085822	0.165041322	0.295621445	0.943842536
TSC22D1	0.488178252	0.388595218	0.216308721	0.28772911	0.229533935	0.297176295	0.943842536
UBA2	0.293525531	0.952287354	0.472799818	0.037603213	0.554587392	0.299501148	0.943842536

UCK1	0.593927314	0.918177914	0.256908507	0.029488445	0.671420569	0.300395215	0.943842536
WDHD1	0.760838545	0.383716856	0.238418475	0.263809032	0.148645435	0.298162518	0.943842536
ZDHHC14	0.080468304	0.909198877	0.082243658	0.513026866	0.886381417	0.298500596	0.943842536
ZNF557	0.050511136	0.975931772	0.544771582	0.82720659	0.121249447	0.296331084	0.943842536
ZNF829	0.711688977	0.517536122	0.755300689	0.026079563	0.378192826	0.298888357	0.943842536
R3HCC1	0.793402552	0.040167482	0.33183523	0.56305735	0.466245332	0.300514396	0.943874021
NDUFA5	0.440668022	0.391059723	0.572527047	0.033317529	0.845180075	0.300615111	0.944009681
CHDH	0.553520593	0.510574381	0.043263394	0.405138795	0.561811667	0.300850411	0.944151521
EFEMP2	0.620690028	0.298826591	0.06940178	0.222705056	0.973872453	0.301295182	0.944151521
KLHDC4	0.110300147	0.499067854	0.052100247	0.99856056	0.971423086	0.300802505	0.944151521
LOC112443216	0.011428878	0.863425654	0.595891558	0.54127089	0.879135175	0.30160589	0.944151521
MARCH1	0.935797712	0.924801032	0.016926797	0.525759757	0.363216558	0.301571234	0.944151521
MEAF6	0.638999816	0.14470924	0.18963813	0.244485156	0.649695234	0.300971059	0.944151521
MSLN	0.318378579	0.11011426	0.605072128	0.577053618	0.227471695	0.300924074	0.944151521
MTG2	0.461064973	0.857409997	0.487437824	0.018354074	0.790314912	0.301457276	0.944151521
NPEPPS	0.694261501	0.217358201	0.31442709	0.062500868	0.943006652	0.301526938	0.944151521
NR4A1	0.814050335	0.00576943	0.928241951	0.684748141	0.938454891	0.301773793	0.944151521
PLIN4	0.679413478	0.529325499	0.550675754	0.061716423	0.229256867	0.301801498	0.944151521
PODN	0.59425438	0.579414759	0.470493799	0.025099961	0.683915487	0.300749071	0.944151521
PRKCB	0.333118671	0.416468373	0.036149043	0.91051946	0.611476649	0.301311248	0.944151521
RHOT1	0.356198661	0.578493988	0.864605635	0.028741327	0.547254526	0.301810912	0.944151521
RMI1	0.192371286	0.740478961	0.319711629	0.360346506	0.169915224	0.301124508	0.944151521
SERINC2	0.600072535	0.029657241	0.39978332	0.573226393	0.683567028	0.301093259	0.944151521
SPOCK2	0.104503801	0.293011602	0.507793365	0.284740687	0.631653586	0.30153096	0.944151521
TRMT10C	0.59520659	0.726591714	0.658877842	0.375234557	0.026178448	0.301651961	0.944151521
WDR53	0.796256385	0.40687575	0.021529985	0.910655682	0.439279948	0.301217411	0.944151521
ZBTB33	0.03903837	0.726239966	0.896464974	0.154237154	0.713428626	0.30153489	0.944151521
LOC104975635	0.125385725	0.938340827	0.671840315	0.11190034	0.317079178	0.30192915	0.944161449
SIRT7	0.524634476	0.010596097	0.704779191	0.903290004	0.792279031	0.301892987	0.944161449
LOC100848315	0.431010946	0.106042137	0.167771554	0.456950282	0.80247858	0.302286633	0.944379581
MECR	0.542277092	0.965606998	0.580678794	0.047522472	0.194470274	0.302197486	0.944379581
SH3GLB1	0.957145435	0.057670358	0.339150368	0.244974308	0.61282818	0.302220689	0.944379581
STARD9	0.78668659	0.149763842	0.395162048	0.660533933	0.091430229	0.302280658	0.944379581
TYW3	0.112049569	0.627658165	0.265322707	0.313787111	0.480021035	0.302227439	0.944379581
LOC619131	0.867422919	0.457279349	0.91145702	0.013391557	0.58127885	0.302407655	0.944442987
POP5	0.738403726	0.712298092	0.397923645	0.086949137	0.154663682	0.302422028	0.944442987
TTPAL	0.381632867	0.156497604	0.869967795	0.559802191	0.096823392	0.302506363	0.944526621
ASB10	0.53090216	0.978357087	0.720924868	0.045262088	0.166365891	0.302676446	0.944754957
RNASE10	0.102248936	0.638509681	0.369984925	0.845155038	0.138137197	0.302694629	0.944754957
DPP6	0.538550658	0.987516408	0.953967959	0.065088679	0.085518737	0.302892066	0.945120278
GPI	0.992682123	0.653884715	0.49303198	0.01170217	0.75470328	0.303007118	0.945120278
IQCA1	0.138238906	0.603113599	0.095188324	0.485119949	0.734366472	0.303054068	0.945120278
ROGDI	0.755763049	0.48181768	0.254620332	0.033533569	0.908636397	0.302943513	0.945120278
STPG1	0.754325805	0.093567581	0.075853458	0.695416742	0.759646925	0.30309963	0.945120278
KIFC1	0.937180927	0.16451103	0.10704381	0.207893224	0.825059153	0.30322473	0.945195663

SPATA16	0.77728121	0.554596292	0.666833374	0.035624368	0.276458627	0.303238996	0.945195663
ACSL5	0.345920193	0.538455114	0.755645394	0.426368934	0.047291787	0.303581885	0.94524378
EIF2AK2	0.932579946	0.97275568	0.165342446	0.433344963	0.043768201	0.303920835	0.94524378
HTRA2	0.285630559	0.218898745	0.687704861	0.067330715	0.982346535	0.303876137	0.94524378
LOC100299201	0.372598809	0.570645909	0.90112099	0.027603243	0.538012199	0.303945611	0.94524378
LOC101902656	0.447214469	0.888999161	0.044460666	0.282487747	0.568347438	0.30357929	0.94524378
LOC101906526	0.023998279	0.826518884	0.415748419	0.569743283	0.603143002	0.303372011	0.94524378
LOC112444921	0.221388271	0.09940358	0.263184571	0.895003338	0.548258107	0.303779067	0.94524378
MPPED2	0.439334696	0.412817363	0.398385571	0.052386285	0.751009769	0.303809187	0.94524378
MYD88	0.57385591	0.899031178	0.085775479	0.082011655	0.783183043	0.303796095	0.94524378
NDUFS6	0.276031693	0.641525682	0.780632649	0.024548675	0.838484773	0.30394543	0.94524378
NEO1	0.231342949	0.622546782	0.054767308	0.688804073	0.522363337	0.303582954	0.94524378
NIPA1	0.534963165	0.392761206	0.036914784	0.367492017	0.997126021	0.303786794	0.94524378
NPEPL1	0.400862263	0.402850076	0.997588287	0.025164501	0.704150207	0.304397181	0.946468765
SASS6	0.252138839	0.190351424	0.080298103	0.797875328	0.929090633	0.304510358	0.946641313
ADAM22	0.160401916	0.085698571	0.986313467	0.525099605	0.414148149	0.308954909	0.947297366
ALS2	0.382124622	0.367565624	0.186885237	0.117679051	0.949719758	0.308242835	0.947297366
ANKRD29	0.762457741	0.701747231	0.273417333	0.109494826	0.18246678	0.307719231	0.947297366
ANKRD39	0.607624617	0.919585358	0.690413407	0.012384605	0.610842023	0.307507228	0.947297366
ANXA3	0.704517706	0.762606265	0.524161645	0.010352526	0.98983157	0.305923426	0.947297366
AQP1	0.500747867	0.47789194	0.619342524	0.020893766	0.945353049	0.307943788	0.947297366
ARF3	0.189385422	0.882663421	0.337217621	0.363631847	0.142471296	0.307602625	0.947297366
ATP6V0B	0.476457062	0.042064182	0.199233276	0.825059726	0.893509354	0.30872255	0.947297366
BTBD7	0.434645301	0.499776598	0.752841538	0.023279708	0.762608713	0.306775405	0.947297366
C23H6orf106	0.733405815	0.890792669	0.145391361	0.046462416	0.656235954	0.306427631	0.947297366
CARM1	0.241331515	0.494672824	0.11236738	0.774250042	0.27843842	0.306220646	0.947297366
CASP7	0.374227391	0.522385426	0.980822311	0.020291413	0.746083762	0.306750443	0.947297366
CEBPG	0.825430827	0.14008193	0.385259902	0.116422164	0.553116142	0.305083442	0.947297366
CRTC1	0.759752585	0.367615687	0.067365867	0.22761223	0.676860791	0.306550732	0.947297366
CRYBG3	0.773441378	0.97783164	0.350219835	0.030598847	0.361714209	0.308143025	0.947297366
CYHR1	0.453019641	0.745206033	0.189715332	0.058986844	0.766732656	0.306451475	0.947297366
DNAJB12	0.621574105	0.808254597	0.093803945	0.238875522	0.261224198	0.308580727	0.947297366
EFNB1	0.130678405	0.341486929	0.121285708	0.755344299	0.714483427	0.307629745	0.947297366
ELF2	0.052884049	0.644293847	0.923665032	0.159328112	0.588196998	0.309001494	0.947297366
EMC6	0.266032133	0.439694412	0.448705866	0.067117554	0.828075876	0.307443983	0.947297366
EPB41L3	0.324762269	0.965480626	0.105490151	0.109235243	0.801518883	0.306419912	0.947297366
FUBP3	0.154607279	0.612112761	0.313320264	0.1278399	0.761093096	0.305887453	0.947297366
GSDMB	0.322576313	0.709763526	0.414458783	0.68378874	0.044299877	0.305369029	0.947297366
IRF2	0.879601634	0.18649952	0.777071126	0.995243307	0.023180494	0.308590244	0.947297366
KLHL15	0.402988541	0.712401832	0.985825897	0.014721773	0.70641352	0.308707716	0.947297366
KMT2E	0.472445615	0.69228544	0.660283138	0.041603361	0.328001747	0.308881984	0.947297366
LAMTOR1	0.364515527	0.554417266	0.466588176	0.033869821	0.923578775	0.30901289	0.947297366
LMF2	0.60778221	0.302226574	0.032676642	0.651657173	0.739098247	0.306174202	0.947297366
LOC100141070	0.709086262	0.417890406	0.372460345	0.533240378	0.05007802	0.308894986	0.947297366
LOC100335990	0.267678851	0.901075706	0.355014755	0.108797361	0.30839909	0.30530475	0.947297366

LOC100337457	0.397472832	0.850639183	0.15353064	0.469275896	0.118748971	0.306260584	0.947297366
LOC101902490	0.750599201	0.334752098	0.562294028	0.137894217	0.147888683	0.305700286	0.947297366
LOC101902757	0.400419612	0.442107416	0.273776648	0.169753066	0.357895137	0.308765037	0.947297366
LOC101904265	0.134994613	0.197959779	0.361774206	0.521466632	0.573288585	0.306139023	0.947297366
LOC101906426	0.289817677	0.221016158	0.230072635	0.196544266	0.990490944	0.305101585	0.947297366
LOC112442049	0.268967069	0.18969782	0.507840392	0.213141736	0.524098857	0.306346273	0.947297366
LOC112445002	0.486928478	0.201921646	0.373902367	0.306266251	0.258979682	0.307384874	0.947297366
LOC112445242	0.05845836	0.600297326	0.156242896	0.695335441	0.766082879	0.307616348	0.947297366
LOC112448454	0.149391998	0.237629815	0.803071299	0.644874491	0.156222585	0.305255665	0.947297366
LOC112449531	0.245400311	0.537063158	0.351187245	0.142967058	0.445875853	0.309050643	0.947297366
LOC530102	0.560318545	0.217774778	0.37253888	0.089943845	0.719816732	0.308698109	0.947297366
MAF	0.312568111	0.485843297	0.226579218	0.122888608	0.688088769	0.307075039	0.947297366
MIF	0.503483006	0.979943335	0.536248642	0.019627338	0.554403179	0.305591304	0.947297366
MIGA2	0.486663364	0.941473972	0.242076209	0.027789032	0.93987093	0.306463074	0.947297366
MRPL34	0.529614198	0.452812347	0.846190038	0.028574536	0.497204888	0.305792372	0.947297366
NEGR1	0.224061667	0.413534856	0.876372306	0.324321045	0.111109334	0.307879889	0.947297366
PAK6	0.325104474	0.956672692	0.506179198	0.045252437	0.409466532	0.307443321	0.947297366
PAX5	0.314142237	0.444025621	0.191622756	0.9714249	0.11352849	0.308922318	0.947297366
PEF1	0.618851774	0.791130996	0.461540866	0.017398833	0.734585565	0.306034364	0.947297366
PKHD1L1	0.159514556	0.978603512	0.076152104	0.63930074	0.386365099	0.30836737	0.947297366
PPP1R7	0.240000803	0.637481588	0.211933697	0.345248179	0.255784876	0.304830776	0.947297366
RAB11B	0.148202403	0.597122442	0.137557259	0.79443537	0.30408307	0.308582588	0.947297366
RAB40C	0.139542526	0.46755373	0.154263649	0.532701624	0.542781162	0.307105375	0.947297366
RARRES1	0.56324016	0.24518012	0.223641028	0.908311658	0.103766704	0.307141987	0.947297366
RNF34	0.559568265	0.134121075	0.768307546	0.657924875	0.075792616	0.305413156	0.947297366
RPS15A	0.604106916	0.366882404	0.815566475	0.037426196	0.434638	0.308566456	0.947297366
RPS18	0.507463009	0.404489586	0.985355271	0.041434011	0.349864692	0.308162167	0.947297366
RSL24D1	0.323476223	0.736072195	0.064406606	0.467933983	0.399743832	0.305080513	0.947297366
SCO1	0.276339742	0.369023427	0.065060106	0.53962717	0.813579962	0.307233835	0.947297366
SEPT10	0.17944211	0.547669902	0.526667201	0.77724856	0.071606735	0.305673569	0.947297366
SH3BP1	0.339173098	0.662960509	0.184602753	0.155445337	0.446711797	0.305757407	0.947297366
SLC2A1	0.872403004	0.172081821	0.062404479	0.418958496	0.751265647	0.308966948	0.947297366
SLC44A3	0.615732209	0.866133027	0.221543797	0.180681902	0.135557078	0.306314678	0.947297366
SMARCAL1	0.169294846	0.188090001	0.437229942	0.4194416	0.503422907	0.308540521	0.947297366
SP7	0.844501956	0.520825839	0.165426553	0.074085865	0.544610025	0.3083437	0.947297366
SUSD3	0.203554787	0.096468908	0.508143017	0.6560748	0.448452364	0.308345305	0.947297366
TAF9	0.554923583	0.716478865	0.585648842	0.180451131	0.068980549	0.306537448	0.947297366
TDP1	0.723696978	0.039539066	0.277243795	0.677243478	0.547517876	0.308626619	0.947297366
THEMIS2	0.92913753	0.838589868	0.014495288	0.851306879	0.306697765	0.30897372	0.947297366
TLN1	0.415116228	0.589447795	0.099715774	0.675919077	0.174675074	0.305677208	0.947297366
TMEM140	0.358641339	0.5362587	0.90223969	0.023262075	0.718374334	0.306600935	0.947297366
TONSL	0.606613761	0.02283465	0.355510681	0.727634895	0.805402715	0.305930139	0.947297366
TRIM13	0.935336079	0.319885827	0.434887869	0.174991818	0.128461586	0.307826878	0.947297366
UBE2M	0.225550438	0.332333994	0.369964183	0.218763867	0.482904243	0.308049276	0.947297366
ZNF692	0.087065377	0.085858656	0.517903905	0.788021819	0.950398739	0.30659022	0.947297366

BTBD6	0.365167228	0.413980938	0.093736417	0.416238317	0.50065963	0.309173256	0.94749623
C28H1orf131	0.975451757	0.004592427	0.824314983	0.949994718	0.843946814	0.30953621	0.947607196
HIGD1B	0.207974688	0.11070353	0.903739278	0.959237577	0.148674307	0.309862665	0.947607196
LOC100848331	0.74288845	0.912494688	0.059892201	0.447004965	0.1635278	0.309879309	0.947607196
LOC101905319	0.700579993	0.918278755	0.045119748	0.128143466	0.79511423	0.30938851	0.947607196
LOC104969140	0.663517981	0.027610292	0.668836784	0.247616966	0.97807507	0.309868943	0.947607196
MON1A	0.347312	0.763857499	0.134142447	0.221290689	0.376014325	0.309565071	0.947607196
NDUFAF8	0.40872903	0.456029285	0.520457944	0.091456099	0.334072254	0.30969601	0.947607196
REEP3	0.763686273	0.089939351	0.498305482	0.086990104	0.993905062	0.309470171	0.947607196
SLC25A10	0.368778888	0.908462358	0.027499597	0.593152271	0.542388734	0.309699025	0.947607196
SMARCC2	0.602551539	0.637654251	0.55025533	0.126201711	0.111231384	0.309881967	0.947607196
TEP1	0.59159902	0.065308341	0.08800405	0.881789668	0.987588594	0.309556563	0.947607196
WDR35	0.52582626	0.814219699	0.104831634	0.976815809	0.067703302	0.309902372	0.947607196
CAPN15	0.776905573	0.156520167	0.764830812	0.050260551	0.635706555	0.310062178	0.947919225
ATP5F1E	0.925164046	0.221996889	0.952312049	0.01642628	0.93109791	0.311008152	0.948453115
ATP5MF	0.774738089	0.342879221	0.973504924	0.013170818	0.884249539	0.311972727	0.948453115
ATXN7L2	0.927863854	0.410201558	0.056995611	0.971979179	0.143069745	0.312204452	0.948453115
B3GALT1	0.088656458	0.085386699	0.828561838	0.822873126	0.579478523	0.310980733	0.948453115
BEAN1	0.930170553	0.502824982	0.034954659	0.348634251	0.526775825	0.31153272	0.948453115
C18H19orf18	0.99704044	0.291289397	0.213229909	0.254276709	0.191456437	0.31211725	0.948453115
C7	0.044445466	0.498281699	0.356358858	0.553885397	0.688931305	0.311960908	0.948453115
CCT7	0.428109089	0.521541482	0.267508666	0.576502246	0.087623651	0.312229332	0.948453115
CGAS	0.922039294	0.375293063	0.02894416	0.33857194	0.885558958	0.311555334	0.948453115
EYA3	0.732280925	0.789373845	0.168587	0.032123287	0.961693898	0.311913519	0.948453115
FARP1	0.258014377	0.079381521	0.198221399	0.74751079	0.99150707	0.311843226	0.948453115
FBXL22	0.153567336	0.56937645	0.452895955	0.351320457	0.215178614	0.31111291	0.948453115
FOXJ2	0.275602431	0.119249578	0.793067729	0.680686535	0.167931231	0.310435196	0.948453115
GNL1	0.07305042	0.860213215	0.374091499	0.320995031	0.397948497	0.311549591	0.948453115
GOLGA4	0.603731292	0.700632651	0.306778278	0.023263229	0.987463207	0.310507412	0.948453115
GPM6A	0.280119763	0.165916501	0.628614408	0.802318502	0.128874083	0.31240226	0.948453115
IQGAP1	0.702295819	0.212413533	0.457906819	0.068442707	0.644164082	0.311966574	0.948453115
IRAK4	0.427929507	0.101169326	0.367794936	0.50910793	0.371800505	0.312079351	0.948453115
KIF3C	0.362866471	0.891375876	0.039212377	0.666221569	0.354252944	0.311101022	0.948453115
LOC107132852	0.067383511	0.727490651	0.288159272	0.44358937	0.477408453	0.311009921	0.948453115
LOC615183	0.020415626	0.587419594	0.719092108	0.405273166	0.854798419	0.310820784	0.948453115
LRP11	0.04955937	0.289675553	0.63169323	0.429166699	0.766035909	0.310530116	0.948453115
MBD3	0.335816219	0.444253326	0.452792802	0.181138859	0.243343301	0.310348233	0.948453115
MRPL18	0.385004302	0.36380903	0.55290319	0.044780929	0.868180781	0.311929938	0.948453115
OLR1	0.703317292	0.736768694	0.020534431	0.61058197	0.459067352	0.310584596	0.948453115
PDCD7	0.150013411	0.037818351	0.810590408	0.811646964	0.802687298	0.311227032	0.948453115
PER2	0.360868999	0.605888468	0.51186447	0.124725333	0.213985639	0.31079765	0.948453115
PHF24	0.841400336	0.706933316	0.258822356	0.762303807	0.025709138	0.312227691	0.948453115
RRAS	0.528293731	0.324220743	0.186568949	0.654274594	0.143256738	0.311188711	0.948453115
SELENON	0.163008858	0.371595529	0.084981832	0.752825687	0.775489642	0.311663166	0.948453115
SEMA3D	0.022492859	0.899507019	0.227131488	0.896088466	0.733435164	0.312372409	0.948453115

SLC9A9	0.540852617	0.841076511	0.191240432	0.118101369	0.293676547	0.312234207	0.948453115
SNED1	0.934143313	0.215756881	0.089525703	0.265597333	0.630483812	0.312432974	0.948453115
SRFBP1	0.399695823	0.148900246	0.50089811	0.125607355	0.80577371	0.312229027	0.948453115
TIMM10B	0.811313629	0.466221604	0.10935137	0.333462605	0.21789373	0.311669418	0.948453115
TRIM28	0.750298265	0.782036781	0.009451776	0.583459011	0.926752772	0.311358469	0.948453115
ZNF407	0.537406723	0.575661781	0.693023507	0.120863169	0.116485738	0.312288991	0.948453115
ZNF582	0.897777384	0.786469644	0.830755696	0.012568926	0.406474166	0.31126221	0.948453115
RGN	0.254042408	0.864519598	0.309063701	0.056330041	0.790806475	0.312536723	0.948592596
ANO8	0.269856746	0.217349534	0.244606565	0.227773647	0.927935391	0.312944987	0.949047684
GCNT2	0.538934619	0.335994345	0.633916189	0.04368275	0.60487437	0.312975813	0.949047684
HSPA8	0.453964991	0.33847189	0.866134874	0.023922524	0.952593077	0.3129659	0.949047684
NPW	0.441490858	0.992844822	0.12358959	0.329338167	0.169727327	0.312746886	0.949047684
TRDMT1	0.831124551	0.311455918	0.418233194	0.93066579	0.030075637	0.312849119	0.949047684
SNX24	0.065138311	0.403374919	0.127344227	0.914976253	0.992445013	0.313227771	0.949636238
ARGLU1	0.338066779	0.260064221	0.051687379	0.728808611	0.921381875	0.31384679	0.949865807
ASNA1	0.301695263	0.529091289	0.474611171	0.137463719	0.295111457	0.314866248	0.949865807
AUTS2	0.892316495	0.438276336	0.43025662	0.020830117	0.874593476	0.314496933	0.949865807
CDKL2	0.576054198	0.85075149	0.688265958	0.027942235	0.324892096	0.314341937	0.949865807
CEP162	0.29399225	0.077602198	0.347802391	0.486799346	0.793146453	0.314415569	0.949865807
ELMO2	0.932278041	0.285080653	0.471982451	0.050772668	0.482165548	0.314751996	0.949865807
IDE	0.668925378	0.62410455	0.542419281	0.840865885	0.016113494	0.314627014	0.949865807
IRAK1	0.524797649	0.119554471	0.355707952	0.346966215	0.395915822	0.314513063	0.949865807
LOC100296952	0.325871873	0.61321307	0.193782433	0.109722292	0.720457595	0.314293561	0.949865807
LOC104974050	0.185371929	0.605006705	0.417797073	0.37763448	0.173218356	0.314478014	0.949865807
LOC112444498	0.180882753	0.594107094	0.992648401	0.092673643	0.309429053	0.314194189	0.949865807
LOC617905	0.52852816	0.091024691	0.643287007	0.876191539	0.112587508	0.313912898	0.949865807
LZTS1	0.782185349	0.829686785	0.310002792	0.033879376	0.447936134	0.313919349	0.949865807
MAF1	0.715477404	0.280350866	0.325667911	0.117049923	0.39886719	0.313764902	0.949865807
MSRB3	0.473566077	0.451818862	0.087203202	0.492722849	0.33384474	0.31467257	0.949865807
NUF2	0.565975104	0.120657043	0.06096038	0.821570321	0.892682694	0.313918877	0.949865807
PDIA6	0.119704111	0.527488693	0.08108941	0.730448691	0.820672107	0.314679546	0.949865807
PPIL6	0.443920116	0.720757565	0.438207971	0.150844341	0.145302422	0.314854678	0.949865807
PRRG4	0.152588087	0.481730406	0.593324189	0.196400155	0.358010784	0.31455047	0.949865807
PSMD1	0.950226011	0.697263965	0.2959507	0.833773355	0.018716658	0.314242061	0.949865807
RIC8A	0.299443719	0.687035672	0.098849709	0.233932572	0.645247654	0.314693171	0.949865807
SEL1L3	0.71718878	0.242009619	0.19153938	0.855178745	0.107338544	0.313852067	0.949865807
SNRNP48	0.310109805	0.498249328	0.875048564	0.266656508	0.085102408	0.314627611	0.949865807
SNRNP70	0.49307833	0.199895586	0.060315696	0.539807554	0.948095369	0.313425098	0.949865807
SNRPD2	0.05749057	0.478173052	0.723045563	0.470991013	0.328148499	0.314806546	0.949865807
SQSTM1	0.551876749	0.179448125	0.082551046	0.559699491	0.666383541	0.31373543	0.949865807
TMED9	0.757032746	0.631717801	0.379556009	0.020147361	0.838649756	0.314569537	0.949865807
KIF27	0.590874244	0.58219274	0.655943578	0.032281942	0.422390736	0.315028473	0.950180532
ASB11	0.101774702	0.115115796	0.554432268	0.850169492	0.558415466	0.3153536	0.950565514
DBN1	0.359033606	0.89308936	0.041192194	0.440000602	0.53192333	0.315704493	0.950565514
ENSA	0.348459708	0.428073859	0.375774148	0.410566004	0.134028211	0.31538374	0.950565514

IFI35	0.35360948	0.543868726	0.746611565	0.164025346	0.131188968	0.315629641	0.950565514
LIMK1	0.379656347	0.408411876	0.16407117	0.683583333	0.177956639	0.315863225	0.950565514
LRRC51	0.859440627	0.625323905	0.021320144	0.825404122	0.326677333	0.315621607	0.950565514
NCAPH	0.251314763	0.555268758	0.401379242	0.239308323	0.230830186	0.315829997	0.950565514
NES	0.194177575	0.228400013	0.875096158	0.100138952	0.795784236	0.315771358	0.950565514
OARD1	0.124552023	0.380998935	0.662791219	0.099317964	0.991343198	0.315954632	0.950565514
PPARGC1B	0.853110039	0.112227225	0.176316673	0.278966195	0.656921192	0.315808474	0.950565514
SLC38A9	0.13691243	0.970160832	0.589166318	0.083976291	0.471448585	0.316024949	0.950565514
SNX21	0.672738788	0.723817484	0.058293476	0.140661273	0.772910282	0.315457052	0.950565514
TGFB3	0.864092748	0.574667354	0.217278387	0.033080349	0.867646582	0.315955701	0.950565514
TNIK	0.950436288	0.927905696	0.692593012	0.029998719	0.169044858	0.315989882	0.950565514
ZNF667	0.276551477	0.929992017	0.280405221	0.15936523	0.268870592	0.315648562	0.950565514
IRAK3	0.339436858	0.150677267	0.573131706	0.106491199	0.993326568	0.316141292	0.950741203
BAG6	0.095035467	0.730757177	0.485773417	0.868162021	0.107058025	0.317748629	0.95135226
BORCS6	0.190122823	0.943743947	0.931034201	0.104452639	0.178863229	0.31707857	0.95135226
CBR1	0.187468473	0.374359534	0.493159605	0.10689762	0.841519757	0.316727797	0.95135226
CCR3	0.023421389	0.748898389	0.244409799	0.804215711	0.901794001	0.316527931	0.95135226
CDC42BPG	0.031430203	0.556496589	0.368750602	0.654068277	0.743141825	0.3177236	0.95135226
CLEC11A	0.238266237	0.337957201	0.059947284	0.829010128	0.78354015	0.31774938	0.95135226
FAM189B	0.851402142	0.540409681	0.493385616	0.021272529	0.646965602	0.317227941	0.95135226
FES	0.65291016	0.626224889	0.189228796	0.065619498	0.619036713	0.318083593	0.95135226
GIPC2	0.438815666	0.32417851	0.198589865	0.120805007	0.918851837	0.31776235	0.95135226
GLRX5	0.456979785	0.422985568	0.941299981	0.023257004	0.736121117	0.316799633	0.95135226
GRHL1	0.049196116	0.675691364	0.364607335	0.318539281	0.813340856	0.317957217	0.95135226
HPRT1	0.767528332	0.39939007	0.034319945	0.958626645	0.309635177	0.317159372	0.95135226
HTRA3	0.937794638	0.377083711	0.08380109	0.819299599	0.128756645	0.317315215	0.95135226
KDR	0.555494649	0.540742968	0.306754706	0.036001439	0.947403026	0.318082375	0.95135226
KLHL8	0.9585968	0.795934718	0.292844428	0.21285309	0.065781365	0.317423333	0.95135226
LOC100126043	0.724002732	0.209709496	0.252230048	0.115005182	0.712856513	0.317935439	0.95135226
LOC100139891	0.452479369	0.455524418	0.95011772	0.054607368	0.291439297	0.316877097	0.95135226
LOC100848226	0.259071904	0.583617498	0.892357451	0.068848173	0.335208639	0.316747065	0.95135226
LOC112447353	0.227867595	0.962915675	0.422249971	0.077784987	0.433585626	0.317249999	0.95135226
LOC112447435	0.615107472	0.043450832	0.201126221	0.949994389	0.613553593	0.317641909	0.95135226
LOC510454	0.420365739	0.985673806	0.188109837	0.259830824	0.15483744	0.317757313	0.95135226
MYNN	0.015464087	0.994679503	0.346104324	0.639978605	0.920087033	0.317714168	0.95135226
NR1H2	0.408412702	0.684512699	0.058083587	0.970038098	0.19945817	0.31803522	0.95135226
RBKS	0.482955764	0.880672121	0.151689938	0.160935602	0.302012763	0.317762999	0.95135226
RUNX1T1	0.941721136	0.519270617	0.563295135	0.439116727	0.025979229	0.318062986	0.95135226
SMARCA1	0.81893565	0.848780551	0.711947819	0.014545605	0.432563005	0.316740241	0.95135226
TJP2	0.063103431	0.935619571	0.32184256	0.645620538	0.254828382	0.317319631	0.95135226
TMBIM4	0.400910714	0.094696446	0.158544312	0.660286274	0.785362622	0.317091827	0.95135226
VSIG2	0.359277237	0.277498428	0.895205385	0.269170121	0.130783931	0.318041654	0.95135226
WDR87	0.65401576	0.271103088	0.526423843	0.650418354	0.051700861	0.317893603	0.95135226
IL6ST	0.637517378	0.537593921	0.125981063	0.44564302	0.163443811	0.318179017	0.951361135
ZNF839	0.337008447	0.182902163	0.728310043	0.091329606	0.767167751	0.318202502	0.951361135

DHCR7	0.057561211	0.607272601	0.458805592	0.287558483	0.682933581	0.318391828	0.951361348
DNM2	0.818093049	0.506515891	0.436635297	0.261390717	0.066702903	0.318625644	0.951361348
HIPK2	0.596176847	0.857306538	0.032502126	0.536713992	0.354195947	0.318778803	0.951361348
LOC100848575	0.467376015	0.520077912	0.040965742	0.400747209	0.79094247	0.318699401	0.951361348
LOC101906226	0.223394792	0.865085575	0.284713493	0.432257941	0.132475122	0.318448564	0.951361348
LOC104971374	0.410410016	0.089882181	0.361120246	0.605042428	0.391823747	0.318782283	0.951361348
PFKFB4	0.092097621	0.386692432	0.164195671	0.699917583	0.769669569	0.318418461	0.951361348
RBM10	0.731774044	0.053027216	0.328257822	0.298014347	0.831366318	0.31868257	0.951361348
XCL2	0.368497833	0.786030243	0.602120844	0.157033382	0.115226924	0.318677011	0.951361348
ZC3H11A	0.100942585	0.633879534	0.26325027	0.376001074	0.497520095	0.318459285	0.951361348
CAMK4	0.716841378	0.136892982	0.492027386	0.138680529	0.476518202	0.320272663	0.951520164
CASD1	0.028925333	0.996900802	0.158003312	0.976040829	0.717851398	0.320342996	0.951520164
CDIP1	0.151420458	0.253996736	0.587393488	0.47941032	0.294477725	0.320210245	0.951520164
CYP51A1	0.57076074	0.441968521	0.016217567	0.854911341	0.904981047	0.319105978	0.951520164
DISP1	0.804010071	0.289659728	0.085999577	0.853986426	0.186286111	0.320068554	0.951520164
HOXC5	0.467427121	0.390499172	0.81703535	0.021947483	0.966706012	0.319060105	0.951520164
IL6R	0.711660372	0.982164224	0.148973757	0.032447361	0.944246165	0.320253544	0.951520164
LOC101902207	0.09283616	0.530069513	0.575492413	0.153086388	0.735293321	0.320138489	0.951520164
LOC112442664	0.471416712	0.606742297	0.783294192	0.343424141	0.04120312	0.319340429	0.951520164
LOC782479	0.283429273	0.68784726	0.237613354	0.12093094	0.564222909	0.318907419	0.951520164
MBNL2	0.711781353	0.897040816	0.053614893	0.146702311	0.634664828	0.320118065	0.951520164
MSANTD2	0.312153832	0.788643321	0.87421911	0.091932804	0.161101779	0.320123008	0.951520164
MYOZ1	0.54124615	0.97809618	0.049839963	0.812956591	0.148268389	0.319799053	0.951520164
NUDT16L1	0.307004909	0.551997334	0.262409849	0.501932843	0.143006514	0.320331244	0.951520164
PROS1	0.623727473	0.185744315	0.094922895	0.291193996	0.988543941	0.319128668	0.951520164
RABL6	0.184709908	0.851726022	0.331284807	0.095961568	0.637347391	0.320131268	0.951520164
RETREG3	0.182124738	0.676380292	0.990610994	0.681132203	0.038141272	0.319338606	0.951520164
RFFL	0.594216441	0.562264447	0.838810778	0.054426688	0.208244758	0.319620631	0.951520164
RNF150	0.731236261	0.609001967	0.024225293	0.9593549	0.308226948	0.320241547	0.951520164
RPS19BP1	0.188957388	0.553487901	0.635720647	0.129502994	0.368245039	0.31936042	0.951520164
SCN2B	0.22257257	0.044217306	0.697300279	0.484667383	0.955663581	0.319719823	0.951520164
SENP7	0.126787331	0.446313535	0.571435326	0.228206302	0.430290144	0.319566178	0.951520164
SNF8	0.702949496	0.094706887	0.13884333	0.517734699	0.662752209	0.319405141	0.951520164
SNHG3	0.16793948	0.706102943	0.330702396	0.286274921	0.282983064	0.319642861	0.951520164
TRAF6	0.285347159	0.53706811	0.091507316	0.924468451	0.245466278	0.319890049	0.951520164
ULK4	0.252267476	0.379785138	0.284988007	0.513761109	0.227142764	0.320071418	0.951520164
NOX5	0.298759658	0.865825821	0.089528543	0.187942943	0.73375711	0.320407585	0.951531461
TMEM86A	0.715865988	0.821998656	0.120592076	0.065646125	0.685846255	0.320462762	0.951531461
TTLL3	0.195338667	0.28529519	0.279646956	0.318558941	0.643835046	0.320529506	0.951557474
FOXM1	0.956171199	0.078618659	0.115955579	0.849544814	0.431917503	0.320624055	0.951666009
APCDD1	0.792364525	0.466206389	0.074262435	0.306690168	0.383560078	0.32191807	0.951891432
C22H3orf22	0.317441082	0.042148303	0.863334724	0.876196664	0.317675328	0.321382663	0.951891432
CDC42EP5	0.444873953	0.583121177	0.028002676	0.888682819	0.497915755	0.321345993	0.951891432
HAGH	0.86034541	0.711295621	0.638710244	0.013031592	0.629604114	0.321009265	0.951891432
HEYL	0.297359356	0.142731992	0.509545284	0.620411346	0.24021314	0.321735935	0.951891432

LOC112442295	0.267459517	0.061435647	0.382498544	0.913788766	0.561400933	0.321791229	0.951891432
LOC615899	0.594376541	0.064005443	0.302649535	0.432805436	0.646004117	0.321563188	0.951891432
LOC781261	0.116619306	0.767200924	0.298545584	0.395424054	0.305302391	0.321810092	0.951891432
LOC786139	0.774328304	0.840722847	0.822381634	0.02331124	0.257821483	0.321492616	0.951891432
NR2C2	0.795017735	0.825738258	0.887417213	0.02045651	0.269546115	0.321250154	0.951891432
NUDT14	0.146167583	0.63132278	0.401157413	0.094524668	0.919266797	0.321448484	0.951891432
PDGFRA	0.109879029	0.642003308	0.386974069	0.447793343	0.263532824	0.321663592	0.951891432
PODXL2	0.68484594	0.926302838	0.076218617	0.274097819	0.243305945	0.321803969	0.951891432
RUSC1	0.325840883	0.549852932	0.579913109	0.091502063	0.336813882	0.320789853	0.951891432
SLC38A6	0.151131165	0.829870304	0.110509441	0.373234197	0.623440583	0.321829068	0.951891432
SORCS2	0.537258228	0.37570651	0.104463967	0.30513367	0.50106684	0.32177678	0.951891432
SREK1IP1	0.168401817	0.290777374	0.113823573	0.886926517	0.651281617	0.321579764	0.951891432
TGFB1I1	0.362547612	0.339086289	0.617361449	0.699612615	0.060761032	0.32188183	0.951891432
TSFM	0.711848335	0.485866776	0.743189653	0.059659055	0.210130793	0.321705172	0.951891432
TVP23B	0.724764654	0.351600066	0.221113925	0.058041961	0.980611982	0.321012426	0.951891432
USP24	0.571642326	0.627172091	0.194068086	0.063113356	0.730387386	0.321025287	0.951891432
ARHGEF17	0.261491989	0.5473986	0.245762918	0.360746892	0.254520816	0.322051716	0.951903985
C26H10orf88	0.028940471	0.19655239	0.828551053	0.885608719	0.776296011	0.322510649	0.951903985
CD247	0.527840188	0.793132755	0.08187599	0.581150017	0.162439617	0.322313579	0.951903985
CNKSR2	0.760773951	0.310820586	0.934860088	0.701820933	0.020925377	0.322792376	0.951903985
DLAT	0.788144577	0.633069482	0.581742787	0.036153087	0.308057043	0.322172596	0.951903985
FAM219A	0.096046218	0.38867613	0.669519425	0.51172703	0.253313149	0.32249554	0.951903985
HMBS	0.216911888	0.649081476	0.921497686	0.082957894	0.301504102	0.322730637	0.951903985
KRT23	0.547926647	0.95355545	0.032372706	0.200183779	0.958067231	0.322678424	0.951903985
LOC112442979	0.668793413	0.122757333	0.602776533	0.44361599	0.147660382	0.322575936	0.951903985
LOC112449406	0.768221741	0.049136596	0.165616508	0.627520826	0.82478649	0.322306818	0.951903985
RIPK3	0.481227772	0.229683169	0.092938336	0.52012349	0.607161152	0.322682768	0.951903985
ROM1	0.850959752	0.292828879	0.231128548	0.064570373	0.872752042	0.322755121	0.951903985
SFXN1	0.473642853	0.537173239	0.453510954	0.054440825	0.515424546	0.322400079	0.951903985
SHC2	0.886675831	0.306301151	0.122596018	0.262782212	0.370428444	0.32255016	0.951903985
UQCR10	0.81849361	0.591674196	0.603223473	0.01287926	0.86138126	0.322540868	0.951903985
EEF1G	0.4922503	0.31198552	0.648971529	0.093267522	0.350001224	0.323106271	0.952045493
EVA1A	0.944164527	0.613902743	0.028419742	0.619695099	0.318380485	0.322953676	0.952045493
GALNT18	0.675520191	0.109598959	0.2795279	0.285848112	0.550195824	0.323165574	0.952045493
GJC1	0.418851573	0.814015642	0.254224694	0.143830878	0.261112586	0.323188437	0.952045493
GOLGB1	0.542556886	0.384051034	0.188350031	0.224353218	0.36966014	0.323169707	0.952045493
ZNF143	0.323287986	0.23156789	0.503072646	0.210037646	0.410874388	0.322958023	0.952045493
C2H2orf88	0.713720002	0.535999576	0.92923689	0.058128995	0.157722669	0.323361589	0.952155323
LOC100848639	0.927930848	0.025923759	0.230407549	0.935135488	0.628976536	0.323399779	0.952155323
NDC80	0.717751118	0.043643699	0.159833418	0.740483731	0.878775734	0.323311467	0.952155323
EGFR	0.951830543	0.470213152	0.210383341	0.079487306	0.436015535	0.323549837	0.952255447
MTCH2	0.839268032	0.418717636	0.612981758	0.074265449	0.203955027	0.323524682	0.952255447
HNRNPD	0.818911664	0.520015721	0.043531061	0.496943762	0.355038809	0.32387631	0.952362322
KIAA0930	0.427921217	0.210568852	0.056942436	0.834547687	0.762886855	0.323697774	0.952362322
LOC100848504	0.810681306	0.957708783	0.04554017	0.177134052	0.522179927	0.323864413	0.952362322

LOC112442053	0.396056656	0.176298592	0.084501576	0.629917905	0.879903337	0.323860839	0.952362322
STX2	0.816242684	0.586082128	0.320138935	0.044607895	0.4785497	0.323815463	0.952362322
CCDC34	0.510723466	0.118905946	0.968823031	0.321990484	0.17278951	0.323996947	0.95254638
MGRN1	0.923638855	0.165426582	0.261416383	0.223921786	0.366157227	0.324067648	0.952583588
STAC2	0.076942616	0.804078063	0.514812907	0.134625325	0.764956863	0.324294648	0.952909485
TTL	0.100812444	0.541740491	0.261323977	0.270481736	0.849677449	0.324294121	0.952909485
FAM3C	0.284565835	0.848519017	0.238560095	0.087525573	0.651789629	0.324566307	0.953059472
HAS2	0.717798313	0.987515322	0.012371641	0.409183735	0.915302973	0.324489559	0.953059472
LOC107133473	0.881999546	0.507396415	0.726548523	0.055854112	0.180880253	0.324513106	0.953059472
PET117	0.459104573	0.976262718	0.015604031	0.582092183	0.807257932	0.32457799	0.953059472
LOC112447385	0.332080603	0.674775975	0.183042206	0.123678357	0.648134421	0.324643365	0.953080906
IQSEC2	0.644471618	0.842126217	0.071501336	0.555508098	0.153196722	0.325291635	0.953266583
LOC101908214	0.343641042	0.764081642	0.708050672	0.243156152	0.072996941	0.325178252	0.953266583
LOC104968476	0.258552957	0.155779103	0.938228733	0.625025347	0.139683339	0.32514809	0.953266583
LOC104971683	0.634818276	0.147504591	0.199906766	0.195344995	0.902236644	0.325146581	0.953266583
MT1E	0.769755084	0.288358658	0.854745723	0.228678791	0.076105039	0.325266924	0.953266583
NBN	0.070368811	0.63640327	0.669834196	0.736141478	0.1491513	0.324897971	0.953266583
RASSF8	0.72368158	0.32268315	0.809244175	0.023006093	0.758149933	0.32501033	0.953266583
SH3GL2	0.77980867	0.378684038	0.278310489	0.292719753	0.137135658	0.325143832	0.953266583
SPPL2B	0.350930171	0.480932322	0.670484738	0.126503665	0.230779966	0.325345569	0.953266583
THNSL2	0.692682714	0.538417496	0.04233319	0.242175052	0.863426693	0.325242484	0.953266583
ZNF628	0.409242035	0.797989609	0.066227551	0.161417902	0.94526763	0.325186214	0.953266583
ACVR2A	0.3870962	0.396933875	0.180860397	0.858577048	0.141920794	0.328974735	0.953284346
ADCY2	0.816009897	0.457360771	0.011942883	0.838490862	0.904277195	0.32868834	0.953284346
AKIRIN1	0.302550849	0.226307417	0.291818412	0.272485096	0.611610953	0.32650587	0.953284346
ANAPC11	0.168109322	0.524884504	0.375952587	0.175758563	0.580027392	0.328786972	0.953284346
APBB1	0.744082692	0.174168079	0.111710091	0.870563267	0.265509695	0.327228844	0.953284346
APH1A	0.349247281	0.705308993	0.587632064	0.030955067	0.739526517	0.325787539	0.953284346
ARMC2	0.630893955	0.336687824	0.491814186	0.940324329	0.034262036	0.328081091	0.953284346
BCAS4	0.256604669	0.398376038	0.160532557	0.492621321	0.420272043	0.329471054	0.953284346
BPTF	0.494605252	0.788277209	0.688262566	0.029197982	0.42618057	0.326915633	0.953284346
BUB3	0.585409797	0.684592117	0.624074443	0.291134837	0.046380527	0.328584582	0.953284346
C5H22orf23	0.513855306	0.24127267	0.5695189	0.068815215	0.701766601	0.330005677	0.953284346
CACYBP	0.819582872	0.312006729	0.64392676	0.040787602	0.503517871	0.328781108	0.953284346
CCDC174	0.200647111	0.98080815	0.570699078	0.065824694	0.451853932	0.326974135	0.953284346
CCND1	0.328921763	0.225923813	0.328528329	0.177834599	0.774788857	0.327996711	0.953284346
CCNI	0.309383651	0.42672425	0.663866656	0.045619632	0.833818657	0.326682219	0.953284346
CCR10	0.525309179	0.371886069	0.66212133	0.056625714	0.464655592	0.329723956	0.953284346
CDCP1	0.655774803	0.030303499	0.3948103	0.854146517	0.506321547	0.329277116	0.953284346
CDK7	0.658969625	0.785251561	0.672097975	0.011794397	0.829736006	0.329729107	0.953284346
CENPK	0.761977755	0.196309125	0.148782013	0.35861438	0.420502616	0.327658289	0.953284346
CREB3L4	0.305089967	0.111799819	0.320484928	0.414453759	0.754232826	0.330320604	0.953284346
CSRNP2	0.316739216	0.411883245	0.932111838	0.047717847	0.585152591	0.329378937	0.953284346
CTSF	0.377299575	0.802062392	0.03152348	0.707808768	0.506019342	0.330304758	0.953284346
DERL3	0.388206443	0.105964322	0.729569946	0.8112353	0.13987801	0.329818742	0.953284346

DNM1	0.698710271	0.166185001	0.346579081	0.23141131	0.357030312	0.326287025	0.953284346
DOK6	0.395848649	0.325829186	0.766041479	0.035767849	0.944795577	0.326903369	0.953284346
EMC8	0.469113039	0.630601838	0.416460341	0.344157512	0.079650324	0.328582222	0.953284346
EXTL3	0.154727712	0.575552363	0.190820866	0.365238531	0.55087253	0.330405299	0.953284346
FANCE	0.611487084	0.713724359	0.817554693	0.038141245	0.249037442	0.329106795	0.953284346
FBL	0.339024539	0.929171781	0.617037365	0.444410312	0.039499088	0.330099574	0.953284346
FBXO30	0.05136409	0.912986124	0.535104167	0.145722569	0.927988822	0.329289678	0.953284346
FHOD3	0.291096257	0.816422772	0.075269693	0.448287501	0.415945551	0.326755359	0.953284346
GARNL3	0.160170412	0.289235017	0.349822875	0.54138536	0.378279459	0.326023166	0.953284346
GPLD1	0.02768497	0.88095472	0.531549652	0.511299247	0.502201018	0.326460652	0.953284346
GYS1	0.391189063	0.77589143	0.345582496	0.056490536	0.557921744	0.32544469	0.953284346
HYPK	0.102846132	0.218532961	0.513894855	0.742323572	0.387999482	0.326362324	0.953284346
IER2	0.305963735	0.11743896	0.464375298	0.243723976	0.830622687	0.328616923	0.953284346
IFFO2	0.589850433	0.192681013	0.636587282	0.325959866	0.14494823	0.330374373	0.953284346
KBTBD4	0.95612395	0.961308525	0.319655681	0.048303548	0.236215915	0.327494834	0.953284346
LOC100296832	0.565872219	0.816792581	0.480914271	0.054104101	0.283396871	0.329933701	0.953284346
LOC104975034	0.229444887	0.705248433	0.223695482	0.131713142	0.694347594	0.325645763	0.953284346
LOC112444778	0.860982284	0.730110887	0.05237228	0.719656307	0.142190495	0.32821844	0.953284346
LOC112445995	0.202708755	0.358731426	0.573524531	0.13640196	0.591625498	0.328076257	0.953284346
LOC112446689	0.227499062	0.859806318	0.247880841	0.072151112	0.955669556	0.327097072	0.953284346
LOC112448582	0.233373374	0.943980524	0.788871439	0.420953055	0.046420201	0.329401842	0.953284346
LOC781224	0.825534405	0.409286651	0.878180583	0.01179782	0.962929662	0.328307642	0.953284346
LOC783838	0.100682944	0.677599802	0.81995744	0.163567388	0.369473937	0.328735097	0.953284346
MAP3K9	0.39392214	0.685870392	0.619517843	0.303579361	0.066543944	0.328764341	0.953284346
MGAT5B	0.243788846	0.678562618	0.09821242	0.817230205	0.255951757	0.329507857	0.953284346
MICAL2	0.338590395	0.54745255	0.437512119	0.310863169	0.131456636	0.325807662	0.953284346
MRPS23	0.904218995	0.44250023	0.442107923	0.046269634	0.415583376	0.32964301	0.953284346
NKRF	0.739331999	0.296181408	0.728824863	0.077489691	0.26918016	0.326465127	0.953284346
NRIP2	0.542951082	0.227029871	0.567380923	0.413682687	0.115389625	0.326886582	0.953284346
NSMCE2	0.043029926	0.770534108	0.322587949	0.325709393	0.974405481	0.329340358	0.953284346
NUDT21	0.528702879	0.517530313	0.304786524	0.29784237	0.13641903	0.329076361	0.953284346
NUP62CL	0.830611769	0.209706999	0.647552357	0.065376483	0.453590565	0.327163977	0.953284346
P4HA1	0.535569516	0.135431222	0.596185229	0.145565469	0.526902993	0.325923201	0.953284346
PARD3B	0.940856201	0.222285821	0.835415733	0.16179099	0.119762129	0.328942651	0.953284346
PAWR	0.091993022	0.50145329	0.234621336	0.996908641	0.313458674	0.328798839	0.953284346
PIK3R2	0.562772813	0.513086193	0.021189184	0.959494836	0.577046679	0.329037785	0.953284346
PITPNA	0.199609723	0.340858981	0.141376383	0.544322995	0.651740787	0.330118348	0.953284346
PLEKHO2	0.490416469	0.812639103	0.024361941	0.364640276	0.961606219	0.329767964	0.953284346
PMM2	0.36709828	0.735531312	0.86935683	0.066493468	0.215263807	0.327828574	0.953284346
POGLUT1	0.046099796	0.740848479	0.883521319	0.115052727	0.963110649	0.327112163	0.953284346
POLE3	0.330413869	0.258828582	0.29848395	0.391078976	0.340203401	0.329413192	0.953284346
POLR2E	0.594526031	0.760868973	0.527275588	0.025191071	0.558965087	0.327766877	0.953284346
PPP1R11	0.47780582	0.09481243	0.251179085	0.411619336	0.724284114	0.329246545	0.953284346
PROZ	0.909006562	0.04580203	0.789875837	0.309205163	0.334465041	0.329622031	0.953284346
RANGAP1	0.612362235	0.660559149	0.167806056	0.476647215	0.104845666	0.329236463	0.953284346

RHBDD1	0.385284808	0.102998867	0.103473574	0.824573206	0.97994147	0.325980069	0.953284346
RNF4	0.162511682	0.681910659	0.714618628	0.042752772	0.993364706	0.327973745	0.953284346
RPUSD3	0.223730175	0.141177702	0.9786263	0.163389047	0.667154939	0.328244579	0.953284346
RYR2	0.160561879	0.676578494	0.800552457	0.471739208	0.081677087	0.327428735	0.953284346
SCN3A	0.291440565	0.955850041	0.020514881	0.814775931	0.724284944	0.328380162	0.953284346
SPAST	0.739641047	0.658735599	0.680168373	0.010389832	0.97377237	0.32751753	0.953284346
SPATA2	0.386610898	0.824921918	0.150191038	0.526002189	0.135476702	0.330158651	0.953284346
STAB1	0.539232468	0.472071326	0.043698864	0.700368576	0.437259369	0.329864376	0.953284346
STARD10	0.259063185	0.357329586	0.968656561	0.11083471	0.333801394	0.3259582	0.953284346
STK4	0.616450955	0.747626951	0.119112927	0.206565421	0.301425561	0.330362181	0.953284346
TCIRG1	0.719869835	0.915533279	0.007493155	0.798635965	0.853732998	0.328144945	0.953284346
TM4SF18	0.735754544	0.183990015	0.076662146	0.498401182	0.647208047	0.327285867	0.953284346
TMEM198	0.217105624	0.617393807	0.340863405	0.545073773	0.134775827	0.327675723	0.953284346
TMEM232	0.688078466	0.214119249	0.187686228	0.987510026	0.124978368	0.330131153	0.953284346
TMEM238	0.345933238	0.356315092	0.934142745	0.039029534	0.746584689	0.327618469	0.953284346
TSN	0.568385321	0.227493033	0.649455055	0.943130522	0.041758054	0.325506729	0.953284346
WDR4	0.285334328	0.887247266	0.352048913	0.194978426	0.191163231	0.326155694	0.953284346
YPEL3	0.938050211	0.898975586	0.599522579	0.644980481	0.010409383	0.329330309	0.953284346
ZFP37	0.040138787	0.452046789	0.424210161	0.816107851	0.533349212	0.327406793	0.953284346
CSTF2T	0.534993204	0.601529975	0.670443153	0.46362255	0.034362338	0.331193132	0.953772179
CYP2S1	0.234178576	0.076677663	0.744794134	0.818061815	0.314221937	0.331213677	0.953772179
DEDD	0.225958331	0.558932179	0.337333219	0.128444553	0.627458646	0.331033514	0.953772179
GPR132	0.069994303	0.69944801	0.596906911	0.170667135	0.687881882	0.330910983	0.953772179
LOC100847304	0.528631622	0.402730154	0.755837638	0.059146451	0.360075699	0.330750485	0.953772179
NAA60	0.572465166	0.908756268	0.460309861	0.029329844	0.48851952	0.330928027	0.953772179
NDE1	0.052799143	0.820861485	0.107394292	0.868164994	0.849036661	0.330917181	0.953772179
NUAK2	0.714710369	0.505077771	0.063758808	0.159448428	0.93577095	0.33105774	0.953772179
PLOD2	0.527735791	0.491583096	0.068029711	0.226639489	0.85855844	0.331057478	0.953772179
REXO2	0.240905299	0.654774622	0.777988158	0.315827188	0.088674038	0.331173466	0.953772179
RNF208	0.341263907	0.601777323	0.674620084	0.024747585	0.99963258	0.330764262	0.953772179
MRPS34	0.535962461	0.564214462	0.466850647	0.072196974	0.337669942	0.33138213	0.954089847
CXCL5	0.77205047	0.852570001	0.13637265	0.075262227	0.510035161	0.3315572	0.954426453
C22H3orf62	0.437604822	0.558068878	0.152152769	0.215239163	0.432136768	0.332005904	0.954955212
KAZALD1	0.086276262	0.254717128	0.348445066	0.582729121	0.773910348	0.331885792	0.954955212
MAZ	0.472526786	0.253228425	0.053261634	0.721049296	0.75222842	0.332031835	0.954955212
SLC26A11	0.423528703	0.553572941	0.289566256	0.377474789	0.134684801	0.331807478	0.954955212
STK26	0.736526522	0.564650658	0.235194253	0.089106905	0.396388921	0.331949636	0.954955212
HGF	0.267309136	0.469321373	0.091457439	0.94637442	0.318741389	0.332215451	0.955315887
MROH1	0.214727997	0.027249611	0.69806199	0.984188939	0.862112545	0.33241428	0.95555421
TRAPPC3	0.474220506	0.077518334	0.664775726	0.164767714	0.860706037	0.332414782	0.95555421
MEIS3	0.328709433	0.318084494	0.076223304	0.672350557	0.647114762	0.332494443	0.955615814
BBIP1	0.069889391	0.629468243	0.733533874	0.179528506	0.602535558	0.33348919	0.95596351
CCHCR1	0.153438761	0.238309949	0.498858384	0.523233868	0.365674254	0.333462911	0.95596351
EXOSC2	0.967092576	0.056564678	0.757684685	0.140340033	0.599173818	0.333254172	0.95596351
FAM3A	0.299620981	0.654602899	0.359194228	0.218710116	0.22581838	0.333004333	0.95596351

LOC101905866	0.448394378	0.837772395	0.19541084	0.275147568	0.172679783	0.333359048	0.95596351
LOC112442228	0.168978387	0.848269333	0.514502323	0.108630722	0.435094934	0.33327224	0.95596351
LOC112448744	0.819274555	0.654201875	0.949370498	0.334207652	0.020476175	0.333119029	0.95596351
MBOAT2	0.642485744	0.747314381	0.371928478	0.098055043	0.199090309	0.333291917	0.95596351
PDRG1	0.777922974	0.286904559	0.253691098	0.135347908	0.454295967	0.333094173	0.95596351
PSMA5	0.822412331	0.566265189	0.926114516	0.039420882	0.204461908	0.33286903	0.95596351
RPP30	0.564067561	0.286632228	0.660420135	0.139936115	0.233556533	0.333446963	0.95596351
STK24	0.234358852	0.658773316	0.119231352	0.28291581	0.667238063	0.332811782	0.95596351
TEX9	0.031408587	0.719791935	0.26655439	0.632428954	0.912723665	0.332964778	0.95596351
TLK1	0.069186845	0.901806065	0.776253702	0.082463697	0.872005061	0.333146451	0.95596351
TMEM258	0.052813494	0.530332958	0.515176302	0.701464077	0.344452349	0.333304834	0.95596351
CAMSAP2	0.465070702	0.267680213	0.362107998	0.248765355	0.312666055	0.334149345	0.95608998
CIAO1	0.806322897	0.572299778	0.314443526	0.032747223	0.736979357	0.333964201	0.95608998
DYNLL2	0.462367745	0.132640903	0.111622636	0.531421368	0.963962041	0.334174159	0.95608998
LY6G5B	0.55402637	0.142936613	0.151574849	0.860264521	0.338586104	0.33372287	0.95608998
NPNT	0.101653623	0.88138213	0.283738782	0.857806455	0.160279373	0.333678429	0.95608998
ORAI2	0.35151086	0.844835925	0.723240131	0.816155365	0.019984403	0.33401662	0.95608998
PAK4	0.829052842	0.72676096	0.008550556	0.886513628	0.766743459	0.33396373	0.95608998
RIPOR2	0.15382498	0.743319558	0.434382395	0.50148622	0.140751923	0.334130101	0.95608998
STS	0.964100998	0.426443361	0.380991348	0.1008428	0.221727241	0.333984093	0.95608998
USP1	0.244239749	0.422452658	0.978771341	0.03861787	0.898501346	0.334059572	0.95608998
ZFYVE19	0.294868925	0.390957066	0.129640603	0.260612116	0.896887157	0.333595632	0.95608998
ATP6V0E1	0.847323596	0.038148032	0.190084117	0.700671706	0.816929834	0.334603968	0.956707172
MASTL	0.875248683	0.175432004	0.110708438	0.663781191	0.311728271	0.334623068	0.956707172
MRPL51	0.904087834	0.362007073	0.832733953	0.016564443	0.77856888	0.334514985	0.956707172
TECPR1	0.553102593	0.060716544	0.187212614	0.647633007	0.863019023	0.334476947	0.956707172
ALKBH4	0.738009942	0.200716717	0.265058607	0.313710047	0.285891593	0.334793441	0.956860879
WDR55	0.69857604	0.30858181	0.594343338	0.056754532	0.484128096	0.334746919	0.956860879
ZNF852	0.134942517	0.748408845	0.235085089	0.507510116	0.292431077	0.334884113	0.956953366
LOC101904749	0.226814514	0.126542621	0.646338498	0.247274381	0.768475952	0.33495158	0.956979522
TTPA	0.821802004	0.753393761	0.014359387	0.425370582	0.932727588	0.335044603	0.957078674
HRC	0.753058699	0.432013567	0.18241183	0.088424093	0.672695377	0.335155055	0.957227569
AGO3	0.188075639	0.994750451	0.868122815	0.036318447	0.599027687	0.335304399	0.957487471
IFI30	0.422930122	0.833682399	0.192107293	0.097870322	0.534114847	0.335613293	0.957553075
KDELC1	0.534208018	0.451882829	0.059157967	0.328860133	0.75426557	0.335677463	0.957553075
LLGL2	0.192346354	0.548347985	0.543553744	0.130279132	0.474114746	0.33562785	0.957553075
NABP1	0.585138611	0.408522339	0.221682342	0.963449321	0.06937617	0.335663632	0.957553075
PAICS	0.660271055	0.097359652	0.578311699	0.287258641	0.331204142	0.335452522	0.957553075
UFSP2	0.207647536	0.037835164	0.914572824	0.56144394	0.878060848	0.335672882	0.957553075
CRTAC1	0.157374572	0.974654604	0.05504248	0.712069915	0.589797238	0.335823482	0.957569435
LAGE3	0.858272625	0.476098999	0.265846411	0.088290983	0.369763549	0.335852823	0.957569435
LOC104973139	0.529110782	0.057768501	0.631562774	0.447035044	0.411217769	0.33594725	0.957569435
RPL13A	0.505842188	0.584594627	0.983210166	0.031831501	0.383406496	0.335935229	0.957569435
SPTY2D1	0.390611947	0.407711974	0.526229051	0.070573942	0.600108549	0.335974944	0.957569435
NR1D1	0.570452649	0.674623541	0.832324665	0.998900109	0.011104251	0.336125712	0.957832794

COLGALT1	0.297335825	0.778525971	0.107035073	0.254247658	0.564648806	0.336297036	0.958154628
LOC104971817	0.593295225	0.103756475	0.166904268	0.882949313	0.392356314	0.336396444	0.958271488
LOC112442215	0.173919948	0.814747783	0.123519495	0.847350535	0.240099958	0.336463175	0.958295239
PPP1R15A	0.240065151	0.045855834	0.795772577	0.736320159	0.55257452	0.336603644	0.958528961
FAM180A	0.374806878	0.965350295	0.094666867	0.838962269	0.124440905	0.33709612	0.958796275
GTF2B	0.633344322	0.050954449	0.508538794	0.231817063	0.938995711	0.336943755	0.958796275
IFITM1	0.560925756	0.923277046	0.084949006	0.354793428	0.228982147	0.337019009	0.958796275
KCNA6	0.571172034	0.642177146	0.023471505	0.498454815	0.832186393	0.336893336	0.958796275
PNPLA3	0.887920539	0.872923821	0.90360546	0.054704767	0.093214299	0.336902963	0.958796275
POLI	0.008671023	0.962070265	0.816314013	0.92464848	0.567235717	0.336915949	0.958796275
RNF13	0.246253799	0.818580782	0.40535991	0.101218423	0.432395562	0.337106484	0.958796275
NDUFA11	0.590204592	0.538196017	0.976509825	0.013225206	0.87253688	0.337237764	0.959003455
COL15A1	0.565225742	0.977602152	0.009478626	0.720703194	0.949159145	0.337382628	0.959083025
LOC616199	0.253030757	0.789544402	0.049374055	0.517758768	0.701430242	0.337360378	0.959083025
POLR3D	0.478078107	0.893483989	0.427004877	0.128956095	0.152518055	0.337575114	0.959464012
KIAA2012	0.233569355	0.970550922	0.101942632	0.801784762	0.193882268	0.337784177	0.959559655
RPS29	0.590582599	0.567010935	0.52239153	0.042441895	0.483858472	0.337782588	0.959559655
SNHG12	0.278233854	0.528984706	0.040167018	0.767117883	0.791889458	0.337736922	0.959559655
LOC112442401	0.621689392	0.40205958	0.06800068	0.569966474	0.371909969	0.338226689	0.960650431
ADTRP	0.359462005	0.394640981	0.161404921	0.408745086	0.385557328	0.338451559	0.960956494
GLRX	0.256773357	0.380345913	0.229742212	0.309303487	0.519796877	0.338408095	0.960956494
SLC25A12	0.683006093	0.884520615	0.014765837	0.534976224	0.756479828	0.338523968	0.96099582
AOAH	0.889135381	0.68263165	0.527178814	0.203512311	0.055576945	0.338896659	0.961020136
CTPS1	0.597240499	0.511590796	0.514536105	0.44196565	0.052137272	0.339044756	0.961020136
MRPL20	0.459282648	0.333470114	0.343513865	0.246947848	0.278499326	0.338866495	0.961020136
NTS	0.144992901	0.490989745	0.618215594	0.448369087	0.183241917	0.338765599	0.961020136
P2RY8	0.763770949	0.885552717	0.070329868	0.392040499	0.194090796	0.338915944	0.961020136
TESK2	0.830753029	0.620999097	0.452891029	0.62959236	0.02462866	0.339055928	0.961020136
TMEM161B	0.367540969	0.335933522	0.37364884	0.112952059	0.695206653	0.339046893	0.961020136
TMOD4	0.752351408	0.685869143	0.424578104	0.383488586	0.043118126	0.339047275	0.961020136
VAV2	0.982679701	0.163229477	0.127786655	0.290627909	0.608182563	0.339059569	0.961020136
ASCL2	0.601985547	0.129759821	0.348288144	0.218337094	0.610925683	0.339307131	0.961057875
LOC617875	0.250296027	0.59389818	0.097418496	0.364019673	0.688396008	0.339303545	0.961057875
PDK2	0.671929838	0.672007591	0.365971806	0.029253483	0.750538296	0.339277726	0.961057875
SLC2A8	0.864195006	0.117328922	0.082135878	0.556899294	0.781833673	0.339189111	0.961057875
AHNAK2	0.67278618	0.37489587	0.166271766	0.932647258	0.092945371	0.339574586	0.961160491
PRDX3	0.364610335	0.6177339	0.942619938	0.028138291	0.607851641	0.339404806	0.961160491
RNF19A	0.4529473	0.177608096	0.271684406	0.388758197	0.427539425	0.339463306	0.961160491
SLC7A10	0.738797982	0.298720601	0.030024424	0.909509397	0.603236253	0.339577633	0.961160491
CSNK2B	0.278502889	0.488246432	0.139261815	0.754062865	0.254919376	0.339768996	0.961266996
GRHPR	0.951624069	0.741336121	0.710140516	0.052816527	0.137681285	0.33989349	0.961266996
LOC505479	0.288414388	0.830229214	0.323691306	0.660228741	0.071187036	0.339883652	0.961266996
MTRF1	0.610577274	0.768714135	0.593153945	0.298518952	0.043839636	0.339908133	0.961266996
STT3A	0.147284153	0.485551144	0.574463176	0.268609543	0.330083895	0.339868945	0.961266996
NDUFA13	0.818291783	0.454062157	0.47768684	0.022223784	0.924441063	0.340030522	0.961447432

ACTR5	0.279701051	0.149012581	0.880996568	0.102718371	0.970562087	0.340621161	0.961602012
ANKHD1	0.256279671	0.852564373	0.787142957	0.022520834	0.945396729	0.340666676	0.961602012
CDK2	0.950450784	0.649107706	0.298800578	0.219571582	0.090436223	0.340615485	0.961602012
CDK5RAP2	0.504968589	0.442510579	0.966073244	0.053442591	0.31793354	0.340920169	0.961602012
CYP2R1	0.901427805	0.182147276	0.128055571	0.192592887	0.906312474	0.341006637	0.961602012
IQANK1	0.903929525	0.027809441	0.67978416	0.22823717	0.93968922	0.340797156	0.961602012
ITIH3	0.154971934	0.744611172	0.153470971	0.458492439	0.452038533	0.341022711	0.961602012
LOC790101	0.746882509	0.690296251	0.015482618	0.773083852	0.593438991	0.340681065	0.961602012
PKIG	0.02529309	0.475172856	0.68794187	0.66874508	0.66054523	0.340275367	0.961602012
PPP1R15B	0.463351616	0.562620474	0.250324682	0.135401885	0.413124802	0.340194524	0.961602012
RHOA	0.23618774	0.297768756	0.168026453	0.404622347	0.766710854	0.340841397	0.961602012
RPLP0	0.505501406	0.425052378	0.793343343	0.078592753	0.272558758	0.340240681	0.961602012
SLC12A2	0.66817589	0.800720173	0.9763	0.007875026	0.892202988	0.34100671	0.961602012
SP140L	0.842986077	0.092314157	0.37028408	0.147468944	0.860452766	0.340443297	0.961602012
TLR8	0.975109475	0.33990717	0.019045246	0.647542276	0.896695424	0.340812774	0.961602012
ZRANB2	0.033489166	0.751488434	0.667118335	0.639397566	0.340695521	0.340483487	0.961602012
BTLA	0.041379605	0.471980425	0.770770483	0.882122031	0.277058385	0.341377571	0.961815029
GATAD2B	0.066365655	0.53426185	0.591676916	0.209728777	0.836328971	0.341406129	0.961815029
PHTF1	0.49184622	0.594717974	0.057901507	0.244673255	0.887054928	0.341249345	0.961815029
PIAS1	0.080542788	0.786829129	0.501716621	0.153046684	0.755531822	0.341275462	0.961815029
RAD51C	0.013142291	0.732986019	0.743374679	0.918667654	0.558840256	0.341267932	0.961815029
SLC6A2	0.951301076	0.199420229	0.917138021	0.053474455	0.39561678	0.341449903	0.961815029
ATP6V1A	0.810573075	0.22430876	0.048095216	0.930294289	0.455280231	0.342390306	0.961888239
BRAT1	0.763199566	0.093454372	0.490967556	0.189387257	0.558209926	0.342320289	0.961888239
CALM2	0.759537047	0.009807899	0.827758639	0.906772861	0.662018379	0.342305768	0.961888239
CDK2AP1	0.791940782	0.580793633	0.106543055	0.098790251	0.768947416	0.343162643	0.961888239
CPT1A	0.678674707	0.783635641	0.392135368	0.092535626	0.192932764	0.343189345	0.961888239
CSE1L	0.150882314	0.318815319	0.755759455	0.572663128	0.178411808	0.342824887	0.961888239
CTTN	0.942137963	0.479390388	0.086583784	0.364272359	0.259903085	0.34233397	0.961888239
DDX17	0.413418786	0.466698886	0.864506743	0.0227842	0.977612462	0.342863999	0.961888239
FGD4	0.136871695	0.960539254	0.056434198	0.936280968	0.5345486	0.342783175	0.961888239
FSBP	0.74273153	0.824600541	0.336868908	0.225063121	0.080114995	0.343059222	0.961888239
HTT	0.665796867	0.872705138	0.792404891	0.040483868	0.19917653	0.342752463	0.961888239
LOC100847604	0.139268401	0.075207216	0.680355282	0.594024421	0.87458724	0.342326034	0.961888239
LOC101909003	0.53858315	0.209940433	0.579965912	0.513175536	0.10957704	0.341726591	0.961888239
LOC104974812	0.982066152	0.920121756	0.022177214	0.412814073	0.448566958	0.342681979	0.961888239
LOC112448082	0.328334825	0.425690574	0.158044442	0.191617608	0.874681696	0.342333141	0.961888239
MEA1	0.369936069	0.035054956	0.410176405	0.746943821	0.934020406	0.342687668	0.961888239
NARF	0.349169712	0.50400908	0.147192026	0.195646192	0.728558626	0.341921258	0.961888239
NCDN	0.70802396	0.409267785	0.251501177	0.133947259	0.380275486	0.342735383	0.961888239
NFYC	0.103421151	0.281886142	0.721499378	0.814954134	0.216976363	0.3430278	0.961888239
PDE6G	0.433530051	0.027387931	0.962419863	0.36703228	0.87906456	0.341702609	0.961888239
PGM2	0.553421597	0.432467634	0.307020025	0.057301269	0.881776883	0.342760417	0.961888239
RALB	0.6609296	0.645191563	0.390139892	0.046667576	0.477744618	0.342612304	0.961888239
RBM25	0.355113924	0.626468499	0.257433384	0.113602463	0.571802469	0.343063076	0.961888239

SACM1L	0.293662846	0.389976504	0.181915592	0.264931183	0.668685583	0.341857544	0.961888239
SCYL1	0.108063489	0.436185245	0.792037435	0.101725844	0.971720579	0.341842185	0.961888239
THAP7	0.346646416	0.883082656	0.223488946	0.078593071	0.688529447	0.342324497	0.961888239
TPM1	0.129625651	0.389155686	0.595520852	0.760467894	0.161263607	0.341584275	0.961888239
TRIP4	0.21075743	0.394554206	0.191123407	0.389337061	0.601008945	0.34300875	0.961888239
WDR83	0.080020221	0.92257709	0.321361119	0.736484876	0.212050152	0.342445714	0.961888239
ZC3H8	0.039920613	0.872883782	0.620555795	0.303483972	0.567525107	0.343234265	0.961888239
EIF4G3	0.40825672	0.612796745	0.561535481	0.063532361	0.41746878	0.343300937	0.961910821
AJAP1	0.309824715	0.309776276	0.986126422	0.044686387	0.882780503	0.343608304	0.961940521
AP1B1	0.292437494	0.796775406	0.992388542	0.104454374	0.154572616	0.343603099	0.961940521
EFHC1	0.193469385	0.705747035	0.058557209	0.481438304	0.969371878	0.343520131	0.961940521
MAK16	0.521526674	0.415852678	0.712783754	0.457495824	0.052810565	0.34366323	0.961940521
PLCL1	0.100898222	0.376092196	0.713083125	0.147604944	0.934637443	0.343586365	0.961940521
RGS7BP	0.904577416	0.742930312	0.943549381	0.021855132	0.269088967	0.343427458	0.961940521
INPP5D	0.856024633	0.813135378	0.069589151	0.415901564	0.18565088	0.343871969	0.962032542
LOC107132247	0.337958719	0.263727181	0.226839236	0.58002062	0.318724047	0.343772838	0.962032542
OFD1	0.062667742	0.18313725	0.820055158	0.47289428	0.840171755	0.343843167	0.962032542
WSB2	0.821027525	0.172148194	0.09100271	0.339975843	0.855672708	0.343939192	0.962056602
COPS7B	0.65196391	0.151751121	0.764790368	0.071764201	0.690488261	0.344251474	0.962298218
HID1	0.392478126	0.163277015	0.252359147	0.299785165	0.772547361	0.344087841	0.962298218
PHLDA3	0.216463692	0.820829061	0.316113272	0.118568694	0.562762724	0.344186273	0.962298218
RPAP2	0.095824846	0.205297221	0.267930608	0.894430348	0.79565362	0.344318758	0.962298218
SPDL1	0.511276268	0.996508861	0.059890191	0.221322144	0.555316037	0.344284948	0.962298218
CCNE2	0.235385317	0.348966736	0.680833213	0.42479322	0.157995313	0.344413991	0.962400479
ANKRD52	0.708938208	0.696262614	0.368146226	0.022871361	0.905802092	0.34487012	0.962447471
BEGAIN	0.230308711	0.560358482	0.243851788	0.19332548	0.617905603	0.344654964	0.962447471
BOD1	0.723755469	0.947569926	0.073682739	0.153465152	0.484681663	0.34462754	0.962447471
C7H19orf44	0.117679556	0.478579782	0.805057882	0.179635839	0.462320656	0.344902251	0.962447471
CD27	0.974357621	0.115234666	0.231792768	0.581421762	0.248996123	0.344995731	0.962447471
FAM149B1	0.066912877	0.52332205	0.161127116	0.816116204	0.816666351	0.344700728	0.962447471
HMGB1	0.336970014	0.079876323	0.940730393	0.206423833	0.721989067	0.345234192	0.962447471
IP6K1	0.53778077	0.731552587	0.834564452	0.027363757	0.419991586	0.34522088	0.962447471
LOC783541	0.354001524	0.549463707	0.08249716	0.810258531	0.28996101	0.345087317	0.962447471
MAPK8IP2	0.107219171	0.099080996	0.935441929	0.461251625	0.823375126	0.345251859	0.962447471
PDE7B	0.03276892	0.812459398	0.89492143	0.184749477	0.856533581	0.345097827	0.962447471
SOX6	0.099751291	0.272026474	0.372540931	0.601897244	0.620279149	0.345251133	0.962447471
TATDN3	0.586367754	0.533376988	0.256525142	0.237702672	0.197351086	0.344828298	0.962447471
TMEM160	0.35119852	0.541474102	0.497479484	0.06512268	0.610934203	0.344836844	0.962447471
LOC112442374	0.990385545	0.095151009	0.594416729	0.088583256	0.761375815	0.345407746	0.962718498
BFAR	0.57702789	0.349930137	0.899964483	0.118125496	0.176116094	0.345508425	0.962835586
CCNY	0.706019864	0.182076196	0.212619719	0.425829653	0.325179008	0.345679027	0.962950198
EFNA4	0.75863428	0.287759487	0.416388931	0.374334221	0.111475343	0.34601897	0.962950198
GEM	0.376483385	0.52197335	0.427331393	0.094858301	0.475738971	0.3458796	0.962950198
LOC112442652	0.252225258	0.991877456	0.369078993	0.043694061	0.938566302	0.34575674	0.962950198
LYPD3	0.825073265	0.047151922	0.228532517	0.496510576	0.858332077	0.345851757	0.962950198

NPDC1	0.286838894	0.486595018	0.077723336	0.837295942	0.417464364	0.345968326	0.962950198
SETD2	0.463067486	0.944106486	0.847419243	0.015052242	0.679946594	0.345962728	0.962950198
WFIKKN1	0.963636497	0.124437376	0.283152877	0.141588392	0.788676166	0.345952831	0.962950198
CDC42EP1	0.260649637	0.747626696	0.138000238	0.200207172	0.70566821	0.346265842	0.963310517
SEC16A	0.81909615	0.700713982	0.188791673	0.297314583	0.11790198	0.346228528	0.963310517
ZDHHC17	0.062454105	0.523865865	0.655812565	0.254038909	0.697725694	0.346421915	0.963581363
LOC508933	0.309673243	0.196081471	0.120886764	0.614191355	0.844656141	0.346617796	0.963636227
MGAT2	0.436565155	0.614232483	0.629788093	0.400140762	0.056352017	0.346616361	0.963636227
ZFYVE16	0.390675093	0.109202051	0.840728546	0.111539453	0.951432274	0.34654957	0.963636227
ATP5PD	0.764717162	0.209002556	0.724002852	0.035249643	0.941273184	0.347871912	0.963745367
BTF3	0.889027906	0.728561875	0.055929619	0.242772806	0.434096142	0.347007414	0.963745367
C16H1orf74	0.664668516	0.489540077	0.98458196	0.150039253	0.080636592	0.34932642	0.963745367
CCL24	0.773187566	0.415875599	0.069168967	0.25464426	0.683294622	0.349085757	0.963745367
CD24	0.690374603	0.9439592	0.056758853	0.358577669	0.288245624	0.3472215	0.963745367
CHST3	0.088844315	0.302342045	0.239991859	0.725960459	0.824144717	0.348569884	0.963745367
CTTNBP2	0.222156022	0.396172959	0.400830529	0.256295801	0.424242232	0.347729457	0.963745367
CXCL14	0.227660172	0.41517654	0.163387023	0.647020024	0.385063769	0.348198087	0.963745367
DNAJA2	0.87023398	0.165611512	0.144684463	0.757090007	0.245664792	0.349417155	0.963745367
DPAGT1	0.805590886	0.469791163	0.696796574	0.060432641	0.241635301	0.348328496	0.963745367
DYNC2H1	0.236831477	0.846999306	0.403804848	0.14693106	0.323300848	0.348207494	0.963745367
EIF3L	0.151833474	0.938868275	0.355483572	0.685403706	0.110094025	0.347251202	0.963745367
GFOD2	0.072771807	0.851588994	0.763141357	0.158475391	0.513161577	0.348136817	0.963745367
GPBAR1	0.215631235	0.115647385	0.721385066	0.63626393	0.338641769	0.349330016	0.963745367
HSD17B10	0.888472039	0.709667438	0.403415445	0.02343998	0.650401476	0.349399837	0.963745367
IL17RD	0.975758895	0.827741736	0.283955454	0.144521022	0.116606529	0.348887875	0.963745367
KDM4C	0.385032367	0.816041754	0.469921527	0.105091474	0.247026129	0.347618852	0.963745367
LOC100847947	0.168590645	0.417595864	0.776580178	0.407901487	0.173503412	0.349063769	0.963745367
LOC101902407	0.775387371	0.269101608	0.031831257	0.92231589	0.630711553	0.348837639	0.963745367
LOC101903526	0.195053528	0.287337491	0.836812419	0.398660733	0.20692035	0.349043136	0.963745367
LOC101907255	0.034263573	0.284311095	0.908744723	0.966408326	0.45152721	0.348807143	0.963745367
LOC504773	0.291132957	0.912012851	0.090956887	0.38212429	0.419990787	0.349322897	0.963745367
LRRC1	0.206420873	0.677868954	0.257465544	0.464344302	0.229314833	0.347740612	0.963745367
MAPK1	0.176471228	0.539640157	0.123824597	0.653139374	0.503288088	0.349335372	0.963745367
MCM3	0.252713497	0.328495156	0.217336761	0.255438861	0.830280199	0.347356996	0.963745367
MED12L	0.293831306	0.979589908	0.587531768	0.816677213	0.027944022	0.348665906	0.963745367
MRC2	0.61479384	0.125766287	0.103920728	0.509960481	0.930894607	0.346874804	0.963745367
MYL5	0.227162592	0.10198824	0.371334223	0.753160708	0.593963576	0.34823745	0.963745367
NDUFA9	0.76144841	0.557208596	0.818245822	0.017306637	0.640091458	0.348130699	0.963745367
NMRAL1	0.42044848	0.292450513	0.733387978	0.11800623	0.36250323	0.348596236	0.963745367
NOXA1	0.423042029	0.110681382	0.148654068	0.691089016	0.797325558	0.347710231	0.963745367
NUP50	0.632985083	0.189877645	0.319796758	0.10657754	0.944007503	0.348973034	0.963745367
PDE3A	0.017651286	0.462499421	0.93878975	0.983742652	0.510963707	0.348388529	0.963745367
PDXP	0.595657288	0.464312466	0.475212213	0.029973316	0.979337424	0.348612396	0.963745367
PGD	0.756539205	0.837299023	0.874914144	0.025236518	0.272685101	0.346852047	0.963745367
PITPNM2	0.592323883	0.632373995	0.076022135	0.563713111	0.23880328	0.347628155	0.963745367

PPRC1	0.745194607	0.764613709	0.57194791	0.038228397	0.309825258	0.348686425	0.963745367
PRDX4	0.738072132	0.235356404	0.037557108	0.629399289	0.938889488	0.348505114	0.963745367
PSMC3IP	0.449771441	0.21654778	0.32117621	0.308348289	0.395898195	0.347044168	0.963745367
PSMG2	0.802267285	0.633951568	0.595893374	0.401987186	0.031522875	0.347913745	0.963745367
RER1	0.586271298	0.374663426	0.340479924	0.345228592	0.149370516	0.348556444	0.963745367
RHOBTB2	0.506199621	0.639658731	0.062992421	0.509047296	0.37217506	0.348860449	0.963745367
RPH3AL	0.198316184	0.732045134	0.189612956	0.167080604	0.830770356	0.347134668	0.963745367
SELENOI	0.852069847	0.030165173	0.422318269	0.899295226	0.396905828	0.349265734	0.963745367
SHROOM2	0.776108913	0.181752723	0.198239418	0.322036133	0.429842895	0.349123391	0.963745367
UBE2G1	0.205652656	0.175482053	0.461220443	0.259289589	0.889229589	0.347805422	0.963745367
ZC3H12C	0.285416204	0.965926764	0.656760135	0.049565134	0.425007334	0.346864017	0.963745367
F2R	0.556911646	0.510010296	0.306503622	0.057169089	0.781204896	0.349802111	0.963893333
LOC101902366	0.614292013	0.15993635	0.103978215	0.411748603	0.923283367	0.349626781	0.963893333
LOC101906607	0.350054132	0.605009649	0.657717139	0.291482711	0.095771707	0.349823209	0.963893333
LOC101908104	0.215652868	0.157056452	0.496041513	0.400952021	0.57706884	0.349774507	0.963893333
LOC783963	0.3915185	0.15145697	0.911688076	0.128433841	0.559535545	0.349684599	0.963893333
NKIRAS2	0.895528689	0.373296712	0.043398877	0.349966523	0.765767406	0.349805107	0.963893333
DNMBP	0.480372588	0.658605	0.866571365	0.057079959	0.24893229	0.350101359	0.964104266
ELOVL1	0.638573484	0.040222305	0.218650543	0.959052245	0.722804086	0.350001801	0.964104266
FHL3	0.382357098	0.498236682	0.206236184	0.188693583	0.526183911	0.350310995	0.964104266
GSTK1	0.767085142	0.055569104	0.244515718	0.389415017	0.960577796	0.35022761	0.964104266
MAPK15	0.199611224	0.282895092	0.71950499	0.586003872	0.163801178	0.350274609	0.964104266
NRN1	0.223951313	0.8277571	0.072640541	0.393747207	0.734998974	0.350160618	0.964104266
PPIL2	0.59896181	0.110698807	0.384157521	0.208836204	0.732036391	0.350036714	0.964104266
ADAM11	0.120089985	0.823169685	0.549875699	0.079223377	0.907201857	0.350542538	0.964276178
FBRSL1	0.889804846	0.67165995	0.456897312	0.015946296	0.899091988	0.350864677	0.964276178
IRF2BPL	0.833828364	0.353495842	0.179545332	0.179079069	0.413305011	0.350944038	0.964276178
KDELR2	0.881271714	0.06570741	0.210330172	0.565607056	0.5681614	0.350824087	0.964276178
LOC104975460	0.920563652	0.616685369	0.086192977	0.743582594	0.107524198	0.350757607	0.964276178
LOC107131772	0.624509103	0.021040053	0.4937077	0.931253329	0.64720918	0.350666589	0.964276178
LOC112442080	0.656506998	0.983866337	0.101851187	0.349316865	0.170040952	0.350576795	0.964276178
LOC112449324	0.818791397	0.49915346	0.928234598	0.010975878	0.937729783	0.35045896	0.964276178
RAB14	0.894688609	0.103042732	0.311026829	0.189520258	0.721143099	0.351019797	0.964276178
ROCK1	0.13982165	0.398639662	0.331104617	0.365836975	0.579174559	0.350684041	0.964276178
ZNF280B	0.328003555	0.549774175	0.467881752	0.453083455	0.102495957	0.350991325	0.964276178
LOC101903326	0.603589904	0.558750907	0.082947955	0.97390935	0.143904471	0.351088785	0.964304275
C17H4orf46	0.813415667	0.091682972	0.396155331	0.901704883	0.147431491	0.35135938	0.96440187
GDF9	0.079366534	0.74474273	0.649975928	0.734465134	0.139144632	0.351308706	0.96440187
LOC512005	0.459345949	0.06700118	0.50319776	0.937955282	0.270247819	0.351282721	0.96440187
TBX21	0.817940538	0.729461318	0.028411378	0.839938226	0.275602894	0.351226675	0.96440187
LOC104974678	0.04617331	0.305640347	0.580060206	0.674774129	0.712032202	0.351576406	0.964674869
PTPN13	0.959316677	0.17110333	0.43542602	0.132206415	0.416098046	0.351523232	0.964674869
AGO1	0.757607515	0.890433922	0.248486457	0.922002054	0.025507657	0.35193805	0.964699404
IKZF3	0.321087718	0.404770353	0.483262725	0.633473045	0.09900915	0.351820123	0.964699404
KCNK5	0.917817001	0.434965926	0.813448429	0.060564123	0.200259066	0.351795196	0.964699404

LOC112446383	0.904066828	0.145847312	0.126467351	0.359012234	0.658489565	0.351932501	0.964699404
LOC112447351	0.143768961	0.106165186	0.951948464	0.82962712	0.326965354	0.351900475	0.964699404
SCAND1	0.214167445	0.897961551	0.143335681	0.244899724	0.583411644	0.351788066	0.964699404
CITED2	0.489424338	0.51843879	0.071026239	0.393499162	0.55624958	0.352031926	0.96479558
EDF1	0.448251283	0.694896035	0.343937277	0.054775929	0.673183925	0.352255367	0.965079971
RAB22A	0.638084794	0.151868884	0.06102849	0.716856296	0.931609631	0.352220456	0.965079971
RAD51	0.887453597	0.180455752	0.220906122	0.20377443	0.548188507	0.352312114	0.965079971
LOC100297676	0.958236396	0.714369922	0.02164123	0.590289116	0.452270535	0.352431958	0.96524714
LOC100297056	0.765042542	0.431375979	0.127316365	0.167041754	0.564046126	0.352582952	0.965338475
ZNF383	0.021806343	0.784433692	0.514623485	0.565124896	0.795648932	0.352557761	0.965338475
FRMPD1	0.950446278	0.881970272	0.81662009	0.0180313	0.320974773	0.352701789	0.965341739
GORASP1	0.241331445	0.772005561	0.353202512	0.102297852	0.588461344	0.35268153	0.965341739
CCNT1	0.14809886	0.261464488	0.546641757	0.191757162	0.977122787	0.352868455	0.965475861
LOC101904601	0.290964672	0.767344573	0.727484263	0.031125872	0.784276011	0.352824613	0.965475861
PDE8A	0.855968089	0.984400856	0.142753656	0.055714323	0.592594772	0.35307257	0.965873304
C13H20orf96	0.229882208	0.128845291	0.159266031	0.899419512	0.936752386	0.353194581	0.966046045
LOC101903713	0.239459629	0.143171738	0.779692488	0.526505504	0.282557111	0.353279303	0.966116755
LOC786948	0.727522228	0.474051484	0.237275755	0.360963527	0.134751685	0.353421962	0.966345855
DNAJC19	0.482501206	0.745553323	0.456585972	0.0325692	0.745109681	0.353637254	0.966493671
SAP25	0.679562039	0.160225126	0.633581081	0.532934359	0.108371718	0.353574771	0.966493671
VAMP5	0.429207025	0.578454766	0.08112541	0.224223823	0.88266405	0.353652702	0.966493671
CPT1B	0.070412353	0.90825059	0.150885788	0.876633029	0.471862596	0.35385441	0.966572312
ISM1	0.845391158	0.358000845	0.184452887	0.182450682	0.391898965	0.353858171	0.966572312
SLC22A16	0.216012459	0.264286331	0.615722471	0.731103271	0.15523756	0.353775198	0.966572312
LOC782922	0.216675093	0.337384228	0.135553156	0.545271912	0.739665184	0.354052443	0.966830547
SMARCA4	0.811197831	0.388970532	0.142693172	0.325877167	0.272421788	0.354070537	0.966830547
GLDN	0.644837964	0.196370715	0.206550385	0.931980207	0.164141071	0.354225625	0.967093118
KIAA0391	0.146328779	0.805278416	0.915320124	0.446319559	0.083159922	0.354308219	0.967157715
AGFG1	0.644503179	0.513428454	0.510383067	0.57445902	0.041338629	0.3545964	0.967274992
CDC14A	0.500292584	0.891187214	0.0691672	0.344495174	0.378038545	0.3548092	0.967274992
JAKMIP3	0.060225562	0.535267845	0.957692101	0.38490762	0.337584717	0.354632394	0.967274992
LOC112445965	0.163058885	0.958656104	0.427031576	0.865756247	0.069634877	0.355122556	0.967274992
LRRC2	0.446349128	0.921262451	0.641985465	0.208661079	0.073110634	0.355235292	0.967274992
MINOS1	0.625371045	0.398643684	0.930895677	0.01899951	0.913170962	0.355204171	0.967274992
NEURL1	0.044340622	0.736734526	0.579968671	0.821224685	0.258444066	0.35499972	0.967274992
NFIA	0.971342612	0.116844745	0.925401569	0.063006272	0.607271787	0.354904301	0.967274992
NIPSNAP1	0.853845986	0.899540569	0.468563864	0.016907276	0.659148404	0.354599814	0.967274992
NOP56	0.898134228	0.3948156	0.765436733	0.215131716	0.068847329	0.354960605	0.967274992
RFC1	0.125153973	0.381222742	0.235224028	0.507093027	0.705690388	0.354807246	0.967274992
RNF115	0.1782571	0.441810275	0.113834229	0.530440434	0.846810189	0.355225628	0.967274992
RTN4RL1	0.041598983	0.588329779	0.590821215	0.535171245	0.518569426	0.354683117	0.967274992
SLC41A1	0.756137646	0.377989715	0.332963043	0.058796675	0.718301237	0.354924842	0.967274992
SYAP1	0.977802629	0.122188273	0.2559414	0.190264832	0.688662566	0.354442898	0.967274992
ZFAND5	0.518928787	0.691990705	0.021486648	0.893008334	0.585093099	0.35539681	0.967554255
LOC112442288	0.64839004	0.546836823	0.666489308	0.039726111	0.429604191	0.355459243	0.967563715

LEF1	0.636952469	0.493801292	0.539559819	0.209413378	0.113599812	0.355619793	0.967679726
PKNOX2	0.50848137	0.844822043	0.223202602	0.084762596	0.496613725	0.355577463	0.967679726
CD40	0.791418977	0.81005185	0.111290977	0.795407201	0.071254657	0.355870563	0.967880645
DCP1A	0.615488216	0.116609468	0.683163044	0.096425109	0.855253966	0.355863822	0.967880645
LOC112442243	0.118005061	0.310900282	0.740725915	0.204105964	0.729011753	0.355866095	0.967880645
BVES	0.199659067	0.457013425	0.380042561	0.797635725	0.146428431	0.356120411	0.967918528
CSNK2A2	0.763694217	0.633121014	0.1886503	0.066049375	0.672264019	0.356117574	0.967918528
DHRS13	0.105358043	0.615107386	0.903790326	0.211817407	0.326138572	0.355966497	0.967918528
UNC5B	0.12495957	0.750315837	0.334892483	0.467514936	0.275716666	0.356012085	0.967918528
VWA5A	0.882622157	0.234399223	0.607751807	0.060784115	0.530161338	0.356183108	0.967928629
ABO	0.351126375	0.198685241	0.215513913	0.933708023	0.292991905	0.358520157	0.967955603
ADGRA2	0.167551848	0.954970069	0.452808048	0.631534921	0.088666675	0.356381985	0.967955603
AP5B1	0.077254043	0.493656938	0.188008883	0.807064283	0.70797106	0.357901356	0.967955603
AURKAIP1	0.640736924	0.339131244	0.756930511	0.090163105	0.276650684	0.358122454	0.967955603
C1S	0.460007369	0.635151444	0.593369801	0.033509515	0.701450401	0.357070513	0.967955603
CBLB	0.407397203	0.632913309	0.779366192	0.033914419	0.603620525	0.358549315	0.967955603
CD1A	0.52413179	0.173126063	0.187588329	0.26418343	0.912746192	0.358194363	0.967955603
CPQ	0.100456585	0.377895018	0.41851783	0.304958905	0.841471762	0.357146442	0.967955603
ETS1	0.438589598	0.305549345	0.402610473	0.099244843	0.768139246	0.358520722	0.967955603
FASTKD3	0.884906397	0.169133429	0.828821895	0.099320054	0.330961748	0.357167363	0.967955603
FZD10	0.10323943	0.400532602	0.316076269	0.339742043	0.924901617	0.358286263	0.967955603
IQGAP2	0.928355349	0.666618262	0.809072524	0.020506167	0.397432568	0.357283583	0.967955603
JAM3	0.457305171	0.115423032	0.41839814	0.975282281	0.190127697	0.357835154	0.967955603
KATNB1	0.128391944	0.799955908	0.606670777	0.084632749	0.77096129	0.356710435	0.967955603
KLF10	0.318822715	0.18872589	0.896739584	0.145415591	0.524327358	0.358552319	0.967955603
KPNA4	0.761018501	0.218569546	0.167196811	0.235919048	0.623757259	0.357737308	0.967955603
LOC101903905	0.120060107	0.47282709	0.873467115	0.206178025	0.400273677	0.357721359	0.967955603
LOC101906779	0.796848815	0.031531293	0.677526701	0.705802543	0.337616667	0.356360613	0.967955603
LOC107131699	0.800682809	0.111881321	0.960846079	0.201758894	0.236470205	0.358272194	0.967955603
LOC107131728	0.088049373	0.748493993	0.818100962	0.269557187	0.282255145	0.358104533	0.967955603
LOC112446757	0.824797833	0.370994068	0.855179144	0.016292263	0.964232682	0.358435249	0.967955603
LOC786733	0.243121664	0.617412353	0.188507609	0.898555972	0.161499013	0.358258485	0.967955603
NAA50	0.119164095	0.163025647	0.586026201	0.49955004	0.717192711	0.357214156	0.967955603
NDUFB1	0.574963637	0.301547124	0.967863423	0.02495541	0.968240631	0.356291312	0.967955603
NECAB3	0.571203136	0.539987497	0.089751863	0.77803965	0.188951692	0.356868943	0.967955603
PARD6G	0.066890535	0.914106231	0.08962818	0.986161746	0.751925682	0.356639165	0.967955603
PCGF5	0.302341742	0.144036001	0.411060093	0.522122617	0.436100192	0.357106593	0.967955603
PDP2	0.74152174	0.388453271	0.891814791	0.231783394	0.068979841	0.358294044	0.967955603
PHF8	0.778386429	0.81448948	0.706903624	0.01127964	0.810682157	0.357951017	0.967955603
POMT2	0.079228632	0.446134622	0.51142039	0.950487476	0.236648258	0.356727602	0.967955603
SIGLEC10	0.757285836	0.191503854	0.205183207	0.281558495	0.485730754	0.356859312	0.967955603
SNTB1	0.862092375	0.134482584	0.230262042	0.298207405	0.51144533	0.356936883	0.967955603
TMEM178B	0.56441076	0.623825345	0.629059961	0.066284182	0.279013513	0.357878744	0.967955603
TMEM208	0.512620508	0.818477464	0.493800048	0.105260711	0.187632974	0.35771499	0.967955603
TMEM251	0.757276035	0.344276088	0.023335384	0.772099323	0.874053542	0.358238498	0.967955603

TOR1AIP2	0.273549506	0.468212412	0.132600797	0.344342863	0.699687405	0.357711702	0.967955603
UQCC3	0.593147447	0.934714626	0.024640534	0.312579679	0.958703714	0.35778953	0.967955603
USP43	0.056199679	0.835534386	0.161927061	0.585602173	0.915810598	0.357175677	0.967955603
XG	0.999478359	0.528543637	0.930469479	0.058162767	0.14346682	0.358082492	0.967955603
ZGPAT	0.639316213	0.870257155	0.28577295	0.037228258	0.694515376	0.358436755	0.967955603
SCD	0.996794391	0.926062673	0.447928161	0.087641958	0.113613914	0.358673589	0.96812373
DAG1	0.471697334	0.570994757	0.713201339	0.028236965	0.759434474	0.358751999	0.968176132
AK8	0.213193235	0.577994587	0.809651452	0.05450159	0.764074905	0.360092698	0.968448958
AKAP11	0.434252042	0.217878441	0.570497643	0.092102596	0.833022776	0.359587761	0.968448958
CUTA	0.46764619	0.476883496	0.563040923	0.043741966	0.755517915	0.359902555	0.968448958
DONSON	0.088194323	0.20574841	0.807680349	0.504025564	0.561009181	0.359695954	0.968448958
DZANK1	0.193672615	0.80881503	0.170332418	0.804440369	0.193194665	0.359791242	0.968448958
GPRIN3	0.162729179	0.708007978	0.129670363	0.674064597	0.411723918	0.359772583	0.968448958
HSPB2	0.630679832	0.151397405	0.610704647	0.334618187	0.212400783	0.359704987	0.968448958
KANSL1	0.354321497	0.506307579	0.905667963	0.027742053	0.921424835	0.360034693	0.968448958
LOC100847791	0.356856841	0.625885889	0.038656884	0.966257896	0.497074618	0.359800951	0.968448958
LOC101904449	0.5314353	0.056051919	0.499449771	0.286042594	0.9745222	0.359809764	0.968448958
LOC513767	0.671701432	0.891994186	0.045554062	0.2731988	0.556236719	0.359827309	0.968448958
LOC787397	0.09586112	0.728922578	0.23699053	0.272572498	0.918323973	0.359729694	0.968448958
LOC788736	0.819711471	0.6701167	0.026097831	0.772206699	0.374669495	0.359825119	0.968448958
MARS	0.638963288	0.866042252	0.583101822	0.68265177	0.01878254	0.359434456	0.968448958
MCM5	0.684495575	0.917432988	0.059160669	0.245039709	0.455733927	0.359871277	0.968448958
MGAT4C	0.616758319	0.629812603	0.251561542	0.297755534	0.141799415	0.358999746	0.968448958
NAXD	0.214990836	0.787947424	0.618489121	0.04139885	0.958021852	0.360118842	0.968448958
PSMD9	0.419754815	0.200701204	0.375345296	0.149261949	0.880270575	0.360093251	0.968448958
SCAF11	0.303692376	0.858808634	0.984835758	0.016325078	0.989457473	0.359878695	0.968448958
TMEM225B	0.265137289	0.285341478	0.333596365	0.701476248	0.233999631	0.359640494	0.968448958
TRIM59	0.669537055	0.476230886	0.331391158	0.051769307	0.759797264	0.360151361	0.968448958
USP38	0.739488007	0.182619829	0.140506494	0.235365537	0.924831681	0.359171046	0.968448958
ADIG	0.34923931	0.621349274	0.337884415	0.147135309	0.390948748	0.362448483	0.968567705
AGAP2	0.444680232	0.673425507	0.034493443	0.412737398	0.984678094	0.361717342	0.968567705
AKAP5	0.49298776	0.627284056	0.094182529	0.406321997	0.362485023	0.365120477	0.968567705
ALAS1	0.373179753	0.661329934	0.806515682	0.061438978	0.344069625	0.362077903	0.968567705
ARMC4	0.079236945	0.701336625	0.346959795	0.427799318	0.513938517	0.363252664	0.968567705
BAG1	0.787064434	0.11281837	0.421622378	0.425753516	0.268598746	0.364809707	0.968567705
BCKDK	0.359869167	0.612781268	0.916590215	0.025478605	0.817537637	0.362175315	0.968567705
BRB	0.345277025	0.311229081	0.525041051	0.111016337	0.678396653	0.36362494	0.968567705
BZW1	0.439130804	0.320566783	0.613815138	0.732745926	0.066163364	0.361384573	0.968567705
CCL26	0.532250131	0.131888487	0.356439482	0.68997692	0.24522319	0.363042506	0.968567705
CCNB1IP1	0.541129168	0.133240366	0.139538395	0.751819158	0.5580902	0.362587619	0.968567705
CCND3	0.371703953	0.906350034	0.089215239	0.824199077	0.171776347	0.36384832	0.968567705
CFDP1	0.362809783	0.057217856	0.58237947	0.501354006	0.687623737	0.360587219	0.968567705
CHST11	0.101645538	0.968423984	0.134412682	0.430523146	0.734884965	0.361272238	0.968567705
COPS2	0.118065777	0.21901771	0.22077657	0.995188867	0.733892775	0.360653012	0.968567705
COX5B	0.84421913	0.463795946	0.727127461	0.01541365	0.973534608	0.364473228	0.968567705

DUS4L	0.495136354	0.684181182	0.109449667	0.401419063	0.28691298	0.364403877	0.968567705
EIF5B	0.619856642	0.885687863	0.401667824	0.439391492	0.044194697	0.364840524	0.968567705
ERICH3	0.428247761	0.187062164	0.988986421	0.557201057	0.094774531	0.361187375	0.968567705
EXOSC9	0.447406556	0.214224731	0.794543547	0.139241307	0.393073841	0.360594402	0.968567705
FAM193B	0.293602527	0.256506344	0.428621731	0.376172454	0.345473264	0.361605855	0.968567705
FKTN	0.035893508	0.525629909	0.909752049	0.930389129	0.264106347	0.362448002	0.968567705
GART	0.980048989	0.681112744	0.543015584	0.37219248	0.031283192	0.362554855	0.968567705
GPR18	0.571963183	0.714101385	0.021452264	0.998868128	0.4830589	0.362826934	0.968567705
HEATR3	0.482488517	0.982443582	0.321667604	0.05547999	0.501630389	0.36341121	0.968567705
IDH3B	0.865115043	0.699304632	0.742650694	0.011048301	0.845637822	0.361703878	0.968567705
IQCC	0.32175474	0.91892252	0.033869534	0.722551321	0.592978921	0.365153153	0.968567705
ISLR2	0.633448298	0.292102632	0.05234423	0.886188282	0.493098488	0.362995622	0.968567705
KCNQ1	0.938154041	0.302686331	0.67410391	0.067083083	0.331757064	0.364029468	0.968567705
KCNT1	0.822256829	0.108760343	0.247601945	0.840972357	0.226470834	0.362434841	0.968567705
LIG4	0.387285469	0.078829479	0.774742535	0.30434869	0.588903655	0.363255669	0.968567705
LOC101902204	0.255921973	0.713828583	0.800336762	0.126518691	0.225430965	0.36067039	0.968567705
LOC101903616	0.267698848	0.680866248	0.587036012	0.163853619	0.239191097	0.361548254	0.968567705
LOC101903832	0.570073279	0.966250067	0.53014987	0.029653625	0.48296087	0.361127316	0.968567705
LOC101903877	0.532916181	0.476830888	0.437967949	0.486993087	0.078582661	0.363988885	0.968567705
LOC101906012	0.570170493	0.73122951	0.750086342	0.023090428	0.593383239	0.364940873	0.968567705
LOC101908048	0.350745113	0.239998038	0.088704642	0.867753067	0.644620952	0.360924311	0.968567705
LOC107131224	0.903548184	0.449154399	0.375397575	0.626309013	0.044777452	0.364486624	0.968567705
LOC107131367	0.737097777	0.100882986	0.612970576	0.342290814	0.274361029	0.36478222	0.968567705
LOC107132335	0.177809477	0.446397888	0.116323296	0.483441799	0.94594326	0.362625533	0.968567705
LOC107132398	0.031844869	0.402490263	0.981896229	0.630850153	0.533177714	0.363025872	0.968567705
LOC112444314	0.696111787	0.195915221	0.902157382	0.84710793	0.039943012	0.360405835	0.968567705
LOC112447031	0.130518077	0.979140764	0.544585999	0.250815515	0.245495991	0.364956899	0.968567705
LOC112448381	0.823667769	0.285326282	0.031517589	0.816358497	0.703588057	0.363818777	0.968567705
LOC527388	0.622722577	0.231058079	0.267279879	0.862478171	0.129217733	0.364982771	0.968567705
LOC783466	0.573238506	0.624032746	0.084005635	0.240819662	0.591477283	0.364775259	0.968567705
LY6D	0.28039536	0.60190209	0.614527121	0.046251157	0.886417003	0.363728904	0.968567705
MALT1	0.478794721	0.389415002	0.627985534	0.413832618	0.087639018	0.363524218	0.968567705
MANBAL	0.301467747	0.143407515	0.1860087	0.542328044	0.98349989	0.365102762	0.968567705
MFSD4A	0.651972079	0.629689319	0.067881082	0.405508089	0.379626847	0.365131157	0.968567705
MKNK2	0.117665906	0.649183112	0.744984733	0.314533328	0.239552065	0.365048028	0.968567705
MUC1	0.528640707	0.55342813	0.076697599	0.308182681	0.614615408	0.363662494	0.968567705
MYBL1	0.323837052	0.619499214	0.538575652	0.063570281	0.624110629	0.365010921	0.968567705
NAV3	0.512300382	0.188753225	0.150668086	0.872824379	0.328453325	0.360922369	0.968567705
NBEA	0.317649482	0.995940258	0.532476336	0.055080708	0.459947775	0.364306298	0.968567705
NCAM1	0.499541344	0.179246015	0.15845507	0.36568082	0.813454943	0.362556674	0.968567705
NUP155	0.268915843	0.46555125	0.748866314	0.248081467	0.183457136	0.364279917	0.968567705
PARD6A	0.37605821	0.126471032	0.566332151	0.167229294	0.946570606	0.364158551	0.968567705
PDCD6	0.667105352	0.299849082	0.680090533	0.08046672	0.38215077	0.361165694	0.968567705
PIBF1	0.134425688	0.708155515	0.795512932	0.215622096	0.258384846	0.362504445	0.968567705
PITPNM1	0.071632609	0.975541023	0.122438521	0.495818462	0.999025784	0.363212488	0.968567705

PSMC1	0.38376612	0.624012933	0.174723361	0.819203034	0.121781604	0.36082983	0.968567705
PYM1	0.361267334	0.763053788	0.058912935	0.758709179	0.339803141	0.361303752	0.968567705
RBM17	0.852196711	0.207120193	0.203905179	0.421176225	0.280756389	0.363868772	0.968567705
SBF2	0.46031188	0.748368925	0.191127184	0.115336674	0.562859058	0.364548719	0.968567705
SH2D1A	0.368493846	0.687014046	0.790627958	0.151600853	0.13931206	0.362808487	0.968567705
SIGMAR1	0.398512687	0.703295518	0.32197213	0.294841428	0.158071077	0.362005515	0.968567705
SLC35A3	0.22916265	0.326336627	0.326197778	0.238643462	0.727067415	0.363010019	0.968567705
STXBP2	0.19612913	0.555714856	0.068495683	0.874202128	0.654209944	0.364377641	0.968567705
TEAD3	0.102368266	0.559358647	0.087682853	0.947243048	0.882951889	0.361763174	0.968567705
TESC	0.933512203	0.569860739	0.051156336	0.168024975	0.918175817	0.361733904	0.968567705
TMC8	0.108678655	0.618871916	0.299729128	0.749560686	0.282540838	0.364368818	0.968567705
TMEM127	0.110052624	0.386310888	0.324851515	0.467898444	0.645110181	0.360621764	0.968567705
TMEM132C	0.938483364	0.509811117	0.410512839	0.033219102	0.652930392	0.364025982	0.968567705
TNFSF13B	0.684736118	0.473123823	0.212453976	0.329417266	0.18474137	0.36136739	0.968567705
TOMM20	0.626470211	0.105047338	0.329259226	0.982479827	0.200758389	0.364535394	0.968567705
TRAK1	0.38077197	0.05721459	0.31695105	0.796892025	0.764722887	0.362088078	0.968567705
TUBGCP6	0.581370801	0.083877265	0.446935282	0.217539794	0.878391392	0.360463485	0.968567705
UBE2G2	0.859258223	0.044297519	0.23052054	0.78255765	0.624751016	0.365122261	0.968567705
WDR24	0.556016013	0.334498598	0.347829208	0.230588697	0.282369807	0.362245917	0.968567705
YBEY	0.196384589	0.934296872	0.252077722	0.260579146	0.351252114	0.363035551	0.968567705
ZDHHC9	0.10201154	0.690656828	0.546491386	0.125990115	0.86109821	0.360937972	0.968567705
ZNF706	0.622578383	0.281239252	0.087788374	0.704575531	0.385608249	0.360900711	0.968567705
ZRSR2	0.061193126	0.382448769	0.667092913	0.475882234	0.571226672	0.363428633	0.968567705
DSTN	0.14033084	0.260741023	0.609634195	0.70970062	0.271976822	0.365705189	0.968649469
EME1	0.44818683	0.704329618	0.042687343	0.523672325	0.608473322	0.365267421	0.968649469
GPR158	0.562559723	0.627271222	0.045116611	0.918833588	0.293624434	0.365323487	0.968649469
HMOX2	0.133518836	0.724549223	0.244997088	0.29362316	0.618966206	0.365774222	0.968649469
KLF12	0.761173672	0.972552188	0.373498676	0.055464737	0.280277007	0.36543282	0.968649469
PORCN	0.490625432	0.690335511	0.378476122	0.083409315	0.401949741	0.365412778	0.968649469
TMEM33	0.083982803	0.185542143	0.826047038	0.369589769	0.905400156	0.365763165	0.968649469
TOPBP1	0.714862497	0.772872047	0.849072271	0.0229478	0.40013665	0.365772602	0.968649469
TOX	0.434852843	0.222389553	0.152147928	0.412212075	0.709846986	0.365694018	0.968649469
TXNDC11	0.05450192	0.296581116	0.56493291	0.600030032	0.786078793	0.365760626	0.968649469
AKR7A2	0.648471094	0.942496911	0.677693188	0.045503535	0.229131927	0.366176769	0.968682467
ASIC2	0.536132827	0.445105522	0.824073721	0.758876455	0.028877184	0.365846556	0.968682467
CMTM8	0.658446446	0.631868914	0.234200448	0.085144443	0.520602648	0.366199868	0.968682467
LRRC49	0.703089651	0.558012729	0.831399253	0.030403923	0.435081509	0.366042148	0.968682467
OGT	0.339284733	0.0935446	0.377879861	0.899940941	0.400166528	0.366197576	0.968682467
RPL7L1	0.864467856	0.523642693	0.3538186	0.231807003	0.116275828	0.366120739	0.968682467
SCUBE2	0.200519858	0.845996222	0.502640577	0.299625735	0.169021826	0.366166151	0.968682467
OCSTAMP	0.888905304	0.32621086	0.030507723	0.775044351	0.630269678	0.366279564	0.968685816
ROMO1	0.477905253	0.396491123	0.381655327	0.07226472	0.827089565	0.366319187	0.968685816
LOC112446663	0.314258067	0.631571415	0.380210764	0.204411216	0.280367596	0.366406749	0.968761263
SELENOM	0.928313031	0.62966388	0.014223135	0.542514866	0.959716908	0.366547929	0.968978426
LOC101907729	0.539402642	0.553976559	0.427993704	0.03454653	0.981132943	0.366774169	0.969279614

RGS22	0.573343927	0.87782718	0.038694668	0.751291845	0.296393089	0.366839052	0.969279614
SAMD11	0.978707094	0.773347659	0.036743344	0.604407529	0.257924973	0.366794335	0.969279614
CARD14	0.11844887	0.297251341	0.545531912	0.280269031	0.80751975	0.367222341	0.969419787
CASK	0.086125272	0.510834116	0.281237712	0.521655577	0.673240712	0.367162194	0.969419787
GSR	0.652656832	0.624617781	0.101301505	0.177445046	0.59332203	0.367246531	0.969419787
LOC100336589	0.15283495	0.822892906	0.067971002	0.612806386	0.829895378	0.367234425	0.969419787
LOC104974020	0.050925946	0.229852651	0.913710231	0.962994691	0.422087419	0.367229723	0.969419787
PITHD1	0.938662077	0.103332051	0.055936891	0.965171784	0.828993214	0.36700171	0.969419787
RPGRIP1L	0.160910016	0.857969284	0.562053747	0.171914834	0.326257444	0.367405958	0.969684653
C7H5orf30	0.962112107	0.19816941	0.406100156	0.063244293	0.889540341	0.367543173	0.969890822
LOC518775	0.849675153	0.211185969	0.567282512	0.243184853	0.17614379	0.367703347	0.970001548
VSTM4	0.186754323	0.214911811	0.259449735	0.488543618	0.856871533	0.367660271	0.970001548
ZADH2	0.697248581	0.270851641	0.798646332	0.401020993	0.072140651	0.367812715	0.970134115
MDH2	0.92378084	0.604563773	0.760134893	0.012959221	0.794018142	0.367991766	0.970335314
PITPNM3	0.987134639	0.794030637	0.296275058	0.255648687	0.073587008	0.368007251	0.970335314
ARPC1B	0.248667446	0.944215209	0.376656078	0.101428734	0.488069835	0.368345006	0.970549544
ATXN2L	0.83070285	0.137906622	0.926561014	0.088568284	0.465976314	0.36844334	0.970549544
HSD17B12	0.787755678	0.742952864	0.907455805	0.053510165	0.154117116	0.368413562	0.970549544
PSENEN	0.353632875	0.323039692	0.34274823	0.196764279	0.568236225	0.368338076	0.970549544
RBM19	0.030751009	0.953482651	0.854557231	0.579776801	0.301296967	0.368305186	0.970549544
SARDH	0.614792576	0.496043957	0.078900817	0.652874531	0.278381161	0.368170326	0.970549544
COPS8	0.505929375	0.218590478	0.224224045	0.259148605	0.682629659	0.368659199	0.970650749
NUDT3	0.161349974	0.217914421	0.269207813	0.93221575	0.497043928	0.368628628	0.970650749
PHF21A	0.88151637	0.237337128	0.147652582	0.497124586	0.285521812	0.368587642	0.970650749
ANAPC4	0.058610132	0.744046818	0.854712277	0.357703199	0.329618609	0.368948177	0.970944281
PREPL	0.700679757	0.467699581	0.173104233	0.085964047	0.90077945	0.368875787	0.970944281
TFB2M	0.492556366	0.567370524	0.206581983	0.275651377	0.276051737	0.368889043	0.970944281
COX7B2	0.203325782	0.899976239	0.095900625	0.601137916	0.417167065	0.369168311	0.971367829
FJX1	0.987019044	0.068812644	0.097565596	0.954390299	0.696575318	0.369335036	0.971650735
CMAS	0.128016963	0.206045556	0.496381593	0.842753938	0.399605033	0.369479141	0.971874047
HIST1H2BI	0.093552217	0.491307408	0.956427152	0.819399713	0.122505064	0.369600337	0.971877582
LOC539166	0.217245238	0.093164463	0.273124958	0.99522395	0.802387642	0.369658148	0.971877582
PVALB	0.208610562	0.947287993	0.090785517	0.704269064	0.34913844	0.369549812	0.971877582
LOC107132045	0.641918741	0.71262377	0.84700825	0.021054966	0.541652506	0.369817979	0.971986362
TMED2	0.382141265	0.079143863	0.589396487	0.616369863	0.402133426	0.369802219	0.971986362
ABCC3	0.709320893	0.260423824	0.320396014	0.101484074	0.738363849	0.370395484	0.972117595
ARMC6	0.533038732	0.852819735	0.21138835	0.188756489	0.245413614	0.370989372	0.972117595
ATP6V0A4	0.660285607	0.993158067	0.020154967	0.751935655	0.446129262	0.370356908	0.972117595
ATP6V0D1	0.152095805	0.376122511	0.323247951	0.792094159	0.304704329	0.371408038	0.972117595
CHMP1A	0.23511065	0.532569818	0.480641323	0.076026364	0.97411889	0.371190759	0.972117595
CSKMT	0.804147555	0.609789559	0.172909311	0.54477057	0.096248328	0.370785076	0.972117595
FNBP1L	0.184801385	0.76379559	0.372652542	0.115076562	0.734107198	0.37070871	0.972117595
KCNN3	0.599535229	0.456975951	0.148273693	0.266959548	0.411340548	0.371327733	0.972117595
LBR	0.3160712	0.183369912	0.763590026	0.172882297	0.582269277	0.371117877	0.972117595
LGALS1	0.318290755	0.464281413	0.315651174	0.337287412	0.281986627	0.370455073	0.972117595

LOC101906469	0.279687035	0.394257477	0.362031082	0.158285078	0.700432482	0.37007488	0.972117595
LOC112447313	0.637171536	0.3208453	0.531232575	0.100163758	0.40892468	0.370876722	0.972117595
LOC783920	0.438749237	0.948579795	0.6889037	0.0161373	0.963534644	0.37122778	0.972117595
MCHR1	0.382833458	0.395172106	0.074279551	0.868069295	0.454213793	0.37024928	0.972117595
MCPH1	0.02875568	0.460899104	0.703566104	0.614681499	0.775923424	0.370844695	0.972117595
MKL2	0.02380581	0.94328165	0.335613002	0.851193875	0.69019668	0.370133559	0.972117595
MTHFD1	0.440035841	0.770983556	0.952578834	0.095552833	0.143575296	0.370350929	0.972117595
NHEJ1	0.294985158	0.23353895	0.669989058	0.300255522	0.32071563	0.370747423	0.972117595
NPR1	0.056610249	0.645140164	0.564491358	0.309888043	0.698289423	0.371337867	0.972117595
SEMA4A	0.284653439	0.598015683	0.381861038	0.448748531	0.1529785	0.371382333	0.972117595
SLC35F2	0.380107457	0.198938847	0.381040232	0.232705952	0.660002324	0.370054865	0.972117595
SLC48A1	0.572494119	0.330696276	0.098507084	0.300070504	0.79289312	0.370478684	0.972117595
TFIP11	0.7187673	0.881233204	0.138922226	0.269320748	0.187785027	0.370946819	0.972117595
TSEN54	0.494933874	0.827569706	0.328353177	0.036819919	0.89932472	0.371060859	0.972117595
XRCC4	0.057516394	0.42867626	0.926989453	0.572085041	0.339879368	0.370726488	0.972117595
ZBTB25	0.630517368	0.346245061	0.343090557	0.40121313	0.147416587	0.370223891	0.972117595
URB2	0.213762779	0.822305979	0.597203057	0.338315958	0.125776173	0.371544196	0.972318897
PRR33	0.983833339	0.079382899	0.263152338	0.304436441	0.714299393	0.371626582	0.972379439
PIK3C2A	0.615069841	0.442295606	0.186113635	0.091324449	0.967138855	0.371721576	0.972382318
TMEM43	0.371990763	0.77101174	0.719719104	0.597501488	0.036262934	0.371746186	0.972382318
ADCYAP1R1	0.159663298	0.853606075	0.489760287	0.11509476	0.58373285	0.372171831	0.972710357
ARL8A	0.222789513	0.147230139	0.243362285	0.680448853	0.825627761	0.372175018	0.972710357
GRO1	0.121826873	0.774789471	0.486999433	0.540441263	0.180577112	0.372227228	0.972710357
HOXC9	0.251674924	0.286524746	0.406082028	0.18246419	0.838955164	0.37210431	0.972710357
SLC10A1	0.190123574	0.642673342	0.236110611	0.230925641	0.672491559	0.372019188	0.972710357
TTC39B	0.811567521	0.702796585	0.294945708	0.36002399	0.074029153	0.37214048	0.972710357
ABL2	0.76945938	0.747785133	0.05313779	0.206849935	0.710123924	0.372407143	0.972719953
C24H18orf25	0.43710712	0.239904598	0.672636106	0.40394294	0.157979052	0.372764352	0.972719953
GABPB2	0.883684377	0.89564827	0.968788322	0.952068271	0.006163585	0.372705053	0.972719953
GNA14	0.197532162	0.308645042	0.29717827	0.712697856	0.348266082	0.372618827	0.972719953
KIAA2026	0.473571102	0.650601902	0.817721763	0.060153511	0.296804449	0.372658085	0.972719953
MYO5B	0.105807041	0.287164815	0.654691132	0.683908946	0.330645505	0.372660182	0.972719953
RAB40B	0.143310855	0.214584407	0.784036469	0.997890049	0.186937869	0.372641899	0.972719953
RPL7A	0.477612877	0.655551725	0.365389294	0.085961151	0.457496814	0.372691817	0.972719953
VSIR	0.056030788	0.799828297	0.902232217	0.144793314	0.768710015	0.372737374	0.972719953
ACIN1	0.523725493	0.080182317	0.338631762	0.502669754	0.632455197	0.373462863	0.9728811
GRIP2	0.327363947	0.485063288	0.470116296	0.380850525	0.159145179	0.373595585	0.9728811
LIFR	0.770813454	0.333479269	0.090709595	0.257582345	0.752229432	0.373357926	0.9728811
LOC100847870	0.189240538	0.254352754	0.580027216	0.187531666	0.863849422	0.373532568	0.9728811
LOC101906280	0.590017829	0.623709741	0.044582878	0.530362055	0.51908114	0.373315804	0.9728811
LOC107132952	0.943979469	0.17510889	0.155193162	0.223606699	0.787227772	0.373280833	0.9728811
LOC516442	0.712649849	0.185386653	0.984403771	0.901583572	0.038463992	0.37308181	0.9728811
LOC784738	0.129497571	0.632423912	0.314731554	0.737523578	0.238058564	0.373627768	0.9728811
SDC3	0.26167529	0.829628015	0.078864314	0.292283021	0.904079418	0.373578709	0.9728811
SGIP1	0.714500413	0.377907998	0.881023613	0.048178634	0.393144865	0.37293263	0.9728811

SMIM13	0.113697402	0.394205	0.381633095	0.550620358	0.480591055	0.373656058	0.9728811
SMNDC1	0.084879682	0.824470479	0.88529422	0.086845374	0.84016351	0.373446081	0.9728811
USP25	0.101480211	0.804150961	0.27621425	0.260951234	0.769235407	0.373595969	0.9728811
ZNF527	0.128813156	0.803199426	0.978772613	0.342066563	0.130331677	0.373243063	0.9728811
ARPC4	0.177739228	0.182509753	0.57909185	0.980273929	0.248466626	0.375385919	0.973418737
ASGR2	0.221790488	0.788695933	0.058239966	0.472438498	0.94574576	0.374558717	0.973418737
CRK	0.788022362	0.253834967	0.027674402	0.869457407	0.948268898	0.374985195	0.973418737
GAS1	0.956408782	0.292352799	0.082939699	0.241628993	0.816830595	0.375445801	0.973418737
HORMAD2	0.909412547	0.185428139	0.566777848	0.055083154	0.867847027	0.375156417	0.973418737
JAM2	0.374016158	0.674012054	0.477599232	0.066226582	0.570076606	0.374334981	0.973418737
KLHL41	0.04347168	0.76992917	0.705785741	0.201284019	0.960162787	0.375035512	0.973418737
LIPT2	0.264771625	0.311477007	0.361817625	0.564934995	0.271252742	0.37528516	0.973418737
LOC100139764	0.216027121	0.186112484	0.787249538	0.211164363	0.678673913	0.373999504	0.973418737
LOC100847951	0.290172712	0.219852719	0.551318519	0.323451825	0.401140709	0.374966458	0.973418737
LOC112444936	0.71155147	0.596407775	0.452014913	0.736937299	0.032262459	0.374867843	0.973418737
LOC614226	0.503507698	0.981495681	0.354561371	0.027721924	0.938963468	0.374878038	0.973418737
LOC783854	0.98635484	0.383218624	0.551618541	0.610773352	0.035897168	0.375247882	0.973418737
LRRC4C	0.698362978	0.711808353	0.177997514	0.868501714	0.059437767	0.375112057	0.973418737
MMAA	0.88184143	0.467958631	0.911582778	0.048626878	0.250249498	0.375464055	0.973418737
PDE1A	0.121604405	0.412002873	0.2364771	0.649833497	0.591789748	0.374711903	0.973418737
POLR1E	0.578418841	0.820436572	0.212452529	0.276944102	0.163774253	0.375295262	0.973418737
PTGDR	0.677430664	0.852994782	0.176932135	0.209522412	0.21282759	0.374810949	0.973418737
RHOG	0.276664228	0.877580007	0.075893209	0.425591404	0.578685492	0.374073323	0.973418737
SCRN1	0.246813327	0.120042233	0.402124344	0.933639659	0.409420179	0.374639304	0.973418737
TAF1A	0.149778517	0.076625894	0.774134795	0.766318668	0.672308833	0.375455262	0.973418737
TEKT3	0.685046749	0.264735478	0.401973776	0.555852593	0.112652797	0.375016098	0.973418737
TNFAIP2	0.492783609	0.416549394	0.543944693	0.734727236	0.055701446	0.375178104	0.973418737
TRPM7	0.153058118	0.819422144	0.875701495	0.116849926	0.354630541	0.374533396	0.973418737
UBL7	0.603351773	0.958089671	0.767119247	0.011131128	0.921798457	0.374492806	0.973418737
WDR36	0.932790555	0.335994025	0.937625562	0.081419571	0.190327324	0.37462583	0.973418737
YWHAH	0.553558524	0.575634903	0.114887992	0.163279211	0.762666	0.374801428	0.973418737
COPA	0.615790748	0.696506694	0.428670989	0.026167254	0.952463856	0.375628807	0.973568813
ERAL1	0.836614431	0.977128981	0.239090288	0.029282902	0.801205303	0.375742694	0.973568813
FAM83F	0.336026389	0.836175736	0.035609506	0.752463909	0.609143023	0.375759238	0.973568813
KCNJ15	0.205693393	0.249372	0.283814577	0.85115614	0.369892765	0.375665272	0.973568813
ARAP2	0.943037959	0.365128361	0.190619092	0.234051027	0.298708779	0.375854623	0.973616693
EFS	0.878653768	0.36408017	0.028459346	0.693599434	0.726882261	0.375896372	0.973616693
HIVEP3	0.197427166	0.474289627	0.873207739	0.356090034	0.15775008	0.376001831	0.973620481
ZNF532	0.558506493	0.746787394	0.072192158	0.728986206	0.209267886	0.37601649	0.973620481
FEM1C	0.818801514	0.174802082	0.261878976	0.262611396	0.467059253	0.376155017	0.973646299
KPNB1	0.604246976	0.329817434	0.893649697	0.063525443	0.406482496	0.376204447	0.973646299
WBP4	0.227634144	0.488318515	0.653261139	0.120924106	0.52339417	0.376102372	0.973646299
COX8A	0.886898999	0.490044828	0.641094304	0.017151428	0.963180222	0.376350603	0.973870978
LOC101908339	0.108982499	0.291286609	0.458001019	0.555836446	0.570112454	0.376503	0.974111733
SERPINB8	0.76480932	0.997885874	0.011146424	0.791426614	0.684833119	0.376619201	0.974258781

B4GALT3	0.587201775	0.960111902	0.757443872	0.194007275	0.055746995	0.376891205	0.974348072
LOC101904468	0.686991442	0.01646238	0.849334729	0.5074084	0.947537857	0.376883017	0.974348072
NCOA5	0.213869331	0.354977252	0.486142216	0.190675127	0.656257548	0.376884378	0.974348072
RPS6KA4	0.10759864	0.800425916	0.229537625	0.405032649	0.576271526	0.376742933	0.974348072
FGFR1OP	0.041238679	0.642269361	0.736166753	0.241902674	0.98072811	0.377146329	0.974377272
HAVCR2	0.465272851	0.754968607	0.030063478	0.564739763	0.775252601	0.377064765	0.974377272
LITAF	0.459816603	0.457215273	0.30355517	0.130032855	0.557753774	0.377239458	0.974377272
PTCD1	0.716665651	0.117891087	0.37860113	0.477008125	0.303289574	0.377211314	0.974377272
TMEM50A	0.803110313	0.018278575	0.90357066	0.402183418	0.867366764	0.377190504	0.974377272
ZBTB38	0.278263206	0.66036621	0.485448069	0.840934184	0.061708533	0.37725874	0.974377272
OSTF1	0.655218366	0.410111511	0.06062913	0.398647405	0.713389818	0.377405064	0.974601811
ACBD3	0.663736151	0.561932542	0.955549176	0.013709169	0.953687879	0.378319919	0.97465367
BMF	0.97443	0.800589048	0.077158125	0.272827763	0.282702969	0.377729927	0.97465367
CHD2	0.541267349	0.462356203	0.359549283	0.280497401	0.184227103	0.377978014	0.97465367
DUSP12	0.701561848	0.274166255	0.686401416	0.040092685	0.880295329	0.378320511	0.97465367
GLA	0.854728827	0.648074899	0.024577904	0.390896962	0.874707817	0.378161568	0.97465367
LIPT1	0.870431992	0.364912002	0.270748798	0.132009283	0.409532144	0.377960449	0.97465367
LOC101902084	0.113039033	0.308686155	0.412979308	0.555731363	0.579726811	0.377730403	0.97465367
LOC516599	0.913849051	0.865509629	0.034354038	0.603472633	0.284261261	0.37837539	0.97465367
MAB21L2	0.014484427	0.904121167	0.648714132	0.577425925	0.946327263	0.37771436	0.97465367
MATN4	0.611429955	0.368096449	0.56652248	0.129265809	0.28215572	0.378003518	0.97465367
MYOCD	0.120016523	0.431560784	0.785377298	0.34260991	0.333409534	0.377870361	0.97465367
SLC39A3	0.401120688	0.532488346	0.276781173	0.252748654	0.311187926	0.377979496	0.97465367
SPTLC3	0.579369149	0.342292479	0.49657226	0.628683963	0.075005001	0.377765884	0.97465367
SRGAP1	0.899611619	0.172209192	0.541821886	0.063001308	0.879496528	0.378023421	0.97465367
TIPARP	0.963695512	0.855989677	0.226976643	0.024914779	0.997774453	0.378144955	0.97465367
ZMYND15	0.682461815	0.324922948	0.046271683	0.688400988	0.659392145	0.378249057	0.97465367
RNF139	0.255414544	0.049142534	0.817008904	0.472394608	0.963226269	0.378547689	0.974944464
PIH1D2	0.290317805	0.082115302	0.518472719	0.830912076	0.45462525	0.378647681	0.975048971
LOC107132225	0.45099386	0.158802231	0.096073954	0.921879349	0.736398813	0.378717459	0.975075653
CEP89	0.446791287	0.181199191	0.455818851	0.640521352	0.197820023	0.378879907	0.975278132
VPS16	0.724590243	0.04340746	0.294271599	0.550786202	0.917409254	0.378914958	0.975278132
SH3PXD2A	0.606732904	0.825094379	0.019521472	0.868953535	0.551021239	0.378999455	0.975342646
PRADC1	0.608975931	0.780888301	0.541601543	0.030460012	0.596975189	0.379139519	0.975397185
ZNF394	0.072568431	0.351443506	0.381735358	0.841292017	0.571700993	0.379112023	0.975397185
ACVR2B	0.111834606	0.635549711	0.454402447	0.236576704	0.616100859	0.37997123	0.975766355
C15H11orf58	0.380559385	0.095725988	0.237526577	0.8936019	0.608503282	0.379889803	0.975766355
FAM214B	0.383698214	0.112923876	0.234937232	0.770914814	0.599173972	0.379783774	0.975766355
GPATCH3	0.127571735	0.907074875	0.74662248	0.122096196	0.445350431	0.379640812	0.975766355
GSK3A	0.505458082	0.69866459	0.151863199	0.360461679	0.243386615	0.37988634	0.975766355
LOC100300483	0.1024945	0.949785486	0.925475796	0.632749459	0.082367938	0.379558361	0.975766355
LOC100336369	0.035927178	0.69034908	0.664183034	0.595861794	0.478496856	0.379604187	0.975766355
LOC112444309	0.278952907	0.686610132	0.776840878	0.763062926	0.041355186	0.379551802	0.975766355
LOC112447305	0.767503514	0.733419485	0.526171531	0.741955195	0.021424971	0.379996513	0.975766355
LYZ2	0.311567148	0.783813658	0.440406353	0.646292055	0.067520429	0.379484286	0.975766355

MFAP2	0.386845594	0.210552945	0.084695235	0.782076561	0.871641017	0.379805423	0.975766355
TTC1	0.955292457	0.416486909	0.372740795	0.063633094	0.498900185	0.379990279	0.975766355
AHCYL2	0.750310456	0.135019357	0.109137914	0.595232498	0.728419191	0.382916343	0.976389077
AKAP12	0.38721656	0.65074707	0.248711243	0.36571147	0.209081251	0.382854445	0.976389077
ANXA6	0.547053892	0.84879816	0.438381741	0.312162081	0.074425757	0.380716529	0.976389077
BCO2	0.625372991	0.699615735	0.724124986	0.059309919	0.251779079	0.38077946	0.976389077
BIRC2	0.223731631	0.177516863	0.473791876	0.47708189	0.526532367	0.380635337	0.976389077
C10H15orf48	0.714315226	0.659738091	0.21497503	0.223132885	0.209012868	0.380565807	0.976389077
CCL4	0.305281929	0.777866233	0.207970599	0.447179602	0.215686929	0.381882058	0.976389077
CDH4	0.863278334	0.222713527	0.120777392	0.626469946	0.326068346	0.381201969	0.976389077
CFAP298	0.342176328	0.742201209	0.155842924	0.122892397	0.976312189	0.381381259	0.976389077
CHD5	0.165049017	0.932165005	0.761123111	0.302117136	0.13458333	0.381812405	0.976389077
CLCN7	0.326931085	0.867896319	0.030248092	0.909203778	0.612699754	0.382486378	0.976389077
COX5A	0.681390166	0.508145362	0.897553197	0.018531923	0.826521404	0.381771826	0.976389077
EPAS1	0.830132826	0.161921331	0.347268982	0.211508205	0.485504112	0.382900287	0.976389077
F2RL2	0.237284929	0.337806503	0.469290639	0.394550099	0.32102462	0.381921941	0.976389077
FADS3	0.233556378	0.517436608	0.259419281	0.211387504	0.716365946	0.381341305	0.976389077
HTR2A	0.320274661	0.31697082	0.409198277	0.184391332	0.623947407	0.382424005	0.976389077
IRF3	0.392539314	0.733462389	0.06156239	0.29386872	0.910724799	0.381211309	0.976389077
JAGN1	0.484262009	0.367450768	0.192879009	0.368429658	0.378266956	0.382556113	0.976389077
KCTD8	0.631290829	0.858799328	0.332962303	0.045464376	0.577192943	0.380984305	0.976389077
KIF2A	0.963959255	0.763699639	0.411767478	0.049643204	0.31450864	0.380841544	0.976389077
KIF9	0.328001342	0.03366452	0.892932023	0.547607855	0.885780232	0.382535394	0.976389077
LAMA5	0.921376926	0.726977205	0.111167503	0.66969913	0.095628044	0.38206406	0.976389077
LOC100337108	0.039629628	0.821185853	0.151187464	0.982908948	0.980271262	0.381106767	0.976389077
LOC112441605	0.310279584	0.850458107	0.02937565	0.896220265	0.679182022	0.380345358	0.976389077
LOC112444352	0.058244787	0.226433864	0.93772321	0.960781632	0.400420799	0.381695579	0.976389077
LOC112447080	0.484256169	0.51226339	0.058465024	0.436104168	0.751579538	0.381551977	0.976389077
LOC112447474	0.487966786	0.352371865	0.567524977	0.578485785	0.084269759	0.381668129	0.976389077
LOC112449172	0.460670851	0.309322677	0.672992936	0.292211211	0.169446018	0.381369618	0.976389077
LOC112449302	0.814805153	0.622426518	0.898639489	0.506819243	0.020546488	0.381286323	0.976389077
LURAP1L	0.390210533	0.624489174	0.056554752	0.767213778	0.448167858	0.381037126	0.976389077
LYRM9	0.624511688	0.795723678	0.642540126	0.021217368	0.706378855	0.382636425	0.976389077
MEX3B	0.761764722	0.38302621	0.104300592	0.192526647	0.813989485	0.382080789	0.976389077
PBRM1	0.053237603	0.727108406	0.860258751	0.1816451	0.78195883	0.380740362	0.976389077
PDIA3	0.38311286	0.404788538	0.078879212	0.483141743	0.808659775	0.382421672	0.976389077
PDZD4	0.363960533	0.529919383	0.280378822	0.24754389	0.356968801	0.382396953	0.976389077
PFAS	0.848975021	0.361959533	0.211609619	0.477190021	0.154461603	0.382886988	0.976389077
PPT2	0.4969273	0.054989506	0.42158662	0.550560309	0.754181348	0.38256459	0.976389077
PRRC2A	0.375708038	0.633960056	0.220308238	0.127986175	0.711467335	0.382385502	0.976389077
QPCT	0.329598107	0.049213177	0.32953919	0.994147578	0.894220431	0.381491814	0.976389077
RCSD1	0.43086252	0.157375462	0.349348311	0.719292237	0.279472499	0.381831661	0.976389077
SLC3A2	0.233016681	0.453213031	0.901842123	0.074691509	0.669742699	0.381913945	0.976389077
SREBF1	0.36542449	0.743358063	0.908449303	0.256572087	0.075369809	0.382176988	0.976389077
STIL	0.166064497	0.751179054	0.22889569	0.696384646	0.23820923	0.380968327	0.976389077

TPM2	0.081239412	0.466232514	0.552388357	0.812904276	0.281557064	0.382743897	0.976389077
TXNRD1	0.667862938	0.280274743	0.145776793	0.253597754	0.683244787	0.3806759	0.976389077
AGFG2	0.873925606	0.933194015	0.229397275	0.033481542	0.76561525	0.382979938	0.976399529
NAE1	0.253511632	0.989347748	0.654229731	0.094462618	0.309576247	0.383074999	0.976490185
CHAF1B	0.66213816	0.661344151	0.928824558	0.018013937	0.656657126	0.383506357	0.976700483
LOC790312	0.060303814	0.851884371	0.891764568	0.132787285	0.790946259	0.383514589	0.976700483
MCM2	0.120649434	0.435090873	0.17743811	0.716223801	0.720776639	0.383410689	0.976700483
MS4A8	0.15893044	0.26115036	0.182704584	0.728568416	0.870738098	0.383486855	0.976700483
VNN1	0.704866657	0.992647107	0.450321606	0.085248734	0.178973444	0.383373084	0.976700483
ZCCHC14	0.231666639	0.400952673	0.171504017	0.305931027	0.987086747	0.383488619	0.976700483
LOC112447301	0.556254848	0.716704072	0.041017618	0.360478495	0.816941647	0.383655171	0.976756998
MEF2D	0.575468059	0.492247037	0.49799594	0.337506968	0.101144752	0.383655817	0.976756998
PGGT1B	0.176427011	0.435591852	0.237795685	0.329497294	0.800194359	0.383745672	0.97683422
DIRAS1	0.952294343	0.565912359	0.253683315	0.048392429	0.729460497	0.384006192	0.977194234
LOC104974850	0.655294508	0.772247541	0.879584713	0.97689632	0.011095984	0.383966302	0.977194234
PRR16	0.666356108	0.176039987	0.272393844	0.477385542	0.316752274	0.384198274	0.977434443
PTP4A1	0.122371323	0.14295819	0.405054506	0.99108962	0.688091329	0.384219706	0.977434443
DUSP15	0.828704983	0.882404147	0.161152472	0.044387871	0.924833733	0.384396383	0.977614555
EHD1	0.136864697	0.672361825	0.249981195	0.43392547	0.484675248	0.384409647	0.977614555
ACSS2	0.852033654	0.950484515	0.907055773	0.019568899	0.337764209	0.384990344	0.97777313
ALG9	0.69439762	0.237879459	0.613176949	0.076136708	0.628295403	0.3846501	0.97777313
GPX8	0.489177672	0.098550187	0.270036233	0.798025552	0.467320276	0.384975985	0.97777313
HES2	0.18723853	0.993915673	0.127518489	0.641004301	0.319566148	0.385186965	0.97777313
KDM5B	0.897111684	0.83526643	0.194377609	0.039361688	0.845492479	0.384722225	0.97777313
MAP2K3	0.370142911	0.23175212	0.40559245	0.571147655	0.2437533	0.384602446	0.97777313
NET1	0.896396886	0.445375504	0.203958941	0.499031963	0.119557462	0.385087427	0.97777313
RORC	0.762471841	0.307409811	0.881634344	0.854316931	0.027503557	0.384998642	0.97777313
SLC6A20	0.806745656	0.781023613	0.079057812	0.642821243	0.151807251	0.385182722	0.97777313
TES	0.264255852	0.252915072	0.871392264	0.463071823	0.180199485	0.385140151	0.97777313
UBXN10	0.93887881	0.519574547	0.102507728	0.700840847	0.138674499	0.385145819	0.97777313
ZNF7	0.092118742	0.220488271	0.949582581	0.840019761	0.29986921	0.385092359	0.97777313
ZFP64	0.177360548	0.761019994	0.16389413	0.748924407	0.293723176	0.385357068	0.978053641
CLIC2	0.772389977	0.093803156	0.271877432	0.263248044	0.940005709	0.385631319	0.978193909
COLQ	0.461137082	0.795676296	0.101276386	0.151497221	0.868805754	0.386187212	0.978193909
DNM1L	0.72977775	0.723441685	0.843773196	0.46143666	0.02378155	0.386100781	0.978193909
ICA1	0.26079694	0.069707234	0.422674393	0.763348006	0.833339342	0.386085224	0.978193909
LOC100847695	0.639647694	0.806738263	0.046399418	0.814526142	0.250507172	0.386002706	0.978193909
LOC101907661	0.583723325	0.729343942	0.014748492	0.990325935	0.783141037	0.385474425	0.978193909
LOC104974749	0.925256548	0.698717302	0.594021584	0.013071575	0.97409839	0.386147586	0.978193909
MRPL41	0.363470505	0.292637464	0.722928104	0.072501657	0.875464121	0.385840827	0.978193909
PLEKHA4	0.567325856	0.211291836	0.384965418	0.134398298	0.78812196	0.386082769	0.978193909
PRKAG3	0.253229151	0.57741681	0.1610314	0.406163491	0.509903368	0.385698781	0.978193909
SINHCAF	0.528190136	0.828211388	0.077208449	0.408836392	0.354086512	0.38613203	0.978193909
TRARG1	0.349510169	0.875904049	0.096865844	0.38457052	0.428295779	0.385963459	0.978193909
ZMYM5	0.183376643	0.775344644	0.777510466	0.076218595	0.579819813	0.38599813	0.978193909

ACD	0.805597582	0.047418813	0.31500695	0.456416728	0.891260933	0.386320088	0.978228509
SLC45A2	0.019049696	0.955574391	0.518529173	0.67981076	0.762656288	0.386277685	0.978228509
C29H11orf98	0.771893395	0.537048606	0.416042824	0.84435539	0.033700032	0.386737328	0.978392897
CACNG5	0.361060326	0.495806489	0.539653019	0.106595241	0.476819178	0.386825224	0.978392897
CLPP	0.546224045	0.509443021	0.307959295	0.164510034	0.349105449	0.387205687	0.978392897
COPZ1	0.236573406	0.669774416	0.202197708	0.615186526	0.250103666	0.387465117	0.978392897
CYFIP2	0.096795695	0.774095841	0.655630561	0.487130738	0.205404685	0.387001274	0.978392897
DSE	0.513394187	0.263291782	0.242815471	0.176952226	0.849291694	0.387570581	0.978392897
HOPX	0.732298586	0.335979033	0.063857454	0.580772444	0.540798882	0.387636988	0.978392897
HOXD8	0.426979743	0.651842128	0.308668739	0.068666842	0.834493211	0.387244648	0.978392897
KIAA1109	0.465988975	0.838538203	0.66637084	0.031482825	0.601180865	0.387425846	0.978392897
LOC100848011	0.398559224	0.14213389	0.213071096	0.422375437	0.96267244	0.386747746	0.978392897
LOC104975974	0.599837528	0.054886665	0.510016604	0.834124237	0.351921267	0.387451355	0.978392897
LOC510536	0.140381739	0.539258691	0.495347976	0.735883861	0.178542405	0.387378949	0.978392897
LOC782343	0.734400084	0.2058454	0.25263006	0.16812937	0.764395556	0.386758437	0.978392897
METAP1	0.960282026	0.314316754	0.137208109	0.203611033	0.583982238	0.387295423	0.978392897
MTDH	0.553960481	0.043605248	0.561741876	0.714494786	0.508023887	0.387329502	0.978392897
NXPH3	0.45821847	0.837984784	0.184715993	0.442566014	0.157171951	0.387604044	0.978392897
RAD21	0.683495617	0.455536849	0.533366917	0.043628734	0.67977702	0.387324556	0.978392897
SAT1	0.116509204	0.693200453	0.122157274	0.72002567	0.691808516	0.386966056	0.978392897
TSSK4	0.62576066	0.253982009	0.490143972	0.309810492	0.20387323	0.387161417	0.978392897
UMAD1	0.540986576	0.637161922	0.039549648	0.503375203	0.714415359	0.386570639	0.978392897
ZNHIT3	0.75563627	0.517330078	0.436744773	0.059629742	0.482697897	0.386956377	0.978392897
CD320	0.712525722	0.126666893	0.104308858	0.917660719	0.572043139	0.38787895	0.978702561
FAM102A	0.344674886	0.748140216	0.336378253	0.066094972	0.861942921	0.387868285	0.978702561
NTHL1	0.742336746	0.123319101	0.492625329	0.111132976	0.988028004	0.388204888	0.979098365
SS18L2	0.589641903	0.336143548	0.129133241	0.218606124	0.886028216	0.388393782	0.979098365
TNFRSF17	0.219755248	0.355231258	0.115111135	0.715130837	0.771405363	0.388384305	0.979098365
TRAF4	0.440590342	0.493407255	0.097685448	0.391698803	0.595471291	0.388251353	0.979098365
ZCCHC11	0.404367822	0.448597607	0.690901831	0.081411364	0.485111836	0.388135704	0.979098365
ZSCAN26	0.284741064	0.298040637	0.292171648	0.88811911	0.225075774	0.388357032	0.979098365
VCAN	0.996266991	0.244457261	0.073109396	0.323211075	0.86200998	0.388502948	0.979112371
WNT5A	0.618720373	0.384443113	0.030843301	0.996903277	0.678346631	0.388518662	0.979112371
ADCK2	0.887713862	0.711313185	0.079289783	0.351264113	0.282384838	0.388682507	0.979224534
RNF223	0.644671129	0.314563102	0.666888508	0.625838557	0.058667977	0.388658511	0.979224534
CCDC93	0.475021709	0.722456776	0.842158483	0.109198976	0.157774786	0.389116311	0.979415303
CLEC4G	0.626530993	0.517561914	0.295786624	0.2694437	0.192669768	0.389112416	0.979415303
FIBIN	0.350160581	0.057184906	0.447073659	0.605587707	0.917602171	0.388959195	0.979415303
LOC112441457	0.475314093	0.286442176	0.592132619	0.209480876	0.294802443	0.389092436	0.979415303
SPAG7	0.565522019	0.67354752	0.825997585	0.31983784	0.049426525	0.388931719	0.979415303
UBE3A	0.584918778	0.402688702	0.327371979	0.224514679	0.287422738	0.389001914	0.979415303
ABHD18	0.313886228	0.187347822	0.320098119	0.306611333	0.866745203	0.389875192	0.9794645
ADCY3	0.617325476	0.532377737	0.078474256	0.273439006	0.7076826	0.389488379	0.9794645
CDH11	0.63234302	0.27080278	0.065886884	0.881095904	0.502059441	0.389497018	0.9794645
CEP112	0.321069571	0.498113199	0.774343948	0.199469871	0.201753521	0.389261425	0.9794645

HDAC7	0.902493192	0.201263917	0.150932444	0.312760675	0.583217057	0.389818913	0.9794645
LOC104968411	0.736284992	0.192539448	0.670266531	0.057521179	0.916181334	0.39004109	0.9794645
LOC112446018	0.483795291	0.575432014	0.669644269	0.069222051	0.387862107	0.389965162	0.9794645
LOC112446798	0.418180249	0.235550403	0.399210885	0.509351825	0.249654611	0.389808473	0.9794645
LOC508628	0.408367761	0.574320765	0.916738416	0.043783101	0.531103159	0.389781859	0.9794645
PPIA	0.902072319	0.571694905	0.785205268	0.029262864	0.420600197	0.389267336	0.9794645
PPID	0.936846053	0.550227847	0.526211407	0.020277649	0.91067873	0.390090791	0.9794645
SNAI2	0.269416422	0.927013518	0.715655906	0.044057135	0.63554799	0.389949038	0.9794645
ST5	0.358278259	0.89487715	0.257267439	0.536480801	0.113061035	0.389894912	0.9794645
STARD3	0.575585781	0.023277644	0.688334206	0.553352101	0.98067792	0.389948056	0.9794645
STEAP4	0.640155354	0.494821453	0.024602727	0.757727132	0.844805865	0.389422874	0.9794645
TOE1	0.132222664	0.438081358	0.409487074	0.324003051	0.651359368	0.389983692	0.9794645
ANXA11	0.255781197	0.320704145	0.422053283	0.354146966	0.409349388	0.390417747	0.979536102
COX7ALP1	0.768709547	0.204504988	0.388149801	0.089153396	0.922265654	0.390357712	0.979536102
DOPEY2	0.755653422	0.502765677	0.510567703	0.110506039	0.234131945	0.390406455	0.979536102
FKBP5	0.961066151	0.379491915	0.109964097	0.373115849	0.334933476	0.390187289	0.979536102
SMAD9	0.295763724	0.927492834	0.068673661	0.441622927	0.602814211	0.390290101	0.979536102
MRPL35	0.643754439	0.755402782	0.889359793	0.021412936	0.54263336	0.390622434	0.979899842
ACYP1	0.688848798	0.150017966	0.43843617	0.142580111	0.77942261	0.390942902	0.979954782
DNAAF3	0.571189243	0.509349915	0.766999036	0.061550997	0.366534541	0.390918505	0.979954782
FZD1	0.930059769	0.796084394	0.05746084	0.337994913	0.349870252	0.39081175	0.979954782
LIN9	0.538363167	0.692422273	0.338574104	0.194446689	0.205128922	0.390913388	0.979954782
TMEM237	0.930476774	0.68562076	0.072039036	0.28262762	0.38741944	0.390847443	0.979954782
CDC37	0.087492826	0.93906093	0.177619764	0.651971378	0.530909094	0.391473522	0.980199122
COX6A1	0.975192566	0.49592424	0.541125145	0.01986307	0.971005838	0.391346751	0.980199122
FNIP2	0.951711271	0.215523898	0.248406559	0.124687217	0.795260007	0.391507093	0.980199122
LOC101907133	0.376475474	0.337157106	0.732722508	0.218567669	0.248321995	0.391361146	0.980199122
LOC112443837	0.442186481	0.756002069	0.697375556	0.059404622	0.364845245	0.391518204	0.980199122
LOC782057	0.030718604	0.775539236	0.570708177	0.607479047	0.610562476	0.391198652	0.980199122
ZBTB21	0.655481689	0.132768387	0.600875087	0.159286463	0.605917587	0.391331676	0.980199122
ZMYM1	0.021109327	0.962427884	0.480933688	0.711602189	0.725483155	0.391240943	0.980199122
COASY	0.900518739	0.624019848	0.633486305	0.020071349	0.70801624	0.39171686	0.980397345
SKAP1	0.788556823	0.419370241	0.558822857	0.056676302	0.48289975	0.391684664	0.980397345
ADPRH	0.741486565	0.105030326	0.490534798	0.157492818	0.853912183	0.394266841	0.980493243
AKAP9	0.751387189	0.354827742	0.786951455	0.034626352	0.705198196	0.393804818	0.980493243
ASCC2	0.082114753	0.826441843	0.298217211	0.639758267	0.392737107	0.392565029	0.980493243
C29H11orf80	0.123028072	0.319928991	0.488611325	0.847863762	0.315612957	0.394548667	0.980493243
CCER2	0.030945507	0.509293908	0.382841957	0.950523717	0.895257981	0.394166003	0.980493243
COPS6	0.59053415	0.521474738	0.686704768	0.221825098	0.108346338	0.392485031	0.980493243
DDX46	0.422139263	0.434943905	0.442932932	0.064844383	0.961678956	0.392126903	0.980493243
DPP3	0.233749037	0.835443558	0.780375253	0.386837129	0.086305672	0.392661408	0.980493243
EXOC7	0.708885082	0.602961212	0.028294337	0.706642111	0.602795273	0.394713088	0.980493243
FZD9	0.206472794	0.250698688	0.289360101	0.66872708	0.513197645	0.394352589	0.980493243
GATA3	0.381776774	0.863082873	0.939535524	0.038508046	0.429208577	0.393594884	0.980493243
GPER1	0.638759784	0.897441297	0.715621363	0.015856527	0.781986594	0.392622061	0.980493243

IGIP	0.142366066	0.607359703	0.333483701	0.278241326	0.642101014	0.394719932	0.980493243
INTS1	0.826451755	0.392695897	0.287088113	0.078022929	0.702024515	0.393164876	0.980493243
IRF9	0.570936745	0.118623792	0.287005358	0.419610683	0.624152404	0.392755968	0.980493243
KLF14	0.308796647	0.481361341	0.510299321	0.306105602	0.221180886	0.394200579	0.980493243
LGALS7	0.295676014	0.136824316	0.961678854	0.49026692	0.267046709	0.392847967	0.980493243
LOC100335744	0.725723924	0.52394623	0.662582538	0.023670762	0.858941171	0.393776897	0.980493243
LOC100847374	0.059889848	0.851562722	0.155989433	0.761313958	0.839362234	0.392524874	0.980493243
LOC100847876	0.268168296	0.978789507	0.370361266	0.098851229	0.530865007	0.393098341	0.980493243
LOC101902786	0.36314674	0.565602434	0.316544639	0.090681907	0.873901025	0.394742481	0.980493243
LOC104970450	0.675317785	0.22321683	0.304684465	0.129282854	0.855130796	0.392327783	0.980493243
LOC107131642	0.417953676	0.634798886	0.225695084	0.524462249	0.163271296	0.393942968	0.980493243
LOC112443415	0.62837744	0.190659111	0.58039626	0.139037124	0.52742363	0.393024133	0.980493243
LOC112447392	0.067262125	0.208100631	0.852530669	0.862155775	0.497547575	0.393663036	0.980493243
LOC112449284	0.802820788	0.104057407	0.694817267	0.298286659	0.296856641	0.394335732	0.980493243
LOC518961	0.438162338	0.321933669	0.070693241	0.623497313	0.820724117	0.393144935	0.980493243
LOC784451	0.97600401	0.21246994	0.073591706	0.370767989	0.908423585	0.394345547	0.980493243
LOC785503	0.062693857	0.277575755	0.975697129	0.621400467	0.482034681	0.392598195	0.980493243
NLRP12	0.72064461	0.661171255	0.031256118	0.619393984	0.549962262	0.39218023	0.980493243
PAFAH2	0.404183334	0.406049763	0.041997467	0.871207947	0.844230409	0.392063986	0.980493243
PATL1	0.633292739	0.152515218	0.773957314	0.136653634	0.503533532	0.394465698	0.980493243
PGLS	0.367479576	0.927129212	0.510411931	0.146276501	0.199722678	0.392417841	0.980493243
PLEKHG6	0.067906146	0.814634642	0.181457304	0.741092962	0.692568794	0.39473134	0.980493243
POLDIP3	0.351849105	0.144260982	0.248195804	0.571685914	0.711566888	0.393852645	0.980493243
POU2F2	0.652246006	0.426952573	0.145821006	0.99899105	0.125803944	0.393166168	0.980493243
PPP2R1B	0.392124873	0.812909004	0.100929288	0.86947991	0.183281852	0.393924739	0.980493243
RSU1	0.376935627	0.031546188	0.515555697	0.91167306	0.917948937	0.394033052	0.980493243
SCAF8	0.448700551	0.77016007	0.545450447	0.027964926	0.967476806	0.393044653	0.980493243
SENP8	0.490029685	0.52600906	0.115044221	0.213709945	0.808384568	0.393795668	0.980493243
SF3B3	0.443637141	0.835999795	0.461180908	0.068128241	0.439431474	0.393719831	0.980493243
SHANK1	0.917433204	0.407578088	0.052609374	0.744769505	0.346438187	0.392265989	0.980493243
SHMT1	0.718171214	0.603856586	0.071628889	0.296551709	0.556284369	0.393842877	0.980493243
SLC35A4	0.522906592	0.650361065	0.535346794	0.076873266	0.366690457	0.394086878	0.980493243
TMEM229A	0.945193585	0.873968922	0.155356652	0.83050896	0.047696929	0.392526052	0.980493243
TMEM61	0.021088988	0.835983722	0.332320514	0.883516576	0.993096273	0.394363881	0.980493243
TNXB	0.378427714	0.404703405	0.39163005	0.086047143	0.9897432	0.393313508	0.980493243
UBN2	0.047900778	0.57051369	0.822035323	0.621251466	0.368133824	0.39427083	0.980493243
URM1	0.272793459	0.706522313	0.702591503	0.169594238	0.224328925	0.39472261	0.980493243
YME1L1	0.639944513	0.761036383	0.988005202	0.262967086	0.040495533	0.393830775	0.980493243
MT2A	0.962750153	0.797357194	0.436606536	0.113092375	0.13599236	0.394816094	0.980527682
MMP16	0.360203273	0.55592524	0.317579452	0.266141457	0.304741487	0.394913586	0.980621403
MAML2	0.230924934	0.887296642	0.535271712	0.080815004	0.582945988	0.395208352	0.981039619
PUS3	0.681763031	0.502961425	0.506457144	0.08813195	0.337542892	0.39518507	0.981039619
ST7L	0.020398621	0.714251344	0.722231793	0.558707429	0.879144798	0.395261347	0.981039619
LOC101902570	0.286719607	0.129532157	0.855374336	0.382891927	0.425529919	0.395499252	0.981481661
NTMT1	0.467054626	0.47000549	0.161640724	0.395870742	0.368723404	0.395605234	0.981596235

ADCY8	0.8338098	0.644040535	0.672101981	0.861048408	0.016708545	0.396027079	0.981796772
BTF3L4	0.038072204	0.380074711	0.683096384	0.927560745	0.565926414	0.395904903	0.981796772
DDX10	0.238467376	0.778579902	0.390187896	0.55561642	0.128984506	0.396003043	0.981796772
MMP25	0.080268484	0.50974147	0.434644425	0.630306222	0.463076342	0.395970525	0.981796772
MRPL49	0.761214002	0.515360022	0.492110335	0.046396458	0.579572522	0.395985502	0.981796772
RAB18	0.304840616	0.289793073	0.13485047	0.659991497	0.660506257	0.396045008	0.981796772
E2F4	0.403823543	0.276657751	0.380078221	0.394106978	0.310593259	0.396191924	0.981918074
PAQR3	0.997325293	0.801800293	0.101444312	0.124430693	0.51500585	0.396213606	0.981918074
WHRN	0.62280219	0.376939202	0.822734291	0.058060039	0.463852387	0.39631578	0.98202299
ACO1	0.593439045	0.381379814	0.057816441	0.940513123	0.423522764	0.396655028	0.982059138
IL17RA	0.758819131	0.29555355	0.175148203	0.349534154	0.379176569	0.396458192	0.982059138
KPTN	0.351533567	0.85540991	0.298821083	0.111216494	0.521669367	0.396689417	0.982059138
PRR22	0.617398888	0.212532479	0.652288147	0.077866776	0.781435158	0.396521003	0.982059138
RHOC	0.095915805	0.750910159	0.792251343	0.531588315	0.171805227	0.396626826	0.982059138
TGFBR1	0.707569253	0.042698271	0.983051695	0.370304022	0.473373812	0.396460868	0.982059138
CFAP161	0.191798277	0.60596219	0.918523601	0.164280308	0.297594754	0.39687063	0.982303812
DSP	0.317369705	0.163073445	0.444204626	0.876260903	0.259185707	0.396941279	0.982303812
VAPA	0.948916378	0.247198586	0.084628828	0.301398429	0.872795528	0.396967819	0.982303812
GABBR2	0.230630785	0.999001824	0.236391329	0.497326564	0.193251122	0.397362576	0.983069595
GAS2L1	0.322253478	0.994405755	0.273519748	0.10265454	0.581890127	0.397397093	0.983069595
CCDC136	0.905186565	0.743223472	0.171363191	0.597682279	0.076014701	0.397463591	0.983085906
BREH1	0.650197598	0.570807294	0.022806752	0.745027212	0.83180658	0.397712046	0.98309071
CBLN2	0.042852603	0.84955675	0.524523409	0.373142465	0.736863281	0.397866282	0.98309071
DDIT4	0.727774058	0.044492	0.272899331	0.820574811	0.723506455	0.397730688	0.98309071
DNTTIP2	0.810990156	0.301229756	0.496549684	0.507972173	0.085113307	0.397681734	0.98309071
LOC100335553	0.268506211	0.174021487	0.847574673	0.302911959	0.437464084	0.397788994	0.98309071
MET	0.05718497	0.683116098	0.88320834	0.636512305	0.239110289	0.397884863	0.98309071
RAB8A	0.08975348	0.494696637	0.785009629	0.58903811	0.255426341	0.39766673	0.98309071
ANKRD27	0.578439961	0.81772436	0.405666704	0.253883974	0.107941211	0.398119209	0.983225633
LOC101905887	0.539195089	0.718559475	0.6952522	0.022200374	0.879243633	0.3981052	0.983225633
POLG	0.530279086	0.038079947	0.500242056	0.540570766	0.962638086	0.398057784	0.983225633
LEP	0.180430192	0.700210598	0.345548239	0.378623134	0.318335646	0.398227062	0.983344013
METTL21A	0.046176506	0.628561665	0.603746455	0.41067743	0.732037636	0.398425644	0.983686362
CERS6	0.424318517	0.88910193	0.243906332	0.179406069	0.31960012	0.398675629	0.983991834
GLRX3	0.786733506	0.22754555	0.315977048	0.906902588	0.102952907	0.398844194	0.983991834
SGCA	0.119001657	0.107774454	0.758605669	0.703622201	0.770607365	0.398655308	0.983991834
TRAPPC6B	0.091851231	0.623301764	0.488859885	0.288397432	0.654023548	0.398768248	0.983991834
UTP14A	0.219140586	0.740451934	0.651900808	0.217616928	0.229440396	0.398849167	0.983991834
ADO	0.63626971	0.371284234	0.441445792	0.965092197	0.05252768	0.399009455	0.984106161
SIDT1	0.547536576	0.328193782	0.445710917	0.288363977	0.228907501	0.399015441	0.984106161
MAPK11	0.686580856	0.106586673	0.579670944	0.125490745	0.994665637	0.399271105	0.984588747
ARL6IP1	0.891238225	0.02827404	0.943574627	0.717815753	0.310581973	0.399455959	0.984685153
C1GALT1C1	0.650605151	0.061889091	0.312876956	0.70841687	0.594458504	0.399597236	0.984685153
LOC101902390	0.227842942	0.804646404	0.199297676	0.285484907	0.508389594	0.39952326	0.984685153
OXR1	0.366165627	0.784396484	0.052967397	0.48480806	0.718352852	0.399372512	0.984685153

PLXDC2	0.771415229	0.405511044	0.361647301	0.046904026	0.999915281	0.399610208	0.984685153
MSMO1	0.905793899	0.405851304	0.081564934	0.673397829	0.26309597	0.399815889	0.98504407
TENM3	0.791677649	0.267109923	0.163398066	0.177513984	0.866417007	0.399876929	0.985046574
FUS	0.603252246	0.693569078	0.189144886	0.638581326	0.105235567	0.399998614	0.985198447
FYN	0.330228664	0.728030098	0.22702809	0.1121405	0.86971358	0.400159933	0.985300025
UXT	0.662576144	0.969337748	0.687213793	0.022882968	0.526924848	0.400114415	0.985300025
LOC100335751	0.66383048	0.078841419	0.1388274	0.88273256	0.830420797	0.400248872	0.985371173
ATF4	0.622798654	0.300510498	0.586631253	0.067469941	0.719513534	0.400366959	0.98551405
LOC107132911	0.821995696	0.59684984	0.282885035	0.076915725	0.499734751	0.400511881	0.98554345
POLR2I	0.236195138	0.094409203	0.59287902	0.533796799	0.756403761	0.400620061	0.98554345
RICTOR	0.112309565	0.953177026	0.734523059	0.386951197	0.175449537	0.400628953	0.98554345
SRM	0.108848625	0.765586166	0.651027048	0.588644715	0.167211302	0.400679173	0.98554345
TNFSF12	0.118024724	0.792028707	0.611755068	0.404214932	0.230994422	0.400667806	0.98554345
C29H11orf68	0.174329248	0.549197649	0.56115074	0.153973744	0.64610282	0.400829675	0.985765893
IFT140	0.581540563	0.214756333	0.090506893	0.653723168	0.724088358	0.401008827	0.985910991
KIAA1755	0.777006831	0.321581978	0.622135884	0.331906352	0.103696785	0.401004814	0.985910991
PDXDC1	0.324090293	0.764073511	0.420317969	0.342158413	0.150481439	0.401276376	0.986125578
PLPPR4	0.687715662	0.758551067	0.065367759	0.241038396	0.651683439	0.401196033	0.986125578
SEMA4D	0.313998527	0.859491524	0.301276902	0.289540143	0.227590543	0.401241307	0.986125578
PDIA4	0.187059786	0.499802196	0.194443645	0.537015576	0.549567254	0.401465051	0.986441525
ADAMTS3	0.662655767	0.661005891	0.23102256	0.074208022	0.715995608	0.40182226	0.986580657
LOC100848995	0.136532463	0.725693673	0.473913279	0.846050782	0.135300628	0.401774357	0.986580657
PRR19	0.86446343	0.83533411	0.388236245	0.027687916	0.692417404	0.401765427	0.986580657
SYT5	0.329344998	0.837797092	0.061818651	0.396899664	0.793486176	0.401676339	0.986580657
TTC32	0.893125446	0.460800608	0.190706338	0.074368537	0.920177888	0.401646389	0.986580657
TIMP3	0.352259846	0.541667913	0.347114923	0.214304466	0.378956513	0.401891972	0.986604213
MSS51	0.27821517	0.671507304	0.375689789	0.737786257	0.103915843	0.401962428	0.986629585
ABHD17A	0.24067039	0.306376749	0.255768655	0.503671517	0.571678309	0.403484425	0.98667857
ACTRT3	0.089038985	0.926241327	0.928269002	0.286280683	0.248220038	0.403786075	0.98667857
COPS7A	0.19073125	0.658038316	0.455907168	0.17293705	0.544242551	0.402099693	0.98667857
DMAC1	0.492836107	0.350268008	0.282986175	0.356564214	0.310214877	0.402653507	0.98667857
DMAC2	0.640641627	0.665009969	0.32251621	0.050505438	0.781222158	0.403207897	0.98667857
EID1	0.245475356	0.755895668	0.202406615	0.281300006	0.512055801	0.402851064	0.98667857
GBE1	0.191533968	0.569599577	0.119869492	0.661625528	0.624035366	0.402529124	0.98667857
HES6	0.960114252	0.091839614	0.37827545	0.280735378	0.579888781	0.403475975	0.98667857
IFT20	0.084156478	0.40296629	0.669189203	0.279626771	0.851355833	0.40262519	0.98667857
KLK12	0.897060261	0.593117504	0.434232795	0.460932288	0.050639927	0.40232492	0.98667857
LOC112442298	0.173622886	0.923821841	0.401909164	0.438194399	0.191893432	0.40318573	0.98667857
LOC112448833	0.088950158	0.279979975	0.437572519	0.903661472	0.552008322	0.403658041	0.98667857
LOC783504	0.613256082	0.05436595	0.785414696	0.282338337	0.733732383	0.40331114	0.98667857
LRMDA	0.138445042	0.959540398	0.043841426	0.944824337	0.98796893	0.403675233	0.98667857
LRRCC1	0.031820845	0.273447078	0.646963885	0.958659731	0.99947041	0.402357362	0.98667857
MARVELD2	0.418724786	0.410488307	0.195749245	0.192920383	0.835794659	0.403323418	0.98667857
NEDD9	0.234692406	0.58942734	0.825409429	0.07459642	0.637788249	0.403549337	0.98667857
PAN3	0.477017201	0.926459736	0.783713346	0.088161004	0.177699934	0.403352085	0.98667857

PARP3	0.435809597	0.065839129	0.691460682	0.297754023	0.919107405	0.403464133	0.98667857
PNN	0.368218099	0.245615998	0.31729834	0.308422641	0.608728727	0.402164975	0.98667857
PNPLA4	0.154971825	0.993475554	0.262262797	0.357958199	0.374063308	0.402751897	0.98667857
PRKACB	0.555713771	0.256664566	0.911231947	0.209174749	0.199068955	0.402919375	0.98667857
RCN1	0.893215806	0.06654002	0.143650669	0.823365483	0.769284569	0.402791421	0.98667857
SEMA4C	0.395501895	0.59539431	0.205243574	0.947094507	0.118136865	0.40278176	0.98667857
SERPINB9	0.043918224	0.579276161	0.419508059	0.527879075	0.961734827	0.403112692	0.98667857
SETD3	0.663856539	0.172475844	0.306822404	0.276080282	0.560591698	0.403695262	0.98667857
SUV39H2	0.179394811	0.740954294	0.396487988	0.471279718	0.218688797	0.403527504	0.98667857
THAP4	0.415322505	0.618846904	0.169540636	0.158813527	0.779905142	0.4024622	0.98667857
TRIL	0.528069468	0.657245555	0.754360688	0.404222801	0.051401801	0.403782148	0.98667857
TYMS	0.319234075	0.21237601	0.674099228	0.128700814	0.92308717	0.403460384	0.98667857
EIF4G2	0.89454092	0.039656237	0.424801533	0.47841165	0.754872975	0.403850236	0.986688437
B3GALNT2	0.694201097	0.099254482	0.191343319	0.533251818	0.776156468	0.404296678	0.986814658
BLOC1S1	0.243577014	0.665955273	0.790814638	0.064037917	0.66499431	0.404482306	0.986814658
CHN2	0.22629314	0.584162894	0.078139258	0.857941901	0.616216139	0.40442609	0.986814658
CRB1	0.568512162	0.162874005	0.98538507	0.235287836	0.254419526	0.404457689	0.986814658
ERMARD	0.062654601	0.482722872	0.400837955	0.466456727	0.968554616	0.404924131	0.986814658
KIAA1324L	0.251416245	0.57460696	0.098055707	0.594629342	0.649612402	0.404762881	0.986814658
KIF13A	0.202032757	0.539350979	0.147803256	0.884411861	0.382401578	0.403996102	0.986814658
LAT2	0.937744811	0.889619249	0.010869448	0.979606056	0.615513599	0.404626826	0.986814658
LOC104975673	0.537564722	0.099798826	0.967352335	0.283494632	0.370521898	0.404130424	0.986814658
LOC112448816	0.527615739	0.178106381	0.376199353	0.295165068	0.523069094	0.404338727	0.986814658
MEDAG	0.431541273	0.481465878	0.995774914	0.051854892	0.509183286	0.404482971	0.986814658
NFKBIA	0.836767882	0.861095346	0.076322084	0.168442507	0.591205516	0.40490152	0.986814658
PSMD14	0.782363662	0.515602365	0.793357916	0.35873539	0.04764495	0.404703438	0.986814658
SCPEP1	0.889066891	0.136901944	0.130813174	0.403372509	0.848458046	0.404065829	0.986814658
UBA3	0.465683082	0.836905281	0.730072406	0.179207042	0.107314058	0.404765452	0.986814658
WDR1	0.171403453	0.360540618	0.331594018	0.361221782	0.738242163	0.404537149	0.986814658
ZYG11B	0.339214803	0.144551798	0.628054716	0.228216515	0.779287842	0.40491856	0.986814658
CADPS2	0.160297427	0.251263447	0.921845401	0.314503549	0.469272542	0.405005069	0.986858878
PRICKLE1	0.957454617	0.746817928	0.228114123	0.04886671	0.688079229	0.405148818	0.986858878
WFDC1	0.282404751	0.219903172	0.529727253	0.583334983	0.285858286	0.405182812	0.986858878
ZBTB5	0.072054393	0.785459471	0.249535857	0.819145321	0.473951259	0.405100584	0.986858878
GOT1	0.685067734	0.463754039	0.344872311	0.091241907	0.549343396	0.405373443	0.987059173
LOC112447032	0.610732314	0.261818628	0.274581531	0.72954275	0.171464838	0.405385341	0.987059173
CBX5	0.900630451	0.015991991	0.967932873	0.452461199	0.871775904	0.405591041	0.987137566
DCSTAMP	0.506234371	0.629502562	0.029730496	0.774216443	0.749441827	0.405541155	0.987137566
PHIP	0.293349338	0.745882395	0.762506949	0.048791624	0.675603737	0.40561269	0.987137566
TGFBR3L	0.82307452	0.233075222	0.235298442	0.293664677	0.414999998	0.405658141	0.987137566
LOC107133049	0.093413091	0.720297905	0.186728601	0.551651576	0.795368752	0.40601015	0.987847676
NEXMIF	0.098762416	0.673358761	0.128859651	0.722603464	0.890616671	0.406080535	0.987872466
LOC100140121	0.945143856	0.015469254	0.978359213	0.611601337	0.630671172	0.406154444	0.987905821
CCDC14	0.191454821	0.375727069	0.220029611	0.500447932	0.699803062	0.406935672	0.988002001
FOXC1	0.389688737	0.096700834	0.875485814	0.226186586	0.743180741	0.407014637	0.988002001

KIF16B	0.863126568	0.89290179	0.197140137	0.230874037	0.158120233	0.407036837	0.988002001
LOC101904768	0.674779148	0.134764964	0.582129951	0.850119013	0.123243347	0.407031426	0.988002001
LOC101906828	0.404502045	0.384941875	0.56278961	0.485472356	0.129981955	0.40653089	0.988002001
LYRM1	0.939234567	0.601194844	0.455184766	0.14293478	0.150678918	0.406708216	0.988002001
MTFMT	0.038373096	0.402314837	0.513889665	0.698710004	0.998190939	0.406633051	0.988002001
NAA25	0.480944881	0.282707224	0.66616166	0.083802409	0.727917148	0.406391816	0.988002001
PLCH2	0.133762921	0.726882714	0.349070385	0.385572844	0.422202677	0.406388026	0.988002001
RPS25	0.531665478	0.524635307	0.424830018	0.074095751	0.629912756	0.406560069	0.988002001
RUBCN	0.46743711	0.318751602	0.193410668	0.896060054	0.214554888	0.406849231	0.988002001
SLC25A25	0.362220193	0.076166051	0.346628176	0.878275082	0.658274905	0.406502353	0.988002001
SLC2A4	0.892335949	0.769932312	0.496990199	0.020306587	0.79917711	0.406879138	0.988002001
TFB1M	0.828002896	0.999691462	0.760457676	0.053831131	0.163634514	0.406984734	0.988002001
LOC112447459	0.449374776	0.7482312	0.363514837	0.139691848	0.325095155	0.407165651	0.988115831
LOC112448777	0.538869525	0.431906238	0.590577584	0.964875073	0.041887292	0.407303323	0.988115831
SHOX2	0.051420204	0.645561754	0.341399677	0.619845642	0.790869094	0.407310852	0.988115831
TTLL5	0.507990619	0.574256028	0.572648974	0.243104207	0.136809432	0.407324575	0.988115831
LOC101906317	0.206162427	0.642317995	0.794226732	0.262474745	0.201461845	0.407489787	0.988370514
MYLK3	0.043567478	0.6382953	0.611149645	0.574807327	0.569670342	0.407603432	0.988500062
ANKRD54	0.914621372	0.459941504	0.47128383	0.034710709	0.809942103	0.407861885	0.988980701
LOC104974057	0.33098018	0.742823973	0.520969923	0.098877241	0.440397967	0.407977582	0.989014814
LOC786614	0.66155065	0.72459984	0.847512097	0.03011812	0.45588554	0.407996484	0.989014814
HAUS8	0.941080678	0.532024453	0.078948484	0.232584902	0.607257427	0.40813837	0.989212641
MRPS5	0.881066074	0.4082738	0.892217205	0.032668357	0.532733713	0.40822043	0.989265428
CASP8AP2	0.25269985	0.779966752	0.456114139	0.247376574	0.251324034	0.40832877	0.989381875
CALML4	0.093467303	0.954724726	0.073384161	0.880829128	0.969970153	0.408500758	0.989492231
RAB1A	0.452670633	0.497650055	0.261937872	0.49905806	0.190053001	0.408555198	0.989492231
SLC20A1	0.599712325	0.183581016	0.79258284	0.431888567	0.14850231	0.408551258	0.989492231
MAP1A	0.692548307	0.95129117	0.01580336	0.994571006	0.540801309	0.408654605	0.989586945
ABCC1	0.417655903	0.76880268	0.977836056	0.018886139	0.947418736	0.40919775	0.989588016
FKBPL	0.201955453	0.919053873	0.560824364	0.06620205	0.814698975	0.409084029	0.989588016
KRTCAP3	0.42715938	0.081323578	0.692879517	0.642154584	0.363408944	0.409164583	0.989588016
LOC101907916	0.103865708	0.876933424	0.368643587	0.889922099	0.187583942	0.408812532	0.989588016
MAPKAPK2	0.224181899	0.296167531	0.723501814	0.165119304	0.706696229	0.408818394	0.989588016
NAB1	0.149383441	0.9721737	0.893334477	0.057904504	0.747008341	0.409008729	0.989588016
RILPL2	0.762056998	0.806985771	0.027358592	0.522897675	0.638131879	0.409077109	0.989588016
TIMM22	0.69971049	0.661270909	0.446025879	0.033812558	0.80506811	0.40919146	0.989588016
TMPRSS6	0.034946954	0.334181792	0.972693348	0.611032409	0.807673009	0.408841556	0.989588016
ACOT9	0.448568034	0.727903606	0.247680868	0.074323347	0.937600693	0.409723763	0.989695039
ADAM1A	0.855404378	0.111873249	0.080020433	0.922388583	0.798145665	0.409784766	0.989695039
CC2D2A	0.510470416	0.474778481	0.11226366	0.533107893	0.388262679	0.409608559	0.989695039
EGFL8	0.707451397	0.073008266	0.695270781	0.227537171	0.689505202	0.409675034	0.989695039
HEXDC	0.275834584	0.245545128	0.95177759	0.091778548	0.950285791	0.409324534	0.989695039
KIAA0100	0.541781113	0.924070878	0.80872566	0.361970188	0.038452368	0.409719263	0.989695039
LOC104975244	0.7260725	0.279922825	0.770074536	0.071267094	0.504403041	0.409443818	0.989695039
LOC782938	0.361820306	0.077616394	0.266497413	0.79212785	0.950751198	0.409748546	0.989695039

PTPRR	0.234594086	0.355853567	0.595120473	0.311215131	0.364109042	0.409547861	0.989695039
MTMR7	0.027168116	0.777822981	0.781049733	0.664638908	0.514467782	0.40996689	0.989843553
NELFA	0.691402923	0.367560213	0.326645014	0.075168895	0.904195375	0.409917586	0.989843553
RABL3	0.342435059	0.382658929	0.318098221	0.216546724	0.625687943	0.410082738	0.989977613
CAPNS1	0.287995467	0.507916342	0.440228993	0.151509208	0.580430107	0.410543813	0.990273182
LOC107132994	0.272290449	0.90187968	0.665028708	0.071122495	0.487373861	0.410483545	0.990273182
SPART	0.479262603	0.47081429	0.135175399	0.713933121	0.260104498	0.410575266	0.990273182
SRP68	0.445912608	0.368482288	0.133233769	0.776546754	0.332704078	0.410333998	0.990273182
TRIP10	0.742424004	0.221497957	0.904382594	0.105404628	0.361432297	0.410627567	0.990273182
USP18	0.973684956	0.464004515	0.092316019	0.176076141	0.770548883	0.410416522	0.990273182
ZNF503	0.112445555	0.707336901	0.400199216	0.442270356	0.401812398	0.410353298	0.990273182
ABCB4	0.442933571	0.960496438	0.672659493	0.116680944	0.171198547	0.412135506	0.991311503
ADPGK	0.906740168	0.10434245	0.397279821	0.631251555	0.242885117	0.413507731	0.991311503
ANKRD11	0.816538072	0.245388683	0.699610602	0.0600607	0.692673254	0.415528111	0.991311503
ANP32A	0.83510335	0.539717854	0.871691964	0.021058946	0.693929771	0.412874219	0.991311503
APOA1	0.261855173	0.793958054	0.429359435	0.419768594	0.154500472	0.414280324	0.991311503
APRT	0.535424624	0.820856786	0.557349799	0.223046057	0.106440956	0.415054368	0.991311503
ARHGAP5	0.461243897	0.699452977	0.97535315	0.047539987	0.382306739	0.412210578	0.991311503
ARHGEF25	0.929435915	0.866779393	0.117527597	0.801265185	0.075976068	0.41353791	0.991311503
ATP5MPL	0.723240974	0.336719325	0.596440855	0.041129994	0.956041309	0.411988872	0.991311503
CARNMT1	0.081785213	0.34580953	0.325461265	0.892733427	0.697971603	0.412697886	0.991311503
CCDC62	0.947654149	0.269793446	0.85402452	0.032916713	0.8110234	0.41544789	0.991311503
CCDC80	0.421483007	0.090312935	0.382178524	0.654183092	0.610090249	0.414777453	0.991311503
CMPK2	0.433076817	0.946254844	0.95851411	0.019529082	0.74482802	0.412049588	0.991311503
COL8A2	0.744114753	0.543134063	0.070431486	0.373445209	0.548378326	0.415455547	0.991311503
CREB3	0.606465764	0.915529333	0.191231715	0.160807205	0.335549127	0.412514206	0.991311503
CSTF3	0.551174114	0.317528638	0.608958241	0.900029617	0.059568757	0.412059406	0.991311503
DBP	0.776187607	0.64780946	0.725760196	0.168509003	0.094473352	0.414875281	0.991311503
DDA1	0.236005625	0.251942684	0.588879786	0.286953319	0.568929439	0.412131906	0.991311503
DENND5A	0.432326667	0.575282667	0.206063299	0.12336688	0.913191757	0.413824682	0.991311503
DSC1	0.808407793	0.685704793	0.054135817	0.309853195	0.622038008	0.414126109	0.991311503
EFHD2	0.368889429	0.659128969	0.112234944	0.26406943	0.80757298	0.415170973	0.991311503
EPHA3	0.769489466	0.538371713	0.630498738	0.474394476	0.04692272	0.415013261	0.991311503
FAHD1	0.659747156	0.577093062	0.305536342	0.093748563	0.524258027	0.412161381	0.991311503
FPGS	0.870328014	0.724744022	0.461318559	0.031072468	0.644313591	0.41534607	0.991311503
FRYL	0.678943151	0.959396583	0.180301138	0.105305294	0.467240661	0.413967942	0.991311503
GATA6	0.440108545	0.830293673	0.266487356	0.197110572	0.296246363	0.411239297	0.991311503
GIPC3	0.370485092	0.14551813	0.391643522	0.273355097	0.992007624	0.412405268	0.991311503
GLS	0.503587769	0.957216806	0.717258646	0.037070006	0.452931949	0.414748948	0.991311503
GLYR1	0.224872417	0.030609637	0.985455137	0.914927896	0.934026838	0.414498659	0.991311503
GOLM1	0.818573356	0.538776632	0.060261696	0.249704672	0.87216894	0.41424727	0.991311503
LOC101902922	0.122436701	0.499819517	0.592212793	0.195706898	0.813607455	0.413734419	0.991311503
LOC101904574	0.872147575	0.67299894	0.475887455	0.146976467	0.142003331	0.415468856	0.991311503
LOC101906717	0.451018105	0.531821369	0.277163475	0.629706026	0.137311248	0.413076348	0.991311503
LOC101907843	0.933257729	0.399012173	0.053811433	0.305732123	0.943129332	0.413949851	0.991311503

LOC101907944	0.363847357	0.682250082	0.039689489	0.595206778	0.973559515	0.411916698	0.991311503
LOC104969159	0.412380021	0.101812805	0.777610718	0.281659682	0.629484591	0.414261767	0.991311503
LOC107132283	0.141253422	0.316683265	0.662434214	0.491891343	0.392899404	0.412442899	0.991311503
LOC112445051	0.602144376	0.453915396	0.588943662	0.709838221	0.050470321	0.413625381	0.991311503
LOC112446454	0.064053843	0.343270553	0.828558679	0.510857818	0.617116611	0.412932835	0.991311503
LOC518495	0.226806066	0.709880563	0.177076861	0.979127938	0.206018211	0.413157249	0.991311503
LOC782950	0.287316296	0.152272442	0.792845294	0.751841808	0.220647727	0.413254584	0.991311503
LOC786512	0.730334018	0.922540082	0.032815725	0.578937392	0.447997976	0.412668925	0.991311503
ME1	0.843489062	0.827967938	0.461738219	0.019843676	0.90455956	0.414252392	0.991311503
MFSD13A	0.351440792	0.68275373	0.725042921	0.04444597	0.741936379	0.412740216	0.991311503
MOB3C	0.353645987	0.720359638	0.498987159	0.106283095	0.423174959	0.412159691	0.991311503
MSN	0.419265391	0.254743574	0.695650923	0.09852794	0.789385209	0.413972293	0.991311503
MTRR	0.129475903	0.581570203	0.127581072	0.832824642	0.718149103	0.413000498	0.991311503
NEK9	0.857430961	0.049898243	0.26362483	0.562916278	0.90530059	0.413063697	0.991311503
NIPSNAP2	0.89025101	0.435152741	0.56071575	0.040968537	0.648705878	0.413801501	0.991311503
ORC6	0.850528249	0.501853817	0.280595237	0.858316681	0.055353343	0.411358775	0.991311503
ORM1	0.45235181	0.495625993	0.19158573	0.206564726	0.644149187	0.412099049	0.991311503
PKMYT1	0.159766588	0.331980167	0.618783744	0.266167858	0.66639084	0.415220726	0.991311503
POLR2D	0.956676548	0.755314688	0.49899739	0.185065081	0.087031466	0.414817547	0.991311503
PRPF4B	0.326995921	0.616938269	0.287089747	0.273000157	0.361410559	0.412071457	0.991311503
RFLNB	0.743583295	0.356124715	0.121591861	0.18298431	0.9860396	0.414877194	0.991311503
RPL30	0.565688654	0.69471955	0.482488897	0.070922174	0.432362867	0.415018275	0.991311503
RPLP2	0.455768139	0.532570093	0.534745891	0.125900483	0.348615114	0.411555274	0.991311503
RPS11	0.436518967	0.618182864	0.37323314	0.103824491	0.549945193	0.413146577	0.991311503
RPS6KA6	0.331932462	0.450489205	0.122419926	0.688724561	0.45652853	0.413295129	0.991311503
RUBCNL	0.189161795	0.623858937	0.539835116	0.463314791	0.197359309	0.415335556	0.991311503
S100A10	0.520370808	0.562209832	0.490169129	0.048375757	0.83639035	0.414661735	0.991311503
S100A11	0.455084038	0.446475806	0.495434114	0.211690572	0.269982914	0.413222265	0.991311503
SACS	0.483306613	0.676033237	0.955214486	0.182236602	0.100073542	0.411401214	0.991311503
SELENOF	0.532156139	0.486799452	0.997309104	0.452002318	0.04983584	0.415173627	0.991311503
SPATA13	0.483088126	0.357551571	0.64934028	0.148214325	0.350778147	0.415510064	0.991311503
TDRD12	0.597773704	0.160450195	0.170624929	0.911379876	0.387381631	0.413943351	0.991311503
TMC6	0.907513203	0.379191534	0.128364547	0.606162554	0.21488186	0.413234884	0.991311503
TTYH3	0.327986876	0.129201714	0.62490963	0.339490375	0.641266305	0.413571867	0.991311503
XPA	0.329413667	0.125845116	0.436975517	0.619430119	0.514492894	0.413806014	0.991311503
ZBTB45	0.232766319	0.770346763	0.158359998	0.602124239	0.339757739	0.414862667	0.991311503
ZFAND2A	0.901205485	0.064165137	0.666550292	0.274252427	0.547662195	0.414280183	0.991311503
ZFP57	0.128322	0.382593172	0.763045266	0.918044077	0.167803841	0.413746305	0.991311503
ZNF729	0.554029956	0.109476312	0.220628304	0.642905382	0.674008102	0.414557113	0.991311503
ZNF74	0.500516697	0.08771543	0.530351811	0.761501471	0.322995124	0.412445997	0.991311503
EIF3J	0.831070539	0.35837146	0.16681963	0.575009377	0.204242361	0.415619443	0.991352912
GPC1	0.413593833	0.401332825	0.667839419	0.088399526	0.595667737	0.415684347	0.991352912
LOC784251	0.263932078	0.553388193	0.246190237	0.522441269	0.310801038	0.415726692	0.991352912
NGFR	0.97417176	0.205069709	0.397211869	0.27238764	0.270505389	0.415965745	0.991778852
DUS3L	0.79211703	0.262504069	0.604146806	0.157945338	0.294952963	0.416124511	0.99201327

ZNF835	0.344154127	0.180918907	0.813889749	0.525314081	0.219981847	0.416234705	0.992131844
CWF19L2	0.195360332	0.401517916	0.562708006	0.143691525	0.92368891	0.416302522	0.99214939
ARSE	0.247286222	0.289596714	0.559008917	0.690629419	0.212171653	0.416523576	0.992231526
CLCN5	0.759619483	0.105824333	0.939669907	0.411027946	0.188911109	0.416500457	0.992231526
LOC101905262	0.573165076	0.133218186	0.922698888	0.151151833	0.551328575	0.416673655	0.992231526
LOC112444348	0.348528194	0.50066941	0.299085791	0.456631739	0.246401192	0.416699755	0.992231526
RGL2	0.9096171	0.046060375	0.157876518	0.970053681	0.914625804	0.416601324	0.992231526
SERBP1	0.764386289	0.587929369	0.450186724	0.681434748	0.042530683	0.416450062	0.992231526
LOC107131273	0.26157451	0.659976762	0.267848256	0.135857406	0.935789198	0.416888396	0.992536699
PARP11	0.92899624	0.405931344	0.166381288	0.3315791	0.282913779	0.417099648	0.992895609
CPPED1	0.486510198	0.75614294	0.078474271	0.379289557	0.538617663	0.417438353	0.993276405
LOC104973229	0.40146813	0.257725032	0.60737542	0.454712292	0.206491895	0.417528707	0.993276405
NPR2	0.734818244	0.312442012	0.495624984	0.184505206	0.280927049	0.417450182	0.993276405
POLR1D	0.607586961	0.975586302	0.071962535	0.259584412	0.5327223	0.417471928	0.993276405
PTK2B	0.71539985	0.604433572	0.921095562	0.028166989	0.526074678	0.41756224	0.993276405
ANKRD31	0.318930103	0.892924707	0.488912679	0.251632857	0.168703899	0.417815393	0.993590554
RTKN2	0.397101785	0.801134831	0.081754648	0.58373067	0.389242451	0.417783359	0.993590554
LOC112441602	0.436641141	0.469890645	0.079588722	0.863593695	0.419586706	0.418000167	0.993741959
MGAT4B	0.289107161	0.43018062	0.060927886	0.792047194	0.985659756	0.417960744	0.993741959
PPP2R5D	0.269355055	0.528238493	0.066064596	0.68642335	0.917553389	0.418095778	0.993825293
DAP	0.288568482	0.335046614	0.390856987	0.194023942	0.80806314	0.418223686	0.993890341
HAUS3	0.242350267	0.869518734	0.522828853	0.056412755	0.95337919	0.418244269	0.993890341
DTD2	0.47330752	0.725935276	0.965444855	0.021342159	0.837310412	0.418310596	0.993904039
FAM171A1	0.739940091	0.248589507	0.510286415	0.10933063	0.57830468	0.418506602	0.994020709
GREM1	0.319385773	0.33246942	0.159951611	0.72758332	0.480920304	0.418750479	0.994020709
LOC112447844	0.736150873	0.409564084	0.647757755	0.592340066	0.051405433	0.418856623	0.994020709
LOC512248	0.457329823	0.563367271	0.458897228	0.862662085	0.058208019	0.418572768	0.994020709
SH2B3	0.583905127	0.532867952	0.911185857	0.047114995	0.444973997	0.41877051	0.994020709
SH2D3A	0.137498177	0.884253765	0.740976724	0.960876893	0.068651878	0.418745306	0.994020709
SPECC1L	0.881943267	0.197500232	0.746926695	0.052046633	0.878466215	0.418904834	0.994020709
TUB	0.801267581	0.831491213	0.186442391	0.078430611	0.609565297	0.418622198	0.994020709
TWISTNB	0.346907987	0.925924394	0.21586655	0.616348409	0.139187695	0.418904188	0.994020709
ARMCX6	0.743047579	0.884971771	0.130230942	0.294763507	0.235748383	0.418975182	0.994043908
BCL2L14	0.318831621	0.616120102	0.258958955	0.464805304	0.25402719	0.420561699	0.994675174
BICDL1	0.301889182	0.300957165	0.738552433	0.478194114	0.187490259	0.420840822	0.994675174
C17H5orf52	0.175413612	0.97104127	0.780319615	0.424622089	0.106525589	0.420727795	0.994675174
CCDC30	0.421996878	0.165243952	0.937782872	0.159646054	0.576295173	0.420850202	0.994675174
CDC7	0.645478802	0.672925927	0.45156378	0.033182731	0.918542456	0.41976169	0.994675174
CPNE2	0.234911636	0.65930473	0.149683562	0.682255388	0.379371922	0.420391324	0.994675174
DDX11	0.47645366	0.235091387	0.338270067	0.446838102	0.355287835	0.420815169	0.994675174
FIGN	0.491125281	0.554486625	0.978868314	0.412574965	0.054714134	0.420877729	0.994675174
GGA1	0.709297495	0.451259292	0.028312479	0.753703993	0.875916888	0.419886406	0.994675174
LOC101904579	0.31532237	0.507573881	0.222615547	0.178999557	0.938025719	0.419878543	0.994675174
LOC104972888	0.205893399	0.739366627	0.425478387	0.15317237	0.603892977	0.420132356	0.994675174
LOC112444926	0.173518343	0.44877177	0.836578119	0.903377353	0.101886172	0.420267066	0.994675174

LOC112448753	0.782112184	0.585594258	0.046200123	0.326731323	0.864316902	0.419680152	0.994675174
MCM9	0.677987762	0.420410748	0.206837288	0.266964675	0.381752825	0.420621489	0.994675174
MIR3064	0.16748011	0.388645004	0.507782347	0.73246264	0.247714843	0.420294496	0.994675174
NCKAP1	0.097821973	0.486036158	0.235397366	0.549899649	0.972644962	0.41998422	0.994675174
NOLC1	0.392482801	0.775881469	0.192026814	0.354480634	0.287683685	0.419330706	0.994675174
PRDM2	0.457577126	0.658319635	0.366191997	0.147111372	0.367748219	0.419457192	0.994675174
RNASEH1	0.876792554	0.756897915	0.232267454	0.195444757	0.198328027	0.419663553	0.994675174
RNF114	0.587539609	0.489825499	0.080009063	0.275695272	0.94296488	0.419983503	0.994675174
SBNO2	0.585904049	0.229985078	0.851085938	0.18352313	0.285674817	0.42074054	0.994675174
SMPDL3A	0.775559142	0.161501143	0.572909308	0.437744993	0.190415162	0.419848147	0.994675174
THUMPD3	0.50841447	0.571957913	0.369055355	0.298404362	0.187390472	0.420410715	0.994675174
TM9SF4	0.601965971	0.461612441	0.319594864	0.094852525	0.711264372	0.420135905	0.994675174
TMEM69	0.748189413	0.526640935	0.163585441	0.179517926	0.519197404	0.420602002	0.994675174
UBXN7	0.127420809	0.757688206	0.430199844	0.237863677	0.607461914	0.420419454	0.994675174
VEPH1	0.912247815	0.338458168	0.058272139	0.464686456	0.7173793	0.420316661	0.994675174
LOC107132949	0.386618468	0.334659575	0.314952338	0.292507448	0.505035554	0.420948574	0.994699359
ADCK1	0.846328947	0.239628763	0.37991994	0.170961087	0.45753201	0.421145681	0.994847277
ADRB3	0.211750198	0.74484321	0.532270013	0.11028793	0.65193645	0.421408202	0.994847277
CDC42	0.628330447	0.062509769	0.328529013	0.868432706	0.539499692	0.421678	0.994847277
CDC42SE1	0.310025046	0.573923351	0.32731016	0.12453687	0.832997977	0.421565418	0.994847277
EEF2KMT	0.180261969	0.719522271	0.361203758	0.157740301	0.817935514	0.421648473	0.994847277
FOXN2	0.375469769	0.477685161	0.874687911	0.188413073	0.204457548	0.421617542	0.994847277
IMMP1L	0.121663641	0.7004132	0.985414851	0.11012235	0.651988835	0.421209189	0.994847277
LOC100847345	0.093881865	0.678972267	0.520608089	0.769313793	0.236429467	0.421406558	0.994847277
LOC101910045	0.457814627	0.520662212	0.1663857	0.774023057	0.196695052	0.421469453	0.994847277
METTL15	0.055512941	0.943189663	0.248482031	0.825503433	0.562339962	0.421507771	0.994847277
PRR5L	0.20971923	0.673657334	0.08141513	0.831755669	0.631736633	0.421629477	0.994847277
AFF2	0.137273071	0.219156817	0.453570756	0.556862946	0.800675212	0.422766163	0.99513775
BZW2	0.992643779	0.543702789	0.428594032	0.223227434	0.118061582	0.423109241	0.99513775
CFLAR	0.794245675	0.89709392	0.029066137	0.329935655	0.891180674	0.422918465	0.99513775
CLDN15	0.871254857	0.758725422	0.698576852	0.014369797	0.91436577	0.422301358	0.99513775
COLGALT2	0.118099325	0.733379708	0.565550469	0.729133065	0.169795724	0.422209097	0.99513775
CXCL2	0.742764723	0.376370062	0.320955741	0.366193045	0.184499509	0.422144111	0.99513775
DNAJC21	0.800464501	0.043271944	0.661402152	0.993238436	0.267782428	0.423027098	0.99513775
ESD	0.464637669	0.070820978	0.651907002	0.320247905	0.885865235	0.422815944	0.99513775
FCRL1	0.647348186	0.488848498	0.023214633	0.831470008	0.997036369	0.422940832	0.99513775
GNAI2	0.091925654	0.445515646	0.686557162	0.422664529	0.510393888	0.422247219	0.99513775
GTF2IRD2	0.870017934	0.699223638	0.019912587	0.788648936	0.634763415	0.422203722	0.99513775
GTF3C5	0.761281241	0.501789847	0.230562743	0.137676186	0.501933568	0.422833847	0.99513775
ID3	0.309007782	0.138243022	0.298542992	0.498810634	0.958445814	0.423135167	0.99513775
LOC101902907	0.551087539	0.752517442	0.661145285	0.022534275	0.980676888	0.422059858	0.99513775
LOC112448014	0.394598315	0.211646696	0.297951059	0.309445511	0.788761153	0.422470067	0.99513775
PPP1R3D	0.702375759	0.253593255	0.768532023	0.152006033	0.292645412	0.42291713	0.99513775
RPL29	0.362026433	0.802008882	0.916123492	0.029159703	0.782085756	0.422260377	0.99513775
SLC25A39	0.845641395	0.867395076	0.604988811	0.03070102	0.445848789	0.422489666	0.99513775

THAP2	0.021740315	0.374405909	0.979241383	0.796406932	0.95670966	0.422459046	0.99513775
TMEM104	0.486601377	0.617820499	0.954747634	0.069595001	0.305159538	0.423098146	0.99513775
TTC14	0.087463796	0.341396194	0.894934395	0.230099775	0.99102643	0.423038949	0.99513775
TUBB4A	0.503115596	0.91880276	0.656118325	0.064875118	0.308563574	0.422412224	0.99513775
CIP2A	0.180706041	0.667159516	0.100040948	0.640670285	0.790210176	0.423386318	0.995443105
PCDHB8	0.508673249	0.279639787	0.825850998	0.062668293	0.829131039	0.423328227	0.995443105
ERC1	0.567997847	0.941993051	0.508203082	0.026136495	0.859555539	0.423463759	0.99548256
GPATCH1	0.094739725	0.24184206	0.47621516	0.685516106	0.817308496	0.423589607	0.99554559
LOC782054	0.119068208	0.52666163	0.265377212	0.626285395	0.586629276	0.423611898	0.99554559
AIFM2	0.484750214	0.612288658	0.565008183	0.037115637	0.983667652	0.42385233	0.99577943
AIFM3	0.873085344	0.51036084	0.417511717	0.04304963	0.764232062	0.423798114	0.99577943
LOC101907803	0.746829664	0.966266199	0.013058973	0.809974917	0.80230423	0.423893431	0.99577943
TCF23	0.153668831	0.183392918	0.793525023	0.313705128	0.873387888	0.423980466	0.995841337
LOC788648	0.928617384	0.219267726	0.046689896	0.992927639	0.649356838	0.424050858	0.995864144
ABCC11	0.90795202	0.872628886	0.013284646	0.710002653	0.861549864	0.432552441	0.995923644
AFF1	0.629661126	0.7193903	0.25195399	0.283860714	0.19134031	0.425982157	0.995923644
AGO4	0.909344829	0.34843208	0.212896811	0.252177872	0.373369229	0.430185403	0.995923644
AKTIP	0.941125526	0.299363291	0.036077054	0.853257236	0.717893855	0.426742184	0.995923644
ALCAM	0.639142913	0.345682385	0.312434707	0.397531664	0.225793098	0.42590767	0.995923644
ALG8	0.765293247	0.589769674	0.362647407	0.708204129	0.054309872	0.428658288	0.995923644
ANXA8L1	0.516468474	0.149952664	0.269687638	0.446889815	0.670574883	0.427652725	0.995923644
AP1S2	0.131698281	0.787455046	0.633720875	0.097857582	0.961961739	0.425645348	0.995923644
ARID3A	0.946297307	0.282011614	0.072999735	0.575766407	0.570769547	0.431568325	0.995923644
ARPP19	0.152842074	0.738678692	0.158404609	0.614349873	0.561915806	0.425287036	0.995923644
ARRDC4	0.374765601	0.892476064	0.522562656	0.046161215	0.796595371	0.432242993	0.995923644
ASIP	0.318792785	0.027004603	0.85820373	0.929389031	0.918400323	0.428950837	0.995923644
ATP5IF1	0.874875052	0.605193492	0.605284115	0.021640968	0.919986907	0.430982728	0.995923644
B3GNTL1	0.695647184	0.222840596	0.696676573	0.071882286	0.803545405	0.427071964	0.995923644
BHLHE22	0.492210681	0.844751587	0.167838579	0.878328819	0.100599133	0.425076723	0.995923644
BRD2	0.43756288	0.575778478	0.64873327	0.084290111	0.456035877	0.428301999	0.995923644
BUD23	0.555805645	0.941470077	0.636031522	0.16804955	0.114375902	0.431431195	0.995923644
CCDC157	0.602000966	0.416993132	0.063302255	0.436436598	0.914321834	0.429907679	0.995923644
CEBPB	0.605208839	0.13804761	0.145648026	0.93236171	0.565379958	0.431904494	0.995923644
COPG2	0.174811734	0.692922693	0.30798481	0.168905118	0.996681216	0.428240684	0.995923644
CWC22	0.249765589	0.273209227	0.772262726	0.231440405	0.519593488	0.429800105	0.995923644
CXADR	0.417513535	0.449776484	0.146345016	0.714592029	0.318792921	0.427694628	0.995923644
CXCR2	0.389820891	0.437288449	0.959873772	0.17109024	0.229458805	0.432149971	0.995923644
DOCK5	0.822741777	0.036635694	0.662694309	0.394998332	0.814762546	0.432282151	0.995923644
EDA	0.049987394	0.683285056	0.736964038	0.59364671	0.411654177	0.424658766	0.995923644
ELMO3	0.506581279	0.051635108	0.93931133	0.713606514	0.358905332	0.428582739	0.995923644
EPOP	0.402022521	0.337519536	0.435696652	0.413685844	0.253831353	0.426238337	0.995923644
FABP2	0.062586726	0.480401669	0.912633411	0.470987046	0.490501865	0.429854692	0.995923644
GABARAPL2	0.670886927	0.59567614	0.493823868	0.081931282	0.393365074	0.430431235	0.995923644
GGN	0.734132015	0.205176414	0.650654508	0.096102917	0.658357614	0.426040144	0.995923644
GGTA1	0.233068968	0.192319839	0.272210925	0.629463595	0.812123422	0.427056015	0.995923644

GJD3	0.58334846	0.634739958	0.487316478	0.038574691	0.912937703	0.430272106	0.995923644
GLP2R	0.994443624	0.042207961	0.604705402	0.725356035	0.340651466	0.428000245	0.995923644
GOSR2	0.782827051	0.94291778	0.667733365	0.061443827	0.209877129	0.430315028	0.995923644
GPD2	0.440256091	0.556780773	0.67950427	0.136728587	0.281282656	0.43167272	0.995923644
HDHD5	0.331506027	0.960167611	0.722754059	0.035386164	0.78448238	0.431137696	0.995923644
HSPA5	0.149712386	0.900570522	0.312516244	0.19318823	0.760926398	0.425849888	0.995923644
ISOC2	0.591688428	0.442173062	0.52965474	0.053403063	0.862964555	0.431134356	0.995923644
ISY1	0.407218048	0.028628739	0.816147557	0.94531423	0.703427198	0.42951981	0.995923644
ITGB1BP1	0.344976409	0.585431989	0.558463269	0.147672597	0.382686104	0.430801022	0.995923644
ITPK1	0.10516437	0.92193094	0.083976117	0.794505166	0.970826305	0.428231654	0.995923644
JADE2	0.375604653	0.920166798	0.713265841	0.875569135	0.029467816	0.4304353	0.995923644
JOSD1	0.392555478	0.263527734	0.602052995	0.187939865	0.540464413	0.429501792	0.995923644
KCNIP3	0.748581268	0.518766017	0.142891897	0.96040197	0.118654173	0.429424433	0.995923644
KCNN1	0.492132431	0.025804609	0.669340484	0.739042326	0.999818941	0.42825394	0.995923644
KLHL2	0.451849216	0.873691301	0.644075398	0.029575801	0.827995939	0.426756333	0.995923644
KRT24	0.380779953	0.751608611	0.098694086	0.244269733	0.9018786	0.426645721	0.995923644
LARGE1	0.930715901	0.208509548	0.109450825	0.318074172	0.942458903	0.430621234	0.995923644
LIN54	0.406136788	0.598169619	0.503294905	0.060417843	0.854290655	0.42907992	0.995923644
LOC100139115	0.306548394	0.640345585	0.102572068	0.323952981	0.956237537	0.427049493	0.995923644
LOC100139144	0.133073971	0.35563772	0.790697254	0.30957365	0.539441948	0.427379419	0.995923644
LOC101902428	0.31510973	0.922601879	0.316450074	0.162895284	0.424877957	0.43062168	0.995923644
LOC101904057	0.411085489	0.204436418	0.747008439	0.12378678	0.827859334	0.432417901	0.995923644
LOC101904268	0.6025257	0.349269434	0.246753081	0.250279752	0.482165394	0.42785718	0.995923644
LOC101905127	0.349234422	0.309013889	0.521086025	0.310139924	0.362934463	0.429598417	0.995923644
LOC101907581	0.578798246	0.066560505	0.798475559	0.60816778	0.342396813	0.431662518	0.995923644
LOC104972065	0.673998463	0.496392094	0.104544721	0.448966844	0.409780539	0.432459821	0.995923644
LOC104972216	0.387877791	0.110069025	0.876520409	0.372089483	0.453768697	0.429284807	0.995923644
LOC107131331	0.081479368	0.28995223	0.744723058	0.497789535	0.715287959	0.42780746	0.995923644
LOC112442598	0.280682929	0.830726442	0.660634332	0.610869306	0.067674744	0.430644062	0.995923644
LOC112443614	0.439599635	0.493235453	0.375550141	0.299673249	0.26316608	0.432102047	0.995923644
LOC112443850	0.68772549	0.507216517	0.795852108	0.052330895	0.424174738	0.424965909	0.995923644
LOC112444206	0.369401424	0.59207079	0.352997038	0.687207493	0.119214641	0.42946695	0.995923644
LOC112444341	0.72972607	0.410021947	0.131926368	0.341253118	0.471368061	0.430135093	0.995923644
LOC112444622	0.725340793	0.430254898	0.194816428	0.735589737	0.142255613	0.430479717	0.995923644
LOC112444889	0.418728632	0.214003012	0.632656044	0.208295433	0.543344134	0.431949031	0.995923644
LOC112446042	0.361196648	0.712256816	0.169941207	0.390166723	0.36553292	0.426995971	0.995923644
LOC522174	0.077682565	0.719166497	0.309750195	0.544617154	0.678450288	0.431348822	0.995923644
LOC531462	0.597856205	0.38056321	0.157408789	0.606831373	0.289897794	0.428791107	0.995923644
LOC787102	0.138460781	0.65989464	0.681188072	0.126884952	0.815047071	0.432503754	0.995923644
LOC788467	0.486790043	0.340047301	0.834677715	0.106472048	0.436106392	0.431930215	0.995923644
LRRK2	0.184214233	0.527604123	0.686811127	0.141878717	0.678918511	0.432320591	0.995923644
LYPLA1	0.168522085	0.727018596	0.351136404	0.436358966	0.337862644	0.42994629	0.995923644
LYSMD1	0.587166817	0.038728077	0.631399873	0.53555395	0.829605611	0.430946347	0.995923644
MCL1	0.604021274	0.291273694	0.070330297	0.614287198	0.829568835	0.428932342	0.995923644
MCTS1	0.90904308	0.76270753	0.620279332	0.057508729	0.249602632	0.42527093	0.995923644

MGAT1	0.57618728	0.674104635	0.344148693	0.053001129	0.897079502	0.430301876	0.995923644
MPG	0.845294821	0.8321808	0.207128853	0.052002475	0.833433445	0.429188097	0.995923644
MRPL44	0.435116921	0.502268006	0.410073323	0.112942037	0.629356905	0.430702184	0.995923644
MRPS22	0.901449621	0.685331875	0.643648737	0.033662725	0.478077963	0.431494882	0.995923644
MXD4	0.742894971	0.250225691	0.264685761	0.89500028	0.142776172	0.42843387	0.995923644
MYL6	0.43456556	0.309409789	0.903357763	0.097156962	0.537826476	0.430067196	0.995923644
NAXE	0.704586339	0.450848047	0.688269423	0.055827603	0.510140534	0.426759801	0.995923644
NDUFB11	0.457170121	0.354376567	0.867179937	0.050677468	0.900223506	0.431765993	0.995923644
NDUFB8	0.892701165	0.575325355	0.447511634	0.036617327	0.760776238	0.431586126	0.995923644
NOL12	0.431729342	0.660770275	0.204359123	0.53077894	0.20516207	0.430107751	0.995923644
NOP2	0.728513665	0.890961782	0.566477759	0.05208326	0.327854187	0.428190822	0.995923644
NOTCH3	0.990188274	0.733130885	0.27073777	0.224391112	0.145481209	0.431943334	0.995923644
NUDCD1	0.879787428	0.514173669	0.20424353	0.110335069	0.604225795	0.424888523	0.995923644
P2RY1	0.88722601	0.165490943	0.401937074	0.737354981	0.145564481	0.42972138	0.995923644
PDK1	0.914957738	0.741177265	0.07661287	0.385214362	0.310289888	0.426296141	0.995923644
PEA15	0.120087889	0.359975849	0.874135247	0.307173493	0.535260609	0.426377698	0.995923644
PELI3	0.401229566	0.778802267	0.042268457	0.497529282	0.969329088	0.430690522	0.995923644
PER1	0.479812179	0.885819826	0.763755576	0.119660955	0.159825567	0.426246415	0.995923644
PFDN2	0.372122594	0.462485291	0.266268449	0.430267089	0.316509202	0.427144058	0.995923644
PKD1L2	0.210564246	0.894930162	0.394104988	0.084963555	0.996086953	0.428372862	0.995923644
PLCB1	0.354246879	0.139729985	0.579863664	0.464608352	0.476979734	0.430443742	0.995923644
PNKD	0.206328449	0.422320998	0.174329069	0.767595308	0.548267263	0.431317758	0.995923644
POU6F1	0.472209232	0.651036489	0.640922353	0.602131481	0.052388972	0.426447712	0.995923644
PSMB4	0.445818203	0.435423392	0.506259019	0.42951289	0.151961472	0.431899864	0.995923644
PSMD2	0.766657831	0.791793262	0.294258772	0.942563076	0.03720676	0.427799296	0.995923644
QTRT2	0.741410191	0.236340163	0.91165652	0.167027672	0.241119291	0.432418293	0.995923644
RAB26	0.461027492	0.154944937	0.861496973	0.194637935	0.51984415	0.426758575	0.995923644
RLIM	0.139871284	0.944109089	0.140761294	0.611307413	0.556058612	0.429288887	0.995923644
RNF39	0.764563917	0.334381226	0.793356754	0.87325292	0.034633445	0.424179427	0.995923644
RNH1	0.492810839	0.755068879	0.387286885	0.054634089	0.803680605	0.42954116	0.995923644
SAMD4B	0.661660983	0.686574434	0.858900267	0.027652428	0.569057184	0.424336004	0.995923644
SCLY	0.51164721	0.328962509	0.137936467	0.574043662	0.47638539	0.430120773	0.995923644
SEMA3G	0.921458455	0.15738165	0.428993629	0.194659595	0.510438786	0.425504046	0.995923644
SF3B5	0.399338715	0.259059442	0.470816659	0.294132785	0.434002331	0.426508003	0.995923644
SFRP4	0.346195413	0.081058203	0.833338449	0.410823757	0.639797511	0.424527341	0.995923644
SLC10A5	0.1314313	0.868725251	0.955198098	0.171494727	0.339629046	0.430212908	0.995923644
SLC15A4	0.904727289	0.788523573	0.506781143	0.023363419	0.742928147	0.428102225	0.995923644
SLC25A43	0.958709825	0.539784471	0.076795516	0.638648284	0.248142626	0.428727787	0.995923644
SLC26A9	0.774703718	0.358448006	0.559716857	0.42911777	0.093256871	0.426571338	0.995923644
SLC35G1	0.776994508	0.987548594	0.1019297	0.282003544	0.289143492	0.430897737	0.995923644
SNCAIP	0.164218187	0.960771843	0.190116804	0.518230847	0.404547242	0.428468018	0.995923644
SNRPD3	0.356352815	0.327754006	0.578620318	0.457533768	0.206466903	0.431077664	0.995923644
SOWAHC	0.922814049	0.140731764	0.162451149	0.774867106	0.389584949	0.430663396	0.995923644
SPEN	0.379337204	0.755816067	0.239969563	0.132229529	0.680646012	0.425800696	0.995923644
SUMO1	0.701784679	0.115516134	0.706234913	0.109895808	0.980509165	0.425158158	0.995923644

SVIP	0.261071145	0.692788528	0.776093643	0.049906539	0.889577738	0.426900272	0.995923644
TMCC3	0.648444648	0.679204568	0.314305448	0.071867208	0.627629865	0.427236191	0.995923644
TMEM115	0.289668927	0.931810243	0.096863196	0.8161575	0.298009468	0.430397322	0.995923644
TMEM60	0.273167611	0.651288973	0.99396389	0.046923042	0.745266293	0.425571917	0.995923644
TOR2A	0.864690778	0.650038913	0.024596308	0.703953728	0.654218476	0.430614874	0.995923644
TRPM4	0.525621205	0.457479321	0.280342486	0.104409235	0.912652239	0.432150091	0.995923644
TSACC	0.128421616	0.826257697	0.840539955	0.472265971	0.147387254	0.426241971	0.995923644
TSC2	0.849420851	0.090948083	0.095652559	0.849151159	0.981176767	0.42480786	0.995923644
TSC22D2	0.5906263	0.657420802	0.547775104	0.036442506	0.799020997	0.425831455	0.995923644
TTC9B	0.066775109	0.937842827	0.26846008	0.439980063	0.867047638	0.431879193	0.995923644
TUBA8	0.816847976	0.307410449	0.073695462	0.789066281	0.436564842	0.430824904	0.995923644
TUBD1	0.25982258	0.981428681	0.063123855	0.440598686	0.865370122	0.424264344	0.995923644
TUSC3	0.962947074	0.566767699	0.042935125	0.878538352	0.301937531	0.426456306	0.995923644
TXN2	0.942745334	0.554942493	0.561830522	0.025041077	0.849308658	0.427437655	0.995923644
UBASH3B	0.352840636	0.648318967	0.522293276	0.236432686	0.227951862	0.432572283	0.995923644
ULK3	0.427830754	0.077321088	0.663645356	0.899916669	0.315282896	0.426818246	0.995923644
VIRMA	0.352951728	0.998303705	0.848669898	0.027582601	0.777137823	0.431779087	0.995923644
YAF2	0.115666264	0.586405675	0.170950667	0.793461963	0.675372968	0.426395671	0.995923644
ZNF248	0.123157353	0.715397304	0.361141324	0.812676332	0.24601422	0.430465374	0.995923644
CARD19	0.503271086	0.378445384	0.14566576	0.273212999	0.84988511	0.432649079	0.995960728
PREP	0.842868972	0.446681984	0.576454802	0.285097594	0.104161757	0.432729699	0.996006604
HMGCL	0.908681108	0.629845292	0.097563151	0.406548345	0.284118525	0.432856769	0.996019692
ZNF345	0.20349898	0.975200221	0.40095264	0.660199992	0.122764409	0.432838405	0.996019692
ALG12	0.292431907	0.944245267	0.658281137	0.061816977	0.57465301	0.433053604	0.996046327
COG8	0.651698993	0.277260472	0.164528046	0.677926979	0.320353927	0.433034974	0.996046327
DGKB	0.293443031	0.967205986	0.807765746	0.380225087	0.074112901	0.433145077	0.996046327
ERMP1	0.356199997	0.901462942	0.276065251	0.212805079	0.342946993	0.433385357	0.996046327
GUK1	0.729676772	0.607869578	0.783225771	0.029088127	0.640312255	0.433414589	0.996046327
LOC112446659	0.606750474	0.354358882	0.5053901	0.133574454	0.445327249	0.433233237	0.996046327
PDS5A	0.2210826	0.470392399	0.789664085	0.11337998	0.693391803	0.433028727	0.996046327
WARS2	0.855904098	0.457418009	0.186362109	0.158352724	0.559581171	0.433274005	0.996046327
ZBTB43	0.264439876	0.677825954	0.16949163	0.937710251	0.22703838	0.433344814	0.996046327
VIPR2	0.590298155	0.265153104	0.10798178	0.491405661	0.780326943	0.4336951	0.996551427
COX18	0.957083868	0.678299931	0.094315172	0.297499412	0.356964074	0.434267936	0.997030091
KIZ	0.233378216	0.704748807	0.784973855	0.058388299	0.861606639	0.434075725	0.997030091
MRM1	0.824420156	0.499887561	0.704273434	0.161464928	0.138666265	0.434166581	0.997030091
OSGIN2	0.95423338	0.267154849	0.047870703	0.858900485	0.620285031	0.434250955	0.997030091
PDCL	0.466538164	0.337540169	0.73303937	0.095871481	0.586531595	0.43396989	0.997030091
SIMC1	0.531579224	0.506433863	0.331645659	0.216638829	0.336126321	0.434243838	0.997030091
LOC101902122	0.82883684	0.762102143	0.500352702	0.586323673	0.035145861	0.434551232	0.997122522
LOC112441682	0.187933805	0.534821249	0.869630242	0.18171649	0.409695733	0.434404338	0.997122522
LRWD1	0.670375393	0.069293686	0.297760003	0.507248833	0.927643592	0.434434957	0.997122522
TUBA3E	0.552509391	0.1366964	0.200612423	0.735907984	0.583955513	0.434506191	0.997122522
ZRANB1	0.812187684	0.230678685	0.351410935	0.212728866	0.465185536	0.434616106	0.99713196
FAXDC2	0.54709259	0.229163955	0.668062638	0.122431108	0.636407747	0.434905	0.997359712

GTF2H3	0.293485056	0.498071881	0.335235604	0.452816439	0.294502195	0.435140792	0.997359712
LOC104976061	0.403673639	0.585725976	0.044367755	0.669862596	0.929799699	0.435112117	0.997359712
LOC107132360	0.525027654	0.862695464	0.052707106	0.337043183	0.811367362	0.434969383	0.997359712
SAMD5	0.450598745	0.517860253	0.11912085	0.475559327	0.494298138	0.435118767	0.997359712
TOM1	0.490039251	0.059071254	0.778071757	0.294754287	0.982526094	0.434816438	0.997359712
USP47	0.784874963	0.161266396	0.119961234	0.501120367	0.857774338	0.434924598	0.997359712
ADGRG2	0.459326766	0.702333317	0.646112156	0.192969238	0.162550913	0.435225743	0.99741512
CEP192	0.751487998	0.133692332	0.222624163	0.30302913	0.965755615	0.435427372	0.997609322
LOC101904308	0.148913558	0.429071399	0.170789522	0.794515264	0.75499086	0.435434001	0.997609322
LOC788745	0.723051404	0.207583133	0.765582202	0.54010592	0.105507535	0.435492852	0.997609322
ZCCHC2	0.562801788	0.540714425	0.155542193	0.489247179	0.282879392	0.435567492	0.997641049
PML	0.733734712	0.587065527	0.184101428	0.188043973	0.43949065	0.435644057	0.997677173
LOC781741	0.805714265	0.563088441	0.926147595	0.722184633	0.021610737	0.435750061	0.99774582
LOXL3	0.79871405	0.384076734	0.161215155	0.241499255	0.549592338	0.435917222	0.99774582
NDUFS5	0.519685205	0.395071871	0.706627781	0.049927622	0.90589942	0.435859504	0.99774582
PRKCA	0.149667337	0.810967377	0.302162013	0.787268505	0.227251993	0.435848582	0.99774582
CDKN1C	0.262092408	0.62522585	0.274101543	0.147259408	0.993036809	0.436029596	0.997863854
LOC112444773	0.110798603	0.737006951	0.635119483	0.5502156	0.230297632	0.436123027	0.997938511
C23H6orf47	0.595654581	0.628196905	0.094325802	0.465309505	0.400831364	0.436420851	0.997945777
KDM4B	0.732321231	0.491786017	0.280685249	0.069131406	0.941452985	0.436319122	0.997945777
LOC112443130	0.725952009	0.113861969	0.79194637	0.210309329	0.478197025	0.43643025	0.997945777
RPL3	0.695492154	0.976562532	0.49376951	0.13825846	0.141833604	0.436245138	0.997945777
TAF11	0.39660688	0.199639746	0.30565561	0.349932799	0.777113017	0.436374225	0.997945777
CCDC183	0.130200262	0.15055617	0.678269791	0.878817774	0.564063715	0.436628168	0.998120193
PHF7	0.188534953	0.375639957	0.227991909	0.804628657	0.507233891	0.436606836	0.998120193
MLEC	0.81144279	0.643760787	0.375380897	0.063781181	0.528165696	0.437021423	0.998880023
LOC789231	0.251502217	0.642093417	0.053349945	0.773289316	0.992159625	0.4371342	0.998998658
SMC5	0.117455857	0.463801969	0.756582328	0.161788889	0.992245235	0.437308729	0.999258362
ATP6V1E2	0.0619573	0.798678215	0.277995193	0.730572165	0.658871756	0.437443371	0.999287746
RNF121	0.581776761	0.551340544	0.166174889	0.188963289	0.657244003	0.437395119	0.999287746
CACNA1H	0.10645197	0.89175753	0.344897883	0.758329516	0.26686275	0.437552613	0.999398181
FITM2	0.995721216	0.684200165	0.155092245	0.093152491	0.673662376	0.437677792	0.999405905
STAR	0.385783878	0.361160252	0.168063477	0.434874667	0.651086424	0.437666647	0.999405905
MYRIP	0.23920172	0.136909067	0.433481119	0.966764288	0.483828092	0.437931783	0.999568635
PLP2	0.325857525	0.674219709	0.37792447	0.133853159	0.597235438	0.437863314	0.999568635
SYT11	0.054501076	0.446652459	0.785510257	0.705172989	0.4924198	0.43792351	0.999568635
ANGPT2	0.052967573	0.360482263	0.777317418	0.653091555	0.691449509	0.439564241	0.999593577
ANO6	0.86981637	0.259859971	0.071914393	0.457348732	0.900424587	0.439342566	0.999593577
ARL10	0.468200124	0.816739338	0.042380998	0.907870549	0.457276638	0.440235361	0.999593577
BMT2	0.022045649	0.964875608	0.660477025	0.622728292	0.782201192	0.443221222	0.999593577
BTRC	0.550024218	0.83881649	0.081294945	0.431811885	0.42273027	0.443302788	0.999593577
C23H6orf136	0.198041505	0.368316991	0.988327907	0.293754686	0.321720702	0.442440936	0.999593577
CCDC153	0.385146079	0.850735779	0.542044912	0.046783339	0.801544763	0.438454053	0.999593577
CCM2	0.149605157	0.365047526	0.914558789	0.837451794	0.160302689	0.43963742	0.999593577
CLUH	0.898347522	0.867473321	0.32812253	0.185495391	0.142864145	0.441489915	0.999593577

CMC2	0.563294275	0.338187835	0.844085426	0.057221739	0.725474456	0.438852573	0.999593577
CMYA5	0.680045034	0.477710364	0.379533013	0.124743235	0.437457084	0.440241515	0.999593577
CPEB3	0.283155449	0.966268847	0.657224562	0.76572305	0.049090347	0.441049775	0.999593577
DCAF17	0.516342381	0.57180753	0.996494488	0.59191881	0.03866323	0.440370157	0.999593577
DHX36	0.401328834	0.773808273	0.880784192	0.090446673	0.274072037	0.441598181	0.999593577
DYRK2	0.959675068	0.758369572	0.234343538	0.660483953	0.06057756	0.442720093	0.999593577
EVC2	0.786937248	0.158451631	0.226948238	0.498259163	0.483435013	0.442527668	0.999593577
FAM120A	0.511247271	0.276872864	0.743217573	0.559411978	0.114045778	0.439811044	0.999593577
FCGRT	0.106752885	0.398152179	0.964623616	0.236248691	0.695207205	0.440389332	0.999593577
FNBP4	0.34337695	0.098497169	0.51230313	0.531810478	0.728194741	0.439765824	0.999593577
GAPDHS	0.363299084	0.052077556	0.951649795	0.572241923	0.661382146	0.442473877	0.999593577
GCNT3	0.940764965	0.605823077	0.449487324	0.05771322	0.458577682	0.441586873	0.999593577
GFRA4	0.194923237	0.35247135	0.249263675	0.50163117	0.785387585	0.440730638	0.999593577
GGCT	0.411066327	0.416644392	0.064194287	0.621627081	0.980093265	0.439461021	0.999593577
GNPTG	0.452240285	0.398289562	0.90727611	0.369738453	0.110820884	0.439401588	0.999593577
GRAMD1B	0.613449566	0.430263606	0.251280844	0.937167612	0.108985542	0.441435753	0.999593577
GRK4	0.772127012	0.102599429	0.704757008	0.189552823	0.64666066	0.443225595	0.999593577
GUCA1A	0.244963448	0.580943567	0.122912062	0.609105514	0.636636347	0.441660129	0.999593577
H3F3A	0.629077041	0.350111192	0.983453247	0.038500577	0.817568327	0.442567281	0.999593577
HOXB9	0.319838356	0.839147912	0.131244774	0.361311515	0.526567763	0.439548191	0.999593577
HSPA9	0.993456132	0.575243401	0.279125917	0.211224679	0.202341477	0.442557251	0.999593577
HSPB6	0.60314368	0.50409534	0.122157798	0.294283883	0.625523761	0.443057485	0.999593577
IFNAR2	0.857445937	0.840699524	0.073494561	0.142075259	0.908895482	0.443166977	0.999593577
LCMT1	0.638193254	0.389140167	0.768808771	0.035901168	0.987397145	0.441281271	0.999593577
LOC100138864	0.719387651	0.20078838	0.105499084	0.445589357	0.997193308	0.441357044	0.999593577
LOC100296205	0.244993676	0.886945489	0.773539594	0.495714568	0.081400974	0.441652821	0.999593577
LOC100336381	0.3421329	0.868768345	0.460641172	0.126137734	0.389325725	0.44012655	0.999593577
LOC100850276	0.700002701	0.441702812	0.439020064	0.198776418	0.24709147	0.438640465	0.999593577
LOC101904258	0.675479021	0.16114291	0.880505979	0.097824065	0.71692624	0.440068338	0.999593577
LOC101904477	0.85038921	0.94892638	0.013101535	0.684021149	0.923048042	0.438853943	0.999593577
LOC101905367	0.239193823	0.997138232	0.234136648	0.172837631	0.704611372	0.442125088	0.999593577
LOC104969496	0.046397678	0.525708845	0.473180377	0.898373405	0.647499789	0.439862091	0.999593577
LOC104969833	0.242003551	0.29103759	0.454345954	0.361653642	0.585535107	0.4414941	0.999593577
LOC112443147	0.478628831	0.426124866	0.139906078	0.903056787	0.261118475	0.440249846	0.999593577
LOC112448856	0.98414125	0.109795513	0.226099809	0.467050247	0.585438693	0.438983386	0.999593577
LOC510860	0.276884374	0.999837044	0.286545251	0.22689373	0.378304464	0.442336475	0.999593577
LOC522540	0.580685347	0.865759832	0.533012029	0.031713985	0.797362387	0.441485446	0.999593577
LOC613460	0.187924586	0.272176424	0.162304031	0.958661534	0.852842178	0.441774906	0.999593577
LOC614424	0.330556846	0.12093138	0.309531096	0.61459961	0.881689146	0.439632979	0.999593577
LOC616868	0.235748563	0.487331779	0.336946492	0.42387585	0.416756403	0.44309425	0.999593577
MAATS1	0.561538174	0.057477292	0.892695495	0.639291512	0.369530974	0.442273263	0.999593577
MAMDC2	0.437358973	0.945332273	0.481202494	0.096357134	0.35120341	0.440358108	0.999593577
MED31	0.55687016	0.388854191	0.147065466	0.597616729	0.352725904	0.439840596	0.999593577
MEF2C	0.159590614	0.333102052	0.92338543	0.155322586	0.893055133	0.442334737	0.999593577
MFAP4	0.265225032	0.579309004	0.058894811	0.760556095	0.993936392	0.443149355	0.999593577

MKRN1	0.966988532	0.777167652	0.126409863	0.191962284	0.365113309	0.438408152	0.999593577
MPHOSPH8	0.190947996	0.457031412	0.568619642	0.147332409	0.92223153	0.440611948	0.999593577
NCK2	0.511233229	0.906547882	0.726544006	0.075788848	0.267978893	0.443103148	0.999593577
NUB1	0.824911337	0.2812351	0.130397469	0.411643169	0.542926033	0.441091429	0.999593577
PEPD	0.563570249	0.562616911	0.173083235	0.231709184	0.530547335	0.440717808	0.999593577
PGAP3	0.461431328	0.509961392	0.741656531	0.043527024	0.900703989	0.4431885	0.999593577
PODNL1	0.674508857	0.082151011	0.198923358	0.990775277	0.613527997	0.439511363	0.999593577
PP2D1	0.41410521	0.317990795	0.187934079	0.50977344	0.530844204	0.439422245	0.999593577
PPP3CA	0.381030644	0.139021804	0.940228518	0.152258327	0.891243492	0.441029222	0.999593577
PPP4R3B	0.39306443	0.280745153	0.271490813	0.858734629	0.259398635	0.438810197	0.999593577
PRDM15	0.82405382	0.139402253	0.916820779	0.192400558	0.329324624	0.438803154	0.999593577
RAB29	0.729675225	0.777141175	0.058473169	0.223408411	0.920685398	0.442624825	0.999593577
RBM18	0.391988609	0.160843161	0.66175834	0.230383914	0.708745617	0.442430881	0.999593577
RHBG	0.098075816	0.597110393	0.179549725	0.648057267	0.986496583	0.440082357	0.999593577
RINL	0.580127834	0.208629426	0.646437113	0.24529775	0.356324831	0.443098107	0.999593577
RPS15	0.523250335	0.93390571	0.933190536	0.030617348	0.48247752	0.440452857	0.999593577
RPS28	0.271759531	0.859429449	0.504230099	0.127503417	0.45294746	0.442137538	0.999593577
RWDD1	0.840196533	0.525903216	0.209715702	0.325529336	0.225944539	0.44250848	0.999593577
RXFP4	0.27015906	0.073909301	0.695672428	0.738026372	0.664652853	0.442460104	0.999593577
SERP1	0.713995868	0.061688472	0.828877669	0.711029799	0.259504091	0.440450689	0.999593577
SLC22A31	0.051362838	0.917296012	0.569791487	0.338440935	0.751359312	0.442790123	0.999593577
SLC2A6	0.973062167	0.976705217	0.032820584	0.620228759	0.345301069	0.438988617	0.999593577
SOGA3	0.925374951	0.476441063	0.313533187	0.139251404	0.34952916	0.440237107	0.999593577
SORCS1	0.732871887	0.149306239	0.778686894	0.239661679	0.332488336	0.441834189	0.999593577
SOX12	0.873962538	0.229583377	0.161221047	0.917660607	0.22990895	0.442744728	0.999593577
TLE4	0.970694182	0.985538701	0.465003207	0.187199846	0.080012133	0.438534247	0.999593577
TPK1	0.078422128	0.695693839	0.345673846	0.589448129	0.6100002	0.441613675	0.999593577
TRIM37	0.652942484	0.821446771	0.10593649	0.226763515	0.526959381	0.441836761	0.999593577
WASF2	0.583973621	0.805885342	0.137574287	0.341343521	0.30666373	0.441516171	0.999593577
WNT16	0.281029038	0.568369191	0.510956922	0.530592178	0.15769689	0.442848843	0.999593577
WTAP	0.923196764	0.88176925	0.789785681	0.014940389	0.698220932	0.439679807	0.999593577
ZBTB17	0.597962948	0.254846012	0.071582645	0.870609083	0.705688734	0.439551857	0.999593577
ZFR	0.753989886	0.552198321	0.227587309	0.341211538	0.211730616	0.443280805	0.999593577
ZNF34	0.419542412	0.214440708	0.901457	0.917196573	0.090863108	0.441039112	0.999593577
ARHGAP1	0.32430933	0.642340075	0.230458333	0.283302514	0.5035991	0.443376559	0.999622572
CDK12	0.203715039	0.50943327	0.582980864	0.137163637	0.826456144	0.443608143	0.999839779
NDUFA4L2	0.923348974	0.998999985	0.39408379	0.05338961	0.353448241	0.443640174	0.999839779
ZNF555	0.661997188	0.778646946	0.677622248	0.083927763	0.234018771	0.443655674	0.999839779
ATP5F1D	0.917066043	0.559232895	0.717468341	0.022227023	0.839394662	0.443778474	0.999892635
CCDC137	0.863543288	0.669443217	0.496910146	0.120740554	0.198161585	0.443983206	0.999892635
CD36	0.281419743	0.437381382	0.321775789	0.247683345	0.703839914	0.444790987	0.999892635
CELSR1	0.113554579	0.805251724	0.097547517	0.949639403	0.815624884	0.444897692	0.999892635
ITGB1	0.639579173	0.157591879	0.535305869	0.202884127	0.629278585	0.444377892	0.999892635
KALRN	0.1721955	0.808504427	0.841755523	0.079142518	0.742353825	0.444292602	0.999892635
KANSL3	0.235002827	0.883235655	0.274509401	0.202176032	0.597468472	0.444227914	0.999892635

KCNS2	0.035853942	0.674465536	0.591321063	0.739499014	0.653335273	0.444894444	0.999892635
LOC100848205	0.381784771	0.700507165	0.298738795	0.862942214	0.099962873	0.444468015	0.999892635
LOC100848443	0.078175076	0.923793804	0.4828814	0.260889771	0.756194055	0.444155932	0.999892635
LOC101904592	0.145452291	0.465731737	0.390825381	0.959554184	0.270960474	0.444253494	0.999892635
LOC780968	0.315327004	0.661173195	0.5620749	0.193163861	0.305142644	0.444856135	0.999892635
MPZL1	0.820828341	0.484264819	0.087878576	0.359667278	0.549646211	0.444816778	0.999892635
NBDY	0.533948049	0.294898031	0.551214122	0.292575866	0.271061484	0.444246563	0.999892635
P2RX3	0.085979533	0.555497112	0.247145523	0.844670184	0.690576329	0.444299701	0.999892635
RAF1	0.524740624	0.691371645	0.225476526	0.610075866	0.138134874	0.444509215	0.999892635
THOC3	0.869222527	0.459037562	0.278622143	0.992142983	0.062344676	0.444072491	0.999892635
TMEM185A	0.279580379	0.413756732	0.676699995	0.532208414	0.165612651	0.444662812	0.999892635
TRIM63	0.713563352	0.446106981	0.277182535	0.844020806	0.092456307	0.444299144	0.999892635
UBQLN1	0.64538111	0.362161856	0.095380002	0.467905989	0.66224894	0.44487919	0.999892635
BRK1	0.516496882	0.38964192	0.623500726	0.089304759	0.616792542	0.444971728	0.999922091
ADAM10	0.725794676	0.692182672	0.282770202	0.070030313	0.695814507	0.445241248	0.999939598
ARHGAP42	0.103264769	0.69268113	0.589953722	0.226198368	0.724973702	0.445187434	0.999939598
CYP1A1	0.342721745	0.837840017	0.053106865	0.848024198	0.53470546	0.445049791	0.999939598
ILDR2	0.05269283	0.363377826	0.540276475	0.834335239	0.802261515	0.44529603	0.999939598
KCTD9	0.999806008	0.124684658	0.629546588	0.104894052	0.840313408	0.445119848	0.999939598
MAPK14	0.891343608	0.836620175	0.544923399	0.042619141	0.399935287	0.445345105	0.999939598
ACACA	0.966634426	0.965994156	0.5968835	0.040364611	0.308381805	0.445634593	0.999964829
CLEC14A	0.459732478	0.532197135	0.943820031	0.037816739	0.794889518	0.4457329	0.999964829
IER3IP1	0.234671018	0.254317714	0.656255057	0.223895857	0.790479151	0.445483988	0.999964829
LOC100141185	0.216580677	0.278584273	0.305184061	0.872983239	0.431953154	0.445782871	0.999964829
LOC112442757	0.12375784	0.421923527	0.433401915	0.504995825	0.607486744	0.445758963	0.999964829
STYX	0.444687735	0.765390964	0.353525428	0.096616929	0.596083069	0.445432775	0.999964829
VIT	0.218606339	0.172118022	0.756699392	0.246489344	0.988557825	0.445633316	0.999964829
A2M	0.203874023	0.820240129	0.901859611	0.326460966	0.703388736	0.75103458	0.999996191
A4GALT	0.150788045	0.948184102	0.78715874	0.459622192	0.409887826	0.657405965	0.999996191
AAAS	0.160413281	0.261372197	0.620215043	0.93354806	0.75900707	0.630007636	0.999996191
AADACL3	0.46049241	0.594333511	0.595461031	0.765859762	0.68312083	0.89619242	0.999996191
AAED1	0.307425165	0.611519523	0.757621908	0.259214563	0.82096574	0.726147933	0.999996191
AAK1	0.774327769	0.945046411	0.703235724	0.37240014	0.775653261	0.955414929	0.999996191
AAMDC	0.663882637	0.625200955	0.461375824	0.44421392	0.752868257	0.855654363	0.999996191
AAR2	0.49963426	0.519241291	0.443355374	0.312655783	0.910613728	0.740656676	0.999996191
AARS	0.724925142	0.533138258	0.67194412	0.225751123	0.282615789	0.60926331	0.999996191
AATF	0.839854828	0.569724932	0.679553631	0.403571131	0.068472226	0.492359085	0.999996191
ABCA1	0.652935083	0.822492423	0.297399118	0.81161654	0.916901525	0.934871242	0.999996191
ABCA10	0.920755025	0.502731291	0.644195555	0.672897509	0.529131872	0.92280592	0.999996191
ABCA13	0.773610302	0.742572816	0.307533383	0.938325152	0.582413788	0.911777782	0.999996191
ABCA4	0.079273839	0.869975346	0.839421221	0.713480996	0.968170616	0.777198128	0.999996191
ABCA5	0.747780208	0.204035144	0.205149744	0.568050718	0.923150416	0.60742906	0.999996191
ABCA7	0.353994212	0.566705	0.327261342	0.461557728	0.958302724	0.718037219	0.999996191
ABCA9	0.877571619	0.823102119	0.593239429	0.535313185	0.717303695	0.963260674	0.999996191
ABCB1	0.182433737	0.263792648	0.689438032	0.288191983	0.756125851	0.452947113	0.999996191

ABCB10	0.058350998	0.786597318	0.867294982	0.481950981	0.922825946	0.622210159	0.999996191
ABCB6	0.778295632	0.90716578	0.264616605	0.34530359	0.735241699	0.807059253	0.999996191
ABCB7	0.967688712	0.315219934	0.637610219	0.452619441	0.960276024	0.895071674	0.999996191
ABCB9	0.40573365	0.500998505	0.497477921	0.820976359	0.365166077	0.72618515	0.999996191
ABCC10	0.690217985	0.26764876	0.506088535	0.96304831	0.83655232	0.879397437	0.999996191
ABCC9	0.423758411	0.541017494	0.185302376	0.361786344	0.984248474	0.591530502	0.999996191
ABCD1	0.995570357	0.747651338	0.569149831	0.352355164	0.287896017	0.789962661	0.999996191
ABCD2	0.166464935	0.741309138	0.444381673	0.791711882	0.278937053	0.548557435	0.999996191
ABCD3	0.494690341	0.440667657	0.417257668	0.648682351	0.502971098	0.72216058	0.999996191
ABCD4	0.585265213	0.672337199	0.338941731	0.594493321	0.288399477	0.672103914	0.999996191
ABCG1	0.352279646	0.634463188	0.892031137	0.771247803	0.074089455	0.536895645	0.999996191
ABCG2	0.489644032	0.390040781	0.798304872	0.56790192	0.237053232	0.651072647	0.999996191
ABCG4	0.767093245	0.634917116	0.742800774	0.964973957	0.721131051	0.986547251	0.999996191
ABHD1	0.947446846	0.900891099	0.215720235	0.115971885	0.943404114	0.647429473	0.999996191
ABHD11	0.677206297	0.701724703	0.412656758	0.539345594	0.687801797	0.874467526	0.999996191
ABHD12	0.538975847	0.786861169	0.485700538	0.265495165	0.653942285	0.756950553	0.999996191
ABHD14A	0.243478717	0.986201824	0.079950861	0.509783844	0.821000567	0.471905563	0.999996191
ABHD14B	0.471348005	0.938253018	0.595525562	0.379193683	0.970654518	0.912261007	0.999996191
ABHD15	0.521573632	0.836688601	0.422115117	0.507141916	0.235965813	0.664981929	0.999996191
ABHD16A	0.075782176	0.65813973	0.877524858	0.458580424	0.620839175	0.554026658	0.999996191
ABHD17C	0.566399808	0.569295756	0.188044286	0.872319318	0.902483436	0.808141425	0.999996191
ABHD2	0.427196317	0.979674883	0.759979723	0.086033307	0.601901141	0.608100065	0.999996191
ABHD4	0.673142352	0.890525009	0.5691254	0.548782614	0.151001795	0.712943839	0.999996191
ABHD8	0.289547822	0.818738079	0.358196921	0.332284353	0.48340435	0.571450279	0.999996191
ABI2	0.084534469	0.962914413	0.637608894	0.442520813	0.577572781	0.566072355	0.999996191
ABI3	0.812241275	0.67409197	0.194047484	0.323957367	0.990731393	0.748177689	0.999996191
ABI3BP	0.862184291	0.462819705	0.166564597	0.322131533	0.994875048	0.658306936	0.999996191
ABLIM3	0.479877739	0.60844962	0.700129485	0.122468826	0.815510096	0.650044123	0.999996191
ABR	0.893463569	0.874404084	0.405122287	0.241475344	0.176326819	0.569117774	0.999996191
ABRAXAS1	0.818028132	0.073697211	0.634965575	0.897881375	0.567377086	0.641087961	0.999996191
ABRAXAS2	0.996088639	0.154901391	0.296751761	0.375989898	0.96493038	0.609769346	0.999996191
ABTB2	0.829579848	0.431221792	0.414344555	0.399141287	0.58949386	0.752333313	0.999996191
ACAA1	0.674072489	0.472471954	0.118763443	0.861155177	0.775343692	0.691319247	0.999996191
ACAA2	0.898818253	0.558572487	0.271275588	0.550666922	0.977008468	0.875500184	0.999996191
ACACB	0.826152455	0.911946113	0.856915232	0.852861744	0.239686268	0.945049434	0.999996191
ACAD10	0.960820432	0.615819803	0.129585794	0.63407182	0.608557235	0.721580437	0.999996191
ACAD11	0.501874683	0.971873468	0.563368661	0.978984278	0.978890031	0.988183405	0.999996191
ACAD8	0.440832669	0.605437054	0.901840992	0.697720118	0.995716947	0.964423167	0.999996191
ACAD9	0.52865021	0.875766206	0.360638919	0.164032616	0.345693863	0.502054645	0.999996191
ACADL	0.742386946	0.623007689	0.563020066	0.856594805	0.98055472	0.980469501	0.999996191
ACADM	0.818664456	0.194727502	0.382265102	0.702551633	0.744012867	0.735485848	0.999996191
ACADS	0.666732419	0.509917174	0.556748213	0.073913541	0.763381769	0.524647193	0.999996191
ACADSB	0.497879936	0.855966878	0.627613131	0.156152766	0.696719771	0.718432622	0.999996191
ACADVL	0.937030347	0.414070841	0.372618395	0.570207987	0.474062161	0.773070824	0.999996191
ACAP2	0.635319447	0.586167625	0.856529496	0.469295745	0.470866079	0.869917177	0.999996191

ACAT1	0.751873296	0.808006387	0.511095891	0.042617034	0.690084144	0.49534615	0.999996191
ACBD4	0.943479971	0.824064967	0.204896088	0.394155957	0.316104676	0.644534522	0.999996191
ACBD5	0.398275561	0.810366718	0.376714748	0.672561345	0.979749694	0.887912434	0.999996191
ACBD6	0.976997386	0.846157255	0.726872976	0.717554768	0.97113676	0.99796724	0.999996191
ACCS	0.920368621	0.367622536	0.339900218	0.641500123	0.523048158	0.770785898	0.999996191
ACE	0.38582103	0.239883344	0.946674255	0.940589141	0.39798459	0.740947994	0.999996191
ACE2	0.942613442	0.891329977	0.207788348	0.829446628	0.840797102	0.937299201	0.999996191
ACER1	0.362955388	0.281437622	0.999135066	0.515177098	0.896447953	0.805982024	0.999996191
ACHE	0.66105204	0.890382819	0.266242409	0.084559071	0.815076014	0.526768337	0.999996191
ACKR1	0.203690353	0.850113619	0.680224011	0.525858666	0.304700124	0.634693289	0.999996191
ACKR2	0.615852642	0.894379579	0.988402706	0.475845221	0.717593401	0.971481349	0.999996191
ACMSD	0.415049839	0.836559929	0.965674107	0.916672058	0.965916966	0.99187246	0.999996191
ACO2	0.666776867	0.585879552	0.875388901	0.0376206	0.56970822	0.455378767	0.999996191
ACOT11	0.481346894	0.77758502	0.998274183	0.557866856	0.751579874	0.959585228	0.999996191
ACOT13	0.408631369	0.611574311	0.533445179	0.945244392	0.938452695	0.934356705	0.999996191
ACOT2	0.880621166	0.783434754	0.682244886	0.016752865	0.991651375	0.466991587	0.999996191
ACOT6	0.480874554	0.796670007	0.705492294	0.059854179	0.485891881	0.467929477	0.999996191
ACOT7	0.327993255	0.51008051	0.135126706	0.537659072	0.579856553	0.448425151	0.999996191
ACOT8	0.496124917	0.76066559	0.460161524	0.123413442	0.73210184	0.598639804	0.999996191
ACOX1	0.225623853	0.958827814	0.291338557	0.822064659	0.373241903	0.639451107	0.999996191
ACOX2	0.775215659	0.46659381	0.515161795	0.493495709	0.830750238	0.881373952	0.999996191
ACOX3	0.149135057	0.28753718	0.633942824	0.916498714	0.507152452	0.556705037	0.999996191
ACP1	0.962560992	0.856387193	0.868850394	0.221148673	0.739633714	0.933390497	0.999996191
ACP2	0.394794379	0.452129286	0.917480662	0.374593257	0.673362634	0.782979519	0.999996191
ACP6	0.641211197	0.628098797	0.956614943	0.598453136	0.519442238	0.935647305	0.999996191
ACRBP	0.280000318	0.530814516	0.277325279	0.896505082	0.376216217	0.575136102	0.999996191
ACSBG1	0.689481387	0.46061807	0.82804616	0.978807155	0.658039174	0.96533128	0.999996191
ACSF2	0.699737918	0.357726084	0.542334611	0.751047844	0.441045994	0.797877133	0.999996191
ACSF3	0.71995098	0.751393208	0.206146121	0.334770868	0.810489777	0.725823679	0.999996191
ACSM1	0.690774751	0.552699873	0.615563366	0.203378283	0.752636522	0.758029569	0.999996191
ACSM2B	0.551313688	0.474125273	0.99957287	0.969754993	0.827696014	0.978354219	0.999996191
ACSS1	0.547736632	0.345904676	0.821321651	0.200744258	0.513067602	0.602781442	0.999996191
ACSS3	0.713956064	0.623783914	0.690436517	0.624572057	0.545055517	0.9212103	0.999996191
ACTL6A	0.683989709	0.09449273	0.614091647	0.987224234	0.717148043	0.711786627	0.999996191
ACTN3	0.427615545	0.352683874	0.166822916	0.634750833	0.881419611	0.577544149	0.999996191
ACTN4	0.3377748	0.452802203	0.128828255	0.5309281	0.692161734	0.453213856	0.999996191
ACTR10	0.900422147	0.364982191	0.496035482	0.488236227	0.97282337	0.883238199	0.999996191
ACTR1B	0.441647099	0.883723434	0.207825267	0.521059807	0.978622804	0.783205704	0.999996191
ACTR6	0.061446771	0.981528337	0.800208902	0.900842582	0.829890511	0.758572107	0.999996191
ACTR8	0.990868632	0.539954446	0.931720795	0.662486718	0.683465969	0.981960196	0.999996191
ACVR1	0.826204001	0.888097235	0.874741362	0.358000667	0.223245517	0.82025907	0.999996191
ACVR1B	0.735969663	0.406710137	0.981690605	0.200575612	0.620315304	0.760986642	0.999996191
ACVR1C	0.709234461	0.993279621	0.401880063	0.307577804	0.97373278	0.895469762	0.999996191
ACVRL1	0.486944853	0.474868047	0.508727994	0.70711819	0.795535406	0.860598355	0.999996191
ACY1	0.856146755	0.840655177	0.410618888	0.661703355	0.752249894	0.954557898	0.999996191

ACYP2	0.61733122	0.725516393	0.230330646	0.100942664	0.806774758	0.480019701	0.999996191
ADAL	0.715872811	0.965339939	0.581940288	0.302808905	0.93660454	0.930596187	0.999996191
ADAM15	0.736362683	0.747185491	0.502894731	0.69818845	0.804711331	0.958987813	0.999996191
ADAM17	0.552491356	0.598582249	0.207736683	0.31721873	0.353370685	0.464241236	0.999996191
ADAM20	0.199741555	0.414125433	0.568722937	0.490634195	0.50120809	0.539813484	0.999996191
ADAM9	0.83598298	0.584948817	0.288908887	0.938480663	0.772982256	0.918798558	0.999996191
ADAMTS1	0.505446119	0.082808054	0.926671171	0.950012051	0.834308668	0.72881415	0.999996191
ADAMTS14	0.733830781	0.680229311	0.58332899	0.860016176	0.611664444	0.9578276	0.999996191
ADAMTS18	0.348242963	0.845287071	0.53819332	0.941404073	0.219459223	0.74056254	0.999996191
ADAMTS20	0.804483791	0.9637444	0.523609	0.276482391	0.789215558	0.901120836	0.999996191
ADAMTS4	0.530914179	0.959813459	0.520377275	0.510835945	0.349941843	0.806952045	0.999996191
ADAMTSL1	0.052952465	0.858104562	0.709257314	0.385390971	0.782720725	0.506983833	0.999996191
ADAMTSL3	0.649206374	0.329529734	0.8995582	0.909273605	0.710965252	0.939431913	0.999996191
ADAMTSL4	0.694778329	0.729507898	0.26029236	0.759427157	0.851864994	0.896325153	0.999996191
ADAMTSL5	0.594711424	0.592989118	0.630825086	0.781892038	0.147725836	0.694655549	0.999996191
ADAP1	0.675444646	0.614267196	0.274919778	0.486303669	0.428284974	0.679509226	0.999996191
ADAP2	0.842432465	0.778216939	0.244989458	0.676971551	0.735194032	0.887609718	0.999996191
ADAR	0.587061807	0.439938669	0.415601245	0.340633629	0.784715272	0.715752014	0.999996191
ADAT1	0.472405809	0.762969706	0.744204206	0.703324152	0.204951094	0.771145889	0.999996191
ADCY4	0.730013374	0.365445458	0.118786819	0.70365892	0.712501704	0.601080705	0.999996191
ADCY7	0.810415762	0.575281021	0.837116928	0.100542933	0.494647074	0.640171058	0.999996191
ADCY9	0.668165051	0.921353042	0.695750197	0.70801525	0.492120866	0.955738302	0.999996191
ADD1	0.94259587	0.714173817	0.387652519	0.609972547	0.379910373	0.846810103	0.999996191
ADD3	0.966902441	0.081212868	0.880371383	0.340200614	0.483630869	0.536588107	0.999996191
ADGB	0.365073351	0.085326561	0.747445824	0.572054495	0.783897048	0.520373926	0.999996191
ADGRA1	0.379734127	0.760797116	0.439800955	0.314069836	0.599438233	0.680838484	0.999996191
ADGRA3	0.307416519	0.671856227	0.70321767	0.843428719	0.50710564	0.850983121	0.999996191
ADGRD1	0.646440634	0.450038063	0.082994182	0.469256277	0.698256745	0.469100428	0.999996191
ADGRE5	0.354375279	0.273854816	0.550145495	0.915592025	0.896633445	0.793417316	0.999996191
ADGRF1	0.593205066	0.8164182	0.791558287	0.640281055	0.73295628	0.969401592	0.999996191
ADGRF3	0.352456816	0.161821851	0.610456977	0.75107125	0.513055904	0.568262768	0.999996191
ADGRF5	0.243102271	0.564580169	0.722892382	0.244329598	0.779761431	0.635001423	0.999996191
ADGRG1	0.228189826	0.598964845	0.605313112	0.425723264	0.842521458	0.722144019	0.999996191
ADGRG5	0.180813252	0.807816775	0.611186585	0.505651024	0.777087347	0.753398523	0.999996191
ADGRG6	0.109745799	0.784894391	0.499781981	0.590430761	0.321604764	0.475037523	0.999996191
ADGRL2	0.552182781	0.968603416	0.337877559	0.175915645	0.931562709	0.721759885	0.999996191
ADGRL3	0.675538356	0.384180759	0.687084173	0.63908888	0.13763824	0.598580088	0.999996191
ADH5	0.695917511	0.663666555	0.980928347	0.43920349	0.993886749	0.975159876	0.999996191
ADHFE1	0.541376903	0.790816461	0.541604063	0.096987203	0.974457318	0.663837759	0.999996191
ADI1	0.620143721	0.771396592	0.12466115	0.296456368	0.710195323	0.555486232	0.999996191
ADIPOQ	0.993938836	0.594675282	0.570334222	0.799638214	0.767177301	0.977617922	0.999996191
ADIPOR1	0.569419259	0.771863705	0.182294005	0.344190307	0.775267109	0.65902397	0.999996191
ADIPOR2	0.739749619	0.95852413	0.10348024	0.813790152	0.28511628	0.614560807	0.999996191
ADIRF	0.28034454	0.545554784	0.661191233	0.403174415	0.639181557	0.697360694	0.999996191
ADK	0.6356027	0.50189189	0.666399362	0.431525515	0.757860823	0.867910882	0.999996191

ADNP2	0.23421807	0.326422035	0.547514394	0.972724099	0.678547137	0.708561706	0.999996191
ADORA1	0.462035727	0.839429158	0.389109429	0.119810965	0.65191184	0.54338987	0.999996191
ADORA2A	0.949065897	0.442098476	0.580814324	0.694500786	0.761704801	0.942857291	0.999996191
ADORA2B	0.563285527	0.890398464	0.389039918	0.15029999	0.657284795	0.638821415	0.999996191
ADPRHL2	0.93845827	0.97970877	0.236977139	0.604085333	0.993150136	0.944159891	0.999996191
ADPRM	0.408264584	0.746291623	0.757348471	0.302948215	0.24303695	0.61415939	0.999996191
ADRA1A	0.456074348	0.616394721	0.946042845	0.209093712	0.168635026	0.500275449	0.999996191
ADRB1	0.737674159	0.412584015	0.531923039	0.265771196	0.809628732	0.75211816	0.999996191
ADRB2	0.242662831	0.707717699	0.130389495	0.733259416	0.726128108	0.545572424	0.999996191
ADSS	0.811842788	0.414820769	0.957797872	0.036206407	0.842474274	0.509225223	0.999996191
ADSSL1	0.657343694	0.923254692	0.986861246	0.474440982	0.797408376	0.98213821	0.999996191
AES	0.596047026	0.487132729	0.176598774	0.582575448	0.776839695	0.674960096	0.999996191
AFAP1L1	0.20009579	0.561506381	0.403287751	0.978391547	0.677684667	0.724478884	0.999996191
AFAP1L2	0.139989023	0.534066376	0.759647154	0.974331802	0.217366494	0.547256659	0.999996191
AFDN	0.933819453	0.840237795	0.416104138	0.374230948	0.914465346	0.928402584	0.999996191
AFG1L	0.784922925	0.499672663	0.163462155	0.706791588	0.725431229	0.741361922	0.999996191
AFMID	0.474912316	0.736509369	0.644079704	0.48844752	0.59662269	0.859408343	0.999996191
AGA	0.893097121	0.835516009	0.572735092	0.879963908	0.835260731	0.993294883	0.999996191
AGAP3	0.809398123	0.672525432	0.099614683	0.670848597	0.658048706	0.680972849	0.999996191
AGBL5	0.51690758	0.87473325	0.873661988	0.242563333	0.502208461	0.809514499	0.999996191
AGER	0.423923906	0.363427183	0.701527098	0.414242803	0.542612247	0.683831268	0.999996191
AGK	0.945482215	0.571508672	0.822969436	0.490308336	0.598102831	0.943938966	0.999996191
AGL	0.835292883	0.643056125	0.896960428	0.683204922	0.632611438	0.97798204	0.999996191
AGO2	0.762748717	0.999886732	0.740953446	0.080724495	0.937557873	0.789117849	0.999996191
AGPAT1	0.551740874	0.643312098	0.571435783	0.526692217	0.245829087	0.698837889	0.999996191
AGPAT2	0.606366458	0.844606761	0.603261606	0.058582995	0.520775125	0.501226643	0.999996191
AGPAT3	0.833012687	0.797403474	0.573905496	0.397804495	0.732313558	0.927748692	0.999996191
AGPAT4	0.991335397	0.558156261	0.937785601	0.273409292	0.169485032	0.681840735	0.999996191
AGPAT5	0.6765353	0.248025738	0.283795703	0.85272352	0.746141949	0.726076683	0.999996191
AGT	0.633593318	0.344897034	0.912662275	0.251945259	0.158661347	0.470490534	0.999996191
AGTPBP1	0.111966696	0.422093092	0.655084686	0.681635293	0.345139952	0.45425989	0.999996191
AGTR2	0.488184439	0.6311466	0.445377848	0.072136295	0.885190453	0.487737513	0.999996191
AGTRAP	0.856366048	0.708629284	0.130136002	0.70973655	0.954558667	0.827209102	0.999996191
AHCTF1	0.53020976	0.935436525	0.958398923	0.158818725	0.858866084	0.857527167	0.999996191
AHCYL1	0.530819982	0.787104203	0.131888482	0.540624492	0.877737651	0.698011936	0.999996191
AHDC1	0.510588495	0.6646964	0.532132	0.470524408	0.927015687	0.885609845	0.999996191
AHNAK	0.969910497	0.65950801	0.631543628	0.494755537	0.26739117	0.827026841	0.999996191
AHSA1	0.954071167	0.375925681	0.821828073	0.149133824	0.794413154	0.752570895	0.999996191
AHSP	0.362678559	0.886514244	0.857771631	0.201220506	0.608704962	0.746429685	0.999996191
AIDA	0.886371418	0.693234072	0.552669495	0.168481654	0.953359769	0.830365117	0.999996191
AIF1L	0.242001481	0.851695284	0.695112047	0.77473651	0.45444253	0.817453566	0.999996191
AIFM1	0.819488281	0.484268085	0.849732872	0.059194137	0.632058325	0.556418524	0.999996191
AIG1	0.895322008	0.784052652	0.352805724	0.866821521	0.472391221	0.91758114	0.999996191
AIMP1	0.371000363	0.893847962	0.179680066	0.31613135	0.454184734	0.483344415	0.999996191
AIP	0.087085023	0.602003156	0.492221531	0.867881161	0.813385662	0.627774189	0.999996191

AJM1	0.811167071	0.936162773	0.19694974	0.746207364	0.83918228	0.908085191	0.999996191
AJUBA	0.795423711	0.765880313	0.934650301	0.139860506	0.291280683	0.674878041	0.999996191
AK1	0.505871231	0.961806608	0.351222135	0.389845997	0.295954801	0.643231511	0.999996191
AK3	0.826239549	0.841692438	0.38245642	0.591205795	0.75374729	0.934587117	0.999996191
AK4	0.379446492	0.718805815	0.35621267	0.307176947	0.874395757	0.697623238	0.999996191
AK7	0.931480515	0.829286258	0.496407468	0.497423601	0.51286502	0.913349313	0.999996191
AK9	0.949580177	0.913542053	0.797171948	0.945990079	0.788339881	0.999386163	0.999996191
AKAIN1	0.577742842	0.946177523	0.548283019	0.103979539	0.948928106	0.72149325	0.999996191
AKAP1	0.465514553	0.936489641	0.559509158	0.715916196	0.977071406	0.965840185	0.999996191
AKAP10	0.803627936	0.17912906	0.806114859	0.216967317	0.744377312	0.633327844	0.999996191
AKAP13	0.970513155	0.72172621	0.943551066	0.210538481	0.181922896	0.691770533	0.999996191
AKAP17A	0.720168816	0.809260234	0.45825547	0.672761136	0.711654563	0.942080559	0.999996191
AKAP2	0.434527483	0.419466631	0.542271021	0.265987911	0.980201132	0.695206186	0.999996191
AKAP8	0.411824367	0.855755504	0.995075025	0.447840998	0.832435818	0.944172909	0.999996191
AKIRIN2	0.827227596	0.36586156	0.76166018	0.90497356	0.791841987	0.963544628	0.999996191
AKNA	0.532155585	0.538063504	0.424733359	0.349905335	0.647278866	0.707974716	0.999996191
AKNAD1	0.372653935	0.290491433	0.451530633	0.532221373	0.770527798	0.646458126	0.999996191
AKR1A1	0.536078028	0.842839302	0.971029586	0.138582466	0.212432622	0.560931597	0.999996191
AKT2	0.671773187	0.831678801	0.597543508	0.304641319	0.534924808	0.829937414	0.999996191
AKT3	0.591983104	0.738618131	0.964992471	0.04260841	0.478717123	0.484437189	0.999996191
ALAD	0.884807848	0.478732598	0.761400796	0.547754652	0.391563314	0.867174253	0.999996191
ALDH16A1	0.363715398	0.961731246	0.476784132	0.449397165	0.322523971	0.682867397	0.999996191
ALDH1A1	0.79511662	0.992226597	0.079657099	0.933871195	0.740769122	0.791990718	0.999996191
ALDH1A3	0.948542034	0.095181792	0.170803584	0.795966463	0.90576893	0.532254276	0.999996191
ALDH1L1	0.198431873	0.796014773	0.44816368	0.94595287	0.379576018	0.692559813	0.999996191
ALDH1L2	0.21112917	0.719906489	0.159454935	0.935482725	0.370254358	0.479874189	0.999996191
ALDH2	0.246938992	0.953389218	0.478181812	0.691702958	0.994278286	0.883233732	0.999996191
ALDH3B1	0.419300713	0.505788325	0.590721214	0.426110282	0.188049369	0.512985945	0.999996191
ALDH4A1	0.883247968	0.541932694	0.725048224	0.107627336	0.623682128	0.67571101	0.999996191
ALDH6A1	0.585386821	0.773399747	0.327838629	0.921267618	0.926828731	0.941227851	0.999996191
ALDH7A1	0.93057676	0.769022906	0.273223482	0.262791601	0.453518813	0.675771508	0.999996191
ALDH9A1	0.871480855	0.363543947	0.494607268	0.405357581	0.374426312	0.679726127	0.999996191
ALDOA	0.810274471	0.820662038	0.596200286	0.203725424	0.481450403	0.772168955	0.999996191
ALDOC	0.806032853	0.777788942	0.359008321	0.229939199	0.438626114	0.67068229	0.999996191
ALG1	0.694092535	0.549958341	0.394026404	0.510455931	0.913485852	0.869190761	0.999996191
ALG10	0.367776545	0.414745141	0.661487139	0.785012516	0.463895193	0.761899766	0.999996191
ALG14	0.712248431	0.236268833	0.828115303	0.31668968	0.767381722	0.746905948	0.999996191
ALG2	0.644780662	0.52750153	0.999881834	0.698623113	0.22405179	0.826378282	0.999996191
ALG6	0.2600398	0.973372667	0.951751787	0.91114627	0.291284795	0.855398995	0.999996191
ALKAL2	0.221354562	0.297043577	0.545803521	0.957651841	0.236421317	0.473936189	0.999996191
ALKBH2	0.366809562	0.965787943	0.245088466	0.904050965	0.930817413	0.875090939	0.999996191
ALKBH3	0.961679163	0.507598684	0.235490606	0.560346993	0.583510218	0.76602522	0.999996191
ALKBH6	0.518736542	0.83094054	0.686146103	0.303456521	0.998945168	0.902646635	0.999996191
ALKBH7	0.383250457	0.635928257	0.670436324	0.19273177	0.909830267	0.71549354	0.999996191
ALMS1	0.390955438	0.447898255	0.571582219	0.432001629	0.174512681	0.460579063	0.999996191

ALOX12	0.128422784	0.43365974	0.559258072	0.763893692	0.9617506	0.672234651	0.999996191
ALOX5	0.579743318	0.943440634	0.149464565	0.720086315	0.948642668	0.834171011	0.999996191
ALPK2	0.867138316	0.892253107	0.818348658	0.800799363	0.501191046	0.986900299	0.999996191
ALPL	0.561927674	0.827655679	0.31341184	0.426109595	0.241354242	0.589763718	0.999996191
ALS2CL	0.422741389	0.195787845	0.533063265	0.603686201	0.518167543	0.573727623	0.999996191
ALYREF	0.259288704	0.244392783	0.83465298	0.965748796	0.509811144	0.697217621	0.999996191
AMACR	0.421110613	0.957044429	0.28485144	0.766141458	0.814487491	0.872263492	0.999996191
AMBRA1	0.582921587	0.902991808	0.651115053	0.390554228	0.585348606	0.884872713	0.999996191
AMDHD2	0.693136069	0.893232586	0.149823365	0.752081828	0.723624644	0.817588746	0.999996191
AMER1	0.571334614	0.084251169	0.556586194	0.610270073	0.518446985	0.481653127	0.999996191
AMFR	0.87969013	0.354855999	0.365007686	0.53966483	0.934294746	0.838718267	0.999996191
AMH	0.196552717	0.832329546	0.780357266	0.448593016	0.508645761	0.718631147	0.999996191
AMHR2	0.646681462	0.118761777	0.468314029	0.747342309	0.949239836	0.693296928	0.999996191
AMIGO1	0.453297925	0.359408888	0.170005137	0.714697101	0.992738208	0.64258562	0.999996191
AMMECR1L	0.787081407	0.779998333	0.774852511	0.02998568	0.887553871	0.557079848	0.999996191
AMN1	0.541127297	0.902054217	0.215904957	0.923864036	0.938761673	0.905071819	0.999996191
AMOT	0.849757837	0.760190137	0.360098628	0.079872687	0.60852404	0.53544589	0.999996191
AMPH	0.845950471	0.305714526	0.667463756	0.653407504	0.691389532	0.884223148	0.999996191
AMY2B	0.420764249	0.322017913	0.119391343	0.697201035	0.687083963	0.465363832	0.999996191
AMZ2	0.780308012	0.440575797	0.13557333	0.533928618	0.544063116	0.570019665	0.999996191
ANAPC1	0.737721208	0.843490689	0.61543248	0.068868259	0.641029468	0.613198932	0.999996191
ANAPC13	0.511951048	0.692989571	0.695366412	0.07585619	0.578617885	0.527249257	0.999996191
ANAPC15	0.258199622	0.423089802	0.444117722	0.278183081	0.975896148	0.564697208	0.999996191
ANAPC16	0.866680857	0.412896736	0.403425174	0.726793813	0.956774516	0.916400426	0.999996191
ANAPC5	0.734811136	0.789663997	0.466934556	0.420387775	0.824478292	0.908415648	0.999996191
ANAPC7	0.54876668	0.841331377	0.837027642	0.611186607	0.474136468	0.928649106	0.999996191
ANGEL1	0.576572329	0.397756543	0.571149369	0.880415923	0.940884204	0.925216307	0.999996191
ANGEL2	0.212260228	0.886832641	0.386400366	0.380691638	0.852862188	0.678352608	0.999996191
ANGPT1	0.653256172	0.359433279	0.913524722	0.696990043	0.549930299	0.891390962	0.999996191
ANGPT4	0.420373653	0.877598385	0.214342398	0.492945463	0.72795269	0.713644485	0.999996191
ANGPTL1	0.400928471	0.710553174	0.627329547	0.681619551	0.892886998	0.925482731	0.999996191
ANGPTL2	0.477499746	0.150606835	0.287148636	0.771631591	0.573625123	0.495523248	0.999996191
ANGPTL6	0.58359056	0.37981946	0.351426235	0.546504652	0.262519735	0.533240558	0.999996191
ANK1	0.13352278	0.771153156	0.737195257	0.502327669	0.523382498	0.645594345	0.999996191
ANK2	0.894796981	0.854523878	0.812014432	0.295190459	0.798669791	0.95414764	0.999996191
ANKAR	0.107007971	0.269988622	0.908012181	0.930800911	0.953041514	0.675506116	0.999996191
ANKEF1	0.866353955	0.973272772	0.342986954	0.386728446	0.382957136	0.789375854	0.999996191
ANKFY1	0.971283413	0.957812853	0.860610092	0.488530263	0.663171425	0.987650316	0.999996191
ANKH	0.671759424	0.585124867	0.617030125	0.957842179	0.920253796	0.979337142	0.999996191
ANKIB1	0.822655893	0.36973795	0.446135802	0.530289461	0.66717039	0.809118409	0.999996191
ANKLE1	0.990089536	0.86184444	0.577389956	0.281241746	0.158178642	0.663867153	0.999996191
ANKRD10	0.828848826	0.529865319	0.784791486	0.777019791	0.524524226	0.950646067	0.999996191
ANKRD13A	0.773646367	0.964489847	0.676805431	0.621022101	0.888965823	0.990057407	0.999996191
ANKRD13D	0.71790189	0.253218893	0.85442367	0.937634944	0.491774722	0.872228431	0.999996191
ANKRD16	0.604950689	0.659516435	0.609867133	0.162660156	0.697770154	0.708477841	0.999996191

ANKRD2	0.712027125	0.754086184	0.539996933	0.123728969	0.762829978	0.706732591	0.999996191
ANKRD24	0.452330277	0.900553514	0.944410859	0.019554725	0.927421852	0.44662641	0.999996191
ANKRD26	0.646979209	0.354031814	0.298043932	0.831968132	0.578760531	0.741362373	0.999996191
ANKRD28	0.983461929	0.690937514	0.69644236	0.875480259	0.853280947	0.995692835	0.999996191
ANKRD33	0.214966481	0.554791746	0.175096505	0.707015113	0.605912288	0.491547103	0.999996191
ANKRD34A	0.443073832	0.20922022	0.404592512	0.858466201	0.907086112	0.719126311	0.999996191
ANKRD35	0.783709545	0.591479108	0.903142601	0.342742723	0.637007007	0.905073319	0.999996191
ANKRD37	0.445683529	0.892151077	0.115472948	0.707253737	0.758301627	0.686442191	0.999996191
ANKRD40	0.858377692	0.39999122	0.091040861	0.786302754	0.988149151	0.683779275	0.999996191
ANKRD42	0.194289695	0.673382439	0.597754497	0.235829871	0.452156724	0.478665444	0.999996191
ANKRD46	0.792834039	0.595392068	0.735317732	0.754826394	0.39206285	0.919061025	0.999996191
ANKRD49	0.747893502	0.944655694	0.73969795	0.509921257	0.686895412	0.97051122	0.999996191
ANKRD50	0.937445765	0.69664465	0.408725861	0.439029267	0.386979628	0.799339524	0.999996191
ANKRD6	0.92537872	0.942720618	0.34163054	0.750756517	0.059422972	0.566515109	0.999996191
ANKRD61	0.721342193	0.229148122	0.929184572	0.736517197	0.152578347	0.617238537	0.999996191
ANKRD9	0.309718299	0.994924643	0.790878949	0.836618248	0.972333036	0.975298614	0.999996191
ANKS1A	0.507049783	0.93818775	0.584540735	0.809951397	0.408845415	0.905993271	0.999996191
ANKS3	0.523206265	0.572218733	0.125983043	0.258592066	0.785209715	0.463245633	0.999996191
ANO10	0.931542211	0.8487608	0.237250576	0.94691531	0.487845061	0.898296249	0.999996191
ANO3	0.725464677	0.808951178	0.22598246	0.17390209	0.986635029	0.671150949	0.999996191
ANTXR1	0.62710108	0.905607974	0.492337624	0.844757445	0.835563729	0.975042511	0.999996191
ANTXR2	0.840203319	0.17015808	0.683653596	0.648472918	0.818141125	0.822055408	0.999996191
ANXA2	0.621131063	0.959551878	0.636854011	0.866289148	0.663896644	0.980375281	0.999996191
ANXA4	0.97598941	0.710372855	0.19893684	0.220759888	0.253144014	0.464399807	0.999996191
ANXA5	0.575376009	0.723048728	0.27972619	0.496531655	0.633702138	0.76127013	0.999996191
AOC1	0.7375043	0.816803043	0.332825407	0.437908919	0.471959845	0.783530114	0.999996191
AOC2	0.656422225	0.188681871	0.7298797	0.114979707	0.853786906	0.490069361	0.999996191
AOC3	0.47115945	0.418515173	0.705541532	0.91740255	0.843489347	0.924351487	0.999996191
AOX2	0.568724699	0.081818234	0.408451888	0.923530795	0.457169271	0.471672667	0.999996191
AP1AR	0.790596206	0.577545889	0.078046833	0.883846109	0.478244487	0.590704544	0.999996191
AP1G1	0.807409174	0.229064201	0.673397176	0.382946752	0.518687751	0.687333238	0.999996191
AP1S1	0.329932726	0.570192341	0.29986481	0.334883617	0.539902553	0.515972539	0.999996191
AP1S3	0.95288336	0.515200156	0.909164619	0.072499104	0.549655351	0.623105745	0.999996191
AP2A1	0.419514934	0.850548704	0.844127805	0.40915253	0.67237919	0.892432826	0.999996191
AP2A2	0.412232012	0.89490544	0.961442863	0.310933963	0.066658361	0.455911539	0.999996191
AP2S1	0.594935496	0.52164138	0.738894956	0.11424433	0.606740491	0.601167093	0.999996191
AP3B1	0.689335914	0.233555873	0.932672483	0.217509353	0.635739599	0.653331066	0.999996191
AP3D1	0.472399576	0.716132567	0.582759326	0.121669939	0.536070845	0.560072817	0.999996191
AP3M1	0.365152604	0.409799657	0.400475029	0.956353562	0.778033579	0.796411066	0.999996191
AP3S1	0.107094117	0.164460774	0.885885909	0.88493637	0.854794578	0.54363737	0.999996191
AP3S2	0.620123851	0.527694345	0.289996319	0.168521505	0.697776237	0.532956047	0.999996191
AP4M1	0.625017165	0.070442778	0.850961598	0.686778031	0.311576792	0.471505057	0.999996191
AP4S1	0.982043424	0.097563982	0.740473608	0.923183377	0.95039193	0.851295771	0.999996191
AP5M1	0.249711154	0.824991915	0.898050813	0.364100356	0.76887352	0.821853682	0.999996191
AP5S1	0.749563686	0.860621375	0.327191322	0.161706801	0.890198301	0.726604449	0.999996191

APBA1	0.591353799	0.944964675	0.445543379	0.677682381	0.265007148	0.796890669	0.999996191
APBB2	0.782643368	0.806509867	0.526217991	0.90397727	0.980481027	0.991644414	0.999996191
APC	0.719689186	0.674464656	0.133292041	0.707221568	0.974156477	0.796352756	0.999996191
APCDD1L	0.879587623	0.339614813	0.402823451	0.888778979	0.256151603	0.706936568	0.999996191
APEH	0.948032423	0.979162618	0.524095308	0.300339239	0.731012677	0.923478105	0.999996191
APEX2	0.44625953	0.587070379	0.583919069	0.071149296	0.855300532	0.498918413	0.999996191
APIP	0.54707451	0.199210466	0.264685043	0.907879194	0.785368737	0.651481871	0.999996191
APLN	0.894119314	0.64261551	0.312624202	0.434044366	0.663555167	0.821668851	0.999996191
APLNR	0.805993059	0.965682143	0.124818206	0.117468635	0.952098859	0.527901172	0.999996191
APMAP	0.246628098	0.956905072	0.504932307	0.634571045	0.240521864	0.627468188	0.999996191
APOBEC3Z2	0.502426159	0.494339554	0.474415135	0.935865823	0.515049438	0.83688819	0.999996191
APOBR	0.154880247	0.760133247	0.240090525	0.884399976	0.366195071	0.495804198	0.999996191
APOD	0.096606555	0.416984379	0.380032992	0.890514139	0.703286547	0.504399398	0.999996191
APOE	0.825496487	0.337279784	0.243502442	0.325310937	0.537921928	0.544626041	0.999996191
APOL3	0.838899777	0.603395084	0.620452203	0.676711053	0.447262724	0.909899476	0.999996191
APOL3	0.141327209	0.894105604	0.987418668	0.934731815	0.105211091	0.551077944	0.999996191
APOM	0.466291785	0.901623317	0.418721499	0.180350852	0.834723871	0.700585646	0.999996191
APOO	0.947048364	0.356501181	0.728539853	0.112964849	0.630865114	0.620265397	0.999996191
APOOL	0.764709642	0.78081216	0.916869467	0.1564664	0.885383762	0.880359994	0.999996191
APOPT1	0.35636742	0.604258113	0.11442992	0.719528888	0.8795027	0.597436254	0.999996191
APP	0.380743437	0.40055305	0.750285965	0.482834815	0.53503032	0.721407381	0.999996191
APPBP2	0.24864082	0.363705266	0.789927638	0.587635963	0.803066442	0.746049975	0.999996191
APPL1	0.800680595	0.27258839	0.935600315	0.243392189	0.795521417	0.775161841	0.999996191
APPL2	0.653103775	0.78591788	0.336717944	0.407377463	0.564589773	0.776128002	0.999996191
APTX	0.695324012	0.880315613	0.943967463	0.125915589	0.779885213	0.836731116	0.999996191
AQP11	0.326564708	0.899808542	0.246505237	0.984387865	0.295623625	0.656267698	0.999996191
AQP7	0.847626854	0.924813279	0.480774777	0.669243667	0.453833416	0.930975289	0.999996191
AQR	0.804644899	0.923655363	0.801744257	0.209731673	0.762922095	0.910264967	0.999996191
AR	0.94262586	0.119510231	0.204098078	0.585897755	0.551744075	0.457876602	0.999996191
ARAF	0.390507337	0.858587951	0.469707723	0.27066139	0.645176572	0.707673818	0.999996191
ARAP1	0.659978861	0.56537004	0.505442323	0.920685281	0.941642014	0.962799208	0.999996191
ARAP3	0.599140233	0.334748889	0.741918837	0.984806583	0.489305362	0.872394517	0.999996191
ARCN1	0.619252101	0.17826773	0.415146335	0.502084616	0.457763682	0.522031887	0.999996191
ARF2	0.101592999	0.718099329	0.521059658	0.838317732	0.687583445	0.663808738	0.999996191
ARF4	0.70603738	0.736233029	0.645255242	0.943572642	0.832471616	0.988200374	0.999996191
ARF5	0.112593394	0.971628882	0.838174649	0.101083993	0.921029342	0.482950401	0.999996191
ARF6	0.557054273	0.343113234	0.950381998	0.436848554	0.310446527	0.686525975	0.999996191
ARFGAP3	0.94639019	0.253121042	0.463766134	0.453285359	0.71115596	0.757204769	0.999996191
ARFGEF2	0.735321507	0.944427966	0.367964919	0.477649161	0.968267506	0.934292975	0.999996191
ARFGEF3	0.638167944	0.543937678	0.257372999	0.962337967	0.284549335	0.685183302	0.999996191
ARHGAP10	0.936266502	0.333645281	0.267872016	0.398687142	0.599459684	0.646007447	0.999996191
ARHGAP12	0.784022542	0.625985817	0.94907729	0.32689978	0.455913126	0.86769678	0.999996191
ARHGAP15	0.119112019	0.746656829	0.348463107	0.906725665	0.929563532	0.697809165	0.999996191
ARHGAP17	0.901195287	0.584200333	0.784627194	0.651316161	0.690329611	0.971426379	0.999996191
ARHGAP18	0.979003314	0.907411197	0.136918147	0.775929433	0.453702312	0.789326748	0.999996191

ARHGAP21	0.930280375	0.408863646	0.957291312	0.246875019	0.290331976	0.697641086	0.999996191
ARHGAP22	0.347931765	0.937233476	0.284143656	0.439468573	0.484526993	0.643365272	0.999996191
ARHGAP24	0.64093942	0.43403201	0.509788273	0.155466672	0.635802466	0.576744479	0.999996191
ARHGAP29	0.265232215	0.860296182	0.996614072	0.058702287	0.58650761	0.467219964	0.999996191
ARHGAP31	0.325211017	0.953546505	0.622387419	0.597527792	0.597153131	0.866520933	0.999996191
ARHGAP32	0.903875034	0.940000167	0.241924887	0.878890524	0.995475485	0.969376642	0.999996191
ARHGAP35	0.989570835	0.98602615	0.077738285	0.246042342	0.957865878	0.624096886	0.999996191
ARHGAP39	0.681618265	0.817935268	0.778927411	0.460365428	0.864252491	0.966708512	0.999996191
ARHGAP44	0.168533194	0.438493296	0.53246357	0.335633454	0.832263477	0.530089786	0.999996191
ARHGAP6	0.571890405	0.789629658	0.838959652	0.746531492	0.24746028	0.868888093	0.999996191
ARHGEF1	0.760569599	0.174405944	0.146053791	0.774178	0.885713645	0.566352711	0.999996191
ARHGEF10	0.486725079	0.971376803	0.060171449	0.960146462	0.935890739	0.693662558	0.999996191
ARHGEF11	0.763825225	0.23041873	0.732501023	0.284509787	0.702902695	0.695296664	0.999996191
ARHGEF12	0.761208991	0.9524002	0.436408394	0.205268594	0.948991982	0.849759272	0.999996191
ARHGEF12	0.524476385	0.37485145	0.477689707	0.807544882	0.107317336	0.474240841	0.999996191
ARHGEF15	0.961149152	0.672893597	0.936459339	0.995089782	0.77550287	0.998867723	0.999996191
ARHGEF18	0.581223515	0.426450441	0.381316666	0.470876982	0.700639712	0.731487101	0.999996191
ARHGEF28	0.163264078	0.633882295	0.538234801	0.637369539	0.739993547	0.698915614	0.999996191
ARHGEF3	0.394007164	0.906028174	0.936485051	0.548940032	0.289917168	0.826291235	0.999996191
ARHGEF37	0.648657787	0.721536789	0.893791911	0.061965527	0.472878821	0.550877555	0.999996191
ARHGEF38	0.221182386	0.849919207	0.575314431	0.915377971	0.411069089	0.780321738	0.999996191
ARHGEF39	0.809304771	0.296794229	0.128740831	0.624371773	0.897845546	0.618089902	0.999996191
ARHGEF4	0.303670125	0.846314787	0.061708225	0.807435222	0.780023638	0.512045805	0.999996191
ARHGEF40	0.913054107	0.713626529	0.070817041	0.412374318	0.861806244	0.607248049	0.999996191
ARHGEF6	0.791135434	0.442595472	0.904449453	0.173610723	0.228745462	0.555809778	0.999996191
ARHGEF7	0.516208053	0.454134562	0.560901137	0.820717202	0.718159401	0.883370386	0.999996191
ARID1A	0.655523297	0.34947848	0.291693043	0.958040032	0.472916116	0.725937482	0.999996191
ARID1B	0.44820762	0.249390957	0.768779257	0.283780288	0.99368101	0.683339973	0.999996191
ARID2	0.680101349	0.797240935	0.753942815	0.065524052	0.620134016	0.609755491	0.999996191
ARID3B	0.560578437	0.535364124	0.773608925	0.213585169	0.895084725	0.795611522	0.999996191
ARID5B	0.913977208	0.743087061	0.329984859	0.972213824	0.678374798	0.954951106	0.999996191
ARIH2	0.288272807	0.834026078	0.272055638	0.710144506	0.204319329	0.502502787	0.999996191
ARL1	0.617777456	0.476926048	0.259978123	0.710404074	0.478137545	0.697052457	0.999996191
ARL13A	0.483165066	0.356595748	0.870971232	0.985461522	0.405214809	0.845382819	0.999996191
ARL13B	0.198252569	0.88279783	0.871233971	0.542042951	0.479278862	0.775501086	0.999996191
ARL15	0.071209895	0.629358054	0.865412339	0.247483316	0.913513948	0.487859876	0.999996191
ARL16	0.289154077	0.987587036	0.723506162	0.89915163	0.991612275	0.970912471	0.999996191
ARL2	0.154694926	0.68645235	0.740344194	0.18143229	0.609932406	0.48641856	0.999996191
ARL2BP	0.786000194	0.624912135	0.1491004	0.462377951	0.909102593	0.729074935	0.999996191
ARL3	0.988841704	0.240135475	0.542744731	0.400695536	0.997636635	0.820975328	0.999996191
ARL4A	0.259907399	0.21267761	0.738799705	0.785835845	0.419211822	0.56879135	0.999996191
ARL4D	0.486030468	0.992646751	0.63738748	0.680373712	0.107656468	0.669171813	0.999996191
ARL5A	0.838495613	0.652723607	0.647408724	0.591929594	0.796346195	0.96433952	0.999996191
ARL6	0.226108929	0.760238549	0.908723569	0.752614642	0.509623274	0.84535425	0.999996191
ARL6IP4	0.255338042	0.945029558	0.287404176	0.437889364	0.405329179	0.551679206	0.999996191

ARL6IP5	0.948594088	0.328346728	0.347236788	0.580198013	0.779474434	0.812275467	0.999996191
ARL6IP6	0.341067817	0.944638366	0.999686255	0.147255128	0.976555582	0.8029798	0.999996191
ARMC5	0.724370922	0.848735861	0.39575661	0.860202367	0.778019756	0.962498434	0.999996191
ARMC7	0.488487228	0.645415865	0.308874445	0.477136115	0.37321004	0.618153615	0.999996191
ARMCX5	0.4512059	0.941635837	0.887577848	0.523181257	0.838874149	0.963683712	0.999996191
ARMH3	0.994826302	0.468726124	0.367605447	0.04663456	0.940713143	0.459966474	0.999996191
ARMH4	0.410634056	0.518132948	0.384991295	0.826232278	0.230446548	0.597469419	0.999996191
ARMT1	0.977550663	0.385457084	0.779075069	0.725238978	0.433401487	0.906248363	0.999996191
ARNT	0.371767838	0.630208698	0.9292845	0.227733301	0.957953287	0.807293512	0.999996191
ARNT2	0.369768888	0.86762449	0.185149189	0.701342857	0.347492531	0.582982132	0.999996191
ARPC1A	0.748885766	0.641647393	0.564736749	0.119664093	0.466378905	0.59175142	0.999996191
ARPC2	0.684307981	0.258746525	0.392783061	0.867968896	0.418433792	0.691349736	0.999996191
ARPC5L	0.916395694	0.282064411	0.241952389	0.744190997	0.351606035	0.606841762	0.999996191
ARPIN	0.550500245	0.863516244	0.535163371	0.843053479	0.730264973	0.959565422	0.999996191
ARRB1	0.464294143	0.571944295	0.528905243	0.566709609	0.147618415	0.542778285	0.999996191
ARRDC1	0.425203456	0.667656399	0.547427728	0.462100188	0.769957993	0.832573023	0.999996191
ARRDC2	0.862053505	0.885723445	0.226497307	0.410938557	0.914443834	0.857873334	0.999996191
ARRDC3	0.724459915	0.946063955	0.971999266	0.131176845	0.119043434	0.519689798	0.999996191
ARSB	0.549261764	0.257942169	0.320056397	0.476461648	0.89357384	0.639121328	0.999996191
ARSG	0.250869518	0.7687464	0.162384708	0.658579427	0.986156046	0.649299096	0.999996191
ARSJ	0.374813751	0.654203186	0.231098275	0.978208748	0.922230845	0.819685001	0.999996191
ARSK	0.162952688	0.866497503	0.642554666	0.528193209	0.928687629	0.796075953	0.999996191
ART4	0.384630215	0.807264752	0.514022202	0.445374095	0.386690186	0.707578243	0.999996191
ARV1	0.413817156	0.965696756	0.330625811	0.294133253	0.849840844	0.742241156	0.999996191
AS3MT	0.878811151	0.941080441	0.334353127	0.842287174	0.670880789	0.95938469	0.999996191
ASAH1	0.933229777	0.975375416	0.15941883	0.64591173	0.697299213	0.858729674	0.999996191
ASAP1	0.451471636	0.06325425	0.741649757	0.955011872	0.826370919	0.610975443	0.999996191
ASAP2	0.608154092	0.694343827	0.203462429	0.698426599	0.198528749	0.545413842	0.999996191
ASB12	0.736534524	0.988375738	0.277682202	0.28611866	0.172610028	0.511952155	0.999996191
ASB14	0.315448772	0.708598248	0.89857596	0.778198668	0.336386668	0.824346657	0.999996191
ASB16	0.158058127	0.675164691	0.509646402	0.892054867	0.723338871	0.753479588	0.999996191
ASB2	0.544414036	0.607276929	0.966763689	0.357525599	0.689514215	0.885642563	0.999996191
ASB3	0.49150197	0.785811777	0.491411336	0.110987637	0.518152753	0.528761957	0.999996191
ASB4	0.50158017	0.925994295	0.805511348	0.070895185	0.865306068	0.67281922	0.999996191
ASB5	0.678133003	0.938829783	0.877834895	0.629155073	0.778687925	0.989486982	0.999996191
ASB6	0.449954154	0.674986771	0.153199167	0.615484988	0.670698418	0.638122105	0.999996191
ASB7	0.406811296	0.308464677	0.952252985	0.657948842	0.921441497	0.873875376	0.999996191
ASB8	0.71722114	0.393634239	0.744115949	0.118368178	0.750920488	0.632612639	0.999996191
ASB9	0.829246312	0.970638994	0.771602639	0.987943283	0.058480373	0.75756066	0.999996191
ASCC3	0.825498261	0.504763767	0.760868424	0.176972473	0.662770543	0.76408077	0.999996191
ASCL4	0.620043841	0.454323018	0.97919569	0.080305454	0.622552001	0.573574619	0.999996191
ASF1A	0.905747868	0.153433292	0.645783951	0.221004431	0.409268842	0.473762457	0.999996191
ASH1L	0.554710633	0.960416294	0.977305245	0.145680174	0.692038372	0.824070889	0.999996191
ASH2L	0.561283349	0.261375697	0.925393521	0.166452151	0.997441875	0.669306658	0.999996191
ASL	0.395748171	0.926086	0.400418371	0.244236426	0.395833266	0.579073034	0.999996191

ASMT	0.555107128	0.501331145	0.269724885	0.522749657	0.665650107	0.697797597	0.999996191
ASNS	0.595768408	0.951340182	0.382430334	0.527099058	0.081392019	0.498713355	0.999996191
ASNSD1	0.581232975	0.573977141	0.815090068	0.172462252	0.567844497	0.701515157	0.999996191
ASPH	0.937369085	0.793461752	0.405946298	0.83721168	0.883208892	0.981450673	0.999996191
ASPN	0.721400714	0.780035067	0.754108966	0.781794	0.9182792	0.992546615	0.999996191
ASRGL1	0.896242186	0.439403668	0.353770607	0.325809555	0.249117749	0.535474084	0.999996191
ASXL1	0.550884703	0.300329954	0.694110096	0.553564215	0.850648451	0.828952566	0.999996191
ASXL2	0.563392885	0.478593688	0.79100426	0.455615878	0.182373497	0.622402432	0.999996191
ASXL3	0.472576469	0.380088553	0.253444036	0.814049235	0.719201876	0.701676978	0.999996191
ATAD1	0.744654794	0.079842122	0.483703816	0.675018375	0.676630299	0.564170143	0.999996191
ATAD2	0.875503263	0.983105343	0.59316733	0.231555187	0.831334586	0.913901802	0.999996191
ATAD5	0.747386798	0.763850564	0.431120824	0.885111684	0.906934627	0.975104757	0.999996191
ATAT1	0.633787426	0.99211048	0.503848317	0.485418183	0.316153787	0.811263003	0.999996191
ATF2	0.178150848	0.874386409	0.285652772	0.649432695	0.804156938	0.67523053	0.999996191
ATF5	0.644755625	0.55607198	0.879267606	0.394514904	0.381079989	0.80692059	0.999996191
ATF6B	0.993747493	0.473744545	0.378266631	0.782427855	0.744198216	0.920137782	0.999996191
ATF7	0.114244869	0.908917646	0.488390158	0.332317401	0.675535828	0.536766489	0.999996191
ATF7IP	0.073356085	0.746420626	0.483161309	0.561799487	0.834270296	0.553081186	0.999996191
ATF7IP2	0.940160718	0.223305459	0.848821858	0.965834807	0.388631712	0.862206048	0.999996191
ATG10	0.84420742	0.765124593	0.48084091	0.705978662	0.958932843	0.978488483	0.999996191
ATG101	0.735313897	0.890325622	0.659538047	0.219658249	0.88628047	0.894328951	0.999996191
ATG12	0.931116286	0.203939763	0.426176409	0.947466658	0.974722003	0.878303049	0.999996191
ATG16L2	0.680841415	0.623229796	0.891408724	0.323707526	0.726545059	0.901658944	0.999996191
ATG2A	0.261333447	0.159732233	0.530821926	0.711344869	0.901887887	0.579457901	0.999996191
ATG2B	0.545694333	0.662839937	0.490351386	0.434305492	0.560453445	0.790765596	0.999996191
ATG4A	0.836218774	0.968787004	0.536608515	0.391260074	0.334307869	0.837072287	0.999996191
ATG4C	0.692840018	0.508105544	0.477290614	0.573305587	0.950479266	0.905284455	0.999996191
ATG4D	0.271736425	0.309490551	0.443124982	0.820397759	0.964887491	0.721026379	0.999996191
ATG5	0.199536786	0.395641221	0.485279827	0.559812486	0.88530714	0.635868238	0.999996191
ATG7	0.942755088	0.8305239	0.646361737	0.359641995	0.613251631	0.928294124	0.999996191
ATG9A	0.330752228	0.419904436	0.359286852	0.956559027	0.246092055	0.542724653	0.999996191
ATL1	0.664828207	0.343011935	0.907402012	0.908112147	0.634237539	0.935151232	0.999996191
ATL3	0.738468826	0.220750077	0.495361153	0.713431992	0.965676025	0.833562894	0.999996191
ATM	0.65430294	0.623731775	0.464311297	0.778755511	0.93465724	0.94904023	0.999996191
ATMIN	0.455754674	0.218297176	0.815765391	0.301312881	0.889441758	0.662379373	0.999996191
ATN1	0.857095485	0.868301379	0.151070553	0.547784363	0.560972386	0.75059339	0.999996191
ATOH8	0.289146583	0.627325019	0.228997905	0.631011977	0.634754214	0.610068874	0.999996191
ATOX1	0.625200644	0.647397627	0.4787749	0.299080178	0.98182279	0.83719198	0.999996191
ATP10A	0.845595054	0.702867273	0.401822439	0.977636585	0.842188722	0.974832448	0.999996191
ATP11A	0.350814907	0.67863393	0.718783343	0.962969866	0.490984399	0.889240289	0.999996191
ATP11B	0.433538557	0.901622666	0.838456759	0.177176187	0.812669452	0.806183314	0.999996191
ATP12A	0.919403031	0.84221814	0.407298677	0.324620067	0.168990909	0.617709242	0.999996191
ATP13A1	0.249020581	0.798636236	0.871267995	0.293722443	0.85423318	0.791996182	0.999996191
ATP13A3	0.884007729	0.833879222	0.210509714	0.239257162	0.530112424	0.642888148	0.999996191
ATP1B2	0.704039066	0.379862846	0.736444649	0.736589318	0.136141603	0.643569504	0.999996191

ATP1B3	0.276558859	0.360292812	0.666939527	0.86394931	0.448005784	0.694848851	0.999996191
ATP23	0.648219319	0.562701319	0.567406862	0.890404113	0.905582948	0.964278549	0.999996191
ATP2A1	0.817848103	0.62673372	0.159752701	0.842734072	0.426237492	0.720469493	0.999996191
ATP2A3	0.525982874	0.900967023	0.368223398	0.845667591	0.994637205	0.954372137	0.999996191
ATP2B1	0.74431448	0.65854816	0.648506015	0.719373773	0.968245691	0.981057564	0.999996191
ATP2B4	0.503601011	0.753250259	0.578054944	0.740709855	0.9713441	0.96012509	0.999996191
ATP2C1	0.909224114	0.224149582	0.164813925	0.694186222	0.804960125	0.633619187	0.999996191
ATP2C2	0.841380727	0.38477972	0.388850341	0.703717869	0.951411016	0.894684569	0.999996191
ATP5F1A	0.962819147	0.671508745	0.862295667	0.20664624	0.550071261	0.854046054	0.999996191
ATP5F1B	0.871469062	0.51833192	0.758521817	0.083905752	0.520296719	0.589338031	0.999996191
ATP5F1C	0.669171523	0.532881946	0.727058962	0.065410345	0.635467788	0.526345857	0.999996191
ATP5MC2	0.870282139	0.561697047	0.664580474	0.47608074	0.549096858	0.895676786	0.999996191
ATP5MG	0.920486039	0.441408633	0.888042119	0.082048374	0.954728221	0.712899049	0.999996191
ATP5PB	0.789664848	0.45575807	0.741184669	0.193766647	0.488629843	0.691328799	0.999996191
ATP5PF	0.73459409	0.19891771	0.859344398	0.074479967	0.834151979	0.466573043	0.999996191
ATP5PO	0.905814062	0.526448308	0.756034034	0.037432658	0.878958766	0.544594593	0.999996191
ATP5S	0.595521875	0.886002515	0.26786366	0.673658202	0.709144052	0.863597771	0.999996191
ATP6AP1L	0.474573214	0.835196823	0.658056463	0.639495964	0.348459585	0.840569314	0.999996191
ATP6AP2	0.508615206	0.447732378	0.12758225	0.788134128	0.962408809	0.664923188	0.999996191
ATP6V0A2	0.896066974	0.947441168	0.828423181	0.992515257	0.216707749	0.956832199	0.999996191
ATP6V0D2	0.867723783	0.6153569	0.210034814	0.198578357	0.956247114	0.658264544	0.999996191
ATP6V1D	0.959323977	0.742732441	0.490275549	0.329510276	0.599928664	0.866925197	0.999996191
ATP6V1E1	0.297109233	0.364680646	0.404203039	0.578059392	0.493125172	0.554388676	0.999996191
ATP6V1F	0.472422187	0.548230898	0.42471511	0.151398067	0.918415411	0.593673345	0.999996191
ATP6V1G1	0.530214335	0.111871244	0.548798645	0.928991206	0.96142026	0.718269369	0.999996191
ATP6V1H	0.793341021	0.63908912	0.196678663	0.53467248	0.163349593	0.486616521	0.999996191
ATP8A1	0.852563668	0.653248134	0.975835205	0.375545858	0.472707079	0.911694216	0.999996191
ATP8B1	0.496881844	0.601265106	0.819509909	0.232118746	0.400169546	0.671040485	0.999996191
ATP9A	0.889608341	0.790551786	0.323690167	0.307145692	0.440591519	0.729208807	0.999996191
ATP9B	0.987851858	0.914340079	0.361188076	0.753626507	0.116478089	0.715393427	0.999996191
ATPAF1	0.922637306	0.619570928	0.413677259	0.063624607	0.678415565	0.516112301	0.999996191
ATR	0.16328179	0.996669091	0.959273433	0.594581399	0.950100057	0.90055167	0.999996191
ATRAID	0.78029443	0.733669523	0.302632353	0.780028828	0.678382556	0.905445399	0.999996191
ATRN	0.726026011	0.264783671	0.874529418	0.271214217	0.822261384	0.765569682	0.999996191
ATRX	0.501774316	0.983103052	0.492499995	0.768627059	0.933192612	0.96728818	0.999996191
ATXN1	0.522086582	0.972816158	0.380748105	0.579412594	0.741154644	0.892724295	0.999996191
ATXN10	0.54839871	0.880162934	0.526352748	0.530112823	0.745039352	0.916346485	0.999996191
ATXN2	0.703916171	0.962272689	0.870117164	0.284241021	0.28721104	0.809492422	0.999996191
ATXN3	0.138878725	0.317910708	0.609424321	0.71035933	0.737524244	0.577827126	0.999996191
ATXN7	0.056772362	0.963357621	0.985878537	0.688318895	0.582685499	0.661255325	0.999996191
ATXN7L1	0.897909015	0.984393509	0.538109578	0.940661326	0.462785402	0.977682301	0.999996191
ATXN7L3	0.5903333	0.635388768	0.331238511	0.502503681	0.688446794	0.789992303	0.999996191
ATXN7L3B	0.832239914	0.20479249	0.946819613	0.503721007	0.588798593	0.808592574	0.999996191
AUH	0.602454615	0.973608187	0.801830169	0.432121449	0.981825324	0.975664874	0.999996191
AUNIP	0.938181804	0.706864221	0.551606549	0.115633606	0.389537063	0.608181829	0.999996191

AUP1	0.532093823	0.488512053	0.468264862	0.228785613	0.338421858	0.501194531	0.999996191
AURKA	0.581628707	0.514954554	0.07077983	0.917243216	0.980593808	0.636697883	0.999996191
AURKC	0.990972412	0.376850295	0.547145383	0.189276662	0.976413496	0.766874973	0.999996191
AVEN	0.597896206	0.540692396	0.21935817	0.782715886	0.619809765	0.749811511	0.999996191
AVPR1A	0.968114073	0.957070466	0.90412846	0.047278396	0.812899811	0.737486747	0.999996191
AVPR2	0.520984256	0.837385825	0.955161681	0.988231229	0.438626289	0.969659589	0.999996191
AXIN1	0.99171832	0.498233785	0.771982257	0.216931646	0.795250442	0.859756951	0.999996191
AXL	0.931317902	0.711759591	0.868858233	0.739461256	0.164298372	0.868853803	0.999996191
AZGP1	0.278651366	0.838368507	0.179923739	0.350584302	0.548052916	0.472832731	0.999996191
AZI2	0.767459704	0.65186135	0.038122942	0.954240142	0.399374646	0.45388816	0.999996191
B3GALNT1	0.473779022	0.950529054	0.482018259	0.877462946	0.325076421	0.850477819	0.999996191
B3GALT2	0.872916634	0.84774356	0.375984052	0.856667478	0.033967339	0.473284452	0.999996191
B3GALT6	0.444288015	0.452849975	0.90134403	0.202972544	0.335529225	0.552320019	0.999996191
B3GAT3	0.496556728	0.458590935	0.769109179	0.119601617	0.712721047	0.588965689	0.999996191
B3GNT2	0.667298796	0.489212994	0.946655446	0.275085488	0.548460663	0.804121173	0.999996191
B3GNT3	0.385491147	0.688649315	0.321195095	0.363228104	0.686113313	0.657840571	0.999996191
B3GNT9	0.576977095	0.772164843	0.275563406	0.663714932	0.425385065	0.751198381	0.999996191
B4GALNT1	0.483957973	0.527406519	0.30852095	0.611128373	0.16064634	0.46494144	0.999996191
B4GALNT3	0.641244486	0.41533375	0.878029562	0.17586303	0.593932637	0.684876036	0.999996191
B4GALT1	0.493747934	0.790049623	0.881229878	0.875089782	0.574234757	0.966691258	0.999996191
B4GALT4	0.398806483	0.74695684	0.243630595	0.622301852	0.770616388	0.751952281	0.999996191
B4GALT6	0.545103708	0.436153201	0.487205857	0.657277015	0.950287876	0.873685131	0.999996191
B4GAT1	0.526896291	0.675940444	0.317904627	0.216225102	0.380808465	0.499184817	0.999996191
B9D1	0.424577954	0.460154401	0.152732729	0.59659973	0.772357183	0.573003071	0.999996191
B9D2	0.52791461	0.588678662	0.500126922	0.276896576	0.311615842	0.568180659	0.999996191
BAAT	0.48116938	0.184543057	0.576317309	0.461906204	0.720908456	0.614745843	0.999996191
BABAM1	0.617907741	0.906532092	0.864252777	0.443685012	0.700538582	0.956405797	0.999996191
BABAM2	0.890669497	0.521007968	0.204992956	0.418575608	0.917332361	0.760814396	0.999996191
BACE1	0.714819274	0.748647578	0.2626691	0.951633444	0.916317873	0.937969107	0.999996191
BACH1	0.61191757	0.538944729	0.889631705	0.402722631	0.641540425	0.880287279	0.999996191
BACH2	0.447447224	0.582355604	0.464875975	0.2989571	0.737531783	0.702081486	0.999996191
BAD	0.20683433	0.711185422	0.389222891	0.829792898	0.689608229	0.740743355	0.999996191
BAG4	0.95564274	0.09159178	0.280642238	0.632183121	0.979143745	0.592527958	0.999996191
BAG5	0.488659164	0.674667261	0.219006786	0.357605483	0.785217946	0.648678121	0.999996191
BAHD1	0.694887623	0.655032962	0.585610499	0.748622487	0.716355493	0.952149446	0.999996191
BAIAP2	0.737555941	0.757516547	0.937535296	0.220982146	0.60448402	0.86884972	0.999996191
BAIAP2L1	0.585473568	0.60751469	0.855426525	0.869812044	0.964559777	0.98706784	0.999996191
BAMBI	0.803088127	0.837590438	0.249663842	0.889689356	0.416652515	0.851310842	0.999996191
BANF1	0.471578737	0.437259479	0.514018035	0.149171175	0.895377425	0.578651668	0.999996191
BANP	0.974122885	0.787815419	0.865135822	0.41266117	0.480427895	0.944805335	0.999996191
BAP1	0.788777231	0.461926621	0.639877015	0.193193327	0.832054118	0.765497508	0.999996191
BARD1	0.99915514	0.931168831	0.64656187	0.166274187	0.863154263	0.89781864	0.999996191
BASP1	0.214224042	0.422034032	0.754547532	0.398458265	0.35217185	0.504107223	0.999996191
BATF	0.950042251	0.776431502	0.079673102	0.595347691	0.325855293	0.537039797	0.999996191
BAX	0.608291399	0.701891954	0.353998914	0.958213115	0.642405912	0.907270198	0.999996191

BAZ1B	0.466687662	0.86558465	0.585527075	0.044024434	0.714873217	0.458149522	0.999996191
BAZ2A	0.895724695	0.873243227	0.968431842	0.04071102	0.690232373	0.65816776	0.999996191
BBOF1	0.356516213	0.910885476	0.992515094	0.86985017	0.202464036	0.836797406	0.999996191
BBS1	0.68776352	0.595319057	0.273280205	0.683618498	0.8184496	0.852175838	0.999996191
BBS10	0.703702327	0.437887695	0.965926294	0.339083889	0.59902881	0.846769509	0.999996191
BBS12	0.85088763	0.658443457	0.239650568	0.814625711	0.812746348	0.901569967	0.999996191
BBS2	0.682687796	0.987369795	0.25765754	0.540513953	0.606147838	0.837187435	0.999996191
BBS4	0.568764354	0.878391061	0.449885143	0.051138188	0.940208434	0.526874392	0.999996191
BBS7	0.206386956	0.646826939	0.712602792	0.335878097	0.55386996	0.622127899	0.999996191
BBX	0.922685244	0.571333016	0.616031976	0.929868602	0.211697097	0.855375863	0.999996191
BCAM	0.378553179	0.874045115	0.393565633	0.424990683	0.639351024	0.754990807	0.999996191
BCAP29	0.383279537	0.497123961	0.735080771	0.512094576	0.686013586	0.813279078	0.999996191
BCAP31	0.892302472	0.71427981	0.427283784	0.501990709	0.613171244	0.893962998	0.999996191
BCAR1	0.565921866	0.955129441	0.789243137	0.51003225	0.987784397	0.979605476	0.999996191
BCAR3	0.470299946	0.401089843	0.691842031	0.082040002	0.799775818	0.483504492	0.999996191
BCAS1	0.56802464	0.921068059	0.617602965	0.1600961	0.283800927	0.585710385	0.999996191
BCAS2	0.509259087	0.795834924	0.903672115	0.861942274	0.326539962	0.919463342	0.999996191
BCAS3	0.573356491	0.630493455	0.518631691	0.968925898	0.404724781	0.875980367	0.999996191
BCAT1	0.83694485	0.859905111	0.825779835	0.92973677	0.384434151	0.979012844	0.999996191
BCDIN3D	0.475806511	0.411795075	0.798928274	0.781147606	0.371489732	0.799629508	0.999996191
BCHE	0.796275331	0.714573702	0.662173754	0.603699716	0.250241347	0.837236077	0.999996191
BCKDHA	0.975422998	0.914401588	0.428533953	0.726451625	0.933113844	0.987609522	0.999996191
BCKDHB	0.877106824	0.857972534	0.602348687	0.226929389	0.621870333	0.855474692	0.999996191
BCL10	0.657309058	0.743465009	0.349103397	0.143752334	0.976797947	0.681118595	0.999996191
BCL2L10	0.367571639	0.530020427	0.456353482	0.562853041	0.336624274	0.61249161	0.999996191
BCL2L11	0.905756812	0.799387004	0.163457368	0.176861336	0.866076769	0.626830804	0.999996191
BCL2L12	0.127843421	0.596115744	0.402258455	0.341012335	0.842232595	0.488619753	0.999996191
BCL2L13	0.490866922	0.721525977	0.349683911	0.330386102	0.962030117	0.774370806	0.999996191
BCL2L2	0.143376954	0.77233975	0.396998029	0.720310591	0.701712135	0.666533864	0.999996191
BCL6	0.134045425	0.352701222	0.437965595	0.506126958	0.966095679	0.514587539	0.999996191
BCL7C	0.341918588	0.806244591	0.515616368	0.653719098	0.946018691	0.900138003	0.999996191
BCL9	0.939774136	0.695840838	0.17526156	0.772579155	0.30718677	0.705568937	0.999996191
BCL9L	0.772058278	0.526254102	0.187706686	0.869596962	0.95152224	0.853398599	0.999996191
BCLAF1	0.473282446	0.624151392	0.573699956	0.469958938	0.95427727	0.880658064	0.999996191
BCLAF3	0.650642273	0.908994833	0.217246469	0.132228782	0.665688015	0.535506427	0.999996191
BCOR	0.339768563	0.938438536	0.311922364	0.329873763	0.857481722	0.71200711	0.999996191
BCS1L	0.713468344	0.711866539	0.781318771	0.043410016	0.436624212	0.460001897	0.999996191
BDH2	0.371471953	0.888434194	0.489700534	0.559400008	0.956235523	0.897992872	0.999996191
BDKRB1	0.910345567	0.298508592	0.257168916	0.338610386	0.619000406	0.585267667	0.999996191
BDKRB2	0.103590856	0.959762727	0.489929592	0.461927229	0.637753069	0.581277883	0.999996191
BECN1	0.750839799	0.646402564	0.329721539	0.538598887	0.925114013	0.887266608	0.999996191
BEND5	0.917086927	0.825058051	0.246858879	0.694756379	0.084570253	0.529797139	0.999996191
BET1L	0.516483361	0.596461301	0.082354161	0.759941697	0.943722415	0.627544354	0.999996191
BEX2	0.665625628	0.6052624	0.65803859	0.092753974	0.721490484	0.622614059	0.999996191
BEX3	0.914968694	0.646654175	0.25778437	0.776959679	0.257069936	0.727102658	0.999996191

BEX5	0.896800263	0.700585237	0.65614111	0.443669879	0.536663176	0.91375159	0.999996191
BFSP1	0.829135545	0.847936288	0.687951602	0.882092952	0.887634624	0.996765493	0.999996191
BGN	0.840364073	0.531135371	0.031350021	0.652243149	0.862193838	0.468132574	0.999996191
BHLHE41	0.608896587	0.327901026	0.965869792	0.165584095	0.916382715	0.719489249	0.999996191
BICD1	0.928124559	0.739803966	0.243629719	0.86103544	0.906217531	0.944025461	0.999996191
BICD2	0.910211342	0.795760746	0.093293779	0.426733291	0.748648674	0.660914732	0.999996191
BICRA	0.989051151	0.749869422	0.682281586	0.189503848	0.280620632	0.703514934	0.999996191
BICRAL	0.635947996	0.826349701	0.821945111	0.894966623	0.264880984	0.918696542	0.999996191
BIN1	0.31505341	0.531568942	0.911186923	0.340367517	0.927613804	0.809720013	0.999996191
BIN3	0.42892119	0.747500152	0.926611445	0.900710768	0.633235856	0.96535879	0.999996191
BIRC3	0.619285625	0.604560447	0.141621374	0.342003373	0.516035301	0.499879967	0.999996191
BIRC6	0.495454091	0.846675761	0.975980548	0.405242701	0.929613152	0.958370742	0.999996191
BIVM	0.246873713	0.953156292	0.637328029	0.575209472	0.844608724	0.874690153	0.999996191
BLCAP	0.880025642	0.698119411	0.820339694	0.561366468	0.589947542	0.96428986	0.999996191
BLK	0.988069652	0.103814005	0.304086504	0.847001477	0.752506994	0.644852994	0.999996191
BLMH	0.980874329	0.72225141	0.354055477	0.772087065	0.223144705	0.790937486	0.999996191
BLOC1S2	0.716938126	0.204100269	0.418904893	0.736112039	0.3003946	0.570234725	0.999996191
BLOC1S3	0.86685205	0.834723262	0.497750406	0.906168691	0.612001945	0.97572038	0.999996191
BLOC1S4	0.616608686	0.740270771	0.661585785	0.40038465	0.367475861	0.795791992	0.999996191
BLOC1S5	0.876569342	0.297794917	0.673160437	0.760121149	0.486908372	0.857985716	0.999996191
BLVRA	0.455708408	0.843795669	0.151095688	0.636139101	0.853449089	0.733651406	0.999996191
BLVRB	0.857001299	0.608125078	0.342924631	0.093363071	0.61739616	0.517847183	0.999996191
BLZF1	0.382351856	0.672255985	0.604149388	0.78061325	0.378413346	0.801317498	0.999996191
BMI1	0.408876667	0.306623398	0.262371933	0.393155366	0.817921917	0.522827546	0.999996191
BMP3	0.378786359	0.566344746	0.986505257	0.825385677	0.445017533	0.883783115	0.999996191
BMP4	0.7510078	0.375620779	0.555066349	0.327249574	0.45959683	0.677815063	0.999996191
BMP5	0.646512689	0.50201868	0.840558071	0.067342975	0.651223233	0.546237361	0.999996191
BMP7	0.942358797	0.968956193	0.52688859	0.865017793	0.345716254	0.952698622	0.999996191
BMPR1A	0.95423079	0.532771675	0.245300454	0.77293244	0.26098945	0.69057197	0.999996191
BMPR2	0.834369123	0.413130222	0.129278764	0.274110658	0.921995244	0.53470692	0.999996191
BMX	0.567263573	0.374407491	0.979244813	0.693956357	0.151774843	0.663756252	0.999996191
BNC2	0.663824063	0.326008618	0.654370699	0.666088864	0.614102766	0.840033123	0.999996191
BNIP1	0.423168324	0.156169337	0.52513602	0.967200584	0.797111421	0.702416694	0.999996191
BNIP3L	0.514443906	0.488595053	0.215584451	0.525601535	0.973452937	0.709226044	0.999996191
BOC	0.748294765	0.443416307	0.202241716	0.135337018	0.929821586	0.480956721	0.999996191
BOD1L1	0.62490353	0.45559888	0.398336188	0.277572245	0.704872921	0.666255116	0.999996191
BOK	0.2600173	0.491640362	0.337663479	0.341830068	0.567211413	0.47932381	0.999996191
BOLA	0.630357613	0.734727338	0.339133816	0.277945396	0.701638097	0.7281323	0.999996191
BOLA1	0.161911269	0.950697825	0.682503205	0.377126668	0.637476514	0.691334608	0.999996191
BOLA2B	0.649837816	0.079910492	0.313366566	0.747993461	0.586579217	0.450716155	0.999996191
BORCS5	0.411204115	0.77325591	0.429308715	0.277102431	0.385646527	0.584466179	0.999996191
BORCS7	0.3166124	0.610542663	0.548611762	0.296876055	0.75062761	0.678490321	0.999996191
BORCS8	0.38703854	0.700103036	0.972957265	0.5299748	0.51312882	0.872380126	0.999996191
BPGM	0.489001038	0.376648	0.546273913	0.867196964	0.477983582	0.784672373	0.999996191
BPHL	0.617357401	0.795098845	0.283893026	0.091142884	0.882931955	0.533891017	0.999996191

BPI	0.2410524	0.679618801	0.763670395	0.998511381	0.404313517	0.817670779	0.999996191
BPNT1	0.766105181	0.379579959	0.585649643	0.116402825	0.495185705	0.508800225	0.999996191
BRAP	0.916753089	0.482179927	0.307083851	0.515383864	0.963447171	0.863343526	0.999996191
BRCA1	0.535994787	0.162772676	0.459688129	0.490287522	0.453938609	0.491137992	0.999996191
BRCC3	0.624882325	0.705437467	0.691915613	0.290306799	0.894879104	0.886411662	0.999996191
BRD1	0.930893817	0.326328616	0.77998211	0.321049622	0.716988507	0.830347783	0.999996191
BRD3	0.764816648	0.855426764	0.210771812	0.84903448	0.591329959	0.867297389	0.999996191
BRD4	0.366499434	0.961615855	0.81453017	0.077092867	0.914687472	0.648373242	0.999996191
BRD7	0.982822663	0.792630509	0.410260925	0.294586298	0.673167909	0.854061193	0.999996191
BRINP1	0.308699722	0.720655473	0.739643752	0.746682689	0.158082689	0.640299604	0.999996191
BRMS1	0.768634175	0.707313405	0.873758137	0.419462137	0.838250303	0.964340946	0.999996191
BRMS1L	0.29166747	0.719997221	0.35938945	0.421090299	0.830815049	0.699876317	0.999996191
BRPF1	0.486844582	0.840619113	0.887320889	0.524254936	0.673229508	0.942303287	0.999996191
BRPF3	0.351182272	0.745828989	0.600822505	0.178694918	0.681409897	0.637660684	0.999996191
BRSK1	0.873275748	0.799730071	0.23054758	0.884415589	0.639363553	0.90458319	0.999996191
BRSK2	0.524806777	0.443940928	0.682960294	0.49463661	0.130040895	0.516623645	0.999996191
BRWD1	0.547897081	0.769016014	0.308066638	0.704189226	0.906087879	0.892366871	0.999996191
BRWD3	0.492473025	0.642442473	0.55429292	0.067025724	0.947072927	0.532499645	0.999996191
BSCL2	0.445982482	0.41956222	0.843461956	0.717345349	0.520437836	0.842730774	0.999996191
BSDC1	0.643337584	0.952151304	0.444965011	0.752120539	0.1338595	0.707260539	0.999996191
BTBD1	0.455640249	0.711117711	0.800419614	0.729026326	0.818573324	0.958641741	0.999996191
BTBD19	0.956107915	0.893178415	0.719388355	0.411678204	0.427954836	0.924942342	0.999996191
BTBD2	0.364093487	0.723083784	0.159598162	0.945936154	0.717226791	0.714523739	0.999996191
BTBD3	0.76991536	0.394772932	0.865563431	0.986373998	0.17909344	0.803562683	0.999996191
BTBD8	0.454889979	0.395638724	0.46734546	0.864997937	0.471027481	0.749093002	0.999996191
BTC	0.315210957	0.994029653	0.678336158	0.814733775	0.747064237	0.943185058	0.999996191
BTD	0.861464567	0.247078013	0.650140536	0.724795943	0.631497235	0.85396396	0.999996191
BTG1	0.774127329	0.662908069	0.059687289	0.486745446	0.955708376	0.579906863	0.999996191
BTN3A3	0.957092771	0.364166724	0.814744979	0.170121333	0.577988956	0.710578593	0.999996191
BUD13	0.99175157	0.211593742	0.660133645	0.568279967	0.935402477	0.876204133	0.999996191
BUD31	0.510364137	0.427099885	0.700409104	0.528959351	0.937373791	0.88010011	0.999996191
C10H14orf1	0.470246229	0.952547226	0.92784591	0.388168101	0.128887993	0.653605787	0.999996191
C10H14orf119	0.076715818	0.408043291	0.919264319	0.473893611	0.611624368	0.478696943	0.999996191
C10H14orf93	0.895065543	0.782419424	0.500738431	0.247778817	0.469103326	0.78060448	0.999996191
C10H15orf41	0.670891848	0.77839906	0.8506397	0.694290749	0.430044014	0.94550955	0.999996191
C10H15orf59	0.983582679	0.319047317	0.087165824	0.875519334	0.58852764	0.577795788	0.999996191
C10H15orf61	0.967428135	0.596136281	0.668307898	0.072240227	0.873740048	0.684101858	0.999996191
C11H2orf40	0.595182362	0.692323608	0.589628908	0.12787731	0.940953862	0.719314308	0.999996191
C11H2orf42	0.953987655	0.627131334	0.885705096	0.786785075	0.121783468	0.818548183	0.999996191
C11H2orf49	0.513695146	0.984752127	0.202778729	0.86552606	0.515170144	0.800817432	0.999996191
C11H2orf68	0.892723524	0.287993474	0.862369537	0.893600459	0.842071568	0.964258066	0.999996191
C11H2orf92	0.871514761	0.977739387	0.439377373	0.934355643	0.3700392	0.943245009	0.999996191
C11H9orf50	0.203281836	0.808167294	0.367303068	0.364822458	0.993716572	0.663494543	0.999996191
C11H9orf78	0.915736122	0.787933941	0.05315952	0.49962325	0.917661682	0.620895116	0.999996191
C13H10orf113	0.842718728	0.894826112	0.071526876	0.959954515	0.827673141	0.789471489	0.999996191

C13H20orf194	0.365901366	0.714671463	0.276768118	0.360960043	0.830934133	0.661990604	0.999996191
C14H8orf33	0.690766585	0.480391229	0.878331293	0.227226821	0.23009707	0.592958461	0.999996191
C14H8orf59	0.944975147	0.341829101	0.723370032	0.270743214	0.343528669	0.662213898	0.999996191
C14H8orf76	0.398452364	0.143714589	0.838306163	0.721181381	0.931245567	0.737739983	0.999996191
C14H8orf82	0.913573427	0.701981168	0.998878742	0.750900466	0.614367714	0.991748571	0.999996191
C14H8orf88	0.63024569	0.890693095	0.747478803	0.446789108	0.309044295	0.840062768	0.999996191
C14H8orf89	0.208423704	0.800605195	0.564917451	0.88453807	0.901870492	0.879168539	0.999996191
C15H11orf49	0.228834725	0.996346402	0.313043596	0.47101296	0.582381663	0.641860135	0.999996191
C15H11orf71	0.787352392	0.411716336	0.40640251	0.081072554	0.903754605	0.505655914	0.999996191
C15H11orf74	0.449180516	0.857629414	0.54151434	0.217878049	0.656208873	0.72310252	0.999996191
C15H11orf94	0.299214819	0.642542527	0.091815028	0.487382495	0.917802731	0.468753782	0.999996191
C15H11orf96	0.429277094	0.183547456	0.752623505	0.859220709	0.840019263	0.789251972	0.999996191
C16H1orf112	0.944566815	0.428703238	0.530020793	0.952709898	0.780546328	0.961003679	0.999996191
C16H1orf116	0.050920691	0.602002025	0.588966654	0.812160632	0.521135711	0.462843244	0.999996191
C16H1orf159	0.910868648	0.888233668	0.502618456	0.734431121	0.77654228	0.983184429	0.999996191
C16H1orf174	0.643257241	0.057127931	0.717027183	0.894162821	0.65234335	0.594619182	0.999996191
C16H1orf21	0.945254124	0.763461928	0.888922525	0.481571482	0.969547102	0.992108437	0.999996191
C16H1orf53	0.836597073	0.827165041	0.343114282	0.382529491	0.692995865	0.853003413	0.999996191
C17H12orf49	0.506280882	0.421803467	0.197989629	0.61270503	0.89879757	0.675611296	0.999996191
C17H4orf33	0.292520721	0.804425206	0.546806307	0.396458459	0.64299442	0.740959985	0.999996191
C18H16orf70	0.89091857	0.933984727	0.3874349	0.71137538	0.868908221	0.975589648	0.999996191
C18H19orf12	0.941411781	0.469796504	0.833528705	0.411054842	0.52348035	0.886561956	0.999996191
C18H19orf33	0.035856451	0.943947181	0.779695247	0.601526478	0.834572466	0.565824603	0.999996191
C18H19orf47	0.607487426	0.420021726	0.483525494	0.693226696	0.120364132	0.517712332	0.999996191
C18H19orf54	0.818497406	0.971864871	0.66586176	0.304775951	0.926900381	0.955952351	0.999996191
C18H19orf81	0.77490085	0.264187211	0.153665571	0.840390637	0.301686247	0.470567137	0.999996191
C18H19orf84	0.608550257	0.188280132	0.574167078	0.451885675	0.296254419	0.488669054	0.999996191
C19H17orf100	0.573742236	0.638241443	0.289631563	0.77998275	0.313883743	0.696664457	0.999996191
C19H17orf49	0.395202326	0.800384693	0.886795495	0.134272497	0.663721461	0.689354132	0.999996191
C19H17orf75	0.138351574	0.954120403	0.985267832	0.578265032	0.86140833	0.857402404	0.999996191
C19H17orf80	0.44870994	0.125071018	0.744795586	0.542113363	0.557546592	0.556676937	0.999996191
C1D	0.39207434	0.520282183	0.520300323	0.922019713	0.534584922	0.823512666	0.999996191
C1GALT1	0.414072521	0.435740096	0.653297747	0.557082997	0.247518965	0.605513081	0.999996191
C1H21orf58	0.839734626	0.678442267	0.794673678	0.910425159	0.786866646	0.994018664	0.999996191
C1H3orf33	0.588306854	0.920396706	0.757014595	0.582710143	0.730274309	0.967353756	0.999996191
C1H3orf38	0.777876983	0.841192687	0.869185939	0.266217722	0.311182052	0.805914833	0.999996191
C1H3orf58	0.771399081	0.670734883	0.804573517	0.960704015	0.637489155	0.987021541	0.999996191
C1H3orf70	0.774444128	0.780686645	0.755607086	0.494703422	0.864419113	0.974457809	0.999996191
C1QA	0.872403016	0.931148463	0.246122771	0.549070832	0.357620699	0.773889705	0.999996191
C1QB	0.994514336	0.734433719	0.174964926	0.440203015	0.382812942	0.660440531	0.999996191
C1QC	0.927942567	0.912644805	0.182435248	0.330866095	0.292275064	0.589116392	0.999996191
C1QTNF1	0.824595892	0.293937295	0.138874796	0.293771853	0.988851434	0.508068383	0.999996191
C1QTNF5	0.435867438	0.930835952	0.175595091	0.813715615	0.588228234	0.748178734	0.999996191
C1QTNF7	0.731898215	0.808083799	0.767300514	0.283606244	0.38254598	0.813385386	0.999996191
C1QTNF9	0.838752004	0.774027568	0.715874556	0.603432911	0.205074734	0.838893054	0.999996191

C1RL	0.982480932	0.348627455	0.632373156	0.568934032	0.11272933	0.574992091	0.999996191
C20H5orf22	0.11112631	0.73024388	0.748930191	0.70941537	0.365210886	0.599331222	0.999996191
C20H5orf34	0.14521137	0.44021923	0.262763847	0.469203176	0.933953183	0.45614008	0.999996191
C20H5orf51	0.226553267	0.529634931	0.333382922	0.737483404	0.727222776	0.659703295	0.999996191
C21H14orf132	0.440825801	0.811940939	0.541649953	0.591309133	0.463226614	0.825978369	0.999996191
C21H14orf28	0.174983733	0.710779295	0.783858994	0.869406971	0.228478876	0.63972972	0.999996191
C21H15orf39	0.211025455	0.29095533	0.942041831	0.541695583	0.862729122	0.704377143	0.999996191
C21H15orf40	0.402231807	0.97730341	0.294169083	0.833048409	0.648173667	0.851772525	0.999996191
C22H3orf14	0.76682583	0.700067373	0.837210662	0.878405714	0.304744229	0.936105155	0.999996191
C22H3orf18	0.999517322	0.790237363	0.325727487	0.798664826	0.564548134	0.932369946	0.999996191
C22H3orf20	0.957595099	0.640953887	0.605112408	0.804863717	0.382924393	0.930975037	0.999996191
C22H3orf49	0.458326058	0.709507918	0.885139854	0.489142115	0.329657972	0.803337922	0.999996191
C23H6orf132	0.374272499	0.613349202	0.49291861	0.10025374	0.656844997	0.458326115	0.999996191
C23H6orf226	0.729764528	0.363890954	0.887166308	0.082577952	0.701086502	0.571444569	0.999996191
C23H6orf52	0.583435133	0.275892851	0.568761349	0.539844472	0.751064337	0.7637565	0.999996191
C23H6orf89	0.470321446	0.566269751	0.674367293	0.193516667	0.562318145	0.641517296	0.999996191
C24H18orf21	0.255334676	0.620104321	0.744798426	0.611586828	0.288073497	0.653450786	0.999996191
C24H18orf32	0.274528863	0.212956067	0.603430107	0.639522253	0.759006887	0.615696751	0.999996191
C24H18orf54	0.36794311	0.563390856	0.738943833	0.529360995	0.643217811	0.823015461	0.999996191
C25H16orf58	0.897706437	0.273088278	0.826632264	0.937696192	0.647999541	0.938422024	0.999996191
C25H16orf71	0.52085021	0.798564473	0.700310128	0.932615117	0.553125034	0.956291384	0.999996191
C25H16orf89	0.649538413	0.715229311	0.507850512	0.232772721	0.333611317	0.628898485	0.999996191
C25H16orf91	0.097786949	0.440537255	0.334879577	0.909538982	0.784394992	0.517672284	0.999996191
C25H7orf26	0.67259671	0.63870223	0.194572814	0.977157349	0.975521192	0.887165866	0.999996191
C25H7orf43	0.921073213	0.447074284	0.203392698	0.851989967	0.910824076	0.857890339	0.999996191
C25H7orf50	0.471137776	0.740076857	0.214309072	0.243752744	0.439404124	0.471195923	0.999996191
C26H10orf143	0.418603425	0.314807414	0.875650979	0.879283188	0.156156864	0.600541739	0.999996191
C27H4orf47	0.796305169	0.800848356	0.166480432	0.607952063	0.723007107	0.804271877	0.999996191
C27H8orf48	0.358697067	0.602168365	0.253996346	0.900515713	0.686943124	0.74729473	0.999996191
C28H10orf71	0.334064349	0.960667182	0.141539273	0.66845763	0.911798565	0.708952043	0.999996191
C29H11orf54	0.805337567	0.545610713	0.581905083	0.859790021	0.648470994	0.951916967	0.999996191
C2CD2	0.888908742	0.768576082	0.403554974	0.989440409	0.609161993	0.963974883	0.999996191
C2CD3	0.389871827	0.590637462	0.954806738	0.445126493	0.785944895	0.882326055	0.999996191
C2H2orf76	0.517970798	0.909929423	0.603431714	0.115875013	0.617115966	0.649284619	0.999996191
C3H1orf109	0.302984404	0.575634789	0.593178771	0.633974842	0.179626883	0.543292885	0.999996191
C3H1orf123	0.716926575	0.71211019	0.378623922	0.139738159	0.702616103	0.635782669	0.999996191
C3H1orf216	0.582653388	0.846998691	0.939253938	0.870854299	0.987331248	0.997426794	0.999996191
C3H1orf226	0.850022855	0.993952507	0.850384557	0.733826248	0.052591299	0.709244784	0.999996191
C3H1orf43	0.959192158	0.162608664	0.407604294	0.714090061	0.91630075	0.78422068	0.999996191
C3H1orf50	0.958867612	0.753995521	0.264219308	0.984399662	0.834813456	0.959743202	0.999996191
C5AR2	0.701879146	0.367513845	0.928468218	0.843187236	0.860510661	0.967098178	0.999996191
C5H12orf10	0.758972865	0.889346613	0.784097316	0.809043828	0.492104206	0.978599697	0.999996191
C5H12orf4	0.889190566	0.712642015	0.815955272	0.34022535	0.302420844	0.826278194	0.999996191
C5H12orf45	0.238826418	0.166560972	0.759218675	0.761240213	0.541276748	0.553772196	0.999996191
C5H12orf56	0.847876676	0.198577344	0.861367978	0.930117073	0.838279434	0.929683164	0.999996191

C5H12orf66	0.529921821	0.386472369	0.872685997	0.800828828	0.313506282	0.797505366	0.999996191
C5H12orf73	0.407776124	0.553955953	0.866233097	0.2253685	0.319107299	0.577491046	0.999996191
C5H12orf75	0.150394262	0.569559375	0.291282037	0.86994662	0.765526225	0.609822315	0.999996191
C6	0.587590191	0.307568577	0.47280894	0.551604111	0.333603028	0.599057223	0.999996191
C6H4orf3	0.097360934	0.791391273	0.511028693	0.502607291	0.855207512	0.613410866	0.999996191
C7H19orf38	0.869452034	0.950774357	0.016226121	0.863050618	0.891264568	0.518136813	0.999996191
C7H19orf53	0.721254077	0.435703039	0.398648183	0.317714241	0.963087109	0.76958718	0.999996191
C7H19orf66	0.628295653	0.719848124	0.281556946	0.491704887	0.95499363	0.84504914	0.999996191
C7H19orf71	0.45897503	0.889976048	0.334710987	0.479198466	0.110809979	0.453681638	0.999996191
C7H1orf35	0.655115042	0.543427498	0.386811642	0.06351207	0.946528026	0.477333099	0.999996191
C7H5orf15	0.949043519	0.540039528	0.718802211	0.77023583	0.736210707	0.978150915	0.999996191
C7H5orf24	0.658095335	0.056594642	0.980063083	0.697271656	0.503117435	0.559271512	0.999996191
C8G	0.617336909	0.079183442	0.738981841	0.590442905	0.883227724	0.634333417	0.999996191
C8H8orf58	0.527729395	0.698933976	0.312416216	0.296885084	0.728455838	0.688757986	0.999996191
C8H9orf152	0.897231632	0.240774946	0.868581979	0.311715202	0.578244112	0.746659159	0.999996191
C8H9orf3	0.644240778	0.548673469	0.162963662	0.726492507	0.299037492	0.554853711	0.999996191
C8H9orf40	0.52655276	0.679328914	0.513973109	0.839393645	0.546865179	0.894852319	0.999996191
C8H9orf64	0.347082818	0.840188029	0.811130656	0.419444196	0.986201517	0.913375143	0.999996191
C8H9orf85	0.952825484	0.184953769	0.456002113	0.617133077	0.997038522	0.814108022	0.999996191
C9H6orf120	0.810747317	0.880007822	0.255242479	0.379360224	0.757959431	0.823666452	0.999996191
C9H6orf163	0.758697598	0.565031329	0.506797336	0.614410458	0.994005028	0.945545146	0.999996191
C9H6orf203	0.702758519	0.201163229	0.957240194	0.594084162	0.882719914	0.870901508	0.999996191
CA10	0.437377379	0.843222015	0.891144687	0.552937998	0.673217807	0.937781747	0.999996191
CA2	0.63971891	0.720076806	0.945439019	0.166159593	0.908574862	0.85962956	0.999996191
CA5B	0.855274923	0.909742311	0.355676804	0.254160744	0.767638615	0.828704127	0.999996191
CA9	0.27992206	0.345060072	0.720581495	0.71105055	0.163492165	0.47317238	0.999996191
CAAP1	0.775044091	0.307699999	0.144513061	0.542809803	0.925031651	0.617746588	0.999996191
CAB39L	0.993442049	0.995222637	0.355927132	0.670262457	0.685661396	0.961992056	0.999996191
CABCOCO1	0.515827387	0.68411743	0.882532731	0.06178156	0.661844479	0.558204399	0.999996191
CABLES1	0.758526576	0.750530954	0.931329381	0.737966897	0.068147319	0.701759942	0.999996191
CABLES2	0.583978081	0.998095194	0.421174632	0.742572752	0.907877415	0.96368101	0.999996191
CABP1	0.175262197	0.51191091	0.220981789	0.773927573	0.564187828	0.485508248	0.999996191
CABP7	0.655957747	0.578523946	0.604519193	0.872237937	0.661288436	0.945292797	0.999996191
CABYR	0.938396921	0.809892176	0.459806884	0.948272895	0.059959738	0.644739256	0.999996191
CACNA1C	0.660300267	0.710363129	0.240083243	0.894737912	0.371335944	0.765195923	0.999996191
CACNA2D1	0.096532342	0.80939228	0.419834526	0.828165961	0.834734536	0.670480739	0.999996191
CACNG4	0.356370979	0.189994826	0.640513309	0.881414608	0.820520686	0.732586647	0.999996191
CACNG7	0.895922134	0.888513322	0.584693248	0.306519844	0.812880456	0.932341123	0.999996191
CACTIN	0.942795836	0.502198378	0.457040407	0.621369188	0.957240232	0.942706014	0.999996191
CADM2	0.636485946	0.116417493	0.648346917	0.911021146	0.317386805	0.574980438	0.999996191
CADM3	0.227935835	0.906130883	0.926703434	0.447749828	0.39588661	0.747237244	0.999996191
CALCOCO2	0.764893982	0.977267144	0.146412292	0.758474985	0.14247903	0.544035355	0.999996191
CALCRL	0.658318807	0.405581927	0.236569317	0.677637692	0.605326109	0.696247766	0.999996191
CALD1	0.7879534	0.970233829	0.367170602	0.474229103	0.665059793	0.901045835	0.999996191
CALHM2	0.847003926	0.936315331	0.56347265	0.613936134	0.770656578	0.978773769	0.999996191

CALHM5	0.267294144	0.601775633	0.58171483	0.203698461	0.617069821	0.542967476	0.999996191
CALHM6	0.322836616	0.729532929	0.501899851	0.447459713	0.385687772	0.649885144	0.999996191
CALML6	0.653448393	0.738903813	0.434134241	0.920203366	0.451998846	0.899084958	0.999996191
CALN1	0.346713532	0.281148948	0.995635522	0.498401961	0.342879257	0.609459241	0.999996191
CALR3	0.668338767	0.988169729	0.168896376	0.556769238	0.776408125	0.809855799	0.999996191
CALU	0.330871763	0.259048611	0.291171463	0.439930382	0.900827942	0.510201772	0.999996191
CALY	0.148801344	0.540794975	0.321718191	0.985892389	0.553943098	0.578404852	0.999996191
CAMK1	0.624438408	0.482117578	0.336123065	0.939161932	0.578510183	0.831645022	0.999996191
CAMK1D	0.410670809	0.79066203	0.547947748	0.696176464	0.234064772	0.717732361	0.999996191
CAMK2A	0.897774718	0.757701604	0.112668246	0.867569018	0.354441193	0.677957948	0.999996191
CAMK2B	0.734705744	0.113422722	0.994741447	0.954198929	0.105306933	0.47845434	0.999996191
CAMK2D	0.450101277	0.306180212	0.595556672	0.35329308	0.817013253	0.678965742	0.999996191
CAMK2N1	0.381435971	0.731963606	0.686340267	0.216318883	0.529299066	0.664066932	0.999996191
CAMKK1	0.714488416	0.773705854	0.704745078	0.757608356	0.497046551	0.954331784	0.999996191
CAMKK2	0.099091234	0.321286222	0.706645214	0.971610181	0.798472411	0.619417407	0.999996191
CAMKMT	0.988966894	0.415125548	0.850346065	0.443111845	0.91596306	0.951393829	0.999996191
CAMLG	0.539949703	0.496701805	0.38218951	0.979017276	0.461849073	0.803090955	0.999996191
CAMSAP1	0.944218259	0.810585121	0.217643113	0.460638358	0.81701041	0.852388505	0.999996191
CAMSAP3	0.03073214	0.588900839	0.794514046	0.944477212	0.841646812	0.537524021	0.999996191
CAMTA1	0.548221414	0.567409556	0.924080495	0.467442724	0.185853019	0.689152391	0.999996191
CAMTA2	0.946742149	0.394472169	0.589281737	0.193470543	0.708557541	0.725267441	0.999996191
CANX	0.893532505	0.20174767	0.80546834	0.676111773	0.094449433	0.498177554	0.999996191
CAPN1	0.126020242	0.547755395	0.565871131	0.507757363	0.833069851	0.608719526	0.999996191
CAPN2	0.865899516	0.864513724	0.241673994	0.410472854	0.786198658	0.841277822	0.999996191
CAPN3	0.95565761	0.541642479	0.850637797	0.291839722	0.300766659	0.771068388	0.999996191
CAPN5	0.181229314	0.723332386	0.342248291	0.90307141	0.882404122	0.756893633	0.999996191
CAPRIN1	0.369611519	0.292185779	0.760172113	0.496044895	0.522640593	0.658147346	0.999996191
CAPRIN2	0.777479853	0.813655023	0.060868578	0.967166445	0.990579599	0.762626047	0.999996191
CAPZA1	0.616807878	0.168807121	0.541685726	0.771748906	0.195307355	0.482182819	0.999996191
CARD10	0.753795768	0.257757391	0.806169391	0.810506	0.245937718	0.731734906	0.999996191
CARD11	0.177912502	0.732498137	0.84913294	0.882981989	0.608316791	0.844106808	0.999996191
CARHSP1	0.371886066	0.834380713	0.95276524	0.361044243	0.343041434	0.76126278	0.999996191
CARMIL1	0.713101005	0.8850054	0.721989479	0.136693895	0.966250566	0.846055631	0.999996191
CARS	0.369447718	0.976363184	0.895071637	0.422443126	0.627688953	0.89672736	0.999996191
CARS2	0.918454315	0.670112513	0.648490107	0.071098984	0.523766269	0.588102215	0.999996191
CASC1	0.940653466	0.432241764	0.531072292	0.748535444	0.523916044	0.895298235	0.999996191
CASC3	0.895503055	0.90750951	0.745997631	0.827261885	0.635598392	0.993634795	0.999996191
CASC4	0.260015964	0.93440519	0.427438002	0.189298627	0.494669848	0.507040879	0.999996191
CASKIN1	0.948207953	0.450717833	0.377689033	0.893966942	0.985474888	0.951702755	0.999996191
CASKIN2	0.351308891	0.746996606	0.648973055	0.963102659	0.765337154	0.940308909	0.999996191
CASP2	0.076964286	0.967153739	0.622780326	0.700573418	0.314784155	0.516407207	0.999996191
CASP3	0.627513346	0.526674765	0.365751904	0.36660852	0.417141698	0.630643593	0.999996191
CASP4	0.901493354	0.979804348	0.62771851	0.803768433	0.143027163	0.854931367	0.999996191
CASP6	0.490432803	0.971564656	0.916460012	0.557620431	0.772003303	0.972168698	0.999996191
CASP9	0.5365043	0.906393483	0.605515269	0.596907988	0.464750351	0.890524397	0.999996191

CAST	0.989234737	0.678863739	0.689181164	0.640894692	0.963863664	0.990814225	0.999996191
CASTOR1	0.613013931	0.936841838	0.839916567	0.723886423	0.597075019	0.978045334	0.999996191
CAT	0.799377213	0.849824179	0.194862316	0.958377422	0.98443729	0.93981044	0.999996191
CATSPER2	0.790083753	0.784955494	0.073020858	0.919891955	0.983947944	0.781602714	0.999996191
CATSPERE	0.973698581	0.182621249	0.400581698	0.539185782	0.334715042	0.560020947	0.999996191
CAV1	0.970757666	0.398711756	0.384809645	0.328629344	0.915391275	0.797247451	0.999996191
CAV3	0.187593688	0.637937817	0.911955807	0.226202062	0.376898802	0.498821	0.999996191
CAVIN2	0.326771247	0.515580153	0.504191789	0.434843258	0.776208269	0.715618217	0.999996191
CAVIN4	0.575875676	0.482157413	0.181608331	0.485171088	0.943793823	0.673989205	0.999996191
CBFA2T2	0.98627316	0.867853167	0.307194771	0.215740776	0.280964565	0.601696109	0.999996191
CBFA2T3	0.826514116	0.536692407	0.665990224	0.846133487	0.112408581	0.711776159	0.999996191
CBLN3	0.835409539	0.620508971	0.63174031	0.969270949	0.061377253	0.640898443	0.999996191
CBLN4	0.528667189	0.572074507	0.4499008	0.162687847	0.935213557	0.652751701	0.999996191
CBS	0.767372398	0.730553939	0.597520144	0.365705058	0.335170089	0.781902895	0.999996191
CBWD2	0.127715857	0.682376847	0.794525721	0.192767611	0.980174692	0.563407034	0.999996191
CBX1	0.810129992	0.41204478	0.41653805	0.812395058	0.74152848	0.893864131	0.999996191
CBY1	0.824730395	0.765736163	0.135352579	0.369559366	0.893681173	0.71267237	0.999996191
CC2D1A	0.897495295	0.387259832	0.434923824	0.095357466	0.920591495	0.566140421	0.999996191
CC2D2B	0.333682867	0.365976055	0.966602855	0.971029318	0.487060298	0.834124208	0.999996191
CCDC102B	0.982885598	0.382203832	0.696695752	0.462866195	0.197293272	0.680675179	0.999996191
CCDC106	0.594479524	0.685316254	0.216888368	0.622325582	0.972192229	0.827080124	0.999996191
CCDC107	0.401675748	0.679458104	0.749495968	0.793486412	0.79221832	0.942612977	0.999996191
CCDC112	0.639334127	0.238814692	0.383298339	0.462947885	0.285044816	0.464746585	0.999996191
CCDC115	0.729346864	0.792914526	0.529021323	0.127845895	0.239034151	0.499713681	0.999996191
CCDC120	0.321004248	0.62713972	0.406213473	0.726149823	0.644534111	0.7693086	0.999996191
CCDC126	0.608444878	0.967355716	0.464304877	0.227096553	0.281969304	0.619931142	0.999996191
CCDC127	0.710981244	0.654690876	0.600969243	0.584531298	0.568842253	0.907239191	0.999996191
CCDC130	0.956095994	0.92184895	0.491683829	0.785936314	0.894989826	0.992561817	0.999996191
CCDC134	0.400469278	0.864384121	0.458248713	0.412303142	0.279740253	0.628624055	0.999996191
CCDC138	0.580319474	0.714171439	0.618989362	0.824861134	0.639614507	0.947355483	0.999996191
CCDC142	0.878951783	0.235015063	0.990335693	0.710595422	0.132861831	0.639201912	0.999996191
CCDC149	0.804262022	0.789334846	0.728844159	0.249183565	0.182571193	0.65599589	0.999996191
CCDC150	0.544340693	0.367344931	0.887214153	0.351866925	0.450291402	0.711847909	0.999996191
CCDC151	0.636192185	0.739621518	0.439065291	0.371415425	0.634667451	0.811541821	0.999996191
CCDC160	0.798095234	0.45480995	0.123759617	0.665731585	0.637137044	0.636561941	0.999996191
CCDC162P	0.137077905	0.772093691	0.818035374	0.512287715	0.446333697	0.644020458	0.999996191
CCDC166	0.845227725	0.970123258	0.972051742	0.081968209	0.834754155	0.830336457	0.999996191
CCDC167	0.5990404	0.602982966	0.824086895	0.678997403	0.852353764	0.966506776	0.999996191
CCDC17	0.150046345	0.505904643	0.191016528	0.649773102	0.797448571	0.459808047	0.999996191
CCDC170	0.651010927	0.531233861	0.411817895	0.980938957	0.610763543	0.896295039	0.999996191
CCDC184	0.314215347	0.844128761	0.480028884	0.441340211	0.824712509	0.803075638	0.999996191
CCDC191	0.915755592	0.155939379	0.314730919	0.686039931	0.537135483	0.609180423	0.999996191
CCDC194	0.785262411	0.883916107	0.056408434	0.464602681	0.495217995	0.492835338	0.999996191
CCDC197	0.574983358	0.823757069	0.764895553	0.328054927	0.919457313	0.925996697	0.999996191
CCDC22	0.350118021	0.657235872	0.806777723	0.778041407	0.337458757	0.811689855	0.999996191

CCDC25	0.880018546	0.76034934	0.551796881	0.443209896	0.104873235	0.616124763	0.999996191
CCDC28A	0.901479228	0.39220762	0.561384157	0.20167874	0.36933793	0.58707599	0.999996191
CCDC32	0.328297899	0.794732652	0.278958551	0.791887078	0.716437875	0.782910505	0.999996191
CCDC36	0.703848156	0.702069254	0.802391538	0.96852222	0.941276902	0.996063567	0.999996191
CCDC43	0.971047557	0.293355551	0.817919194	0.231373297	0.870543145	0.805236315	0.999996191
CCDC47	0.791952477	0.315610467	0.210580224	0.725904944	0.3399501	0.562008565	0.999996191
CCDC50	0.69632616	0.926534599	0.710265056	0.8518813	0.774711396	0.992359602	0.999996191
CCDC51	0.989660678	0.490611885	0.59197478	0.061636865	0.562797919	0.511712817	0.999996191
CCDC57	0.651765932	0.073538776	0.494858234	0.457676777	0.98437779	0.524748874	0.999996191
CCDC58	0.921865615	0.361797806	0.644005636	0.108911361	0.377510803	0.48917473	0.999996191
CCDC59	0.868161772	0.345977526	0.343384726	0.820424228	0.551952118	0.804415822	0.999996191
CCDC6	0.865824693	0.24573834	0.315906965	0.58962886	0.696654042	0.708426642	0.999996191
CCDC69	0.742154548	0.770917307	0.748329452	0.312519475	0.715587022	0.910777818	0.999996191
CCDC71	0.209361796	0.93799215	0.606091647	0.561684478	0.731553302	0.812258493	0.999996191
CCDC8	0.320629139	0.936483399	0.139334842	0.860359899	0.804971507	0.717620534	0.999996191
CCDC85A	0.590593779	0.946496779	0.74532184	0.856107288	0.616689193	0.980742805	0.999996191
CCDC85B	0.242233829	0.881316709	0.509110083	0.408821402	0.700875792	0.731250584	0.999996191
CCDC85C	0.723307384	0.828362817	0.346877512	0.10474704	0.540614495	0.543096243	0.999996191
CCDC88A	0.736931993	0.784315466	0.982385917	0.741437051	0.336562522	0.951392267	0.999996191
CCDC88C	0.717467085	0.574122798	0.089670234	0.87187433	0.949534556	0.727812419	0.999996191
CCDC89	0.418571518	0.991623492	0.517348387	0.135458349	0.250726042	0.45449082	0.999996191
CCDC9	0.362862721	0.662188194	0.958026795	0.198730369	0.376161307	0.616655969	0.999996191
CCDC90B	0.841473021	0.355933832	0.931984652	0.71450078	0.826876562	0.963425235	0.999996191
CCDC91	0.349987552	0.234298536	0.382258705	0.82714796	0.977455239	0.691993011	0.999996191
CCDC92	0.999326958	0.826706022	0.70539146	0.518052438	0.763248296	0.982897758	0.999996191
CCDC96	0.499958736	0.449567289	0.993129414	0.698261661	0.927087764	0.953066342	0.999996191
CCDC97	0.654392344	0.698449165	0.590966155	0.710085566	0.941471073	0.969640703	0.999996191
CCDC9B	0.531088244	0.768028704	0.545155576	0.058754684	0.958996499	0.555081206	0.999996191
CCL16	0.807090304	0.710284508	0.388987067	0.246983595	0.427120237	0.677597698	0.999996191
CCL25	0.497044038	0.286520538	0.501833561	0.255256657	0.735846054	0.56836523	0.999996191
CCL28	0.795860817	0.827825192	0.896382548	0.820400694	0.844494167	0.997725664	0.999996191
CCM2L	0.50492932	0.508897317	0.556227864	0.745598449	0.98150978	0.921121797	0.999996191
CCNB1	0.584129768	0.32318459	0.162168893	0.69851035	0.819455906	0.620202292	0.999996191
CCNB3	0.204961952	0.396016104	0.626750268	0.610898645	0.967956174	0.724721394	0.999996191
CCNC	0.387283907	0.266218336	0.584211588	0.41652947	0.875784601	0.664351131	0.999996191
CCND2	0.794995037	0.391411744	0.830160343	0.19273809	0.931033277	0.803120548	0.999996191
CCNE1	0.723300907	0.444206469	0.562902932	0.601962806	0.969019372	0.922090064	0.999996191
CCNF	0.628348734	0.322562243	0.194286769	0.892536971	0.288268903	0.514718479	0.999996191
CCNG1	0.574539499	0.530547205	0.246351756	0.645788233	0.644138713	0.731819959	0.999996191
CCNH	0.111297598	0.405712344	0.718197155	0.857762226	0.526165835	0.585117632	0.999996191
CCNI2	0.440064881	0.96954471	0.502386728	0.137072869	0.931434697	0.706740807	0.999996191
CCNK	0.873557748	0.536694993	0.539952617	0.861348553	0.276021598	0.84606647	0.999996191
CCNL1	0.823031381	0.215641805	0.919306943	0.355017259	0.520674752	0.725208671	0.999996191
CCNL2	0.51304963	0.153494496	0.680126501	0.92836942	0.543499504	0.704333394	0.999996191
CCNQ	0.988392779	0.756790416	0.053688382	0.93674212	0.658346124	0.687552496	0.999996191

CCNT2	0.244574619	0.982350895	0.897273337	0.267758919	0.956446018	0.8323223	0.999996191
CCNYL1	0.978320934	0.41354873	0.413203046	0.170628952	0.936375891	0.702089302	0.999996191
CCP110	0.73674454	0.43031899	0.872264894	0.472166747	0.644808933	0.894541731	0.999996191
CCPG1	0.711620862	0.343776506	0.34966041	0.763145093	0.789413649	0.821020248	0.999996191
CCR7	0.399957889	0.898771327	0.891881235	0.153105126	0.340117539	0.610744654	0.999996191
CCR8	0.970185022	0.968588725	0.266034404	0.568918819	0.923747054	0.944632401	0.999996191
CCRL2	0.893456297	0.859585687	0.183136815	0.304729872	0.22486754	0.50536904	0.999996191
CCS	0.463058596	0.496497471	0.977012089	0.723581461	0.880922198	0.952286771	0.999996191
CCSER1	0.278963879	0.930500109	0.673949322	0.164773587	0.847333979	0.684872527	0.999996191
CCSER2	0.365945935	0.798933213	0.139494944	0.456338146	0.999518378	0.631869495	0.999996191
CCT2	0.98198522	0.571752361	0.315103674	0.280471119	0.479115953	0.679643752	0.999996191
CCT3	0.700829331	0.432306762	0.685963654	0.472682279	0.361550981	0.755687851	0.999996191
CCT4	0.894424355	0.309057319	0.920293637	0.059917946	0.89651423	0.571813415	0.999996191
CCT6A	0.962611095	0.46127714	0.446161053	0.208448607	0.338800188	0.576369402	0.999996191
CCT6B	0.356380671	0.649377293	0.241138836	0.581055316	0.402845399	0.56310508	0.999996191
CCZ1	0.314505241	0.663235232	0.181679734	0.842110118	0.750237389	0.681014175	0.999996191
CD109	0.833339038	0.80888972	0.832912427	0.933896125	0.117346615	0.849501762	0.999996191
CD160	0.552310208	0.375273381	0.984359687	0.811241111	0.069426352	0.538533069	0.999996191
CD163	0.722906699	0.764323836	0.152233668	0.232285429	0.357436677	0.446803899	0.999996191
CD19	0.929935369	0.244318464	0.326161313	0.774842127	0.14983132	0.484365351	0.999996191
CD200	0.36934771	0.602147522	0.503551147	0.919302572	0.124963335	0.560172707	0.999996191
CD209	0.497629274	0.83137214	0.511855442	0.654174806	0.720150923	0.915667696	0.999996191
CD274	0.990536926	0.981204905	0.045892068	0.428640413	0.856968736	0.607077177	0.999996191
CD276	0.129325046	0.728117597	0.488768584	0.832397089	0.991562804	0.767949049	0.999996191
CD2AP	0.635285114	0.738050108	0.698101423	0.485472751	0.60953919	0.912164906	0.999996191
CD2BP2	0.563391662	0.567497661	0.80276113	0.451791504	0.756905025	0.899941326	0.999996191
CD302	0.36519311	0.967499418	0.753428857	0.360891033	0.820119657	0.88563691	0.999996191
CD34	0.628531943	0.516160399	0.73485979	0.547159641	0.533918698	0.868173388	0.999996191
CD38	0.516851018	0.618900957	0.660165252	0.742284395	0.482119853	0.879864105	0.999996191
CD44	0.136320511	0.699982885	0.295326942	0.605806485	0.892062646	0.592835502	0.999996191
CD46	0.977200712	0.339562234	0.740209639	0.91755663	0.830063543	0.97186923	0.999996191
CD55	0.392321736	0.305040046	0.268189997	0.350198228	0.665380654	0.458981881	0.999996191
CD58	0.9432467	0.582968186	0.289370072	0.754669105	0.436160278	0.823705875	0.999996191
CD59	0.83822085	0.071433086	0.319732769	0.682391302	0.996872633	0.562521052	0.999996191
CD7	0.630643584	0.871857241	0.407270741	0.142303036	0.743548922	0.67899321	0.999996191
CD72	0.225002296	0.885995063	0.347242593	0.852509823	0.561083885	0.742717154	0.999996191
CD80	0.974607749	0.551776094	0.315420389	0.652813249	0.418970899	0.80326393	0.999996191
CD81	0.390397498	0.191697751	0.557057617	0.352831526	0.818574947	0.547454687	0.999996191
CD9	0.655838937	0.607673916	0.216372323	0.784426494	0.910222945	0.849605218	0.999996191
CD99L2	0.903326678	0.893084744	0.376227738	0.238250037	0.79558117	0.838943589	0.999996191
CDADC1	0.772961488	0.316511439	0.289995875	0.76589713	0.340665848	0.630913452	0.999996191
CDC123	0.381981701	0.70918118	0.785631758	0.786960875	0.282132339	0.80640854	0.999996191
CDC14B	0.215126451	0.967935971	0.694919004	0.601773793	0.088729335	0.464831166	0.999996191
CDC23	0.922937495	0.854663215	0.22243558	0.487578306	0.463960899	0.775856328	0.999996191
CDC26	0.508655936	0.338999314	0.413634795	0.634672321	0.96610524	0.793029278	0.999996191

CDC34	0.503684912	0.406348369	0.831335284	0.311770935	0.722826569	0.769642313	0.999996191
CDC40	0.146510253	0.498839747	0.578878004	0.996929162	0.263500903	0.532185609	0.999996191
CDC42BPA	0.825493681	0.481341346	0.589456553	0.556184163	0.743148317	0.912102323	0.999996191
CDC42EP4	0.189996191	0.966919057	0.215108585	0.326360185	0.671518125	0.485586225	0.999996191
CDC42SE2	0.413792361	0.72294276	0.240549669	0.798794173	0.960520486	0.832331795	0.999996191
CDC5L	0.193927225	0.571945554	0.984499058	0.144242303	0.999374976	0.599268163	0.999996191
CDCA2	0.539087741	0.439537311	0.108101809	0.868722234	0.938979739	0.654550843	0.999996191
CDCA5	0.831266867	0.383578762	0.279544141	0.378570961	0.909927508	0.728584545	0.999996191
CDCA7L	0.829296169	0.939369163	0.901631654	0.755773982	0.775066624	0.997785526	0.999996191
CDH1	0.991788888	0.36262699	0.811313476	0.619928284	0.325480449	0.842606324	0.999996191
CDH13	0.800133281	0.866076365	0.297696929	0.74768423	0.826336253	0.941774418	0.999996191
CDH22	0.084706762	0.973921945	0.646803431	0.773238347	0.881343957	0.760003632	0.999996191
CDH23	0.42941287	0.748411311	0.872259938	0.941839013	0.588154588	0.958901715	0.999996191
CDH24	0.882167748	0.181958526	0.286956241	0.93055541	0.710318556	0.726994096	0.999996191
CDH3	0.415154543	0.554662488	0.339823953	0.40497584	0.704135503	0.66734993	0.999996191
CDH5	0.250279186	0.906806101	0.512138593	0.125107726	0.936498769	0.571143289	0.999996191
CDH6	0.252778989	0.800313802	0.096101725	0.561488107	0.643832461	0.447925352	0.999996191
CDH7	0.761884813	0.815667923	0.490239739	0.595247898	0.778404438	0.951069339	0.999996191
CDK10	0.425207736	0.506731637	0.357058624	0.173995254	0.900114693	0.547588646	0.999996191
CDK11B	0.390089335	0.981392169	0.485972129	0.546370438	0.469265995	0.808019192	0.999996191
CDK13	0.795853343	0.96331204	0.728921649	0.039483121	0.92086594	0.649100916	0.999996191
CDK14	0.480189362	0.990749979	0.429153374	0.231783556	0.797583441	0.766784736	0.999996191
CDK17	0.41071973	0.500229448	0.611060358	0.102917908	0.910639832	0.543046748	0.999996191
CDK18	0.539124183	0.8580932	0.642795151	0.133258946	0.777677363	0.729274895	0.999996191
CDK19	0.773024314	0.498938149	0.849601467	0.183014302	0.599104054	0.757799202	0.999996191
CDK20	0.784613383	0.772540038	0.920544593	0.162237133	0.571523408	0.821678547	0.999996191
CDK4	0.231336211	0.44027093	0.560257335	0.753632325	0.9327025	0.777734679	0.999996191
CDK5	0.635963207	0.69941061	0.615603476	0.046049798	0.9050785	0.537226515	0.999996191
CDK5R1	0.793520664	0.978297023	0.611151765	0.380992295	0.574563004	0.920316312	0.999996191
CDK5RAP1	0.401942593	0.951755304	0.847752515	0.120059173	0.951254403	0.76335474	0.999996191
CDK5RAP3	0.93491023	0.944483352	0.555355965	0.536928303	0.519302158	0.948268994	0.999996191
CDK6	0.479749533	0.364226672	0.193640081	0.76702284	0.409735028	0.523828208	0.999996191
CDKAL1	0.404758804	0.503064562	0.80352557	0.960776142	0.790415298	0.939307446	0.999996191
CDKL4	0.620019283	0.225826067	0.278962919	0.958369484	0.251331182	0.500879083	0.999996191
CDKL5	0.660646474	0.623477134	0.757218742	0.170029502	0.334163233	0.62239174	0.999996191
CDKN2AIP	0.301323426	0.524793194	0.388806007	0.193213158	0.659614841	0.467365934	0.999996191
CDKN2AIPNL	0.444327615	0.620005449	0.496669752	0.607085248	0.672499898	0.834220253	0.999996191
CDKN2C	0.662180635	0.804763993	0.947171674	0.162230638	0.58936562	0.810003391	0.999996191
CDKN2D	0.503634643	0.882465369	0.907757614	0.5679116	0.257563631	0.842974757	0.999996191
CDKN3	0.945621037	0.192627078	0.298450231	0.442473046	0.882393191	0.657615662	0.999996191
CDO1	0.888559459	0.755331104	0.65089655	0.278899978	0.734414106	0.902400211	0.999996191
CDON	0.681235951	0.1622302	0.508245627	0.232923042	0.586867374	0.463704143	0.999996191
CDPF1	0.197721078	0.506239607	0.583998656	0.656185971	0.745751183	0.715178178	0.999996191
CDR2L	0.630961179	0.624882118	0.542027922	0.79227487	0.32082844	0.829690525	0.999996191
CDRT1	0.846023538	0.441697201	0.826635825	0.142142644	0.276962145	0.549357656	0.999996191

CDRT4	0.634063337	0.556165033	0.31663676	0.341659	0.283173281	0.526809647	0.999996191
CDV3	0.322708009	0.611595075	0.75921226	0.638193466	0.137559973	0.564457764	0.999996191
CDYL	0.555435659	0.256184799	0.572480565	0.914269728	0.657510371	0.812473075	0.999996191
CDYL2	0.428665192	0.944987071	0.183043961	0.95885647	0.212461343	0.591246331	0.999996191
CEACAM1	0.642683014	0.723141483	0.81913991	0.107915706	0.4539369	0.632362016	0.999996191
CEACAM19	0.936159397	0.905823176	0.37204957	0.93583685	0.152540119	0.798146016	0.999996191
CEBPA	0.789359901	0.760018267	0.588879224	0.630547491	0.205294963	0.800791919	0.999996191
CEBPD	0.757569488	0.545096865	0.215695578	0.223363331	0.606959935	0.548009729	0.999996191
CEBPZOS	0.369347681	0.770396891	0.667752608	0.230648824	0.728530103	0.735920322	0.999996191
CECR2	0.993162791	0.933656334	0.960858952	0.132691267	0.974710589	0.931677161	0.999996191
CELA1	0.90970587	0.940326119	0.290948995	0.083750177	0.502278046	0.520888494	0.999996191
CELF2	0.874144044	0.997748755	0.315808091	0.947990193	0.749363742	0.974549896	0.999996191
CELF6	0.792413127	0.668334801	0.531173655	0.717227201	0.556419657	0.928912894	0.999996191
CELSR2	0.545430422	0.809347306	0.264517652	0.333448251	0.821984826	0.736375873	0.999996191
CEND1	0.61829204	0.822319668	0.20241353	0.53574282	0.956256404	0.824798867	0.999996191
CENPH	0.668336717	0.583534674	0.046819888	0.52408724	0.925505643	0.489704407	0.999996191
CENPJ	0.843826791	0.357821518	0.747161034	0.572505823	0.941746435	0.937201518	0.999996191
CENPL	0.490309385	0.602339975	0.775311949	0.300327928	0.675251781	0.803420009	0.999996191
CENPM	0.576979953	0.553780692	0.855907994	0.768485572	0.649011202	0.948051072	0.999996191
CENPN	0.479353162	0.432503828	0.331299184	0.48038207	0.533925408	0.621240017	0.999996191
CENPO	0.809782596	0.094798943	0.359973636	0.521331926	0.848982994	0.550456213	0.999996191
CENPP	0.396666371	0.952860271	0.811510583	0.146191845	0.356301713	0.602166806	0.999996191
CENPQ	0.746486688	0.32536692	0.69233412	0.163285183	0.70375085	0.639296835	0.999996191
CENPS	0.791735966	0.253682926	0.715812295	0.280210849	0.936062033	0.766622896	0.999996191
CENPT	0.937999316	0.170272987	0.756903194	0.359412715	0.924979461	0.778088789	0.999996191
CENPU	0.104869691	0.864906901	0.837444408	0.242858339	0.951243494	0.620469478	0.999996191
CENPV	0.552474448	0.957569314	0.348952193	0.952645682	0.5274538	0.906903239	0.999996191
CEP104	0.820960073	0.95990503	0.064550066	0.256634325	0.981167777	0.55932268	0.999996191
CEP135	0.50974737	0.225948898	0.691925874	0.491046375	0.549320545	0.660090308	0.999996191
CEP152	0.354595564	0.631436192	0.330026054	0.22662389	0.628037464	0.521746352	0.999996191
CEP164	0.642918324	0.246158919	0.276565561	0.611638472	0.41373134	0.531538943	0.999996191
CEP170	0.77319411	0.0915757	0.255767029	0.930797777	0.511883835	0.484907029	0.999996191
CEP19	0.799440117	0.277027615	0.666066654	0.182998873	0.928930671	0.689950869	0.999996191
CEP250	0.905780561	0.055843726	0.311864694	0.705122597	0.780664409	0.4860702	0.999996191
CEP290	0.119959989	0.340067457	0.811766025	0.929139105	0.9477087	0.718828697	0.999996191
CEP295NL	0.587891724	0.515439492	0.369800006	0.938712032	0.296836206	0.731742999	0.999996191
CEP41	0.885574371	0.95693729	0.990784483	0.662181757	0.561260441	0.993138382	0.999996191
CEP44	0.26521579	0.622680715	0.895243493	0.399361273	0.316913834	0.633016417	0.999996191
CEP63	0.587963043	0.348746087	0.999631631	0.659509051	0.417375166	0.835827592	0.999996191
CEP70	0.56648398	0.946102854	0.604313526	0.042427465	0.681981706	0.500153123	0.999996191
CEP76	0.908632974	0.379085244	0.359626885	0.190982241	0.801229866	0.635541854	0.999996191
CEP78	0.949580189	0.300820996	0.089489193	0.581570099	0.956169477	0.57944815	0.999996191
CEP83	0.300117568	0.800235156	0.756094482	0.54602231	0.504405208	0.816020435	0.999996191
CEP85	0.706090324	0.226102978	0.30305375	0.465327563	0.69734781	0.598758825	0.999996191
CEP97	0.061092165	0.709046355	0.806101217	0.690916112	0.630017955	0.592453379	0.999996191

CERCAM	0.648517574	0.445591011	0.116671395	0.878665272	0.485811926	0.581844763	0.999996191
CERK	0.626419419	0.78869729	0.508590426	0.790186845	0.107496506	0.658697802	0.999996191
CERS1	0.911965893	0.529445663	0.434538524	0.969090157	0.284057969	0.839564564	0.999996191
CERS2	0.684481278	0.336122109	0.497843154	0.793450444	0.448994941	0.780798984	0.999996191
CERS5	0.969792164	0.697592286	0.329663064	0.560730755	0.355306321	0.795798124	0.999996191
CES2	0.090521345	0.988863483	0.640580479	0.815358003	0.173172723	0.473287947	0.999996191
CETN2	0.906382273	0.302613827	0.844126219	0.781317045	0.246306921	0.796280231	0.999996191
CETN3	0.293668947	0.534318476	0.610065553	0.911104163	0.679038265	0.843536713	0.999996191
CETN4	0.442519112	0.582511466	0.193268376	0.542713699	0.819315398	0.665935862	0.999996191
CFAP126	0.81876207	0.809384052	0.555673279	0.843682956	0.955922631	0.991881886	0.999996191
CFAP300	0.73443388	0.578949359	0.262046017	0.960779273	0.144618653	0.596034331	0.999996191
CFAP53	0.268604939	0.895980701	0.664955882	0.403052625	0.388406189	0.689771015	0.999996191
CFAP54	0.583849944	0.758322002	0.990986911	0.285214259	0.617872079	0.883046544	0.999996191
CFAP70	0.944696571	0.808782823	0.131246754	0.276202974	0.973819174	0.703962012	0.999996191
CFAP97	0.689206761	0.5622583	0.282959171	0.623360862	0.455595794	0.731239867	0.999996191
CFD	0.073770211	0.502134794	0.950594923	0.656109239	0.587460237	0.570490504	0.999996191
CFDP2	0.990408641	0.339145513	0.527367686	0.170690753	0.385988633	0.541491711	0.999996191
CFL2	0.943344448	0.127872768	0.12589822	0.891422607	0.804640499	0.528383442	0.999996191
CGGBP1	0.448958177	0.988265174	0.806587209	0.846238187	0.044083471	0.567309124	0.999996191
CGN	0.539201432	0.305985906	0.9982501	0.733582065	0.37010882	0.796903958	0.999996191
CGNL1	0.781961273	0.552852616	0.161142742	0.913476062	0.958295292	0.848115593	0.999996191
CGRRF1	0.239756405	0.743552668	0.424265388	0.708524771	0.675607219	0.759201674	0.999996191
CHAC2	0.664090487	0.114143346	0.211725101	0.487709333	0.959436222	0.459724694	0.999996191
CHAF1A	0.464509615	0.991692668	0.369149401	0.455147435	0.555078749	0.789908743	0.999996191
CHAMP1	0.763338975	0.829697953	0.832960351	0.807201511	0.987848543	0.998012902	0.999996191
CHCHD1	0.906083802	0.816136133	0.273537776	0.41758422	0.382738462	0.738258857	0.999996191
CHCHD10	0.65684629	0.429457334	0.426693453	0.065393573	0.887888473	0.446926704	0.999996191
CHCHD2	0.985857473	0.600496496	0.444544419	0.044040508	0.755535748	0.48761633	0.999996191
CHCHD3	0.713402792	0.527685002	0.690286575	0.173504729	0.675025601	0.726922828	0.999996191
CHCHD5	0.287494901	0.234955504	0.99843195	0.598034312	0.530063	0.659020687	0.999996191
CHCHD7	0.711816164	0.090511615	0.56282385	0.73864966	0.573855026	0.594629801	0.999996191
CHCHD8	0.993638377	0.998532474	0.854958685	0.570153247	0.066310031	0.736757313	0.999996191
CHD1L	0.446726463	0.285711184	0.435810581	0.711068207	0.679592022	0.703303464	0.999996191
CHD3	0.185617788	0.748591605	0.518284049	0.725667099	0.573096653	0.723891261	0.999996191
CHD4	0.738080513	0.799872675	0.082159751	0.41759381	0.704582531	0.580217071	0.999996191
CHD6	0.783800627	0.790059361	0.452680174	0.929559585	0.445561972	0.932459619	0.999996191
CHD7	0.423825664	0.86295598	0.746860247	0.633965424	0.487534341	0.894906057	0.999996191
CHD8	0.745316358	0.806569801	0.48638719	0.494360442	0.988510428	0.952112449	0.999996191
CHD9	0.902135657	0.972572543	0.952826821	0.293707514	0.842873038	0.977657896	0.999996191
CHEK1	0.74103974	0.423400082	0.400220333	0.87498468	0.863985217	0.909734994	0.999996191
CHEK2	0.479757195	0.631319641	0.555132505	0.762913755	0.425055232	0.830296819	0.999996191
CHERP	0.513099788	0.605170517	0.294694045	0.491904544	0.994133573	0.797026575	0.999996191
CHFR	0.600433593	0.228901229	0.988268669	0.74851749	0.972852	0.914657024	0.999996191
CHIC1	0.599587522	0.207520269	0.927138798	0.194742156	0.384072184	0.484903058	0.999996191
CHIC2	0.906284351	0.979643995	0.950330806	0.90655616	0.454509747	0.995397479	0.999996191

CHKA	0.551231555	0.914455503	0.919751722	0.202840059	0.079843141	0.459696323	0.999996191
CHKB	0.282372236	0.341221797	0.340489727	0.26986207	0.824328153	0.454613886	0.999996191
CHL1	0.889699431	0.855124742	0.0798188	0.404712344	0.480342517	0.543679557	0.999996191
CHM	0.587539052	0.822408547	0.826064584	0.789161308	0.235629236	0.877325444	0.999996191
CHML	0.431127357	0.792981032	0.759120436	0.790214344	0.961668644	0.975002709	0.999996191
CHMP1B	0.772283777	0.540644867	0.101588074	0.485146595	0.412843555	0.482059588	0.999996191
CHMP3	0.717510001	0.158489181	0.236456054	0.516439512	0.796102561	0.531182383	0.999996191
CHMP4A	0.807565855	0.395538628	0.149375983	0.29244594	0.725050252	0.514450436	0.999996191
CHMP4B	0.269166546	0.142280334	0.630625037	0.971173225	0.486027919	0.537014305	0.999996191
CHMP4C	0.746233324	0.253147611	0.887598081	0.170883713	0.645350001	0.630698736	0.999996191
CHMP5	0.858436475	0.521341138	0.350647093	0.574036065	0.406224127	0.761152749	0.999996191
CHMP7	0.317861128	0.299538063	0.368848732	0.326432632	0.613152615	0.447947348	0.999996191
CHN1	0.901594813	0.730484583	0.070732892	0.620808502	0.924343826	0.702248446	0.999996191
CHP1	0.717644666	0.3860052	0.265732298	0.629756491	0.425591347	0.643359322	0.999996191
CHPT1	0.566690294	0.854111324	0.311289517	0.751308946	0.715084437	0.889305016	0.999996191
CHRAC1	0.493618412	0.745325774	0.975952177	0.257766658	0.238095745	0.664917178	0.999996191
CHRDL1	0.930850897	0.644196472	0.315207825	0.976298192	0.048304451	0.490888255	0.999996191
CHRM3	0.752035944	0.990091648	0.510488948	0.874182146	0.213266634	0.870694197	0.999996191
CHRNA3	0.524722919	0.502863655	0.305328565	0.52010571	0.253049685	0.523285694	0.999996191
CHRNA3	0.540643894	0.751463634	0.430742115	0.547366303	0.697091737	0.861946823	0.999996191
CHRNA5	0.960871224	0.882105977	0.990852404	0.547647766	0.571354037	0.988110711	0.999996191
CHRNA7	0.383578749	0.779699741	0.451555206	0.846639823	0.755132258	0.897827527	0.999996191
CHRNB4	0.487207735	0.325665428	0.818719207	0.988940008	0.791094011	0.917828421	0.999996191
CHRNE	0.155745132	0.953918263	0.406545931	0.476421411	0.779801028	0.668438482	0.999996191
CHST1	0.448182347	0.491846193	0.689526485	0.761725247	0.817137614	0.909323032	0.999996191
CHST12	0.214594304	0.310015738	0.316701925	0.751356316	0.955362184	0.591485301	0.999996191
CHST14	0.235703994	0.858150651	0.49549277	0.880694772	0.953208838	0.894450533	0.999996191
CHST15	0.755166021	0.897014244	0.668948755	0.883545563	0.951957587	0.996854581	0.999996191
CHST2	0.514533886	0.694986806	0.766008916	0.97808115	0.734314608	0.974862087	0.999996191
CHST8	0.163756724	0.586388398	0.940981354	0.787772458	0.893127398	0.854531925	0.999996191
CHST9	0.222239291	0.821583576	0.637649626	0.402732627	0.189129928	0.489940551	0.999996191
CHSY1	0.807288295	0.757550188	0.616019986	0.121042601	0.460660066	0.655595834	0.999996191
CHSY3	0.495470214	0.874439654	0.843933085	0.790881389	0.52001668	0.956355552	0.999996191
CHTF8	0.57156599	0.533676801	0.847725883	0.415697811	0.321998858	0.750934151	0.999996191
CHTOP	0.89266284	0.54274785	0.906654792	0.491218312	0.321271674	0.867488677	0.999996191
CHURC1	0.444826891	0.214407316	0.524853678	0.63656476	0.814517222	0.696580449	0.999996191
CIAPIN1	0.465769562	0.824925315	0.50199139	0.428129389	0.231924319	0.637554843	0.999996191
CIB1	0.228765752	0.433822356	0.321721785	0.508243456	0.933264162	0.591750962	0.999996191
CIB2	0.339010257	0.931600216	0.366403916	0.663330812	0.782357792	0.845721004	0.999996191
CIB4	0.489866926	0.858619838	0.543166714	0.64002299	0.886617934	0.943385683	0.999996191
CIC	0.407134787	0.547948775	0.286929378	0.762124715	0.71555292	0.752500743	0.999996191
CIDEC	0.874178778	0.878961805	0.169635471	0.471685306	0.559770222	0.749878159	0.999996191
CILP	0.270700759	0.750599277	0.15283102	0.326885784	0.892848943	0.493956288	0.999996191
CINP	0.985355755	0.826471606	0.910887419	0.623024068	0.338057539	0.959375418	0.999996191
CIPC	0.534432266	0.750605062	0.601649191	0.644995398	0.601815799	0.908123272	0.999996191

CIR1	0.900063759	0.520874501	0.620366513	0.435121895	0.514382568	0.858124788	0.999996191
CIRBP	0.469979118	0.880389536	0.895923661	0.296262982	0.611684374	0.862847657	0.999996191
CISD1	0.967111299	0.814042467	0.792254316	0.060368567	0.925497338	0.75218378	0.999996191
CISD3	0.805294831	0.320957546	0.37168892	0.285830047	0.797659214	0.66373597	0.999996191
CITED1	0.681118288	0.822007286	0.881783402	0.030811175	0.794754247	0.548226598	0.999996191
CITED4	0.814917785	0.811819817	0.395752957	0.322020731	0.457707847	0.770792549	0.999996191
CIZ1	0.63564623	0.293537615	0.173763366	0.684616783	0.754216945	0.611280333	0.999996191
CKAP5	0.74988551	0.963292676	0.75167341	0.290607872	0.389208839	0.849213701	0.999996191
CKS1B	0.903167473	0.478587766	0.357055871	0.720226355	0.456625569	0.818494543	0.999996191
CLASP2	0.541157678	0.491752446	0.8896826	0.105265833	0.329589954	0.475915266	0.999996191
CLCA3	0.675991353	0.954155991	0.946742854	0.977280645	0.672765649	0.997513111	0.999996191
CLCC1	0.433206865	0.662499918	0.153942829	0.590358476	0.510114223	0.566653403	0.999996191
CLCF1	0.565048146	0.659494491	0.301363276	0.771768938	0.864507705	0.878660828	0.999996191
CLCN2	0.826549914	0.40639165	0.695143183	0.655893226	0.44718312	0.86570546	0.999996191
CLCN3	0.827347713	0.627253204	0.550777794	0.665316609	0.153426958	0.718937667	0.999996191
CLCN4	0.317023486	0.639696182	0.268123502	0.430164106	0.65053733	0.592667733	0.999996191
CLCN6	0.770500161	0.654894599	0.694823546	0.551777961	0.312402176	0.846714319	0.999996191
CLDN1	0.389584669	0.196160686	0.698715531	0.454374239	0.683543566	0.609443756	0.999996191
CLDN12	0.926778249	0.880890047	0.766575241	0.794041027	0.303237204	0.956521497	0.999996191
CLDN12	0.336948647	0.548402277	0.970172798	0.860369588	0.399765124	0.849830742	0.999996191
CLDN20	0.566264267	0.109622752	0.670942491	0.789767428	0.736978347	0.683414904	0.999996191
CLDN5	0.435836245	0.135602139	0.956146337	0.481800789	0.573569004	0.597715159	0.999996191
CLEC16A	0.736269956	0.431848603	0.997213613	0.128018532	0.359571004	0.584570852	0.999996191
CLEC18C	0.366553861	0.507448277	0.369636624	0.145234392	0.812166917	0.473593323	0.999996191
CLEC1A	0.676530091	0.6362956	0.808357466	0.486080944	0.844535594	0.952090115	0.999996191
CLEC3B	0.342467633	0.662881256	0.831835452	0.454935864	0.548468338	0.805924217	0.999996191
CLEC9A	0.485081863	0.867370402	0.226482273	0.580231373	0.713624929	0.774793869	0.999996191
CLIC1	0.277126799	0.903443406	0.519524586	0.924053421	0.785873856	0.909128437	0.999996191
CLIC3	0.551290914	0.667193628	0.454088909	0.430308222	0.523521925	0.766216761	0.999996191
CLIP1	0.774798924	0.757256918	0.972540215	0.854923995	0.424710201	0.977715916	0.999996191
CLIP3	0.597432066	0.68602538	0.151962023	0.81022889	0.793511072	0.777434339	0.999996191
CLIP4	0.720306669	0.814968064	0.367183805	0.252859363	0.687151077	0.765374232	0.999996191
CLK2	0.549772072	0.476207827	0.589811207	0.339794236	0.822213529	0.790641352	0.999996191
CLK3	0.865853446	0.859651976	0.428292865	0.584883387	0.249240627	0.803556937	0.999996191
CLMN	0.463235219	0.966006359	0.929783702	0.302871847	0.099171619	0.554588413	0.999996191
CLMP	0.438080324	0.345449101	0.291146114	0.576526486	0.795902206	0.64813007	0.999996191
CLN6	0.933691567	0.761858477	0.713395607	0.369277294	0.26108309	0.812323166	0.999996191
CLN8	0.226271754	0.930246134	0.990809472	0.15156865	0.990198383	0.732200935	0.999996191
CLNK	0.499769644	0.783205045	0.700387837	0.106547339	0.642412966	0.633568677	0.999996191
CLNS1A	0.7953982	0.238120931	0.472914664	0.589789108	0.736011634	0.772152746	0.999996191
CLOCK	0.66608892	0.643469025	0.694931301	0.780622797	0.969937062	0.981921202	0.999996191
CLP1	0.744053861	0.31060864	0.454588626	0.25565206	0.904956922	0.68392891	0.999996191
CLPB	0.858860685	0.755384099	0.821659834	0.338234734	0.427090024	0.88248138	0.999996191
CLPTM1	0.447864284	0.389126167	0.451452653	0.926525962	0.232928379	0.614043565	0.999996191
CLSPN	0.931659451	0.268666086	0.149986044	0.944872147	0.279602105	0.510727019	0.999996191

CLSTN1	0.701077652	0.990766243	0.516484351	0.644396797	0.958304071	0.981085325	0.999996191
CLSTN2	0.705724365	0.592381088	0.871556344	0.842847133	0.170939472	0.824084593	0.999996191
CLSTN3	0.190609801	0.711335963	0.877861789	0.172943468	0.54716228	0.534724197	0.999996191
CLTA	0.69008063	0.139371733	0.188396089	0.929023323	0.791628855	0.566951539	0.999996191
CLTC	0.952280606	0.277317706	0.699497214	0.496439896	0.699845136	0.855976732	0.999996191
CLTRN	0.61241558	0.418127073	0.940202791	0.926265749	0.472109155	0.921860577	0.999996191
CLUAP1	0.987829702	0.238078702	0.578719493	0.678779551	0.447326113	0.783054818	0.999996191
CLYBL	0.964757918	0.826351883	0.213081789	0.579726575	0.480991944	0.806827785	0.999996191
CMBL	0.745449525	0.812316235	0.665356716	0.189699206	0.729448256	0.833904411	0.999996191
CMC1	0.790302941	0.522330592	0.971762486	0.104214443	0.672502571	0.711881103	0.999996191
CMC4	0.464539036	0.071644114	0.710849755	0.468834304	0.871595026	0.505942249	0.999996191
CMIP	0.875594106	0.885355363	0.766449618	0.588172437	0.586585498	0.97714742	0.999996191
CMKLR1	0.948622933	0.137093786	0.793374524	0.152081162	0.836041443	0.563930068	0.999996191
CMPK1	0.467162138	0.401222336	0.830445546	0.746272466	0.237486067	0.708268958	0.999996191
CMTM3	0.441977108	0.829914708	0.050641484	0.636652631	0.964519686	0.537127475	0.999996191
CMTM4	0.549165445	0.295135916	0.654415583	0.934506707	0.353187185	0.753029021	0.999996191
CMTM7	0.997349693	0.969095164	0.394090763	0.928391622	0.94934749	0.994735339	0.999996191
CMTR1	0.743341535	0.686910638	0.435695929	0.384047985	0.782807389	0.862190066	0.999996191
CMTR2	0.611426263	0.85530742	0.532415796	0.522918468	0.105712225	0.594894728	0.999996191
CNBP	0.962498628	0.103499875	0.141377799	0.929547446	0.880172418	0.53906019	0.999996191
CNEP1R1	0.332142336	0.199188826	0.943893187	0.506626301	0.836907679	0.700412543	0.999996191
CNIH3	0.492814513	0.669467557	0.04523475	0.620611691	0.764688953	0.449288726	0.999996191
CNIH4	0.424728495	0.993993243	0.777310812	0.427264035	0.62032336	0.898776406	0.999996191
CNMD	0.893393836	0.22243775	0.723330437	0.921772613	0.876824726	0.932527279	0.999996191
CNN3	0.792694708	0.816876341	0.991123219	0.115726237	0.432985003	0.737269005	0.999996191
CNNM2	0.738056472	0.622265775	0.90630806	0.429758205	0.509159131	0.904629104	0.999996191
CNNM3	0.764261659	0.47723666	0.467610405	0.461398431	0.158212222	0.55386617	0.999996191
CNNM4	0.556137403	0.598294712	0.614875915	0.162999721	0.425403489	0.579057726	0.999996191
CNOT10	0.14016075	0.469083418	0.676118357	0.744902007	0.343459356	0.53656672	0.999996191
CNOT2	0.747302192	0.9000258	0.759411152	0.084024422	0.918322864	0.774588845	0.999996191
CNOT3	0.969362092	0.746855512	0.427119498	0.332231835	0.915220471	0.90856666	0.999996191
CNOT8	0.977427607	0.788449763	0.950904231	0.360989794	0.909288606	0.984742725	0.999996191
CNOT9	0.645427628	0.833056336	0.683447061	0.922585181	0.754067234	0.987122415	0.999996191
CNPY2	0.556675793	0.732942759	0.304069849	0.337237045	0.592982206	0.687891553	0.999996191
CNPY3	0.729832688	0.884594264	0.85425752	0.901445863	0.943878642	0.998893619	0.999996191
CNPY4	0.975692356	0.78824812	0.363937947	0.534968746	0.73867552	0.92731259	0.999996191
CNR1	0.774366465	0.711226322	0.542756024	0.961995027	0.41968695	0.936419347	0.999996191
CNRIP1	0.68405486	0.878220178	0.793670799	0.134280924	0.581254342	0.764220033	0.999996191
CNST	0.782097263	0.479908174	0.290731991	0.294886292	0.884720586	0.714271673	0.999996191
CNTF	0.845455298	0.976197551	0.902441596	0.194519791	0.978145618	0.951407017	0.999996191
CNTN1	0.405847281	0.979596397	0.593743358	0.815021759	0.681349451	0.944420947	0.999996191
CNTNAP3	0.842108739	0.717114033	0.944027682	0.676791693	0.341700817	0.944954217	0.999996191
COA1	0.427893238	0.561838991	0.893104589	0.166331404	0.58465998	0.654417045	0.999996191
COA3	0.954502483	0.639133214	0.706950872	0.025300651	0.95268907	0.519549784	0.999996191
COA5	0.222577239	0.490192163	0.360000803	0.30273972	0.811332875	0.505557595	0.999996191

COA6	0.433380895	0.533396865	0.454405932	0.233683338	0.796509333	0.6415927	0.999996191
COA7	0.495370993	0.742080799	0.371374252	0.300952264	0.282608328	0.54051557	0.999996191
COBLL1	0.783522002	0.572717259	0.884830402	0.127680976	0.270634538	0.572587288	0.999996191
COCH	0.509794347	0.571062325	0.426539423	0.83292547	0.975189386	0.916950304	0.999996191
COG1	0.353083508	0.225385212	0.626536195	0.918217667	0.923773717	0.787144048	0.999996191
COG3	0.455834468	0.949324512	0.460772003	0.155674867	0.986190003	0.728015193	0.999996191
COG4	0.805867046	0.765229739	0.638195612	0.129349541	0.638953035	0.739397649	0.999996191
COG5	0.669444097	0.998306875	0.624070074	0.980581419	0.373786889	0.957669873	0.999996191
COG7	0.942613446	0.673307487	0.174015446	0.477115156	0.795197159	0.785506116	0.999996191
COIL	0.251935323	0.284546033	0.667803676	0.526105145	0.440876221	0.532018266	0.999996191
COL11A2	0.330407155	0.558447425	0.828126372	0.579826652	0.775090532	0.866103215	0.999996191
COL13A1	0.944235748	0.217666406	0.086489242	0.857896151	0.614611947	0.500179447	0.999996191
COL18A1	0.870841907	0.456785583	0.304730028	0.94784436	0.835596227	0.911101956	0.999996191
COL23A1	0.56057969	0.458332243	0.129786479	0.408168275	0.735597445	0.512492059	0.999996191
COL27A1	0.501574075	0.342452378	0.360862183	0.988189427	0.205605369	0.556066472	0.999996191
COL28A1	0.874175937	0.165461254	0.440418611	0.834289166	0.41488321	0.665033123	0.999996191
COL4A1	0.313186527	0.594981915	0.260495753	0.564996185	0.579064296	0.600995466	0.999996191
COL4A2	0.429384278	0.551036131	0.230364174	0.51892983	0.728765291	0.651907478	0.999996191
COL4A3BP	0.297680765	0.537003719	0.202492976	0.981120405	0.874274383	0.709503825	0.999996191
COL4A5	0.256387805	0.805110341	0.339699538	0.693920171	0.16691116	0.473853948	0.999996191
COL4A6	0.189497705	0.833997868	0.577846022	0.422271273	0.212084254	0.475125368	0.999996191
COL5A3	0.789448787	0.740274019	0.221443306	0.943826116	0.263223355	0.737226301	0.999996191
COLEC10	0.622015771	0.721295837	0.317806158	0.566221367	0.700551417	0.836218141	0.999996191
COMMD1	0.354031443	0.538561162	0.836009765	0.533450637	0.984670204	0.893810744	0.999996191
COMMD10	0.913900731	0.873318361	0.706023714	0.450510879	0.444694188	0.929506754	0.999996191
COMMD2	0.864209486	0.23536853	0.778072938	0.375134304	0.501856773	0.722914446	0.999996191
COMMD3	0.860606331	0.753733711	0.635777376	0.04846826	0.532786243	0.524108724	0.999996191
COMMD4	0.23673532	0.591421707	0.276252561	0.443620667	0.921725087	0.600187816	0.999996191
COMMD6	0.432349123	0.757140694	0.67640083	0.065244646	0.565927431	0.475058353	0.999996191
COMMD7	0.739567553	0.279260505	0.978187211	0.233443387	0.82466508	0.772203406	0.999996191
COMMD8	0.246861306	0.502037223	0.279434775	0.803364744	0.863495923	0.681670484	0.999996191
COMMD9	0.961851959	0.983657389	0.374250044	0.759387805	0.607931348	0.962782447	0.999996191
COMT	0.421746169	0.164414881	0.151534771	0.746636558	0.96613778	0.461386585	0.999996191
COMTD1	0.566730296	0.491574699	0.505119177	0.121569376	0.968480998	0.609255487	0.999996191
COP1	0.954996865	0.94801205	0.177173828	0.454039923	0.793458491	0.839650354	0.999996191
COPB1	0.273341578	0.503593947	0.254953506	0.693122215	0.509859772	0.553128782	0.999996191
COPB2	0.810149121	0.700960136	0.313171605	0.848988735	0.451203095	0.864926253	0.999996191
COPE	0.249140064	0.883937256	0.502599391	0.107346993	0.661178332	0.467829177	0.999996191
COPRS	0.873587712	0.58672911	0.52627113	0.203336437	0.964733734	0.825394665	0.999996191
COPS4	0.444991295	0.69693709	0.632523146	0.779844254	0.383808598	0.842173389	0.999996191
COPS5	0.759751239	0.750907897	0.562421376	0.125486154	0.288740657	0.540756013	0.999996191
COPS9	0.680878061	0.283984134	0.497512574	0.162963982	0.970115971	0.592567518	0.999996191
COPZ2	0.173928172	0.278957299	0.461374306	0.460239125	0.839886619	0.485424624	0.999996191
COQ10A	0.361899831	0.499622731	0.2141625	0.334852499	0.575298996	0.458526627	0.999996191
COQ2	0.850043564	0.488693151	0.604818956	0.302973841	0.98599153	0.878899908	0.999996191

COQ3	0.835897491	0.771661842	0.70089983	0.452853912	0.519410767	0.92298477	0.999996191
COQ4	0.528434203	0.8585569	0.965627935	0.103780226	0.557362059	0.691976241	0.999996191
COQ5	0.883772276	0.612242062	0.314894342	0.245124271	0.842562961	0.753983138	0.999996191
COQ6	0.725035322	0.619825976	0.9103488	0.047507501	0.720693128	0.57659339	0.999996191
COQ7	0.367039296	0.518078789	0.484698967	0.144926509	0.98290122	0.564083372	0.999996191
COQ8B	0.397932453	0.452240417	0.679862876	0.516578872	0.87826116	0.83319888	0.999996191
CORIN	0.28143277	0.919249686	0.786072322	0.283529707	0.278829888	0.603387149	0.999996191
CORO1B	0.501680748	0.862238479	0.706080359	0.384545198	0.918878138	0.924624714	0.999996191
CORO6	0.568303253	0.403558586	0.524380311	0.247658394	0.394375948	0.542721422	0.999996191
COX14	0.518569476	0.852545239	0.772903695	0.21109192	0.918136967	0.860715344	0.999996191
COX15	0.886467148	0.70538838	0.968092351	0.418503995	0.679212391	0.966426204	0.999996191
COX16	0.244777117	0.6650484	0.956913152	0.553640555	0.67051063	0.839758952	0.999996191
COX17	0.636041035	0.335324725	0.617818927	0.096299565	0.755974703	0.504494951	0.999996191
COX19	0.22045268	0.690146833	0.486665148	0.699323837	0.734143708	0.768077592	0.999996191
COX20	0.328393979	0.656115652	0.704305154	0.13615518	0.836021143	0.617397019	0.999996191
COX4I1	0.882607825	0.299876116	0.505927339	0.209746545	0.872100904	0.685417786	0.999996191
COX4I2	0.209388447	0.963729835	0.574980071	0.203882853	0.51000473	0.54783624	0.999996191
COX7A1	0.848878204	0.48571613	0.180044407	0.117072284	0.917854873	0.470594	0.999996191
COX7A2	0.863816033	0.439553817	0.761150405	0.040105295	0.983727659	0.537051697	0.999996191
COX7A2L	0.73220655	0.270320059	0.657042606	0.358036473	0.461471781	0.660002737	0.999996191
COX7B	0.783024275	0.515826549	0.9528741	0.021803578	0.868150698	0.454299051	0.999996191
COX8B	0.604808509	0.655412184	0.346176302	0.072789056	0.777719705	0.465803389	0.999996191
CPD	0.249954912	0.674559845	0.778079496	0.256912035	0.366459486	0.552337409	0.999996191
CPEB1	0.652289566	0.672996676	0.266554947	0.782629807	0.310813633	0.714238409	0.999996191
CPEB2	0.89115441	0.108090805	0.114332333	0.794075226	0.990947994	0.485702713	0.999996191
CPED1	0.993313042	0.697157241	0.501976512	0.756332526	0.960277073	0.986655563	0.999996191
CPLANE1	0.37574057	0.214134244	0.990456992	0.165583936	0.636669396	0.480018756	0.999996191
CPLANE2	0.199357587	0.712603899	0.406774948	0.801401718	0.519531032	0.681962056	0.999996191
CPN2	0.793547791	0.997397665	0.610044707	0.488545592	0.814375819	0.973473269	0.999996191
CPNE1	0.365132151	0.905268711	0.874645283	0.245761859	0.105698367	0.459730849	0.999996191
CPNE3	0.820419216	0.580328294	0.090763936	0.338154621	0.806751703	0.543416833	0.999996191
CPNE5	0.377072106	0.193034573	0.555489015	0.744393962	0.609824754	0.629251156	0.999996191
CPNE8	0.398819174	0.830780037	0.793688433	0.349949321	0.425735498	0.77352472	0.999996191
CPOX	0.861146558	0.972289902	0.252252018	0.370884612	0.249511171	0.641526821	0.999996191
CPSF2	0.787402709	0.330466905	0.484512591	0.493671305	0.267691187	0.610345906	0.999996191
CPSF3	0.987982659	0.68863189	0.887986765	0.766706839	0.030440883	0.5778782	0.999996191
CPSF4	0.798286048	0.987888057	0.596052953	0.556472073	0.902227402	0.983941326	0.999996191
CPSF6	0.270325187	0.667945209	0.944810025	0.105202454	0.841225768	0.591146584	0.999996191
CPSF7	0.637882514	0.844607552	0.787456794	0.151104742	0.454496275	0.718672668	0.999996191
CPT2	0.900510198	0.344414207	0.57260599	0.106286482	0.704802825	0.566630021	0.999996191
CPTP	0.85245313	0.656955681	0.789904051	0.291518601	0.716624897	0.906440377	0.999996191
CPXM1	0.234593578	0.758956555	0.261865572	0.297851004	0.636593922	0.489364322	0.999996191
CPXM2	0.801333895	0.591934876	0.058968113	0.393924862	0.676645269	0.458426912	0.999996191
CRACR2A	0.690368395	0.773373771	0.569566024	0.404713225	0.644730311	0.886602688	0.999996191
CRACR2B	0.827809113	0.160734025	0.677147193	0.348436804	0.827818746	0.696832141	0.999996191

CRADD	0.907023537	0.860278577	0.992585625	0.873416752	0.976400953	0.999927487	0.999996191
CRAMP1	0.977283934	0.359023439	0.784607962	0.923211642	0.905013962	0.982818038	0.999996191
CRAT	0.745030995	0.726022912	0.966508082	0.15102186	0.749723985	0.843454888	0.999996191
CRBN	0.495954838	0.778505381	0.053711808	0.484598267	0.837314881	0.480314103	0.999996191
CRCP	0.870188339	0.284373718	0.393648669	0.75601472	0.667532526	0.813129866	0.999996191
CREB3L2	0.710084451	0.550779402	0.27471831	0.284637792	0.636418383	0.64070593	0.999996191
CREBBP	0.504019679	0.981000644	0.936291816	0.088743678	0.598976331	0.686314391	0.999996191
CREBL2	0.936897399	0.37070796	0.653995933	0.684995044	0.521110559	0.889526904	0.999996191
CREBZF	0.390281048	0.677103568	0.917605197	0.76263736	0.197039978	0.760377099	0.999996191
CREG1	0.928916664	0.352536621	0.159709765	0.890101651	0.977173128	0.79987806	0.999996191
CREG2	0.685552421	0.430268409	0.743999026	0.108036552	0.796576483	0.634832562	0.999996191
CRELD1	0.576034533	0.305189426	0.582097501	0.884967108	0.776527463	0.869581421	0.999996191
CRIP2	0.743705289	0.68086328	0.706280563	0.107959556	0.209875066	0.473443079	0.999996191
CRIPT	0.147274064	0.708818873	0.50774464	0.637243403	0.890914553	0.724783515	0.999996191
CRKL	0.245564535	0.791961523	0.265630858	0.730166014	0.855400579	0.737890941	0.999996191
CRLF3	0.296759591	0.685645895	0.400092886	0.976140943	0.543340092	0.790785541	0.999996191
CRNKL1	0.858385123	0.692922137	0.899087601	0.253592259	0.992344135	0.946837797	0.999996191
CROCC	0.732898043	0.127658532	0.134352977	0.939480396	0.714586676	0.480835506	0.999996191
CROT	0.49092827	0.909548432	0.976086891	0.41762633	0.882344725	0.961473685	0.999996191
CRTAM	0.713819482	0.152446215	0.449900467	0.447497413	0.49059436	0.525847896	0.999996191
CRTAP	0.299232582	0.462164005	0.226211119	0.820795315	0.823202079	0.656810332	0.999996191
CRTC2	0.725544712	0.750046099	0.901232423	0.391806315	0.464067568	0.901967364	0.999996191
CRTC3	0.847363793	0.57133389	0.840817224	0.804998614	0.132290639	0.79148641	0.999996191
CRY1	0.695248352	0.833606121	0.381231252	0.674421058	0.900022132	0.946525297	0.999996191
CRY2	0.399594325	0.920508658	0.89874514	0.413981156	0.548745903	0.878982289	0.999996191
CRYBA4	0.820583437	0.358326991	0.456984266	0.902282437	0.544755648	0.860307522	0.999996191
CRYBG1	0.917306458	0.7880133	0.469632129	0.555018029	0.975327976	0.970759057	0.999996191
CRYZ	0.482336091	0.863948406	0.428839256	0.384921947	0.659091288	0.799291396	0.999996191
CRYZL1	0.391524464	0.743180634	0.09869556	0.533821249	0.741838667	0.536559056	0.999996191
CS	0.950808688	0.586228759	0.8586283	0.116542539	0.511156531	0.714548317	0.999996191
CSAD	0.485621604	0.790648033	0.786155909	0.423376176	0.960575163	0.938117196	0.999996191
CSDC2	0.644171044	0.485812145	0.783067656	0.500720197	0.569984659	0.868786022	0.999996191
CSDE1	0.889457464	0.522977212	0.24029075	0.992447232	0.803336478	0.901882158	0.999996191
CSF2RA	0.461643122	0.493709464	0.123646447	0.598237482	0.742407454	0.554882757	0.999996191
CSF2RB	0.856285718	0.84291914	0.798125179	0.759723087	0.432720097	0.972618934	0.999996191
CSF3	0.444719104	0.910323484	0.894747424	0.444057908	0.720434419	0.932261632	0.999996191
CSGALNACT1	0.609136236	0.324473717	0.69183868	0.731576915	0.208040182	0.653779077	0.999996191
CSK	0.317580149	0.922849253	0.424317145	0.410006282	0.834704425	0.788255169	0.999996191
CSNK1A1	0.616116004	0.537003145	0.984590067	0.932360613	0.878927851	0.988651452	0.999996191
CSNK1D	0.382927019	0.957765435	0.725165626	0.055445931	0.784409292	0.539790585	0.999996191
CSNK1E	0.177781679	0.983751797	0.775859446	0.626765669	0.09787158	0.478327428	0.999996191
CSNK1G1	0.594831784	0.959471475	0.783298913	0.93473342	0.856506141	0.995901338	0.999996191
CSNK1G3	0.071566855	0.68921738	0.599084229	0.833034364	0.981737725	0.682816139	0.999996191
CSNK2A1	0.721614619	0.351443574	0.892723218	0.514794414	0.181976251	0.657468048	0.999996191
CSRNP3	0.578551668	0.481995557	0.738343721	0.32576679	0.733268422	0.813207324	0.999996191

CSRP1	0.380680809	0.266354704	0.826774698	0.308913623	0.27028462	0.447200901	0.999996191
CST3	0.232162608	0.413009538	0.971018535	0.914316935	0.740589017	0.853254483	0.999996191
CST6	0.259318337	0.840352866	0.391647099	0.569552481	0.869859425	0.787108458	0.999996191
CST7	0.947815888	0.963346989	0.103934618	0.883521555	0.787745918	0.860315296	0.999996191
CSTF1	0.927737223	0.27839485	0.870579426	0.370779161	0.103358378	0.484660174	0.999996191
CSTF2	0.398824595	0.959221445	0.85492076	0.278563294	0.711474579	0.857483404	0.999996191
CTBP2	0.899639278	0.639084438	0.603629955	0.166206991	0.816327618	0.805812668	0.999996191
CTC1	0.467982201	0.178003409	0.531161715	0.553019177	0.446696197	0.52903044	0.999996191
CTCF	0.338579799	0.936795521	0.482312596	0.77138644	0.802132885	0.909385937	0.999996191
CTDNEP1	0.313340269	0.935636803	0.747280286	0.31721835	0.919526825	0.855322225	0.999996191
CTDP1	0.875157778	0.799000221	0.903507919	0.859321404	0.845614719	0.998746462	0.999996191
CTDSP1	0.173587222	0.950540589	0.986848197	0.624095638	0.670815312	0.865020947	0.999996191
CTDSP2	0.948273519	0.641779533	0.8987895	0.94176436	0.565273494	0.991340204	0.999996191
CTDSPL	0.708655876	0.602913929	0.43459273	0.496271372	0.206928446	0.636704486	0.999996191
CTDSPL2	0.764818973	0.47712524	0.978253748	0.587026635	0.53096324	0.927954329	0.999996191
CTF1	0.893439905	0.882630296	0.501656217	0.711376534	0.37678317	0.922659174	0.999996191
CTH	0.702319537	0.123352081	0.26582217	0.441866936	0.723468235	0.456165605	0.999996191
CTIF	0.688728998	0.737923193	0.360847359	0.462892699	0.249806392	0.657441418	0.999996191
CTNNA1	0.552099474	0.726748289	0.509461233	0.778975006	0.892986948	0.951697107	0.999996191
CTNNA3	0.722772364	0.525043136	0.659278452	0.439716412	0.365576233	0.778213011	0.999996191
CTNNAL1	0.283148936	0.693479042	0.181478157	0.707106076	0.96869148	0.684742956	0.999996191
CTNNB1	0.400902677	0.650796608	0.151001917	0.315269599	0.941404534	0.54185489	0.999996191
CTNNBIP1	0.364223563	0.308633098	0.391766683	0.574150617	0.96960209	0.68559487	0.999996191
CTNNBL1	0.466749702	0.852242002	0.34711474	0.233869273	0.550328334	0.622940993	0.999996191
CTNND1	0.756885191	0.927889536	0.847467285	0.390770426	0.595944514	0.949474733	0.999996191
CTR9	0.632801941	0.91542395	0.850122554	0.526840017	0.747641923	0.974044167	0.999996191
CTSD	0.36399114	0.724115181	0.346745543	0.967846527	0.960646029	0.89574905	0.999996191
CTSL	0.276590474	0.263187047	0.470420338	0.894741734	0.559692053	0.615979225	0.999996191
CTSW	0.847451692	0.9128053	0.424379814	0.055984299	0.786371722	0.582661353	0.999996191
CTTNBP2NL	0.627624022	0.723668041	0.732020482	0.031488797	0.829806793	0.486155878	0.999996191
CTU1	0.558884001	0.549194294	0.689846977	0.268245937	0.590898032	0.745228308	0.999996191
CTU2	0.572914408	0.572860136	0.995253809	0.49329578	0.738766572	0.93502874	0.999996191
CTXN1	0.641442657	0.778025533	0.330934637	0.222097313	0.503080129	0.630299978	0.999996191
CUL2	0.325603188	0.780010864	0.525633425	0.950086758	0.937428983	0.934908485	0.999996191
CUL4B	0.18871689	0.895204926	0.403590264	0.713716958	0.425317379	0.652704754	0.999996191
CUL5	0.13023043	0.462969906	0.360092218	0.878523084	0.443917772	0.481446498	0.999996191
CUL9	0.439705373	0.122151799	0.300649028	0.512330066	0.971748111	0.472008057	0.999996191
CUTC	0.865412666	0.889726127	0.566251724	0.827968219	0.765580414	0.98978418	0.999996191
CUX1	0.947792368	0.797575209	0.407910251	0.476940662	0.445805787	0.859204033	0.999996191
CWC15	0.96783174	0.378554736	0.365991358	0.911053513	0.568953244	0.867874961	0.999996191
CWC25	0.136869652	0.949934963	0.882030404	0.183532621	0.443744659	0.499521557	0.999996191
CWC27	0.799565416	0.624812813	0.587585878	0.314573625	0.643160697	0.843978884	0.999996191
CXCL12	0.585372546	0.840548624	0.662462236	0.070756515	0.809046794	0.632469105	0.999996191
CXCL9	0.312588236	0.566171073	0.517691537	0.883621193	0.22301384	0.62603316	0.999996191
CXHXorf36	0.756591784	0.919687498	0.041731405	0.360629551	0.823319057	0.484761024	0.999996191

CXHXorf56	0.887087719	0.896146931	0.260632541	0.097393446	0.547713769	0.531134911	0.999996191
CXHXorf58	0.93124429	0.282300798	0.329334214	0.73448457	0.965401795	0.849153308	0.999996191
CXXC1	0.778392961	0.086324283	0.123061796	0.914240938	0.94103167	0.450076513	0.999996191
CYB561	0.740509562	0.741208892	0.07537593	0.718730975	0.754450057	0.668389482	0.999996191
CYB561D1	0.576062246	0.479196153	0.176951096	0.716257694	0.813776449	0.714288305	0.999996191
CYB5A	0.283527286	0.973043158	0.42108541	0.223612047	0.773781333	0.646999042	0.999996191
CYB5B	0.395648666	0.603206315	0.586116655	0.140354963	0.364417523	0.451084301	0.999996191
CYB5D1	0.878990301	0.831829733	0.460135359	0.770713123	0.751660975	0.974322556	0.999996191
CYB5R1	0.565708167	0.733833365	0.298773102	0.616144814	0.803490917	0.849186095	0.999996191
CYBA	0.622089395	0.848971931	0.040708713	0.794250744	0.953600846	0.605878753	0.999996191
CYBRD1	0.930750479	0.845836678	0.833437519	0.159816611	0.652223225	0.865503147	0.999996191
CYFIP1	0.624521097	0.963581711	0.451740756	0.80030472	0.947971063	0.977472839	0.999996191
CYGB	0.869551881	0.67283162	0.426855428	0.051733697	0.751452881	0.506734262	0.999996191
CYLD	0.558712561	0.804956262	0.275368382	0.555653219	0.749972963	0.821265355	0.999996191
CYP26B1	0.723752375	0.816315605	0.641426394	0.600523502	0.410960063	0.907913186	0.999996191
CYP27B1	0.148637238	0.715686319	0.289624612	0.443946717	0.842928939	0.53916863	0.999996191
CYP2D14	0.614100561	0.495912855	0.165093541	0.561688921	0.926972194	0.698228974	0.999996191
CYP2U1	0.78519242	0.963082937	0.612491954	0.789186785	0.740198805	0.989100043	0.999996191
CYP39A1	0.680207346	0.856224192	0.9021892	0.64128052	0.113493007	0.769159577	0.999996191
CYP4A11	0.431147685	0.593806711	0.91945252	0.219787765	0.286734853	0.587733017	0.999996191
CYP7B1	0.273475016	0.573205765	0.280043827	0.972072575	0.843917	0.758225355	0.999996191
CYP8B1	0.446836405	0.907920033	0.851260748	0.892892156	0.436248738	0.946801233	0.999996191
CYS1	0.655805334	0.497810005	0.705112037	0.853245213	0.462422387	0.904280399	0.999996191
CYSRT1	0.868390272	0.911489035	0.44027264	0.756542978	0.232599459	0.848984359	0.999996191
CYSTM1	0.668817053	0.548656356	0.070462727	0.319641913	0.902243618	0.458458067	0.999996191
CYTH1	0.51662154	0.905223029	0.396430948	0.421759485	0.502563533	0.774060423	0.999996191
CYTH3	0.763524225	0.41457122	0.851439574	0.865648479	0.424106644	0.914699317	0.999996191
CYYR1	0.360140897	0.835013096	0.610516059	0.739928168	0.651899562	0.901086715	0.999996191
D2HGDH	0.600244589	0.314701792	0.362861075	0.213715073	0.873756107	0.559184531	0.999996191
DAAM1	0.269554581	0.742112254	0.35617353	0.981750833	0.403203506	0.712487703	0.999996191
DAAM2	0.313296992	0.387995372	0.970497241	0.835160434	0.667750361	0.859724228	0.999996191
DAB1	0.871097153	0.668457137	0.758581259	0.800808969	0.556308633	0.97487601	0.999996191
DAB2	0.569682798	0.489018522	0.925277352	0.141829006	0.806132446	0.720842816	0.999996191
DAB2IP	0.182643496	0.906568768	0.617777264	0.292997224	0.80426116	0.682318671	0.999996191
DACT1	0.661011482	0.65491152	0.122928943	0.673460977	0.890994453	0.735950059	0.999996191
DACT2	0.852546357	0.979365652	0.230910216	0.600361405	0.087078965	0.513746489	0.999996191
DAD1	0.756674601	0.428302501	0.569718725	0.241523763	0.771541626	0.749831532	0.999996191
DAGLA	0.839323393	0.941811043	0.189995404	0.066582485	0.951160827	0.502911128	0.999996191
DAGLB	0.511687452	0.299809431	0.686693343	0.600432634	0.302718034	0.637515331	0.999996191
DAP3	0.512569983	0.389127391	0.649819939	0.322250516	0.195250293	0.47459891	0.999996191
DAPK1	0.643464586	0.992075265	0.815739391	0.603818634	0.18288088	0.838866425	0.999996191
DARS2	0.807227687	0.683942026	0.453846975	0.198523072	0.963206111	0.808776091	0.999996191
DAXX	0.570045812	0.749647098	0.984328859	0.6257247	0.124166134	0.740278513	0.999996191
DAZAP1	0.884282909	0.341949342	0.142839799	0.890480595	0.635414344	0.684983702	0.999996191
DBI	0.936284283	0.504455171	0.88411488	0.112694534	0.490081334	0.673758971	0.999996191

DBNDD1	0.884396771	0.71539675	0.408334409	0.060978372	0.731492729	0.539075743	0.999996191
DBNDD2	0.454942666	0.384914651	0.802475862	0.703111595	0.109425791	0.526963986	0.999996191
DBNL	0.133832433	0.60337792	0.857718424	0.720202619	0.269521537	0.568662906	0.999996191
DBR1	0.954568782	0.287201853	0.995219186	0.416345337	0.17476456	0.64457653	0.999996191
DBT	0.935130762	0.432917321	0.2778661	0.404450068	0.624203362	0.713803371	0.999996191
DCAF12	0.832078308	0.884123619	0.288668349	0.297175546	0.464052762	0.719644855	0.999996191
DCAF15	0.622759187	0.724411381	0.560496946	0.995594558	0.657964511	0.963741937	0.999996191
DCAF16	0.660170115	0.544281753	0.617093289	0.837749132	0.545885583	0.917568984	0.999996191
DCAF4	0.993355522	0.871088844	0.4119495	0.169005789	0.480602818	0.717478682	0.999996191
DCAF6	0.60109593	0.487890594	0.069088957	0.463878653	0.998026816	0.500329919	0.999996191
DCAF7	0.81621207	0.983824543	0.768841895	0.51803797	0.933857261	0.992033544	0.999996191
DCAF8	0.952067586	0.673788969	0.037662831	0.597000891	0.797280015	0.538678509	0.999996191
DCBLD1	0.433922353	0.626019619	0.780799597	0.083131744	0.651113597	0.538359489	0.999996191
DCBLD2	0.721220042	0.677131188	0.574521847	0.726309079	0.914888485	0.971661099	0.999996191
DCDC2B	0.676955366	0.393406473	0.21940159	0.729374108	0.588600713	0.690019258	0.999996191
DCHS1	0.183302199	0.587905154	0.395662046	0.733288274	0.993923323	0.730849668	0.999996191
DCHS2	0.432476922	0.186948522	0.247206686	0.865953459	0.840092628	0.583834439	0.999996191
DCK	0.158179188	0.75357667	0.598065351	0.604123459	0.320515681	0.573763138	0.999996191
DCLK1	0.387125942	0.882941324	0.494799485	0.978038209	0.618433435	0.918584215	0.999996191
DCLK3	0.802524239	0.78362806	0.60896501	0.105608733	0.922284383	0.764642916	0.999996191
DCLRE1A	0.446974931	0.756295069	0.928121585	0.85850345	0.820810285	0.980987877	0.999996191
DCLRE1C	0.990384633	0.874588391	0.898821891	0.979245843	0.298887711	0.982395507	0.999996191
DCN	0.448518566	0.52799975	0.631391543	0.204384399	0.950295982	0.71805596	0.999996191
DCP1B	0.673862152	0.218389102	0.730438422	0.879641343	0.195939672	0.631082376	0.999996191
DCP2	0.411215332	0.838010154	0.648346318	0.830358855	0.263768794	0.812352484	0.999996191
DCPS	0.470701697	0.964997543	0.420532482	0.757165673	0.171092561	0.687389687	0.999996191
DCTN1	0.873701365	0.112839944	0.489668538	0.540748062	0.3024333	0.468726579	0.999996191
DCTN2	0.950191489	0.94397855	0.189756817	0.692356488	0.281226875	0.742879448	0.999996191
DCTN3	0.77047565	0.625893626	0.568850152	0.1185537	0.739138457	0.681784441	0.999996191
DCTN4	0.147533926	0.415316437	0.328235103	0.511821898	0.804435195	0.4773824	0.999996191
DCTN5	0.814048524	0.399131654	0.760387364	0.067297876	0.532374853	0.489596366	0.999996191
DCUN1D1	0.113388547	0.59001981	0.552433153	0.679820771	0.537583259	0.569557913	0.999996191
DCUN1D2	0.593303724	0.763021706	0.363585868	0.332672831	0.743371494	0.780360903	0.999996191
DCUN1D3	0.987374569	0.542215271	0.662585191	0.746141886	0.652913897	0.966719892	0.999996191
DCUN1D4	0.405296007	0.278558564	0.287101904	0.793891704	0.830361358	0.658915507	0.999996191
DCUN1D5	0.224480098	0.354856534	0.897794299	0.824791096	0.453855876	0.70252839	0.999996191
DCX	0.606157538	0.200856489	0.459771085	0.929675797	0.99947042	0.822559306	0.999996191
DDAH1	0.782910962	0.634530458	0.292241397	0.148758207	0.938758736	0.648675366	0.999996191
DDAH2	0.506267509	0.59829369	0.685349167	0.533277911	0.672727274	0.877802467	0.999996191
DDB1	0.728503475	0.85397542	0.438103605	0.211766363	0.541999421	0.732096999	0.999996191
DDB2	0.647042126	0.729448377	0.831584564	0.653107887	0.477200656	0.937768252	0.999996191
DDHD1	0.297052737	0.596606879	0.4709246	0.659064296	0.329582306	0.626831876	0.999996191
DDHD2	0.52696405	0.994708527	0.844356552	0.91635996	0.827015688	0.994717764	0.999996191
DDIT3	0.806129645	0.427318822	0.891033355	0.100504056	0.43521965	0.568395733	0.999996191
DDIT4L	0.299477878	0.724601706	0.825528631	0.181011153	0.56809073	0.629960298	0.999996191

DDO	0.377510581	0.890268083	0.059170482	0.796648199	0.692253396	0.529663058	0.999996191
DDOST	0.252830604	0.648054191	0.167365924	0.773054395	0.824654292	0.619736373	0.999996191
DDR2	0.445267784	0.697284209	0.156735709	0.658406115	0.483893104	0.596313887	0.999996191
DDRGK1	0.653459641	0.681671635	0.198997863	0.112971171	0.894416883	0.491773662	0.999996191
DDT	0.879781362	0.921741537	0.509930549	0.0538183	0.59760968	0.566572822	0.999996191
DDX1	0.980871333	0.617231611	0.593030599	0.348960945	0.173315378	0.662052885	0.999996191
DDX19B	0.539156544	0.726374229	0.686888757	0.701736309	0.899624412	0.965513502	0.999996191
DDX20	0.220622627	0.573663399	0.729694737	0.69506114	0.8400819	0.828496543	0.999996191
DDX23	0.520536547	0.938789664	0.195130363	0.676061384	0.385691113	0.688312586	0.999996191
DDX28	0.688082073	0.410971299	0.390054322	0.804420441	0.939844834	0.893275985	0.999996191
DDX39A	0.151917398	0.762950374	0.705674407	0.413255144	0.368686599	0.554046358	0.999996191
DDX39B	0.476584833	0.329560923	0.242238604	0.533280469	0.949409348	0.638690446	0.999996191
DDX42	0.509256294	0.525857193	0.777109958	0.99537084	0.895029319	0.971314102	0.999996191
DDX5	0.351297873	0.30801314	0.961270486	0.412204481	0.247858403	0.52370375	0.999996191
DDX50	0.925507755	0.540196594	0.855820309	0.255979737	0.298433434	0.740310401	0.999996191
DDX52	0.344228706	0.139044857	0.790021088	0.680252749	0.575611742	0.587355476	0.999996191
DDX55	0.274004903	0.345683972	0.954718779	0.418042026	0.432629743	0.6067283	0.999996191
DDX59	0.998544629	0.799711832	0.93459221	0.297280491	0.826437771	0.970618878	0.999996191
DEAF1	0.971357451	0.455343879	0.264306693	0.152230744	0.670447446	0.54571201	0.999996191
DECR1	0.918956287	0.912441752	0.535070099	0.047945255	0.607648923	0.563227027	0.999996191
DECR2	0.245849589	0.239638153	0.449809839	0.838014455	0.835317547	0.631327031	0.999996191
DEF8	0.074870739	0.835329667	0.803160229	0.939352211	0.939867648	0.795461191	0.999996191
DEGS1	0.62297149	0.726576337	0.562381001	0.258445888	0.561590055	0.762901234	0.999996191
DEK	0.16768555	0.926310196	0.512187177	0.13178638	0.665065384	0.446528967	0.999996191
DENND1B	0.706573381	0.817869545	0.91706582	0.257963618	0.346767086	0.806964364	0.999996191
DENND2A	0.365029185	0.689658127	0.483094127	0.307796716	0.713545333	0.70209118	0.999996191
DENND2C	0.440021674	0.996163384	0.639161205	0.078694285	0.587874149	0.561600293	0.999996191
DENND3	0.294829522	0.405715144	0.928916971	0.671976445	0.948742199	0.870642848	0.999996191
DENND4A	0.822974949	0.68917039	0.561199147	0.342800393	0.628047442	0.865792812	0.999996191
DENND4B	0.870587786	0.201945919	0.346769578	0.676556602	0.822772087	0.747289193	0.999996191
DENND4C	0.529440392	0.593710092	0.682500236	0.758948457	0.775806536	0.940910267	0.999996191
DEPDC1B	0.807331879	0.514089868	0.154859504	0.642808582	0.731574146	0.72561872	0.999996191
DEPDC5	0.761168144	0.339615294	0.741424796	0.969016893	0.836142084	0.958904743	0.999996191
DEPDC7	0.367737734	0.784586445	0.638789611	0.239499181	0.496612291	0.663893194	0.999996191
DEPP1	0.921288976	0.635076811	0.497276316	0.306855014	0.940297057	0.894158476	0.999996191
DEPTOR	0.721244969	0.834736646	0.038351802	0.4751945	0.99365429	0.528545358	0.999996191
DERA	0.830533738	0.145617885	0.567300065	0.994724882	0.350094349	0.680614566	0.999996191
DERL2	0.394730042	0.313105611	0.646201054	0.985827936	0.990894246	0.884283843	0.999996191
DESI2	0.468736586	0.669182256	0.604388511	0.079817459	0.979659636	0.587588994	0.999996191
DET1	0.923402858	0.906193645	0.742443864	0.440664818	0.597040931	0.962775059	0.999996191
DFFA	0.222459908	0.344331054	0.269455101	0.980183445	0.440472196	0.490835245	0.999996191
DGAT1	0.349654212	0.937666529	0.774497039	0.655520438	0.203105919	0.746582266	0.999996191
DGAT2	0.926762888	0.776063303	0.955907214	0.131166186	0.604161811	0.83017257	0.999996191
DGCR2	0.91846236	0.468546871	0.187338873	0.200405882	0.950159592	0.594389581	0.999996191
DGCR6L	0.645397633	0.971126958	0.27709303	0.061786028	0.865767923	0.49853439	0.999996191

DGKA	0.717818439	0.606263049	0.379133358	0.207540797	0.863675627	0.721505746	0.999996191
DGKD	0.353424034	0.557517289	0.689862093	0.278901968	0.840296593	0.735504957	0.999996191
DGKE	0.54674854	0.580221702	0.411790346	0.628130261	0.396853398	0.739610238	0.999996191
DGKG	0.403489837	0.606517304	0.898769146	0.891032305	0.079497416	0.59726806	0.999996191
DGKH	0.175921072	0.964561103	0.829795405	0.94835181	0.411488834	0.831553055	0.999996191
DGKZ	0.367697671	0.259665968	0.817624038	0.912394555	0.601049316	0.78929024	0.999996191
DGUOK	0.548396612	0.648001245	0.663133339	0.221061839	0.730009804	0.768147376	0.999996191
DHCR24	0.391620823	0.871053849	0.383023957	0.238396582	0.256209194	0.470675638	0.999996191
DHDH	0.143286143	0.940816629	0.434027786	0.613270894	0.979028006	0.753668334	0.999996191
DHFR	0.538931201	0.848809722	0.668086424	0.418880154	0.898096066	0.931436821	0.999996191
DHH	0.538871839	0.262854169	0.606018929	0.457573208	0.93217413	0.76125316	0.999996191
DHRS12	0.645214373	0.896624692	0.496420327	0.934191642	0.699076351	0.972028048	0.999996191
DHRS3	0.771007587	0.785302916	0.3770047	0.057298325	0.57307591	0.459382106	0.999996191
DHRS4	0.385514961	0.600085565	0.584059395	0.300917724	0.754649441	0.728456572	0.999996191
DHRS7	0.790030079	0.957508526	0.176306375	0.475099003	0.582986786	0.76287104	0.999996191
DHRS7B	0.690922217	0.914094926	0.569970739	0.118171482	0.554923763	0.678271867	0.999996191
DHTKD1	0.570157291	0.548309896	0.530147828	0.897256809	0.798972639	0.934836333	0.999996191
DHX15	0.474685142	0.937840741	0.969142639	0.048390506	0.35175918	0.455731962	0.999996191
DHX16	0.930884347	0.779738839	0.584037326	0.447969306	0.712716735	0.947355225	0.999996191
DHX32	0.275327867	0.892959995	0.569902492	0.299782155	0.601881086	0.691522528	0.999996191
DHX33	0.554077782	0.782995389	0.307264745	0.742597236	0.314767309	0.731351176	0.999996191
DHX34	0.195953184	0.891829984	0.312160837	0.896510108	0.960950858	0.805480899	0.999996191
DHX38	0.652872567	0.231525739	0.099155284	0.880793597	0.768089773	0.514868986	0.999996191
DHX8	0.638014768	0.855343342	0.933740912	0.371975739	0.894066667	0.965366025	0.999996191
DHX9	0.424988694	0.973229187	0.848286939	0.268352476	0.769548384	0.8738972	0.999996191
DIABLO	0.752965471	0.459383548	0.587152847	0.137884062	0.354736472	0.511023261	0.999996191
DIAPH1	0.578259678	0.25790354	0.305812556	0.768597653	0.406429288	0.579882186	0.999996191
DIAPH2	0.747568558	0.410626165	0.320981635	0.501880946	0.660727553	0.740238857	0.999996191
DIAPH2	0.372876311	0.718613757	0.593139292	0.582883808	0.902839343	0.893669733	0.999996191
DICER1	0.777873622	0.642322795	0.510609776	0.493867243	0.601471302	0.880254806	0.999996191
DIDO1	0.571058277	0.373440253	0.649998162	0.370592328	0.455122125	0.67640621	0.999996191
DIMT1	0.87505333	0.609945751	0.849952887	0.41698171	0.13691408	0.696170496	0.999996191
DIO1	0.440327888	0.642361994	0.880532649	0.629048111	0.93209935	0.953949318	0.999996191
DIO2	0.861989135	0.798748316	0.617088332	0.580964047	0.812494834	0.975947753	0.999996191
DIO3	0.35691508	0.367307799	0.926817791	0.568475494	0.855479245	0.843178866	0.999996191
DIP2A	0.928618771	0.78904412	0.113284456	0.58905333	0.679418011	0.743326394	0.999996191
DIS3L	0.347311816	0.652178781	0.998604124	0.982491149	0.768382609	0.965896787	0.999996191
DIS3L2	0.828895521	0.355842077	0.824663757	0.840095462	0.122656195	0.689859271	0.999996191
DISC1	0.690908102	0.649178025	0.122600379	0.326903611	0.572191959	0.517554937	0.999996191
DIXDC1	0.309848792	0.693962287	0.738504597	0.656086511	0.379924469	0.775360833	0.999996191
DKK1	0.232475193	0.48364578	0.525125493	0.492988166	0.571359609	0.609991486	0.999996191
DKK3	0.653870522	0.480446296	0.303412459	0.521747207	0.615893623	0.728123152	0.999996191
DKKL1	0.240550867	0.989765212	0.268929188	0.266427671	0.826247353	0.577805448	0.999996191
DLC1	0.697329451	0.900578862	0.938346346	0.018339187	0.995829314	0.526088841	0.999996191
DLD	0.497850572	0.730512587	0.730844946	0.282486864	0.293321696	0.664805212	0.999996191

DLEC1	0.133745715	0.775018295	0.529828951	0.97534178	0.737738437	0.77506732	0.999996191
DLG2	0.964447203	0.829795495	0.543884559	0.179068593	0.926888226	0.873476598	0.999996191
DLG3	0.588909292	0.763716438	0.423990035	0.353046408	0.700469789	0.80606714	0.999996191
DLG4	0.392375031	0.472755136	0.226260689	0.364935774	0.793120704	0.549153139	0.999996191
DLG5	0.535463697	0.636924639	0.314731958	0.263073846	0.641641229	0.62672571	0.999996191
DLGAP1	0.513802399	0.786277013	0.16832889	0.234172317	0.57474925	0.495771096	0.999996191
DLGAP3	0.33877767	0.509999216	0.167397141	0.552028166	0.909506297	0.583580727	0.999996191
DLGAP4	0.520285853	0.913142197	0.139499622	0.951938885	0.72676211	0.801242145	0.999996191
DLL1	0.542523945	0.220110802	0.879291591	0.539680988	0.9826758	0.833712169	0.999996191
DLL4	0.167185907	0.245338398	0.547394642	0.915142452	0.962793243	0.643887541	0.999996191
DLST	0.881564683	0.575949818	0.865093086	0.115833807	0.838437914	0.788664616	0.999996191
DMAP1	0.50052914	0.940859366	0.20345928	0.152492345	0.608218634	0.490325588	0.999996191
DMKN	0.555862515	0.613628678	0.949096071	0.917675514	0.027333779	0.473824434	0.999996191
DMTF1	0.181933883	0.618469789	0.873563425	0.408778415	0.683303805	0.70735776	0.999996191
DMTN	0.581252563	0.263169508	0.936818182	0.280058972	0.571897051	0.672826498	0.999996191
DMXL1	0.537511919	0.55605199	0.095720124	0.739754896	0.568999354	0.547479534	0.999996191
DMXL2	0.601747511	0.921589897	0.977473611	0.920605719	0.909364772	0.998663201	0.999996191
DNAAF2	0.579309592	0.367353714	0.713616418	0.333335204	0.164771683	0.478708095	0.999996191
DNAAF4	0.323657334	0.377801211	0.83140623	0.656933341	0.864142075	0.839443314	0.999996191
DNAH1	0.97505499	0.210512922	0.953805726	0.414452555	0.390859922	0.734669286	0.999996191
DNAH12	0.436063872	0.659904759	0.567205752	0.276273157	0.190843806	0.484420835	0.999996191
DNAH5	0.243612599	0.991695859	0.843759369	0.831919554	0.257367248	0.792673932	0.999996191
DNAI1	0.48811982	0.677045019	0.526748174	0.888688323	0.045226142	0.447127273	0.999996191
DNAJA4	0.7413043	0.069031919	0.881507228	0.462559741	0.632791006	0.565176822	0.999996191
DNAJB11	0.375918801	0.75495792	0.666032744	0.244461222	0.73051547	0.746298778	0.999996191
DNAJB2	0.737190539	0.455303083	0.293730429	0.269221829	0.953590696	0.691745421	0.999996191
DNAJB5	0.631314503	0.587650052	0.995056382	0.990994482	0.376043111	0.94881199	0.999996191
DNAJB6	0.756334684	0.278687219	0.624486534	0.500678883	0.898350097	0.843486682	0.999996191
DNAJC1	0.602909186	0.498520944	0.981534755	0.105077272	0.825421432	0.693840289	0.999996191
DNAJC10	0.477082788	0.968829879	0.431283273	0.479805471	0.635747202	0.847668199	0.999996191
DNAJC12	0.252549063	0.951734615	0.861965076	0.59315346	0.699280439	0.897216887	0.999996191
DNAJC13	0.548852201	0.432082621	0.870108436	0.872625708	0.589762047	0.922829066	0.999996191
DNAJC15	0.190997732	0.945014994	0.615089922	0.858972932	0.405378521	0.771114873	0.999996191
DNAJC17	0.492832504	0.335943266	0.615314773	0.983389139	0.689549553	0.866974903	0.999996191
DNAJC18	0.986849443	0.272779986	0.447585124	0.56536155	0.79469533	0.829125454	0.999996191
DNAJC24	0.349455545	0.844396136	0.845582107	0.068678022	0.653984209	0.533766986	0.999996191
DNAJC4	0.860191543	0.446315423	0.449174316	0.441217534	0.896352699	0.865085864	0.999996191
DNAJC6	0.560058492	0.117674499	0.907913883	0.888739834	0.841905856	0.797115371	0.999996191
DNAJC7	0.16596676	0.282012966	0.637578271	0.456613439	0.587322097	0.471183317	0.999996191
DNAJC9	0.473694998	0.528993596	0.900812523	0.494089179	0.805463512	0.902897724	0.999996191
DNAL1	0.822676847	0.764114809	0.718993593	0.584119222	0.86028002	0.982245839	0.999996191
DNAL4	0.681994371	0.555756206	0.96575616	0.138846303	0.927856513	0.806063336	0.999996191
DNASE1	0.553001126	0.381605563	0.704459047	0.175044621	0.514440044	0.567834128	0.999996191
DNASE1L1	0.266676796	0.507775211	0.461442286	0.569067363	0.92793544	0.742064453	0.999996191
DND1	0.892712386	0.856157592	0.876138055	0.901928473	0.654736202	0.997332235	0.999996191

DNHD1	0.899401547	0.526992111	0.627038346	0.385320109	0.128414204	0.586034063	0.999996191
DNM3	0.413801596	0.675199174	0.947927492	0.744767147	0.186559798	0.76217463	0.999996191
DNMT1	0.59433706	0.354289604	0.164646165	0.838624642	0.757676088	0.664849836	0.999996191
DNMT3B	0.51875848	0.135287007	0.40526748	0.452781739	0.599843508	0.464795839	0.999996191
DNPEP	0.861658369	0.935209705	0.357691571	0.201171024	0.303772296	0.621209935	0.999996191
DNPH1	0.203666137	0.782252229	0.882968406	0.620112778	0.929099319	0.889473387	0.999996191
DNTTIP1	0.863961873	0.739565685	0.312076422	0.941357876	0.337021646	0.853780346	0.999996191
DOC2B	0.320457123	0.872954242	0.920279664	0.666382434	0.71981878	0.938703869	0.999996191
DOC2G	0.939311714	0.767945086	0.105797622	0.992393067	0.595479786	0.798380807	0.999996191
DOCK1	0.409808928	0.909642444	0.620846227	0.343736045	0.769325696	0.848677336	0.999996191
DOCK10	0.751600283	0.762764772	0.485663766	0.081157319	0.559578514	0.556841255	0.999996191
DOCK11	0.718684587	0.670140312	0.393803126	0.912795638	0.844153982	0.954012812	0.999996191
DOCK3	0.657638066	0.948295667	0.717004985	0.394331265	0.734706244	0.943317094	0.999996191
DOCK6	0.956431947	0.244195658	0.245783716	0.233238733	0.640928266	0.483900159	0.999996191
DOCK7	0.150156633	0.787178463	0.631305958	0.226620059	0.933891363	0.599906289	0.999996191
DOCK9	0.382417345	0.606604469	0.892777391	0.776265754	0.775631161	0.939657438	0.999996191
DOK1	0.680174807	0.583979782	0.492633341	0.546920525	0.391395853	0.785444909	0.999996191
DOK4	0.329478724	0.471817156	0.086979161	0.527032039	0.976841279	0.446228691	0.999996191
DOLPP1	0.237742586	0.725602553	0.788738344	0.140885555	0.371537761	0.450275862	0.999996191
DOPEY1	0.56893541	0.940632713	0.618819989	0.473204705	0.996150831	0.95931137	0.999996191
DOT1L	0.794440213	0.248698875	0.527029186	0.929721159	0.759891684	0.87606528	0.999996191
DPCD	0.40939975	0.601009617	0.54246187	0.185839058	0.640758539	0.601152445	0.999996191
DPEP3	0.077640826	0.920783896	0.188762228	0.823699625	0.977869888	0.527973322	0.999996191
DPF1	0.841367276	0.445029512	0.872923828	0.608389121	0.143588917	0.714832749	0.999996191
DPF2	0.839093597	0.42475078	0.511636937	0.437861558	0.203884089	0.605819868	0.999996191
DPF3	0.622318893	0.849185082	0.768792295	0.295637517	0.669708257	0.888456269	0.999996191
DPH2	0.990720896	0.379302238	0.391176269	0.329338909	0.862558027	0.78489818	0.999996191
DPH5	0.340224641	0.934718516	0.629475053	0.397776835	0.17319283	0.573582294	0.999996191
DPH6	0.801851692	0.620368713	0.970116901	0.876724998	0.263752941	0.92826762	0.999996191
DPM3	0.172689366	0.896168647	0.476704052	0.661675012	0.928276675	0.799202149	0.999996191
DPP7	0.229850121	0.904591512	0.250594083	0.982976212	0.748817044	0.769677005	0.999996191
DPP8	0.203664322	0.81951073	0.99374178	0.944993894	0.634250145	0.915252909	0.999996191
DPY19L1	0.204069805	0.34360655	0.865334459	0.78350982	0.189646992	0.492991207	0.999996191
DPY19L3	0.479944839	0.982103163	0.355845283	0.945851717	0.743500513	0.934094033	0.999996191
DPY19L4	0.937166402	0.948633137	0.539937948	0.534842291	0.675032486	0.96691427	0.999996191
DPY30	0.994893934	0.226844511	0.752679964	0.940300287	0.742915009	0.934708268	0.999996191
DPYD	0.906221059	0.771175199	0.095956161	0.658020774	0.890643296	0.774081933	0.999996191
DPYSL2	0.303706963	0.816625029	0.98503266	0.031886348	0.89700258	0.446899564	0.999996191
DPYSL3	0.604682561	0.791590173	0.334165728	0.650241976	0.698279646	0.874232654	0.999996191
DQX1	0.351391882	0.611816066	0.712392597	0.695701005	0.313003419	0.744055395	0.999996191
DR1	0.106603872	0.646304	0.841067678	0.343624713	0.683547388	0.571043471	0.999996191
DRD1	0.304068354	0.312516901	0.894012296	0.863306538	0.618114317	0.799280125	0.999996191
DRD2	0.967573656	0.362148065	0.993466012	0.224912184	0.827694298	0.857447227	0.999996191
DROSHA	0.673971112	0.63541182	0.272327425	0.10547854	0.838614937	0.518108195	0.999996191
DSB	0.616835328	0.370249398	0.1435807	0.502479157	0.936950001	0.595485346	0.999996191

DSC2	0.980807299	0.999906586	0.076481198	0.630533221	0.915051533	0.79119	0.999996191
DSCC1	0.902701021	0.688594663	0.483275858	0.393831421	0.181780063	0.660173774	0.999996191
DSEL	0.610195127	0.228764972	0.811188305	0.736161318	0.640226351	0.826797924	0.999996191
DSN1	0.959339561	0.945097335	0.70940994	0.575675807	0.730775481	0.989103056	0.999996191
DST	0.609958566	0.587167459	0.076908657	0.554751148	0.622794334	0.503009808	0.999996191
DSTYK	0.379679077	0.578983567	0.473785639	0.502844688	0.417778609	0.663528756	0.999996191
DTD1	0.490010075	0.540625796	0.79969927	0.261614703	0.876031952	0.811026136	0.999996191
DTNA	0.75807749	0.423008441	0.861663173	0.69858551	0.254837779	0.813234512	0.999996191
DTNB	0.993169022	0.102526529	0.863384534	0.324014768	0.801406932	0.671779944	0.999996191
DTWD1	0.204905846	0.91520692	0.931798056	0.785041181	0.594178426	0.890234983	0.999996191
DTWD2	0.617277097	0.308215603	0.716349922	0.583275778	0.252727592	0.646898385	0.999996191
DTX2	0.493454386	0.17153465	0.787608675	0.585456827	0.608002201	0.67929338	0.999996191
DTX3	0.9935221	0.852398887	0.051800485	0.493065576	0.752701059	0.60584448	0.999996191
DUS1L	0.832564265	0.702494812	0.641784622	0.119346205	0.499173096	0.667767076	0.999996191
DUS2	0.94050522	0.984129208	0.808344122	0.208249467	0.369713789	0.83914659	0.999996191
DUSP1	0.820126748	0.223615709	0.154611601	0.828835268	0.928074409	0.662915605	0.999996191
DUSP10	0.742032361	0.708996589	0.998136335	0.218329723	0.522945729	0.845464311	0.999996191
DUSP18	0.716873923	0.29598169	0.89663385	0.367455164	0.269435125	0.63430658	0.999996191
DUSP22	0.635131728	0.440514801	0.343885425	0.906291014	0.525402225	0.801100312	0.999996191
DUSP23	0.21384485	0.80951159	0.649369031	0.20130959	0.619441337	0.576740016	0.999996191
DUSP28	0.809477804	0.905740456	0.217101175	0.16094413	0.989315286	0.692002374	0.999996191
DUSP5	0.211729475	0.711359962	0.692356331	0.882401268	0.471555544	0.7916533	0.999996191
DUSP6	0.78554558	0.709567362	0.511445022	0.448116737	0.91837526	0.933540309	0.999996191
DUSP7	0.680006988	0.955562652	0.831122892	0.850670407	0.858736932	0.997303538	0.999996191
DUT	0.721541946	0.61239351	0.866352746	0.415563696	0.984571285	0.95956927	0.999996191
DVL2	0.96103606	0.500491658	0.671030338	0.142133837	0.860324008	0.774840631	0.999996191
DVL3	0.886185678	0.94445899	0.015303323	0.834315227	0.73201739	0.467060058	0.999996191
DXO	0.763693497	0.449705376	0.658711392	0.753288453	0.113196928	0.638964689	0.999996191
DYM	0.40905934	0.333546146	0.394359585	0.45707393	0.829329223	0.649846289	0.999996191
DYNC1H1	0.774363626	0.930796618	0.630621373	0.70766788	0.923530304	0.991889014	0.999996191
DYNC1I1	0.718082365	0.727582733	0.109820938	0.69537029	0.900106897	0.757718864	0.999996191
DYNC1LI2	0.624248081	0.647202662	0.266527499	0.419055553	0.810564875	0.761065994	0.999996191
DYNLL1	0.544510004	0.215428021	0.979375757	0.672834825	0.896613976	0.867453864	0.999996191
DYNLRB1	0.580874113	0.424297016	0.589778554	0.100863694	0.685935173	0.513326207	0.999996191
DYNLT1	0.821113701	0.206812093	0.457193807	0.422518218	0.726379129	0.680086551	0.999996191
DYRK1B	0.403450044	0.211167953	0.617765452	0.858743644	0.542764711	0.685710137	0.999996191
DYSF	0.196056595	0.752668344	0.801789626	0.149000435	0.797961921	0.577425684	0.999996191
DZIP1	0.486678136	0.898672367	0.579300171	0.783831644	0.948856315	0.972315891	0.999996191
DZIP1L	0.686223065	0.782098172	0.533012986	0.931564375	0.539319318	0.952608686	0.999996191
DZIP3	0.54790456	0.574507374	0.544969248	0.982340696	0.604184144	0.918037492	0.999996191
E2F1	0.671521328	0.221198648	0.126117904	0.637192467	0.939137674	0.533828198	0.999996191
E2F3	0.712675019	0.388338618	0.83463239	0.330208273	0.108880057	0.477914401	0.999996191
E2F5	0.158242992	0.580438425	0.783848783	0.754397033	0.743317605	0.778900321	0.999996191
E2F7	0.670962884	0.147328963	0.201291825	0.678950457	0.797493832	0.526302352	0.999996191
E2F8	0.943040719	0.447945765	0.161213382	0.262711962	0.788584661	0.577992254	0.999996191

E4F1	0.886507693	0.421684926	0.735825627	0.46256755	0.634054744	0.888853784	0.999996191
EAF1	0.173413982	0.565208511	0.490579843	0.201945065	0.899158234	0.487076577	0.999996191
EAF2	0.38434548	0.077969808	0.733023805	0.983833537	0.37942255	0.475598643	0.999996191
EBAG9	0.226583086	0.310860915	0.68903748	0.862972282	0.464122052	0.640460842	0.999996191
EBD	0.094956751	0.95057519	0.440132697	0.79649814	0.292409318	0.497787628	0.999996191
EBF1	0.700155365	0.913099302	0.426115509	0.152478504	0.444261122	0.630305522	0.999996191
EBF2	0.900206934	0.464473823	0.454543288	0.138583443	0.483976243	0.558396422	0.999996191
EBF3	0.773934783	0.494615176	0.360056722	0.761419239	0.835408866	0.899801223	0.999996191
EBI3	0.885328597	0.949334523	0.310590397	0.154699524	0.37687701	0.592709818	0.999996191
EBNA1BP2	0.566600995	0.743472597	0.225272527	0.757617931	0.224789053	0.60440509	0.999996191
ECE1	0.316114514	0.945469287	0.62060963	0.565347439	0.658232652	0.866857129	0.999996191
ECH1	0.897181496	0.628508127	0.52235491	0.530405001	0.343259062	0.827590274	0.999996191
ECHDC1	0.538383047	0.556382391	0.973188315	0.652051512	0.43380276	0.891783856	0.999996191
ECHDC2	0.92855231	0.240769271	0.321721933	0.647697038	0.941615723	0.793560277	0.999996191
ECHDC3	0.648359136	0.72259615	0.386720344	0.177067355	0.743275083	0.680223855	0.999996191
ECHS1	0.944738176	0.663800012	0.32542365	0.191323833	0.886662018	0.750973591	0.999996191
ECI1	0.919524686	0.265048994	0.494819327	0.235763452	0.907871483	0.695529027	0.999996191
ECI2	0.466176921	0.801773643	0.489044267	0.949122386	0.676097631	0.933518243	0.999996191
ECM2	0.650810398	0.712825903	0.636226061	0.499500561	0.810542344	0.935420409	0.999996191
ECPAS	0.981660182	0.893893588	0.416135456	0.960952789	0.805394944	0.990471125	0.999996191
ECSCR	0.50842919	0.551066999	0.43249031	0.907845408	0.776066492	0.896363451	0.999996191
ECT2	0.788065048	0.6033358	0.133268853	0.758460451	0.391785054	0.634238795	0.999996191
EDC3	0.45981748	0.124228288	0.396945086	0.993947738	0.692518983	0.597609419	0.999996191
EDC4	0.591723295	0.714246746	0.483616602	0.608518762	0.952653468	0.93455683	0.999996191
EDEM1	0.588415858	0.419674638	0.471941149	0.568691529	0.45413257	0.724825296	0.999996191
EDEM2	0.907463286	0.89970322	0.301844444	0.713424614	0.865491226	0.957304438	0.999996191
EDEM3	0.477865207	0.850894804	0.996718953	0.561295119	0.792667684	0.969548893	0.999996191
EDN1	0.947371639	0.554705426	0.726984207	0.73265402	0.999982202	0.990178125	0.999996191
EDN3	0.443657706	0.886955033	0.186697271	0.891908326	0.405819335	0.701235493	0.999996191
EDNRA	0.482601586	0.6288466	0.460849987	0.725371399	0.117382812	0.545344005	0.999996191
EDRF1	0.226797489	0.347593425	0.972835578	0.334220629	0.91126051	0.676221846	0.999996191
EEA1	0.647097696	0.957312601	0.171243756	0.299826689	0.37282956	0.54453461	0.999996191
EEF1A1	0.844827286	0.902930795	0.389061687	0.300207584	0.748056214	0.861669026	0.999996191
EEF1AKMT1	0.774878135	0.401059365	0.876504808	0.865173005	0.813592854	0.97336027	0.999996191
EEF1AKMT2	0.428206119	0.684379706	0.777069639	0.200710338	0.862454193	0.774624648	0.999996191
EEF1AKMT3	0.759385225	0.270664451	0.819914433	0.367440585	0.181369819	0.534176086	0.999996191
EEF1B2	0.792532844	0.769870361	0.280826053	0.830180892	0.464517278	0.86037631	0.999996191
EEF1D	0.870353757	0.373750456	0.550069353	0.376422883	0.534749059	0.758254201	0.999996191
EEF2	0.445234082	0.758926162	0.802975892	0.677239559	0.366735982	0.863312408	0.999996191
EEFSEC	0.503663251	0.851774589	0.854608353	0.447212189	0.17880461	0.719849006	0.999996191
EEPD1	0.757382858	0.598689568	0.59748792	0.157841958	0.482993628	0.652299343	0.999996191
EFCAB14	0.861227216	0.13040801	0.539866714	0.709734855	0.855515947	0.762256519	0.999996191
EFCAB2	0.189728649	0.217607419	0.728613786	0.934854389	0.678689413	0.636887292	0.999996191
EFCAB5	0.592064566	0.322171745	0.742669578	0.989624242	0.331697956	0.803663415	0.999996191
EFCAB6	0.745526441	0.793821908	0.908684028	0.978483981	0.461312238	0.985115905	0.999996191

EFCAB7	0.864543679	0.585796797	0.417448135	0.81712172	0.889231195	0.958055427	0.999996191
EFCAB8	0.64608403	0.5970678	0.472043202	0.718249555	0.774555	0.91745576	0.999996191
EFCC1	0.463518837	0.963571069	0.427876366	0.34097559	0.885696502	0.839446659	0.999996191
EFEMP1	0.536818295	0.645806583	0.266085115	0.828369491	0.669824895	0.819891897	0.999996191
EFHD1	0.76305837	0.519626392	0.409773017	0.953046799	0.948441832	0.954421943	0.999996191
EFL1	0.927764273	0.924548888	0.227912533	0.132263824	0.406719791	0.52173327	0.999996191
EFNA1	0.280429825	0.835773226	0.75370883	0.795233339	0.772897735	0.92528923	0.999996191
EFNA2	0.594063069	0.652832253	0.173637178	0.993627058	0.115811365	0.465360484	0.999996191
EFNA5	0.712930529	0.885306056	0.326143268	0.783324268	0.808798695	0.943944581	0.999996191
EFR3A	0.710530027	0.060336729	0.72987623	0.649782002	0.951633227	0.639554856	0.999996191
EFTUD2	0.263181243	0.428257252	0.50380349	0.945906348	0.325769645	0.61991159	0.999996191
EGFL7	0.540305015	0.497390886	0.871318939	0.591943924	0.463159099	0.856021404	0.999996191
EGFLAM	0.42526094	0.759715623	0.585038235	0.071308885	0.702107423	0.501963399	0.999996191
EGLN1	0.40078015	0.557439718	0.223710196	0.518679233	0.658771251	0.615164894	0.999996191
EGLN2	0.617394315	0.430866219	0.229316854	0.931742906	0.832566042	0.806656776	0.999996191
EGLN3	0.870096153	0.491229671	0.760079958	0.79085629	0.985064873	0.986747855	0.999996191
EGR1	0.75215793	0.205849199	0.41876812	0.680730276	0.212332796	0.500159911	0.999996191
EGR2	0.961771014	0.153311161	0.511868252	0.273531611	0.58554547	0.548213614	0.999996191
EGR3	0.748455183	0.033557141	0.447393013	0.875901617	0.794380772	0.466968171	0.999996191
EHBP1	0.744251975	0.483761995	0.292108605	0.149989266	0.7494648	0.543957586	0.999996191
EHBP1L1	0.679789281	0.807930356	0.936918925	0.765548357	0.450794475	0.968550361	0.999996191
EHD2	0.219301314	0.978015486	0.347007022	0.732084588	0.796538734	0.791691222	0.999996191
EHD3	0.666177186	0.529994036	0.29006078	0.132871664	0.616772583	0.479835896	0.999996191
EHD4	0.713701871	0.544951829	0.971259767	0.551357103	0.761142665	0.960492479	0.999996191
EHHADH	0.721827733	0.881960541	0.642762952	0.110560292	0.549571108	0.68830658	0.999996191
EHMT1	0.955680822	0.187660569	0.467803576	0.21654852	0.947387223	0.616697505	0.999996191
EHMT2	0.580546364	0.10326305	0.310145871	0.739841262	0.589097327	0.473448718	0.999996191
EID2	0.473546559	0.492349461	0.153695955	0.566141382	0.35598332	0.4527481	0.999996191
EID3	0.349880985	0.667160103	0.740380621	0.280607617	0.42206437	0.650535997	0.999996191
EIF1AD	0.738962236	0.149970096	0.283627842	0.642201259	0.626803153	0.556963475	0.999996191
EIF1AX	0.667593258	0.319080829	0.51088713	0.487382187	0.55478095	0.720549205	0.999996191
EIF1B	0.825248831	0.716456598	0.231641047	0.159397926	0.741653062	0.604763327	0.999996191
EIF2A	0.881770527	0.873490546	0.884180779	0.344010532	0.042886801	0.513149743	0.999996191
EIF2AK1	0.74990527	0.981799865	0.811661726	0.768745981	0.969782398	0.998522185	0.999996191
EIF2AK3	0.608920507	0.982180973	0.368257186	0.057444092	0.969369177	0.550977928	0.999996191
EIF2AK4	0.566502935	0.62790549	0.35764632	0.857817894	0.730754866	0.887284959	0.999996191
EIF2B3	0.954167874	0.794946165	0.885179242	0.152271705	0.207655241	0.657660739	0.999996191
EIF2B4	0.257863951	0.754780432	0.637005166	0.09435369	0.671249474	0.467746683	0.999996191
EIF2B5	0.823568707	0.722855053	0.294251579	0.607641103	0.247676074	0.699583456	0.999996191
EIF2D	0.104442247	0.451841517	0.701096824	0.846918627	0.57306495	0.603152514	0.999996191
EIF2S2	0.96026552	0.662933015	0.289881568	0.510161089	0.11968774	0.53479954	0.999996191
EIF2S3	0.348256466	0.843330393	0.602887986	0.220539405	0.404375712	0.599884984	0.999996191
EIF3D	0.704439141	0.804236685	0.805032794	0.269929272	0.29187839	0.757820487	0.999996191
EIF3F	0.634949935	0.876548134	0.576356906	0.160259808	0.242327625	0.553980271	0.999996191
EIF3G	0.358580348	0.846928896	0.191787944	0.974164291	0.423046784	0.681506918	0.999996191

EIF3H	0.731175252	0.881498909	0.15678519	0.220772993	0.588138405	0.56396599	0.999996191
EIF3I	0.715287676	0.552739589	0.332259414	0.738371184	0.072500926	0.448027617	0.999996191
EIF4B	0.305534454	0.816778165	0.633357286	0.174781562	0.597286889	0.60845425	0.999996191
EIF4E3	0.758404477	0.372615605	0.572248578	0.456328692	0.551421877	0.780305201	0.999996191
EIF4EBP2	0.619443264	0.845029414	0.097311527	0.876976958	0.928980064	0.783793467	0.999996191
EIF4ENIF1	0.979827428	0.918354658	0.995628251	0.330208259	0.743663293	0.980751141	0.999996191
EIF5	0.574554085	0.606100978	0.431020162	0.705263031	0.614028742	0.857902567	0.999996191
EIF5A2	0.848086197	0.927669507	0.836902647	0.934882835	0.871348988	0.999532961	0.999996191
ELAC1	0.93790855	0.437970982	0.892629479	0.064926359	0.763851766	0.627435714	0.999996191
ELF4	0.922788387	0.706717772	0.829766434	0.726996259	0.243466754	0.910817135	0.999996191
ELFN2	0.273620374	0.753877554	0.556645799	0.892411223	0.677373845	0.867673265	0.999996191
ELK3	0.966909912	0.907143005	0.078777623	0.845212069	0.786906346	0.801638529	0.999996191
ELK4	0.543664417	0.876448325	0.962019672	0.055191716	0.744695892	0.634357275	0.999996191
ELL	0.220432324	0.506931968	0.764720014	0.398805438	0.317179091	0.526918672	0.999996191
ELMO1	0.429896067	0.856957667	0.618012429	0.603780787	0.566222279	0.883966475	0.999996191
ELMOD3	0.280742682	0.609501115	0.664558738	0.973827466	0.538705025	0.844678105	0.999996191
ELMSAN1	0.590912783	0.701409133	0.959401733	0.327951167	0.13481858	0.620844648	0.999996191
ELOA	0.885689344	0.792706611	0.74383014	0.724804641	0.436799328	0.963610096	0.999996191
ELOC	0.303024081	0.508249518	0.869319997	0.376857297	0.559726723	0.712743534	0.999996191
ELOVL4	0.984490916	0.860951247	0.970381616	0.533294495	0.524244977	0.982805825	0.999996191
ELOVL7	0.790988784	0.623188755	0.184826288	0.580633317	0.988322899	0.823412279	0.999996191
ELP2	0.77322047	0.418887843	0.454538667	0.242181063	0.373132706	0.566631881	0.999996191
EMC1	0.479769974	0.558393164	0.452782278	0.266343579	0.256542422	0.477549412	0.999996191
EMC2	0.534412468	0.30444403	0.088912325	0.70992569	0.894197552	0.49638841	0.999996191
EMC3	0.698821088	0.557263987	0.355684448	0.384961902	0.394050878	0.655644403	0.999996191
EMC4	0.998231644	0.556605709	0.246676625	0.41622541	0.284753413	0.605405773	0.999996191
EMC7	0.978311191	0.225208122	0.184996289	0.417194744	0.551300869	0.50021449	0.999996191
EMCN	0.601344755	0.851253629	0.897810423	0.05798436	0.668168292	0.623322786	0.999996191
EMD	0.915144606	0.694050945	0.100985077	0.933339797	0.175421736	0.521468108	0.999996191
EMILIN2	0.141440222	0.904692973	0.988393708	0.882197699	0.643315513	0.872546788	0.999996191
EML1	0.526338042	0.59242151	0.04258064	0.90715742	0.819693663	0.509868491	0.999996191
EML2	0.632693085	0.692772669	0.660017561	0.214329496	0.521538388	0.738307502	0.999996191
EML3	0.93589457	0.188911978	0.554161967	0.37585177	0.772056547	0.714015924	0.999996191
EML4	0.332840598	0.438394468	0.495067757	0.305278543	0.784346762	0.617658951	0.999996191
EML5	0.359719821	0.576294424	0.634775954	0.433176099	0.216486893	0.552164611	0.999996191
EMP2	0.350286946	0.464337107	0.430004277	0.664505988	0.88256451	0.781726143	0.999996191
EMSY	0.242926994	0.640067732	0.936982943	0.096500549	0.709057888	0.511680149	0.999996191
EMX2	0.569890556	0.885480918	0.075604016	0.729871489	0.772385349	0.660190209	0.999996191
ENDOD1	0.676193356	0.107086634	0.681043463	0.935698888	0.889163904	0.781776688	0.999996191
ENDOU	0.924002434	0.472599916	0.522005148	0.908582037	0.405899755	0.894342378	0.999996191
ENGASE	0.439985197	0.840010247	0.581495868	0.316731043	0.755530205	0.820686948	0.999996191
ENKUR	0.63491846	0.591002243	0.232103167	0.326143489	0.453026299	0.560215833	0.999996191
ENO1	0.416158793	0.772395642	0.736759968	0.326822489	0.216450087	0.611421142	0.999996191
ENO2	0.261683504	0.899739206	0.550309472	0.929002565	0.086485648	0.519818834	0.999996191
ENO3	0.895169294	0.376263683	0.865591995	0.616950965	0.324209162	0.841095614	0.999996191

ENO4	0.933174486	0.742836069	0.727104521	0.412531716	0.053134105	0.531058724	0.999996191
ENOPH1	0.411635604	0.777952344	0.913727669	0.952480477	0.379696916	0.922435729	0.999996191
ENOSF1	0.75217904	0.826248035	0.168235468	0.781068492	0.194979193	0.601510109	0.999996191
ENOX1	0.887481076	0.423024444	0.611547585	0.248885956	0.640970262	0.761315744	0.999996191
ENOX2	0.409842748	0.229077696	0.853746674	0.871396002	0.463720513	0.738605761	0.999996191
ENPEP	0.164731939	0.282921804	0.740193909	0.77888376	0.769958621	0.652622458	0.999996191
ENPP1	0.648817473	0.493635486	0.924607671	0.028852573	0.922609383	0.468450334	0.999996191
ENPP6	0.695436206	0.209523302	0.893842228	0.690917871	0.433200922	0.772617107	0.999996191
ENTPD3	0.284953706	0.793341398	0.906870784	0.587914419	0.72390613	0.899183268	0.999996191
ENTPD4	0.8678271	0.53639742	0.242476041	0.31665872	0.68181725	0.684433871	0.999996191
ENTPD5	0.878697245	0.57954784	0.723134112	0.863291772	0.94027492	0.992055523	0.999996191
ENTPD6	0.691611108	0.600001648	0.318266327	0.106561387	0.709055759	0.511870153	0.999996191
ENTPD7	0.384487376	0.533551158	0.815959829	0.967122259	0.329914092	0.826919769	0.999996191
ENTR1	0.587402653	0.842364534	0.273131215	0.509398061	0.641933183	0.794854263	0.999996191
EOGT	0.169646422	0.661439207	0.429717672	0.67296635	0.735305722	0.680349905	0.999996191
EP300	0.609689363	0.777332714	0.744457666	0.073272247	0.720350452	0.632083385	0.999996191
EP400	0.83325557	0.924546132	0.658503908	0.310504289	0.808335286	0.941676693	0.999996191
EPB41	0.877367207	0.681903286	0.235801383	0.769685263	0.267828117	0.718318328	0.999996191
EPB41L1	0.314919069	0.773526413	0.745417879	0.800020734	0.671242732	0.912967595	0.999996191
EPB41L2	0.984219992	0.634382024	0.899599792	0.392774691	0.273700397	0.846575597	0.999996191
EPB41L4A	0.71766821	0.498167011	0.253583884	0.795098384	0.497811361	0.757572841	0.999996191
EPC1	0.51965478	0.671619773	0.979830967	0.109619964	0.782599617	0.719977528	0.999996191
EPG5	0.292605287	0.714419842	0.807448803	0.804210698	0.841029359	0.930696361	0.999996191
EPHA1	0.565551097	0.833866338	0.136903857	0.677006412	0.551030803	0.68216506	0.999996191
EPHA2	0.17568051	0.80035461	0.753348037	0.533586908	0.440395358	0.688525618	0.999996191
EPHA7	0.430889161	0.970696818	0.483608775	0.551269851	0.334277713	0.764513065	0.999996191
EPHB2	0.296350056	0.752467418	0.176884224	0.864186766	0.260981491	0.490520699	0.999996191
EPHB4	0.283479186	0.559127547	0.608260475	0.965329894	0.522386144	0.811251762	0.999996191
EPHX1	0.101808269	0.654006804	0.371782362	0.89365425	0.447541434	0.510391927	0.999996191
EPHX2	0.969741856	0.948670254	0.240305087	0.074308166	0.982838572	0.604215258	0.999996191
EPHX3	0.453664554	0.702329091	0.827931421	0.616546959	0.558125382	0.904210676	0.999996191
EPM2A	0.89901199	0.45889729	0.722601446	0.066740279	0.651369501	0.561579283	0.999996191
EPN2	0.273796792	0.775644347	0.583819576	0.515944185	0.799040064	0.819663856	0.999996191
EPN3	0.678041337	0.731594847	0.267369105	0.610496471	0.518547829	0.785863175	0.999996191
EPS15	0.66068327	0.804520179	0.27471055	0.056571601	0.967829731	0.470997401	0.999996191
EPS15L1	0.843458925	0.722444173	0.756471488	0.018725413	0.8902886	0.463856431	0.999996191
EPS8	0.985899781	0.805303421	0.78584526	0.066618117	0.803741345	0.744369577	0.999996191
EPS8L2	0.950046313	0.162632071	0.461093376	0.860160772	0.305234843	0.632944662	0.999996191
ERAS	0.519935969	0.575104844	0.916021388	0.818390674	0.108416052	0.683903576	0.999996191
ERC2	0.172820326	0.737253294	0.992419253	0.969184555	0.131612249	0.604016093	0.999996191
ERCC1	0.231998064	0.551224406	0.404799092	0.393971303	0.51613508	0.521910541	0.999996191
ERCC2	0.982947103	0.660545655	0.720554459	0.177083341	0.555698926	0.801941488	0.999996191
ERCC3	0.859117146	0.252903079	0.68735832	0.399927336	0.818861708	0.812261613	0.999996191
ERCC4	0.95869739	0.21481044	0.936957133	0.415587723	0.719006907	0.839290792	0.999996191
ERCC5	0.429358283	0.413024135	0.512442849	0.542562775	0.663870146	0.740570721	0.999996191

ERCC6	0.926550453	0.64999961	0.851386239	0.869744744	0.132051118	0.842645683	0.999996191
ERCC6L2	0.483422303	0.569911085	0.385655368	0.208224353	0.58667555	0.56187533	0.999996191
ERCC8	0.316843497	0.522789382	0.673912952	0.783117791	0.747192776	0.858640341	0.999996191
ERF	0.221662194	0.720868614	0.941952605	0.114856273	0.743277344	0.559933098	0.999996191
ERFE	0.715277196	0.30201516	0.808472441	0.404856507	0.339108016	0.681298468	0.999996191
ERG	0.280889199	0.769393941	0.826899259	0.143867885	0.794116878	0.650042414	0.999996191
ERGIC2	0.627821282	0.499279727	0.421952449	0.152176876	0.387728705	0.466634223	0.999996191
ERGIC3	0.086505954	0.945088327	0.857899394	0.214883167	0.849718941	0.55929053	0.999996191
ERI1	0.323361514	0.358268823	0.514734438	0.325225518	0.888386054	0.61689298	0.999996191
ERI2	0.253346481	0.831053277	0.417799743	0.833251141	0.665713491	0.811869883	0.999996191
ERICH1	0.41566303	0.248140339	0.886615198	0.939785196	0.45636765	0.773715249	0.999996191
ERLEC1	0.935793965	0.868188153	0.109184647	0.513686934	0.81285405	0.763361579	0.999996191
ERLIN2	0.628145251	0.827179923	0.538751978	0.416155027	0.867719827	0.917204892	0.999996191
ERMAP	0.917413058	0.87338169	0.556927637	0.785073755	0.846680274	0.991848645	0.999996191
ERN1	0.669822423	0.71699616	0.532591727	0.072220353	0.994634948	0.629454207	0.999996191
ERO1B	0.063997137	0.642145752	0.831020525	0.671243872	0.703155724	0.603894087	0.999996191
ERP44	0.945224111	0.542748207	0.989309958	0.179897121	0.817600788	0.87813785	0.999996191
ERRFI1	0.818990372	0.916735512	0.621390868	0.30982809	0.309383528	0.796918251	0.999996191
ESCO2	0.798637983	0.601339989	0.183131116	0.581505674	0.918963058	0.805486818	0.999996191
ESF1	0.296568589	0.643026098	0.862223035	0.088063598	0.683232849	0.510254748	0.999996191
ESR1	0.668271902	0.943570764	0.903636813	0.224882044	0.412074136	0.825044266	0.999996191
ESRP2	0.136840441	0.638975642	0.958952204	0.936920024	0.173591584	0.571414364	0.999996191
ESRRA	0.787340724	0.644480424	0.962163785	0.279864489	0.386885564	0.825233794	0.999996191
ESYT1	0.629972628	0.850779751	0.442841376	0.705336093	0.726763484	0.93723102	0.999996191
ETFA	0.677161798	0.641953655	0.991562119	0.018338655	0.936443733	0.457144993	0.999996191
ETFB	0.831567617	0.343068252	0.595348124	0.267396129	0.679154569	0.729440092	0.999996191
ETFBKMT	0.269898993	0.113930874	0.931848984	0.523185639	0.650094544	0.507449028	0.999996191
ETFDH	0.937629657	0.558664644	0.654132828	0.116148212	0.752672859	0.723921537	0.999996191
ETFRF1	0.694198396	0.465962677	0.233346508	0.336160557	0.449865984	0.537264521	0.999996191
ETHE1	0.265922443	0.239154509	0.98158418	0.256234099	0.716414209	0.538008214	0.999996191
ETNK1	0.30275525	0.946380573	0.452687697	0.147640795	0.7629948	0.584780907	0.999996191
ETNPPL	0.724100189	0.8248623	0.420823688	0.60248481	0.157046409	0.679714751	0.999996191
ETS2	0.90324492	0.058860097	0.47896623	0.569805814	0.607892434	0.488945513	0.999996191
ETV1	0.86052761	0.718474651	0.758146985	0.579418356	0.155128571	0.786474876	0.999996191
ETV2	0.68615575	0.627429587	0.664741139	0.517217806	0.56114208	0.892748348	0.999996191
ETV3	0.478585571	0.60229778	0.802386608	0.618068514	0.179942107	0.694858795	0.999996191
ETV4	0.759438378	0.318648745	0.406058203	0.52600957	0.961731198	0.815001565	0.999996191
ETV5	0.08496445	0.788529581	0.777234453	0.349139736	0.972536623	0.621950311	0.999996191
ETV6	0.293632239	0.833758306	0.54773236	0.958897911	0.402437162	0.821707924	0.999996191
EVA1C	0.415883021	0.539462808	0.563974726	0.267701676	0.969185951	0.74111869	0.999996191
EVC	0.345985735	0.614724647	0.476585607	0.537343492	0.896687856	0.812024808	0.999996191
EVI5	0.486553222	0.436080553	0.318342787	0.176634728	0.966721834	0.539239756	0.999996191
EVI5L	0.547444043	0.569416733	0.277770819	0.276351484	0.846841172	0.648576005	0.999996191
EWSR1	0.85051806	0.472337422	0.379422425	0.944551019	0.987580039	0.951692266	0.999996191
EXD2	0.386590856	0.78991938	0.110087208	0.891054168	0.533384716	0.602178488	0.999996191

EXO5	0.352848419	0.605768734	0.066811237	0.840931895	0.849254712	0.515956003	0.999996191
EXOC1	0.752729332	0.851994398	0.794384151	0.359638172	0.188491432	0.750522346	0.999996191
EXOC1L	0.439108066	0.408058921	0.877702549	0.291847391	0.73900308	0.747191664	0.999996191
EXOC2	0.984233972	0.598652945	0.305967612	0.062809067	0.986182973	0.53308894	0.999996191
EXOC3L1	0.428300568	0.193064119	0.260216741	0.575306771	0.691756611	0.483513262	0.999996191
EXOC3L2	0.336523203	0.995561119	0.111937187	0.443984114	0.920014812	0.593972933	0.999996191
EXOC4	0.857695985	0.741988814	0.933051917	0.277935729	0.672015616	0.927605293	0.999996191
EXOC5	0.176573363	0.748183377	0.736233213	0.745265539	0.660846896	0.808739084	0.999996191
EXOC6B	0.644890026	0.477707114	0.661037341	0.1642291	0.686263209	0.672822513	0.999996191
EXOC8	0.449102701	0.235009609	0.385433274	0.862670143	0.402472668	0.578204876	0.999996191
EXOG	0.507257397	0.767817289	0.903800361	0.254796132	0.237942427	0.658677897	0.999996191
EXOSC1	0.882160642	0.495888802	0.634526319	0.734039515	0.519551629	0.922475488	0.999996191
EXOSC10	0.594832839	0.944597648	0.464337188	0.651839739	0.342759224	0.841029412	0.999996191
EXOSC3	0.747283367	0.38870753	0.10234049	0.757016041	0.848663101	0.637011928	0.999996191
EXOSC5	0.790972582	0.879995417	0.48969962	0.312521279	0.453676815	0.810240355	0.999996191
EXOSC6	0.253185175	0.776591182	0.233187554	0.441484503	0.702693489	0.57956875	0.999996191
EXOSC7	0.737076223	0.343106768	0.238173961	0.619703275	0.589893604	0.664759992	0.999996191
EXOSC8	0.346470684	0.360902308	0.47463529	0.998631316	0.539678909	0.736261939	0.999996191
EXT1	0.66485819	0.792805669	0.78829015	0.709038476	0.197337305	0.840611492	0.999996191
EZH1	0.620234167	0.805773989	0.703746318	0.994445774	0.31319469	0.926259838	0.999996191
EZR	0.45908469	0.53286277	0.480049043	0.885241777	0.114351893	0.545009937	0.999996191
F12	0.727754788	0.847944354	0.263986639	0.521000503	0.451265175	0.769434626	0.999996191
F8A1	0.86733744	0.765654058	0.657088673	0.161238781	0.970517334	0.865267421	0.999996191
FA2H	0.70199328	0.537538296	0.933786609	0.931461049	0.520207927	0.965888139	0.999996191
FAAP100	0.477625701	0.645415042	0.3227719	0.873554131	0.500938627	0.792257091	0.999996191
FAAP20	0.42048755	0.8875849	0.452971313	0.092383747	0.950667409	0.587900122	0.999996191
FAAP24	0.780819976	0.461821213	0.829003848	0.534126816	0.170653572	0.705910548	0.999996191
FABP5	0.251954145	0.236327483	0.64108958	0.340129076	0.648800198	0.480509736	0.999996191
FABP9	0.056810758	0.994314677	0.705088336	0.91381	0.792046072	0.71664998	0.999996191
FADD	0.342044634	0.788211109	0.967913641	0.46262802	0.23231071	0.71141675	0.999996191
FAF1	0.987270849	0.521735263	0.241144838	0.637218894	0.148309311	0.542600507	0.999996191
FAF2	0.369045954	0.784694944	0.558766951	0.458480405	0.589690204	0.79308672	0.999996191
FAH	0.466865656	0.929206636	0.394149825	0.832901703	0.272247056	0.77164496	0.999996191
FAM104A	0.405887392	0.633977029	0.542237765	0.167465759	0.857059481	0.646277898	0.999996191
FAM107A	0.634165195	0.582297146	0.1916205	0.993929615	0.20881429	0.58577391	0.999996191
FAM110A	0.391971149	0.836403246	0.850846019	0.535792345	0.919463373	0.94871602	0.999996191
FAM110B	0.252307039	0.694059279	0.838716129	0.488627692	0.695034511	0.815576084	0.999996191
FAM110C	0.318703216	0.388683729	0.526774985	0.550742954	0.407576492	0.585264638	0.999996191
FAM110D	0.617344504	0.770839345	0.786044069	0.597738032	0.947374908	0.97886902	0.999996191
FAM111B	0.508142903	0.421856258	0.912786139	0.706674284	0.569821712	0.885639691	0.999996191
FAM114A1	0.625458546	0.980235435	0.485426409	0.684740056	0.796841641	0.962294154	0.999996191
FAM117A	0.543560884	0.773717617	0.490497611	0.87833063	0.136245643	0.686924971	0.999996191
FAM117B	0.377123766	0.987906729	0.666210273	0.421713648	0.783634605	0.891079136	0.999996191
FAM118B	0.902235095	0.518074924	0.570570883	0.352107717	0.528442822	0.814713107	0.999996191
FAM120C	0.265486206	0.930072368	0.447403289	0.737674751	0.984560476	0.888112167	0.999996191

FAM122A	0.303923071	0.392086689	0.67388778	0.262223522	0.602079123	0.557353251	0.999996191
FAM122B	0.820073026	0.934391726	0.870665974	0.928345324	0.385458403	0.984429369	0.999996191
FAM124A	0.757007238	0.226452982	0.420273918	0.410403173	0.442590347	0.563455212	0.999996191
FAM126A	0.956796275	0.981266718	0.369860147	0.890400528	0.18083861	0.83437325	0.999996191
FAM129B	0.985444417	0.877497482	0.242914108	0.280876561	0.828406946	0.812148205	0.999996191
FAM129C	0.18836062	0.747649185	0.303045104	0.694914337	0.964670285	0.715206025	0.999996191
FAM131B	0.762707594	0.653259941	0.404329916	0.316753066	0.197672628	0.55637448	0.999996191
FAM133A	0.834861957	0.641654732	0.855854147	0.621146727	0.998064075	0.990641251	0.999996191
FAM133B	0.075745291	0.892391358	0.472045679	0.555727764	0.650815541	0.539347408	0.999996191
FAM13A	0.887441808	0.390134669	0.67861671	0.782133344	0.209820423	0.770642019	0.999996191
FAM13B	0.652304608	0.649177575	0.628403089	0.776979081	0.040545741	0.479625525	0.999996191
FAM149A	0.654235403	0.830908474	0.900555806	0.955555984	0.142006631	0.861175255	0.999996191
FAM151A	0.771186266	0.975156501	0.237490343	0.872397039	0.804607431	0.940178831	0.999996191
FAM151B	0.365084392	0.624895733	0.366247448	0.7776969	0.795238507	0.821478939	0.999996191
FAM155B	0.537729484	0.808028255	0.812055643	0.718827131	0.594513473	0.956574261	0.999996191
FAM160A1	0.158671023	0.879777182	0.57297276	0.581552794	0.764733871	0.755966718	0.999996191
FAM160B1	0.343656317	0.484378607	0.514703953	0.22001357	0.895943668	0.612995264	0.999996191
FAM161A	0.565638498	0.70847126	0.07037906	0.861122469	0.347233505	0.480712563	0.999996191
FAM161B	0.363639318	0.199744334	0.466329648	0.947337722	0.240232559	0.4644166	0.999996191
FAM162A	0.776961866	0.748109376	0.816725461	0.216192136	0.993227069	0.918177231	0.999996191
FAM162B	0.324788743	0.919865897	0.501083066	0.306846411	0.636528434	0.719346749	0.999996191
FAM166A	0.372601599	0.661985932	0.947127667	0.357225401	0.755885862	0.853341533	0.999996191
FAM167B	0.966653289	0.641530412	0.130748621	0.805402915	0.789610086	0.821122682	0.999996191
FAM168A	0.835880455	0.651746386	0.694056894	0.622907573	0.10392461	0.685284557	0.999996191
FAM168B	0.498269193	0.212935531	0.906105738	0.369345921	0.925718445	0.741355844	0.999996191
FAM171A2	0.776612743	0.601227823	0.028426092	0.711227513	0.965887086	0.495068622	0.999996191
FAM171B	0.835991535	0.522545726	0.112733171	0.574988338	0.623901364	0.621788547	0.999996191
FAM172A	0.809356079	0.215622023	0.290047404	0.82232486	0.934842385	0.772292576	0.999996191
FAM173A	0.821179767	0.871742913	0.501920674	0.061636415	0.959041037	0.657741699	0.999996191
FAM173B	0.676976169	0.871124726	0.384713458	0.274158349	0.441471905	0.70738739	0.999996191
FAM174B	0.413123879	0.846555802	0.312319493	0.454145649	0.278605919	0.573994873	0.999996191
FAM177A1	0.56528767	0.243454553	0.292877506	0.442873905	0.614817265	0.529799603	0.999996191
FAM181B	0.623480664	0.993460033	0.668500922	0.811632389	0.098825702	0.743285256	0.999996191
FAM184B	0.805323301	0.329987146	0.640126219	0.761570507	0.929071702	0.936149321	0.999996191
FAM185A	0.211002726	0.777054467	0.969419671	0.692511694	0.681112991	0.878743301	0.999996191
FAM186B	0.085615473	0.230691668	0.747745853	0.947058616	0.505347152	0.448927668	0.999996191
FAM192A	0.791006334	0.539250525	0.987304973	0.146241223	0.712491882	0.793615728	0.999996191
FAM193A	0.599067099	0.896213604	0.694964503	0.470791874	0.714896795	0.940345112	0.999996191
FAM196A	0.441566706	0.523886005	0.908232491	0.464427388	0.970542279	0.909444741	0.999996191
FAM199X	0.668775918	0.499686707	0.515158097	0.325008349	0.857987855	0.809104915	0.999996191
FAM200A	0.528439145	0.104453509	0.720458282	0.997897051	0.398203891	0.600025527	0.999996191
FAM204A	0.652860929	0.683754599	0.439079905	0.167355611	0.903856247	0.721977567	0.999996191
FAM205C	0.512991934	0.960228407	0.619944047	0.596586992	0.137885363	0.690293239	0.999996191
FAM208A	0.934000148	0.842700987	0.866280763	0.157448955	0.485434989	0.822877632	0.999996191
FAM208B	0.767299227	0.565132336	0.698390981	0.068409383	0.820505429	0.614260061	0.999996191

FAM20B	0.800822317	0.296264434	0.707801487	0.370110707	0.523876606	0.739588851	0.999996191
FAM20C	0.436370091	0.878951798	0.897189158	0.299652555	0.953722769	0.913977928	0.999996191
FAM210A	0.617174132	0.930596164	0.432117308	0.698282286	0.114199487	0.643967708	0.999996191
FAM212B	0.143550324	0.562061591	0.584693294	0.652455007	0.895610412	0.708131041	0.999996191
FAM213A	0.906500094	0.405820968	0.416958438	0.306521199	0.652307572	0.728371374	0.999996191
FAM213B	0.57722748	0.639663571	0.837119782	0.060002174	0.857435514	0.601253442	0.999996191
FAM214A	0.213500062	0.736930562	0.816581709	0.854952182	0.08447384	0.498306977	0.999996191
FAM216A	0.315607345	0.354121613	0.86570472	0.39304363	0.806407123	0.728352461	0.999996191
FAM21A	0.868944087	0.544015069	0.188854809	0.225574333	0.705418788	0.579323246	0.999996191
FAM221A	0.118924271	0.37679966	0.763702485	0.472773418	0.577997024	0.499757907	0.999996191
FAM222B	0.505685357	0.948193826	0.640487952	0.571892852	0.18642886	0.740631868	0.999996191
FAM228B	0.556091199	0.243186177	0.412871029	0.956969401	0.880401629	0.805642085	0.999996191
FAM229A	0.979943092	0.907600035	0.570157666	0.188991919	0.445900203	0.788973898	0.999996191
FAM229B	0.309262693	0.749659297	0.226745424	0.692488848	0.803757442	0.719475006	0.999996191
FAM234B	0.266191291	0.378915089	0.295238073	0.701297324	0.65267507	0.571316595	0.999996191
FAM241B	0.824810922	0.817270069	0.555823408	0.959097294	0.490877689	0.968106238	0.999996191
FAM25A	0.799020497	0.849398378	0.832007072	0.149334404	0.41741465	0.75402841	0.999996191
FAM32A	0.234655926	0.842087466	0.734449245	0.475108149	0.702432539	0.810611311	0.999996191
FAM43A	0.085733363	0.820612955	0.406904587	0.839197065	0.424868565	0.516110034	0.999996191
FAM45A	0.839180829	0.928832727	0.242315557	0.717018177	0.873327098	0.934369487	0.999996191
FAM46A	0.54960413	0.643584357	0.208097402	0.509965056	0.215011132	0.472717393	0.999996191
FAM49A	0.899538236	0.698346091	0.182388788	0.677497442	0.387575783	0.724741963	0.999996191
FAM50A	0.275884495	0.602318595	0.770398863	0.643491551	0.511856525	0.786615784	0.999996191
FAM53C	0.396790209	0.688914017	0.986534637	0.263244307	0.517355523	0.761817104	0.999996191
FAM57A	0.377921277	0.193052564	0.622727152	0.38588278	0.873877119	0.594000226	0.999996191
FAM57B	0.529431964	0.640048519	0.994234877	0.201141858	0.242766774	0.607875675	0.999996191
FAM69B	0.882799216	0.34379748	0.173181417	0.588965196	0.350180502	0.527465325	0.999996191
FAM69C	0.341634341	0.682985655	0.738953937	0.582844171	0.123977958	0.554003748	0.999996191
FAM71F2	0.596780937	0.882690163	0.848501719	0.700438905	0.477913353	0.955947558	0.999996191
FAM76A	0.664347624	0.530406149	0.185022418	0.942100333	0.680304721	0.785015685	0.999996191
FAM76B	0.083147029	0.843315173	0.825380485	0.416904672	0.469968757	0.536007216	0.999996191
FAM78B	0.722263507	0.35514085	0.501210647	0.476560992	0.455180702	0.710343428	0.999996191
FAM81A	0.230170012	0.971901144	0.768588702	0.451146303	0.131853076	0.516487845	0.999996191
FAM83G	0.195422399	0.791404517	0.880848208	0.739851823	0.466589561	0.805593028	0.999996191
FAM83H	0.864176378	0.938024618	0.241312932	0.087870024	0.76956375	0.56552423	0.999996191
FAM84B	0.44413488	0.979464478	0.46439859	0.307674673	0.134030268	0.478483527	0.999996191
FAM89A	0.863647731	0.622730071	0.609525953	0.063262744	0.886382101	0.62954572	0.999996191
FAM89B	0.17990039	0.571010096	0.420093998	0.376859392	0.934703569	0.592474723	0.999996191
FAM8A1	0.957518437	0.889421584	0.685731935	0.226866314	0.921881299	0.937615989	0.999996191
FAM92A	0.985387203	0.441875868	0.276571061	0.98440207	0.938567786	0.927951171	0.999996191
FAM92B	0.766393605	0.991895457	0.977657248	0.093476375	0.953911836	0.860813897	0.999996191
FAM96A	0.310336695	0.473428239	0.582574154	0.705936577	0.460868723	0.710063365	0.999996191
FAM96B	0.297218029	0.584353866	0.635157652	0.183542616	0.960535772	0.640558668	0.999996191
FAM98B	0.152229708	0.793165063	0.704543089	0.911178203	0.417673064	0.738523778	0.999996191
FAM98C	0.157502198	0.420863219	0.472529436	0.504905471	0.951543445	0.590512912	0.999996191

FANCA	0.343605172	0.534115322	0.073605597	0.967046398	0.540512483	0.448745685	0.999996191
FANCD2	0.385778805	0.496510442	0.216753066	0.309817675	0.62341061	0.471543659	0.999996191
FANCL	0.476635129	0.702395284	0.137354969	0.251486443	0.879441367	0.515432703	0.999996191
FANCM	0.243347703	0.529686899	0.174486684	0.409704382	0.769634807	0.449522984	0.999996191
FAR1	0.132501801	0.908895686	0.867746223	0.988466673	0.740489174	0.881550346	0.999996191
FAR2	0.358194103	0.523264469	0.216386962	0.58099668	0.78463607	0.630677086	0.999996191
FARP2	0.813272877	0.268274964	0.908499733	0.321057521	0.123055906	0.467260107	0.999996191
FAS	0.659343609	0.290575847	0.279772379	0.417613782	0.48007472	0.525815347	0.999996191
FASN	0.950651501	0.912666881	0.546153295	0.062009369	0.482359305	0.578880756	0.999996191
FASTKD1	0.181087928	0.477101788	0.731567403	0.296010938	0.476028147	0.490731276	0.999996191
FASTKD5	0.518141863	0.170361493	0.340705147	0.889369925	0.6770048	0.626610614	0.999996191
FAT1	0.699577964	0.578706192	0.60241095	0.039696833	0.997252957	0.505698985	0.999996191
FAT4	0.801141304	0.979568015	0.451398966	0.842323706	0.51875797	0.958653615	0.999996191
FAU	0.391324151	0.90733651	0.975501507	0.244692444	0.735203847	0.851452212	0.999996191
FBF1	0.911982338	0.400327447	0.970531792	0.278704544	0.580562689	0.838392414	0.999996191
FBLN2	0.40985513	0.831240514	0.656188763	0.890334327	0.169842636	0.746569437	0.999996191
FBN1	0.82813408	0.838353452	0.210041027	0.502649601	0.491894126	0.758443068	0.999996191
FBP1	0.68222463	0.930173424	0.694370861	0.915362846	0.05446125	0.664298755	0.999996191
FBP2	0.679079277	0.078956827	0.466897439	0.903219288	0.849643655	0.638165728	0.999996191
FBRS	0.819380062	0.813112146	0.730798966	0.290842491	0.18436299	0.697710488	0.999996191
FBXL12	0.980640583	0.390310542	0.641574549	0.922353889	0.919681142	0.978000584	0.999996191
FBXL15	0.902834672	0.988062969	0.514247708	0.060821976	0.915771009	0.693568614	0.999996191
FBXL17	0.666669883	0.932263786	0.47779088	0.973402116	0.899321613	0.987728088	0.999996191
FBXL19	0.736240347	0.271939317	0.156922747	0.569707873	0.435856883	0.466573398	0.999996191
FBXL2	0.658062656	0.507273458	0.254204507	0.691911263	0.613949009	0.758403606	0.999996191
FBXL20	0.895154235	0.710257668	0.811759795	0.352463606	0.96702115	0.967921691	0.999996191
FBXL21	0.137192226	0.877812869	0.528692727	0.241925888	0.620152822	0.503710959	0.999996191
FBXL4	0.589079734	0.775226187	0.588484888	0.27261009	0.811343434	0.844111932	0.999996191
FBXL8	0.916637694	0.109275752	0.542854996	0.443497252	0.993923595	0.681227959	0.999996191
FBXO10	0.874334807	0.603752594	0.806712978	0.190918794	0.335615406	0.706177121	0.999996191
FBXO15	0.663977299	0.168626841	0.304782965	0.781973418	0.499232521	0.566894125	0.999996191
FBXO17	0.339193345	0.360168683	0.451336079	0.887264775	0.870480203	0.788363654	0.999996191
FBXO2	0.904678654	0.548166511	0.331112002	0.381419398	0.941654622	0.842875871	0.999996191
FBXO21	0.822907347	0.999331011	0.892819196	0.160185713	0.281576327	0.742745137	0.999996191
FBXO22	0.115442899	0.735490483	0.882195816	0.964183695	0.48646523	0.753688119	0.999996191
FBXO24	0.778595928	0.042766709	0.622714838	0.849133579	0.991532849	0.61946668	0.999996191
FBXO25	0.546973257	0.956285302	0.709149282	0.717808192	0.737677781	0.974767653	0.999996191
FBXO31	0.730016636	0.8589302	0.333309944	0.23372511	0.985603802	0.809594886	0.999996191
FBXO32	0.622976623	0.779300087	0.820301499	0.11857248	0.389298033	0.629554002	0.999996191
FBXO34	0.790216099	0.481182716	0.982552474	0.520572386	0.658369053	0.942212604	0.999996191
FBXO38	0.531076034	0.634348894	0.339042146	0.496492956	0.862464124	0.812266033	0.999996191
FBXO4	0.621866767	0.75037258	0.689858802	0.478452747	0.842919773	0.943518964	0.999996191
FBXO42	0.478586509	0.660710964	0.500102393	0.918574319	0.477155371	0.86746522	0.999996191
FBXO44	0.96790069	0.385369862	0.499694405	0.826121846	0.159541823	0.685983761	0.999996191
FBXO45	0.785830958	0.323868533	0.386779982	0.247349696	0.860408566	0.655068194	0.999996191

FBXO46	0.649985153	0.394823313	0.382184662	0.386707016	0.418299663	0.600802181	0.999996191
FBXO47	0.426639344	0.970305142	0.310328643	0.912339002	0.743807753	0.899074186	0.999996191
FBXO48	0.266543584	0.799221822	0.858714789	0.370079384	0.799237781	0.829046343	0.999996191
FBXO5	0.632729332	0.579237895	0.563334881	0.801745322	0.874746345	0.953238241	0.999996191
FBXO6	0.436410016	0.267995139	0.363640091	0.24880052	0.745687482	0.468622065	0.999996191
FBXO8	0.982594487	0.227628727	0.127231126	0.73896144	0.618208026	0.562180758	0.999996191
FBXO9	0.43623625	0.850148889	0.544756282	0.190226012	0.881780839	0.747023486	0.999996191
FBXW2	0.928617626	0.67274808	0.113964122	0.926503511	0.684399071	0.798559328	0.999996191
FBXW4	0.728181319	0.579199975	0.47752149	0.978919989	0.973842932	0.973441043	0.999996191
FBXW5	0.303141508	0.90205155	0.874099041	0.085808233	0.896764261	0.629656823	0.999996191
FBXW7	0.644658107	0.272314638	0.710588133	0.169936123	0.446498288	0.502000176	0.999996191
FBXW8	0.481848244	0.684336207	0.561949515	0.482427569	0.635765965	0.83699434	0.999996191
FBXW9	0.309088148	0.381979269	0.643222806	0.447052192	0.635598053	0.660830607	0.999996191
FCAR	0.620985786	0.799578903	0.054534846	0.957238335	0.607989624	0.599494003	0.999996191
FCER2	0.40405091	0.854292868	0.080529753	0.70872431	0.38461842	0.461326165	0.999996191
FCHSD1	0.870332923	0.200148	0.724981521	0.586581865	0.484322345	0.757539391	0.999996191
FCHSD2	0.73117755	0.809260963	0.533800407	0.916006854	0.847065393	0.985501569	0.999996191
FCRLA	0.872240627	0.929476041	0.124544374	0.67068093	0.162571651	0.530393205	0.999996191
FDFT1	0.987500965	0.667190897	0.805477128	0.33856439	0.137174458	0.686616909	0.999996191
FDX1	0.889946841	0.379382629	0.636517266	0.79161102	0.875867583	0.955611545	0.999996191
FDXR	0.612183636	0.739664801	0.514431671	0.547345109	0.298302948	0.768167893	0.999996191
FECH	0.709715221	0.897580463	0.824148805	0.257787944	0.897168369	0.937029392	0.999996191
FEM1B	0.277010663	0.622714609	0.69070555	0.198866171	0.878972105	0.653917553	0.999996191
FEN1	0.698908087	0.789938978	0.536187958	0.602195501	0.381758627	0.864770433	0.999996191
FER	0.628283348	0.937098521	0.872938801	0.353385153	0.677397244	0.938337812	0.999996191
FER1L5	0.714473509	0.524520183	0.508305171	0.692609584	0.738495589	0.912872669	0.999996191
FEZ1	0.797252943	0.503389946	0.802647631	0.452358969	0.220164364	0.736821633	0.999996191
FEZ2	0.325345114	0.389555206	0.929772068	0.777796632	0.352535125	0.738157228	0.999996191
FFAR4	0.52619786	0.42417593	0.693604887	0.984836643	0.476609248	0.874311659	0.999996191
FGD1	0.878700525	0.468964345	0.758527588	0.587613244	0.954090914	0.967665843	0.999996191
FGD5	0.845121848	0.725666342	0.116576267	0.705972789	0.936823847	0.80652167	0.999996191
FGF12	0.655622556	0.525516734	0.907386259	0.054770622	0.775557898	0.566284854	0.999996191
FGF16	0.912346663	0.838545126	0.467446931	0.517708714	0.337474267	0.851871877	0.999996191
FGF18	0.787135519	0.447106071	0.158472733	0.345075949	0.490374715	0.501457872	0.999996191
FGF9	0.864250256	0.407718224	0.236217	0.890687463	0.89548478	0.8610828	0.999996191
FGFR1OP2	0.373482985	0.846627869	0.861795133	0.071177643	0.808578048	0.598551842	0.999996191
FGFR2	0.257148156	0.904464203	0.157262076	0.334269318	0.596220191	0.454407618	0.999996191
FGFRL1	0.527132997	0.662532403	0.487792104	0.736010457	0.79749684	0.915940059	0.999996191
FGGY	0.375231298	0.761925317	0.185185441	0.999163481	0.895964217	0.806927954	0.999996191
FGL2	0.691621121	0.7785641	0.970627726	0.712492647	0.95125596	0.995727356	0.999996191
FGR	0.656930766	0.477262597	0.460515188	0.756627879	0.425719709	0.803687843	0.999996191
FH	0.841397925	0.410853697	0.58525753	0.252518314	0.905741297	0.802819547	0.999996191
FHIT	0.756099143	0.576006139	0.938489185	0.998702309	0.901697545	0.996348143	0.999996191
FHOD1	0.973556206	0.665741399	0.338873096	0.291221602	0.205210101	0.564033571	0.999996191
FIG4	0.35587693	0.852238052	0.729566764	0.160484724	0.332350689	0.54362705	0.999996191

FIGNL1	0.808602272	0.596893814	0.339895458	0.84946225	0.946126692	0.944960317	0.999996191
FIGNL2	0.880430446	0.71290507	0.948066483	0.608766326	0.370052478	0.946484582	0.999996191
FIS1	0.264607497	0.673496161	0.446291536	0.440933278	0.937094856	0.741316835	0.999996191
FITM1	0.837206765	0.663325868	0.969574557	0.220638133	0.114106091	0.570258555	0.999996191
FIZ1	0.475846965	0.540729671	0.930476307	0.87057212	0.528308855	0.926827938	0.999996191
FKBP11	0.697417558	0.832340434	0.084010285	0.780191133	0.260666972	0.510717953	0.999996191
FKBP15	0.499795231	0.93878512	0.038906311	0.967443972	0.930378652	0.607633177	0.999996191
FKBP1A	0.763024651	0.266829656	0.769191066	0.145024143	0.645546291	0.585447334	0.999996191
FKBP2	0.527740911	0.480879382	0.584086365	0.524549696	0.928900929	0.873439247	0.999996191
FKBP3	0.520517123	0.639072962	0.670544885	0.131270952	0.312847201	0.495928757	0.999996191
FKBP4	0.600914205	0.209947402	0.587168677	0.180366715	0.819747806	0.52941685	0.999996191
FKBP8	0.817961652	0.778233436	0.525322869	0.383640977	0.643910287	0.892024137	0.999996191
FLAD1	0.912564214	0.68683429	0.319119358	0.740133879	0.148741118	0.664767353	0.999996191
FLII	0.342960377	0.898682064	0.101809452	0.937661453	0.547569642	0.603797917	0.999996191
FLNB	0.581071364	0.876259101	0.327501052	0.555058005	0.296486039	0.70726491	0.999996191
FLOT2	0.387555061	0.761698726	0.431206055	0.527411922	0.826508511	0.833138737	0.999996191
FLRT1	0.93655991	0.775728732	0.785272823	0.433758355	0.646090536	0.961136467	0.999996191
FLRT2	0.601396048	0.436040338	0.448303343	0.895544366	0.796814714	0.894063004	0.999996191
FLRT3	0.498161719	0.680957695	0.569441487	0.226130817	0.199301688	0.486542858	0.999996191
FLT1	0.440097038	0.470681482	0.815392517	0.603219588	0.905183539	0.906187892	0.999996191
FLT3LG	0.772917078	0.5023953	0.799319482	0.76444872	0.803758867	0.97303963	0.999996191
FLVCR1	0.056648637	0.825210837	0.640091339	0.425498463	0.98839313	0.55592168	0.999996191
FLYWCH1	0.380499032	0.343427342	0.392735877	0.748884986	0.372811571	0.58098687	0.999996191
FLYWCH2	0.97089455	0.799775557	0.697465557	0.121299474	0.449696547	0.721296255	0.999996191
FMC1	0.576359578	0.781614972	0.612437469	0.196108417	0.54329792	0.720354421	0.999996191
FMN1	0.211332286	0.935703577	0.805209921	0.225906923	0.950864966	0.74873402	0.999996191
FMNL2	0.852457859	0.796950818	0.895345864	0.059368687	0.498124726	0.625314102	0.999996191
FMNL3	0.876329071	0.357566319	0.168572497	0.561314082	0.648310835	0.638272799	0.999996191
FMO5	0.90116589	0.974970277	0.977992225	0.970069169	0.959983999	0.999996191	0.999996191
FMR1	0.337564954	0.830111164	0.531266671	0.928436246	0.943751951	0.943962899	0.999996191
FN3K	0.859752979	0.777027403	0.380805726	0.112022322	0.948638375	0.704402557	0.999996191
FN3KRP	0.484649897	0.96327552	0.388183328	0.803351307	0.922908464	0.946694102	0.999996191
FNBP1	0.447166832	0.395118035	0.900745019	0.760890709	0.147611084	0.624076521	0.999996191
FNDC3A	0.591992544	0.712781401	0.453403841	0.58279163	0.152411278	0.614205005	0.999996191
FNDC3B	0.965229093	0.859801491	0.607321065	0.155488116	0.963699323	0.879774141	0.999996191
FNTA	0.920958925	0.822291138	0.307776222	0.200356204	0.93099437	0.791996666	0.999996191
FOCAD	0.949368547	0.983563514	0.956314715	0.922785839	0.159382937	0.944599387	0.999996191
FOLH1B	0.652856378	0.993334344	0.985550398	0.438258125	0.431665321	0.936607113	0.999996191
FOS	0.739984041	0.138978129	0.856660337	0.359408726	0.672668208	0.658293735	0.999996191
FOSL2	0.518281062	0.770981994	0.303540367	0.568299686	0.873371009	0.846104434	0.999996191
FOXC2	0.502239656	0.163674938	0.749376493	0.447443815	0.986427536	0.705494653	0.999996191
FOXD2	0.768231959	0.11301783	0.620819145	0.545531368	0.950464545	0.710756807	0.999996191
FOXF1	0.906937088	0.413990722	0.191668097	0.278710146	0.650673807	0.562927305	0.999996191
FOXF2	0.918291584	0.702189632	0.738523872	0.726319608	0.788989835	0.989380336	0.999996191
FOXK1	0.651148572	0.620571673	0.498765818	0.989519619	0.397066768	0.886325336	0.999996191

FOXL1	0.851561246	0.702527182	0.602243957	0.228167887	0.747025934	0.849202701	0.999996191
FOXN3	0.686135193	0.942587199	0.524852416	0.940104998	0.338585085	0.924751424	0.999996191
FOXO1	0.894607418	0.391740078	0.294797917	0.406227827	0.376924889	0.600232498	0.999996191
FOXP1	0.906909382	0.716242728	0.117469551	0.920766316	0.713578336	0.816431871	0.999996191
FOXP2	0.251439141	0.830786869	0.55797786	0.281521475	0.715133706	0.677120399	0.999996191
FOXP4	0.392271845	0.46240266	0.112099021	0.933543891	0.820337304	0.597163162	0.999996191
FOXQ1	0.303595342	0.726694445	0.92327923	0.297632574	0.8365626	0.818365223	0.999996191
FPGT	0.607561566	0.524114778	0.473461433	0.275647851	0.954524569	0.775751362	0.999996191
FRAT1	0.584251266	0.628959158	0.534251493	0.316588805	0.367132866	0.671692811	0.999996191
FRAT2	0.563858046	0.996636471	0.798265407	0.248141419	0.559259911	0.85131221	0.999996191
FRG1	0.226320851	0.349149255	0.681791515	0.700884821	0.613197283	0.674529137	0.999996191
FRK	0.687771272	0.959070639	0.487941729	0.869951628	0.778083941	0.980277285	0.999996191
FRMD3	0.816529211	0.454256697	0.574392804	0.569117704	0.915083539	0.927651221	0.999996191
FRMD4A	0.655688652	0.950828638	0.628940487	0.60336197	0.599113958	0.951423279	0.999996191
FRMD4B	0.815156252	0.620128351	0.101198205	0.692664637	0.474672876	0.612193297	0.999996191
FRMD5	0.163258849	0.850376403	0.831438407	0.49593421	0.773416153	0.795175617	0.999996191
FRMD6	0.753552672	0.547918546	0.805694828	0.337660432	0.73839991	0.89256055	0.999996191
FRMD8	0.709755241	0.480032391	0.893304212	0.592877415	0.298214911	0.828150208	0.999996191
FRMPD3	0.161585372	0.881852092	0.799496307	0.76196384	0.559867872	0.811191355	0.999996191
FRMPD4	0.46956852	0.367246048	0.646703235	0.809271902	0.964202652	0.898841666	0.999996191
FRS2	0.646647625	0.465363475	0.754945941	0.152874397	0.797485457	0.709031411	0.999996191
FRS3	0.850314559	0.309196225	0.41295084	0.298102216	0.516961933	0.611170068	0.999996191
FRY	0.389206004	0.230285134	0.829873483	0.480093077	0.654946981	0.676473544	0.999996191
FSD1L	0.834067369	0.533137876	0.362178649	0.281380355	0.279176038	0.556944584	0.999996191
FSD2	0.473643347	0.867265285	0.421887239	0.970184298	0.773429613	0.943673558	0.999996191
FSTL1	0.857457255	0.863793881	0.16856754	0.436539331	0.580091895	0.734087831	0.999996191
FSTL3	0.201821797	0.463968751	0.411949869	0.997292777	0.360637627	0.574741096	0.999996191
FTCDNL1	0.837910718	0.727632502	0.117970252	0.468815422	0.817772878	0.708188678	0.999996191
FTO	0.625990946	0.347396181	0.680282553	0.659906212	0.19255427	0.633918158	0.999996191
FTSJ1	0.722247465	0.95273907	0.342466536	0.516632957	0.866047557	0.922028538	0.999996191
FUCA2	0.811205757	0.888179413	0.657666201	0.422600376	0.804458865	0.961699494	0.999996191
FUK	0.584346436	0.145387106	0.798507116	0.199997588	0.906360773	0.551491399	0.999996191
FUNDC1	0.468940555	0.307884945	0.847781888	0.182656174	0.959050935	0.659594057	0.999996191
FUNDC2	0.85631625	0.540843205	0.438493472	0.119679124	0.784960613	0.636804501	0.999996191
FUOM	0.855926926	0.484358955	0.761151758	0.636803732	0.272135364	0.830775215	0.999996191
FURIN	0.206330257	0.751053623	0.523476654	0.391003946	0.980008884	0.730899617	0.999996191
FUT10	0.778177545	0.438835586	0.365611578	0.440789725	0.980810533	0.828656029	0.999996191
FUT11	0.726844503	0.979535386	0.487311391	0.086958542	0.857448071	0.695950256	0.999996191
FUT7	0.96404398	0.489672728	0.085330448	0.8552895	0.525150793	0.626444603	0.999996191
FUT8	0.7176733	0.836562271	0.185130275	0.601257251	0.754082313	0.817296189	0.999996191
FUZ	0.647848999	0.273627868	0.469932892	0.414054283	0.916511509	0.734065569	0.999996191
FXR2	0.729306566	0.970655625	0.54003211	0.27620495	0.957661468	0.917246288	0.999996191
FXYD1	0.257756025	0.560047014	0.863631786	0.598135931	0.5506974	0.781930571	0.999996191
FXYD6	0.962984893	0.520513744	0.395786224	0.317608592	0.969024328	0.848308015	0.999996191
FXYD7	0.340757289	0.614922022	0.390447353	0.877692734	0.989125576	0.871026576	0.999996191

FYCO1	0.95291821	0.634838797	0.17510141	0.800285505	0.987520664	0.893788439	0.999996191
FYTTD1	0.546019572	0.130293809	0.302956039	0.96173999	0.583439947	0.548298983	0.999996191
FZD3	0.689635197	0.396339245	0.502854015	0.357748064	0.291777898	0.581239028	0.999996191
FZD4	0.800090438	0.954219981	0.455355749	0.494158233	0.218373808	0.765680427	0.999996191
FZD6	0.687942495	0.585915589	0.619059577	0.648996908	0.826526539	0.946345972	0.999996191
FZD7	0.726519214	0.817442018	0.293844474	0.274418527	0.663245853	0.734947951	0.999996191
FZD8	0.805593344	0.536718207	0.201172915	0.256127779	0.323401017	0.45232911	0.999996191
FZR1	0.50134955	0.8984536	0.793993605	0.297471894	0.879560541	0.907981177	0.999996191
G0S2	0.235488035	0.413409928	0.712260974	0.218845958	0.641500323	0.507235643	0.999996191
G3BP1	0.874821343	0.17067637	0.669267269	0.230811111	0.675987899	0.597410467	0.999996191
G3BP2	0.953050603	0.111437076	0.168851859	0.877411152	0.720875688	0.536059101	0.999996191
G6PC	0.516864997	0.724024678	0.52356164	0.868460982	0.962759702	0.962939925	0.999996191
G6PC3	0.536028057	0.90134812	0.848378241	0.048141349	0.828301609	0.606605684	0.999996191
GAA	0.569274221	0.692492975	0.760405278	0.946637386	0.755812006	0.979500469	0.999996191
GAB1	0.488308389	0.314207669	0.807994761	0.5308721	0.783840233	0.821193777	0.999996191
GAB2	0.618830484	0.917220562	0.92547705	0.915168202	0.570255479	0.989527296	0.999996191
GAB3	0.634500366	0.453361597	0.507553632	0.12830648	0.867070971	0.605386583	0.999996191
GABPA	0.875901145	0.841097628	0.521599881	0.947885874	0.511315322	0.971596567	0.999996191
GABPB1	0.078874008	0.409582028	0.956646574	0.259190197	0.87283015	0.447000527	0.999996191
GABRB1	0.14186296	0.886169285	0.393495933	0.706706804	0.300203817	0.521346529	0.999996191
GABRG3	0.966561006	0.785013304	0.906734289	0.598857628	0.530374386	0.980424396	0.999996191
GADD45B	0.304969158	0.80696988	0.454082264	0.3902121	0.508910253	0.666280843	0.999996191
GADD45GIP1	0.839430887	0.994136254	0.14641499	0.113909428	0.715291809	0.511427607	0.999996191
GAL3ST4	0.502898427	0.818964252	0.08921984	0.959138884	0.882192578	0.730946899	0.999996191
GALK1	0.964837197	0.690020792	0.682348476	0.071831309	0.97548728	0.735357008	0.999996191
GALM	0.726001936	0.631756125	0.267569078	0.919590519	0.539481479	0.847862028	0.999996191
GALNS	0.427856951	0.370845042	0.241992592	0.67675403	0.891960201	0.67472356	0.999996191
GALNT11	0.695294568	0.868049867	0.135084739	0.118075427	0.799642612	0.464168061	0.999996191
GALNT14	0.192474054	0.453038814	0.796583132	0.942601038	0.437387547	0.715393275	0.999996191
GALNT15	0.854156506	0.528083221	0.231066191	0.793621411	0.154073758	0.558353918	0.999996191
GALNT16	0.236942875	0.969021355	0.694253656	0.358610618	0.623939788	0.756455852	0.999996191
GALNT2	0.4512234	0.898788233	0.647036072	0.076622354	0.885714695	0.623351098	0.999996191
GALNT7	0.943004648	0.856893209	0.976439601	0.038318301	0.666377802	0.647449318	0.999996191
GALT	0.164564513	0.853313064	0.872861381	0.594670503	0.707473469	0.821132405	0.999996191
GAMT	0.847778064	0.65031134	0.320681976	0.296689955	0.305728459	0.602897048	0.999996191
GAN	0.302426328	0.59022198	0.710334371	0.200801298	0.957400802	0.684482804	0.999996191
GANAB	0.974605015	0.310037943	0.172583071	0.517811394	0.282679614	0.462649633	0.999996191
GAPDH	0.403793711	0.379125108	0.923503353	0.219259448	0.324029201	0.51309482	0.999996191
GAPVD1	0.433446944	0.821989974	0.961033957	0.075958899	0.710431259	0.630555283	0.999996191
GAR1	0.754463591	0.712207081	0.772143098	0.068599948	0.249736953	0.449925002	0.999996191
GAREM1	0.445767744	0.841269459	0.99484639	0.510794679	0.574280866	0.926156727	0.999996191
GAREM2	0.482468497	0.206980939	0.654784622	0.701688989	0.901259048	0.783164469	0.999996191
GAS6	0.28698575	0.63040259	0.195072365	0.753823784	0.523314942	0.575415174	0.999996191
GAS7	0.390202944	0.537267025	0.436632176	0.506405426	0.954605673	0.795080743	0.999996191
GAT	0.780379846	0.914425918	0.537277673	0.750448676	0.881446329	0.986825307	0.999996191

GATA2	0.610435765	0.68169613	0.591668529	0.701818823	0.891772833	0.958300127	0.999996191
GATAD1	0.532861005	0.326239627	0.878028028	0.427852594	0.608354528	0.776026623	0.999996191
GATB	0.640745663	0.254993791	0.611468143	0.897111044	0.422266533	0.767274846	0.999996191
GATC	0.723714557	0.426174407	0.386109401	0.259961844	0.685469491	0.657574139	0.999996191
GATD3A	0.805932867	0.737935198	0.162861743	0.648637435	0.939232583	0.842961279	0.999996191
GATM	0.558433644	0.816888344	0.939330654	0.845951235	0.541011691	0.974680077	0.999996191
GBA	0.14463203	0.60240467	0.835378717	0.87941232	0.783716003	0.816527194	0.999996191
GBF1	0.57812273	0.293715284	0.706588234	0.273879909	0.761537498	0.689553895	0.999996191
GBP4	0.808048974	0.442333734	0.127167767	0.275477769	0.949096558	0.544949501	0.999996191
GBP6	0.229384858	0.524113737	0.486875847	0.695536584	0.571375479	0.675429413	0.999996191
GBX1	0.896309318	0.59026791	0.54979227	0.742921722	0.927680777	0.975924459	0.999996191
GCC2	0.696564059	0.543245829	0.98789373	0.56795716	0.576877824	0.937893425	0.999996191
GCDH	0.579934562	0.637779805	0.745177186	0.04162693	0.944034612	0.527301498	0.999996191
GCGR	0.651578873	0.57582577	0.644868673	0.837898718	0.733610868	0.955458279	0.999996191
GCH1	0.290802838	0.575164707	0.17102122	0.531591933	0.834553519	0.557536939	0.999996191
GCK	0.914800633	0.984094794	0.411432079	0.486376016	0.297454958	0.827465252	0.999996191
GCLC	0.941929151	0.640887947	0.40146263	0.458671267	0.402353471	0.796937874	0.999996191
GCLM	0.660278401	0.67339139	0.098975243	0.742177237	0.473697435	0.595904821	0.999996191
GCNT1	0.902818997	0.212914103	0.52427942	0.175642259	0.571191056	0.514328143	0.999996191
GCNT4	0.868145245	0.725394379	0.327394063	0.120276604	0.424754278	0.522030416	0.999996191
GCNT7	0.603417614	0.793653637	0.331169012	0.575212871	0.335566128	0.72802316	0.999996191
GDAP1	0.257211986	0.159080592	0.30420438	0.850256492	0.778756514	0.476526626	0.999996191
GDAP2	0.851640676	0.486481852	0.68575313	0.733091454	0.99106622	0.977519056	0.999996191
GDE1	0.245502807	0.455180017	0.274384511	0.329666337	0.886014628	0.491758672	0.999996191
GDF1	0.774941568	0.291234403	0.069639998	0.910262683	0.873557889	0.554596824	0.999996191
GDF5	0.49838279	0.42713557	0.599465947	0.364557307	0.308209167	0.581125831	0.999996191
GDI2	0.859191748	0.261219273	0.574011288	0.948108084	0.138554528	0.613398619	0.999996191
GDNF	0.524771305	0.915327998	0.826307433	0.702964761	0.216971349	0.846975745	0.999996191
GDPD1	0.959755596	0.747261626	0.170404081	0.570906345	0.568000428	0.775579542	0.999996191
GDPD3	0.550105501	0.40705118	0.541573649	0.555454848	0.437297198	0.720745176	0.999996191
GDPD4	0.503170867	0.770917719	0.189788831	0.326366878	0.804458666	0.639353977	0.999996191
GDPD5	0.871233412	0.490959357	0.554575306	0.375146415	0.483511276	0.790178618	0.999996191
GDPGP1	0.237075008	0.782523776	0.858965747	0.687125095	0.380173684	0.784342218	0.999996191
GEMIN7	0.765824597	0.723068335	0.528997831	0.454823085	0.423033964	0.835655074	0.999996191
GEMIN8	0.823368172	0.506910093	0.328909956	0.761703997	0.686337644	0.872524249	0.999996191
GEN1	0.581875878	0.584193377	0.257642875	0.345545077	0.672665343	0.649469331	0.999996191
GFER	0.557097685	0.564120749	0.409798357	0.204676994	0.343900793	0.49399574	0.999996191
GFI1	0.114383622	0.636980235	0.65553281	0.511810859	0.513994722	0.555624521	0.999996191
GFOD1	0.532447571	0.425047863	0.245771622	0.323334836	0.594196574	0.524757368	0.999996191
GFPT1	0.12967273	0.791351652	0.366350225	0.520083516	0.548512089	0.525429686	0.999996191
GFRA3	0.90243885	0.548848287	0.355523217	0.809477871	0.45304704	0.856917004	0.999996191
GGACT	0.947896823	0.744106076	0.555575521	0.167993383	0.975828559	0.856119409	0.999996191
GGCX	0.88264057	0.526081921	0.359152968	0.222935078	0.402364865	0.589357885	0.999996191
GGH	0.629918941	0.859478658	0.289614161	0.79630548	0.951448444	0.934821803	0.999996191
GGNBP2	0.219517731	0.531868322	0.509373301	0.778611849	0.188703427	0.487219965	0.999996191

GGPS1	0.482868061	0.807920799	0.285892231	0.546901459	0.891179265	0.829804056	0.999996191
GGT1	0.17923966	0.733743401	0.164672129	0.78836796	0.422914328	0.452718062	0.999996191
GGT5	0.894984135	0.82884964	0.424786878	0.828518063	0.585572516	0.957674507	0.999996191
GGT7	0.294187114	0.416322224	0.913331713	0.757605583	0.305076915	0.695837789	0.999996191
GHDC	0.675896782	0.648769973	0.366531778	0.408073505	0.699245601	0.801280299	0.999996191
GHITM	0.721187321	0.868584697	0.237289925	0.21891238	0.661759733	0.660420777	0.999996191
GHR	0.790260576	0.797652205	0.050700038	0.567694592	0.917003786	0.610065586	0.999996191
GHSR	0.41196521	0.425674264	0.305389445	0.322402237	0.441879338	0.462561071	0.999996191
GIGYF1	0.218643944	0.425923964	0.970223353	0.868128686	0.109529539	0.484111772	0.999996191
GIGYF2	0.799211797	0.930791677	0.68051786	0.13899553	0.933322126	0.859455914	0.999996191
GIN1	0.887388413	0.894503739	0.708597974	0.388979915	0.278017801	0.84771914	0.999996191
GINM1	0.472142209	0.277857946	0.53295625	0.281383656	0.845542193	0.610040108	0.999996191
GINS1	0.824358131	0.899370892	0.725642268	0.102007369	0.574469798	0.733552042	0.999996191
GINS2	0.959925758	0.517008734	0.21863547	0.821579187	0.409476123	0.760702382	0.999996191
GINS3	0.591375849	0.286737027	0.802911937	0.648530629	0.883524791	0.884276677	0.999996191
GINS4	0.651944581	0.422778941	0.50271517	0.322138937	0.96844414	0.790993162	0.999996191
GIPR	0.691531091	0.931851108	0.583423283	0.109440074	0.699378039	0.716311339	0.999996191
GIT1	0.269687534	0.834808714	0.750940601	0.785165522	0.83764993	0.92788293	0.999996191
GIT2	0.971223911	0.906128788	0.797571401	0.187482004	0.893554246	0.93377362	0.999996191
GJA10	0.413210248	0.41565473	0.658281096	0.500267706	0.218926983	0.552823545	0.999996191
GJA4	0.784864244	0.932094884	0.741626478	0.60014757	0.735900088	0.984582813	0.999996191
GJB2	0.635043992	0.243505002	0.788288467	0.773644697	0.374179061	0.754491776	0.999996191
GJB6	0.980424331	0.903144925	0.790272023	0.992562652	0.739671314	0.999370145	0.999996191
GJC2	0.435196359	0.560589118	0.238800314	0.167969211	0.86555396	0.481511331	0.999996191
GKAP1	0.441781321	0.71060045	0.320496724	0.756234553	0.559651061	0.788349386	0.999996191
GLB1	0.282228587	0.476703685	0.955547386	0.984051143	0.947449848	0.935726992	0.999996191
GLB1L	0.955351231	0.735558391	0.451225512	0.420479267	0.499231236	0.861470613	0.999996191
GLB1L3	0.669818293	0.870017117	0.63232468	0.977529422	0.482528643	0.967112954	0.999996191
GLCCI1	0.604536697	0.7660462	0.418599973	0.257962534	0.204050378	0.516054437	0.999996191
GLDC	0.562275902	0.286071634	0.138319304	0.80795807	0.933297614	0.611700424	0.999996191
GLE1	0.761259798	0.865986179	0.847784536	0.82195852	0.471157298	0.979955575	0.999996191
GLG1	0.501668155	0.562325942	0.247051677	0.55295937	0.356470214	0.572831377	0.999996191
GLI1	0.590429289	0.635673981	0.373006843	0.31024234	0.879067968	0.768867695	0.999996191
GLI2	0.318960218	0.48638788	0.668731058	0.491876733	0.414673895	0.657022754	0.999996191
GLIPR1	0.466847661	0.773451793	0.118015066	0.242109806	0.801408991	0.477108252	0.999996191
GLIPR2	0.500006443	0.901173321	0.787846028	0.114144858	0.521225481	0.656651948	0.999996191
GLIS2	0.996513759	0.339116071	0.172913906	0.277115044	0.565949931	0.496007822	0.999996191
GLMN	0.501524423	0.898308414	0.985778513	0.869836594	0.512224753	0.975192257	0.999996191
GLMP	0.348262358	0.527593952	0.214594741	0.990791471	0.817564419	0.735991633	0.999996191
GLOD4	0.854565486	0.772200194	0.265575155	0.391338779	0.500285808	0.74931835	0.999996191
GLRB	0.028143336	0.966419883	0.680278345	0.795046273	0.501956488	0.45670133	0.999996191
GLRX2	0.863417292	0.42785495	0.720171788	0.776944475	0.930305607	0.973532742	0.999996191
GLS2	0.801356288	0.259037579	0.846477239	0.628874015	0.900299641	0.915340025	0.999996191
GLT8D1	0.663103137	0.472032088	0.205173071	0.435509629	0.630451451	0.621415011	0.999996191
GLTP	0.096517922	0.94433234	0.839390558	0.153040673	0.828313583	0.506536821	0.999996191

GLUD1	0.467880826	0.849995843	0.367566845	0.639162296	0.553829927	0.821705323	0.999996191
GLYAT	0.568880198	0.962791309	0.725919562	0.140834343	0.440195438	0.686634785	0.999996191
GLYCTK	0.725521412	0.810821243	0.747692975	0.095165322	0.868849227	0.760024016	0.999996191
GMDS	0.423373729	0.79016651	0.658471674	0.116091204	0.966806702	0.687222999	0.999996191
GMEB1	0.243341479	0.6789092	0.705028218	0.663760253	0.918760336	0.87103531	0.999996191
GMEB2	0.15169574	0.517532909	0.872089868	0.222425726	0.567781328	0.485286647	0.999996191
GMFB	0.121085434	0.73662307	0.743961434	0.728801682	0.309711918	0.589599496	0.999996191
GMNN	0.609618953	0.612569554	0.811302498	0.756374619	0.326459139	0.878440136	0.999996191
GMPR2	0.872467683	0.919191168	0.449964175	0.765385679	0.564590968	0.95922683	0.999996191
GMPS	0.30015436	0.196326352	0.650756096	0.402049743	0.997091171	0.594661539	0.999996191
GNAI1	0.164185257	0.899006738	0.113771769	0.805911473	0.584400531	0.469049962	0.999996191
GNAO1	0.662100153	0.661080503	0.299491576	0.738946226	0.980660311	0.909817768	0.999996191
GNAQ	0.846855301	0.72417265	0.61153223	0.546702345	0.966186575	0.975255962	0.999996191
GNAS	0.968596961	0.253662529	0.796691744	0.768010258	0.828624846	0.939559106	0.999996191
GNAT2	0.416607301	0.688273253	0.575427097	0.990157875	0.957593409	0.959479037	0.999996191
GNB1	0.391751736	0.407780521	0.173558614	0.812555274	0.934994625	0.656130708	0.999996191
GNB2	0.176294588	0.485116395	0.549339265	0.743728565	0.285122595	0.511562315	0.999996191
GNB4	0.853315252	0.574179017	0.908901838	0.988383886	0.329792524	0.953447202	0.999996191
GNB5	0.910767015	0.325686392	0.259107521	0.94084327	0.839421793	0.847392292	0.999996191
GNE	0.403536974	0.685242199	0.6544615	0.229573805	0.208771728	0.485864294	0.999996191
GNG10	0.980743168	0.46082224	0.713827224	0.886657948	0.947890342	0.989170213	0.999996191
GNG12	0.564680713	0.271761968	0.302028183	0.794891995	0.447497434	0.608295261	0.999996191
GNG2	0.546103655	0.468265651	0.446437691	0.853566142	0.55485388	0.828929339	0.999996191
GNG3	0.441456385	0.790320457	0.703191624	0.560965519	0.508001049	0.868731707	0.999996191
GNG5	0.695614098	0.275634622	0.883374026	0.334144239	0.568861738	0.737481202	0.999996191
GNL2	0.605108324	0.405249165	0.299136206	0.488012248	0.234925342	0.480205004	0.999996191
GNPAT	0.889541713	0.398118006	0.379852096	0.843974282	0.921594183	0.921160503	0.999996191
GNPDA2	0.191235403	0.711261028	0.27656327	0.339217368	0.704251182	0.492388731	0.999996191
GNPNAT1	0.366679045	0.362732012	0.208742805	0.993484918	0.363605624	0.512815208	0.999996191
GNRH2	0.385262588	0.273893529	0.940678925	0.783587682	0.384166912	0.723451526	0.999996191
GOLGA1	0.360165811	0.276984287	0.244271504	0.886632115	0.793376381	0.615897618	0.999996191
GOLGA5	0.433469914	0.978130901	0.234527088	0.543467256	0.324232357	0.620181027	0.999996191
GOLGA7	0.827596141	0.597568306	0.294053627	0.073972371	0.69977752	0.460151591	0.999996191
GOLIM4	0.684033818	0.942290301	0.370903878	0.845275147	0.553923835	0.928599728	0.999996191
GOLPH3	0.730835869	0.512291268	0.641914373	0.188336949	0.977159476	0.794998916	0.999996191
GOLPH3L	0.81265154	0.703448779	0.196385995	0.395623329	0.454802489	0.647961268	0.999996191
GOLT1B	0.76344443	0.093877546	0.659715028	0.857522202	0.994663502	0.778707007	0.999996191
GON4L	0.33135537	0.328722892	0.532020615	0.720700964	0.854715163	0.756611313	0.999996191
GON7	0.311047962	0.743889072	0.142845237	0.975145728	0.51683917	0.61031191	0.999996191
GOPC	0.19799424	0.826737222	0.694674896	0.520424328	0.973809051	0.839210357	0.999996191
GORAB	0.845674236	0.927775845	0.426291837	0.241636838	0.442471824	0.756937842	0.999996191
GOSR1	0.367777361	0.885778067	0.152679882	0.502216496	0.6713306	0.611611935	0.999996191
GOT2	0.882926057	0.708118664	0.473634348	0.112040372	0.537370084	0.623573648	0.999996191
GPAA1	0.787663709	0.992716827	0.524973775	0.347455856	0.71720963	0.918580883	0.999996191
GPAM	0.656660058	0.944646733	0.983784362	0.637901275	0.501617818	0.974431713	0.999996191

GPANK1	0.529486502	0.872501353	0.309282624	0.730400838	0.674297179	0.869679058	0.999996191
GPAT2	0.125551744	0.713574581	0.633371315	0.407298551	0.34428477	0.470141954	0.999996191
GPAT3	0.62539612	0.835179379	0.563136869	0.509344652	0.501854735	0.879145078	0.999996191
GPAT4	0.515995711	0.611532513	0.171284903	0.694006889	0.341463399	0.559317212	0.999996191
GPATCH11	0.92155273	0.768781114	0.433434593	0.783688129	0.903320009	0.980169696	0.999996191
GPATCH2	0.458112739	0.910817185	0.619003488	0.170113932	0.902788426	0.775742907	0.999996191
GPATCH2L	0.696046883	0.430814284	0.529165813	0.161309305	0.504550518	0.560909294	0.999996191
GPC4	0.442060878	0.400499873	0.193054467	0.602510763	0.965579937	0.644889849	0.999996191
GPC6	0.345024915	0.82842453	0.903995229	0.595870562	0.506021245	0.884094614	0.999996191
GPCPD1	0.653132312	0.819649479	0.868943256	0.695034858	0.069849012	0.669680969	0.999996191
GPD1	0.837680565	0.86817003	0.950435356	0.408475227	0.626809693	0.968322696	0.999996191
GPHN	0.160526628	0.51523746	0.202780249	0.823862105	0.905804517	0.554880001	0.999996191
GPKOW	0.689951606	0.852691357	0.294023863	0.815244762	0.905353749	0.941935363	0.999996191
GPM6B	0.219736785	0.501716583	0.786561153	0.260697031	0.662575874	0.589604193	0.999996191
GPN1	0.413841409	0.537441406	0.105765896	0.865086552	0.519579148	0.522754081	0.999996191
GPN2	0.447524789	0.843615296	0.261069894	0.788416806	0.987663971	0.882022262	0.999996191
GPR1	0.60232472	0.569785257	0.351202656	0.423257386	0.955856665	0.811761527	0.999996191
GPR107	0.875052529	0.49140931	0.205759003	0.349643694	0.747263067	0.674217102	0.999996191
GPR108	0.911183029	0.748216674	0.922301347	0.720207018	0.745893749	0.994856567	0.999996191
GPR135	0.278761347	0.930433089	0.288556967	0.391811642	0.927349089	0.705526771	0.999996191
GPR137	0.644015837	0.342727168	0.733197349	0.15065616	0.992056303	0.682983023	0.999996191
GPR137C	0.993697871	0.637204421	0.505274674	0.178422701	0.571210966	0.73985502	0.999996191
GPR143	0.731250902	0.808031537	0.154541402	0.819074375	0.991472637	0.877198476	0.999996191
GPR146	0.15502311	0.673874712	0.892117765	0.6558968	0.665584917	0.78027581	0.999996191
GPR153	0.24915861	0.554491242	0.617605108	0.669757861	0.868046687	0.814653816	0.999996191
GPR156	0.729332041	0.487775677	0.171193255	0.86852295	0.217425738	0.538693352	0.999996191
GPR157	0.77363563	0.963066702	0.912848324	0.226442935	0.950366228	0.954139825	0.999996191
GPR160	0.765721746	0.928349931	0.098207642	0.119885557	0.963310564	0.472524266	0.999996191
GPR161	0.842897849	0.869415263	0.209599435	0.619610643	0.685817192	0.858530722	0.999996191
GPR17	0.220391933	0.631691996	0.514406441	0.823222682	0.2984329	0.62098734	0.999996191
GPR174	0.585642357	0.371124252	0.661526985	0.513329308	0.137707369	0.515311249	0.999996191
GPR176	0.65492357	0.828522302	0.498927074	0.289453578	0.248263314	0.640621752	0.999996191
GPR180	0.24635766	0.640748256	0.573891894	0.935279635	0.965432518	0.890710917	0.999996191
GPR182	0.661720863	0.868715283	0.359594671	0.132515072	0.853724733	0.676455586	0.999996191
GPR19	0.513456677	0.985619458	0.228266088	0.867974706	0.956288804	0.91094917	0.999996191
GPR21	0.565174604	0.732524189	0.173576511	0.986105193	0.429165114	0.726780675	0.999996191
GPR27	0.922308865	0.445906306	0.838535493	0.894520744	0.749924357	0.983073752	0.999996191
GPR31	0.80838432	0.884157084	0.900930667	0.775002629	0.400171326	0.975710943	0.999996191
GPR39	0.748262331	0.786402744	0.182398288	0.259100324	0.892744679	0.688021409	0.999996191
GPR52	0.61381783	0.733691549	0.714167051	0.923976179	0.865898021	0.987361723	0.999996191
GPR55	0.888954951	0.867885298	0.650891182	0.091872007	0.205224594	0.502059791	0.999996191
GPR62	0.957859476	0.886520412	0.132062056	0.194350947	0.949939359	0.652766749	0.999996191
GPR63	0.5366395	0.935196625	0.443752658	0.468364329	0.880790605	0.905709968	0.999996191
GPR89A	0.358436208	0.92980517	0.3078836	0.689175826	0.621757796	0.793965529	0.999996191
GPRASP1	0.288803622	0.522259106	0.440996905	0.225506991	0.577803491	0.485720077	0.999996191

GPRC5B	0.503507151	0.84283541	0.200093478	0.902333594	0.620723289	0.807517672	0.999996191
GPS2	0.983313553	0.888762693	0.521398041	0.089262605	0.756815825	0.729064775	0.999996191
GPSM1	0.754212122	0.518326975	0.161637875	0.31910501	0.86222046	0.61865815	0.999996191
GPSM2	0.507122283	0.524872863	0.449883713	0.263107658	0.715188067	0.669248505	0.999996191
GRAMD2A	0.956307516	0.105920269	0.758687249	0.33958321	0.310416469	0.47338891	0.999996191
GRAMD2B	0.760756057	0.584636652	0.715981988	0.238313047	0.133862338	0.515219218	0.999996191
GRAP	0.735555624	0.874778916	0.601593173	0.136721615	0.652090786	0.750394103	0.999996191
GRAP2	0.39421813	0.924433862	0.364821828	0.390111646	0.561602344	0.718620356	0.999996191
GRASP	0.206752295	0.441468618	0.604688608	0.964304793	0.711011397	0.767254343	0.999996191
GRB14	0.978662366	0.845401447	0.747614604	0.957612294	0.427845991	0.986797754	0.999996191
GRB2	0.089408014	0.508967033	0.2613021	0.913916947	0.90058126	0.508231063	0.999996191
GREB1	0.733228985	0.720826702	0.611356895	0.57173216	0.579488169	0.923728514	0.999996191
GRIA1	0.93134719	0.119285938	0.107251628	0.960221539	0.787917728	0.492966747	0.999996191
GRIA3	0.218222478	0.57229036	0.49441997	0.881399207	0.770235659	0.785577489	0.999996191
GRID1	0.415837091	0.488260506	0.11561886	0.631473662	0.99823082	0.587243213	0.999996191
GRID2IP	0.0979486	0.454076715	0.872070872	0.452825003	0.971359791	0.614969948	0.999996191
GRIK5	0.869125818	0.440566474	0.216871122	0.945205983	0.498881269	0.773426312	0.999996191
GRIN2D	0.832529009	0.641283211	0.08885827	0.596646565	0.968281407	0.707022183	0.999996191
GRIN3A	0.075177846	0.899389644	0.44901604	0.694825727	0.539798168	0.536800362	0.999996191
GRINA	0.450204377	0.842441135	0.535722729	0.555472981	0.508584153	0.838579907	0.999996191
GRIP1	0.490244771	0.961805	0.353110488	0.095039162	0.456382754	0.452743718	0.999996191
GRIPAP1	0.477838346	0.5659297	0.956130651	0.418044657	0.642553182	0.867764604	0.999996191
GRK2	0.432085439	0.972778608	0.909260804	0.7297571	0.666641464	0.971490783	0.999996191
GRK5	0.756649459	0.833289149	0.853850819	0.760633176	0.437439944	0.96911941	0.999996191
GRK6	0.339539216	0.942042251	0.407474072	0.770777942	0.919828474	0.906428211	0.999996191
GRM8	0.455163352	0.673657069	0.830276009	0.729067777	0.196261653	0.760322886	0.999996191
GRSF1	0.219474113	0.262680916	0.291086569	0.670552225	0.652209736	0.455619344	0.999996191
GSC	0.187657282	0.570486683	0.538720015	0.324254764	0.520052465	0.507056475	0.999996191
GSC2	0.49069102	0.638195596	0.638264765	0.920673164	0.211879536	0.772654821	0.999996191
GSK3B	0.786263042	0.75443127	0.698931872	0.529554636	0.343964614	0.87976163	0.999996191
GSKIP	0.186985532	0.308166289	0.379081481	0.905761644	0.527665127	0.520355482	0.999996191
GSN	0.268197433	0.964059042	0.28240288	0.32707432	0.669924485	0.602440514	0.999996191
GSPT2	0.858923357	0.906172962	0.760747822	0.600753123	0.235659329	0.893966814	0.999996191
GSTA1	0.924327523	0.466417952	0.409087713	0.134668107	0.835846945	0.644571503	0.999996191
GSTA2	0.031867266	0.922627783	0.493642578	0.84563878	0.822322188	0.513996526	0.999996191
GSTCD	0.897489748	0.457603915	0.35413665	0.989840016	0.850313601	0.937840211	0.999996191
GSTM1	0.879674007	0.967750851	0.20018757	0.197687238	0.878247697	0.721591303	0.999996191
GSTM2	0.468435251	0.655630245	0.594344171	0.7390051	0.256398228	0.750796768	0.999996191
GSTP1	0.507544082	0.577834369	0.905466802	0.974388756	0.20215177	0.823490263	0.999996191
GSTZ1	0.615401619	0.742623075	0.866509878	0.96783909	0.277210804	0.922884061	0.999996191
GTDC1	0.434164141	0.69920414	0.432018122	0.715852927	0.810789119	0.880870847	0.999996191
GTF2A1	0.794244388	0.685437711	0.845666808	0.188084379	0.830402544	0.872801572	0.999996191
GTF2A2	0.578496623	0.882860055	0.334797801	0.59887651	0.238857621	0.685147427	0.999996191
GTF2E2	0.989436041	0.453583853	0.444092508	0.557619804	0.978364767	0.925447331	0.999996191
GTF2F2	0.603948394	0.758327734	0.795019174	0.881025996	0.345164968	0.927428381	0.999996191

GTF2H2	0.039728596	0.734672369	0.719522349	0.52022136	0.706534366	0.464660176	0.999996191
GTF2H5	0.969201619	0.520701981	0.758410028	0.051879358	0.59645354	0.544296844	0.999996191
GTF2I	0.997141896	0.983999483	0.410015325	0.211737405	0.647102532	0.832066942	0.999996191
GTF2IRD1	0.887791024	0.600953267	0.682079506	0.436855081	0.894873693	0.951737473	0.999996191
GTF3A	0.331998526	0.988095629	0.161416526	0.776889564	0.977544354	0.778197728	0.999996191
GTF3C2	0.7785708	0.563433868	0.67932933	0.295224396	0.942238835	0.892487985	0.999996191
GTF3C3	0.050926372	0.821855294	0.940742384	0.542353253	0.572469238	0.550362326	0.999996191
GTF3C4	0.750367516	0.473100078	0.724092978	0.71734268	0.550512967	0.917694261	0.999996191
GTPBP1	0.405913256	0.714768796	0.311472355	0.346849788	0.377852397	0.544298296	0.999996191
GTPBP10	0.155936926	0.802586139	0.890603045	0.60332465	0.998077931	0.862714344	0.999996191
GTPBP2	0.555562503	0.733171664	0.335808913	0.847568818	0.829444977	0.911294299	0.999996191
GTPBP8	0.136222824	0.868247942	0.232756926	0.995796051	0.950889397	0.697414632	0.999996191
GUCY1B1	0.878101579	0.542473562	0.416595734	0.683249865	0.400284075	0.829546014	0.999996191
GULO	0.555281034	0.880497071	0.785506277	0.703312013	0.766888816	0.977705031	0.999996191
GULP1	0.997946176	0.704768648	0.674575106	0.700116576	0.97971857	0.994091702	0.999996191
GUSB	0.333652888	0.074725415	0.572117832	0.82531153	0.93712857	0.530788991	0.999996191
GVQW3	0.317540881	0.443270212	0.424078335	0.47624235	0.376978066	0.525292336	0.999996191
GXYLT1	0.759627309	0.465044781	0.47419627	0.209542034	0.554763637	0.64080577	0.999996191
GYG2	0.594764539	0.958091406	0.839103566	0.692438397	0.338486161	0.928727541	0.999996191
GYPC	0.634717177	0.625102503	0.307529139	0.360062382	0.995916124	0.793111501	0.999996191
GZF1	0.741106201	0.684499846	0.353088916	0.078153543	0.857569955	0.546884567	0.999996191
H2AFV	0.508356921	0.76964057	0.974169961	0.052845724	0.957933794	0.639008971	0.999996191
H2AFX	0.094167014	0.987369112	0.803905902	0.808554198	0.593873804	0.757606968	0.999996191
H2AFZ	0.280441923	0.266345138	0.474336924	0.490171928	0.449463377	0.466674633	0.999996191
H2B	0.596783388	0.750570181	0.850794831	0.946271835	0.3149244	0.93014211	0.999996191
H3F3B	0.642200866	0.225288181	0.729031553	0.452219097	0.949681571	0.799144835	0.999996191
H3F3C	0.379476166	0.59398778	0.934159323	0.146178568	0.921892478	0.713646907	0.999996191
HACD1	0.540062954	0.5160027	0.712299594	0.909654827	0.608670495	0.926620649	0.999996191
HACD2	0.657987907	0.561618722	0.431448638	0.497455713	0.160191163	0.557761933	0.999996191
HACD3	0.77673406	0.089535539	0.541604807	0.983006941	0.489701483	0.626864915	0.999996191
HACL1	0.725017074	0.99162802	0.729796446	0.489863934	0.819426191	0.978574445	0.999996191
HADH	0.884087466	0.754672907	0.260802549	0.804702402	0.766906544	0.92407143	0.999996191
HADHA	0.945806671	0.793691483	0.638903777	0.613953622	0.625086939	0.970860883	0.999996191
HADHB	0.830573804	0.781332615	0.219774264	0.875506469	0.465703589	0.840647699	0.999996191
HAGHL	0.686886488	0.660762119	0.569952973	0.145658823	0.969302143	0.760799505	0.999996191
HAND1	0.989457269	0.864917735	0.58622864	0.561259456	0.286256549	0.888732711	0.999996191
HAP1	0.872573731	0.490355643	0.418635915	0.260122578	0.688207646	0.736731822	0.999996191
HARS	0.644628709	0.549227397	0.568182327	0.574969429	0.21526155	0.688575016	0.999996191
HARS2	0.642388946	0.59921544	0.404192459	0.509713347	0.987218003	0.884765433	0.999996191
HASPIN	0.79750982	0.269224115	0.349966373	0.820521572	0.722481106	0.796232715	0.999996191
HAT1	0.390566336	0.29479659	0.722028939	0.545517337	0.476851307	0.661251555	0.999996191
HAUS1	0.576367045	0.597418659	0.29943178	0.59476364	0.809808432	0.814832034	0.999996191
HAUS2	0.536681559	0.068957587	0.661692675	0.940033444	0.611634126	0.577592905	0.999996191
HAUS4	0.554370955	0.709648708	0.862517204	0.414895923	0.574885047	0.889283672	0.999996191
HAUS5	0.199165367	0.614425063	0.465432187	0.77453047	0.839004648	0.763225417	0.999996191

HAUS6	0.089384543	0.799986125	0.971032317	0.410670256	0.960479372	0.706887498	0.999996191
HAUS7	0.702122002	0.902065747	0.645429319	0.417287302	0.871214675	0.95539675	0.999996191
HAX1	0.287587023	0.319961808	0.450347223	0.786014473	0.955387308	0.731108514	0.999996191
HBA	0.570837645	0.964957221	0.566794683	0.262961909	0.861530126	0.870421901	0.999996191
HBA1	0.632452379	0.567212453	0.722120186	0.668128582	0.57784389	0.915961257	0.999996191
HBS1L	0.245872755	0.967364563	0.328699988	0.889059022	0.531692269	0.762952458	0.999996191
HCAR1	0.7257635	0.929267941	0.590235921	0.286961655	0.709622136	0.88949481	0.999996191
HCFC1	0.671986858	0.498067718	0.616471249	0.234564141	0.850763951	0.782404332	0.999996191
HCFC1R1	0.339771916	0.712587104	0.811840479	0.04237465	0.986891263	0.476042954	0.999996191
HCFC2	0.338094425	0.866934397	0.904662395	0.48209693	0.826417337	0.922246673	0.999996191
HCST	0.495634366	0.961283609	0.349493579	0.745346022	0.62592484	0.883695333	0.999996191
HDAC1	0.735249957	0.876679326	0.166291533	0.135506213	0.916915598	0.5668359	0.999996191
HDAC3	0.20981224	0.585321347	0.162918129	0.414758097	0.8802685	0.45477798	0.999996191
HDAC4	0.852956219	0.253642174	0.467691936	0.56739884	0.847323001	0.811351756	0.999996191
HDAC5	0.382663976	0.557181049	0.626479182	0.605153252	0.139296215	0.534662389	0.999996191
HDAC6	0.893374494	0.59604453	0.081025851	0.741151971	0.857815318	0.707185511	0.999996191
HDAC8	0.69682763	0.749974357	0.877130162	0.348277962	0.946695841	0.956761193	0.999996191
HDAC9	0.032767788	0.634461841	0.956634401	0.753253002	0.509888078	0.462776479	0.999996191
HDDC2	0.70923996	0.506157245	0.331386895	0.845033003	0.089137006	0.491856792	0.999996191
HDDC3	0.789697586	0.912190481	0.770985339	0.535345321	0.807019947	0.984639634	0.999996191
HDGF	0.45976084	0.801162138	0.570682999	0.178084821	0.527925522	0.643689495	0.999996191
HDGFL3	0.58817431	0.552092781	0.803831938	0.085806794	0.715946445	0.602883056	0.999996191
HDHD2	0.672465987	0.768212563	0.497980674	0.220683061	0.683075109	0.771677816	0.999996191
HEATR1	0.400773023	0.495781478	0.988659342	0.331472144	0.326898824	0.658172115	0.999996191
HEATR5A	0.570507758	0.898596597	0.523594833	0.055267561	0.867656742	0.56026887	0.999996191
HEATR5B	0.581906067	0.408143765	0.266999402	0.884003946	0.467461843	0.698423217	0.999996191
HEATR6	0.57843989	0.855295896	0.116420578	0.380065913	0.886821899	0.640206428	0.999996191
HEBP1	0.840205784	0.316156249	0.068689437	0.393468825	0.984942834	0.449006745	0.999996191
HECTD2	0.714330259	0.536969898	0.738635396	0.571785951	0.69751285	0.929606557	0.999996191
HECTD4	0.627441103	0.824905181	0.492532736	0.710864753	0.241772082	0.79334769	0.999996191
HECW1	0.616031382	0.49918567	0.659962797	0.255770339	0.716596387	0.764133711	0.999996191
HECW2	0.700972631	0.147462448	0.89255084	0.630849326	0.391828568	0.67158129	0.999996191
HEG1	0.456696968	0.406574496	0.672458	0.323537278	0.922875893	0.764549091	0.999996191
HELQ	0.116285993	0.615754233	0.858426592	0.696982285	0.494204176	0.657127016	0.999996191
HELZ	0.612914652	0.964994958	0.452989954	0.078681937	0.558112526	0.543035277	0.999996191
HEPACAM	0.964989441	0.132053199	0.235431486	0.973335717	0.248109566	0.453317111	0.999996191
HEPH	0.957285194	0.204128774	0.246353495	0.494059881	0.503012843	0.546226844	0.999996191
HERC1	0.596646166	0.906084541	0.825930249	0.067854908	0.886397545	0.703132547	0.999996191
HERC2	0.960594746	0.973610205	0.834213302	0.268547614	0.989554307	0.977752919	0.999996191
HERC3	0.755672458	0.551872788	0.530216901	0.927622487	0.865990858	0.968566967	0.999996191
HERC4	0.306926878	0.694753479	0.181709646	0.490642667	0.963412437	0.628836541	0.999996191
HERPUD2	0.862066995	0.841280772	0.05264716	0.729991199	0.992509995	0.708801065	0.999996191
HES1	0.364890375	0.073819733	0.877842329	0.750839179	0.983970297	0.619597106	0.999996191
HES4	0.864410338	0.269977566	0.746357249	0.307651705	0.970284212	0.822497623	0.999996191
HES7	0.97217431	0.850696383	0.563517716	0.261791742	0.81424671	0.915173128	0.999996191

HESX1	0.330323717	0.43850763	0.469082608	0.78084561	0.775782654	0.782338385	0.999996191
HEXA	0.698395998	0.829698118	0.73613242	0.870292333	0.762990039	0.990537972	0.999996191
HEXB	0.928480401	0.627381978	0.635013469	0.078280459	0.850324807	0.686425938	0.999996191
HEY1	0.911300141	0.88122383	0.422459418	0.354249066	0.478312626	0.838814199	0.999996191
HFE	0.166162584	0.431697544	0.968997432	0.892584515	0.971684643	0.846323304	0.999996191
HGFAC	0.879565744	0.060632037	0.743006285	0.913255718	0.589725181	0.658669608	0.999996191
HGS	0.520086829	0.588490522	0.556928318	0.370382207	0.748457297	0.806411282	0.999996191
HGSNAT	0.684855365	0.580082983	0.559489782	0.727336239	0.948572479	0.957918988	0.999996191
HHAT	0.790584954	0.925915336	0.25924663	0.943940211	0.697817536	0.939897021	0.999996191
HIBADH	0.868077602	0.675045769	0.450078874	0.065129504	0.960709349	0.608480536	0.999996191
HIBCH	0.640985561	0.682638974	0.666150926	0.14231639	0.962692208	0.776952221	0.999996191
HIC1	0.219354565	0.621610189	0.334485073	0.38700109	0.498503017	0.488494863	0.999996191
HIC2	0.389551423	0.688524849	0.155450476	0.674561852	0.582145548	0.606944101	0.999996191
HIF3A	0.587801871	0.68472018	0.503514898	0.637907115	0.077690346	0.513076634	0.999996191
HIGD1A	0.556341351	0.492435997	0.412335613	0.301128774	0.604322534	0.651380073	0.999996191
HIKESHI	0.963608515	0.43456939	0.548325493	0.623038298	0.676148556	0.91200351	0.999996191
HILPDA	0.586839739	0.604244106	0.390693406	0.647812041	0.473562214	0.78801025	0.999996191
HINFP	0.561376771	0.866601698	0.188716088	0.573399602	0.95680608	0.817212418	0.999996191
HINT1	0.959013055	0.476188068	0.627456645	0.198953561	0.980688013	0.834354207	0.999996191
HINT2	0.660271553	0.562052522	0.276881764	0.353103589	0.899194428	0.739959091	0.999996191
HINT3	0.486332603	0.602839232	0.613910557	0.11079893	0.537901455	0.525475111	0.999996191
HIP1	0.331592852	0.716323698	0.827900723	0.55898001	0.966630673	0.922893252	0.999996191
HIP1R	0.971600725	0.25203852	0.121747255	0.406907749	0.896182412	0.528014046	0.999996191
HIPK1	0.740534247	0.817358719	0.839847977	0.256135413	0.977401838	0.941627529	0.999996191
HIRIP3	0.94290623	0.261643762	0.408136006	0.818304274	0.634055623	0.823287282	0.999996191
HIST1H1C	0.234202052	0.932046256	0.275727922	0.758441335	0.778970509	0.755902519	0.999996191
HIST1H1D	0.849131296	0.64124022	0.644924932	0.563200852	0.990885348	0.974638542	0.999996191
HIST1H1E	0.613785083	0.754908004	0.874429564	0.140875708	0.288722085	0.608211495	0.999996191
HIST1H1E	0.730006464	0.36651985	0.900292069	0.708394231	0.155671209	0.701036941	0.999996191
HIST1H2AC	0.776071855	0.716788986	0.859583478	0.752320122	0.588663375	0.978854854	0.999996191
HIST1H2AK	0.61171959	0.312967603	0.655651375	0.103751723	0.973319233	0.557316419	0.999996191
HIST1H2BB	0.628503535	0.877651591	0.715390282	0.944090098	0.994492779	0.996447794	0.999996191
HIST1H2BD	0.393582677	0.298763544	0.320254955	0.303544509	0.754487006	0.484819807	0.999996191
HIST1H2BL	0.793895747	0.984598783	0.791490917	0.386853767	0.989299199	0.984081924	0.999996191
HIST2H2AA4	0.569456269	0.142250021	0.54669059	0.167516815	0.995737643	0.456771692	0.999996191
HIST2H2AC	0.859479134	0.597061281	0.398799937	0.830595377	0.612467943	0.920591373	0.999996191
HIST2H2BE	0.943522567	0.720152426	0.527076856	0.283744454	0.932743334	0.909550644	0.999996191
HIST3H2A	0.278313629	0.1075452	0.957060103	0.609832584	0.51634933	0.49307812	0.999996191
HIVEP1	0.432801771	0.91284936	0.682530854	0.896201504	0.259562012	0.852478231	0.999996191
HIVEP2	0.898145408	0.915665596	0.290837759	0.75201189	0.852324061	0.957896599	0.999996191
HLCS	0.951674108	0.868274185	0.440060831	0.414263853	0.600307316	0.903731131	0.999996191
HLF	0.80923938	0.345742156	0.174460484	0.897312059	0.617492986	0.704483311	0.999996191
HLTF	0.472555968	0.776448434	0.706127312	0.937259258	0.858908129	0.978067475	0.999996191
HMBOX1	0.622366991	0.523086462	0.918564213	0.457121827	0.766129293	0.921265234	0.999996191
HMCES	0.269106742	0.287554947	0.865639913	0.199662252	0.816635849	0.528886586	0.999996191

HMCN2	0.084102926	0.799893465	0.673581007	0.738264782	0.659010375	0.665003655	0.999996191
HMG20A	0.805620153	0.083695483	0.682955079	0.93756401	0.954209508	0.78250104	0.999996191
HMG20B	0.892305167	0.610780582	0.459016899	0.114234972	0.743497788	0.657817846	0.999996191
HMGN3	0.198329145	0.747652998	0.802121528	0.474447507	0.973295384	0.831483363	0.999996191
HMGN4	0.195868306	0.527126576	0.960048828	0.352119573	0.5646761	0.643156838	0.999996191
HMGXB4	0.429928497	0.226717277	0.61753495	0.914069731	0.209433698	0.539063613	0.999996191
HNF4A	0.623889065	0.700792852	0.203775286	0.487861794	0.864948382	0.766069419	0.999996191
HNRNPA0	0.862251057	0.768499846	0.570032036	0.411301664	0.790542342	0.938166141	0.999996191
HNRNPA1	0.881120345	0.965729093	0.805944651	0.300638046	0.897587191	0.971200255	0.999996191
HNRNPA2B1	0.311257106	0.114800829	0.693839788	0.398346312	0.927795829	0.495980969	0.999996191
HNRNPA3	0.605003794	0.243935589	0.939511661	0.443070045	0.883598349	0.8295748	0.999996191
HNRNPC	0.932590365	0.625167954	0.920248209	0.787532845	0.298191266	0.940664858	0.999996191
HNRNPDL	0.48477695	0.849335534	0.690890163	0.335759408	0.722674728	0.866855811	0.999996191
HNRNPH1	0.624710973	0.410508331	0.456035148	0.39056668	0.685961786	0.732393174	0.999996191
HNRNPH2	0.817478673	0.293906188	0.64484196	0.933819212	0.088101806	0.558383944	0.999996191
HNRNPL	0.129993538	0.841339973	0.679794135	0.972483164	0.841297001	0.847721482	0.999996191
HNRNPLL	0.759188498	0.704243018	0.806564202	0.031337962	0.953599727	0.560494098	0.999996191
HNRNPM	0.276275633	0.760861748	0.561510344	0.434357556	0.742262449	0.768271187	0.999996191
HNRNPR	0.925716003	0.262138623	0.813578457	0.844392977	0.648607341	0.924833255	0.999996191
HNRNPU	0.627441912	0.403611643	0.923157618	0.104254057	0.898876319	0.663780137	0.999996191
HNRNPUL1	0.262437031	0.808632487	0.431789354	0.424703875	0.88060806	0.749098368	0.999996191
HNRNPUL2	0.633501746	0.853079111	0.941751682	0.123338814	0.88681502	0.833662277	0.999996191
HOMER2	0.912419349	0.554040219	0.930324371	0.989256552	0.255299776	0.934804201	0.999996191
HOMER3	0.583830213	0.510229125	0.222082354	0.579910008	0.923134331	0.755157833	0.999996191
HOMEZ	0.415867157	0.509718246	0.585731208	0.259645849	0.881436354	0.713913658	0.999996191
HOOK2	0.299252865	0.917282917	0.740920999	0.889528472	0.630383916	0.930586227	0.999996191
HOOK3	0.828675495	0.36674008	0.84669249	0.141860877	0.994130491	0.759625269	0.999996191
HOXA2	0.773582787	0.899414571	0.692977706	0.195390022	0.681727311	0.856082424	0.999996191
HOXA3	0.358949269	0.994292062	0.56186634	0.248811188	0.627913005	0.732373676	0.999996191
HOXA4	0.50210637	0.865547406	0.735731554	0.107738732	0.821170918	0.713061339	0.999996191
HOXA5	0.815474987	0.798294286	0.80140019	0.717019664	0.285090571	0.923301479	0.999996191
HOXB2	0.835592951	0.932899909	0.899225971	0.500590494	0.500637828	0.967831315	0.999996191
HOXB4	0.899855199	0.292245965	0.26965584	0.284986913	0.621262325	0.555482309	0.999996191
HOXB5	0.156981921	0.922163289	0.330024047	0.937774717	0.587608848	0.699313322	0.999996191
HOXB6	0.331133551	0.335887632	0.243272452	0.972364725	0.787860118	0.652999009	0.999996191
HOXB7	0.867894382	0.874848629	0.594381318	0.808786926	0.449074942	0.962982217	0.999996191
HOXB8	0.948232966	0.2735656	0.829688313	0.822336733	0.276245848	0.812205414	0.999996191
HOXC10	0.478882594	0.565799985	0.454296652	0.495018484	0.452094042	0.707997237	0.999996191
HOXC4	0.987224238	0.914016461	0.630246165	0.386075369	0.980321672	0.97967814	0.999996191
HOXC6	0.916786809	0.82977387	0.491311842	0.83534427	0.234958984	0.875661985	0.999996191
HOXC8	0.838154137	0.331308641	0.955210571	0.44897448	0.387132183	0.80218719	0.999996191
HP1BP3	0.918526984	0.55098922	0.919704999	0.598551129	0.518730088	0.953076535	0.999996191
HPCAL1	0.424672079	0.797732861	0.601177678	0.515047355	0.276666371	0.717923971	0.999996191
HPCAL4	0.757134744	0.997014472	0.702175074	0.500807533	0.323066429	0.896951427	0.999996191
HPDL	0.654671066	0.638134423	0.559426981	0.63232876	0.121733391	0.62533235	0.999996191

HPF1	0.713182129	0.387836783	0.811389465	0.304990399	0.643307615	0.794223517	0.999996191
HPGD	0.975858404	0.306223538	0.306865489	0.289006802	0.628648698	0.610340338	0.999996191
HPS1	0.99386002	0.93832172	0.064299597	0.681699894	0.962833716	0.774340997	0.999996191
HPS3	0.954183761	0.936429813	0.708551283	0.926385206	0.671445876	0.997281431	0.999996191
HPS4	0.633285315	0.652098293	0.170336341	0.596556089	0.616603259	0.695989493	0.999996191
HPS5	0.846329948	0.706462678	0.49985029	0.324292504	0.874977127	0.895484809	0.999996191
HRASLS	0.967776526	0.669231161	0.864817666	0.4672446	0.283592898	0.877319618	0.999996191
HRCT1	0.448297346	0.565392986	0.629457709	0.793520063	0.308795733	0.773133443	0.999996191
HRH1	0.809237565	0.902382338	0.230007959	0.969665058	0.559033735	0.904585952	0.999996191
HRH2	0.83060358	0.599481566	0.295927097	0.833539318	0.730943169	0.902819007	0.999996191
HRH4	0.867022926	0.293347212	0.487867991	0.636701292	0.201963971	0.601913071	0.999996191
HS1BP3	0.640939646	0.532197244	0.468703702	0.862171261	0.614397121	0.895312348	0.999996191
HS2ST1	0.502736217	0.718674527	0.706287985	0.343339152	0.900585708	0.88583654	0.999996191
HS3ST1	0.473599757	0.40358785	0.792856661	0.95818492	0.306446732	0.796054788	0.999996191
HS3ST2	0.624657988	0.564101427	0.633138976	0.42042721	0.964657282	0.903804582	0.999996191
HS3ST3A1	0.301814357	0.88418267	0.86004048	0.680015934	0.668746024	0.920878612	0.999996191
HS3ST3B1	0.366331348	0.448467684	0.110091028	0.950682302	0.997178702	0.615948163	0.999996191
HS3ST4	0.426769844	0.94031936	0.571544387	0.681353147	0.561546855	0.899922752	0.999996191
HS3ST6	0.86190907	0.71498702	0.352995171	0.663135432	0.584759822	0.894791752	0.999996191
HSBP1	0.417417179	0.693836642	0.739410544	0.072416259	0.733666152	0.536642981	0.999996191
HSCB	0.843116939	0.697792203	0.696168902	0.374060372	0.825184226	0.940990432	0.999996191
HSD11B1L	0.68826859	0.968310903	0.164491463	0.954543649	0.667515305	0.86859983	0.999996191
HSD17B1	0.449690621	0.986718105	0.705162702	0.851411217	0.099392857	0.700418488	0.999996191
HSD17B4	0.652260283	0.204759565	0.323418673	0.454071869	0.374051175	0.455550325	0.999996191
HSD17B7	0.515547029	0.374851732	0.226361513	0.508454307	0.477168214	0.523463362	0.999996191
HSD17B8	0.938775454	0.92925163	0.932340942	0.348770617	0.555313787	0.960007395	0.999996191
HSD3B7	0.270779402	0.854898066	0.082904111	0.738303447	0.607995153	0.484611804	0.999996191
HSDL1	0.940195144	0.063825305	0.353396934	0.785949325	0.658926444	0.529932983	0.999996191
HSDL2	0.363963469	0.944503159	0.589971734	0.955150598	0.955409797	0.971205664	0.999996191
HSF1	0.341790696	0.597231703	0.425802138	0.412725857	0.342306558	0.551220694	0.999996191
HSF5	0.436257239	0.84018923	0.489525367	0.824090457	0.121151509	0.624507949	0.999996191
HSP90B1	0.674955114	0.464849213	0.141324535	0.323101023	0.91864265	0.564550181	0.999996191
HSPA12A	0.865492865	0.744293747	0.574753107	0.481285846	0.975671018	0.967131367	0.999996191
HSPA12B	0.618595944	0.279147564	0.74851276	0.400131925	0.809122139	0.785271735	0.999996191
HSPA14	0.906103923	0.874777658	0.544969964	0.200338758	0.889200753	0.882383292	0.999996191
HSPA4	0.97981915	0.426584188	0.857079924	0.125301747	0.944614216	0.787600198	0.999996191
HSPB1	0.287789917	0.604605625	0.267869758	0.96712746	0.463952691	0.654731965	0.999996191
HSPB7	0.069238915	0.811000799	0.26773121	0.903896248	0.532352395	0.453049218	0.999996191
HSPBAP1	0.476810265	0.543396175	0.763915071	0.91831841	0.252254846	0.80123629	0.999996191
HSPD1	0.875932322	0.336593695	0.301363964	0.236928965	0.95320789	0.646667874	0.999996191
HSPE1	0.863388661	0.448245244	0.76003239	0.228416142	0.713330554	0.808821826	0.999996191
HTATIP2	0.942517962	0.772450735	0.438877822	0.86228569	0.371943318	0.918788216	0.999996191
HTR1E	0.815310743	0.232264598	0.69893514	0.850173286	0.568218267	0.855405	0.999996191
HTRA1	0.235342992	0.607226669	0.389276867	0.522016387	0.61240281	0.623083552	0.999996191
HTRA4	0.998264795	0.840873768	0.895742236	0.302882612	0.778424976	0.968436103	0.999996191

HUNK	0.810228691	0.525994317	0.030338415	0.912206286	0.603003098	0.450025119	0.999996191
HUS1	0.254674172	0.768111402	0.970045085	0.108567851	0.81369287	0.611541665	0.999996191
HUWE1	0.752424089	0.693618104	0.772594389	0.109172437	0.694366043	0.72773274	0.999996191
HVCN1	0.332882141	0.729482568	0.707675574	0.245733565	0.785857053	0.743132356	0.999996191
HYAL1	0.382209815	0.358246267	0.397598547	0.686223557	0.978179629	0.760903548	0.999996191
HYAL2	0.436389159	0.920463472	0.127187813	0.453694549	0.8224047	0.636642675	0.999996191
HYAL3	0.845634481	0.707781096	0.253377013	0.828306717	0.278705124	0.753036965	0.999996191
HYI	0.22449952	0.724956342	0.872079495	0.794553766	0.191202467	0.660685248	0.999996191
HYKK	0.794512433	0.91099756	0.198320694	0.117148091	0.877787511	0.586760443	0.999996191
HYLS1	0.430052958	0.587750673	0.129461338	0.703003067	0.99777521	0.672836323	0.999996191
HYOU1	0.407326387	0.804024006	0.244347244	0.373514109	0.662139249	0.643971994	0.999996191
IAH1	0.817359803	0.569934004	0.72932393	0.695273891	0.85094619	0.976074138	0.999996191
IARS	0.874446303	0.461045898	0.978744022	0.86652279	0.218886005	0.878499151	0.999996191
IARS2	0.549428517	0.594843985	0.552132247	0.770762733	0.804073427	0.928501399	0.999996191
IBA57	0.889743529	0.584695806	0.966895031	0.036370266	0.997199142	0.628063399	0.999996191
IBTK	0.383810061	0.544814985	0.362226522	0.992201678	0.610205021	0.80126858	0.999996191
ICA1L	0.143090353	0.627170203	0.552131875	0.341251849	0.539418417	0.495129844	0.999996191
ICAM1	0.841035077	0.186575367	0.894149672	0.366580285	0.413724952	0.658110935	0.999996191
ICE1	0.654835924	0.55705086	0.803085273	0.538078555	0.935296473	0.954739265	0.999996191
ICK	0.546988746	0.960746714	0.537022578	0.842834159	0.712153559	0.965335911	0.999996191
ICMT	0.679546601	0.395205536	0.297426056	0.355158656	0.41797175	0.544522117	0.999996191
ICOS	0.476044277	0.649254221	0.478184945	0.82621476	0.156084476	0.636613681	0.999996191
ID4	0.173105788	0.519791085	0.521381896	0.984668017	0.314170515	0.583470044	0.999996191
IDH1	0.507416172	0.804029587	0.309710993	0.595515483	0.674392599	0.818457453	0.999996191
IDH3G	0.951038291	0.517724564	0.705507356	0.279951284	0.728642704	0.870684256	0.999996191
IDS	0.989639702	0.39510142	0.69607292	0.890169005	0.728207605	0.968117789	0.999996191
IER5L	0.476008738	0.675003991	0.198389203	0.206790646	0.825945528	0.528282321	0.999996191
IFI47	0.944009757	0.769685925	0.924601021	0.561968389	0.457757467	0.966722881	0.999996191
IFITM2	0.826194041	0.452967882	0.139641914	0.282124865	0.511496999	0.460476681	0.999996191
IFITM3	0.417782239	0.593417846	0.210130944	0.249687149	0.98562224	0.559500932	0.999996191
IFNAR1	0.799055856	0.715374769	0.521347141	0.073516851	0.945831113	0.652940553	0.999996191
IFNGR1	0.936348788	0.769673275	0.175019056	0.17262953	0.749633078	0.606343785	0.999996191
IFT172	0.787195227	0.021887581	0.762027602	0.735001285	0.947555331	0.495600313	0.999996191
IFT27	0.589484141	0.847935073	0.508410467	0.139005092	0.814875269	0.716372903	0.999996191
IFT46	0.914977317	0.790739528	0.940134726	0.44180178	0.617562712	0.971376257	0.999996191
IFT52	0.990533801	0.86292127	0.522899127	0.7600327	0.692178024	0.983783134	0.999996191
IFT74	0.442855626	0.26904328	0.432791406	0.570099819	0.854592322	0.690312466	0.999996191
IFT80	0.865766142	0.122715815	0.536598111	0.283266421	0.524570136	0.481536722	0.999996191
IFT81	0.747525636	0.581584429	0.758255015	0.076229329	0.327890197	0.476475969	0.999996191
IFT88	0.647682189	0.936235577	0.116715959	0.846684091	0.691706651	0.783584618	0.999996191
IGBP1	0.908114556	0.899326356	0.434851126	0.24321622	0.397835946	0.749599866	0.999996191
IGDCC4	0.061027621	0.806305979	0.216512707	0.980802046	0.99592328	0.519756387	0.999996191
IGF1R	0.300476737	0.514935959	0.63011077	0.502519404	0.158191614	0.465387782	0.999996191
IGF2	0.885723853	0.708470808	0.764819052	0.253667822	0.692343435	0.894688197	0.999996191
IGF2BP2	0.953420555	0.912905224	0.838866883	0.106733471	0.113042781	0.488717392	0.999996191

IGF2R	0.555662406	0.812475499	0.040647307	0.981573687	0.399860802	0.452269771	0.999996191
IGFALS	0.694536672	0.362304989	0.948671055	0.125457579	0.854480389	0.693866273	0.999996191
IGFBP3	0.641894553	0.830879708	0.179155286	0.162722154	0.668677529	0.519573342	0.999996191
IGFBP4	0.377904844	0.857532677	0.265409138	0.397577907	0.239431967	0.475323179	0.999996191
IGFBP5	0.246236989	0.481719698	0.650476944	0.659093739	0.530880305	0.704140097	0.999996191
IGFBP6	0.401222669	0.596482525	0.459932812	0.236756833	0.689012511	0.624961614	0.999996191
IGFBP7	0.869127853	0.700795502	0.176017332	0.672182677	0.780544174	0.83533493	0.999996191
IGFLR1	0.779304881	0.148531077	0.513116165	0.391555659	0.509111266	0.544238764	0.999996191
IGHMBP2	0.302626277	0.120405626	0.819521523	0.807075817	0.637948385	0.594687307	0.999996191
IGSF1	0.868545187	0.487998906	0.355280512	0.94625343	0.815403916	0.932536406	0.999996191
IGSF3	0.117610441	0.27664363	0.885730906	0.503073694	0.510532752	0.457127862	0.999996191
IGSF8	0.790784451	0.394060456	0.390799141	0.944327568	0.34672729	0.776676415	0.999996191
IGSF9	0.241759933	0.356659558	0.494893712	0.378314118	0.659930759	0.524179649	0.999996191
IKBIP	0.279545688	0.660561103	0.447578704	0.409007623	0.962280697	0.739408611	0.999996191
IKBKB	0.623607943	0.863107096	0.33734724	0.756229744	0.835418454	0.931200528	0.999996191
IKZF2	0.500693093	0.527245557	0.583206098	0.986105115	0.112201617	0.614672853	0.999996191
IKZF4	0.482413488	0.573319823	0.412692505	0.688296394	0.654198803	0.820578444	0.999996191
IL10RB	0.994122284	0.97104468	0.236121137	0.167651415	0.757396486	0.717411065	0.999996191
IL12RB2	0.605102696	0.498673294	0.255707992	0.885910324	0.67043737	0.801155841	0.999996191
IL13RA1	0.845675955	0.688122212	0.870233765	0.576259002	0.606646182	0.968345817	0.999996191
IL15	0.957889996	0.965719015	0.268693	0.06958224	0.772958111	0.567566092	0.999996191
IL15RA	0.409435011	0.293493778	0.37458363	0.76217927	0.396944858	0.571144165	0.999996191
IL17RC	0.939851458	0.711278995	0.68691133	0.929557656	0.78683344	0.994744133	0.999996191
IL17RE	0.200920017	0.633691491	0.122841929	0.898063376	0.645646577	0.494069668	0.999996191
IL17REL	0.458349389	0.196098995	0.966689612	0.350400946	0.618944863	0.634394285	0.999996191
IL18	0.90118408	0.770181775	0.021387742	0.929365515	0.689561337	0.502945833	0.999996191
IL1R1	0.829740945	0.449234337	0.781960712	0.407237406	0.889884267	0.92223038	0.999996191
IL20RB	0.992456036	0.449084501	0.780230291	0.667145559	0.106466373	0.687034777	0.999996191
IL27RA	0.159704398	0.672993022	0.986870789	0.726145846	0.603231998	0.803517365	0.999996191
IL2RB	0.827646797	0.969025642	0.162713631	0.449678754	0.933924976	0.831131627	0.999996191
IL3RA	0.367729411	0.275246027	0.369487136	0.996042404	0.378758903	0.577994165	0.999996191
IL4I1	0.789284301	0.056662523	0.984848153	0.810281924	0.441713435	0.599557741	0.999996191
IL4R	0.792363325	0.821101399	0.521149662	0.092665185	0.96487033	0.726183769	0.999996191
IL5RA	0.927485314	0.805403854	0.77849454	0.329251663	0.228121293	0.792810313	0.999996191
ILF2	0.884788854	0.540371079	0.590493281	0.806230248	0.473368246	0.924444148	0.999996191
ILF3	0.721163887	0.809539356	0.808459204	0.961602393	0.586457641	0.988552227	0.999996191
ILKAP	0.616466235	0.828599709	0.401800482	0.912889584	0.570104454	0.923481167	0.999996191
IMMT	0.912287831	0.678305017	0.850540675	0.080744857	0.290002008	0.551918393	0.999996191
IMPACT	0.482853193	0.945745553	0.249846658	0.544485371	0.601913478	0.765085753	0.999996191
IMPAD1	0.228804086	0.501967328	0.854722562	0.709013391	0.803220359	0.834348346	0.999996191
IMPDH1	0.902142329	0.9573258	0.268137241	0.930642245	0.535968649	0.931924898	0.999996191
IMPDH2	0.664749497	0.924287245	0.498636297	0.551539089	0.27060989	0.80077102	0.999996191
INAFM1	0.734066153	0.756394496	0.888269173	0.722614107	0.555382072	0.975209427	0.999996191
INCA1	0.777699599	0.727950285	0.280153366	0.858947857	0.157162153	0.659306075	0.999996191
INF2	0.71762389	0.507965156	0.336829077	0.288232928	0.700726805	0.68780712	0.999996191

ING1	0.808030735	0.543167049	0.725157106	0.461365533	0.970717986	0.951904297	0.999996191
ING3	0.243014523	0.886967594	0.959592835	0.570717474	0.577643584	0.865060153	0.999996191
ING4	0.497844147	0.994437676	0.657252472	0.854162647	0.277094682	0.882497891	0.999996191
ING5	0.784953537	0.666507595	0.812836185	0.601878008	0.727367293	0.971572391	0.999996191
INHBA	0.354574809	0.322092022	0.180526794	0.576292747	0.902419573	0.525389313	0.999996191
INHBB	0.693358553	0.268977534	0.203985238	0.637492658	0.786142286	0.636675873	0.999996191
INIP	0.362642868	0.627497653	0.155288932	0.521446536	0.503717012	0.498366711	0.999996191
INO80	0.867189273	0.812808172	0.967106638	0.04846717	0.954398263	0.73358554	0.999996191
INO80C	0.556872996	0.505856054	0.875929649	0.510313001	0.832167187	0.921326681	0.999996191
INO80D	0.15049102	0.987286242	0.427145764	0.720584091	0.65363281	0.723521497	0.999996191
INO80E	0.919279857	0.777370553	0.837274362	0.946458244	0.803325642	0.998681228	0.999996191
INPP4A	0.287834827	0.898862014	0.485064332	0.588248799	0.351561108	0.696574181	0.999996191
INPP5E	0.783039057	0.412223231	0.721506288	0.805704089	0.643779938	0.936514617	0.999996191
INPP5K	0.291640907	0.636483569	0.71110336	0.919753897	0.654138199	0.886718659	0.999996191
INPPL1	0.871169218	0.784149442	0.881442192	0.579818466	0.708704354	0.985879402	0.999996191
INSL3	0.823994828	0.724018561	0.939711903	0.72668689	0.106548251	0.791719124	0.999996191
INTS10	0.35415129	0.883193809	0.437549301	0.262398561	0.202142534	0.453664977	0.999996191
INTS11	0.677130047	0.783629629	0.799338456	0.099432258	0.350283983	0.586917308	0.999996191
INTS12	0.505889008	0.134856978	0.892129077	0.718202259	0.264000456	0.539344239	0.999996191
INTS14	0.550286592	0.539736903	0.86360435	0.62645412	0.464424168	0.878092012	0.999996191
INTS2	0.25869859	0.743131183	0.975584493	0.984062841	0.474669343	0.899704579	0.999996191
INTS3	0.706306832	0.474986945	0.44578178	0.412679154	0.47903522	0.721442748	0.999996191
INTS4	0.271430378	0.918944398	0.922714827	0.321101516	0.627493674	0.803189366	0.999996191
INTS5	0.122334266	0.885737259	0.221447651	0.925855319	0.467683828	0.519453963	0.999996191
INTS6L	0.625756045	0.804796744	0.283085116	0.633482113	0.282406229	0.693215853	0.999996191
INTS7	0.964152254	0.270099101	0.639556226	0.28056095	0.888551494	0.783885347	0.999996191
INTS8	0.469637294	0.876317909	0.573726211	0.447115885	0.236348094	0.688992787	0.999996191
INTS9	0.520658769	0.824729961	0.932504503	0.056471134	0.591970408	0.567817413	0.999996191
INTU	0.984208149	0.2230993	0.386693188	0.590867919	0.527609475	0.700358353	0.999996191
INVS	0.885615868	0.951033656	0.537852841	0.357435855	0.907482644	0.954464654	0.999996191
IP6K2	0.883543568	0.650020302	0.86913421	0.160202148	0.142536645	0.536988674	0.999996191
IP6K3	0.764951102	0.759889363	0.458311313	0.243181499	0.614175047	0.776297846	0.999996191
IPMK	0.766770437	0.769420045	0.329859157	0.810490265	0.720454398	0.930204704	0.999996191
IPO11	0.433513993	0.492844597	0.129452787	0.461769725	0.91938897	0.54265666	0.999996191
IPO5	0.590436863	0.926703691	0.215505778	0.465821103	0.29838797	0.607143491	0.999996191
IPO8	0.213541506	0.402262634	0.980583498	0.625313093	0.901498871	0.807239891	0.999996191
IPO9	0.88499641	0.701627885	0.165766771	0.464828903	0.47759516	0.671966476	0.999996191
IPP	0.070334649	0.58267114	0.919032811	0.893097551	0.318238919	0.525082413	0.999996191
IPPK	0.935879071	0.459457857	0.568096421	0.42538623	0.229301774	0.68008207	0.999996191
IQCA1L	0.153703156	0.103863008	0.840073006	0.935748945	0.787608986	0.510079035	0.999996191
IQCE	0.752897149	0.757348114	0.01657665	0.808967505	0.942420681	0.452359439	0.999996191
IQCG	0.670288785	0.655434824	0.924303283	0.82916687	0.573889719	0.973819021	0.999996191
IQCH	0.837545118	0.95559181	0.222856582	0.420996326	0.248539978	0.632507011	0.999996191
IQCK	0.898822201	0.804976085	0.180200461	0.847837623	0.293083499	0.738655547	0.999996191
IRAK1BP1	0.902950788	0.565823609	0.802941314	0.151478019	0.902522978	0.834861376	0.999996191

IREB2	0.55112952	0.471079763	0.572455847	0.948420913	0.445470934	0.852638588	0.999996191
IRF2BP1	0.250294572	0.470735577	0.187807961	0.61661693	0.521696212	0.450180871	0.999996191
IRF2BP2	0.858054399	0.964933984	0.774118286	0.334805544	0.594492085	0.941860383	0.999996191
IRS1	0.722579377	0.857671539	0.181652613	0.421187804	0.695022592	0.741836073	0.999996191
IRX5	0.536096987	0.409797833	0.107195456	0.449952062	0.685593566	0.453800826	0.999996191
ISCA1	0.72051196	0.20182998	0.169370452	0.593755493	0.901910622	0.564970372	0.999996191
ISCU	0.687579344	0.806938999	0.248946847	0.090619831	0.974463733	0.549928063	0.999996191
ISG20	0.487158432	0.519317882	0.108928741	0.667926624	0.683786719	0.555953248	0.999996191
ISG20L2	0.56558471	0.919845601	0.879324497	0.207817395	0.329184011	0.732171718	0.999996191
ISL2	0.949997681	0.753788106	0.490074373	0.092780034	0.33106165	0.526397751	0.999996191
ISLR	0.182200659	0.634247224	0.83694245	0.987935543	0.80540402	0.882393978	0.999996191
ISOC1	0.818337123	0.722666264	0.63957735	0.15558651	0.957732402	0.835654684	0.999996191
ISPD	0.934453037	0.448438001	0.630623438	0.145943886	0.279468326	0.526376529	0.999996191
IST1	0.742703328	0.569903499	0.448970305	0.681469057	0.681110183	0.900576969	0.999996191
ISYNA1	0.91982237	0.117866383	0.387153866	0.70176427	0.406108365	0.546203831	0.999996191
ITCH	0.382388912	0.967780872	0.324681235	0.337973424	0.786649145	0.736023307	0.999996191
ITFG1	0.773976453	0.549113983	0.745559886	0.816869874	0.721490321	0.9717634	0.999996191
ITFG2	0.894986842	0.674963513	0.233464039	0.544651603	0.45706343	0.753557293	0.999996191
ITGA1	0.985713693	0.180841731	0.799304077	0.267581372	0.771593143	0.720495493	0.999996191
ITGA11	0.779206785	0.85349718	0.815804874	0.220507991	0.633314705	0.880225294	0.999996191
ITGA2	0.601172313	0.350076416	0.849184748	0.112586479	0.952667224	0.637729401	0.999996191
ITGA5	0.444081306	0.520346561	0.3692315	0.444452668	0.660182137	0.689633278	0.999996191
ITGA6	0.555861813	0.574348708	0.669307437	0.498917526	0.305440233	0.739604033	0.999996191
ITGA7	0.97813579	0.331852787	0.463429752	0.724907163	0.72404823	0.885923359	0.999996191
ITGA8	0.081299268	0.551545119	0.611493099	0.924302206	0.500063411	0.557282302	0.999996191
ITGAE	0.853162222	0.803037866	0.45337133	0.524615244	0.941792802	0.957979169	0.999996191
ITGAV	0.860688813	0.881081215	0.246132367	0.887242495	0.056930461	0.501269725	0.999996191
ITGB4	0.517808518	0.388823647	0.801665609	0.899042362	0.40126681	0.840855364	0.999996191
ITGB5	0.628098912	0.715846211	0.819215014	0.758413152	0.467607598	0.9440957	0.999996191
ITIH5	0.604377249	0.621229323	0.338225375	0.480882633	0.45645351	0.710248086	0.999996191
ITM2B	0.875311797	0.906391558	0.559543503	0.61470526	0.589271366	0.961566044	0.999996191
ITM2C	0.385256975	0.802079283	0.102422513	0.502748896	0.855811861	0.571132004	0.999996191
ITPA	0.542590369	0.795373731	0.657505727	0.033250271	0.786513154	0.457588932	0.999996191
ITPKB	0.328227613	0.71766037	0.37992347	0.59921922	0.666361226	0.756804252	0.999996191
ITPR1	0.56649155	0.813661111	0.817740771	0.95545036	0.570987806	0.977314197	0.999996191
ITPR2	0.767246366	0.859664934	0.699894505	0.927511031	0.633959248	0.989206476	0.999996191
ITPR3	0.931636693	0.090274458	0.456629576	0.460628228	0.41095426	0.453922664	0.999996191
ITPRIP	0.246959528	0.660084077	0.501985934	0.328525557	0.645616998	0.618329113	0.999996191
ITPRIPL1	0.42696953	0.270814941	0.512488748	0.538402168	0.852322711	0.7055245	0.999996191
ITPRIPL2	0.967726499	0.67720353	0.178089899	0.60904849	0.404820695	0.716309303	0.999996191
ITSN1	0.856752167	0.918660397	0.952126453	0.019101378	0.597577318	0.48331306	0.999996191
IVD	0.829951662	0.605836079	0.599342741	0.125841931	0.840328665	0.735572596	0.999996191
IVNS1ABP	0.348350251	0.589154089	0.815958033	0.612134541	0.787299164	0.888901056	0.999996191
IWS1	0.91641815	0.633790684	0.586035943	0.539215934	0.664734317	0.937506094	0.999996191
IZUMO1	0.876324325	0.999686018	0.614004344	0.414024264	0.15581692	0.751405027	0.999996191

JADE1	0.50575236	0.324278721	0.868243517	0.358000191	0.520290702	0.700743805	0.999996191
JADE3	0.407854029	0.415643053	0.739287093	0.17959228	0.567943817	0.558935627	0.999996191
JAG1	0.893922289	0.959015012	0.520183192	0.328808003	0.821563918	0.936235835	0.999996191
JAG2	0.818207886	0.676959205	0.20774327	0.444847162	0.971111406	0.814998704	0.999996191
JAK2	0.258116371	0.840922771	0.660089319	0.086728236	0.82741749	0.517477938	0.999996191
JAK3	0.453239663	0.896169999	0.289277337	0.311515463	0.775269564	0.71365485	0.999996191
JARID2	0.9149271	0.723411319	0.069192223	0.711432972	0.49837644	0.605323172	0.999996191
JAZF1	0.400525775	0.998816201	0.814545816	0.8324229	0.927721765	0.986532033	0.999996191
JCAD	0.668488309	0.714419316	0.529196367	0.219066509	0.150972105	0.479091605	0.999996191
JKAMP	0.31007437	0.448966115	0.904416377	0.765077658	0.353660291	0.748000222	0.999996191
JMJD1C	0.33586143	0.801589166	0.49168963	0.495187306	0.888944756	0.840971755	0.999996191
JMJD4	0.134509835	0.396282307	0.926776971	0.646247642	0.86260282	0.707936565	0.999996191
JMJD7	0.940517168	0.835084835	0.552936964	0.685478583	0.771203443	0.982733799	0.999996191
JMJD8	0.68910912	0.81020586	0.734313434	0.649571745	0.517387319	0.948952192	0.999996191
JOSD2	0.353398451	0.386458592	0.477803191	0.180528367	0.784178246	0.497490964	0.999996191
JPH1	0.389823219	0.520013861	0.555054616	0.751614203	0.363075862	0.728589397	0.999996191
JPH4	0.509111624	0.971148132	0.45594909	0.365044771	0.461278972	0.767818277	0.999996191
JPT1	0.440898637	0.5227348	0.073069397	0.695239851	0.821999965	0.505103807	0.999996191
JRK	0.704045445	0.818057831	0.562719844	0.057416984	0.970238418	0.626036068	0.999996191
JRKL	0.287008769	0.820907914	0.419037008	0.158399974	0.823556757	0.560380008	0.999996191
JSRP1	0.369820621	0.776525524	0.742650968	0.15450364	0.417577387	0.573142471	0.999996191
JTB	0.837579203	0.79272953	0.359074447	0.620441093	0.754997112	0.928354878	0.999996191
JUN	0.521200524	0.290100531	0.932262049	0.961651588	0.956632243	0.943409486	0.999996191
JUP	0.647817374	0.879069846	0.420295764	0.420925787	0.741716218	0.878281742	0.999996191
KANK1	0.796703611	0.608125579	0.569010302	0.621773295	0.144253419	0.687245126	0.999996191
KANK3	0.487927721	0.845642881	0.506506086	0.977938616	0.736227973	0.956405174	0.999996191
KANSL2	0.345357748	0.543539219	0.926213299	0.724898369	0.539308138	0.864589247	0.999996191
KANTR	0.691931343	0.787816189	0.635926631	0.279988455	0.965082448	0.908103991	0.999996191
KARS	0.31469561	0.964340321	0.324661188	0.212826613	0.382142402	0.471414372	0.999996191
KAT2A	0.767196901	0.744953408	0.943502863	0.195539784	0.507816551	0.827339386	0.999996191
KAT2B	0.343000952	0.99181508	0.463554006	0.967365044	0.952303129	0.953515123	0.999996191
KAT5	0.674167824	0.778502717	0.84705922	0.979873559	0.674976627	0.991610562	0.999996191
KAT6A	0.930920766	0.395182171	0.753188363	0.637148231	0.080279069	0.578871968	0.999996191
KAT6B	0.723382539	0.990057769	0.971052089	0.454326156	0.47985643	0.957015441	0.999996191
KAT7	0.177784494	0.836155705	0.378487798	0.575488401	0.362174533	0.542409671	0.999996191
KAT8	0.794768594	0.326893584	0.687207275	0.768694007	0.766684475	0.921795955	0.999996191
KATNA1	0.962709121	0.407317274	0.747025794	0.13809816	0.630075928	0.693094972	0.999996191
KATNBL1	0.858875634	0.724674508	0.739463795	0.231966116	0.60887193	0.857912097	0.999996191
KBTBD11	0.117865164	0.793644212	0.956278152	0.205545496	0.426873061	0.467665688	0.999996191
KBTBD3	0.186232471	0.487769662	0.760147807	0.747038235	0.684162405	0.754513952	0.999996191
KCMF1	0.933274099	0.477431712	0.143269781	0.73847347	0.678366048	0.736226285	0.999996191
KCNAB1	0.621501837	0.412642403	0.853914294	0.339052892	0.995769697	0.876778776	0.999996191
KCNAB2	0.187550214	0.833408616	0.296537252	0.986662047	0.589073327	0.703729668	0.999996191
KCNB2	0.913443284	0.600462586	0.621790516	0.502576482	0.265742251	0.800097253	0.999996191
KCNC2	0.50265441	0.421277055	0.308569126	0.653545165	0.669024356	0.714944079	0.999996191

KCNC3	0.878265334	0.604465247	0.979029707	0.050041991	0.703155038	0.62854748	0.999996191
KCNC4	0.264520624	0.934726133	0.273230862	0.732095496	0.482741435	0.680473306	0.999996191
KCND3	0.743186318	0.481490292	0.132697122	0.181888922	0.998822586	0.484865323	0.999996191
KCNH4	0.148763271	0.76597117	0.303765273	0.775979163	0.570508698	0.594035227	0.999996191
KCNIP2	0.388006409	0.664591431	0.873955293	0.770106002	0.383828972	0.861591389	0.999996191
KCNJ11	0.738124249	0.651747824	0.715411969	0.865621839	0.374053539	0.928118621	0.999996191
KCNJ12	0.418915381	0.476293481	0.821658363	0.350151658	0.974035412	0.83437418	0.999996191
KCNJ13	0.237932817	0.735017549	0.127216273	0.552429074	0.93757992	0.539068556	0.999996191
KCNJ16	0.814283041	0.292300807	0.859235617	0.17605005	0.50078878	0.625771853	0.999996191
KCNK13	0.418652643	0.415090687	0.83544434	0.35245488	0.581321441	0.722600758	0.999996191
KCNK6	0.43150392	0.807745409	0.851001785	0.726224616	0.427793603	0.906085227	0.999996191
KCNK7	0.702050578	0.695370712	0.252349505	0.770445841	0.473484879	0.797769559	0.999996191
KCNMB2	0.768765599	0.276237832	0.653568529	0.157794571	0.98381121	0.660538167	0.999996191
KCNMB3	0.206568765	0.292944539	0.9800028	0.845675409	0.195515918	0.50858589	0.999996191
KCNMB4	0.831599143	0.594228206	0.595850303	0.905430344	0.765734096	0.976923094	0.999996191
KCNQ4	0.987146769	0.731207464	0.1466536	0.602223106	0.175397381	0.533336829	0.999996191
KCNQ5	0.159081428	0.581830252	0.74913803	0.908267553	0.313509888	0.643507785	0.999996191
KCTD12	0.628159773	0.988599	0.03204472	0.452730313	0.863808836	0.466129243	0.999996191
KCTD13	0.262061399	0.917154029	0.424529802	0.452713607	0.257090957	0.544818399	0.999996191
KCTD18	0.433822808	0.556715037	0.495318839	0.611185844	0.867928661	0.854253215	0.999996191
KCTD2	0.354260426	0.461869199	0.178716136	0.335032994	0.961811682	0.501169212	0.999996191
KCTD20	0.224302908	0.845399946	0.33384917	0.406308352	0.80056844	0.651711764	0.999996191
KCTD21	0.52577977	0.58816191	0.610473089	0.050527568	0.816463612	0.466265643	0.999996191
KCTD6	0.654262749	0.752919787	0.933006228	0.252690537	0.22290271	0.696083749	0.999996191
KCTD7	0.217315571	0.85982108	0.525414092	0.651601118	0.150078567	0.504648416	0.999996191
KDELR1	0.705622317	0.755214434	0.885332678	0.08565988	0.821810295	0.743282449	0.999996191
KDM1A	0.841536547	0.939060699	0.717094153	0.166080216	0.808408405	0.880806555	0.999996191
KDM1B	0.521472864	0.829087019	0.608407362	0.580859905	0.695926637	0.922973465	0.999996191
KDM2A	0.577529817	0.563658125	0.444224863	0.159866333	0.715908752	0.609045504	0.999996191
KDM2B	0.958597245	0.872003215	0.113292046	0.645624543	0.393853978	0.682127784	0.999996191
KDM3A	0.806602589	0.496740724	0.429563792	0.182615519	0.366854801	0.539186759	0.999996191
KDM3B	0.576925996	0.691566142	0.852565557	0.528669841	0.51626283	0.907009263	0.999996191
KDM4A	0.557649226	0.134627654	0.825456586	0.666572516	0.510291591	0.656269733	0.999996191
KDM5A	0.469857747	0.675204488	0.72251803	0.811018241	0.901815797	0.964604898	0.999996191
KDM5C	0.93476092	0.208225305	0.458906822	0.477497989	0.56466432	0.682150729	0.999996191
KDM6B	0.455511364	0.729053624	0.789937339	0.841367948	0.137934923	0.726984054	0.999996191
KDM8	0.964503863	0.550834842	0.088574956	0.434416855	0.931037123	0.636343508	0.999996191
KDSR	0.770525757	0.841292182	0.541270171	0.612604503	0.74095174	0.96084312	0.999996191
KHDRBS1	0.529802763	0.200798194	0.889990336	0.12555001	0.712161844	0.481412846	0.999996191
KHK	0.947400469	0.61425832	0.559442236	0.344220939	0.841461203	0.908924862	0.999996191
KHNYN	0.720219456	0.933903785	0.794259514	0.489268711	0.931482299	0.985237551	0.999996191
KHSRP	0.571683206	0.866357151	0.72290563	0.349545237	0.925569659	0.932220957	0.999996191
KIAA0141	0.587985452	0.587010868	0.178704984	0.665262679	0.603270204	0.687461191	0.999996191
KIAA0232	0.982801515	0.90003302	0.227824689	0.243261175	0.933013393	0.800817472	0.999996191
KIAA0319L	0.89743415	0.134002781	0.709136462	0.845268402	0.513424964	0.763215271	0.999996191

KIAA0355	0.889969451	0.458712762	0.28703012	0.579978052	0.927972426	0.853302527	0.999996191
KIAA0408	0.748163046	0.58425529	0.48759136	0.483206081	0.321059965	0.74245791	0.999996191
KIAA0513	0.757372447	0.948492918	0.197705567	0.282379763	0.824009197	0.742352763	0.999996191
KIAA0556	0.872061374	0.656671826	0.283879203	0.41593739	0.874981694	0.843376741	0.999996191
KIAA0753	0.668795255	0.357503446	0.963794964	0.115829859	0.969778617	0.69606667	0.999996191
KIAA0754	0.607723466	0.370161872	0.389839188	0.388001322	0.421263881	0.581065965	0.999996191
KIAA0825	0.607764213	0.702655692	0.344130638	0.391713315	0.463860232	0.702037221	0.999996191
KIAA0895	0.956435775	0.850754	0.753309742	0.881532996	0.353439373	0.973124222	0.999996191
KIAA1211	0.427331062	0.200530174	0.667752365	0.329749249	0.46592474	0.488341375	0.999996191
KIAA1324	0.08235492	0.703121419	0.718861147	0.325756867	0.625968455	0.48189979	0.999996191
KIAA1328	0.48694809	0.526169889	0.696636694	0.346766893	0.734432196	0.799750981	0.999996191
KIAA1468	0.343987017	0.956297719	0.135014373	0.327614483	0.829175558	0.547840226	0.999996191
KIAA1522	0.594794815	0.64145179	0.721459722	0.097804563	0.500570277	0.569118277	0.999996191
KIAA1549	0.881883579	0.290410584	0.217111348	0.715268964	0.85180928	0.746967338	0.999996191
KIAA1614	0.778312696	0.227000429	0.316920254	0.988085815	0.563008859	0.731288452	0.999996191
KIAA1671	0.826858967	0.625141396	0.737759808	0.638364075	0.310427366	0.879860425	0.999996191
KIAA2013	0.780500084	0.378251103	0.250513896	0.194262732	0.753056042	0.527096761	0.999996191
KIDINS220	0.947779279	0.767016232	0.484004446	0.054306492	0.431987087	0.47680257	0.999996191
KIF13B	0.445184315	0.475535622	0.75612346	0.439249112	0.43597141	0.728274416	0.999996191
KIF17	0.756266166	0.374213695	0.971259083	0.537973232	0.068474837	0.51460593	0.999996191
KIF1C	0.698513125	0.954263652	0.922496342	0.415882456	0.575876258	0.954648661	0.999996191
KIF21A	0.695673542	0.836777805	0.387082388	0.714296288	0.446286939	0.872652061	0.999996191
KIF24	0.503880675	0.471108066	0.685747113	0.655297884	0.456463155	0.811512041	0.999996191
KIF25	0.562720428	0.452449236	0.72132271	0.092741765	0.711397294	0.548658007	0.999996191
KIF26A	0.969883656	0.377404587	0.133026709	0.460231157	0.720498344	0.60422493	0.999996191
KIF26B	0.401830461	0.575051026	0.169847801	0.773338202	0.559382226	0.614024784	0.999996191
KIF3B	0.813659829	0.596878585	0.607567223	0.966929986	0.474751597	0.947424571	0.999996191
KIF5B	0.915445225	0.882653051	0.183011637	0.12372969	0.778042454	0.579729833	0.999996191
KIF7	0.885970207	0.280671457	0.303057622	0.968968249	0.526251091	0.770034059	0.999996191
KIFC3	0.431901166	0.970405492	0.956217791	0.843503072	0.237228908	0.888044659	0.999996191
KIN	0.710584453	0.656985121	0.827040939	0.549481044	0.898613406	0.973019052	0.999996191
KIRREL1	0.756255457	0.369457807	0.515975769	0.415789895	0.774451733	0.803372987	0.999996191
KIRREL3	0.705329417	0.270775345	0.63506113	0.361652017	0.651092395	0.714875247	0.999996191
KL	0.67148975	0.425678848	0.910995575	0.232246442	0.926634611	0.834734772	0.999996191
KLB	0.523916373	0.759287582	0.144195789	0.610788982	0.97683421	0.748849564	0.999996191
KLC1	0.698859662	0.507252899	0.773653829	0.061373392	0.960338745	0.604445428	0.999996191
KLC4	0.862895399	0.329392098	0.257891591	0.230651722	0.877699764	0.58778972	0.999996191
KLF13	0.394262677	0.978253651	0.278282466	0.746336629	0.358306595	0.715822106	0.999996191
KLF16	0.117204672	0.861399468	0.285600355	0.989004547	0.492436131	0.577087203	0.999996191
KLF2	0.788323993	0.28381811	0.99032709	0.879649881	0.212142609	0.783151876	0.999996191
KLF3	0.58189522	0.876264498	0.838581275	0.500266835	0.75857898	0.962239287	0.999996191
KLF6	0.847311501	0.59015015	0.381192797	0.364403635	0.980971589	0.864951706	0.999996191
KLF8	0.343541334	0.754187786	0.753492841	0.952928097	0.792998545	0.954793612	0.999996191
KLF9	0.545580324	0.628305211	0.082664239	0.812210499	0.44217162	0.515550776	0.999996191
KLHDC1	0.211575765	0.621649622	0.932727971	0.912308366	0.7353035	0.89151956	0.999996191

KLHDC10	0.453454329	0.396119605	0.744525889	0.885740226	0.766512762	0.90423886	0.999996191
KLHDC3	0.684907616	0.957979701	0.716799381	0.710351354	0.936477611	0.993200259	0.999996191
KLHDC8B	0.920503041	0.771421941	0.061219403	0.689540972	0.676303086	0.6486602	0.999996191
KLHDC9	0.788837735	0.557672256	0.772482019	0.203116835	0.578102699	0.776808646	0.999996191
KLHL11	0.293951988	0.740281559	0.779517177	0.139173778	0.564098376	0.566825592	0.999996191
KLHL12	0.750865907	0.845048606	0.396831353	0.959690276	0.782195403	0.972501539	0.999996191
KLHL18	0.930885957	0.332099943	0.915618562	0.304372056	0.72029922	0.850825847	0.999996191
KLHL20	0.557993322	0.887962612	0.778598988	0.766230814	0.759715151	0.981724774	0.999996191
KLHL22	0.633454665	0.399802488	0.407228983	0.68412342	0.885913291	0.851935879	0.999996191
KLHL23	0.451706478	0.792576116	0.670162624	0.944043474	0.560609177	0.941413049	0.999996191
KLHL26	0.439771868	0.67970608	0.231568875	0.34840959	0.869543562	0.655262187	0.999996191
KLHL28	0.702809831	0.89833951	0.693765067	0.2456332	0.206400176	0.666414335	0.999996191
KLHL29	0.507106313	0.094228376	0.93020425	0.635310246	0.443583585	0.555035321	0.999996191
KLHL30	0.270903415	0.562826819	0.400774517	0.82924707	0.196643487	0.511597337	0.999996191
KLHL32	0.304152724	0.885789169	0.555763132	0.398333094	0.68194631	0.780223132	0.999996191
KLHL33	0.665462311	0.557015786	0.560902746	0.225121129	0.220516644	0.518204166	0.999996191
KLHL36	0.179620236	0.390588496	0.583680687	0.622638684	0.360937634	0.496783297	0.999996191
KLHL38	0.879955395	0.91302873	0.838505412	0.151368452	0.654639029	0.861906823	0.999996191
KLHL4	0.384049661	0.649798504	0.258595096	0.267988665	0.928227663	0.603094804	0.999996191
KLHL42	0.787335769	0.557390578	0.187748271	0.406555139	0.490770624	0.607736337	0.999996191
KLHL5	0.753102333	0.826895083	0.773035773	0.428061159	0.687189513	0.951341901	0.999996191
KLHL7	0.670314445	0.813849283	0.471515143	0.247626164	0.939679184	0.845200138	0.999996191
KLHL9	0.374477697	0.836779077	0.660057595	0.947724832	0.66386354	0.943739395	0.999996191
KLK10	0.929857469	0.085861663	0.70290319	0.498735227	0.366515602	0.517051066	0.999996191
KLK4	0.379990731	0.904795644	0.693279736	0.882235368	0.758604527	0.960967236	0.999996191
KLKB1	0.803063671	0.467863913	0.775188238	0.103591343	0.507808991	0.594009218	0.999996191
KLRD1	0.929820195	0.92721446	0.150468374	0.12170297	0.781111668	0.552038005	0.999996191
KLRF2	0.994981822	0.927241436	0.850351841	0.983345578	0.989848258	0.9999905	0.999996191
KLRG1	0.766171122	0.760865071	0.833757607	0.353448249	0.899915539	0.958555077	0.999996191
KLRK1	0.329526168	0.691883646	0.352064656	0.509856227	0.975048916	0.776814219	0.999996191
KMT2A	0.761863342	0.883111693	0.708948812	0.150615584	0.271094222	0.640835059	0.999996191
KMT2B	0.766540547	0.766802772	0.555679596	0.349404801	0.827994045	0.909174458	0.999996191
KMT2C	0.816213958	0.630681594	0.80194398	0.468011221	0.229555109	0.795474843	0.999996191
KMT2D	0.865711463	0.681734063	0.780470138	0.556956206	0.613023521	0.959882873	0.999996191
KMT5A	0.881493579	0.86845338	0.915338243	0.804780457	0.322457816	0.970091247	0.999996191
KNSTRN	0.946340913	0.392114907	0.731453191	0.614936568	0.792890102	0.945305037	0.999996191
KPNA3	0.311199492	0.144670008	0.716018795	0.576470513	0.83421265	0.596292964	0.999996191
KPNA5	0.219914685	0.968168058	0.2757844	0.768362815	0.470482247	0.657628629	0.999996191
KRAS	0.552779412	0.40819149	0.52598242	0.163411973	0.517012416	0.512770539	0.999996191
KRBA1	0.427378977	0.403876901	0.451094032	0.743753319	0.729552541	0.786963051	0.999996191
KRCC1	0.234331437	0.671887662	0.332991692	0.632764513	0.673274822	0.667536646	0.999996191
KREMEN1	0.326745617	0.51510525	0.584924684	0.49586961	0.557858333	0.705803081	0.999996191
KRT10	0.526078414	0.512641069	0.540691738	0.393656031	0.35867541	0.651680548	0.999996191
KRT18	0.501496469	0.599802295	0.512578062	0.755795231	0.62086737	0.873686671	0.999996191
KRT7	0.462636284	0.61301165	0.524897595	0.324874469	0.24878973	0.54731413	0.999996191

KRT8	0.247492562	0.36078789	0.956680361	0.80333293	0.605851177	0.784124151	0.999996191
KRT80	0.855518068	0.962984756	0.749191434	0.380524626	0.15754144	0.763173816	0.999996191
KRTCAP2	0.971915	0.62867596	0.416730399	0.299713285	0.866575499	0.860506116	0.999996191
KSR1	0.297775304	0.667892902	0.763979384	0.267892204	0.978498793	0.776475286	0.999996191
KTI12	0.303930427	0.393812388	0.619998964	0.436720094	0.808662941	0.698444675	0.999996191
KTN1	0.42486917	0.491265637	0.386655209	0.837353838	0.601398214	0.780082894	0.999996191
KXD1	0.468164533	0.710060723	0.501188884	0.742632876	0.718071672	0.901498281	0.999996191
KYAT1	0.959758613	0.900439046	0.345354892	0.424910205	0.181773741	0.673669722	0.999996191
KYAT3	0.989700235	0.753442668	0.567489785	0.115368351	0.917616061	0.797219746	0.999996191
L2HGDH	0.750205566	0.653035015	0.838104595	0.081618274	0.308057983	0.51824692	0.999996191
L3HYPDH	0.696691549	0.934120644	0.906592749	0.694241617	0.767156235	0.993303335	0.999996191
L3MBTL1	0.6112357	0.262923969	0.646974812	0.446718401	0.7185698	0.744194615	0.999996191
L3MBTL3	0.316555113	0.870962479	0.365478514	0.337589669	0.236504319	0.472139098	0.999996191
LACC1	0.03484075	0.598122328	0.717179711	0.871470016	0.54057246	0.448237461	0.999996191
LACTB	0.982730325	0.764150552	0.438700081	0.320159322	0.409863038	0.791002085	0.999996191
LACTB2	0.760108361	0.806494145	0.344685426	0.337220662	0.91057359	0.857629388	0.999996191
LAMA4	0.7952438	0.428170713	0.534144988	0.242579969	0.85417293	0.766504613	0.999996191
LAMC1	0.315871165	0.187550751	0.537176787	0.694192847	0.892918741	0.64332592	0.999996191
LAMP1	0.419264306	0.643140112	0.203033845	0.78951132	0.902382322	0.77271848	0.999996191
LAMTOR2	0.494399907	0.372370894	0.720869005	0.070839483	0.795403051	0.458960111	0.999996191
LAMTOR4	0.485620917	0.50413968	0.654272481	0.09701382	0.908836649	0.578189127	0.999996191
LAMTOR5	0.870268689	0.627141327	0.220050178	0.312239372	0.921989757	0.750730984	0.999996191
LANCL1	0.284658597	0.521201622	0.946443124	0.506384638	0.727653886	0.821687763	0.999996191
LANCL2	0.665366789	0.557605044	0.077019567	0.710371739	0.585043747	0.544817974	0.999996191
LANCL3	0.616244427	0.847637647	0.17389102	0.238055578	0.451168337	0.507636877	0.999996191
LAP3	0.587009627	0.692527409	0.048586711	0.549114302	0.836409042	0.494125691	0.999996191
LAPTM4A	0.602693159	0.091577718	0.521546067	0.714585562	0.773493362	0.60135886	0.999996191
LAPTM4B	0.811094247	0.724323638	0.045232064	0.8433029	0.682893596	0.593779102	0.999996191
LARP1	0.812383888	0.840680219	0.215190794	0.169921409	0.320903015	0.471429047	0.999996191
LARP1B	0.841569786	0.662361427	0.381367692	0.093755586	0.592403411	0.543709604	0.999996191
LARP6	0.583641292	0.787991559	0.821961768	0.980015742	0.374664357	0.949599941	0.999996191
LASP1	0.377104636	0.704515892	0.974203168	0.20044938	0.575772283	0.723394964	0.999996191
LATS1	0.519569084	0.144720202	0.866573892	0.588535591	0.742873012	0.714400022	0.999996191
LATS2	0.503567182	0.374600219	0.424644019	0.575849076	0.479429598	0.665606643	0.999996191
LAYN	0.159115486	0.902867671	0.581374966	0.580422723	0.954455784	0.802803866	0.999996191
LBH	0.51126689	0.495908643	0.676113729	0.671624637	0.100433334	0.539724221	0.999996191
LCA5	0.698866528	0.607101282	0.95470003	0.648866972	0.580458929	0.957510923	0.999996191
LCAT	0.476534025	0.287717682	0.965592465	0.266864092	0.762133717	0.703633999	0.999996191
LCLAT1	0.704322666	0.351730058	0.957923631	0.808580997	0.554556623	0.923055885	0.999996191
LCN6	0.756750759	0.733052074	0.766393235	0.809328081	0.352416981	0.936896025	0.999996191
LCOR	0.225650977	0.982293318	0.455871865	0.516834362	0.514140105	0.703095185	0.999996191
LCTL	0.72150538	0.885604337	0.24679831	0.368819977	0.158714105	0.497352075	0.999996191
LDAH	0.577492894	0.969061546	0.461016888	0.569394077	0.782895351	0.931471251	0.999996191
LDB1	0.65034031	0.812555643	0.494127259	0.499977267	0.610100557	0.887118189	0.999996191
LDB2	0.078108469	0.518968373	0.539340429	0.853957042	0.648686341	0.548566758	0.999996191

LDHA	0.937552976	0.268635791	0.051544434	0.812109819	0.826303416	0.486663604	0.999996191
LDHAL6B	0.741904334	0.578931928	0.884447538	0.710642124	0.856367293	0.983044095	0.999996191
LDHC	0.396815394	0.260052251	0.887322484	0.187696236	0.71562285	0.55152206	0.999996191
LDHD	0.881067987	0.710840651	0.53597557	0.965625848	0.717595241	0.983313739	0.999996191
LDLR	0.552085924	0.714485184	0.316574471	0.522469634	0.24393269	0.601411485	0.999996191
LDLRAD3	0.871646339	0.602957449	0.429989514	0.317255682	0.72187806	0.821736687	0.999996191
LDLRAD4	0.431800092	0.757904649	0.344534247	0.689914574	0.232905398	0.626714784	0.999996191
LEMD2	0.910901967	0.443824628	0.62001893	0.386783625	0.902504567	0.899547056	0.999996191
LEMD3	0.421813219	0.609787772	0.86352865	0.060593175	0.879238697	0.544132958	0.999996191
LENG1	0.243060907	0.360489316	0.411869604	0.371381027	0.81484457	0.528866801	0.999996191
LEO1	0.646287117	0.492938917	0.835033962	0.366467885	0.347347999	0.746884336	0.999996191
LEPR	0.99403691	0.89278717	0.503277385	0.357429579	0.446232245	0.871455531	0.999996191
LEPROT	0.57322714	0.302377342	0.914420972	0.214430299	0.996211385	0.746857172	0.999996191
LEPROTL1	0.559205509	0.107617288	0.347261956	0.728493545	0.799497536	0.549528549	0.999996191
LETM1	0.736618973	0.936891864	0.828692951	0.031381835	0.570875335	0.516822463	0.999996191
LETM2	0.285782883	0.674205284	0.687834932	0.913711405	0.850789896	0.919399209	0.999996191
LETMD1	0.712565989	0.372236677	0.265536787	0.765234836	0.947662064	0.819539695	0.999996191
LFNG	0.629047119	0.743180994	0.973272371	0.711607248	0.933088915	0.992333559	0.999996191
LGALS12	0.786513463	0.480289176	0.57638898	0.624933001	0.373226742	0.818585078	0.999996191
LGALS3BP	0.430347604	0.589388532	0.804188865	0.297174108	0.700668974	0.787892149	0.999996191
LGALS4	0.575592952	0.724056684	0.552987459	0.816336228	0.727189086	0.9483199	0.999996191
LGALSL	0.663819886	0.339177752	0.222672572	0.714227585	0.72724759	0.697222855	0.999996191
LGI4	0.769479526	0.14255327	0.808790131	0.179035364	0.492368109	0.467015885	0.999996191
LGR4	0.28376393	0.76040229	0.846683737	0.671109965	0.300897911	0.762635057	0.999996191
LGR6	0.1361764	0.513156677	0.340136762	0.917027268	0.413004356	0.492705347	0.999996191
LHCGR	0.802475287	0.875302109	0.152845042	0.45119371	0.675855568	0.740606819	0.999996191
LHFPL1	0.921021388	0.898128689	0.285153645	0.450621088	0.440452981	0.804821958	0.999996191
LHFPL2	0.32867713	0.495490837	0.313053225	0.688977839	0.22967962	0.472645339	0.999996191
LHFPL3	0.866863858	0.905062021	0.810971441	0.078158212	0.484454649	0.682214019	0.999996191
LHFPL6	0.617871102	0.848416038	0.308365645	0.269319408	0.977555008	0.788248549	0.999996191
LHPP	0.806557821	0.617355589	0.636994259	0.387936183	0.628470439	0.883065313	0.999996191
LIAS	0.841596183	0.680210564	0.684973547	0.275462898	0.879788228	0.909864801	0.999996191
LIMA1	0.942989264	0.847775727	0.484661092	0.815036937	0.49421426	0.959292999	0.999996191
LIMCH1	0.654385833	0.675334402	0.378981669	0.08606821	0.852053534	0.551261662	0.999996191
LIMD1	0.521113827	0.933975964	0.722695735	0.741163539	0.231824686	0.846714478	0.999996191
LIN37	0.919272082	0.968333969	0.368021109	0.750419493	0.954532301	0.983696618	0.999996191
LIN52	0.788221088	0.062887446	0.86736001	0.650815113	0.824791738	0.67389447	0.999996191
LIN7B	0.376000058	0.815074157	0.236924888	0.317684827	0.972377073	0.668356403	0.999996191
LIN7C	0.220525186	0.126531038	0.565394861	0.696338678	0.952693127	0.520825296	0.999996191
LINGO1	0.876959718	0.412807526	0.345205686	0.697536106	0.915455523	0.887376349	0.999996191
LINS1	0.257172573	0.441119344	0.708514044	0.107614638	0.895787194	0.465341381	0.999996191
LIPC	0.965139643	0.854641716	0.184923673	0.449638266	0.745620275	0.819743154	0.999996191
LIPE	0.69731717	0.821434852	0.115582569	0.858287803	0.364315067	0.652746439	0.999996191
LIX1L	0.176413849	0.918248071	0.987949264	0.671145941	0.386640362	0.783923788	0.999996191
LLGL1	0.455655152	0.295115053	0.276818115	0.732235683	0.543386511	0.587418671	0.999996191

LMAN2L	0.128718695	0.746243199	0.438239085	0.258565001	0.838943425	0.495341218	0.999996191
LMBR1	0.978733494	0.425586104	0.049066804	0.508270973	0.784720521	0.47452637	0.999996191
LMBRD1	0.540637973	0.358334717	0.445320927	0.489647425	0.848830269	0.757432235	0.999996191
LMBRD2	0.083014694	0.910569349	0.608632991	0.635501079	0.761785142	0.66698931	0.999996191
LMF1	0.664998295	0.296900272	0.863693106	0.491642329	0.101545549	0.482443279	0.999996191
LMLN	0.858224066	0.908657052	0.970855729	0.127337353	0.8961849	0.897915276	0.999996191
LMNA	0.193715303	0.791613425	0.135889255	0.447619087	0.855736822	0.470708813	0.999996191
LMNB2	0.210825835	0.167637521	0.436134698	0.768919349	0.661292935	0.46741132	0.999996191
LMO1	0.891936671	0.319750705	0.570165506	0.326923596	0.358618698	0.636665394	0.999996191
LMO2	0.555575434	0.808493575	0.783135511	0.995091366	0.953402667	0.994616887	0.999996191
LMO4	0.403441962	0.345129587	0.224382904	0.644301011	0.584360651	0.542996746	0.999996191
LMOD3	0.976556038	0.300744018	0.559447981	0.584563947	0.315974288	0.726387314	0.999996191
LMTK2	0.847427394	0.923554981	0.557865161	0.153548335	0.562837942	0.766732575	0.999996191
LMX1A	0.722273325	0.819754705	0.063408721	0.811974388	0.713749739	0.662443475	0.999996191
LNP1	0.957024675	0.848044171	0.397746026	0.975777661	0.581634019	0.970567637	0.999996191
LNPEP	0.632385776	0.890808854	0.50285938	0.358421291	0.776159964	0.885665067	0.999996191
LNPK	0.323396977	0.934640177	0.742570304	0.52070504	0.53592575	0.852250733	0.999996191
LNX1	0.524479969	0.552011672	0.258734205	0.51538911	0.870227425	0.745421866	0.999996191
LOC100124497	0.349214093	0.801167799	0.909583003	0.619554103	0.459830875	0.873982357	0.999996191
LOC100138078	0.650123365	0.942024023	0.84014139	0.720544578	0.641019238	0.984237859	0.999996191
LOC100138131	0.520594183	0.862182624	0.982637356	0.886221159	0.403469717	0.960094927	0.999996191
LOC100138449	0.597736642	0.828491598	0.98560206	0.991100207	0.378847081	0.970587023	0.999996191
LOC100138633	0.871134227	0.498248302	0.446146773	0.807238526	0.901032344	0.950877393	0.999996191
LOC100138645	0.548900458	0.876746751	0.930069273	0.2446315	0.806058887	0.900654719	0.999996191
LOC100138933	0.756464917	0.537000593	0.685105334	0.316273158	0.725629096	0.855240889	0.999996191
LOC100139325	0.919170633	0.432023441	0.595406114	0.166887291	0.520010167	0.651015773	0.999996191
LOC100139345	0.747459959	0.246930703	0.825539359	0.869570289	0.118870653	0.599380427	0.999996191
LOC100139360	0.316771672	0.522965861	0.65941363	0.862752614	0.913592861	0.897470003	0.999996191
LOC100139363	0.765612202	0.836101712	0.061107419	0.292244215	0.631443702	0.452661828	0.999996191
LOC100139638	0.76540607	0.994212407	0.565042467	0.859313771	0.127507268	0.80590234	0.999996191
LOC100139732	0.646160622	0.568145114	0.821533295	0.894926615	0.727393523	0.974743242	0.999996191
LOC100139990	0.606815281	0.534778811	0.530322665	0.717216472	0.369387305	0.800267795	0.999996191
LOC100139996	0.735883396	0.53328295	0.385979901	0.117393666	0.894871386	0.601379995	0.999996191
LOC100140207	0.262480408	0.881925347	0.42497986	0.960898367	0.570386594	0.828478029	0.999996191
LOC100140372	0.478422022	0.326907071	0.952368505	0.914324139	0.06004163	0.475088597	0.999996191
LOC100140533	0.804931349	0.658851211	0.540816948	0.386888	0.852125381	0.909254738	0.999996191
LOC100140873	0.583419866	0.804396128	0.932824729	0.806067636	0.864193437	0.992572358	0.999996191
LOC100140915	0.60199148	0.869288892	0.921164517	0.476157669	0.905005525	0.977854002	0.999996191
LOC100140958	0.605179455	0.425495169	0.460487336	0.584166617	0.677519989	0.805238918	0.999996191
LOC100141168	0.729823663	0.848042847	0.56946754	0.295679234	0.368959391	0.770141754	0.999996191
LOC100141253	0.195240325	0.192420417	0.830767029	0.855221011	0.580031557	0.596039321	0.999996191
LOC100174924	0.416873949	0.154582991	0.375713549	0.471709394	0.759922543	0.485974891	0.999996191
LOC100196898	0.36137132	0.598230711	0.719986615	0.805073463	0.611392672	0.88177239	0.999996191
LOC100294792	0.366347644	0.760604653	0.919622877	0.99161583	0.858888961	0.980362658	0.999996191
LOC100294994	0.325142368	0.752385055	0.141644563	0.655300648	0.913712813	0.653176574	0.999996191

LOC100295130	0.575856809	0.711780502	0.281512021	0.708377435	0.517946047	0.7873254	0.999996191
LOC100295347	0.914107665	0.518109376	0.859126734	0.035447804	0.696867089	0.513221024	0.999996191
LOC100295687	0.836736272	0.315825157	0.201706546	0.70031441	0.533376826	0.645143862	0.999996191
LOC100295750	0.310642586	0.463217046	0.996080615	0.138102344	0.881550829	0.619375893	0.999996191
LOC100295848	0.653774174	0.864245737	0.215174845	0.315809026	0.736178716	0.712909122	0.999996191
LOC100296121	0.447118918	0.118737122	0.974999283	0.38848807	0.544813753	0.529467946	0.999996191
LOC100296211	0.447775481	0.483943852	0.456818704	0.467775088	0.692361873	0.736698246	0.999996191
LOC100296324	0.594460643	0.529695593	0.651235412	0.047221174	0.991905163	0.504731353	0.999996191
LOC100296627	0.330508652	0.625771294	0.827889056	0.348149413	0.582779886	0.751617628	0.999996191
LOC100296900	0.56321855	0.924322854	0.603627406	0.394501647	0.634256686	0.885357745	0.999996191
LOC100297097	0.630053813	0.855099313	0.502076976	0.975864297	0.734997631	0.974057071	0.999996191
LOC100297152	0.421515083	0.566659655	0.43958284	0.535744201	0.317035218	0.623627417	0.999996191
LOC100297170	0.393799684	0.820606811	0.672581543	0.160221596	0.56708264	0.643543822	0.999996191
LOC100297420	0.864359247	0.787561416	0.410259857	0.273922363	0.639103437	0.81220519	0.999996191
LOC100297498	0.981861824	0.566173313	0.412964941	0.347021859	0.980656374	0.884473755	0.999996191
LOC100297513	0.563832783	0.977330761	0.794176898	0.717545428	0.867355115	0.989317412	0.999996191
LOC100297616	0.862803774	0.936274812	0.991541952	0.964850953	0.234488155	0.969871265	0.999996191
LOC100297725	0.171806074	0.326683562	0.984712162	0.151636019	0.994144862	0.478499506	0.999996191
LOC100298453	0.422404679	0.229667084	0.407441802	0.31492101	0.864223068	0.526015963	0.999996191
LOC100298774	0.687887824	0.49748746	0.920979555	0.768381055	0.621546311	0.956432815	0.999996191
LOC100298868	0.761716883	0.688157745	0.305496523	0.317943116	0.717176104	0.760756606	0.999996191
LOC100298923	0.359432972	0.545385943	0.688569493	0.284772766	0.454659282	0.619675285	0.999996191
LOC100299025	0.646012649	0.962123273	0.804953172	0.618423729	0.373127173	0.931869871	0.999996191
LOC100299281	0.320375857	0.638930019	0.871975378	0.781757902	0.957128503	0.946144273	0.999996191
LOC100299303	0.163454465	0.926266336	0.832375914	0.388491363	0.931052534	0.800229857	0.999996191
LOC100299503	0.313796201	0.383814524	0.554066997	0.14878023	0.926509688	0.496701513	0.999996191
LOC100299705	0.030738312	0.69048415	0.514434466	0.938407503	0.840531522	0.48455616	0.999996191
LOC100299757	0.862949782	0.433588422	0.448316167	0.256578679	0.768486832	0.742514112	0.999996191
LOC100299845	0.664837343	0.77592487	0.732169054	0.404640739	0.600428805	0.905565189	0.999996191
LOC100300095	0.789491689	0.419397345	0.879040803	0.977800525	0.374724997	0.923303579	0.999996191
LOC100300881	0.334841201	0.497762243	0.536903949	0.901524778	0.088655468	0.451024605	0.999996191
LOC100300896	0.726613802	0.764204348	0.391845769	0.468272729	0.719238915	0.875516878	0.999996191
LOC100300938	0.900258978	0.542536848	0.617170924	0.221851992	0.886094039	0.843626743	0.999996191
LOC100335177	0.984079654	0.92419498	0.545961918	0.927554116	0.552026222	0.98691861	0.999996191
LOC100335190	0.74208337	0.46779677	0.394373319	0.633694572	0.887606416	0.882480618	0.999996191
LOC100335205	0.745654848	0.89371683	0.785895614	0.175055536	0.99267564	0.904533117	0.999996191
LOC100335268	0.135702294	0.574121069	0.145802268	0.788819149	0.804202593	0.452356984	0.999996191
LOC100335340	0.396911397	0.670754264	0.248675695	0.372147419	0.870170362	0.659568605	0.999996191
LOC100335404	0.725217486	0.62446892	0.770777122	0.411016725	0.965984836	0.949466818	0.999996191
LOC100335467	0.611746904	0.730244774	0.14818243	0.314477506	0.938345396	0.64141575	0.999996191
LOC100335642	0.936373804	0.74086593	0.180374202	0.537748975	0.229000459	0.595120084	0.999996191
LOC100335822	0.939096025	0.513499219	0.253978951	0.817015465	0.15590707	0.597525095	0.999996191
LOC100335828	0.211686019	0.665609736	0.093224897	0.592174791	0.923220706	0.451743717	0.999996191
LOC100335936	0.67974961	0.777001648	0.423585638	0.130970107	0.895228246	0.698618403	0.999996191
LOC100336013	0.757981762	0.186915519	0.684243357	0.445361099	0.837653824	0.759000568	0.999996191

LOC100336104	0.659087014	0.372913055	0.823557941	0.868980086	0.133623035	0.677431607	0.999996191
LOC100336161	0.285624039	0.691220168	0.723694454	0.355370405	0.686665047	0.752276495	0.999996191
LOC100336368	0.330508198	0.8020017	0.044624414	0.936343922	0.873736023	0.506127403	0.999996191
LOC100336448	0.252504233	0.542763548	0.874542873	0.24164739	0.777725337	0.669180491	0.999996191
LOC100336532	0.915179018	0.791405578	0.842806863	0.74355287	0.971992571	0.998442976	0.999996191
LOC100336564	0.635061684	0.165926895	0.390589982	0.933772299	0.823387496	0.734252893	0.999996191
LOC100336602	0.605005824	0.856462824	0.255898219	0.858682622	0.502452654	0.838046286	0.999996191
LOC100336644	0.963882105	0.595776389	0.42328118	0.699141168	0.604151603	0.919003874	0.999996191
LOC100336734	0.975927707	0.1424662	0.276614548	0.676655873	0.656635942	0.615287908	0.999996191
LOC100336777	0.12679931	0.997504883	0.562202185	0.940220637	0.805416107	0.828263799	0.999996191
LOC100336897	0.943216145	0.405639239	0.539637382	0.081802384	0.488921576	0.47687585	0.999996191
LOC100336909	0.240755137	0.564879517	0.423309997	0.929467473	0.608679482	0.739642546	0.999996191
LOC100336941	0.921950657	0.149530671	0.335634349	0.699208671	0.221121198	0.451065854	0.999996191
LOC100336976	0.776978535	0.795696629	0.583437478	0.158336468	0.759433278	0.791581512	0.999996191
LOC100337044	0.905709985	0.151164194	0.207901376	0.630122888	0.705305191	0.556927924	0.999996191
LOC100337081	0.97863821	0.838002002	0.054726216	0.988840017	0.471581466	0.654874043	0.999996191
LOC100337293	0.327732222	0.772531848	0.652726837	0.773921116	0.717383708	0.905547318	0.999996191
LOC100337323	0.677998986	0.721447423	0.869117811	0.056874072	0.465653949	0.534646959	0.999996191
LOC100337355	0.380960149	0.505057985	0.678144873	0.249015097	0.429301529	0.575782705	0.999996191
LOC100337390	0.368285257	0.865942906	0.15931551	0.240467902	0.670117846	0.475317553	0.999996191
LOC100337495	0.529046303	0.484967157	0.434598405	0.891125841	0.389668355	0.771398309	0.999996191
LOC100337507	0.614950781	0.86946465	0.24060113	0.875525883	0.408913854	0.802010375	0.999996191
LOC100462699	0.65240648	0.97132811	0.625472722	0.975169746	0.470089687	0.97004054	0.999996191
LOC100616098	0.769207051	0.779350308	0.745612004	0.104811567	0.944542023	0.795080956	0.999996191
LOC100847118	0.666022259	0.289690052	0.574445599	0.776943107	0.750986275	0.85712856	0.999996191
LOC100847122	0.804044656	0.054819231	0.351099078	0.887950125	0.698147848	0.504510083	0.999996191
LOC100847143	0.28248811	0.682685716	0.946775092	0.966032786	0.272482722	0.809302218	0.999996191
LOC100847156	0.710895095	0.70835478	0.4606347	0.779435299	0.910314306	0.963278607	0.999996191
LOC100847180	0.2017472	0.461249921	0.631944925	0.870174785	0.385234585	0.643198573	0.999996191
LOC100847182	0.981329353	0.096594072	0.588966035	0.860902637	0.951802931	0.800845633	0.999996191
LOC100847190	0.361134328	0.443830296	0.34553853	0.28390521	0.963074344	0.59173107	0.999996191
LOC100847236	0.707315019	0.237740897	0.712144601	0.65068376	0.823863794	0.856016364	0.999996191
LOC100847269	0.735565514	0.589903445	0.082187956	0.420995684	0.658518468	0.510133044	0.999996191
LOC100847284	0.646831928	0.848109899	0.868926828	0.424968152	0.398416433	0.888907548	0.999996191
LOC100847320	0.25122426	0.587734425	0.748657647	0.832698932	0.962502253	0.901140296	0.999996191
LOC100847326	0.769823449	0.790277627	0.592200031	0.869195458	0.099814674	0.731941931	0.999996191
LOC100847357	0.562355072	0.896768608	0.669921113	0.700215004	0.323558717	0.881643222	0.999996191
LOC100847363	0.167409922	0.965700088	0.751383176	0.652142588	0.874325112	0.867362405	0.999996191
LOC100847453	0.535397597	0.959771724	0.887776067	0.9674855	0.942725487	0.997902813	0.999996191
LOC100847490	0.40363865	0.855401217	0.636388328	0.879821304	0.550731155	0.923116959	0.999996191
LOC100847546	0.798037944	0.635449653	0.683598546	0.835318116	0.182663045	0.825331035	0.999996191
LOC100847554	0.599477233	0.447873378	0.247483275	0.494570273	0.573242615	0.634333096	0.999996191
LOC100847567	0.613416483	0.355437705	0.820630226	0.590604746	0.93396528	0.914400517	0.999996191
LOC100847573	0.702393759	0.493081743	0.770424092	0.631770309	0.939245892	0.960397642	0.999996191
LOC100847609	0.709773972	0.717197993	0.190536678	0.857492406	0.415645652	0.750704194	0.999996191

LOC100847612	0.681393143	0.920200977	0.686284547	0.630058863	0.200872066	0.830108353	0.999996191
LOC100847613	0.413431877	0.714437161	0.670328535	0.976460219	0.305004865	0.84285438	0.999996191
LOC100847745	0.965286093	0.890530796	0.705725556	0.965639466	0.625872614	0.99628823	0.999996191
LOC100847759	0.607718189	0.664031225	0.553749788	0.466133111	0.496066119	0.821467717	0.999996191
LOC100847765	0.285741946	0.947980183	0.997315635	0.948562406	0.122457769	0.732680061	0.999996191
LOC100847773	0.565450472	0.210195607	0.973969389	0.932764582	0.536290682	0.83998067	0.999996191
LOC100847780	0.046687017	0.755928796	0.625896918	0.988697047	0.781343254	0.615012733	0.999996191
LOC100847782	0.349202201	0.425294339	0.896372002	0.279985409	0.584940701	0.66283751	0.999996191
LOC100847802	0.307561917	0.156119261	0.865170721	0.758555859	0.545275217	0.616365683	0.999996191
LOC100847819	0.290285835	0.835738287	0.132181398	0.94479243	0.796813213	0.682613494	0.999996191
LOC100847825	0.769795231	0.176969146	0.79099874	0.622163733	0.84824851	0.837093834	0.999996191
LOC100847831	0.940815463	0.720876873	0.46938245	0.256740367	0.429376597	0.753477903	0.999996191
LOC100847835	0.597061437	0.860041047	0.590255684	0.095462029	0.813389803	0.677686877	0.999996191
LOC100847839	0.853136693	0.643242307	0.688024148	0.13154051	0.953683573	0.806815992	0.999996191
LOC100847841	0.46235374	0.72467848	0.689429587	0.938070724	0.673967623	0.953956365	0.999996191
LOC100847890	0.234477433	0.683142277	0.857203725	0.698619804	0.838023733	0.888370293	0.999996191
LOC100847934	0.278132211	0.614756126	0.642021617	0.829751199	0.774993502	0.870134445	0.999996191
LOC100847941	0.928767399	0.907168673	0.623120989	0.059666856	0.986275089	0.729754612	0.999996191
LOC100847946	0.248531775	0.359431833	0.428263715	0.404648115	0.924046595	0.580668734	0.999996191
LOC100847999	0.644768965	0.618549849	0.19705803	0.74351824	0.234849287	0.572628675	0.999996191
LOC100848007	0.797595422	0.942175319	0.753795461	0.637564262	0.727766258	0.988117323	0.999996191
LOC100848077	0.695738875	0.998146354	0.849124655	0.693760028	0.241917784	0.914725562	0.999996191
LOC100848105	0.578989125	0.736353647	0.965885149	0.577617118	0.556889471	0.94539008	0.999996191
LOC100848122	0.347146289	0.600729149	0.881254248	0.297218043	0.659158411	0.7581852	0.999996191
LOC100848138	0.801302645	0.912871845	0.992914366	0.964560908	0.983630609	0.999956191	0.999996191
LOC100848148	0.482119882	0.344577989	0.433128797	0.331505912	0.7176502	0.615632611	0.999996191
LOC100848171	0.886409691	0.727147772	0.863488488	0.745332108	0.986066927	0.997722611	0.999996191
LOC100848177	0.625167772	0.18549873	0.551334709	0.978437591	0.697415923	0.792612058	0.999996191
LOC100848208	0.684284882	0.482228287	0.303000231	0.234503762	0.315112378	0.456808792	0.999996191
LOC100848212	0.273232911	0.354741843	0.366999552	0.579896732	0.696422106	0.581496331	0.999996191
LOC100848264	0.508726395	0.960015922	0.727702899	0.06907724	0.612301802	0.590298969	0.999996191
LOC100848324	0.24240213	0.447269637	0.758129087	0.772375895	0.314150798	0.645473471	0.999996191
LOC100848325	0.543080708	0.772913456	0.886834062	0.923260927	0.806749086	0.989885867	0.999996191
LOC100848339	0.37550889	0.417016108	0.358814329	0.314074996	0.574780114	0.514933067	0.999996191
LOC100848353	0.841322195	0.556732271	0.921712759	0.140608206	0.9111016	0.832611052	0.999996191
LOC100848357	0.611468055	0.717874528	0.663331601	0.923343474	0.493108536	0.945468014	0.999996191
LOC100848369	0.749469402	0.842890845	0.835183707	0.124552516	0.470151309	0.729754094	0.999996191
LOC100848407	0.455882335	0.320186625	0.215359296	0.841858664	0.703056317	0.631907931	0.999996191
LOC100848439	0.495397506	0.602916094	0.748221096	0.958216544	0.966011667	0.977633636	0.999996191
LOC100848469	0.866214484	0.547629467	0.443343421	0.527400813	0.948060803	0.921724444	0.999996191
LOC100848472	0.740928121	0.761046987	0.715271592	0.813306677	0.820269207	0.98891714	0.999996191
LOC100848478	0.503405431	0.852137674	0.489446192	0.23360667	0.832082351	0.780828386	0.999996191
LOC100848492	0.893671244	0.281001579	0.493537015	0.911017675	0.834023402	0.908756038	0.999996191
LOC100848495	0.551063332	0.503645982	0.907949914	0.878419422	0.998617562	0.980979707	0.999996191
LOC100848507	0.303983475	0.866246128	0.611982442	0.928913953	0.14400798	0.660636904	0.999996191

LOC100848527	0.562439761	0.996044123	0.128659286	0.338204318	0.95624921	0.675829404	0.999996191
LOC100848538	0.854164202	0.163674987	0.851049351	0.605678859	0.833309438	0.845717051	0.999996191
LOC100848568	0.41003311	0.5449782	0.621429224	0.430848654	0.384298239	0.673166003	0.999996191
LOC100848569	0.287744844	0.129335718	0.951989375	0.251416445	0.990957461	0.489081916	0.999996191
LOC100848570	0.427095838	0.725644099	0.838356299	0.406828501	0.90492085	0.910656748	0.999996191
LOC100848581	0.850903474	0.986787822	0.828560073	0.785682856	0.696335986	0.99683596	0.999996191
LOC100848642	0.031796487	0.936270743	0.688429957	0.927992001	0.996435896	0.635499712	0.999996191
LOC100848665	0.629901353	0.570346343	0.564142097	0.035951797	0.990218775	0.452583625	0.999996191
LOC100848689	0.270945593	0.708569344	0.429970089	0.754894258	0.209753304	0.563221956	0.999996191
LOC100848699	0.282582413	0.482180608	0.938817555	0.412965478	0.984004599	0.822457318	0.999996191
LOC100848721	0.97395878	0.954423849	0.86554077	0.3611068	0.45636958	0.945481357	0.999996191
LOC100848766	0.83121357	0.875010414	0.95667767	0.620587741	0.183854653	0.886674672	0.999996191
LOC100848815	0.898135488	0.889415346	0.935089059	0.795416604	0.911358089	0.999564118	0.999996191
LOC100848869	0.582741941	0.88080246	0.935638023	0.639540184	0.193177043	0.843822717	0.999996191
LOC100848872	0.758060032	0.611379043	0.666217977	0.174936243	0.442497761	0.680680732	0.999996191
LOC100848886	0.753944879	0.785055014	0.700276845	0.043537259	0.878929805	0.600746399	0.999996191
LOC100848906	0.109485633	0.853849403	0.858518457	0.288081283	0.527177115	0.549795945	0.999996191
LOC100848912	0.90667658	0.552073695	0.533685792	0.911303815	0.917504717	0.981472607	0.999996191
LOC100848939	0.288228553	0.619874096	0.49346821	0.207246736	0.678771147	0.553130102	0.999996191
LOC100848985	0.249766778	0.794997887	0.743064914	0.713024083	0.149059685	0.598533378	0.999996191
LOC100848991	0.249285556	0.698337812	0.794264753	0.648400457	0.951448362	0.896253628	0.999996191
LOC100849023	0.24448885	0.802540983	0.691227631	0.634401256	0.987017821	0.895675893	0.999996191
LOC100849046	0.174489891	0.93590784	0.31843662	0.986482198	0.544771668	0.710743651	0.999996191
LOC100849067	0.804391888	0.858878792	0.347697578	0.923152232	0.986789413	0.980492358	0.999996191
LOC100849069	0.395146405	0.533742211	0.718694849	0.136580226	0.88286768	0.628430043	0.999996191
LOC100849681	0.726364085	0.453906497	0.828850056	0.480298879	0.563844023	0.876912703	0.999996191
LOC100850437	0.664410445	0.771373955	0.06797468	0.971537275	0.836196158	0.713151164	0.999996191
LOC100850875	0.507248298	0.948162661	0.166093984	0.456746693	0.815170637	0.722577698	0.999996191
LOC100851323	0.616908881	0.369279085	0.67034776	0.990799634	0.567404607	0.897092685	0.999996191
LOC100852077	0.267645213	0.904595243	0.322231637	0.748544011	0.398340414	0.675431008	0.999996191
LOC101901950	0.901769679	0.521405032	0.92574395	0.924970852	0.179107737	0.873212462	0.999996191
LOC101901960	0.376026342	0.806814157	0.728295524	0.771803311	0.869655844	0.955225863	0.999996191
LOC101901983	0.546271313	0.567069333	0.683645401	0.504386799	0.457534491	0.812137702	0.999996191
LOC101902029	0.236990685	0.985401499	0.314681485	0.133591117	0.846441256	0.478022056	0.999996191
LOC101902030	0.750025694	0.213978983	0.999341324	0.139080987	0.96098555	0.65953994	0.999996191
LOC101902036	0.970692935	0.951216949	0.899997142	0.353286333	0.566863003	0.964080372	0.999996191
LOC101902048	0.888003132	0.79600128	0.136418904	0.731994764	0.637686093	0.798042012	0.999996191
LOC101902067	0.735124473	0.49598592	0.67903471	0.738513691	0.937708481	0.966177929	0.999996191
LOC101902083	0.045068821	0.687198391	0.976502591	0.713654112	0.323440441	0.446731629	0.999996191
LOC101902106	0.413584055	0.517496596	0.630710145	0.932558162	0.567900484	0.871967666	0.999996191
LOC101902124	0.814668982	0.68995924	0.489872896	0.67930966	0.747041063	0.950186302	0.999996191
LOC101902141	0.679046561	0.527830246	0.99745794	0.237594931	0.162794919	0.574104478	0.999996191
LOC101902154	0.770639641	0.4961485	0.938924575	0.165543861	0.725034235	0.790428176	0.999996191
LOC101902172	0.989373109	0.446239825	0.869676397	0.413382799	0.767973612	0.937416697	0.999996191
LOC101902174	0.285701541	0.975755632	0.41310967	0.736139521	0.464141604	0.774299592	0.999996191

LOC101902221	0.552595694	0.106229332	0.726072525	0.721753502	0.925792408	0.714342663	0.999996191
LOC101902232	0.975001635	0.241301297	0.482198745	0.921245292	0.574907279	0.845802492	0.999996191
LOC101902288	0.169695103	0.247730638	0.824365725	0.783323097	0.262170786	0.450147631	0.999996191
LOC101902290	0.990570849	0.874451049	0.892232474	0.983121054	0.505336764	0.996954893	0.999996191
LOC101902301	0.369706365	0.060784701	0.822342497	0.95691751	0.518920877	0.49625504	0.999996191
LOC101902345	0.855627247	0.524407474	0.644516678	0.837918343	0.821691195	0.975544148	0.999996191
LOC101902346	0.441261598	0.141380628	0.566030723	0.40682368	0.952445976	0.572058078	0.999996191
LOC101902360	0.56077066	0.762723161	0.693201558	0.927087685	0.923637755	0.986865265	0.999996191
LOC101902361	0.271167549	0.968668502	0.669072297	0.731881601	0.087645491	0.534894865	0.999996191
LOC101902385	0.865239923	0.398870714	0.953883571	0.116911303	0.683522034	0.699173275	0.999996191
LOC101902430	0.81311228	0.954011821	0.397015728	0.452937291	0.878644295	0.937961035	0.999996191
LOC101902440	0.671883082	0.941692634	0.824134985	0.374713726	0.63929204	0.939825428	0.999996191
LOC101902444	0.60368057	0.449922179	0.876571333	0.772382342	0.498062912	0.905327438	0.999996191
LOC101902449	0.576278879	0.939306895	0.147349643	0.73537654	0.272765459	0.602435476	0.999996191
LOC101902458	0.143321832	0.286287569	0.752974371	0.727123939	0.704533248	0.600333708	0.999996191
LOC101902469	0.181798204	0.667044847	0.827998236	0.626996926	0.57471771	0.759085876	0.999996191
LOC101902475	0.166634494	0.403557943	0.903070365	0.66945074	0.284773041	0.539959632	0.999996191
LOC101902531	0.331969671	0.913027405	0.104813046	0.692022824	0.534590865	0.542828226	0.999996191
LOC101902542	0.828890101	0.838896619	0.841543414	0.108843697	0.381343218	0.683789825	0.999996191
LOC101902551	0.333280114	0.54256762	0.945716302	0.239574012	0.896089231	0.761745163	0.999996191
LOC101902644	0.711958081	0.864117372	0.514716489	0.445098724	0.741998408	0.921106531	0.999996191
LOC101902663	0.940866933	0.93205383	0.804741866	0.927832414	0.680989281	0.998529005	0.999996191
LOC101902664	0.973448712	0.646598757	0.704586623	0.835573776	0.40893721	0.956973731	0.999996191
LOC101902668	0.273420251	0.982240395	0.846146989	0.860880909	0.579759172	0.930002929	0.999996191
LOC101902681	0.547460774	0.582873942	0.893724803	0.092498003	0.711901148	0.63372281	0.999996191
LOC101902705	0.222520438	0.862535433	0.487253246	0.596850606	0.156636913	0.487326043	0.999996191
LOC101902760	0.456750555	0.645634092	0.658381368	0.215588014	0.678782778	0.713888669	0.999996191
LOC101902768	0.747281473	0.406098829	0.561743464	0.851520356	0.995930445	0.953105989	0.999996191
LOC101902807	0.521580882	0.592622054	0.094634854	0.869607354	0.378622395	0.5052403	0.999996191
LOC101902808	0.551326756	0.746627464	0.697367824	0.159308781	0.413528708	0.635088774	0.999996191
LOC101902809	0.787949208	0.463392161	0.077359726	0.488021479	0.982315483	0.57004561	0.999996191
LOC101902812	0.657210998	0.24670078	0.25158931	0.357137589	0.967712939	0.577842559	0.999996191
LOC101902831	0.956763176	0.893829139	0.505825517	0.974455803	0.925277771	0.997160214	0.999996191
LOC101902838	0.361453524	0.981997057	0.798935798	0.453236363	0.89723464	0.931754297	0.999996191
LOC101902839	0.908236849	0.442246845	0.815063932	0.115171544	0.4739754	0.624039174	0.999996191
LOC101902840	0.640664466	0.447690474	0.583216622	0.717510783	0.388144842	0.803977819	0.999996191
LOC101902854	0.868123739	0.845066669	0.588565129	0.025053895	0.938362366	0.515077915	0.999996191
LOC101902856	0.453079272	0.884765373	0.557653898	0.539161421	0.344692676	0.783991708	0.999996191
LOC101902861	0.894476909	0.786451242	0.344879339	0.744518526	0.851098474	0.958114485	0.999996191
LOC101902895	0.692229361	0.3165012	0.264476145	0.796475599	0.945164464	0.792577376	0.999996191
LOC101902918	0.754002868	0.875370514	0.76839283	0.750409259	0.079742007	0.726385537	0.999996191
LOC101902926	0.564995648	0.226220973	0.593200426	0.316262518	0.924509589	0.666078852	0.999996191
LOC101902930	0.694523832	0.749524592	0.875181586	0.819022805	0.903204303	0.994812048	0.999996191
LOC101902937	0.828980942	0.354742565	0.846789581	0.037682337	0.843514972	0.469189195	0.999996191
LOC101902959	0.626404653	0.91779526	0.131584348	0.802808765	0.761330671	0.802684001	0.999996191

LOC101902968	0.354977822	0.28683915	0.930184754	0.257462768	0.63757657	0.596857879	0.999996191
LOC101902983	0.917324559	0.676521616	0.267516961	0.906230146	0.72248065	0.925412683	0.999996191
LOC101902991	0.321926739	0.645768923	0.328921857	0.998168296	0.555957955	0.76775489	0.999996191
LOC101902994	0.388363441	0.691786328	0.789239933	0.453929349	0.979099877	0.908847035	0.999996191
LOC101902998	0.340974445	0.725878351	0.863542101	0.63714376	0.058463123	0.470240916	0.999996191
LOC101903015	0.347458114	0.104660992	0.676430652	0.93462872	0.637761346	0.585462226	0.999996191
LOC101903056	0.849170827	0.175903963	0.631337128	0.929835355	0.90308913	0.886329112	0.999996191
LOC101903097	0.878599391	0.676888307	0.794255213	0.399528666	0.722974983	0.948076972	0.999996191
LOC101903098	0.943997231	0.991451172	0.531226066	0.636983066	0.229965936	0.874633971	0.999996191
LOC101903114	0.786874322	0.94421356	0.712832148	0.907731577	0.143264701	0.866537107	0.999996191
LOC101903165	0.741732556	0.417427147	0.844486026	0.530494914	0.519775146	0.87318372	0.999996191
LOC101903200	0.9030566	0.433865703	0.294100851	0.185517513	0.396710285	0.481737204	0.999996191
LOC101903205	0.434486169	0.645985031	0.99655511	0.665583746	0.39885876	0.877386763	0.999996191
LOC101903232	0.995766526	0.854203296	0.087671234	0.622558003	0.387049505	0.625103314	0.999996191
LOC101903253	0.13332018	0.737866067	0.465551037	0.518424106	0.862042594	0.650522991	0.999996191
LOC101903281	0.402004097	0.40847103	0.762156137	0.998132782	0.786958116	0.913931903	0.999996191
LOC101903301	0.523413193	0.583440744	0.988031194	0.096813679	0.549576464	0.603103608	0.999996191
LOC101903356	0.945744222	0.814474252	0.697165535	0.942336644	0.955992714	0.999076473	0.999996191
LOC101903375	0.766260174	0.493911315	0.445777901	0.854138383	0.692153154	0.915642881	0.999996191
LOC101903385	0.817020696	0.272152395	0.889001708	0.318192223	0.794327091	0.815851075	0.999996191
LOC101903397	0.602940309	0.955755016	0.798949845	0.402577018	0.227691224	0.786768377	0.999996191
LOC101903400	0.310275176	0.762519058	0.589650426	0.619711363	0.354275869	0.728117738	0.999996191
LOC101903402	0.714299386	0.29468456	0.507461903	0.66025259	0.804819216	0.836788932	0.999996191
LOC101903413	0.541985872	0.750127195	0.523341683	0.798155045	0.443362555	0.879344202	0.999996191
LOC101903424	0.691339214	0.349215318	0.39569859	0.098994317	0.88307052	0.478931124	0.999996191
LOC101903438	0.438464109	0.647290918	0.953237994	0.294966911	0.304455387	0.683847979	0.999996191
LOC101903478	0.631877923	0.475491498	0.592934943	0.125532052	0.677363567	0.591795596	0.999996191
LOC101903545	0.298286023	0.993437133	0.9290804	0.151638691	0.543012593	0.670422921	0.999996191
LOC101903557	0.155066806	0.899849175	0.652600691	0.289469009	0.488356167	0.560284351	0.999996191
LOC101903567	0.584058328	0.96526756	0.262879381	0.317793491	0.682237118	0.737116647	0.999996191
LOC101903572	0.622798125	0.949511013	0.981806413	0.801077968	0.931338967	0.998286588	0.999996191
LOC101903574	0.621474519	0.83629419	0.080132186	0.509007789	0.515257089	0.528899978	0.999996191
LOC101903586	0.805803349	0.800773699	0.172218317	0.605963603	0.957662546	0.85670427	0.999996191
LOC101903600	0.818469634	0.228711524	0.778655495	0.891983879	0.530464882	0.866736833	0.999996191
LOC101903615	0.805223669	0.899104521	0.064294923	0.256402113	0.829448486	0.510373627	0.999996191
LOC101903629	0.765568791	0.765458471	0.334387423	0.256283192	0.251902258	0.556932432	0.999996191
LOC101903645	0.489890467	0.103780249	0.52526675	0.688787418	0.764745146	0.577416896	0.999996191
LOC101903649	0.775316902	0.319290715	0.734734902	0.348022476	0.223310151	0.578361199	0.999996191
LOC101903678	0.674914955	0.412560848	0.89890954	0.703822972	0.727260191	0.942266113	0.999996191
LOC101903682	0.456075128	0.441782774	0.419213824	0.830464871	0.940465748	0.860133767	0.999996191
LOC101903793	0.602355521	0.311157661	0.584937136	0.963148665	0.296540466	0.732274413	0.999996191
LOC101903806	0.689563446	0.751328165	0.408531768	0.922842826	0.76776178	0.956132125	0.999996191
LOC101903820	0.326898002	0.755194889	0.364032077	0.439248347	0.729472679	0.716442509	0.999996191
LOC101903831	0.39777563	0.848572671	0.379673471	0.95290551	0.854144518	0.920810067	0.999996191
LOC101903851	0.4384324	0.24475844	0.534457387	0.590642099	0.452143783	0.593943709	0.999996191

LOC101903853	0.937158265	0.65244267	0.020100537	0.721554768	0.88013709	0.466660179	0.999996191
LOC101903900	0.71884023	0.583356802	0.308528262	0.782824804	0.628065846	0.854620395	0.999996191
LOC101903913	0.158876327	0.137587197	0.727340305	0.981602665	0.837443191	0.563207898	0.999996191
LOC101903928	0.607051106	0.674361263	0.588723001	0.923482646	0.225389744	0.816528847	0.999996191
LOC101903976	0.61455383	0.461642984	0.820260921	0.874925379	0.610167498	0.939294141	0.999996191
LOC101903988	0.980900063	0.612783056	0.199283384	0.909031507	0.580177822	0.85356805	0.999996191
LOC101903992	0.415991486	0.77894587	0.223737198	0.766028424	0.605108478	0.745468457	0.999996191
LOC101903997	0.341245427	0.573537716	0.670854818	0.802586666	0.73831698	0.883904867	0.999996191
LOC101904039	0.177063362	0.597548625	0.841750006	0.605920072	0.225854254	0.549782295	0.999996191
LOC101904062	0.427630701	0.352270421	0.626172941	0.194567285	0.396721354	0.454199551	0.999996191
LOC101904069	0.267166302	0.731686377	0.303541308	0.390044248	0.773093978	0.62427042	0.999996191
LOC101904084	0.585693847	0.337600451	0.856289907	0.939624675	0.768844253	0.937760886	0.999996191
LOC101904097	0.885692165	0.560223623	0.143757866	0.79502788	0.178554547	0.514609385	0.999996191
LOC101904098	0.407011269	0.871838989	0.554044971	0.792027909	0.602688657	0.908336046	0.999996191
LOC101904103	0.229251818	0.248653067	0.758623967	0.369480856	0.644777295	0.517857023	0.999996191
LOC101904133	0.573429118	0.869014634	0.995183828	0.809970054	0.762494283	0.992681328	0.999996191
LOC101904156	0.652095186	0.917355806	0.544287595	0.958993299	0.810089289	0.986726396	0.999996191
LOC101904173	0.828735467	0.713136369	0.673939515	0.171738175	0.279867469	0.637476302	0.999996191
LOC101904187	0.564026518	0.339572947	0.43315542	0.995559043	0.417440816	0.750213909	0.999996191
LOC101904227	0.403136579	0.375286331	0.227359517	0.694689076	0.380729768	0.494659654	0.999996191
LOC101904248	0.8891393	0.865407905	0.332257281	0.952760387	0.996382182	0.985109083	0.999996191
LOC101904270	0.481781869	0.480157507	0.621548355	0.698930368	0.53153869	0.82694571	0.999996191
LOC101904290	0.871375937	0.034799231	0.682579166	0.953062012	0.445034857	0.488080549	0.999996191
LOC101904314	0.56658598	0.9516739	0.934203645	0.282132486	0.699636016	0.915276762	0.999996191
LOC101904332	0.796739613	0.771462385	0.780792738	0.017773411	0.940838803	0.471685782	0.999996191
LOC101904339	0.520098395	0.962021416	0.624613809	0.401388111	0.41316743	0.821971185	0.999996191
LOC101904355	0.557138529	0.294049783	0.829484391	0.398527848	0.511889285	0.709204451	0.999996191
LOC101904377	0.642316273	0.68316873	0.928716762	0.545619837	0.867003221	0.9736827	0.999996191
LOC101904393	0.838897166	0.959596516	0.739864213	0.69294317	0.841932541	0.99538828	0.999996191
LOC101904396	0.626820884	0.807119299	0.788105305	0.419838931	0.62482874	0.921120761	0.999996191
LOC101904413	0.731156521	0.859628057	0.538870078	0.464125841	0.842743506	0.94539958	0.999996191
LOC101904435	0.177293862	0.499908237	0.75527102	0.311778355	0.637814681	0.566743813	0.999996191
LOC101904442	0.92774152	0.635687768	0.248807747	0.63862646	0.309393122	0.717738068	0.999996191
LOC101904447	0.441399844	0.704523874	0.346529536	0.279775298	0.511110735	0.595126031	0.999996191
LOC101904492	0.183792982	0.952143492	0.18149643	0.57292373	0.815654703	0.587830606	0.999996191
LOC101904498	0.798785866	0.826153811	0.389983445	0.420332129	0.779871129	0.894805292	0.999996191
LOC101904520	0.340684624	0.924999823	0.928818117	0.283081157	0.551370457	0.800616203	0.999996191
LOC101904526	0.604734229	0.862837907	0.112484662	0.294784143	0.538505033	0.499072783	0.999996191
LOC101904529	0.557560457	0.860356899	0.509607046	0.184733748	0.176739465	0.470698304	0.999996191
LOC101904536	0.660786498	0.378478118	0.23701306	0.627015884	0.895475466	0.743671361	0.999996191
LOC101904614	0.985501034	0.755116717	0.740073578	0.312872547	0.662341168	0.930663222	0.999996191
LOC101904622	0.95923966	0.759465752	0.184322244	0.874014551	0.296871146	0.752152175	0.999996191
LOC101904642	0.93839645	0.406844151	0.183600383	0.815578832	0.435299572	0.688477903	0.999996191
LOC101904667	0.840958385	0.271886674	0.994652225	0.710524692	0.567665108	0.905515749	0.999996191
LOC101904691	0.927482361	0.639975147	0.61578785	0.637301042	0.035168753	0.475427083	0.999996191

LOC101904698	0.740650279	0.210947034	0.929601108	0.282031487	0.276782146	0.535973191	0.999996191
LOC101904701	0.470503984	0.296469027	0.162150281	0.922922731	0.493444486	0.517826329	0.999996191
LOC101904705	0.823210583	0.459363225	0.802157207	0.139846038	0.585081699	0.687967393	0.999996191
LOC101904753	0.689726488	0.97916654	0.234048159	0.69678438	0.680753544	0.878752821	0.999996191
LOC101904757	0.472074223	0.746902488	0.32313689	0.108123689	0.801737718	0.509942545	0.999996191
LOC101904769	0.117700715	0.736212627	0.96202598	0.685271973	0.918427006	0.823991068	0.999996191
LOC101904796	0.657105343	0.192457836	0.85626627	0.478105192	0.957581811	0.814551697	0.999996191
LOC101904810	0.641934567	0.254222897	0.656893967	0.412302389	0.985425025	0.792314843	0.999996191
LOC101904822	0.860965959	0.903596484	0.422772998	0.699704542	0.654973357	0.956545294	0.999996191
LOC101904840	0.776090368	0.128577496	0.304524762	0.675695637	0.882405249	0.626721305	0.999996191
LOC101904849	0.921351812	0.835787067	0.439938682	0.838858156	0.11392614	0.738530171	0.999996191
LOC101904855	0.223976882	0.474096674	0.94428695	0.711729549	0.811552226	0.840006196	0.999996191
LOC101904871	0.89804093	0.644140929	0.997226061	0.46373676	0.763151381	0.976925013	0.999996191
LOC101904891	0.409031785	0.781360692	0.372257918	0.709444787	0.238707444	0.647460301	0.999996191
LOC101904902	0.617691657	0.776258717	0.442017889	0.879013751	0.9909556	0.971049213	0.999996191
LOC101904916	0.612324327	0.481159253	0.50883177	0.805795687	0.784254246	0.909490713	0.999996191
LOC101904947	0.922925361	0.991141816	0.749368892	0.769821083	0.842124	0.998502034	0.999996191
LOC101904962	0.19992371	0.927042679	0.981480398	0.186788841	0.494787055	0.612104408	0.999996191
LOC101904963	0.867791702	0.759554794	0.746285509	0.990793741	0.455602283	0.981194519	0.999996191
LOC101905010	0.772172971	0.883979794	0.843258858	0.051805898	0.438223031	0.563175185	0.999996191
LOC101905014	0.121542682	0.802730392	0.534570459	0.647164894	0.550092301	0.631505381	0.999996191
LOC101905033	0.141780152	0.987956788	0.822488545	0.728378587	0.901011568	0.879930627	0.999996191
LOC101905046	0.90176726	0.252855339	0.241437296	0.571779331	0.90767169	0.714953226	0.999996191
LOC101905049	0.829392011	0.985906833	0.766846185	0.75718317	0.907584042	0.998240036	0.999996191
LOC101905053	0.232042407	0.526715767	0.395899226	0.376672593	0.684744176	0.55432794	0.999996191
LOC101905114	0.598948467	0.897696848	0.498668875	0.342326184	0.537987209	0.813878912	0.999996191
LOC101905141	0.770236945	0.661581017	0.242942937	0.996553512	0.43558463	0.827928787	0.999996191
LOC101905167	0.561703504	0.076870856	0.38343411	0.769762557	0.952312574	0.548973834	0.999996191
LOC101905179	0.969421798	0.51940981	0.958268964	0.390900843	0.585611413	0.92716317	0.999996191
LOC101905188	0.923886082	0.312017118	0.772716738	0.758260325	0.939821246	0.960592405	0.999996191
LOC101905199	0.994212243	0.7129238	0.59189158	0.76475893	0.176490431	0.836405288	0.999996191
LOC101905203	0.281750894	0.426056688	0.696049012	0.74653733	0.966651536	0.846353485	0.999996191
LOC101905219	0.219956215	0.265243419	0.837311178	0.231251124	0.803490662	0.494232641	0.999996191
LOC101905228	0.743114822	0.824870498	0.475937197	0.588489721	0.212757828	0.760820812	0.999996191
LOC101905232	0.64746123	0.374756824	0.607096567	0.813399299	0.991526802	0.93482763	0.999996191
LOC101905239	0.462782676	0.912148828	0.483724803	0.790978366	0.67230266	0.925298778	0.999996191
LOC101905254	0.773207006	0.732748487	0.435625464	0.841914904	0.348878549	0.873972951	0.999996191
LOC101905265	0.13044071	0.960561132	0.498371653	0.835856889	0.673438342	0.753773046	0.999996191
LOC101905293	0.895663427	0.887717388	0.151926343	0.317451518	0.182824889	0.447485264	0.999996191
LOC101905343	0.267144581	0.426980847	0.51029959	0.904003903	0.536976005	0.712839329	0.999996191
LOC101905357	0.406409092	0.43772074	0.312387647	0.957139053	0.976175889	0.822270694	0.999996191
LOC101905365	0.34424577	0.581660781	0.726841753	0.458506359	0.214430632	0.580726484	0.999996191
LOC101905390	0.437424824	0.419018076	0.590543341	0.93837556	0.856037951	0.898732965	0.999996191
LOC101905399	0.790612599	0.621126712	0.322711027	0.126211524	0.888105174	0.622853767	0.999996191
LOC101905453	0.559933454	0.849563211	0.639078183	0.613204379	0.384423807	0.872317103	0.999996191

LOC101905493	0.972105799	0.6637028	0.911074676	0.64754284	0.726139701	0.989786808	0.999996191
LOC101905498	0.738450601	0.842778448	0.988405467	0.844370053	0.656630422	0.995042281	0.999996191
LOC101905510	0.840425914	0.079434786	0.333631093	0.780096193	0.426135663	0.457189224	0.999996191
LOC101905513	0.841197639	0.89125065	0.771963205	0.933511263	0.835481914	0.99862345	0.999996191
LOC101905525	0.326765617	0.970940549	0.305032764	0.743544861	0.227650757	0.607043782	0.999996191
LOC101905533	0.771710637	0.450043697	0.457346768	0.945705164	0.062300148	0.499892918	0.999996191
LOC101905571	0.819765986	0.831511784	0.333895204	0.535328031	0.514293283	0.852316196	0.999996191
LOC101905586	0.198303526	0.72034794	0.43557352	0.960211372	0.960210144	0.838488903	0.999996191
LOC101905588	0.705105929	0.369896999	0.375871584	0.601385264	0.54092268	0.73570407	0.999996191
LOC101905593	0.088566802	0.692365954	0.934713321	0.399585581	0.332218871	0.462075331	0.999996191
LOC101905595	0.700519081	0.170655914	0.944944159	0.507252305	0.968669909	0.833194012	0.999996191
LOC101905648	0.358594769	0.472497425	0.911754954	0.160269481	0.359195207	0.490462881	0.999996191
LOC101905666	0.495046445	0.690843274	0.842242886	0.322260301	0.184526926	0.615752054	0.999996191
LOC101905668	0.942404717	0.740020083	0.53908916	0.534836982	0.667860921	0.946645829	0.999996191
LOC101905686	0.5662945	0.926891022	0.329955567	0.368438492	0.340566104	0.66220601	0.999996191
LOC101905687	0.539563616	0.992899735	0.709151116	0.284434849	0.525438488	0.836841305	0.999996191
LOC101905706	0.768021512	0.932354839	0.063133388	0.986334822	0.54470404	0.683788683	0.999996191
LOC101905708	0.169629336	0.398564298	0.192893389	0.912860785	0.949867689	0.535476304	0.999996191
LOC101905711	0.351267515	0.690458317	0.538920288	0.354101348	0.949219555	0.793825877	0.999996191
LOC101905723	0.374459987	0.934818358	0.969439083	0.093393823	0.988191788	0.732314492	0.999996191
LOC101905734	0.37069516	0.949279694	0.998681098	0.421777669	0.148578015	0.664797	0.999996191
LOC101905770	0.883794894	0.590070258	0.217805519	0.48786849	0.287608829	0.601690725	0.999996191
LOC101905771	0.788792557	0.446426755	0.896737496	0.446728873	0.983264179	0.949538759	0.999996191
LOC101905779	0.934987831	0.750330673	0.034167519	0.708327135	0.42122253	0.451015178	0.999996191
LOC101905786	0.346716361	0.757465687	0.604333014	0.540729049	0.54700717	0.805290565	0.999996191
LOC101905801	0.49454657	0.563485875	0.620636827	0.426825928	0.277693807	0.650832945	0.999996191
LOC101905813	0.87149999	0.101811522	0.694154271	0.806646134	0.254953406	0.557178656	0.999996191
LOC101905818	0.35448484	0.339912272	0.479252192	0.600904422	0.568448619	0.643320138	0.999996191
LOC101905821	0.936192968	0.677132526	0.467453826	0.799824235	0.93214752	0.980954057	0.999996191
LOC101905845	0.51675848	0.512395197	0.187102117	0.774164086	0.407588648	0.597921021	0.999996191
LOC101905875	0.640296332	0.730803218	0.088956068	0.254362644	0.783413616	0.477691042	0.999996191
LOC101905894	0.386224091	0.777914948	0.979143923	0.180591614	0.263048541	0.57614839	0.999996191
LOC101905908	0.121843779	0.740743871	0.678892108	0.371769035	0.68353311	0.597149026	0.999996191
LOC101905951	0.974983768	0.933615624	0.729384399	0.556074848	0.835207247	0.992848967	0.999996191
LOC101905977	0.598246263	0.763305455	0.369207345	0.678649788	0.888256179	0.917832096	0.999996191
LOC101905997	0.997458031	0.42795589	0.752437856	0.051280611	0.926971292	0.59332957	0.999996191
LOC101906008	0.7128018	0.55274781	0.0577722	0.634305303	0.678993675	0.508549564	0.999996191
LOC101906009	0.119357344	0.934524997	0.853637089	0.320643166	0.631537778	0.638873116	0.999996191
LOC101906018	0.983259474	0.739016878	0.334979872	0.434375961	0.258572622	0.706548375	0.999996191
LOC101906021	0.994459821	0.284495409	0.216413171	0.317798551	0.464928716	0.493616434	0.999996191
LOC101906067	0.502242597	0.103896342	0.554718558	0.786274928	0.818212406	0.632078018	0.999996191
LOC101906077	0.36857188	0.281559862	0.580345139	0.502142857	0.833487676	0.690948536	0.999996191
LOC101906086	0.600575609	0.555852232	0.514939348	0.117479158	0.588072757	0.544823302	0.999996191
LOC101906110	0.927738856	0.256607585	0.573542134	0.669430137	0.911589163	0.893168974	0.999996191
LOC101906120	0.413885395	0.866445171	0.031724313	0.955967406	0.871494977	0.502256099	0.999996191

LOC101906131	0.87994974	0.451101276	0.20936336	0.749242994	0.80022593	0.815399169	0.999996191
LOC101906134	0.626020735	0.432759446	0.450083689	0.078611734	0.75418563	0.452928729	0.999996191
LOC101906135	0.539066197	0.463927671	0.946669189	0.105039321	0.8490708	0.65659773	0.999996191
LOC101906167	0.84962006	0.605323941	0.727180983	0.561638155	0.574386467	0.936386614	0.999996191
LOC101906177	0.153679441	0.280321391	0.752545814	0.558290073	0.886456165	0.602989232	0.999996191
LOC101906200	0.766743816	0.56350657	0.473017837	0.592836213	0.565927628	0.865880512	0.999996191
LOC101906206	0.895605737	0.406593526	0.7327666	0.362210022	0.30238311	0.719253693	0.999996191
LOC101906218	0.978275105	0.189911169	0.729785174	0.422711351	0.804125629	0.802122826	0.999996191
LOC101906221	0.908524674	0.85279186	0.968709173	0.840380278	0.470754087	0.991876267	0.999996191
LOC101906235	0.319319522	0.14958043	0.443928069	0.96318482	0.944881642	0.639037358	0.999996191
LOC101906266	0.25417513	0.836060438	0.29002108	0.847708838	0.299658543	0.598211284	0.999996191
LOC101906273	0.226739868	0.511197571	0.474618248	0.779759106	0.877079141	0.766205145	0.999996191
LOC101906276	0.930663359	0.26035473	0.642162012	0.802957079	0.980021603	0.937862252	0.999996191
LOC101906283	0.776080776	0.37413317	0.577768777	0.84582804	0.619336387	0.90010521	0.999996191
LOC101906315	0.595341018	0.384826176	0.911496142	0.887950811	0.556180729	0.919516825	0.999996191
LOC101906347	0.906712854	0.190330661	0.291121562	0.705910242	0.489483901	0.618365129	0.999996191
LOC101906358	0.578071354	0.318816339	0.685828867	0.566106507	0.911697117	0.858449746	0.999996191
LOC101906363	0.699949222	0.362553976	0.55571164	0.682486387	0.621703426	0.845153784	0.999996191
LOC101906364	0.620088058	0.614042182	0.654127648	0.862363006	0.700586055	0.95640789	0.999996191
LOC101906366	0.830814887	0.755596516	0.40077163	0.230781794	0.755847789	0.793638041	0.999996191
LOC101906367	0.630247904	0.822759459	0.650133482	0.854039767	0.13966753	0.778190108	0.999996191
LOC101906392	0.866416242	0.459417098	0.389054056	0.902444639	0.51667914	0.873404594	0.999996191
LOC101906398	0.966550151	0.223449369	0.739286722	0.694953613	0.474566847	0.824595916	0.999996191
LOC101906408	0.763646773	0.176276386	0.589551683	0.695177661	0.227552453	0.555462382	0.999996191
LOC101906411	0.872757359	0.230038781	0.546745974	0.452881362	0.849413257	0.786869149	0.999996191
LOC101906451	0.551981937	0.978695356	0.817822299	0.447155576	0.810018103	0.961201842	0.999996191
LOC101906457	0.565539736	0.89874732	0.717823627	0.874324527	0.657920997	0.978393201	0.999996191
LOC101906460	0.59394	0.517815722	0.731875033	0.243408643	0.499412096	0.706706982	0.999996191
LOC101906472	0.960511891	0.516845978	0.789432167	0.935724225	0.667852237	0.98547406	0.999996191
LOC101906477	0.420710609	0.654509059	0.537678503	0.142724748	0.67864735	0.581152891	0.999996191
LOC101906484	0.861992111	0.883311958	0.077529307	0.455675499	0.998276009	0.70311087	0.999996191
LOC101906512	0.918468061	0.172694609	0.615442452	0.855922364	0.129397985	0.526961866	0.999996191
LOC101906513	0.800470367	0.878218158	0.4945826	0.433640558	0.629672388	0.909744149	0.999996191
LOC101906522	0.992737289	0.856598109	0.149795481	0.949265607	0.638221067	0.882784085	0.999996191
LOC101906545	0.514477806	0.620689327	0.783393833	0.591909124	0.433438411	0.855978954	0.999996191
LOC101906546	0.611637059	0.917524162	0.659821285	0.527744311	0.365070815	0.871664824	0.999996191
LOC101906588	0.124941577	0.749591916	0.960342437	0.598452371	0.954435996	0.820504256	0.999996191
LOC101906606	0.698646679	0.551792495	0.672888843	0.260648087	0.600950671	0.780043275	0.999996191
LOC101906632	0.433702653	0.754178045	0.163416657	0.846347739	0.62036986	0.711547871	0.999996191
LOC101906656	0.173959754	0.576249887	0.843624422	0.806822091	0.877251328	0.845206006	0.999996191
LOC101906676	0.923412652	0.935034993	0.078272932	0.735922269	0.673737269	0.744934032	0.999996191
LOC101906688	0.661041218	0.608944525	0.603738869	0.921034583	0.538657628	0.936323666	0.999996191
LOC101906730	0.729155979	0.512890082	0.165827549	0.825279061	0.287981762	0.586472677	0.999996191
LOC101906756	0.713770753	0.931452793	0.991119336	0.038645741	0.611626593	0.597206684	0.999996191
LOC101906836	0.624998159	0.75976575	0.568433506	0.744378438	0.781465494	0.959758929	0.999996191

LOC101906837	0.70537542	0.252104991	0.057804347	0.923501292	0.735412174	0.446738449	0.999996191
LOC101906850	0.86891151	0.284607013	0.601126017	0.39237878	0.846114509	0.813792714	0.999996191
LOC101906855	0.73053748	0.701948488	0.900345502	0.593379197	0.285498097	0.884633876	0.999996191
LOC101906914	0.934382569	0.473640238	0.898082302	0.030265857	0.665549072	0.471255814	0.999996191
LOC101906923	0.799166801	0.303952367	0.068624052	0.734317687	0.709457575	0.4860859	0.999996191
LOC101906966	0.511032627	0.903284319	0.816894179	0.320715733	0.639323402	0.88304245	0.999996191
LOC101906989	0.756647608	0.344913252	0.39663524	0.375856681	0.211480235	0.476217014	0.999996191
LOC101907000	0.74360617	0.962320051	0.877438638	0.070976112	0.483979993	0.66073849	0.999996191
LOC101907005	0.821368547	0.665060966	0.306528174	0.70090663	0.818735605	0.91120561	0.999996191
LOC101907006	0.671293667	0.408088744	0.73883502	0.759229611	0.697301026	0.923832311	0.999996191
LOC101907017	0.186913989	0.646858725	0.412084941	0.427719749	0.944715509	0.647308073	0.999996191
LOC101907084	0.43235376	0.433125972	0.730066625	0.260835913	0.408736512	0.584308203	0.999996191
LOC101907126	0.798912649	0.5547224	0.844814347	0.147505415	0.819746214	0.799041665	0.999996191
LOC101907132	0.429521329	0.203145263	0.84639854	0.856415775	0.766829085	0.810846269	0.999996191
LOC101907138	0.640956199	0.413257195	0.907218685	0.653321778	0.558999143	0.899929158	0.999996191
LOC101907140	0.36993838	0.231129429	0.478173278	0.681467516	0.610313656	0.614330869	0.999996191
LOC101907174	0.688254297	0.914555851	0.36804605	0.324980777	0.986493851	0.877416661	0.999996191
LOC101907189	0.22117939	0.709191975	0.381667963	0.356992447	0.798233345	0.614966938	0.999996191
LOC101907195	0.920892638	0.440077451	0.422449852	0.966335146	0.520552267	0.897496533	0.999996191
LOC101907213	0.682377107	0.658353385	0.989786643	0.160509522	0.64727517	0.802535214	0.999996191
LOC101907247	0.60023615	0.060141427	0.946089586	0.717117863	0.606723297	0.588057375	0.999996191
LOC101907250	0.981211146	0.868948328	0.526412727	0.699394821	0.91064789	0.990810048	0.999996191
LOC101907294	0.083441911	0.511560025	0.672110896	0.469084399	0.893575619	0.547214123	0.999996191
LOC101907320	0.065391465	0.58068945	0.452358497	0.445348155	0.937574377	0.451520363	0.999996191
LOC101907322	0.386012167	0.819725927	0.611914789	0.867073675	0.454093556	0.881086136	0.999996191
LOC101907327	0.917993876	0.979213049	0.814728279	0.381484061	0.662836969	0.971242887	0.999996191
LOC101907348	0.579619306	0.455365749	0.1972547	0.256104497	0.540864632	0.452495552	0.999996191
LOC101907353	0.793695989	0.529519681	0.738412398	0.982738525	0.329949393	0.916678813	0.999996191
LOC101907383	0.509173741	0.690190333	0.504413425	0.628064738	0.729203298	0.889699837	0.999996191
LOC101907404	0.126069139	0.459058006	0.834221845	0.773446815	0.70217603	0.698537159	0.999996191
LOC101907487	0.194659225	0.470240596	0.68167634	0.261152322	0.866459335	0.578139619	0.999996191
LOC101907491	0.591888855	0.858808539	0.635520498	0.049397451	0.739571144	0.543624629	0.999996191
LOC101907503	0.604088418	0.874926923	0.959467506	0.166224931	0.883754266	0.877845141	0.999996191
LOC101907514	0.542916656	0.572631606	0.916075525	0.532200324	0.958733949	0.953538482	0.999996191
LOC101907518	0.830832285	0.47602388	0.547479397	0.084221105	0.435075845	0.469618901	0.999996191
LOC101907523	0.961019236	0.17086334	0.244557484	0.233399822	0.744790049	0.446724505	0.999996191
LOC101907544	0.316278714	0.957377253	0.953728005	0.974588247	0.665714343	0.971966134	0.999996191
LOC101907545	0.460757388	0.926035679	0.242521434	0.098660552	0.916580189	0.499877756	0.999996191
LOC101907549	0.867890857	0.897503896	0.234119864	0.66230514	0.763017855	0.906095965	0.999996191
LOC101907570	0.152869574	0.306251562	0.987527144	0.508419676	0.619316771	0.584065392	0.999996191
LOC101907577	0.84132635	0.957047112	0.723169749	0.269982657	0.492812436	0.883321251	0.999996191
LOC101907606	0.857538593	0.977693894	0.261753243	0.059387541	0.715001076	0.499102581	0.999996191
LOC101907613	0.981786064	0.192184174	0.743416787	0.781421459	0.200989346	0.664863799	0.999996191
LOC101907615	0.270088055	0.648914916	0.110959927	0.812919112	0.792107358	0.554977963	0.999996191
LOC101907622	0.125841665	0.774274275	0.899316041	0.458726311	0.770814523	0.7302903	0.999996191

LOC101907641	0.820184194	0.826899562	0.641035477	0.429316393	0.868468015	0.962162597	0.999996191
LOC101907648	0.570419311	0.53474251	0.270288118	0.6208256	0.579267433	0.721982133	0.999996191
LOC101907653	0.976935743	0.829414183	0.845337576	0.864813128	0.427591544	0.986777732	0.999996191
LOC101907658	0.218312879	0.331474548	0.616058922	0.860663751	0.527341033	0.64828691	0.999996191
LOC101907688	0.775672593	0.430750636	0.76327624	0.617305372	0.480199	0.879910194	0.999996191
LOC101907697	0.628742526	0.737062412	0.364104569	0.900956089	0.891024526	0.947427029	0.999996191
LOC101907713	0.129594417	0.368148906	0.472796958	0.639056742	0.946604284	0.571532548	0.999996191
LOC101907744	0.210500162	0.887100598	0.594799535	0.838273667	0.283849221	0.700055601	0.999996191
LOC101907747	0.790386528	0.374663583	0.974315552	0.533382925	0.275315446	0.787464901	0.999996191
LOC101907749	0.578897205	0.873414112	0.485157274	0.272021019	0.330735274	0.66520505	0.999996191
LOC101907797	0.8067739	0.469293106	0.889558221	0.147930999	0.345381191	0.616650782	0.999996191
LOC101907800	0.726095248	0.876252355	0.632700703	0.896904076	0.292847572	0.92234085	0.999996191
LOC101907835	0.745569967	0.629576799	0.982770826	0.689763946	0.444401065	0.951218564	0.999996191
LOC101907883	0.813957391	0.874346523	0.916365335	0.94214174	0.637833359	0.997221006	0.999996191
LOC101907893	0.798844828	0.144376546	0.947541618	0.583427342	0.887689701	0.836329667	0.999996191
LOC101907920	0.999787822	0.300896137	0.800642558	0.835126987	0.788179424	0.960497965	0.999996191
LOC101907941	0.107689215	0.88743818	0.537685299	0.817831237	0.290787948	0.550289675	0.999996191
LOC101907943	0.339695689	0.930901426	0.2471255	0.162189498	0.812314241	0.517737147	0.999996191
LOC101907965	0.748464892	0.574963096	0.949909832	0.240071441	0.773769187	0.880534571	0.999996191
LOC101907998	0.431132236	0.620693332	0.751091113	0.538541155	0.325843748	0.754403094	0.999996191
LOC101908034	0.588005714	0.570464042	0.789745167	0.569840585	0.565109922	0.896261624	0.999996191
LOC101908046	0.075209051	0.730483133	0.887951621	0.278637074	0.708066939	0.505112683	0.999996191
LOC101908075	0.452916386	0.93008936	0.181639744	0.849584873	0.227205637	0.586880733	0.999996191
LOC101908111	0.199672877	0.074874432	0.892712301	0.75955478	0.826999993	0.479634734	0.999996191
LOC101908123	0.275122789	0.690853532	0.68180227	0.882916169	0.459211142	0.824228907	0.999996191
LOC101908149	0.814174755	0.097758252	0.520921921	0.365530883	0.550213197	0.478655597	0.999996191
LOC101908154	0.465361584	0.148460803	0.572620691	0.306098036	0.72667558	0.488515605	0.999996191
LOC101908166	0.174885213	0.78502775	0.508726832	0.392464856	0.338539672	0.49832611	0.999996191
LOC101908185	0.372929566	0.779382345	0.585627502	0.64514916	0.240716411	0.700097675	0.999996191
LOC101908204	0.27640569	0.967621497	0.613829607	0.515921559	0.643004458	0.83011309	0.999996191
LOC101908205	0.496908596	0.736993198	0.358244542	0.976487782	0.582031692	0.877976054	0.999996191
LOC101908535	0.90414361	0.813148894	0.072738066	0.863908241	0.67296142	0.730936281	0.999996191
LOC101908577	0.931337088	0.603485933	0.765723093	0.024596236	0.995540503	0.522124713	0.999996191
LOC101908759	0.273002558	0.639108877	0.196292867	0.402819052	0.788917615	0.528223955	0.999996191
LOC101909083	0.779595868	0.233228765	0.932662421	0.75095863	0.937641971	0.935342865	0.999996191
LOC101909140	0.557427508	0.608212913	0.143267296	0.632838189	0.253495471	0.466357594	0.999996191
LOC101909173	0.264214709	0.328522933	0.673243941	0.616172556	0.329967092	0.544908292	0.999996191
LOC101909384	0.721976632	0.641933521	0.031152445	0.810216904	0.684418664	0.471258302	0.999996191
LOC101909432	0.160872106	0.712212423	0.56438903	0.447843839	0.959190891	0.70958839	0.999996191
LOC101909718	0.377141679	0.988746646	0.66502483	0.22682935	0.152443163	0.483764871	0.999996191
LOC101909754	0.359875886	0.64436639	0.174168348	0.405887125	0.777412998	0.558351108	0.999996191
LOC101910094	0.610850929	0.064492326	0.978908738	0.510435185	0.677932195	0.567226103	0.999996191
LOC101910153	0.034387868	0.505207584	0.687631855	0.884276063	0.793886692	0.47969727	0.999996191
LOC104968434	0.542294415	0.720914261	0.651896835	0.482663815	0.522447578	0.856182528	0.999996191
LOC104968435	0.37369247	0.559289768	0.855519544	0.52596421	0.120078342	0.535221321	0.999996191

LOC104968479	0.479162041	0.19059422	0.709926872	0.926819189	0.429210493	0.695366941	0.999996191
LOC104968518	0.735230534	0.629279385	0.415004639	0.479477661	0.431517016	0.776016864	0.999996191
LOC104968522	0.351645119	0.524661385	0.547674302	0.203990103	0.355627908	0.455396376	0.999996191
LOC104968634	0.541966965	0.753145795	0.411828837	0.634397298	0.909852626	0.912374943	0.999996191
LOC104968656	0.489490655	0.664767521	0.799841607	0.69492032	0.922172053	0.964238267	0.999996191
LOC104968671	0.711647486	0.819052952	0.932151142	0.613304416	0.545181615	0.970029773	0.999996191
LOC104968751	0.561719362	0.97016889	0.443540379	0.668247338	0.738545174	0.935245849	0.999996191
LOC104968807	0.653144586	0.973382109	0.179232067	0.933383683	0.220093409	0.676646161	0.999996191
LOC104968820	0.685522163	0.550272293	0.226509508	0.224895741	0.899761568	0.617579749	0.999996191
LOC104968873	0.165607533	0.874978166	0.359900489	0.93366287	0.65607396	0.736023086	0.999996191
LOC104968964	0.263828954	0.341921074	0.915022413	0.499366013	0.916933859	0.767030709	0.999996191
LOC104969024	0.432788476	0.39100384	0.773689855	0.299967204	0.652542052	0.694141832	0.999996191
LOC104969028	0.142784559	0.865010408	0.154272076	0.675746427	0.679640765	0.487491855	0.999996191
LOC104969067	0.967213158	0.915293893	0.112049671	0.502745927	0.881730393	0.793980021	0.999996191
LOC104969177	0.714635674	0.676038057	0.197911133	0.638356301	0.468583723	0.715149528	0.999996191
LOC104969192	0.518109078	0.211129873	0.439864914	0.276666618	0.964007429	0.559687095	0.999996191
LOC104969238	0.680181457	0.672433868	0.576970232	0.737212514	0.856972091	0.96420895	0.999996191
LOC104969259	0.111112511	0.496456896	0.896004563	0.995706622	0.899803756	0.79520715	0.999996191
LOC104969299	0.977589823	0.577310565	0.64063195	0.951800444	0.569604409	0.974652142	0.999996191
LOC104969340	0.852083002	0.391019402	0.689230234	0.519198822	0.801630313	0.910560024	0.999996191
LOC104969353	0.141731005	0.923418417	0.638371767	0.3487269	0.330102677	0.504979071	0.999996191
LOC104969378	0.26600888	0.908704694	0.560299296	0.781805179	0.377735844	0.777228878	0.999996191
LOC104969425	0.432977306	0.292859527	0.413153604	0.760107675	0.465253144	0.631076449	0.999996191
LOC104969611	0.454278108	0.906422581	0.351620894	0.6691045	0.396573291	0.769992252	0.999996191
LOC104969648	0.987791669	0.492185912	0.878501293	0.102011587	0.795354238	0.751152947	0.999996191
LOC104969916	0.230861247	0.656883748	0.512055726	0.616682939	0.473680174	0.670538031	0.999996191
LOC104969981	0.736002414	0.688311214	0.154894906	0.200507164	0.746050154	0.54259066	0.999996191
LOC104970103	0.251316281	0.50079239	0.277455026	0.862261197	0.804694689	0.683317759	0.999996191
LOC104970105	0.33540813	0.801590786	0.386053751	0.430502205	0.862933032	0.770649921	0.999996191
LOC104970145	0.750907431	0.465462284	0.616279737	0.379830739	0.7307688	0.84502845	0.999996191
LOC104970162	0.356852967	0.126031299	0.956178194	0.640838617	0.649606553	0.624376134	0.999996191
LOC104970173	0.763428237	0.460072541	0.560599134	0.320363261	0.863966978	0.830221333	0.999996191
LOC104970503	0.16869188	0.086646602	0.945805263	0.884752011	0.710309456	0.486164906	0.999996191
LOC104970589	0.610939089	0.094558748	0.433869248	0.730018652	0.653046603	0.545995048	0.999996191
LOC104970628	0.711812053	0.879909233	0.395974074	0.411564826	0.94695442	0.911915694	0.999996191
LOC104970645	0.664226453	0.941300643	0.437500354	0.700761754	0.56431836	0.924879679	0.999996191
LOC104970698	0.404813574	0.973555096	0.566797759	0.496639657	0.89624684	0.915274586	0.999996191
LOC104970779	0.291077043	0.224245461	0.579571492	0.850892501	0.678000858	0.663035327	0.999996191
LOC104970809	0.847893442	0.304801296	0.840957647	0.79795656	0.738653034	0.9422542	0.999996191
LOC104970815	0.926658922	0.84940085	0.90956316	0.346943325	0.192317944	0.808262174	0.999996191
LOC104970852	0.776712762	0.698875183	0.024615492	0.819471903	0.984342976	0.526376791	0.999996191
LOC104970908	0.56651801	0.760870004	0.768165042	0.07818094	0.483476684	0.554873798	0.999996191
LOC104970913	0.698643563	0.281079054	0.750638605	0.976513452	0.395212055	0.837148055	0.999996191
LOC104970966	0.551015953	0.896416821	0.876567922	0.130572958	0.257557951	0.584112035	0.999996191
LOC104971021	0.243704843	0.620906573	0.922367556	0.669271353	0.695505255	0.857827291	0.999996191

LOC104971030	0.980646409	0.193252002	0.821399406	0.870941223	0.571173787	0.883255181	0.999996191
LOC104971057	0.48199696	0.877408476	0.779677612	0.594346155	0.502488749	0.914136957	0.999996191
LOC104971220	0.402473697	0.403332655	0.844827156	0.704439295	0.904819725	0.899419556	0.999996191
LOC104971266	0.982796951	0.704949634	0.478879716	0.610371094	0.22885531	0.803085035	0.999996191
LOC104971296	0.718363837	0.999842388	0.597252664	0.443260238	0.811956788	0.958451387	0.999996191
LOC104971307	0.449706206	0.811454157	0.693514838	0.450397159	0.900983059	0.919033995	0.999996191
LOC104971464	0.405791191	0.987869944	0.527599869	0.417316297	0.377063139	0.743662817	0.999996191
LOC104971501	0.321053012	0.628296914	0.990440485	0.660263264	0.382610491	0.817551397	0.999996191
LOC104971503	0.64906015	0.533481306	0.763823616	0.815316155	0.436360416	0.908659536	0.999996191
LOC104971510	0.261013538	0.566589763	0.327084909	0.622710086	0.361129365	0.528116356	0.999996191
LOC104971613	0.694543183	0.826645592	0.290018506	0.785301302	0.559477361	0.875274981	0.999996191
LOC104971814	0.325727797	0.649581315	0.420542428	0.718910276	0.778357124	0.815276866	0.999996191
LOC104971845	0.935398917	0.292098459	0.892800654	0.532388953	0.891418271	0.932159683	0.999996191
LOC104971852	0.833153349	0.079002823	0.414461821	0.559522904	0.918912028	0.57685857	0.999996191
LOC104971926	0.868863193	0.70348112	0.719360712	0.620699749	0.777772796	0.978976583	0.999996191
LOC104972026	0.620430827	0.989406413	0.981582159	0.540527165	0.738086269	0.984716772	0.999996191
LOC104972045	0.572287189	0.964073453	0.150747475	0.953758321	0.333561566	0.700284951	0.999996191
LOC104972290	0.823405104	0.760566761	0.442906685	0.051322111	0.897285463	0.558788027	0.999996191
LOC104972346	0.498308755	0.617868559	0.883736478	0.84356162	0.488233228	0.928720609	0.999996191
LOC104972390	0.824931306	0.388835115	0.595455976	0.163149138	0.447778387	0.575849342	0.999996191
LOC104972400	0.485818427	0.960613809	0.82362965	0.292371626	0.742182106	0.8933017	0.999996191
LOC104972407	0.824079552	0.431340918	0.388053965	0.232177851	0.691223697	0.665802482	0.999996191
LOC104972409	0.121560285	0.768602998	0.732694991	0.428518364	0.857965582	0.69066027	0.999996191
LOC104972417	0.536009361	0.595848461	0.989620992	0.226972883	0.816704315	0.841835461	0.999996191
LOC104972526	0.277581493	0.680916876	0.704021026	0.941354261	0.799284454	0.916088231	0.999996191
LOC104972542	0.835134879	0.90491304	0.34945584	0.998457575	0.710525513	0.971963076	0.999996191
LOC104972545	0.671752083	0.873699601	0.234482656	0.949644789	0.455220461	0.844249626	0.999996191
LOC104972567	0.4282892	0.248070264	0.771521565	0.889022725	0.782472648	0.837523273	0.999996191
LOC104972578	0.135121202	0.849075561	0.978904765	0.982203763	0.789259957	0.898903378	0.999996191
LOC104972584	0.320270197	0.089440787	0.953062556	0.445271109	0.979172488	0.545255648	0.999996191
LOC104972595	0.558167022	0.460778449	0.533310749	0.683072324	0.766151833	0.872554793	0.999996191
LOC104972622	0.703682986	0.62081221	0.34580858	0.06660992	0.882584898	0.490210656	0.999996191
LOC104972724	0.725981292	0.248252764	0.898622155	0.957614643	0.072104763	0.533361226	0.999996191
LOC104972830	0.212021407	0.959952917	0.519862614	0.788441871	0.843614744	0.869693442	0.999996191
LOC104973050	0.990250169	0.700397136	0.811604104	0.814473487	0.641484139	0.99161662	0.999996191
LOC104973054	0.915867957	0.57232028	0.06875223	0.328674903	0.608812999	0.452484455	0.999996191
LOC104973058	0.885629977	0.621841674	0.97653945	0.902778069	0.054818012	0.701405784	0.999996191
LOC104973073	0.038707027	0.972367337	0.863521941	0.812432939	0.842652342	0.666791107	0.999996191
LOC104973100	0.854093551	0.362083107	0.437710389	0.422980398	0.807230061	0.802615743	0.999996191
LOC104973105	0.669271936	0.165946355	0.258048584	0.503027727	0.530730234	0.463075761	0.999996191
LOC104973145	0.715457137	0.815006174	0.294338364	0.34339957	0.841524027	0.814620999	0.999996191
LOC104973224	0.69866158	0.992514446	0.638506565	0.849688219	0.713398447	0.988831931	0.999996191
LOC104973252	0.727165198	0.584110101	0.500446052	0.755973691	0.408738468	0.859473569	0.999996191
LOC104973285	0.756115609	0.995136762	0.355464242	0.723217506	0.867083268	0.964637246	0.999996191
LOC104973382	0.284714813	0.525959592	0.527785827	0.996146308	0.956012706	0.879296515	0.999996191

LOC104973390	0.243199263	0.588573452	0.29624273	0.776574126	0.962563468	0.734565346	0.999996191
LOC104973431	0.979776483	0.859849265	0.234698676	0.769807652	0.399091532	0.847510798	0.999996191
LOC104973485	0.44748594	0.300114901	0.676025949	0.191870787	0.857943293	0.589171041	0.999996191
LOC104973517	0.743576923	0.436387553	0.55508476	0.340468286	0.854819155	0.823851101	0.999996191
LOC104973551	0.107589974	0.923690777	0.844874481	0.928237828	0.380838934	0.72218985	0.999996191
LOC104973746	0.53840604	0.803743966	0.682908041	0.387815179	0.423848985	0.811110102	0.999996191
LOC104973760	0.67125115	0.499638636	0.811410993	0.816985812	0.814851503	0.969851627	0.999996191
LOC104973767	0.594002657	0.47456713	0.737330574	0.836372841	0.150911079	0.69864106	0.999996191
LOC104973803	0.315645518	0.818259874	0.607006421	0.565436624	0.709168721	0.852817843	0.999996191
LOC104973826	0.555720181	0.991071237	0.28613445	0.954938298	0.175363252	0.699779697	0.999996191
LOC104973848	0.891879245	0.88047026	0.2131486	0.617857664	0.983155126	0.917880338	0.999996191
LOC104974034	0.911720719	0.870001433	0.913933589	0.667083884	0.240852863	0.932791197	0.999996191
LOC104974070	0.78001555	0.651174814	0.273288267	0.137491507	0.796852427	0.592564317	0.999996191
LOC104974113	0.143988244	0.936288916	0.595953708	0.325609179	0.5914903	0.595933432	0.999996191
LOC104974137	0.914803483	0.814496734	0.104365836	0.894397592	0.100301972	0.446610303	0.999996191
LOC104974144	0.258814233	0.707283882	0.937976254	0.880333054	0.852385559	0.942801665	0.999996191
LOC104974260	0.846976993	0.488200136	0.394510577	0.240031049	0.341532123	0.567632783	0.999996191
LOC104974269	0.364218878	0.510934887	0.3476238	0.796071301	0.184324636	0.502535195	0.999996191
LOC104974272	0.513719066	0.711129623	0.458787587	0.196273687	0.246681451	0.473704271	0.999996191
LOC104974330	0.350981362	0.145444464	0.873806863	0.490663111	0.917299341	0.646765639	0.999996191
LOC104974345	0.482467923	0.748361261	0.865484476	0.056444567	0.744609373	0.564150633	0.999996191
LOC104974443	0.792813984	0.145159528	0.407102571	0.941631568	0.92150761	0.780137887	0.999996191
LOC104974459	0.469167405	0.947364432	0.356336352	0.279601784	0.525238553	0.675407746	0.999996191
LOC104974473	0.813669549	0.525244306	0.582078836	0.99369856	0.656774176	0.96227916	0.999996191
LOC104974542	0.860501596	0.531199381	0.372921207	0.605205847	0.65224821	0.863091965	0.999996191
LOC104974666	0.424316923	0.656269489	0.13272814	0.750061393	0.472042533	0.563450932	0.999996191
LOC104974667	0.872842983	0.743972582	0.037993775	0.804127952	0.883197451	0.620185426	0.999996191
LOC104974758	0.837239325	0.572163074	0.799845431	0.739031717	0.924168166	0.987964385	0.999996191
LOC104974837	0.98618418	0.419043006	0.638692215	0.363252707	0.933605315	0.902445403	0.999996191
LOC104974883	0.257768447	0.253138023	0.847061328	0.673938029	0.912167578	0.747512816	0.999996191
LOC104974890	0.393062964	0.964192468	0.662354621	0.727136725	0.972612382	0.968530723	0.999996191
LOC104974891	0.636877694	0.856865117	0.560370866	0.269852182	0.235551388	0.640456412	0.999996191
LOC104974912	0.631546631	0.893780688	0.292411567	0.230060873	0.585771713	0.666734072	0.999996191
LOC104974934	0.5484919	0.496371249	0.642674167	0.879386344	0.485058932	0.878108887	0.999996191
LOC104974937	0.455725982	0.85907925	0.670661818	0.159691268	0.308103692	0.560968812	0.999996191
LOC104975004	0.387336148	0.791371757	0.549385318	0.981499445	0.581021028	0.911137247	0.999996191
LOC104975006	0.607278067	0.678553307	0.180072102	0.551032716	0.554125886	0.670314386	0.999996191
LOC104975007	0.304982122	0.411527892	0.607249723	0.859204892	0.281670392	0.630212052	0.999996191
LOC104975022	0.634494493	0.501037663	0.328230836	0.88402485	0.8569353	0.886084356	0.999996191
LOC104975027	0.856080309	0.748697897	0.942561654	0.190449927	0.545803049	0.852651687	0.999996191
LOC104975044	0.889307387	0.41391671	0.778400204	0.470239447	0.900066956	0.936905449	0.999996191
LOC104975073	0.840628905	0.713025793	0.597050937	0.836934505	0.272111204	0.890220796	0.999996191
LOC104975091	0.453003274	0.359021359	0.13571659	0.491064632	0.975373939	0.522729041	0.999996191
LOC104975099	0.918835113	0.048021234	0.44584079	0.632314735	0.927493688	0.539294719	0.999996191
LOC104975111	0.169668278	0.412506812	0.777212738	0.801254933	0.631248799	0.707760222	0.999996191

LOC104975162	0.723215046	0.627749671	0.805338726	0.725359875	0.356868472	0.909369517	0.999996191
LOC104975196	0.20123452	0.408004509	0.637689915	0.374494524	0.898504289	0.62124421	0.999996191
LOC104975222	0.156279998	0.84323355	0.602006048	0.934885784	0.096665276	0.451450035	0.999996191
LOC104975283	0.744003575	0.802751303	0.962112666	0.819619717	0.97397344	0.998740759	0.999996191
LOC104975286	0.590123527	0.215344831	0.704210341	0.976650206	0.292536745	0.693695667	0.999996191
LOC104975290	0.797221719	0.816347697	0.749625318	0.129570659	0.708824895	0.797253546	0.999996191
LOC104975299	0.865015367	0.819220756	0.483308265	0.201105382	0.64371161	0.795419747	0.999996191
LOC104975324	0.833323235	0.343042789	0.968570728	0.685608712	0.485569624	0.906121226	0.999996191
LOC104975415	0.994798641	0.476193305	0.113109052	0.870519449	0.814687853	0.768010473	0.999996191
LOC104975610	0.945060532	0.829073863	0.820347061	0.890138537	0.264078137	0.956736833	0.999996191
LOC104975612	0.759751749	0.163560495	0.783327465	0.638815356	0.953758746	0.843757978	0.999996191
LOC104975666	0.514337082	0.89962877	0.694527	0.591069397	0.608723775	0.932031868	0.999996191
LOC104975684	0.638448683	0.300312109	0.907295571	0.516251992	0.478810964	0.790067602	0.999996191
LOC104975686	0.481489426	0.189624719	0.765421917	0.550183457	0.84032359	0.738147948	0.999996191
LOC104975749	0.384795615	0.441646608	0.632673848	0.1470023	0.617871942	0.507830482	0.999996191
LOC104975782	0.995228909	0.736187335	0.717468621	0.347105134	0.065058412	0.544736921	0.999996191
LOC104975788	0.641216653	0.632060567	0.825808149	0.660856273	0.727897852	0.961656395	0.999996191
LOC104975811	0.499532386	0.916081097	0.65489229	0.531578063	0.208081969	0.742927304	0.999996191
LOC104975849	0.786649496	0.833585252	0.792008889	0.318826659	0.905968951	0.956159218	0.999996191
LOC104975861	0.986973601	0.583431801	0.741141194	0.712990073	0.090450201	0.707824735	0.999996191
LOC104975890	0.953833911	0.833304441	0.464143713	0.926488959	0.182265841	0.851421393	0.999996191
LOC104975911	0.514557952	0.673027787	0.731731489	0.96004169	0.916554581	0.981392529	0.999996191
LOC104975925	0.375904834	0.73497068	0.609931109	0.351940836	0.714011755	0.787364772	0.999996191
LOC104975960	0.317334073	0.736092173	0.624042246	0.791097658	0.246842785	0.714245811	0.999996191
LOC104975977	0.976882965	0.420765239	0.383814363	0.845244417	0.580923269	0.883305061	0.999996191
LOC104975979	0.730993881	0.744461128	0.150569272	0.54622033	0.6191903	0.709141648	0.999996191
LOC104976062	0.603178398	0.826801314	0.722569281	0.671159702	0.842865181	0.976844723	0.999996191
LOC104976078	0.871611582	0.204206292	0.66714489	0.678002916	0.787028563	0.85402326	0.999996191
LOC104976281	0.310486921	0.252904746	0.745603384	0.625060857	0.964915868	0.754619654	0.999996191
LOC104976293	0.987974658	0.674418287	0.266859511	0.047090182	0.933521798	0.466921914	0.999996191
LOC104976321	0.596309576	0.791093316	0.786581004	0.098928624	0.829742178	0.727069403	0.999996191
LOC104976344	0.31207118	0.866060159	0.192234904	0.259186092	0.8921508	0.547028998	0.999996191
LOC104976448	0.370272702	0.684379928	0.651877212	0.832319372	0.207117769	0.714324109	0.999996191
LOC104976573	0.117271888	0.754395245	0.634460177	0.906703379	0.7767869	0.775140698	0.999996191
LOC104976574	0.865278033	0.535209709	0.089718756	0.586496809	0.375020556	0.495489535	0.999996191
LOC104976575	0.45822947	0.945265444	0.983473562	0.956896438	0.845437307	0.995237619	0.999996191
LOC104976614	0.822195151	0.951703645	0.743502524	0.831426552	0.969272901	0.998887957	0.999996191
LOC104976664	0.10308083	0.964217336	0.786806922	0.615596518	0.661146375	0.735338445	0.999996191
LOC104976804	0.281436547	0.126785785	0.770149995	0.982682168	0.270021122	0.454463079	0.999996191
LOC107131225	0.341935654	0.249166274	0.8178382	0.790812714	0.938934544	0.82168014	0.999996191
LOC107131289	0.277969361	0.192691749	0.674837275	0.277135126	0.967434503	0.506396607	0.999996191
LOC107131293	0.996802139	0.707487183	0.447596132	0.035149078	0.745867865	0.47726649	0.999996191
LOC107131296	0.388464558	0.822137121	0.974094858	0.12737346	0.463803882	0.629506383	0.999996191
LOC107131311	0.666814598	0.493810843	0.319961575	0.66046111	0.561507514	0.773031544	0.999996191
LOC107131323	0.967148402	0.985088108	0.278113479	0.754255256	0.162451162	0.739048843	0.999996191

LOC107131341	0.855429038	0.802073355	0.750844489	0.464160474	0.45742016	0.926096601	0.999996191
LOC107131356	0.879401467	0.81712913	0.781770549	0.518030316	0.722880767	0.978514301	0.999996191
LOC107131357	0.490146704	0.774121197	0.574154257	0.9935495	0.48392164	0.92128206	0.999996191
LOC107131368	0.206089376	0.521348639	0.56596317	0.86943479	0.743608706	0.774144	0.999996191
LOC107131424	0.515631967	0.826425326	0.332989543	0.722133971	0.089083672	0.495279185	0.999996191
LOC107131452	0.968685048	0.646254769	0.888782637	0.787227693	0.928560608	0.997659908	0.999996191
LOC107131455	0.968301356	0.829640619	0.842095686	0.969339045	0.827339452	0.999570252	0.999996191
LOC107131458	0.324042255	0.390436805	0.939411428	0.318440998	0.283251417	0.525358029	0.999996191
LOC107131489	0.365305247	0.960594532	0.797756777	0.113326414	0.338077422	0.525447059	0.999996191
LOC107131494	0.720112365	0.611360872	0.894694937	0.812001096	0.415393832	0.94566515	0.999996191
LOC107131498	0.394160607	0.97106885	0.087019834	0.395394494	0.792951796	0.520407928	0.999996191
LOC107131510	0.56304718	0.239167422	0.452117005	0.820096578	0.153233211	0.46306665	0.999996191
LOC107131525	0.971607241	0.875060216	0.922225885	0.74140204	0.854598067	0.999216611	0.999996191
LOC107131531	0.170101997	0.765641433	0.503922028	0.485685144	0.538206891	0.61605458	0.999996191
LOC107131566	0.672986645	0.92359897	0.172015631	0.558870022	0.504333658	0.725061118	0.999996191
LOC107131573	0.05094652	0.534592762	0.845066465	0.631376147	0.695517205	0.514261672	0.999996191
LOC107131607	0.902454243	0.638000094	0.344770657	0.18641122	0.409628878	0.591920738	0.999996191
LOC107131615	0.387186592	0.877282481	0.907870652	0.086719032	0.264549665	0.449091029	0.999996191
LOC107131619	0.590405201	0.294065441	0.908544264	0.953624612	0.621605326	0.907887126	0.999996191
LOC107131623	0.869516079	0.74216853	0.247263824	0.282511424	0.566410147	0.693433467	0.999996191
LOC107131643	0.433101818	0.663463778	0.196430965	0.753909217	0.766435124	0.73989983	0.999996191
LOC107131649	0.124737653	0.85089854	0.602586028	0.838897911	0.266737013	0.580760579	0.999996191
LOC107131651	0.707537203	0.679728036	0.768298637	0.543402931	0.967520211	0.974131925	0.999996191
LOC107131652	0.768301696	0.783613614	0.797917099	0.463793629	0.675260623	0.956393409	0.999996191
LOC107131660	0.892044743	0.896600992	0.304430157	0.545602635	0.364019462	0.810348487	0.999996191
LOC107131684	0.641368907	0.799436068	0.293287436	0.396294836	0.680537161	0.779708834	0.999996191
LOC107131703	0.45488699	0.760341647	0.426500413	0.07588854	0.766971929	0.483998154	0.999996191
LOC107131710	0.104769808	0.946004113	0.785769035	0.705516486	0.23031545	0.556997215	0.999996191
LOC107131749	0.79595057	0.799159326	0.611746376	0.855005885	0.979231775	0.994115954	0.999996191
LOC107131769	0.959739008	0.864201387	0.929460418	0.030627223	0.794019023	0.633387258	0.999996191
LOC107131792	0.859108006	0.390501722	0.602772916	0.235025687	0.331116546	0.599219163	0.999996191
LOC107131834	0.974988798	0.780482696	0.584680577	0.987436765	0.880043718	0.997046904	0.999996191
LOC107131846	0.455947878	0.888865141	0.596746623	0.865032456	0.417035568	0.899174482	0.999996191
LOC107131848	0.928354158	0.264560392	0.264275322	0.852280434	0.432559073	0.68090433	0.999996191
LOC107131906	0.775803786	0.940451864	0.718084786	0.479143936	0.572952995	0.952672309	0.999996191
LOC107131919	0.83223342	0.881177152	0.68362385	0.355621789	0.866455835	0.958493706	0.999996191
LOC107131939	0.909175437	0.440826626	0.30819482	0.293398465	0.80412473	0.718712551	0.999996191
LOC107131940	0.224150539	0.888461818	0.44325612	0.2106873	0.499474677	0.498519176	0.999996191
LOC107131941	0.623403892	0.943826732	0.968730353	0.662712641	0.81950693	0.992944484	0.999996191
LOC107131974	0.908627159	0.066499297	0.59249182	0.31638961	0.8544647	0.506151364	0.999996191
LOC107131992	0.505125264	0.658475971	0.086365502	0.762762344	0.620491394	0.5708256	0.999996191
LOC107132032	0.130116309	0.355163212	0.797969487	0.341295995	0.95455242	0.54702593	0.999996191
LOC107132070	0.476745435	0.21056976	0.925247761	0.568290629	0.396257049	0.65475855	0.999996191
LOC107132092	0.670510167	0.360975986	0.785704626	0.752162196	0.236249947	0.746501348	0.999996191
LOC107132093	0.738573105	0.393100658	0.907704852	0.516604161	0.847641293	0.931828125	0.999996191

LOC107132098	0.423161259	0.685008513	0.538663983	0.758580577	0.815918347	0.911896603	0.999996191
LOC107132121	0.26645348	0.516982859	0.776200291	0.878143965	0.781660937	0.875731659	0.999996191
LOC107132175	0.917379764	0.858678519	0.308469593	0.252448918	0.627894136	0.770452713	0.999996191
LOC107132189	0.724583518	0.758785701	0.634961618	0.339869353	0.256923788	0.727227144	0.999996191
LOC107132192	0.183938506	0.604941626	0.792461834	0.923007756	0.894334842	0.874554049	0.999996191
LOC107132196	0.742919072	0.468218485	0.970356835	0.306089491	0.531686097	0.831507885	0.999996191
LOC107132228	0.9247063	0.112177145	0.226452049	0.304658083	0.977343932	0.447069693	0.999996191
LOC107132237	0.151427926	0.972683303	0.811529668	0.469083638	0.831107089	0.804028589	0.999996191
LOC107132243	0.962414588	0.6421333	0.775673644	0.451906411	0.978313616	0.978895174	0.999996191
LOC107132251	0.864292248	0.854541094	0.722674839	0.941209961	0.594504064	0.992032203	0.999996191
LOC107132255	0.679046399	0.959649398	0.934245048	0.26071462	0.959155281	0.957342012	0.999996191
LOC107132262	0.710229629	0.98910511	0.43524791	0.552616319	0.868856666	0.954389985	0.999996191
LOC107132270	0.932626612	0.923517837	0.123972102	0.271687898	0.676118256	0.64221637	0.999996191
LOC107132278	0.513489996	0.764156364	0.45080153	0.573038981	0.241846615	0.685579909	0.999996191
LOC107132288	0.532917877	0.31783974	0.071734101	0.719867142	0.888129626	0.46580502	0.999996191
LOC107132296	0.363266716	0.773720083	0.686512001	0.727220209	0.397367904	0.833926111	0.999996191
LOC107132300	0.586554069	0.491615208	0.508544584	0.386657385	0.247454542	0.576921124	0.999996191
LOC107132302	0.722504304	0.944968542	0.50446406	0.211097356	0.478884241	0.752025513	0.999996191
LOC107132308	0.766001747	0.819743806	0.208710928	0.924519399	0.927198468	0.928986865	0.999996191
LOC107132317	0.473616078	0.749480617	0.886252743	0.131616942	0.979751489	0.779756791	0.999996191
LOC107132327	0.937231605	0.728772801	0.704943421	0.931280151	0.788362292	0.995692027	0.999996191
LOC107132374	0.871262772	0.840212806	0.298136724	0.866274181	0.750941367	0.951565518	0.999996191
LOC107132386	0.694804277	0.884442519	0.696197303	0.644984346	0.820852329	0.982121892	0.999996191
LOC107132395	0.866370301	0.498573024	0.597196433	0.397668541	0.890873477	0.905051331	0.999996191
LOC107132410	0.893861142	0.612457641	0.025884829	0.803315416	0.725983986	0.477021039	0.999996191
LOC107132431	0.906918523	0.710645886	0.273087027	0.13398976	0.590979643	0.575620114	0.999996191
LOC107132450	0.609853465	0.614856789	0.441376867	0.473454907	0.936081042	0.875648286	0.999996191
LOC107132465	0.875021909	0.925170629	0.06683433	0.390359568	0.587461331	0.553206875	0.999996191
LOC107132486	0.644999535	0.884689548	0.629675458	0.902124913	0.562624184	0.97027568	0.999996191
LOC107132487	0.94861433	0.229699089	0.402811033	0.575214874	0.392431486	0.644182772	0.999996191
LOC107132490	0.714198664	0.514602673	0.906825987	0.552207757	0.67521393	0.939321345	0.999996191
LOC107132515	0.944543862	0.236151343	0.633715914	0.337906122	0.428486251	0.650516468	0.999996191
LOC107132532	0.483605243	0.599005723	0.506669137	0.55161761	0.499864351	0.779331547	0.999996191
LOC107132546	0.186470785	0.990291766	0.838689884	0.446108126	0.33387896	0.673801203	0.999996191
LOC107132556	0.966935433	0.705453091	0.856787723	0.898179159	0.426897414	0.981625683	0.999996191
LOC107132577	0.240670546	0.996414637	0.617298413	0.868176301	0.552911058	0.871093172	0.999996191
LOC107132589	0.935578529	0.132922143	0.279767182	0.789663691	0.718883072	0.643567172	0.999996191
LOC107132606	0.792782805	0.275753228	0.478869489	0.282212048	0.912907809	0.703950974	0.999996191
LOC107132610	0.959317333	0.151523962	0.446564534	0.666113179	0.700223669	0.725941933	0.999996191
LOC107132617	0.486510462	0.276620918	0.505511884	0.111066485	0.935735082	0.448984624	0.999996191
LOC107132664	0.753432474	0.777398553	0.876096246	0.894945081	0.778846249	0.995890459	0.999996191
LOC107132672	0.996278655	0.762774227	0.726260483	0.843207573	0.755279688	0.995592562	0.999996191
LOC107132713	0.886668685	0.633411996	0.496544343	0.567377998	0.966697462	0.957715356	0.999996191
LOC107132735	0.870751901	0.313552073	0.534461886	0.64089915	0.755636655	0.870292852	0.999996191
LOC107132748	0.27335121	0.958353468	0.46614535	0.501047583	0.508527928	0.731081216	0.999996191

LOC107132757	0.310277732	0.962310864	0.323110272	0.991058731	0.567570446	0.829527213	0.999996191
LOC107132767	0.802828875	0.39877825	0.402020105	0.192034055	0.398564159	0.509453594	0.999996191
LOC107132783	0.200559821	0.50175571	0.631197369	0.879050069	0.720303437	0.778218977	0.999996191
LOC107132784	0.352153741	0.72209268	0.468416364	0.397777531	0.790094378	0.765292126	0.999996191
LOC107132793	0.730983892	0.586672853	0.927289051	0.685171001	0.963930696	0.988091575	0.999996191
LOC107132798	0.463880192	0.607940992	0.74487535	0.221690128	0.498397601	0.674993165	0.999996191
LOC107132799	0.981169338	0.845258979	0.235650309	0.917297171	0.565026759	0.917444758	0.999996191
LOC107132849	0.70276865	0.683593762	0.095257469	0.604094214	0.872338432	0.682408513	0.999996191
LOC107132870	0.212376035	0.720861331	0.191743227	0.861444129	0.477183504	0.547868897	0.999996191
LOC107132877	0.66134877	0.407908643	0.775133109	0.814757271	0.864612493	0.954669985	0.999996191
LOC107132883	0.266576634	0.514993	0.670459225	0.175867611	0.77441132	0.555182588	0.999996191
LOC107132924	0.075613858	0.910687126	0.991070358	0.663067123	0.329439889	0.58868363	0.999996191
LOC107132942	0.697490829	0.86670648	0.874415817	0.860721128	0.194656486	0.901094584	0.999996191
LOC107132944	0.719056971	0.513484368	0.930920859	0.597875443	0.498089547	0.918652953	0.999996191
LOC107132958	0.897582974	0.647228619	0.674282311	0.053633619	0.501164686	0.521958904	0.999996191
LOC107132967	0.468109598	0.563622636	0.586467399	0.097819401	0.78164605	0.544092851	0.999996191
LOC107132971	0.990253605	0.66013264	0.368100078	0.268650636	0.9683469	0.852161513	0.999996191
LOC107132987	0.903018348	0.647377185	0.597276074	0.445846244	0.994966206	0.958702976	0.999996191
LOC107133032	0.626491301	0.904421495	0.142571932	0.256524977	0.441674433	0.49577431	0.999996191
LOC107133048	0.299616839	0.921885895	0.532671637	0.781109712	0.408290019	0.80521069	0.999996191
LOC107133071	0.416531534	0.425563292	0.214830305	0.504646925	0.969944246	0.632264703	0.999996191
LOC107133075	0.57751282	0.465855103	0.329944339	0.865352849	0.767323374	0.842784552	0.999996191
LOC107133095	0.121929322	0.451154129	0.969906995	0.282875939	0.60503111	0.4953418	0.999996191
LOC107133150	0.581772369	0.934610062	0.119970789	0.495904148	0.848173505	0.707231536	0.999996191
LOC107133166	0.831059555	0.759984678	0.525801736	0.452287779	0.275503632	0.783290751	0.999996191
LOC107133180	0.369190114	0.698725773	0.678718954	0.747832096	0.873032586	0.930829819	0.999996191
LOC107133190	0.64285203	0.700028185	0.150855585	0.747751503	0.166460915	0.481077185	0.999996191
LOC107133209	0.358661682	0.363709722	0.814344301	0.122590098	0.894847412	0.541207833	0.999996191
LOC107133226	0.362024871	0.615941293	0.443924366	0.502599007	0.691681262	0.749863385	0.999996191
LOC107133289	0.348801096	0.297250583	0.932606151	0.99529439	0.806603949	0.883593168	0.999996191
LOC107133294	0.61808545	0.803555708	0.914642298	0.793549691	0.584517108	0.978598597	0.999996191
LOC107133302	0.867972006	0.790322198	0.354639722	0.217524263	0.291023704	0.595009867	0.999996191
LOC107133343	0.658225305	0.067147701	0.497569203	0.596064245	0.782915763	0.517134891	0.999996191
LOC107133459	0.508095535	0.932472684	0.172073449	0.556051694	0.657172394	0.722886367	0.999996191
LOC112441455	0.235584944	0.109874865	0.797286099	0.771869886	0.487006685	0.465562786	0.999996191
LOC112441456	0.94326881	0.307269425	0.86046032	0.808392076	0.419719766	0.895202541	0.999996191
LOC112441469	0.133577988	0.827203658	0.890100702	0.723705404	0.889302674	0.85386952	0.999996191
LOC112441472	0.670570932	0.848574121	0.165692698	0.624688678	0.377176785	0.66648533	0.999996191
LOC112441473	0.563639159	0.477964007	0.748321039	0.333523236	0.979912989	0.859944578	0.999996191
LOC112441481	0.872564076	0.346592299	0.443241169	0.149655857	0.931725105	0.632802631	0.999996191
LOC112441491	0.346544859	0.201344287	0.858913893	0.402446562	0.782266123	0.634634854	0.999996191
LOC112441493	0.498164025	0.212770655	0.694698628	0.319890351	0.869265563	0.650603616	0.999996191
LOC112441500	0.810477505	0.927400714	0.87648547	0.691805724	0.189181905	0.897649237	0.999996191
LOC112441506	0.750839265	0.934225744	0.657814676	0.386239813	0.275055731	0.812650164	0.999996191
LOC112441511	0.721083327	0.934479906	0.401717977	0.823760893	0.869907638	0.974045108	0.999996191

LOC112441525	0.586668616	0.597230677	0.303925146	0.318929067	0.611746808	0.653447499	0.999996191
LOC112441530	0.213020121	0.559122037	0.484698822	0.996264964	0.893035035	0.820469096	0.999996191
LOC112441543	0.948783148	0.795691804	0.783977272	0.395542357	0.286212679	0.862459436	0.999996191
LOC112441554	0.728071021	0.847350615	0.749909584	0.054384253	0.658547691	0.60926946	0.999996191
LOC112441566	0.927279547	0.948901057	0.229759297	0.076035636	0.675052699	0.519211145	0.999996191
LOC112441568	0.984280391	0.163866485	0.727053027	0.85635311	0.771852886	0.883387063	0.999996191
LOC112441603	0.391176521	0.882833799	0.41624896	0.871773644	0.762630209	0.910551741	0.999996191
LOC112441607	0.551091752	0.904226058	0.18996877	0.251364922	0.889374844	0.657040215	0.999996191
LOC112441611	0.142950804	0.572323477	0.701194853	0.833944212	0.551465238	0.699725309	0.999996191
LOC112441619	0.948071149	0.838340124	0.939590496	0.556247848	0.445464502	0.971195206	0.999996191
LOC112441629	0.808578274	0.907475796	0.99157524	0.6934326	0.366851668	0.971209057	0.999996191
LOC112441638	0.476368276	0.932819488	0.551314144	0.584089096	0.666598547	0.910317639	0.999996191
LOC112441639	0.368042718	0.801618127	0.935952869	0.672111443	0.20120813	0.764844421	0.999996191
LOC112441644	0.723349498	0.315118888	0.249031383	0.731107552	0.988157296	0.78168871	0.999996191
LOC112441645	0.791407925	0.703390785	0.369803009	0.122308633	0.77041105	0.640047888	0.999996191
LOC112441654	0.545080837	0.321857634	0.200429649	0.910806582	0.819453417	0.698715255	0.999996191
LOC112441659	0.574741137	0.073629699	0.91616357	0.952519251	0.733232337	0.704708873	0.999996191
LOC112441718	0.09589344	0.966679782	0.330157333	0.468456136	0.519330385	0.458190496	0.999996191
LOC112441770	0.487335685	0.807379796	0.733749462	0.86820468	0.316303323	0.886489637	0.999996191
LOC112441778	0.99558441	0.768999391	0.314824209	0.143156056	0.962643	0.74330346	0.999996191
LOC112441810	0.929360098	0.898209272	0.991005458	0.682138728	0.718977462	0.99763252	0.999996191
LOC112441827	0.69890892	0.978372066	0.531464464	0.739218092	0.908359637	0.985328349	0.999996191
LOC112441834	0.295296768	0.629841729	0.773152769	0.559555711	0.594222502	0.808420595	0.999996191
LOC112441843	0.935615987	0.522762352	0.183519382	0.817240459	0.288427435	0.656994295	0.999996191
LOC112441846	0.60338414	0.725038868	0.939364561	0.688897304	0.99334441	0.990321744	0.999996191
LOC112441859	0.845711187	0.378408578	0.483688913	0.861669691	0.822047979	0.926362002	0.999996191
LOC112441868	0.507268361	0.893702249	0.378514993	0.315601187	0.170316826	0.497206329	0.999996191
LOC112441879	0.823755487	0.935141633	0.208257682	0.870400051	0.817208405	0.93064699	0.999996191
LOC112441880	0.396592492	0.229534291	0.403560832	0.514804159	0.728631299	0.573429214	0.999996191
LOC112441885	0.820608362	0.831516991	0.691359534	0.102159773	0.182264333	0.488186097	0.999996191
LOC112441886	0.237446885	0.986871969	0.298985167	0.538387355	0.31988282	0.547856949	0.999996191
LOC112441887	0.894810449	0.478085015	0.43003565	0.109856978	0.683844728	0.573997241	0.999996191
LOC112441888	0.520837475	0.930577017	0.865339514	0.675315589	0.865422622	0.985508012	0.999996191
LOC112442038	0.790048931	0.565996498	0.395908878	0.516860397	0.890744965	0.890230301	0.999996191
LOC112442039	0.782377	0.919466768	0.221077384	0.567165366	0.944427364	0.896078739	0.999996191
LOC112442040	0.86158088	0.807162356	0.441685628	0.732453356	0.346184836	0.884052749	0.999996191
LOC112442048	0.679512644	0.133574208	0.889643583	0.553994862	0.439349245	0.642612809	0.999996191
LOC112442071	0.493240564	0.323034116	0.703577875	0.368001624	0.732212655	0.725504516	0.999996191
LOC112442079	0.708590933	0.406225697	0.210374435	0.32596136	0.495675665	0.508179873	0.999996191
LOC112442081	0.76630836	0.816964532	0.654272128	0.111549806	0.634556093	0.717743189	0.999996191
LOC112442082	0.85754874	0.73840572	0.329958816	0.622402606	0.197411882	0.694475251	0.999996191
LOC112442083	0.851091473	0.900099331	0.320599219	0.585843011	0.720881607	0.920170087	0.999996191
LOC112442091	0.548984005	0.347296338	0.892694501	0.996545594	0.425253326	0.873246703	0.999996191
LOC112442191	0.669829745	0.276451486	0.229726768	0.697693672	0.680062639	0.647806967	0.999996191
LOC112442208	0.454292069	0.711699934	0.879545253	0.722045187	0.754278048	0.95869643	0.999996191

LOC112442214	0.142127762	0.376069248	0.802871821	0.92984407	0.543132627	0.661675392	0.999996191
LOC112442221	0.688184929	0.606999892	0.619205317	0.42268863	0.759005049	0.89262831	0.999996191
LOC112442223	0.700982035	0.947715354	0.502693199	0.610231719	0.909939031	0.97132644	0.999996191
LOC112442227	0.340153275	0.470917612	0.973860872	0.26405351	0.879029637	0.759219827	0.999996191
LOC112442244	0.790579389	0.651311794	0.576167976	0.275385605	0.798550214	0.858463822	0.999996191
LOC112442245	0.797033138	0.698521353	0.367866158	0.804542768	0.53829986	0.901287938	0.999996191
LOC112442246	0.694345548	0.343318141	0.578650433	0.466514428	0.666357371	0.789577087	0.999996191
LOC112442248	0.626599115	0.797365008	0.295600427	0.715353462	0.486924801	0.820733951	0.999996191
LOC112442254	0.739777441	0.410579198	0.533706186	0.568300978	0.935404482	0.89757797	0.999996191
LOC112442257	0.996270708	0.830708615	0.385127779	0.740251108	0.77624161	0.97054704	0.999996191
LOC112442263	0.942057791	0.668670693	0.193907667	0.4856281	0.594949796	0.754513389	0.999996191
LOC112442264	0.524913596	0.246174612	0.337721739	0.539875357	0.652183712	0.594570737	0.999996191
LOC112442265	0.086486466	0.777468645	0.400277966	0.961144464	0.696476879	0.625627758	0.999996191
LOC112442278	0.260844929	0.508945256	0.548264246	0.944254596	0.981171075	0.863411963	0.999996191
LOC112442280	0.087952628	0.62263281	0.570467399	0.654823674	0.922301304	0.634633822	0.999996191
LOC112442292	0.412712291	0.155516928	0.840280072	0.316988012	0.49376384	0.480889344	0.999996191
LOC112442296	0.507926312	0.493770431	0.674967501	0.306665918	0.590385167	0.728241912	0.999996191
LOC112442307	0.801713579	0.280707386	0.219289528	0.592654871	0.403058206	0.543409131	0.999996191
LOC112442312	0.609690767	0.429958221	0.728720978	0.656668158	0.733427745	0.905886666	0.999996191
LOC112442323	0.347013201	0.520688566	0.831619411	0.314286089	0.999542552	0.806231569	0.999996191
LOC112442347	0.496640277	0.721694777	0.198085234	0.246540151	0.632802885	0.531548126	0.999996191
LOC112442349	0.436411862	0.986774674	0.123531336	0.176572179	0.856202694	0.472077176	0.999996191
LOC112442352	0.611907537	0.875693891	0.609898174	0.147073342	0.170475078	0.475464288	0.999996191
LOC112442365	0.836347765	0.290598651	0.680561316	0.45859571	0.852937415	0.857199196	0.999996191
LOC112442367	0.72282233	0.628713098	0.889217555	0.4524802	0.828674248	0.956964839	0.999996191
LOC112442382	0.206069607	0.300092749	0.385014226	0.947814057	0.682387615	0.594995147	0.999996191
LOC112442383	0.417152118	0.606830076	0.824307224	0.713743802	0.439789607	0.859057842	0.999996191
LOC112442384	0.366756577	0.51052179	0.894293586	0.661599563	0.471296698	0.823186783	0.999996191
LOC112442386	0.50417821	0.448381879	0.306594152	0.516152384	0.385720467	0.57369461	0.999996191
LOC112442392	0.80984584	0.492554635	0.562336699	0.627306685	0.407858547	0.838553302	0.999996191
LOC112442408	0.204693615	0.52120066	0.502592986	0.906520794	0.667171039	0.738837839	0.999996191
LOC112442414	0.795043658	0.533936401	0.507014962	0.218181289	0.389315217	0.628476198	0.999996191
LOC112442538	0.778795469	0.507470659	0.572337659	0.783047051	0.959859758	0.965591602	0.999996191
LOC112442544	0.549690724	0.554363758	0.334039508	0.654047103	0.796972301	0.825844075	0.999996191
LOC112442547	0.321332085	0.895893252	0.877253953	0.244353268	0.478263397	0.721112894	0.999996191
LOC112442559	0.543364224	0.382202489	0.673960777	0.897375183	0.752547313	0.909210319	0.999996191
LOC112442585	0.374748883	0.625957907	0.914067753	0.738222981	0.799540876	0.941094874	0.999996191
LOC112442593	0.576024235	0.165816844	0.757785665	0.133007994	0.796544313	0.463475617	0.999996191
LOC112442597	0.700254937	0.477924808	0.850892828	0.932343609	0.520710138	0.949248509	0.999996191
LOC112442602	0.647094812	0.971549238	0.846176281	0.738196245	0.837202383	0.99430901	0.999996191
LOC112442611	0.37086008	0.890074395	0.551914195	0.874184441	0.403191988	0.856055775	0.999996191
LOC112442613	0.908416392	0.202176122	0.959233882	0.392705395	0.611475818	0.787194913	0.999996191
LOC112442619	0.717266394	0.918876686	0.965078836	0.726874621	0.71348397	0.994378661	0.999996191
LOC112442623	0.869048674	0.311990813	0.199184109	0.222314198	0.859370277	0.518140025	0.999996191
LOC112442625	0.476730062	0.73728837	0.511342231	0.795297919	0.613114671	0.899750274	0.999996191

LOC112442630	0.597813681	0.203453107	0.431000104	0.510871761	0.62164165	0.610192071	0.999996191
LOC112442633	0.87826081	0.386274202	0.937774459	0.37409664	0.32864511	0.773223016	0.999996191
LOC112442636	0.336732063	0.898443895	0.205650368	0.13802605	0.963384038	0.47721436	0.999996191
LOC112442649	0.560093708	0.768127769	0.118961485	0.496609277	0.74068109	0.634201009	0.999996191
LOC112442656	0.727581189	0.564483735	0.988050466	0.618432888	0.88133405	0.981007788	0.999996191
LOC112442657	0.344544074	0.943196693	0.612803529	0.06925824	0.928116263	0.559206204	0.999996191
LOC112442676	0.818915987	0.310284583	0.54264184	0.896884969	0.871228208	0.924438184	0.999996191
LOC112442683	0.295203856	0.953283734	0.481874449	0.262990143	0.896304333	0.736139656	0.999996191
LOC112442687	0.534866758	0.766662721	0.480346417	0.579180699	0.735565991	0.89410446	0.999996191
LOC112442693	0.879777351	0.725184389	0.363818316	0.931772244	0.251487313	0.829900763	0.999996191
LOC112442702	0.877282106	0.932209022	0.972627697	0.840119956	0.587900174	0.997251764	0.999996191
LOC112442704	0.963659794	0.296897825	0.607645018	0.494540079	0.93074938	0.887753227	0.999996191
LOC112442708	0.936873807	0.67864031	0.592325749	0.78641807	0.902864914	0.988707902	0.999996191
LOC112442715	0.97065223	0.856639015	0.840079901	0.210083952	0.902336367	0.945358677	0.999996191
LOC112442721	0.972116467	0.413374646	0.981770446	0.654709106	0.715366545	0.971104184	0.999996191
LOC112442754	0.944681152	0.326605536	0.258591323	0.219952098	0.963548315	0.613232317	0.999996191
LOC112442787	0.310037707	0.801298019	0.076826161	0.88841711	0.436542741	0.457143427	0.999996191
LOC112442802	0.850385474	0.991888031	0.69866819	0.698985642	0.831773958	0.995129327	0.999996191
LOC112442805	0.724733515	0.21569583	0.664112187	0.775445395	0.85319035	0.866130253	0.999996191
LOC112442843	0.794898975	0.965122346	0.582090697	0.707802832	0.070225585	0.666326919	0.999996191
LOC112442849	0.857263544	0.708358949	0.405602507	0.915394955	0.14094446	0.735040853	0.999996191
LOC112442865	0.360928912	0.945705648	0.16805663	0.828664071	0.567742644	0.704069521	0.999996191
LOC112442866	0.348316409	0.901123533	0.082270688	0.870082198	0.464312169	0.520214792	0.999996191
LOC112442867	0.240436111	0.868734953	0.985834545	0.367912084	0.985162928	0.878109288	0.999996191
LOC112442949	0.130135386	0.542025878	0.867550533	0.648069223	0.604088799	0.681131303	0.999996191
LOC112442952	0.320962382	0.611270544	0.999067125	0.215443591	0.269001391	0.536346186	0.999996191
LOC112442967	0.790609923	0.537038133	0.641810589	0.144859267	0.373127932	0.586343444	0.999996191
LOC112442987	0.909694017	0.916908774	0.135898346	0.905042834	0.629522452	0.856927358	0.999996191
LOC112442997	0.886391864	0.973397595	0.997806482	0.012497072	0.683363932	0.455935175	0.999996191
LOC112443001	0.241128045	0.770031619	0.443662722	0.563621481	0.695229904	0.737970896	0.999996191
LOC112443004	0.187187066	0.405496393	0.888115822	0.364017341	0.987624596	0.683364728	0.999996191
LOC112443007	0.922553878	0.771913318	0.961272654	0.127138077	0.869925674	0.880122075	0.999996191
LOC112443011	0.159923044	0.328992392	0.74177723	0.497509277	0.814646347	0.600215135	0.999996191
LOC112443015	0.585702993	0.30378458	0.202725238	0.379874041	0.749330195	0.517219975	0.999996191
LOC112443139	0.926089663	0.739357313	0.447020348	0.044067533	0.539727837	0.454172281	0.999996191
LOC112443140	0.47729987	0.656966305	0.806067085	0.054260023	0.936339599	0.559816334	0.999996191
LOC112443141	0.87102117	0.716582494	0.22112788	0.152628904	0.922440687	0.640392558	0.999996191
LOC112443142	0.909243051	0.141230799	0.509224088	0.805695737	0.751117546	0.775319555	0.999996191
LOC112443143	0.428754205	0.813489642	0.199285613	0.371949193	0.310118069	0.4715164	0.999996191
LOC112443144	0.249577233	0.529472983	0.97407874	0.633320398	0.131224406	0.524953989	0.999996191
LOC112443151	0.575951307	0.526468273	0.422537222	0.489608432	0.857361604	0.828060996	0.999996191
LOC112443159	0.292957491	0.59617258	0.878102702	0.297085939	0.168497823	0.463680527	0.999996191
LOC112443163	0.703930309	0.980612867	0.668003954	0.594599908	0.622123952	0.965820353	0.999996191
LOC112443170	0.446411031	0.443324795	0.751176016	0.847881412	0.471586677	0.844115847	0.999996191
LOC112443176	0.88374095	0.6265071	0.059852755	0.960228687	0.241395369	0.4637801	0.999996191

LOC112443177	0.97814588	0.502601392	0.887719174	0.655028759	0.987681474	0.990442577	0.999996191
LOC112443178	0.925303359	0.702234695	0.623887492	0.58025357	0.603654278	0.951578073	0.999996191
LOC112443193	0.923014937	0.705215565	0.601657189	0.040948536	0.911778745	0.584925511	0.999996191
LOC112443199	0.539614971	0.555041741	0.977003972	0.725168237	0.678791245	0.952795481	0.999996191
LOC112443213	0.35766891	0.263256755	0.250806119	0.875420651	0.420541397	0.486295054	0.999996191
LOC112443214	0.218485013	0.921205954	0.540746317	0.216163108	0.368474728	0.485761022	0.999996191
LOC112443215	0.800736959	0.791373602	0.797444026	0.737891408	0.369645449	0.948981097	0.999996191
LOC112443223	0.679892323	0.131641096	0.8851607	0.933575103	0.439901758	0.739447429	0.999996191
LOC112443225	0.677586299	0.549041341	0.765295706	0.534706566	0.554221106	0.894818795	0.999996191
LOC112443240	0.972144873	0.307997698	0.522009173	0.884943158	0.87657375	0.93688211	0.999996191
LOC112443243	0.24578021	0.483750211	0.517418474	0.887480908	0.971459483	0.825779885	0.999996191
LOC112443244	0.420387045	0.9847129	0.185777666	0.241701864	0.500381599	0.498753545	0.999996191
LOC112443328	0.644954101	0.089893107	0.487980699	0.416378541	0.62185391	0.455282863	0.999996191
LOC112443339	0.442615297	0.745553037	0.43004072	0.332305763	0.163966296	0.464968773	0.999996191
LOC112443416	0.804731065	0.433470037	0.463563607	0.702209508	0.270290474	0.728505226	0.999996191
LOC112443419	0.613408519	0.722023454	0.847132673	0.643075178	0.453980268	0.926252473	0.999996191
LOC112443422	0.623984818	0.951517143	0.822118826	0.727407624	0.137133747	0.811508417	0.999996191
LOC112443425	0.482602817	0.912696299	0.202099973	0.763461344	0.560334874	0.768398776	0.999996191
LOC112443431	0.976559268	0.134251425	0.630879958	0.935138236	0.374705772	0.717667301	0.999996191
LOC112443437	0.845244833	0.336380288	0.41098286	0.919597048	0.693455682	0.877885064	0.999996191
LOC112443452	0.479081093	0.470549621	0.887243628	0.687865656	0.793777702	0.925927464	0.999996191
LOC112443463	0.331797141	0.898737646	0.543538311	0.126459137	0.76296761	0.597995282	0.999996191
LOC112443469	0.703390015	0.939696197	0.506114467	0.224705549	0.391480793	0.720561364	0.999996191
LOC112443475	0.234623384	0.700739737	0.270318954	0.805280577	0.843646648	0.725420187	0.999996191
LOC112443476	0.780171424	0.99847392	0.188528474	0.97673752	0.536991201	0.882522401	0.999996191
LOC112443499	0.658608925	0.458521809	0.313172235	0.28769139	0.925476649	0.690752099	0.999996191
LOC112443502	0.304287726	0.364415349	0.538386665	0.241393959	0.914049875	0.564719901	0.999996191
LOC112443503	0.86134201	0.636391562	0.772989351	0.519102226	0.073264579	0.603841444	0.999996191
LOC112443509	0.277087442	0.882054235	0.457306027	0.456216393	0.430274971	0.664062388	0.999996191
LOC112443512	0.708498672	0.94283902	0.837548383	0.289193206	0.613213005	0.915023379	0.999996191
LOC112443728	0.289641077	0.716455351	0.709919823	0.280307748	0.364479018	0.590544731	0.999996191
LOC112443767	0.155727477	0.465439876	0.502014596	0.77236526	0.554917647	0.597459104	0.999996191
LOC112443783	0.46496321	0.870003256	0.971669039	0.796535492	0.757239622	0.984135983	0.999996191
LOC112443816	0.557577177	0.250069529	0.340350574	0.74735796	0.440665851	0.5978779	0.999996191
LOC112443849	0.667650008	0.62623938	0.553746287	0.888662166	0.546546126	0.929090662	0.999996191
LOC112443853	0.308506615	0.541316762	0.779487846	0.973006553	0.390389093	0.814109797	0.999996191
LOC112443859	0.565657565	0.869725791	0.806865174	0.312113058	0.464745024	0.839074863	0.999996191
LOC112443864	0.990850719	0.769819301	0.967924713	0.970830466	0.459964467	0.994367259	0.999996191
LOC112443877	0.96179428	0.909411427	0.641590443	0.97085681	0.344569587	0.972085582	0.999996191
LOC112444147	0.863309121	0.260056875	0.688454163	0.685083905	0.435224822	0.802119955	0.999996191
LOC112444171	0.465062404	0.359021803	0.932827905	0.586005639	0.588793546	0.827933906	0.999996191
LOC112444190	0.717828532	0.875067441	0.983625553	0.563276471	0.901279588	0.993261173	0.999996191
LOC112444194	0.835659621	0.96641756	0.932985427	0.404385405	0.548143598	0.964335951	0.999996191
LOC112444198	0.5902053	0.924990373	0.967380403	0.564239768	0.852251113	0.986876844	0.999996191
LOC112444207	0.235439008	0.912333009	0.863370344	0.273194681	0.739205482	0.765371529	0.999996191

LOC112444215	0.477632068	0.921821781	0.520500668	0.586462549	0.630902717	0.895473327	0.999996191
LOC112444276	0.103638281	0.464380121	0.419970243	0.521978277	0.836156003	0.488973941	0.999996191
LOC112444278	0.999293693	0.085085619	0.818627286	0.856275254	0.600005004	0.756938622	0.999996191
LOC112444279	0.287163269	0.932945761	0.249934847	0.425033727	0.347747674	0.510324206	0.999996191
LOC112444281	0.934401294	0.867358369	0.882530448	0.661498772	0.790447581	0.996586614	0.999996191
LOC112444285	0.690084518	0.575954471	0.873551902	0.857141637	0.893293406	0.988510134	0.999996191
LOC112444287	0.684052848	0.553498671	0.628886088	0.116333241	0.319824544	0.489754804	0.999996191
LOC112444288	0.995477942	0.461339972	0.230705819	0.586357278	0.256870521	0.601941506	0.999996191
LOC112444289	0.817000988	0.572641755	0.982704339	0.56413219	0.528506716	0.948492372	0.999996191
LOC112444300	0.733996054	0.807181881	0.614746274	0.078052149	0.697939664	0.64446124	0.999996191
LOC112444326	0.590233076	0.461144083	0.866217639	0.67343817	0.051780344	0.476075765	0.999996191
LOC112444328	0.342568451	0.575291837	0.562061484	0.595232637	0.656278958	0.791167106	0.999996191
LOC112444333	0.816878201	0.822046116	0.405544603	0.182304049	0.93785878	0.803885039	0.999996191
LOC112444339	0.924501646	0.681267266	0.952537139	0.67853563	0.953510037	0.997098303	0.999996191
LOC112444340	0.656242008	0.188724789	0.548738996	0.256991359	0.849623243	0.58778637	0.999996191
LOC112444350	0.821590672	0.221111302	0.869569039	0.214059417	0.719383169	0.68408535	0.999996191
LOC112444351	0.09519961	0.371228253	0.957484015	0.229548819	0.901546754	0.447283995	0.999996191
LOC112444463	0.377525366	0.665271553	0.875514979	0.94505287	0.748007347	0.958980755	0.999996191
LOC112444464	0.718132473	0.991779143	0.289605629	0.828162113	0.872559802	0.955636914	0.999996191
LOC112444473	0.70653999	0.991501193	0.572623203	0.200812804	0.56816556	0.800928533	0.999996191
LOC112444474	0.653485488	0.864305274	0.350240591	0.590887729	0.704236518	0.891555868	0.999996191
LOC112444484	0.942688128	0.56065859	0.37568685	0.940624658	0.757650423	0.951280671	0.999996191
LOC112444502	0.787328528	0.676878473	0.922786953	0.22388172	0.325493485	0.75732932	0.999996191
LOC112444520	0.432137794	0.671718699	0.33485007	0.830034921	0.52786124	0.788368017	0.999996191
LOC112444521	0.787058341	0.476066372	0.719921361	0.275560383	0.567456945	0.786676311	0.999996191
LOC112444531	0.734035684	0.398783113	0.80292115	0.90856682	0.500831139	0.923619031	0.999996191
LOC112444588	0.375233028	0.888739962	0.098178327	0.907468041	0.702428469	0.654326195	0.999996191
LOC112444593	0.839941967	0.680676043	0.560856906	0.392874141	0.078771153	0.510825337	0.999996191
LOC112444613	0.766810078	0.661175775	0.674225645	0.78316359	0.830619266	0.981261712	0.999996191
LOC112444616	0.423849076	0.447058693	0.395523927	0.181257179	0.584675806	0.469811815	0.999996191
LOC112444626	0.603823931	0.540394877	0.88537301	0.268006439	0.546653006	0.787283983	0.999996191
LOC112444635	0.436574892	0.699498391	0.943038669	0.271914668	0.921870702	0.873369039	0.999996191
LOC112444653	0.710289711	0.145688469	0.158239476	0.91605836	0.701571871	0.521863007	0.999996191
LOC112444681	0.114732698	0.588676016	0.43181909	0.915859618	0.556662751	0.588181376	0.999996191
LOC112444726	0.954779499	0.928218379	0.700365952	0.030659825	0.68689068	0.563238793	0.999996191
LOC112444752	0.826738303	0.253661496	0.476208	0.78092018	0.804210661	0.852456485	0.999996191
LOC112444763	0.803844684	0.684611386	0.747967955	0.979606632	0.176619695	0.871415849	0.999996191
LOC112444770	0.581281522	0.956025082	0.831641657	0.885691849	0.149345281	0.84849659	0.999996191
LOC112444842	0.85290517	0.153654192	0.90104907	0.619543568	0.95111031	0.868035522	0.999996191
LOC112444843	0.870155399	0.894996677	0.758679578	0.612091245	0.984577431	0.995815385	0.999996191
LOC112444846	0.323725515	0.804637982	0.769244871	0.686631449	0.5952362	0.890867497	0.999996191
LOC112444847	0.298145265	0.458228594	0.387209653	0.20709243	0.8513672	0.49926785	0.999996191
LOC112444867	0.946059088	0.419597223	0.515822022	0.434876237	0.705207845	0.852646702	0.999996191
LOC112444869	0.834583503	0.34048103	0.302079827	0.638087021	0.996137915	0.830407537	0.999996191
LOC112444871	0.732395104	0.43623835	0.974636604	0.588206516	0.843906802	0.958543452	0.999996191

LOC112444888	0.425121742	0.446216477	0.930634696	0.965964119	0.306864719	0.823561249	0.999996191
LOC112444897	0.813415684	0.127120445	0.551734891	0.786983546	0.592989373	0.701469698	0.999996191
LOC112444904	0.699409578	0.621886097	0.943063451	0.567031613	0.058165465	0.569869427	0.999996191
LOC112444907	0.966704267	0.26090971	0.738830941	0.267120456	0.24146632	0.547120967	0.999996191
LOC112444909	0.719703045	0.164509268	0.938366742	0.563147419	0.723437388	0.799008065	0.999996191
LOC112444920	0.24203752	0.236279631	0.713540866	0.658564361	0.502179441	0.569392988	0.999996191
LOC112444924	0.545088041	0.839406625	0.39505005	0.891435136	0.532172931	0.896936742	0.999996191
LOC112444967	0.991915652	0.383975472	0.825610041	0.765405167	0.794224966	0.973179417	0.999996191
LOC112445001	0.894270115	0.683011761	0.371132321	0.091193496	0.863851693	0.623891726	0.999996191
LOC112445007	0.848420279	0.317361931	0.414743716	0.918781288	0.689516933	0.870452975	0.999996191
LOC112445029	0.646458881	0.177932818	0.161462791	0.827866973	0.799051633	0.551316496	0.999996191
LOC112445033	0.563166313	0.693326378	0.888136507	0.240110376	0.653689146	0.830015003	0.999996191
LOC112445035	0.997155437	0.836836214	0.743339467	0.71851477	0.342404166	0.957532095	0.999996191
LOC112445041	0.995507151	0.481208261	0.561244789	0.325187907	0.619919553	0.829325835	0.999996191
LOC112445052	0.809556258	0.835505251	0.754072088	0.622531526	0.501954411	0.96089819	0.999996191
LOC112445060	0.830491285	0.281557022	0.359630053	0.34229227	0.946492425	0.705879959	0.999996191
LOC112445063	0.418318609	0.724150965	0.099863047	0.656950108	0.586518993	0.541253188	0.999996191
LOC112445065	0.700249817	0.706502654	0.519232474	0.205735293	0.439677	0.675215569	0.999996191
LOC112445076	0.522261793	0.949669597	0.777034371	0.062515883	0.643957947	0.596451326	0.999996191
LOC112445088	0.946499836	0.572812571	0.882499103	0.362381963	0.343625953	0.844479401	0.999996191
LOC112445150	0.979656648	0.871846187	0.087988215	0.826507384	0.351336095	0.663019287	0.999996191
LOC112445177	0.929858612	0.206594528	0.517543309	0.114358664	0.987320993	0.534086752	0.999996191
LOC112445178	0.615902752	0.494635608	0.976995324	0.161842468	0.636907897	0.728436913	0.999996191
LOC112445190	0.944054269	0.880808377	0.757052828	0.476247499	0.427480489	0.942298382	0.999996191
LOC112445193	0.66633375	0.436895906	0.750862343	0.058138647	0.868833479	0.530948739	0.999996191
LOC112445194	0.844612406	0.796775897	0.221453747	0.709102612	0.368568923	0.772467112	0.999996191
LOC112445889	0.44466708	0.533637529	0.382133161	0.305559797	0.357400682	0.510431049	0.999996191
LOC112445912	0.821240472	0.469735796	0.486833639	0.066722217	0.796849075	0.511984808	0.999996191
LOC112445915	0.992798012	0.890415723	0.617315014	0.340366346	0.990475842	0.970829795	0.999996191
LOC112445927	0.234550657	0.852724486	0.908610055	0.902028993	0.111964033	0.629241747	0.999996191
LOC112445938	0.207963523	0.797406871	0.823574292	0.507744005	0.614530826	0.788482417	0.999996191
LOC112445939	0.981441269	0.172135026	0.847700218	0.094522656	0.855249626	0.539957338	0.999996191
LOC112445944	0.799526299	0.833456544	0.415598331	0.261856831	0.855539369	0.850788816	0.999996191
LOC112445951	0.782692798	0.992883419	0.604243203	0.291591827	0.996610335	0.948089933	0.999996191
LOC112445952	0.507658419	0.51615759	0.24023003	0.488611297	0.80073605	0.686474037	0.999996191
LOC112445968	0.765433409	0.104627392	0.91868295	0.368260455	0.580778519	0.599202761	0.999996191
LOC112445971	0.501182251	0.931801326	0.835489669	0.86133599	0.921027181	0.992942381	0.999996191
LOC112445972	0.537440784	0.720906266	0.410665172	0.78556948	0.725732915	0.904121439	0.999996191
LOC112445980	0.766930702	0.205576265	0.461828541	0.70612936	0.1976758	0.515309096	0.999996191
LOC112445982	0.877371712	0.761441967	0.633272239	0.462784183	0.446415676	0.899406078	0.999996191
LOC112445985	0.519709559	0.527964985	0.872842938	0.469155137	0.920433915	0.919838238	0.999996191
LOC112445988	0.499231196	0.839949998	0.919472364	0.506789258	0.737229069	0.952803801	0.999996191
LOC112445989	0.135665789	0.449756036	0.899552258	0.619056314	0.541907309	0.629874661	0.999996191
LOC112445996	0.592526436	0.764544363	0.66559994	0.30391734	0.96652743	0.901104401	0.999996191
LOC112446001	0.288226077	0.614946122	0.84635613	0.693837633	0.607428261	0.853685699	0.999996191

LOC112446004	0.907603981	0.90044904	0.823456333	0.101355931	0.510968849	0.752211594	0.999996191
LOC112446007	0.593478199	0.844740498	0.232360655	0.733110392	0.867858022	0.877121904	0.999996191
LOC112446010	0.622783891	0.629601927	0.418897778	0.426579027	0.251152695	0.621022117	0.999996191
LOC112446012	0.584610289	0.276563905	0.245034982	0.42417912	0.994109638	0.610872435	0.999996191
LOC112446021	0.980009336	0.829244841	0.127300942	0.523990685	0.859465597	0.803992062	0.999996191
LOC112446022	0.475735077	0.915730088	0.772868946	0.365572694	0.785960778	0.912019553	0.999996191
LOC112446024	0.455437868	0.347993029	0.558873717	0.291678347	0.357234207	0.497317572	0.999996191
LOC112446029	0.499540797	0.782318818	0.745674456	0.81797521	0.315134348	0.879016041	0.999996191
LOC112446033	0.249282142	0.740860125	0.582929154	0.167826876	0.615901055	0.532427369	0.999996191
LOC112446034	0.925160738	0.659351025	0.891816692	0.429735057	0.936809818	0.980532917	0.999996191
LOC112446036	0.08574427	0.65147808	0.985708306	0.276225266	0.519505373	0.468873968	0.999996191
LOC112446039	0.612208434	0.840533895	0.676285647	0.946720652	0.660602176	0.98022792	0.999996191
LOC112446044	0.914532548	0.985472978	0.617531869	0.112542973	0.463119589	0.717835332	0.999996191
LOC112446053	0.611025634	0.846598765	0.893821121	0.392264877	0.820634756	0.955520417	0.999996191
LOC112446127	0.426582872	0.431957596	0.659105064	0.353549476	0.869877699	0.7648867	0.999996191
LOC112446129	0.49456954	0.532446276	0.340523899	0.379917091	0.258036401	0.488325003	0.999996191
LOC112446357	0.905313917	0.939212089	0.893563348	0.831165444	0.604707366	0.996881085	0.999996191
LOC112446360	0.752428626	0.557313666	0.592862858	0.242107029	0.84027124	0.817898695	0.999996191
LOC112446369	0.923587874	0.405271109	0.232824463	0.586820566	0.504200655	0.69531827	0.999996191
LOC112446375	0.572961265	0.201108876	0.563687421	0.22811193	0.998051376	0.587111693	0.999996191
LOC112446381	0.340314676	0.39225214	0.917053643	0.770003852	0.50424656	0.807411667	0.999996191
LOC112446388	0.180584497	0.711493339	0.91352707	0.480841483	0.155392251	0.487894461	0.999996191
LOC112446390	0.426504142	0.805987337	0.550899572	0.161093352	0.482554823	0.586240517	0.999996191
LOC112446402	0.553382304	0.440559843	0.256012763	0.949113509	0.480440817	0.714217948	0.999996191
LOC112446406	0.760112291	0.64794658	0.939651024	0.169823871	0.717873472	0.835821476	0.999996191
LOC112446407	0.49529652	0.929161391	0.926082879	0.535915103	0.2431002	0.833246221	0.999996191
LOC112446417	0.957807345	0.591912658	0.855476495	0.929562629	0.930294198	0.997983443	0.999996191
LOC112446427	0.869047409	0.889041806	0.378081967	0.089604691	0.981136529	0.694543273	0.999996191
LOC112446452	0.802354551	0.931393121	0.691691824	0.468068001	0.764004449	0.971128353	0.999996191
LOC112446457	0.702636071	0.052408248	0.385356533	0.72602785	0.753074865	0.465581517	0.999996191
LOC112446462	0.632603079	0.868031097	0.988987317	0.903220488	0.534506087	0.988030283	0.999996191
LOC112446639	0.582522236	0.490065104	0.892805956	0.453456329	0.473087934	0.830750831	0.999996191
LOC112446642	0.744195611	0.911756118	0.893625583	0.838041739	0.620933975	0.993399341	0.999996191
LOC112446645	0.291282065	0.819757523	0.58728867	0.847680335	0.930642983	0.927332711	0.999996191
LOC112446667	0.202273663	0.696817831	0.85488479	0.577035976	0.866809578	0.846281063	0.999996191
LOC112446668	0.583986021	0.48096937	0.283857115	0.8184044	0.928706435	0.847134387	0.999996191
LOC112446676	0.820792401	0.505525122	0.538727342	0.676874335	0.862330043	0.943986703	0.999996191
LOC112446691	0.618519303	0.315672279	0.772045517	0.13496902	0.48685524	0.510482287	0.999996191
LOC112446696	0.998456066	0.898295566	0.029027329	0.410266895	0.655442062	0.447237192	0.999996191
LOC112446699	0.8802872	0.295970971	0.09841981	0.584599796	0.697681659	0.520691329	0.999996191
LOC112446701	0.15157475	0.865278447	0.73454513	0.707857202	0.672190037	0.801196141	0.999996191
LOC112446708	0.624933876	0.932342257	0.478255017	0.331833155	0.515960806	0.808051028	0.999996191
LOC112446709	0.446606236	0.336674995	0.992187741	0.86974524	0.337596002	0.79331294	0.999996191
LOC112446716	0.255270158	0.924091566	0.394503606	0.931710805	0.964619287	0.893667794	0.999996191
LOC112446725	0.925219236	0.989968864	0.986692214	0.526978152	0.831334854	0.997347389	0.999996191

LOC112446733	0.607333719	0.964834377	0.314108421	0.395492137	0.389207568	0.713354021	0.999996191
LOC112446740	0.900214347	0.859123427	0.879544139	0.113813289	0.476405157	0.762590522	0.999996191
LOC112446753	0.500027982	0.92081037	0.459616753	0.861298123	0.449240228	0.890848213	0.999996191
LOC112446760	0.620592884	0.726009611	0.147207381	0.391840923	0.528696639	0.572869756	0.999996191
LOC112446761	0.817570648	0.631636736	0.631227055	0.554914619	0.096657447	0.619758761	0.999996191
LOC112446771	0.559229489	0.602718786	0.694156707	0.596000752	0.317715583	0.795292869	0.999996191
LOC112446775	0.406847524	0.868424195	0.276898883	0.897513446	0.763218942	0.862486103	0.999996191
LOC112446777	0.770085434	0.66742068	0.654332257	0.419662207	0.482940499	0.864999677	0.999996191
LOC112446779	0.932663649	0.612340925	0.748587915	0.608399469	0.809862214	0.978583609	0.999996191
LOC112446793	0.8787508	0.546636212	0.689991226	0.487699286	0.598376727	0.911997832	0.999996191
LOC112446795	0.517870408	0.957659506	0.435001473	0.346540275	0.733092856	0.831138001	0.999996191
LOC112446799	0.199841224	0.946981099	0.568080299	0.651326794	0.961909967	0.863238214	0.999996191
LOC112446822	0.652428457	0.992961558	0.993636199	0.986270752	0.879869639	0.999655377	0.999996191
LOC112446855	0.889491507	0.433320729	0.36135852	0.394694384	0.740506013	0.780375223	0.999996191
LOC112446879	0.364240474	0.178953142	0.510017998	0.86659708	0.95628451	0.708012922	0.999996191
LOC112446882	0.950630608	0.155506846	0.653774663	0.835207311	0.668179008	0.828527941	0.999996191
LOC112447026	0.232597247	0.710981819	0.603093478	0.899900287	0.796213894	0.871908708	0.999996191
LOC112447027	0.997787366	0.605286426	0.599247129	0.391585053	0.576598716	0.89057452	0.999996191
LOC112447029	0.314957737	0.595055663	0.929938158	0.995261577	0.735851289	0.941911582	0.999996191
LOC112447030	0.715282589	0.397088358	0.782969824	0.875144738	0.323795029	0.853185995	0.999996191
LOC112447041	0.471526836	0.569131125	0.548281903	0.722638943	0.350189498	0.764317144	0.999996191
LOC112447066	0.434389559	0.648821278	0.684341514	0.20712706	0.568243978	0.670692356	0.999996191
LOC112447085	0.381245475	0.345105857	0.6214903	0.737445027	0.422395266	0.69296008	0.999996191
LOC112447087	0.76367688	0.718475414	0.204946732	0.559573884	0.727992457	0.801080136	0.999996191
LOC112447103	0.866629087	0.6991292	0.255832787	0.987689072	0.080760386	0.552535751	0.999996191
LOC112447118	0.516950015	0.567394067	0.689780516	0.682211532	0.0602813	0.47825543	0.999996191
LOC112447140	0.229108775	0.741633183	0.299758998	0.962746779	0.414550135	0.649192324	0.999996191
LOC112447290	0.395025967	0.367751637	0.551325298	0.409385438	0.871737257	0.71503118	0.999996191
LOC112447291	0.498893851	0.983349827	0.837297111	0.191891196	0.98472838	0.883579363	0.999996191
LOC112447303	0.083334742	0.897482388	0.811957468	0.95119564	0.868237242	0.81649095	0.999996191
LOC112447309	0.281841532	0.995386842	0.916446027	0.814069334	0.052632168	0.530505431	0.999996191
LOC112447322	0.682916424	0.28505445	0.150692368	0.41204269	0.63848349	0.464625291	0.999996191
LOC112447323	0.930356744	0.998464526	0.929193137	0.720372807	0.395783272	0.985666131	0.999996191
LOC112447324	0.774491724	0.31845258	0.254984455	0.180796731	0.98678795	0.533990404	0.999996191
LOC112447328	0.80182803	0.432647701	0.909741201	0.349040459	0.71551243	0.885686568	0.999996191
LOC112447340	0.534828101	0.576360265	0.805738311	0.501365104	0.78985232	0.913993782	0.999996191
LOC112447342	0.398676092	0.812192831	0.423469844	0.808332887	0.700659274	0.883652978	0.999996191
LOC112447346	0.788060881	0.945961751	0.787418372	0.621397299	0.632547795	0.982956507	0.999996191
LOC112447347	0.841029235	0.374735882	0.838833857	0.851245041	0.947798212	0.979222189	0.999996191
LOC112447350	0.177679441	0.549581122	0.204133407	0.951605593	0.795602959	0.591068737	0.999996191
LOC112447360	0.639614496	0.78484825	0.14566649	0.099807855	0.969246482	0.449073579	0.999996191
LOC112447370	0.180011943	0.698861664	0.544373236	0.9753595	0.604609859	0.778958657	0.999996191
LOC112447399	0.719634751	0.402010523	0.978081543	0.98328318	0.906550203	0.986619486	0.999996191
LOC112447411	0.932213087	0.787967809	0.808726008	0.565772802	0.430091714	0.953096325	0.999996191
LOC112447418	0.458337838	0.779483537	0.445694956	0.074793744	0.699678826	0.478527745	0.999996191

LOC112447420	0.721427239	0.819218184	0.974337232	0.931973399	0.613160183	0.994327488	0.999996191
LOC112447433	0.78952403	0.515914297	0.804265263	0.629682439	0.816058328	0.964896391	0.999996191
LOC112447438	0.474829344	0.88660592	0.285505112	0.84683871	0.341152173	0.751528463	0.999996191
LOC112447443	0.39984038	0.528844054	0.739070376	0.973046666	0.670493363	0.918203521	0.999996191
LOC112447460	0.205235392	0.313097184	0.849842707	0.585969046	0.249625334	0.470843207	0.999996191
LOC112447461	0.650956131	0.794060264	0.779135762	0.587055421	0.94194241	0.981333263	0.999996191
LOC112447462	0.859171994	0.786440152	0.53433268	0.217631724	0.996809361	0.884823462	0.999996191
LOC112447469	0.72445226	0.302873686	0.88379684	0.981204273	0.752343572	0.952271378	0.999996191
LOC112447473	0.439735148	0.536273109	0.778708965	0.806332379	0.698300311	0.919811333	0.999996191
LOC112447495	0.881322811	0.33544978	0.393347677	0.199173104	0.76770997	0.6230543	0.999996191
LOC112447499	0.756326812	0.747425366	0.853774806	0.381022551	0.998833827	0.970730152	0.999996191
LOC112447506	0.694565704	0.918395105	0.876769755	0.280918359	0.752274141	0.934299534	0.999996191
LOC112447508	0.75949459	0.846870548	0.836772226	0.968861929	0.711962969	0.996478113	0.999996191
LOC112447510	0.502794341	0.997291446	0.518030869	0.556451953	0.913753145	0.94512089	0.999996191
LOC112447523	0.344777477	0.462219954	0.481437622	0.966613396	0.765631677	0.836844542	0.999996191
LOC112447599	0.691713228	0.077364227	0.497923879	0.729706443	0.841768034	0.60687224	0.999996191
LOC112447727	0.4217327	0.671597583	0.516266725	0.432922183	0.196688274	0.553880608	0.999996191
LOC112447731	0.875049392	0.62044207	0.770323278	0.990131416	0.861811949	0.995852921	0.999996191
LOC112447735	0.79556568	0.348174524	0.95918093	0.947944499	0.765560365	0.973691509	0.999996191
LOC112447762	0.490332546	0.126755935	0.872920747	0.698220661	0.510059724	0.639283641	0.999996191
LOC112447769	0.414703673	0.925394378	0.638020217	0.982152612	0.281251064	0.863854696	0.999996191
LOC112447797	0.120180675	0.870555877	0.999448855	0.363396969	0.8905454	0.746758759	0.999996191
LOC112447802	0.231024725	0.731961486	0.912879177	0.7442465	0.53319566	0.848817459	0.999996191
LOC112447811	0.19413356	0.944878351	0.947965018	0.060349101	0.847354617	0.490438243	0.999996191
LOC112447817	0.908336444	0.774297452	0.949650917	0.616864004	0.799790076	0.994356594	0.999996191
LOC112447824	0.673172488	0.55350925	0.876122667	0.430704071	0.213830292	0.724616505	0.999996191
LOC112447832	0.847662946	0.805063216	0.944581198	0.558710677	0.290476634	0.921142009	0.999996191
LOC112447838	0.41784829	0.883040106	0.868077387	0.976979495	0.379279086	0.934727236	0.999996191
LOC112447842	0.56733118	0.721684454	0.987645399	0.786333308	0.668454255	0.9790445	0.999996191
LOC112447845	0.122617344	0.787665983	0.618132416	0.645403855	0.540667104	0.653972554	0.999996191
LOC112447846	0.904815417	0.586051181	0.576048017	0.150723324	0.29033446	0.567546592	0.999996191
LOC112447858	0.459897083	0.77016815	0.970638693	0.597099176	0.048649694	0.512019008	0.999996191
LOC112448021	0.628211582	0.35366677	0.68353887	0.730909658	0.127931446	0.579250444	0.999996191
LOC112448022	0.781822871	0.748098538	0.496773681	0.510661657	0.896685129	0.945794007	0.999996191
LOC112448030	0.467473648	0.756618018	0.460748957	0.967071703	0.751237893	0.934476164	0.999996191
LOC112448032	0.452230496	0.923262674	0.046413425	0.882140575	0.961413033	0.607683769	0.999996191
LOC112448038	0.176232494	0.944511197	0.716050692	0.972515503	0.184299801	0.658872884	0.999996191
LOC112448045	0.715749744	0.794074186	0.481385248	0.829407474	0.503548359	0.930791674	0.999996191
LOC112448056	0.466228523	0.836958135	0.136786662	0.845103285	0.916162977	0.783056908	0.999996191
LOC112448057	0.659279364	0.928909385	0.890818383	0.633524951	0.82862702	0.990863832	0.999996191
LOC112448062	0.94454292	0.80104203	0.890007573	0.42959638	0.179195272	0.82200379	0.999996191
LOC112448075	0.900778957	0.620971798	0.160216172	0.599103239	0.761694263	0.781195051	0.999996191
LOC112448078	0.469248409	0.629679111	0.955795721	0.39334374	0.533677179	0.843696614	0.999996191
LOC112448088	0.410006897	0.121404409	0.710973193	0.938630321	0.946370372	0.733015643	0.999996191
LOC112448090	0.542543044	0.793612363	0.659490907	0.396238426	0.725574609	0.890446604	0.999996191

LOC112448103	0.603983664	0.534400662	0.804959077	0.674935608	0.417705634	0.875450717	0.999996191
LOC112448155	0.609490524	0.940018837	0.480686661	0.064405326	0.52641016	0.499470536	0.999996191
LOC112448169	0.818558162	0.699756261	0.699657199	0.874800988	0.785989232	0.989690617	0.999996191
LOC112448253	0.715770255	0.248604656	0.892389948	0.330952196	0.737316627	0.771534702	0.999996191
LOC112448260	0.795635955	0.767179085	0.550880773	0.630275907	0.367702369	0.884128127	0.999996191
LOC112448271	0.83971093	0.25543034	0.532101658	0.909528552	0.593084889	0.849591152	0.999996191
LOC112448304	0.473974633	0.375687381	0.725346199	0.460963155	0.864430057	0.820806081	0.999996191
LOC112448335	0.811074519	0.271506289	0.777214657	0.651591838	0.239946124	0.702440834	0.999996191
LOC112448364	0.655883388	0.67676634	0.67156991	0.276516753	0.689740581	0.837052353	0.999996191
LOC112448366	0.876010528	0.621838421	0.601435503	0.167531756	0.162692082	0.491217046	0.999996191
LOC112448368	0.702713382	0.188871049	0.889410738	0.80510205	0.936653993	0.901743669	0.999996191
LOC112448373	0.471899602	0.927965514	0.154091637	0.895145031	0.930101142	0.835138255	0.999996191
LOC112448387	0.169202998	0.948535465	0.746471241	0.360943756	0.959445694	0.78375363	0.999996191
LOC112448390	0.527094646	0.260912722	0.927579086	0.435531872	0.806828403	0.797330201	0.999996191
LOC112448430	0.988135089	0.483201136	0.673287207	0.546864345	0.646618374	0.930243859	0.999996191
LOC112448453	0.319939874	0.420146764	0.946897627	0.705045818	0.525690818	0.806131214	0.999996191
LOC112448474	0.647842597	0.954335403	0.36583805	0.74336687	0.507494225	0.896294854	0.999996191
LOC112448488	0.528868143	0.692562333	0.534327875	0.997472011	0.625568237	0.937601172	0.999996191
LOC112448507	0.71115668	0.856421523	0.65999436	0.48084777	0.86379933	0.964312053	0.999996191
LOC112448511	0.496561591	0.602691384	0.633081238	0.457476147	0.933641601	0.889266413	0.999996191
LOC112448518	0.341366463	0.743719256	0.963292599	0.046496589	0.633313666	0.452246547	0.999996191
LOC112448524	0.464590531	0.600781544	0.280042955	0.748368582	0.31328346	0.628946037	0.999996191
LOC112448531	0.726460486	0.918910924	0.742356546	0.533459095	0.966377036	0.98709713	0.999996191
LOC112448540	0.992469545	0.787573314	0.519528402	0.653951168	0.434310975	0.931768336	0.999996191
LOC112448579	0.313940975	0.732207867	0.457469326	0.482311052	0.64589779	0.740725924	0.999996191
LOC112448627	0.546790956	0.606966821	0.38513302	0.403744408	0.315053099	0.605577669	0.999996191
LOC112448760	0.6929872	0.405799187	0.74069708	0.760841572	0.975374195	0.95854457	0.999996191
LOC112448762	0.086708923	0.753813088	0.603065029	0.783833056	0.556195254	0.61638739	0.999996191
LOC112448764	0.588734891	0.864703842	0.51930008	0.62680462	0.946336995	0.959660066	0.999996191
LOC112448770	0.890994965	0.368111745	0.984392709	0.725660624	0.614257217	0.952722623	0.999996191
LOC112448772	0.643310211	0.538542564	0.747121362	0.860853652	0.716273173	0.961003319	0.999996191
LOC112448773	0.611152816	0.252492001	0.66699805	0.755461837	0.642549763	0.815852834	0.999996191
LOC112448776	0.248644722	0.590143255	0.929314226	0.961842673	0.507366753	0.861437949	0.999996191
LOC112448832	0.773172908	0.45079865	0.836453297	0.404612662	0.992297102	0.933304367	0.999996191
LOC112448847	0.875629022	0.640029832	0.859908868	0.90946973	0.778647774	0.995054462	0.999996191
LOC112448848	0.178995164	0.919468271	0.694003196	0.877906811	0.666900234	0.862168171	0.999996191
LOC112448853	0.684633325	0.570237668	0.554254883	0.173772406	0.698497742	0.698862122	0.999996191
LOC112448863	0.698690167	0.408788717	0.665079187	0.71100811	0.558078348	0.87949677	0.999996191
LOC112448889	0.498839773	0.359440218	0.374755466	0.295252951	0.720583516	0.580549575	0.999996191
LOC112448894	0.149021193	0.587479291	0.754166607	0.879226596	0.522797085	0.726389148	0.999996191
LOC112449052	0.544919234	0.488402571	0.92837213	0.861756613	0.478365675	0.918083477	0.999996191
LOC112449053	0.859316392	0.656914927	0.38291675	0.70936378	0.972336756	0.955658538	0.999996191
LOC112449056	0.643898733	0.861229627	0.974989078	0.879037546	0.458061162	0.980241535	0.999996191
LOC112449059	0.28840539	0.376254647	0.808335299	0.837840106	0.414497831	0.72709203	0.999996191
LOC112449073	0.25498199	0.609628582	0.564478691	0.537606941	0.594379047	0.711367419	0.999996191

LOC112449075	0.169008234	0.594997613	0.638700535	0.197273998	0.817530183	0.518879272	0.999996191
LOC112449080	0.688695614	0.953607175	0.49629479	0.773259216	0.185713246	0.804784964	0.999996191
LOC112449086	0.601729659	0.352271213	0.594686483	0.107628262	0.795483113	0.526627176	0.999996191
LOC112449087	0.377667218	0.750822784	0.294315576	0.555972919	0.165107026	0.46329995	0.999996191
LOC112449092	0.923978794	0.919201292	0.709259464	0.567492605	0.370493054	0.941167101	0.999996191
LOC112449099	0.805609928	0.918497737	0.957754551	0.18606916	0.964291567	0.9415478	0.999996191
LOC112449102	0.193407978	0.414459314	0.87425284	0.879903806	0.146160836	0.492925282	0.999996191
LOC112449106	0.053858023	0.6193834	0.745088172	0.92443329	0.963710546	0.665855541	0.999996191
LOC112449111	0.782442107	0.985194112	0.685862003	0.33990532	0.117861034	0.657204562	0.999996191
LOC112449115	0.527362777	0.678101737	0.510548177	0.381998339	0.249093867	0.618511175	0.999996191
LOC112449245	0.57555239	0.964772245	0.606990699	0.676925228	0.78064382	0.968745154	0.999996191
LOC112449247	0.462963208	0.759154512	0.993594811	0.972843671	0.825382373	0.990233452	0.999996191
LOC112449254	0.676462543	0.743534593	0.579084143	0.272668506	0.362003246	0.716137903	0.999996191
LOC112449258	0.549613302	0.479497323	0.429662412	0.801737583	0.08475243	0.464076883	0.999996191
LOC112449266	0.477733187	0.457027006	0.955691864	0.717686146	0.835484719	0.939986435	0.999996191
LOC112449275	0.842598386	0.98620765	0.414967877	0.750388944	0.653898569	0.965255849	0.999996191
LOC112449282	0.908132766	0.863903572	0.495581911	0.318629203	0.711403252	0.900469184	0.999996191
LOC112449318	0.183385457	0.66210987	0.609683017	0.848387456	0.394285699	0.687527819	0.999996191
LOC112449338	0.873184132	0.657415411	0.61760719	0.886237587	0.336860391	0.922456995	0.999996191
LOC112449346	0.844876038	0.749464108	0.853381679	0.931497453	0.744871223	0.996622942	0.999996191
LOC112449358	0.162232701	0.36307929	0.577121099	0.405370767	0.742363781	0.51653265	0.999996191
LOC112449360	0.549366571	0.823854332	0.571052566	0.719827005	0.839331632	0.959334341	0.999996191
LOC112449363	0.515356848	0.890121491	0.945671418	0.338209487	0.786295479	0.931793757	0.999996191
LOC112449367	0.768566801	0.229191517	0.461373955	0.362060868	0.619411177	0.627879563	0.999996191
LOC112449505	0.355794905	0.614168753	0.844958138	0.307431409	0.742737171	0.786593725	0.999996191
LOC112449510	0.777989207	0.755986407	0.209294685	0.949131732	0.793953804	0.90690418	0.999996191
LOC112449516	0.536047382	0.338534512	0.795161706	0.641919249	0.262641924	0.684103562	0.999996191
LOC112449548	0.689231094	0.879565949	0.998320431	0.169428758	0.427742616	0.793535808	0.999996191
LOC112449552	0.899984805	0.814212061	0.158122907	0.668852914	0.259477964	0.64708352	0.999996191
LOC112449558	0.868846932	0.843504758	0.968735129	0.56416897	0.129106987	0.821597959	0.999996191
LOC112449560	0.040009854	0.63524728	0.852938608	0.955413651	0.824185956	0.615084339	0.999996191
LOC112449561	0.998278758	0.46555135	0.21157488	0.622856649	0.126931588	0.465937202	0.999996191
LOC112449563	0.340301141	0.870654596	0.115970973	0.71929131	0.488762267	0.548076639	0.999996191
LOC112449590	0.608485815	0.836746165	0.607285993	0.094848361	0.967462793	0.713627011	0.999996191
LOC112449596	0.597989211	0.265356144	0.789170606	0.255730713	0.440466032	0.577949227	0.999996191
LOC112449602	0.232823083	0.875597463	0.844081764	0.468297285	0.142239784	0.538050111	0.999996191
LOC112449614	0.742013497	0.988013433	0.17314247	0.917102411	0.178481663	0.653457506	0.999996191
LOC112449615	0.71438852	0.921810492	0.234359809	0.81977937	0.569460661	0.873085363	0.999996191
LOC112449618	0.296952353	0.329129915	0.392608536	0.515855852	0.726900655	0.581801474	0.999996191
LOC407171	0.333280256	0.804428686	0.714031861	0.335937236	0.206954051	0.566708043	0.999996191
LOC504858	0.898616408	0.638942843	0.733661962	0.609504221	0.272777633	0.868984427	0.999996191
LOC505099	0.191038424	0.142006633	0.980743638	0.606017275	0.561223973	0.493668172	0.999996191
LOC505199	0.50969328	0.601669528	0.504020785	0.425808165	0.369549103	0.684057035	0.999996191
LOC505600	0.28345072	0.795387273	0.616840908	0.451092587	0.402246722	0.691162367	0.999996191
LOC505918	0.254444745	0.873082237	0.897816333	0.982197411	0.49980682	0.913456456	0.999996191

LOC506181	0.423386581	0.935828527	0.624091885	0.566316768	0.648289182	0.904222884	0.999996191
LOC506408	0.689183134	0.045524874	0.385626887	0.930555046	0.75691926	0.482629409	0.999996191
LOC507443	0.433518013	0.68696516	0.667869489	0.607452047	0.935829974	0.929674563	0.999996191
LOC507696	0.676721699	0.203156649	0.346956145	0.907364422	0.623651425	0.704103055	0.999996191
LOC507930	0.967422543	0.718547166	0.431521104	0.672428768	0.226437367	0.800571915	0.999996191
LOC508131	0.11327943	0.442180734	0.570323133	0.790321711	0.87580424	0.643795547	0.999996191
LOC508455	0.337849587	0.946861462	0.508915225	0.852812	0.83845618	0.932733929	0.999996191
LOC509006	0.308640903	0.795638241	0.758160954	0.725586025	0.223801831	0.725668302	0.999996191
LOC509034	0.670226103	0.734868734	0.570316588	0.878284536	0.095824595	0.678557488	0.999996191
LOC509155	0.205052015	0.697482078	0.838105749	0.688624464	0.444458813	0.761616938	0.999996191
LOC509184	0.954984218	0.888346202	0.460342732	0.900178164	0.296440776	0.920707485	0.999996191
LOC509810	0.776647223	0.253068201	0.989797371	0.499912044	0.517247411	0.816994767	0.999996191
LOC509941	0.523541028	0.810425826	0.207700942	0.785917934	0.692750415	0.809011663	0.999996191
LOC509972	0.607633883	0.392220966	0.300040011	0.390727519	0.800239531	0.667738427	0.999996191
LOC510185	0.553186912	0.49570649	0.142861501	0.885828364	0.609823678	0.65703704	0.999996191
LOC510193	0.774904793	0.906611374	0.904910122	0.196820438	0.428811693	0.827675183	0.999996191
LOC510362	0.122319449	0.662053847	0.620549346	0.700801246	0.672699663	0.678968656	0.999996191
LOC510382	0.25866428	0.985180965	0.960883885	0.434820143	0.822801661	0.899701549	0.999996191
LOC510613	0.94327392	0.862893101	0.124610387	0.82732049	0.921710579	0.88308629	0.999996191
LOC510798	0.396920723	0.973625577	0.640827456	0.513388261	0.621222754	0.88597111	0.999996191
LOC510913	0.535290639	0.761685157	0.911850987	0.245929346	0.912497735	0.893341511	0.999996191
LOC511161	0.843820119	0.343888102	0.573166607	0.427209188	0.446692947	0.734813341	0.999996191
LOC511409	0.893870132	0.895722154	0.68713798	0.563172574	0.276629996	0.896876467	0.999996191
LOC511531	0.49422828	0.9719313	0.325786892	0.363643223	0.200087537	0.536793963	0.999996191
LOC511847	0.222118241	0.613056164	0.774565845	0.207894684	0.615838119	0.569512132	0.999996191
LOC511937	0.172396956	0.976585546	0.693808187	0.800455944	0.756073803	0.870345446	0.999996191
LOC512149	0.687854803	0.146902314	0.516484342	0.300294515	0.848458884	0.566536482	0.999996191
LOC512165	0.361574651	0.430368345	0.520838017	0.632765622	0.515989349	0.700300876	0.999996191
LOC512175	0.818519379	0.329329899	0.596686973	0.253726127	0.931872678	0.768153427	0.999996191
LOC512323	0.636357302	0.248437781	0.176884156	0.982554914	0.679322545	0.632533914	0.999996191
LOC512440	0.61647912	0.231692586	0.676634455	0.915044499	0.998370124	0.900700641	0.999996191
LOC512464	0.441788476	0.964505551	0.129390002	0.690380822	0.200051239	0.462212031	0.999996191
LOC512541	0.875164348	0.364308807	0.73375047	0.432079445	0.753129064	0.880886613	0.999996191
LOC512617	0.471555107	0.854139824	0.837828079	0.36537014	0.560334668	0.866989564	0.999996191
LOC512684	0.646072608	0.02700359	0.733015876	0.920303353	0.593849044	0.446938932	0.999996191
LOC512863	0.852327069	0.903926891	0.552916221	0.214955229	0.212894662	0.64102118	0.999996191
LOC512953	0.983997942	0.625815947	0.900591642	0.068365424	0.473445266	0.62490215	0.999996191
LOC512978	0.776234527	0.227179285	0.899982103	0.523397153	0.777945532	0.85701816	0.999996191
LOC513573	0.455096736	0.82427015	0.807269742	0.505839343	0.238248586	0.760660639	0.999996191
LOC513779	0.482060432	0.658051254	0.160040945	0.161811243	0.966536321	0.469753617	0.999996191
LOC513969	0.243070663	0.489754866	0.835186126	0.259729111	0.851529113	0.664500776	0.999996191
LOC514011	0.438581137	0.219701263	0.921517603	0.436527631	0.569062414	0.665102312	0.999996191
LOC514680	0.809768186	0.953511893	0.279659273	0.705297504	0.483545706	0.876213123	0.999996191
LOC515042	0.826977338	0.061116619	0.609956689	0.589394457	0.766535877	0.575496945	0.999996191
LOC515089	0.275248924	0.822212777	0.922402455	0.137611467	0.86804884	0.688871925	0.999996191

LOC515150	0.844953813	0.991338948	0.046436425	0.51297932	0.491947602	0.508787933	0.999996191
LOC515227	0.157313605	0.697702166	0.737444056	0.337370196	0.328808517	0.492226263	0.999996191
LOC515333	0.277145748	0.932644393	0.220983569	0.536645525	0.987238428	0.725846841	0.999996191
LOC515547	0.327500718	0.979228777	0.37344738	0.470613387	0.833599233	0.80543291	0.999996191
LOC515578	0.269454174	0.656021598	0.300802771	0.450295943	0.701562075	0.611939961	0.999996191
LOC515736	0.577471375	0.403108119	0.268594987	0.357872534	0.562015697	0.555790129	0.999996191
LOC515823	0.243477996	0.328223441	0.942174288	0.680625353	0.581634747	0.722986194	0.999996191
LOC516355	0.435823928	0.902424423	0.509856157	0.2999911	0.938612671	0.835945031	0.999996191
LOC516742	0.957402721	0.167976336	0.083635683	0.811099054	0.952165039	0.519410994	0.999996191
LOC516849	0.711596885	0.809915872	0.546827533	0.744656002	0.492218448	0.93193062	0.999996191
LOC518623	0.441016634	0.78902406	0.133379781	0.62722326	0.675520031	0.642722404	0.999996191
LOC518768	0.548150693	0.605827669	0.917623051	0.417883211	0.799766669	0.918071452	0.999996191
LOC518980	0.565887151	0.703141224	0.87947616	0.927891453	0.385632012	0.940064479	0.999996191
LOC519145	0.783365144	0.464463448	0.952305964	0.461128423	0.674501402	0.924467612	0.999996191
LOC519208	0.916000376	0.784874938	0.972688154	0.755128777	0.186043376	0.913857976	0.999996191
LOC520104	0.726767347	0.987839402	0.763076841	0.416239241	0.522388851	0.935099045	0.999996191
LOC520336	0.819943762	0.282609739	0.602050277	0.748866182	0.54897985	0.838451142	0.999996191
LOC521224	0.088348317	0.695686234	0.37135289	0.481329191	0.98811454	0.527727056	0.999996191
LOC521580	0.456315599	0.801498811	0.817812172	0.557191142	0.710499085	0.93448915	0.999996191
LOC521656	0.698727107	0.291671593	0.800859039	0.693421247	0.373951634	0.78727001	0.999996191
LOC522610	0.276808442	0.369507733	0.190158478	0.875722918	0.415543201	0.449171012	0.999996191
LOC523461	0.68383059	0.506227289	0.95601427	0.428019737	0.980841638	0.949686265	0.999996191
LOC523963	0.889664138	0.307626205	0.401399227	0.986433661	0.876604379	0.909818714	0.999996191
LOC524181	0.607435256	0.361748765	0.973256751	0.206807207	0.574515444	0.692499661	0.999996191
LOC525426	0.30301317	0.797010226	0.365646827	0.443533467	0.209907495	0.47607228	0.999996191
LOC526769	0.560476576	0.744222194	0.216478892	0.262446333	0.959833049	0.67107816	0.999996191
LOC527186	0.182849698	0.263448076	0.894356036	0.741344494	0.301178712	0.505010189	0.999996191
LOC527744	0.964322436	0.333008103	0.812406068	0.671950137	0.407153161	0.871733598	0.999996191
LOC528767	0.778385525	0.92827837	0.656902215	0.452054309	0.93672508	0.976069045	0.999996191
LOC528802	0.285934867	0.637757907	0.726570861	0.616561274	0.096740213	0.468906208	0.999996191
LOC529125	0.998916469	0.785271976	0.939574417	0.069083492	0.625750752	0.735528775	0.999996191
LOC529399	0.630228184	0.571488269	0.138321032	0.466739197	0.785519867	0.628298347	0.999996191
LOC529930	0.403447066	0.501566134	0.925605296	0.945216996	0.870262852	0.958288198	0.999996191
LOC530348	0.907792696	0.797116434	0.822885261	0.487356984	0.22351901	0.857587757	0.999996191
LOC531038	0.931449949	0.3597552	0.676937322	0.814010687	0.214383915	0.775377401	0.999996191
LOC531090	0.285992806	0.809426153	0.230710532	0.739795924	0.413408903	0.606477756	0.999996191
LOC531152	0.278195331	0.499122058	0.320995255	0.449743062	0.668481542	0.568024926	0.999996191
LOC531679	0.655541819	0.78750122	0.225366851	0.870381745	0.531995262	0.828333304	0.999996191
LOC532875	0.91571784	0.972415319	0.411733419	0.054778744	0.711998361	0.580597072	0.999996191
LOC533093	0.582654227	0.991970319	0.632438027	0.881919226	0.674979981	0.980216706	0.999996191
LOC533308	0.347028125	0.905066115	0.314247511	0.628636831	0.22937638	0.579679397	0.999996191
LOC533597	0.595928021	0.331572422	0.080438435	0.83450461	0.808268211	0.525362711	0.999996191
LOC534155	0.630115529	0.870388433	0.436850328	0.583188287	0.925388738	0.943137212	0.999996191
LOC534391	0.873520439	0.824394491	0.90818985	0.387621463	0.879911692	0.981410343	0.999996191
LOC534520	0.975287355	0.454726198	0.39574165	0.931715409	0.801519309	0.944408919	0.999996191

LOC534627	0.588395678	0.458005283	0.471557301	0.275550461	0.78401473	0.70734521	0.999996191
LOC534630	0.846169889	0.318066248	0.316562647	0.824159453	0.568312725	0.776819954	0.999996191
LOC534742	0.809465914	0.532281957	0.481074289	0.230351993	0.893997283	0.788775134	0.999996191
LOC535280	0.201154092	0.714924795	0.900138085	0.106976572	0.921809353	0.558668363	0.999996191
LOC537017	0.764107042	0.735372773	0.591560611	0.061414789	0.949215699	0.639846326	0.999996191
LOC539009	0.81838637	0.741641145	0.315286008	0.186129884	0.637026948	0.670594774	0.999996191
LOC539069	0.905564855	0.969295626	0.983226432	0.383796384	0.400542574	0.945535862	0.999996191
LOC540014	0.266809519	0.45788835	0.831981028	0.895611965	0.870479436	0.886418344	0.999996191
LOC540707	0.42647916	0.984182751	0.244043227	0.77263523	0.608919386	0.80976227	0.999996191
LOC541276	0.204696974	0.558814033	0.940297125	0.527745426	0.798101983	0.799162437	0.999996191
LOC548613	0.110977572	0.537998646	0.563231576	0.503741651	0.77481212	0.564025428	0.999996191
LOC613401	0.792924924	0.879387919	0.540402922	0.230146387	0.436092951	0.767146095	0.999996191
LOC613444	0.303124629	0.680824178	0.982673965	0.672449807	0.16741296	0.6717947	0.999996191
LOC613519	0.825526431	0.469980394	0.512705409	0.19553703	0.306047967	0.5452732	0.999996191
LOC613570	0.884838505	0.864212705	0.345398639	0.98306351	0.396439814	0.919298996	0.999996191
LOC613664	0.559484465	0.508721049	0.593803905	0.53877842	0.3593089	0.740491342	0.999996191
LOC613677	0.574252427	0.525744479	0.466857015	0.806899274	0.225060469	0.693908755	0.999996191
LOC614091	0.670567193	0.825425452	0.713239553	0.486889262	0.063044647	0.548681942	0.999996191
LOC614129	0.402589066	0.769118136	0.382249766	0.11838532	0.700716678	0.508835257	0.999996191
LOC614141	0.335371666	0.549173914	0.10351205	0.722833194	0.522890343	0.452347987	0.999996191
LOC614208	0.315122707	0.583812128	0.69769881	0.548467691	0.83463282	0.842290993	0.999996191
LOC614376	0.876709779	0.078488972	0.99524773	0.4226358	0.582295376	0.612594488	0.999996191
LOC614402	0.076473065	0.569499326	0.846144638	0.91096097	0.936451172	0.733014201	0.999996191
LOC614423	0.909699991	0.580847377	0.500237439	0.28177025	0.196076996	0.58467989	0.999996191
LOC614531	0.619004115	0.904163447	0.288589625	0.319849899	0.732609189	0.767282271	0.999996191
LOC614614	0.497547348	0.829892506	0.763820862	0.698057864	0.375952159	0.892285173	0.999996191
LOC614617	0.499164827	0.961513775	0.545475141	0.466509043	0.705901743	0.897637869	0.999996191
LOC614643	0.849006139	0.853935019	0.94510612	0.088499868	0.380470501	0.673834836	0.999996191
LOC614695	0.735487777	0.998480511	0.616760018	0.641100779	0.452282627	0.944596528	0.999996191
LOC614741	0.791218284	0.394323177	0.156210916	0.36548299	0.480187123	0.483302106	0.999996191
LOC614785	0.12959758	0.912868258	0.611108128	0.17080968	0.591419895	0.454747953	0.999996191
LOC614882	0.359572659	0.780254777	0.643451203	0.245390891	0.312777389	0.574490443	0.999996191
LOC614914	0.522953752	0.72454165	0.844758802	0.869561125	0.728875969	0.976581358	0.999996191
LOC614922	0.837206217	0.466762684	0.941811366	0.143837313	0.888292233	0.805579717	0.999996191
LOC614923	0.438430857	0.955465966	0.587369688	0.086998262	0.521425718	0.533002409	0.999996191
LOC615112	0.139025947	0.957096288	0.660685146	0.829052648	0.121953744	0.490361206	0.999996191
LOC615223	0.421613834	0.275875019	0.97832493	0.890019065	0.463019867	0.805102448	0.999996191
LOC615258	0.84558792	0.349679812	0.345987466	0.456580153	0.310344309	0.583246094	0.999996191
LOC615271	0.854627971	0.249489618	0.969346417	0.802378126	0.925665521	0.958001658	0.999996191
LOC615454	0.216649975	0.557605701	0.551949448	0.564883684	0.651692866	0.685830512	0.999996191
LOC615514	0.936448366	0.858982209	0.154300753	0.396176524	0.99323161	0.812025703	0.999996191
LOC615521	0.982673878	0.602195294	0.741261668	0.854269804	0.9255434	0.995354304	0.999996191
LOC615663	0.539397133	0.291883631	0.461030313	0.891091664	0.940671866	0.84775958	0.999996191
LOC615768	0.574288528	0.176603855	0.477968827	0.897013831	0.375273843	0.606291732	0.999996191
LOC615792	0.437986702	0.810690979	0.993652128	0.346521928	0.865477805	0.922425765	0.999996191

LOC615959	0.549579613	0.327336596	0.210990424	0.863754598	0.98661234	0.738357958	0.999996191
LOC615989	0.165388381	0.95274646	0.996599797	0.754116675	0.735392299	0.898941582	0.999996191
LOC616051	0.266125129	0.629621301	0.611860239	0.705598714	0.518051264	0.765489347	0.999996191
LOC616094	0.376559998	0.494568616	0.84492087	0.446218116	0.422668596	0.72216054	0.999996191
LOC616200	0.621238539	0.724596634	0.542872746	0.083950264	0.931843458	0.637201542	0.999996191
LOC616254	0.353021354	0.935284645	0.645164797	0.204246238	0.91734825	0.776848932	0.999996191
LOC616281	0.610660853	0.451410079	0.432096883	0.475507802	0.665664255	0.766583222	0.999996191
LOC616295	0.665912474	0.809052538	0.979188025	0.803159141	0.910313994	0.997015253	0.999996191
LOC616304	0.31561553	0.674944909	0.913113115	0.660688573	0.782828517	0.916650387	0.999996191
LOC616400	0.314850872	0.591885242	0.223665677	0.521858915	0.596351969	0.561757713	0.999996191
LOC616427	0.633280842	0.844320333	0.316833216	0.609081084	0.591583436	0.848267257	0.999996191
LOC616538	0.753742811	0.968020401	0.693620353	0.891952537	0.978865043	0.998455957	0.999996191
LOC616720	0.577309232	0.629592936	0.873679032	0.063206966	0.474197065	0.503037097	0.999996191
LOC616942	0.70415276	0.359762234	0.229761393	0.874561813	0.260728625	0.56617119	0.999996191
LOC616948	0.973045372	0.752688368	0.381602572	0.049708388	0.607738719	0.480929294	0.999996191
LOC616957	0.913157342	0.889488727	0.250653534	0.8488726	0.850203215	0.954461598	0.999996191
LOC617224	0.923859562	0.884844608	0.874214647	0.386835014	0.508136933	0.950647037	0.999996191
LOC617648	0.405866215	0.953638967	0.715731743	0.877456849	0.994050673	0.984928347	0.999996191
LOC617654	0.867145333	0.734671697	0.771014938	0.356953815	0.808906226	0.951475048	0.999996191
LOC617692	0.374373349	0.442888	0.765639909	0.124584129	0.927297669	0.585505776	0.999996191
LOC617698	0.800306351	0.659737253	0.703324653	0.120862563	0.876826013	0.774323868	0.999996191
LOC618220	0.96221364	0.955589673	0.286003572	0.262027638	0.177427722	0.550379885	0.999996191
LOC618256	0.835606486	0.81283888	0.6275212	0.501465797	0.288473205	0.849823419	0.999996191
LOC618409	0.794797711	0.94991018	0.415155548	0.511218279	0.586369715	0.908478965	0.999996191
LOC618633	0.754428834	0.884950461	0.979117656	0.784787606	0.909738144	0.998858222	0.999996191
LOC618787	0.638964102	0.26526351	0.351243834	0.894602962	0.750124855	0.777023202	0.999996191
LOC618939	0.959106365	0.013826364	0.790845387	0.934630555	0.801673044	0.467874142	0.999996191
LOC619026	0.745810318	0.818519257	0.396171162	0.678349462	0.801678395	0.944730462	0.999996191
LOC619156	0.836158752	0.469077399	0.691070152	0.932233632	0.75335296	0.97292992	0.999996191
LOC619159	0.152220628	0.949406738	0.175477497	0.841306984	0.741318031	0.600199705	0.999996191
LOC751811	0.86122057	0.870693982	0.230447681	0.763821456	0.824302202	0.925516306	0.999996191
LOC780963	0.744068931	0.945042085	0.517453466	0.564813019	0.803528718	0.963522007	0.999996191
LOC781004	0.607451458	0.403478115	0.716482388	0.880921168	0.267943644	0.783583351	0.999996191
LOC781059	0.738784745	0.664410789	0.648837849	0.852864414	0.853954692	0.983191329	0.999996191
LOC781064	0.683637885	0.501794089	0.287954323	0.097475902	0.771543035	0.457790072	0.999996191
LOC781100	0.791372001	0.281369871	0.809973733	0.194084174	0.338992333	0.544662316	0.999996191
LOC781108	0.921256207	0.142234576	0.426574176	0.364208146	0.738015888	0.590199667	0.999996191
LOC781158	0.751086476	0.756526085	0.459492413	0.766402818	0.405072187	0.889485194	0.999996191
LOC781218	0.470142449	0.684887925	0.09763072	0.656735562	0.555018113	0.537995465	0.999996191
LOC781254	0.859839343	0.274115482	0.194797854	0.507149198	0.950054927	0.665667332	0.999996191
LOC781280	0.523987613	0.522456332	0.641138087	0.795663229	0.174655092	0.684605202	0.999996191
LOC781298	0.698867241	0.355936354	0.637013702	0.214020374	0.851831383	0.717052529	0.999996191
LOC781379	0.931909594	0.218880959	0.873843683	0.730138104	0.962759614	0.940125283	0.999996191
LOC781421	0.73722033	0.908361504	0.322904708	0.168281858	0.329129457	0.546434509	0.999996191
LOC781439	0.522447272	0.884515203	0.58275286	0.065488178	0.869390115	0.594147791	0.999996191

LOC781499	0.694683887	0.753317403	0.847233434	0.254787886	0.704747505	0.88705441	0.999996191
LOC781533	0.921522529	0.86430891	0.258450228	0.819432825	0.172782298	0.71873106	0.999996191
LOC781565	0.333990091	0.781519009	0.26009384	0.179713826	0.86533046	0.522469338	0.999996191
LOC781576	0.857169306	0.39827734	0.908048439	0.539811921	0.704453731	0.934033119	0.999996191
LOC781612	0.338751252	0.896185152	0.278947366	0.30914823	0.552446327	0.582802469	0.999996191
LOC781646	0.959989556	0.263625709	0.470894741	0.98447084	0.195515097	0.672709483	0.999996191
LOC781688	0.835836203	0.398520297	0.87671157	0.728816581	0.143096704	0.727055574	0.999996191
LOC781692	0.488425094	0.617385574	0.992386612	0.330584239	0.884857706	0.899601881	0.999996191
LOC781728	0.870057797	0.517506529	0.630915734	0.943809683	0.643113298	0.966567622	0.999996191
LOC781770	0.738484607	0.790784556	0.38757476	0.891335048	0.719787666	0.953478152	0.999996191
LOC781799	0.760153894	0.556394058	0.62899828	0.460515095	0.500532666	0.848976984	0.999996191
LOC781813	0.443424139	0.892502175	0.539096656	0.304997884	0.573458082	0.764713493	0.999996191
LOC781913	0.278802089	0.33973535	0.356634066	0.568692683	0.977275492	0.633664662	0.999996191
LOC781982	0.793863795	0.712963807	0.48436614	0.566239232	0.813162548	0.940844638	0.999996191
LOC782024	0.515367463	0.418722643	0.557305445	0.906693899	0.686122345	0.878451984	0.999996191
LOC782032	0.71851444	0.919093414	0.694573487	0.985931137	0.719940943	0.994101669	0.999996191
LOC782101	0.058787927	0.550804171	0.484119471	0.816249118	0.549495567	0.447998255	0.999996191
LOC782114	0.806884392	0.411106769	0.277895033	0.387149041	0.451304489	0.603740013	0.999996191
LOC782120	0.566562977	0.656462573	0.913622556	0.336852827	0.930317906	0.923136915	0.999996191
LOC782159	0.302133627	0.563226857	0.974016597	0.691528267	0.915293921	0.921461926	0.999996191
LOC782202	0.82256492	0.88640642	0.490685911	0.946715238	0.675111848	0.982554107	0.999996191
LOC782258	0.983594842	0.886160446	0.716560785	0.919391676	0.464822826	0.9886466	0.999996191
LOC782293	0.507381518	0.759484981	0.426035994	0.282246382	0.48638082	0.669286135	0.999996191
LOC782305	0.886430425	0.971539768	0.681427833	0.555265667	0.768630753	0.986351901	0.999996191
LOC782385	0.488516107	0.944817308	0.647464313	0.792597162	0.635084581	0.956382057	0.999996191
LOC782418	0.861258071	0.27783074	0.288828318	0.480697788	0.292423895	0.506854775	0.999996191
LOC782437	0.933834133	0.772331486	0.848982601	0.222846917	0.324620588	0.795255698	0.999996191
LOC782470	0.746579076	0.653222372	0.964023121	0.670962999	0.993922323	0.993250408	0.999996191
LOC782525	0.28195813	0.632612303	0.991407521	0.36498391	0.222799069	0.581687237	0.999996191
LOC782527	0.901184049	0.389801268	0.257335777	0.89470619	0.102275563	0.477190785	0.999996191
LOC782560	0.986546861	0.102137018	0.506254555	0.601596206	0.355841636	0.528853691	0.999996191
LOC782566	0.429716463	0.602283726	0.301699648	0.856642109	0.258698613	0.617740366	0.999996191
LOC782609	0.15151912	0.532473385	0.38596131	0.269320365	0.984840468	0.476911842	0.999996191
LOC782673	0.298054278	0.169182381	0.928529827	0.551670482	0.526783968	0.570983315	0.999996191
LOC782688	0.723631006	0.707924119	0.685283607	0.449186693	0.273126581	0.790347231	0.999996191
LOC782755	0.564584871	0.697207869	0.705525802	0.662052198	0.951913699	0.967582373	0.999996191
LOC782776	0.517728687	0.954831472	0.74674201	0.30004456	0.709284512	0.885238558	0.999996191
LOC782799	0.535687772	0.679581855	0.578618787	0.232907153	0.146594073	0.452008047	0.999996191
LOC782812	0.714433361	0.638107632	0.951526944	0.047899017	0.697329147	0.583152543	0.999996191
LOC782954	0.272090199	0.973792974	0.792728571	0.13576492	0.404791747	0.539394907	0.999996191
LOC782987	0.817607843	0.444726361	0.303986259	0.988159968	0.572124934	0.851894994	0.999996191
LOC783022	0.952694102	0.907582319	0.849885294	0.761773077	0.199200274	0.928190019	0.999996191
LOC783060	0.868086106	0.724012148	0.944077968	0.916938171	0.606548226	0.994387163	0.999996191
LOC783142	0.303079027	0.413506096	0.71047072	0.704023839	0.262950275	0.608253574	0.999996191
LOC783163	0.720455515	0.970707759	0.560219784	0.098851968	0.664782553	0.69503382	0.999996191

LOC783185	0.833971808	0.630347371	0.448515793	0.939976557	0.59782436	0.945413056	0.999996191
LOC783195	0.697915501	0.89626706	0.842608653	0.223847332	0.588645954	0.867757793	0.999996191
LOC783202	0.836086499	0.736169028	0.517692278	0.031277227	0.845795304	0.480629556	0.999996191
LOC783255	0.75646256	0.618741314	0.671234422	0.283229656	0.483651915	0.79021679	0.999996191
LOC783261	0.811706272	0.54564792	0.392613543	0.450972243	0.457356548	0.757478044	0.999996191
LOC783294	0.57207323	0.853548986	0.879497898	0.425783317	0.75786139	0.949457811	0.999996191
LOC783301	0.776324771	0.95953171	0.915977322	0.352373747	0.915866854	0.980795689	0.999996191
LOC783376	0.775148089	0.432098594	0.664937504	0.582823646	0.666646618	0.898115589	0.999996191
LOC783378	0.322105681	0.178886291	0.227981362	0.625672237	0.876730357	0.452352829	0.999996191
LOC783396	0.03840462	0.943128984	0.550671555	0.91211006	0.84618174	0.594931324	0.999996191
LOC783421	0.752939806	0.271920595	0.662130337	0.148683389	0.822041653	0.609267629	0.999996191
LOC783461	0.764687194	0.630853855	0.665625926	0.501812994	0.870492825	0.950517397	0.999996191
LOC783533	0.462875628	0.573283443	0.658131845	0.395497257	0.966856722	0.861963189	0.999996191
LOC783539	0.654545621	0.922491497	0.706074699	0.895289884	0.459138579	0.967670016	0.999996191
LOC783612	0.810322323	0.807952689	0.615650905	0.555650439	0.382617499	0.896850187	0.999996191
LOC783657	0.194740262	0.455430562	0.99511451	0.114586488	0.889263212	0.492523577	0.999996191
LOC783730	0.287182823	0.624215923	0.482570754	0.166276599	0.648883238	0.499402298	0.999996191
LOC783803	0.350588186	0.517171439	0.610690179	0.633418388	0.635206847	0.796259052	0.999996191
LOC783926	0.814352309	0.851564402	0.775311726	0.928442885	0.130430836	0.858156009	0.999996191
LOC783942	0.173414387	0.696562953	0.439149135	0.993590608	0.522279491	0.707859216	0.999996191
LOC783988	0.905084796	0.259085483	0.640512295	0.380572174	0.320235247	0.628721734	0.999996191
LOC784054	0.410675395	0.719281547	0.830492256	0.452601849	0.69285522	0.88234463	0.999996191
LOC784058	0.931165987	0.23218916	0.876661817	0.642660055	0.731798195	0.901918345	0.999996191
LOC784088	0.54187809	0.902777951	0.510677581	0.749194584	0.967552609	0.969825508	0.999996191
LOC784127	0.619598791	0.128767352	0.351831519	0.947320833	0.860698859	0.672279592	0.999996191
LOC784208	0.543953207	0.777172566	0.619488849	0.212482747	0.754041722	0.785749836	0.999996191
LOC784243	0.964658115	0.335374107	0.368068144	0.273435737	0.893574202	0.718404506	0.999996191
LOC784297	0.626417966	0.25938433	0.367749354	0.959710166	0.867493354	0.815127637	0.999996191
LOC784322	0.915478295	0.088855007	0.433620504	0.681035065	0.686979437	0.608481788	0.999996191
LOC784354	0.966802771	0.912435957	0.407214821	0.537834917	0.546286781	0.922140303	0.999996191
LOC784357	0.96389309	0.418123143	0.611522594	0.96081665	0.507560913	0.936005827	0.999996191
LOC784464	0.91236858	0.989988302	0.896139688	0.480443067	0.621766448	0.984956054	0.999996191
LOC784473	0.742879106	0.569540301	0.920561664	0.375339582	0.891297021	0.943860787	0.999996191
LOC784488	0.732677771	0.412927834	0.394265787	0.786370731	0.380772243	0.756712685	0.999996191
LOC784521	0.778912372	0.707448302	0.575063638	0.857715241	0.301905996	0.891131958	0.999996191
LOC784522	0.990012136	0.177855581	0.831083488	0.811746502	0.62420872	0.877185204	0.999996191
LOC784659	0.641359702	0.985576437	0.68095224	0.799241297	0.516341798	0.96856896	0.999996191
LOC784735	0.717653133	0.646481625	0.554212003	0.689828763	0.856443617	0.95716938	0.999996191
LOC784808	0.377004741	0.375397977	0.995054263	0.794027299	0.896721128	0.916264259	0.999996191
LOC784841	0.46856319	0.504723951	0.169915201	0.395625986	0.914096429	0.583729057	0.999996191
LOC784866	0.925637775	0.811681298	0.325642415	0.661374405	0.331446251	0.827605942	0.999996191
LOC784966	0.618236163	0.555343695	0.418846379	0.966047066	0.913286249	0.941334907	0.999996191
LOC784980	0.782990305	0.733776399	0.234755786	0.479389212	0.396400282	0.694165209	0.999996191
LOC785087	0.195564673	0.905102995	0.215460442	0.693013773	0.401475476	0.523421195	0.999996191
LOC785216	0.844143795	0.969763379	0.709447937	0.795553228	0.688881138	0.99359997	0.999996191

LOC785386	0.482663667	0.97704256	0.710049973	0.532836127	0.487263354	0.898717157	0.999996191
LOC785403	0.374716463	0.26965439	0.704791231	0.833409964	0.740415838	0.793870462	0.999996191
LOC785445	0.678556715	0.916909705	0.994203167	0.894992007	0.912149902	0.999294499	0.999996191
LOC785477	0.838961552	0.212799672	0.22944899	0.775364335	0.304816218	0.506212193	0.999996191
LOC785605	0.688096899	0.89960784	0.433103187	0.934013165	0.869868379	0.980267883	0.999996191
LOC785630	0.393601134	0.555186823	0.265718643	0.59294757	0.392400899	0.569606017	0.999996191
LOC785693	0.606459786	0.753378881	0.481222473	0.517468552	0.543049272	0.850145189	0.999996191
LOC785760	0.185262023	0.468472189	0.608920337	0.742936026	0.578467626	0.670787218	0.999996191
LOC785761	0.540591191	0.718865787	0.892871588	0.274512254	0.905717894	0.89772193	0.999996191
LOC785804	0.813822353	0.680612859	0.312874117	0.060443978	0.842377014	0.489019235	0.999996191
LOC785843	0.879778214	0.785177818	0.206361444	0.898564492	0.77750474	0.915466576	0.999996191
LOC785873	0.526470549	0.560889934	0.338776198	0.76236114	0.170748836	0.562505497	0.999996191
LOC786015	0.830590101	0.6676879	0.665373566	0.565408694	0.220285575	0.801646846	0.999996191
LOC786055	0.442079989	0.421381081	0.763996024	0.433090175	0.552353153	0.747881912	0.999996191
LOC786173	0.419452493	0.858504417	0.98937842	0.186553436	0.938646282	0.851640802	0.999996191
LOC786252	0.765187376	0.746682639	0.473099555	0.09339508	0.968122792	0.68499577	0.999996191
LOC786256	0.717111441	0.596223496	0.741971972	0.795783304	0.260976978	0.85993847	0.999996191
LOC786258	0.912559738	0.404990471	0.580762352	0.495742592	0.262776225	0.710838833	0.999996191
LOC786303	0.899238745	0.558506829	0.871201258	0.785494412	0.84614568	0.991303374	0.999996191
LOC786332	0.95403284	0.798400411	0.584337579	0.72838515	0.868934538	0.990374223	0.999996191
LOC786363	0.684944049	0.334032674	0.450822155	0.889048258	0.502700759	0.802166363	0.999996191
LOC786372	0.86964796	0.351407302	0.152244881	0.862845661	0.593763046	0.680155595	0.999996191
LOC786417	0.683856194	0.797610274	0.665237772	0.575412067	0.977805018	0.976926359	0.999996191
LOC786435	0.429115592	0.485427041	0.670290822	0.87016333	0.948223806	0.931650168	0.999996191
LOC786489	0.976429993	0.707040725	0.829875305	0.594073951	0.882865782	0.992193344	0.999996191
LOC786553	0.894054476	0.427262489	0.192481274	0.378315014	0.968619006	0.703756658	0.999996191
LOC786616	0.458735678	0.789469315	0.448026404	0.204205242	0.830296637	0.707741998	0.999996191
LOC786783	0.289018647	0.650923544	0.744005967	0.977575396	0.643665396	0.900384172	0.999996191
LOC786914	0.780894881	0.212119039	0.529835262	0.761406309	0.154522868	0.518284819	0.999996191
LOC786930	0.484697326	0.692501493	0.959568298	0.521390505	0.640535634	0.924257762	0.999996191
LOC786978	0.838998023	0.594668234	0.082643426	0.982259769	0.644007729	0.697531778	0.999996191
LOC787057	0.662634427	0.268122168	0.720253971	0.868459602	0.34433755	0.769277309	0.999996191
LOC787074	0.816623225	0.322065455	0.534696537	0.964277423	0.429827549	0.841016359	0.999996191
LOC787237	0.890661337	0.302903834	0.820050574	0.580933956	0.902574311	0.932369925	0.999996191
LOC787250	0.466872268	0.959561541	0.360134117	0.918403173	0.751137879	0.927984948	0.999996191
LOC787257	0.997724044	0.67864897	0.322118227	0.169369101	0.807439321	0.723115103	0.999996191
LOC787269	0.723423223	0.273943688	0.178372889	0.724945573	0.807305779	0.652620789	0.999996191
LOC787287	0.594810812	0.711777499	0.325700877	0.401455784	0.337721691	0.632848003	0.999996191
LOC787497	0.729603637	0.997877145	0.912854698	0.417206508	0.633584018	0.967833796	0.999996191
LOC787530	0.69051282	0.975999086	0.960885105	0.172553154	0.370188848	0.783224435	0.999996191
LOC787550	0.083389868	0.799741967	0.182361595	0.843889964	0.840548814	0.484866999	0.999996191
LOC787679	0.513771575	0.600004702	0.421958745	0.708237071	0.598907653	0.832220127	0.999996191
LOC787714	0.628874398	0.456438616	0.610622205	0.92840657	0.495158081	0.888683878	0.999996191
LOC787812	0.338892427	0.221767786	0.985608066	0.828764898	0.836427882	0.82042387	0.999996191
LOC787858	0.964833337	0.148343533	0.318874132	0.298020085	0.925099709	0.555899661	0.999996191

LOC787875	0.949780553	0.66304271	0.991313998	0.922635162	0.342150037	0.974959731	0.999996191
LOC787905	0.241804762	0.421688826	0.554474425	0.520933856	0.390857376	0.538875423	0.999996191
LOC788142	0.440770712	0.572023604	0.708486437	0.868285938	0.869511412	0.947031746	0.999996191
LOC788183	0.964520092	0.98997167	0.758443075	0.965478003	0.498238159	0.995434403	0.999996191
LOC788201	0.108128086	0.93206219	0.360871079	0.504265372	0.453453554	0.478163688	0.999996191
LOC788205	0.717163646	0.714181333	0.751808409	0.700658802	0.378258947	0.918310213	0.999996191
LOC788293	0.902584595	0.975494233	0.972648887	0.945448706	0.979164973	0.999995363	0.999996191
LOC788405	0.989137511	0.637322204	0.641839471	0.128236606	0.740245011	0.769944437	0.999996191
LOC788414	0.859983994	0.891883381	0.459765253	0.226833164	0.340326349	0.705732427	0.999996191
LOC788541	0.832926775	0.872164449	0.802631982	0.19794667	0.48609337	0.834917979	0.999996191
LOC788634	0.821524682	0.93486441	0.274658487	0.35935712	0.193220008	0.585253276	0.999996191
LOC788672	0.802969272	0.838867992	0.124610451	0.824072513	0.4495443	0.73096135	0.999996191
LOC788724	0.69266644	0.329816628	0.603856954	0.107920072	0.639019971	0.502952813	0.999996191
LOC789018	0.617846796	0.718142147	0.69781477	0.05856033	0.401435368	0.454140399	0.999996191
LOC789157	0.273159588	0.455992133	0.115645352	0.714569202	0.867919541	0.491296133	0.999996191
LOC789192	0.455743609	0.966696577	0.594487554	0.558718623	0.593909781	0.898676401	0.999996191
LOC789258	0.253274253	0.894415118	0.984854404	0.230428032	0.840889645	0.790998293	0.999996191
LOC789352	0.679902146	0.900068594	0.470744769	0.229750612	0.339852415	0.668905694	0.999996191
LOC789384	0.628773636	0.957233736	0.882689963	0.957387332	0.532543154	0.989137496	0.999996191
LOC789494	0.813894035	0.720031904	0.695028695	0.692197187	0.907219793	0.987141194	0.999996191
LOC789551	0.489191981	0.554948518	0.103965677	0.401965508	0.922771206	0.520879182	0.999996191
LOC789569	0.380818472	0.876781992	0.89102051	0.190452459	0.273296022	0.596078464	0.999996191
LOC789587	0.152354551	0.603440488	0.546622325	0.731821829	0.603362194	0.66626785	0.999996191
LOC789626	0.855522904	0.93430141	0.915434692	0.813353166	0.881172416	0.999452497	0.999996191
LOC789694	0.553375723	0.558294048	0.91722589	0.062004279	0.822196704	0.582578622	0.999996191
LOC789715	0.277277597	0.669446428	0.697945656	0.450264323	0.293326115	0.615545734	0.999996191
LOC789733	0.478319828	0.651728583	0.438555537	0.819398109	0.716259234	0.888115208	0.999996191
LOC789764	0.536888449	0.413436209	0.221859765	0.701991309	0.710804726	0.686037788	0.999996191
LOC789895	0.469282307	0.812265389	0.490710615	0.100680287	0.653543702	0.551657305	0.999996191
LOC789960	0.672135349	0.456825848	0.669124545	0.624258126	0.825627209	0.922509784	0.999996191
LOC789996	0.396287747	0.536797965	0.980467316	0.901959037	0.648371219	0.937481003	0.999996191
LOC789997	0.731288881	0.799079129	0.693363325	0.995962418	0.792503183	0.993707759	0.999996191
LOC790037	0.116833966	0.575281989	0.963880831	0.702968922	0.970540038	0.794881713	0.999996191
LOC790098	0.52280271	0.908841744	0.370895559	0.841267365	0.740834583	0.926549206	0.999996191
LOC790218	0.736504645	0.301792149	0.502098118	0.809085237	0.201663183	0.627699199	0.999996191
LOC790266	0.709185781	0.619692912	0.978676706	0.610401529	0.917789195	0.984813223	0.999996191
LOC790271	0.512177408	0.542310133	0.541060494	0.408520092	0.322353113	0.643964759	0.999996191
LOC790871	0.999716282	0.834616183	0.699466757	0.705026211	0.794012042	0.994175938	0.999996191
LOC790886	0.791277221	0.924841362	0.566853162	0.666670458	0.73012315	0.976323432	0.999996191
LONP1	0.940259773	0.626258755	0.346437867	0.310329504	0.702395304	0.795927847	0.999996191
LONP2	0.825025451	0.932815853	0.430345903	0.478213189	0.44369424	0.869477059	0.999996191
LONRF1	0.169146884	0.822239349	0.870082469	0.342662056	0.725938864	0.724844604	0.999996191
LONRF2	0.647850036	0.609154859	0.818543468	0.443526819	0.099285404	0.579582924	0.999996191
LOX	0.838064013	0.516460449	0.233601114	0.266982508	0.570063713	0.594858389	0.999996191
LPAR1	0.196084025	0.936745986	0.57347641	0.8013631	0.969427286	0.890765536	0.999996191

LPAR4	0.759177627	0.860901422	0.674739326	0.412928632	0.526031748	0.910829366	0.999996191
LPAR5	0.372048078	0.516164108	0.407390409	0.982000421	0.196036739	0.590670369	0.999996191
LPCAT1	0.141599346	0.464932086	0.839309344	0.943916831	0.155842165	0.473999876	0.999996191
LPCAT3	0.978544974	0.941079635	0.616333281	0.685349718	0.029607238	0.538960345	0.999996191
LPCAT4	0.938600436	0.632176937	0.218158936	0.948185561	0.555579298	0.865068215	0.999996191
LPIN2	0.785721892	0.642658541	0.309783972	0.953657209	0.08855914	0.565280447	0.999996191
LPIN3	0.977213783	0.025471414	0.878870109	0.977568974	0.610293089	0.562935958	0.999996191
LPL	0.446874973	0.632282554	0.862893102	0.742426285	0.235723087	0.788705864	0.999996191
LRAT	0.475731057	0.366051321	0.596585751	0.220317986	0.34239271	0.467394881	0.999996191
LRCH1	0.650362538	0.442030225	0.421838271	0.306791778	0.77742098	0.717283829	0.999996191
LRCH2	0.381669143	0.618538142	0.847887933	0.311448156	0.776625076	0.810549444	0.999996191
LRCH3	0.398525139	0.660189749	0.923780252	0.776160705	0.236461116	0.796477639	0.999996191
LRCH4	0.726418103	0.844919961	0.791964495	0.184796038	0.81719811	0.875755411	0.999996191
LRFN1	0.70110725	0.88299773	0.677034043	0.201914896	0.208347016	0.621409535	0.999996191
LRFN3	0.672314814	0.87963197	0.528563555	0.06097695	0.431520301	0.476154536	0.999996191
LRG1	0.95649849	0.49001666	0.233491801	0.30753957	0.421946339	0.579259628	0.999996191
LRIG1	0.614365652	0.711725636	0.097177383	0.399975099	0.694911974	0.543764023	0.999996191
LRIG2	0.137921425	0.945638573	0.918772583	0.732443714	0.290218965	0.692971125	0.999996191
LRMP	0.753526155	0.947467937	0.07315569	0.540044363	0.297616667	0.47987394	0.999996191
LRP1	0.495497602	0.720354079	0.104621964	0.386525996	0.845762787	0.550095135	0.999996191
LRP10	0.231104031	0.894470277	0.500506284	0.293291007	0.440573844	0.567576447	0.999996191
LRP12	0.923460057	0.646435887	0.891784938	0.476554406	0.909995341	0.982982477	0.999996191
LRP2BP	0.717683605	0.120969453	0.979749969	0.19889683	0.819699977	0.574657259	0.999996191
LRP5	0.094963259	0.744436468	0.554426076	0.661951672	0.520433654	0.569496904	0.999996191
LRP8	0.433090785	0.797551811	0.488550941	0.825492618	0.711849989	0.9149592	0.999996191
LRPAP1	0.483042103	0.919204892	0.148116493	0.833404011	0.797974354	0.793041859	0.999996191
LRRC14	0.687321396	0.165222264	0.640657024	0.218868274	0.50755178	0.472965449	0.999996191
LRRC20	0.687110768	0.373715487	0.266787111	0.226176129	0.584219125	0.493731636	0.999996191
LRRC27	0.765257604	0.683139683	0.427674162	0.121048959	0.405630111	0.529852784	0.999996191
LRRC28	0.934292353	0.67428696	0.48453263	0.785329353	0.887158712	0.979072671	0.999996191
LRRC32	0.625060568	0.901987039	0.616499494	0.956513146	0.86349247	0.990934158	0.999996191
LRRC3B	0.31640568	0.402750163	0.409421078	0.385880426	0.377124312	0.461690157	0.999996191
LRRC40	0.074956248	0.861111823	0.885600816	0.756655975	0.410341402	0.622686465	0.999996191
LRRC42	0.976576145	0.731124007	0.065959563	0.544718138	0.912530161	0.676654347	0.999996191
LRRC55	0.555923269	0.29186226	0.617363603	0.738966936	0.688096598	0.819075761	0.999996191
LRRC58	0.842724426	0.81009578	0.641599061	0.158011171	0.90242864	0.851815721	0.999996191
LRRC69	0.854233047	0.889567627	0.63189726	0.293846612	0.691212689	0.912989417	0.999996191
LRRC7	0.808662899	0.147258182	0.705207939	0.376280592	0.903114221	0.714730032	0.999996191
LRRC74B	0.125802066	0.261700416	0.92379298	0.813936868	0.592609135	0.585559715	0.999996191
LRRC75A	0.325626159	0.931859837	0.53554177	0.871457265	0.989240593	0.950409427	0.999996191
LRRC8A	0.075284592	0.967474322	0.695968396	0.621749451	0.861344982	0.705199752	0.999996191
LRRC8C	0.310725314	0.855715606	0.341603486	0.21649965	0.513105315	0.513945223	0.999996191
LRRC8E	0.979115551	0.924926847	0.494945015	0.24886039	0.424717343	0.806852688	0.999996191
LRRFIP1	0.755771398	0.654051273	0.674509073	0.452442718	0.922963247	0.949871546	0.999996191
LRRFIP2	0.491605188	0.343000939	0.547617498	0.444616303	0.756219878	0.730674583	0.999996191

LRRK1	0.466380138	0.40920434	0.892015729	0.31921226	0.50331836	0.706630087	0.999996191
LRRN1	0.40437492	0.262287459	0.255029174	0.553882064	0.863385869	0.561214996	0.999996191
LRRN4CL	0.833369433	0.911037815	0.609122194	0.682362231	0.505976225	0.961035233	0.999996191
LRSAM1	0.902436768	0.823541357	0.578572011	0.243477362	0.897645497	0.908505794	0.999996191
LSM1	0.542401425	0.533206961	0.472473021	0.493959878	0.269103954	0.627209984	0.999996191
LSM11	0.139454316	0.981463713	0.828845826	0.320228923	0.896391829	0.739610593	0.999996191
LSM12	0.839290489	0.208083787	0.768596973	0.606729391	0.161323996	0.564218809	0.999996191
LSM14A	0.208833244	0.797453508	0.915652944	0.097703385	0.986312564	0.58588804	0.999996191
LSM2	0.36414216	0.350371431	0.769669146	0.411645017	0.904656753	0.761028791	0.999996191
LSM3	0.731021082	0.800791486	0.465542954	0.906146779	0.671663728	0.963840813	0.999996191
LSM4	0.523284767	0.78638323	0.877669086	0.070989691	0.468027841	0.546802623	0.999996191
LSM6	0.958585255	0.085766383	0.841836326	0.359043012	0.492776503	0.550683966	0.999996191
LSM7	0.518891197	0.765645347	0.291096416	0.924133179	0.985074334	0.921858059	0.999996191
LSM8	0.872368815	0.346802698	0.731476023	0.323106273	0.779941696	0.833953558	0.999996191
LSR	0.757733908	0.829053073	0.583023388	0.450303514	0.599765247	0.914666486	0.999996191
LTB	0.613396891	0.468175395	0.660899143	0.733527134	0.151922661	0.656928695	0.999996191
LTB4R	0.379942324	0.93605885	0.267688739	0.136507829	0.64547624	0.479744052	0.999996191
LTB4R2	0.4511905	0.724512858	0.657557475	0.487708893	0.558717139	0.841790479	0.999996191
LTBP1	0.687858171	0.962482588	0.072963161	0.687055653	0.776826103	0.695296097	0.999996191
LTBP4	0.063456969	0.603917721	0.740452013	0.915275191	0.588322609	0.593480785	0.999996191
LTBR	0.363310953	0.796648218	0.482201045	0.422052759	0.21241296	0.554814465	0.999996191
LTC4S	0.813598639	0.275217687	0.152541006	0.42878401	0.798257515	0.541823988	0.999996191
LUZP6	0.66433418	0.249943982	0.293489964	0.811000366	0.692255072	0.706689348	0.999996191
LVRN	0.532061785	0.29302072	0.513805946	0.288247191	0.723669099	0.610913338	0.999996191
LY96	0.655473116	0.879435896	0.228432877	0.081296657	0.764769437	0.475309573	0.999996191
LYL1	0.784379854	0.553180833	0.325928892	0.256000831	0.358441409	0.561836866	0.999996191
LYPD1	0.786145909	0.779297538	0.475241127	0.149674733	0.266040161	0.540225797	0.999996191
LYPD6	0.101140027	0.966846938	0.242073251	0.448159789	0.965711177	0.516806062	0.999996191
LYPLAL1	0.79121276	0.847535721	0.957755802	0.4573709	0.99283232	0.991383641	0.999996191
LYRM2	0.970816992	0.397625106	0.184960114	0.90148322	0.929019587	0.845047548	0.999996191
LYRM7	0.132398501	0.828252169	0.473696617	0.475230899	0.593087354	0.585176659	0.999996191
LYSMD2	0.914717765	0.786265611	0.756964481	0.074913513	0.790817095	0.737817221	0.999996191
LYSMD4	0.549801507	0.629101003	0.813997799	0.337908512	0.89953247	0.896676569	0.999996191
LYST	0.393298862	0.709125765	0.575415421	0.273285577	0.894301428	0.773719676	0.999996191
LYVE1	0.930542761	0.896387282	0.669566432	0.788915708	0.838032189	0.996396714	0.999996191
LYZ1	0.423806547	0.832820782	0.587208145	0.673625867	0.458557929	0.855600848	0.999996191
LZIC	0.912747494	0.252061843	0.628319525	0.620699744	0.391895273	0.753844512	0.999996191
LZTFL1	0.504573049	0.559023329	0.562399578	0.597923081	0.528084337	0.816280173	0.999996191
LZTR1	0.51153554	0.701582614	0.410776798	0.285732282	0.618288259	0.697243916	0.999996191
LZTS2	0.830617028	0.398075913	0.313817984	0.368236623	0.709089528	0.704822984	0.999996191
LZTS3	0.721585291	0.915998243	0.643684043	0.91819394	0.868070978	0.994932816	0.999996191
M1AP	0.393900683	0.550238258	0.909053929	0.305249256	0.653811423	0.774177419	0.999996191
MACO1	0.993051559	0.769381938	0.353002639	0.644033922	0.639735277	0.927814271	0.999996191
MACROD1	0.602575596	0.836307004	0.53452224	0.695310797	0.70871798	0.945581734	0.999996191
MACROD2	0.164765942	0.55223447	0.289369564	0.59411723	0.627186908	0.508693375	0.999996191

MAD1L1	0.495756246	0.132617792	0.954887094	0.561751142	0.636204795	0.668417261	0.999996191
MAD2L1BP	0.551873848	0.082939718	0.797763691	0.646609841	0.920975128	0.662328781	0.999996191
MAD2L2	0.590994881	0.589330844	0.443289601	0.676562618	0.625537208	0.85869468	0.999996191
MADD	0.67462896	0.789251254	0.673203441	0.368555191	0.61577233	0.889970549	0.999996191
MAEA	0.919824713	0.831892561	0.297220817	0.08557851	0.759841842	0.587131253	0.999996191
MAFA	0.426711387	0.658828719	0.785410758	0.668932205	0.65462921	0.911955437	0.999996191
MAGED2	0.374309987	0.684974837	0.44475193	0.656362281	0.858902203	0.85622666	0.999996191
MAGEE2	0.814048941	0.977751411	0.200611326	0.200329723	0.898501103	0.716078544	0.999996191
MAGEH1	0.11859004	0.666601847	0.692156186	0.201543581	0.857968828	0.501929694	0.999996191
MAGI1	0.177432078	0.723291566	0.809709231	0.481112192	0.393479505	0.642788716	0.999996191
MAGI2	0.500697149	0.762125227	0.163991795	0.462139837	0.548882659	0.600904242	0.999996191
MAGOH	0.517894386	0.342083662	0.178607413	0.503085431	0.745005193	0.544558837	0.999996191
MAGOHB	0.627156528	0.562257333	0.809330402	0.945339517	0.505650408	0.948063764	0.999996191
MAGT1	0.450956019	0.129006665	0.67580535	0.400742761	0.666978587	0.521591456	0.999996191
MAL	0.534321975	0.625527036	0.396005701	0.882288	0.21077294	0.686359168	0.999996191
MAL2	0.208363296	0.802765163	0.386980828	0.900829188	0.650832854	0.76777093	0.999996191
MALL	0.810624984	0.791825769	0.890446663	0.974825386	0.798044212	0.998506593	0.999996191
MALSU1	0.948272125	0.938836142	0.971769118	0.082262876	0.763885748	0.829820707	0.999996191
MAML1	0.681545274	0.620458363	0.789131189	0.378874733	0.961688538	0.937164449	0.999996191
MAMLD1	0.62487812	0.686689826	0.404559131	0.125522074	0.762389165	0.609776532	0.999996191
MAMSTR	0.56479563	0.873326865	0.22911178	0.740692131	0.08381812	0.447617953	0.999996191
MAN1A1	0.878778489	0.738076916	0.819178646	0.038400608	0.750230639	0.593826397	0.999996191
MAN1A2	0.739410531	0.67257885	0.939549455	0.125394087	0.925448941	0.829392856	0.999996191
MAN1B1	0.80140555	0.183447045	0.249785343	0.534382966	0.479019733	0.500721163	0.999996191
MAN1B1	0.360363404	0.894685038	0.485446849	0.648252958	0.564475746	0.838222472	0.999996191
MAN2A1	0.716717341	0.888585191	0.800053665	0.379628758	0.983068313	0.97286491	0.999996191
MAN2B1	0.461072844	0.94159323	0.79754036	0.259049248	0.70221712	0.853108102	0.999996191
MAN2C1	0.528210529	0.599647718	0.775233874	0.742225417	0.473432526	0.897742642	0.999996191
MANEA	0.866802217	0.960283931	0.34377651	0.691968814	0.931257045	0.970974694	0.999996191
MANF	0.124135005	0.756922275	0.547224355	0.292240878	0.986545453	0.587591881	0.999996191
MANSC4	0.281599889	0.662223826	0.811964482	0.760796719	0.804993893	0.90686664	0.999996191
MAOA	0.889814489	0.70339032	0.613629188	0.047825539	0.999257912	0.629249565	0.999996191
MAP10	0.490943683	0.840884628	0.878945672	0.963915783	0.96616663	0.994864705	0.999996191
MAP1B	0.741021512	0.934960889	0.078231053	0.994558675	0.928455829	0.816144054	0.999996191
MAP1LC3A	0.660987619	0.912608227	0.228106577	0.209633082	0.570510729	0.607936496	0.999996191
MAP1LC3B	0.615617643	0.597361698	0.39614741	0.910608174	0.756927937	0.916429161	0.999996191
MAP1S	0.296790384	0.759333178	0.495783472	0.24841212	0.497142781	0.57368639	0.999996191
MAP2K1	0.982717277	0.871671363	0.318639663	0.073001619	0.622235509	0.553067242	0.999996191
MAP2K2	0.191129767	0.999424147	0.529222191	0.214180421	0.84260742	0.628071828	0.999996191
MAP2K5	0.804894315	0.509840543	0.397495809	0.984645941	0.901906088	0.953274467	0.999996191
MAP2K7	0.245269833	0.854168679	0.703521679	0.493120413	0.644963085	0.805040847	0.999996191
MAP3K11	0.917033967	0.93273052	0.174317138	0.292019108	0.282795939	0.551740977	0.999996191
MAP3K13	0.105350932	0.883920964	0.583406933	0.352950772	0.929150089	0.623439524	0.999996191
MAP3K14	0.930010785	0.304315975	0.529780154	0.718802709	0.358999699	0.771267062	0.999996191
MAP3K2	0.580372925	0.818806595	0.649251104	0.128778215	0.752377516	0.723535035	0.999996191

MAP3K20	0.713424695	0.485945765	0.950308851	0.971250845	0.559145116	0.969042779	0.999996191
MAP3K21	0.332899739	0.330997469	0.652031851	0.824358603	0.641121677	0.767876218	0.999996191
MAP3K3	0.531758446	0.955953221	0.838056443	0.403870512	0.706681989	0.937165329	0.999996191
MAP3K4	0.558416393	0.845716651	0.364407447	0.62583274	0.773795972	0.893196424	0.999996191
MAP3K5	0.227738495	0.692110422	0.187118089	0.330532911	0.937873361	0.49557599	0.999996191
MAP3K7	0.492856904	0.996438802	0.436527231	0.730007773	0.191148418	0.72366635	0.999996191
MAP3K7CL	0.397432374	0.715884074	0.493973156	0.935179096	0.403679292	0.825835456	0.999996191
MAP3K8	0.294890195	0.628935934	0.986921498	0.79669472	0.968968881	0.951156201	0.999996191
MAP4	0.456423196	0.703429548	0.509554958	0.497374204	0.644837088	0.824004683	0.999996191
MAP6D1	0.998786651	0.074353549	0.587659644	0.784768107	0.286809491	0.508918167	0.999996191
MAP7	0.34018051	0.827618099	0.079607512	0.77684939	0.917816198	0.602209963	0.999996191
MAP7D1	0.652477244	0.897404462	0.185312092	0.332600494	0.800880003	0.717150119	0.999996191
MAP9	0.881757954	0.755685506	0.443459707	0.198361706	0.326002785	0.637114302	0.999996191
MAPK12	0.854439352	0.704614036	0.99320558	0.383451846	0.822393986	0.972356877	0.999996191
MAPK1IP1L	0.879468087	0.139009271	0.605557795	0.395258479	0.834971553	0.68493414	0.999996191
MAPK6	0.665445486	0.125446038	0.631258342	0.789681305	0.76102834	0.734395433	0.999996191
MAPK7	0.516991458	0.84932525	0.191897878	0.563905062	0.687447129	0.74018385	0.999996191
MAPK8	0.217160905	0.34982882	0.454233708	0.633610611	0.322302446	0.448396721	0.999996191
MAPK8IP1	0.901478354	0.716733589	0.163197746	0.709461001	0.901757069	0.863469452	0.999996191
MAPK8IP3	0.810777585	0.838146693	0.43415419	0.78703534	0.658927817	0.957738894	0.999996191
MAPKAP1	0.943132604	0.68869312	0.616184661	0.409671214	0.773071813	0.941243853	0.999996191
MAPKAPK5	0.612425473	0.414088592	0.615109793	0.183748275	0.784843699	0.668928529	0.999996191
MAPKBP1	0.673738822	0.590213357	0.685847634	0.55834333	0.307560353	0.804885744	0.999996191
MAPRE2	0.099021962	0.589786175	0.951402793	0.838393049	0.684949387	0.73580498	0.999996191
MAPRE3	0.808308296	0.526649771	0.289747116	0.653396589	0.604651978	0.811645969	0.999996191
MARC2	0.559988315	0.692592904	0.422813915	0.875337604	0.906933394	0.943777724	0.999996191
MARCH2	0.857029109	0.874351769	0.313481982	0.213848418	0.967433367	0.811186399	0.999996191
MARCH3	0.315584411	0.495402935	0.687072688	0.385742296	0.246941102	0.516572212	0.999996191
MARCH5	0.970486004	0.367877108	0.350636047	0.499571879	0.596023993	0.764511104	0.999996191
MARCH7	0.330049573	0.424934425	0.955421956	0.460717706	0.978083878	0.846574155	0.999996191
MARCH8	0.444591748	0.200154654	0.792441322	0.646511395	0.58101956	0.700493739	0.999996191
MARCH9	0.825797966	0.868779779	0.382725881	0.892609995	0.763805584	0.971913677	0.999996191
MARF1	0.69295048	0.680535629	0.489630822	0.259301138	0.482674544	0.717120791	0.999996191
MARK1	0.529704361	0.818606032	0.585914708	0.24259461	0.222853454	0.572802192	0.999996191
MARK2	0.502825483	0.566208211	0.587000644	0.582519042	0.389412959	0.767579663	0.999996191
MARK3	0.856515426	0.590860436	0.508727867	0.177872637	0.334320642	0.593864664	0.999996191
MARK4	0.390716609	0.660754785	0.320762498	0.175180307	0.750295886	0.528230882	0.999996191
MARS2	0.991784099	0.610521656	0.759030401	0.595785067	0.358041445	0.913610628	0.999996191
MAS1	0.527204295	0.077712716	0.68640808	0.506095234	0.733188221	0.520270063	0.999996191
MASP1	0.314958309	0.371675658	0.827229519	0.446045395	0.716978562	0.730200761	0.999996191
MAST2	0.514635007	0.790361881	0.057931192	0.682106073	0.798995849	0.559823094	0.999996191
MAST3	0.901097609	0.66501239	0.503945619	0.946004054	0.427137552	0.93752246	0.999996191
MATN2	0.296817311	0.363029869	0.177969783	0.498557907	0.752102399	0.451977423	0.999996191
MATR3	0.483582246	0.671844015	0.764674479	0.113595402	0.633545363	0.62412794	0.999996191
MAU2	0.422628496	0.611080202	0.688967114	0.882969117	0.204839457	0.737406784	0.999996191

MAVS	0.323950418	0.928349528	0.966389861	0.553699742	0.515900979	0.892686193	0.999996191
MB	0.99198894	0.47838109	0.677475751	0.197063329	0.196908047	0.554249757	0.999996191
MB21D2	0.727520881	0.969861309	0.243694837	0.2309376	0.570609521	0.670328366	0.999996191
MBD1	0.95174602	0.806578346	0.570675724	0.7686514	0.919092528	0.992939042	0.999996191
MBD2	0.871509109	0.83558976	0.724399488	0.726190465	0.561723136	0.979666101	0.999996191
MBD4	0.97211821	0.992459327	0.638061257	0.501698277	0.860809782	0.988511557	0.999996191
MBD5	0.677439207	0.992751066	0.989670908	0.276490449	0.794368787	0.954036847	0.999996191
MBD6	0.883818512	0.273426128	0.674122631	0.754238706	0.169833263	0.654303187	0.999996191
MBIP	0.423342097	0.433540487	0.284297756	0.518526153	0.930183475	0.690650294	0.999996191
MBLAC1	0.561401284	0.911678219	0.360853641	0.538853113	0.76449598	0.880806595	0.999996191
MBNL3	0.250883416	0.383755121	0.464532125	0.829724825	0.78853639	0.719488074	0.999996191
MBOAT1	0.173670479	0.798589039	0.614749861	0.266079622	0.555862461	0.556321114	0.999996191
MBOAT7	0.879723869	0.736967389	0.732957662	0.533595023	0.269535447	0.865397868	0.999996191
MBTPS1	0.362045092	0.913301806	0.107026465	0.906163653	0.866654963	0.709685303	0.999996191
MCAM	0.905896695	0.5851467	0.470181734	0.430949183	0.232091575	0.688812191	0.999996191
MCC	0.145969143	0.818847537	0.816878813	0.617638576	0.785711041	0.806878221	0.999996191
MCCC1	0.828673543	0.712602635	0.326829212	0.406807283	0.297804345	0.676421143	0.999996191
MCCC2	0.93583572	0.816716048	0.547207715	0.656200909	0.576416169	0.960332399	0.999996191
MCCD1	0.23653037	0.662783659	0.879014132	0.51499527	0.304534277	0.661130784	0.999996191
MCEE	0.747503445	0.866463128	0.340738518	0.162112707	0.876005573	0.732442422	0.999996191
MCF2	0.209138182	0.669389145	0.729368614	0.96143139	0.843776653	0.892387104	0.999996191
MCF2L	0.926778653	0.189748969	0.804190282	0.841501359	0.279260065	0.743402266	0.999996191
MCM10	0.171260899	0.901349826	0.779535279	0.263535864	0.873644063	0.7090878	0.999996191
MCM3AP	0.969766523	0.126722033	0.637154664	0.16051238	0.976205436	0.551055632	0.999996191
MCM4	0.840133217	0.704552114	0.359140413	0.132022587	0.514991638	0.582675659	0.999996191
MCM7	0.995705867	0.770888621	0.555949414	0.58232622	0.953159518	0.984096276	0.999996191
MCOLN1	0.16879192	0.888447553	0.261273097	0.491323082	0.665925544	0.559484851	0.999996191
MCOLN2	0.953284347	0.752950563	0.751442923	0.258227123	0.296095662	0.782684924	0.999996191
MCRIP1	0.639422088	0.668182212	0.250494437	0.169383787	0.768338511	0.57550346	0.999996191
MCTP1	0.189691287	0.656130784	0.877720621	0.998840336	0.815238021	0.90165549	0.999996191
MCU	0.078674743	0.826849051	0.526877707	0.333510348	0.88502059	0.514438656	0.999996191
MCUB	0.925555836	0.845542053	0.333060146	0.53809233	0.696098862	0.913114068	0.999996191
MDC1	0.382242793	0.844060803	0.413520306	0.457768791	0.235370974	0.581619076	0.999996191
MDFI	0.408500681	0.610710294	0.89995553	0.433440606	0.857054246	0.893297586	0.999996191
MDFIC	0.683672895	0.845364984	0.101647972	0.253087577	0.933070001	0.574732854	0.999996191
MDGA2	0.142669593	0.571138647	0.84185483	0.308211258	0.807389014	0.615083127	0.999996191
MDH1	0.740789921	0.576247952	0.93540643	0.087556567	0.515473423	0.625678015	0.999996191
MDH1B	0.066089751	0.774430869	0.337386806	0.814598038	0.654327262	0.496810998	0.999996191
MDM1	0.996274804	0.247047889	0.378647127	0.195648387	0.405467103	0.456923538	0.999996191
MDN1	0.678595264	0.780975508	0.662383656	0.073325658	0.554829618	0.580352728	0.999996191
MDP1	0.803276723	0.718578474	0.665814965	0.304173555	0.297788726	0.751992092	0.999996191
ME3	0.911455366	0.176305548	0.727009558	0.375261884	0.483283516	0.657266907	0.999996191
MECOM	0.482046496	0.77862086	0.115361303	0.451693602	0.656960461	0.559922407	0.999996191
MECP2	0.515946534	0.956619019	0.654087593	0.794926814	0.216500146	0.83334941	0.999996191
MED1	0.473527163	0.809472541	0.765011343	0.074124283	0.970013299	0.656313551	0.999996191

MED10	0.819567348	0.143857607	0.127500132	0.828313965	0.778049261	0.506335563	0.999996191
MED13L	0.864375163	0.935522106	0.510621576	0.520837427	0.70880312	0.957443909	0.999996191
MED14	0.830399028	0.724067685	0.988539386	0.30742917	0.916130881	0.964500697	0.999996191
MED15	0.814935259	0.784578768	0.917826322	0.468963954	0.970187009	0.988657931	0.999996191
MED16	0.322384301	0.705239691	0.422461982	0.970169914	0.889896628	0.892534145	0.999996191
MED18	0.296882426	0.470460025	0.980741419	0.116571761	0.707674192	0.535346729	0.999996191
MED20	0.406703579	0.715854198	0.728424156	0.51704313	0.938865073	0.919312915	0.999996191
MED22	0.652622926	0.884874527	0.480131705	0.433279394	0.84862883	0.918193418	0.999996191
MED23	0.105984458	0.499296187	0.833401341	0.406020651	0.823913933	0.586659366	0.999996191
MED24	0.45680558	0.617126848	0.769964678	0.115397298	0.921961539	0.674015871	0.999996191
MED25	0.64824216	0.775976846	0.560155366	0.6297412	0.786052106	0.950026815	0.999996191
MED26	0.267802405	0.997484683	0.77888465	0.948709398	0.732224397	0.953085268	0.999996191
MED28	0.673681838	0.266422592	0.13961466	0.569790167	0.902648087	0.56051287	0.999996191
MED4	0.716487463	0.394309669	0.858909221	0.438169078	0.71485203	0.880664983	0.999996191
MED7	0.975645775	0.823923566	0.692666199	0.173789778	0.904440167	0.899577119	0.999996191
MEFV	0.624537871	0.100412382	0.708740974	0.854193457	0.270292007	0.517115714	0.999996191
MEGF9	0.45941638	0.050338311	0.502965675	0.744612933	0.943443979	0.474961738	0.999996191
MEIOB	0.437835121	0.690080473	0.993849182	0.275625587	0.32361451	0.702620578	0.999996191
MEIS1	0.568074022	0.973769838	0.068748765	0.524858942	0.806604023	0.603665742	0.999996191
MEIS2	0.852297099	0.421516112	0.06975519	0.553557013	0.780901056	0.527331342	0.999996191
MELK	0.531706433	0.708164908	0.336391377	0.887664753	0.702108975	0.885900147	0.999996191
MELTF	0.903343844	0.631792034	0.808909207	0.778444694	0.660477535	0.984186357	0.999996191
MEMO1	0.73379654	0.617772032	0.972893095	0.135879061	0.223758357	0.568154645	0.999996191
MEN1	0.203389728	0.986146328	0.961413192	0.506660609	0.613061804	0.84531114	0.999996191
MEPCE	0.304751168	0.351625019	0.389339874	0.413414149	0.637031689	0.530018204	0.999996191
MESP1	0.16105796	0.449748646	0.609022958	0.666209958	0.721479361	0.6574207	0.999996191
MESP2	0.212996208	0.909061033	0.577807369	0.472922897	0.857840077	0.799488305	0.999996191
MEST	0.912113427	0.4830798	0.334348052	0.380634821	0.277323027	0.596905104	0.999996191
METAP1D	0.72472539	0.497708802	0.788225647	0.093002716	0.930119121	0.686206973	0.999996191
METRN	0.274493775	0.271855904	0.731268432	0.693762476	0.904030697	0.748853473	0.999996191
METRNL	0.391747099	0.924858158	0.353550377	0.518936005	0.439734088	0.719286525	0.999996191
METTL14	0.29612485	0.44313329	0.723919703	0.8324279	0.824738601	0.858406675	0.999996191
METTL17	0.607541358	0.981786874	0.966242162	0.109870509	0.665594462	0.786538377	0.999996191
METTL18	0.065076212	0.816024477	0.640693981	0.744363816	0.612035834	0.596267194	0.999996191
METTL22	0.974630926	0.210522038	0.635551707	0.082873685	0.877109986	0.502272357	0.999996191
METTL23	0.80102581	0.238570696	0.318186048	0.747497224	0.862112619	0.773550048	0.999996191
METTL25	0.823168478	0.98031175	0.461952845	0.990606657	0.309135832	0.930688952	0.999996191
METTL26	0.370749309	0.539706758	0.925482115	0.351755391	0.90666118	0.843099508	0.999996191
METTL27	0.979358153	0.477030602	0.280288457	0.934245357	0.833902422	0.918267558	0.999996191
METTL2A	0.84714687	0.458719981	0.930635403	0.960452679	0.788960908	0.989515184	0.999996191
METTL5	0.709567158	0.540336557	0.107267866	0.713437272	0.948580385	0.709964951	0.999996191
METTL6	0.216712824	0.475534	0.83302976	0.410843894	0.203365313	0.451531517	0.999996191
METTL7A	0.250423689	0.639521934	0.999426831	0.516824649	0.679865248	0.835310016	0.999996191
METTL8	0.365694684	0.576579074	0.346207368	0.679659558	0.577128599	0.715369112	0.999996191
METTL9	0.804519581	0.363498958	0.268582799	0.184445498	0.641655592	0.498648559	0.999996191

MEX3A	0.775986111	0.652779891	0.126261572	0.4567939	0.455800265	0.566814698	0.999996191
MEX3C	0.973459379	0.744032066	0.63609236	0.211983193	0.80944129	0.886092639	0.999996191
MEX3D	0.317622865	0.90688958	0.19659582	0.88684229	0.744588929	0.765094194	0.999996191
MFAP1	0.759644033	0.111461189	0.517267478	0.458762143	0.718409403	0.582423026	0.999996191
MFAP3	0.451598129	0.493174806	0.434051535	0.779148004	0.48990704	0.762674195	0.999996191
MFAP3L	0.601259662	0.791697082	0.250027102	0.887155137	0.961520424	0.917707777	0.999996191
MFAP5	0.724406083	0.285320287	0.232498904	0.715502314	0.364467868	0.555117396	0.999996191
MFF	0.865117081	0.504681346	0.741929835	0.291306462	0.986599431	0.907352631	0.999996191
MFN2	0.872248428	0.930014852	0.523559162	0.296311926	0.371383038	0.804533341	0.999996191
MFNG	0.076056667	0.583899146	0.964846177	0.234707672	0.712148426	0.45126596	0.999996191
MFSD1	0.602584632	0.550944862	0.387609377	0.501784658	0.965145447	0.851476799	0.999996191
MFSD10	0.548837184	0.321882623	0.753620388	0.796594143	0.768885992	0.890293052	0.999996191
MFSD12	0.446161629	0.873011271	0.178795991	0.808035308	0.412056158	0.674807132	0.999996191
MFSD14A	0.73843239	0.320643302	0.375536292	0.248054331	0.7778421	0.616063943	0.999996191
MFSD14B	0.357892668	0.26630144	0.351950797	0.348043146	0.838038714	0.508173751	0.999996191
MFSD2A	0.823089263	0.3154705	0.41281661	0.628595756	0.287027818	0.639468335	0.999996191
MFSD2B	0.380363858	0.619459071	0.209640226	0.740459778	0.374995366	0.572521787	0.999996191
MFSD3	0.558792981	0.699075081	0.326677064	0.191865047	0.661680391	0.604881755	0.999996191
MFSD4B	0.476303404	0.744940287	0.841496041	0.131422958	0.322858626	0.557212596	0.999996191
MFSD5	0.270426065	0.731817903	0.770076748	0.567046237	0.360356208	0.731243178	0.999996191
MFSD6	0.108259342	0.80273428	0.762314681	0.566418039	0.280398832	0.521825615	0.999996191
MGA	0.725401819	0.643756281	0.998160887	0.158976969	0.958323902	0.871001766	0.999996191
MGARP	0.735730608	0.442712332	0.900718703	0.891492779	0.595589227	0.959145944	0.999996191
MGAT5	0.622529541	0.561819307	0.574639328	0.946452938	0.42366628	0.888706872	0.999996191
MGC127055	0.628976986	0.872667044	0.289389727	0.568836569	0.981547323	0.901272078	0.999996191
MGC137036	0.612537626	0.784302411	0.144981183	0.590535985	0.863532852	0.755691625	0.999996191
MGC148714	0.74539592	0.325234059	0.899138178	0.044364585	0.970436388	0.500411829	0.999996191
MGLL	0.910514678	0.930451489	0.238199956	0.53705865	0.905175231	0.91368625	0.999996191
MGME1	0.998437021	0.171246008	0.866441336	0.79148058	0.557069739	0.858638609	0.999996191
MGMT	0.663907651	0.756887213	0.331407984	0.338538364	0.900230674	0.818482324	0.999996191
MGP	0.259647324	0.881380347	0.156019333	0.288004904	0.914306063	0.500755857	0.999996191
MGST1	0.826606757	0.66413171	0.525516554	0.347621272	0.292687381	0.720078383	0.999996191
MGST3	0.6528643	0.571278077	0.656820429	0.399191731	0.684515127	0.862316833	0.999996191
MIA	0.190007106	0.16001037	0.896587066	0.504271047	0.941268925	0.561265709	0.999996191
MIA2	0.250456149	0.53769443	0.849324272	0.157167025	0.546797016	0.509044378	0.999996191
MIA3	0.907238317	0.522735377	0.987861263	0.030278797	0.939649238	0.566999968	0.999996191
MIB1	0.745789805	0.454654251	0.071199687	0.753870343	0.649681783	0.543986452	0.999996191
MIB2	0.666314864	0.233556196	0.641437977	0.489621384	0.958177768	0.804874734	0.999996191
MIC1	0.462396713	0.561717564	0.436534457	0.27126314	0.364694688	0.533938699	0.999996191
MICAL1	0.285556263	0.415150447	0.752500846	0.564473914	0.242607226	0.550233729	0.999996191
MICAL3	0.763249521	0.980092346	0.616997462	0.594784613	0.281983002	0.883207958	0.999996191
MICU1	0.494403865	0.498368193	0.354327427	0.480852252	0.988840189	0.783851629	0.999996191
MICU2	0.596485596	0.975178464	0.972177965	0.457435354	0.97996314	0.986807979	0.999996191
MID1	0.372636279	0.961956487	0.053523572	0.500228728	0.897736661	0.484638072	0.999996191
MID2	0.577551943	0.673349471	0.639818963	0.035064763	0.903040334	0.468357939	0.999996191

MIDN	0.154633911	0.662314022	0.421048222	0.941962237	0.97939685	0.776267074	0.999996191
MIEN1	0.182980363	0.582021538	0.627495287	0.34014286	0.935348808	0.657944749	0.999996191
MIER2	0.373134201	0.155366839	0.40147339	0.359481984	0.984455833	0.476412601	0.999996191
MIER3	0.616898525	0.346092117	0.694541751	0.280170382	0.949373125	0.774726857	0.999996191
MIF4GD	0.293846786	0.770349543	0.392552692	0.51416688	0.858130533	0.773651831	0.999996191
MIGA1	0.398692742	0.323960683	0.892727909	0.96124227	0.970396077	0.924247049	0.999996191
MINDY1	0.324499602	0.735371051	0.508637903	0.985776071	0.646324457	0.883067455	0.999996191
MINDY3	0.948046088	0.396707149	0.333620859	0.61174628	0.601095535	0.802318318	0.999996191
MINDY4	0.127248549	0.707619893	0.417587319	0.531334179	0.971516057	0.640169649	0.999996191
MINK1	0.927287366	0.634817775	0.102294225	0.822763899	0.261544477	0.561552707	0.999996191
MIOS	0.933349781	0.537175763	0.466546428	0.396423609	0.463239546	0.789884539	0.999996191
MIPEP	0.655734179	0.17612352	0.650789222	0.937340516	0.643937711	0.799401519	0.999996191
MIPOL1	0.980066821	0.669692702	0.821069209	0.657385383	0.071365071	0.691530321	0.999996191
MIS12	0.840673466	0.722966475	0.56360359	0.352657906	0.425681828	0.820666376	0.999996191
MIS18A	0.439562629	0.699149174	0.634394734	0.78937357	0.948618067	0.953925625	0.999996191
MISP3	0.70378808	0.540394887	0.771048668	0.669404494	0.627786691	0.938501351	0.999996191
MITD1	0.421635824	0.250498385	0.154204923	0.826311471	0.541805781	0.454459347	0.999996191
MKKS	0.89210171	0.086479615	0.41683672	0.779899975	0.434853993	0.528611514	0.999996191
MKL1	0.427277144	0.287759991	0.465336963	0.884888581	0.931171803	0.806015416	0.999996191
MKLN1	0.960783606	0.829293739	0.838985081	0.864240392	0.169469615	0.913450296	0.999996191
MKRN2	0.67106938	0.605356802	0.534216828	0.870631808	0.808414848	0.957605195	0.999996191
MKRN3	0.832281362	0.522969021	0.787000635	0.83821438	0.876674385	0.986542644	0.999996191
MKS1	0.542483525	0.838684189	0.690080528	0.256713745	0.259147484	0.654486018	0.999996191
MLC1	0.837683583	0.778469967	0.438851685	0.111844328	0.46492883	0.588339621	0.999996191
MLF2	0.36574155	0.588428247	0.555636402	0.163590366	0.620697628	0.549062223	0.999996191
MLH1	0.929677773	0.922014382	0.638881808	0.420782119	0.873107852	0.976125154	0.999996191
MLH3	0.442825761	0.766443373	0.675476389	0.947235182	0.667890335	0.953378746	0.999996191
MLKL	0.735203035	0.73406765	0.392632302	0.563029144	0.83380619	0.915332164	0.999996191
MLLT10	0.426029808	0.351525326	0.307635648	0.883647284	0.59349224	0.682777315	0.999996191
MLLT11	0.965859118	0.374087902	0.948371352	0.180542344	0.225102192	0.575467618	0.999996191
MLLT6	0.906620811	0.306237963	0.97548	0.443956512	0.755209615	0.90425201	0.999996191
MLPH	0.490547057	0.66467389	0.77964843	0.827479131	0.867475137	0.970312355	0.999996191
MLST8	0.482224625	0.747262648	0.628532171	0.114301832	0.63973645	0.60917882	0.999996191
MLXIP	0.44424462	0.929018418	0.608967874	0.493690833	0.371264325	0.80204543	0.999996191
MMAB	0.735836463	0.879570243	0.575685029	0.068529427	0.399199646	0.515852475	0.999996191
MMADHC	0.970904485	0.470479417	0.734904081	0.289521179	0.273919974	0.701461254	0.999996191
MMD	0.726136703	0.972599734	0.761929113	0.419324508	0.338703692	0.88143111	0.999996191
MME	0.967513661	0.594633434	0.920828333	0.50658233	0.619793094	0.964043103	0.999996191
MMP14	0.565720395	0.510101673	0.505514042	0.051848377	0.97598828	0.456654052	0.999996191
MMP17	0.870070691	0.099223987	0.815396115	0.416103105	0.968100188	0.713521604	0.999996191
MMP24	0.917323487	0.147949707	0.707448988	0.366508962	0.465610134	0.607082868	0.999996191
MMP24OS	0.979106424	0.893715851	0.434164773	0.115848977	0.790867902	0.751974028	0.999996191
MMRN1	0.420435165	0.947178848	0.024352531	0.908929871	0.966360415	0.482546082	0.999996191
MMRN2	0.343846177	0.485193382	0.590500751	0.988653816	0.479447801	0.804382532	0.999996191
MMS19	0.998291277	0.609274857	0.497509861	0.922788555	0.47816937	0.946122453	0.999996191

MMS22L	0.967915854	0.056515499	0.882399708	0.462917253	0.963304193	0.66034327	0.999996191
MNAT1	0.194821362	0.535941996	0.238398598	0.778792399	0.459579684	0.490792955	0.999996191
MNF1	0.545654385	0.678854614	0.701845754	0.475779349	0.609470699	0.879518704	0.999996191
MNT	0.990985076	0.995922006	0.826036595	0.771216018	0.475788723	0.992075146	0.999996191
MOB1A	0.415772452	0.513969779	0.76150339	0.144012279	0.723808856	0.613845538	0.999996191
MOB1B	0.348870539	0.647332073	0.268870418	0.59874658	0.57128351	0.65338544	0.999996191
MOB2	0.945485227	0.417104827	0.865114836	0.541190886	0.276978583	0.819751843	0.999996191
MOB3B	0.524274562	0.956328805	0.716981627	0.735294504	0.503847143	0.945885915	0.999996191
MOB4	0.396885793	0.09335603	0.315317315	0.778872839	0.94089912	0.483482516	0.999996191
MOCOS	0.514876403	0.875309444	0.853654421	0.191936111	0.545262148	0.7784157	0.999996191
MOCS1	0.966213995	0.903867755	0.291903881	0.512598478	0.847291984	0.92742307	0.999996191
MOCS2	0.262509811	0.573788331	0.853128162	0.845034663	0.713196573	0.883270252	0.999996191
MOCS3	0.421170971	0.924247133	0.463392558	0.672622049	0.473440212	0.838696214	0.999996191
MOGAT1	0.627484748	0.934623697	0.883249981	0.692048889	0.804063474	0.991050302	0.999996191
MOGS	0.349704347	0.546300161	0.292843796	0.538526257	0.57432003	0.617730378	0.999996191
MORC2	0.825791761	0.84507775	0.624695259	0.697377408	0.554429716	0.964988253	0.999996191
MORC3	0.33524265	0.180102259	0.414257531	0.419472841	0.686513708	0.45227677	0.999996191
MORC4	0.932365878	0.877346924	0.659304384	0.124145347	0.760914437	0.819114817	0.999996191
MORF4L2	0.901373647	0.872500388	0.907057308	0.814922962	0.758009893	0.998433247	0.999996191
MORN1	0.982342689	0.092373773	0.633995947	0.684859114	0.650713375	0.694223047	0.999996191
MORN2	0.979698771	0.438028827	0.20308491	0.938362428	0.618306558	0.817860681	0.999996191
MORN4	0.693388122	0.936219763	0.907849913	0.694478239	0.585470864	0.984584286	0.999996191
MOSMO	0.423846393	0.468856742	0.772421022	0.698995678	0.856307953	0.905716894	0.999996191
MOSPD2	0.588656302	0.850279822	0.078051897	0.377800439	0.670198195	0.510226444	0.999996191
MOSPD3	0.336964033	0.171387717	0.957748748	0.981296967	0.534280332	0.717778362	0.999996191
MOV10	0.538475516	0.129203226	0.562922774	0.233497538	0.991653257	0.49406147	0.999996191
MPC2	0.684439209	0.5958081	0.493832248	0.051838368	0.720543253	0.460015432	0.999996191
MPDU1	0.692592921	0.884953389	0.620791936	0.26828064	0.595072712	0.847506912	0.999996191
MPDZ	0.529841758	0.974575179	0.573482934	0.793831641	0.234347638	0.831973056	0.999996191
MPHOSPH6	0.777164021	0.722894714	0.754837614	0.465465805	0.390669417	0.88267866	0.999996191
MPI	0.777225306	0.824833925	0.413045372	0.103819298	0.418213755	0.538633374	0.999996191
MPLKIP	0.828841302	0.760607255	0.245732971	0.538709778	0.542269729	0.79897862	0.999996191
MPP1	0.823906407	0.716829367	0.073227449	0.90622519	0.833354302	0.740166891	0.999996191
MPP2	0.482153871	0.683856901	0.082478385	0.62402708	0.753727418	0.559058504	0.999996191
MPP6	0.892206884	0.276755619	0.811341091	0.194309689	0.679517715	0.700228481	0.999996191
MPST	0.415298577	0.850831217	0.689290734	0.106988154	0.825026239	0.660106723	0.999996191
MPV17	0.833960377	0.716823158	0.58221034	0.06338915	0.561657841	0.55295926	0.999996191
MPV17L	0.554356508	0.79866928	0.582274093	0.097642589	0.591811065	0.588548117	0.999996191
MPZL2	0.296265286	0.205193603	0.56487589	0.619682985	0.569491751	0.548691043	0.999996191
MR1	0.772955763	0.915622931	0.504188269	0.036103436	0.733365703	0.501661573	0.999996191
MRAS	0.518949943	0.665950004	0.628862656	0.808170954	0.606881528	0.923248321	0.999996191
MRC1	0.549133256	0.696099095	0.321544152	0.365226056	0.288314248	0.561322958	0.999996191
MRE11	0.448193568	0.49503839	0.985497097	0.98486212	0.913629372	0.974865241	0.999996191
MRFAP1L1	0.550629199	0.261362112	0.26065244	0.686246846	0.795144626	0.650538941	0.999996191
MRGBP	0.647369697	0.566521638	0.466630604	0.4317975	0.733019654	0.829227568	0.999996191

MRGPRF	0.452418587	0.837951326	0.636256899	0.932930376	0.051383478	0.539720211	0.999996191
MRI1	0.411251151	0.819963592	0.727487376	0.918645588	0.997255357	0.98176052	0.999996191
MRM2	0.084351834	0.541383431	0.547028525	0.40909616	0.837777905	0.483481838	0.999996191
MRNIP	0.936158747	0.815603981	0.990492197	0.906372099	0.509502908	0.995479565	0.999996191
MRPL10	0.75334491	0.912409077	0.616866237	0.881226503	0.66362955	0.985963716	0.999996191
MRPL13	0.896815536	0.490677142	0.740017318	0.135840913	0.7991818	0.754830102	0.999996191
MRPL17	0.62208484	0.642114938	0.282007381	0.61040088	0.109393964	0.46001513	0.999996191
MRPL22	0.472393723	0.695601234	0.667575002	0.986726998	0.360095178	0.884154427	0.999996191
MRPL24	0.571368148	0.571837443	0.402920477	0.19510734	0.478948929	0.551566388	0.999996191
MRPL27	0.523981896	0.492800726	0.625307103	0.114141846	0.714131193	0.564556569	0.999996191
MRPL28	0.789767029	0.63964591	0.793707763	0.157996561	0.933097785	0.843238175	0.999996191
MRPL3	0.936285753	0.555150867	0.633677393	0.98471443	0.443969598	0.952770194	0.999996191
MRPL30	0.794877169	0.508096261	0.60474296	0.178627997	0.78451509	0.748864376	0.999996191
MRPL32	0.396748152	0.740552157	0.735194596	0.11278286	0.402471614	0.508580738	0.999996191
MRPL33	0.68198729	0.110373416	0.632198236	0.19042202	0.989423968	0.491962136	0.999996191
MRPL37	0.572744296	0.526270867	0.77818909	0.127448588	0.245530978	0.455636876	0.999996191
MRPL38	0.948823682	0.350934802	0.569600978	0.397595816	0.841795724	0.854303788	0.999996191
MRPL39	0.924671703	0.646176136	0.634402498	0.328694272	0.771045767	0.911178374	0.999996191
MRPL40	0.23787398	0.660980231	0.358416076	0.297402616	0.560671573	0.500652786	0.999996191
MRPL42	0.306540587	0.213845372	0.757627165	0.458488862	0.53684791	0.550353602	0.999996191
MRPL45	0.982303665	0.681121553	0.622277678	0.62547585	0.538380206	0.950478192	0.999996191
MRPL47	0.148955849	0.850760433	0.435127845	0.795714054	0.699970896	0.728636251	0.999996191
MRPL50	0.531672171	0.200653927	0.862277136	0.91675634	0.212745604	0.624802271	0.999996191
MRPL52	0.373073329	0.307803254	0.858742263	0.724533026	0.515066158	0.762180379	0.999996191
MRPL53	0.849810295	0.543551822	0.480098126	0.246890718	0.252997972	0.574437968	0.999996191
MRPL58	0.724794033	0.715084077	0.46770108	0.58966565	0.584017311	0.893414751	0.999996191
MRPL9	0.728042629	0.900444042	0.534429327	0.19921802	0.417229109	0.7185749	0.999996191
MRPS10	0.48881249	0.656019329	0.413964412	0.758184787	0.617924112	0.851157883	0.999996191
MRPS11	0.492615764	0.550378958	0.925094562	0.111584006	0.642649603	0.625287847	0.999996191
MRPS14	0.675061174	0.613107184	0.692458976	0.131154318	0.338903058	0.558271264	0.999996191
MRPS15	0.752360875	0.598365155	0.281093848	0.174039019	0.937319145	0.652195079	0.999996191
MRPS17	0.750478751	0.824616249	0.176707784	0.799475727	0.256464038	0.668289651	0.999996191
MRPS18A	0.717378923	0.568706717	0.540640692	0.172563299	0.263479084	0.512800065	0.999996191
MRPS2	0.468234458	0.62637427	0.292191986	0.108554927	0.778802526	0.453317221	0.999996191
MRPS25	0.397815173	0.657882467	0.608915504	0.054189516	0.849831746	0.455611552	0.999996191
MRPS28	0.858189709	0.753036037	0.78132469	0.325823482	0.697619936	0.931253881	0.999996191
MRPS31	0.794546283	0.357438627	0.839629592	0.218275005	0.205496054	0.524925698	0.999996191
MRPS33	0.916198538	0.33677032	0.39664012	0.523667298	0.918593492	0.842593402	0.999996191
MRPS35	0.541512596	0.900402819	0.604095723	0.428130732	0.325334301	0.781759696	0.999996191
MRPS36	0.536812345	0.548350555	0.852266522	0.116735869	0.879128758	0.69503115	0.999996191
MRPS6	0.774623285	0.555485013	0.154282185	0.371475128	0.648099859	0.602238839	0.999996191
MRPS9	0.837137622	0.822703173	0.313729861	0.064011991	0.645663514	0.491227224	0.999996191
MRRF	0.985510486	0.624346325	0.566613992	0.89462567	0.043013465	0.568252063	0.999996191
MRS2	0.54917689	0.676656582	0.399270507	0.213968244	0.626137763	0.644822274	0.999996191
MS4A13	0.258649039	0.609180247	0.987923978	0.907864791	0.853081922	0.936311608	0.999996191

MS4A7	0.273183226	0.438371953	0.092899547	0.969656337	0.96500313	0.519817103	0.999996191
MSANTD1	0.521200033	0.603673956	0.212719419	0.463100292	0.799253252	0.68760301	0.999996191
MSANTD3	0.827136833	0.773266571	0.404401418	0.541475418	0.345902316	0.810651859	0.999996191
MSANTD4	0.391786179	0.978984637	0.754625159	0.648574737	0.145396828	0.706232095	0.999996191
MSC	0.955339686	0.614599815	0.210840848	0.948815096	0.17278333	0.648876144	0.999996191
MSH2	0.468311696	0.97438668	0.926116876	0.822751457	0.998029884	0.995364095	0.999996191
MSH3	0.892259051	0.906161638	0.850733892	0.896116447	0.503159744	0.992990319	0.999996191
MSH6	0.27568594	0.946049135	0.996952368	0.363762184	0.942210052	0.901887443	0.999996191
MSL3	0.770681168	0.995085581	0.545073086	0.64693822	0.905948941	0.985487816	0.999996191
MSRA	0.398447284	0.599439909	0.352590786	0.104754048	0.953674172	0.480277983	0.999996191
MSRB1	0.417502311	0.673102199	0.772697486	0.108740756	0.644696433	0.59275309	0.999996191
MSRB2	0.764551156	0.87770877	0.319586383	0.602636565	0.411691147	0.82630135	0.999996191
MST1	0.184022918	0.241026378	0.848536228	0.446758387	0.496433783	0.478841337	0.999996191
MST1R	0.843442415	0.381522052	0.861037955	0.106928283	0.513667949	0.592697286	0.999996191
MSX2	0.986145513	0.795549199	0.647847504	0.775003492	0.304440186	0.935776087	0.999996191
MT1E	0.736063455	0.642526953	0.857870976	0.923837895	0.337367448	0.94101403	0.999996191
MT3	0.021327079	0.981563512	0.666854742	0.738887997	0.790553039	0.474585235	0.999996191
MTA2	0.578255928	0.949275433	0.681501584	0.483896566	0.655850963	0.934758963	0.999996191
MTA3	0.057116094	0.962933565	0.621109111	0.764761828	0.494127321	0.560822098	0.999996191
MTCH1	0.194790371	0.414899927	0.411459668	0.329971257	0.704347914	0.464883545	0.999996191
MTERF3	0.319980843	0.410391112	0.41771075	0.47937825	0.396709428	0.520203808	0.999996191
MTERF4	0.615677792	0.839297671	0.626187866	0.263413536	0.144062027	0.551209188	0.999996191
MTF1	0.578171856	0.742135153	0.724453876	0.541708348	0.947267755	0.960959777	0.999996191
MTF2	0.239217865	0.975676738	0.415799692	0.686278417	0.592727047	0.774882211	0.999996191
MTFR1	0.741869906	0.461648128	0.161029007	0.64468848	0.940087688	0.744465061	0.999996191
MTFR2	0.324079604	0.601719954	0.373725304	0.383072666	0.714786772	0.645581515	0.999996191
MTHFD2L	0.352030064	0.891728305	0.570493694	0.541358597	0.588553213	0.837630784	0.999996191
MTHFR	0.823081369	0.99893377	0.877008358	0.887596648	0.589678806	0.996717408	0.999996191
MTHFS	0.558813161	0.648517737	0.513633413	0.765437952	0.756446154	0.924475946	0.999996191
MTHFSD	0.128396983	0.711178909	0.77853427	0.434498329	0.386159274	0.545655718	0.999996191
MTIF2	0.16819781	0.214297925	0.680900415	0.751048593	0.754333189	0.575168249	0.999996191
MTIF3	0.709980044	0.624719717	0.885788863	0.146259633	0.284732116	0.606806067	0.999996191
MTM1	0.654968828	0.201140422	0.481109883	0.813393457	0.601060925	0.730311206	0.999996191
MTMR10	0.906710922	0.724061769	0.143678674	0.846720949	0.906088877	0.873722621	0.999996191
MTMR11	0.944206245	0.45809055	0.996471884	0.805097242	0.407070404	0.951126377	0.999996191
MTMR3	0.822871214	0.44593108	0.535734521	0.981063934	0.645334447	0.939472847	0.999996191
MTMR4	0.738089433	0.39229642	0.900860998	0.674410392	0.092500938	0.605741222	0.999996191
MTMR6	0.205870472	0.737026678	0.068493598	0.855972506	0.795486828	0.449137806	0.999996191
MTMR9	0.885787365	0.445473427	0.536954746	0.386837693	0.543972208	0.796393536	0.999996191
MTMR9	0.528833603	0.716026338	0.670810845	0.625922849	0.677698229	0.92444424	0.999996191
MTO1	0.25138332	0.870621923	0.475831575	0.588495792	0.78356188	0.809160259	0.999996191
MTOR	0.744041341	0.979097673	0.147026096	0.358079646	0.799274193	0.728278064	0.999996191
MTPN	0.774511481	0.362178625	0.039761076	0.953884101	0.690843342	0.4558782	0.999996191
MTREX	0.12071014	0.655580979	0.958579592	0.428261182	0.360357809	0.54208158	0.999996191
MTRF1L	0.992402805	0.348410421	0.752901876	0.87356875	0.94096248	0.979385518	0.999996191

MTSS1L	0.648399017	0.858570288	0.112269012	0.391976305	0.897197188	0.664416171	0.999996191
MTUS1	0.573505491	0.274594127	0.483753506	0.782892603	0.598218126	0.7565208	0.999996191
MTUS2	0.831410237	0.552431344	0.357848193	0.127667288	0.874898438	0.629290252	0.999996191
MTX2	0.948088607	0.592035072	0.674802422	0.782494899	0.603562231	0.969030721	0.999996191
MTX3	0.713600076	0.669283049	0.695096397	0.322150455	0.370349532	0.775477729	0.999996191
MUC20	0.923679361	0.840016218	0.775357203	0.764135536	0.895309443	0.997789163	0.999996191
MUM1	0.941896475	0.956053948	0.087180975	0.20181065	0.56768239	0.492541466	0.999996191
MUM1L1	0.547746815	0.764438553	0.823910225	0.698704879	0.060172135	0.583353806	0.999996191
MUS81	0.845362923	0.334702552	0.825811145	0.582509307	0.98491248	0.946485417	0.999996191
MUT	0.971942327	0.964433857	0.505193048	0.147275582	0.479637016	0.744615847	0.999996191
MUTYH	0.519859258	0.070400518	0.692692823	0.981082207	0.805858002	0.646439866	0.999996191
MVB12A	0.904198253	0.715956496	0.225804756	0.560096905	0.89348827	0.875265874	0.999996191
MXD1	0.995115984	0.49964533	0.77440826	0.325468806	0.368506321	0.802473787	0.999996191
MXD3	0.786826229	0.448182049	0.26227806	0.97516469	0.396972019	0.757162149	0.999996191
MXI1	0.534130422	0.479250009	0.714518229	0.886948728	0.066552977	0.526698979	0.999996191
MXRA7	0.616191269	0.717093845	0.318248018	0.756477624	0.496411488	0.825060727	0.999996191
MXRA8	0.390846662	0.760960063	0.415993603	0.911102603	0.817315166	0.906061453	0.999996191
MYBPHL	0.989973097	0.878632728	0.679235981	0.411152928	0.373131492	0.904022659	0.999996191
MYC	0.352964473	0.848221391	0.262619365	0.464419735	0.87142722	0.735292708	0.999996191
MYCBP	0.906749517	0.789741504	0.416167761	0.403631688	0.701454766	0.894827307	0.999996191
MYCBPAP	0.513286789	0.649908905	0.584558947	0.599696956	0.989795709	0.932142182	0.999996191
MYDGF	0.185975017	0.686630132	0.333984035	0.823647304	0.948448128	0.74386466	0.999996191
MYEF2	0.616834106	0.282662239	0.317243082	0.681854146	0.676657752	0.693336425	0.999996191
MYF6	0.179906159	0.986497063	0.844768052	0.333376794	0.338732725	0.613478412	0.999996191
MYH14	0.844530512	0.90884468	0.277985111	0.379283561	0.554257743	0.797436682	0.999996191
MYH6	0.40475662	0.548788151	0.402401319	0.984621338	0.629120057	0.832789524	0.999996191
MYH9	0.328202218	0.776277178	0.970162	0.06608225	0.617728495	0.51394218	0.999996191
MYL4	0.950631448	0.583009706	0.754002875	0.994515541	0.090714	0.766575497	0.999996191
MYL6B	0.995875865	0.811125438	0.026334602	0.769253604	0.921115643	0.590830169	0.999996191
MYLIP	0.273825233	0.632118714	0.808632215	0.210161706	0.863377066	0.692400041	0.999996191
MYLK2	0.60724958	0.297501503	0.748496527	0.537742079	0.954110744	0.86760491	0.999996191
MYMK	0.271628948	0.435879238	0.635178818	0.969596613	0.906948444	0.860502391	0.999996191
MYO10	0.60851768	0.382193179	0.619509958	0.952344459	0.674407091	0.906606144	0.999996191
MYO18A	0.746019014	0.465109585	0.475626685	0.878716412	0.148818509	0.660854241	0.999996191
MYO19	0.848419358	0.713513315	0.459097069	0.081892409	0.462259669	0.521809142	0.999996191
MYO1B	0.539613483	0.836580477	0.724522646	0.582636448	0.827763011	0.960112753	0.999996191
MYO1C	0.253637029	0.851059123	0.434426416	0.265250311	0.527280397	0.563882686	0.999996191
MYO1D	0.841158601	0.592326718	0.347750182	0.682908113	0.159421064	0.634591408	0.999996191
MYO1H	0.968073475	0.506727288	0.839175048	0.444651182	0.791697481	0.953306737	0.999996191
MYO5A	0.047734674	0.984912581	0.835091561	0.586135375	0.663650135	0.59338294	0.999996191
MYO6	0.498296213	0.764631209	0.622953216	0.259170771	0.984076082	0.846970466	0.999996191
MYO7A	0.361639751	0.428442388	0.361041633	0.233029712	0.861030659	0.534063088	0.999996191
MYO9A	0.333735394	0.886120248	0.974313265	0.244269338	0.430988895	0.726296481	0.999996191
MYO9B	0.999077977	0.983942121	0.514147479	0.737146262	0.646305011	0.984786037	0.999996191
MYOF	0.889053268	0.915817575	0.424993171	0.741813877	0.753531305	0.973878357	0.999996191

MYORG	0.227817277	0.206441297	0.838011205	0.800025333	0.767249396	0.683020507	0.999996191
MYOZ3	0.300577484	0.908456657	0.59067846	0.987681972	0.216238602	0.750053902	0.999996191
MYSM1	0.249976232	0.399992138	0.878149889	0.759245796	0.393805268	0.698779641	0.999996191
MYZAP	0.06306333	0.903098349	0.874784174	0.833750141	0.729751835	0.726164134	0.999996191
MZT1	0.338893607	0.095467286	0.428591171	0.875185004	0.939485635	0.537038891	0.999996191
N4BP2	0.774449343	0.979936803	0.782188745	0.015096463	0.986910795	0.489441816	0.999996191
N4BP2L1	0.350734386	0.768939084	0.331464643	0.662052275	0.773749687	0.801021901	0.999996191
N4BP2L2	0.243185651	0.28587735	0.738721691	0.727659113	0.949713118	0.755550262	0.999996191
N4BP3	0.883924717	0.918850192	0.379612439	0.19848209	0.541603874	0.742899271	0.999996191
N6AMT1	0.402144445	0.452965912	0.407031751	0.219141405	0.653232422	0.523469545	0.999996191
NAA16	0.458552195	0.533608	0.690421311	0.751807176	0.091379171	0.540429	0.999996191
NAA20	0.772512462	0.430512198	0.630555677	0.107481412	0.757195913	0.615044691	0.999996191
NAA30	0.070229451	0.465934054	0.394493266	0.782748446	0.925778215	0.499812906	0.999996191
NAA35	0.624646369	0.281856547	0.896258453	0.878878444	0.317104688	0.793999863	0.999996191
NAA38	0.414662005	0.451907995	0.368475081	0.182125836	0.905124963	0.53672393	0.999996191
NAALAD2	0.56708753	0.605548033	0.753028552	0.46585545	0.738045351	0.90158813	0.999996191
NAALADL2	0.373593742	0.882620816	0.669225594	0.848123756	0.987449248	0.971114447	0.999996191
NAB2	0.593391166	0.805984877	0.354909259	0.379275181	0.987712623	0.854563782	0.999996191
NABP2	0.623377187	0.96377046	0.60300323	0.060063024	0.843342811	0.629211849	0.999996191
NACA	0.839176784	0.746715783	0.939330526	0.181529125	0.426612418	0.800229581	0.999996191
NACAD	0.713872122	0.781779203	0.048231321	0.861816221	0.592856391	0.573049171	0.999996191
NADK	0.542616616	0.985084078	0.359959683	0.183321262	0.733211731	0.695894558	0.999996191
NADK2	0.38350686	0.761441436	0.929720309	0.88859062	0.872483502	0.978543119	0.999996191
NADSYN1	0.404639065	0.729962295	0.605923453	0.273624313	0.865050789	0.787437274	0.999996191
NAGK	0.464431183	0.418561968	0.088107248	0.541218994	0.850275848	0.468424052	0.999996191
NAGLU	0.34897514	0.745712428	0.788539736	0.26576099	0.243784628	0.566503699	0.999996191
NAGPA	0.684312388	0.474949273	0.451883998	0.312322369	0.941349898	0.790798874	0.999996191
NAGS	0.197306016	0.59798586	0.914530015	0.650297883	0.78098702	0.831119197	0.999996191
NAIF1	0.373448991	0.752321017	0.552948926	0.26253051	0.770575787	0.732963819	0.999996191
NALCN	0.98832589	0.392618993	0.229882027	0.572061529	0.992843069	0.818188681	0.999996191
NANP	0.527809109	0.577298978	0.738031192	0.786795508	0.209945338	0.763887073	0.999996191
NANS	0.272469329	0.808961613	0.168393886	0.520588342	0.581010479	0.534104423	0.999996191
NAP1L3	0.578723119	0.793960057	0.906189964	0.893552434	0.472207776	0.967837018	0.999996191
NAP1L5	0.327949508	0.723745655	0.557522586	0.120183498	0.439156614	0.446815391	0.999996191
NAPA	0.521831699	0.883286679	0.408415255	0.075238886	0.611535125	0.485607435	0.999996191
NAPB	0.3542417	0.155348291	0.603047134	0.436656966	0.80041011	0.540311064	0.999996191
NAPEPLD	0.847124346	0.242472242	0.755676425	0.619568785	0.270526459	0.697037476	0.999996191
NAPG	0.875757562	0.674800436	0.372441928	0.346017461	0.911771413	0.867734028	0.999996191
NAPRT	0.588437054	0.39858008	0.572317468	0.266365063	0.91380246	0.740230797	0.999996191
NARS	0.711875396	0.387205483	0.370072505	0.488194145	0.63338358	0.733645166	0.999996191
NARS2	0.467750333	0.575635386	0.829567178	0.592582517	0.193167491	0.693694236	0.999996191
NASP	0.91930037	0.848062394	0.465456883	0.430450779	0.971646846	0.957097638	0.999996191
NATD1	0.576802927	0.926823137	0.217494046	0.480008139	0.988174069	0.832153175	0.999996191
NBAS	0.746259643	0.578889844	0.612056993	0.175905489	0.607101124	0.712713454	0.999996191
NBEAL1	0.634068923	0.624021935	0.867711051	0.30853966	0.550898212	0.841207694	0.999996191

NBEAL2	0.831588267	0.636112614	0.597271788	0.967462004	0.294195017	0.903028107	0.999996191
NBR1	0.744939118	0.469956537	0.198749938	0.778741744	0.631034117	0.748678696	0.999996191
NCALD	0.51446683	0.424910779	0.46074329	0.380905415	0.74328039	0.714583418	0.999996191
NCAPD3	0.948358508	0.043933939	0.765338546	0.783875285	0.69759218	0.619234878	0.999996191
NCAPG2	0.989352746	0.027482364	0.438340581	0.782826693	0.898179777	0.479557835	0.999996191
NCAPH2	0.830821149	0.48851859	0.288938281	0.392952205	0.667461586	0.728914212	0.999996191
NCBP1	0.089558316	0.929355705	0.737277171	0.290779614	0.645221146	0.538898179	0.999996191
NCBP2	0.356666323	0.803011141	0.72501318	0.772625206	0.152197402	0.684815492	0.999996191
NCBP3	0.723826055	0.743979999	0.777221216	0.189353508	0.735878062	0.841107543	0.999996191
NCKAP5	0.803976512	0.714678541	0.372003461	0.901305916	0.182325873	0.753646051	0.999996191
NCKAP5L	0.782358866	0.508184349	0.063009873	0.905288243	0.751331557	0.614729272	0.999996191
NCKIPSD	0.740079465	0.788027345	0.42919089	0.084152052	0.607228344	0.559048798	0.999996191
NCL	0.773727972	0.499403328	0.579021737	0.196319336	0.329534496	0.58295475	0.999996191
NCOA1	0.523113131	0.961566644	0.742057255	0.072514835	0.795834452	0.66048884	0.999996191
NCOA3	0.51518855	0.396870785	0.163155528	0.852840783	0.34135997	0.50679431	0.999996191
NCOA4	0.967595942	0.871938477	0.138369649	0.80721867	0.69221823	0.85843878	0.999996191
NCOA6	0.511809083	0.901094098	0.790307317	0.135988921	0.199679132	0.510328783	0.999996191
NCOR1	0.62234465	0.672176129	0.773456561	0.551618026	0.6715195	0.935720333	0.999996191
NCR3	0.419194851	0.948486828	0.723699969	0.188977433	0.674240869	0.761499687	0.999996191
NCR3LG1	0.851251282	0.576109471	0.546545403	0.95150316	0.444054286	0.929844145	0.999996191
NCSTN	0.273494211	0.460114389	0.859034719	0.386602545	0.680914737	0.714188266	0.999996191
NDC1	0.360121095	0.857487882	0.955315653	0.570652377	0.588682691	0.914885092	0.999996191
NDFIP1	0.721934387	0.375250898	0.884773807	0.836730593	0.894731043	0.969234057	0.999996191
NDFIP2	0.841071639	0.851001862	0.751979799	0.183003818	0.71308038	0.869403989	0.999996191
NDN	0.132841421	0.367874017	0.931624612	0.881599217	0.301419665	0.548365521	0.999996191
NDNF	0.289976711	0.867423266	0.463384204	0.457229603	0.740373274	0.774791974	0.999996191
NDP	0.525184428	0.506239702	0.741164736	0.079937977	0.912087207	0.581513366	0.999996191
NDRG1	0.674969158	0.817840548	0.114734665	0.597461026	0.398133537	0.590732496	0.999996191
NDRG2	0.833793657	0.80852429	0.498117857	0.877841821	0.621466495	0.970563208	0.999996191
NDRG3	0.348806149	0.315693379	0.891138113	0.676946752	0.914279239	0.847479684	0.999996191
NDST2	0.277046522	0.240126581	0.715400986	0.770395176	0.885479914	0.73905028	0.999996191
NDST3	0.885984715	0.918677366	0.902136462	0.187291264	0.797804083	0.926434341	0.999996191
NDUFA10	0.971797295	0.554174634	0.629552611	0.032840347	0.84602848	0.501111025	0.999996191
NDUFA12	0.631925304	0.858884808	0.772408156	0.505222907	0.900562183	0.973049457	0.999996191
NDUFA2	0.794636248	0.262200295	0.820856766	0.05911399	0.741779004	0.45947985	0.999996191
NDUFA4	0.876732508	0.453354635	0.854605774	0.05191675	0.964774552	0.614437028	0.999996191
NDUFA6	0.765174435	0.412681037	0.82483915	0.036359825	0.908647067	0.484409221	0.999996191
NDUFA7	0.736516747	0.57208727	0.961089953	0.025413691	0.802908225	0.476995051	0.999996191
NDUFA8	0.552139968	0.465660762	0.652917892	0.057710558	0.815486808	0.468850177	0.999996191
NDUFAF1	0.892987443	0.674685299	0.735584097	0.249667398	0.249333912	0.708278926	0.999996191
NDUFAF2	0.778600196	0.077090056	0.478398652	0.466440801	0.822756781	0.530572843	0.999996191
NDUFAF3	0.997765933	0.537504505	0.839915775	0.043656933	0.823304684	0.604757284	0.999996191
NDUFAF7	0.738060795	0.706556233	0.853640944	0.03241079	0.8098353	0.541712231	0.999996191
NDUFB10	0.594572556	0.649700718	0.492163406	0.107322806	0.665472553	0.570578732	0.999996191
NDUFB2	0.496939463	0.650464892	0.524249273	0.089068391	0.897236416	0.570065158	0.999996191

NDUFB4	0.498349797	0.252767236	0.868007544	0.125323249	0.67294965	0.497156359	0.999996191
NDUFB5	0.613497086	0.61882719	0.74830924	0.178891316	0.749185635	0.768367352	0.999996191
NDUFB6	0.7332479	0.483702275	0.893986971	0.174825851	0.824670385	0.800722283	0.999996191
NDUFB9	0.698508142	0.987060541	0.947226845	0.095347968	0.606186711	0.766801116	0.999996191
NDUFC1	0.51006899	0.498464926	0.984000642	0.046337605	0.903692896	0.521013098	0.999996191
NDUFS1	0.927594627	0.58439739	0.926228789	0.072827633	0.580884325	0.657757088	0.999996191
NDUFS2	0.971261262	0.387015149	0.785777971	0.126621045	0.788227531	0.720894974	0.999996191
NDUFS4	0.597500945	0.608773121	0.939723067	0.092979628	0.874992931	0.709801875	0.999996191
NDUFS7	0.595305114	0.660053404	0.721248123	0.047628015	0.88661676	0.546287994	0.999996191
NDUFV1	0.918899324	0.451595917	0.841884213	0.025802884	0.863848632	0.466242258	0.999996191
NDUFV2	0.953696806	0.452320152	0.732268061	0.058596207	0.883155669	0.606631178	0.999996191
NDUFV3	0.897883478	0.417299351	0.442883519	0.294611387	0.927013142	0.799228556	0.999996191
NEBL	0.800819096	0.723834756	0.891725218	0.942137894	0.798512935	0.997122017	0.999996191
NECAB2	0.540090197	0.515485801	0.478614803	0.085815244	0.864296103	0.510064131	0.999996191
NECAP1	0.920448555	0.556652607	0.395634507	0.77074487	0.549691299	0.897138421	0.999996191
NECAP2	0.582651998	0.799519979	0.363046229	0.876106844	0.858298958	0.941560215	0.999996191
NECTIN1	0.785026146	0.682088071	0.590517804	0.583094187	0.461147661	0.89582646	0.999996191
NECTIN2	0.157116075	0.968621304	0.627495549	0.205978057	0.70252453	0.573971373	0.999996191
NECTIN3	0.599788543	0.749665935	0.482139052	0.505742387	0.516992569	0.836568395	0.999996191
NECTIN4	0.196485379	0.703534879	0.457919314	0.526516242	0.619959017	0.652375422	0.999996191
NEDD1	0.143733848	0.455686163	0.829133978	0.989381223	0.940884566	0.817823053	0.999996191
NEDD4L	0.209300117	0.876109375	0.950501876	0.667881765	0.869531445	0.917360537	0.999996191
NEDD8	0.669470221	0.597916833	0.672076329	0.067751187	0.786263294	0.581023512	0.999996191
NEIL2	0.392635366	0.986839756	0.46796277	0.34214054	0.299873703	0.631881572	0.999996191
NEK1	0.878362127	0.711951198	0.835344706	0.389691149	0.629051045	0.942220014	0.999996191
NEK3	0.573059908	0.905213226	0.72688795	0.658702292	0.544258199	0.947243362	0.999996191
NEK6	0.848468873	0.525644294	0.970563829	0.478115595	0.158835614	0.741368496	0.999996191
NEK7	0.920677577	0.310856635	0.212583426	0.890535638	0.193781003	0.521423886	0.999996191
NEK8	0.754337297	0.200301076	0.582262712	0.528770521	0.933943304	0.791877636	0.999996191
NELFB	0.81790643	0.258158118	0.674392385	0.104757241	0.982771677	0.585431304	0.999996191
NELFCD	0.659333496	0.667368955	0.838483334	0.953193515	0.75823189	0.988613758	0.999996191
NEMP1	0.268488478	0.674402153	0.701306873	0.217924041	0.397703526	0.530331157	0.999996191
NEU1	0.701387949	0.969423744	0.346255006	0.152646735	0.61634293	0.665917387	0.999996191
NEURL1B	0.718743213	0.407742529	0.286398256	0.767006847	0.475511477	0.728016645	0.999996191
NEURL2	0.642171708	0.792872005	0.833300947	0.462091364	0.111316422	0.663035168	0.999996191
NEURL3	0.915328177	0.149725648	0.146389432	0.7500448	0.896540449	0.569328424	0.999996191
NEURL4	0.315721465	0.091433065	0.507835128	0.536914534	0.903221405	0.449957887	0.999996191
NF1	0.92080627	0.312797911	0.943971329	0.056096225	0.937438383	0.580573239	0.999996191
NFAT5	0.200805451	0.91273758	0.526161866	0.231504028	0.343015938	0.46323143	0.999996191
NFATC2	0.583800223	0.761497855	0.315089408	0.643043315	0.345021181	0.730860826	0.999996191
NFATC2IP	0.697659747	0.571201935	0.984146898	0.754745497	0.353303497	0.921103185	0.999996191
NFATC4	0.497423266	0.169696539	0.495779786	0.437202859	0.982823446	0.625249138	0.999996191
NFIB	0.499089969	0.534481644	0.383355397	0.5081065	0.285719828	0.587877937	0.999996191
NFIL3	0.603282536	0.160811466	0.584105349	0.514929655	0.641722468	0.633156304	0.999996191
NFIX	0.55329385	0.573965177	0.070759651	0.887515163	0.574324254	0.537919536	0.999996191

NFKBIB	0.598029884	0.636909075	0.914910703	0.911766379	0.147313112	0.80478476	0.999996191
NFKBIE	0.301538411	0.829377737	0.385358576	0.5296407	0.911899502	0.803830687	0.999996191
NFKBIL1	0.398184901	0.581145203	0.755284659	0.612773211	0.098942776	0.523163472	0.999996191
NFRKB	0.638854411	0.105171292	0.675889964	0.414050683	0.615734154	0.539964881	0.999996191
NFS1	0.959504626	0.738577731	0.662301845	0.247715783	0.894211594	0.92043719	0.999996191
NFU1	0.613705705	0.68782049	0.076397871	0.474127774	0.911309319	0.575580439	0.999996191
NFX1	0.554821253	0.857553444	0.736977305	0.147733932	0.598142902	0.730298288	0.999996191
NFYA	0.835917836	0.622140986	0.145806733	0.688572463	0.519352252	0.704985619	0.999996191
NGDN	0.895675978	0.389133355	0.683949981	0.635958573	0.497516567	0.879588331	0.999996191
NGEF	0.137434562	0.935120001	0.622429516	0.142365573	0.65433764	0.458336806	0.999996191
NGLY1	0.934477763	0.830915681	0.821023472	0.018897298	0.662896562	0.470798866	0.999996191
NGRN	0.48492703	0.44537172	0.537792272	0.500990116	0.805954145	0.80511972	0.999996191
NHLH1	0.640031188	0.803123368	0.463050809	0.23337201	0.134874261	0.459296828	0.999996191
NHLRC2	0.507920181	0.579823828	0.875993133	0.509260212	0.884921553	0.932601922	0.999996191
NHLRC3	0.266715349	0.593528838	0.407589067	0.253195514	0.794745553	0.56193468	0.999996191
NHS	0.760271137	0.674721526	0.646486379	0.849936056	0.317235857	0.902311501	0.999996191
NHSL1	0.1470091	0.90448901	0.143568592	0.950519646	0.598740427	0.527883398	0.999996191
NHSL2	0.034295378	0.96306469	0.311018424	0.913575952	0.998286574	0.50009708	0.999996191
NICN1	0.466368253	0.167222622	0.991848954	0.160967771	0.561375623	0.446954699	0.999996191
NID1	0.103633747	0.678616647	0.812425955	0.240583737	0.789061617	0.527569569	0.999996191
NIM1K	0.854842018	0.66365303	0.568553575	0.249807816	0.670822589	0.828879724	0.999996191
NINJ1	0.602929186	0.184800393	0.713842666	0.52055124	0.980675752	0.779916262	0.999996191
NIPAL1	0.743552616	0.620479096	0.633823986	0.870091393	0.415252934	0.922257162	0.999996191
NIPAL3	0.778843694	0.975452534	0.798735182	0.197737251	0.77334733	0.906948226	0.999996191
NIPAL4	0.653931704	0.328409586	0.608117739	0.497398706	0.657372856	0.788844696	0.999996191
NIPBL	0.445909577	0.883400944	0.991988442	0.072232033	0.852119993	0.68189298	0.999996191
NIPSNAP3A	0.773749043	0.957192171	0.226195675	0.420028868	0.603091221	0.787747169	0.999996191
NISCH	0.953754889	0.505412084	0.684763352	0.095536971	0.904510046	0.714637654	0.999996191
NIT1	0.941638819	0.21528405	0.426917034	0.141528638	0.917327521	0.534261154	0.999996191
NIT2	0.764165773	0.28925961	0.779696239	0.563379255	0.699091714	0.86438833	0.999996191
NKAIN2	0.759156338	0.275470159	0.521528953	0.313240289	0.301253177	0.517665843	0.999996191
NKAIN3	0.278034781	0.664635286	0.766118765	0.303106703	0.950181648	0.780663006	0.999996191
NKAP	0.621574855	0.168919476	0.240988967	0.451799721	0.839311694	0.504536164	0.999996191
NKAPL	0.423230368	0.439911447	0.829327741	0.39757246	0.501398926	0.729047855	0.999996191
NKIRAS1	0.729791336	0.877427575	0.775198481	0.066435944	0.806941938	0.701380211	0.999996191
NKPD1	0.744762513	0.049202438	0.656451653	0.986433062	0.71474975	0.613818682	0.999996191
NLGN1	0.37545537	0.701876027	0.722073088	0.617119676	0.391528521	0.801709675	0.999996191
NLGN2	0.888143732	0.726718348	0.030566685	0.829900466	0.947443349	0.596419084	0.999996191
NMB	0.821015357	0.650987827	0.130704569	0.985605364	0.251152002	0.617606894	0.999996191
NME1	0.84511392	0.39173262	0.756581119	0.15604546	0.778026612	0.726764545	0.999996191
NME3	0.889049413	0.864956495	0.193190834	0.332574558	0.596717769	0.720913862	0.999996191
NME4	0.426974399	0.149856573	0.309283186	0.590110574	0.862905046	0.513702564	0.999996191
NME6	0.746873672	0.937637097	0.490165516	0.256488886	0.700590367	0.849886126	0.999996191
NME7	0.926890857	0.961079494	0.980052644	0.351698657	0.388251505	0.935177291	0.999996191
NMI	0.263457219	0.952092134	0.302255071	0.479465193	0.885454462	0.737437515	0.999996191

NMNAT1	0.932199334	0.256731912	0.718229964	0.152958539	0.714827866	0.633877481	0.999996191
NMNAT2	0.344149484	0.589156444	0.960413826	0.399814019	0.343455521	0.702305646	0.999996191
NMRK1	0.099569666	0.49756653	0.644694447	0.428999193	0.632960384	0.485847212	0.999996191
NMUR1	0.759422071	0.751701888	0.676586504	0.136324004	0.576440418	0.726405254	0.999996191
NNAT	0.5990411	0.814332103	0.998499881	0.205733044	0.73852629	0.876915612	0.999996191
NNT	0.986407734	0.391315907	0.958140366	0.915243577	0.46941181	0.960666293	0.999996191
NOBOX	0.74765313	0.666510869	0.747887874	0.909261803	0.402230273	0.947987989	0.999996191
NOCT	0.341956474	0.749102117	0.893157927	0.488213779	0.372589928	0.784303154	0.999996191
NOD1	0.471365669	0.705816253	0.215717913	0.609508812	0.829779387	0.759668988	0.999996191
NODAL	0.706761513	0.779627062	0.325272922	0.088289514	0.68937422	0.52865313	0.999996191
NOL10	0.281214748	0.350758006	0.800634625	0.557128513	0.34415384	0.591718836	0.999996191
NOL3	0.718646713	0.622857841	0.38618194	0.784914403	0.904791297	0.93812162	0.999996191
NOL4L	0.746996942	0.619214321	0.18958669	0.827483269	0.849152171	0.849727494	0.999996191
NOL7	0.74541004	0.60650829	0.48459955	0.112674696	0.868274243	0.659518672	0.999996191
NOL8	0.235221603	0.505957602	0.717946561	0.594114324	0.814456307	0.783135533	0.999996191
NOL9	0.649645044	0.119553587	0.956042017	0.542620499	0.242052486	0.507578427	0.999996191
NONO	0.460953445	0.755980108	0.564156649	0.163346183	0.695807144	0.667612692	0.999996191
NOP53	0.335244142	0.705245592	0.338603136	0.219064828	0.885221697	0.596571646	0.999996191
NOP58	0.952090001	0.973693649	0.946541369	0.275235216	0.270394036	0.85860767	0.999996191
NOP9	0.605362278	0.545509531	0.797020447	0.29146316	0.610863595	0.8049864	0.999996191
NOS1	0.713705875	0.623363855	0.731460952	0.300106506	0.29683896	0.717718849	0.999996191
NOS1AP	0.978051355	0.553903673	0.992244634	0.508913904	0.645556546	0.968182934	0.999996191
NOS2	0.814744598	0.987683929	0.328357189	0.037140804	0.789874043	0.465420092	0.999996191
NOS3	0.543724756	0.641882132	0.307020287	0.981182509	0.537148287	0.835975009	0.999996191
NOSIP	0.932861867	0.508560206	0.398470938	0.494486013	0.889793478	0.892935516	0.999996191
NOSTRIN	0.548407893	0.484006145	0.34491055	0.413741089	0.918422584	0.751868795	0.999996191
NOTCH1	0.366024292	0.991109434	0.394717132	0.750706889	0.957625478	0.919304134	0.999996191
NOTCH2	0.965045627	0.915911507	0.385413837	0.544019818	0.856054315	0.960551141	0.999996191
NOTCH4	0.444386025	0.634860409	0.777874906	0.666726319	0.465745944	0.864970645	0.999996191
NOV	0.578522681	0.264837204	0.742760826	0.095645147	0.945623263	0.517681437	0.999996191
NOVA1	0.444663017	0.986100596	0.881238852	0.650638779	0.337308157	0.895487875	0.999996191
NOVA2	0.751035666	0.791293493	0.962042542	0.82509584	0.763014424	0.995994624	0.999996191
NOX4	0.997040388	0.820908079	0.941558459	0.290534922	0.080411792	0.625485863	0.999996191
NPAS2	0.917845823	0.713505623	0.295266316	0.915508622	0.551281061	0.913066947	0.999996191
NPAT	0.800519694	0.090706512	0.423616457	0.652028247	0.68419054	0.572616626	0.999996191
NPC1	0.98805243	0.686991768	0.097061519	0.724393956	0.612725789	0.719366625	0.999996191
NPHP1	0.996043045	0.908945862	0.903021048	0.33634347	0.378153766	0.920456101	0.999996191
NPHP3	0.552512341	0.669955433	0.661986772	0.231330446	0.75339883	0.788861969	0.999996191
NPHP4	0.815759323	0.470261539	0.14401539	0.983696537	0.154740391	0.480200972	0.999996191
NPL	0.342001511	0.718861077	0.158715831	0.531696046	0.586132568	0.549351659	0.999996191
NPLOC4	0.802493915	0.532040102	0.290724613	0.635028929	0.241743081	0.63657086	0.999996191
NPM1	0.854132064	0.74162715	0.711106637	0.109960895	0.225390274	0.533040857	0.999996191
NPR3	0.620755027	0.235799881	0.921228958	0.29160549	0.726358177	0.714887413	0.999996191
NPRL2	0.836970226	0.876869778	0.689472444	0.111869057	0.88784916	0.816844988	0.999996191
NPRL3	0.682262455	0.84528927	0.371895264	0.631327256	0.866457559	0.933541256	0.999996191

NPTN	0.657025847	0.394612897	0.67414905	0.999613191	0.967040857	0.965156477	0.999996191
NQO1	0.458490745	0.609517768	0.594209718	0.28033557	0.913083988	0.788031162	0.999996191
NQO2	0.766226963	0.342477774	0.425263611	0.425668315	0.614153254	0.718919628	0.999996191
NR1D2	0.419992192	0.834801252	0.717048124	0.300421632	0.504570066	0.7685272	0.999996191
NR1I3	0.42997309	0.258700595	0.985507685	0.166122528	0.706771191	0.560253871	0.999996191
NR2C1	0.382175729	0.806796874	0.993352434	0.947279584	0.764818596	0.981163751	0.999996191
NR2F1	0.648822321	0.112227696	0.396725688	0.453837268	0.907707143	0.545213739	0.999996191
NR3C1	0.856776853	0.475856882	0.226736736	0.504182831	0.742504788	0.7509006	0.999996191
NR5A2	0.890929149	0.989891011	0.101939039	0.475972361	0.724391266	0.730372961	0.999996191
NR6A1	0.534728267	0.904350653	0.973164147	0.396615645	0.969956475	0.969807677	0.999996191
NRADD	0.963273116	0.211813102	0.321277289	0.213992226	0.603759824	0.481492741	0.999996191
NRAP	0.654091123	0.173347411	0.424908134	0.299563541	0.919686101	0.566189185	0.999996191
NRARP	0.533267167	0.941383217	0.754001657	0.026289135	0.949836961	0.501736841	0.999996191
NRBF2	0.363689776	0.63682811	0.369568025	0.830628353	0.981087074	0.868394284	0.999996191
NRBP1	0.542610018	0.949951517	0.494250051	0.892147302	0.358139569	0.890055759	0.999996191
NRDC	0.873999801	0.732035348	0.620008134	0.888670433	0.385775946	0.947783358	0.999996191
NRDE2	0.843680816	0.68908034	0.939556536	0.743748672	0.046746209	0.635908835	0.999996191
NRGN	0.363253531	0.326707558	0.295428095	0.517535119	0.674925309	0.550703559	0.999996191
NRIP3	0.38547353	0.610299406	0.494264534	0.29890504	0.862224328	0.724001281	0.999996191
NRK	0.748292208	0.817860167	0.922653346	0.314289899	0.127936186	0.670720811	0.999996191
NRP1	0.589340151	0.676475746	0.949035764	0.76573721	0.542767058	0.959874293	0.999996191
NRTN	0.850295712	0.394480093	0.248734922	0.714066172	0.290477484	0.617754456	0.999996191
NRXN2	0.244524281	0.534893105	0.507345348	0.591505826	0.742998859	0.71885136	0.999996191
NSD1	0.99986249	0.326829591	0.088001431	0.962701111	0.889066543	0.686359761	0.999996191
NSD2	0.927921391	0.085316228	0.554096694	0.543932915	0.789816651	0.634404948	0.999996191
NSD3	0.541720286	0.944821164	0.926476697	0.159340695	0.751546697	0.836859881	0.999996191
NSF	0.731350382	0.368886208	0.787620292	0.167585744	0.684740317	0.684544964	0.999996191
NSG1	0.266382593	0.537314949	0.733350107	0.569473985	0.336267182	0.646996298	0.999996191
NSMAF	0.816330824	0.686907994	0.820119499	0.863595662	0.25916078	0.919287782	0.999996191
NSMCE1	0.342088981	0.50800935	0.340879462	0.367868275	0.708777309	0.5955838	0.999996191
NSMCE3	0.568770822	0.920847211	0.544013992	0.414963908	0.819103034	0.912149524	0.999996191
NSMF	0.813956413	0.891086434	0.584547058	0.084515011	0.454523989	0.605911701	0.999996191
NSRP1	0.078098668	0.808178755	0.498986087	0.465904442	0.614012466	0.49286319	0.999996191
NSUN3	0.332198189	0.874293308	0.944364563	0.377775003	0.40609318	0.786246849	0.999996191
NSUN4	0.642783894	0.754233253	0.879518145	0.576737299	0.747805282	0.97080528	0.999996191
NSUN6	0.308365123	0.979356446	0.163421204	0.272807225	0.775392358	0.520352796	0.999996191
NT5C	0.844466916	0.459994221	0.176208745	0.907797204	0.347822046	0.661137256	0.999996191
NT5C2	0.781049387	0.84476132	0.50843007	0.502797655	0.558805711	0.908864817	0.999996191
NT5DC1	0.677544957	0.847664103	0.274829186	0.505481227	0.432599791	0.750417122	0.999996191
NT5M	0.448026698	0.902911762	0.445027591	0.118879205	0.389745399	0.478707793	0.999996191
NTAN1	0.490234926	0.882671939	0.551002214	0.234498786	0.693559277	0.771669275	0.999996191
NTM	0.548998748	0.409755331	0.38479882	0.937425691	0.942295385	0.881500454	0.999996191
NTN3	0.943338049	0.934755432	0.032144545	0.912791209	0.556235126	0.581839533	0.999996191
NTN4	0.510998381	0.28901964	0.630939148	0.522558362	0.170294853	0.477636146	0.999996191
NTN5	0.766931495	0.628683247	0.631674041	0.261405002	0.939216103	0.878374752	0.999996191

NTNG2	0.837428215	0.969768931	0.472756652	0.644003263	0.650307318	0.961560098	0.999996191
NTPCR	0.856744549	0.538937455	0.629775666	0.568296921	0.758597249	0.940175337	0.999996191
NTRK2	0.222790312	0.825951945	0.725700913	0.813924826	0.92543465	0.916630006	0.999996191
NTSR2	0.345049052	0.664760747	0.849193866	0.192397578	0.649960241	0.684340981	0.999996191
NUBP1	0.527136182	0.870438571	0.966164673	0.235303027	0.636717928	0.861149094	0.999996191
NUBPL	0.674458562	0.686535232	0.181205777	0.996903711	0.549045323	0.801518746	0.999996191
NUCB1	0.244676829	0.414247108	0.163658947	0.619417513	0.899854462	0.497649284	0.999996191
NUCKS1	0.531157313	0.126488969	0.860940612	0.99353107	0.735508832	0.787045777	0.999996191
NUDC	0.449117421	0.52131189	0.102091036	0.947053242	0.855824591	0.639803798	0.999996191
NUDCD2	0.656725298	0.683965464	0.793975063	0.370523047	0.804865607	0.922999775	0.999996191
NUDT1	0.596690764	0.670202368	0.794125518	0.808140794	0.973755667	0.98626678	0.999996191
NUDT12	0.28281215	0.693562486	0.633292905	0.262771175	0.833312936	0.705572708	0.999996191
NUDT13	0.411766814	0.606187137	0.522061456	0.082434159	0.91173852	0.508368138	0.999996191
NUDT15	0.451455916	0.632476206	0.232945997	0.87110014	0.299889964	0.618546809	0.999996191
NUDT2	0.752488961	0.650421959	0.687801104	0.621914471	0.697801102	0.95398251	0.999996191
NUDT22	0.372989574	0.703029696	0.456647918	0.368158052	0.502125663	0.66579229	0.999996191
NUDT5	0.332472786	0.279770369	0.943530497	0.605930942	0.181676979	0.50582289	0.999996191
NUDT6	0.162116744	0.804850656	0.581207802	0.842145094	0.494998404	0.734065641	0.999996191
NUDT7	0.646300307	0.776166563	0.920068773	0.546363516	0.307613954	0.883493541	0.999996191
NUFIP1	0.877377858	0.975980909	0.918262905	0.806146756	0.838685052	0.999502405	0.999996191
NUP107	0.214133109	0.730093743	0.871222021	0.334442146	0.888251057	0.779295114	0.999996191
NUP133	0.561308964	0.965975507	0.875638173	0.210422297	0.669607462	0.862221755	0.999996191
NUP160	0.36722044	0.550638893	0.9057186	0.073909322	0.842921322	0.537180063	0.999996191
NUP188	0.579619277	0.679066508	0.958937511	0.433459063	0.160010918	0.698231468	0.999996191
NUP205	0.848573953	0.912305836	0.833821919	0.753645686	0.102445353	0.815435923	0.999996191
NUP210L	0.415395967	0.247496456	0.662701267	0.987210701	0.840022949	0.836050633	0.999996191
NUP214	0.414672431	0.950224878	0.710293571	0.197678487	0.995160458	0.8318801	0.999996191
NUP35	0.376615677	0.652225121	0.494223146	0.282433689	0.407023286	0.575882301	0.999996191
NUP37	0.463582747	0.871845079	0.982268013	0.641942745	0.382480603	0.912925714	0.999996191
NUP43	0.324629079	0.897007959	0.534985981	0.520645819	0.939177495	0.880975108	0.999996191
NUP54	0.49499953	0.593426945	0.356537866	0.22547276	0.968029203	0.672038612	0.999996191
NUP58	0.890570695	0.548282491	0.664942401	0.41695281	0.946288672	0.942257577	0.999996191
NUP62	0.341126754	0.60534868	0.342193513	0.271116255	0.611091157	0.542087638	0.999996191
NUP85	0.634858791	0.580290871	0.553606537	0.586203539	0.442821294	0.825479756	0.999996191
NUP88	0.860395289	0.912393495	0.851899615	0.293763357	0.322893726	0.854196322	0.999996191
NUP93	0.600026458	0.63317928	0.910253503	0.783775384	0.053010676	0.581532176	0.999996191
NUPL2	0.493278923	0.19638549	0.732994277	0.15049509	0.954229724	0.515927657	0.999996191
NUPR2	0.63523423	0.916611458	0.143452866	0.531578076	0.845294927	0.765763517	0.999996191
NUTF2	0.650374659	0.44511143	0.241681028	0.130439351	0.910744615	0.478059869	0.999996191
NVL	0.393785143	0.445990613	0.32633061	0.777867756	0.882114755	0.77419373	0.999996191
NXF1	0.49734365	0.430812525	0.530723596	0.23988345	0.588365985	0.603051633	0.999996191
NXN	0.296306888	0.901496503	0.654514895	0.434504292	0.870717823	0.860531707	0.999996191
NXPE2	0.575733336	0.874239982	0.415821499	0.4238731	0.399440642	0.755266034	0.999996191
NXPE3	0.822278902	0.426857349	0.23547536	0.428092456	0.542540349	0.638010718	0.999996191
NXPE4	0.357959522	0.946184758	0.885998503	0.953149919	0.875280157	0.986334898	0.999996191

NXPH2	0.369808439	0.481712938	0.480994912	0.677521039	0.556482088	0.738123383	0.999996191
NXT1	0.406878252	0.647253005	0.273174015	0.742741609	0.684428057	0.761042696	0.999996191
NXT2	0.79378874	0.419933147	0.17724131	0.900271314	0.730423218	0.772008245	0.999996191
NYAP1	0.849115118	0.099401718	0.552760206	0.533198003	0.554249853	0.573536316	0.999996191
NYX	0.787265956	0.986594941	0.130743013	0.917275816	0.745524481	0.867747144	0.999996191
OAF	0.092195172	0.800746012	0.5275772	0.321610325	0.795389923	0.511574123	0.999996191
OAT	0.443758143	0.691649052	0.715504182	0.902118268	0.46865668	0.907016535	0.999996191
OAZ1	0.576642784	0.34543965	0.243010282	0.550634467	0.732246585	0.64125172	0.999996191
OCEL1	0.288334419	0.823386039	0.649268565	0.483910444	0.431878932	0.73759481	0.999996191
OCIAD1	0.038058962	0.415700869	0.98290241	0.600216697	0.982909699	0.496208302	0.999996191
OCIAD2	0.889973283	0.230687913	0.530416316	0.817843837	0.313450136	0.710536089	0.999996191
OCLN	0.59354506	0.228976535	0.480006284	0.470678541	0.859856738	0.699866756	0.999996191
OCRL	0.656991019	0.625713972	0.257713296	0.33921635	0.523712159	0.634153745	0.999996191
ODF2	0.516963638	0.385930232	0.387407966	0.638200845	0.188190006	0.498393669	0.999996191
ODF2L	0.119941379	0.773699895	0.941712468	0.90045936	0.935949927	0.876229708	0.999996191
ODR4	0.060113228	0.579650032	0.881306555	0.628235627	0.618990633	0.545877441	0.999996191
OGA	0.534472689	0.346983917	0.614738614	0.826600454	0.24224721	0.671779138	0.999996191
OGDH	0.855023211	0.500004594	0.61991725	0.425086573	0.45176647	0.818948573	0.999996191
OGFOD3	0.613676582	0.724716783	0.874025083	0.15956576	0.707358953	0.79359149	0.999996191
OGFR	0.238419186	0.646875398	0.840723039	0.856597289	0.583132864	0.857361056	0.999996191
OGFRL1	0.790185414	0.205060804	0.342862879	0.410857691	0.443183333	0.514426944	0.999996191
OGG1	0.575408222	0.145976145	0.763899373	0.348132536	0.495610734	0.531450137	0.999996191
OGN	0.327090255	0.587165939	0.702880094	0.292671465	0.774405997	0.727916155	0.999996191
OLA1	0.842257628	0.680821444	0.829816058	0.452217133	0.704784874	0.957036337	0.999996191
OLFML2A	0.526107684	0.407630719	0.212942801	0.56452633	0.419257337	0.526908368	0.999996191
OMA1	0.989303739	0.78825226	0.691779474	0.862792506	0.506433006	0.983890132	0.999996191
OMD	0.358567678	0.28850766	0.200274829	0.950469041	0.39297933	0.465116213	0.999996191
OMP	0.902616146	0.419495145	0.055870814	0.974298801	0.965524689	0.645048611	0.999996191
ONECUT2	0.152875535	0.87377962	0.715114622	0.578454236	0.66906668	0.763020729	0.999996191
OOEP	0.608229888	0.868157352	0.341806796	0.131167042	0.397671901	0.501003796	0.999996191
OPA1	0.772913761	0.710922018	0.681498279	0.582860847	0.334816689	0.875119527	0.999996191
OPHN1	0.980330357	0.205928097	0.292693086	0.591883753	0.505550567	0.62194537	0.999996191
OPLAH	0.689610096	0.458856698	0.222981893	0.359959131	0.810307468	0.651600254	0.999996191
OPRL1	0.123577784	0.912848375	0.889543267	0.858652737	0.234797449	0.648260038	0.999996191
OPTN	0.939010727	0.913330152	0.235073329	0.720422672	0.917278724	0.945918887	0.999996191
OR51E1	0.661655174	0.819563208	0.878818796	0.184559834	0.615161928	0.829040609	0.999996191
ORAI1	0.516888916	0.846906908	0.450872602	0.901328556	0.770866088	0.948531116	0.999996191
ORAI3	0.56942027	0.696085507	0.418116166	0.442281139	0.81510137	0.844914161	0.999996191
ORAOV1	0.790944206	0.763282944	0.140501594	0.767908216	0.688819177	0.797486583	0.999996191
ORC1	0.886706391	0.933923419	0.22574977	0.468262789	0.62713142	0.831411249	0.999996191
ORC2	0.973744289	0.519283647	0.369168125	0.988914816	0.989175818	0.970356897	0.999996191
ORC3	0.371815796	0.112362517	0.783127717	0.82024413	0.472687991	0.557459963	0.999996191
ORC4	0.279829532	0.77220346	0.19124258	0.271213436	0.896051215	0.513064976	0.999996191
ORC5	0.474166566	0.868060797	0.83875502	0.786611454	0.576242251	0.959499988	0.999996191
ORMDL1	0.109075333	0.422827669	0.98131192	0.915609949	0.551315261	0.67192565	0.999996191

ORMDL2	0.306921879	0.840886002	0.543220666	0.396292301	0.593329128	0.741891664	0.999996191
ORMDL3	0.993360982	0.549770008	0.443228969	0.485455112	0.97064486	0.930597706	0.999996191
OS9	0.31959735	0.438039607	0.474108739	0.533445214	0.391077058	0.574363057	0.999996191
OSBP	0.883171281	0.306313199	0.432803208	0.333639897	0.213659096	0.47882435	0.999996191
OSBP2	0.172410944	0.753523961	0.543486417	0.214369425	0.628144079	0.502835078	0.999996191
OSBPL10	0.542254864	0.738168992	0.634879106	0.357762562	0.816741879	0.87738951	0.999996191
OSBPL2	0.970664843	0.598303673	0.312917123	0.636375079	0.578626612	0.862265382	0.999996191
OSBPL3	0.986355379	0.890476687	0.79582153	0.38453334	0.626679327	0.964939677	0.999996191
OSBPL5	0.987496841	0.477780529	0.180555814	0.727292399	0.604566773	0.765397717	0.999996191
OSBPL6	0.946187567	0.403176149	0.043406524	0.543806951	0.844532698	0.461979861	0.999996191
OSGEP	0.912410963	0.805560164	0.969767942	0.766790358	0.68217737	0.996541631	0.999996191
OSGEPL1	0.395619209	0.814868881	0.980988098	0.615096001	0.835890542	0.962391219	0.999996191
OSMR	0.422482655	0.956481295	0.810654932	0.639868386	0.266731899	0.834360764	0.999996191
OSTC	0.219024762	0.623146079	0.544040377	0.657609143	0.633712118	0.730047258	0.999996191
OSTM1	0.636719283	0.550088398	0.121983251	0.384775003	0.801436447	0.564757627	0.999996191
OTOGL	0.946479602	0.783275452	0.670818742	0.72324223	0.571852695	0.977327937	0.999996191
OTOP1	0.44666801	0.281740168	0.643279486	0.458501323	0.628718612	0.676045599	0.999996191
OTOR	0.824568715	0.801247436	0.445056916	0.557414263	0.648644172	0.922956093	0.999996191
OTUB1	0.381420075	0.876156129	0.712917791	0.533864473	0.737488608	0.908276662	0.999996191
OTUB2	0.566177629	0.719039718	0.1686596	0.542355291	0.821816913	0.727966843	0.999996191
OTUD3	0.225532329	0.341463543	0.514305567	0.75441712	0.654457257	0.641630631	0.999996191
OTUD4	0.525340239	0.656143108	0.665579796	0.354064223	0.974180472	0.886232363	0.999996191
OTUD5	0.409930114	0.488606195	0.451052304	0.419104825	0.329412976	0.554212262	0.999996191
OTUD7A	0.988495783	0.987969441	0.87305944	0.394604959	0.312111424	0.921569842	0.999996191
OTUD7B	0.61625895	0.699267466	0.235293716	0.863077076	0.995159403	0.898941098	0.999996191
OTULIN	0.542710519	0.467864139	0.339017007	0.716673001	0.82500131	0.818951708	0.999996191
OTULINL	0.798276098	0.772531167	0.231132349	0.225460375	0.770329953	0.687471173	0.999996191
OVCA2	0.893252912	0.97235462	0.501142806	0.254550197	0.068923075	0.462728627	0.999996191
OVGP1	0.854681754	0.722962539	0.905219683	0.484015324	0.968754385	0.988041853	0.999996191
OXA1L	0.994298776	0.457963491	0.968375582	0.635298919	0.665431899	0.971652063	0.999996191
OXCT1	0.818123244	0.961742053	0.420847692	0.901080518	0.598647633	0.968934358	0.999996191
OXNAD1	0.426356655	0.961203496	0.711672213	0.068219873	0.867084072	0.617150893	0.999996191
OXSR1	0.948338817	0.83550765	0.50462537	0.806801803	0.425449627	0.948602681	0.999996191
P2RX4	0.380028795	0.387738531	0.891968408	0.762187449	0.949822911	0.910017807	0.999996191
P2RX6	0.673467881	0.962137581	0.048956722	0.538415552	0.829020158	0.578690151	0.999996191
P2RY14	0.710815344	0.973470004	0.18041103	0.985165634	0.316979218	0.772623824	0.999996191
P2RY2	0.569549983	0.949461843	0.163023835	0.939699092	0.104956299	0.486309177	0.999996191
P3H2	0.976366792	0.507168061	0.86674809	0.114094655	0.211722382	0.519050865	0.999996191
P4HA3	0.531189609	0.426844895	0.224913347	0.798728668	0.31517231	0.559755729	0.999996191
PAAF1	0.95645432	0.488280164	0.582607831	0.209283902	0.71920992	0.781449989	0.999996191
PABPC4L	0.619474454	0.738937151	0.891143738	0.902082093	0.4337756	0.961012035	0.999996191
PABPC5	0.221809667	0.951717688	0.602323	0.97462978	0.896959018	0.927846454	0.999996191
PACS1	0.280944824	0.893915607	0.283429066	0.731859062	0.767970439	0.777273445	0.999996191
PACSIN1	0.774425148	0.048022105	0.826213919	0.675001721	0.850969077	0.62159916	0.999996191
PACSIN2	0.736534145	0.665446945	0.467234613	0.745382875	0.655013978	0.928476066	0.999996191

PACSIN3	0.499250413	0.825687864	0.660678751	0.830550166	0.250165795	0.836297587	0.999996191
PAF1	0.358898079	0.586324667	0.747830532	0.436885154	0.611929945	0.786217756	0.999996191
PAFAH1B1	0.74851258	0.551678994	0.304842876	0.90085777	0.752211868	0.896254083	0.999996191
PAFAH1B2	0.764973233	0.288843919	0.681429803	0.537532578	0.997460871	0.888941986	0.999996191
PAFAH1B3	0.320451852	0.763857163	0.232407965	0.547543322	0.729313049	0.670830702	0.999996191
PAG1	0.927428368	0.298082079	0.454100149	0.233721463	0.367073106	0.526233047	0.999996191
PAICSP	0.58663891	0.581016295	0.448233862	0.673340062	0.75068678	0.882875904	0.999996191
PAIP1	0.868368479	0.469580342	0.246375111	0.86789034	0.357276114	0.731305307	0.999996191
PAIP2	0.296527294	0.498456707	0.364646856	0.546028326	0.779535713	0.672733846	0.999996191
PAIP2B	0.859623856	0.464015362	0.175535392	0.702518939	0.84729266	0.784554277	0.999996191
PAK1	0.942364634	0.634562309	0.398128279	0.244140525	0.70921879	0.782610326	0.999996191
PAK1IP1	0.299087949	0.707176902	0.12290363	0.658343949	0.484607931	0.477663163	0.999996191
PALM	0.469333641	0.384529934	0.161654311	0.743268544	0.63963901	0.574689815	0.999996191
PALM2	0.884031633	0.122640576	0.707818196	0.271831614	0.909401759	0.635700099	0.999996191
PALMD	0.768303253	0.681038843	0.906803472	0.699921606	0.677732263	0.981829367	0.999996191
PAM	0.222281588	0.671737738	0.397397499	0.224122589	0.785798784	0.520540904	0.999996191
PAM16	0.689856655	0.525450972	0.778335507	0.217225877	0.676892256	0.783735	0.999996191
PANK2	0.73346564	0.358490936	0.523929791	0.167267699	0.895847173	0.652194676	0.999996191
PANK3	0.789216393	0.381996939	0.36825813	0.542072105	0.246130974	0.587440933	0.999996191
PANK4	0.498674446	0.619797272	0.448094142	0.778414745	0.458309643	0.813980781	0.999996191
PANX1	0.925657304	0.48180787	0.20523518	0.566636971	0.519363443	0.703711862	0.999996191
PANX2	0.115630681	0.826967367	0.186299312	0.97355777	0.74755185	0.561659349	0.999996191
PAPD4	0.06777192	0.75791352	0.36612155	0.651741708	0.739839228	0.494051816	0.999996191
PAPD7	0.844492846	0.915359557	0.194931806	0.372104532	0.935891373	0.824022995	0.999996191
PAPLN	0.978959524	0.25579517	0.368328357	0.214409674	0.509179774	0.513563228	0.999996191
PAPOLB	0.890599911	0.347115698	0.48236639	0.065959728	0.90152495	0.489923066	0.999996191
PAPOLG	0.37559359	0.887146008	0.813026239	0.106704417	0.712956738	0.651874221	0.999996191
PAPPA	0.627938523	0.218624327	0.512957879	0.775061849	0.214106728	0.541740164	0.999996191
PAPSS1	0.935661061	0.591938893	0.308721775	0.9682251	0.478519519	0.88638252	0.999996191
PAPSS2	0.586782675	0.798038511	0.25889202	0.994156598	0.22687845	0.706582854	0.999996191
PAQR4	0.272598134	0.849109433	0.513805719	0.369875948	0.894300932	0.774256414	0.999996191
PAQR5	0.595451037	0.531885185	0.231975239	0.928620048	0.849212117	0.840062643	0.999996191
PAQR6	0.300449261	0.322030351	0.567138786	0.714226605	0.824086496	0.73807456	0.999996191
PARG	0.8796637	0.927362273	0.983771089	0.951547278	0.509753958	0.99713535	0.999996191
PARK7	0.574544139	0.612338914	0.706853313	0.166886609	0.628137177	0.697425201	0.999996191
PARL	0.967707096	0.276073057	0.242881884	0.719828883	0.323200452	0.591125992	0.999996191
PARN	0.612902497	0.583618561	0.512598977	0.658190864	0.590049976	0.871398793	0.999996191
PARP1	0.666236231	0.991395098	0.476637951	0.760861501	0.752332312	0.969510523	0.999996191
PARP16	0.912258913	0.849673484	0.462051598	0.574929711	0.484895013	0.915764137	0.999996191
PARP2	0.534614228	0.435519363	0.702574135	0.866333366	0.722219203	0.918645739	0.999996191
PARP4	0.493815174	0.574446383	0.647349474	0.523842543	0.912036002	0.89988974	0.999996191
PARP8	0.82395489	0.338079424	0.693979969	0.889420148	0.81038782	0.949938317	0.999996191
PARPBP	0.750472205	0.838365095	0.173899738	0.631544431	0.622369936	0.790086118	0.999996191
PARS2	0.986148426	0.688260467	0.333128134	0.062582247	0.576632369	0.47469654	0.999996191
PARVA	0.593740102	0.280077664	0.333768864	0.671841402	0.629751228	0.677262402	0.999996191

PARVB	0.636491044	0.758160076	0.595244939	0.923485076	0.96388811	0.987127683	0.999996191
PASK	0.422890351	0.147220529	0.740863251	0.410130784	0.627615508	0.544768391	0.999996191
PATJ	0.640210827	0.855523495	0.740905971	0.75225165	0.641500815	0.974596991	0.999996191
PATL2	0.582787499	0.249420723	0.242298558	0.697680703	0.37871583	0.498851797	0.999996191
PATZ1	0.910624259	0.394265762	0.958212637	0.770343836	0.376700707	0.915750122	0.999996191
PAXBP1	0.235942858	0.38612793	0.32090385	0.497937487	0.83836622	0.550043883	0.999996191
PAXIP1	0.794243339	0.775090777	0.81399176	0.686933536	0.501204054	0.966606812	0.999996191
PAXX	0.213607594	0.730612827	0.676566541	0.651718685	0.839159835	0.839535226	0.999996191
PBDC1	0.767105309	0.89536009	0.545869901	0.462456274	0.274817523	0.807835819	0.999996191
PBK	0.945393796	0.424044628	0.048273845	0.691104549	0.570944882	0.462718465	0.999996191
PBX1	0.903467035	0.945825698	0.122834421	0.764337245	0.771838804	0.850490687	0.999996191
PBX2	0.661420344	0.715202478	0.142540438	0.567072308	0.671594711	0.694533767	0.999996191
PBX3	0.706758557	0.764026625	0.787826271	0.724687369	0.529430974	0.962670624	0.999996191
PBX4	0.795641342	0.871375682	0.037342613	0.896246472	0.773086586	0.624770826	0.999996191
PC	0.804918561	0.732056204	0.500020871	0.398947119	0.309904721	0.760321212	0.999996191
PCBD1	0.310788724	0.623596913	0.467195028	0.178727486	0.957891286	0.59628369	0.999996191
PCBD2	0.418675094	0.668714389	0.643829698	0.047080528	0.878208289	0.45836452	0.999996191
PCBP3	0.428660734	0.262621024	0.977389276	0.169711646	0.985172514	0.629697325	0.999996191
PCBP4	0.81479066	0.471822552	0.244043975	0.747325338	0.528951759	0.763592919	0.999996191
PCCA	0.736837581	0.502732312	0.995904763	0.543896863	0.63725814	0.942080124	0.999996191
PCCB	0.925618851	0.491448028	0.868309596	0.408000603	0.847804726	0.948200897	0.999996191
PCDH11X	0.236157695	0.787114274	0.360952852	0.867296389	0.662218397	0.770538789	0.999996191
PCDH17	0.906082815	0.641677603	0.043566786	0.887721664	0.727787586	0.606850645	0.999996191
PCDH18	0.362271398	0.927041459	0.524927348	0.126689333	0.822382915	0.629387757	0.999996191
PCDH9	0.846523831	0.634326185	0.847735491	0.509395873	0.642693173	0.955624562	0.999996191
PCDHA13	0.065982335	0.301670332	0.988326411	0.463213495	0.999709501	0.494906789	0.999996191
PCDHB11	0.842113229	0.829410508	0.384671592	0.988233679	0.308513436	0.890901004	0.999996191
PCDHB14	0.903409097	0.583987614	0.115810403	0.434244304	0.849731374	0.669351673	0.999996191
PCDHGA8	0.617811594	0.796367967	0.50159944	0.072079709	0.682264219	0.548972335	0.999996191
PCDHGB4	0.934523132	0.676299482	0.808162424	0.633675209	0.68228721	0.980932537	0.999996191
PCDHGC3	0.665472117	0.111308448	0.786066879	0.872493523	0.180021728	0.495626322	0.999996191
PCED1A	0.780863135	0.031788294	0.740084881	0.976994604	0.705259366	0.557046076	0.999996191
PCGF1	0.90130178	0.265860427	0.499736213	0.50728719	0.706248405	0.789664153	0.999996191
PCGF2	0.609160555	0.902627858	0.755840191	0.176023196	0.500506552	0.761255671	0.999996191
PCGF3	0.554543462	0.879264682	0.145618612	0.888985131	0.41477463	0.698247232	0.999996191
PCGF6	0.208861035	0.770013433	0.616187897	0.613934145	0.341754518	0.653599028	0.999996191
PCID2	0.50645369	0.769130171	0.664748703	0.136196764	0.632599803	0.667311882	0.999996191
PCIF1	0.262025181	0.797103153	0.718819381	0.823257375	0.614742646	0.880618397	0.999996191
PCK1	0.089215747	0.501829209	0.909405751	0.638112824	0.91375574	0.679370678	0.999996191
PCK2	0.648512454	0.622844802	0.568414035	0.566056183	0.986426074	0.942327611	0.999996191
PCM1	0.187860536	0.788959399	0.76600186	0.133282383	0.616485203	0.499301196	0.999996191
PCMT1	0.531437675	0.477419145	0.353878967	0.790693147	0.924608344	0.859381907	0.999996191
PCMTD1	0.992813253	0.588856392	0.242115498	0.478263876	0.385832608	0.697799273	0.999996191
PCNA	0.548507355	0.667909479	0.645098116	0.200039501	0.912759115	0.790683746	0.999996191
PCNP	0.44952448	0.291817898	0.567207131	0.460134741	0.975775421	0.744371219	0.999996191

PCNT	0.976243693	0.863561843	0.82567096	0.849450217	0.35975999	0.979085059	0.999996191
PCNX1	0.56811812	0.615690677	0.698304921	0.131778327	0.826096473	0.701226893	0.999996191
PCOLCE2	0.272907834	0.832784359	0.737384582	0.532652813	0.868147705	0.883360978	0.999996191
PCP4L1	0.581362645	0.583857107	0.571805519	0.209475476	0.914551437	0.764064241	0.999996191
PCSK4	0.889104083	0.17923478	0.500650439	0.765210707	0.277216355	0.613405389	0.999996191
PCSK5	0.68245986	0.9122428	0.200486853	0.489491295	0.418092301	0.693514517	0.999996191
PCSK6	0.303684614	0.944321596	0.999874873	0.845604881	0.912384672	0.981017538	0.999996191
PCSK7	0.073398692	0.762119331	0.78159893	0.517811085	0.462223998	0.520798565	0.999996191
PCYOX1	0.967943665	0.908794605	0.356084114	0.977750109	0.812241885	0.986088735	0.999996191
PCYOX1L	0.528975251	0.665775067	0.437236874	0.784241032	0.928826216	0.928819789	0.999996191
PCYT1B	0.990709021	0.149637388	0.457193443	0.953108012	0.802094434	0.821925935	0.999996191
PDAP1	0.209267554	0.787018565	0.597246215	0.722360452	0.339635574	0.682545795	0.999996191
PDCD10	0.486029521	0.760044097	0.37895446	0.707440304	0.925538716	0.905420504	0.999996191
PDCD2	0.391307003	0.925648637	0.308893831	0.761942637	0.901750512	0.882245491	0.999996191
PDCD2L	0.953341428	0.945721918	0.453930167	0.650557147	0.811975828	0.979896776	0.999996191
PDCD4	0.593613348	0.871238465	0.489998145	0.233552749	0.861787818	0.819310763	0.999996191
PDCD5	0.66027954	0.468233982	0.729961687	0.248653033	0.925797806	0.822363449	0.999996191
PDCD6IP	0.448819454	0.304681582	0.926272768	0.66892914	0.81361391	0.866671062	0.999996191
PDE10A	0.89098068	0.322226608	0.846305768	0.570507034	0.74738363	0.920035484	0.999996191
PDE1C	0.141839453	0.333296365	0.571183421	0.528590743	0.802905073	0.538019064	0.999996191
PDE2A	0.141778386	0.941784597	0.943814596	0.353082811	0.40119961	0.623827245	0.999996191
PDE3B	0.841277328	0.779705728	0.098245715	0.925870129	0.8697791	0.822188714	0.999996191
PDE4C	0.745957057	0.13843537	0.572985479	0.380042254	0.99931743	0.668720153	0.999996191
PDE4DIP	0.94226131	0.795500923	0.348163819	0.708250111	0.241913848	0.796893199	0.999996191
PDE5A	0.751541429	0.335947107	0.333740141	0.508158001	0.904315231	0.771407682	0.999996191
PDE6A	0.879188991	0.900062929	0.347045782	0.538731887	0.884512015	0.944264454	0.999996191
PDE6C	0.030403127	0.832909181	0.881768232	0.95903953	0.678873474	0.583802607	0.999996191
PDE6D	0.359124213	0.560137008	0.961118996	0.173707959	0.684495593	0.673131875	0.999996191
PDE8B	0.827818273	0.355331193	0.266358262	0.579635576	0.614732392	0.710544045	0.999996191
PDE9A	0.964890974	0.706970857	0.883934764	0.885593438	0.462715079	0.985824749	0.999996191
PDGFC	0.731617882	0.710347392	0.435622244	0.977197257	0.328144505	0.874172801	0.999996191
PDGFD	0.648109549	0.835274549	0.090768596	0.378534813	0.844275756	0.59880772	0.999996191
PDGFRB	0.743771758	0.44964389	0.157003113	0.854905142	0.678200219	0.726975788	0.999996191
PDGFRL	0.304519707	0.851153604	0.159803309	0.769283646	0.99857831	0.735279334	0.999996191
PDHA1	0.892862739	0.745024971	0.471108467	0.267329955	0.964485312	0.889062637	0.999996191
PDHB	0.84595379	0.517582588	0.959402042	0.289064624	0.71806084	0.899096438	0.999996191
PDIA5	0.952824447	0.348581182	0.042692676	0.541512965	0.995395857	0.462884286	0.999996191
PDIK1L	0.490708502	0.457967177	0.69231059	0.861035941	0.175918724	0.677947651	0.999996191
PDK3	0.139446617	0.956050494	0.716140465	0.676059519	0.524603066	0.746875508	0.999996191
PDK4	0.480086708	0.962713652	0.279775364	0.211257638	0.634133181	0.617950694	0.999996191
PDLIM1	0.568669562	0.183136966	0.923020247	0.267262395	0.942919098	0.683281513	0.999996191
PDLIM2	0.336227914	0.573507612	0.780391883	0.257672275	0.56396775	0.663422482	0.999996191
PDLIM4	0.801681664	0.94776682	0.178600219	0.731556295	0.881189969	0.899516677	0.999996191
PDPN	0.869775613	0.156814772	0.336388872	0.52635222	0.364081681	0.488363358	0.999996191
PDPR	0.686508751	0.379738254	0.354554971	0.636523265	0.342925419	0.647725778	0.999996191

PDS5B	0.586225558	0.946419141	0.618840205	0.200365829	0.978213396	0.863106251	0.999996191
PDSS2	0.797388423	0.626813767	0.57827805	0.765272147	0.680304334	0.956407633	0.999996191
PDZD11	0.630536118	0.515068965	0.352979644	0.18459085	0.699579995	0.587325015	0.999996191
PDZD2	0.767852369	0.912655635	0.757563424	0.822115285	0.858592204	0.996615384	0.999996191
PDZD8	0.797865593	0.480365315	0.237448385	0.616850274	0.633419691	0.755898291	0.999996191
PDZD9	0.981221937	0.509225699	0.443209333	0.417382373	0.616950144	0.837533843	0.999996191
PEAK1	0.944174985	0.763470089	0.484513852	0.106552424	0.58794729	0.663532153	0.999996191
PEAR1	0.83363835	0.923275788	0.467845014	0.625394594	0.778850587	0.967725513	0.999996191
PEBP1	0.939640713	0.745635567	0.576684573	0.142551998	0.64261407	0.763228891	0.999996191
PECAM1	0.551178413	0.754461362	0.879694161	0.72003406	0.756915834	0.975617116	0.999996191
PECR	0.709089328	0.619300171	0.849203513	0.345358454	0.449722663	0.840014699	0.999996191
PEG10	0.837057499	0.951773223	0.95137763	0.573698376	0.712056073	0.992949926	0.999996191
PELO	0.995132399	0.171031854	0.316645064	0.951617311	0.835578295	0.789463395	0.999996191
PELP1	0.447710928	0.676431864	0.290464249	0.309824121	0.44480224	0.548753659	0.999996191
PEMT	0.273211129	0.869844871	0.580784261	0.14876746	0.907901338	0.632291532	0.999996191
PEX1	0.997942534	0.99650947	0.773598839	0.717738963	0.335437055	0.971252577	0.999996191
PEX10	0.591895094	0.218808837	0.861782217	0.878992445	0.206419169	0.648451086	0.999996191
PEX11A	0.767151316	0.690419106	0.167221901	0.690129709	0.854223148	0.823197111	0.999996191
PEX11G	0.471450644	0.862312485	0.663538999	0.074531664	0.927917551	0.632433648	0.999996191
PEX12	0.648126466	0.606935551	0.877039331	0.473190676	0.758181887	0.938931069	0.999996191
PEX13	0.292166291	0.372578416	0.620044079	0.76262186	0.561064559	0.716994745	0.999996191
PEX14	0.92782165	0.910728264	0.174634907	0.351491308	0.900416452	0.804405154	0.999996191
PEX16	0.817907008	0.870378114	0.958014506	0.044441774	0.527026883	0.60213013	0.999996191
PEX19	0.425272438	0.475941643	0.609809652	0.266943519	0.631277426	0.653666336	0.999996191
PEX2	0.950703221	0.62528703	0.65272295	0.243512529	0.238369457	0.669160982	0.999996191
PEX26	0.656290041	0.867068569	0.541992145	0.137759953	0.590765296	0.690138152	0.999996191
PEX3	0.627045991	0.925992357	0.784597172	0.739488089	0.37052264	0.939757943	0.999996191
PEX7	0.842922432	0.67229751	0.985143695	0.43427222	0.588531818	0.951992706	0.999996191
PFDN1	0.830885527	0.653405824	0.740018862	0.268228799	0.630310701	0.864487632	0.999996191
PFDN4	0.710952065	0.485179409	0.68429101	0.083911253	0.926861145	0.629285131	0.999996191
PFDN5	0.414444831	0.426261606	0.377973959	0.264796722	0.832996518	0.58633553	0.999996191
PFDN6	0.238505487	0.612534601	0.756197212	0.213308183	0.346339023	0.474745267	0.999996191
PFKFB1	0.226443651	0.987427352	0.890805578	0.056929472	0.82276623	0.499321029	0.999996191
PFKFB2	0.65108505	0.569520774	0.872316453	0.877570589	0.496708081	0.950967841	0.999996191
PFKL	0.861387666	0.882011683	0.887708283	0.954454693	0.803623027	0.999398801	0.999996191
PFKM	0.656129825	0.941328951	0.304011648	0.667762889	0.893128876	0.928645655	0.999996191
PFKP	0.580548686	0.787315043	0.333162341	0.806827696	0.104827104	0.560383048	0.999996191
PGAM1	0.862292747	0.66542571	0.646208862	0.228394872	0.578651495	0.812592039	0.999996191
PGAM2	0.670783289	0.971598357	0.65059352	0.751697479	0.378029122	0.936254816	0.999996191
PGAP1	0.435708864	0.729628155	0.805541443	0.649588784	0.82953925	0.949085429	0.999996191
PGAP2	0.983224599	0.61758984	0.593741685	0.906720764	0.791032667	0.987540074	0.999996191
PGBD1	0.803495415	0.591816718	0.488366972	0.969852745	0.816941846	0.9708389	0.999996191
PGBD2	0.330308341	0.533324679	0.954313208	0.652284211	0.451979845	0.814504934	0.999996191
PGK1	0.804113675	0.786657615	0.764621563	0.1475843	0.227920127	0.605705777	0.999996191
PGM1	0.741534745	0.801275574	0.719808983	0.728221473	0.436650959	0.947785822	0.999996191

PGM3	0.131570405	0.711182606	0.650762934	0.75060122	0.6606496	0.725433334	0.999996191
PGPEP1	0.712367366	0.260430793	0.728737319	0.88017625	0.410663876	0.812122328	0.999996191
PGR	0.520383028	0.292390284	0.928802533	0.879109874	0.072016415	0.491575746	0.999996191
PGRMC1	0.927339248	0.790392359	0.76948897	0.241895719	0.620501065	0.895259109	0.999996191
PGRMC2	0.217348066	0.798468665	0.321794213	0.974701181	0.838635223	0.800479905	0.999996191
PGS1	0.293324546	0.507094621	0.317927831	0.450034426	0.329170417	0.447349725	0.999996191
PHACTR1	0.751002788	0.87609935	0.367707273	0.588007453	0.769223447	0.92614716	0.999996191
PHACTR2	0.908694175	0.813402412	0.359563072	0.093994284	0.847960486	0.657220877	0.999996191
PHACTR3	0.575608127	0.699483611	0.708973201	0.30088148	0.324829642	0.710416552	0.999996191
PHAX	0.86193681	0.205446022	0.106502528	0.938050408	0.943808725	0.610768912	0.999996191
PHB	0.338774128	0.792234585	0.620429401	0.286769251	0.335999178	0.602991509	0.999996191
PHC1	0.22541594	0.902052593	0.483521906	0.217515971	0.963080568	0.651749832	0.999996191
PHC2	0.623248475	0.75623288	0.882706117	0.313422128	0.535635573	0.868585988	0.999996191
PHETA2	0.578214882	0.898181984	0.232770723	0.787199178	0.815380524	0.883534748	0.999996191
PHF1	0.490591735	0.776137087	0.790149029	0.895496096	0.502959014	0.947461779	0.999996191
PHF10	0.514316077	0.910915654	0.438245258	0.135752696	0.291869153	0.474154907	0.999996191
PHF12	0.736834691	0.857019252	0.71463848	0.375388658	0.940497958	0.960872328	0.999996191
PHF19	0.626438426	0.640779296	0.615247251	0.755722516	0.640460075	0.935450733	0.999996191
PHF2	0.436295802	0.974574568	0.776243888	0.433797733	0.826336801	0.93440653	0.999996191
PHF20	0.993589537	0.488625692	0.139041917	0.260863932	0.547866318	0.505557008	0.999996191
PHF20L1	0.201088228	0.625861269	0.859363871	0.593621346	0.523111155	0.745356172	0.999996191
PHF23	0.638155467	0.316534511	0.864311179	0.201239017	0.599833238	0.656227092	0.999996191
PHF3	0.428303	0.859493136	0.953010857	0.208140011	0.886067215	0.857205723	0.999996191
PHF5A	0.940273353	0.599891859	0.683747884	0.936005441	0.919457227	0.994506364	0.999996191
PHF6	0.390114885	0.846221506	0.56671257	0.731886618	0.894739372	0.93791916	0.999996191
PHKA1	0.085216642	0.78441651	0.396286341	0.955881681	0.615192266	0.597234356	0.999996191
PHKA2	0.847817359	0.864200161	0.846446536	0.465173521	0.323109881	0.907504501	0.999996191
PHKB	0.813930748	0.9932461	0.770663088	0.426941985	0.320730551	0.89627213	0.999996191
PHKG2	0.491021456	0.747348663	0.78855562	0.299569432	0.842205686	0.874982187	0.999996191
PHLDA1	0.299973474	0.535718487	0.492789516	0.141089638	0.977689587	0.528917101	0.999996191
PHLDB3	0.871070333	0.647189952	0.076344991	0.690209418	0.880051761	0.697971163	0.999996191
PHLPP2	0.16501602	0.34097158	0.484513035	0.575193405	0.69803916	0.52929508	0.999996191
PHOSPHO2	0.481095299	0.855803487	0.589509015	0.784355177	0.769659373	0.954228953	0.999996191
PHRF1	0.823536442	0.796102585	0.717538457	0.306689071	0.747998917	0.924620142	0.999996191
PHTF2	0.614567874	0.830786816	0.7677492	0.125015453	0.814898091	0.776948711	0.999996191
PHYH	0.819479891	0.457811337	0.115261209	0.420269921	0.62295403	0.535698751	0.999996191
PHYKPL	0.514801422	0.75394433	0.520666165	0.310756566	0.745144948	0.804743496	0.999996191
PI15	0.478128618	0.698623147	0.948769483	0.733064438	0.141216865	0.741001365	0.999996191
PI3	0.389945819	0.258182884	0.499802139	0.227287666	0.626435574	0.451327739	0.999996191
PI4K2B	0.88658607	0.841344747	0.460919456	0.157311287	0.923533741	0.815810817	0.999996191
PI4KA	0.796205815	0.921802219	0.489313452	0.19141356	0.737868492	0.818381757	0.999996191
PI4KB	0.969593189	0.181812909	0.89143342	0.470802188	0.7654893	0.836431386	0.999996191
PIAS3	0.439447863	0.840460496	0.168767619	0.638849574	0.511171681	0.649455989	0.999996191
PIAS4	0.249781454	0.976477916	0.948633421	0.949095705	0.70273781	0.958415027	0.999996191
PICK1	0.591216855	0.512948794	0.97611542	0.606034671	0.863283394	0.958694045	0.999996191

PID1	0.171474552	0.768139695	0.579616467	0.409557526	0.865040692	0.704496579	0.999996191
PIDD1	0.960067748	0.136503897	0.659101668	0.533025921	0.83281857	0.769640092	0.999996191
PIEZO1	0.558920632	0.64540883	0.628972474	0.57433529	0.915718384	0.935276231	0.999996191
PIGB	0.68595891	0.919911267	0.357051548	0.687214731	0.068996051	0.524697582	0.999996191
PIGC	0.864335349	0.286303933	0.197576587	0.209221133	0.835471504	0.483152207	0.999996191
PIGF	0.132067499	0.917127349	0.836163193	0.974348245	0.265655128	0.698499433	0.999996191
PIGG	0.954769384	0.191835119	0.497926731	0.780310114	0.836912444	0.844421311	0.999996191
PIGH	0.568602408	0.343613657	0.927970723	0.534857999	0.770209589	0.87821244	0.999996191
PIGK	0.798142016	0.871633529	0.759424293	0.58585337	0.792245914	0.985523761	0.999996191
PIGL	0.960317549	0.168893483	0.853096905	0.559930025	0.444635489	0.750054694	0.999996191
PIGP	0.909421259	0.728582783	0.7786488	0.463089702	0.915983063	0.98049674	0.999996191
PIGS	0.965170264	0.98660585	0.753236235	0.969678979	0.914232354	0.999891042	0.999996191
PIGT	0.069868896	0.794043912	0.718444827	0.435045524	0.542030623	0.500697342	0.999996191
PIGU	0.975399507	0.336223784	0.121747917	0.93080794	0.761658775	0.713184841	0.999996191
PIGV	0.753684342	0.61206773	0.619872544	0.959035082	0.696003132	0.973089817	0.999996191
PIGX	0.783657796	0.493818101	0.215066936	0.203633648	0.651663491	0.530996112	0.999996191
PIGY	0.959329743	0.318957857	0.326364984	0.403439174	0.510400703	0.651438937	0.999996191
PIH1D1	0.946887819	0.977140815	0.515068662	0.693159329	0.671892589	0.981173204	0.999996191
PIK3C2B	0.715188436	0.878820136	0.082779765	0.370301079	0.749373612	0.582463008	0.999996191
PIK3CB	0.79289181	0.500731617	0.330954391	0.716452277	0.749441313	0.87005492	0.999996191
PIK3IP1	0.792085511	0.914687237	0.869028423	0.537766996	0.49135394	0.964057208	0.999996191
PIK3R3	0.682984457	0.425287918	0.956489947	0.349964078	0.267247312	0.696801472	0.999996191
PIM1	0.527375664	0.516407672	0.869455396	0.411946266	0.212496219	0.65299156	0.999996191
PIM2	0.99280333	0.388160362	0.768300254	0.842380602	0.992944301	0.985914263	0.999996191
PIN1	0.492068442	0.697317303	0.627041178	0.533255139	0.612303265	0.869432597	0.999996191
PIN4	0.351646121	0.450839722	0.670545388	0.104606482	0.816253931	0.494235893	0.999996191
PINK1	0.582103498	0.825619187	0.478403371	0.768490757	0.615793972	0.925520781	0.999996191
PIP4K2B	0.797061542	0.901039367	0.818282857	0.648358636	0.88491832	0.994821288	0.999996191
PIP4K2C	0.183813775	0.907428888	0.984166422	0.419164024	0.348261957	0.681183373	0.999996191
PIP4P1	0.645377788	0.664969376	0.602851765	0.750792787	0.985517887	0.973264935	0.999996191
PIP4P2	0.885143468	0.62614757	0.571775886	0.934657524	0.585437047	0.966951859	0.999996191
PIP5K1A	0.358283157	0.837765406	0.936781067	0.3960541	0.48021466	0.827134035	0.999996191
PIP5K1B	0.578517305	0.393840152	0.812067298	0.732338498	0.42353594	0.83854883	0.999996191
PITPNB	0.18734405	0.482870676	0.658852141	0.766582349	0.456912584	0.654382659	0.999996191
PITPNC1	0.104555694	0.372464129	0.76996971	0.446958208	0.601348087	0.472456381	0.999996191
PITRM1	0.758573226	0.760808552	0.631525194	0.75741705	0.695021442	0.973400003	0.999996191
PITX3	0.752618345	0.650343467	0.333735807	0.158156939	0.920887182	0.679786382	0.999996191
PJA1	0.913766451	0.67141621	0.721772084	0.188483165	0.138919813	0.540246737	0.999996191
PJA2	0.648211849	0.469836658	0.522210279	0.965750684	0.674396478	0.92001658	0.999996191
PJVK	0.127325896	0.947195434	0.565065204	0.926696321	0.985676915	0.851298049	0.999996191
PKD2	0.527906716	0.813429152	0.481870168	0.741665476	0.377857096	0.84020268	0.999996191
PKDREJ	0.661532502	0.555945016	0.827582809	0.27226073	0.817140057	0.864026703	0.999996191
PKIA	0.248252313	0.991096076	0.286225491	0.2409786	0.804819597	0.571711545	0.999996191
PKM	0.5098845	0.944522805	0.324461344	0.494660798	0.673310362	0.822656681	0.999996191
PKN1	0.65333205	0.776878704	0.535724929	0.746561465	0.714410999	0.95337036	0.999996191

PKN3	0.888308548	0.164385245	0.37183416	0.767981517	0.745254694	0.730851612	0.999996191
PKP1	0.816293887	0.619453453	0.142673436	0.901128027	0.157685866	0.516922882	0.999996191
PKP2	0.201244066	0.560301559	0.599345384	0.288697107	0.60152932	0.542556449	0.999996191
PKP4	0.191773026	0.630334259	0.874991421	0.30179947	0.622486837	0.644752093	0.999996191
PLA1A	0.229701569	0.799270099	0.839410835	0.19821504	0.495833853	0.591771157	0.999996191
PLA2G12A	0.645580069	0.580984744	0.504604326	0.684790943	0.755763086	0.913503663	0.999996191
PLA2G15	0.901058324	0.379066726	0.347586682	0.733232192	0.700586616	0.848127472	0.999996191
PLA2G16	0.838902531	0.338007309	0.621165391	0.61962961	0.8017902	0.8995566	0.999996191
PLA2G4A	0.662466731	0.759934567	0.082438303	0.873806447	0.782125058	0.713565289	0.999996191
PLA2G5	0.509782287	0.87903346	0.713078688	0.221324381	0.398003019	0.712110248	0.999996191
PLA2G6	0.504960596	0.80797526	0.818059481	0.294088671	0.23523931	0.673990649	0.999996191
PLAA	0.590379814	0.577173378	0.391624844	0.89368992	0.192179188	0.672548584	0.999996191
PLAC9	0.944193345	0.51767405	0.315049065	0.468278513	0.8176823	0.842842511	0.999996191
PLAGL1	0.933683767	0.255095824	0.886825942	0.103323092	0.552570808	0.547753006	0.999996191
PLAGL2	0.641560958	0.894682935	0.786420981	0.196564889	0.804141325	0.871685512	0.999996191
PLB1	0.133858896	0.839995776	0.806352479	0.796443031	0.934639321	0.863538786	0.999996191
PLBD1	0.992487097	0.141207403	0.346609039	0.763536502	0.884417765	0.740972331	0.999996191
PLBD2	0.344056519	0.678691819	0.445861992	0.951176095	0.745069159	0.876485954	0.999996191
PLCB3	0.460102147	0.680202173	0.385918661	0.95526268	0.703759018	0.889718507	0.999996191
PLCD1	0.670229745	0.350289953	0.343081943	0.810964031	0.629183963	0.782075299	0.999996191
PLCD3	0.687822492	0.495399023	0.14603146	0.494485484	0.943240559	0.674984592	0.999996191
PLCD4	0.214451801	0.949919178	0.875298048	0.988712574	0.487599392	0.897257493	0.999996191
PLCE1	0.280260687	0.641231907	0.257662367	0.951443294	0.164458823	0.453327841	0.999996191
PLCG1	0.592642522	0.941835692	0.623277938	0.758619672	0.518050961	0.94826369	0.999996191
PLCG2	0.321524055	0.161856387	0.846699415	0.858585431	0.876209604	0.742925226	0.999996191
PLCH1	0.67888123	0.891286803	0.159194109	0.618853183	0.799614727	0.807894572	0.999996191
PLCXD3	0.469257935	0.77191851	0.532652937	0.391947297	0.630893782	0.80805333	0.999996191
PLD1	0.634587638	0.61777577	0.35471787	0.687525187	0.247388755	0.678653284	0.999996191
PLD5	0.505857747	0.920010207	0.082474072	0.998414492	0.390839274	0.589596199	0.999996191
PLEC	0.587123957	0.459201572	0.43650993	0.464826507	0.168056012	0.496593044	0.999996191
PLEKHA2	0.97273171	0.933488177	0.329451179	0.774475317	0.368137957	0.896239999	0.999996191
PLEKHA3	0.406446371	0.526152604	0.255671875	0.979318841	0.737251013	0.774881636	0.999996191
PLEKHA5	0.956216274	0.724048216	0.851788964	0.243656708	0.735927745	0.922356475	0.999996191
PLEKHA7	0.788934411	0.273716342	0.86460502	0.647599701	0.921082292	0.928053469	0.999996191
PLEKHA8	0.438072957	0.82356588	0.480132108	0.717354147	0.597388352	0.877345831	0.999996191
PLEKHB1	0.280867443	0.333991506	0.191396607	0.679528829	0.88386349	0.526470454	0.999996191
PLEKHD1	0.595548832	0.926706985	0.53666336	0.419064538	0.289721594	0.75796	0.999996191
PLEKHG1	0.138016501	0.984392909	0.791922118	0.649454402	0.75142957	0.824121049	0.999996191
PLEKHG3	0.72457259	0.604070187	0.990347173	0.406461535	0.97800602	0.966521887	0.999996191
PLEKHG5	0.579783531	0.682586693	0.609079468	0.340613288	0.39239806	0.737609752	0.999996191
PLEKHG7	0.969320206	0.20876126	0.432911279	0.372215241	0.645419912	0.655954348	0.999996191
PLEKHH2	0.799005665	0.681255874	0.069396154	0.732277861	0.670356195	0.631245852	0.999996191
PLEKHM1	0.751733522	0.858137374	0.206985565	0.705553645	0.982753351	0.906667322	0.999996191
PLEKHM3	0.873800269	0.798286192	0.354921861	0.832676949	0.147259169	0.726441523	0.999996191
PLEKHO1	0.388406174	0.80917887	0.585565369	0.428377373	0.315374321	0.688307588	0.999996191

PLGRKT	0.455064406	0.715709544	0.783726138	0.608049358	0.877141149	0.94787958	0.999996191
PLIN1	0.836917021	0.90481407	0.182008312	0.979154923	0.538346551	0.874282801	0.999996191
PLK1	0.628903638	0.18965996	0.270123786	0.880220443	0.250707766	0.449979249	0.999996191
PLK2	0.57849437	0.354157007	0.96744378	0.84058923	0.8714728	0.953470016	0.999996191
PLK3	0.154830082	0.371509623	0.828892427	0.47396658	0.392718939	0.49007688	0.999996191
PLOD3	0.805091942	0.191567409	0.141445216	0.741970661	0.774248525	0.555124716	0.999996191
PLPP2	0.297329264	0.581078657	0.88411173	0.563441895	0.425439245	0.761262505	0.999996191
PLPP3	0.477278554	0.661251994	0.901724018	0.69095513	0.799801195	0.959883193	0.999996191
PLPP4	0.772615603	0.867016856	0.472674139	0.479522051	0.999690546	0.957103228	0.999996191
PLPP5	0.596204234	0.865511013	0.507075672	0.324164207	0.830601573	0.869849536	0.999996191
PLPP6	0.559814092	0.726733736	0.78158616	0.31319359	0.563696254	0.835015976	0.999996191
PLPP7	0.190374526	0.74483085	0.361135546	0.787231513	0.83405396	0.745564739	0.999996191
PLPPR1	0.572700583	0.930248485	0.508144408	0.537263373	0.102153675	0.588033043	0.999996191
PLPPR3	0.907994738	0.804298149	0.674129447	0.772334089	0.879415587	0.99465613	0.999996191
PLPPR5	0.662230464	0.593382698	0.415806147	0.771047086	0.976042887	0.93828511	0.999996191
PLSCR2	0.645452087	0.876253046	0.552134354	0.259855557	0.569793636	0.802683001	0.999996191
PLSCR4	0.08347435	0.864831121	0.881675467	0.222737248	0.723796628	0.517106833	0.999996191
PLTP	0.104987437	0.840052444	0.590466437	0.707129853	0.544945859	0.646675893	0.999996191
PLVAP	0.34552657	0.848336316	0.795972576	0.712735765	0.386208748	0.85608215	0.999996191
PLXNA2	0.298573044	0.662838369	0.48499739	0.816858876	0.776097063	0.847778337	0.999996191
PLXNA4	0.34148078	0.237009895	0.664239962	0.969282354	0.396378358	0.652302858	0.999996191
PLXNB1	0.360236719	0.632548351	0.48152671	0.733840711	0.444697342	0.757177058	0.999996191
PLXND1	0.594982975	0.925753853	0.063115052	0.36310144	0.767525449	0.506345693	0.999996191
PM20D2	0.972111216	0.672879571	0.692544803	0.073195902	0.298088974	0.510080384	0.999996191
PMAIP1	0.750612117	0.57777984	0.723690493	0.150643519	0.297672925	0.577517303	0.999996191
PMEL	0.378871269	0.91825688	0.738121809	0.978294861	0.7239467	0.970100477	0.999996191
PMEPA1	0.347086549	0.231644055	0.756883189	0.85970323	0.661473769	0.750898775	0.999996191
PMP22	0.388505848	0.441144233	0.735314747	0.494427934	0.823849437	0.820377107	0.999996191
PMPCA	0.776368967	0.349676707	0.63358815	0.058751376	0.930737445	0.50082861	0.999996191
PMPCB	0.633125231	0.700576368	0.28267431	0.334556331	0.560644043	0.677544164	0.999996191
PMVK	0.83150372	0.765752544	0.06125792	0.422025838	0.813903551	0.567988362	0.999996191
PNKP	0.760042593	0.138400185	0.843146861	0.295840153	0.960073835	0.690830393	0.999996191
PNLDC1	0.163518767	0.804985193	0.5309915	0.832130673	0.741652586	0.790618023	0.999996191
PNMA2	0.754995247	0.379459832	0.540235048	0.491467224	0.610709993	0.803488259	0.999996191
PNMA8B	0.601631285	0.190790989	0.26609401	0.3986852	0.583135259	0.44975102	0.999996191
PNP	0.404291925	0.702147909	0.867953118	0.062547681	0.879581453	0.570248295	0.999996191
PNPLA2	0.671009989	0.888709001	0.228983279	0.348166103	0.898954751	0.788992297	0.999996191
PNPLA6	0.938762781	0.53874944	0.318428241	0.667770751	0.74112859	0.887209284	0.999996191
PNPLA8	0.310628998	0.492583572	0.666548098	0.915815631	0.99276079	0.906858261	0.999996191
PNRC1	0.517432977	0.911973349	0.313949567	0.181318445	0.298754985	0.471685929	0.999996191
POC5	0.602365262	0.345003091	0.143855194	0.306735531	0.985039091	0.49333631	0.999996191
PODXL	0.189971736	0.743266714	0.464502683	0.342028409	0.401826518	0.492952123	0.999996191
POFUT1	0.482897	0.943132573	0.505390779	0.749952269	0.760834564	0.944598871	0.999996191
POGK	0.659508644	0.925407541	0.479575244	0.917122585	0.821144954	0.980843737	0.999996191
POGZ	0.611056139	0.877206377	0.993342453	0.713142303	0.518045951	0.974854825	0.999996191

POLA1	0.142977842	0.399559323	0.384865989	0.401035458	0.975053538	0.484244381	0.999996191
POLA2	0.841714189	0.097330223	0.866669822	0.829374643	0.255620285	0.590565278	0.999996191
POLB	0.772506269	0.788388936	0.62619304	0.846044301	0.547209249	0.968168237	0.999996191
POLD1	0.924202516	0.058829673	0.454459079	0.57655251	0.762180263	0.527773672	0.999996191
POLD2	0.936622048	0.477277879	0.496497391	0.320778377	0.826133345	0.84245125	0.999996191
POLD3	0.524096008	0.926800694	0.882593119	0.677931004	0.883202275	0.987271238	0.999996191
POLDIP2	0.744695787	0.194039913	0.440071258	0.532246162	0.729703398	0.687015514	0.999996191
POLE2	0.373506869	0.518479688	0.863233244	0.993902973	0.735474977	0.937665489	0.999996191
POLE4	0.979754562	0.634475898	0.677837671	0.409416607	0.587100168	0.91743272	0.999996191
POLH	0.657829475	0.307922231	0.811103989	0.2138291	0.435781125	0.593859363	0.999996191
POLK	0.17135967	0.935262045	0.464352358	0.982948509	0.622872219	0.800154904	0.999996191
POLL	0.420167014	0.071500095	0.916433707	0.700919314	0.496203414	0.504157687	0.999996191
POLM	0.270056563	0.381767366	0.954940424	0.274591395	0.846943119	0.672356287	0.999996191
POLN	0.776350025	0.791672435	0.290662845	0.108244916	0.996537951	0.638764871	0.999996191
POLQ	0.959242941	0.065198521	0.288424378	0.82446526	0.90435511	0.56873673	0.999996191
POLR1C	0.719661718	0.749291381	0.43105436	0.356732867	0.172027122	0.580119554	0.999996191
POLR2B	0.337520781	0.510673273	0.291111641	0.398433416	0.579534654	0.540103838	0.999996191
POLR2G	0.370883615	0.510563004	0.972416887	0.816640549	0.552411491	0.89276257	0.999996191
POLR2H	0.973363335	0.566189394	0.718459188	0.124588042	0.8802351	0.791780148	0.999996191
POLR2J	0.674631061	0.778943337	0.912264645	0.044404099	0.686507872	0.584815547	0.999996191
POLR2M	0.89059705	0.574288304	0.134771173	0.95024179	0.950688052	0.851352522	0.999996191
POLR3C	0.59897835	0.700181938	0.675312723	0.323178122	0.895796074	0.891028545	0.999996191
POLR3E	0.749723368	0.982834235	0.220265372	0.170762089	0.510305196	0.578466975	0.999996191
POLR3GL	0.747739081	0.634673579	0.339911395	0.422950349	0.615218253	0.785812225	0.999996191
POMC	0.78259995	0.87498036	0.647585264	0.525777457	0.578942676	0.947110166	0.999996191
POMGNT1	0.603083354	0.62287128	0.599795454	0.262228106	0.691319536	0.780972969	0.999996191
POMP	0.933007652	0.279284169	0.367574182	0.848157276	0.825697357	0.86262295	0.999996191
PON2	0.973572202	0.652419319	0.43909639	0.923141266	0.353229404	0.904444923	0.999996191
PON3	0.919501984	0.96237492	0.333332806	0.201267338	0.65762598	0.772891052	0.999996191
POP7	0.716957542	0.720382943	0.47275289	0.431902565	0.717421286	0.88002101	0.999996191
POPDC3	0.803209205	0.682237653	0.570334913	0.497372039	0.59450825	0.906438804	0.999996191
POU2F1	0.220092629	0.859574992	0.941048511	0.407308795	0.626112279	0.799541125	0.999996191
POU2F3	0.819558542	0.138981396	0.728460085	0.661123142	0.914675326	0.816567427	0.999996191
PPA2	0.90819468	0.820395672	0.325342425	0.821023398	0.719236206	0.952265629	0.999996191
PPARA	0.618056633	0.773284156	0.441422966	0.62280917	0.295008654	0.771599119	0.999996191
PPARG	0.716192798	0.902802586	0.978101601	0.405386521	0.84805637	0.980177543	0.999996191
PPARGC1A	0.945959542	0.762704883	0.237219454	0.535356364	0.192880383	0.621859407	0.999996191
PPCDC	0.809151003	0.750849548	0.808939045	0.113158761	0.633213616	0.754118562	0.999996191
PPCS	0.852583566	0.960174333	0.649742023	0.142381402	0.10470684	0.469519156	0.999996191
PPEF1	0.237335153	0.599772755	0.58036291	0.195517887	0.685874107	0.531581176	0.999996191
PPFIA1	0.460198211	0.936159925	0.666470855	0.885294323	0.898204232	0.982485093	0.999996191
PPFIA3	0.816155694	0.628769707	0.94130604	0.886989174	0.289847568	0.939259221	0.999996191
PPFIA4	0.413003728	0.544711236	0.368972329	0.997142319	0.586041271	0.810865733	0.999996191
PPFIBP1	0.883472361	0.700308468	0.178941047	0.716517435	0.809351192	0.856032378	0.999996191
PPFIBP2	0.761234956	0.693737638	0.854009813	0.16722157	0.157284126	0.54459403	0.999996191

PPHLN1	0.5501429	0.843604645	0.221094168	0.362834854	0.982659359	0.761110066	0.999996191
PPIB	0.473085341	0.453707491	0.465916119	0.705253026	0.464954876	0.740916308	0.999996191
PPIC	0.994069457	0.578797296	0.035274646	0.64225101	0.807317588	0.521856164	0.999996191
PPIH	0.550466776	0.255034568	0.731765339	0.450551229	0.31531819	0.584562312	0.999996191
PPIL1	0.88810093	0.596459862	0.862335277	0.175778008	0.254330502	0.650086432	0.999996191
PPIL4	0.381721345	0.946387978	0.813057903	0.112547634	0.825101102	0.706104141	0.999996191
PPIP5K1	0.704980506	0.952422256	0.64564465	0.065503266	0.718207566	0.649830598	0.999996191
PPIP5K2	0.47472997	0.183822585	0.321754175	0.814188823	0.738177738	0.612841913	0.999996191
PPM1A	0.683059731	0.989410877	0.075505173	0.704071267	0.902537724	0.738817812	0.999996191
PPM1D	0.424986797	0.955690557	0.830500316	0.276597706	0.950208192	0.901223619	0.999996191
PPM1H	0.945038489	0.581338732	0.641094423	0.472462831	0.993065002	0.963572716	0.999996191
PPM1J	0.972444959	0.793784845	0.502220242	0.240885947	0.15858761	0.587401163	0.999996191
PPM1K	0.284419965	0.945471713	0.392754389	0.454112588	0.147800351	0.44944324	0.999996191
PPM1L	0.779011459	0.931340615	0.265454605	0.202804142	0.599872066	0.67682579	0.999996191
PPM1M	0.7625526	0.72278518	0.87870887	0.161364734	0.896878229	0.869101361	0.999996191
PPM1N	0.638760724	0.839712653	0.886322274	0.829578306	0.732941533	0.991131931	0.999996191
PPOX	0.566579211	0.798619525	0.430433206	0.778900603	0.594083821	0.90330456	0.999996191
PPP1R10	0.956215162	0.502538997	0.767511658	0.105512812	0.88527574	0.750067513	0.999996191
PPP1R12A	0.346119428	0.564327369	0.977577954	0.495032905	0.597101061	0.835880716	0.999996191
PPP1R12C	0.642135775	0.736943125	0.381637314	0.608288454	0.744034794	0.890608391	0.999996191
PPP1R13B	0.25664019	0.897634512	0.909783406	0.667086021	0.61373277	0.897023479	0.999996191
PPP1R13L	0.271036081	0.957791559	0.827759678	0.59068565	0.248986916	0.734009507	0.999996191
PPP1R14A	0.507551694	0.181239925	0.860729886	0.590310637	0.181653754	0.481948281	0.999996191
PPP1R16B	0.849520255	0.64009158	0.745207738	0.544304317	0.792253974	0.967474616	0.999996191
PPP1R18	0.404907474	0.775090228	0.63228405	0.133830793	0.565182386	0.59000689	0.999996191
PPP1R1B	0.980078436	0.832142349	0.199783413	0.805406348	0.194936433	0.693796153	0.999996191
PPP1R21	0.675304304	0.643670793	0.903916374	0.652057543	0.934764646	0.984560097	0.999996191
PPP1R35	0.599594391	0.924927287	0.276043173	0.056345306	0.989042005	0.482828526	0.999996191
PPP1R36	0.993750772	0.56982616	0.048828694	0.34607677	0.995994537	0.503285851	0.999996191
PPP1R37	0.201087227	0.743065562	0.403952665	0.808863513	0.230380745	0.534460306	0.999996191
PPP1R3E	0.9976044	0.059948286	0.568143694	0.479588603	0.917543697	0.58925661	0.999996191
PPP1R3G	0.540612344	0.695259242	0.934572647	0.991817018	0.034624292	0.547810911	0.999996191
PPP1R42	0.32399571	0.126628106	0.340582898	0.987171681	0.619710826	0.483190887	0.999996191
PPP1R8	0.980434542	0.369024818	0.279913886	0.566757953	0.40686947	0.676188949	0.999996191
PPP1R9B	0.63297382	0.568583223	0.323749255	0.388197367	0.810004631	0.761372715	0.999996191
PPP2CA	0.699977837	0.130960691	0.579548442	0.563297663	0.500524563	0.589609975	0.999996191
PPP2R2B	0.997336835	0.262853953	0.747770912	0.103150138	0.379006512	0.463367121	0.999996191
PPP2R3A	0.465163202	0.5600431	0.093562161	0.511088836	0.828888767	0.518283684	0.999996191
PPP2R3B	0.974140902	0.628358589	0.845193233	0.586982183	0.915450134	0.989968066	0.999996191
PPP2R3C	0.167234618	0.803956048	0.396147749	0.956527287	0.270759236	0.573627648	0.999996191
PPP2R5A	0.843047062	0.652092724	0.157440226	0.406116638	0.697420325	0.685579232	0.999996191
PPP2R5B	0.589824842	0.934339421	0.499857603	0.177264633	0.72119073	0.754122603	0.999996191
PPP2R5C	0.376715008	0.251943875	0.958348747	0.641823217	0.932672451	0.830070696	0.999996191
PPP3CB	0.473214168	0.491247886	0.978038427	0.986949859	0.556900394	0.939867342	0.999996191
PPP3CC	0.768827134	0.570820328	0.541367253	0.710060986	0.163687051	0.708458354	0.999996191

PPP3R1	0.930647705	0.116083822	0.912626708	0.722461225	0.241637268	0.616695915	0.999996191
PPP4C	0.420596129	0.580599825	0.989616994	0.677649	0.72119605	0.9342247	0.999996191
PPP4R1	0.638818159	0.893466474	0.785447326	0.858401514	0.91705948	0.995662653	0.999996191
PPP4R3A	0.539325622	0.902932817	0.990568918	0.041137858	0.782171894	0.596534793	0.999996191
PPP4R4	0.766537391	0.376654671	0.214191774	0.969515048	0.238433191	0.580543532	0.999996191
PPP5C	0.933989899	0.565735666	0.406254207	0.666386547	0.374342199	0.827349744	0.999996191
PPP6C	0.656965914	0.1438941	0.482307344	0.630541984	0.778139523	0.667843696	0.999996191
PPP6R1	0.840846033	0.252135173	0.735250854	0.701669597	0.69498446	0.8806785	0.999996191
PPP6R3	0.708304066	0.802545149	0.593624633	0.180044674	0.974498885	0.843488027	0.999996191
PPT1	0.987836968	0.630680696	0.387089859	0.942470576	0.718243307	0.962683459	0.999996191
PPWD1	0.355493421	0.577948665	0.622762457	0.598484117	0.87515278	0.862487771	0.999996191
PPYR1	0.443244558	0.539496104	0.985284111	0.478160963	0.504977866	0.837154029	0.999996191
PQLC1	0.114913416	0.634269996	0.937755533	0.430368991	0.405683277	0.545742289	0.999996191
PQLC3	0.611732682	0.706927076	0.553201945	0.396271116	0.523495134	0.814724581	0.999996191
PRAF2	0.450865086	0.759999456	0.330671501	0.60475361	0.826117449	0.836356106	0.999996191
PRAM1	0.395192429	0.196341761	0.668681062	0.502257923	0.480404604	0.554926484	0.999996191
PRCC	0.682963861	0.531576188	0.243689407	0.550408958	0.93478525	0.799986967	0.999996191
PRDM10	0.897613818	0.392332369	0.418614059	0.989514733	0.79396217	0.932204892	0.999996191
PRDM11	0.984497929	0.985076967	0.091480618	0.793449887	0.329306871	0.67475254	0.999996191
PRDM16	0.757124201	0.339033542	0.779999534	0.427411918	0.417441672	0.756745499	0.999996191
PRDM4	0.198886307	0.915765556	0.854860983	0.304891326	0.301654252	0.580873299	0.999996191
PRDM6	0.749699543	0.442743506	0.521446349	0.734383883	0.297844335	0.767333091	0.999996191
PRDM8	0.852274404	0.495625596	0.883010666	0.646342241	0.522186727	0.940582753	0.999996191
PRDX1	0.759244656	0.538248154	0.447322172	0.586063162	0.834534654	0.902297666	0.999996191
PRDX2	0.503584982	0.718638366	0.601382088	0.169575131	0.649614172	0.681273419	0.999996191
PRDX5	0.383231108	0.239368232	0.849140882	0.167817856	0.900118519	0.543050424	0.999996191
PRDX6	0.99022432	0.669729101	0.230472602	0.944017723	0.978133703	0.951052632	0.999996191
PREB	0.125347604	0.543048025	0.677651951	0.439097028	0.381953246	0.46506194	0.999996191
PRELID2	0.623317867	0.327597606	0.22627599	0.268044564	0.698777128	0.485453861	0.999996191
PRELID3A	0.853728699	0.779778652	0.754400015	0.025133993	0.956572851	0.547993486	0.999996191
PRELID3B	0.617492642	0.450704665	0.693897479	0.906225149	0.556565159	0.91283474	0.999996191
PRELP	0.91837919	0.800426762	0.19715023	0.150981102	0.939535295	0.651386176	0.999996191
PREX2	0.560525265	0.896301988	0.574924347	0.349651278	0.519385907	0.823956397	0.999996191
PRICKLE3	0.723693927	0.758291437	0.70826171	0.98315759	0.541279141	0.977626612	0.999996191
PRIM1	0.397139803	0.595278371	0.567191658	0.602229073	0.245953725	0.644661121	0.999996191
PRIM2	0.952180757	0.819079469	0.713447778	0.874289232	0.607806862	0.991763331	0.999996191
PRIMA1	0.638167769	0.508908088	0.21263753	0.989954061	0.642656829	0.793831531	0.999996191
PRIMPOL	0.884259837	0.259911039	0.247372648	0.426284855	0.673649339	0.60638729	0.999996191
PRKAA1	0.430123511	0.813423233	0.820960412	0.393962816	0.97827558	0.927403114	0.999996191
PRKAA2	0.847421297	0.064235406	0.431070618	0.737803624	0.845040095	0.585029598	0.999996191
PRKAB1	0.531551815	0.205809446	0.476199234	0.95086276	0.888650128	0.794169107	0.999996191
PRKAB2	0.219500419	0.478774548	0.520502763	0.836302332	0.203215487	0.498657757	0.999996191
PRKACA	0.90220394	0.573807497	0.201938552	0.342868798	0.945478538	0.747032133	0.999996191
PRKAG1	0.646686852	0.657212561	0.519591792	0.498920434	0.306223512	0.746204928	0.999996191
PRKAR1B	0.680929341	0.533439159	0.092936924	0.842126804	0.609808722	0.618097819	0.999996191

PRKAR2A	0.782663216	0.817665377	0.250418941	0.732717528	0.700586389	0.891470747	0.999996191
PRKAR2B	0.77832912	0.519308637	0.594555356	0.900966914	0.954376533	0.977575235	0.999996191
PRKCD	0.844981225	0.956311759	0.163327157	0.789652716	0.942280044	0.913807729	0.999996191
PRKCE	0.201361143	0.944601555	0.914931248	0.811169816	0.868109732	0.937946429	0.999996191
PRKCG	0.769892518	0.420201314	0.803046783	0.675242917	0.544568372	0.910501069	0.999996191
PRKCH	0.580094488	0.828948742	0.346066452	0.661243576	0.399282981	0.79383952	0.999996191
PRKD1	0.533471542	0.766165846	0.497526609	0.744339042	0.517481692	0.884831148	0.999996191
PRKD3	0.894364802	0.974484099	0.451860972	0.953213862	0.99705623	0.996598031	0.999996191
PRKDC	0.840757082	0.481684072	0.648151393	0.598358401	0.417709404	0.859302968	0.999996191
PRKG1	0.334608381	0.53193187	0.36537503	0.787878647	0.450303075	0.673842024	0.999996191
PRKN	0.71947479	0.718865049	0.68017623	0.628690845	0.97103442	0.979567067	0.999996191
PRKRA	0.875819058	0.888294989	0.177929477	0.755601933	0.854489349	0.902252674	0.999996191
PRL	0.953669753	0.887764678	0.994615272	0.202814022	0.698213249	0.935204408	0.999996191
PRMT6	0.450202031	0.235482642	0.606013954	0.581308735	0.391822404	0.58507642	0.999996191
PRMT9	0.638343858	0.635698052	0.815835413	0.077641148	0.78472645	0.647676529	0.999996191
PRND	0.335110442	0.654836276	0.373021954	0.561551536	0.837059517	0.77026522	0.999996191
PRNP	0.562351588	0.337522097	0.499537375	0.235350926	0.684039564	0.593280961	0.999996191
PROB1	0.789181464	0.700966488	0.433383004	0.967592585	0.770510177	0.968976543	0.999996191
PROCA1	0.250435643	0.864218706	0.510254542	0.204412914	0.430229163	0.506801693	0.999996191
PROCR	0.618285096	0.723831156	0.721357516	0.724407554	0.205691478	0.809449838	0.999996191
PRODH	0.715138879	0.256871273	0.199979607	0.558844038	0.727864257	0.589141899	0.999996191
PROKR2	0.743882736	0.412996053	0.639403474	0.970144197	0.357949573	0.865119831	0.999996191
PROM1	0.196225628	0.921891295	0.62411595	0.413158059	0.463174295	0.661071351	0.999996191
PRORSD1	0.531039463	0.498331974	0.126491708	0.405651356	0.82076822	0.53271828	0.999996191
PROSER1	0.47135052	0.96965627	0.902521393	0.61582603	0.55920499	0.951611604	0.999996191
PROSER3	0.80713298	0.303662798	0.562042819	0.826690799	0.564744785	0.856293205	0.999996191
PROX1	0.432736574	0.877999002	0.064130096	0.938329774	0.626291263	0.580860864	0.999996191
PROX2	0.887330091	0.321341949	0.730005014	0.219630037	0.518184386	0.678956751	0.999996191
PRPF18	0.980955632	0.545622859	0.084158607	0.378922361	0.55359272	0.501682079	0.999996191
PRPF3	0.457327373	0.182360944	0.644647255	0.590450288	0.718565749	0.671623229	0.999996191
PRPF31	0.164576512	0.709283699	0.857685244	0.325744562	0.605245056	0.643454531	0.999996191
PRPF38A	0.589483611	0.656018858	0.907108327	0.230709487	0.886023042	0.87240206	0.999996191
PRPF38B	0.589952841	0.858326536	0.594487586	0.039648594	0.638460107	0.462346183	0.999996191
PRPF39	0.205843101	0.363433085	0.445192666	0.331577402	0.906522695	0.512468626	0.999996191
PRPF4	0.918229163	0.66446605	0.418387472	0.288659901	0.665967665	0.81282982	0.999996191
PRPF6	0.586822193	0.801597161	0.581738674	0.928246566	0.944844495	0.984649529	0.999996191
PRPS2	0.573135708	0.279311212	0.471640637	0.54938821	0.365569316	0.591995664	0.999996191
PRPSAP1	0.79468638	0.823794587	0.088119091	0.674602795	0.348872211	0.570556271	0.999996191
PRR13	0.246970673	0.789780583	0.422872116	0.380487577	0.570884416	0.624530838	0.999996191
PRR14	0.711726831	0.549124916	0.402802548	0.811601173	0.796562017	0.917993572	0.999996191
PRR14L	0.667482064	0.647699401	0.32566563	0.662523261	0.717990901	0.862393511	0.999996191
PRR15	0.428802425	0.727727245	0.587760993	0.251857401	0.28366167	0.563703276	0.999996191
PRR18	0.307490431	0.943878871	0.43558197	0.783141028	0.590306694	0.841441712	0.999996191
PRR29	0.946041956	0.756258228	0.574868077	0.689910492	0.797291838	0.982066899	0.999996191
PRR3	0.224612781	0.641643178	0.480093481	0.590384068	0.621105869	0.69221236	0.999996191

PRR5	0.667209349	0.619958946	0.2430508	0.839721213	0.55586441	0.805227386	0.999996191
PRR7	0.204551633	0.404712826	0.783699717	0.499434131	0.511910277	0.609478599	0.999996191
PRRC1	0.579063345	0.972187177	0.181300495	0.22092864	0.481483651	0.52775344	0.999996191
PRRC2B	0.755598085	0.880173236	0.488501211	0.712010067	0.439765303	0.917938406	0.999996191
PRRG1	0.794081697	0.170680981	0.374522594	0.227973233	0.990578863	0.538069178	0.999996191
PRRG2	0.358784702	0.376402295	0.835029324	0.075462228	0.997920264	0.48198508	0.999996191
PRRG3	0.794244806	0.386594899	0.797214096	0.070059584	0.693333433	0.545052241	0.999996191
PRRT1	0.277367819	0.332603831	0.491415756	0.829590103	0.358697012	0.569321245	0.999996191
PRRT1B	0.821924924	0.429523489	0.844790229	0.123592918	0.930771731	0.749305984	0.999996191
PRRX1	0.22560959	0.958338856	0.27898622	0.53832803	0.525969077	0.615184325	0.999996191
PRRX2	0.677618204	0.52160718	0.050632109	0.920382422	0.639180603	0.521939313	0.999996191
PRSS2	0.883550637	0.80116211	0.383561851	0.819849311	0.597364123	0.945743589	0.999996191
PRSS23	0.399828276	0.65148385	0.660230611	0.24534208	0.287669365	0.54899261	0.999996191
PRSS33	0.566889847	0.499302276	0.398184906	0.961943402	0.806388989	0.899439301	0.999996191
PRSS42	0.992905968	0.545749672	0.447059771	0.733032536	0.934887442	0.963905469	0.999996191
PRSS48	0.765577093	0.82591362	0.887222799	0.75757276	0.149822033	0.854769755	0.999996191
PRTFDC1	0.970943214	0.968688243	0.529971904	0.281990639	0.746213085	0.921438246	0.999996191
PRUNE1	0.556689467	0.300418319	0.488170667	0.598831854	0.715244813	0.752818828	0.999996191
PRX	0.704262546	0.423677747	0.972374248	0.459333697	0.268408264	0.756991031	0.999996191
PSCA	0.150781627	0.306890969	0.551213387	0.815104973	0.407438844	0.481528018	0.999996191
PSD3	0.559162437	0.495416084	0.979874338	0.701836587	0.659466232	0.940386435	0.999996191
PSEN1	0.939268871	0.619928538	0.117424611	0.899765421	0.294559841	0.626754798	0.999996191
PSEN2	0.553304149	0.873761419	0.459601443	0.152224398	0.92259982	0.731632506	0.999996191
PSMA2	0.849967642	0.5346188	0.654040474	0.469444003	0.514165602	0.872461892	0.999996191
PSMA3	0.909530333	0.565922302	0.590863146	0.194298407	0.388480361	0.672860505	0.999996191
PSMA4	0.956305164	0.430609774	0.810050048	0.179865396	0.28154858	0.613038519	0.999996191
PSMA6	0.97031157	0.376863339	0.240856902	0.516992898	0.290119245	0.56527225	0.999996191
PSMB1	0.669218999	0.479112162	0.56015993	0.738927544	0.451957636	0.845533117	0.999996191
PSMB3	0.459396728	0.737526133	0.632865678	0.384927599	0.180979694	0.589076148	0.999996191
PSMB5	0.662062898	0.645143974	0.411226881	0.115171407	0.458141433	0.498090626	0.999996191
PSMB7	0.551635252	0.584845858	0.700430095	0.300010271	0.177914438	0.547787679	0.999996191
PSMC2	0.856319379	0.434220736	0.429873841	0.680699548	0.423286878	0.802005162	0.999996191
PSMC3	0.185359527	0.430350343	0.642201853	0.475270804	0.745939921	0.627186686	0.999996191
PSMC4	0.684365603	0.740017295	0.424346674	0.239611738	0.199897211	0.517697118	0.999996191
PSMC5	0.624770303	0.674179523	0.302968718	0.3687632	0.148885061	0.447374125	0.999996191
PSMC6	0.461863728	0.650833136	0.258922121	0.405489066	0.519864955	0.607344997	0.999996191
PSMD10	0.634211907	0.737539994	0.74423272	0.28432385	0.542446959	0.827783538	0.999996191
PSMD11	0.883257184	0.521608209	0.351475943	0.782174155	0.431388776	0.830638087	0.999996191
PSMD13	0.716443671	0.910943506	0.457100986	0.275736858	0.634351175	0.823090971	0.999996191
PSMD3	0.786932213	0.714427291	0.32423466	0.311728504	0.18054437	0.517069058	0.999996191
PSMD4	0.843082515	0.741577749	0.826021158	0.151444786	0.188158001	0.586171986	0.999996191
PSMD5	0.928733988	0.529913118	0.809453155	0.658788708	0.269471337	0.870400755	0.999996191
PSMD7	0.994565266	0.276577924	0.353008329	0.53317176	0.311222834	0.603820722	0.999996191
PSME4	0.369488037	0.854823926	0.435500855	0.14620576	0.580018823	0.541392472	0.999996191
PSMG1	0.6512565	0.401245575	0.688213235	0.193583341	0.896366421	0.731633848	0.999996191

PSMG3	0.681890073	0.978627044	0.314942198	0.42929584	0.653993647	0.842954492	0.999996191
PSMG4	0.130038199	0.753142915	0.204501908	0.47757325	0.744572325	0.450269501	0.999996191
PSPC1	0.809854151	0.654572908	0.791287105	0.657229924	0.399102502	0.926741617	0.999996191
PSPN	0.786234963	0.302251554	0.799953746	0.721871899	0.359756058	0.813844202	0.999996191
PSTK	0.549667822	0.336875033	0.826099015	0.903386976	0.434103513	0.845551616	0.999996191
PSTPIP2	0.639175197	0.556691117	0.448259787	0.387253721	0.561493786	0.751304848	0.999996191
PTAR1	0.098112519	0.970975598	0.715593814	0.793186464	0.626435014	0.746938605	0.999996191
PTBP1	0.537759132	0.791549386	0.981428176	0.16647685	0.774611517	0.828334403	0.999996191
PTBP2	0.618771618	0.821183098	0.785685729	0.973815857	0.319997699	0.939430413	0.999996191
PTCH1	0.86982663	0.660485609	0.675503374	0.365564449	0.658863306	0.907844805	0.999996191
PTDSS1	0.876025952	0.535029909	0.132583084	0.93696987	0.463446316	0.704045218	0.999996191
PTDSS2	0.805363773	0.832691985	0.221124393	0.114764019	0.863935093	0.585995335	0.999996191
PTEN	0.519526553	0.762747748	0.548411516	0.23958086	0.259385557	0.569530095	0.999996191
PTER	0.974073388	0.929010173	0.652397374	0.394289072	0.701297137	0.962682642	0.999996191
PTGDR2	0.892254626	0.690361002	0.912635272	0.420444573	0.670126311	0.960425644	0.999996191
PTGDS	0.838259612	0.862153548	0.022577325	0.994269077	0.568109452	0.497061988	0.999996191
PTGER1	0.657172768	0.763399425	0.068735488	0.899910609	0.467446931	0.583377296	0.999996191
PTGER3	0.54443655	0.940164906	0.106665148	0.656336283	0.941019117	0.7461053	0.999996191
PTGER4	0.91315101	0.111774925	0.66758626	0.780938391	0.699624916	0.764287525	0.999996191
PTGES	0.932234637	0.237195128	0.23831414	0.542598256	0.28585151	0.475007598	0.999996191
PTGFRN	0.241757286	0.766938569	0.301745748	0.574650173	0.577696989	0.631564293	0.999996191
PTGIS	0.677762034	0.553414243	0.325227358	0.852739243	0.440780409	0.801241497	0.999996191
PTGR2	0.53516684	0.847682632	0.666864101	0.537413651	0.580730657	0.909074424	0.999996191
PTGS1	0.914036994	0.591435892	0.899171531	0.4655398	0.103430956	0.676621928	0.999996191
PTH1R	0.758704222	0.261403026	0.133948173	0.787477354	0.694844625	0.583780253	0.999996191
PTK2	0.605437951	0.822384201	0.493479901	0.980578794	0.928116965	0.981525839	0.999996191
PTMA	0.47124353	0.488647349	0.624890252	0.221517568	0.872914586	0.709906595	0.999996191
PTMA	0.512286243	0.605107246	0.988734491	0.683325684	0.808217754	0.965285049	0.999996191
PTP4A2	0.538646208	0.76980554	0.261433721	0.507415113	0.202726464	0.532822783	0.999996191
PTPA	0.390583306	0.89138523	0.696739212	0.236536932	0.335242952	0.638411409	0.999996191
PTPDC1	0.477711609	0.547553271	0.409843337	0.315243037	0.268881051	0.494438398	0.999996191
PTPN1	0.659454891	0.747713868	0.714309822	0.70911943	0.561169117	0.950451345	0.999996191
PTPN11	0.808905164	0.674140555	0.137603862	0.768453105	0.436152581	0.690517418	0.999996191
PTPN14	0.375343863	0.998909548	0.210210541	0.51438832	0.699033	0.713406581	0.999996191
PTPN18	0.212764078	0.949254209	0.534990996	0.623291566	0.864541352	0.840846535	0.999996191
PTPN21	0.424283078	0.49699085	0.171394163	0.624906507	0.482617163	0.528498768	0.999996191
PTPN23	0.84553114	0.607259454	0.476362257	0.04045992	0.77712353	0.463994492	0.999996191
PTPN3	0.641674533	0.696993931	0.053594313	0.894824317	0.550533899	0.543727454	0.999996191
PTPN4	0.335116483	0.621055916	0.628551035	0.295960291	0.311358084	0.547679733	0.999996191
PTPN9	0.819127071	0.608341563	0.440976152	0.131625576	0.934070486	0.704276941	0.999996191
PTPRA	0.445954639	0.953797601	0.416919825	0.720267809	0.857698167	0.926271748	0.999996191
PTPRB	0.368376375	0.439837615	0.665745396	0.4904203	0.707206832	0.765179453	0.999996191
PTPRCAP	0.736714165	0.752378984	0.074790592	0.791306615	0.2737233	0.492236465	0.999996191
PTPRD	0.975271393	0.519538974	0.107567504	0.514481018	0.262814457	0.456354643	0.999996191
PTPRH	0.877305216	0.777257698	0.811921512	0.730312133	0.702389848	0.990617196	0.999996191

PTPRM	0.41167215	0.911471627	0.467986292	0.194717268	0.837525828	0.715392143	0.999996191
PTPRU	0.187410094	0.870737856	0.184050283	0.984485853	0.75000436	0.666146726	0.999996191
PTRHD1	0.346876504	0.840209653	0.691369225	0.546699145	0.677599743	0.878125879	0.999996191
PTS	0.76579852	0.864809249	0.184836329	0.460532813	0.834938226	0.805744086	0.999996191
PTTG1	0.21680111	0.527939014	0.16995357	0.941294095	0.819081558	0.589856057	0.999996191
PTTG1IP	0.347504697	0.520283716	0.45261122	0.405232778	0.738808106	0.685463651	0.999996191
PUDP	0.563596009	0.931341304	0.237858535	0.141910962	0.49910584	0.489419912	0.999996191
PUF60	0.462245145	0.889236387	0.521374433	0.98046616	0.150617131	0.734273288	0.999996191
PUM1	0.547814236	0.996524368	0.847574589	0.061894749	0.83669492	0.681170392	0.999996191
PUM3	0.349085897	0.856489504	0.959364578	0.42400179	0.121448643	0.586889198	0.999996191
PURA	0.94569656	0.071300726	0.27245979	0.941839819	0.752998172	0.562610224	0.999996191
PUS7L	0.144853684	0.941975811	0.738549341	0.275848411	0.420592426	0.541835408	0.999996191
PVRIG	0.290866229	0.975329408	0.822840598	0.867044176	0.208805607	0.787015451	0.999996191
PWP1	0.808893757	0.484150326	0.262726816	0.202115759	0.338936262	0.448434098	0.999996191
PWWP2A	0.256029199	0.674708843	0.203921205	0.390109244	0.99924766	0.57275064	0.999996191
PWWP2B	0.586520631	0.866204048	0.291484537	0.368683486	0.711382666	0.771957617	0.999996191
PWWP2B	0.734001281	0.3638799	0.914905365	0.659455061	0.796250104	0.942410516	0.999996191
PXDN	0.78423173	0.504236095	0.104413707	0.493817739	0.421987731	0.484384364	0.999996191
PXK	0.882965791	0.206521221	0.921276878	0.753990345	0.547019758	0.867418813	0.999996191
PXMP2	0.996661207	0.761970988	0.314351616	0.044600392	0.712280593	0.461485175	0.999996191
PXN	0.228396195	0.632551091	0.431643596	0.45042944	0.547858353	0.594862627	0.999996191
PXYLP1	0.617192638	0.965119154	0.297958938	0.453428666	0.677051586	0.830184615	0.999996191
PYCR1	0.760340149	0.771902179	0.097675411	0.247155178	0.77755704	0.530523324	0.999996191
PYCR3	0.896736063	0.868001491	0.585972997	0.593896251	0.505932037	0.948473012	0.999996191
PYGB	0.230109648	0.826716326	0.433583519	0.991341831	0.469490732	0.769854972	0.999996191
PYGL	0.326240465	0.77029717	0.540957765	0.609789031	0.158598311	0.564349543	0.999996191
PYGO1	0.355159406	0.782491817	0.153859495	0.625775349	0.718284419	0.638246969	0.999996191
PYGO2	0.861579555	0.662951984	0.037001582	0.818241214	0.749350276	0.561565417	0.999996191
PYROXD1	0.073012823	0.717242063	0.930340738	0.637940731	0.442524698	0.573061161	0.999996191
PYROXD2	0.455846516	0.083442897	0.565751351	0.827327463	0.510632901	0.494525294	0.999996191
PYURF	0.446666448	0.90420634	0.997676527	0.639151469	0.527710204	0.947726128	0.999996191
QARS	0.689397745	0.464577204	0.848594247	0.996233857	0.230532695	0.851721895	0.999996191
QKI	0.929209281	0.76145267	0.205492753	0.771577136	0.33442908	0.765693537	0.999996191
QPCTL	0.774770093	0.400724073	0.787341957	0.309524539	0.594788026	0.798010566	0.999996191
QPRT	0.792556841	0.882137863	0.359663997	0.05231564	0.803541209	0.522702611	0.999996191
QRICH1	0.325590378	0.728004247	0.414573931	0.894780875	0.860166549	0.879974458	0.999996191
QRSL1	0.344270174	0.964358306	0.976545202	0.145036713	0.359401514	0.613126093	0.999996191
QSER1	0.406984295	0.683128999	0.551696226	0.313766204	0.412674595	0.644654742	0.999996191
QSOX1	0.150199688	0.908441212	0.399667917	0.479750714	0.701059367	0.629112917	0.999996191
QSOX2	0.416342874	0.739852151	0.425367796	0.723948098	0.827868668	0.885182931	0.999996191
R3HCC1L	0.225691347	0.893730947	0.881010713	0.095156282	0.762553124	0.560610464	0.999996191
R3HDM1	0.826158583	0.975767275	0.356787122	0.635448472	0.539422229	0.914273551	0.999996191
R3HDM2	0.889536112	0.363070127	0.511802258	0.419509142	0.56228123	0.772652348	0.999996191
R3HDM4	0.287906853	0.830184878	0.613460786	0.735925329	0.915262721	0.914481925	0.999996191
RAB10	0.970192063	0.35582813	0.132308133	0.884868906	0.262146906	0.523139235	0.999996191

RAB11A	0.854002355	0.489508447	0.260019157	0.558133138	0.569911656	0.750737654	0.999996191
RAB11FIP1	0.694779735	0.871985412	0.51827651	0.203936733	0.775160289	0.814755487	0.999996191
RAB11FIP2	0.579327204	0.448987669	0.914489802	0.852113181	0.531101798	0.924344099	0.999996191
RAB11FIP3	0.908402671	0.758541373	0.19974423	0.457973625	0.875943457	0.832337684	0.999996191
RAB11FIP4	0.24422183	0.287102895	0.336678726	0.944194391	0.360052232	0.471690673	0.999996191
RAB11FIP5	0.544004169	0.797522397	0.143999281	0.413991761	0.522984989	0.569838523	0.999996191
RAB15	0.821001995	0.957745553	0.43928782	0.735072849	0.443194006	0.92916548	0.999996191
RAB19	0.664064621	0.750012504	0.581662564	0.850657974	0.032215706	0.469734207	0.999996191
RAB1B	0.418244277	0.792251066	0.290618686	0.630937864	0.607523021	0.762731994	0.999996191
RAB20	0.939267137	0.37970304	0.258734963	0.769973566	0.282500693	0.646717314	0.999996191
RAB23	0.856681833	0.661942414	0.385661665	0.170512705	0.592150016	0.665315907	0.999996191
RAB24	0.354120888	0.722693286	0.571134421	0.270116606	0.580451696	0.67253072	0.999996191
RAB27A	0.481200295	0.975063779	0.834322346	0.755764521	0.525887782	0.959052651	0.999996191
RAB27B	0.518981697	0.139666978	0.665152627	0.485972041	0.877474705	0.651402506	0.999996191
RAB28	0.112772551	0.687959361	0.44426897	0.488414992	0.8461371	0.579847505	0.999996191
RAB2A	0.362569623	0.206621756	0.51821309	0.388477215	0.652085009	0.509137921	0.999996191
RAB2B	0.425609548	0.03856082	0.808468579	0.803628014	0.913244954	0.507295325	0.999996191
RAB32	0.826004431	0.984502703	0.299038446	0.066274024	0.657701946	0.523222073	0.999996191
RAB33A	0.996548766	0.659345843	0.379832117	0.9150481	0.779881373	0.96874374	0.999996191
RAB33B	0.799299262	0.606629951	0.373918742	0.833576309	0.359845045	0.829878169	0.999996191
RAB34	0.931771937	0.741412848	0.244940706	0.399804929	0.920786173	0.851408325	0.999996191
RAB35	0.508623406	0.311967309	0.382282457	0.543657667	0.997494934	0.741494292	0.999996191
RAB36	0.776225845	0.326919096	0.874603463	0.046108389	0.69584238	0.450244102	0.999996191
RAB37	0.824762022	0.780804702	0.125939426	0.396505925	0.448859756	0.582416135	0.999996191
RAB38	0.381970341	0.884806961	0.662212468	0.152000266	0.737120943	0.689950963	0.999996191
RAB39A	0.737850535	0.84651005	0.404603798	0.228222015	0.274950999	0.600709864	0.999996191
RAB3B	0.61822194	0.415046862	0.143364329	0.618367845	0.822448825	0.632982828	0.999996191
RAB3C	0.443407317	0.779315884	0.677843921	0.957510142	0.980376866	0.980724616	0.999996191
RAB3D	0.69547543	0.988144549	0.362881159	0.541504874	0.572158888	0.88294877	0.999996191
RAB3GAP1	0.84331276	0.494145821	0.176747106	0.698320743	0.352128798	0.62664642	0.999996191
RAB3IL1	0.675561031	0.707281886	0.260773394	0.701752366	0.898337178	0.885218952	0.999996191
RAB3IP	0.579347294	0.927422736	0.126804577	0.819094684	0.919481405	0.820310638	0.999996191
RAB42	0.701298886	0.954994019	0.085476018	0.332718409	0.893287313	0.614442309	0.999996191
RAB4A	0.592275766	0.642104374	0.710985834	0.096024334	0.797012937	0.6526707	0.999996191
RAB4B	0.655008849	0.595627164	0.934174625	0.646844211	0.843050578	0.975440991	0.999996191
RAB5A	0.721151559	0.5318249	0.288007537	0.456566102	0.862999799	0.79218317	0.999996191
RAB5B	0.859004048	0.71891764	0.552912922	0.313789985	0.443756292	0.807465906	0.999996191
RAB6A	0.788272465	0.069286893	0.371146886	0.945117473	0.993430449	0.636337452	0.999996191
RAB6B	0.277557893	0.742641029	0.583433924	0.642082415	0.146096884	0.535026012	0.999996191
RAB8B	0.32848009	0.969250608	0.263849221	0.172833081	0.562351848	0.474813466	0.999996191
RAB9B	0.864160159	0.600957899	0.442310852	0.231432029	0.628036639	0.744256765	0.999996191
RABAC1	0.344514985	0.571225488	0.69002132	0.234898135	0.526174115	0.61177737	0.999996191
RABEPK	0.749838884	0.169348027	0.671756328	0.778235208	0.558289098	0.763471893	0.999996191
RABGAP1	0.426434916	0.392649812	0.834080292	0.233442001	0.378399092	0.552107081	0.999996191
RABGAP1L	0.462498185	0.895329457	0.909744949	0.114855871	0.30427191	0.564611518	0.999996191

RABGAP1L	0.607056594	0.247443729	0.96241919	0.236941704	0.824859394	0.712833264	0.999996191
RABGGTB	0.194557978	0.401694843	0.679499615	0.998233169	0.671348938	0.756055945	0.999996191
RABIF	0.085544338	0.758150082	0.702277282	0.403040894	0.683302124	0.555299194	0.999996191
RABL2B	0.619550311	0.68262511	0.970383957	0.964074331	0.847283755	0.994706725	0.999996191
RAC3	0.968691493	0.594892512	0.876434606	0.033571745	0.55445257	0.500740757	0.999996191
RACGAP1	0.660381178	0.438015421	0.519837622	0.783555038	0.121974666	0.581566357	0.999996191
RACK1	0.459228556	0.764870053	0.842402451	0.272482889	0.648303774	0.823374028	0.999996191
RAD1	0.329532748	0.7723154	0.898086642	0.441106466	0.072155516	0.454047698	0.999996191
RAD18	0.17531829	0.659886618	0.83087939	0.884008331	0.952472526	0.889287245	0.999996191
RAD23B	0.345630898	0.446312462	0.23636785	0.730504981	0.302331743	0.47230949	0.999996191
RAD51B	0.273590857	0.444911108	0.19957353	0.751254753	0.840041605	0.594128078	0.999996191
RAD51D	0.934751644	0.978224464	0.381802865	0.033034655	0.672720471	0.465579079	0.999996191
RAD52	0.270335505	0.594008234	0.344743775	0.307100386	0.773328613	0.564348999	0.999996191
RAD54B	0.678382472	0.073601707	0.954290716	0.817992517	0.426523556	0.609911688	0.999996191
RAD54L	0.784379309	0.80257295	0.071602309	0.753174332	0.84223537	0.715101464	0.999996191
RAD54L2	0.654581025	0.280961086	0.756621138	0.451657261	0.623034222	0.773421953	0.999996191
RAD9A	0.590334484	0.618736392	0.860944534	0.626222519	0.957233114	0.972337664	0.999996191
RAD9B	0.802272814	0.820100393	0.125043515	0.361595047	0.45175093	0.568586452	0.999996191
RADIL	0.560895402	0.481040095	0.392107066	0.450053021	0.311692769	0.587810768	0.999996191
RAE1	0.813422382	0.73217538	0.328924197	0.176050894	0.550585271	0.635884697	0.999996191
RAI14	0.953528261	0.601329921	0.401030375	0.181472312	0.410535424	0.615776906	0.999996191
RAI2	0.475998807	0.879884415	0.428777199	0.647412574	0.978235791	0.930296403	0.999996191
RALA	0.236017644	0.389033606	0.762983805	0.44760679	0.300547914	0.501200997	0.999996191
RALGAPA2	0.924012481	0.195527251	0.403727022	0.692627034	0.741294205	0.765370124	0.999996191
RALGDS	0.162628533	0.370609974	0.28182002	0.45105731	0.909820317	0.446470958	0.999996191
RALGPS1	0.161505251	0.803900009	0.94491103	0.977785418	0.83343181	0.91591799	0.999996191
RALGPS2	0.520550706	0.413559445	0.104476065	0.952868522	0.601097449	0.560426566	0.999996191
RALY	0.428689878	0.886743863	0.630074102	0.140083883	0.75082697	0.690839937	0.999996191
RAMMET	0.831066155	0.358166561	0.858054509	0.889117753	0.51007068	0.932215834	0.999996191
RAMP2	0.514338932	0.91785877	0.807847107	0.57830953	0.680835015	0.956238835	0.999996191
RAMP3	0.043398188	0.640125434	0.840504924	0.682800272	0.779992535	0.553638482	0.999996191
RAN	0.848656677	0.330986273	0.868188655	0.886139817	0.419368705	0.904004642	0.999996191
RANBP10	0.594777093	0.920622779	0.978014147	0.887958837	0.938842615	0.99853865	0.999996191
RANBP17	0.639828836	0.926468828	0.356857708	0.203192767	0.707419195	0.726750953	0.999996191
RANBP3	0.726849352	0.924478415	0.851439283	0.319990417	0.390663735	0.872029349	0.999996191
RANBP9	0.967309594	0.291204385	0.240846485	0.772286311	0.273563312	0.581052252	0.999996191
RAP1B	0.880389252	0.325819634	0.427885604	0.829491727	0.481349179	0.812600326	0.999996191
RAP1GAP	0.574703082	0.323880389	0.828998456	0.681692993	0.212530111	0.667713919	0.999996191
RAP1GDS1	0.802199156	0.763001143	0.81474968	0.933548851	0.587702961	0.989464096	0.999996191
RAP2A	0.987975383	0.073112663	0.771166473	0.658527084	0.391236714	0.581302455	0.999996191
RAP2C	0.145245455	0.328231296	0.352370276	0.794534431	0.85023597	0.536155015	0.999996191
RAPGEF1	0.355851382	0.084783261	0.646099091	0.6388319	0.832430727	0.519017375	0.999996191
RAPGEF2	0.601310249	0.282918206	0.721648275	0.389692273	0.965776307	0.802562377	0.999996191
RAPGEF3	0.415996565	0.857114553	0.412952453	0.993114893	0.858778667	0.940342316	0.999996191
RAPGEF4	0.667385008	0.236294707	0.877742094	0.138395262	0.418583885	0.471538948	0.999996191

RAPGEF5	0.645216851	0.296411578	0.070221099	0.876356465	0.972256693	0.537731089	0.999996191
RAPH1	0.207250139	0.572389441	0.95699313	0.763446882	0.257281935	0.66721809	0.999996191
RARA	0.626524007	0.965339008	0.348945203	0.2336074	0.909081995	0.797301631	0.999996191
RARB	0.263102485	0.859927879	0.653223937	0.649506051	0.748801501	0.872747287	0.999996191
RARG	0.479811009	0.882565161	0.77631988	0.300450778	0.110149077	0.528147281	0.999996191
RARS	0.730490929	0.762025207	0.841000892	0.153112476	0.205494355	0.586349164	0.999996191
RARS2	0.932847465	0.914450366	0.492730542	0.411182749	0.840386779	0.953496277	0.999996191
RASA1	0.514677948	0.407703365	0.441158856	0.555181937	0.882869599	0.799433374	0.999996191
RASA2	0.55564626	0.995294866	0.978306224	0.263597336	0.602880061	0.897284818	0.999996191
RASA3	0.719669469	0.998676851	0.956722947	0.223027776	0.792881031	0.937170103	0.999996191
RASAL1	0.962754608	0.892269898	0.4057085	0.996744175	0.439371566	0.957546055	0.999996191
RASEF	0.697983432	0.512404302	0.78822656	0.962897765	0.40961317	0.92787941	0.999996191
RASGEF1B	0.641976951	0.663298803	0.093792399	0.434028842	0.490009125	0.482029604	0.999996191
RASGRF2	0.72299337	0.415567861	0.759773874	0.364850019	0.650780777	0.82932967	0.999996191
RASGRP2	0.376246892	0.609277738	0.616448094	0.54308141	0.971611312	0.877979052	0.999996191
RASIP1	0.276050294	0.986513326	0.901982438	0.476629158	0.194883675	0.671674326	0.999996191
RASL11A	0.824313883	0.025127256	0.893753185	0.76683067	0.528788313	0.459646001	0.999996191
RASL11B	0.88380012	0.22929181	0.084159193	0.794194395	0.862676037	0.541720267	0.999996191
RASSF1	0.640226496	0.803653859	0.732070462	0.384035406	0.285509155	0.782941664	0.999996191
RASSF2	0.758168979	0.9635792	0.132611652	0.203962288	0.644749114	0.558290786	0.999996191
RASSF3	0.647556075	0.217012586	0.274692648	0.742228074	0.784798982	0.668838847	0.999996191
RASSF7	0.543398369	0.226148995	0.856511973	0.7283056	0.938818167	0.872927004	0.999996191
RAVER1	0.670084448	0.664923895	0.472676351	0.65010188	0.946694729	0.943366715	0.999996191
RB1	0.38754103	0.908282257	0.583695671	0.368478915	0.280998051	0.658057776	0.999996191
RB1CC1	0.610185403	0.759885733	0.192977639	0.411168026	0.674178613	0.687842092	0.999996191
RBAK	0.933527303	0.961877961	0.988391017	0.243450978	0.848811719	0.970633289	0.999996191
RBBP4	0.972409918	0.221461986	0.356620202	0.713823021	0.215901742	0.54417478	0.999996191
RBBP7	0.70086443	0.88586516	0.08587349	0.695202945	0.385010669	0.580206126	0.999996191
RBBP8	0.695937308	0.861092062	0.941079218	0.527070694	0.535123761	0.960747442	0.999996191
RBBP9	0.725089359	0.950247073	0.732543883	0.245315166	0.402127645	0.815276203	0.999996191
RBCK1	0.476339789	0.356059536	0.515691087	0.193087274	0.883670775	0.588891518	0.999996191
RBFA	0.575935739	0.978584654	0.816690685	0.07642737	0.418184696	0.586105845	0.999996191
RBFOX3	0.517044652	0.83308079	0.393938586	0.875823434	0.44334967	0.859947596	0.999996191
RBL1	0.84055954	0.578456233	0.131028192	0.691426792	0.217686134	0.504425096	0.999996191
RBM11	0.978023056	0.933399348	0.179067714	0.516194908	0.735738732	0.850887556	0.999996191
RBM12B	0.339815682	0.871293309	0.784566329	0.559952544	0.41457878	0.828497562	0.999996191
RBM14	0.729218791	0.836647009	0.659533548	0.188462147	0.757588328	0.838719189	0.999996191
RBM15	0.208041511	0.867037574	0.636979053	0.688527308	0.449436639	0.755881713	0.999996191
RBM15B	0.382998663	0.824008897	0.640814628	0.576068266	0.981658798	0.930881916	0.999996191
RBM20	0.190912351	0.85319337	0.340866806	0.542929017	0.399911834	0.547685413	0.999996191
RBM22	0.8776417	0.486335614	0.271641158	0.201152161	0.924795952	0.660738774	0.999996191
RBM23	0.43390423	0.721447434	0.227099088	0.858063868	0.779889658	0.80756488	0.999996191
RBM24	0.429484403	0.226502095	0.812284946	0.924190735	0.223908367	0.606688146	0.999996191
RBM26	0.26985373	0.948957266	0.649653734	0.266180274	0.956414165	0.787395289	0.999996191
RBM27	0.849360141	0.849394212	0.798925643	0.181669235	0.841310046	0.900414293	0.999996191

RBM33	0.219881726	0.294256816	0.560733589	0.565798379	0.371273369	0.462368333	0.999996191
RBM38	0.420943934	0.373745359	0.87605254	0.486181446	0.152011219	0.515722804	0.999996191
RBM4	0.458372375	0.409377214	0.800882558	0.076475372	0.698281802	0.471690093	0.999996191
RBM41	0.070233417	0.809190063	0.963053586	0.667074535	0.364145491	0.56650685	0.999996191
RBM42	0.312863042	0.441117451	0.359973521	0.629361333	0.532856576	0.610340309	0.999996191
RBM43	0.552048273	0.538632709	0.15761803	0.318118754	0.737204708	0.530084835	0.999996191
RBM45	0.931224234	0.96699918	0.93483359	0.214934004	0.210965204	0.768821044	0.999996191
RBM4B	0.469967902	0.440269123	0.579749566	0.519900235	0.344716102	0.660106135	0.999996191
RBM6	0.470537764	0.371917452	0.653309872	0.792540989	0.932189521	0.894966586	0.999996191
RBM7	0.923364804	0.480118817	0.704607535	0.262088795	0.997242461	0.890455382	0.999996191
RBM8A	0.934220864	0.657718683	0.582516756	0.102121935	0.489027976	0.624081626	0.999996191
RBMS1	0.937634635	0.906465986	0.142210036	0.152940352	0.659980117	0.549976269	0.999996191
RBMS2	0.36476407	0.839752456	0.27028417	0.439263533	0.234037585	0.48239859	0.999996191
RBMS3	0.891935408	0.79440546	0.840967771	0.324313754	0.940444304	0.970055266	0.999996191
RBMX2	0.463509381	0.164588769	0.43924132	0.646717774	0.353686876	0.463389574	0.999996191
RBP1	0.434248574	0.403042238	0.502105298	0.326385847	0.987531922	0.713304632	0.999996191
RBP2	0.088379879	0.282026693	0.947014786	0.684117956	0.96067946	0.596433829	0.999996191
RBP4	0.41863709	0.808319715	0.650804831	0.862073915	0.535183451	0.917801763	0.999996191
RBPJ	0.291173167	0.811800854	0.775813896	0.253242957	0.902046503	0.785462059	0.999996191
RBPMS2	0.423495569	0.119057121	0.685453226	0.563774176	0.901673665	0.620701809	0.999996191
RBSN	0.549973929	0.873175464	0.298798971	0.242973137	0.523552589	0.628170811	0.999996191
RBX1	0.927057423	0.404094948	0.887808856	0.058514394	0.86656003	0.612713859	0.999996191
RC3H1	0.2627501	0.498931063	0.790058199	0.069255585	0.994356788	0.450533781	0.999996191
RC3H2	0.458762586	0.625066199	0.959083102	0.687475062	0.677340495	0.942228401	0.999996191
RCAN1	0.701271066	0.617623969	0.088227115	0.398765475	0.566050998	0.484841629	0.999996191
RCAN2	0.863468493	0.786263946	0.849843669	0.9047125	0.163405964	0.896246386	0.999996191
RCAN3	0.380088868	0.904095935	0.5751339	0.215534125	0.382121469	0.60580167	0.999996191
RCBTB1	0.529184451	0.643331354	0.768104861	0.324688953	0.193583642	0.607693621	0.999996191
RCBTB2	0.918667022	0.928938423	0.675647618	0.14803487	0.574900579	0.812822015	0.999996191
RCC1L	0.821450457	0.695451216	0.240731241	0.533505127	0.484930045	0.75600664	0.999996191
RCC2	0.45832052	0.905170901	0.714591929	0.690934673	0.899153192	0.970898995	0.999996191
RCCD1	0.350615686	0.295067843	0.681925698	0.407591404	0.585088401	0.61224907	0.999996191
RCE1	0.413045532	0.948854127	0.685845836	0.700151119	0.752613484	0.951361995	0.999996191
RCN2	0.260308064	0.895147393	0.080680279	0.660308571	0.641273038	0.470220427	0.999996191
RCOR3	0.742389838	0.926496632	0.968718242	0.453693591	0.586905273	0.968489902	0.999996191
RDH11	0.627040363	0.226957696	0.455824183	0.793474378	0.512855336	0.699832934	0.999996191
RDH14	0.838537684	0.761825228	0.345975837	0.969786751	0.722858064	0.958726428	0.999996191
RDH5	0.272412038	0.081941408	0.675228817	0.752259074	0.694461561	0.468245447	0.999996191
RDH8	0.891608451	0.584040471	0.173254112	0.551554969	0.885088096	0.794260598	0.999996191
RDM1	0.583861178	0.172674187	0.79349051	0.879319978	0.97582177	0.86604124	0.999996191
RDX	0.602026623	0.350251162	0.381717245	0.536346163	0.789766088	0.748145599	0.999996191
RECK	0.642708535	0.836587507	0.612535889	0.415319207	0.798479609	0.925938288	0.999996191
RECQL5	0.322608853	0.249907412	0.624019111	0.680907432	0.577593627	0.643921842	0.999996191
REEP5	0.297355607	0.768748882	0.151922904	0.981676755	0.712414213	0.683782582	0.999996191
REEP6	0.817862461	0.803488758	0.596395284	0.251310798	0.491167842	0.810411279	0.999996191

REL	0.953709138	0.968976321	0.315904874	0.22730349	0.961381404	0.855061638	0.999996191
RELA	0.358898068	0.582127957	0.754176994	0.731508905	0.924840143	0.923253225	0.999996191
RELB	0.398204995	0.905162998	0.115234724	0.498515719	0.494242448	0.516601778	0.999996191
RELL1	0.586493381	0.471018149	0.303791924	0.105238748	0.862980243	0.46237899	0.999996191
RELN	0.128295107	0.372338453	0.268285334	0.829893757	0.923603978	0.508925933	0.999996191
RELT	0.507571848	0.574379291	0.196013695	0.467321642	0.805201677	0.660148657	0.999996191
REM1	0.992315352	0.526361277	0.308176216	0.840628491	0.609885479	0.891890993	0.999996191
REPIN1	0.973412856	0.903260551	0.369001069	0.2406625	0.092337067	0.452448836	0.999996191
RERG	0.84789133	0.773664352	0.426713202	0.457749713	0.896553514	0.931350888	0.999996191
RESP18	0.425933389	0.909966552	0.325833469	0.346613302	0.860177144	0.766345363	0.999996191
REST	0.381266586	0.772219538	0.957357889	0.518715157	0.942198697	0.948933325	0.999996191
RETREG1	0.693626946	0.613568199	0.079446819	0.943379813	0.660333241	0.656116995	0.999996191
RETREG2	0.937459521	0.72203534	0.06544757	0.803013466	0.454360044	0.60442823	0.999996191
RETSAT	0.590823764	0.873899444	0.637925181	0.601993283	0.03748414	0.457870633	0.999996191
REV1	0.490539676	0.519169563	0.943776479	0.097225647	0.726615787	0.614047661	0.999996191
REV3L	0.640216208	0.667580642	0.959857079	0.376120223	0.659284175	0.91793962	0.999996191
REX1BD	0.494431239	0.843599605	0.589478236	0.206111216	0.658441245	0.744151668	0.999996191
REXO1	0.791197491	0.310446376	0.702723769	0.476880858	0.802369883	0.860305064	0.999996191
REXO5	0.499477666	0.756710096	0.491203164	0.655238425	0.882944692	0.924095959	0.999996191
RFC2	0.607808558	0.823301429	0.47735401	0.261540743	0.944770135	0.843005014	0.999996191
RFC3	0.935438141	0.825679276	0.371433143	0.063505892	0.866468712	0.599826296	0.999996191
RFC4	0.690376593	0.343371296	0.946198385	0.246664271	0.625201494	0.750817806	0.999996191
RFESD	0.637837709	0.881144378	0.298540787	0.64953216	0.248892234	0.705043475	0.999996191
RFK	0.971298049	0.595685611	0.926028851	0.101092197	0.833248651	0.798508418	0.999996191
RFTN1	0.58051214	0.198612179	0.596778285	0.261927454	0.72861341	0.56411454	0.999996191
RFTN2	0.923388394	0.960082748	0.155563191	0.681108183	0.836651285	0.885287392	0.999996191
RFWD3	0.358925716	0.993612515	0.201644265	0.237825211	0.543487528	0.498634441	0.999996191
RFX1	0.172920563	0.737659856	0.519668872	0.592822438	0.199234567	0.467215335	0.999996191
RFX2	0.485756767	0.932148419	0.428257855	0.450327725	0.900914949	0.885432907	0.999996191
RFX3	0.510319403	0.974425575	0.675725636	0.500802162	0.367571283	0.850317652	0.999996191
RFX5	0.806621495	0.934928138	0.030076363	0.969782239	0.917056588	0.647687915	0.999996191
RFX7	0.49512704	0.357684454	0.494026578	0.462010503	0.485895539	0.642482416	0.999996191
RGL1	0.755275953	0.990711983	0.468363947	0.440848064	0.250053364	0.770995434	0.999996191
RGMB	0.994190241	0.979111492	0.507276206	0.324247966	0.220410343	0.754507639	0.999996191
RGP1	0.701213216	0.891058025	0.935107465	0.415878524	0.41892889	0.918016316	0.999996191
RGS11	0.964929703	0.486610706	0.984087771	0.393130391	0.472683414	0.897111705	0.999996191
RGS13	0.301189391	0.859853976	0.810878094	0.938455672	0.931658872	0.970704868	0.999996191
RGS19	0.7500594	0.729214796	0.40320665	0.781869862	0.67543528	0.932783635	0.999996191
RGS2	0.270505938	0.427682506	0.526428129	0.4007702	0.522961195	0.558656892	0.999996191
RGS3	0.971241466	0.115755265	0.6723653	0.470790566	0.209835609	0.458715767	0.999996191
RGS6	0.254215146	0.820833015	0.683876392	0.219423793	0.370375591	0.540288911	0.999996191
RHBDD2	0.621697032	0.527446314	0.936779436	0.878275766	0.374369827	0.917109225	0.999996191
RHBDD3	0.328300959	0.946111561	0.687549108	0.105199368	0.86705974	0.640872206	0.999996191
RHBDF2	0.924109336	0.99424449	0.118863099	0.985119682	0.573558027	0.849947621	0.999996191
RHBDL1	0.382786296	0.519675534	0.374540172	0.762641402	0.863402923	0.8127827	0.999996191

RHBDL2	0.544306332	0.77543133	0.239685865	0.943147549	0.580889584	0.832953955	0.999996191
RHBDL3	0.48482124	0.768060184	0.464852767	0.607887218	0.588267416	0.850431969	0.999996191
RHNO1	0.835148512	0.881189794	0.790157963	0.810934261	0.713124154	0.994768698	0.999996191
RHOB	0.938221404	0.672826629	0.553096229	0.751433824	0.712914346	0.971860045	0.999996191
RHOBTB1	0.639353542	0.906410141	0.439019442	0.458351823	0.413156086	0.8097185	0.999996191
RHOBTB3	0.277248407	0.92334727	0.151729919	0.471929396	0.794221841	0.584083545	0.999996191
RHOJ	0.926063623	0.783721943	0.079412404	0.448508239	0.878564191	0.670775558	0.999996191
RHOQ	0.79909306	0.679496199	0.243662756	0.689239033	0.605609872	0.832370457	0.999996191
RHOV	0.289676801	0.169982079	0.4353453	0.623031006	0.678417698	0.493902833	0.999996191
RHPN2	0.899549416	0.636970942	0.809229728	0.530322106	0.29840708	0.875702116	0.999996191
RIBC1	0.599431653	0.921975639	0.154518856	0.463690645	0.392655494	0.596869073	0.999996191
RIC3	0.97766329	0.420532919	0.395663055	0.780059381	0.963742139	0.937741678	0.999996191
RIDA	0.744065682	0.906879954	0.212977572	0.458522244	0.388964328	0.694168086	0.999996191
RIF1	0.293076187	0.708812298	0.718769362	0.247881668	0.925660254	0.749047373	0.999996191
RILP	0.306765339	0.804409407	0.770261743	0.527939216	0.517795379	0.822387512	0.999996191
RILPL1	0.577969988	0.415585821	0.299530159	0.232692206	0.803035524	0.568653772	0.999996191
RIMBP2	0.478709798	0.420825598	0.954992073	0.575513238	0.236841232	0.698560372	0.999996191
RIMKLB	0.514421699	0.304979873	0.193801911	0.749361888	0.639930528	0.584373543	0.999996191
RIMS3	0.964353422	0.463070073	0.977059523	0.387714427	0.344387663	0.840941168	0.999996191
RIN1	0.889343206	0.301333521	0.70397995	0.088846878	0.766469908	0.559902958	0.999996191
RIN2	0.575085304	0.888759186	0.855248354	0.218754148	0.375961341	0.757911303	0.999996191
RIN3	0.511236732	0.85056551	0.047156783	0.436458199	0.803964167	0.452093188	0.999996191
RING1	0.896102184	0.973265059	0.219271607	0.474650473	0.905212207	0.89131075	0.999996191
RINT1	0.391150213	0.59157551	0.502661945	0.111010945	0.875079921	0.535326951	0.999996191
RIOK1	0.858422559	0.707313246	0.506913264	0.141493526	0.382517204	0.610315444	0.999996191
RIOX2	0.632520506	0.707436553	0.580917546	0.15126285	0.599344289	0.677944525	0.999996191
RIPK2	0.334788039	0.345497634	0.430693347	0.496629885	0.916088588	0.670381321	0.999996191
RIPOR1	0.868481759	0.486506861	0.379832681	0.804148623	0.632529813	0.890437104	0.999996191
RIPPLY3	0.595020343	0.857602551	0.913020324	0.440729563	0.911219322	0.971882169	0.999996191
RMC1	0.639783865	0.995312517	0.277449449	0.447036001	0.271374683	0.659514253	0.999996191
RMDN1	0.840948858	0.962270746	0.631283571	0.243621979	0.364264574	0.799281201	0.999996191
RMDN2	0.370407505	0.298982968	0.663612619	0.47851161	0.943669872	0.743134829	0.999996191
RMDN3	0.711099033	0.571771913	0.454679961	0.49980142	0.454387931	0.78585218	0.999996191
RMI2	0.352153769	0.76036298	0.627947749	0.442959638	0.940663226	0.869037239	0.999996191
RMND1	0.667823797	0.936511749	0.973625834	0.022177468	0.629368337	0.482141262	0.999996191
RMND5B	0.359170577	0.3331675	0.601563116	0.74764384	0.646858153	0.752003027	0.999996191
RN18S1	0.433834202	0.533712274	0.23173506	0.882809697	0.489045678	0.674620736	0.999996191
RN7SL1	0.690481838	0.094333347	0.272813642	0.746835112	0.628650502	0.478747512	0.999996191
RNASE12	0.577706613	0.326360754	0.211783164	0.499984636	0.483071508	0.505491345	0.999996191
RNASE13	0.920579814	0.482525891	0.757792504	0.475731429	0.87612665	0.950539669	0.999996191
RNASE4	0.946589782	0.968268314	0.656548323	0.632931241	0.50303136	0.973314986	0.999996191
RNASEH2A	0.75452771	0.868873985	0.695529	0.734976678	0.698878438	0.983615532	0.999996191
RNASEH2B	0.784206245	0.676485036	0.719653315	0.090972627	0.571465787	0.644528218	0.999996191
RNASEK	0.322435688	0.262481124	0.638374489	0.641922862	0.795666496	0.708326647	0.999996191
RNASEL	0.519491398	0.831387186	0.154324108	0.752935641	0.782785014	0.774004292	0.999996191

RND3	0.61949717	0.370880808	0.751141702	0.938464001	0.358458472	0.840388967	0.999996191
RNF10	0.781893695	0.780738507	0.778690749	0.334817365	0.539514156	0.897114995	0.999996191
RNF11	0.258818691	0.355984177	0.318046714	0.73252681	0.973046391	0.654483061	0.999996191
RNF111	0.681388609	0.782485001	0.662545011	0.425734916	0.95154672	0.952243187	0.999996191
RNF113A	0.988032058	0.708033855	0.632761851	0.679630585	0.476992451	0.952477077	0.999996191
RNF123	0.886727862	0.359424499	0.852723706	0.209886729	0.345468674	0.643128987	0.999996191
RNF125	0.546737509	0.226086688	0.076049282	0.915340835	0.880580114	0.461323705	0.999996191
RNF126	0.820155249	0.81133625	0.21581714	0.455752774	0.737435524	0.810020928	0.999996191
RNF128	0.810219089	0.840905364	0.068346257	0.661758083	0.903517615	0.710027681	0.999996191
RNF130	0.809711328	0.832519288	0.461025934	0.930977401	0.783131888	0.98213724	0.999996191
RNF135	0.775923884	0.875186105	0.402313079	0.098879201	0.69666183	0.634139848	0.999996191
RNF14	0.426083802	0.589900296	0.635495463	0.199349526	0.419253359	0.567299675	0.999996191
RNF141	0.664973052	0.590155418	0.627716761	0.801708705	0.997870236	0.974960024	0.999996191
RNF144A	0.779693502	0.909175175	0.330340498	0.837240515	0.925586736	0.969958944	0.999996191
RNF144B	0.617470662	0.48569441	0.083128435	0.685138621	0.733471976	0.555066565	0.999996191
RNF145	0.258024751	0.818020672	0.927497481	0.714570728	0.56831007	0.886862733	0.999996191
RNF149	0.801002897	0.842536135	0.76111689	0.999355521	0.919581056	0.998931084	0.999996191
RNF152	0.827229651	0.585199773	0.148688085	0.328142761	0.427828537	0.514224716	0.999996191
RNF157	0.675493216	0.842001673	0.3416487	0.87470166	0.65372313	0.927806008	0.999996191
RNF166	0.715206603	0.481572319	0.114865724	0.973033868	0.503849682	0.640032288	0.999996191
RNF169	0.696498342	0.901604893	0.61474846	0.388843291	0.65243448	0.913486089	0.999996191
RNF170	0.100818064	0.872334631	0.504407753	0.65521711	0.486852105	0.578572518	0.999996191
RNF180	0.854863356	0.771350897	0.927642873	0.753900924	0.420634674	0.974044818	0.999996191
RNF181	0.58677445	0.427729944	0.539478835	0.525470319	0.840645901	0.84508539	0.999996191
RNF187	0.6904444	0.45525044	0.798391452	0.086558402	0.793683425	0.617020941	0.999996191
RNF20	0.418508243	0.627110791	0.985273117	0.114428973	0.718523633	0.657941616	0.999996191
RNF215	0.830324082	0.629266677	0.921622529	0.225787078	0.987356007	0.924036882	0.999996191
RNF216	0.406631211	0.493271562	0.440917007	0.644916173	0.8474073	0.810256498	0.999996191
RNF217	0.715147924	0.944506256	0.881182574	0.109633737	0.657413358	0.789653325	0.999996191
RNF219	0.73376252	0.429472113	0.755042237	0.807839088	0.349990235	0.863057786	0.999996191
RNF220	0.519534151	0.945626903	0.689543568	0.08772092	0.549597141	0.606456085	0.999996191
RNF227	0.263547093	0.579811226	0.585419003	0.290393283	0.58394443	0.592068955	0.999996191
RNF24	0.891824399	0.747955813	0.151784208	0.661879447	0.756060467	0.818195397	0.999996191
RNF25	0.333665583	0.543481369	0.876933865	0.515343652	0.19768483	0.604879803	0.999996191
RNF26	0.940822777	0.694044109	0.617693247	0.427333445	0.769311899	0.945484608	0.999996191
RNF31	0.543135154	0.145316836	0.529155797	0.497228213	0.581629558	0.548053958	0.999996191
RNF38	0.878115162	0.086859902	0.253718142	0.478949945	0.995518242	0.497271097	0.999996191
RNF41	0.284656035	0.499193813	0.931251314	0.149612926	0.812531793	0.603502708	0.999996191
RNF43	0.154367384	0.544303111	0.806853469	0.403490999	0.696508769	0.636542071	0.999996191
RNF44	0.660400429	0.289203401	0.777095983	0.559299798	0.558094716	0.803019602	0.999996191
RNF5	0.749039456	0.089182312	0.732505631	0.47804071	0.574531109	0.568586908	0.999996191
RNF6	0.360668752	0.658398258	0.868260079	0.840026724	0.944257303	0.962815913	0.999996191
RNF8	0.748561086	0.984944044	0.79367065	0.348831	0.689576294	0.950822645	0.999996191
RNFT1	0.310017287	0.822479921	0.999585461	0.778738958	0.137380892	0.706046319	0.999996191
RNFT2	0.677453921	0.957499531	0.696743277	0.898804596	0.819607453	0.994568737	0.999996191

RNGTT	0.682584255	0.856101369	0.992717307	0.871785057	0.335936978	0.965542055	0.999996191
RNLS	0.930695761	0.800187988	0.968337321	0.113869481	0.218824021	0.625106259	0.999996191
RNMT	0.542055989	0.54237875	0.421650092	0.648962814	0.169276203	0.571141828	0.999996191
RNPEP	0.736682952	0.86454848	0.723441401	0.045637527	0.449461306	0.501727757	0.999996191
RNPEPL1	0.577366524	0.951073743	0.541825047	0.316862951	0.881705178	0.892850086	0.999996191
RNPS1	0.548633294	0.574354693	0.44075351	0.077642059	0.669184958	0.452604039	0.999996191
ROBO1	0.958374736	0.612754558	0.27433061	0.268391608	0.644519919	0.710203964	0.999996191
ROBO4	0.450822915	0.756556701	0.979417799	0.338350343	0.526111888	0.844175547	0.999996191
ROR1	0.982629793	0.297727104	0.35395485	0.677405031	0.45991735	0.73786726	0.999996191
RORA	0.81359554	0.707614349	0.504224117	0.536391293	0.442983434	0.866753207	0.999996191
RORB	0.417155648	0.321484316	0.790222102	0.921161355	0.876137754	0.896601495	0.999996191
RP9	0.500579761	0.932905922	0.960522685	0.720352573	0.324464134	0.921386811	0.999996191
RPA1	0.472723979	0.222886487	0.836745267	0.655748488	0.188726957	0.528689766	0.999996191
RPA2	0.394716827	0.238415035	0.264210885	0.777384411	0.441626011	0.482932168	0.999996191
RPA3	0.705773671	0.547480623	0.101660252	0.205377454	0.888898007	0.451494573	0.999996191
RPA4	0.037784289	0.876067142	0.926578483	0.508121131	0.547074925	0.482714815	0.999996191
RPAIN	0.821892115	0.633988148	0.864128586	0.940997335	0.726104182	0.992792824	0.999996191
RPAP1	0.697431094	0.178267675	0.588676123	0.44323797	0.531276872	0.616956503	0.999996191
RPAP3	0.118984802	0.460496834	0.838057374	0.386470445	0.819440473	0.583860667	0.999996191
RPE	0.510016083	0.284577326	0.782186328	0.522494267	0.680729111	0.778926183	0.999996191
RPGR	0.409914087	0.477233394	0.550652826	0.643964168	0.876608016	0.84767363	0.999996191
RPGRIP1	0.772946531	0.508163783	0.749240433	0.52991636	0.420692984	0.859303675	0.999996191
RPIA	0.980893932	0.341464032	0.765137429	0.646726876	0.197935714	0.740990377	0.999996191
RPL10	0.309727815	0.965019607	0.97780712	0.215263001	0.700888331	0.794466169	0.999996191
RPL10A	0.284067369	0.832925278	0.919635611	0.064572921	0.618748309	0.486287968	0.999996191
RPL11	0.654251027	0.641251178	0.8873035	0.138424361	0.608632734	0.732573927	0.999996191
RPL13	0.555858917	0.96897638	0.87893449	0.528675066	0.406059953	0.917826931	0.999996191
RPL14	0.319810624	0.986094159	0.992145365	0.086749143	0.761912134	0.652544733	0.999996191
RPL15	0.484404763	0.781814667	0.648951416	0.203724317	0.586912829	0.720292655	0.999996191
RPL17	0.510793891	0.85950583	0.548918541	0.328244043	0.470124177	0.764092937	0.999996191
RPL18	0.453962609	0.782302739	0.709046985	0.385001117	0.781662798	0.880246671	0.999996191
RPL18A	0.263086896	0.646337571	0.88251608	0.199447748	0.484370138	0.583262732	0.999996191
RPL19	0.36752011	0.925410163	0.758486803	0.247530198	0.579972264	0.76333309	0.999996191
RPL21	0.920594479	0.448945768	0.773107875	0.085950253	0.821787319	0.669556319	0.999996191
RPL22	0.5179889	0.403289491	0.632261495	0.197797614	0.83241874	0.66234229	0.999996191
RPL23	0.572067103	0.806628747	0.311199777	0.10420959	0.567705896	0.482059934	0.999996191
RPL23A	0.641113471	0.653798641	0.45418633	0.187622957	0.337392011	0.54762381	0.999996191
RPL24	0.754333961	0.757472213	0.987410817	0.15581708	0.292921235	0.695067097	0.999996191
RPL26	0.496896729	0.959664937	0.378724848	0.164888039	0.354442176	0.522416088	0.999996191
RPL26L1	0.619480453	0.681850743	0.276743297	0.784975868	0.798721229	0.875531708	0.999996191
RPL27	0.683348264	0.687123	0.699161194	0.474226359	0.660294608	0.919144054	0.999996191
RPL27A	0.565756252	0.859532762	0.797283995	0.038457263	0.479849037	0.45108478	0.999996191
RPL28	0.544961606	0.565665842	0.672870153	0.270103938	0.428534017	0.681550583	0.999996191
RPL31	0.368186391	0.630096742	0.504568222	0.184152749	0.721755598	0.596995793	0.999996191
RPL32	0.194936597	0.992247528	0.836137586	0.269448668	0.989661862	0.79058526	0.999996191

RPL34	0.568111753	0.761873232	0.843456801	0.144752194	0.688887232	0.76020533	0.999996191
RPL35	0.545731734	0.735813803	0.691133062	0.20561204	0.60815496	0.751418309	0.999996191
RPL35A	0.664270676	0.851045169	0.593496934	0.091071949	0.855023729	0.697849222	0.999996191
RPL36	0.251690756	0.761214284	0.978838389	0.155459652	0.794095595	0.674503013	0.999996191
RPL36AL	0.633139563	0.543965067	0.827452965	0.306632557	0.576476437	0.817231453	0.999996191
RPL37	0.798483636	0.919241036	0.848843679	0.096986975	0.486052954	0.720196324	0.999996191
RPL37A	0.675847967	0.510421612	0.240976435	0.411917106	0.433250735	0.587740763	0.999996191
RPL38	0.873086027	0.670351558	0.177040444	0.154741162	0.516520992	0.477407579	0.999996191
RPL4	0.530738974	0.89742632	0.731672393	0.149477399	0.432160608	0.669067917	0.999996191
RPL5	0.637398069	0.755211124	0.765273784	0.143136344	0.588515277	0.730579784	0.999996191
RPL6	0.67423121	0.671606462	0.872849547	0.099088706	0.305591081	0.546299505	0.999996191
RPL7	0.82651075	0.592125913	0.9453193	0.153926824	0.545638119	0.772034001	0.999996191
RPL9	0.457035969	0.622184234	0.768012583	0.103787748	0.724388657	0.607494615	0.999996191
RPN1	0.2897669	0.573997958	0.278540778	0.565738437	0.342361248	0.492112792	0.999996191
RPN2	0.184920965	0.592735001	0.229399711	0.651482574	0.494165867	0.473257133	0.999996191
RPP14	0.3888892	0.527731645	0.43309761	0.744907586	0.971301214	0.85628611	0.999996191
RPP21	0.576730904	0.54445484	0.275064015	0.518384142	0.497993681	0.667200643	0.999996191
RPP25	0.71064593	0.94152275	0.881426482	0.431810367	0.639533449	0.962510897	0.999996191
RPP25L	0.532846754	0.696005351	0.81962572	0.856149243	0.964716998	0.986443169	0.999996191
RPRD1A	0.461884346	0.810486182	0.502552918	0.604866918	0.506742173	0.839310998	0.999996191
RPRM	0.840620425	0.272807232	0.36234038	0.380485078	0.873010832	0.708370273	0.999996191
RPRML	0.616076971	0.274369091	0.964017641	0.760006376	0.834164899	0.919710188	0.999996191
RPS10	0.450827302	0.248065041	0.937280615	0.319205668	0.685982831	0.672829243	0.999996191
RPS13	0.723361159	0.753234379	0.364809455	0.212291745	0.493178978	0.653771468	0.999996191
RPS14	0.17308205	0.931474792	0.971774911	0.108064206	0.551490944	0.49946944	0.999996191
RPS16	0.371405282	0.920576899	0.828686922	0.205207521	0.405327148	0.677950036	0.999996191
RPS17	0.927594652	0.420648499	0.711827265	0.139861509	0.55689227	0.661322466	0.999996191
RPS19	0.343159305	0.838399724	0.994046879	0.070777188	0.630717392	0.558692162	0.999996191
RPS2	0.531356596	0.684753061	0.670876672	0.117727282	0.348509204	0.512547059	0.999996191
RPS21	0.560849798	0.958275004	0.975594234	0.112999437	0.901654367	0.826962483	0.999996191
RPS23	0.470500298	0.747648904	0.141189426	0.683650389	0.584471917	0.644503361	0.999996191
RPS24	0.566002424	0.909251583	0.922699673	0.025941172	0.707916455	0.486849129	0.999996191
RPS27	0.557893676	0.822628916	0.753403803	0.03670654	0.904558717	0.538359589	0.999996191
RPS27A	0.938896204	0.555228376	0.96285555	0.046744996	0.744246329	0.619517007	0.999996191
RPS27L	0.981770528	0.42241812	0.455668929	0.206563517	0.989988899	0.771048485	0.999996191
RPS27P	0.473322016	0.966410112	0.645062144	0.134904202	0.859530031	0.748795623	0.999996191
RPS3	0.371513728	0.731144593	0.674626507	0.101928773	0.49054089	0.495971095	0.999996191
RPS3A	0.86733876	0.693588194	0.780984716	0.195464707	0.703099648	0.856893217	0.999996191
RPS4X	0.498267828	0.860530973	0.645392338	0.134830092	0.667715692	0.688695327	0.999996191
RPS5	0.273314726	0.95799197	0.851572073	0.268054058	0.761368934	0.799933884	0.999996191
RPS6KA2	0.658691509	0.763920547	0.459144488	0.262445598	0.443820495	0.703524019	0.999996191
RPS6KA5	0.933091527	0.874136226	0.566895629	0.080773644	0.26278198	0.508761723	0.999996191
RPS6KB1	0.798938512	0.92601675	0.263398591	0.634737821	0.271338017	0.745230237	0.999996191
RPS6KC1	0.696980471	0.74427746	0.797246281	0.329010993	0.318875501	0.791645296	0.999996191
RPS7	0.286287357	0.936857468	0.606227054	0.251188269	0.736992486	0.724837941	0.999996191

RPS8	0.75162682	0.95653427	0.970541243	0.199339955	0.67296915	0.908021139	0.999996191
RPS9	0.462341526	0.684526457	0.9020876	0.326726896	0.287282115	0.702716325	0.999996191
RPUSD2	0.432772933	0.682032248	0.652330225	0.164560544	0.62529459	0.644182154	0.999996191
RPUSD4	0.848578474	0.988106983	0.681233877	0.622698743	0.113581625	0.779019912	0.999996191
RRAGA	0.998919584	0.178841134	0.472113316	0.546418093	0.317915619	0.585316105	0.999996191
RRAS2	0.724020434	0.846146763	0.681315033	0.684050811	0.709411377	0.976495289	0.999996191
RREB1	0.866456409	0.870872993	0.753206917	0.596357603	0.747300794	0.986777649	0.999996191
RRH	0.704018303	0.648538253	0.887377574	0.722753293	0.89765481	0.988120833	0.999996191
RRM1	0.948537829	0.477621427	0.979227602	0.755884087	0.66761875	0.981580507	0.999996191
RRM2B	0.083661628	0.431213749	0.412435954	0.662228253	0.806999906	0.470019201	0.999996191
RRN3	0.693827614	0.484039215	0.698279485	0.318421149	0.646336882	0.810015456	0.999996191
RRNAD1	0.625474438	0.813199848	0.910794682	0.72154881	0.335528357	0.928809582	0.999996191
RRP1	0.475599238	0.73720744	0.608332198	0.232769685	0.14971688	0.457887302	0.999996191
RRP1B	0.999177313	0.775815302	0.696837589	0.402707296	0.774921798	0.964993137	0.999996191
RSBN1	0.424021613	0.836771135	0.901012818	0.046388852	0.83753307	0.553408733	0.999996191
RSBN1L	0.232685516	0.78544229	0.59959631	0.360493729	0.481033568	0.636031231	0.999996191
RSF1	0.306975332	0.663450241	0.660953418	0.089933663	0.848653545	0.517337559	0.999996191
RSPH3	0.851647335	0.801250039	0.923035915	0.969602996	0.70870129	0.998279407	0.999996191
RSPO1	0.151639976	0.191372967	0.957948496	0.930858934	0.325623305	0.480563012	0.999996191
RSRC1	0.952404632	0.927556524	0.812098566	0.526317921	0.41341252	0.959307962	0.999996191
RTCA	0.781318797	0.631205265	0.459263279	0.160138515	0.632366984	0.67269354	0.999996191
RTF1	0.867623339	0.799262418	0.98303894	0.03931268	0.481824585	0.560877592	0.999996191
RTF2	0.743951769	0.553549606	0.714201019	0.288274139	0.425971203	0.758754516	0.999996191
RTKN	0.882766578	0.368055652	0.794192988	0.830022118	0.263411375	0.835813868	0.999996191
RTL5	0.862199863	0.155122782	0.222545456	0.287281048	0.860737318	0.456123057	0.999996191
RTL8C	0.770400165	0.610347504	0.421554667	0.119187711	0.99014637	0.676513452	0.999996191
RTL8C	0.213252702	0.671839077	0.765015656	0.179672549	0.471094763	0.498277872	0.999996191
RTL9	0.955250963	0.997080201	0.635444261	0.745680456	0.552374474	0.986171703	0.999996191
RTN1	0.921458219	0.295432932	0.602816219	0.408810999	0.523828841	0.75373499	0.999996191
RTN2	0.649292168	0.175005277	0.424636136	0.530013759	0.355681147	0.494625952	0.999996191
RTN3	0.99875852	0.605299796	0.470069728	0.730250119	0.718053056	0.955615207	0.999996191
RTN4	0.841558629	0.839716134	0.567248325	0.659579998	0.488863205	0.943103746	0.999996191
RTN4IP1	0.804610697	0.520565735	0.718618554	0.294640683	0.856668983	0.880605292	0.999996191
RTN4R	0.907158863	0.774757204	0.409391823	0.279228093	0.553511648	0.795944803	0.999996191
RTN4RL2	0.956542057	0.2163407	0.373549661	0.827117737	0.689148286	0.794338807	0.999996191
RTRAF	0.668120175	0.686373652	0.502354864	0.058580608	0.768655442	0.519146902	0.999996191
RTTN	0.518971047	0.460744404	0.765497054	0.601818969	0.511243582	0.835529345	0.999996191
RUFY1	0.985891692	0.958470493	0.28016446	0.165740972	0.744016164	0.740080935	0.999996191
RUFY2	0.127740684	0.349145438	0.427195963	0.918518905	0.911247157	0.601806495	0.999996191
RUNDC1	0.191149193	0.467825388	0.860811748	0.538307042	0.944078432	0.773253661	0.999996191
RUNDC3A	0.420198659	0.549270819	0.881325895	0.534090069	0.586350347	0.854837613	0.999996191
RUNX2	0.300318981	0.868934766	0.980014293	0.209190393	0.325258497	0.618829195	0.999996191
RUSC2	0.23701935	0.88021997	0.860807718	0.828036186	0.103678113	0.595226908	0.999996191
RUVBL2	0.238826037	0.860478767	0.44502815	0.990434988	0.420102933	0.768262866	0.999996191
RWDD2A	0.387175174	0.865287982	0.307891881	0.870133028	0.674524354	0.846984824	0.999996191

RWDD2B	0.410463083	0.455159637	0.233614079	0.626281283	0.66723497	0.628010571	0.999996191
RWDD3	0.324196298	0.199701079	0.818271456	0.625367773	0.868801677	0.716360195	0.999996191
RWDD4	0.091194227	0.505519589	0.449209581	0.480562316	0.927015018	0.497240862	0.999996191
RXFP3	0.852651928	0.550250635	0.077499543	0.372945129	0.914492994	0.553106262	0.999996191
RXRB	0.433426716	0.54652828	0.328599563	0.877364065	0.704344893	0.809443824	0.999996191
RXRG	0.859147615	0.582425948	0.5752951	0.847172841	0.198985749	0.810941323	0.999996191
RYK	0.971040156	0.33039333	0.103964177	0.513976237	0.598456695	0.51707455	0.999996191
RYR1	0.275333274	0.935353312	0.64781846	0.936590682	0.674670378	0.922009673	0.999996191
RYR3	0.506713796	0.834535883	0.037855767	0.675283529	0.647488887	0.447197379	0.999996191
S100A13	0.593170023	0.533755516	0.799578047	0.299679598	0.283163509	0.659957478	0.999996191
S100A14	0.421767871	0.904265098	0.341815406	0.194490915	0.841001151	0.658513428	0.999996191
S100A16	0.301936526	0.685000175	0.156812967	0.400887944	0.550960123	0.451307034	0.999996191
S100A2	0.541928115	0.072208695	0.372774901	0.659387735	0.898212347	0.485142583	0.999996191
S100A3	0.608216444	0.828318732	0.407539992	0.457794652	0.356287397	0.744822534	0.999996191
S100A4	0.990163415	0.523812695	0.842307457	0.534032208	0.22444973	0.823673037	0.999996191
S100A9	0.742887002	0.174365105	0.877303714	0.523449539	0.297192317	0.621919806	0.999996191
S100G	0.68386393	0.422259014	0.164684559	0.918085941	0.604456798	0.699781915	0.999996191
S100PBP	0.289838702	0.638712429	0.158650929	0.676447119	0.351407871	0.446746226	0.999996191
S1PR1	0.799224312	0.721615429	0.743962962	0.165828605	0.917304574	0.858523519	0.999996191
S1PR2	0.752045419	0.774360192	0.361369877	0.338532318	0.927548669	0.86038633	0.999996191
SAAL1	0.612794026	0.908056029	0.390084406	0.943848123	0.774711807	0.960583194	0.999996191
SAFB2	0.806393459	0.338381376	0.904050545	0.045199853	0.919719433	0.516992552	0.999996191
SALL2	0.780212392	0.999088406	0.06094969	0.967996637	0.264667632	0.549532703	0.999996191
SAMD1	0.942729566	0.670542933	0.238217374	0.456871266	0.510401243	0.753591421	0.999996191
SAMD10	0.306004441	0.763126237	0.392426325	0.9427186	0.135176538	0.541609073	0.999996191
SAMD12	0.557221333	0.862549968	0.569930188	0.727361263	0.280858973	0.834503657	0.999996191
SAMD13	0.367507671	0.905505954	0.900647654	0.399024564	0.972002239	0.932588237	0.999996191
SAMD14	0.292383445	0.776516834	0.247618066	0.648892227	0.479397237	0.619810173	0.999996191
SAMD4A	0.864840144	0.95813332	0.804593425	0.690225516	0.864449864	0.997404397	0.999996191
SAMD8	0.197990144	0.580002603	0.951242746	0.138700751	0.663874702	0.513356819	0.999996191
SAMM50	0.979431472	0.576271579	0.285714288	0.832554871	0.484952926	0.858157404	0.999996191
SAO	0.986556792	0.884927646	0.201064957	0.056085508	0.72167741	0.449847858	0.999996191
SAP30	0.239833641	0.382722094	0.943469956	0.922684668	0.731914356	0.841551018	0.999996191
SAPCD2	0.632791213	0.110011293	0.851270283	0.241750215	0.522911963	0.459284762	0.999996191
SAR1A	0.867600679	0.140400584	0.374054268	0.596675514	0.512013284	0.575386558	0.999996191
SARAF	0.782027732	0.179791003	0.203036455	0.502903374	0.805392458	0.539716602	0.999996191
SARM1	0.633872651	0.388302891	0.412116896	0.582989303	0.907305936	0.827673535	0.999996191
SARNP	0.876437536	0.221753632	0.777374886	0.991698559	0.749965415	0.929011806	0.999996191
SARS	0.513460781	0.621095696	0.516467449	0.634853947	0.216793809	0.670415288	0.999996191
SARS2	0.93341739	0.378196084	0.329738231	0.146430749	0.660667759	0.534685311	0.999996191
SART1	0.704638449	0.917458011	0.100483635	0.465660707	0.996027368	0.725018572	0.999996191
SART3	0.941363856	0.761274999	0.745539106	0.435676002	0.94008979	0.980492745	0.999996191
SASH1	0.841007898	0.495419212	0.412443286	0.603240238	0.832553279	0.897775336	0.999996191
SATB1	0.808234088	0.676522168	0.251687034	0.118245581	0.63062317	0.517120844	0.999996191
SATB2	0.212448442	0.678183996	0.806393395	0.114760045	0.684514505	0.495249993	0.999996191

SAXO2	0.64370524	0.854365506	0.399227015	0.13936736	0.703063091	0.660239569	0.999996191
SAYSD1	0.569473491	0.554125607	0.732121658	0.059016091	0.662616829	0.493366889	0.999996191
SBDS	0.74495351	0.703231886	0.540901881	0.858055149	0.591047951	0.952601054	0.999996191
SBK1	0.300295277	0.671405009	0.829453977	0.742830144	0.305256055	0.767632486	0.999996191
SBNO1	0.467508579	0.790604798	0.888018593	0.3406531	0.652747961	0.874935264	0.999996191
SC5D	0.520093871	0.619203044	0.770881088	0.260374841	0.901993873	0.841065751	0.999996191
SCAF1	0.435447049	0.622391599	0.079118047	0.458686035	0.925206133	0.494699367	0.999996191
SCAF4	0.661304515	0.867611534	0.97888013	0.023434247	0.840853053	0.531383731	0.999996191
SCAI	0.606403405	0.787147681	0.452439641	0.476424668	0.565726333	0.840800815	0.999996191
SCAMP1	0.123255103	0.79340299	0.753475145	0.524229137	0.853620333	0.74193498	0.999996191
SCAMP2	0.373884605	0.390785409	0.249654462	0.953493696	0.712283725	0.687608621	0.999996191
SCAMP3	0.518260126	0.613211156	0.803329815	0.165192805	0.471035044	0.644700496	0.999996191
SCAMP4	0.631566403	0.788298304	0.277334107	0.527785836	0.816101954	0.844195402	0.999996191
SCAMP5	0.303682237	0.840440622	0.05309977	0.798499303	0.996140098	0.526405072	0.999996191
SCAP	0.896828123	0.877468886	0.615032695	0.385711901	0.59493213	0.927756827	0.999996191
SCARA3	0.826890005	0.607724214	0.832806899	0.492733022	0.372954122	0.882304275	0.999996191
SCARA5	0.877361452	0.122532945	0.62780198	0.639731949	0.462461534	0.645703521	0.999996191
SCARB1	0.450864812	0.749853498	0.668124214	0.420695952	0.771036865	0.875491654	0.999996191
SCARF1	0.90511161	0.628661558	0.54915553	0.836903511	0.55062767	0.952769539	0.999996191
SCCPDH	0.891536641	0.846844347	0.409879739	0.242181805	0.509355307	0.768833932	0.999996191
SCD5	0.471621657	0.697354	0.533782371	0.450173874	0.995092254	0.885380279	0.999996191
SCFD1	0.3777905	0.775553632	0.896317377	0.622891934	0.312430587	0.81964469	0.999996191
SCFD2	0.940350931	0.34585892	0.944003027	0.731437334	0.345806584	0.883644638	0.999996191
SCG5	0.787547767	0.837857799	0.275688238	0.769496499	0.527847836	0.876688646	0.999996191
SCHIP1	0.349690833	0.549112557	0.689557248	0.888993936	0.101403939	0.545789383	0.999996191
SCLT1	0.983894555	0.983502979	0.900063266	0.304939297	0.42390513	0.929217487	0.999996191
SCML1	0.175536955	0.383658355	0.927313383	0.379930505	0.526630361	0.554561888	0.999996191
SCML2	0.658322427	0.981659649	0.97150973	0.107016836	0.416766377	0.711121906	0.999996191
SCN11A	0.145568202	0.594448318	0.550583836	0.823201919	0.7730012	0.72619309	0.999996191
SCN2A	0.52008669	0.539413665	0.123586086	0.798616744	0.704649162	0.641186151	0.999996191
SCN3B	0.287200539	0.698794128	0.915185939	0.835504398	0.405345579	0.851188376	0.999996191
SCN9A	0.38532607	0.17995814	0.740170956	0.163271385	0.946781473	0.469617219	0.999996191
SCNM1	0.453007182	0.987643769	0.846325209	0.718215708	0.759542761	0.977555634	0.999996191
SCNN1A	0.722098224	0.45560885	0.561185549	0.803540851	0.315869593	0.80498467	0.999996191
SCOC	0.145242796	0.645550731	0.477803917	0.97409556	0.833297686	0.760004921	0.999996191
SCP2D1	0.84277716	0.600996772	0.538210086	0.732439831	0.698468413	0.950016231	0.999996191
SCRN2	0.361086778	0.268693087	0.847175243	0.884427646	0.523415475	0.768245861	0.999996191
SCRN3	0.637842302	0.69238305	0.353363282	0.610963542	0.366016617	0.752448237	0.999996191
SCUBE3	0.54692693	0.591938193	0.878142891	0.421295329	0.987344924	0.934356511	0.999996191
SCYL3	0.183289227	0.886689552	0.422217096	0.3723574	0.91894557	0.677234582	0.999996191
SDAD1	0.862170786	0.836852626	0.465501302	0.23886749	0.125511933	0.513562147	0.999996191
SDC2	0.42619417	0.179513119	0.827874551	0.154388672	0.953077449	0.499128718	0.999996191
SDCCAG8	0.902157747	0.266956828	0.989397948	0.965300444	0.293343746	0.863499742	0.999996191
SDE2	0.210834877	0.563027039	0.946650396	0.64080783	0.993246083	0.872033024	0.999996191
SDF2	0.300962079	0.512440994	0.678029177	0.26771449	0.954449793	0.702157491	0.999996191

SDF2L1	0.23217945	0.728241419	0.513589773	0.416387261	0.328125085	0.544637313	0.999996191
SDF4	0.71943037	0.606950721	0.247190373	0.498321021	0.515876362	0.709380978	0.999996191
SDHA	0.762746205	0.529036586	0.612723526	0.064810496	0.874668513	0.576713731	0.999996191
SDHAF1	0.254541455	0.683805209	0.721427693	0.582079906	0.636094412	0.803632829	0.999996191
SDHAF2	0.59120632	0.49799546	0.866500865	0.131702876	0.623060772	0.654925082	0.999996191
SDHAF3	0.893771799	0.994040101	0.976226284	0.477105416	0.202320853	0.893799434	0.999996191
SDHB	0.884323001	0.561507833	0.415416857	0.318979556	0.889137041	0.841604643	0.999996191
SDHC	0.8782749	0.950897089	0.530502832	0.278941685	0.792981807	0.91356357	0.999996191
SDHD	0.583302947	0.558037931	0.645431751	0.115977022	0.995725649	0.683575522	0.999996191
SDR39U1	0.84503117	0.863815171	0.322083864	0.470399846	0.954023755	0.922102848	0.999996191
SDR42E1	0.70263953	0.633174677	0.214627345	0.849882697	0.581626026	0.806219323	0.999996191
SDSL	0.822583092	0.978401987	0.571630701	0.647558766	0.675893958	0.976170372	0.999996191
SEC11A	0.622603463	0.420589475	0.260532388	0.803178583	0.666124301	0.760687617	0.999996191
SEC11C	0.602359949	0.335245298	0.922652975	0.312065726	0.159238617	0.497907038	0.999996191
SEC14L2	0.084539502	0.804141931	0.655470312	0.404201359	0.525807005	0.502105394	0.999996191
SEC22B	0.601186575	0.708788729	0.319253406	0.192442425	0.314436539	0.476304422	0.999996191
SEC22C	0.837155837	0.936567813	0.642418799	0.874681138	0.364219079	0.96140776	0.999996191
SEC23A	0.400787037	0.794041525	0.153821303	0.830233953	0.819585789	0.743826241	0.999996191
SEC23IP	0.442173187	0.867084382	0.331399838	0.203176609	0.732447835	0.635060055	0.999996191
SEC24A	0.695397142	0.93152617	0.370060533	0.148176699	0.344771143	0.550701438	0.999996191
SEC24C	0.867699804	0.715275044	0.778932595	0.179635993	0.226216048	0.64252622	0.999996191
SEC24D	0.842054523	0.660634342	0.430798554	0.102270968	0.837647158	0.65112232	0.999996191
SEC31B	0.597840129	0.91779859	0.820927792	0.06632771	0.937640447	0.711199716	0.999996191
SEC61A2	0.801599468	0.549108457	0.351957122	0.388761739	0.516607069	0.731075725	0.999996191
SEC61B	0.257156693	0.215483157	0.544153296	0.743977818	0.318224798	0.450691849	0.999996191
SEC61G	0.863591112	0.169830673	0.730900787	0.832775868	0.69128735	0.8499617	0.999996191
SEC62	0.437205248	0.691500694	0.113034876	0.266832828	0.951008781	0.485825692	0.999996191
SECISBP2L	0.406248444	0.610278576	0.164795574	0.813658194	0.619937112	0.651870452	0.999996191
SEH1L	0.693620387	0.634484405	0.330518543	0.314505972	0.238685751	0.528837261	0.999996191
SEL1L	0.969842572	0.439182138	0.656885619	0.825697483	0.950143658	0.980642709	0.999996191
SELENOH	0.354547385	0.45403027	0.753391144	0.190984008	0.775411541	0.625007343	0.999996191
SELENOV	0.169235209	0.820965081	0.459246332	0.196763011	0.993582207	0.554234062	0.999996191
SELENOW	0.671930132	0.866936257	0.284951689	0.064212801	0.836160318	0.490857943	0.999996191
SEM1	0.312988372	0.207597779	0.556462857	0.982569185	0.628491968	0.667471682	0.999996191
SEMA3A	0.938138266	0.682283396	0.055864986	0.684983017	0.868882878	0.65813468	0.999996191
SEMA3B	0.962165614	0.052577039	0.324947961	0.930642347	0.819027533	0.555088712	0.999996191
SEMA3C	0.869800273	0.9714531	0.741404801	0.82076781	0.962691212	0.999198447	0.999996191
SEMA3F	0.124401971	0.869213875	0.723095725	0.683931954	0.14020472	0.459435105	0.999996191
SEMA4G	0.106417952	0.428952903	0.62543563	0.896983632	0.417736361	0.524959644	0.999996191
SEMA5A	0.819031539	0.934871145	0.643270264	0.786462399	0.944605806	0.996257214	0.999996191
SEMA5B	0.503208796	0.26449692	0.539809037	0.985612187	0.147282627	0.520168301	0.999996191
SEMA6D	0.994210493	0.739906233	0.606823872	0.652397084	0.475119421	0.949323672	0.999996191
SEMA7A	0.363776333	0.605190338	0.635539169	0.383414102	0.790373518	0.787594356	0.999996191
SENP1	0.172598452	0.671709992	0.624355932	0.477600834	0.63757079	0.66496198	0.999996191
SENP2	0.683223727	0.821553263	0.700913037	0.47889272	0.600420585	0.929727008	0.999996191

SENP3	0.831608413	0.473839661	0.740005831	0.445669016	0.952761141	0.938965212	0.999996191
SENP5	0.800532641	0.606858728	0.600338133	0.484488542	0.549002146	0.883500797	0.999996191
SEPSECS	0.138882037	0.580559205	0.689259474	0.735300217	0.643819233	0.69918693	0.999996191
SEPT11	0.207391798	0.955854044	0.705235773	0.597344109	0.817441261	0.865227012	0.999996191
SEPT2	0.609572496	0.742168709	0.607863061	0.765602135	0.811375783	0.96592447	0.999996191
SEPT3	0.887049376	0.599371329	0.239978481	0.593733028	0.382523272	0.717638748	0.999996191
SEPT4	0.44787283	0.635170003	0.489439635	0.109847876	0.871003977	0.566889749	0.999996191
SEPT7	0.776593236	0.056375444	0.519733637	0.425854511	0.76799281	0.458098933	0.999996191
SEPT8	0.901122524	0.362822065	0.758925473	0.394133266	0.072181412	0.448699416	0.999996191
SERAC1	0.709592	0.941712625	0.089914099	0.682459701	0.576079991	0.678403764	0.999996191
SERF1A	0.966832942	0.56605246	0.726941009	0.650961196	0.578359537	0.956034414	0.999996191
SERF2	0.611519034	0.647804515	0.562860558	0.102253043	0.738995501	0.612534931	0.999996191
SERINC1	0.449990794	0.632403075	0.353951125	0.92015653	0.417807122	0.771419123	0.999996191
SERINC4	0.172456817	0.617372307	0.936444211	0.40418363	0.442547602	0.623630567	0.999996191
SERINC5	0.640708218	0.569896009	0.286250668	0.366478536	0.664913097	0.692949338	0.999996191
SERP2	0.927292525	0.706218941	0.883005172	0.620472535	0.92063973	0.994406736	0.999996191
SERPINA5	0.277788561	0.253025622	0.762858415	0.44455305	0.328228812	0.467092447	0.999996191
SERPINB1	0.77012649	0.971710346	0.357590921	0.208208606	0.72406345	0.778766039	0.999996191
SERPING1	0.423425966	0.852375347	0.585328325	0.545926824	0.124886493	0.581998267	0.999996191
SERPINH1	0.518383873	0.411243266	0.243156846	0.598297431	0.832698923	0.695619753	0.999996191
SERTAD2	0.419688006	0.659308357	0.286476998	0.162214531	0.858288361	0.530859774	0.999996191
SERTAD3	0.505241991	0.51294129	0.77731133	0.873191781	0.192575106	0.746947671	0.999996191
SERTAD4	0.947434764	0.782852266	0.393650394	0.852000989	0.202494344	0.817223442	0.999996191
SESN2	0.062946947	0.948349758	0.918365335	0.308900125	0.918884729	0.597029236	0.999996191
SESN3	0.322782017	0.620459344	0.544660749	0.525628039	0.673044207	0.770792986	0.999996191
SESTD1	0.657261138	0.745226667	0.980599594	0.177582456	0.361511596	0.729382669	0.999996191
SET	0.886968045	0.292075295	0.882632317	0.695142377	0.188203996	0.723668186	0.999996191
SETBP1	0.8397227	0.806001041	0.5833013	0.662640855	0.811643198	0.978991025	0.999996191
SETD1A	0.619719554	0.801476409	0.832742853	0.266638627	0.755662539	0.893194225	0.999996191
SETD1B	0.664025346	0.64673436	0.882999345	0.467544764	0.882744947	0.959508489	0.999996191
SETD4	0.183507457	0.572813911	0.695605827	0.272304599	0.897933436	0.624117026	0.999996191
SETD5	0.282918901	0.759766699	0.854417283	0.257480685	0.157806123	0.458592378	0.999996191
SETD6	0.408631395	0.907183135	0.862038904	0.623100655	0.659382939	0.944571275	0.999996191
SETD9	0.544757631	0.088267494	0.565793083	0.505494683	0.88812629	0.55019016	0.999996191
SETDB1	0.501750272	0.364952384	0.115060032	0.918437556	0.459189789	0.490304722	0.999996191
SETX	0.503217249	0.656569867	0.377157909	0.812598468	0.372671639	0.766748883	0.999996191
SF1	0.525808909	0.618412841	0.731380305	0.380466503	0.643851278	0.840936852	0.999996191
SF3A1	0.361159576	0.854782109	0.784614474	0.636805661	0.886335456	0.948257443	0.999996191
SF3A2	0.265282656	0.79512055	0.716100747	0.914530001	0.671176636	0.906842517	0.999996191
SF3A3	0.692707696	0.677382003	0.287027727	0.549667024	0.33711975	0.689032871	0.999996191
SF3B2	0.372771273	0.979521618	0.318976337	0.834043141	0.942116277	0.90522973	0.999996191
SF3B4	0.063089358	0.37398209	0.944045513	0.890632251	0.754031159	0.589345518	0.999996191
SF3B6	0.930203916	0.19353283	0.886289611	0.226177213	0.85717294	0.729983012	0.999996191
SFMBT1	0.827034491	0.887686601	0.857340913	0.986998973	0.479238924	0.991948043	0.999996191
SFPQ	0.870946625	0.746892422	0.886038905	0.409434849	0.660889039	0.959239024	0.999996191

SFRS18	0.502735243	0.503977512	0.742554404	0.603542281	0.21694194	0.686516681	0.999996191
SFSWAP	0.516114412	0.16411878	0.368323068	0.554576324	0.527843509	0.495368554	0.999996191
SFT2D1	0.784976385	0.48098311	0.655367384	0.331855889	0.709542248	0.840954136	0.999996191
SFT2D2	0.727911267	0.213883296	0.343290709	0.988315464	0.777246358	0.781888327	0.999996191
SFT2D3	0.587182615	0.985290271	0.255399666	0.472923878	0.807991411	0.835941826	0.999996191
SFXN2	0.837929096	0.555756123	0.445576242	0.037728344	0.972278043	0.462138414	0.999996191
SFXN3	0.561646108	0.708920763	0.253978401	0.89104482	0.727735113	0.859228506	0.999996191
SFXN4	0.880864011	0.716763394	0.775921487	0.322249729	0.376643937	0.844163169	0.999996191
SFXN5	0.791966768	0.593289917	0.634385007	0.121446007	0.948192592	0.749391942	0.999996191
SGCB	0.615900833	0.224353105	0.448866524	0.963025188	0.448204332	0.702530318	0.999996191
SGCE	0.225886376	0.793918591	0.475463242	0.362171045	0.577002383	0.62346241	0.999996191
SGF29	0.666404158	0.769248657	0.04725119	0.870637402	0.362936575	0.463136886	0.999996191
SGK1	0.234378209	0.780642667	0.323843876	0.353188043	0.825026203	0.617303067	0.999996191
SGK3	0.591211755	0.921688714	0.419572162	0.448558383	0.330149681	0.746858895	0.999996191
SGMS2	0.496085766	0.585436397	0.888847796	0.361221327	0.947434683	0.900779447	0.999996191
SGO1	0.745230875	0.292056345	0.398538992	0.903190616	0.366118857	0.715698339	0.999996191
SGO2	0.446493153	0.559715157	0.701265529	0.760388123	0.90485244	0.936331379	0.999996191
SGPP1	0.768444028	0.924512473	0.703904631	0.865977237	0.03187507	0.573761375	0.999996191
SGSM1	0.612176902	0.640843482	0.929612953	0.549456537	0.496328571	0.915307336	0.999996191
SGSM2	0.267956093	0.703148561	0.747742992	0.924544809	0.527185716	0.866094983	0.999996191
SGSM3	0.961452422	0.799317537	0.881982046	0.030034457	0.515602546	0.521372701	0.999996191
SGTA	0.66322364	0.833027301	0.874149849	0.843375834	0.684860265	0.990073923	0.999996191
SH2D3C	0.483109431	0.490520776	0.759217924	0.520307085	0.566344406	0.825710241	0.999996191
SH2D7	0.639788222	0.811293222	0.728554004	0.497605847	0.827234361	0.959091546	0.999996191
SH3BGRL	0.255414683	0.76617738	0.220971802	0.854719515	0.898022779	0.743164632	0.999996191
SH3BGRL2	0.493769676	0.85408819	0.831168702	0.421775126	0.544123079	0.88846401	0.999996191
SH3BP2	0.348386877	0.954385819	0.078381737	0.825427244	0.489127798	0.521833015	0.999996191
SH3BP4	0.686612076	0.839583058	0.558390571	0.658110676	0.719288523	0.957413134	0.999996191
SH3BP5	0.661247413	0.663657475	0.545830633	0.062932447	0.883905007	0.566929954	0.999996191
SH3D19	0.885269849	0.465362546	0.444439895	0.176427619	0.761084027	0.686138374	0.999996191
SH3D21	0.660922073	0.560112324	0.247407504	0.641808375	0.670150242	0.774500649	0.999996191
SH3GLB2	0.643216363	0.674796361	0.546617068	0.271481953	0.288426054	0.631610852	0.999996191
SH3KBP1	0.968197419	0.485629174	0.239405349	0.268241536	0.90911416	0.70732197	0.999996191
SH3PXD2B	0.687477612	0.669687567	0.273284042	0.164625672	0.940575558	0.640897359	0.999996191
SH3RF1	0.198822281	0.79499752	0.734531251	0.577798463	0.688712093	0.802551195	0.999996191
SH3RF3	0.639655385	0.708807431	0.110415238	0.658882735	0.893609607	0.720867892	0.999996191
SHANK3	0.884271353	0.576982172	0.7971091	0.788591883	0.946296628	0.992450125	0.999996191
SHB	0.863712703	0.664942487	0.340494528	0.405941782	0.652839329	0.821956367	0.999996191
SHBG	0.95123901	0.950962477	0.194039384	0.424612781	0.24774325	0.630418483	0.999996191
SHC1	0.648841957	0.968200005	0.319077011	0.241494907	0.944169436	0.800687004	0.999996191
SHCBP1L	0.21077687	0.179213454	0.462190181	0.698001976	0.596484188	0.453902292	0.999996191
SHE	0.142826223	0.774822959	0.736403632	0.506205279	0.743516422	0.728386795	0.999996191
SHF	0.503654765	0.783775092	0.427817735	0.710462148	0.655289089	0.885349417	0.999996191
SHISA5	0.283308484	0.783985315	0.18333723	0.361330087	0.496778638	0.454893134	0.999996191
SHISA6	0.625186056	0.333752956	0.375289489	0.90876354	0.974058321	0.867475901	0.999996191

SHISA7	0.43708754	0.608027949	0.255557703	0.309409077	0.337696754	0.449635953	0.999996191
SHISAL1	0.275064115	0.816228464	0.643163967	0.673794616	0.216075439	0.655751392	0.999996191
SHISAL2A	0.514359747	0.260727826	0.906417081	0.750878756	0.863604914	0.885699455	0.999996191
SHLD1	0.476642612	0.818075899	0.970960412	0.194298763	0.223267292	0.607551968	0.999996191
SHLD2	0.284098424	0.991050778	0.209420472	0.594895897	0.667584613	0.676715571	0.999996191
SHMT2	0.551818569	0.52830099	0.877054891	0.376077635	0.195966085	0.634388268	0.999996191
SHPK	0.804596682	0.155766867	0.428841544	0.489621649	0.634446527	0.610752089	0.999996191
SHPRH	0.192352536	0.384544348	0.983118894	0.300091955	0.793302965	0.617826873	0.999996191
SHQ1	0.629002354	0.804958454	0.830223055	0.578298737	0.711255751	0.966755443	0.999996191
SHROOM1	0.835405591	0.817147009	0.703908067	0.856876477	0.547387659	0.981893534	0.999996191
SHROOM4	0.897065004	0.707158786	0.858614455	0.997016423	0.401193534	0.980278541	0.999996191
SIAH2	0.639530979	0.346017447	0.945280134	0.304475405	0.58825827	0.765444315	0.999996191
SIDT2	0.568208054	0.929974671	0.612102238	0.536480585	0.302671249	0.824162355	0.999996191
SIGIRR	0.719681393	0.552009511	0.043512963	0.582481584	0.819284145	0.476692963	0.999996191
SIGLEC5	0.712378256	0.996894783	0.03806233	0.927557032	0.44045689	0.530978351	0.999996191
SIGLEC8	0.085912302	0.492035215	0.766221739	0.94426011	0.731277636	0.667798389	0.999996191
SIGLECL1	0.790086866	0.911213158	0.364047826	0.333870518	0.386845469	0.74681835	0.999996191
SIK1	0.48049346	0.491894487	0.302300133	0.412928237	0.319735336	0.501374654	0.999996191
SIK2	0.443612216	0.987116353	0.081267792	0.869083671	0.842161375	0.697260008	0.999996191
SIK3	0.835097195	0.736354043	0.248769888	0.562759472	0.376147041	0.73856319	0.999996191
SIKE1	0.653079681	0.722806536	0.953662752	0.97983415	0.760786039	0.994727637	0.999996191
SIL1	0.676294496	0.925224007	0.394849632	0.772888038	0.747757224	0.952053875	0.999996191
SIN3A	0.72901596	0.737025281	0.874536693	0.45771539	0.98098232	0.978665939	0.999996191
SIN3B	0.909393607	0.863501005	0.722582738	0.925853535	0.474374993	0.986158785	0.999996191
SIPA1	0.718157942	0.194353831	0.453073056	0.852770854	0.685701293	0.763060431	0.999996191
SIPA1L1	0.391517377	0.810705611	0.775303085	0.856576849	0.275640134	0.840514159	0.999996191
SIRT2	0.915733167	0.532493179	0.415454645	0.564748889	0.294436215	0.745915021	0.999996191
SIRT3	0.848384454	0.759627607	0.30216622	0.485573391	0.753506702	0.871479512	0.999996191
SIRT5	0.661596944	0.404326026	0.593691252	0.200056015	0.530410259	0.612569345	0.999996191
SIRT6	0.517409594	0.840181158	0.361167484	0.4126711	0.673659495	0.792685646	0.999996191
SIX1	0.820440247	0.524512348	0.13125772	0.93709349	0.601298896	0.735332435	0.999996191
SKA1	0.856291497	0.870017797	0.647157865	0.440063127	0.861485993	0.970417947	0.999996191
SKA2	0.323981757	0.572532077	0.207827908	0.296111136	0.973600043	0.532184319	0.999996191
SKAP2	0.808456794	0.533333359	0.417705799	0.544508725	0.605798734	0.844030382	0.999996191
SKI	0.195895353	0.994968332	0.539706191	0.90828562	0.834329534	0.887232952	0.999996191
SKIDA1	0.873368137	0.662676657	0.506460061	0.155021241	0.656997446	0.723282374	0.999996191
SKP1	0.625852682	0.413728115	0.342494049	0.716991127	0.898292279	0.837791889	0.999996191
SLAIN1	0.697670403	0.405550251	0.805446541	0.191385057	0.693252019	0.725689068	0.999996191
SLC10A3	0.604709114	0.870581989	0.44214457	0.282455131	0.517398991	0.747725593	0.999996191
SLC10A6	0.900699719	0.829433028	0.458963308	0.782109715	0.386633826	0.920126134	0.999996191
SLC10A7	0.262628651	0.47843962	0.908816111	0.782244598	0.852726688	0.880969167	0.999996191
SLC11A2	0.954829055	0.26317247	0.710527836	0.879530802	0.161553609	0.692194962	0.999996191
SLC12A4	0.478499682	0.975185736	0.270548737	0.251135822	0.554165294	0.62071344	0.999996191
SLC12A6	0.640539718	0.740610992	0.86025122	0.589602672	0.124226672	0.723516063	0.999996191
SLC12A7	0.759357907	0.660750395	0.998373999	0.518852133	0.812433704	0.978707984	0.999996191

SLC12A9	0.863306513	0.076932623	0.682811011	0.862686213	0.666586452	0.697498147	0.999996191
SLC16A10	0.830428389	0.89432262	0.961994578	0.394116107	0.911879902	0.987281968	0.999996191
SLC16A12	0.677881958	0.98252664	0.199685892	0.374494699	0.246799197	0.551418221	0.999996191
SLC16A2	0.987777082	0.945281951	0.479950382	0.991850287	0.510841921	0.982234798	0.999996191
SLC17A9	0.814879852	0.155538902	0.318443601	0.383397237	0.459881183	0.450133571	0.999996191
SLC19A3	0.283546039	0.362419519	0.460672657	0.347193219	0.686794337	0.535144048	0.999996191
SLC1A1	0.815602363	0.166778231	0.297950406	0.665225108	0.318420862	0.48397475	0.999996191
SLC1A3	0.428505231	0.944992228	0.677879284	0.121882199	0.967866084	0.73855902	0.999996191
SLC1A5	0.509790575	0.599584531	0.728530905	0.202129291	0.243904323	0.529860434	0.999996191
SLC1A7	0.328330304	0.998971085	0.517919806	0.471287704	0.472572302	0.767215918	0.999996191
SLC20A2	0.669901376	0.78197623	0.581687871	0.195125687	0.86695066	0.821066202	0.999996191
SLC22A15	0.575989066	0.786551976	0.17946334	0.879298192	0.333343316	0.680111951	0.999996191
SLC22A17	0.603496385	0.64115043	0.824604707	0.113629001	0.50900016	0.630305893	0.999996191
SLC22A3	0.768371261	0.751660053	0.937227921	0.613286558	0.291183034	0.911923993	0.999996191
SLC22A4	0.717547397	0.813019967	0.486901339	0.800217594	0.388403485	0.900690522	0.999996191
SLC22A5	0.61151111	0.861924728	0.669360144	0.680647062	0.307920438	0.876790893	0.999996191
SLC23A3	0.838366783	0.693453098	0.721168404	0.485628425	0.546227784	0.927904909	0.999996191
SLC24A3	0.531216193	0.808472993	0.354058112	0.196791532	0.574691825	0.616526923	0.999996191
SLC25A11	0.219031157	0.314185044	0.91464378	0.279836685	0.704975774	0.553356136	0.999996191
SLC25A14	0.420928191	0.592132694	0.907389994	0.257336288	0.451848472	0.699103339	0.999996191
SLC25A16	0.186713654	0.502254799	0.487496216	0.741074842	0.536261613	0.627257129	0.999996191
SLC25A17	0.745248641	0.6593131	0.64648787	0.065578224	0.784509934	0.606576498	0.999996191
SLC25A19	0.850490048	0.949856451	0.73573845	0.252680179	0.285457333	0.789537453	0.999996191
SLC25A20	0.869958806	0.929567918	0.614086457	0.278871974	0.570754319	0.886075957	0.999996191
SLC25A21	0.865852718	0.775465521	0.31885669	0.988624491	0.383635776	0.889723981	0.999996191
SLC25A23	0.944372919	0.655258714	0.303177844	0.156299563	0.840077235	0.686517028	0.999996191
SLC25A28	0.379597971	0.705912681	0.836972544	0.512411923	0.487484854	0.834686986	0.999996191
SLC25A29	0.956372944	0.834189118	0.93002655	0.658767935	0.299485089	0.954150085	0.999996191
SLC25A3	0.880220444	0.71319096	0.868140303	0.134744362	0.703761376	0.82149423	0.999996191
SLC25A32	0.264836491	0.794862974	0.319222231	0.578502205	0.974671057	0.767485281	0.999996191
SLC25A34	0.217399222	0.972426586	0.431228127	0.418479788	0.412300975	0.599124993	0.999996191
SLC25A35	0.950191617	0.851478222	0.311465525	0.309808113	0.498006064	0.77214269	0.999996191
SLC25A36	0.13370506	0.371485129	0.621321194	0.46122299	0.996429407	0.579008747	0.999996191
SLC25A37	0.749713613	0.602665574	0.351316769	0.517667125	0.909977972	0.878371901	0.999996191
SLC25A37	0.68979804	0.567573471	0.359654211	0.651678221	0.840589442	0.882713065	0.999996191
SLC25A38	0.561042988	0.410157287	0.40316789	0.811014847	0.443257939	0.744061438	0.999996191
SLC25A40	0.1393823	0.772965245	0.997023109	0.686599277	0.772730146	0.837435653	0.999996191
SLC25A41	0.82445775	0.160977667	0.682520242	0.177027522	0.832260725	0.567242734	0.999996191
SLC25A42	0.986356775	0.418185356	0.927274469	0.086115701	0.609341389	0.646704066	0.999996191
SLC25A44	0.752318294	0.568634327	0.534921837	0.801478057	0.738351471	0.947402801	0.999996191
SLC25A46	0.566148187	0.366709281	0.166125629	0.99232992	0.414301488	0.578963362	0.999996191
SLC25A53	0.613565481	0.460806246	0.348912722	0.733147025	0.543034954	0.77396255	0.999996191
SLC25A6	0.424074482	0.428391799	0.987628125	0.363689819	0.618176085	0.778749351	0.999996191
SLC26A1	0.947772184	0.209640209	0.499918098	0.862394272	0.36708759	0.73306681	0.999996191
SLC26A10	0.834684937	0.696350739	0.415990088	0.29140405	0.554988853	0.773175321	0.999996191

SLC26A6	0.638208478	0.409719551	0.388466643	0.961632198	0.578237631	0.836001947	0.999996191
SLC26A7	0.311414084	0.508046558	0.125411492	0.829441986	0.600324424	0.51000166	0.999996191
SLC27A1	0.965909935	0.300867245	0.540553481	0.772080178	0.693279756	0.894371682	0.999996191
SLC27A3	0.057822841	0.583764943	0.984702969	0.333136453	0.998329244	0.531170211	0.999996191
SLC27A4	0.301440863	0.800290651	0.805937951	0.998422755	0.953544449	0.971213276	0.999996191
SLC27A5	0.721400343	0.565702885	0.659069697	0.7274441	0.420310076	0.891424513	0.999996191
SLC27A6	0.270021543	0.959025232	0.719340561	0.692389941	0.962484858	0.939219557	0.999996191
SLC29A1	0.186136097	0.537013788	0.766820361	0.617408656	0.389850553	0.630257767	0.999996191
SLC29A3	0.511811586	0.577447859	0.475764036	0.111464648	0.780700692	0.550535772	0.999996191
SLC2A12	0.565750076	0.912519808	0.58930373	0.115258181	0.874941817	0.728436008	0.999996191
SLC2A13	0.91372009	0.175051989	0.404104208	0.242649013	0.75089626	0.543220678	0.999996191
SLC2A4RG	0.635298162	0.43771859	0.794905569	0.327811742	0.839090936	0.847656769	0.999996191
SLC30A1	0.889502896	0.551779419	0.501826652	0.040179956	0.822784103	0.474322161	0.999996191
SLC30A6	0.205172283	0.745539219	0.299482807	0.87038493	0.236094962	0.500987806	0.999996191
SLC30A7	0.445589972	0.823373673	0.974902437	0.600041539	0.241077678	0.821688487	0.999996191
SLC30A9	0.614864236	0.630693997	0.665043712	0.652648145	0.568033506	0.910601485	0.999996191
SLC33A1	0.859920745	0.42492294	0.518106489	0.433736491	0.295256407	0.683438869	0.999996191
SLC35A1	0.379158789	0.685927694	0.216348285	0.264302197	0.891292165	0.565920168	0.999996191
SLC35A2	0.424164258	0.671514492	0.967288936	0.899421523	0.892241283	0.980990862	0.999996191
SLC35B2	0.683259275	0.735730486	0.96118701	0.365316868	0.273211582	0.809882064	0.999996191
SLC35B3	0.428179361	0.714349696	0.743134629	0.778730825	0.864348112	0.957735668	0.999996191
SLC35B4	0.602495234	0.937000114	0.90292348	0.552297336	0.963299748	0.989176443	0.999996191
SLC35C1	0.522118407	0.606393827	0.279879295	0.616944287	0.361671547	0.64378297	0.999996191
SLC35C2	0.759257128	0.533297614	0.354091032	0.061403649	0.85321723	0.459765887	0.999996191
SLC35D1	0.801634888	0.880668454	0.962137881	0.953331761	0.196772111	0.941744922	0.999996191
SLC35D2	0.539041711	0.701682684	0.31666353	0.700311888	0.977000125	0.890957494	0.999996191
SLC35E1	0.70184447	0.276229029	0.185285202	0.651326516	0.559837828	0.563627782	0.999996191
SLC35E2	0.68450192	0.875708117	0.892314016	0.709248035	0.674123236	0.987134792	0.999996191
SLC35E3	0.560538675	0.771498373	0.709359073	0.907553394	0.253205089	0.869934251	0.999996191
SLC35E4	0.843833553	0.146016971	0.971250137	0.549828741	0.82532149	0.829642002	0.999996191
SLC35F5	0.238351101	0.993173279	0.254602811	0.631920928	0.822850536	0.73243405	0.999996191
SLC35F6	0.557823288	0.534307602	0.42769801	0.932380548	0.558108921	0.860959747	0.999996191
SLC36A1	0.625610022	0.907793751	0.442678583	0.689529226	0.939818009	0.962536203	0.999996191
SLC37A1	0.270770371	0.883290954	0.47654343	0.997617725	0.709253926	0.888799665	0.999996191
SLC37A3	0.920307399	0.696474997	0.161758899	0.152558225	0.727501789	0.538803896	0.999996191
SLC37A4	0.574756728	0.529471515	0.821598058	0.230158962	0.367891219	0.657112324	0.999996191
SLC38A1	0.905850184	0.447760356	0.770372024	0.083956037	0.950401503	0.688841928	0.999996191
SLC38A10	0.90278443	0.676792676	0.086593169	0.608131222	0.992984991	0.736049252	0.999996191
SLC38A3	0.508258222	0.78536486	0.102045017	0.476735295	0.565504318	0.529913666	0.999996191
SLC38A5	0.545119943	0.233707671	0.173283789	0.598833524	0.707862181	0.499883581	0.999996191
SLC38A7	0.558392477	0.911068472	0.898068739	0.965247144	0.654594802	0.991093968	0.999996191
SLC39A1	0.855891441	0.948390001	0.644316861	0.66831146	0.260980659	0.904822916	0.999996191
SLC39A10	0.661515256	0.876776788	0.356283896	0.080711775	0.642716765	0.525345985	0.999996191
SLC39A11	0.31805117	0.431453401	0.832937317	0.920746285	0.631625166	0.861271718	0.999996191
SLC39A13	0.62405801	0.474670267	0.046270131	0.705078095	0.953336108	0.4969892	0.999996191

SLC39A2	0.729142828	0.329044876	0.142958142	0.33785815	0.879990682	0.515932544	0.999996191
SLC39A6	0.663105866	0.275988608	0.832921869	0.109182262	0.589911249	0.508824278	0.999996191
SLC39A9	0.836052981	0.834884797	0.804653614	0.980724158	0.722000362	0.997401214	0.999996191
SLC40A1	0.223051387	0.466097659	0.789681076	0.57735624	0.578104261	0.706985992	0.999996191
SLC41A2	0.331803027	0.659927545	0.902762197	0.160000092	0.865348927	0.706756141	0.999996191
SLC43A3	0.838424859	0.784487589	0.538333953	0.513991418	0.59418878	0.924845348	0.999996191
SLC44A1	0.898989478	0.403239492	0.697621001	0.162804815	0.566132047	0.675818793	0.999996191
SLC44A2	0.260728568	0.506466397	0.70507913	0.162299068	0.709104633	0.525268438	0.999996191
SLC45A4	0.778787558	0.284459281	0.554419434	0.591029326	0.192464929	0.576097868	0.999996191
SLC46A1	0.7885458	0.654559906	0.247562355	0.621192158	0.107976754	0.483673119	0.999996191
SLC46A3	0.799747832	0.852914921	0.177664119	0.486992557	0.230915023	0.571283523	0.999996191
SLC4A10	0.657057373	0.388611383	0.492625921	0.843858367	0.5709181	0.847139191	0.999996191
SLC4A1AP	0.585262952	0.917011238	0.198145599	0.995230469	0.486746639	0.820964292	0.999996191
SLC4A3	0.809785929	0.842415011	0.093788388	0.833629929	0.311380418	0.60972047	0.999996191
SLC4A4	0.520612649	0.977651455	0.832438587	0.969989837	0.33032121	0.947625676	0.999996191
SLC4A5	0.614240973	0.261573763	0.67374968	0.534944035	0.452487432	0.698409814	0.999996191
SLC4A7	0.935534372	0.745417358	0.844165262	0.857254416	0.786140384	0.997371942	0.999996191
SLC50A1	0.173545612	0.610618301	0.666994811	0.189720127	0.885107205	0.544708958	0.999996191
SLC52A2	0.571875958	0.480871993	0.404937529	0.786521071	0.916833833	0.888222882	0.999996191
SLC5A2	0.859937677	0.770741105	0.203934241	0.072427545	0.998920707	0.50808555	0.999996191
SLC6A1	0.244604401	0.758214257	0.189402163	0.99824995	0.219962263	0.464521706	0.999996191
SLC6A14	0.508137908	0.217649842	0.788162481	0.417716511	0.780814276	0.71401428	0.999996191
SLC6A3	0.418285622	0.423272886	0.90290637	0.22735646	0.933644133	0.747268907	0.999996191
SLC6A9	0.907895121	0.894486734	0.830672393	0.91750322	0.15379725	0.910069831	0.999996191
SLC7A1	0.169549825	0.407679745	0.936141671	0.252892365	0.890232375	0.584205556	0.999996191
SLC7A2	0.212338841	0.391023202	0.476069489	0.426658869	0.582432672	0.508915438	0.999996191
SLC7A6OS	0.336325659	0.839688852	0.143766986	0.700610854	0.77265074	0.664403548	0.999996191
SLC8A3	0.132068781	0.739951911	0.892162992	0.314066194	0.418196027	0.537870846	0.999996191
SLC8B1	0.495390114	0.649027821	0.218746015	0.586605306	0.770786937	0.735172959	0.999996191
SLC9A3R2	0.461434497	0.890721804	0.334633593	0.464889658	0.843959421	0.828609421	0.999996191
SLC9A7	0.875446418	0.634446538	0.088932486	0.540904163	0.963917232	0.695089662	0.999996191
SLC9A8	0.15917114	0.797642425	0.913243017	0.943394241	0.513940128	0.835242169	0.999996191
SLC9C2	0.4195546	0.50676838	0.222279019	0.972598437	0.941770013	0.791239882	0.999996191
SLCO2A1	0.397093276	0.716762305	0.515110721	0.678258666	0.610093775	0.847311813	0.999996191
SLCO3A1	0.752805397	0.511889427	0.493448345	0.446066101	0.725798742	0.849586128	0.999996191
SLCO4C1	0.392005137	0.274223722	0.837233702	0.425427429	0.423288336	0.604957071	0.999996191
SLCO5A1	0.655227967	0.416074758	0.731438334	0.630418897	0.550570747	0.867255497	0.999996191
SLFNL1	0.44365782	0.596761417	0.969781177	0.800405193	0.737680047	0.957006308	0.999996191
SLIRP	0.972021378	0.541905015	0.52697999	0.559084974	0.819291015	0.94154101	0.999996191
SLIT3	0.504778542	0.769352529	0.036338424	0.928643437	0.688172513	0.493043978	0.999996191
SLITRK4	0.847628372	0.864164959	0.185506567	0.129624941	0.605956666	0.524523517	0.999996191
SLK	0.457004383	0.200966964	0.346702516	0.915802929	0.836447204	0.684609411	0.999996191
SLU7	0.951993583	0.786111836	0.483768397	0.830599122	0.311610325	0.90814911	0.999996191
SLX4IP	0.54087015	0.81503941	0.510088108	0.859256236	0.903895954	0.967437117	0.999996191
SMAD2	0.90477841	0.02758599	0.471954764	0.921752373	0.875142705	0.502728639	0.999996191

SMAD3	0.941007453	0.870559173	0.504251579	0.451732933	0.546857825	0.918300936	0.999996191
SMAD4	0.409101403	0.422328179	0.803832452	0.35010398	0.408477631	0.644662655	0.999996191
SMAGP	0.133938737	0.948135274	0.975445131	0.886439367	0.46988065	0.821223966	0.999996191
SMAP1	0.963243152	0.36643793	0.80402713	0.849872934	0.532830577	0.942560166	0.999996191
SMARCA2	0.814877157	0.486698097	0.118956053	0.525227433	0.475188192	0.543185197	0.999996191
SMARCB1	0.359651212	0.588957577	0.234288793	0.384324657	0.522666145	0.511677907	0.999996191
SMARCD1	0.692364205	0.841056347	0.888454203	0.876143379	0.960896698	0.998334764	0.999996191
SMARCD2	0.210468084	0.819991061	0.44188873	0.391537689	0.636086975	0.635933489	0.999996191
SMARCD3	0.991405773	0.478020577	0.519673259	0.633055792	0.177708174	0.709094906	0.999996191
SMARCE1	0.937563734	0.345977263	0.534854916	0.832852117	0.610308788	0.900548742	0.999996191
SMC1A	0.787043395	0.725560413	0.804960415	0.077706379	0.920707834	0.741451087	0.999996191
SMC2	0.974024722	0.27463556	0.224241867	0.130749732	0.978886895	0.463688173	0.999996191
SMC3	0.185141425	0.76605575	0.355946137	0.318944347	0.485250135	0.466845545	0.999996191
SMC6	0.418596604	0.684770923	0.797230692	0.402315615	0.630786331	0.84021423	0.999996191
SMCHD1	0.251275815	0.908236534	0.571466359	0.12618221	0.838128891	0.573606681	0.999996191
SMCO3	0.541236834	0.863157566	0.909845527	0.704978974	0.385393157	0.931903525	0.999996191
SMCR8	0.676821232	0.92082431	0.834960376	0.083411961	0.665017287	0.716900892	0.999996191
SMDT1	0.678898608	0.310549648	0.531419751	0.112735998	0.817483972	0.518281871	0.999996191
SMG1	0.352862641	0.761746026	0.974052536	0.0903212	0.686903386	0.605396387	0.999996191
SMG5	0.426882489	0.790686179	0.413979355	0.125672682	0.410969309	0.452620465	0.999996191
SMG6	0.559444003	0.933519849	0.506700418	0.312680163	0.339432827	0.711689996	0.999996191
SMG7	0.880640004	0.131628191	0.721639646	0.938991955	0.581804733	0.800666889	0.999996191
SMG8	0.65492398	0.237127515	0.362931829	0.270047211	0.942413855	0.581203814	0.999996191
SMG9	0.199854371	0.388631676	0.961675568	0.41985308	0.565500997	0.622534411	0.999996191
SMIM1	0.931127932	0.664488527	0.193958304	0.857298739	0.449708508	0.802794127	0.999996191
SMIM11A	0.563309898	0.492245246	0.378212556	0.1564933	0.705596259	0.540006594	0.999996191
SMIM14	0.302212587	0.225337985	0.365869087	0.8043301	0.939592339	0.634245917	0.999996191
SMIM15	0.403948928	0.385252328	0.501634468	0.906003261	0.950556484	0.862963332	0.999996191
SMIM17	0.276764459	0.80189855	0.762463366	0.453123456	0.635194299	0.811556914	0.999996191
SMIM19	0.976346137	0.385979909	0.275205494	0.832302953	0.639275991	0.832243328	0.999996191
SMIM20	0.78495148	0.65981858	0.675031584	0.212720773	0.640198601	0.807702021	0.999996191
SMIM26	0.777462071	0.371172631	0.216290067	0.305304733	0.850102422	0.604863338	0.999996191
SMIM30	0.276986616	0.461223997	0.445114091	0.560095506	0.778471905	0.68776905	0.999996191
SMIM33	0.887191949	0.965061568	0.13240323	0.904797464	0.781721169	0.888021489	0.999996191
SMIM37	0.135487305	0.800240978	0.665969383	0.211432566	0.8058573	0.55158092	0.999996191
SMIM4	0.322301397	0.912730383	0.387188325	0.123428504	0.740652385	0.51986043	0.999996191
SMIM7	0.992436648	0.173490472	0.680867959	0.867783316	0.728255059	0.877065625	0.999996191
SMIM8	0.78296367	0.436490668	0.983507097	0.670535383	0.574286064	0.943233846	0.999996191
SMKR1	0.685571328	0.86692981	0.556459083	0.813344752	0.320366036	0.897581804	0.999996191
SMN2	0.888546207	0.740119645	0.63652654	0.489610448	0.071152529	0.584403287	0.999996191
SMO	0.91297485	0.588241746	0.266398352	0.933242781	0.540137605	0.873226064	0.999996191
SMOC1	0.765511263	0.582394936	0.899553292	0.653388549	0.975708575	0.98712618	0.999996191
SMOX	0.609025655	0.956326729	0.78737649	0.290239898	0.25225829	0.745305558	0.999996191
SMPD1	0.302787958	0.452022097	0.51272718	0.761258866	0.936627658	0.816101124	0.999996191
SMPD2	0.976346705	0.593946208	0.633108224	0.997826603	0.858255289	0.993316887	0.999996191

SMPD4	0.600837821	0.172515161	0.698166818	0.122233045	0.959355421	0.481858077	0.999996191
SMPD5	0.664813434	0.441811602	0.969317794	0.743216199	0.773846605	0.962907366	0.999996191
SMPDL3B	0.40917037	0.653876263	0.733208488	0.594592446	0.30237851	0.754398457	0.999996191
SMPX	0.126921348	0.939387128	0.618393915	0.903785955	0.496097981	0.742416802	0.999996191
SMS	0.819588787	0.616912507	0.85508396	0.361929324	0.610164271	0.910433629	0.999996191
SMTNL1	0.71272012	0.739105428	0.950989337	0.124286886	0.314719968	0.642027837	0.999996191
SMURF1	0.970115226	0.396398156	0.946034451	0.685893454	0.665163553	0.963888447	0.999996191
SMYD2	0.600911424	0.823369183	0.172074498	0.997149663	0.490108805	0.784259578	0.999996191
SMYD3	0.136099986	0.94975887	0.370027232	0.234058986	0.728056082	0.474503481	0.999996191
SMYD4	0.902606899	0.972381446	0.298133136	0.368987926	0.884009535	0.896331186	0.999996191
SNAI1	0.064746917	0.944060019	0.755621684	0.99579264	0.277071742	0.558338852	0.999996191
SNAP91	0.82901123	0.758106302	0.280208017	0.104902497	0.627777873	0.540290222	0.999996191
SNAPC1	0.804979452	0.435757902	0.49012098	0.437846689	0.483800612	0.760276535	0.999996191
SNAPC3	0.29464433	0.769285258	0.465561909	0.822544933	0.864017985	0.878791157	0.999996191
SNAPIN	0.849695171	0.628634778	0.385188804	0.146501621	0.826971598	0.688800467	0.999996191
SND1	0.386924334	0.793699475	0.956481226	0.705846603	0.056567518	0.542431052	0.999996191
SNHG4	0.954917931	0.37287275	0.534125533	0.148983397	0.494715417	0.576733443	0.999996191
SNIP1	0.39782578	0.52664841	0.820648225	0.31092452	0.301992432	0.60420118	0.999996191
SNN	0.940268754	0.961858637	0.145780141	0.340893227	0.540208999	0.683718851	0.999996191
SNRNP200	0.300511381	0.827997954	0.590842945	0.781916618	0.71203458	0.890796224	0.999996191
SNRNP27	0.333916092	0.943098216	0.60252399	0.101298294	0.910482195	0.619939752	0.999996191
SNRNP35	0.282380368	0.918934854	0.484314453	0.829235049	0.079341027	0.477112296	0.999996191
SNRNP40	0.533316399	0.895710448	0.556772092	0.135049085	0.994610315	0.756757359	0.999996191
SNRPA1	0.779044923	0.656217173	0.362292257	0.188398099	0.22564175	0.468232463	0.999996191
SNRPB2	0.958370902	0.238771413	0.892549303	0.393772608	0.182168682	0.58530996	0.999996191
SNRPC	0.617941007	0.613456752	0.954273952	0.83550214	0.463582774	0.950422992	0.999996191
SNRPD1	0.262011057	0.67253506	0.622394581	0.694020645	0.205945202	0.598458303	0.999996191
SNRPE	0.999742354	0.925102005	0.887500302	0.768837293	0.558961678	0.995654764	0.999996191
SNRPF	0.675842503	0.172085259	0.706304933	0.872015479	0.777383051	0.833712208	0.999996191
SNRPG	0.947119062	0.795663031	0.933027218	0.973985462	0.859380816	0.999775028	0.999996191
SNRPN	0.886097963	0.410865233	0.898815387	0.649341084	0.753409955	0.961231461	0.999996191
SNTB2	0.642785713	0.58900912	0.716542856	0.364951755	0.659432261	0.858571197	0.999996191
SNUPN	0.678857101	0.837064785	0.840783243	0.300743349	0.934803209	0.946664395	0.999996191
SNURF	0.228326929	0.90378812	0.486121632	0.521701637	0.429974986	0.668986019	0.999996191
SNW1	0.490539716	0.931155691	0.995354967	0.056059579	0.868699973	0.66583507	0.999996191
SNX1	0.814322435	0.684047209	0.656747441	0.557464231	0.942524636	0.973508409	0.999996191
SNX12	0.509211357	0.303993223	0.401855181	0.855574908	0.831183793	0.795027716	0.999996191
SNX13	0.186972396	0.35302569	0.34299084	0.952039777	0.377013598	0.473952575	0.999996191
SNX14	0.278326081	0.702004626	0.835273093	0.724708304	0.601141828	0.871173535	0.999996191
SNX15	0.685966418	0.332779598	0.911768321	0.117793383	0.899519025	0.665065371	0.999996191
SNX18	0.755625642	0.321520386	0.580275523	0.270236426	0.935577345	0.756334162	0.999996191
SNX2	0.456734156	0.679847072	0.141357697	0.220089188	0.752665156	0.453953479	0.999996191
SNX25	0.880974182	0.695965982	0.311134394	0.570722241	0.928833693	0.917252605	0.999996191
SNX27	0.701252022	0.970769904	0.884452465	0.731069594	0.438304137	0.97372725	0.999996191
SNX29	0.816758838	0.524242889	0.749601948	0.204153155	0.60908065	0.77684244	0.999996191

SNX3	0.563518775	0.376098767	0.631141099	0.559162556	0.874106374	0.858780632	0.999996191
SNX30	0.760995167	0.356272111	0.710085992	0.115564686	0.918067526	0.650127645	0.999996191
SNX31	0.793522342	0.877123021	0.569919174	0.307249251	0.92532344	0.929398737	0.999996191
SNX33	0.461122185	0.828562299	0.401130064	0.847636102	0.374026051	0.811153734	0.999996191
SNX6	0.345210938	0.89148517	0.345346679	0.530606972	0.579457785	0.740259364	0.999996191
SNX7	0.354285041	0.598253106	0.151445904	0.854403035	0.974726851	0.702251072	0.999996191
SNX8	0.996588964	0.322139059	0.204116606	0.768960606	0.748888637	0.766747674	0.999996191
SNX9	0.625004099	0.772169572	0.292685334	0.292819517	0.636284982	0.699250686	0.999996191
SOCS1	0.760377509	0.791252719	0.849936473	0.108653943	0.431446286	0.681251968	0.999996191
SOCS4	0.547655904	0.798649349	0.806492927	0.392947639	0.457012344	0.85398549	0.999996191
SOCS5	0.382838181	0.420663975	0.457927611	0.237491041	0.70384006	0.551962883	0.999996191
SOCS7	0.376870735	0.896334578	0.987012919	0.026254366	0.906203287	0.469584239	0.999996191
SOD1	0.798480382	0.77046765	0.345702347	0.331388003	0.867864731	0.848583743	0.999996191
SOD2	0.721551526	0.935380226	0.5200027	0.409251127	0.31845992	0.80082545	0.999996191
SOD3	0.364091859	0.170878087	0.383891158	0.464441734	0.916811979	0.515425564	0.999996191
SOGA1	0.776371023	0.812757217	0.129384063	0.96004788	0.544286969	0.788674286	0.999996191
SORBS1	0.641959667	0.940101472	0.270014163	0.678389631	0.743160077	0.891290828	0.999996191
SORBS3	0.75089983	0.91214317	0.794732261	0.867001967	0.093680905	0.794927542	0.999996191
SORD	0.85052877	0.738083764	0.470837107	0.254614315	0.318046153	0.680955687	0.999996191
SORL1	0.800048765	0.809462392	0.034265659	0.575743376	0.988451142	0.556599762	0.999996191
SOSTDC1	0.861523738	0.82610098	0.106478648	0.275860298	0.968148406	0.648339556	0.999996191
SOX13	0.920583357	0.38160741	0.745604904	0.896853958	0.166857923	0.773607556	0.999996191
SOX15	0.682156188	0.938282019	0.731093499	0.950705456	0.10894893	0.8107329	0.999996191
SOX17	0.382882595	0.036030292	0.970085991	0.890526487	0.91183623	0.527927295	0.999996191
SOX18	0.712237973	0.921571732	0.2227893	0.471476282	0.345427154	0.67998694	0.999996191
SP1	0.726396074	0.885247615	0.564812304	0.119391361	0.781420068	0.747002285	0.999996191
SP2	0.288053432	0.563759657	0.739528237	0.969964116	0.209675218	0.684868397	0.999996191
SP3	0.289549162	0.506042216	0.941125404	0.148877637	0.68039477	0.576057202	0.999996191
SPA17	0.681148786	0.539910244	0.725276466	0.84188217	0.548060123	0.938367461	0.999996191
SPAAR	0.746489752	0.640895814	0.270247668	0.775533696	0.432533688	0.791570381	0.999996191
SPAG1	0.672604172	0.410368017	0.809148001	0.786353271	0.230897094	0.779684952	0.999996191
SPAG16	0.887417036	0.851006732	0.860764013	0.281940861	0.679213879	0.939490413	0.999996191
SPAG9	0.6746889	0.357740945	0.339246745	0.361101086	0.918451061	0.705263692	0.999996191
SPAM1	0.997741626	0.686224615	0.780555799	0.441226472	0.887615677	0.978250533	0.999996191
SPATA21	0.968612056	0.482502835	0.782165748	0.593636533	0.604410701	0.944475298	0.999996191
SPATA22	0.409875352	0.782276799	0.4545116	0.691488063	0.689595777	0.867846872	0.999996191
SPATA24	0.922887946	0.601147887	0.163650378	0.684406644	0.904841775	0.8352679	0.999996191
SPATA2L	0.292386581	0.8994066	0.688123192	0.816198758	0.668037698	0.914368516	0.999996191
SPATA6	0.153911192	0.892193611	0.473158413	0.407869503	0.912418998	0.682921681	0.999996191
SPATA6L	0.452884875	0.906621278	0.990599624	0.645871024	0.335572372	0.90050226	0.999996191
SPATS2	0.789253502	0.370168287	0.498657885	0.997481179	0.723302133	0.921675981	0.999996191
SPATS2L	0.808323971	0.941088116	0.619342615	0.600960814	0.45991101	0.943801335	0.999996191
SPC24	0.471286942	0.40561466	0.160867945	0.76576403	0.35727779	0.480282705	0.999996191
SPC25	0.824524953	0.928060729	0.932740529	0.878091337	0.473403878	0.991855775	0.999996191
SPCS1	0.681875843	0.443333966	0.575954447	0.151887069	0.914437707	0.682943031	0.999996191

SPCS2	0.380850343	0.315456694	0.839449332	0.960772876	0.613951175	0.844241775	0.999996191
SPEF1	0.469731296	0.852602742	0.481759577	0.751708791	0.444580731	0.856686658	0.999996191
SPEF2	0.464312231	0.547716512	0.705977904	0.346188536	0.593491253	0.762613818	0.999996191
SPEG	0.241766979	0.76744411	0.258339333	0.730878078	0.228874841	0.471529005	0.999996191
SPESP1	0.502435379	0.365930114	0.439059591	0.585713287	0.566071171	0.702479199	0.999996191
SPG11	0.283703007	0.643668786	0.202488077	0.697401194	0.943881344	0.68420144	0.999996191
SPG21	0.940611127	0.486708152	0.101949452	0.79647677	0.735892463	0.706663532	0.999996191
SPG7	0.940781498	0.929182675	0.657492283	0.299454163	0.555745815	0.910653262	0.999996191
SPHK2	0.65796559	0.997617293	0.391048979	0.031543277	0.887389073	0.451833713	0.999996191
SPICE1	0.653290843	0.621511661	0.351155848	0.424460477	0.364482746	0.665107781	0.999996191
SPIN1	0.889173248	0.590694777	0.364122894	0.244342578	0.445363067	0.653781248	0.999996191
SPIN2	0.852755723	0.800957589	0.861130998	0.956278117	0.979418016	0.999616535	0.999996191
SPIN2B	0.355472469	0.253098514	0.492316102	0.353945879	0.795795115	0.55426473	0.999996191
SPIN4	0.763218372	0.895378332	0.467826343	0.998806484	0.988286807	0.993403198	0.999996191
SPINDOC	0.996825926	0.637648784	0.534294477	0.854045057	0.428787312	0.939399171	0.999996191
SPIRE1	0.343531048	0.409037049	0.398275989	0.68677337	0.654808089	0.690653139	0.999996191
SPNS1	0.314708138	0.594117169	0.474344678	0.910205389	0.401097256	0.738546514	0.999996191
SPPL2A	0.706655407	0.454309649	0.302557092	0.275795867	0.850846322	0.671477867	0.999996191
SPPL3	0.911410673	0.626810598	0.997443314	0.363338263	0.271072985	0.83497226	0.999996191
SPR	0.714314861	0.516051593	0.462368116	0.813029108	0.978962189	0.947561053	0.999996191
SPRED1	0.664983843	0.626186522	0.739866543	0.035093246	0.660987843	0.450879331	0.999996191
SPRED3	0.632661129	0.447282366	0.165135373	0.870273994	0.848837699	0.75044162	0.999996191
SPRTN	0.865684959	0.795210947	0.983502219	0.567465447	0.516322935	0.975333679	0.999996191
SPRY1	0.553067226	0.110929217	0.778942812	0.793512826	0.298407315	0.535611399	0.999996191
SPRY4	0.440264031	0.475400068	0.774920713	0.920840918	0.694116054	0.920110271	0.999996191
SPSB2	0.205207553	0.941166695	0.724054133	0.254293142	0.538892447	0.637673726	0.999996191
SPSB3	0.751884874	0.619019681	0.775545668	0.340558727	0.935703069	0.931486158	0.999996191
SPTA1	0.210593944	0.47838307	0.989181978	0.457186997	0.507510376	0.674262263	0.999996191
SPTAN1	0.682792385	0.513136364	0.094498888	0.499442783	0.651337558	0.52624218	0.999996191
SPTBN1	0.776731668	0.990740467	0.530287466	0.94524932	0.280669949	0.924972849	0.999996191
SPTBN4	0.651468054	0.750918648	0.693406182	0.059281787	0.626916891	0.556267228	0.999996191
SPTLC2	0.399953105	0.725143995	0.647675495	0.273532882	0.140154781	0.452238439	0.999996191
SPTSSA	0.730713955	0.434433251	0.541077151	0.262780946	0.186441784	0.480323607	0.999996191
SPTY2D1OS	0.686096545	0.071282341	0.539936904	0.92793032	0.683085904	0.611244202	0.999996191
SPX	0.966719389	0.466200087	0.467587698	0.179742851	0.43620496	0.608717694	0.999996191
SQLE	0.333994963	0.51149299	0.141521095	0.621523784	0.548189767	0.476427667	0.999996191
SRA1	0.541392478	0.30469217	0.437298307	0.975137297	0.434890742	0.727889443	0.999996191
SRBD1	0.959540894	0.951940028	0.086963008	0.41368628	0.588970688	0.639609598	0.999996191
SRC	0.860464294	0.870463075	0.350863734	0.493268465	0.697125507	0.903646463	0.999996191
SRCIN1	0.300947404	0.143619857	0.630061923	0.997460334	0.981578633	0.701751417	0.999996191
SRD5A1	0.453836298	0.884736245	0.451963961	0.109364902	0.914170503	0.626992542	0.999996191
SRD5A3	0.858045014	0.549604884	0.605877407	0.836945558	0.921719327	0.980841947	0.999996191
SREBF2	0.032545992	0.95676374	0.835729644	0.868848046	0.47024663	0.523804117	0.999996191
SRI	0.8285633	0.679374998	0.403766407	0.646905081	0.982593401	0.953048107	0.999996191
SRL	0.153556783	0.721484091	0.827059234	0.373909812	0.371849186	0.558286505	0.999996191

SRMS	0.875963242	0.333884166	0.821820862	0.970937205	0.294508165	0.866228102	0.999996191
SRP14	0.815090078	0.171258786	0.470218291	0.361378967	0.829273387	0.642777692	0.999996191
SRP54	0.683596281	0.6022672	0.623943255	0.344499221	0.38443442	0.747746475	0.999996191
SRP72	0.548941728	0.91080336	0.482065888	0.085313181	0.545207839	0.533835451	0.999996191
SRP9	0.273058635	0.651225545	0.71604653	0.82212828	0.68501797	0.872406193	0.999996191
SRPK1	0.76001881	0.881856552	0.624931577	0.101761387	0.341418087	0.583994185	0.999996191
SRPK2	0.588494258	0.674767255	0.307057913	0.233927723	0.818447438	0.67611648	0.999996191
SRR	0.993145414	0.502392546	0.759525371	0.62557961	0.230464992	0.830632981	0.999996191
SRRM1	0.892612397	0.244158101	0.456859325	0.245032675	0.749344261	0.628476993	0.999996191
SRRM2	0.57881519	0.494632393	0.403859918	0.304638102	0.486159929	0.615701349	0.999996191
SRRM5	0.072444305	0.720965095	0.742228839	0.563778437	0.354726863	0.465446519	0.999996191
SRRT	0.878512698	0.718080671	0.509770294	0.367542474	0.95635066	0.929644703	0.999996191
SRSF1	0.289403714	0.463908012	0.759523085	0.977397558	0.525298274	0.823640372	0.999996191
SRSF10	0.086142553	0.287685326	0.663867335	0.752701727	0.645438074	0.470952032	0.999996191
SRSF2	0.652460398	0.820666254	0.169445078	0.26767417	0.602201095	0.584965807	0.999996191
SRSF3	0.851932495	0.083585104	0.961263286	0.520848726	0.327793188	0.541750695	0.999996191
SRSF4	0.618384781	0.505948029	0.471155782	0.691828289	0.444754588	0.79937013	0.999996191
SRSF5	0.50739313	0.657204438	0.854843108	0.055974313	0.571251995	0.495005723	0.999996191
SRSF7	0.866717768	0.888721817	0.774836926	0.314653097	0.459744948	0.897824046	0.999996191
SRSF9	0.393376417	0.455982488	0.364918766	0.815558358	0.498890542	0.70153241	0.999996191
SS18	0.446086934	0.866886511	0.349759831	0.479730028	0.210597408	0.571803809	0.999996191
SS18L1	0.839083079	0.657706076	0.430280898	0.254309221	0.854273136	0.821197897	0.999996191
SSB	0.844801858	0.929495399	0.246624436	0.18273431	0.490755759	0.618447384	0.999996191
SSBP2	0.909710815	0.793962531	0.883095422	0.136056755	0.522413779	0.799289556	0.999996191
SSBP3	0.841658047	0.772774977	0.729080439	0.273784637	0.686212756	0.901847713	0.999996191
SSBP4	0.944311983	0.296495547	0.34328757	0.861224641	0.778397586	0.856575825	0.999996191
SSH1	0.917536228	0.512260973	0.871109336	0.356143984	0.055485462	0.473163862	0.999996191
SSH3	0.710871995	0.695459011	0.36866269	0.932263909	0.552269832	0.908325063	0.999996191
SSNA1	0.649679816	0.34221513	0.166187164	0.330545064	0.826981915	0.514131283	0.999996191
SSPN	0.311618233	0.453919802	0.592553784	0.366915342	0.860218543	0.700247793	0.999996191
SSR1	0.831525989	0.216009794	0.675503668	0.158546756	0.379649394	0.454742039	0.999996191
SSR2	0.302599964	0.785171769	0.689503074	0.292257717	0.840376937	0.778292757	0.999996191
SSRP1	0.29679921	0.938687054	0.274412941	0.830337232	0.544700619	0.750750655	0.999996191
SSU72	0.894581879	0.643282948	0.723066792	0.187375941	0.239789987	0.632851023	0.999996191
SSUH2	0.882469869	0.519680402	0.143506576	0.488768603	0.805593879	0.696277541	0.999996191
SSX2IP	0.946140123	0.969715627	0.879033231	0.949966047	0.530082035	0.997643604	0.999996191
ST3GAL1	0.076060864	0.521537179	0.701958924	0.673091972	0.420043995	0.46821678	0.999996191
ST3GAL2	0.775610412	0.767986784	0.413362848	0.34113006	0.90048281	0.87998011	0.999996191
ST3GAL3	0.29976086	0.707275486	0.243927095	0.483263152	0.823890284	0.651700539	0.999996191
ST3GAL4	0.654807289	0.480161596	0.62612902	0.217175827	0.297914245	0.558242851	0.999996191
ST6GAL2	0.585872494	0.598733312	0.10928561	0.419001858	0.626682369	0.513500684	0.999996191
ST6GALNAC2	0.293879593	0.754915833	0.698757179	0.61368489	0.610882782	0.840551819	0.999996191
ST6GALNAC3	0.68332855	0.719279972	0.449357047	0.871300903	0.038204184	0.455925118	0.999996191
ST6GALNAC5	0.808898833	0.877395504	0.703097262	0.726323403	0.699044896	0.986788195	0.999996191
ST6GALNAC6	0.764406687	0.57519977	0.431749098	0.976017938	0.470424164	0.899048697	0.999996191

ST7	0.454459017	0.594257729	0.862110989	0.839246815	0.74612032	0.953811705	0.999996191
ST8SIA1	0.254479664	0.881633871	0.740293044	0.233171052	0.242546198	0.50058344	0.999996191
ST8SIA5	0.57801457	0.554424746	0.11213835	0.407334326	0.575334847	0.480466843	0.999996191
STAG1	0.278588223	0.832457573	0.984493837	0.207521717	0.722344201	0.748854976	0.999996191
STAG3	0.736745006	0.502780504	0.602456953	0.960019909	0.856192152	0.970644129	0.999996191
STAM2	0.55430601	0.627358333	0.827886136	0.601471574	0.891727328	0.958461719	0.999996191
STAMBP	0.937456112	0.593216747	0.146876238	0.450501033	0.193403381	0.450140099	0.999996191
STAMBPL1	0.84495076	0.852235532	0.152384522	0.625330975	0.549067087	0.766458706	0.999996191
STARD3NL	0.692085743	0.994435501	0.879992961	0.533002829	0.702981671	0.982207416	0.999996191
STARD8	0.680315967	0.467422247	0.543897146	0.33451595	0.647878805	0.765530907	0.999996191
STAT1	0.94391593	0.426605216	0.136930255	0.259639153	0.77892992	0.532826009	0.999996191
STAT2	0.93199611	0.452493247	0.552261501	0.315029012	0.379804812	0.710195412	0.999996191
STAT3	0.485150158	0.754200126	0.98792387	0.176313403	0.575532846	0.761588292	0.999996191
STAT5B	0.299317005	0.395209049	0.475474676	0.75906265	0.558130262	0.680091227	0.999996191
STAT6	0.527403508	0.877722847	0.880651459	0.059940159	0.9243416	0.669711053	0.999996191
STAU1	0.217394551	0.925697942	0.3766761	0.481598627	0.92715606	0.746799249	0.999996191
STAU2	0.970215907	0.773337541	0.493488134	0.553966283	0.682259486	0.950316281	0.999996191
STC1	0.251301547	0.64657033	0.697177848	0.493056958	0.406602261	0.67076943	0.999996191
STIM1	0.520277663	0.882933244	0.561829789	0.545595153	0.073067456	0.517609041	0.999996191
STIM2	0.35914308	0.654752303	0.893206579	0.507556519	0.487711238	0.822494172	0.999996191
STIP1	0.825565469	0.335337242	0.948071694	0.031509781	0.926998836	0.463432389	0.999996191
STK10	0.576124195	0.931875186	0.346379458	0.317243467	0.688991016	0.780109821	0.999996191
STK11	0.89736636	0.970562068	0.329912433	0.243228681	0.826088293	0.839503596	0.999996191
STK11IP	0.923771374	0.780066289	0.581441607	0.989887931	0.943492601	0.99720212	0.999996191
STK16	0.526802522	0.655967702	0.525554286	0.413782272	0.60859704	0.800803118	0.999996191
STK17A	0.969032303	0.068048134	0.133124422	0.978790427	0.857500174	0.456310587	0.999996191
STK25	0.866758117	0.667279332	0.162902147	0.153755048	0.728842175	0.522481765	0.999996191
STK3	0.251987819	0.777982494	0.833859262	0.160702893	0.765531425	0.64709711	0.999996191
STK35	0.931669098	0.504640601	0.85453443	0.16373313	0.343801404	0.669962778	0.999996191
STK39	0.758357755	0.771914749	0.880566307	0.706921781	0.123779621	0.798404544	0.999996191
STMN1	0.328343661	0.717813245	0.594450482	0.656314078	0.339169921	0.731523921	0.999996191
STMN3	0.35005561	0.516317541	0.764091286	0.530062163	0.192362946	0.577618478	0.999996191
STMP1	0.894302447	0.73719926	0.402732122	0.448787099	0.959072583	0.930804595	0.999996191
STN1	0.988859043	0.625742056	0.674387802	0.748449518	0.683340387	0.979252058	0.999996191
STOM	0.450529564	0.242101031	0.641948668	0.416272702	0.571266485	0.610226833	0.999996191
STOML1	0.887627523	0.64155315	0.668948216	0.8755472	0.861875339	0.990972539	0.999996191
STON1	0.340932561	0.100548792	0.369086942	0.972166323	0.757509464	0.499082055	0.999996191
STON2	0.791434391	0.878387583	0.135670576	0.977510109	0.089857733	0.477465276	0.999996191
STRADA	0.64718455	0.119687663	0.860997381	0.710858241	0.939661957	0.79624921	0.999996191
STRADB	0.660914303	0.705906738	0.361040344	0.999489159	0.978331699	0.963333475	0.999996191
STRAP	0.900648034	0.684681518	0.231364785	0.509701829	0.150597244	0.529395762	0.999996191
STRIP1	0.842533404	0.625704008	0.244534074	0.232061185	0.620363792	0.631411096	0.999996191
STRN	0.499057465	0.554914193	0.903058584	0.340885585	0.96521484	0.891504219	0.999996191
STRN4	0.394486668	0.109668992	0.774992346	0.944194297	0.911565561	0.716850585	0.999996191
STUM	0.87159736	0.816663279	0.301175865	0.677894518	0.505470754	0.875856748	0.999996191

STX10	0.570291277	0.88881006	0.849456057	0.495060941	0.785054813	0.964474905	0.999996191
STX11	0.866767423	0.495370141	0.812577172	0.633474658	0.56893722	0.940472007	0.999996191
STX16	0.19866944	0.93922142	0.55793725	0.294963534	0.924787492	0.713799339	0.999996191
STX17	0.707394007	0.135331701	0.216285165	0.464563909	0.83462678	0.471757778	0.999996191
STX3	0.968850889	0.684163129	0.908540794	0.277763286	0.789967449	0.945167783	0.999996191
STX4	0.994634478	0.978305223	0.160688197	0.459968056	0.884431084	0.85461413	0.999996191
STX5	0.338736444	0.394402982	0.090328432	0.696960042	0.968289142	0.474354622	0.999996191
STX6	0.852089057	0.625906247	0.651857042	0.908276077	0.183461275	0.840044018	0.999996191
STX8	0.869419918	0.439741149	0.75216252	0.113090021	0.647515386	0.656071094	0.999996191
STXBP1	0.341589338	0.623084674	0.597227496	0.251687599	0.618822848	0.644036714	0.999996191
STXBP4	0.982929566	0.755311797	0.753435905	0.599810737	0.796907243	0.988704747	0.999996191
STXBP6	0.251845535	0.714567867	0.464147304	0.458246073	0.247904232	0.502468101	0.999996191
STYXL1	0.580371541	0.954431866	0.892366987	0.731563069	0.870937807	0.99335642	0.999996191
SUB1	0.317328395	0.143199721	0.565581141	0.953635884	0.838830538	0.651397955	0.999996191
SUCLA2	0.707657026	0.472171581	0.198676678	0.486996911	0.325240388	0.521702056	0.999996191
SUCLG1	0.781094457	0.708459248	0.469448649	0.099358938	0.416600268	0.525934301	0.999996191
SUCLG2	0.818555281	0.193037323	0.337386829	0.588941853	0.592815339	0.631980085	0.999996191
SUCNR1	0.365443717	0.978505451	0.28941781	0.32748899	0.725780522	0.686241419	0.999996191
SUCO	0.461853931	0.489405771	0.682468222	0.143390978	0.879766235	0.64067657	0.999996191
SUDS3	0.505327793	0.692447073	0.250457176	0.181785748	0.992427628	0.600130439	0.999996191
SUGCT	0.678626471	0.720056777	0.374596879	0.839842996	0.48382329	0.877622774	0.999996191
SUGP1	0.98426483	0.480128129	0.925609536	0.066976454	0.792711149	0.675109673	0.999996191
SULF1	0.70661874	0.878821178	0.825863314	0.39001937	0.501381597	0.916283116	0.999996191
SULF2	0.804337795	0.645578474	0.190499307	0.632698828	0.371327983	0.675244341	0.999996191
SULT1A1	0.740325447	0.63030254	0.088800425	0.533654819	0.524134139	0.540170254	0.999996191
SULT1B1	0.885996754	0.222893957	0.845303991	0.810792806	0.930894335	0.940664282	0.999996191
SULT1C4	0.881881117	0.611009574	0.791536723	0.356679309	0.184429215	0.711492978	0.999996191
SUMF1	0.263333863	0.91692996	0.218342621	0.985188188	0.986574036	0.820081409	0.999996191
SUMF2	0.128810331	0.699154806	0.7438722	0.466602838	0.72316346	0.669868211	0.999996191
SUMO2	0.476223452	0.479447838	0.804565409	0.112775882	0.741301691	0.594468184	0.999996191
SUMO3	0.536467696	0.403235589	0.566329773	0.324395188	0.850045848	0.746442588	0.999996191
SUN1	0.861138651	0.648743591	0.63305356	0.560841725	0.891453711	0.968264404	0.999996191
SUPT16H	0.475629161	0.747385779	0.755021047	0.095077901	0.90011322	0.67297217	0.999996191
SUPT20H	0.311430586	0.308741632	0.878695645	0.70177409	0.548095041	0.739227986	0.999996191
SUPT5H	0.748349605	0.689791851	0.846231213	0.760888695	0.691265866	0.982769102	0.999996191
SUPT6H	0.68293996	0.59725654	0.169669334	0.132726919	0.797660725	0.455319598	0.999996191
SUPT7L	0.23874256	0.548287104	0.49893929	0.487555643	0.837258019	0.701733771	0.999996191
SUSD6	0.203694932	0.936547741	0.843173723	0.944401943	0.369746067	0.835102525	0.999996191
SUV39H1	0.638756125	0.680784817	0.597357219	0.64675314	0.672167059	0.92954039	0.999996191
SV2A	0.375009124	0.862325704	0.467075232	0.835250261	0.46500686	0.842039404	0.999996191
SVBP	0.507984064	0.760102815	0.727683578	0.16504919	0.884634502	0.781753492	0.999996191
SWAP70	0.267851415	0.577853591	0.812238325	0.3750387	0.966247319	0.800131159	0.999996191
SWSAP1	0.982874114	0.496611284	0.460643124	0.621985544	0.376411112	0.824539809	0.999996191
SWT1	0.325750911	0.741756818	0.973423202	0.547799601	0.366740706	0.806410471	0.999996191
SYBU	0.214981527	0.948334038	0.869242393	0.163425159	0.616807555	0.623956162	0.999996191

SYCE1L	0.894874483	0.953919685	0.397688338	0.732479648	0.810090314	0.976192719	0.999996191
SYCE2	0.615823443	0.743052569	0.992114924	0.204838081	0.407765049	0.767623834	0.999996191
SYCP2	0.603229088	0.760776932	0.776402482	0.867329083	0.394469248	0.937425593	0.999996191
SYDE1	0.259569298	0.901417488	0.381575645	0.845626191	0.622309869	0.805431979	0.999996191
SYF2	0.76565136	0.244844518	0.161564133	0.471431312	0.572439074	0.475013626	0.999996191
SYMPK	0.573992068	0.963947996	0.488434497	0.040684467	0.968747332	0.524137864	0.999996191
SYN1	0.627377342	0.874866933	0.491437142	0.60845064	0.5659939	0.907077169	0.999996191
SYN2	0.305838549	0.644954132	0.954050547	0.628994736	0.800509106	0.909512953	0.999996191
SYN3	0.906447756	0.327113844	0.18095304	0.905703076	0.860134718	0.785069055	0.999996191
SYNDIG1	0.540316107	0.744784753	0.620926163	0.161688825	0.913328868	0.762673308	0.999996191
SYNE1	0.519341634	0.467048565	0.686214952	0.217739583	0.214284409	0.465755413	0.999996191
SYNE3	0.713490186	0.517841706	0.669830187	0.702749766	0.317976684	0.832597604	0.999996191
SYNGR2	0.103291108	0.776521504	0.738968889	0.5075681	0.550834929	0.6092929	0.999996191
SYNGR3	0.687413551	0.777474635	0.910133241	0.67721188	0.355458934	0.933337932	0.999996191
SYNJ1	0.880179862	0.76219016	0.416948505	0.710152565	0.665728701	0.945236048	0.999996191
SYNJ2BP	0.182933167	0.602772898	0.92152173	0.728152695	0.49773633	0.762315605	0.999996191
SYNPO	0.446161381	0.360644799	0.714977239	0.906161496	0.116186116	0.548589216	0.999996191
SYNPO2	0.072287328	0.51436288	0.684723462	0.994899063	0.293560999	0.457951339	0.999996191
SYNRG	0.909080038	0.89638507	0.640832587	0.969927152	0.937461758	0.998967293	0.999996191
SYT12	0.811899756	0.871688874	0.731381823	0.374081566	0.987769628	0.973212665	0.999996191
SYT15	0.873654933	0.638139243	0.85723829	0.454179741	0.321833072	0.868614025	0.999996191
SYT3	0.10690784	0.686308911	0.546038264	0.769577213	0.414350399	0.558820788	0.999996191
SYT4	0.905840239	0.50455374	0.755912001	0.992287808	0.968539152	0.994513527	0.999996191
SYT7	0.862770178	0.38421416	0.797048174	0.890525573	0.436725782	0.919099387	0.999996191
SYTL2	0.889939402	0.978904629	0.715185574	0.987397332	0.179643007	0.927221913	0.999996191
SYTL4	0.781789968	0.938794483	0.510922102	0.45710983	0.456372241	0.884653688	0.999996191
SZT2	0.99946761	0.591620805	0.133431791	0.350598758	0.336038157	0.49864105	0.999996191
TAAR1	0.93195536	0.744459161	0.689947999	0.140093415	0.744258977	0.815680002	0.999996191
TAC3	0.831029896	0.114763788	0.266610083	0.996787554	0.314425851	0.470420277	0.999996191
TACC2	0.217432066	0.782650519	0.258525272	0.26051386	0.686687735	0.46815737	0.999996191
TACR2	0.934695412	0.223121914	0.47087683	0.266275551	0.85538768	0.667813604	0.999996191
TADA1	0.758346173	0.558699018	0.931021669	0.590988907	0.080773398	0.634248805	0.999996191
TADA2A	0.9838703	0.855607109	0.900779459	0.153871893	0.6687379	0.884302483	0.999996191
TADA2B	0.628814702	0.452279909	0.458027808	0.531286328	0.816809328	0.836132336	0.999996191
TADA3	0.397341218	0.124421617	0.7051875	0.805077265	0.641266444	0.625426257	0.999996191
TAF1	0.395489725	0.612068918	0.642693267	0.225304719	0.762352371	0.702173527	0.999996191
TAF10	0.370813842	0.334418566	0.384555978	0.675576908	0.547407687	0.621446135	0.999996191
TAF12	0.857346685	0.801367236	0.987290339	0.961466871	0.613521977	0.997473392	0.999996191
TAF13	0.377127547	0.654819192	0.936059869	0.395431367	0.635269023	0.840421887	0.999996191
TAF1B	0.743722821	0.692411327	0.545267213	0.153801914	0.3837549	0.609311752	0.999996191
TAF1C	0.680344097	0.360794435	0.219898099	0.609507681	0.700218368	0.673541208	0.999996191
TAF2	0.638490977	0.358471739	0.173411988	0.301104695	0.960256849	0.538285164	0.999996191
TAF3	0.898805973	0.996335995	0.740052113	0.228755731	0.774023884	0.933560504	0.999996191
TAF4	0.804216529	0.9170415	0.97934278	0.214132671	0.303881589	0.805486753	0.999996191
TAF5	0.045862985	0.719195948	0.602826327	0.522660113	0.98817372	0.517259193	0.999996191

TAF5L	0.607494208	0.670692376	0.588447738	0.296014014	0.431286295	0.727999704	0.999996191
TAF6	0.953896154	0.251348671	0.618417009	0.48891539	0.877082338	0.854550723	0.999996191
TAF6L	0.838231667	0.967123501	0.263841543	0.058666923	0.878690219	0.530680901	0.999996191
TAF7	0.600613442	0.629467766	0.961762139	0.494037012	0.93056214	0.964398739	0.999996191
TAF8	0.517048965	0.784740354	0.061601933	0.991889912	0.342977553	0.482224318	0.999996191
TAF9B	0.802860144	0.344365323	0.873791479	0.632751857	0.896026222	0.948422901	0.999996191
TAL1	0.760761306	0.580116414	0.513081644	0.979077816	0.567067323	0.940452217	0.999996191
TALDO1	0.581528053	0.926819803	0.285133447	0.065538315	0.868207139	0.487356269	0.999996191
TAMM41	0.219772951	0.380227948	0.760904045	0.487083959	0.666956204	0.652316926	0.999996191
TANC2	0.94689961	0.605497667	0.516996454	0.480974327	0.378869197	0.828768124	0.999996191
TANGO2	0.740495941	0.817642108	0.73902065	0.661477224	0.991090762	0.991545165	0.999996191
TANGO6	0.385843407	0.623319283	0.79143028	0.438195426	0.263399241	0.664322484	0.999996191
TANK	0.746641526	0.334801131	0.46315273	0.14244041	0.9668744	0.601779355	0.999996191
TAOK1	0.165658329	0.575258236	0.794361207	0.415715304	0.782393134	0.686421162	0.999996191
TAOK2	0.566821408	0.141449712	0.321892939	0.838660733	0.546133444	0.543930018	0.999996191
TAPBPL	0.827783688	0.677388655	0.415446629	0.799446443	0.863979283	0.961612146	0.999996191
TARBP2	0.740584398	0.31647238	0.235246418	0.34694341	0.494032667	0.501710611	0.999996191
TARS	0.574384248	0.743937776	0.340797657	0.784435947	0.251098321	0.715701496	0.999996191
TARS2	0.783722498	0.413740876	0.262407432	0.2916053	0.996615549	0.68725472	0.999996191
TARSL2	0.925917634	0.198223493	0.583501571	0.218287213	0.457841205	0.525054842	0.999996191
TASP1	0.76778533	0.590156853	0.902460517	0.879030751	0.903346024	0.994043861	0.999996191
TATDN2	0.515221197	0.750805854	0.710645974	0.298515454	0.39058669	0.736650656	0.999996191
TAX1BP3	0.056151658	0.392839692	0.963642475	0.676366254	0.779570324	0.533789785	0.999996191
TAZ	0.970502106	0.494122527	0.561865406	0.058054546	0.940317122	0.586073163	0.999996191
TBC1D1	0.57677803	0.482235184	0.298440697	0.893631942	0.668356225	0.814556947	0.999996191
TBC1D10A	0.787303954	0.805458694	0.94622141	0.680077979	0.231670928	0.909233256	0.999996191
TBC1D10B	0.494660929	0.919168753	0.563078529	0.311280492	0.744198496	0.843759849	0.999996191
TBC1D10C	0.196500648	0.890701105	0.790079544	0.227840564	0.233966657	0.456399242	0.999996191
TBC1D12	0.186735263	0.850118857	0.788292183	0.332179437	0.406035495	0.612875248	0.999996191
TBC1D13	0.927775189	0.446993113	0.16228044	0.203729968	0.613491845	0.480242292	0.999996191
TBC1D16	0.681367703	0.963955321	0.389381057	0.3678978	0.369704163	0.75185289	0.999996191
TBC1D17	0.905673797	0.921002901	0.346953876	0.108675264	0.763642742	0.681618367	0.999996191
TBC1D19	0.150075871	0.418332854	0.821528635	0.298544788	0.687118756	0.522873524	0.999996191
TBC1D2	0.626823806	0.560003653	0.216711184	0.937611152	0.629902428	0.797720919	0.999996191
TBC1D20	0.830027561	0.656055108	0.847958062	0.89521832	0.738078042	0.992584269	0.999996191
TBC1D22A	0.791094575	0.836021136	0.130831312	0.427101694	0.548551334	0.648659382	0.999996191
TBC1D25	0.380275056	0.630691528	0.249502078	0.947491072	0.670844236	0.768177342	0.999996191
TBC1D2B	0.848179781	0.763228529	0.989903909	0.705092122	0.743430104	0.994746988	0.999996191
TBC1D30	0.435495043	0.540521249	0.340088107	0.918674505	0.862386099	0.854169916	0.999996191
TBC1D31	0.585007377	0.17496949	0.976548782	0.979295235	0.471759879	0.802470788	0.999996191
TBC1D5	0.466811932	0.871880155	0.889081115	0.799213757	0.861917023	0.986167885	0.999996191
TBC1D7	0.942223156	0.988209255	0.400655902	0.709234428	0.996761453	0.988235221	0.999996191
TBC1D8	0.695479285	0.887950677	0.498030102	0.815787395	0.786331814	0.975024001	0.999996191
TBC1D8B	0.713226001	0.412886394	0.16848066	0.43976198	0.826868743	0.625885945	0.999996191
TBC1D9	0.74819056	0.209249901	0.330119774	0.876218579	0.736375421	0.744037017	0.999996191

TBC1D9B	0.634881629	0.93705398	0.262862162	0.823923561	0.374495771	0.809975208	0.999996191
TBCA	0.666782183	0.368038398	0.297891245	0.752157805	0.592550963	0.7397099	0.999996191
TBCB	0.240887261	0.914446092	0.781999116	0.350408766	0.661986625	0.777054444	0.999996191
TBCC	0.926093211	0.792113519	0.671527439	0.218626027	0.392599844	0.787102456	0.999996191
TBCCD1	0.849913551	0.938568363	0.829818025	0.522150146	0.764870782	0.988318997	0.999996191
TBCD	0.563069736	0.41738603	0.601715172	0.521937819	0.637716088	0.805744121	0.999996191
TBCE	0.977401291	0.814817768	0.97431688	0.1724989	0.66400162	0.901542688	0.999996191
TBCEL	0.66191896	0.515714081	0.933061441	0.872184112	0.775560552	0.979727206	0.999996191
TBCK	0.907493236	0.978380847	0.423929743	0.756439755	0.683994526	0.974276174	0.999996191
TBKBP1	0.847438534	0.514247158	0.717645484	0.564002829	0.257590987	0.799670428	0.999996191
TBP	0.66828467	0.853353238	0.6143369	0.095331002	0.598729991	0.64598861	0.999996191
TBPL1	0.258445599	0.9947709	0.734350675	0.138303509	0.726513431	0.635696993	0.999996191
TBX18	0.949291467	0.276598153	0.905158055	0.642156265	0.711420402	0.925291114	0.999996191
TBX2	0.967042914	0.095475565	0.96921855	0.396828501	0.812610867	0.716842845	0.999996191
TBX6	0.628669863	0.905798336	0.824135942	0.444702	0.458851366	0.910794401	0.999996191
TC2N	0.248270518	0.324507037	0.999430211	0.555063663	0.892347477	0.77671487	0.999996191
TCAIM	0.552062979	0.551026311	0.21200524	0.58528139	0.883301713	0.744003029	0.999996191
TCAM1	0.990715722	0.765369297	0.9538297	0.034323627	0.662305071	0.607758176	0.999996191
TCEA3	0.285254975	0.891194054	0.667987133	0.28873625	0.280494863	0.573050048	0.999996191
TCEAL1	0.760079493	0.546998659	0.41097574	0.452109744	0.238745129	0.630194476	0.999996191
TCEAL4	0.810557659	0.489864293	0.824205462	0.668406596	0.03505441	0.463465306	0.999996191
TCEAL8	0.848499308	0.693625861	0.152906504	0.795902667	0.259047667	0.631365203	0.999996191
TCEAL9	0.740431871	0.818404061	0.134854281	0.360841554	0.692951124	0.650200555	0.999996191
TCEANC	0.274942886	0.699962543	0.76949674	0.110661698	0.520778752	0.482895991	0.999996191
TCEANC2	0.681671885	0.675677384	0.394423771	0.865002177	0.866059285	0.947850734	0.999996191
TCERG1	0.740663389	0.42928817	0.462828774	0.351360928	0.410107915	0.657430126	0.999996191
TCF12	0.432213109	0.994842047	0.368090478	0.247318884	0.334389807	0.563496847	0.999996191
TCF20	0.894433688	0.2121737	0.113261977	0.843090686	0.697288311	0.556712242	0.999996191
TCF24	0.322976413	0.669516916	0.105797996	0.450159141	0.854836308	0.488595409	0.999996191
TCF25	0.703383147	0.679688169	0.391055015	0.64617543	0.849809852	0.918994554	0.999996191
TCF3	0.783215872	0.544819933	0.646302819	0.031796639	0.950577697	0.478588635	0.999996191
TCF4	0.810806055	0.703836978	0.627375822	0.123929902	0.93858576	0.784419666	0.999996191
TCF7	0.973275471	0.92577581	0.478123295	0.054012435	0.817407562	0.636210345	0.999996191
TCF7L1	0.358746062	0.780772148	0.674097137	0.500300597	0.395403024	0.7648856	0.999996191
TCF7L2	0.991851119	0.558602381	0.667234572	0.279283337	0.29357206	0.726148307	0.999996191
TCFL5	0.75933065	0.562512294	0.153691537	0.48388225	0.479368556	0.592809147	0.999996191
TCHP	0.679219519	0.789021129	0.731449987	0.585279932	0.191585493	0.793912481	0.999996191
TCIM	0.471999195	0.313799291	0.231228131	0.787716763	0.270498781	0.454599334	0.999996191
TCN2	0.426087818	0.836966638	0.61157534	0.124669634	0.680795721	0.630911924	0.999996191
TCOF1	0.761200883	0.245357961	0.459808926	0.298073102	0.989922867	0.691966987	0.999996191
TCP1	0.785680795	0.295672484	0.493436171	0.498430557	0.196199349	0.533815657	0.999996191
TCP11L1	0.409091793	0.959199683	0.379126455	0.6313767	0.278386299	0.698013477	0.999996191
TCP11L2	0.418442424	0.824633554	0.239821044	0.606479544	0.421281714	0.656860804	0.999996191
TCTA	0.639182897	0.861564639	0.626089379	0.092667444	0.978822879	0.732041216	0.999996191
TCTEX1D1	0.154796908	0.633562787	0.615486881	0.991640001	0.234677738	0.577149746	0.999996191

TCTEX1D2	0.903012779	0.225654116	0.990714665	0.344217918	0.516058711	0.757451427	0.999996191
TCTN1	0.448503056	0.62487029	0.443998886	0.824265656	0.838953494	0.897388043	0.999996191
TCTN2	0.939999073	0.624755663	0.410498999	0.647305962	0.529648837	0.892093958	0.999996191
TCTN3	0.863370827	0.70433307	0.669842964	0.58987766	0.703092137	0.965146135	0.999996191
TDG	0.537151288	0.890868461	0.4780745	0.971517795	0.553237149	0.938281271	0.999996191
TDP2	0.606328006	0.563445177	0.335001211	0.29519069	0.577037602	0.641021512	0.999996191
TDRD10	0.519757901	0.431034758	0.17269577	0.52532791	0.640699303	0.562504032	0.999996191
TDRD6	0.733572659	0.189870205	0.604199886	0.861606005	0.983296309	0.871576806	0.999996191
TDRD7	0.76955163	0.785518803	0.467212142	0.12883699	0.717590857	0.697737284	0.999996191
TDRD9	0.245373252	0.900997149	0.415071622	0.755112491	0.860180952	0.844542793	0.999996191
TDRKH	0.453787896	0.246625217	0.298652704	0.925711501	0.727335315	0.669000815	0.999996191
TDRP	0.978479338	0.590691264	0.664100947	0.544875797	0.689041477	0.952836033	0.999996191
TEAD4	0.334578117	0.434122718	0.986891835	0.546660751	0.581610357	0.800198789	0.999996191
TECPR2	0.978513732	0.664083407	0.739840215	0.725808544	0.086444405	0.72523501	0.999996191
TECR	0.756385176	0.729302982	0.738355414	0.101626026	0.216727119	0.49206455	0.999996191
TEDC1	0.998542846	0.652231306	0.317210376	0.440273994	0.728146348	0.860725209	0.999996191
TEF	0.416049091	0.998281508	0.855196068	0.580444093	0.044394405	0.495774335	0.999996191
TEK	0.513037673	0.786191644	0.865288052	0.27901393	0.910040343	0.901172318	0.999996191
TEN1	0.950965502	0.881004737	0.632998303	0.335050273	0.992940337	0.968119409	0.999996191
TENM1	0.718578722	0.72079938	0.684724537	0.935423863	0.69859205	0.98315388	0.999996191
TENM4	0.834555656	0.90484949	0.559412934	0.063759437	0.436878519	0.543061168	0.999996191
TESK1	0.532616215	0.959128741	0.13635781	0.837951706	0.191796897	0.533571393	0.999996191
TEX10	0.773377164	0.874673228	0.996148309	0.259595673	0.599304642	0.921380487	0.999996191
TEX12	0.907085671	0.678895735	0.59813184	0.807083489	0.137797487	0.781493792	0.999996191
TEX14	0.648520814	0.554313317	0.216529498	0.520082313	0.494257857	0.64610539	0.999996191
TEX2	0.928455785	0.47284584	0.306028306	0.502395559	0.94689623	0.855343568	0.999996191
TEX261	0.84053323	0.929969102	0.418006901	0.556317004	0.913346261	0.963907932	0.999996191
TEX30	0.750712153	0.725910356	0.606645499	0.402056212	0.865433156	0.931490617	0.999996191
TEX35	0.894718747	0.877078082	0.367248848	0.431158944	0.850691954	0.922311434	0.999996191
TFAP4	0.746682133	0.512670644	0.42608125	0.870950115	0.388691987	0.832344039	0.999996191
TFDP2	0.620284352	0.967191688	0.859109982	0.263578569	0.534192871	0.87412263	0.999996191
TFE3	0.244192163	0.796693097	0.775321147	0.400471648	0.177402893	0.52528298	0.999996191
TFEB	0.200521388	0.579762618	0.978348445	0.641174064	0.812986794	0.843705986	0.999996191
TFPI	0.257358586	0.860609254	0.261746698	0.870123757	0.300558272	0.591964073	0.999996191
TFPI2	0.864741534	0.403523455	0.073745518	0.799575647	0.547550834	0.534771592	0.999996191
TFPT	0.638647312	0.694087777	0.681219034	0.733069675	0.91003256	0.976195095	0.999996191
TG	0.991521079	0.336749434	0.39279844	0.656218816	0.918174001	0.886040989	0.999996191
TGDS	0.051551617	0.702988924	0.696723502	0.874905342	0.682953792	0.591009259	0.999996191
TGFB1	0.696969929	0.531655227	0.165799497	0.444997198	0.768693744	0.655678532	0.999996191
TGFBR2	0.985714607	0.228434698	0.70905147	0.298837864	0.367240612	0.620180426	0.999996191
TGFBR3	0.92216746	0.960466513	0.958185848	0.293928868	0.36881173	0.905883319	0.999996191
TGFBRAP1	0.459075137	0.546031049	0.988165626	0.314905194	0.810078254	0.853601548	0.999996191
TGIF1	0.690313066	0.763500903	0.777904643	0.175579524	0.918377238	0.860455792	0.999996191
TGIF2	0.719881247	0.843200105	0.952287587	0.099363012	0.684713981	0.774201948	0.999996191
TGM2	0.234662899	0.27006267	0.274466202	0.788182647	0.954574475	0.563460266	0.999996191

TGM3	0.890236604	0.738796835	0.44556638	0.067719551	0.572410024	0.536343477	0.999996191
TGOLN2	0.777958437	0.411395971	0.522906371	0.470018454	0.961412764	0.87996106	0.999996191
TGS1	0.909178868	0.549014066	0.661941598	0.758024408	0.88704918	0.981220139	0.999996191
THADA	0.914640123	0.868715698	0.85466706	0.912425547	0.371465269	0.982847638	0.999996191
THAP1	0.854396282	0.227439169	0.530787033	0.366810649	0.840673509	0.735208797	0.999996191
THAP11	0.857520063	0.710324038	0.408183124	0.421396447	0.790276602	0.892332324	0.999996191
THAP12	0.512768093	0.1096324	0.269173512	0.864284322	0.880931178	0.539029639	0.999996191
THAP5	0.671028176	0.906235678	0.655275448	0.287655593	0.990653587	0.930128774	0.999996191
THAP6	0.406000529	0.862725836	0.448085184	0.720968636	0.614768443	0.867998997	0.999996191
THAP8	0.866016832	0.144704096	0.375658357	0.62036787	0.814434334	0.679737568	0.999996191
THBD	0.271676911	0.7714494	0.190847006	0.518976762	0.462728979	0.504742397	0.999996191
THEM4	0.704158334	0.905408002	0.861966891	0.25474122	0.847377152	0.934675388	0.999996191
THG1L	0.071926679	0.939346452	0.356917081	0.563083888	0.901394747	0.550595381	0.999996191
THNSL1	0.141503968	0.996361753	0.914032345	0.793456144	0.219321743	0.668322816	0.999996191
THOC1	0.409164601	0.624224237	0.262163107	0.654321359	0.925682864	0.779712209	0.999996191
THOC2	0.548320341	0.631815823	0.989269504	0.070954931	0.979348837	0.679984509	0.999996191
THOC5	0.300033579	0.983298902	0.71859962	0.500043534	0.905691246	0.911110268	0.999996191
THOC6	0.633364243	0.417221879	0.868082626	0.515673922	0.838581016	0.915000576	0.999996191
THOC7	0.261568804	0.213506925	0.627498909	0.458137947	0.623371119	0.512417463	0.999996191
THRA	0.151149365	0.911928182	0.719643474	0.326900244	0.512450125	0.609828853	0.999996191
THRAP3	0.8701112	0.917656533	0.424542033	0.876269049	0.900819004	0.98873064	0.999996191
THRB	0.915856393	0.789419305	0.135792194	0.377640888	0.855591739	0.734708887	0.999996191
THRSP	0.763492614	0.664068969	0.333527824	0.18418466	0.505821481	0.599434559	0.999996191
THSD1	0.532538157	0.286868839	0.142327725	0.520740214	0.946153255	0.525226112	0.999996191
THSD7A	0.341112302	0.87475751	0.374379781	0.341591304	0.736388224	0.711788957	0.999996191
THTPA	0.945409233	0.694387062	0.511011504	0.069910809	0.84114451	0.643338949	0.999996191
THUMPD2	0.451820534	0.432316356	0.93635255	0.896006387	0.125840851	0.651998325	0.999996191
THYN1	0.65064038	0.678303212	0.330770286	0.318562715	0.775749029	0.75854368	0.999996191
TIAM2	0.948447224	0.773379127	0.511470477	0.462954168	0.731064311	0.941410854	0.999996191
TICAM1	0.181261473	0.745204277	0.888288303	0.413288646	0.534114772	0.700476824	0.999996191
TICAM2	0.621875896	0.856939397	0.471078209	0.471788084	0.779097688	0.906253955	0.999996191
TICRR	0.922533214	0.153859854	0.702197825	0.901129028	0.537290441	0.809992257	0.999996191
TIE1	0.71567161	0.469595216	0.187666725	0.678628012	0.358931412	0.594532384	0.999996191
TIFA	0.501237284	0.50521093	0.486310925	0.353500243	0.231370027	0.513614708	0.999996191
TIGAR	0.801573081	0.147511959	0.584848174	0.580186618	0.611774081	0.685823894	0.999996191
TIGD2	0.351592926	0.861175437	0.901322263	0.135236632	0.389421502	0.581582078	0.999996191
TIGD7	0.857480533	0.587469431	0.433220935	0.46969237	0.82233903	0.894693473	0.999996191
TIMELESS	0.790989072	0.197761537	0.689995055	0.809981476	0.346270777	0.725914365	0.999996191
TIMP4	0.500145288	0.690732449	0.874433596	0.386170367	0.393871915	0.801604185	0.999996191
TINF2	0.847772041	0.85451397	0.214661991	0.594655277	0.634320386	0.842021701	0.999996191
TIPIN	0.896238159	0.622525487	0.384172467	0.605203415	0.633897051	0.891412651	0.999996191
TIPRL	0.390206413	0.094865848	0.362921921	0.941297624	0.842421393	0.524167817	0.999996191
TJAP1	0.431992641	0.336597108	0.346875691	0.777237507	0.99114894	0.772031161	0.999996191
TJP1	0.497643883	0.679002086	0.936722591	0.149886404	0.384903046	0.628248664	0.999996191
TKFC	0.502798269	0.588952185	0.986976691	0.631788373	0.923875006	0.96582956	0.999996191

TLCD1	0.545016467	0.405950358	0.52223995	0.742785819	0.384247641	0.741966658	0.999996191
TLDC1	0.708044002	0.273910477	0.778675504	0.729749901	0.971060113	0.92368833	0.999996191
TLDC2	0.710850688	0.704821588	0.168119433	0.403125068	0.207819635	0.44864101	0.999996191
TLE1	0.331674599	0.695999267	0.284328734	0.785191312	0.431101703	0.666507941	0.999996191
TLE2	0.891570106	0.448414031	0.139390117	0.685370025	0.583345369	0.667054331	0.999996191
TLE6	0.397208075	0.473699214	0.581669245	0.541430331	0.670847113	0.77613103	0.999996191
TLK2	0.640721115	0.734105702	0.929061552	0.42588373	0.739720027	0.9488752	0.999996191
TLL1	0.145417165	0.58979573	0.848229349	0.277673628	0.755804339	0.593325475	0.999996191
TLN2	0.949289566	0.738162415	0.490304921	0.961739217	0.693805666	0.982669304	0.999996191
TLNRD1	0.481339528	0.562769731	0.557872665	0.508562864	0.450592717	0.751025077	0.999996191
TLR3	0.19902657	0.754312328	0.149761957	0.453787608	0.892686901	0.494864139	0.999996191
TLR5	0.588027215	0.637936467	0.294068371	0.804709947	0.099947735	0.49002697	0.999996191
TLR9	0.592936285	0.760638417	0.591832602	0.632783497	0.17069104	0.71667042	0.999996191
TM2D2	0.59207876	0.506302877	0.914673971	0.981451465	0.762097957	0.977171698	0.999996191
TM2D3	0.918877838	0.690559494	0.898977243	0.211654828	0.864323752	0.920860622	0.999996191
TM4SF1	0.791102172	0.634072031	0.771526859	0.390833665	0.801661357	0.936892416	0.999996191
TM4SF5	0.129957679	0.918481459	0.913941193	0.526316751	0.189447264	0.528109883	0.999996191
TM6SF1	0.929649188	0.340457551	0.68203231	0.958165655	0.695221255	0.952653084	0.999996191
TM6SF2	0.117300858	0.91965171	0.833020426	0.32060885	0.968319549	0.710411738	0.999996191
TM7SF3	0.751657486	0.805353663	0.667684774	0.904461567	0.716090241	0.987976251	0.999996191
TM9SF1	0.530603775	0.930002065	0.480420493	0.63637444	0.813742736	0.938123574	0.999996191
TMA7	0.190869057	0.74883129	0.893796507	0.104497205	0.709737364	0.502187184	0.999996191
TMBIM6	0.975150585	0.564238366	0.518935636	0.858482945	0.869640256	0.97919142	0.999996191
TMC7	0.840112116	0.182873403	0.830596675	0.349348292	0.533842156	0.679846305	0.999996191
TMCO1	0.590589287	0.783652708	0.879052695	0.307694616	0.056235749	0.448214892	0.999996191
TMCO3	0.341058705	0.685941784	0.643861147	0.600480566	0.872515914	0.885861193	0.999996191
TMCO4	0.962279273	0.378086052	0.601818572	0.104122681	0.451842844	0.517837652	0.999996191
TMCO6	0.897769346	0.310200349	0.283211049	0.426905523	0.702782262	0.678740569	0.999996191
TMED8	0.927883107	0.256301951	0.946589285	0.264992477	0.175096984	0.520451353	0.999996191
TMEFF1	0.395243899	0.611754195	0.269253434	0.614106164	0.846458368	0.746768665	0.999996191
TMEFF2	0.580989775	0.434007051	0.584949562	0.132392677	0.979972516	0.637401131	0.999996191
TMEM101	0.637202123	0.180206129	0.606341768	0.512840924	0.750515962	0.702721414	0.999996191
TMEM102	0.776456946	0.821612596	0.612915137	0.641425088	0.199961146	0.816484314	0.999996191
TMEM106B	0.253616812	0.719485854	0.147448507	0.726082439	0.799523972	0.597755822	0.999996191
TMEM11	0.811084261	0.314994048	0.773768224	0.533926483	0.502824959	0.825887281	0.999996191
TMEM110	0.734238911	0.898534433	0.098878526	0.779606668	0.90334344	0.801578671	0.999996191
TMEM116	0.867403392	0.805242729	0.428668874	0.517717321	0.912618843	0.95125571	0.999996191
TMEM117	0.829449377	0.578563835	0.670342181	0.621693421	0.454231961	0.904304136	0.999996191
TMEM120B	0.689963478	0.182052041	0.633719984	0.464370224	0.99854432	0.762725727	0.999996191
TMEM121	0.537625235	0.686247397	0.713826061	0.641457732	0.097276005	0.607661303	0.999996191
TMEM121B	0.444793267	0.444711923	0.640593229	0.095172041	0.823416519	0.510948548	0.999996191
TMEM123	0.670423646	0.773138524	0.735315177	0.737236669	0.787081121	0.981003548	0.999996191
TMEM131	0.954759883	0.319324775	0.465525944	0.073184328	0.919991914	0.503778251	0.999996191
TMEM131L	0.886469142	0.91616067	0.489444705	0.963184347	0.123048164	0.805896263	0.999996191
TMEM132B	0.821354133	0.847828876	0.109005713	0.369409517	0.688969518	0.639260451	0.999996191

TMEM134	0.930965065	0.893102984	0.725403276	0.473838257	0.53870951	0.958229681	0.999996191
TMEM135	0.433180996	0.83146542	0.216607512	0.761979545	0.367780518	0.663382821	0.999996191
TMEM136	0.618143419	0.333442925	0.691330817	0.473049357	0.327370495	0.665185112	0.999996191
TMEM141	0.35613563	0.867698452	0.846661855	0.413718184	0.685692231	0.877323739	0.999996191
TMEM143	0.98551701	0.905474051	0.84542144	0.08177593	0.608010397	0.765657386	0.999996191
TMEM145	0.826037244	0.810047483	0.109754173	0.132008347	0.886611234	0.484200055	0.999996191
TMEM147	0.634730455	0.706086484	0.418930813	0.069297233	0.839304417	0.528850748	0.999996191
TMEM14A	0.492743761	0.722539418	0.187768644	0.741865684	0.418749261	0.653365645	0.999996191
TMEM14C	0.586044554	0.69277505	0.406682926	0.278726368	0.964394807	0.795599243	0.999996191
TMEM150A	0.154944331	0.804311694	0.960269808	0.580943366	0.449062547	0.731718243	0.999996191
TMEM150B	0.76307477	0.921133194	0.172857242	0.509854166	0.707041433	0.793292454	0.999996191
TMEM151A	0.471576049	0.203316445	0.85927661	0.808674458	0.244752954	0.606148726	0.999996191
TMEM154	0.590741838	0.706575781	0.659507781	0.075210826	0.64806391	0.56827639	0.999996191
TMEM158	0.814235967	0.705204697	0.254835781	0.449369381	0.684837375	0.798121488	0.999996191
TMEM159	0.738559037	0.990323188	0.514713661	0.12544131	0.921073719	0.792082155	0.999996191
TMEM161A	0.746219357	0.989305065	0.322602961	0.06430094	0.691590076	0.523062285	0.999996191
TMEM163	0.812358897	0.89214121	0.649850794	0.236357629	0.786709629	0.89965755	0.999996191
TMEM164	0.991542335	0.828323392	0.529375409	0.96730896	0.08962782	0.766548824	0.999996191
TMEM167A	0.308039736	0.622509424	0.737579808	0.821106063	0.640695069	0.877676707	0.999996191
TMEM167B	0.631717098	0.069080375	0.410521807	0.984377728	0.693889795	0.550548846	0.999996191
TMEM169	0.870499889	0.512698301	0.610400866	0.557879771	0.081526321	0.552941625	0.999996191
TMEM170B	0.720977344	0.171149047	0.293120344	0.904279879	0.821327683	0.703186646	0.999996191
TMEM175	0.80102885	0.780321332	0.777380486	0.21899251	0.845152553	0.903038578	0.999996191
TMEM178A	0.679934089	0.268818127	0.885196018	0.238000442	0.959632754	0.762934184	0.999996191
TMEM179B	0.4980679	0.535876641	0.924053547	0.355786064	0.858883018	0.879479599	0.999996191
TMEM18	0.329070484	0.698555295	0.825059484	0.421300732	0.220216456	0.62100779	0.999996191
TMEM183A	0.736743504	0.891302665	0.790935681	0.914071448	0.218765464	0.920318854	0.999996191
TMEM184A	0.956442983	0.134183598	0.667433079	0.579451505	0.271477225	0.569095715	0.999996191
TMEM184B	0.261359402	0.93347867	0.535517647	0.165053258	0.944653803	0.649605984	0.999996191
TMEM184C	0.302192849	0.526049518	0.329876064	0.944941912	0.801493286	0.775967475	0.999996191
TMEM185B	0.613866962	0.455675097	0.200472851	0.348488951	0.498043892	0.507200149	0.999996191
TMEM187	0.265130758	0.443877364	0.752020818	0.832155474	0.263283201	0.639973008	0.999996191
TMEM19	0.411224442	0.734205071	0.665341781	0.826092318	0.345705672	0.838492145	0.999996191
TMEM192	0.103695093	0.639016779	0.401092988	0.58796561	0.492037765	0.463957038	0.999996191
TMEM199	0.662498713	0.026825369	0.798600703	0.838258682	0.787213627	0.500034657	0.999996191
TMEM200B	0.14264631	0.843591279	0.730400143	0.470807103	0.245575233	0.5152803	0.999996191
TMEM201	0.635806562	0.808181045	0.747401329	0.980551001	0.07975018	0.724409864	0.999996191
TMEM205	0.480311503	0.894765794	0.368364716	0.715328789	0.30450976	0.750247517	0.999996191
TMEM214	0.606767935	0.838065183	0.735578256	0.046149228	0.86822616	0.589721119	0.999996191
TMEM218	0.795642885	0.152318304	0.618661346	0.63438026	0.2813993	0.567796752	0.999996191
TMEM219	0.310701232	0.629067133	0.423608468	0.263745407	0.802340048	0.620167216	0.999996191
TMEM220	0.768136966	0.309231288	0.127150934	0.817941851	0.943219809	0.675753964	0.999996191
TMEM229B	0.338744571	0.811063658	0.519139481	0.895668164	0.086191271	0.530420436	0.999996191
TMEM230	0.446995964	0.171403368	0.860953238	0.510540838	0.342174736	0.539069169	0.999996191
TMEM233	0.859641185	0.541845024	0.518655937	0.859296465	0.493525842	0.918759767	0.999996191

TMEM234	0.455260116	0.959057871	0.843007426	0.285658226	0.240011972	0.691174742	0.999996191
TMEM240	0.989671016	0.833566927	0.960402691	0.946393053	0.125784385	0.908945025	0.999996191
TMEM241	0.577679627	0.644213369	0.9266819	0.477823394	0.953029326	0.959772986	0.999996191
TMEM243	0.211633378	0.754414531	0.264694036	0.62279934	0.976127984	0.694623677	0.999996191
TMEM246	0.957913855	0.825372896	0.647769486	0.754955884	0.737424407	0.990734336	0.999996191
TMEM249	0.890375505	0.813765601	0.413575446	0.349219043	0.13740702	0.581674981	0.999996191
TMEM254	0.482843615	0.711532799	0.932453106	0.095018061	0.477000311	0.583560157	0.999996191
TMEM255A	0.953749779	0.710238289	0.776894127	0.63731509	0.270676356	0.904221381	0.999996191
TMEM256	0.941062517	0.953259718	0.240744855	0.131614004	0.688339497	0.641731491	0.999996191
TMEM259	0.709185257	0.68483486	0.229493114	0.321144029	0.543664416	0.640676883	0.999996191
TMEM260	0.943402252	0.759653979	0.467149507	0.838762938	0.818481011	0.98278339	0.999996191
TMEM265	0.889260387	0.148513517	0.329805308	0.518729518	0.339661674	0.463614999	0.999996191
TMEM267	0.607008411	0.163091387	0.573052125	0.725606461	0.283733491	0.541638055	0.999996191
TMEM268	0.510443621	0.957470441	0.651758614	0.127472393	0.474099097	0.638563061	0.999996191
TMEM273	0.587036006	0.642737364	0.637287298	0.786933549	0.210238764	0.776263643	0.999996191
TMEM38A	0.701012942	0.994100427	0.224640377	0.452049094	0.569331847	0.778533519	0.999996191
TMEM39A	0.892341124	0.522025036	0.407296463	0.295902907	0.892702548	0.81637334	0.999996191
TMEM39B	0.081205359	0.608261943	0.973241599	0.475849887	0.494601094	0.535579947	0.999996191
TMEM41A	0.886307422	0.321825558	0.656761889	0.179178287	0.435476671	0.584860933	0.999996191
TMEM41B	0.538056286	0.347494996	0.274384769	0.987227655	0.700295275	0.755430052	0.999996191
TMEM42	0.771689032	0.327659183	0.2981911	0.349681857	0.884329507	0.675874316	0.999996191
TMEM44	0.566910119	0.70717064	0.731362307	0.946547755	0.555229204	0.958299641	0.999996191
TMEM47	0.516192114	0.864139096	0.548132276	0.971664048	0.967247466	0.982774903	0.999996191
TMEM50B	0.45174309	0.229907523	0.780495215	0.995790117	0.29456787	0.679676285	0.999996191
TMEM51	0.680206713	0.501496994	0.502515079	0.600926722	0.086371731	0.490542916	0.999996191
TMEM53	0.590960491	0.79513841	0.319204833	0.490797167	0.865070491	0.854793613	0.999996191
TMEM63B	0.890435478	0.994748227	0.608590395	0.45956723	0.635864356	0.96000847	0.999996191
TMEM65	0.295612403	0.691340867	0.468957338	0.97751008	0.426159594	0.776905809	0.999996191
TMEM67	0.638500187	0.688019875	0.096348078	0.396475239	0.678942468	0.536908696	0.999996191
TMEM68	0.705839638	0.824126497	0.377373144	0.516473288	0.792796475	0.902969106	0.999996191
TMEM74	0.554360414	0.809058413	0.520550312	0.987200337	0.939688091	0.979987301	0.999996191
TMEM74B	0.887159204	0.864333898	0.920890057	0.772110564	0.578300831	0.993382806	0.999996191
TMEM80	0.410760319	0.283678158	0.643795616	0.104170455	0.896552276	0.447370114	0.999996191
TMEM81	0.791586695	0.818995661	0.315680104	0.705341743	0.511754121	0.8766584	0.999996191
TMEM86B	0.784388684	0.709037748	0.812554984	0.609244997	0.450639175	0.939166896	0.999996191
TMEM87B	0.955701061	0.311445232	0.972375723	0.797010942	0.506637389	0.933141905	0.999996191
TMEM88B	0.584593381	0.575049538	0.50979384	0.666339914	0.604363978	0.866837821	0.999996191
TMEM8A	0.075647941	0.839903557	0.435448305	0.981592155	0.452322442	0.551288916	0.999996191
TMEM8B	0.403213868	0.396983222	0.740057592	0.633592832	0.270163524	0.648706181	0.999996191
TMEM9	0.234593597	0.990333856	0.28329476	0.486565255	0.837859197	0.702960366	0.999996191
TMEM98	0.953219279	0.925592921	0.319723483	0.50199235	0.313283952	0.795523431	0.999996191
TMEM9B	0.99488184	0.197035802	0.355494019	0.98002246	0.395753728	0.704354611	0.999996191
TMLHE	0.84937209	0.739459725	0.644556154	0.336439025	0.964941453	0.944663509	0.999996191
TMOD1	0.632466563	0.618800556	0.626536453	0.617577012	0.432936096	0.859200566	0.999996191
TMOD2	0.903465214	0.124964815	0.991493189	0.229375496	0.476257327	0.550421643	0.999996191

TMPO	0.351529083	0.775903808	0.782846185	0.095387783	0.732987186	0.588963788	0.999996191
TMPPE	0.790604046	0.802945864	0.917973991	0.148946715	0.157691952	0.572121985	0.999996191
TMTC1	0.38823623	0.628327369	0.147476212	0.973969925	0.635059926	0.666806668	0.999996191
TMTC2	0.286709931	0.432864529	0.502584089	0.958313779	0.35977071	0.660163779	0.999996191
TMTC3	0.304909592	0.586656768	0.655754446	0.689769343	0.64608288	0.823387188	0.999996191
TMTC4	0.957243182	0.822448591	0.203072547	0.851680012	0.693115971	0.909024214	0.999996191
TMX1	0.268140859	0.930593879	0.673883286	0.193934014	0.872014161	0.714058237	0.999996191
TMX2	0.721373946	0.490866361	0.523199267	0.12392789	0.553356814	0.55775467	0.999996191
TMX4	0.844297883	0.337612878	0.122761222	0.849838411	0.730984366	0.66226313	0.999996191
TNFAIP1	0.51187624	0.321041771	0.583984579	0.323279537	0.39981608	0.553154681	0.999996191
TNFAIP3	0.821461195	0.553089733	0.805007578	0.027313026	0.925645514	0.497670325	0.999996191
TNFAIP6	0.284738892	0.845903337	0.400779067	0.19451614	0.476374631	0.491530179	0.999996191
TNFRSF11A	0.964633273	0.884733609	0.037815504	0.472121641	0.705150915	0.525780877	0.999996191
TNFRSF1A	0.401698889	0.910619579	0.727739727	0.86167347	0.504880291	0.932196561	0.999996191
TNFRSF4	0.313907391	0.495197565	0.886165077	0.751162183	0.164487126	0.614507142	0.999996191
TNFRSF8	0.983547314	0.159674799	0.896392916	0.67312629	0.139906122	0.565959951	0.999996191
TNFSF15	0.373304297	0.413593879	0.199715742	0.69843696	0.586606024	0.556673563	0.999996191
TNFSF18	0.914143218	0.759784639	0.047803599	0.482579916	0.847505424	0.570590918	0.999996191
TNFSF4	0.959847609	0.943451517	0.268044073	0.338852413	0.554278505	0.800253315	0.999996191
TNFSF9	0.683573972	0.694134457	0.295304906	0.398705497	0.261729806	0.584924447	0.999996191
TNIP1	0.828845821	0.846359081	0.344709919	0.953757742	0.589989605	0.94783461	0.999996191
TNIP2	0.796276035	0.139458825	0.91037946	0.240361094	0.584361333	0.579238536	0.999996191
TNK1	0.115858242	0.515665934	0.634903906	0.978851656	0.329299775	0.550392549	0.999996191
TNK2	0.436294969	0.821551615	0.463975253	0.528082825	0.57550915	0.817790155	0.999996191
TNKS	0.195682541	0.945015978	0.800918711	0.055504279	0.874715774	0.451978511	0.999996191
TNKS1BP1	0.731649818	0.42218817	0.547373031	0.697004594	0.68058581	0.888064163	0.999996191
TNKS2	0.570128631	0.565056018	0.15838895	0.885863745	0.671087514	0.726299411	0.999996191
TNNI3	0.616769749	0.944191374	0.82188653	0.518681206	0.999973709	0.986008316	0.999996191
TNNT1	0.345258835	0.959423117	0.61712531	0.474583503	0.703131761	0.86511785	0.999996191
TNPO1	0.539665311	0.513322584	0.243814195	0.745797112	0.904727107	0.800191842	0.999996191
TNPO3	0.974367017	0.866701115	0.511584038	0.437583268	0.452909396	0.896741162	0.999996191
TNRC6A	0.403608101	0.863062628	0.931779069	0.94493579	0.325426667	0.915722942	0.999996191
TNRC6B	0.78431842	0.207222213	0.499162495	0.700519339	0.69428373	0.774794991	0.999996191
TNRC6C	0.794154958	0.672496026	0.581753078	0.970878283	0.242699305	0.875374702	0.999996191
TNS1	0.686753126	0.513200272	0.432595455	0.955841137	0.367950087	0.827574288	0.999996191
TNS2	0.856381796	0.535915045	0.939725984	0.89967027	0.331078161	0.94252265	0.999996191
TNS3	0.730569693	0.820983959	0.742928252	0.386198688	0.76730395	0.945098916	0.999996191
TOGARAM1	0.447368727	0.833751599	0.748243321	0.107598956	0.561630614	0.612728732	0.999996191
TOM1L1	0.619231555	0.934520529	0.162856807	0.357603298	0.591566006	0.645400068	0.999996191
TOMM22	0.690297538	0.390825206	0.415814282	0.587894312	0.335119364	0.665487127	0.999996191
TOMM40L	0.460011218	0.675506839	0.404977375	0.309633418	0.194810428	0.461649442	0.999996191
TOMM6	0.565037994	0.370987988	0.664743983	0.149965239	0.467363937	0.50784348	0.999996191
TOMM7	0.506288035	0.205521512	0.362375729	0.303348852	0.724676485	0.477564762	0.999996191
TOMT	0.997459495	0.536934841	0.635434094	0.051615573	0.972080024	0.615140952	0.999996191
TOP2B	0.585577773	0.995701024	0.881169595	0.034822119	0.768740232	0.573053619	0.999996191

TOP3A	0.907736281	0.433850889	0.657990715	0.950463303	0.920976767	0.982187894	0.999996191
TOPORS	0.438580382	0.959427857	0.970554603	0.127231626	0.960345538	0.815645188	0.999996191
TOR1AIP1	0.705274703	0.794613168	0.176145574	0.614817869	0.610157589	0.763322811	0.999996191
TOR1B	0.975700597	0.471724695	0.610961331	0.293563577	0.318158787	0.698860227	0.999996191
TOR3A	0.549603223	0.846488947	0.324030649	0.72350201	0.728425106	0.886772539	0.999996191
TOR4A	0.116456338	0.942942487	0.98017117	0.082755314	0.808332858	0.452209024	0.999996191
TOX2	0.479882828	0.635538889	0.62090896	0.476750868	0.425948651	0.770162505	0.999996191
TP53	0.598694527	0.888586714	0.325364479	0.637350106	0.403426905	0.796082667	0.999996191
TP53I11	0.989256586	0.338732812	0.543866234	0.145684908	0.692719849	0.629650458	0.999996191
TP53I13	0.79315994	0.852887297	0.21756092	0.409272577	0.959280301	0.839634914	0.999996191
TP53I3	0.27962886	0.56107836	0.253781013	0.961079638	0.82276827	0.733303847	0.999996191
TP53INP2	0.768822289	0.521321308	0.98118937	0.445298805	0.75350743	0.945035445	0.999996191
TP53RK	0.849560928	0.665537413	0.638987802	0.287186097	0.654964795	0.864560531	0.999996191
TPBGL	0.439538989	0.714553443	0.18321262	0.400922575	0.700319795	0.604346043	0.999996191
TPC3	0.137733465	0.304933917	0.957081014	0.674448147	0.457896919	0.553306623	0.999996191
TPCN1	0.067125382	0.943861504	0.654201169	0.386202285	0.934058892	0.589259532	0.999996191
TPCN2	0.257158159	0.589591075	0.220842318	0.796438581	0.346419597	0.497495808	0.999996191
TPD52	0.253375101	0.194972344	0.6121336	0.938985735	0.828604287	0.677634038	0.999996191
TPGS1	0.890792277	0.808194774	0.284534006	0.501766176	0.861649568	0.901094238	0.999996191
TPM3	0.839229247	0.188117501	0.68079388	0.338009457	0.617027366	0.668236403	0.999996191
TPM4	0.580277966	0.791081755	0.981732012	0.139936073	0.6022983	0.767929653	0.999996191
TPMT	0.319510867	0.717139114	0.211641678	0.493138194	0.917051983	0.663980323	0.999996191
TPO	0.768774678	0.97335306	0.592107582	0.864159243	0.216121784	0.892251694	0.999996191
TPP2	0.801124263	0.6566832	0.322558887	0.181199109	0.499821661	0.594607657	0.999996191
TPR	0.879039303	0.466843781	0.426862669	0.305850939	0.918359797	0.813276143	0.999996191
TPRA1	0.612137119	0.720823778	0.344594894	0.642985814	0.9422856	0.906049688	0.999996191
TPRG1	0.135282316	0.689248379	0.596965882	0.976633306	0.873604253	0.80726868	0.999996191
TPRG1L	0.634706134	0.611599747	0.072648461	0.518946222	0.908925114	0.566605853	0.999996191
TPRKB	0.030886942	0.716000584	0.742377312	0.976432724	0.729194671	0.541799393	0.999996191
TPST2	0.535391683	0.842808661	0.693151242	0.62014128	0.654710044	0.94142157	0.999996191
TPT1	0.879366993	0.917165798	0.292478147	0.371603779	0.492162615	0.790644313	0.999996191
TPX2	0.66703089	0.802379351	0.260284784	0.130507533	0.773763585	0.577427066	0.999996191
TRADD	0.71176147	0.318453135	0.906102053	0.587331905	0.31249457	0.766548905	0.999996191
TRAF1	0.725172888	0.471094544	0.395696385	0.384525723	0.748931137	0.772373626	0.999996191
TRAF2	0.744902912	0.755737082	0.982864153	0.156691574	0.482592331	0.785244261	0.999996191
TRAF3IP1	0.662848151	0.647021073	0.843620986	0.336838394	0.155942125	0.636053362	0.999996191
TRAF3IP2	0.67071635	0.692633995	0.358429404	0.389821427	0.624725535	0.779686634	0.999996191
TRAF7	0.477103862	0.254312974	0.689492755	0.143646733	0.753764364	0.493853881	0.999996191
TRAFD1	0.683428368	0.863619174	0.105373458	0.973003187	0.989349743	0.845242114	0.999996191
TRAIP	0.654585646	0.572281586	0.176032887	0.182933449	0.722057015	0.486637758	0.999996191
TRAM1L1	0.441962171	0.889010377	0.559141092	0.720524361	0.914053174	0.953174931	0.999996191
TRAP1	0.991707192	0.527557905	0.592291015	0.072735849	0.786669185	0.622497639	0.999996191
TRAPPC1	0.301922706	0.685464457	0.771133662	0.158253	0.861173175	0.66236731	0.999996191
TRAPPC10	0.974759985	0.589274056	0.160139412	0.443323213	0.991953947	0.779244661	0.999996191
TRAPPC11	0.491221182	0.841527581	0.92060468	0.745754073	0.837977034	0.984267423	0.999996191

TRAPPC12	0.601951937	0.904094307	0.465418896	0.291881885	0.297522669	0.664559926	0.999996191
TRAPPC13	0.791343146	0.860762203	0.742166244	0.092765945	0.494294309	0.674747653	0.999996191
TRAPPC2	0.555540075	0.593602871	0.823017769	0.412123865	0.367444648	0.782080133	0.999996191
TRAPPC2L	0.765293229	0.507334109	0.442455193	0.583088967	0.882877901	0.900908738	0.999996191
TRAPPC4	0.731591499	0.551780322	0.556744237	0.510004453	0.507494495	0.840697358	0.999996191
TRAPPC5	0.575081868	0.610982732	0.39166649	0.080780786	0.847047759	0.501044226	0.999996191
TRAPPC6A	0.169139468	0.916895546	0.902803801	0.379259252	0.86828878	0.802195859	0.999996191
TRDN	0.14496284	0.985855723	0.931251972	0.800186891	0.817117199	0.898838437	0.999996191
TRERF1	0.980249952	0.86691054	0.122386642	0.14556861	0.533172061	0.472741105	0.999996191
TRIAP1	0.69575624	0.552067297	0.61163367	0.537064088	0.60245665	0.880678252	0.999996191
TRIB1	0.525281681	0.985386195	0.200335707	0.84933632	0.086539417	0.462377852	0.999996191
TRIM11	0.824505895	0.361004127	0.675032478	0.073949265	0.936965349	0.57540668	0.999996191
TRIM14	0.548494876	0.716668989	0.125855044	0.972732701	0.629526286	0.726053422	0.999996191
TRIM16	0.612934777	0.555169533	0.8044166	0.482725869	0.840737453	0.927784728	0.999996191
TRIM17	0.456042153	0.673584918	0.622738494	0.308614444	0.881810898	0.822704217	0.999996191
TRIM2	0.874543187	0.87338126	0.883518628	0.914157832	0.863454793	0.999509307	0.999996191
TRIM23	0.572297781	0.610587712	0.494418	0.476490866	0.42326977	0.752167672	0.999996191
TRIM27	0.4274114	0.911784474	0.835751806	0.33821246	0.716473985	0.885868975	0.999996191
TRIM3	0.801029913	0.796501615	0.815862088	0.748600396	0.512924534	0.975758376	0.999996191
TRIM31	0.125292532	0.585682551	0.340102791	0.874171519	0.53537063	0.541645637	0.999996191
TRIM32	0.447960915	0.177741955	0.982359308	0.406925668	0.955709366	0.726823301	0.999996191
TRIM33	0.136233191	0.472650504	0.902793173	0.34412518	0.81317909	0.60567946	0.999996191
TRIM35	0.996642226	0.653544374	0.613874402	0.536294356	0.062534318	0.568161185	0.999996191
TRIM38	0.818922388	0.886413609	0.210803322	0.393707569	0.35598217	0.659635405	0.999996191
TRIM4	0.898356317	0.773019415	0.370042322	0.323622335	0.564137105	0.805183015	0.999996191
TRIM41	0.650011029	0.900939145	0.33532195	0.270025013	0.717278314	0.76817069	0.999996191
TRIM45	0.536667968	0.719713382	0.98730231	0.599776596	0.642543374	0.954477273	0.999996191
TRIM47	0.41674455	0.92082389	0.938868711	0.621411241	0.380817452	0.896190767	0.999996191
TRIM52	0.292467099	0.73106249	0.937495752	0.753120469	0.332091138	0.816424945	0.999996191
TRIM56	0.894963263	0.975946292	0.706031262	0.087212106	0.26232092	0.577983159	0.999996191
TRIM62	0.498388596	0.303984575	0.824522115	0.754736249	0.243326195	0.672729487	0.999996191
TRIM65	0.237009174	0.378055654	0.826283704	0.169230238	0.609246558	0.462655159	0.999996191
TRIM68	0.595771804	0.79254275	0.925011784	0.084039814	0.372759944	0.572054341	0.999996191
TRIM8	0.576235638	0.644204928	0.956415471	0.151404137	0.683804975	0.761965934	0.999996191
TRIM9	0.774736947	0.24844185	0.347648597	0.244418704	0.810962395	0.566044963	0.999996191
TRIO	0.768603417	0.43485263	0.372226458	0.85804106	0.808706622	0.897808508	0.999996191
TRIP11	0.848487129	0.540354689	0.534589913	0.087040987	0.890391253	0.635956548	0.999996191
TRIP13	0.472611852	0.166020126	0.596365504	0.637729144	0.865010362	0.695528305	0.999996191
TRIP6	0.424046101	0.687605766	0.386369328	0.561658435	0.806084735	0.819306058	0.999996191
TRIQK	0.490865187	0.891868161	0.986258209	0.1499712	0.923349161	0.845031504	0.999996191
TRIR	0.553125622	0.767350319	0.208514846	0.503280361	0.717553713	0.736116278	0.999996191
TRIT1	0.981058286	0.890495362	0.369501831	0.348260082	0.488209289	0.831368711	0.999996191
TRMO	0.999322521	0.808819168	0.849892949	0.927703082	0.836694564	0.999512801	0.999996191
TRMT10A	0.648728106	0.381935489	0.309098678	0.220237575	0.593512919	0.512468232	0.999996191
TRMT10B	0.936082015	0.543840091	0.820685686	0.238932923	0.070735725	0.448753098	0.999996191

TRMT11	0.556270995	0.618286173	0.908801131	0.330071513	0.899950795	0.907019102	0.999996191
TRMT112	0.167359883	0.625215864	0.501877228	0.703037801	0.773637723	0.714894956	0.999996191
TRMT12	0.876062169	0.621310449	0.032250853	0.755064796	0.594994184	0.468530609	0.999996191
TRMT13	0.137122676	0.274436211	0.484049986	0.904738461	0.96616603	0.60150522	0.999996191
TRMT1L	0.539353876	0.970736237	0.482609051	0.543556269	0.707562733	0.912562003	0.999996191
TRMT2B	0.112992222	0.943616401	0.50534771	0.993588693	0.697205434	0.764758693	0.999996191
TRMT44	0.512247371	0.171232843	0.592627733	0.624098249	0.433104832	0.577193504	0.999996191
TRMT61B	0.152097566	0.600163394	0.816082878	0.818079635	0.457704999	0.710382237	0.999996191
TRMU	0.205403705	0.7415629	0.484300446	0.386459133	0.550872446	0.598817737	0.999996191
TRNAU1AP	0.507738837	0.659395378	0.702324726	0.913296003	0.267933743	0.838964657	0.999996191
TRNP1	0.906927576	0.233571457	0.412769861	0.520655483	0.872882851	0.776066576	0.999996191
TRNT1	0.839665293	0.654314882	0.794146466	0.304110558	0.17017948	0.66965623	0.999996191
TRPC2	0.651537792	0.285801887	0.4685052	0.787507882	0.566920311	0.772463617	0.999996191
TRPC3	0.668216552	0.851826824	0.255252074	0.594276787	0.569687575	0.813226659	0.999996191
TRPC4	0.196511511	0.781155153	0.79536764	0.646817378	0.872425128	0.866585701	0.999996191
TRPC4AP	0.557362964	0.764222822	0.225321621	0.450886401	0.390598463	0.613157835	0.999996191
TRPC6	0.336197682	0.794924045	0.816277232	0.100213376	0.53437518	0.54168129	0.999996191
TRPM2	0.998462859	0.161389689	0.845143218	0.211699938	0.326424861	0.500937654	0.999996191
TRPM6	0.833219523	0.525599678	0.72699423	0.674206159	0.541154166	0.932511586	0.999996191
TRPS1	0.955232269	0.266972954	0.74823644	0.752428613	0.55961348	0.888300235	0.999996191
TRPV4	0.76783477	0.226332335	0.216007261	0.762267875	0.600164649	0.616260728	0.999996191
TRRAP	0.49271936	0.749426829	0.892025615	0.312970404	0.448127947	0.802533878	0.999996191
TRUB1	0.096903571	0.689137579	0.592773545	0.522349018	0.706541142	0.584757937	0.999996191
TSC1	0.40399077	0.494766396	0.891814848	0.854678866	0.124696118	0.635980206	0.999996191
TSC22D4	0.981664849	0.485086702	0.770931046	0.092515367	0.805555062	0.706688255	0.999996191
TSEN15	0.493029962	0.560573012	0.579274253	0.871581505	0.972421848	0.947583794	0.999996191
TSEN2	0.844019028	0.876321898	0.814909378	0.429185344	0.899564448	0.98333264	0.999996191
TSEN34	0.281701986	0.89123327	0.256556631	0.989388531	0.516650816	0.741666941	0.999996191
TSG101	0.384931096	0.379199255	0.391986317	0.618523555	0.757115883	0.702691903	0.999996191
TSGA10	0.620147783	0.811550439	0.495939013	0.6407118	0.816608244	0.944070396	0.999996191
TSGA10IP	0.14540796	0.413274131	0.647911276	0.941574669	0.407022208	0.588865364	0.999996191
TSKS	0.618669545	0.783636072	0.446368374	0.071667876	0.825162391	0.55915675	0.999996191
TSNARE1	0.64622434	0.057336295	0.956444752	0.427780884	0.470930207	0.450701784	0.999996191
TSNAX	0.545013606	0.891152816	0.751470229	0.672782091	0.528219675	0.943432481	0.999996191
TSPAN11	0.511847622	0.833822335	0.309775425	0.192732286	0.45828325	0.541602039	0.999996191
TSPAN12	0.575465145	0.795866454	0.785449682	0.687216739	0.486556857	0.936082625	0.999996191
TSPAN13	0.643926167	0.299378684	0.124271155	0.501325049	0.953683663	0.537915601	0.999996191
TSPAN14	0.315421878	0.465444314	0.879636062	0.075547771	0.984851561	0.504800277	0.999996191
TSPAN15	0.558751104	0.567060214	0.727449042	0.273526895	0.961717875	0.847217626	0.999996191
TSPAN17	0.889471438	0.922479993	0.250229807	0.679644502	0.959430183	0.946367839	0.999996191
TSPAN2	0.546271777	0.403294687	0.516902059	0.630997934	0.827061863	0.844083319	0.999996191
TSPAN3	0.39895698	0.308721232	0.876772743	0.961527799	0.853170915	0.901129081	0.999996191
TSPAN31	0.389430703	0.753192093	0.183579818	0.590643861	0.400511558	0.558257959	0.999996191
TSPAN32	0.992223831	0.699213647	0.077729812	0.95987684	0.390894492	0.648289317	0.999996191
TSPAN33	0.854848108	0.586661272	0.413746685	0.824769268	0.990945588	0.965416217	0.999996191

TSPAN4	0.804698865	0.090829327	0.384418301	0.326925631	0.824430601	0.461226998	0.999996191
TSPAN5	0.547588795	0.740223645	0.331113326	0.943563673	0.880082476	0.928133867	0.999996191
TSPAN6	0.159106543	0.702779959	0.693304923	0.405704299	0.752381575	0.67874487	0.999996191
TSPEAR	0.099207145	0.924579923	0.888786645	0.578655236	0.42400271	0.646038647	0.999996191
TSPOAP1	0.785215931	0.794918944	0.338338828	0.592745538	0.257640642	0.737807902	0.999996191
TSPYL2	0.659901954	0.580165607	0.400862866	0.526715328	0.687999245	0.833507914	0.999996191
TSPYL5	0.975530624	0.982763856	0.29714116	0.603530688	0.739528936	0.941540635	0.999996191
TSSK1B	0.183040986	0.579329834	0.336344191	0.813775169	0.576246898	0.611096045	0.999996191
TST	0.713408901	0.65869828	0.562076526	0.224802314	0.955760693	0.836759747	0.999996191
TSTA3	0.588112303	0.668881081	0.605953705	0.14950257	0.486165532	0.617978693	0.999996191
TSTD1	0.786979964	0.110462185	0.906266789	0.302365376	0.511747524	0.549823096	0.999996191
TSTD2	0.70732135	0.552341622	0.973903773	0.432428205	0.224348488	0.762737046	0.999996191
TSTD3	0.792015343	0.381677667	0.457796775	0.67940505	0.66287439	0.851488321	0.999996191
TTBK2	0.404853884	0.921264075	0.575970411	0.247081048	0.357550322	0.635780952	0.999996191
TTC12	0.619604351	0.596803814	0.160803241	0.895421994	0.929190433	0.81420076	0.999996191
TTC13	0.240083418	0.590481153	0.614184971	0.159766274	0.594923521	0.477276535	0.999996191
TTC17	0.527320635	0.90759492	0.516543169	0.38758157	0.734532222	0.869698581	0.999996191
TTC21B	0.23962826	0.733358132	0.508066115	0.776308475	0.630308704	0.792847313	0.999996191
TTC23	0.840905868	0.769209815	0.476637826	0.114026691	0.900821186	0.734393951	0.999996191
TTC26	0.964254407	0.746400706	0.384439862	0.392849857	0.719881444	0.884693101	0.999996191
TTC27	0.617602562	0.83201609	0.551498523	0.323495501	0.410510246	0.766254064	0.999996191
TTC28	0.826999636	0.449106946	0.862911045	0.507579249	0.294643626	0.808841521	0.999996191
TTC3	0.657902544	0.823248636	0.20413286	0.965921418	0.669164538	0.871911493	0.999996191
TTC30A	0.374973723	0.649879719	0.302326886	0.687773046	0.435218278	0.665059806	0.999996191
TTC30B	0.895414379	0.701718076	0.970849368	0.070110863	0.551357414	0.678065811	0.999996191
TTC31	0.274982958	0.490388883	0.6294098	0.854147491	0.964563264	0.868757916	0.999996191
TTC36	0.532574567	0.930287329	0.248336106	0.187673971	0.404230825	0.499410367	0.999996191
TTC37	0.711100257	0.742033591	0.582338845	0.509397997	0.200576999	0.732771003	0.999996191
TTC39A	0.289957583	0.674161286	0.860441885	0.855904465	0.398477877	0.83848228	0.999996191
TTC5	0.82056711	0.80081833	0.761919895	0.887503861	0.410247347	0.970249456	0.999996191
TTC7A	0.484388712	0.957778462	0.237175313	0.824321451	0.859971195	0.884259107	0.999996191
TTC7B	0.792723922	0.839147678	0.456118353	0.532371691	0.736869301	0.935018545	0.999996191
TTC8	0.389975986	0.584340606	0.830626763	0.725599294	0.321246587	0.794569558	0.999996191
TTC9	0.845927575	0.813914839	0.123935056	0.742684748	0.388887814	0.686607913	0.999996191
TTF1	0.462665894	0.430403957	0.637370241	0.360730703	0.444459348	0.649398263	0.999996191
TTF2	0.674794038	0.442930977	0.808611935	0.920123209	0.761863668	0.965348034	0.999996191
TTI1	0.968332697	0.797923532	0.361070782	0.942087979	0.465225553	0.937725645	0.999996191
TTI2	0.624517668	0.940786576	0.17975042	0.688269607	0.686705886	0.815695838	0.999996191
TTK	0.950743642	0.346498964	0.073056427	0.58211817	0.715381308	0.512684495	0.999996191
TTLL1	0.340214079	0.79614802	0.829897494	0.588274323	0.427269433	0.836051449	0.999996191
TTLL4	0.718062754	0.255636512	0.828871869	0.32468832	0.889703673	0.793902452	0.999996191
TTLL7	0.564549805	0.619693462	0.599283832	0.3855664	0.419915118	0.747331895	0.999996191
TTLL9	0.349089086	0.919274377	0.617096232	0.70421195	0.986785975	0.948840507	0.999996191
TUBB1	0.646132827	0.421336997	0.591005955	0.666736975	0.682471044	0.87537964	0.999996191
TUBB3	0.864663644	0.47357946	0.759999082	0.267741632	0.660554915	0.831826342	0.999996191

TUBE1	0.342819486	0.43930272	0.599217048	0.385342152	0.311944799	0.527591857	0.999996191
TUBGCP2	0.694899515	0.951184655	0.493425922	0.167323334	0.719981313	0.774033553	0.999996191
TUBGCP3	0.727988752	0.381721612	0.104692408	0.284861195	0.860544237	0.450514799	0.999996191
TUBGCP4	0.93722014	0.780605927	0.815008257	0.172260954	0.827545026	0.895789619	0.999996191
TUFT1	0.614787124	0.13751504	0.531588928	0.664379722	0.660749799	0.643355678	0.999996191
TULP3	0.832511061	0.774719536	0.81234981	0.545081311	0.413471366	0.934205085	0.999996191
TULP4	0.574662224	0.941669946	0.208147347	0.809567993	0.950313205	0.89830046	0.999996191
TUSC1	0.865420649	0.904266812	0.800828441	0.32134143	0.184437209	0.763869517	0.999996191
TUSC2	0.630809814	0.132082517	0.460936408	0.201283868	0.988286217	0.46280219	0.999996191
TUT1	0.814108656	0.182250467	0.975845099	0.190884915	0.264968319	0.455226576	0.999996191
TWF1	0.468232904	0.702259872	0.518851391	0.629328136	0.107068684	0.538613533	0.999996191
TWIST1	0.596123227	0.934076972	0.262454898	0.206520215	0.459914918	0.574838522	0.999996191
TWSG1	0.832583385	0.625593368	0.803794511	0.394415804	0.949406545	0.959640189	0.999996191
TXLNB	0.17357223	0.864824804	0.422192947	0.713801846	0.877967604	0.775971146	0.999996191
TXLNG	0.720991291	0.758777156	0.274149602	0.702702256	0.546326003	0.839071745	0.999996191
TXN	0.375193878	0.428459874	0.767883246	0.479031688	0.196662458	0.540810094	0.999996191
TXNDC12	0.795223084	0.765137649	0.411879746	0.551124361	0.304756782	0.786307437	0.999996191
TXNDC15	0.69467789	0.459436463	0.908149065	0.550011478	0.35327577	0.835533496	0.999996191
TXNDC16	0.870225092	0.952944995	0.258036263	0.328259066	0.451287617	0.734577176	0.999996191
TXNDC17	0.363951602	0.31444605	0.743902896	0.771956429	0.5549918	0.760555688	0.999996191
TXNDC9	0.895962969	0.428718964	0.250320724	0.350152866	0.933417651	0.732954575	0.999996191
TXNIP	0.945272003	0.943100477	0.134153227	0.314902573	0.459551586	0.617774054	0.999996191
TXNL1	0.95023627	0.220261592	0.18994052	0.828233408	0.6359835	0.654982295	0.999996191
TXNL4A	0.78766834	0.6559262	0.448255767	0.107678849	0.476675633	0.545000498	0.999996191
TXNL4B	0.338365541	0.472748629	0.616000583	0.478431566	0.290408295	0.572171078	0.999996191
TXNRD2	0.821452188	0.251405592	0.940324027	0.052064405	0.977652946	0.510091815	0.999996191
TYRO3	0.76236913	0.652642841	0.716498863	0.669934971	0.661177147	0.960193811	0.999996191
TYW1	0.909839783	0.980745375	0.866231904	0.760766882	0.736176944	0.998281154	0.999996191
TYW5	0.203620369	0.675449382	0.449109606	0.488574062	0.826148775	0.688839032	0.999996191
U2AF1	0.844315543	0.679022316	0.157724716	0.83740227	0.161980688	0.550999132	0.999996191
U2AF1L4	0.673616463	0.181105519	0.920934661	0.14469295	0.966569894	0.598920039	0.999996191
U2SURP	0.211036234	0.858976134	0.898265565	0.690319713	0.718120163	0.888929254	0.999996191
UACA	0.593423297	0.432482653	0.94204075	0.132867442	0.728285361	0.676533453	0.999996191
UAP1	0.467099609	0.677843543	0.33759278	0.341145841	0.506919525	0.630631441	0.999996191
UAP1L1	0.489196484	0.93790735	0.230007983	0.956202978	0.523586099	0.825147615	0.999996191
UBA1	0.509647523	0.380089236	0.488769288	0.723557512	0.152411868	0.520378574	0.999996191
UBA5	0.537023622	0.204993064	0.512226268	0.441562092	0.380960938	0.502403996	0.999996191
UBA52	0.518055306	0.900142039	0.406224934	0.289444166	0.451837921	0.687615342	0.999996191
UBAC1	0.63726392	0.798154442	0.552247426	0.07006744	0.721468392	0.579236655	0.999996191
UBAC2	0.659778273	0.935066476	0.697749291	0.983007865	0.788441669	0.994610876	0.999996191
UBALD1	0.330458875	0.367106414	0.949721761	0.97619326	0.59560891	0.862427036	0.999996191
UBALD2	0.467627471	0.952243164	0.672022624	0.424620189	0.897989147	0.93064092	0.999996191
UBAP1	0.555309976	0.399892071	0.515508299	0.511863837	0.307770076	0.625810696	0.999996191
UBAP1L	0.30645033	0.962988454	0.859391141	0.997576187	0.456265517	0.931857932	0.999996191
UBAP2L	0.63902217	0.627913041	0.647170024	0.369240295	0.978943097	0.908358371	0.999996191

UBB	0.624402212	0.531640565	0.398567785	0.634185817	0.584563429	0.812748468	0.999996191
UBC	0.529345817	0.536262234	0.828009074	0.757895438	0.520591816	0.906873154	0.999996191
UBE2A	0.558459673	0.701457544	0.833364473	0.711679977	0.420410178	0.913168379	0.999996191
UBE2B	0.185200096	0.474217051	0.412117312	0.991878972	0.648707037	0.675651066	0.999996191
UBE2D1	0.678159694	0.455382167	0.958579108	0.852568366	0.871910043	0.980763933	0.999996191
UBE2D2	0.52973227	0.169449288	0.205093032	0.967447089	0.525580702	0.499941704	0.999996191
UBE2D4	0.624783273	0.938017323	0.737932156	0.812170965	0.582973012	0.977086896	0.999996191
UBE2E1	0.542099693	0.374681195	0.72020159	0.911141402	0.800528567	0.923358148	0.999996191
UBE2E2	0.732001415	0.492940411	0.560207922	0.576283358	0.805002261	0.908242199	0.999996191
UBE2E3	0.75793713	0.728867815	0.178298403	0.62627773	0.888961364	0.831229086	0.999996191
UBE2F	0.728306378	0.175436912	0.44316298	0.966946468	0.562896929	0.729291442	0.999996191
UBE2I	0.594979801	0.351398697	0.856822522	0.24795472	0.936304397	0.784181353	0.999996191
UBE2J1	0.649177719	0.264479485	0.937423134	0.607074419	0.794334844	0.883569674	0.999996191
UBE2J2	0.615364832	0.402508516	0.52736707	0.885201432	0.3305792	0.769074139	0.999996191
UBE2K	0.759969781	0.328530172	0.557237064	0.401851208	0.646036966	0.758765689	0.999996191
UBE2L3	0.731335267	0.578602446	0.696353345	0.093480241	0.772855639	0.658194005	0.999996191
UBE2L6	0.598704923	0.833431359	0.73911142	0.81394852	0.743407695	0.98143038	0.999996191
UBE2N	0.971899487	0.245876504	0.340059979	0.569110842	0.44617009	0.652111229	0.999996191
UBE2O	0.837038551	0.709507364	0.235263433	0.584395243	0.934320429	0.881181579	0.999996191
UBE2Q1	0.917099074	0.534408168	0.189901288	0.470961846	0.55970322	0.685729821	0.999996191
UBE2Q2	0.304601178	0.471809539	0.269065496	0.988322052	0.974964228	0.764441237	0.999996191
UBE2QL1	0.662928864	0.397535908	0.092776089	0.576936801	0.788781817	0.532405015	0.999996191
UBE2R2	0.689803048	0.671827206	0.207942302	0.218486848	0.521340991	0.529831525	0.999996191
UBE2S	0.068557031	0.638765693	0.701401395	0.856530292	0.395435578	0.519696416	0.999996191
UBE2T	0.555492314	0.467030123	0.539966805	0.47003599	0.471189334	0.730542091	0.999996191
UBE2V1	0.633093146	0.499959839	0.752482563	0.264088092	0.426365168	0.702863858	0.999996191
UBE2Z	0.645366004	0.46232596	0.113859031	0.261544112	0.839493413	0.458511355	0.999996191
UBE3C	0.918017635	0.442901364	0.828545603	0.116631954	0.895850865	0.754030019	0.999996191
UBE3D	0.177798635	0.86855969	0.890629309	0.653420604	0.415311385	0.764754507	0.999996191
UBE4A	0.847073308	0.994713098	0.191715777	0.896786213	0.336420862	0.811664723	0.999996191
UBE4B	0.65885952	0.613756975	0.586235106	0.321804211	0.172857581	0.564928439	0.999996191
UBOX5	0.540174971	0.366711862	0.400420575	0.485051219	0.780697432	0.72443456	0.999996191
UBP1	0.308631411	0.760737617	0.999151961	0.323732138	0.695353274	0.825061761	0.999996191
UBQLN2	0.365312256	0.788892547	0.275832177	0.850862373	0.108913994	0.456283411	0.999996191
UBQLN4	0.463034209	0.718646235	0.910388109	0.200995794	0.223276689	0.570817066	0.999996191
UBR1	0.234005261	0.952178671	0.707108362	0.104717657	0.864421981	0.580085858	0.999996191
UBR2	0.431735152	0.962552158	0.384264829	0.101266291	0.529792618	0.483599692	0.999996191
UBR4	0.846063142	0.567717491	0.633085368	0.079202216	0.969023419	0.676062876	0.999996191
UBTD1	0.683065462	0.797947162	0.298245102	0.59056773	0.63355414	0.847707532	0.999996191
UBTD2	0.790623047	0.668849101	0.740500597	0.351616925	0.960378681	0.945229498	0.999996191
UBTF	0.36484632	0.51131587	0.587159316	0.868412018	0.924370376	0.900173355	0.999996191
UBXN1	0.885104042	0.750788654	0.319492936	0.339892701	0.317431797	0.672443248	0.999996191
UBXN11	0.534445186	0.690022758	0.15162015	0.405048495	0.518054999	0.542505935	0.999996191
UBXN2A	0.977578758	0.364412003	0.451268422	0.712968618	0.854493084	0.913489656	0.999996191
UBXN2B	0.392677469	0.661467639	0.825752772	0.901862928	0.614341388	0.93485476	0.999996191

UBXN4	0.857950191	0.881472051	0.571758828	0.390929948	0.20029743	0.746856319	0.999996191
UBXN6	0.976086148	0.926441596	0.357456929	0.79456063	0.843442877	0.979997407	0.999996191
UBXN8	0.766334762	0.416900519	0.493163479	0.505424679	0.883499519	0.869650753	0.999996191
UCHL5	0.744421918	0.761792418	0.429179012	0.731515238	0.703106058	0.940035769	0.999996191
UFC1	0.746996189	0.40408371	0.713780826	0.295309557	0.501787136	0.735914888	0.999996191
UFD1	0.987191069	0.778553859	0.278476807	0.581025774	0.750157973	0.907602466	0.999996191
UFL1	0.800105515	0.977289343	0.476341716	0.15869863	0.436945991	0.695641034	0.999996191
UGCG	0.915997736	0.941991862	0.366515875	0.154501617	0.561905786	0.707358885	0.999996191
UGDH	0.489037879	0.697831022	0.608223522	0.482647472	0.89295433	0.902368137	0.999996191
UGGT1	0.832497123	0.519720958	0.750710342	0.731834891	0.208637418	0.814610548	0.999996191
UGP2	0.807230145	0.397706067	0.772089076	0.388306361	0.389077158	0.76535935	0.999996191
UHMK1	0.976573973	0.708117119	0.153664656	0.755498085	0.515703457	0.783379172	0.999996191
UHRF1BP1	0.955862488	0.705920363	0.03210845	0.569076389	0.865250338	0.524432704	0.999996191
ULBP21	0.895114201	0.892261182	0.645495619	0.974241361	0.558862729	0.990265148	0.999996191
ULK1	0.816618646	0.422938449	0.102207572	0.689546299	0.359590796	0.487533531	0.999996191
ULK2	0.636099392	0.99474292	0.30854223	0.379041386	0.414020225	0.72817642	0.999996191
UNC119	0.474871342	0.999732356	0.106709943	0.2860027	0.688186966	0.511721687	0.999996191
UNC119B	0.502974336	0.66452011	0.415793157	0.308506821	0.595044411	0.693272832	0.999996191
UNC13C	0.789088258	0.604110906	0.836658928	0.179710929	0.433064305	0.730629132	0.999996191
UNC45A	0.211263485	0.481846799	0.289342801	0.282154489	0.851353163	0.449106298	0.999996191
UNC45B	0.775171458	0.852581531	0.922739427	0.707650942	0.513124865	0.981063791	0.999996191
UNC50	0.382342583	0.855352871	0.828141602	0.291088981	0.724066348	0.837694391	0.999996191
UNC80	0.562239655	0.609232413	0.786036664	0.245993277	0.117049965	0.465439445	0.999996191
UNG	0.873088077	0.215360388	0.837132524	0.102925543	0.786383803	0.558290823	0.999996191
UNK	0.962279074	0.134273303	0.517446037	0.932472756	0.904847336	0.835798199	0.999996191
UNKL	0.837081735	0.474294973	0.452704796	0.528729933	0.15068581	0.580872643	0.999996191
UPF3A	0.626328515	0.786990072	0.218199189	0.881541652	0.696065665	0.860193432	0.999996191
UPK1B	0.286890893	0.215907668	0.806376247	0.918055893	0.308388034	0.578440287	0.999996191
UPP1	0.736605359	0.46000376	0.35783202	0.88708102	0.54409711	0.841653023	0.999996191
UPRT	0.705684561	0.495483245	0.684152009	0.960444998	0.379147575	0.898974861	0.999996191
UQCC1	0.895139163	0.777409121	0.767522226	0.054465569	0.906407394	0.699616859	0.999996191
UQCR11	0.852278249	0.503304632	0.8945797	0.022518656	0.875270633	0.460999634	0.999996191
UQCRB	0.845413357	0.470226242	0.482902738	0.098188563	0.995043528	0.633477711	0.999996191
UQCRC2	0.958813897	0.543888312	0.871343225	0.093239815	0.49642485	0.655835714	0.999996191
UQCRFS1	0.918508229	0.636783877	0.769567211	0.025750247	0.670620566	0.465911712	0.999996191
UQCRH	0.826105164	0.425568594	0.535667328	0.099028021	0.767319996	0.580737305	0.999996191
URB1	0.674846125	0.476259026	0.247176339	0.628611983	0.316020205	0.599771723	0.999996191
URGCP	0.040256893	0.710226687	0.59022344	0.866071457	0.950355855	0.574963428	0.999996191
URI1	0.678795728	0.770398624	0.384377241	0.564814891	0.70692862	0.888152318	0.999996191
UROC1	0.590635237	0.947180799	0.633052038	0.590259995	0.887731566	0.97137257	0.999996191
UROD	0.879811489	0.73406671	0.750801208	0.051081154	0.792880787	0.642463054	0.999996191
UROS	0.949985652	0.961143828	0.800392337	0.333971014	0.954441596	0.983379417	0.999996191
USB1	0.219911476	0.891122669	0.813380767	0.344056454	0.410687793	0.669159013	0.999996191
USE1	0.494291722	0.727232324	0.422141931	0.744043298	0.373068675	0.78642945	0.999996191
USF1	0.584800434	0.576891166	0.988010007	0.57069073	0.868072604	0.963518351	0.999996191

USF2	0.247922926	0.671867178	0.203925402	0.622153569	0.503845709	0.524077681	0.999996191
USF3	0.655039148	0.652426169	0.691155261	0.056849092	0.441804176	0.457541002	0.999996191
USO1	0.816275444	0.412104972	0.836204609	0.529137661	0.218735945	0.739570874	0.999996191
USP11	0.594740494	0.686440716	0.862856635	0.109746114	0.546523326	0.656723636	0.999996191
USP14	0.776586087	0.587622301	0.243168078	0.965314597	0.349806366	0.765465496	0.999996191
USP16	0.379765317	0.992854996	0.608880878	0.708582974	0.367668133	0.845086694	0.999996191
USP20	0.729488404	0.224866556	0.426563231	0.17507725	0.619133362	0.461505476	0.999996191
USP27X	0.737673267	0.729568654	0.921238113	0.917943757	0.650657109	0.991803407	0.999996191
USP3	0.458185996	0.878709693	0.466235775	0.352679662	0.16013706	0.523250895	0.999996191
USP30	0.976504723	0.986378179	0.688706001	0.088134449	0.164634299	0.505127647	0.999996191
USP31	0.980932635	0.981202855	0.816233962	0.170368261	0.976350758	0.944133145	0.999996191
USP33	0.399240392	0.735292278	0.816502777	0.825991797	0.538508434	0.923271524	0.999996191
USP36	0.848625015	0.866407641	0.579482727	0.567026871	0.435034825	0.921666472	0.999996191
USP37	0.675672484	0.51775971	0.831384518	0.059939239	0.983470282	0.615936165	0.999996191
USP39	0.570318925	0.987289386	0.381908031	0.161118112	0.660656796	0.672298332	0.999996191
USP40	0.316181278	0.602339803	0.502734897	0.628528321	0.642926851	0.771263304	0.999996191
USP45	0.775020881	0.617642813	0.949853752	0.804087567	0.277415087	0.91759431	0.999996191
USP46	0.924834506	0.339324578	0.81511651	0.144183476	0.724589778	0.702191801	0.999996191
USP48	0.480454444	0.9483399	0.541703139	0.054375965	0.905172712	0.549159125	0.999996191
USP49	0.894895912	0.844343096	0.231825278	0.738264215	0.516938733	0.862118143	0.999996191
USP8	0.651133506	0.643489398	0.729165211	0.192775015	0.373445162	0.664545682	0.999996191
USP9X	0.48012005	0.208388525	0.518818969	0.937526359	0.894417845	0.792203325	0.999996191
UST	0.348817116	0.784811744	0.907054141	0.178499763	0.968047607	0.789686299	0.999996191
UTP11	0.643271942	0.476869971	0.888017979	0.810391641	0.116501878	0.694823469	0.999996191
UTP20	0.892525091	0.73115262	0.80589223	0.172656707	0.21673134	0.642864018	0.999996191
UTP23	0.883761822	0.647587192	0.733042332	0.520902966	0.770502042	0.964913994	0.999996191
UTP25	0.442849602	0.152329977	0.176118657	0.638852842	0.962426127	0.454782332	0.999996191
UTP3	0.630678007	0.460537214	0.963730103	0.826581857	0.404792433	0.908088912	0.999996191
UVRAG	0.560061922	0.412245766	0.465978187	0.369659043	0.932572449	0.763602623	0.999996191
UVSSA	0.877786198	0.271771278	0.673404493	0.197824704	0.953029447	0.726003864	0.999996191
UXS1	0.989311481	0.955882918	0.030356381	0.536145342	0.837579025	0.560559749	0.999996191
VAC14	0.175943127	0.877298992	0.286170567	0.673011299	0.915533304	0.705690446	0.999996191
VAMP1	0.930491366	0.541416002	0.507678702	0.193094753	0.921939349	0.80002993	0.999996191
VAMP2	0.120107317	0.594546614	0.514422372	0.509190243	0.817306254	0.593578855	0.999996191
VAMP3	0.97545681	0.249256071	0.219128529	0.669177066	0.861210737	0.728586145	0.999996191
VAMP4	0.556530146	0.470445231	0.472865725	0.994397209	0.751711671	0.906613602	0.999996191
VAMP7	0.961086886	0.336510457	0.212539399	0.671031385	0.806139743	0.764068627	0.999996191
VAMP8	0.295609798	0.698914895	0.945267697	0.097663055	0.956548636	0.628078142	0.999996191
VAPB	0.842669204	0.473762103	0.20576928	0.97682839	0.260187022	0.654405664	0.999996191
VARS2	0.587669688	0.710389004	0.547364312	0.434664174	0.23687633	0.677630546	0.999996191
VASH1	0.707930586	0.363385604	0.086653838	0.540041369	0.718071535	0.485245334	0.999996191
VASH2	0.798146854	0.872888418	0.37219228	0.574199311	0.57841024	0.897497173	0.999996191
VAV3	0.600116363	0.611765702	0.579254627	0.559396572	0.327843517	0.772704238	0.999996191
VBP1	0.977965823	0.359336499	0.565063253	0.591748888	0.497353481	0.841437789	0.999996191
VCAM1	0.618610816	0.400721329	0.346417473	0.233459959	0.610862749	0.550703314	0.999996191

VCP	0.48229818	0.460384807	0.453503513	0.795858813	0.379404583	0.726742734	0.999996191
VDAC1	0.798809951	0.575820998	0.477466019	0.348285727	0.634192423	0.810877715	0.999996191
VDAC2	0.82257215	0.598285496	0.827266078	0.106695608	0.226179068	0.508957547	0.999996191
VDAC3	0.873568205	0.646511514	0.652503819	0.358583251	0.702962537	0.907077854	0.999996191
VEGFA	0.463613118	0.864248876	0.076828717	0.780845925	0.728326443	0.620015012	0.999996191
VEGFB	0.961744133	0.436769656	0.708450843	0.633427306	0.483283969	0.904658429	0.999996191
VEZF1	0.144805461	0.925472217	0.830283525	0.164944928	0.707673065	0.562001177	0.999996191
VGLL3	0.652375337	0.250352962	0.340034554	0.82939374	0.221113569	0.515699114	0.999996191
VGLL4	0.442725031	0.667408995	0.224828216	0.667027894	0.31807321	0.577797225	0.999996191
VIM	0.974651525	0.279274489	0.330068948	0.874550754	0.184693261	0.583456525	0.999996191
VIPAS39	0.776510984	0.520141855	0.753094133	0.087423427	0.721521583	0.637911251	0.999996191
VKORC1	0.491109963	0.637127447	0.057037769	0.938662608	0.957373855	0.602915861	0.999996191
VKORC1L1	0.890434124	0.370807948	0.547391402	0.944281236	0.637217001	0.925467499	0.999996191
VMAC	0.469018877	0.978950666	0.811266109	0.364581792	0.368086448	0.81593706	0.999996191
VN1R1	0.13926269	0.636924643	0.880262249	0.877370257	0.777254882	0.826418159	0.999996191
VPS11	0.585862652	0.713138392	0.465391857	0.910989902	0.674296752	0.935371809	0.999996191
VPS13A	0.30812273	0.508937237	0.478743097	0.204076983	0.631374856	0.5060527	0.999996191
VPS13B	0.620645518	0.863289003	0.962350976	0.223932485	0.619211456	0.871981002	0.999996191
VPS13D	0.494547066	0.989810673	0.863074548	0.239339972	0.753599168	0.881022488	0.999996191
VPS25	0.993348711	0.514112427	0.542987288	0.394913071	0.632772334	0.867429263	0.999996191
VPS26A	0.250138503	0.411507997	0.36670237	0.832999813	0.996097211	0.732317348	0.999996191
VPS26C	0.421757244	0.667310055	0.878655913	0.296773597	0.290949514	0.658780355	0.999996191
VPS28	0.273410267	0.834919316	0.60245898	0.189089038	0.636775085	0.609149511	0.999996191
VPS29	0.359589046	0.564396511	0.444869314	0.658828031	0.733821261	0.792695729	0.999996191
VPS33A	0.893657589	0.431541966	0.280346038	0.726730171	0.994289836	0.884470005	0.999996191
VPS33B	0.271955572	0.644561188	0.922113497	0.499302385	0.793075196	0.85556588	0.999996191
VPS35	0.695966011	0.403322147	0.240049734	0.924210411	0.582197443	0.759460135	0.999996191
VPS35L	0.603400488	0.982586053	0.584313668	0.478931364	0.83762355	0.949711206	0.999996191
VPS36	0.673271459	0.91582555	0.606753245	0.485520147	0.799016063	0.953435138	0.999996191
VPS37B	0.510983975	0.811786315	0.786609901	0.406185052	0.698364152	0.906632833	0.999996191
VPS37D	0.88595908	0.86629591	0.230234425	0.6176071	0.729402749	0.887038507	0.999996191
VPS39	0.720352313	0.511474621	0.496908083	0.736409318	0.702418267	0.909444108	0.999996191
VPS41	0.885740682	0.554045838	0.031917437	0.635793996	0.86295155	0.484165176	0.999996191
VPS45	0.411254912	0.955755905	0.971432772	0.264571602	0.457272339	0.802524967	0.999996191
VPS4A	0.708591183	0.678701808	0.469493748	0.225998403	0.254669559	0.562107909	0.999996191
VPS4B	0.64719663	0.741086293	0.360892215	0.834608642	0.815221225	0.933934933	0.999996191
VPS50	0.14546356	0.472370001	0.875733042	0.929630224	0.717014277	0.777731574	0.999996191
VPS51	0.951825686	0.692536431	0.572662986	0.099222062	0.680353482	0.693049624	0.999996191
VPS52	0.993907922	0.162250035	0.580106631	0.372879688	0.548209086	0.637262693	0.999996191
VPS53	0.771803865	0.574100592	0.884504496	0.690876398	0.385309434	0.920832618	0.999996191
VPS54	0.204344356	0.797276316	0.967517091	0.551056919	0.790802562	0.866142452	0.999996191
VPS72	0.795308999	0.667131015	0.465470473	0.809944048	0.297219494	0.844143428	0.999996191
VPS9D1	0.945868507	0.468623739	0.124084421	0.15805863	0.896191284	0.466331541	0.999996191
VRK1	0.54856953	0.576332924	0.808290247	0.251331264	0.868949324	0.834073591	0.999996191
VRK2	0.142496073	0.997860826	0.621063328	0.345436358	0.702756188	0.659555973	0.999996191

VRK3	0.686915856	0.797562995	0.369908224	0.611721508	0.925151962	0.931180697	0.999996191
VTA1	0.217046291	0.516293786	0.767720125	0.466239163	0.507587714	0.649497704	0.999996191
VTI1A	0.234966387	0.328910767	0.774816381	0.649620388	0.217204196	0.481057386	0.999996191
VWA1	0.495209564	0.796536636	0.830137693	0.596274343	0.962932548	0.972177751	0.999996191
VWA2	0.856203026	0.681695437	0.847925911	0.826161708	0.375447166	0.958001147	0.999996191
VWA5B2	0.750061	0.392745515	0.900115402	0.762056157	0.44779266	0.903806766	0.999996191
VWA8	0.944310933	0.775198775	0.077237591	0.905269557	0.893006957	0.800699778	0.999996191
WASF3	0.49107153	0.426150004	0.317311652	0.737734023	0.403179616	0.643574759	0.999996191
WASHC1	0.315470743	0.817765964	0.213110953	0.427631397	0.859013226	0.64792139	0.999996191
WASHC3	0.706318487	0.915069234	0.591698679	0.929039809	0.832531799	0.991773452	0.999996191
WASHC4	0.27415765	0.888708616	0.326860726	0.650637431	0.554801095	0.7161225	0.999996191
WASHC5	0.898300993	0.865663209	0.575349486	0.771025458	0.66232412	0.982516657	0.999996191
WASL	0.361346928	0.773344352	0.457259816	0.610772948	0.962883737	0.879072375	0.999996191
WBP1	0.101689579	0.737493637	0.923630176	0.306732996	0.98977859	0.655810213	0.999996191
WBP11	0.760074121	0.287637058	0.29855771	0.503463921	0.32614419	0.525313534	0.999996191
WBP1L	0.776585915	0.043374839	0.565143244	0.421045883	0.992162739	0.470036553	0.999996191
WDCP	0.718321742	0.636330535	0.640388919	0.992316796	0.06803926	0.643693369	0.999996191
WDFY1	0.682760264	0.568841783	0.883220901	0.297262438	0.52558117	0.827485611	0.999996191
WDFY2	0.372606753	0.249769448	0.242918914	0.803080521	0.894393762	0.605331546	0.999996191
WDFY3	0.875950664	0.871234777	0.460471074	0.307461299	0.133013588	0.581572042	0.999996191
WDPCP	0.789892714	0.397198173	0.557658011	0.825344787	0.594052939	0.896987697	0.999996191
WDR11	0.388140729	0.608534725	0.121729282	0.830672771	0.570148009	0.571129584	0.999996191
WDR17	0.848487521	0.207732847	0.477705307	0.94845799	0.471393793	0.766310571	0.999996191
WDR20	0.474895564	0.825004925	0.941928106	0.034194486	0.715596241	0.493281243	0.999996191
WDR25	0.909648864	0.949044195	0.686708193	0.513050733	0.988518953	0.992208046	0.999996191
WDR26	0.421175007	0.766853948	0.907963681	0.426442181	0.828206417	0.920004202	0.999996191
WDR27	0.406891706	0.339605103	0.81934152	0.75021746	0.118316252	0.513192633	0.999996191
WDR31	0.443795499	0.110354155	0.577970435	0.896462391	0.385604682	0.508193605	0.999996191
WDR33	0.128798546	0.993606184	0.884023741	0.251112469	0.54823286	0.597201741	0.999996191
WDR34	0.364102718	0.937140557	0.199791772	0.687535865	0.965498534	0.798973729	0.999996191
WDR37	0.882274298	0.736445277	0.516509149	0.768760277	0.355603358	0.905536917	0.999996191
WDR41	0.934423633	0.957719892	0.775105689	0.654417688	0.430967075	0.974539045	0.999996191
WDR45	0.787658072	0.835982626	0.300148756	0.874835913	0.919617679	0.960718923	0.999996191
WDR45B	0.383478425	0.451683523	0.63803972	0.683228005	0.436742229	0.741960778	0.999996191
WDR47	0.649464642	0.211162841	0.517662055	0.162962098	0.659440242	0.462555981	0.999996191
WDR48	0.570795977	0.64815265	0.107626756	0.885641969	0.380768229	0.56841992	0.999996191
WDR5	0.829126419	0.463645872	0.095439849	0.406011837	0.772039711	0.538689357	0.999996191
WDR54	0.953074299	0.305348661	0.129589448	0.459205811	0.525508719	0.494721113	0.999996191
WDR59	0.198536809	0.562965851	0.685913638	0.953084733	0.829837027	0.847224441	0.999996191
WDR60	0.893226394	0.777901127	0.973684957	0.863454605	0.772121897	0.998617871	0.999996191
WDR61	0.944731566	0.688734029	0.572596568	0.452421712	0.41179222	0.867676185	0.999996191
WDR62	0.940887943	0.057920465	0.411686208	0.450335217	0.811393103	0.475553572	0.999996191
WDR7	0.848872668	0.527543476	0.190232859	0.418356277	0.853685273	0.726861976	0.999996191
WDR70	0.723668367	0.656302347	0.711640527	0.704681156	0.428629006	0.918349709	0.999996191
WDR73	0.721876837	0.62231626	0.611030453	0.184644157	0.372395008	0.634709245	0.999996191

WDR75	0.466302785	0.517719566	0.889888015	0.104024728	0.554953828	0.553121549	0.999996191
WDR78	0.656748023	0.422704826	0.902900065	0.802146895	0.668876317	0.94677687	0.999996191
WDR81	0.898896631	0.680305808	0.704304185	0.272047103	0.537549102	0.853107098	0.999996191
WDR82	0.535316545	0.667768878	0.953433582	0.495747027	0.700278162	0.93441108	0.999996191
WDR83OS	0.826149774	0.764145587	0.781192562	0.101758995	0.670393345	0.745676753	0.999996191
WDR89	0.90116108	0.969415693	0.904264716	0.112592821	0.163867928	0.584301917	0.999996191
WDR91	0.725765339	0.928992635	0.065854966	0.857174395	0.67645268	0.695026994	0.999996191
WDR92	0.915709543	0.73069247	0.631188405	0.547171636	0.140023061	0.738422349	0.999996191
WDR93	0.540821487	0.799996445	0.419693253	0.778507237	0.79480719	0.929000799	0.999996191
WDSUB1	0.15508105	0.600259879	0.376846136	0.555869051	0.769810655	0.590030877	0.999996191
WDYHV1	0.94742117	0.571517746	0.893732123	0.755109319	0.428121037	0.959477775	0.999996191
WHAMM	0.86829141	0.970909861	0.847769212	0.917717192	0.24130413	0.960367442	0.999996191
WIF1	0.645109749	0.46199973	0.86472492	0.289000642	0.152244212	0.536005598	0.999996191
WIPF2	0.742875494	0.379353922	0.501298609	0.693415767	0.607086632	0.844190497	0.999996191
WIPF3	0.747120669	0.851469362	0.519000943	0.839346982	0.613659767	0.965610054	0.999996191
WIPI1	0.946321875	0.346860791	0.081424712	0.416973629	0.911844488	0.515279713	0.999996191
WIPI2	0.454856889	0.614225549	0.222808553	0.374628703	0.976810824	0.671361699	0.999996191
WISP2	0.750836811	0.120750303	0.34257984	0.425550809	0.889821179	0.542964624	0.999996191
WLS	0.541747159	0.985899631	0.421551128	0.434144402	0.912620237	0.902014761	0.999996191
WNT10B	0.958994965	0.562298269	0.187206105	0.481437631	0.493805854	0.681473058	0.999996191
WNT11	0.065188467	0.938344874	0.36836099	0.739981534	0.89071006	0.587947643	0.999996191
WNT2B	0.258973364	0.747731028	0.545196255	0.886117898	0.217020362	0.648946645	0.999996191
WNT5B	0.042265576	0.672200085	0.526443001	0.641985168	0.907254139	0.486661442	0.999996191
WNT7B	0.991301546	0.968913669	0.851032267	0.983968989	0.097879609	0.885523969	0.999996191
WNT8B	0.944747393	0.850123844	0.468989255	0.44465209	0.985298255	0.96347317	0.999996191
WNT9A	0.539397943	0.37087441	0.348357569	0.662199687	0.194816564	0.492464723	0.999996191
WRAP73	0.903411877	0.047942659	0.760651516	0.918149985	0.950710055	0.716190996	0.999996191
WRN	0.167947837	0.879186634	0.617243054	0.453669821	0.44949902	0.63169717	0.999996191
WRNIP1	0.773068091	0.857361775	0.091556543	0.7240951	0.766918138	0.745984495	0.999996191
WSCD2	0.874135288	0.541024833	0.146207479	0.273969045	0.445057585	0.480667088	0.999996191
WTIP	0.822843523	0.30142334	0.066038	0.554875207	0.904723549	0.476096464	0.999996191
WWC2	0.828955174	0.764116654	0.193552381	0.271903092	0.608070081	0.648637405	0.999996191
WWC3	0.462112694	0.661526235	0.204071783	0.897864721	0.94037	0.824640675	0.999996191
WWP2	0.420880116	0.158582267	0.979247566	0.442154693	0.747894667	0.66114254	0.999996191
WWTR1	0.51944607	0.579947766	0.382414906	0.873450373	0.114954445	0.539792064	0.999996191
XAB2	0.772537766	0.737172936	0.487045895	0.808377035	0.681645147	0.957653783	0.999996191
XBP1	0.486269873	0.435283122	0.62214683	0.083295637	0.879676146	0.505586836	0.999996191
XDH	0.772257724	0.835611614	0.221822525	0.264272328	0.539200433	0.649858073	0.999996191
XIAP	0.595377338	0.610109672	0.769927864	0.151092026	0.540965885	0.672039247	0.999996191
XIRP1	0.37250248	0.792409881	0.811273554	0.329341107	0.535842114	0.787009536	0.999996191
XKR4	0.92335792	0.58343968	0.773844183	0.328025124	0.283630394	0.771710089	0.999996191
XKR6	0.083266942	0.593525601	0.781626538	0.857702519	0.829199942	0.707480534	0.999996191
XKR8	0.810179158	0.694263057	0.893937493	0.929905104	0.436886435	0.976958279	0.999996191
XKRX	0.281326318	0.255611357	0.400923595	0.76447431	0.696096208	0.594270803	0.999996191
XPC	0.606564826	0.583915064	0.970093346	0.722954485	0.643690121	0.961140103	0.999996191

XPNPEP1	0.807564502	0.723168172	0.585019405	0.684753804	0.160906339	0.766302945	0.999996191
XPNPEP2	0.827762619	0.902189168	0.629743584	0.275405687	0.497238656	0.856504263	0.999996191
XPNPEP3	0.954422052	0.95277185	0.81442933	0.733791965	0.30284741	0.963277798	0.999996191
XPO4	0.225325969	0.504625652	0.688699337	0.540669916	0.230456696	0.507668876	0.999996191
XPO5	0.638437548	0.806576389	0.403646211	0.561821616	0.198697271	0.674940467	0.999996191
XPO6	0.682850621	0.959693816	0.957621389	0.242054581	0.628315866	0.910390318	0.999996191
XPO7	0.942277628	0.68088587	0.308489823	0.648621139	0.432593242	0.833274971	0.999996191
XRCC1	0.806761559	0.138509899	0.269943233	0.922258463	0.329945539	0.496303551	0.999996191
XRCC2	0.660068986	0.879212319	0.556626581	0.852338238	0.125416268	0.750500802	0.999996191
XRCC6	0.898754194	0.399987029	0.312532602	0.773598626	0.728757515	0.85396922	0.999996191
XRN1	0.370585092	0.841478873	0.777992778	0.154941032	0.793228475	0.723052587	0.999996191
XRN2	0.719230425	0.520135583	0.630865479	0.794963206	0.349644756	0.859285811	0.999996191
XRRA1	0.833347693	0.084670498	0.504702316	0.941056637	0.665691289	0.667307117	0.999996191
XXYLT1	0.540829379	0.779629458	0.132900249	0.343901637	0.945895111	0.627905507	0.999996191
XYLT1	0.439653916	0.972299221	0.753676148	0.260835641	0.706383426	0.843901367	0.999996191
YAE1D1	0.810651613	0.572845169	0.694894051	0.901103106	0.895183597	0.987775836	0.999996191
YAP1	0.639456998	0.695091362	0.275948427	0.914751079	0.393578242	0.794718087	0.999996191
YARS2	0.655314355	0.839375815	0.490931469	0.140258666	0.796351749	0.725224223	0.999996191
YBX2	0.688205234	0.629755158	0.382756588	0.629239577	0.226910645	0.678924973	0.999996191
YEATS4	0.9654291	0.668996691	0.690053962	0.957023696	0.299149302	0.9418779	0.999996191
YES1	0.42565072	0.538384718	0.433758858	0.514942255	0.88221664	0.798604885	0.999996191
YIF1A	0.391448178	0.586544074	0.626132556	0.25404091	0.53626861	0.641927392	0.999996191
YIF1B	0.385767673	0.901965218	0.953561282	0.171368923	0.423357582	0.682054143	0.999996191
YIPF1	0.346588319	0.679632518	0.236124372	0.943170043	0.979947231	0.820614173	0.999996191
YIPF3	0.155894102	0.906389443	0.205110271	0.746696855	0.857379616	0.631368367	0.999996191
YIPF4	0.09789453	0.699746044	0.275689064	0.873490854	0.604841573	0.511841278	0.999996191
YIPF5	0.347306879	0.390789943	0.857375834	0.747332942	0.158592349	0.573595584	0.999996191
YIPF6	0.153185463	0.684401752	0.450807759	0.965337266	0.780905731	0.756260071	0.999996191
YJEFN3	0.873691751	0.672816768	0.58407265	0.312136947	0.678718588	0.874450956	0.999996191
YLPM1	0.740428755	0.805977481	0.37729667	0.421315483	0.986301942	0.907964635	0.999996191
YOD1	0.628387419	0.554313438	0.857071778	0.24279191	0.753634184	0.830598919	0.999996191
YPEL2	0.535208574	0.833303327	0.798476078	0.741735659	0.746101499	0.974961382	0.999996191
YPEL4	0.92900543	0.44544701	0.901606186	0.19459452	0.39599264	0.716144404	0.999996191
YPEL5	0.28248294	0.152282302	0.45225343	0.829263807	0.625928804	0.514096039	0.999996191
YRDC	0.159502735	0.596412059	0.349204695	0.545460996	0.682954537	0.552705654	0.999996191
YTHDC1	0.329239685	0.912069816	0.995252618	0.057813725	0.752721192	0.562263945	0.999996191
YTHDC2	0.1339289	0.607401749	0.728851849	0.472272445	0.63876216	0.624203293	0.999996191
YWHAB	0.748053657	0.326834571	0.929038014	0.65024353	0.669782413	0.914675276	0.999996191
YWHAE	0.721571911	0.611189692	0.444869972	0.51849454	0.111087553	0.53535105	0.999996191
YWHAG	0.898410191	0.581168342	0.207532245	0.598505341	0.93280287	0.846866389	0.999996191
YWHAZ	0.59291313	0.376448407	0.472947982	0.168167986	0.689582344	0.550625437	0.999996191
YY1	0.954236605	0.979025905	0.151541366	0.370552923	0.655832478	0.749825203	0.999996191
ZAP70	0.857687035	0.99609148	0.759758297	0.680230329	0.152054451	0.862754914	0.999996191
ZBED1	0.923680204	0.832285476	0.834156487	0.323841415	0.513648357	0.923327862	0.999996191
ZBED3	0.742988152	0.919884733	0.535857175	0.813987988	0.489942255	0.953964612	0.999996191

ZBED4	0.501454042	0.409620994	0.481843549	0.769409415	0.563226603	0.789617182	0.999996191
ZBED8	0.440739947	0.41716524	0.405264755	0.361814914	0.741810797	0.646009672	0.999996191
ZBTB12	0.969349572	0.812022172	0.170816836	0.837294987	0.616444114	0.867649505	0.999996191
ZBTB2	0.35045525	0.752745003	0.559436053	0.064492722	0.902804494	0.484144992	0.999996191
ZBTB24	0.405392638	0.649606749	0.72331654	0.828201899	0.373032234	0.84253469	0.999996191
ZBTB26	0.139680639	0.782279686	0.715562578	0.367384457	0.476773345	0.572238885	0.999996191
ZBTB3	0.99991098	0.924015426	0.542669564	0.987354934	0.491695987	0.985227735	0.999996191
ZBTB34	0.542394791	0.914279735	0.375813722	0.243090098	0.239396602	0.527554664	0.999996191
ZBTB39	0.697963883	0.99593781	0.777254633	0.860437938	0.431680577	0.975983842	0.999996191
ZBTB4	0.804370289	0.853192835	0.438646307	0.673311722	0.205298637	0.784278142	0.999996191
ZBTB41	0.231933316	0.853451806	0.83488482	0.630412614	0.518874336	0.828896168	0.999996191
ZBTB42	0.468875695	0.839567013	0.670754397	0.517006557	0.703991946	0.911224091	0.999996191
ZBTB44	0.156978953	0.624276328	0.461565627	0.308236227	0.748334596	0.520239746	0.999996191
ZBTB46	0.400070929	0.242667367	0.496050222	0.180892406	0.917706431	0.470995361	0.999996191
ZBTB48	0.934114988	0.482666396	0.97139796	0.837164805	0.306898282	0.929161112	0.999996191
ZBTB49	0.656330579	0.22944428	0.401205241	0.622580666	0.71253386	0.702748217	0.999996191
ZBTB6	0.766980894	0.522545422	0.889854925	0.098131428	0.50647011	0.622434825	0.999996191
ZBTB7A	0.812775593	0.511615036	0.559238951	0.115521154	0.745745758	0.646348832	0.999996191
ZBTB8A	0.389879018	0.722211881	0.752560822	0.452777811	0.792132785	0.88065471	0.999996191
ZBTB9	0.323654826	0.540169122	0.760766192	0.679174989	0.32445035	0.719791626	0.999996191
ZC2HC1C	0.626720799	0.331696784	0.558328625	0.837618263	0.516364525	0.8166508	0.999996191
ZC3H10	0.803826208	0.423460615	0.571171368	0.059831367	0.661016118	0.463963952	0.999996191
ZC3H12A	0.376409176	0.265869217	0.861413605	0.553146289	0.236154106	0.53468438	0.999996191
ZC3H13	0.399628802	0.739517399	0.693286752	0.110866706	0.820757897	0.632306223	0.999996191
ZC3H15	0.311997386	0.584387584	0.747591016	0.619241365	0.129026855	0.528340965	0.999996191
ZC3H18	0.600521276	0.852277359	0.350804581	0.207557664	0.348444402	0.561957036	0.999996191
ZC3H3	0.547423236	0.538197513	0.50295155	0.564002007	0.781454237	0.858619554	0.999996191
ZC3H4	0.441146638	0.896574468	0.608917327	0.844549317	0.693043381	0.950949602	0.999996191
ZC3H6	0.141626321	0.881919408	0.984341106	0.884986416	0.318081086	0.750917459	0.999996191
ZC3H7A	0.426492048	0.893229272	0.793168085	0.930338991	0.966831369	0.989248139	0.999996191
ZC3HAV1	0.979225915	0.650906426	0.393394636	0.181100658	0.62628822	0.714075226	0.999996191
ZC3HAV1L	0.963031228	0.974237831	0.680579555	0.989593511	0.7853642	0.999211238	0.999996191
ZC3HC1	0.948956674	0.466144942	0.851986846	0.966734091	0.780368145	0.990650696	0.999996191
ZC4H2	0.73683139	0.653044756	0.496973872	0.849449745	0.106138447	0.660665037	0.999996191
ZCCHC10	0.305231677	0.352497827	0.617761173	0.365998284	0.898489174	0.663328796	0.999996191
ZCCHC24	0.404861629	0.861234063	0.45067535	0.377653543	0.941450167	0.834247758	0.999996191
ZCCHC3	0.768515716	0.848968985	0.893038479	0.263079612	0.573452416	0.900135709	0.999996191
ZCCHC4	0.162638433	0.856293307	0.892235168	0.682605125	0.5770658	0.812393373	0.999996191
ZCCHC7	0.345732367	0.705882061	0.710136406	0.834882187	0.709374613	0.918968919	0.999996191
ZCCHC8	0.536844092	0.450680964	0.994635802	0.836868631	0.416491286	0.894045098	0.999996191
ZCCHC9	0.45108633	0.926582762	0.408058007	0.254379014	0.467870963	0.64891421	0.999996191
ZCRB1	0.794720487	0.163477598	0.603744097	0.748528699	0.833833373	0.812429687	0.999996191
ZCWPW1	0.730819676	0.917732735	0.108226705	0.73231458	0.878950002	0.80447637	0.999996191
ZCWPW2	0.559575015	0.947019101	0.84384892	0.387760715	0.77936904	0.947217603	0.999996191
ZDBF2	0.21108309	0.546035197	0.871829897	0.246164452	0.633420368	0.598368053	0.999996191

ZDHHC1	0.606246587	0.446614811	0.800130255	0.243068774	0.854935073	0.79807702	0.999996191
ZDHHC12	0.311876	0.784679421	0.526971161	0.889668855	0.554894008	0.854749795	0.999996191
ZDHHC15	0.713953506	0.835109813	0.392717959	0.725322029	0.868786496	0.954806533	0.999996191
ZDHHC16	0.154745814	0.258413296	0.464091993	0.847414261	0.96663785	0.592484486	0.999996191
ZDHHC19	0.376116528	0.967826698	0.945335193	0.542217216	0.690548036	0.942805192	0.999996191
ZDHHC21	0.210747007	0.752925398	0.113425418	0.994172509	0.735705387	0.564594562	0.999996191
ZDHHC24	0.084199557	0.722082309	0.798334141	0.870994147	0.985825042	0.784553993	0.999996191
ZDHHC3	0.599336218	0.579637217	0.598445792	0.465509082	0.717195074	0.867671724	0.999996191
ZDHHC4	0.674968422	0.235027482	0.70011062	0.129725725	0.76884744	0.531561817	0.999996191
ZDHHC5	0.271855953	0.858282746	0.327083253	0.883967472	0.760424628	0.820269004	0.999996191
ZDHHC6	0.964562796	0.460322787	0.541328243	0.529611436	0.15887259	0.648189471	0.999996191
ZDHHC7	0.815826523	0.503357164	0.09683187	0.631752691	0.631003831	0.600632769	0.999996191
ZDHHC8	0.569540403	0.96880548	0.094449905	0.515054333	0.860571872	0.674068474	0.999996191
ZEB1	0.809745979	0.423097251	0.624040255	0.511066262	0.786390542	0.897201142	0.999996191
ZEB2	0.921721516	0.74022019	0.275118318	0.693174545	0.758876069	0.914454835	0.999996191
ZER1	0.306744402	0.995301054	0.567888397	0.8231556	0.643963995	0.905754231	0.999996191
ZFAND1	0.402115726	0.625597087	0.388165361	0.938360961	0.881305729	0.888981697	0.999996191
ZFAND2B	0.926056803	0.556176839	0.760940016	0.898146846	0.323334951	0.930372682	0.999996191
ZFAND3	0.684036059	0.673839508	0.023335516	0.997811562	0.723872892	0.46582198	0.999996191
ZFAND4	0.914728338	0.376938217	0.532360174	0.706724426	0.991319077	0.942621594	0.999996191
ZFAND6	0.315118964	0.881693549	0.545797605	0.867077139	0.405031416	0.826451796	0.999996191
ZFAT	0.799620986	0.298946691	0.893587676	0.100547202	0.596798481	0.55945907	0.999996191
ZFHX2	0.669192889	0.777319168	0.697633397	0.581254645	0.971455634	0.97712599	0.999996191
ZFHX3	0.660533789	0.698350542	0.505490133	0.594366526	0.570959328	0.886226038	0.999996191
ZFHX4	0.819591525	0.796097709	0.08043262	0.540637534	0.930603236	0.699877674	0.999996191
ZFP1	0.523979123	0.701762539	0.70434265	0.88897476	0.093855433	0.66110524	0.999996191
ZFP28	0.494866057	0.680205201	0.764474042	0.472616798	0.837089457	0.918028818	0.999996191
ZFP3	0.87996676	0.183131422	0.656488355	0.806173	0.731248629	0.851589913	0.999996191
ZFP30	0.294851	0.944442372	0.365739443	0.631021397	0.717542651	0.802228801	0.999996191
ZFP36	0.485281771	0.114530776	0.723513732	0.865520214	0.404197291	0.577433862	0.999996191
ZFP36L2	0.940389494	0.744765864	0.84052212	0.440002902	0.942317913	0.98533765	0.999996191
ZFP62	0.188850384	0.781475461	0.687222288	0.552427295	0.645860465	0.759107052	0.999996191
ZFP69	0.286602637	0.949338063	0.788292259	0.498953593	0.910220687	0.912841314	0.999996191
ZFP91	0.890206956	0.677188812	0.86228337	0.95089424	0.641426282	0.99351108	0.999996191
ZFPM1	0.781939504	0.622655604	0.258755968	0.294044956	0.364462405	0.569478817	0.999996191
ZFYVE1	0.77792792	0.889402189	0.592468668	0.441874704	0.094397669	0.61540765	0.999996191
ZFYVE26	0.783376586	0.877917102	0.613418572	0.609236431	0.664488036	0.965907706	0.999996191
ZFYVE27	0.533569139	0.310743047	0.966244286	0.107417754	0.889937991	0.593926013	0.999996191
ZFYVE9	0.250479245	0.307925198	0.349149972	0.74659263	0.749910907	0.590883307	0.999996191
ZHX1	0.431423108	0.914020456	0.605334482	0.941224269	0.488999467	0.926580106	0.999996191
ZHX2	0.591477576	0.654588493	0.464382614	0.835322178	0.732143009	0.926674435	0.999996191
ZHX3	0.797521457	0.217462572	0.574071461	0.533399705	0.591850264	0.732982326	0.999996191
ZKSCAN1	0.426528592	0.857774179	0.769953819	0.126033599	0.670089781	0.679781951	0.999996191
ZKSCAN2	0.405520393	0.300880865	0.691016804	0.357539897	0.54855468	0.608880432	0.999996191
ZKSCAN4	0.786778955	0.922858433	0.422175968	0.99979914	0.766818989	0.983761178	0.999996191

ZKSCAN7	0.708253031	0.816653615	0.133854943	0.360748592	0.563673002	0.599296302	0.999996191
ZKSCAN8	0.883154834	0.621902536	0.633311735	0.617791784	0.416027828	0.902288251	0.999996191
ZMAT3	0.330908274	0.644137897	0.423701353	0.313089213	0.328462393	0.498482853	0.999996191
ZMIZ1	0.761129375	0.640369927	0.042014751	0.69245244	0.89658632	0.557891102	0.999996191
ZMIZ2	0.553324142	0.693070696	0.612270432	0.680515276	0.191617356	0.728052563	0.999996191
ZMYM2	0.932096521	0.676656344	0.85344611	0.051513714	0.765588328	0.657646151	0.999996191
ZMYM3	0.617736866	0.667138463	0.231432997	0.661584413	0.596805041	0.766373185	0.999996191
ZMYND11	0.736792266	0.321619392	0.285389183	0.929362012	0.714994007	0.797760224	0.999996191
ZMYND12	0.352143576	0.643585701	0.615877209	0.475786086	0.478983082	0.735225184	0.999996191
ZMYND19	0.196291804	0.266538708	0.591328144	0.63841539	0.487158971	0.505062128	0.999996191
ZNF10	0.555738467	0.5034748	0.830470907	0.23761502	0.404067243	0.667315115	0.999996191
ZNF106	0.631724088	0.706400742	0.371169002	0.475672881	0.188844158	0.588304783	0.999996191
ZNF112	0.295523987	0.656921479	0.323774173	0.534114907	0.511849283	0.616379628	0.999996191
ZNF132	0.446690661	0.438154372	0.977078738	0.951771992	0.71254619	0.943421484	0.999996191
ZNF133	0.946245839	0.550650979	0.670567141	0.488218062	0.320636396	0.830806452	0.999996191
ZNF140	0.218860784	0.822188573	0.217549055	0.725908245	0.476513863	0.570046547	0.999996191
ZNF142	0.822122136	0.537741012	0.480285038	0.565462571	0.985964846	0.934461922	0.999996191
ZNF146	0.068199418	0.971376713	0.910311342	0.169304261	0.829050304	0.481392766	0.999996191
ZNF154	0.363405335	0.945673867	0.918042912	0.668177295	0.578793917	0.937514515	0.999996191
ZNF16	0.996151348	0.760007796	0.611422193	0.572904599	0.240683269	0.855140812	0.999996191
ZNF165	0.367325001	0.152182622	0.590740011	0.594428137	0.566494851	0.53229258	0.999996191
ZNF169	0.685730823	0.932590473	0.708919494	0.932428102	0.71923176	0.992495968	0.999996191
ZNF174	0.497382737	0.530131265	0.357422879	0.479782175	0.343963294	0.596928291	0.999996191
ZNF175	0.202528075	0.360828629	0.473040159	0.447358176	0.539348576	0.478701962	0.999996191
ZNF177	0.545375228	0.665610874	0.419314555	0.922807156	0.716975677	0.916772886	0.999996191
ZNF18	0.530176807	0.378135306	0.922833573	0.061129372	0.967944641	0.529316179	0.999996191
ZNF180	0.273136976	0.635671871	0.578530184	0.853384482	0.85730946	0.875917215	0.999996191
ZNF189	0.569738357	0.6097532	0.558833102	0.691927426	0.610162561	0.890979194	0.999996191
ZNF197	0.658234256	0.688479703	0.778027091	0.39981988	0.831125762	0.933403093	0.999996191
ZNF2	0.933831406	0.502011165	0.914289647	0.694373277	0.354533943	0.922109993	0.999996191
ZNF200	0.779443904	0.461163193	0.719150053	0.577220231	0.95176278	0.951588055	0.999996191
ZNF202	0.920694637	0.779848275	0.814416419	0.206925448	0.479931875	0.84043054	0.999996191
ZNF205	0.732576453	0.54801427	0.525508085	0.458675524	0.978638114	0.909441792	0.999996191
ZNF212	0.839438283	0.783337808	0.75753014	0.977693497	0.339538678	0.963619941	0.999996191
ZNF214	0.684997781	0.415477245	0.96520842	0.497787626	0.495213767	0.864032958	0.999996191
ZNF22	0.576142763	0.259245151	0.529843437	0.717560982	0.737193591	0.785341497	0.999996191
ZNF226	0.463042689	0.953529243	0.828614756	0.480767595	0.321979068	0.836427603	0.999996191
ZNF227	0.605772949	0.959934054	0.486793694	0.954137566	0.089691176	0.683280362	0.999996191
ZNF23	0.675126215	0.68883212	0.819063586	0.862052837	0.832793968	0.989446377	0.999996191
ZNF235	0.887454306	0.833249713	0.920696134	0.835825754	0.45240288	0.987378333	0.999996191
ZNF236	0.690073173	0.449974899	0.938586965	0.343590492	0.78957134	0.886115916	0.999996191
ZNF239	0.363743228	0.561291138	0.495537504	0.332071708	0.568421057	0.636996411	0.999996191
ZNF24	0.941078933	0.472701777	0.433569026	0.991848745	0.740804467	0.951408166	0.999996191
ZNF25	0.214749446	0.75155809	0.335828248	0.700903733	0.701680031	0.701707386	0.999996191
ZNF260	0.237597044	0.743984123	0.861088967	0.304499421	0.902692104	0.785239731	0.999996191

ZNF263	0.624726986	0.833802204	0.760215886	0.510365843	0.192228058	0.772004347	0.999996191
ZNF266	0.768355001	0.427342752	0.263239247	0.264036527	0.307853959	0.447864965	0.999996191
ZNF268	0.170720383	0.716922	0.808523822	0.541395362	0.699681936	0.765538381	0.999996191
ZNF274	0.399161191	0.199036603	0.680113979	0.909708531	0.833685621	0.781559102	0.999996191
ZNF275	0.837739486	0.370766111	0.325211144	0.245556274	0.633958728	0.599069244	0.999996191
ZNF280C	0.704260795	0.401919083	0.497468483	0.945723489	0.420935835	0.834780178	0.999996191
ZNF280D	0.075271842	0.610237497	0.73336184	0.512463684	0.746752591	0.560556869	0.999996191
ZNF281	0.954017372	0.844944424	0.737502054	0.57467973	0.747975364	0.987106915	0.999996191
ZNF282	0.795510536	0.418714244	0.496277486	0.51865594	0.618468678	0.825738851	0.999996191
ZNF283	0.138104198	0.556538388	0.96125443	0.942799337	0.363716337	0.691932144	0.999996191
ZNF286A	0.228202329	0.824325951	0.96011525	0.49725088	0.198367626	0.623423941	0.999996191
ZNF287	0.609455663	0.333027847	0.328618786	0.452016102	0.440155736	0.566143013	0.999996191
ZNF292	0.170550751	0.461766155	0.46880599	0.823989804	0.314625802	0.504083757	0.999996191
ZNF3	0.813595004	0.685578833	0.910804379	0.354852284	0.880947344	0.960628671	0.999996191
ZNF300	0.834802435	0.316268899	0.826854919	0.583749745	0.698382592	0.901718379	0.999996191
ZNF304	0.379084557	0.225007735	0.96270117	0.194763458	0.505610255	0.473062062	0.999996191
ZNF316	0.926775043	0.825432107	0.662952216	0.489206434	0.890046246	0.980930435	0.999996191
ZNF317	0.675667638	0.678555399	0.752878282	0.699891802	0.773336014	0.971789836	0.999996191
ZNF319	0.487433914	0.521666608	0.651927951	0.898206399	0.477799589	0.871262734	0.999996191
ZNF32	0.85522461	0.765188488	0.222682828	0.538088194	0.935521272	0.875661847	0.999996191
ZNF322	0.292622817	0.930703209	0.858197987	0.48869752	0.944416966	0.924572945	0.999996191
ZNF329	0.743827031	0.689195766	0.716013971	0.462929102	0.566476929	0.911416158	0.999996191
ZNF330	0.373549657	0.816385147	0.6240475	0.706223427	0.373032824	0.816435632	0.999996191
ZNF331	0.960913596	0.603166255	0.740154522	0.97573463	0.519241306	0.980159893	0.999996191
ZNF333	0.717309876	0.744709484	0.674698847	0.973731096	0.349093505	0.937920232	0.999996191
ZNF33B	0.205200924	0.417045384	0.869513848	0.773606246	0.143621628	0.477094186	0.999996191
ZNF346	0.881544496	0.510807932	0.753888843	0.278098493	0.756209919	0.871768546	0.999996191
ZNF35	0.434127632	0.471975994	0.943543206	0.919627542	0.835384564	0.95534734	0.999996191
ZNF350	0.902702134	0.167673064	0.252034937	0.840469613	0.438401753	0.577268466	0.999996191
ZNF358	0.746019272	0.992581245	0.157181336	0.748978476	0.502020852	0.793147684	0.999996191
ZNF366	0.283698526	0.901776172	0.952218214	0.507370309	0.872394176	0.924526959	0.999996191
ZNF367	0.436612388	0.614441672	0.422905351	0.801198606	0.70743108	0.856273195	0.999996191
ZNF382	0.445149547	0.441128425	0.88818346	0.720662708	0.469238922	0.842884426	0.999996191
ZNF384	0.837243857	0.616468626	0.602082026	0.510690969	0.20429598	0.73879311	0.999996191
ZNF389	0.906747398	0.081285865	0.473064763	0.318011394	0.983685085	0.528630817	0.999996191
ZNF397	0.210269516	0.828264678	0.841150311	0.931595606	0.834474201	0.930435614	0.999996191
ZNF398	0.791124123	0.835615742	0.794768197	0.562077373	0.572574243	0.965210436	0.999996191
ZNF408	0.807706148	0.767645938	0.610479104	0.080482402	0.242765942	0.456983993	0.999996191
ZNF41	0.204137343	0.48122045	0.605129157	0.84378696	0.472680795	0.679117675	0.999996191
ZNF419	0.679830014	0.275658315	0.289715376	0.801061561	0.967723625	0.786290453	0.999996191
ZNF420	0.127052419	0.582784569	0.624178764	0.572390463	0.636366331	0.612367171	0.999996191
ZNF423	0.683116928	0.671710067	0.199456371	0.335513834	0.463180678	0.579554553	0.999996191
ZNF444	0.749024847	0.595932417	0.242623581	0.925646617	0.275920531	0.708777335	0.999996191
ZNF445	0.418018601	0.604988575	0.758114539	0.53363527	0.704504837	0.873146957	0.999996191
ZNF449	0.754557205	0.825642159	0.912408389	0.539592144	0.907838537	0.990018302	0.999996191

ZNF45	0.661473745	0.841210182	0.394845912	0.914633239	0.193905624	0.772540778	0.999996191
ZNF462	0.689246952	0.341842136	0.783107615	0.250935904	0.901399114	0.784800862	0.999996191
ZNF467	0.847396189	0.272827993	0.523225822	0.992347525	0.439932241	0.825067942	0.999996191
ZNF473	0.527780697	0.272342712	0.632380581	0.202921036	0.645297952	0.54524591	0.999996191
ZNF48	0.981459352	0.39180693	0.545015864	0.54280464	0.727547312	0.892281067	0.999996191
ZNF496	0.76302029	0.736774357	0.769219046	0.569234706	0.69635114	0.966160695	0.999996191
ZNF500	0.546686685	0.130543543	0.866062666	0.7960204	0.75048742	0.762792882	0.999996191
ZNF507	0.569889391	0.697990879	0.853845872	0.052618635	0.896123232	0.602632374	0.999996191
ZNF511	0.619549284	0.464842706	0.408876606	0.327335652	0.898302846	0.751001309	0.999996191
ZNF512	0.836747885	0.780789423	0.739058393	0.631488236	0.339898858	0.92007768	0.999996191
ZNF513	0.529110254	0.99491192	0.873006405	0.240690462	0.638687007	0.870249948	0.999996191
ZNF514	0.41794604	0.790525207	0.847075686	0.920606611	0.647330574	0.964237148	0.999996191
ZNF518A	0.629703909	0.909893008	0.356869488	0.118533423	0.532541114	0.56079652	0.999996191
ZNF518B	0.717953021	0.675069884	0.178881079	0.420894335	0.963485611	0.75381806	0.999996191
ZNF521	0.539531605	0.456219214	0.220210939	0.459356325	0.727094004	0.626564106	0.999996191
ZNF524	0.447578349	0.879969964	0.987343279	0.375577122	0.779054617	0.930341304	0.999996191
ZNF526	0.54828707	0.626538216	0.470065236	0.782729101	0.754336983	0.910263932	0.999996191
ZNF529	0.130979601	0.27749448	0.683824309	0.295088038	0.991684272	0.454007965	0.999996191
ZNF543	0.957322369	0.975070025	0.470367473	0.087978006	0.535149086	0.652465498	0.999996191
ZNF548	0.305535443	0.848650372	0.654996478	0.96433122	0.25392055	0.784169422	0.999996191
ZNF554	0.831353193	0.624474467	0.946701894	0.78723244	0.582438293	0.981887104	0.999996191
ZNF565	0.528492002	0.917481588	0.759996525	0.608777728	0.080144033	0.625218071	0.999996191
ZNF567	0.048931504	0.308366555	0.897843492	0.786890672	0.712085703	0.461655302	0.999996191
ZNF568	0.156921592	0.830428114	0.998686986	0.966594382	0.353801799	0.79608278	0.999996191
ZNF569	0.042791241	0.968524453	0.512072047	0.676909745	0.774942451	0.53250655	0.999996191
ZNF570	0.041401952	0.631233342	0.92689469	0.742089022	0.868392035	0.597645894	0.999996191
ZNF572	0.260055945	0.362344974	0.259962418	0.680511961	0.928051124	0.595894677	0.999996191
ZNF574	0.488082854	0.776432748	0.898200171	0.4762995	0.968332304	0.959747134	0.999996191
ZNF575	0.499591341	0.525823586	0.445108582	0.873941268	0.546812771	0.834269772	0.999996191
ZNF576	0.838001429	0.729631366	0.822042249	0.151919691	0.563991357	0.790332614	0.999996191
ZNF579	0.611256294	0.863326788	0.894044741	0.575917332	0.53463797	0.953512379	0.999996191
ZNF580	0.722118567	0.965942292	0.109362639	0.787778366	0.721058452	0.791412749	0.999996191
ZNF581	0.30345984	0.663203017	0.381470395	0.764290348	0.626782863	0.762068103	0.999996191
ZNF583	0.31537186	0.26964971	0.735409157	0.934050516	0.870948439	0.818886255	0.999996191
ZNF584	0.483896783	0.98554529	0.506549438	0.664454962	0.241666848	0.771732642	0.999996191
ZNF585A	0.51218274	0.674466275	0.820973312	0.533410011	0.305346171	0.802516592	0.999996191
ZNF592	0.677049097	0.761149103	0.687950094	0.558860254	0.595615328	0.934141726	0.999996191
ZNF596	0.783578598	0.827776603	0.567628294	0.727523522	0.587039963	0.959870826	0.999996191
ZNF597	0.933092389	0.641214835	0.508330264	0.31160356	0.900931409	0.896377331	0.999996191
ZNF598	0.794908188	0.451169414	0.367673617	0.289846113	0.85566354	0.740405744	0.999996191
ZNF606	0.589600406	0.733051712	0.904129877	0.972777194	0.678256608	0.987432856	0.999996191
ZNF609	0.764971244	0.434003268	0.751154622	0.038365922	0.811557351	0.465725946	0.999996191
ZNF613	0.819363908	0.504102379	0.805397448	0.303182332	0.527307965	0.826226407	0.999996191
ZNF614	0.937109823	0.519971624	0.742438357	0.782061131	0.895170182	0.986774088	0.999996191
ZNF618	0.882014447	0.802461476	0.482941633	0.046569373	0.507390538	0.472848494	0.999996191

ZNF621	0.864721772	0.377729037	0.796750114	0.03222504	0.951272829	0.470611431	0.999996191
ZNF622	0.540133553	0.606973878	0.553695261	0.147146647	0.550787859	0.586119089	0.999996191
ZNF623	0.993007523	0.335173714	0.64788812	0.7848704	0.826617865	0.950291807	0.999996191
ZNF629	0.585774006	0.766147543	0.281181571	0.935350144	0.692273419	0.89056729	0.999996191
ZNF639	0.230714096	0.296201302	0.915434988	0.367576652	0.625465129	0.581722109	0.999996191
ZNF641	0.752975398	0.180369745	0.914549795	0.461889926	0.306529002	0.620893073	0.999996191
ZNF646	0.720115403	0.706581346	0.628506425	0.709266835	0.446707545	0.917478435	0.999996191
ZNF653	0.593719034	0.53157131	0.608729315	0.518465788	0.523566276	0.822995125	0.999996191
ZNF655	0.76177518	0.721330483	0.417402793	0.753998891	0.918342193	0.960629258	0.999996191
ZNF664	0.66134007	0.760923241	0.500429162	0.551206706	0.948851676	0.944864414	0.999996191
ZNF668	0.302300159	0.93055645	0.403960355	0.204413598	0.70803324	0.607821554	0.999996191
ZNF672	0.689617533	0.478990073	0.737424998	0.307522252	0.981908609	0.876041112	0.999996191
ZNF677	0.207889911	0.318878888	0.992289875	0.668653536	0.972445383	0.789133024	0.999996191
ZNF683	0.918028149	0.995867366	0.285485142	0.617483918	0.6108496	0.91410258	0.999996191
ZNF687	0.724982327	0.711535204	0.667410828	0.909235734	0.905339748	0.990554704	0.999996191
ZNF689	0.914865763	0.943576725	0.67464231	0.304728593	0.20395224	0.759151531	0.999996191
ZNF691	0.873849171	0.725596786	0.920971107	0.997534399	0.941097399	0.999602207	0.999996191
ZNF696	0.678388611	0.238002351	0.564424696	0.691195873	0.13754719	0.485658492	0.999996191
ZNF697	0.657613378	0.814202942	0.426422198	0.316388318	0.577531042	0.78473444	0.999996191
ZNF699	0.165139221	0.755044257	0.621121102	0.836608743	0.352076495	0.671635449	0.999996191
ZNF70	0.543064284	0.817303206	0.515279605	0.898409982	0.542685516	0.92818626	0.999996191
ZNF703	0.514815289	0.324194727	0.858488265	0.683740705	0.57878769	0.836623964	0.999996191
ZNF710	0.555565414	0.876671212	0.142159954	0.697297526	0.202988838	0.508489619	0.999996191
ZNF713	0.646909503	0.651813482	0.976268401	0.249711635	0.840333711	0.897890785	0.999996191
ZNF740	0.403138995	0.19988537	0.243007621	0.904881748	0.942124913	0.610729718	0.999996191
ZNF746	0.309132065	0.917979334	0.080559119	0.874777135	0.699478865	0.576332207	0.999996191
ZNF75A	0.590285558	0.453175061	0.590732774	0.316204363	0.621033634	0.730579171	0.999996191
ZNF75D	0.275759873	0.351114731	0.24298581	0.358217639	0.871312699	0.455713068	0.999996191
ZNF76	0.837173835	0.121049412	0.738863704	0.889094339	0.593354907	0.774992237	0.999996191
ZNF771	0.624922715	0.732190783	0.159254996	0.458310488	0.510460215	0.614824092	0.999996191
ZNF774	0.508123616	0.525214189	0.881491732	0.696420776	0.902462346	0.954974652	0.999996191
ZNF777	0.378542552	0.513064649	0.246848494	0.157477604	0.933358578	0.448389372	0.999996191
ZNF782	0.071125429	0.594116303	0.874647686	0.825716575	0.568131719	0.618126239	0.999996191
ZNF783	0.936954542	0.625960775	0.874014242	0.153198078	0.859231812	0.863504514	0.999996191
ZNF784	0.546197928	0.273328002	0.773614936	0.639613392	0.790886112	0.841388865	0.999996191
ZNF787	0.674649411	0.656996183	0.754052941	0.62024572	0.676405999	0.95049018	0.999996191
ZNF789	0.865460751	0.363810729	0.414843868	0.357828703	0.425674708	0.644999165	0.999996191
ZNF792	0.418077439	0.782548323	0.245351304	0.442376391	0.389539986	0.574164111	0.999996191
ZNF793	0.453257041	0.519581006	0.485266451	0.49592775	0.507519547	0.716232094	0.999996191
ZNF8	0.509494133	0.933703534	0.84640783	0.873377136	0.442253611	0.959022003	0.999996191
ZNF81	0.789450947	0.683754585	0.655650608	0.295372862	0.969580561	0.917517271	0.999996191
ZNF814	0.480158391	0.944073546	0.921716828	0.380852048	0.489834788	0.884159164	0.999996191
ZNF821	0.86024523	0.624223533	0.905173729	0.750489182	0.818682029	0.992030748	0.999996191
ZNF830	0.397771593	0.902035553	0.445189524	0.8251711	0.816126815	0.924265508	0.999996191
ZNF845	0.453787911	0.888129325	0.175131246	0.497433732	0.970751199	0.748083886	0.999996191

ZNF853	0.430808508	0.656650786	0.845384937	0.77793883	0.535495179	0.915507736	0.999996191
ZNF865	0.525008118	0.982148877	0.58920875	0.282051108	0.463590565	0.776012174	0.999996191
ZNF891	0.718088316	0.727064557	0.867436986	0.794472961	0.681537604	0.985524697	0.999996191
ZNF93	0.13927886	0.481166831	0.288813751	0.744209549	0.881721193	0.557692722	0.999996191
ZNFX1	0.470280116	0.999292092	0.512656349	0.083637855	0.71320729	0.581567754	0.999996191
ZNHIT1	0.214484906	0.759607071	0.735675691	0.404760847	0.831909586	0.778842542	0.999996191
ZNHIT6	0.50044912	0.369492767	0.821310183	0.519178813	0.23749742	0.633167478	0.999996191
ZNRF1	0.56283671	0.484215746	0.129184035	0.429921251	0.645297023	0.507861127	0.999996191
ZNRF3	0.910621713	0.510982712	0.862575597	0.649770926	0.431539112	0.929178766	0.999996191
ZPBP	0.99609573	0.719146497	0.623800294	0.797226298	0.02908071	0.518903824	0.999996191
ZRANB3	0.247061003	0.614369247	0.671890772	0.949782089	0.851148443	0.891761084	0.999996191
ZSCAN12	0.318422693	0.99785643	0.092707967	0.469587252	0.645793602	0.491284847	0.999996191
ZSCAN16	0.496194944	0.346931405	0.727183883	0.527091897	0.15722705	0.519165291	0.999996191
ZSCAN2	0.601278433	0.54165044	0.098269435	0.536938179	0.488822224	0.479996214	0.999996191
ZSCAN20	0.686078879	0.763545651	0.526281035	0.579802304	0.731054006	0.933131616	0.999996191
ZSCAN23	0.25327473	0.426075672	0.418926968	0.301152745	0.642098669	0.487302413	0.999996191
ZSCAN25	0.784054993	0.158793552	0.565559482	0.990401547	0.418318602	0.718910908	0.999996191
ZSCAN30	0.308681241	0.144022798	0.796799398	0.838041883	0.695374877	0.652193843	0.999996191
ZSCAN31	0.939748236	0.832988234	0.373719891	0.449273303	0.144262407	0.635601562	0.999996191
ZSCAN4	0.883821214	0.638226478	0.450683951	0.72722022	0.373979307	0.867100419	0.999996191
ZSWIM1	0.677184219	0.262534361	0.469763748	0.151344604	0.907249379	0.538141471	0.999996191
ZSWIM3	0.714311407	0.773748883	0.516336981	0.768919985	0.621459989	0.948030797	0.999996191
ZSWIM5	0.882721483	0.520449271	0.541723639	0.434068329	0.241366962	0.697467352	0.999996191
ZSWIM8	0.52325715	0.723148567	0.902016925	0.646578714	0.32858477	0.87401246	0.999996191
ZSWIM9	0.265007419	0.916077628	0.883859413	0.801711468	0.777416384	0.946267429	0.999996191
ZW10	0.833966259	0.535606407	0.348444491	0.260498396	0.49814261	0.647931844	0.999996191
ZWINT	0.87038222	0.250415307	0.360808833	0.14034102	0.921817653	0.51549507	0.999996191
ZXDB	0.375210848	0.690325538	0.701157587	0.731208588	0.853021351	0.929871242	0.999996191
ZXDC	0.839427234	0.697110216	0.388792552	0.757291452	0.816873912	0.950811467	0.999996191
ZYX	0.086480724	0.233376901	0.964960032	0.803294808	0.573884711	0.492213903	0.999996191
ZZEF1	0.664820343	0.965729915	0.84695909	0.323090744	0.991500154	0.967263098	0.999996191
ZZZ3	0.095566128	0.955349603	0.612695895	0.567254261	0.86604062	0.707533339	0.999996191
A2ML1	0.185537229	NaN	0.842790247	0.41564882	0.956534954	NaN	NaN
ABAT	0.783640238	0.705058511	NaN	0.105184449	0.279342873	NaN	NaN
ACBD7	0.125085365	0.175795245	0.015255881	NaN	0.025319867	NaN	NaN
ACOT2	0.841874288	0.856812879	NaN	0.06067322	0.889053092	NaN	NaN
ACP5	0.358371638	0.572946265	0.127626711	0.426416387	NaN	NaN	NaN
ACTA1	0.659948246	NaN	0.890446452	0.186180895	0.560229562	NaN	NaN
ACTN2	0.479272288	NaN	0.713964472	NaN	0.675895452	NaN	NaN
ADAM8	0.035795375	0.939470121	0.000356758	NaN	0.044369542	NaN	NaN
ADAMTS19	0.00171207	0.516703261	0.372611948	NaN	0.387189337	NaN	NaN
ADRA2C	0.826158995	NaN	0.220161326	0.622283849	0.509131561	NaN	NaN
ALB	0.564341508	0.215373427	NaN	0.918837501	0.530400758	NaN	NaN
ALDOB	NaN	0.63559002	0.00433842	0.519458229	0.554023345	NaN	NaN
AMZ1	0.623467855	0.072235141	0.003105897	0.763367128	NaN	NaN	NaN

APOA4	NaN	0.274364804	0.315843437	0.076429	0.450666095	NaN	NaN
ARHGAP11A	0.628899054	0.01095011	NaN	0.37849109	0.63207472	NaN	NaN
ART3	0.613301493	0.000897834	0.092387806	NaN	0.022438126	NaN	NaN
ATP10B	0.124428478	0.046628665	NaN	0.243136314	0.483534217	NaN	NaN
BAIAP2L2	NaN	0.426485549	0.873423902	0.797201845	0.345433836	NaN	NaN
BCL2L15	NaN	0.447804484	0.002392762	0.061333423	0.17424859	NaN	NaN
BIRC5	0.683922207	0.027837359	NaN	0.347158135	0.633419681	NaN	NaN
BOLA	0.353795577	0.436785216	0.975492379	NaN	0.53971893	NaN	NaN
BOLA3	0.882917806	0.774127666	NaN	0.011687259	0.644071865	NaN	NaN
BSG	0.670861617	0.705990267	NaN	0.187942731	0.709004853	NaN	NaN
BUB1B	0.530357186	0.230520681	NaN	0.717968103	0.820800689	NaN	NaN
C10H15orf62	0.983154404	NaN	0.353598352	0.753922737	0.907306227	NaN	NaN
C19H17orf58	0.818486002	0.75223976	0.135001842	NaN	0.594332441	NaN	NaN
C4BPA	NaN	0.244390096	NaN	0.730071658	0.618805279	NaN	NaN
CA12	NaN	0.030987226	0.193922551	0.041347259	0.637847593	NaN	NaN
CA13	NaN	0.456107834	0.986511385	0.175511478	NaN	NaN	NaN
CADM4	0.182905971	0.049357666	0.39070545	NaN	0.84275494	NaN	NaN
CATHL5	0.107748481	0.525718681	NaN	0.098680802	0.901115404	NaN	NaN
CCDC73	0.591636889	0.588052433	NaN	0.109429313	0.871825713	NaN	NaN
CCL17	0.342817121	0.155069144	0.384934548	NaN	0.938360205	NaN	NaN
CCL5	NaN	0.734152832	0.397667003	0.264325565	0.499585582	NaN	NaN
CCR9	0.45288744	0.324190111	0.902021761	0.415014152	NaN	NaN	NaN
CD5L	0.976144723	0.362583883	0.163053419	0.08216137	NaN	NaN	NaN
CDC6	0.36697437	0.11446171	NaN	0.566871537	0.412130936	NaN	NaN
CDCA8	0.757619959	0.170302561	NaN	0.253197126	0.988226335	NaN	NaN
CDH17	NaN	0.17099671	0.049540025	0.045247308	0.366185074	NaN	NaN
CDH19	0.883132998	0.008576372	0.190186504	NaN	0.028236	NaN	NaN
CDKN2A	0.464923831	0.497755006	NaN	0.891963394	0.664721466	NaN	NaN
CERS3	0.144743901	0.397347806	NaN	0.006350915	0.541116233	NaN	NaN
CFHR5	0.452970731	NaN	NaN	0.96017754	0.740719966	NaN	NaN
CHAD	NaN	0.236152046	0.035936794	0.085661886	0.102469286	NaN	NaN
CHGA	NaN	0.066734678	0.828258873	0.406392042	0.529208089	NaN	NaN
CKB	0.886619606	0.895388315	NaN	0.180475884	0.874002101	NaN	NaN
CKMT1A	NaN	0.016312239	0.207218382	0.014434426	0.413994204	NaN	NaN
CLDN3	NaN	0.892050428	0.782305244	0.236831326	0.148807663	NaN	NaN
COBL	0.70804504	0.242826676	NaN	0.562488173	0.100812688	NaN	NaN
COL24A1	0.469593763	NaN	0.008568196	0.794179014	0.541963688	NaN	NaN
COL2A1	0.523192466	0.165552748	0.05951265	NaN	0.020023434	NaN	NaN
CR2	0.451208141	0.620714845	0.244111208	0.47978067	NaN	NaN	NaN
CRB2	NaN	0.106818298	0.673664034	0.820001315	0.233469743	NaN	NaN
CXCL11	0.773413933	0.003503758	0.873457252	0.347247942	NaN	NaN	NaN
CXCL8	0.674006026	0.070289196	0.114773611	NaN	0.001819823	NaN	NaN
CXHXorf57	0.014402253	0.389223061	0.716347113	NaN	0.263438928	NaN	NaN
CYP21	0.54858348	0.622531211	NaN	0.463920502	0.692212713	NaN	NaN
CYP46A1	0.885558745	0.582850779	NaN	0.172930755	0.910917369	NaN	NaN

DDX58	0.799013219	0.136612189	0.115417486	NaN	0.254738049	NaN	NaN
DEFB7	0.419125178	0.851571306	0.255382327	NaN	0.433974046	NaN	NaN
DMRT3	0.453593499	NaN	0.558393227	0.292502025	0.634559143	NaN	NaN
DNAH9	0.97438056	0.452282287	0.344597407	NaN	0.587301253	NaN	NaN
DNAJB13	NaN	0.357182004	0.872291561	0.014152203	0.465500993	NaN	NaN
DPP10	0.04010694	0.013027325	0.048845675	NaN	0.11313183	NaN	NaN
DPP4	0.737587782	0.071335562	NaN	0.39442082	0.032534243	NaN	NaN
DSG2	NaN	0.734885366	0.91472014	0.369761932	0.5088269	NaN	NaN
EEF1A2	0.808286648	NaN	0.082233035	0.175271332	0.004065315	NaN	NaN
ELOVL6	0.788415929	0.889955182	NaN	0.087244979	0.263097881	NaN	NaN
FAM3B	NaN	0.756942247	0.290328068	0.089180295	0.768449603	NaN	NaN
FAM49B	0.892825599	0.862802579	NaN	0.126539351	0.218952654	NaN	NaN
FAM83D	0.04721699	NaN	0.401174952	0.01032883	0.071668145	NaN	NaN
FCGBP	NaN	0.468718323	0.223063686	NaN	0.495685404	NaN	NaN
FCGR2A	0.756863885	0.829896219	NaN	0.025626517	0.026134436	NaN	NaN
FGB	0.652438584	0.522698734	NaN	0.958043576	0.272324143	NaN	NaN
FOSL1	0.039238983	0.923625162	0.833599848	0.569883603	NaN	NaN	NaN
FOXD1	0.690083605	0.157754572	0.01044906	NaN	0.965323653	NaN	NaN
FOXD3	0.977694414	0.078429062	0.730982177	NaN	0.228389758	NaN	NaN
FREM2	0.36557003	0.531334372	0.378298263	NaN	0.066253469	NaN	NaN
FZD5	NaN	0.328578144	0.671773006	0.035046231	0.608623999	NaN	NaN
G6PD	0.812580325	0.608263558	NaN	0.003813093	0.569874515	NaN	NaN
GABRB3	0.85044541	0.526319503	0.222557172	NaN	0.786764086	NaN	NaN
GOLGA7B	0.268857415	0.275839688	0.277865973	0.020066917	NaN	NaN	NaN
GPT2	0.548005886	0.799624887	NaN	0.611460624	0.522723564	NaN	NaN
GRIA2	NaN	0.469445507	0.462228238	0.566985523	0.380743202	NaN	NaN
GRIK4	0.360616793	0.593576554	0.477623167	NaN	0.14485031	NaN	NaN
GSS	0.732366603	0.869967361	NaN	0.036528001	0.372055936	NaN	NaN
GSTA3	NaN	0.444422709	NaN	0.129197171	0.493029546	NaN	NaN
GSTM1	0.672817364	0.667420904	NaN	0.495199498	0.559956536	NaN	NaN
GUCY2C	NaN	0.893051676	0.327067614	0.545363121	0.550166175	NaN	NaN
GYS2	0.833303266	0.865891735	0.208148283	NaN	0.518623691	NaN	NaN
HAND2	0.136489587	NaN	0.977554927	0.061575764	0.23117058	NaN	NaN
HMOX1	0.99413728	0.132039493	NaN	0.022292663	0.951355807	NaN	NaN
HNF4G	NaN	0.877006754	0.169769154	0.100482985	0.593844466	NaN	NaN
HP	0.645185211	NaN	0.249336464	0.3358411	0.04205075	NaN	NaN
HPSE2	0.051823218	NaN	0.765959107	0.757216579	0.213414971	NaN	NaN
IFIH1	0.786824172	0.225697209	0.106306728	NaN	0.545099468	NaN	NaN
IGLL1	0.089382026	NaN	NaN	0.063343823	0.344183632	NaN	NaN
IGSF11	0.324583229	0.431485425	0.885357756	NaN	0.7737428	NaN	NaN
IGSF5	NaN	0.080384424	NaN	0.967984147	0.470273428	NaN	NaN
IGSF9B	0.148397907	0.336119917	0.408358835	NaN	0.280724055	NaN	NaN
IL1B	0.078340266	0.28859329	0.004162495	NaN	0.33603994	NaN	NaN
IRX1	0.997462269	0.551581708	NaN	0.726463114	0.487571341	NaN	NaN
ISG15	0.366091861	0.28704958	0.077283522	NaN	0.530107181	NaN	NaN

JAKMIP2	0.608841436	0.230018188	0.851435732	NaN	0.599330673	NaN	NaN
KCNA4	0.927289989	0.964568976	NaN	0.896148425	0.827528492	NaN	NaN
KCNK2	0.552937794	0.240122112	NaN	0.37060702	0.843822931	NaN	NaN
KCNN2	0.681778715	0.375800672	NaN	0.069244913	0.971889631	NaN	NaN
KIAA1211L	NaN	0.212031164	0.749868061	0.628492387	0.125826236	NaN	NaN
KIF11	0.862532666	0.105737316	NaN	0.23803431	0.875664988	NaN	NaN
KIF20A	0.643714292	0.121642831	NaN	0.297830454	0.588568047	NaN	NaN
KIF2C	0.949411428	0.030665441	NaN	0.140602573	0.468731211	NaN	NaN
KLHL31	0.774674537	0.780682661	NaN	0.202533494	0.282974547	NaN	NaN
LAMC3	0.018848082	0.423982708	NaN	0.015322924	0.349682264	NaN	NaN
LCN2	0.702447407	0.095086185	0.698808019	0.418279228	NaN	NaN	NaN
LDHB	0.951034921	0.853916229	NaN	0.348302278	0.582501553	NaN	NaN
LGI1	0.608870163	0.313697841	0.190501841	NaN	0.123532433	NaN	NaN
LGR5	0.470589212	0.312725141	0.65040151	0.156168413	NaN	NaN	NaN
LIN7A	0.681562226	0.802110169	NaN	0.487383166	0.750320555	NaN	NaN
LOC100138641	0.980598961	0.83000256	NaN	0.323138088	0.047560584	NaN	NaN
LOC100139670	NaN	0.584694615	0.012638766	NaN	0.166335752	NaN	NaN
LOC100139885	NaN	0.776413776	0.055905655	0.199681798	0.637938444	NaN	NaN
LOC100140226	NaN	0.864578199	0.456895349	0.549712302	0.919731103	NaN	NaN
LOC100297192	0.016728831	NaN	0.001104286	0.018668563	0.433430242	NaN	NaN
LOC100297779	NaN	0.52768639	0.156600256	0.030478615	NaN	NaN	NaN
LOC100298356	0.963624451	0.209503483	0.012722065	NaN	0.106441744	NaN	NaN
LOC100300115	0.038045348	0.933837316	0.130782783	0.483526947	NaN	NaN	NaN
LOC100301224	0.53593368	0.517033193	NaN	0.038525747	0.728166207	NaN	NaN
LOC100847119	0.023231423	NaN	3.75E-06	0.278666466	0.18720255	NaN	NaN
LOC100847415	0.653835944	NaN	0.866985329	0.420259659	0.186350163	NaN	NaN
LOC100847724	0.901026438	NaN	0.011232576	0.137873949	0.478113732	NaN	NaN
LOC100847981	0.647859112	0.581418695	0.013963845	NaN	0.304295525	NaN	NaN
LOC100848536	0.72497132	0.002983559	0.307483343	NaN	0.633308004	NaN	NaN
LOC100851369	0.161675103	0.462977593	NaN	0.145120042	0.979214511	NaN	NaN
LOC101901948	0.938755104	0.57010104	0.446994558	0.472847333	NaN	NaN	NaN
LOC101902787	0.878755129	0.624153191	0.014848853	NaN	0.059730644	NaN	NaN
LOC101903284	0.944047628	0.271335592	NaN	NaN	NaN	NaN	NaN
LOC101903734	NaN	0.248471734	0.218074361	0.97314069	0.726381045	NaN	NaN
LOC101905242	0.303592741	0.192333569	NaN	0.309752762	0.029481404	NaN	NaN
LOC101905509	0.405465437	0.81920883	0.432267002	NaN	0.226124004	NaN	NaN
LOC101906743	NaN	0.543997068	0.035799454	0.087430677	0.024173162	NaN	NaN
LOC101907335	0.764606087	0.870842758	NaN	0.259386232	0.733143525	NaN	NaN
LOC104968484	0.120761056	0.038284042	NaN	0.018255791	0.764423278	NaN	NaN
LOC104973965	0.648950246	0.741746155	NaN	0.054739811	0.916937218	NaN	NaN
LOC104974214	NaN	0.907979028	0.652436478	0.027377842	0.30155235	NaN	NaN
LOC104974444	0.719985041	0.772324708	NaN	0.39086625	0.782612545	NaN	NaN
LOC104974455	0.181601742	NaN	0.441570183	0.179238812	0.717454626	NaN	NaN
LOC104976942	0.919765044	NaN	0.961076561	0.222338297	0.188016087	NaN	NaN
LOC107131864	0.898144395	0.719448421	NaN	NaN	0.647839785	NaN	NaN

LOC107131942	0.000161926	NaN	NaN	0.793302358	NaN	NaN	NaN
LOC112441507	0.701689838	0.263531496	0.003094768	NaN	0.195405469	NaN	NaN
LOC112441557	0.862630032	0.144545044	0.9594185	NaN	0.605753261	NaN	NaN
LOC112441777	0.187223861	NaN	0.763697573	0.286632156	0.247556546	NaN	NaN
LOC112442062	0.069515519	NaN	0.002936256	0.066959752	0.50982267	NaN	NaN
LOC112443013	0.750664351	0.719726218	NaN	0.065362934	0.677624964	NaN	NaN
LOC112443862	0.254374049	0.995678088	0.046570683	0.955179051	NaN	NaN	NaN
LOC112444652	0.887773433	0.080958782	0.100584716	0.097228148	NaN	NaN	NaN
LOC112445090	0.177644573	0.964942474	NaN	0.671530087	0.517734239	NaN	NaN
LOC112446680	0.884457219	0.94662127	0.976704558	NaN	NaN	NaN	NaN
LOC112446726	0.362887088	NaN	0.827891107	0.944605838	0.113478018	NaN	NaN
LOC112447079	NaN	0.892987997	0.78611338	0.877761705	0.210713448	NaN	NaN
LOC112447816	0.789199864	NaN	0.129936322	0.484936446	0.376963151	NaN	NaN
LOC112448034	0.807583926	0.634760803	NaN	0.797720283	0.254037673	NaN	NaN
LOC505033	0.424423469	0.072283709	0.620297104	NaN	0.22641628	NaN	NaN
LOC507055	0.809362869	0.136563641	0.029243861	NaN	0.445452093	NaN	NaN
LOC508459	0.171555458	0.020859008	0.027824019	0.218644594	NaN	NaN	NaN
LOC509283	0.958307342	0.320451889	0.053507725	NaN	0.237360457	NaN	NaN
LOC509911	0.260502135	NaN	0.233518244	0.113404484	0.479252482	NaN	NaN
LOC511683	0.677939106	NaN	0.384345217	0.985824335	0.036259956	NaN	NaN
LOC513210	0.931688212	0.274908489	NaN	0.918194968	0.692891351	NaN	NaN
LOC514978	NaN	0.815752928	0.017905335	0.156380522	2.00E-07	NaN	NaN
LOC515676	0.263699482	0.284670853	0.603967582	NaN	NaN	NaN	NaN
LOC516421	0.725745768	NaN	0.637395081	0.843272267	0.2967891	NaN	NaN
LOC519274	0.052618009	NaN	0.016537318	0.902078052	NaN	NaN	NaN
LOC530653	NaN	0.083019518	0.129336318	0.850547155	0.314692558	NaN	NaN
LOC615051	0.368245598	0.01650331	NaN	NaN	NaN	NaN	NaN
LOC616782	NaN	0.285089986	0.566873397	0.060979639	0.382062421	NaN	NaN
LOC616830	0.006281439	0.215806639	NaN	0.052759327	0.111374207	NaN	NaN
LOC618297	0.999738614	0.892719637	NaN	0.007449387	0.974248876	NaN	NaN
LOC781736	NaN	0.457224461	0.916308371	0.933890071	0.145955985	NaN	NaN
LOC781796	NaN	0.228695602	0.465745408	0.046172657	0.04284943	NaN	NaN
LOC783106	0.12210612	0.211293924	NaN	0.090885898	0.318491793	NaN	NaN
LOC784266	0.020623591	0.858701323	0.348436529	0.469259901	NaN	NaN	NaN
LOC785161	0.844328496	NaN	0.264869215	0.991173516	0.624312061	NaN	NaN
LOC789829	NaN	0.424999965	0.086796385	0.01319733	0.034733496	NaN	NaN
LTF	NaN	0.628023696	0.977638601	0.103392076	0.092044812	NaN	NaN
LUZP2	0.077234519	0.036315614	0.079747008	NaN	0.761702154	NaN	NaN
MAPK4	0.177591377	NaN	0.793458219	0.019591585	0.191243063	NaN	NaN
MARCO	0.09795188	0.304002515	NaN	0.079731947	0.857472916	NaN	NaN
MEF2B	NaN	0.645428969	0.519343725	NaN	0.060301713	NaN	NaN
MEGF11	NaN	0.926579978	NaN	0.339199379	0.829937803	NaN	NaN
MID1IP1	0.714669879	0.734080352	NaN	0.109982753	0.023490981	NaN	NaN
MISP	NaN	0.516908507	0.445813244	0.206457054	0.125839301	NaN	NaN
MLXIPL	0.41119573	0.884501845	NaN	0.040850913	0.271312198	NaN	NaN

MMP12	0.698663438	0.645416435	0.002970523	NaN	0.140820982	NaN	NaN
MOXD1	0.927898998	NaN	0.142645171	NaN	0.935083635	NaN	NaN
MPC1	0.625367589	0.751586336	NaN	0.011246618	0.582155584	NaN	NaN
MPZ	0.88315827	0.045939589	0.069001786	NaN	0.140241863	NaN	NaN
MT1A	0.354366398	0.777080517	0.400200385	0.072177908	NaN	NaN	NaN
MUSK	0.236314099	NaN	0.395213491	0.002271224	0.464497938	NaN	NaN
MX1	0.876562968	0.074127315	0.007575712	NaN	0.733364942	NaN	NaN
MX2	NaN	0.334465738	0.001215743	NaN	0.273072166	NaN	NaN
MYO1A	NaN	0.910243117	0.278161847	0.198023177	0.086441191	NaN	NaN
NAALADL1	NaN	NaN	0.512842281	0.996594008	0.770563426	NaN	NaN
NPFFR2	0.159187628	NaN	0.152190341	0.544201948	0.624938423	NaN	NaN
OAS1Y	0.800739011	0.128948467	0.02026514	NaN	0.358059831	NaN	NaN
OAS1Z	NaN	0.50976385	0.006061362	0.125757734	0.331288259	NaN	NaN
OPCML	0.485456487	NaN	0.270971646	0.285358101	0.128417929	NaN	NaN
OSM	0.386612018	0.024804711	0.146957767	NaN	0.054416054	NaN	NaN
P2RX1	0.170691961	NaN	0.817285631	0.003668398	0.244660339	NaN	NaN
PARD6B	0.30297654	0.128495452	NaN	0.894009215	0.101957799	NaN	NaN
PBLD	0.757381523	0.786708152	NaN	0.518481638	0.193356736	NaN	NaN
PCDH10	0.594917307	0.024906906	0.158484903	NaN	0.132130486	NaN	NaN
PCLAF	0.696259158	0.113390747	NaN	0.049281604	0.912436194	NaN	NaN
PDZK1	NaN	0.644215158	0.411893161	0.206679798	0.526405553	NaN	NaN
PDZRN4	0.056693044	0.306737019	0.877036646	NaN	0.172720032	NaN	NaN
PEBP4	0.019227423	NaN	0.810687476	NaN	0.531240671	NaN	NaN
PHF21B	0.349359708	NaN	0.275559342	0.753067696	0.409650678	NaN	NaN
PIR	0.846513637	0.991171267	NaN	0.222314519	0.957635713	NaN	NaN
PKD2L1	0.836490143	0.746781918	NaN	NaN	0.841436228	NaN	NaN
PLA2G2D1	NaN	0.921752982	0.016626058	0.106149711	0.006979645	NaN	NaN
PLEK2	NaN	0.227481843	0.725834882	0.281976612	0.731416561	NaN	NaN
PLP1	0.529671282	0.023298885	0.030619744	NaN	0.18398855	NaN	NaN
PPDPFL	0.909273202	NaN	0.108502996	0.014293748	0.913007813	NaN	NaN
PPM1E	0.001483229	0.162644034	NaN	3.76E-05	0.688522282	NaN	NaN
PRSS35	0.023956758	NaN	0.000167879	0.637389851	0.939358353	NaN	NaN
PTCH2	0.878663476	0.968145964	NaN	0.373914775	0.956035984	NaN	NaN
PTGIR	0.888153747	NaN	0.0010197	0.435923979	0.91744346	NaN	NaN
PTPRQ	0.975316893	0.725422003	NaN	0.387790927	0.212569003	NaN	NaN
RASL12	0.267051218	NaN	0.790382958	0.747683911	0.293473098	NaN	NaN
REC8	0.842011358	0.039263443	0.238310931	0.047563265	NaN	NaN	NaN
RHOD	0.866075794	0.409845247	3.64E-05	0.948264715	NaN	NaN	NaN
RIPK4	NaN	0.796726682	0.808073161	0.00924951	0.551949911	NaN	NaN
RRM2	0.577182375	0.281110015	NaN	0.477301917	0.761044049	NaN	NaN
RSAD2	0.799748214	0.565492169	0.173227032	NaN	0.554431799	NaN	NaN
SAMD9	0.849327302	0.117594879	0.015725962	NaN	0.003215141	NaN	NaN
SCP2	0.791297397	0.805465639	NaN	0.337193899	0.725607912	NaN	NaN
SCUBE1	0.484776174	0.024673952	0.085836106	NaN	0.376622599	NaN	NaN
SECTM1A	0.430778134	0.411445499	0.975518538	0.769582029	NaN	NaN	NaN

SERHL2	0.506487641	0.015887386	NaN	0.173227824	0.870878949	NaN	NaN
SGCG	0.60854066	0.565331109	0.850421005	NaN	0.920074005	NaN	NaN
SH3RF2	0.076868878	0.659511627	NaN	0.490752479	0.793397146	NaN	NaN
SIX2	0.460020912	NaN	0.072950967	0.046590511	0.356658401	NaN	NaN
SLC13A3	0.994952498	0.817146208	NaN	0.34403186	0.641749859	NaN	NaN
SLC15A1	NaN	0.581576491	0.897912888	0.836487669	0.294740004	NaN	NaN
SLC16A1	0.089702036	0.639180979	NaN	0.272320136	0.137693625	NaN	NaN
SLC16A7	0.847431739	0.988294316	NaN	0.548667882	0.29846495	NaN	NaN
SLC25A4	0.880035458	0.408758265	NaN	0.009051048	0.524947535	NaN	NaN
SLC35F1	0.750152146	0.037927615	0.206728215	NaN	0.319895551	NaN	NaN
SLC51B	NaN	0.439257099	0.374422699	0.907815963	0.418887667	NaN	NaN
SLC5A9	0.928935459	0.740788451	NaN	0.371170249	0.966510652	NaN	NaN
SLC6A8	NaN	0.46154995	0.219841326	0.144451789	0.358627065	NaN	NaN
SLC9A2	0.229211428	0.936560282	0.9146453	0.098357264	NaN	NaN	NaN
SLC9A3	NaN	0.237068522	0.260409419	0.366418849	0.604129179	NaN	NaN
SNAP25	0.91086688	0.945312027	0.052578679	NaN	0.354262583	NaN	NaN
SNCA	0.626202073	0.168049061	0.676725315	NaN	0.858604858	NaN	NaN
SNRNP25	0.56636173	0.449209527	NaN	0.036850873	0.225232923	NaN	NaN
SOCS3	0.086540532	0.462857665	0.662841238	0.199758103	NaN	NaN	NaN
SOX10	0.827926117	0.0057558	0.248174042	NaN	0.079400998	NaN	NaN
SPCS3	0.046065225	0.100993268	0.793071477	0.318180383	NaN	NaN	NaN
SPHAR	0.010160137	0.469391584	0.12924109	NaN	NaN	NaN	NaN
SPOCK1	0.848945173	0.079270855	0.746621731	NaN	0.062663831	NaN	NaN
SPRY3	0.802318096	0.730730232	NaN	0.047059284	0.061449001	NaN	NaN
SPTB	0.901291585	0.496581363	0.493392095	0.709597799	NaN	NaN	NaN
SRXN1	0.085809639	0.529869876	NaN	0.437641272	0.506288605	NaN	NaN
ST14	NaN	0.723612421	0.169645984	0.344221945	0.459705135	NaN	NaN
ST8SIA2	0.847912945	0.114970701	0.062168244	0.100211447	NaN	NaN	NaN
SVOP	0.973151966	0.479571367	0.003151929	0.195311232	NaN	NaN	NaN
TDH	0.685179767	0.459768689	NaN	0.266960544	0.840406685	NaN	NaN
TECTB	0.659133938	0.640315244	NaN	0.613878689	0.236376023	NaN	NaN
TGM5	0.27261209	0.673990668	NaN	0.000789343	0.463797011	NaN	NaN
TIMD4	0.501335484	0.560208521	0.279542195	0.454755193	NaN	NaN	NaN
TKT	0.848497501	0.822900916	NaN	0.348176122	0.44387205	NaN	NaN
TMEM179	0.898469948	0.038361581	NaN	0.628392887	0.942280196	NaN	NaN
TMEM216	0.383812059	0.6446952	0.163594278	0.148848304	NaN	NaN	NaN
TMEM45B	NaN	0.890020627	0.961750471	0.559300368	0.516942086	NaN	NaN
TMPRSS2	NaN	0.703303655	0.057919886	0.014700652	0.002405044	NaN	NaN
TMPRSS5	0.542187285	0.085210371	0.336003663	NaN	0.076754088	NaN	NaN
TNFRSF11B	0.484172878	0.498515834	0.043786104	NaN	0.229084241	NaN	NaN
TRIB3	0.013948581	0.135886991	0.243811253	NaN	0.036266632	NaN	NaN
TRIM44	0.07525931	0.785287726	NaN	0.362439098	0.599405518	NaN	NaN
TTYH1	0.780240735	0.004352238	0.102029268	NaN	0.629906868	NaN	NaN
TUBA1D	NaN	0.164248729	0.418985562	0.391743532	0.1729496	NaN	NaN
UBE2C	0.338593195	0.096452266	NaN	0.317191385	0.979086192	NaN	NaN

UCHL3	0.67548157	0.459177648	NaN	0.122959232	0.500691274	NaN	NaN
UCMA	0.857113896	0.583553928	NaN	0.275027649	0.968334963	NaN	NaN
UCP1	NaN	0.242531344	0.344972078	0.000552125	0.651622497	NaN	NaN
UHRF1	0.706299115	0.164192374	NaN	0.160398319	0.417640604	NaN	NaN
VIL1	NaN	0.77047861	0.402737824	0.000751207	0.903721916	NaN	NaN
WNT6	0.812897072	0.39138195	0.487983207	NaN	0.05058816	NaN	NaN
YDJC	0.966039725	0.680375138	NaN	0.014555842	0.336043633	NaN	NaN
ZBTB37	NaN	0.000859789	NaN	0.693251385	0.010069699	NaN	NaN
ZDHHC23	0.082848885	0.957802879	0.653879388	NaN	NaN	NaN	NaN
