# Supplementary material for: Genes associated with body weight gain and feed intake identified by meta-analysis of the mesenteric fat from crossbred beef steers
Source: PLoS One. 2020 Jan 7;15(1):e0227154. doi: 10.1371/journal.pone.0227154 (PMC6946124; doi:10.1371/journal.pone.0227154)
Supplement: S3 Table — Genes are ordered by adjusted meta-P-value. The individual cohort cells for DEGs identified in the meta-analysis are colored according to the sign of their log2 fold change, where green indicates up-regulation and red indicates down-regulation in low gain, low intake. (PDF) [file pone.0227154.s003.pdf]

Supplemental Table 3. Differentially expressed genes associated with the gain x intake interaction for each individual cohort and the meta analyses.
Green indicates up-regulation in animals with low gain, low intake. Red indicates down-regulation in animals with low gain, low intake.

Gene	Group 1 Raw P-value	Group 2 Raw P-value	Group 3 Raw P-value	Group 4 Raw P-value	Group 5 Raw P-value	Meta-P-value	Adjusted Meta-P-value
LOC100300716	7.12E-05	0.000508957	1.31E-05	0.804942937	0.591051152	7.81E-09	4.27E-05
PTAFR	0.100477672	0.64893609	9.23E-06	0.009610866	0.000878887	1.14E-07	0.000466231
FST	0.000145542	0.025717358	0.125167387	0.161340979	0.000669829	7.94E-07	0.002606736
AACS	0.359842406	0.925965698	0.028867064	1.05E-05	0.005847554	6.13E-06	0.016756576
H19	0.000854847	0.003172897	0.020932687	0.020329357	0.901353428	9.71E-06	0.02277232
MRPL14	0.000149776	0.151026567	0.468066066	0.000439747	0.411836029	1.60E-05	0.026224822
PDLIM5	0.259653831	3.67E-07	0.689605662	0.032237978	0.865533522	1.54E-05	0.026224822
RCOR2	0.106032816	0.329329769	0.270299075	2.67E-05	0.005920085	1.30E-05	0.026224822
HPS6	0.579517997	0.198383224	0.685985116	1.61E-06	0.025612063	2.44E-05	0.036476044
IL18BP	0.662117801	0.131346771	0.005723558	0.835996083	1.06E-05	3.13E-05	0.042765245
GLCE	0.522326899	0.406129069	8.09E-07	0.035172729	0.941997027	3.84E-05	0.048500326
JCHAIN	0.000721429	0.012557281	0.00484065	0.186538652	0.814205948	4.36E-05	0.05109403
CREB3L1	0.219539242	0.042019136	0.009773103	0.000885834	0.154763993	7.13E-05	0.062160292
FCER1A	0.048481379	0.041064488	0.041209641	0.022633792	0.006059421	6.62E-05	0.062160292
OLFML3	0.193327992	0.118424143	0.012640346	0.000159026	0.248404324	6.71E-05	0.062160292
PDE4B	0.490985292	0.645511101	1.01E-06	0.27167138	0.143593442	7.17E-05	0.062160292
RASSF9	0.490525096	0.659972066	0.000110593	0.001621923	0.215213883	7.20E-05	0.062160292
CD5	0.002052085	0.623915466	0.756178084	0.002841077	0.004864112	7.60E-05	0.06233403
TRIP12	0.365376136	0.236814261	0.000162829	0.001893367	0.679664029	9.66E-05	0.075500256
BTAF1	0.160124891	0.882741046	0.96729281	2.45E-07	0.912917615	0.000145872	0.07610185
CHI3L1	0.216449611	3.44E-05	0.072831971	0.13512182	0.424195638	0.000147573	0.07610185
FAM210B	0.762363365	0.282532902	4.14E-06	0.220908777	0.11836701	0.000117784	0.07610185
KCNK12	0.308964792	0.00046466	0.004190012	0.246648625	0.161960223	0.000120622	0.07610185
KPNA1	0.270437735	0.00024171	0.009662748	0.087772391	0.543603426	0.000144093	0.07610185
LOC101906455	0.751803997	3.18E-06	0.296117674	0.471155937	0.093727105	0.000148392	0.07610185
LOC782367	0.001538704	0.068272464	0.316197191	0.051327186	0.017706457	0.000144284	0.07610185
PDE4D	0.048583699	0.406776545	0.276647109	1.33E-05	0.35505108	0.000127703	0.07610185
PTK7	0.408877815	0.006594096	0.000962209	0.020417705	0.492381638	0.000128644	0.07610185
SPP1	0.657210721	0.297199358	0.010964149	0.869703686	1.22E-05	0.000115246	0.07610185
SRPX	0.094924528	0.051282396	0.049340992	0.000132918	0.746024778	0.000119783	0.07610185
LOC112445144	0.576346869	0.790824629	0.0055123	3.39E-05	0.410650025	0.000162021	0.080573338
CAB39	0.808342123	0.00019977	0.016990025	0.061664456	0.233028655	0.000177785	0.081045291
LOC789607	0.097960307	0.047412911	0.416689445	2.24E-05	0.893780624	0.000175698	0.081045291
TNNT3	0.159514619	0.019098203	0.003714493	0.018825909	0.182795123	0.000176084	0.081045291
LOC100138922	0.514946515	0.925364639	4.30E-05	0.00284354	0.838674153	0.000210306	0.093279052
CCDC117	0.141208274	0.421774808	0.817600344	2.87E-06	0.48170269	0.00026965	0.094153623
ELOVL3	0.18745347	0.431419564	0.001924716	0.013354889	0.031297723	0.000262458	0.094153623
EPM2AIP1	0.445270675	0.732994584	0.000178382	0.011118606	0.097492597	0.000256344	0.094153623
FAM124B	0.736679314	0.558908983	8.25E-05	0.005132206	0.322307793	0.000234332	0.094153623
INSIG1	0.026226289	0.424558858	0.567781679	0.002562312	0.003191436	0.00021958	0.094153623
KCNA3	0.110324335	0.874416649	0.120916782	0.287936998	1.95E-05	0.000263819	0.094153623

LOC107131807	0.541706135	0.576606177	8.07E-07	0.960526928	0.254543894	0.00025174	0.094153623
MXRA5	0.503573607	0.008510204	5.30E-05	0.360327136	0.69853116	0.000237289	0.094153623
RNF165	0.644900241	0.34663062	0.011698131	0.000163297	0.154792711	0.000265703	0.094153623
SYS1	0.551890777	2.04E-06	0.101012615	0.56861597	0.94670829	0.000250219	0.094153623
LOC107132475	0.887928473	0.189971026	0.377064379	0.000445911	0.002724147	0.000299773	0.102491005
CBARP	0.222931978	0.995757933	0.000636939	0.002887273	0.1971218	0.000309348	0.103606391
TIMM13	0.042291436	0.260526296	0.036626441	0.006275875	0.045056486	0.000404735	0.132842195
SH2B2	0.156891367	0.961025901	0.000117081	0.018269302	0.422317021	0.00046349	0.149143959
EMB	0.02325917	0.042497472	0.000615599	0.675696909	0.371449196	0.000505863	0.156636123
WAPL	0.419373648	0.013207966	0.324486993	0.00015948	0.521727604	0.000497816	0.156636123
ACSL6	0.388286147	0.205682241	0.196574918	0.017858678	0.000585479	0.000534479	0.162432078
ADAM12	0.420101243	0.002156625	0.005284192	0.138276522	0.315889563	0.000642546	0.170925284
ADAMTS17	0.22187747	0.005263358	0.001130439	0.751429331	0.216029451	0.000654593	0.170925284
CDCA4	0.605761076	0.012849291	0.017625762	0.737043956	0.001840776	0.000588153	0.170925284
COL5A1	0.201640659	0.0053572	0.001244877	0.350109037	0.467472162	0.00066796	0.170925284
DDX3X	0.144047446	0.046464324	0.044414532	0.001174286	0.624240532	0.000662935	0.170925284
HSPH1	0.074002015	0.597632663	0.675689468	1.44E-05	0.47635649	0.000633343	0.170925284
INTS6	0.699669753	0.363686566	0.844417442	1.31E-06	0.811613441	0.000687409	0.170925284
LOC101906101	0.091783236	0.225043448	0.00114556	0.0424841	0.226000845	0.00068422	0.170925284
NID2	0.117922315	0.495008305	0.154925486	0.104284786	0.000211944	0.000620886	0.170925284
SVEP1	0.106461535	0.032504934	0.088292699	0.001581691	0.456818558	0.000669513	0.170925284
UNC5A	0.405521262	0.07966127	0.000330711	0.023479273	0.851059062	0.000652686	0.170925284
WDR13	0.368969504	0.000133342	0.408948624	0.023639015	0.462632118	0.000667832	0.170925284
MZF1	0.598076016	0.000371808	0.001543611	0.862155519	0.807191775	0.000710718	0.174083393
HSD11B2	0.014677371	0.912020465	0.008641395	0.003685977	0.61824061	0.000765668	0.184784934
MAPRE1	0.778615544	4.76E-05	0.030086631	0.324653312	0.835634117	0.000849692	0.202091222
CREM	0.062585716	0.718704407	0.443706622	2.11E-05	0.740966465	0.000869821	0.203923321
ZNF684	0.826902083	0.15905081	0.023446569	0.000449475	0.234769817	0.000897427	0.207432027
MAT2A	0.18608199	0.078427774	0.000365925	0.074277535	0.846174886	0.000918603	0.209377655
GALNT5	0.681276951	0.000306016	0.02395989	0.083192099	0.955193465	0.001041839	0.212125102
GPT	0.520050201	0.651359781	3.32E-05	0.127586793	0.272551717	0.001029398	0.212125102
KCTD10	0.532209687	3.12E-06	0.298477524	0.842118591	0.928999696	0.001024259	0.212125102
LOC101902413	0.281224761	0.11375779	0.002299871	0.483993857	0.010205226	0.00097512	0.212125102
LOC101905979	0.744699594	0.000322291	0.104594171	0.143914884	0.109610406	0.001039986	0.212125102
LOC101906565	0.721554701	1.67E-05	0.25802486	0.411297785	0.31280475	0.001046989	0.212125102
PAK5	0.005091504	0.501959958	0.034848035	0.026826375	0.160083917	0.001013247	0.212125102
PUS10	0.962984849	6.14E-06	0.446749894	0.271298999	0.508159469	0.000977094	0.212125102
TTC38	0.242469456	0.033037289	0.000275996	0.987918502	0.163312314	0.000961568	0.212125102
CCDC188	0.702227545	0.000602045	0.537233099	0.006151998	0.30220176	0.001091216	0.213411088
KBTBD8	0.467566578	0.508764533	0.000453817	0.875618142	0.004495161	0.001096339	0.213411088
KCP	0.005273407	0.423493766	0.007249333	0.086533925	0.311703459	0.00111897	0.213411088
LYZ	0.300754611	0.08800736	0.001912865	0.020799809	0.420810071	0.001131361	0.213411088
NTNG1	1.80E-05	0.620690825	0.115617854	0.490656716	0.677545564	0.001105644	0.213411088
PTGS2	0.151913222	0.022069307	0.022916984	0.120723697	0.047296387	0.001122826	0.213411088

ADCK5	0.558827587	0.000234612	0.016441915	0.231966985	0.94234718	0.001184446	0.213603781
COL21A1	0.391319447	0.044128641	0.019864986	0.008714708	0.153332661	0.001160277	0.213603781
MAP2K6	0.276043165	0.971103855	2.73E-05	0.200784909	0.312484941	0.001162587	0.213603781
SLC30A4	0.621660585	0.276964677	0.060818429	0.000185732	0.240846269	0.001179208	0.213603781
CXCL13	0.04548451	0.740622513	0.001720298	0.303349861	0.027617195	0.001211144	0.216044443
GZMB	0.070317824	0.363885674	0.00027919	0.083860786	0.853076814	0.001258398	0.222059928
NEIL1	0.141913761	0.681932827	0.114312907	6.40E-05	0.734985234	0.001275599	0.222700534
LOC112441594	0.539715071	0.000119037	0.124102726	0.361736776	0.183643326	0.001292347	0.223249614
COL6A5	0.040099076	0.45986417	0.018618138	0.042874375	0.037118588	0.00132259	0.226093936
ANXA9	0.079503585	0.638102978	0.000313751	0.372269942	0.09483206	0.001350493	0.226388048
CLN5	0.403617819	0.189264973	1.19E-05	0.75829745	0.848260591	0.001393285	0.226388048
GIMAP7	0.179558415	0.212395367	0.048773374	0.099878644	0.003028874	0.001351907	0.226388048
MTFP1	0.046149341	0.657841813	0.078535811	0.015199984	0.016169316	0.001393262	0.226388048
SMU1	0.211264689	0.094404974	0.000712595	0.055478096	0.7296836	0.00137439	0.226388048
HOGA1	0.032750438	0.968376114	0.161680732	0.000299888	0.39598412	0.00143355	0.229493737
LOC112447770	0.00822176	0.279295363	0.230452877	0.10777097	0.010745312	0.001440367	0.229493737
GCAT	0.464427697	0.525320217	0.916429458	1.24E-05	0.22737564	0.001473001	0.230223033
LOC506828	0.239093947	0.021328347	0.00068749	0.984960788	0.182757721	0.001472106	0.230223033
CACNA1G	0.743100965	0.093904108	3.84E-05	0.40831738	0.594522304	0.001506003	0.233160518
MVD	0.582643255	0.562705773	0.959633928	0.000857424	0.002518793	0.00155499	0.238494849
TPBG	0.010302047	0.022618338	0.015352778	0.285190071	0.689518183	0.001595468	0.24243725
CACNA1A	0.393662033	0.002116331	0.428982361	0.008397533	0.24755096	0.001661217	0.24872476
KHDC4	0.332580264	0.342725646	0.886045299	5.49E-05	0.13458325	0.001667158	0.24872476
TRAF3	0.32917025	0.569361311	0.033780512	0.005328406	0.022604391	0.001693472	0.250374562
SDS	0.849804857	0.091070003	0.008003897	0.011664047	0.10695394	0.001710194	0.250589278
RYBP	0.403995984	0.03699465	0.120948626	0.001153104	0.387955365	0.001768502	0.254586668
SLC14A1	0.06296528	0.018882862	0.346429906	0.041033819	0.047483342	0.001758616	0.254586668
CYP4V2	0.001866692	0.041403183	0.071690215	0.20720946	0.719178131	0.001795879	0.25627978
HSPB3	0.76113462	0.38276724	0.00053503	0.006163961	0.912179999	0.00187657	0.26548619
JAML	0.013403611	0.19499965	0.090937282	0.061080553	0.067185894	0.002030197	0.276226636
KLK7	0.288799105	0.022687654	0.002814048	0.179981576	0.285455651	0.001987054	0.276226636
LOC112445943	0.015350275	0.395295384	0.577097131	0.009076526	0.029789766	0.001986348	0.276226636
SH3TC1	0.318160582	0.13370806	0.002235464	0.021234553	0.480331486	0.002021894	0.276226636
WT1	0.497148827	0.000845941	0.112898819	0.038539526	0.535343002	0.002036648	0.276226636
SNX16	0.790695265	0.310654396	0.000313245	0.055658725	0.231443736	0.002054271	0.276333119
ANKRD17	0.23561351	0.645130376	0.527648439	1.41E-05	0.926288754	0.002139004	0.28370758
NIF3L1	0.484551632	0.811451959	0.54637034	0.00187365	0.002638401	0.002160956	0.28370758
NIP7	0.387824131	0.001141363	0.411779603	0.089107149	0.064759505	0.002145762	0.28370758
ADAM19	0.630091656	0.253508983	0.000163757	0.908725551	0.053811889	0.002475924	0.284142595
ALKBH1	0.070116125	0.576499854	0.897589379	4.91E-05	0.643517633	0.002284784	0.284142595
C4H7orf25	0.792732644	0.110225934	0.190641557	0.001615042	0.045110774	0.00238274	0.284142595
CD93	0.301305058	0.925008196	0.002395573	0.003648195	0.515157488	0.00244151	0.284142595
ECM1	0.875991971	0.028004571	0.008914297	0.523628438	0.011126523	0.002468827	0.284142595
ICAM3	0.413513935	0.426033168	0.005522637	0.050999841	0.02194267	0.002200771	0.284142595

IMPA2	0.982910552	0.402891427	0.002949558	0.393001634	0.00238087	0.002206907	0.284142595
IPO7	0.335945706	0.006425099	0.072675663	0.018536036	0.430735662	0.002438172	0.284142595
ITGAD	0.959008438	0.037548709	0.055199113	0.107702852	0.005417774	0.002305035	0.284142595
MYLK	0.000109189	0.110742417	0.423568695	0.693306187	0.348016871	0.002414418	0.284142595
PRMT7	0.690241599	0.966425699	0.615735211	2.38E-05	0.123103503	0.002370447	0.284142595
SCYL2	0.681411581	0.000696371	0.025315681	0.272333263	0.359587622	0.002329022	0.284142595
SLC32A1	0.9017763	0.007115468	0.701052191	0.001599915	0.175797142	0.002456255	0.284142595
SLC6A16	6.74E-05	0.985918145	0.037655025	0.827362447	0.588594312	0.002388559	0.284142595
TMEM236	0.748713923	0.578455685	0.001108264	0.079307325	0.029007348	0.002223576	0.284142595
TOP1	0.304106458	0.328302127	0.688563029	7.19E-05	0.236605517	0.002318983	0.284142595
TP53INP1	0.213313477	0.481909588	0.000448579	0.262323556	0.092886307	0.002252106	0.284142595
WDR90	0.810408877	0.000146615	0.10323555	0.709120087	0.14011457	0.002390062	0.284142595
TFRC	0.014105775	0.004384914	0.533427056	0.257929232	0.154914391	0.002531088	0.288456178
CLINT1	0.00098238	0.399210702	0.310885641	0.347340672	0.036471215	0.00284023	0.28915433
GSE1	0.792687205	0.22354509	8.80E-05	0.675803523	0.155131865	0.002960083	0.28915433
GSTT2	0.274441221	0.276341639	0.714599058	9.50E-05	0.311642662	0.002919285	0.28915433
H1F0	0.561488299	0.945228257	2.28E-05	0.309779549	0.420048278	0.002878739	0.28915433
HAS3	0.608907982	0.782918458	7.48E-06	0.623796591	0.725257194	0.002932889	0.28915433
IL7R	0.109620771	0.289827807	0.154747419	0.02949407	0.009793091	0.002671957	0.28915433
L3MBTL2	0.925985933	0.131166862	0.222748577	0.023930309	0.002442282	0.00288908	0.28915433
LOC101907985	0.926075886	0.684523372	6.48E-05	0.039743562	0.842001388	0.002608142	0.28915433
LOC615278	0.108138428	0.828860417	0.014941182	0.314492317	0.003301108	0.002631086	0.28915433
LOC784052	0.29464035	0.162326396	0.228307764	0.000211913	0.642385967	0.002762271	0.28915433
PKNOX1	0.088036436	0.700401326	0.779967819	3.51E-05	0.838920371	0.002668877	0.28915433
PLEKHA6	0.648238628	0.002138024	0.179730334	0.028618938	0.204717007	0.002725608	0.28915433
POSTN	0.058624987	0.076290983	0.000699873	0.695653877	0.729647128	0.002899208	0.28915433
PTN	0.164369125	0.033585928	0.012401106	0.026719553	0.765582143	0.002644987	0.28915433
PUM2	0.119516202	0.211642543	0.00642828	0.014662078	0.632134769	0.002790031	0.28915433
PYCR2	0.747081974	0.336082401	0.403612468	1.89E-05	0.776693422	0.002768118	0.28915433
SMTNL2	0.504296376	0.752499812	0.000109999	0.09173872	0.414133758	0.0028953	0.28915433
SOX4	0.631320968	0.638999882	4.61E-06	0.954373005	0.838624568	0.002762983	0.28915433
TAS1R3	0.042463391	0.007901043	0.011818411	0.66068001	0.620683643	0.002948244	0.28915433
TPST1	0.902631515	0.435850966	0.449152342	1.45E-05	0.6304177	0.002935156	0.28915433
TUBB	0.137036121	0.498717246	0.00192193	0.299321554	0.039947233	0.002874936	0.28915433
TYSND1	0.743501799	0.748723109	0.918901377	1.28E-05	0.215395803	0.002665499	0.28915433
WDR86	0.075646491	0.02557195	0.032354458	0.051257124	0.508292654	0.002954333	0.28915433
XK	0.27723663	0.014435399	0.001107713	0.622258873	0.545624906	0.002787428	0.28915433
NCR1	0.253153067	0.697620242	0.001458595	0.075283659	0.086039637	0.003003983	0.29170627
DCAF1	0.277766	0.908200699	0.581763613	1.28E-05	0.912865298	0.003057237	0.294388099
PIEZO2	0.36824804	0.070014866	0.000567067	0.12465337	0.94232817	0.003067477	0.294388099
C7H19orf25	0.271957294	0.75291258	0.043471073	0.000372603	0.533848518	0.003135939	0.294902391
CCR2	0.180357224	0.548334095	0.172973336	0.02067084	0.00518606	0.003216594	0.294902391
IFRD1	0.209047043	0.224214989	0.102226862	0.004297543	0.088449599	0.003200758	0.294902391
IGFBP2	0.835525324	0.000650416	0.211445363	0.030300184	0.520749336	0.003190291	0.294902391

JAK1	0.968558328	0.332119992	0.000544881	0.010828794	0.943004657	0.00316063	0.294902391
MZB1	0.142748321	0.015975852	0.005212562	0.410702488	0.368690165	0.003173604	0.294902391
SMARCA5	0.440827485	0.321403224	0.032618017	0.000532413	0.724663938	0.00315193	0.294902391
SMPD3	0.781320088	0.226689544	0.00023892	0.312990133	0.132022938	0.003107748	0.294902391
ARL11	0.790291217	0.004501001	0.002820869	0.278486948	0.71689919	0.00342867	0.305803826
LOC100139916	0.012337905	0.49567309	0.073692402	0.011574697	0.378198755	0.003390976	0.305803826
PLEKHJ1	0.545216722	0.002734242	0.101495963	0.366139797	0.035201434	0.003362759	0.305803826
PLIN5	0.059041516	0.828002501	0.02828227	0.022078402	0.065032751	0.003406304	0.305803826
RXFP1	0.084497782	0.503976814	0.20629119	0.108664734	0.002091849	0.003420781	0.305803826
ESRP1	0.593039767	0.12072007	0.099600727	0.000302477	0.940917447	0.003460871	0.305977787
KCNMA1	9.10E-05	0.332714973	0.172778491	0.407516732	0.954941532	0.00346791	0.305977787
GNAI3	0.196875018	0.001615389	0.033863651	0.216676934	0.891373965	0.003522926	0.309169727
ELP3	0.158828779	0.043187635	0.806833706	0.231739251	0.00169326	0.003634043	0.317224879
LOC112441505	0.876138592	0.805902563	7.24E-05	0.415269551	0.105743099	0.003720482	0.323051977
LOC107131896	0.938840425	0.009061935	0.051612341	0.024378932	0.214582787	0.00378381	0.326821624
NUDT9	0.379448111	0.066374758	0.002447213	0.09029299	0.418244169	0.003819895	0.328211021
ACTR3	0.824382498	0.000401073	0.11889733	0.071567611	0.857040319	0.003917991	0.329734104
KYNU	0.231829777	0.210309617	0.032399634	0.945958791	0.001602461	0.003898516	0.329734104
LOC100850659	0.205140454	0.08434475	0.126364368	0.006089282	0.181106743	0.003917943	0.329734104
SLC23A2	0.757912263	0.03143185	0.00188967	0.090311145	0.582733594	0.003868706	0.329734104
COL5A2	0.196446905	0.021472793	0.005557773	0.675254412	0.156149728	0.003988551	0.330371651
CYP2B6	0.103808316	0.137938369	0.012275323	0.015038537	0.960738967	0.004066484	0.330371651
HK2	0.05976358	0.167944378	0.403238581	0.710702634	0.000878187	0.004050926	0.330371651
KCNG1	0.038296676	0.602275996	0.001574926	0.236033342	0.284065705	0.003946373	0.330371651
LOC112441499	0.058449089	0.008997767	0.010933785	0.742373231	0.591610816	0.004050272	0.330371651
LRRC46	0.528851989	0.31095062	0.143121258	0.000426753	0.249901499	0.004032479	0.330371651
TMED10	0.958157336	0.000183548	0.179603747	0.140094452	0.554377049	0.003966772	0.330371651
CABIN1	0.776121574	0.191672947	0.002636332	0.006920696	0.943193689	0.004089911	0.330638071
ELOF1	0.018364944	0.622531214	0.225938215	0.001753782	0.572432089	0.004127871	0.330999554
MARC1	0.455716527	0.111800298	0.000154034	0.376836589	0.884793677	0.004154573	0.330999554
NR3C2	0.001349398	0.690608837	0.178004614	0.431202306	0.036585643	0.004154891	0.330999554
BCL11B	0.104445895	0.642335139	0.45852324	0.026637531	0.003368253	0.004316257	0.332872069
C2CD2L	0.003589844	0.083864546	0.329038486	0.301435402	0.090054751	0.004236562	0.332872069
DIAPH3	0.44203018	0.004444466	0.007428383	0.22132267	0.851579499	0.004305633	0.332872069
GAL	0.350695784	0.835075739	0.019378124	0.005055454	0.095631648	0.004297932	0.332872069
LOC100847365	0.189133308	0.264311133	0.130834752	0.057312146	0.00735349	0.004312233	0.332872069
PPP1CB	0.544408348	0.008775788	0.023272465	0.025059345	0.970649568	0.004253941	0.332872069
PRLR	0.000143822	0.225328354	0.2680728	0.952802094	0.333885343	0.00432038	0.332872069
C1QTNF6	0.346047387	0.058462072	0.002280185	0.541637582	0.116472933	0.004482774	0.335539476
CCDC84	0.226099944	0.000482912	0.24775478	0.224682817	0.468538291	0.00441393	0.335539476
CEBPZ	0.132421715	0.620940686	0.012549704	0.008345706	0.331421331	0.004421059	0.335539476
NUDT18	0.999908425	0.063620776	0.738500152	0.000596721	0.103496215	0.004473139	0.335539476
PLEKHG4	0.444365218	0.018062566	0.005001276	0.090304649	0.799771874	0.004470721	0.335539476
SLC31A2	0.175892491	0.367336365	0.00570545	0.43201217	0.018040651	0.004441933	0.335539476

TRPC1	0.530561238	0.677423036	0.490827962	0.013444122	0.00123292	0.004498122	0.335539476
CSPG5	0.335056528	0.520251155	0.2068373	0.000819792	0.101789153	0.004590537	0.338588731
DLX3	0.217863448	0.161085164	0.000155191	0.694129855	0.797527189	0.004597547	0.338588731
NUP98	0.275710067	0.256598674	0.630087119	7.00E-05	0.966841965	0.004600895	0.338588731
SFRP1	0.007735163	0.437590328	0.251867493	0.55738707	0.006451118	0.004652297	0.340843069
CALM3	0.016003131	0.423077599	0.523042199	0.001656523	0.529440245	0.004695847	0.342504635
AKR1B1	0.031375685	0.037353823	0.007228056	0.543632835	0.690264334	0.004774284	0.343592771
APLP1	0.003479268	0.56716686	0.199273864	0.008595375	0.946094639	0.004794513	0.343592771
LOC101907041	0.996651704	0.2528505	0.02808434	0.002549559	0.175631563	0.004763863	0.343592771
TMEM203	0.591063214	0.022212738	0.063410091	0.005891831	0.642790326	0.004746504	0.343592771
CKS2	0.838062788	0.00172372	0.021065921	0.608002605	0.17706056	0.004877788	0.348040759
CPM	0.404729108	0.127623304	0.01247997	0.982549126	0.005221389	0.004910652	0.348868896
FAM136A	0.26060078	0.294275418	0.28965682	0.175533869	0.0008615	0.004965513	0.349738334
LOC100297044	0.516604372	0.014208973	0.001860357	0.258860083	0.948067353	0.004957317	0.349738334
ATP7A	0.102403127	0.065303113	0.050766259	0.037229894	0.284930443	0.005217239	0.353324284
COL3A1	0.317274832	0.020566487	0.00667535	0.320826885	0.257686432	0.005217062	0.353324284
HAS1	0.961762516	0.006496479	0.001094114	0.711851081	0.733615173	0.005185039	0.353324284
LCMT2	0.8621394	0.015530973	0.101054086	0.297248311	0.008590119	0.005065846	0.353324284
LOC783540	0.067908399	0.34636157	0.510301692	0.150382825	0.001916572	0.005070449	0.353324284
MAGEL2	0.141840345	0.163438239	0.002508934	0.305871674	0.203220287	0.005231723	0.353324284
SLC1A2	0.464644514	0.483953408	0.000310378	0.205224665	0.247237395	0.005155389	0.353324284
THBS4	0.672426998	0.798650897	0.091198523	0.00890565	0.008164883	0.005176057	0.353324284
TMEM245	0.005916748	0.050800284	0.08571356	0.26847626	0.504713928	0.005103297	0.353324284
TRAPPC8	0.44001591	0.166979058	0.00096083	0.053147455	0.958061556	0.00521042	0.353324284
TCAF1	0.433146875	0.260511835	0.001268092	0.426378518	0.061424478	0.005366768	0.359485848
TROAP	0.11781519	0.005993125	0.060439836	0.11777831	0.742096615	0.005348865	0.359485848
LOC513659	0.87575043	0.47475249	0.51940413	3.25E-05	0.53993825	0.005408436	0.360804263
SLC16A3	0.100592526	0.922027402	0.025779309	0.01064514	0.151860825	0.00548567	0.364475038
C18H16orf86	0.914516458	0.001472584	0.022992474	0.819042675	0.156310698	0.005584722	0.369559939
TMEM100	0.858182914	0.000866706	0.757142575	0.025423896	0.285694826	0.005710045	0.37633555
TPPP	0.3843244	0.160957091	0.815739298	0.000448958	0.183265592	0.005770534	0.378800931
ADM5	0.636721504	0.14420397	0.002847665	0.038148966	0.437776773	0.005979842	0.380977595
BBS9	0.138091897	0.748486071	0.284815449	0.030626682	0.004959587	0.006080853	0.380977595
LAMB3	0.034136679	0.75988208	0.006227286	0.057694955	0.459398161	0.005897219	0.380977595
LOC100300806	0.373356618	0.113463194	0.000708036	0.234673679	0.628703295	0.0060364	0.380977595
LOC101908206	0.869517476	0.552556516	3.30E-05	0.561304213	0.500679951	0.006063992	0.380977595
LOC514181	0.00671333	0.499209153	0.0449163	0.374315295	0.075018342	0.00584409	0.380977595
ME2	0.877309304	0.284671107	0.00235644	0.056268916	0.135077617	0.00608227	0.380977595
NUAK1	0.955710916	0.169899962	0.088479752	0.001280862	0.242747608	0.006076508	0.380977595
P3H1	0.51283008	0.009125789	0.003451151	0.278283356	0.957941386	0.005920671	0.380977595
PTI	0.13522607	0.191831092	0.021041028	0.4294322	0.018524368	0.005955913	0.380977595
RFXANK	0.506510581	0.004738275	0.018237155	0.144800032	0.687986731	0.005973659	0.380977595
ZMYND8	0.233077443	0.11355095	0.002521349	0.974612693	0.066952158	0.005967894	0.380977595
LOC617565	0.515848196	0.234076136	0.00022398	0.643640753	0.260114092	0.006134762	0.38217746

SIRPA	0.004156241	0.635627769	0.995687738	0.275362332	0.006298097	0.006167157	0.38217746
SMUG1	0.330249065	0.010283322	0.711827854	0.981482983	0.001924512	0.006171289	0.38217746
APC2	0.006186942	0.04760316	0.047198013	0.398430617	0.852286304	0.006317366	0.386010235
CLASRP	0.983544223	0.000718597	0.025877273	0.650367367	0.394677134	0.00629308	0.386010235
FNDC1	0.927577305	0.074369261	0.001874005	0.097430216	0.379577835	0.006374308	0.386010235
LOC112441884	0.005230664	0.215039396	0.16830394	0.128646176	0.194740697	0.006338388	0.386010235
LOC781001	0.495736415	0.000540972	0.111485525	0.233077252	0.671604287	0.006279404	0.386010235
SLIT1	0.292501327	0.156241942	0.66769184	0.001944113	0.080291205	0.006357615	0.386010235
CENPB	0.584684701	0.002832423	0.318335869	0.04200297	0.221204997	0.006484014	0.389777135
ZNF428	0.193216473	0.353993757	0.000207656	0.422163908	0.815322114	0.006475132	0.389777135
RRS1	0.390694001	0.026521971	0.048939614	0.104052923	0.09412462	0.006547534	0.392159065
ANTXRL	0.609976092	0.001389943	0.76156972	0.299429988	0.026012912	0.006605477	0.392368099
BEND6	0.345087372	0.945789011	0.011143496	0.006446998	0.217048301	0.006660805	0.392368099
CD248	0.404214326	0.569069831	0.000136172	0.229051074	0.714338262	0.006693677	0.392368099
CIITA	0.966293857	0.368398873	0.055459359	0.001196857	0.218901194	0.006737017	0.392368099
HDC	0.032032323	0.066635078	0.011877752	0.812444059	0.252666456	0.006766204	0.392368099
INAFM2	0.363892952	0.00381267	0.428371206	0.013290169	0.658076158	0.006760334	0.392368099
SAP30L	0.000361883	0.360114526	0.563330783	0.32329277	0.213011453	0.006629758	0.392368099
SIX4	0.52052836	0.053117653	0.114988785	0.001979812	0.818482645	0.006718297	0.392368099
TMCC1	0.776325463	0.989570998	0.000435781	0.069284364	0.222737023	0.0067315	0.392368099
CCL2	0.803572705	0.512416293	6.59E-05	0.735947505	0.262276867	0.006800223	0.392952328
FAM118A	0.255910806	0.832141942	0.116947127	0.089531537	0.002363036	0.006825027	0.393001841
ACAN	0.431342998	0.091255153	0.00910837	0.071171112	0.207685345	0.006852748	0.393218332
CKAP4	0.50380518	0.368340229	0.001060308	0.289002317	0.094605616	0.006925434	0.395189872
EHF	0.162287739	0.693231102	0.989447383	0.0002426	0.200420379	0.006954886	0.395189872
GCC1	0.073884422	0.820833762	0.018093097	0.0720358	0.068874312	0.00698343	0.395189872
TARBP1	0.68982168	0.019501187	0.002282241	0.431809481	0.410302612	0.006979251	0.395189872
AFF4	0.521824689	0.075849841	0.463933433	0.000342474	0.970100331	0.007562747	0.395569854
AP2B1	0.677343587	0.048989198	0.071032788	0.005398966	0.491118684	0.007691348	0.395569854
ARMCX3	0.198207722	0.2899739	0.303400374	0.000546331	0.674702514	0.007843577	0.395569854
BMP1	0.225269402	0.030104755	0.001332291	0.946746063	0.738666181	0.007750324	0.395569854
BMPER	0.06230715	0.501746606	0.016893043	0.777114079	0.015108054	0.00764891	0.395569854
CA3	0.039480618	0.112868656	0.008751294	0.305838986	0.493541493	0.007376011	0.395569854
CCAR1	0.556257257	0.04043412	0.465186721	0.000960401	0.617204408	0.007650249	0.395569854
CD4	0.41624221	0.292124593	0.184008917	0.176329906	0.001568854	0.007639547	0.395569854
CLASP1	0.604738641	0.000596613	0.397669974	0.094816688	0.433718229	0.007388256	0.395569854
CNOT7	0.127424987	0.016387482	0.047154697	0.07888501	0.768409731	0.00744801	0.395569854
CTHRC1	0.436221796	0.013742458	0.011090731	0.120244048	0.749600661	0.007468976	0.395569854
DNAJB4	0.018252623	0.383727626	0.4348437	0.035428597	0.054453855	0.007366728	0.395569854
ECD	0.78314937	0.276540892	0.923119162	0.002003263	0.014435255	0.007283652	0.395569854
ELF3	0.053389686	0.115405441	0.479928238	0.002260152	0.944430544	0.007744714	0.395569854
HOXD3	0.254252021	0.281321357	0.009554816	0.026489629	0.32333243	0.007347182	0.395569854
IFI27	0.253324852	0.000923514	0.340084215	0.515145559	0.155214421	0.007787167	0.395569854
KCNH1	0.080574246	0.598084119	0.374455614	0.004044051	0.086056666	0.007717287	0.395569854

LAT	0.20161124	0.093978057	0.095400457	0.063227759	0.055286266	0.007750305	0.395569854
LOC100139881	0.163718259	0.204990898	0.024088413	0.013352373	0.578238598	0.00768443	0.395569854
LOC104976020	0.124002496	0.798225228	0.199141403	0.176341734	0.001842569	0.007823858	0.395569854
LOC512867	0.338296657	0.341422785	0.016419396	0.429555897	0.006881402	0.007128221	0.395569854
LRRC8B	0.301498022	0.276675361	0.006415301	0.066773202	0.155930463	0.007097943	0.395569854
MYO1E	0.741884667	0.574536773	0.052466154	0.006617573	0.040914635	0.007523084	0.395569854
PIK3R6	0.70686365	0.445157555	0.003370609	0.007176655	0.850405824	0.00788199	0.395569854
PPP1R3B	0.008475659	0.144760357	0.286045942	0.065685933	0.244203038	0.007149387	0.395569854
PTPRT	0.089057479	0.48813027	0.674579321	0.069195032	0.002771412	0.007144003	0.395569854
RABGEF1	0.623421703	0.039578237	0.022888835	0.018213066	0.565247663	0.007312577	0.395569854
RRAGD	0.657925884	0.044396424	0.002012952	0.413620598	0.243954172	0.007416775	0.395569854
SBF1	0.733629772	0.001105006	0.418605389	0.027799377	0.685235375	0.007874591	0.395569854
SCIN	0.21965093	0.085601819	0.004801602	0.928986806	0.066474334	0.007100928	0.395569854
SIAE	0.077438165	0.885945118	0.023256431	0.102010378	0.037245377	0.007529269	0.395569854
SRGAP3	0.000465567	0.714108996	0.835022834	0.087571181	0.258268925	0.007716274	0.395569854
SYNM	0.00039498	0.194833201	0.292268965	0.651688237	0.380750554	0.007105919	0.395569854
TSR1	0.08744058	0.327630258	0.085628277	0.37060036	0.007063426	0.007838186	0.395569854
WNT4	0.380886829	0.013826083	0.00216635	0.542183363	0.979289219	0.007525143	0.395569854
ZFX	0.062822128	0.104702627	0.268893189	0.00583115	0.564450516	0.007319015	0.395569854
ZNF182	0.659819744	0.082265912	0.001433801	0.541975966	0.144901683	0.007572587	0.395569854
KIF14	0.001383	0.565791176	0.209213142	0.124718986	0.320675459	0.007944995	0.397516223
PIPOX	0.158569809	0.79308595	0.049630336	0.001704162	0.622375732	0.008006247	0.399274067
TENM2	0.014296627	0.091595507	0.152704222	0.088227193	0.376740834	0.008028788	0.399274067
KCNMB1	0.014564354	0.045244007	0.779682456	0.125306757	0.105572948	0.008154567	0.400856805
RASGRP3	0.851150773	0.527382767	0.006528201	0.044828487	0.051773092	0.008158319	0.400856805
TMEM120A	0.213277392	0.701774635	0.040038858	0.007972642	0.14093688	0.008101838	0.400856805
TSHZ3	0.398116917	0.275021343	0.007105715	0.0276478	0.314211701	0.008122868	0.400856805
BAHCC1	0.893886899	0.110151592	0.006254095	0.031457388	0.354683795	0.00821629	0.401114753
CCSAP	0.081982279	0.659909932	0.654296593	0.71631342	0.000269782	0.008191229	0.401114753
NDST1	0.172086377	0.878997604	0.009048963	0.23271675	0.021647324	0.008236894	0.401114753
RAB7B	0.745230847	0.687421637	0.377656654	0.000271597	0.132466478	0.008290879	0.402303612
STX12	0.214741073	0.173729543	0.046613618	0.024469876	0.164124158	0.008310336	0.402303612
ALPK3	0.376105216	0.828365059	0.541002151	0.645373146	6.54E-05	0.008420585	0.405622048
ZNF131	0.314446629	0.801552319	0.995607726	4.57E-05	0.621058429	0.008428317	0.405622048
CHST13	0.270427303	0.566590373	0.004569398	0.030747275	0.333151578	0.008465045	0.406198422
CNOT1	0.136053801	0.503262788	0.774758609	0.000136896	0.994961858	0.00850909	0.4070887
TBL1XR1	0.334398515	0.030446628	0.001357483	0.588214595	0.896150255	0.008558016	0.4070887
ZNF469	0.910480279	0.016440628	0.013066397	0.153982711	0.241819074	0.008555999	0.4070887
TCEA1	0.699618444	0.223754317	0.158891907	0.22240035	0.00132929	0.008613418	0.408539878
AQP9	0.854289826	0.731234462	0.145687713	0.00514744	0.016886858	0.009061219	0.420935737
CENPA	0.19253454	0.018165967	0.010810762	0.303260773	0.672635935	0.008903476	0.420935737
COL1A1	0.642006892	0.015370493	0.009279128	0.137667256	0.624102967	0.009026545	0.420935737
DGKI	0.304375081	0.17412355	0.00164384	0.104757329	0.864928131	0.009047724	0.420935737
LOC104969384	0.320490521	0.085701188	0.014864741	0.138325027	0.140997355	0.009102445	0.420935737

LOC107131975	0.951083204	0.000131425	0.456252133	0.606012056	0.232397043	0.00915691	0.420935737
LSAMP	0.000621861	0.269572097	0.820980166	0.074605787	0.775705302	0.009103886	0.420935737
PAQR7	0.761475945	0.235512166	0.027186892	0.007096486	0.227569933	0.009031814	0.420935737
PPIF	0.692335369	0.184764797	0.423212522	0.018178374	0.007869063	0.00892842	0.420935737
PTGES3	0.086856554	0.212133848	0.169947594	0.004584103	0.558795113	0.009148478	0.420935737
TMEM64	0.352320072	0.0635632	0.292707274	0.001653482	0.738619344	0.009136266	0.420935737
TYRP1	0.269655466	0.840748304	0.171818996	0.049602459	0.004174783	0.009184265	0.421013884
CFI	0.003498162	0.08048151	0.037111518	0.862280362	0.912547172	0.009306031	0.424359175
MFSD9	0.07762929	0.621685229	0.392623438	0.030774532	0.01412557	0.009318213	0.424359175
PRAG1	0.059863225	0.801208039	0.034463563	0.024393419	0.204809054	0.009334816	0.424359175
KIF4A	0.410735101	0.025375783	0.033661615	0.102528609	0.231882305	0.009399581	0.424949106
RAP1A	0.335302862	0.05986051	0.001155982	0.505063399	0.710003921	0.009383229	0.424949106
ATP13A2	0.160916905	0.038503954	0.117597148	0.032039076	0.361550099	0.00947656	0.42503764
CCNJ	0.011850417	0.773315726	0.009070056	0.13057992	0.774005265	0.009445965	0.42503764
FAM160A2	0.992689243	0.434236207	0.015094818	0.00458193	0.283213579	0.009479238	0.42503764
GPBP1	0.172573562	0.62136118	0.746897325	0.00037049	0.287081989	0.009537227	0.426472581
LOC516494	0.90535615	0.074497163	0.024057146	0.009852119	0.544258012	0.009677441	0.429233744
LOC788801	0.258375593	0.371261401	0.007027967	0.095741419	0.134764487	0.00967588	0.429233744
LRRC24	0.088865251	0.185022364	0.015330321	0.035685415	0.964451896	0.009658087	0.429233744
FLNC	0.001304772	0.221453166	0.21014193	0.71642316	0.206029072	0.009877722	0.436189841
LOC112442610	0.807127721	0.024659892	0.035065589	0.302164829	0.042558595	0.00988743	0.436189841
CENPF	0.638079387	0.004398792	0.01181531	0.285414275	0.952237895	0.009916189	0.436285732
ACAP1	0.563812716	0.005061434	0.023100185	0.192707697	0.716629362	0.00998481	0.436307929
HGH1	0.326634705	0.54776405	0.751353853	0.037034852	0.001831642	0.009996452	0.436307929
TIMP1	0.764382982	0.08122748	0.028350107	0.017665646	0.293184803	0.009994676	0.436307929
CPZ	0.27435407	0.028745017	0.021682213	0.234231823	0.229453486	0.010050013	0.437482113
STOX2	0.35997957	0.888416828	0.002832512	0.020062607	0.508359726	0.01008687	0.437924932
ARMCX4	0.711831156	0.666266747	0.000992782	0.262560647	0.076281029	0.010230282	0.440653954
CFB	0.054649163	0.011418959	0.107422207	0.196873378	0.714416184	0.010228941	0.440653954
USPL1	0.259512907	0.460894524	0.918072715	9.52E-05	0.89578024	0.010178091	0.440653954
IGF1	0.540917801	0.031873139	0.022823606	0.428353126	0.056350686	0.010280956	0.441677425
DIRAS2	0.891211682	0.840413639	0.012154098	0.778206114	0.001352076	0.010340624	0.443080868
AOX1	0.225091654	0.864222315	0.272923789	0.018482635	0.009804963	0.010372523	0.443290302
GDF6	0.825022352	0.008897758	0.041801197	0.324016557	0.097892423	0.010455265	0.444009844
KLRG2	0.150669861	0.000186219	0.625418283	0.932589985	0.601103898	0.010531879	0.444009844
PAN2	0.836605619	9.63E-05	0.455993378	0.956930673	0.28164363	0.010578749	0.444009844
PSRC1	0.918235499	0.414771969	0.003880903	0.008164113	0.809404806	0.010480461	0.444009844
RHOH	0.595258173	0.770313518	0.034393479	0.080867038	0.007699275	0.010518693	0.444009844
VASN	0.89204445	0.50488536	0.000210617	0.111072757	0.934969157	0.010542148	0.444009844
ZAR1L	0.324572271	0.018598681	0.164459681	0.277779567	0.03577806	0.010553721	0.444009844
ADAMTS2	0.104289614	0.009545976	0.054720081	0.291216742	0.674395625	0.011157144	0.447299164
ARFGEF1	0.162847451	0.9215758	0.013319862	0.007179106	0.729904376	0.01099636	0.447299164
CYP20A1	0.006053007	0.680020279	0.022157981	0.164438664	0.70746865	0.011093819	0.447299164
EMC9	0.273223883	0.252914095	0.763650409	0.000203798	0.980479675	0.011046447	0.447299164

FLT3	0.760693346	0.138279316	0.066966874	0.122613494	0.011816021	0.010801496	0.447299164
GLIS3	0.062146896	0.257881964	0.069623462	0.016583263	0.551553179	0.010801787	0.447299164
GTF2H1	0.226460639	0.716424403	0.192792244	0.000612385	0.560206349	0.011179815	0.447299164
LOC404103	0.062354923	0.171311486	0.073926153	0.310933226	0.043985726	0.011229496	0.447299164
LOC783577	0.302228446	0.212333085	0.083448372	0.002012418	0.979605347	0.011055442	0.447299164
PALB2	0.268117675	0.000554756	0.23293186	0.449612761	0.650008983	0.010743198	0.447299164
PCDH19	0.686484328	0.560059619	0.271496376	0.125139268	0.000810655	0.011078487	0.447299164
PCF11	0.1437188	0.490042011	0.896938494	0.003902707	0.042567971	0.011010447	0.447299164
PICALM	0.62346556	0.166586528	0.000492826	0.248047032	0.810472416	0.010862836	0.447299164
PREX1	0.055399291	0.453690795	0.937074924	0.847959654	0.000531328	0.011094551	0.447299164
RRAGC	0.431050134	0.054834366	0.001991546	0.23222674	0.926925911	0.010748528	0.447299164
SEC16B	0.647485243	0.029988093	0.013974122	0.213652785	0.181678026	0.011037539	0.447299164
SH3BGR	0.016556177	0.095506328	0.903600857	0.406724253	0.018473062	0.01118304	0.447299164
TTLL11	0.696617891	0.004489973	0.043060916	0.334605811	0.239646067	0.011229339	0.447299164
ZBTB40	0.523557757	0.039737464	0.083340245	0.367771864	0.016055995	0.010825415	0.447299164
ZBTB8OS	0.025853813	0.343158742	0.566724289	0.003094008	0.664958788	0.010902226	0.447299164
ZNF451	0.228408851	0.568066755	0.419414782	0.000243185	0.794599694	0.011025804	0.447299164
ACTR2	0.3693044	0.007224912	0.088884716	0.235262767	0.206161413	0.011724637	0.448524169
ARFGAP1	0.007270331	0.650274208	0.538436587	0.041922508	0.10398981	0.011440283	0.448524169
CAPN8	0.113630562	0.005142231	0.039695129	0.602632011	0.83062314	0.011799367	0.448524169
CBX2	0.653236018	0.791026018	0.016185576	0.003170826	0.418884718	0.01144809	0.448524169
CDK8	0.15686531	0.75673622	0.011598642	0.013683535	0.591368683	0.011471201	0.448524169
CENPC	0.243067783	0.193968622	0.820105142	0.001341011	0.216264106	0.011522124	0.448524169
CHMP6	0.102840422	0.37267089	0.090612868	0.243892872	0.013811115	0.011860195	0.448524169
CKAP2L	0.757971865	0.002384651	0.051633326	0.199809274	0.593660341	0.011421149	0.448524169
DAZAP2	0.400971345	0.00051593	0.252772582	0.232945869	0.937976996	0.011670778	0.448524169
EN1	0.334331893	0.000205524	0.367040984	0.858055976	0.537256905	0.011810733	0.448524169
EZH2	0.359284233	0.012243627	0.055216056	0.087180334	0.525946854	0.011468287	0.448524169
FAM91A1	0.994285119	0.003408146	0.216261188	0.036031641	0.414236197	0.011327473	0.448524169
FARSB	0.557772001	0.712969047	0.280761028	0.583022554	0.000174332	0.01161658	0.448524169
FHL5	0.004277257	0.26010301	0.961225818	0.40615666	0.027374436	0.011992814	0.448524169
GLULP	0.841003782	0.336841404	0.000255324	0.426877025	0.385506184	0.012001762	0.448524169
GPR162	0.924387438	0.106945546	0.000860324	0.212686249	0.65200787	0.011926899	0.448524169
LOC101903647	0.393983662	0.013427546	0.186221112	0.013844886	0.834903372	0.011644119	0.448524169
LOC112444603	0.288252903	0.442660359	0.720512039	0.00022026	0.555550935	0.011547589	0.448524169
LOC514457	0.614369571	0.008160462	0.169098103	0.162477404	0.079199459	0.011307092	0.448524169
POLR1B	0.87411575	0.042160895	0.284481927	0.370230713	0.003063436	0.011993565	0.448524169
RALGAPB	0.52403685	0.032154486	0.006281373	0.113008893	0.937476598	0.011521867	0.448524169
SGK2	0.192120114	0.850469458	0.049653795	0.00241834	0.608421452	0.012025509	0.448524169
SIGLEC11	0.006347459	0.8252667	0.372278885	0.030725096	0.190043756	0.011643754	0.448524169
SLC16A14	0.294894468	0.748571283	0.200929344	0.000876149	0.302394892	0.011897367	0.448524169
SNTA1	0.846969252	0.314134954	0.104784431	0.147242233	0.002906952	0.012022578	0.448524169
TM9SF2	0.327591792	0.101175832	0.015939948	0.023011497	0.948203636	0.011741976	0.448524169
TRAF3IP3	0.340867584	0.390129609	0.068008974	0.126851387	0.010083518	0.011770202	0.448524169

VASP	0.384562661	0.045136603	0.311590956	0.09135024	0.023790355	0.011899161	0.448524169
CCR4	0.026613994	0.870685447	0.73228912	0.004141163	0.170793492	0.01206987	0.449157916
LOC112442226	0.157245804	0.176203345	0.113031144	0.02967624	0.129652647	0.01210282	0.44936511
ATP6V0A1	0.998716102	0.048787882	0.001594013	0.933880437	0.168437923	0.012217406	0.451597191
TRAM2	0.553566046	0.359711991	0.000718655	0.214509495	0.398029495	0.012217973	0.451597191
LOC107131704	0.169928085	0.002024132	0.400613378	0.929504847	0.096378599	0.012303784	0.451716774
MYB	0.073510916	0.516150227	0.014642386	0.029189687	0.758872633	0.012278231	0.451716774
RGS7	0.004469426	0.454001937	0.205599423	0.865809761	0.034040187	0.012270666	0.451716774
PLD4	0.044349118	0.137903689	0.084038892	0.137121759	0.175834627	0.012336492	0.451906632
CAPZB	0.332727484	0.001884193	0.065152713	0.56672285	0.546756226	0.012515156	0.454714738
LOC100847509	0.170095122	0.318420368	0.847092779	0.00061552	0.447477665	0.012501926	0.454714738
MRPL16	0.265625214	0.869264139	0.582152375	0.054552821	0.001721319	0.012492108	0.454714738
PRKAR1A	0.546093496	0.023979462	0.100719586	0.013720776	0.706931639	0.012607105	0.454714738
PTCD2	0.26064185	0.344191387	0.288508552	0.049097971	0.010062391	0.012602975	0.454714738
SLAIN2	0.600323276	0.01213223	0.16608057	0.014438251	0.731810039	0.012598824	0.454714738
TRA2A	0.534197332	0.217820064	0.325007628	0.003970574	0.084699933	0.012556831	0.454714738
CHST6	0.799454891	0.034936531	0.028361013	0.060561219	0.267901973	0.01264651	0.45513569
EIF2S1	0.837150403	0.004828906	0.038792296	0.454128311	0.181522264	0.012696991	0.455952571
GUCD1	0.424032037	0.002039999	0.080995709	0.586070554	0.317820202	0.012779187	0.457902267
RIPOR3	0.013676962	0.194139538	0.938446376	0.117531743	0.04521585	0.012906728	0.461464748
MEG3	0.1292833	0.023454489	0.010169179	0.523878768	0.831338793	0.013030723	0.464885191
AARSD1	0.338672785	0.328170202	0.273017024	0.347311338	0.001278964	0.013063066	0.465001625
IL18R1	0.557901438	0.110217482	0.60183703	0.018301022	0.019963273	0.013090656	0.465001625
LOC782951	0.17489934	0.547803306	0.674233615	0.000306195	0.690612826	0.013182399	0.467249149
LOC782348	0.744296667	0.012076102	0.740999322	0.364571619	0.005651743	0.013223722	0.467703654
CAMK2G	0.129487861	0.212294516	0.038729605	0.086546295	0.150345722	0.013308698	0.468688936
LOC112444310	0.310819815	0.012673371	0.108189095	0.045439765	0.715206199	0.013306642	0.468688936
DHRS1	0.042912757	0.035910431	0.910437371	0.311116782	0.032219797	0.01344577	0.468763014
KIFC2	0.655436166	0.056568603	0.000902181	0.624477133	0.669229569	0.013390975	0.468763014
M6PR	0.776488504	0.015892225	0.006319286	0.216648931	0.822792345	0.013339625	0.468763014
PCDH1	0.792850313	0.999847235	0.001825991	0.019154628	0.505508846	0.013414751	0.468763014
PCYT2	0.425456569	0.645630038	0.660556137	0.00126179	0.061481032	0.013453621	0.468763014
KCNH8	0.186970167	0.23811146	0.033070098	0.206871224	0.04682472	0.013573719	0.471945547
CCL22	0.094552086	0.336477262	0.002221178	0.20482143	0.997793622	0.013690047	0.473109108
EFNB2	0.635563548	0.43655243	0.000185735	0.315715586	0.883540033	0.013647037	0.473109108
ZNF391	0.914164621	0.023650954	0.017805515	0.308076315	0.121817546	0.01369367	0.473109108
CDKN1A	0.925871039	0.113628206	0.005701616	0.093053007	0.262096352	0.013810265	0.473448064
DUSP16	0.485860278	0.887095622	0.506010499	0.000988191	0.067942739	0.013818879	0.473448064
MRPS24	0.370900579	0.588948826	0.973319273	0.000323277	0.212092315	0.013777198	0.473448064
STPG4	0.018734791	0.042227836	0.123821411	0.409141444	0.36310038	0.013761145	0.473448064
TMEM35A	0.011494832	0.539802902	0.14584561	0.44592955	0.036467427	0.013865915	0.474069858
SELL	0.248317092	0.082684935	0.119568737	0.185143523	0.032723304	0.013966095	0.476502244
COL1A2	0.912482024	0.020520195	0.020275109	0.070855976	0.555260744	0.014005935	0.47687011
ABT1	0.522000655	0.010950125	0.082790783	0.371687927	0.08808574	0.014357692	0.477194245

ATG4B	0.732101316	0.99424235	0.002546581	0.240648745	0.034376439	0.014257591	0.477194245
CBL	0.705935849	0.134743475	0.106980787	0.002522968	0.591901659	0.014170634	0.477194245
CBX6	0.408937605	0.289945549	0.013392852	0.017966667	0.532833303	0.014174289	0.477194245
DCAF13	0.985992753	0.1021405	0.002612486	0.51734842	0.111775447	0.014182021	0.477194245
DDX21	0.789816317	0.138476129	0.30063211	0.001683274	0.279212901	0.014332454	0.477194245
DNAJC27	0.151235978	0.219893414	0.394300337	0.002691338	0.435735272	0.01428472	0.477194245
FAM167A	0.61835469	0.564620716	0.059182287	0.582963508	0.001275153	0.014273693	0.477194245
FSCN1	0.770476872	0.407589669	0.00037666	0.815319821	0.160766322	0.014364387	0.477194245
GIMAP7	0.09196316	0.958774383	0.04982665	0.111547212	0.03153443	0.014332654	0.477194245
IL2RA	0.303356175	0.677165531	0.426293282	0.00318387	0.055281249	0.01430708	0.477194245
PBXIP1	0.207047915	0.183774325	0.614496116	0.61416418	0.001069697	0.014274366	0.477194245
FABP1	0.006729493	0.218868438	0.694953708	0.028273096	0.537982643	0.014404882	0.477572768
DLGAP5	0.451372717	0.009594713	0.033179808	0.187055293	0.589450458	0.014576228	0.477694159
LOC112441663	0.57828644	0.931412791	0.00190477	0.016452487	0.942453918	0.014616229	0.477694159
MAP3K12	0.429777013	0.041003312	0.004277753	0.818120877	0.258433409	0.014634993	0.477694159
MTAP	0.977652508	0.21371854	0.051251342	0.007528836	0.197818059	0.014641409	0.477694159
NFYB	0.139579979	0.797992756	0.763846307	0.000306188	0.607053489	0.01455774	0.477694159
PAK2	0.510850471	0.015856821	0.063804305	0.041482551	0.739157538	0.014578585	0.477694159
STEAP2	0.07134185	0.424641076	0.009048858	0.20377785	0.28214006	0.014524669	0.477694159
TARDBP	0.689577081	0.921752739	0.467029462	0.01181221	0.004507192	0.014551753	0.477694159
HOXA9	0.793984992	0.001069974	0.126210526	0.182284638	0.818602485	0.014672845	0.477769975
GPR35	0.175047892	0.024546547	0.468351704	0.031740467	0.252598303	0.014756592	0.479056366
ODC1	0.684159663	0.049040782	0.031673374	0.041248595	0.368604608	0.014770734	0.479056366
GMPR	0.121592993	0.013828237	0.031309599	0.47223458	0.653467489	0.014824937	0.479865957
ACLY	0.650023451	0.583855046	0.298626209	0.002837723	0.050732378	0.014868391	0.480197593
CAPZA2	0.251532649	0.062787932	0.002629347	0.75133089	0.527332905	0.014952225	0.480197593
GPR171	0.062413219	0.509384398	0.026639602	0.068678887	0.282074432	0.01492442	0.480197593
GPX3	0.676982971	0.294171944	0.04635496	0.060473345	0.029417015	0.014933721	0.480197593
LOC112442023	0.358943668	0.002726655	0.208700278	0.12415728	0.655203147	0.015052297	0.481526798
MUSTN1	0.035633797	0.062576747	0.81150769	0.427804413	0.021439741	0.015040724	0.481526798
CDK3	0.674777015	0.00173034	0.213538191	0.29234671	0.230760209	0.015176688	0.483620623
TTR	0.064696551	0.140215675	0.931953509	0.487675918	0.004069964	0.015152369	0.483620623
HOXA6	0.163539044	0.254160712	0.041555826	0.084903722	0.117344316	0.015412266	0.485560133
LOC100848703	0.946641523	0.484872258	0.931825468	0.000133187	0.301594589	0.015395091	0.485560133
LOC101907219	0.479750603	0.147760752	0.294454594	0.172173333	0.004831725	0.015506037	0.485560133
LOC782706	0.397933796	0.769175631	0.001593259	0.508679939	0.06876292	0.015320968	0.485560133
LUZP1	0.04528929	0.31678149	0.823158308	0.010764138	0.137111801	0.015545324	0.485560133
MTCL1	0.505142706	0.096705762	0.001614394	0.696392501	0.317904231	0.015563014	0.485560133
MTR	0.632796256	0.227397344	0.001147768	0.516264019	0.20263026	0.015453663	0.485560133
P2RY6	0.544737048	0.160180911	0.1983682	0.088889383	0.011344431	0.015560024	0.485560133
SELENOK	0.456030084	0.02587297	0.008321192	0.334397342	0.531722096	0.015561726	0.485560133
SPATA46	0.602065164	0.000145635	0.656221375	0.550958397	0.541583354	0.015388196	0.485560133
STK32B	0.201892964	0.035547064	0.07645339	0.172962026	0.179741977	0.015320976	0.485560133
FAIM2	0.914708155	0.883585645	0.009567883	0.104087476	0.021883994	0.015656081	0.487536897

ABL1	0.714222051	0.777776267	0.451781156	0.000184404	0.387041258	0.015833481	0.488699389
DEPDC1	0.330423345	0.005300854	0.062563315	0.40120788	0.405983477	0.015796011	0.488699389
FBN2	0.382396706	0.16573428	0.021648744	0.926220241	0.014106843	0.015842305	0.488699389
LHX6	0.222543595	0.100479078	0.008469348	0.622775525	0.151773924	0.015826758	0.488699389
NDOR1	0.107324152	0.053304119	0.250219612	0.017981171	0.693653497	0.015799094	0.488699389
KIF21B	0.437744787	0.147915445	0.129398558	0.151611026	0.01419825	0.015906876	0.488853455
SNPH	0.489087307	0.17488204	0.00931188	0.510997134	0.044305309	0.015904749	0.488853455
PSTPIP1	0.418984628	0.21057793	0.320299242	0.014070137	0.045490069	0.015937751	0.488886791
FGG	0.116474589	0.036964243	0.080363503	0.815381265	0.064474933	0.015998117	0.489822932
UTP15	0.739847596	0.018577044	0.015828003	0.346396537	0.244892948	0.01615415	0.493679232
JPH2	0.002537528	0.309356973	0.120694392	0.670549962	0.292770219	0.016239868	0.494278403
LOC107131817	0.459917445	0.817755541	0.064430519	0.747281892	0.001030083	0.016270939	0.494278403
MAP1LC3C	0.016709528	0.691367915	0.005119357	0.530851324	0.595408295	0.01629423	0.494278403
RGS16	0.070054402	0.922759379	0.002079296	0.232803474	0.594355802	0.016238874	0.494278403
PMS2	0.924338416	0.743538032	0.263602013	0.000115777	0.894364982	0.01633314	0.494544578
PGF	0.029614151	0.18915827	0.028096319	0.175403365	0.68510817	0.016423082	0.496352109
ASGR1	0.09178466	0.823567538	0.15228799	0.025236101	0.065427036	0.016477578	0.497083705
PLCB4	0.025430852	0.397918703	0.763396625	0.524744046	0.004714708	0.016538632	0.498010064
CLIC4	0.72548819	0.162256472	0.000480642	0.359364469	0.95494164	0.016714757	0.499158885
TMEM176B	0.223842575	0.270907396	0.002442268	0.924369642	0.140472483	0.016607403	0.499158885
UNC5C	0.005603985	0.079105141	0.116464506	0.529684798	0.709350095	0.016704633	0.499158885
USP2	0.59261074	0.321639041	0.035505441	0.019100975	0.150391527	0.016728864	0.499158885
VAT1	0.58797402	0.20059311	0.006366866	0.183597195	0.140674474	0.016702255	0.499158885
C5H12orf29	0.870539348	0.335921236	0.099982462	0.084562148	0.007924002	0.016815821	0.499904283
INO80B	0.029120615	0.458852543	0.409908741	0.004594714	0.776485579	0.016786836	0.499904283
SNRPA	0.128901085	0.789978119	0.005591759	0.127026723	0.271573336	0.01684523	0.499904283
UTP4	0.963707926	0.751166735	0.221740952	0.000674518	0.182026655	0.016883073	0.500122946
BID	0.293081607	0.793573193	0.00089458	0.459712925	0.211922391	0.017203827	0.502933971
C2	0.193100991	0.025886713	0.101653853	0.045075027	0.876982331	0.017099471	0.502933971
CHD1	0.356280411	0.898810558	0.947958209	0.000103918	0.639063572	0.017140918	0.502933971
FKBP10	0.791400612	0.037603658	0.002122687	0.530580142	0.606966737	0.017245515	0.502933971
KBTBD7	0.881798464	0.131428845	0.048611163	0.219730475	0.016335995	0.017176627	0.502933971
NKTR	0.837391739	0.069594867	0.022392825	0.236942817	0.065838676	0.017253783	0.502933971
RAB21	0.847469642	0.000293546	0.266056943	0.328047867	0.936466357	0.01723957	0.502933971
TGFBI	0.818378644	0.132876418	0.022920703	0.015992725	0.51000506	0.017237506	0.502933971
TMEM126B	0.581691832	0.389265177	0.86587522	0.192551794	0.000537128	0.017208093	0.502933971
ACER2	0.445574937	0.582614095	0.001334517	0.263061975	0.224845922	0.017329183	0.503317583
BHLHE40	0.271870784	0.325376911	0.320270754	0.001461324	0.496183161	0.017358205	0.503317583
CD79A	0.085261123	0.637672494	0.007455156	0.70485827	0.072161084	0.017399848	0.503317583
CDC42EP3	0.014915518	0.042601813	0.957143309	0.408004396	0.08290743	0.017375508	0.503317583
KCTD17	0.811068159	0.351192561	0.000624019	0.501262824	0.234124112	0.017537104	0.503317583
LOC530077	0.697550856	0.512136105	0.007083487	0.0755466	0.109456199	0.017573638	0.503317583
LOC781339	0.010215521	0.175768401	0.414253897	0.731055668	0.03842624	0.017556941	0.503317583
MCMBP	0.301599879	0.552220806	0.029626884	0.007528598	0.562887115	0.017565568	0.503317583

MMP23	0.254562567	0.042909959	0.020810102	0.12304568	0.741738942	0.017473277	0.503317583
NOA1	0.528486758	0.527038308	0.659352786	0.088078882	0.001281398	0.017462666	0.503317583
ITK	0.354660343	0.451344284	0.597621893	0.044611448	0.004961472	0.017713233	0.505622778
LSMEM1	0.132407355	0.169329181	0.020522063	0.934490737	0.04925566	0.017715745	0.505622778
POLR2K	0.364508574	0.001010937	0.202283505	0.636122259	0.448144126	0.01775544	0.505875917
P2RY13	0.02587914	0.593308573	0.047692223	0.380879158	0.076779149	0.0178474	0.507614692
EBPL	0.959356403	0.200589943	0.005289809	0.111416648	0.189751084	0.017906651	0.507771216
HJURP	0.159313552	0.018930362	0.046254815	0.665816165	0.232003803	0.017921928	0.507771216
LIF	0.593796003	0.432449315	0.002618845	0.033622554	0.954922121	0.017945726	0.507771216
ZNF19	0.277493214	0.579110725	0.554690945	0.00381375	0.063946485	0.018027385	0.509203815
SMAD1	0.288301016	0.293139302	0.001736273	0.46960208	0.316415268	0.018063218	0.509339286
ZNF831	0.050870502	0.802789169	0.945312249	0.103877998	0.005477902	0.0181538	0.511015458
NPC2	0.518381446	0.005322027	0.009908878	0.825917887	0.985676737	0.018311821	0.514580973
CASQ1	0.030255912	0.478553478	0.42068975	0.045563908	0.082291416	0.018630951	0.516866083
CCNDBP1	0.836331989	0.014848063	0.063442104	0.217916601	0.132966529	0.01862468	0.516866083
DMRT2	0.029193824	0.225866066	0.149779443	0.410865519	0.056607391	0.018702219	0.516866083
ERO1A	0.445578111	0.004405381	0.295339676	0.050665369	0.779074997	0.018655015	0.516866083
GBP5	0.944976281	0.155588606	0.054554426	0.004099024	0.707830012	0.018865565	0.516866083
GJA1	0.151676394	0.468669227	0.002768996	0.287216394	0.401560472	0.018556456	0.516866083
LOC509118	0.364347356	0.336730041	0.001283275	0.147660497	0.975563068	0.018544207	0.516866083
LOC782177	0.150815745	0.255564321	0.009469111	0.575912407	0.108536393	0.018616944	0.516866083
PKDCC	0.782260454	0.161412121	0.002664107	0.317493437	0.217063935	0.018817136	0.516866083
RBM39	0.54028796	0.387358589	0.419926589	0.005064434	0.052195853	0.018843617	0.516866083
RGS12	0.486735991	0.183614498	0.001657092	0.157485818	0.970547203	0.018520557	0.516866083
RRBP1	0.158616362	0.140612994	0.006051988	0.34878516	0.478829129	0.018469597	0.516866083
TSC22D3	0.375316588	0.448871549	0.001517526	0.302938206	0.298711003	0.018791177	0.516866083
TSR2	0.654257717	0.001208667	0.044811238	0.924107205	0.703433842	0.018737272	0.516866083
ZNF75D	0.96091143	0.001392693	0.093384901	0.212906137	0.873564411	0.018849963	0.516866083
GMPPB	0.028813707	0.823615046	0.197575981	0.263219474	0.01896472	0.018937531	0.517958653
KNTC1	0.96439254	0.015205718	0.114751783	0.015664854	0.89011044	0.018968567	0.517958653
H2AFY2	0.418001541	0.074249213	0.582694205	0.001375771	0.949513581	0.019055005	0.519454621
APOC3	0.227036781	0.000783433	0.547360702	0.403892438	0.609717821	0.019242964	0.520257462
ASAH2	0.97282875	0.771464844	0.000302311	0.260924035	0.404145134	0.019216216	0.520257462
CENPX	0.438843024	0.836674639	0.458029069	0.000207284	0.682465713	0.019144189	0.520257462
LOC112448166	0.36785766	0.716949647	0.155411387	0.007122558	0.081852275	0.019200334	0.520257462
TUBA1A	0.044475491	0.176041763	0.025662116	0.531918235	0.222757779	0.019153011	0.520257462
DAPK3	0.093279847	0.553358129	0.149686763	0.056610855	0.055406542	0.019381078	0.522270737
MOB3A	0.40207882	0.159337196	0.327553466	0.247919512	0.004650304	0.019359267	0.522270737
DPYSL5	0.071514993	0.127592367	0.249659551	0.039703087	0.270695773	0.019513124	0.522872531
NAV1	0.085980418	0.007717934	0.091874124	0.541955504	0.736906072	0.01944142	0.522872531
ZDHHC2	0.856937046	0.066457072	0.002152286	0.53074766	0.375233456	0.019474473	0.522872531
ZNF711	0.523608432	0.026263033	0.047223996	0.105997575	0.356171594	0.019530855	0.522872531
ARL4C	0.275541787	0.259848111	0.004630804	0.475243983	0.163410817	0.020176792	0.528533644
ATAD3A	0.281320268	0.906525532	0.875957477	0.035764831	0.003238163	0.020240281	0.528533644

ATP6V1C2	0.008906217	0.910954996	0.005643541	0.882411517	0.619844692	0.019808347	0.528533644
CD2	0.087491321	0.704585692	0.283047498	0.199925999	0.007586909	0.020547466	0.528533644
CUL3	0.099684617	0.027139787	0.26997565	0.058764043	0.604283489	0.020274061	0.528533644
CYP1B1	0.10505095	0.861801613	0.515181282	0.01050062	0.053234718	0.02034428	0.528533644
DTX4	0.472517732	0.000594638	0.130189363	0.917365031	0.78813836	0.020538032	0.528533644
ILVBL	0.770861716	0.551755334	0.901332043	0.001727543	0.03857504	0.020071769	0.528533644
KIAA1217	0.206853331	0.106552175	0.082507868	0.025281936	0.565303759	0.020301953	0.528533644
LGALS3	0.087413674	0.106403562	0.0482883	0.972231605	0.0600043	0.020411196	0.528533644
LOC101906178	0.422399423	0.231483578	0.468592559	0.000656509	0.87856931	0.020527521	0.528533644
LOC112447626	0.000988166	0.129311415	0.772529222	0.340134832	0.784428071	0.020481573	0.528533644
LOC533307	0.271060698	0.371034089	0.004565046	0.37101681	0.151149601	0.020175719	0.528533644
MFSD8	0.807582677	0.666569484	0.042285825	0.007470158	0.154659324	0.020461184	0.528533644
NDUFA1	0.96759843	0.73014402	0.180732365	0.000355765	0.58155113	0.02052237	0.528533644
PIM3	0.817707791	0.395543418	0.555269741	0.000495124	0.284477542	0.019940836	0.528533644
PLXNA3	0.228691643	0.045498682	0.019096317	0.364763098	0.346426832	0.019842442	0.528533644
POU2AF1	0.175260467	0.160510047	0.029018981	0.714625165	0.043154499	0.019877392	0.528533644
RASAL3	0.052964553	0.581985558	0.394971528	0.03747843	0.055540736	0.019965159	0.528533644
RIMS1	0.013647132	0.589375	0.093746372	0.168618874	0.200480264	0.020041822	0.528533644
SEMA4F	0.033015733	0.095931869	0.019203308	0.542796362	0.770529544	0.020014926	0.528533644
SEPT1	0.299034588	0.098424008	0.336467562	0.108328394	0.023296583	0.019781248	0.528533644
TIMM10	0.965635266	0.705469035	0.091698012	0.001275153	0.321062189	0.020085862	0.528533644
TSSC4	0.109517971	0.964460061	0.879846208	0.016904559	0.016493352	0.020260996	0.528533644
VPS18	0.454253108	0.453751955	0.162973412	0.333408731	0.002334131	0.020380259	0.528533644
BOP1	0.177710208	0.988925608	0.61176802	0.089016598	0.002811796	0.020775027	0.5296719
CD28	0.582322492	0.147104241	0.006163587	0.621878646	0.082311427	0.020834321	0.5296719
CDC20	0.599544299	0.036951048	0.01725102	0.096519428	0.733502751	0.020849921	0.5296719
LOC100140586	0.002039931	0.091477082	0.326343511	0.45378912	0.966412674	0.020670862	0.5296719
LOC101906024	0.742816076	0.241167095	0.000465951	0.964693186	0.332100045	0.020689077	0.5296719
SLC18B1	0.714931641	0.009942595	0.030243529	0.96170181	0.130501667	0.020811009	0.5296719
SUPT4H1	0.731185793	0.006661257	0.266992474	0.094327497	0.22055765	0.02084878	0.5296719
ZNF410	0.074833701	0.651101921	0.041185254	0.129428076	0.102783874	0.020665253	0.5296719
MED11	0.104061309	0.379729673	0.081029754	0.020646363	0.413900392	0.021005104	0.532789424
LSM14B	0.131567586	0.130797989	0.175214884	0.020399303	0.447012926	0.021072653	0.532855626
NEXN	0.115983929	0.210010276	0.15175435	0.41166041	0.018033548	0.021045188	0.532855626
ACAP3	0.353240915	0.048619831	0.002695055	0.921860858	0.649590638	0.021185246	0.533114487
IFFO1	0.003328527	0.335794605	0.303664103	0.104368419	0.779357462	0.021129469	0.533114487
PFN2	0.137009916	0.040156524	0.337240767	0.026624594	0.562187229	0.02121283	0.533114487
SAC3D1	0.086621809	0.125592771	0.18879773	0.084276882	0.16029391	0.021199985	0.533114487
B4GALT7	0.249671	0.931009806	0.485018066	0.046074929	0.005411794	0.02138373	0.534137589
LOC101904923	0.467635381	0.352530034	0.040158985	0.011750105	0.360829355	0.02136239	0.534137589
MYH3	0.874923423	0.112852562	0.000530818	0.674251482	0.792898277	0.021337605	0.534137589
PHACTR4	0.272678004	0.341104226	0.268540587	0.530515812	0.002117327	0.02135601	0.534137589
ZNF335	0.48658934	0.002531703	0.445817529	0.080772797	0.635172158	0.02141637	0.5341399
PSMD12	0.90871779	0.046174551	0.307072778	0.009696091	0.227342686	0.021529401	0.536144169

UBN1	0.764211619	0.025600765	0.650819106	0.002291195	0.977150524	0.021581823	0.536635304
MED12	0.171694388	0.361487031	0.249735098	0.037512871	0.049144985	0.021615887	0.536669175
MPV17L2	0.279705497	0.344055881	0.076752869	0.072096693	0.05433942	0.021796211	0.540328736
RASAL2	0.825253113	0.760498696	0.308367952	0.01360211	0.011039663	0.021857949	0.541041939
AKAP7	0.172550649	0.347699357	0.54849801	0.00123363	0.723937288	0.022020009	0.541533002
COL16A1	0.218122714	0.022194021	0.053306517	0.195263381	0.584733503	0.022057297	0.541533002
ELL2	0.489140025	0.615278269	0.000400575	0.259235871	0.940185526	0.022017201	0.541533002
FZD2	0.54460764	0.042735931	0.00667025	0.223866253	0.845427599	0.022016752	0.541533002
IMPA1	0.479557644	0.518664865	0.437046754	0.238080523	0.001139572	0.022071534	0.541533002
TCF19	0.351500528	0.011593904	0.049167112	0.148700881	0.990156349	0.022075777	0.541533002
ARHGDIA	0.028458312	0.201994775	0.218139502	0.149355967	0.158838722	0.02219722	0.542628559
FBLN7	0.403632432	0.117794175	0.004075063	0.437152291	0.353459617	0.022290169	0.542628559
GMPPA	0.187823385	0.034229376	0.007883972	0.793584282	0.745054822	0.022305668	0.542628559
LMOD1	0.036121062	0.135963028	0.490836166	0.107570259	0.115563253	0.022304141	0.542628559
MKX	0.010106766	0.350840341	0.101307378	0.599322255	0.139328704	0.022318827	0.542628559
WDR19	0.427490881	0.011426757	0.055402305	0.250525804	0.439470526	0.02222072	0.542628559
LOC112445197	0.505214195	0.018308814	0.196181872	0.020984748	0.789686913	0.022355741	0.542721982
AFTPH	0.377993208	0.595543582	0.017071207	0.013488039	0.587750031	0.02254808	0.543384952
DHX35	0.247326677	0.010855428	0.516137746	0.041100481	0.533762767	0.0225165	0.543384952
POLR3B	0.13768633	0.630636441	0.022705393	0.09786581	0.157132287	0.02247614	0.543384952
SCN8A	0.354119891	0.540089058	0.350116582	0.003650418	0.124893324	0.022579099	0.543384952
SERPINE2	0.539873225	0.081702008	0.026564044	0.029408197	0.878913127	0.022460392	0.543384952
TRIM36	0.887087667	0.33813616	0.402380874	0.008094979	0.031251831	0.022581716	0.543384952
AMT	0.997884549	0.01204411	0.079967609	0.584617421	0.054752175	0.022693401	0.545272914
C2CD4B	0.834381136	0.295905413	0.002392229	0.066204218	0.790698296	0.022768281	0.546272319
ACSM5	0.415850815	0.084492564	0.029145192	0.095375535	0.323193128	0.023080877	0.546851594
GP9	0.483479002	0.25739907	0.028775631	0.02518147	0.34586668	0.022899099	0.546851594
HBB	0.176391551	0.106711101	0.734314849	0.052118649	0.043751002	0.02305744	0.546851594
LOC101903289	0.725395714	0.425053186	0.001573454	0.835949037	0.077755382	0.02306553	0.546851594
LOC107131398	0.180422107	0.004875686	0.306628654	0.254837561	0.456409002	0.022987914	0.546851594
LOC112449261	0.16949825	0.762261755	0.00716659	0.092751335	0.367498818	0.023078639	0.546851594
NKD1	0.455864805	0.875137451	0.002010678	0.124291442	0.316849934	0.023092326	0.546851594
NLRC3	0.081894617	0.170399744	0.020667745	0.357671408	0.302521879	0.022908087	0.546851594
UFSP1	0.122031287	0.736168338	0.383810381	0.014581191	0.062453634	0.023000441	0.546851594
CXXC5	0.152019062	0.266693051	0.142438793	0.262722758	0.021434892	0.023536892	0.548615111
DCXR	0.190930975	0.340390994	0.560482104	0.001822566	0.488710113	0.023501119	0.548615111
FBXO28	0.273430309	0.015224533	0.053519552	0.173051186	0.830537249	0.023298934	0.548615111
HIPK3	0.656777207	0.223027926	0.060134226	0.005979183	0.61423108	0.023455751	0.548615111
HSPB11	0.271922657	0.570386613	0.446850258	0.273852022	0.001681779	0.023250521	0.548615111
MED9	0.241409768	0.056169781	0.024048614	0.242374063	0.411689045	0.023545647	0.548615111
NDRG4	0.132745466	0.16541941	0.954915918	0.162157706	0.009507027	0.023444439	0.548615111
OIT3	0.133333412	0.808129307	0.001509501	0.539103469	0.363715441	0.023237352	0.548615111
RAPGEFL1	0.141775404	0.027169303	0.051808022	0.21936975	0.745957717	0.023601381	0.548615111
SCARB2	0.788498692	0.139658447	0.000699344	0.546670366	0.774625004	0.023580299	0.548615111

SLMAP	0.004642266	0.172118644	0.607523592	0.863659193	0.077281213	0.023479336	0.548615111
STAM	0.741462384	0.052224613	0.027853514	0.055615619	0.536799653	0.023384243	0.548615111
UCK2	0.314819178	0.710579159	0.620910271	0.236583741	0.000975173	0.02331064	0.548615111
MEG8	0.523390278	0.005550483	0.031136649	0.688097849	0.528638952	0.023718297	0.548999961
PTPN2	0.577637376	0.15844786	0.06492883	0.010196525	0.54092033	0.023658422	0.548999961
SFRP2	0.107026547	0.102906494	0.006777347	0.712436711	0.618158491	0.023704058	0.548999961
ATXN1L	0.278245116	0.474216382	0.00207111	0.827988801	0.149432622	0.024146106	0.54932821
CSNK1B	0.933215786	0.028980579	0.204990932	0.31271039	0.019595602	0.024220819	0.54932821
DENR	0.14249341	0.174206722	0.248130153	0.147179724	0.036900433	0.023976925	0.54932821
DERL1	0.292980987	0.003553307	0.162835127	0.587656125	0.334462305	0.023914451	0.54932821
GDF11	0.671716463	0.556089205	0.004096511	0.239787132	0.092863612	0.024268049	0.54932821
ISCA2	0.89992119	0.001685892	0.0907723	0.33256141	0.742782462	0.024242621	0.54932821
LOC101909196	0.203949752	0.003445168	0.419225616	0.292790631	0.390544039	0.024085384	0.54932821
LOC112447082	0.007642004	0.475365206	0.421193559	0.413612134	0.053773985	0.024248502	0.54932821
MED6	0.135990102	0.976512587	0.73223844	0.009742763	0.035617424	0.024113522	0.54932821
NUDT8	0.600057102	0.297829566	0.728658174	0.00042229	0.616616264	0.024191068	0.54932821
P4HB	0.081078435	0.467437421	0.080554208	0.532780543	0.020415739	0.023861779	0.54932821
PADI1	0.242684326	0.70971806	0.083043731	0.004771481	0.495483408	0.024147548	0.54932821
STXBP3	0.181147836	0.32993132	0.03239227	0.117380706	0.147835458	0.024044149	0.54932821
TMEM242	0.00292676	0.032151445	0.69679021	0.51464971	0.986987125	0.023908028	0.54932821
TNFSF10	0.281196238	0.929897192	0.023988621	0.005813245	0.925972239	0.024123858	0.54932821
ZSWIM6	0.63012729	0.97122612	0.556670246	0.000141494	0.694221276	0.023982842	0.54932821
BCL7A	0.393547786	0.089512221	0.446203069	0.002372577	0.916570506	0.024318712	0.549716787
CAND1	0.364232724	0.081553402	0.391751394	0.013931307	0.213933634	0.024550961	0.550187372
CARD9	0.157254315	0.004214907	0.229047395	0.752154239	0.304051013	0.02456824	0.550187372
CLDN10	0.056871842	0.19096639	0.310765112	0.021005876	0.485378199	0.024425627	0.550187372
CXCL16	0.285707651	0.417259985	0.001384518	0.349512114	0.609093274	0.024761649	0.550187372
FFAR2	0.3127771	0.350359588	0.847728697	0.005173184	0.072239006	0.02456699	0.550187372
KCNS3	0.214545021	0.509836344	0.703807086	0.241344431	0.001897055	0.024811932	0.550187372
LAIR1	0.282046274	0.232502266	0.028493558	0.19217473	0.097550926	0.024711289	0.550187372
LENG8	0.880321115	0.005680486	0.094278577	0.847492029	0.087477694	0.024675968	0.550187372
LOC107133024	0.850958119	0.677889049	0.139022049	0.000693504	0.634158825	0.0248223	0.550187372
MYOM1	0.137315585	0.323544242	0.852877333	0.309752665	0.002997334	0.024780944	0.550187372
P2RY10	0.658981579	0.202984204	0.022969193	0.182445161	0.063127267	0.024875939	0.550187372
PRPF19	0.419767818	0.121855955	0.277465102	0.224796492	0.010773852	0.024407854	0.550187372
SLC4A11	0.060321044	0.178972921	0.219731353	0.452774644	0.032940518	0.024873322	0.550187372
TMSB4X	0.903398822	0.000559884	0.382253309	0.632643429	0.282015799	0.024464463	0.550187372
TNNT2	0.212985093	0.052256632	0.048121898	0.122185295	0.534903597	0.024700343	0.550187372
USP4	0.730375939	0.037632131	0.001660141	0.885836033	0.866369527	0.024707006	0.550187372
GTF2H4	0.601951155	0.133753758	0.587649591	0.005701918	0.131722237	0.024944776	0.550226773
LOC534967	0.234591881	0.681069943	0.576075348	0.001215299	0.317581433	0.024939388	0.550226773
CDS1	0.085145218	0.235576046	0.009710674	0.566087728	0.323265418	0.024994325	0.550579691
PLEKHA6	0.972342524	0.012841389	0.194461375	0.040097442	0.37013616	0.025174	0.553466743
WFDC3	0.394292737	0.002739988	0.173394755	0.244136175	0.788867987	0.025192838	0.553466743

GALNT10	0.009140563	0.019528137	0.579393352	0.935545363	0.375802569	0.025321721	0.554921566
HSD17B11	0.489849484	0.332111922	0.003403244	0.171735705	0.384020055	0.02539124	0.554921566
LOC101905514	0.52020388	0.121627847	0.145767165	0.0761292	0.052013692	0.025394315	0.554921566
MYH11	0.001353631	0.328795872	0.385498015	0.672593811	0.31621837	0.025381082	0.554921566
C13H20orf202	0.358767881	0.469244576	0.000991489	0.782278719	0.286103201	0.025773079	0.557606549
DUSP26	0.306975648	0.608212333	0.03495733	0.013260695	0.432048608	0.025789006	0.557606549
FGF2	0.490364041	0.638912262	0.008732293	0.127986344	0.106780184	0.02578692	0.557606549
LOC783686	0.793610458	0.878400352	0.119870486	0.012261786	0.036105338	0.025609094	0.557606549
PLPPR2	0.490945892	0.686716411	0.000730783	0.430374165	0.35203351	0.025759311	0.557606549
RNF185	0.76099933	0.031660654	0.008252872	0.281708491	0.666791231	0.025769749	0.557606549
SHANK2	0.017185137	0.61622583	0.561962033	0.027332546	0.229235419	0.025741215	0.557606549
TMEM200A	0.376501432	0.534548586	0.815613121	0.000270034	0.841989328	0.025756717	0.557606549
HOXD4	0.070849663	0.23274821	0.10601091	0.082930621	0.258835328	0.025847835	0.55814318
ANKRD13B	0.308121448	0.016007768	0.056064507	0.282914061	0.496368599	0.026431842	0.559223374
CCDC61	0.580657187	0.189474323	0.392950168	0.002082095	0.431692709	0.026443077	0.559223374
CENPW	0.205354703	0.013075297	0.100312412	0.143895218	0.974027419	0.025949557	0.559223374
CHAC1	0.001542588	0.31806247	0.517007327	0.157323051	0.964788135	0.026284913	0.559223374
FCHO2	0.069918951	0.641942644	0.01316085	0.202192318	0.324821212	0.026415379	0.559223374
GLI3	0.25567368	0.031136795	0.140437067	0.036177684	0.95757151	0.026386455	0.559223374
GPR4	0.042309279	0.415154359	0.301302179	0.133879358	0.054551722	0.026351487	0.559223374
IL17B	0.025724557	0.161897936	0.677690938	0.127585343	0.106604642	0.026234262	0.559223374
NLRP3	0.338331409	0.605997495	0.448910353	0.009446777	0.04416875	0.026241102	0.559223374
PHYHD1	0.44375426	0.651401525	0.000897589	0.158010484	0.933096944	0.02617464	0.559223374
PIGZ	0.858907057	0.153027399	0.878907713	0.877757066	0.000379725	0.026285646	0.559223374
SMARCC1	0.814342204	0.096708443	0.086075281	0.043546195	0.13044058	0.026286071	0.559223374
SPOUT1	0.44648695	0.963786038	0.56028448	0.016601631	0.009587837	0.026229209	0.559223374
TMEM119	0.810955217	0.196972346	0.001221984	0.766600311	0.254207339	0.026078296	0.559223374
TMEM138	0.910592116	0.06706221	0.432810003	0.302914224	0.004810335	0.026289278	0.559223374
ZNF862	0.089921451	0.073530719	0.359949386	0.032027098	0.499421496	0.026091362	0.559223374
DDX18	0.449161673	0.055736467	0.067996048	0.041184744	0.559022584	0.026590527	0.560896062
LY75	0.334994366	0.63204757	0.34101873	0.069723643	0.007781557	0.026583375	0.560896062
ANP32E	0.611565363	0.000326576	0.418499758	0.896091154	0.526448424	0.026695916	0.562396249
C25H16orf72	0.214878592	0.319262047	0.137016134	0.028282895	0.151163115	0.027027945	0.562439494
CADM1	0.381949294	0.163761391	0.295528629	0.400923037	0.005340378	0.026760772	0.562439494
CCNJL	0.011670942	0.10520616	0.070614652	0.576493953	0.80666625	0.027086635	0.562439494
GPX7	0.704023147	0.050787117	0.004927138	0.926298643	0.245392362	0.026965904	0.562439494
LOC100140431	0.676776706	0.023463315	0.011883757	0.919424573	0.23239512	0.027086679	0.562439494
OSBPL11	0.879098436	0.981743576	0.003804278	0.01370439	0.893436079	0.027033982	0.562439494
RBBP6	0.349184692	0.251810055	0.18586404	0.032685166	0.07558111	0.027109234	0.562439494
RPL22L1	0.90211213	0.082150269	0.110213399	0.195199039	0.025016887	0.026896026	0.562439494
RSPH10B	0.5092603	0.003710777	0.509297411	0.100795133	0.412604582	0.02695793	0.562439494
SAV1	0.218794493	0.653062621	0.001815209	0.29457178	0.526853862	0.027056995	0.562439494
SNX17	0.583619513	0.493865557	0.266455785	0.004274277	0.121503508	0.026896039	0.562439494
TP53BP2	0.117114443	0.670441638	0.015152039	0.425091873	0.078592639	0.026835349	0.562439494

GPR137B	0.271452872	0.102222404	0.009497806	0.793453309	0.194100952	0.027203756	0.562977102
LSMEM2	0.805185055	0.079648167	0.020354281	0.053705013	0.57875275	0.027196521	0.562977102
ACAT2	0.354844735	0.884916523	0.607122077	0.03006986	0.007254379	0.027635879	0.563476436
AEBP2	0.186345658	0.755376686	0.976153613	0.00228959	0.129776559	0.027307443	0.563476436
ICOSLG	0.048777478	0.379902092	0.282559563	0.019526101	0.402934566	0.027467074	0.563476436
KLF15	0.682999345	0.156634017	0.000771938	0.762773704	0.658281623	0.027584183	0.563476436
LOC789374	0.76888047	0.005537517	0.781951784	0.832102305	0.014785844	0.027365595	0.563476436
MSTO1	0.45070331	0.685324534	0.591501209	0.024917796	0.009090075	0.027548061	0.563476436
PNO1	0.55696821	0.049488894	0.032401746	0.088391682	0.524460461	0.027556754	0.563476436
RND2	0.59085492	0.348505828	0.344373999	0.071415704	0.008057228	0.027297055	0.563476436
SLIT2	0.698063376	0.064364498	0.074565159	0.01266279	0.979577124	0.027623358	0.563476436
TFDP1	0.046499331	0.055281732	0.099022446	0.484261306	0.333044614	0.027405231	0.563476436
TPD52L2	0.090600162	0.806140571	0.168388183	0.035011119	0.09660253	0.027639908	0.563476436
ZNF608	0.94524601	0.022539631	0.072120706	0.092139259	0.291464154	0.027497095	0.563476436
CASTOR2	0.870411926	0.014989587	0.056509322	0.098871932	0.572277467	0.027692369	0.563845487
MOG	0.493392644	0.049230808	0.999736029	0.593097927	0.002912435	0.027791192	0.565156448
LOC104970180	0.070408393	0.810126796	0.658596827	0.390895043	0.002879734	0.027937412	0.565327835
RRP7A	0.04604461	0.821402723	0.123027703	0.580540251	0.01564676	0.027928439	0.565327835
TM7SF2	0.262868641	0.503197156	0.615399982	0.005932396	0.087434201	0.027909725	0.565327835
TMEM206	0.221125722	0.78372142	0.390346327	0.011859124	0.052610419	0.027902616	0.565327835
RERE	0.995937127	0.457018151	0.954598489	0.672503201	0.000145104	0.027985504	0.565603572
ABCC5	0.818844732	0.099465219	0.091992195	0.932779194	0.006156889	0.028254357	0.567407781
ACKR4	0.010158814	0.756789479	0.012791399	0.751748629	0.578881707	0.02815476	0.567407781
FBXW12	0.437102376	0.005644615	0.30904563	0.386828677	0.146380825	0.028316798	0.567407781
GPR183	0.948662176	0.535499835	0.248945612	0.020626112	0.016500517	0.028259685	0.567407781
ITGB7	0.14444927	0.184952019	0.287664114	0.178158183	0.031508712	0.028302161	0.567407781
STX18	0.544668228	0.041849258	0.049487715	0.055712005	0.679810358	0.028123465	0.567407781
ZC3H12D	0.704226526	0.023967665	0.058144622	0.798266077	0.054946481	0.028261579	0.567407781
CLECL1	0.273858379	0.56521223	0.012458058	0.263872938	0.085420561	0.028439699	0.56848222
SEMA6B	0.354929734	0.183957315	0.001279976	0.684156448	0.758819593	0.028406202	0.56848222
CDC73	0.410452702	0.339580098	0.041109325	0.01992651	0.38197271	0.028501886	0.568924228
SDK1	0.586424434	0.038104278	0.027853483	0.440892988	0.15918371	0.028531146	0.568924228
TMEM176A	0.128575605	0.175502746	0.017441889	0.982403892	0.113442208	0.028608004	0.569764505
BDH1	0.801211244	0.836168806	0.193169988	0.001819693	0.188433321	0.028823605	0.570919148
BDP1	0.529822582	0.608252109	0.742825205	0.000356756	0.520560649	0.028858311	0.570919148
CBX7	0.195255048	0.37186446	0.535215141	0.238196202	0.004802368	0.028856669	0.570919148
DUOXA2	0.964805595	0.025044069	0.010068768	0.231382228	0.790449187	0.028874712	0.570919148
IDI1	0.671399264	0.925492688	0.083544452	0.017584774	0.048703118	0.028859224	0.570919148
SMAP2	0.983850213	0.542696419	0.382661441	0.022706423	0.009523191	0.028741883	0.570919148
ADGRF2	0.008207471	0.525871577	0.093376394	0.13471547	0.82873054	0.029083349	0.5710197
KMT5C	0.806136031	0.017445339	0.012205766	0.854207326	0.307433294	0.029118461	0.5710197
NF2	0.465812106	0.715289362	0.284148821	0.001159277	0.408867195	0.029033614	0.5710197
NIN	0.521155845	0.469258276	0.018331354	0.048976049	0.205362952	0.029123362	0.5710197
RIOX1	0.409758872	0.528529322	0.374844628	0.0026437	0.209945678	0.029109826	0.5710197

SEPHS2	0.60688496	0.339000307	0.205304453	0.009628922	0.11083149	0.029117537	0.5710197
SLC16A6	0.044629827	0.341003135	0.294286628	0.970465153	0.010354917	0.02908871	0.5710197
ACSM3	0.918690631	0.001314457	0.585272247	0.189008058	0.342440251	0.029396041	0.575678311
OXTR	0.254248379	0.069898586	0.023846487	0.11509959	0.944922781	0.029539981	0.577807657
ADAMTS7	0.162574422	0.121234853	0.019863127	0.233682222	0.524226935	0.030307331	0.580365932
AFAP1	0.386770354	0.883967165	0.029375192	0.103950557	0.044837391	0.029836551	0.580365932
ARHGEF26	0.003197789	0.129696753	0.835974752	0.691532949	0.196709492	0.029982086	0.580365932
CNTNAP1	0.052044415	0.082314442	0.145590746	0.282012243	0.266742318	0.02988124	0.580365932
DNAJA1	0.205762722	0.60399172	0.810882793	0.000817797	0.576032048	0.030108792	0.580365932
DOLK	0.269144232	0.182992086	0.72840299	0.072308808	0.018195693	0.029997177	0.580365932
FARSA	0.11078181	0.459163551	0.449380354	0.731122092	0.002862176	0.03025623	0.580365932
GZMK	0.159394148	0.941437747	0.718565986	0.018017967	0.024219096	0.029936842	0.580365932
HECTD3	0.237693216	0.003817376	0.73894887	0.079382962	0.900717647	0.030300121	0.580365932
KIF22	0.128410609	0.024088391	0.061777642	0.30338657	0.818510121	0.0301004	0.580365932
LOC112442284	0.379227566	0.078728491	0.369951686	0.017567717	0.246058001	0.030220004	0.580365932
LOC112442634	0.072492026	0.830499652	0.008079118	0.258952411	0.373531733	0.029934473	0.580365932
MMP28	0.265401679	0.780821399	0.315723196	0.000852243	0.852633133	0.03013741	0.580365932
PROKR1	0.711326087	0.282199765	0.048696324	0.367801966	0.013082341	0.029929132	0.580365932
SAE1	0.270947819	0.058058441	0.633037507	0.572654576	0.008175275	0.029758495	0.580365932
SPRN	0.506364415	0.012240028	0.038557073	0.781728467	0.255810376	0.0302377	0.580365932
TUBG2	0.247799742	0.647453201	0.04929639	0.009937816	0.60918164	0.030275379	0.580365932
YTHDF2	0.519608039	0.237363872	0.144793957	0.008023751	0.325955506	0.029793968	0.580365932
RSRC2	0.612367917	0.124668188	0.270988128	0.007315294	0.323130746	0.030690173	0.586328793
SEC24B	0.111883639	0.508613565	0.860437634	0.001325146	0.753385119	0.030682258	0.586328793
FILIP1L	0.38507884	0.949666693	0.112672526	0.205491136	0.005892686	0.031089511	0.590986672
GNGT2	0.016603384	0.824306702	0.096258943	0.090422211	0.41768859	0.031034666	0.590986672
LTN1	0.215519734	0.075968154	0.131522141	0.033110525	0.699222969	0.031073567	0.590986672
TLL2	0.643995845	0.193820613	0.003398919	0.23179327	0.507987032	0.031114038	0.590986672
VSIG10	0.468020307	0.547921251	0.138763898	0.467448209	0.00299806	0.031079734	0.590986672
ARL14EP	0.251979176	0.033137393	0.00913955	0.666659763	0.985120442	0.031179783	0.591457684
ZNF318	0.957419408	0.101934934	0.079313221	0.922824404	0.007027241	0.031210917	0.591457684
KIF1BP	0.018156222	0.192817666	0.324788115	0.533100705	0.083187966	0.031302038	0.591817674
TAOK3	0.780685384	0.743895628	0.299629963	0.008335616	0.034737538	0.031286637	0.591817674
INPP5J	0.098398175	0.030177184	0.031985396	0.570726363	0.934049731	0.031384377	0.592691607
LOC107132469	0.067583506	0.239029735	0.625506516	0.463702436	0.010880573	0.031524148	0.594646879
RGS18	0.97421862	0.254524178	0.005193605	0.654805085	0.060596392	0.03157075	0.59484223
CCDC158	0.052715399	0.766352721	0.009722922	0.27906939	0.475089078	0.031957985	0.595962414
DBF4B	0.90848239	0.690110677	0.871129279	0.000233143	0.408900437	0.031953636	0.595962414
ELFN1	0.608799603	0.176423694	0.007483054	0.260841181	0.245223823	0.031694185	0.595962414
FAM198A	0.914759961	0.04456115	0.050060227	0.175683212	0.145946812	0.032054138	0.595962414
IL33	0.065426848	0.484287776	0.018767089	0.230232843	0.378413302	0.031851298	0.595962414
LNX2	0.272696659	0.88580742	0.36189003	0.004945781	0.120287181	0.031929618	0.595962414
LOC782779	0.253043452	0.189887369	0.531387699	0.004461217	0.453460302	0.031790325	0.595962414
MAGED1	0.688641356	0.260357652	0.002032182	0.15156947	0.948954085	0.032087426	0.595962414

NPTX1	0.647136239	0.200322921	0.003469071	0.577299385	0.201105503	0.03201071	0.595962414
OPTC	0.999797237	0.178003459	0.461930184	0.966848384	0.000650547	0.031811888	0.595962414
PKLR	0.06440772	0.146971705	0.213452601	0.284078903	0.091526899	0.03213861	0.595962414
RBM12	0.160583135	0.012171265	0.236206618	0.458252588	0.244401353	0.031811077	0.595962414
SMIM3	0.168316285	0.842702839	0.078761453	0.628320559	0.007475115	0.032112607	0.595962414
WDR6	0.834189864	0.728878774	0.026891138	0.013760905	0.229958331	0.031824699	0.595962414
ARIH1	0.284463872	0.390223565	0.421204916	0.001880445	0.61069257	0.03259094	0.596630518
ASPSCR1	0.189374501	0.037307118	0.702028153	0.021138046	0.548612535	0.0340592	0.596630518
BCL11A	0.835650391	0.499579988	0.060860145	0.042833565	0.052131401	0.03376195	0.596630518
BHLHA15	0.352878498	0.045712501	0.03220452	0.740684342	0.149619256	0.034079231	0.596630518
C6H4orf48	0.08825975	0.270204846	0.008501162	0.631262755	0.429983078	0.03310879	0.596630518
CDC42EP2	0.376562929	0.178394753	0.008520316	0.147898744	0.66589618	0.033622951	0.596630518
CHUK	0.062244812	0.160363533	0.109376299	0.116408385	0.420881388	0.032512012	0.596630518
COL6A1	0.109539852	0.151076559	0.043706209	0.102542455	0.767787284	0.033842155	0.596630518
COL7A1	0.599162918	0.134346539	0.126289675	0.117290152	0.046873123	0.03343901	0.596630518
CX3CR1	0.515071439	0.176283361	0.001764581	0.905614182	0.37334843	0.032777298	0.596630518
DCTN6	0.632510751	0.02120108	0.043335586	0.650446295	0.142808023	0.032702679	0.596630518
DNAH10	0.438630172	0.010912762	0.41836263	0.292618737	0.096254826	0.033636052	0.596630518
EBP	0.317583948	0.492570885	0.245855234	0.011752449	0.127657367	0.034128597	0.596630518
EXTL1	0.211468783	0.596804737	0.024125094	0.127528629	0.139392597	0.032758761	0.596630518
FCRL5	0.121132839	0.588687027	0.121130341	0.272607512	0.023382478	0.033120124	0.596630518
FN1	0.393535126	0.629012544	0.001175974	0.352755301	0.535844221	0.033106684	0.596630518
FTSJ3	0.071751949	0.189291265	0.361927695	0.048945013	0.225892446	0.032846001	0.596630518
GIMAP4	0.382963307	0.59528437	0.184126566	0.167273992	0.007951714	0.033417452	0.596630518
HDAC10	0.381267002	0.053045941	0.380950334	0.00874491	0.810401233	0.032943549	0.596630518
INSR	0.6320945	0.362892507	0.010582571	0.175404985	0.125013606	0.032410059	0.596630518
IQGAP3	0.109507972	0.04570769	0.058607431	0.328751829	0.54705738	0.03222559	0.596630518
ITIH4	0.016074726	0.169163293	0.082286103	0.813670403	0.306849153	0.033430381	0.596630518
LMNB1	0.291630478	0.076820127	0.03528284	0.289341126	0.240041213	0.033058597	0.596630518
LOC100300510	0.01606115	0.852930972	0.487838464	0.161047821	0.051354747	0.033202035	0.596630518
LOC101903026	0.823122259	0.005432616	0.022244493	0.701641164	0.789897623	0.033147209	0.596630518
LOC104970537	0.868983409	0.007733613	0.181722918	0.887330744	0.052115783	0.03366321	0.596630518
LOC112441484	0.301818284	0.001636753	0.25775302	0.729419197	0.615969308	0.033942574	0.596630518
LOC112443504	0.39248033	0.240769159	0.787941425	0.008965322	0.083684603	0.033429125	0.596630518
LOC112445030	0.383216841	0.142654245	0.380969346	0.009291064	0.289373818	0.033479425	0.596630518
LOC112449613	0.359603979	0.73854159	0.43831017	0.474993229	0.001038615	0.034025352	0.596630518
LOC784769	0.420737195	0.132332805	0.037095862	0.038779581	0.675120668	0.032739102	0.596630518
MYRF	0.508958627	0.009465098	0.068839494	0.24267521	0.71426002	0.0340456	0.596630518
NAPSA	0.470763177	0.322766457	0.205070603	0.092903627	0.019612336	0.033777374	0.596630518
NCEH1	0.7336383	0.069112234	0.024015171	0.286524085	0.15304734	0.032475214	0.596630518
NINJ2	0.10164561	0.017926857	0.652339789	0.126935257	0.357070698	0.032662545	0.596630518
PAPPA2	0.801164994	0.017604565	0.242947285	0.19155446	0.082443343	0.032754516	0.596630518
PDCD1	0.847067028	0.061003325	0.124604628	0.02819741	0.297803562	0.032736983	0.596630518
PDGFA	0.476705309	0.406692958	0.167259268	0.027355008	0.064335104	0.033888777	0.596630518

PDLIM7	0.571114582	0.861748193	0.002630628	0.070167423	0.625627581	0.033800177	0.596630518
PDZD3	0.011008244	0.242137357	0.920841936	0.055908035	0.394165223	0.032745342	0.596630518
PPP1R2	0.59847845	0.006434712	0.120382176	0.579536152	0.201627786	0.032776943	0.596630518
PSPH	0.303183908	0.286063681	0.657312639	0.004760347	0.206868419	0.033535219	0.596630518
RANBP6	0.610298285	0.228674658	0.312049086	0.017710513	0.07212589	0.033339599	0.596630518
RGS4	0.037981268	0.475781042	0.00671529	0.646018491	0.681280894	0.032480158	0.596630518
SEPT5	0.086283196	0.031623323	0.127578274	0.267202667	0.573127005	0.032441285	0.596630518
SHISA4	0.547642297	0.092004247	0.062017451	0.027542406	0.632192272	0.032868973	0.596630518
SOBP	0.062326083	0.241071762	0.354137711	0.019391105	0.545391765	0.033585963	0.596630518
TBC1D22B	0.90151269	0.720320025	0.000789488	0.842647319	0.133621536	0.034137838	0.596630518
TESPA1	0.648859437	0.332174087	0.031593249	0.789974	0.010690211	0.03405476	0.596630518
TET3	0.336274331	0.256645138	0.026456576	0.292814718	0.085419783	0.033904756	0.596630518
TUBB2A	0.286664305	0.003367491	0.934868661	0.454272874	0.133025647	0.032918163	0.596630518
USP53	0.526834674	0.405769845	0.034003488	0.01269245	0.601301375	0.033281132	0.596630518
VEGFC	0.396975499	0.873531068	0.004712105	0.046383531	0.733676762	0.033330758	0.596630518
ZNF341	0.964146807	0.017290175	0.023419512	0.160454199	0.875767631	0.033043622	0.596630518
C17H22orf39	0.828109371	0.797350445	0.564617468	0.000263279	0.591466817	0.034262289	0.5972934
CCDC114	0.479429574	0.055606824	0.003519856	0.947163967	0.657754333	0.034415337	0.5972934
CHRNA1	0.008059403	0.349949975	0.562805074	0.162156022	0.226467146	0.034351526	0.5972934
DES	0.002277925	0.203405127	0.575435913	0.971831171	0.223833639	0.034240967	0.5972934
HSPA13	0.627481524	0.011920336	0.09595366	0.199283948	0.407009809	0.034322377	0.5972934
PPDPF	0.730508363	0.134841613	0.001663417	0.792311971	0.450630373	0.034430538	0.5972934
SLC9A1	0.523075084	0.093814938	0.095715877	0.021182812	0.586042826	0.034357986	0.5972934
CSTB	0.185669132	0.379298914	0.13174943	0.447722114	0.014124038	0.034494948	0.597434784
OGFOD2	0.112420464	0.57894043	0.784821932	0.004117283	0.279191681	0.034511497	0.597434784
AKAP8L	0.854308177	0.049402576	0.211511838	0.067269649	0.098555452	0.034686259	0.597937176
LOC100298890	0.348583523	0.036818684	0.22209334	0.296523231	0.070003336	0.034680622	0.597937176
PLEKHA1	0.226375691	0.048273017	0.016286015	0.529203799	0.625916769	0.034599156	0.597937176
RGS14	0.075557426	0.191349058	0.759861074	0.073157362	0.073473906	0.034636836	0.597937176
EIF1	0.976445585	0.049335253	0.010634044	0.289164326	0.404319715	0.034951076	0.601870002
MON1B	0.865539428	0.013012689	0.088408025	0.972961138	0.062052685	0.035034781	0.602679027
CCBE1	0.797350461	0.011933646	0.389117745	0.025427187	0.642274535	0.035164741	0.604281217
POLD4	0.506521579	0.054911762	0.020133283	0.315365528	0.342970506	0.035202276	0.604293466
GPR173	0.577594251	0.933990983	0.002780247	0.060967841	0.66458608	0.035277411	0.604342459
RABEP1	0.800625141	0.407238223	0.125514236	0.002958666	0.501950308	0.035278781	0.604342459
AKAP4	0.254216504	0.547506591	0.024309242	0.207797047	0.090805058	0.036402725	0.604356254
BNIP2	0.685925868	0.0242149	0.077033587	0.054305465	0.901072721	0.035953675	0.604356254
C15H11orf87	0.78438199	0.714924243	0.20366257	0.000706826	0.765753349	0.035662591	0.604356254
C18H19orf48	0.049633379	0.697443745	0.524427536	0.010752449	0.335786942	0.037016623	0.604356254
CD69	0.933802426	0.531526985	0.170387857	0.005925801	0.128729968	0.036645081	0.604356254
CNKSR3	0.033496714	0.31529847	0.684443279	0.028224572	0.326140375	0.037373039	0.604356254
CNOT4	0.335882485	0.325224506	0.382456307	0.001812053	0.855022613	0.036723222	0.604356254
CNTFR	0.004893835	0.725622422	0.594241813	0.063651461	0.474705571	0.036372985	0.604356254
COL6A2	0.184631904	0.12545947	0.030919788	0.09778392	0.91757405	0.036554506	0.604356254

COQ10B	0.146098569	0.196294623	0.171910776	0.042209458	0.316022857	0.037094994	0.604356254
CPSF1	0.816126618	0.003431512	0.036661153	0.762023928	0.809360258	0.036213677	0.604356254
DFFB	0.566985897	0.00205803	0.202453717	0.850528333	0.320974281	0.036637732	0.604356254
DTL	0.019996033	0.551340989	0.109150877	0.207707892	0.25155726	0.03605067	0.604356254
EOMES	0.238324042	0.441746574	0.093332847	0.148117569	0.042767591	0.035819682	0.604356254
GCHFR	0.769184871	0.337419342	0.048048056	0.013255312	0.387820919	0.036497944	0.604356254
HENMT1	0.661292416	0.013181428	0.66860084	0.193435219	0.058909894	0.037326813	0.604356254
KIAA1024	0.388247683	0.797063401	0.302081272	0.001320667	0.52436092	0.036725818	0.604356254
LIG1	0.901609857	0.00275157	0.060242422	0.63441869	0.696107527	0.037180161	0.604356254
LOC100848246	0.687534242	0.235763861	0.774790173	0.0254867	0.020321959	0.036838084	0.604356254
LOC101902869	0.026093064	0.167422763	0.314755437	0.731171019	0.065751135	0.037217202	0.604356254
LOC101906739	0.202411871	0.15340018	0.007847374	0.281871559	0.90955455	0.035902204	0.604356254
LOC112441542	0.254633381	0.564419872	0.176647535	0.111484176	0.021866877	0.035690061	0.604356254
LOC112448523	0.784067474	0.047050018	0.005098204	0.927905041	0.361101391	0.036102592	0.604356254
MAP4K3	0.266252125	0.129098542	0.02083966	0.243542876	0.381314443	0.037365953	0.604356254
MFSD6L	0.25401593	0.348194991	0.002352562	0.379311985	0.781171363	0.035603372	0.604356254
MKI67	0.444649829	0.022279499	0.044737011	0.337738139	0.424201206	0.036276492	0.604356254
MPPE1	0.04945039	0.140892879	0.297780823	0.137039147	0.22720815	0.036676119	0.604356254
MYH10	0.235629069	0.52933265	0.025690171	0.024957939	0.763391361	0.03538008	0.604356254
NRM	0.896662663	0.722244487	0.24210743	0.000821418	0.507948375	0.036971458	0.604356254
PANK1	0.697669186	0.504953501	0.732558847	0.030358442	0.008137069	0.036369524	0.604356254
PI4K2A	0.566393041	0.034902329	0.399295951	0.019880299	0.418431259	0.037058583	0.604356254
PIGO	0.382861943	0.005281448	0.065294343	0.511771788	0.937025718	0.036210439	0.604356254
PLIN2	0.837364569	0.017332345	0.032986473	0.915473585	0.145223087	0.036332161	0.604356254
PLN	0.001816961	0.123293425	0.777720082	0.373566326	0.985412706	0.036508477	0.604356254
PMM1	0.347648666	0.832128118	0.410964283	0.001018989	0.514888421	0.035868079	0.604356254
PPL	0.569804331	0.006114322	0.297495877	0.094080973	0.671453211	0.036991338	0.604356254
PPM1F	0.91682168	0.194270206	0.32338238	0.045108708	0.024350298	0.036193118	0.604356254
PTPN12	0.202590975	0.762538121	0.647634707	0.004697297	0.134238044	0.036127585	0.604356254
RCN3	0.376223488	0.121172154	0.007383774	0.47210925	0.397581214	0.036162552	0.604356254
RERGL	0.476662389	0.344505272	0.334830068	0.009496086	0.127473806	0.037378685	0.604356254
SCG3	0.547208851	0.300039918	0.003083182	0.140535269	0.928614202	0.037201799	0.604356254
SDC1	0.458695494	0.524716062	0.001128543	0.396338286	0.606253611	0.036916456	0.604356254
SLC25A51	0.044641726	0.034610571	0.49691454	0.135017902	0.637298274	0.037202541	0.604356254
SRGN	0.050591213	0.513506852	0.538721206	0.080133882	0.054733776	0.035503702	0.604356254
STAP2	0.834880811	0.244833252	0.005681574	0.161898072	0.334296693	0.036043065	0.604356254
STBD1	0.037395621	0.22796589	0.50714445	0.097966143	0.151056963	0.03645208	0.604356254
STK38	0.222148779	0.911484161	0.562396372	0.002510192	0.213564783	0.035379644	0.604356254
THAP3	0.261496657	0.003530329	0.957712907	0.271400924	0.276728599	0.037323286	0.604356254
TMEM109	0.810100358	0.002705119	0.10111538	0.462127944	0.60502131	0.035713636	0.604356254
TMEM35B	0.044151	0.081269908	0.058919072	0.484524683	0.615266325	0.036104819	0.604356254
TMEM82	0.783497676	0.578932555	0.545025161	0.00455481	0.05621424	0.036205145	0.604356254
TPGS2	0.346805686	0.048124601	0.628830724	0.232479641	0.025363168	0.035687461	0.604356254
TRIM66	0.643713968	0.232558234	0.38761765	0.050577381	0.021633074	0.036274379	0.604356254

XYLB	0.326787089	0.519491514	0.04566178	0.442489475	0.019286169	0.037234077	0.604356254
ZNF296	0.095800534	0.057160925	0.125796494	0.211183539	0.456617953	0.037332381	0.604356254
ZNF436	0.323145359	0.314747854	0.00077809	0.978919605	0.793493729	0.035536243	0.604356254
ZNF484	0.624567082	0.790100843	0.645104319	0.017807654	0.011459609	0.036807544	0.604356254
C25H16orf45	0.233618515	0.010934194	0.076478909	0.955661189	0.357253169	0.037428919	0.604572826
ABTB1	0.644430158	0.016630733	0.065930954	0.104299413	0.935676536	0.038228066	0.604731088
AHR	0.573258938	0.456148765	0.003880932	0.915088532	0.072503675	0.037653846	0.604731088
ATG9B	0.011710831	0.812353756	0.699046004	0.015044105	0.684957719	0.038076896	0.604731088
BMP2	0.144076514	0.040238249	0.087855477	0.19107142	0.702253515	0.038011487	0.604731088
CP	0.56107823	0.026743636	0.394147492	0.253248802	0.044869623	0.037609095	0.604731088
DEDD2	0.188383521	0.403458088	0.936725736	0.003993823	0.244054782	0.038381483	0.604731088
GPX4	0.488032372	0.764325098	0.853131005	0.000874088	0.245866247	0.038028453	0.604731088
HCN4	0.070070325	0.741598838	0.665348999	0.004105223	0.484503178	0.038161295	0.604731088
HPSE	0.85415997	0.13281523	0.4996827	0.018872641	0.064260163	0.038154051	0.604731088
LDB3	0.001129928	0.372210882	0.349967844	0.929413996	0.508256428	0.03842798	0.604731088
LOC112448395	0.729334285	0.081306267	0.175899184	0.032883325	0.197903749	0.037848332	0.604731088
LOC782675	0.002353581	0.908087339	0.378684493	0.537635266	0.157629332	0.038098595	0.604731088
LOC785629	0.468490769	0.796630519	0.138136456	0.006684517	0.198762223	0.03806586	0.604731088
LPGAT1	0.695366585	0.206879397	0.114411554	0.00856289	0.492784599	0.038400997	0.604731088
MROH6	0.222508958	0.004606733	0.135833264	0.592946447	0.81396724	0.037607259	0.604731088
MYBL2	0.764385713	0.000495977	0.460395959	0.564666847	0.699505481	0.038222779	0.604731088
MZT2B	0.16886273	0.783167999	0.970787587	0.001004107	0.528722627	0.03794662	0.604731088
PABPN1	0.730138151	0.01185273	0.083905755	0.261288261	0.366543963	0.038433644	0.604731088
PADI2	0.924578748	0.126260625	0.066505744	0.12918607	0.068635485	0.038186324	0.604731088
PGLYRP2	0.660335369	0.146721418	0.124068937	0.161620485	0.035713753	0.038377179	0.604731088
PPP2CB	0.349346584	0.22483599	0.011734463	0.291833399	0.253015928	0.037910505	0.604731088
SMIM10L1	0.269702058	0.165977366	0.016541136	0.110908006	0.824485869	0.037787608	0.604731088
SPIB	0.236813746	0.720436652	0.00660938	0.757067986	0.079819841	0.037940445	0.604731088
TAPT1	0.267788306	0.374955372	0.043662604	0.050459259	0.312066578	0.03825511	0.604731088
UNC13B	0.138800359	0.109989067	0.486056419	0.104302657	0.088238545	0.037994277	0.604731088
WDR43	0.742991569	0.257978364	0.175828532	0.005962734	0.342188739	0.038160274	0.604731088
ZBTB11	0.286615359	0.627834144	0.524940589	0.001575589	0.464961161	0.038313477	0.604731088
PCOLCE	0.242223003	0.097411385	0.047355351	0.06744956	0.927947674	0.038570376	0.606301196
BUB1	0.275316997	0.039240595	0.056154188	0.129915805	0.89097149	0.038670575	0.607294552
ANPEP	0.151764626	0.517344513	0.680628375	0.020530046	0.065229338	0.039136273	0.609480904
ASPHD2	0.6617788	0.035336355	0.147569736	0.111561931	0.184495688	0.03895094	0.609480904
CARNS1	0.066221874	0.389014319	0.20828562	0.114372273	0.116356973	0.039081949	0.609480904
DHX58	0.654822679	0.020073378	0.375471975	0.052451608	0.273700415	0.038889807	0.609480904
ESM1	0.435056681	0.011180836	0.106457605	0.181604079	0.762357248	0.039181181	0.609480904
HHEX	0.250938413	0.803996938	0.003393847	0.165575322	0.627503261	0.038990182	0.609480904
ITGB8	0.47852872	0.585506772	0.010561541	0.422556933	0.057291737	0.039161987	0.609480904
RIC1	0.592199663	0.597856056	0.741697893	0.000526663	0.512033774	0.038876644	0.609480904
RIOK2	0.131942413	0.327671918	0.055540881	0.045510784	0.65385053	0.039098441	0.609480904
SLC7A8	0.935642163	0.087458142	0.018165185	0.115331245	0.416519223	0.039081516	0.609480904

ELP1	0.27750377	0.007345645	0.111623464	0.513186375	0.61558167	0.039245932	0.609520737
PET100	0.017409585	0.315104028	0.249574046	0.090508471	0.580357613	0.039258023	0.609520737
C5AR1	0.112662551	0.716472062	0.963498936	0.00508272	0.184468398	0.039603552	0.611259489
CENPE	0.401379662	0.024105273	0.017291742	0.542647322	0.803917912	0.039625869	0.611259489
DENND1A	0.446954769	0.703376763	0.731966033	0.700634287	0.00044881	0.039410897	0.611259489
ENKD1	0.912730886	0.010346724	0.041850039	0.970677934	0.19126813	0.039760023	0.611259489
ERI3	0.936275344	0.025214194	0.625227796	0.01141877	0.43570614	0.039779729	0.611259489
GTF3C1	0.651092499	0.048759784	0.01746839	0.57127574	0.229087328	0.039485947	0.611259489
RCOR1	0.766470439	0.60111792	0.081829872	0.003462032	0.558052187	0.039576167	0.611259489
RTEL1	0.342165215	0.002334963	0.114029482	0.92960666	0.860748311	0.039595497	0.611259489
SLC49A3	0.404675468	0.007851758	0.081683607	0.379149447	0.745167287	0.039743468	0.611259489
TMEM87A	0.206355751	0.118593739	0.006549147	0.567058375	0.805057002	0.039688282	0.611259489
TXK	0.358291078	0.637506658	0.086497525	0.331125641	0.011170424	0.039657662	0.611259489
LOC615733	0.191227383	0.191087067	0.240209462	0.26935915	0.031217305	0.039907105	0.612643118
ATF6	0.384544097	0.015294508	0.300510168	0.191797729	0.224865344	0.040727475	0.612799716
ATP6AP1	0.180745131	0.051196115	0.040673513	0.72333803	0.273214427	0.040102576	0.612799716
C11H9orf116	0.985775518	0.489600585	0.011408894	0.077795239	0.176622285	0.040536083	0.612799716
C29H11orf24	0.105786945	0.350994045	0.116962976	0.480449588	0.036947734	0.041018608	0.612799716
C7H5orf63	0.48512886	0.56350937	0.878468879	0.155335455	0.002024348	0.040487752	0.612799716
CDCA3	0.108910442	0.076583706	0.085868162	0.130257186	0.807362329	0.040421239	0.612799716
CEP95	0.939345543	0.005721387	0.048335773	0.477462939	0.623753322	0.041109857	0.612799716
CEPT1	0.154157217	0.108845248	0.045900961	0.263073839	0.382153214	0.041131467	0.612799716
CXCR4	0.271605086	0.78285802	0.127068314	0.026575574	0.108037605	0.041179539	0.612799716
DNAJC25	0.550323968	0.088104417	0.502803789	0.00679533	0.472442889	0.041410548	0.612799716
ENDOG	0.733282151	0.545505219	0.471744338	0.002976876	0.132783325	0.040173782	0.612799716
FANCF	0.886254689	0.701596455	0.420119323	0.00504684	0.058488711	0.041024221	0.612799716
FASLG	0.027243254	0.905365177	0.259862988	0.148439661	0.080241297	0.040766995	0.612799716
FGF11	0.000881699	0.358205525	0.61036779	0.481152438	0.846417769	0.041490889	0.612799716
FREM1	0.080101331	0.064087332	0.971213279	0.39360446	0.040169709	0.041597641	0.612799716
GPBP1L1	0.419643813	0.058996987	0.213644999	0.156489835	0.093276044	0.041056928	0.612799716
HEATR4	0.365617495	0.067265612	0.164556682	0.382249994	0.050922737	0.041580141	0.612799716
ITPKC	0.656594479	0.457643163	0.072607924	0.035399177	0.098097649	0.040571291	0.612799716
LOC100847995	0.675301657	0.034657112	0.507740951	0.008838237	0.730150115	0.040881632	0.612799716
LOC100848263	0.953829197	0.047567023	0.028250425	0.364116312	0.165089424	0.041003443	0.612799716
LOC107131209	0.458402671	0.103615222	0.01192397	0.20847722	0.658399279	0.041234733	0.612799716
LOC617475	0.537771474	0.216226174	0.847002856	0.000953743	0.825308325	0.041162992	0.612799716
LOC781663	0.245570453	0.090499923	0.04236041	0.101617964	0.791946775	0.040570759	0.612799716
LOC788425	0.605476944	0.030269187	0.681401838	0.064657141	0.094976972	0.040883191	0.612799716
MED13	0.404187861	0.407585762	0.95266787	0.000637104	0.763871813	0.040778814	0.612799716
MERTK	0.738550459	0.117987711	0.298331527	0.045114023	0.065736553	0.041019722	0.612799716
MRPL57	0.25826235	0.546233261	0.927112924	0.006924989	0.086132279	0.041325543	0.612799716
MRPS21	0.924929281	0.160053005	0.495355831	0.002776425	0.386877195	0.041577362	0.612799716
NDEL1	0.391703122	0.426333363	0.305973433	0.00794428	0.192009015	0.041302146	0.612799716
NOL11	0.29694549	0.212522944	0.983805161	0.014542306	0.084577154	0.040773083	0.612799716

NSMCE4A	0.520444119	0.449030012	0.002934719	0.14066933	0.803592714	0.041163757	0.612799716
P3H4	0.762464149	0.20459715	0.000684403	0.871569781	0.83965655	0.04136616	0.612799716
PKN2	0.582694857	0.174790773	0.010108712	0.108299677	0.688350762	0.040904315	0.612799716
PPP1R12B	0.238974494	0.187015464	0.203454998	0.072039574	0.113645216	0.040123533	0.612799716
PRR11	0.092672539	0.162312612	0.600001186	0.025958728	0.333498772	0.04136607	0.612799716
RANBP2	0.358651237	0.203739139	0.167991901	0.007451459	0.836220734	0.040815672	0.612799716
RBM47	0.522063485	0.129401781	0.051953638	0.877074926	0.02556954	0.041558719	0.612799716
SCML4	0.174822957	0.845377326	0.394232776	0.010552448	0.123964742	0.040724305	0.612799716
SDCBP	0.505346983	0.123821052	0.010362131	0.12417434	0.939582902	0.040532444	0.612799716
SLC7A6	0.324729966	0.457589241	0.098602968	0.033356553	0.15965287	0.041330884	0.612799716
TCAF2	0.606832776	0.080848952	0.009966698	0.940390902	0.161764297	0.040104068	0.612799716
TMF1	0.251116882	0.991986243	0.807718186	0.000580047	0.653579769	0.040745001	0.612799716
TP53BP1	0.307109612	0.342859898	0.029862449	0.086549813	0.289535553	0.041586693	0.612799716
TRAPPC9	0.423683671	0.665608901	0.250727967	0.10861943	0.010093885	0.041162447	0.612799716
TRMT1	0.108181259	0.490765031	0.153984557	0.185749914	0.050555147	0.040910506	0.612799716
AASDHPPT	0.728540999	0.286974161	0.096677825	0.143827415	0.027310884	0.041785819	0.615019795
POP4	0.481747353	0.046958547	0.101740022	0.438239032	0.078911072	0.041850844	0.615424913
DARS	0.904739195	0.173622823	0.188918092	0.577258764	0.004682245	0.042054543	0.616776139
FKBP7	0.782111554	0.125875624	0.006007118	0.207445649	0.653827221	0.042055481	0.616776139
FNDC10	0.23411109	0.419791883	0.079181854	0.020733404	0.496275004	0.042008682	0.616776139
AAGAB	0.071354916	0.274775391	0.22523178	0.339916205	0.055977586	0.043300125	0.617871977
ADM2	0.683496841	0.038509803	0.19961855	0.124324397	0.124254851	0.04236912	0.617871977
ANLN	0.96766541	0.067277352	0.040492455	0.239249999	0.130767954	0.042796636	0.617871977
ARPC5	0.52540725	0.002215906	0.127117446	0.883424668	0.648808711	0.043558509	0.617871977
BYSL	0.401443447	0.127620773	0.188982567	0.196718614	0.043478417	0.042905954	0.617871977
CBX4	0.81070599	0.068114027	0.183863581	0.017270463	0.470935555	0.042830028	0.617871977
CCDC168	0.946771107	0.011929735	0.147338076	0.609906826	0.081820786	0.042982357	0.617871977
CTSB	0.197580967	0.155231049	0.007801573	0.77182284	0.440546099	0.042432688	0.617871977
DRAM2	0.635512731	0.152532044	0.258340458	0.19628122	0.016456228	0.042277787	0.617871977
EMILIN1	0.117915631	0.868009846	0.044540945	0.121898987	0.151284123	0.043314599	0.617871977
FAM166B	0.207638402	0.980013465	0.034128455	0.981017662	0.012452129	0.043560897	0.617871977
GPRC5A	0.194902123	0.220127158	0.115118376	0.0620287	0.276821047	0.043551459	0.617871977
JMY	0.041630544	0.670462145	0.237132376	0.047553309	0.264056916	0.043003325	0.617871977
JPT2	0.602504008	0.069387894	0.102874248	0.289583671	0.065557051	0.042526625	0.617871977
KPNA2	0.32994462	0.365450836	0.22163459	0.363075512	0.00868951	0.043392898	0.617871977
LOC112446791	0.003329894	0.674153171	0.489051004	0.823328222	0.092392262	0.04313375	0.617871977
LOC527388	0.338466691	0.087463519	0.104664076	0.620401927	0.043112008	0.042926125	0.617871977
LOC781022	0.210737352	0.016185459	0.115194684	0.36452025	0.577642961	0.042880753	0.617871977
MACF1	0.127833909	0.804118856	0.439433231	0.016732488	0.111660213	0.043419115	0.617871977
MGC152281	0.319544062	0.090135075	0.419489893	0.009980989	0.689545337	0.043017432	0.617871977
MIS18BP1	0.838933342	0.009471039	0.038133472	0.524944311	0.519661035	0.042855025	0.617871977
NEK2	0.606460136	0.005865252	0.047815878	0.578725255	0.837672387	0.042789599	0.617871977
NPPC	0.058425198	0.164394712	0.522531205	0.497870736	0.032793918	0.042623057	0.617871977
NSDHL	0.249786903	0.907696791	0.362039351	0.031450252	0.032815404	0.043522647	0.617871977

PCNX3	0.998928994	0.003583152	0.086827694	0.36651367	0.739483754	0.043366412	0.617871977
POLR2F	0.108887293	0.468573557	0.939522404	0.003881388	0.436651623	0.042393999	0.617871977
PRKD2	0.024110851	0.727787091	0.722893885	0.030957397	0.212795308	0.043150336	0.617871977
PURG	0.149416707	0.524844688	0.178093802	0.040729542	0.142727152	0.04237626	0.617871977
RAD23A	0.204818445	0.082699463	0.094254973	0.266385643	0.192881872	0.042651913	0.617871977
RAD50	0.185334831	0.043942999	0.087347011	0.40860252	0.29094941	0.043475256	0.617871977
SIRT1	0.151927188	0.404500304	0.221261223	0.012628542	0.48136772	0.042856575	0.617871977
SLX1A	0.372582072	0.019932824	0.12960406	0.300706856	0.286748995	0.042966009	0.617871977
SMTN	0.003745315	0.280873994	0.407832428	0.958545639	0.202506181	0.04305786	0.617871977
SRRM3	0.284303929	0.720244366	0.020654843	0.243841448	0.078560187	0.042321107	0.617871977
TMED3	0.067144168	0.052672718	0.071384749	0.985029296	0.339425419	0.043423803	0.617871977
TUBB4B	0.336772824	0.259857775	0.930672729	0.127744815	0.00805262	0.043220962	0.617871977
VHL	0.879276991	0.154889143	0.942846411	0.002109756	0.306974106	0.043019862	0.617871977
ZNRD1	0.98791959	0.002251713	0.485476383	0.646894316	0.121253446	0.043520261	0.617871977
PYGM	0.008295188	0.284774034	0.421901555	0.462294709	0.185568657	0.043774151	0.620360611
AGPS	0.551694581	0.121259437	0.016903293	0.733376531	0.106085634	0.044564143	0.620571756
ANKRD13C	0.689278419	0.006081872	0.722176838	0.788696948	0.036495702	0.044299005	0.620571756
BAG3	0.022435703	0.534284916	0.555940676	0.029533202	0.447894425	0.044619254	0.620571756
CCL11	0.255519971	0.151180284	0.01917868	0.529197115	0.225157225	0.04465878	0.620571756
GID8	0.848010498	0.200830624	0.424844061	0.021656306	0.056200818	0.044591018	0.620571756
HMGCS1	0.328683576	0.388328714	0.148688258	0.221954865	0.020871897	0.044545634	0.620571756
HNRNPH3	0.667642305	0.516239658	0.013110341	0.156192503	0.125072194	0.044658015	0.620571756
KBTBD2	0.067598894	0.293000868	0.458252144	0.157245422	0.061785245	0.044628801	0.620571756
KCNH2	0.028757132	0.243703804	0.259278376	0.137753987	0.349864417	0.044436504	0.620571756
LOC101903383	0.296872797	0.454498494	0.024479175	0.567511503	0.046120447	0.044078643	0.620571756
LOC104971345	0.361325345	0.010622269	0.20349686	0.941814792	0.117733239	0.044127615	0.620571756
LOC783797	0.092812669	0.760092129	0.188946322	0.513315281	0.012650784	0.04411334	0.620571756
MLF1	0.35500316	0.329751872	0.518721175	0.013674415	0.105440039	0.044429441	0.620571756
NR2C2AP	0.080231188	0.334164121	0.206138238	0.978164648	0.016149171	0.044349874	0.620571756
PDGFRA	0.038125685	0.411126473	0.213658016	0.247918311	0.103682825	0.043961845	0.620571756
PRG4	0.181583531	0.016287695	0.205992273	0.606165461	0.23822082	0.044563421	0.620571756
PTGFR	0.499150612	0.637677154	0.034369192	0.032481411	0.244210463	0.044182542	0.620571756
RLF	0.190983884	0.720281288	0.558252209	0.00314723	0.365175352	0.044653452	0.620571756
TACO1	0.232715482	0.229024433	0.461095571	0.043376515	0.081359861	0.044167383	0.620571756
UBLCP1	0.915019988	0.404699477	0.256997645	0.124747131	0.007401035	0.044528609	0.620571756
VWA3B	0.772896413	0.298104095	0.431993564	0.007923718	0.110047546	0.044187315	0.620571756
WFIKKN2	0.397382268	0.038759188	0.583863686	0.062142465	0.15424939	0.043998555	0.620571756
ZNF12	0.867443852	0.668482849	0.235210703	0.039338606	0.016040344	0.043955103	0.620571756
SLC16A9	0.273064503	0.360070189	0.131022102	0.019304742	0.356068705	0.044745858	0.621255732
MOK	0.898831941	0.014332199	0.209609839	0.818762249	0.04010842	0.044784632	0.62126847
ANAPC10	0.522280901	0.013523568	0.687659196	0.030652819	0.59713058	0.044856392	0.621738391
PRRC2C	0.520936195	0.605759285	0.561517955	0.002613577	0.192361258	0.044914076	0.622012575
LIPA	0.300703582	0.129218223	0.063381896	0.784486909	0.046434035	0.045111561	0.623819155
SLFN11	0.290174284	0.219095229	0.0472223	0.118767462	0.251813627	0.045135322	0.623819155

TAGLN	0.214203297	0.138516233	0.773681109	0.368295779	0.0106369	0.045179824	0.623819155
ZNF638	0.368776208	0.171013496	0.647748615	0.004430313	0.497191932	0.045196574	0.623819155
NUP210	0.961637321	0.062063833	0.136027356	0.139951682	0.07958712	0.045335837	0.624166466
PIK3C3	0.015995161	0.386821161	0.194290643	0.188697309	0.398438232	0.045321518	0.624166466
RALBP1	0.495441651	0.020023468	0.11212086	0.249262591	0.3258086	0.045305905	0.624166466
UGGT2	0.964840045	0.100878421	0.032835665	0.032844647	0.863031181	0.0453876	0.62435533
THOP1	0.406018554	0.998264585	0.546893543	0.083164304	0.004923729	0.045442127	0.624581868
EXD1	0.712346208	0.177890541	0.030918508	0.499587236	0.046591137	0.045576377	0.62537954
FLCN	0.187631205	0.489173889	0.53454276	0.11155077	0.016642407	0.045541163	0.62537954
CATSPERD	0.816385102	0.017122102	0.070847075	0.710233405	0.130739471	0.045813123	0.625490156
FCN1	0.257862773	0.85428323	0.530098552	0.036371296	0.021632134	0.045788336	0.625490156
GPNMB	0.59558861	0.223864468	0.049410268	0.832769405	0.016672409	0.045661282	0.625490156
MYL9	0.057877893	0.154236471	0.814084058	0.269833687	0.046759395	0.045731046	0.625490156
OTUD6B	0.863947156	0.153405855	0.003401231	0.483362286	0.420952155	0.045740029	0.625490156
SPAG5	0.818127324	0.01445468	0.016411188	0.507858008	0.927323734	0.045639707	0.625490156
CD8B	0.017959631	0.578020035	0.392319972	0.059186794	0.382422594	0.045883427	0.625929275
LOC101904976	0.201536105	0.033895745	0.760153328	0.022807274	0.78098918	0.045980333	0.626210164
PRKCQ	0.324555701	0.703479152	0.109577373	0.313351777	0.011798257	0.045979919	0.626210164
LRRC66	0.045272041	0.385936431	0.033779591	0.774466531	0.203510261	0.046143846	0.627597656
RAB12	0.784518903	0.001799439	0.323744975	0.3599162	0.565807093	0.046158697	0.627597656
CELF1	0.445029395	0.003015011	0.726970865	0.68279228	0.140154852	0.046243485	0.628229988
SNCG	0.590140064	0.1541773	0.264796366	0.043181854	0.089880664	0.04629422	0.628399042
BAK1	0.19124213	0.4904287	0.4750302	0.057343084	0.036929356	0.046552638	0.628835775
CRABP2	0.821003677	0.177689321	0.009995246	0.251541688	0.256800676	0.046504072	0.628835775
EIF3A	0.807625249	0.178624139	0.254720218	0.011826706	0.217050147	0.046546219	0.628835775
JMJD6	0.425585571	0.293263052	0.982565856	0.00083612	0.921718058	0.046602028	0.628835775
LOC100337213	0.026455852	0.811232813	0.889298471	0.015476139	0.319608221	0.046570138	0.628835775
LSP1	0.32320178	0.012036386	0.164485123	0.155710782	0.9481371	0.046589582	0.628835775
PTP4A3	0.766819394	0.694785599	0.006414682	0.04158938	0.665630294	0.046632938	0.628835775
UGT8	0.057767812	0.951661486	0.379116132	0.244221848	0.018417414	0.046367296	0.628835775
LOC518526	0.487770324	0.507240562	0.906556855	0.834042234	0.000507314	0.046723647	0.629083513
NEK4	0.209971393	0.297016349	0.345095194	0.303482314	0.014551663	0.046766308	0.629083513
SMARCC2	0.314741891	0.34280689	0.292848317	0.059541571	0.05049514	0.046752309	0.629083513
CFAP100	0.336727132	0.031337115	0.09092219	0.172308641	0.576122592	0.046826955	0.629383427
GSDME	0.124165999	0.609991622	0.010117212	0.176328409	0.709821108	0.047030827	0.630303845
IKZF1	0.094226358	0.934956425	0.347000561	0.219902978	0.014251613	0.046999178	0.630303845
METTL13	0.105154983	0.340235326	0.1088151	0.126155623	0.195210031	0.047020617	0.630303845
NFE2	0.030804834	0.09116425	0.892710964	0.273250155	0.140092322	0.047049065	0.630303845
ANOS1	0.619105081	0.125441352	0.168533658	0.047135988	0.155843283	0.047103282	0.63047349
HSF2	0.335413193	0.547792821	0.507528702	0.001083809	0.95245592	0.047138564	0.63047349
CIT	0.259385264	0.069034907	0.012857378	0.601342082	0.69896189	0.047293304	0.630511054
CLTB	0.422124902	0.104195778	0.180762896	0.096449896	0.126439101	0.047350396	0.630511054
DBF4	0.354732891	0.020881507	0.032828425	0.595310756	0.667725815	0.047260357	0.630511054
LOC112448070	0.267983152	0.036306215	0.733436598	0.890653921	0.015273619	0.047385581	0.630511054

TAB2	0.306012945	0.573336618	0.914481666	0.00079929	0.754328991	0.047283028	0.630511054
WEE1	0.479632441	0.774696991	0.295220877	0.005265988	0.168002722	0.047377549	0.630511054
ZFC3H1	0.398442765	0.064715126	0.354600364	0.03536872	0.300424914	0.047410313	0.630511054
FARS2	0.644435877	0.920219218	0.448598566	0.060737757	0.006026592	0.047477817	0.630738085
LOC112443175	0.753492874	0.846938244	0.008646156	0.919176836	0.019217415	0.047504252	0.630738085
ARL8B	0.97984009	0.091223676	0.006737949	0.476840696	0.342066188	0.047737888	0.630778165
CARMIL2	0.873099535	0.020446511	0.640505581	0.064778431	0.131838063	0.047560699	0.630778165
HBQ1	0.337410523	0.448599371	0.834847241	0.00193987	0.400445884	0.047715116	0.630778165
LOC782598	0.752368048	0.103118098	0.019377991	0.679857493	0.096017847	0.0477085	0.630778165
LPP	0.030028653	0.288190093	0.04213363	0.894380495	0.301049837	0.047719476	0.630778165
MEG9	0.993760728	0.018216166	0.019512214	0.404550165	0.687316145	0.047731078	0.630778165
ADRA1D	0.367846643	0.604335133	0.021015954	0.23851258	0.088325031	0.047793617	0.63100647
CCDC78	0.492265638	0.019686858	0.297436509	0.412751069	0.082886852	0.047852165	0.631271609
COLEC12	0.303288698	0.455706951	0.118535627	0.010372399	0.584167698	0.048048674	0.63143858
LRRC3	0.898898227	0.100620003	0.063237132	0.058083882	0.299251559	0.048093489	0.63143858
NOG	0.92577698	0.005323171	0.587229573	0.114335779	0.300872816	0.048134158	0.63143858
OVOS2	0.223152729	0.735580942	0.000897897	0.736548481	0.913464676	0.048017389	0.63143858
S1PR5	0.150159614	0.023230202	0.112360489	0.572363864	0.44296251	0.048079718	0.63143858
TOP2A	0.391640409	0.033602823	0.034903174	0.300185868	0.719178781	0.04801759	0.63143858
ZNF276	0.986988214	0.002575697	0.292238868	0.254829776	0.525680217	0.04812498	0.63143858
ABCF2	0.421864497	0.905845881	0.166746779	0.484917864	0.003252662	0.048420175	0.631461993
CD22	0.025090721	0.340634252	0.068563809	0.856508047	0.19946287	0.048302461	0.631461993
LOC104974460	0.060264236	0.230028295	0.839000104	0.595824414	0.014412914	0.048232256	0.631461993
MAP4K1	0.455048428	0.066833554	0.371146628	0.17765238	0.050160729	0.048443766	0.631461993
NCAPG	0.755881224	0.011418326	0.041195907	0.308923906	0.913199069	0.048360328	0.631461993
PSMF1	0.657144402	0.008123161	0.056647178	0.408461469	0.814242687	0.048439372	0.631461993
RPRD2	0.248713125	0.177571199	0.102185717	0.026016868	0.849127584	0.048178219	0.631461993
SMAD7	0.627089557	0.000703833	0.729765329	0.365257444	0.850994068	0.048303711	0.631461993
VSTM2L	0.79190368	0.058232738	0.276155922	0.019197241	0.412006416	0.048485633	0.631506134
LOC101907540	0.914939734	0.017704205	0.437920153	0.015470049	0.921238121	0.048596022	0.632441964
CD79B	0.177079014	0.210950568	0.238241598	0.781886739	0.014589358	0.048722461	0.632582515
LOC112444164	0.352936785	0.041366095	0.505552206	0.024603148	0.558442841	0.048689844	0.632582515
SLC2A3	0.169283706	0.707287749	0.002158462	0.644393818	0.608794297	0.048682946	0.632582515
NFKBID	0.728899109	0.018777037	0.045905487	0.25817862	0.628317348	0.048841872	0.633631594
ABCD4	0.340967581	0.27227293	0.109367072	0.026202795	0.391936241	0.049537849	0.634013938
ACSL3	0.711458288	0.16640988	0.012266848	0.15686463	0.449177199	0.048963848	0.634013938
ATRNL1	0.297934177	0.04248998	0.561134167	0.045694069	0.319801486	0.049399942	0.634013938
C2CD5	0.351539781	0.629363065	0.403270026	0.101985233	0.011444202	0.049497379	0.634013938
CD164L2	0.006678287	0.764061035	0.395370339	0.30138896	0.168311764	0.048966281	0.634013938
E2F2	0.179419231	0.022357973	0.155112152	0.552291851	0.301202376	0.049312605	0.634013938
EPDR1	0.09212633	0.345357758	0.58842334	0.011124463	0.503396311	0.049705276	0.634013938
ERH	0.707310445	0.063615238	0.286033565	0.059779056	0.133107995	0.048987437	0.634013938
FABP7	0.234484268	0.877142335	0.639947739	0.992892113	0.000798775	0.049572341	0.634013938
FTL	0.31378388	0.09272423	0.023137859	0.268820995	0.578073804	0.049638771	0.634013938

GIMAP7	0.063412816	0.619219883	0.307262573	0.138875528	0.061720653	0.049285406	0.634013938
ITGA2B	0.800239991	0.003361282	0.129245534	0.794107161	0.378357624	0.04959126	0.634013938
LOC104974923	0.334575014	0.340084872	0.040777331	0.033891408	0.667065419	0.049721281	0.634013938
MEGF8	0.479179258	0.104848107	0.17524472	0.03103164	0.380927566	0.04948016	0.634013938
MRPL19	0.503590976	0.481911974	0.35832949	0.151617374	0.007934759	0.049640004	0.634013938
RUVBL1	0.177099194	0.543661352	0.487685967	0.351012381	0.006353074	0.049666978	0.634013938
SECISBP2	0.826809276	0.405196688	0.281965245	0.006126942	0.180569817	0.049607775	0.634013938
SF3B1	0.486692035	0.107339146	0.291237086	0.068792475	0.098753076	0.049268718	0.634013938
SUPV3L1	0.123829995	0.324169824	0.658196014	0.085762968	0.04624631	0.049690997	0.634013938
TPPP3	0.562066591	0.511259867	0.221020582	0.003394707	0.478860721	0.049235185	0.634013938
VCPIP1	0.605767512	0.055558482	0.006527053	0.853845583	0.549285336	0.049170344	0.634013938
ZSCAN29	0.719406958	0.425984035	0.039111919	0.328529392	0.02608174	0.04907473	0.634013938
HOXA7	0.09040664	0.238530078	0.674108084	0.009374652	0.773041417	0.049854313	0.635216713
DNAJC2	0.592481093	0.60042752	0.205658499	0.880828198	0.001636995	0.049896238	0.635257691
AVPI1	0.674507109	0.52007165	0.262649108	0.162316168	0.007082116	0.050019483	0.636333126
CNDP2	0.065995098	0.143658932	0.027491144	0.736251493	0.554473728	0.05016186	0.637156567
FAHD2A	0.155363186	0.406136225	0.46986771	0.050356256	0.071219876	0.05014083	0.637156567
EPC2	0.015004152	0.798029598	0.744130822	0.047172436	0.253498271	0.050204689	0.637207387
THPO	0.41049326	0.043788564	0.230554984	0.079190894	0.325663789	0.050300867	0.63793472
CIDEA	0.267791447	0.355841059	0.417894197	0.005023905	0.536952604	0.050459545	0.638799684
SHOC2	0.52560333	0.22067143	0.172863725	0.007106126	0.753151823	0.050425555	0.638799684
TIMM8B	0.52548616	0.352731147	0.452181238	0.002413135	0.531573057	0.050485844	0.638799684
LOC784007	0.07160057	0.122259953	0.021933405	0.96116639	0.584140144	0.050569346	0.639363277
AMPD3	0.599872013	0.296458543	0.291482086	0.005925582	0.360134113	0.051382473	0.641121069
C3H1orf162	0.554504111	0.04532394	0.135956405	0.152455387	0.21306928	0.051489706	0.641121069
CAMK1G	0.180026629	0.854387141	0.78645935	0.033006727	0.027395781	0.051027265	0.641121069
CNN1	0.011254214	0.187538706	0.925962877	0.798026482	0.069983438	0.050958925	0.641121069
CYP4B1	0.864537156	0.794649386	0.005301037	0.031327167	0.969022695	0.051363866	0.641121069
EIF4A3	0.701545951	0.006505646	0.082153043	0.933695895	0.315007322	0.05128511	0.641121069
EIF5A	0.16525179	0.368114953	0.887987086	0.234198004	0.008720172	0.051295922	0.641121069
KIF18A	0.257048931	0.040879828	0.230170849	0.767483215	0.059621711	0.051398461	0.641121069
KIF20B	0.446224338	0.066983766	0.019478151	0.366479227	0.516104762	0.051238545	0.641121069
LOC101907276	0.494701932	0.047781635	0.074067509	0.127007593	0.495725893	0.051271055	0.641121069
LOC104968422	0.6938097	0.131534522	0.060101385	0.187169153	0.106933917	0.051140703	0.641121069
MAGEF1	0.39903163	0.745728545	0.003398107	0.150570576	0.714115046	0.050837289	0.641121069
MED19	0.374131136	0.562821887	0.927528623	0.003855684	0.145724923	0.05112928	0.641121069
PAQR8	0.213243697	0.523613211	0.724871324	0.388800706	0.003525434	0.05147445	0.641121069
PIF1	0.301844289	0.114050658	0.085835835	0.821111153	0.045040723	0.050998429	0.641121069
RBM3	0.224439485	0.714484794	0.001477309	0.750838727	0.611372446	0.050843275	0.641121069
RRP15	0.284742761	0.062754338	0.119760841	0.492053255	0.103740733	0.050985076	0.641121069
SGPL1	0.393855118	0.36049754	0.008055592	0.13205046	0.719183797	0.050807092	0.641121069
TMEM209	0.941649535	0.412716576	0.664603428	0.108667194	0.003952912	0.051476799	0.641121069
YTHDF3	0.294825793	0.254085751	0.014757654	0.142813747	0.702864878	0.051483063	0.641121069
ADNP	0.150191462	0.975826881	0.135677389	0.036172776	0.157844777	0.052214903	0.641450308

ALG9	0.393577949	0.09096277	0.333231257	0.02802738	0.335372819	0.05181682	0.641450308
ATIC	0.219353493	0.193545303	0.465448985	0.369895964	0.015488943	0.052123256	0.641450308
C29H11orf95	0.5558874	0.419992657	0.019246344	0.170714317	0.1472135	0.052041408	0.641450308
CLEC10A	0.324882806	0.329984864	0.06417881	0.076700713	0.212720332	0.051851461	0.641450308
FANCG	0.83677833	0.00516995	0.184791125	0.895896339	0.156815264	0.051866433	0.641450308
FBXO33	0.451043681	0.743308717	0.002034032	0.647130697	0.257482297	0.05224067	0.641450308
GSDMD	0.114040622	0.04931124	0.105546994	0.35561928	0.535146628	0.052049919	0.641450308
GTF2E1	0.110903101	0.020811569	0.255353358	0.529096605	0.365272165	0.052319115	0.641450308
LOC100336414	0.454489737	0.610475579	0.681621758	0.001008706	0.58304359	0.05155607	0.641450308
LOC112441502	0.447395652	0.00960075	0.665782644	0.480742694	0.081898959	0.051947306	0.641450308
MNS1	0.96762804	0.052813472	0.074434156	0.095950893	0.307912938	0.051886958	0.641450308
PKIB	0.270011183	0.567670611	0.612317985	0.04919518	0.024583802	0.052206689	0.641450308
PLD2	0.710849702	0.108470128	0.002624445	0.991778152	0.566239056	0.052244742	0.641450308
PLEKHG2	0.508877482	0.11105027	0.003832244	0.79503775	0.6619241	0.052336967	0.641450308
PPP1R14B	0.061255516	0.506612137	0.558384608	0.606716416	0.010615551	0.051664733	0.641450308
RGS1	0.453656748	0.33354355	0.454008582	0.001703367	0.972703813	0.05229618	0.641450308
SELENOP	0.767921387	0.967160383	0.004006031	0.069737397	0.54360503	0.052003305	0.641450308
TMEM97	0.280045875	0.508100397	0.409804871	0.053636006	0.03620312	0.052127521	0.641450308
TOMM70	0.159473837	0.104193397	0.195476777	0.73480019	0.047199318	0.05196276	0.641450308
TRAPPC3	0.223234635	0.039407409	0.371569742	0.058240917	0.59219588	0.051988132	0.641450308
ANKRD23	0.367095871	0.00196187	0.646437601	0.594395668	0.412799335	0.052411995	0.64169626
CLK4	0.15336375	0.483549505	0.858200658	0.020916192	0.085875266	0.052435238	0.64169626
SCEL	0.147000022	0.011582029	0.907958313	0.109556176	0.677117603	0.052536989	0.642462389
ADAM33	0.297750949	0.04361349	0.108841587	0.546184787	0.151467986	0.053171312	0.643615661
ANKRD63	0.398053677	0.292363708	0.044198203	0.235103368	0.095536428	0.052778243	0.643615661
API5	0.336856479	0.022576372	0.142940216	0.63007661	0.172362187	0.053485715	0.643615661
BRIX1	0.618888925	0.250396928	0.842455617	0.006209122	0.143911004	0.053094741	0.643615661
FICD	0.032098989	0.023502877	0.591071634	0.865246081	0.298852821	0.052714775	0.643615661
GIMAP8	0.446136178	0.50571545	0.025579804	0.108710556	0.184264231	0.052799668	0.643615661
KDM7A	0.243900436	0.857439698	0.178747385	0.008819258	0.357177272	0.053401113	0.643615661
KIF1B	0.996636836	0.314446297	0.001634937	0.493648286	0.461500117	0.053114396	0.643615661
LOC100298530	0.516079747	0.133335249	0.142043664	0.59165345	0.020120522	0.053010285	0.643615661
MRPS18C	0.717096381	0.713272542	0.134520498	0.002634261	0.642987509	0.053062376	0.643615661
PEX11B	0.989394032	0.379091817	0.340752087	0.002145421	0.426293682	0.053159571	0.643615661
PLEKHF1	0.258159449	0.290102129	0.041587639	0.610742078	0.061975594	0.053439722	0.643615661
PLPBP	0.257143429	0.363447352	0.27294425	0.016786775	0.272720162	0.05312957	0.643615661
PLXDC1	0.022229778	0.504642846	0.248723387	0.052917758	0.798895563	0.053458322	0.643615661
RAET1L	0.512020199	0.972463335	0.001023451	0.363301733	0.637066287	0.053454708	0.643615661
SLAMF1	0.418668771	0.447811995	0.609922498	0.003678711	0.280713245	0.053494105	0.643615661
SLC25A13	0.336122918	0.718032062	0.260536471	0.047994411	0.039017725	0.053400459	0.643615661
SOX5	0.753559128	0.243724473	0.260965889	0.182052285	0.013400938	0.05317148	0.643615661
TMEM30A	0.227334932	0.025549068	0.359872011	0.065679024	0.85157786	0.053164606	0.643615661
USP24	0.328539306	0.334074867	0.124831852	0.019599516	0.435685104	0.053189806	0.643615661
WBP2	0.468965599	0.200331983	0.02194069	0.078290025	0.713765433	0.052681426	0.643615661

ZNF566	0.212987079	0.078406293	0.180815724	0.279395983	0.139112232	0.05329179	0.643615661
CACNB3	0.269289798	0.684320775	0.570005672	0.017546536	0.064469532	0.053699513	0.64372732
LOC104970450	0.418991235	0.086113572	0.127258839	0.043476209	0.595166611	0.05369571	0.64372732
LONRF3	0.573137678	0.080446726	0.035165524	0.093130769	0.786499532	0.053682124	0.64372732
PLSCR3	0.34172981	0.246668404	0.002825796	0.933574237	0.534051014	0.053681574	0.64372732
ZNF207	0.717693278	0.158481169	0.630922225	0.002066562	0.800650588	0.053675103	0.64372732
ADAMTS10	0.907804117	0.008374938	0.16517952	0.322489532	0.297736728	0.054186266	0.64675873
ANGPTL8	0.505026021	0.696505677	0.376626427	0.002853224	0.320272982	0.054318879	0.64675873
CACNB2	0.002382636	0.314299229	0.714003249	0.394121134	0.571767347	0.054161137	0.64675873
CMTM6	0.876633676	0.006468712	0.169370482	0.135341245	0.925961204	0.054126382	0.64675873
DMD	0.251286977	0.211486099	0.289424723	0.199863992	0.039250092	0.054208104	0.64675873
METTL1	0.303179843	0.301928125	0.118454537	0.855366615	0.012975088	0.054120371	0.64675873
NT5E	0.232713033	0.083933046	0.724531047	0.008624517	0.992113965	0.054326699	0.64675873
STXBP5	0.040077872	0.478218851	0.991248923	0.007302551	0.866697337	0.054092642	0.64675873
TATDN1	0.141379525	0.652770812	0.004286074	0.747326527	0.409870976	0.054346493	0.64675873
TMED4	0.7993941	0.011718789	0.086812721	0.20453471	0.727014792	0.054282921	0.64675873
ABI1	0.642985171	0.140143388	0.26811927	0.008111421	0.618996933	0.054386683	0.646768009
ADAMTS9	0.175559119	0.886213578	0.750181162	0.001731151	0.604663243	0.05462487	0.646836729
CD53	0.288012124	0.289294957	0.011979401	0.440190565	0.280352151	0.054900106	0.646836729
CDK1	0.887484951	0.726621787	0.048930576	0.009448433	0.412180719	0.054819908	0.646836729
CRYL1	0.605497415	0.1713557	0.003281144	0.941104015	0.381727574	0.054659704	0.646836729
CUBN	0.386433964	0.23831883	0.090304585	0.067525881	0.218733203	0.054806829	0.646836729
ENTPD2	0.5888269	0.966539593	0.000352495	0.619884964	0.981777269	0.05460227	0.646836729
FAM207A	0.075033541	0.374228355	0.406614783	0.101753752	0.106087418	0.05492015	0.646836729
FEM1A	0.619410291	0.243664209	0.031883034	0.216275506	0.119082927	0.05510651	0.646836729
GABARAPL1	0.802125128	0.914354386	0.004110785	0.358604064	0.112961223	0.054613252	0.646836729
LOC101903248	0.192090079	0.096510065	0.049959523	0.147373366	0.898479417	0.054752193	0.646836729
LOC107132921	0.900263988	0.030117458	0.075518986	0.174441051	0.340844397	0.054507004	0.646836729
LYAR	0.397552295	0.912358664	0.502886131	0.000918789	0.734593597	0.05488156	0.646836729
MTHFD1L	0.098133562	0.4575327	0.276968643	0.584153923	0.017028624	0.055043512	0.646836729
PARP6	0.666679211	0.01396109	0.216402586	0.168021472	0.36146146	0.054667292	0.646836729
PROS1	0.340933255	0.076464933	0.035114663	0.152227914	0.889891068	0.055127124	0.646836729
TACC3	0.213570459	0.041992459	0.109570072	0.371943427	0.339107915	0.05510905	0.646836729
TERC	0.873679382	0.26899917	0.781629852	0.00876869	0.077018486	0.055141343	0.646836729
TRANK1	0.9634141	0.019769019	0.02955435	0.363329547	0.604947715	0.055048251	0.646836729
UBR3	0.331421643	0.246310485	0.026943694	0.067839143	0.829839294	0.055076043	0.646836729
USP42	0.369874015	0.762695998	0.637262935	0.000753939	0.918277779	0.055250556	0.647654911
HNRNPK	0.799858174	0.087104674	0.040336383	0.215269849	0.206862806	0.055436802	0.648563835
LOC101903038	0.413172058	0.594357012	0.042988726	0.027340928	0.434506698	0.05550931	0.648563835
LOC516108	0.078677017	0.591735129	0.444244055	0.022057762	0.274965459	0.055516665	0.648563835
POMT1	0.218465026	0.01514769	0.68145877	0.309466194	0.179287178	0.05543002	0.648563835
STYK1	0.538569762	0.104128465	0.020187235	0.775748548	0.142870454	0.055525695	0.648563835
EFCAB11	0.026317616	0.227745117	0.11679664	0.355410578	0.505628303	0.055614827	0.648565299
FASTKD2	0.444946503	0.960702662	0.623915954	0.033346481	0.014141486	0.055605426	0.648565299

FGF10	0.22416089	0.553723936	0.882928693	0.180588558	0.006374975	0.055713818	0.648565299
FOXK2	0.58441305	0.098861495	0.824247036	0.00938273	0.281976584	0.055666595	0.648565299
MCRIP2	0.301482316	0.686236303	0.798173348	0.002559303	0.298617387	0.055723422	0.648565299
IMP3	0.233572127	0.060401982	0.860722324	0.326941645	0.031828013	0.055766339	0.648604814
ATPAF2	0.148364406	0.667272098	0.545654187	0.023275425	0.101857968	0.056226743	0.649572411
CD63	0.717864611	0.029625088	0.041211333	0.288423097	0.50625146	0.056200768	0.649572411
ECSIT	0.374730538	0.978520357	0.897193337	0.001595835	0.243108715	0.056109561	0.649572411
FAAH	0.503674253	0.014900326	0.841874631	0.076297464	0.264395424	0.056061565	0.649572411
FLNA	0.040662525	0.210289087	0.361949988	0.362364202	0.113285277	0.055952636	0.649572411
LSS	0.767608921	0.971514358	0.715067708	0.008572545	0.027892106	0.056075052	0.649572411
MDM4	0.003758505	0.747993001	0.129651095	0.666376764	0.524401059	0.056039051	0.649572411
MYL12A	0.406284822	0.051394047	0.038084735	0.561762607	0.286748557	0.056235118	0.649572411
RAB9A	0.312833275	0.219022435	0.11966505	0.037162017	0.420541885	0.056245347	0.649572411
SLC39A8	0.017057378	0.934381122	0.244204823	0.125962612	0.260128158	0.056082336	0.649572411
PRPF40B	0.961838929	0.097522558	0.031193063	0.534021288	0.082304765	0.056370132	0.650098551
SMARCAL1	0.068832479	0.069727554	0.349746103	0.187756521	0.407637009	0.056335993	0.650098551
CCDC77	0.991076088	0.074305796	0.045566815	0.670691378	0.057291092	0.056460738	0.650686215
LOC618456	0.884594923	0.111873027	0.00373177	0.376948491	0.927522123	0.056508732	0.650782322
MEIS3	0.221382139	0.130224021	0.032739906	0.381513695	0.359636613	0.056611907	0.651056764
TAF4B	0.92627259	0.674934121	0.012687909	0.044172078	0.369211087	0.056574233	0.651056764
NUCB2	0.909568541	0.326758973	0.014296596	0.13500605	0.226128559	0.056669308	0.651260515
ABCA6	0.067609821	0.472487703	0.68774474	0.008558793	0.703706052	0.057362065	0.652056023
ARHGAP40	0.86932305	0.152488011	0.862720447	0.011024171	0.103576547	0.056900537	0.652056023
C18H16orf87	0.415153704	0.346759926	0.025880275	0.037471358	0.942139829	0.057151688	0.652056023
CRELD2	0.023399171	0.904654287	0.121318298	0.718883781	0.072340544	0.057687354	0.652056023
DNMBP	0.251124619	0.471726189	0.627155348	0.017182092	0.102198992	0.056867097	0.652056023
FCMR	0.045767274	0.465849232	0.730790677	0.747884338	0.011440648	0.057624783	0.652056023
GDI1	0.118031238	0.061586319	0.177853589	0.188755059	0.54457705	0.057513262	0.652056023
GEMIN4	0.307209417	0.826125565	0.015442199	0.33717661	0.098934414	0.056940641	0.652056023
GPR141	0.589722277	0.490126015	0.427458558	0.028760008	0.036702557	0.056855688	0.652056023
GTSE1	0.078016075	0.29506148	0.079248322	0.548780061	0.132420123	0.057427306	0.652056023
IDH3A	0.28856292	0.606586561	0.419566273	0.163967266	0.011069214	0.057619052	0.652056023
IRF8	0.618120528	0.995502917	0.128477964	0.386255226	0.004371061	0.057667526	0.652056023
KCNE4	0.223944699	0.122377978	0.216843887	0.041357151	0.5401699	0.057478507	0.652056023
LOC101904942	0.645852723	0.058349214	0.411429707	0.207996383	0.041239229	0.05753987	0.652056023
LOC104976082	0.309452604	0.252269824	0.027752231	0.812426415	0.075668338	0.057590503	0.652056023
LOC112442253	0.592228422	0.14461613	0.099567357	0.022644887	0.685290585	0.057365012	0.652056023
LOC540403	0.395176352	0.014761532	0.482706872	0.193003199	0.241805218	0.057120676	0.652056023
MICALL1	0.063223865	0.614641816	0.127716624	0.143039648	0.184506976	0.057006866	0.652056023
NDUFAF6	0.799198912	0.250887023	0.06515635	0.458257881	0.022267864	0.057624769	0.652056023
NMD3	0.809811854	0.331933566	0.286335908	0.202149572	0.008513363	0.057398709	0.652056023
PGGHG	0.743874412	0.021432245	0.089053667	0.146125558	0.643818306	0.057692118	0.652056023
RDH13	0.17821329	0.540369525	0.90401704	0.074124298	0.020633321	0.057580917	0.652056023
TROVE2	0.279101186	0.075124131	0.125872558	0.050796569	0.996025595	0.057681445	0.652056023

VMA21	0.735200325	0.13273778	0.272073598	0.269899223	0.018543308	0.057511134	0.652056023
C22H3orf67	0.172117791	0.050092068	0.062853626	0.346696217	0.712720873	0.057780242	0.652602583
CD1D	0.178685799	0.513828047	0.026059393	0.135494715	0.413739281	0.057839404	0.652821499
ACTB	0.989417162	0.000550887	0.379215032	0.824810241	0.790467579	0.058005897	0.653128282
DNM1	0.414067004	0.059641948	0.147658415	0.124671518	0.298197677	0.058217225	0.653128282
IL9R	0.174336723	0.145077459	0.027361218	0.351704451	0.557735617	0.058264567	0.653128282
KCNJ8	0.251724182	0.995198708	0.00906004	0.481617933	0.124109001	0.058243304	0.653128282
LOC101904794	0.833834991	0.045534623	0.007362833	0.937887303	0.517080603	0.058219155	0.653128282
NUFIP2	0.309128183	0.624904365	0.641050756	0.002011797	0.54090719	0.058004764	0.653128282
PEX6	0.996290654	0.017747009	0.276948135	0.043247409	0.638854061	0.058145199	0.653128282
SAFB	0.717936654	0.293736054	0.311181334	0.009826267	0.20949413	0.058091757	0.653128282
SPDYA	0.153478065	0.19093738	0.993487067	0.062181082	0.07444626	0.058008781	0.653128282
TMED6	0.902381678	0.442646725	0.079872962	0.134497491	0.031535145	0.058152138	0.653128282
CDC45	0.330426451	0.019036185	0.038949493	0.82272257	0.674679944	0.058328245	0.653395783
SLC38A8	0.686436867	0.003882207	0.372795669	0.777911487	0.177061651	0.058549694	0.655429075
TSPYL1	0.345616026	0.013479272	0.161598642	0.505756182	0.360466041	0.058656606	0.656178292
PTX3	0.431920949	0.441072863	0.780298613	0.037294879	0.024868519	0.058819187	0.657548822
SMIM12	0.211520164	0.169326052	0.356580149	0.014393231	0.751278546	0.058878467	0.657763462
DTX1	0.546866608	0.238387575	0.007279577	0.717804556	0.202971576	0.058921518	0.657796623
BLOC1S6	0.579136228	0.00723709	0.102657463	0.422993603	0.763127571	0.059083314	0.657821662
LOC107131239	0.651780617	0.381462874	0.047515306	0.062606798	0.187781776	0.059082984	0.657821662
MRPL4	0.090405579	0.508376384	0.438179006	0.025944819	0.265116359	0.058988283	0.657821662
RXYLT1	0.310074844	0.163828369	0.089809753	0.075506479	0.403641194	0.059124182	0.657821662
TEAD2	0.2021079	0.672705407	0.144843557	0.234228419	0.030128142	0.05910431	0.657821662
ABCC2	0.082603859	0.463761856	0.279357214	0.124124154	0.106130602	0.059623375	0.658226805
ABHD16B	0.275246159	0.764002436	0.0101063	0.195744678	0.368609406	0.062747422	0.658226805
ACOT4	0.803622919	0.605981347	0.081844754	0.017810296	0.198439339	0.059594047	0.658226805
AMD1	0.012371176	0.284813851	0.473362336	0.493537793	0.17405004	0.060211909	0.658226805
ARHGAP28	0.756741729	0.325272704	0.002810849	0.335472645	0.634604331	0.061234611	0.658226805
ARHGEF5	0.333170493	0.004107784	0.182165598	0.754101104	0.799729521	0.062002839	0.658226805
ARMCX2	0.455665517	0.893924511	0.022269451	0.065460037	0.248023967	0.06122891	0.658226805
ASPM	0.182118983	0.068221592	0.052155068	0.305743581	0.766332073	0.062370565	0.658226805
C3	0.246316384	0.021640467	0.177141576	0.149658683	0.99702232	0.059601228	0.658226805
CA11	0.24820784	0.17906001	0.008011906	0.815561822	0.507780882	0.061276603	0.658226805
CALCOCO1	0.342981144	0.462136938	0.081674447	0.675770455	0.016719002	0.060973374	0.658226805
CNTN4	0.007803911	0.993691568	0.623261299	0.035301891	0.887065231	0.062252227	0.658226805
COL22A1	0.764339899	0.824292823	0.906302842	0.000425679	0.633077436	0.062880225	0.658226805
COMMD5	0.168060776	0.573836543	0.00940577	0.275211472	0.603147411	0.062057049	0.658226805
CTNS	0.090515758	0.479393987	0.037598688	0.144756722	0.630445146	0.061636804	0.658226805
DHODH	0.377792028	0.663603682	0.479940425	0.315405142	0.004033728	0.06268265	0.658226805
DNAJC16	0.138934523	0.380584977	0.73360272	0.333451587	0.011270888	0.060852085	0.658226805
DUOX2	0.500119515	0.031096256	0.020671641	0.604121968	0.796636285	0.063087643	0.658226805
EIF4EBP1	0.851446042	0.916651912	0.000537766	0.496627841	0.71995052	0.06193143	0.658226805
EIF6	0.769118522	0.042543116	0.03533549	0.497300089	0.26871162	0.063034627	0.658226805

EMP1	0.719594055	0.091716929	0.003461276	0.805076091	0.83039121	0.062592508	0.658226805
EXD3	0.805960737	0.021671751	0.039842423	0.312962121	0.686426356	0.061788921	0.658226805
FBXO16	0.696961488	0.319307857	0.139705317	0.013908825	0.350887376	0.062348069	0.658226805
FGD6	0.072253107	0.570656314	0.18184904	0.542646036	0.037425703	0.062482174	0.658226805
FKBP1B	0.411945788	0.105264082	0.005592216	0.725845887	0.79701234	0.05944482	0.658226805
FUBP1	0.884221959	0.666389179	0.771305365	0.000423389	0.730105334	0.059497129	0.658226805
GADD45A	0.918773116	0.086608824	0.008862575	0.254559402	0.817452093	0.06109677	0.658226805
GLUL	0.958368252	0.75520224	0.000921374	0.395660806	0.550564448	0.06072016	0.658226805
GOLGB1	0.303587405	0.207410344	0.096236078	0.109024846	0.213063933	0.059567826	0.658226805
GOT1L1	0.062752231	0.203050334	0.628414638	0.026657896	0.665087503	0.05987733	0.658226805
GPC5	0.649539954	0.432165675	0.096594064	0.7446744	0.007346056	0.061494958	0.658226805
GPR155	0.318976006	0.225375	0.011262357	0.560611977	0.317340501	0.060407941	0.658226805
GPR68	0.601730367	0.026280424	0.016263649	0.635123224	0.893566026	0.060896659	0.658226805
GRAMD4	0.905597257	0.014425273	0.037961829	0.989867567	0.302803902	0.061573888	0.658226805
HABP4	0.176714949	0.804651532	0.132480964	0.013999832	0.545814663	0.060384402	0.658226805
HMBS	0.080758278	0.356784934	0.767716733	0.049335365	0.130546931	0.060006347	0.658226805
HMGB3	0.985796427	0.219324264	0.628445666	0.003074245	0.338085184	0.059686456	0.658226805
HRAS	0.315691498	0.52543298	0.12584341	0.016133743	0.423659865	0.060060489	0.658226805
INCENP	0.718439543	0.006174277	0.576066656	0.254662997	0.215517211	0.059435082	0.658226805
KCND1	0.569499399	0.326405498	0.001409877	0.922202506	0.62820055	0.062371323	0.658226805
KIF18B	0.641545803	0.044230219	0.071590864	0.079042668	0.927110022	0.061630061	0.658226805
KLHL17	0.41888393	0.010655558	0.072207018	0.709816059	0.623138376	0.060028219	0.658226805
KLK8	0.684335865	0.05225031	0.017794451	0.587237383	0.413415982	0.063026282	0.658226805
KPNA4	0.463464608	0.094426943	0.061992802	0.100117628	0.523703693	0.059950062	0.658226805
LMAN1	0.515175319	0.957092522	0.182662974	0.843418081	0.001944244	0.061334042	0.658226805
LOC100849865	0.095155741	0.367682207	0.884521003	0.004890496	0.956300683	0.060584324	0.658226805
LOC101903752	0.629585279	0.235835263	0.016123527	0.078667702	0.748186426	0.059604773	0.658226805
LOC101904265	0.14246903	0.075683281	0.1539677	0.279640029	0.30697238	0.060017205	0.658226805
LOC101905925	0.095824486	0.036624847	0.35376145	0.481915692	0.236332927	0.059732652	0.658226805
LOC101907574	0.636008734	0.780455783	0.024835561	0.820857196	0.01451859	0.061139141	0.658226805
LOC104973519	0.33585548	0.033237461	0.023695543	0.927223747	0.576149636	0.059708258	0.658226805
LOC104973739	0.677263532	0.09758525	0.055748348	0.534005403	0.078177393	0.062864487	0.658226805
LOC107131416	0.968435188	0.528757851	0.568863349	0.035014597	0.015102854	0.062920874	0.658226805
LOC112446470	0.240689136	0.356814822	0.282712667	0.01232146	0.511591714	0.062674429	0.658226805
LOC530973	0.884618184	0.030967702	0.464782597	0.2045799	0.057260827	0.061702287	0.658226805
LOC784289	0.360714824	0.880513965	0.007817293	0.132414138	0.433560755	0.060024968	0.658226805
LOC786352	0.600947706	0.544850412	0.950527843	0.002477555	0.188953474	0.060830232	0.658226805
LOC789148	0.129250322	0.047333231	0.145386728	0.260605171	0.623451899	0.060528822	0.658226805
LOXL1	0.343600301	0.511089263	0.001448389	0.682771946	0.840747493	0.060908587	0.658226805
LRFN4	0.389198335	0.073643882	0.095426779	0.35727178	0.147052895	0.0603208	0.658226805
MAFG	0.467145014	0.224175759	0.0256718	0.833008803	0.063395716	0.059879206	0.658226805
MEGF6	0.119475702	0.03023504	0.3874107	0.137280274	0.775749691	0.061672649	0.658226805
MSX1	0.305400556	0.384827836	0.018211089	0.285969061	0.244691281	0.061855467	0.658226805
MTHFD2	0.104255212	0.692579935	0.150767675	0.196681722	0.066673692	0.060079955	0.658226805

MYCBP2	0.336740311	0.091419426	0.828902221	0.023868394	0.246749107	0.061985733	0.658226805
NFE2L1	0.675189287	0.502381723	0.003904949	0.638380458	0.170048127	0.060344124	0.658226805
NOD2	0.687482908	0.894193873	0.29247855	0.277690227	0.00303588	0.062308333	0.658226805
NUSAP1	0.877209046	0.02176056	0.019149101	0.461181014	0.900674438	0.062371687	0.658226805
PDSS1	0.443854368	0.330970969	0.171060078	0.352872975	0.01616361	0.060226714	0.658226805
PHF21A	0.882295634	0.090234739	0.050539933	0.247171464	0.145927506	0.060685752	0.658226805
PIK3R5	0.983527991	0.290870694	0.12942709	0.114306056	0.036553538	0.063084913	0.658226805
PIMREG	0.853797057	0.022732422	0.053706714	0.201763021	0.715873606	0.062054271	0.658226805
PNCK	0.005804631	0.883918296	0.07651258	0.692303028	0.526536943	0.06016843	0.658226805
PPA1	0.642215016	0.355129668	0.657155397	0.119422777	0.008644903	0.063091266	0.658226805
PPP1CC	0.087477074	0.917318616	0.461259453	0.00404495	0.987450891	0.061371381	0.658226805
PPP1R1A	0.727317176	0.844858066	0.132895308	0.007666315	0.241870477	0.062269541	0.658226805
PPTC7	0.671880317	0.604759552	0.055135725	0.007284069	0.909260836	0.061507258	0.658226805
PTPRG	0.856770929	0.039652379	0.157003665	0.859363577	0.032351704	0.061485558	0.658226805
PXMP4	0.37989947	0.41718664	0.6654404	0.108124133	0.013020917	0.061533245	0.658226805
RALGAPA1	0.189677812	0.687712056	0.724381531	0.004525296	0.360443008	0.06294112	0.658226805
RIOK3	0.493288165	0.073503069	0.229566815	0.023354955	0.795157739	0.063052994	0.658226805
RIPK1	0.271530578	0.721778645	0.535676384	0.001694058	0.859694545	0.062636606	0.658226805
SH2D1A	0.165109119	0.400694954	0.510256053	0.084092118	0.05082616	0.060470129	0.658226805
SH2D2A	0.144981945	0.282756564	0.346927582	0.971288944	0.010652104	0.061196624	0.658226805
SIT1	0.251130222	0.975925985	0.621988716	0.842881704	0.001174968	0.062156973	0.658226805
SLC2A9	0.332881539	0.009940214	0.255035011	0.488079143	0.358980029	0.061376303	0.658226805
SLC31A1	0.101380825	0.225025422	0.287465111	0.831989935	0.027881017	0.062444725	0.658226805
SLTM	0.897912764	0.190833653	0.195546164	0.020549924	0.20801349	0.060201772	0.658226805
TFCP2	0.918198684	0.533572935	0.02136762	0.090258935	0.163180969	0.062956233	0.658226805
THY1	0.764577387	0.039284785	0.029349473	0.216810463	0.762525605	0.06084077	0.658226805
TMEM156	0.204502306	0.18423931	0.654519789	0.077798303	0.080367381	0.06295684	0.658226805
TSC22D1	0.30430699	0.219784781	0.084299974	0.303715718	0.086272478	0.061344622	0.658226805
UBASH3A	0.247517662	0.922088909	0.748000857	0.407064853	0.00210237	0.060932203	0.658226805
WDR74	0.392947594	0.159105424	0.057068516	0.407877784	0.095984519	0.059289395	0.658226805
XYLT2	0.340375176	0.469978068	0.033516136	0.035796995	0.805073256	0.063037503	0.658226805
ZCCHC13	0.219203931	0.976479361	0.009526492	0.182493348	0.404375349	0.062034277	0.658226805
ZNF148	0.629213395	0.010440341	0.876167874	0.042883994	0.584130552	0.060443836	0.658226805
ZNF470	0.237456248	0.146567532	0.068615081	0.264933768	0.228293953	0.060508837	0.658226805
AASDH	0.064510405	0.205375283	0.191901832	0.162076495	0.375982343	0.063140705	0.658324081
AKAP6	0.032559754	0.316763512	0.749376475	0.426083555	0.047148546	0.06322305	0.658325317
HIST1H3G	0.764509593	0.251331358	0.116613856	0.734729413	0.009440708	0.063261168	0.658325317
LARS	0.384334235	0.083324875	0.466096826	0.073714758	0.141125926	0.06322673	0.658325317
PTPRS	0.384598032	0.095573618	0.141295369	0.031527397	0.952559785	0.063397244	0.659323304
MRPL23	0.229054659	0.507556439	0.139872563	0.118211278	0.081391464	0.063515774	0.660137662
ACTA2	0.07322534	0.281194702	0.706806861	0.403838716	0.026743415	0.06369303	0.660980075
CPAMD8	0.662508523	0.075972048	0.591404566	0.062707763	0.084361208	0.063764043	0.660980075
LAMP3	0.53041839	0.636072324	0.541331847	0.360849539	0.002391464	0.063798211	0.660980075
LOC107131330	0.681085698	0.085858054	0.069884592	0.077901085	0.494288017	0.063736848	0.660980075

USP12	0.768333305	0.061603091	0.017826034	0.745290576	0.250552233	0.06378507	0.660980075
GFPT2	0.528574574	0.04282972	0.253611797	0.036956594	0.744398275	0.063881745	0.661427959
CCNA2	0.649569905	0.079335657	0.033230859	0.321403191	0.287915691	0.064009353	0.661497164
MIIP	0.687973038	0.004928355	0.933904425	0.225902266	0.22122227	0.063953577	0.661497164
TMEM250	0.742117597	0.416485613	0.882712016	0.006745136	0.086105494	0.064005959	0.661497164
SELENBP1	0.062748055	0.288358292	0.591445312	0.017334379	0.85659682	0.06411465	0.662168356
CAPN10	0.110471967	0.02111577	0.206381763	0.41742738	0.792475751	0.064200846	0.662641564
CEP57	0.085784285	0.746183059	0.744382268	0.149856945	0.022347512	0.064277554	0.66288407
CHTF18	0.715940439	0.007480096	0.058823338	0.770994581	0.658157051	0.06434552	0.66288407
TBXA2R	0.554569815	0.797227517	0.955712123	0.000552641	0.684300772	0.064331303	0.66288407
EVPL	0.837136855	0.002652313	0.110987701	0.801001384	0.812387527	0.064469019	0.663477159
SLC6A17	0.015094044	0.213021555	0.244896522	0.823398506	0.247421405	0.064483948	0.663477159
GLI4	0.926797301	0.676608501	0.149163274	0.003204809	0.539169309	0.06477631	0.665234058
RPP38	0.061302071	0.450491094	0.189701475	0.136555309	0.225674337	0.064732317	0.665234058
SERPINF1	0.673690732	0.095323719	0.038162217	0.073077505	0.902290581	0.064768556	0.665234058
EVL	0.222000247	0.237512219	0.016348349	0.351919044	0.534893814	0.064930715	0.665860751
METAP2	0.389209069	0.432967433	0.180698033	0.022118612	0.241094933	0.064959056	0.665860751
SHMT1	0.438597686	0.441947677	0.023559174	0.127404982	0.278958186	0.064939662	0.665860751
GDF9	0.027569865	0.562582207	0.366953865	0.433388621	0.066052898	0.065091377	0.665969198
HECTD1	0.371574671	0.17853271	0.017099393	0.146155128	0.981650381	0.065047422	0.665969198
RNASE1	0.505760568	0.031463258	0.318023791	0.563417191	0.057116429	0.065073098	0.665969198
ETF1	0.385236894	0.090574531	0.048148278	0.48969031	0.198524516	0.065186334	0.666110165
SUSD4	0.947830397	0.008126137	0.200704261	0.402310887	0.262417992	0.065157025	0.666110165
ABCA2	0.424798095	0.043893409	0.025705943	0.462730016	0.739015362	0.065326534	0.667127415
BAZ1A	0.476935856	0.132919294	0.643725468	0.007497945	0.536604376	0.06539451	0.667218427
EPB41L5	0.402592479	0.69274038	0.251947899	0.177311394	0.013185799	0.06541676	0.667218427
CKAP2	0.867556388	0.032347586	0.032695387	0.276929177	0.650055536	0.065631109	0.668562525
DNAJC30	0.384059597	0.772466281	0.222120312	0.007181962	0.349357929	0.065670757	0.668562525
SLF1	0.723549192	0.021300029	0.474060719	0.685125791	0.033022924	0.065660537	0.668562525
GRHL2	0.721083986	0.143935619	0.021793866	0.534070249	0.137117735	0.065743774	0.668652441
NAT14	0.848808072	0.1955103	0.38759525	0.523782008	0.00491883	0.065761077	0.668652441
CEMIP	0.313320216	0.267001167	0.044460716	0.152790311	0.292373125	0.065865361	0.668883934
PRDM5	0.89853171	0.027807062	0.098303743	0.077527806	0.871860944	0.065833547	0.668883934
CFAP46	0.196708912	0.459569294	0.161551978	0.022534378	0.506471475	0.065991337	0.669748817
FYN	0.13677979	0.425937683	0.110666755	0.038034683	0.6809521	0.066064065	0.67007254
ADAMTS12	0.267426344	0.543582008	0.002786399	0.502209491	0.824968162	0.066261738	0.670640586
DHX40	0.142187979	0.491960535	0.06590234	0.193026546	0.188154173	0.066168962	0.670640586
RBM5	0.738810515	0.246666822	0.569206424	0.564703446	0.002865033	0.066264403	0.670640586
STAB1	0.267301522	0.229425208	0.012800712	0.832898446	0.256804733	0.066283531	0.670640586
ACSL1	0.457432406	0.440422028	0.954856175	0.081517037	0.010719596	0.066328508	0.67068216
C17H12orf65	0.091296341	0.290915842	0.656074464	0.035308899	0.27500295	0.066589788	0.671337619
GFRA1	0.217989467	0.043821467	0.155806613	0.221877411	0.510850762	0.066471696	0.671337619
MLYCD	0.300360833	0.682577731	0.975074043	0.002922247	0.289689663	0.06659787	0.671337619
STARD9	0.692255038	0.079078663	0.256924948	0.405781196	0.029619561	0.066553494	0.671337619

SYNE1	0.255173125	0.247408886	0.41304223	0.081382376	0.079518193	0.066483322	0.671337619
CLK1	0.649275618	0.12316471	0.392616712	0.121678792	0.044404592	0.06669434	0.671485166
PENK	0.647772313	0.500937347	0.065101198	0.008109106	0.990015364	0.066683499	0.671485166
SENP6	0.653516668	0.744312518	0.316340903	0.003016863	0.36700201	0.066866934	0.672810087
TRIP10	0.470304021	0.086001813	0.664397738	0.033896886	0.187373921	0.066940375	0.673136335
DACT3	0.297281773	0.328092826	0.040677394	0.05160129	0.836370591	0.067069814	0.674024934
DEFB4A	0.245160856	0.059243576	0.086166018	0.73479886	0.188451927	0.067556491	0.674189627
DUSP11	0.275746824	0.251758752	0.257057862	0.132037952	0.073410696	0.067480451	0.674189627
LOC506495	0.318271136	0.02091092	0.036198701	0.779834444	0.919857769	0.067443304	0.674189627
LOC617905	0.264578671	0.031935884	0.432003686	0.724110794	0.064977743	0.067191972	0.674189627
LOC788175	0.00407399	0.16792128	0.97787711	0.832547902	0.310761418	0.06750495	0.674189627
NAA25	0.244840899	0.117068108	0.387450887	0.028153741	0.552644792	0.067437229	0.674189627
PODXL2	0.656207924	0.732141642	0.023305496	0.148500882	0.103598668	0.067311889	0.674189627
PPP4R2	0.838743145	0.632622706	0.270110311	0.006655704	0.181536293	0.067526089	0.674189627
RAB3GAP2	0.407906705	0.581195741	0.002514776	0.354629692	0.816817256	0.06741493	0.674189627
TENT5C	0.71855095	0.615150367	0.740202012	0.000622774	0.842805417	0.067187857	0.674189627
TMED5	0.856953962	0.065829712	0.049050162	0.143244795	0.434468397	0.06730059	0.674189627
WSB2	0.630414354	0.065251495	0.034436386	0.144524	0.84696592	0.067579181	0.674189627
PCYT1A	0.510391365	0.17077403	0.055802658	0.057621938	0.620273972	0.06768324	0.674225816
RECQL4	0.092233976	0.044287278	0.052396706	0.911851765	0.889586468	0.06763031	0.674225816
SAMD3	0.630306942	0.260045933	0.914088715	0.278506274	0.004168401	0.06770606	0.674225816
ANKRD12	0.295789055	0.458999687	0.986612195	0.00930346	0.144820742	0.069222547	0.674601376
ATP6V1C1	0.944260143	0.298412227	0.00435312	0.454141455	0.32162156	0.068920006	0.674601376
BPTF	0.273601547	0.495187061	0.440612396	0.0150931	0.196717165	0.068475662	0.674601376
C7H19orf70	0.363739444	0.47864743	0.79068161	0.001640497	0.793078887	0.068906135	0.674601376
CCDC141	0.349105838	0.392285469	0.072201851	0.450360929	0.039756872	0.068429789	0.674601376
CCDC186	0.012439705	0.466313365	0.498125727	0.154736655	0.403905601	0.069249677	0.674601376
CCL1	0.031502012	0.947301868	0.363849552	0.080017187	0.202927093	0.068258847	0.674601376
CNOT11	0.5649871	0.011006158	0.141825867	0.781529937	0.262108995	0.06926472	0.674601376
DNER	0.566683471	0.729987522	0.017007784	0.497268315	0.050264154	0.068153286	0.674601376
EPHA4	0.988208881	0.990039877	0.006841046	0.170077166	0.158467732	0.069202912	0.674601376
ESPNL	0.930231235	0.017526974	0.068407964	0.232673552	0.680059301	0.068299121	0.674601376
FABP4	0.06237067	0.354199181	0.029652709	0.491639482	0.553667933	0.06872441	0.674601376
FBXL5	0.338597179	0.409220479	0.05364917	0.152988339	0.154226004	0.068046526	0.674601376
FDX2	0.094186388	0.396241915	0.374239233	0.052815641	0.241292946	0.068650037	0.674601376
GATAD2A	0.397247854	0.526663762	0.004073258	0.952154143	0.220599351	0.06888223	0.674601376
GCNA	0.826715787	0.076840459	0.264185624	0.793230231	0.013281843	0.068376102	0.674601376
GDF10	0.353421845	0.188804453	0.189338335	0.514893319	0.027241829	0.068469266	0.674601376
IL11RA	0.001144529	0.699785815	0.321797228	0.820619347	0.832279455	0.068194057	0.674601376
LOC786039	0.47183434	0.494992296	0.08998942	0.278228721	0.030427752	0.068635446	0.674601376
LRP6	0.361087768	0.618967284	0.51452205	0.001578365	0.989541867	0.069023009	0.674601376
MS4A1	0.091841681	0.802656048	0.277933303	0.834350614	0.01050605	0.069020316	0.674601376
MTBP	0.023819499	0.742233809	0.750473892	0.679041254	0.019579764	0.068281574	0.674601376
PDPK1	0.936128494	0.088842158	0.012180906	0.295601367	0.597164717	0.06884287	0.674601376

PRCP	0.673227889	0.824620397	0.001800604	0.664388287	0.271760627	0.069225105	0.674601376
PTHLH	0.059958402	0.693689477	0.01122898	0.590717887	0.642142936	0.068456924	0.674601376
RBPMS	0.289929273	0.147491557	0.347561342	0.200579501	0.059646745	0.068608197	0.674601376
RET	0.893376252	0.261504674	0.018308742	0.077917403	0.52859813	0.068226815	0.674601376
RHOF	0.215766455	0.049321446	0.255398677	0.668180147	0.098325909	0.068782506	0.674601376
SLC43A2	0.149858074	0.165766379	0.877882608	0.114018905	0.071488133	0.06859491	0.674601376
SPNS2	0.702232981	0.28129365	0.019342965	0.054756592	0.841259894	0.068188922	0.674601376
SPRYD7	0.954396701	0.054420748	0.140964732	0.075965754	0.321631706	0.068856748	0.674601376
TOMM5	0.306997751	0.411665052	0.566655658	0.01730452	0.14469749	0.068955605	0.674601376
TRPM1	0.249447774	0.277834239	0.00682379	0.774760015	0.484910482	0.068575564	0.674601376
TSR3	0.164666319	0.700651713	0.432750377	0.0128229	0.276777421	0.068465824	0.674601376
UHRF1BP1L	0.463474318	0.294360634	0.313162349	0.005469946	0.748056698	0.067912291	0.674601376
ZCCHC17	0.212094373	0.253577625	0.212485464	0.070687673	0.222970973	0.069140864	0.674601376
ZFYVE21	0.406300734	0.876443585	0.010477734	0.098095836	0.487444891	0.068745934	0.674601376
HS6ST1	0.697906901	0.71212291	0.806546874	0.260714671	0.001731686	0.069337387	0.674908571
ARHGAP20	0.148767718	0.013294601	0.899214012	0.12543394	0.814358455	0.069496575	0.674981982
ATP6V0E1	0.566771402	0.011317349	0.06856893	0.739975685	0.561867058	0.06977078	0.674981982
CNTLN	0.325682068	0.060189197	0.282729955	0.062658379	0.523853375	0.069553713	0.674981982
CXCR6	0.173166885	0.987281715	0.400947502	0.842958949	0.003179798	0.069970154	0.674981982
FAT3	0.027189872	0.295714012	0.167665765	0.63993504	0.213192057	0.070011766	0.674981982
FBLIM1	0.698145792	0.63393908	0.003782728	0.617983535	0.176558446	0.069725849	0.674981982
KIF23	0.601508225	0.674396845	0.059297193	0.023715881	0.31908137	0.069578564	0.674981982
LOC100336282	0.05971853	0.838513209	0.072649042	0.937935262	0.053568944	0.069751898	0.674981982
LOC101904177	0.474270707	0.153525408	0.887763691	0.153952416	0.018281644	0.069556839	0.674981982
LOC104970249	0.009603013	0.054849457	0.457733765	0.8558183	0.87957478	0.069455278	0.674981982
MAF1	0.955101674	0.121759234	0.228241375	0.03909713	0.175977395	0.069714638	0.674981982
RUNX1	0.611780764	0.113841543	0.142005949	0.175558699	0.106197806	0.070119092	0.674981982
SOX7	0.110447029	0.512334993	0.350444787	0.0115715	0.798981333	0.06987896	0.674981982
TAX1BP1	0.339881188	0.015124774	0.108831238	0.706021324	0.46546513	0.069996721	0.674981982
TMEM107	0.089226925	0.747553714	0.030367813	0.322127713	0.281647974	0.069978572	0.674981982
TMEM202	0.143785343	0.805919228	0.013715839	0.520307801	0.22268657	0.070065139	0.674981982
USP15	0.122160442	0.171255808	0.083298509	0.131023418	0.807708195	0.070126396	0.674981982
USP34	0.322427256	0.818860412	0.414253468	0.003982221	0.418169598	0.069602922	0.674981982
VCL	0.481551512	0.091071087	0.025341495	0.739488589	0.223321562	0.069923675	0.674981982
CUL7	0.478873378	0.003641169	0.197852093	0.915574289	0.585396649	0.070235696	0.675258953
LMBR1L	0.931832699	0.004952764	0.107034795	0.392397952	0.95395282	0.070237465	0.675258953
ACTG2	0.0116842	0.238464648	0.654576623	0.635261483	0.177185316	0.074758943	0.675653448
ADAMTS6	0.654621572	0.02882852	0.026813868	0.55360537	0.689132987	0.072070566	0.675653448
ALDH1A2	0.543307578	0.00624221	0.20718722	0.426476383	0.670205847	0.073790567	0.675653448
ANKRD11	0.605855726	0.093745417	0.434780521	0.019467272	0.402486009	0.072166726	0.675653448
C16H1orf105	0.187475531	0.083592567	0.975614324	0.666830656	0.019221333	0.072718671	0.675653448
C1R	0.529914316	0.413212128	0.508024755	0.00186798	0.907329978	0.071058794	0.675653448
CALM	0.7877178	0.002704539	0.567792673	0.76454247	0.204849828	0.071263558	0.675653448
CARD6	0.945249972	0.073267328	0.260342921	0.473577322	0.023712511	0.074147655	0.675653448

CCDC18	0.35866886	0.16020458	0.159022038	0.972601688	0.022446986	0.073494439	0.675653448
CD1B	0.191149833	0.003284499	0.571461997	0.794985482	0.662694985	0.071166726	0.675653448
CD3E	0.072702606	0.529361815	0.694626087	0.290104996	0.024018927	0.070547813	0.675653448
CD3EAP	0.707304975	0.279463055	0.103825144	0.435039061	0.022625208	0.074044378	0.675653448
CDC27	0.846958752	0.034764446	0.29535025	0.725163579	0.0318952	0.073855958	0.675653448
CENPBD1	0.079245	0.294001661	0.341056331	0.111874372	0.215416471	0.071722676	0.675653448
CHD2	0.524807802	0.214942154	0.152782494	0.129966416	0.08476418	0.071356624	0.675653448
CNTN3	0.51116698	0.028250075	0.520899577	0.038092027	0.710459371	0.07438567	0.675653448
COX7C	0.404457879	0.909601648	0.516600906	0.001248091	0.831577954	0.07300281	0.675653448
CRYBG2	0.226844547	0.122582834	0.366519765	0.122628649	0.159576074	0.073483968	0.675653448
CYB5R2	0.04722739	0.928663855	0.219564176	0.183173373	0.108545424	0.071715822	0.675653448
CYSLTR2	0.101038038	0.633999638	0.011067712	0.720269249	0.384363426	0.072786546	0.675653448
DALRD3	0.133519877	0.127872578	0.507307383	0.262583942	0.08913035	0.074200223	0.675653448
DCAF11	0.800292284	0.610833243	0.001198985	0.331534343	0.991768031	0.071996111	0.675653448
DCAF5	0.643303943	0.410062559	0.72934513	0.025503231	0.040114986	0.072909867	0.675653448
DNAJC3	0.840343768	0.500384376	0.825595771	0.004465757	0.126946276	0.072904051	0.675653448
DNMT3A	0.541191654	0.054660296	0.017334107	0.856162136	0.450167418	0.073085609	0.675653448
E2F6	0.032365473	0.124395392	0.145104809	0.439068245	0.779438304	0.073591591	0.675653448
ERCC6L	0.297059975	0.042157973	0.110275999	0.152001065	0.940811493	0.07305546	0.675653448
FGD2	0.144887169	0.104969882	0.20977904	0.347578758	0.172853393	0.071765173	0.675653448
GALNT1	0.003945464	0.445345542	0.440190891	0.55237513	0.447796283	0.071682577	0.675653448
GALNT6	0.249821927	0.282035227	0.194149503	0.27810323	0.04955085	0.071051576	0.675653448
GNAT1	0.721071392	0.099683418	0.247356186	0.752504608	0.014406773	0.072003644	0.675653448
GNS	0.656407623	0.088950377	0.020434291	0.634296942	0.251560834	0.071472377	0.675653448
GPR65	0.301398521	0.457947716	0.443471108	0.011064837	0.288114854	0.072533439	0.675653448
H2AFY	0.064006203	0.860204928	0.550447928	0.006563767	0.945265798	0.070945971	0.675653448
HELB	0.359927914	0.615538942	0.002196359	0.680243307	0.612538718	0.07420875	0.675653448
HHIPL1	0.135094257	0.773940511	0.177764563	0.01370395	0.767539384	0.072613514	0.675653448
HMGCLL1	0.205589182	0.051721757	0.371150091	0.282409063	0.177162352	0.073047342	0.675653448
KCTD1	0.201961267	0.548575808	0.017224458	0.122746614	0.829930087	0.072370981	0.675653448
LHFPL4	0.083437433	0.734266703	0.812428441	0.135566215	0.029564073	0.073494102	0.675653448
LOC100196897	0.233920033	0.72777943	0.013298376	0.103426283	0.860404606	0.073927211	0.675653448
LOC100295712	0.968468825	0.00305586	0.290035412	0.439488059	0.524334064	0.073122806	0.675653448
LOC101902059	0.444010434	0.424253381	0.12598336	0.68814411	0.012238343	0.073576639	0.675653448
LOC101902537	0.57731107	0.009783776	0.927895933	0.770346257	0.045900073	0.070329596	0.675653448
LOC101903604	0.392101318	0.004748807	0.421735293	0.711804682	0.339720819	0.071363182	0.675653448
LOC101905099	0.886130541	0.009814655	0.570779421	0.688844312	0.058763571	0.073812832	0.675653448
LOC101908760	0.49973566	0.025759383	0.232186504	0.448398944	0.152840454	0.074661934	0.675653448
LOC104974348	0.993714171	0.032542839	0.155198553	0.302047081	0.131283888	0.073390811	0.675653448
LOC112446717	0.925139267	0.089431812	0.009380512	0.600860564	0.423782497	0.073084384	0.675653448
LOC112449100	0.083735553	0.020143284	0.646071232	0.304239838	0.616792288	0.074586284	0.675653448
LOC513508	0.9734512	0.001756158	0.600766346	0.805459021	0.232022883	0.071821447	0.675653448
LOC515551	0.465303343	0.537522673	0.00310708	0.303893524	0.843463039	0.073429318	0.675653448
LOC536097	0.039071467	0.721280627	0.486957458	0.413666115	0.034931562	0.073233074	0.675653448

LOC614625	0.29628849	0.484448355	0.002411535	0.653754069	0.855720116	0.072202135	0.675653448
LRRC45	0.017988719	0.069787855	0.210179599	0.824533551	0.884096883	0.071912501	0.675653448
LRRC51	0.663582765	0.511103591	0.006274644	0.551523007	0.165228428	0.072265633	0.675653448
LRRTM2	0.130077585	0.102987547	0.489230156	0.050559503	0.595921821	0.073049279	0.675653448
MADCAM1	0.356425926	0.997049907	0.00171952	0.775952415	0.413569119	0.072747363	0.675653448
MAFK	0.190830987	0.111779473	0.060234759	0.622013078	0.250638554	0.073674792	0.675653448
MAIP1	0.5624303	0.362376723	0.38789618	0.04429624	0.057520639	0.073920686	0.675653448
MAST4	0.160239304	0.827144858	0.899496594	0.007868476	0.203655518	0.071622062	0.675653448
MEOX2	0.575206092	0.328298391	0.02225738	0.097991255	0.491143772	0.074106093	0.675653448
MMP15	0.05392289	0.87523087	0.509002826	0.026753346	0.291712548	0.070819223	0.675653448
MMP19	0.382623416	0.214702153	0.315446593	0.710423612	0.011019328	0.07423257	0.675653448
MSL2	0.329052158	0.292429012	0.381758417	0.040690782	0.124862849	0.070629586	0.675653448
MSMO1	0.659047035	0.202481126	0.0380477	0.376786455	0.102403682	0.072703763	0.675653448
MYL12B	0.291341982	0.090426073	0.528408157	0.016517286	0.83949444	0.072065346	0.675653448
NDUFA3	0.787184245	0.96360985	0.470214802	0.000830283	0.682935879	0.074097727	0.675653448
NDUFAF4	0.392567227	0.085033969	0.92604987	0.395057945	0.01544091	0.071065982	0.675653448
NDUFB3	0.55191268	0.730714103	0.46330533	0.001303676	0.777105687	0.071228629	0.675653448
NOXO1	0.405256242	0.537179516	0.83493841	0.058733545	0.019232863	0.074766112	0.675653448
NUP153	0.202962482	0.475386333	0.886452735	0.003369482	0.667750819	0.071933945	0.675653448
OAS2	0.676884458	0.085584816	0.116838972	0.575599413	0.052251259	0.074386318	0.675653448
OLFML2B	0.444875365	0.291184999	0.11478914	0.035709661	0.386209381	0.074713187	0.675653448
OSER1	0.678895169	0.039000637	0.095453373	0.117486679	0.629335425	0.070681518	0.675653448
PACRGL	0.960011546	0.247632887	0.495386446	0.171587794	0.009900159	0.073619424	0.675653448
PAWR	0.0311369	0.279130619	0.096690682	0.937747362	0.258425154	0.074403854	0.675653448
PDE12	0.794738964	0.587123398	0.576955303	0.261256122	0.002736616	0.071941939	0.675653448
PIK3CA	0.302139339	0.198242835	0.220626732	0.019791463	0.782288971	0.074610035	0.675653448
PNPLA3	0.62612955	0.69347699	0.672338948	0.021829371	0.029372519	0.070752688	0.675653448
PRUNE2	0.304540517	0.124482472	0.074505713	0.262327653	0.255247394	0.07119073	0.675653448
PSMA7	0.221265153	0.538530927	0.489551516	0.075963995	0.0449804	0.073457562	0.675653448
RAB43	0.46356028	0.982905323	0.027810523	0.039528138	0.371317098	0.070481326	0.675653448
RABEP2	0.888757711	0.109007705	0.005163037	0.759123263	0.534982636	0.074292773	0.675653448
RHBDF1	0.6352453	0.006652878	0.198714952	0.294322395	0.776848508	0.071839576	0.675653448
RNF19B	0.886330054	0.609345085	0.017899067	0.063776649	0.319569763	0.072951456	0.675653448
SH3GL3	0.034351462	0.101039477	0.262109729	0.313078608	0.710144812	0.07410178	0.675653448
SLC25A15	0.85631216	0.482144569	0.976079763	0.001117265	0.443220662	0.073509753	0.675653448
SLC35B1	0.112982822	0.440451717	0.847048096	0.55368066	0.008113012	0.071240931	0.675653448
SNX11	0.891311263	0.130019175	0.540814405	0.069898798	0.045716106	0.073666553	0.675653448
SNX19	0.771350575	0.628099374	0.304827566	0.036015035	0.036681039	0.072526149	0.675653448
SNX5	0.154356845	0.422597868	0.034684399	0.468156602	0.17665668	0.070737254	0.675653448
SPTLC1	0.75185219	0.0460763	0.022875215	0.944054863	0.273056247	0.074540218	0.675653448
STT3B	0.94102969	0.184324858	0.008330603	0.168124271	0.805520791	0.072656897	0.675653448
SUGT1	0.204357704	0.207433203	0.227974865	0.021519509	0.894291301	0.070480281	0.675653448
TBRG4	0.106668314	0.647017532	0.920907567	0.423950984	0.007449586	0.073766786	0.675653448
TEAD1	0.06150342	0.455038042	0.031635923	0.340807769	0.635694794	0.071794574	0.675653448

THUMPD1	0.348769052	0.185454616	0.132908485	0.138186027	0.171725023	0.074479116	0.675653448
TNFAIP8L3	0.196108305	0.603223926	0.004606871	0.617462235	0.556579471	0.070777181	0.675653448
TONSL	0.368666275	0.005983156	0.150873244	0.896322147	0.654153157	0.072532091	0.675653448
TPSB2	0.674548666	0.328398842	0.026663578	0.129101333	0.248548418	0.071281626	0.675653448
UQCRQ	0.325045431	0.718384188	0.936242026	0.001549338	0.568422791	0.071954829	0.675653448
VWCE	0.260913782	0.909536938	0.002235527	0.880986419	0.438703874	0.07470514	0.675653448
WNK2	0.033029843	0.52496013	0.429940045	0.42895332	0.063221271	0.074080879	0.675653448
ZBTB14	0.381746051	0.095121524	0.057479553	0.101149808	0.966279205	0.074479975	0.675653448
ZFPM2	0.31097508	0.224275741	0.091235721	0.043200943	0.718737	0.07307371	0.675653448
ZNF432	0.004231009	0.98878558	0.740813816	0.68014737	0.095819116	0.074039893	0.675653448
ZNF512B	0.723467085	0.152935731	0.002856055	0.899528155	0.718545723	0.074533977	0.675653448
ZNF652	0.97467715	0.00846827	0.147526025	0.589658527	0.277552309	0.073449134	0.675653448
ZNF800	0.261182705	0.563695012	0.947339809	0.003104891	0.46919971	0.074303022	0.675653448
EPHB1	0.032613583	0.502826696	0.428515015	0.081146649	0.361703882	0.074968359	0.67710828
TWIST2	0.005001529	0.909603715	0.177704233	0.349256095	0.731210107	0.075012742	0.677136473
ARF1	0.649156544	0.008806039	0.064886344	0.890732223	0.636774228	0.075856639	0.677303215
B3GALT1	0.027898697	0.035423069	0.64771535	0.535657622	0.607383294	0.075400309	0.677303215
CD99	0.782033403	0.087861825	0.537660832	0.4245321	0.013217019	0.075191513	0.677303215
CNGA3	0.016454271	0.229506625	0.421370965	0.424356571	0.310480186	0.075699892	0.677303215
DNAJC11	0.409479117	0.547419026	0.548855785	0.034099386	0.04995458	0.075682135	0.677303215
EBF4	0.01047812	0.534373718	0.151888483	0.835716023	0.292405691	0.075306837	0.677303215
HAPLN1	0.368116785	0.124261876	0.100586931	0.114974978	0.397514608	0.075836471	0.677303215
HERPUD1	0.730278897	0.71081778	0.610454147	0.000797188	0.831161241	0.075766484	0.677303215
MINDY2	0.426513077	0.038188358	0.138419057	0.179617042	0.518933235	0.075805031	0.677303215
NFIC	0.663107041	0.179265001	0.050428755	0.272207069	0.126707389	0.075076412	0.677303215
NOX5	0.128621479	0.729359109	0.035623948	0.135828396	0.457336652	0.075257973	0.677303215
PLAU	0.873493618	0.891111021	0.000498747	0.562844607	0.957838527	0.075622233	0.677303215
PPP1R3D	0.472051324	0.1020765	0.503609651	0.054551139	0.157738108	0.075518519	0.677303215
PRDM2	0.238416354	0.360537536	0.20884467	0.073664155	0.157593523	0.075431271	0.677303215
PRPS1	0.756567179	0.018721375	0.038270724	0.971296056	0.393942318	0.075218088	0.677303215
SEC31A	0.65852924	0.668796251	0.126996931	0.018879275	0.198817291	0.075761853	0.677303215
SPON1	0.513720767	0.072244958	0.151744085	0.059557244	0.622444866	0.075511539	0.677303215
TIMP3	0.313072802	0.269884541	0.158741303	0.084531866	0.183503304	0.075356291	0.677303215
TMEM94	0.459050622	0.16745366	0.70677083	0.009564252	0.402907086	0.075636397	0.677303215
USP54	0.108713356	0.707698114	0.226515562	0.059562729	0.199707539	0.075193577	0.677303215
LIMS2	0.201157357	0.208357644	0.312428566	0.265495352	0.060783177	0.076056052	0.677985198
LOC526488	0.28483119	0.385001521	0.695954801	0.089892389	0.030831061	0.076098272	0.677985198
STK36	0.210692373	0.083036074	0.45049265	0.085157379	0.314974786	0.076073207	0.677985198
XPOT	0.705526947	0.857726093	0.778138115	0.614219175	0.000730658	0.076058153	0.677985198
ASS1	0.221131774	0.121642302	0.811940664	0.024515258	0.397345965	0.076361083	0.679588796
MPP5	0.502113842	0.494082612	0.006335709	0.42292389	0.319848084	0.076333717	0.679588796
ATP5MD	0.36182506	0.437493764	0.62997282	0.002637704	0.812239288	0.076552946	0.680558178
WWP1	0.803858879	0.004013792	0.770540372	0.446555367	0.192369577	0.07653616	0.680558178
ANGPTL5	0.054485727	0.882241391	0.167165924	0.322023698	0.082842811	0.076705563	0.680694097

ANKRD22	0.733462756	0.095289282	0.041158029	0.947161009	0.079831602	0.077369553	0.680694097
ANXA7	0.681131447	0.011068388	0.073764282	0.957223264	0.40736788	0.07723098	0.680694097
ATF1	0.543865359	0.474493141	0.630732855	0.001541349	0.854648166	0.076715457	0.680694097
GLDN	0.349899153	0.073274313	0.138106737	0.733411751	0.083807999	0.077397793	0.680694097
GNB1L	0.674773313	0.00862955	0.547094915	0.422250869	0.161745683	0.077383978	0.680694097
HECA	0.415276601	0.533798112	0.12254719	0.033438408	0.236533844	0.076809969	0.680694097
HSP90AA1	0.292876702	0.334707138	0.312269374	0.011785057	0.601903241	0.077291715	0.680694097
KIAA1257	0.393399735	0.024636755	0.063598936	0.676924006	0.514541469	0.076775471	0.680694097
LOC506989	0.778631778	0.46194617	0.811552225	0.351175879	0.002121411	0.077360495	0.680694097
LOC618541	0.253481866	0.610616698	0.995929342	0.576382527	0.002421635	0.076873631	0.680694097
LOC781494	0.062562459	0.842409755	0.059067315	0.949180135	0.073418342	0.07724955	0.680694097
MAP2K3	0.245535846	0.087325412	0.184119802	0.449149429	0.121564907	0.076956748	0.680694097
POMGNT2	0.323847947	0.090474271	0.133530292	0.710227936	0.078018168	0.077218133	0.680694097
RANGRF	0.09832218	0.697347412	0.572541725	0.087550729	0.063040093	0.077191259	0.680694097
SLC45A3	0.638078064	0.189193512	0.050226069	0.101375176	0.353125606	0.077274052	0.680694097
SPOPL	0.422765123	0.288317439	0.075372454	0.029986247	0.785844984	0.077155164	0.680694097
SYTL3	0.139643073	0.654428243	0.355546846	0.033928686	0.197107974	0.077324616	0.680694097
TUBB6	0.077485355	0.177268949	0.348646534	0.120356037	0.375960443	0.07719792	0.680694097
WRB	0.462759971	0.007236334	0.145808367	0.47585208	0.925235829	0.07683352	0.680694097
GBGT1	0.394550966	0.657963268	0.068640873	0.101524415	0.120657898	0.077532092	0.681509991
CYP2R1	0.996448502	0.085053789	0.049776644	0.075636736	0.685299775	0.077613947	0.681864285
ACIN1	0.719661237	0.02467059	0.14508182	0.251480036	0.338683308	0.077765486	0.682579715
MSL1	0.929217658	0.225251062	0.226776281	0.06524241	0.070864606	0.077778567	0.682579715
ARIH2OS	0.227226204	0.2298219	0.182850429	0.048256339	0.477730031	0.077920936	0.683431255
SLX4	0.934138629	0.0196755	0.233832974	0.157910909	0.324627237	0.077958888	0.683431255
SIX5	0.755940483	0.344142599	0.007123229	0.128365476	0.929814855	0.078140544	0.684191005
TAB3	0.865690802	0.158397088	0.025784574	0.882763253	0.070911374	0.078170625	0.684191005
TEC	0.99239265	0.328628585	0.376929865	0.001953446	0.921237754	0.078148525	0.684191005
CDK16	0.639415777	0.162175592	0.021973231	0.10790242	0.901774849	0.078251826	0.684536632
LOC529792	0.296673946	0.067113749	0.137905794	0.60612302	0.13338303	0.078309709	0.684678013
NDUFS3	0.357529001	0.908607186	0.421621135	0.002934403	0.554813273	0.078517107	0.685760639
USP10	0.636613819	0.124994678	0.186294591	0.441323247	0.034066321	0.078492879	0.685760639
NLRX1	0.329868785	0.776158441	0.354561946	0.050487763	0.048810315	0.078667484	0.686343481
TMEM70	0.95013181	0.443527914	0.280256278	0.089292388	0.021202738	0.078644465	0.686343481
SYNE2	0.088904203	0.295993513	0.401092436	0.53845593	0.039401468	0.078713564	0.686380606
ALDH18A1	0.611073195	0.924456328	0.143866484	0.030971748	0.089187167	0.078831087	0.687040344
ABCB8	0.460775658	0.736536068	0.98193987	0.00861842	0.078740447	0.079173725	0.687310604
CHP2	0.479429513	0.185672258	0.023700896	0.450529396	0.238415274	0.079270713	0.687310604
HARBI1	0.312726922	0.344716008	0.285380832	0.024741142	0.297700474	0.079266081	0.687310604
LOC100336208	0.548458227	0.432818833	0.156897923	0.007713293	0.784484629	0.079011974	0.687310604
LOC104973322	0.406353052	0.805380479	0.590720807	0.364135582	0.003221292	0.079301597	0.687310604
LOC112445176	0.357771671	0.54698978	0.039892845	0.04189282	0.694296093	0.07936467	0.687310604
NPTXR	0.230815833	0.963105539	0.012219721	0.093699386	0.89094371	0.079302427	0.687310604
ROBO2	0.381653552	0.743885801	0.937585993	0.002646191	0.322160802	0.079334017	0.687310604

RSPRY1	0.84883902	0.361106888	0.165576843	0.008537237	0.520980974	0.079088223	0.687310604
SLC25A33	0.88597226	0.73093076	0.000854256	0.845522651	0.483399777	0.079165312	0.687310604
UBE2H	0.760467123	0.351289576	0.181141072	0.146712448	0.031684455	0.078924341	0.687310604
VAV2	0.9674531	0.059705307	0.061950333	0.195088768	0.324463998	0.079248233	0.687310604
NUS1	0.826331913	0.028122024	0.421087921	0.051447189	0.451951966	0.079457924	0.687755267
GCA	0.855087436	0.12903415	0.074496911	0.450196269	0.061623138	0.079562961	0.688301397
WDR47	0.372784558	0.110361278	0.269237975	0.056800346	0.36293417	0.07962689	0.688491515
DNAJA3	0.5059855	0.669586678	0.226169611	0.096434346	0.031051093	0.079854338	0.689575365
KCNJ2	0.004585681	0.803349015	0.087802703	0.757843126	0.93521279	0.079812885	0.689575365
KLHL15	0.251714064	0.513366069	0.869120867	0.004721451	0.433131711	0.079900221	0.689575365
LOC101906754	0.998489449	0.159601298	0.090807941	0.648322162	0.024490676	0.079920318	0.689575365
MED29	0.749842552	0.371925916	0.546756996	0.001634404	0.923126061	0.07997963	0.689724496
PPARD	0.513757951	0.952257567	0.26880025	0.040058047	0.043771477	0.080086487	0.690283268
BEND7	0.273258566	0.868834489	0.0846605	0.039048713	0.296337796	0.080496811	0.690692119
CCR5	0.083200915	0.971604694	0.568832352	0.641642221	0.007895351	0.080571285	0.690692119
CHRNB1	0.216110661	0.349312527	0.050639283	0.620769659	0.097474592	0.08023638	0.690692119
CXCR1	0.5674828	0.046901458	0.916783553	0.020280951	0.470805009	0.08057882	0.690692119
EPHB3	0.667957153	0.296439068	0.001574984	0.924410992	0.804493292	0.0803616	0.690692119
MAPK8IP2	0.037987229	0.04744132	0.993448227	0.223306976	0.582128589	0.080526924	0.690692119
MRPL55	0.104563552	0.873570514	0.555638366	0.02698499	0.169902894	0.080519566	0.690692119
MTDH	0.414029496	0.015062329	0.365869912	0.415069834	0.244565342	0.080298144	0.690692119
SCAPER	0.123024001	0.562876794	0.632249689	0.048527458	0.109703612	0.080596881	0.690692119
TMEM92	0.100108018	0.194523377	0.046106608	0.767773993	0.337773096	0.080548957	0.690692119
ZSWIM2	0.470452264	0.496351869	0.270774112	0.090590265	0.040684247	0.080588114	0.690692119
LRRN3	0.468179483	0.035375216	0.371481317	0.552395772	0.068679038	0.080665058	0.690915592
ASB10	0.326062866	0.862494008	0.599469236	0.021287021	0.065106977	0.080713671	0.690965496
ATG3	0.215134784	0.477220433	0.189221248	0.078518707	0.154407843	0.081095307	0.690965496
C4A	0.253476765	0.192776743	0.048050996	0.119450466	0.843598734	0.081313106	0.690965496
CDAN1	0.799288485	0.025892477	0.49143136	0.920233587	0.02531914	0.08138683	0.690965496
DEFB13	0.555146372	0.517664811	0.618726042	0.028781963	0.045937626	0.081007473	0.690965496
LOC104970821	0.540275289	0.091562059	0.028612568	0.80344684	0.206946396	0.081057949	0.690965496
LOC107132820	0.078913325	0.734083149	0.758130418	0.185151979	0.029010487	0.081170257	0.690965496
LOC112448454	0.051507894	0.134756296	0.833884095	0.52557347	0.077423752	0.081094661	0.690965496
LOC539166	0.110442751	0.029589793	0.153380605	0.922647864	0.512513554	0.081398296	0.690965496
NIPAL2	0.11989311	0.477281474	0.008207209	0.641276874	0.78751148	0.081428753	0.690965496
NUAK2	0.446915802	0.242956578	0.022399825	0.109036321	0.886913701	0.081030348	0.690965496
PELI2	0.414799578	0.437641871	0.538726268	0.049297015	0.049086602	0.081323029	0.690965496
SH3GLB1	0.776157804	0.029764936	0.15775029	0.099461182	0.646623612	0.080863221	0.690965496
STK40	0.330782228	0.181637961	0.46876795	0.351030046	0.023841132	0.081132265	0.690965496
SYNGAP1	0.143848621	0.190844518	0.035256321	0.249591193	0.979109292	0.08129809	0.690965496
SYT9	0.563969575	0.217100503	0.053358495	0.114085791	0.315562709	0.081028783	0.690965496
TGM1	0.003142829	0.319943317	0.908858672	0.312741643	0.825562497	0.081181597	0.690965496
TM4SF19	0.151024103	0.388712234	0.072635131	0.620700087	0.089594906	0.081419871	0.690965496
KCNK1	0.157752779	0.978779797	0.22169631	0.071384158	0.097479385	0.081635329	0.691515499

LOC100335514	0.622413412	0.06168703	0.867523324	0.349805767	0.020437965	0.081622432	0.691515499
MARCH6	0.361322142	0.074652217	0.292885611	0.057708996	0.522755073	0.081662119	0.691515499
TOMM40	0.203801464	0.575649154	0.788107334	0.038665241	0.066530332	0.081564056	0.691515499
ACOT9	0.224352195	0.589886575	0.107447455	0.022612734	0.743312878	0.081799805	0.691572345
AP5Z1	0.19692157	0.031917568	0.2324011	0.171600888	0.971348604	0.082695215	0.691572345
ARRDC5	0.515793007	0.072861348	0.353560495	0.891458149	0.020518103	0.082607776	0.691572345
ATE1	0.079326975	0.720810733	0.33301791	0.037902871	0.334992601	0.082355606	0.691572345
AVIL	0.568126999	0.562131335	0.004745215	0.470980606	0.34091513	0.08266527	0.691572345
AZGP1	0.110172312	0.552684971	0.076638887	0.149655851	0.343330399	0.081953573	0.691572345
BMP6	0.49032297	0.07269834	0.029799738	0.598217941	0.377409981	0.081963207	0.691572345
CMAS	0.07180699	0.086382821	0.28467435	0.783459298	0.175624703	0.082593228	0.691572345
CXCR2	0.29582097	0.198371843	0.780660874	0.061166139	0.086814457	0.082652524	0.691572345
DNASE1L3	0.47894442	0.743937566	0.89964854	0.008492868	0.087739981	0.081769506	0.691572345
HOXB3	0.892903987	0.143385858	0.016605963	0.530090303	0.21496778	0.082453974	0.691572345
LLGL2	0.105729207	0.442512487	0.450546456	0.047542305	0.239939378	0.082091023	0.691572345
LOC100140403	0.561777914	0.925027247	0.119973957	0.054123863	0.071117179	0.081994035	0.691572345
LOC783033	0.875172397	0.132861732	0.009036382	0.826398953	0.280089437	0.082641345	0.691572345
MEAF6	0.418582284	0.097694409	0.086690529	0.164444659	0.41045596	0.081854027	0.691572345
OIP5	0.140338339	0.013577946	0.242795217	0.531119664	0.987297205	0.082520155	0.691572345
QPCT	0.159262904	0.017673593	0.136990268	0.915122598	0.677102465	0.081782417	0.691572345
RNF121	0.298302239	0.294093045	0.080397835	0.068234097	0.506187242	0.082722352	0.691572345
SBF2	0.415081457	0.446466245	0.071110229	0.061780165	0.297280253	0.082406054	0.691572345
SPEN	0.167239625	0.466295277	0.092146148	0.072042142	0.466803608	0.082331197	0.691572345
TRAM1	0.998183053	0.025922472	0.544154494	0.053352337	0.320336148	0.082127336	0.691572345
TTC4	0.575926735	0.12852836	0.131624226	0.924941842	0.026812541	0.082326316	0.691572345
VNN2	0.461477712	0.650319134	0.160820438	0.022064391	0.228325474	0.08262906	0.691572345
ZNF768	0.556298336	0.436657741	0.064821264	0.031141235	0.495545319	0.08259783	0.691572345
ZNF775	0.202083662	0.856331899	0.16272478	0.082736486	0.104331634	0.082615107	0.691572345
LRRC59	0.249185335	0.549899714	0.443910514	0.800659188	0.005014647	0.082844634	0.692065367
PPIL3	0.440343743	0.309530269	0.473025975	0.004726369	0.801806212	0.082865666	0.692065367
BCL7B	0.729695667	0.005384342	0.155851495	0.769642727	0.524962762	0.083476239	0.6926671
FGF1	0.033251505	0.072947386	0.117721562	0.970508641	0.889211747	0.08328211	0.6926671
IFI6	0.338582442	0.111927604	0.032410413	0.342167323	0.584671342	0.083141942	0.6926671
LAMTOR1	0.23395099	0.282075404	0.218169692	0.022229088	0.769301151	0.083239411	0.6926671
LRRC8D	0.694673699	0.873919659	0.848506852	0.933702862	0.000511868	0.083236277	0.6926671
MRPL54	0.287136927	0.53722375	0.341496055	0.014085159	0.333557827	0.083494282	0.6926671
NEGR1	0.152036056	0.284485488	0.88814116	0.134500165	0.047871028	0.083462835	0.6926671
NME2	0.13542751	0.579789444	0.631461753	0.18327461	0.027058783	0.083175501	0.6926671
NT5DC2	0.013946826	0.095053183	0.694861266	0.495368757	0.53980545	0.083262326	0.6926671
PRC1	0.434399216	0.030985952	0.10960989	0.409702123	0.408704151	0.083406031	0.6926671
TRIB2	0.246797597	0.861041179	0.00551989	0.740511656	0.285127894	0.08352862	0.6926671
UBL3	0.93564149	0.547627642	0.030018061	0.016196917	0.99405351	0.083523278	0.6926671
USP35	0.597275356	0.069951231	0.015338057	0.970358785	0.395849921	0.083228225	0.6926671
WDR18	0.792683776	0.79015074	0.817369238	0.540335101	0.000894847	0.083503038	0.6926671

LOC104971817	0.414651312	0.034588059	0.083951664	0.618625134	0.333423511	0.083663745	0.693087189
SLC7A11	0.247446647	0.457425115	0.032212063	0.891600664	0.076373081	0.083648871	0.693087189
LOC519309	0.447074512	0.045160674	0.489581246	0.833717062	0.030223735	0.083807465	0.693269946
PER3	0.455546673	0.033027552	0.132439616	0.896012792	0.139334439	0.083747208	0.693269946
TIRAP	0.235382207	0.29991226	0.130347164	0.037687036	0.718315586	0.083812539	0.693269946
ACTG1	0.220439944	0.009533122	0.287994022	0.537939793	0.845404778	0.088850129	0.693702098
AMIGO2	0.081477782	0.25196858	0.052905279	0.457191277	0.512675743	0.084889043	0.693702098
BAALC	0.433961034	0.024607432	0.825128378	0.59108058	0.051845276	0.087863533	0.693702098
BCL2L1	0.674112357	0.144679445	0.017119739	0.50825708	0.314515026	0.087270853	0.693702098
BICC1	0.782287151	0.098043058	0.148011675	0.033250076	0.672234596	0.084725022	0.693702098
BOLA	0.809651177	0.058219542	0.308967064	0.025310076	0.683225961	0.084353348	0.693702098
C11H2orf50	0.572002254	0.031087581	0.063903771	0.737534006	0.325985176	0.088467268	0.693702098
C16H1orf115	0.621441629	0.672284199	0.069448621	0.012760175	0.743230639	0.088836991	0.693702098
C17H4orf46	0.70080157	0.03971034	0.179208554	0.912205264	0.059184564	0.087717666	0.693702098
C1QL3	0.488686101	0.67871164	0.547308708	0.36989812	0.004123702	0.089161861	0.693702098
C2H2orf72	0.692347535	0.07266524	0.024953514	0.59562647	0.37096111	0.089253772	0.693702098
C7H19orf57	0.299256462	0.997080582	0.005826186	0.210697442	0.690536064	0.084566311	0.693702098
CALM2	0.460781097	0.002706345	0.887590442	0.674519841	0.368284274	0.088797788	0.693702098
CBX8	0.522368446	0.555205311	0.641764547	0.003045066	0.457739704	0.085832713	0.693702098
CCDC15	0.403640877	0.188003151	0.127429007	0.028381977	0.936060236	0.085342644	0.693702098
CCDC93	0.225319547	0.772966061	0.765900084	0.035627477	0.05563296	0.086789221	0.693702098
CCNI	0.25584493	0.198564671	0.382165067	0.025224152	0.547930247	0.087542483	0.693702098
CDK5RAP2	0.245378201	0.280885555	0.797572901	0.036038624	0.131137573	0.085903267	0.693702098
CPNE9	0.384780993	0.059924999	0.795171797	0.1579024	0.093706296	0.088105424	0.693702098
CRYAB	0.456874445	0.833801514	0.015690242	0.04663663	0.980490877	0.088487331	0.693702098
CYREN	0.517197106	0.625830136	0.87063392	0.001066526	0.897631845	0.087819088	0.693702098
DDX56	0.414713894	0.260733164	0.151007756	0.161981738	0.099352103	0.086478776	0.693702098
DDX6	0.642171485	0.001573568	0.424547066	0.782017075	0.755106492	0.084644336	0.693702098
DEXI	0.499514294	0.940612323	0.343788769	0.026539791	0.062144783	0.087175962	0.693702098
DGKQ	0.591537463	0.012291074	0.499573591	0.082216026	0.865204091	0.08562758	0.693702098
DMPK	0.853118281	0.203723207	0.796554443	0.002036663	0.952574487	0.087590959	0.693702098
DNAJC5	0.366250661	0.149115042	0.060211032	0.323072345	0.244586559	0.085912205	0.693702098
DOK7	0.280540371	0.957432586	0.797930201	0.00381957	0.334390445	0.088567804	0.693702098
DPH3	0.918409158	0.234325702	0.00171368	0.721887792	0.986312304	0.086441832	0.693702098
EGR1	0.450681932	0.086149261	0.221212639	0.393977052	0.079718434	0.087812396	0.693702098
EI24	0.364464623	0.098468724	0.074488792	0.422417522	0.226627725	0.085149971	0.693702098
EIF4E	0.728314554	0.007387346	0.438667972	0.575507903	0.20038383	0.088273345	0.693702098
EPAS1	0.703582883	0.05712597	0.247812207	0.079008986	0.332395747	0.086248016	0.693702098
EPB42	0.02724141	0.732263329	0.216730155	0.277406301	0.222734845	0.087313079	0.693702098
EVC2	0.503054778	0.095266386	0.093209464	0.2544435	0.236733081	0.087683102	0.693702098
FLVCR2	0.033889887	0.328308121	0.083629113	0.387157557	0.750492857	0.087928405	0.693702098
FMOD	0.18265681	0.125590594	0.092930527	0.206424462	0.602509452	0.086932766	0.693702098
GATA5	0.279245044	0.06678045	0.061234223	0.378945528	0.610252922	0.086726975	0.693702098
GNA12	0.643443155	0.397535655	0.152286749	0.034860824	0.193195123	0.0863967	0.693702098

GRK3	0.414345362	0.5812232	0.057296302	0.599527992	0.030191657	0.083943109	0.693702098
GSDMB	0.149168378	0.913441638	0.318788995	0.38729696	0.015409497	0.085794762	0.693702098
GXYLT2	0.503204962	0.175782064	0.014362595	0.210254391	0.938176836	0.084108176	0.693702098
GYG1	0.254396277	0.038801005	0.24556193	0.799817182	0.138126699	0.087437941	0.693702098
HAPLN3	0.560508171	0.942149404	0.001734302	0.415514982	0.68449498	0.086036778	0.693702098
HR	0.202993668	0.018162137	0.178394784	0.916340282	0.434423287	0.086294266	0.693702098
HSPA2	0.5216088	0.552625464	0.031091327	0.049820684	0.62154099	0.08927887	0.693702098
IL1A	0.200123037	0.471016857	0.046607881	0.068939663	0.876759947	0.087010759	0.693702098
KIAA0040	0.403567568	0.17661908	0.056353636	0.685284925	0.100126858	0.088920556	0.693702098
KIAA1147	0.324846339	0.657508083	0.178536197	0.015417461	0.426378364	0.084123087	0.693702098
KLK12	0.707661989	0.309742321	0.27206258	0.224494117	0.01958424	0.086364673	0.693702098
KRT23	0.326156938	0.760317096	0.008941932	0.159731211	0.769811301	0.088364633	0.693702098
LIPG	0.127217611	0.945111975	0.526255962	0.069879789	0.060189908	0.087123237	0.693702098
LIPJ	0.788914497	0.887345095	0.155922975	0.338339289	0.007099171	0.086363017	0.693702098
LLPH	0.421557466	0.042157013	0.422507653	0.531377441	0.06694907	0.087311689	0.693702098
LOC100847410	0.377896991	0.430236032	0.162291354	0.029051767	0.353154433	0.087995703	0.693702098
LOC101905312	0.558098049	0.041829145	0.993043385	0.012231711	0.931977246	0.086766043	0.693702098
LOC104975244	0.464362854	0.116648693	0.527422027	0.029303984	0.313939742	0.086488251	0.693702098
LOC107132301	0.002357876	0.570725416	0.357550914	0.725322022	0.797073708	0.089401215	0.693702098
LOC107132853	0.564462562	0.76247193	0.540490948	0.129067469	0.008721229	0.086298969	0.693702098
LOC112441476	0.883081659	0.054258669	0.848598051	0.180998496	0.034238592	0.084379119	0.693702098
LOC112441478	0.444982186	0.025957949	0.113353859	0.297512492	0.704683548	0.088712037	0.693702098
LOC112442053	0.661551978	0.069408681	0.026375747	0.341982303	0.626987675	0.085881741	0.693702098
LOC112443510	0.213501954	0.288771094	0.330536908	0.060969127	0.216491301	0.087666678	0.693702098
LOC112445242	0.017899464	0.430318069	0.054020212	0.857499294	0.732986823	0.086237832	0.693702098
LOC112446002	0.751856673	0.73153768	0.033839747	0.368641217	0.039203134	0.087666393	0.693702098
LOC112446426	0.342726752	0.900000372	0.040286127	0.023466962	0.864226355	0.084386833	0.693702098
LOC112446756	0.626654775	0.771552458	0.162716187	0.055868332	0.061989848	0.088327139	0.693702098
LOC524810	0.331161044	0.01196753	0.146160242	0.643184603	0.698306472	0.085975497	0.693702098
LOC618367	0.974009163	0.197793924	0.043897638	0.768413284	0.040324428	0.086338306	0.693702098
LOC787234	0.791107299	0.332463214	0.627147822	0.067342385	0.024290333	0.087825328	0.693702098
LRRC17	0.826574032	0.190718565	0.002114438	0.916606183	0.84346697	0.085497658	0.693702098
LURAP1	0.414410827	0.817316605	0.044152774	0.065144021	0.258683871	0.084386006	0.693702098
MAGI3	0.568233692	0.234966579	0.421837586	0.118904294	0.038597174	0.085648915	0.693702098
MANBAP1	0.955152641	0.058891275	0.067386609	0.304298653	0.236133077	0.088308555	0.693702098
MBLAC2	0.501037348	0.483143221	0.430736508	0.057673659	0.045033618	0.088014532	0.693702098
MCHR1	0.330755023	0.224394789	0.023733844	0.628012784	0.232839965	0.085473768	0.693702098
MTX1	0.451357243	0.787862753	0.82167643	0.00567364	0.167242987	0.089229419	0.693702098
MVK	0.263493192	0.839410634	0.690135767	0.013880535	0.121648717	0.085505915	0.693702098
MYBPC2	0.185177041	0.095767852	0.057714349	0.473954209	0.554163517	0.087635924	0.693702098
MYCT1	0.447131769	0.836959808	0.008646504	0.084925888	0.950933712	0.086197783	0.693702098
NAT9	0.919685866	0.599323794	0.106113603	0.004956494	0.892976232	0.085724601	0.693702098
NCAPD2	0.691629977	0.064000952	0.083683014	0.448246227	0.157936363	0.086375074	0.693702098
NDUFS8	0.303194213	0.932483581	0.984200742	0.00297104	0.319854353	0.086795992	0.693702098

NEDD4	0.200832542	0.562448925	0.430283682	0.007736922	0.733406164	0.088954847	0.693702098
PHOSPHO1	0.10721783	0.102805227	0.147436627	0.544500518	0.311138426	0.088865904	0.693702098
PI16	0.742444825	0.039366515	0.072762748	0.122711552	0.975739027	0.084899822	0.693702098
POLR2A	0.368491219	0.829067801	0.381118887	0.003621342	0.621288969	0.086322101	0.693702098
PPP1R15B	0.27023093	0.284680915	0.09619489	0.128530149	0.280010387	0.087160964	0.693702098
PQBP1	0.155158516	0.201574117	0.668756523	0.144813158	0.090081596	0.088399739	0.693702098
PRR12	0.400060904	0.127953259	0.0182564	0.371533978	0.802485842	0.089486767	0.693702098
PRR33	0.880597017	0.026542014	0.150371217	0.191836804	0.412861757	0.089436835	0.693702098
PTGES2	0.182301131	0.830547701	0.627354543	0.00742793	0.383216835	0.087932642	0.693702098
RBFOX2	0.157390406	0.479861863	0.173930803	0.410570681	0.048271277	0.086009448	0.693702098
REEP2	0.230313961	0.265627601	0.187239298	0.45985408	0.050408492	0.087007418	0.693702098
RNF146	0.793483603	0.393034425	0.027788732	0.063462686	0.487304111	0.087481777	0.693702098
ROBO3	0.437584326	0.347872117	0.066648295	0.59876165	0.043253158	0.086473535	0.693702098
RPS6KB2	0.486095231	0.157597693	0.878546757	0.008172816	0.485014332	0.087247525	0.693702098
RRP8	0.872984158	0.444021104	0.451498047	0.009333708	0.169918062	0.089286667	0.693702098
RSAD1	0.973648444	0.006150312	0.077459146	0.853631459	0.672299759	0.087135266	0.693702098
SAMD15	0.561308169	0.071481635	0.065403384	0.614481324	0.17233727	0.089349492	0.693702098
SDHAF4	0.95756763	0.74955626	0.101025474	0.004067009	0.889372821	0.086382898	0.693702098
SEMA3E	0.085085838	0.954908667	0.071703061	0.264210976	0.163812074	0.084412253	0.693702098
SH3PXD2A	0.337278422	0.553143014	0.007294158	0.607865957	0.332788503	0.088858475	0.693702098
SIGIRR	0.433745728	0.276766609	0.012294852	0.322910039	0.544348467	0.085833781	0.693702098
SIRPB1	0.051249619	0.956663316	0.662455233	0.343293452	0.02444408	0.088343119	0.693702098
SIRT4	0.632358861	0.209668069	0.208670783	0.018271623	0.547768808	0.089163651	0.693702098
SLAMF8	0.760532008	0.284107048	0.03595226	0.269071377	0.130816338	0.088510541	0.693702098
SLC25A45	0.830518379	0.2938483	0.112312811	0.020520686	0.490711741	0.088994874	0.693702098
SLC2A10	0.242452448	0.984446906	0.009434444	0.669385472	0.170443693	0.085344645	0.693702098
SLC38A4	0.125275839	0.909863153	0.057961133	0.287462473	0.133948929	0.084851855	0.693702098
SLC39A14	0.681829684	0.893772859	0.031461086	0.078924633	0.184117864	0.089481576	0.693702098
SLC39A3	0.376730943	0.270064497	0.213907975	0.09938103	0.126828905	0.088676175	0.693702098
SLC9A5	0.91042357	0.064180379	0.018506964	0.559185633	0.443348687	0.087496671	0.693702098
SMYD5	0.361090363	0.380104274	0.46672139	0.6577585	0.006074573	0.085156751	0.693702098
SNRK	0.075298832	0.831598659	0.640175977	0.057482942	0.111540978	0.085365649	0.693702098
SOCS6	0.654175615	0.043280997	0.027118598	0.451685158	0.729163173	0.084556674	0.693702098
SP100	0.962429026	0.83860661	0.849475206	0.686077456	0.000561908	0.086773754	0.693702098
SPRYD3	0.076143746	0.287888432	0.565027112	0.026043552	0.786258749	0.084702255	0.693702098
ST8SIA4	0.596253692	0.208858354	0.232491251	0.010607788	0.882795404	0.088073974	0.693702098
STARD3	0.357193071	0.006371095	0.400126916	0.324796806	0.895765246	0.086891175	0.693702098
STRN3	0.613120364	0.234637186	0.242920802	0.008447031	0.852544451	0.084318223	0.693702098
STX1A	0.038645284	0.890129921	0.077684164	0.174243097	0.550603014	0.085239276	0.693702098
SYT2	0.870878608	0.252415847	0.004626701	0.563101212	0.480970472	0.088891074	0.693702098
TECPR1	0.665226208	0.020265003	0.083366023	0.394025022	0.598877951	0.086943393	0.693702098
TK2	0.051452919	0.769396465	0.718359548	0.010653681	0.846980819	0.085285141	0.693702098
TRMT6	0.727212897	0.369378556	0.305867315	0.583314873	0.005530023	0.086911694	0.693702098
TSHZ2	0.914089529	0.062617908	0.063801599	0.105339164	0.652310678	0.084174231	0.693702098

UBAP2	0.971503585	0.870923944	0.029724091	0.02446368	0.446309195	0.088728973	0.693702098
UBE2W	0.20092994	0.045657877	0.108252243	0.391741073	0.714954895	0.089396375	0.693702098
UBR5	0.495002271	0.82727513	0.58000806	0.002183971	0.50902099	0.086722086	0.693702098
VANGL1	0.407657802	0.889472941	0.231395428	0.004660588	0.652368041	0.084991222	0.693702098
VANGL2	0.427648135	0.179951832	0.006734644	0.867623379	0.57271543	0.085463893	0.693702098
WAC	0.43953238	0.541276176	0.575880083	0.002712337	0.708399165	0.08656922	0.693702098
LOC101902293	0.727456762	0.063495471	0.200530922	0.370175781	0.081408146	0.089580257	0.693771403
UBFD1	0.045542644	0.332847931	0.665251794	0.318351602	0.08689426	0.089549166	0.693771403
KLHL25	0.061088071	0.900168984	0.173305419	0.233051959	0.125967333	0.089699991	0.693887307
KRIT1	0.03121399	0.213874076	0.898803274	0.080060809	0.583104254	0.08976435	0.693887307
LOC782021	0.250510191	0.626894596	0.166114987	0.186618633	0.057504788	0.089734207	0.693887307
VCAN	0.987195451	0.105224009	0.028057367	0.154469973	0.622021436	0.089749436	0.693887307
STX7	0.793872109	0.022004483	0.034080353	0.519702465	0.906393106	0.089824406	0.694024636
CAPS2	0.18388217	0.749998304	0.007113208	0.294212088	0.977563083	0.090142014	0.694516714
CXCL5	0.828279675	0.655375687	0.052937799	0.036053681	0.271827424	0.09004608	0.694516714
LOC107131516	0.6720865	0.073331217	0.810614554	0.022585162	0.311768175	0.089986647	0.694516714
LOC782264	0.598023867	0.934452783	0.00132194	0.536736768	0.711509466	0.090137036	0.694516714
PHF14	0.336398217	0.397593357	0.005931976	0.680536393	0.522048021	0.09009169	0.694516714
SLC25A5	0.166523581	0.502521305	0.71653207	0.537432724	0.008721919	0.089940655	0.694516714
CERS4	0.233100078	0.601155724	0.099890629	0.047965297	0.420937605	0.090230021	0.69481038
MRPL43	0.219906783	0.26556681	0.504992835	0.097670627	0.098259628	0.090307144	0.69481038
SEC61B	0.100949655	0.085399234	0.426282359	0.514913453	0.149554034	0.090301314	0.69481038
FERMT1	0.421238637	0.148583716	0.423437315	0.229545247	0.046649563	0.09044851	0.695246138
LOC104976061	0.178181577	0.302828605	0.013795086	0.527135115	0.723247082	0.090447093	0.695246138
PATL1	0.497266015	0.055632469	0.866550255	0.046057306	0.257563135	0.090556228	0.695748245
CCL8	0.179769548	0.137312107	0.925586363	0.019941556	0.625281585	0.090651287	0.69582707
MED27	0.622983272	0.326551656	0.130295356	0.110147264	0.097556292	0.090640318	0.69582707
GIMAP6	0.557367587	0.54832998	0.187168176	0.174006121	0.028692866	0.090782172	0.69618048
RAB7A	0.043696228	0.224015649	0.868373436	0.055793321	0.601910245	0.090756485	0.69618048
F5	0.893590154	0.619091987	0.402598127	0.009129006	0.140603466	0.090834528	0.696256628
ADIG	0.246921315	0.387003792	0.15318689	0.076979668	0.254550759	0.09101234	0.696968505
MMP2	0.824797972	0.224544843	0.066311133	0.126399969	0.184679206	0.090982653	0.696968505
APRT	0.357043933	0.721641207	0.31424585	0.083411989	0.042561753	0.091123817	0.69719848
YBX3	0.919575684	0.278806231	0.014318373	0.150649835	0.519800982	0.091127338	0.69719848
ASB13	0.931634374	0.93843029	0.427534689	0.0711586	0.010887297	0.091515472	0.697242531
ATP6V0B	0.227099756	0.021401679	0.072823449	0.897024148	0.906557486	0.091193922	0.697242531
EPYC	0.705567183	0.343852693	0.002186794	0.633059015	0.861689248	0.091484529	0.697242531
FRRS1L	0.675957101	0.097022049	0.034910578	0.669215236	0.188665886	0.091422425	0.697242531
MPHOSPH9	0.590740316	0.001277501	0.586963163	0.651962552	0.998608672	0.091297877	0.697242531
NUP50	0.366103696	0.12892529	0.2050263	0.034396476	0.868847007	0.091447283	0.697242531
SFI1	0.398800653	0.011435518	0.081606834	0.870793434	0.892798977	0.091471505	0.697242531
SNAP23	0.864763276	0.026873838	0.358682285	0.079680053	0.434258466	0.091303743	0.697242531
SREK1	0.871014029	0.184538468	0.08386454	0.072595097	0.294598427	0.091278507	0.697242531
DSE	0.281260966	0.12781725	0.141385468	0.065020522	0.877259448	0.091578633	0.697399974

LOXL2	0.883226002	0.150090633	0.011971661	0.426310705	0.429669314	0.091720578	0.697958695
NAP1L1	0.015489966	0.830409368	0.318289873	0.135418385	0.524483282	0.091737061	0.697958695
FAM222A	0.409067436	0.823899379	0.01122051	0.547472229	0.140597034	0.091791677	0.698050606
SERP1	0.476071167	0.018258211	0.64908373	0.490504116	0.105847154	0.092127582	0.700280567
PTPRO	0.180332845	0.088187783	0.324469111	0.06192422	0.918781078	0.092247878	0.700870335
UBR7	0.996086431	0.012040482	0.120926351	0.285155655	0.710791948	0.092316569	0.701067658
HELLS	0.329722606	0.440876005	0.003820546	0.783534803	0.67662822	0.092404514	0.701086678
VEGFD	0.017755645	0.215678762	0.660625039	0.183355062	0.634640728	0.092394718	0.701086678
CCDC189	0.200192834	0.022456427	0.650494046	0.122258664	0.825973329	0.092562527	0.701212349
CORO1C	0.989382595	0.018410103	0.0869792	0.201695093	0.923965137	0.092551233	0.701212349
PLEKHH3	0.178099818	0.22500013	0.063553715	0.263980102	0.439324735	0.092570669	0.701212349
PRPF8	0.584845102	0.383172369	0.336114665	0.005081643	0.771951186	0.092591991	0.701212349
LOC786726	0.651603777	0.094655756	0.06807555	0.433255801	0.162632391	0.092660858	0.701410215
SQSTM1	0.325992246	0.140370924	0.028244647	0.622605381	0.368371716	0.092765658	0.701879764
DNLZ	0.132229645	0.417166874	0.667230124	0.059264695	0.136304564	0.092926928	0.701932336
F3	0.743798238	0.466659089	0.018304345	0.595671786	0.078443801	0.09284682	0.701932336
SRRD	0.645562595	0.156774198	0.174659077	0.024361108	0.690644785	0.092943694	0.701932336
THNSL2	0.532263197	0.33958967	0.029280231	0.095134954	0.5901166	0.092891283	0.701932336
ANKMY2	0.150140449	0.172749299	0.175846671	0.067412183	0.97807241	0.093541114	0.702617699
BTBD11	0.007661721	0.744373401	0.661625089	0.95008884	0.0838538	0.09352347	0.702617699
CHKB	0.111970287	0.269918706	0.163412816	0.107664274	0.563374972	0.09333339	0.702617699
CNTROB	0.91370222	0.00645717	0.080293813	0.932272848	0.678469547	0.093347253	0.702617699
DMAC1	0.415384619	0.164601158	0.150688625	0.223988363	0.130433958	0.093593823	0.702617699
ELAVL1	0.32274094	0.014250153	0.298619213	0.597021426	0.367215665	0.093609123	0.702617699
KIAA0586	0.075009796	0.114266251	0.378369223	0.330380792	0.280141698	0.093439696	0.702617699
LDLRAP1	0.110692894	0.074051439	0.897475335	0.097087361	0.421665648	0.093622137	0.702617699
LOC104973229	0.197073598	0.113153576	0.381863814	0.331608607	0.10562288	0.093096802	0.702617699
LOC112449072	0.031012435	0.079410155	0.161461207	0.966413141	0.783531834	0.093608763	0.702617699
MKL2	0.011487408	0.769540263	0.140020886	0.582728928	0.414705163	0.093253871	0.702617699
PSAT1	0.144564307	0.413021699	0.446124126	0.316088394	0.035776857	0.093633838	0.702617699
SELP	0.530773266	0.293791064	0.024754664	0.656494948	0.118178237	0.093318759	0.702617699
ZNRF2	0.34614132	0.061214048	0.056555401	0.591079139	0.422437341	0.093270587	0.702617699
SKP2	0.586148968	0.446042434	0.065886893	0.273279672	0.064239876	0.093845693	0.703885589
GPATCH3	0.043627237	0.870070263	0.872564308	0.041031441	0.222923301	0.093944146	0.70430214
GCSH	0.339798164	0.811953463	0.496510639	0.007828848	0.28271439	0.093987331	0.70430415
AKIP1	0.969144022	0.113668608	0.336283969	0.008462205	0.980333221	0.094724771	0.704625304
ASCL2	0.32985868	0.069166167	0.323204036	0.090665418	0.454305251	0.094082762	0.704625304
ASNA1	0.238454753	0.316034682	0.371543563	0.048248281	0.225151685	0.0941605	0.704625304
B3GNT6	0.189790191	0.06178091	0.041843187	0.985482525	0.635301404	0.094698189	0.704625304
BBS5	0.712468114	0.713966382	0.028552025	0.745575407	0.028360161	0.094685427	0.704625304
DPM1	0.445959347	0.965683988	0.172094704	0.091208746	0.045448432	0.094707224	0.704625304
F2RL2	0.103761915	0.432239122	0.218684682	0.226499241	0.137960552	0.094574731	0.704625304
GPC3	0.92850139	0.02709553	0.2383027	0.053597292	0.951342261	0.094434579	0.704625304
GSPT1	0.659678563	0.152687527	0.05222116	0.938861765	0.061895733	0.094429044	0.704625304

LUC7L	0.748636649	0.041338662	0.134119599	0.291275162	0.251534603	0.094149635	0.704625304
MVP	0.367103138	0.411542545	0.134640706	0.266154305	0.056666564	0.094629859	0.704625304
NAV2	0.455192531	0.831169502	0.007349336	0.399907792	0.27655381	0.094760103	0.704625304
NBEA	0.130179132	0.945856084	0.275931415	0.040512307	0.222104056	0.094437377	0.704625304
RABL6	0.08171671	0.981044186	0.237230786	0.030736216	0.523880049	0.094530733	0.704625304
RBBP5	0.221023867	0.017766608	0.343225604	0.307532198	0.738653194	0.094518366	0.704625304
TTC33	0.788016025	0.888336646	0.005339769	0.904458734	0.090430749	0.09444102	0.704625304
ZNF705A	0.902564883	0.346040543	0.021920966	0.200854511	0.221975578	0.094354477	0.704625304
LPAR6	0.979898357	0.956890307	0.006050704	0.088096685	0.616174773	0.094840919	0.704770739
UTP18	0.381228706	0.202613153	0.039375353	0.746619244	0.135684647	0.094865551	0.704770739
STK19	0.846946463	0.023300541	0.128815011	0.497901535	0.244041035	0.095002962	0.705153147
SYDE2	0.019170671	0.922823547	0.307610373	0.254701945	0.222724381	0.094972893	0.705153147
ASTE1	0.356839787	0.249504496	0.655403525	0.010507703	0.506110482	0.095258579	0.706101732
CBR4	0.43182815	0.507938608	0.132644926	0.025683906	0.414859149	0.095202944	0.706101732
FAHD1	0.361758106	0.353903155	0.132235059	0.069326553	0.264806079	0.095342702	0.706101732
FAP	0.758892609	0.279155123	0.124737722	0.21051915	0.055884793	0.095359464	0.706101732
NKAIN1	0.563239002	0.762393628	0.002992736	0.250157545	0.967580345	0.095388918	0.706101732
PLEKHB2	0.354045802	0.086946828	0.076857611	0.691065513	0.190175258	0.095367624	0.706101732
ELAVL3	0.208742316	0.031533996	0.404313211	0.632232912	0.186871633	0.095985393	0.706437616
FBXO3	0.22536201	0.149598626	0.177469261	0.171493908	0.304854253	0.095697937	0.706437616
FERMT2	0.178844923	0.034260516	0.235807869	0.264090571	0.821364754	0.095805586	0.706437616
HSPA4L	0.303079056	0.329935479	0.593023528	0.02324581	0.227037243	0.095726737	0.706437616
LOC515697	0.103208016	0.454622583	0.821326291	0.010627564	0.767465644	0.095965603	0.706437616
MRVI1	0.025655465	0.291270048	0.291788238	0.871148257	0.165285248	0.095901423	0.706437616
NFKB1	0.321267343	0.536554721	0.664587955	0.003710596	0.734464833	0.095593018	0.706437616
PAQR3	0.941924285	0.922246808	0.033600097	0.041403076	0.259369396	0.095810334	0.706437616
RPRD1B	0.358660218	0.094251723	0.243520427	0.055399874	0.68881974	0.095933638	0.706437616
SIVA1	0.112391861	0.690026232	0.07153376	0.058367284	0.96240801	0.095490392	0.706437616
SPC24	0.250826236	0.196341644	0.068997367	0.574988871	0.159741984	0.095573784	0.706437616
TAGLN2	0.213342508	0.525879665	0.125487688	0.044318968	0.504011041	0.095993899	0.706437616
ZNF385D	0.743187554	0.561878642	0.04540234	0.021573313	0.765968624	0.095783834	0.706437616
TMEM223	0.57130588	0.569777719	0.270631311	0.00954294	0.374778654	0.096097916	0.706886108
AMPD2	0.167185558	0.911230152	0.339458604	0.397822342	0.015408048	0.096435832	0.707004579
CHAF1B	0.391312388	0.402692309	0.742644986	0.004606921	0.587231333	0.096365088	0.707004579
DIRAS1	0.756588022	0.293143741	0.14360648	0.019594852	0.505952818	0.096220221	0.707004579
FAM234A	0.139070885	0.536973503	0.448359019	0.009806251	0.964592606	0.096386056	0.707004579
LAMP2	0.685187651	0.433936869	0.004185919	0.496705371	0.510940183	0.096236812	0.707004579
MAP3K1	0.254150186	0.050536533	0.080741339	0.899453	0.339582717	0.096392969	0.707004579
MYL6	0.434185262	0.12572059	0.670525915	0.032037125	0.270444455	0.096458671	0.707004579
TMBIM4	0.430919803	0.031199614	0.056266874	0.522689619	0.799717264	0.096298602	0.707004579
PCDHGA2	0.024996534	0.888632881	0.122357296	0.178876342	0.653024606	0.096520951	0.707145235
IFNLR1	0.064797009	0.513934684	0.076710974	0.20785914	0.599088668	0.096632074	0.707327817
LOC112445051	0.385445649	0.288139337	0.351228939	0.559706667	0.01456399	0.096608485	0.707327817
DCTN4	0.051306599	0.195725054	0.145900677	0.380634832	0.572216549	0.096807463	0.707349635

MCM8	0.00388208	0.428137793	0.454636384	0.531833766	0.793892042	0.096795083	0.707349635
VPS16	0.480243283	0.019308132	0.138197864	0.345695693	0.719171813	0.096715399	0.707349635
WDR87	0.484496343	0.144889846	0.745132997	0.403431722	0.015103256	0.096737671	0.707349635
ARHGEF19	0.462421586	0.432656021	0.390111837	0.004331349	0.951242911	0.097237534	0.707756556
ASZ1	0.208614105	0.544091188	0.181240213	0.015718506	0.998384672	0.097456451	0.707756556
CFDP1	0.255527554	0.018120223	0.315141354	0.253814692	0.869480715	0.097314847	0.707756556
EFEMP2	0.873685104	0.12379541	0.043655033	0.083124313	0.820961103	0.097348568	0.707756556
GSTO1	0.57818118	0.433275995	0.56193714	0.200513829	0.011439301	0.097466932	0.707756556
LOC100126544	0.088930876	0.463709615	0.278563952	0.035839759	0.78348184	0.097409714	0.707756556
LOC104971162	0.360161047	0.059706885	0.209243924	0.206282181	0.345232563	0.097039327	0.707756556
LOC514507	0.038959164	0.091980431	0.995292474	0.253437134	0.356029915	0.09727987	0.707756556
NDUFS6	0.119421354	0.730271897	0.660520718	0.009827687	0.565335425	0.096970172	0.707756556
PIP5K1C	0.144564425	0.377420189	0.341765828	0.302466647	0.057044396	0.097266074	0.707756556
SERPINB5	0.03801526	0.031487156	0.708693843	0.496017571	0.762538379	0.097111672	0.707756556
TNRC18	0.963891514	0.636482055	0.029410986	0.323420375	0.054866925	0.096994814	0.707756556
TTPA	0.578692848	0.557708239	0.004807991	0.292486919	0.709017376	0.097275953	0.707756556
ZBTB7B	0.229653099	0.423876798	0.023103001	0.178483153	0.797459408	0.09697991	0.707756556
LOC107131843	0.678647753	0.829751935	0.609169124	0.007009061	0.134492318	0.097548145	0.708032997
BNIP3	0.166641391	0.996711148	0.017799266	0.696232974	0.157246659	0.097600243	0.70806699
RPF1	0.122097583	0.19433826	0.219502335	0.587663517	0.105817443	0.09763912	0.70806699
CFH	0.856086483	0.111743932	0.112116072	0.053306517	0.568319363	0.097819532	0.708901945
MAT2B	0.897000772	0.014764724	0.245767529	0.663518591	0.15050441	0.09784065	0.708901945
ARFGAP2	0.748893394	0.135612204	0.063501862	0.543150799	0.09426094	0.098728128	0.709148354
ATP7B	0.030601767	0.198421067	0.245037855	0.327160668	0.676731726	0.098595806	0.709148354
BCL2	0.539506582	0.37429059	0.001990673	0.958887194	0.854917394	0.098615678	0.709148354
C1H21orf91	0.159562297	0.014813839	0.784786107	0.882130809	0.20177184	0.098726208	0.709148354
EPHB2	0.181769346	0.452869759	0.0646031	0.613379824	0.101450675	0.098855629	0.709148354
GABBR1	0.784798123	0.067126041	0.482218556	0.858241497	0.01515161	0.098754782	0.709148354
GIPC3	0.239412033	0.078520899	0.170948274	0.108349928	0.944467539	0.098500031	0.709148354
HDLBP	0.162933714	0.717935748	0.258655215	0.644137944	0.01682393	0.098332105	0.709148354
LGALS8	0.574097818	0.251378784	0.004439664	0.938642495	0.547778537	0.098599043	0.709148354
LITAF	0.445763223	0.212129804	0.138223274	0.054471279	0.464380195	0.098802823	0.709148354
LOC101903501	0.169919432	0.82615097	0.139917496	0.056369008	0.297814921	0.098650194	0.709148354
LOC112445999	0.706511258	0.294675143	0.049047903	0.210966978	0.151483355	0.098063729	0.709148354
LOC615559	0.215675289	0.197685114	0.012193508	0.90010488	0.702225516	0.098455809	0.709148354
NAA15	0.383695836	0.124156471	0.113982869	0.376364485	0.160030445	0.098186644	0.709148354
NKRF	0.616158471	0.118869262	0.460265647	0.033297286	0.291824223	0.09827694	0.709148354
PSMB6	0.275645594	0.42429516	0.283494141	0.119400049	0.083603972	0.098863862	0.709148354
RGS7BP	0.724820265	0.442982943	0.75248717	0.013025334	0.105247391	0.098906035	0.709148354
SLC25A1	0.624935135	0.999751655	0.914328233	0.002785297	0.205990436	0.098309117	0.709148354
SLC46A2	0.213790922	0.714053059	0.058366424	0.123667325	0.299249436	0.098651476	0.709148354
SPRYD4	0.581515119	0.506337385	0.509055978	0.015628909	0.138987691	0.097934969	0.709148354
TUBA1B	0.108862123	0.304435843	0.211915878	0.617323336	0.075731466	0.098410465	0.709148354
WNT2	0.004922236	0.47091736	0.530008543	0.819321463	0.329090873	0.098911741	0.709148354

YEATS2	0.610260331	0.478878301	0.541426902	0.028246857	0.073421228	0.098377442	0.709148354
ZSWIM4	0.455400637	0.842706511	0.003349845	0.489815651	0.524580661	0.098751965	0.709148354
ASMTL	0.276620809	0.260940276	0.448499797	0.077186596	0.133388428	0.099264416	0.709507115
CYSLTR1	0.086813141	0.872184926	0.011579626	0.641936676	0.590970352	0.099145976	0.709507115
EIF4EBP3	0.276695649	0.677407781	0.301442271	0.510365978	0.011553852	0.09924036	0.709507115
LOC101907152	0.086579976	0.550669582	0.846034249	0.333997107	0.024683406	0.099132565	0.709507115
LOC112449523	0.272379533	0.133598141	0.038984926	0.644492966	0.36292159	0.099009033	0.709507115
PRKCI	0.407115594	0.890989746	0.543886136	0.00172113	0.979908487	0.099165827	0.709507115
SLC26A2	0.519258881	0.981551421	0.002713307	0.292448628	0.823009747	0.099185892	0.709507115
AQP1	0.249322376	0.242761483	0.385895205	0.019465856	0.744003194	0.100109896	0.709790194
CAPN11	0.008753927	0.545520939	0.120811801	0.705973182	0.826216935	0.099812072	0.709790194
CCL21	0.440449056	0.649172647	0.006601135	0.824785112	0.216713068	0.099956663	0.709790194
IDUA	0.018330552	0.591698708	0.109064276	0.488793434	0.584293777	0.100037983	0.709790194
IFT20	0.027295822	0.236696515	0.604886333	0.128367255	0.674282594	0.100109804	0.709790194
LOC101903832	0.485777994	0.876763859	0.285208909	0.010836574	0.256570047	0.100020283	0.709790194
LOC101906280	0.306173274	0.545285871	0.012650268	0.517994438	0.308453926	0.099970712	0.709790194
LOC783224	0.012070704	0.408381415	0.364177076	0.358191257	0.522225993	0.099690485	0.709790194
MIEF2	0.214926616	0.273541519	0.768546149	0.018024369	0.413783885	0.099892781	0.709790194
MRPS7	0.637639524	0.848001641	0.248012857	0.036187608	0.069774926	0.100169038	0.709790194
NLRP12	0.555153227	0.446643002	0.008493058	0.424873304	0.378396875	0.100160976	0.709790194
PGP	0.247042585	0.643848337	0.711841873	0.0704977	0.042290912	0.099991111	0.709790194
POLR3H	0.25875169	0.295882243	0.62798358	0.045041353	0.155220999	0.099746904	0.709790194
RPS19BP1	0.090942315	0.478423193	0.39864253	0.120157925	0.161891113	0.09996154	0.709790194
SAP18	0.230745777	0.126370933	0.303171479	0.125086931	0.302989589	0.099561867	0.709790194
SAPCD1	0.483080377	0.009982816	0.745319082	0.176942805	0.52776418	0.099664492	0.709790194
ST3GAL5	0.693750027	0.604129616	0.006430543	0.16572423	0.756575899	0.100051744	0.709790194
TK1	0.070076324	0.901681267	0.653060892	0.010541561	0.774713136	0.099893682	0.709790194
TMEM165	0.931169565	0.914412302	0.687821841	0.44701749	0.001285051	0.099796959	0.709790194
YARS	0.477175287	0.280964472	0.689587918	0.490676884	0.007417001	0.099803748	0.709790194
UMAD1	0.272151324	0.406527142	0.011039216	0.621884972	0.447952887	0.100444227	0.71143298
CC2D2A	0.24647425	0.224123316	0.050721993	0.432164472	0.281379283	0.100525963	0.711704739
ADO	0.341667503	0.221736162	0.251166725	0.966351731	0.018589892	0.10071455	0.71212465
LOC112447408	0.356893292	0.105304801	0.046463115	0.695182678	0.281438505	0.100683006	0.71212465
LOC112447819	0.005443469	0.537007754	0.50018388	0.244227646	0.957283066	0.100715454	0.71212465
ZNF438	0.475697052	0.425281423	0.038062162	0.852269114	0.052383663	0.101042175	0.714127103
ATXN2L	0.91674221	0.066879621	0.696111921	0.030528731	0.264532864	0.101193812	0.714354343
FOXO4	0.083495786	0.775183006	0.098637366	0.536364966	0.100673385	0.101204082	0.714354343
OSR1	0.100295011	0.729156992	0.103649305	0.062813161	0.724059332	0.101204914	0.714354343
DCP1A	0.327609696	0.087481279	0.474473539	0.044356418	0.576185669	0.101675083	0.714599482
HOXD9	0.372282138	0.675251512	0.149999849	0.028402531	0.323177214	0.101436974	0.714599482
LOC101906511	0.582154827	0.257289296	0.985140415	0.018455837	0.12677316	0.101288848	0.714599482
LOC107131675	0.056273788	0.067903033	0.765296352	0.296711287	0.398349535	0.101356258	0.714599482
LOC112442664	0.240070344	0.850271402	0.496816911	0.217878324	0.015718173	0.10163561	0.714599482
MITF	0.768207821	0.999596834	0.01655775	0.640417044	0.042678964	0.101672252	0.714599482

MYOM3	0.046380163	0.436529656	0.20798429	0.43749035	0.188079846	0.101498711	0.714599482
POLR2I	0.118713317	0.037146139	0.309180289	0.28145436	0.901278583	0.101392204	0.714599482
SSC5D	0.195384182	0.120445626	0.038552158	0.422767689	0.903498738	0.101508245	0.714599482
TAB1	0.966374721	0.780758977	0.701599178	0.000763045	0.860308164	0.101668795	0.714599482
ADRM1	0.45977642	0.282258248	0.278350004	0.109432107	0.088124082	0.101812176	0.714931146
CRYM	0.099588288	0.853182641	0.109574439	0.475263588	0.07878317	0.101852966	0.714931146
DHX36	0.314605108	0.476450984	0.735590816	0.028414593	0.111130089	0.101781212	0.714931146
ACTR5	0.168908012	0.092342257	0.817527375	0.032936604	0.831048274	0.101924286	0.715125893
AK6	0.070721554	0.726421981	0.265564933	0.271764112	0.095049834	0.102489955	0.715457387
AMOTL2	0.046140289	0.021704894	0.704042295	0.705362636	0.709586068	0.102570953	0.715457387
CCNE2	0.153836181	0.376523652	0.438420077	0.237780514	0.058118377	0.102243769	0.715457387
DBN1	0.252680538	0.652124612	0.015675063	0.256098735	0.534052485	0.102631644	0.715457387
DCAF10	0.077098408	0.356575138	0.779077102	0.019303375	0.845806562	0.102035013	0.715457387
GMEB2	0.052536588	0.254740364	0.932001901	0.084879007	0.333702563	0.102636136	0.715457387
GUCA1A	0.096911822	0.442486789	0.07255181	0.331084625	0.341951347	0.102459382	0.715457387
LOC101902527	0.222849512	0.063577577	0.926620736	0.117326077	0.227707932	0.102211343	0.715457387
LOC104970711	0.761323316	0.100154821	0.017204016	0.792070301	0.337778107	0.102248507	0.715457387
LOC104976232	0.405546252	0.02630791	0.874800875	0.159790083	0.237024878	0.102669072	0.715457387
LRRC4C	0.410123011	0.790595589	0.084212785	0.664047124	0.01937712	0.102311708	0.715457387
OSBPL7	0.6366809	0.019102752	0.312058592	0.289545894	0.32132964	0.102607169	0.715457387
RASSF5	0.553512195	0.941968667	0.063242255	0.181478365	0.058798946	0.102396825	0.715457387
SETD2	0.286122141	0.922438965	0.566416646	0.004294313	0.547786743	0.102365097	0.715457387
SOX9	0.629792655	0.523255971	0.02591007	0.056479986	0.732785927	0.102651803	0.715457387
SUZ12	0.201417058	0.182746668	0.062584942	0.248559995	0.611031749	0.102065886	0.715457387
NOC4L	0.503527593	0.326684063	0.278000458	0.629141731	0.012314011	0.102799729	0.716063816
SELENOO	0.324945	0.42476251	0.720604314	0.026680409	0.133661406	0.102868379	0.716238004
EXTL2	0.111714965	0.374409003	0.114456762	0.285213484	0.260189219	0.102964252	0.716364169
LPIN1	0.881273994	0.54269071	0.015660661	0.2907699	0.1631571	0.102973803	0.716364169
ANXA8L1	0.271189377	0.052717331	0.105570234	0.559758214	0.423954852	0.103443958	0.716899825
APH1B	0.668680093	0.51045615	0.491758952	0.452165575	0.004710118	0.10333107	0.716899825
ENY2	0.50282838	0.356844572	0.1863047	0.017193999	0.622770141	0.10340851	0.716899825
LOC100848799	0.438677093	0.441120448	0.161681548	0.890846334	0.012823014	0.10331707	0.716899825
LOC613460	0.068607098	0.148105657	0.059198016	0.91217798	0.649319482	0.103130869	0.716899825
PNMA1	0.161720816	0.090631699	0.864502297	0.77887785	0.036251714	0.103378932	0.716899825
SHKBP1	0.52262907	0.823077197	0.669645355	0.003780461	0.328237605	0.103324768	0.716899825
TM9SF3	0.828369408	0.553706558	0.281477793	0.025025009	0.110300432	0.10314628	0.716899825
TSLP	0.710737369	0.341631317	0.669268294	0.01413363	0.155310623	0.103203716	0.716899825
FDPS	0.168657192	0.995153611	0.673111595	0.095490236	0.033372404	0.103749551	0.718519787
POU3F1	0.130453032	0.372729011	0.369628844	0.392991927	0.050986708	0.103766662	0.718519787
SLC25A26	0.631696096	0.708053412	0.472664774	0.001844942	0.923961806	0.103809056	0.718519787
ADGRL1	0.095266041	0.844806617	0.025950758	0.48254424	0.368443891	0.105596852	0.71859068
ANO9	0.785054489	0.965137616	0.35036848	0.020044204	0.070644727	0.106337915	0.71859068
ATP13A4	0.112221808	0.937154366	0.01578274	0.64597113	0.341255849	0.104714088	0.71859068
BATF3	0.011539569	0.58526787	0.657597574	0.914138452	0.09285062	0.106508019	0.71859068

CALM1	0.846407599	0.044435572	0.130943249	0.207805593	0.371722429	0.107063566	0.71859068
CBLL1	0.001582192	0.471495878	0.688799478	0.845227398	0.837737761	0.104377004	0.71859068
CCDC174	0.079197184	0.924709717	0.478012004	0.049463561	0.217363464	0.106413718	0.71859068
CHDH	0.733008841	0.247943273	0.034030458	0.189283547	0.311082878	0.104432834	0.71859068
CHORDC1	0.984235015	0.645280299	0.842054629	0.000997326	0.691376054	0.105179556	0.71859068
CRIP1	0.103608844	0.641398636	0.376799937	0.023622335	0.631729064	0.105976706	0.71859068
CYR61	0.685957863	0.071054255	0.021650009	0.757138476	0.451909748	0.10391955	0.71859068
CYTH2	0.735352087	0.575540633	0.924560797	0.434299209	0.002223471	0.106651437	0.71859068
DNAJC28	0.958197497	0.50779984	0.220620891	0.024772295	0.139589518	0.105577346	0.71859068
DSTN	0.055154241	0.151268373	0.640707681	0.541196708	0.131828007	0.107215251	0.71859068
DYNLRB2	0.35467547	0.187167783	0.023211014	0.572377243	0.42341538	0.105937212	0.71859068
EIPR1	0.203604586	0.269950309	0.727845265	0.032442038	0.287499727	0.10588865	0.71859068
EPB41L3	0.66216718	0.874871784	0.034376404	0.037487832	0.512735533	0.10744114	0.71859068
F13A1	0.29947566	0.658589629	0.121778282	0.416532925	0.038185101	0.107319218	0.71859068
FBXL6	0.728363642	0.033853109	0.310371074	0.092990686	0.532697713	0.106850317	0.71859068
FOXP3	0.166543244	0.844611465	0.42814032	0.022427658	0.27208626	0.104975439	0.71859068
HMGXB3	0.524662029	0.024032927	0.572515569	0.062620968	0.835279098	0.106609156	0.71859068
HSP90AB1	0.498215166	0.631520706	0.543961027	0.002359798	0.939826421	0.106926875	0.71859068
HSPA1A	0.353090647	0.901746967	0.482359583	0.025580461	0.096929871	0.107125093	0.71859068
IDE	0.946808776	0.523474786	0.300909538	0.588401996	0.004121367	0.104020343	0.71859068
IL17RB	0.40909544	0.561628843	0.066302086	0.160268351	0.151398431	0.105322135	0.71859068
KIF15	0.58757271	0.058549937	0.035902628	0.493067076	0.627047756	0.107295508	0.71859068
LAMA2	0.082000305	0.215402416	0.544833912	0.041161715	0.919919903	0.104467903	0.71859068
LOC100297099	0.237306335	0.493547867	0.153239263	0.043801019	0.468573093	0.105114783	0.71859068
LOC100335608	0.828489239	0.126375438	0.259222846	0.074638841	0.186326609	0.106586455	0.71859068
LOC101902288	0.119020962	0.099607255	0.536922827	0.486408925	0.120065327	0.105665013	0.71859068
LOC101904344	0.145229589	0.069831824	0.434067431	0.366227703	0.225336681	0.104286049	0.71859068
LOC107133049	0.03237154	0.668376795	0.071192138	0.281717575	0.873655598	0.106853486	0.71859068
LOC107133276	0.25458296	0.055784927	0.038114194	0.705290308	0.957959359	0.104684353	0.71859068
LOC112443415	0.570159911	0.075271228	0.312101499	0.047596477	0.584158262	0.105773466	0.71859068
LOC112444309	0.316545137	0.412869381	0.506219468	0.472521606	0.011925873	0.105839042	0.71859068
LOC112445044	0.563839309	0.289697081	0.014661713	0.245512967	0.614412106	0.10395316	0.71859068
LOC112448084	0.87342511	0.505401861	0.149134425	0.434383593	0.013206018	0.106617645	0.71859068
LOC112449284	0.507636266	0.051308513	0.865571888	0.121070367	0.134468874	0.104898791	0.71859068
LOC508933	0.145736056	0.071060347	0.051844122	0.768558152	0.918815375	0.106857391	0.71859068
LOC515358	0.351745822	0.459392837	0.024826053	0.253435895	0.362073561	0.105075506	0.71859068
LOC530102	0.291778043	0.088869603	0.644288553	0.037197763	0.612203742	0.107067724	0.71859068
LOC618076	0.859722234	0.904439555	0.567403036	0.001068002	0.784530878	0.105327839	0.71859068
LOC618169	0.673190004	0.383608443	0.011304946	0.325157013	0.402733127	0.107363551	0.71859068
LOC788648	0.75037714	0.083803486	0.014615548	0.928942744	0.435912694	0.105734369	0.71859068
LRRC34	0.291550499	0.210916539	0.53290995	0.014531752	0.779066725	0.105543746	0.71859068
MANBAL	0.233183556	0.06746684	0.083112426	0.337596802	0.8585196	0.106829931	0.71859068
MPZL3	0.520574506	0.326719269	0.070957595	0.093975724	0.33318604	0.106655861	0.71859068
NDUFA5	0.260676424	0.492457085	0.304423215	0.016866083	0.575150072	0.106849615	0.71859068

NUBP2	0.050522137	0.695550644	0.520923656	0.15241738	0.13723991	0.107461299	0.71859068
PAMR1	0.129863114	0.013471334	0.430876348	0.496585527	0.99891251	0.106015826	0.71859068
PLXDC2	0.483489588	0.182386043	0.323018379	0.01337135	0.998954604	0.107071641	0.71859068
PPCS	0.57294644	0.988883401	0.362425986	0.049390257	0.036888357	0.106049004	0.71859068
PTPRR	0.095550397	0.171324971	0.374093871	0.1620398	0.372290769	0.105289953	0.71859068
RASGRP1	0.120641519	0.909819739	0.946935924	0.145034589	0.025409988	0.10748232	0.71859068
RDH10	0.166085673	0.419675351	0.550878227	0.045062119	0.210763298	0.104513818	0.71859068
RENBP	0.581553116	0.300181438	0.049547039	0.081248093	0.522860547	0.104965688	0.71859068
RFFL	0.454730846	0.284950562	0.668228996	0.05181432	0.082603256	0.105477981	0.71859068
RGL3	0.84825709	0.020733004	0.301410223	0.758845011	0.091430083	0.105020867	0.71859068
RHPN1	0.8822904	0.196046638	0.02092065	0.130612204	0.781359993	0.105268289	0.71859068
RPF2	0.497595618	0.053901974	0.180228743	0.712451584	0.105350883	0.104210639	0.71859068
RPP40	0.971643219	0.341366617	0.349509642	0.899727814	0.003663076	0.107326887	0.71859068
RTCB	0.780281232	0.185130349	0.034101911	0.964350974	0.080599477	0.107457833	0.71859068
SCRG1	0.439157579	0.327105176	0.614264531	0.004885362	0.888092925	0.107449892	0.71859068
SDR16C5	0.576958493	0.994388932	0.184242621	0.048050523	0.072234381	0.104875108	0.71859068
SEC63	0.880158283	0.041398747	0.214912577	0.090202774	0.515724322	0.10445055	0.71859068
SERINC2	0.429901292	0.008035955	0.592425577	0.29557369	0.631025996	0.10727192	0.71859068
SLC29A2	0.431522506	0.975179591	0.006245466	0.325096732	0.42342063	0.104037877	0.71859068
SLC48A1	0.591529952	0.229264099	0.036225352	0.129928046	0.574340844	0.104828942	0.71859068
SNU13	0.939438765	0.046126437	0.064134403	0.206230251	0.65223146	0.106000935	0.71859068
SOST	0.890808669	0.171683043	0.734583936	0.051034899	0.064174791	0.105047787	0.71859068
SPATA5L1	0.339127272	0.011376609	0.267403394	0.449256625	0.792768606	0.104964783	0.71859068
SRGAP1	0.842918098	0.113642452	0.273089492	0.019193978	0.744702311	0.106016949	0.71859068
TBL2	0.875878587	0.30256585	0.198943966	0.029589233	0.242858493	0.106812958	0.71859068
TIMM23	0.35953607	0.446395134	0.460921716	0.061840613	0.079248765	0.104163573	0.71859068
TMEM248	0.996654165	0.214522937	0.006587875	0.806554044	0.322410906	0.104775045	0.71859068
TMEM263	0.785383804	0.270845413	0.117137802	0.070367665	0.216151525	0.106834013	0.71859068
TMEM38B	0.913184595	0.071714993	0.118927834	0.065370113	0.726191004	0.105337597	0.71859068
TOP3B	0.583264278	0.022446491	0.2817935	0.165773008	0.612845576	0.106160477	0.71859068
TRIM25	0.141332753	0.00853686	0.399265002	0.837611	0.940881641	0.106938773	0.71859068
TRIM34	0.773893109	0.799812639	0.144456416	0.004308314	0.987552047	0.107064123	0.71859068
TXNDC5	0.113822984	0.266131559	0.015973449	0.933998051	0.806454977	0.104478548	0.71859068
USP38	0.441910031	0.066043054	0.051881242	0.306824759	0.818402178	0.10702977	0.71859068
ZFAND5	0.262079361	0.47330988	0.005644719	0.64523903	0.848036783	0.107497418	0.71859068
ZNF414	0.857834641	0.163640015	0.027260018	0.104558938	0.915246662	0.1047629	0.71859068
AP4E1	0.346029674	0.184355409	0.012177653	0.752224545	0.663838095	0.108258181	0.718852434
C8H9orf131	0.606038766	0.132578023	0.43464796	0.203222796	0.054497515	0.108076445	0.718852434
CAV2	0.715421634	0.574121511	0.099081109	0.030363481	0.312868182	0.108049711	0.718852434
CDK2	0.762404777	0.405941769	0.286258407	0.155487707	0.028545195	0.109095977	0.718852434
CHCHD6	0.181841177	0.042262492	0.249514304	0.70967818	0.288627547	0.109025452	0.718852434
CSGALNACT2	0.852145194	0.498822289	0.982824106	0.002425219	0.386479694	0.108836155	0.718852434
DPM2	0.405282154	0.195179423	0.357761382	0.032722959	0.414776103	0.10765173	0.718852434
FABP3	0.126582587	0.221263462	0.147676696	0.520922135	0.182077558	0.108951763	0.718852434

FBXW11	0.457650588	0.168920202	0.171300633	0.056492575	0.522824636	0.108766306	0.718852434
GET4	0.134390852	0.718162329	0.281444913	0.073615881	0.1938341	0.108207892	0.718852434
GID4	0.335551076	0.395845178	0.120623324	0.132776291	0.183507284	0.108647894	0.718852434
GIMAP5	0.421383141	0.629226376	0.086940377	0.415245469	0.041005444	0.108984125	0.718852434
GJC1	0.584158807	0.661774736	0.100025487	0.077536223	0.128794103	0.107976571	0.718852434
HIF1A	0.752219209	0.564800126	0.117441757	0.038918841	0.202552585	0.109113486	0.718852434
HSF4	0.549760221	0.011822596	0.185620714	0.45695784	0.700605745	0.107992121	0.718852434
LOC100848598	0.799253801	0.919799361	0.139527595	0.116373965	0.032882626	0.108985298	0.718852434
LOC100848684	0.163352801	0.392793779	0.346342759	0.045827286	0.38184846	0.108409856	0.718852434
LOC100848883	0.192739891	0.754440411	0.760759274	0.733707033	0.00473474	0.107681536	0.718852434
LOC101906134	0.338893299	0.238069893	0.228098906	0.02636368	0.804819217	0.108662945	0.718852434
LOC112446013	0.283985722	0.081471784	0.611598149	0.708874269	0.038849567	0.108539394	0.718852434
LOC785745	0.599919382	0.01548736	0.066835642	0.972589278	0.649605746	0.108956449	0.718852434
MAN2A2	0.336609478	0.16072326	0.109650355	0.216922967	0.303464465	0.1086677	0.718852434
MBNL2	0.561241258	0.80296674	0.015860958	0.071759702	0.757714484	0.108374444	0.718852434
METAP1	0.861087191	0.178784126	0.047534129	0.07494395	0.703942058	0.10796282	0.718852434
MFAP2	0.242354203	0.081720334	0.059425918	0.543620914	0.610703455	0.108703896	0.718852434
NEU3	0.977422659	0.239357839	0.426015537	0.499984811	0.007845968	0.108743101	0.718852434
PHF11	0.937095614	0.036545926	0.208674346	0.654353566	0.084061631	0.109076804	0.718852434
ROR2	0.749188437	0.193344255	0.010742064	0.530786093	0.466946555	0.107898059	0.718852434
RSU1	0.317794596	0.008594821	0.254319538	0.725389321	0.765547317	0.107913525	0.718852434
SCN1B	0.145700197	0.180940354	0.118887712	0.177805249	0.700977208	0.10868938	0.718852434
SELENOW	0.372562213	0.678618218	0.119439387	0.02357379	0.550903695	0.108930698	0.718852434
SEMA4A	0.388365852	0.369340267	0.16678157	0.271419068	0.06053339	0.109069515	0.718852434
SLC16A13	0.121585092	0.408123724	0.91782751	0.021994499	0.392529348	0.109093519	0.718852434
TH	0.96280572	0.147009334	0.210919866	0.175296644	0.07380986	0.107995885	0.718852434
TRHDE	0.104567301	0.203691354	0.523621664	0.218927027	0.158157557	0.107979824	0.718852434
UPF2	0.62428987	0.21171824	0.704024094	0.006365778	0.657609635	0.108514759	0.718852434
LOC101906006	0.821029302	0.485609955	0.575126735	0.002718992	0.631363076	0.10916164	0.718881088
GCSAML	0.72457249	0.199926205	0.039229556	0.203935166	0.340026973	0.109229215	0.719037563
LOC104974850	0.421778351	0.594026778	0.687960667	0.848530264	0.002703164	0.109432565	0.719823254
LTV1	0.229915491	0.280544777	0.601710627	0.291837413	0.034939858	0.109493567	0.719823254
PA2G4	0.703951692	0.508722208	0.116355041	0.053598658	0.177042275	0.109439137	0.719823254
SETD7	0.915818764	0.187061858	0.011750537	0.805135964	0.244293168	0.109524018	0.719823254
CEP128	0.26879025	0.734445114	0.898335956	0.029537786	0.07587507	0.109761154	0.720228029
CXCL10	0.074323078	0.148472195	0.30676508	0.962123012	0.121793393	0.109637978	0.720228029
LOC509513	0.08348955	0.605334598	0.57875801	0.566419026	0.023970212	0.109710366	0.720228029
ZMYM4	0.036080165	0.918686221	0.536240555	0.024994488	0.89441434	0.109745342	0.720228029
ANKHD1	0.110060283	0.958064094	0.543052301	0.008601191	0.814283443	0.11032328	0.72075596
C1S	0.466785382	0.488646256	0.316997257	0.009443594	0.589786257	0.110582479	0.72075596
CHID1	0.209680176	0.876152101	0.463204692	0.048254905	0.097335882	0.110110804	0.72075596
FOXN2	0.225841543	0.225644934	0.610998242	0.170626413	0.075764305	0.110550882	0.72075596
HNRNPF	0.448736889	0.990270833	0.367084926	0.00251826	0.973568436	0.110147491	0.72075596
KRTCAP3	0.695538174	0.025980181	0.392624089	0.348064011	0.162908181	0.110516527	0.72075596

LIG3	0.526683711	0.98071277	0.009063825	0.778138641	0.110507781	0.110561385	0.72075596
LOC104975460	0.791440028	0.35692708	0.044182079	0.540041298	0.059747479	0.110581168	0.72075596
LOC112442295	0.479737046	0.018353716	0.21917908	0.732239564	0.283517949	0.110259802	0.72075596
NDUFB7	0.333607073	0.599190298	0.76944084	0.002817635	0.926379847	0.110387808	0.72075596
NFE2L2	0.873773923	0.965534485	0.010322661	0.052298084	0.884910191	0.110632153	0.72075596
PLXNA1	0.286627237	0.392844921	0.496653565	0.370563853	0.019399557	0.110473981	0.72075596
PRPF40A	0.397011872	0.155470863	0.889989958	0.030111547	0.241625406	0.110109225	0.72075596
SREK1IP1	0.186346607	0.223549447	0.037987373	0.625546901	0.404688534	0.110253326	0.72075596
TMEM132E	0.991070968	0.456738591	0.02464459	0.850614355	0.042367755	0.110476171	0.72075596
TSPYL6	0.412066079	0.954844475	0.456809839	0.005122729	0.435088096	0.110253096	0.72075596
ZNF184	0.166186423	0.5264817	0.060014831	0.950871218	0.080664321	0.110588437	0.72075596
ZNF609	0.651189484	0.197293999	0.458477782	0.013074341	0.523030249	0.110595014	0.72075596
FADS2	0.145230632	0.247199647	0.129719047	0.734379347	0.118220926	0.110831663	0.721482914
SNF8	0.419815303	0.070018535	0.07944061	0.262243466	0.659811779	0.110790318	0.721482914
ATP6V0A4	0.538457014	0.922880219	0.008634007	0.463374945	0.204367096	0.111139622	0.722480767
IKBKE	0.056111738	0.167137855	0.19502356	0.482266523	0.46009072	0.111066304	0.722480767
NR4A2	0.26628473	0.104321399	0.585063525	0.080559986	0.310196033	0.11111441	0.722480767
TUBG1	0.128252416	0.247796883	0.319818707	0.235815607	0.169575201	0.111161047	0.722480767
LOC112443526	0.360220683	0.024434751	0.099961837	0.489504827	0.944728527	0.111229859	0.722641813
TEX22	0.940216335	0.002150707	0.457613421	0.517956487	0.851394095	0.11141226	0.723540403
CAND2	0.552136309	0.943395546	0.093913254	0.373772978	0.022366681	0.111549526	0.723652314
CUX2	0.110606414	0.581883878	0.009203163	0.815399614	0.846868662	0.11155881	0.723652314
RGCC	0.730956732	0.141570517	0.005771383	0.764540349	0.895810023	0.111561779	0.723652314
MGC139164	0.407059249	0.004567372	0.25690455	0.877280968	0.978511613	0.111713035	0.724347143
YKT6	0.428397783	0.416475943	0.330450441	0.394021036	0.017698483	0.11188759	0.725192432
HSD11B1	0.223388732	0.28549571	0.043547066	0.374043631	0.396164436	0.111948613	0.725301494
SIRPB2	0.572894714	0.460413832	0.563302252	0.012714659	0.218250451	0.11206635	0.725777771
MRPL12	0.245740922	0.690208185	0.888275695	0.013241732	0.206894201	0.112135542	0.725939399
ITGA3	0.632932762	0.582793557	0.395900984	0.118873375	0.023828308	0.11227262	0.72603936
LOC101906664	0.392816267	0.391356916	0.124900309	0.154964901	0.139111515	0.112314393	0.72603936
LOC101907369	0.743224159	0.568263986	0.029675661	0.707045502	0.046661419	0.112249081	0.72603936
LOC104969670	0.020979364	0.157456271	0.233195039	0.555562463	0.968084048	0.112372188	0.72603936
MCUR1	0.58999357	0.006351953	0.615606269	0.336261781	0.53396834	0.112362397	0.72603936
ANXA11	0.351167355	0.138843731	0.190242991	0.23903049	0.187211582	0.112491844	0.726240619
HSPA1L	0.710262104	0.985433408	0.572438915	0.004107197	0.252075554	0.112450294	0.726240619
AKAP11	0.205944507	0.084987431	0.82171802	0.052137876	0.555175042	0.112678807	0.726659186
CDS2	0.313387175	0.058050262	0.069641377	0.973948694	0.337654678	0.11273023	0.726659186
LOC783854	0.868775352	0.248486768	0.309983305	0.321529197	0.019347665	0.112676906	0.726659186
TRAF4	0.380677611	0.234638108	0.031029831	0.487175062	0.30857842	0.112733794	0.726659186
AHI1	0.643394065	0.98313007	0.957603225	0.001067965	0.648349986	0.113153168	0.727409899
B3GNTL1	0.734016245	0.083137803	0.575092849	0.02284669	0.523206695	0.113167694	0.727409899
FOPNL	0.903875809	0.189996858	0.210121838	0.016850926	0.690305053	0.113204855	0.727409899
LOC518134	0.373560892	0.526887464	0.0852162	0.049902677	0.501338473	0.113184997	0.727409899
RPS6KA3	0.593792656	0.235248561	0.011900431	0.575379869	0.437895795	0.113066348	0.727409899

SOX8	0.766743124	0.811503798	0.003900427	0.305212393	0.565886271	0.113115485	0.727409899
VTI1B	0.133363412	0.153824598	0.117772174	0.864301726	0.200572633	0.113065217	0.727409899
ZMPSTE24	0.396523478	0.060798249	0.514140716	0.796832903	0.042343493	0.112970197	0.727409899
ADGRE3	0.04883385	0.631480541	0.030176934	0.465788497	0.972143372	0.113453008	0.727547589
CX3CL1	0.047583492	0.458065139	0.030691103	0.699724478	0.900924749	0.113502654	0.727547589
IQCA1	0.156718075	0.668925968	0.036118662	0.234967291	0.473234452	0.11339954	0.727547589
LOC112446759	0.760142255	0.144930935	0.061075698	0.347501449	0.180563482	0.113576354	0.727547589
LYPLA2	0.18555928	0.456102859	0.988876256	0.324513087	0.015546135	0.113580947	0.727547589
RPS6KA4	0.146722552	0.532017253	0.089516967	0.190738766	0.316317216	0.113484669	0.727547589
TDRD12	0.420939657	0.101142768	0.06315349	0.848121659	0.184524691	0.113362994	0.727547589
ZKSCAN5	0.929021503	0.25049172	0.915028725	0.662597126	0.0029815	0.113344365	0.727547589
BRD3OS	0.245303649	0.134233384	0.319553568	0.550203283	0.073204159	0.113821105	0.728801464
ARHGEF10L	0.140256574	0.119731234	0.365886742	0.079282901	0.873036873	0.114046472	0.729390744
LOC100847495	0.865892476	0.090756143	0.10744358	0.117088379	0.429980684	0.114015913	0.729390744
SLC4A8	0.235083374	0.971149014	0.08410452	0.025116149	0.880903335	0.113975216	0.729390744
ANKRD33B	0.904789851	0.041158122	0.020498171	0.794353779	0.709708913	0.114807723	0.729466082
ARMCX1	0.828018223	0.360613141	0.080517474	0.459838424	0.03860897	0.114280126	0.729466082
CCNT1	0.088474834	0.188031942	0.295588012	0.090735108	0.965247531	0.114858348	0.729466082
CYTIP	0.909078155	0.880454806	0.072169457	0.24437133	0.030265318	0.114338346	0.729466082
FUBP3	0.094374579	0.322382086	0.223911091	0.128000504	0.49381548	0.114847265	0.729466082
IFI44	0.909737214	0.057212577	0.222503182	0.065721629	0.56203337	0.114421067	0.729466082
INPP1	0.090434757	0.225919647	0.14997364	0.696390867	0.201122101	0.114629704	0.729466082
KIAA1109	0.260261709	0.616824695	0.428854404	0.018962144	0.329048732	0.11469109	0.729466082
LOC104975814	0.225800679	0.281122173	0.014162222	0.689605811	0.689050093	0.114330363	0.729466082
LOC508666	0.488058303	0.81077056	0.318255027	0.051849357	0.065218089	0.114130497	0.729466082
LOC782343	0.439668968	0.109054308	0.224502712	0.085875658	0.463713933	0.114554317	0.729466082
NHP2	0.178920084	0.570190481	0.802213383	0.271889849	0.019329089	0.114772074	0.729466082
PDE3A	0.005454636	0.258545378	0.817973412	0.857124517	0.434957931	0.114766049	0.729466082
PXDC1	0.621389826	0.544667839	0.034819167	0.408153324	0.089136235	0.114566428	0.729466082
RAD51	0.843264565	0.067552477	0.266189905	0.095401414	0.295675117	0.114413963	0.729466082
REC114	0.17277377	0.775301403	0.376590834	0.05810192	0.146099895	0.114487114	0.729466082
SLAMF6	0.462950528	0.597522323	0.035370107	0.352400373	0.123575635	0.114165773	0.729466082
TIGIT	0.150347464	0.386459744	0.97862716	0.565976041	0.013263557	0.114281706	0.729466082
ARVCF	0.078383573	0.749334462	0.866467883	0.040825377	0.210937437	0.115993825	0.729778769
C1QTNF3	0.734088833	0.037372117	0.687190727	0.032624763	0.713081848	0.116042431	0.729778769
CAPS	0.012920896	0.21269687	0.645665178	0.607599092	0.403480475	0.115508156	0.729778769
CCL19	0.603912743	0.351225104	0.072650586	0.044673524	0.630568909	0.11537055	0.729778769
CD3D	0.079004517	0.54008032	0.921993678	0.339610257	0.032559556	0.11550767	0.729778769
CD6	0.024417041	0.969568018	0.689807793	0.178955249	0.14895191	0.115551485	0.729778769
CLEC5A	0.152698673	0.530759806	0.059296236	0.129517797	0.695960083	0.115234827	0.729778769
CNN2	0.387334291	0.597149064	0.013629692	0.18998982	0.727065724	0.115576782	0.729778769
CYB5RL	0.28341577	0.733845173	0.011748278	0.4852638	0.36995577	0.11605296	0.729778769
DAPK2	0.819511505	0.513792178	0.14838018	0.007586392	0.912004943	0.115096873	0.729778769
DUSP4	0.629180398	0.013459368	0.93002625	0.25755309	0.215706145	0.115886716	0.729778769

DYNC1LI1	0.974484326	0.206311685	0.315415681	0.008006063	0.863006698	0.11597549	0.729778769
ELP5	0.215144186	0.423850282	0.028516303	0.422991134	0.394291454	0.115311051	0.729778769
FAIM	0.962429147	0.005694685	0.686879718	0.363346304	0.321424088	0.11620163	0.729778769
FAM120B	0.958143497	0.441523487	0.319974374	0.010818114	0.298990671	0.115929446	0.729778769
GALNT17	0.540032768	0.160619591	0.030060321	0.245028742	0.688580606	0.116241649	0.729778769
GPR132	0.052485179	0.466574009	0.464244492	0.088785363	0.430193957	0.115389711	0.729778769
KBTBD6	0.465234154	0.310130628	0.43393757	0.015957394	0.433002111	0.115148343	0.729778769
LOC101909003	0.418360948	0.078172245	0.423453567	0.311006977	0.101775774	0.116006899	0.729778769
LOC104969028	0.049922041	0.593222208	0.061319458	0.42232954	0.56519265	0.115276963	0.729778769
LOC112446383	0.818577861	0.051277187	0.055466719	0.487062072	0.386974775	0.116076014	0.729778769
LOC112447005	0.029102221	0.354419319	0.380724109	0.342425836	0.326678089	0.116144746	0.729778769
LOC112449547	0.923259915	0.721975707	0.003462263	0.483180611	0.387334818	0.115044875	0.729778769
MRPS18A	0.430239786	0.308008595	0.366641396	0.078375558	0.113460352	0.115065332	0.729778769
NPEPL1	0.668485682	0.218123353	0.948796966	0.007177915	0.439272834	0.115687955	0.729778769
PLCXD2	0.088950199	0.892891145	0.153503834	0.244732135	0.144857701	0.115088862	0.729778769
RAC1	0.920669015	0.019776643	0.04119832	0.62302733	0.934138168	0.115741005	0.729778769
SORBS2	0.133180279	0.362681409	0.397956455	0.319007384	0.071187112	0.11573336	0.729778769
TBC1D15	0.396863032	0.429867644	0.027565898	0.129816527	0.720309045	0.11621339	0.729778769
TMEM177	0.874370611	0.897604953	0.932507234	0.002133585	0.280719741	0.116005667	0.729778769
LOC112443428	0.723035361	0.075678116	0.160836352	0.088884401	0.563258966	0.116341642	0.730120637
NEMP2	0.399832133	0.007656346	0.901924357	0.223047078	0.71592909	0.116385082	0.730120637
LTBP2	0.028558626	0.633232531	0.297069792	0.426647588	0.192733638	0.116513289	0.730366536
WASF1	0.014929047	0.530941798	0.289488953	0.963114995	0.199799066	0.116482621	0.730366536
CSTF2T	0.55343575	0.329842358	0.631163119	0.294980357	0.013016769	0.116607747	0.730679549
ADCY5	0.209300361	0.116311116	0.328059643	0.092609186	0.603861461	0.117231951	0.731265941
APBB3	0.470990181	0.053416751	0.25071072	0.383838482	0.185184006	0.117486883	0.731265941
EPOR	0.767200672	0.188704904	0.021967007	0.148099472	0.949246706	0.117301795	0.731265941
HDAC2	0.891538867	0.358685957	0.536699263	0.06385846	0.040665959	0.117095438	0.731265941
IRF4	0.158827104	0.722995227	0.05082518	0.756904511	0.100734083	0.116992627	0.731265941
KCNG2	0.500912111	0.361170869	0.012742256	0.299211617	0.649582428	0.117444191	0.731265941
LOC101903540	0.33036548	0.5069987	0.846740009	0.033585368	0.093195488	0.116832201	0.731265941
MASTL	0.606148272	0.072178576	0.098051056	0.426517184	0.243596047	0.117097122	0.731265941
MIER1	0.202445103	0.59742076	0.539318428	0.058384824	0.117368179	0.117284725	0.731265941
NECAB3	0.606539678	0.320656082	0.051214766	0.552764871	0.081159541	0.117268142	0.731265941
PDLIM3	0.033723527	0.318580249	0.334285858	0.991313885	0.125635944	0.117332047	0.731265941
PLCB1	0.283452344	0.087457192	0.29849956	0.269128121	0.224072632	0.1171761	0.731265941
RABL3	0.426071775	0.168608823	0.1784698	0.104207144	0.334534165	0.117282083	0.731265941
RBM18	0.287880039	0.056267603	0.499579947	0.104551345	0.527492383	0.11718423	0.731265941
SAP130	0.9712157	0.847106296	0.001290527	0.825879303	0.510620779	0.11739898	0.731265941
SLC9B2	0.089482805	0.967055989	0.130335191	0.212932943	0.186735596	0.117503399	0.731265941
SPTSSA	0.508846656	0.231273834	0.409845448	0.135402116	0.067951575	0.116811319	0.731265941
SSBP1	0.409307238	0.337901113	0.661272415	0.017539074	0.277232833	0.116949364	0.731265941
CRLF1	0.254845361	0.129092579	0.176852798	0.142987029	0.544779069	0.11820201	0.731809802
DYNLL2	0.225232523	0.047159034	0.125409037	0.428550303	0.789653144	0.117844349	0.731809802

LOC509415	0.171124696	0.403267541	0.039262629	0.520533432	0.321466514	0.118226871	0.731809802
LOC513329	0.710278954	0.320447764	0.225375892	0.129020498	0.068044133	0.117780366	0.731809802
LOC781989	0.793249738	0.208160118	0.018670295	0.242779449	0.601308858	0.117738503	0.731809802
LOC786586	0.760624006	0.067919941	0.016337876	0.77599607	0.691439768	0.118151572	0.731809802
MARCKS	0.276051062	0.628923408	0.024319544	0.11610385	0.924985738	0.11823521	0.731809802
MDM2	0.985418745	0.664220495	0.015182829	0.115856821	0.394535068	0.118352739	0.731809802
POR	0.559280721	0.870615552	0.895580572	0.174070438	0.005987882	0.118393457	0.731809802
RMND5A	0.166881478	0.404758687	0.208526817	0.224315605	0.143042478	0.11801631	0.731809802
TEP1	0.669476583	0.020640687	0.036214194	0.989808462	0.914307689	0.118152348	0.731809802
THBS3	0.467828449	0.091669436	0.018680034	0.667970902	0.847380316	0.118235286	0.731809802
TRIP4	0.095704346	0.369434457	0.0935247	0.18732294	0.725678302	0.117656654	0.731809802
UNC80	0.476559193	0.320102228	0.604881726	0.111120506	0.043997008	0.1178947	0.731809802
ZMAT2	0.740501308	0.012245042	0.484424569	0.269197392	0.3810327	0.117811184	0.731809802
ZMYND15	0.41062449	0.20894094	0.013335942	0.82386102	0.48140447	0.11828594	0.731809802
ZNF605	0.567222968	0.146290786	0.272105935	0.848609816	0.02364155	0.118169286	0.731809802
ZNF827	0.022071487	0.207253091	0.228643835	0.691521851	0.627616784	0.118306793	0.731809802
KY	0.604476482	0.275362556	0.021087407	0.35910286	0.361353667	0.118531166	0.732385151
ADSL	0.648874427	0.977572699	0.962225109	0.023920558	0.031441967	0.119054175	0.732585002
AHSA2	0.645782671	0.862735808	0.918864176	0.00495428	0.182847784	0.11973505	0.732585002
ASCC1	0.28681625	0.730810318	0.508026064	0.008026608	0.535195731	0.118819431	0.732585002
BASP1	0.079715462	0.243611863	0.488985162	0.177287536	0.275586782	0.119764018	0.732585002
BCAT2	0.229463775	0.223317154	0.660819233	0.201552381	0.06811372	0.119898221	0.732585002
CCNB2	0.29922124	0.115186001	0.080195626	0.210092963	0.800417369	0.1198873	0.732585002
CLIP2	0.732615244	0.19367816	0.005838433	0.766884893	0.725625842	0.119335537	0.732585002
CSPP1	0.584910138	0.04549866	0.677440063	0.27495336	0.092047531	0.118649293	0.732585002
DKK2	0.093104587	0.980215004	0.290923325	0.273055796	0.0640301	0.119800066	0.732585002
DOK5	0.332589778	0.531350724	0.331343627	0.015458851	0.507900251	0.119155209	0.732585002
GK	0.190989427	0.296273959	0.030367727	0.777911434	0.346702088	0.119691175	0.732585002
GRP	0.008130552	0.193303334	0.66164768	0.465074118	0.960007614	0.119811882	0.732585002
IL6	0.026242687	0.677822034	0.980696915	0.338568269	0.077983299	0.119275596	0.732585002
ITPKA	0.002734346	0.688758633	0.869647719	0.895277639	0.315145483	0.119495337	0.732585002
KIAA1143	0.646823857	0.435422033	0.168488178	0.037266366	0.260858415	0.119380508	0.732585002
LOC104969027	0.269667915	0.218042794	0.507165412	0.049009988	0.318101115	0.119902706	0.732585002
LOC112444864	0.751975063	0.759339284	0.203575579	0.008178023	0.481872874	0.118912567	0.732585002
LOC112447316	0.076175254	0.113350627	0.236856077	0.66891683	0.336679712	0.119276335	0.732585002
MND1	0.74237063	0.038876716	0.337572157	0.813681908	0.058310929	0.119518304	0.732585002
NIPA1	0.362244813	0.194093533	0.025512144	0.258836934	0.985598117	0.118842022	0.732585002
PDIA6	0.161329411	0.429187904	0.029025874	0.428670983	0.532022859	0.118951721	0.732585002
PDZD7	0.103932586	0.278541527	0.325152847	0.371175556	0.130854119	0.118782128	0.732585002
RAB29	0.436992775	0.692729798	0.017324744	0.094291732	0.934620613	0.119507866	0.732585002
SPOP	0.914501532	0.320300463	0.490283737	0.008248083	0.392477542	0.119900678	0.732585002
SUSD5	0.260239408	0.597205059	0.589549921	0.014101832	0.353371768	0.118694472	0.732585002
TGFBR1	0.630716244	0.013796452	0.879256069	0.226447834	0.268226117	0.119874191	0.732585002
TMEM50A	0.843073097	0.006144872	0.652618023	0.219334535	0.620667776	0.119228911	0.732585002

TMEM59	0.156043131	0.414444714	0.040994561	0.369690073	0.471827274	0.119545567	0.732585002
TMEM59L	0.190447131	0.386216995	0.053378614	0.125269514	0.934658723	0.119147079	0.732585002
ZFP2	0.339593722	0.145824477	0.512545267	0.557537871	0.032374202	0.118920239	0.732585002
HDAC7	0.888291782	0.078353198	0.052368534	0.411676114	0.310105642	0.119961135	0.732669216
ADH6	0.687306452	0.9880342	0.012714594	0.401172233	0.134478091	0.120032198	0.732830507
TMEM71	0.768678391	0.974183111	0.070426022	0.49557128	0.017836162	0.120081538	0.732859098
CUL1	0.194723262	0.129223479	0.148313679	0.147739583	0.846981515	0.120203205	0.732904351
FUT1	0.656085943	0.890803899	0.003304253	0.587593414	0.412212797	0.120312249	0.732904351
GNB3	0.68260189	0.081759608	0.022438979	0.637867368	0.585146298	0.120264122	0.732904351
NCOA6	0.262751357	0.694886266	0.584385269	0.050563445	0.086687184	0.120302162	0.732904351
TNFRSF25	0.618997008	0.171041799	0.172906434	0.313132118	0.081456783	0.120194766	0.732904351
CAVIN3	0.477518347	0.07519578	0.385835539	0.052189173	0.649824102	0.12061465	0.733113711
FLT4	0.686607567	0.392335964	0.00755324	0.390617709	0.590027481	0.120484474	0.733113711
KIAA1551	0.300763511	0.168436854	0.232016253	0.066471564	0.600721695	0.120541282	0.733113711
LOC100336941	0.779910919	0.081000496	0.141802639	0.462375556	0.113305259	0.120536937	0.733113711
SCNN1D	0.882285428	0.00150152	0.978313233	0.457065644	0.792905451	0.120592299	0.733113711
TMEM132A	0.989161778	0.114981946	0.560598543	0.457158547	0.016099426	0.120531385	0.733113711
CDK2AP2	0.151135771	0.204481186	0.354393485	0.05952671	0.723021864	0.120833638	0.733308661
CMSS1	0.634549463	0.20281299	0.171530855	0.207579619	0.102841959	0.12081634	0.733308661
DIP2B	0.723654576	0.95061965	0.002090062	0.443607133	0.739731204	0.120895928	0.733308661
LOC100849050	0.237205831	0.325259409	0.272405657	0.030389833	0.739335125	0.120953776	0.733308661
LOC531747	0.766617996	0.152185484	0.877542383	0.398185113	0.011592021	0.121004196	0.733308661
MLX	0.815209357	0.539208655	0.316235607	0.01817418	0.186968154	0.120972204	0.733308661
SH3GL2	0.872163485	0.456073874	0.109946357	0.231816419	0.046423598	0.12072894	0.733308661
UBE2QL1	0.36502302	0.176991735	0.034485762	0.418752977	0.506162979	0.120956949	0.733308661
ABCA3	0.2498656	0.762886945	0.128641234	0.980788785	0.020669031	0.124471654	0.734205678
ACYP1	0.495587988	0.096460778	0.353143411	0.060254448	0.484341669	0.123853608	0.734205678
AKR7A2	0.720007835	0.730761402	0.689103238	0.015074979	0.090847905	0.124396034	0.734205678
AMMECR1	0.462492311	0.263628565	0.041524003	0.389542667	0.242111457	0.121707696	0.734205678
ATP5MC3	0.246557618	0.694610211	0.84700957	0.013669962	0.243337424	0.122423418	0.734205678
BTBD9	0.991200815	0.19909928	0.34618239	0.706865511	0.009996975	0.122457676	0.734205678
BTLA	0.018367552	0.296561594	0.815475955	0.742764906	0.146185112	0.122393156	0.734205678
C13H20orf27	0.095176598	0.388965321	0.711819304	0.043698787	0.424555646	0.123322128	0.734205678
C3H1orf109	0.164927189	0.33414328	0.316328109	0.406151546	0.068434295	0.122707168	0.734205678
CCDC102A	0.913314924	0.173865264	0.004055017	0.820859814	0.917550727	0.122770353	0.734205678
CEP192	0.781734053	0.06379066	0.083187906	0.133024532	0.883894402	0.123162786	0.734205678
CEP295	0.379908651	0.020383157	0.175870486	0.560019252	0.652530956	0.124552349	0.734205678
CHCHD4	0.19658515	0.201400514	0.365423752	0.30873775	0.109074536	0.123085698	0.734205678
COG2	0.505675223	0.496254773	0.755319248	0.00400657	0.649454247	0.1239275	0.734205678
COMMD4	0.089638025	0.307489496	0.120911777	0.202513395	0.726835937	0.123554634	0.734205678
CROCC2	0.722348038	0.01109445	0.161263611	0.616769843	0.598100196	0.121601084	0.734205678
DDX11	0.468045739	0.094750519	0.145894585	0.460537724	0.159904978	0.121562038	0.734205678
ELF1	0.916758377	0.96108205	0.678018421	0.001566044	0.519029127	0.122853619	0.734205678
FOXS1	0.589044532	0.379567781	0.442487122	0.074011861	0.065321221	0.121821649	0.734205678

FYB1	0.392758238	0.832292157	0.381147974	0.106000534	0.037386933	0.124006172	0.734205678
GOLGA7	0.608644033	0.357473536	0.134543793	0.022592569	0.750381869	0.124356164	0.734205678
GPR83	0.365001313	0.651400537	0.751070105	0.158887929	0.016821266	0.121677313	0.734205678
HK3	0.424600136	0.180946749	0.066285595	0.881456726	0.105885904	0.121398281	0.734205678
IDH2	0.258593562	0.493404438	0.199587755	0.035198657	0.552080187	0.1241591	0.734205678
IL6R	0.736192568	0.858766078	0.064403756	0.012994469	0.907211564	0.122064995	0.734205678
INPP5A	0.205602701	0.277360296	0.637434718	0.045519303	0.287046035	0.121346202	0.734205678
KANSL1	0.153396206	0.589601344	0.723195295	0.010876374	0.687395899	0.123338753	0.734205678
KATNAL1	0.368146626	0.031216552	0.846579939	0.706172689	0.069213143	0.121427046	0.734205678
KCNK17	0.9151905	0.718030293	0.89282312	0.001226847	0.688282407	0.124237822	0.734205678
KIAA1191	0.965271949	0.147906858	0.147706281	0.046946453	0.496926988	0.123753272	0.734205678
LARP4	0.714410177	0.39414364	0.025290704	0.320900364	0.215843899	0.123934636	0.734205678
LOC100848895	0.500956516	0.05782175	0.225891342	0.914234356	0.08083184	0.12256624	0.734205678
LOC101905499	0.366334833	0.291983825	0.138912229	0.288247617	0.111182762	0.121522047	0.734205678
LOC101905956	0.152586341	0.136085557	0.189716015	0.235991525	0.528154764	0.123619384	0.734205678
LOC101907302	0.655244231	0.5839614	0.002858993	0.776138054	0.568212549	0.122411775	0.734205678
LOC112443226	0.844878434	0.57349246	0.029230531	0.260958951	0.130490385	0.12238961	0.734205678
LOC112445031	0.208400219	0.146800497	0.023029917	0.793669323	0.850399406	0.121428201	0.734205678
LOC539893	0.823129762	0.034592293	0.34083558	0.548598692	0.093231697	0.124370853	0.734205678
LOC783466	0.978463244	0.348393384	0.033018797	0.096808394	0.440119392	0.12200448	0.734205678
LXN	0.258489795	0.76236543	0.01932388	0.434578803	0.295228562	0.12327667	0.734205678
MAFF	0.288136525	0.076903253	0.071055641	0.385550508	0.815492004	0.124184541	0.734205678
MAMDC4	0.738360713	0.017497627	0.15325852	0.683709838	0.364809471	0.124020131	0.734205678
MOSPD2	0.374628085	0.599430195	0.029332855	0.164265005	0.455006584	0.123804711	0.734205678
NAIP	0.451686161	0.21542988	0.598944479	0.126530921	0.067261337	0.124319883	0.734205678
NKD2	0.580460044	0.358100218	0.506415107	0.007410571	0.616959564	0.122245285	0.734205678
NUDT14	0.054377324	0.499612976	0.530674733	0.042430579	0.801976484	0.123560859	0.734205678
NUMA1	0.709769591	0.173814712	0.728325098	0.531384591	0.010180907	0.122928172	0.734205678
PCDH12	0.754873732	0.84082759	0.007421534	0.173636596	0.586634984	0.122039797	0.734205678
PIK3C2A	0.32448623	0.823344173	0.066698717	0.034285588	0.814298978	0.124527266	0.734205678
PROCA1	0.144688791	0.698360942	0.246100771	0.091719706	0.212251066	0.122646552	0.734205678
PSMA1	0.747402451	0.541317851	0.225405545	0.198842252	0.026747081	0.122775307	0.734205678
RCAN1	0.826929304	0.371896423	0.027601681	0.199464123	0.28632921	0.122743719	0.734205678
RETREG3	0.114406289	0.597995322	0.929864368	0.546065572	0.01390252	0.122483489	0.734205678
RGL2	0.705181335	0.013662747	0.076994207	0.818119446	0.806826319	0.123429709	0.734205678
RPGRIP1L	0.068948743	0.586531017	0.518788904	0.073188255	0.317615977	0.123153815	0.734205678
RPLP1	0.312134816	0.390623189	0.010132291	0.923912192	0.42717672	0.123137111	0.734205678
RSRP1	0.585401922	0.160678703	0.587049789	0.03828413	0.228988687	0.122643379	0.734205678
RUNX3	0.786046978	0.664196817	0.220019056	0.11256281	0.038004443	0.123674255	0.734205678
SAT2	0.141494281	0.167623528	0.070157009	0.996477838	0.299082026	0.124305757	0.734205678
SLA2	0.132613426	0.573078858	0.135216501	0.241762067	0.192355805	0.121763859	0.734205678
SNRNP48	0.951686837	0.239216967	0.725626116	0.110448899	0.02645572	0.122447949	0.734205678
SNRNP70	0.482233369	0.074275014	0.018183306	0.92701475	0.806360828	0.123033161	0.734205678
STK24	0.365652409	0.386683672	0.039573051	0.135645388	0.638398539	0.122706544	0.734205678

SYNGR1	0.038112481	0.387282762	0.732576972	0.09408792	0.484809767	0.123931584	0.734205678
TBC1D32	0.036837684	0.071261037	0.907687887	0.208397304	0.964193186	0.121891182	0.734205678
TDP1	0.421798921	0.011483616	0.20392238	0.7268254	0.670773655	0.122286669	0.734205678
TGFB1I1	0.354922126	0.296850426	0.375245259	0.648500903	0.018698625	0.121980469	0.734205678
TIMM17A	0.658425568	0.551100882	0.252347928	0.044686708	0.120828763	0.124095909	0.734205678
TMBIM7	0.210582676	0.835671817	0.495113897	0.01189899	0.479888121	0.12453093	0.734205678
TMUB1	0.587124011	0.341261028	0.037843365	0.104482672	0.620107043	0.123655569	0.734205678
TREX1	0.398820432	0.374783677	0.098274753	0.053244536	0.606004307	0.121204835	0.734205678
UBE2V2	0.43598839	0.165640165	0.096361489	0.37309776	0.186187823	0.122548456	0.734205678
UBQLN1	0.59501991	0.154087498	0.035341455	0.285201452	0.533309824	0.123877627	0.734205678
USP28	0.407658795	0.105083058	0.296376404	0.416568848	0.09058174	0.12193251	0.734205678
XRCC3	0.305688932	0.076634866	0.941104037	0.023947085	0.924728623	0.123226457	0.734205678
ZNF143	0.376879776	0.097875882	0.753380211	0.096141884	0.184631202	0.123940585	0.734205678
ACD	0.882601284	0.013809158	0.133738225	0.422014806	0.733526601	0.125511368	0.73470691
ADA	0.339157771	0.653156786	0.236280889	0.009846466	0.973057682	0.125083583	0.73470691
ANO1	0.085042475	0.08701404	0.518608923	0.264561356	0.49322102	0.124982025	0.73470691
ANO6	0.642514731	0.10115578	0.055228805	0.214504187	0.652441875	0.125203929	0.73470691
BAZ1B	0.217483251	0.652032659	0.344516847	0.025044374	0.412604127	0.125546512	0.73470691
DLX4	0.212054319	0.016643114	0.406734399	0.476098987	0.740434747	0.12571184	0.73470691
EIF4G2	0.682251177	0.013485189	0.263581791	0.246266586	0.837109551	0.124865313	0.73470691
GCH1	0.140681743	0.35090417	0.063607862	0.260988559	0.616508332	0.125602515	0.73470691
KIF5C	0.82808065	0.686944279	0.012213097	0.087079318	0.835552375	0.125636499	0.73470691
LOC100848315	0.409706779	0.034206025	0.178627031	0.210751875	0.957992063	0.12562807	0.73470691
LOC112448105	0.272246219	0.552351682	0.097869311	0.179725695	0.189323074	0.124982761	0.73470691
LOC522763	0.773497369	0.471117345	0.30970852	0.813629552	0.00543169	0.124705025	0.73470691
MRC2	0.470439823	0.055932972	0.073198575	0.355562459	0.73642792	0.125477585	0.73470691
MYOZ1	0.791979907	0.885320534	0.015102446	0.536629237	0.089011577	0.125680628	0.73470691
POLR3A	0.68671029	0.833480557	0.657721739	0.080336178	0.016725711	0.12568452	0.73470691
PVR	0.327632639	0.508761169	0.466389372	0.098122637	0.065819209	0.125164553	0.73470691
RBM28	0.893527641	0.611839766	0.181749712	0.026727048	0.189355673	0.125273221	0.73470691
ROCK2	0.24155122	0.233869942	0.300107254	0.051707362	0.576373415	0.125605568	0.73470691
SLBP	0.643556114	0.627996695	0.063125196	0.081457345	0.242490276	0.12542138	0.73470691
STARD5	0.076447672	0.97436316	0.468420901	0.018111963	0.797222059	0.125404523	0.73470691
TCF15	0.920303995	0.694730106	0.213973462	0.004285305	0.851943919	0.124800577	0.73470691
TIMM10B	0.601776293	0.303599253	0.075053041	0.227220447	0.161815568	0.125453636	0.73470691
TMEM72	0.011808554	0.51415099	0.83479935	0.107589244	0.917955314	0.124954262	0.73470691
TNFAIP3	0.540530158	0.285499678	0.61877263	0.007479524	0.700525437	0.124921359	0.73470691
LOC100848307	0.345591679	0.593709662	0.090404989	0.293706465	0.0930116	0.125809092	0.735013532
OVCA2	0.855759881	0.86101869	0.306875455	0.0990651	0.022638687	0.125860366	0.735051413
BORCS6	0.358072137	0.757844861	0.737517227	0.039767525	0.064018505	0.126192551	0.735160081
CCDC80	0.20649375	0.028548631	0.178120162	0.882181408	0.548867213	0.12604293	0.735160081
KCTD12	0.385295283	0.940214038	0.008711541	0.265422528	0.607289781	0.126078226	0.735160081
LANCL3	0.561066178	0.743252899	0.063370979	0.091886128	0.209145432	0.125963706	0.735160081
MTFR1L	0.492700439	0.143636389	0.603437264	0.057531101	0.207374794	0.126189499	0.735160081

PNISR	0.958539794	0.130008103	0.08167272	0.518462971	0.096250292	0.125969826	0.735160081
SAP25	0.420906655	0.055674764	0.593798283	0.676666188	0.054099597	0.126175512	0.735160081
ANO8	0.171684437	0.081246494	0.12234377	0.358929438	0.833180022	0.126307012	0.735394176
ARC	0.028553078	0.062657452	0.873568555	0.484009702	0.681789648	0.127046371	0.735394176
CDH2	0.747950796	0.352159565	0.01518355	0.761124065	0.169841994	0.127218577	0.735394176
COLGALT1	0.460407219	0.707709184	0.036046833	0.148622067	0.294719745	0.126872533	0.735394176
CRK	0.930036806	0.144199322	0.007737242	0.605215732	0.819642835	0.126909506	0.735394176
EFS	0.670373868	0.241874743	0.008468602	0.507867928	0.738462209	0.126944248	0.735394176
ESPN	0.113668596	0.599685135	0.389252387	0.026204372	0.735121655	0.126414193	0.735394176
IFRD2	0.388932671	0.847526351	0.596057392	0.039720918	0.065887724	0.126837305	0.735394176
LOC101902122	0.77543744	0.85884465	0.248318134	0.309760809	0.010012091	0.126655947	0.735394176
LOC101903193	0.631248551	0.757900929	0.401329495	0.015823994	0.170124406	0.127203979	0.735394176
LOC101907405	0.986250911	0.67728388	0.312254046	0.017188627	0.143424923	0.126836094	0.735394176
LOC112448743	0.39987873	0.027099891	0.637021325	0.397901982	0.186492591	0.126569508	0.735394176
MAGED4B	0.48977953	0.192414319	0.009130251	0.758583877	0.785053613	0.126591562	0.735394176
MIF	0.307220123	0.84144899	0.775015658	0.005595948	0.455757493	0.126392734	0.735394176
MYCN	0.778957713	0.169202285	0.016753927	0.784990065	0.298116714	0.12718618	0.735394176
RAB5C	0.007785404	0.336437517	0.609651963	0.787161151	0.410233303	0.127035805	0.735394176
TBL3	0.325324865	0.069096532	0.906191143	0.157170022	0.160233332	0.126671317	0.735394176
UBA2	0.261645304	0.795478001	0.513449715	0.01506674	0.321055445	0.127210764	0.735394176
UBE2G2	0.801015694	0.012661883	0.095309822	0.533646991	0.990449104	0.126387528	0.735394176
ZBTB5	0.026445181	0.681349931	0.150144176	0.69177324	0.275080568	0.126919981	0.735394176
ZMAT5	0.025657856	0.744461653	0.897560108	0.032126305	0.936894412	0.127087083	0.735394176
ZNF706	0.455698958	0.119964999	0.027482023	0.402677431	0.85191356	0.12699877	0.735394176
BGLAP	0.559923062	0.986356206	0.001496451	0.719958095	0.869753078	0.127290382	0.735505242
PAX5	0.29474992	0.829050233	0.069351561	0.809824833	0.037728838	0.127327426	0.735505242
DNAJB9	0.080713262	0.199740281	0.905120544	0.088429594	0.401699729	0.127402462	0.735562107
MID2	0.454713536	0.397667086	0.395778174	0.010345896	0.700307524	0.127426913	0.735562107
RRAS	0.491673595	0.137856254	0.111991766	0.621742674	0.110051168	0.12754563	0.735988512
CCDC86	0.085252185	0.895718385	0.143668758	0.507143644	0.093529072	0.12767972	0.736244515
LSG1	0.149227157	0.605199631	0.259745391	0.033690227	0.658437689	0.127679073	0.736244515
LOC101902083	0.013263694	0.455139293	0.828223081	0.668057392	0.155939687	0.12774657	0.7363336
THBS2	0.840007692	0.070935774	0.056900537	0.788637048	0.194903335	0.127784906	0.7363336
SNX4	0.481942019	0.408403462	0.011716974	0.237232073	0.953698459	0.127870286	0.736566956
APOLD1	0.884196592	0.403246007	0.006044921	0.494793968	0.490820192	0.128094636	0.737341662
FNBP4	0.510383733	0.036760744	0.248618355	0.263511225	0.425759386	0.128081759	0.737341662
ABHD13	0.328901332	0.063253976	0.133929455	0.355809666	0.54644405	0.130558351	0.737365203
ABHD17B	0.419584866	0.399267017	0.224905571	0.981483831	0.014476761	0.129703565	0.737365203
ACSL5	0.301993472	0.991442642	0.526014855	0.248529247	0.01372987	0.129979809	0.737365203
ADRA2A	0.321592901	0.099993783	0.20893199	0.797024983	0.101143211	0.130541734	0.737365203
ALAS1	0.387464957	0.52403709	0.543178622	0.031877359	0.151495316	0.129337024	0.737365203
AP1M1	0.540333401	0.280192226	0.69663664	0.343361338	0.014894582	0.130244558	0.737365203
ARFIP1	0.189583573	0.43361592	0.212629189	0.323202759	0.094572781	0.129560107	0.737365203
C22H3orf22	0.866340675	0.012567382	0.590411897	0.639770635	0.130096461	0.129660645	0.737365203

CDC25B	0.434866348	0.284533732	0.108192797	0.183907006	0.219869988	0.130501682	0.737365203
CENPI	0.156415903	0.486699008	0.939747692	0.237018849	0.032025706	0.13073158	0.737365203
CLEC4A	0.119101453	0.247625518	0.202445636	0.255566388	0.350292746	0.129590793	0.737365203
CLPP	0.312991577	0.586316085	0.211813098	0.080178819	0.173925789	0.130599577	0.737365203
COL6A3	0.652240425	0.152556266	0.033365677	0.297299297	0.534126057	0.128605812	0.737365203
DYNC1I2	0.750756877	0.953903579	0.195750975	0.004247586	0.905593125	0.130224502	0.737365203
EIF2B2	0.186891636	0.37974445	0.526896959	0.201497836	0.069957958	0.128595824	0.737365203
FAM126B	0.030501424	0.4557145	0.648051534	0.084475297	0.706685273	0.130024736	0.737365203
FAM84A	0.069509996	0.137808159	0.207472977	0.348081124	0.775580385	0.129861234	0.737365203
FASTK	0.210032718	0.166767614	0.419628148	0.037964029	0.954447212	0.129331881	0.737365203
FGF7	0.555363878	0.123497887	0.018287278	0.973462451	0.437345466	0.12952047	0.737365203
GIMAP7	0.052489436	0.732044648	0.725560167	0.196263476	0.095880986	0.128258098	0.737365203
GLO1	0.351626568	0.616227577	0.162754387	0.032316581	0.476836976	0.130784296	0.737365203
HNRNPAB	0.480341484	0.029115749	0.508950096	0.270435929	0.277702637	0.129597717	0.737365203
IMPG2	0.798658884	0.734042493	0.512846917	0.007164265	0.244453611	0.128517684	0.737365203
INPP5F	0.488363629	0.199140652	0.880364954	0.503749577	0.01243449	0.129831052	0.737365203
LEP	0.065251957	0.409264751	0.157539819	0.839727715	0.153500009	0.130631881	0.737365203
LGI2	0.352608777	0.421430291	0.017485895	0.299808285	0.697689723	0.130794595	0.737365203
LOC100297240	0.857487493	0.004452947	0.18442611	0.917803263	0.840467095	0.130753858	0.737365203
LOC100336476	0.314941525	0.015752141	0.771397905	0.331652722	0.425588305	0.130347028	0.737365203
LOC100847708	0.387019399	0.230031812	0.645041653	0.233884158	0.039133551	0.128389774	0.737365203
LOC104974666	0.193129424	0.376912747	0.044477642	0.742594001	0.221769667	0.129413905	0.737365203
LOC107132382	0.516275101	0.561154183	0.015420631	0.554514918	0.21608936	0.129699289	0.737365203
LOC112447305	0.677101306	0.657688264	0.27793459	0.732776449	0.005964714	0.130456029	0.737365203
LOC112447381	0.512225698	0.044591406	0.900721592	0.318848723	0.081094565	0.129248115	0.737365203
LOC112448354	0.116771114	0.137250019	0.152754444	0.504428474	0.433718565	0.129738507	0.737365203
LOC783604	0.277064953	0.025927426	0.287280697	0.270399093	0.956980998	0.129524751	0.737365203
MORF4L1	0.920256161	0.089589725	0.191045268	0.046780902	0.712245777	0.128282146	0.737365203
MRPS34	0.280559656	0.655274411	0.709489874	0.028156291	0.143693057	0.128676506	0.737365203
MTMR14	0.876684576	0.658450447	0.040576658	0.100287163	0.225989148	0.129099173	0.737365203
NPAS3	0.272558922	0.956581273	0.059137013	0.040321613	0.847638947	0.128575464	0.737365203
OLFML2A	0.324162447	0.20238391	0.086864631	0.504805729	0.187777637	0.130351772	0.737365203
PARP10	0.689804209	0.016867399	0.67900314	0.12025832	0.567018197	0.13015397	0.737365203
PPIA	0.742647561	0.407084164	0.48688037	0.011220344	0.31727103	0.128171463	0.737365203
PPP1R7	0.37227737	0.402975265	0.079879339	0.172238853	0.254616972	0.128379857	0.737365203
PVALB	0.256371794	0.749919013	0.030035054	0.607793999	0.152996393	0.129920769	0.737365203
RAC2	0.165229554	0.456842338	0.768679948	0.162829453	0.057309576	0.130519576	0.737365203
RAD21	0.386475371	0.219443286	0.275603797	0.044132102	0.516803927	0.129401549	0.737365203
RBM25	0.999672433	0.333724969	0.099843881	0.055288814	0.293159495	0.130311808	0.737365203
RNF182	0.072759562	0.561022934	0.173138088	0.112453909	0.662758109	0.128543042	0.737365203
RNF208	0.267899936	0.433029192	0.681371282	0.00685967	0.979456091	0.129129829	0.737365203
SAG	0.332550479	0.971276292	0.340744961	0.005136163	0.956301278	0.130403848	0.737365203
SGCA	0.042379859	0.062780685	0.681593114	0.488263948	0.60770652	0.130071312	0.737365203
SIMC1	0.307958148	0.269541738	0.239661803	0.124806205	0.212678765	0.128721022	0.737365203

SLA	0.424793751	0.468850316	0.959703496	0.018782601	0.147244988	0.128798063	0.737365203
SNX22	0.606135057	0.003445974	0.479739455	0.643180701	0.820037711	0.128782945	0.737365203
SORT1	0.303838341	0.285257457	0.079828815	0.096652664	0.812083519	0.130734836	0.737365203
TARBP2	0.534636324	0.154287375	0.12934604	0.214947738	0.235179358	0.130239603	0.737365203
TIMM44	0.381646719	0.877875707	0.58045608	0.013454673	0.206662744	0.130426426	0.737365203
TREML2	0.553436636	0.24381012	0.516142103	0.500155229	0.01553508	0.130477598	0.737365203
UMPS	0.746988862	0.402705967	0.369157398	0.677606011	0.00715971	0.130158709	0.737365203
WAS	0.135313866	0.397518661	0.621060489	0.242316774	0.065841835	0.129386596	0.737365203
CCHCR1	0.103102014	0.151084496	0.378357171	0.59091886	0.156645504	0.131064965	0.737369607
LOC101902851	0.544156703	0.406714697	0.724614617	0.288327796	0.011791175	0.131019886	0.737369607
LOC112444461	0.64529853	0.581339362	0.8002869	0.07645756	0.023758588	0.131038671	0.737369607
PHF10	0.249136538	0.772060325	0.258419012	0.083341977	0.13151484	0.130967717	0.737369607
RIC8B	0.368238086	0.037430647	0.233788558	0.171772073	0.983581004	0.130916092	0.737369607
RTN4RL1	0.013217079	0.423242781	0.324219049	0.356581817	0.842731482	0.130994442	0.737369607
DYRK1A	0.252005224	0.148090433	0.704650714	0.024088144	0.862918583	0.131206629	0.737547477
ISPD	0.915553425	0.206548862	0.34530652	0.074089893	0.11316179	0.131321292	0.737547477
LOC112446690	0.694388288	0.959160702	0.161750212	0.019135252	0.26550536	0.131300581	0.737547477
LOC787851	0.178128344	0.055481167	0.455935827	0.401654149	0.302477841	0.131314293	0.737547477
VPS37A	0.937249206	0.006925213	0.105326536	0.857529883	0.933525073	0.131293088	0.737547477
ALG11	0.563695043	0.065729653	0.107089885	0.663723372	0.211523987	0.132584778	0.737582099
CDIPT	0.240744698	0.569408396	0.579409587	0.061288492	0.112903083	0.131602434	0.737582099
CTPS1	0.310268522	0.295674072	0.507203714	0.673227914	0.01766697	0.132106555	0.737582099
EFR3B	0.301589309	0.290978428	0.070532692	0.126572632	0.702767029	0.131730947	0.737582099
FES	0.367888806	0.346263559	0.552653237	0.019713916	0.397504135	0.131877152	0.737582099
FOSB	0.631333886	0.009950257	0.235186513	0.464591848	0.799933911	0.131532221	0.737582099
GABRA1	0.166107684	0.640330519	0.037954132	0.896746656	0.153880874	0.132585899	0.737582099
IRF3	0.179475277	0.445803384	0.059704171	0.14566767	0.800064539	0.132541005	0.737582099
KDM4C	0.179158599	0.529976228	0.651416998	0.058284123	0.152374706	0.131563146	0.737582099
LOC107131542	0.712383651	0.584207099	0.013805468	0.118059664	0.812728981	0.131824938	0.737582099
LOC539973	0.625341043	0.097645291	0.102239927	0.868891883	0.102199912	0.132232137	0.737582099
MAML2	0.118836198	0.887817601	0.26639764	0.029651897	0.667294092	0.132462171	0.737582099
MANEAL	0.273667823	0.188197981	0.436207785	0.109086766	0.22724377	0.132567162	0.737582099
MAX	0.580108601	0.4009077	0.043602843	0.057272888	0.954912328	0.132261731	0.737582099
NLRC5	0.755908798	0.00457398	0.986586113	0.291152384	0.559035741	0.132342018	0.737582099
OST4	0.373102073	0.09435981	0.737159767	0.028171092	0.759243382	0.132325617	0.737582099
P4HA2	0.448685016	0.276277736	0.018876909	0.242993909	0.967652709	0.131683183	0.737582099
PCNX4	0.482643356	0.535744009	0.828469289	0.059520554	0.043639926	0.132502306	0.737582099
PLSCR4	0.032338231	0.676447199	0.701956606	0.083294386	0.433133515	0.132180529	0.737582099
POU6F1	0.251865021	0.442244467	0.465826172	0.653094891	0.016285119	0.13189908	0.737582099
RRP12	0.516652201	0.716193983	0.421187737	0.308290454	0.011481731	0.131874045	0.737582099
SCARF2	0.338496548	0.646103857	0.006521798	0.429821672	0.90781875	0.132519205	0.737582099
SLC2A4	0.641446497	0.502466879	0.501224234	0.006823397	0.504421013	0.132448893	0.737582099
SLC43A1	0.250427153	0.024285277	0.215209476	0.475173321	0.883305461	0.131568593	0.737582099
SSTR1	0.356442351	0.14904526	0.236491405	0.058913924	0.749390809	0.132273171	0.737582099

STK38L	0.533862458	0.73508338	0.004664167	0.311453188	0.976523423	0.132536744	0.737582099
UBE3A	0.41832552	0.178233127	0.188773329	0.319429556	0.123506639	0.132350473	0.737582099
UTP6	0.291218498	0.113030084	0.05371636	0.783875795	0.40033936	0.132297716	0.737582099
EXT2	0.847887895	0.098804477	0.044226073	0.274572484	0.549247773	0.132806964	0.737811472
HSF2BP	0.788723279	0.527746005	0.174705451	0.010412715	0.73706722	0.132724058	0.737811472
LOC783776	0.016392778	0.148025235	0.514618769	0.880459529	0.508032081	0.132782628	0.737811472
TIA1	0.798618834	0.118274998	0.118882381	0.122821412	0.40460506	0.132711956	0.737811472
AGPAT2	0.320017952	0.67395394	0.322025402	0.031133373	0.259173691	0.133025243	0.737827825
FAM71E1	0.468995129	0.067609016	0.109587464	0.849392851	0.189699048	0.132956978	0.737827825
GNG7	0.91682756	0.549268267	0.02755627	0.130773442	0.308486983	0.132947085	0.737827825
GPR88	0.632664006	0.637280756	0.221434065	0.007324094	0.857692444	0.133079664	0.737827825
LOC101902786	0.154830524	0.714579893	0.275463761	0.029833509	0.616161731	0.133001448	0.737827825
TPK1	0.029086743	0.526499854	0.193613313	0.31018341	0.609717549	0.133069855	0.737827825
NFKBIA	0.974575564	0.588091567	0.032119796	0.063796465	0.478084268	0.133164258	0.737835279
TBC1D14	0.21992195	0.937897706	0.330971472	0.47248171	0.017409013	0.133170928	0.737835279
CD226	0.280378666	0.218856276	0.691243982	0.139728584	0.095197523	0.133521744	0.739279807
LOC112446659	0.52108392	0.233904354	0.360682334	0.053778754	0.238616645	0.133510913	0.739279807
FAM20A	0.119926164	0.554067737	0.1807318	0.20654622	0.227871956	0.133652839	0.73944838
MREG	0.15072797	0.25521841	0.160001052	0.297886535	0.308155503	0.133624006	0.73944838
SETD5	0.121086051	0.473772772	0.580391632	0.150391828	0.112931075	0.133687365	0.73944838
CNTN2	0.42699769	0.249481597	0.011193554	0.816767627	0.583009733	0.133990172	0.740238183
DERL3	0.643148828	0.036511632	0.443333275	0.590657111	0.092398921	0.134035906	0.740238183
EIF3B	0.477243562	0.353357587	0.088616924	0.608132157	0.062581123	0.134110236	0.740238183
EPHA5	0.053079774	0.619117336	0.081300795	0.284025262	0.74983957	0.134145899	0.740238183
LOC112442377	0.40213711	0.83811933	0.953280417	0.052716179	0.033576604	0.134104943	0.740238183
MFSD14B	0.202981254	0.106930753	0.220131537	0.153681925	0.774499997	0.134105566	0.740238183
MUC16	0.169102532	0.049484216	0.280853352	0.570713257	0.422864007	0.133906698	0.740238183
MRPL36	0.143962033	0.406792328	0.566359925	0.072749681	0.236054866	0.13421995	0.740397848
ATP6V0C	0.11789224	0.138052622	0.06222372	0.568922765	0.995072911	0.13470353	0.740945322
CBR1	0.072022954	0.798654012	0.3369889	0.037175621	0.792641076	0.134428205	0.740945322
CD68	0.290994305	0.196853761	0.120479328	0.840227441	0.098526541	0.134447094	0.740945322
EID2	0.352364801	0.391938705	0.054449504	0.311752241	0.244629481	0.13472554	0.740945322
NAGK	0.239898792	0.187603048	0.073258468	0.282041734	0.615418203	0.134569639	0.740945322
PGD	0.45622561	0.671963593	0.683620022	0.023918203	0.114303831	0.134658371	0.740945322
STK17A	0.802242065	0.020443403	0.04665185	0.972957673	0.767938084	0.134490875	0.740945322
TBC1D10C	0.147895347	0.893109468	0.546545762	0.085739791	0.092612157	0.134693643	0.740945322
TPM1	0.05763148	0.224066113	0.999290068	0.747258922	0.059406595	0.134641948	0.740945322
LOC781726	0.516167693	0.003795384	0.528930864	0.838477589	0.660808352	0.134809138	0.741156706
LOC101904449	0.782040939	0.022039429	0.317996657	0.117173138	0.896664732	0.135031058	0.741578249
LOC107132531	0.883041475	0.048949748	0.457522014	0.123603654	0.235691107	0.135066564	0.741578249
LOC112442080	0.532366238	0.870288396	0.032833685	0.632758265	0.059789184	0.13498714	0.741578249
NIFK	0.537907432	0.305761468	0.440494914	0.065386317	0.121426636	0.134949219	0.741578249
ATP6V1E1	0.126244446	0.177913553	0.17919443	0.378155313	0.379116992	0.135181461	0.741960853
LOC527796	0.678667651	0.354672732	0.210604963	0.181931199	0.06268354	0.135322788	0.742488225

ERICH3	0.664669584	0.072673819	0.905199675	0.435132861	0.03045096	0.135483426	0.742624753
PDK1	0.677432641	0.502209316	0.033548933	0.175508768	0.28918994	0.135476658	0.742624753
RAB10	0.829165859	0.159758038	0.044302901	0.679334851	0.145208915	0.135425522	0.742624753
ATF3	0.059648251	0.108468997	0.210228631	0.669461049	0.638641546	0.135762733	0.743890857
C23H6orf201	0.007839896	0.551487658	0.200346475	0.862205044	0.77935664	0.135830961	0.743890857
RBM34	0.326013922	0.115270122	0.148640706	0.355580459	0.293129218	0.135850399	0.743890857
COX6B1	0.5273086	0.688483102	0.78882408	0.003125846	0.653996097	0.136262468	0.744042401
EFHB	0.85059747	0.938609311	0.176964724	0.007706824	0.537468276	0.136235387	0.744042401
ICA1	0.125156915	0.033319914	0.276401339	0.70981224	0.713738887	0.136073066	0.744042401
LOC100140121	0.907183889	0.004320961	0.857686256	0.322644831	0.539870239	0.136286116	0.744042401
LOC112445995	0.128649375	0.175530426	0.401827349	0.102684567	0.62804254	0.136230197	0.744042401
MPHOSPH10	0.585964911	0.377544699	0.207869792	0.045341682	0.279707277	0.135978572	0.744042401
MRPL11	0.334040052	0.16272092	0.62922275	0.04006204	0.426576862	0.136141096	0.744042401
NDUFA11	0.340751998	0.718022451	0.900399174	0.004095443	0.647542537	0.136107341	0.744042401
RRAD	0.010033317	0.906794883	0.262631803	0.635795491	0.384694109	0.13613372	0.744042401
LOC782418	0.692068617	0.230521409	0.129770883	0.229814792	0.123173797	0.136340573	0.744092166
CRB3	0.072172419	0.101704792	0.344708417	0.239403236	0.969271988	0.13647969	0.744433069
PLOD1	0.471517234	0.223868395	0.036357719	0.176677275	0.866072146	0.136493761	0.744433069
AVL9	0.730242417	0.094430644	0.013071871	0.765101225	0.852183015	0.136553813	0.744513165
TRAT1	0.997174342	0.975653915	0.358845777	0.030676036	0.054912082	0.136600853	0.744522283
LOC112447526	0.228282287	0.03363903	0.144481633	0.546580538	0.971193917	0.136712584	0.744636649
TIPARP	0.793292051	0.731756023	0.151302299	0.007080821	0.946983285	0.136709543	0.744636649
CLEC4D	0.057362683	0.886088217	0.107162429	0.118387257	0.916275079	0.136953287	0.745185815
FCHO1	0.9435576	0.093882857	0.826034445	0.025138012	0.321192532	0.136947994	0.745185815
LOC104975034	0.086259412	0.485991069	0.31466098	0.113963262	0.393258757	0.13699504	0.745185815
RSL1D1	0.762544603	0.198911854	0.746106963	0.032343973	0.161471397	0.136976864	0.745185815
CASZ1	0.725844195	0.469482352	0.010873848	0.433616266	0.370311237	0.137480193	0.745234861
CLPX	0.73568269	0.364084249	0.007726419	0.391937578	0.731905843	0.137310841	0.745234861
HSD17B12	0.677100949	0.640016367	0.744613957	0.032949194	0.055776791	0.137229516	0.745234861
LOC107131642	0.200438128	0.340520694	0.13961476	0.814000815	0.076593666	0.137368117	0.745234861
MAZ	0.722913377	0.157227031	0.024521965	0.471863702	0.450775789	0.137207303	0.745234861
MOB4	0.277568182	0.033466442	0.138030879	0.514811185	0.902759826	0.137594396	0.745234861
PDIA5	0.8320737	0.147314717	0.012494466	0.412224477	0.943679809	0.137578475	0.745234861
RAB5IF	0.260680628	0.439272729	0.455652401	0.090031082	0.125950576	0.137055374	0.745234861
TMEM251	0.606015928	0.145111994	0.021271979	0.505441993	0.629124759	0.137460212	0.745234861
TOX4	0.486862596	0.033877501	0.67125753	0.059015383	0.911293782	0.137534427	0.745234861
TRAIP	0.467621406	0.311154922	0.062717869	0.134496368	0.483837449	0.137333323	0.745234861
ZCCHC11	0.231721671	0.399959367	0.453516087	0.061827225	0.229058731	0.137512058	0.745234861
ZNF688	0.636429446	0.014307237	0.160712408	0.655214478	0.61882165	0.137269483	0.745234861
DNTTIP2	0.658054722	0.142232766	0.375319777	0.402429347	0.04232386	0.137900992	0.746402766
TAF6L	0.557058729	0.833215822	0.110057581	0.019110349	0.612852961	0.137894954	0.746402766
HINT3	0.275608226	0.31853454	0.382981392	0.0638448	0.279099286	0.138000732	0.746667842
MRPL34	0.271957399	0.522208316	0.623341796	0.027613318	0.245217807	0.138040962	0.746667842
RAB14	0.800807639	0.033145084	0.138054635	0.246686859	0.664059505	0.138147278	0.746996699

LOC112449531	0.259317666	0.352813157	0.468917062	0.055360025	0.253030022	0.138232738	0.747212605
ACOD1	0.156633715	0.237654073	0.063839918	0.995700124	0.254873261	0.138500689	0.747602448
EMC8	0.258857159	0.461670645	0.467805347	0.332308439	0.032495259	0.138578188	0.747602448
GLYR1	0.152862938	0.008282522	0.869500141	0.680284133	0.805359807	0.138507517	0.747602448
HOXB4	0.646148122	0.151044263	0.107304333	0.113801698	0.506356084	0.138550101	0.747602448
LOC782479	0.112958944	0.394674346	0.099271405	0.14752467	0.923345537	0.138472192	0.747602448
TSN	0.354783111	0.224575907	0.514928545	0.956923002	0.015375775	0.138572605	0.747602448
ARRDC3	0.513541299	0.757684227	0.812248451	0.044716891	0.042773483	0.138679585	0.74765791
CPA3	0.507251638	0.638659882	0.685587541	0.059822437	0.045483122	0.13865713	0.74765791
ABCB4	0.302169542	0.815068502	0.90131171	0.045191696	0.060345977	0.138789503	0.747712517
CA4	0.203910008	0.612596293	0.970551156	0.335777558	0.014905094	0.138963084	0.747712517
CNTRL	0.659624178	0.048808623	0.11786953	0.179938331	0.887274399	0.138850711	0.747712517
GNA13	0.974107342	0.063479553	0.019487705	0.904838279	0.556111816	0.138913449	0.747712517
SLC26A11	0.22451488	0.336894402	0.145744572	0.332262303	0.165234897	0.138769789	0.747712517
TLE3	0.911477357	0.607647368	0.159477467	0.016071264	0.427250375	0.13893007	0.747712517
BAZ2B	0.310179304	0.077234326	0.121487281	0.623314285	0.3352598	0.139142793	0.747943783
LOC112444505	0.699740168	0.619868462	0.501366211	0.006438862	0.434313438	0.139135992	0.747943783
TPM2	0.040169943	0.257186041	0.603060945	0.589927577	0.165464813	0.139137812	0.747943783
ARMC1	0.45444547	0.090066506	0.25142051	0.535029114	0.113374802	0.141136761	0.748194726
ATP2A2	0.884767937	0.045616031	0.280120738	0.095585428	0.572068466	0.140391276	0.748194726
CHMP1B	0.486965176	0.344125611	0.043026907	0.354883129	0.243727377	0.141066631	0.748194726
COA5	0.156701237	0.289810542	0.156079917	0.159614396	0.551396601	0.141088831	0.748194726
CYB5R4	0.306333412	0.025395919	0.173892073	0.978538417	0.463218995	0.139768529	0.748194726
DNAJC14	0.34678792	0.228408696	0.580986572	0.019376398	0.688009918	0.139805184	0.748194726
ENPP4	0.092058929	0.031055657	0.519098797	0.982456077	0.427362416	0.140999457	0.748194726
FAM160B1	0.151725998	0.247166137	0.272554372	0.091995943	0.650179324	0.139540029	0.748194726
FBXO8	0.873099903	0.085384492	0.057758	0.439308041	0.326978876	0.140428773	0.748194726
GMPS	0.122713164	0.072129802	0.369197348	0.199782355	0.943728232	0.140132811	0.748194726
GPR45	0.024077329	0.538148667	0.918894796	0.082833823	0.625271447	0.140200622	0.748194726
GUCY1A1	0.44645155	0.089976119	0.256264523	0.978626851	0.061953173	0.141124511	0.748194726
KNL1	0.867997836	0.099420584	0.049836301	0.248119813	0.57392625	0.139672745	0.748194726
LBR	0.173866504	0.068037069	0.498041797	0.302419921	0.350311426	0.141128283	0.748194726
LOC100336868	0.313390214	0.083334602	0.686264724	0.045036639	0.769348609	0.140738064	0.748194726
LOC107131567	0.019940183	0.097847989	0.953633065	0.811202655	0.41085585	0.14062997	0.748194726
LOC112442386	0.354093932	0.209493455	0.125100338	0.390216232	0.168546714	0.139411534	0.748194726
LOC112443250	0.763970843	0.327543635	0.419781205	0.104176678	0.056738197	0.140724488	0.748194726
LOC112444622	0.428022783	0.209750774	0.131012396	0.92856188	0.056933258	0.140838728	0.748194726
LOC511386	0.855759848	0.029923856	0.634119048	0.098298138	0.382417122	0.139420291	0.748194726
LOC526966	0.602528728	0.475880715	0.352021719	0.542612275	0.011384277	0.141048118	0.748194726
MAB21L2	0.005954691	0.700923717	0.370279206	0.507406015	0.791806751	0.14072788	0.748194726
MB	0.955082553	0.2390592	0.508886466	0.074816144	0.071598812	0.140911178	0.748194726
ME1	0.561482694	0.542024248	0.215742851	0.012381234	0.756920182	0.140033469	0.748194726
MGAT4A	0.188042515	0.220580223	0.024371853	0.947868949	0.645627211	0.140445987	0.748194726
MGAT4C	0.343677946	0.468561664	0.267541213	0.247707759	0.058501228	0.141149892	0.748194726

MTMR12	0.192378513	0.433960613	0.297743637	0.451048183	0.055566799	0.140985538	0.748194726
MYNN	0.00612968	0.989250848	0.219961276	0.575852702	0.80085265	0.140006836	0.748194726
PABPC4	0.833483524	0.867868244	0.807894244	0.007350511	0.144399483	0.140648971	0.748194726
SAR1B	0.472436752	0.329816892	0.033575983	0.654386433	0.179210112	0.139810763	0.748194726
SCAF8	0.228601874	0.884832358	0.38434059	0.009992163	0.803574874	0.141137317	0.748194726
SGIP1	0.467316236	0.163528133	0.716117065	0.05251066	0.215647347	0.14057648	0.748194726
SLAMF9	0.450507286	0.952290931	0.473956729	0.426514063	0.007094507	0.140026456	0.748194726
SMYD3	0.056356393	0.904332589	0.158541005	0.105584155	0.728550257	0.140805322	0.748194726
SOGA3	0.710084812	0.735583756	0.130733564	0.049929575	0.179225029	0.139501699	0.748194726
SPTBN5	0.821132815	0.069365842	0.133310476	0.694646564	0.116585948	0.139985486	0.748194726
SURF4	0.088815555	0.407732867	0.091613691	0.955430991	0.193839097	0.139920673	0.748194726
TMEM126A	0.583547849	0.430799304	0.621431094	0.026790279	0.147138533	0.140093909	0.748194726
TNIP2	0.538140053	0.047969701	0.68886185	0.096282393	0.362014297	0.140592069	0.748194726
TRMT61A	0.248824641	0.753562799	0.923226282	0.157342441	0.022662953	0.140276688	0.748194726
USP32	0.228709867	0.453205187	0.312463893	0.085497465	0.220425526	0.139415346	0.748194726
WBP4	0.406329362	0.27697415	0.368738341	0.054536923	0.27190165	0.140039237	0.748194726
ZNF555	0.464026613	0.479661522	0.492134223	0.026207228	0.213629525	0.139775816	0.748194726
ADA2	0.514797533	0.546578648	0.197586859	0.11711167	0.096751949	0.141842656	0.748560516
ALKBH8	0.01285654	0.868365634	0.744073597	0.128163622	0.605597579	0.143650276	0.748560516
ALMS1	0.265102301	0.550905539	0.291085469	0.197978264	0.075675142	0.142696151	0.748560516
AP1G2	0.432908075	0.027345995	0.111871672	0.851523209	0.559295268	0.141938983	0.748560516
C26H10orf82	0.020371513	0.68650184	0.836475389	0.367946426	0.148558622	0.143004415	0.748560516
CLEC12A	0.382644229	0.308403476	0.789483959	0.163379948	0.042556174	0.144016027	0.748560516
CREBRF	0.086546787	0.636037692	0.021086532	0.665893267	0.83540606	0.14376739	0.748560516
CSE1L	0.106311309	0.145224857	0.454441906	0.351733582	0.261253507	0.143647255	0.748560516
DEDD	0.244056663	0.403088577	0.151586232	0.078960141	0.533980558	0.141696015	0.748560516
DENND1C	0.215621502	0.180577119	0.312367659	0.15715722	0.33167787	0.142336303	0.748560516
DGCR8	0.446011468	0.009274179	0.22961246	0.759355628	0.898893731	0.144079509	0.748560516
DHX57	0.726916584	0.161179088	0.284977909	0.896133954	0.021271252	0.142640345	0.748560516
DPH7	0.231417982	0.627384205	0.621846772	0.03437244	0.204792183	0.142527052	0.748560516
EEF2KMT	0.21193985	0.446676552	0.167915219	0.058835858	0.68667196	0.143343442	0.748560516
ELF2	0.057678759	0.367412543	0.690256075	0.076151742	0.577059545	0.143414212	0.748560516
ERBIN	0.716078654	0.923148185	0.577094987	0.015737189	0.105497274	0.142260527	0.748560516
FBXL7	0.049033935	0.765499554	0.345693285	0.246244235	0.196977739	0.141772916	0.748560516
FBXO41	0.364600369	0.092822268	0.899812271	0.422252411	0.049125126	0.142054954	0.748560516
GRB10	0.271939554	0.428147475	0.326965445	0.224058161	0.075867171	0.143937169	0.748560516
GRPEL1	0.241709871	0.252888693	0.1357042	0.533690449	0.145918193	0.143798904	0.748560516
IL1RAP	0.496507075	0.73587248	0.844330676	0.002052242	0.991599901	0.141575142	0.748560516
KIT	0.222270705	0.53026566	0.285137891	0.084716472	0.225696475	0.143385908	0.748560516
KLF11	0.210590382	0.859599993	0.722808896	0.151261895	0.031905811	0.142030095	0.748560516
KMT5B	0.029471005	0.907628881	0.688201862	0.207237496	0.167086101	0.142758135	0.748560516
KPNB1	0.783254297	0.279160339	0.762215879	0.018930502	0.203188899	0.143201281	0.748560516
LEF1	0.515176553	0.259693701	0.424383605	0.174085946	0.063455089	0.141504083	0.748560516
LIX1	0.240390575	0.907147757	0.382523765	0.042545984	0.181793439	0.143704189	0.748560516

LOC100299503	0.190602471	0.208590196	0.300990989	0.069951955	0.754424224	0.1420361	0.748560516
LOC101903877	0.823840656	0.358496459	0.241433132	0.335125555	0.026697626	0.1428258	0.748560516
LOC101905875	0.348172854	0.917136769	0.032097653	0.102223297	0.615062199	0.143610581	0.748560516
LOC101906120	0.257388401	0.608531771	0.008622504	0.790034956	0.600783999	0.143196553	0.748560516
LOC112446351	0.43138038	0.501298797	0.018299575	0.178999832	0.907985084	0.143458987	0.748560516
LOC781977	0.024545742	0.179171544	0.263911007	0.648042188	0.833939029	0.141510134	0.748560516
LOC784148	0.617134737	0.48710375	0.056837874	0.377002652	0.099874873	0.143478887	0.748560516
LOC788736	0.570188893	0.637759333	0.020786979	0.492250096	0.171279667	0.142745736	0.748560516
LOC789694	0.280222565	0.290545094	0.677715542	0.02186304	0.533296924	0.14347957	0.748560516
LSM4	0.255943913	0.695645162	0.609812303	0.027118133	0.217947238	0.143280976	0.748560516
MMP16	0.390607969	0.283301997	0.174448518	0.192884942	0.168801974	0.141669222	0.748560516
MTCH2	0.568607652	0.43336658	0.325784222	0.059566797	0.133559966	0.142910748	0.748560516
NCLN	0.126978523	0.95202505	0.462491957	0.20850908	0.055619657	0.144091355	0.748560516
NDC80	0.646095721	0.031809631	0.069131217	0.501498633	0.910003841	0.14409254	0.748560516
P4HA3	0.42939813	0.225461809	0.084155389	0.576735908	0.13644002	0.143207973	0.748560516
PGLS	0.208561667	0.833871277	0.649470637	0.07300838	0.076845751	0.14230303	0.748560516
PGM2	0.288589153	0.36870704	0.197591464	0.039576009	0.754316925	0.14155911	0.748560516
PITPNA	0.568130167	0.187904297	0.062665136	0.271706667	0.355619454	0.143849482	0.748560516
PTPN13	0.204940464	0.212672564	0.490772011	0.051161129	0.578370144	0.142209974	0.748560516
RANBP1	0.126206852	0.498889019	0.479640656	0.543857825	0.038285755	0.141703353	0.748560516
RBKS	0.229769222	0.785603985	0.052187219	0.097704283	0.687018331	0.142134345	0.748560516
REEP1	0.383851067	0.009063449	0.253582701	0.736325409	0.992211167	0.143624736	0.748560516
RFWD3	0.308623514	0.974758746	0.073808732	0.092483294	0.314269389	0.143723767	0.748560516
RWDD1	0.590545455	0.32638049	0.114589158	0.134122645	0.218016937	0.143780406	0.748560516
S100A1	0.825162345	0.213837118	0.029963878	0.658111872	0.184212926	0.14319117	0.748560516
SERTM1	0.032031278	0.963595209	0.777187974	0.484439336	0.055009409	0.14298121	0.748560516
ST6GALNAC4	0.878514862	0.014457711	0.204467033	0.416198929	0.596465289	0.143644249	0.748560516
TIMM21	0.668065932	0.806475924	0.381563576	0.010500127	0.29053547	0.141497871	0.748560516
TMEM168	0.870498969	0.22455282	0.680616241	0.062123631	0.075748232	0.141363948	0.748560516
TMEM88	0.823788417	0.861782163	0.149510057	0.01291345	0.464744364	0.142706562	0.748560516
TOR2A	0.614103629	0.370506475	0.007484613	0.478681955	0.790442013	0.14360205	0.748560516
TRPT1	0.415661118	0.754689956	0.31497203	0.010249092	0.638735489	0.143901812	0.748560516
TYK2	0.642193355	0.432859981	0.3452398	0.056689506	0.115881953	0.14190427	0.748560516
VPS8	0.390221565	0.27841549	0.184043313	0.145335496	0.219936152	0.142967152	0.748560516
ZBTB16	0.463862068	0.886947892	0.047926141	0.187047345	0.174605029	0.143555913	0.748560516
ZFP41	0.920004676	0.321119937	0.560404914	0.055382422	0.070679231	0.144052858	0.748560516
CENPN	0.246911688	0.251952266	0.175241245	0.226640339	0.263334713	0.144363547	0.74898516
DUSP14	0.722138177	0.029220624	0.254730176	0.137908792	0.878161826	0.144402477	0.74898516
LCK	0.066305934	0.926095706	0.361413668	0.880882924	0.033221691	0.144220874	0.74898516
RECQL	0.80407214	0.031241475	0.147237178	0.182684905	0.962812518	0.144353931	0.74898516
TUB	0.959470741	0.72063987	0.082519828	0.024071105	0.473404059	0.144308326	0.74898516
GAS8	0.109197899	0.309868814	0.542565655	0.086648522	0.410273527	0.144605739	0.749565631
NR2C2	0.82187815	0.547696373	0.625076548	0.005317043	0.436069829	0.144574499	0.749565631
LOC616840	0.043418916	0.735329218	0.055360614	0.781702806	0.473399021	0.144778259	0.749986114

PHPT1	0.323285113	0.472108236	0.836945605	0.005587674	0.916190707	0.144762076	0.749986114
ANKRD55	0.623595472	0.195978396	0.020415941	0.337622996	0.777117204	0.144845089	0.750020181
ATP5ME	0.230677477	0.673875667	0.923993628	0.00482368	0.946229709	0.144959428	0.750020181
BZW1	0.838885367	0.131862933	0.323176398	0.541480699	0.034398462	0.146190817	0.750020181
C29H11orf80	0.167287593	0.206736413	0.234211003	0.635899836	0.12896316	0.145999908	0.750020181
CACUL1	0.861127547	0.01990907	0.059877241	0.654560379	0.978226852	0.14516663	0.750020181
CC2D1B	0.078992975	0.158874113	0.087937095	0.62312347	0.969556154	0.146295773	0.750020181
CCDC62	0.748228302	0.159386059	0.731040495	0.010661819	0.707749659	0.145233424	0.750020181
COX5B	0.565367405	0.639320918	0.527292938	0.004233844	0.82553107	0.14622427	0.750020181
CPEB4	0.140609857	0.070157376	0.735108834	0.118003846	0.778887533	0.146268488	0.750020181
DNAJB14	0.885038875	0.125311057	0.728491834	0.012018878	0.674887724	0.144930896	0.750020181
GPS1	0.408064144	0.59322513	0.326048443	0.068080385	0.122655977	0.145379689	0.750020181
HDAC9	0.010107538	0.365872325	0.934213413	0.522150529	0.369949504	0.146368686	0.750020181
HSDL1	0.904615693	0.021334554	0.149263935	0.622456255	0.372218203	0.146377801	0.750020181
LARS2	0.498456413	0.885353078	0.90531549	0.012044606	0.137108276	0.145463268	0.750020181
LAX1	0.661316622	0.9698256	0.014548064	0.118463993	0.600177579	0.145896195	0.750020181
LOC101902656	0.281677415	0.640318383	0.015933609	0.752245537	0.306044424	0.145683075	0.750020181
LOC104973054	0.675065518	0.42276316	0.031611904	0.206046706	0.353596498	0.145166234	0.750020181
LOC112444279	0.114198656	0.966463215	0.114380746	0.191172034	0.276580487	0.146384415	0.750020181
LOC112447360	0.356278067	0.841470674	0.053483335	0.050517036	0.814796254	0.145487584	0.750020181
LURAP1L	0.364760243	0.336894776	0.035311289	0.46869367	0.324191058	0.145409658	0.750020181
MYOCD	0.068057895	0.474704515	0.943316872	0.153059594	0.141905091	0.145721691	0.750020181
NIPSNAP1	0.574391808	0.699846348	0.255206318	0.013073758	0.492489545	0.145554324	0.750020181
NT5DC3	0.593789535	0.115916475	0.048700891	0.522909628	0.379236138	0.146056281	0.750020181
OGT	0.933335885	0.03154358	0.167593169	0.6956694	0.192875682	0.145734024	0.750020181
OSCAR	0.069453232	0.278787044	0.106828106	0.328050761	0.976983493	0.145842249	0.750020181
PDHX	0.84674362	0.950280436	0.124704846	0.029889393	0.221716016	0.146083737	0.750020181
PURB	0.614088699	0.16622307	0.049412941	0.168414002	0.772137046	0.144996955	0.750020181
SMC4	0.574171776	0.056493431	0.057484183	0.373818656	0.949099817	0.145674829	0.750020181
SYAP1	0.916964557	0.040955932	0.100476659	0.199736885	0.875565827	0.145478148	0.750020181
TRUB2	0.329534932	0.464063302	0.432559338	0.012453143	0.808993891	0.146256952	0.750020181
TTC19	0.783601007	0.381521379	0.423781202	0.15078549	0.034601319	0.145610436	0.750020181
TUFM	0.517675285	0.86663132	0.625384506	0.019741715	0.1184473	0.145017528	0.750020181
USF3	0.366414525	0.381163242	0.592442593	0.035604469	0.22347959	0.145294443	0.750020181
WFS1	0.188301401	0.107900531	0.294184312	0.272434112	0.408694228	0.146148699	0.750020181
ZCCHC6	0.41464162	0.147659709	0.378257404	0.035641491	0.803663523	0.145892187	0.750020181
CAP2	0.010809201	0.248859134	0.434491343	0.861363934	0.664739783	0.146589839	0.750048694
CD300LB	0.113237327	0.491006485	0.092143693	0.789399545	0.165372699	0.146541549	0.750048694
LOC512869	0.576980269	0.901846222	0.119734359	0.014080825	0.762918681	0.146599359	0.750048694
RBM48	0.127490773	0.832186119	0.019260819	0.868645249	0.377360058	0.146664204	0.750048694
TECR	0.462731898	0.947651104	0.466323238	0.033615473	0.097280139	0.146527851	0.750048694
TRA2B	0.664190387	0.980939017	0.434215126	0.004543906	0.520837428	0.1466273	0.750048694
MRPL33	0.474511048	0.041053883	0.412549844	0.083451737	0.999463479	0.146720496	0.750102821
ACAD9	0.260568627	0.606641875	0.292323615	0.095671176	0.1524171	0.14713437	0.750581954

LOC617785	0.113092633	0.75244297	0.025890656	0.387703694	0.787813864	0.147031688	0.750581954
PLCL2	0.939736163	0.077733033	0.20095484	0.095631874	0.478424637	0.146875898	0.750581954
RINL	0.322057094	0.076800528	0.352754491	0.224469964	0.343581279	0.147028535	0.750581954
SUOX	0.700318484	0.789998257	0.42792372	0.009345574	0.303858035	0.146956425	0.750581954
TRAK1	0.878193603	0.018302834	0.140231873	0.624026443	0.479020847	0.147130608	0.750581954
ZBED5	0.95627833	0.189227821	0.152683307	0.031908489	0.763664826	0.147067588	0.750581954
GLT8D2	0.078529644	0.367095383	0.047811683	0.654991055	0.748282571	0.147339355	0.750851131
LOC789748	0.248703437	0.212904226	0.662453324	0.92152785	0.020898765	0.14733988	0.750851131
PNN	0.931192891	0.095462997	0.130801006	0.146181287	0.397591683	0.147370148	0.750851131
SON	0.31316082	0.720665441	0.391001268	0.013406463	0.571053192	0.147343493	0.750851131
NR2F6	0.037999198	0.713332126	0.480763945	0.080694186	0.643349415	0.147457128	0.75106112
LOC101904357	0.5906005	0.568080338	0.727208217	0.003383135	0.822236617	0.147713541	0.752133701
LOC101907843	0.74743321	0.180200192	0.015631226	0.417581173	0.773495022	0.147868662	0.752456624
LOC104969050	0.543670332	0.164239399	0.959706919	0.578658235	0.013707088	0.147831716	0.752456624
LOC100337457	0.738003307	0.647862749	0.136773026	0.247070464	0.042164449	0.148014543	0.752567287
METTL21A	0.015073886	0.456118232	0.31808597	0.323663607	0.963056378	0.148066971	0.752567287
ROCK1	0.133376535	0.372359018	0.137799406	0.297103471	0.33489326	0.147976442	0.752567287
THBS1	0.308038735	0.618354786	0.528303656	0.020496913	0.33053297	0.148073839	0.752567287
LOC107131471	0.652516742	0.122204904	0.326678279	0.628123731	0.041729652	0.148195972	0.752954828
LOC112448737	0.967399023	0.234802581	0.461109973	0.484624098	0.013464893	0.148275596	0.753126215
FHOD3	0.126619645	0.525419685	0.030246806	0.341739255	0.996060099	0.148450794	0.753316627
TTC25	0.127658285	0.862821546	0.118422242	0.071688465	0.731806755	0.148373318	0.753316627
XKR5	0.60306719	0.737573876	0.003364465	0.675296814	0.677665399	0.148437818	0.753316627
ABCF3	0.318069828	0.463755386	0.614174974	0.0895378	0.085915924	0.149848321	0.753326567
ANAPC15	0.131241494	0.245167761	0.204231643	0.114788139	0.928412957	0.150242999	0.753326567
AURKB	0.022084817	0.765704755	0.28983418	0.710805603	0.198098794	0.14905747	0.753326567
BCL6B	0.017218264	0.811594092	0.208280917	0.255693394	0.931444388	0.149414026	0.753326567
CSNK2A2	0.539863466	0.790898366	0.16649817	0.026406181	0.373045679	0.150240365	0.753326567
DENND2D	0.825926271	0.174755731	0.413442137	0.128003784	0.090900815	0.149548869	0.753326567
DIRC2	0.874433418	0.86996511	0.015666859	0.126034944	0.461191294	0.149363464	0.753326567
EMC6	0.122866014	0.332770774	0.303759371	0.097583598	0.567835323	0.148828743	0.753326567
FXR1	0.174008805	0.604726522	0.209263702	0.056698784	0.551375524	0.148854638	0.753326567
IZUMO4	0.995400372	0.283849147	0.046943186	0.71827419	0.073122484	0.149813889	0.753326567
KCNT1	0.561376421	0.075598606	0.096581487	0.672081118	0.251987519	0.149527354	0.753326567
KCTD15	0.936453757	0.290964308	0.148959076	0.027879813	0.617967865	0.150122009	0.753326567
KIAA1211	0.207780185	0.144327374	0.409973982	0.143768023	0.396088647	0.15021832	0.753326567
LCP1	0.187447576	0.221071991	0.20646019	0.166502278	0.487923702	0.149631616	0.753326567
LOC112447844	0.440937399	0.468678988	0.397365854	0.315069796	0.027035442	0.150147142	0.753326567
LOC787858	0.789577728	0.05268424	0.139753079	0.16085944	0.747847808	0.150130981	0.753326567
MFSD13A	0.152907895	0.447789923	0.575415569	0.039279516	0.448288253	0.149480174	0.753326567
MRPL15	0.234116454	0.107599261	0.200688131	0.625211008	0.217093762	0.148593372	0.753326567
NEO1	0.350948892	0.341981315	0.024938922	0.403508597	0.574109628	0.14943589	0.753326567
NPHP4	0.752107246	0.21936589	0.058748889	0.857004263	0.082929461	0.148909404	0.753326567
NUP160	0.171407997	0.287844549	0.673231525	0.027190148	0.762330037	0.148866288	0.753326567

PER1	0.456682154	0.687376896	0.462882558	0.054596341	0.086516212	0.14861264	0.753326567
PGM5	0.041013579	0.13787015	0.442279184	0.653820655	0.424601861	0.149540842	0.753326567
PLAC8	0.953291288	0.12330778	0.416600165	0.181014241	0.078951742	0.15018955	0.753326567
PNPO	0.695957572	0.8745461	0.358341351	0.069362773	0.046139045	0.149974136	0.753326567
PTPRCAP	0.573707846	0.454641037	0.041048657	0.591951956	0.108357769	0.1486616	0.753326567
REPS1	0.850841044	0.147138945	0.022890927	0.422858116	0.569803525	0.149098697	0.753326567
SEC11C	0.41523032	0.188441898	0.932415986	0.169886019	0.055888498	0.149358575	0.753326567
SHCBP1	0.971224302	0.114572426	0.099184941	0.314178469	0.201844404	0.150194883	0.753326567
SLC1A5	0.25741824	0.355233991	0.426115166	0.177810427	0.099494373	0.148964415	0.753326567
SNAI2	0.113609527	0.78211024	0.527458328	0.015961404	0.930823825	0.14977849	0.753326567
TMEM45A	0.969751389	0.350452551	0.00902188	0.288296949	0.786847995	0.149686732	0.753326567
TMEM69	0.44624727	0.489893005	0.057591174	0.107168491	0.511800478	0.149106374	0.753326567
TNC	0.021199807	0.636761224	0.277537962	0.759846938	0.244017418	0.14958611	0.753326567
TTLL12	0.472158808	0.928273829	0.010665916	0.501061601	0.293528089	0.148754018	0.753326567
WISP1	0.656035604	0.027898653	0.113417471	0.384146443	0.87666153	0.150097818	0.753326567
ZBTB47	0.401876266	0.416526786	0.440102504	0.032758441	0.285796057	0.149007516	0.753326567
ZNF385B	0.994579829	0.201985474	0.591608196	0.117467257	0.04957936	0.149294438	0.753326567
ZNHIT2	0.590408669	0.148494808	0.560711239	0.02893922	0.489133283	0.149723984	0.753326567
PTGES	0.834461339	0.08983259	0.090673788	0.339584518	0.304146425	0.150440787	0.754087891
LOC104970976	0.538381645	0.385001399	0.075921873	0.06107987	0.730971712	0.150509274	0.754200825
LOC112444585	0.014986378	0.385431405	0.79688272	0.581601764	0.262670871	0.150576297	0.754234909
LOXL3	0.751200514	0.226684017	0.0580865	0.092135567	0.772320718	0.150651493	0.754234909
PITHD1	0.933558621	0.033131266	0.028855475	0.793722901	0.993604024	0.150653954	0.754234909
LOC112444206	0.765040837	0.350093493	0.149215567	0.449304185	0.039227984	0.150715676	0.754313804
CEBPG	0.727277095	0.04836039	0.167457457	0.4327958	0.276617321	0.150797723	0.754449572
DYNC2H1	0.14686415	0.717313403	0.290300757	0.051026653	0.452606913	0.150935612	0.754449572
HEBP1	0.957944886	0.171703109	0.024333839	0.186494473	0.946058207	0.150920957	0.754449572
SCRIB	0.779769054	0.010494077	0.128981803	0.763425268	0.875544422	0.150840287	0.754449572
SIGLEC1	0.465217586	0.771362013	0.983020361	0.009815476	0.20407989	0.150972664	0.754449572
TSSK2	0.712158552	0.962252138	0.378909454	0.006994008	0.389561318	0.151069321	0.754702779
LY6G5B	0.783799124	0.073645876	0.10216605	0.671924219	0.178697862	0.151143848	0.754845309
CD27	0.96419829	0.170152227	0.114054818	0.352596965	0.107509547	0.151283729	0.75531405
LOC101903868	0.242273359	0.874739887	0.017168744	0.390060043	0.500983519	0.151478521	0.756056574
CATSPERG	0.965204019	0.09793909	0.04117771	0.271699291	0.673069944	0.151574236	0.756165541
RBMX2	0.602535339	0.058164686	0.216310904	0.602390061	0.155913926	0.151592507	0.756165541
FMO3	0.60840493	0.315584538	0.080162061	0.163324858	0.283555737	0.151683937	0.7563917
CKMT2	0.244211125	0.773812681	0.738881363	0.834018796	0.006127781	0.151775787	0.756619817
ASF1A	0.705624187	0.059936111	0.802374652	0.083719835	0.251664527	0.151933291	0.7567154
HAGH	0.583816362	0.871084774	0.467266629	0.005646481	0.532681512	0.151906354	0.7567154
METTL16	0.868185637	0.230390736	0.144916995	0.049387891	0.499203945	0.151896634	0.7567154
MRPS23	0.6539076	0.352089798	0.40308895	0.031187614	0.247358605	0.152044781	0.757040929
FBXO11	0.361799558	0.848469151	0.087611721	0.069493443	0.383389591	0.152114804	0.757159855
DGKB	0.178392434	0.82041036	0.831952841	0.188853815	0.03123362	0.152304641	0.757415594
MINOS1	0.332737541	0.483180256	0.775591266	0.006631165	0.868360805	0.152281859	0.757415594

PALLD	0.220330976	0.425109817	0.025981081	0.651805609	0.452556104	0.152261146	0.757415594
ARID4B	0.559848413	0.296667193	0.096732156	0.065337876	0.685438705	0.152454189	0.757929626
CBR3	0.158788202	0.536261503	0.32481564	0.287230734	0.090882967	0.152738568	0.758016905
LOC101904087	0.377808859	0.236985505	0.037011033	0.272000336	0.799928118	0.152624883	0.758016905
NAMPT	0.267051248	0.061242959	0.084504976	0.975292022	0.535064088	0.152649144	0.758016905
NR2F2	0.265900369	0.870853924	0.005052135	0.776270861	0.794975461	0.152731179	0.758016905
NUPR1	0.963582617	0.438341343	0.027009221	0.131971429	0.479629849	0.152748882	0.758016905
PAICS	0.45082053	0.053381714	0.692189441	0.115026324	0.376497476	0.152669416	0.758016905
DMBT1	0.493458844	0.674707989	0.10564834	0.758477989	0.027103742	0.152863113	0.758215884
FANCM	0.367219803	0.268975581	0.065995565	0.231039015	0.480779232	0.152973785	0.758215884
KCNE3	0.181614014	0.180481414	0.209211452	0.203320499	0.519005159	0.152923552	0.758215884
PSD	0.723413494	0.189533825	0.706968103	0.024223854	0.308307272	0.152957729	0.758215884
ANKLE2	0.419341898	0.037898206	0.210169447	0.280997064	0.773645918	0.153204791	0.758219404
GLRX	0.100488945	0.187044418	0.738503352	0.192376641	0.271790411	0.153167048	0.758219404
KAZN	0.853550271	0.892204032	0.060198673	0.170963919	0.092644492	0.153205505	0.758219404
LOC100847454	0.490932146	0.428803456	0.186463135	0.071230976	0.259558618	0.153162314	0.758219404
USP7	0.81908799	0.481095999	0.031201636	0.096001653	0.61456092	0.15312549	0.758219404
ARHGEF12	0.463319544	0.164287279	0.235751623	0.51516182	0.079423166	0.154125286	0.758402943
ATXN7L2	0.788469764	0.186312055	0.024299403	0.872057577	0.234013887	0.153472653	0.758402943
BRI3BP	0.280763053	0.373763226	0.420125731	0.12483744	0.133662252	0.154285723	0.758402943
CABP1	0.063524896	0.731525006	0.082331359	0.668780448	0.287216612	0.154202002	0.758402943
CCT5	0.569491767	0.332014419	0.269180972	0.137982614	0.104179275	0.153832102	0.758402943
CLU	0.239380135	0.529190152	0.721019098	0.016103969	0.498434505	0.154002574	0.758402943
CXXC1	0.741690675	0.027676677	0.046887686	0.99964164	0.761752851	0.153977054	0.758402943
DDX54	0.15479555	0.985870928	0.12857782	0.409750277	0.09172145	0.154489558	0.758402943
DLX5	0.012487368	0.193500057	0.684058443	0.906247317	0.492537015	0.154527099	0.758402943
FTH1	0.19215801	0.031849244	0.217810163	0.833386712	0.654545962	0.153323078	0.758402943
HNRNPD	0.643686177	0.27285439	0.014826723	0.860601801	0.325579764	0.15360628	0.758402943
ITGB1BP2	0.798351959	0.441526778	0.237580082	0.07503701	0.115932648	0.153479195	0.758402943
LOC100297399	0.296354491	0.613875456	0.09811247	0.051923103	0.787248359	0.153602169	0.758402943
LOC112442745	0.065131882	0.754049448	0.108602417	0.345394257	0.400770942	0.154587128	0.758402943
LOC112446481	0.783585894	0.164386417	0.873825262	0.844646325	0.007769816	0.154628984	0.758402943
LOC768255	0.525331249	0.80797463	0.193004652	0.386088415	0.023110023	0.153754177	0.758402943
LOC786065	0.705336276	0.224461067	0.041235998	0.358859093	0.312171195	0.153800836	0.758402943
MMAB	0.483352406	0.750904385	0.351991191	0.02072999	0.278863934	0.154611546	0.758402943
MTG1	0.854266552	0.099980189	0.209545498	0.045570889	0.893892672	0.153539338	0.758402943
NGF	0.820306872	0.218736143	0.097202229	0.068080107	0.618189429	0.154103279	0.758402943
NPR2	0.719071108	0.143344783	0.845328806	0.073558604	0.114628	0.154177547	0.758402943
NUMB	0.850689951	0.693213585	0.808652634	0.053945996	0.028484748	0.153961395	0.758402943
PDXK	0.294823222	0.956419562	0.039188707	0.549912756	0.120758694	0.15407813	0.758402943
PLA2G2C	0.617814126	0.255401514	0.019293132	0.456877726	0.53059023	0.154548632	0.758402943
PMM2	0.176020328	0.507943931	0.621523714	0.054401917	0.243185305	0.15423188	0.758402943
RBM10	0.59720745	0.015411531	0.263384139	0.548706684	0.553914199	0.154414648	0.758402943
SCMH1	0.04647381	0.817396787	0.594123471	0.337282198	0.095538051	0.153335487	0.758402943

STARD7	0.727966507	0.18243369	0.067999071	0.122934111	0.659460663	0.15388672	0.758402943
TMEM170A	0.697398125	0.196424252	0.059481042	0.117885332	0.765132663	0.154205714	0.758402943
WDR53	0.892938182	0.181860674	0.011296679	0.67759451	0.593558355	0.154529676	0.758402943
FAM189A2	0.271681535	0.336552024	0.036471653	0.240124652	0.925428463	0.154894441	0.75858032
FBXL22	0.060785332	0.327359495	0.401134669	0.155859001	0.597993028	0.155219835	0.75858032
LAP3	0.666089774	0.506543752	0.013923516	0.281418294	0.560408647	0.154876871	0.75858032
LOC101903261	0.779712143	0.770802328	0.07391072	0.480282346	0.034866186	0.155209049	0.75858032
LOC529196	0.308884233	0.262151175	0.299729047	0.052106974	0.587248747	0.155076208	0.75858032
MCFD2	0.799186364	0.031374863	0.154576918	0.686270901	0.279520192	0.155170137	0.75858032
REEP4	0.038792697	0.696805672	0.242536062	0.157734797	0.716760563	0.154912133	0.75858032
S100A2	0.270040029	0.06215693	0.162641534	0.413724675	0.658047032	0.155138284	0.75858032
SINHCAF	0.259165879	0.552270569	0.083391289	0.182735623	0.341021644	0.155203237	0.75858032
SQOR	0.498334082	0.635548479	0.069937761	0.572612384	0.058585596	0.155121969	0.75858032
TMEM189	0.534730713	0.299350803	0.03562343	0.140672526	0.924839534	0.154986473	0.75858032
TNFAIP2	0.277563103	0.301671723	0.300829939	0.690876459	0.042661893	0.155050011	0.75858032
MT1E	0.490589711	0.523720743	0.626623479	0.182354976	0.025389183	0.15538313	0.759152291
EFNA4	0.485303245	0.153173375	0.26468674	0.937434776	0.040489286	0.155540662	0.759469742
RUSC1	0.145703589	0.274197515	0.299508445	0.071722061	0.869785133	0.155501727	0.759469742
ATP5PD	0.472493687	0.388043827	0.446488381	0.009762426	0.939267737	0.15596936	0.759573248
LOC786474	0.178970944	0.872663826	0.849004558	0.043677281	0.12962415	0.15597842	0.759573248
NT5C3B	0.471282857	0.159524876	0.106271506	0.205974871	0.455615359	0.155873947	0.759573248
SUSD3	0.090077983	0.095505045	0.250557139	0.365731577	0.951309119	0.155892535	0.759573248
TMED7	0.4142062	0.161975493	0.275093836	0.681085261	0.059661599	0.155893936	0.759573248
TRIM11	0.686486145	0.160567792	0.381476935	0.024477543	0.727398699	0.155750433	0.759573248
UEVLD	0.490127283	0.086359517	0.117012546	0.806447667	0.1877486	0.155886467	0.759573248
USF2	0.167548342	0.374457824	0.075228801	0.573259562	0.276483063	0.155682638	0.759573248
ZPBP2	0.273381722	0.702696029	0.786945264	0.384962392	0.012885708	0.155887176	0.759573248
ADM	0.672409926	0.745344735	0.132346805	0.043982308	0.260804261	0.157104131	0.75965071
AGBL2	0.43620531	0.03656875	0.091545489	0.772974269	0.670402785	0.156646841	0.75965071
AK2	0.948032333	0.99497077	0.174857488	0.559665143	0.008168763	0.156349511	0.75965071
ATP1B1	0.382323719	0.860020687	0.354227035	0.017857601	0.364779637	0.156867088	0.75965071
BAG1	0.8224595	0.055652903	0.191138655	0.308361492	0.279193829	0.156255679	0.75965071
BLOC1S1	0.238853437	0.64459879	0.507415982	0.019101562	0.503472206	0.156045674	0.75965071
BZW2	0.996578362	0.452529855	0.32368455	0.085869013	0.060310887	0.156564362	0.75965071
DHX29	0.74999607	0.519998145	0.571738986	0.461332723	0.007367407	0.156772496	0.75965071
DNAJC21	0.960125729	0.017584345	0.435884964	0.963773112	0.106992155	0.156882089	0.75965071
LOC100335990	0.350756151	0.763707646	0.152569014	0.052837917	0.351653419	0.156941502	0.75965071
LOC100847700	0.128479206	0.546314502	0.543456241	0.090625507	0.217787298	0.156218255	0.75965071
LOC785842	0.457138414	0.814534364	0.003491709	0.816826579	0.716487394	0.157111146	0.75965071
LOC787122	0.390599608	0.841004437	0.515078678	0.005136502	0.874646362	0.157027691	0.75965071
LYN	0.653858641	0.37114526	0.429397227	0.015319359	0.474660896	0.156757682	0.75965071
NACC1	0.349648573	0.933367041	0.786018155	0.011643316	0.251845089	0.156141728	0.75965071
ORAI2	0.228442795	0.75224549	0.971147314	0.823897355	0.005508421	0.156721128	0.75965071
PCED1B	0.390605137	0.314390364	0.33455157	0.107909782	0.170027984	0.156320361	0.75965071

POLG	0.675600435	0.01320483	0.240489991	0.442192495	0.802436419	0.157151555	0.75965071
POP1	0.583330205	0.388890326	0.698392416	0.217806681	0.021835975	0.156288162	0.75965071
PRPS2	0.331094722	0.128491498	0.244382161	0.323663626	0.224828288	0.156627891	0.75965071
SCPEP1	0.628425783	0.105995137	0.076756943	0.261151069	0.569106398	0.156996509	0.75965071
SLC35G2	0.538523754	0.085647062	0.055738693	0.422616788	0.699576083	0.15701842	0.75965071
TERF2IP	0.206489117	0.088606833	0.544800493	0.245015605	0.310652333	0.156866058	0.75965071
TIE1	0.414848859	0.380884222	0.067517889	0.462901454	0.153289385	0.156678505	0.75965071
TUBB4A	0.900820141	0.69412155	0.377502198	0.021228079	0.150691962	0.156463933	0.75965071
KDELC1	0.328325777	0.378331512	0.05282713	0.1866146	0.62270182	0.157291119	0.759654078
LOC100141145	0.105277605	0.048822079	0.464160376	0.988999415	0.322837055	0.157201657	0.759654078
PRAP1	0.815179117	0.520437501	0.163469891	0.018965742	0.579425518	0.157245773	0.759654078
SEC23B	0.227827074	0.034484783	0.945872896	0.444826006	0.2309725	0.157399143	0.759952144
PPP1R11	0.961545512	0.030323665	0.137652488	0.193638288	0.982943633	0.157445691	0.759953306
CREB1	0.349378962	0.32458554	0.406003547	0.017173336	0.966944978	0.157515075	0.760064658
ADD2	0.017622247	0.517000991	0.332335432	0.85735904	0.295776618	0.157874075	0.761036221
ADGRD1	0.839826384	0.391222299	0.025694323	0.220671925	0.412288604	0.157901915	0.761036221
CLBA1	0.541073961	0.20584154	0.162868273	0.182831597	0.231377329	0.157823861	0.761036221
ZNF326	0.889184993	0.78172682	0.220452719	0.011805133	0.424468187	0.157878509	0.761036221
RGN	0.098555487	0.849739162	0.130418604	0.122663319	0.573748898	0.157968954	0.761118113
UBA6	0.046988756	0.62293832	0.558823025	0.114775826	0.409635756	0.158011664	0.761118113
ARHGAP27	0.713919198	0.141996952	0.175998536	0.259758642	0.166414443	0.158253	0.761163536
DENND5A	0.718653498	0.300606097	0.092211865	0.05709477	0.677772134	0.158211215	0.761163536
LOC107131728	0.169791941	0.526223924	0.557416239	0.119616867	0.129402682	0.158214888	0.761163536
SPSB1	0.059670706	0.235878207	0.328694053	0.456747943	0.364671175	0.158178946	0.761163536
TDRD3	0.122253014	0.445951221	0.170139722	0.230259818	0.36063912	0.158144741	0.761163536
CCDC136	0.881600914	0.454844024	0.0652929	0.500135011	0.059126177	0.158579284	0.76157528
CFL2	0.941955556	0.046478666	0.045861191	0.632949304	0.611400584	0.15888403	0.76157528
CYSTM1	0.86295424	0.299408509	0.03072356	0.13235818	0.740716552	0.159021642	0.76157528
DNAAF3	0.45541771	0.443490091	0.506554793	0.047185271	0.160731011	0.158766944	0.76157528
ESAM	0.223688745	0.255241128	0.336905983	0.375802173	0.107764427	0.159102427	0.76157528
IQCC	0.240976634	0.727059798	0.009796718	0.458765717	0.989235564	0.159098133	0.76157528
LOC516442	0.956572274	0.107727291	0.861109804	0.800929678	0.010969497	0.159170097	0.76157528
MICAL2	0.158423797	0.288112326	0.205869106	0.164886879	0.501233551	0.158840049	0.76157528
NEDD9	0.300704784	0.436310249	0.536423539	0.030489051	0.360571854	0.158522909	0.76157528
OXSM	0.849204929	0.986154654	0.500687582	0.002852491	0.64608911	0.158417687	0.76157528
SAMD4B	0.855815486	0.434574331	0.726755845	0.008635549	0.333986174	0.159163823	0.76157528
SEMA6C	0.54076593	0.323601318	0.015503582	0.473378887	0.605523828	0.158956069	0.76157528
SHARPIN	0.753574463	0.040976783	0.445313909	0.071370563	0.790071285	0.158706118	0.76157528
SHLD1	0.234329529	0.537366841	0.865223675	0.082363727	0.086475439	0.158772555	0.76157528
SLC12A2	0.427858056	0.604849502	0.844261517	0.003819266	0.930565204	0.158830467	0.76157528
TNFRSF21	0.007358925	0.846799285	0.388377569	0.619674187	0.516158588	0.158565206	0.76157528
UQCR10	0.558891587	0.675737987	0.423468294	0.008097428	0.602044719	0.159173921	0.76157528
USP19	0.408235216	0.070852191	0.169116373	0.263240167	0.600639393	0.158490853	0.76157528
LMX1B	0.31584211	0.741885059	0.246328039	0.73086521	0.01853954	0.159439809	0.762171579

LOC104970812	0.33758063	0.644073091	0.810277847	0.022573135	0.196656264	0.159437757	0.762171579
LOC107132228	0.918519331	0.040375213	0.202780923	0.125349744	0.830550132	0.159530764	0.762171579
LOC510520	0.246363852	0.280957806	0.081296063	0.545664241	0.254964932	0.159525657	0.762171579
STK26	0.723266315	0.288728128	0.384142313	0.052676723	0.185191305	0.159491312	0.762171579
SYNJ2	0.144603898	0.252783466	0.849131578	0.05358439	0.471060158	0.15958951	0.762230341
ADRB2	0.092520897	0.583403514	0.059841551	0.499641698	0.486121055	0.159706845	0.762471011
BTG3	0.501323037	0.039541918	0.399041569	0.477913664	0.207588771	0.159732822	0.762471011
TEPSIN	0.438839058	0.504752644	0.919696272	0.01051445	0.367318362	0.159951514	0.763292903
CHRNA3	0.578293709	0.240982282	0.209453826	0.276141564	0.098096672	0.160375634	0.76331918
LOC112444773	0.10605734	0.441149904	0.358341287	0.408708346	0.115061815	0.160131072	0.76331918
LOC504773	0.135970761	0.997328474	0.062738399	0.493586829	0.188013836	0.160249829	0.76331918
LRRC10B	0.606705048	0.183948404	0.453916417	0.02512936	0.619718567	0.160181749	0.76331918
PPP2R5E	0.129293916	0.054218048	0.836872255	0.155842122	0.863455589	0.160237091	0.76331918
PPP6R2	0.426285126	0.105601619	0.057535914	0.33658321	0.906920547	0.160368433	0.76331918
RHEB	0.682973647	0.008308771	0.780666716	0.720846875	0.247291313	0.160267144	0.76331918
SYCP3	0.765958001	0.046974506	0.260597751	0.623740712	0.135123472	0.160329201	0.76331918
ZNF521	0.282151282	0.210969431	0.118042331	0.26384212	0.426453172	0.160365146	0.76331918
DENND6A	0.454905684	0.233758293	0.039054723	0.370822274	0.514437057	0.160543943	0.763677287
RXRA	0.417437931	0.556048959	0.560114526	0.010738021	0.567300759	0.160516152	0.763677287
GARS	0.216569112	0.456002349	0.939428349	0.580371686	0.014757598	0.16079886	0.763782949
LOC100337355	0.218796003	0.333134251	0.4178872	0.110315614	0.236207011	0.160699623	0.763782949
LOC515042	0.865784297	0.018671853	0.325365542	0.320313023	0.471607512	0.160793881	0.763782949
RNASE6	0.404267359	0.545661698	0.356099098	0.091387261	0.110451903	0.160615466	0.763782949
TET1	0.904277716	0.536715777	0.013509289	0.26174703	0.462979849	0.160793593	0.763782949
SPARCL1	0.141701294	0.271342244	0.314642237	0.694564604	0.094730719	0.160949807	0.764278729
TLR10	0.281457301	0.482530329	0.669635755	0.013785754	0.635604039	0.16104512	0.764510112
GPR50	0.140967966	0.228901626	0.072803687	0.765470071	0.443564184	0.161127246	0.764678782
CAGE1	0.315878705	0.243957536	0.174731698	0.116722688	0.507803964	0.161176857	0.764693092
CDKL1	0.041430762	0.142088812	0.340104626	0.594066511	0.672739935	0.16139902	0.765243395
LOC101906989	0.797849546	0.145475975	0.365055353	0.226345629	0.083465365	0.161433119	0.765243395
LOC785568	0.155944117	0.076141764	0.269465241	0.300031533	0.834063393	0.161455201	0.765243395
PNPT1	0.648613347	0.534421655	0.441539763	0.024483608	0.213846427	0.161525996	0.765243395
ZNF624	0.1183272	0.416247211	0.303456251	0.261787614	0.204724095	0.161492812	0.765243395
NAA40	0.24361259	0.308681002	0.547636998	0.132061907	0.147474177	0.16160124	0.765378919
LOC112449324	0.99846704	0.35815145	0.750794662	0.003971172	0.75354583	0.161751111	0.765405322
PET117	0.97666	0.906046435	0.004996181	0.298376062	0.609258779	0.161780495	0.765405322
SYT1	0.833419873	0.806608158	0.191766437	0.016014813	0.389351272	0.161793374	0.765405322
WDR12	0.663626672	0.283830428	0.108232077	0.59262236	0.066525897	0.161782439	0.765405322
PLRG1	0.239711392	0.27834294	0.039303356	0.99383382	0.308713628	0.161874046	0.765566274
C26H10orf62	0.786601521	0.226605595	0.017461741	0.575793964	0.449206937	0.161925467	0.765588835
C15H11orf58	0.863929921	0.035960906	0.127970656	0.635413188	0.318987883	0.162009754	0.765697437
CDR2	0.265970613	0.251768334	0.849691963	0.038427092	0.369068588	0.162127439	0.765697437
FOXM1	0.868732864	0.025650587	0.063782291	0.595385179	0.954812082	0.162238773	0.765697437
LOC104972216	0.603910389	0.042680143	0.705408655	0.160281651	0.277369416	0.16227504	0.765697437

OTUD1	0.590533509	0.076211725	0.092506301	0.337749482	0.574229646	0.162182644	0.765697437
PTPDC1	0.461101975	0.28402551	0.181744648	0.136173683	0.248753889	0.162055366	0.765697437
SRP72	0.462906872	0.668299062	0.265686141	0.032797477	0.299380891	0.162138796	0.765697437
BVES	0.09664666	0.419386266	0.751133155	0.510000424	0.052098508	0.162341296	0.765789883
TUBA1C	0.167577658	0.073334959	0.218805485	0.423787042	0.710399541	0.162405504	0.765872624
SLAMF7	0.278916599	0.197835003	0.179773016	0.42856605	0.190527357	0.162453666	0.765879663
NDUFAF5	0.138379275	0.731038289	0.956298635	0.028924466	0.289964781	0.162601023	0.766354217
METTL3	0.232956376	0.365047713	0.787244846	0.012823191	0.946226337	0.162702871	0.766614075
LOC100138864	0.420030129	0.080485302	0.07165685	0.339959494	0.987442764	0.162796522	0.766835168
LOC112441605	0.300965422	0.585143917	0.010873489	0.640700186	0.665658883	0.163168854	0.7676442
LOC112441885	0.642842159	0.73781415	0.523738149	0.037448092	0.087916949	0.163291663	0.7676442
PFKFB4	0.038514365	0.731131845	0.138305679	0.429356364	0.487776822	0.163057284	0.7676442
POLR3K	0.381699279	0.37130066	0.651149171	0.016180651	0.546322044	0.163072243	0.7676442
THEM6	0.340021054	0.899899162	0.782979255	0.007388703	0.462029731	0.16329571	0.7676442
TTLL3	0.279596296	0.133438512	0.120099845	0.474164372	0.384743468	0.163249061	0.7676442
VSIG2	0.313830805	0.257599604	0.645137331	0.337997821	0.046389224	0.163282865	0.7676442
CXHXorf36	0.47163992	0.684031772	0.014537989	0.30543899	0.571816351	0.163432856	0.767849013
SEPT9	0.217102153	0.570755838	0.724324875	0.093181915	0.097901966	0.1633925	0.767849013
DMAC2	0.671359165	0.494176764	0.163953073	0.028094986	0.536578352	0.163522427	0.768049958
IL1RN	0.01574501	0.393033206	0.58631919	0.466409103	0.485214853	0.163640549	0.768384851
NLK	0.894206059	0.178298869	0.084834775	0.233383535	0.260308597	0.163703077	0.768458581
IMMP2L	0.957782298	0.12608255	0.135994042	0.161547993	0.310063835	0.163798585	0.768687039
POLA1	0.048951585	0.349502109	0.21636695	0.268802433	0.827617284	0.16389421	0.768848811
RASSF10	0.244944934	0.830214675	0.031524271	0.630442315	0.203836929	0.163926756	0.768848811
TMEM37	0.187571883	0.085235539	0.199390968	0.335578105	0.770949764	0.164023571	0.769083092
CAPN7	0.605538399	0.219842451	0.383958477	0.049260096	0.328948802	0.164395479	0.769727306
FBXW7	0.609073746	0.108962491	0.425631558	0.06077424	0.482299341	0.164366438	0.769727306
MCEMP1	0.246583384	0.23745016	0.569646308	0.442316265	0.056088112	0.164311719	0.769727306
TMEM25	0.256282452	0.069644472	0.291074101	0.191808603	0.829801434	0.164252523	0.769727306
ZRANB1	0.692838099	0.142323063	0.161350926	0.198234011	0.26252666	0.164369088	0.769727306
AZIN1	0.675111586	0.025712221	0.316895715	0.47317193	0.318830175	0.164566589	0.770308696
CDC37L1	0.63464873	0.190447499	0.047089757	0.173740142	0.841101314	0.164763053	0.770449132
CYP51A1	0.319450142	0.393869165	0.00908551	0.948149884	0.767963201	0.164831327	0.770449132
FCRLA	0.660485903	0.95739874	0.041338113	0.481403575	0.066133448	0.164814315	0.770449132
LOC101904768	0.838045799	0.057905107	0.649643105	0.575218629	0.04586627	0.164763496	0.770449132
TMEM160	0.783035911	0.335162065	0.443314014	0.020449597	0.349168588	0.164658977	0.770449132
LOC533921	0.899560562	0.180820495	0.553737626	0.010137635	0.91448105	0.165108503	0.771305335
SPAG8	0.527935263	0.126811421	0.223239962	0.097784109	0.571333079	0.165103487	0.771305335
RGS20	0.861897757	0.144317237	0.102847177	0.167007328	0.391390488	0.165233648	0.771670291
CIART	0.427947396	0.459812237	0.065126524	0.177455631	0.368617105	0.165452688	0.771700661
FBXL14	0.028467313	0.665031339	0.305052249	0.354533748	0.40913674	0.165390877	0.771700661
SURF6	0.318973502	0.768344312	0.51393336	0.236755918	0.028118193	0.165475268	0.771700661
WDR3	0.944358539	0.429997676	0.348129394	0.098138675	0.060365787	0.165367772	0.771700661
WDR97	0.337334529	0.213112129	0.290547577	0.357543631	0.11203895	0.165288726	0.771700661

CPT1A	0.723366919	0.712445045	0.254865767	0.083268857	0.076726132	0.165544538	0.771766759
QDPR	0.172235737	0.531611663	0.670974218	0.058564565	0.23333567	0.165583496	0.771766759
ATP1A1	0.864883733	0.658030632	0.126813736	0.017996031	0.647883018	0.165787061	0.772233278
CHST10	0.035365698	0.337267026	0.475791141	0.251039572	0.591894839	0.165972941	0.772233278
COL12A1	0.314486282	0.479524303	0.085035527	0.10874699	0.60457366	0.165956695	0.772233278
DPP3	0.160592496	0.873522788	0.609773578	0.278625942	0.035472593	0.166201203	0.772233278
GPN3	0.315137796	0.142862908	0.151591753	0.138966905	0.890919897	0.166153029	0.772233278
IL1RL1	0.543201238	0.284456313	0.28121863	0.020801895	0.934820318	0.166154219	0.772233278
LOC101905267	0.22452822	0.087554455	0.652565607	0.912614838	0.072189791	0.166171542	0.772233278
NFKBIZ	0.23747928	0.164833798	0.113248065	0.946393813	0.200925806	0.165942723	0.772233278
TMEM208	0.423964833	0.641382669	0.328799344	0.093039835	0.101538117	0.166118567	0.772233278
TMEM266	0.648350494	0.729611617	0.900113121	0.007589325	0.261285073	0.166086139	0.772233278
USP6NL	0.224461369	0.886807466	0.580757106	0.009693699	0.753243842	0.166060569	0.772233278
LOC107132949	0.183996275	0.145322207	0.225162484	0.224823699	0.62610037	0.166412858	0.772779122
SHOX2	0.026870595	0.462088528	0.321524253	0.404037189	0.525139498	0.166369949	0.772779122
EME2	0.394075176	0.021486741	0.44385428	0.241303297	0.936426728	0.166596923	0.772917043
LOC101907566	0.114738595	0.318226606	0.586768246	0.046804078	0.847124259	0.166620623	0.772917043
LPCAT3	0.86896275	0.739872103	0.429973985	0.385393564	0.007974247	0.166630949	0.772917043
SLC5A6	0.871116288	0.422911371	0.817957308	0.210682385	0.013381129	0.166627483	0.772917043
CILP2	0.648878992	0.210188428	0.659097628	0.531365893	0.017795807	0.166679754	0.772924961
SH3TC2	0.589170116	0.192461073	0.369689308	0.437625406	0.046384222	0.16677345	0.773140985
GLP1R	0.055959634	0.493912573	0.244089865	0.774912948	0.163071525	0.166939156	0.773472188
PHLDB2	0.278933936	0.927652653	0.573915484	0.869022184	0.006604717	0.166921607	0.773472188
C24H18orf25	0.205251809	0.119856029	0.562902499	0.232777314	0.264770649	0.167038103	0.773595357
LOC100847870	0.312672742	0.152721533	0.307616664	0.067323335	0.863241105	0.167060017	0.773595357
F11R	0.127456514	0.80680223	0.474353808	0.172019653	0.101829735	0.167138903	0.773635663
TMBIM1	0.762538861	0.163730396	0.034858014	0.265887346	0.738602176	0.167163004	0.773635663
LOC101905262	0.380221808	0.115294418	0.771436946	0.052004415	0.486679685	0.167291118	0.773851315
MCRS1	0.289471612	0.292733986	0.810672501	0.077384963	0.161031414	0.16730391	0.773851315
ABHD5	0.881101034	0.42269543	0.0502562	0.220392292	0.212519117	0.16942959	0.773926933
ACYP2	0.563476866	0.656551155	0.089896546	0.049929669	0.530763591	0.169907251	0.773926933
ANGPTL4	0.041709342	0.806593943	0.332132356	0.235491138	0.334970114	0.169914005	0.773926933
ASAP3	0.627950629	0.013279345	0.47069058	0.561003739	0.399288926	0.169688606	0.773926933
C5H12orf71	0.386253176	0.921819202	0.007264722	0.539149463	0.631919851	0.16989908	0.773926933
CACNA1D	0.017417068	0.737030552	0.101325684	0.708933659	0.949349595	0.169300455	0.773926933
CD59	0.769890537	0.023257396	0.136452006	0.38199508	0.944239069	0.169899437	0.773926933
CYFIP2	0.125898696	0.51422946	0.740843629	0.241751425	0.075911146	0.169789674	0.773926933
DUSP15	0.543868984	0.891726352	0.101998781	0.023483285	0.745287181	0.168310357	0.773926933
FAM193B	0.79688183	0.11357383	0.196053791	0.20988689	0.231230343	0.16783316	0.773926933
FNIP1	0.280297679	0.898570812	0.145900208	0.033555674	0.711917789	0.169550695	0.773926933
HAS2	0.416843496	0.962115266	0.010259247	0.23376513	0.908906772	0.169177241	0.773926933
ID2	0.290378995	0.859207662	0.015806006	0.227699797	0.969862087	0.168835802	0.773926933
IFITM3	0.354710971	0.026137995	0.207330146	0.465454164	0.961306758	0.16772471	0.773926933
IQANK1	0.664804507	0.008345338	0.713815286	0.273620379	0.803715956	0.16883923	0.773926933

IQGAP1	0.93887418	0.083052104	0.301918937	0.102900103	0.359164816	0.16875342	0.773926933
IRAK3	0.316150667	0.134912646	0.322816704	0.070070243	0.909009526	0.169464187	0.773926933
KLF7	0.449710034	0.857175742	0.554297304	0.069583594	0.057908299	0.167815803	0.773926933
LIPT1	0.815406133	0.5256094	0.125767146	0.068010094	0.238293035	0.169110071	0.773926933
LOC100848226	0.241697041	0.39752428	0.761391728	0.048653574	0.242909034	0.168186774	0.773926933
LOC101902407	0.560699504	0.212592289	0.016602436	0.998819213	0.439994549	0.16871754	0.773926933
LOC101904275	0.703791229	0.601401706	0.640006312	0.161818698	0.020034384	0.169586641	0.773926933
LOC101908014	0.617982426	0.128708246	0.506811145	0.087092415	0.246477183	0.168265651	0.773926933
LOC104976276	0.020530301	0.751853774	0.187488934	0.933874474	0.321283155	0.168572927	0.773926933
LOC112442047	0.487094964	0.190920411	0.601329109	0.390069946	0.040137713	0.169313145	0.773926933
LOC112446699	0.703878965	0.139758521	0.032777715	0.461691823	0.585064406	0.168846659	0.773926933
LOC112447371	0.381003313	0.030714337	0.137778135	0.571413883	0.938943112	0.168235785	0.773926933
LOC524576	0.956209219	0.836946009	0.041871496	0.030727829	0.854462846	0.16975132	0.773926933
LOC616063	0.615505918	0.811782572	0.559071869	0.06494969	0.047230523	0.16739544	0.773926933
LOC616944	0.614893623	0.920472502	0.00220274	0.979988792	0.712242278	0.168766802	0.773926933
LOC781064	0.384605912	0.824544124	0.167997747	0.034536668	0.476473998	0.16943282	0.773926933
MARK4	0.180523493	0.371041232	0.312545583	0.070825012	0.58700149	0.168781653	0.773926933
MFAP5	0.422101776	0.136030551	0.088917763	0.509543785	0.338511088	0.169834625	0.773926933
MFSD4B	0.319631047	0.444234514	0.702477689	0.057752292	0.151310819	0.168913022	0.773926933
MTPN	0.929056632	0.154945321	0.01118138	0.820861576	0.658645242	0.168769799	0.773926933
NDE1	0.098924404	0.534493535	0.040365073	0.717828785	0.567250087	0.168648274	0.773926933
NRAS	0.639359965	0.033501827	0.144905987	0.290462132	0.973059868	0.169489909	0.773926933
NSUN5	0.245454849	0.054037594	0.310828057	0.695533193	0.304550203	0.169084412	0.773926933
PHETA1	0.01703771	0.475254888	0.755389614	0.153938705	0.920029559	0.168362757	0.773926933
PIAS1	0.025168851	0.586333105	0.83756105	0.076344287	0.933780044	0.169886125	0.773926933
PIGC	0.69193478	0.155251165	0.104451144	0.083841637	0.923740713	0.168643333	0.773926933
POLE	0.782459138	0.161370045	0.194321063	0.19185688	0.184498112	0.168592048	0.773926933
POU2F2	0.356628049	0.267150733	0.18143045	0.967030908	0.051693302	0.168136508	0.773926933
PSMC5	0.378564622	0.651390291	0.184012626	0.164105383	0.115626185	0.167818892	0.773926933
SASH3	0.266371682	0.854646816	0.315068364	0.173486943	0.070622416	0.169646884	0.773926933
SLC23A1	0.113132674	0.406714765	0.378867567	0.665292113	0.074305113	0.167898528	0.773926933
SLC8A1	0.012096934	0.251320657	0.4109089	0.71166699	0.984228752	0.169261255	0.773926933
SLIT3	0.244873099	0.57245045	0.01628239	0.89455012	0.425558992	0.168630683	0.773926933
SMARCAD1	0.82116387	0.263313877	0.120887128	0.901960041	0.037240832	0.169564496	0.773926933
STEAP4	0.669820729	0.465596976	0.00732378	0.461703458	0.817975542	0.167982797	0.773926933
STRIP2	0.53924527	0.741756199	0.141448926	0.026263142	0.585272414	0.168711003	0.773926933
TMEM182	0.99530644	0.074579659	0.91313217	0.895121221	0.014291288	0.168446119	0.773926933
TXNIP	0.737311507	0.817668864	0.047099879	0.135725254	0.227914948	0.169604059	0.773926933
ZBTB22	0.307939665	0.384608867	0.338882165	0.024968864	0.859846428	0.167889677	0.773926933
ZYG11B	0.4197075	0.04921841	0.519418076	0.094249884	0.860017986	0.168716728	0.773926933
MYO7A	0.154840843	0.218651375	0.424993984	0.088281251	0.694312209	0.1699686	0.773960792
COX5A	0.390367773	0.690756434	0.642348776	0.00877158	0.580825144	0.170020216	0.77398107
ADAM17	0.300813756	0.37080566	0.116432564	0.370712057	0.185978825	0.171334474	0.774341876
ADAMTS15	0.222062893	0.479713858	0.24702912	0.157047751	0.216988072	0.17147109	0.774341876

AGAP2	0.323244957	0.383119426	0.012871836	0.570420886	0.987985033	0.171633456	0.774341876
AKTIP	0.762834349	0.121146939	0.023090296	0.667513885	0.631847763	0.171798973	0.774341876
ATP5IF1	0.613773753	0.813019669	0.359308958	0.007017155	0.714946536	0.171750328	0.774341876
CCDC157	0.396958479	0.436664433	0.019536733	0.34078464	0.771753775	0.170852517	0.774341876
DDX46	0.464054004	0.358431796	0.202078761	0.027656503	0.964147461	0.171421554	0.774341876
DHRS11	0.126815555	0.891235327	0.34538065	0.088681284	0.256357628	0.170527868	0.774341876
FAM46A	0.720823109	0.370795931	0.123445651	0.255909523	0.106269922	0.171525795	0.774341876
FBXO6	0.29357467	0.119594529	0.393629224	0.13611882	0.478273205	0.171771091	0.774341876
FOXRED1	0.350294274	0.678070671	0.800562244	0.007321663	0.644307271	0.17149859	0.774341876
GGA1	0.682124859	0.213626831	0.007750385	0.811811917	0.977843986	0.171448979	0.774341876
IRGQ	0.589906818	0.262617135	0.011224322	0.717520441	0.721210933	0.171781893	0.774341876
LOC100139549	0.313941762	0.169678721	0.169718831	0.266382943	0.367841375	0.170368552	0.774341876
LOC101905779	0.995742094	0.669900267	0.009957612	0.408124693	0.32923561	0.171041469	0.774341876
LOC112442218	0.726352037	0.237525451	0.181878669	0.125563338	0.227213374	0.171317198	0.774341876
LOC112442677	0.754728522	0.003739592	0.953867808	0.37005756	0.888737501	0.170321352	0.774341876
LOC112444348	0.290077143	0.552703609	0.131778663	0.399495427	0.10542222	0.170767542	0.774341876
LOC783963	0.178057185	0.145081839	0.751540193	0.080504394	0.566658013	0.170346125	0.774341876
LOC788745	0.550575657	0.145202124	0.621029718	0.312419614	0.057888536	0.171587751	0.774341876
MRPL20	0.287900585	0.169561037	0.384495969	0.294737152	0.160596002	0.170629392	0.774341876
MTRF1	0.974613791	0.867015146	0.352497701	0.226033808	0.013236035	0.170903101	0.774341876
N4BP1	0.401844611	0.354208709	0.076147547	0.114786687	0.722175971	0.171645066	0.774341876
NOP10	0.283225805	0.221984646	0.212384272	0.209688411	0.319871456	0.171357257	0.774341876
NYNRIN	0.87666315	0.157629486	0.022427683	0.333483642	0.86438335	0.171129664	0.774341876
OSBPL1A	0.884063892	0.834990383	0.009570465	0.308631491	0.406734009	0.170467202	0.774341876
PDRG1	0.478676054	0.122955601	0.147871573	0.476746674	0.21548953	0.171203179	0.774341876
POLDIP3	0.409124775	0.05539763	0.120067749	0.501261367	0.659995947	0.171826941	0.774341876
PSMB2	0.25944952	0.344466709	0.381894004	0.31176887	0.084623497	0.171845294	0.774341876
S100A5	0.738748112	0.704355215	0.396649503	0.457008425	0.009482351	0.171233734	0.774341876
SLC4A2	0.562532552	0.250745635	0.158839484	0.056403208	0.706945382	0.171128337	0.774341876
ST6GALNAC3	0.575449865	0.437618424	0.230555488	0.780546145	0.019547306	0.170366594	0.774341876
UIMC1	0.862520304	0.129230636	0.575192975	0.035096759	0.398929192	0.171562294	0.774341876
XBP1	0.468307877	0.220678454	0.332825044	0.031184993	0.833931878	0.171243812	0.774341876
ZDHHC13	0.002555507	0.517780226	0.850147168	0.906354478	0.870562193	0.170543116	0.774341876
ZFP90	0.684157452	0.758667937	0.310634013	0.017999481	0.308384078	0.171290713	0.774341876
ZNF215	0.834180056	0.747631322	0.180427208	0.018457165	0.42965713	0.171025746	0.774341876
CLCN7	0.221681262	0.655097902	0.024899508	0.762221658	0.327411685	0.172040672	0.775009461
CEP350	0.387229236	0.973225669	0.571676066	0.00580369	0.722637549	0.172156761	0.775319596
SHTN1	0.216999054	0.353353156	0.172253683	0.482630736	0.142203912	0.172451029	0.776218825
TIMM17B	0.881876541	0.333553689	0.539824755	0.007380262	0.773387997	0.172437129	0.776218825
LOC104973139	0.376554443	0.017558873	0.46376722	0.680032411	0.435740059	0.172664788	0.776452405
NDUFAF8	0.266922224	0.608540218	0.670912168	0.060148371	0.138659163	0.172692175	0.776452405
PRRX2	0.408033736	0.287385473	0.024885214	0.904642297	0.344184107	0.172662354	0.776452405
ZNF644	0.185600055	0.968536785	0.938122649	0.023542761	0.228760532	0.172625103	0.776452405
C7H19orf71	0.547433667	0.68137027	0.146467652	0.459288874	0.036503585	0.173399342	0.77649915

CDKN2B	0.300623904	0.34531617	0.245991904	0.042292676	0.848721428	0.173465379	0.77649915
CYCS	0.181361339	0.529038967	0.685443626	0.065559018	0.213763257	0.173966599	0.77649915
DRG1	0.860437452	0.232720614	0.327663512	0.994180955	0.014034701	0.173351574	0.77649915
EMC2	0.342818554	0.132152581	0.034372367	0.753301621	0.785583427	0.173954152	0.77649915
ESD	0.26647023	0.021405852	0.586998207	0.287760023	0.955947814	0.17390605	0.77649915
IGF2BP2	0.927591748	0.670903138	0.579217172	0.058249225	0.043828607	0.173826601	0.77649915
LOC101906512	0.716657988	0.150046595	0.330572446	0.595766922	0.043173198	0.173234724	0.77649915
LOC101906916	0.694751088	0.523865716	0.003228717	0.898458159	0.86648145	0.173285395	0.77649915
LOC104976247	0.204333934	0.143185616	0.767296341	0.426905592	0.095106745	0.172951187	0.77649915
LOC107131699	0.627187717	0.053918596	0.87733309	0.304263771	0.102007225	0.173884781	0.77649915
LOC112444290	0.982360758	0.312560568	0.549506388	0.158129585	0.034425407	0.17365042	0.77649915
LOC614129	0.199066074	0.530543088	0.261291724	0.077057072	0.433091806	0.173896221	0.77649915
LOXL4	0.039358295	0.816109842	0.103626243	0.936369843	0.29451993	0.173597034	0.77649915
MACROD2	0.132451617	0.415663712	0.11847777	0.3798443	0.370303551	0.173551324	0.77649915
METTL6	0.13648056	0.257634544	0.699290589	0.232444527	0.159471257	0.172948695	0.77649915
NT5M	0.224805748	0.723396641	0.637802161	0.051134707	0.173072826	0.173596569	0.77649915
NUF2	0.926855515	0.046902041	0.046309243	0.571213891	0.794117385	0.173121105	0.77649915
OLFM1	0.841923108	0.212107626	0.023688538	0.531209141	0.410205117	0.173980097	0.77649915
PPM1G	0.42510655	0.017727909	0.468370431	0.983103152	0.265564496	0.173954837	0.77649915
RAB2B	0.200551218	0.016803444	0.583524332	0.520616739	0.895131457	0.173443477	0.77649915
RNF34	0.859856475	0.090375156	0.632234656	0.761857403	0.024442856	0.173294374	0.77649915
SLC25A12	0.756465595	0.653291476	0.003728134	0.57001296	0.874383704	0.173630253	0.77649915
TMEM225B	0.110457782	0.834029562	0.138596607	0.412185665	0.173229156	0.172971781	0.77649915
TRAF6	0.113974299	0.369762548	0.119233672	0.703406917	0.258791833	0.173274813	0.77649915
YTHDF1	0.621761733	0.315936985	0.140370806	0.039056567	0.851341828	0.173488058	0.77649915
ZBTB18	0.912521438	0.114601996	0.086102333	0.132192442	0.772620655	0.173766965	0.77649915
ATP1A2	0.019343519	0.597428637	0.497204518	0.525236437	0.305806175	0.174090729	0.776570521
CHST7	0.479214991	0.934459799	0.025976251	0.08143458	0.973923487	0.174057689	0.776570521
PSENEN	0.795073234	0.150695841	0.221234126	0.071408726	0.488142383	0.174196459	0.776831004
RAB22A	0.985515544	0.088398278	0.020698145	0.652607073	0.785636592	0.174250093	0.776859081
A1CF	0.023934633	0.547948463	0.510400646	0.879103961	0.157559026	0.174514174	0.777167739
ANKZF1	0.708177584	0.027258115	0.641035012	0.142171823	0.528773656	0.174819423	0.777167739
AZI2	0.664136236	0.444611526	0.012136485	0.94692061	0.273341968	0.174554258	0.777167739
CSRP1	0.200610818	0.308346364	0.901955133	0.151499342	0.110010714	0.174781454	0.777167739
DHX30	0.479360234	0.24656806	0.398618267	0.036934707	0.535081432	0.174905767	0.777167739
FAM171A2	0.710160615	0.31632714	0.008195262	0.634458072	0.797227582	0.174912241	0.777167739
GAK	0.660749719	0.018755995	0.476150937	0.195115009	0.806812153	0.174688486	0.777167739
HSPB7	0.021742321	0.646033863	0.277405688	0.809777166	0.293935826	0.17454239	0.777167739
ITGA10	0.842028802	0.044109745	0.428617529	0.277691659	0.210077008	0.174664759	0.777167739
LIMS1	0.156248343	0.83552259	0.13362407	0.160169554	0.333528847	0.174982316	0.777167739
LOC112446757	0.783895781	0.412806978	0.638978195	0.005436991	0.827282785	0.17479843	0.777167739
NCS1	0.494110388	0.136969422	0.078035902	0.427431839	0.412321085	0.174870569	0.777167739
TCF3	0.609001647	0.27054811	0.593403432	0.012527864	0.755917577	0.17438797	0.777167739
ZMAT1	0.304159833	0.992821393	0.673910222	0.2135362	0.021439334	0.174958178	0.777167739

MCOLN3	0.272452244	0.377933865	0.081566718	0.998388461	0.111257799	0.175083582	0.777196827
TOMM7	0.502203928	0.088876082	0.179323115	0.1265576	0.92089275	0.175072842	0.777196827
NINL	0.990773513	0.015833643	0.406494418	0.178516812	0.820669184	0.175212562	0.777559047
FGFR1OP	0.021236677	0.825333922	0.472978722	0.132839569	0.849374564	0.175324548	0.777635446
NAV3	0.284047471	0.071897366	0.064828601	0.759196915	0.930477146	0.175313108	0.777635446
FAM160B2	0.790855147	0.748971347	0.231069138	0.01075236	0.635918219	0.175372247	0.777636841
ADAM1A	0.66930439	0.056190346	0.061950365	0.755550863	0.532053363	0.175444257	0.777692089
LOC112444314	0.888342266	0.091025092	0.984866469	0.999525517	0.011770672	0.175479484	0.777692089
CD40	0.495213315	0.784716988	0.063837013	0.548909799	0.068936935	0.175653158	0.777733316
EPHA3	0.649259882	0.912894843	0.34428594	0.34171799	0.013472159	0.175723942	0.777733316
LOC104974272	0.450674244	0.413133462	0.646593202	0.07550477	0.103222356	0.175610952	0.777733316
SPART	0.229206412	0.338163131	0.107871432	0.461739705	0.243035176	0.175609553	0.777733316
VLDLR	0.871526883	0.059613396	0.802194934	0.041812063	0.539095793	0.175725741	0.777733316
MAP2K4	0.682704483	0.003044951	0.668816045	0.763989479	0.885211242	0.175807022	0.777756208
MRPL41	0.207311229	0.569770247	0.567093321	0.022008711	0.637925702	0.175825698	0.777756208
MOB3C	0.328969054	0.525111909	0.288550025	0.040388984	0.468297389	0.176053744	0.778555104
ADCK2	0.675305014	0.429001642	0.150892264	0.164318739	0.131385835	0.176149401	0.778628853
SAMSN1	0.362286463	0.735599509	0.196416445	0.048262732	0.373639596	0.176165312	0.778628853
TMEM128	0.518093608	0.068101796	0.091252891	0.506310917	0.579661248	0.176265274	0.778860907
FCF1	0.56147389	0.011059011	0.332333405	0.493984609	0.927658348	0.176332754	0.778861113
UFM1	0.638075364	0.136145264	0.63828479	0.901185765	0.018929835	0.17636024	0.778861113
LOC514257	0.06973407	0.440944681	0.423900084	0.273473064	0.265683394	0.176471523	0.778886545
OSGIN2	0.76384094	0.116056641	0.014152782	0.962305838	0.78485077	0.176523484	0.778886545
RNF115	0.531349152	0.202291692	0.04011911	0.262868831	0.835419258	0.176467534	0.778886545
TRMT10B	0.812435128	0.28894065	0.681677014	0.213489865	0.027746822	0.176555844	0.778886545
HSPG2	0.994987414	0.360804505	0.00825554	0.349608	0.916543664	0.176727412	0.778989069
MYADM	0.747434042	0.01790484	0.934876037	0.236473035	0.321297213	0.176816421	0.778989069
NDUFB2	0.264786088	0.546347595	0.335273449	0.029674329	0.659739469	0.176716123	0.778989069
SULT1A1	0.508951976	0.962658176	0.027763605	0.2628011	0.265847813	0.17679485	0.778989069
TRIM24	0.101314005	0.873094964	0.071985184	0.268340751	0.555715985	0.176715455	0.778989069
COQ7	0.18154435	0.361556191	0.287329121	0.058118569	0.869327512	0.177041169	0.779012442
EPRS	0.73866199	0.434506322	0.88008762	0.431335528	0.007835162	0.177211276	0.779012442
GABRE	0.526452477	0.010262079	0.951125386	0.321682734	0.575611789	0.176902775	0.779012442
KDM5B	0.780393603	0.643743155	0.081625281	0.041184617	0.565773129	0.177296415	0.779012442
LOC101905887	0.306094698	0.904695998	0.742854754	0.007156303	0.646504189	0.176931166	0.779012442
LOC112446663	0.452232282	0.536792743	0.171791106	0.175419826	0.130427604	0.177165463	0.779012442
NDUFS5	0.277609189	0.646191374	0.418063263	0.014658105	0.867376738	0.177102407	0.779012442
PKNOX2	0.258175709	0.928231204	0.304417589	0.051747726	0.253063921	0.177282364	0.779012442
SCAF11	0.141748634	0.581237493	0.862420879	0.014840201	0.905339293	0.177213833	0.779012442
SLC6A1	0.31019142	0.486847391	0.068232299	0.959006195	0.096482249	0.177093602	0.779012442
COX8A	0.631819364	0.62138223	0.462069556	0.005687524	0.927026734	0.177392067	0.779179328
IRAK1	0.257044291	0.077193797	0.493695964	0.547198735	0.178506297	0.177429355	0.779179328
KCNG3	0.684109323	0.786435178	0.006781359	0.287998075	0.912758067	0.177644277	0.77928902
LOC100847345	0.061588318	0.548684195	0.597911418	0.522831582	0.090751044	0.177605788	0.77928902

SOS1	0.25184056	0.247716181	0.878449125	0.021483257	0.814149733	0.177590765	0.77928902
WDR27	0.291183851	0.181739543	0.777522433	0.504560678	0.046138599	0.17752777	0.77928902
GGA2	0.157048611	0.078883564	0.925762682	0.738268056	0.113371038	0.177727151	0.779444219
LMCD1	0.259070448	0.809880795	0.411739822	0.197913097	0.056192983	0.177809178	0.779595625
LOC112446689	0.460504473	0.776241462	0.100460667	0.027970505	0.957373329	0.177892939	0.779754548
CDC42	0.969817959	0.024503957	0.209128041	0.62172517	0.312926693	0.178403068	0.779808017
CRIP2	0.922223618	0.627846215	0.505754883	0.034862381	0.094777936	0.178470732	0.779808017
DYM	0.181274097	0.15027643	0.2002036	0.263010084	0.672332977	0.178161031	0.779808017
LOC112443751	0.858352735	0.053962173	0.156760021	0.73750237	0.180884933	0.178570381	0.779808017
LOC112448014	0.688275193	0.154979658	0.12144057	0.143135119	0.522087871	0.178513071	0.779808017
MINPP1	0.120573085	0.722887105	0.892093844	0.288516675	0.04301504	0.178218548	0.779808017
PALM3	0.247046582	0.097703612	0.105091578	0.836288802	0.454185589	0.17807321	0.779808017
PIAS2	0.43534949	0.653663675	0.221448134	0.025349446	0.603547514	0.178137162	0.779808017
ROM1	0.726365339	0.11706132	0.284081755	0.042530291	0.937561529	0.178043749	0.779808017
SNAPC5	0.519166146	0.588217563	0.353420511	0.013795927	0.649540997	0.178427578	0.779808017
TAGAP	0.372212871	0.677452988	0.498539891	0.224883735	0.034253912	0.178545023	0.779808017
TPI1	0.197463559	0.937749763	0.961911108	0.055734726	0.097413546	0.178419073	0.779808017
TUBGCP5	0.899421015	0.058553351	0.027378061	0.998460074	0.670070124	0.178185367	0.779808017
ZNF446	0.345594308	0.660815196	0.474990269	0.017192493	0.51861457	0.17843217	0.779808017
ACSS2	0.571859817	0.78852051	0.95108537	0.015311394	0.147768485	0.178734733	0.779953915
GMCL1	0.342388315	0.832988022	0.027566393	0.291319451	0.423483783	0.17869705	0.779953915
GNG11	0.175041952	0.614648133	0.126253985	0.695712767	0.102694904	0.178750707	0.779953915
LOC100295797	0.255563803	0.217635996	0.201320127	0.953849368	0.090906684	0.178793896	0.779953915
ADAT2	0.052589776	0.396892128	0.873972936	0.085054207	0.62794372	0.179116767	0.780117664
AGMO	0.448412871	0.560790353	0.005272682	0.911211066	0.80446104	0.178889135	0.780117664
DYNLRB1	0.615604751	0.207561887	0.305499032	0.037049339	0.672782442	0.178992826	0.780117664
EIF2B1	0.4554511	0.48998955	0.376826027	0.306996787	0.037704517	0.179032164	0.780117664
GNAI1	0.089309283	0.964491882	0.055606937	0.656016752	0.310219205	0.179164187	0.780117664
LOC101905167	0.424554796	0.031695634	0.185885528	0.50964212	0.764460475	0.179140709	0.780117664
LOC112444355	0.67876199	0.625894816	0.634046755	0.005155772	0.700657957	0.178998317	0.780117664
WDR77	0.376905331	0.530944791	0.131466634	0.963788755	0.038467584	0.179221721	0.780161183
NFKB2	0.248987138	0.898029324	0.194338027	0.024878838	0.903488521	0.17935232	0.780522655
ALDH5A1	0.270188063	0.761937702	0.15084361	0.077178915	0.408517449	0.179578215	0.780776808
AMMECR1L	0.493419425	0.485441669	0.475880022	0.010071547	0.854099623	0.179715449	0.780776808
CACYBP	0.565932903	0.428867723	0.348442588	0.028660787	0.403656177	0.179512717	0.780776808
CCDC47	0.496278241	0.186380186	0.129867303	0.563434478	0.144915901	0.179743353	0.780776808
EDF1	0.346856534	0.566800469	0.298102177	0.036629297	0.45672934	0.179710524	0.780776808
HK1	0.359936747	0.260208867	0.054398993	0.247616786	0.777440025	0.179743756	0.780776808
NUDCD1	0.645366382	0.260076912	0.253689029	0.059393438	0.387403567	0.179641031	0.780776808
IMP4	0.204864043	0.651556103	0.989382967	0.190277154	0.039265099	0.180305473	0.781097287
IRAK4	0.90768957	0.036338908	0.186909961	0.32188623	0.49550159	0.179982875	0.781097287
LOC101902570	0.133288076	0.054795984	0.881131486	0.16871358	0.909093369	0.18034109	0.781097287
NANOS1	0.95295112	0.436003883	0.02137089	0.575987058	0.192107187	0.179907148	0.781097287
PEMT	0.107245315	0.750561604	0.300265635	0.051831334	0.787323456	0.180270843	0.781097287

POLR3G	0.796880998	0.476765366	0.10791828	0.466465328	0.051579897	0.180287596	0.781097287
RHOT1	0.157745747	0.29622785	0.604310952	0.04914599	0.710779133	0.180281894	0.781097287
ROGDI	0.457043726	0.228123464	0.809794729	0.017434824	0.66861873	0.180071744	0.781097287
STAC2	0.144854686	0.562037009	0.318357099	0.04801915	0.790262159	0.180006419	0.781097287
STAMBP	0.886613197	0.348572527	0.074471064	0.20753164	0.20663743	0.180334341	0.781097287
TMEM108	0.03457917	0.302726713	0.299973398	0.929834025	0.337715406	0.180246622	0.781097287
CDH11	0.452977493	0.129551925	0.038751856	0.617392072	0.70482527	0.180583727	0.781235782
CDK15	0.373395681	0.006460723	0.713784799	0.723890519	0.795329407	0.180753901	0.781235782
GDE1	0.103225146	0.329401065	0.178523474	0.259121614	0.630168642	0.180738361	0.781235782
LOC112446456	0.358009785	0.022339989	0.375131676	0.784268995	0.421083011	0.180700951	0.781235782
MTUS2	0.654747895	0.278532436	0.153977957	0.051881597	0.679985122	0.180685029	0.781235782
PSMC3IP	0.259879983	0.129278635	0.160265839	0.909829078	0.201908778	0.180540244	0.781235782
RNF112	0.458298952	0.088228454	0.149861202	0.314458625	0.519214102	0.180562244	0.781235782
RNF2	0.166762916	0.745015801	0.184569567	0.084653379	0.510669202	0.180747667	0.781235782
LOC101902366	0.678572824	0.067711235	0.03936035	0.796184216	0.68956675	0.180899419	0.781262921
NOC2L	0.131966775	0.938325492	0.503660528	0.42906824	0.037101547	0.180892538	0.781262921
RRP9	0.155132536	0.264635526	0.59401556	0.744928455	0.054658666	0.180902998	0.781262921
AQP3	0.717737362	0.164311335	0.164197397	0.138869057	0.370900153	0.181325793	0.781477095
C14H8orf37	0.019909726	0.551366164	0.433460762	0.440042951	0.476375914	0.18133413	0.781477095
CFAP20	0.79234263	0.021733311	0.340157622	0.218774941	0.778825223	0.181390142	0.781477095
FGD3	0.577608765	0.390641327	0.915763978	0.03726402	0.129439918	0.18125851	0.781477095
GNA15	0.017013622	0.760949738	0.376953526	0.616662768	0.331630844	0.181387199	0.781477095
LOC100335822	0.77487661	0.248579739	0.130123945	0.739104463	0.053897967	0.181428781	0.781477095
MSRA	0.202957695	0.676091405	0.15930095	0.046475933	0.979277493	0.181086184	0.781477095
NOL6	0.454775493	0.200580524	0.825749613	0.821982843	0.016087703	0.18120126	0.781477095
PACS2	0.845695886	0.934531734	0.319346199	0.030650376	0.128745778	0.181189885	0.781477095
PSD4	0.261636205	0.676569446	0.047840828	0.382789698	0.307981657	0.181420114	0.781477095
WNT5A	0.436204036	0.184598616	0.015187552	0.938371445	0.871518119	0.181586759	0.781952322
FITM1	0.617939643	0.421280118	0.957834919	0.091791172	0.043816075	0.18184578	0.782575154
LOC104975673	0.36690661	0.056187826	0.892283386	0.127038966	0.429275041	0.181874452	0.782575154
PPP1CA	0.490729724	0.211151584	0.728017006	0.048316199	0.275068076	0.181817155	0.782575154
NDUFB8	0.689289814	0.808008075	0.205855619	0.013407617	0.65331465	0.181979715	0.782822831
LOC112449102	0.084480656	0.187552209	0.864540571	0.869401381	0.084668471	0.182363589	0.782861261
MEF2D	0.658081301	0.456416562	0.578545628	0.155513062	0.037257116	0.182220657	0.782861261
NR1H2	0.455656463	0.579289554	0.035830443	0.997385515	0.106609182	0.182108608	0.782861261
PLPPR4	0.810301489	0.543589317	0.019511383	0.14272578	0.822428848	0.182407319	0.782861261
SERF2	0.377738024	0.423793713	0.305437737	0.036075387	0.571588316	0.182353804	0.782861261
SZT2	0.974498643	0.351610955	0.090970029	0.218473918	0.148154009	0.18241798	0.782861261
YPEL3	0.788111677	0.95005558	0.799268962	0.661744676	0.00254302	0.182245926	0.782861261
ZNF618	0.634575241	0.600956714	0.27642231	0.016932942	0.564994253	0.182378709	0.782861261
ZPR1	0.983054493	0.292176978	0.037347001	0.128667252	0.728747299	0.182126393	0.782861261
ADD3	0.846080941	0.025720795	0.648717593	0.206395676	0.351911537	0.183964919	0.783454088
AKIRIN1	0.228565786	0.097977368	0.167791716	0.784389656	0.347728872	0.183919456	0.783454088
ARMH3	0.928270456	0.3414466	0.166504818	0.026589852	0.733153628	0.184285017	0.783454088

BUD23	0.355620033	0.87358119	0.39801177	0.076528067	0.107907314	0.183561493	0.783454088
CCNY	0.619484843	0.07301686	0.087343826	0.993029498	0.261218001	0.183913095	0.783454088
CD96	0.525848745	0.629371356	0.080099044	0.657990584	0.058225748	0.183049234	0.783454088
CHRM1	0.602131733	0.060301821	0.041724787	0.749886884	0.904881335	0.184211726	0.783454088
CYC1	0.459609091	0.951095355	0.933968929	0.003797731	0.661405057	0.183976108	0.783454088
DCP1B	0.446826158	0.081088241	0.501668833	0.772636578	0.073378371	0.184444659	0.783454088
DHX15	0.332167993	0.743917953	0.807401781	0.013852318	0.369677289	0.183618952	0.783454088
DTYMK	0.360141817	0.94136793	0.073533109	0.040711326	0.996968135	0.182693861	0.783454088
FAM13C	0.541630608	0.956531491	0.048736154	0.161127705	0.251454021	0.183743482	0.783454088
FOXJ3	0.342702261	0.319574818	0.858118595	0.014185581	0.77128195	0.184232428	0.783454088
KANK2	0.436520912	0.872881948	0.008722516	0.309642829	0.987535427	0.183112161	0.783454088
LOC101905876	0.639367572	0.322328246	0.303877437	0.20880671	0.078338449	0.183872387	0.783454088
LOC101906818	0.719988587	0.143250105	0.082601266	0.264876815	0.454769185	0.18404356	0.783454088
LOC101907803	0.465230641	0.798162333	0.009575369	0.523604593	0.544606382	0.182889068	0.783454088
MCM6	0.732344432	0.960741498	0.012708682	0.831642633	0.137971935	0.184023182	0.783454088
MFGE8	0.711781424	0.429044051	0.705036155	0.008060775	0.591046429	0.184002952	0.783454088
MMP25	0.12900608	0.269999433	0.26858883	0.344302192	0.316056925	0.183277179	0.783454088
MRPL27	0.336771419	0.341354959	0.415940075	0.046451334	0.463799537	0.184410105	0.783454088
NFATC1	0.624672499	0.3344306	0.012032302	0.680423439	0.602650687	0.1844657	0.783454088
NPNT	0.058381568	0.630968352	0.177123033	0.59170084	0.26223721	0.182747097	0.783454088
OGFRL1	0.929362262	0.079928251	0.143810082	0.239195109	0.402821991	0.184330364	0.783454088
PAPOLA	0.67170615	0.504603111	0.263540369	0.037526058	0.305463222	0.183828574	0.783454088
PPFIA2	0.635107684	0.171315056	0.301729857	0.041125727	0.760734415	0.184124311	0.783454088
PPP4R3B	0.556070728	0.226705512	0.10824351	0.583097478	0.129340109	0.184314495	0.783454088
PRDX4	0.96572664	0.146073401	0.029447898	0.341022075	0.722795332	0.183830604	0.783454088
PRSS53	0.655549828	0.10963247	0.374428436	0.32106558	0.118970285	0.184199025	0.783454088
REEP3	0.492552478	0.031469341	0.237979927	0.276237787	0.999867321	0.183351995	0.783454088
REPIN1	0.931800781	0.817243446	0.362513869	0.092227093	0.039777965	0.182779017	0.783454088
RYR2	0.205768149	0.512538094	0.516697148	0.222424506	0.084225792	0.183541896	0.783454088
SELENON	0.673435944	0.199828412	0.033697667	0.455030257	0.49601062	0.183788057	0.783454088
SLC16A5	0.800384852	0.331042799	0.632457731	0.017372674	0.347999664	0.182814109	0.783454088
SMIM11A	0.285424378	0.234032491	0.318699638	0.05719455	0.840338617	0.183760073	0.783454088
SRRM1	0.772834545	0.095097971	0.210977112	0.145927611	0.448515226	0.182978418	0.783454088
STK17B	0.424166524	0.891066887	0.468687987	0.238020611	0.024246638	0.183680433	0.783454088
TOPBP1	0.681972469	0.571226968	0.62529366	0.006056644	0.69057984	0.183351905	0.783454088
TOR1AIP2	0.609384232	0.323170913	0.0672079	0.192055679	0.403484836	0.183989667	0.783454088
ZDHHC18	0.664797647	0.725925908	0.22469292	0.019901766	0.468572824	0.18263404	0.783454088
LOC101906526	0.022730184	0.563627257	0.658329789	0.388833472	0.314856286	0.184634445	0.783967886
ALPK1	0.528378857	0.101958002	0.124501393	0.321147656	0.480852597	0.184932036	0.784823266
LOC104974345	0.39637232	0.535812065	0.591101856	0.01727351	0.478005229	0.185006025	0.784823266
TMEM173	0.753562434	0.107157778	0.033852756	0.98939618	0.383220895	0.184998266	0.784823266
ZFR	0.717217692	0.281347101	0.105764772	0.165289858	0.293904421	0.18502719	0.784823266
JADE3	0.192862053	0.220996705	0.576092739	0.067807299	0.623901024	0.185211417	0.785327499
SPTLC2	0.246333849	0.435577537	0.452638728	0.118362829	0.180757893	0.185241774	0.785327499

LOC789337	0.502807409	0.535374328	0.512806227	0.016719373	0.450624103	0.185329038	0.785494535
C18H16orf46	0.572918282	0.129292962	0.645935283	0.389441719	0.055953786	0.185569363	0.785695107
LRRN2	0.628818186	0.045111064	0.196818456	0.918921783	0.203158368	0.185537958	0.785695107
PTH1R	0.488386044	0.171593054	0.044965197	0.581985658	0.475647691	0.185615741	0.785695107
SHROOM2	0.787552827	0.214633222	0.072062722	0.160161709	0.534091785	0.185510096	0.785695107
TMEM198	0.080514837	0.473884152	0.14397439	0.697816101	0.272091511	0.185605113	0.785695107
CAMK2N1	0.171290134	0.449581894	0.392250212	0.108355527	0.320043867	0.18602251	0.787010935
DLAT	0.568416964	0.528286599	0.639425329	0.040724166	0.133949018	0.1860121	0.787010935
DCTD	0.963983659	0.523123416	0.939768893	0.164629758	0.01344713	0.186171332	0.787060793
GALNT12	0.035694044	0.171728652	0.36623834	0.56041814	0.833527911	0.186127163	0.787060793
MGC157082	0.971222593	0.080694974	0.856773385	0.3560846	0.043946514	0.186322051	0.787060793
NOL12	0.233988551	0.448879309	0.102042189	0.877941971	0.111664524	0.18631673	0.787060793
PTPRE	0.808474541	0.689333311	0.111567068	0.771550285	0.021887687	0.186252231	0.787060793
REV1	0.236710083	0.373731878	0.888750511	0.031458221	0.424584265	0.186265402	0.787060793
ASF1B	0.494990843	0.789903736	0.037235654	0.091806535	0.788961395	0.186668186	0.787204173
BTBD10	0.161963404	0.293180481	0.791476545	0.050480377	0.556601783	0.186801685	0.787204173
HSD17B14	0.174889791	0.027715208	0.498936662	0.786463158	0.554663975	0.186707655	0.787204173
KDM2A	0.299489347	0.319673337	0.203123084	0.091058956	0.596417307	0.186815721	0.787204173
LOC112444633	0.006942204	0.22960977	0.829196608	0.813865005	0.981158937	0.18675248	0.787204173
LOC112448808	0.837640986	0.05138996	0.1605167	0.61977382	0.246117649	0.18661743	0.787204173
MAPK9	0.042263193	0.856697399	0.447406284	0.301202463	0.216500501	0.186835675	0.787204173
MIEF1	0.928145605	0.276452482	0.325668543	0.119120248	0.105732195	0.186477422	0.787204173
TMCO1	0.39950182	0.487185575	0.702784784	0.433369227	0.017751755	0.186461719	0.787204173
ZDHHC7	0.61063523	0.261719609	0.035664552	0.480282139	0.384910257	0.186590278	0.787204173
HPGDS	0.471559309	0.344457693	0.264013335	0.40011827	0.061616914	0.18691999	0.787246686
SAFB2	0.968779818	0.142032356	0.708053015	0.013532027	0.802522104	0.186989677	0.787246686
TESK2	0.654449547	0.842901176	0.435870098	0.537841484	0.008180942	0.18698286	0.787246686
ATP6V1B2	0.914604777	0.235935914	0.018986005	0.774576518	0.333601804	0.187046901	0.787285636
AEN	0.570436828	0.276225045	0.212170067	0.081943585	0.387153848	0.187225824	0.78741467
LOC107131703	0.26039357	0.459262972	0.309426296	0.02917019	0.981597905	0.187129239	0.78741467
LOC112444896	0.422642615	0.011687035	0.392353181	0.787676089	0.695097563	0.187269481	0.78741467
NRBP2	0.325920719	0.051386441	0.675490182	0.180090462	0.520313357	0.187177015	0.78741467
BRF2	0.816514124	0.102818787	0.681260565	0.073110723	0.254386066	0.187510471	0.787671173
CAVIN1	0.619070517	0.089399212	0.167724826	0.937413596	0.122696276	0.18787165	0.787671173
G3BP1	0.685250795	0.061018224	0.572290141	0.099478223	0.448586501	0.187886425	0.787671173
ILK	0.661474973	0.170558882	0.248922638	0.466002927	0.081604869	0.187899286	0.787671173
LOC112446798	0.956365473	0.091651575	0.264584658	0.428492893	0.107407707	0.187843309	0.787671173
MDFIC2	0.39671685	0.976134545	0.993512955	0.177748329	0.015611521	0.187867345	0.787671173
OARD1	0.049875201	0.581088847	0.791910772	0.048009869	0.96455789	0.187429433	0.787671173
PARD3	0.447229598	0.810897041	0.891436159	0.003797831	0.86548356	0.187411224	0.787671173
RHOT2	0.123046631	0.046888091	0.732237661	0.453381099	0.557623402	0.187906443	0.787671173
SLC15A2	0.262264254	0.802188455	0.334494436	0.052723052	0.286737481	0.187525175	0.787671173
SOX6	0.113512816	0.155503628	0.25821024	0.356600821	0.655023704	0.187594417	0.787671173
VPS9D1	0.898653507	0.224541175	0.048514191	0.116866438	0.931363013	0.187676868	0.787671173

ATAD2B	0.575449946	0.554371996	0.251010819	0.017581296	0.760502244	0.188145463	0.788471704
PARD3B	0.76300447	0.283858277	0.551159846	0.069817645	0.128676869	0.188307186	0.788947979
PRMT5	0.726445043	0.731493912	0.628735845	0.010288447	0.312312971	0.188408344	0.789170324
LRRC71	0.131431092	0.661656085	0.177285207	0.709212616	0.098295999	0.188519462	0.789434268
GTPBP3	0.491256361	0.024435328	0.51512345	0.252744416	0.688079923	0.188574157	0.789461858
CEP250	0.656467127	0.025271926	0.168976056	0.771409389	0.499335087	0.188978794	0.789655213
ENKUR	0.353348586	0.319740646	0.260343197	0.150832871	0.243430022	0.188993247	0.789655213
GNA14	0.190394137	0.333211892	0.154369317	0.709640934	0.155126175	0.188822138	0.789655213
LOC100847304	0.318798131	0.467689078	0.506397984	0.075041841	0.190299952	0.188833035	0.789655213
LOC101906235	0.160076939	0.051858388	0.202645752	0.785537169	0.817370369	0.189005282	0.789655213
PODN	0.469630165	0.339221927	0.841422109	0.017886589	0.450272244	0.18895692	0.789655213
TTC32	0.98812102	0.214154402	0.11762053	0.053107245	0.815010822	0.188749707	0.789655213
VSIG4	0.256434897	0.216552882	0.200090086	0.28492976	0.340860357	0.188917327	0.789655213
ARPC4	0.286084215	0.081956689	0.405556582	0.851299676	0.133714601	0.189212952	0.789919551
KCNQ1	0.933662631	0.219867167	0.741604967	0.027302742	0.260408891	0.189211599	0.789919551
SUV39H2	0.703801163	0.440787629	0.18160452	0.22894288	0.083890075	0.189179695	0.789919551
BANK1	0.950174993	0.873115872	0.035605276	0.248537401	0.148420471	0.189865118	0.790075842
DDR1	0.941130622	0.613057168	0.184202246	0.131187182	0.078019082	0.189698226	0.790075842
DNAJA2	0.859784196	0.066891103	0.102245936	0.752778342	0.245932813	0.1897773	0.790075842
DUSP3	0.070253136	0.052680072	0.947907299	0.798230976	0.3897227	0.190020678	0.790075842
ESS2	0.252635824	0.47904872	0.895600702	0.432707819	0.023230473	0.189857521	0.790075842
GPALPP1	0.073165132	0.158454209	0.202020248	0.475167499	0.978072276	0.189762147	0.790075842
LIME1	0.806888305	0.392750528	0.73088423	0.013516151	0.348417923	0.189968595	0.790075842
LOC112444479	0.372893814	0.558359584	0.508531237	0.347317387	0.029528102	0.189526516	0.790075842
LOC782456	0.08222728	0.555856436	0.136569865	0.80087704	0.217559549	0.189684515	0.790075842
MMAA	0.779029553	0.557214685	0.749962861	0.026773746	0.12481252	0.18970852	0.790075842
NYAP1	0.603299055	0.036620979	0.298408139	0.573727441	0.287962393	0.189828725	0.790075842
PDE1A	0.3883043	0.233037595	0.094154145	0.414479509	0.30749455	0.189526352	0.790075842
PPID	0.867620365	0.960283515	0.26594703	0.006257166	0.786541834	0.189944032	0.790075842
RNF19A	0.359017437	0.325594661	0.118299229	0.175419761	0.44981225	0.190003074	0.790075842
SLC5A3	0.889282203	0.495866883	0.222831254	0.024200059	0.457157197	0.189635958	0.790075842
THUMPD3	0.607471905	0.729993357	0.221357474	0.141801691	0.078024382	0.189542839	0.790075842
ASL	0.187582868	0.711368942	0.195132374	0.229488568	0.182942249	0.190186183	0.790304609
CEP55	0.165852172	0.292577388	0.04900791	0.562637252	0.819823051	0.190522931	0.790304609
CFAP44	0.856761819	0.102793793	0.477888549	0.733644286	0.035422389	0.190237096	0.790304609
GNL2	0.432004147	0.198694041	0.142410518	0.250677322	0.358269736	0.190605426	0.790304609
HRH4	0.719748569	0.129484303	0.231267402	0.40338781	0.126196236	0.190546729	0.790304609
LOC101906408	0.535203656	0.065793439	0.500120898	0.406366455	0.152751334	0.190183248	0.790304609
MGC126945	0.207562599	0.571633793	0.239694942	0.044509341	0.86599844	0.190458784	0.790304609
MTMR1	0.961190554	0.843652015	0.012163131	0.251604276	0.442332244	0.190592897	0.790304609
POLG2	0.724170529	0.116336136	0.068138779	0.255907605	0.74729629	0.190602212	0.790304609
SLC46A1	0.562152893	0.37817555	0.417055473	0.329154237	0.037602547	0.190564194	0.790304609
YWHAH	0.556960225	0.691537077	0.095298017	0.057576578	0.518672276	0.190451579	0.790304609
LOC112444926	0.266460771	0.206710337	0.713046275	0.75880747	0.03689253	0.190751562	0.79052169

NFRKB	0.347722976	0.048990341	0.48566994	0.346829454	0.383545842	0.190850462	0.79052169
PNMA8B	0.368934589	0.218216714	0.127423119	0.194573536	0.550947332	0.190773435	0.79052169
STAC	0.559277767	0.512673382	0.065247351	0.111606375	0.526908778	0.190813479	0.79052169
ABCC1	0.362875802	0.505037444	0.954098383	0.007964048	0.802904155	0.192413825	0.791404333
ADPGK	0.722633543	0.227931182	0.189330421	0.374045128	0.095217609	0.191753669	0.791404333
BDKRB2	0.033410392	0.966164359	0.326494516	0.271069772	0.389798236	0.192015317	0.791404333
BTBD7	0.201612308	0.349504035	0.452175418	0.05319376	0.658806279	0.192281659	0.791404333
C18H19orf18	0.947978365	0.46716868	0.234219635	0.13048764	0.082579147	0.192382453	0.791404333
DCAKD	0.272010204	0.838861417	0.493279501	0.064798162	0.152386393	0.191820733	0.791404333
DST	0.320180593	0.497493835	0.054277085	0.357639915	0.358617508	0.191592413	0.791404333
ENO4	0.827015914	0.445739216	0.50054645	0.190559613	0.03175844	0.192289555	0.791404333
FAM186B	0.134322411	0.094614517	0.47283471	0.742205885	0.249335658	0.191877129	0.791404333
GFI1	0.055232398	0.351417023	0.422582154	0.525268943	0.258923202	0.192186976	0.791404333
IPCEF1	0.263759718	0.985907673	0.811894493	0.018580794	0.282798103	0.191639942	0.791404333
LOC112447857	0.622726431	0.628741535	0.04066702	0.187689825	0.372401311	0.191954624	0.791404333
LRRN4	0.58065706	0.022583637	0.28086253	0.440088944	0.681929534	0.191275771	0.791404333
NMT1	0.586556212	0.459422445	0.629563566	0.273199643	0.024070255	0.192196513	0.791404333
NSA2	0.776651565	0.134309016	0.073872073	0.33408801	0.434163044	0.192380212	0.791404333
PDE1C	0.080084846	0.218746942	0.290160961	0.313795247	0.6940152	0.19142598	0.791404333
PKD1L2	0.178060409	0.644031451	0.200641059	0.051580117	0.935266146	0.191691661	0.791404333
PRPF18	0.845488348	0.397038572	0.026162751	0.456735367	0.277555057	0.191994322	0.791404333
PRPSAP1	0.509223907	0.533547505	0.028356237	0.973652991	0.148724034	0.192195046	0.791404333
PSMB10	0.804891103	0.192767826	0.182881815	0.129506979	0.303069227	0.192026234	0.791404333
PTPN13	0.861753766	0.082528342	0.425187586	0.044575325	0.827587064	0.192185732	0.791404333
RAB3A	0.876342492	0.312728835	0.162691498	0.089875727	0.276828775	0.19163429	0.791404333
RAPGEF4	0.389518736	0.180202482	0.871866188	0.065090353	0.277310184	0.191215206	0.791404333
RHOBTB2	0.352155651	0.345497975	0.160499859	0.245753033	0.231187851	0.191647615	0.791404333
SCHIP1	0.148227051	0.34419017	0.615741505	0.797549829	0.044534557	0.192209562	0.791404333
STAR	0.171635972	0.345273676	0.210756955	0.224021518	0.399137935	0.19229809	0.791404333
TXN	0.169961669	0.495811791	0.639429837	0.228086305	0.090507514	0.191904574	0.791404333
ZMYM5	0.454669485	0.496617035	0.534337378	0.025611482	0.359860239	0.191871914	0.791404333
PSMD9	0.299696072	0.2026534	0.211385415	0.121093322	0.720841488	0.192642225	0.792145214
AOAH	0.997770413	0.436325195	0.985847551	0.134923946	0.019391187	0.192842538	0.792571725
ZNF770	0.967763732	0.542032416	0.420062863	0.069480804	0.073321353	0.19281047	0.792571725
LOC101902346	0.201242536	0.063117876	0.58705101	0.186247302	0.809455835	0.192954216	0.79283216
LOC112442307	0.961704561	0.203040912	0.083055055	0.37923649	0.182948999	0.193046045	0.792882615
LOC112444726	0.794143572	0.821745619	0.529559287	0.008334511	0.390728183	0.193063124	0.792882615
NDUFB1	0.313571868	0.530223146	0.946379592	0.008555952	0.836734332	0.193157482	0.792978648
TMPRSS6	0.025789828	0.176323208	0.81841926	0.350675411	0.863355722	0.193183148	0.792978648
UQCRC1	0.887212085	0.688200229	0.554177172	0.006275961	0.531246905	0.193306851	0.793288004
SERPINE1	0.58111118	0.205273744	0.143030328	0.204623169	0.32348181	0.193412038	0.793521237
CEP164	0.683451037	0.119231196	0.134001215	0.495231676	0.209157274	0.193564199	0.793558522
LOC101903758	0.285453694	0.387929222	0.193690716	0.057044756	0.924694786	0.193592636	0.793558522
MECR	0.592673895	0.814119348	0.323962464	0.067678285	0.106969131	0.193614546	0.793558522

RNH1	0.246785521	0.564254883	0.793147366	0.018653107	0.54855164	0.193479177	0.793558522
LOC112444931	0.608508158	0.205749954	0.773459789	0.026085907	0.449049287	0.193853252	0.793920632
LOC781197	0.121300918	0.815099272	0.564524014	0.109025057	0.186709021	0.194015549	0.793920632
NUDT17	0.501813274	0.316234622	0.300939022	0.17247047	0.137980157	0.194041536	0.793920632
SELENOI	0.689600574	0.008457535	0.204740492	0.995026126	0.954763155	0.193860488	0.793920632
SFSWAP	0.971440924	0.057605433	0.251450274	0.297455978	0.271391153	0.193992907	0.793920632
TMEM104	0.255382996	0.493635567	0.822068045	0.063584189	0.172286123	0.193936548	0.793920632
ZUP1	0.547799142	0.268602508	0.488530468	0.096445845	0.163486041	0.193771182	0.793920632
HTR6	0.785964555	0.2282847	0.605175647	0.017304239	0.605144603	0.194090252	0.793922017
SORL1	0.507860229	0.569459062	0.012444854	0.359675818	0.878900026	0.19415348	0.793982744
BREH1	0.429114754	0.718266811	0.008250714	0.574240583	0.78112199	0.194410898	0.794310028
CRBN	0.495506495	0.568733727	0.017877695	0.244742957	0.9253486	0.194439027	0.794310028
IL10RA	0.281102525	0.767407939	0.825807672	0.043372014	0.147728407	0.194475516	0.794310028
MRPS16	0.158488866	0.597412965	0.83638757	0.025595672	0.562054873	0.194286961	0.794310028
VARS	0.663951285	0.595021725	0.827280196	0.742228439	0.004703867	0.194445692	0.794310028
ADARB1	0.95093314	0.430992167	0.012958952	0.543290408	0.396059658	0.194599789	0.79461984
CERCAM	0.72281875	0.230019651	0.0392871	0.611267718	0.286689569	0.19476281	0.794705446
KCNK3	0.06610802	0.304931612	0.60393229	0.175498671	0.536292839	0.194862879	0.794705446
LOC112448582	0.365687146	0.822685417	0.495234932	0.263659318	0.029132263	0.194736111	0.794705446
LOC112448744	0.750677781	0.527562976	0.99170981	0.13919775	0.02095668	0.194850514	0.794705446
PUSL1	0.357696533	0.877466822	0.458090764	0.716658899	0.011116801	0.194832515	0.794705446
CORO2B	0.082305218	0.429896508	0.211750208	0.269285355	0.568321952	0.19493261	0.794760941
SELENOT	0.736263567	0.461865309	0.672025352	0.627453711	0.007999861	0.194973344	0.794760941
LOC100848148	0.275653531	0.208374921	0.257062827	0.137313245	0.56756343	0.195291912	0.795861823
ALG5	0.283173691	0.418928612	0.661933054	0.167089836	0.088105467	0.195751924	0.795957589
CYP1A1	0.200369969	0.775185685	0.018640333	0.69060699	0.577973293	0.195721974	0.795957589
EFNB1	0.35529593	0.150345323	0.05711735	0.901029513	0.420216303	0.195682392	0.795957589
EIF4E2	0.418030258	0.046812879	0.539908979	0.60190552	0.181088698	0.195370229	0.795957589
GDPD3	0.426703752	0.180769329	0.268116299	0.279165309	0.199852313	0.195562964	0.795957589
MRPL21	0.36905232	0.547224872	0.873262161	0.0158731	0.412005321	0.195520761	0.795957589
PDE1B	0.381950194	0.671488604	0.661541783	0.012373312	0.549596044	0.195559992	0.795957589
PGBD5	0.00860323	0.392347297	0.952435403	0.896985327	0.400155269	0.195571433	0.795957589
RPA3	0.546512867	0.528231761	0.034320305	0.121546159	0.959008515	0.195654474	0.795957589
S1PR4	0.698590566	0.54561127	0.914716018	0.072192643	0.045974947	0.195855559	0.796181715
LOC112441481	0.723930186	0.177919772	0.22704495	0.051875907	0.764189362	0.196039653	0.796732724
C25H7orf50	0.244478333	0.577249328	0.079747253	0.319920028	0.322319854	0.196143865	0.796761627
CXHXorf56	0.783754928	0.643731311	0.127608747	0.031612766	0.57014688	0.1961361	0.796761627
ETFBKMT	0.177823232	0.03720835	0.727589588	0.569669387	0.42348048	0.196219077	0.796807581
MAGEH1	0.039161077	0.592768675	0.708612422	0.074021907	0.954337735	0.196274709	0.796807581
SFN	0.458940296	0.160527629	0.544930706	0.209193031	0.13839771	0.196300838	0.796807581
COMMD3	0.690393276	0.801539331	0.341228966	0.023471551	0.262616194	0.196442054	0.797001734
SLC22A16	0.080273358	0.109681474	0.812086021	0.483772894	0.336510332	0.196445799	0.797001734
INSL6	0.463046125	0.571262515	0.675271069	0.04889072	0.133475815	0.196590186	0.797048707
S100A12	0.53242953	0.624818313	0.056541892	0.119556009	0.51839989	0.196603081	0.797048707

SLC25A39	0.597300289	0.822287958	0.345370457	0.023851593	0.287908411	0.196521864	0.797048707
IL12RB1	0.976249105	0.00832722	0.555967787	0.788091917	0.327809873	0.19676298	0.797438844
LOC100141070	0.687438038	0.208072539	0.217096953	0.465420109	0.080924893	0.196930784	0.797438844
LOC112442189	0.04867693	0.035732912	0.899654122	0.932305487	0.801371263	0.196891122	0.797438844
NUDCD3	0.42676738	0.135265471	0.126321771	0.30559253	0.524239888	0.196814225	0.797438844
NUMBL	0.434049534	0.230593214	0.560897787	0.024433782	0.852743433	0.196942273	0.797438844
FHDC1	0.709957507	0.400976434	0.00730357	0.697280266	0.811376908	0.197511189	0.797970487
KIZ	0.159169497	0.664157759	0.810321684	0.019600354	0.699529556	0.197357529	0.797970487
LOC101902468	0.489664787	0.182542475	0.683237032	0.055692822	0.345700767	0.197468522	0.797970487
LOC101907944	0.184774645	0.733862989	0.02645202	0.395529387	0.826706495	0.197214617	0.797970487
PLK1	0.365541244	0.234080168	0.160060459	0.618525803	0.138782689	0.197455535	0.797970487
SELENOM	0.796907373	0.552817736	0.010172945	0.269071842	0.974157775	0.197375163	0.797970487
SREBF1	0.182567765	0.448138199	0.688374922	0.280906392	0.074301263	0.197441694	0.797970487
SYNC	0.019813508	0.573147053	0.453153626	0.370773148	0.616216905	0.197464083	0.797970487
TRAF7	0.247736282	0.127744511	0.868764011	0.094257907	0.453022898	0.197313893	0.797970487
ADCK1	0.621164968	0.116062083	0.456758044	0.171008822	0.213135864	0.199562088	0.798388698
ANAPC11	0.106623323	0.631478229	0.692247466	0.068771957	0.371811824	0.198844184	0.798388698
ANKS6	0.261529663	0.144605358	0.154647379	0.432578635	0.471880613	0.199017719	0.798388698
APEX1	0.304067162	0.645127968	0.496464761	0.82686379	0.015242739	0.201867399	0.798388698
APOBEC3H	0.41777697	0.650166144	0.041419714	0.714123083	0.149731537	0.199798635	0.798388698
ARMC4	0.027000877	0.535637822	0.160641161	0.686345234	0.762025041	0.20082766	0.798388698
ASNS	0.30910372	0.826503306	0.216593058	0.490948202	0.044191474	0.199587753	0.798388698
ATP5MF	0.476696042	0.895753675	0.888469356	0.004189191	0.742239181	0.197798783	0.798388698
ATP5MPL	0.425075316	0.782674396	0.332647982	0.011844828	0.920180087	0.200074925	0.798388698
BEND3	0.594549526	0.260518936	0.398619365	0.028497084	0.677325308	0.198838759	0.798388698
BOD1L1	0.456130556	0.210403135	0.243108455	0.127382173	0.405365625	0.199948331	0.798388698
BRD2	0.480677579	0.362807791	0.383105148	0.0801144	0.227304593	0.200957767	0.798388698
CACNA1B	0.01283432	0.801145619	0.305944578	0.498153721	0.770723321	0.200206073	0.798388698
CCDC171	0.053326373	0.174063418	0.924497814	0.981973855	0.140712533	0.198323646	0.798388698
CEMIP2	0.841290823	0.512059783	0.090779327	0.032672564	0.961241859	0.201931315	0.798388698
CHCHD7	0.413746027	0.033631555	0.326436625	0.865881367	0.308190965	0.200576878	0.798388698
CHMP3	0.631440402	0.069469994	0.090502445	0.357610888	0.866439349	0.202090467	0.798388698
CNBP	0.996932038	0.033767937	0.082593598	0.703263678	0.614617603	0.199699005	0.798388698
CPSF2	0.690564653	0.136727522	0.322974875	0.285459016	0.137271698	0.199113802	0.798388698
DIDO1	0.321143057	0.161338186	0.395966036	0.164394915	0.354450717	0.199157091	0.798388698
DOCK10	0.753342386	0.645166451	0.240151113	0.033580858	0.312100851	0.201519331	0.798388698
EIF2AK3	0.447157312	0.943099387	0.184333338	0.017713005	0.878160932	0.200323965	0.798388698
ELN	0.79884294	0.590919337	0.016242179	0.203181533	0.768681224	0.199328013	0.798388698
ERBB2	0.22600848	0.693604504	0.578216882	0.211407733	0.063360606	0.200744766	0.798388698
FAM131B	0.787753256	0.479166511	0.275821044	0.15615928	0.073173356	0.198660634	0.798388698
FBXO32	0.458735217	0.557889919	0.608293114	0.042168854	0.185177566	0.200871662	0.798388698
FFAR3	0.393329696	0.259057053	0.217130577	0.160099546	0.347064674	0.202027385	0.798388698
FIBP	0.341151791	0.959349306	0.427820033	0.059354942	0.147670283	0.20185188	0.798388698
FLOT1	0.330649876	0.389616225	0.24947425	0.611332374	0.061706501	0.200596148	0.798388698

GOLGA4	0.864112785	0.399259681	0.329043441	0.012171093	0.882558788	0.201191204	0.798388698
GYS1	0.259371538	0.765651923	0.799679786	0.022059944	0.337720917	0.198099669	0.798388698
HEXIM1	0.909137447	0.184542631	0.143939557	0.060694817	0.837801092	0.201914499	0.798388698
HSPB8	0.06014681	0.192113284	0.545352287	0.599455809	0.317743767	0.199567453	0.798388698
IER2	0.429094553	0.136381274	0.238797263	0.143199704	0.593060984	0.198415178	0.798388698
IGDCC4	0.032020306	0.535619832	0.080726408	0.90362407	0.965844219	0.200254176	0.798388698
INPP5D	0.650095648	0.591893525	0.21478073	0.21770893	0.067086918	0.200144983	0.798388698
IQCN	0.786672691	0.239422522	0.486604757	0.689104906	0.019296165	0.201129431	0.798388698
KAT14	0.873821082	0.412115341	0.124721454	0.052542239	0.518512432	0.201546558	0.798388698
KIAA1958	0.578641369	0.91914241	0.647237803	0.007471359	0.459817919	0.198055046	0.798388698
KIF16B	0.798149651	0.800806389	0.188924106	0.087293473	0.115153266	0.200718655	0.798388698
LAMA3	0.037508645	0.910525162	0.270559305	0.459330125	0.281554907	0.199118513	0.798388698
LEMD3	0.262448317	0.485996279	0.608228417	0.022318809	0.708508175	0.201810003	0.798388698
LOC100847791	0.214364944	0.821861883	0.031781163	0.877730135	0.244022617	0.199480811	0.798388698
LOC100848208	0.391934429	0.457244607	0.174946471	0.103161459	0.365890809	0.198124022	0.798388698
LOC100848504	0.882706592	0.88900783	0.031603864	0.169415699	0.289775477	0.201031245	0.798388698
LOC100848639	0.932794772	0.044993338	0.089608985	0.854314066	0.375472551	0.20008629	0.798388698
LOC101904121	0.27370947	0.810344324	0.293828738	0.084948619	0.220021379	0.201078034	0.798388698
LOC101905510	0.845821182	0.028541994	0.403195972	0.513346443	0.24269459	0.200622231	0.798388698
LOC104972821	0.520427004	0.542244988	0.190508422	0.311153049	0.072103117	0.200066469	0.798388698
LOC107131418	0.533140148	0.321546233	0.230079254	0.593508837	0.050865303	0.198751313	0.798388698
LOC112442298	0.064377819	0.69068894	0.539250187	0.202924487	0.244654867	0.198724615	0.798388698
LOC112447459	0.293299989	0.450235925	0.175577669	0.080914497	0.636686041	0.199071325	0.798388698
LOC522610	0.109984096	0.326216823	0.128360323	0.884733415	0.297130704	0.200451336	0.798388698
LOC782938	0.377404701	0.044035226	0.103982139	0.882456572	0.793080557	0.200344965	0.798388698
LOC788599	0.193040039	0.524291115	0.926524238	0.217363145	0.060315655	0.202031901	0.798388698
MID1	0.160860984	0.780679869	0.052441093	0.241253312	0.771169589	0.201681535	0.798388698
MNAT1	0.093076212	0.352121934	0.097364344	0.815523836	0.469499208	0.201393008	0.798388698
MRPL51	0.888511277	0.318388138	0.558581015	0.015465993	0.490941904	0.199528012	0.798388698
NDUFA13	0.563826104	0.916282084	0.413725134	0.007122167	0.801633347	0.201267519	0.798388698
NDUFC2	0.742630455	0.474173771	0.441660401	0.015889149	0.484874202	0.199390162	0.798388698
NEK9	0.807995123	0.016146349	0.12805642	0.932139085	0.75789548	0.197852081	0.798388698
NOTCH3	0.89721204	0.966874529	0.106052252	0.088326015	0.150622662	0.201573586	0.798388698
OFD1	0.105245439	0.076739061	0.552112268	0.280122786	0.971010658	0.200638762	0.798388698
OTUD5	0.418571235	0.254890796	0.240371279	0.260799911	0.18218238	0.201112402	0.798388698
PEAK1	0.876091699	0.472367681	0.229228547	0.039837318	0.315136476	0.198769185	0.798388698
PFKFB3	0.215196354	0.041392157	0.278324948	0.527057885	0.919470642	0.199666395	0.798388698
PHLDA2	0.702868166	0.396049373	0.469063718	0.042492882	0.212878245	0.197928845	0.798388698
PHTF1	0.532898411	0.470473223	0.0327932	0.178974457	0.835148747	0.201990371	0.798388698
PIGQ	0.91828956	0.679093937	0.852040885	0.005396175	0.42891506	0.202063916	0.798388698
PIK3C2B	0.415658035	0.648102565	0.027009206	0.244946468	0.663455542	0.198039385	0.798388698
PLEKHM2	0.485415491	0.963233582	0.235885349	0.031036421	0.345631253	0.198099355	0.798388698
POLR2E	0.34919654	0.540954182	0.54228381	0.037227274	0.320677317	0.201483965	0.798388698
PPP2R1B	0.192702904	0.595443318	0.043030188	0.729521076	0.330393568	0.198695028	0.798388698

PSMC1	0.32375698	0.396864899	0.124620169	0.52881499	0.142002838	0.199748808	0.798388698
RSF1	0.13086142	0.827822473	0.424490874	0.045501344	0.573632031	0.199565914	0.798388698
SCLY	0.277330999	0.337649135	0.047184261	0.696484991	0.390479336	0.199682831	0.798388698
SCO1	0.594274263	0.386840262	0.021727896	0.289152578	0.81995725	0.1981986	0.798388698
SEMA6A	0.224029419	0.950354162	0.150830366	0.139354701	0.268576794	0.199706022	0.798388698
SLC25A43	0.773439609	0.288461901	0.035495509	0.410880018	0.367401413	0.199158559	0.798388698
SLC6A6	0.048018135	0.717367482	0.063127998	0.693615992	0.785060734	0.198183794	0.798388698
SMDT1	0.40915187	0.288679909	0.269967565	0.038274028	0.983082817	0.199526379	0.798388698
SYNPO2	0.028540027	0.260195486	0.903034319	0.996128425	0.182965388	0.201426089	0.798388698
TAF5	0.017310194	0.482414156	0.509038527	0.298026367	0.94759592	0.199583747	0.798388698
TMEM80	0.987419685	0.116307403	0.417439522	0.037787829	0.673356004	0.201226063	0.798388698
TNFRSF18	0.026786833	0.895472166	0.689273539	0.442449886	0.164044042	0.199545848	0.798388698
TRPV4	0.476707133	0.103226462	0.149637272	0.517690386	0.31713423	0.200302146	0.798388698
UBL4A	0.238701115	0.319078211	0.770237343	0.028524066	0.715048347	0.199247681	0.798388698
UBL5	0.537516678	0.132472014	0.24154196	0.118062265	0.601024521	0.201276474	0.798388698
UCP2	0.466995395	0.209472873	0.160378324	0.198900404	0.392855883	0.201737685	0.798388698
UTRN	0.414592155	0.834190231	0.691058312	0.018576313	0.268749423	0.198961978	0.798388698
XRCC4	0.039877483	0.58747472	0.699497541	0.364583971	0.201784878	0.200017297	0.798388698
ZNF550	0.725229417	0.004735081	0.547404477	0.968348769	0.666629959	0.200688023	0.798388698
CD84	0.24960616	0.143254879	0.160344156	0.478523982	0.448902344	0.202217686	0.798582069
RTRAF	0.84741994	0.407897609	0.307414326	0.02257069	0.514303121	0.202375907	0.798582069
TALDO1	0.299063521	0.943001563	0.233061835	0.028161338	0.665523759	0.202239212	0.798582069
TMEM186	0.716289378	0.753527065	0.854578164	0.04263693	0.062719463	0.2023734	0.798582069
TP63	0.328379339	0.654326651	0.190905958	0.117529268	0.25587662	0.20238272	0.798582069
RPP30	0.841955708	0.1304144	0.382947564	0.072056988	0.407545938	0.202487596	0.798803829
OXLD1	0.850160849	0.918203758	0.147739283	0.034402983	0.311472024	0.202569702	0.798935683
EFL1	0.703354095	0.780134704	0.087075125	0.074904882	0.345878517	0.202741407	0.799420767
ERMP1	0.259879439	0.981242662	0.124887106	0.272437219	0.143540702	0.203370903	0.801362722
LRP2BP	0.567036473	0.040712372	0.873183678	0.077804396	0.794145384	0.2033804	0.801362722
SRRM2	0.645932254	0.261004646	0.207178822	0.15236229	0.233941588	0.203336964	0.801362722
ARFRP1	0.240174651	0.941540956	0.378755814	0.085597668	0.170645721	0.203843691	0.801828329
CWF19L1	0.344692212	0.117553299	0.360171058	0.191075053	0.449054272	0.2039383	0.801828329
EXOSC4	0.166281135	0.885770428	0.934245143	0.089881589	0.101013939	0.20369858	0.801828329
FAM169A	0.267768364	0.195328959	0.623234151	0.168865943	0.227100677	0.203760973	0.801828329
GDF1	0.476009313	0.187618094	0.02767698	0.837114418	0.60314722	0.20358847	0.801828329
LOC533597	0.849583114	0.137938451	0.025687212	0.695444408	0.597666357	0.203854804	0.801828329
MATN4	0.920758596	0.603032572	0.304780971	0.056884376	0.129866303	0.203767255	0.801828329
TLCD2	0.19476877	0.096928128	0.12261931	0.712691632	0.758426156	0.203858051	0.801828329
ZFYVE9	0.124091799	0.193879995	0.158465475	0.615096035	0.53378067	0.203899624	0.801828329
C23H6orf132	0.77218592	0.338720416	0.326422674	0.040524142	0.36397771	0.204527065	0.801868931
CD164	0.698748362	0.237930917	0.062195564	0.892464141	0.137814117	0.205557825	0.801868931
COPZ1	0.51608572	0.553177215	0.079340819	0.406215997	0.138374277	0.205673494	0.801868931
ELP4	0.845769563	0.906072434	0.187078222	0.27310306	0.032339448	0.205095722	0.801868931
ENPP1	0.422751705	0.600981513	0.783980814	0.008632279	0.729188334	0.204067525	0.801868931

ENTPD1	0.647789513	0.693814674	0.815044388	0.148993315	0.023400538	0.206000817	0.801868931
EP300	0.357974244	0.641206864	0.443452618	0.02472135	0.50468477	0.205406925	0.801868931
FAM3C	0.133891475	0.926167205	0.563218575	0.045501217	0.400655439	0.205677876	0.801868931
FBXO32	0.063221299	0.23745821	0.850749148	0.253344793	0.389400571	0.204581949	0.801868931
FDFT1	0.874684827	0.380446976	0.546537591	0.146902531	0.04753161	0.205404037	0.801868931
GLT8D1	0.505976482	0.247512452	0.089311805	0.304965303	0.372054874	0.205335635	0.801868931
GPR17	0.114032836	0.357751626	0.290806171	0.850425789	0.124479	0.204242739	0.801868931
HID1	0.177758134	0.337327968	0.124733766	0.347071426	0.487498157	0.205038043	0.801868931
HIGD1A	0.279940064	0.292540283	0.247652948	0.133386571	0.46917068	0.205346121	0.801868931
HOPX	0.581406356	0.433018379	0.020539146	0.777163319	0.313055792	0.204423893	0.801868931
HSH2D	0.204211589	0.1879023	0.296786329	0.664872361	0.168199394	0.20570338	0.801868931
IL3RA	0.339259999	0.135821958	0.164331891	0.93087056	0.178718441	0.204563325	0.801868931
ISY1	0.191483877	0.02342101	0.739656758	0.882168388	0.43608482	0.205913701	0.801868931
KCNJ15	0.110408879	0.12725251	0.875905552	0.589671339	0.174212765	0.204932166	0.801868931
LIFR	0.539566501	0.346698819	0.028459439	0.278027248	0.857005911	0.205287739	0.801868931
LOC101904595	0.927389859	0.019234376	0.357604894	0.402120015	0.489426508	0.204204214	0.801868931
LOC101907348	0.298539807	0.22028242	0.097487381	0.591745735	0.336602818	0.205984418	0.801868931
LOC104974070	0.653025598	0.415537655	0.107503324	0.062416962	0.694635501	0.204981349	0.801868931
LOC104976574	0.686752956	0.431875551	0.028365915	0.372512569	0.402612387	0.204731725	0.801868931
LOC112442652	0.212818641	0.920670477	0.158381932	0.054132435	0.756021837	0.205412194	0.801868931
LOC511229	0.854294506	0.384604729	0.013854863	0.727063041	0.38084613	0.204626486	0.801868931
LOC782609	0.052898319	0.474963158	0.210858602	0.245541244	0.98173847	0.205991665	0.801868931
LRRC3B	0.144980888	0.212610556	0.677594355	0.312517255	0.194947872	0.20561625	0.801868931
MCM5	0.754124359	0.828744303	0.021565953	0.315851039	0.29946814	0.205811011	0.801868931
NUB1	0.536736402	0.18941569	0.051901926	0.884042116	0.271839422	0.205251857	0.801868931
PHGDH	0.408874824	0.29645741	0.066912078	0.413210755	0.379633104	0.205600583	0.801868931
PRRG1	0.497775979	0.151470925	0.190179386	0.093277851	0.949780481	0.205440391	0.801868931
PSMD14	0.536404212	0.515795684	0.573752782	0.317624016	0.0250381	0.204785902	0.801868931
RBMS2	0.441095668	0.680628052	0.10688906	0.244808916	0.159588706	0.204064697	0.801868931
RHOBTB3	0.128370011	0.959849227	0.065693487	0.220432152	0.705413884	0.204473193	0.801868931
SEPHS1	0.576022693	0.398378331	0.03238935	0.381338567	0.44525935	0.204750303	0.801868931
SETDB1	0.976387635	0.160236404	0.037962041	0.690031623	0.311375164	0.205912605	0.801868931
STUB1	0.211607741	0.953895661	0.334920146	0.040030248	0.47029259	0.205633678	0.801868931
TMEM144	0.071576986	0.994720765	0.141064512	0.235060619	0.534864397	0.204811514	0.801868931
TMEM265	0.926499006	0.106276627	0.158111497	0.558724369	0.144668968	0.204451898	0.801868931
ZC3H13	0.257603175	0.473589414	0.446529496	0.043622134	0.529947931	0.204529772	0.801868931
ZNF354A	0.470059551	0.089301926	0.043748529	0.905236737	0.767734542	0.205928163	0.801868931
TM4SF18	0.90386726	0.568566549	0.026586883	0.257448358	0.363373644	0.206084318	0.802003733
SUFU	0.272439182	0.880659429	0.242804883	0.402153197	0.054659842	0.206277169	0.802563923
CCDC57	0.967127911	0.027341305	0.243392201	0.228642879	0.87459524	0.206804713	0.802965871
COPZ2	0.089735329	0.286334925	0.407751045	0.214430977	0.572745762	0.206782345	0.802965871
COX11	0.11589011	0.983561163	0.216707022	0.145776545	0.356817877	0.206630432	0.802965871
EDA	0.051348348	0.948656015	0.449312176	0.307402617	0.191170078	0.206740835	0.802965871
GBA2	0.6882172	0.94796268	0.085513414	0.029653433	0.776253608	0.206575877	0.802965871

ISCA1	0.591746354	0.110830858	0.064208886	0.441662239	0.691782763	0.206774873	0.802965871
LOC104974678	0.031023963	0.332816124	0.456252983	0.664560284	0.411149177	0.206820836	0.802965871
SCRN1	0.123849762	0.081653869	0.265460351	0.874021046	0.548241567	0.206754049	0.802965871
TRMU	0.086665513	0.650776116	0.252746753	0.222749158	0.40477335	0.206663481	0.802965871
IGIP	0.502845067	0.339130029	0.167614992	0.128669179	0.350410948	0.206947903	0.803269166
DNAJC8	0.480506184	0.006785932	0.602200002	0.808198376	0.812739163	0.207033458	0.803411228
POLR2J	0.440538175	0.486678336	0.839410177	0.012692444	0.564915019	0.20708433	0.803418662
ARHGEF17	0.108411074	0.437598108	0.262803014	0.192220242	0.538824443	0.207156527	0.803508808
MFNG	0.025217191	0.674249277	0.829808833	0.099964363	0.916664506	0.207283927	0.803812977
ALDH1A3	0.778768124	0.060182371	0.065866453	0.612097161	0.702500807	0.210079485	0.803942707
ATF4	0.330471844	0.307196811	0.304349329	0.077966173	0.543377409	0.208593006	0.803942707
BRAT1	0.781576675	0.031015909	0.94514698	0.195943786	0.29101958	0.208392555	0.803942707
CCDC153	0.167911898	0.58164127	0.973906805	0.027041219	0.517185333	0.210304639	0.803942707
CCR10	0.660371222	0.239932217	0.767164505	0.033764624	0.316706032	0.20785066	0.803942707
CTSZ	0.069976513	0.335239033	0.434922312	0.492568509	0.260863594	0.20875583	0.803942707
DUSP2	0.89604538	0.247239925	0.531882057	0.088333218	0.125625943	0.208479864	0.803942707
EHMT2	0.478895219	0.06214189	0.148681347	0.488473733	0.611423597	0.209603101	0.803942707
ESPL1	0.459281163	0.073664712	0.065090141	0.622459591	0.953235209	0.208406062	0.803942707
FAM219B	0.115836781	0.914312325	0.917570819	0.027151657	0.497509611	0.208897363	0.803942707
GNPTG	0.307315352	0.258694061	0.94754745	0.298350343	0.058879213	0.209748903	0.803942707
HBP1	0.242158634	0.653892755	0.317058893	0.67278422	0.039344675	0.210201962	0.803942707
HDHD5	0.150428502	0.873082956	0.660901581	0.023523089	0.641159702	0.208605615	0.803942707
HNRNPH2	0.595496475	0.118430747	0.374298327	0.81919881	0.060329484	0.208239376	0.803942707
ICE2	0.062605142	0.369674245	0.302889643	0.943602078	0.19730808	0.208280035	0.803942707
IFI27L2	0.064584177	0.680054271	0.29915087	0.191917655	0.524995692	0.209790652	0.803942707
IL17RE	0.076310354	0.543790985	0.048677642	0.84789593	0.763614071	0.208502817	0.803942707
KCTD3	0.726382452	0.918364469	0.657719354	0.436508586	0.00693558	0.210150056	0.803942707
KDM6A	0.267890712	0.996445294	0.753436717	0.027622321	0.238986288	0.210099628	0.803942707
KRT19	0.204123103	0.032586307	0.801541207	0.259793792	0.947736472	0.208895182	0.803942707
LOC101904477	0.649616204	0.799857352	0.004947543	0.730544092	0.689790582	0.207495601	0.803942707
LOC101906086	0.998814075	0.285019506	0.372270777	0.03889876	0.322536952	0.210257234	0.803942707
LOC101906870	0.219174981	0.133510814	0.885803381	0.428552362	0.119702699	0.210261939	0.803942707
LOC101907084	0.237546463	0.251574525	0.498500725	0.138118801	0.323441997	0.210354639	0.803942707
LOC104971374	0.969397622	0.042438401	0.332009261	0.336181251	0.287226972	0.20939386	0.803942707
LOC104975626	0.922692189	0.656553304	0.023691614	0.333912744	0.276167487	0.209765265	0.803942707
LOC112443235	0.611515676	0.706171794	0.48460356	0.233778757	0.026481497	0.207502757	0.803942707
MET	0.017028471	0.409110401	0.666971259	0.345192719	0.814542336	0.208389975	0.803942707
MLLT1	0.69670724	0.796199806	0.053072407	0.967025362	0.046508654	0.209810784	0.803942707
MPC2	0.45533002	0.756875359	0.298203823	0.025897357	0.49830164	0.209982058	0.803942707
MRPL18	0.174845222	0.510302142	0.43837633	0.055426058	0.598526102	0.207666332	0.803942707
MS4A2	0.76261226	0.303101031	0.161310831	0.555884796	0.063872033	0.209795459	0.803942707
NDUFAB1	0.613630243	0.632030757	0.738678033	0.005899999	0.781773735	0.209595345	0.803942707
NOC3L	0.641669508	0.751922701	0.121515405	0.07827079	0.287489616	0.209425162	0.803942707
ORM1	0.323818414	0.73933077	0.170352475	0.084071409	0.381705148	0.208576861	0.803942707

PER2	0.897057808	0.906173597	0.277733969	0.072514628	0.079341642	0.20777872	0.803942707
PPIL2	0.361741495	0.035900512	0.302377246	0.672072196	0.496543822	0.208712614	0.803942707
PRMT1	0.499475976	0.209641207	0.143143771	0.63437571	0.136880911	0.207989257	0.803942707
PTPN7	0.136561012	0.729318529	0.364507846	0.37675877	0.096701903	0.209697227	0.803942707
RAB44	0.919211401	0.332915942	0.322094886	0.257062327	0.052147171	0.20958796	0.803942707
RAP1GAP2	0.234969235	0.422842733	0.019770326	0.982327244	0.677705062	0.208487982	0.803942707
RBBP4	0.944633849	0.08285638	0.349452135	0.55676172	0.086569318	0.209345096	0.803942707
RCC1	0.385873245	0.640943254	0.652854913	0.608374031	0.013410614	0.209269215	0.803942707
RIPOR2	0.053006548	0.741568959	0.999126586	0.267901509	0.126455024	0.210325817	0.803942707
S100A10	0.337820668	0.582127637	0.880836098	0.013860326	0.551578299	0.209827284	0.803942707
SARAF	0.484239218	0.20555974	0.083642427	0.258167685	0.608713438	0.208546205	0.803942707
SCN3A	0.551683824	0.864173719	0.008611332	0.702546341	0.449027851	0.207468204	0.803942707
SELENOF	0.638278439	0.284872141	0.989011869	0.509780631	0.014353744	0.20914941	0.803942707
SRP14	0.55729652	0.071688616	0.219308396	0.200132903	0.754683426	0.209752545	0.803942707
ST13	0.888105345	0.348474575	0.119627141	0.03875506	0.90954002	0.208272276	0.803942707
THRSP	0.464951128	0.412260862	0.24489008	0.068147849	0.41247043	0.209439293	0.803942707
TMEM219	0.171928888	0.597553015	0.202837691	0.115824904	0.54513157	0.209141359	0.803942707
TMEM222	0.241961909	0.962067868	0.847567156	0.087329851	0.076388521	0.209175679	0.803942707
TMEM63A	0.836837649	0.064233617	0.756006425	0.05181054	0.630265278	0.210045554	0.803942707
TOMM20	0.657388937	0.036422283	0.143011137	0.886751119	0.43593401	0.209778715	0.803942707
TRABD	0.444261948	0.014048282	0.268972294	0.977466995	0.797427121	0.208551956	0.803942707
TSPAN4	0.568088005	0.039657086	0.533698759	0.152158779	0.713960699	0.208366911	0.803942707
UBL7	0.334862081	0.843439579	0.680157592	0.007876152	0.867235772	0.208849091	0.803942707
VAPA	0.991463098	0.101969906	0.058117148	0.29958917	0.742968734	0.208499901	0.803942707
YPEL1	0.369328518	0.588350722	0.022855448	0.382091139	0.685281625	0.207897232	0.803942707
ZNF383	0.132511098	0.745365517	0.187183828	0.311280829	0.225218461	0.207549908	0.803942707
ZNF503	0.039854585	0.411754521	0.485769694	0.787515266	0.209043633	0.208864292	0.803942707
BOLA2B	0.67261928	0.048038735	0.12880961	0.454749207	0.704872765	0.2106154	0.804358341
DFFA	0.092245123	0.267736776	0.106733944	0.842108467	0.600749457	0.210572052	0.804358341
LOC107132465	0.612640932	0.992145913	0.02020278	0.347085764	0.313114179	0.210649906	0.804358341
LPIN3	0.97751148	0.007963777	0.61472146	0.868204808	0.321387455	0.210708458	0.804358341
ZFAT	0.503664772	0.224902145	0.870682073	0.035252945	0.383996949	0.210695556	0.804358341
EARS2	0.258393736	0.5537377	0.247278335	0.41790304	0.090601202	0.211055605	0.804601856
GABARAP	0.640788764	0.040054389	0.074927494	0.758936054	0.919601745	0.211259876	0.804601856
LOC104975006	0.319928978	0.663670484	0.065527155	0.298448592	0.322670073	0.211073817	0.804601856
LOC112447402	0.40438488	0.856506791	0.024628266	0.50045833	0.314693471	0.211359423	0.804601856
LOC506408	0.75643282	0.026866586	0.183381527	0.751098525	0.478281561	0.21099096	0.804601856
NHLRC1	0.643722991	0.41266988	0.022433308	0.702932819	0.319658933	0.211009862	0.804601856
PDS5A	0.718576675	0.227384316	0.502085874	0.038450456	0.425551598	0.211273219	0.804601856
PPP1R3C	0.075462367	0.156433747	0.246088616	0.827566023	0.556261235	0.210871597	0.804601856
RELT	0.286614417	0.440829604	0.094030815	0.218979553	0.516158917	0.211312796	0.804601856
SEC24A	0.422355326	0.908359744	0.226224867	0.098058645	0.157759206	0.21129575	0.804601856
TCTEX1D1	0.063652831	0.545857566	0.454286294	0.939696988	0.090233627	0.210957485	0.804601856
USP5	0.202724333	0.992128935	0.813093968	0.070173802	0.117065842	0.211360588	0.804601856

CHMP1A	0.753406536	0.273486537	0.300064596	0.026398984	0.82433021	0.211520464	0.804650519
PCBD2	0.246398543	0.41752532	0.348257585	0.044784721	0.838010024	0.211454415	0.804650519
TTC21A	0.847185261	0.021794306	0.405334689	0.545657452	0.329463576	0.211519029	0.804650519
PEG3	0.14383693	0.592727771	0.403484462	0.042730443	0.916595318	0.211668204	0.805025931
C11H9orf50	0.108783015	0.530775718	0.157899301	0.162303991	0.912553854	0.211908239	0.805353359
COPS8	0.770865332	0.081533674	0.107777018	0.204397436	0.97565082	0.211950591	0.805353359
STRA6	0.582673009	0.052195975	0.114532254	0.584587707	0.662516213	0.211808465	0.805353359
TERF2	0.580352287	0.362054514	0.71025945	0.042343566	0.213696425	0.211914986	0.805353359
ACOT8	0.334180704	0.622290396	0.350865073	0.04229349	0.438634964	0.212167964	0.80543337
LOC100335467	0.427412435	0.524876312	0.051189739	0.128378734	0.91761348	0.21210582	0.80543337
POT1	0.044703203	0.841178809	0.613506678	0.062568169	0.936829879	0.212061473	0.80543337
TTC22	0.29557509	0.41307472	0.047418612	0.603380322	0.387382474	0.212139548	0.80543337
PMVK	0.546128221	0.604999435	0.025812434	0.294146252	0.540017942	0.212256632	0.805583624
ADGB	0.315263923	0.033422818	0.576825198	0.291348344	0.76778538	0.212643571	0.8055922
AEBP1	0.216826635	0.440734881	0.125473445	0.19718345	0.594011897	0.216155396	0.8055922
ALDH1B1	0.567792587	0.016885651	0.238707341	0.696960314	0.854099073	0.212860463	0.8055922
AP3S2	0.413106237	0.292957307	0.208100638	0.129365414	0.432554677	0.216529596	0.8055922
ARF5	0.07030516	0.816804579	0.554018007	0.051799968	0.842333458	0.214888225	0.8055922
BATF	0.965013366	0.502223075	0.046568943	0.429626131	0.141227123	0.21341746	0.8055922
BCCIP	0.591601424	0.311148165	0.272527235	0.46394122	0.058817302	0.213377551	0.8055922
CCDC71L	0.079149652	0.097407511	0.971822843	0.39669067	0.466430661	0.21474469	0.8055922
CD101	0.167622488	0.534458968	0.438327186	0.050374958	0.694059575	0.213696025	0.8055922
CFAP69	0.265023324	0.119361322	0.252425486	0.240551311	0.714833249	0.213705193	0.8055922
CORO1A	0.198235771	0.917117568	0.546254461	0.412466786	0.033810141	0.214637517	0.8055922
COX7B2	0.079681898	0.705854285	0.181223878	0.644823183	0.210710367	0.214632204	0.8055922
CPT1C	0.269753222	0.16746212	0.134169153	0.359022323	0.639581018	0.215165483	0.8055922
CROCC	0.843994698	0.06465477	0.056119599	0.963233014	0.470025241	0.214754609	0.8055922
CTSV	0.076149665	0.758076399	0.247916725	0.10037205	0.978761334	0.21627301	0.8055922
CUEDC2	0.283881153	0.901697139	0.806480615	0.009004632	0.747084974	0.214934257	0.8055922
CYP2J2	0.096695543	0.208533128	0.439350872	0.190427583	0.811237915	0.213351901	0.8055922
DDX52	0.153023552	0.062343418	0.992108915	0.380081925	0.389726103	0.215964243	0.8055922
DHPS	0.853534201	0.368392317	0.167136334	0.034941689	0.759388273	0.215379766	0.8055922
DUSP12	0.447481713	0.734740317	0.478535495	0.014089063	0.625010078	0.214676038	0.8055922
EIF4A1	0.322575272	0.15566073	0.856138422	0.300936987	0.107091128	0.214673729	0.8055922
ELL3	0.778723023	0.19414538	0.242168215	0.270847043	0.137807177	0.213191103	0.8055922
ELP6	0.608367344	0.86193227	0.242471701	0.132161872	0.080960905	0.212709852	0.8055922
FAM43B	0.848877379	0.978709775	0.099134147	0.950864193	0.01739091	0.212829082	0.8055922
FAM46B	0.582108998	0.360292283	0.255217848	0.047110569	0.556186438	0.216005372	0.8055922
FNBP1L	0.07520068	0.477892345	0.163027182	0.496842317	0.48106938	0.215837272	0.8055922
GALNT9	0.135214467	0.140474638	0.928085236	0.249995791	0.314331823	0.214660462	0.8055922
GFER	0.290018949	0.538495747	0.308617839	0.157328349	0.183728988	0.215280805	0.8055922
GPN1	0.196730935	0.294050232	0.048717722	0.654480802	0.737796984	0.212742869	0.8055922
GRAMD1C	0.671020223	0.80302402	0.057451876	0.1191672	0.378618661	0.215559113	0.8055922
HSPA6	0.172625522	0.327755771	0.493394829	0.134148487	0.371023897	0.2149868	0.8055922

HSPA8	0.371417086	0.48117946	0.608704017	0.016596082	0.768810591	0.214877562	0.8055922
IRAK2	0.535564652	0.830035413	0.172934338	0.019664881	0.930775909	0.216360787	0.8055922
JSRP1	0.182999574	0.807740369	0.63247812	0.063008798	0.235865462	0.214985629	0.8055922
KIF3A	0.383928728	0.839779456	0.090205941	0.165825945	0.288916451	0.215296155	0.8055922
KMT2A	0.465844501	0.637749712	0.407400474	0.066654269	0.173603361	0.21585248	0.8055922
LAMA5	0.775306242	0.797463727	0.049467567	0.472606503	0.095426629	0.214197939	0.8055922
LOC100141266	0.118610465	0.288909371	0.125172652	0.764037321	0.421788396	0.21442942	0.8055922
LOC101902839	0.688831444	0.208860523	0.824171899	0.03990753	0.28977434	0.213558215	0.8055922
LOC101905743	0.875855885	0.492088072	0.017029846	0.204608416	0.904409684	0.212534883	0.8055922
LOC101907729	0.399077223	0.281249865	0.671195153	0.018881815	0.990559213	0.216510402	0.8055922
LOC104968411	0.663214419	0.069550649	0.48700145	0.08488258	0.717788351	0.213359168	0.8055922
LOC104971683	0.73628646	0.195119896	0.098397056	0.124344694	0.789437424	0.214846455	0.8055922
LOC104974667	0.678944976	0.45511965	0.013626075	0.539382218	0.618308893	0.216136734	0.8055922
LOC107133284	0.281426177	0.360227441	0.466390896	0.059590261	0.496677327	0.215762841	0.8055922
LOC112444616	0.328864655	0.513080726	0.245419484	0.106140206	0.310800079	0.213153851	0.8055922
LOC512005	0.521996331	0.081492787	0.353478872	0.778718517	0.116769293	0.21324968	0.8055922
LOC515578	0.251979816	0.421561322	0.153852744	0.207376293	0.403125118	0.213168174	0.8055922
MFSD12	0.246566076	0.6030195	0.092358283	0.518199852	0.197709303	0.216345403	0.8055922
MSRB3	0.222913142	0.271567588	0.238153025	0.6805985	0.141630478	0.215006731	0.8055922
PAK3	0.128716858	0.765242121	0.338984442	0.045173187	0.929955232	0.216017346	0.8055922
PCSK4	0.780011341	0.063943033	0.410012101	0.553421003	0.124461211	0.216474809	0.8055922
PDF	0.448915712	0.713030181	0.922800582	0.150294835	0.031447528	0.215505077	0.8055922
PFDN2	0.265347492	0.216726599	0.142605294	0.270090194	0.626953066	0.214929145	0.8055922
PHLPP2	0.143681324	0.155088882	0.234432108	0.293360043	0.886178992	0.212521418	0.8055922
PIK3R2	0.463201659	0.248019425	0.016574855	0.978983639	0.754813371	0.216359364	0.8055922
PKMYT1	0.156743564	0.140911321	0.552506997	0.224346553	0.513693508	0.216303235	0.8055922
PNPLA7	0.569273128	0.024623899	0.263723832	0.760673915	0.496443562	0.215501109	0.8055922
POLQ	0.772995149	0.019468871	0.19630089	0.539188213	0.870305194	0.214740733	0.8055922
PRAM1	0.181388452	0.076794592	0.375356722	0.299867087	0.881993461	0.214473122	0.8055922
PRDM15	0.722573851	0.091128473	0.820151289	0.155878988	0.163549807	0.213997284	0.8055922
PRRX1	0.084432774	0.963479904	0.111312243	0.281413114	0.550207759	0.215969944	0.8055922
PSAP	0.537844108	0.362328373	0.010332914	0.938233262	0.745840291	0.21651442	0.8055922
RACGAP1	0.393142555	0.204613194	0.43906256	0.818672963	0.048545466	0.216095654	0.8055922
RAD51D	0.720508833	0.864725543	0.186291664	0.014077701	0.829715746	0.212336267	0.8055922
RBMS1	0.762589841	0.696119052	0.057742536	0.092183744	0.486814282	0.213902779	0.8055922
RTP4	0.915787466	0.07216457	0.31818556	0.097478631	0.668343049	0.213460388	0.8055922
SBNO2	0.303849925	0.133821181	0.594000989	0.270180057	0.213511961	0.215288883	0.8055922
SH3YL1	0.762128828	0.042356875	0.099572188	0.830683723	0.525102154	0.215970483	0.8055922
SLC25A17	0.47860889	0.363091288	0.379821428	0.039916018	0.532828896	0.216106137	0.8055922
SMARCA1	0.663409944	0.581957946	0.920592104	0.011463168	0.332975399	0.212406888	0.8055922
SPRED2	0.516691404	0.508936074	0.235807759	0.181306476	0.124725877	0.215983664	0.8055922
TAF1	0.264861439	0.366006726	0.363815789	0.084369824	0.461335008	0.213680216	0.8055922
TBC1D13	0.698829286	0.318125316	0.094135216	0.075535803	0.875702276	0.214585843	0.8055922
TFE3	0.15129113	0.540104332	0.501878472	0.400226809	0.083148867	0.213049493	0.8055922

THAP9	0.599413529	0.113258365	0.603566744	0.038953234	0.861947513	0.213917313	0.8055922
TIFA	0.24157314	0.311108356	0.379549963	0.364073271	0.131243745	0.212911779	0.8055922
TRIM13	0.826221418	0.232849547	0.512124347	0.068894678	0.200482555	0.212741531	0.8055922
TSEN54	0.306676292	0.543023955	0.155581561	0.058748419	0.900324949	0.213496334	0.8055922
TSTD1	0.493400133	0.073893829	0.739702905	0.122672097	0.42086355	0.215215196	0.8055922
TTC39B	0.92416887	0.401793086	0.331810551	0.381339483	0.028910465	0.212543779	0.8055922
UBE2D3	0.569036655	0.135254673	0.174160278	0.182098734	0.575685509	0.21621217	0.8055922
UBE2Z	0.562921099	0.392557768	0.056681962	0.129389002	0.866659754	0.216164046	0.8055922
ULK3	0.916803903	0.023851162	0.580197099	0.83840017	0.129843051	0.214336813	0.8055922
VASH1	0.435133046	0.225588771	0.027014456	0.535056864	0.992394821	0.216434569	0.8055922
ZBTB17	0.686289207	0.146517643	0.021801984	0.973063105	0.63878592	0.212885204	0.8055922
ZNHIT3	0.705391092	0.435134407	0.592061604	0.0191246	0.402062758	0.215604006	0.8055922
C3H1orf52	0.953859944	0.789029897	0.006993743	0.480097281	0.558754622	0.216740459	0.806193943
WRAP53	0.58018308	0.29335026	0.040309132	0.477564964	0.431429495	0.216855866	0.806440431
PANK3	0.887471057	0.169313572	0.193714243	0.329752711	0.147336011	0.216908045	0.806451729
MEF2A	0.3950091	0.186991125	0.184383056	0.192094258	0.540889113	0.21697517	0.806518575
FCGR2B	0.041477754	0.749559249	0.883494817	0.671040245	0.076927503	0.217197508	0.806614235
PFDN6	0.093935726	0.681489497	0.516290212	0.270143384	0.158794404	0.217186931	0.806614235
PRKAB2	0.288415493	0.231371165	0.25423492	0.782178788	0.106826854	0.21717085	0.806614235
TMEM167B	0.600641688	0.020831904	0.273614295	0.950894583	0.43524687	0.217120962	0.806614235
ALG13	0.486936611	0.258757558	0.305023696	0.918789206	0.04035215	0.217733197	0.80676408
CCAR2	0.659512528	0.014101424	0.283231317	0.586527755	0.921690721	0.217662455	0.80676408
CCDC191	0.911378309	0.073155535	0.128676348	0.40095199	0.413009329	0.21740938	0.80676408
DLGAP1	0.453410087	0.983365989	0.059561419	0.103619664	0.516219923	0.217397165	0.80676408
LOC107132225	0.287824705	0.206841378	0.067645468	0.72340492	0.488639331	0.21763044	0.80676408
LOC107132852	0.248939484	0.476842576	0.126115511	0.424434407	0.224227014	0.217721651	0.80676408
LOC112442049	0.967028229	0.202227594	0.25009092	0.087173241	0.333386046	0.217462602	0.80676408
LOC511617	0.411277754	0.989116887	0.386277438	0.48711953	0.018631116	0.217827776	0.80676408
POLR1A	0.440809071	0.954193476	0.891390485	0.10841429	0.034979488	0.217499598	0.80676408
SDK2	0.821217971	0.02240022	0.199323741	0.799125961	0.484720653	0.217379077	0.80676408
SPATA9	0.206015931	0.106993353	0.228203221	0.387006934	0.732347507	0.217792143	0.80676408
YWHAQ	0.702261201	0.214230466	0.763096093	0.012903158	0.961619919	0.217702178	0.80676408
INTS13	0.085863782	0.611376284	0.24589411	0.115091324	0.960953278	0.217942582	0.806825111
LOC783497	0.536463645	0.068941236	0.212572872	0.350531286	0.517819072	0.217897271	0.806825111
ARL2	0.226214448	0.569064914	0.521524976	0.064844927	0.32807745	0.217992572	0.80682817
LOC101907133	0.251104009	0.371176095	0.534499518	0.299137067	0.095970415	0.218138741	0.807187121
LOC112441525	0.613019628	0.341215531	0.145809751	0.141666053	0.331408923	0.218272919	0.807501549
DLG4	0.385432164	0.225196542	0.098180631	0.19007285	0.885600674	0.218468498	0.807640593
KCNJ10	0.471269674	0.176084484	0.136795086	0.400998832	0.315382504	0.21855657	0.807640593
LOC112444841	0.508039456	0.049779744	0.407116241	0.148561528	0.938387565	0.218535159	0.807640593
MCL1	0.507386805	0.191203621	0.04431787	0.580218549	0.575477445	0.21855462	0.807640593
RBM4	0.287589174	0.476206233	0.614230231	0.04098808	0.41635249	0.218550437	0.807640593
SSU72	0.643236879	0.427723468	0.842942018	0.068029555	0.091039181	0.218611183	0.807660542
GABPB1	0.039107716	0.375020784	0.903307811	0.179839196	0.603351026	0.218699808	0.807690507

NADK	0.26883431	0.90054045	0.155199453	0.082511288	0.464245818	0.21883434	0.807690507
SUPT6H	0.556669795	0.316501418	0.203837405	0.044825143	0.893128904	0.218721333	0.807690507
SYBU	0.117017571	0.814872567	0.79272526	0.058105871	0.327777071	0.218865376	0.807690507
TBX1	0.340510483	0.297294211	0.021357512	0.955670434	0.69671806	0.218857974	0.807690507
BTF3	0.640920176	0.427456698	0.016363078	0.353122988	0.910160256	0.218952206	0.807784037
MEX3A	0.47770898	0.659993743	0.090409859	0.240415194	0.210314839	0.218989165	0.807784037
AP3B2	0.471414787	0.820294545	0.149782691	0.569744284	0.044077693	0.219999812	0.807942711
ARMC6	0.466838595	0.862042676	0.255490313	0.147618762	0.095935319	0.220115899	0.807942711
ASCC2	0.025824588	0.825884537	0.253888337	0.706777688	0.379427562	0.21981524	0.807942711
C19H17orf80	0.618566336	0.063795727	0.456820757	0.269923006	0.297585263	0.219502377	0.807942711
CMC2	0.299043484	0.622158277	0.874841	0.020897228	0.424241413	0.219119713	0.807942711
GATD1	0.640263912	0.739095591	0.285387614	0.029092899	0.371332847	0.220333444	0.807942711
HGF	0.153355279	0.218813935	0.289768591	0.921211319	0.161483038	0.219384695	0.807942711
LOC100848025	0.582505559	0.93244429	0.007020296	0.381436633	0.999099707	0.219892088	0.807942711
LOC107133095	0.050190079	0.557998811	0.825322409	0.164485704	0.382937403	0.220100818	0.807942711
LOC112448833	0.10684599	0.114177851	0.204217464	0.719095647	0.812838435	0.220124501	0.807942711
LOC789231	0.539407397	0.349439729	0.016167129	0.481973441	0.984139839	0.219306272	0.807942711
LY86	0.008338157	0.77586287	0.575631178	0.819126273	0.475451765	0.219675414	0.807942711
MAGT1	0.393469885	0.043857369	0.3855622	0.55144181	0.395308649	0.219682486	0.807942711
MRPL46	0.355765256	0.608087317	0.343456354	0.056917902	0.34256301	0.219557034	0.807942711
NDUFB11	0.331236853	0.469260384	0.685997201	0.014637886	0.933079985	0.220136776	0.807942711
NMT2	0.055243216	0.168068364	0.380281604	0.623069486	0.659401001	0.219700719	0.807942711
NPAT	0.693239043	0.064650965	0.194456406	0.35524635	0.466369068	0.219189048	0.807942711
RBCK1	0.4112343	0.152119699	0.42077562	0.084116784	0.655273129	0.219719524	0.807942711
RHBG	0.031875376	0.374656697	0.325526276	0.416624916	0.89455091	0.219565862	0.807942711
SORCS2	0.472869136	0.168767511	0.402878592	0.135119107	0.335949436	0.220371515	0.807942711
TOLLIP	0.455766296	0.756735996	0.036008208	0.22999797	0.507903453	0.219710103	0.807942711
TSSK1B	0.158958322	0.334291332	0.145504145	0.639679641	0.294606294	0.220191762	0.807942711
VPS41	0.902954584	0.281020239	0.008853172	0.899325651	0.718762407	0.219814613	0.807942711
YJU2	0.598078968	0.2474553	0.89524294	0.014276735	0.77083309	0.220267361	0.807942711
ZC3H11A	0.44682802	0.602611735	0.105367411	0.19124961	0.267729467	0.219859404	0.807942711
ZNF106	0.400749366	0.523535802	0.161194426	0.631438528	0.06789841	0.219650849	0.807942711
ZNF385A	0.019391745	0.701381019	0.577557416	0.213327739	0.870038535	0.22025882	0.807942711
ZNF507	0.407287643	0.408386057	0.815684509	0.015408438	0.698386019	0.220410671	0.807942711
COX17	0.411875348	0.334286497	0.34437203	0.033172973	0.92930548	0.220539747	0.808232164
KIAA0930	0.243347252	0.088737788	0.140210119	0.803909624	0.601393945	0.220699047	0.808232164
LOC101905525	0.155303868	0.863407649	0.127056127	0.748983101	0.114750188	0.22073435	0.808232164
RNF7	0.198476643	0.084914216	0.31411803	0.426779438	0.647444916	0.220624723	0.808232164
SH2B3	0.51183762	0.431421264	0.836187251	0.019312435	0.410630197	0.220735882	0.808232164
KAT7	0.078718196	0.557759167	0.369366281	0.505145887	0.179042176	0.220920992	0.808595131
LOC112442032	0.361589176	0.376514145	0.023531335	0.651064451	0.703288067	0.220933555	0.808595131
AATF	0.675707949	0.34441194	0.443715977	0.387583568	0.037188521	0.222548982	0.808939503
AP4M1	0.692389119	0.021709267	0.720925733	0.724490314	0.188490871	0.221906505	0.808939503
ARHGAP33	0.713810458	0.246602523	0.181957868	0.094649284	0.494054804	0.223249305	0.808939503

ATP13A3	0.631436074	0.725836665	0.109288597	0.093098261	0.317551821	0.221980854	0.808939503
ATP5PF	0.43227046	0.23755528	0.610173159	0.026785354	0.876806388	0.221284183	0.808939503
CLASP2	0.343390599	0.591898343	0.640429846	0.033962523	0.338875054	0.223275765	0.808939503
CRTAM	0.438342878	0.066016427	0.304223773	0.718023837	0.232705377	0.221240766	0.808939503
EIF4H	0.897587449	0.054033337	0.098646096	0.829502605	0.373704875	0.2221513	0.808939503
FAM98A	0.56322853	0.302521762	0.936542931	0.430761333	0.021419793	0.221347138	0.808939503
FDXACB1	0.39613259	0.461189584	0.208698445	0.401231213	0.096821987	0.222008671	0.808939503
GRAMD1A	0.271388007	0.243611385	0.976541487	0.024544723	0.944643367	0.223187921	0.808939503
GTF2H5	0.813569674	0.386541123	0.599820324	0.021575051	0.363826028	0.221970082	0.808939503
GZMA	0.969954981	0.043559918	0.684165015	0.122449873	0.422638917	0.223116564	0.808939503
HLX	0.510022009	0.342501198	0.029630455	0.633222778	0.456218789	0.223063041	0.808939503
HMGA1	0.158493453	0.183117408	0.072913027	0.924713122	0.765717791	0.223295104	0.808939503
JAM3	0.768272007	0.133081369	0.24518298	0.856901132	0.06946221	0.22282749	0.808939503
LMBR1	0.96902209	0.247103256	0.01683694	0.528034286	0.702001263	0.223000429	0.808939503
LOC100336368	0.141780789	0.816780127	0.029060848	0.724098153	0.605391663	0.221562731	0.808939503
LOC101904332	0.923523889	0.72356868	0.639267609	0.004585876	0.764871497	0.223294986	0.808939503
LOC104973099	0.016698957	0.944301053	0.541600805	0.396575025	0.435427566	0.221525849	0.808939503
LOC104975663	0.869891319	0.317069977	0.418122435	0.02285273	0.566619861	0.222917765	0.808939503
LOC112443850	0.930570532	0.789770515	0.670016766	0.015352428	0.197745678	0.223037585	0.808939503
LOC112444889	0.231414413	0.356850261	0.840822301	0.079218108	0.271762334	0.223031014	0.808939503
LOC112446734	0.315027392	0.154613126	0.300807698	0.230103183	0.442308406	0.222758063	0.808939503
LOC112447010	0.515077066	0.623639552	0.852824536	0.938591301	0.005721694	0.221256679	0.808939503
MEDAG	0.194858043	0.297363847	0.950937888	0.067686564	0.395743587	0.22161649	0.808939503
MRPL2	0.249281199	0.694907248	0.502068317	0.038518413	0.447230805	0.223284159	0.808939503
NELFB	0.543191079	0.100325116	0.448289027	0.064463982	0.949720851	0.223092457	0.808939503
NUDT3	0.624753904	0.096652985	0.105549483	0.709288539	0.33024185	0.222887445	0.808939503
PCM1	0.157041335	0.580879264	0.524684967	0.088197374	0.353365024	0.222795602	0.808939503
PINX1	0.073594986	0.715564005	0.824216662	0.590270457	0.058177134	0.222709308	0.808939503
POLR2C	0.850584212	0.034772634	0.068720691	0.982315478	0.746708154	0.222735699	0.808939503
POLR3F	0.099216549	0.817187156	0.109444557	0.413068372	0.403832248	0.22193566	0.808939503
PRELID1	0.190313686	0.586695318	0.468210677	0.271172299	0.104264009	0.221777263	0.808939503
PRPF4B	0.581539373	0.363214129	0.151421782	0.132084969	0.351334179	0.22223815	0.808939503
PRPSAP2	0.026464634	0.299652473	0.235308215	0.827960536	0.957330021	0.221851062	0.808939503
PUS7	0.355958344	0.157327621	0.53809628	0.538173102	0.09184517	0.222632581	0.808939503
RPP21	0.308942508	0.288883846	0.163658503	0.363886021	0.279083139	0.222170124	0.808939503
SEC24D	0.611431918	0.543885326	0.197631243	0.041137529	0.552611354	0.222973133	0.808939503
SMARCA2	0.522709786	0.351730382	0.048537479	0.413509557	0.399791479	0.221563269	0.808939503
TAC3	0.553570208	0.132705362	0.150462095	0.956435568	0.140931774	0.222660924	0.808939503
THAP12	0.476652649	0.067582643	0.115281246	0.621875558	0.637094122	0.221265924	0.808939503
TNFSF9	0.459012846	0.51416483	0.119016099	0.402186729	0.130874481	0.221805896	0.808939503
TRIM7	0.130454499	0.331759094	0.749728942	0.099850323	0.455337602	0.22156387	0.808939503
TSC22D2	0.330927733	0.389690623	0.839813824	0.01647217	0.829496849	0.22190468	0.808939503
UBE2D2	0.991586868	0.063045661	0.075067822	0.867739175	0.365900166	0.222670848	0.808939503
ARHGEF39	0.717606268	0.126161489	0.053884362	0.347001573	0.886980139	0.22352644	0.809061805

COL25A1	0.196636845	0.435373984	0.473271218	0.724625111	0.051231916	0.223724752	0.809061805
HOXB9	0.246104986	0.961821946	0.063705418	0.313186909	0.317527301	0.223386223	0.809061805
LOC101902361	0.372123429	0.833719574	0.38190844	0.432271318	0.029317371	0.223533286	0.809061805
LOC101904103	0.126284489	0.208351596	0.512510959	0.244843551	0.455090128	0.22360751	0.809061805
LOC616199	0.177104864	0.632162311	0.024812069	0.886653118	0.610019751	0.223603294	0.809061805
MOSPD1	0.521547918	0.098404613	0.165823998	0.951950884	0.185783843	0.223798955	0.809061805
MSN	0.49291412	0.13039983	0.398754182	0.113558544	0.51724359	0.223821863	0.809061805
PFDN4	0.837381725	0.232104134	0.41762941	0.026085666	0.710678917	0.223771458	0.809061805
SPP2	0.393348903	0.229260045	0.281898101	0.23522914	0.251502635	0.223709478	0.809061805
CCND1	0.324808621	0.324487588	0.205246072	0.146970796	0.474999055	0.224171349	0.809093178
CDC42EP5	0.629867915	0.313145261	0.045192711	0.656902241	0.257571029	0.224026225	0.809093178
CLEC4G	0.405952237	0.251098461	0.389372377	0.307653906	0.123524121	0.224035727	0.809093178
LARP1B	0.622902332	0.96415519	0.200180995	0.034242538	0.366847272	0.224175655	0.809093178
LOC100848940	0.258471041	0.388674499	0.201335272	0.118688995	0.627848983	0.22395456	0.809093178
LOC112443339	0.214137561	0.491544269	0.498833889	0.497941777	0.057655006	0.223965791	0.809093178
LOC112444588	0.171699982	0.632591816	0.032279299	0.665188577	0.647300746	0.22413125	0.809093178
BFAR	0.97817763	0.320195457	0.669605994	0.112720205	0.064053601	0.224474116	0.809525904
CD160	0.560214623	0.23132261	0.914305606	0.556125446	0.023041133	0.224769368	0.809525904
CUL9	0.707822815	0.040724185	0.125425618	0.516343231	0.814491454	0.224936818	0.809525904
DNMT1	0.308345878	0.152441238	0.060194051	0.563366135	0.952273214	0.224745071	0.809525904
GREB1L	0.774506689	0.108510909	0.897056573	0.03820425	0.527536144	0.224857718	0.809525904
IL34	0.385593605	0.06470334	0.746131887	0.158026072	0.516856159	0.224932251	0.809525904
LOC101905866	0.381305492	0.731262519	0.074099333	0.108168251	0.677124987	0.224404872	0.809525904
NDUFA7	0.444405768	0.842136103	0.815235337	0.00914282	0.544987663	0.224917062	0.809525904
PLAG1	0.163489345	0.62528687	0.86428069	0.111082553	0.154700672	0.224774825	0.809525904
PPP1R14C	0.808726833	0.042744523	0.153588072	0.522649276	0.546035026	0.224544643	0.809525904
PRELID3A	0.593410125	0.58374443	0.458946693	0.009581563	0.994945657	0.22457176	0.809525904
PRICKLE1	0.773977531	0.466590961	0.19459389	0.030768946	0.700998419	0.224584124	0.809525904
WDHD1	0.544776181	0.231540417	0.129659611	0.199524965	0.46587295	0.224917721	0.809525904
CCDC34	0.317444471	0.1342736	0.936442542	0.625354087	0.061110513	0.225297328	0.810106112
CDC16	0.814706911	0.943234478	0.816622457	0.03230911	0.075277404	0.22536221	0.810106112
LOC112447756	0.588583062	0.153272228	0.122769508	0.364774164	0.378189467	0.225482198	0.810106112
MRPL17	0.406947817	0.389628428	0.112232208	0.906503157	0.094724111	0.225492945	0.810106112
NFX1	0.277847828	0.618557255	0.440962992	0.058479138	0.3444994	0.225400144	0.810106112
PISD	0.153603069	0.348637641	0.310321939	0.175485678	0.523331882	0.225355347	0.810106112
SNAPC4	0.967867276	0.014691321	0.168942437	0.895975729	0.70884025	0.22531767	0.810106112
STYX	0.904603226	0.810843491	0.213085685	0.030879544	0.315904697	0.225244241	0.810106112
ACADS	0.485039434	0.599425098	0.318686029	0.035599948	0.464287192	0.225745496	0.810487451
ATP6V1F	0.310585222	0.445355397	0.257800063	0.05260147	0.817663178	0.22590937	0.810487451
CYP27A1	0.042642395	0.884069416	0.350744159	0.446823777	0.259906463	0.226046629	0.810487451
EXOC3L2	0.170402388	0.958076397	0.049876658	0.208572345	0.903891214	0.226014003	0.810487451
IGSF10	0.424035829	0.76667579	0.050952449	0.45539093	0.203206932	0.225846955	0.810487451
MPG	0.56231234	0.796568295	0.302890206	0.018404957	0.615218723	0.226092959	0.810487451
PHLPP1	0.846996686	0.802337242	0.082899228	0.146833912	0.185647998	0.226055465	0.810487451

RABGGTA	0.434698455	0.608069711	0.572630896	0.013806716	0.734610953	0.226018673	0.810487451
SHC2	0.683132931	0.940974097	0.051506386	0.115472985	0.401241912	0.225932151	0.810487451
TTK	0.914949108	0.156710908	0.074651295	0.299408548	0.478115647	0.225802445	0.810487451
MAP3K6	0.98604571	0.012558954	0.482783269	0.522313461	0.492170222	0.226144641	0.810495678
BORCS5	0.313378459	0.572927121	0.466306774	0.109142361	0.168764924	0.226525687	0.810673803
EHD3	0.558381685	0.512128225	0.138989807	0.051856389	0.746851437	0.22632128	0.810673803
ENSA	0.650411947	0.223685527	0.187573951	0.754715533	0.074884586	0.226540993	0.810673803
LOC100847376	0.308841087	0.581029698	0.907831863	0.141378919	0.066810787	0.226279904	0.810673803
LOC101902084	0.041924638	0.370704285	0.184367568	0.605126643	0.889249542	0.226508943	0.810673803
LOC101906756	0.425907766	0.825960428	0.89392982	0.010917534	0.448677246	0.226401189	0.810673803
LOC112448816	0.94523536	0.063895702	0.221485104	0.365593942	0.315373494	0.226542466	0.810673803
VDAC2	0.580716624	0.55901999	0.607895008	0.053016405	0.147479226	0.226589527	0.810673803
AP1AR	0.493103872	0.401673369	0.024652323	0.855834489	0.371116229	0.227164221	0.810779413
EMC10	0.456149558	0.887991823	0.420853973	0.014006035	0.646989045	0.226719196	0.810779413
FAM177A1	0.676046006	0.092829145	0.121567809	0.619202154	0.328406803	0.227204548	0.810779413
IER5L	0.382396652	0.388938945	0.151875321	0.128041613	0.536354565	0.227194657	0.810779413
LOC100139548	0.215722975	0.371857688	0.968687441	0.062652612	0.317836972	0.226911948	0.810779413
NOX4	0.968139516	0.615152833	0.734835195	0.119102132	0.029764575	0.227206204	0.810779413
PATL2	0.565892334	0.099426194	0.121550803	0.46443971	0.487222492	0.226924627	0.810779413
PMS1	0.414650449	0.384148811	0.720139743	0.423534606	0.031934805	0.227211791	0.810779413
QPRT	0.510724617	0.659197555	0.346979961	0.025159253	0.527894933	0.227211902	0.810779413
RPS6KA5	0.726935717	0.772814955	0.312822806	0.052739392	0.166735363	0.226764201	0.810779413
SPIRE1	0.154781962	0.184699271	0.195930641	0.451438387	0.613294207	0.227160626	0.810779413
WDR36	0.955295584	0.351356679	0.732735949	0.033439892	0.188436408	0.227084534	0.810779413
AP2A2	0.219721436	0.771325494	0.790906176	0.37285834	0.031085259	0.22736346	0.81112644
APOBEC2	0.477411162	0.044950453	0.173763538	0.579335896	0.719429968	0.227408004	0.81112644
GOSR2	0.585881087	0.954787315	0.734368825	0.041776568	0.090640856	0.22750786	0.811130023
SRPK3	0.520547828	0.498951857	0.236793327	0.479528832	0.052734664	0.227485045	0.811130023
LOC101905403	0.867663787	0.12274445	0.348501342	0.405343637	0.103588253	0.227717812	0.811349762
NUDT4	0.328781206	0.691265746	0.025576841	0.371568682	0.721081141	0.22764747	0.811349762
TGFB3	0.628610136	0.421135502	0.226991268	0.032151678	0.80641082	0.227686675	0.811349762
HSD17B10	0.637772962	0.877170508	0.265726913	0.017605514	0.59707678	0.228024152	0.811522083
LOC112446033	0.108520206	0.498326032	0.486482999	0.062526594	0.948340594	0.227829902	0.811522083
LOC781261	0.045876287	0.471845179	0.179705782	0.815266345	0.492727191	0.228021882	0.811522083
NACAD	0.708219042	0.49506103	0.024355305	0.586787932	0.312018012	0.228080653	0.811522083
RNF13	0.1452598	0.757336405	0.216996501	0.071329297	0.918431954	0.228112325	0.811522083
TRMT5	0.513311543	0.810202179	0.377153171	0.279669084	0.035579867	0.227887363	0.811522083
ZNRF1	0.325137007	0.738889796	0.048770136	0.195545758	0.681417445	0.227918891	0.811522083
ARID1B	0.293931645	0.097712774	0.491637427	0.112518673	0.985197152	0.228213879	0.811707405
LMO3	0.087192787	0.283980635	0.415499904	0.427016294	0.357349915	0.228550849	0.812553724
TOP2B	0.413582367	0.935871707	0.618057229	0.012763269	0.513984692	0.228509175	0.812553724
IER3	0.617164107	0.64149167	0.030957459	0.495127226	0.258875486	0.228625828	0.812644243
CLN3	0.087402569	0.032605584	0.956816847	0.902603939	0.639400417	0.228822383	0.813123334
FLRT3	0.55973828	0.941825824	0.421998237	0.09098962	0.077801605	0.228909256	0.813123334

TMEM150C	0.345215697	0.841649913	0.039515963	0.283652751	0.483508671	0.228893324	0.813123334
COPG1	0.226259763	0.981308009	0.223851596	0.227728615	0.139375188	0.229100458	0.813564568
GHR	0.506425838	0.681084934	0.016094664	0.340590689	0.834940918	0.229182195	0.813564568
NDUFB4	0.256492264	0.508485281	0.599463298	0.044920822	0.449328161	0.229139971	0.813564568
ARGLU1	0.636308524	0.103534657	0.056567638	0.616945715	0.694607158	0.230502037	0.813626742
B3GALT4	0.511791993	0.320659719	0.657888989	0.027371051	0.540146585	0.230445623	0.813626742
CDK9	0.942620293	0.028090469	0.186754183	0.817884927	0.394054583	0.230266827	0.813626742
CDYL2	0.368170421	0.860692365	0.071470579	0.885589676	0.078964223	0.229549551	0.813626742
COMMD6	0.340096464	0.523947637	0.442765916	0.029257671	0.687000319	0.229700305	0.813626742
COX6A1	0.825407443	0.925545875	0.32028619	0.00656742	0.983384445	0.229296354	0.813626742
EIF4A2	0.074855481	0.454530571	0.549834118	0.218279095	0.38777216	0.229528567	0.813626742
FBXO36	0.028489754	0.70139773	0.129799999	0.661352014	0.927057445	0.230017439	0.813626742
GTF3C5	0.460162809	0.578781011	0.114369667	0.20645602	0.252638647	0.229911421	0.813626742
HSPA5	0.25956114	0.848488515	0.203310708	0.074919477	0.47322243	0.229817285	0.813626742
IQSEC2	0.748799336	0.563262771	0.021628014	0.434587716	0.398650709	0.229306976	0.813626742
LCORL	0.297844016	0.448702435	0.07749682	0.473786864	0.324023922	0.229997501	0.813626742
LOC101906240	0.179777786	0.26743586	0.100205243	0.660407381	0.499949029	0.230048137	0.813626742
MED1	0.317682646	0.519685666	0.511890063	0.023408185	0.807054617	0.230468802	0.813626742
NCAPD3	0.761954385	0.015448291	0.571245375	0.486447949	0.486093614	0.229996747	0.813626742
NCAPH	0.139393682	0.336457312	0.198083063	0.190725857	0.897708602	0.230043298	0.813626742
OGG1	0.29653521	0.087388239	0.472435727	0.368350523	0.354134443	0.230500377	0.813626742
PKHD1L1	0.172601167	0.982866486	0.079753438	0.37621179	0.313852337	0.230538319	0.813626742
RIBC1	0.317570425	0.816686675	0.075025187	0.47319708	0.173065262	0.230250995	0.813626742
RPA2	0.206975749	0.433600108	0.115467066	0.565851347	0.270231354	0.229606817	0.813626742
SLC10A1	0.455523123	0.751761003	0.126159805	0.097907441	0.373784506	0.229355557	0.813626742
SPEG	0.172188872	0.89553225	0.12347029	0.442997395	0.189325663	0.230489309	0.813626742
TAOK2	0.973469788	0.047965219	0.139907333	0.847916415	0.287321662	0.230107874	0.813626742
TOR1A	0.390452468	0.532512337	0.921989805	0.069766493	0.118720526	0.229841134	0.813626742
USP21	0.874991337	0.156127547	0.036811376	0.956446121	0.33153159	0.230328983	0.813626742
USP47	0.533203575	0.066313752	0.129288678	0.366831058	0.951784438	0.230437253	0.813626742
ZFP36L1	0.304255351	0.766405615	0.028291375	0.559812615	0.430453993	0.229978855	0.813626742
HACD2	0.508354502	0.316115316	0.241403128	0.636264043	0.064816375	0.230704851	0.813772295
NHSL2	0.013654516	0.934956272	0.135151846	0.969623933	0.956474349	0.230728322	0.813772295
SRSF6	0.564409357	0.134757222	0.55161854	0.077803656	0.489943173	0.230666253	0.813772295
LOC785477	0.984305844	0.137781271	0.132800268	0.557479694	0.159679105	0.230947283	0.814019731
SEC61A1	0.174711878	0.51603656	0.617326157	0.587921993	0.048980208	0.230910757	0.814019731
WDR48	0.592929605	0.64639623	0.03495331	0.698456051	0.171322736	0.230933562	0.814019731
DRG2	0.471129254	0.726375375	0.114708632	0.405307446	0.101137575	0.231369259	0.814108258
LIN9	0.41969862	0.839945244	0.180950391	0.327746018	0.076884688	0.231245335	0.814108258
LOC100139764	0.401008335	0.230995861	0.576316759	0.077812881	0.386954864	0.231246742	0.814108258
LOC107132767	0.524653217	0.181998041	0.181545125	0.164431022	0.563914128	0.231244028	0.814108258
LOC783641	0.941472254	0.210980522	0.304971947	0.035833068	0.741115922	0.23133901	0.814108258
NAA10	0.395511546	0.896007174	0.796973798	0.038961596	0.146205716	0.231348487	0.814108258
PDK4	0.272961986	0.811035163	0.113283139	0.08311615	0.769679974	0.231028141	0.814108258

PIGW	0.962406327	0.079169561	0.459928607	0.273733585	0.167335263	0.231086201	0.814108258
TMEM17	0.04450689	0.183133791	0.263569025	0.829027004	0.904570755	0.231503238	0.814262367
VEGFA	0.23411286	0.653875863	0.038470765	0.510017016	0.536413261	0.231512291	0.814262367
ACBD3	0.817942595	0.290935647	0.871204541	0.01023701	0.76203688	0.231949712	0.814386802
BACE2	0.22779139	0.348516088	0.038915403	0.898118963	0.583167099	0.232007842	0.814386802
DNAJC19	0.268041671	0.71916844	0.427686975	0.033091972	0.591828087	0.231761319	0.814386802
DPEP3	0.053080684	0.694935303	0.068366162	0.68584852	0.936228481	0.232093539	0.814386802
IL17D	0.642038145	0.02173967	0.872519478	0.532650087	0.24962813	0.232091499	0.814386802
LMNB2	0.191231847	0.214134093	0.209351999	0.5192335	0.36350413	0.232004309	0.814386802
LOC100141253	0.085402276	0.06954617	0.803446676	0.607343691	0.558435541	0.232033991	0.814386802
LOC112444326	0.779754474	0.213433023	0.767847913	0.555517627	0.022791196	0.231994898	0.814386802
MTERF4	0.894368129	0.653486318	0.374574461	0.13078628	0.056433927	0.231845007	0.814386802
RAB19	0.654318662	0.500157541	0.348155085	0.922955416	0.015359897	0.231803846	0.814386802
SNRPD1	0.113850771	0.438077187	0.330150969	0.632367774	0.155148664	0.231824768	0.814386802
DISP1	0.522211219	0.138238145	0.037858048	0.66456066	0.892140899	0.232164601	0.814462006
S100A8	0.445737988	0.561850678	0.317001685	0.04969844	0.410972669	0.23224871	0.814582942
N4BP2	0.492439827	0.942228083	0.483918803	0.008158445	0.887055633	0.232493172	0.815266121
ANXA6	0.303622504	0.631821875	0.945490469	0.242701851	0.037182921	0.233333052	0.815487738
ARHGEF1	0.961095113	0.06259841	0.049844927	0.625473427	0.868109302	0.23273036	0.815487738
BCAS1	0.429063505	0.726950888	0.411974427	0.111364615	0.114383567	0.233334487	0.815487738
BLVRB	0.58032732	0.942240464	0.160022788	0.030872328	0.603373813	0.232846778	0.815487738
CHCHD2	0.867469854	0.457905713	0.30021274	0.02864171	0.476442157	0.232659974	0.815487738
CIDEB	0.322058075	0.116826727	0.1105889	0.688309328	0.570786615	0.23318496	0.815487738
COX7ALP1	0.613463546	0.514151972	0.205198078	0.032810721	0.76747544	0.232836522	0.815487738
DISC1	0.398934667	0.35701016	0.048375706	0.246390538	0.964356917	0.233351436	0.815487738
EIF3CL	0.822099882	0.740342847	0.08633247	0.162796139	0.190708862	0.232946146	0.815487738
GTPBP6	0.781624657	0.805537348	0.731047595	0.025253369	0.140380212	0.232974882	0.815487738
LOC101902531	0.197590202	0.670780404	0.049572987	0.435759083	0.571635268	0.233320807	0.815487738
LOC101906828	0.52219043	0.468367148	0.313887537	0.366434804	0.058024893	0.233013581	0.815487738
MIC1	0.362220317	0.378303862	0.215864917	0.108269691	0.510945016	0.233299637	0.815487738
POPDC2	0.138068319	0.474553255	0.903999836	0.758195598	0.036322445	0.232934743	0.815487738
RGS2	0.503924303	0.17998182	0.083087985	0.441754667	0.491683112	0.233332053	0.815487738
SFXN2	0.884480299	0.802445177	0.26695398	0.010538524	0.818331461	0.233132755	0.815487738
GLP2R	0.984805721	0.011942042	0.405258925	0.485518407	0.708141732	0.233462129	0.815501688
TTC1	0.842724424	0.22609122	0.190285853	0.097160147	0.465353446	0.233504505	0.815501688
ZDHHC4	0.564471027	0.109517007	0.731522053	0.043278527	0.837325634	0.233470082	0.815501688
DCAF8	0.755441619	0.374202359	0.01113844	0.794442754	0.656113667	0.233644666	0.815644036
HIPK2	0.757265748	0.675045915	0.009627256	0.40007907	0.833371294	0.233616718	0.815644036
ANKRA2	0.479287413	0.457561679	0.080905953	0.46084473	0.200958002	0.233781154	0.81593866
LOC112447474	0.551567122	0.369074492	0.299597577	0.616843423	0.043714431	0.233878219	0.81593866
LRRN1	0.275277781	0.104333033	0.290915265	0.281304178	0.699655723	0.233870122	0.81593866
BEND5	0.967446511	0.536575648	0.105383021	0.845851845	0.035606744	0.234092671	0.816322841
IL17RA	0.626206076	0.310816506	0.065224598	0.792001683	0.163916494	0.234126368	0.816322841
SLC38A5	0.442022456	0.090044642	0.081639248	0.571249132	0.887966879	0.234137567	0.816322841

UQCR11	0.581974211	0.586000799	0.970853718	0.008059862	0.618351121	0.234269572	0.816609591
ACOT6	0.261699191	0.68682906	0.403588397	0.082876477	0.275478098	0.234693898	0.817914963
CDON	0.38101933	0.087653023	0.296224125	0.208661392	0.803044793	0.234802939	0.818033367
PDK2	0.81035316	0.423132999	0.394775529	0.012453269	0.98361138	0.234827566	0.818033367
ALOX15	0.663093406	0.174502938	0.035255397	0.456212439	0.892218415	0.234996774	0.818101836
G0S2	0.092633474	0.188272429	0.435142437	0.321495298	0.680490732	0.234981158	0.818101836
POLR2B	0.194112782	0.260702607	0.247916984	0.212044555	0.624123803	0.234986693	0.818101836
AIMP2	0.280816475	0.211124186	0.899685113	0.847888174	0.036764415	0.23515082	0.818464499
WDR35	0.275703966	0.623555712	0.042273352	0.884391298	0.258899999	0.235242894	0.818611351
ACOT2	0.948645001	0.541746753	0.502930875	0.006897605	0.936082639	0.235580081	0.819250974
CCDC30	0.682056909	0.057978546	0.887995598	0.056352963	0.844739568	0.23577235	0.819250974
CDC5L	0.117161816	0.294335413	0.868873489	0.057002126	0.978556032	0.235751864	0.819250974
EIF3E	0.023934835	0.873847485	0.31320789	0.429189239	0.5931043	0.235488434	0.819250974
OAZ1	0.595539331	0.20123664	0.092982333	0.276709585	0.5414753	0.235633653	0.819250974
TLR7	0.150506821	0.727353707	0.958179175	0.0308677	0.516297071	0.235776147	0.819250974
ZNF792	0.382651493	0.798004001	0.114412074	0.201939049	0.236928187	0.235766732	0.819250974
NDUFA9	0.553113512	0.851631223	0.582398438	0.011667105	0.522781203	0.235887848	0.819396416
PIN4	0.239037132	0.429148155	0.660672069	0.034393186	0.718335265	0.235965361	0.819396416
ZNF384	0.703743357	0.325325183	0.316204454	0.293933468	0.078689759	0.235967794	0.819396416
TAF8	0.427379401	0.534769276	0.053803841	0.904754497	0.150620269	0.236059071	0.819539966
CLSTN3	0.099432804	0.937666324	0.615394702	0.073200723	0.399331279	0.236159158	0.819540694
NHLH1	0.682165039	0.746622824	0.219869535	0.329965198	0.045372039	0.236114005	0.819540694
QTRT1	0.373028588	0.106835761	0.353631868	0.211751559	0.562747187	0.236312083	0.819898011
IKZF5	0.238916339	0.95198429	0.29109552	0.796912715	0.031871415	0.236466022	0.820167994
SETD3	0.698751844	0.266273452	0.137777618	0.172341575	0.380860126	0.236536366	0.820167994
TIMM22	0.413774809	0.589229001	0.853132243	0.012772387	0.633375638	0.236539828	0.820167994
CNP	0.540147121	0.428636467	0.175610827	0.433460902	0.095677842	0.23678602	0.820622893
LOC112443141	0.824462107	0.472244297	0.082495219	0.055653696	0.942855927	0.236730027	0.820622893
RMI1	0.396810326	0.516132953	0.228028096	0.306308391	0.117910414	0.236821036	0.820622893
GART	0.898631046	0.453394706	0.518109436	0.339210224	0.023597403	0.237027807	0.820667343
MSS51	0.956964015	0.376605654	0.178410862	0.444694054	0.059064119	0.236967602	0.820667343
UNC79	0.081451353	0.892773131	0.442848822	0.06070537	0.864396709	0.237033892	0.820667343
ZGPAT	0.482706581	0.605085972	0.160089064	0.091626181	0.394313902	0.237003891	0.820667343
CAPG	0.318366629	0.374220124	0.459845838	0.86655565	0.035659277	0.237249496	0.821051893
NOX1	0.67560845	0.099494682	0.036568017	0.790733771	0.871694189	0.237345084	0.821051893
PNPLA4	0.053491811	0.914611494	0.305523699	0.164401076	0.688657101	0.237206574	0.821051893
RASSF8	0.466185779	0.319971853	0.518755929	0.041338557	0.5295458	0.237318663	0.821051893
ANAPC13	0.250444374	0.542520699	0.408672235	0.077674158	0.393645441	0.237585951	0.821538779
ENO2	0.149181971	0.977953367	0.276214376	0.949648665	0.044358769	0.237569426	0.821538779
TNFRSF6B	0.395541419	0.5035551	0.06428695	0.954367673	0.139073734	0.237704097	0.821774158
CDK2AP1	0.527059907	0.936709895	0.07955976	0.091890931	0.471956201	0.237976701	0.822492944
MTA3	0.028985378	0.885219427	0.343229025	0.76514052	0.252877072	0.238012247	0.822492944
AUP1	0.280728887	0.433714144	0.299385798	0.210517619	0.22349834	0.238774531	0.82252914
B4GALT3	0.533253198	0.780838618	0.482141991	0.472797275	0.018044692	0.238614769	0.82252914

CHN2	0.164888059	0.488819677	0.062648971	0.862541987	0.392815525	0.238486729	0.82252914
IFT80	0.833773092	0.047900373	0.308122827	0.193715788	0.715727807	0.238163197	0.82252914
LOC101907189	0.083659355	0.433771571	0.192051096	0.271335545	0.905937522	0.238642677	0.82252914
LOC112443479	0.053887448	0.317846001	0.144310802	0.733182319	0.945761612	0.238696663	0.82252914
MTERF2	0.700127541	0.652590921	0.729839804	0.013174242	0.38903481	0.238362134	0.82252914
NCKAP1	0.116665081	0.261372059	0.192371882	0.344240126	0.845689544	0.238268705	0.82252914
NELL1	0.906722586	0.695147815	0.102490854	0.050124598	0.529262088	0.238686252	0.82252914
NRF1	0.289224074	0.437025401	0.860925829	0.029017886	0.542256429	0.238583172	0.82252914
OGFOD1	0.271663	0.392935802	0.742339566	0.701816096	0.0308354	0.238759099	0.82252914
PCBD1	0.196634351	0.396766245	0.245518874	0.112377619	0.794630085	0.238460531	0.82252914
PHLDA3	0.173682922	0.550151409	0.304406198	0.197618914	0.297523067	0.238438408	0.82252914
TXNRD3	0.443873003	0.150949352	0.896342837	0.030152781	0.944755768	0.23848412	0.82252914
UBIAD1	0.566089755	0.183994203	0.106768024	0.788681926	0.194679115	0.238251898	0.82252914
RIDA	0.572261513	0.793991173	0.095383073	0.227645642	0.173966848	0.238861883	0.822657369
ERF	0.139213083	0.644666252	0.769724384	0.056679175	0.441175009	0.239614616	0.82285846
ETFRF1	0.707268388	0.329933124	0.124487121	0.266026035	0.223349601	0.239522167	0.82285846
KLHL42	0.499347119	0.311647013	0.103177544	0.204619962	0.523751625	0.239164334	0.82285846
LOC100336669	0.602324256	0.478756206	0.511766342	0.02129701	0.546848564	0.239022955	0.82285846
LOC786948	0.968711393	0.510312666	0.131873119	0.214386038	0.123134877	0.239175316	0.82285846
LRRC8C	0.127349876	0.928301917	0.207420895	0.252614294	0.278200067	0.239335455	0.82285846
LSM1	0.347910025	0.307052687	0.471362347	0.308233256	0.111061465	0.239368585	0.82285846
PPP3CA	0.231503409	0.180696901	0.795577343	0.079429664	0.652070119	0.239365846	0.82285846
RND1	0.457568582	0.043924623	0.858197165	0.16814323	0.594746062	0.239445776	0.82285846
SDC2	0.852917199	0.086958606	0.560762713	0.054515962	0.757702402	0.238973856	0.82285846
SEC22B	0.449698712	0.406876832	0.173893639	0.310324136	0.174987202	0.23964324	0.82285846
TEFM	0.675076238	0.133119953	0.61152962	0.88942636	0.035371932	0.239722521	0.82285846
TNFSF8	0.24150572	0.810016548	0.450111682	0.054339534	0.360995196	0.239607634	0.82285846
TRPA1	0.032504036	0.637430313	0.376124085	0.71087286	0.311836611	0.239624257	0.82285846
ZNF365	0.756881118	0.814489396	0.012291842	0.263810953	0.864546793	0.239676146	0.82285846
ZNF667	0.946070622	0.724230769	0.132278296	0.084156149	0.226066964	0.239405043	0.82285846
ACO2	0.398032495	0.966151394	0.742697257	0.017576469	0.344647045	0.239803825	0.822965407
MRO	0.399284118	0.540698859	0.143268471	0.582073802	0.096142433	0.239858008	0.822979254
AARS2	0.511800485	0.014275296	0.669662472	0.534716401	0.665504469	0.240547152	0.822991492
ADAMTS16	0.360842659	0.63864179	0.289455484	0.125970752	0.209684084	0.241958883	0.822991492
AKAP9	0.623378855	0.162280185	0.604010583	0.07076455	0.407083051	0.241841009	0.822991492
C10H14orf1	0.305408687	0.872644241	0.701418097	0.179142527	0.052491451	0.241683102	0.822991492
CACHD1	0.144840723	0.369474605	0.452594039	0.100870034	0.711187235	0.240306113	0.822991492
CEP170	0.504749477	0.052964604	0.24359332	0.70854153	0.381603266	0.241880222	0.822991492
CFDP2	0.906663977	0.141523843	0.502488772	0.060942534	0.443245294	0.240588174	0.822991492
CPEB2	0.877301647	0.102268226	0.040312878	0.518344125	0.925346081	0.240122773	0.822991492
CRYBG3	0.480315375	0.953905076	0.711633655	0.017860168	0.302757113	0.242034293	0.822991492
CSNK2B	0.189511681	0.375912808	0.137954654	0.4536622	0.389702197	0.240304667	0.822991492
CXHXorf38	0.496632665	0.246531906	0.353486401	0.822828624	0.049633917	0.242335476	0.822991492
DDA1	0.261704846	0.109152261	0.326718991	0.26249112	0.722020543	0.242421417	0.822991492

DTX2	0.25667704	0.097384448	0.615454175	0.323969781	0.353675701	0.242010797	0.822991492
F2RL1	0.812388169	0.282912821	0.278835557	0.028124653	0.969204803	0.240942243	0.822991492
FAM122A	0.126008775	0.290831811	0.382948104	0.213963701	0.581243647	0.240837038	0.822991492
FAM96A	0.143143615	0.419431037	0.305869856	0.451065101	0.214281079	0.242834822	0.822991492
FBLN1	0.78139778	0.034289367	0.507416435	0.143849794	0.892929188	0.240902826	0.822991492
FBN1	0.622173962	0.553302518	0.084854585	0.25825788	0.233676198	0.242020577	0.822991492
FBXL19	0.764565929	0.112612456	0.058048596	0.787009004	0.440360557	0.239939467	0.822991492
GEM	0.196870145	0.861663073	0.246557563	0.187220998	0.222873671	0.240828335	0.822991492
GPR176	0.733492793	0.540550851	0.239829284	0.174397076	0.104839879	0.240379574	0.822991492
HCRTR1	0.295297851	0.531827346	0.02597931	0.656157625	0.658303706	0.241986456	0.822991492
IGSF9	0.157665974	0.151289983	0.324079486	0.229430079	0.999022005	0.242624612	0.822991492
ITGB1	0.396117989	0.086607983	0.490576571	0.307843214	0.337682938	0.241122378	0.822991492
LENG9	0.777029439	0.824782505	0.068726003	0.117549723	0.341817185	0.242484508	0.822991492
LOC100849237	0.968686711	0.992970175	0.068354388	0.688461253	0.03889129	0.241856563	0.822991492
LOC101903200	0.969813715	0.236800921	0.150373718	0.139968438	0.359201276	0.240219485	0.822991492
LOC101903478	0.908884193	0.267923701	0.313923268	0.056119026	0.407725847	0.24109336	0.822991492
LOC101903616	0.557942821	0.558809907	0.339146382	0.075456602	0.221512804	0.242326718	0.822991492
LOC101907545	0.492722784	0.988065655	0.148739255	0.034278822	0.711061634	0.242165332	0.822991492
LOC104969833	0.123987762	0.12614455	0.283020974	0.473703266	0.845604478	0.242710767	0.822991492
LOC112442288	0.904482085	0.564362653	0.505175462	0.030184282	0.227996013	0.242811885	0.822991492
LOC112446761	0.739463933	0.424110655	0.512629493	0.325067195	0.033287587	0.240448915	0.822991492
LOC782054	0.460344606	0.258270161	0.133441817	0.339164599	0.328034158	0.242173985	0.822991492
LOC785804	0.522927953	0.885728598	0.129708468	0.052762161	0.558898278	0.242608479	0.822991492
LOC790218	0.98167179	0.125817216	0.24270961	0.561036308	0.104383059	0.24152787	0.822991492
MICU3	0.368245694	0.636237953	0.036380618	0.452570658	0.454840651	0.241461992	0.822991492
MMACHC	0.498781306	0.479054273	0.964346896	0.052517751	0.14464305	0.241177966	0.822991492
MTG2	0.852471672	0.755702524	0.616893671	0.008897521	0.493409788	0.240792632	0.822991492
NDUFA8	0.319536648	0.654430032	0.510828618	0.021691347	0.754835892	0.241086988	0.822991492
NKAPD1	0.445165574	0.034865498	0.298806886	0.528684311	0.716011502	0.241530635	0.822991492
NRARP	0.616430532	0.75587486	0.632245714	0.007056364	0.854355014	0.242902531	0.822991492
PBK	0.752918076	0.2285541	0.034843049	0.50370971	0.578333587	0.240927127	0.822991492
PCBP2	0.729637492	0.175046217	0.160799033	0.098883922	0.869566296	0.242226743	0.822991492
PDCD6	0.553139779	0.22223343	0.779055213	0.102025594	0.179184593	0.24120263	0.822991492
PLCL1	0.090391121	0.191535691	0.440769743	0.318566375	0.717734726	0.240801685	0.822991492
PLXNB3	0.030742421	0.199813835	0.70179109	0.749914247	0.544554044	0.241858275	0.822991492
POLR2L	0.302271287	0.792662271	0.50316227	0.038917051	0.377162979	0.242470442	0.822991492
PRR19	0.992165151	0.700824951	0.173120493	0.014965534	0.985986888	0.24292065	0.822991492
PRRT1	0.233608217	0.185685439	0.30513203	0.560529547	0.239012408	0.242719845	0.822991492
PSMA4	0.773591455	0.209635141	0.751081298	0.100819473	0.14401546	0.242403127	0.822991492
SAA3	0.042058332	0.466076072	0.392724585	0.919509806	0.250911728	0.242912122	0.822991492
SLC44A3	0.639698123	0.746681347	0.095972321	0.287915462	0.133511172	0.241971675	0.822991492
SPG11	0.18554976	0.356228412	0.090545423	0.396668522	0.734358412	0.240703679	0.822991492
SSR1	0.850647856	0.120773279	0.430260766	0.224062442	0.177918521	0.241972677	0.822991492
STON2	0.748115124	0.630085547	0.087940076	0.883406895	0.047883758	0.241388531	0.822991492

STX17	0.925856237	0.048104296	0.19281404	0.219620265	0.934379503	0.241977913	0.822991492
SYF2	0.805960008	0.155075907	0.057539053	0.756042142	0.319615634	0.240323606	0.822991492
TFB1M	0.933205841	0.990985535	0.942321373	0.033384947	0.06041119	0.241663721	0.822991492
TMEM129	0.294976063	0.224930592	0.487654345	0.892107099	0.061378281	0.242612017	0.822991492
TMEM230	0.66477606	0.093211912	0.59222513	0.249579737	0.19349799	0.242650443	0.822991492
DCLK2	0.963705864	0.839254041	0.233426037	0.719696123	0.013085528	0.243036449	0.823056616
DPF2	0.632431387	0.226812603	0.543342916	0.1993741	0.114711499	0.243339008	0.823056616
FYTTD1	0.66710902	0.05828989	0.158215613	0.954169389	0.303363958	0.243228819	0.823056616
GALNT15	0.814511249	0.463160623	0.107725347	0.679466013	0.064547464	0.243328916	0.823056616
MINK1	0.751182706	0.405417932	0.039793338	0.708351916	0.207195659	0.243080602	0.823056616
MS4A8	0.283172644	0.250580402	0.074894733	0.454647732	0.737756767	0.243341094	0.823056616
PDZD11	0.492973465	0.312572721	0.242445897	0.096076554	0.49601906	0.243194899	0.823056616
VAT1L	0.472630368	0.422674156	0.04109532	0.233644897	0.928071308	0.243181753	0.823056616
PDZD4	0.205388812	0.324366125	0.117863127	0.670708364	0.338716858	0.243429741	0.823186788
CD86	0.339552983	0.944228663	0.44660926	0.289319135	0.043124305	0.243608542	0.8233769
GPATCH2L	0.510662709	0.24702196	0.317632907	0.151024977	0.295319628	0.243642768	0.8233769
KRI1	0.541570174	0.117279449	0.47216924	0.523538759	0.113878103	0.243705183	0.8233769
OSBPL8	0.989455719	0.240484833	0.057052982	0.208923508	0.630541325	0.243732627	0.8233769
PTPN22	0.374814853	0.827124837	0.861184694	0.370825458	0.018064454	0.243736822	0.8233769
CEP126	0.251437215	0.487354878	0.664945966	0.138096993	0.159132576	0.243881435	0.823391562
CTSC	0.273348618	0.170505934	0.983765269	0.043273577	0.904733868	0.244180467	0.823391562
DUSP8	0.346329491	0.748943172	0.729367685	0.67399611	0.014079712	0.24419272	0.823391562
FARP2	0.702704157	0.285278118	0.759617673	0.25074987	0.046956466	0.244037557	0.823391562
LOC100196901	0.041249358	0.98758422	0.526428758	0.101796348	0.821267273	0.244030816	0.823391562
LPAR2	0.22002828	0.271123698	0.321901247	0.315564027	0.295982936	0.244079003	0.823391562
NLGN3	0.280423847	0.990679738	0.346602584	0.460712279	0.040386529	0.243947931	0.823391562
PYM1	0.267501931	0.581930415	0.123107829	0.610455943	0.153253856	0.244031291	0.823391562
ZBTB34	0.47423148	0.706856615	0.270190187	0.098367188	0.201417307	0.244138307	0.823391562
LOC786733	0.236681979	0.606093173	0.228241024	0.736023978	0.074560105	0.244292027	0.823557201
IL7	0.904579027	0.72710707	0.010858525	0.894936554	0.281421126	0.244420877	0.823653185
LOC101905801	0.273771499	0.432397942	0.673941784	0.193635116	0.116428547	0.244413489	0.823653185
MLLT10	0.327246672	0.168120431	0.157750891	0.671493691	0.308831914	0.244494428	0.823731895
ASIC2	0.914605907	0.503914862	0.915297849	0.495147038	0.008622661	0.244576379	0.823756645
GADD45G	0.751062302	0.269893424	0.065865451	0.508020048	0.265703937	0.24465236	0.823756645
HMGN1	0.328466453	0.129065768	0.752218066	0.496662747	0.113760338	0.244623016	0.823756645
UBE2N	0.949111239	0.110266226	0.204126874	0.413856365	0.20397631	0.24473076	0.82385159
CDKL4	0.415860413	0.361861731	0.134850407	0.919148006	0.096889547	0.244983314	0.823885749
GJD3	0.689627459	0.382962478	0.345316522	0.029068946	0.682597005	0.245142533	0.823885749
KIAA2026	0.226395394	0.478951371	0.71605555	0.118214118	0.196609918	0.244811085	0.823885749
KMT2E	0.236363019	0.908721877	0.390867991	0.130527868	0.164985581	0.245034356	0.823885749
NIPSNAP2	0.646328171	0.389706753	0.333063016	0.045265891	0.476380279	0.245104021	0.823885749
PFKP	0.938648307	0.694444728	0.147692737	0.542310197	0.034626389	0.245024657	0.823885749
RHOA	0.492325156	0.131973937	0.08562527	0.450391072	0.72133165	0.245000432	0.823885749
RINT1	0.870589022	0.356929836	0.263129114	0.036124346	0.612319022	0.245076533	0.823885749

BRK1	0.297141396	0.309261479	0.336565655	0.067706685	0.864570819	0.245200264	0.823911041
STAT5A	0.96512536	0.569484617	0.924685428	0.232193491	0.015352661	0.245283802	0.824023023
BLOC1S2	0.696453371	0.222154641	0.195164996	0.474159756	0.126658506	0.245398424	0.824239363
ANKRD40	0.613052515	0.211990567	0.02923722	0.499028856	0.958503326	0.245664066	0.824243478
APLNR	0.812907319	0.952566676	0.043097456	0.055812977	0.974937805	0.245561939	0.824243478
NDUFB10	0.514928	0.600568291	0.388098622	0.035227157	0.42986906	0.245662743	0.824243478
RGS6	0.149176001	0.607618993	0.769516597	0.082594764	0.31557482	0.245700999	0.824243478
TOMM40L	0.27557872	0.402974099	0.680971417	0.21932259	0.109529131	0.245607425	0.824243478
ZGRF1	0.760708887	0.056810745	0.155823246	0.708961718	0.380486927	0.245601632	0.824243478
SPATA20	0.043773879	0.106085563	0.89776494	0.694744253	0.627994997	0.245759614	0.824271618
ABRAXAS2	0.945782543	0.055535356	0.169971841	0.208490784	0.979775721	0.246074018	0.824575734
COX10	0.571982073	0.612531923	0.868098445	0.334976627	0.017895172	0.246040563	0.824575734
LOC107132870	0.082259291	0.947108783	0.13716134	0.67551762	0.252695473	0.246101514	0.824575734
LRWD1	0.803878214	0.020950279	0.166097665	0.93087534	0.699531093	0.245933081	0.824575734
PPM1L	0.540017812	0.706045932	0.121885019	0.124762334	0.3144163	0.246026557	0.824575734
TRMT10C	0.858158294	0.482114511	0.370019795	0.770657535	0.015468994	0.246161149	0.824607188
CAAP1	0.856860225	0.190330308	0.049386488	0.316458732	0.718485969	0.246575491	0.824823227
KPTN	0.544224584	0.797599966	0.290371396	0.036189354	0.401811628	0.246675038	0.824823227
LOC104975590	0.668255662	0.191086788	0.536099317	0.900088669	0.029690745	0.246454489	0.824823227
LOC104975593	0.48196791	0.337585197	0.015307904	0.904460123	0.813755582	0.246697282	0.824823227
LOC112447302	0.308076501	0.049031941	0.77741604	0.225054854	0.692143472	0.246440832	0.824823227
MRPL53	0.832145476	0.37531286	0.291698581	0.185767768	0.10839586	0.246783116	0.824823227
PGR	0.491549864	0.315147764	0.874072257	0.612990632	0.022110147	0.246828765	0.824823227
RASGEF1B	0.41483009	0.434824844	0.044717954	0.23173754	0.977923882	0.246355567	0.824823227
SMIM37	0.049100947	0.774211269	0.926160467	0.094444622	0.551217317	0.246679266	0.824823227
TCEAL4	0.517936343	0.372006831	0.558129592	0.787422916	0.021619558	0.246535502	0.824823227
TCF20	0.637251766	0.078284896	0.081937525	0.752173387	0.595021753	0.246453859	0.824823227
ZNF407	0.622789638	0.29434295	0.392270394	0.618857854	0.041234584	0.246817239	0.824823227
EVA1A	0.750449354	0.348002672	0.017466272	0.478869781	0.84128712	0.246992856	0.825203536
CLTA	0.620282273	0.075036036	0.084862144	0.890350825	0.52340321	0.247188611	0.82535347
MGAT2	0.590297131	0.504524948	0.489607842	0.765588443	0.0164841	0.247158474	0.82535347
ZDHHC17	0.134343877	0.834088044	0.364460981	0.111728767	0.403123561	0.247108957	0.82535347
NXF1	0.911995556	0.232276612	0.279409399	0.091094956	0.342017674	0.247414044	0.825602816
PPP6C	0.359813528	0.050346922	0.243388157	0.595523555	0.702318175	0.247414213	0.825602816
SIGMAR1	0.346558463	0.709607584	0.830000067	0.164969277	0.054764139	0.247411299	0.825602816
PRMT6	0.446061167	0.093564906	0.654108504	0.392417191	0.172282404	0.247515251	0.825772063
MESP1	0.068082537	0.362954978	0.407075092	0.409809476	0.448168575	0.247637687	0.82587231
POC5	0.772129782	0.148499372	0.048926531	0.343681174	0.958298969	0.247645947	0.82587231
GHSR	0.813934727	0.206050776	0.192823611	0.21242413	0.269484058	0.247881032	0.826036743
GIPC2	0.565862434	0.137672818	0.089526368	0.35641858	0.745692779	0.248039545	0.826036743
IL4I1	0.589198499	0.01967471	0.882537338	0.818193971	0.221210089	0.247909787	0.826036743
LOC104968435	0.25390466	0.360116256	0.740231286	0.258554703	0.105931697	0.248047594	0.826036743
NOV	0.296748239	0.216376776	0.569410434	0.060605736	0.835475586	0.247884022	0.826036743
RNF31	0.432357585	0.064176315	0.463001656	0.237270037	0.607835868	0.247983664	0.826036743

SNED1	0.992513865	0.080949616	0.177362568	0.385374877	0.337051393	0.247862076	0.826036743
LOC101902922	0.042546747	0.42089388	0.324213969	0.610987592	0.523266145	0.2482054	0.826387577
LOC112444904	0.64666118	0.342644546	0.732108775	0.348656786	0.032832546	0.248253656	0.826387577
CEP131	0.665202934	0.394801496	0.035134204	0.373571312	0.539561333	0.248442782	0.826849421
FAM174A	0.306742742	0.138013295	0.330535126	0.260570665	0.511554997	0.248792412	0.827254022
GDPD4	0.247722847	0.568488208	0.122926752	0.211376415	0.50948766	0.248734762	0.827254022
LOC100848941	0.4515106	0.363839261	0.527846418	0.03357944	0.640971388	0.248866803	0.827254022
METTL9	0.50963508	0.278176687	0.13661849	0.102773151	0.937491274	0.248849843	0.827254022
TMA7	0.110965298	0.691465236	0.664299904	0.037662862	0.971257605	0.248747417	0.827254022
TTC13	0.154673339	0.701528295	0.756404675	0.063419542	0.357917893	0.248650282	0.827254022
ACO1	0.488158963	0.261760977	0.021032254	0.940577089	0.741436206	0.249376131	0.827384261
ARL8A	0.270066281	0.061777809	0.212981174	0.742278562	0.709964404	0.249271964	0.827384261
ARMCX6	0.45005395	0.95357498	0.048845187	0.150487379	0.59361015	0.249270573	0.827384261
BNC1	0.508645913	0.022560312	0.491502962	0.513159191	0.645662632	0.249019651	0.827384261
C3H1orf54	0.021162665	0.523200688	0.892826607	0.326747211	0.57849387	0.249011799	0.827384261
GLRX5	0.372324738	0.519755007	0.917263699	0.015053939	0.700557902	0.249233226	0.827384261
KALRN	0.141823494	0.76924603	0.750225904	0.050329172	0.45511018	0.249410148	0.827384261
LOC104968476	0.523870815	0.065625314	0.737217702	0.761219688	0.097046287	0.249253158	0.827384261
NDUFA2	0.520907802	0.509449451	0.676748882	0.017885913	0.58321499	0.249322763	0.827384261
TRARG1	0.147430951	0.606794492	0.080033315	0.439668175	0.593931297	0.249080487	0.827384261
C25H16orf54	0.421687656	0.584108831	0.38182631	0.097271658	0.205036441	0.249473363	0.827426711
ARFIP2	0.285683714	0.913412647	0.407785516	0.112544767	0.157070869	0.249819615	0.827758338
CHCHD10	0.365646627	0.674717263	0.465938265	0.024274216	0.673533081	0.249710626	0.827758338
DNM2	0.598483415	0.800063588	0.340056227	0.184862927	0.062478638	0.249792593	0.827758338
LOC101902128	0.344557349	0.152713624	0.405984991	0.97470201	0.090420218	0.249926425	0.827758338
LOC101908113	0.046182978	0.809611393	0.605696065	0.830782275	0.100029119	0.249881694	0.827758338
SCD	0.936531699	0.696796315	0.928312172	0.055176703	0.056309218	0.249890278	0.827758338
SEMA5B	0.596768391	0.124439479	0.519912264	0.948030885	0.051389679	0.249816032	0.827758338
REPS2	0.522234218	0.374675523	0.207679649	0.517974091	0.089612676	0.250152057	0.828338461
BRD9	0.063613472	0.624373316	0.446477092	0.349499558	0.304735854	0.250312044	0.828701019
KRT24	0.185460291	0.81634287	0.160097077	0.093240482	0.836683068	0.250454968	0.829006953
AGFG2	0.711125673	0.882416446	0.214321905	0.028940842	0.48666635	0.250667254	0.829336355
CHPF2	0.684651242	0.839676088	0.135763986	0.529781548	0.045902023	0.250910249	0.829336355
HM13	0.206141506	0.849994359	0.20825225	0.870605452	0.059782016	0.250985735	0.829336355
LMX1A	0.509116507	0.538803141	0.023271891	0.630866307	0.471198217	0.250889331	0.829336355
LOC112444288	0.973518517	0.314617215	0.129286635	0.424309263	0.112986824	0.250937279	0.829336355
MEIOB	0.226853427	0.464931103	0.911877163	0.130591686	0.15123471	0.25100657	0.829336355
NT5C	0.561200066	0.342686053	0.084240049	0.800536145	0.146138065	0.250736886	0.829336355
SCN11A	0.053751031	0.382174547	0.315904314	0.618173904	0.473242579	0.250939511	0.829336355
TLE4	0.883746549	0.92059252	0.460160798	0.20212205	0.025103168	0.251009303	0.829336355
SLC39A7	0.0788982	0.881608773	0.442873583	0.552514464	0.11170854	0.251123333	0.829386515
SPON2	0.078800655	0.432114772	0.151770551	0.790094118	0.465656263	0.251125562	0.829386515
ZNF829	0.817053004	0.334509458	0.632926326	0.015920712	0.691303563	0.25128928	0.829760236
KDELC2	0.060286795	0.239134335	0.93511026	0.970149437	0.145783392	0.251466638	0.830178837

ARSI	0.391120951	0.136490236	0.148362688	0.789906823	0.304907501	0.251525076	0.83020475
FKBP9	0.763138271	0.906388745	0.61785197	0.014710665	0.303564474	0.251582513	0.830220249
TSPAN9	0.107501416	0.651239236	0.066517145	0.489712204	0.837198132	0.25163095	0.830220249
PPP1R16A	0.722535204	0.086847857	0.519662964	0.099868769	0.58650147	0.251681949	0.830221601
LOC101904601	0.936496364	0.535171462	0.439102069	0.011057081	0.786355755	0.251903137	0.830317149
NCAPG2	0.963562919	0.01668327	0.302563775	0.611734884	0.64314622	0.251910755	0.830317149
SLC15A4	0.724738032	0.994145546	0.335347997	0.008058826	0.982792059	0.251913295	0.830317149
TEX9	0.034727406	0.42875602	0.196683616	0.750064919	0.870802	0.251859453	0.830317149
NCK1	0.924080947	0.977559561	0.024293968	0.25530105	0.341759634	0.251987931	0.830396371
DGKA	0.610356858	0.326799388	0.167673932	0.096521454	0.59374235	0.252108393	0.830626548
PRPF38B	0.564274112	0.606024219	0.307854517	0.035110566	0.51894524	0.252202134	0.83076861
CENPO	0.600207098	0.038830413	0.232882097	0.515821007	0.686408589	0.252429461	0.831350568
LOC112448269	0.914779663	0.616076641	0.879142331	0.011083082	0.350264049	0.252535447	0.831415381
UBA7	0.773975569	0.105512365	0.342274832	0.092926098	0.740587692	0.252550465	0.831415381
BCORL1	0.893442138	0.612853786	0.097352603	0.141552271	0.255989251	0.253055971	0.831744751
CCR3	0.006723445	0.717495388	0.99053222	0.576005848	0.701393259	0.252987746	0.831744751
HTRA2	0.327254695	0.18541329	0.388530362	0.094192199	0.868103866	0.252809826	0.831744751
LOC112445029	0.767030027	0.069266563	0.076796545	0.769016506	0.615593489	0.253055383	0.831744751
PANX1	0.718830426	0.316360588	0.082906182	0.353741302	0.289259593	0.252903319	0.831744751
RAD9B	0.668804856	0.718826876	0.044719274	0.430337986	0.208290913	0.252770323	0.831744751
RPRM	0.602360911	0.107327394	0.272761473	0.165120698	0.663094294	0.253004459	0.831744751
RRAGA	0.988777879	0.086041657	0.52258376	0.333286907	0.13008473	0.252801256	0.831744751
LRFN3	0.7296224	0.888586769	0.513020137	0.024008964	0.242012244	0.253122042	0.831755962
METTL22	0.822297959	0.251683196	0.504485567	0.02710686	0.683263527	0.253194558	0.831755962
ZBTB2	0.15603584	0.451038597	0.56669649	0.050950251	0.95175327	0.25321143	0.831755962
ABHD8	0.183009851	0.527455945	0.154567466	0.137858003	0.941939291	0.253430482	0.832021742
BBS7	0.120932134	0.507490683	0.468622432	0.215145951	0.31293741	0.253359712	0.832021742
DECR1	0.792040595	0.962392413	0.267459736	0.028554771	0.332848216	0.253444439	0.832021742
SSR4	0.649576189	0.1654169	0.080011132	0.453403844	0.497353472	0.253508903	0.832066921
FUNDC2	0.608082172	0.356885861	0.204202328	0.050895366	0.860766702	0.25368026	0.832462856
RETREG2	0.92766025	0.450228886	0.020117301	0.858745966	0.269319622	0.253796023	0.832657429
TXNRD2	0.808529082	0.110868734	0.90710307	0.028724091	0.832292399	0.253841028	0.832657429
LRRC41	0.04855002	0.710830777	0.188713553	0.492142229	0.607438422	0.254030344	0.832992128
VIPR2	0.339957617	0.127906825	0.224768086	0.251903298	0.790887733	0.25404458	0.832992128
APEX2	0.71341017	0.304371105	0.311834102	0.048126924	0.599020424	0.254354917	0.833177354
NID1	0.050897054	0.541840851	0.932673176	0.147573483	0.514225674	0.25434354	0.833177354
RMRP	0.924465399	0.775941735	0.114295527	0.426989537	0.055746374	0.2543228	0.833177354
RPS4Y1	0.672057972	0.17099212	0.216271253	0.753108238	0.104182221	0.25422345	0.833177354
SEC14L1	0.238344114	0.608216789	0.247910526	0.109185212	0.496750999	0.254174593	0.833177354
ATP5F1D	0.697576246	0.955439246	0.643998354	0.008045374	0.566426613	0.254602817	0.833347167
EIF3J	0.547171617	0.181683731	0.069619027	0.815796191	0.346423436	0.254599143	0.833347167
GTF2B	0.944952507	0.050156255	0.374231461	0.122133464	0.901908152	0.254461707	0.833347167
ZDHHC20	0.885130722	0.173351993	0.060940479	0.560738117	0.373071521	0.254609877	0.833347167
CLP1	0.444700829	0.287615553	0.216827876	0.102412848	0.689197063	0.254694787	0.833458852

ZSCAN2	0.413832197	0.268189208	0.031312267	0.616619009	0.913857178	0.254746492	0.833461859
BTG1	0.696802353	0.365881685	0.031062835	0.327413278	0.76546933	0.256409467	0.833758773
CDK5	0.519850049	0.70140479	0.480526759	0.017282647	0.656383853	0.256585653	0.833758773
CEBPD	0.921219858	0.349015251	0.079917968	0.118842697	0.64500816	0.255461519	0.833758773
COL8A1	0.055434972	0.752860348	0.085175293	0.947480645	0.587422168	0.25601497	0.833758773
CYB5R3	0.527523124	0.57425911	0.058408707	0.300859566	0.37094988	0.255779399	0.833758773
DNASE1	0.744073353	0.186764923	0.404888336	0.112437283	0.313122485	0.256168495	0.833758773
FKBP1A	0.708155422	0.109036688	0.476550514	0.067561872	0.797033427	0.256202001	0.833758773
GEMIN5	0.435669578	0.039083518	0.733366256	0.285957772	0.555373509	0.256307704	0.833758773
GGN	0.704649989	0.234754442	0.7119873	0.030460097	0.546597421	0.254912159	0.833758773
GIPC1	0.178434162	0.739393453	0.089006292	0.378984298	0.441632169	0.255195866	0.833758773
HDAC3	0.113934522	0.415611514	0.058356763	0.796385397	0.89968708	0.256105162	0.833758773
ISCU	0.393409871	0.582139598	0.137910551	0.066055578	0.952894254	0.256611796	0.833758773
LAGE3	0.588635235	0.264298501	0.193647428	0.082256283	0.802239056	0.25661222	0.833758773
LOC100139345	0.756923575	0.155310825	0.654845507	0.600172799	0.042699051	0.255660067	0.833758773
LOC101902854	0.648731769	0.616790778	0.908770043	0.007518552	0.723508984	0.255987195	0.833758773
LOC101904574	0.650718516	0.598781543	0.287475115	0.062216812	0.28495219	0.256474019	0.833758773
LOC104973105	0.379889141	0.207888034	0.10168681	0.756208717	0.327373924	0.256615414	0.833758773
LOC112446010	0.404829676	0.697768958	0.200616463	0.307391889	0.113098592	0.255492924	0.833758773
LRRC47	0.850206629	0.225127516	0.174312516	0.368873971	0.160626645	0.255913675	0.833758773
MLLT3	0.11106112	0.805933984	0.384011746	0.234171816	0.24410005	0.255153854	0.833758773
NCKAP5L	0.86313928	0.249120847	0.022166156	0.923757422	0.449538905	0.256064583	0.833758773
PARD6A	0.1620912	0.250605248	0.993847767	0.058630144	0.834606517	0.255826742	0.833758773
PARS2	0.880769332	0.389122804	0.772753673	0.02032477	0.367926463	0.256141595	0.833758773
PP2D1	0.266688233	0.47717324	0.234493799	0.255129789	0.260818237	0.256465124	0.833758773
PRRC1	0.506637583	0.880711941	0.151556053	0.100103597	0.291887316	0.255854464	0.833758773
PTPRF	0.618590667	0.111700222	0.327363757	0.12135456	0.718533375	0.255632697	0.833758773
RBX1	0.698361537	0.257309909	0.634250098	0.018453148	0.936467734	0.25545321	0.833758773
REP15	0.882860333	0.672312981	0.079475533	0.498779031	0.083781011	0.255564265	0.833758773
RER1	0.586461188	0.316681166	0.357538082	0.295755955	0.100724485	0.255992104	0.833758773
SLC39A6	0.516483391	0.230130964	0.569507792	0.035354952	0.828659718	0.256307329	0.833758773
SLC7A4	0.974112783	0.34294276	0.191804755	0.524489117	0.05908324	0.256459588	0.833758773
SLC8A2	0.122388936	0.485239345	0.664846601	0.064735019	0.775172381	0.256193101	0.833758773
VIPR1	0.101511548	0.172806413	0.498800781	0.446875953	0.505662005	0.255934341	0.833758773
VSIG10L	0.459022594	0.01568238	0.475525382	0.935145301	0.616193508	0.255639794	0.833758773
ZC4H2	0.782277455	0.378693021	0.242713917	0.807454684	0.034173851	0.256363635	0.833758773
PAPOLB	0.976564125	0.217166245	0.550821461	0.019979135	0.852280984	0.256680159	0.833804056
LOC100141258	0.081006219	0.703690447	0.60215629	0.066813321	0.868805846	0.256889212	0.83384748
LOC101906779	0.828122504	0.04754561	0.56057385	0.539765256	0.167323311	0.256947578	0.83384748
LOC107131792	0.581606218	0.175415597	0.315346092	0.151050611	0.410067968	0.256908647	0.83384748
OSR2	0.640243125	0.823613174	0.011313487	0.374606526	0.89151343	0.256881459	0.83384748
PDXP	0.660752946	0.306936116	0.617265044	0.018738299	0.84857091	0.25676977	0.83384748
NAT10	0.120671767	0.690377222	0.65727276	0.965566565	0.037737731	0.257060959	0.833885631
RTF1	0.619683629	0.884331576	0.875272115	0.018284341	0.227440724	0.25702665	0.833885631

CDK12	0.33366557	0.249709428	0.481805937	0.0867015	0.57379784	0.257177722	0.833965712
LIG4	0.301539311	0.026371375	0.643522976	0.7064516	0.55254926	0.257203519	0.833965712
NMRAL1	0.464371918	0.62850303	0.439132987	0.057157599	0.272754343	0.257238098	0.833965712
DOPEY2	0.45856413	0.274303528	0.293816681	0.381067519	0.141971499	0.257322794	0.834061336
MYCL	0.192866254	0.395032053	0.553540344	0.089775781	0.528294702	0.25736924	0.834061336
KLHL21	0.159318566	0.76181927	0.135471686	0.884894532	0.137585687	0.25747182	0.834064358
LOC112445912	0.564902361	0.220226416	0.231453886	0.074367444	0.934528681	0.257429198	0.834064358
LOC104970589	0.505681113	0.029874365	0.67132712	0.430232095	0.45899217	0.257525092	0.834072288
ARHGEF9	0.723603252	0.360158328	0.04255855	0.260698967	0.694326625	0.25783054	0.834664888
CRIM1	0.179582575	0.200559024	0.437841965	0.300172897	0.424395836	0.257911501	0.834664888
LOC112443614	0.204380035	0.450853306	0.508859261	0.418668622	0.102262518	0.257823199	0.834664888
VTA1	0.228031251	0.316833349	0.469717808	0.227735793	0.259938486	0.257910866	0.834664888
COX20	0.155375054	0.421819676	0.455913864	0.075556967	0.890637186	0.258026155	0.83473563
DEPDC7	0.29422464	0.48656512	0.370568298	0.091924991	0.412359065	0.258035089	0.83473563
MANBA	0.535117293	0.543621016	0.1660836	0.0417873	0.996732746	0.258120441	0.834847173
NAP1L5	0.319408354	0.573978212	0.550730272	0.039648276	0.503358217	0.258288881	0.834944948
NFYC	0.342065081	0.184345988	0.488109672	0.606759166	0.107908626	0.258303303	0.834944948
RABGAP1	0.192710128	0.264135529	0.969492306	0.103852727	0.392987949	0.25822884	0.834944948
ANKRD27	0.380267885	0.932415883	0.53827946	0.10768596	0.098111457	0.258376082	0.835015733
BOLA1	0.058146638	0.812747135	0.576508339	0.162585593	0.456381882	0.258694902	0.835148307
CCDC28A	0.784979651	0.391418543	0.282593644	0.09378091	0.248727362	0.258926	0.835148307
CDH15	0.895991698	0.396204662	0.90293787	0.048714853	0.129571321	0.258797089	0.835148307
FOXJ2	0.122215436	0.271643295	0.591667986	0.407447227	0.252210411	0.258505729	0.835148307
HIGD2A	0.644605654	0.71086128	0.282357675	0.019741599	0.791726049	0.258735047	0.835148307
LOC101903526	0.470344302	0.241977292	0.555051618	0.364690639	0.08789005	0.258893803	0.835148307
LTB	0.493306134	0.219476882	0.364268746	0.815652242	0.06277093	0.258549754	0.835148307
NUDT16L1	0.211377695	0.562534793	0.543380408	0.320973649	0.097464085	0.258674908	0.835148307
STX8	0.709213343	0.234553806	0.534105611	0.03686334	0.617947394	0.258837611	0.835148307
ZFYVE1	0.574202353	0.636327798	0.355043896	0.240531622	0.064861654	0.258836289	0.835148307
ADGRB2	0.734300348	0.48154863	0.009211457	0.714489762	0.870936649	0.259019338	0.835275014
BCKDK	0.398619446	0.866643739	0.790574861	0.011944364	0.621826019	0.259119776	0.835275014
CAD	0.987165845	0.073299746	0.147743176	0.322011016	0.589486593	0.259168873	0.835275014
NRIP3	0.172170254	0.340277433	0.318005264	0.149192549	0.729776494	0.259116487	0.835275014
ATP5F1E	0.725720093	0.771256565	0.822584205	0.006001059	0.737483786	0.259682513	0.835741152
DCTN3	0.495348923	0.338698538	0.403584913	0.047490509	0.633066535	0.259563517	0.835741152
LOC101903564	0.559571644	0.040081998	0.303875779	0.692169111	0.431600057	0.25958633	0.835741152
LOC101908104	0.83222473	0.075979235	0.279919076	0.383743688	0.299933952	0.25965548	0.835741152
LOC107133071	0.403870012	0.276750896	0.090585763	0.244079504	0.824783757	0.259720911	0.835741152
LOC112442851	0.526724935	0.066011176	0.411037132	0.801737386	0.177479417	0.259433035	0.835741152
PEX5	0.183767437	0.769488804	0.220990614	0.693480318	0.093960628	0.259594939	0.835741152
SPTLC3	0.529249287	0.338745889	0.816651993	0.34254981	0.040554644	0.25945409	0.835741152
KLF12	0.986493577	0.81828769	0.338130284	0.0291833	0.256187168	0.259869084	0.836054017
KLK13	0.513609769	0.201606009	0.953258945	0.957211544	0.021635822	0.260086175	0.83629246
PARP12	0.65053626	0.017752744	0.681124171	0.276954497	0.937692036	0.260000511	0.83629246

REXO2	0.093060228	0.459681667	0.572861486	0.373135487	0.223576331	0.260096077	0.83629246
VAV1	0.32977638	0.987480459	0.565942295	0.072635271	0.152829961	0.260186365	0.836418891
SLCO1C1	0.82567696	0.518270404	0.315088612	0.020338188	0.747092842	0.260362364	0.836820752
SENP8	0.902209722	0.598714121	0.038505967	0.15998525	0.616525622	0.260535608	0.837213602
MED10	0.677570238	0.137001217	0.054079051	0.552931665	0.739482914	0.260599531	0.837255071
PSMG2	0.512409229	0.584119023	0.343541531	0.743560294	0.026868732	0.260701653	0.837255347
SLC2A6	0.906519003	0.936646714	0.014220249	0.827783663	0.20552453	0.260695743	0.837255347
FBXL3	0.082582577	0.154342985	0.595469371	0.897954289	0.301662894	0.260802794	0.837416289
DCTPP1	0.306321161	0.956004507	0.40853852	0.098297821	0.175046543	0.260963675	0.83755349
LOC107131424	0.380136402	0.707240951	0.365398755	0.699547885	0.029975978	0.261049668	0.83755349
PGS1	0.1234006	0.967560991	0.258431887	0.377495278	0.176707982	0.260946914	0.83755349
UBAC1	0.368003481	0.842047541	0.320040902	0.032187175	0.645214867	0.261025523	0.83755349
GNL3L	0.432784892	0.485942071	0.509009776	0.108322753	0.177794342	0.261152536	0.837719755
KIF13A	0.353016392	0.410222048	0.063797813	0.738302232	0.302467062	0.261242695	0.837845196
COX7A1	0.619744088	0.803282599	0.128688099	0.038379017	0.840647452	0.261472819	0.838080776
FBXW5	0.126997843	0.730245258	0.914100321	0.027618649	0.882531028	0.261433579	0.838080776
IQCH	0.604790259	0.938923306	0.20029279	0.189076728	0.096150815	0.261520423	0.838080776
TTC39C	0.539662663	0.323100061	0.219853067	0.49353789	0.109211523	0.261430549	0.838080776
FCAR	0.415784692	0.589235507	0.022686565	0.818924835	0.455315056	0.261807523	0.838317703
GPAT2	0.167687802	0.790032939	0.500434338	0.185319709	0.168737867	0.261849768	0.838317703
ITFG2	0.67208122	0.511294283	0.088199021	0.315540837	0.216657092	0.261779337	0.838317703
LOC614785	0.052921198	0.993610633	0.439549409	0.224927522	0.398200284	0.261667141	0.838317703
PFN1	0.06183244	0.256958184	0.65617719	0.47521235	0.418148906	0.261760124	0.838317703
CACNA1H	0.034684977	0.665452466	0.339465319	0.622587675	0.425392713	0.261970522	0.83837719
LGALS1	0.20715127	0.258329811	0.219587964	0.176749796	0.998969198	0.261952326	0.83837719
ADGRV1	0.159312436	0.763876201	0.026184449	0.757844831	0.861495917	0.262291143	0.838470663
COLQ	0.255960745	0.61430997	0.033058224	0.625552668	0.639878372	0.262306281	0.838470663
LAMC2	0.34964572	0.134185456	0.769912549	0.459120837	0.125440356	0.262288165	0.838470663
MRPL24	0.304601161	0.945814478	0.252507816	0.073217989	0.390188085	0.262161875	0.838470663
SLCO4A1	0.601942584	0.791814905	0.196638751	0.0244295	0.907548825	0.26214107	0.838470663
ZNF219	0.477085939	0.290320649	0.175512954	0.360781587	0.23712378	0.26224865	0.838470663
RTL5	0.615525507	0.467859383	0.089188997	0.138638049	0.584755112	0.262401317	0.838611102
AP1G1	0.513120514	0.107565977	0.511100304	0.281569807	0.2623783	0.262513196	0.838731164
RAF1	0.620419443	0.596514032	0.089786918	0.754198695	0.083177452	0.2625411	0.838731164
LOC100850276	0.398669773	0.29333662	0.200261921	0.723771905	0.123115382	0.262680972	0.83901468
SDF2L1	0.111260484	0.798624669	0.376472925	0.347956672	0.17940955	0.262767024	0.839126218
TM2D1	0.143522293	0.902239534	0.439255992	0.055988596	0.656217143	0.262859433	0.839258006
BCLAF3	0.50216168	0.79415642	0.09304644	0.126980248	0.446572266	0.263724208	0.839350509
C1QBP	0.607379783	0.748719142	0.727037567	0.093183591	0.067908111	0.263002238	0.839350509
C7H5orf30	0.783667049	0.198019873	0.206440933	0.097340434	0.671312878	0.263077049	0.839350509
CCDC51	0.97347473	0.456201112	0.434271363	0.030345245	0.359696413	0.263780063	0.839350509
CD52	0.147723358	0.847746762	0.428273001	0.577883878	0.067878728	0.26370468	0.839350509
COL26A1	0.064753478	0.994073142	0.268709944	0.32110265	0.378143665	0.263487843	0.839350509
DOLPP1	0.100667679	0.97808176	0.808898783	0.156179263	0.16924953	0.263792092	0.839350509

FSTL1	0.584375732	0.631732688	0.059934421	0.279303358	0.340795974	0.263839372	0.839350509
LIN7C	0.432772348	0.042531412	0.285739269	0.444237152	0.901116584	0.263799337	0.839350509
LOC112447103	0.651240542	0.806686722	0.099846835	0.944363874	0.04237635	0.263423102	0.839350509
LOC112447118	0.261345128	0.565748447	0.615446563	0.390016403	0.059447611	0.264064754	0.839350509
LYRM9	0.798112877	0.519608377	0.347286915	0.036097167	0.404399642	0.263617997	0.839350509
MAN1A1	0.663612172	0.45783872	0.615419958	0.019238033	0.5831338	0.263331038	0.839350509
MIB1	0.994280443	0.314039863	0.021801182	0.479975581	0.644730782	0.263868176	0.839350509
NPEPPS	0.437940026	0.479923224	0.226795972	0.051185417	0.85826267	0.263115964	0.839350509
OSBPL6	0.771601184	0.676107687	0.015069648	0.406659674	0.656705222	0.263443454	0.839350509
RAC3	0.952562856	0.409271532	0.674996311	0.009311301	0.856664667	0.263418508	0.839350509
SEC13	0.034187529	0.692486278	0.286932576	0.355040593	0.873780421	0.263917096	0.839350509
SLC35A5	0.287509877	0.235029473	0.052800778	0.941113591	0.625877948	0.263569746	0.839350509
TPRN	0.618168929	0.439368409	0.29585749	0.064268908	0.408426314	0.264032029	0.839350509
TXNL1	0.893676801	0.111547885	0.090741509	0.54778328	0.42416553	0.26358281	0.839350509
UPF3B	0.615591888	0.07066006	0.98566823	0.427109754	0.114791781	0.263599113	0.839350509
WHRN	0.650345672	0.286270633	0.598080185	0.047963944	0.394859185	0.264004147	0.839350509
ERGIC2	0.739788428	0.614617342	0.19465947	0.131303258	0.181715267	0.264184183	0.839517409
LOC101908166	0.071551646	0.91971658	0.319102345	0.577452077	0.17420335	0.264219573	0.839517409
BARX1	0.556684989	0.145729004	0.601386879	0.045560445	0.952859345	0.264555332	0.839518675
COA3	0.760372893	0.44200398	0.430836019	0.018784915	0.779428066	0.264679156	0.839518675
COL4A6	0.068201963	0.756475731	0.434878846	0.189173236	0.499652451	0.264719415	0.839518675
FADS1	0.344407469	0.891440583	0.495962295	0.257761591	0.053975331	0.264584814	0.839518675
LOC112448378	0.891855275	0.748809385	0.103032748	0.248646033	0.123631325	0.264387372	0.839518675
LOC785087	0.088824972	0.901779822	0.079770105	0.629916316	0.525146983	0.264296442	0.839518675
MRPL49	0.6808204	0.260657651	0.262653084	0.103762043	0.437266279	0.264363736	0.839518675
NCOA3	0.465314877	0.256599544	0.112236165	0.901341439	0.175591852	0.26473153	0.839518675
SLC6A2	0.896041265	0.130880679	0.678060085	0.050898426	0.523987044	0.264721976	0.839518675
SP140L	0.664332773	0.029069078	0.339688507	0.408639595	0.790406353	0.26460278	0.839518675
ADCY4	0.71482291	0.328139876	0.039664324	0.49528409	0.461461875	0.265058489	0.839703607
ADTRP	0.319833588	0.222108518	0.121416025	0.27658217	0.894088173	0.265445373	0.839703607
ARID4A	0.426892709	0.416736506	0.422163971	0.046829461	0.605835338	0.265318146	0.839703607
ATP6V1H	0.549969264	0.59197555	0.075290205	0.488918502	0.177895039	0.265390085	0.839703607
C5H12orf75	0.056507921	0.29220106	0.428098535	0.598893261	0.50594764	0.265978646	0.839703607
CALU	0.198440593	0.386864791	0.168099047	0.230975078	0.720156478	0.266259394	0.839703607
CARD19	0.247407204	0.206574634	0.169484671	0.3922984	0.633774395	0.266677499	0.839703607
CCR6	0.508858759	0.348033162	0.318226525	0.183272797	0.208214869	0.266499226	0.839703607
COQ8A	0.293796542	0.779769665	0.643913615	0.106659866	0.136122164	0.26597297	0.839703607
DHRS9	0.23843236	0.474112597	0.289160106	0.099708452	0.660997087	0.266720277	0.839703607
DIS3L2	0.627397279	0.151244148	0.558936102	0.984341195	0.041143968	0.266343985	0.839703607
FBRS	0.601007685	0.564855559	0.664706489	0.122353584	0.077690589	0.266167117	0.839703607
HDGFL3	0.317707363	0.289419995	0.575355302	0.089166659	0.456107158	0.266557167	0.839703607
IPO5	0.304730285	0.840386609	0.085318106	0.243893332	0.402953001	0.266301448	0.839703607
KLF14	0.543307175	0.337723726	0.441971053	0.217194356	0.121361895	0.265727114	0.839703607
LAMTOR5	0.598093673	0.335011573	0.087313223	0.149737234	0.818987368	0.266191639	0.839703607

LOC101903232	0.982359903	0.883424668	0.029013014	0.349542519	0.242050234	0.265289846	0.839703607
LOC112441452	0.714436197	0.851567902	0.005728452	0.985372089	0.62492893	0.2662312	0.839703607
LOC112447309	0.245498721	0.92404734	0.856930912	0.712941964	0.015464745	0.266065547	0.839703607
LOC112448853	0.616036354	0.289587438	0.420768423	0.066187737	0.433154097	0.266582967	0.839703607
LOC112449602	0.087830774	0.999693018	0.609489154	0.565597002	0.070876801	0.266184161	0.839703607
LOC531462	0.310442746	0.962453068	0.056159131	0.493253001	0.259566686	0.266363046	0.839703607
MAOB	0.9493339	0.069287969	0.738634058	0.059926077	0.734140922	0.265718239	0.839703607
MSI2	0.691802676	0.889395039	0.462201465	0.036089325	0.209821449	0.266667488	0.839703607
OTULINL	0.998116658	0.512954619	0.093557417	0.08761716	0.510828911	0.266099839	0.839703607
PIGY	0.993995031	0.149664407	0.227509262	0.181037852	0.348475434	0.265584051	0.839703607
RBM33	0.26781064	0.131002616	0.737824933	0.41599255	0.197444811	0.265042304	0.839703607
RBM4B	0.393587319	0.229668756	0.301082965	0.498231732	0.156629348	0.264908184	0.839703607
RCCD1	0.272803248	0.132580617	0.38303807	0.484269808	0.320969625	0.266663356	0.839703607
RRAGB	0.57784411	0.468989528	0.644228916	0.059105601	0.206539151	0.265350303	0.839703607
RUM1	0.898662164	0.241806679	0.839605187	0.035300313	0.331041371	0.265395858	0.839703607
SBSPON	0.714602324	0.436731882	0.080438981	0.789252722	0.107922209	0.265768453	0.839703607
SMPD4	0.350998273	0.075011996	0.622842893	0.166299399	0.781552277	0.265355528	0.839703607
TMEM229B	0.599408967	0.529783704	0.252299145	0.677390925	0.039432973	0.265875052	0.839703607
TSPAN13	0.348134841	0.167015759	0.043702681	0.92381112	0.906665199	0.265174157	0.839703607
TSPYL4	0.021077874	0.59757338	0.210858951	0.939224067	0.852806972	0.265111812	0.839703607
URM1	0.217548091	0.791734008	0.459408664	0.206484448	0.131527273	0.266403195	0.839703607
VAMP8	0.128204778	0.621272968	0.790568991	0.044271395	0.772883993	0.266734197	0.839703607
CLMP	0.267286655	0.227748539	0.186641012	0.370502744	0.513023126	0.267029421	0.839852237
DANCR	0.671196317	0.178071406	0.194517284	0.617417771	0.15041815	0.267003837	0.839852237
HERPUD2	0.588306186	0.556666292	0.015788411	0.436965181	0.955305102	0.266958076	0.839852237
LRP11	0.353407521	0.116406774	0.52672225	0.205574642	0.484830398	0.267037291	0.839852237
TSPAN18	0.84262819	0.514658764	0.029733165	0.173956508	0.961633035	0.266876018	0.839852237
MRPL44	0.280928637	0.810286208	0.391817161	0.072213326	0.335881325	0.267250854	0.840362858
ABHD10	0.325512613	0.52273957	0.38660334	0.105336768	0.314296087	0.268110043	0.840443133
BPNT1	0.466085195	0.475309321	0.340635912	0.080718603	0.356263081	0.267649883	0.840443133
C8H9orf72	0.583521484	0.540821225	0.431776048	0.03504788	0.456404519	0.268211233	0.840443133
CDH6	0.120216142	0.530089076	0.192174546	0.484405523	0.369102383	0.268796583	0.840443133
COA6	0.281436627	0.562176202	0.23490922	0.095964275	0.615182195	0.269063506	0.840443133
COPS9	0.382621196	0.395748511	0.237385131	0.060812355	0.996207235	0.268093994	0.840443133
COQ10A	0.546251691	0.246712535	0.270129749	0.191915029	0.314064613	0.269068809	0.840443133
FAM114A2	0.323201225	0.503914341	0.141385242	0.131775996	0.716490377	0.267886765	0.840443133
FZD8	0.658026981	0.852885872	0.101120747	0.09884997	0.387645888	0.267918054	0.840443133
HHIP	0.980720352	0.01828834	0.66910149	0.833126173	0.219165085	0.268894157	0.840443133
HUWE1	0.457925158	0.419971736	0.668651298	0.038251222	0.445803639	0.268985658	0.840443133
ITSN2	0.361325971	0.282342916	0.248204441	0.109266131	0.788405908	0.26831162	0.840443133
KCNMB3	0.084704286	0.127919731	0.979861163	0.889063272	0.230312408	0.267879988	0.840443133
KDR	0.424554616	0.311681784	0.146455002	0.144767933	0.771724826	0.267357655	0.840443133
KIF3C	0.357807253	0.710609436	0.064069009	0.890242428	0.15041143	0.268310496	0.840443133
LOC100335744	0.965768831	0.793476526	0.409205655	0.011178442	0.623814043	0.268625801	0.840443133

LOC104970387	0.311217932	0.528499953	0.029836716	0.467922768	0.955317639	0.269038691	0.840443133
LOC112445980	0.776286978	0.165540812	0.256976203	0.466893291	0.141438808	0.268277819	0.840443133
LOC615610	0.982494081	0.435674647	0.109769441	0.047628988	0.971208275	0.267850497	0.840443133
LOC619159	0.054109835	0.808646848	0.163754612	0.595745059	0.513415677	0.268913251	0.840443133
MED30	0.953793117	0.396967122	0.179356253	0.166372287	0.19200287	0.267601998	0.840443133
MMS22L	0.837743303	0.01703327	0.623230088	0.266665262	0.922355749	0.268666264	0.840443133
MTMR7	0.009753041	0.69136551	0.518142853	0.664592071	0.937509939	0.26804828	0.840443133
MYC	0.211069931	0.684309473	0.11636242	0.216892945	0.600587313	0.268781753	0.840443133
OLFML1	0.336747523	0.678456449	0.061788093	0.231244929	0.670417808	0.268733209	0.840443133
PAK6	0.440805078	0.772934699	0.928485081	0.032047988	0.21593623	0.268775064	0.840443133
POLD1	0.935841467	0.019614177	0.22062614	0.667418227	0.811500472	0.269019068	0.840443133
PPIH	0.428471926	0.152842786	0.767318389	0.210175393	0.206372153	0.268209779	0.840443133
PPP1R14A	0.39611778	0.133852004	0.973740848	0.368949173	0.114377993	0.268158372	0.840443133
SELENOV	0.061261736	0.566050263	0.73936474	0.086436217	0.980371812	0.267801041	0.840443133
SEMA3F	0.048028567	0.869415373	0.648166852	0.47155796	0.171417646	0.268690502	0.840443133
SGSH	0.295435135	0.587118483	0.03421447	0.491513799	0.746271358	0.268048832	0.840443133
SIAH1	0.107590164	0.873907744	0.817956418	0.96751092	0.029391677	0.268644482	0.840443133
TP53I3	0.145818492	0.348761293	0.098046771	0.784353196	0.558317387	0.268443235	0.840443133
VOPP1	0.10233459	0.079008443	0.526458827	0.526840474	0.977209285	0.268904047	0.840443133
APOL3	0.048122143	0.645207763	0.910721597	0.85481113	0.091271384	0.269764504	0.840452639
ARID1A	0.3833872	0.157555252	0.192145112	0.814872026	0.232155977	0.269153	0.840452639
B4GALNT1	0.391837849	0.80841354	0.210342831	0.410555749	0.08063411	0.269741879	0.840452639
BTG2	0.279992394	0.116445691	0.620500079	0.285420275	0.380365308	0.26919089	0.840452639
COLGALT2	0.292440513	0.777692148	0.303930832	0.427332461	0.074615094	0.269639391	0.840452639
EEF1E1	0.54331934	0.419697407	0.229508351	0.590449725	0.071240236	0.269487094	0.840452639
GRHL1	0.150139822	0.879942151	0.158142689	0.138524695	0.762717319	0.269840044	0.840452639
LOC613401	0.532867615	0.612497884	0.267277414	0.087282331	0.288548637	0.269230624	0.840452639
MED28	0.410669932	0.134414251	0.070100799	0.866231933	0.656978271	0.269530958	0.840452639
MMP9	0.768796662	0.127952533	0.153074466	0.558483115	0.261683425	0.26944304	0.840452639
MYEF2	0.60280112	0.130207522	0.131052385	0.497950485	0.430285325	0.269634997	0.840452639
NFYA	0.653868893	0.341286278	0.06423236	0.552730053	0.278005726	0.269555773	0.840452639
RELL1	0.503203591	0.525640778	0.235256904	0.04418044	0.79990134	0.269351953	0.840452639
SRD5A1	0.257044825	0.815530963	0.224791442	0.06724877	0.693586622	0.269284646	0.840452639
TSSK3	0.885353322	0.015367059	0.346012815	0.884625499	0.529886634	0.269796203	0.840452639
NLRC4	0.889139211	0.550980703	0.655244487	0.069211334	0.099428318	0.26993099	0.840576373
BICDL1	0.129111918	0.211804265	0.545758076	0.383189468	0.387312207	0.270280617	0.840965416
DBNDD1	0.691627543	0.435700082	0.32024332	0.026199855	0.877044296	0.270424367	0.840965416
LOC100849652	0.106902881	0.876321325	0.652272297	0.667785884	0.054236484	0.270172215	0.840965416
LOC107132534	0.506582155	0.569949543	0.746470764	0.484701499	0.02119385	0.270223476	0.840965416
LOC515169	0.586262324	0.531288214	0.072911929	0.901037611	0.108364666	0.270421681	0.840965416
LOC515828	0.820482227	0.127649133	0.667523174	0.231691342	0.136822384	0.270353128	0.840965416
SELENOS	0.691448973	0.009534928	0.757863689	0.468708459	0.944871466	0.270152551	0.840965416
TMEM67	0.493322789	0.824142548	0.041281883	0.219234363	0.602835251	0.270465875	0.840965416
ADPRH	0.514012154	0.106267757	0.417585065	0.150621626	0.649391955	0.271212734	0.841172283

ARHGAP45	0.080575782	0.673228017	0.948239118	0.223205227	0.194198587	0.271129741	0.841172283
ATP2C1	0.681688254	0.08869571	0.062166749	0.852940393	0.694580503	0.27096709	0.841172283
C17H12orf49	0.313157491	0.193131376	0.120046768	0.417418773	0.734448678	0.270911129	0.841172283
DZANK1	0.308712052	0.634566789	0.203450205	0.55597831	0.100452029	0.270912934	0.841172283
EPN1	0.104314862	0.600355562	0.711507997	0.154683546	0.323222454	0.271024248	0.841172283
FLNB	0.499857503	0.693561961	0.178757342	0.296515234	0.121541643	0.271347594	0.841172283
GEMIN6	0.837189014	0.268101056	0.409111617	0.778606214	0.031177528	0.271096558	0.841172283
HSPBP1	0.52455764	0.392303323	0.025638122	0.896115398	0.471080288	0.270988619	0.841172283
LOC100847818	0.969357467	0.161976707	0.153221063	0.203610351	0.454420314	0.270915004	0.841172283
LRG1	0.906506578	0.232561558	0.178650392	0.311361524	0.190323219	0.271258977	0.841172283
MKNK1	0.407294166	0.119876531	0.677184793	0.270425674	0.248923599	0.270900432	0.841172283
PSMA3	0.676830892	0.406587936	0.333255718	0.138865319	0.175379142	0.271352511	0.841172283
PSMB4	0.352322397	0.530392479	0.294171973	0.212653289	0.190331798	0.270858479	0.841172283
SEC16A	0.680559579	0.524308518	0.129936452	0.895190444	0.053728645	0.271150671	0.841172283
TWISTNB	0.875821437	0.750916363	0.120836088	0.351527805	0.079566552	0.270731234	0.841172283
ABLIM1	0.327867938	0.652702914	0.228220644	0.698046929	0.065781979	0.27188155	0.84122325
BCS1L	0.413573812	0.759712763	0.738845093	0.041305906	0.233535814	0.271691076	0.84122325
CEP85	0.426159473	0.180647056	0.263953593	0.277938539	0.396248169	0.271609243	0.84122325
CSF1	0.880266556	0.751188624	0.011665227	0.462831712	0.628084685	0.271862705	0.84122325
LOC101902937	0.560143733	0.775932541	0.594828831	0.011844117	0.73224692	0.271856144	0.84122325
MAF	0.157586911	0.307304157	0.907405989	0.074737946	0.682531642	0.271819939	0.84122325
MCEE	0.480045737	0.842651909	0.159334602	0.057328207	0.60692318	0.271876195	0.84122325
NOL9	0.376218031	0.061462713	0.839372967	0.746872826	0.154608063	0.271799368	0.84122325
PLK4	0.654907128	0.053251965	0.199426477	0.722222743	0.445773895	0.271679902	0.84122325
RAB18	0.55052638	0.116384076	0.056870964	0.685105732	0.897546029	0.271767054	0.84122325
PPARGC1A	0.747483296	0.993216163	0.110457368	0.341629041	0.080132775	0.272013837	0.841361337
TLR6	0.034247849	0.401505646	0.762543579	0.911512605	0.234910559	0.272028716	0.841361337
CENPK	0.929177578	0.073308192	0.086813173	0.973476086	0.390589971	0.272216051	0.841443173
COX7B	0.59019275	0.832752825	0.902020671	0.007800598	0.650503583	0.272281913	0.841443173
PXMP2	0.941685454	0.928929821	0.164270207	0.030031814	0.520699803	0.272136536	0.841443173
SRGAP2	0.384218937	0.271768173	0.047894611	0.586216602	0.766978525	0.272222639	0.841443173
TESC	0.71096707	0.749314204	0.017663954	0.306243443	0.7807918	0.27231154	0.841443173
DCAF17	0.616129666	0.431863132	0.951973	0.807281774	0.011043147	0.27277637	0.841719673
LAPTM4A	0.316698878	0.030349701	0.559141789	0.780695402	0.537607913	0.272629851	0.841719673
LOC101907916	0.161990761	0.700740211	0.161109958	0.965675881	0.127698786	0.272605271	0.841719673
LOC407171	0.327123975	0.66383059	0.606297281	0.18357492	0.093370257	0.272691649	0.841719673
PPIL1	0.709311169	0.418532013	0.956250661	0.077523878	0.102637311	0.272811342	0.841719673
RNF125	0.899401007	0.141255033	0.033595219	0.85400355	0.619295971	0.272726149	0.841719673
SLC7A5	0.065564021	0.107150091	0.921358152	0.602444266	0.578667692	0.272678381	0.841719673
STAG2	0.197464506	0.566869053	0.51138402	0.057514894	0.68590412	0.272776907	0.841719673
MCM2	0.345045092	0.271193442	0.074543867	0.570007412	0.568400223	0.272878165	0.841767588
COPS2	0.411786465	0.085747314	0.13093591	0.980748875	0.498824557	0.272984317	0.841936785
GPER1	0.414123179	0.814778281	0.421090025	0.020012352	0.796548183	0.273162776	0.842328884
ABHD6	0.160825611	0.797354212	0.056344494	0.78685389	0.401350201	0.274127033	0.842426761

AP1B1	0.154469757	0.862518425	0.919818966	0.299001116	0.062144624	0.273861889	0.842426761
ATP6V1G1	0.262494955	0.03964959	0.27646381	0.983734913	0.803383922	0.273684808	0.842426761
CSNK1G2	0.053796441	0.385292868	0.664571442	0.262435996	0.629120258	0.273698006	0.842426761
IGFLR1	0.904854189	0.051213765	0.595154701	0.178285023	0.464379216	0.274219125	0.842426761
KCNA5	0.451766979	0.476728197	0.140395325	0.328890042	0.229399271	0.27409861	0.842426761
LOC112441839	0.540300072	0.326871467	0.06971136	0.473895027	0.390128534	0.273806453	0.842426761
LOC404051	0.329661722	0.420711604	0.047389622	0.711358056	0.486585732	0.273740107	0.842426761
LOC531090	0.173003661	0.556658194	0.250907646	0.438468758	0.215220546	0.274038513	0.842426761
LOC781710	0.212451	0.655811441	0.998564872	0.02798293	0.586325934	0.274178217	0.842426761
MESD	0.158465191	0.076644767	0.481183613	0.644212103	0.606368329	0.274192085	0.842426761
NKPD1	0.510985147	0.014257744	0.457040862	0.993497945	0.687886484	0.273774237	0.842426761
PPIL6	0.203055832	0.585750534	0.442103612	0.181284665	0.238549638	0.273682677	0.842426761
SETDB2	0.772518285	0.642761488	0.010170394	0.550686024	0.819022845	0.273893602	0.842426761
SFR1	0.299059621	0.448414588	0.040167541	0.765820524	0.551904302	0.273835823	0.842426761
SH2D5	0.205641935	0.782053591	0.347471581	0.777451986	0.052502113	0.274079764	0.842426761
SNAPIN	0.805243104	0.335961151	0.203511172	0.060126989	0.686739217	0.273644834	0.842426761
TMEM8A	0.080398714	0.556120285	0.209113621	0.939232584	0.25836884	0.273389841	0.842426761
VEZF1	0.101370404	0.741837955	0.838113932	0.058862336	0.612766456	0.27364188	0.842426761
ZCCHC14	0.442720314	0.520291191	0.061961477	0.170384758	0.938990574	0.274221178	0.842426761
AP3M2	0.918035814	0.074275116	0.880378711	0.072702808	0.525218537	0.274723358	0.84257676
BAAT	0.805876305	0.073670625	0.311046624	0.291496981	0.425401854	0.274590689	0.84257676
CCDC9B	0.430808771	0.658776713	0.564367956	0.018294913	0.781976915	0.274677037	0.84257676
LIMD2	0.539071507	0.838008935	0.451028863	0.096757374	0.116281162	0.274732085	0.84257676
NEXMIF	0.049086661	0.663178283	0.261390497	0.423150977	0.635599935	0.27451301	0.84257676
PM20D1	0.761580723	0.828660314	0.065745482	0.141657222	0.389434107	0.27453351	0.84257676
PTGER1	0.677451491	0.463007268	0.020770137	0.807120602	0.435934895	0.274723876	0.84257676
SUN2	0.79222017	0.287287655	0.017799863	0.614943914	0.920154378	0.274726962	0.84257676
ZNF582	0.839781214	0.641355954	0.542552841	0.012171034	0.64445931	0.274713331	0.84257676
CEP89	0.656247682	0.06458487	0.21757529	0.485349761	0.513181979	0.274984791	0.84272183
FASTKD5	0.267694141	0.162965314	0.182927249	0.702051608	0.409552149	0.274851545	0.84272183
MRPS14	0.384589086	0.593367696	0.403879553	0.07017344	0.355003626	0.274937909	0.84272183
MRPS30	0.9017409	0.747942113	0.49813624	0.237603616	0.028770969	0.2749761	0.84272183
ITIH1	0.519823616	0.311703103	0.228869702	0.166344722	0.372535236	0.27505312	0.842773853
ABHD18	0.348937632	0.153971097	0.140340085	0.447904749	0.681736878	0.275296364	0.843361701
CYP2S1	0.188074137	0.139636094	0.797996351	0.531058146	0.207040204	0.275402569	0.84347097
RPAP1	0.402095278	0.065123531	0.303313959	0.952669597	0.304600129	0.275434826	0.84347097
ATP10D	0.839968746	0.249174024	0.044945181	0.444271824	0.552749863	0.275734094	0.844090906
PTPMT1	0.467298885	0.115885679	0.597049238	0.132969915	0.537351486	0.275740135	0.844090906
CAVIN4	0.70372307	0.229403904	0.069150345	0.237112078	0.873508428	0.275851876	0.844275481
SFMBT2	0.389185277	0.66850229	0.010808636	0.896233125	0.918092374	0.275948176	0.844412738
FAM69B	0.74617753	0.212555275	0.068504594	0.410027314	0.520206882	0.276154535	0.844571762
NACC2	0.482698393	0.624125757	0.130881905	0.133035891	0.441626647	0.276103024	0.844571762
XPO4	0.090941852	0.991875714	0.704659931	0.272867482	0.133605868	0.27614107	0.844571762
EMSY	0.12743758	0.951257208	0.730941526	0.061719167	0.424744885	0.276457065	0.84463075

IL17RD	0.881103776	0.941161831	0.37784424	0.174270994	0.042553811	0.276499711	0.84463075
LOC101906195	0.690555839	0.072673095	0.162421032	0.418404036	0.681648303	0.276560996	0.84463075
NSD2	0.878893602	0.030349572	0.277234913	0.630196372	0.497700858	0.27626255	0.84463075
SRSF3	0.640126552	0.049353389	0.922214526	0.435171541	0.183050763	0.276342646	0.84463075
THYN1	0.361586469	0.49588074	0.184607718	0.137106608	0.511915788	0.276478129	0.84463075
WSCD2	0.876012729	0.531026657	0.088303302	0.266188084	0.212313773	0.276379921	0.84463075
WWOX	0.567062623	0.536566395	0.014326971	0.906458809	0.588430853	0.276585562	0.84463075
AMZ2	0.487257016	0.29245623	0.073815257	0.29886931	0.739928359	0.276640981	0.844636208
GPM6A	0.112611282	0.097899748	0.432037953	0.523600387	0.933204059	0.27670912	0.844636208
MPV17	0.565154027	0.70997595	0.342222205	0.022292067	0.7605033	0.276741752	0.844636208
ABCD3	0.708673643	0.203602563	0.186289815	0.360118766	0.241101007	0.277071811	0.8449611
ARHGAP30	0.348356886	0.608403565	0.638582395	0.190894313	0.091465345	0.278711991	0.8449611
ATG13	0.775030733	0.511092335	0.315821153	0.432552389	0.043124677	0.277059931	0.8449611
BCNT2	0.358007376	0.458771352	0.363656235	0.077879925	0.506941738	0.278433122	0.8449611
C16H1orf74	0.416284631	0.326256352	0.867196778	0.112500527	0.17775342	0.278274538	0.8449611
CHD1L	0.417913231	0.113578251	0.231845793	0.541584172	0.396274776	0.278639282	0.8449611
DSC1	0.550165182	0.490573811	0.034281879	0.53634259	0.471986555	0.277546446	0.8449611
FAM129A	0.108277432	0.09483024	0.943564836	0.645070119	0.377926316	0.278647681	0.8449611
FBXO46	0.663000664	0.205242756	0.170058414	0.508755788	0.199955996	0.278209162	0.8449611
G3BP2	0.911352269	0.081435322	0.103404263	0.697871557	0.439058844	0.278061386	0.8449611
GRIK3	0.309520781	0.766638849	0.526763091	0.339790186	0.054975332	0.277135619	0.8449611
GTF3C6	0.884428306	0.61937729	0.171510388	0.487745486	0.051129643	0.277588893	0.8449611
HDHD3	0.73585173	0.12954675	0.256803535	0.142685327	0.675108635	0.278435527	0.8449611
LOC100299242	0.807835797	0.43254779	0.043146305	0.859935931	0.180916908	0.27773157	0.8449611
LOC101906317	0.667844735	0.360314944	0.565640878	0.23072748	0.075311631	0.278826438	0.8449611
LOC104969159	0.799599053	0.047296005	0.493975902	0.201640955	0.628270303	0.278907701	0.8449611
LOC107131367	0.714028361	0.038861648	0.806989052	0.977743176	0.107822526	0.278577335	0.8449611
LOC112443444	0.215000972	0.641659609	0.318238756	0.786495014	0.067689775	0.277269328	0.8449611
LOC112447599	0.587232885	0.036251796	0.241457758	0.561753075	0.812897791	0.277827307	0.8449611
LOC514189	0.764872096	0.576079991	0.223473208	0.120644894	0.199107348	0.278836559	0.8449611
LRIG3	0.449360194	0.884717345	0.068784816	0.228925828	0.37516054	0.277900304	0.8449611
LUC7L3	0.844124784	0.226625017	0.093141775	0.145488393	0.912417783	0.278832857	0.8449611
NCF4	0.335932836	0.089895456	0.688873567	0.613688525	0.183082101	0.277271254	0.8449611
PPP1R37	0.087662214	0.664728338	0.782092508	0.590415434	0.086934096	0.2773737	0.8449611
PPRC1	0.629865134	0.572273857	0.876031131	0.014623275	0.508603956	0.277897799	0.8449611
PRR5L	0.731543067	0.37840503	0.029343206	0.563354093	0.514462925	0.278214065	0.8449611
RETN	0.268836043	0.555027388	0.367679954	0.087804752	0.489795665	0.278506457	0.8449611
RGS9	0.252690671	0.233296822	0.191122102	0.917122178	0.227082856	0.277784346	0.8449611
RMND1	0.646351262	0.881397658	0.835694775	0.011467792	0.427023307	0.276937783	0.8449611
SERPINA5	0.269136029	0.613106261	0.472575573	0.173470334	0.173496481	0.277806815	0.8449611
SF3B3	0.759136515	0.595309528	0.73284629	0.020701783	0.344152236	0.278515292	0.8449611
SLC25A22	0.149327892	0.903793901	0.125939732	0.93498189	0.146736133	0.276966108	0.8449611
SMAD6	0.783740527	0.214676697	0.448078495	0.043758281	0.71155567	0.27783275	0.8449611
SNN	0.729536805	0.782206736	0.049855038	0.144922227	0.570164699	0.278024256	0.8449611

SVIL	0.088985805	0.307725746	0.148430174	0.678997565	0.857462122	0.278896218	0.8449611
TES	0.25733839	0.4251323	0.614342219	0.51216414	0.068640293	0.278695213	0.8449611
TMCC3	0.468808123	0.552975293	0.13008898	0.103836697	0.668868333	0.277546105	0.8449611
TPX2	0.621291749	0.722339444	0.146185683	0.075926429	0.474487319	0.27873525	0.8449611
TRAPPC1	0.174728541	0.558085908	0.472824462	0.060013073	0.852871954	0.278533656	0.8449611
VAMP3	0.962025449	0.095882202	0.090697383	0.370718918	0.75362841	0.277270564	0.8449611
PLAT	0.941287338	0.808757383	0.859140781	0.019397179	0.186722147	0.279032576	0.845183391
MED17	0.982268853	0.294456008	0.711440526	0.265051246	0.0434577	0.27910749	0.845254293
ZNF654	0.418312805	0.694610734	0.663022673	0.018695324	0.65839416	0.279169433	0.845285898
AXIN2	0.050650353	0.233584669	0.625261421	0.331526522	0.967351187	0.279230441	0.84531466
AGER	0.732960102	0.154815681	0.404335793	0.193483571	0.268858832	0.280025427	0.84605889
ATP8B4	0.604137681	0.078351959	0.590347039	0.089500719	0.970963443	0.282319602	0.84605889
BCO2	0.752964269	0.403851004	0.514217676	0.141336218	0.109686786	0.282083284	0.84605889
BORCS7	0.202750831	0.321139783	0.27507545	0.261196022	0.51145022	0.280351991	0.84605889
CADM3	0.093498166	0.66225905	0.70140889	0.206299271	0.269421089	0.281527283	0.84605889
CCZ1	0.191865054	0.549521208	0.083320115	0.612108493	0.449557031	0.28171424	0.84605889
CD72	0.117414097	0.624711305	0.155113454	0.57324584	0.366124456	0.280090209	0.84605889
COPS6	0.430473467	0.439240023	0.608450464	0.106261032	0.197518857	0.28156534	0.84605889
CYP3A4	0.340652295	0.706503425	0.237868002	0.048281671	0.86525208	0.280293531	0.84605889
DBR1	0.820243761	0.148748822	0.984376047	0.292528585	0.067984724	0.280126258	0.84605889
DTD2	0.362019235	0.703502613	0.911885595	0.013865933	0.753382761	0.282191033	0.84605889
EME1	0.602308897	0.408654745	0.029700369	0.976051003	0.340451481	0.282363326	0.84605889
EXOC6B	0.349567392	0.290724662	0.482577406	0.071772961	0.685518044	0.281474739	0.84605889
FARP1	0.748798336	0.047654753	0.161391457	0.445686363	0.938051219	0.281184063	0.84605889
FTO	0.661142032	0.155058012	0.442357313	0.649286171	0.081588778	0.280885439	0.84605889
IL18	0.928199133	0.618232737	0.005577065	0.783471409	0.967555637	0.282189659	0.84605889
IL32	0.1497435	0.856688701	0.508172235	0.045393392	0.8069068	0.28008389	0.84605889
IRF1	0.671508171	0.538643629	0.444534517	0.142048829	0.105818238	0.281687684	0.84605889
ISOC2	0.547842616	0.277945415	0.631033185	0.034857233	0.720440463	0.281474436	0.84605889
KDM2B	0.794465971	0.664793429	0.05776017	0.385544264	0.204476533	0.281031685	0.84605889
LOC100296952	0.162806631	0.416065397	0.070974399	0.524897618	0.954584989	0.281250325	0.84605889
LOC101904435	0.174425986	0.659076652	0.454752275	0.133065403	0.343363286	0.280127428	0.84605889
LOC104969545	0.377495332	0.497427458	0.749415198	0.018503093	0.923972751	0.281080239	0.84605889
LOC104975749	0.616293647	0.204029789	0.398406753	0.068620354	0.702332128	0.281549252	0.84605889
LOC512464	0.321828553	0.792309484	0.054131558	0.481177621	0.363685547	0.281609734	0.84605889
LRRC40	0.042985124	0.635442512	0.715556504	0.598929373	0.203446714	0.279739105	0.84605889
MAPK11	0.863806605	0.151813033	0.358701688	0.053923874	0.956094299	0.282141564	0.84605889
MATR3	0.228357345	0.401202334	0.807348377	0.09580285	0.34241841	0.282213208	0.84605889
MIGA2	0.245489361	0.845073067	0.874597231	0.018362466	0.725617776	0.281723576	0.84605889
MSLN	0.469550486	0.051511941	0.326426386	0.364861731	0.826850521	0.27975921	0.84605889
MT2A	0.918088101	0.931347389	0.348138224	0.138536274	0.057911536	0.28010775	0.84605889
MUL1	0.523186985	0.338591635	0.186239284	0.245290919	0.296545276	0.280747656	0.84605889
NDUFC1	0.372040478	0.545027335	0.880704989	0.020708269	0.654835257	0.281950069	0.84605889
NOB1	0.246373135	0.847288023	0.461463769	0.458772079	0.054466714	0.281148245	0.84605889

PAN3	0.325633022	0.942686791	0.967851705	0.102858752	0.078012634	0.279874817	0.84605889
PARP11	0.825844229	0.223207016	0.399985031	0.144667359	0.226817651	0.281822364	0.84605889
PLEKHH2	0.776732295	0.409723286	0.021073478	0.946585903	0.37769167	0.280631431	0.84605889
PPP2CA	0.700728839	0.047590359	0.305375062	0.353291002	0.670980153	0.28153133	0.84605889
RB1CC1	0.405799729	0.468008537	0.130576464	0.18937548	0.506645337	0.279615524	0.84605889
RHOU	0.124796954	0.195752005	0.383864416	0.352310416	0.727122865	0.280883174	0.84605889
RNF14	0.798168182	0.362565244	0.341535907	0.110168073	0.223074832	0.282350429	0.84605889
SH3BP1	0.46401911	0.672304812	0.478694147	0.055171255	0.292073415	0.281110073	0.84605889
SP7	0.909719836	0.802463997	0.097728225	0.073683895	0.45338139	0.279837897	0.84605889
SYVN1	0.106315093	0.764174384	0.22348958	0.258890693	0.514732928	0.281836412	0.84605889
TANC1	0.39838609	0.299927518	0.777091739	0.026451668	0.985008983	0.281818971	0.84605889
TCF21	0.117452991	0.818049734	0.514982169	0.068497588	0.703650763	0.279922747	0.84605889
TOGARAM1	0.21489637	0.672177352	0.463467092	0.067201635	0.536837969	0.281595528	0.84605889
TREM2	0.233618029	0.238573914	0.380674364	0.626693097	0.180283662	0.280600515	0.84605889
TRIM28	0.967441022	0.579392715	0.009102549	0.67337809	0.705373073	0.28204906	0.84605889
TRIM35	0.941216131	0.371249568	0.336401994	0.27138622	0.075002113	0.280350973	0.84605889
TSTA3	0.303806274	0.540885021	0.588973477	0.056119318	0.444098149	0.281422586	0.84605889
UBE2R2	0.740039683	0.397439176	0.076415444	0.346779465	0.310422736	0.281827438	0.84605889
USP43	0.017985229	0.623047937	0.611928501	0.411477786	0.857819212	0.281878359	0.84605889
WDR76	0.98134757	0.651152009	0.032826537	0.762618048	0.149721364	0.280486832	0.84605889
ZCCHC10	0.145182986	0.273076263	0.346280906	0.256938187	0.684097105	0.281479351	0.84605889
ZNF641	0.489554121	0.165519518	0.672884777	0.225919551	0.196684087	0.282010897	0.84605889
LOC112445002	0.527677142	0.111376599	0.314704312	0.66016945	0.199084166	0.282452623	0.846171961
LOC112441554	0.42911785	0.799737914	0.767407444	0.019255759	0.47965563	0.282539409	0.846277467
ABCC9	0.291145847	0.462295129	0.100213475	0.19876605	0.908529899	0.282722296	0.846607594
PALD1	0.223947912	0.180816002	0.66069614	0.623360836	0.146126538	0.282788727	0.846607594
SCYL1	0.037227854	0.201046984	0.502769703	0.738497916	0.877044105	0.282804389	0.846607594
ADCY3	0.576073534	0.270027406	0.105642483	0.256948713	0.577806602	0.282941697	0.84686416
CLHC1	0.418388843	0.788622007	0.252939743	0.358435974	0.081686108	0.283147938	0.847326916
CPXM1	0.214169596	0.945466652	0.103972937	0.147714826	0.786091964	0.283208044	0.847344217
KPNA6	0.978217587	0.20624452	0.09331795	0.38373204	0.338569959	0.283280995	0.847344217
LARP4B	0.737734677	0.738110381	0.01080189	0.725310133	0.573489521	0.28331503	0.847344217
LOC101907487	0.090721392	0.26385052	0.934829067	0.123419704	0.886203496	0.28336025	0.847344217
ABRAXAS1	0.614590921	0.047882262	0.34153125	0.861382386	0.288745997	0.286189243	0.847367254
ACOT7	0.356858436	0.768877887	0.054795266	0.382043178	0.430014056	0.28458164	0.847367254
ACTL6A	0.385365486	0.030444025	0.511903923	0.898681986	0.462677155	0.2860484	0.847367254
ATRIP	0.738599334	0.188320819	0.731668794	0.506539926	0.048091759	0.285079004	0.847367254
B3GAT1	0.032660475	0.665553966	0.219211563	0.598326976	0.869822208	0.285119747	0.847367254
BUB3	0.792345808	0.427896148	0.34332347	0.520351007	0.040899386	0.284975362	0.847367254
CDC42EP4	0.227539934	0.80704621	0.089261757	0.395179191	0.380614003	0.284338154	0.847367254
CYHR1	0.208289996	0.954150962	0.838455948	0.031281685	0.478000865	0.285751017	0.847367254
DAGLA	0.644898842	0.806604147	0.082447737	0.073655004	0.788435735	0.285693835	0.847367254
DYRK2	0.795869979	0.457325249	0.088545814	0.53641196	0.144001623	0.285632666	0.847367254
DYRK3	0.099805755	0.251007742	0.463991087	0.327127515	0.656595771	0.286023703	0.847367254

EML6	0.036486944	0.480214663	0.353148818	0.751336172	0.531075923	0.28452934	0.847367254
FAM120A	0.253880399	0.523841001	0.494240993	0.968235863	0.039085658	0.285529231	0.847367254
FAM212A	0.955049602	0.759207927	0.087008064	0.097206301	0.401119548	0.284034958	0.847367254
FANCC	0.670668014	0.525531208	0.515558866	0.331666529	0.040801853	0.283989303	0.847367254
GAPDH	0.24641896	0.989911206	0.706372915	0.097915647	0.145833947	0.28406592	0.847367254
GFM1	0.982330753	0.975357951	0.588486676	0.076264938	0.057285258	0.284223077	0.847367254
H1FX	0.296915525	0.336034097	0.037908782	0.85266875	0.770723733	0.285427153	0.847367254
HCLS1	0.520655541	0.990552456	0.726106094	0.102230268	0.064637849	0.284828904	0.847367254
HEMK1	0.364711022	0.182710326	0.737877643	0.567671668	0.089457992	0.286037668	0.847367254
KIAA0408	0.483559176	0.656411067	0.239755793	0.227969714	0.14293969	0.285115531	0.847367254
KIAA1324	0.02545558	0.44645313	0.55481462	0.516443391	0.758039765	0.284498529	0.847367254
LIMK2	0.611462051	0.755085032	0.188289335	0.045467852	0.630653096	0.28581323	0.847367254
LOC101902561	0.417516291	0.71493942	0.043997082	0.545663102	0.346049282	0.285116906	0.847367254
LOC101906001	0.292321882	0.24835806	0.393831773	0.151894997	0.569733037	0.284819898	0.847367254
LOC101906837	0.751643393	0.220970337	0.026193663	0.793801878	0.718305755	0.285158351	0.847367254
LOC101906923	0.675834691	0.537732606	0.020624936	0.457930564	0.71533547	0.283787387	0.847367254
LOC101907483	0.471955485	0.158772568	0.707611415	0.561108018	0.082791112	0.284215491	0.847367254
LOC613664	0.365135572	0.247877532	0.328408268	0.306257799	0.273207884	0.285504472	0.847367254
LOC784322	0.674330477	0.028002273	0.705673538	0.401520926	0.467186751	0.286178548	0.847367254
LOC788672	0.50825541	0.593761074	0.043825075	0.624726411	0.299710438	0.284925541	0.847367254
LOC790271	0.979332439	0.273011091	0.269339783	0.226100018	0.152395532	0.285195421	0.847367254
MARF1	0.421101372	0.396672199	0.235102966	0.241969427	0.25967261	0.28444981	0.847367254
MDH1	0.47147543	0.502287059	0.843438906	0.048610627	0.255357415	0.285090796	0.847367254
MDK	0.11712678	0.226502993	0.106067828	0.92268971	0.947623361	0.284062888	0.847367254
MEIS2	0.655839441	0.215056727	0.078313509	0.344051551	0.648007123	0.284181359	0.847367254
MFAP1	0.62848169	0.038737062	0.357716138	0.587737366	0.483781157	0.284919982	0.847367254
MIER2	0.320566518	0.058626578	0.326402795	0.468664314	0.866482981	0.285721356	0.847367254
N6AMT1	0.552211017	0.550661222	0.18060818	0.120172744	0.37781	0.285850391	0.847367254
NAE1	0.710948212	0.943989355	0.455005231	0.064279174	0.125702984	0.284441856	0.847367254
NES	0.313479822	0.544550763	0.721678458	0.038903408	0.514437608	0.284341774	0.847367254
NOP2	0.972463804	0.732320978	0.804641708	0.020442918	0.211981214	0.285298396	0.847367254
OPA3	0.486626211	0.933210749	0.137723091	0.040062254	0.985471232	0.284541575	0.847367254
PTMS	0.059848739	0.362351401	0.609128829	0.566974425	0.332217424	0.285564398	0.847367254
PUDP	0.551513502	0.77094055	0.141216863	0.051809262	0.787738463	0.283524548	0.847367254
PWWP2A	0.240104178	0.54550847	0.089196937	0.213598597	0.984653491	0.283886981	0.847367254
RAB6A	0.784894314	0.021502966	0.203237055	0.799685093	0.910057746	0.286002972	0.847367254
RNF214	0.959030141	0.712721961	0.945431344	0.514039154	0.007410006	0.284121879	0.847367254
RPS6KA6	0.45844577	0.209863731	0.040523002	0.969327655	0.658986572	0.285686055	0.847367254
SIGLEC8	0.031470708	0.244617417	0.961480686	0.763965024	0.434396697	0.28384441	0.847367254
SLC38A1	0.72410954	0.252157703	0.521481288	0.033689488	0.779612656	0.286246527	0.847367254
SNHG12	0.705821277	0.403716123	0.018842536	0.527628806	0.881987856	0.286126846	0.847367254
TIMM9	0.582914968	0.758526772	0.663684664	0.218129329	0.03830199	0.283592175	0.847367254
TMEM131	0.829203244	0.66949053	0.21871193	0.025507932	0.802945294	0.285490073	0.847367254
UBXN11	0.318982196	0.96453782	0.108883163	0.27390046	0.272578242	0.286259464	0.847367254

ZFP1	0.54480151	0.416741706	0.446548267	0.792854725	0.030529235	0.283718073	0.847367254
ATP6V0D1	0.148113408	0.32824657	0.271350792	0.50105096	0.379729959	0.28673953	0.84756528
BIRC2	0.535005741	0.097590971	0.243692561	0.696357	0.283000729	0.286599485	0.84756528
FAM198B	0.824653464	0.175605968	0.828075018	0.130921078	0.15984433	0.286710075	0.84756528
INTS10	0.328343457	0.620869965	0.433164797	0.271318598	0.10471157	0.286669585	0.84756528
LOC101903424	0.504686413	0.315008626	0.312082752	0.072688171	0.695550636	0.286654085	0.84756528
LOC614923	0.295432545	0.840193502	0.869277621	0.034803385	0.333423364	0.28640889	0.84756528
RGS22	0.344492238	0.92159112	0.109160109	0.454341317	0.159358431	0.286695132	0.84756528
ZNF839	0.251015364	0.297605507	0.568391932	0.074659649	0.790772138	0.286567503	0.84756528
LOC112441457	0.348129389	0.187422791	0.531549929	0.319009162	0.226995981	0.28681569	0.847637724
RBM43	0.28923071	0.47958512	0.108184196	0.208546856	0.803562884	0.286991854	0.847782396
SIRT5	0.923296996	0.233269342	0.308509101	0.145479235	0.260207029	0.287019621	0.847782396
ZNF502	0.438272733	0.888248855	0.440031369	0.01637282	0.896430822	0.286962609	0.847782396
EDNRB	0.387324235	0.790585314	0.314463025	0.027723686	0.943337108	0.287181878	0.847803886
LOC101905041	0.150552635	0.197573077	0.17110868	0.641548776	0.771179993	0.287170323	0.847803886
MPPED2	0.230276001	0.283001858	0.328812259	0.215880344	0.543998762	0.287084628	0.847803886
FAM47E	0.046392358	0.588545462	0.697027659	0.581650999	0.227639005	0.287267009	0.847872845
LOC112443006	0.805001766	0.504646221	0.37995856	0.463782653	0.035211549	0.287308567	0.847872845
BMS1	0.995100515	0.543679331	0.730809347	0.019123452	0.333954598	0.287540419	0.848154481
CAMTA1	0.489585843	0.315639791	0.691092588	0.276238318	0.08560259	0.287559048	0.848154481
SPAG7	0.484471772	0.37796607	0.736621617	0.136341883	0.137216233	0.287457611	0.848154481
CABCOCO1	0.558836864	0.388388496	0.758664762	0.033816535	0.453803638	0.287643017	0.848249695
PCED1A	0.577538057	0.010093686	0.500167434	0.965297041	0.898266417	0.287709263	0.84829262
TMED2	0.940709883	0.025182288	0.714878186	0.723681374	0.206536663	0.287869234	0.848611821
LOC112442683	0.139437025	0.989340662	0.261085262	0.110038358	0.640266176	0.288205646	0.849450944
C2H2orf69	0.327508413	0.151348685	0.064992186	0.831066218	0.948333157	0.288282634	0.849525284
LRFN1	0.422030183	0.868194594	0.524665117	0.07646757	0.172933044	0.288450195	0.849866453
PTPRC	0.428505976	0.587067182	0.879530561	0.114991357	0.100015906	0.288584432	0.850109337
ARRDC4	0.608342809	0.805844959	0.288007515	0.036083568	0.50082961	0.28894937	0.850297488
BRD4	0.258163086	0.925732984	0.581249846	0.026450099	0.693502571	0.288767352	0.850297488
BRPF3	0.206321907	0.722455569	0.47668068	0.089552312	0.401933131	0.289264243	0.850297488
GATAD2B	0.342479561	0.353771691	0.487882114	0.077300309	0.559124178	0.289123346	0.850297488
HOMER3	0.345282557	0.254584162	0.100760691	0.402646085	0.716916864	0.289225251	0.850297488
LOC104976804	0.703915522	0.063007159	0.546295649	0.986346408	0.106804012	0.288996686	0.850297488
NDRG1	0.853852004	0.615660709	0.044487036	0.408165798	0.268068964	0.289334633	0.850297488
OGA	0.647938114	0.161696567	0.334534767	0.695514426	0.104966139	0.28933161	0.850297488
PDIA4	0.250397018	0.990136749	0.133042592	0.266476705	0.291200244	0.28937368	0.850297488
PRR15L	0.047116015	0.866864155	0.448706897	0.302673464	0.460295364	0.28903966	0.850297488
PSMD1	0.973881158	0.454392929	0.229809943	0.725049586	0.034652355	0.289137252	0.850297488
TIPRL	0.97936742	0.032597875	0.154841629	0.731268695	0.704808986	0.288752727	0.850297488
TMEM170B	0.533041692	0.06073476	0.126873244	0.780245277	0.795630037	0.28885842	0.850297488
YAF2	0.374709528	0.327565059	0.060213575	0.887573628	0.389771619	0.289228952	0.850297488
MYL5	0.135846996	0.150897408	0.282688332	0.777240349	0.56851306	0.289426475	0.850300372
EVA1C	0.284607053	0.273879463	0.291509183	0.14039419	0.803287687	0.289533075	0.850406272

ZRSR2	0.095266195	0.339050543	0.719928397	0.224801534	0.49033392	0.28956616	0.850406272
ANKRD42	0.813287823	0.373835525	0.316242719	0.099590272	0.268288478	0.289871857	0.850543009
LOC101907491	0.333305532	0.730964119	0.712589999	0.016407865	0.901112929	0.289761243	0.850543009
POLRMT	0.472882727	0.042698449	0.572878479	0.974218197	0.227841664	0.289794871	0.850543009
PQLC2	0.262533968	0.778339552	0.971811164	0.10844262	0.119272258	0.289844293	0.850543009
SCAMP1	0.045059539	0.505020811	0.454898728	0.290220613	0.854244721	0.289733712	0.850543009
CYB5B	0.728691614	0.320123907	0.303963629	0.126844127	0.287510926	0.290759772	0.850931729
EFEMP1	0.279436475	0.403652607	0.113986755	0.539483099	0.372226972	0.290548138	0.850931729
EMC3	0.413552396	0.436490057	0.172542885	0.18300425	0.455549978	0.291319916	0.850931729
ESF1	0.542575605	0.394352227	0.58827097	0.027859872	0.738841845	0.291023763	0.850931729
EXOC8	0.707624518	0.106577167	0.297753581	0.651053213	0.17734517	0.291119888	0.850931729
GLG1	0.701500002	0.284240615	0.154629904	0.276359093	0.302810895	0.290461184	0.850931729
INTS1	0.668312519	0.276461205	0.71696407	0.047296605	0.410947877	0.290172488	0.850931729
JDP2	0.706345009	0.452435111	0.623598215	0.08336176	0.155957211	0.291023076	0.850931729
LOC100847190	0.173136517	0.608547543	0.145982185	0.210934376	0.797770525	0.29088487	0.850931729
LSM12	0.557487505	0.142809728	0.539037353	0.98199749	0.061533389	0.291142761	0.850931729
MCM3	0.784426852	0.144738249	0.092533346	0.451207859	0.543588657	0.290282733	0.850931729
MFSD11	0.141705822	0.717736115	0.70140136	0.45009734	0.080714746	0.291063126	0.850931729
MYBBP1A	0.542012049	0.400196329	0.529264942	0.750736871	0.029989429	0.29069817	0.850931729
OBSCN	0.361327469	0.101734696	0.568508358	0.209352777	0.588392505	0.290149047	0.850931729
PLAUR	0.649070909	0.496544485	0.076966889	0.147431615	0.706393154	0.290629497	0.850931729
POLA2	0.882493549	0.081809591	0.607213471	0.555394213	0.106606535	0.291270837	0.850931729
PROCR	0.38672613	0.469836215	0.422283469	0.437405762	0.07718389	0.290996454	0.850931729
SCN4B	0.158370558	0.747908041	0.08861276	0.864606763	0.285793932	0.291160763	0.850931729
SERBP1	0.761473394	0.335618919	0.267903374	0.386182521	0.098192962	0.291305259	0.850931729
SERTAD2	0.25409793	0.692661339	0.153119618	0.117321924	0.813949797	0.290110639	0.850931729
SLC25A46	0.881708142	0.240259826	0.063016022	0.961126849	0.201692454	0.290859531	0.850931729
SLC4A3	0.716711187	0.667227481	0.051866828	0.720303152	0.145371761	0.29135247	0.850931729
TECPR2	0.993861289	0.370228266	0.492059888	0.521992995	0.027324168	0.290577506	0.850931729
TRIM65	0.811685946	0.16367391	0.545734557	0.107228413	0.333948547	0.291300893	0.850931729
XDH	0.596961854	0.629489283	0.094792357	0.118275083	0.614546348	0.290931065	0.850931729
ZSWIM1	0.413095247	0.140169775	0.979544818	0.064369853	0.706690796	0.290457526	0.850931729
PSMC4	0.663931529	0.558325011	0.465799548	0.122075625	0.123388899	0.291543857	0.851187732
RBM8A	0.739007935	0.365860435	0.404814873	0.101434582	0.234194895	0.291503148	0.851187732
DUSP23	0.200286977	0.735699975	0.369453909	0.09517792	0.502180978	0.291605145	0.851215232
RAB11A	0.803444075	0.25795483	0.103873771	0.299983295	0.403293003	0.291734446	0.85122941
TMEM258	0.072804162	0.272100489	0.415778674	0.595013804	0.531536628	0.291761079	0.85122941
TMSB10	0.084956565	0.223044387	0.163072116	0.936189851	0.900514193	0.29176561	0.85122941
COPS7B	0.650186348	0.059827059	0.567411701	0.189235913	0.624352133	0.291904904	0.851370176
DENND2A	0.405480928	0.457388405	0.235944841	0.144553482	0.413025767	0.292157228	0.851370176
DUS4L	0.816433001	0.65049459	0.035484489	0.956429295	0.144970342	0.292177005	0.851370176
JAK2	0.211087738	0.913583223	0.793363933	0.031541687	0.540781753	0.29200633	0.851370176
SACS	0.29209323	0.900556902	0.992941453	0.099425697	0.100530516	0.292057222	0.851370176
SGCB	0.43719866	0.097238788	0.373257123	0.784435058	0.209737704	0.292059454	0.851370176

SRF	0.067668923	0.185281048	0.793434725	0.436890345	0.600974298	0.292122116	0.851370176
LOC505099	0.083313192	0.232822717	0.864100986	0.316946513	0.492112123	0.292245642	0.851419
ALDH3B1	0.221782496	0.451689702	0.306576338	0.345219941	0.250879644	0.294609184	0.851952321
ARHGEF2	0.823592893	0.302267397	0.247689803	0.105629942	0.402157881	0.292509835	0.851952321
ARHGEF40	0.828400426	0.69701441	0.03539426	0.185080808	0.707889049	0.295516801	0.851952321
ATP2B2	0.208777395	0.373729684	0.210465544	0.388872938	0.414846646	0.294057504	0.851952321
ATP5PO	0.685914265	0.834655201	0.493468734	0.011413639	0.828138084	0.295144554	0.851952321
C19H17orf53	0.136281297	0.113730135	0.248696429	0.789859307	0.863108662	0.292949783	0.851952321
CANX	0.663732468	0.177279372	0.560379512	0.874732042	0.045963286	0.294152733	0.851952321
CCNB1IP1	0.683114199	0.063120838	0.143145127	0.743312257	0.577674579	0.294114155	0.851952321
CCR1	0.142973662	0.343726866	0.262660741	0.266549934	0.765756909	0.293307378	0.851952321
CD19	0.841150452	0.094038068	0.849456921	0.495841713	0.079245471	0.293591152	0.851952321
CDC40	0.081884319	0.238263915	0.304807413	0.992693958	0.445714586	0.293125833	0.851952321
CLCN4	0.41444124	0.845984883	0.105590404	0.203933922	0.355690223	0.295918456	0.851952321
CRYBB1	0.383004877	0.284114905	0.080508716	0.638679545	0.474223798	0.294274997	0.851952321
CSRNP1	0.269589945	0.385529676	0.034012875	0.810237635	0.918371172	0.293087718	0.851952321
DDX19A	0.58431361	0.091952614	0.378851195	0.226796379	0.569910372	0.293114723	0.851952321
DIRAS3	0.053077933	0.474517233	0.331463854	0.711730341	0.451617378	0.29581622	0.851952321
DNAJA4	0.718937844	0.035656211	0.638161362	0.256900182	0.6351534	0.295092952	0.851952321
FAM107A	0.912385573	0.30075707	0.073904698	0.982596671	0.13216936	0.293256496	0.851952321
FAM204A	0.439458111	0.615317096	0.203899297	0.065752839	0.72911139	0.293749891	0.851952321
FAM213B	0.493099952	0.37422078	0.829683215	0.018487669	0.929280774	0.293077901	0.851952321
FRMD4B	0.675461966	0.33610202	0.036117059	0.503931304	0.639533402	0.293711606	0.851952321
GALNS	0.224682633	0.159335275	0.292171699	0.393306642	0.652377139	0.295835617	0.851952321
GRHPR	0.754329817	0.716015634	0.961647769	0.041414114	0.123115335	0.294008033	0.851952321
GSAP	0.25327895	0.381922258	0.248931828	0.465848779	0.23501921	0.293391865	0.851952321
HAUS3	0.288346652	0.64445616	0.315818996	0.057454742	0.790464194	0.294889158	0.851952321
HEY2	0.581720727	0.459849311	0.359227331	0.090564053	0.306311639	0.294909417	0.851952321
IFI35	0.673456723	0.285126069	0.596450064	0.3779678	0.061573788	0.294894449	0.851952321
KCTD2	0.307362989	0.317831457	0.097828391	0.348813506	0.804103254	0.295667618	0.851952321
L2HGDH	0.613274212	0.753335825	0.630448909	0.069914707	0.130101904	0.294066337	0.851952321
LAMTOR4	0.371527267	0.578971461	0.473755958	0.030836728	0.845254765	0.294417141	0.851952321
LDHC	0.174499309	0.412998847	0.844622806	0.090252416	0.48669005	0.295319319	0.851952321
LIMCH1	0.626260187	0.375852725	0.178682193	0.077006145	0.82652691	0.295483506	0.851952321
LOC101906067	0.334148227	0.037035237	0.794555082	0.503204308	0.538818283	0.294923993	0.851952321
LOC112444340	0.427980157	0.068347486	0.664124175	0.157342785	0.869366375	0.294476009	0.851952321
LOC112446129	0.240227378	0.510904325	0.510925938	0.426427694	0.100304504	0.295752188	0.851952321
LOC527186	0.178696297	0.11773928	0.817179349	0.546093018	0.279840463	0.292929318	0.851952321
LOC615768	0.298686836	0.279378813	0.239505529	0.693157895	0.192856074	0.2952163	0.851952321
LOC616903	0.468561342	0.12489432	0.301931985	0.632538739	0.236514517	0.293757007	0.851952321
LRIG1	0.486369136	0.72072689	0.098569835	0.194090213	0.393854611	0.293649084	0.851952321
LRRK2	0.068576078	0.692882094	0.533616395	0.116117418	0.897320897	0.293676142	0.851952321
LTB4R	0.321455142	0.989549389	0.259174809	0.050583026	0.632338196	0.293423962	0.851952321
LTC4S	0.718068435	0.197140082	0.060215793	0.571758517	0.54927654	0.29548942	0.851952321

MBNL1	0.877370075	0.803393912	0.012067925	0.906868653	0.344122637	0.294336158	0.851952321
MED31	0.510944811	0.323124841	0.065779198	0.622233372	0.393248871	0.294478705	0.851952321
MFSD6	0.131031839	0.507389162	0.532218638	0.655080971	0.115416585	0.295399792	0.851952321
N4BP3	0.621038207	0.680767662	0.258999058	0.075478482	0.321205737	0.29434255	0.851952321
NCAM1	0.256891486	0.443529294	0.057374157	0.787493962	0.521959564	0.296001056	0.851952321
NCOR2	0.825823595	0.166960368	0.124312255	0.599771213	0.259382581	0.294948009	0.851952321
NDUFS7	0.314044298	0.914100726	0.547905692	0.019947561	0.841338495	0.293564434	0.851952321
NPC1	0.959393918	0.606171261	0.031146176	0.422971243	0.348167601	0.294997006	0.851952321
NTN4	0.321990595	0.310844322	0.782460331	0.289087945	0.117253944	0.294337514	0.851952321
OGN	0.336083466	0.304377478	0.443297213	0.117408503	0.496083009	0.29364538	0.851952321
PGAM5	0.700520006	0.674295596	0.217065752	0.147390824	0.176817573	0.29523761	0.851952321
PHACTR3	0.378878527	0.707209381	0.587988297	0.121177029	0.139106282	0.294393579	0.851952321
PITPNM2	0.566328374	0.851405697	0.051650969	0.890721718	0.119588134	0.294245341	0.851952321
PRKAG3	0.218907379	0.714740193	0.081534064	0.26519432	0.784783717	0.294355168	0.851952321
R3HCC1L	0.251109657	0.986298124	0.631395804	0.035825525	0.474451454	0.294511493	0.851952321
RAB31	0.372916995	0.143683126	0.330098855	0.262917226	0.570262152	0.294195568	0.851952321
RP2	0.289466269	0.473254104	0.07784945	0.496709975	0.506431804	0.295779357	0.851952321
RPA1	0.952636321	0.084663682	0.892869682	0.362277595	0.101631039	0.294171823	0.851952321
SARS2	0.715842408	0.204046289	0.698215097	0.069466093	0.376578612	0.295018365	0.851952321
SLC35G1	0.479857509	0.990148507	0.08190353	0.479877935	0.143872441	0.295985617	0.851952321
SMG1	0.37428304	0.554419401	0.864435875	0.030548739	0.488758848	0.295554039	0.851952321
SNAP91	0.833268492	0.496504202	0.236012072	0.038467265	0.707625016	0.29450485	0.851952321
SRP19	0.451046018	0.312942537	0.594671515	0.261169745	0.121998343	0.295358101	0.851952321
STK16	0.275406405	0.395388104	0.356048156	0.216937503	0.319493472	0.296010733	0.851952321
TOMM22	0.591801958	0.17834747	0.256109777	0.320198112	0.303792876	0.293034401	0.851952321
UBA5	0.496496691	0.178680241	0.248164994	0.7239991	0.168265876	0.295746374	0.851952321
WWC2	0.773990124	0.675420562	0.09052344	0.166018706	0.339530724	0.295000357	0.851952321
ASB8	0.414953342	0.251289115	0.443660691	0.119460843	0.48724461	0.296293623	0.852224036
PIH1D2	0.512407904	0.14799701	0.252023208	0.578066133	0.243747375	0.296303206	0.852224036
ROMO1	0.506428425	0.470730939	0.470985493	0.026222994	0.914695683	0.29631286	0.852224036
SLC25A30	0.353802502	0.250515261	0.240642413	0.58309462	0.216535121	0.2963057	0.852224036
ATP6V1A	0.59578171	0.108595105	0.134420779	0.704101921	0.440300145	0.29646838	0.852521916
SUGP1	0.898134444	0.269433879	0.794706825	0.02362493	0.593755261	0.29654136	0.852582385
ABCA5	0.456152585	0.176334243	0.097878145	0.377728246	0.909354284	0.296879497	0.852688984
ABHD3	0.826285983	0.160630729	0.041858403	0.89833737	0.540889903	0.296640088	0.852688984
LOC101907642	0.022737138	0.684743686	0.731135385	0.694916737	0.341783536	0.296847923	0.852688984
LOC107132606	0.947408878	0.108492922	0.271042003	0.111989076	0.866817555	0.296890187	0.852688984
PGM2L1	0.773073327	0.023468093	0.376155285	0.568698917	0.696212984	0.296766947	0.852688984
SP4	0.120913913	0.317847407	0.353200653	0.230392028	0.863866608	0.296748051	0.852688984
ZNF33B	0.075244458	0.243655973	0.725027701	0.916070312	0.222415816	0.297087454	0.853106249
AURKAIP1	0.414126835	0.925617708	0.939363678	0.030583659	0.246117001	0.297191919	0.853129468
P4HA1	0.30079496	0.985854101	0.309405042	0.049881335	0.592473443	0.297251496	0.853129468
TRPC6	0.61932671	0.949370102	0.535412915	0.032683266	0.263515551	0.297238275	0.853129468
AFF2	0.297083299	0.081517156	0.537370891	0.412968419	0.505091874	0.297401052	0.853409454

ACVR2A	0.667424796	0.562906671	0.068485162	0.61116485	0.173567408	0.298155278	0.853657599
ARID3A	0.909935241	0.14785802	0.094089255	0.642787185	0.335145919	0.298039119	0.853657599
CAPZA1	0.827323765	0.104196984	0.542971244	0.805086181	0.072435219	0.298167321	0.853657599
CNIH3	0.322570819	0.593537285	0.035865888	0.6155763	0.64583857	0.298186698	0.853657599
CSRNP3	0.295834637	0.289793499	0.446105436	0.161990463	0.439028447	0.297676974	0.853657599
FUNDC1	0.224449976	0.129232257	0.605311693	0.20079869	0.772615854	0.297879348	0.853657599
LOC101902029	0.162980318	0.907389086	0.149850888	0.150438341	0.816474085	0.297782379	0.853657599
LOC522540	0.307096656	0.655471012	0.93264555	0.016141721	0.897911966	0.297731473	0.853657599
MZT1	0.97285629	0.030198783	0.204612663	0.626176605	0.724112741	0.297968246	0.853657599
PRF1	0.64493043	0.327023522	0.570369207	0.653725388	0.034722313	0.298215771	0.853657599
RAB3GAP1	0.561363801	0.240178202	0.124630385	0.88700347	0.182813018	0.297923957	0.853657599
SGSM3	0.997248402	0.503882255	0.657261005	0.033061977	0.249779447	0.298057961	0.853657599
WDR7	0.588974678	0.322100875	0.078655555	0.318627171	0.573882415	0.298111347	0.853657599
YWHAE	0.818798333	0.424865426	0.204735329	0.286780498	0.133577233	0.298104837	0.853657599
ATP8B1	0.249645559	0.543478381	0.989289714	0.097013758	0.210642583	0.298838886	0.85375852
ATPAF1	0.940801901	0.442003859	0.234442771	0.047744724	0.59084953	0.299206797	0.85375852
AUTS2	0.951454929	0.693510674	0.751404712	0.006574021	0.84283725	0.299055899	0.85375852
CCDC137	0.940773122	0.476812997	0.239271883	0.334408596	0.076670252	0.299291498	0.85375852
CD48	0.193864402	0.941029082	0.940623348	0.10380363	0.153712351	0.298593332	0.85375852
DNPEP	0.993318509	0.879259613	0.176459127	0.112302273	0.157953006	0.298379421	0.85375852
GPATCH8	0.158890881	0.078828402	0.869816698	0.459064779	0.548910283	0.29895859	0.85375852
GREM1	0.384797686	0.162553049	0.237042434	0.785858884	0.235470125	0.298878693	0.85375852
INHA	0.642655809	0.031135647	0.447228421	0.833806342	0.368073371	0.299016132	0.85375852
INTS11	0.377714763	0.489596455	0.503686242	0.078837762	0.372140611	0.298327689	0.85375852
KIAA0895L	0.687836464	0.991058093	0.030840354	0.173382852	0.751901052	0.298731016	0.85375852
LRRC57	0.118592087	0.112502879	0.272395083	0.838798224	0.897110683	0.298429048	0.85375852
NAPB	0.908327434	0.054011075	0.321397739	0.217023284	0.802487853	0.298999414	0.85375852
P2RY1	0.750230322	0.284832958	0.177754329	0.706032183	0.102121461	0.298627846	0.85375852
RALGDS	0.227305754	0.158906102	0.134098821	0.583328543	0.972877049	0.299137853	0.85375852
RNF103	0.134593893	0.522608862	0.529063388	0.311893092	0.23569694	0.298475965	0.85375852
SLC38A2	0.12335146	0.927885321	0.694150611	0.116795638	0.295668221	0.298875359	0.85375852
TIMM50	0.453068373	0.784650218	0.357925261	0.202077744	0.106465017	0.298567679	0.85375852
TTC9B	0.030779876	0.868183764	0.688001715	0.250185259	0.598098711	0.299251202	0.85375852
UBASH3B	0.219435948	0.844714665	0.790829093	0.175875825	0.106720944	0.299268305	0.85375852
STAMBPL1	0.562236139	0.576042056	0.05277757	0.392027957	0.410925155	0.299378618	0.853829209
WRAP73	0.714784907	0.013736996	0.463155884	0.757563992	0.799501367	0.299420334	0.853829209
LOC112449302	0.875032425	0.554550678	0.659946893	0.541434476	0.015894032	0.299491004	0.85388236
CCRL2	0.635369775	0.582533642	0.138980282	0.216243852	0.247885469	0.299567984	0.85395348
ABCB1	0.093227294	0.14406657	0.689188092	0.376376618	0.79455073	0.300108548	0.854159103
GALNT3	0.136311882	0.937600794	0.137293753	0.709537049	0.221912007	0.299843411	0.854159103
GPR174	0.544895697	0.306289391	0.433029895	0.74547924	0.051313631	0.299930659	0.854159103
LOC101903713	0.946522174	0.086034288	0.64804974	0.285728154	0.183450577	0.300010936	0.854159103
LYRM1	0.757337301	0.322398422	0.215653009	0.972097413	0.054009881	0.299927233	0.854159103
NPM3	0.655835113	0.86291367	0.649126664	0.465045944	0.016159024	0.299729772	0.854159103

SEPT10	0.253526276	0.414263861	0.378875113	0.655817935	0.105828192	0.299786711	0.854159103
SH3BP5L	0.416916685	0.114435043	0.784916133	0.093419899	0.790467211	0.299970047	0.854159103
SMURF2	0.503936579	0.642497037	0.52640694	0.077315029	0.210017048	0.300076201	0.854159103
LOC104969496	0.02364152	0.776610914	0.594561307	0.721848373	0.351539595	0.300206799	0.854214693
LOC112443484	0.331632868	0.027920302	0.393257805	0.890318427	0.854623415	0.300232182	0.854214693
PGK1	0.517149121	0.736674942	0.522636321	0.091589769	0.152100465	0.300389984	0.854405785
YME1L1	0.584384185	0.639086637	0.94888661	0.243589736	0.032135086	0.300403472	0.854405785
UBR2	0.418526531	0.848248066	0.260570885	0.037890143	0.792181261	0.300535107	0.854632063
DHRS13	0.323728329	0.783336764	0.985676882	0.079790272	0.139850718	0.301161157	0.854846456
GAS1	0.780716979	0.132108955	0.192704798	0.181145942	0.774403662	0.301108623	0.854846456
GSTP1	0.511196747	0.201537858	0.659465028	0.057635232	0.710779647	0.30086691	0.854846456
GUK1	0.553662859	0.872701644	0.918617477	0.014078775	0.446130874	0.301095368	0.854846456
HEBP2	0.572856259	0.745232911	0.145983161	0.056032449	0.799374534	0.301273903	0.854846456
LAPTM5	0.206195023	0.277916063	0.149051072	0.4157391	0.785014558	0.30108015	0.854846456
LETM1	0.434372926	0.842303629	0.650482639	0.019484895	0.602156598	0.301320138	0.854846456
MED23	0.043397785	0.352596559	0.901301982	0.203180508	0.995883192	0.301234187	0.854846456
MRPL30	0.539072359	0.310363914	0.412210063	0.071251644	0.565588314	0.300666148	0.854846456
RCSD1	0.850155161	0.230765867	0.153637494	0.542160025	0.170205545	0.300773662	0.854846456
RNF38	0.9450627	0.035267609	0.105330355	0.859993135	0.925088555	0.301350204	0.854846456
SLC12A9	0.682953515	0.034332193	0.441891287	0.684030291	0.3935438	0.30116055	0.854846456
TBRG1	0.756212063	0.025732394	0.536360753	0.74405462	0.358173023	0.300776956	0.854846456
TMEM132C	0.721610935	0.38784916	0.736684412	0.017162794	0.789519744	0.301391847	0.854846456
ZNF628	0.20727207	0.76311701	0.319552197	0.058695566	0.938502124	0.300916453	0.854846456
ICOS	0.606612074	0.451290606	0.253367588	0.646886908	0.062325615	0.301524842	0.855047481
SMIM4	0.272853723	0.836750796	0.291985935	0.095405982	0.439826148	0.301566926	0.855047481
LOC107132410	0.884235919	0.328935857	0.0198653	0.67966389	0.713630292	0.30182265	0.855300474
LRBA	0.681082198	0.91379847	0.468950278	0.01727671	0.556496758	0.302001845	0.855300474
MEST	0.694869355	0.434273673	0.168429333	0.227981273	0.241812944	0.301797354	0.855300474
MPP2	0.273036528	0.783288403	0.029781773	0.556174206	0.791728996	0.301931232	0.855300474
SLC22A17	0.315325389	0.480235301	0.60209215	0.115011056	0.267448872	0.301923518	0.855300474
TMED1	0.182192993	0.857746126	0.319668678	0.139095045	0.40388512	0.302020977	0.855300474
ULK4	0.121153565	0.749389133	0.115343585	0.712621664	0.375292897	0.301734256	0.855300474
ACAD10	0.79378011	0.477035353	0.043477499	0.36041291	0.473390725	0.302141472	0.855445719
ADGRG6	0.038138419	0.998236071	0.274128757	0.831560742	0.323874351	0.302233192	0.855445719
BLK	0.903532268	0.045715296	0.165652951	0.905684937	0.456345131	0.303086201	0.855445719
CEP112	0.560546656	0.747889007	0.511193242	0.122589752	0.107075076	0.302347361	0.855445719
DOK6	0.772598258	0.834342237	0.465363153	0.012780125	0.736117468	0.302795848	0.855445719
EEA1	0.629097936	0.916241787	0.067278838	0.163251495	0.4483732	0.303610526	0.855445719
FAM131A	0.30630408	0.495735212	0.82999534	0.480511958	0.046764645	0.303287363	0.855445719
GPR18	0.798228905	0.9886756	0.015940784	0.97732966	0.229668939	0.302880131	0.855445719
GPR37L1	0.776520985	0.360070107	0.114489796	0.508503425	0.173985935	0.3032922	0.855445719
IK	0.735101303	0.119682619	0.25596976	0.164975683	0.75817688	0.302533959	0.855445719
IQCK	0.994268358	0.513411447	0.072907495	0.597446977	0.127577973	0.30351801	0.855445719
KCNB2	0.723730767	0.367756621	0.331522249	0.280789718	0.114377975	0.303375496	0.855445719

LOC101904062	0.810542878	0.163553933	0.812844203	0.14128915	0.185122296	0.302615722	0.855445719
LOC101906366	0.724342579	0.492092977	0.191167159	0.091318188	0.455514468	0.303404507	0.855445719
LOC101908048	0.467061248	0.23544266	0.050687341	0.778511165	0.651683118	0.303081335	0.855445719
LOC104972065	0.374832757	0.568249566	0.271252014	0.255577506	0.192201196	0.303586265	0.855445719
LOC789764	0.269408337	0.650966828	0.082703627	0.426907264	0.457515909	0.303330545	0.855445719
LRRC61	0.063289107	0.563771009	0.720298682	0.241581572	0.456031134	0.303256008	0.855445719
NDUFV1	0.680878102	0.643865106	0.737464865	0.011558058	0.758171452	0.303338178	0.855445719
NFAT5	0.30283223	0.979522373	0.387959007	0.117160709	0.20841752	0.302200258	0.855445719
NLN	0.098182247	0.732325425	0.902881792	0.194618618	0.223473538	0.302862178	0.855445719
PRKAA2	0.656802511	0.022373638	0.459618914	0.447247133	0.932087323	0.302474859	0.855445719
PTPRD	0.928152523	0.266784998	0.060466783	0.251845773	0.746792726	0.302493781	0.855445719
SENP1	0.111583225	0.494434152	0.427617489	0.233683651	0.512736816	0.303025307	0.855445719
SF3B5	0.274318325	0.105491667	0.746741055	0.418539785	0.312264958	0.302902227	0.855445719
SOCS5	0.461507131	0.200838202	0.329347371	0.130299638	0.713774047	0.303636056	0.855445719
TBC1D8B	0.994224368	0.193594303	0.068819413	0.215031082	0.990661403	0.302777277	0.855445719
TMEM106C	0.183157646	0.719356692	0.991497545	0.625021185	0.034638788	0.303100185	0.855445719
UNG	0.613169932	0.081980925	0.686936964	0.143492769	0.572847176	0.303602696	0.855445719
ZNF572	0.312028447	0.203607454	0.149463968	0.421322511	0.703741778	0.302468078	0.855445719
GRB7	0.965867897	0.551538033	0.353957644	0.063257094	0.238527408	0.303929013	0.856124106
COL14A1	0.99783521	0.271067695	0.26012506	0.051564282	0.784622526	0.304005257	0.856136123
HMMR	0.872602649	0.014366058	0.634049715	0.474878622	0.75434291	0.304037617	0.856136123
SOGA1	0.483348366	0.54328461	0.043185958	0.92300244	0.272190928	0.304127206	0.856241478
LOC104975974	0.957410167	0.06538536	0.271671105	0.688352951	0.243513754	0.304207825	0.856321547
LOC786512	0.688943914	0.706628812	0.010864772	0.677043268	0.79659342	0.304302113	0.856440058
ADIRF	0.115222759	0.329939627	0.399062963	0.192256313	0.978989209	0.304438838	0.856677945
CBLN4	0.383983258	0.382633009	0.213498994	0.117258648	0.776951264	0.304553913	0.856707965
LAMTOR3	0.700276478	0.123259911	0.078871947	0.909517135	0.461413626	0.304517127	0.856707965
DHDDS	0.381985761	0.310937162	0.489539659	0.48442737	0.101747215	0.304951041	0.857051912
DNASE2	0.138152101	0.310656666	0.230057882	0.775801779	0.373629264	0.304759826	0.857051912
IL16	0.408068044	0.829564949	0.477800621	0.617884068	0.028656487	0.304854362	0.857051912
LAG3	0.269665704	0.107585053	0.898072418	0.607111714	0.181225947	0.304989529	0.857051912
LOC529399	0.547379313	0.295982433	0.047484055	0.456633742	0.815989433	0.304981983	0.857051912
NRP2	0.588966761	0.037051888	0.683110965	0.409420651	0.469356596	0.304888419	0.857051912
ABCE1	0.836310746	0.220362894	0.41913621	0.350644996	0.105960743	0.305149816	0.857062062
JUNB	0.75926235	0.086402647	0.877625264	0.280676325	0.177555773	0.305115761	0.857062062
LOC101906410	0.421628397	0.984888762	0.066387061	0.164276547	0.633366565	0.305071344	0.857062062
ACACA	0.869271465	0.920022927	0.913397987	0.030342378	0.129619635	0.305298716	0.857101792
LOC112442787	0.296085276	0.507871953	0.097987881	0.627088434	0.311080161	0.305366619	0.857101792
MECOM	0.249849698	0.978959119	0.037881617	0.642183651	0.483487051	0.305486769	0.857101792
MRPS18B	0.50709075	0.450745009	0.407531217	0.102176108	0.302338161	0.305520361	0.857101792
SATB1	0.514863553	0.601308846	0.353741133	0.070212555	0.37391198	0.305403796	0.857101792
TOMM6	0.34034285	0.41806836	0.650192149	0.139984301	0.222209819	0.305529552	0.857101792
ZKSCAN2	0.215215533	0.1240196	0.40502165	0.776406734	0.342531873	0.305395062	0.857101792
TXLNA	0.317438821	0.667861261	0.445918195	0.141698076	0.214930689	0.30559932	0.857150989

IFT43	0.810381795	0.16538048	0.450371288	0.444212459	0.107503668	0.305759442	0.857241119
PBRM1	0.032337488	0.56951513	0.725387063	0.275021778	0.784110939	0.305684129	0.857241119
TMEM214	0.699464985	0.784945305	0.551117317	0.014748481	0.646026951	0.305788161	0.857241119
CD82	0.47318311	0.35994446	0.04647824	0.810227863	0.450542845	0.306115328	0.857428905
COBLL1	0.512036358	0.450421658	0.827301739	0.055394276	0.273408514	0.306116383	0.857428905
HDDC2	0.452800306	0.292298564	0.765614634	0.837810568	0.034004975	0.305977426	0.857428905
LOC101902449	0.888456625	0.774625228	0.077928298	0.448236712	0.120081165	0.305968832	0.857428905
LOC101905127	0.995927109	0.145266611	0.272584044	0.443589251	0.165149445	0.306081071	0.857428905
FSTL4	0.916847276	0.351462689	0.340482778	0.470747818	0.056031056	0.306319728	0.857655017
PRR16	0.425591718	0.163818584	0.20264101	0.231584268	0.884710482	0.306353891	0.857655017
RAD23B	0.827311841	0.233193666	0.127401545	0.887468816	0.132654794	0.306302953	0.857655017
CBFB	0.612770925	0.090708137	0.443786599	0.119843244	0.979576734	0.306411165	0.857669049
IL17REL	0.216849474	0.074792437	0.86211469	0.230336105	0.900269467	0.306587882	0.857871054
KIDINS220	0.834198544	0.848010612	0.792712721	0.024908399	0.207556266	0.306572502	0.857871054
ALOX5AP	0.11005644	0.410119166	0.270019818	0.880398008	0.270652352	0.306813769	0.857899533
ALS2CL	0.414310052	0.268218496	0.285091272	0.340720629	0.268983057	0.306784389	0.857899533
C13H20orf96	0.584283042	0.098934794	0.061304587	0.907010203	0.904002769	0.306890092	0.857899533
LOC112445078	0.586767953	0.150969503	0.111590232	0.296901111	0.989035742	0.3067468	0.857899533
PCGF6	0.265849091	0.583376842	0.328135854	0.323268749	0.17667532	0.306932417	0.857899533
RIMKLB	0.26365688	0.448876521	0.07079571	0.925580061	0.374876329	0.306963991	0.857899533
SH2B1	0.911439464	0.016730272	0.71402686	0.402062234	0.664050076	0.306952481	0.857899533
AP4B1	0.66729465	0.58664533	0.073480403	0.348543222	0.290295788	0.307122152	0.857903167
EPB41	0.798110604	0.539933715	0.122213105	0.525528755	0.105265279	0.307265284	0.857903167
STT3A	0.481393188	0.433142137	0.702589032	0.116778117	0.170241202	0.307218176	0.857903167
SUMO1	0.796826809	0.046287162	0.423886876	0.220309377	0.845936114	0.307278948	0.857903167
TADA3	0.60072226	0.045458351	0.470466816	0.576308537	0.393295827	0.307196387	0.857903167
TXN2	0.739736228	0.715719852	0.479591716	0.016336899	0.701528146	0.307104028	0.857903167
LOC107131289	0.117667889	0.227278556	0.422476002	0.28259667	0.914699567	0.307610025	0.858140352
NUTF2	0.656949234	0.274990198	0.097122988	0.237632864	0.699813121	0.30747862	0.858140352
PAGR1	0.332276993	0.117978819	0.31570873	0.904739715	0.260548753	0.307460398	0.858140352
SEPT7	0.594088001	0.037101272	0.419687793	0.530247602	0.595479083	0.307625354	0.858140352
ZMYND19	0.117632773	0.104153831	0.606272301	0.664264282	0.5919369	0.307617708	0.858140352
MDGA2	0.153092288	0.452932869	0.650649539	0.125673955	0.515517447	0.307726709	0.858277196
YPEL5	0.645339504	0.064666182	0.248806516	0.556844521	0.505769966	0.3077889	0.85830478
MYBPH	0.080826235	0.505190882	0.780580281	0.223992901	0.410087445	0.30795865	0.858632248
FASN	0.754474888	0.913929179	0.608732856	0.030731261	0.227536189	0.308309695	0.859238414
IDH3B	0.651363988	0.840060148	0.617166121	0.010634944	0.817658687	0.308384091	0.859238414
LCN1	0.721850709	0.943842013	0.039292462	0.204131993	0.537359187	0.308380264	0.859238414
LOC781254	0.999179183	0.108270546	0.100569609	0.32049826	0.842631932	0.308462438	0.859238414
LOC783202	0.806395801	0.629449097	0.599861351	0.015771205	0.612217081	0.308542561	0.859238414
TSACC	0.045505323	0.833304837	0.633366855	0.257327413	0.475221507	0.30840367	0.859238414
ZC2HC1A	0.974910785	0.209488451	0.05064059	0.635477279	0.447192443	0.308506123	0.859238414
MN1	0.86445263	0.115172299	0.337502703	0.290789042	0.301156842	0.308675203	0.859331834
PAK1IP1	0.170612149	0.868744041	0.0497008	0.420839812	0.94965764	0.308744211	0.859331834

PEX11G	0.235277857	0.953001786	0.394634352	0.038869992	0.856232747	0.30878556	0.859331834
TARS	0.546249806	0.54525343	0.145054564	0.662658208	0.1028544	0.308771748	0.859331834
PDIA3	0.828869287	0.659556775	0.029363717	0.35166177	0.522039356	0.308883895	0.85945975
PJA1	0.677197824	0.375294886	0.775899495	0.196889963	0.07593742	0.308947597	0.859491272
MFN1	0.345957799	0.62274432	0.706493463	0.044014114	0.440949918	0.309224517	0.859970098
MKL1	0.231960167	0.115398765	0.216171231	0.646320556	0.789689082	0.309190038	0.859970098
NCKAP5	0.596613998	0.41358907	0.275741609	0.668604306	0.065060603	0.309495269	0.860577238
SDC4	0.732226336	0.062503615	0.803339615	0.189373324	0.425629848	0.309674329	0.860783436
TRRAP	0.318973676	0.447539475	0.77340027	0.127453058	0.210580777	0.309660926	0.860783436
DTNB	0.972271391	0.034290968	0.70273426	0.19240747	0.659143925	0.310053258	0.861613051
HMGB1	0.497407816	0.025725956	0.735311129	0.473081712	0.667642865	0.310077794	0.861613051
SSSCA1	0.755945934	0.424769323	0.371386544	0.254714326	0.097922881	0.310199373	0.861804963
CAP1	0.208303507	0.116387049	0.327001364	0.962671055	0.39151477	0.310843584	0.862280943
LOC100847122	0.633492035	0.022405973	0.684545242	0.772579591	0.39798842	0.310825227	0.862280943
LOC100848939	0.371061021	0.329719456	0.236385799	0.172749966	0.597569275	0.310725963	0.862280943
LOC101903629	0.493512013	0.485294931	0.154683052	0.756378584	0.10655024	0.310733277	0.862280943
LOC104968964	0.132524805	0.153849503	0.677626666	0.255748918	0.844532134	0.310659275	0.862280943
LOC614741	0.513748784	0.238012363	0.080384578	0.492559989	0.616749549	0.310750021	0.862280943
PDE7B	0.009362108	0.99639893	0.690757916	0.580490613	0.796662249	0.310459202	0.862280943
SLC22A18	0.180106558	0.613774384	0.819529088	0.056650916	0.581524405	0.310680152	0.862280943
TCF24	0.430051237	0.466020903	0.051241948	0.476777936	0.610058818	0.310798482	0.862280943
MFSD14A	0.806941642	0.134676247	0.193214594	0.227122144	0.627004865	0.310950508	0.862431771
CD3G	0.06214905	0.943911096	0.638581889	0.620372004	0.128758207	0.311050665	0.862499277
TMEM9B	0.955760146	0.086700066	0.156167679	0.865072058	0.26745296	0.311132516	0.862499277
ZNF181	0.549497916	0.887621521	0.022474803	0.566709824	0.481913463	0.311119536	0.862499277
BEGAIN	0.433090618	0.33262664	0.095941172	0.59637096	0.363574088	0.311260629	0.862563017
PARP9	0.501531588	0.061858539	0.85220422	0.275869525	0.410737943	0.311214739	0.862563017
MRPS25	0.186196683	0.805613211	0.75446455	0.039007075	0.679535956	0.311405789	0.862819585
FUT7	0.957167621	0.283671922	0.026932116	0.711530825	0.577108431	0.311547661	0.862830976
HPD	0.640272887	0.907059831	0.280997313	0.025903459	0.710933173	0.311666952	0.862830976
HSP90B1	0.475492979	0.871819102	0.07602628	0.134465613	0.709172729	0.311669492	0.862830976
RAD52	0.111066024	0.404121376	0.173001155	0.578462086	0.668351816	0.311513604	0.862830976
RASGRP4	0.3540624	0.36030578	0.284174237	0.431600614	0.192083355	0.311672782	0.862830976
EMP3	0.126666148	0.798877877	0.047833701	0.932496075	0.666489275	0.311806474	0.862930545
FBXL2	0.537279134	0.426104419	0.103289553	0.393509631	0.323303211	0.311813913	0.862930545
ATP8B2	0.737537379	0.120133242	0.356993387	0.313261124	0.304284847	0.312127789	0.863073706
FANCD2	0.474375221	0.351249544	0.252543339	0.141838463	0.505719284	0.312286373	0.863073706
HTR2A	0.59613889	0.354357942	0.231332172	0.073435746	0.840716538	0.312222521	0.863073706
MYH15	0.175420025	0.606146512	0.091450372	0.782917516	0.395941764	0.312093435	0.863073706
SIK3	0.56807539	0.531585273	0.162982916	0.318700675	0.1924166	0.312276293	0.863073706
SMARCA4	0.659749477	0.223741293	0.079710511	0.888902242	0.288433241	0.312208513	0.863073706
UHMK1	0.964078182	0.408360989	0.053645077	0.570733316	0.250204397	0.312167285	0.863073706
WNK4	0.792615009	0.395447094	0.414490675	0.108663893	0.213397283	0.312011381	0.863073706
C8H9orf3	0.393696685	0.796559264	0.058023557	0.833781893	0.199146448	0.312428266	0.863175131

LOC104970930	0.975616355	0.542746483	0.066301541	0.238939288	0.360072076	0.31238474	0.863175131
IFIT3	0.740081722	0.456818389	0.341092982	0.032579512	0.806118483	0.312766835	0.863731879
LOC112441654	0.280835861	0.275795325	0.080283031	0.748881108	0.650709097	0.312840308	0.863731879
SOX12	0.617919239	0.185003778	0.058577638	0.94709792	0.477656457	0.312804709	0.863731879
TSFM	0.413950671	0.778747521	0.614583258	0.143424183	0.106551298	0.312723319	0.863731879
IL6ST	0.378379819	0.864296775	0.202622654	0.763619694	0.059962768	0.313029294	0.864029522
MPST	0.191718591	0.960028291	0.539505289	0.053225081	0.574195179	0.313056331	0.864029522
THAP8	0.756645315	0.055856703	0.171800522	0.489010369	0.854989574	0.313106061	0.864029522
CD55	0.379398687	0.201259211	0.699732066	0.151942654	0.374225487	0.313213132	0.864036205
FMNL1	0.254770603	0.552398335	0.599420257	0.068750731	0.52382715	0.313213783	0.864036205
LEPROTL1	0.7355529	0.037159078	0.313609224	0.54684047	0.648638682	0.31332329	0.864193026
KIF1A	0.995944846	0.062416875	0.114706691	0.981197172	0.434731895	0.313380133	0.864204564
AADAT	0.714318189	0.805600818	0.544588697	0.035107139	0.277760711	0.314053015	0.86464359
ARRB1	0.336510393	0.295265332	0.275619318	0.434644128	0.25635657	0.313839569	0.86464359
CDKN2AIP	0.161302594	0.475849352	0.183558385	0.24996138	0.865897533	0.313749166	0.86464359
GLS	0.855719646	0.898782349	0.417302901	0.037285865	0.255288458	0.314008961	0.86464359
IGSF6	0.526270682	0.591388778	0.340191146	0.115627519	0.249256865	0.313844425	0.86464359
JOSD2	0.151224911	0.717673638	0.888856859	0.064611327	0.489593801	0.313848752	0.86464359
PAXBP1	0.91531134	0.171137143	0.133597856	0.238098745	0.612978002	0.31397658	0.86464359
SEC22A	0.169550093	0.316614528	0.547443266	0.169373256	0.61335158	0.313913203	0.86464359
SND1	0.249927156	0.58504977	0.765832458	0.653174692	0.041784683	0.314066202	0.86464359
VSTM1	0.123595631	0.226446948	0.188539861	0.8665584	0.66828774	0.314048933	0.86464359
ATP5PB	0.494016926	0.644980168	0.439410933	0.092791938	0.236262964	0.314685882	0.864753854
FMNL2	0.883445458	0.50125903	0.68570423	0.039672466	0.254687957	0.314623642	0.864753854
IFI30	0.445777841	0.826617056	0.303445777	0.100031544	0.27414072	0.314537198	0.864753854
LOC101909718	0.189222857	0.959580891	0.461571689	0.08704146	0.419687227	0.314316745	0.864753854
LOC112443144	0.621939897	0.326740269	0.820302068	0.339190514	0.054251659	0.314592359	0.864753854
LOC786258	0.71534195	0.208585424	0.367455778	0.239465968	0.2329585	0.314177843	0.864753854
MROH1	0.833988057	0.013155859	0.426497064	0.942896494	0.694025557	0.314345873	0.864753854
PKD1	0.883062661	0.155212709	0.12833274	0.18060734	0.964712712	0.314463021	0.864753854
SIGLEC15	0.403116506	0.677324941	0.179672878	0.063652931	0.981313034	0.314445362	0.864753854
SLC22A23	0.788841461	0.282052713	0.033628771	0.536592174	0.764305045	0.314645323	0.864753854
TTLL5	0.66818805	0.33148687	0.479442115	0.137613604	0.209623644	0.314401385	0.864753854
TMEM185A	0.716712931	0.256428392	0.534809709	0.328244038	0.095218437	0.314805921	0.864938887
MSC	0.841759281	0.349310671	0.079070674	0.884598071	0.149613749	0.315038493	0.865009058
PRDX2	0.249042993	0.882705524	0.550042378	0.071419307	0.356406978	0.315076633	0.865009058
RAB37	0.907445703	0.85457461	0.04296629	0.364492777	0.253464034	0.315095006	0.865009058
SLC30A5	0.352590383	0.249309452	0.8945409	0.088920458	0.440196569	0.315079807	0.865009058
WASF2	0.582150377	0.518148367	0.047627988	0.579665118	0.369492713	0.315039118	0.865009058
CRTAC1	0.168972763	0.859606469	0.048094154	0.448632711	0.983092682	0.315223983	0.865073709
GMFB	0.185432399	0.629738305	0.483618227	0.433594512	0.125785205	0.315180246	0.865073709
NPM1	0.70959201	0.66244985	0.417279605	0.056486793	0.278270756	0.315326215	0.865209582
ANAPC1	0.457512635	0.797930613	0.410142697	0.055659398	0.370832862	0.315662371	0.865359706
LOC100174924	0.844922976	0.079057673	0.386882817	0.256693923	0.46646644	0.315846552	0.865359706

LOC507581	0.645494183	0.27520611	0.072872642	0.322325898	0.740790729	0.315690735	0.865359706
LOC618737	0.25077506	0.183245184	0.451228084	0.845233014	0.17653919	0.315831341	0.865359706
LPCAT2	0.205057844	0.460756635	0.037453127	0.983350814	0.888313156	0.315692384	0.865359706
PLEKHF2	0.612895012	0.401046441	0.028402772	0.633609178	0.698212901	0.315573356	0.865359706
QRICH2	0.431034773	0.147352486	0.434234863	0.986235815	0.113766847	0.315851309	0.865359706
RAB2A	0.317808012	0.089064629	0.453415462	0.526255012	0.458185089	0.315855502	0.865359706
VAMP7	0.860958054	0.157372361	0.080863417	0.37242593	0.757920764	0.31576372	0.865359706
LOC112447858	0.368348617	0.48281396	0.986260282	0.32076228	0.055069319	0.316027548	0.865686545
CHMP4A	0.83380303	0.179928343	0.075624978	0.305501483	0.894511478	0.316128249	0.865731694
LYNX1	0.825301306	0.743770471	0.613634813	0.016945156	0.48586346	0.316159281	0.865731694
MPND	0.119848643	0.141136099	0.418387295	0.815472642	0.537516763	0.316202289	0.865731694
NF1	0.686260882	0.417937988	0.750416351	0.018502028	0.779575993	0.316311606	0.865886534
KANSL1L	0.303022694	0.972386883	0.609601927	0.132243111	0.130751462	0.31637754	0.865922583
CDKN2AIPNL	0.25349979	0.339443481	0.303218577	0.317793978	0.375214628	0.3166248	0.866248643
DMXL1	0.34627493	0.428972309	0.035856547	0.854434911	0.68372262	0.316641839	0.866248643
SSR3	0.531261906	0.228333758	0.044330085	0.769955838	0.751578413	0.316655025	0.866248643
GNL3	0.293907428	0.593116929	0.711932063	0.356988502	0.070338745	0.316860866	0.866352778
HEATR1	0.291718988	0.323850577	0.92120732	0.139069335	0.257722862	0.316997792	0.866352778
MCCC1	0.866937757	0.685818846	0.201618939	0.203976743	0.12740653	0.31681534	0.866352778
NCL	0.912322871	0.240668222	0.451532739	0.073097792	0.430177851	0.316917445	0.866352778
PRNP	0.515749905	0.179280108	0.240975035	0.334856335	0.418105184	0.317009837	0.866352778
SKIDA1	0.602892829	0.658771759	0.316599586	0.06541719	0.37859529	0.316766106	0.866352778
TMX4	0.966684297	0.141282226	0.04054575	0.585593616	0.962562951	0.317094648	0.866440271
CCT4	0.793887255	0.438852184	0.686824362	0.019046137	0.68600233	0.317331049	0.866941876
ARMC10	0.970910371	0.020900942	0.285484932	0.615260636	0.878409423	0.31753992	0.867090343
POLR1E	0.514654756	0.530452693	0.215695112	0.202404524	0.262707892	0.317543901	0.867090343
PSME3	0.27278941	0.460429017	0.431173833	0.954703656	0.0605417	0.317500561	0.867090343
KIF17	0.497789341	0.234479916	0.884513424	0.95005135	0.031939795	0.317623152	0.86716246
ICMT	0.678306941	0.261316358	0.318603342	0.254144137	0.218754536	0.317937331	0.867807999
SPECC1	0.76546598	0.281610118	0.044241631	0.583877946	0.563945731	0.317965358	0.867807999
TAF1A	0.594113329	0.026442069	0.57327024	0.902505648	0.387372104	0.31834373	0.8686962
LOC101904701	0.987238182	0.184506528	0.089251437	0.823985396	0.235177793	0.318431011	0.868789912
CALR	0.435424071	0.296237434	0.160860465	0.245087416	0.619994546	0.318546109	0.868820355
CD1E	0.583268473	0.034029718	0.332206823	0.731698208	0.653941474	0.31864454	0.868820355
CIZ1	0.566492808	0.119467876	0.17911387	0.384082442	0.678223006	0.318766218	0.868820355
EMC7	0.838006359	0.139596189	0.190385092	0.188975889	0.751325462	0.318971582	0.868820355
FKBP14	0.368225124	0.412753344	0.543189014	0.200835162	0.190322702	0.318670862	0.868820355
HIBADH	0.988557542	0.471990651	0.222765792	0.039092008	0.777181405	0.318773712	0.868820355
HSF5	0.7482235	0.578356201	0.233030677	0.556986742	0.056288701	0.318944132	0.868820355
LAMTOR2	0.293900568	0.526486529	0.532483761	0.053976629	0.710285954	0.318819937	0.868820355
PSME4	0.609476521	0.869419257	0.225012618	0.081875538	0.323369277	0.318724363	0.868820355
TMEM106A	0.540786438	0.149274183	0.12802622	0.807292601	0.379004818	0.318970517	0.868820355
ADGRL3	0.425672815	0.222019011	0.387392012	0.51364808	0.16835884	0.319148183	0.86894845
BATF2	0.899380173	0.095301807	0.892746314	0.094667546	0.43717309	0.319184415	0.86894845

CHRD	0.576584481	0.067001695	0.591797398	0.236328197	0.586310544	0.319230407	0.86894845
LOC112443328	0.36114234	0.51665889	0.267558696	0.187212209	0.338877152	0.319201344	0.86894845
PCDH17	0.661313192	0.514378427	0.020628634	0.662790908	0.681841017	0.319382102	0.869217192
DDX17	0.392576272	0.392206282	0.791639675	0.030529716	0.854008345	0.319692607	0.869533314
DHRS3	0.473971294	0.985817034	0.577743867	0.018736611	0.629692398	0.320010401	0.869533314
EPHA2	0.122300025	0.923252693	0.497659076	0.272666663	0.207637013	0.319847728	0.869533314
HACE1	0.464434397	0.972304972	0.541226007	0.230899652	0.056446465	0.320031224	0.869533314
HMGCR	0.98514724	0.150551658	0.060186632	0.479874457	0.7429097	0.319892626	0.869533314
LOC101902435	0.559721203	0.036147014	0.925791812	0.279888435	0.608927504	0.320346013	0.869533314
LOC104972290	0.533265897	0.470654884	0.324274816	0.061111721	0.641525148	0.320275768	0.869533314
LOC112441880	0.339836918	0.141501177	0.234986797	0.294460644	0.955092616	0.319691204	0.869533314
MANF	0.108903839	0.665537906	0.392464163	0.11730397	0.954672385	0.320037066	0.869533314
METRNL	0.293691487	0.867272255	0.17020638	0.266364834	0.274954392	0.319562229	0.869533314
PDCD1LG2	0.367519754	0.364896718	0.935076611	0.182354189	0.139400407	0.320135426	0.869533314
PGAP3	0.937151662	0.580213707	0.498506457	0.01498758	0.783561501	0.319933639	0.869533314
QKI	0.701769139	0.46039202	0.076900711	0.882364334	0.14507775	0.319809445	0.869533314
TRIM21	0.364122432	0.056292867	0.846353054	0.209821614	0.876120943	0.320198558	0.869533314
UBE2V1	0.373576197	0.45351987	0.490297326	0.118473163	0.324366245	0.320340202	0.869533314
UHRF2	0.665839027	0.257134991	0.661018273	0.195526142	0.144033727	0.320113818	0.869533314
ANO3	0.810865844	0.524398596	0.085225514	0.090976792	0.970280899	0.320646862	0.869912035
C17H12orf43	0.279632756	0.867505938	0.965796819	0.348493215	0.039171306	0.320613561	0.869912035
LOC784697	0.334746279	0.356788488	0.391922352	0.078479517	0.870238371	0.320550895	0.869912035
TMEM178B	0.288092882	0.340658983	0.545583282	0.221927572	0.269299309	0.320697569	0.869912035
ASB11	0.687498976	0.064854463	0.291404349	0.56886243	0.436529711	0.321892373	0.870040396
CUL4A	0.494168216	0.552599764	0.178327235	0.420404231	0.157070944	0.321402285	0.870040396
DLK2	0.679409185	0.607063685	0.054001151	0.964164482	0.150164221	0.321811019	0.870040396
FAM104A	0.325897896	0.560668103	0.325899659	0.06000464	0.902084028	0.321749649	0.870040396
FUK	0.588303493	0.06543865	0.50393971	0.237455378	0.697436267	0.321280005	0.870040396
KIFC1	0.866564341	0.058959145	0.119188859	0.690497392	0.763010445	0.321072268	0.870040396
LOC100295347	0.798337494	0.84378045	0.633384873	0.010404858	0.722717094	0.321074942	0.870040396
LOC100847999	0.822955292	0.908503978	0.088322364	0.550000708	0.088727515	0.321713524	0.870040396
LOC101908214	0.210219597	0.878318311	0.530895709	0.101149354	0.323033174	0.320826198	0.870040396
LOC107131992	0.554326588	0.361044555	0.041181776	0.634498021	0.616489011	0.321777815	0.870040396
LOC112441616	0.062051918	0.656643277	0.315222691	0.881756112	0.28492767	0.321911235	0.870040396
LOC112447362	0.753205931	0.26863886	0.05619755	0.949690317	0.297654655	0.321344873	0.870040396
LOC787905	0.452454428	0.202085161	0.28498074	0.642806699	0.192520085	0.321809322	0.870040396
MARCH5	0.973204035	0.181352077	0.167670777	0.253990382	0.428245745	0.321545627	0.870040396
NAPA	0.550167093	0.693846466	0.435748157	0.044596344	0.433607737	0.321443712	0.870040396
PASK	0.779668144	0.058662488	0.76516788	0.265520778	0.345190989	0.32104041	0.870040396
RNF128	0.580719532	0.704436336	0.030646729	0.374563345	0.685490634	0.321553991	0.870040396
SLITRK5	0.43661454	0.312874101	0.739629278	0.263070215	0.121200868	0.321667954	0.870040396
SSB	0.614224269	0.726147209	0.217809484	0.066331532	0.500086797	0.321713027	0.870040396
STRIP1	0.790758814	0.335360423	0.242210287	0.099526082	0.503680946	0.321594774	0.870040396
SURF2	0.331614731	0.181146406	0.170798438	0.82921049	0.379077599	0.321829137	0.870040396

THSD4	0.038908873	0.774650458	0.282772523	0.452382266	0.835934248	0.321738321	0.870040396
COPA	0.706285815	0.453779534	0.709899661	0.01789203	0.793787209	0.322112658	0.870387331
EDNRA	0.228662058	0.335937134	0.21500245	0.423752159	0.461824613	0.322145673	0.870387331
ATAD1	0.747308849	0.034967277	0.279412818	0.673337665	0.659903198	0.322694926	0.87043827
CD244	0.384335679	0.456558944	0.557437971	0.075781053	0.437510689	0.322638528	0.87043827
FNTB	0.12572832	0.917583352	0.555767504	0.09036301	0.558915904	0.322422228	0.87043827
HOXB6	0.438695396	0.148479497	0.097703685	0.818820955	0.62185109	0.322525223	0.87043827
KATNA1	0.838957464	0.257670488	0.446402592	0.099790436	0.336647287	0.322585475	0.87043827
LOC100848872	0.707249551	0.349749476	0.826671505	0.078499627	0.20167264	0.322378055	0.87043827
LOC104971852	0.545817118	0.047098914	0.252627705	0.715017151	0.697845967	0.322524953	0.87043827
LOC782114	0.713025345	0.204801827	0.138151412	0.385113993	0.416549178	0.322335247	0.87043827
LUZP6	0.555199591	0.095867234	0.130471698	0.550545744	0.848349445	0.322655457	0.87043827
NSUN2	0.463552652	0.233416335	0.772459151	0.653476336	0.059347092	0.322565262	0.87043827
AIF1	0.788558794	0.899659158	0.409262182	0.091045892	0.123975397	0.324170454	0.87050614
AMOT	0.886889141	0.4734693	0.152977786	0.062127092	0.815209312	0.323104127	0.87050614
C10H15orf48	0.546149603	0.385582824	0.53198423	0.219473714	0.132112358	0.322873779	0.87050614
CIP2A	0.350777916	0.577565125	0.031964125	0.933155324	0.539396153	0.323379111	0.87050614
CRLF3	0.167145964	0.385214474	0.186267838	0.915002812	0.296933108	0.323331914	0.87050614
CXCL3	0.587899696	0.389762227	0.889242791	0.750391425	0.021336016	0.323502424	0.87050614
DENND4B	0.683725564	0.082345429	0.288606873	0.376693156	0.532429132	0.32335212	0.87050614
EFCAB14	0.586737736	0.050376583	0.323738335	0.420961357	0.808410067	0.323238927	0.87050614
EPC1	0.256616301	0.494692503	0.888500566	0.048347487	0.602249885	0.32447982	0.87050614
FHL2	0.204742293	0.71283547	0.055342892	0.846680648	0.479195088	0.324164106	0.87050614
FSBP	0.832158733	0.916882819	0.173057212	0.100451138	0.247469738	0.324398534	0.87050614
GNPDA1	0.213515323	0.076784993	0.426999992	0.659158757	0.711695811	0.324476379	0.87050614
LOC101904891	0.543704229	0.597818451	0.161163587	0.687982954	0.091078976	0.324401262	0.87050614
LOC101905666	0.806175095	0.49592822	0.630434436	0.150286491	0.086674986	0.324437342	0.87050614
LOC104975007	0.130575282	0.317878186	0.960449091	0.669350189	0.122437569	0.323717575	0.87050614
LOC112442079	0.408046774	0.589570058	0.382072818	0.134350292	0.266124616	0.32457663	0.87050614
LOC511847	0.236019412	0.323945478	0.568621333	0.090114906	0.832194138	0.323414845	0.87050614
LOC525426	0.946448843	0.56602566	0.364502799	0.214197927	0.078039511	0.323582944	0.87050614
MATK	0.288132522	0.204569948	0.386098225	0.598030812	0.239533736	0.323400753	0.87050614
MTURN	0.480256973	0.235381709	0.07680259	0.667932143	0.564472377	0.323997972	0.87050614
PGPEP1	0.473702966	0.105823617	0.449100837	0.64980739	0.223733715	0.323981597	0.87050614
PLEKHA4	0.324112027	0.11237717	0.364811781	0.486313531	0.507707436	0.324326203	0.87050614
PTP4A2	0.316006943	0.572035059	0.260520314	0.897414039	0.076895288	0.322940767	0.87050614
RAB1A	0.404524242	0.87852937	0.202065922	0.377390537	0.120583346	0.323753952	0.87050614
RASSF3	0.939452852	0.0937628	0.112061435	0.597756002	0.553405527	0.323638528	0.87050614
RCHY1	0.66097246	0.235887929	0.134332724	0.698371467	0.224390449	0.324390141	0.87050614
RSL24D1	0.616812235	0.498226929	0.039001388	0.476073481	0.571547313	0.323455981	0.87050614
S100A14	0.383571731	0.746012168	0.155589184	0.078178377	0.933727981	0.322949325	0.87050614
SQLE	0.519711559	0.269929994	0.077441304	0.736613904	0.406688971	0.323154128	0.87050614
TDRD10	0.283864157	0.639271175	0.062471856	0.814032304	0.355980051	0.324520084	0.87050614
TRIM5	0.415901212	0.077812852	0.981706852	0.240965265	0.427344923	0.323916861	0.87050614

UCKL1	0.163329064	0.286032656	0.13805596	0.54673448	0.927613759	0.32389046	0.87050614
UGCG	0.791912678	0.784832699	0.197695005	0.07578318	0.352905087	0.324565053	0.87050614
VGLL4	0.207914265	0.409314335	0.184621625	0.560239167	0.37182616	0.323977749	0.87050614
ZBTB1	0.583739822	0.718682734	0.035985359	0.613309741	0.352545607	0.323587523	0.87050614
GJC2	0.521998662	0.282887119	0.237863407	0.103343109	0.90629809	0.32472752	0.870626258
UBE2M	0.138180163	0.427216563	0.481946793	0.325544078	0.355125758	0.324701245	0.870626258
ACP4	0.645897638	0.792732643	0.737911382	0.027458806	0.319662766	0.325910853	0.870771047
BMPR2	0.725147566	0.201181615	0.050538814	0.463980656	0.965852405	0.325364333	0.870771047
ESYT2	0.777730872	0.122363113	0.669208984	0.180981253	0.287318616	0.325698191	0.870771047
GIGYF1	0.437290861	0.202886706	0.811319051	0.984471882	0.04666636	0.325490117	0.870771047
GLYAT	0.530332598	0.92831314	0.591279671	0.050652803	0.224900403	0.325897719	0.870771047
KRBA2	0.283372938	0.046535095	0.347516885	0.747130361	0.96205459	0.324912382	0.870771047
LOC100335751	0.598027988	0.030333756	0.32395861	0.67324644	0.834545466	0.325266756	0.870771047
LOC101909140	0.28944181	0.592401683	0.089760116	0.450384101	0.478679043	0.325985234	0.870771047
LOC112446760	0.559555436	0.745673474	0.051217656	0.59022215	0.261043653	0.324854768	0.870771047
LOC787257	0.9534248	0.470206043	0.15199846	0.064988363	0.744317671	0.32501249	0.870771047
LYPLA1	0.812177851	0.503588995	0.196865571	0.281413421	0.145707387	0.325254005	0.870771047
MGRN1	0.724264227	0.05932577	0.541693889	0.811556843	0.175052541	0.325476206	0.870771047
NSD1	0.988369589	0.139107012	0.030876588	0.844493295	0.922131489	0.325444145	0.870771047
PDE6D	0.172161708	0.284869612	0.843426012	0.141536949	0.565246714	0.325596375	0.870771047
RNASE12	0.332999696	0.213351864	0.131137586	0.474320815	0.746907976	0.325214593	0.870771047
RNF20	0.283078862	0.754310909	0.863585316	0.042635037	0.420245691	0.325358469	0.870771047
RNPEP	0.645996575	0.71353465	0.544352569	0.016484244	0.802601746	0.326054968	0.870771047
SLC19A2	0.783458005	0.185126359	0.065030583	0.371540613	0.946382567	0.325912984	0.870771047
SLC37A3	0.750886988	0.68674566	0.19052478	0.059813233	0.562390868	0.325400504	0.870771047
STX2	0.640406055	0.308911129	0.265060709	0.281149534	0.224746494	0.325774429	0.870771047
TDP2	0.376631172	0.33510279	0.14699657	0.583976517	0.304935236	0.325348849	0.870771047
VAMP5	0.296047117	0.862077762	0.025381383	0.546538722	0.936704767	0.325903378	0.870771047
WBP1	0.053370701	0.716182626	0.691712871	0.125451716	0.998272759	0.32567772	0.870771047
ZNF529	0.161600348	0.109717491	0.759809791	0.263676681	0.934533171	0.326053204	0.870771047
CREBBP	0.307310591	0.904219177	0.736179141	0.042296437	0.384571908	0.326400386	0.870997248
LOC107131510	0.302446482	0.169717455	0.79378768	0.647947423	0.125971204	0.326324055	0.870997248
PRR15	0.929836501	0.436839372	0.314713566	0.230996891	0.112659122	0.326366636	0.870997248
RPS27L	0.848179754	0.197104357	0.244101292	0.090995703	0.89609645	0.326405038	0.870997248
ZFYVE19	0.214764428	0.961469338	0.11745389	0.187017278	0.732747423	0.326226235	0.870997248
FAM43A	0.064726301	0.938119011	0.205256861	0.915352372	0.292130494	0.326632354	0.871178865
RNF114	0.502517564	0.302342112	0.053312116	0.517801264	0.794518239	0.326612444	0.871178865
YTHDC1	0.22975368	0.881157302	0.938082634	0.037945775	0.462183137	0.326541969	0.871178865
GCLM	0.782053797	0.374135676	0.050682891	0.555102656	0.40512534	0.326729919	0.871259181
LOC531557	0.890232944	0.32621915	0.131592604	0.160792265	0.542868308	0.326768647	0.871259181
LOC112442979	0.442560837	0.970822469	0.391139531	0.296498796	0.066973421	0.326824745	0.871267202
SCAF4	0.925299515	0.620845108	0.871151976	0.010392151	0.642082483	0.326921553	0.871383726
AGFG1	0.68094524	0.709978906	0.24886921	0.448996273	0.062203541	0.327847254	0.87171284
AGPAT5	0.600022363	0.131215123	0.112506184	0.853580431	0.449768911	0.32960622	0.87171284

ARL6IP4	0.098548573	0.919796391	0.465549602	0.341790141	0.232557706	0.32757983	0.87171284
C15H11orf74	0.211363221	0.647121126	0.307026649	0.19201716	0.417867241	0.328248735	0.87171284
C23H6orf62	0.743538288	0.656741249	0.642547298	0.937650517	0.011485014	0.328658367	0.87171284
CAMSAP2	0.856541363	0.133057419	0.689181646	0.235755107	0.18207622	0.328339082	0.87171284
CARS2	0.680020586	0.500458514	0.635967908	0.048067836	0.321598598	0.327205519	0.87171284
CDC42EP1	0.170584929	0.48086879	0.202714127	0.501756294	0.405042711	0.328682042	0.87171284
CFL1	0.208393761	0.157280563	0.680067276	0.330407437	0.458647152	0.328612456	0.87171284
CITED2	0.54462629	0.636130165	0.022007632	0.644043463	0.683694824	0.327714517	0.87171284
CLGN	0.90949044	0.24197794	0.542846396	0.800886341	0.03548623	0.329374737	0.87171284
CSKMT	0.524277499	0.446932941	0.06939119	0.294025681	0.711230048	0.32958636	0.87171284
CUL5	0.467554729	0.214622444	0.218578789	0.761946305	0.20362614	0.329712431	0.87171284
DDX41	0.589398217	0.864807434	0.548894876	0.028208415	0.425446017	0.327729842	0.87171284
EXOSC2	0.991706901	0.092078196	0.974488101	0.101085999	0.371775693	0.327135928	0.87171284
GOSR1	0.399208824	0.715513165	0.052725339	0.294147337	0.762666253	0.328645228	0.87171284
GTF2A2	0.858967609	0.619396121	0.175234829	0.315000946	0.115583478	0.329337878	0.87171284
LOC101903992	0.345663299	0.565561251	0.083708959	0.46895525	0.442324385	0.32933918	0.87171284
LOC101905228	0.454861687	0.540448116	0.268949003	0.313493722	0.163640412	0.329218577	0.87171284
LOC101906426	0.529021222	0.580798071	0.162883284	0.074417845	0.914530544	0.329840701	0.87171284
LOC104972390	0.768903044	0.282050383	0.609226055	0.11216278	0.229382318	0.32954624	0.87171284
LOC107132032	0.258032234	0.154873107	0.506040667	0.173400616	0.95822463	0.32783596	0.87171284
LOC112447087	0.684935485	0.432565535	0.087125459	0.296215327	0.43793545	0.327330991	0.87171284
LRPPRC	0.703833856	0.853874494	0.603271675	0.332762471	0.02801115	0.328681189	0.87171284
MAATS1	0.959844115	0.053996852	0.88842208	0.393550176	0.187653537	0.329600688	0.87171284
NDUFS4	0.339575885	0.613511303	0.768249327	0.032068961	0.658148686	0.328622767	0.87171284
NRK	0.480962646	0.526229037	0.688895897	0.203696424	0.095074004	0.328559713	0.87171284
NUDT22	0.162166036	0.905890619	0.385239525	0.220124968	0.272013792	0.329083867	0.87171284
PIK3CG	0.882963425	0.039122394	0.143505565	0.874176946	0.777022055	0.328146919	0.87171284
PPAN	0.746519799	0.885235792	0.688373839	0.12642581	0.058378963	0.327721772	0.87171284
PRPF39	0.52836669	0.184099795	0.246922303	0.214637809	0.659516391	0.32957873	0.87171284
PRRT4	0.738024216	0.491303043	0.340783057	0.14674789	0.185729821	0.328174757	0.87171284
PTRH2	0.34423637	0.311193302	0.233315805	0.287937067	0.473266041	0.329835248	0.87171284
RANGAP1	0.439212434	0.507144421	0.105382707	0.767411229	0.187177281	0.328346363	0.87171284
RBM22	0.760634804	0.476298191	0.158483765	0.078614224	0.75468629	0.329860261	0.87171284
RFNG	0.41237365	0.438637792	0.26853811	0.334320818	0.207813403	0.328476443	0.87171284
RRP36	0.454795614	0.227006667	0.88838012	0.980089504	0.03783024	0.329605179	0.87171284
SCN9A	0.635525909	0.074098108	0.464319157	0.175078374	0.881673463	0.328495943	0.87171284
SCUBE2	0.088593034	0.601308543	0.376225388	0.186836002	0.906316756	0.329308035	0.87171284
SDHA	0.590303177	0.746481225	0.43535934	0.028921553	0.606298716	0.32800454	0.87171284
SH3PXD2B	0.853674567	0.669459004	0.127339628	0.060472514	0.771989065	0.329465773	0.87171284
SHC4	0.109408497	0.836561908	0.083227327	0.48595555	0.913041746	0.328701534	0.87171284
ST3GAL1	0.07576122	0.293345224	0.539011041	0.375842615	0.744564727	0.327489584	0.87171284
STPG1	0.459545395	0.119316807	0.153156898	0.728632653	0.55416312	0.3291812	0.87171284
SUSD2	0.909651552	0.532035879	0.100294069	0.306324403	0.228165815	0.329252838	0.87171284
SYNJ2BP	0.085123886	0.315136698	0.841614066	0.632064478	0.238355933	0.329636576	0.87171284

TFAM	0.903413281	0.034917837	0.22974312	0.888999693	0.522315251	0.328059017	0.87171284
THBD	0.489432777	0.878904853	0.141702078	0.255222615	0.215594479	0.32756954	0.87171284
TOM1L2	0.339251515	0.967995014	0.789418612	0.024834761	0.528103808	0.329578964	0.87171284
TPP2	0.601123835	0.563187119	0.133552012	0.235394465	0.314406928	0.327226323	0.87171284
ZFP57	0.050904505	0.178046192	0.711421159	0.768626075	0.678963703	0.328047721	0.87171284
ZNF423	0.41494188	0.429025459	0.080160111	0.257241246	0.927476766	0.329780138	0.87171284
ZNF696	0.819818091	0.302898156	0.323106552	0.392578419	0.106437936	0.327506754	0.87171284
COPG2	0.075770797	0.920177269	0.229815207	0.227434912	0.935792655	0.330024283	0.871844663
DNMT3B	0.503418433	0.064826536	0.218436389	0.815000643	0.587160392	0.33006952	0.871844663
HERC4	0.274636546	0.95123009	0.065055895	0.244090878	0.822180929	0.330044945	0.871844663
LOC112446369	0.713196905	0.22903348	0.173405643	0.379430892	0.317544837	0.330133519	0.871873379
ADAM11	0.307544575	0.533475692	0.418423783	0.073749201	0.675283109	0.330397033	0.872024887
PEX19	0.191029829	0.225460106	0.3805258	0.529208401	0.394268029	0.330428076	0.872024887
PHYH	0.877348311	0.420850774	0.073059879	0.344009734	0.368105169	0.330274103	0.872024887
PITPNB	0.086542303	0.949611433	0.383739954	0.513060881	0.211451548	0.330502392	0.872024887
PNKD	0.569106637	0.297175598	0.104035974	0.712233918	0.272948967	0.330469396	0.872024887
SLC7A2	0.08473793	0.292683604	0.307760204	0.60937757	0.735592761	0.330509707	0.872024887
CCNL2	0.853769126	0.053967872	0.388455353	0.70825935	0.270435214	0.330803082	0.872658637
PEX16	0.620027498	0.971501769	0.777522756	0.028166004	0.260224669	0.330997307	0.873010249
TOMT	0.946617481	0.266941351	0.542146448	0.030012195	0.83516006	0.331042763	0.873010249
LOC112444920	0.468726981	0.14021724	0.517350011	0.396914817	0.254708202	0.331203831	0.873154388
PWP2	0.165811845	0.373710552	0.823826609	0.531359903	0.126688666	0.33115847	0.873154388
SKIL	0.184381632	0.628445795	0.369161694	0.411176317	0.195566619	0.331299094	0.873265248
SRP68	0.592925655	0.180423822	0.362378946	0.523220509	0.169734211	0.33143109	0.873472879
CALCRL	0.363687314	0.373868012	0.104950545	0.487007933	0.497051146	0.331929272	0.873825451
CAV1	0.950027388	0.199006988	0.177805294	0.146195685	0.703492857	0.332057606	0.873825451
CCDC89	0.464068455	0.91112836	0.930796812	0.072885628	0.121116453	0.33278289	0.873825451
CD36	0.112800254	0.263753064	0.13597767	0.899969507	0.94947625	0.332038343	0.873825451
CEP85L	0.098718161	0.886420685	0.800618135	0.126664726	0.388732812	0.331724399	0.873825451
CIAO1	0.961137956	0.29254568	0.269061173	0.104609751	0.437514377	0.332280597	0.873825451
GATC	0.52136188	0.227357995	0.494743338	0.152950513	0.387201959	0.332733686	0.873825451
HSPB6	0.96158687	0.751658523	0.047481779	0.179443585	0.564300887	0.332821686	0.873825451
LOC100139891	0.575875949	0.213448005	0.749115375	0.162118703	0.231899222	0.332248838	0.873825451
LOC100616098	0.474833621	0.500922988	0.486989097	0.038190255	0.78427203	0.332573709	0.873825451
LOC112448736	0.749360055	0.101876378	0.907875001	0.601326304	0.083061374	0.332247893	0.873825451
LOC510536	0.142006584	0.272130267	0.948801883	0.597237717	0.158718748	0.332842782	0.873825451
MYH7	0.87691379	0.665687514	0.260516479	0.260627408	0.087082251	0.331807495	0.873825451
NEDD8	0.449121356	0.383665729	0.806212501	0.043147632	0.579164403	0.332667606	0.873825451
NOS2	0.788591063	0.893663176	0.176833514	0.053933607	0.515931235	0.332502002	0.873825451
PSMA5	0.602479436	0.512077359	0.731667896	0.06413287	0.239486611	0.332471911	0.873825451
SH3BP5	0.364340524	0.691089025	0.525439037	0.03757363	0.695838172	0.33213009	0.873825451
SLC35C2	0.493250126	0.972941605	0.196680974	0.043377627	0.846080813	0.33234873	0.873825451
SRSF5	0.87910747	0.713534151	0.942113347	0.017562857	0.334310431	0.332592217	0.873825451
TMEM256	0.736986867	0.799734063	0.093445839	0.079972481	0.78362934	0.331808975	0.873825451

TSC1	0.461037467	0.235505436	0.66871274	0.760873643	0.062452221	0.331747629	0.873825451
TSKU	0.155988168	0.56805736	0.141408439	0.296945145	0.929594364	0.332121083	0.873825451
WDR24	0.288297012	0.176800473	0.896693245	0.474032632	0.160065527	0.332512936	0.873825451
WWC1	0.503339344	0.014089702	0.747697297	0.890359121	0.735695694	0.332745329	0.873825451
LYPD3	0.726516879	0.091350687	0.311963894	0.250377276	0.671279412	0.333022311	0.873883447
PLEK	0.399685485	0.189017109	0.100908478	0.543026052	0.840750016	0.333047887	0.873883447
PRKX	0.233570549	0.334979655	0.26619322	0.891998821	0.187306099	0.333018748	0.873883447
TELO2	0.907133878	0.008349426	0.859295745	0.847437235	0.631166497	0.333077872	0.873883447
SCCPDH	0.672023476	0.682921424	0.242236632	0.120004041	0.261040507	0.33313843	0.873902617
CCDC12	0.60218798	0.181300997	0.14525881	0.345536883	0.636264081	0.333312582	0.874104611
CISD2	0.485757969	0.215112606	0.942135992	0.573099172	0.061802314	0.333321958	0.874104611
ATOH8	0.317153717	0.393409567	0.231063598	0.33746527	0.359367636	0.333727021	0.874354293
ERI1	0.208675641	0.159689327	0.728616773	0.224012212	0.642478615	0.333645626	0.874354293
GORASP1	0.266099006	0.482876808	0.416852237	0.099857488	0.653438785	0.333669687	0.874354293
LRAT	0.232324775	0.302002892	0.354774154	0.925778207	0.151786287	0.333790119	0.874354293
MGAT4B	0.416457906	0.303590336	0.053373456	0.520716777	0.994571576	0.333661008	0.874354293
STIL	0.146869803	0.479505077	0.188559685	0.767170887	0.342618399	0.333474096	0.874354293
TUBD1	0.828605395	0.875784549	0.018903192	0.338750332	0.752713502	0.333787207	0.874354293
METTL8	0.240684998	0.782989524	0.145577471	0.421093739	0.302982658	0.33389231	0.874482396
ACHE	0.697520017	0.676216766	0.368731266	0.02635886	0.768149587	0.334796029	0.874574762
ANGPT2	0.026982348	0.411055098	0.703941441	0.537664554	0.838696292	0.334764186	0.874574762
ARCN1	0.877338817	0.092527188	0.750556839	0.242715366	0.237738181	0.334552352	0.874574762
CD300A	0.352658642	0.489859414	0.91406667	0.128937085	0.173086002	0.334905664	0.874574762
ENDOV	0.495917406	0.538404185	0.067459816	0.566384047	0.343572115	0.334097073	0.874574762
FAM149B1	0.258753562	0.381959191	0.105692807	0.627741266	0.538682146	0.33525618	0.874574762
FAM217B	0.895120145	0.798516895	0.262958118	0.31646811	0.059263787	0.33494961	0.874574762
FIG4	0.270966752	0.575749035	0.801836006	0.060837969	0.46381642	0.335150695	0.874574762
IP6K2	0.969895858	0.382921824	0.873014004	0.057604216	0.188741091	0.334951603	0.874574762
KIF26A	0.922405587	0.36860522	0.051843401	0.212832688	0.941014058	0.335169879	0.874574762
KLKB1	0.737600455	0.226519251	0.49077412	0.142915414	0.301389796	0.335240194	0.874574762
LOC101902760	0.239177688	0.349728738	0.517092126	0.153228412	0.531847708	0.334941051	0.874574762
LOC112444498	0.077551925	0.46398113	0.968080954	0.772504839	0.131063455	0.335023953	0.874574762
LOC112445925	0.705918728	0.564527702	0.015579562	0.852059783	0.667740098	0.335259876	0.874574762
LOC112447313	0.653300232	0.613151983	0.262431292	0.162158762	0.20564096	0.334115886	0.874574762
MRM3	0.344008986	0.313450198	0.576721161	0.858114674	0.066020343	0.334865637	0.874574762
NAXE	0.415495571	0.915197992	0.900488537	0.01705545	0.603921573	0.335029455	0.874574762
POC1A	0.79289375	0.152718785	0.631927724	0.061303486	0.750097288	0.334676542	0.874574762
RANBP9	0.804671144	0.118302564	0.169554352	0.968776614	0.224205382	0.334131208	0.874574762
SECISBP2L	0.487265613	0.33888186	0.063316409	0.985502858	0.342343097	0.335045439	0.874574762
SLC33A1	0.588636751	0.19082813	0.787932094	0.328436156	0.120912964	0.334513116	0.874574762
SMAD2	0.663314819	0.020750773	0.282883552	0.979226057	0.924595179	0.334958172	0.874574762
THTPA	0.737569274	0.619942098	0.321940211	0.033990777	0.705472949	0.335155866	0.874574762
TULP2	0.87704583	0.024071342	0.848098079	0.673982195	0.291406098	0.334586474	0.874574762
ZBTB21	0.510264954	0.511604139	0.332553506	0.081793512	0.495334746	0.334618538	0.874574762

LOC101907813	0.139586827	0.77569452	0.114735273	0.632561203	0.449986594	0.335418813	0.874711288
NCALD	0.450943373	0.191144685	0.377973357	0.200093761	0.542262118	0.335368526	0.874711288
ABCD2	0.096742506	0.934398089	0.45987977	0.498893274	0.170881636	0.335750396	0.874853227
EXO5	0.43131026	0.321894981	0.061788528	0.565286704	0.731802188	0.33594954	0.874853227
FRS3	0.576435535	0.245865206	0.253114506	0.11980215	0.825185414	0.33584842	0.874853227
HIC1	0.922286381	0.356570532	0.167130736	0.168614538	0.382754405	0.335883506	0.874853227
ITGB3	0.175716986	0.347595064	0.525756757	0.969785292	0.113789537	0.335734372	0.874853227
LOC104974050	0.839523862	0.457046036	0.430241089	0.16292431	0.131945216	0.335953021	0.874853227
LOC104975091	0.513244145	0.494228888	0.05026531	0.32932802	0.844978274	0.335922096	0.874853227
LOC614226	0.250948228	0.86516176	0.174498791	0.122064051	0.765796765	0.335639619	0.874853227
PELI1	0.375279874	0.357999949	0.235896488	0.367872186	0.304154142	0.335837869	0.874853227
RTL8C	0.152325549	0.527982545	0.813312579	0.091845418	0.591074269	0.336044378	0.87495229
FXN	0.797784976	0.165278685	0.329388824	0.177041159	0.462217486	0.336176412	0.875018414
HEATR6	0.505143985	0.581435054	0.116713862	0.16457328	0.629908613	0.336156934	0.875018414
EGR2	0.839593876	0.095491681	0.87158594	0.133981685	0.379757205	0.336233087	0.875027148
CARD10	0.458700277	0.144094189	0.520692539	0.647180344	0.159730275	0.336327244	0.875133408
NDP	0.312831099	0.503815454	0.460762291	0.058945841	0.831735074	0.336440132	0.875288364
ADAM22	0.321131595	0.05718004	0.874165688	0.618461273	0.359423087	0.336765753	0.875359474
AKNA	0.76281215	0.293043921	0.304046489	0.149618913	0.350967478	0.336801653	0.875359474
BCL2L13	0.238522466	0.942101083	0.147926664	0.137567087	0.7815065	0.337000863	0.875359474
COPE	0.531015113	0.699208863	0.613320388	0.034738275	0.451178241	0.336806459	0.875359474
GSTA1	0.716609788	0.218619665	0.490762719	0.065767633	0.704745047	0.336575176	0.875359474
MPRIP	0.879772991	0.678501191	0.111149843	0.126549399	0.425492338	0.336953026	0.875359474
NAA20	0.503679743	0.371760874	0.504673832	0.080924133	0.466900102	0.336866341	0.875359474
PRKCB	0.549388564	0.225642215	0.103445499	0.712087131	0.391342046	0.336994237	0.875359474
SCAMP3	0.425436848	0.40125458	0.761385962	0.092504617	0.297052042	0.336909498	0.875359474
TMEM232	0.410521045	0.079245642	0.769381417	0.913581904	0.155889218	0.33661947	0.875359474
AARS	0.590140763	0.317136836	0.650368071	0.186521547	0.159107814	0.338613929	0.875504349
ACTRT3	0.031200787	0.703080146	0.770456849	0.227662786	0.959546799	0.34191491	0.875504349
AFF3	0.470783388	0.63073484	0.638119285	0.500214609	0.038386239	0.33969624	0.875504349
ARFGEF3	0.360785073	0.555521506	0.155739816	0.847614995	0.138508729	0.340781975	0.875504349
ASCL4	0.706081382	0.246725102	0.989422502	0.034924586	0.611542874	0.341472581	0.875504349
ASGR2	0.628132866	0.804815058	0.022439127	0.405109809	0.797975582	0.34088327	0.875504349
BCL9	0.729631353	0.413923828	0.070204374	0.477061432	0.366115467	0.342369723	0.875504349
C23H6orf136	0.980034302	0.176834313	0.910910784	0.148190253	0.154351664	0.33855591	0.875504349
CDH4	0.594811776	0.558584389	0.043938308	0.840144688	0.29590664	0.339320864	0.875504349
CDK10	0.296414565	0.260356261	0.163581125	0.448441673	0.648665022	0.341096082	0.875504349
CDKN3	0.743369874	0.088599839	0.217685686	0.275914118	0.932810777	0.341830634	0.875504349
CISH	0.72839384	0.023686848	0.512369975	0.775888806	0.529403487	0.339398266	0.875504349
COX7A2	0.611100571	0.840896672	0.593528939	0.013444462	0.874074763	0.337439428	0.875504349
CPOX	0.956243229	0.82738747	0.135028207	0.345749814	0.09766466	0.338412673	0.875504349
CSTF3	0.466408587	0.390720219	0.748201972	0.939909269	0.028427339	0.339894808	0.875504349
DAPP1	0.938915103	0.168517069	0.310557418	0.205779171	0.3606241	0.340032012	0.875504349
E2F1	0.375750302	0.164239991	0.12588519	0.480135436	0.985391164	0.341234727	0.875504349

EDIL3	0.655790681	0.82134906	0.976569814	0.072795708	0.095829203	0.340982144	0.875504349
EIF3M	0.197750221	0.897866125	0.082880782	0.246467078	0.998323611	0.338971233	0.875504349
ELP2	0.532927498	0.190549466	0.261731319	0.707116731	0.194712177	0.340570054	0.875504349
EVA1B	0.370790635	0.389066478	0.425636457	0.098225138	0.598815087	0.338587846	0.875504349
EYA3	0.765155405	0.638559423	0.686927324	0.013844983	0.785804138	0.340240634	0.875504349
FAM173B	0.543499042	0.704276308	0.170535922	0.108586441	0.513089424	0.339635346	0.875504349
FAM49A	0.677069378	0.400140648	0.096862551	0.39448988	0.346148245	0.337408481	0.875504349
FAM96B	0.209591856	0.33437454	0.452155458	0.131071009	0.872174692	0.339038116	0.875504349
FCHSD1	0.904542176	0.091746978	0.494085724	0.306812912	0.287259432	0.338679362	0.875504349
GABRB1	0.048439421	0.789958617	0.269157184	0.900076832	0.390295028	0.338856523	0.875504349
GALNT11	0.399092297	0.955309657	0.33097941	0.05374974	0.543707838	0.341734827	0.875504349
GCFC2	0.691937809	0.211127595	0.304266732	0.390188992	0.20880092	0.33899249	0.875504349
GJA10	0.732320239	0.224985251	0.369803382	0.617141204	0.095501764	0.337727427	0.875504349
GOLM1	0.86864243	0.266563787	0.174700124	0.106250512	0.861407593	0.342332956	0.875504349
GSC	0.07376077	0.44934123	0.526614014	0.423933293	0.488810405	0.338804595	0.875504349
HASPIN	0.973011434	0.114363861	0.148646033	0.530522371	0.4204679	0.341817169	0.875504349
KDM3A	0.992180016	0.875441066	0.405195989	0.065199606	0.158259749	0.339417173	0.875504349
LEO1	0.423994569	0.359000501	0.613591399	0.242436077	0.159801255	0.338866988	0.875504349
LIPT2	0.104945731	0.31160341	0.579489866	0.740781179	0.263839672	0.342391501	0.875504349
LOC101906607	0.739627472	0.359260986	0.487282039	0.567336889	0.049857273	0.340694344	0.875504349
LOC101907606	0.57933972	0.863841371	0.109754669	0.082545404	0.793575581	0.338021453	0.875504349
LOC101907648	0.349476713	0.276852898	0.119914326	0.423631586	0.736606832	0.338953823	0.875504349
LOC107132300	0.597253162	0.571322506	0.246345483	0.451686528	0.094814827	0.338099792	0.875504349
LOC112441545	0.011669955	0.826844946	0.553688074	0.992954252	0.693475737	0.341371852	0.875504349
LOC112441887	0.833078562	0.402207664	0.198040982	0.062133545	0.874600254	0.33835236	0.875504349
LOC112442593	0.743209008	0.210706648	0.625303863	0.050088224	0.75466368	0.342296052	0.875504349
LOC112442719	0.700339358	0.028101592	0.693558163	0.885807358	0.301070021	0.339775216	0.875504349
LOC112446796	0.421429573	0.435203306	0.032806715	0.65783875	0.91464323	0.33894989	0.875504349
LOC112447353	0.26616687	0.795721075	0.263303123	0.268500818	0.244838619	0.340841672	0.875504349
LOC516599	0.903284134	0.621717323	0.113380993	0.453508508	0.127845502	0.341898027	0.875504349
LOC537848	0.222820831	0.640871141	0.897513339	0.106420779	0.265755808	0.339132159	0.875504349
LOC616948	0.892813424	0.460852024	0.549173734	0.036834843	0.444116288	0.342093005	0.875504349
LOC783541	0.201020879	0.813367779	0.042076482	0.799053136	0.652946506	0.337657863	0.875504349
LRIF1	0.25776001	0.991698155	0.79512926	0.038965083	0.464615585	0.341400841	0.875504349
MAP3K5	0.113668181	0.908269095	0.277588763	0.150413358	0.858378997	0.342244201	0.875504349
MAPK6	0.390869261	0.074316818	0.375347498	0.55729579	0.59888935	0.33972498	0.875504349
MAPT	0.047864138	0.910388119	0.419182213	0.863079086	0.228128493	0.337951631	0.875504349
MLC1	0.604362283	0.700252978	0.267124958	0.039872466	0.816162243	0.341370955	0.875504349
MTCP1	0.907746617	0.027467392	0.878379247	0.429783466	0.386522936	0.339691849	0.875504349
MYD88	0.871060456	0.709285217	0.083413777	0.118672103	0.604620511	0.342145694	0.875504349
MYO5A	0.039128053	0.986676938	0.611010834	0.381074666	0.400633023	0.338159055	0.875504349
NFXL1	0.414879395	0.202599476	0.931087758	0.230427109	0.200248389	0.338569352	0.875504349
PAFAH2	0.183514154	0.309772861	0.144079247	0.706926361	0.633838064	0.341005435	0.875504349
PAM	0.332601669	0.513718029	0.290397998	0.115953193	0.638230723	0.341087749	0.875504349

PEBP1	0.805963525	0.686160293	0.351006021	0.052613726	0.362055565	0.342142662	0.875504349
PGA5	0.510769629	0.353339884	0.411781285	0.185570621	0.262957945	0.339202667	0.875504349
PHC3	0.058996821	0.407066202	0.954635618	0.871650081	0.182657632	0.340185981	0.875504349
PHLDB1	0.146638788	0.229026134	0.985671729	0.382337173	0.282773763	0.33721907	0.875504349
PSMD8	0.448708008	0.437730223	0.701843273	0.20469056	0.127880487	0.338451261	0.875504349
RARRES2	0.25388353	0.227051183	0.498762815	0.633783232	0.203228479	0.342368748	0.875504349
RBL2	0.402936515	0.812023813	0.032181465	0.482311641	0.716562163	0.339727869	0.875504349
RBP2	0.116884311	0.121868489	0.744138226	0.392174475	0.87448781	0.339568662	0.875504349
RDH16	0.652124275	0.697292538	0.262189045	0.754352125	0.04035713	0.339332608	0.875504349
RNF5	0.884117619	0.039883733	0.590710578	0.225031045	0.768743504	0.338240621	0.875504349
RUBCNL	0.362148841	0.727804366	0.851682893	0.215434582	0.075506197	0.340243828	0.875504349
SAYSD1	0.568764204	0.280539636	0.760135964	0.080662861	0.376771302	0.341668155	0.875504349
SERINC5	0.377287148	0.771652808	0.15869286	0.16086277	0.481436582	0.337182477	0.875504349
SETMAR	0.156487208	0.794633916	0.904603117	0.074861153	0.431158873	0.339382426	0.875504349
SNAP29	0.191020077	0.620555743	0.381104535	0.698209362	0.116082476	0.340653714	0.875504349
SNX10	0.482827109	0.191020471	0.234867688	0.231945465	0.735825818	0.342116815	0.875504349
SNX2	0.688308305	0.420001605	0.150106429	0.102898408	0.817239419	0.340144244	0.875504349
SOD1	0.510527077	0.486629112	0.170779935	0.137312599	0.629987119	0.341015502	0.875504349
SPX	0.853581693	0.756728493	0.308515421	0.064065913	0.288841247	0.341730584	0.875504349
SRPX2	0.770446569	0.765405554	0.090522366	0.320319039	0.210016376	0.337728826	0.875504349
SS18L1	0.753222177	0.371893738	0.208823065	0.100655791	0.616686484	0.339391369	0.875504349
SUCLG1	0.665895806	0.74556375	0.417257348	0.042371727	0.417585627	0.340813955	0.875504349
TCEAL9	0.905220248	0.548056312	0.055761974	0.33628591	0.394012699	0.340820815	0.875504349
TEAD3	0.08658424	0.398108996	0.145671409	0.777761316	0.947044644	0.342178296	0.875504349
TIMM8A	0.435713342	0.632734798	0.346650334	0.335628505	0.114447327	0.341044373	0.875504349
TMEM218	0.66657636	0.053952531	0.670421914	0.433419766	0.352834948	0.34171035	0.875504349
TMEM267	0.828762876	0.05736027	0.349359471	0.431808135	0.501716874	0.33801869	0.875504349
TNFSF4	0.798686353	0.846534641	0.11964029	0.14630594	0.312423088	0.342133491	0.875504349
TRMT12	0.675407008	0.341646903	0.061420136	0.643876144	0.403686388	0.341574414	0.875504349
WBP11	0.78422793	0.116147048	0.121435325	0.338924307	0.960675988	0.338161211	0.875504349
WDSUB1	0.233456733	0.312431151	0.164458159	0.329468144	0.917760987	0.339230191	0.875504349
XG	0.996728658	0.28081279	0.743338466	0.043345386	0.404129623	0.339954074	0.875504349
ZNF135	0.121604279	0.356196512	0.758962592	0.154496118	0.72257539	0.341002919	0.875504349
ZNF304	0.26664344	0.105477851	0.805455763	0.519957765	0.312819707	0.341607796	0.875504349
ZNF420	0.17521234	0.322674818	0.361339323	0.333779411	0.53207594	0.339271475	0.875504349
ZNF518B	0.465309657	0.545852283	0.081399144	0.20215084	0.870486368	0.339686798	0.875504349
ZNF704	0.196914913	0.802806494	0.800903914	0.868027531	0.03262802	0.337510274	0.875504349
ZNF74	0.881281551	0.050735562	0.271680879	0.700474499	0.428752874	0.340107535	0.875504349
ZNHIT6	0.239783711	0.168843566	0.722019593	0.264928695	0.474545769	0.341212078	0.875504349
PLS3	0.413113889	0.052759684	0.953987632	0.384494594	0.46368956	0.342527451	0.875715531
CD163	0.420721657	0.827541956	0.772795469	0.088554764	0.155755958	0.342692896	0.875729229
GPR75	0.780560006	0.326927353	0.54747344	0.035575235	0.746635872	0.342682833	0.875729229
SYNCRIP	0.749976641	0.749534427	0.344208701	0.120587014	0.158989307	0.342631279	0.875729229
CORO2A	0.043310003	0.781954995	0.174937636	0.654242075	0.958918682	0.342926051	0.876188606

FANCL	0.665729988	0.928408261	0.07090845	0.127402316	0.665964859	0.34299254	0.876222069
DLGAP3	0.350920722	0.303217477	0.087522419	0.602571111	0.66425417	0.343363587	0.876249731
FUCA1	0.160080239	0.405356471	0.238081768	0.554664478	0.434995897	0.343360556	0.876249731
GAS2	0.564276707	0.019022803	0.40491785	0.875007586	0.980208572	0.343377126	0.876249731
IGF2BP3	0.178736123	0.360469232	0.942602122	0.940468857	0.065197369	0.343209139	0.876249731
LOC104972797	0.928353762	0.633313295	0.502822616	0.065614248	0.191906481	0.343157147	0.876249731
LOC509034	0.615738321	0.820073167	0.325189473	0.619881318	0.036607356	0.343306596	0.876249731
REX1BD	0.23946949	0.836865767	0.461516202	0.101715843	0.395769975	0.343188498	0.876249731
SRBD1	0.820270395	0.762713647	0.031376477	0.423403419	0.448757428	0.343455773	0.876314163
CTPS2	0.272968072	0.341602663	0.075118605	0.62526629	0.852233503	0.34356613	0.876323246
MRPS2	0.436182539	0.778964866	0.267364024	0.081988362	0.501021736	0.34352893	0.876323246
SLC9A9	0.275278744	0.591112343	0.644866587	0.046349328	0.767960389	0.343667804	0.876446362
BNIP1	0.24621996	0.056224963	0.66443151	0.805965389	0.504604133	0.343904045	0.876741674
CREB3	0.907263409	0.951385062	0.125038474	0.056722253	0.611568136	0.344030731	0.876741674
FABP2	0.198389182	0.277500318	0.793291415	0.271484145	0.315713828	0.344002927	0.876741674
FKRP	0.969567279	0.033242124	0.773550113	0.191316147	0.785022318	0.344050721	0.876741674
PALM2	0.814910603	0.095736588	0.646480531	0.109205779	0.679321089	0.343937031	0.876741674
DGKG	0.19300255	0.490809135	0.648399415	0.980542666	0.062251791	0.344242584	0.877094404
CLEC11A	0.339653613	0.154171322	0.273361312	0.540293767	0.48494154	0.344298485	0.877100659
LARP7	0.309055918	0.819809094	0.600890963	0.05329151	0.462512634	0.344379366	0.877170538
MEX3B	0.879383364	0.192575668	0.052584857	0.454665467	0.927488481	0.344489308	0.877186458
PVRIG	0.120512886	0.918100429	0.672704213	0.653479646	0.07720939	0.344492518	0.877186458
CDCA2	0.334219024	0.200020481	0.080136747	0.70856811	0.98976646	0.344563195	0.877230313
WDR75	0.885775743	0.525524272	0.640368325	0.034595651	0.364658249	0.344704207	0.877453194
AASS	0.276340532	0.179238936	0.795342444	0.287592039	0.332943359	0.345168704	0.877748089
C26H10orf88	0.190669898	0.088209657	0.638993255	0.654436024	0.535807219	0.345025554	0.877748089
CEP135	0.441925124	0.097317308	0.967735046	0.31995933	0.283213422	0.345142576	0.877748089
CHD4	0.695589099	0.943195659	0.033384851	0.318731057	0.541548787	0.345515365	0.877748089
CLYBL	0.796700682	0.777051667	0.081461313	0.296756173	0.252402069	0.345383079	0.877748089
SLC38A9	0.579882316	0.98690588	0.313272598	0.070495039	0.298509303	0.345195412	0.877748089
SUCLA2	0.499109872	0.561076777	0.152314854	0.457105661	0.193397604	0.345116101	0.877748089
TLDC2	0.483005845	0.514243832	0.311724526	0.189829601	0.257130688	0.345460707	0.877748089
TTF1	0.430364878	0.206058159	0.37285024	0.241368048	0.472438389	0.345101232	0.877748089
TWF1	0.505877553	0.402624974	0.609459841	0.690830788	0.04408279	0.345503426	0.877748089
UBE2L3	0.489394869	0.567466496	0.454588638	0.060971747	0.491054993	0.345484119	0.877748089
UFL1	0.646095521	0.853111729	0.294669041	0.055110554	0.42105111	0.345038339	0.877748089
ZMYM2	0.717847455	0.397250284	0.902781162	0.016866175	0.86988579	0.345372035	0.877748089
PARM1	0.853716438	0.58351953	0.415683254	0.053895177	0.33887378	0.34556925	0.877749104
LOC101902428	0.401651726	0.71375167	0.711153688	0.093779123	0.198043413	0.345747439	0.877929943
MSRB1	0.200801764	0.525273699	0.777348443	0.130981564	0.352478036	0.345706812	0.877929943
EML2	0.574762957	0.427566296	0.374817582	0.134782428	0.305377577	0.345942703	0.878289867
ATG5	0.159458053	0.180875813	0.239336839	0.805902649	0.686391205	0.347035983	0.878462787
ATP5MC1	0.777511329	0.47764285	0.835987953	0.02096697	0.586800235	0.347087106	0.878462787
BAG2	0.097731468	0.118105527	0.688350574	0.52601834	0.910620134	0.346529784	0.878462787

CLEC9A	0.366185113	0.673771997	0.097368695	0.310118076	0.514672501	0.347670209	0.878462787
CMKLR1	0.990810355	0.084923595	0.512976762	0.108330352	0.817406023	0.347181696	0.878462787
DDX55	0.384203202	0.230148513	0.790314233	0.208695853	0.262759428	0.347582107	0.878462787
GPI	0.943094284	0.819298902	0.763234966	0.012609253	0.514220143	0.347248478	0.878462787
IRX5	0.652710134	0.751861861	0.063175339	0.313000322	0.391489831	0.346255055	0.878462787
KDM5C	0.757813405	0.0883255	0.263706751	0.50282014	0.429614093	0.346814215	0.878462787
LOC100296324	0.390533088	0.959614024	0.523991143	0.021584624	0.898546858	0.346639243	0.878462787
LOC104973073	0.010799737	0.860067654	0.785822989	0.820160797	0.639308655	0.347384428	0.878462787
LOC107131134	0.516017435	0.799878578	0.039640563	0.595852316	0.392140463	0.347218309	0.878462787
LOC107132247	0.332388796	0.115007548	0.817937799	0.376855726	0.323518835	0.346781438	0.878462787
LOC781612	0.152414041	0.728475966	0.122746614	0.280252251	0.998483435	0.346841978	0.878462787
LOC783045	0.671927362	0.0405966	0.24947388	0.758569595	0.742634964	0.347640902	0.878462787
LOC784841	0.220478343	0.53435165	0.11730544	0.400191855	0.688799881	0.346677581	0.878462787
MEOX1	0.263559159	0.704776722	0.02985036	0.770471262	0.891687602	0.346669088	0.878462787
MRPL37	0.291153484	0.995235238	0.935730896	0.061846291	0.227165768	0.346671494	0.878462787
NUDT16	0.067481414	0.597405617	0.727310794	0.228379708	0.570473633	0.347097885	0.878462787
PDAP1	0.092186576	0.490457594	0.435812079	0.473109032	0.409656941	0.347056817	0.878462787
PPIP5K2	0.731592773	0.067868634	0.271391255	0.521401236	0.545495855	0.347602125	0.878462787
PPP2R2A	0.267812832	0.899343205	0.767245861	0.034173757	0.603865782	0.346836642	0.878462787
RPAP2	0.782102212	0.103974918	0.118256293	0.723401557	0.547473851	0.346637691	0.878462787
S100A16	0.994634325	0.574014604	0.126432105	0.176365005	0.299366172	0.346743151	0.878462787
SLC19A3	0.756545113	0.154257092	0.215052898	0.37032595	0.408573438	0.346187653	0.878462787
SLC25A10	0.176657565	0.713662414	0.097878977	0.451020958	0.687783761	0.347413677	0.878462787
SLC46A3	0.96386763	0.616994711	0.083610265	0.841551555	0.090752862	0.346194666	0.878462787
SNRPD3	0.312779361	0.201007019	0.80069541	0.275658302	0.274677018	0.346761154	0.878462787
TNFRSF19	0.464895566	0.681757238	0.64636267	0.9356934	0.019989275	0.347564822	0.878462787
VIRMA	0.214347311	0.966288156	0.781901382	0.036030118	0.655658083	0.347327943	0.878462787
ZMIZ1	0.980778429	0.367199072	0.020669399	0.797146272	0.64594577	0.347614999	0.878462787
ANKLE1	0.90843229	0.647191473	0.795699822	0.147150176	0.0561343	0.348860397	0.878743405
AP2S1	0.352427681	0.934281183	0.912594116	0.037266132	0.3450987	0.348867187	0.878743405
COIL	0.895520624	0.170526341	0.370301871	0.320047977	0.212837869	0.348374242	0.878743405
COL15A1	0.444064891	0.835923621	0.025428057	0.452755294	0.907348914	0.349390699	0.878743405
COPS5	0.682430805	0.608869541	0.313450419	0.115503492	0.257015008	0.348945762	0.878743405
CPEB1	0.599312977	0.384928234	0.156820763	0.768125366	0.139380908	0.349216864	0.878743405
CTTNBP2	0.752214296	0.175459238	0.17846766	0.165504973	0.990659521	0.348771772	0.878743405
DDX47	0.922015967	0.204714809	0.405366684	0.712429828	0.070899621	0.348880907	0.878743405
EAF2	0.510286868	0.030085486	0.4382302	0.867595015	0.661009003	0.348625321	0.878743405
FAM173A	0.53109409	0.601696077	0.505050296	0.024268126	0.990826942	0.349515631	0.878743405
FOXF1	0.839295878	0.381406466	0.09958866	0.266745134	0.452374423	0.34816978	0.878743405
ITPRIP	0.184854913	0.768235346	0.292091912	0.138467686	0.669906684	0.348205058	0.878743405
LBP	0.898068328	0.536823174	0.891679764	0.283607251	0.031767206	0.349207494	0.878743405
LOC107131531	0.34357105	0.51950342	0.255450391	0.270935277	0.311984475	0.348453002	0.878743405
LOC112449516	0.269022522	0.189302295	0.663680103	0.789299417	0.145336864	0.349375426	0.878743405
MBTPS2	0.324890296	0.379888296	0.376570818	0.161664635	0.512723456	0.34839209	0.878743405

MUC1	0.364518354	0.401295568	0.258531141	0.184610151	0.555611519	0.349447058	0.878743405
NDUFA4	0.693183402	0.772771473	0.577815029	0.015574433	0.796278247	0.34783737	0.878743405
PLOD2	0.919740922	0.306402443	0.242751175	0.085171283	0.666188903	0.349548288	0.878743405
RAPGEF6	0.468435143	0.738757305	0.946912355	0.016624829	0.706953583	0.34834677	0.878743405
RBM20	0.12113187	0.725140014	0.201817627	0.358661798	0.604709783	0.348086492	0.878743405
RELB	0.27171024	0.805259529	0.045301504	0.693558408	0.564161869	0.349417997	0.878743405
RGMA	0.572419398	0.3956343	0.29714679	0.119572962	0.48219548	0.349486667	0.878743405
SCN2A	0.614664992	0.343035826	0.044207094	0.602101117	0.688015373	0.34874517	0.878743405
SERPINA5	0.260266533	0.259245204	0.505195883	0.603075302	0.188317101	0.349138305	0.878743405
SLC35A4	0.256232571	0.487931218	0.98696546	0.19991467	0.156810801	0.34902004	0.878743405
SPAG9	0.497346016	0.272503507	0.177650953	0.235133492	0.680231219	0.348332726	0.878743405
TAF1D	0.871877195	0.060304516	0.386228169	0.329549038	0.574072196	0.347968027	0.878743405
TCIM	0.231493171	0.79994735	0.098798799	0.551664999	0.384260635	0.349421996	0.878743405
TMEM252	0.357934222	0.049056056	0.538470117	0.613963746	0.665371873	0.34879007	0.878743405
UQCRB	0.647595384	0.379031037	0.356451893	0.047877914	0.926489196	0.349528963	0.878743405
ZNF280B	0.332178116	0.58310041	0.724215312	0.306608867	0.090168801	0.349411562	0.878743405
ZNF622	0.751964391	0.394613	0.515546782	0.056110878	0.448323698	0.348229032	0.878743405
AGGF1	0.732177686	0.668767085	0.022721059	0.551086814	0.634065392	0.349783333	0.879064974
ERO1B	0.083593851	0.50613037	0.559327394	0.408931293	0.401707863	0.349779612	0.879064974
GPR34	0.214962562	0.847549433	0.422331395	0.284697692	0.177620035	0.349918924	0.879271085
ACTR1A	0.457712345	0.140784756	0.585181016	0.363353756	0.284933353	0.350433924	0.879304241
CDC7	0.737680443	0.397435476	0.253005529	0.077459701	0.680759708	0.350725632	0.879304241
DCAF4	0.926348901	0.691481279	0.183028732	0.124731293	0.268012796	0.351034349	0.879304241
EIF1B	0.53729649	0.560805391	0.281895756	0.056632526	0.810171841	0.350166987	0.879304241
FAM214B	0.519000519	0.408727569	0.116744652	0.471345013	0.334615843	0.350509289	0.879304241
FAM76A	0.375460494	0.328176235	0.079054491	0.818284894	0.490398013	0.350625178	0.879304241
GALE	0.17302838	0.66076293	0.872106552	0.207277429	0.189232594	0.35070647	0.879304241
GORASP2	0.505701478	0.066559856	0.994004683	0.515359482	0.226205558	0.350291214	0.879304241
ISM1	0.681116167	0.308434861	0.500443075	0.16181376	0.231173547	0.351562239	0.879304241
KIF26B	0.50093455	0.435160248	0.099551859	0.493201933	0.365514417	0.350751965	0.879304241
LOC100297152	0.70770018	0.410994984	0.300978659	0.268844527	0.166813842	0.351300266	0.879304241
LOC101904698	0.452249183	0.162440263	0.702845959	0.213686769	0.356509082	0.351593104	0.879304241
LOC101904769	0.038590493	0.473457686	0.785249458	0.401437971	0.682756561	0.351548237	0.879304241
LOC504548	0.037638788	0.872855704	0.685805886	0.199901779	0.872335347	0.351415464	0.879304241
LOC521224	0.078212644	0.457122761	0.168318338	0.715591897	0.906962127	0.350499253	0.879304241
LYSMD3	0.313706837	0.539722478	0.530396488	0.046416172	0.942524255	0.351407501	0.879304241
MFSD2B	0.505376489	0.327743541	0.253174169	0.438240864	0.21303036	0.350863316	0.879304241
PARL	0.8322925	0.291837522	0.109270757	0.759620677	0.194423845	0.351056383	0.879304241
PSMD7	0.940061523	0.130787091	0.21402411	0.435334783	0.341867974	0.350913916	0.879304241
RHBDD3	0.213153601	0.748726075	0.657761986	0.061176667	0.610543382	0.351098727	0.879304241
TANGO6	0.210317661	0.339995658	0.530177516	0.272733312	0.376651468	0.350057388	0.879304241
TMEM121B	0.248533068	0.347538024	0.426531755	0.141756757	0.750243294	0.350991267	0.879304241
TMX2	0.444410349	0.441405352	0.347529733	0.16106848	0.357444503	0.351257009	0.879304241
TTC9C	0.721710263	0.378041393	0.65647983	0.024440137	0.898242471	0.351536652	0.879304241

TXLNG	0.433099687	0.526370862	0.109064037	0.434899122	0.363095626	0.351305948	0.879304241
WDR20	0.985017971	0.630379077	0.744326741	0.009651127	0.881657574	0.351560332	0.879304241
XIRP1	0.184483882	0.688327929	0.517791112	0.136359	0.438099853	0.35137497	0.879304241
ZBTB44	0.334388255	0.501959046	0.225656556	0.154376431	0.668029414	0.35051627	0.879304241
ZC3H14	0.602792067	0.420586318	0.157134228	0.14204489	0.694092083	0.351364623	0.879304241
ZNF10	0.691273198	0.286223968	0.688378662	0.090056723	0.318532136	0.350554778	0.879304241
ZNF266	0.477463786	0.223191874	0.102412365	0.610102035	0.584827752	0.350040885	0.879304241
DOC2A	0.838152221	0.034194655	0.911336278	0.539454891	0.279664712	0.351867514	0.879722392
EGFLAM	0.663608486	0.469333497	0.420010945	0.031550571	0.954616393	0.351845356	0.879722392
DESI1	0.280537196	0.680965904	0.279745502	0.266134349	0.27736929	0.352040048	0.880019685
PSMD2	0.708088376	0.727105678	0.255685301	0.743977068	0.040311415	0.352160911	0.880187741
LOC783730	0.204803363	0.493101034	0.707607603	0.069126201	0.799579055	0.352228878	0.88022356
COQ9	0.930533794	0.94521981	0.499784759	0.011892482	0.75642014	0.352411595	0.880427087
KMT2C	0.57796243	0.355484451	0.518529845	0.234358393	0.158444401	0.352471267	0.880427087
RAVER2	0.82207221	0.85993516	0.224566072	0.029459154	0.84581101	0.352457768	0.880427087
LOC100336976	0.65126244	0.722179934	0.30282874	0.060344867	0.46108837	0.35274573	0.880710509
PDCL	0.427714299	0.155621959	0.583809045	0.160375463	0.635489282	0.352645617	0.880710509
SYCE2	0.502067821	0.510193588	0.931301227	0.081398387	0.204064351	0.352727037	0.880710509
LOC107131715	0.908561103	0.324986436	0.16564558	0.130054859	0.624391948	0.353087392	0.881429447
NEMF	0.56854992	0.158508201	0.086861229	0.872561727	0.581893756	0.353195288	0.881430638
NKAP	0.330854749	0.060219782	0.330365556	0.919962096	0.656251622	0.353168752	0.881430638
HAUS8	0.925932216	0.261375406	0.299066964	0.088530301	0.620565803	0.35326863	0.881467327
NMB	0.62619183	0.722707531	0.046223902	0.976376165	0.194749063	0.353317414	0.881467327
CWC22	0.117802914	0.897561864	0.795097934	0.100773794	0.469694767	0.35337983	0.881489038
GTSF1	0.768576002	0.252159179	0.089342624	0.294605325	0.780741925	0.353509498	0.881544502
JAKMIP3	0.083034204	0.263636635	0.809065342	0.703960859	0.319323658	0.353458768	0.881544502
ARG2	0.331584963	0.355883298	0.09032865	0.599058038	0.625667237	0.353998444	0.881623699
FAM161A	0.653800187	0.535115114	0.021890359	0.891961521	0.585158377	0.354078472	0.881623699
FBH1	0.6987494	0.047516477	0.572642612	0.694674211	0.302197158	0.353848931	0.881623699
LOC101905706	0.496573204	0.776825865	0.025828828	0.950298494	0.422137969	0.354064717	0.881623699
MRPL13	0.640729962	0.405351171	0.500163076	0.059749925	0.514105397	0.353808552	0.881623699
NODAL	0.42804081	0.722574202	0.1431819	0.112145395	0.803605224	0.353834333	0.881623699
OSTC	0.147697804	0.623219905	0.269934999	0.450715295	0.356856246	0.354044757	0.881623699
PRMT3	0.625321052	0.472794032	0.614569359	0.94796994	0.023163323	0.353784938	0.881623699
TET2	0.841600862	0.555740482	0.027520687	0.891589913	0.347524691	0.35373038	0.881623699
WDR83	0.033759437	0.901971846	0.597770602	0.786021096	0.279151734	0.353947453	0.881623699
CDK20	0.620718836	0.515247466	0.74495506	0.05738915	0.29414766	0.355031925	0.881883762
DCUN1D4	0.46772554	0.119516252	0.171458031	0.714093889	0.587556647	0.355012238	0.881883762
DPCD	0.510395993	0.341398932	0.268975497	0.170646012	0.500622105	0.354336579	0.881883762
KCNK13	0.53199703	0.204235089	0.7800051	0.154721582	0.30597619	0.354653215	0.881883762
KRT7	0.2449607	0.32269366	0.370619293	0.200941841	0.682538631	0.354880638	0.881883762
LOC100336734	0.871689222	0.063819887	0.373961346	0.383436632	0.503487453	0.354814286	0.881883762
LOC101904378	0.043765285	0.683521088	0.332923638	0.446246015	0.904540327	0.354957823	0.881883762
LOC104969409	0.030533675	0.909641946	0.953102932	0.309705255	0.490161493	0.354903722	0.881883762

LOC112443469	0.775481066	0.988558612	0.301177904	0.08399459	0.207310037	0.35497203	0.881883762
LOC615454	0.146831584	0.485906066	0.300766392	0.434821385	0.430952358	0.354998066	0.881883762
MINDY3	0.899085191	0.194963502	0.16559676	0.399011386	0.346651645	0.354763537	0.881883762
NTN1	0.680725206	0.180416268	0.655771645	0.051015457	0.974270913	0.354298539	0.881883762
PMPCB	0.748258753	0.555107615	0.132809771	0.151167868	0.480927735	0.35458996	0.881883762
SNX12	0.428592428	0.125744067	0.212154582	0.579562389	0.606990166	0.355042716	0.881883762
STS	0.867608395	0.935496318	0.607632013	0.07432345	0.109696232	0.354991916	0.881883762
ZWILCH	0.919147628	0.395371414	0.712568843	0.399827325	0.038705037	0.354468255	0.881883762
LOC614208	0.150781887	0.314714734	0.523430823	0.289894261	0.559235863	0.355219182	0.881921633
PID1	0.072144707	0.692180217	0.465071668	0.290296493	0.597220781	0.355204912	0.881921633
RASIP1	0.126982408	0.910881937	0.655587226	0.319866966	0.165911282	0.355119589	0.881921633
MTFR2	0.133511276	0.347243458	0.235536731	0.50540975	0.729973071	0.355290991	0.881949054
PSEN2	0.32357858	0.862105825	0.249289377	0.062489674	0.92734099	0.355337709	0.881949054
PHAX	0.72087295	0.104723093	0.098831574	0.72253922	0.747939554	0.355420989	0.882022357
CDKN1C	0.842087558	0.631048058	0.145301929	0.057727773	0.906390037	0.355730698	0.882657466
YBX2	0.418431317	0.41680191	0.324178444	0.595047725	0.12018028	0.355849465	0.882818681
SERAC1	0.492324526	0.729022458	0.030531008	0.881013832	0.419073864	0.355951	0.8829371
ADORA1	0.266312837	0.641522132	0.344000841	0.130820802	0.526526373	0.356041658	0.882939388
DNAJC1	0.335904353	0.382983577	0.879192825	0.066233396	0.540742596	0.356144495	0.882939388
INPP4B	0.55731402	0.617997459	0.726925208	0.29206534	0.055405688	0.356167129	0.882939388
LOC101903615	0.655228103	0.64476744	0.032058942	0.416343693	0.718252679	0.356118194	0.882939388
ADD1	0.743092992	0.418420762	0.168819693	0.336286971	0.231151674	0.357269143	0.88294185
APLF	0.067982949	0.392008037	0.99380547	0.650227038	0.236152504	0.356755584	0.88294185
B3GAT3	0.278207716	0.929891478	0.762899622	0.045748851	0.449982841	0.356609073	0.88294185
CASP8AP2	0.985703526	0.481113246	0.377758184	0.143802671	0.157953748	0.356845333	0.88294185
CCL25	0.239168999	0.831638597	0.414731463	0.109426209	0.449156451	0.356279797	0.88294185
CNNM3	0.569608256	0.54056909	0.392075807	0.230371252	0.146391046	0.356929888	0.88294185
DYNLT3	0.481888846	0.247715352	0.205196067	0.653667894	0.253858304	0.356670824	0.88294185
GFM2	0.605373567	0.657515648	0.971860277	0.088742634	0.118856215	0.357269504	0.88294185
GPR20	0.400284164	0.613103369	0.33132544	0.093353604	0.537118105	0.357151235	0.88294185
LOC100848484	0.959124024	0.53164617	0.737470289	0.014911366	0.727792423	0.35729796	0.88294185
LOC101906632	0.368514898	0.452551853	0.065560636	0.570517477	0.653183733	0.357048371	0.88294185
LOC786987	0.462606188	0.765823368	0.239703009	0.16614163	0.288404109	0.35684141	0.88294185
MAPK1	0.765672894	0.277292131	0.139888281	0.521275523	0.262761299	0.356804656	0.88294185
PARP3	0.7890404	0.020879702	0.555282843	0.635220147	0.699440146	0.356669125	0.88294185
PHF8	0.495822037	0.597416429	0.406913062	0.063431999	0.533721956	0.357284026	0.88294185
PHLDB3	0.816862087	0.354519446	0.02356076	0.841897443	0.70827847	0.35682273	0.88294185
PLCE1	0.139899896	0.726055239	0.170963764	0.964699706	0.243145505	0.35700487	0.88294185
PPIC	0.988046066	0.299763121	0.03212963	0.478689116	0.89316744	0.356824966	0.88294185
PSIP1	0.430099523	0.610135793	0.076557589	0.406691832	0.498687727	0.357050873	0.88294185
SPG21	0.916978753	0.259158214	0.032978439	0.838126535	0.621135454	0.357256114	0.88294185
TGM3	0.926406307	0.98608572	0.206213828	0.059301377	0.364040627	0.356753668	0.88294185
CNPPD1	0.272225254	0.993530859	0.428238117	0.137686136	0.256290363	0.357531361	0.883385601
LOC512672	0.469244395	0.572135508	0.292199126	0.209359034	0.249033539	0.357642193	0.883393443

SOCS7	0.24980533	0.742338613	0.914480497	0.031643596	0.762096818	0.357623436	0.883393443
PAAF1	0.778348332	0.282928501	0.363812768	0.078603083	0.650384978	0.357861955	0.883803243
C28H10orf71	0.312703175	0.80259278	0.048070054	0.372724169	0.912029558	0.358062997	0.884166692
DUSP19	0.513933767	0.528317149	0.181289853	0.380810129	0.21922395	0.358376683	0.884578392
LOC112449258	0.781915533	0.259502503	0.636944434	0.582936404	0.054604767	0.358550199	0.884578392
MAPK8	0.080957681	0.903138634	0.702019444	0.38360959	0.208779072	0.358436404	0.884578392
MMP14	0.641703141	0.349104614	0.388817985	0.053756304	0.879172687	0.358652653	0.884578392
MTMR9	0.667104882	0.205815627	0.282092977	0.207690859	0.510951717	0.358409072	0.884578392
RADIL	0.773570813	0.313707448	0.22602735	0.231464948	0.32425867	0.358660936	0.884578392
SLC19A1	0.234686692	0.488076215	0.34732208	0.719038009	0.143892877	0.358638258	0.884578392
TCEAL8	0.568478326	0.434437805	0.138853455	0.875939306	0.136890455	0.358474984	0.884578392
BIRC3	0.667423258	0.600253025	0.132491087	0.310508813	0.250026165	0.358811594	0.884626845
DUSP28	0.516307211	0.739388853	0.156299859	0.078135718	0.883684553	0.358777361	0.884626845
INTS6L	0.72516364	0.587212031	0.116669607	0.61032743	0.135930068	0.358842295	0.884626845
ABLIM2	0.799159359	0.073478108	0.622004631	0.763481024	0.149267341	0.360383833	0.884628654
AP3B1	0.476466072	0.192925473	0.709563248	0.168599759	0.381103167	0.361453628	0.884628654
ARHGAP10	0.734019535	0.140835978	0.117047118	0.438176998	0.781950035	0.359757674	0.884628654
C7H19orf44	0.79467536	0.230201754	0.619251355	0.169547315	0.216517058	0.36023975	0.884628654
CCDC6	0.929274201	0.220086269	0.138814419	0.3112136	0.473710506	0.361248051	0.884628654
CCDC88B	0.555324456	0.348078624	0.604886909	0.138056568	0.258336247	0.360669748	0.884628654
CDH24	0.669836814	0.075922565	0.159383908	0.808825845	0.635934352	0.360637606	0.884628654
COA7	0.871759598	0.54574933	0.169487815	0.357282125	0.145652301	0.3616505	0.884628654
CTSA	0.15821434	0.857480188	0.35889521	0.604977774	0.140630318	0.359629982	0.884628654
DCTN5	0.521271219	0.360531117	0.459219858	0.134538372	0.359356825	0.360762999	0.884628654
DDIAS	0.877678489	0.096940499	0.311716782	0.245315063	0.639327539	0.360275849	0.884628654
DDX31	0.803620925	0.103852351	0.288834747	0.961280103	0.178916199	0.359759867	0.884628654
DUS1L	0.66806817	0.586626987	0.453217191	0.098158119	0.240959114	0.361831828	0.884628654
EAF1	0.773979339	0.300294318	0.283134874	0.07556946	0.837310257	0.360439567	0.884628654
EHD1	0.575365605	0.49502806	0.219137957	0.227504969	0.295271819	0.361523002	0.884628654
ERAS	0.934608211	0.318639056	0.740925293	0.53789697	0.035112979	0.360572005	0.884628654
FAM50A	0.108605743	0.329534479	0.908683901	0.347973893	0.364951913	0.359159446	0.884628654
FITM2	0.998677308	0.454593198	0.133371628	0.183285398	0.376458441	0.360964097	0.884628654
FKBP11	0.839810276	0.549620376	0.103467104	0.672092153	0.128749081	0.359256773	0.884628654
FZD10	0.92464091	0.270334716	0.149679936	0.159403249	0.701067471	0.361086477	0.884628654
GINM1	0.36888911	0.1806528	0.291836295	0.286184571	0.75343577	0.361547598	0.884628654
GTF3C3	0.090442491	0.761778682	0.735102412	0.283198967	0.291301095	0.360973657	0.884628654
HPGD	0.889667724	0.303940822	0.128159615	0.125986488	0.947452377	0.359411597	0.884628654
IGFALS	0.71387777	0.178122315	0.958068998	0.058002846	0.588173501	0.360147961	0.884628654
JAGN1	0.76445825	0.160420599	0.077508128	0.435004538	0.997619045	0.358969093	0.884628654
KLF5	0.221418842	0.578275719	0.311745847	0.114085972	0.917875451	0.361039805	0.884628654
LAPTM4B	0.517742127	0.504366924	0.03930841	0.564783935	0.721466191	0.361140052	0.884628654
LOC101903820	0.143034003	0.526561554	0.220535908	0.50705145	0.498909302	0.361861688	0.884628654
LOC101905593	0.535231291	0.395769487	0.789669984	0.178578301	0.138972535	0.359965384	0.884628654
LOC101905770	0.891579618	0.318425091	0.128479199	0.837901962	0.137260717	0.36160809	0.884628654

LOC101909754	0.265652279	0.357131973	0.097377598	0.943385142	0.478477084	0.360673628	0.884628654
LOC104976344	0.23513387	0.641379385	0.396994747	0.10870638	0.639242527	0.36030726	0.884628654
LOC112442392	0.521454901	0.316253175	0.298706184	0.376957985	0.226036953	0.361690678	0.884628654
LOC112442952	0.200545945	0.600914911	0.973692077	0.336888222	0.10519821	0.360237695	0.884628654
LOC112443499	0.820388042	0.314323096	0.131544509	0.158052106	0.782284758	0.361569713	0.884628654
LOC505600	0.356623897	0.51315507	0.330025619	0.332850375	0.208036059	0.361122178	0.884628654
LOC789867	0.63941834	0.787746939	0.296684906	0.141995801	0.196491846	0.360650462	0.884628654
LYZ2	0.138979927	0.533610199	0.800144991	0.505478708	0.139051814	0.36069993	0.884628654
MAML3	0.583465867	0.219057774	0.499250428	0.144121363	0.453816352	0.360799747	0.884628654
NDUFA10	0.840543147	0.85474687	0.533165556	0.018338384	0.592973843	0.360494975	0.884628654
PRKCZ	0.476396451	0.433022467	0.075169516	0.594560232	0.454503946	0.361432691	0.884628654
RARS	0.50581778	0.60570104	0.989293352	0.073374706	0.188766477	0.361719769	0.884628654
RDH11	0.334635576	0.156246808	0.355727743	0.544695051	0.408736082	0.359572882	0.884628654
SLC25A16	0.41620041	0.291667941	0.267277556	0.447222132	0.289232503	0.361674713	0.884628654
SNX15	0.54897197	0.185530433	0.778786373	0.073262915	0.72284105	0.36181444	0.884628654
STAT2	0.949988104	0.243298652	0.675942703	0.133891341	0.19996682	0.361152432	0.884628654
TADA1	0.6506673	0.537166439	0.749194138	0.363201872	0.043854895	0.360701358	0.884628654
TMEFF2	0.707077197	0.205271142	0.55420532	0.061473206	0.840834784	0.360208595	0.884628654
TMEM204	0.90705033	0.210643745	0.29855859	0.342837606	0.214288478	0.361448073	0.884628654
TNFSF15	0.287146989	0.22063118	0.136899235	0.657346274	0.73488182	0.361407285	0.884628654
TRPM2	0.955924147	0.336067993	0.974870938	0.085585071	0.154466644	0.359548768	0.884628654
UBD	0.215848783	0.439997693	0.559952907	0.231309677	0.341437955	0.361795077	0.884628654
WDR92	0.786170158	0.48864374	0.37642332	0.596387882	0.048169594	0.360073529	0.884628654
ZBED8	0.483946402	0.188318659	0.184136001	0.4394051	0.565211399	0.360584822	0.884628654
ZNF34	0.57702628	0.433108024	0.779363735	0.748521768	0.028706208	0.361236958	0.884628654
ZNF354C	0.712185282	0.348186156	0.040765211	0.529999646	0.772082817	0.359405923	0.884628654
EFNA2	0.448369729	0.356819062	0.123590255	0.911158808	0.23345152	0.362012798	0.884866253
SYP	0.120879879	0.569942814	0.796236916	0.232338951	0.330197742	0.362108406	0.884968139
KCNA2	0.46903268	0.956917796	0.264606037	0.058215978	0.608973182	0.362178612	0.885007921
EPOP	0.23176533	0.512627322	0.694261573	0.431653597	0.118298061	0.362239557	0.885025067
GPC1	0.188715318	0.80405829	0.628167597	0.053462323	0.82701261	0.362327767	0.885108809
CSNK2A1	0.60587575	0.152394742	0.659483716	0.798720524	0.086855726	0.362697175	0.885294162
DNASE1L1	0.208830836	0.262926545	0.275906549	0.34421843	0.810017458	0.362685823	0.885294162
GDPD5	0.729194735	0.324680163	0.3462866	0.183376598	0.281128129	0.362781259	0.885294162
H3F3C	0.293014563	0.345464493	0.716839091	0.065999014	0.881627139	0.362619627	0.885294162
HSBP1	0.195776004	0.453936853	0.447636171	0.116743328	0.90866403	0.362539559	0.885294162
LOC104976321	0.409805867	0.580394775	0.645798588	0.037483347	0.733883438	0.362738186	0.885294162
RNMT	0.525723741	0.679840067	0.20376174	0.9488684	0.061154844	0.362759773	0.885294162
TCTA	0.347681958	0.943956416	0.401438148	0.037317766	0.86051144	0.362941258	0.885552926
BHLHE41	0.325646823	0.16756893	0.815732483	0.115851457	0.821212966	0.36309352	0.885574099
SAMD5	0.210540931	0.480309873	0.127724011	0.330688479	0.991420836	0.363079868	0.885574099
ZDBF2	0.100495376	0.720598317	0.678802155	0.169339986	0.508982456	0.363165784	0.885574099
ZNF362	0.675290975	0.260190478	0.318758425	0.081644866	0.926523428	0.363160145	0.885574099
ALDH4A1	0.773203518	0.669480054	0.517260807	0.038441014	0.411917528	0.363274947	0.885708684

TBCA	0.368039065	0.227709853	0.13899865	0.472118234	0.77133144	0.363359018	0.885782064
AKAP2	0.439729969	0.229031843	0.357278906	0.131856885	0.9019685	0.364739776	0.885989412
ARHGAP22	0.169736241	0.8425232	0.294218982	0.208996618	0.485194203	0.364267172	0.885989412
ATP5F1C	0.491709859	0.784846044	0.513339232	0.032743085	0.658249347	0.36438547	0.885989412
CIB1	0.242800183	0.418608754	0.16957347	0.255450192	0.971270966	0.364622949	0.885989412
IP6K1	0.2761761	0.876899442	0.67619286	0.03533441	0.737065222	0.364204829	0.885989412
KRCC1	0.188291541	0.499599806	0.158044665	0.6044769	0.474965694	0.364337181	0.885989412
LAMB2	0.747799805	0.299152736	0.134125045	0.561032975	0.253109699	0.364050653	0.885989412
LOC100296832	0.584498387	0.526364889	0.234890836	0.471658687	0.125529126	0.364712587	0.885989412
LOC112445150	0.91634884	0.968098198	0.036981154	0.822744664	0.157307549	0.363502992	0.885989412
LOC112449406	0.672420535	0.20802824	0.100456585	0.435563144	0.697060543	0.364260383	0.885989412
MICALL2	0.712969699	0.027978483	0.399807887	0.803580855	0.665290442	0.364160271	0.885989412
MRTO4	0.176452193	0.831358654	0.401548298	0.956382241	0.075864057	0.364535415	0.885989412
NT5DC1	0.761760815	0.663467568	0.111353072	0.251504351	0.302089953	0.364608585	0.885989412
PSMA6	0.977086809	0.391898861	0.096684249	0.586565897	0.195774827	0.363706467	0.885989412
RILPL1	0.297534641	0.216830302	0.533699781	0.167980281	0.736429674	0.363999397	0.885989412
SCAND1	0.176740593	0.713156029	0.399502935	0.270307081	0.313695642	0.36438506	0.885989412
SGCE	0.168076387	0.497517639	0.560571254	0.221739405	0.409536629	0.363904173	0.885989412
SPDL1	0.57528333	0.976782696	0.03756472	0.653953664	0.309431167	0.364444671	0.885989412
SRPK1	0.728448246	0.89628054	0.371629454	0.120438401	0.146343906	0.364634048	0.885989412
TRIM59	0.47742013	0.630221208	0.190497259	0.142142608	0.525189814	0.364717818	0.885989412
TRIM68	0.386888865	0.787142288	0.869297374	0.06089495	0.263680277	0.363681165	0.885989412
TSPO	0.78796196	0.487080715	0.504377185	0.023084851	0.952220595	0.363847815	0.885989412
USP50	0.305366362	0.092084323	0.593266604	0.460495401	0.556464995	0.364571407	0.885989412
YIF1B	0.201032866	0.812737712	0.783048785	0.07186371	0.463621298	0.364121021	0.885989412
AOX2	0.628752971	0.026502437	0.310346828	0.847203051	0.98259689	0.365682333	0.886202788
ARHGAP5	0.371362762	0.401553712	0.882634493	0.04948206	0.663247974	0.366217619	0.886202788
EIF3I	0.492959521	0.610962863	0.187107354	0.560283817	0.136235642	0.365551244	0.886202788
HCN2	0.162596831	0.270761458	0.585697859	0.416246609	0.402174145	0.366105034	0.886202788
HEATR5B	0.300357423	0.34764137	0.1553921	0.621358928	0.427073611	0.365707063	0.886202788
HPDL	0.492986759	0.936892362	0.340490496	0.619998032	0.043971697	0.365034094	0.886202788
KRT79	0.317969016	0.386793563	0.884419483	0.057704547	0.688260252	0.366231632	0.886202788
LOC104973485	0.719197842	0.125155574	0.427126336	0.17864843	0.628214386	0.36604117	0.886202788
LOC107131623	0.75950191	0.491274837	0.096010248	0.332272804	0.360848192	0.365323773	0.886202788
LOC112442265	0.031975196	0.511752304	0.695527072	0.788270286	0.478623278	0.365274946	0.886202788
LOC112446036	0.20513273	0.396197844	0.991606423	0.136246883	0.393113388	0.366102629	0.886202788
LOC112449115	0.9082438	0.581939391	0.368348276	0.214668977	0.102965685	0.365618504	0.886202788
LOC534181	0.985023419	0.977502092	0.052638406	0.577022752	0.147302399	0.365789193	0.886202788
LSM10	0.629293909	0.095414747	0.228198808	0.7282558	0.43068496	0.365409505	0.886202788
NDUFAF3	0.949086379	0.721501864	0.585266851	0.012334046	0.873143111	0.366087085	0.886202788
NFATC4	0.241047205	0.074392961	0.30997159	0.856576837	0.906130112	0.366023229	0.886202788
OAZ2	0.730105292	0.284975201	0.221510632	0.357165544	0.260631938	0.365139813	0.886202788
PPP1R3F	0.599547289	0.484007529	0.789056882	0.030164136	0.623459705	0.365721292	0.886202788
RHBDD1	0.384542493	0.272401042	0.05294385	0.921683381	0.844459514	0.366102049	0.886202788

STK32C	0.384493488	0.682464149	0.020144216	0.975858578	0.831622247	0.365120858	0.886202788
TCHP	0.379540228	0.91602822	0.551025743	0.306330416	0.072998717	0.364907053	0.886202788
TMEM269	0.097256621	0.378180974	0.778406539	0.378379242	0.398731907	0.366212895	0.886202788
TNFSF12	0.452634436	0.502520372	0.444628549	0.218805837	0.194398675	0.365563999	0.886202788
UQCC3	0.385952022	0.961978638	0.094500484	0.143529135	0.855791967	0.36585154	0.886202788
ZBTB49	0.367341997	0.124150939	0.176574078	0.570037672	0.939840683	0.36602054	0.886202788
ZNF576	0.692363897	0.427337915	0.876219593	0.057317992	0.288949867	0.365266306	0.886202788
LOC112449596	0.506461152	0.111837433	0.529828303	0.169924195	0.847573451	0.366310292	0.88626245
ADCY7	0.559385977	0.380863071	0.733362623	0.08736714	0.33501718	0.375305358	0.88626422
ADGRG5	0.065094137	0.531092156	0.333632286	0.514867037	0.76413508	0.374061138	0.88626422
AKAP5	0.313328103	0.485427094	0.240629772	0.31060427	0.391967372	0.371148135	0.88626422
ANK1	0.085449777	0.94228776	0.454223095	0.384126642	0.309913514	0.367468794	0.88626422
ANKRD45	0.632485638	0.749143397	0.113990721	0.140300724	0.592761276	0.372432646	0.88626422
ARF3	0.354139418	0.801179337	0.372280899	0.514069822	0.080120831	0.36734607	0.88626422
ARRB2	0.158677883	0.487356692	0.106650651	0.836119136	0.652149667	0.372623052	0.88626422
ARRDC1	0.217711804	0.368828508	0.331720854	0.226183204	0.753230014	0.374069306	0.88626422
ATMIN	0.22259518	0.124537344	0.526187838	0.418524654	0.748900865	0.375263638	0.88626422
ATN1	0.713020332	0.620171518	0.096779476	0.354215658	0.287056923	0.367379077	0.88626422
ATP6AP2	0.510938153	0.265603264	0.061401871	0.669431543	0.782481117	0.367864788	0.88626422
ATXN7L3	0.393875033	0.594945584	0.1639217	0.296785273	0.400303344	0.374968814	0.88626422
BHLHE22	0.711579273	0.578300885	0.064067626	0.610661052	0.28225554	0.374287472	0.88626422
BRIP1	0.582908166	0.092658065	0.670533469	0.99173476	0.127492268	0.375515649	0.88626422
BTRC	0.848051027	0.75054289	0.059390572	0.616330856	0.190320581	0.370371936	0.88626422
C1QTNF2	0.732440349	0.401881682	0.02019607	0.902722511	0.826454043	0.37040701	0.88626422
C22H3orf62	0.416979697	0.493417483	0.083024796	0.940344053	0.27451152	0.369481496	0.88626422
C24H18orf32	0.768576709	0.090652082	0.364621149	0.371825419	0.459373051	0.3669336	0.88626422
C29H11orf68	0.710615792	0.276051927	0.702474276	0.056776516	0.576043081	0.372968188	0.88626422
CBLN2	0.056578364	0.567989514	0.899378748	0.316578104	0.494296016	0.373528757	0.88626422
CDC37	0.256916924	0.73631225	0.155024468	0.464921577	0.319125929	0.367365943	0.88626422
CDK18	0.367875908	0.617562829	0.886440356	0.046915395	0.481509433	0.374469701	0.88626422
CDRT4	0.358830333	0.657220013	0.139145195	0.184717407	0.72388783	0.368699384	0.88626422
CGGBP1	0.267238292	0.902880221	0.560763377	0.862755539	0.038914357	0.374231234	0.88626422
CHMP2A	0.348291201	0.649099337	0.40722802	0.057812193	0.822385787	0.368311562	0.88626422
CLEC18C	0.597347299	0.256095278	0.257938217	0.20297855	0.551324324	0.369707405	0.88626422
CLK2	0.793014466	0.22390163	0.304374837	0.154176327	0.534521261	0.371074608	0.88626422
CNTNAP2	0.906493972	0.128482401	0.163587929	0.448974246	0.532752031	0.374748165	0.88626422
COPS3	0.762124067	0.33540533	0.299686269	0.353500342	0.163136587	0.369781482	0.88626422
COX7A2L	0.536230282	0.462635094	0.367984925	0.156605543	0.308315215	0.369421965	0.88626422
CPSF7	0.766168236	0.565134361	0.884066057	0.052474228	0.222232195	0.371435894	0.88626422
CRYZL1	0.560392643	0.441344602	0.032709559	0.564114296	0.984777827	0.372515596	0.88626422
CXCL2	0.85790129	0.5888327	0.15603413	0.270175883	0.209665661	0.37147598	0.88626422
DACT2	0.844248661	0.838202829	0.206505976	0.871934988	0.035844409	0.37509959	0.88626422
DEF6	0.586307838	0.666035431	0.903784954	0.137272305	0.093132606	0.373149785	0.88626422
DLL4	0.094834153	0.217907888	0.276153228	0.905755141	0.837468382	0.366552929	0.88626422

DNHD1	0.900748093	0.257704353	0.518976859	0.709991958	0.053007649	0.373920973	0.88626422
EIF1AX	0.463426072	0.366128985	0.260736239	0.257400334	0.382645374	0.367593946	0.88626422
EMILIN3	0.049560488	0.426660484	0.452806022	0.796840343	0.579074405	0.369793273	0.88626422
ENC1	0.454965349	0.405853302	0.584213881	0.058407574	0.703629756	0.370341857	0.88626422
EPN3	0.527308537	0.450884558	0.104547331	0.37503178	0.482753246	0.372731307	0.88626422
ERCC3	0.584985224	0.097642535	0.500718221	0.175996713	0.883936343	0.370917902	0.88626422
ERCC4	0.799108137	0.082782316	0.834370157	0.195878678	0.41665485	0.372889286	0.88626422
ESCO1	0.505374371	0.325973448	0.562832049	0.083762833	0.577177699	0.372105381	0.88626422
EXOC6	0.113959157	0.189978559	0.962062238	0.330223615	0.649645325	0.371593068	0.88626422
FADD	0.320967299	0.625125202	0.945374814	0.228955861	0.09987345	0.366868772	0.88626422
FAM107B	0.780121683	0.766034383	0.745112399	0.082768527	0.121826794	0.372364796	0.88626422
FAM161B	0.390190002	0.083407106	0.744349952	0.950498178	0.19059866	0.368728729	0.88626422
FCER2	0.346853727	0.834017843	0.045249468	0.635806446	0.539095126	0.372248949	0.88626422
GLOD4	0.584725434	0.476267084	0.124061375	0.281233753	0.464029576	0.373031708	0.88626422
GNG2	0.340300859	0.327233054	0.224276397	0.654479006	0.278036791	0.374303449	0.88626422
GNPNAT1	0.942036185	0.238936988	0.09642109	0.911309403	0.231159311	0.375263162	0.88626422
GON7	0.220388007	0.484075035	0.060740634	0.824584084	0.829229115	0.370252692	0.88626422
GPATCH4	0.225551884	0.646631672	0.318478441	0.736796046	0.127604357	0.367949836	0.88626422
GPD1L	0.946111671	0.160960968	0.535425667	0.08267046	0.676757214	0.374910333	0.88626422
GPSM1	0.509065843	0.295546877	0.357231522	0.134625639	0.611102373	0.369923699	0.88626422
GZF1	0.439645859	0.675675558	0.712035299	0.02736066	0.791501747	0.375566665	0.88626422
HDHD2	0.38711335	0.502834373	0.29946917	0.151673412	0.508225913	0.372490276	0.88626422
HINT2	0.362250456	0.932518134	0.116954796	0.149772112	0.753173725	0.371177625	0.88626422
HMCN1	0.269474266	0.793291441	0.050000536	0.45021218	0.925526768	0.371075033	0.88626422
HMGCL	0.977581239	0.651560391	0.039306399	0.203075702	0.890784124	0.373749918	0.88626422
HOOK3	0.620870003	0.247333681	0.583507847	0.053069133	0.92835284	0.369665003	0.88626422
HSPA9	0.917368463	0.805581606	0.290291026	0.164466226	0.126926882	0.371951736	0.88626422
HTRA3	0.902805306	0.338882829	0.033829216	0.787429241	0.560663776	0.375170534	0.88626422
IFT57	0.27832898	0.363022336	0.301333838	0.94289454	0.152778767	0.368633038	0.88626422
IL2RG	0.507217003	0.677954988	0.209980336	0.067326444	0.90382377	0.368917182	0.88626422
IPO4	0.217703817	0.604424213	0.399845789	0.750074855	0.111956002	0.369799331	0.88626422
IRS2	0.440151239	0.224174955	0.284664604	0.345559103	0.446118612	0.366599607	0.88626422
KLF9	0.655825899	0.44744522	0.025864426	0.886169233	0.653522377	0.368977797	0.88626422
LMAN2L	0.162482878	0.484035845	0.26263287	0.240077961	0.87531797	0.366984974	0.88626422
LOC100295848	0.418149875	0.92156317	0.083540577	0.145929913	0.960984684	0.373239126	0.88626422
LOC100298923	0.959597875	0.28796672	0.446409687	0.173598659	0.213232746	0.37506552	0.88626422
LOC100299712	0.275085148	0.574556185	0.080737366	0.818360842	0.433568077	0.373703881	0.88626422
LOC100335642	0.777756038	0.521967553	0.064238642	0.266118602	0.647722048	0.372551243	0.88626422
LOC101904442	0.715846421	0.344639816	0.116460965	0.605915177	0.2622048	0.375011097	0.88626422
LOC101906230	0.51392349	0.699939506	0.609787808	0.095410508	0.213472533	0.371568999	0.88626422
LOC104969611	0.251240771	0.725385592	0.160892188	0.405245231	0.36852459	0.36838174	0.88626422
LOC104971464	0.25651008	0.943227333	0.500823615	0.189165727	0.195531511	0.372079516	0.88626422
LOC104973058	0.850381715	0.372001711	0.973784233	0.77354906	0.018364745	0.368277934	0.88626422
LOC107131530	0.469452668	0.506452537	0.945685388	0.870160332	0.022288528	0.367716737	0.88626422

LOC107132487	0.778799044	0.435224155	0.179406807	0.303465916	0.234657052	0.366608965	0.88626422
LOC112441566	0.968831194	0.913347943	0.382728235	0.02321969	0.563322527	0.370215645	0.88626422
LOC112441683	0.947213324	0.02397655	0.88107146	0.958101579	0.227530689	0.367767579	0.88626422
LOC112443193	0.752011997	0.521451994	0.793366711	0.020812133	0.686269209	0.370705543	0.88626422
LOC112444775	0.38143907	0.769674019	0.232091308	0.412127328	0.158554609	0.371027387	0.88626422
LOC112444778	0.628759029	0.442415354	0.529664926	0.548187136	0.05535327	0.371683585	0.88626422
LOC112446018	0.279468466	0.965021001	0.840642988	0.0369063	0.546928258	0.375415387	0.88626422
LOC112449280	0.473554086	0.561415036	0.076109694	0.455455284	0.494339546	0.37469585	0.88626422
LOC618289	0.475058142	0.295012227	0.647058857	0.523147286	0.091893107	0.36767277	0.88626422
LOC782293	0.278286732	0.460140327	0.193485526	0.211648372	0.867979215	0.374544769	0.88626422
LOC787287	0.343053683	0.726681087	0.146099867	0.177179454	0.706643048	0.374845247	0.88626422
LOC789587	0.071751994	0.334445889	0.279773374	0.987400801	0.65384667	0.366760128	0.88626422
MAP2	0.431105842	0.347305855	0.295569126	0.844439765	0.117804583	0.369225726	0.88626422
MBTD1	0.059799993	0.642592398	0.709304032	0.263720893	0.616788394	0.370348177	0.88626422
MDH2	0.861035367	0.917796581	0.94085464	0.010649068	0.558083295	0.369816973	0.88626422
MICU1	0.345583829	0.306678888	0.170308507	0.262880896	0.928449159	0.36933731	0.88626422
MMADHC	0.888083708	0.402676689	0.525434889	0.17538418	0.137712015	0.374078287	0.88626422
MON1A	0.301268291	0.623199584	0.442510221	0.232236685	0.235817988	0.374491645	0.88626422
MRS2	0.479342854	0.42219286	0.213796623	0.292089093	0.353422097	0.371528988	0.88626422
NCBP1	0.091501809	0.70197225	0.851262814	0.121648805	0.681941841	0.373995996	0.88626422
NCOA1	0.339334099	0.957016237	0.453331555	0.048757084	0.604158262	0.366838719	0.88626422
NCOA2	0.385195059	0.804176726	0.790279922	0.230905655	0.077179642	0.367787049	0.88626422
NDUFA6	0.480055543	0.806882989	0.570398225	0.026975507	0.758514787	0.373459677	0.88626422
NEURL1	0.071431252	0.989934802	0.688583225	0.828959837	0.112704456	0.374459984	0.88626422
NEURL4	0.976021954	0.031605182	0.38243314	0.576736042	0.655310326	0.371246338	0.88626422
NSFL1C	0.269401967	0.814254502	0.36477016	0.306415046	0.18109552	0.370585888	0.88626422
NSMF	0.619340854	0.645580446	0.960249033	0.052179832	0.217835472	0.367839911	0.88626422
NUP155	0.136569369	0.991090086	0.681582885	0.095337981	0.500062658	0.369075515	0.88626422
OCEL1	0.330446585	0.585152847	0.405457726	0.269382376	0.21238486	0.372206056	0.88626422
PDP2	0.596860513	0.264471052	0.946140206	0.111681851	0.262754346	0.368513556	0.88626422
PHACTR2	0.859378659	0.629890442	0.458995238	0.031475763	0.572651569	0.371954381	0.88626422
PLPP1	0.689427524	0.253201152	0.11471934	0.260137216	0.878222236	0.375373171	0.88626422
POLH	0.36008072	0.212977997	0.592171245	0.400644438	0.238978106	0.367257356	0.88626422
PPP1R9B	0.773575468	0.289318185	0.138093227	0.284389536	0.521193304	0.375582222	0.88626422
PRELID2	0.643534005	0.581005112	0.086473392	0.154544034	0.893228725	0.371412115	0.88626422
PRKCA	0.507089257	0.996132257	0.178882827	0.498559048	0.101503284	0.375287745	0.88626422
PSMB5	0.539038908	0.62604006	0.496761019	0.082820122	0.317781846	0.369573074	0.88626422
PSMC3	0.137308996	0.482155008	0.48253521	0.282157333	0.497976642	0.372318646	0.88626422
REEP5	0.17348405	0.612629013	0.091760815	0.921208529	0.495136067	0.370879001	0.88626422
RGS3	0.860132691	0.088889217	0.375636226	0.250376687	0.620584778	0.371385718	0.88626422
RNF123	0.796533287	0.243086037	0.862457372	0.079350359	0.3397663	0.372802869	0.88626422
RNF41	0.305051991	0.281861802	0.897138428	0.059205627	0.97344101	0.370785526	0.88626422
RPUSD2	0.408175804	0.446534428	0.610988328	0.110517388	0.354055822	0.367599761	0.88626422
S1PR3	0.43470136	0.480848795	0.317338249	0.071397077	0.914702321	0.366667752	0.88626422

SAR1A	0.603436129	0.064867488	0.819934257	0.32776687	0.42934911	0.373311966	0.88626422
SETD9	0.275222187	0.24891773	0.301492192	0.260743125	0.842400538	0.374024906	0.88626422
SHMT2	0.280105631	0.977510274	0.726423317	0.188221724	0.122176924	0.375333996	0.88626422
SIK1	0.550021229	0.432655214	0.129417389	0.233135681	0.61188378	0.368898523	0.88626422
SIX1	0.813975724	0.437176371	0.047619563	0.761813789	0.342373513	0.369854328	0.88626422
SLC16A11	0.437497756	0.948511844	0.028835741	0.551827569	0.670556729	0.370142411	0.88626422
SLC25A19	0.616368964	0.771165202	0.445014365	0.149261162	0.14342276	0.373722592	0.88626422
SLC7A1	0.49291159	0.197006853	0.727479689	0.097804942	0.634265251	0.368500379	0.88626422
SLCO3A1	0.522079364	0.273826646	0.237578064	0.213869171	0.604972324	0.368938965	0.88626422
SLITRK4	0.706377133	0.651238829	0.083492533	0.149606006	0.769495261	0.369919184	0.88626422
SMAD5	0.847451827	0.659330653	0.185636542	0.086452774	0.506686529	0.374265911	0.88626422
SMCO4	0.235614422	0.242897115	0.251156863	0.44261361	0.701337243	0.371365124	0.88626422
SMIM20	0.893768419	0.48196472	0.375315769	0.079233484	0.346782856	0.370661243	0.88626422
SMNDC1	0.49943812	0.599487593	0.665402001	0.028720925	0.766630783	0.368656756	0.88626422
SNAPC1	0.553541929	0.214012563	0.342731646	0.279031046	0.399815104	0.373770013	0.88626422
SPARC	0.21772221	0.196042775	0.185847415	0.737084635	0.779462248	0.374754141	0.88626422
SPHK1	0.269729274	0.401746364	0.072941365	0.626592827	0.919788311	0.374682011	0.88626422
SPSB4	0.089356347	0.488072129	0.856301434	0.149096144	0.800853643	0.371267692	0.88626422
SRP54	0.666044037	0.326930042	0.652827302	0.145522825	0.221474363	0.375599759	0.88626422
SRSF11	0.458986203	0.194482217	0.23271587	0.944606612	0.230806821	0.373751366	0.88626422
SSPN	0.140484818	0.32109439	0.335586758	0.493942032	0.588572541	0.369173376	0.88626422
STK3	0.330266827	0.618576324	0.5597467	0.056545868	0.680348822	0.3691146	0.88626422
STK35	0.919178508	0.625826277	0.86863554	0.057600845	0.156729717	0.373112446	0.88626422
STRBP	0.235086638	0.744113725	0.518107509	0.093148405	0.53125974	0.372191554	0.88626422
SYPL2	0.110616361	0.374460892	0.562514519	0.247537667	0.757946768	0.36811177	0.88626422
TAF1C	0.578084508	0.217458004	0.222211141	0.346750463	0.464488754	0.372690235	0.88626422
TIMP2	0.623595587	0.39037265	0.032514704	0.805693559	0.69307133	0.369857081	0.88626422
TINAGL1	0.3128007	0.237154266	0.109312301	0.92108245	0.599445047	0.371914341	0.88626422
TMEM147	0.615888559	0.802120302	0.421948461	0.025669464	0.838083339	0.37216786	0.88626422
TMEM187	0.330115583	0.231514879	0.855058419	0.66895195	0.10232276	0.371764484	0.88626422
TMEM200C	0.466245247	0.228932252	0.327978942	0.50661559	0.249686798	0.370161787	0.88626422
TMEM229A	0.971986696	0.837260461	0.36225338	0.542700527	0.028551601	0.37512413	0.88626422
TNFRSF11A	0.937476598	0.622692118	0.017474657	0.966430152	0.457426769	0.373062003	0.88626422
TNKS	0.089976094	0.880642017	0.647460701	0.13155893	0.656523185	0.370260353	0.88626422
TPRG1L	0.829732801	0.459539532	0.029272336	0.532137922	0.746613479	0.370380695	0.88626422
TRERF1	0.941818339	0.599850983	0.076347884	0.129116417	0.815280659	0.374153764	0.88626422
TRPV2	0.318729608	0.219189441	0.327961055	0.882366707	0.220135176	0.370954602	0.88626422
TTYH3	0.535103505	0.06194746	0.334904866	0.583876332	0.672590196	0.367678862	0.88626422
TYROBP	0.202523859	0.25618847	0.234723325	0.528366576	0.689453934	0.37045186	0.88626422
UBXN7	0.369798814	0.499674384	0.225074028	0.111911746	0.981580979	0.375144723	0.88626422
VDAC1	0.511072807	0.554358565	0.250500919	0.185672973	0.346451736	0.375031837	0.88626422
XRCC5	0.394804677	0.203269352	0.410338074	0.342364362	0.396125696	0.371510822	0.88626422
YIPF2	0.35316021	0.280006418	0.776746377	0.058807609	0.978105074	0.369794059	0.88626422
YIPF5	0.918305006	0.190352488	0.726614545	0.587268918	0.061387259	0.375508862	0.88626422

ZBP1	0.916863655	0.075577079	0.771931853	0.111158126	0.736593565	0.368405341	0.88626422
ZFAND3	0.781577785	0.483045275	0.017302948	0.950000797	0.730760956	0.37396316	0.88626422
ZNF740	0.786292468	0.073934654	0.117811165	0.894101458	0.740382413	0.373919659	0.88626422
ZSCAN16	0.311241797	0.153178609	0.819188347	0.268774465	0.427508115	0.372280821	0.88626422
DDX10	0.182519335	0.972476604	0.559427085	0.377291329	0.122443425	0.375796965	0.886533144
ODF2	0.834047262	0.169655364	0.169160957	0.60280685	0.317961334	0.375821771	0.886533144
DCBLD1	0.367304356	0.937445124	0.72280439	0.032154707	0.57348628	0.375877052	0.886536117
ASTN2	0.5006827	0.089761879	0.35813163	0.436663341	0.659253506	0.37740921	0.887222017
DBI	0.777278481	0.622552538	0.799308151	0.043631138	0.274479473	0.377363566	0.887222017
EMC4	0.960801201	0.345613407	0.111628624	0.186730576	0.668110605	0.377100375	0.887222017
FBL	0.330427112	0.861046541	0.45977432	0.36284922	0.097203296	0.376726335	0.887222017
FGF14	0.144604804	0.146368474	0.971547546	0.347029603	0.646610441	0.376744624	0.887222017
GHITM	0.547717912	0.689477881	0.126053014	0.152097212	0.638380464	0.377014329	0.887222017
GSTA2	0.068898973	0.840167517	0.236551453	0.563175339	0.598766215	0.37685638	0.887222017
HEATR5A	0.444847162	0.645985189	0.26027715	0.099426425	0.621488189	0.377004238	0.887222017
LOC101905014	0.126080974	0.511178716	0.26309706	0.351889936	0.776756214	0.377458731	0.887222017
LOC787904	0.380603237	0.193985802	0.609708966	0.126935875	0.805558552	0.376353107	0.887222017
MAOA	0.838244374	0.551416393	0.627012588	0.016490312	0.969435152	0.377400987	0.887222017
MIPOL1	0.96301171	0.703899658	0.532907695	0.483487861	0.026365553	0.376416156	0.887222017
MORN2	0.955863145	0.251127679	0.076902881	0.748232129	0.335779123	0.377567442	0.887222017
MYL4	0.788005253	0.299043379	0.461522037	0.987801212	0.043099107	0.377296911	0.887222017
P4HTM	0.847601998	0.293237216	0.560761288	0.197250184	0.167555803	0.376471409	0.887222017
PDGFD	0.39176609	0.551728672	0.155839539	0.184173791	0.746098364	0.377245145	0.887222017
RGS10	0.290966341	0.947968033	0.158720755	0.245354271	0.43101646	0.377283588	0.887222017
RNF168	0.169513369	0.560723462	0.445052848	0.279700039	0.389555942	0.376567946	0.887222017
RPS6KL1	0.726391831	0.540450736	0.900497275	0.015282915	0.855771287	0.377066969	0.887222017
SHCBP1L	0.084446527	0.358441477	0.418293158	0.732185893	0.50047549	0.377627554	0.887222017
SLC35C1	0.284902548	0.353553499	0.869528455	0.325714587	0.162281979	0.377278075	0.887222017
SOX11	0.47631686	0.390340383	0.236069391	0.877820821	0.120360947	0.377548017	0.887222017
TBPL1	0.112776673	0.944142561	0.432763129	0.234005707	0.42785134	0.376716056	0.887222017
TLL1	0.050739399	0.602338045	0.566685	0.558463361	0.477809876	0.376994233	0.887222017
TNK1	0.125946089	0.258869678	0.425160344	0.979787349	0.34158796	0.377615688	0.887222017
VPS35	0.66988555	0.195336361	0.109409754	0.937044841	0.343498803	0.376530434	0.887222017
XPO7	0.988302344	0.387302456	0.127990224	0.402860962	0.23433649	0.377118353	0.887222017
ALKBH5	0.988474815	0.094740037	0.057252201	0.885626564	0.977971342	0.377769718	0.887428978
LOC101905708	0.244753506	0.225099962	0.140494687	0.712903829	0.841982693	0.377855168	0.887455
NOP16	0.991179559	0.402901698	0.113295944	0.241340888	0.425591228	0.377888949	0.887455
BRCA1	0.52176051	0.059054331	0.376393944	0.744451551	0.538618264	0.377997983	0.887457067
INHBB	0.585206425	0.266651718	0.077903951	0.758272842	0.50435557	0.377957957	0.887457067
APBA3	0.735296831	0.155114993	0.125799128	0.515029071	0.629763914	0.378116981	0.887482519
LOC101904705	0.766116777	0.253616825	0.850595432	0.047339664	0.594681652	0.378080659	0.887482519
GPRIN3	0.786882136	0.414220514	0.055214727	0.568801909	0.454967141	0.378240889	0.88760785
HEPH	0.824199463	0.258002465	0.147407435	0.23749376	0.625974371	0.378332637	0.88760785
JRKL	0.384656214	0.559327396	0.374011117	0.067705954	0.855302165	0.378327237	0.88760785

PIGA	0.916238057	0.095018207	0.653278381	0.098134669	0.835356896	0.378415712	0.88767585
ARHGAP9	0.086413848	0.412760736	0.512088324	0.806395583	0.316912895	0.37860396	0.887741618
INPP5B	0.992583551	0.70297619	0.024440825	0.558984321	0.489914953	0.378689143	0.887741618
LOC100336897	0.732399721	0.783792969	0.823882063	0.039155474	0.252296589	0.378754026	0.887741618
LOC101906008	0.421346034	0.396839416	0.044432686	0.682480417	0.920499476	0.378587376	0.887741618
LOC101907603	0.600386856	0.443036624	0.109420953	0.359493335	0.446575349	0.378768314	0.887741618
YARS2	0.372053651	0.862719137	0.58274907	0.049427142	0.504988429	0.378636984	0.887741618
ANGPTL2	0.836225223	0.065880159	0.427720338	0.521180549	0.382052793	0.379434912	0.887857039
ARSA	0.810179733	0.344239916	0.163902383	0.125084901	0.819747889	0.37927259	0.887857039
C7H19orf53	0.558389411	0.259598086	0.229709478	0.145495409	0.965754125	0.378982647	0.887857039
CCDC32	0.213597744	0.521724265	0.110118905	0.655360959	0.583691705	0.379514649	0.887857039
GGT1	0.104857491	0.582566142	0.148395136	0.646368221	0.801190967	0.379520878	0.887857039
GNB1	0.528561901	0.262318202	0.067873763	0.519387124	0.959848347	0.379423985	0.887857039
LOC100298868	0.487416923	0.489139445	0.150678044	0.307518112	0.423999799	0.379163439	0.887857039
LOC107133032	0.508412235	0.75469775	0.386796938	0.155726111	0.20237016	0.378925595	0.887857039
MEIS1	0.312860381	0.924059143	0.051239053	0.518660282	0.610280345	0.379328666	0.887857039
NREP	0.737842074	0.965695096	0.03247943	0.350906345	0.57682649	0.379174091	0.887857039
PDE6C	0.020365726	0.58131112	0.63514877	0.96347433	0.645621399	0.3789338	0.887857039
PTCD3	0.591429533	0.959475223	0.504436615	0.492450713	0.033295764	0.379489144	0.887857039
RNF139	0.214027493	0.043122417	0.666870353	0.965695519	0.789121329	0.379378615	0.887857039
RNASET2	0.406740163	0.296484867	0.084505854	0.617013391	0.74687408	0.379583382	0.887876693
ABCC3	0.790637538	0.103822195	0.164841191	0.490316393	0.714481508	0.381093334	0.888020655
ADGRL4	0.0626906	0.889593731	0.7258971	0.432549368	0.271573793	0.381612807	0.888020655
ADRB3	0.07928083	0.471696634	0.320104673	0.443317648	0.893156805	0.381080251	0.888020655
ALG3	0.196223331	0.945309379	0.845846999	0.794356051	0.037991835	0.380914713	0.888020655
AOC2	0.429573138	0.224005944	0.455838567	0.187769661	0.57994184	0.382331846	0.888020655
ARAP2	0.753863407	0.732473458	0.153231134	0.195749452	0.28498492	0.380404773	0.888020655
ARAP3	0.327677983	0.153619787	0.444497603	0.866104676	0.246328877	0.382231671	0.888020655
CCNF	0.785609484	0.341303149	0.106560716	0.88213566	0.188009254	0.381040351	0.888020655
CCNQ	0.884887952	0.64695622	0.025810152	0.758948183	0.425411154	0.382128977	0.888020655
CHMP5	0.831073679	0.264578503	0.150174703	0.639819073	0.226764423	0.382819932	0.888020655
COA1	0.242974699	0.45466365	0.634470619	0.093235402	0.729469403	0.382007927	0.888020655
COQ4	0.602728525	0.79168553	0.825074217	0.039766802	0.301054891	0.380173709	0.888020655
DDX39B	0.752145792	0.136482528	0.092293648	0.621635239	0.809053054	0.381937995	0.888020655
DTX3	0.940799079	0.653192807	0.016035568	0.535564331	0.904470654	0.382223898	0.888020655
EIF4G3	0.284245138	0.327340106	0.395614336	0.679501588	0.189042042	0.380687729	0.888020655
ETS1	0.481830578	0.124161202	0.217439336	0.603785496	0.607019367	0.382027678	0.888020655
FAM129B	0.901087807	0.707260659	0.094433953	0.138862751	0.57073614	0.382099531	0.888020655
FXYD3	0.421096347	0.072449807	0.226296361	0.858316873	0.796720109	0.380440436	0.888020655
GDI2	0.762822483	0.233971596	0.332886778	0.782402165	0.102288624	0.381593321	0.888020655
HSPB2	0.3400226	0.149884861	0.785922267	0.298337167	0.399736714	0.382335193	0.888020655
KCTD11	0.555823179	0.85883744	0.18600593	0.819205644	0.065526559	0.381983263	0.888020655
KIAA1549	0.881133025	0.129350206	0.171839518	0.424812157	0.575298687	0.382669957	0.888020655
KIAA1614	0.912391025	0.101472073	0.167290674	0.902877496	0.337258921	0.380269717	0.888020655

KLHDC2	0.76999557	0.293309836	0.977128421	0.330743603	0.064450019	0.379856103	0.888020655
LHFPL2	0.219471148	0.516302188	0.655198106	0.624173605	0.103371781	0.382796916	0.888020655
LOC101904039	0.108058285	0.427621315	0.674260501	0.50491824	0.303206506	0.382102813	0.888020655
LOC101904590	0.625317333	0.804681588	0.325625414	0.248752076	0.116203252	0.380954217	0.888020655
LOC101905757	0.403148285	0.601440367	0.841440969	0.895222434	0.026072067	0.381834881	0.888020655
LOC101905786	0.213225754	0.461897231	0.366476893	0.361231159	0.361550863	0.380193226	0.888020655
LOC112448627	0.273848197	0.639439596	0.474181837	0.443348903	0.130035619	0.382685038	0.888020655
LOC789569	0.235618538	0.808672959	0.631834857	0.345272089	0.113934584	0.380944961	0.888020655
MDC1	0.682935737	0.690242354	0.22132858	0.451746486	0.101213904	0.382121192	0.888020655
MIR3064	0.846925896	0.169349737	0.346737596	0.947098835	0.10000018	0.380059406	0.888020655
MORC4	0.713255667	0.82126167	0.368823273	0.047090071	0.470748917	0.382760392	0.888020655
MRM1	0.912277185	0.702176611	0.418205913	0.254252917	0.069647284	0.381217453	0.888020655
NFASC	0.249423593	0.786618735	0.166143021	0.425098012	0.343177352	0.381612386	0.888020655
NICN1	0.928891837	0.069440193	0.934539318	0.163857129	0.483869689	0.382425223	0.888020655
NIPBL	0.235766528	0.864934449	0.939799459	0.030820247	0.804717109	0.381533343	0.888020655
NOXA1	0.374309148	0.042489969	0.777696765	0.674980302	0.565081077	0.380320935	0.888020655
NSRP1	0.157632637	0.676574797	0.24224291	0.217032696	0.839134856	0.379889224	0.888020655
OSTF1	0.389630943	0.183599304	0.099171665	0.943479895	0.706496823	0.380703306	0.888020655
OSTM1	0.70907076	0.282702178	0.040242564	0.6503118	0.896638933	0.379842176	0.888020655
PHB2	0.463815001	0.605081091	0.908489767	0.152032721	0.12311855	0.382190347	0.888020655
PLVAP	0.145185129	0.588004436	0.78951304	0.420675611	0.168512798	0.382378448	0.888020655
POMK	0.34767458	0.650475077	0.222692691	0.248113626	0.380618447	0.38163419	0.888020655
PPT2	0.484799684	0.119528435	0.409087753	0.307285074	0.647533293	0.380292841	0.888020655
RHOV	0.741543516	0.068317166	0.239373172	0.985278081	0.395929306	0.380764259	0.888020655
RNF144B	0.902545642	0.399289817	0.067967994	0.428373543	0.456652056	0.382837495	0.888020655
RNF150	0.621078471	0.367083604	0.044570819	0.879772029	0.535136356	0.382585714	0.888020655
RPAP3	0.277906554	0.213018246	0.69856287	0.178455114	0.64665342	0.382183881	0.888020655
SLC35A3	0.458625504	0.13829905	0.149796283	0.818727855	0.605665519	0.380105553	0.888020655
STOM	0.741787391	0.092543881	0.391865924	0.292975922	0.600866373	0.380934325	0.888020655
TAMM41	0.110345815	0.723238174	0.467000798	0.230532159	0.551573283	0.381051848	0.888020655
TBL1X	0.169801869	0.764296275	0.930318858	0.041283627	0.953198851	0.381465221	0.888020655
TCP11	0.08004561	0.244013943	0.538389541	0.662547817	0.684706974	0.382127491	0.888020655
TMEM53	0.35230989	0.51692607	0.186529918	0.235192725	0.598659574	0.382551654	0.888020655
TRIM38	0.867377319	0.808083121	0.080386525	0.407950593	0.205784989	0.380745687	0.888020655
XPR1	0.981325778	0.528276006	0.697370805	0.043075204	0.305984096	0.38193819	0.888020655
ZBTB6	0.810522372	0.257870392	0.647834689	0.090719892	0.389473781	0.382591232	0.888020655
CEP57L1	0.212746971	0.344755625	0.456020409	0.784784449	0.182701599	0.382980342	0.888038369
EIF4B	0.127523933	0.613826405	0.42537789	0.174547958	0.825105535	0.382974612	0.888038369
POP5	0.543648718	0.43768021	0.450312156	0.262797465	0.170395753	0.383061581	0.888038369
PSMD6	0.495825499	0.449996717	0.650218203	0.491470909	0.067291247	0.383055905	0.888038369
EOGT	0.160747468	0.382604447	0.195525968	0.409865866	0.973986869	0.383145616	0.888107726
CDH8	0.638997536	0.10904378	0.786782703	0.610615276	0.143579033	0.383340293	0.888139591
ERP29	0.688337146	0.913125669	0.349300811	0.843896291	0.025947056	0.383375837	0.888139591
FKBP3	0.291077916	0.440522834	0.371378544	0.180860312	0.557829755	0.383275111	0.888139591

TCN1	0.483523548	0.100420528	0.180141328	0.748024997	0.734218168	0.383258373	0.888139591
CDT1	0.461869409	0.432292757	0.103143211	0.480832775	0.485993239	0.383545662	0.888407602
FAM234B	0.115200765	0.701931131	0.221384023	0.429240624	0.627015472	0.383737837	0.888475489
GRIP1	0.250055596	0.899394855	0.179732481	0.533082926	0.223820183	0.383899805	0.888475489
LOC112441650	0.746974228	0.045684581	0.422187599	0.376500853	0.888651297	0.383813831	0.888475489
LOC112442997	0.628335251	0.822094496	0.948474308	0.020802881	0.472421401	0.383631679	0.888475489
TMEM220	0.468472924	0.377015894	0.04247136	0.770141815	0.834409031	0.383818301	0.888475489
ZNF532	0.755033172	0.468648157	0.094849488	0.504356983	0.284843483	0.383856606	0.888475489
LOC107131224	0.931290327	0.870926037	0.933684051	0.44746861	0.014238316	0.383967224	0.88850622
ANG	0.374607517	0.719186128	0.067725388	0.32951782	0.803941947	0.384261582	0.888560917
ANKRD61	0.816300048	0.321850998	0.739382443	0.440757032	0.056397306	0.384096109	0.888560917
C16H1orf53	0.599549163	0.722733639	0.143675379	0.197422486	0.393022936	0.384159266	0.888560917
TRIP11	0.619054092	0.431925336	0.887839292	0.027421205	0.742301333	0.384211174	0.888560917
TTC30B	0.806760122	0.489410311	0.991850404	0.042108993	0.292893168	0.38413973	0.888560917
MAPK1IP1L	0.619741558	0.047818337	0.316626816	0.521102375	0.988973806	0.384331962	0.888598455
ITGB2	0.277358933	0.267466702	0.14124401	0.642141996	0.719012057	0.384402656	0.888636707
BRB	0.165153357	0.24941974	0.585611516	0.499156682	0.402381516	0.384647768	0.888912571
HIF1AN	0.752112253	0.254644266	0.193543246	0.30715671	0.425545218	0.384646853	0.888912571
KCND3	0.701212737	0.347134706	0.04510455	0.454397169	0.97202305	0.384789427	0.888912571
MFHAS1	0.570153387	0.999690305	0.123723447	0.852637571	0.08065211	0.384792816	0.888912571
SPTAN1	0.954743321	0.254831791	0.068432802	0.804963605	0.361768036	0.384763401	0.888912571
CDC42SE2	0.219509912	0.491199941	0.091469842	0.541447458	0.909292078	0.385003214	0.888934991
LOC100140372	0.569397064	0.149038324	0.79011599	0.734986842	0.098446929	0.384866495	0.888934991
LOC616868	0.144546721	0.250515584	0.311205481	0.912162985	0.472563319	0.38506799	0.888934991
PTPA	0.318552768	0.690801117	0.587868009	0.155990138	0.240569337	0.384966271	0.888934991
USP14	0.763546054	0.359311715	0.12426346	0.826520424	0.172398441	0.385073356	0.888934991
ADGRB1	0.672765231	0.112389481	0.483075231	0.816149161	0.164589165	0.386703538	0.888946878
AHSP	0.15526893	0.751373529	0.626577627	0.194538775	0.342938943	0.385712255	0.888946878
APPL1	0.528711652	0.19434671	0.760069982	0.10836186	0.575660815	0.385544882	0.888946878
ATRN	0.45568108	0.204508526	0.664085127	0.13226311	0.599232518	0.386649176	0.888946878
CCDC149	0.819340373	0.500939533	0.431069191	0.194765271	0.14133302	0.385491933	0.888946878
CFAP36	0.4542731	0.102821042	0.49358008	0.357687313	0.594806376	0.386651253	0.888946878
COX8B	0.545778128	0.442001129	0.751804496	0.043701436	0.615110997	0.385657895	0.888946878
DECR2	0.107985052	0.343403245	0.261533595	0.595121797	0.850088587	0.386700079	0.888946878
DIAPH2	0.637222274	0.551750894	0.132836863	0.242016384	0.433641151	0.386535404	0.888946878
DYRK1B	0.452856792	0.134482459	0.326781937	0.583599462	0.420693115	0.386022868	0.888946878
EBI3	0.768976268	0.769054531	0.12838839	0.281400611	0.229270636	0.386438144	0.888946878
GNG12	0.842622038	0.116206736	0.148894722	0.895351914	0.375108648	0.386372313	0.888946878
HES6	0.943292561	0.772405428	0.166351129	0.117778837	0.341087675	0.385453986	0.888946878
HUNK	0.672901045	0.731639233	0.014062563	0.846794136	0.836199604	0.386562722	0.888946878
LMO4	0.719743037	0.33104693	0.105495089	0.390167447	0.495694918	0.385196405	0.888946878
MMP24	0.902354559	0.052004827	0.423028307	0.465473522	0.527541052	0.38563833	0.888946878
MRPS6	0.64072816	0.392722511	0.061423366	0.443033836	0.716470863	0.38668619	0.888946878
NSF	0.531160156	0.166313835	0.874643155	0.158952089	0.396884444	0.385628389	0.888946878

NUP210L	0.252295064	0.094869541	0.420439922	0.872965917	0.554903196	0.385643014	0.888946878
PABPC1L2A	0.591419177	0.638966951	0.113365532	0.533034801	0.214343598	0.386307121	0.888946878
PARP14	0.901024924	0.069151953	0.717459404	0.270369892	0.40294742	0.385489488	0.888946878
PDCD11	0.684443107	0.735464914	0.488198831	0.101556813	0.195708899	0.385966799	0.888946878
PIM1	0.354114873	0.771761869	0.965880665	0.18679356	0.099062843	0.38596979	0.888946878
POLL	0.542401731	0.027600087	0.931662616	0.408482204	0.860049098	0.386482253	0.888946878
PPIL4	0.233998501	0.850337752	0.597800724	0.075708001	0.542756089	0.38607647	0.888946878
PRODH	0.488053792	0.883931689	0.072785853	0.282342124	0.551536788	0.386142666	0.888946878
RAMP1	0.593369067	0.628002685	0.831269974	0.018320185	0.86306786	0.386413322	0.888946878
RNF25	0.16616675	0.270281948	0.920955589	0.249569558	0.473546154	0.386093885	0.888946878
SYPL1	0.92716564	0.400586848	0.030738954	0.530511353	0.808842874	0.386450423	0.888946878
WIPF1	0.427802625	0.336196661	0.866961222	0.047227087	0.827397232	0.385563271	0.888946878
ABO	0.278943737	0.285567356	0.293157152	0.742911219	0.283774651	0.387253669	0.888981078
AP1S1	0.476879556	0.473603249	0.46403849	0.145469225	0.323535165	0.38756775	0.888981078
C27H8orf48	0.172013917	0.352829697	0.122056173	0.818391732	0.814296134	0.387704181	0.888981078
COL8A2	0.457259812	0.310621832	0.197446991	0.262195153	0.669586657	0.387269033	0.888981078
COPS7A	0.436855376	0.364935124	0.236207667	0.131203407	0.994654111	0.386963137	0.888981078
CXCL9	0.333375147	0.286746743	0.302483042	0.765836653	0.223937125	0.388444314	0.888981078
DGCR6L	0.441973827	0.865479427	0.385659746	0.055020834	0.607479044	0.387509762	0.888981078
DNAJB1	0.267289527	0.801830211	0.439073815	0.096161805	0.548504541	0.388591365	0.888981078
DYNC2LI1	0.180288548	0.564280051	0.4526115	0.700882132	0.153518448	0.388293043	0.888981078
FAM174B	0.972419649	0.596602845	0.142292671	0.319011582	0.186535468	0.386897234	0.888981078
HPN	0.532672091	0.622139283	0.217108268	0.472812973	0.145133672	0.387722808	0.888981078
KCNC2	0.357595125	0.18909784	0.281002484	0.360531837	0.719986388	0.38756256	0.888981078
LOC100299201	0.511644902	0.295106457	0.695332024	0.057938345	0.814413479	0.388276692	0.888981078
LOC100848527	0.342773448	0.942420256	0.088060729	0.183303223	0.951614127	0.388546202	0.888981078
LOC101903851	0.206789056	0.454690768	0.518099951	0.420005031	0.240022628	0.386849987	0.888981078
LOC104974057	0.284829791	0.682472659	0.786652341	0.051807357	0.625803976	0.388402163	0.888981078
LOC107133459	0.503562418	0.758871039	0.063477001	0.334753548	0.6088296	0.387940344	0.888981078
LOC112441886	0.1174395	0.904935736	0.655688248	0.493042432	0.143743531	0.387768199	0.888981078
LPL	0.277027624	0.526256733	0.809382375	0.448417849	0.093818458	0.38861436	0.888981078
MAGI1	0.063004019	0.61982297	0.854563632	0.833665593	0.176773738	0.387085643	0.888981078
MED21	0.315979746	0.338669469	0.508311879	0.753113964	0.120509038	0.387709382	0.888981078
MMP11	0.375219179	0.411269465	0.036545032	0.989558765	0.889204835	0.388553069	0.888981078
MTHFSD	0.082902336	0.650879952	0.598297004	0.251803587	0.608901235	0.388141915	0.888981078
PRDM1	0.829141914	0.736211089	0.973277262	0.896859553	0.009315822	0.388602194	0.888981078
PSME2	0.413810034	0.636675462	0.520659052	0.459394027	0.078029076	0.387057185	0.888981078
PTGR1	0.656265438	0.036354906	0.261597739	0.855382018	0.92718625	0.388145829	0.888981078
RABGAP1L	0.270041782	0.760146631	0.670602767	0.05028366	0.711362066	0.387281945	0.888981078
SLC15A3	0.417415329	0.65084769	0.561546658	0.131905492	0.245682996	0.387945224	0.888981078
SLC41A3	0.969961055	0.047443542	0.753021567	0.171352599	0.832269048	0.387878002	0.888981078
SPIRE2	0.701577942	0.477218282	0.231903052	0.405223982	0.157522937	0.388347519	0.888981078
TMEM161B	0.57073307	0.399441103	0.327427843	0.101738359	0.64977026	0.387634048	0.888981078
TNFAIP8L1	0.396265146	0.171172815	0.293373988	0.523879497	0.475288385	0.388306959	0.888981078

TSG101	0.196860865	0.181149086	0.196327493	0.717725423	0.986291689	0.388348256	0.888981078
TTLL7	0.363801371	0.501765997	0.408021751	0.338437412	0.19636886	0.388144404	0.888981078
USP39	0.381929908	0.907930579	0.198797561	0.099976817	0.719325568	0.388397977	0.888981078
UQCRFS1	0.694668263	0.780832825	0.650407188	0.029938529	0.470157651	0.388670647	0.888982534
WDR73	0.562933046	0.491673428	0.510491791	0.071899763	0.488972826	0.388723336	0.888982534
CACNB1	0.459559876	0.417644485	0.157061679	0.952457354	0.173230647	0.388932813	0.88908405
DDT	0.885618408	0.788199918	0.731317706	0.01610675	0.604765437	0.38889303	0.88908405
HEATR3	0.35230267	0.932665867	0.137653418	0.41586018	0.264592701	0.389032815	0.88908405
PRKG2	0.299267271	0.312403639	0.290379997	0.270226501	0.678421508	0.389038606	0.88908405
WDR46	0.687016244	0.925385582	0.328701393	0.345830192	0.068795894	0.388867632	0.88908405
ALDH7A1	0.790062136	0.979161653	0.25608109	0.102099706	0.249299809	0.391183061	0.889413418
ARID2	0.415932237	0.925333302	0.453741177	0.032945617	0.870134882	0.389999784	0.889413418
BCL3	0.417425482	0.747735695	0.086986885	0.921715299	0.201699036	0.391350469	0.889413418
C3H1orf226	0.732954697	0.93460867	0.855275829	0.511460329	0.01672055	0.390136861	0.889413418
CCDC112	0.390999381	0.768468862	0.201521351	0.705082652	0.118083318	0.391149875	0.889413418
CCNB3	0.077030754	0.229676992	0.443914308	0.775927034	0.824405173	0.390578287	0.889413418
CCNH	0.142451245	0.18524984	0.420748945	0.897656125	0.506111301	0.391245024	0.889413418
CHMP4B	0.377031221	0.049067915	0.379372373	0.968266653	0.736430457	0.389944632	0.889413418
CUEDC1	0.408177902	0.326511854	0.159215348	0.249743475	0.947147646	0.390427988	0.889413418
DCPS	0.790839108	0.994461898	0.206240103	0.486090388	0.063619332	0.390318391	0.889413418
DHX34	0.088918472	0.633527955	0.140218463	0.663048795	0.958346094	0.390422553	0.889413418
GCNT7	0.534352883	0.509766323	0.14791502	0.343913394	0.364301839	0.391365837	0.889413418
GDF5	0.337992482	0.196943018	0.564558182	0.157342243	0.843545254	0.38939384	0.889413418
GRWD1	0.890070375	0.301316171	0.098813092	0.487391871	0.388866793	0.3905397	0.889413418
GVQW3	0.510386536	0.253926489	0.244950678	0.956784681	0.16492978	0.390107928	0.889413418
HOXD8	0.369457913	0.604968059	0.248441462	0.161275794	0.558454868	0.389835527	0.889413418
HSBP1L1	0.544684693	0.484337711	0.035943148	0.942598359	0.562807874	0.390788008	0.889413418
KDELR2	0.617256086	0.148690156	0.57332369	0.287134339	0.331749931	0.390200332	0.889413418
LLGL1	0.358648109	0.153330309	0.112599878	0.85855713	0.94509968	0.390593016	0.889413418
LOC100847819	0.120119532	0.899622959	0.100241915	0.917789059	0.506176553	0.39085313	0.889413418
LOC104969425	0.361245717	0.129227466	0.589494813	0.473436395	0.386675117	0.391033749	0.889413418
LOC104970145	0.614159167	0.260869971	0.390225228	0.179988387	0.445112412	0.390083498	0.889413418
LOC107132092	0.414747398	0.349380914	0.515556291	0.468868172	0.142351338	0.389341397	0.889413418
LOC112449254	0.387434798	0.449028227	0.315215139	0.138293995	0.665582239	0.391351572	0.889413418
LOC615663	0.275890535	0.116879998	0.250415108	0.782294153	0.797440791	0.391018852	0.889413418
LOC616400	0.729962288	0.492147766	0.083695297	0.448184663	0.370918819	0.389742566	0.889413418
LOC786914	0.649193419	0.177232189	0.578704524	0.883601454	0.085330433	0.39046157	0.889413418
LONRF2	0.806881415	0.319577352	0.779069282	0.202480145	0.124042849	0.39128776	0.889413418
LYSMD1	0.814259854	0.041410539	0.621228998	0.431780214	0.557952604	0.391314171	0.889413418
MPHOSPH8	0.69908766	0.30801627	0.305535879	0.08451225	0.901685617	0.390237726	0.889413418
NEURL1B	0.603005676	0.222111227	0.115290647	0.466388786	0.693182826	0.389534331	0.889413418
POLR3GL	0.514239797	0.372525979	0.15187047	0.302516185	0.570864886	0.390591593	0.889413418
PRICKLE2	0.666397039	0.882330931	0.847567802	0.261632171	0.03867513	0.391191527	0.889413418
SETD4	0.25180682	0.291797877	0.394216615	0.176881977	0.982651045	0.390928491	0.889413418

SMIM13	0.279836122	0.200962632	0.950484918	0.277865165	0.33915001	0.391012866	0.889413418
SNW1	0.31216361	0.91845054	0.966422022	0.018359187	0.992582138	0.391404772	0.889413418
SUMO3	0.734611922	0.192521645	0.426410286	0.143625753	0.582142799	0.391178312	0.889413418
TCAM1	0.919449085	0.586607313	0.7584845	0.032836599	0.371035316	0.389274928	0.889413418
TLE2	0.937033913	0.542558908	0.058610475	0.426303902	0.395633673	0.390634163	0.889413418
TNRC6B	0.504747981	0.084037698	0.305189851	0.652704524	0.596030238	0.390980104	0.889413418
UROD	0.639276877	0.890661772	0.625558507	0.022943321	0.611398991	0.389673734	0.889413418
CPNE1	0.276615348	0.911928539	0.865448817	0.237944743	0.097268253	0.391518812	0.889448537
DSEL	0.710914164	0.088168621	0.539070832	0.433815756	0.345228798	0.391770397	0.889448537
FAM98C	0.160264497	0.207222587	0.444008522	0.455541837	0.753213238	0.391741031	0.889448537
LOC112442191	0.648847502	0.276294849	0.09374453	0.64698018	0.465053298	0.391643653	0.889448537
LOC784251	0.164480223	0.323498226	0.273480177	0.773632902	0.449593554	0.391799615	0.889448537
SPCS1	0.404770028	0.271382933	0.746611802	0.079679151	0.77420494	0.391731337	0.889448537
VPS13C	0.38356547	0.495609094	0.850198988	0.061126921	0.512033777	0.391710152	0.889448537
CCDC82	0.772204017	0.901792185	0.184407827	0.101177464	0.391030006	0.392423728	0.889634728
CREB5	0.491401357	0.475862931	0.88964875	0.132179439	0.184319604	0.39202934	0.889634728
LMBRD1	0.570680166	0.282147107	0.212988998	0.254014583	0.581705182	0.391997065	0.889634728
LOC101903557	0.059719817	0.734184025	0.701668945	0.380815434	0.433500719	0.392366149	0.889634728
LOC101904097	0.714567105	0.756172426	0.089310337	0.743931518	0.141478423	0.392377053	0.889634728
LOC107131948	0.714877778	0.054687852	0.346735059	0.804602408	0.464904341	0.392102589	0.889634728
LOC782560	0.928294276	0.036860079	0.975219504	0.925972243	0.164274433	0.392272137	0.889634728
METTL5	0.897132735	0.321427631	0.043293158	0.489360533	0.830681524	0.392238145	0.889634728
MYH9	0.285744444	0.566178945	0.860002899	0.090386273	0.403911684	0.392389032	0.889634728
PERP	0.537299719	0.269423672	0.587616405	0.132282953	0.450776789	0.392158109	0.889634728
ACER3	0.693811291	0.500586215	0.434050353	0.075183912	0.450059981	0.393085885	0.889982366
BDKRB1	0.665627061	0.971495384	0.101357496	0.227602525	0.341855631	0.393044752	0.889982366
DPP6	0.276796943	0.999857438	0.775295053	0.247862703	0.095695507	0.392711214	0.889982366
EXOC3L1	0.75008752	0.069943279	0.139763577	0.79182907	0.878740167	0.393119382	0.889982366
GNAL	0.828088778	0.593200441	0.107587084	0.286954894	0.335891814	0.392856432	0.889982366
HLF	0.696909863	0.157426586	0.069671951	0.740989149	0.899144984	0.392819475	0.889982366
LOC783838	0.057236286	0.75640225	0.822637349	0.217012034	0.659454995	0.392951415	0.889982366
SLC6A20	0.628553968	0.653407287	0.462319468	0.380431292	0.070540381	0.392906258	0.889982366
TRIAP1	0.42461472	0.340894971	0.36199404	0.293014123	0.331853337	0.392894501	0.889982366
UNC45A	0.210159521	0.295163901	0.750675771	0.120596732	0.90781798	0.392988396	0.889982366
LOC101905533	0.471596705	0.208557242	0.484368646	0.826140402	0.129721949	0.393231661	0.890113764
CCT8	0.630708676	0.566087208	0.439472864	0.825919824	0.039473831	0.393555459	0.890643333
OPHN1	0.949948668	0.267628966	0.117334353	0.322354044	0.532220693	0.393628426	0.890643333
PLCD3	0.418690211	0.530822871	0.125936515	0.236862013	0.7718679	0.393609108	0.890643333
TCP1	0.603756644	0.32577002	0.430318951	0.477523035	0.126724324	0.393755609	0.890808285
ACSS1	0.278925363	0.913113298	0.857129547	0.087851131	0.267496863	0.39402464	0.890981583
B3GNT8	0.733918152	0.443875528	0.05390896	0.53214912	0.550118446	0.394380081	0.890981583
EPHA1	0.682158251	0.678086749	0.046301761	0.535136158	0.448569874	0.39438121	0.890981583
HTR7	0.061681531	0.691759212	0.393827148	0.547220039	0.559355097	0.394458488	0.890981583
IQSEC1	0.297769231	0.768119564	0.219296297	0.114535685	0.89327987	0.394079084	0.890981583

LOC101902895	0.391185361	0.162881102	0.160953886	0.507908905	0.984648865	0.393985376	0.890981583
MFSD4A	0.806664928	0.946992722	0.132852941	0.253252082	0.199751608	0.394149843	0.890981583
MOB1A	0.57942311	0.252818316	0.64706962	0.083814797	0.646332694	0.394179474	0.890981583
PGM3	0.19499656	0.412452292	0.353964982	0.461366966	0.391207519	0.394286963	0.890981583
RAE1	0.715865924	0.440004334	0.153804913	0.314757542	0.337179725	0.394394205	0.890981583
TNXB	0.716853966	0.179887904	0.619374677	0.071894273	0.894025951	0.394140797	0.890981583
ZNF76	0.663085841	0.040849986	0.669891355	0.902551492	0.314118547	0.394483711	0.890981583
APOPT1	0.189073115	0.701584402	0.097993196	0.438761784	0.902576059	0.394592825	0.89098278
HTR2B	0.035884571	0.830180186	0.850378221	0.952702435	0.213268052	0.394576493	0.89098278
CHN1	0.649986241	0.484014144	0.021427136	0.818432138	0.933997952	0.394759106	0.891113023
TUBB2B	0.129681174	0.130424027	0.963683284	0.549166429	0.575521659	0.394713251	0.891113023
JAM2	0.193019181	0.448769544	0.28421322	0.693712318	0.301952606	0.394885307	0.891275308
LOC104972045	0.346508889	0.93728153	0.117714989	0.787642517	0.171334066	0.394962541	0.891327043
SLC25A25	0.783197086	0.056229761	0.411708364	0.62607035	0.45466089	0.395019166	0.891332261
LMTK2	0.759077253	0.898925204	0.361157705	0.06437758	0.325513998	0.395123179	0.891444389
ASAP2	0.780411807	0.447490041	0.129564436	0.833907144	0.137177862	0.395498193	0.891618462
CACFD1	0.49855924	0.762241815	0.057643524	0.372446394	0.634519067	0.395526318	0.891618462
LCP2	0.146521437	0.711173284	0.698539365	0.392410949	0.181127499	0.395421289	0.891618462
LOC112444341	0.56526222	0.733247496	0.150111241	0.142699091	0.582956458	0.395489177	0.891618462
LRP4	0.135658676	0.580685653	0.145006086	0.599195198	0.7560984	0.39547029	0.891618462
TBC1D9B	0.352793485	0.72021939	0.128059277	0.534513719	0.297647591	0.395522172	0.891618462
CAMK2B	0.457527378	0.292163977	0.963319504	0.759598494	0.052975271	0.395679922	0.891777563
CDC42SE1	0.588459571	0.307610393	0.369667021	0.141403947	0.547887366	0.395759917	0.891777563
PIP5K1A	0.154074609	0.937844547	0.832724585	0.18066164	0.238456579	0.395743677	0.891777563
LOC100337044	0.663376279	0.076668808	0.638337327	0.389779778	0.410641928	0.396152954	0.892540655
LOC614423	0.684248182	0.305692191	0.241207292	0.133418661	0.772373739	0.396237296	0.892608135
NLGN4X	0.708284373	0.376943223	0.300731445	0.771769522	0.083936874	0.396302308	0.892632057
EPHX2	0.834902354	0.998986822	0.129840461	0.054548842	0.881881527	0.396568917	0.892987445
NAA50	0.934315272	0.094018617	0.362484932	0.29782917	0.549248463	0.396542984	0.892987445
CPSF3	0.877187478	0.457993437	0.692616767	0.496248126	0.037766085	0.396738554	0.893101986
TMEM18	0.506084395	0.660299622	0.735194505	0.195946725	0.108357197	0.396783047	0.893101986
TPR	0.679384452	0.223560484	0.316588283	0.125219455	0.866015824	0.396722362	0.893101986
ALDH3A2	0.960510217	0.348870474	0.465564866	0.037664474	0.88844411	0.396915359	0.893157967
LHCGR	0.5583453	0.628168987	0.062662726	0.412563933	0.575747699	0.396916766	0.893157967
CARD14	0.065940307	0.650797692	0.386552119	0.354831264	0.888088505	0.397134939	0.893169731
DTNBP1	0.315566898	0.734468859	0.069225898	0.698803155	0.465946091	0.397035902	0.893169731
FZD9	0.524029446	0.096244527	0.50099334	0.748987194	0.276229402	0.397144426	0.893169731
LOC513779	0.991539153	0.363872624	0.067255867	0.261750201	0.823306553	0.39719412	0.893169731
TCAP	0.253270904	0.164009241	0.673786278	0.761678381	0.245263924	0.397172361	0.893169731
ANKRD39	0.394705936	0.861162531	0.642687326	0.04353142	0.552716833	0.398045314	0.893347076
ATF7	0.160625156	0.662264333	0.681960141	0.171315314	0.423201902	0.398153863	0.893347076
CMC4	0.81695937	0.021670004	0.923818614	0.534528662	0.600096524	0.397729427	0.893347076
DNAAF5	0.752318508	0.344434619	0.107456588	0.218917738	0.860989594	0.397799106	0.893347076
DNM1L	0.791644404	0.622588486	0.960669674	0.39133866	0.028452667	0.398549408	0.893347076

DOCK7	0.194066073	0.836937287	0.495390443	0.091984524	0.71153837	0.398366482	0.893347076
ENTPD6	0.390892886	0.937943803	0.997743121	0.035053477	0.411073011	0.398519673	0.893347076
FAM208B	0.689429708	0.304915455	0.534688313	0.082318706	0.569280119	0.398401482	0.893347076
GLI1	0.310956749	0.718043028	0.184398165	0.185509214	0.689199587	0.398295401	0.893347076
ITGA8	0.03647718	0.360613806	0.596824493	0.778098088	0.861121485	0.398177318	0.893347076
LOC101904069	0.131506807	0.560087992	0.211569936	0.407939196	0.825679628	0.397816322	0.893347076
LOC112443143	0.91971982	0.660816909	0.072497966	0.165191108	0.721916303	0.397996336	0.893347076
LOC112449087	0.927067949	0.935867713	0.324203474	0.300726585	0.062179601	0.398159881	0.893347076
MRPS35	0.602864928	0.687818578	0.320632675	0.205220499	0.192057109	0.3975438	0.893347076
MUTYH	0.324399492	0.06099597	0.393044529	0.9751337	0.691477103	0.39766318	0.893347076
NDUFV2	0.842441955	0.881393282	0.494987029	0.020609412	0.69520792	0.398359057	0.893347076
NSD3	0.26967292	0.748471342	0.718599546	0.079534709	0.454067828	0.397476533	0.893347076
PRDM4	0.092330215	0.711905703	0.709218521	0.225310262	0.498718709	0.39747888	0.893347076
RAB20	0.736864479	0.281876877	0.105864336	0.826912516	0.290003466	0.398579446	0.893347076
RASD1	0.564099489	0.822844508	0.027156616	0.99533933	0.420167553	0.398534767	0.893347076
RFC3	0.732206048	0.58816545	0.308889394	0.066562623	0.594292626	0.398237623	0.893347076
TRIM4	0.893981812	0.505622553	0.242118781	0.16299626	0.294588159	0.398011829	0.893347076
TRMT2A	0.496800357	0.422085913	0.591901941	0.158567686	0.266397623	0.397629729	0.893347076
ZMAT3	0.534050096	0.59534492	0.489803006	0.140859486	0.239627954	0.398057418	0.893347076
DDHD1	0.540254893	0.642884361	0.295770937	0.373605821	0.137490325	0.398699435	0.893493982
MIF4GD	0.204156993	0.589877593	0.177966693	0.328189307	0.75105057	0.398885454	0.893788802
ZSCAN21	0.746439147	0.168322598	0.909583777	0.289965362	0.15947776	0.398949543	0.893810369
ARMC12	0.624683156	0.502957726	0.501446837	0.475514274	0.070675908	0.399265814	0.894260164
KCNK7	0.833945897	0.398278112	0.144820066	0.481182303	0.228916018	0.399376114	0.894260164
KRBA1	0.260772947	0.187636736	0.304664073	0.600931635	0.591607762	0.399422765	0.894260164
LOC104974912	0.451367975	0.78849281	0.209072727	0.095084823	0.748392905	0.39927179	0.894260164
LOC509972	0.31836281	0.700261799	0.123888581	0.201997531	0.949676215	0.399374879	0.894260164
GSTT4	0.406340911	0.213113135	0.172770474	0.507341823	0.698520449	0.399495419	0.894300821
AMDHD1	0.10608767	0.098920482	0.776261332	0.664948118	0.979714532	0.399647388	0.894389975
FPGS	0.624636754	0.461462655	0.910701908	0.057230247	0.353505512	0.399768487	0.894389975
LCTL	0.476386048	0.984820357	0.207862915	0.202042806	0.269474311	0.399726938	0.894389975
LY6E	0.915848333	0.371705069	0.754626679	0.051953051	0.397733618	0.399690015	0.894389975
TMEM14C	0.409279201	0.466492225	0.216464805	0.153364248	0.838086774	0.399807742	0.894389975
CDYL	0.608463306	0.098868161	0.300223543	0.675006882	0.436701475	0.400177593	0.894717325
CLN6	0.801688952	0.554520598	0.603357529	0.186588063	0.106547254	0.400444747	0.894717325
FKTN	0.05881557	0.643849221	0.971764263	0.895749445	0.161533635	0.400200034	0.894717325
GAPDHS	0.501363937	0.062271207	0.799425802	0.493292574	0.43236043	0.400154798	0.894717325
LOC101902475	0.340472661	0.182858296	0.653655048	0.959138916	0.136542263	0.400356337	0.894717325
MDGA1	0.291247594	0.35623966	0.069298028	0.82884997	0.893262306	0.400159161	0.894717325
TBX3	0.232825301	0.293096993	0.809121361	0.129196427	0.746814875	0.40028898	0.894717325
TMEM199	0.932523242	0.019288214	0.613205101	0.740371679	0.652260697	0.400254352	0.894717325
TMOD4	0.96673228	0.53361163	0.195336193	0.928268781	0.056997633	0.400414171	0.894717325
ARIH2	0.12651834	0.563456723	0.357236875	0.497794194	0.424659342	0.402034601	0.894767302
C8H9orf152	0.652597528	0.098265475	0.602880197	0.19791414	0.703189763	0.401944536	0.894767302

CDK6	0.257189517	0.193060894	0.641062524	0.591632154	0.285472072	0.401804735	0.894767302
DOCK6	0.774123161	0.136585104	0.223455218	0.621342452	0.364842129	0.401180529	0.894767302
ELOVL1	0.4689403	0.361630771	0.091568964	0.80567114	0.430515623	0.40211815	0.894767302
FAM241A	0.565681202	0.71215551	0.022660591	0.78757344	0.748523997	0.401978334	0.894767302
FANCE	0.789434616	0.707446602	0.911030965	0.075532018	0.139381116	0.401195168	0.894767302
GNG5	0.957284524	0.117398748	0.950385112	0.171729787	0.291944603	0.401146057	0.894767302
IPO11	0.196710061	0.234546707	0.789459779	0.216072711	0.683095485	0.401806235	0.894767302
L3MBTL1	0.969178507	0.109475712	0.553752926	0.20988921	0.433321117	0.400795404	0.894767302
LEPR	0.918385412	0.744605993	0.241696343	0.158454119	0.205337158	0.401854475	0.894767302
LIN54	0.532289015	0.611130807	0.79497087	0.033336446	0.623595774	0.401801658	0.894767302
LOC100139363	0.479343291	0.794214513	0.211828412	0.194685319	0.341235325	0.401225097	0.894767302
LOC100847269	0.434003973	0.854050582	0.031009281	0.777377519	0.601847617	0.401852497	0.894767302
LOC101902301	0.247642815	0.106283242	0.832731819	0.776723066	0.314389488	0.401061397	0.894767302
LOC101903574	0.702762519	0.696245401	0.029059414	0.380580365	0.991068569	0.401398843	0.894767302
LOC101905908	0.048516348	0.499434472	0.677036163	0.338645277	0.963077777	0.401005668	0.894767302
LOC112446127	0.453075658	0.256002314	0.362619657	0.187403872	0.680479877	0.401418813	0.894767302
LOC785403	0.166503311	0.134260822	0.477941408	0.895347522	0.562854584	0.402063013	0.894767302
MATN2	0.983204493	0.349874781	0.115103207	0.298469261	0.452937313	0.401081273	0.894767302
MMP24OS	0.845532361	0.653175691	0.344736688	0.038499894	0.733507816	0.40182346	0.894767302
PPIE	0.882132842	0.10224162	0.830500382	0.220357698	0.323470078	0.400651889	0.894767302
RAI1	0.806111155	0.300139271	0.084981145	0.815366207	0.321461035	0.402211833	0.894767302
RB1	0.47239471	0.929787829	0.515374824	0.198298308	0.119442192	0.401353092	0.894767302
SIPA1	0.468612875	0.103081427	0.210844283	0.815949793	0.642400683	0.400637299	0.894767302
SLC30A2	0.634740655	0.956722619	0.024257379	0.488257257	0.741817605	0.400539829	0.894767302
SNHG4	0.833987458	0.623934564	0.273712567	0.05128754	0.732711823	0.40106465	0.894767302
STIM1	0.897057766	0.619768317	0.779187811	0.459090294	0.027005609	0.401643451	0.894767302
TBCD	0.354585101	0.298102129	0.465835053	0.278004356	0.393672025	0.402205097	0.894767302
XRN1	0.376549156	0.655767916	0.679887772	0.056583366	0.564531225	0.401390368	0.894767302
ZNF274	0.221367665	0.105870552	0.381411609	0.998573633	0.600149533	0.401212402	0.894767302
ZNF782	0.09505183	0.311933597	0.799509973	0.688690214	0.328785275	0.401542996	0.894767302
EHHADH	0.660353349	0.849868082	0.483856253	0.071831802	0.276755366	0.402495043	0.895254816
SRSF12	0.515601582	0.491621448	0.125915571	0.552962094	0.305952622	0.402540082	0.895254816
PSMB7	0.385094587	0.65757657	0.405160103	0.142750216	0.368861093	0.402619407	0.895309904
ALG8	0.510219976	0.304959571	0.154760068	0.795257186	0.28425548	0.403889458	0.895334841
ALKBH3	0.930717573	0.427138019	0.091432891	0.344252776	0.437984162	0.405028174	0.895334841
AP1S3	0.786685544	0.272780662	0.67889197	0.073671711	0.505475338	0.403326845	0.895334841
APOBR	0.053616463	0.459680522	0.931042733	0.767693872	0.310328364	0.404606732	0.895334841
ATP5F1B	0.772488202	0.860456909	0.607782827	0.038683522	0.346872863	0.403191781	0.895334841
C23H6orf47	0.733208826	0.343873686	0.447096574	0.216976179	0.221410781	0.40302725	0.895334841
CIDEC	0.612751075	0.616335596	0.064923785	0.382725303	0.583733854	0.404944241	0.895334841
CPT2	0.902173526	0.548107645	0.345680517	0.076929685	0.412545016	0.403319132	0.895334841
CYTH1	0.346359185	0.722747427	0.239886397	0.189048487	0.483409951	0.405253563	0.895334841
DBNL	0.436082465	0.384162551	0.621102688	0.440992848	0.118843975	0.404190699	0.895334841
DNAH17	0.958272622	0.635815231	0.052927479	0.348947584	0.484171352	0.404038161	0.895334841

DNAJC7	0.101493186	0.600467288	0.769107473	0.238715454	0.490116469	0.405131986	0.895334841
EDC3	0.69720105	0.083548101	0.186865713	0.96317193	0.523210403	0.405175009	0.895334841
EMX2	0.343767812	0.975200988	0.055929252	0.503351771	0.577553919	0.404116106	0.895334841
HSD17B4	0.392709271	0.232324784	0.36627624	0.294870998	0.557358778	0.40538293	0.895334841
LGALSL	0.629284287	0.165063563	0.10519994	0.966114918	0.51860048	0.404854645	0.895334841
LOC101902721	0.464095373	0.321491407	0.558180583	0.155815859	0.4212732	0.404604182	0.895334841
LOC101905046	0.662062861	0.17110993	0.107189872	0.675634387	0.662976663	0.403759498	0.895334841
LOC101905114	0.381033462	0.649553504	0.293959533	0.174916831	0.429228608	0.404472525	0.895334841
LOC101905156	0.039885575	0.16300402	0.855461007	0.99593556	0.977083934	0.402926578	0.895334841
LOC101905997	0.963253633	0.721334049	0.481072125	0.016568428	0.989286733	0.40497652	0.895334841
LOC101907247	0.333863031	0.082134686	0.883585982	0.416830593	0.53945916	0.404038704	0.895334841
LOC112442414	0.510648975	0.269756298	0.946473926	0.194068736	0.214047863	0.403038962	0.895334841
LOC112443509	0.117561375	0.939247767	0.244222926	0.293620847	0.68370067	0.402967667	0.895334841
LTBP1	0.432189545	0.987830971	0.0221406	0.780443521	0.74085192	0.40456261	0.895334841
NDUFAF1	0.779916046	0.500072146	0.619538472	0.222940761	0.100900811	0.403641771	0.895334841
NMUR1	0.803019791	0.452343346	0.384550706	0.081579495	0.476720113	0.403549074	0.895334841
NOP56	0.66230593	0.456554499	0.741229763	0.344475124	0.070703133	0.404362415	0.895334841
NUP93	0.465359454	0.339061162	0.93377758	0.985525824	0.037593717	0.40435958	0.895334841
P2RX5	0.461526725	0.655984619	0.043053139	0.822650408	0.506286217	0.403438877	0.895334841
PAIP2B	0.601014722	0.221613031	0.090309956	0.524954146	0.868494865	0.405136452	0.895334841
PDLIM1	0.540583716	0.152014925	0.695191032	0.1296729	0.740567827	0.405199352	0.895334841
PGGT1B	0.323423507	0.199222326	0.234290256	0.683897036	0.531895018	0.405359005	0.895334841
PHKG1	0.407664569	0.920345072	0.914212979	0.049940697	0.316235852	0.403076556	0.895334841
PPP1R36	0.955149461	0.739643908	0.014154053	0.585786253	0.933064033	0.40456504	0.895334841
PRKAR1B	0.715485897	0.334433276	0.084257682	0.699053265	0.384580919	0.403173409	0.895334841
RAD54L	0.533133499	0.66455022	0.034836097	0.706955152	0.625928271	0.404443125	0.895334841
RIT1	0.137100083	0.614901632	0.632353014	0.306117401	0.333810204	0.404011183	0.895334841
RTTN	0.299881968	0.360276845	0.474874866	0.31378379	0.339717414	0.404676546	0.895334841
SEC62	0.796576778	0.39860909	0.041302971	0.420856932	0.984635395	0.403615982	0.895334841
SOSTDC1	0.680547221	0.56671538	0.082395108	0.21326825	0.800988507	0.403427475	0.895334841
STK25	0.892237404	0.480401821	0.265868001	0.105390413	0.455546185	0.404742381	0.895334841
SUPT3H	0.347877229	0.996340448	0.625646649	0.075572602	0.334285211	0.404958617	0.895334841
TSPAN31	0.571248523	0.632543008	0.096756971	0.549552068	0.285894961	0.405410151	0.895334841
U2AF2	0.483148639	0.314016022	0.061880213	0.589313133	0.992370966	0.405329655	0.895334841
UBXN4	0.819801389	0.647999958	0.554437822	0.220947888	0.083735211	0.404066696	0.895334841
UNC119B	0.534048696	0.469611212	0.41550301	0.163747141	0.318093152	0.403406023	0.895334841
VEPH1	0.777176313	0.393332624	0.070444446	0.606468027	0.415047855	0.403178918	0.895334841
VPS26A	0.270094108	0.183905789	0.157151219	0.702687491	0.997957535	0.404826542	0.895334841
YBEY	0.225157855	0.746901812	0.842179804	0.261427052	0.148141933	0.405167103	0.895334841
ZC2HC1C	0.35665917	0.253937688	0.286450394	0.745737301	0.283925474	0.405413028	0.895334841
CDIP1	0.257101247	0.188418522	0.792871598	0.530568378	0.269683393	0.40549267	0.895390233
TWNK	0.543907722	0.280372146	0.476822321	0.407286625	0.185697886	0.405607733	0.895523814
ARL6IP5	0.900344279	0.217238038	0.155565033	0.297090979	0.614480793	0.40728426	0.895526716
B3GNT7	0.484569141	0.950409685	0.172578838	0.139672475	0.498614688	0.406693732	0.895526716

BOC	0.685808652	0.207550033	0.340016731	0.125644433	0.907865449	0.406252658	0.895526716
CADPS2	0.08150643	0.811635599	0.700236349	0.213260554	0.56169622	0.407111958	0.895526716
FAM206A	0.298321549	0.121535871	0.789951509	0.261217282	0.74056706	0.40685865	0.895526716
FLYWCH1	0.171086283	0.298064199	0.830469926	0.768669212	0.17051588	0.4071676	0.895526716
FNBP1	0.872536812	0.218680047	0.669123174	0.532892392	0.081533754	0.407060151	0.895526716
GAMT	0.844597469	0.638783799	0.523195067	0.140022497	0.139968995	0.406603473	0.895526716
GNL1	0.253059046	0.997120215	0.172809733	0.178377261	0.708719854	0.406005333	0.895526716
HMG20B	0.64557792	0.434681892	0.613710977	0.037286217	0.86580722	0.407439075	0.895526716
HNRNPA3	0.332384581	0.10497948	0.725786553	0.30532877	0.719760131	0.40761591	0.895526716
KIAA0825	0.327828639	0.605068105	0.252465955	0.432258694	0.254937944	0.406191274	0.895526716
LOC101904042	0.43407804	0.243230101	0.959133854	0.098750875	0.556341596	0.407552754	0.895526716
LOC101907682	0.374870757	0.216973102	0.219805142	0.762274551	0.406711951	0.406923664	0.895526716
LOC112443139	0.788082676	0.633134475	0.377082256	0.05410708	0.540814934	0.40579447	0.895526716
LOC112444907	0.878195269	0.168934559	0.439223776	0.603055877	0.141640723	0.407628089	0.895526716
LOC112446740	0.661503393	0.594834972	0.638024566	0.091851473	0.241208929	0.407513452	0.895526716
LOC112448777	0.803225408	0.226784879	0.980753086	0.968733629	0.031906043	0.406290491	0.895526716
LRRC7	0.528467611	0.148106917	0.493852221	0.216289596	0.662432194	0.40678581	0.895526716
LYL1	0.638931381	0.340935347	0.549420507	0.148267454	0.312498152	0.407001433	0.895526716
MANSC1	0.751255402	0.874158605	0.015356698	0.860714311	0.637243388	0.406581435	0.895526716
MGST1	0.647443114	0.575872303	0.390754068	0.286608743	0.132740307	0.406923627	0.895526716
NECAB2	0.56854356	0.254525921	0.527098903	0.088399321	0.819002814	0.406303518	0.895526716
NLRP1	0.532407645	0.189292967	0.252668725	0.353331426	0.616035374	0.406921534	0.895526716
PIK3R4	0.905619996	0.950687768	0.179985427	0.042932082	0.83577592	0.407454358	0.895526716
PLA2G4B	0.789488309	0.200929503	0.170472478	0.481722311	0.426561391	0.407349281	0.895526716
PML	0.893553065	0.309198049	0.250930659	0.166217018	0.481957211	0.407261216	0.895526716
PRPF3	0.766742694	0.065805883	0.660483062	0.30543517	0.543795603	0.40669402	0.895526716
PSMD3	0.595936342	0.505321952	0.362289603	0.374822037	0.134860655	0.406075992	0.895526716
SLC38A3	0.250340562	0.549154148	0.170500077	0.45981054	0.511204359	0.40591707	0.895526716
TMC4	0.590170272	0.698120235	0.122742225	0.164514127	0.66236348	0.405947085	0.895526716
TRAPPC6B	0.581723111	0.331314009	0.237246038	0.337687554	0.360387136	0.407588651	0.895526716
TSNARE1	0.872850273	0.033418163	0.99547941	0.193795196	0.977995631	0.405727915	0.895526716
TSPAN7	0.244692375	0.140138291	0.836024249	0.256141133	0.75660468	0.407319234	0.895526716
ZMYND11	0.463932251	0.140589319	0.159109503	0.829808898	0.640118003	0.406001256	0.895526716
ZNF729	0.721523447	0.060353735	0.307534359	0.80583148	0.513789046	0.406983191	0.895526716
ZSCAN31	0.791320458	0.802734272	0.554210482	0.261464217	0.060251233	0.407021865	0.895526716
SLC39A10	0.411605966	0.711859631	0.803222192	0.06505608	0.363654757	0.40768539	0.895532718
LOC112442071	0.254930866	0.193195069	0.411083556	0.638964543	0.430684494	0.407800059	0.895664718
LDLR	0.732385175	0.45774277	0.338602984	0.267345866	0.18372558	0.407920448	0.895689385
NAA38	0.648511396	0.800840316	0.173300768	0.067974326	0.911299984	0.407910897	0.895689385
CDCA7	0.986015607	0.572996112	0.040424558	0.917494487	0.266350743	0.408092398	0.895827227
LOC101902390	0.200425392	0.575310096	0.741516215	0.251098842	0.259891654	0.40804363	0.895827227
C5H12orf66	0.299213694	0.340953948	0.783087149	0.516501173	0.135748141	0.408695768	0.895869574
DENND6B	0.754956258	0.040721506	0.885600735	0.34737214	0.591322626	0.408428091	0.895869574
ERAL1	0.590474905	0.952085647	0.890830338	0.021532385	0.518963277	0.408543842	0.895869574

ERCC1	0.249351412	0.991315064	0.192551598	0.172340914	0.683592589	0.408875944	0.895869574
FILIP1	0.113010694	0.260678051	0.732648657	0.571908021	0.452530477	0.408230414	0.895869574
LOC112449563	0.698913144	0.740417321	0.038731586	0.417565508	0.669069405	0.40864465	0.895869574
MIER3	0.335244064	0.164385724	0.438625937	0.309944014	0.747506559	0.40866675	0.895869574
MKS1	0.946775948	0.569802435	0.390935159	0.171774627	0.154444387	0.408507411	0.895869574
PIK3R1	0.878209482	0.649573064	0.024742949	0.850683237	0.465232612	0.408238888	0.895869574
SCRN2	0.252293647	0.130051644	0.591917976	0.638348672	0.45110769	0.408435391	0.895869574
SESTD1	0.772777921	0.695850204	0.863614195	0.064135466	0.187811327	0.408469706	0.895869574
SIPA1L1	0.198888574	0.696684096	0.536087246	0.696217241	0.108405716	0.408845553	0.895869574
TMEM30B	0.540619995	0.115883218	0.946951018	0.432777131	0.218334127	0.40882533	0.895869574
UTP25	0.531854861	0.207287289	0.17516825	0.370704147	0.782531076	0.408718192	0.895869574
ARPP19	0.564639474	0.571921538	0.056145555	0.3814561	0.811663815	0.409065295	0.895925605
LOC101904520	0.145339535	0.816434927	0.835578658	0.121788688	0.46468153	0.408991953	0.895925605
RETREG1	0.858858987	0.454774899	0.04113837	0.784305094	0.445412193	0.409053154	0.895925605
DDO	0.72502288	0.761166606	0.029064289	0.755327051	0.46353297	0.409128625	0.895944738
ERC1	0.45795521	0.976356116	0.927847756	0.021448421	0.633602554	0.409794692	0.896068576
FGFR3	0.089434617	0.379029252	0.669940453	0.399170865	0.621959489	0.409800642	0.896068576
FOXO6	0.977947144	0.290196116	0.387853386	0.076995566	0.664155807	0.409518631	0.896068576
GBF1	0.327003931	0.277280476	0.678595105	0.110627815	0.825942956	0.409319265	0.896068576
LOC101905586	0.084777838	0.433638339	0.204925218	0.841678933	0.887916936	0.409562935	0.896068576
LOC112447011	0.246286706	0.768054594	0.0439263	0.738134679	0.916435192	0.409279497	0.896068576
RNASEH1	0.673382681	0.455773041	0.166140683	0.269768026	0.409665749	0.409711656	0.896068576
S100PBP	0.191823187	0.445011591	0.119078963	0.916682376	0.605286222	0.409859612	0.896068576
SLC27A5	0.473323281	0.28691901	0.478078987	0.461266477	0.188099941	0.409653601	0.896068576
SLC8A3	0.075786395	0.618187131	0.855832178	0.233237409	0.602091957	0.40957718	0.896068576
SLCO4C1	0.227660994	0.25884056	0.790587603	0.580709123	0.2079004	0.409392166	0.896068576
TACC1	0.85980974	0.14038704	0.069774517	0.927635443	0.722062121	0.409894997	0.896068576
TIGD5	0.846843903	0.814796456	0.482728533	0.034199595	0.495116309	0.409858259	0.896068576
MED18	0.391561229	0.447104613	0.887942708	0.064982512	0.558915511	0.410034386	0.896147381
MYZAP	0.026609641	0.658467261	0.966545491	0.756429258	0.440745324	0.410040258	0.896147381
PTRH1	0.478322512	0.663951647	0.750659266	0.18992339	0.124778074	0.410143488	0.896162378
TMED8	0.801049765	0.209104074	0.881085004	0.554662096	0.069021054	0.410156335	0.896162378
BEX3	0.797805344	0.52024047	0.170974922	0.707734751	0.113216105	0.411231946	0.896192079
C3H1orf210	0.176550093	0.417274485	0.820696464	0.163213972	0.575723508	0.411088338	0.896192079
CEP120	0.505769435	0.32875943	0.756143647	0.110377963	0.408493395	0.410721778	0.896192079
ELAC1	0.739385427	0.29974489	0.848821025	0.03718109	0.816948673	0.412067648	0.896192079
ENPP5	0.602848872	0.559068369	0.657573709	0.093907493	0.27380523	0.411601276	0.896192079
FZD7	0.917582349	0.527997269	0.121335887	0.10972021	0.878775827	0.410693824	0.896192079
GLIPR1	0.217961342	0.550606779	0.641257326	0.139402656	0.529569528	0.411089805	0.896192079
IFNAR2	0.587340072	0.628400277	0.042019163	0.542900284	0.678075789	0.411917867	0.896192079
ITGAV	0.597120881	0.700386057	0.919297947	0.789335216	0.018744388	0.411300747	0.896192079
ITPRIPL2	0.900792312	0.892966937	0.063747213	0.342370695	0.324665705	0.411638099	0.896192079
JOSD1	0.418674557	0.390774505	0.317598947	0.206687385	0.531847938	0.412000397	0.896192079
KLHL29	0.937173143	0.035416653	0.706942352	0.974261134	0.247767488	0.410577141	0.896192079

LOC100847782	0.251218881	0.313516696	0.934969752	0.185750828	0.417129952	0.411816836	0.896192079
LOC100847946	0.764464478	0.19222546	0.197357212	0.276444385	0.705468436	0.410334062	0.896192079
LOC101905343	0.327722552	0.195897924	0.356332797	0.924993718	0.267734145	0.41061742	0.896192079
LOC101905630	0.897563516	0.392679936	0.293308292	0.064279838	0.853574267	0.410815943	0.896192079
LOC101906469	0.366300829	0.182945039	0.157756685	0.576121667	0.936728945	0.411801945	0.896192079
LOC112441644	0.976275218	0.138831172	0.105584518	0.4458724	0.893834446	0.411744512	0.896192079
LOC112443528	0.646085089	0.323351104	0.693796864	0.187996259	0.208910788	0.411424564	0.896192079
LOC112444600	0.974933411	0.144987287	0.44063933	0.597472048	0.152268989	0.410649615	0.896192079
LOC505479	0.648383852	0.786790069	0.414332597	0.72906731	0.036954508	0.411490336	0.896192079
LOC616200	0.362775368	0.871442689	0.721053301	0.030662748	0.812736153	0.411073147	0.896192079
LVRN	0.278707318	0.117152008	0.6128489	0.394329487	0.719806809	0.411042791	0.896192079
NPW	0.731739673	0.966406334	0.092434446	0.165558972	0.526585711	0.411606034	0.896192079
NSUN6	0.751626391	0.841210121	0.060658024	0.167417305	0.887571521	0.411616353	0.896192079
NUDT21	0.731853989	0.372791493	0.550121411	0.375355401	0.101357249	0.411947028	0.896192079
PNRC2	0.372940602	0.328671851	0.205741945	0.340873936	0.664770527	0.412081252	0.896192079
PPP1R12A	0.263874257	0.29079789	0.908076879	0.239405772	0.33937447	0.410496801	0.896192079
RARRES1	0.451467531	0.236545628	0.5198958	0.683437327	0.149210822	0.410509297	0.896192079
RBP1	0.196513491	0.179401511	0.496707322	0.3330354	0.975999845	0.41140613	0.896192079
SAMHD1	0.335053555	0.42251357	0.474001348	0.322263911	0.263630039	0.411671934	0.896192079
SEC11A	0.345125573	0.323834751	0.100979414	0.526455531	0.951811218	0.410310466	0.896192079
SMIM30	0.664332245	0.257264592	0.206321412	0.310842809	0.518482244	0.411143164	0.896192079
SMPX	0.051531098	0.964361916	0.653071439	0.709754347	0.246340365	0.41088194	0.896192079
SNAPC2	0.197134176	0.314728206	0.798771003	0.158391334	0.720354132	0.410291551	0.896192079
CARNMT1	0.827662352	0.148529485	0.140605976	0.687093857	0.481585675	0.412225102	0.896267375
IKZF3	0.469606211	0.697284156	0.761324062	0.720929804	0.031814581	0.412175589	0.896267375
CBLB	0.716183165	0.63835217	0.849267577	0.041555692	0.354833306	0.412390793	0.896271396
CD8A	0.41038566	0.587296115	0.357353137	0.770218632	0.086292133	0.412370594	0.896271396
TMEM39B	0.027625233	0.479637454	0.822197971	0.534448073	0.983114414	0.412360016	0.896271396
OSBP2	0.115818118	0.724066646	0.868434326	0.089286166	0.880879341	0.412472261	0.896329751
ARHGEF6	0.503182448	0.254296102	0.72894727	0.28884163	0.213398431	0.413104703	0.896495189
BMP2K	0.810580161	0.218068714	0.837808536	0.18364946	0.211312755	0.413041098	0.896495189
CD300E	0.202528904	0.322795037	0.428456857	0.739716256	0.277057391	0.412848314	0.896495189
CHRAC1	0.253103635	0.636145316	0.833336356	0.315672095	0.135904765	0.41331318	0.896495189
CTH	0.517909537	0.061900469	0.415598625	0.549119847	0.786560876	0.413264537	0.896495189
GYPC	0.492344501	0.333416427	0.236856148	0.1573059	0.938786235	0.412886042	0.896495189
LOC100336161	0.175265178	0.948598756	0.591030297	0.150813534	0.387566086	0.412933509	0.896495189
LOC101906312	0.406651612	0.457649003	0.159271226	0.699177984	0.277629814	0.41323514	0.896495189
LOC104974443	0.944638597	0.059017634	0.182258459	0.801513849	0.704956542	0.412868889	0.896495189
LPIN2	0.585116093	0.754128509	0.15044947	0.763769082	0.113496883	0.41326557	0.896495189
NFATC3	0.636478003	0.387062147	0.436925694	0.067832205	0.785106663	0.412606405	0.896495189
NQO2	0.838869826	0.223182807	0.276520605	0.191980635	0.577436633	0.412804853	0.896495189
RBM19	0.034065509	0.797555944	0.994206985	0.50756714	0.419032471	0.412980382	0.896495189
TTC14	0.734971685	0.156702712	0.63856808	0.087413472	0.894691908	0.41318045	0.896495189
GPATCH2	0.549466716	0.671631231	0.338824474	0.070688786	0.651680322	0.413425041	0.896619313

VPS28	0.448746775	0.892200421	0.331245564	0.068111861	0.638112089	0.413542829	0.896756258
SYT11	0.209332278	0.27669375	0.490274043	0.456594131	0.444717944	0.413603756	0.896769882
ARHGEF12	0.460498603	0.807982861	0.200799355	0.095892267	0.806740404	0.414002864	0.89705536
B9D2	0.965234426	0.385067356	0.253554447	0.399329171	0.153422546	0.413827197	0.89705536
CRISPLD2	0.785012849	0.897716522	0.290329383	0.040597799	0.696215395	0.414097874	0.89705536
CTC1	0.670402935	0.069805967	0.897511632	0.672854724	0.204454064	0.413954143	0.89705536
MSI1	0.697758698	0.642323937	0.057908908	0.622161507	0.358176265	0.414118055	0.89705536
PCBP4	0.581117472	0.220362877	0.162756872	0.458479127	0.605014253	0.414048508	0.89705536
TMEM136	0.670178988	0.245505647	0.393746217	0.64878586	0.137592445	0.414102397	0.89705536
C7H5orf24	0.360529862	0.037310167	0.872394638	0.750884925	0.657327951	0.414365396	0.89726198
COPB1	0.195682804	0.243260012	0.829281775	0.39222285	0.374677124	0.414630997	0.89726198
ELK4	0.323508962	0.802552264	0.789746894	0.061559483	0.45971522	0.41467634	0.89726198
FAAP24	0.505627424	0.735560501	0.546450392	0.263339103	0.108237738	0.414387667	0.89726198
IRF7	0.869076749	0.039638172	0.891557727	0.26882336	0.701499184	0.414356976	0.89726198
MPDU1	0.467524315	0.755991384	0.334681394	0.145340099	0.337248797	0.41454211	0.89726198
NIM1K	0.705391841	0.369397741	0.477637048	0.104442032	0.446812924	0.414829918	0.89726198
NPLOC4	0.554030814	0.350993746	0.233998364	0.774980673	0.164666206	0.41479772	0.89726198
SEH1L	0.407197546	0.371793178	0.333550907	0.49870288	0.230576348	0.414791399	0.89726198
TGFBR3L	0.660748917	0.342613139	0.142001222	0.817134336	0.22094831	0.414709724	0.89726198
TWIST1	0.503323702	0.901347439	0.166327615	0.080646109	0.954635319	0.414869533	0.89726198
VCAM1	0.739273204	0.179463341	0.338188814	0.378732093	0.341002349	0.414440731	0.89726198
CCDC17	0.630004279	0.370732699	0.073077831	0.661359107	0.515039754	0.415003939	0.897434397
SMAD4	0.877644091	0.196599779	0.692327917	0.269040195	0.180967886	0.415068109	0.897454907
ACOX1	0.111898815	0.964044612	0.147684178	0.728726796	0.504747989	0.416347493	0.897457197
ADHFE1	0.300448848	0.743465502	0.508686407	0.054154599	0.950410453	0.416007096	0.897457197
APOO	0.746301889	0.345576157	0.681530086	0.078891963	0.421100571	0.415745941	0.897457197
BTBD6	0.208454842	0.342827643	0.389454905	0.87689126	0.240579049	0.416682049	0.897457197
C5H22orf23	0.861018239	0.292604271	0.574465102	0.068377156	0.588630129	0.415333771	0.897457197
CDCA5	0.782667516	0.166967849	0.150477372	0.448720184	0.664233	0.416379461	0.897457197
COMMD2	0.698165568	0.094626983	0.692440985	0.439067465	0.29202393	0.416508337	0.897457197
CTCFL	0.4546486	0.314819836	0.622898761	0.67944353	0.096534649	0.415992964	0.897457197
DEGS1	0.665751711	0.615585406	0.293070824	0.116336525	0.421205429	0.417088247	0.897457197
ELMO3	0.353948652	0.040350199	0.934965081	0.501552617	0.875439016	0.41643687	0.897457197
FAM221A	0.067187815	0.9673048	0.567279746	0.54121944	0.295082141	0.417160587	0.897457197
FUT4	0.654716218	0.123168165	0.42659435	0.426824249	0.396844418	0.415383078	0.897457197
GNE	0.452981406	0.488707487	0.759789617	0.11736362	0.298149678	0.417091123	0.897457197
GON4L	0.184953732	0.261210054	0.289017388	0.424604256	0.988521351	0.41636767	0.897457197
HOXC10	0.500809625	0.421476163	0.238275006	0.352367699	0.332246773	0.417165921	0.897457197
LOC100139115	0.327860997	0.677759625	0.225509483	0.143344695	0.811227802	0.415390396	0.897457197
LOC104971510	0.165226447	0.755817438	0.141542703	0.479040897	0.688867548	0.415561859	0.897457197
LOC107132431	0.713841137	0.553674658	0.773122976	0.049854264	0.382856572	0.415542632	0.897457197
LOC112447727	0.631631305	0.547697451	0.638728687	0.244247176	0.108953573	0.416934559	0.897457197
LOC112448893	0.647833762	0.031105103	0.851330691	0.499042438	0.683538873	0.416111476	0.897457197
NUPR2	0.35307866	0.709088919	0.122969808	0.260933406	0.728354042	0.416091345	0.897457197

PCBP1	0.868447681	0.15831174	0.483540329	0.092172745	0.960096074	0.417018925	0.897457197
PNKP	0.631588361	0.050237368	0.638338775	0.330050657	0.8761484	0.416259062	0.897457197
PRDX1	0.465877817	0.303816072	0.205763267	0.340057308	0.592041319	0.416449038	0.897457197
PRRG4	0.332181647	0.226812937	0.3462933	0.797259529	0.280217422	0.415440427	0.897457197
RBPMS2	0.939238887	0.039104927	0.794921516	0.288867449	0.69034069	0.41524903	0.897457197
SARS	0.275668676	0.811736561	0.253850558	0.564101406	0.182270562	0.415782173	0.897457197
SLC25A27	0.625291051	0.20230308	0.906517351	0.061617415	0.825810874	0.415621675	0.897457197
ST3GAL6	0.315229903	0.301644141	0.520446702	0.861191609	0.138026271	0.417000633	0.897457197
SUPT16H	0.419942198	0.466951552	0.827472484	0.037497866	0.967943088	0.417201364	0.897457197
SYTL1	0.486750587	0.091684585	0.147014943	0.993147333	0.901195513	0.416701064	0.897457197
TAF4	0.511564571	0.751906031	0.839898712	0.083396613	0.21777493	0.416563804	0.897457197
TATDN3	0.778352377	0.338561996	0.307795342	0.509000182	0.141743378	0.416112657	0.897457197
TFB2M	0.97225579	0.456747608	0.123000395	0.64930068	0.16479517	0.415899512	0.897457197
TNFRSF8	0.983693771	0.328065004	0.663700467	0.401539392	0.068478246	0.417201935	0.897457197
TRIL	0.84748521	0.476184177	0.845123428	0.178534442	0.09650645	0.416822189	0.897457197
TRIM41	0.600230167	0.650595159	0.148752919	0.201855508	0.498441715	0.415897733	0.897457197
WDR83OS	0.647945799	0.576053193	0.584716652	0.068717753	0.38900756	0.415593776	0.897457197
ZDHHC9	0.260161574	0.39212591	0.510399877	0.129596311	0.871945684	0.417040375	0.897457197
TMEM135	0.976167977	0.544182781	0.087943935	0.756551603	0.166734289	0.417306405	0.897564275
DEPTOR	0.420109728	0.54781804	0.090401564	0.289035764	0.980483638	0.417396055	0.897576143
ID1	0.198359534	0.855779465	0.242079551	0.179845012	0.799890384	0.417842042	0.897576143
LOC101904498	0.503165626	0.61823076	0.17446535	0.218830645	0.497701836	0.417820905	0.897576143
MAPK3	0.462835313	0.321843103	0.314291863	0.225385832	0.558877042	0.417427391	0.897576143
NUDT15	0.555412763	0.362518954	0.140234612	0.934660677	0.224021108	0.417858859	0.897576143
RAB40B	0.054328175	0.959756771	0.988429899	0.982832556	0.116535462	0.417593624	0.897576143
SYNDIG1	0.267225048	0.461104428	0.329076149	0.179817531	0.809645015	0.417605305	0.897576143
TMTC1	0.555249436	0.419407239	0.070913906	0.888469406	0.402787649	0.417791795	0.897576143
TRAPPC5	0.38452054	0.87759047	0.592552843	0.050312957	0.586661802	0.417568623	0.897576143
TUBE1	0.541783844	0.229617323	0.465305013	0.565034039	0.180530549	0.417642323	0.897576143
MCRIP1	0.345131545	0.574445577	0.914873107	0.062232222	0.52441013	0.418069607	0.89791131
ACAT1	0.575256075	0.886418977	0.313313598	0.057402375	0.645809015	0.418161098	0.897990288
FAM69A	0.176064397	0.64081486	0.838911644	0.093173863	0.671900379	0.41824205	0.898046615
DHCR24	0.751721441	0.901489135	0.191304978	0.410746131	0.111353878	0.418361301	0.898067666
PRRT2	0.710993698	0.731041881	0.026428684	0.576746494	0.748270761	0.418323837	0.898067666
ABCF1	0.713521421	0.593244663	0.793872041	0.339498639	0.052121657	0.418843305	0.898073363
CCDC124	0.733874678	0.352415648	0.212668747	0.211614132	0.511905402	0.419184813	0.898073363
CDC14A	0.256059803	0.683627019	0.117648524	0.426131681	0.677288141	0.418771203	0.898073363
G6PC3	0.275887443	0.754769866	0.876675477	0.040458977	0.805684608	0.418968352	0.898073363
IFT172	0.791690921	0.019327968	0.651591449	0.598866661	0.997365144	0.419100725	0.898073363
ITPA	0.327243903	0.768140995	0.650156367	0.039557979	0.921012139	0.419072464	0.898073363
KANK1	0.763849825	0.485247241	0.426237941	0.375431289	0.100438896	0.419161354	0.898073363
LOC101905813	0.944741113	0.05072204	0.627202705	0.558944655	0.35427096	0.418990592	0.898073363
MRPS33	0.703202101	0.177712591	0.180245035	0.286271911	0.923096157	0.419016392	0.898073363
NARS	0.471567673	0.273665077	0.187400924	0.697515686	0.353015236	0.419093243	0.898073363

PTP4A1	0.822867963	0.063307033	0.210689451	0.961641819	0.56297928	0.418718572	0.898073363
RAB28	0.29449988	0.387094453	0.27024777	0.264414913	0.729953202	0.418842338	0.898073363
SCO2	0.282505837	0.970923223	0.572942402	0.071481356	0.528278045	0.418501373	0.898073363
WNT9B	0.140503596	0.69874156	0.810088628	0.499644829	0.14974041	0.418957195	0.898073363
XIAP	0.369256104	0.927040565	0.482492363	0.133240081	0.269841119	0.418612026	0.898073363
HAP1	0.605914062	0.57786143	0.220477424	0.164084766	0.470626274	0.419274654	0.89814859
EML3	0.717945936	0.083593716	0.415675997	0.331140425	0.721951365	0.419352299	0.898197674
PTPRK	0.164964976	0.62563012	0.519026811	0.477052353	0.233477693	0.419419316	0.898223985
SPOCK2	0.638125518	0.886360763	0.244412833	0.116253998	0.371573743	0.419570151	0.89842977
CCDC58	0.734419779	0.478722467	0.404242072	0.14639851	0.287229664	0.41970492	0.898601102
ADAMTS5	0.732684432	0.486518481	0.109194944	0.66096479	0.233817867	0.420824387	0.89878927
BANF1	0.693737635	0.284147184	0.556460997	0.076311605	0.716965643	0.420424959	0.89878927
BMI1	0.70242541	0.200740652	0.110874854	0.619581664	0.619087355	0.42028963	0.89878927
ECHDC3	0.384576607	0.655916371	0.328313979	0.14963453	0.485916334	0.420997692	0.89878927
FAT1	0.78017159	0.960235163	0.731991972	0.011083595	0.988137726	0.420547188	0.89878927
LOC101904536	0.363635938	0.224174906	0.280571104	0.343469923	0.762935765	0.420192768	0.89878927
LOC101905734	0.202834292	0.931101299	0.962737399	0.282450489	0.116902681	0.420484385	0.89878927
LOC510860	0.99369659	0.987179043	0.304679639	0.108644666	0.185002931	0.420589783	0.89878927
LOC524181	0.533021676	0.196089309	0.833310835	0.12693604	0.541657036	0.420051532	0.89878927
LRCH1	0.367992857	0.498450506	0.211165061	0.319098965	0.484878198	0.4201802	0.89878927
LYPD6	0.080107378	0.832754637	0.244538544	0.368920363	0.997285565	0.420435521	0.89878927
PCK1	0.043099509	0.244002603	0.671616986	0.921042325	0.925389698	0.420949809	0.89878927
PDE2A	0.076447166	0.773629634	0.741964432	0.402748474	0.33901244	0.420135087	0.89878927
PREB	0.108628186	0.284069277	0.983590185	0.430425039	0.460785263	0.420943648	0.89878927
PSKH1	0.358346611	0.497281946	0.137379594	0.27175605	0.903137429	0.420621775	0.89878927
PTGER3	0.675082599	0.910062131	0.034685956	0.386972719	0.728753083	0.420653765	0.89878927
SELPLG	0.132109905	0.628848576	0.918623221	0.14083546	0.560049175	0.420935744	0.89878927
SMIM14	0.528734979	0.120831397	0.157941919	0.815418667	0.728906901	0.4203089	0.89878927
STARD8	0.439314331	0.258459655	0.281491705	0.226782607	0.828356077	0.420503151	0.89878927
TAF11	0.905434556	0.073500946	0.275620475	0.474728925	0.688365366	0.420213567	0.89878927
ZNF311	0.260689717	0.126026541	0.523102871	0.762388106	0.458795008	0.42070325	0.89878927
ZNF516	0.809488118	0.499650211	0.855353445	0.021646807	0.803395017	0.420852119	0.89878927
JPH4	0.344131358	0.936548917	0.334292369	0.24464672	0.228712123	0.421193356	0.898856199
LOC101904810	0.495971303	0.110528499	0.686917167	0.183438171	0.872364133	0.42111952	0.898856199
PPARA	0.566423977	0.495370406	0.46903344	0.339130445	0.135037846	0.421149152	0.898856199
CHTF8	0.291131741	0.705474805	0.600366456	0.347277562	0.141040025	0.421507968	0.89890323
CLCN5	0.540726991	0.440401113	0.725785611	0.35837989	0.097612204	0.421695957	0.89890323
FN3K	0.721729569	0.894635214	0.198574031	0.05933751	0.794770448	0.421708364	0.89890323
LOC101905818	0.340213127	0.143154556	0.978694885	0.429296412	0.295254599	0.421567564	0.89890323
LOC112442082	0.988061611	0.446051429	0.137312497	0.92438638	0.108010586	0.421583913	0.89890323
NXPE3	0.606970606	0.232347354	0.255901294	0.301819677	0.553711823	0.421272098	0.89890323
PLEKHG5	0.300116825	0.384918418	0.565272476	0.539782432	0.171392253	0.421555448	0.89890323
TCOF1	0.593738819	0.120819317	0.2278871	0.413107725	0.8947345	0.421587932	0.89890323
TMEM14A	0.243319989	0.453545013	0.090812074	0.659772563	0.914369148	0.421686097	0.89890323

TCF23	0.053236223	0.467826869	0.950880551	0.350529894	0.728748891	0.421790279	0.898961074
LOC100847609	0.457716568	0.426833373	0.212204417	0.726438796	0.201075893	0.421966936	0.899220801
CDPF1	0.071808435	0.385957354	0.629116189	0.395268778	0.87956888	0.42214064	0.899378839
MLIP	0.115131725	0.493494346	0.933353488	0.216248195	0.528637096	0.422150704	0.899378839
ACBD4	0.781291082	0.857722521	0.121361195	0.231933431	0.322707322	0.422853824	0.89947558
ALS2	0.501013625	0.773844424	0.067851969	0.238375389	0.969553616	0.422650994	0.89947558
ELK3	0.836387893	0.988475018	0.024278359	0.561988	0.538305564	0.422433425	0.89947558
GUCY1B1	0.770605168	0.284315045	0.232540825	0.41451457	0.288189545	0.422829019	0.89947558
KLHL8	0.85932106	0.690853991	0.135233073	0.120056817	0.630743965	0.42263742	0.89947558
LIN52	0.658940803	0.0225754	0.956982355	0.448078484	0.953305946	0.42267998	0.89947558
LOC101908359	0.987364271	0.618364553	0.03851383	0.534184744	0.48436856	0.422772509	0.89947558
LOC112444333	0.622834034	0.699145246	0.179432393	0.106963729	0.728017365	0.422777548	0.89947558
LOC112448038	0.098354603	0.963005514	0.445170273	0.817122048	0.176055937	0.422251	0.89947558
MTMR6	0.91374976	0.522514943	0.026055811	0.88766363	0.550272276	0.422555196	0.89947558
OSBPL5	0.911967351	0.347309857	0.069432483	0.713784053	0.38748025	0.422720845	0.89947558
RIMKLA	0.218189288	0.174217194	0.95987265	0.284283883	0.586019542	0.422612831	0.89947558
ANXA1	0.516377675	0.300763569	0.425892992	0.204250535	0.451145969	0.423076079	0.899831718
SGF29	0.421322121	0.936476683	0.135162384	0.718638275	0.159105948	0.42315141	0.899875313
CCDC88C	0.742032468	0.59492981	0.028118928	0.640898517	0.767398651	0.423362705	0.900097118
CLCC1	0.30979146	0.594504786	0.173299677	0.46069981	0.415206304	0.423365404	0.900097118
TNFAIP1	0.576873357	0.597459734	0.363305077	0.228931443	0.213095672	0.423458883	0.90017924
RICTOR	0.145358869	0.973671776	0.69930924	0.371606979	0.166186972	0.423563227	0.900284434
ARHGAP19	0.572602898	0.8022359	0.140259436	0.286500283	0.331392957	0.423702541	0.90046392
AGTRAP	0.723479795	0.433464469	0.049821536	0.47151603	0.835623885	0.424791605	0.900683673
AMHR2	0.825295214	0.039933815	0.373098917	0.66671624	0.75066115	0.424734012	0.900683673
BOD1	0.713048583	0.892781822	0.128972283	0.137438123	0.543198018	0.424047211	0.900683673
CCSER1	0.110180086	0.868169193	0.376982684	0.221288753	0.770493547	0.424574685	0.900683673
COQ6	0.80143439	0.516090616	0.821405224	0.042930636	0.420720686	0.424239913	0.900683673
FFAR4	0.272660129	0.223968998	0.411723141	0.928689985	0.262425494	0.423993875	0.900683673
GINS1	0.659415546	0.919857802	0.429016703	0.052722219	0.44877581	0.424820499	0.900683673
HOXC9	0.36217166	0.235627708	0.472224572	0.274668166	0.553651771	0.424010838	0.900683673
HTT	0.819497478	0.608344792	0.833296142	0.170105028	0.087005291	0.424575627	0.900683673
LOC104975925	0.608096115	0.467194077	0.34902636	0.150397028	0.412092669	0.424497266	0.900683673
LOC112445177	0.770036406	0.407928265	0.256188496	0.085505839	0.892234229	0.424325805	0.900683673
LOC112447324	0.479531639	0.325747918	0.297661929	0.151809632	0.870629903	0.424494565	0.900683673
MYOC	0.137774753	0.668592056	0.49277921	0.373923264	0.36145502	0.424203215	0.900683673
RAB5B	0.989102533	0.42052131	0.533777631	0.133705762	0.207233105	0.424670518	0.900683673
RIPK2	0.510257887	0.155446315	0.194414842	0.450349665	0.886010099	0.424705861	0.900683673
ST6GAL1	0.682491784	0.883062121	0.015179281	0.832137581	0.804716748	0.423948893	0.900683673
TAF5L	0.578607762	0.68901894	0.487126424	0.15602317	0.202449053	0.424180223	0.900683673
TNFRSF17	0.119282459	0.22424813	0.495569697	0.983349793	0.471676519	0.424576753	0.900683673
WRN	0.100065018	0.627399503	0.886144855	0.527951559	0.209663634	0.424848718	0.900683673
DLK1	0.16375184	0.246070482	0.446904531	0.524092904	0.653763467	0.425182317	0.901144727
IBA57	0.628847727	0.67348011	0.808291453	0.018338975	0.983102037	0.425230928	0.901144727

LOC112445063	0.576979178	0.437385765	0.108491628	0.746870764	0.30176851	0.425202549	0.901144727
LOC112448153	0.710125132	0.495963558	0.329342409	0.561926297	0.094760958	0.425361547	0.90123925
ZBED4	0.308847217	0.352261888	0.227386579	0.582507809	0.428672081	0.425385365	0.90123925
NAP1L4	0.225176749	0.988521416	0.708956385	0.070203975	0.558120026	0.425550989	0.901473769
C15H11orf49	0.097452434	0.977261267	0.393300954	0.220370454	0.750173639	0.425799135	0.901766628
NEPRO	0.720602385	0.404407386	0.793518239	0.840865292	0.031835625	0.425746709	0.901766628
TSPYL2	0.590919132	0.444437549	0.19302425	0.305695481	0.39973821	0.425867352	0.901794724
BRF1	0.699112933	0.186673841	0.380177045	0.18995086	0.657743349	0.425986112	0.901929827
FUS	0.415064621	0.479111967	0.950083629	0.347063621	0.094572858	0.426055913	0.901961247
MOCS2	0.130987302	0.310424564	0.60112759	0.568421629	0.446654632	0.426176738	0.902100664
ACVR2B	0.405879214	0.341212194	0.350949867	0.254285658	0.510717907	0.429111533	0.90210852
AHCY	0.449512532	0.759498379	0.619998741	0.306775773	0.096647418	0.428116061	0.90210852
ARRDC2	0.710571838	0.674393079	0.087730018	0.184225631	0.806471385	0.427295272	0.90210852
C27H4orf47	0.989359313	0.513051277	0.0593386	0.496341891	0.420922989	0.428580024	0.90210852
CADM2	0.381805694	0.19637365	0.351967806	0.963076018	0.246760407	0.42799416	0.90210852
CCDC146	0.268531323	0.7113144	0.098825299	0.339838727	0.985309066	0.429351018	0.90210852
CCER2	0.075419413	0.427362116	0.393194316	0.764066252	0.64688289	0.427786564	0.90210852
CD151	0.313591168	0.748425304	0.183688778	0.295384556	0.495608843	0.429092539	0.90210852
CIAPIN1	0.226216687	0.680299834	0.252202931	0.516044084	0.312225747	0.42750306	0.90210852
CTSH	0.404367585	0.643112654	0.048068702	0.979639492	0.510890678	0.427578421	0.90210852
CXADR	0.816195629	0.471262306	0.140922419	0.822176479	0.139659448	0.426684567	0.90210852
CYBC1	0.109397749	0.961203625	0.951305407	0.394876986	0.1584095	0.427604981	0.90210852
CYP2D14	0.455236054	0.543712584	0.075550077	0.423292288	0.7895131	0.427387683	0.90210852
DNAH2	0.511277759	0.724128766	0.274342216	0.0708085	0.872261912	0.428047129	0.90210852
EBD	0.084156073	0.920676394	0.902031712	0.752969643	0.118727247	0.427348499	0.90210852
EDN3	0.333106407	0.625092928	0.105583592	0.835211705	0.338762033	0.426582376	0.90210852
EEF1AKMT3	0.676436227	0.783106594	0.758652804	0.23397192	0.066782387	0.428213899	0.90210852
EVI5	0.926576649	0.29516327	0.131288033	0.213050008	0.824308515	0.428940433	0.90210852
F2R	0.370443767	0.725615023	0.125616413	0.385765433	0.482534736	0.428377325	0.90210852
FBXO38	0.350938657	0.500299551	0.15801425	0.261755328	0.867419828	0.428758031	0.90210852
FKBP5	0.870516008	0.842447296	0.051254334	0.197680923	0.850158299	0.429249187	0.90210852
GRSF1	0.370194501	0.102640129	0.357647346	0.672402625	0.685619899	0.427815179	0.90210852
GTF2H3	0.43028937	0.311880316	0.759643766	0.326990092	0.187574107	0.427477355	0.90210852
ITGA9	0.084445945	0.842127502	0.10286535	0.96232863	0.889034093	0.427637126	0.90210852
ITGB1BP1	0.83765198	0.487510409	0.355249473	0.260417622	0.166486313	0.428498026	0.90210852
LOC101902665	0.691358749	0.471192101	0.803033056	0.462215365	0.052165007	0.428987619	0.90210852
LOC101903015	0.177691818	0.158561872	0.608481716	0.842779416	0.43702821	0.429178402	0.90210852
LOC101905319	0.417139281	0.984016818	0.535263946	0.042944044	0.66434433	0.427907843	0.90210852
LOC104972407	0.683964085	0.365174341	0.178632902	0.355090169	0.395114706	0.42767265	0.90210852
LOC107132589	0.916121829	0.05635309	0.336975095	0.736789967	0.484468265	0.426289331	0.90210852
LOC112441645	0.832319789	0.441305988	0.433568247	0.077415787	0.506197923	0.427147742	0.90210852
LOC112442401	0.507564878	0.368737706	0.364949871	0.300432455	0.308150612	0.429423664	0.90210852
LOC112444936	0.999412209	0.555023749	0.447145991	0.475564338	0.05334487	0.428568794	0.90210852
LOC112445193	0.779435852	0.986057664	0.547213166	0.019714435	0.755451997	0.427781532	0.90210852

LOC786616	0.458516132	0.797543765	0.225538984	0.09531676	0.804291826	0.429408304	0.90210852
MCM9	0.689823794	0.994010258	0.08922331	0.416354492	0.248029022	0.429272497	0.90210852
MEPCE	0.468809019	0.575083812	0.169697138	0.395885648	0.346499364	0.428117271	0.90210852
MIEN1	0.211405299	0.382751211	0.381211433	0.215583531	0.935786545	0.426652098	0.90210852
MRPL40	0.136166505	0.816758136	0.582664011	0.123872031	0.786022898	0.429040186	0.90210852
NDUFAF7	0.985716874	0.59368108	0.599594071	0.028980875	0.616768515	0.428006826	0.90210852
NIPA2	0.895077649	0.441681075	0.036843337	0.862537513	0.497878549	0.427542537	0.90210852
NRIP1	0.471494493	0.397527311	0.99671772	0.174355548	0.191132711	0.426729934	0.90210852
NT5C3A	0.475071348	0.932591748	0.145448357	0.389528795	0.249966136	0.428079905	0.90210852
PHIP	0.394967233	0.67583815	0.600458229	0.054394948	0.715754503	0.427136536	0.90210852
PPEF1	0.336461274	0.367360085	0.856903356	0.096119269	0.620950692	0.429373522	0.90210852
RUSC2	0.133368674	0.906431705	0.923223564	0.670835048	0.083935668	0.428349948	0.90210852
SGPP1	0.556043783	0.979792911	0.407452272	0.601245896	0.04682441	0.427389204	0.90210852
SLITRK6	0.85323661	0.961463646	0.215720605	0.041426356	0.851563562	0.427207548	0.90210852
SNRNP27	0.885483127	0.732223423	0.386858128	0.037523818	0.668069997	0.428448141	0.90210852
SNRPB	0.702457748	0.096837135	0.721322038	0.814135111	0.156796149	0.427778657	0.90210852
SRFBP1	0.642773002	0.338604379	0.68589068	0.050278602	0.841334845	0.429186395	0.90210852
TMEM158	0.530563364	0.415168095	0.101544494	0.341212073	0.814611462	0.426493838	0.90210852
TMEM192	0.893753621	0.344520032	0.200611174	0.325668961	0.313553058	0.428994112	0.90210852
TMEM42	0.492653592	0.141764868	0.373197063	0.390093074	0.620452729	0.429014283	0.90210852
TRAF2	0.458077216	0.456150895	0.867448808	0.067156038	0.516986654	0.428587907	0.90210852
TRAF3IP2	0.628529003	0.839601786	0.153110386	0.229590952	0.337720843	0.427815572	0.90210852
USHBP1	0.518681857	0.708744081	0.065007222	0.342905821	0.761778178	0.427194709	0.90210852
ZBTB10	0.354853833	0.968514066	0.232601553	0.230170247	0.337824077	0.426460186	0.90210852
ZCCHC24	0.178719454	0.635431963	0.25562787	0.274183876	0.789089183	0.428253963	0.90210852
GPD2	0.218953462	0.430525778	0.758984667	0.186512004	0.474351377	0.429598648	0.902209683
SPAG1	0.408782357	0.330872536	0.605210217	0.48815891	0.158440466	0.429636748	0.902209683
SSH1	0.725470486	0.311643119	0.932246437	0.340489879	0.088209659	0.429613583	0.902209683
NXPH2	0.314064707	0.489163074	0.272179229	0.543010386	0.279070334	0.42978162	0.902282994
ZC3H6	0.048343081	0.773959797	0.963894279	0.925677169	0.189750982	0.429732198	0.902282994
MUT	0.913385268	0.986312675	0.440319495	0.062966547	0.253866594	0.429902269	0.902420841
ANXA5	0.45473228	0.486773563	0.116986143	0.275240526	0.890211506	0.430010338	0.902431755
CSTF1	0.808593265	0.127037996	0.776193512	0.429105594	0.185456752	0.430017447	0.902431755
ASPA	0.040464628	0.674387573	0.523337327	0.564206631	0.787753127	0.430079131	0.902445803
GEMIN8	0.540431531	0.25794494	0.227374241	0.517587465	0.387031492	0.430136553	0.902450905
AIFM1	0.635215091	0.921081216	0.825273504	0.024792676	0.531199255	0.430399432	0.902555386
APP	0.986389802	0.17713557	0.575973086	0.231372896	0.275908343	0.432172639	0.902555386
ARL5B	0.245409106	0.114406444	0.567839247	0.440131709	0.913688264	0.43181797	0.902555386
ARNTL	0.683697381	0.414754468	0.100480375	0.936493004	0.238971894	0.430874741	0.902555386
CGREF1	0.077878795	0.816026324	0.16709688	0.635133471	0.949370179	0.431596043	0.902555386
COMMD8	0.632015397	0.277105345	0.120065829	0.51751008	0.588135009	0.431514283	0.902555386
ENDOD1	0.47931342	0.04556661	0.403506189	0.967523787	0.746164773	0.430485437	0.902555386
FBXO30	0.172451315	0.978538691	0.459096313	0.115855119	0.710236841	0.430826458	0.902555386
FBXO45	0.694842386	0.134779452	0.208820783	0.36509463	0.895678383	0.431375727	0.902555386

FGFR1	0.568655029	0.914130704	0.06186702	0.652677019	0.305155085	0.43165322	0.902555386
FRG1	0.289110573	0.299043309	0.457259481	0.464663494	0.348812759	0.431715758	0.902555386
GALNT2	0.587166781	0.906946713	0.50647041	0.029048539	0.814977472	0.431106335	0.902555386
GLRX3	0.840449631	0.513319978	0.148919872	0.93956525	0.106023191	0.431511284	0.902555386
GLYCTK	0.689632955	0.927316095	0.512764353	0.030107383	0.649149535	0.431751628	0.902555386
GNPDA2	0.581154026	0.502578656	0.136210913	0.39938451	0.404259791	0.432142672	0.902555386
GPC4	0.220848213	0.52011897	0.221275735	0.319024906	0.793057993	0.432343131	0.902555386
HEPACAM	0.802274049	0.044205826	0.325205584	0.915354675	0.607424347	0.431855797	0.902555386
HEXDC	0.841716434	0.097077834	0.800317522	0.111925423	0.876298038	0.431890573	0.902555386
HMGXB4	0.268750102	0.637433199	0.330327924	0.828926998	0.137018097	0.432248252	0.902555386
HYOU1	0.372433235	0.808385475	0.361185951	0.162147338	0.363978463	0.431992375	0.902555386
LGR4	0.202910247	0.702964299	0.791944213	0.390512005	0.145756114	0.432316357	0.902555386
LOC104969097	0.86062733	0.170249699	0.19896197	0.44753402	0.492688827	0.432267261	0.902555386
LOC112445982	0.746235263	0.469901305	0.343674344	0.215884166	0.246216445	0.431666043	0.902555386
LOC112448253	0.454345515	0.11811491	0.643190374	0.304006452	0.609182584	0.431303592	0.902555386
LOC512863	0.761015302	0.959329346	0.897892024	0.080115211	0.121297589	0.430700887	0.902555386
LOC534627	0.303054088	0.576469102	0.221369109	0.30370025	0.544165694	0.431275102	0.902555386
LOC787397	0.481870145	0.468928842	0.097576533	0.429073682	0.679913053	0.432386231	0.902555386
LPCAT1	0.054681554	0.362104317	0.74464805	0.734209773	0.593352615	0.432142539	0.902555386
MAMLD1	0.604614186	0.385925578	0.7669054	0.073967574	0.482176229	0.431027869	0.902555386
MARCH3	0.39992907	0.235963362	0.559879764	0.183243047	0.662009711	0.431764742	0.902555386
MGAT5B	0.097105993	0.644628804	0.932982806	0.72518698	0.15009277	0.430336207	0.902555386
MRPL35	0.450898947	0.778460308	0.644552469	0.077330671	0.365231678	0.431237003	0.902555386
NRN1	0.084305834	0.733255877	0.708842987	0.202024025	0.718936293	0.430542842	0.902555386
ORC4	0.206496492	0.60082526	0.083876837	0.918608088	0.670051852	0.431655729	0.902555386
PDP1	0.752530937	0.351615398	0.619796404	0.114806073	0.339572451	0.431334699	0.902555386
PHF13	0.210180523	0.732686355	0.555580131	0.323767486	0.230801654	0.43133103	0.902555386
PLEKHG6	0.451878474	0.960892815	0.082431763	0.443838188	0.400275986	0.430390602	0.902555386
RAB3B	0.424028004	0.391929184	0.056465033	0.942975569	0.723033019	0.431455302	0.902555386
SOX13	0.946522792	0.26709138	0.504422062	0.837559314	0.060129257	0.432113653	0.902555386
TMEM62	0.452191219	0.911649087	0.929485029	0.02018866	0.828132059	0.431678813	0.902555386
CENPQ	0.67670324	0.192940624	0.693887106	0.165577023	0.429345278	0.432606349	0.90270055
CHTOP	0.634828858	0.346675275	0.744737845	0.291621849	0.134769114	0.432636723	0.90270055
GAPT	0.838019167	0.09369973	0.5746936	0.719484224	0.198430464	0.4326627	0.90270055
MTX3	0.880956168	0.415752965	0.42469976	0.237380672	0.174491374	0.432675798	0.90270055
LOC101902030	0.63483753	0.079084244	0.994167118	0.165763169	0.779679059	0.432885967	0.902794713
LOC614732	0.640477528	0.139083718	0.157309284	0.742830873	0.619369737	0.432789301	0.902794713
PCSK1N	0.362972916	0.70392339	0.431135621	0.070610691	0.829176033	0.432851872	0.902794713
CDC25A	0.350216934	0.641631985	0.231514013	0.129507831	0.960519027	0.433441421	0.902805834
DNAAF2	0.427993468	0.741566827	0.592949923	0.290532602	0.118308999	0.433367559	0.902805834
E2F8	0.853669357	0.312499612	0.10147918	0.328942881	0.725814365	0.433224953	0.902805834
FAM89B	0.152519983	0.332708085	0.380674416	0.468289571	0.715274016	0.433412688	0.902805834
ITGAX	0.378284611	0.185780401	0.255549173	0.926295167	0.388315897	0.433130988	0.902805834
LOC533308	0.206226226	0.895163786	0.14602634	0.76944403	0.311914227	0.433396294	0.902805834

POLE3	0.989132569	0.114180643	0.244870071	0.786539895	0.297259879	0.433296095	0.902805834
RBBP9	0.442475956	0.753330412	0.431780443	0.189865244	0.236408077	0.433138322	0.902805834
SERPIND1	0.403576967	0.226396049	0.518392622	0.256094112	0.533285308	0.43336561	0.902805834
ZNF22	0.503565613	0.127613433	0.505379593	0.416527665	0.477550579	0.433133299	0.902805834
EAPP	0.164486287	0.614319688	0.300383292	0.500467373	0.426743391	0.43373803	0.903082655
MRPS11	0.276309683	0.627041191	0.958366897	0.079311308	0.491973343	0.433641101	0.903082655
RMDN3	0.412742416	0.297500136	0.219230897	0.299790954	0.804027955	0.433904499	0.903082655
RNF181	0.340768597	0.339970795	0.34822832	0.289053914	0.555975684	0.433761056	0.903082655
SIRT2	0.675290564	0.734475185	0.185053139	0.293042633	0.241235853	0.433896168	0.903082655
TBC1D23	0.426181778	0.517936337	0.144267947	0.498148054	0.408943814	0.433866605	0.903082655
ADPRM	0.223332813	0.464184662	0.46943308	0.596875959	0.22350869	0.433999853	0.903166573
BRD8	0.83476343	0.115645907	0.864674025	0.688566898	0.113290318	0.4345171	0.9032591
CD14	0.229962259	0.466815177	0.55171725	0.146637386	0.749264124	0.434401838	0.9032591
DDIT4L	0.265459146	0.475638819	0.668000013	0.143826278	0.536747709	0.434507016	0.9032591
FAM78B	0.689871058	0.306382985	0.247725888	0.579438507	0.214649545	0.434539674	0.9032591
KATNB1	0.223065193	0.916095811	0.671113193	0.081043938	0.585169237	0.434310677	0.9032591
LOC112443837	0.284432744	0.94976437	0.713987673	0.059736155	0.564045753	0.434177217	0.9032591
PRR13	0.446231645	0.531985869	0.191907055	0.165127258	0.863738561	0.434143007	0.9032591
SLC17A9	0.523667558	0.177667999	0.480049729	0.386254735	0.37717667	0.434390606	0.9032591
TRIM63	0.507001016	0.354439572	0.13601318	0.801289378	0.331985992	0.434258799	0.9032591
ABCD4	0.331362007	0.443274259	0.435924381	0.861924867	0.120490953	0.438186359	0.903267193
ACADSB	0.263293007	0.873985599	0.365191407	0.190906107	0.40947744	0.436054802	0.903267193
ACSL4	0.670880399	0.384162044	0.179801899	0.524817453	0.270148474	0.436075168	0.903267193
ACVR1B	0.745113521	0.437967227	0.847568836	0.073171749	0.328465158	0.438130569	0.903267193
AGPAT1	0.667591459	0.997093052	0.377173435	0.26156559	0.101180773	0.438047723	0.903267193
ALKBH7	0.205485149	0.889865519	0.372007147	0.10724976	0.908378039	0.437585682	0.903267193
ATL2	0.792532723	0.142181035	0.954359171	0.43879981	0.140738951	0.437961227	0.903267193
CBS	0.50767179	0.844662926	0.61585429	0.158866008	0.15738031	0.436944353	0.903267193
CCDC68	0.885958548	0.276453271	0.081207132	0.360740062	0.911238222	0.435227823	0.903267193
CDK7	0.635778658	0.963021009	0.963089415	0.020476611	0.545730181	0.436589752	0.903267193
CNIH1	0.68812364	0.111474593	0.597934885	0.759166633	0.190890642	0.438108906	0.903267193
COL4A5	0.104091043	0.874373758	0.370008742	0.442006554	0.445458853	0.437682212	0.903267193
CYP4A11	0.694273086	0.733439056	0.694590202	0.160688911	0.114949592	0.435091599	0.903267193
DIP2A	0.823687267	0.492617645	0.043440682	0.669941747	0.563010329	0.438150948	0.903267193
E2F3	0.535419888	0.590273617	0.574634646	0.732810654	0.049552009	0.436729872	0.903267193
EIF4E3	0.680019307	0.231463756	0.29086752	0.34319822	0.416953436	0.435579852	0.903267193
ERCC5	0.260196086	0.65568191	0.322667391	0.298717349	0.40394741	0.437994602	0.903267193
ETFA	0.827136593	0.577336904	0.954932781	0.015462306	0.936408319	0.436941569	0.903267193
FBXO44	0.810586875	0.533895913	0.468898641	0.53757846	0.060795345	0.437717878	0.903267193
FRRS1	0.336942588	0.935234917	0.379231971	0.110751926	0.501934677	0.43801303	0.903267193
FURIN	0.152209433	0.472182859	0.605591185	0.176200109	0.859598517	0.436665687	0.903267193
GALC	0.702141198	0.899374152	0.262864014	0.208201618	0.190654669	0.436582978	0.903267193
GCDH	0.642866984	0.670127087	0.77552048	0.023848593	0.819781522	0.435057913	0.903267193
GLB1L	0.867541596	0.46395066	0.311145054	0.201805182	0.261720131	0.437253692	0.903267193

GSKIP	0.948917889	0.145929215	0.169872854	0.656688755	0.423917277	0.435500918	0.903267193
GSTM1	0.728979252	0.798439974	0.112308132	0.165055314	0.615576973	0.437972931	0.903267193
HBA	0.327098423	0.910063227	0.328099171	0.115358322	0.585098057	0.436665516	0.903267193
HMGB2	0.303463475	0.825358828	0.084685918	0.492920583	0.636161897	0.43822456	0.903267193
ICAM1	0.717658798	0.070478685	0.650778839	0.243081898	0.817780035	0.43536547	0.903267193
KLC1	0.398739961	0.515793501	0.623079782	0.062733761	0.817422146	0.436112309	0.903267193
LOC100126043	0.444778804	0.40802916	0.112742163	0.773987343	0.415704157	0.43642707	0.903267193
LOC101907965	0.46047603	0.404511739	0.755818309	0.096575711	0.479707124	0.434803881	0.903267193
LOC104969719	0.720343879	0.028176774	0.791196824	0.677601534	0.603854385	0.436098345	0.903267193
LOC107131939	0.938430479	0.64684741	0.157695091	0.124345315	0.555798669	0.437283722	0.903267193
LOC107133473	0.617796923	0.426220309	0.946970405	0.15430323	0.173037445	0.438396392	0.903267193
LOC112442280	0.045139439	0.342607644	0.987574723	0.554341201	0.785905007	0.438291196	0.903267193
LOC112442623	0.77792851	0.882803626	0.119613005	0.138128419	0.582147535	0.43701376	0.903267193
LOC112442843	0.540279025	0.819775632	0.679775293	0.725384899	0.029894147	0.4349779	0.903267193
LOC112443130	0.49759916	0.069564988	0.84425495	0.46973465	0.482342548	0.437437944	0.903267193
LOC112448373	0.226283517	0.9755255	0.053142052	0.642419427	0.86824913	0.435363276	0.903267193
LOC512486	0.652813637	0.324108799	0.194974568	0.173824874	0.923966039	0.437548185	0.903267193
MAU2	0.556592215	0.333651434	0.453421552	0.628075678	0.125188871	0.437420971	0.903267193
METTL18	0.069597366	0.592282592	0.543699107	0.881598239	0.335863071	0.437825029	0.903267193
NBR1	0.448989383	0.848183664	0.075597468	0.609648254	0.374567096	0.436186749	0.903267193
NKIRAS1	0.908706966	0.632606094	0.605089136	0.033676843	0.56208225	0.436454398	0.903267193
OSGIN1	0.261760265	0.534846382	0.12716929	0.564711651	0.653131959	0.435985974	0.903267193
PDPN	0.804850828	0.09191096	0.487085811	0.881172585	0.208080389	0.437046759	0.903267193
PKN3	0.749618562	0.130670669	0.161250189	0.948816666	0.444177093	0.438366463	0.903267193
PORCN	0.334767027	0.49777329	0.279082454	0.375808525	0.380872449	0.438364788	0.903267193
PPIG	0.281245834	0.691605813	0.490199702	0.124809718	0.552968201	0.436357288	0.903267193
PTMA	0.244870805	0.26550099	0.335616432	0.49860246	0.606729944	0.436892206	0.903267193
RNASE10	0.791679967	0.601525923	0.31203936	0.751910431	0.05910872	0.436986696	0.903267193
RNF223	0.571314399	0.946695891	0.84980697	0.801096631	0.017925238	0.43687594	0.903267193
SCAMP2	0.759058461	0.226173012	0.096539426	0.84919991	0.469143384	0.436948553	0.903267193
SEC61G	0.675440211	0.067596174	0.445888274	0.708351917	0.4602069	0.437834664	0.903267193
SFRP5	0.772405671	0.725427545	0.09030344	0.367988431	0.351864575	0.43558847	0.903267193
SLC2A13	0.967403462	0.086769475	0.907081326	0.094750861	0.908451173	0.435649573	0.903267193
SLC35E3	0.469005738	0.473783103	0.42569755	0.690577062	0.101102105	0.436986767	0.903267193
SLF2	0.475025792	0.890072376	0.187976967	0.1109893	0.746434309	0.436459235	0.903267193
SPHK2	0.36637216	0.962479899	0.577422009	0.044082044	0.730071377	0.435621016	0.903267193
SPPL2A	0.912219674	0.216621773	0.131246452	0.282206593	0.898224227	0.436186287	0.903267193
SPRED3	0.648717835	0.235963742	0.063821348	0.920426867	0.72814655	0.435476659	0.903267193
TLR4	0.482849196	0.942498809	0.90261626	0.090309182	0.178484811	0.437428486	0.903267193
TRIO	0.540784093	0.218233923	0.162472658	0.636004389	0.540306596	0.436582696	0.903267193
TUSC2	0.54449291	0.044276105	0.434255852	0.692851422	0.898775606	0.434723232	0.903267193
TYMS	0.296876072	0.121952978	0.512439137	0.433955682	0.824130648	0.437800049	0.903267193
UBE2K	0.526921295	0.306252244	0.279895314	0.180978984	0.806414762	0.436655274	0.903267193
WDR44	0.871121586	0.778443318	0.140751326	0.790858145	0.087769646	0.437538225	0.903267193

WDR62	0.813264831	0.027243292	0.507276636	0.924584068	0.627927976	0.434881363	0.903267193
ZBTB7A	0.738758532	0.417263029	0.559790686	0.082324304	0.459668532	0.435009121	0.903267193
UFC1	0.518427405	0.446827262	0.447541607	0.130893615	0.490874504	0.438484494	0.903335304
LOC101907138	0.346493568	0.186951936	0.888606956	0.396985277	0.291606641	0.43854766	0.903352033
MRPS12	0.412646546	0.881261292	0.632089535	0.350209812	0.082852985	0.438705425	0.903450211
SMOC2	0.461933398	0.790703043	0.130695979	0.656727458	0.212713008	0.43867972	0.903450211
ASH1L	0.277642196	0.823712783	0.844570983	0.069444976	0.498255392	0.439065273	0.903472445
IDNK	0.505461844	0.191402886	0.291063759	0.373617162	0.634441059	0.438844586	0.903472445
LOC104975196	0.23976197	0.217571669	0.515064595	0.379032894	0.655935292	0.438981364	0.903472445
LRRC1	0.937816539	0.404016357	0.19195321	0.220411772	0.416409225	0.438854366	0.903472445
MTM1	0.773893534	0.107472204	0.226813017	0.683949162	0.518097073	0.439101592	0.903472445
TMEM145	0.54826034	0.647129029	0.070674052	0.321056256	0.830186205	0.43906692	0.903472445
ZNF213	0.873520817	0.56533649	0.86203319	0.019246327	0.815789086	0.439081305	0.903472445
CABLES1	0.92526634	0.492962175	0.70604292	0.533948886	0.039039506	0.439842182	0.903562943
CD37	0.323764026	0.667995109	0.660389959	0.185639789	0.252921445	0.439657936	0.903562943
ELOC	0.212065937	0.774219433	0.637446727	0.225541324	0.284421185	0.439862967	0.903562943
KRT10	0.431519229	0.741944564	0.720096262	0.173046268	0.167984174	0.439550459	0.903562943
LOC101903400	0.23654449	0.496672965	0.419376388	0.863105878	0.157654067	0.439616576	0.903562943
LOC112442613	0.753008174	0.078228902	0.780351075	0.199108348	0.733757981	0.439916392	0.903562943
MAP2K1	0.854312397	0.697826323	0.197656874	0.098878688	0.576325957	0.439895229	0.903562943
MEFV	0.77701267	0.033243817	0.422528336	0.874393829	0.701077021	0.439257851	0.903562943
METTL4	0.19788053	0.637512497	0.377110735	0.1455059	0.96894437	0.439690653	0.903562943
PAPLN	0.845221196	0.407509266	0.339118838	0.079287372	0.724311321	0.43971042	0.903562943
PDPR	0.499217597	0.16410967	0.378022766	0.755032253	0.286605835	0.439550368	0.903562943
PGAM1	0.586272043	0.636812626	0.355873168	0.118731172	0.425507004	0.439826881	0.903562943
PSCA	0.922137515	0.125330469	0.339773766	0.889264789	0.191817706	0.439457321	0.903562943
RIPK3	0.277760288	0.086315863	0.955540908	0.452614718	0.645785633	0.439402892	0.903562943
LOC112449505	0.317472062	0.331257602	0.563782976	0.135553135	0.836140894	0.440026661	0.903676327
FOSL2	0.441049773	0.51594983	0.152418113	0.316640582	0.612317938	0.440148968	0.903751423
SLC1A7	0.180554389	0.964332667	0.255413122	0.643849367	0.2348947	0.440173367	0.903751423
C23H6orf52	0.31697609	0.420840883	0.35463133	0.311640469	0.456600753	0.440325391	0.903837397
LOC107133343	0.963734329	0.023774097	0.652954562	0.455630905	0.98725341	0.440274546	0.903837397
CEBPZOS	0.466445372	0.762125736	0.370535965	0.099492016	0.514111685	0.440483547	0.90383981
LOC112444351	0.458904701	0.291142142	0.768189588	0.096910159	0.677122072	0.440414374	0.90383981
ZNF710	0.350553836	0.763958261	0.261033418	0.431016868	0.223619238	0.440491792	0.90383981
MEMO1	0.539767762	0.705135246	0.912114958	0.209014743	0.09294283	0.440652554	0.90405664
CREB3L4	0.838424698	0.047672893	0.730897118	0.274461035	0.842424453	0.440928085	0.904396687
FAN1	0.362680881	0.630023183	0.578089636	0.140507964	0.363937859	0.440928517	0.904396687
CDRT1	0.995953471	0.623462449	0.579685736	0.055032808	0.341333545	0.44110561	0.904646859
AGRN	0.315041843	0.639040168	0.63463112	0.329607069	0.160721615	0.441285933	0.904790665
LOC101907622	0.064181815	0.728891175	0.681049026	0.219485994	0.97013326	0.441691512	0.904790665
LOC104975054	0.569541355	0.637044429	0.539731263	0.505447059	0.068433071	0.441417654	0.904790665
LOC534578	0.922428853	0.262283968	0.10078135	0.522360893	0.532388177	0.4416076	0.904790665
LOC781439	0.308526462	0.653680686	0.356415363	0.117977972	0.799740178	0.441640156	0.904790665

MAP1S	0.783181451	0.463479769	0.309468834	0.206434758	0.292178656	0.441469076	0.904790665
MTPAP	0.736597503	0.896876603	0.132676653	0.168973761	0.457231696	0.441376715	0.904790665
PEX10	0.994870791	0.09195268	0.872043271	0.810191532	0.10498381	0.441727062	0.904790665
RSBN1	0.192275538	0.763619927	0.997341902	0.075137147	0.616328801	0.441619197	0.904790665
ZNF286A	0.137746112	0.844427258	0.827640456	0.321014777	0.219128344	0.441374224	0.904790665
HSPA4	0.847247958	0.287635088	0.584257319	0.049378754	0.965772047	0.441845284	0.904919875
MAK16	0.914492163	0.192467761	0.676250337	0.679418161	0.084010014	0.441943364	0.905007804
IARS	0.620167534	0.219860575	0.922924967	0.653000513	0.082714684	0.442026425	0.905045872
RCL1	0.767894548	0.77295218	0.028586034	0.788793424	0.507996531	0.442072251	0.905045872
CST7	0.8310937	0.79595601	0.033346034	0.624503925	0.494057721	0.442260065	0.905091648
LOC112443244	0.331870919	0.870512134	0.129644727	0.313359711	0.579838719	0.442241096	0.905091648
NXPE2	0.393922419	0.615093061	0.445568127	0.277487306	0.22718646	0.442258079	0.905091648
TUBGCP6	0.951962403	0.050107784	0.24794653	0.905379813	0.63651074	0.4425125	0.905495342
BLOC1S5	0.814612383	0.123078814	0.385623976	0.6716142	0.262840737	0.442751987	0.905569685
CGRRF1	0.315130657	0.508831315	0.196454019	0.53323831	0.406739303	0.442935096	0.905569685
DPH1	0.61078857	0.905389365	0.218578486	0.081740717	0.690625919	0.442713158	0.905569685
FAM53B	0.384127092	0.613855834	0.548150711	0.103734175	0.508637742	0.442614627	0.905569685
LOC112442271	0.453495488	0.703353221	0.20123541	0.310659475	0.34244391	0.442838684	0.905569685
POLR3D	0.230821262	0.654411659	0.898441919	0.616951482	0.081570359	0.442869199	0.905569685
TPMT	0.169868308	0.673383307	0.302306803	0.255241712	0.773971851	0.442909393	0.905569685
KCNT2	0.991688704	0.168532571	0.228543734	0.186747278	0.958669281	0.443092605	0.90562955
LOC785630	0.728599902	0.409048759	0.15422076	0.487621973	0.305178151	0.44312993	0.90562955
SNX24	0.063521708	0.179871897	0.877339636	0.722762778	0.943912549	0.443104417	0.90562955
ARHGAP24	0.436469045	0.742891447	0.400756167	0.133017836	0.396571846	0.44351379	0.905678883
CCT2	0.909609991	0.425853823	0.189375107	0.222110455	0.420706024	0.443512013	0.905678883
EGR3	0.484636124	0.089164399	0.220417847	0.845069122	0.850941983	0.443374109	0.905678883
EMC1	0.53475523	0.712132371	0.217659636	0.313296872	0.263779554	0.443392206	0.905678883
LOC112444300	0.62090615	0.618473095	0.433310958	0.071632135	0.574822906	0.443431749	0.905678883
MRC1	0.278373126	0.817788145	0.781919237	0.220142486	0.174953439	0.44354038	0.905678883
SELENOH	0.159302432	0.914014998	0.824225164	0.119735983	0.476363814	0.443268254	0.905678883
LOC112447041	0.880134251	0.319290838	0.273493032	0.562588939	0.158870172	0.443888638	0.906073265
NRXN1	0.274511445	0.961677474	0.180789379	0.541084751	0.266033873	0.443908854	0.906073265
SMIM26	0.963610876	0.525220466	0.127486126	0.123879589	0.85974813	0.443954368	0.906073265
WDFY2	0.206780176	0.594224404	0.161939352	0.508359098	0.678729514	0.443792892	0.906073265
CD38	0.256416167	0.327709043	0.408957614	0.696281237	0.287617492	0.44421191	0.906402613
DCSTAMP	0.485148001	0.463205021	0.115459331	0.482534276	0.550335032	0.444422813	0.906402613
ELL	0.158569714	0.398559011	0.749214298	0.402410634	0.36134737	0.444293531	0.906402613
MAP7	0.661772827	0.594174823	0.036122173	0.488826305	0.992522067	0.444447128	0.906402613
ST3GAL3	0.371903004	0.434476815	0.126668504	0.630628242	0.533711916	0.444387662	0.906402613
STK4	0.399021534	0.687120499	0.981890492	0.19108944	0.133881503	0.444348831	0.906402613
CBFA2T2	0.875750631	0.769853156	0.12508209	0.483602071	0.169125119	0.444604591	0.906534359
ETNK2	0.882039195	0.365697992	0.126158597	0.306975843	0.552337745	0.444668111	0.906534359
LOC104973100	0.575960292	0.439924988	0.203328597	0.214617102	0.624058454	0.444677447	0.906534359
ADAT1	0.28542891	0.462314116	0.590147314	0.405096238	0.219304148	0.445140891	0.906911337

ADORA2B	0.371505324	0.630485208	0.552198116	0.054074872	0.991654126	0.445583691	0.906911337
CXHXorf21	0.56585805	0.581638098	0.503859089	0.389586393	0.107048808	0.445081779	0.906911337
DACT1	0.741878295	0.758583921	0.040873689	0.476175044	0.633601673	0.445694423	0.906911337
LOC100848205	0.593479689	0.421997175	0.548327248	0.779754812	0.064603262	0.445127545	0.906911337
LOC101903645	0.344590835	0.071114908	0.544378485	0.591108013	0.879790034	0.44563271	0.906911337
LOC104975299	0.707799653	0.662478403	0.236239333	0.16612377	0.377020139	0.44564059	0.906911337
LOC786614	0.385247182	0.529910269	0.687917966	0.052168284	0.946569195	0.445562071	0.906911337
MRPS36	0.380728342	0.496884789	0.658864929	0.077996663	0.711166698	0.445024794	0.906911337
NAXD	0.297238994	0.664383565	0.971055921	0.039541653	0.913132455	0.445285856	0.906911337
PEX11A	0.559955756	0.491432139	0.065757066	0.403398637	0.949716039	0.445501332	0.906911337
RFLNB	0.452311422	0.395866572	0.04091941	0.979854644	0.966972165	0.445746563	0.906911337
SMC5	0.437972508	0.289496102	0.52485304	0.106909794	0.975651888	0.445725551	0.906911337
SNX13	0.862842837	0.313562759	0.203724095	0.753908531	0.166293356	0.444934399	0.906911337
THAP7	0.276232648	0.768602852	0.834286776	0.098748399	0.396455493	0.445553733	0.906911337
TMEM238	0.164109202	0.188924681	0.715641398	0.318398784	0.982143898	0.445654001	0.906911337
CCND3	0.974893608	0.65921947	0.047978963	0.583593208	0.386040037	0.445864153	0.907038133
LOC112447359	0.645157068	0.022679965	0.902934094	0.587545682	0.895532437	0.445988613	0.907178872
ALG12	0.33114678	0.758249016	0.924942512	0.058696485	0.517963947	0.448745747	0.907389006
ARL13B	0.112501981	0.687102317	0.602506164	0.384683522	0.390842516	0.447274802	0.907389006
ATG2B	0.317379713	0.367984301	0.345067734	0.221618642	0.784100174	0.447291976	0.907389006
ATP11C	0.296525954	0.784762462	0.757975467	0.05238808	0.760234657	0.447839941	0.907389006
BAG5	0.376269538	0.429910678	0.089979735	0.978668412	0.491964582	0.447414428	0.907389006
BPHL	0.331463502	0.93681389	0.239109992	0.094780159	0.990495759	0.446464356	0.907389006
C18H19orf47	0.39034063	0.20704852	0.705954263	0.470991092	0.26119125	0.447688232	0.907389006
CCL24	0.609021177	0.485395636	0.38354498	0.111766573	0.550475649	0.446601764	0.907389006
CKAP5	0.551898002	0.791284011	0.44999959	0.186286433	0.192747134	0.448629024	0.907389006
COASY	0.685630098	0.759024342	0.67719018	0.048923016	0.409294191	0.448642294	0.907389006
DIP2C	0.023116106	0.782922706	0.891564314	0.450525954	0.972954001	0.449047379	0.907389006
EIF3K	0.41747681	0.679281077	0.109354366	0.376281027	0.600090672	0.447274286	0.907389006
EPS8L2	0.748987443	0.101761278	0.219364922	0.83580648	0.504658179	0.448529076	0.907389006
FAM222B	0.264014288	0.852093562	0.441447385	0.771853262	0.090774323	0.446153096	0.907389006
FGF18	0.613650965	0.290394721	0.179789768	0.502695596	0.435357197	0.447509917	0.907389006
GAL3ST4	0.272713457	0.93024034	0.055749875	0.800241959	0.620720078	0.447850745	0.907389006
GPR55	0.688291078	0.609372027	0.380022086	0.092549731	0.476060295	0.447784699	0.907389006
GRIA3	0.140706993	0.627940285	0.255585776	0.653297827	0.475337895	0.447533715	0.907389006
GRO1	0.479406631	0.495936611	0.288640622	0.277529451	0.370004475	0.448397162	0.907389006
GSK3B	0.83065188	0.452921501	0.402786094	0.31289618	0.147699151	0.447293633	0.907389006
HECW1	0.674700883	0.399607778	0.3636999	0.100030275	0.718219439	0.44834552	0.907389006
IFITM1	0.365528194	0.794505065	0.677782955	0.399195116	0.089782067	0.448592546	0.907389006
IRF2BPL	0.720569349	0.34233846	0.655487797	0.122679123	0.352115124	0.446828306	0.907389006
LOC101904013	0.049358532	0.796118041	0.297657527	0.691358015	0.87365727	0.448844392	0.907389006
LOC101905588	0.502265881	0.158710332	0.480804702	0.669423988	0.273571933	0.447695001	0.907389006
LOC101906018	0.86020428	0.488726796	0.157278625	0.539849659	0.195482154	0.446651715	0.907389006
LOC104974020	0.048325723	0.299458155	0.788099187	0.821747351	0.744879531	0.446734033	0.907389006

LOC112442215	0.367277436	0.522136421	0.522413045	0.747580438	0.093913238	0.448060251	0.907389006
LOC112442292	0.183433153	0.71458756	0.814958581	0.180502478	0.366707712	0.448995847	0.907389006
LOC112446733	0.763210867	0.886128249	0.180911522	0.277696008	0.205598844	0.446847118	0.907389006
LOC112449614	0.441501492	0.892577437	0.315175648	0.80208153	0.070922806	0.448858458	0.907389006
LOC531038	0.795598955	0.152770421	0.398917858	0.654995422	0.222335446	0.448747854	0.907389006
LTA4H	0.202039464	0.61128102	0.16809894	0.623717803	0.5406069	0.447219176	0.907389006
LYRM4	0.590035669	0.349644389	0.337704509	0.189379991	0.535838128	0.4489703	0.907389006
MRGBP	0.351153516	0.290066674	0.257629507	0.373515375	0.719112148	0.448434603	0.907389006
NANS	0.310030647	0.70834581	0.102357779	0.767181516	0.409471294	0.448759465	0.907389006
NSMCE1	0.271380823	0.5001979	0.151420875	0.720701019	0.47767242	0.449126392	0.907389006
PCP4L1	0.45675792	0.800451076	0.290941324	0.078228284	0.843044072	0.447597248	0.907389006
PIP4K2A	0.616810267	0.859684458	0.742503396	0.022252348	0.801787295	0.447835013	0.907389006
PWWP2B	0.420495206	0.651953855	0.193428748	0.207101828	0.641469986	0.44833826	0.907389006
RAB24	0.251279483	0.458546645	0.457926658	0.30858823	0.434481761	0.449083687	0.907389006
RBM14	0.497915001	0.700218303	0.372462457	0.11081858	0.485435824	0.446854407	0.907389006
RPL7L1	0.884597058	0.321688658	0.431652228	0.542159228	0.104652584	0.446438009	0.907389006
RPUSD1	0.99478979	0.687078983	0.206680152	0.298225755	0.167578018	0.448721361	0.907389006
SMC1A	0.589948485	0.656649503	0.531570886	0.039422684	0.864868974	0.447745917	0.907389006
SPIDR	0.488548905	0.228774512	0.295543763	0.861834737	0.248502246	0.449085486	0.907389006
SUMF2	0.098852991	0.914642639	0.447305672	0.236756616	0.736537007	0.448535206	0.907389006
SYNPR	0.077313572	0.381685235	0.938311378	0.269646012	0.942058633	0.448061482	0.907389006
TBKBP1	0.597244698	0.331932742	0.723655357	0.284520016	0.17203141	0.447766019	0.907389006
TEX264	0.066104764	0.926787224	0.3021417	0.456950691	0.836387387	0.449088947	0.907389006
TLN1	0.451494579	0.565184248	0.056217377	0.547948277	0.88937528	0.446985532	0.907389006
TNFSF18	0.746819585	0.460854515	0.105516406	0.337993442	0.568466461	0.446649786	0.907389006
UBE3B	0.637486288	0.479557894	0.650178627	0.062525876	0.564540632	0.447621359	0.907389006
ZNF835	0.145002089	0.997416096	0.578885345	0.802507554	0.104691414	0.448071002	0.907389006
ZSCAN26	0.807105491	0.14102788	0.238445967	0.875343509	0.297854648	0.449132953	0.907389006
LOC112445888	0.655958986	0.483280505	0.124601	0.277157683	0.64679343	0.449247936	0.907509586
GRN	0.262037014	0.545510057	0.195888886	0.579726167	0.436491953	0.449363945	0.90763221
E2F7	0.873363184	0.113193837	0.227158803	0.382837712	0.824773489	0.44949546	0.907786118
WDR1	0.707754909	0.174140969	0.140529125	0.693152978	0.591233009	0.449675575	0.908038128
ZCCHC9	0.979558929	0.749389332	0.181304671	0.134339677	0.397453297	0.449879484	0.908338117
PABPC1	0.432863827	0.765172203	0.173674952	0.145284051	0.850673884	0.44995756	0.908383997
CHST11	0.033005791	0.800871653	0.461300928	0.715685589	0.815196847	0.450077384	0.908514139
SOWAHA	0.146878505	0.935454273	0.226191265	0.957996641	0.239198963	0.450264324	0.90877971
AAMP	0.392844799	0.421877044	0.077415706	0.611597569	0.922809668	0.453223553	0.908822911
ALDH1L2	0.338914984	0.424228612	0.075062455	0.920143611	0.717641199	0.450385454	0.908822911
ATF5	0.371686328	0.8430474	0.808014376	0.172681011	0.165608272	0.453207333	0.908822911
BCKDHB	0.609167533	0.916830629	0.412328799	0.085563602	0.363497264	0.451277703	0.908822911
C19H17orf113	0.794144308	0.423763502	0.387472192	0.522079482	0.105457838	0.451694546	0.908822911
CDK5RAP1	0.177492413	0.879884865	0.625133936	0.096541817	0.757245163	0.450652729	0.908822911
CNPY2	0.764839771	0.448656936	0.201542001	0.140686118	0.744814105	0.453361101	0.908822911
CWC25	0.44594839	0.761918764	0.632035357	0.113108901	0.29550631	0.451661488	0.908822911

DMWD	0.919060367	0.25055983	0.900668597	0.04857409	0.715276062	0.452357394	0.908822911
DOCK4	0.050557174	0.961327549	0.707716378	0.782026698	0.266148949	0.451195942	0.908822911
DPY19L1	0.878409304	0.176061995	0.590892495	0.954903882	0.082597235	0.452397231	0.908822911
FAM117B	0.201409813	0.908785453	0.402115041	0.19084669	0.51650162	0.453565635	0.908822911
FAM216A	0.149501163	0.162517727	0.915996486	0.622280671	0.520138647	0.452294229	0.908822911
FEM1B	0.111488249	0.812286317	0.416110888	0.192386932	0.994340612	0.452422559	0.908822911
FGF16	0.906798166	0.810077743	0.256299045	0.259058516	0.147381825	0.451918376	0.908822911
FOXP2	0.359142962	0.903501503	0.353647638	0.114047891	0.554841656	0.45371922	0.908822911
FXYD1	0.105822529	0.488646583	0.93169146	0.437906494	0.34194012	0.452552956	0.908822911
GAS2L1	0.402445835	0.98058914	0.449801814	0.106639517	0.381791077	0.452873633	0.908822911
GLIS2	0.944203967	0.147872273	0.17680457	0.42998906	0.678387721	0.452227465	0.908822911
GPR89A	0.323912767	0.706413764	0.125064926	0.621313266	0.4047511	0.452121046	0.908822911
HELZ	0.884365647	0.88278364	0.64208436	0.032325246	0.442210064	0.451354963	0.908822911
ID4	0.061898764	0.270198284	0.717369017	0.994821433	0.608266045	0.453687477	0.908822911
ITGA6	0.527712061	0.295080033	0.370405333	0.354953369	0.349588321	0.451147919	0.908822911
ITGBL1	0.354597812	0.151071876	0.888235295	0.160386163	0.938243236	0.451224676	0.908822911
KCTD7	0.297943101	0.896288605	0.514123454	0.968898272	0.05399772	0.451785863	0.908822911
KLHL11	0.19988664	0.459968008	0.809716486	0.153959914	0.629428581	0.452561936	0.908822911
KLHL13	0.886186604	0.657790889	0.266695831	0.143849824	0.323565025	0.453095034	0.908822911
LOC104970103	0.274198431	0.462034769	0.137200058	0.791910526	0.518951844	0.450802453	0.908822911
LOC107131403	0.526181933	0.840622363	0.218025683	0.10978687	0.685331927	0.453585008	0.908822911
LOC112441770	0.247744691	0.744675355	0.497711314	0.606030319	0.129922487	0.452942389	0.908822911
LOC112442248	0.874062604	0.512985111	0.14838626	0.441194199	0.243011935	0.450557814	0.908822911
LOC112448856	0.909953908	0.055116834	0.267256104	0.541621431	0.997020755	0.453148767	0.908822911
LOC518775	0.809641206	0.112511623	0.287758863	0.891719539	0.308557979	0.452514962	0.908822911
LOC781688	0.925591026	0.354077662	0.631219392	0.626906559	0.055141179	0.450998534	0.908822911
MGAT1	0.707195958	0.602457995	0.225683638	0.106261358	0.70835339	0.453132636	0.908822911
MYBL1	0.138068879	0.338918302	0.367325311	0.64113807	0.648455312	0.450874462	0.908822911
MYO9A	0.142444566	0.953891661	0.831232898	0.172551244	0.370405909	0.45267051	0.908822911
NAT1	0.625240249	0.334765423	0.533291756	0.158945807	0.405483444	0.452060963	0.908822911
NGRN	0.239389417	0.209057387	0.411720708	0.681238181	0.514101508	0.452612108	0.908822911
NRG2	0.063440541	0.768863113	0.379267715	0.467794998	0.834769562	0.452801673	0.908822911
PBDC1	0.642268785	0.638931951	0.433662284	0.264936321	0.152972473	0.452512546	0.908822911
PHF20	0.91300146	0.258369943	0.329468779	0.244510244	0.377960715	0.45177327	0.908822911
PLD3	0.223687131	0.734777356	0.307587366	0.661459264	0.215446222	0.452319888	0.908822911
PRUNE1	0.569376478	0.143699482	0.561845057	0.352149957	0.447757681	0.453398974	0.908822911
PTDSS2	0.592566731	0.551834575	0.279046587	0.1275966	0.61226321	0.450435977	0.908822911
RAP1B	0.619994852	0.135018852	0.223443803	0.96135895	0.398162718	0.451211156	0.908822911
SEC23A	0.18569177	0.50388341	0.20689704	0.695699873	0.53575831	0.452590325	0.908822911
SF3A3	0.530375636	0.377439752	0.132021577	0.275266405	0.983571422	0.451104152	0.908822911
SFXN1	0.298570081	0.742890486	0.210201779	0.194062671	0.800266932	0.453211458	0.908822911
SLC25A37	0.469185157	0.36208302	0.156365266	0.374829127	0.729064951	0.453665498	0.908822911
SPTY2D1OS	0.724670027	0.117227646	0.269372204	0.704035769	0.449660685	0.453300951	0.908822911
TDRKH	0.254283617	0.537313729	0.128478605	0.698575559	0.58547086	0.451701418	0.908822911

TLNRD1	0.838350511	0.349348523	0.292439317	0.359043135	0.234973897	0.452843691	0.908822911
TMEM50B	0.371582743	0.198721614	0.593099726	0.93720037	0.175226282	0.452014097	0.908822911
TOB2	0.815352187	0.663881855	0.924338141	0.815907442	0.017771606	0.453559837	0.908822911
TTPAL	0.266829892	0.086833088	0.934418201	0.770830285	0.431058437	0.452053328	0.908822911
USP13	0.947848291	0.774028316	0.947944724	0.374809738	0.027738599	0.452961455	0.908822911
USP37	0.400028689	0.347564436	0.740809802	0.07934288	0.888071392	0.453623183	0.908822911
VAPB	0.7661792	0.256155877	0.085048175	0.916646267	0.471297613	0.452479315	0.908822911
WFDC1	0.184852711	0.299179489	0.770146716	0.45551491	0.368916455	0.451155865	0.908822911
XRCC1	0.604241247	0.077943661	0.730553693	0.838628875	0.251093131	0.453319467	0.908822911
ZMYND12	0.255406071	0.766608386	0.32529948	0.239338824	0.470925389	0.451684077	0.908822911
ATP5MG	0.73465375	0.745955307	0.660284018	0.026033209	0.775644335	0.45482394	0.908909081
BRCA2	0.924101362	0.062574224	0.296853224	0.601323583	0.704977629	0.454095248	0.908909081
CCDC9	0.181879475	0.762028216	0.830850267	0.155255087	0.413544541	0.456931263	0.908909081
CRYBA4	0.730675332	0.152438413	0.328497244	0.705324741	0.285641167	0.456402818	0.908909081
CSPG4	0.338329263	0.273970231	0.422751107	0.305217069	0.611748049	0.455067133	0.908909081
DDIT3	0.799083825	0.439132993	0.924144573	0.089217745	0.252360929	0.454694696	0.908909081
DNAJC18	0.906752279	0.143881407	0.383970762	0.29151886	0.506187789	0.456900072	0.908909081
EFCAB2	0.290588142	0.086319996	0.450422847	0.735334985	0.881074784	0.455148667	0.908909081
EFHC1	0.5373535	0.585141089	0.058250878	0.462142601	0.860020191	0.454163892	0.908909081
EHBP1	0.651282029	0.623609215	0.200942474	0.16867206	0.530666007	0.454783376	0.908909081
FBP2	0.89169995	0.08133449	0.243376472	0.680528063	0.608588291	0.454916325	0.908909081
FTCDNL1	0.650348777	0.49681582	0.040367525	0.83761785	0.66930306	0.454957737	0.908909081
GATA3	0.470796898	0.705715673	0.978805998	0.105207744	0.215850283	0.45673166	0.908909081
GOT2	0.883148389	0.884741273	0.466821629	0.070232105	0.288556126	0.456898887	0.908909081
GPR21	0.35406251	0.45414464	0.069111387	0.888540907	0.735853967	0.4538297	0.908909081
HPF1	0.511403324	0.184057313	0.785936672	0.148233873	0.672778026	0.456552021	0.908909081
IL20RA	0.452499704	0.11460442	0.502694391	0.409909329	0.692056339	0.456975406	0.908909081
IL23A	0.032319208	0.986072943	0.958830663	0.279824476	0.86364953	0.456721216	0.908909081
KCNS2	0.05578336	0.500271509	0.586865171	0.514964425	0.875846826	0.456770256	0.908909081
KIF7	0.646833453	0.163834551	0.216287457	0.824620522	0.388775066	0.455836232	0.908909081
LEPROT	0.293186061	0.127349297	0.85984159	0.24517052	0.931392155	0.45541615	0.908909081
LOC100335268	0.053749427	0.783379537	0.313266142	0.888804458	0.620520254	0.454046565	0.908909081
LOC101905053	0.124399089	0.25998202	0.510343265	0.992241609	0.449268904	0.456068326	0.908909081
LOC101908185	0.16206885	0.948077652	0.345709825	0.824353915	0.168625663	0.4567038	0.908909081
LOC101909173	0.905165357	0.145064025	0.397056266	0.619960928	0.228403194	0.456668286	0.908909081
LOC107131293	0.936316474	0.641761475	0.218606913	0.066967529	0.830464897	0.454794857	0.908909081
LOC112445007	0.568198194	0.130198547	0.185404819	0.894014646	0.600419257	0.456183215	0.908909081
LOC112448524	0.661958929	0.587839464	0.140868167	0.520107847	0.258325265	0.456239394	0.908909081
LOC112448773	0.340777969	0.15538921	0.377281163	0.45667094	0.799654064	0.454555732	0.908909081
LOC511531	0.235150377	0.849409089	0.562499784	0.161661236	0.402487766	0.454917068	0.908909081
LOC512627	0.523485846	0.130014489	0.427193558	0.479272159	0.524352835	0.454828288	0.908909081
LOC512953	0.862361973	0.981849054	0.847631392	0.0238696	0.431216654	0.456781716	0.908909081
LRRC2	0.209244954	0.981589334	0.395734547	0.148252817	0.607465787	0.455151072	0.908909081
MRPS31	0.500828228	0.612775227	0.602288712	0.524345891	0.075318273	0.454658281	0.908909081

PAPSS2	0.386527042	0.552873354	0.106335251	0.969396897	0.333316226	0.455696161	0.908909081
PCGF5	0.149859125	0.479547304	0.437506632	0.592510572	0.396752017	0.456877339	0.908909081
PCTP	0.814619255	0.42155575	0.492408256	0.057520344	0.747963818	0.454052868	0.908909081
PDCL3	0.343871344	0.118786821	0.546997799	0.849491592	0.387942757	0.456203891	0.908909081
PLA2R1	0.486581161	0.895904384	0.06255188	0.333268561	0.810712196	0.456303283	0.908909081
PPAT	0.27086433	0.487953212	0.522745207	0.851461388	0.125667549	0.456915848	0.908909081
PPP2R3A	0.82491616	0.551788035	0.065172439	0.444487267	0.552791466	0.454393147	0.908909081
PSMC6	0.890972158	0.377743889	0.163546316	0.405201411	0.327007194	0.454501404	0.908909081
RALA	0.402464168	0.200688098	0.581813713	0.24385367	0.639785853	0.455431583	0.908909081
RBFOX1	0.391228654	0.959046733	0.042378631	0.500246988	0.926800108	0.456414078	0.908909081
RDH8	0.697320556	0.304935005	0.063721312	0.706557776	0.761025641	0.454313036	0.908909081
RSPO2	0.068627601	0.933097639	0.246178107	0.716712005	0.650849781	0.455967273	0.908909081
SESN1	0.807468429	0.557868231	0.019986268	0.869805009	0.941131489	0.456361951	0.908909081
SFRS18	0.468747178	0.37830536	0.568533037	0.863226282	0.083856818	0.454611897	0.908909081
SNRPF	0.642008182	0.061005374	0.41364483	0.60612143	0.749408362	0.456096161	0.908909081
SOS2	0.502747521	0.131882903	0.495954586	0.34094227	0.653478696	0.45530671	0.908909081
TMEM106B	0.526442988	0.735647212	0.065947257	0.494291643	0.585982303	0.457029903	0.908909081
TMTC2	0.114538189	0.234429974	0.435105888	0.917661173	0.683818317	0.455425333	0.908909081
TNFAIP8L2	0.319573309	0.719475772	0.824991002	0.135744939	0.284176297	0.455082847	0.908909081
WDTC1	0.458482692	0.651630993	0.753849524	0.97509461	0.03311804	0.454005153	0.908909081
XCR1	0.574142512	0.10343016	0.785839685	0.327760394	0.478874887	0.455260215	0.908909081
ZNF214	0.459173983	0.213918063	0.798491608	0.322951523	0.291241916	0.456536756	0.908909081
ZNF599	0.972978937	0.615210838	0.126846197	0.457179181	0.210231315	0.454607308	0.908909081
ZNF653	0.799856809	0.408370216	0.346203199	0.254858075	0.255953355	0.456524547	0.908909081
ZNF879	0.341071847	0.997500975	0.232894037	0.363503377	0.25553389	0.45612086	0.908909081
ATOX1	0.514727653	0.520349752	0.229815996	0.136692706	0.879781854	0.457148381	0.909034543
PPP3R1	0.705138958	0.050903384	0.947530387	0.434512353	0.50216385	0.457596357	0.909580927
PRKACB	0.547000057	0.295384922	0.763809668	0.753052709	0.07987603	0.457654232	0.909580927
PTOV1	0.23163883	0.831370349	0.766524469	0.092090018	0.546083181	0.457654472	0.909580927
SUB1	0.882839318	0.049456847	0.29647937	0.977495783	0.586282651	0.457539732	0.909580927
ZHX3	0.71348139	0.083137161	0.771392008	0.281870497	0.575720857	0.45770028	0.909580927
LOC107131940	0.361591509	0.861924842	0.249609087	0.399950955	0.238733991	0.45776386	0.909597131
TTC27	0.604909451	0.684541007	0.392054862	0.150768956	0.303592555	0.457834141	0.909626645
UBA3	0.782178303	0.964637886	0.478225543	0.232749211	0.088554245	0.45798266	0.909811576
PIGU	0.83396655	0.228836517	0.095508504	0.864573255	0.472128603	0.458055817	0.909846771
LOC100850875	0.247197015	0.90088576	0.139210558	0.301209213	0.798045729	0.458345364	0.909952445
LOC104968873	0.180616795	0.872101582	0.163070561	0.714546561	0.406115082	0.458386257	0.909952445
LOC112442263	0.729727315	0.664945767	0.142762286	0.281775856	0.381428211	0.458178867	0.909952445
RBSN	0.440111158	0.60782744	0.211901459	0.287969282	0.456450659	0.458318788	0.909952445
RPS23	0.219697038	0.939692126	0.060414965	0.640320254	0.932668438	0.458262032	0.909952445
EIF2B3	0.842213805	0.500360996	0.686893275	0.248142204	0.10381319	0.458455388	0.909979605
CNOT2	0.959516488	0.688302798	0.559218629	0.029430349	0.68699428	0.458708321	0.910151403
EBF2	0.646644387	0.220753285	0.27198307	0.455990697	0.421764478	0.458699423	0.910151403
NAA35	0.691146847	0.118667491	0.653922233	0.616376064	0.225796241	0.458637668	0.910151403

TMA16	0.550364306	0.252633074	0.410714162	0.156563309	0.835794435	0.458835031	0.910292757
CCSER2	0.546682232	0.610135671	0.101013162	0.226119022	0.981390011	0.458937431	0.910353884
ZBTB32	0.581492733	0.07338652	0.323600315	0.840252798	0.644516537	0.458976786	0.910353884
CHCHD3	0.42420846	0.550464742	0.484560287	0.121304425	0.545065842	0.459046326	0.910381783
TRMT44	0.962691751	0.07010381	0.966074867	0.559309023	0.205284	0.459155288	0.910487848
TFG	0.197426751	0.518647358	0.756018311	0.812054126	0.119195065	0.459323152	0.910710673
ANKRD26	0.887640202	0.15022418	0.179336535	0.544361234	0.57672678	0.459673341	0.910887181
CCDC102B	0.855182719	0.202671281	0.514730227	0.385799203	0.218102401	0.459652588	0.910887181
CDK5R1	0.503398773	0.87346357	0.333685504	0.173280964	0.295216873	0.459625262	0.910887181
LRRC49	0.428202422	0.74655683	0.668148377	0.11473595	0.306445923	0.459729185	0.910887181
NFU1	0.818011987	0.581528542	0.040438789	0.553854679	0.704940447	0.459745202	0.910887181
NMNAT2	0.145493464	0.339645654	0.921855499	0.312399386	0.527555675	0.459676632	0.910887181
LOC790009	0.183007309	0.450243681	0.663043864	0.358630051	0.383553204	0.459849783	0.910984402
LHFPL1	0.685752306	0.684288039	0.162122549	0.274733361	0.359845374	0.459992681	0.910992145
LOC100848443	0.052738706	0.893397005	0.459274145	0.553821564	0.628056637	0.460132013	0.910992145
OOEP	0.345859687	0.813045342	0.753567275	0.079669291	0.445903514	0.460154278	0.910992145
RCN1	0.889381412	0.350425394	0.092923376	0.546004788	0.475954918	0.460113953	0.910992145
SMG9	0.073356284	0.186549203	0.898209755	0.740792557	0.826879437	0.460186758	0.910992145
SP3	0.132002943	0.301525865	0.739799827	0.284683687	0.897735466	0.460098481	0.910992145
LOC107131769	0.797168345	0.727494969	0.702554472	0.027486306	0.672904931	0.460344762	0.91111936
SMAD9	0.202554022	0.890402674	0.102068622	0.825625619	0.495946658	0.460389166	0.91111936
WDR17	0.582417674	0.105959019	0.427435415	0.928648619	0.307759977	0.460417577	0.91111936
COQ5	0.876291359	0.609579787	0.149529835	0.120803252	0.782365358	0.46066059	0.911490349
DEK	0.208283258	0.69686518	0.513319333	0.146271995	0.692995571	0.46073789	0.911516727
SPPL2B	0.485807787	0.294325416	0.459022595	0.443633248	0.25944548	0.460785008	0.911516727
STK39	0.487093364	0.471820552	0.713427978	0.924709458	0.049841597	0.460842802	0.911521179
IGFBP4	0.763044061	0.58157108	0.185689585	0.547729687	0.167657061	0.461087157	0.91167486
LMTK3	0.966667199	0.54356664	0.352324093	0.233284451	0.175171908	0.46104349	0.91167486
LOC104974260	0.606469498	0.781277275	0.186248558	0.122237299	0.701300747	0.461040494	0.91167486
OTUD4	0.405231733	0.35898079	0.402514662	0.157805717	0.819454516	0.461201109	0.911790314
FASTKD1	0.577013785	0.245029071	0.593035566	0.118678485	0.762402945	0.461547008	0.911815089
LAMC1	0.220535559	0.158902598	0.482886067	0.66740181	0.670724925	0.46127637	0.911815089
LOC100847171	0.253380674	0.132988234	0.561290929	0.746061377	0.537197967	0.461399298	0.911815089
LOC112448863	0.557641593	0.184076593	0.37400859	0.414322933	0.476605898	0.461419975	0.911815089
THOC7	0.926313873	0.161977788	0.627854197	0.216770413	0.371399469	0.461494367	0.911815089
ZNF567	0.101722293	0.283939847	0.647416063	0.544946945	0.744449869	0.461536483	0.911815089
LOC112444921	0.865256988	0.070557051	0.184299459	0.978145496	0.689568606	0.461608763	0.911827325
HTR1B	0.16812404	0.36861204	0.851252705	0.223574198	0.643791176	0.461705478	0.911906768
RTL8C	0.470525064	0.666065013	0.527892174	0.046933515	0.978203115	0.461760115	0.911906768
INCA1	0.547154336	0.694916843	0.209631892	0.607867906	0.156814577	0.461817553	0.911910464
ACAA1	0.468760527	0.937818512	0.04962217	0.601622752	0.581130567	0.462498963	0.912095712
ACTN4	0.443646124	0.25476759	0.158360457	0.99972297	0.425749789	0.462299675	0.912095712
AGT	0.615544799	0.512832982	0.933805617	0.416753988	0.06203849	0.462370543	0.912095712
ATG12	0.756197144	0.080772731	0.191796386	0.792075529	0.821109314	0.462318727	0.912095712

AURKA	0.298466772	0.25122154	0.115496332	0.913594429	0.963737942	0.46245342	0.912095712
C10H14orf93	0.657432348	0.507667888	0.241340325	0.379877451	0.248656483	0.462069026	0.912095712
HINT1	0.789577031	0.429166023	0.347037011	0.073023962	0.888511048	0.462573547	0.912095712
INSIG2	0.485151429	0.141844498	0.365497787	0.82682897	0.366829177	0.462544698	0.912095712
LOC101904227	0.730206833	0.201305935	0.262770395	0.617244588	0.320138508	0.462635706	0.912095712
LOC104976274	0.982522707	0.041186637	0.73931947	0.456268856	0.55930944	0.462689465	0.912095712
PPP4R4	0.648453302	0.195496809	0.110905045	0.908480486	0.597326128	0.462562353	0.912095712
SERINC3	0.552279084	0.362081305	0.228596582	0.273384934	0.610932395	0.462688682	0.912095712
SERTAD1	0.19726422	0.233474296	0.226898535	0.888986833	0.819536009	0.462184183	0.912095712
ZC3H15	0.671323962	0.340619686	0.592224989	0.440338435	0.127995046	0.462633334	0.912095712
PLCH2	0.565093837	0.540211806	0.521880375	0.167507701	0.286399081	0.462878951	0.912359651
ABHD11	0.377295159	0.914374618	0.196034785	0.289212907	0.391448378	0.463197095	0.912548322
NCAPH2	0.554089927	0.934431149	0.217510221	0.175411853	0.387409311	0.463116594	0.912548322
SLC25A35	0.750265089	0.808842175	0.299954652	0.136912922	0.307181755	0.463173617	0.912548322
TOMM34	0.757950954	0.572065109	0.943639321	0.796211614	0.02349996	0.463179512	0.912548322
FKBP2	0.291124729	0.28539044	0.468444471	0.262714514	0.749304279	0.463316403	0.912673807
ERGIC1	0.523025528	0.783782358	0.090075061	0.71981531	0.288738904	0.463618827	0.912940792
LOC101907797	0.677427552	0.732539068	0.907713604	0.050839849	0.335061222	0.463586358	0.912940792
LOC112442091	0.390742943	0.145849087	0.686886879	0.936825964	0.209149912	0.463514213	0.912940792
CHSY1	0.870109721	0.872333333	0.619385302	0.043560887	0.375259401	0.463865817	0.912988956
LOC100337323	0.6520471	0.429340517	0.93343499	0.052453053	0.560278708	0.463739129	0.912988956
SOX15	0.408786738	0.72692402	0.762259264	0.822090327	0.04126897	0.463860025	0.912988956
TMX3	0.338487985	0.342838162	0.484696417	0.237648468	0.574631267	0.463775033	0.912988956
HMGN5	0.835055671	0.157205933	0.440714671	0.165038004	0.805196518	0.463941223	0.913008195
LTBP3	0.713743345	0.230059203	0.193086876	0.251391758	0.965017247	0.464021105	0.913008195
S100B	0.423901234	0.418222631	0.487391071	0.111250236	0.800242215	0.464042493	0.913008195
SKA3	0.8658995	0.173848181	0.496769921	0.152261907	0.676060927	0.464165897	0.913141517
ARHGAP44	0.590755969	0.355603568	0.268234631	0.247473872	0.555882881	0.465422819	0.913173596
BNIP3L	0.693986023	0.234376068	0.080857908	0.674840479	0.872087193	0.465151458	0.913173596
DHRSX	0.55096867	0.168807573	0.741995168	0.232661238	0.482439981	0.465292359	0.913173596
FLII	0.619513836	0.866637898	0.037687383	0.775800946	0.491849455	0.464703492	0.913173596
GLIPR2	0.478450652	0.738714471	0.491282624	0.052336401	0.848968452	0.464568105	0.913173596
GMDS	0.317342043	0.711827727	0.471031128	0.079493245	0.912707932	0.464681522	0.913173596
HSPD1	0.961313614	0.661648147	0.156317155	0.09276399	0.835649536	0.464386514	0.913173596
INTS4	0.117393697	0.691403175	0.822533359	0.136618345	0.848130951	0.465049469	0.913173596
LOC101905399	0.495563243	0.329466023	0.132834279	0.445006731	0.798144575	0.464289376	0.913173596
LOC101907998	0.248251189	0.370456118	0.811131251	0.59380211	0.175035716	0.465458467	0.913173596
LOC107132278	0.256417574	0.996710668	0.644226887	0.488442312	0.096047402	0.464781212	0.913173596
LOC112441472	0.750136009	0.703645746	0.057992356	0.378379134	0.669289408	0.465424575	0.913173596
LOC112445033	0.446939629	0.425127776	0.715505268	0.131517031	0.433670781	0.46547327	0.913173596
LOC785873	0.281567888	0.871892807	0.87493121	0.599547392	0.060081904	0.465085024	0.913173596
MLST8	0.248243602	0.91638266	0.666348674	0.062154977	0.820340261	0.464893887	0.913173596
MYO19	0.885581041	0.421245925	0.271801412	0.334358463	0.227561741	0.464562606	0.913173596
P2RY14	0.410720143	0.851709226	0.109074326	0.869402751	0.233222187	0.465069858	0.913173596

PPP6R1	0.563657265	0.113807423	0.432878993	0.67934421	0.411138591	0.465517659	0.913173596
QSER1	0.183041044	0.539367491	0.906128211	0.421571551	0.205246244	0.465160538	0.913173596
RBM17	0.574671294	0.10845574	0.280667976	0.486323012	0.909879474	0.465162272	0.913173596
RPL23A	0.349957562	0.394903568	0.291048735	0.596547199	0.321183564	0.46437319	0.913173596
SLC17A5	0.23696794	0.408554867	0.25158199	0.890363975	0.357174391	0.465284142	0.913173596
SPTBN4	0.358652353	0.75557894	0.870479055	0.04418454	0.739917815	0.464495617	0.913173596
TMEM33	0.390903453	0.227991445	0.616479863	0.198818529	0.706636019	0.46465889	0.913173596
LOC508131	0.120923591	0.209262504	0.336532916	0.986840201	0.924443174	0.465819214	0.913655925
KERA	0.35202139	0.13029008	0.927820552	0.263503397	0.693399753	0.465967482	0.913687473
NET1	0.933860002	0.5026884	0.82123467	0.34462832	0.058532808	0.466002325	0.913687473
STAP1	0.118486352	0.690240206	0.57069303	0.624613435	0.266664897	0.4659401	0.913687473
DESI2	0.3204739	0.373516585	0.936365172	0.0810426	0.856844027	0.466153721	0.913765973
LOC107131607	0.75462456	0.343394988	0.144595489	0.29489433	0.70433346	0.466137709	0.913765973
TDRD7	0.836192862	0.668207946	0.219535864	0.1504629	0.422004777	0.466281355	0.913797864
ZBED6CL	0.379233349	0.092086674	0.775556969	0.454315627	0.632949365	0.466270266	0.913797864
LOC100847320	0.105380675	0.333814715	0.44681327	0.557943473	0.888509957	0.466353872	0.913830852
FAM69C	0.652606846	0.385442314	0.742703248	0.307971163	0.13555678	0.466525218	0.913839264
LOC101906266	0.123314284	0.668215491	0.409407201	0.771785176	0.29951428	0.466501019	0.913839264
RNF4	0.320828907	0.385769684	0.586267333	0.113773964	0.944684005	0.466512385	0.913839264
CYB5A	0.816815019	0.852833225	0.259347943	0.083492436	0.517856978	0.46680349	0.914166118
SLC7A10	0.644506952	0.164113337	0.151107651	0.684512284	0.713840175	0.466767172	0.914166118
PKP1	0.525161532	0.653700349	0.144734344	0.818762586	0.192157758	0.466941222	0.914326739
LOC101907213	0.642596439	0.376809848	0.929206099	0.076141306	0.456603624	0.467054715	0.914439863
DENND5B	0.797410736	0.620183494	0.081314802	0.239884775	0.811401603	0.467170294	0.914528411
HSPA14	0.689367493	0.653738032	0.300063306	0.085716157	0.675428223	0.467211395	0.914528411
ACTN1	0.493858006	0.181819226	0.383434299	0.289361367	0.78921977	0.467985499	0.914580227
ADIPOR1	0.803507367	0.58288992	0.118229142	0.158326888	0.897220138	0.46806172	0.914580227
CCDC14	0.749055899	0.28367433	0.097148884	0.956907052	0.398189861	0.468052321	0.914580227
CDKL5	0.46314555	0.858540041	0.456000146	0.177905185	0.242923678	0.467374375	0.914580227
CTBP1	0.572593883	0.737929959	0.997627415	0.053258999	0.349938311	0.467837181	0.914580227
CUTA	0.296879591	0.338273077	0.864222503	0.107764511	0.846323794	0.469198225	0.914580227
DHRS4	0.189378263	0.868665695	0.306924776	0.15664732	0.992138047	0.467627256	0.914580227
DNAJC12	0.11992392	0.756120384	0.609547794	0.349421898	0.40783882	0.468306546	0.914580227
ELOVL7	0.495960406	0.703200499	0.067369438	0.344279845	0.977697258	0.46903978	0.914580227
ENPEP	0.087015799	0.357022358	0.547473606	0.525108683	0.877409988	0.467377286	0.914580227
EPSTI1	0.690531594	0.052115635	0.823027561	0.276555255	0.963104455	0.468588673	0.914580227
ETV2	0.471976754	0.356137854	0.573603035	0.284982775	0.288500216	0.469460273	0.914580227
FAM53C	0.316589586	0.637369033	0.880410529	0.126798581	0.351648925	0.469327657	0.914580227
GAPVD1	0.197873243	0.668780589	0.856346468	0.111249542	0.623544994	0.467950109	0.914580227
GCNT2	0.590166657	0.275023332	0.3537036	0.379148118	0.361741453	0.468243175	0.914580227
GDAP1	0.424900186	0.287349197	0.139494196	0.57547941	0.807298766	0.469128054	0.914580227
GOLGA5	0.842121357	0.836310038	0.189023073	0.271271513	0.217990698	0.468204943	0.914580227
GSTM3	0.976105239	0.175550872	0.184073616	0.634290175	0.395722702	0.469233396	0.914580227
LANCL2	0.510007563	0.525397656	0.044445998	0.748005397	0.885510184	0.468578032	0.914580227

LOC100847604	0.682301548	0.030595257	0.936353874	0.655609115	0.617633207	0.469181751	0.914580227
LOC112443485	0.575093335	0.328687026	0.158156829	0.80824463	0.324710619	0.467601622	0.914580227
LOC112448805	0.402809499	0.776358024	0.030606883	0.968867833	0.850626449	0.468573273	0.914580227
LPCAT4	0.904291874	0.339878455	0.12156425	0.758610936	0.278387625	0.468624413	0.914580227
LSM6	0.900833789	0.040501397	0.940963872	0.271218373	0.848639706	0.468883666	0.914580227
MCAT	0.550178088	0.932134001	0.190122215	0.083947278	0.963981418	0.468617181	0.914580227
NR5A2	0.928689338	0.925627223	0.048458627	0.347180891	0.545135621	0.468470826	0.914580227
P2RY8	0.686183965	0.818260582	0.992865077	0.172137131	0.081824024	0.467738683	0.914580227
PFKFB1	0.407946672	0.875283589	0.656467517	0.063285704	0.534372672	0.469460413	0.914580227
POLR2M	0.743821412	0.297482032	0.055490549	0.765230608	0.837350609	0.468100601	0.914580227
PYCR1	0.468144686	0.475356593	0.236985199	0.258772498	0.579297193	0.468971211	0.914580227
RBM45	0.828036015	0.809042586	0.790951834	0.175458427	0.085066052	0.46903814	0.914580227
RMDN1	0.841192248	0.907997021	0.410205279	0.108019059	0.234233985	0.469467055	0.914580227
SACM1L	0.516737543	0.585378241	0.067324182	0.665394299	0.582368857	0.468644276	0.914580227
SLC11A2	0.794452216	0.118865058	0.706226231	0.951686402	0.124821061	0.469348922	0.914580227
SWSAP1	0.978003316	0.287791594	0.228388196	0.357901965	0.343797629	0.46906224	0.914580227
TEAD4	0.204182799	0.253199957	0.934792238	0.5035276	0.324985783	0.469031631	0.914580227
TMEM132B	0.531531435	0.742660516	0.108582256	0.307010826	0.59776402	0.468064622	0.914580227
TTC12	0.331937628	0.389939352	0.101096034	0.731032466	0.820529716	0.4676725	0.914580227
VPS33A	0.678684045	0.205569615	0.114811917	0.53474795	0.914998586	0.46740829	0.914580227
ZDHHC6	0.838001882	0.274214275	0.352900563	0.876476036	0.110680229	0.46807955	0.914580227
RAP2C	0.647809633	0.216069385	0.172774725	0.542118865	0.604869227	0.469527709	0.91458982
LOC112448271	0.590265802	0.09874396	0.305212175	0.87350451	0.510617484	0.469629338	0.914599381
SYK	0.473170967	0.722919335	0.363615421	0.110543742	0.577127616	0.469644079	0.914599381
CTXN1	0.380964423	0.676709596	0.879281378	0.126507209	0.276881699	0.469755686	0.914599664
SMG7	0.862359484	0.048602102	0.656629042	0.933474956	0.309024512	0.469728579	0.914599664
APELA	0.291554354	0.108760668	0.601829946	0.725328004	0.574058326	0.469893797	0.914648497
FAM102B	0.6321731	0.663876515	0.138993707	0.697351128	0.19551641	0.470059437	0.914648497
HCFC1R1	0.648906409	0.837492132	0.995898565	0.015981121	0.919527389	0.470058031	0.914648497
PLXNA4	0.263291951	0.156412275	0.960165716	0.844312411	0.237992054	0.469879041	0.914648497
SPATA5	0.397459362	0.884461465	0.627058234	0.788804436	0.045722559	0.469987024	0.914648497
EIF3G	0.447720985	0.836337502	0.072383299	0.945201407	0.310723817	0.470214933	0.914734147
GPATCH1	0.146159612	0.391602097	0.226246639	0.969780646	0.63379225	0.470193539	0.914734147
ARNT2	0.205378822	0.957465735	0.265401116	0.965229036	0.158157041	0.470370926	0.914745029
LOC101903997	0.149779605	0.464700556	0.375935894	0.5706966	0.533603479	0.470397209	0.914745029
SLC12A6	0.368186034	0.704558614	0.61676092	0.453640217	0.109815029	0.470443486	0.914745029
VPS4A	0.435295901	0.632816342	0.251721286	0.372292683	0.308747714	0.470441752	0.914745029
DHX37	0.570257224	0.487173631	0.407718529	0.316542396	0.222458787	0.470577664	0.914897529
FOXC1	0.186827305	0.185191431	0.867389545	0.47745277	0.557321558	0.470792488	0.915206766
ARHGAP23	0.749203598	0.614240878	0.705364892	0.15834266	0.15547601	0.470918454	0.915343212
DQX1	0.648478419	0.535296668	0.412101132	0.422769704	0.13242256	0.471313053	0.915407911
EVI2A	0.324183096	0.938479058	0.830138993	0.169824788	0.186648508	0.471242822	0.915407911
LOC112443015	0.952127444	0.782857812	0.099832346	0.187297384	0.574886751	0.47139798	0.915407911
LOC112448894	0.092140618	0.303607725	0.470175009	0.618484581	0.984647426	0.471343675	0.915407911

LOC788201	0.052991585	0.736456765	0.528529266	0.562034691	0.690948349	0.471343176	0.915407911
NEURL2	0.347926515	0.975407599	0.546844259	0.430364686	0.100276223	0.471318144	0.915407911
PTBP3	0.301462664	0.37453451	0.769840303	0.156503686	0.588931172	0.471377898	0.915407911
STIP1	0.997655927	0.73220281	0.815927311	0.017973955	0.747158866	0.471213154	0.915407911
SGK1	0.368869293	0.610455515	0.155329636	0.277271656	0.826837088	0.47154111	0.915577515
LOC104973252	0.567389145	0.314215042	0.408021715	0.53181393	0.207347497	0.471600997	0.915585468
ACKR1	0.10003731	0.572057514	0.382507177	0.628670406	0.584150494	0.471993054	0.915588416
CCDC183	0.384134345	0.101187339	0.440362138	0.611253271	0.767638701	0.471829535	0.915588416
FBXO24	0.779157572	0.018282585	0.716145751	0.842621299	0.93467206	0.471895764	0.915588416
HRC	0.713158898	0.87732848	0.071027201	0.330307262	0.54679307	0.471711418	0.915588416
LOC615899	0.995395561	0.4876112	0.127130197	0.257780962	0.505249997	0.471945544	0.915588416
PRKAG2	0.296026541	0.608349939	0.43719698	0.779510419	0.130964571	0.47197133	0.915588416
TMEM150A	0.054672585	0.954386636	0.785739226	0.306272261	0.639943147	0.471923084	0.915588416
CCNB1	0.776156004	0.150336534	0.115020499	0.677678886	0.884753079	0.472179177	0.915638789
CD2BP2	0.353779385	0.289814215	0.575875698	0.246002501	0.554489872	0.472337548	0.915638789
DUS3L	0.631808986	0.102095582	0.896584895	0.446445669	0.312275357	0.472536517	0.915638789
LOC112442630	0.466814387	0.747440869	0.258340253	0.259931517	0.344109698	0.472527543	0.915638789
LOC112446691	0.355469461	0.212675369	0.475041897	0.340000501	0.658822268	0.472122555	0.915638789
NLE1	0.433777927	0.622116104	0.106496772	0.825466757	0.339651398	0.472418666	0.915638789
PREPL	0.855943094	0.695925467	0.36187624	0.04467436	0.836833151	0.472442296	0.915638789
PTGER2	0.53119446	0.322301634	0.955787019	0.431928404	0.114103348	0.472576963	0.915638789
SDHAF2	0.335314646	0.580417439	0.61030501	0.145017746	0.467406695	0.472271522	0.915638789
WASF3	0.23866074	0.61823264	0.586276945	0.447963559	0.207723811	0.472233401	0.915638789
CIPC	0.263275947	0.49483045	0.532454523	0.370207726	0.314347659	0.472751791	0.915653209
ICA1L	0.128730644	0.345064628	0.863006475	0.779086239	0.270271201	0.47274173	0.915653209
ZNF75D	0.388772498	0.155170268	0.606373311	0.344630894	0.640029193	0.472665015	0.915653209
FBXL21	0.371686443	0.610142102	0.269752468	0.276664756	0.477841979	0.473088343	0.915809113
ITM2C	0.309913436	0.928295471	0.118886043	0.328726016	0.719406971	0.473111307	0.915809113
LOC508153	0.916724869	0.912840547	0.529849027	0.031426962	0.580352035	0.473075268	0.915809113
RPS6KC1	0.574074298	0.47860272	0.571308445	0.137576876	0.374506504	0.473091452	0.915809113
WIF1	0.38573762	0.4283255	0.916019531	0.171319513	0.311885319	0.473072105	0.915809113
ADI1	0.449037934	0.991797497	0.223913612	0.183608639	0.445258957	0.474544085	0.915813314
AFF1	0.906809789	0.848804413	0.12172648	0.179333157	0.484242533	0.474184301	0.915813314
ATP13A1	0.137961534	0.755785983	0.612979644	0.216358392	0.592449134	0.475438068	0.915813314
BRI3	0.507237805	0.464339058	0.05496665	0.954101857	0.664609109	0.475806448	0.915813314
C23H6orf89	0.520075663	0.286423892	0.906659609	0.1148514	0.529517393	0.475902579	0.915813314
CD81	0.286975105	0.551468211	0.318800992	0.168207106	0.959381278	0.474304593	0.915813314
CHURC1	0.897945154	0.098834565	0.285382269	0.466884755	0.685552812	0.473516779	0.915813314
GTF2IRD2	0.601529807	0.416945811	0.133902115	0.623472563	0.389443151	0.474583134	0.915813314
GXYLT1	0.602599911	0.304578217	0.242551448	0.398976852	0.462146225	0.475786236	0.915813314
HDGFL2	0.508963998	0.816925964	0.2174218	0.098246857	0.921109343	0.475176333	0.915813314
KIAA1755	0.49761955	0.404463393	0.5672341	0.944605157	0.076128809	0.475819522	0.915813314
KRAS	0.27632623	0.756272503	0.343816908	0.375281019	0.301698	0.474152619	0.915813314
LAMP1	0.480856088	0.347871206	0.085307036	0.851666262	0.672298035	0.474944819	0.915813314

LGALS12	0.541486648	0.240856968	0.370827937	0.749323452	0.224265148	0.473982727	0.915813314
LOC100847326	0.575147719	0.497379428	0.415785425	0.883282591	0.077145591	0.473479571	0.915813314
LOC100848689	0.644359014	0.406876211	0.208378389	0.902994792	0.16464381	0.47386688	0.915813314
LOC101902808	0.698450525	0.648683485	0.568106372	0.102462707	0.311307156	0.4758257	0.915813314
LOC101906363	0.760813523	0.206245722	0.329673153	0.468890768	0.338098376	0.475619771	0.915813314
LOC107132335	0.131443495	0.765429756	0.371856458	0.295863674	0.742040956	0.475903719	0.915813314
LOC112444352	0.289051014	0.134427785	0.939815816	0.882411714	0.251375804	0.473376633	0.915813314
LOC112448579	0.206668727	0.585085479	0.766463785	0.246637411	0.357603426	0.475027129	0.915813314
LOC514011	0.616843822	0.242323361	0.921176671	0.198875306	0.296488604	0.47379527	0.915813314
MAPK15	0.10568511	0.17770007	0.744798306	0.631556525	0.919805106	0.473939899	0.915813314
MOGS	0.499004991	0.797707049	0.248608352	0.280089928	0.293048706	0.473878505	0.915813314
MYO5C	0.945194301	0.234206462	0.081267036	0.753015482	0.597300668	0.473180293	0.915813314
NDUFB6	0.4873725	0.624613256	0.71504764	0.069456801	0.538130941	0.474174895	0.915813314
NEK8	0.516379107	0.099189024	0.306274548	0.627977107	0.830562785	0.4752001	0.915813314
PDE6G	0.954970481	0.071527937	0.858682199	0.158753271	0.875735771	0.474586589	0.915813314
PIDD1	0.7766049	0.047497627	0.507424096	0.79958345	0.547400092	0.4754314	0.915813314
POU2F1	0.099095483	0.583518705	0.749454968	0.315721775	0.599595314	0.475684881	0.915813314
PSMD13	0.509342561	0.872900969	0.343090726	0.109676941	0.487419835	0.474590176	0.915813314
PTPRJ	0.382936791	0.323196721	0.657721896	0.140971568	0.712991117	0.47519697	0.915813314
RTL6	0.675972623	0.306363817	0.049524632	0.890791847	0.897855169	0.475665363	0.915813314
RUNDC3A	0.188019722	0.365260805	0.843353058	0.310139613	0.456522324	0.47560873	0.915813314
S100A11	0.649530451	0.35512022	0.544311071	0.110237532	0.584893191	0.473262565	0.915813314
SERPINI1	0.100140553	0.605616398	0.817131757	0.222589054	0.738854274	0.474489677	0.915813314
SHROOM3	0.799597318	0.044028867	0.32151799	0.752259622	0.959751815	0.474982947	0.915813314
SLC10A3	0.321515822	0.962262704	0.306927687	0.246688693	0.349012785	0.475059075	0.915813314
SLC25A23	0.775469308	0.393996777	0.197534439	0.154857456	0.870263554	0.474121806	0.915813314
SRCIN1	0.153891597	0.181362422	0.343913698	0.989035037	0.857926596	0.474368835	0.915813314
STMN1	0.259930527	0.664770892	0.317354872	0.613721094	0.24345344	0.475452185	0.915813314
STX6	0.648530237	0.570732877	0.487273906	0.682416266	0.06584897	0.473474289	0.915813314
THNSL1	0.172476304	0.97263569	0.683417433	0.523346409	0.136581268	0.475487644	0.915813314
TIGAR	0.548089529	0.072559914	0.970177306	0.479682809	0.440886566	0.474706502	0.915813314
TMSB15B	0.052418931	0.978292858	0.246569497	0.698882969	0.922615609	0.474556361	0.915813314
TPP1	0.787784967	0.487946717	0.067361583	0.838639701	0.375298407	0.474482237	0.915813314
TRPC4	0.098022278	0.528569915	0.724383293	0.358644065	0.605928008	0.47462277	0.915813314
TTLL1	0.251053264	0.821037767	0.54161303	0.351785026	0.206981764	0.474014941	0.915813314
UBQLN2	0.865139542	0.776283042	0.496128403	0.569902639	0.043019408	0.474909532	0.915813314
UPK1B	0.174277351	0.138478712	0.598291752	0.853145823	0.662174975	0.474644818	0.915813314
AOX4	0.454986256	0.312330854	0.28159925	0.759670478	0.271631781	0.476872544	0.916136101
BORA	0.740065541	0.067060397	0.188761752	0.914667609	0.961564328	0.476470631	0.916136101
CDK13	0.627104044	0.821891515	0.617250491	0.03626038	0.713723828	0.476339062	0.916136101
CEP170B	0.401603587	0.778648273	0.108675739	0.352654654	0.689979798	0.477127138	0.916136101
CIC	0.637170886	0.327855163	0.17475399	0.475237091	0.476096107	0.476922668	0.916136101
ERLEC1	0.726263625	0.599483635	0.103513067	0.348465258	0.527177792	0.477347755	0.916136101
FLYWCH2	0.954539153	0.72564827	0.413520966	0.114551019	0.25175746	0.476934721	0.916136101

GALNT14	0.074152911	0.673704155	0.909763498	0.911056625	0.198937262	0.476426822	0.916136101
H2AFZ	0.201971134	0.200451978	0.314027309	0.90151569	0.721430389	0.47711761	0.916136101
KMT2B	0.635380901	0.467246469	0.323515043	0.15499997	0.552883401	0.476281111	0.916136101
LOC104972888	0.656290238	0.968264606	0.225960678	0.105676359	0.542888956	0.476436998	0.916136101
LOC782950	0.118469379	0.966232544	0.809558147	0.622043038	0.143674093	0.477411245	0.916136101
NEMP1	0.19562566	0.419997804	0.563129622	0.610672024	0.29276893	0.477192878	0.916136101
NUCKS1	0.268436016	0.043139843	0.843902958	0.984080707	0.860285539	0.47722265	0.916136101
OAF	0.080439292	0.718063475	0.383788589	0.687653973	0.541311869	0.476742608	0.916136101
RFX1	0.7954899	0.623064346	0.253988973	0.878504852	0.074691763	0.476934955	0.916136101
RNF187	0.9709465	0.308815392	0.916567365	0.056227826	0.534533897	0.476929026	0.916136101
SDAD1	0.634836009	0.898376124	0.22398233	0.676065652	0.095729519	0.477089704	0.916136101
SLC26A7	0.617466051	0.284935827	0.057919715	0.898241075	0.904539049	0.477357572	0.916136101
STX5	0.427604849	0.398907506	0.09710579	0.547643085	0.911464163	0.477101827	0.916136101
UBE4B	0.764147928	0.323156478	0.312358228	0.304960759	0.350362161	0.47651778	0.916136101
VPS13B	0.328912405	0.81511246	0.788255189	0.085042014	0.460587778	0.477317202	0.916136101
XPO5	0.367907663	0.77706684	0.179870161	0.302519492	0.530047712	0.47661012	0.916136101
ZSWIM8	0.396642526	0.442354136	0.654832791	0.52387037	0.137055784	0.476692438	0.916136101
AAAS	0.294329037	0.172356533	0.331002566	0.812413136	0.632335195	0.484854832	0.916214087
AFMID	0.224122718	0.460514692	0.987076707	0.267063134	0.310110184	0.480804743	0.916214087
AGO3	0.844273025	0.953379059	0.937899258	0.027532318	0.404579704	0.480191019	0.916214087
ANKRD2	0.410200017	0.999035071	0.299074642	0.07380941	0.950872304	0.484336898	0.916214087
AP5S1	0.495337928	0.799145166	0.547842401	0.059037875	0.64836746	0.477830951	0.916214087
APOE	0.660395318	0.934650202	0.093847745	0.165598122	0.877468155	0.480364382	0.916214087
ATXN7	0.054938297	0.891667013	0.990432887	0.395757812	0.443176231	0.482362429	0.916214087
B2M	0.276117383	0.199200884	0.420958848	0.469241961	0.780321867	0.481682585	0.916214087
BAG4	0.765144964	0.09979254	0.13927918	0.904010447	0.89546281	0.484488494	0.916214087
BCDIN3D	0.470780341	0.199152146	0.69278248	0.617313995	0.209290989	0.479814737	0.916214087
BECN1	0.755557619	0.419499487	0.136466348	0.282759905	0.695908854	0.482398623	0.916214087
CAPNS1	0.738773477	0.356809717	0.202911205	0.262050669	0.594962237	0.478668158	0.916214087
CCDC28B	0.349414412	0.05030797	0.712606434	0.746444378	0.9193729	0.484221468	0.916214087
CCNL1	0.532928409	0.266324108	0.88376275	0.1684196	0.40495292	0.483333545	0.916214087
CEP104	0.705896339	0.775407043	0.02374614	0.760167759	0.869462048	0.484097912	0.916214087
CFLAR	0.989303386	0.859087127	0.072365464	0.182375025	0.740814798	0.47801491	0.916214087
CHCHD5	0.121682388	0.555165801	0.956364349	0.326681062	0.393580736	0.477955522	0.916214087
CHD5	0.394275616	0.980805507	0.739600873	0.147529233	0.202890249	0.48346316	0.916214087
CLSPN	0.706720119	0.19993068	0.155479874	0.809227551	0.480867485	0.48320066	0.916214087
CMBL	0.645287182	0.713834223	0.560267914	0.074077691	0.440412052	0.480418689	0.916214087
CNNM4	0.889716048	0.337707716	0.522201181	0.063291869	0.855588477	0.48208187	0.916214087
CSRP2	0.141084063	0.307924813	0.94513233	0.986965523	0.209746535	0.48215305	0.916214087
CTTN	0.780442363	0.62026837	0.901414649	0.184729775	0.106044671	0.483189561	0.916214087
CYTH4	0.271580477	0.692689113	0.923291235	0.257539159	0.191915547	0.483973521	0.916214087
DAG1	0.621526274	0.461601719	0.945744306	0.054859125	0.567357031	0.480971298	0.916214087
DNAJB11	0.442954482	0.91762654	0.474112405	0.095363547	0.463424229	0.482513572	0.916214087
EIF1AD	0.658635361	0.053241865	0.758061078	0.348446347	0.911999894	0.481024859	0.916214087

ELAC2	0.751062224	0.565586651	0.461692637	0.347486073	0.125285943	0.482977416	0.916214087
ELOVL5	0.482931913	0.89740076	0.694590352	0.080197879	0.348967479	0.480526455	0.916214087
EPHX3	0.357299031	0.414329032	0.58283215	0.346411306	0.283826596	0.481796556	0.916214087
ERBB4	0.24310274	0.584002537	0.24311218	0.416460286	0.599861801	0.484778639	0.916214087
ERN1	0.845458871	0.530844851	0.628567252	0.031959146	0.921938898	0.478072038	0.916214087
EXOC1	0.887200173	0.579158812	0.512022675	0.27351612	0.117078498	0.480534712	0.916214087
FGL1	0.654683142	0.820697004	0.113181037	0.308101217	0.454009796	0.482293909	0.916214087
GEMIN7	0.978492979	0.431596394	0.301404774	0.323993645	0.206161338	0.482202488	0.916214087
GK5	0.692424807	0.585673035	0.06310809	0.97726429	0.336499872	0.480341715	0.916214087
GPR37	0.984364884	0.744356881	0.09441727	0.762905412	0.161444085	0.482603807	0.916214087
HOMER1	0.855884371	0.264837026	0.803495232	0.451004853	0.102726942	0.480818392	0.916214087
IL21R	0.0630866	0.496363571	0.99751806	0.577541753	0.473549181	0.483077987	0.916214087
INO80	0.773214625	0.519702023	0.940466289	0.025489388	0.896106762	0.484981691	0.916214087
IQCB1	0.193060476	0.677007118	0.866436918	0.080104745	0.938724424	0.482493509	0.916214087
IRF9	0.472250596	0.051363083	0.978557279	0.354584894	0.984655139	0.47752853	0.916214087
JRK	0.423700199	0.661757425	0.677930324	0.050506106	0.886836015	0.48245551	0.916214087
KCNH3	0.575446072	0.617671552	0.488240753	0.828321743	0.059486937	0.483251178	0.916214087
KCNJ16	0.954182168	0.769909308	0.582559974	0.078017636	0.258490827	0.484953211	0.916214087
KDM4B	0.808232717	0.438006414	0.287131093	0.089246046	0.945162504	0.483747139	0.916214087
KRR1	0.516208258	0.800666956	0.476432996	0.045230304	0.935681115	0.478545249	0.916214087
LATS2	0.47987573	0.319958879	0.24367209	0.890148838	0.250224379	0.478538921	0.916214087
LOC100296205	0.631515978	0.741371872	0.502377119	0.815108659	0.044285598	0.481949249	0.916214087
LOC100296627	0.231862044	0.34229407	0.646177845	0.428581874	0.378105158	0.478037761	0.916214087
LOC100335828	0.21462947	0.398192631	0.234870355	0.599234261	0.714960172	0.484294399	0.916214087
LOC100847831	0.822623281	0.899997756	0.584042399	0.099211536	0.199783971	0.483669934	0.916214087
LOC101902809	0.984003616	0.225040729	0.094632015	0.456430422	0.894590537	0.483368377	0.916214087
LOC101903913	0.415869354	0.047693474	0.889868718	0.875623937	0.554071619	0.483507817	0.916214087
LOC101904691	0.82160523	0.840511278	0.393244149	0.923358596	0.033381079	0.479345115	0.916214087
LOC101905897	0.931188812	0.971267476	0.162315505	0.168331824	0.34339154	0.481849873	0.916214087
LOC104971021	0.09295644	0.399323701	0.693192968	0.449359821	0.73226627	0.481441153	0.916214087
LOC107131331	0.263414898	0.412906403	0.503618364	0.380690864	0.413305734	0.484694721	0.916214087
LOC107132192	0.066121297	0.404214773	0.53872488	0.874391722	0.668097751	0.48023941	0.916214087
LOC112441473	0.284245908	0.537693392	0.452279951	0.14861951	0.840641342	0.485068696	0.916214087
LOC112442598	0.968891938	0.760973204	0.363892632	0.403176966	0.079656614	0.484651702	0.916214087
LOC112443011	0.186573008	0.212971043	0.440261403	0.913351423	0.522604669	0.478904465	0.916214087
LOC112448474	0.364434839	0.822096286	0.15719503	0.529028699	0.338957158	0.480971071	0.916214087
LOC616720	0.303746672	0.606882751	0.629362299	0.304020013	0.235703292	0.478103828	0.916214087
LOC781100	0.56021929	0.395310199	0.572972513	0.110849463	0.596937974	0.479913895	0.916214087
LOC781646	0.77701649	0.42751269	0.226331634	0.901109939	0.127706703	0.485403797	0.916214087
LOC783657	0.180086763	0.379362403	0.926494898	0.163070772	0.822367846	0.481904189	0.916214087
LOC786055	0.208794166	0.68086686	0.529124061	0.202733568	0.566736524	0.485204772	0.916214087
LOC786978	0.899172311	0.403856365	0.044276089	0.981737803	0.532337281	0.480052189	0.916214087
LONRF1	0.093622666	0.815695951	0.901549551	0.236543903	0.516025946	0.480077719	0.916214087
LRRC42	0.928380767	0.562204503	0.028717001	0.838518993	0.677525683	0.482484359	0.916214087

LZTS2	0.844053497	0.669060154	0.182320801	0.18103689	0.448837604	0.47925597	0.916214087
MALT1	0.675463353	0.255453784	0.730593044	0.766359013	0.089564385	0.485421251	0.916214087
MAP1A	0.736924621	0.760671186	0.061479714	0.92640919	0.270480405	0.485055055	0.916214087
MBD3	0.295966899	0.406674817	0.486141282	0.555674624	0.263076054	0.483309331	0.916214087
MGC148714	0.893240761	0.853245149	0.875273095	0.014241242	0.894367335	0.482084663	0.916214087
MGME1	0.989579944	0.069497938	0.794628833	0.52522289	0.297512302	0.48300606	0.916214087
MKRN1	0.851647134	0.559585812	0.117145082	0.541660564	0.279973109	0.481429359	0.916214087
MLF2	0.360977719	0.553583011	0.433403398	0.115492468	0.857193747	0.483745253	0.916214087
MPP7	0.064933669	0.32245339	0.507759896	0.890405954	0.896901303	0.481949112	0.916214087
MPZL1	0.538999275	0.555066282	0.30385612	0.324794568	0.287184247	0.481714224	0.916214087
MSRB2	0.658634128	0.760540339	0.187885682	0.329227591	0.269914886	0.479195265	0.916214087
MTFR1	0.875846784	0.281040909	0.124836869	0.367693764	0.742371356	0.479726925	0.916214087
NANP	0.309770319	0.34450204	0.942751783	0.575450331	0.146392688	0.481624009	0.916214087
NARF	0.593245422	0.430715996	0.454967516	0.135661082	0.535439057	0.480956626	0.916214087
NCOA4	0.883799643	0.632482077	0.047262299	0.517377462	0.623662117	0.482684813	0.916214087
NDUFA4L2	0.822996056	0.964320385	0.233737737	0.282208979	0.160955894	0.480558738	0.916214087
NDUFB5	0.583317402	0.649899861	0.464325772	0.09514468	0.497633956	0.478558953	0.916214087
NELFE	0.697496832	0.309443007	0.174071668	0.277937387	0.803475245	0.479778413	0.916214087
NFIA	0.828240845	0.104808788	0.731529376	0.299617362	0.453647471	0.484962568	0.916214087
NOL7	0.518538867	0.376398055	0.288152657	0.250393539	0.597729376	0.480370831	0.916214087
NR1H3	0.594887086	0.506011171	0.056967858	0.719261834	0.683454738	0.480641068	0.916214087
NUP85	0.404593199	0.334300529	0.279323118	0.671495855	0.339794647	0.484730617	0.916214087
OBSL1	0.619482329	0.506810728	0.042135635	0.74317852	0.877744121	0.484926141	0.916214087
OPLAH	0.887398759	0.214620882	0.430813175	0.185464264	0.559571891	0.482483386	0.916214087
OXNAD1	0.19762035	0.885886787	0.978292876	0.064490003	0.778886688	0.484360339	0.916214087
PAK4	0.883992328	0.606266005	0.021853449	0.809916177	0.875441846	0.477899888	0.916214087
PANX2	0.166072269	0.930587368	0.080656192	0.826516662	0.831179928	0.483513428	0.916214087
PDCD5	0.415640338	0.344596993	0.59418418	0.102590215	0.967814414	0.481073299	0.916214087
PDE7A	0.276747186	0.51923873	0.817222853	0.565249427	0.129658007	0.484438363	0.916214087
PFDN1	0.63751596	0.422656963	0.454737099	0.111551896	0.629370822	0.484350874	0.916214087
PLEKHG1	0.077283667	0.977944642	0.507457767	0.456154514	0.491728539	0.484357733	0.916214087
PLXND1	0.88533268	0.707593323	0.176153582	0.161324352	0.467118289	0.478155736	0.916214087
POFUT2	0.93813297	0.64943688	0.184175695	0.250542476	0.299746897	0.480577284	0.916214087
PPCDC	0.762840013	0.470820893	0.909053984	0.076598278	0.339741651	0.482083372	0.916214087
PPP2R5A	0.714225929	0.369079709	0.05569243	0.882033933	0.647216466	0.479575061	0.916214087
PPP6R3	0.409256671	0.644867604	0.551788071	0.065009148	0.895250319	0.481624598	0.916214087
PSMB3	0.326766039	0.666735988	0.586496234	0.173223616	0.390003819	0.484988088	0.916214087
PUM1	0.397271645	0.955309778	0.826702277	0.027565921	0.992599289	0.483972818	0.916214087
PUS7L	0.610972263	0.950814652	0.510089166	0.149343563	0.195527516	0.485421492	0.916214087
RABEPK	0.603917729	0.16927786	0.381829043	0.59406792	0.372891382	0.485297897	0.916214087
RAD54B	0.884439493	0.026175758	0.82116465	0.620014968	0.724909752	0.483110663	0.916214087
RAD54L2	0.360513678	0.402477727	0.457654474	0.31354776	0.403280418	0.479922087	0.916214087
RASD2	0.896243319	0.995152988	0.076996232	0.622804109	0.194718335	0.478422409	0.916214087
RASL10B	0.460442719	0.854629732	0.550260276	0.08469737	0.460717157	0.481063952	0.916214087

RHOJ	0.726408144	0.771007964	0.030586048	0.794346282	0.635638897	0.485347719	0.916214087
RNF152	0.750660011	0.324370453	0.051013297	0.824718565	0.822135206	0.480470938	0.916214087
RNF167	0.463590588	0.506490303	0.225446302	0.418990085	0.376519013	0.478924776	0.916214087
SALL2	0.755946784	0.965986344	0.019784778	0.838061218	0.70675322	0.483386151	0.916214087
SCNN1A	0.957856122	0.21358725	0.527608976	0.56927986	0.137291436	0.480782354	0.916214087
SDHB	0.634472325	0.767434827	0.185014493	0.142958481	0.65146977	0.479775745	0.916214087
SEC31B	0.45669318	0.756895253	0.592616497	0.054562257	0.773353341	0.485229869	0.916214087
SET	0.823966042	0.208777537	0.628431697	0.692246889	0.112525356	0.480448049	0.916214087
SETX	0.247009049	0.360255843	0.433164651	0.524364169	0.425011402	0.484092304	0.916214087
SH3BGRL	0.371072878	0.540206185	0.106042118	0.603129468	0.649417456	0.478375414	0.916214087
SLC2A12	0.286025153	0.871750987	0.542888049	0.102910433	0.61625566	0.483972775	0.916214087
SLC35E1	0.977833952	0.171501336	0.25891186	0.422408602	0.46957033	0.484559837	0.916214087
SNCAIP	0.462247807	0.840929093	0.09676631	0.84106732	0.271387032	0.483994291	0.916214087
SPATA6	0.062913602	0.83208311	0.304602546	0.778882791	0.673350727	0.479184757	0.916214087
TCEANC	0.470483572	0.531427371	0.57125005	0.177904443	0.340556757	0.485435469	0.916214087
TEX12	0.660492324	0.475774538	0.701860904	0.520219875	0.074925714	0.484230484	0.916214087
TIMM29	0.922221732	0.155932176	0.252216957	0.915298192	0.25241834	0.479550712	0.916214087
TMEM140	0.153350023	0.76904773	0.696813985	0.197106805	0.526607142	0.482798051	0.916214087
TOPORS	0.259615721	0.797076809	0.982105735	0.044332981	0.960374663	0.485421147	0.916214087
TRIP13	0.713035959	0.083429809	0.346153466	0.594050431	0.698714874	0.483170092	0.916214087
URB2	0.975924763	0.583107281	0.4720827	0.182869824	0.17077055	0.479764722	0.916214087
URI1	0.65429703	0.624957836	0.171962411	0.28532449	0.418753731	0.48002503	0.916214087
WDFY4	0.458159559	0.48444035	0.203744114	0.241222492	0.760033431	0.47760339	0.916214087
WDR55	0.397282573	0.903555436	0.730395534	0.097289474	0.336867608	0.484145927	0.916214087
ZBTB26	0.999664598	0.483886824	0.413282916	0.179631879	0.232599763	0.478966256	0.916214087
ZNF146	0.261176603	0.918492478	0.796841225	0.064663007	0.686087991	0.481734017	0.916214087
ZNF275	0.552281387	0.724861767	0.355720686	0.145878569	0.399492506	0.477784789	0.916214087
ZNF639	0.516055142	0.152082991	0.906710243	0.209860728	0.572993199	0.483381507	0.916214087
ABCC11	0.940490946	0.823396595	0.039667519	0.461344957	0.614472671	0.486592246	0.916415387
ANGPT4	0.19513223	0.609742314	0.233312884	0.733582731	0.425794752	0.485804233	0.916415387
BAMBI	0.530451172	0.638232513	0.099988347	0.631233085	0.408612359	0.48707789	0.916415387
C29H11orf98	0.487352087	0.352828049	0.823620706	0.768874329	0.0802071	0.487131373	0.916415387
C8G	0.94300892	0.056958762	0.801347331	0.327960251	0.618358834	0.487028778	0.916415387
CCDC25	0.778934133	0.473695639	0.297416775	0.446863903	0.176892066	0.485882748	0.916415387
CLIC2	0.482380598	0.332059394	0.117604368	0.569829914	0.809829422	0.486271147	0.916415387
DDR2	0.337481583	0.651238694	0.311540158	0.557233331	0.228503975	0.486807174	0.916415387
DNAH1	0.973325484	0.078304054	0.833459015	0.198366906	0.691744545	0.486767575	0.916415387
FAM219A	0.777485679	0.169263592	0.375636357	0.672189386	0.262698741	0.487034828	0.916415387
FBLN5	0.114958109	0.646784573	0.610761647	0.228575756	0.836708316	0.486105151	0.916415387
GJA5	0.952119875	0.117720897	0.459255497	0.537380157	0.314981532	0.486693426	0.916415387
IFT122	0.628517908	0.376241311	0.650389178	0.15118686	0.372674676	0.48569345	0.916415387
IGSF8	0.589392113	0.182014182	0.185841166	0.813562305	0.535403687	0.486083323	0.916415387
LOC100300881	0.351651954	0.238926101	0.694813166	0.818380619	0.182280559	0.486597863	0.916415387
LOC101908339	0.18583352	0.818097379	0.248580756	0.278501964	0.828257982	0.486788261	0.916415387

LOC104975027	0.643592428	0.52013847	0.81126329	0.117851008	0.271586597	0.486256563	0.916415387
LOC107132952	0.779777575	0.223419229	0.32259982	0.30703318	0.503773625	0.486274657	0.916415387
LOC112447523	0.337716004	0.237969439	0.226640962	0.809364314	0.588779951	0.485992277	0.916415387
LOC789352	0.658443669	0.734594178	0.221635982	0.367159786	0.221357148	0.486688671	0.916415387
MEGF10	0.292121118	0.988850415	0.086441977	0.897210513	0.38804851	0.48628416	0.916415387
OXR1	0.787713636	0.711903454	0.090235611	0.348193631	0.494341883	0.486629715	0.916415387
PLAA	0.384456263	0.563404432	0.189768379	0.63835927	0.330529365	0.485847686	0.916415387
PRADC1	0.358002721	0.806641614	0.660341481	0.077711201	0.587853289	0.48666036	0.916415387
RAB34	0.730050599	0.481818395	0.126514603	0.238629165	0.821131136	0.486840123	0.916415387
SNX30	0.645194839	0.2670879	0.727985423	0.070080933	0.992823665	0.487020681	0.916415387
SOAT1	0.551642041	0.293015641	0.136431705	0.949999874	0.414487728	0.486072772	0.916415387
TRNP1	0.667286063	0.131464241	0.258606722	0.501313493	0.761954102	0.4856919	0.916415387
UNC13C	0.873464633	0.418392431	0.91821235	0.084523575	0.307979982	0.487161528	0.916415387
MYO18A	0.774308348	0.247963656	0.256990348	0.75909051	0.23331608	0.487239565	0.916457134
RAD51AP1	0.79092361	0.269461275	0.063061195	0.857426448	0.758822793	0.487355245	0.916569669
LDLRAD3	0.738601165	0.583079393	0.331748133	0.14394691	0.425364063	0.487430999	0.916607095
UXS1	0.898398142	0.936908411	0.069667482	0.270583485	0.551643628	0.48753542	0.916698416
PAIP2	0.52533638	0.238189856	0.161100651	0.904626554	0.48034346	0.48767227	0.916760471
USP22	0.966211118	0.132941823	0.220639103	0.362651817	0.852298755	0.487680148	0.916760471
WDR89	0.792673247	0.823765667	0.671792435	0.285357107	0.070008292	0.487753529	0.9167934
CLDN1	0.305673885	0.180356164	0.546940585	0.301660795	0.966164416	0.488273672	0.91693347
HMGN2	0.393109329	0.168670782	0.270837988	0.783637127	0.623275744	0.487917331	0.91693347
MGAT3	0.154158726	0.612443571	0.255645826	0.489484798	0.743715877	0.48823969	0.91693347
MGP	0.242463604	0.654678869	0.668147035	0.114756982	0.721599412	0.488156272	0.91693347
MTIF3	0.594629897	0.507875824	0.635619994	0.197080401	0.232304346	0.488275035	0.91693347
NPY5R	0.080190919	0.309685451	0.561817146	0.865084143	0.72807099	0.488262521	0.91693347
PLEC	0.609151179	0.270066231	0.22185734	0.318506177	0.755585926	0.488176888	0.91693347
PROX1	0.726703921	0.634969419	0.029234943	0.750383988	0.867005929	0.488027511	0.91693347
GABBR2	0.834626068	0.989898574	0.486097474	0.273481073	0.080061765	0.488383411	0.916938244
SKA2	0.88809817	0.921005953	0.080561316	0.162099526	0.823266977	0.488389324	0.916938244
C3H1orf123	0.749211831	0.474367298	0.53631793	0.053583591	0.861570742	0.488513047	0.917065616
LOC104974269	0.956416196	0.772850948	0.187715342	0.876933569	0.072402492	0.488724142	0.917147158
TNK2	0.314396807	0.611439148	0.313454766	0.377792499	0.386964942	0.488710816	0.917147158
UPF3A	0.402024139	0.685012833	0.08715712	0.654066716	0.560961127	0.488658834	0.917147158
LOC101906012	0.636464946	0.615741867	0.575657159	0.052628813	0.743662333	0.489136858	0.917189227
MRPL9	0.435024153	0.774951959	0.282886542	0.134157424	0.690531329	0.489247964	0.917189227
POLR1D	0.895740335	0.852181007	0.038688367	0.735880094	0.405797486	0.488914057	0.917189227
PRIMPOL	0.795919148	0.203065096	0.368501661	0.192476347	0.768914285	0.488824292	0.917189227
SPATA13	0.869115377	0.533570503	0.440383469	0.271797229	0.159103341	0.48917422	0.917189227
SSBP4	0.742788284	0.125498748	0.184484664	0.603088902	0.850769474	0.48901881	0.917189227
UBE3C	0.918798931	0.456699515	0.59243216	0.053111414	0.66867681	0.489118776	0.917189227
UBXN10	0.75078432	0.468537537	0.054033201	0.474336867	0.97993018	0.489249558	0.917189227
UQCRH	0.873472124	0.913574754	0.337407672	0.033278549	0.98556579	0.489162991	0.917189227
TSKS	0.546460502	0.544386616	0.613936094	0.0507127	0.954275234	0.489324798	0.917225501

SLC10A5	0.788183689	0.608670007	0.785392792	0.115828614	0.202618335	0.489414296	0.917288489
MTRR	0.061830846	0.933140446	0.388529978	0.939320109	0.420358265	0.489592	0.917516765
FNIP2	0.80985978	0.087846164	0.226063732	0.600607143	0.917015674	0.489728058	0.917666952
CYB561A3	0.16067399	0.649642172	0.704774261	0.900543129	0.133849155	0.489922821	0.917781233
ENHO	0.278061672	0.336915487	0.981424082	0.169983457	0.567472283	0.48995682	0.917781233
SUGCT	0.466489587	0.417816593	0.171703224	0.560858679	0.472358041	0.489897741	0.917781233
C7	0.906854728	0.495340096	0.15095498	0.312778893	0.418616188	0.490157044	0.917827635
MGST2	0.543684286	0.701260442	0.041357955	0.779788689	0.722260297	0.490205302	0.917827635
SLC25A28	0.214539255	0.412529459	0.550782594	0.777193813	0.234284991	0.490103436	0.917827635
TBC1D1	0.599277512	0.228362005	0.121921464	0.8134028	0.654169014	0.490150902	0.917827635
ANKRD44	0.281867739	0.948628639	0.616410974	0.105650424	0.51160594	0.490783701	0.917965615
DOK3	0.876627981	0.618606712	0.45245086	0.402834406	0.090045612	0.490601908	0.917965615
FUT8	0.637620331	0.777120027	0.068957079	0.320257179	0.815083438	0.491000742	0.917965615
ISOC1	0.549840465	0.671143805	0.352147569	0.082298656	0.834089111	0.491022867	0.917965615
LOC100849681	0.838717929	0.212177104	0.585309637	0.25139268	0.339742607	0.49052224	0.917965615
LOC112445011	0.103952349	0.32200334	0.937776063	0.668239899	0.424336858	0.490621644	0.917965615
LRRC8A	0.104847754	0.968591915	0.398164495	0.348515425	0.631636404	0.490627872	0.917965615
PRKG1	0.23183379	0.262342948	0.293149676	0.625490005	0.800052809	0.491062101	0.917965615
PROZ	0.663324603	0.05388962	0.770708397	0.348277793	0.927286772	0.490547024	0.917965615
RAB11FIP5	0.485450591	0.936289131	0.103095267	0.389992334	0.486665309	0.490470228	0.917965615
RBP7	0.180291032	0.539964874	0.303689021	0.43980663	0.685027408	0.490749597	0.917965615
STEAP3	0.40453423	0.977640679	0.07102881	0.926450493	0.342799049	0.491043559	0.917965615
TTYH2	0.085606083	0.526056749	0.711609189	0.8979407	0.309792427	0.490901895	0.917965615
ZNF236	0.651457366	0.213793274	0.721839007	0.166397514	0.533080083	0.49097086	0.917965615
CTU1	0.751828846	0.490662427	0.676646533	0.114229387	0.313634917	0.491483065	0.918081971
DIABLO	0.735648545	0.2792346	0.582019522	0.161189158	0.464782567	0.491779099	0.918081971
FAM210A	0.720631779	0.852399711	0.388341239	0.712250182	0.052684274	0.491660565	0.918081971
HECTD4	0.349552334	0.76102741	0.528946641	0.56968871	0.111624155	0.491591062	0.918081971
HORMAD2	0.687699156	0.722759973	0.413740834	0.070875391	0.614589835	0.491795662	0.918081971
KCNH4	0.184682846	0.466120708	0.132170728	0.991580719	0.792554269	0.491462433	0.918081971
MFSD10	0.284815354	0.143538242	0.674835671	0.690464879	0.470056261	0.491718633	0.918081971
OMD	0.986094635	0.443068631	0.14586438	0.816412273	0.171816652	0.491418391	0.918081971
RPS6KB1	0.543211412	0.86620485	0.134596508	0.968126296	0.14599649	0.491666483	0.918081971
UBE4A	0.600761779	0.958555376	0.152293182	0.647785194	0.157468471	0.491550466	0.918081971
USP48	0.978740338	0.799469487	0.821490403	0.015895068	0.875786982	0.491596405	0.918081971
ZNF93	0.843578924	0.251142303	0.116323983	0.486459203	0.74480166	0.491201088	0.918081971
LOC107132724	0.441324095	0.310054619	0.09492033	0.824105509	0.837728064	0.491982245	0.918172905
SKP1	0.919863404	0.217311111	0.143805951	0.473661768	0.658262493	0.491900925	0.918172905
ZNF140	0.683254288	0.580926905	0.083460499	0.640546102	0.422645532	0.492012219	0.918172905
AAMDC	0.81114462	0.421151695	0.227429704	0.202692759	0.5697389	0.492091522	0.918216482
EIF4EBP2	0.847209984	0.562418159	0.034872124	0.639369129	0.845812746	0.492370547	0.918395682
GSTT1	0.381683877	0.68139559	0.883512948	0.066671552	0.586670446	0.492411407	0.918395682
LOC101905367	0.249207857	0.942420846	0.494067131	0.144265755	0.536874761	0.492402053	0.918395682
PDE3B	0.649926211	0.611533911	0.031375596	0.938027528	0.768018236	0.492331871	0.918395682

LOC785408	0.685599623	0.057395758	0.731675538	0.791299497	0.394699276	0.492507172	0.918444555
ZNF692	0.844462854	0.032381129	0.571549322	0.6090053	0.944992739	0.492549542	0.918444555
LOC112444328	0.367744056	0.299871233	0.352121858	0.633539717	0.365744207	0.492612867	0.918458277
CORO6	0.385192318	0.19161955	0.483815437	0.303025054	0.832115378	0.49275507	0.918619045
ATXN7L3B	0.963611306	0.164175359	0.74864957	0.250377846	0.303843791	0.492872194	0.918733028
HOXC5	0.32533102	0.1702598	0.688347122	0.245313267	0.96472223	0.493141682	0.918848141
LOC537017	0.954351421	0.866773744	0.338270159	0.037455806	0.861413099	0.49324374	0.918848141
RAB8A	0.53043384	0.245783904	0.782542759	0.801332863	0.110449602	0.493269888	0.918848141
RILPL2	0.890846117	0.853753595	0.016096348	0.801137559	0.919630839	0.493062911	0.918848141
TINF2	0.638495716	0.58050118	0.094081503	0.389366892	0.664205986	0.493039074	0.918848141
TSPAN6	0.055737944	0.792757169	0.547579941	0.796161763	0.468690102	0.493251131	0.918848141
FAM110B	0.110438825	0.814673916	0.938628875	0.243380526	0.44025364	0.4936606	0.918954363
GGPS1	0.648383885	0.59759844	0.116966273	0.313466313	0.636250613	0.493461973	0.918954363
LOC618071	0.351896502	0.812205676	0.516278767	0.246589743	0.248687191	0.493662889	0.918954363
PDZD8	0.770562735	0.240051432	0.353773796	0.331531998	0.416647391	0.493466513	0.918954363
PRMT2	0.937242318	0.827270668	0.087023758	0.545606612	0.245785059	0.493653866	0.918954363
TFPI	0.335298159	0.628292732	0.143187266	0.651201433	0.460524404	0.493608403	0.918954363
CARM1	0.508965429	0.520638188	0.071262792	0.516038309	0.935376029	0.495008845	0.91902794
DAGLB	0.321765073	0.298032577	0.733329305	0.446941495	0.289989114	0.494999787	0.91902794
DSTYK	0.254514818	0.839933778	0.431225931	0.406571254	0.242473637	0.494459017	0.91902794
DYNLT1	0.865616974	0.075848668	0.469374734	0.514800824	0.574129768	0.494875708	0.91902794
ESRRG	0.297644513	0.78865765	0.80946979	0.318281047	0.150687567	0.49497474	0.91902794
KCNIP3	0.492867967	0.368322981	0.276999133	0.84285707	0.214481618	0.494509107	0.91902794
LOC107133075	0.303795828	0.674126481	0.138823021	0.663989876	0.480332654	0.49404358	0.91902794
LOC112444909	0.790010823	0.120944736	0.778025651	0.289478362	0.421071636	0.493914953	0.91902794
LOC112446716	0.102663644	0.721928194	0.185809264	0.707256639	0.932155959	0.494279988	0.91902794
LOC510454	0.596438946	0.963777069	0.331604346	0.876383878	0.054343375	0.494263014	0.91902794
LOC513767	0.448920244	0.698364196	0.037297621	0.980330106	0.791604831	0.494181832	0.91902794
LOC784127	0.395144583	0.248691167	0.180778458	0.872936547	0.586930033	0.49474341	0.91902794
MAN1B1	0.512762925	0.782152621	0.233695973	0.362198584	0.267085109	0.494029756	0.91902794
MPZL2	0.875267622	0.112440357	0.360092725	0.33674913	0.760884183	0.494305024	0.91902794
NECTIN4	0.074343523	0.739120327	0.945134998	0.534351422	0.328339724	0.494943767	0.91902794
PTPN11	0.556248413	0.378430947	0.051253284	0.858296561	0.983182986	0.494793138	0.91902794
PTS	0.837274437	0.617284902	0.067312475	0.260936893	0.997508866	0.49379379	0.91902794
SAMD11	0.881787675	0.516414418	0.072456508	0.466080339	0.592847501	0.495046431	0.91902794
SH2D3C	0.300777765	0.757254473	0.502566436	0.267076107	0.298123006	0.494990218	0.91902794
SNTB1	0.980092045	0.095815069	0.734575291	0.134774966	0.979306173	0.494799896	0.91902794
TMEM88B	0.457475675	0.29428081	0.363471746	0.506213285	0.366656078	0.494343579	0.91902794
UQCRC2	0.959984061	0.911833995	0.604766506	0.051177234	0.334606123	0.493996751	0.91902794
ZDHHC16	0.28950995	0.218755466	0.308316754	0.565703536	0.82293325	0.494505362	0.91902794
ZNF668	0.649016179	0.743580057	0.245396427	0.175156718	0.438606552	0.494667324	0.91902794
PPP1R8	0.846533116	0.166666282	0.66251845	0.526537048	0.185313653	0.495124158	0.919068268
CCL3	0.865461141	0.14472093	0.312829098	0.330032247	0.705828867	0.495258701	0.919205216
LOC112445927	0.190342027	0.644523199	0.722621619	0.84496055	0.121882552	0.495309958	0.919205216

DPH5	0.219486975	0.843833527	0.395227569	0.830712526	0.15034668	0.495563565	0.919495359
ZNF133	0.761656181	0.274734865	0.522627325	0.414732401	0.201585482	0.495578359	0.919495359
LYPD1	0.501831607	0.981097271	0.655701902	0.058468404	0.485019088	0.495818943	0.919629823
MTMR10	0.952136969	0.443197857	0.048907813	0.566068756	0.783585634	0.495808024	0.919629823
WDPCP	0.788466566	0.23981933	0.288088444	0.54444949	0.308568147	0.495753052	0.919629823
COX18	0.897108509	0.465921439	0.223183827	0.63100265	0.155631067	0.495943097	0.919652221
PRR5	0.956636461	0.355120203	0.124048991	0.555030348	0.391658654	0.495939744	0.919652221
FAM19A5	0.557798466	0.41887169	0.623642391	0.41196528	0.152920108	0.496314551	0.920161006
LOC100336602	0.321309127	0.660884723	0.108586872	0.724967245	0.549340339	0.49638568	0.920161006
PLEKHM3	0.625927346	0.503419974	0.215829344	0.983590368	0.137269458	0.496371637	0.920161006
DDX39A	0.743670323	0.681619006	0.410561907	0.192587929	0.229213524	0.496464193	0.920202607
PEX13	0.80779992	0.159958927	0.451985439	0.501736512	0.313837703	0.496655022	0.92045235
MPI	0.739789682	0.981197657	0.344945974	0.088053178	0.417433975	0.496796255	0.920506192
RIN1	0.972533239	0.140587365	0.609790107	0.229512673	0.480917492	0.496779058	0.920506192
COG1	0.219520755	0.20931225	0.366701263	0.686536965	0.795969852	0.496879223	0.920555987
CASP1	0.060967263	0.800439073	0.745898694	0.792732751	0.319479468	0.497105927	0.920617649
FAM124A	0.868789116	0.663794099	0.284373882	0.27279095	0.206084324	0.497122056	0.920617649
GPR161	0.648723256	0.650866703	0.082217117	0.687643118	0.386173533	0.497099439	0.920617649
HYI	0.083903665	0.54885716	0.664031728	0.544417508	0.554167555	0.497245748	0.920617649
PDGFRB	0.66761579	0.609560227	0.059249503	0.632001916	0.605422117	0.497246744	0.920617649
TUBGCP2	0.454558955	0.827609049	0.481960634	0.12125823	0.419635163	0.497249091	0.920617649
SLCO2B1	0.18233947	0.914106379	0.251332393	0.799672082	0.275527372	0.49733122	0.920665837
ENOX1	0.638799538	0.371925579	0.481889828	0.222775468	0.362005684	0.497394623	0.920679354
KLB	0.423668851	0.784465669	0.060737305	0.510866467	0.896451406	0.49762466	0.921001274
LOC782688	0.507832035	0.405880774	0.411921167	0.446615114	0.243925847	0.497724446	0.92108208
AHCTF1	0.279864145	0.715066279	0.778176646	0.090801545	0.655000193	0.497973118	0.921379809
BFSP2	0.514555428	0.6715995	0.22817939	0.79410418	0.149479418	0.499926356	0.921379809
CA5B	0.657732451	0.701463273	0.263595534	0.164941579	0.467549937	0.500299523	0.921379809
CLDN4	0.280735332	0.187388682	0.935658373	0.391666272	0.486373774	0.500254039	0.921379809
DCUN1D1	0.797217179	0.307100171	0.324013537	0.404958122	0.290289939	0.499235601	0.921379809
E4F1	0.731339692	0.24535449	0.538904368	0.282932617	0.340013678	0.498783654	0.921379809
EGFL7	0.267390346	0.564883575	0.646983857	0.326727373	0.291065031	0.498599709	0.921379809
ERLIN1	0.550048069	0.200010668	0.234748772	0.824544904	0.43936048	0.499847087	0.921379809
IQCA1L	0.470855408	0.037833575	0.983629619	0.974188464	0.547263434	0.499566427	0.921379809
ITSN1	0.604632303	0.715787897	0.781090431	0.041185873	0.672568088	0.500004917	0.921379809
KLHL3	0.14204298	0.49707558	0.203275498	0.84674665	0.766434266	0.499019233	0.921379809
LOC100337108	0.042678524	0.795787125	0.311618815	0.881833554	0.996275258	0.498694655	0.921379809
LOC101902918	0.526187602	0.864449617	0.678254979	0.579026261	0.052362051	0.499802517	0.921379809
LOC101904749	0.147284669	0.355637639	0.443028406	0.774605494	0.521644601	0.500257488	0.921379809
LOC112441506	0.934928414	0.721930054	0.379031808	0.182392	0.199840632	0.499225668	0.921379809
LOC112448082	0.866182897	0.755966655	0.055135179	0.270551146	0.955260169	0.499342497	0.921379809
LOC783680	0.184335028	0.303255808	0.414676511	0.651124389	0.618118038	0.499321015	0.921379809
LOC789157	0.961359792	0.227468069	0.079277373	0.612161419	0.874393602	0.498324181	0.921379809
LOC789258	0.239068388	0.661659291	0.886557703	0.110334393	0.59970232	0.498316342	0.921379809

LRRFIP2	0.400854138	0.15028569	0.512266664	0.41799311	0.725026952	0.499774801	0.921379809
LSM8	0.764168484	0.223599192	0.532208879	0.147352973	0.695155077	0.499030527	0.921379809
MAP4K5	0.196320173	0.896408697	0.263354738	0.586642565	0.341112574	0.498219492	0.921379809
MARK1	0.327263866	0.611556397	0.334404943	0.500659702	0.279269663	0.499881815	0.921379809
MCOLN1	0.174580094	0.669889335	0.287786059	0.737679636	0.376839801	0.499846943	0.921379809
MLEC	0.681528108	0.644201278	0.795320673	0.080323959	0.333492201	0.499798335	0.921379809
MYO1F	0.301348104	0.998243106	0.931556849	0.128011195	0.261359674	0.500235483	0.921379809
NDUFS1	0.814682186	0.954204655	0.82997656	0.048716668	0.297990057	0.500053405	0.921379809
PAXX	0.120885607	0.853214793	0.403513908	0.395600159	0.567904112	0.499731868	0.921379809
PEF1	0.824040448	0.49365998	0.270707619	0.104597818	0.809367199	0.499185683	0.921379809
PHC2	0.80541789	0.513961032	0.63624937	0.132726839	0.265486563	0.498346199	0.921379809
PLA2G6	0.251091218	0.749877203	0.599792288	0.697494533	0.118921194	0.500073799	0.921379809
PSTPIP2	0.475821106	0.59547279	0.486456074	0.232624367	0.291174332	0.499447417	0.921379809
PXK	0.821405593	0.076795836	0.80521331	0.578411476	0.318893827	0.500102308	0.921379809
RNF219	0.723297016	0.19700535	0.460095395	0.800255018	0.176696523	0.498140737	0.921379809
SIPA1L3	0.845601495	0.051787702	0.623923342	0.80862166	0.42255572	0.499445461	0.921379809
SNRNP35	0.792979924	0.866974558	0.532012239	0.960087229	0.026452222	0.498509732	0.921379809
SORD	0.69524319	0.889850019	0.222540957	0.155757205	0.433504522	0.498655933	0.921379809
SPATA16	0.507589427	0.850776696	0.388538899	0.510363608	0.10928794	0.49989932	0.921379809
SPIN1	0.641231688	0.304851824	0.605799585	0.173706528	0.453733843	0.499401473	0.921379809
TMEM234	0.900284614	0.772675562	0.673118965	0.212849347	0.093979108	0.500052352	0.921379809
UBE2G1	0.926243439	0.064635873	0.303924351	0.621635531	0.823529723	0.499029373	0.921379809
UCHL1	0.757836884	0.664697439	0.765303252	0.035038621	0.687443353	0.498447463	0.921379809
WDR54	0.781243259	0.359037566	0.182159951	0.70066244	0.259677485	0.498663161	0.921379809
LOC784451	0.922035436	0.958496256	0.027998526	0.53469996	0.709319288	0.500417028	0.921492802
USP11	0.507587003	0.913043128	0.625074804	0.105721543	0.30655904	0.500496715	0.921536136
ULK1	0.769594138	0.370061193	0.282701233	0.476290958	0.244932591	0.50056966	0.92156705
PI15	0.435706252	0.397035946	0.815310323	0.666372296	0.10004838	0.500780007	0.921747497
SH3GLB2	0.362959923	0.935597906	0.91041727	0.118906632	0.255722123	0.500732611	0.921747497
C2H2orf76	0.252053895	0.973200885	0.336271376	0.31885765	0.357894773	0.500978751	0.921880014
FNTA	0.692077676	0.610054216	0.135538939	0.192048535	0.85630328	0.500930803	0.921880014
LOC112446044	0.724650914	0.926303315	0.643855601	0.100022481	0.217870643	0.5010767	0.921880014
TMEM43	0.39934866	0.475407137	0.488042287	0.3427076	0.2965265	0.501032085	0.921880014
ADAMTSL5	0.730242579	0.550478794	0.350021057	0.841597129	0.079624491	0.501284693	0.921991708
C11H2orf42	0.861660668	0.744220759	0.729325464	0.502542203	0.040162886	0.501500465	0.921991708
CAPN6	0.07164854	0.66683338	0.70936627	0.923134633	0.302419231	0.501934125	0.921991708
CCDC85C	0.442934926	0.61158768	0.548731191	0.064937431	0.978711919	0.501650915	0.921991708
FAM76B	0.239366076	0.565649646	0.611730764	0.296687355	0.384641227	0.501743843	0.921991708
IVNS1ABP	0.151669996	0.312247898	0.661126255	0.585320169	0.515136444	0.501518656	0.921991708
LOC101904396	0.450389483	0.567794596	0.537572163	0.187846016	0.366487214	0.50198013	0.921991708
LOC112448518	0.89431898	0.562512979	0.910754543	0.023843925	0.865199187	0.50174165	0.921991708
LOC789035	0.884916348	0.95285443	0.897754402	0.040049167	0.311615874	0.501647105	0.921991708
MIA3	0.869517091	0.668534915	0.87629918	0.025509809	0.725796165	0.501337628	0.921991708
NIPAL3	0.628989462	0.825851091	0.526040481	0.072075417	0.478892492	0.501343184	0.921991708

PIP5K1B	0.333196442	0.268036516	0.589852493	0.945311262	0.189999118	0.501931768	0.921991708
PRKCSH	0.96130827	0.070890724	0.334090461	0.632416568	0.655478582	0.501466658	0.921991708
VPS13A	0.400897498	0.437059093	0.612877364	0.120574426	0.730129058	0.501776648	0.921991708
WDR4	0.226389276	0.99104836	0.612709258	0.797020658	0.086362561	0.501947217	0.921991708
ADNP2	0.152766182	0.375917965	0.451462491	0.946850564	0.386163672	0.502287561	0.922168352
GTDC1	0.322003909	0.398239387	0.253520489	0.482014454	0.604639918	0.502193519	0.922168352
HINFP	0.817718322	0.708226752	0.071738282	0.297710778	0.766487002	0.502301072	0.922168352
LOC112445065	0.708863391	0.41472226	0.316013398	0.284682239	0.358194573	0.502163307	0.922168352
VRK2	0.240269868	0.98129919	0.336704456	0.21248759	0.562197907	0.502363631	0.922180039
TMEM41B	0.876366186	0.149856275	0.111236336	0.948001504	0.685212851	0.502479018	0.922288689
ABCB10	0.186664666	0.489411835	0.598363185	0.232288432	0.748892629	0.502867599	0.922507101
DEPDC1B	0.53777281	0.330074459	0.143273439	0.421825137	0.88655078	0.502894998	0.922507101
LOC101905357	0.211165427	0.32320793	0.163584962	0.864632277	0.984673161	0.502791813	0.922507101
SHBG	0.85725207	0.757623913	0.089265299	0.267110653	0.613637585	0.502738802	0.922507101
TCP11L1	0.652824148	0.998149266	0.168388533	0.383087397	0.226378725	0.502991502	0.922507101
UBN2	0.468959276	0.293400315	0.779180558	0.388726961	0.228284797	0.502956262	0.922507101
USO1	0.895925607	0.184364053	0.90519823	0.687598809	0.092412132	0.50269936	0.922507101
KLHL41	0.069381657	0.608167137	0.725452709	0.365133016	0.851968834	0.503126023	0.922547615
OPRL1	0.193382406	0.830126588	0.976085741	0.581020097	0.104593751	0.503123712	0.922547615
LOC510613	0.872914937	0.636644745	0.044309204	0.564008427	0.686724707	0.503417433	0.922978828
ZADH2	0.519617096	0.635755847	0.64253129	0.259380504	0.173308899	0.503501027	0.923028971
LOC100847182	0.864731059	0.0464588	0.327657206	0.885275355	0.819446127	0.503647364	0.923194112
ATP6V1E2	0.033891581	0.566577578	0.831888935	0.63450119	0.945642111	0.504337184	0.923684192
DAP3	0.36422922	0.780263962	0.431336043	0.380982961	0.205445456	0.504533855	0.923684192
GUF1	0.437196836	0.483273346	0.542362618	0.166417124	0.502838432	0.5044274	0.923684192
HIGD1B	0.160422896	0.617148783	0.797745996	0.876445145	0.138240013	0.504038421	0.923684192
LOC615989	0.058368173	0.791154126	0.939794484	0.453793917	0.487197271	0.50453361	0.923684192
LZTS1	0.675357838	0.885680785	0.125969092	0.561219518	0.226292735	0.504038068	0.923684192
PTPN9	0.933870093	0.932247833	0.228892534	0.050443787	0.953695129	0.504376191	0.923684192
SIRT3	0.5664054	0.597960925	0.188763592	0.331577113	0.451896869	0.504237006	0.923684192
SMG8	0.568076719	0.491125538	0.400425684	0.111137176	0.772688385	0.50451283	0.923684192
ZBTB38	0.111107246	0.525062879	0.652770195	0.601518377	0.41885031	0.504528525	0.923684192
ZSCAN25	0.617759258	0.055121624	0.345681015	0.894281471	0.909286762	0.50408669	0.923684192
BLZF1	0.238847004	0.383039127	0.317734177	0.484263965	0.682067704	0.504663592	0.923698487
CPT1B	0.260886879	0.891536045	0.052812974	0.941886456	0.829781345	0.504645344	0.923698487
MARCKSL1	0.977880422	0.949420024	0.019922933	0.767013307	0.6769328	0.504710519	0.923698487
LOC101904057	0.951645912	0.076852434	0.446659992	0.461172217	0.638232881	0.504927912	0.923962888
LOC107132317	0.594466519	0.452919439	0.919379195	0.045559519	0.852751821	0.504967592	0.923962888
TCF7L2	0.917510025	0.721642389	0.633593516	0.119829149	0.191369009	0.505024613	0.923964206
LOC515227	0.365320361	0.49240742	0.591855361	0.567154478	0.159423481	0.505149121	0.924088979
LOC786553	0.695054579	0.26381255	0.121147919	0.46174433	0.939029761	0.505253677	0.924177228
APOD	0.080629259	0.545140105	0.54585791	0.889247653	0.451878908	0.505431413	0.924254343
C21H14orf28	0.58763359	0.421596328	0.522026765	0.669734735	0.111383039	0.505557178	0.924254343
FKBP4	0.97502379	0.425988785	0.357335259	0.093141946	0.698784377	0.505797529	0.924254343

GINS2	0.91134055	0.29474408	0.150331705	0.995456484	0.240255947	0.505752453	0.924254343
HYAL3	0.705161196	0.586608059	0.372204871	0.559073435	0.112093382	0.505578316	0.924254343
LOC101907000	0.910271	0.96939459	0.774002933	0.049210383	0.287351601	0.505757384	0.924254343
LOC107132283	0.330726359	0.197286295	0.87282137	0.834392394	0.203295783	0.505802709	0.924254343
PPP4R3A	0.416893425	0.863858603	0.981427536	0.053154123	0.513157923	0.505426576	0.924254343
TMEM185B	0.693773821	0.217449856	0.241954451	0.88601037	0.298542739	0.505702278	0.924254343
CEP152	0.288995769	0.923161794	0.496536845	0.217943013	0.3347848	0.50590565	0.92432369
MGA	0.423174736	0.43955821	0.981216562	0.060537926	0.875271932	0.50600963	0.92432369
TCERG1	0.972005429	0.200295572	0.214538252	0.430220507	0.538134176	0.505990266	0.92432369
ATXN10	0.27367394	0.73843756	0.335450498	0.313510493	0.458291624	0.50734114	0.924512171
BSDC1	0.723731726	0.9719555	0.218855882	0.783871628	0.08033457	0.506464233	0.924512171
CRB1	0.395350923	0.062791645	0.871159029	0.738205923	0.609810277	0.507248548	0.924512171
EGLN1	0.293063447	0.598724873	0.261372769	0.292370572	0.725687003	0.507153089	0.924512171
EID1	0.61077475	0.457454511	0.313073185	0.447002633	0.249260961	0.507457045	0.924512171
EIF2S2	0.821226493	0.365506099	0.19970106	0.918824401	0.176767116	0.507256126	0.924512171
EML4	0.14483509	0.33906285	0.249882985	0.977419385	0.812821323	0.507511881	0.924512171
FAM20B	0.62763168	0.125566167	0.439098951	0.709596584	0.395986031	0.507023677	0.924512171
FAM81A	0.086683035	0.964396871	0.583862713	0.31471644	0.632492328	0.506870671	0.924512171
FOLR2	0.33010566	0.187904303	0.204154413	0.869033813	0.884816212	0.507284619	0.924512171
KIFAP3	0.536251701	0.317686061	0.776151178	0.670580493	0.109437084	0.506635679	0.924512171
LOC104969140	0.717751884	0.090859878	0.912500058	0.178035996	0.91549575	0.506556154	0.924512171
LOC787679	0.248734089	0.572272857	0.192990514	0.511101573	0.693006463	0.507147339	0.924512171
NCF1	0.219140098	0.513435266	0.983013883	0.214222521	0.410588633	0.507113084	0.924512171
NDST2	0.529700017	0.092382473	0.414828122	0.584979746	0.820943939	0.507501289	0.924512171
RRM2B	0.930261108	0.256353542	0.183215758	0.384162674	0.580310802	0.507346801	0.924512171
RRP1	0.268622917	0.877826602	0.562602307	0.229314331	0.319897377	0.507180126	0.924512171
SAMD8	0.362383824	0.325968548	0.774470054	0.128281409	0.827167416	0.506712042	0.924512171
SATB2	0.157529771	0.864731225	0.964990035	0.184660816	0.401421373	0.507415181	0.924512171
SDC3	0.655699938	0.63783763	0.07453696	0.410042502	0.759652856	0.506765652	0.924512171
SLC22A31	0.060983801	0.729662357	0.448610469	0.602836557	0.809728936	0.507416567	0.924512171
SLC25A11	0.154450345	0.79325966	0.672735921	0.131832199	0.894838934	0.507016783	0.924512171
SUCO	0.22885135	0.308504502	0.408723058	0.548408768	0.6155788	0.507368015	0.924512171
TGOLN2	0.602614535	0.183732898	0.272955038	0.37353268	0.861034342	0.506952939	0.924512171
TTN	0.653595595	0.031625881	0.966835546	0.556781303	0.876192154	0.507521184	0.924512171
DUSP10	0.477102644	0.693985163	0.96128724	0.081354228	0.376786344	0.507651113	0.924646216
CLPTM1L	0.495731471	0.747563169	0.504026532	0.156951525	0.333075662	0.507808427	0.924727486
LOC112449080	0.726255612	0.902280373	0.366460124	0.499903164	0.081325849	0.507772297	0.924727486
C20H5orf22	0.257501333	0.581151007	0.450165763	0.486962649	0.297947762	0.507989308	0.924749034
NBEAL1	0.398164002	0.993606974	0.618426119	0.127892817	0.312301834	0.507949911	0.924749034
PMPCA	0.684304797	0.593379481	0.561287995	0.04668131	0.918454557	0.507942783	0.924749034
RFX5	0.53321641	0.795123042	0.030677137	0.90828989	0.827739251	0.508072763	0.924798371
LOC112446882	0.768037675	0.058559766	0.898653791	0.56128621	0.43150149	0.508275082	0.925064031
MEF2C	0.115392791	0.171399503	0.89697004	0.561193704	0.984055089	0.508428886	0.925241345
CAMK1	0.341977895	0.324074521	0.261458248	0.997660769	0.339185952	0.508587104	0.925309588

KCTD21	0.28481606	0.338947363	0.624113423	0.198688176	0.819380883	0.508652561	0.925309588
LOC112443416	0.615945629	0.266627722	0.715963298	0.449741869	0.185527288	0.50869192	0.925309588
MYPOP	0.456145053	0.843437551	0.628228418	0.055479129	0.731540976	0.508662746	0.925309588
CS	0.922072334	0.839760336	0.844790143	0.055435556	0.270735177	0.508817919	0.925333651
PTCD1	0.416030318	0.056405917	0.859120773	0.681628734	0.714264608	0.508778577	0.925333651
CDKN1B	0.10711002	0.581381761	0.951351386	0.323228545	0.513453518	0.509093871	0.925372895
EEPD1	0.757771247	0.363740954	0.99534573	0.055958655	0.639922454	0.508945601	0.925372895
LOC107131944	0.370121399	0.461214889	0.184297471	0.742772516	0.420641445	0.509047149	0.925372895
LOC112443159	0.654823163	0.616231592	0.621683579	0.119252117	0.328798739	0.509175818	0.925372895
MUM1L1	0.950500131	0.477810282	0.955368254	0.62442698	0.036305979	0.509177822	0.925372895
SLC35F2	0.240977743	0.343050481	0.397731727	0.56538943	0.528897146	0.509094221	0.925372895
C11H2orf40	0.412109478	0.779234153	0.422542803	0.076365749	0.951070603	0.509532813	0.92551859
CRTC3	0.60264096	0.395940436	0.868786797	0.871392294	0.054567316	0.509569575	0.92551859
EFR3A	0.740833966	0.032138226	0.558250665	0.971763545	0.763077132	0.509549356	0.92551859
LOC100336381	0.487271247	0.66473271	0.218039962	0.526805907	0.264975464	0.509596367	0.92551859
MTREX	0.226270726	0.773137729	0.948264273	0.240364425	0.247178177	0.509546715	0.92551859
NBN	0.364476904	0.368536457	0.462790751	0.862876435	0.183555759	0.509355861	0.92551859
ACTN3	0.916798055	0.2707938	0.066998291	0.912660618	0.64987065	0.509726498	0.925545254
PRR14L	0.563776516	0.352244902	0.136269275	0.390180088	0.9348745	0.50983664	0.925545254
PTDSS1	0.640103902	0.445489641	0.052965651	0.778477189	0.839417724	0.50981009	0.925545254
WNT5B	0.076289755	0.450654295	0.324863109	0.94272947	0.936746044	0.50968632	0.925545254
LOC787812	0.159112165	0.118897802	0.897975443	0.693572473	0.838473712	0.509989531	0.925720406
ANAPC16	0.677180545	0.213955444	0.181988336	0.485078299	0.775263435	0.510683114	0.925721999
AR	0.971030673	0.066739903	0.899787244	0.329210828	0.516339619	0.510612169	0.925721999
C1QTNF9	0.967279294	0.540711523	0.453032727	0.398918488	0.104823357	0.510535196	0.925721999
CAMK2A	0.75866202	0.838749523	0.082042271	0.628858284	0.302103413	0.51072372	0.925721999
COL11A2	0.167382799	0.431659637	0.592880796	0.307496367	0.752081656	0.510507318	0.925721999
FAM168B	0.336994052	0.269517135	0.880764376	0.176621047	0.70168968	0.510650092	0.925721999
HUS1	0.171946677	0.636887561	0.871927757	0.141150876	0.733746456	0.510180687	0.925721999
HYAL2	0.555147733	0.69702625	0.101579236	0.391777428	0.643472971	0.510553748	0.925721999
ISLR2	0.53943757	0.287301483	0.085149344	0.766733117	0.977858644	0.510272903	0.925721999
LOC100299757	0.680239235	0.291029633	0.233195716	0.40138076	0.534983089	0.51063292	0.925721999
LOC112447392	0.456482293	0.186850315	0.572538387	0.589347756	0.343694557	0.510223783	0.925721999
SARM1	0.366411161	0.296918233	0.210413095	0.466198075	0.927396352	0.510330436	0.925721999
TP53	0.759928115	0.945750211	0.137406095	0.348112218	0.287684671	0.510191155	0.925721999
EFCC1	0.484949314	0.785369909	0.215761043	0.159791757	0.758113813	0.511416006	0.925958361
GPX8	0.510826903	0.426211268	0.174710608	0.545006732	0.479166026	0.51101648	0.925958361
IFNGR2	0.460893809	0.650817031	0.708503698	0.076101729	0.615449122	0.511397103	0.925958361
IGSF3	0.872442859	0.183829688	0.70589655	0.259455401	0.338379708	0.511126368	0.925958361
L3MBTL3	0.651987078	0.650720078	0.178640194	0.180911905	0.72545962	0.511269538	0.925958361
LOC100295130	0.32919101	0.999920121	0.197291662	0.544992795	0.280800927	0.511105058	0.925958361
LOC615514	0.726488859	0.593631735	0.09576311	0.24121286	0.99929357	0.511418352	0.925958361
PTPN21	0.5648882	0.57527799	0.102453624	0.456599827	0.654017454	0.511183257	0.925958361
RNF43	0.103835916	0.841535564	0.742665271	0.178275649	0.860080703	0.511334931	0.925958361

ZNF527	0.727794885	0.50829231	0.91024782	0.178604976	0.165418976	0.51129899	0.925958361
NME4	0.228378873	0.816512489	0.180254245	0.38080227	0.778008482	0.511483152	0.925973525
VIM	0.844782543	0.824041207	0.138183711	0.792438333	0.130713861	0.51159085	0.926066339
ABCD1	0.964356823	0.695433773	0.790486166	0.151190252	0.124524966	0.51190582	0.926082144
AJUBA	0.675914179	0.467650395	0.74844064	0.047415181	0.897329481	0.513490593	0.926082144
BAHD1	0.393636847	0.395219243	0.316141336	0.484596048	0.42121622	0.512998552	0.926082144
BDH2	0.177490955	0.692941791	0.325370921	0.288088758	0.872952675	0.513459998	0.926082144
C9H6orf120	0.839085892	0.627559978	0.200547066	0.20643655	0.461185895	0.513275107	0.926082144
CDC23	0.950242713	0.729819349	0.095999089	0.230839099	0.655211286	0.513563237	0.926082144
CHST8	0.066672733	0.306311439	0.984722812	0.528791907	0.945292178	0.513247367	0.926082144
DDX20	0.123942807	0.305017242	0.815503647	0.396728238	0.819117431	0.51261473	0.926082144
DPT	0.102873806	0.748098759	0.155206403	0.952907842	0.878445003	0.512238391	0.926082144
ENGASE	0.205728987	0.591374567	0.306034841	0.5875844	0.457498384	0.512432566	0.926082144
FMNL3	0.746430583	0.505518157	0.062700773	0.567470123	0.749670821	0.513478983	0.926082144
FOXO3	0.933498394	0.569500435	0.134486921	0.207376467	0.678781163	0.513463544	0.926082144
KCMF1	0.768072168	0.523543151	0.053608074	0.519034317	0.896412149	0.512820888	0.926082144
LHFPL6	0.500701647	0.739000044	0.2631965	0.122858787	0.840581412	0.513339458	0.926082144
LOC101906347	0.811811855	0.186789385	0.491486401	0.559522349	0.23954899	0.512062357	0.926082144
LOC101906392	0.838904388	0.253856836	0.191037194	0.761759999	0.324333023	0.513226751	0.926082144
LOC101906411	0.93577942	0.12013191	0.465740379	0.31212502	0.614619704	0.513089546	0.926082144
LOC112446402	0.276674533	0.45078849	0.160718124	0.976260299	0.510390213	0.512038177	0.926082144
LOC112446708	0.59672328	0.908705655	0.456221219	0.15732018	0.256739357	0.512113142	0.926082144
LOC507696	0.622054394	0.420249516	0.156602797	0.673917531	0.363019849	0.512554873	0.926082144
MED24	0.271798371	0.342842803	0.512344518	0.242225082	0.865684545	0.512473102	0.926082144
MGC137036	0.773702117	0.492342707	0.0493838	0.552475943	0.961077235	0.512047941	0.926082144
PDIK1L	0.836390629	0.211872704	0.605540255	0.859034712	0.108821972	0.512849367	0.926082144
PIN1	0.589111108	0.413771757	0.334113827	0.275625819	0.447812219	0.513243914	0.926082144
RAPH1	0.088069422	0.93191237	0.780160817	0.743839474	0.210697432	0.512922033	0.926082144
RBM38	0.312141412	0.232765771	0.676521228	0.239770676	0.846106949	0.51173553	0.926082144
SLC6A4	0.487246645	0.977065722	0.331006337	0.095559857	0.664581498	0.512408336	0.926082144
SMC3	0.495109848	0.620328172	0.34427111	0.176970421	0.535610776	0.512684969	0.926082144
TAOK1	0.132459797	0.824979195	0.843839672	0.219169639	0.496118241	0.51276222	0.926082144
TSPEAR	0.115571481	0.700785577	0.871666989	0.721222485	0.196102192	0.511979207	0.926082144
TXNDC15	0.559529186	0.226228687	0.71918259	0.359749109	0.307485675	0.513574651	0.926082144
UBQLN4	0.423003444	0.763146874	0.773062038	0.312659079	0.128712978	0.513067499	0.926082144
USP20	0.763800579	0.451913424	0.749654248	0.102520816	0.378029237	0.512797214	0.926082144
YPEL4	0.701188131	0.294001258	0.935208254	0.070426812	0.73608005	0.512159127	0.926082144
ZNF793	0.650543492	0.29955144	0.56163317	0.236487667	0.386337022	0.512254126	0.926082144
LOC513580	0.74756703	0.859626867	0.430493376	0.491899753	0.07404047	0.513677517	0.926165869
CTF1	0.78987854	0.622383177	0.259908498	0.41011696	0.192767945	0.514156532	0.926598759
EVI2B	0.505979722	0.82964254	0.785724878	0.164111442	0.186571613	0.514112406	0.926598759
GRID1	0.209557207	0.231329595	0.300845505	0.7247687	0.954820698	0.513991228	0.926598759
LOC112448791	0.927324939	0.418939943	0.689872653	0.045250952	0.832943693	0.514162933	0.926598759
RPTOR	0.944700331	0.939694066	0.486651021	0.111155997	0.210402315	0.514199921	0.926598759

COL4A3BP	0.558850796	0.311339127	0.085987041	0.85747779	0.788047182	0.514310573	0.926696401
C10H15orf65	0.576398535	0.739583009	0.985442584	0.482003359	0.049985558	0.514525807	0.926706214
HSPA12B	0.681901562	0.161716691	0.798004525	0.183206118	0.627474389	0.51443165	0.926706214
LOC100141185	0.613610908	0.279867293	0.250610676	0.805861422	0.291855447	0.514541894	0.926706214
RAB26	0.309706489	0.426356756	0.639232384	0.145199947	0.825873776	0.514537314	0.926706214
FAF1	0.880698756	0.257146377	0.727828468	0.463899694	0.132611792	0.514874764	0.927203967
ARMT1	0.85370002	0.171000242	0.741541048	0.472457339	0.198955987	0.515529292	0.927279837
C23H6orf141	0.685680692	0.91215285	0.161339914	0.519864357	0.193979822	0.515538433	0.927279837
DOCK2	0.452342849	0.894654244	0.992871442	0.150824355	0.167856909	0.515473006	0.927279837
GABARAPL2	0.550930211	0.350560526	0.235992671	0.770161999	0.289641478	0.515375395	0.927279837
LOC101902551	0.342726171	0.40235403	0.918496006	0.111578743	0.718693826	0.515183975	0.927279837
LPAR1	0.073871251	0.772441968	0.301930421	0.687544411	0.856579433	0.51499301	0.927279837
MARS	0.347395018	0.815135218	0.642912589	0.924671466	0.060377293	0.515317151	0.927279837
MS4A7	0.108751892	0.219688365	0.578169603	0.856822108	0.859362233	0.515446793	0.927279837
SLC20A1	0.794532435	0.224382356	0.52060259	0.306289265	0.357394663	0.515239418	0.927279837
TBCC	0.862525196	0.513464601	0.373201177	0.247517902	0.248326119	0.515225504	0.927279837
ZNF287	0.368575427	0.333494512	0.376790453	0.858464603	0.255480245	0.515199714	0.927279837
ACOX3	0.586254343	0.114724075	0.681062318	0.850939884	0.261617871	0.515934564	0.927408499
ACSM1	0.397652937	0.478769573	0.325842469	0.223780782	0.735942334	0.51628554	0.927408499
AGBL5	0.290387861	0.606245946	0.619113892	0.325788999	0.286916798	0.515760006	0.927408499
AKT2	0.488914377	0.572196049	0.424064475	0.22407521	0.384338724	0.516290955	0.927408499
C18H16orf74	0.447976963	0.338496174	0.269709836	0.510509474	0.490252873	0.516642645	0.927408499
CENPT	0.729721694	0.06223713	0.970230703	0.31364244	0.740836566	0.516689768	0.927408499
IDH1	0.912204969	0.538267392	0.160990759	0.316461882	0.408662412	0.516403481	0.927408499
LAS1L	0.402655242	0.360837144	0.517271065	0.187838483	0.723247005	0.516168282	0.927408499
LOC100337495	0.324763147	0.380167892	0.229522841	0.64207573	0.561222491	0.516192257	0.927408499
LOC100847835	0.523649732	0.811757783	0.49016889	0.073662674	0.666388579	0.516494602	0.927408499
LOC104975666	0.254232358	0.858040008	0.430297088	0.320754587	0.338240354	0.515679909	0.927408499
LOC107131458	0.143907126	0.687872573	0.975522441	0.850848203	0.124588578	0.516654629	0.927408499
LOC112449172	0.586538684	0.230266831	0.541659723	0.411202149	0.339450635	0.516191037	0.927408499
MAGOH	0.986045762	0.156133765	0.322541565	0.241761562	0.852924598	0.516706398	0.927408499
MRPS10	0.566765512	0.46481904	0.206069644	0.524480571	0.359687058	0.516740194	0.927408499
NFKBIL1	0.185517447	0.366992875	0.48887435	0.841392641	0.364587399	0.516170882	0.927408499
RAB35	0.653763444	0.14883356	0.165727945	0.672341307	0.943565019	0.51653388	0.927408499
RAB5A	0.521546095	0.289516517	0.141432489	0.681402693	0.703532208	0.516674186	0.927408499
SLC2A8	0.613168071	0.094566803	0.371300356	0.853929448	0.554244697	0.515793268	0.927408499
SYT15	0.86445868	0.582667615	0.679329507	0.217362301	0.137397526	0.516330429	0.927408499
FBXL8	0.934827142	0.19386324	0.271124871	0.214957758	0.970717711	0.51695085	0.927573797
MRPL42	0.556057853	0.37655312	0.608843306	0.30114646	0.267134831	0.51700186	0.927573797
WDR33	0.244517438	0.988646235	0.830917756	0.177955191	0.286879349	0.516983979	0.927573797
B4GALT5	0.918944142	0.644081737	0.690253609	0.036957701	0.679571487	0.517096601	0.927581474
PKIG	0.142073795	0.427878048	0.65377599	0.675496089	0.382248023	0.517119183	0.927581474
BRD1	0.720944009	0.352376658	0.618026898	0.140782928	0.464411729	0.517179007	0.927587398
LOC100336589	0.081594141	0.853297064	0.811632678	0.331605321	0.548169812	0.517302899	0.927607971

LOC101908149	0.630070959	0.460614169	0.313687422	0.242841912	0.464792234	0.517368289	0.927607971
RBM24	0.255700392	0.24754939	0.898009036	0.949740446	0.19034705	0.517376377	0.927607971
SYN2	0.13063129	0.388853226	0.898419877	0.37003554	0.60864233	0.517416572	0.927607971
ARHGAP1	0.38079053	0.464972269	0.240524386	0.939986031	0.257426436	0.51790554	0.927652411
ATP2C2	0.642053676	0.264766186	0.173164502	0.402736788	0.871565142	0.518411155	0.927652411
CDK17	0.183186674	0.385938478	0.811803271	0.265372338	0.675854614	0.517699726	0.927652411
CPXM2	0.505704135	0.3412248	0.200238853	0.737095785	0.405598068	0.518364083	0.927652411
FBXL18	0.595226904	0.202358583	0.416280828	0.898069107	0.229274856	0.518256424	0.927652411
HGFAC	0.986516822	0.018745732	0.962680787	0.710339543	0.814498518	0.51781832	0.927652411
LOC100848212	0.428187884	0.659940786	0.199607003	0.358602033	0.51013252	0.518149882	0.927652411
LOC101904614	0.871639885	0.512265984	0.480106393	0.128216144	0.375489539	0.518193113	0.927652411
LOC511713	0.402177559	0.69388809	0.492241835	0.357514215	0.210446051	0.518457089	0.927652411
LOC614617	0.262815246	0.77935306	0.499862461	0.222407255	0.452221245	0.517770562	0.927652411
MSANTD1	0.549240353	0.42280987	0.081796039	0.869854183	0.623595431	0.517880572	0.927652411
NTAN1	0.861580503	0.915416491	0.31394233	0.093509039	0.444248921	0.517557999	0.927652411
POLD2	0.752071657	0.450698553	0.405396989	0.136474598	0.550129628	0.518120373	0.927652411
RNF44	0.84880067	0.135855251	0.491400514	0.572606361	0.31732094	0.517743454	0.927652411
RTKN2	0.878733163	0.746496135	0.066197275	0.372219844	0.638324899	0.518130442	0.927652411
SUPT20H	0.668886143	0.125436155	0.754199651	0.587812206	0.276863063	0.517783402	0.927652411
TAP1	0.564688201	0.454406275	0.791976701	0.137865981	0.367994775	0.517999697	0.927652411
TRAPPC6A	0.166556442	0.719421125	0.732458378	0.164384486	0.716582091	0.518515359	0.927652411
UBE2Q1	0.871350691	0.308747174	0.365686693	0.346756813	0.302998851	0.518479289	0.927652411
TRIM8	0.754192718	0.354698849	0.952580118	0.10310674	0.393683008	0.518612002	0.927724174
CSRNP2	0.275855433	0.536825258	0.861759724	0.21892963	0.37048412	0.518740922	0.927853654
DOHH	0.909965702	0.233594971	0.386234819	0.420466787	0.299987277	0.518827456	0.9279073
LOC101906367	0.485395262	0.556099492	0.985178812	0.751074489	0.051909349	0.519051658	0.928207123
KLHDC9	0.965195058	0.689998696	0.474992145	0.084091143	0.39006349	0.519200643	0.928268418
LOC100337507	0.447226209	0.631214637	0.13984033	0.841796301	0.312290495	0.519229771	0.928268418
PITPNC1	0.36083193	0.880681162	0.469647189	0.222004347	0.313542672	0.519425317	0.928268418
POLR2D	0.836236802	0.459433194	0.302998501	0.228218635	0.390927365	0.519375789	0.928268418
QTRT2	0.571231587	0.716778249	0.785939448	0.233841092	0.138022736	0.519383715	0.928268418
SDHD	0.953128129	0.386192315	0.447579562	0.065836228	0.956978007	0.519268587	0.928268418
DRAP1	0.448293112	0.216934294	0.62604127	0.301127116	0.567404165	0.519677745	0.928474371
IQGAP2	0.824548823	0.679012015	0.933476495	0.023001657	0.865474581	0.51971029	0.928474371
PIGH	0.362973652	0.312114505	0.71741186	0.263477823	0.485575836	0.519601033	0.928474371
ASCC3	0.711774555	0.953207554	0.61581999	0.066154442	0.377377849	0.520191099	0.928512485
DCTN1	0.647656532	0.231905124	0.598894219	0.72204664	0.160442383	0.520004754	0.928512485
GGTA1	0.670668418	0.313595074	0.110982183	0.501930608	0.891174924	0.52037239	0.928512485
HAUS4	0.388872094	0.424175584	0.813993186	0.251956928	0.307897258	0.519924841	0.928512485
ITPKB	0.215462152	0.834932932	0.173717397	0.429048797	0.778271853	0.520271265	0.928512485
LOC104971814	0.670469737	0.381374107	0.190161323	0.426424982	0.503438554	0.52033104	0.928512485
LOC112446001	0.210354359	0.326740116	0.823659884	0.39281658	0.46925066	0.52026809	0.928512485
N4BP2L2	0.16073233	0.287864301	0.519018866	0.573899852	0.757957871	0.520467147	0.928512485
PPIP5K1	0.422996779	0.794947485	0.400507817	0.116956147	0.661199004	0.519897255	0.928512485

PRRC2A	0.928351664	0.453680315	0.241051117	0.168104077	0.610893097	0.520102574	0.928512485
SSH2	0.595617378	0.631053606	0.717895541	0.151735151	0.254824072	0.52023552	0.928512485
UAP1	0.583533981	0.490069045	0.552094858	0.183037751	0.361439947	0.520449171	0.928512485
ZDHHC3	0.336076445	0.736046565	0.318536268	0.2711546	0.487676427	0.51998802	0.928512485
LOC104973382	0.114151288	0.392791665	0.276232109	0.936899907	0.901094915	0.520651829	0.928740997
ABLIM3	0.284647093	0.58196747	0.398459301	0.290234309	0.547124073	0.521104532	0.928789262
DAZAP1	0.627782152	0.801452368	0.071042873	0.79593097	0.369135432	0.521471093	0.928789262
GORAB	0.621919454	0.711976321	0.192806038	0.19143857	0.642310017	0.521395598	0.928789262
GSTK1	0.715380182	0.652594494	0.119932897	0.193617514	0.966395919	0.521015267	0.928789262
KAT6A	0.769522681	0.427090432	0.461522706	0.900430733	0.076629122	0.520824349	0.928789262
KIF21A	0.637348922	0.611134775	0.205746145	0.633358046	0.206233601	0.520857413	0.928789262
LOC101904840	0.972063991	0.100412384	0.153757155	0.876827399	0.7991547	0.52172907	0.928789262
LOC107132360	0.256824477	0.877585253	0.407100189	0.168014985	0.680014303	0.521134526	0.928789262
LOC788405	0.924929043	0.366996162	0.356989734	0.10126323	0.856408792	0.52159711	0.928789262
MARCH2	0.69021876	0.717849486	0.288095758	0.091223731	0.806777717	0.521536399	0.928789262
MBOAT2	0.347376405	0.889995998	0.166343487	0.25245087	0.808307669	0.521331899	0.928789262
MPV17L	0.872886176	0.64500988	0.388163499	0.117195	0.410361288	0.521621248	0.928789262
PLCG2	0.241083179	0.076927404	0.954819896	0.913609992	0.650151838	0.52176496	0.928789262
RBL1	0.556396686	0.336642408	0.180550416	0.421178344	0.736682251	0.521310343	0.928789262
SHISA7	0.730692417	0.64138427	0.501745439	0.136195871	0.3273929	0.521167707	0.928789262
SIRT7	0.301193029	0.102502364	0.769963051	0.892073961	0.495166838	0.521441897	0.928789262
SRPK2	0.787519984	0.379720824	0.242930233	0.273644675	0.528830224	0.521663234	0.928789262
U2AF1	0.839183036	0.416991001	0.089703462	0.701484671	0.47779309	0.521810797	0.928789262
UST	0.35411549	0.504028219	0.8324133	0.072576295	0.973357799	0.521357233	0.928789262
ZMYM3	0.587082546	0.395714468	0.087477497	0.983167465	0.52483413	0.521192659	0.928789262
MSR1	0.402534132	0.385537818	0.121047465	0.930612026	0.602253747	0.521951051	0.928837421
TAZ	0.875196861	0.243852036	0.535030988	0.120740492	0.763597216	0.521931413	0.928837421
C25H7orf43	0.688397291	0.209207534	0.07781185	0.958410245	0.980641733	0.522016404	0.928852999
ABR	0.913682408	0.866684461	0.19737805	0.131203745	0.51394851	0.522145382	0.92888107
GNPTAB	0.763562671	0.254976335	0.41088682	0.288314782	0.456879556	0.522108217	0.92888107
RAB3IL1	0.389230126	0.469979996	0.13157474	0.644567171	0.679822124	0.522276071	0.929012855
GOLT1B	0.818957697	0.033503385	0.653522704	0.636080444	0.925091476	0.522356762	0.929055687
LOC787550	0.776941689	0.506177682	0.085761467	0.560811663	0.558134572	0.522457565	0.929134277
PIGM	0.5127259	0.849362401	0.309085451	0.335055706	0.234233262	0.522580463	0.929252137
AKAP10	0.508459346	0.122839702	0.941886502	0.398244247	0.451513027	0.522837242	0.929305965
KCTD20	0.126581108	0.939606299	0.332732622	0.338751222	0.789078501	0.522836337	0.929305965
MAP3K7	0.814307959	0.932720143	0.210927848	0.944868136	0.069868511	0.522799561	0.929305965
TUBB1	0.430319783	0.189806666	0.661475321	0.371746735	0.526362965	0.522722322	0.929305965
SUCNR1	0.584205095	0.842924467	0.336862911	0.146948275	0.434095442	0.522899629	0.929316203
CORO7	0.039087483	0.494684066	0.724187335	0.868395197	0.870855923	0.523040167	0.929465314
ALDOA	0.517629783	0.782757958	0.402023161	0.13907245	0.467620906	0.523106479	0.929482506
STOML2	0.589060039	0.785114558	0.55518863	0.10834536	0.380940542	0.523179424	0.929511479
ABI3BP	0.804410017	0.223215086	0.304485254	0.211420779	0.921675731	0.52417619	0.929579651
ATP2A1	0.68242152	0.913092213	0.131303844	0.68337272	0.191599622	0.525222134	0.929579651

BRINP1	0.129362914	0.701503274	0.984765847	0.764237807	0.156175493	0.524402715	0.929579651
CCDC134	0.223293126	0.681302025	0.698891656	0.733313513	0.136161959	0.523514026	0.929579651
CCNG1	0.602093769	0.424117663	0.13401943	0.683251296	0.457991715	0.525160883	0.929579651
CHST3	0.649932201	0.122901216	0.499886228	0.473959721	0.562435335	0.524009328	0.929579651
CSMD2	0.042104171	0.753677852	0.983318125	0.420304729	0.814273257	0.524631323	0.929579651
ELOB	0.953089439	0.582564901	0.912669605	0.025531107	0.820790045	0.523567466	0.929579651
IFFO2	0.518415174	0.913032344	0.432285155	0.611475844	0.085215044	0.52432263	0.929579651
KITLG	0.989788828	0.174362492	0.247495356	0.315064433	0.791168482	0.524062208	0.929579651
LOC100140533	0.887770788	0.361784134	0.307803031	0.168304621	0.640429704	0.524218672	0.929579651
LOC100297420	0.965884819	0.675127438	0.182114465	0.13083818	0.689100939	0.525124557	0.929579651
LOC101906077	0.653558119	0.304668646	0.371634144	0.240630492	0.601860049	0.525299033	0.929579651
LOC101907615	0.853562555	0.401715848	0.08055695	0.751665185	0.515529304	0.52506461	0.929579651
LOC112446423	0.690991417	0.373245584	0.519454284	0.112423864	0.706244701	0.523887098	0.929579651
LOC780968	0.964743986	0.368540856	0.330296136	0.101565349	0.897096515	0.524999691	0.929579651
LOC783255	0.798074622	0.332096415	0.372233688	0.223445462	0.485415356	0.525009466	0.929579651
MAD1L1	0.561738588	0.075772036	0.795872044	0.912206494	0.343335987	0.523396028	0.929579651
MAN1A2	0.64655056	0.452748919	0.833133773	0.050951585	0.859072298	0.524554191	0.929579651
METTL26	0.159459176	0.503788337	0.789623533	0.230120387	0.730634221	0.524384854	0.929579651
MFSD2A	0.691263721	0.210243633	0.183573016	0.780096308	0.512706526	0.524480434	0.929579651
MRPL47	0.24656196	0.775262897	0.226784895	0.532802902	0.463696454	0.525175337	0.929579651
MXD4	0.731839457	0.349002417	0.230893709	0.735788036	0.244848399	0.523660102	0.929579651
NHSL1	0.260350653	0.984257046	0.139245273	0.917117444	0.326584964	0.52477466	0.929579651
NUBPL	0.386300798	0.391901835	0.252148007	0.987867989	0.282133026	0.523923152	0.929579651
RPN2	0.369284876	0.562203072	0.105209318	0.80136046	0.608200609	0.524041626	0.929579651
SERINC4	0.374802885	0.352321957	0.743544922	0.439420297	0.24633006	0.523721423	0.929579651
SP110	0.765381482	0.098853707	0.450750145	0.471543967	0.662257027	0.524116366	0.929579651
SPATA24	0.708457408	0.818029698	0.062003862	0.399064725	0.741309037	0.523763521	0.929579651
TEX2	0.737055849	0.221686512	0.128439582	0.635020346	0.803207446	0.525072785	0.929579651
TIMMDC1	0.797265471	0.205898206	0.320954003	0.31885368	0.634736601	0.524345912	0.929579651
TOX	0.200726291	0.606004677	0.282037766	0.76271472	0.409597809	0.525313612	0.929579651
UBE2B	0.621163541	0.265085814	0.183029804	0.903283189	0.39335322	0.525145456	0.929579651
UGP2	0.737405503	0.193912538	0.889935128	0.243263758	0.346209778	0.52530495	0.929579651
WDFY3	0.683076448	0.853047087	0.597757385	0.274188016	0.111702856	0.524431487	0.929579651
WDR11	0.851440775	0.395297401	0.125247238	0.617614368	0.410813945	0.524922746	0.929579651
ZNF280D	0.243482689	0.538122426	0.432733861	0.400583151	0.467088042	0.523379741	0.929579651
GCN1	0.719323979	0.273257269	0.563349167	0.697769392	0.138907524	0.52557668	0.929788777
GHDC	0.937093228	0.364336562	0.183232274	0.431042158	0.398270673	0.525695964	0.929788777
MB21D2	0.439597446	0.895362874	0.09322129	0.815256181	0.359059186	0.525715073	0.929788777
RPL37A	0.62454251	0.382504824	0.091823128	0.542395509	0.90258368	0.525682452	0.929788777
SERPINH1	0.618611526	0.491614348	0.174354239	0.329662705	0.613742427	0.525498074	0.929788777
B3GNT5	0.608779557	0.475032159	0.142349141	0.550197352	0.474594292	0.525867212	0.929789182
LOC100300938	0.757047277	0.698783072	0.339194576	0.093624787	0.639904682	0.525885272	0.929789182
OAT	0.206189743	0.418903794	0.413631234	0.651531564	0.461621282	0.525796255	0.929789182
ARHGAP29	0.103439995	0.953304098	0.95190518	0.348905388	0.328759047	0.526180778	0.929910915

KIAA2012	0.90813402	0.896484596	0.040856721	0.57939814	0.558632788	0.526163697	0.929910915
KPNA3	0.551104781	0.052881074	0.64559026	0.677940843	0.843446206	0.526024695	0.929910915
MYL6B	0.972208293	0.776467835	0.04242291	0.490646451	0.68519783	0.526167216	0.929910915
ADGRG2	0.617110005	0.541240151	0.61547419	0.073533513	0.71412527	0.526669964	0.929914182
CABP7	0.441978511	0.295473163	0.321232976	0.646303738	0.397266554	0.526250005	0.929914182
FRMD5	0.121018629	0.679601975	0.597911499	0.450497074	0.486475366	0.526353924	0.929914182
LOC100141168	0.446254634	0.601740134	0.398099424	0.123364397	0.818452367	0.526645726	0.929914182
LOC112442611	0.15903809	0.720827294	0.337365725	0.79608426	0.350655535	0.526692142	0.929914182
LOC781499	0.71294124	0.467205017	0.803361042	0.0981942	0.410448924	0.526493461	0.929914182
OCRL	0.955782603	0.796294837	0.308441434	0.151769393	0.302776131	0.526534669	0.929914182
TAAR1	0.712991553	0.468335847	0.801823523	0.058019457	0.695200312	0.526749268	0.929914182
TRAPPC2	0.629976317	0.435252796	0.659383323	0.320572186	0.185917762	0.526333836	0.929914182
ZKSCAN1	0.195984992	0.656507335	0.769954939	0.118809436	0.917404196	0.526721024	0.929914182
CD300LG	0.085612509	0.286443133	0.590543523	0.815400361	0.915764128	0.527001036	0.930158529
KLHDC7A	0.456721493	0.212108938	0.44065443	0.360060531	0.70337373	0.5269523	0.930158529
ANKRD37	0.845632086	0.632858819	0.072118687	0.557407783	0.503601127	0.527355634	0.930206641
ARHGAP31	0.196322067	0.957621653	0.348933023	0.50786519	0.325215691	0.527368386	0.930206641
CHST9	0.787636351	0.78601571	0.748190945	0.232996157	0.100346084	0.527278405	0.930206641
JAG2	0.573719109	0.826530318	0.118895317	0.2302509	0.833554348	0.527121313	0.930206641
LOC101902440	0.399150344	0.795122204	0.552470782	0.171519834	0.359960736	0.527204573	0.930206641
TRAPPC13	0.804994314	0.592099421	0.508069057	0.178501117	0.250423574	0.52719462	0.930206641
ZNF282	0.536070317	0.222043555	0.260600258	0.464618988	0.752021548	0.527428062	0.930211922
CRTAP	0.501817831	0.381946776	0.187108791	0.548700478	0.551349442	0.527621316	0.930277011
PMEPA1	0.173920911	0.171098157	0.631515772	0.59497416	0.970154104	0.527584162	0.930277011
SUSD1	0.194655297	0.715581307	0.423436484	0.27952443	0.658117749	0.527635027	0.930277011
BCL2L14	0.402418392	0.329313604	0.20264313	0.451141662	0.897864306	0.528117251	0.930313971
COG4	0.720923812	0.76109637	0.804890655	0.069878743	0.352386539	0.528068105	0.930313971
EHMT1	0.787420324	0.068088799	0.378973022	0.576609563	0.927885348	0.527996798	0.930313971
LOC101906656	0.064248516	0.321695704	0.848675413	0.991059951	0.625119594	0.527929778	0.930313971
LOC107133289	0.228694041	0.124524461	0.789123002	0.931900263	0.518851502	0.527909599	0.930313971
MAGI2	0.748539876	0.594917855	0.22951926	0.240943031	0.441824508	0.528166185	0.930313971
NPR3	0.328975108	0.156500571	0.685893186	0.689929683	0.446531963	0.528138971	0.930313971
RECQL5	0.347938771	0.104096232	0.880138863	0.860599792	0.395786098	0.527771874	0.930313971
RHOQ	0.777929105	0.403559385	0.098800224	0.733237276	0.478174816	0.528070867	0.930313971
LOC112441718	0.093273073	0.808691907	0.410131369	0.973765909	0.361430236	0.528290964	0.930387182
PINK1	0.299833121	0.555398696	0.243645417	0.46813779	0.573316177	0.528321135	0.930387182
ADK	0.629257848	0.30450966	0.39275222	0.300021663	0.485709429	0.529659042	0.930804814
AKAIN1	0.298507183	0.786660202	0.331485012	0.185064828	0.758985843	0.529085653	0.930804814
ASB12	0.879324642	0.968150704	0.261849154	0.113872061	0.433309722	0.530218397	0.930804814
B3GNT3	0.475571991	0.590415546	0.136330593	0.677436106	0.423431292	0.529895227	0.930804814
BMF	0.989737634	0.509500704	0.041929835	0.857426134	0.602507109	0.528903954	0.930804814
CEP295NL	0.39474739	0.75376967	0.16544312	0.941735326	0.237239734	0.530202649	0.930804814
CSF2RA	0.33526587	0.267325677	0.411225311	0.491865287	0.605071275	0.529696162	0.930804814
DMKN	0.334509464	0.75506031	0.746535715	0.810475189	0.071867675	0.529936822	0.930804814

GANAB	0.827023282	0.33618296	0.167249254	0.379159181	0.622398421	0.529776952	0.930804814
HSPB1	0.655431075	0.58474709	0.116139893	0.895030787	0.274841534	0.529362019	0.930804814
JMJD1C	0.237019564	0.819443018	0.257381356	0.276997394	0.794939239	0.530362756	0.930804814
KIF27	0.348189863	0.321173449	0.366353996	0.758486458	0.352654661	0.529517929	0.930804814
LDHA	0.804888985	0.242842924	0.145345295	0.639339688	0.603167694	0.529464692	0.930804814
LOC100848324	0.85221318	0.23551561	0.470465751	0.727194229	0.160349877	0.530417486	0.930804814
LOC101904849	0.698724997	0.855732659	0.40729168	0.55719365	0.080670219	0.529306373	0.930804814
LOC101907943	0.776334563	0.708593364	0.497459748	0.056508048	0.710235531	0.529937996	0.930804814
LOC104975044	0.628176341	0.196731541	0.481575334	0.250843611	0.732619633	0.529145848	0.930804814
LOC107132515	0.747950864	0.430343606	0.397297525	0.173101527	0.49656014	0.530095306	0.930804814
LOC112441494	0.294644576	0.665232564	0.350066398	0.299731116	0.535013825	0.530288341	0.930804814
LOC512165	0.522999588	0.194882945	0.350690121	0.371603429	0.828206512	0.530243747	0.930804814
LOC784054	0.218296447	0.511996782	0.635775493	0.375188921	0.411981627	0.529950604	0.930804814
LRMP	0.592110659	0.816491322	0.337452726	0.564441171	0.119615506	0.530486712	0.930804814
MIA2	0.328971951	0.349053781	0.619825878	0.553644069	0.277431175	0.529060965	0.930804814
MYH14	0.561170625	0.675852611	0.160467659	0.256741868	0.704793997	0.530451684	0.930804814
NDUFS2	0.912850296	0.636931066	0.613600923	0.052418343	0.5840605	0.528891829	0.930804814
PAK1	0.948844184	0.366047755	0.187650322	0.334896825	0.503867699	0.530196792	0.930804814
PCMTD2	0.316150189	0.308852816	0.566214899	0.639876513	0.310990734	0.530266823	0.930804814
SNX7	0.327490612	0.365799957	0.106880414	0.975626903	0.874373989	0.528892925	0.930804814
SUV39H1	0.354142074	0.38318797	0.454741065	0.379881722	0.468672788	0.530005676	0.930804814
TLCD1	0.278917937	0.179931435	0.507608641	0.478718646	0.899676238	0.529747015	0.930804814
TMEM273	0.667862735	0.348111438	0.57150163	0.674722299	0.122070458	0.52925807	0.930804814
TNFRSF12A	0.228078358	0.98477906	0.924017122	0.232908027	0.226040017	0.52895882	0.930804814
ZNF395	0.238433875	0.061143023	0.894929754	0.92233865	0.911694999	0.52973161	0.930804814
ZNF462	0.402064937	0.577994572	0.586049874	0.104940701	0.767494308	0.529701004	0.930804814
CHL1	0.82117342	0.727650222	0.031502998	0.944818759	0.619674244	0.530594926	0.930895161
CEACAM1	0.349757164	0.608460225	0.625713575	0.361218459	0.229281346	0.530722286	0.931019073
FKBPL	0.091731796	0.868309076	0.294381669	0.691217117	0.68180284	0.531100173	0.931131647
NEK6	0.879761489	0.402975009	0.840713802	0.393802763	0.094160651	0.531126887	0.931131647
PI4KB	0.881406164	0.09426425	0.917785809	0.226002022	0.641271654	0.53111787	0.931131647
TRIM33	0.271783116	0.38816086	0.769672716	0.178763661	0.761163548	0.531064897	0.931131647
UNKL	0.915240782	0.409411739	0.254152439	0.273612054	0.423604967	0.530885249	0.931131647
VCPKMT	0.795599581	0.02275712	0.838251223	0.916363288	0.794237709	0.531024146	0.931131647
BNC2	0.392515584	0.148302922	0.360285979	0.687893651	0.767924937	0.531587593	0.931236539
CISD1	0.821651075	0.811579783	0.65137727	0.036784739	0.693994997	0.531754166	0.931236539
DNAJC13	0.288820855	0.196385902	0.711458615	0.853686317	0.3211292	0.531313483	0.931236539
FGD5	0.67812281	0.472065606	0.050124316	0.961496264	0.718547856	0.531704876	0.931236539
GTPBP4	0.593136081	0.741697791	0.283049554	0.294511795	0.302137773	0.531612706	0.931236539
LOC100295750	0.825799627	0.225364382	0.961868823	0.063221867	0.979621657	0.531721932	0.931236539
LOC617692	0.543423882	0.537554948	0.646221916	0.082738619	0.709128094	0.531536118	0.931236539
MBOAT1	0.541891131	0.601491202	0.39287154	0.113240588	0.764164762	0.531625593	0.931236539
PITX3	0.504717853	0.535380605	0.155681483	0.354278081	0.743146175	0.531531996	0.931236539
ZNF227	0.609908186	0.802006679	0.91055624	0.865160521	0.028740352	0.531519414	0.931236539

FBXO2	0.691886136	0.512086386	0.171230231	0.237004765	0.771464944	0.531822465	0.931256772
CTDSPL	0.584696755	0.540536731	0.322363057	0.746517656	0.1459439	0.531951648	0.931342001
LBH	0.288469622	0.798713458	0.513568132	0.608597545	0.154214435	0.532046563	0.931342001
RASL11A	0.574804755	0.10000703	0.739221393	0.586269424	0.445812486	0.532059895	0.931342001
VDR	0.948367209	0.198730871	0.674818461	0.168910974	0.517101395	0.532098141	0.931342001
BABAM2	0.837684683	0.452106498	0.091128191	0.355755724	0.907178708	0.532603627	0.931809502
DYSF	0.516592425	0.965677069	0.55727398	0.059480693	0.676268388	0.533357922	0.931809502
FGFR2	0.126815343	0.967699444	0.445558091	0.333052777	0.614322126	0.533437534	0.931809502
LOC100336564	0.391932517	0.06258949	0.859112397	0.849797089	0.624687479	0.533444048	0.931809502
LOC101906358	0.911011236	0.14765291	0.416060612	0.290522344	0.687550486	0.533297855	0.931809502
LOC101906850	0.829545319	0.114859896	0.797171898	0.257755688	0.570640971	0.533177694	0.931809502
LOC505199	0.247766898	0.328520266	0.279337033	0.536057725	0.917834844	0.533430633	0.931809502
LOC512323	0.36194822	0.096327529	0.391685974	0.851299932	0.957611166	0.532510889	0.931809502
LOC786173	0.311183475	0.594670034	0.954360813	0.083820217	0.7554042	0.533353344	0.931809502
LOC786252	0.469815024	0.782786794	0.223392096	0.167005889	0.815094935	0.533372947	0.931809502
NDFIP2	0.654520596	0.605043678	0.805261183	0.083205363	0.420576594	0.532964078	0.931809502
NLGN1	0.718156135	0.587340572	0.429178692	0.33462673	0.184262992	0.533008918	0.931809502
PARPBP	0.761869365	0.719115695	0.170789557	0.344920579	0.346134679	0.533165923	0.931809502
RNASEH2B	0.690218126	0.42008269	0.826276598	0.078030256	0.598165612	0.533352345	0.931809502
RYR1	0.10866037	0.723178692	0.362078644	0.870057088	0.451083247	0.533086923	0.931809502
SCRN3	0.380259178	0.391202114	0.207236307	0.432591716	0.836349779	0.532863359	0.931809502
SHLD2	0.681402483	0.894019356	0.112166076	0.314985393	0.518439266	0.532945377	0.931809502
SLC11A1	0.819866644	0.454176483	0.191636097	0.521589563	0.299755444	0.532919441	0.931809502
TIMP4	0.675211037	0.444664669	0.838645451	0.191781219	0.231447643	0.533255392	0.931809502
RNF135	0.941038291	0.665040768	0.306609131	0.058690408	0.994316544	0.533615385	0.931987312
SIK2	0.331337243	0.919939321	0.046770177	0.911330803	0.862101927	0.533659422	0.931987312
NRXN2	0.503910451	0.280338867	0.261548164	0.598049908	0.507297587	0.533814925	0.932159687
MAP3K9	0.22876432	0.390880145	0.894994213	0.989388251	0.141705177	0.533996934	0.932279115
TMCC2	0.205595084	0.687204925	0.146650616	0.623070537	0.868977341	0.533963663	0.932279115
CYYR1	0.158719028	0.563629071	0.325268435	0.766950517	0.503313228	0.534200569	0.932337077
LOC101903678	0.420460211	0.215082282	0.6565405	0.430059433	0.439792982	0.534155869	0.932337077
XCL2	0.454084141	0.803321074	0.886425157	0.634943312	0.054705217	0.534183623	0.932337077
ADIPOR2	0.867793329	0.771029917	0.043014502	0.920580118	0.424371725	0.534393323	0.93240381
LOC788414	0.607397881	0.8249633	0.565807077	0.099599194	0.398155145	0.53438193	0.93240381
SLC16A12	0.388715763	0.930097633	0.405683203	0.778867219	0.098461295	0.534466068	0.93240381
ZNF358	0.812091089	0.925242627	0.073731824	0.563435758	0.360290401	0.534437957	0.93240381
BICD2	0.69150761	0.563667794	0.075245433	0.488951543	0.785468677	0.534736701	0.93246758
LOC101908154	0.216130363	0.379184403	0.914217002	0.352847477	0.42624664	0.534810459	0.93246758
MEA1	0.605721605	0.125792448	0.306340716	0.645666995	0.746893301	0.534607354	0.93246758
MTSS1L	0.738062076	0.745985785	0.164244272	0.18797608	0.663014207	0.53484354	0.93246758
TGIF2	0.456125631	0.88889768	0.945822001	0.062814963	0.467744489	0.53479029	0.93246758
TOM1	0.766652535	0.158643285	0.894671974	0.118089807	0.876723863	0.534764855	0.93246758
LOC112447032	0.405404643	0.238329609	0.993585155	0.831724207	0.141503936	0.535316614	0.933094101
ROR1	0.976784351	0.521887083	0.149598008	0.682610767	0.216978232	0.53525999	0.933094101

BROX	0.449911507	0.414574743	0.096517819	0.862536055	0.732104351	0.53648521	0.933268401
C14H8orf59	0.846937067	0.145957795	0.511601533	0.183151229	0.981326872	0.536461601	0.933268401
CLK3	0.621223655	0.769302899	0.424995331	0.307588117	0.182064051	0.536588431	0.933268401
CLNS1A	0.637665021	0.119263977	0.339233987	0.485453459	0.907412213	0.536426399	0.933268401
CMYA5	0.959786819	0.365224395	0.361088723	0.147408805	0.609733376	0.53662726	0.933268401
COX4I1	0.709659359	0.761847604	0.312080084	0.080120698	0.841329524	0.536576022	0.933268401
GRIN1	0.801406507	0.793428972	0.421998483	0.403344809	0.104949983	0.536328783	0.933268401
GTPBP2	0.309539742	0.526109049	0.179507187	0.711682004	0.546553691	0.536531285	0.933268401
LOC107133190	0.506067726	0.710920452	0.055359482	0.692795617	0.824371104	0.536599951	0.933268401
PALM	0.841555679	0.226569391	0.139073552	0.98531271	0.435260919	0.53655738	0.933268401
PLA2G12A	0.365813739	0.342463073	0.364146109	0.467113273	0.533989313	0.536667717	0.933268401
PODNL1	0.376490844	0.49003247	0.081642806	0.895978388	0.841507575	0.53629466	0.933268401
RMC1	0.51845448	0.964220117	0.126046321	0.720586705	0.249865133	0.536100213	0.933268401
RPN1	0.836336907	0.616048514	0.262605154	0.581402519	0.14391245	0.535691033	0.933268401
SLC27A3	0.063918935	0.462885467	0.991776952	0.39678158	0.976507667	0.536509992	0.933268401
SLC9A8	0.148916371	0.536942742	0.745849091	0.743166694	0.255890921	0.536036085	0.933268401
SPRY2	0.553844705	0.187529757	0.492841084	0.356380756	0.62351911	0.536590995	0.933268401
TGIF1	0.410552646	0.806861165	0.594091669	0.080971589	0.712131121	0.536146147	0.933268401
THOC1	0.541033685	0.523381326	0.144711342	0.359103274	0.773055307	0.536611112	0.933268401
TMEM110	0.481706821	0.803918279	0.070416055	0.495376483	0.841411371	0.536451239	0.933268401
TRDMT1	0.712243959	0.920860394	0.211720153	0.771890708	0.106158228	0.536663349	0.933268401
VAMP2	0.830747213	0.315384959	0.257078152	0.315299679	0.533220357	0.535748227	0.933268401
FBXW2	0.891331326	0.395283527	0.04704518	0.826087556	0.831796077	0.536843771	0.933283578
LOC112446645	0.136577757	0.643183131	0.303204986	0.570540418	0.749334461	0.536803067	0.933283578
PRKCH	0.735054891	0.71959696	0.241682371	0.452432914	0.196985819	0.536903922	0.933283578
TMEM181	0.707999762	0.613261019	0.945526563	0.235853293	0.117631088	0.536848285	0.933283578
FAM101A	0.345709686	0.906697888	0.927228963	0.050191343	0.781367502	0.536992486	0.933286163
PUS1	0.264850381	0.081921144	0.712907273	0.890638669	0.827513202	0.537019148	0.933286163
ARMC9	0.33934856	0.560084494	0.457761986	0.585154348	0.224590167	0.53758651	0.93332433
CEP68	0.943713113	0.976589471	0.068408426	0.228845241	0.79147929	0.537342156	0.93332433
LOC100337053	0.528637396	0.17856508	0.818425868	0.477792272	0.309100882	0.53718	0.93332433
LOC101902232	0.940689169	0.094093161	0.521504747	0.818749517	0.302310047	0.537439929	0.93332433
MAPKBP1	0.466325043	0.519688857	0.411247354	0.280340783	0.408205401	0.537104243	0.93332433
PRKRIP1	0.178080516	0.574480078	0.668329578	0.24937557	0.670290898	0.537498315	0.93332433
PUS3	0.494979136	0.629319248	0.342599735	0.484918119	0.220607666	0.537294322	0.93332433
SDF2	0.720319407	0.247583913	0.66661302	0.125300529	0.76813187	0.53772223	0.93332433
SMC2	0.937801707	0.108184893	0.176340745	0.682747071	0.936597497	0.537692912	0.93332433
UBTD1	0.604527839	0.525864261	0.150457377	0.616093267	0.388296227	0.537723572	0.93332433
VDAC3	0.616908593	0.499270362	0.501313861	0.153981001	0.481140773	0.537676456	0.93332433
VPS45	0.384944812	0.880507344	0.814675194	0.193312681	0.213961445	0.537372292	0.93332433
FRMD6	0.500542534	0.560148879	0.550423467	0.165854656	0.447720997	0.538014484	0.93373051
LOC104974669	0.804429022	0.941116779	0.18435036	0.600087788	0.136896965	0.538105398	0.933740119
NSG1	0.10386606	0.658081466	0.431744597	0.467199032	0.831948076	0.538190713	0.933740119
ZNF593	0.341369161	0.379160007	0.744875182	0.927893186	0.128217169	0.538189002	0.933740119

EBAG9	0.452495666	0.126358638	0.389840981	0.93278663	0.551883878	0.53826022	0.933761995
LOC100848264	0.36000066	0.795499232	0.454691608	0.273636365	0.322305604	0.53842022	0.933793144
LOC107131311	0.559708461	0.446449159	0.204232046	0.389359256	0.578169746	0.538491262	0.933793144
LOC112442296	0.280762738	0.752180443	0.48561382	0.150888421	0.742771387	0.538578985	0.933793144
MXD3	0.868113669	0.211448336	0.207080833	0.910730345	0.331977095	0.538559279	0.933793144
PIK3CD	0.468208921	0.996143989	0.825875788	0.121155586	0.2460583	0.538401065	0.933793144
ZNF613	0.548933251	0.423643184	0.72187094	0.174639035	0.392134921	0.538619579	0.933793144
NRROS	0.579632637	0.643279125	0.226035163	0.156659393	0.871089717	0.538703566	0.9338401
CSDE1	0.778026349	0.261363042	0.091316065	0.902680688	0.687540233	0.539085401	0.933945226
EMP2	0.380266762	0.249935513	0.315366216	0.443959999	0.865240414	0.538907356	0.933945226
LMO1	0.979399742	0.234256748	0.331371093	0.193027155	0.785378491	0.539105668	0.933945226
LOC100140958	0.38222336	0.192473414	0.231619921	0.769881481	0.878486461	0.539087548	0.933945226
MRPS9	0.699159946	0.84979706	0.584415828	0.059314328	0.559483584	0.539060286	0.933945226
PHKB	0.521300805	0.977581178	0.480623902	0.291082876	0.161436787	0.538843132	0.933945226
PLAGL1	0.949532172	0.220878484	0.644300872	0.086631711	0.985065124	0.539206382	0.934021103
LOC112446007	0.531129588	0.580030146	0.132639354	0.432305399	0.653007031	0.53926708	0.934027657
BLVRA	0.239163053	0.806992165	0.111570996	0.920775515	0.583783751	0.53992038	0.93403125
EPB41L2	0.877092343	0.356481237	0.853539791	0.318871051	0.136090505	0.540020559	0.93403125
GBE1	0.11466745	0.925397873	0.150905484	0.838499589	0.862591598	0.540035074	0.93403125
HAX1	0.114476192	0.927910657	0.208007398	0.527837775	0.989659206	0.539378666	0.93403125
HIVEP3	0.972658501	0.837092167	0.981270491	0.260300681	0.055679379	0.539995716	0.93403125
IFIT2	0.672608158	0.480379216	0.711371026	0.159370274	0.315527383	0.539642269	0.93403125
LOC104974891	0.549482613	0.859786424	0.386278453	0.568732928	0.111608872	0.540065964	0.93403125
LOC618787	0.858313875	0.135263603	0.222309016	0.718513614	0.623632548	0.539756849	0.93403125
MGST3	0.421018933	0.428269485	0.379397007	0.184959133	0.915149052	0.539990526	0.93403125
MILR1	0.696396395	0.316446117	0.754960344	0.161716875	0.429376794	0.539548981	0.93403125
RIMBP2	0.394639711	0.274762113	0.937047483	0.868908908	0.13096681	0.539715938	0.93403125
SERTAD4	0.743167269	0.491756503	0.236195471	0.602012321	0.222564445	0.539766278	0.93403125
SLC50A1	0.068023603	0.520292156	0.577833067	0.859711626	0.658171589	0.539868033	0.93403125
ZSCAN30	0.849401503	0.062862777	0.582361978	0.934379179	0.398393082	0.539926554	0.93403125
ABCB9	0.524966922	0.754017813	0.242046116	0.530013851	0.229586016	0.541292169	0.934044388
ANKRD34A	0.83409028	0.087189769	0.279147725	0.612630869	0.941522349	0.542126085	0.934044388
ATP1B3	0.109905038	0.552222424	0.465294153	0.649153812	0.637510028	0.54175329	0.934044388
BTN3A3	0.886635832	0.157770663	0.685943557	0.306747974	0.394199227	0.540375609	0.934044388
CDC26	0.397526513	0.502077887	0.207203529	0.344788658	0.819180621	0.5416536	0.934044388
CDNF	0.228858119	0.260228881	0.735476042	0.338600136	0.786639839	0.541427961	0.934044388
CETN2	0.95268834	0.126816282	0.792963646	0.482561476	0.253784573	0.542501809	0.934044388
CYB561	0.50139885	0.832350212	0.087496284	0.654028592	0.488956985	0.541600629	0.934044388
DHRS7	0.545366669	0.887174969	0.072479425	0.444714004	0.746730949	0.541078354	0.934044388
FBXO17	0.163022196	0.861375963	0.213654393	0.625548206	0.619508625	0.540771735	0.934044388
GALM	0.558837944	0.834874994	0.108068251	0.852351864	0.270435967	0.540696802	0.934044388
GOLPH3	0.683614615	0.248986215	0.832466113	0.088236251	0.928800674	0.540536053	0.934044388
HYPK	0.101499002	0.390426002	0.418237156	0.820829893	0.861782222	0.542359742	0.934044388
KCNN4	0.524344166	0.286792691	0.225655388	0.702247438	0.487285772	0.540527862	0.934044388

KIAA0141	0.796154767	0.451295442	0.07699618	0.714919501	0.588164525	0.540870175	0.934044388
KIAA0513	0.735266258	0.8851842	0.078873855	0.239052895	0.956157045	0.54251833	0.934044388
KYAT3	0.92472186	0.541293999	0.844607463	0.04073633	0.679687104	0.542059281	0.934044388
LMF1	0.854541032	0.303137767	0.845623772	0.586356427	0.091026004	0.541833903	0.934044388
LOC101902174	0.209267653	0.825648149	0.20764515	0.555581774	0.585422975	0.541464533	0.934044388
LOC101902807	0.600788456	0.962068126	0.038274789	0.599743403	0.882210015	0.542053781	0.934044388
LOC101906226	0.346556806	0.944512174	0.364990679	0.28604766	0.342439446	0.542013474	0.934044388
LOC101908075	0.752174121	0.747646786	0.113304776	0.879088579	0.208777858	0.54187909	0.934044388
LOC104975635	0.474677701	0.731563307	0.729176426	0.32710898	0.140298082	0.540669898	0.934044388
LOC112444869	0.679896748	0.148963574	0.355185625	0.348628506	0.935010753	0.542396715	0.934044388
LOC786015	0.667219009	0.593961998	0.423559462	0.331846765	0.208421084	0.540491229	0.934044388
LRRC20	0.964764575	0.31049507	0.249427282	0.509536533	0.308261953	0.542553791	0.934044388
NAB1	0.051674354	0.818789918	0.771982724	0.734498868	0.484323545	0.540649814	0.934044388
NR1D2	0.287920564	0.906022788	0.772893019	0.19692397	0.294236636	0.541679758	0.934044388
NTRK3	0.71032713	0.648372232	0.760012513	0.336639506	0.09835175	0.540153158	0.934044388
NUP54	0.531208295	0.832646275	0.186180888	0.170009156	0.829906152	0.540641011	0.934044388
NXPH3	0.634192893	0.58576097	0.395437286	0.491668987	0.161259468	0.54110466	0.934044388
PAPD5	0.435413414	0.837452023	0.592464411	0.1130368	0.479728106	0.542212594	0.934044388
PHB	0.416969272	0.593522639	0.538189664	0.295854172	0.297640465	0.542435589	0.934044388
PQLC1	0.264135631	0.761683716	0.751763778	0.390500742	0.197069638	0.540976044	0.934044388
RHOC	0.688708552	0.520156174	0.638426292	0.490483709	0.104147096	0.541690846	0.934044388
RPL22	0.31201604	0.190498423	0.361736972	0.910679406	0.596989407	0.541797003	0.934044388
SCAI	0.410627527	0.774650401	0.214474215	0.341868226	0.502595353	0.542329981	0.934044388
SFXN3	0.282827836	0.40921559	0.134413354	0.935215082	0.80364154	0.541839747	0.934044388
SGK3	0.615558531	0.959818026	0.438460793	0.296269629	0.151817052	0.5411834	0.934044388
TAL1	0.466689203	0.299092203	0.332927376	0.837736369	0.301222115	0.542404913	0.934044388
TBC1D24	0.693487422	0.133264049	0.64957842	0.833761579	0.23311655	0.541447623	0.934044388
TMEM11	0.938542401	0.130451292	0.474142572	0.825653558	0.244884283	0.542577853	0.934044388
VMP1	0.343314903	0.682225383	0.229032483	0.88795006	0.246058254	0.542302725	0.934044388
WDR34	0.41957894	0.720288601	0.094722087	0.473455129	0.855903901	0.540339575	0.934044388
ERCC8	0.130012365	0.846429554	0.487004567	0.485087111	0.452116767	0.542845523	0.934160237
FIS1	0.413994569	0.40995436	0.376695296	0.206771596	0.889262059	0.54287284	0.934160237
POMP	0.898885365	0.143158173	0.189081977	0.589375456	0.819088251	0.542719993	0.934160237
SIDT1	0.485931241	0.503195199	0.579359829	0.509453037	0.162858871	0.542843943	0.934160237
EFTUD2	0.611583896	0.276881683	0.400703595	0.992178107	0.17467689	0.542942134	0.934181522
HOXB5	0.286769627	0.852632233	0.146479678	0.949501148	0.34596937	0.543033072	0.93419712
SPAST	0.443752415	0.866555846	0.959422743	0.037746119	0.845003559	0.543065049	0.93419712
AIMP1	0.561808595	0.71680678	0.069314947	0.66478229	0.634510057	0.54317814	0.934239804
SMIM17	0.125486521	0.549824556	0.83794654	0.551450223	0.369359824	0.543203717	0.934239804
TOR1AIP1	0.635974775	0.880478188	0.143705482	0.356062602	0.411222912	0.543310136	0.934324912
ADAMTSL4	0.425972887	0.498079117	0.101912086	0.898425289	0.606942852	0.543442182	0.934379159
LOC101906606	0.788014935	0.286888186	0.847985314	0.15369919	0.40030276	0.543512489	0.934379159
PPP1R15A	0.09739515	0.742010733	0.889018793	0.663954021	0.276426991	0.543460285	0.934379159
INIP	0.309955493	0.338339439	0.994479456	0.372447405	0.303830924	0.543622047	0.934392963

USP46	0.85351919	0.447286383	0.606857413	0.062493891	0.815172608	0.543634392	0.934392963
ASXL3	0.245124692	0.701089325	0.245470806	0.538090634	0.520128353	0.543701887	0.934403723
SNRPD2	0.294639958	0.268653747	0.420763849	0.59151284	0.599457996	0.543754527	0.934403723
CAV3	0.316436711	0.795100181	0.693870967	0.085275862	0.794950456	0.544153839	0.934861228
EIF2S3	0.17914038	0.767633235	0.345326138	0.263676896	0.946227113	0.544369679	0.934861228
ENO1	0.564801945	0.763411631	0.556448419	0.235817388	0.209331288	0.544301771	0.934861228
FBLL1	0.382499375	0.3846973	0.444129251	0.469583414	0.385977239	0.544321064	0.934861228
GRAP	0.947605975	0.608196726	0.562978998	0.070540358	0.517938939	0.544473569	0.934861228
PSMA2	0.768923801	0.402118174	0.387407946	0.261229366	0.37872039	0.544414577	0.934861228
RWDD3	0.502486103	0.144762201	0.645316326	0.339132006	0.743400485	0.544149393	0.934861228
VKORC1L1	0.845117592	0.160105138	0.272514622	0.877590615	0.366338632	0.544476486	0.934861228
COX14	0.529326244	0.625969612	0.649259661	0.080658597	0.6838934	0.544673305	0.934992759
LOC530929	0.797209655	0.754413748	0.067778136	0.844857501	0.344662608	0.544724012	0.934992759
SAMD1	0.762459941	0.987204939	0.242475092	0.21094442	0.308170783	0.544636697	0.934992759
AK4	0.375331133	0.43457546	0.196770505	0.55102125	0.672297945	0.545041129	0.935257695
EXOSC3	0.649791457	0.390408965	0.1720062	0.479501464	0.569198574	0.545358815	0.935257695
LGALS3BP	0.211685055	0.887488008	0.704708379	0.137821497	0.652652321	0.545345324	0.935257695
NADSYN1	0.662285068	0.445044388	0.319031251	0.166622498	0.760465706	0.54544826	0.935257695
POU2F3	0.760515046	0.128972352	0.446679859	0.400948659	0.678194972	0.545425956	0.935257695
PPP1R10	0.800121707	0.907701203	0.634252781	0.034174698	0.755882077	0.545195261	0.935257695
TENM4	0.731083577	0.791011508	0.781867942	0.127139838	0.206912225	0.545120851	0.935257695
TMC6	0.924501184	0.184915624	0.761835367	0.329400127	0.277475445	0.54527029	0.935257695
YWHAB	0.660963432	0.172901187	0.724134973	0.386649831	0.372195581	0.545355446	0.935257695
ZNF180	0.110450903	0.373931297	0.40033203	0.73278004	0.981640029	0.545101605	0.935257695
GTF3A	0.338696409	0.890479932	0.065204631	0.721830009	0.839645317	0.545513938	0.93527259
ABI3	0.738779986	0.696403899	0.157358243	0.15502816	0.950674194	0.545718289	0.935331099
ALDH1A1	0.583942903	0.945953037	0.048038057	0.858537848	0.528311694	0.5473776	0.935331099
C8H9orf40	0.261796666	0.796522095	0.249183223	0.802041713	0.286527762	0.545866366	0.935331099
D2HGDH	0.806290655	0.186855548	0.875040255	0.103231057	0.88514422	0.54754286	0.935331099
DGAT2	0.704084794	0.723796923	0.939868361	0.077087264	0.325928433	0.547350517	0.935331099
DHX9	0.229038202	0.842152534	0.905001648	0.117367205	0.584732959	0.546486458	0.935331099
GATA6	0.703170786	0.558862411	0.434807813	0.072291324	0.965905408	0.545707601	0.935331099
HAGHL	0.430019196	0.84714867	0.37630757	0.102919702	0.848519867	0.546353691	0.935331099
HTATIP2	0.849878783	0.475011305	0.300494532	0.595602902	0.165450802	0.546077207	0.935331099
IFT140	0.378495044	0.090574318	0.993927414	0.647179381	0.54526207	0.547190248	0.935331099
LOC100139325	0.961518953	0.196144731	0.311012788	0.247589112	0.828727813	0.547369286	0.935331099
LOC104970779	0.256141163	0.420257839	0.318883737	0.788300641	0.443442265	0.546796642	0.935331099
LOC112442704	0.834089795	0.133678708	0.483825075	0.27502069	0.806576643	0.546275937	0.935331099
LOC112443243	0.194095964	0.291922516	0.27023956	0.895132488	0.878044787	0.54736083	0.935331099
LOC512617	0.310724391	0.638282638	0.576238985	0.169848057	0.62017773	0.54741845	0.935331099
LOC786974	0.794089144	0.494380359	0.322664959	0.845783284	0.111390898	0.54575482	0.935331099
MCM3AP	0.887580681	0.04341495	0.560845089	0.606185693	0.913979628	0.5463895	0.935331099
MOSPD3	0.194210001	0.230361216	0.769778821	0.852035125	0.410138454	0.547359899	0.935331099
MTHFD1	0.230528029	0.738509497	0.972525816	0.301432419	0.240811673	0.547101488	0.935331099

MTHFS	0.299226795	0.495767302	0.273216841	0.630901399	0.467054157	0.545897259	0.935331099
NCKAP1L	0.252570078	0.610106423	0.862940796	0.207942842	0.433897847	0.546771721	0.935331099
PHKA1	0.136302699	0.548435325	0.203133529	0.985195436	0.802545775	0.546905529	0.935331099
PLD5	0.287700049	0.699187195	0.232128915	0.970047551	0.263995648	0.546134126	0.935331099
PNMA8A	0.733496057	0.255578278	0.665194905	0.200736277	0.481115058	0.547496755	0.935331099
PROKR2	0.672741422	0.398579454	0.358614454	0.807687145	0.153741236	0.545857561	0.935331099
PTPRN2	0.483870703	0.202886884	0.239616667	0.545276435	0.933039099	0.546294641	0.935331099
ROBO1	0.888765555	0.53009628	0.122141285	0.324890347	0.643989343	0.547441446	0.935331099
RPGR	0.183003899	0.226523179	0.741769337	0.401579802	0.972607077	0.546970498	0.935331099
SLC18A2	0.756280525	0.242985156	0.936220098	0.446442574	0.155626868	0.546062944	0.935331099
SLC20A2	0.631163913	0.757517859	0.307212125	0.08731091	0.936642576	0.546998979	0.935331099
STMN3	0.31436507	0.775667533	0.465856556	0.284949642	0.370495771	0.546689568	0.935331099
SYT17	0.462065768	0.539853443	0.783374237	0.340152505	0.180181116	0.546433386	0.935331099
SZRD1	0.992555534	0.460309157	0.64086484	0.049542691	0.828153932	0.54701913	0.935331099
TRPM7	0.579068228	0.529394671	0.919627746	0.043780038	0.974151191	0.54717878	0.935331099
TTI1	0.840457191	0.543578958	0.158169833	0.767893735	0.216334033	0.54687212	0.935331099
AGO2	0.858362183	0.992797003	0.604153594	0.030912872	0.757130545	0.547603876	0.935337969
CLEC2B	0.698839364	0.915969226	0.028527302	0.799315747	0.827685598	0.548094987	0.935551653
MAP3K13	0.328749976	0.790695811	0.322112428	0.179934894	0.801371366	0.547975593	0.935551653
NTRK2	0.083145856	0.74338784	0.447250062	0.526653263	0.82985736	0.54810909	0.935551653
PTEN	0.686586255	0.700610713	0.273475605	0.102197133	0.898711728	0.548115655	0.935551653
PTTG1	0.371179231	0.399880382	0.109736802	0.760267879	0.975004785	0.54797894	0.935551653
VPS33B	0.173947149	0.433606128	0.694334066	0.252409547	0.914093651	0.548128032	0.935551653
YIPF4	0.516480894	0.436297711	0.116105054	0.985593228	0.467918326	0.547854033	0.935551653
LOC101907174	0.390397022	0.674447034	0.346845088	0.133928782	0.988601891	0.548263629	0.935685775
ADORA3	0.177781765	0.631951388	0.273411169	0.927692896	0.428081042	0.549953777	0.93576533
BACH1	0.326384898	0.297198572	0.946496746	0.315536506	0.422666125	0.550671159	0.93576533
BCL2L12	0.561598966	0.337907272	0.520628097	0.201094089	0.612093121	0.549358592	0.93576533
BGN	0.585209681	0.766033298	0.095406608	0.440559982	0.649311741	0.550517986	0.93576533
CDKL2	0.738081111	0.622479889	0.587019857	0.194195811	0.233394827	0.550349126	0.93576533
CDKN2C	0.895027652	0.519188342	0.748547152	0.109353387	0.318220205	0.54846436	0.93576533
CNOT6L	0.362309762	0.826567109	0.757448999	0.077343514	0.698046611	0.55070511	0.93576533
DGKD	0.769040468	0.293329827	0.531477224	0.155063509	0.656146342	0.549946292	0.93576533
DIO3	0.175602924	0.383943662	0.821986137	0.291918383	0.755869717	0.550419223	0.93576533
EIF3H	0.429744708	0.617251169	0.09082133	0.561649566	0.902126923	0.550075571	0.93576533
EPM2A	0.757534441	0.246816526	0.666071209	0.112592901	0.872591291	0.550528248	0.93576533
ETFB	0.805561022	0.696045866	0.341182385	0.127495712	0.50156718	0.550496662	0.93576533
FABP5	0.311397504	0.17087607	0.733618366	0.38508508	0.805788348	0.548598273	0.93576533
FAM166A	0.234213922	0.377503616	0.996854856	0.236994895	0.583633737	0.549833663	0.93576533
GGACT	0.778759827	0.582687319	0.280162518	0.115365795	0.826542994	0.548751464	0.93576533
GIGYF2	0.859081303	0.705005304	0.447166817	0.047098327	0.951663467	0.549013173	0.93576533
GRIA1	0.907152335	0.806313287	0.034849334	0.968819011	0.489990009	0.548406101	0.93576533
HDAC4	0.911512198	0.100123677	0.64459966	0.299625391	0.687085006	0.548569254	0.93576533
IGHMBP2	0.785804798	0.041870945	0.567674657	0.671984138	0.97509298	0.550578046	0.93576533

LNX1	0.296483552	0.691816092	0.142122781	0.570581905	0.730569976	0.549211504	0.93576533
LOC101906484	0.70195885	0.951055911	0.02871801	0.65434211	0.970031383	0.549488482	0.93576533
LOC104975811	0.572208351	0.687510684	0.397989555	0.991393992	0.078508275	0.54975509	0.93576533
LOC107132175	0.862186469	0.798690011	0.531092975	0.099859168	0.334715522	0.550349353	0.93576533
LOC112443816	0.518865009	0.306786201	0.454860557	0.574084185	0.292031969	0.549008484	0.93576533
LOC515823	0.338920323	0.149287014	0.749031033	0.399704933	0.804404316	0.549740229	0.93576533
LOC541276	0.194123744	0.379040845	0.764453791	0.42697129	0.508450845	0.55015066	0.93576533
MAP4K2	0.709167266	0.57600735	0.050431115	0.73101647	0.803913221	0.548495699	0.93576533
MRPS5	0.756259201	0.938031172	0.892363842	0.062091418	0.309094201	0.549175146	0.93576533
NXT2	0.908486664	0.458438032	0.072398533	0.871826894	0.46246137	0.549299681	0.93576533
PLXNC1	0.178874167	0.723837884	0.558631325	0.246943922	0.681766272	0.549614371	0.93576533
PPM1A	0.920293599	0.884990222	0.026610292	0.860520383	0.650768244	0.548976536	0.93576533
R3HDM2	0.994449459	0.290384513	0.511836216	0.201303395	0.408585748	0.54929386	0.93576533
RARA	0.901946902	0.922157766	0.151501778	0.135510025	0.711941841	0.549293147	0.93576533
RIC8A	0.275035327	0.99799808	0.42704282	0.199694607	0.52180874	0.550196439	0.93576533
STON1	0.421778358	0.233087124	0.312631602	0.823325148	0.480452195	0.549309529	0.93576533
SYTL4	0.495485364	0.890741511	0.25241779	0.236913518	0.462068597	0.549901455	0.93576533
TAX1BP3	0.398680555	0.171635441	0.806590978	0.386246062	0.574154763	0.550598539	0.93576533
THSD1	0.73263017	0.380808181	0.051315924	0.960656177	0.890276538	0.550668146	0.93576533
TMEM175	0.50794049	0.526094204	0.477991654	0.147218921	0.649526437	0.55019195	0.93576533
TMEM39A	0.708634859	0.255080087	0.421110819	0.163578149	0.9813083	0.550266574	0.93576533
TXNDC9	0.640444832	0.200498603	0.34123152	0.385180801	0.723760024	0.550211505	0.93576533
XRN2	0.812563947	0.253042863	0.410570171	0.965600348	0.150166661	0.550612464	0.93576533
COMMD10	0.885519031	0.610807002	0.404064073	0.253705027	0.221245699	0.551030076	0.935833031
MAPKAPK2	0.913416826	0.121532612	0.563785262	0.290978512	0.673622389	0.551028725	0.935833031
NELFA	0.664891058	0.177550112	0.422374913	0.250100285	0.982893891	0.550868654	0.935833031
UBR1	0.211616042	0.776157374	0.895509081	0.141345585	0.589537409	0.550856119	0.935833031
ZNF830	0.242161784	0.65024582	0.215959674	0.688570844	0.523854336	0.551011958	0.935833031
LOC512978	0.476614101	0.203257875	0.647029148	0.346171644	0.566198582	0.551313655	0.935999613
MFSD3	0.284809635	0.405424737	0.71914942	0.342214026	0.432269264	0.551286209	0.935999613
MRPL32	0.231566651	0.781749633	0.56127358	0.314786326	0.384203053	0.551356301	0.935999613
PRDX5	0.336744849	0.420219098	0.579195609	0.229173055	0.654161922	0.551336809	0.935999613
ATP6V1D	0.774599559	0.475355251	0.232639987	0.252235267	0.569078037	0.551472235	0.936008325
PEX2	0.801191723	0.357294361	0.619194025	0.675068486	0.102760861	0.551475504	0.936008325
TBX6	0.380644663	0.89341174	0.596356742	0.234337533	0.258824769	0.551546276	0.936031638
NPRL3	0.418712846	0.703116491	0.164992475	0.37820525	0.66977703	0.551608223	0.93603997
CTNNAL1	0.461401186	0.955029828	0.069016189	0.431287088	0.939435766	0.551879658	0.936306943
FAM3A	0.198821745	0.632135923	0.539609043	0.409413111	0.443702074	0.551847247	0.936306943
EPPK1	0.899003805	0.066626036	0.655288621	0.337463366	0.930889075	0.552004437	0.936325046
LOC781913	0.165268871	0.42344052	0.695160448	0.297823676	0.850958632	0.551992733	0.936325046
INPP4A	0.167238176	0.883240284	0.261615135	0.553396101	0.57695705	0.552134306	0.936448543
RUFY3	0.725757262	0.94779594	0.642627934	0.048079342	0.580934908	0.552264644	0.93657281
ARL1	0.563265266	0.246296154	0.288444587	0.773024045	0.400074582	0.552713092	0.937045637
LOC104973803	0.163297829	0.527974197	0.328256182	0.509160463	0.858835553	0.55271475	0.937045637

WDR61	0.957927049	0.388006297	0.352074688	0.389898695	0.242544771	0.552705473	0.937045637
HAUS2	0.423661907	0.040663707	0.899940842	0.903154252	0.884106917	0.552774992	0.937050965
DERA	0.857936793	0.126180942	0.711222186	0.966513458	0.166434861	0.552863134	0.937103584
CRCP	0.771903364	0.162184365	0.4726256	0.503263922	0.416096286	0.552939237	0.937135786
AGO1	0.72071617	0.921086739	0.106497918	0.752848626	0.234739923	0.5545401	0.937139078
ATG4D	0.562555778	0.175341036	0.239208974	0.665064443	0.794060976	0.554028859	0.937139078
ATL3	0.54057211	0.111124383	0.304045376	0.804406628	0.84698347	0.553767063	0.937139078
CLUH	0.654262664	0.797786347	0.437589149	0.303402769	0.180239507	0.55448451	0.937139078
COL28A1	0.627531954	0.711687847	0.270222849	0.552932772	0.186927146	0.5542269	0.937139078
CRTC2	0.756463037	0.47151571	0.933519856	0.171074943	0.217960717	0.553333968	0.937139078
DLD	0.510281036	0.796188028	0.438097368	0.195477061	0.357665941	0.553774634	0.937139078
DNAJC17	0.592738836	0.143313191	0.33795813	0.856232053	0.506601007	0.553907632	0.937139078
DPP9	0.222144522	0.831504029	0.81721576	0.884960313	0.093122235	0.553706027	0.937139078
FAM160A1	0.059004607	0.974502341	0.470613002	0.562279964	0.819160416	0.554077527	0.937139078
FAM25A	0.945120936	0.567837711	0.635054572	0.195858897	0.187086138	0.554454781	0.937139078
GRAMD2A	0.93265369	0.064451805	0.526910838	0.512922795	0.766674679	0.553945547	0.937139078
KLHL33	0.464502935	0.496186698	0.289777678	0.318137071	0.58511342	0.553590647	0.937139078
KRT8	0.246185239	0.170423452	0.768510583	0.508960442	0.755582972	0.553084442	0.937139078
LOC101902757	0.730134978	0.256996337	0.191560973	0.758399247	0.455422511	0.553323997	0.937139078
LOC112446777	0.671615078	0.368517005	0.38781645	0.383219096	0.338229533	0.553727675	0.937139078
LOC531679	0.628515686	0.911110991	0.091555163	0.883727493	0.267722118	0.553157553	0.937139078
NHLRC2	0.244803247	0.29718308	0.630631409	0.345509233	0.787356194	0.554339901	0.937139078
NRGN	0.394596823	0.366567056	0.316441847	0.415960498	0.654173502	0.553941222	0.937139078
PHETA2	0.620535772	0.645887659	0.1048741	0.557517035	0.529887889	0.553361423	0.937139078
RAB4A	0.884775268	0.39012372	0.725907894	0.057061981	0.871789829	0.554086898	0.937139078
RFC5	0.723977598	0.174367731	0.225150656	0.602272542	0.725746676	0.553453519	0.937139078
SLC12A4	0.900845411	0.863575866	0.40406515	0.098006905	0.404014165	0.553812792	0.937139078
SNX20	0.551529538	0.464066955	0.651921029	0.304203406	0.244608079	0.553337154	0.937139078
SPATA7	0.954954719	0.512237744	0.750253415	0.59573275	0.057117728	0.554444681	0.937139078
ST6GAL2	0.873160823	0.338210936	0.11214468	0.931195162	0.405011012	0.554481544	0.937139078
THRA	0.275417791	0.854536798	0.672272365	0.237422127	0.331393338	0.553847075	0.937139078
TMEM60	0.720145472	0.385364218	0.917021158	0.052659854	0.931105075	0.554296378	0.937139078
RNLS	0.705222118	0.953238331	0.859612615	0.094778937	0.228223765	0.554629584	0.937193791
LOC100848575	0.644977181	0.262676038	0.337903574	0.358742682	0.609182368	0.554799865	0.93732181
QPCTL	0.778418965	0.409168537	0.785748991	0.137458783	0.363714465	0.554819576	0.93732181
EIF5B	0.640677387	0.801785954	0.482935918	0.357001313	0.141428564	0.555023788	0.937377316
LOC104970105	0.828197379	0.506091619	0.196504868	0.232733252	0.65323682	0.554966785	0.937377316
MYH6	0.207644978	0.581128831	0.184953581	0.886902724	0.632616377	0.554970151	0.937377316
ANKIB1	0.593137151	0.631622473	0.336901969	0.263635239	0.376888163	0.555261707	0.937497742
LOC101903649	0.814989724	0.131369293	0.714410076	0.260478148	0.630007472	0.555429068	0.937497742
LOC104969981	0.969568388	0.501742101	0.081116348	0.495518104	0.64214432	0.555494976	0.937497742
LOC112443502	0.928316481	0.381421145	0.454019311	0.112067634	0.696542446	0.555381461	0.937497742
MAPK8IP1	0.757986837	0.426153762	0.111818696	0.407938159	0.851936945	0.555443947	0.937497742
SMAD3	0.748006221	0.599676501	0.256930886	0.381862589	0.284811338	0.5551603	0.937497742

USP1	0.232732922	0.829774166	0.956059766	0.103213095	0.658239059	0.555301081	0.937497742
TMEM41A	0.791186938	0.154442512	0.59150332	0.655540775	0.265132688	0.555586363	0.937555558
CHM	0.524847878	0.861216709	0.585687903	0.533199607	0.089128039	0.555874013	0.937578734
CYBRD1	0.789975548	0.753286409	0.811338991	0.060915748	0.428524721	0.556212626	0.937578734
EXOC1L	0.583428543	0.440651677	0.616766834	0.181241548	0.43910873	0.556457063	0.937578734
FH	0.65750831	0.6273745	0.365300149	0.107910971	0.775279924	0.556265953	0.937578734
GRAMD2B	0.53428097	0.337513646	0.413684244	0.173325169	0.974051226	0.556076277	0.937578734
LPAR3	0.765260935	0.475526867	0.386210667	0.10468439	0.85660696	0.556208558	0.937578734
NAGPA	0.572940488	0.37147009	0.436263159	0.14107902	0.961840954	0.556153338	0.937578734
NGLY1	0.959465108	0.980292786	0.601917725	0.024982314	0.889064793	0.555773089	0.937578734
PIGX	0.548991435	0.62120004	0.09779255	0.414300896	0.909940612	0.555748105	0.937578734
PKP4	0.294895547	0.649566359	0.727645932	0.273929736	0.330258433	0.556311464	0.937578734
PPFIBP2	0.828963717	0.521420321	0.754370902	0.160276285	0.240692048	0.555839986	0.937578734
SLC22A15	0.300265164	0.565567205	0.812075431	0.661203968	0.138265155	0.556279953	0.937578734
STAU1	0.535829384	0.711465347	0.162677843	0.256973019	0.791451327	0.556362568	0.937578734
TFEC	0.277378793	0.380003368	0.628320407	0.467628459	0.407363351	0.55640874	0.937578734
VAC14	0.448231006	0.63380596	0.116953403	0.432621297	0.876940765	0.55624256	0.937578734
AP3M1	0.502821347	0.181952178	0.204621756	0.773193193	0.875920397	0.55736072	0.937884572
APBA1	0.573033075	0.790350571	0.475741098	0.408666403	0.144059466	0.557451675	0.937884572
BCL6	0.20541798	0.354229548	0.290746321	0.754435491	0.793402369	0.557129464	0.937884572
CD200R1L	0.104207425	0.374720387	0.567694352	0.577836401	0.988964461	0.557197229	0.937884572
CD58	0.952329167	0.299618567	0.212813632	0.7054326	0.295735515	0.557200822	0.937884572
DCP2	0.311243334	0.609880358	0.442316957	0.612582918	0.24696722	0.55771806	0.937884572
DTWD2	0.33649396	0.391971856	0.903487184	0.886283137	0.120322494	0.557802625	0.937884572
EXOSC5	0.494649239	0.928034614	0.241342696	0.504562841	0.226952719	0.557480659	0.937884572
FOXO1	0.931030547	0.875134135	0.127348452	0.180080578	0.676841333	0.556878429	0.937884572
FOXP1	0.69900791	0.527218184	0.063343814	0.689845971	0.786208024	0.557091046	0.937884572
GPRASP1	0.323027017	0.765178272	0.299069027	0.339865372	0.50433598	0.557238684	0.937884572
LOC107131772	0.424624233	0.065714348	0.478927992	0.954061404	0.99384507	0.557252155	0.937884572
LOC112442081	0.690638833	0.983045897	0.518831351	0.079375984	0.454350493	0.557739595	0.937884572
LOC614207	0.393000798	0.748142704	0.81666486	0.201379711	0.261657322	0.556960045	0.937884572
MTA2	0.422589928	0.796571324	0.412753875	0.253836615	0.360140476	0.557708619	0.937884572
NEURL3	0.972854272	0.744429162	0.055210847	0.493792949	0.643173611	0.557667663	0.937884572
PIK3CB	0.721163911	0.239771062	0.237326691	0.637433073	0.484450615	0.55726598	0.937884572
PSMB9	0.400033562	0.796631274	0.281864068	0.471742616	0.299082176	0.557278243	0.937884572
SH3D19	0.621566978	0.218367821	0.217112243	0.679709007	0.633537413	0.557529295	0.937884572
TXNDC17	0.15893177	0.668647017	0.908038895	0.472568613	0.278726351	0.557838725	0.937884572
ZNF543	0.778200156	0.827289752	0.89988917	0.043405143	0.504410838	0.55744402	0.937884572
AMER1	0.305989303	0.073913919	0.89142327	0.642512411	0.984256083	0.558437097	0.93802457
AQP11	0.135396542	0.985622628	0.342423035	0.919109343	0.303358771	0.558303722	0.93802457
BLOC1S4	0.386019786	0.478378993	0.479454594	0.176283201	0.822138288	0.559668305	0.93802457
BPI	0.27931648	0.388431514	0.558513935	0.95709813	0.220675477	0.559167846	0.93802457
CERS2	0.391680438	0.286127729	0.237824495	0.998913934	0.480989586	0.559281535	0.93802457
CTSD	0.284800437	0.423291978	0.151516633	0.837329074	0.839288509	0.559740763	0.93802457

DIAPH1	0.382190637	0.100418796	0.535878999	0.83583921	0.745881033	0.559521682	0.93802457
ERRFI1	0.732398632	0.713251008	0.405090359	0.127693815	0.472671244	0.558775885	0.93802457
FAM171B	0.982435246	0.54697192	0.187854442	0.295375957	0.426617702	0.557993482	0.93802457
FIGN	0.629980117	0.278464422	0.951761592	0.245129419	0.313860838	0.559877646	0.93802457
FOS	0.587968807	0.548635042	0.618648923	0.170482603	0.375544151	0.558843351	0.93802457
GPR160	0.465370747	0.846543188	0.061755253	0.613610021	0.860083003	0.559787948	0.93802457
HDAC5	0.747250703	0.737043273	0.614538999	0.345360627	0.108961743	0.55823759	0.93802457
IPO13	0.53140442	0.463607051	0.54267714	0.650803773	0.146955884	0.558989819	0.93802457
KYAT1	0.902295711	0.96679017	0.460787344	0.483671227	0.066108244	0.559979691	0.93802457
LOC101902991	0.901154359	0.350468857	0.144251564	0.987280121	0.285235231	0.559636289	0.93802457
LOC101904447	0.634235138	0.633487999	0.557555333	0.136188886	0.420762629	0.559743885	0.93802457
LOC104974137	0.859794942	0.626660125	0.630000179	0.830752867	0.045565295	0.559929216	0.93802457
LOC107131566	0.460124822	0.727073044	0.097699945	0.481482055	0.816040423	0.55982557	0.93802457
LOC787891	0.350967595	0.284436045	0.5324031	0.636633373	0.377010366	0.558538467	0.93802457
MBD6	0.859076049	0.117653846	0.376143563	0.984784011	0.340059766	0.558163175	0.93802457
MCTS1	0.874782746	0.903788312	0.377341086	0.121906711	0.352583594	0.55953792	0.93802457
NKAIN3	0.110460727	0.551371017	0.838627503	0.308678347	0.810161237	0.558788149	0.93802457
PARP16	0.752722489	0.632836271	0.292775371	0.386441483	0.238471202	0.559976563	0.93802457
PDGFB	0.297314523	0.72493223	0.433126783	0.151087884	0.907877839	0.55926978	0.93802457
PSMD11	0.74019587	0.319537385	0.154698093	0.607237517	0.575556399	0.559010205	0.93802457
RBM23	0.489943331	0.595998047	0.091842849	0.968988716	0.49095388	0.558565279	0.93802457
RNF11	0.890788435	0.156738031	0.130383034	0.8516705	0.827340832	0.559597908	0.93802457
RNFT1	0.843406787	0.623179824	0.977510254	0.479861867	0.051873322	0.559024526	0.93802457
TMEM159	0.449423622	0.935915162	0.749763199	0.042184508	0.960146293	0.558791843	0.93802457
USP3	0.514079642	0.887349778	0.292728677	0.156611092	0.611344211	0.558965486	0.93802457
VGF	0.637348811	0.437376259	0.093987821	0.938251131	0.520706211	0.55919454	0.93802457
WDR45B	0.786290619	0.310833602	0.347340638	0.455758485	0.329127873	0.558197926	0.93802457
YTHDC2	0.146133708	0.839050874	0.649981555	0.433221114	0.369223649	0.558407326	0.93802457
ZFP30	0.484615511	0.901487472	0.165692232	0.403748773	0.437808829	0.559123233	0.93802457
ZNF500	0.853262139	0.045347861	0.830137525	0.612707123	0.650760719	0.559304327	0.93802457
ETHE1	0.204940682	0.904583177	0.978618966	0.1532238	0.463129106	0.560303754	0.938375846
MTA1	0.963248003	0.0514836	0.376141113	0.80476312	0.857604007	0.56030113	0.938375846
CERS6	0.424672749	0.63219985	0.181603412	0.543029164	0.486706461	0.560483044	0.93858033
SSNA1	0.441580333	0.516304138	0.458120676	0.13946004	0.885084271	0.560574628	0.938637917
LOC518961	0.904863809	0.650553339	0.050477179	0.686021658	0.633034469	0.560750536	0.938805212
LOC614141	0.149003192	0.427113982	0.263111327	0.789899065	0.97580385	0.560788952	0.938805212
ACTR3B	0.747057766	0.24618685	0.931376647	0.102129472	0.741469424	0.561753807	0.938924585
AHSA1	0.982644656	0.665783056	0.604392864	0.051241022	0.642729012	0.562510435	0.938924585
AKAP12	0.450302602	0.717713003	0.37674211	0.202968233	0.52424454	0.561519933	0.938924585
ALKBH4	0.436508487	0.509718651	0.462280789	0.141733347	0.893927856	0.562647687	0.938924585
ASB4	0.436503096	0.733381525	0.561041994	0.108927777	0.66685566	0.562860491	0.938924585
AXIN1	0.906139673	0.326919301	0.727978446	0.10116217	0.598569455	0.563039676	0.938924585
C1RL	0.996647552	0.382963399	0.951992043	0.629712022	0.057172038	0.563381837	0.938924585
C3H1orf43	0.957110781	0.241009537	0.195725456	0.423188407	0.683461023	0.563041607	0.938924585

CDCP1	0.760122378	0.133114599	0.226310507	0.63083906	0.904625483	0.56317921	0.938924585
DNAH12	0.459294687	0.875143133	0.28908361	0.827580585	0.135281902	0.562310656	0.938924585
DNAJB2	0.923313018	0.318119641	0.214295861	0.270885737	0.761851441	0.562029249	0.938924585
DPF3	0.432935177	0.61148254	0.616608314	0.150691679	0.528294542	0.562105575	0.938924585
ESR1	0.549117093	0.742184931	0.715056795	0.084133528	0.529189131	0.561802128	0.938924585
INPP5K	0.148639949	0.372079585	0.429957031	0.787813593	0.691659707	0.561542538	0.938924585
LMNA	0.765230619	0.807953534	0.079452025	0.286501986	0.921092273	0.561632166	0.938924585
LOC100335553	0.649666871	0.29681521	0.796462819	0.365285671	0.232139295	0.562523491	0.938924585
LOC101903402	0.670048992	0.187224573	0.315639841	0.364412119	0.899722524	0.561919666	0.938924585
LOC101906513	0.730708683	0.621403131	0.242052512	0.254925513	0.462980655	0.561760342	0.938924585
LOC101910153	0.258197988	0.26254163	0.558557586	0.630515208	0.546934286	0.563023161	0.938924585
LOC104975684	0.369280567	0.238647751	0.89965597	0.444691115	0.370433074	0.563071434	0.938924585
LOC107132883	0.799684532	0.587203461	0.373872755	0.078258088	0.941283165	0.561173668	0.938924585
LOC107133226	0.527771114	0.412388947	0.241155615	0.628409354	0.395391872	0.562788268	0.938924585
LOC112449086	0.783130496	0.979035044	0.524244676	0.049446538	0.652525878	0.561714472	0.938924585
LOC613822	0.280662763	0.155310477	0.410666872	0.931121725	0.780064697	0.562207875	0.938924585
METTL14	0.121019772	0.605891372	0.462713879	0.547661266	0.701180964	0.562604287	0.938924585
MPP6	0.682313613	0.52367715	0.661224842	0.097471276	0.567747664	0.563280188	0.938924585
MYRIP	0.098568053	0.626765273	0.491829704	0.964748181	0.443615896	0.562236544	0.938924585
NCOA7	0.725507197	0.261774057	0.601511634	0.284493159	0.400655191	0.562493002	0.938924585
NUDT19	0.889644048	0.447735711	0.391490256	0.217179259	0.382865483	0.561680539	0.938924585
PCDH20	0.566253994	0.668242268	0.419350355	0.531021924	0.154958568	0.563023418	0.938924585
RALGAPA2	0.837028734	0.077713294	0.735968921	0.430430845	0.630858315	0.562171528	0.938924585
RNF227	0.333696159	0.795632416	0.323174948	0.216371125	0.698908022	0.561812349	0.938924585
SAP30BP	0.688783477	0.44589197	0.239035037	0.985107547	0.180987508	0.563492063	0.938924585
SBNO1	0.552347729	0.612557415	0.627425426	0.144887179	0.421851449	0.561808045	0.938924585
SEL1L3	0.449488928	0.449053741	0.59680846	0.579681268	0.187407669	0.563457766	0.938924585
SGMS2	0.24160986	0.30379821	0.655041613	0.322271874	0.836225193	0.561543432	0.938924585
SLC25A34	0.319047715	0.856070879	0.254822386	0.340653683	0.549690437	0.56266128	0.938924585
SLC35F6	0.33537623	0.286030955	0.274121546	0.950676966	0.521119066	0.562583279	0.938924585
SP140	0.862631665	0.110190552	0.572425408	0.47615633	0.501393527	0.562032481	0.938924585
SPINT2	0.425401405	0.137593254	0.568974451	0.80241067	0.488990873	0.563172535	0.938924585
SPPL3	0.679752725	0.59700486	0.950250792	0.282714385	0.119218464	0.562139638	0.938924585
TBC1D10B	0.79719525	0.759903782	0.304795625	0.151926634	0.466125859	0.563298083	0.938924585
TNFAIP6	0.664107695	0.99871123	0.255465904	0.142095658	0.541563066	0.562753144	0.938924585
TNFSF13B	0.617892063	0.499351859	0.325606411	0.640690672	0.203307675	0.563451328	0.938924585
TOM1L1	0.500044004	0.728414313	0.190572203	0.397832248	0.468042959	0.561063385	0.938924585
ZBTB33	0.282301769	0.464513915	0.978928641	0.209494078	0.481777063	0.561528719	0.938924585
LOC786363	0.385811523	0.314562062	0.230911733	0.629900684	0.741864354	0.563588802	0.93899044
XKR6	0.063798309	0.770777717	0.549416083	0.626332003	0.774721609	0.563795593	0.939239618
KNOP1	0.440451374	0.666014616	0.248502976	0.838553949	0.21465474	0.563970887	0.939340935
LOC107132757	0.152339322	0.975191678	0.318808435	0.921923057	0.300456765	0.563936994	0.939340935
SBDS	0.565422089	0.447464569	0.274749815	0.585826909	0.322403995	0.564083544	0.939433229
ARHGEF28	0.109181886	0.634543738	0.271142353	0.928239166	0.753928329	0.564332587	0.93947879

DKK3	0.541631354	0.376042135	0.274943263	0.480599244	0.488760049	0.564449237	0.93947879
HMOX2	0.740555057	0.856468415	0.099454726	0.238231551	0.874215393	0.564204883	0.93947879
PKDREJ	0.399204836	0.340085822	0.683488616	0.262384573	0.5402812	0.564454383	0.93947879
PLEKHH1	0.555288433	0.998842079	0.071251921	0.668190524	0.497719682	0.564287162	0.93947879
PRDX3	0.980549468	0.65186542	0.73953566	0.038823758	0.71671995	0.564436565	0.93947879
LOC112446042	0.206959794	0.775808237	0.504337333	0.546422616	0.297385726	0.564515002	0.939484402
LOC101902204	0.268005114	0.987700176	0.597831827	0.12502608	0.665402921	0.564613712	0.9395534
ALG10	0.221415267	0.380648176	0.733035645	0.488351194	0.437027414	0.564908942	0.939800432
CARF	0.171642376	0.555595553	0.567796166	0.247045656	0.987104453	0.565183748	0.939800432
CASP2	0.2564084	0.823893104	0.38529691	0.588351131	0.2755657	0.565070187	0.939800432
LOC529125	0.963136512	0.847512085	0.724132933	0.067044677	0.333158461	0.565162042	0.939800432
PPME1	0.287871597	0.803788016	0.798231656	0.144910645	0.493051632	0.565070893	0.939800432
SMARCB1	0.211153746	0.395430887	0.332435432	0.673293951	0.706001402	0.565037572	0.939800432
TIAL1	0.226869691	0.173657998	0.617474281	0.900622272	0.602594589	0.56515798	0.939800432
TPC3	0.049371984	0.827199144	0.855284611	0.733222844	0.515657156	0.565220295	0.939800432
PSPN	0.649206297	0.266840871	0.810982678	0.527230105	0.178426849	0.565357421	0.9398649
RNF122	0.313764581	0.585474753	0.749920601	0.098254295	0.976772689	0.565430879	0.9398649
RRN3	0.63337564	0.258581648	0.6941922	0.130530329	0.890849943	0.565423039	0.9398649
UTP14A	0.085646818	0.750701547	0.413910124	0.692280853	0.717921397	0.565507012	0.93989625
ABHD14B	0.240055361	0.952148658	0.310529917	0.230328237	0.810368425	0.565820436	0.940130041
C16H1orf174	0.830537533	0.095240988	0.48044016	0.780946863	0.446393998	0.565826203	0.940130041
FBXO31	0.472484368	0.587943284	0.327387688	0.160805219	0.906526598	0.565961102	0.940130041
GSN	0.612112848	0.801319769	0.306978006	0.228943316	0.384610083	0.565973246	0.940130041
LOC100294723	0.963734343	0.907411183	0.467253521	0.387366269	0.083781417	0.566013368	0.940130041
LOC107132911	0.611042495	0.37831195	0.746911278	0.082011303	0.935307403	0.565762352	0.940130041
NUP35	0.371677103	0.559395308	0.860550081	0.411853843	0.179994456	0.566048683	0.940130041
C8H9orf85	0.883000021	0.165084463	0.262384166	0.356964457	0.973115784	0.566373505	0.940574335
ABHD1	0.762991761	0.732219914	0.104106514	0.236008897	0.974290227	0.567645619	0.94090711
ADAMTS3	0.677160315	0.910225987	0.887391082	0.027022894	0.905909401	0.567875215	0.94090711
C19H17orf49	0.297196191	0.922068411	0.662286755	0.196392827	0.373508493	0.566766598	0.94090711
C25H16orf89	0.461111453	0.827247682	0.275866032	0.129958988	0.978920075	0.56784002	0.94090711
C8H8orf58	0.898182553	0.436414923	0.527013105	0.123783518	0.521752192	0.56718182	0.94090711
CARMIL1	0.438517544	0.690212811	0.764127009	0.060770247	0.950507727	0.567434968	0.94090711
EXTL3	0.823827495	0.448955868	0.398461809	0.20379441	0.445288076	0.567647378	0.94090711
FAM172A	0.84054126	0.08008055	0.453427772	0.582774535	0.750784357	0.567358414	0.94090711
GPBAR1	0.110999086	0.615830364	0.421266762	0.830124787	0.559813221	0.567764869	0.94090711
IPO8	0.086000795	0.245710888	0.844455717	0.900971749	0.83015861	0.567254397	0.94090711
LOC101905390	0.234132732	0.316104704	0.305341607	0.722984964	0.815273955	0.566870315	0.94090711
LOC112445889	0.897921959	1	0.182681045	0.410703647	0.198153574	0.567292873	0.94090711
LOC112445972	0.272653434	0.57438982	0.18672858	0.763703741	0.599595014	0.567892567	0.94090711
LOC783378	0.140612179	0.805243273	0.325837662	0.593212795	0.609625593	0.567187287	0.94090711
MAP3K11	0.914590488	0.734234834	0.067191915	0.652495182	0.452362567	0.566839531	0.94090711
MESP2	0.116827908	0.70012736	0.500412044	0.353892701	0.922940113	0.567574716	0.94090711
MRFAP1L1	0.453006661	0.222253005	0.496758681	0.407133704	0.655362943	0.567228008	0.94090711

MYMK	0.222136329	0.197939782	0.364745405	0.895371403	0.92956515	0.567276431	0.94090711
NPRL2	0.624208669	0.857408548	0.601299478	0.062198444	0.668115325	0.567638228	0.94090711
PAXIP1	0.497783701	0.478004726	0.535684594	0.388910123	0.269296644	0.567292722	0.94090711
PTBP2	0.352679066	0.550135665	0.621638335	0.840400136	0.131692959	0.567281045	0.94090711
TTLL4	0.884430727	0.128172223	0.654551935	0.226990349	0.79498885	0.56787427	0.94090711
VPS26C	0.461585997	0.850797888	0.631464712	0.138708568	0.388565921	0.567530071	0.94090711
ALAD	0.669671849	0.578706693	0.575647847	0.272738791	0.220280213	0.568065269	0.940995995
ANKRD31	0.378361368	0.837143256	0.595789384	0.164332692	0.434628179	0.569151071	0.940995995
ATG2A	0.827611405	0.10986074	0.269145277	0.846684125	0.650465475	0.569133381	0.940995995
CATSPERE	0.9580128	0.093076921	0.234461754	0.927825115	0.691805804	0.568303542	0.940995995
CCT7	0.549040578	0.514727476	0.389644581	0.524369239	0.233486377	0.569199118	0.940995995
CHP1	0.602444276	0.953491968	0.105098257	0.915401149	0.243353156	0.568722581	0.940995995
DCAF12	0.715863771	0.783285026	0.312647289	0.278142247	0.276055614	0.568896293	0.940995995
FXYD5	0.211689005	0.871543814	0.787042689	0.382598997	0.242022902	0.568681767	0.940995995
IVD	0.654877224	0.94052512	0.847237733	0.046474551	0.556091874	0.56926502	0.940995995
KIF2A	0.809107379	0.641773483	0.298609957	0.11295778	0.767656167	0.568676876	0.940995995
LOC112442967	0.996880801	0.267907274	0.388211299	0.600454023	0.216195663	0.568875933	0.940995995
LOC783612	0.53696243	0.753008846	0.715025153	0.279921364	0.165720295	0.568188537	0.940995995
LTB4R2	0.264426189	0.66314262	0.57993854	0.269845019	0.489605107	0.568533939	0.940995995
NOP9	0.38971618	0.450667404	0.889500829	0.11675316	0.7384671	0.569021872	0.940995995
PAPD7	0.828033432	0.749361287	0.071376449	0.345368534	0.877655947	0.56837713	0.940995995
PRR3	0.205699632	0.924551547	0.414073917	0.498715398	0.343018768	0.569049647	0.940995995
PSEN1	0.846854995	0.363021908	0.073940377	0.797460523	0.743800537	0.56921693	0.940995995
SCN2B	0.09477969	0.566877771	0.488006207	0.6090097	0.841616372	0.56858496	0.940995995
SLITRK2	0.928977311	0.42060261	0.575454989	0.181885683	0.328178884	0.56833108	0.940995995
SMG6	0.504292289	0.740390898	0.320079971	0.738416058	0.152265348	0.568556348	0.940995995
SNTB2	0.673553619	0.462743505	0.4605961	0.15621521	0.601353674	0.56926	0.940995995
ZNF419	0.799034395	0.201727939	0.118475697	0.755239356	0.932211374	0.568670071	0.940995995
ZNF789	0.756895498	0.499312383	0.488565646	0.227715248	0.320443698	0.569077162	0.940995995
ATG16L2	0.441245578	0.3317769	0.790487322	0.194531595	0.599961053	0.569548544	0.941198191
CLEC7A	0.446785902	0.852256651	0.139684286	0.259850344	0.977568531	0.569616747	0.941198191
ING2	0.336084194	0.60465069	0.672978273	0.507725291	0.194423937	0.569458491	0.941198191
STC1	0.097073575	0.403266207	0.458653632	0.766151559	0.98207311	0.569593736	0.941198191
ARHGEF18	0.4509582	0.214713662	0.850893009	0.244678966	0.670768424	0.569773475	0.941212402
LZIC	0.723839326	0.102007907	0.361697838	0.714915607	0.70850093	0.569852211	0.941212402
SH2D3A	0.161267983	0.801015075	0.515794581	0.781114663	0.259750049	0.569727146	0.941212402
ZCWPW2	0.318174455	0.741446792	0.647328005	0.182061817	0.486557621	0.569854758	0.941212402
GLI2	0.385879873	0.25038558	0.626693216	0.785799486	0.284522061	0.569997715	0.941259056
PSMD4	0.572556234	0.670819724	0.542558776	0.330370235	0.196599753	0.569957899	0.941259056
ACAA2	0.645819045	0.791869626	0.111036504	0.280342165	0.850836944	0.570096037	0.941317606
TCP11L2	0.938535023	0.654910065	0.091320307	0.319240017	0.756074795	0.570147889	0.941317606
ASXL1	0.984525598	0.123711112	0.447907144	0.436858695	0.570205051	0.570732761	0.941650441
BOK	0.298951271	0.70280745	0.489387925	0.209288829	0.635658175	0.572007792	0.941650441
CNOT10	0.842520856	0.542135662	0.411007041	0.502926113	0.144457174	0.571438531	0.941650441

CTSO	0.980513425	0.639795775	0.518514485	0.285883954	0.146304681	0.570958904	0.941650441
DGKE	0.774662083	0.348863222	0.336374908	0.740553795	0.202131903	0.570993958	0.941650441
FBXO21	0.563389848	0.998855723	0.72142228	0.070360881	0.477846598	0.571589862	0.941650441
FCGR3A	0.359860443	0.725807914	0.151799124	0.91108238	0.377295254	0.571297343	0.941650441
IFNGR1	0.729555939	0.632671908	0.217449929	0.169233435	0.804726709	0.571861428	0.941650441
IMMT	0.729195778	0.7292317	0.571966032	0.172233753	0.259775388	0.571000027	0.941650441
ITGA5	0.685149294	0.45763323	0.163093326	0.470048988	0.568429301	0.571784632	0.941650441
KCNQ4	0.926013246	0.858506935	0.447036724	0.454972987	0.084537232	0.571864474	0.941650441
LOC100298774	0.388310107	0.252999746	0.77599384	0.475522565	0.376608952	0.571633374	0.941650441
LOC100848569	0.40110998	0.280772654	0.794645838	0.17009223	0.89507507	0.571240251	0.941650441
LOC101902469	0.200561995	0.745428418	0.631173001	0.382415944	0.378760193	0.571849101	0.941650441
LOC101902754	0.90792232	0.92042459	0.040758728	0.50176348	0.797677887	0.571350796	0.941650441
LOC101907383	0.353872793	0.552723837	0.261162483	0.532321584	0.500518917	0.571028757	0.941650441
LOC112443476	0.562582346	0.958610403	0.110353042	0.864230716	0.265928114	0.571984103	0.941650441
LOC112445041	0.944699387	0.228299954	0.584976778	0.245293262	0.439711927	0.570998139	0.941650441
LOC112447303	0.056916483	0.673097146	0.738361952	0.780344796	0.615537301	0.570701724	0.941650441
LOC783803	0.385457773	0.506344841	0.347382019	0.362908727	0.553366369	0.571108874	0.941650441
MOV10	0.50335548	0.049554284	0.737634966	0.80012629	0.925925007	0.571327522	0.941650441
PCDHGA8	0.697524473	0.85946877	0.426699501	0.079235117	0.673396625	0.571580614	0.941650441
PRKCE	0.077802583	0.738407483	0.72185237	0.533561911	0.616174282	0.571369827	0.941650441
SASS6	0.840019875	0.096531458	0.24300949	0.985823508	0.702078775	0.571432066	0.941650441
SAXO2	0.521659461	0.812005161	0.379757487	0.095832565	0.887374899	0.572013481	0.941650441
SLC2A1	0.693598807	0.950008944	0.027124781	0.908003873	0.841839068	0.571767054	0.941650441
TCTEX1D2	0.651966698	0.522323439	0.992787673	0.144231995	0.280428429	0.571938885	0.941650441
TPRA1	0.349913896	0.60993051	0.23817591	0.362254628	0.739613048	0.571161039	0.941650441
ZNF48	0.893410568	0.259522149	0.274227477	0.346041515	0.618443537	0.570986028	0.941650441
NCSTN	0.490981255	0.51146586	0.58939816	0.196250317	0.471129792	0.57208865	0.941673877
PSMB1	0.51658962	0.288935723	0.478542934	0.715518101	0.267838932	0.572142479	0.941673877
CACNB4	0.611666859	0.723354057	0.159094139	0.389243481	0.501274608	0.572790478	0.941731581
CHCHD8	0.923547449	0.956790971	0.580366255	0.541467513	0.049405236	0.572570765	0.941731581
DMAP1	0.385098123	0.756809771	0.928567111	0.121928527	0.416276031	0.572808765	0.941731581
LOC112446639	0.300739892	0.61079807	0.685868515	0.390243633	0.279016986	0.572558388	0.941731581
MSANTD4	0.952102222	0.850119391	0.457654865	0.358273675	0.103409922	0.572639745	0.941731581
MYF6	0.106219239	0.895462312	0.926973136	0.249490159	0.623698413	0.572580512	0.941731581
PLCH1	0.760439871	0.947425773	0.093963777	0.352772268	0.57430411	0.572518691	0.941731581
RECK	0.386946381	0.570687642	0.595974119	0.18824201	0.554431314	0.572801103	0.941731581
SLC44A1	0.676479638	0.190203287	0.42691116	0.871829265	0.286394178	0.572518237	0.941731581
TMEM52	0.481014102	0.138466902	0.392961655	0.913460421	0.574030736	0.572639809	0.941731581
TUBGCP3	0.624775379	0.183790369	0.429597032	0.418438024	0.665059434	0.572693565	0.941731581
ABCC10	0.949803177	0.111200252	0.258884404	0.871765581	0.579665668	0.573951249	0.94188748
ACP2	0.224180014	0.262662376	0.682145153	0.905537658	0.3793609	0.573687494	0.94188748
DDX27	0.839967192	0.51023712	0.703203943	0.101167062	0.453156466	0.573942521	0.94188748
ERAP1	0.949163815	0.094250462	0.724676096	0.241772879	0.88136793	0.573911523	0.94188748
HIST2H2BE	0.804241873	0.60965697	0.281100476	0.13871907	0.720909356	0.573473629	0.94188748

LOC100847951	0.126362511	0.617167658	0.278947224	0.968653701	0.653748182	0.573371984	0.94188748
LOC107132524	0.543324688	0.496376294	0.216022701	0.43882405	0.540362434	0.573916875	0.94188748
LOC112446024	0.907183347	0.170555452	0.925346292	0.610564413	0.157349641	0.573077837	0.94188748
MORN1	0.856357256	0.198586275	0.359020822	0.408566366	0.551349491	0.573056111	0.94188748
NECAP1	0.684958843	0.308749137	0.225351709	0.576120956	0.501288708	0.573198325	0.94188748
NGDN	0.835579091	0.170551067	0.981549956	0.414095997	0.238666809	0.574051464	0.94188748
NOP14	0.536322186	0.990186639	0.738873182	0.684770575	0.051327925	0.573589867	0.94188748
PNPLA2	0.384217632	0.800718803	0.256023361	0.217314437	0.805533341	0.573544191	0.94188748
PPP1R3E	0.944923279	0.138272271	0.291006018	0.4192341	0.865303049	0.573610773	0.94188748
PRKACA	0.751619386	0.584008773	0.141956626	0.265444914	0.834260849	0.5736958	0.94188748
RAB11B	0.970375753	0.531733758	0.119709759	0.513423183	0.435870273	0.574028729	0.94188748
RASGRP2	0.16879195	0.436784909	0.544134993	0.345167265	0.998148835	0.574008439	0.94188748
ROBO4	0.32598842	0.711373023	0.933489768	0.220305526	0.289399698	0.573732919	0.94188748
SCML2	0.708820332	0.996838823	0.983336971	0.034884143	0.568019985	0.573252884	0.94188748
TIGD4	0.395070706	0.838807991	0.225567441	0.904692586	0.203965658	0.573616757	0.94188748
FADS6	0.301179206	0.663284984	0.252703898	0.364559026	0.752129904	0.574296711	0.942126225
HNRNPLL	0.72579583	0.816786479	0.576244466	0.048687995	0.833659581	0.574627677	0.942126225
LACC1	0.066049913	0.6300839	0.572540371	0.900162725	0.646129624	0.574528291	0.942126225
LOC107133048	0.172438116	0.954530772	0.336653096	0.913043134	0.274161253	0.574700226	0.942126225
LOC112444276	0.868891944	0.487155739	0.197347496	0.286416845	0.579605042	0.574652419	0.942126225
PREX2	0.318043108	0.734514715	0.760362976	0.259005296	0.301090457	0.574437222	0.942126225
RTN2	0.623865673	0.587182937	0.19885536	0.496582293	0.383477816	0.574713645	0.942126225
UGGT1	0.695332864	0.497370613	0.449352409	0.981523619	0.090771042	0.574345833	0.942126225
ZNHIT1	0.234764396	0.565622673	0.441114263	0.243254414	0.972248834	0.574452918	0.942126225
LOC104969378	0.447212237	0.747944189	0.282281444	0.888599447	0.165557981	0.57497577	0.942461781
PAPPA	0.502136553	0.368562169	0.338264326	0.513819052	0.432142534	0.57511157	0.942590231
LOC784808	0.197986564	0.162946301	0.987697599	0.5950676	0.733568414	0.575238683	0.942704416
ADAMTSL2	0.896766231	0.063653338	0.844455284	0.402865837	0.716852046	0.575397347	0.942870281
GMIP	0.170721312	0.807233044	0.974804448	0.289950686	0.357801957	0.575622033	0.94297429
TJAP1	0.407030625	0.140755202	0.56447255	0.478297913	0.901079716	0.575633199	0.94297429
TSGA10IP	0.819096358	0.189104741	0.354330338	0.987950773	0.256929514	0.575543577	0.94297429
DDX24	0.894972219	0.233183604	0.413046359	0.581465078	0.278399058	0.575856889	0.94316589
LOC101908034	0.753856174	0.338138724	0.533077491	0.314653493	0.326369733	0.575865104	0.94316589
HIP1R	0.812253065	0.599136324	0.046611529	0.637863149	0.965335144	0.576043375	0.943269589
LOC112446457	0.831571622	0.057359509	0.748556907	0.682528974	0.572986208	0.575989502	0.943269589
SRSF2	0.619328682	0.755880879	0.433256051	0.212938574	0.323588703	0.576155256	0.943358665
ADCY6	0.61117276	0.816680698	0.494992713	0.107161919	0.528574678	0.576420614	0.943698991
CWC15	0.83530437	0.166411308	0.199343064	0.787779307	0.641344535	0.5764932	0.943723682
TSC22D4	0.930711461	0.445374168	0.57890203	0.105260855	0.554856174	0.576701832	0.943971052
ATP5S	0.69072855	0.898356822	0.105162593	0.405023854	0.530840295	0.576911272	0.944077404
NEK7	0.699153383	0.155609601	0.353601632	0.903375214	0.40382892	0.576967766	0.944077404
NHEJ1	0.449194041	0.615006279	0.948864931	0.375921186	0.142404695	0.576945615	0.944077404
PCDH7	0.389457786	0.5922561	0.264676032	0.271063766	0.848703453	0.577111969	0.944077404
PSME1	0.311691731	0.72184285	0.343443939	0.80631494	0.22535786	0.577062558	0.944077404

ZC3H8	0.808575207	0.63330511	0.635716923	0.145942058	0.295551353	0.577066746	0.944077404
NR2F1	0.434424322	0.95942962	0.174018995	0.272230879	0.71153202	0.577176095	0.944088199
PLA2G16	0.861752138	0.155564984	0.357363204	0.335909866	0.873411483	0.577260835	0.944132705
FGGY	0.285820047	0.755117391	0.072429962	0.998489887	0.903201984	0.577844491	0.94440559
GGNBP2	0.410757073	0.346453317	0.246836846	0.846641147	0.474309321	0.577954618	0.94440559
GKAP1	0.750536727	0.490023047	0.13831219	0.526981526	0.525529871	0.577705172	0.94440559
GPLD1	0.223548096	0.756761899	0.322623864	0.60708674	0.424789216	0.57753084	0.94440559
LOC100848991	0.209708902	0.402273368	0.538005624	0.408598665	0.760017971	0.577796214	0.94440559
LOC112444763	0.768565273	0.504593032	0.621487563	0.915534627	0.063868369	0.57778251	0.94440559
PHF23	0.988406041	0.246046439	0.965883524	0.089754577	0.669018599	0.577941785	0.94440559
SLC35F5	0.562381194	0.921075625	0.101777996	0.468311443	0.571471826	0.578003153	0.94440559
SNX21	0.549211904	0.787160181	0.624328971	0.070147038	0.743987394	0.577682755	0.94440559
SNX25	0.642771196	0.497703683	0.198826609	0.312622811	0.708327898	0.57766728	0.94440559
LOC107131619	0.307762835	0.222224955	0.661728534	0.935828283	0.333463904	0.578192475	0.944438795
NOP53	0.395529863	0.404093878	0.289667001	0.463142167	0.658433091	0.578126741	0.944438795
RMND5B	0.155672798	0.274360165	0.778833958	0.555462726	0.764378476	0.578196123	0.944438795
NRADD	0.890488494	0.118700557	0.490982891	0.482969626	0.563700105	0.578273466	0.944469361
VPS37C	0.225196577	0.22125284	0.533104675	0.702498847	0.757413718	0.578329938	0.944469361
ANKAR	0.210413491	0.135698281	0.692558894	0.882724964	0.817413979	0.580173526	0.944472546
APAF1	0.643710731	0.94847871	0.481655307	0.095980112	0.504217621	0.579674315	0.944472546
ARPC1A	0.938647243	0.360250214	0.37984286	0.396350453	0.278878898	0.57920635	0.944472546
C26H10orf143	0.461735843	0.441130632	0.678117171	0.946493914	0.108747752	0.579473296	0.944472546
CDKN2D	0.651512402	0.840927261	0.781810365	0.296524808	0.112189199	0.579915691	0.944472546
COG6	0.225806159	0.350189996	0.924429757	0.273794275	0.707184347	0.578610423	0.944472546
DLG1	0.825566782	0.41930761	0.979396765	0.085314356	0.49261218	0.579905093	0.944472546
DVL1	0.316953805	0.739871148	0.576290735	0.150487154	0.701022773	0.58001866	0.944472546
FAM151A	0.471790233	0.832824059	0.10883544	0.646325685	0.51557229	0.579924132	0.944472546
HMG20A	0.604509314	0.093117703	0.401664177	0.733670672	0.858135587	0.579719613	0.944472546
IFITM3	0.29469281	0.307525887	0.255195774	0.675096464	0.913705151	0.580139813	0.944472546
IKZF2	0.581893236	0.258695371	0.437895869	0.86906397	0.248746769	0.579923453	0.944472546
LOC100851323	0.376942998	0.166197223	0.399849635	0.938680221	0.605191991	0.579653346	0.944472546
LOC101901950	0.654734448	0.412273377	0.854418398	0.935300276	0.065987664	0.579710444	0.944472546
LOC101906021	0.924765852	0.123780848	0.577394937	0.694029221	0.30962081	0.57927798	0.944472546
LOC104974934	0.564591613	0.236720614	0.605223596	0.649636084	0.269921927	0.579022911	0.944472546
LOC112442687	0.445353599	0.544605507	0.295933556	0.363935655	0.542669861	0.578909925	0.944472546
LOC784087	0.937079595	0.138526068	0.878595898	0.42227593	0.295216525	0.579486908	0.944472546
MAP3K10	0.273333974	0.504744865	0.562637806	0.23736088	0.772990964	0.579818977	0.944472546
MLLT11	0.793171682	0.408412519	0.865296979	0.148960517	0.341537595	0.580070392	0.944472546
MYLK2	0.382324839	0.306908905	0.459743918	0.301984651	0.869803735	0.578830758	0.944472546
NHLRC3	0.108204333	0.671962105	0.778719702	0.331621978	0.758353773	0.579777797	0.944472546
PCYT1B	0.939708614	0.145561818	0.224850151	0.824740556	0.560343099	0.579429362	0.944472546
PLCXD3	0.220656276	0.634450806	0.346142463	0.357208476	0.822611601	0.579777774	0.944472546
RPS6KA2	0.505859326	0.893776165	0.465799125	0.117821355	0.572374243	0.579275831	0.944472546
RTF2	0.734663443	0.295242165	0.693958705	0.118683575	0.793372821	0.578877282	0.944472546

SRSF4	0.934912007	0.372218413	0.480959098	0.400134183	0.211497449	0.578751935	0.944472546
TFIP11	0.747027523	0.895257052	0.731267307	0.422668411	0.068745391	0.57938412	0.944472546
THOC6	0.805379709	0.188118507	0.595539386	0.262083903	0.599884428	0.579047894	0.944472546
THSD7A	0.148467915	0.856172681	0.343708682	0.357872895	0.911739275	0.580000219	0.944472546
TMEM184A	0.816344938	0.173379308	0.375180634	0.315775194	0.8433863	0.57845222	0.944472546
VIT	0.315520199	0.603363773	0.815038468	0.095599502	0.958860036	0.579558869	0.944472546
BBS12	0.993559885	0.363560637	0.119526477	0.637802307	0.521603677	0.581463985	0.944476598
CCDC115	0.502402791	0.885410221	0.555748139	0.190389504	0.305421203	0.581622567	0.944476598
CRTC1	0.5249777	0.521744284	0.095004626	0.980860029	0.563142155	0.581600949	0.944476598
DNAJC24	0.734314067	0.731234889	0.564567138	0.114209393	0.413645754	0.580893636	0.944476598
EIF5	0.685932926	0.31796179	0.224697566	0.787114	0.372338079	0.581449821	0.944476598
EPHX1	0.236526603	0.447778546	0.172451003	0.915280256	0.857869752	0.581161471	0.944476598
ETS2	0.745032796	0.160359687	0.244601429	0.495568326	0.987440225	0.580607469	0.944476598
GPR63	0.42275379	0.773799679	0.322804217	0.220471707	0.614985949	0.580843872	0.944476598
IBTK	0.89830879	0.296721221	0.16740667	0.944326752	0.340167885	0.581062395	0.944476598
ITPR3	0.819005714	0.114958084	0.250956071	0.76681602	0.791084198	0.581051419	0.944476598
LOC101902705	0.606176094	0.866967384	0.498465597	0.366202052	0.149889326	0.581672352	0.944476598
LOC104975222	0.910268426	0.588064407	0.332421541	0.764496555	0.105333778	0.581001705	0.944476598
LOC107132783	0.257057784	0.256275295	0.724091931	0.621796526	0.481428491	0.580324971	0.944476598
LOC107132971	0.978908426	0.772126158	0.166609408	0.113932586	0.995156991	0.580306271	0.944476598
LOC112448368	0.439217798	0.072214577	0.874739026	0.659221194	0.785892137	0.58160498	0.944476598
LOC520336	0.826591107	0.169899909	0.32600295	0.900613326	0.346594876	0.580483319	0.944476598
LOC616281	0.611900637	0.208244703	0.789817871	0.241780857	0.587563411	0.580569309	0.944476598
LOC784521	0.480163946	0.604735064	0.293474623	0.967700297	0.173718336	0.580950372	0.944476598
MAP1B	0.47207016	0.796755994	0.050725309	0.991505345	0.757351923	0.580967163	0.944476598
MTHFD2L	0.680630648	0.681267024	0.334324001	0.30077071	0.308053721	0.581462498	0.944476598
PHKA2	0.577604195	0.89984059	0.781977329	0.217296348	0.161813363	0.580479291	0.944476598
RCBTB1	0.909794352	0.449742455	0.838324245	0.156876857	0.266973663	0.581501816	0.944476598
SLC40A1	0.323128417	0.307982089	0.535568288	0.888745605	0.301858836	0.580586432	0.944476598
TMEM19	0.801802452	0.471353731	0.375890174	0.538136958	0.187918622	0.581498016	0.944476598
TOR4A	0.766996476	0.916663804	0.843439728	0.034802846	0.695306188	0.581279343	0.944476598
TPRG1	0.055560884	0.975778553	0.330334405	0.953999631	0.841526277	0.581655324	0.944476598
CD200	0.779397609	0.36077856	0.268936661	0.899427342	0.21184966	0.582081291	0.944600876
FRS2	0.489135386	0.289623546	0.715116503	0.274587496	0.518407193	0.582236704	0.944600876
IMPACT	0.446665591	0.805044958	0.118329256	0.433335016	0.783130404	0.5824874	0.944600876
LOC101904642	0.788144021	0.686632292	0.065629784	0.840787994	0.482329507	0.581999743	0.944600876
LOC101905199	0.934522295	0.691467853	0.326363809	0.760399259	0.089828148	0.582025058	0.944600876
LPXN	0.257921309	0.905022984	0.695925123	0.495977055	0.178835577	0.582072891	0.944600876
MINDY1	0.18006947	0.46092644	0.274414243	0.937000582	0.676019032	0.582319632	0.944600876
NENF	0.649597953	0.212331089	0.708911857	0.991976121	0.148614977	0.582157514	0.944600876
PWP1	0.662438323	0.234629861	0.498269438	0.839448795	0.221876792	0.582284752	0.944600876
SYT7	0.809149058	0.206346706	0.571592516	0.652769867	0.231787028	0.582491508	0.944600876
TIMELESS	0.843953292	0.298652715	0.536495257	0.724556184	0.147329164	0.582424366	0.944600876
WSB1	0.828097041	0.391093914	0.342307762	0.494548405	0.26331692	0.582448963	0.944600876

ZMYM1	0.303668897	0.81008975	0.2269903	0.546952114	0.472807573	0.582497158	0.944600876
MAP6	0.747883375	0.992788322	0.438209285	0.127038872	0.349621597	0.58264573	0.944655116
TMEM249	0.73843216	0.782233972	0.611579754	0.186327608	0.219495787	0.58259961	0.944655116
LOC104975977	0.921325943	0.192845703	0.167078149	0.796512151	0.611370174	0.582704971	0.944657837
SEPSECS	0.646205116	0.312891738	0.417690859	0.440374195	0.388832842	0.582778558	0.944683812
APCDD1	0.826703627	0.35318923	0.351599408	0.144829897	0.974018582	0.583058573	0.944884038
DNAJC4	0.969054933	0.204027848	0.406509171	0.267736478	0.673742048	0.583272408	0.944884038
INHBA	0.152433535	0.884744776	0.322435608	0.418611725	0.796811915	0.583361494	0.944884038
NUBP1	0.296705714	0.698514828	0.856609051	0.235491339	0.346838121	0.583306626	0.944884038
PAM16	0.451161836	0.562118209	0.961516842	0.150771233	0.394406475	0.583303707	0.944884038
PCNP	0.957556253	0.158316199	0.289725192	0.356689191	0.925860479	0.583362688	0.944884038
TLR5	0.468897771	0.356246786	0.149182405	0.660749238	0.880672581	0.58331195	0.944884038
ZAP70	0.637544855	0.93621267	0.512870466	0.576148371	0.082106763	0.583047545	0.944884038
MAP1LC3A	0.913970867	0.702520495	0.43247512	0.080668327	0.647941514	0.583487795	0.944900159
PAOX	0.737821117	0.404397865	0.216791531	0.449669736	0.498937716	0.583465853	0.944900159
B4GALT2	0.242647445	0.807912342	0.099060384	0.766763126	0.975358602	0.583610334	0.944975834
RDM1	0.568726812	0.06122835	0.671217461	0.721505782	0.861378926	0.58364969	0.944975834
ZNF112	0.374023022	0.977813927	0.473837998	0.293806997	0.285417785	0.583727477	0.945008546
ARHGEF25	0.844772538	0.821291578	0.085912046	0.759896875	0.321002782	0.583828969	0.945035234
GPR156	0.587551047	0.532673781	0.0810108	0.775781626	0.73967679	0.583952627	0.945035234
MST1R	0.64230125	0.706655234	0.818106275	0.066267616	0.591223227	0.583942605	0.945035234
NAPRT	0.70193578	0.337924348	0.73205144	0.110015674	0.761669904	0.583974304	0.945035234
KCNAB1	0.822297103	0.18613877	0.575048373	0.17231422	0.96083052	0.584268464	0.945233029
KLHDC4	0.133675448	0.249268999	0.487324579	0.972191547	0.923375994	0.58432692	0.945233029
NUP205	0.566894112	0.942978377	0.802613575	0.498183627	0.068138875	0.584159706	0.945233029
POC1B	0.694729619	0.588621465	0.817409294	0.304179946	0.143365994	0.584326722	0.945233029
HRASLS	0.844417916	0.42068355	0.863318164	0.290094239	0.163949383	0.584444977	0.94527195
RPL15	0.355782198	0.49136593	0.406463406	0.458037053	0.4481969	0.58446618	0.94527195
ARHGAP21	0.906925662	0.618165982	0.770425587	0.12900242	0.262105034	0.584689955	0.945354369
CNEP1R1	0.775175356	0.072462991	0.741382988	0.604827298	0.579669626	0.584640797	0.945354369
DAP	0.688029309	0.185722576	0.650670273	0.181118443	0.969779524	0.584684402	0.945354369
LOC104973848	0.632380262	0.750779849	0.102542682	0.339599896	0.883620001	0.584756107	0.945368188
PFAS	0.704109891	0.161594321	0.577322078	0.669265069	0.332693412	0.584978938	0.945542141
ZNF45	0.372009536	0.929087458	0.720830065	0.712369639	0.082392861	0.58493936	0.945542141
ABHD17A	0.564091549	0.472428718	0.741360096	0.246576718	0.302245932	0.586276046	0.945583722
ALDH1L1	0.099552724	0.543554859	0.301471451	0.999096933	0.913942675	0.58853171	0.945583722
AMFR	0.614562983	0.153817783	0.259003717	0.749214274	0.802797285	0.58630434	0.945583722
AQR	0.512529163	0.870289611	0.868084789	0.082589895	0.463139641	0.587419341	0.945583722
C2CD3	0.192593863	0.304800082	0.861084854	0.29469137	0.989993754	0.58657829	0.945583722
C5H12orf73	0.57709752	0.428353519	0.991444108	0.106629018	0.568376253	0.587977619	0.945583722
CCDC66	0.98667607	0.063733333	0.982351873	0.313826388	0.76346968	0.587287878	0.945583722
CCM2L	0.242752857	0.248881889	0.471131616	0.580195249	0.900442115	0.588200798	0.945583722
CDADC1	0.636648297	0.257667067	0.259887553	0.467305315	0.735681761	0.585384771	0.945583722
CHMP2B	0.490332913	0.43792194	0.13146111	0.665454312	0.786359937	0.586900956	0.945583722

CMPK1	0.601444142	0.73645434	0.603393511	0.476401983	0.115421042	0.585906033	0.945583722
CPNE2	0.737669295	0.417845839	0.131693914	0.552967209	0.65970289	0.587378119	0.945583722
CTSK	0.224716159	0.474877024	0.723202901	0.493199856	0.386193061	0.58595197	0.945583722
CUL4B	0.406948771	0.914882893	0.483774638	0.416532748	0.195910487	0.585930572	0.945583722
DPYD	0.711759749	0.604295036	0.048585988	0.811538587	0.869601647	0.586587089	0.945583722
EDRF1	0.142287489	0.968312565	0.920470013	0.165863661	0.705309529	0.587750226	0.945583722
ERFE	0.600671571	0.294873394	0.985176719	0.306405618	0.277641491	0.587859838	0.945583722
FBXL15	0.65347723	0.970747028	0.628364374	0.050038666	0.743833769	0.587753255	0.945583722
FOXRED2	0.741873724	0.597422133	0.508718918	0.125669922	0.525991586	0.588634654	0.945583722
GAN	0.129587306	0.802223969	0.431102322	0.340101987	0.97410858	0.58789881	0.945583722
GCNT3	0.821437061	0.50420701	0.207037214	0.772086276	0.224819401	0.588380403	0.945583722
GNAI2	0.736030322	0.230100463	0.551763178	0.476160012	0.334253768	0.588230012	0.945583722
GOLPH3L	0.96589798	0.910781829	0.074811916	0.325042852	0.696609471	0.588610961	0.945583722
HIVEP1	0.421302138	0.82522707	0.592483576	0.694921151	0.103009328	0.586556902	0.945583722
HLCS	0.757808296	0.61795473	0.334296302	0.296738396	0.315192008	0.585188848	0.945583722
HMGN4	0.461701836	0.259743279	0.901486014	0.473807124	0.290305236	0.588196997	0.945583722
HNRNPL	0.061635812	0.887966501	0.387594646	0.827880416	0.847556546	0.588386023	0.945583722
HPS4	0.339414347	0.760267611	0.093182067	0.622738505	0.988706505	0.587338247	0.945583722
HS3ST1	0.716611966	0.183876804	0.543243353	0.925321465	0.224726673	0.588390014	0.945583722
IDO1	0.965291982	0.428515829	0.130900547	0.337337481	0.806339209	0.586330516	0.945583722
KIAA0319L	0.867291958	0.062573338	0.706250229	0.643321829	0.603278401	0.588258726	0.945583722
KLF6	0.916475611	0.616975099	0.198974049	0.155360402	0.849406623	0.587892532	0.945583722
LOC101903385	0.778216689	0.230219968	0.682283054	0.216983354	0.553256606	0.585620189	0.945583722
LOC104970628	0.425243719	0.717662879	0.188374656	0.274317262	0.932134588	0.585956262	0.945583722
LOC104971266	0.968302201	0.42246789	0.950817281	0.406456994	0.093418058	0.586866268	0.945583722
LOC112442805	0.741385067	0.080176904	0.615585768	0.477140727	0.846508525	0.587006012	0.945583722
LOC112442849	0.578923292	0.820708398	0.788791085	0.687948009	0.057187809	0.586545914	0.945583722
LOC112446021	0.948154705	0.825394313	0.087738313	0.368252093	0.582753083	0.586424604	0.945583722
LOC112447085	0.448661422	0.591207849	0.332856673	0.880321226	0.191371726	0.588247908	0.945583722
LRP8	0.301009211	0.57270768	0.234382628	0.714725383	0.513653867	0.587716691	0.945583722
MAP9	0.901568846	0.456813707	0.715758484	0.102849543	0.488707876	0.587496671	0.945583722
MARCH1	0.79842483	0.99828032	0.375821712	0.29382795	0.168009677	0.587111726	0.945583722
MLXIP	0.668064973	0.742300234	0.360643586	0.23549013	0.351032008	0.587067046	0.945583722
NAGA	0.400539588	0.829947734	0.529669637	0.274013386	0.303784062	0.585386267	0.945583722
NIT1	0.776664466	0.951100273	0.304173613	0.097402558	0.677946359	0.587760986	0.945583722
PABPC5	0.089890393	0.753200061	0.368366552	0.821098395	0.718603696	0.586169081	0.945583722
PBX1	0.652588599	0.775873929	0.102429032	0.46357771	0.618451072	0.588179203	0.945583722
PDLIM2	0.480510586	0.605963658	0.613548442	0.099590553	0.835907914	0.588220987	0.945583722
PIK3AP1	0.596078778	0.452211519	0.635899434	0.34281258	0.251062254	0.586653683	0.945583722
POLR2G	0.250671872	0.444868394	0.895362512	0.524461356	0.282602698	0.587259382	0.945583722
PRKAB1	0.889805723	0.11587575	0.295765048	0.750906558	0.647373056	0.587594605	0.945583722
RNPC3	0.698908107	0.29714728	0.757165602	0.482608625	0.193662644	0.585916469	0.945583722
SLC6A14	0.297691114	0.119058643	0.555338834	0.794872335	0.93532444	0.58507616	0.945583722
SNHG3	0.918493723	0.426544253	0.200340915	0.240311809	0.779196859	0.585920315	0.945583722

SNX3	0.49543178	0.242083368	0.532922789	0.286017177	0.813600814	0.588239611	0.945583722
SYNJ1	0.613617782	0.461720905	0.264930252	0.490682622	0.404460594	0.588539145	0.945583722
TBCB	0.259631262	0.896815299	0.761639584	0.152931823	0.544672865	0.586909204	0.945583722
TMEM164	0.910145817	0.761699167	0.259600058	0.98611168	0.083295602	0.587045339	0.945583722
TRAPPC2L	0.464518296	0.609358213	0.246337596	0.307097648	0.685476052	0.585671093	0.945583722
TTC37	0.943381639	0.643792573	0.787721563	0.371031752	0.083906396	0.588506179	0.945583722
WARS2	0.931328934	0.215928978	0.420608331	0.173889295	0.996417826	0.585372046	0.945583722
XRRA1	0.550677648	0.264043686	0.260157905	0.855473676	0.457073933	0.587159842	0.945583722
YLPM1	0.579270401	0.662354236	0.202412477	0.1905282	0.998567735	0.586955113	0.945583722
HPS5	0.678420867	0.643210656	0.421528693	0.133449922	0.607759144	0.588827133	0.945627747
KLHL23	0.225766098	0.554446139	0.378462421	0.99890377	0.315175256	0.588776659	0.945627747
ST3GAL4	0.630158943	0.275745528	0.715255512	0.881394132	0.136193648	0.588834924	0.945627747
HAUS7	0.428952757	0.679066705	0.349443329	0.227690596	0.644269232	0.588997992	0.945704534
IER5	0.685832155	0.523103578	0.148323321	0.630351529	0.445154729	0.588996935	0.945704534
LOC782566	0.357879572	0.588942233	0.127872043	0.610114577	0.908359043	0.589062004	0.945714787
ABHD4	0.770723492	0.913335136	0.289197586	0.550112315	0.133432886	0.589144146	0.94575414
BANP	0.847665624	0.589023693	0.601353289	0.189869701	0.262413566	0.58936406	0.94580165
DSB	0.333191545	0.382481234	0.678074655	0.240890108	0.718205316	0.589238825	0.94580165
LOC107131848	0.915986418	0.103826213	0.389712964	0.980023598	0.411983508	0.589421487	0.94580165
SPRED1	0.619682158	0.394998749	0.625819311	0.266203406	0.367045812	0.589461902	0.94580165
TESK1	0.936514018	0.878042115	0.2413972	0.778322443	0.096802417	0.589309735	0.94580165
ABCG2	0.551314106	0.647930925	0.523518015	0.769021653	0.104268183	0.589821042	0.945808303
ANKRD46	0.616523051	0.312675364	0.459265741	0.45534078	0.37354368	0.590642679	0.945808303
ARHGEF37	0.464586959	0.442944464	0.801765504	0.180350367	0.505727184	0.590513731	0.945808303
ASMT	0.476834322	0.486839699	0.664238702	0.265139254	0.368153614	0.590551244	0.945808303
ATAT1	0.735462218	0.906690298	0.261396095	0.668647749	0.129123074	0.590523889	0.945808303
CARHSP1	0.416232966	0.854160572	0.964549193	0.159910464	0.27435307	0.590464522	0.945808303
CEP162	0.805246666	0.679700316	0.146379695	0.252603571	0.744008637	0.590624649	0.945808303
CORIN	0.43205989	0.889143519	0.644361234	0.116943685	0.518519188	0.590016686	0.945808303
DARS2	0.899860984	0.6545847	0.209345323	0.130399033	0.932531955	0.589817822	0.945808303
FEN1	0.411021308	0.793105142	0.69434314	0.402467577	0.165210374	0.590531854	0.945808303
GAREM2	0.364953955	0.080052942	0.744334635	0.827910736	0.831848326	0.589579949	0.945808303
GPCPD1	0.596422073	0.565991527	0.600251392	0.742786421	0.099538355	0.589642606	0.945808303
LOC104970852	0.499523417	0.925291555	0.052207722	0.664016547	0.936389538	0.589935125	0.945808303
MCTP2	0.708647472	0.64505171	0.317567154	0.835042986	0.123833859	0.590026427	0.945808303
MNF1	0.272444433	0.975110007	0.477076835	0.225992674	0.523032178	0.589638766	0.945808303
MOB2	0.897425184	0.358780659	0.890690606	0.270406222	0.194217923	0.590676333	0.945808303
NAA60	0.661761038	0.771734616	0.877133727	0.040263504	0.831725404	0.589900148	0.945808303
NTMT1	0.740446578	0.855742965	0.186904483	0.210530057	0.604072162	0.590676146	0.945808303
PYURF	0.221935897	0.670499068	0.950200387	0.391581119	0.271523809	0.590322818	0.945808303
SLC25A37	0.983305266	0.287430948	0.240426749	0.378969608	0.583755851	0.590309256	0.945808303
USP9X	0.441935669	0.204374482	0.360793736	0.724874318	0.637383458	0.59060831	0.945808303
COQ2	0.621266976	0.45285219	0.379156609	0.144396234	0.980969625	0.591305345	0.946261533
CYP3A5	0.654156868	0.777411238	0.11944728	0.425932635	0.583394918	0.591102814	0.946261533

EPS15	0.745326013	0.759064333	0.672558992	0.039716488	0.998379403	0.591017351	0.946261533
LOC615183	0.089737323	0.595312549	0.942143949	0.486374913	0.616841279	0.591177807	0.946261533
MARK3	0.600809141	0.681641082	0.820261916	0.07142831	0.629677327	0.591291226	0.946261533
TSC2	0.599179104	0.031371347	0.996928349	0.905075171	0.890635828	0.591248821	0.946261533
SEMA7A	0.811988181	0.384204334	0.364609355	0.185323371	0.717449716	0.591482977	0.946453505
RAB1B	0.572052326	0.653294426	0.207888746	0.343370689	0.567622744	0.5917236	0.946746222
IMMP1L	0.729430761	0.609266604	0.94073875	0.069629948	0.520700661	0.591923351	0.946757877
LOC104975004	0.341346338	0.511354332	0.293783451	0.933306409	0.316867504	0.592013733	0.946757877
MFSD5	0.924107904	0.624014911	0.486430496	0.352476153	0.153309808	0.591920417	0.946757877
PI3	0.924240648	0.123784411	0.597704276	0.664792647	0.333607179	0.592019337	0.946757877
YIF1A	0.582353515	0.456014719	0.881168467	0.104648668	0.618811729	0.591866053	0.946757877
ZFP3	0.615086497	0.083341093	0.380527	0.80135283	0.970672473	0.592117151	0.946822037
HIST3H2A	0.596029215	0.058191126	0.782941287	0.81340088	0.687988137	0.592413566	0.947091546
LOC518080	0.471420442	0.533146937	0.248392222	0.53015914	0.459255678	0.592465091	0.947091546
SLC35A1	0.253507285	0.521074015	0.796898621	0.226975043	0.636352736	0.592516538	0.947091546
TBC1D16	0.384739044	0.882993809	0.373511938	0.368999898	0.324651533	0.592473154	0.947091546
ACP6	0.346006845	0.334964762	0.765851183	0.530075974	0.324867781	0.593548765	0.947139038
ARSE	0.162353505	0.784724441	0.345189667	0.392989644	0.881716142	0.592954476	0.947139038
BTBD8	0.392925669	0.281893886	0.304645291	0.907524864	0.501609386	0.594508514	0.947139038
C1H21orf62	0.082554013	0.923812409	0.784418465	0.478275419	0.535428508	0.59398627	0.947139038
CCDC142	0.61615503	0.112534526	0.899815493	0.408874037	0.597541986	0.593017502	0.947139038
CLCN2	0.540792161	0.184368602	0.433651894	0.611412166	0.580609589	0.594357112	0.947139038
DCX	0.423070911	0.228206178	0.227912049	0.70678409	0.983554942	0.593692272	0.947139038
DNAJC10	0.495704876	0.815947961	0.394873043	0.231383585	0.412465051	0.593006583	0.947139038
EED	0.185471291	0.233639383	0.655003083	0.552790943	0.977405461	0.594189011	0.947139038
EPS15L1	0.562063963	0.686440866	0.90499735	0.069240729	0.631792786	0.593414127	0.947139038
FAM167B	0.821390246	0.684034417	0.058637857	0.554911653	0.835598003	0.593438481	0.947139038
GRINA	0.658807612	0.939770552	0.266257484	0.327588535	0.283251888	0.59368803	0.947139038
HTR4	0.625180417	0.698836005	0.198957404	0.206135853	0.855296348	0.594058664	0.947139038
KCNN3	0.968313939	0.566976463	0.125738518	0.965756651	0.229296533	0.593567488	0.947139038
LOC101904239	0.412788162	0.298786122	0.477233272	0.408531288	0.63419392	0.593097498	0.947139038
LOC112445939	0.914043114	0.743332866	0.86898409	0.034464223	0.754362611	0.594372172	0.947139038
LOC112446012	0.854038534	0.206244702	0.36813814	0.244795685	0.958911181	0.592733475	0.947139038
LOC112448848	0.098629896	0.749570537	0.588194927	0.615672041	0.571486884	0.593739239	0.947139038
LOC515418	0.293560239	0.151018142	0.385503061	0.917402524	0.979283097	0.594422316	0.947139038
LOC785693	0.513180537	0.839658968	0.226604917	0.269637403	0.582039204	0.594042493	0.947139038
MARK2	0.448624032	0.651045201	0.956056854	0.30967015	0.176642039	0.593413383	0.947139038
NDUFB9	0.41102522	0.890723556	0.944416676	0.061147029	0.726505994	0.594497266	0.947139038
PRPF4	0.789949757	0.861700118	0.230694005	0.187330374	0.521663698	0.594318513	0.947139038
SLC26A6	0.343296232	0.184538095	0.82481651	0.782758842	0.374366587	0.593891332	0.947139038
SLC30A1	0.62848454	0.645754479	0.241050238	0.204292324	0.768498012	0.594485959	0.947139038
SMTNL1	0.410609058	0.441257919	0.761394739	0.199641217	0.556407032	0.594042009	0.947139038
SS18L2	0.556226476	0.188175979	0.284166761	0.747307263	0.686491785	0.593213043	0.947139038
TAF15	0.629538866	0.905878539	0.814143777	0.435036062	0.075743093	0.593723049	0.947139038

TMEM86A	0.53278261	0.534405925	0.314581537	0.38944675	0.437556531	0.593263727	0.947139038
WFIKKN1	0.797839606	0.5992782	0.126394822	0.333327902	0.759996175	0.593855013	0.947139038
XAF1	0.903915581	0.088912472	0.622403941	0.348984703	0.879064901	0.594318278	0.947139038
YOD1	0.350092729	0.926580524	0.592387432	0.120582247	0.662170768	0.594289259	0.947139038
ZBTB12	0.980213259	0.55661258	0.060189297	0.560462616	0.832403196	0.593996981	0.947139038
ZNF32	0.76437526	0.559670573	0.084605281	0.581734794	0.726972353	0.593820544	0.947139038
BUD13	0.925758339	0.099400677	0.773388458	0.300960617	0.717851049	0.594694036	0.947342634
CPNE8	0.39111323	0.628798453	0.662682356	0.165598634	0.570066504	0.594816383	0.947445566
FAAP20	0.188403711	0.814945559	0.902591087	0.138822821	0.800196544	0.594935143	0.947542763
TMEM151A	0.337080351	0.139412808	0.685551041	0.526347712	0.908587109	0.595091385	0.947699633
UBE2J2	0.747211465	0.407048863	0.362049416	0.780314667	0.179500082	0.595305247	0.947948225
ARL10	0.820343153	0.587045443	0.195098229	0.71655144	0.229559652	0.595695762	0.948202052
HSD3B7	0.605055593	0.869355544	0.06950139	0.792290488	0.533210866	0.595564013	0.948202052
MMRN1	0.266227072	0.976055207	0.093269257	0.707559109	0.901063927	0.59565986	0.948202052
TRADD	0.455877496	0.673499848	0.819421289	0.383332972	0.160197046	0.595633024	0.948202052
B4GALT6	0.490043226	0.220279581	0.231023596	0.792800736	0.782573333	0.595915657	0.948216225
LOC100848886	0.637667445	0.930171313	0.449559069	0.07283203	0.796388749	0.595842775	0.948216225
NIPSNAP3A	0.481263815	0.788374085	0.619725047	0.202616304	0.324795473	0.595935783	0.948216225
THAP4	0.223183536	0.863233165	0.740936386	0.204877898	0.528929458	0.595875311	0.948216225
APTX	0.432204621	0.614181253	0.880211927	0.096399057	0.687295403	0.596021177	0.948260158
SNX18	0.950457284	0.335956696	0.322833532	0.171986994	0.873892657	0.596182032	0.94842413
BRAF	0.766367875	0.575008561	0.997304078	0.132929181	0.265690746	0.596535191	0.948481201
CPE	0.705900421	0.977910809	0.045281388	0.871398045	0.570270012	0.59668027	0.948481201
DVL2	0.880742807	0.267292318	0.372313608	0.259732578	0.681595877	0.596473284	0.948481201
GRID2	0.130486492	0.931690075	0.713288516	0.4920888	0.363902887	0.596622749	0.948481201
HDAC11	0.615486321	0.424455144	0.569191733	0.154951238	0.673021334	0.596354383	0.948481201
KIF25	0.318909621	0.521431814	0.772796372	0.246346024	0.489936714	0.596391636	0.948481201
MAPK14	0.632174828	0.658349486	0.82188506	0.145844459	0.311186934	0.596571115	0.948481201
PELI3	0.346713863	0.481760838	0.386968645	0.24756293	0.969013422	0.596336993	0.948481201
ADAP1	0.375954903	0.636188289	0.222683753	0.346448665	0.843493341	0.597070801	0.948667577
COX19	0.278134184	0.396551323	0.344504807	0.909425602	0.449995243	0.596888597	0.948667577
CYB561D2	0.912077636	0.838405913	0.40542552	0.111367866	0.450472222	0.596933874	0.948667577
GGCX	0.904257564	0.39208291	0.315566124	0.478163321	0.290957084	0.597086552	0.948667577
LOC112441834	0.44244445	0.397001612	0.593230127	0.325562567	0.458686417	0.597023297	0.948667577
AGAP3	0.680836914	0.412091993	0.116968444	0.480865448	0.986661334	0.597145755	0.948669795
EIF2A	0.676044719	0.736089607	0.868260085	0.989677703	0.036439944	0.597293631	0.948812871
ZNF565	0.840598508	0.763226166	0.70186791	0.332433362	0.104200806	0.597493404	0.949038352
ALKBH6	0.277322064	0.731824774	0.567076663	0.140219129	0.973067953	0.598798888	0.949281437
APIP	0.758975488	0.128698604	0.239297717	0.81919175	0.819453373	0.59864917	0.949281437
CCDC8	0.470814696	0.801891183	0.106801141	0.589061996	0.661935081	0.599038421	0.949281437
CENPJ	0.748897596	0.164922996	0.480900693	0.292912021	0.899482064	0.598128353	0.949281437
CNOT3	0.923804767	0.460617512	0.236274656	0.173916729	0.893697058	0.597850614	0.949281437
COG8	0.90725965	0.122008222	0.536418241	0.878286493	0.30031063	0.598281913	0.949281437
CYB5D2	0.961067787	0.498153744	0.499884199	0.133919476	0.490807981	0.599139101	0.949281437

ELF4	0.984677888	0.529147676	0.716701545	0.449430264	0.093733672	0.599150394	0.949281437
GATD3A	0.544950071	0.443218846	0.202619173	0.35439301	0.904721239	0.598651613	0.949281437
GCLC	0.743848937	0.79952328	0.287991557	0.213663585	0.429163203	0.598827983	0.949281437
GM2A	0.113748579	0.400649335	0.762309494	0.557981197	0.811086549	0.599043246	0.949281437
GSTM2	0.413144676	0.88073059	0.321818299	0.834125833	0.160305563	0.598239304	0.949281437
LACTB2	0.988488898	0.984629451	0.145763064	0.146567748	0.755878071	0.59897765	0.949281437
LOC112441639	0.163551429	0.550539972	0.79161225	0.84168015	0.261730542	0.59878698	0.949281437
MED12L	0.181325796	0.961575982	0.323025714	0.54244794	0.514858792	0.599132061	0.949281437
MYO1C	0.549473415	0.741541212	0.441052479	0.277710159	0.314352828	0.598618413	0.949281437
NUP214	0.352459716	0.749494689	0.432487206	0.143852601	0.956050807	0.598918321	0.949281437
PROSER3	0.589986866	0.12264492	0.284483094	0.892210196	0.854561908	0.598698118	0.949281437
PTTG1IP	0.443245909	0.25451513	0.241802006	0.985998003	0.581552558	0.598036183	0.949281437
RDX	0.918395836	0.153031572	0.204775439	0.582958753	0.931072588	0.597779177	0.949281437
RPL26	0.24795898	0.815723164	0.164307266	0.642852419	0.73235124	0.59809308	0.949281437
SLC25A14	0.270607696	0.545976994	0.740868039	0.68691698	0.208645061	0.598612278	0.949281437
TMEM154	0.340255178	0.459621579	0.77045946	0.282055106	0.461144641	0.598414195	0.949281437
TMEM8B	0.434893342	0.81569569	0.501368263	0.68436732	0.128880066	0.598601097	0.949281437
TVP23B	0.733286456	0.213102371	0.652481817	0.168557249	0.913760022	0.598810642	0.949281437
WIPF3	0.445455842	0.586801217	0.283426336	0.568960428	0.371574025	0.598296324	0.949281437
LRRC58	0.625393636	0.724434965	0.413985346	0.104067606	0.806370228	0.599249315	0.949311964
LY6D	0.404638607	0.403383889	0.925502416	0.118162592	0.881911048	0.599285354	0.949311964
DYNC1LI2	0.390096319	0.393611214	0.22260042	0.526935005	0.874762193	0.599439731	0.949373232
SYMPK	0.445365955	0.789017112	0.636856396	0.075059538	0.937638504	0.599383173	0.949373232
HS3ST3B1	0.817217466	0.612306396	0.043991726	0.751760898	0.952840284	0.599603446	0.949540881
FAM126A	0.998865921	0.919068166	0.348256907	0.656198862	0.07519475	0.599693335	0.949591597
NAGLU	0.541620793	0.596684301	0.589546595	0.159222628	0.520335304	0.599813795	0.949690708
IL4R	0.8001106	0.749715516	0.580989798	0.053772205	0.843207685	0.600020743	0.949889107
LOC112448260	0.525791622	0.510671213	0.287714424	0.427759804	0.478263055	0.600054864	0.949889107
C7H19orf24	0.769915791	0.987996829	0.843523508	0.374708976	0.065821944	0.600313876	0.950016086
FAM92B	0.465832196	0.986148752	0.842588692	0.042682269	0.957846154	0.600300437	0.950016086
LOC786372	0.707000226	0.44052761	0.12870281	0.607744629	0.650374592	0.600540301	0.950016086
RPL12	0.658114663	0.412131276	0.297049401	0.332270277	0.591834161	0.600536682	0.950016086
SORBS1	0.350434759	0.7254253	0.194396237	0.648732974	0.493878517	0.600409475	0.950016086
UVRAG	0.71171233	0.452960785	0.25085958	0.229211187	0.854214259	0.600420501	0.950016086
XXYLT1	0.452437927	0.729890875	0.195663376	0.329614499	0.743623114	0.600459651	0.950016086
LOC782101	0.844612289	0.279339563	0.267485273	0.916456112	0.274065217	0.600625511	0.950059302
DUSP6	0.493766129	0.409985859	0.277484032	0.332322405	0.849799527	0.600782093	0.950076057
STRN4	0.298352108	0.185605492	0.482843112	0.75831353	0.782512352	0.600809781	0.950076057
SYT3	0.07306195	0.806901575	0.994280996	0.671090871	0.403313493	0.600800386	0.950076057
AK3	0.684230983	0.722920034	0.165776181	0.336081449	0.576102158	0.600941342	0.950130117
CREG1	0.734469309	0.501971007	0.080669192	0.641134205	0.832705197	0.600959759	0.950130117
ETNPPL	0.87483008	0.594201912	0.188359553	0.448147841	0.361985987	0.601030775	0.950150857
ASB1	0.782855365	0.63592459	0.658401386	0.060618267	0.799899782	0.601146779	0.950242708
CLNK	0.388908142	0.830710255	0.990491431	0.13845238	0.359000997	0.601293263	0.950291193

SUCLG2	0.653161525	0.852298408	0.164292463	0.478582477	0.363327351	0.60126696	0.950291193
MAN2C1	0.707785204	0.407756165	0.477006484	0.444531348	0.260035452	0.601391023	0.950354172
LOC101905595	0.39987759	0.079637925	0.805724555	0.766890466	0.809145671	0.601495317	0.950376013
PSMC2	0.57807572	0.466449275	0.592761452	0.457016871	0.21799614	0.601520666	0.950376013
VASH2	0.78871318	0.746111338	0.18218784	0.340782451	0.436167587	0.60166544	0.95051324
ETFDH	0.799320161	0.828815331	0.457201176	0.061887092	0.851243518	0.601919925	0.950613649
LOC101901983	0.621702862	0.286898354	0.383274986	0.2782235	0.839103276	0.601960699	0.950613649
LOC112447460	0.451086864	0.259308288	0.986449502	0.30523815	0.45310851	0.601943574	0.950613649
RABAC1	0.253996095	0.704264232	0.978053064	0.10813341	0.84337681	0.601905055	0.950613649
REV3L	0.640492014	0.371985387	0.92233608	0.163067922	0.445564251	0.60204016	0.950647653
LOC107132748	0.113386573	0.907870774	0.228892962	0.759494709	0.892654389	0.602138239	0.950711049
EPS8	0.881299826	0.510723886	0.754222814	0.073856035	0.637327048	0.602197331	0.950712881
HIKESHI	0.790496621	0.484131954	0.293301294	0.366016112	0.389304091	0.602380929	0.950911256
CCNE1	0.422493679	0.208155788	0.293466897	0.701730691	0.884127093	0.602599105	0.950974438
DKK1	0.154059378	0.885238388	0.268809666	0.456969658	0.95699662	0.602840609	0.950974438
F12	0.542411246	0.774201993	0.171213936	0.258537361	0.863559339	0.603085946	0.950974438
HEYL	0.957881143	0.256555957	0.422697708	0.652064565	0.237023292	0.603116321	0.950974438
KLHL38	0.663432478	0.732795871	0.687270082	0.053738187	0.893754801	0.603028039	0.950974438
LDB1	0.505462758	0.575381235	0.23995289	0.609138836	0.37738115	0.602964637	0.950974438
LOC101903326	0.889382309	0.348187627	0.170007996	0.899276324	0.33891903	0.60300636	0.950974438
LOC107132121	0.182559011	0.287953009	0.595519266	0.845050646	0.605390887	0.602637856	0.950974438
LOC112444847	0.123351025	0.565832213	0.436428298	0.904330014	0.581243898	0.602588192	0.950974438
PSTK	0.281380076	0.334245513	0.554109804	0.733973542	0.419692438	0.603098844	0.950974438
ZDHHC1	0.318240054	0.207771415	0.700829232	0.534880567	0.646679188	0.602799097	0.950974438
ZNF277	0.236479371	0.667607845	0.450565986	0.413355044	0.545025933	0.602760924	0.950974438
LOC107132532	0.239951091	0.478056274	0.887102348	0.600603598	0.262912994	0.603282511	0.951094414
NEFH	0.897598151	0.631986425	0.404583669	0.073754793	0.949390011	0.603308321	0.951094414
GCNT4	0.722874093	0.972109524	0.149567423	0.78158225	0.195804235	0.603478318	0.951160871
LOC104970645	0.48452636	0.95516865	0.231544132	0.444413452	0.338028991	0.60364027	0.951160871
LOC104975073	0.572013553	0.796397216	0.310880378	0.871637827	0.130394995	0.603619602	0.951160871
LOC104975099	0.743351756	0.359904601	0.204986145	0.344555937	0.851625609	0.60356949	0.951160871
TRPC2	0.412714573	0.297036814	0.218852862	0.97479995	0.614993016	0.603472192	0.951160871
ALG14	0.44640563	0.483278124	0.784985863	0.190047048	0.500876195	0.60391312	0.95131678
MITD1	0.801262634	0.096444727	0.971961434	0.637879229	0.336380369	0.603864347	0.95131678
PES1	0.33848506	0.934709597	0.419026063	0.777896105	0.15623723	0.603815795	0.95131678
CHAC2	0.365831942	0.536397894	0.285842335	0.359629459	0.79985012	0.604083256	0.951486783
ERAP2	0.726785446	0.862499941	0.260311794	0.146016884	0.677549503	0.604192865	0.951486783
LOC112449059	0.703404903	0.357709065	0.569916297	0.577761282	0.194913643	0.604252956	0.951486783
SNX9	0.75799037	0.511817769	0.220192728	0.483917225	0.390584523	0.604221996	0.951486783
LOC104974113	0.596674638	0.863256307	0.590332506	0.157372494	0.337966481	0.604540041	0.951792344
PRPF31	0.094512813	0.888523772	0.659267516	0.338813188	0.862275217	0.604563	0.951792344
EVC	0.427859179	0.364115648	0.438038989	0.36008566	0.658815343	0.604740297	0.951868815
PLCB3	0.236541157	0.445718517	0.168068493	0.989286954	0.924284588	0.604901583	0.951868815
SP2	0.542690801	0.413339547	0.954593531	0.827232533	0.09136119	0.604670595	0.951868815

TOB1	0.374119599	0.382235325	0.181689658	0.784095634	0.795089464	0.604845378	0.951868815
YWHAZ	0.654929151	0.367366127	0.234804699	0.42148008	0.68015516	0.604813383	0.951868815
HAUS1	0.314032355	0.894367678	0.120484644	0.916182158	0.524054181	0.605439764	0.95189423
HIF3A	0.618566889	0.989880474	0.243634671	0.461556005	0.235775217	0.605282985	0.95189423
HRH2	0.952871888	0.385638134	0.169107453	0.564636647	0.462522625	0.605214821	0.95189423
KHDRBS3	0.935288165	0.970896072	0.423865322	0.054999554	0.767422349	0.605421564	0.95189423
LOC112447385	0.634539032	0.870860888	0.599297105	0.067780657	0.723576278	0.605376534	0.95189423
LRRC55	0.896464848	0.152868212	0.410415933	0.512161192	0.563852137	0.605379748	0.95189423
NTF3	0.78644307	0.794357874	0.139346301	0.857700523	0.217359861	0.605221414	0.95189423
SMIM7	0.932204147	0.07623615	0.470226682	0.693006563	0.700042126	0.605017917	0.95189423
SNAP47	0.50042467	0.37394481	0.436312971	0.541739582	0.36677002	0.605147224	0.95189423
PPP2R5D	0.913370826	0.452308668	0.134430229	0.406419777	0.720045924	0.60549985	0.951897504
CTNND2	0.560722211	0.747942105	0.545022519	0.241176359	0.29496178	0.605597142	0.951908522
ZC3H7B	0.886982361	0.408304335	0.423348772	0.150542581	0.704581632	0.605622867	0.951908522
ARSB	0.619550735	0.743508016	0.131177326	0.339857323	0.793377574	0.60598658	0.951922726
CASK	0.784589361	0.322795231	0.348523838	0.31265479	0.591303105	0.606291702	0.951922726
CD320	0.494761238	0.385624189	0.104687963	0.947900702	0.860239534	0.605915205	0.951922726
CPTP	0.736100355	0.747723911	0.59241024	0.119131771	0.422174912	0.607254493	0.951922726
KXD1	0.338417311	0.448817705	0.262208114	0.446178152	0.922325502	0.607137854	0.951922726
LOC100296900	0.386420999	0.734138893	0.389113706	0.432075819	0.342977776	0.606769648	0.951922726
LOC100297056	0.773388453	0.36386491	0.07270667	0.855169579	0.933377515	0.606446393	0.951922726
LOC101907893	0.574177787	0.119559318	0.839043285	0.426642415	0.667338411	0.607256049	0.951922726
LOC107132098	0.291675442	0.657856285	0.275619288	0.497384902	0.622348624	0.606915806	0.951922726
LOC107133150	0.573510737	0.885540687	0.156646286	0.243169561	0.845149379	0.606666993	0.951922726
LOC112443463	0.976637832	0.975878379	0.269496162	0.129809613	0.490009522	0.606523421	0.951922726
LOC509941	0.262586918	0.996531863	0.092009181	0.799990278	0.846255576	0.60606914	0.951922726
LOC515570	0.677115103	0.948993938	0.12765794	0.839544563	0.237355308	0.6066237	0.951922726
LOC784866	0.696744353	0.845259569	0.159819917	0.67743675	0.255799484	0.606195418	0.951922726
LOC786139	0.929708745	0.945597298	0.575056217	0.043369499	0.746869939	0.606972395	0.951922726
LONP1	0.732491767	0.511926491	0.357974648	0.168983022	0.719307562	0.60626613	0.951922726
PHF1	0.237896361	0.857723443	0.492530764	0.653298861	0.249681163	0.607184984	0.951922726
PPP1R42	0.673980826	0.380309072	0.188819139	0.968716617	0.348056732	0.606291598	0.951922726
SAMD14	0.860070556	0.476940413	0.110235881	0.41289272	0.876929687	0.606941115	0.951922726
SARDH	0.828072996	0.492500049	0.97589671	0.368596173	0.110979524	0.605840435	0.951922726
SLC24A3	0.457704631	0.530367919	0.201960544	0.996446652	0.333555616	0.606010624	0.951922726
SLC7A7	0.331695608	0.993311511	0.160952384	0.33371258	0.925344455	0.606974994	0.951922726
SYTL2	0.646203441	0.948365181	0.422733546	0.979007952	0.06445904	0.606652966	0.951922726
THAP5	0.426226582	0.956852339	0.372708511	0.119989921	0.897254159	0.606847615	0.951922726
TIPIN	0.673487827	0.350010218	0.185485709	0.569991301	0.654675862	0.606263957	0.951922726
TNKS2	0.875277613	0.368518336	0.132975655	0.623770484	0.612230514	0.607028105	0.951922726
ZCRB1	0.972687159	0.078629679	0.434554231	0.527404224	0.932384732	0.606590444	0.951922726
ZNF784	0.884018543	0.107270322	0.765068643	0.400487714	0.562496296	0.606593948	0.951922726
APLN	0.787731663	0.355251421	0.231892529	0.278426744	0.90797837	0.607329265	0.951937945
LOC104975022	0.454334856	0.330695255	0.248084586	0.67538504	0.651851976	0.60738177	0.951937945

HNRNPA2B1	0.189917371	0.788942571	0.445999928	0.351154965	0.700081231	0.607600926	0.952044962
RBM6	0.765590426	0.168540843	0.356163148	0.502729708	0.710864403	0.607546949	0.952044962
SULF2	0.601942933	0.956482624	0.069868918	0.676477702	0.60377312	0.60762409	0.952044962
POLK	0.12266189	0.959769839	0.268393691	0.872713069	0.596138879	0.607724843	0.952111923
AKT3	0.894560447	0.478371875	0.839710143	0.127272494	0.361685164	0.608938688	0.952188938
ARAF	0.470779538	0.879967403	0.219558266	0.247807546	0.73050441	0.608040795	0.952188938
CALCOCO2	0.471642002	0.942090597	0.073925372	0.526328549	0.952817456	0.608127931	0.952188938
CISD3	0.717300461	0.529834126	0.351268683	0.163795711	0.755295435	0.608637624	0.952188938
GALK2	0.467093767	0.262238239	0.741946989	0.499319293	0.365021558	0.609208191	0.952188938
KANSL2	0.553799244	0.279803159	0.799274064	0.422472864	0.315753687	0.608704153	0.952188938
LOC100847947	0.768048072	0.533971171	0.513574838	0.19327296	0.407099085	0.609303147	0.952188938
LOC100848507	0.311580743	0.619308677	0.380627019	0.878521394	0.256594038	0.609121236	0.952188938
LOC101904579	0.844960509	0.256926471	0.989004567	0.086603725	0.891689165	0.6093986	0.952188938
LOC101906546	0.544409138	0.688284066	0.485338442	0.523484558	0.173982123	0.609197626	0.952188938
LOC104972584	0.408474255	0.23287914	0.978886493	0.208331438	0.851218336	0.608603274	0.952188938
LOC112444897	0.579846424	0.217261369	0.693830605	0.544798102	0.346698262	0.608564101	0.952188938
LOC112446375	0.35258304	0.073833245	0.694821332	0.951012316	0.962955477	0.60921225	0.952188938
LOC112449510	0.977974045	0.467432073	0.080060172	0.797461606	0.56780742	0.609299565	0.952188938
MAGED2	0.304564619	0.560859139	0.210226788	0.767097341	0.601451805	0.609254371	0.952188938
MEX3C	0.872145108	0.444143092	0.458216539	0.174774619	0.531591266	0.608340566	0.952188938
MPHOSPH6	0.912514501	0.420615963	0.608304619	0.230567473	0.307051981	0.608797889	0.952188938
MTSS1	0.140626722	0.921532732	0.793569451	0.228451623	0.702332207	0.608458164	0.952188938
NSUN3	0.880611348	0.659172784	0.810231802	0.182412982	0.192581616	0.608711098	0.952188938
PFDN5	0.558185233	0.194563939	0.197860626	0.832417625	0.921704809	0.608293202	0.952188938
PGRMC1	0.840129593	0.643774825	0.637358731	0.127205605	0.377188666	0.60892032	0.952188938
POGLUT1	0.603514741	0.902102688	0.779099605	0.049541045	0.788861271	0.60935772	0.952188938
RPSA	0.832635836	0.611994799	0.311243378	0.585010157	0.177608609	0.608202087	0.952188938
SPINDOC	0.946010214	0.343863015	0.374211856	0.617611831	0.219298737	0.608301011	0.952188938
TBC1D9	0.455270895	0.501110305	0.2069845	0.649206808	0.53654644	0.607843186	0.952188938
THG1L	0.706705402	0.726965814	0.166124028	0.283874585	0.679890417	0.608121188	0.952188938
TXNL4A	0.495352516	0.50382233	0.829396378	0.082247846	0.968005835	0.608213795	0.952188938
ZDHHC21	0.818450724	0.496014686	0.102425326	0.914038463	0.436057227	0.609312677	0.952188938
CLTC	0.754551189	0.114337119	0.642528079	0.696202084	0.429772409	0.609465227	0.952202385
ADGRF3	0.998380649	0.057810396	0.590377748	0.711115936	0.691689048	0.611505983	0.952257661
AKT1S1	0.466931905	0.939856708	0.650310864	0.115715105	0.502855497	0.609702259	0.952257661
ALOX12	0.16118022	0.453723544	0.452935395	0.543626184	0.93200341	0.611763858	0.952257661
ANAPC5	0.851414489	0.534055846	0.245485619	0.218565357	0.681481973	0.60993591	0.952257661
C20H5orf34	0.594048602	0.237303004	0.390207594	0.38911419	0.776232418	0.609801252	0.952257661
CACNG4	0.31443257	0.157397256	0.813097785	0.780826948	0.533155557	0.611412866	0.952257661
CASP3	0.78730534	0.343589465	0.338753255	0.308019648	0.593052462	0.611260902	0.952257661
COG3	0.988238639	0.932980462	0.223505384	0.088137436	0.919549387	0.610820409	0.952257661
DDOST	0.303582155	0.760241785	0.137109965	0.903835172	0.586473685	0.611663895	0.952257661
DNAL4	0.557106608	0.785995288	0.816495263	0.046962448	0.992207549	0.610329403	0.952257661
EWSR1	0.626443072	0.221401626	0.164499237	0.76747819	0.954486155	0.610957083	0.952257661

FER1L5	0.927772997	0.377619896	0.260677537	0.395185712	0.462914629	0.610885345	0.952257661
FHL3	0.70048266	0.457818983	0.260009296	0.212441107	0.944061653	0.611072573	0.952257661
FRY	0.180370799	0.427835778	0.603261332	0.722602732	0.49460922	0.610080218	0.952257661
GALNT18	0.956431495	0.456834608	0.33178827	0.339437909	0.339763	0.611023745	0.952257661
HTR1E	0.536753539	0.247385601	0.68581159	0.587939902	0.312044584	0.610885972	0.952257661
KLHL6	0.227385527	0.902159712	0.728645532	0.375237105	0.297928355	0.610920318	0.952257661
LOC101905219	0.869942718	0.178727818	0.616228137	0.339965778	0.515230776	0.611765721	0.952257661
LOC101907835	0.448213012	0.336520525	0.985418213	0.469746648	0.238795424	0.610482686	0.952257661
LOC104976664	0.074261126	0.800412405	0.840788355	0.407753428	0.82031191	0.610991545	0.952257661
LOC112444350	0.576569597	0.159760578	0.634391988	0.624228287	0.459385262	0.611468305	0.952257661
LOC112447301	0.502093443	0.50566878	0.724041527	0.162948993	0.558538416	0.611162416	0.952257661
LOC112448090	0.576899307	0.529432822	0.439703213	0.189071411	0.655156932	0.610049258	0.952257661
LOC112449615	0.427107978	0.949660458	0.221828359	0.626993835	0.297576997	0.61182163	0.952257661
LOC618268	0.99445682	0.978517148	0.739598238	0.696567155	0.033244068	0.610402295	0.952257661
LOC781280	0.437448974	0.300305691	0.887418186	0.590359093	0.242643501	0.610796307	0.952257661
NUDT7	0.705030874	0.547426427	0.772165757	0.426453325	0.131533055	0.610997018	0.952257661
NUP133	0.298127685	0.80153681	0.844516912	0.13818833	0.600483021	0.611336484	0.952257661
ORC1	0.624097953	0.733889126	0.155689995	0.679145767	0.345250493	0.611037632	0.952257661
PAICSP	0.335878006	0.529858348	0.514390076	0.396634939	0.460303275	0.61096022	0.952257661
SKAP1	0.855458468	0.187397855	0.379578349	0.41830411	0.658883785	0.61163296	0.952257661
SLC28A1	0.869112327	0.694005524	0.72021455	0.043486328	0.88576881	0.611187112	0.952257661
SPAAR	0.941350119	0.347480173	0.111508365	0.562533956	0.808975339	0.609613844	0.952257661
TAF1B	0.757683504	0.402080748	0.270970872	0.592967298	0.339622464	0.609918569	0.952257661
TIGD7	0.588570732	0.392941591	0.247335986	0.348935685	0.831838	0.6096702	0.952257661
TNPO1	0.890819524	0.283195696	0.10004182	0.864450808	0.76329576	0.610251179	0.952257661
UPK3B	0.963859718	0.022506926	0.892458785	0.925569093	0.928730314	0.610127858	0.952257661
VSIR	0.901332951	0.700217322	0.789208958	0.071429141	0.468843662	0.610575835	0.952257661
WDCP	0.842582185	0.397217472	0.371640813	0.929236046	0.145087551	0.611612416	0.952257661
ZNF189	0.502869976	0.330619702	0.66771688	0.424645119	0.354385011	0.610874528	0.952257661
NOCT	0.163399091	0.635769561	0.841632965	0.328813781	0.58419526	0.611909004	0.952303334
ARSH	0.286080597	0.362198856	0.781972591	0.220736086	0.941200069	0.612359415	0.952375528
CTSF	0.977957534	0.871278848	0.143814149	0.456584749	0.300932673	0.612398621	0.952375528
CYLD	0.310551114	0.568361578	0.206798003	0.890166517	0.517648113	0.612192101	0.952375528
DONSON	0.744338575	0.182743559	0.541585013	0.254318069	0.898793376	0.612419654	0.952375528
KIAA1328	0.230948021	0.305901301	0.444130888	0.942899284	0.568706374	0.61225948	0.952375528
KRTCAP2	0.943358255	0.360008135	0.491230762	0.126020336	0.799457566	0.612057511	0.952375528
LOC107131974	0.964773721	0.07752895	0.885190057	0.400238704	0.635180393	0.612341332	0.952375528
TNIK	0.83025772	0.79224313	0.732619568	0.216480826	0.161183421	0.612138028	0.952375528
ADRA1A	0.236927289	0.345030242	0.816710866	0.389023404	0.650192645	0.612977914	0.952382028
CASP4	0.727740076	0.879296058	0.748661587	0.607310244	0.057974363	0.612746523	0.952382028
CLEC4E	0.547412762	0.935116698	0.538249727	0.082525839	0.741836873	0.612755221	0.952382028
DNM3	0.273384808	0.997278487	0.797623119	0.760739959	0.10187832	0.612595053	0.952382028
DUS2	0.978759514	0.88098179	0.762177452	0.123181405	0.208400306	0.612791006	0.952382028
KATNBL1	0.755762598	0.763190492	0.723066692	0.116702181	0.346724557	0.612843931	0.952382028

LOC100847612	0.520134546	0.712686932	0.990269467	0.441832788	0.103872057	0.612513247	0.952382028
LOC101902968	0.251700269	0.124463987	0.704160211	0.986928079	0.774346737	0.612645969	0.952382028
MAD2L2	0.886574823	0.310038263	0.204937762	0.896360488	0.334369288	0.612934494	0.952382028
UBE2E2	0.45391406	0.709316221	0.299582293	0.315031928	0.5558179	0.613004166	0.952382028
LYST	0.224201832	0.902068272	0.365705566	0.340026398	0.671914031	0.613102924	0.952445294
ACVR1C	0.460476751	0.989639803	0.223651129	0.172440587	0.973063006	0.615440498	0.952475553
ADGRL2	0.397837989	0.874192013	0.157284191	0.38993334	0.792801515	0.613245197	0.952475553
ARMC7	0.506935891	0.873648337	0.13181758	0.83327189	0.353539204	0.616542455	0.952475553
BAD	0.209411075	0.467991634	0.3493029	0.736426069	0.683968959	0.617045208	0.952475553
BCHE	0.501412304	0.807154899	0.537510429	0.505116493	0.156641171	0.616699331	0.952475553
C2H2orf88	0.42271841	0.570762573	0.959073898	0.996798688	0.074120018	0.615379931	0.952475553
CACNG5	0.296128213	0.273418506	0.367200115	0.589496946	0.978551051	0.61599761	0.952475553
CALR3	0.655730107	0.929708348	0.071909545	0.700103636	0.56016179	0.616473604	0.952475553
CCT6A	0.966965227	0.368091728	0.334747745	0.75177718	0.189873529	0.614362315	0.952475553
CDC34	0.316369371	0.975548854	0.687373565	0.192976643	0.420856719	0.616896745	0.952475553
CELSR1	0.453025185	0.560462462	0.0914396	0.770507408	0.954557566	0.615146348	0.952475553
CHCHD1	0.812323189	0.524791096	0.35063729	0.186385414	0.620047714	0.617407165	0.952475553
CHRNA7	0.374893395	0.507203908	0.217909872	0.838990322	0.487576856	0.613701138	0.952475553
CNMD	0.677127549	0.101449658	0.462439095	0.706224772	0.76033762	0.614940849	0.952475553
CSF1R	0.16050226	0.964390436	0.884967692	0.403749318	0.312248058	0.617345367	0.952475553
DCAF6	0.436307328	0.972008389	0.101599078	0.418944123	0.953861739	0.616770184	0.952475553
DCHS2	0.196577031	0.492087944	0.404424807	0.684272325	0.645869289	0.61757644	0.952475553
DKKL1	0.091405407	0.889831646	0.35831769	0.932518833	0.629615497	0.615550464	0.952475553
DNAAF4	0.394874449	0.178683699	0.58714409	0.556970996	0.733586368	0.613433759	0.952475553
DXO	0.465766987	0.229165442	0.624874075	0.537888816	0.48154083	0.617418642	0.952475553
EBNA1BP2	0.455861592	0.492541952	0.390207484	0.780734529	0.248202845	0.614022389	0.952475553
FAM133B	0.734353865	0.634744621	0.267411108	0.278825965	0.496154043	0.617057859	0.952475553
GPR137	0.356028104	0.376633684	0.839956865	0.166879251	0.918007706	0.617181731	0.952475553
GRB2	0.889906601	0.247262513	0.177303766	0.672837848	0.651577785	0.615467894	0.952475553
H4	0.886703881	0.588288651	0.173164809	0.989804497	0.193069252	0.61726364	0.952475553
HDAC1	0.862742378	0.608603697	0.061170756	0.740487873	0.722859098	0.616472234	0.952475553
IPPK	0.756180942	0.276823703	0.940081417	0.92133872	0.094853628	0.616533642	0.952475553
IQCD	0.828322066	0.096127488	0.49788233	0.605542938	0.715935391	0.616409803	0.952475553
ITCH	0.611040481	0.918234845	0.159027072	0.229076475	0.835257334	0.615107445	0.952475553
ITPK1	0.279270509	0.763678466	0.188957675	0.50708005	0.828143412	0.613393188	0.952475553
JCAD	0.581072936	0.586273572	0.404838159	0.383173404	0.324381951	0.615902697	0.952475553
LCMT1	0.357740402	0.169487189	0.470000591	0.658953869	0.908103223	0.614881714	0.952475553
LOC100139144	0.339210345	0.599065249	0.493682414	0.302432367	0.568450163	0.6170929	0.952475553
LOC100847546	0.539123408	0.886293712	0.52449415	0.78490076	0.086295041	0.613988732	0.952475553
LOC101904468	0.413164401	0.287606063	0.600279557	0.299852257	0.794405488	0.614176163	0.952475553
LOC107133209	0.459414073	0.158797962	0.522734951	0.627566616	0.718459794	0.616500064	0.952475553
LOC112446668	0.32263311	0.374251019	0.166766946	0.911842747	0.940928914	0.617428848	0.952475553
LOC112449073	0.136769012	0.463090189	0.795520871	0.918098094	0.371846336	0.616573982	0.952475553
LOC511937	0.120634391	0.878828019	0.392915899	0.689003671	0.597393947	0.615944305	0.952475553

LOC781412	0.431998573	0.166340427	0.959589252	0.892393457	0.277216752	0.61494818	0.952475553
LOC788724	0.734017888	0.240640345	0.403525489	0.428042364	0.560697046	0.615496547	0.952475553
LRMDA	0.046894738	0.908986203	0.467878158	0.96644082	0.878124317	0.613421601	0.952475553
MAP2K7	0.099458332	0.590144323	0.756279083	0.586383516	0.653429718	0.614373102	0.952475553
MSANTD3	0.561626095	0.770803744	0.237724798	0.858725311	0.192485095	0.614397745	0.952475553
NAT8L	0.771024547	0.564365203	0.186550792	0.324252628	0.643291203	0.613498557	0.952475553
NISCH	0.79562461	0.273549322	0.764608185	0.115351287	0.883665755	0.613847656	0.952475553
NOL4L	0.730653665	0.949717061	0.071002088	0.586280856	0.58901768	0.614440013	0.952475553
OAS1X	0.461076819	0.997029812	0.534263184	0.190442673	0.369220259	0.61735142	0.952475553
PAPD4	0.061770761	0.61919787	0.644041722	0.728602063	0.949828173	0.614822528	0.952475553
PARN	0.957589766	0.369967974	0.335305714	0.401675711	0.356542496	0.614422247	0.952475553
PHF3	0.39484673	0.685235868	0.996719201	0.080732203	0.778087921	0.613588587	0.952475553
PIGN	0.238502242	0.508491867	0.89306299	0.389962585	0.407806712	0.616834059	0.952475553
PTPRM	0.393981816	0.668399424	0.642280292	0.177872762	0.565462026	0.614412483	0.952475553
RBM15	0.713507051	0.642879278	0.379104992	0.456011763	0.217753239	0.617325069	0.952475553
RNF113A	0.971934485	0.416389618	0.366711612	0.467825762	0.2439396	0.613548605	0.952475553
RNF166	0.830239786	0.226738544	0.223221325	0.880748181	0.465525595	0.616889822	0.952475553
S100G	0.662199635	0.619609759	0.080246822	0.686211877	0.760822914	0.616447809	0.952475553
SCARA5	0.861086845	0.242951976	0.957845256	0.373626793	0.229912108	0.616711182	0.952475553
SCP2D1	0.812724388	0.313025239	0.270242204	0.554709801	0.450490502	0.61633561	0.952475553
SEMA4C	0.908795177	0.884845293	0.076099567	0.8230119	0.336988089	0.613956868	0.952475553
SGO2	0.229773439	0.326712923	0.405512658	0.854306642	0.656177479	0.61502205	0.952475553
SH3D21	0.414485708	0.301731855	0.692521383	0.347535731	0.57061666	0.616281889	0.952475553
SH3RF1	0.187729094	0.646797204	0.579862858	0.299051149	0.815632956	0.616262749	0.952475553
SHPK	0.615026007	0.74604998	0.193479729	0.246099086	0.78563089	0.61615492	0.952475553
SLC23A3	0.76241569	0.446729871	0.419086772	0.23321645	0.51936218	0.617568966	0.952475553
SLC25A32	0.519684304	0.715702981	0.168873479	0.310894137	0.879330418	0.616232738	0.952475553
SLC30A7	0.852678016	0.614358833	0.850662077	0.367273757	0.10437733	0.615226457	0.952475553
SMO	0.792259425	0.314544995	0.148153472	0.789195322	0.59343563	0.617591393	0.952475553
SMPDL3B	0.596870911	0.580809082	0.455252924	0.407249759	0.265789589	0.615228719	0.952475553
SOD2	0.736765066	0.83589911	0.313666097	0.656024106	0.135610124	0.616396344	0.952475553
SOWAHC	0.719771379	0.659589476	0.101128426	0.477658294	0.741360075	0.614294847	0.952475553
SRPRA	0.617446738	0.217180546	0.313098226	0.893449453	0.452497257	0.613979683	0.952475553
STRADA	0.805784878	0.056428816	0.664064967	0.786092423	0.728314232	0.617547722	0.952475553
TICRR	0.733929624	0.097680637	0.796541333	0.757735755	0.39188175	0.613780577	0.952475553
TRIM23	0.319542325	0.558032505	0.674590046	0.225304904	0.635373963	0.616785497	0.952475553
UAP1L1	0.560780677	0.738710045	0.087031078	0.81333534	0.58916353	0.617423562	0.952475553
UNK	0.789863124	0.123311881	0.35891494	0.742157405	0.659792966	0.615627343	0.952475553
RNF24	0.835598384	0.721167947	0.123475627	0.364673852	0.63771647	0.617738595	0.952606555
YEATS4	0.803850183	0.480865746	0.394714242	0.873218212	0.129916014	0.61779243	0.952606555
LOC112442559	0.403175354	0.216281868	0.401885932	0.820530402	0.603096783	0.61816627	0.953003914
LOC112447762	0.404476045	0.294120059	0.603514646	0.967413794	0.249618117	0.618119081	0.953003914
ARPC5L	0.980265796	0.842957069	0.193839991	0.721253578	0.150287425	0.61839324	0.953163949
RETSAT	0.556542125	0.864333128	0.386966633	0.329627871	0.28303507	0.618444319	0.953163949

UPRT	0.607060203	0.253948546	0.839460577	0.811068534	0.165403497	0.618382776	0.953163949
ABHD12	0.308358227	0.850936116	0.985419102	0.135854767	0.495014215	0.61869126	0.953216701
IMPDH2	0.594762406	0.692156235	0.501947528	0.402556752	0.209062713	0.618710883	0.953216701
ITGA1	0.86947743	0.070402392	0.604816247	0.526917868	0.890895615	0.618589131	0.953216701
ZNF280C	0.407484829	0.36272147	0.813359945	0.74350604	0.194467712	0.618616371	0.953216701
SERINC1	0.674586516	0.37596304	0.149517369	0.68331171	0.671485397	0.61880992	0.95327979
EPHB4	0.355631519	0.300092148	0.788080274	0.806924637	0.256500067	0.618907012	0.953339869
ABCA7	0.289397353	0.313132075	0.229523979	0.923288821	0.907532096	0.619133643	0.953358659
LOC104974144	0.130612763	0.428787318	0.790814552	0.614251804	0.640676665	0.619150643	0.953358659
NGFR	0.989938253	0.358073206	0.373646712	0.549878815	0.239392602	0.619209673	0.953358659
PARK7	0.445636568	0.710678992	0.483337473	0.280449256	0.406078117	0.619187113	0.953358659
RFC1	0.538817657	0.412969736	0.645683595	0.257720203	0.470427558	0.619030782	0.953358659
AP2A1	0.427628988	0.570258812	0.772917135	0.196672566	0.470669629	0.619350536	0.953486084
LOC101903600	0.664803163	0.094251567	0.510687015	0.643941933	0.847270902	0.619473505	0.953496501
NR4A3	0.218271439	0.237809666	0.693328507	0.519928993	0.933017985	0.61947083	0.953496501
ZBTB9	0.138209669	0.939968562	0.990199336	0.99256627	0.136816625	0.619593716	0.953592092
ANGPTL6	0.659886119	0.446306696	0.918819823	0.544546607	0.11894192	0.620236824	0.953598138
C3AR1	0.236312732	0.983054482	0.404133547	0.885456303	0.210571451	0.619991035	0.953598138
CASD1	0.438523749	0.953195758	0.065072496	0.856291562	0.751358378	0.619939426	0.953598138
DPYSL3	0.364715027	0.640686973	0.311148878	0.366404887	0.656390251	0.619781881	0.953598138
GOLIM4	0.547729477	0.767753081	0.167379986	0.727519733	0.342184682	0.620189062	0.953598138
LOC101902221	0.838209734	0.041797188	0.966159218	0.675695886	0.764262322	0.61971488	0.953598138
LOC101904355	0.783736885	0.457333347	0.932290875	0.185340433	0.282859921	0.62014392	0.953598138
LOC112443151	0.841442153	0.2910218	0.194381768	0.620198979	0.593072132	0.620030451	0.953598138
LOC751811	0.685384729	0.600838236	0.091835596	0.866180898	0.534688606	0.620106316	0.953598138
PRPF38A	0.305895536	0.491757798	0.753734157	0.247492356	0.623392103	0.619860362	0.953598138
TSSK4	0.48103883	0.632455405	0.665666825	0.889694241	0.097099008	0.619885931	0.953598138
AHNAK2	0.433628209	0.528277905	0.117820785	0.783870387	0.82974273	0.620545729	0.953702581
CFAP53	0.772345881	0.882684573	0.79534343	0.17903749	0.180877197	0.620595324	0.953702581
LOC100124497	0.389786797	0.601303343	0.707130679	0.333173771	0.317847632	0.620510024	0.953702581
NUDCD2	0.616273843	0.481121469	0.551914378	0.17306087	0.619290336	0.620367526	0.953702581
SERPING1	0.448534802	0.659093743	0.301028791	0.338400682	0.582610927	0.620443019	0.953702581
ASXL2	0.733542054	0.470162125	0.508556931	0.629233311	0.159207446	0.620724604	0.953716791
COP1	0.763055428	0.802715194	0.10093057	0.325266317	0.873929613	0.620755423	0.953716791
STRN	0.363682002	0.492220803	0.68404107	0.177721073	0.807797864	0.620822767	0.953716791
TICAM2	0.510135899	0.617693174	0.245772216	0.26332658	0.862087042	0.620837029	0.953716791
PLA2G4A	0.377532811	0.667614499	0.156330011	0.633933434	0.704091269	0.620908158	0.953736782
ATP12A	0.702777684	0.8171223	0.350702223	0.396946309	0.221154609	0.621931771	0.953813527
BLNK	0.177519562	0.668950745	0.289181729	0.677748462	0.756751088	0.62119482	0.953813527
BRAP	0.676855661	0.235220924	0.470345702	0.281276763	0.847051326	0.623713622	0.953813527
CANT1	0.398590487	0.307616564	0.980002819	0.436714864	0.339293528	0.623310929	0.953813527
CDH7	0.577169046	0.525694492	0.250967624	0.332250241	0.706510921	0.624075975	0.953813527
CEP78	0.954783153	0.282146908	0.173689444	0.498007439	0.764635056	0.623448562	0.953813527
CHPT1	0.622729379	0.688847726	0.136635168	0.712267458	0.427210938	0.623640649	0.953813527

DMTN	0.511303988	0.829463815	0.870058693	0.166356804	0.291010682	0.623957373	0.953813527
DSCC1	0.704407008	0.663782985	0.630677004	0.768377061	0.078675829	0.623551566	0.953813527
DUSP1	0.65302673	0.763622063	0.079973668	0.576481657	0.771298419	0.622513374	0.953813527
DYNC1I1	0.417737267	0.461855047	0.230984396	0.408678412	0.978199222	0.623429249	0.953813527
ECHDC1	0.926406792	0.280042112	0.816121928	0.426072122	0.196755229	0.622704601	0.953813527
ERBB3	0.568005009	0.531154236	0.152139511	0.490346713	0.783443373	0.621416511	0.953813527
ERCC6L2	0.43786841	0.453846845	0.221350358	0.966111103	0.417382117	0.622571711	0.953813527
FAM118B	0.923413798	0.280197263	0.319325288	0.282925439	0.76075659	0.623074226	0.953813527
HNRNPH1	0.912354093	0.230123623	0.669621805	0.170354314	0.737236257	0.621681124	0.953813527
KIAA0753	0.444576454	0.213254901	0.879977964	0.218976411	0.978180982	0.624028961	0.953813527
KLF4	0.341411852	0.699779095	0.113626231	0.686763071	0.954980605	0.623302888	0.953813527
LAMB1	0.859816854	0.440620312	0.642394759	0.079534946	0.913738908	0.622013298	0.953813527
LOC101902458	0.754920713	0.117481107	0.679507641	0.556649097	0.530083083	0.62306428	0.953813527
LOC101903545	0.555706226	0.947636943	0.705058199	0.176850441	0.269996076	0.622473538	0.953813527
LOC107132958	0.675714384	0.351719867	0.978540966	0.31613106	0.240074161	0.621608092	0.953813527
LOC112441868	0.400107824	0.770425944	0.60614237	0.384683281	0.248901232	0.624244477	0.953813527
LOC112444463	0.164588673	0.368535642	0.728154875	0.769361263	0.524349379	0.623453407	0.953813527
LOC112444502	0.529499313	0.382157138	0.762332932	0.692307876	0.166027606	0.622498647	0.953813527
LOC784357	0.786777815	0.29189529	0.321381405	0.986082184	0.244589071	0.623271606	0.953813527
MACROD1	0.317084269	0.87258873	0.305092471	0.393928438	0.533558575	0.622626203	0.953813527
NAPG	0.60811817	0.391956356	0.185774942	0.406463862	0.980630988	0.621603017	0.953813527
NOSTRIN	0.299990438	0.771928877	0.14747001	0.721313099	0.71647589	0.621591067	0.953813527
NQO1	0.929976552	0.406411353	0.425563842	0.16084117	0.68189379	0.621502327	0.953813527
PAPSS1	0.729004815	0.800800821	0.131736033	0.892608907	0.258444548	0.622613993	0.953813527
PDE8B	0.987919102	0.551274248	0.229231404	0.344540491	0.412923036	0.62283192	0.953813527
PLA2G15	0.801644125	0.222036242	0.185404505	0.643335536	0.837988285	0.623159675	0.953813527
PYGO2	0.602121407	0.638989915	0.095028276	0.723873066	0.675293803	0.624052477	0.953813527
PYROXD2	0.321676632	0.269351843	0.726157067	0.904146089	0.311304714	0.622256978	0.953813527
RBFA	0.543173786	0.95313955	0.524741603	0.3223701	0.202632388	0.622666317	0.953813527
RGMB	0.949104966	0.954299045	0.258615019	0.851315395	0.089665262	0.624133931	0.953813527
S100A13	0.417459147	0.804815126	0.55309144	0.386727289	0.248802976	0.624134045	0.953813527
SDHAF1	0.454437682	0.409635265	0.540104434	0.340756603	0.522203704	0.624253789	0.953813527
SERPINB6	0.471073164	0.719762386	0.74401013	0.152888056	0.462597629	0.623713866	0.953813527
SF1	0.822700881	0.431853255	0.591745934	0.172873088	0.489828803	0.623287373	0.953813527
SIL1	0.381642463	0.978358872	0.192814402	0.500752117	0.49599308	0.624145434	0.953813527
SLC41A2	0.683421351	0.761966208	0.680617145	0.06065975	0.831655539	0.624135756	0.953813527
SMG5	0.486545536	0.925352896	0.765746985	0.184044419	0.280370522	0.623146147	0.953813527
SPA17	0.644046282	0.272632459	0.554773912	0.671352575	0.2728969	0.623770816	0.953813527
SPATS2	0.846504789	0.190852908	0.238124163	0.962630649	0.478705218	0.622466208	0.953813527
STRAP	0.820348404	0.496337204	0.43056991	0.274595049	0.371783004	0.624329103	0.953813527
SUDS3	0.782120833	0.466982934	0.096292149	0.548084821	0.923104905	0.623188157	0.953813527
TEX35	0.637989824	0.823043357	0.163107297	0.35750093	0.576625947	0.621666361	0.953813527
THEMIS2	0.911166842	0.762634455	0.285017762	0.610609153	0.147529224	0.623711304	0.953813527
TMEM81	0.60553305	0.698479637	0.163305617	0.614458971	0.415902157	0.621619392	0.953813527

TRIM62	0.276293821	0.576446557	0.902587536	0.584371669	0.213029542	0.624305471	0.953813527
TSPAN11	0.43861442	0.938600492	0.519029919	0.316698526	0.262972771	0.623209894	0.953813527
ULK2	0.34855185	0.983671762	0.289431177	0.846503479	0.211127638	0.622545924	0.953813527
WDR81	0.866945889	0.387293235	0.623656304	0.202703424	0.419861615	0.62349398	0.953813527
ZFP64	0.813014092	0.55858259	0.549264325	0.479263704	0.14916194	0.623608658	0.953813527
ZFPM1	0.728404025	0.333191326	0.164588674	0.487402123	0.913678459	0.62313517	0.953813527
ZW10	0.971256659	0.294816896	0.90885181	0.286469184	0.239379064	0.62376344	0.953813527
ANKS1A	0.336940086	0.815496963	0.350336546	0.557671831	0.333707306	0.624510749	0.953832839
HSD17B7	0.544527675	0.89939748	0.54833332	0.291925184	0.228846459	0.624792844	0.953832839
LOC112449318	0.100974651	0.956814426	0.325381112	0.798615519	0.713692891	0.62454522	0.953832839
PCNX1	0.331375807	0.77159029	0.992155719	0.104094889	0.679262401	0.624757916	0.953832839
RDH5	0.865281196	0.113420966	0.433066586	0.46429982	0.908642518	0.62468706	0.953832839
ST8SIA1	0.115670951	0.651780704	0.844466771	0.469269211	0.599959413	0.624622038	0.953832839
TACC2	0.918218865	0.522300773	0.623915238	0.12401505	0.483269799	0.624713899	0.953832839
ZBTB45	0.54385457	0.622197837	0.439280345	0.320573847	0.376514511	0.624806716	0.953832839
HS3ST6	0.632116686	0.925616246	0.166934678	0.479050733	0.383681069	0.624925383	0.953925259
LOC617141	0.324483788	0.845634698	0.304021892	0.361932989	0.594962451	0.625047608	0.954023093
CWF19L2	0.099249446	0.9128936	0.742299469	0.366538262	0.729444082	0.625245688	0.954111346
PLBD2	0.210264246	0.933555894	0.203738634	0.989207381	0.454754923	0.625337983	0.954111346
SGO1	0.520445136	0.209709765	0.2395143	0.789132576	0.8714417	0.625189297	0.954111346
YJEFN3	0.610916008	0.44815622	0.947371833	0.182360306	0.380336753	0.625331732	0.954111346
ZC3H18	0.736801946	0.979732197	0.438337223	0.076504309	0.743427914	0.625404268	0.954123775
ADAM10	0.48998782	0.446012266	0.706082224	0.294568731	0.396361042	0.625617358	0.954162565
KIAA0391	0.756605723	0.855188108	0.890694398	0.958854365	0.032592078	0.625554261	0.954162565
LOC104972827	0.341263567	0.397071842	0.769762597	0.815987494	0.211784176	0.625719858	0.954162565
LOC112446454	0.217637988	0.406220028	0.605489522	0.936718319	0.359140648	0.625532462	0.954162565
PIKFYVE	0.49104527	0.137616605	0.367416768	0.854301065	0.849829395	0.625720402	0.954162565
BRCC3	0.770833025	0.739681717	0.411517045	0.116146335	0.661847346	0.625838406	0.954165187
RAD51B	0.905234649	0.339507521	0.111395207	0.915002417	0.575674451	0.625802375	0.954165187
PARVA	0.700333384	0.126899465	0.560828787	0.574416066	0.630214414	0.625907438	0.954181789
ACCS	0.683775968	0.224875354	0.59453436	0.563304484	0.351135085	0.626330401	0.954649226
B4GAT1	0.874775356	0.392660588	0.632529497	0.128226234	0.648925791	0.626292828	0.954649226
ATG4A	0.661656288	0.912829805	0.325542691	0.661879826	0.139428608	0.627010182	0.954975787
C6	0.515572252	0.947763976	0.236005645	0.4931654	0.318987469	0.626972893	0.954975787
CDC42BPA	0.61655669	0.243511857	0.323020212	0.659913868	0.566430267	0.626828126	0.954975787
ETAA1	0.33470337	0.2312034	0.5572565	0.890626458	0.472307376	0.626951038	0.954975787
GADD45B	0.791606584	0.803114103	0.38887071	0.196411003	0.373460355	0.626893655	0.954975787
IL1R1	0.57616469	0.208070901	0.486692139	0.414539055	0.748774094	0.626635616	0.954975787
PQLC3	0.440644819	0.438424851	0.74767834	0.47276314	0.265463788	0.626824075	0.954975787
TMOD2	0.899041462	0.42179903	0.913550343	0.228138583	0.229489966	0.626928753	0.954975787
HEY1	0.978831102	0.887077275	0.2072118	0.307259966	0.328477276	0.627160324	0.955027195
LOC107131429	0.789873609	0.395473426	0.882481133	0.484987556	0.135796987	0.627118067	0.955027195
METTL17	0.410703518	0.847962006	0.847560686	0.166798772	0.369157337	0.627333499	0.955202269
ANKRD13D	0.750045831	0.163333854	0.58540598	0.778293372	0.325771351	0.627422306	0.955212285

GIN1	0.726251248	0.787447666	0.413638529	0.256623508	0.299590033	0.627456489	0.955212285
ACTR10	0.749371103	0.163224191	0.377428953	0.45744864	0.862167757	0.627680253	0.955296177
FAM117A	0.269591051	0.92931153	0.266624594	0.667082516	0.40873979	0.627744438	0.955296177
NCOA5	0.577678889	0.758257083	0.954192419	0.080608036	0.540365609	0.627659639	0.955296177
TCEAL1	0.612708682	0.51050911	0.599944947	0.908474416	0.106809102	0.62769784	0.955296177
TDRP	0.845640219	0.327335727	0.442771439	0.290807335	0.511169591	0.627805402	0.955300367
ACP1	0.782517241	0.819708438	0.618082956	0.083177482	0.55300133	0.627986703	0.95531051
KIF13B	0.364459832	0.431547244	0.871285058	0.206630208	0.643688609	0.627885888	0.95531051
SMARCD3	0.89956096	0.399373587	0.818131706	0.395777427	0.156732808	0.627947146	0.95531051
AGMAT	0.923956576	0.864110818	0.053273778	0.487463271	0.880446305	0.628187895	0.955350899
FADS3	0.736001504	0.59934934	0.168007439	0.370568815	0.664345413	0.628084781	0.955350899
SMN2	0.760864928	0.550189452	0.377367808	0.30259375	0.38178788	0.628137283	0.955350899
ARHGEF3	0.19028283	0.783571031	0.717231471	0.277658358	0.615388755	0.628376086	0.955548549
B4GALT1	0.321218266	0.513768881	0.615067738	0.609315915	0.295741016	0.628577367	0.955575855
BBC3	0.682461981	0.909755068	0.057841226	0.972627677	0.523387632	0.628471857	0.955575855
LHPP	0.535949477	0.605449093	0.47276381	0.200582189	0.594944004	0.628743409	0.955575855
LOC616427	0.592990749	0.754925394	0.211472739	0.539091543	0.358324701	0.628532629	0.955575855
NFATC2	0.991869169	0.479872268	0.129880655	0.920143239	0.321780605	0.628709963	0.955575855
ZNF175	0.908764278	0.153410031	0.241681187	0.681519325	0.796972343	0.62867823	0.955575855
BCLAF1	0.39719618	0.803779353	0.293812607	0.221745224	0.88092629	0.628919756	0.955755359
UBE2Q2	0.80492099	0.242429791	0.107179495	0.977829524	0.896472793	0.629029981	0.955834353
SPICE1	0.479292982	0.504009061	0.376465885	0.676506214	0.298134942	0.629119278	0.955881535
CBX5	0.647683805	0.191833944	0.798773867	0.21654576	0.853801526	0.629195411	0.95588212
PWWP2B	0.530285323	0.186190636	0.677953095	0.478624097	0.572859177	0.629236155	0.95588212
LARP1	0.895512518	0.861995113	0.340516683	0.271680035	0.257141809	0.629342207	0.955954735
CTSS	0.279775713	0.261592392	0.559831576	0.525386705	0.853499889	0.629445574	0.956023259
ASIP	0.734201267	0.034590909	0.907827328	0.896582719	0.890777549	0.62987964	0.956401591
ATRAID	0.664954781	0.712445942	0.12960666	0.714433823	0.420262742	0.630110982	0.956401591
CFAP161	0.762402813	0.745957307	0.706500724	0.094011343	0.487892143	0.630048413	0.956401591
HSPBAP1	0.943966016	0.440829201	0.465893025	0.835163789	0.113956662	0.630277448	0.956401591
MCC	0.10703442	0.847290433	0.530471601	0.443485188	0.863759635	0.630037064	0.956401591
NUDT5	0.170370059	0.670177612	0.764536939	0.353859895	0.596407408	0.629976834	0.956401591
SIAH2	0.552681505	0.167790692	0.776611135	0.304272166	0.841656973	0.630196235	0.956401591
TCTN2	0.98634623	0.436898287	0.381532074	0.422756112	0.265199027	0.630087752	0.956401591
TPM3	0.625789652	0.179451751	0.68015007	0.682649644	0.353745197	0.630207699	0.956401591
ZNF25	0.912242758	0.632217887	0.141343838	0.498079058	0.454380677	0.630254021	0.956401591
LOC101905711	0.603624923	0.674332487	0.307857937	0.189039966	0.779332133	0.630386994	0.956479378
ZZZ3	0.277711777	0.872101026	0.339046526	0.291583043	0.771386401	0.630471034	0.956518456
CPD	0.101718109	0.667898581	0.572990882	0.711358703	0.667591746	0.630651638	0.956615587
LOC789551	0.593356685	0.644610859	0.073363209	0.956555275	0.688564269	0.630602252	0.956615587
ATP9A	0.798098301	0.733883113	0.696114384	0.220392099	0.205941618	0.630853129	0.956803411
LOC112443012	0.780259917	0.37207363	0.704743379	0.206779296	0.437685236	0.630971939	0.956803411
RAPGEF1	0.938724928	0.151788612	0.409465277	0.416637689	0.761897639	0.631008672	0.956803411
ZNF367	0.708770691	0.488003599	0.210089364	0.594169278	0.428871608	0.630972922	0.956803411

SCOC	0.349853489	0.396314884	0.288790456	0.818785582	0.565452486	0.631200273	0.956964649
SGMS1	0.880530972	0.997817394	0.072711598	0.366840402	0.791168326	0.631231633	0.956964649
FBP1	0.988694919	0.770099544	0.718717676	0.681120496	0.049792026	0.631414177	0.956976179
KIAA0100	0.984741723	0.726202526	0.905791481	0.644979563	0.044419749	0.631404791	0.956976179
TPM4	0.319658322	0.598543184	0.850742948	0.114900358	0.991996312	0.631351226	0.956976179
FJX1	0.871942284	0.472396769	0.089105762	0.767981746	0.658730528	0.631507694	0.957029529
TTC7A	0.396876171	0.988563438	0.092814194	0.871490162	0.585283832	0.631572402	0.957039214
GPR158	0.334649137	0.336171632	0.300371915	0.811524566	0.678113643	0.631802388	0.957210948
MRPS27	0.756291338	0.908238019	0.842414475	0.328187479	0.097913222	0.631786072	0.957210948
SEC23IP	0.67173842	0.621888022	0.502802121	0.177968695	0.497644205	0.631873373	0.957230123
SLC4A10	0.735205453	0.182331657	0.850179	0.56257481	0.29032157	0.631994248	0.957324867
RPS6KA1	0.126274803	0.821791911	0.631759051	0.776821331	0.365645864	0.632071207	0.957353077
LOC112447492	0.267838983	0.24068295	0.534881942	0.82270365	0.656631495	0.632129838	0.957353523
RAB13	0.553800626	0.91506737	0.042275014	0.889307712	0.977985479	0.632189467	0.957355481
NRBF2	0.171718064	0.343678011	0.54500619	0.685715079	0.845270132	0.632294569	0.957426294
RUNDC1	0.16232244	0.260831305	0.79883254	0.595000995	0.927089525	0.632440766	0.957559314
CLN8	0.528897492	0.704856804	0.949252579	0.057933848	0.910630474	0.632573623	0.957564304
FOXC2	0.246345249	0.83592424	0.501241255	0.207167302	0.873274398	0.632619108	0.957564304
KIF5B	0.678113294	0.635499266	0.164835486	0.507993143	0.517298239	0.632545794	0.957564304
MRPS15	0.918502266	0.879243602	0.44477398	0.071742788	0.725138693	0.632753287	0.957679073
TRPM4	0.649428854	0.434887155	0.114570754	0.74085824	0.780152371	0.632918971	0.957781673
ZNF197	0.394323087	0.556110304	0.481089886	0.183139248	0.968094023	0.632937801	0.957781673
NBEAL2	0.611100097	0.382768226	0.648672696	0.798556302	0.154457142	0.633049827	0.957862872
KDM4A	0.792531888	0.170494937	0.556291511	0.705370898	0.353253782	0.633206201	0.958011152
IMPAD1	0.297777031	0.242426843	0.902420697	0.536654378	0.53619399	0.633369281	0.958169549
LATS1	0.264548791	0.311368394	0.697081628	0.565673227	0.578291192	0.63376448	0.958502339
NCOR1	0.371008662	0.382556746	0.514280627	0.365011646	0.704760021	0.633697869	0.958502339
SEMA4D	0.266084576	0.605358741	0.770806681	0.770064101	0.1964003	0.633706394	0.958502339
AFG3L2	0.83549323	0.646721972	0.924517117	0.0496502	0.758145623	0.63397724	0.958572482
DDIT4	0.874923642	0.375434614	0.10863093	0.551764559	0.95548853	0.634061846	0.958572482
LOC101903253	0.655569606	0.522336011	0.22625091	0.268578999	0.904935275	0.634246916	0.958572482
LOC101907017	0.625190992	0.877685461	0.191955281	0.233040371	0.767057918	0.634229968	0.958572482
MBP	0.289402912	0.661513028	0.378997444	0.297497338	0.872090795	0.634190099	0.958572482
NUDT13	0.456364215	0.325449899	0.506636487	0.329825087	0.758826043	0.634278142	0.958572482
PRR7	0.47281836	0.189202981	0.929686397	0.273134346	0.828551523	0.634159626	0.958572482
TIGD2	0.708981398	0.972616207	0.753699384	0.089795777	0.403162579	0.634094859	0.958572482
HPRT1	0.60834727	0.349368578	0.84768519	0.771493871	0.135727298	0.634616206	0.958995079
HCFC2	0.228348054	0.622605958	0.706763228	0.259551125	0.723836292	0.634741302	0.959095802
ART4	0.400738286	0.601604849	0.415217192	0.3226571	0.586095344	0.635285185	0.959362657
CLDND1	0.729215035	0.346486701	0.725615708	0.347568735	0.29732849	0.635451201	0.959362657
DUSP5	0.443703546	0.461428038	0.497282668	0.84508744	0.220153488	0.635406821	0.959362657
ERLIN2	0.66306542	0.581640945	0.309624566	0.201450457	0.787470205	0.635416595	0.959362657
LUM	0.799828616	0.77261601	0.067818447	0.531759578	0.849883575	0.635386949	0.959362657
MAMSTR	0.329416409	0.646747842	0.130483489	0.880599346	0.774151564	0.635502495	0.959362657

MGMT	0.727700263	0.602440843	0.160579454	0.301558011	0.891200422	0.635171021	0.959362657
PKM	0.631895013	0.747524691	0.140291711	0.310481248	0.919133763	0.635087093	0.959362657
ST8SIA5	0.712742357	0.771759551	0.043497827	0.880020912	0.898824067	0.635236987	0.959362657
UBAP1	0.911750202	0.294895331	0.563029767	0.36193464	0.34557659	0.635326834	0.959362657
ABI2	0.126910932	0.783685502	0.402879034	0.540290458	0.884153351	0.637450089	0.959439617
ADAM20	0.663121579	0.557817733	0.610687311	0.232926409	0.360995188	0.63594525	0.959439617
ARSK	0.057828117	0.700998518	0.717726894	0.767002801	0.851361216	0.635990978	0.959439617
BABAM1	0.423515675	0.671993559	0.649087579	0.258088069	0.400248558	0.636849764	0.959439617
C10H14orf119	0.186887675	0.970242105	0.692077671	0.441579709	0.342897467	0.636019231	0.959439617
C13H20orf194	0.351611487	0.546676731	0.583265193	0.310714542	0.547550127	0.636764115	0.959439617
C14H8orf33	0.401780889	0.819151867	0.829374299	0.225796851	0.311043992	0.637753013	0.959439617
C1QC	0.88510379	0.856510102	0.368711953	0.161512622	0.422053231	0.636558322	0.959439617
CA9	0.627324133	0.145210271	0.445707448	0.685848334	0.688061864	0.637640952	0.959439617
CAPN15	0.809113559	0.095210334	0.777484902	0.905143428	0.353335085	0.637593685	0.959439617
CCNYL1	0.961266474	0.608398409	0.507816709	0.069320146	0.925741739	0.636603551	0.959439617
CFAP54	0.339049524	0.505522122	0.906024925	0.191293092	0.644864094	0.637603234	0.959439617
CTNNA3	0.893412566	0.746162544	0.688469544	0.225398754	0.185303754	0.63773592	0.959439617
DCTN2	0.864968484	0.735421066	0.069225302	0.606714985	0.711042214	0.635970798	0.959439617
DKC1	0.310718698	0.38913622	0.760644818	0.658398255	0.316517536	0.637705085	0.959439617
DOT1L	0.638669888	0.107589112	0.682002868	0.882805287	0.460722529	0.636624767	0.959439617
DROSHA	0.661429281	0.654094567	0.128598914	0.620875469	0.555135603	0.637807372	0.959439617
FAM205C	0.63864892	0.78238436	0.507904553	0.734615902	0.102205331	0.636563143	0.959439617
FCRL1	0.356385289	0.234939707	0.446714724	0.545841954	0.939679286	0.637892003	0.959439617
GLRB	0.180762714	0.998253033	0.395089646	0.702376808	0.382891516	0.637774185	0.959439617
IGF1R	0.610123852	0.525425114	0.90249444	0.904786351	0.072664733	0.636223466	0.959439617
IL5RA	0.756869829	0.687930166	0.479490215	0.444546055	0.171733274	0.636616297	0.959439617
KDM6B	0.630151966	0.720290068	0.494034036	0.557337828	0.153135102	0.637419711	0.959439617
KLC4	0.795897159	0.953535379	0.288647883	0.105458118	0.828244594	0.637373794	0.959439617
KMT2D	0.596539331	0.397754671	0.532848748	0.458229523	0.328216402	0.636159859	0.959439617
LOC101906508	0.373649888	0.458209178	0.32694065	0.50631711	0.675995981	0.637627226	0.959439617
LOC104974330	0.799286834	0.137886401	0.745228573	0.240436563	0.967607946	0.637109687	0.959439617
LOC112441469	0.06783539	0.949319255	0.667910963	0.517216156	0.861887787	0.637782706	0.959439617
LOC783261	0.743973921	0.781221223	0.174222914	0.224290625	0.844621536	0.637872783	0.959439617
MPLKIP	0.656281603	0.869366798	0.100568534	0.611656472	0.542711704	0.63648841	0.959439617
MYCBP	0.68507384	0.492030021	0.192600527	0.500904509	0.586438035	0.636727398	0.959439617
POLR3C	0.639993921	0.400559615	0.391027902	0.29738781	0.641651581	0.637316417	0.959439617
QSOX2	0.189857511	0.935829727	0.45904066	0.42224587	0.555601247	0.637375427	0.959439617
SLC25A36	0.739601622	0.290512086	0.331709012	0.286928504	0.934042643	0.637040516	0.959439617
SLC3A2	0.673621841	0.342549887	0.674693766	0.210180533	0.583969096	0.637116663	0.959439617
TMEM143	0.976499781	0.878279021	0.804530716	0.054154278	0.510143737	0.636641535	0.959439617
TRIB1	0.386255088	0.871855401	0.236407726	0.611553008	0.390184534	0.635972229	0.959439617
UCHL5	0.68173608	0.482396801	0.229195384	0.464191608	0.545557517	0.636907689	0.959439617
ZDHHC12	0.146972083	0.521679759	0.297652388	0.906343744	0.924445924	0.637248717	0.959439617
ZNF81	0.494487601	0.385120666	0.36272289	0.315454816	0.877042358	0.637141796	0.959439617

NOS1	0.411630823	0.990182809	0.722553811	0.217624404	0.299565371	0.638044749	0.959493482
RSPO3	0.399207387	0.438650891	0.187627499	0.630717629	0.926277669	0.637998683	0.959493482
STAT5B	0.806170503	0.17312785	0.643582302	0.467481065	0.457494066	0.638159318	0.959577842
ADGRE5	0.296843778	0.125227064	0.635056157	0.84617309	0.962576477	0.638332598	0.959662539
MCOLN2	0.919746051	0.495962175	0.469946981	0.10123809	0.885792295	0.63829143	0.959662539
IRF2	0.637163387	0.069252084	0.618499117	0.987174454	0.714410231	0.638526685	0.959690579
ISYNA1	0.696774277	0.365109264	0.285382856	0.879854293	0.301181443	0.638444611	0.959690579
WIPI2	0.296866548	0.972568244	0.258649334	0.311000964	0.828555162	0.638486913	0.959690579
ARHGEF16	0.154216644	0.424952625	0.544380645	0.541311885	0.998392401	0.638867991	0.95978808
CLIP3	0.935697927	0.554936217	0.107111996	0.632236895	0.548185539	0.638823466	0.95978808
KCTD6	0.553755255	0.577695602	0.713717194	0.997110136	0.08475998	0.639027088	0.95978808
LOC100296211	0.290900663	0.367658576	0.826599328	0.231655991	0.941805981	0.638942718	0.95978808
LOC781224	0.746910102	0.189275764	0.645140825	0.269126245	0.785565771	0.638882921	0.95978808
LONP2	0.772629332	0.842248778	0.339592377	0.273186439	0.319418289	0.638897293	0.95978808
OCSTAMP	0.627567151	0.168297144	0.4576401	0.840595889	0.475007758	0.639059433	0.95978808
ZSCAN23	0.238764748	0.547775768	0.793659768	0.531008517	0.34989952	0.638925981	0.95978808
CARS	0.316165248	0.956285173	0.754963662	0.239364409	0.353368526	0.639134109	0.959812395
C22H3orf49	0.727904349	0.419841443	0.807074809	0.248621367	0.318557215	0.641420265	0.959867285
CCR7	0.538053472	0.702546474	0.853561855	0.337928621	0.177537919	0.639645198	0.959867285
CFAP97	0.440409321	0.51976656	0.495414929	0.714541311	0.23909939	0.639819734	0.959867285
COPRS	0.783896169	0.476072265	0.556016713	0.11914875	0.789543868	0.641278959	0.959867285
COQ8B	0.405728327	0.225177196	0.866232502	0.252061047	0.97148267	0.639862572	0.959867285
EID3	0.555552736	0.495034393	0.501358974	0.214759432	0.65348279	0.639575166	0.959867285
FAM162A	0.493649947	0.942223435	0.5729549	0.080127513	0.908862013	0.640149477	0.959867285
FIGNL2	0.613871734	0.643066054	0.925285287	0.328179868	0.162527563	0.640902538	0.959867285
GLDC	0.399815452	0.369639638	0.208175398	0.817685253	0.770582081	0.639924438	0.959867285
HPCAL4	0.827032892	0.946563346	0.467824669	0.370934075	0.143483046	0.640994741	0.959867285
IGFBP7	0.728751626	0.429069464	0.106435015	0.712330497	0.823935419	0.641405461	0.959867285
LOC112443004	0.227129955	0.61130556	0.97268813	0.15533006	0.923911216	0.639888259	0.959867285
LOC112444888	0.957588902	0.227410041	0.839671891	0.852233236	0.124823834	0.640590717	0.959867285
LOC112447351	0.912079605	0.095789734	0.766823836	0.736926485	0.393643025	0.640419716	0.959867285
LOC516355	0.595108357	0.675277774	0.342633162	0.158092576	0.89233321	0.640317371	0.959867285
LOC781298	0.410222911	0.556047489	0.48085939	0.301677553	0.587812422	0.640580514	0.959867285
LOC783942	0.531465777	0.430485829	0.199592545	0.995522607	0.427568579	0.6404473	0.959867285
MAML1	0.470626619	0.378300312	0.535998331	0.207665612	0.974733694	0.639229509	0.959867285
NATD1	0.295590119	0.733373859	0.231813336	0.406262369	0.948491628	0.639709167	0.959867285
NPHP3	0.685737965	0.499386666	0.5340965	0.23425938	0.452175183	0.63981022	0.959867285
NUP107	0.312030914	0.432549254	0.678307585	0.216976602	0.980980606	0.640940915	0.959867285
PLK3	0.174713736	0.539771175	0.718233961	0.382313902	0.750767928	0.640489158	0.959867285
PLXNB1	0.156517531	0.99057412	0.394583045	0.49009283	0.647847943	0.640314397	0.959867285
RYR3	0.600345299	0.628832218	0.08702648	0.940763955	0.63086211	0.641061973	0.959867285
SFT2D1	0.497322512	0.300776096	0.55130326	0.571761782	0.410628599	0.639683292	0.959867285
SLC30A9	0.792042091	0.395828061	0.377783469	0.477883509	0.34182638	0.639543487	0.959867285
STXBP1	0.648268219	0.741479398	0.314175167	0.1507827	0.858009552	0.641451741	0.959867285

TCF7L1	0.530575394	0.763482523	0.602791208	0.340284697	0.234755953	0.641138282	0.959867285
TMEM134	0.728590113	0.705853415	0.466605487	0.289629971	0.279655376	0.640437822	0.959867285
TNFRSF4	0.607277896	0.245762222	0.869154802	0.982708177	0.152315779	0.640237677	0.959867285
TNS1	0.838968127	0.326275236	0.315049078	0.853755276	0.264424424	0.640765363	0.959867285
TREML1	0.793737982	0.481690138	0.459718383	0.761852406	0.145742466	0.641237917	0.959867285
TRIM9	0.478107968	0.860044382	0.178745907	0.482150932	0.54916207	0.640686108	0.959867285
TRIOBP	0.69252933	0.831464349	0.096603121	0.585963108	0.599276712	0.641407375	0.959867285
TRIR	0.430855348	0.549444496	0.304585503	0.284793185	0.947663275	0.640677877	0.959867285
VCP	0.65742421	0.379381909	0.410508184	0.781945372	0.243450635	0.640985098	0.959867285
VTI1A	0.114202712	0.980225832	0.528071838	0.366082428	0.89592021	0.639956454	0.959867285
WASHC4	0.467176648	0.627186905	0.210589073	0.886742093	0.356297606	0.641026786	0.959867285
ZFAND6	0.65927226	0.799513988	0.272054521	0.59329366	0.228274681	0.640284922	0.959867285
ATG9A	0.72511216	0.584926331	0.487532139	0.944131144	0.10025842	0.641808492	0.960201206
EIF3L	0.488147595	0.817783334	0.834455901	0.954012548	0.06159315	0.641815915	0.960201206
SH2D1B	0.884558801	0.393085508	0.702360458	0.337759338	0.237342158	0.641850419	0.960201206
HSD11B1L	0.907923389	0.999762127	0.072802671	0.760640447	0.389690224	0.64195656	0.960272455
LOC615959	0.747168805	0.552991557	0.084632372	0.608498601	0.921411176	0.642134002	0.960440686
MRPL22	0.412359535	0.636767588	0.379395606	0.873370731	0.225400411	0.642186073	0.960440686
LGMN	0.222778181	0.323478392	0.416125775	0.656441163	0.996760245	0.642287874	0.960505403
MAP3K14	0.724681741	0.212834866	0.285547802	0.58910394	0.757240647	0.642542199	0.960515498
REEP6	0.590663977	0.926060335	0.81332887	0.128229581	0.344477477	0.642587268	0.960515498
REXO1	0.997319715	0.148644337	0.516894435	0.434488811	0.590228795	0.642582147	0.960515498
RFT1	0.949190899	0.377184397	0.749932806	0.091351386	0.800438552	0.642397443	0.960515498
SAT1	0.351546056	0.680957598	0.305593196	0.686454279	0.391122643	0.64248636	0.960515498
LOC782922	0.586691974	0.159977859	0.840531477	0.422851771	0.590042202	0.642902061	0.960831744
SLAIN1	0.646382662	0.428376288	0.678674728	0.267215028	0.392002119	0.642915934	0.960831744
C24H18orf21	0.177235905	0.415951943	0.837686252	0.343558651	0.928290779	0.643021615	0.960902178
ARL2BP	0.514173893	0.43511907	0.246781085	0.426790304	0.838938036	0.643746829	0.961176291
C18H19orf54	0.536580865	0.837334491	0.442709666	0.132765983	0.750093957	0.644142941	0.961176291
C22H3orf14	0.508694482	0.761365963	0.587505976	0.679041213	0.128095564	0.643979927	0.961176291
ECT2	0.937023705	0.47942662	0.097950359	0.471545552	0.951327949	0.643458337	0.961176291
FZD4	0.732346749	0.837451127	0.215681465	0.449974061	0.331603694	0.643441807	0.961176291
LOC101906569	0.634451474	0.279516355	0.416204549	0.418391528	0.641707392	0.644222382	0.961176291
LOC101906717	0.948915104	0.325045551	0.175400272	0.638173924	0.571907251	0.643518503	0.961176291
LOC112443425	0.303091672	0.698694082	0.456497204	0.604862804	0.338934402	0.644240064	0.961176291
LOC112447824	0.395012659	0.497773807	0.687786721	0.336782879	0.435178715	0.644259289	0.961176291
NDST3	0.625089814	0.68044279	0.719119646	0.104590167	0.618573626	0.643945967	0.961176291
NME9	0.53464685	0.957225658	0.459242093	0.168129572	0.499610145	0.643487881	0.961176291
NOLC1	0.964173178	0.476453445	0.491113956	0.26115736	0.335870037	0.643952206	0.961176291
NPR1	0.368867042	0.558492529	0.969060166	0.209624335	0.473092036	0.644039398	0.961176291
POLDIP2	0.960757912	0.635387633	0.242070727	0.261771063	0.511832745	0.644048121	0.961176291
TBK1	0.751893838	0.742842526	0.283594506	0.394599028	0.316583951	0.643936328	0.961176291
UBE2E1	0.498876549	0.199868955	0.534207641	0.697393022	0.53278855	0.643974538	0.961176291
VSTM4	0.951107891	0.708603203	0.13181633	0.254587536	0.874519028	0.64385205	0.961176291

WWP2	0.852362625	0.266861055	0.837885277	0.233124564	0.446057117	0.644240489	0.961176291
MFSD1	0.586482172	0.365388824	0.172966545	0.554223801	0.965584498	0.644409037	0.961312308
RFK	0.816365617	0.488470697	0.710042231	0.085977129	0.815622874	0.644603257	0.961427252
TMEM259	0.447186492	0.705830449	0.313869955	0.576343104	0.347648499	0.644549559	0.961427252
MASP1	0.139227429	0.566464905	0.56241768	0.736879297	0.607938502	0.64475295	0.961537564
NR1I3	0.205024153	0.498732775	0.985787234	0.441574521	0.446658108	0.644852991	0.961537564
RNF220	0.370279382	0.738850008	0.768001143	0.228285767	0.414450097	0.644836543	0.961537564
NYX	0.780744205	0.906241574	0.044206689	0.823593888	0.772611499	0.645068583	0.961771646
SGCD	0.479912364	0.66414047	0.105505121	0.672942313	0.879777526	0.645127506	0.961772121
CMTR2	0.567407364	0.697222729	0.813371415	0.384764086	0.160872003	0.645210333	0.961808227
CAPRIN1	0.980707044	0.441080021	0.499298735	0.305316285	0.302295802	0.645376099	0.961967952
AHNAK	0.946655547	0.450064751	0.337821191	0.719032603	0.192820873	0.645581611	0.962012151
ORAOV1	0.553620356	0.62128401	0.108811097	0.539983186	0.98709636	0.645518106	0.962012151
TTC23	0.754603128	0.655087577	0.381044544	0.153684955	0.689322379	0.645577541	0.962012151
APOOL	0.862114876	0.655175778	0.803784022	0.06010771	0.735087614	0.64660406	0.962194944
CEP72	0.123921074	0.941508287	0.299704398	0.607094691	0.947231998	0.647073016	0.962194944
CWC27	0.57166609	0.443270375	0.878817847	0.134225249	0.669980497	0.646278148	0.962194944
FANCA	0.793435028	0.284600018	0.295408702	0.817542728	0.366879258	0.646096954	0.962194944
HNRNPU	0.77934663	0.248026232	0.985930662	0.162793202	0.644256779	0.645902234	0.962194944
JUND	0.472947723	0.384150921	0.794252418	0.295902982	0.471340227	0.647244749	0.962194944
KANK3	0.287566785	0.580704238	0.27399343	0.914136614	0.481077616	0.647201032	0.962194944
KREMEN1	0.393135202	0.500558604	0.515520009	0.452908314	0.435200813	0.645979743	0.962194944
LOC100847490	0.252040599	0.942855019	0.346722061	0.796901122	0.306428008	0.647187404	0.962194944
LOC101904855	0.502889197	0.223296245	0.78196032	0.409618519	0.559458632	0.647212663	0.962194944
LOC101906364	0.351996141	0.552854563	0.356952296	0.586348342	0.492741299	0.646694842	0.962194944
LOC104973826	0.53741716	0.907663067	0.82558752	0.774078643	0.064575496	0.647287318	0.962194944
LOC104975861	0.918960899	0.440764432	0.545990628	0.951101817	0.095362375	0.646586626	0.962194944
LOC112442214	0.253462978	0.354118405	0.570405151	0.725939437	0.541158943	0.647116367	0.962194944
LOC112447802	0.108130457	0.701830291	0.718196747	0.511205419	0.720537552	0.646758653	0.962194944
LOC617654	0.957426176	0.439173908	0.472615022	0.155328243	0.652088554	0.647266742	0.962194944
MRPS17	0.937022972	0.708127022	0.209711531	0.506858275	0.284912174	0.646940922	0.962194944
PACSIN2	0.750796195	0.409494963	0.2278621	0.454652723	0.629253383	0.6464317	0.962194944
PELO	0.921310316	0.086927618	0.393078174	0.942226341	0.67490077	0.646202389	0.962194944
PTRHD1	0.18551608	0.741774782	0.737774174	0.275722422	0.716054494	0.64645289	0.962194944
RAPGEF5	0.497097357	0.66100434	0.094817955	0.765705565	0.837773384	0.64588192	0.962194944
RNPEPL1	0.403508981	0.885988428	0.400357589	0.1694723	0.828697085	0.647004996	0.962194944
SLC9C2	0.296801202	0.847208189	0.097269191	0.861113341	0.954036795	0.646929816	0.962194944
TLE6	0.613365835	0.342308193	0.331940233	0.295670376	0.974947183	0.646897967	0.962194944
TMEM127	0.588295241	0.180388129	0.541242218	0.565621243	0.618070912	0.646796096	0.962194944
TMOD3	0.773058529	0.643834957	0.484011123	0.159913475	0.520703757	0.646597955	0.962194944
ZNF217	0.649612014	0.649004669	0.884099591	0.126657016	0.425647734	0.646941539	0.962194944
BCOR	0.286183499	0.7301171	0.942842594	0.142039942	0.720833846	0.647678148	0.962310591
C21H15orf39	0.355124431	0.193114307	0.94757762	0.527069367	0.588560604	0.647564381	0.962310591
EML5	0.714721916	0.913514099	0.763039557	0.272198306	0.148817494	0.647775583	0.962310591

LCAT	0.239332641	0.946511744	0.901545433	0.132010291	0.748489365	0.647762179	0.962310591
PRSS2	0.662059668	0.590896029	0.177747433	0.530429535	0.546946724	0.647706144	0.962310591
RAN	0.576376796	0.227155464	0.621584437	0.886018865	0.279595223	0.647579552	0.962310591
WDR59	0.124754911	0.461595323	0.749288684	0.76834373	0.607988752	0.647542166	0.962310591
CHIC1	0.969546707	0.866373403	0.731994708	0.162285745	0.202306641	0.647835562	0.962312583
ARID5A	0.47503615	0.734916688	0.649067157	0.130250061	0.684350732	0.647942843	0.962384831
LOC101907294	0.491733184	0.672233742	0.426949055	0.220857925	0.648278474	0.648027939	0.962424119
HP1BP3	0.757276179	0.285431712	0.803469032	0.324304731	0.359263572	0.648293765	0.962731786
KCNC4	0.864496064	0.726716206	0.267853147	0.512836928	0.234772431	0.648544854	0.962857989
NIPAL4	0.804649186	0.67803957	0.335996748	0.303585686	0.364151147	0.64859367	0.962857989
NOTCH4	0.406256137	0.861936735	0.537441235	0.36881341	0.292032113	0.64863139	0.962857989
PDE8A	0.844233131	0.978596225	0.149297537	0.232797256	0.706010435	0.648660108	0.962857989
PROSER2	0.883855838	0.102335076	0.397701548	0.702022085	0.802823809	0.648672106	0.962857989
CMPK2	0.711723913	0.748683525	0.816837557	0.09232319	0.505932078	0.649217621	0.963128329
DERL2	0.175051874	0.393121409	0.364407546	0.898348654	0.901876153	0.649093677	0.963128329
DNAJC9	0.222752201	0.295819639	0.757250712	0.755501069	0.540258215	0.649566485	0.963128329
INTS8	0.397253594	0.884449204	0.655911169	0.749429221	0.117960589	0.64962405	0.963128329
KANTR	0.659892275	0.898616711	0.353963064	0.122768036	0.791148526	0.649756788	0.963128329
LDLRAD4	0.198458738	0.616902956	0.899409195	0.396927239	0.465637962	0.649422074	0.963128329
LOC100139996	0.996309351	0.784611861	0.213885935	0.152499823	0.799232935	0.64967827	0.963128329
LOC101902840	0.883904231	0.262497485	0.303773218	0.916502733	0.314820121	0.649274913	0.963128329
LOC101904339	0.536151648	0.871344879	0.750311639	0.19002768	0.305881388	0.64964023	0.963128329
LOC104968434	0.493893956	0.508167098	0.44460995	0.352458352	0.516909953	0.649214737	0.963128329
LOC107132270	0.774494797	0.969051969	0.043676035	0.796767126	0.780782065	0.649811232	0.963128329
LOC112444626	0.342356883	0.999128617	0.803150564	0.188041679	0.39475932	0.649817488	0.963128329
LOC112445035	0.942476738	0.658806367	0.537037981	0.424601415	0.144060776	0.649851928	0.963128329
NUDT6	0.46787737	0.516556251	0.627562053	0.562050272	0.238315703	0.649076385	0.963128329
PHF19	0.405705519	0.356929749	0.324594855	0.590458432	0.733997982	0.649608857	0.963128329
PRORSD1	0.700339432	0.813720794	0.159590275	0.336304146	0.666287399	0.649682863	0.963128329
RPE	0.728583539	0.143647087	0.533949491	0.518790845	0.702774158	0.649638034	0.963128329
SV2B	0.507091355	0.807253835	0.067202825	0.829094257	0.894560817	0.649913474	0.963132565
CELA1	0.745587159	0.802592971	0.220986059	0.54112871	0.285558354	0.650208339	0.963243869
GNPAT	0.833998097	0.239161241	0.265593113	0.562019774	0.686250949	0.650187286	0.963243869
ITGA2	0.383437934	0.352705571	0.588391113	0.280570839	0.915592415	0.650282056	0.963243869
LOC101907744	0.371012734	0.625659204	0.589001168	0.896678561	0.166555297	0.65006638	0.963243869
LOC112448453	0.157464431	0.858372727	0.741624765	0.787055078	0.259059029	0.650249826	0.963243869
COMMD1	0.40044507	0.336055908	0.559236223	0.296445113	0.921110842	0.651309542	0.963342613
CRAT	0.522860951	0.751552679	0.838001883	0.090677838	0.686116051	0.650718243	0.963342613
ECH1	0.982428676	0.726072225	0.304165736	0.270127949	0.351090896	0.651567432	0.963342613
HVCN1	0.472607302	0.427679846	0.872506875	0.132091675	0.881032364	0.651062273	0.963342613
IREB2	0.951468824	0.342058114	0.29212589	0.919093632	0.234966132	0.651140486	0.963342613
LOC100140207	0.332708168	0.967777961	0.200358542	0.859652575	0.370944326	0.651521861	0.963342613
LOC104968522	0.542241512	0.572386028	0.279129527	0.948390022	0.249170611	0.650575414	0.963342613
LOC112444613	0.469521561	0.39977318	0.374594774	0.505507098	0.578912802	0.65156343	0.963342613

LOC781158	0.601335149	0.851205955	0.420475326	0.465766201	0.20465208	0.650981664	0.963342613
MPP1	0.919130406	0.878766229	0.045159391	0.93086007	0.60508616	0.651262035	0.963342613
NABP1	0.526248459	0.568079095	0.620614588	0.933827389	0.118278979	0.650766045	0.963342613
NXT1	0.887945257	0.446900219	0.265762055	0.468549295	0.415002381	0.650900696	0.963342613
REXO4	0.1463306	0.761548515	0.812569527	0.741836333	0.304641133	0.650493914	0.963342613
RNF10	0.534921509	0.61002558	0.544531703	0.239317289	0.483258243	0.651313427	0.963342613
SS18	0.597832576	0.728150794	0.48995121	0.283368371	0.338961852	0.650703227	0.963342613
TMEM101	0.482117735	0.420591587	0.3449886	0.298443899	0.982439285	0.650941677	0.963342613
TP53INP2	0.670620355	0.255252662	0.900007029	0.233387688	0.572195596	0.651538302	0.963342613
TPCN1	0.047245489	0.73936245	0.763577126	0.794750295	0.966371662	0.650698553	0.963342613
UBE2J1	0.624843092	0.107081771	0.85738034	0.613378706	0.584820637	0.65158144	0.963342613
ZFP62	0.324639216	0.48272228	0.695859384	0.349032788	0.538213869	0.650696485	0.963342613
ZWINT	0.95819156	0.157351436	0.296216523	0.613937835	0.749295163	0.651266289	0.963342613
ACAP2	0.373409951	0.47273279	0.578209101	0.235885011	0.856776948	0.652049908	0.96337463
AIP	0.854585909	0.324564481	0.239656169	0.595147265	0.523138517	0.652692815	0.96337463
ANGEL2	0.239646213	0.665870596	0.167944028	0.896809136	0.857769839	0.651933657	0.96337463
BCAP31	0.710162219	0.674770933	0.242863664	0.273510184	0.647946641	0.652020524	0.96337463
C18H19orf84	0.790523987	0.77327793	0.882991443	0.209140267	0.182710276	0.652028865	0.96337463
CCL16	0.919999689	0.539973953	0.245422266	0.298170605	0.567061297	0.651921765	0.96337463
CRACR2B	0.756821049	0.429879273	0.379054632	0.258972562	0.647818753	0.652630995	0.96337463
DDX1	0.936728759	0.642147535	0.664958626	0.446514433	0.115895019	0.652718452	0.96337463
EGFR	0.769745898	0.99127415	0.087547403	0.464311967	0.665590095	0.6522055	0.96337463
FAM21A	0.608655891	0.583825937	0.179790375	0.620527839	0.519904781	0.651893676	0.96337463
GNB4	0.704525641	0.357996488	0.66821692	0.881808831	0.139133101	0.652518369	0.96337463
HES4	0.713256189	0.454775876	0.475566208	0.164472506	0.815703449	0.652689714	0.96337463
LOC104973517	0.924568794	0.24906834	0.317218553	0.437644128	0.645796233	0.652219775	0.96337463
LOC107132395	0.603752573	0.240865563	0.398086824	0.425544164	0.838776329	0.652385627	0.96337463
MED20	0.19450583	0.622812441	0.776673093	0.253992055	0.864116545	0.652259138	0.96337463
MYCBPAP	0.535396322	0.364020286	0.30046357	0.397323153	0.887298132	0.652207778	0.96337463
PCDHB8	0.336315148	0.184511874	0.884077281	0.495790878	0.759447222	0.652320427	0.96337463
PTGDR	0.379331121	0.644035811	0.979296386	0.439196	0.196451572	0.652185459	0.96337463
VPS52	0.98819517	0.516762946	0.32365405	0.163769728	0.763674078	0.652454999	0.96337463
LOC613677	0.758682665	0.260572415	0.322114955	0.623998965	0.521423478	0.652911681	0.963573166
ECE2	0.62933649	0.208231995	0.288548543	0.63063044	0.871216077	0.653439714	0.963608879
FAM129C	0.130615299	0.447754564	0.377106825	0.960945122	0.979299056	0.653243218	0.963608879
HERC6	0.791503103	0.112454679	0.880993595	0.4231216	0.62565021	0.653282415	0.963608879
LINS1	0.272635564	0.479554651	0.529195284	0.466775344	0.642980117	0.653346316	0.963608879
LOC781379	0.886230833	0.08474135	0.603546393	0.470505672	0.973815951	0.653369935	0.963608879
SLC25A3	0.694376215	0.654795122	0.710064914	0.087567045	0.734963712	0.653464336	0.963608879
ST5	0.425037062	0.668074928	0.16001607	0.973468305	0.468988087	0.653147507	0.963608879
TSPAN3	0.175480337	0.224918465	0.672700657	0.857707617	0.91127164	0.653220841	0.963608879
VPS25	0.944447067	0.323552952	0.563444562	0.17286829	0.697170628	0.653205601	0.963608879
FMN1	0.18662395	0.942182187	0.560449754	0.234853543	0.898082255	0.653530448	0.963619783
UBE2F	0.445013667	0.216333123	0.293724225	0.800531637	0.918914357	0.653681956	0.963756587

BIVM	0.247474517	0.77585245	0.615784137	0.309334232	0.56996661	0.654097571	0.963849801
C15H11orf96	0.291545489	0.213908622	0.53353101	0.772370502	0.810810013	0.654018363	0.963849801
GPX1	0.627338739	0.318625693	0.671565934	0.437905653	0.354207551	0.653870612	0.963849801
LOC101902856	0.471558943	0.620919028	0.350089435	0.81132638	0.250532126	0.654004858	0.963849801
LOC101905571	0.840213801	0.892571605	0.14617852	0.313475223	0.606473988	0.654060007	0.963849801
YWHAG	0.70936439	0.299532864	0.132293911	0.990509593	0.748093876	0.653941798	0.963849801
DNAH5	0.231965766	0.897245487	0.970942114	0.984998276	0.104804932	0.654245788	0.963890334
NOL10	0.787514838	0.149908293	0.559434106	0.322101277	0.980453136	0.654204007	0.963890334
PCSK7	0.743883866	0.634619404	0.744862104	0.275471711	0.215490839	0.654360015	0.963890334
PPM1K	0.545308023	0.878042448	0.288603802	0.233354921	0.647248282	0.654336285	0.963890334
F8A1	0.919065944	0.622228649	0.363065752	0.105294827	0.955617345	0.654527366	0.963963798
LOC112443142	0.697368158	0.07943623	0.998239626	0.625124383	0.604344909	0.654524596	0.963963798
KCNMB2	0.996587461	0.39597942	0.62666218	0.093982648	0.899551875	0.654672071	0.964012028
PREP	0.969801564	0.419202403	0.364384713	0.865633463	0.163044779	0.654677598	0.964012028
ETV5	0.152927072	0.985517515	0.501423582	0.280095372	0.988338777	0.654793182	0.964067773
LOC112447473	0.235314694	0.36650578	0.721961388	0.547059941	0.614477467	0.654891691	0.964067773
LOC509810	0.638316004	0.56973877	0.886419457	0.244172942	0.265844321	0.654842763	0.964067773
RBPJ	0.622347043	0.563070911	0.629683121	0.138800026	0.683721192	0.654982192	0.964114517
CAVIN2	0.654680268	0.397987946	0.283637601	0.284529305	0.996203215	0.65505021	0.964128162
NPY1R	0.292369518	0.886419072	0.684600641	0.699965001	0.16882303	0.655220121	0.964215149
RGS5	0.804440868	0.292789217	0.576588326	0.178950903	0.86274506	0.65522682	0.964215149
GTPBP8	0.31048503	0.876976846	0.088952462	0.945984919	0.915388045	0.655291802	0.964224313
LOC112442365	0.621610712	0.499764554	0.425373632	0.258474118	0.614424003	0.655410068	0.964311873
AFAP1L1	0.343607024	0.389901734	0.367751571	0.890563046	0.478779287	0.655603948	0.964328974
LOC101904529	0.413176127	0.759396456	0.908790745	0.191059901	0.385433992	0.655521142	0.964328974
LOC515736	0.502365054	0.391293488	0.200758314	0.834026357	0.638816189	0.655774258	0.964328974
SIDT2	0.688249559	0.72998681	0.362885324	0.283291759	0.406850834	0.655661278	0.964328974
ZBED3	0.833421115	0.708105434	0.264508566	0.574174878	0.23443128	0.655644406	0.964328974
ZNF583	0.374089349	0.115882353	0.646778147	0.783539688	0.95693528	0.655747459	0.964328974
ABL2	0.937975969	0.633827352	0.708198549	0.097502289	0.512789015	0.656007593	0.964330401
MCU	0.386652471	0.591927354	0.291190979	0.328729675	0.960705326	0.655973929	0.964330401
NASP	0.811192579	0.680539611	0.217980926	0.206238236	0.848314503	0.656030346	0.964330401
RALY	0.990258675	0.62980616	0.362907611	0.165058095	0.563663227	0.656069034	0.964330401
RFXAP	0.173071275	0.784562122	0.88632936	0.231041647	0.75724128	0.656052814	0.964330401
SMYD4	0.812904561	0.898390668	0.233223932	0.158061532	0.782434454	0.656133602	0.964338935
SP1	0.464588856	0.785663793	0.285732365	0.281801277	0.716957493	0.656200584	0.964351014
TNNT1	0.147674564	0.976311912	0.940355157	0.347707795	0.4475356	0.656437	0.964525705
ZNF16	0.962955761	0.624764065	0.430353962	0.29805226	0.273341678	0.656400317	0.964525705
FEM1C	0.565778932	0.229877328	0.573830543	0.832864744	0.33960786	0.65654943	0.964604538
NTPCR	0.58362526	0.420653829	0.353829302	0.357122814	0.681055207	0.656717171	0.964678258
SHANK1	0.761552274	0.251310632	0.589757127	0.967257255	0.193496638	0.656693195	0.964678258
HAT1	0.204666512	0.303797366	0.818605657	0.43995449	0.943932498	0.656807619	0.964724769
MRPL52	0.204080183	0.446445261	0.655357524	0.56901154	0.622960191	0.657065365	0.964764765
OTOR	0.534525736	0.527260089	0.70582089	0.280386477	0.379450381	0.657052522	0.964764765

SNAI1	0.108232144	0.993736383	0.488839533	0.927882293	0.433797541	0.657040706	0.964764765
ZSCAN12	0.706817377	0.948657178	0.236063518	0.25718274	0.51993833	0.657070001	0.964764765
ACVR1	0.562110857	0.897138775	0.625112575	0.334033534	0.202350857	0.658369071	0.964783133
AGPAT3	0.728200412	0.720264675	0.297846507	0.276833316	0.492073774	0.658121999	0.964783133
AMY2B	0.707981019	0.132896295	0.816672623	0.591560959	0.466301613	0.657343605	0.964783133
C14H8orf88	0.808758681	0.636346103	0.496182621	0.20452814	0.406591172	0.657709667	0.964783133
CALN1	0.160106175	0.400747083	0.984433078	0.401138322	0.836187315	0.657259924	0.964783133
CCDC184	0.210955161	0.691273353	0.591138946	0.311411487	0.791408691	0.65779942	0.964783133
CEP76	0.807969789	0.413167383	0.897209477	0.109397084	0.649876686	0.658242024	0.964783133
COTL1	0.311983994	0.657066105	0.820553693	0.176261012	0.718761699	0.658393567	0.964783133
DPY30	0.97343542	0.106681426	0.456063967	0.767120802	0.585184069	0.657939817	0.964783133
EIF2AK2	0.97164371	0.883524007	0.858683795	0.199857747	0.144085402	0.657635528	0.964783133
FUT11	0.56903098	0.891337185	0.340370823	0.183037312	0.673688287	0.658186934	0.964783133
FZD1	0.932695139	0.620294478	0.75984276	0.194204256	0.248516745	0.657535096	0.964783133
GPS2	0.958245982	0.735039578	0.688805147	0.056492438	0.776723866	0.658192405	0.964783133
HCFC1	0.694746039	0.389474945	0.448738151	0.223133235	0.781929225	0.657245535	0.964783133
ITGA4	0.992113709	0.301925854	0.886164583	0.335614547	0.238043863	0.657444545	0.964783133
KLHDC8B	0.973426928	0.528270419	0.129088745	0.389477984	0.824418307	0.658433485	0.964783133
LOC100139638	0.465663208	0.942310665	0.964163516	0.90443821	0.055655429	0.658265105	0.964783133
LOC107131615	0.170436716	0.696135808	0.664160466	0.853203673	0.315846584	0.657707041	0.964783133
LOC614424	0.992628602	0.246661342	0.18470286	0.598141696	0.787434649	0.658299611	0.964783133
MKKS	0.635880898	0.781635692	0.374519248	0.510736369	0.223131935	0.657506863	0.964783133
PRKAG1	0.845648262	0.363608153	0.673730557	0.466607723	0.220341437	0.658289574	0.964783133
PRR14	0.635716723	0.349136135	0.177648179	0.740028848	0.729083206	0.658059569	0.964783133
VPS53	0.748065211	0.39910786	0.620779115	0.408366895	0.281620202	0.658434653	0.964783133
CCDC22	0.87937621	0.523810737	0.52498515	0.627640078	0.140624403	0.658697623	0.964823875
POMGNT1	0.595639956	0.528043983	0.808273165	0.142245931	0.59015209	0.658674478	0.964823875
RIF1	0.423890043	0.546881458	0.594033622	0.175694762	0.881873914	0.658632708	0.964823875
RWDD2A	0.184103106	0.991643793	0.139280317	0.992625229	0.845054931	0.658567252	0.964823875
TUSC1	0.917333185	0.722880114	0.899254199	0.439998059	0.081427814	0.658891329	0.964935355
UBE2T	0.403748862	0.629585903	0.976259255	0.33516905	0.256825883	0.658862077	0.964935355
C1QTNF7	0.521051463	0.898693478	0.611354324	0.117117884	0.637681815	0.659031725	0.964968741
CSTB	0.267157411	0.358433818	0.436403976	0.750951323	0.681102641	0.658976477	0.964968741
ARPC3	0.810119572	0.179571265	0.280224751	0.896829394	0.585839911	0.659376498	0.965015974
CENPU	0.397047691	0.590092228	0.854223306	0.138786916	0.770711251	0.659283653	0.965015974
LOC107131846	0.232873104	0.896561813	0.676511886	0.744346622	0.203655868	0.659316362	0.965015974
RFX7	0.797742932	0.2011859	0.331261428	0.497550909	0.809139113	0.659246828	0.965015974
SHPRH	0.191108214	0.259768638	0.856085832	0.508448469	0.99138148	0.659416802	0.965015974
SPATA2	0.36109133	0.557310313	0.919831495	0.3093387	0.374028511	0.659367588	0.965015974
MAPKAPK5	0.616982352	0.302122499	0.40009381	0.521357483	0.551749332	0.65969659	0.965081186
PGAM2	0.532640781	0.851354334	0.530186326	0.451638622	0.197559702	0.659682087	0.965081186
PRKD1	0.326839472	0.560563154	0.639571526	0.640833869	0.285548662	0.65959735	0.965081186
RBBP7	0.988370392	0.661335316	0.173325199	0.560281282	0.337893631	0.659649149	0.965081186
WIPF2	0.942168091	0.190610941	0.476149328	0.395330623	0.634821021	0.659757609	0.96508442

ANTXR2	0.572620631	0.124531516	0.812166729	0.709221821	0.527005052	0.661442026	0.96520886
ANXA4	0.977000353	0.408377882	0.311729769	0.736383006	0.236730306	0.661757797	0.96520886
ATF2	0.437249183	0.687040519	0.140372105	0.905510872	0.565191167	0.660856087	0.96520886
BTNL9	0.34456432	0.369532705	0.918911006	0.482261856	0.383836451	0.661548453	0.96520886
CCDC160	0.590985574	0.224207956	0.233426739	0.7622835	0.917806286	0.661378251	0.96520886
CD276	0.236298866	0.521100616	0.23324119	0.834004764	0.903783738	0.661456907	0.96520886
DAB2	0.781227404	0.778653374	0.792708896	0.057067451	0.784559789	0.660931706	0.96520886
DAD1	0.491485682	0.379902569	0.925527543	0.209660045	0.593943011	0.660296444	0.96520886
DNAJC15	0.236231724	0.85145778	0.334475423	0.983535613	0.326555741	0.661094465	0.96520886
DPF1	0.558761679	0.766506007	0.709643448	0.465944709	0.152135397	0.660526949	0.96520886
ETV3	0.400277209	0.800447504	0.901959415	0.39389935	0.188922392	0.660169342	0.96520886
EXOC3	0.291050351	0.625746055	0.735215332	0.484846503	0.331109657	0.660083033	0.96520886
GALNT4	0.222183701	0.301990289	0.680382643	0.598256085	0.79246603	0.661414169	0.96520886
GAR1	0.673312007	0.424345072	0.871537139	0.723135262	0.120422977	0.661783565	0.96520886
INTS12	0.541516915	0.833580742	0.694733697	0.669340798	0.102808233	0.660843557	0.96520886
INTU	0.878484689	0.098549494	0.966570974	0.534244833	0.48461116	0.661606865	0.96520886
KLC2	0.231246203	0.571564307	0.325925815	0.532875248	0.938811454	0.660578671	0.96520886
LOC101907570	0.293994317	0.153214857	0.956148741	0.521837585	0.961541289	0.66111939	0.96520886
LOC112442374	0.996187392	0.03106863	0.903170155	0.862707116	0.893992941	0.660653895	0.96520886
LOC531152	0.813446623	0.757001663	0.131784646	0.470148566	0.563942883	0.660264183	0.96520886
LRCH4	0.434500213	0.962446042	0.973915384	0.071963153	0.734771233	0.660438586	0.96520886
MOSMO	0.28494314	0.405332896	0.676698188	0.474250698	0.584272031	0.661531592	0.96520886
NACA	0.572042965	0.449921149	0.785897375	0.149662566	0.714796901	0.661369597	0.96520886
PRELID3B	0.365139232	0.607926415	0.404656003	0.65948793	0.364126561	0.660754549	0.96520886
PTPRB	0.35326063	0.210190527	0.977352151	0.663750512	0.446928439	0.660372399	0.96520886
RNASEH2A	0.465120241	0.615748751	0.396927523	0.459180515	0.412379909	0.660353852	0.96520886
RPIA	0.967978283	0.20590271	0.574894934	0.351240903	0.53873101	0.661757972	0.96520886
SCN7A	0.800698506	0.848418925	0.068786327	0.886858305	0.519428755	0.660354196	0.96520886
SNX6	0.320101073	0.717392996	0.683061796	0.463875713	0.297056359	0.66115354	0.96520886
TCEANC2	0.382992267	0.439286075	0.235961299	0.782374068	0.692282829	0.660136528	0.96520886
TXNDC16	0.645501348	0.989893009	0.100159773	0.909099593	0.371356127	0.661080047	0.96520886
WDFY1	0.962494791	0.746656596	0.892756773	0.124099902	0.269731677	0.659903197	0.96520886
ZFP28	0.701843343	0.473570038	0.485813841	0.225750509	0.59456412	0.661681106	0.96520886
APPL2	0.802616716	0.708067462	0.714875592	0.185786293	0.287656828	0.662030672	0.965359888
LRRC14	0.999775011	0.763431552	0.992513279	0.108888677	0.263259273	0.662063588	0.965359888
SMS	0.59936608	0.338622619	0.577690933	0.249041066	0.7434444	0.661995479	0.965359888
ANKS3	0.451148343	0.303107798	0.855676461	0.323506247	0.574533302	0.662354368	0.965540735
DCAF15	0.391061627	0.531293822	0.29466654	0.932331271	0.381495853	0.662599461	0.965540735
LOC104975960	0.843861684	0.857698848	0.512885489	0.605955751	0.096703814	0.662393392	0.965540735
LOC112444474	0.825623862	0.589165823	0.226117167	0.367064949	0.539203993	0.662545	0.965540735
LRP10	0.643801316	0.694106512	0.319716283	0.742836145	0.204936873	0.662368675	0.965540735
NUP62	0.785537795	0.772277648	0.802522964	0.112501744	0.397218105	0.662427131	0.965540735
PRX	0.578885674	0.36810066	0.81675612	0.760620631	0.16440567	0.662494521	0.965540735
CEP70	0.781319066	0.739401765	0.348546431	0.237231819	0.456275171	0.662778013	0.965550625

FLRT1	0.720390251	0.477832182	0.557165761	0.196842756	0.577233676	0.662746386	0.965550625
LOC101908759	0.108770824	0.804705644	0.839148377	0.597788768	0.496487611	0.662811651	0.965550625
SLC39A2	0.952436792	0.573126553	0.233840168	0.174120412	0.98096996	0.66284159	0.965550625
PXYLP1	0.328522054	0.868686118	0.924417404	0.209022544	0.395660241	0.662975954	0.965660636
A4GALT	0.094995442	0.912646999	0.80184716	0.93112479	0.337560622	0.663265312	0.965740631
C11H9orf16	0.367415024	0.322155757	0.770016431	0.29916485	0.801310705	0.663255208	0.965740631
LOC101907195	0.761296244	0.202077759	0.189807878	0.797916958	0.93780999	0.663266264	0.965740631
LOC112443163	0.404494524	0.871013551	0.411583506	0.404242451	0.372743857	0.663261311	0.965740631
ADCYAP1R1	0.055748877	0.914437463	0.88579524	0.71557753	0.680415156	0.664473651	0.965795164
ARFGAP3	0.939551203	0.189100138	0.644108911	0.334317727	0.577990488	0.665592193	0.965795164
C15H11orf71	0.741492568	0.80659329	0.28371387	0.138035269	0.937743538	0.664277447	0.965795164
CBX3	0.766005783	0.549324829	0.185197053	0.590301833	0.478689147	0.664773258	0.965795164
CCM2	0.092271108	0.843251511	0.722957617	0.808391262	0.486894698	0.665834287	0.965795164
DCN	0.856759961	0.263954462	0.609984451	0.1649261	0.964108992	0.664011513	0.965795164
DDRGK1	0.531334694	0.389264795	0.1170914	0.913106586	0.994063855	0.664437778	0.965795164
EFHD2	0.392089663	0.377177741	0.573544198	0.363839305	0.71688521	0.665684378	0.965795164
ETV4	0.634296513	0.13086681	0.337844807	0.844211631	0.934021052	0.665590126	0.965795164
FAM162B	0.901338328	0.696184461	0.316702254	0.310785558	0.35654644	0.664780957	0.965795164
FLAD1	0.765839349	0.631352178	0.160694389	0.869056222	0.326100631	0.664767222	0.965795164
GANC	0.984427473	0.511998029	0.317055964	0.19948385	0.687792757	0.663935726	0.965795164
GNAZ	0.967125695	0.10892127	0.420252296	0.656680954	0.759082118	0.665187903	0.965795164
GPAT3	0.603833349	0.698328902	0.379072379	0.433889127	0.31884096	0.665592383	0.965795164
GREB1	0.433111417	0.993134616	0.341897101	0.503813799	0.298610855	0.665693893	0.965795164
HARS	0.453397996	0.547583503	0.536772795	0.846509518	0.195743812	0.665318418	0.965795164
KCTD14	0.339310825	0.936134638	0.262364995	0.626210115	0.42236311	0.664963566	0.965795164
LOC100848665	0.574578461	0.934047129	0.285227395	0.162086602	0.888528629	0.664999364	0.965795164
LOC100848766	0.806368072	0.750653381	0.850153864	0.646847349	0.065705747	0.663452861	0.965795164
LOC101906167	0.856584455	0.71449926	0.428654733	0.283010776	0.29751828	0.665388227	0.965795164
LOC101910045	0.449641161	0.27101356	0.748641817	0.482044533	0.500015946	0.664495165	0.965795164
LOC112442757	0.514394658	0.189688014	0.709460647	0.578570547	0.550373405	0.664977197	0.965795164
LOC112448515	0.532139672	0.327322604	0.978313076	0.987162525	0.130804961	0.664625144	0.965795164
LOC112449552	0.660509723	0.526312153	0.353385263	0.493404885	0.363393852	0.664831037	0.965795164
LOC789895	0.602984227	0.623650922	0.335964085	0.244959128	0.710321921	0.664444567	0.965795164
LRRK1	0.515202889	0.311808313	0.762065121	0.198875589	0.908741625	0.665694405	0.965795164
LY96	0.378232099	0.941939432	0.594211336	0.216020379	0.483159571	0.665438106	0.965795164
MBIP	0.824613864	0.264456226	0.240624289	0.526893964	0.79131544	0.663516163	0.965795164
MYLK3	0.713652526	0.481405204	0.691205978	0.318126136	0.290481262	0.664101145	0.965795164
NRIP2	0.401645195	0.685301556	0.885142745	0.5105398	0.177962954	0.665791507	0.965795164
PDXDC1	0.460608475	0.700251635	0.325955372	0.739739563	0.281976304	0.663972629	0.965795164
QSOX1	0.44846803	0.672182439	0.309616968	0.483633898	0.485175014	0.663714099	0.965795164
RANBP3	0.782306838	0.733344895	0.585597511	0.308283346	0.212530091	0.664698036	0.965795164
SDR39U1	0.568822123	0.654507207	0.274584361	0.276798996	0.78039718	0.665321363	0.965795164
SFXN5	0.594609811	0.766273137	0.429797877	0.117876883	0.958538791	0.665711561	0.965795164
SIGLEC10	0.556340261	0.60967259	0.975276658	0.153631353	0.43339981	0.664822749	0.965795164

TACR2	0.713556744	0.085350962	0.810232232	0.465587458	0.953272976	0.663716985	0.965795164
TCHH	0.324210381	0.694377041	0.302228883	0.67938129	0.475842505	0.664554679	0.965795164
TEX10	0.707595847	0.850019366	0.932509322	0.101427448	0.389060767	0.665766161	0.965795164
TGFB2	0.677263628	0.755928726	0.232275853	0.227204261	0.817711496	0.665416447	0.965795164
TTC9	0.711593816	0.545827881	0.175924728	0.818478782	0.393656491	0.664735881	0.965795164
ZFP36	0.575057287	0.282026075	0.449340436	0.627140296	0.484304574	0.665774642	0.965795164
ZHX2	0.458575143	0.559552718	0.345295839	0.563297177	0.440564304	0.664489595	0.965795164
ABCB6	0.647666823	0.99432493	0.390888384	0.160637859	0.577650712	0.676231185	0.965871699
ABHD15	0.94294413	0.550749652	0.693943895	0.343147819	0.184254916	0.671414837	0.965871699
ACER1	0.210192327	0.395211621	0.976247852	0.319662816	0.883499616	0.672416249	0.965871699
ACRBP	0.21171427	0.433967806	0.975821677	0.785082628	0.332216104	0.676440176	0.965871699
ADAM15	0.881599937	0.463528809	0.247740702	0.437445626	0.52733704	0.676191919	0.965871699
ADRA2B	0.536478215	0.330768493	0.469941813	0.719143499	0.384744009	0.673847298	0.965871699
ALDH9A1	0.830512096	0.611991438	0.285553945	0.27724133	0.564571473	0.670829789	0.965871699
AMH	0.835303902	0.546773951	0.678375185	0.209979565	0.348731386	0.670576679	0.965871699
ANP32B	0.479515757	0.653612806	0.701217718	0.167144355	0.639333134	0.677279705	0.965871699
AP3S1	0.220179731	0.296068344	0.624099082	0.654664292	0.885432139	0.678083511	0.965871699
ARF2	0.502025393	0.429463501	0.2536757	0.841342815	0.509451054	0.676927615	0.965871699
ARL6IP1	0.809968683	0.171382341	0.824582219	0.52568882	0.372718329	0.66833441	0.965871699
ARSJ	0.251308168	0.436333963	0.364150521	0.841923824	0.692075821	0.675466445	0.965871699
ATF6B	0.934504133	0.224846189	0.202339764	0.583973061	0.911441442	0.670075454	0.965871699
ATF7IP	0.188370412	0.457419647	0.343745777	0.835821112	0.931606276	0.673759234	0.965871699
ATP11A	0.263511694	0.528919899	0.420752364	0.940534674	0.420725372	0.674955975	0.965871699
B3GALNT1	0.704721111	0.940098501	0.255675015	0.72526994	0.189840896	0.675928702	0.965871699
B4GALT4	0.792986645	0.613736777	0.118418159	0.416696792	0.981897564	0.678063826	0.965871699
BMX	0.616822734	0.622634457	0.844940215	0.941321715	0.076102385	0.675298026	0.965871699
BOLA	0.543739654	0.297518187	0.738775922	0.423948198	0.440973259	0.667602526	0.965871699
BoLA	0.574528095	0.573647773	0.801912722	0.587250214	0.150673238	0.676453047	0.965871699
BRMS1L	0.608179162	0.483768032	0.630312316	0.190556068	0.642764594	0.670804233	0.965871699
BTF3L4	0.614389373	0.164244232	0.411846659	0.917341217	0.595858216	0.670823901	0.965871699
C11H2orf92	0.638246704	0.895177885	0.202219637	0.921193566	0.210671953	0.668290162	0.965871699
C25H16orf71	0.294086366	0.545260397	0.488904485	0.709245118	0.412343258	0.672618697	0.965871699
CAMLG	0.517786196	0.237792879	0.221396211	0.929044219	0.889764076	0.669252942	0.965871699
CCL4	0.304224227	0.857796214	0.952389124	0.995506779	0.090141531	0.667253056	0.965871699
CLDN11	0.440105608	0.727485595	0.226650599	0.403527425	0.784141715	0.672908132	0.965871699
COL27A1	0.655607361	0.242220857	0.194024718	0.879304007	0.847354486	0.67286828	0.965871699
COLEC10	0.966996031	0.82880444	0.134394104	0.469178901	0.451440215	0.671652142	0.965871699
COX4I2	0.094751315	0.79119818	0.513034206	0.622213367	0.928850896	0.666599813	0.965871699
CRYZ	0.668359506	0.979219027	0.284236728	0.227264019	0.537884859	0.671023755	0.965871699
CTCF	0.210604383	0.766432468	0.267573194	0.564114695	0.923559846	0.668978081	0.965871699
CTNNBIP1	0.533295705	0.627847525	0.176303703	0.48928279	0.805832827	0.675535907	0.965871699
CYP39A1	0.987669629	0.584663903	0.72018905	0.647041907	0.085212133	0.67263667	0.965871699
DDAH1	0.594915218	0.993428305	0.136521981	0.352310953	0.801317837	0.671352366	0.965871699
DDX49	0.289130169	0.707124337	0.648177312	0.765621707	0.227636987	0.67404044	0.965871699

DLG5	0.38811983	0.365398204	0.720329006	0.263003766	0.855094603	0.673012471	0.965871699
DTNA	0.461400798	0.210395737	0.960311377	0.397471639	0.609832231	0.669795594	0.965871699
DUT	0.439207298	0.416373677	0.607653576	0.232338332	0.90081994	0.675392635	0.965871699
DVL3	0.75555347	0.745138399	0.079046205	0.652782532	0.811906093	0.678110593	0.965871699
DZIP1L	0.516478609	0.511767974	0.274278246	0.924863535	0.337737789	0.670211655	0.965871699
EIF2B5	0.543347155	0.802017184	0.230162646	0.321836413	0.724890263	0.676569262	0.965871699
EIF2D	0.49663417	0.295745515	0.50618744	0.566212692	0.555700265	0.676517029	0.965871699
ELK1	0.105662625	0.850488375	0.898869436	0.317365333	0.88510977	0.670600229	0.965871699
EPHA7	0.845584384	0.88329358	0.382377067	0.350097795	0.230256263	0.673421506	0.965871699
ERCC6	0.776989567	0.50483967	0.570608501	0.673446162	0.153567674	0.6744735	0.965871699
ERP44	0.960863306	0.532309407	0.973041955	0.079201142	0.595984967	0.67733719	0.965871699
ESCO2	0.677037048	0.32821497	0.147757941	0.753177889	0.942278025	0.675763466	0.965871699
ETNK1	0.641422352	0.762582808	0.365599244	0.159095787	0.815545995	0.674935276	0.965871699
EXOC7	0.42962303	0.595591758	0.4441952	0.608488825	0.329255879	0.671294733	0.965871699
EZH1	0.456741598	0.773204363	0.531066966	0.959695259	0.127835456	0.673307182	0.965871699
FAH	0.553481823	0.919918634	0.173216497	0.56110943	0.476186736	0.677936335	0.965871699
FAM193A	0.549574743	0.646997702	0.431599923	0.23012757	0.660721648	0.676031233	0.965871699
FAM228B	0.594869884	0.202912087	0.244021368	0.838404823	0.938617957	0.674737368	0.965871699
FAM78A	0.22456691	0.51606051	0.299539118	0.734298805	0.922561064	0.677535226	0.965871699
FLRT2	0.914098117	0.242284027	0.207852843	0.659979696	0.756162879	0.673003632	0.965871699
GFPT1	0.511780426	0.995580582	0.557103928	0.253592879	0.325472353	0.676812113	0.965871699
GLCCI1	0.532176592	0.51921029	0.319158602	0.489451217	0.532218305	0.67299708	0.965871699
GLUD1	0.54476265	0.764206946	0.389559041	0.360139493	0.382377684	0.667518642	0.965871699
GNA11	0.798900794	0.12242188	0.298652009	0.976353645	0.783503756	0.66761294	0.965871699
GPR1	0.901357818	0.310826302	0.520850215	0.191108037	0.823192694	0.672864568	0.965871699
GPR39	0.466369261	0.490942242	0.178371942	0.587740282	0.981041903	0.67779936	0.965871699
GRAP2	0.237744108	0.691953193	0.648377589	0.603825125	0.344647746	0.66633071	0.965871699
GRK4	0.705129294	0.128171531	0.766185247	0.870103978	0.37661616	0.670609713	0.965871699
GRM8	0.993741045	0.47268074	0.555974902	0.726464357	0.119565222	0.670543212	0.965871699
GRPEL2	0.794149583	0.847601928	0.582101842	0.098513826	0.577940265	0.667303124	0.965871699
GTF2E2	0.898509713	0.208606836	0.370427089	0.346893203	0.92682539	0.667425943	0.965871699
GTPBP1	0.945462178	0.424242605	0.767769453	0.415357289	0.176109237	0.669191427	0.965871699
HBEGF	0.806570682	0.628370645	0.611658921	0.13406227	0.564492379	0.67707311	0.965871699
HERC3	0.467543301	0.38143214	0.272794399	0.734982801	0.6277996	0.668511168	0.965871699
HTRA1	0.866491507	0.439186402	0.246421761	0.264338022	0.907742747	0.668977328	0.965871699
IFT88	0.82335305	0.782080842	0.039247876	0.998452121	0.880188855	0.666445285	0.965871699
IL13RA1	0.816569683	0.417683154	0.59821191	0.295454234	0.378796094	0.671828845	0.965871699
ING3	0.260368135	0.914223104	0.787651355	0.386785772	0.315586283	0.672263176	0.965871699
IPO9	0.980212483	0.437907737	0.223053956	0.446900625	0.522065126	0.667560508	0.965871699
JADE2	0.18403175	0.871383496	0.449409834	0.758081284	0.411707249	0.668903182	0.965871699
KCNIP2	0.388621388	0.437916758	0.978872564	0.674291944	0.202707557	0.671279572	0.965871699
KCNJ12	0.298894648	0.22864955	0.90701185	0.411064314	0.904056109	0.673534061	0.965871699
KCNK4	0.231978392	0.929330409	0.74713096	0.191071715	0.723134556	0.666836064	0.965871699
KLHL32	0.159602022	0.850805969	0.284636384	0.914508452	0.636764115	0.669026971	0.965871699

KLK4	0.225894583	0.660017625	0.400224847	0.619044682	0.635492969	0.677191752	0.965871699
LOC100847567	0.610583844	0.162088019	0.863789606	0.306587136	0.889256028	0.67580216	0.965871699
LOC100847765	0.194349832	0.767056327	0.952075648	0.982074031	0.166580534	0.675072746	0.965871699
LOC100847825	0.942591065	0.125720448	0.498186308	0.575101717	0.681419124	0.674370956	0.965871699
LOC100847839	0.790516457	0.834483306	0.541752145	0.077223023	0.816090892	0.669156216	0.965871699
LOC100848331	0.706729686	0.974811692	0.418977847	0.280399203	0.286161184	0.674582049	0.965871699
LOC101902211	0.576064371	0.187040136	0.332843202	0.824720553	0.760899151	0.6690063	0.965871699
LOC101902861	0.762540022	0.594312038	0.151183979	0.509637318	0.671932887	0.677089967	0.965871699
LOC101903114	0.778860093	0.959360151	0.585877104	0.978615103	0.054582539	0.676439793	0.965871699
LOC101903900	0.494740734	0.308120597	0.703062463	0.620894585	0.335079006	0.66720646	0.965871699
LOC101904187	0.755771978	0.218753718	0.239533596	0.9562842	0.593382143	0.668717583	0.965871699
LOC101904308	0.666736157	0.30982716	0.217405231	0.633715047	0.793947716	0.669789124	0.965871699
LOC101906273	0.158106006	0.753654734	0.367402631	0.69301282	0.760532027	0.673853596	0.965871699
LOC101906545	0.697849252	0.434827196	0.825605555	0.330724798	0.283830511	0.677539189	0.965871699
LOC101906588	0.111643992	0.682284332	0.841590856	0.444155866	0.826734659	0.677729507	0.965871699
LOC101907749	0.457009433	0.604317808	0.467778784	0.488988665	0.352925463	0.667186524	0.965871699
LOC104972567	0.231289802	0.307738349	0.902399783	0.734959392	0.498155495	0.6775341	0.965871699
LOC104976293	0.882269544	0.92290611	0.20128661	0.144464207	0.96383863	0.671715884	0.965871699
LOC107131919	0.558215564	0.615109946	0.622144463	0.155633084	0.694980703	0.674121076	0.965871699
LOC107132302	0.854178859	0.974958686	0.29432742	0.261248307	0.36054843	0.673970199	0.965871699
LOC107132784	0.772403767	0.420027329	0.290468955	0.303670516	0.788291048	0.669467721	0.965871699
LOC112442257	0.98303078	0.542623454	0.182543317	0.490773204	0.493762444	0.678187776	0.965871699
LOC112442585	0.249958923	0.479500317	0.754021843	0.485818233	0.527536211	0.674588719	0.965871699
LOC112442633	0.875905744	0.980358326	0.722020318	0.247945979	0.152405999	0.676813585	0.965871699
LOC112443417	0.528507765	0.586083945	0.922989274	0.22860327	0.3513137	0.672899012	0.965871699
LOC112444871	0.467620151	0.320969889	0.884315953	0.311733272	0.560211463	0.67473725	0.965871699
LOC112445178	0.408565149	0.681988737	0.992573768	0.096686528	0.85682357	0.672484873	0.965871699
LOC112446795	0.252290529	0.975311048	0.729486707	0.279405476	0.463341662	0.675227423	0.965871699
LOC112446879	0.87800975	0.064645407	0.828825607	0.592608035	0.823623961	0.672904063	0.965871699
LOC112449106	0.169462667	0.330076749	0.537756777	0.800801606	0.953975412	0.673056131	0.965871699
LOC513969	0.665207674	0.910955873	0.558952454	0.118666729	0.571584706	0.673012361	0.965871699
LOC518495	0.595812423	0.882207988	0.069197239	0.871690057	0.717211097	0.671018492	0.965871699
LOC532875	0.685234552	0.903382808	0.348419289	0.124684304	0.860631536	0.674443447	0.965871699
LOC614376	0.827146972	0.19930267	0.932076522	0.494766502	0.299353534	0.671176613	0.965871699
LOC781218	0.539559	0.536664906	0.459884158	0.480154006	0.36708291	0.677163712	0.965871699
LOC781770	0.486779928	0.89093767	0.170247906	0.631745347	0.478888808	0.667556068	0.965871699
LOC782987	0.716763593	0.525267153	0.122913373	0.937601242	0.532432195	0.67408409	0.965871699
LOC789018	0.340267449	0.481304585	0.496169148	0.405192875	0.678736151	0.667643091	0.965871699
LOC789960	0.41396504	0.236727333	0.465267277	0.934463912	0.549461633	0.676663497	0.965871699
LSR	0.680580315	0.568550509	0.364489819	0.39001482	0.423126672	0.675534455	0.965871699
LY9	0.527063339	0.867446809	0.989479295	0.114394094	0.447972122	0.674767103	0.965871699
LYSMD2	0.886325946	0.683664803	0.823761747	0.05086185	0.889105558	0.669589799	0.965871699
MAP3K2	0.457156589	0.552713564	0.812045205	0.198429042	0.552547238	0.668934954	0.965871699
MAS1	0.265604803	0.215986811	0.877279536	0.859140287	0.544716448	0.677833203	0.965871699

MMRN2	0.368128222	0.349228398	0.809327272	0.899659607	0.23774874	0.666835774	0.965871699
MPDZ	0.324828183	0.824304894	0.482276915	0.499986032	0.360969353	0.675793818	0.965871699
MRAS	0.289845002	0.561428368	0.366234291	0.517385211	0.725420663	0.667817705	0.965871699
MRPS22	0.847241949	0.948878803	0.453759419	0.14842005	0.429548892	0.675384283	0.965871699
MYORG	0.493846952	0.185707945	0.580689649	0.811697465	0.531375146	0.672978107	0.965871699
NABP2	0.330983004	0.840315374	0.493147894	0.307466318	0.559413935	0.678154542	0.965871699
NFIB	0.283626598	0.867706201	0.469235053	0.512072044	0.385810543	0.67166089	0.965871699
NFIX	0.936521771	0.342015783	0.163981134	0.800801143	0.534667313	0.668866175	0.965871699
NOP58	0.913801206	0.892072922	0.74127251	0.121903591	0.311707806	0.672902935	0.965871699
NPL	0.147577667	0.623057287	0.458287164	0.541390981	0.974434911	0.66662129	0.965871699
OSBPL10	0.558260891	0.441571262	0.979611746	0.173993806	0.533837109	0.668359819	0.965871699
PANK2	0.611593206	0.613096195	0.68323877	0.137468291	0.64349898	0.670363734	0.965871699
PARD6G	0.893654849	0.672357501	0.071383281	0.943805821	0.54859583	0.666420532	0.965871699
PCDH18	0.184473669	0.884678332	0.257827369	0.912743986	0.61092654	0.677097423	0.965871699
PCDHGC3	0.900721919	0.399495483	0.552686231	0.642246816	0.177675812	0.670630864	0.965871699
PGM1	0.505765072	0.751199957	0.42327339	0.434408414	0.32945222	0.673359546	0.965871699
PLAC8	0.373930856	0.724029022	0.587602478	0.186633254	0.788522165	0.676672132	0.965871699
PLPP6	0.56534799	0.619423233	0.856401096	0.127568461	0.602751369	0.67373755	0.965871699
POLN	0.75169698	0.89387258	0.74640717	0.046343078	0.964367701	0.668219856	0.965871699
PPFIBP1	0.675815076	0.421653495	0.380925574	0.414413905	0.515732427	0.674908007	0.965871699
PPP2R5C	0.884141789	0.103460097	0.974289586	0.354795419	0.718939248	0.670962448	0.965871699
PRKAA1	0.238993004	0.912457041	0.586634007	0.200232597	0.91960302	0.677861886	0.965871699
PRRG3	0.896740663	0.70975882	0.579235643	0.080968886	0.783883495	0.676568935	0.965871699
PRRT1B	0.943683649	0.610379986	0.586610646	0.093953186	0.721543585	0.672436627	0.965871699
PRSS48	0.795428268	0.857001318	0.696911268	0.77601619	0.063966339	0.678077035	0.965871699
PSD3	0.281007871	0.464989471	0.861573769	0.445926853	0.457250625	0.672848349	0.965871699
PTGFRN	0.845812312	0.591839699	0.164246879	0.416535982	0.661255278	0.670221841	0.965871699
RCBTB2	0.792085538	0.861165221	0.794157814	0.118729106	0.358494126	0.673711881	0.965871699
RFX3	0.397740956	0.90359061	0.389039865	0.497503491	0.331813226	0.673913512	0.965871699
RNPS1	0.671341497	0.832185862	0.216061749	0.454774135	0.413399754	0.67062915	0.965871699
RORA	0.74541806	0.405705873	0.294450798	0.296959772	0.855674159	0.670057742	0.965871699
RRNAD1	0.759440141	0.569416905	0.846335156	0.419295801	0.146780468	0.66917435	0.965871699
RSBN1L	0.216542092	0.593265151	0.473585972	0.566779594	0.651414405	0.668640378	0.965871699
RTN1	0.918535113	0.174275008	0.508587929	0.318313857	0.86487484	0.668212686	0.965871699
RUNX1T1	0.977448611	0.553683453	0.802693845	0.212251635	0.242053757	0.667390368	0.965871699
RXFP4	0.11180397	0.494738357	0.927152955	0.811232367	0.559594433	0.675586917	0.965871699
S100A9	0.553228813	0.83877855	0.854876727	0.299119006	0.198371151	0.67771827	0.965871699
SDCCAG8	0.761494861	0.133948938	0.921797591	0.794594714	0.311696161	0.675638753	0.965871699
SENP3	0.573714768	0.349433872	0.493680996	0.230566607	0.990366522	0.66982061	0.965871699
SESN2	0.24862176	0.982475844	0.683351054	0.139620761	0.994245326	0.674669509	0.965871699
SLC2A4RG	0.397662914	0.677576324	0.900201827	0.139944378	0.674746551	0.672418122	0.965871699
SLC5A2	0.636996232	0.544758067	0.305029105	0.21569355	0.975740574	0.667025595	0.965871699
SLCO5A1	0.62112676	0.399575951	0.465373337	0.621570092	0.322690048	0.67462856	0.965871699
SLFNL1	0.259101704	0.329869633	0.843642089	0.512950745	0.63766755	0.678103156	0.965871699

SOWAHD	0.615785477	0.863353895	0.755414559	0.137583895	0.413377404	0.671885617	0.965871699
SPI1	0.243711576	0.49293991	0.935657539	0.401252178	0.514672894	0.675021123	0.965871699
SPRTN	0.604483415	0.520972502	0.938507464	0.313131923	0.253193233	0.67684286	0.965871699
SPTA1	0.91877238	0.257612413	0.90232564	0.211517561	0.493086624	0.667005152	0.965871699
SRSF7	0.631673927	0.688545312	0.47589985	0.132779505	0.826532428	0.670818884	0.965871699
SRSF9	0.183605686	0.463538389	0.959513969	0.890840349	0.310099813	0.669473824	0.965871699
STARD13	0.777736248	0.987645112	0.559462635	0.139932073	0.374935223	0.669363483	0.965871699
STX4	0.994561885	0.8583739	0.130229447	0.262032727	0.774439794	0.669490013	0.965871699
TAF2	0.652978547	0.972340104	0.078627398	0.601619357	0.775856251	0.675761593	0.965871699
TBP	0.463712608	0.805432464	0.377580586	0.209417099	0.79599473	0.67746479	0.965871699
TBX18	0.766714437	0.113053513	0.655828746	0.6540151	0.63280537	0.677623663	0.965871699
TCAIM	0.971085782	0.484185571	0.1658612	0.454414245	0.639227902	0.670277748	0.965871699
TENM3	0.77910988	0.410579478	0.767422805	0.115764653	0.785142312	0.667339668	0.965871699
THAP6	0.900461243	0.611643934	0.228662146	0.440041735	0.405943476	0.668933812	0.965871699
THRB	0.692131711	0.494754113	0.200371797	0.408995478	0.80462156	0.669652932	0.965871699
TIAM2	0.810684644	0.515795332	0.515790027	0.215407014	0.497657034	0.674242435	0.965871699
TMEM184C	0.258013922	0.715306935	0.193809908	0.742912757	0.844101777	0.668364133	0.965871699
TMPPE	0.846716172	0.57964764	0.987777946	0.05317853	0.900664931	0.675076976	0.965871699
TPD52	0.920714209	0.116462703	0.402895004	0.892377365	0.574818563	0.666009165	0.965871699
TPT1	0.614552707	0.702123208	0.117045314	0.97028687	0.45326889	0.666456721	0.965871699
TRIM27	0.388938504	0.825094051	0.588362508	0.14297547	0.838684613	0.670173853	0.965871699
TSPAN14	0.38496448	0.236244084	0.730590787	0.407900762	0.869963368	0.678045362	0.965871699
TUG1	0.278772279	0.511662167	0.538057978	0.331845821	0.894091895	0.671287686	0.965871699
UBA1	0.56235608	0.896088002	0.362930718	0.859005324	0.141365349	0.666430777	0.965871699
UBXN8	0.561169112	0.214657902	0.341636919	0.641544219	0.858468472	0.670382611	0.965871699
VAMP4	0.745343342	0.251167762	0.268437045	0.987083863	0.456849461	0.670352876	0.965871699
VNN1	0.779548285	0.91444219	0.795560678	0.110203788	0.370081714	0.67432036	0.965871699
VPS26B	0.453871897	0.947291796	0.134947642	0.433983811	0.883469752	0.666754435	0.965871699
WDR31	0.22571341	0.377433338	0.388700131	0.780858107	0.879012908	0.670929639	0.965871699
WIPI1	0.868561487	0.176037408	0.293727872	0.540191134	0.946460237	0.672905778	0.965871699
WLS	0.306222976	0.866792801	0.367225537	0.282111364	0.817779864	0.668855754	0.965871699
XPA	0.397414879	0.972310536	0.202081696	0.328521739	0.875829654	0.668684356	0.965871699
ZFAND4	0.69735225	0.278052537	0.303053685	0.433691723	0.906560446	0.674101394	0.965871699
ZFHX3	0.540390595	0.642029408	0.246401716	0.318913929	0.841345109	0.672706278	0.965871699
ZKSCAN8	0.774403078	0.342822735	0.398074308	0.951841178	0.227989874	0.67267356	0.965871699
ZNF383	0.090044314	0.709233543	0.857317618	0.84863035	0.498975832	0.674775327	0.965871699
ZNF592	0.674126383	0.471546232	0.735251151	0.288907505	0.344420078	0.675384093	0.965871699
ZNF689	0.999251577	0.733259753	0.522631699	0.815288491	0.07472217	0.67598265	0.965871699
ZNF771	0.682005274	0.762942375	0.59211981	0.242838277	0.297125751	0.666619648	0.965871699
ZNF852	0.52085986	0.964333092	0.48531862	0.269924905	0.356712404	0.677164056	0.965871699
LOC783142	0.122769611	0.726961689	0.423071805	0.728207586	0.858618785	0.678295533	0.965941339
TMEM150B	0.47405868	0.861880649	0.283211304	0.253849736	0.804213842	0.678414189	0.966026486
BCL2L2	0.167673259	0.520728242	0.582751121	0.575321386	0.807418687	0.678516466	0.966030633
TMTC3	0.755585487	0.320456172	0.362730027	0.680980788	0.3952191	0.67853483	0.966030633

AGTR2	0.271562825	0.9368883	0.435053612	0.235478882	0.913101211	0.679856257	0.966077441
AMDHD2	0.619749378	0.950961706	0.196128664	0.487977385	0.421977734	0.679873036	0.966077441
CCDC69	0.461622151	0.739976597	0.446868233	0.167397809	0.932105381	0.680002084	0.966077441
CHEK2	0.242580671	0.495010513	0.92659703	0.465523961	0.459733904	0.679962495	0.966077441
CTDNEP1	0.335680811	0.882702361	0.508791153	0.229927162	0.686161016	0.679733597	0.966077441
ETV1	0.651609487	0.489927458	0.760481726	0.297633486	0.328791793	0.679516925	0.966077441
FCGR1A	0.259814312	0.688178587	0.578570823	0.662132241	0.345773987	0.678913613	0.966077441
FLVCR1	0.315971136	0.939915649	0.456396833	0.191541421	0.911081356	0.678664285	0.966077441
GADD45GIP1	0.74139448	0.932418712	0.287579898	0.256328138	0.467674999	0.680118169	0.966077441
ITGB3BP	0.219670562	0.29131396	0.59193237	0.794424296	0.789818403	0.679594639	0.966077441
JPT1	0.355462198	0.295585485	0.399675316	0.99403525	0.568226511	0.679203185	0.966077441
LOC100138645	0.455764098	0.926548059	0.703430742	0.119797826	0.668983654	0.679910673	0.966077441
LOC101903301	0.669626039	0.399599198	0.941106106	0.134475121	0.69866335	0.678711858	0.966077441
LOC107132924	0.217181823	0.74282205	0.930419522	0.618349097	0.256770855	0.680121398	0.966077441
LOC112443140	0.557443866	0.749440731	0.570336637	0.110501849	0.905606458	0.680216003	0.966077441
LOC112447027	0.992415403	0.447237866	0.324579167	0.172456872	0.95888876	0.680047798	0.966077441
LOC618733	0.649469991	0.500717003	0.132788129	0.721132034	0.76454802	0.679926895	0.966077441
METAP1D	0.528793937	0.301045626	0.517739432	0.35855932	0.805537996	0.679903194	0.966077441
NAA30	0.977174197	0.241865111	0.176628105	0.816297185	0.699196705	0.680070651	0.966077441
PCDH11X	0.660170148	0.504696299	0.1791592	0.596025077	0.668872874	0.679839065	0.966077441
PLPP7	0.213403465	0.594231577	0.217026784	0.932165417	0.92254945	0.678777747	0.966077441
RHBDL1	0.357962325	0.261246629	0.4006568	0.695326095	0.910863615	0.679290269	0.966077441
SEMA3D	0.317629365	0.911684535	0.210081062	0.641581436	0.609193536	0.679672309	0.966077441
SPESP1	0.876659337	0.491605404	0.358388004	0.324962971	0.472860296	0.679319381	0.966077441
STMP1	0.976234975	0.45775764	0.248333333	0.227847963	0.939688758	0.679536295	0.966077441
ZNF329	0.75737034	0.548782396	0.75501507	0.223874918	0.338061821	0.679452239	0.966077441
ZNF585A	0.423899219	0.40290217	0.549600769	0.515121795	0.493028101	0.680176951	0.966077441
ZNF672	0.388797964	0.255334686	0.574477074	0.475132917	0.878984471	0.680003835	0.966077441
C13H10orf113	0.946178325	0.651468742	0.060015809	0.774977527	0.832047217	0.680300952	0.966114479
LOC107132944	0.602157061	0.288695683	0.944543925	0.531640406	0.273379017	0.680383891	0.966148658
LNPK	0.450054211	0.87019184	0.476859305	0.317445327	0.402701568	0.680458315	0.966170739
LOC104975607	0.228786799	0.779527168	0.777470889	0.438835492	0.392917649	0.680737901	0.966484098
CSGALNACT1	0.323857891	0.812776805	0.392426371	0.657659773	0.352202034	0.680883846	0.966536319
OSBPL3	0.86991769	0.649544829	0.499171066	0.186125777	0.455787722	0.680896653	0.966536319
RUFY2	0.977185935	0.187263068	0.259158303	0.739478818	0.682509044	0.680951369	0.966536319
BET1	0.559388751	0.489272581	0.626330908	0.173664621	0.808407999	0.682012611	0.966836906
BTBD3	0.574476878	0.188824438	0.998554853	0.906823978	0.245921748	0.682730712	0.966836906
CHEK1	0.961660855	0.198506183	0.330750897	0.620864951	0.6162563	0.682745369	0.966836906
CREBL2	0.72406032	0.159847609	0.358647636	0.941898335	0.616832301	0.682417783	0.966836906
DUSP22	0.498918835	0.495796247	0.164179124	0.669215132	0.88882057	0.682734411	0.966836906
EBF1	0.877319293	0.906325075	0.61232295	0.234914348	0.210606169	0.682187426	0.966836906
GOLGA1	0.538840744	0.111125756	0.952803893	0.795062752	0.530074273	0.681837268	0.966836906
GRAMD1B	0.880274949	0.322562011	0.128122421	0.719649997	0.919628032	0.682092715	0.966836906
KDM3B	0.295431288	0.882386185	0.706932422	0.391761354	0.332080705	0.681276544	0.966836906

KIAA0232	0.930413276	0.736589636	0.323475148	0.118770956	0.917491063	0.682744921	0.966836906
LOC100852077	0.180456267	0.821809425	0.856125359	0.525418303	0.360972584	0.682123735	0.966836906
LOC104971503	0.376955719	0.377700013	0.818451068	0.818069975	0.252834344	0.682300448	0.966836906
LOC112447328	0.739791985	0.329241759	0.693622131	0.148909597	0.957704482	0.682231455	0.966836906
LOC112449075	0.23857355	0.563671627	0.37665143	0.906729354	0.525578573	0.682588552	0.966836906
LOC782799	0.476784971	0.523792627	0.849340674	0.13225956	0.858849182	0.682234196	0.966836906
MARCH8	0.893310878	0.23270376	0.875196986	0.416961334	0.317931972	0.682427502	0.966836906
MDFI	0.180862406	0.921189417	0.777246201	0.321463334	0.579061642	0.682325177	0.966836906
NUP58	0.74406609	0.2874297	0.419096057	0.357125899	0.753600749	0.682462053	0.966836906
OCLN	0.317940395	0.468459164	0.63100138	0.375106904	0.684436087	0.682513861	0.966836906
PCMT1	0.933257647	0.290859887	0.149831921	0.856560895	0.692152694	0.682386555	0.966836906
R3HDM1	0.644222036	0.827366106	0.197160046	0.771158759	0.29636343	0.681616203	0.966836906
RPS4X	0.33548724	0.723114285	0.349360748	0.396868159	0.714558369	0.681758952	0.966836906
SLC44A2	0.345374498	0.263787485	0.811346586	0.421887783	0.769797941	0.681528848	0.966836906
TMEFF1	0.25109619	0.923339486	0.161455831	0.968264782	0.666538276	0.682753818	0.966836906
TRMT13	0.49924678	0.111510658	0.541900076	0.944668789	0.843933018	0.681891438	0.966836906
TTC8	0.397438823	0.491024431	0.958643333	0.547006216	0.234725909	0.681644324	0.966836906
ZYX	0.903369885	0.129639329	0.933618741	0.510747445	0.43076635	0.681927751	0.966836906
CTBP2	0.647680011	0.449277557	0.517973911	0.299674505	0.53538344	0.682942346	0.966937006
LPAR4	0.484774087	0.743491561	0.82948068	0.23576632	0.343073154	0.682938953	0.966937006
DIMT1	0.923218575	0.354409912	0.613597785	0.877708325	0.137329388	0.683080411	0.966965636
STAU2	0.871676068	0.53740215	0.305210085	0.342388694	0.494254207	0.683042699	0.966965636
NONO	0.486278809	0.609573557	0.484894459	0.222884699	0.755943206	0.683223002	0.967084069
ADCY2	0.527546581	0.252482887	0.33937766	0.822625055	0.655686896	0.684534581	0.967261665
ANKRD35	0.497477685	0.902024581	0.824829614	0.162169262	0.404266494	0.683607866	0.967261665
AQP4	0.549538224	0.771300012	0.307821145	0.595772748	0.3142757	0.684906909	0.967261665
BCAR3	0.236070282	0.630442294	0.416563313	0.561939331	0.699744801	0.684500019	0.967261665
BTK	0.501991305	0.974255438	0.48093768	0.207283424	0.502044756	0.685286762	0.967261665
C1H21orf2	0.96762368	0.538756226	0.276516146	0.409261795	0.411961574	0.683912673	0.967261665
C9H6orf203	0.604929095	0.209457946	0.805373501	0.384184522	0.619069817	0.683645989	0.967261665
CCDC59	0.607906686	0.145273522	0.419956067	0.845803343	0.775388649	0.684063521	0.967261665
CFAP300	0.509035507	0.871503849	0.107583263	0.906693665	0.565681217	0.685299927	0.967261665
COL13A1	0.760011912	0.728009002	0.152319795	0.729268542	0.396029885	0.684202777	0.967261665
CSNK1D	0.804257369	0.833301924	0.782941497	0.054610365	0.853555439	0.685139366	0.967261665
DBP	0.495007063	0.411483092	0.655170757	0.865701317	0.210231271	0.683781835	0.967261665
ENOSF1	0.88672854	0.630429556	0.064064168	0.836566009	0.816443975	0.685155154	0.967261665
FAM171A1	0.96581652	0.323442812	0.519416869	0.451173487	0.331472373	0.683610796	0.967261665
FAM241B	0.536445863	0.607424533	0.308546915	0.913657752	0.265933964	0.684900528	0.967261665
GATAD1	0.330607024	0.622633054	0.781727717	0.366156194	0.414423156	0.684817481	0.967261665
KLHL4	0.566225019	0.439915237	0.107407989	0.968879953	0.941448827	0.684706755	0.967261665
LOC101904962	0.931375073	0.86104311	0.879213808	0.08804145	0.393905194	0.68509013	0.967261665
LOC101907713	0.529805375	0.54419177	0.331313551	0.344914187	0.741086472	0.684807387	0.967261665
LOC112449099	0.618754155	0.6880249	0.769514885	0.094555811	0.790199654	0.685286549	0.967261665
MARVELD1	0.883585234	0.132552403	0.822520328	0.462228411	0.546218769	0.684059012	0.967261665

MBOAT7	0.624952748	0.647243531	0.529576925	0.348417777	0.326997603	0.68471899	0.967261665
MTMR4	0.43734155	0.383176597	0.778168665	0.390095118	0.479469585	0.684601596	0.967261665
MTMR9	0.260728339	0.536430216	0.512181559	0.343196861	0.98985749	0.684163809	0.967261665
PRND	0.667335109	0.934448391	0.171793731	0.318892686	0.710275299	0.683601351	0.967261665
RFTN1	0.8198833	0.295188571	0.711040891	0.300128894	0.471100158	0.684130794	0.967261665
SPR	0.424655146	0.479617184	0.238045562	0.60152976	0.836954711	0.684746341	0.967261665
STARD10	0.70645107	0.287739086	0.873555199	0.952363275	0.1438694	0.684119736	0.967261665
TBC1D31	0.665828125	0.157094691	0.870208149	0.995142214	0.270321442	0.685352425	0.967261665
TNPO3	0.876566139	0.596688355	0.37280623	0.414785796	0.30238674	0.68512534	0.967261665
TSEN34	0.368768751	0.643387029	0.229115254	0.924161992	0.487255873	0.685295997	0.967261665
TTBK2	0.311715641	0.802741434	0.71509468	0.247529422	0.550975244	0.684704708	0.967261665
TTC3	0.83518126	0.704562927	0.102947709	0.794603279	0.505336769	0.684077937	0.967261665
ZC3H3	0.423237374	0.414570325	0.331169514	0.624273394	0.672069402	0.684511028	0.967261665
CREB3L2	0.953401525	0.840621257	0.20397727	0.269890556	0.55545039	0.685520818	0.967332944
SDE2	0.099944406	0.750681354	0.740675705	0.458774185	0.961012399	0.685467093	0.967332944
C22H3orf20	0.802540924	0.525756556	0.474625302	0.534773487	0.228936834	0.685608614	0.967352229
GRIK2	0.29179041	0.570732938	0.325812954	0.723426182	0.624769991	0.685652375	0.967352229
LOC510382	0.145816012	0.863094245	0.834193945	0.308851123	0.757131159	0.685858813	0.967502994
PRR22	0.60903274	0.344609191	0.671994628	0.216682431	0.803405859	0.685877145	0.967502994
BCAP29	0.249331897	0.441129113	0.433135627	0.549126068	0.940256639	0.686230267	0.967585266
LOC534742	0.648132314	0.338209129	0.323490017	0.45180305	0.7675451	0.686175567	0.967585266
PRDM10	0.779080326	0.281902305	0.188492986	0.888291068	0.668332778	0.686069346	0.967585266
TBC1D10A	0.84526306	0.640176897	0.931519358	0.4551705	0.107171791	0.686164635	0.967585266
TRIM56	0.643134142	0.825561569	0.436830195	0.65803453	0.161087795	0.686136455	0.967585266
ARL4D	0.233506624	0.93856365	0.669776362	0.380275902	0.441493863	0.686599497	0.96763119
SRL	0.23248061	0.824084816	0.947616018	0.700263139	0.193780388	0.686530973	0.96763119
TCFL5	0.787516802	0.520680872	0.207080003	0.448519915	0.647147641	0.686616611	0.96763119
TLK1	0.452609362	0.792921357	0.79530163	0.128684257	0.670629068	0.686500372	0.96763119
WNT16	0.603669036	0.335902667	0.281148887	0.607135185	0.711958289	0.686587245	0.96763119
XPNPEP2	0.542841551	0.686311558	0.363707188	0.339416146	0.535868673	0.686608798	0.96763119
EFNA1	0.131219668	0.680502219	0.463007127	0.642137228	0.929167243	0.686786991	0.967705101
TRAF5	0.963979676	0.341584048	0.652847105	0.286973664	0.399868936	0.686786438	0.967705101
AFAP1L2	0.249397832	0.389018377	0.463665875	0.882179003	0.622020533	0.686916582	0.967721524
KIRREL1	0.485089655	0.577772673	0.250221568	0.740653961	0.475171171	0.686889827	0.967721524
EXOC2	0.926008168	0.441424396	0.282127626	0.232544856	0.92191146	0.687220386	0.967949735
GDPD1	0.836036347	0.451586893	0.075957959	0.933396204	0.925015922	0.687505903	0.967949735
ING4	0.652066825	0.922414913	0.379106264	0.96032104	0.113011221	0.687400279	0.967949735
LOC104974837	0.974048193	0.187758775	0.951809401	0.192520827	0.739156275	0.687590203	0.967949735
MIA	0.581424392	0.099107121	0.640323767	0.902976536	0.743260473	0.68753267	0.967949735
SPTSSB	0.801076814	0.99024549	0.995896912	0.035611679	0.879197326	0.68730819	0.967949735
ST7	0.525677707	0.32449299	0.58677884	0.554740886	0.445929609	0.687506258	0.967949735
TBC1D19	0.459359136	0.18682626	0.535384792	0.849684587	0.63456166	0.687609409	0.967949735
ZBTB7C	0.978848859	0.956112366	0.359669713	0.085756815	0.856722434	0.687275737	0.967949735
ACE	0.232713015	0.327626135	0.821044116	0.990696878	0.408906171	0.692113017	0.96801054

ADAM9	0.560443644	0.702904714	0.118291981	0.948799456	0.574715403	0.692503705	0.96801054
ALDH6A1	0.609103653	0.547506337	0.154435324	0.686190558	0.701767213	0.687821386	0.96801054
ALPL	0.984763371	0.620547874	0.169961714	0.826356977	0.292926735	0.690451046	0.96801054
ANKRD50	0.741812578	0.689777584	0.502903257	0.273724047	0.356273327	0.690095426	0.96801054
ARMC2	0.349334447	0.162737747	0.690911427	0.780858572	0.81513776	0.689372257	0.96801054
ARNT	0.363926771	0.611769902	0.872838105	0.169439917	0.769471786	0.691941815	0.96801054
ASNSD1	0.829617582	0.335115153	0.526180864	0.49753896	0.343118199	0.689160139	0.96801054
BCR	0.521751831	0.751746489	0.485754309	0.546868802	0.239226494	0.688789912	0.96801054
BST1	0.764975463	0.595072794	0.572974366	0.442193984	0.216828425	0.689428377	0.96801054
C1QTNF1	0.651305009	0.120502075	0.716111506	0.459263281	0.981633653	0.691953581	0.96801054
C24H18orf54	0.280469304	0.331737841	0.803073073	0.67673297	0.501911283	0.692268068	0.96801054
CALHM5	0.245616923	0.87012169	0.885490845	0.324490779	0.415035956	0.693079961	0.96801054
CALHM6	0.869513459	0.848760724	0.404857363	0.428495141	0.1945138	0.688619077	0.96801054
CAPN3	0.769368691	0.442455947	0.912920052	0.422061665	0.189712119	0.688462829	0.96801054
CCDC150	0.85256347	0.231762628	0.747823077	0.741164079	0.227717614	0.688894532	0.96801054
CCDC166	0.566509115	0.841263836	0.819863554	0.076641222	0.844277097	0.691535366	0.96801054
CCT6B	0.411512743	0.620675092	0.179661335	0.763815684	0.725886818	0.692747064	0.96801054
CDH5	0.44558664	0.880800458	0.25669693	0.351767248	0.717195942	0.692553653	0.96801054
CHD3	0.377441898	0.82823304	0.32715129	0.424412359	0.579709891	0.690613091	0.96801054
DCBLD2	0.743643425	0.389107338	0.301098748	0.430314577	0.678628653	0.692747798	0.96801054
DGUOK	0.533665482	0.884279337	0.477360472	0.180283321	0.625943471	0.692585637	0.96801054
ECHDC2	0.864939657	0.446212937	0.136732002	0.614223931	0.785296612	0.692835262	0.96801054
ENG	0.694323984	0.629583655	0.257867701	0.267792407	0.843573221	0.692912867	0.96801054
ESRRA	0.515725544	0.639136674	0.933075782	0.174074091	0.47197877	0.69142788	0.96801054
EXOSC9	0.867558581	0.082112763	0.855721508	0.610484101	0.682788958	0.692500067	0.96801054
FAM114A1	0.351585991	0.933366873	0.34303229	0.444165698	0.505992328	0.691659497	0.96801054
FRYL	0.385311521	0.831834784	0.285016967	0.484719485	0.573424897	0.692359928	0.96801054
GDPGP1	0.089769131	0.521172753	0.753810017	0.980588285	0.732121817	0.691807143	0.96801054
GGT5	0.892000317	0.630748668	0.196514035	0.696234799	0.32809854	0.691334272	0.96801054
GSTP1	0.821833342	0.314466012	0.678107714	0.866656368	0.164354593	0.689074419	0.96801054
H3F3B	0.558170542	0.153304926	0.574025793	0.674477542	0.764697253	0.691927271	0.96801054
HACD3	0.490436217	0.272670324	0.724897327	0.876430634	0.300086061	0.693146399	0.96801054
HNRNPR	0.763517501	0.187584563	0.816508645	0.608502929	0.352545119	0.690037829	0.96801054
KIAA0355	0.705981918	0.784186547	0.114601975	0.4623642	0.869065633	0.693138226	0.96801054
LOC100847118	0.397915359	0.142044914	0.707965731	0.87632403	0.714359337	0.689751785	0.96801054
LOC101903905	0.80239452	0.325227453	0.700328191	0.43063409	0.320659242	0.69118072	0.96801054
LOC101905151	0.075894388	0.484742217	0.824978223	0.978363949	0.851850031	0.691623415	0.96801054
LOC101905845	0.291684124	0.503033184	0.945415429	0.590500963	0.311130483	0.693073243	0.96801054
LOC104971845	0.909856991	0.153703974	0.633932511	0.29109392	0.985270157	0.69262733	0.96801054
LOC104972031	0.710331249	0.273892723	0.539171662	0.993018483	0.243547266	0.692192374	0.96801054
LOC104973154	0.428866153	0.998896895	0.935060951	0.549720282	0.113919516	0.690023616	0.96801054
LOC107132798	0.613869956	0.795770673	0.442795437	0.442416132	0.261561364	0.68960165	0.96801054
LOC112443176	0.662306821	0.462165218	0.790975136	0.988414737	0.106532363	0.693137448	0.96801054
LOC112443240	0.836905121	0.195275906	0.254310836	0.627513052	0.956784033	0.689004015	0.96801054

LOC112444681	0.407039702	0.580610587	0.283649268	0.745740241	0.499478058	0.689128812	0.96801054
LOC112444924	0.300718046	0.949121322	0.302904075	0.650099638	0.45110514	0.692074811	0.96801054
LOC112445968	0.661945413	0.789203291	0.878423108	0.170111203	0.319632946	0.688991947	0.96801054
LOC112448366	0.962049022	0.808250335	0.44275759	0.093652587	0.772947687	0.68876082	0.96801054
LOC112448520	0.144557187	0.741389699	0.697373727	0.969946226	0.348946042	0.691637248	0.96801054
LOC112449590	0.911288328	0.595795667	0.841386766	0.057634765	0.956415489	0.690759538	0.96801054
LOC513894	0.269592387	0.564092435	0.997293471	0.787425781	0.207807901	0.68794927	0.96801054
LOC518623	0.341263814	0.955518077	0.204793726	0.433735456	0.869575181	0.690802996	0.96801054
LOC527744	0.789686674	0.16750012	0.576067062	0.505626299	0.647789989	0.689039741	0.96801054
LOC614614	0.529369138	0.558133424	0.611384713	0.396748133	0.349945076	0.689980881	0.96801054
LOC781256	0.864455561	0.413653634	0.742229262	0.264786885	0.355602525	0.689293827	0.96801054
LOC782527	0.934120092	0.61405511	0.76628632	0.652499419	0.088194708	0.691622179	0.96801054
LOC784243	0.799247924	0.473473068	0.168696331	0.546203836	0.714684196	0.688748545	0.96801054
MAN2B1	0.560633158	0.929421663	0.617889397	0.104725189	0.746789789	0.690747059	0.96801054
MAPK13	0.838342886	0.249521666	0.187946154	0.888707178	0.712025114	0.688422446	0.96801054
MFN2	0.63280701	0.808075234	0.536937985	0.27537208	0.334194946	0.69141949	0.96801054
MIS18A	0.247975471	0.741438004	0.357137515	0.493706951	0.775362284	0.690410188	0.96801054
MUM1	0.907353024	0.84552547	0.176929578	0.198370249	0.94370936	0.692506923	0.96801054
N4BP2L1	0.434377696	0.612903061	0.234557399	0.57619826	0.702965703	0.691618068	0.96801054
NAAA	0.571892291	0.973286986	0.144529612	0.451136323	0.692719622	0.690447623	0.96801054
NBAS	0.452203305	0.38006116	0.37826417	0.970136365	0.394964163	0.688669262	0.96801054
NFIL3	0.781289371	0.190532721	0.447845146	0.385719675	0.990745697	0.693005871	0.96801054
PAFAH1B2	0.469543895	0.134945962	0.5863395	0.702579744	0.960835703	0.689982656	0.96801054
PCGF1	0.682195954	0.65094509	0.304673327	0.461356618	0.406263934	0.692114255	0.96801054
PDS5B	0.308882134	0.740055524	0.362811949	0.341489918	0.89036893	0.691028776	0.96801054
PMP2	0.47963858	0.977596202	0.081452084	0.70738964	0.938573863	0.692100952	0.96801054
PSMD5	0.731513543	0.346378172	0.653125215	0.854964262	0.177424225	0.690160354	0.96801054
PTCH1	0.738857951	0.846031132	0.376775098	0.159694344	0.667736663	0.690246499	0.96801054
PTPN4	0.515271252	0.512809566	0.401146239	0.626838881	0.38263112	0.692601276	0.96801054
RASSF2	0.478622895	0.85931291	0.601938852	0.111964948	0.918567589	0.692893701	0.96801054
RTN4R	0.801396042	0.476986016	0.73732693	0.110193701	0.808484399	0.690209696	0.96801054
SCARF1	0.676793452	0.604935891	0.277256254	0.775690416	0.284510145	0.689761981	0.96801054
SEC22C	0.602647613	0.725033587	0.427398552	0.671334212	0.201236933	0.691123982	0.96801054
SEM1	0.299337525	0.164824692	0.801434379	0.929636222	0.686260606	0.691102871	0.96801054
SENP2	0.779886779	0.659670504	0.579331981	0.26555341	0.322079091	0.693119913	0.96801054
SLK	0.332868948	0.279988109	0.508043892	0.766739183	0.698806285	0.69219659	0.96801054
SORBS3	0.909879693	0.942520183	0.832962484	0.640340758	0.055300091	0.691628069	0.96801054
SRR	0.952644499	0.394396594	0.510267883	0.768102926	0.172189743	0.692094735	0.96801054
SRRT	0.640434322	0.425228616	0.629564427	0.164026957	0.903964247	0.6925892	0.96801054
SSBP3	0.66125105	0.493110344	0.936297708	0.107504135	0.761907133	0.689414973	0.96801054
SSUH2	0.69374918	0.475438932	0.279011795	0.400705036	0.676931487	0.689076361	0.96801054
SVBP	0.254556013	0.690381125	0.44608112	0.504885879	0.631167933	0.689225145	0.96801054
TMEM120B	0.570943277	0.346568339	0.339582388	0.394853591	0.961195754	0.693197238	0.96801054
TMEM200B	0.808574777	0.671471236	0.435885057	0.320137028	0.334255507	0.691848574	0.96801054

TTC5	0.551044813	0.566738379	0.545271447	0.81535122	0.182415316	0.691872646	0.96801054
VPS50	0.084046134	0.469019662	0.659094107	0.957088377	0.998107621	0.687963968	0.96801054
VPS51	0.915929064	0.667008548	0.898604499	0.086705474	0.522871949	0.688505223	0.96801054
ZNF226	0.394360144	0.976086909	0.655213572	0.710452228	0.14094026	0.691316252	0.96801054
ZNF511	0.327840757	0.718214555	0.507087644	0.281336828	0.741248777	0.688588335	0.96801054
TSPAN2	0.810865474	0.44975643	0.384520913	0.338456004	0.538313605	0.693555803	0.96834646
ZNF330	0.5489743	0.912388497	0.359136487	0.527259308	0.269299345	0.693496917	0.96834646
ADCY9	0.369357266	0.722936141	0.394339903	0.43698341	0.555883011	0.693769429	0.968562323
DHDH	0.200518321	0.767617741	0.222667353	0.817330757	0.913981318	0.693957853	0.96866057
EEF1AKMT2	0.462064848	0.834512411	0.699825476	0.154440857	0.614163905	0.693905198	0.96866057
RNASE4	0.821450036	0.868463183	0.391426486	0.378829959	0.242141746	0.694049149	0.968705611
ABHD2	0.248606099	0.988140831	0.936497445	0.328221384	0.34109566	0.695105247	0.968835486
ACY1	0.583103038	0.80380707	0.229744789	0.496781696	0.480856977	0.694858948	0.968835486
AKAP13	0.970730139	0.729061767	0.755071334	0.113294745	0.425022582	0.694924643	0.968835486
CHMP4C	0.747296367	0.503477732	0.805218561	0.127381294	0.6682819	0.695358603	0.968835486
CXCL14	0.244300551	0.912305841	0.343600055	0.787335183	0.427709802	0.695347405	0.968835486
FAM192A	0.927702806	0.272027932	0.915775514	0.116544045	0.952260492	0.69429754	0.968835486
GMFG	0.324477607	0.845292166	0.792090615	0.359355527	0.329402482	0.694811692	0.968835486
HNF4A	0.597069501	0.814306734	0.137471408	0.419590172	0.919999682	0.695440986	0.968835486
LOC100848568	0.982025978	0.531184882	0.591092577	0.426252684	0.195281869	0.6944287	0.968835486
LOC107132713	0.720109063	0.434423699	0.273779839	0.351931704	0.854177842	0.695034218	0.968835486
LOC112441456	0.737593141	0.178561411	0.60464255	0.819985348	0.393414243	0.694608649	0.968835486
LOC112442538	0.599477987	0.254770518	0.29191681	0.608742805	0.950396015	0.695388584	0.968835486
LOC112445076	0.318193353	0.762297724	0.593407405	0.203222692	0.882019702	0.695432525	0.968835486
LOC112446799	0.265550232	0.981934612	0.330764385	0.358074496	0.832915506	0.694858075	0.968835486
LOC511161	0.997636703	0.653975189	0.306365296	0.248376843	0.516625119	0.694298705	0.968835486
LOC781799	0.787339755	0.307204037	0.525275867	0.843959774	0.239678132	0.694682796	0.968835486
PDCD4	0.596750896	0.599558645	0.591229146	0.12540347	0.972033594	0.695322788	0.968835486
RBM12B	0.256057014	0.606041961	0.699314726	0.33367207	0.710085593	0.694778449	0.968835486
RBM42	0.732525039	0.238268275	0.700743359	0.346336045	0.608270103	0.695176813	0.968835486
SERTAD3	0.330357504	0.701399203	0.891146619	0.940501701	0.132186944	0.694470139	0.968835486
SPG7	0.878620438	0.771806681	0.664729821	0.159026716	0.358800491	0.694838486	0.968835486
UBTD2	0.499352128	0.37231775	0.457697561	0.321926615	0.939106963	0.694879719	0.968835486
SRC	0.583533229	0.792232195	0.150823414	0.405649846	0.912591815	0.695519136	0.968862112
LOC107132196	0.593498638	0.217996094	0.806237908	0.873002814	0.283662637	0.695667363	0.968914023
RPS26	0.968861139	0.718373278	0.553303388	0.070406225	0.952750997	0.695674482	0.968914023
CRABP1	0.832110417	0.425953516	0.283658616	0.710725536	0.361892816	0.695876198	0.968948267
CRNKL1	0.87263621	0.429420457	0.714038943	0.098111078	0.984759736	0.69581649	0.968948267
RAB36	0.510000328	0.818688193	0.966102338	0.117880374	0.543698452	0.695827794	0.968948267
DCDC2B	0.409619954	0.283615859	0.809583163	0.890487378	0.309586726	0.696390738	0.969387422
KCNA6	0.831569383	0.731199997	0.274633177	0.269267736	0.577114436	0.696546004	0.969387422
LOC101902644	0.812143474	0.714976808	0.287582179	0.205305653	0.756172324	0.696357618	0.969387422
LOC101902998	0.194865457	0.842824417	0.650450915	0.345590937	0.702832829	0.696531375	0.969387422
LOC104974890	0.172195662	0.813754665	0.470478609	0.467213705	0.841462391	0.696312239	0.969387422

LOC112446771	0.305304704	0.322546539	0.394476145	0.819045631	0.815592591	0.696543379	0.969387422
LOC112448762	0.541383499	0.452176845	0.434732215	0.489282881	0.498818125	0.696724598	0.969538579
VGLL3	0.439447485	0.591367849	0.245097763	0.540768284	0.754277232	0.696772774	0.969538579
PITPNM3	0.954824433	0.690184321	0.147745523	0.355046352	0.752604153	0.69704202	0.969830995
ADGRA1	0.184107868	0.549839195	0.795506359	0.959803756	0.33762831	0.697625384	0.969936127
ANKEF1	0.823196468	0.926347934	0.173272122	0.391816925	0.507210971	0.698821832	0.969936127
ARL3	0.880983052	0.094688481	0.97030064	0.335585344	0.9686235	0.699197994	0.969936127
ATF7IP2	0.921993958	0.251748751	0.582184962	0.931313853	0.208683847	0.698849739	0.969936127
CEP63	0.559235784	0.256078036	0.978755486	0.871648217	0.215487326	0.699319762	0.969936127
CPNE3	0.575165555	0.40522505	0.319685169	0.580468675	0.606506418	0.698621824	0.969936127
DOPEY1	0.30785485	0.817818391	0.491620309	0.226856922	0.936094434	0.699010277	0.969936127
ECHS1	0.969544014	0.736600458	0.401367302	0.131686047	0.694755039	0.698571917	0.969936127
EFCAB8	0.384756808	0.420600614	0.357010106	0.803969826	0.562270477	0.697779675	0.969936127
FAT2	0.862370178	0.691312016	0.189337531	0.488248322	0.475185983	0.698304788	0.969936127
FBXO15	0.645470183	0.698713486	0.163240143	0.597556073	0.598188385	0.69923774	0.969936127
FOCAD	0.903953413	0.863131504	0.767077191	0.694278428	0.062986642	0.698187713	0.969936127
FUT10	0.852865872	0.260641502	0.19042441	0.633703482	0.981417026	0.699311865	0.969936127
GFOD1	0.677587539	0.511177186	0.097182552	0.873929062	0.887207763	0.697649137	0.969936127
HIST1H2BD	0.603527097	0.592968425	0.714875482	0.217335883	0.471706408	0.698593579	0.969936127
ING1	0.592340867	0.292076886	0.803578837	0.23459345	0.807402577	0.699363487	0.969936127
KCNAB2	0.142656076	0.82657367	0.82533531	0.870874639	0.308875427	0.698230638	0.969936127
KHSRP	0.616213391	0.652749831	0.615520056	0.153041637	0.694113701	0.699123426	0.969936127
KLF13	0.320900978	0.996424025	0.125942184	0.742621706	0.876439797	0.698467205	0.969936127
LAMA4	0.506876929	0.564827257	0.281332073	0.358080539	0.903726399	0.697396245	0.969936127
LOC100335936	0.42165477	0.526293685	0.1899511	0.788709878	0.784690064	0.697567615	0.969936127
LOC101909384	0.441016189	0.98111105	0.206231733	0.534394476	0.549610241	0.698453402	0.969936127
LOC104972622	0.401830158	0.436347669	0.265544919	0.707632901	0.793599357	0.698001186	0.969936127
LOC112444171	0.613221266	0.254572458	0.879484833	0.56104332	0.338249217	0.697323267	0.969936127
LOC784738	0.867395005	0.401502035	0.198695078	0.935963825	0.405893398	0.699036114	0.969936127
LRRC8E	0.953372697	0.761430697	0.478211628	0.11658659	0.644633685	0.697581947	0.969936127
MTIF2	0.222776438	0.48549118	0.754693307	0.602988429	0.533558409	0.698836814	0.969936127
NARFL	0.491183316	0.492273063	0.755541951	0.197293343	0.72672332	0.698340551	0.969936127
NOSIP	0.732468101	0.492194969	0.223671968	0.436432413	0.745340183	0.698614373	0.969936127
OLFM2	0.071433748	0.916222554	0.971830072	0.719171633	0.572767608	0.698391834	0.969936127
OSBPL2	0.810369752	0.313618137	0.340681813	0.935310372	0.324999648	0.699261198	0.969936127
R3HCC1	0.79628063	0.260324643	0.289594271	0.610674548	0.713725369	0.69812939	0.969936127
RPL30	0.382893119	0.401095561	0.256576475	0.812527707	0.819526087	0.69867373	0.969936127
SMIM33	0.917035192	0.968092413	0.044865369	0.953231958	0.688324842	0.697905459	0.969936127
TEDC2	0.933495375	0.077662038	0.534786348	0.947414958	0.714851827	0.698812712	0.969936127
TMEM26	0.52070891	0.86774452	0.748981857	0.086225455	0.892330049	0.697203362	0.969936127
UBXN2A	0.860202019	0.182382212	0.217080473	0.993791328	0.776518836	0.698986627	0.969936127
WTAP	0.708786076	0.924903237	0.600873665	0.125166374	0.531765419	0.698523026	0.969936127
CXHXorf58	0.753775416	0.199575237	0.454697795	0.434466507	0.88685621	0.699530386	0.970036834
LYRM7	0.795523946	0.552832442	0.241757671	0.326696634	0.758863094	0.699554319	0.970036834

LOC782120	0.287498462	0.748075832	0.841336821	0.16217482	0.899719217	0.699866478	0.970167268
LRRC4	0.745986502	0.346677634	0.820270432	0.96822204	0.128481924	0.699773401	0.970167268
TMBIM6	0.915138222	0.284718605	0.279887712	0.605980186	0.597500461	0.699884851	0.970167268
U2AF1L4	0.967644222	0.277770566	0.686955436	0.160612743	0.890120274	0.699830119	0.970167268
AMBRA1	0.491915922	0.779128147	0.520189733	0.29176357	0.456451425	0.70094695	0.970183324
C17H4orf33	0.899304965	0.514228058	0.3896747	0.291530127	0.505991811	0.701169849	0.970183324
CAMSAP3	0.176190461	0.423728431	0.751488947	0.743384759	0.636293732	0.70084833	0.970183324
CSF2RB	0.673383148	0.558847218	0.564926961	0.622338212	0.201281379	0.701516829	0.970183324
DHCR7	0.672831237	0.869012416	0.72994853	0.162013136	0.382956244	0.700433043	0.970183324
DPP7	0.09636696	0.743844404	0.451000961	0.865525717	0.946950914	0.700552947	0.970183324
ENAH	0.353636842	0.862696715	0.349643854	0.816161025	0.30408295	0.700382556	0.970183324
ENTR1	0.3876207	0.761698915	0.421726775	0.306835987	0.697369412	0.701610852	0.970183324
EPN2	0.989740863	0.66228931	0.300069275	0.262954708	0.514861106	0.701509812	0.970183324
ERMARD	0.217158399	0.463847069	0.428732438	0.737454465	0.835912954	0.701453404	0.970183324
FASTKD3	0.997856465	0.161121769	0.950039025	0.849517412	0.203880463	0.700250974	0.970183324
GAB3	0.930749992	0.28981911	0.244189122	0.607861832	0.664820975	0.701436583	0.970183324
GBA	0.189844795	0.627819159	0.550135001	0.719371833	0.562313435	0.700746977	0.970183324
GINS4	0.73662813	0.987615555	0.319232458	0.135471453	0.843777134	0.70091675	0.970183324
ITGAM	0.291065851	0.482308834	0.417321098	0.503959977	0.896780194	0.700408799	0.970183324
LOC100299705	0.212141414	0.570119547	0.315797214	0.733435151	0.947939161	0.700971104	0.970183324
LOC100336104	0.652106849	0.643456156	0.857348792	0.935711211	0.078859796	0.70090524	0.970183324
MRPL58	0.430699103	0.734643497	0.390698077	0.311267544	0.691057761	0.701235278	0.970183324
MXI1	0.72734973	0.890279027	0.414758402	0.695231716	0.141583384	0.700116145	0.970183324
NDNF	0.135651122	0.812886814	0.972866965	0.353248734	0.700196468	0.700823404	0.970183324
PACSIN3	0.239440914	0.912075293	0.507950289	0.608506917	0.393520876	0.70103368	0.970183324
PPP1R13L	0.404911388	0.777907112	0.955084991	0.855348618	0.103015694	0.700632962	0.970183324
RAB39A	0.442330171	0.665938175	0.858223841	0.968889905	0.108274714	0.700724054	0.970183324
RWDD2B	0.793339356	0.764438147	0.269861876	0.440990784	0.368519351	0.701276181	0.970183324
SAPCD2	0.734835814	0.627003681	0.654522713	0.09204996	0.959778989	0.701606132	0.970183324
SERPINB1	0.527090235	0.818388245	0.239945892	0.322140039	0.796948956	0.701099626	0.970183324
TM2D3	0.725379371	0.542059752	0.689023765	0.151642533	0.643481505	0.7001149	0.970183324
TRPV1	0.864397752	0.62967671	0.269154511	0.744967623	0.242768446	0.700538542	0.970183324
XRCC6	0.716998978	0.342924854	0.230167285	0.582166265	0.804405892	0.70059191	0.970183324
IER3IP1	0.5184917	0.216748761	0.524621251	0.460906468	0.981833566	0.701877642	0.970225234
KDM1A	0.735614111	0.917147336	0.508182997	0.098544611	0.789187746	0.701757354	0.970225234
SLC45A4	0.563346418	0.432136734	0.360383092	0.305240547	0.995957953	0.701811146	0.970225234
UQCC1	0.742909515	0.799887617	0.596035445	0.099867434	0.754119204	0.701836094	0.970225234
SETD1A	0.352383586	0.51767526	0.620712733	0.337797488	0.697833818	0.701955851	0.970251618
ATP1B2	0.425112708	0.980354469	0.436500913	0.875559502	0.168542745	0.703057267	0.970281421
BIN2	0.53339744	0.638350234	0.903014038	0.310545731	0.28026918	0.702455991	0.970281421
BSN	0.452567173	0.553514309	0.43859442	0.56214754	0.43469742	0.703076799	0.970281421
C18H19orf81	0.885590124	0.218978438	0.857201051	0.916925067	0.17567215	0.702565526	0.970281421
CCT3	0.639874928	0.597622695	0.644465782	0.274459477	0.395561775	0.702415935	0.970281421
CD80	0.820587514	0.630973048	0.749410548	0.368752496	0.187491971	0.702927147	0.970281421

GGA3	0.599893823	0.157936901	0.952414866	0.305735228	0.971552269	0.702760665	0.970281421
IP6K3	0.571908358	0.86758962	0.321727648	0.264162933	0.635932386	0.702856044	0.970281421
LOC107131368	0.127293537	0.548551159	0.485487374	0.994193808	0.79510715	0.702717566	0.970281421
LOC107132308	0.993988857	0.585955333	0.078338724	0.839805741	0.697723306	0.702270299	0.970281421
LOC112442597	0.99330368	0.225582898	0.652403137	0.720867935	0.254402475	0.702798379	0.970281421
LOC781692	0.285105506	0.335889022	0.904155179	0.381021879	0.809579291	0.702080118	0.970281421
LOC784058	0.838761848	0.114765209	0.637473996	0.988193013	0.442739139	0.703071818	0.970281421
NSMCE2	0.385583997	0.597322301	0.155368585	0.913073115	0.820510806	0.702798159	0.970281421
SF3B6	0.737012833	0.070402873	0.889414332	0.743446993	0.782535323	0.703081711	0.970281421
TBC1D4	0.360843751	0.601548657	0.24595221	0.709267912	0.708583834	0.70295762	0.970281421
VMAC	0.484081008	0.99013524	0.519776491	0.191554114	0.562659986	0.703100765	0.970281421
ZBTB46	0.292310414	0.57780733	0.241126633	0.934401819	0.703172401	0.70243951	0.970281421
ZCCHC18	0.406974859	0.260712678	0.583769776	0.606958783	0.713217963	0.702829931	0.970281421
SDHC	0.891952412	0.752733756	0.492175631	0.16395508	0.495810412	0.703178843	0.970307575
ZNF177	0.489919247	0.53525614	0.199842777	0.783891176	0.654134231	0.703246582	0.97031946
EMD	0.879785399	0.438843695	0.112575718	0.811562035	0.762620911	0.703451465	0.97043897
NECTIN1	0.551287461	0.981750348	0.305248705	0.397120723	0.409922803	0.703405535	0.97043897
IKBKB	0.609445058	0.872762021	0.141239065	0.456355125	0.785336505	0.703620018	0.97050833
KANK4	0.740648495	0.620402491	0.569932406	0.107520363	0.956168675	0.703615746	0.97050833
AXL	0.771658531	0.830113018	0.630831365	0.720281569	0.092578235	0.703771241	0.97052428
IDH3G	0.843416349	0.425766861	0.76117627	0.133446163	0.739210173	0.703897476	0.97052428
LOC101905977	0.566716931	0.801259464	0.161666807	0.547859241	0.670611816	0.703953596	0.97052428
TMCO6	0.671796083	0.129660018	0.753982506	0.981103176	0.418654095	0.703986413	0.97052428
TPBGL	0.371478439	0.603032456	0.573894451	0.418524838	0.501120119	0.703895828	0.97052428
WTIP	0.850051874	0.695055643	0.094124502	0.723528821	0.67025679	0.703937872	0.97052428
DPH6	0.506422401	0.331254341	0.946912963	0.654600542	0.260042399	0.704440607	0.970742699
LOC101902664	0.843948074	0.506896527	0.403620734	0.574640473	0.27248964	0.704418409	0.970742699
LOC101907549	0.759456651	0.74042583	0.157741601	0.517109658	0.589245042	0.704354343	0.970742699
PLIN4	0.847690698	0.642700583	0.443669713	0.171048769	0.653455472	0.704280486	0.970742699
RGS2	0.937342817	0.208737967	0.291047415	0.757463722	0.626378691	0.704289576	0.970742699
SLC38A6	0.868525642	0.573416407	0.171813902	0.941840483	0.33566739	0.704525458	0.970778111
PBX2	0.364768347	0.456625818	0.407939669	0.896652681	0.444490961	0.704729605	0.970977881
DHFR	0.61189644	0.605897507	0.527353826	0.196249418	0.706252824	0.704856107	0.97107065
TRPC4AP	0.46319759	0.463429277	0.98305144	0.730283856	0.175928332	0.704946766	0.971114025
PXN	0.71601236	0.903582612	0.263555287	0.215056126	0.740187719	0.705167237	0.971336203
PDE11A	0.989526843	0.703560901	0.098106606	0.830971314	0.478792469	0.705390647	0.971480858
SLC25A53	0.533989671	0.947341617	0.236624008	0.454587279	0.499395565	0.70539037	0.971480858
ARPIN	0.358149758	0.838377091	0.320691224	0.657135812	0.429996563	0.705633116	0.971547447
ILF2	0.76935892	0.463715494	0.381354898	0.856126273	0.233716106	0.705732288	0.971547447
LOC101902172	0.893106115	0.204786879	0.752148951	0.316748201	0.624113444	0.70553421	0.971547447
PAIP1	0.81726426	0.388291308	0.159623208	0.925837696	0.580485648	0.705735002	0.971547447
SYT5	0.821161389	0.57335376	0.563697633	0.203557362	0.503651988	0.70563466	0.971547447
ZNF18	0.291514774	0.695237978	0.721449873	0.221326486	0.841534429	0.705806773	0.97156475
BAG6	0.975573704	0.585620268	0.281865483	0.729026944	0.232128443	0.705932091	0.971655754

KANSL3	0.17531967	0.964870401	0.508039724	0.991115855	0.320631843	0.706345327	0.971691053
LHFPL3	0.844373075	0.809232168	0.524758624	0.313425069	0.243121328	0.706431415	0.971691053
LOC104969648	0.888813601	0.791783147	0.850975936	0.072962018	0.624470357	0.706175578	0.971691053
LOC112444147	0.679868912	0.978367565	0.476928361	0.406548455	0.211813432	0.706397204	0.971691053
PRTFDC1	0.808291637	0.82322936	0.298724617	0.237472769	0.577917471	0.706130086	0.971691053
STXBP2	0.191665203	0.790483035	0.341899014	0.610844051	0.862698124	0.706254838	0.971691053
TFAP4	0.656534487	0.94257507	0.230165981	0.711939131	0.269430424	0.706422713	0.971691053
VPS13D	0.291751528	0.928796267	0.749555307	0.255300359	0.526258082	0.706195218	0.971691053
DNTTIP1	0.600260564	0.810461003	0.290110055	0.817804948	0.236980597	0.706639822	0.971747047
LOC112443475	0.544711144	0.753240836	0.137299777	0.544203002	0.891893211	0.706572242	0.971747047
M1AP	0.360121657	0.708130733	0.747645153	0.265192907	0.541003044	0.706649763	0.971747047
CLIC5	0.819998213	0.277594929	0.149688982	0.845151107	0.950237443	0.706720621	0.97176306
C8H9orf64	0.253548193	0.828429747	0.59461795	0.227477077	0.963897197	0.706871088	0.971807107
FDX1	0.752877806	0.204384491	0.342918103	0.525533545	0.987501461	0.706861606	0.971807107
ADAMTSL3	0.427012404	0.288999419	0.691229393	0.751888211	0.42751738	0.707110596	0.971973532
ALDH16A1	0.679618531	0.81908206	0.455221262	0.218012304	0.496270115	0.707087526	0.971973532
RPGRIP1	0.474081861	0.260103236	0.801610433	0.391105712	0.709754051	0.707241656	0.972072262
CASP8	0.496399716	0.417942241	0.587853262	0.239390574	0.942326286	0.70775235	0.972095436
CCDC91	0.999181846	0.167531843	0.19931065	0.899287205	0.917156662	0.707791625	0.972095436
FRMPD3	0.358294772	0.620077288	0.50350233	0.461511689	0.532134319	0.707473687	0.972095436
LOC112449561	0.992572766	0.744062824	0.10607571	0.514096242	0.681897371	0.707411905	0.972095436
LOC539069	0.660995589	0.804242689	0.883925111	0.222511149	0.262946905	0.707618559	0.972095436
LOC781304	0.293804345	0.899540922	0.464502388	0.235810815	0.948966112	0.70746918	0.972095436
MDM1	0.948062512	0.221679175	0.538103715	0.762388753	0.319153484	0.707787043	0.972095436
PSMD10	0.444639412	0.930354801	0.636790059	0.345337576	0.302224864	0.70762114	0.972095436
TGFB1	0.803922498	0.274493345	0.618327872	0.273271478	0.737482841	0.707658184	0.972095436
ZSCAN20	0.389580624	0.741805402	0.38035806	0.300813676	0.832535554	0.707864649	0.972114373
EEF1B2	0.52659998	0.738237341	0.119958437	0.780019829	0.757254334	0.707985507	0.97211572
NRDC	0.762891745	0.462034894	0.404255501	0.63855077	0.302828995	0.708043337	0.97211572
PRKAR2A	0.797588842	0.625039785	0.106689966	0.771639395	0.671190069	0.707992179	0.97211572
AGPAT4	0.926180571	0.94551263	0.741374722	0.208546051	0.20603068	0.71039426	0.972420433
ARPC2	0.436166187	0.287361043	0.765400657	0.968237415	0.29806155	0.708953669	0.972420433
B4GALNT3	0.880798299	0.597292502	0.67985767	0.200695888	0.385641158	0.708930302	0.972420433
CXCL12	0.402075082	0.581010306	0.364179598	0.392118654	0.82931261	0.708815545	0.972420433
DOCK8	0.355072063	0.5181078	0.480882149	0.45647159	0.692177531	0.710777209	0.972420433
FAM180A	0.291905497	0.860257945	0.135821804	0.856461522	0.953703193	0.710142504	0.972420433
FBXL12	0.859758044	0.170878987	0.398249221	0.6887422	0.688162968	0.709266363	0.972420433
FBXO40	0.875551861	0.786703013	0.748775167	0.702196386	0.077049438	0.710454678	0.972420433
FNDC3A	0.714031352	0.460791988	0.259883451	0.425331347	0.767480949	0.710508982	0.972420433
GPIHBP1	0.115677989	0.431519639	0.824435902	0.81493552	0.824364444	0.708687289	0.972420433
GSR	0.357795165	0.5393136	0.602368891	0.635010329	0.379182239	0.711023388	0.972420433
HADH	0.777956055	0.762189165	0.108643316	0.534980435	0.809734227	0.710467442	0.972420433
HSPE1	0.843986939	0.729441989	0.463047244	0.149742016	0.650514017	0.709523178	0.972420433
KCTD8	0.729676958	0.757945553	0.354752653	0.437178128	0.322042926	0.708518336	0.972420433

KLHL7	0.456767717	0.524754746	0.466050239	0.331896676	0.749657943	0.709698281	0.972420433
LGR6	0.965114136	0.478435155	0.318103643	0.823828411	0.229985829	0.709943338	0.972420433
LOC107131684	0.956773927	0.526870135	0.134719637	0.820445734	0.502662301	0.711156359	0.972420433
LOC107133180	0.201809061	0.596928343	0.380048117	0.631130914	0.96641705	0.710592287	0.972420433
LOC112443147	0.325767131	0.222136457	0.700204196	0.651586183	0.840018213	0.709285945	0.972420433
LOC613660	0.258485258	0.669138568	0.850354943	0.592688503	0.320886563	0.710919305	0.972420433
LOC614531	0.372862348	0.815504627	0.221889048	0.451648654	0.914749327	0.710253252	0.972420433
LRP1	0.868938544	0.419353503	0.521849081	0.216501256	0.67275617	0.709030974	0.972420433
LRRC74B	0.092384832	0.699735796	0.761891512	0.981812669	0.579429921	0.711238661	0.972420433
MAPKAPK3	0.315302205	0.657726073	0.428696675	0.652096436	0.481032143	0.710338676	0.972420433
METTL27	0.838529513	0.236359462	0.240593502	0.819031985	0.71557166	0.710743258	0.972420433
MT1E	0.445988067	0.774040273	0.827603843	0.698214338	0.140497968	0.711287239	0.972420433
MXRA7	0.571728158	0.461719276	0.420951847	0.652276311	0.384203874	0.710068605	0.972420433
MYO1B	0.320890246	0.552771341	0.521317296	0.426686754	0.708801543	0.710878847	0.972420433
NAB2	0.367579289	0.773645374	0.151965328	0.734539229	0.878352307	0.710300508	0.972420433
NMI	0.130984796	0.756322351	0.476767364	0.938087081	0.625306896	0.709091794	0.972420433
NMNAT1	0.768760877	0.458968429	0.41632879	0.329894762	0.576842679	0.710793368	0.972420433
PDE5A	0.468975753	0.156009571	0.582391619	0.933512658	0.703655224	0.71103644	0.972420433
PEAR1	0.571522165	0.932633831	0.2711928	0.336908505	0.573062438	0.710482846	0.972420433
PLEKHB1	0.278134635	0.287393888	0.750304431	0.493178939	0.939713152	0.709706797	0.972420433
PLIN1	0.560940624	0.665925788	0.14330866	0.851174446	0.612548138	0.710499382	0.972420433
PYCARD	0.226335102	0.712707634	0.935785974	0.57036836	0.321292816	0.708795283	0.972420433
RPL38	0.837179437	0.593417515	0.189629563	0.355350846	0.834216209	0.710607858	0.972420433
SAAL1	0.406931148	0.978607626	0.180817602	0.742455892	0.523084645	0.710867816	0.972420433
SC5D	0.902925168	0.446266363	0.976252185	0.10531732	0.668917111	0.709139852	0.972420433
SCML1	0.792112491	0.181884478	0.909436164	0.825700324	0.25841226	0.710813988	0.972420433
SEMA3B	0.812303669	0.157567232	0.417019826	0.952367569	0.544173869	0.708789571	0.972420433
SH2D4A	0.190353618	0.58763327	0.490727455	0.91337724	0.558656842	0.711170928	0.972420433
SH3GL1	0.93643979	0.631909552	0.928174064	0.467447194	0.108478122	0.710088626	0.972420433
SLC9A3R1	0.208361119	0.716332725	0.395046069	0.764417118	0.613819972	0.708819176	0.972420433
SYNGR3	0.393628985	0.494581683	0.692529824	0.749853566	0.276998536	0.711133317	0.972420433
TAF13	0.649392126	0.534641844	0.771572483	0.248269884	0.419881886	0.710597729	0.972420433
TMEM68	0.488549818	0.593358835	0.421905416	0.427768642	0.532750115	0.710235777	0.972420433
TP53I13	0.980568292	0.592055872	0.238971628	0.203552401	0.987541406	0.710343209	0.972420433
TRAPPC10	0.823809157	0.754944633	0.086774457	0.557369917	0.929683256	0.710867506	0.972420433
ZNF569	0.122913295	0.950369352	0.488169642	0.715295637	0.682677465	0.710055355	0.972420433
ZSWIM5	0.620464194	0.691959911	0.817471964	0.335442906	0.235745853	0.709426957	0.972420433
ARHGAP25	0.201325105	0.977718246	0.939838281	0.624994633	0.242573235	0.7114259	0.972488054
HRCT1	0.317769781	0.65462302	0.425574683	0.959862555	0.330310274	0.711569367	0.972488054
LCLAT1	0.402537957	0.160418778	0.871710248	0.567237909	0.878909349	0.711539843	0.972488054
NCBP3	0.506467543	0.778888748	0.660004911	0.161421587	0.667859188	0.711573734	0.972488054
RASL11B	0.712643484	0.26983324	0.369362147	0.600343846	0.659041601	0.711798594	0.972714358
CENPH	0.427870603	0.3155262	0.905050518	0.265420715	0.868026188	0.712130043	0.973086273
CAMTA2	0.74396459	0.172733864	0.512592982	0.958985802	0.446052749	0.712311018	0.97314149

FAM8A1	0.769540584	0.947893483	0.385251576	0.129741345	0.773578938	0.712494276	0.97314149
HADHB	0.853649211	0.638809619	0.08551436	0.657112693	0.920580122	0.712526242	0.97314149
LOC784980	0.644036549	0.635390652	0.215319896	0.865791	0.369639059	0.712453871	0.97314149
NEIL2	0.689948168	0.87828842	0.761025326	0.471590779	0.129681231	0.712484027	0.97314149
PLXNB2	0.799183004	0.334855627	0.159296767	0.734884781	0.899679253	0.71236233	0.97314149
DSC2	0.894688836	0.998952984	0.050937791	0.759968623	0.816205901	0.712728536	0.973275624
HGSNAT	0.384396722	0.318027745	0.33398722	0.916749619	0.75450204	0.712743066	0.973275624
IGFBP6	0.537810894	0.330686605	0.284410219	0.580841479	0.962271968	0.712945327	0.973427213
OR51E1	0.536250651	0.572019794	0.846765466	0.259822799	0.418976825	0.712972707	0.973427213
CTXND1	0.495831662	0.610199718	0.822991952	0.849670729	0.134242	0.713819602	0.973700405
DAXX	0.964594462	0.448188552	0.862230473	0.34318219	0.22168159	0.713531314	0.973700405
GLS2	0.547627839	0.187722115	0.720314373	0.589205318	0.650975404	0.713825457	0.973700405
GPR19	0.623183033	0.939581985	0.104322299	0.605499071	0.766555792	0.713488106	0.973700405
HOXA2	0.512895695	0.717142289	0.71442378	0.114077142	0.945736751	0.713476919	0.973700405
LOC534155	0.487515595	0.636301011	0.256013788	0.377974808	0.946096332	0.713807232	0.973700405
LYRM2	0.809037178	0.806196001	0.086380759	0.673988377	0.745707309	0.713252329	0.973700405
NOD1	0.33408287	0.447988796	0.3813922	0.917692857	0.541388539	0.713538467	0.973700405
RTN4IP1	0.521817491	0.70391572	0.976501917	0.1256315	0.630177204	0.713790109	0.973700405
SMCHD1	0.09653265	0.662253803	0.597900506	0.820643875	0.904668359	0.713657779	0.973700405
TKFC	0.250713366	0.352062703	0.995601702	0.349005259	0.924526097	0.713509842	0.973700405
RTN4	0.716524017	0.637492413	0.330306426	0.805761124	0.233705012	0.713888072	0.973704882
AMOTL1	0.53185661	0.381566158	0.66926966	0.988482121	0.212321753	0.714515463	0.973738857
BRMS1	0.50813034	0.410176897	0.879665102	0.281201377	0.552885906	0.714510114	0.973738857
DNAJB6	0.640982488	0.17974517	0.356923283	0.772270929	0.895551477	0.714081515	0.973738857
FLI1	0.274414199	0.938469554	0.331738039	0.570319252	0.584320058	0.714279742	0.973738857
GTF2A1	0.586392784	0.393063422	0.754584514	0.270151813	0.605722027	0.714213804	0.973738857
LOC104972545	0.44282323	0.67231347	0.134575205	0.932147609	0.763469142	0.714565661	0.973738857
NDN	0.763559159	0.202724285	0.79984741	0.919139882	0.250393829	0.714441852	0.973738857
SEMA3G	0.870306455	0.431266317	0.233545266	0.70316997	0.461533399	0.714131364	0.973738857
SLC10A7	0.532778801	0.272289186	0.661963571	0.516974505	0.573735941	0.714368137	0.973738857
SMIM19	0.846562907	0.194814282	0.402520234	0.616114701	0.695067062	0.714001538	0.973738857
TNS3	0.783587684	0.698251206	0.455689119	0.181197179	0.630684049	0.714428878	0.973738857
LOC524650	0.337269477	0.838532882	0.924763891	0.130528979	0.836027158	0.714746101	0.973823019
PRMT9	0.793213315	0.648744062	0.523464915	0.129155861	0.820295962	0.714737486	0.973823019
LOC100848570	0.621217201	0.444111459	0.562184308	0.194478114	0.947337787	0.714980193	0.97398024
PIGB	0.513268274	0.880098125	0.233323947	0.513503083	0.527822978	0.714926064	0.97398024
DDX51	0.618658715	0.570299914	0.598993826	0.450081209	0.300585569	0.715087947	0.974046174
HDAC6	0.730758712	0.359220846	0.390064422	0.482245811	0.579860531	0.715362996	0.974126131
PMEL	0.177252862	0.705516419	0.64064708	0.838204379	0.42615865	0.715265164	0.974126131
PSMG3	0.382580681	0.953896525	0.649051759	0.304996889	0.396381151	0.71538408	0.974126131
TARSL2	0.706682273	0.908928277	0.316834206	0.651977122	0.215794218	0.715360434	0.974126131
APMAP	0.693015963	0.938599967	0.892296155	0.396807915	0.12458216	0.715759259	0.974232827
COL4A1	0.216583325	0.855197695	0.502672938	0.587084929	0.524892805	0.715750336	0.974232827
LOC104970908	0.28796791	0.461092611	0.519381455	0.614022623	0.677102353	0.715622854	0.974232827

MSH6	0.145548139	0.814963051	0.974639121	0.269938033	0.919111281	0.7156943	0.974232827
OPTN	0.747795712	0.757694963	0.124041331	0.418488397	0.975219918	0.71569832	0.974232827
EPB41L4A	0.675000642	0.313847962	0.80369168	0.499328874	0.338120388	0.716110314	0.974468189
LOC107131710	0.540606668	0.89429722	0.936473089	0.68314807	0.092924095	0.716080008	0.974468189
USP16	0.805287674	0.923792216	0.434613178	0.406789465	0.218450638	0.716014134	0.974468189
GCK	0.89268837	0.973584539	0.203263182	0.254418167	0.640190082	0.716293382	0.974476309
LOC101907006	0.435831599	0.180624782	0.920908951	0.462118271	0.858880841	0.716297526	0.974476309
LOC112442693	0.65624787	0.423190734	0.527028729	0.82611774	0.238112832	0.716413178	0.974476309
PPP2R3C	0.815605645	0.683468337	0.224019388	0.912371486	0.252450593	0.716224677	0.974476309
ZNF568	0.299615538	0.704235617	0.993380573	0.896748567	0.153147906	0.716376702	0.974476309
AHCYL2	0.856648736	0.596338607	0.163773906	0.633728491	0.543664952	0.716637145	0.974557145
EGLN2	0.590186893	0.300113956	0.295572746	0.707015052	0.778792745	0.71664402	0.974557145
FBLN2	0.532386791	0.553724436	0.358821412	0.671833954	0.405706908	0.716680318	0.974557145
LOC515547	0.988208586	0.849244097	0.207001453	0.244955364	0.677853867	0.716769529	0.974557145
SMKR1	0.54330437	0.703554946	0.899117364	0.611572126	0.137199213	0.716716735	0.974557145
LOC104975111	0.208138744	0.858791405	0.489428864	0.553615352	0.596743305	0.717140577	0.974980865
PCOLCE2	0.225633645	0.905277098	0.574964535	0.412748511	0.596472448	0.717217758	0.975005022
AMIGO1	0.708002012	0.30323969	0.176583012	0.816415209	0.936060272	0.717603433	0.97502316
C18H16orf70	0.795332637	0.726037276	0.174454426	0.456023803	0.631907604	0.717973467	0.97502316
COMT	0.979144783	0.154472642	0.469670613	0.45313862	0.899627663	0.7175166	0.97502316
GGH	0.457751045	0.623669706	0.126107394	0.999441676	0.806134389	0.71782425	0.97502316
KCNC3	0.703427131	0.589253008	0.993630771	0.113351821	0.622042876	0.718045137	0.97502316
LOC104968518	0.809109908	0.518304948	0.231078592	0.753848778	0.396635278	0.717622585	0.97502316
MAPRE2	0.151471158	0.392186894	0.92065224	0.766468635	0.691900192	0.717809836	0.97502316
MST1	0.144838291	0.772109055	0.669385745	0.418661509	0.926121963	0.717946763	0.97502316
NUP188	0.4579595	0.418139077	0.911399719	0.825507289	0.201070074	0.717577512	0.97502316
SKAP2	0.57573794	0.485209674	0.273328305	0.408250369	0.927940609	0.717297928	0.97502316
TBCE	0.830858689	0.765007593	0.826064664	0.107335993	0.514499659	0.717757792	0.97502316
UPP1	0.541332907	0.645631393	0.231517384	0.668099425	0.537231659	0.718062879	0.97502316
USB1	0.117246104	0.920478601	0.809697969	0.374285258	0.887567398	0.717977149	0.97502316
ZNF292	0.188859906	0.973616113	0.228287374	0.84519649	0.818354857	0.718007541	0.97502316
CHST2	0.2806689	0.409817528	0.552396694	0.980655597	0.46650565	0.718226311	0.975164391
LOX	0.676699158	0.308536068	0.763468371	0.438185488	0.416367707	0.718321731	0.975168018
MRPL1	0.930339147	0.556798889	0.721297553	0.294254333	0.264551598	0.718347826	0.975168018
LOC107133294	0.46894865	0.509160798	0.71759152	0.543780282	0.312544237	0.718568545	0.975306294
LOC787309	0.437797719	0.728168761	0.144425627	0.974836731	0.648743741	0.718552103	0.975306294
ABHD14A	0.409798495	0.869037911	0.537079575	0.250358001	0.611699892	0.7196845	0.975345262
ARHGEF7	0.470297178	0.767533018	0.283047778	0.684915958	0.421574926	0.721034499	0.975345262
BICRA	0.974112989	0.591263221	0.73986182	0.154975288	0.446764092	0.721052069	0.975345262
CENPL	0.293241673	0.664863985	0.577884725	0.676698304	0.383158763	0.719171841	0.975345262
CHRNB4	0.409810252	0.225859331	0.560171727	0.904849765	0.627912616	0.720762514	0.975345262
CIR1	0.665348367	0.968621805	0.508358418	0.323212332	0.278657928	0.72107438	0.975345262
CLEC6A	0.429097101	0.673604063	0.929612451	0.250480236	0.43732294	0.720596817	0.975345262
CYP7B1	0.755694183	0.593297687	0.110652339	0.885339013	0.671811818	0.721076077	0.975345262

ENDOU	0.695921619	0.334161816	0.340624904	0.667167936	0.556377318	0.720404916	0.975345262
FAM19A3	0.152137213	0.964327199	0.583009688	0.376185871	0.917145428	0.721093417	0.975345262
GOLGA2	0.36517707	0.555666071	0.20498684	0.726482316	0.975657234	0.720914298	0.975345262
KTI12	0.764888675	0.207832744	0.501157027	0.67869284	0.543725399	0.720379077	0.975345262
LMF2	0.434600788	0.122666973	0.964515585	0.874593154	0.650537618	0.719447982	0.975345262
LOC101906397	0.965931521	0.706958328	0.205224901	0.6814496	0.30818996	0.720590056	0.975345262
LOC107131273	0.109810682	0.553479266	0.722097415	0.877467932	0.763892122	0.720495193	0.975345262
LOC112441778	0.97643038	0.504500283	0.991533208	0.076158664	0.791169946	0.720579856	0.975345262
LOC112442352	0.506901053	0.800044293	0.345002281	0.346846115	0.604606608	0.719999382	0.975345262
LOC521580	0.212643692	0.523803942	0.827485882	0.725417037	0.436691146	0.719071576	0.975345262
LYZ1	0.230996317	0.674065599	0.652495544	0.494226335	0.586122345	0.7205798	0.975345262
MCM4	0.564929642	0.403252709	0.189124275	0.934306057	0.730794183	0.720494389	0.975345262
MFAP3L	0.955828284	0.61395403	0.096499218	0.647266252	0.801910491	0.720339571	0.975345262
MSANTD2	0.705365828	0.93801734	0.610420837	0.922944863	0.07880756	0.720230421	0.975345262
NBDY	0.864982942	0.243878066	0.896898867	0.528269192	0.291703589	0.718799363	0.975345262
NKIRAS2	0.841816834	0.177849249	0.316144146	0.915016488	0.678296828	0.7202327	0.975345262
PECR	0.504636554	0.522838529	0.636633348	0.471544303	0.369554032	0.719548507	0.975345262
PGBD2	0.292695816	0.76748312	0.767349459	0.474638489	0.357510949	0.719415206	0.975345262
PLEKHA8	0.379099419	0.734629743	0.256018353	0.502111872	0.815504144	0.719060828	0.975345262
POLR1C	0.776733024	0.950487318	0.88035685	0.307178049	0.146269135	0.719104649	0.975345262
PYROXD1	0.68990547	0.549518144	0.713160554	0.516659532	0.210695343	0.72058826	0.975345262
RASSF1	0.485388236	0.633270394	0.4414447	0.597238617	0.362198369	0.720078032	0.975345262
RIOX2	0.775838816	0.734022897	0.887343578	0.164286101	0.35394583	0.720279418	0.975345262
SLC35D1	0.509758684	0.619573943	0.781646979	0.974433455	0.12225868	0.720449508	0.975345262
SLC4A5	0.398479159	0.876451022	0.377251367	0.483210953	0.459499935	0.719439429	0.975345262
SLC8B1	0.679550325	0.493462125	0.108145114	0.98374569	0.819126982	0.719234234	0.975345262
SOCS1	0.526256912	0.816873845	0.95802217	0.363096483	0.195087008	0.71891204	0.975345262
SYCP2	0.793805759	0.459896776	0.681902582	0.683460344	0.173067488	0.720678762	0.975345262
TGFBRAP1	0.748734222	0.507859669	0.960832485	0.129442792	0.623426139	0.720921404	0.975345262
TRAP1	0.914109587	0.484168712	0.667137879	0.107285967	0.931193081	0.721013562	0.975345262
TTC31	0.747932209	0.235919997	0.336480424	0.575514612	0.860363175	0.720372987	0.975345262
URB1	0.781573307	0.466505229	0.499746217	0.953210038	0.169749001	0.720916876	0.975345262
YBX1	0.70999823	0.522583008	0.177173013	0.839484235	0.531798502	0.720043406	0.975345262
ZNF703	0.425558724	0.939817778	0.596634738	0.401339836	0.306950146	0.720358517	0.975345262
C1GALT1C1	0.428756437	0.546984936	0.916314911	0.431127576	0.319114881	0.721445478	0.97548302
FAM151B	0.716314491	0.969586438	0.156641407	0.514956363	0.528513509	0.721724399	0.97548302
HIST1H1E	0.477936234	0.416745819	0.791567579	0.462483452	0.405848546	0.721622397	0.97548302
LOC101906009	0.285454032	0.742601161	0.791086055	0.484248262	0.364105007	0.721457087	0.97548302
LOC112443216	0.474106292	0.622155553	0.359166705	0.44065566	0.633144176	0.721397124	0.97548302
MAL	0.522979423	0.915136391	0.192644002	0.809549478	0.396829343	0.721789672	0.97548302
SWI5	0.687811958	0.889327002	0.513553864	0.253435902	0.37188676	0.721712494	0.97548302
TNS2	0.972222869	0.270050785	0.759979403	0.824432196	0.180037558	0.721771391	0.97548302
TUBA3E	0.584765724	0.682604892	0.31266182	0.435169049	0.544717149	0.721563889	0.97548302
VARS2	0.314000673	0.489294073	0.408015855	0.648786724	0.727782302	0.721662341	0.97548302

ABCA1	0.711622259	0.618992118	0.160456322	0.518220345	0.85985069	0.733358901	0.97564491
ACADVL	0.719859453	0.651240369	0.225494664	0.301879324	0.990450303	0.734033699	0.97564491
ACE2	0.860997765	0.759188449	0.11594865	0.543034466	0.734765976	0.725716401	0.97564491
ADAMTS14	0.595014446	0.40973683	0.299143667	0.587775397	0.73162611	0.732574061	0.97564491
ANGPTL7	0.502287836	0.914897813	0.165039957	0.722930756	0.572919821	0.732873171	0.97564491
AP2M1	0.324516158	0.184183118	0.951795094	0.542251069	0.973259133	0.724352918	0.97564491
APOA1	0.780106471	0.500547325	0.996944181	0.300625229	0.271558103	0.73505516	0.97564491
ATG14	0.458530274	0.515751556	0.849009964	0.937732656	0.170218887	0.736628773	0.97564491
ATG4C	0.838861669	0.375137485	0.384286225	0.30882318	0.851300884	0.735128733	0.97564491
ATXN2	0.602757086	0.917879433	0.79342594	0.117696863	0.606524902	0.732416238	0.97564491
BCAM	0.667739018	0.704743037	0.236018278	0.391499088	0.728717461	0.734500693	0.97564491
BCAS2	0.628573365	0.53514075	0.79542965	0.652081546	0.181495914	0.734380448	0.97564491
BCL2L10	0.878119791	0.31843773	0.232988642	0.865493606	0.564146919	0.735232482	0.97564491
BIN1	0.413692051	0.626750053	0.707457606	0.240989788	0.702238191	0.730645358	0.97564491
BRWD3	0.70928523	0.676690419	0.397054149	0.205719393	0.806536337	0.734106453	0.97564491
BTBD2	0.556543707	0.78738383	0.107165404	0.782251107	0.83273017	0.727886675	0.97564491
CALML4	0.488820834	0.769993327	0.118158902	0.811527739	0.831883034	0.724357374	0.97564491
CAMKMT	0.881613222	0.29218135	0.613476962	0.236157243	0.811082769	0.725890928	0.97564491
CCL14	0.814280282	0.789908919	0.628695016	0.17028	0.461476303	0.735031837	0.97564491
CCNI2	0.499401799	0.803597256	0.691631903	0.155428079	0.732241546	0.733928363	0.97564491
CD1A	0.899014615	0.963784952	0.071472275	0.53332627	0.926583418	0.727960937	0.97564491
CD83	0.252100125	0.629244868	0.797635553	0.883879285	0.280993467	0.732951624	0.97564491
CDH13	0.72146769	0.697674342	0.146321006	0.608256395	0.713868478	0.736229358	0.97564491
CDV3	0.403568073	0.59500103	0.478078298	0.357350916	0.729691791	0.723791241	0.97564491
CHMP7	0.906868473	0.150333066	0.281272049	0.966233272	0.824117677	0.727541974	0.97564491
CHRNE	0.236237759	0.79808302	0.645389148	0.475173191	0.520571353	0.724828293	0.97564491
CLCF1	0.791150692	0.508338211	0.266672805	0.474316514	0.589797768	0.724223924	0.97564491
CLPTM1	0.576010125	0.567652634	0.220968597	0.703899802	0.590384283	0.724366167	0.97564491
CPLANE1	0.990206585	0.101952922	0.911298975	0.512683586	0.639474977	0.72522322	0.97564491
CPPED1	0.533854059	0.572029358	0.310680487	0.95833378	0.342707014	0.731355076	0.97564491
CTNNB1	0.68943873	0.355425289	0.556363612	0.25832516	0.850922541	0.724005192	0.97564491
CYB561D1	0.336846728	0.922712862	0.350822417	0.482288203	0.59107909	0.730897948	0.97564491
CYBB	0.202805487	0.440406377	0.498869422	0.766353674	0.929972983	0.734909205	0.97564491
CYFIP1	0.354723894	0.802303642	0.234443904	0.578062019	0.828889938	0.736168267	0.97564491
CYP8B1	0.346147811	0.868260063	0.748007027	0.679599138	0.199500612	0.727202291	0.97564491
DAAM1	0.140945324	0.441042908	0.549287159	0.939389964	0.943018785	0.725761378	0.97564491
DCLK1	0.201487446	0.832903083	0.263052416	0.978098055	0.72301357	0.731709426	0.97564491
DNAI1	0.285714652	0.876388209	0.25798619	0.672545377	0.68580178	0.722910697	0.97564491
DOCK9	0.258283298	0.692981592	0.672380802	0.515174117	0.49367989	0.727991659	0.97564491
DOK1	0.881435871	0.414507469	0.354441089	0.451157963	0.543456295	0.734883591	0.97564491
EIF4G1	0.446794349	0.984700028	0.864037084	0.943382429	0.087042423	0.731687431	0.97564491
ENPP2	0.225525228	0.393876509	0.595432386	0.790177907	0.743666421	0.730877272	0.97564491
ENTPD4	0.594429951	0.264404746	0.307202813	0.804370283	0.801019826	0.731052018	0.97564491
ERICH1	0.250259958	0.885584327	0.871050416	0.727477571	0.219219057	0.729088836	0.97564491

EXOC4	0.654997466	0.477693487	0.78557707	0.205145206	0.607942761	0.72828191	0.97564491
FBXO7	0.493583174	0.740035875	0.526762396	0.474121562	0.33465087	0.727504181	0.97564491
FBXW8	0.36211172	0.64134209	0.369313447	0.394871982	0.939779428	0.735336249	0.97564491
FDXR	0.535044649	0.936102678	0.252863744	0.98507463	0.257077867	0.736769111	0.97564491
FIP1L1	0.838005344	0.782562316	0.602104016	0.098596594	0.762396166	0.722184904	0.97564491
GALR3	0.329824909	0.8887077	0.701080832	0.798698102	0.190838888	0.732335009	0.97564491
GAREM1	0.235865088	0.72098551	0.940964454	0.650141867	0.307944809	0.736558246	0.97564491
GCC2	0.740971672	0.337048138	0.900353459	0.320921658	0.411297369	0.722176918	0.97564491
GDNF	0.559827801	0.99333675	0.650949177	0.542995152	0.160942105	0.73419445	0.97564491
GEMIN2	0.834074703	0.366368237	0.763553174	0.29879958	0.439076399	0.728013063	0.97564491
GLMP	0.168743406	0.782391081	0.259300034	0.960932413	0.913285063	0.724480818	0.97564491
GNB2	0.439436824	0.233152636	0.636497518	0.852431875	0.553619033	0.729020202	0.97564491
GNRH2	0.852266588	0.109400446	0.978149455	0.486072816	0.688626774	0.72749353	0.97564491
GOT1	0.95437576	0.258446433	0.987086608	0.177870736	0.73706451	0.735873319	0.97564491
GPAA1	0.709628446	0.954416551	0.549059169	0.155747358	0.545445061	0.733933725	0.97564491
GRIPAP1	0.326935965	0.285962676	0.972407362	0.451893151	0.757723892	0.731170019	0.97564491
HES7	0.827792816	0.898310003	0.534214673	0.115028141	0.6825641	0.731535347	0.97564491
HNRNPM	0.924174004	0.492798275	0.711769549	0.219784133	0.440098556	0.732528963	0.97564491
ID3	0.188049649	0.452652756	0.687023798	0.542091534	0.948697861	0.72467987	0.97564491
IGFBP5	0.423387441	0.450063769	0.39969595	0.757985104	0.555725561	0.736824643	0.97564491
IL12RB2	0.342389637	0.238999109	0.984892396	0.664060615	0.569459303	0.72718577	0.97564491
IL15	0.890061584	0.913784265	0.572244866	0.065526545	0.981983348	0.723877916	0.97564491
ITIH3	0.687168506	0.773939826	0.626453241	0.394037323	0.227143952	0.723063987	0.97564491
ITPRIPL1	0.625687943	0.208801782	0.379356938	0.734240136	0.868424147	0.733998584	0.97564491
JAG1	0.640595441	0.778190467	0.357708025	0.209257408	0.832193719	0.73070972	0.97564491
JAK3	0.914783884	0.648324449	0.424559936	0.246924096	0.5057298	0.73305772	0.97564491
KARS	0.48517482	0.966668877	0.242622329	0.430293433	0.639695133	0.73232831	0.97564491
KDELR3	0.915736028	0.957213773	0.302932108	0.133878887	0.86652431	0.729199336	0.97564491
KDSR	0.779945623	0.55698489	0.32684009	0.355793717	0.597072897	0.725227287	0.97564491
KHDRBS1	0.506347698	0.232321677	0.971932665	0.620564611	0.436076698	0.730025842	0.97564491
KIRREL3	0.54180161	0.439511827	0.376470799	0.839849418	0.415599379	0.732144071	0.97564491
LANCL1	0.764896429	0.262543811	0.879820224	0.246296815	0.68986214	0.724335929	0.97564491
LIPE	0.773263749	0.759498964	0.108145561	0.593763389	0.791943948	0.723357395	0.97564491
LIX1L	0.065726108	0.937776122	0.973620197	0.665829716	0.798557687	0.735804739	0.97564491
LMAN2	0.23911041	0.77340914	0.345818766	0.590049538	0.832054727	0.732783185	0.97564491
LOC100138933	0.844505403	0.500371836	0.399565022	0.428259404	0.424148393	0.728371134	0.97564491
LOC100297170	0.692708167	0.624468227	0.373711207	0.203483555	0.931041083	0.728105398	0.97564491
LOC100297498	0.918156397	0.847565147	0.200769451	0.208031352	0.949990069	0.72964049	0.97564491
LOC100299303	0.099515136	0.698805758	0.86615859	0.698310475	0.761564764	0.736538422	0.97564491
LOC100300483	0.147127759	0.755280729	0.942955379	0.625601535	0.464503568	0.72701423	0.97564491
LOC100848077	0.579790546	0.953790235	0.772462194	0.776789233	0.093724466	0.730992719	0.97564491
LOC100849046	0.516724932	0.788374771	0.2474807	0.988768794	0.302085168	0.724918059	0.97564491
LOC101902385	0.975595824	0.595388992	0.762443983	0.101253891	0.694198551	0.731173079	0.97564491
LOC101902444	0.45606713	0.256167278	0.671028505	0.558103482	0.686933358	0.724554891	0.97564491

LOC101902490	0.477926862	0.390761254	0.314195916	0.90705719	0.570351763	0.726436526	0.97564491
LOC101902812	0.647034323	0.610420776	0.141057289	0.559514476	0.954644152	0.722675162	0.97564491
LOC101902983	0.691010573	0.640491053	0.184814921	0.717337955	0.527637744	0.730142724	0.97564491
LOC101903438	0.199283472	0.868323376	0.933235566	0.385756643	0.506957516	0.733877246	0.97564491
LOC101904753	0.749358693	0.96061523	0.176416284	0.52612648	0.452685279	0.72574712	0.97564491
LOC101905894	0.17994966	0.536420427	0.892136819	0.708258628	0.52205366	0.73541609	0.97564491
LOC101906131	0.953980557	0.238002091	0.366268119	0.463833581	0.817199424	0.733523351	0.97564491
LOC101906200	0.627456951	0.301332744	0.435921393	0.975125362	0.378623135	0.726894818	0.97564491
LOC101906283	0.482526362	0.423758282	0.784822103	0.563489382	0.335403295	0.726268219	0.97564491
LOC101908123	0.61686389	0.750937009	0.415346789	0.645085067	0.253456652	0.733140888	0.97564491
LOC104968751	0.283292514	0.919080066	0.215065458	0.787803606	0.716212727	0.733959323	0.97564491
LOC104970966	0.338483696	0.642880135	0.757294832	0.21348927	0.858716382	0.725527224	0.97564491
LOC104972595	0.296011812	0.559196539	0.478016797	0.406974123	0.995496763	0.736678397	0.97564491
LOC104974459	0.382190087	0.778020086	0.646843099	0.167101371	0.941054513	0.725746925	0.97564491
LOC107132450	0.821875719	0.944201223	0.24640683	0.22557309	0.727788305	0.732748219	0.97564491
LOC107132617	0.586309949	0.694179787	0.321175788	0.30141445	0.760648669	0.724018575	0.97564491
LOC107133166	0.988271995	0.893554651	0.393631625	0.215770268	0.403915193	0.726053501	0.97564491
LOC107133268	0.295887213	0.588695168	0.555857005	0.417610322	0.787960932	0.735528579	0.97564491
LOC112441500	0.914462007	0.932512298	0.815801807	0.439694651	0.1008182	0.729406368	0.97564491
LOC112441607	0.324686229	0.741442871	0.637627297	0.295252992	0.660911466	0.723912313	0.97564491
LOC112442323	0.26777923	0.323387857	0.579527617	0.614542721	0.993826549	0.728253974	0.97564491
LOC112442347	0.595367559	0.825366763	0.103735077	0.666844421	0.920957157	0.732231969	0.97564491
LOC112442866	0.276975928	0.761306133	0.737641815	0.598114519	0.331010036	0.729136477	0.97564491
LOC112443223	0.755274409	0.406193586	0.695110511	0.746250903	0.201404	0.736644503	0.97564491
LOC112444846	0.259449031	0.572658246	0.494155022	0.498620344	0.84512165	0.730015173	0.97564491
LOC112445060	0.675598591	0.843223973	0.375851811	0.191605665	0.752281159	0.72955452	0.97564491
LOC112447070	0.681117697	0.552663639	0.691914025	0.135486684	0.841732784	0.722327052	0.97564491
LOC112448381	0.596279904	0.302671282	0.29188946	0.66141589	0.912579158	0.735153048	0.97564491
LOC112449618	0.91113841	0.431899336	0.373802409	0.267843608	0.799786117	0.733460491	0.97564491
LOC510362	0.526312785	0.646756234	0.415746	0.495590108	0.436427339	0.727997908	0.97564491
LOC516849	0.415489559	0.599545631	0.329521629	0.798354936	0.466237239	0.72766031	0.97564491
LOC528767	0.666198029	0.714745399	0.384341589	0.226398141	0.743982058	0.729325051	0.97564491
LOC614091	0.52160819	0.53584621	0.432142715	0.299874886	0.846707295	0.728361449	0.97564491
LOC618256	0.553452961	0.910595815	0.584913284	0.579335741	0.174334933	0.722765596	0.97564491
LOC618939	0.905085968	0.098325726	0.694206877	0.78500018	0.646675406	0.73256934	0.97564491
LOC781533	0.787005724	0.827772221	0.120144418	0.6739774	0.582011353	0.728571368	0.97564491
LOC784354	0.80706365	0.685700069	0.222173149	0.916019405	0.272180621	0.728279144	0.97564491
LOC787237	0.636343512	0.236288069	0.968700634	0.329608607	0.655213498	0.733132238	0.97564491
LOC787269	0.838971276	0.910437007	0.118842994	0.468338615	0.734842345	0.731844014	0.97564491
LOC788467	0.244682197	0.993688274	0.877655635	0.710881244	0.19949616	0.725853111	0.97564491
LSM11	0.416463513	0.940724925	0.581105003	0.139233542	0.973471986	0.729521884	0.97564491
MAFB	0.447853569	0.78533497	0.63897081	0.646693869	0.209327979	0.726847904	0.97564491
MAGEE2	0.902420079	0.851525612	0.126474346	0.361005016	0.889843073	0.731718929	0.97564491
MAN1C1	0.479253503	0.771104785	0.528655528	0.684169859	0.232794882	0.731092893	0.97564491

MAP2K5	0.612783545	0.247049382	0.314627368	0.978495495	0.659277882	0.728721237	0.97564491
MAP4K4	0.685206565	0.869713202	0.316353309	0.217169566	0.73096148	0.723745755	0.97564491
MBD5	0.421219539	0.986812665	0.885928871	0.144369632	0.565978982	0.724770439	0.97564491
MBNL3	0.306545276	0.859831935	0.21989361	0.551136237	0.970849724	0.730462071	0.97564491
MED16	0.536523422	0.417716383	0.219644	0.842158266	0.770487911	0.736000005	0.97564491
MIB2	0.724056092	0.176528851	0.551844875	0.481248404	0.90851509	0.729411286	0.97564491
MRPL28	0.914390752	0.859477657	0.579071662	0.095565871	0.729722075	0.734796707	0.97564491
MTCH1	0.595019471	0.269784952	0.520179362	0.908268865	0.422715258	0.736696079	0.97564491
MTO1	0.174407365	0.792195074	0.576988151	0.636867877	0.62973818	0.736182819	0.97564491
MYO1G	0.61877286	0.915913068	0.218898001	0.359970716	0.689905721	0.729228309	0.97564491
MYO5B	0.984142854	0.180835698	0.374936469	0.573561605	0.799983396	0.728047302	0.97564491
NAA16	0.320040761	0.342905267	0.389370634	0.967035736	0.773123816	0.736036704	0.97564491
NAIF1	0.166370772	0.619063429	0.628801769	0.815900611	0.586808904	0.730430934	0.97564491
NDRG3	0.162533964	0.44764088	0.64194144	0.678425484	0.982596405	0.731204459	0.97564491
NDUFA12	0.342067698	0.794241805	0.6040973	0.265599301	0.702336635	0.728039888	0.97564491
NEK1	0.971048564	0.925830009	0.575979209	0.172207657	0.336424887	0.724205437	0.97564491
NFE2L3	0.114510198	0.913526305	0.41390239	0.844632188	0.832399652	0.726962837	0.97564491
NME1	0.932084731	0.497426067	0.663389488	0.169408713	0.600497004	0.732135698	0.97564491
ODR4	0.221817977	0.473699678	0.860475993	0.913977498	0.379147175	0.732387716	0.97564491
ORMDL3	0.958278571	0.296758898	0.247295505	0.519516329	0.817246718	0.72331069	0.97564491
OS9	0.965683335	0.478468111	0.318737909	0.586393119	0.344617233	0.722693594	0.97564491
OTUB1	0.317491149	0.733368043	0.792608705	0.268255107	0.607762	0.724759892	0.97564491
PAQR6	0.170892872	0.483149373	0.791985355	0.886654754	0.537512258	0.731385715	0.97564491
PCBP3	0.213122343	0.301706856	0.840196562	0.649708885	0.899145559	0.733754094	0.97564491
PCDHA13	0.038191872	0.8369669	0.979995662	0.973926555	0.981999841	0.72395364	0.97564491
PCDHB14	0.917140163	0.977961393	0.104213946	0.542747027	0.611197185	0.730431876	0.97564491
PDCD2	0.177749803	0.694253467	0.540454426	0.537223786	0.852029702	0.7274984	0.97564491
PDCD6IP	0.596161772	0.216134692	0.695666459	0.370116986	0.960154881	0.735491631	0.97564491
PDHA1	0.892965129	0.990801086	0.249886659	0.156515551	0.867176753	0.724253122	0.97564491
PELP1	0.873115787	0.537704561	0.242669623	0.380771088	0.697309794	0.725771586	0.97564491
PHF7	0.393765811	0.792016694	0.134819653	0.963094739	0.769791697	0.731430001	0.97564491
PJA2	0.715024969	0.22438476	0.542257883	0.908475112	0.381345281	0.725089508	0.97564491
PJVK	0.226358664	0.789209958	0.285483913	0.712214744	0.867865085	0.733534082	0.97564491
PKIA	0.147990925	0.925701167	0.475225843	0.872627697	0.557257265	0.734335616	0.97564491
PLA2G5	0.68563519	0.755731518	0.655345518	0.33781442	0.264386134	0.726261395	0.97564491
PLEKHA5	0.775061195	0.671334655	0.575826417	0.174407102	0.600792419	0.732765268	0.97564491
PLPP2	0.730123823	0.322290275	0.623619455	0.308244218	0.669210153	0.725902031	0.97564491
POFUT1	0.632447251	0.759737887	0.267769087	0.44822073	0.521027838	0.724502103	0.97564491
POLE4	0.869800772	0.340204613	0.907596301	0.393982869	0.302217571	0.736215803	0.97564491
POLM	0.386825424	0.87807694	0.776251643	0.204300637	0.575618438	0.730428614	0.97564491
PON3	0.879706727	0.7819439	0.17275309	0.729458676	0.367051633	0.735274782	0.97564491
PPP1R13B	0.161572018	0.9975751	0.854671644	0.647401735	0.349657583	0.731499265	0.97564491
PROM1	0.174707196	0.84046994	0.432814744	0.990567399	0.489671629	0.729332242	0.97564491
PUM3	0.70201022	0.578555547	0.817874243	0.541728866	0.174640817	0.732958682	0.97564491

PXDN	0.487097973	0.686780258	0.443826654	0.250540257	0.856626798	0.735554893	0.97564491
RAB27B	0.498587647	0.331050182	0.368295255	0.741821414	0.690105296	0.73111772	0.97564491
RAB3IP	0.933585334	0.946630147	0.044675649	0.867066275	0.904375889	0.730147939	0.97564491
RAB9B	0.627135917	0.894986071	0.844700851	0.196279456	0.335802632	0.731893803	0.97564491
RAMP3	0.125532229	0.639343019	0.799577748	0.576137959	0.811936281	0.724326721	0.97564491
RAP2B	0.857319397	0.25764017	0.591906515	0.330450596	0.697864623	0.72514831	0.97564491
RARS2	0.980831704	0.861011126	0.239911145	0.204874606	0.734886467	0.727353339	0.97564491
RASSF4	0.226275229	0.302837095	0.791241493	0.734991922	0.787055208	0.732587019	0.97564491
RHBDL2	0.66493839	0.862091492	0.095288456	0.736080208	0.797955583	0.736831577	0.97564491
RIN3	0.861174368	0.715712937	0.110611872	0.652498343	0.678140726	0.725254357	0.97564491
RNASEK	0.383444045	0.249131836	0.454499274	0.735667149	0.975128465	0.731274143	0.97564491
RNF180	0.800750334	0.526550749	0.772233961	0.463863222	0.201263755	0.72669318	0.97564491
RNF40	0.937006315	0.256940472	0.750672065	0.639386588	0.264247642	0.727543414	0.97564491
RPL7A	0.866896748	0.648566055	0.165025853	0.411235657	0.830244119	0.73445625	0.97564491
RPL9	0.340209588	0.334487303	0.501953879	0.769176615	0.718863524	0.733891705	0.97564491
S100A3	0.522670131	0.540948778	0.747576261	0.82104204	0.184720262	0.736676646	0.97564491
SCAMP5	0.123540866	0.556911721	0.718882976	0.658452405	0.978353567	0.735537099	0.97564491
SEMA4G	0.919766099	0.288236393	0.436333655	0.742308457	0.367966223	0.733967174	0.97564491
SENP7	0.447268689	0.655960758	0.854391209	0.164081394	0.741506519	0.727316601	0.97564491
SERPINB8	0.631493631	0.962738613	0.104175232	0.524103973	0.935997186	0.730809354	0.97564491
SH3BGRL3	0.173646977	0.599446703	0.919474779	0.612631799	0.516676157	0.726056195	0.97564491
SH3KBP1	0.805550642	0.494062062	0.912135804	0.127126778	0.66868511	0.729535317	0.97564491
SHISAL2A	0.295737771	0.197199256	0.806920239	0.718498551	0.934452384	0.733962191	0.97564491
SLC25A20	0.602224269	0.807174415	0.453869756	0.196273806	0.707900869	0.728277516	0.97564491
SLC36A4	0.49850827	0.317447973	0.426848481	0.89773651	0.4931409	0.723604343	0.97564491
SMAGP	0.265756319	0.911050199	0.93663822	0.623923305	0.225590138	0.735870687	0.97564491
SMARCD2	0.249263329	0.607442201	0.99361617	0.29961012	0.683854077	0.729322672	0.97564491
SMARCE1	0.829271788	0.292298811	0.59684367	0.646076184	0.321268753	0.72438696	0.97564491
SMYD2	0.83593232	0.73840126	0.095399187	0.940106226	0.57235256	0.734490188	0.97564491
SPECC1L	0.969798299	0.115872134	0.478672278	0.935311816	0.612834535	0.72936447	0.97564491
SRSF1	0.614115769	0.319470495	0.620066672	0.952098388	0.257352189	0.722990576	0.97564491
SUPT7L	0.174284404	0.786789523	0.929543866	0.423756034	0.560913477	0.726066265	0.97564491
TBC1D30	0.546621436	0.623057322	0.144592174	0.687178941	0.883089971	0.723471843	0.97564491
TFDP2	0.362866448	0.897765391	0.585511472	0.552121295	0.286602767	0.725353932	0.97564491
THADA	0.688716959	0.598752599	0.685798019	0.706706985	0.159394178	0.73550296	0.97564491
THOC5	0.120758983	0.911319738	0.478132926	0.675806923	0.8536048	0.726422377	0.97564491
TLK2	0.734356643	0.570991313	0.736722869	0.231684736	0.437804208	0.732403333	0.97564491
TLR9	0.964488985	0.598599234	0.407711558	0.820365696	0.165048849	0.7355936	0.97564491
TMEM231	0.981368795	0.72573827	0.146514755	0.330130881	0.926527625	0.735865969	0.97564491
TMEM254	0.868962671	0.410070058	0.787694336	0.483284823	0.236167565	0.736556467	0.97564491
TMOD1	0.5765301	0.327261775	0.672262227	0.357017242	0.698777602	0.734246458	0.97564491
TMTC4	0.960170529	0.881995038	0.074383012	0.726234224	0.66273732	0.726199952	0.97564491
TMUB2	0.995871224	0.944922264	0.528508769	0.260643492	0.243327516	0.73364426	0.97564491
TRAPPC12	0.426565036	0.869798613	0.931806604	0.4906733	0.188203093	0.735913725	0.97564491

TRIM17	0.466697049	0.391939553	0.647070255	0.353879495	0.714562222	0.723762131	0.97564491
TRMT10A	0.998924661	0.244638997	0.952613485	0.229750062	0.571002148	0.727573187	0.97564491
TSNAX	0.286197088	0.798483513	0.999086965	0.374849749	0.364399356	0.73151719	0.97564491
TSPOAP1	0.585334772	0.512496394	0.179697119	0.86520978	0.635901598	0.722040091	0.97564491
TTC17	0.333621698	0.78240029	0.71684718	0.284875321	0.558528112	0.722764352	0.97564491
UBE2O	0.623747589	0.411332702	0.386629597	0.434420881	0.713522587	0.728851732	0.97564491
UTP20	0.634306392	0.84686961	0.792708772	0.377311683	0.196413438	0.733735471	0.97564491
VIPAS39	0.545739269	0.317401084	0.480761355	0.844296856	0.442467097	0.731054144	0.97564491
VPS29	0.732107898	0.412670195	0.254280918	0.899177756	0.432262257	0.723319704	0.97564491
VRK3	0.50602405	0.714987251	0.251107478	0.375370683	0.900696842	0.728657657	0.97564491
VWF	0.138189008	0.951004473	0.900307453	0.7317156	0.343206614	0.722387543	0.97564491
WARS	0.349500756	0.777048634	0.909433839	0.387779126	0.321193278	0.72893961	0.97564491
WBP1L	0.905935255	0.143946118	0.298083581	0.805178904	0.952604075	0.723038881	0.97564491
WNT9A	0.754023036	0.167350562	0.347209262	0.724624775	0.939216962	0.723057261	0.97564491
WWC3	0.734139709	0.381170929	0.18528066	0.829458641	0.72723618	0.732049064	0.97564491
XPNPEP1	0.860250657	0.535630351	0.801828594	0.604020049	0.142570474	0.735268524	0.97564491
XPO6	0.602128882	0.99706991	0.793850157	0.153204505	0.406865225	0.722357409	0.97564491
YIPF6	0.942491958	0.385101181	0.21717273	0.795610935	0.482603427	0.725870783	0.97564491
ZBTB48	0.724699776	0.255634123	0.81245767	0.629628523	0.318314583	0.725248999	0.97564491
ZCCHC3	0.565270032	0.77689399	0.940194948	0.238092529	0.316275771	0.730945068	0.97564491
ZCCHC8	0.581272189	0.291174955	0.999606543	0.892288636	0.202870906	0.728102293	0.97564491
ZNF165	0.579212336	0.383949813	0.748733507	0.338654152	0.542425939	0.727863037	0.97564491
ZNF260	0.718714279	0.441882645	0.745227875	0.166619139	0.784501279	0.730002541	0.97564491
ZNF345	0.393552433	0.991270537	0.641946285	0.372129435	0.318976421	0.722475224	0.97564491
ZNF346	0.790797614	0.680563643	0.569671752	0.119552169	0.813258914	0.722997538	0.97564491
ZNF382	0.893206217	0.212236694	0.719526986	0.501724506	0.438668988	0.724336832	0.97564491
ZNF398	0.534572866	0.76312461	0.762857971	0.345386801	0.292064729	0.732754745	0.97564491
ZNF518A	0.811542896	0.943790883	0.88804278	0.04957964	0.921521329	0.730850947	0.97564491
ZNF536	0.669236273	0.879261826	0.234399054	0.46583162	0.495808242	0.735502959	0.97564491
ZNF623	0.91069144	0.152223131	0.408249783	0.820805424	0.667197321	0.730349727	0.97564491
ZNF697	0.427201112	0.851452466	0.237631796	0.971738963	0.363726447	0.727639726	0.97564491
AOC1	0.561899077	0.920611718	0.858739181	0.219625344	0.329381699	0.737132676	0.975728694
LOC781565	0.44586929	0.482655226	0.698339367	0.242861192	0.879963933	0.737027628	0.975728694
SENP5	0.639437817	0.974286706	0.313566013	0.228647273	0.719240439	0.737079355	0.975728694
VBP1	0.83714775	0.249421152	0.381533018	0.926510664	0.435034307	0.73698773	0.975728694
EEF1D	0.706606863	0.825281618	0.883871769	0.205883689	0.303145008	0.737332315	0.975914236
LOC101906206	0.77930949	0.63942956	0.645295827	0.289455046	0.345754194	0.737404847	0.975925449
TNNI2	0.789024074	0.84683443	0.135353901	0.437991712	0.812666102	0.737459722	0.975925449
LOC100847719	0.527814338	0.408003073	0.782259894	0.336247999	0.569057467	0.737706784	0.976173684
ARL14EPL	0.92835584	0.341434573	0.771912349	0.744217328	0.178672555	0.739442647	0.976409895
ASB2	0.337677807	0.335382889	0.960006654	0.750370122	0.396500059	0.738362955	0.976409895
ASB7	0.738260315	0.127441229	0.754916606	0.565735124	0.810672724	0.73967021	0.976409895
ATP5F1A	0.788966776	0.980996257	0.865805304	0.08308828	0.583864305	0.739292401	0.976409895
CD34	0.567338997	0.790909215	0.5387037	0.284466509	0.471943058	0.738966545	0.976409895

FAM84B	0.203326374	0.843760204	0.715825958	0.403000401	0.655155888	0.738807607	0.976409895
HNRNPUL2	0.893643446	0.586547801	0.818157176	0.120860019	0.626678709	0.739135091	0.976409895
ISG20	0.632262038	0.254551479	0.796904634	0.371153889	0.683754611	0.739522486	0.976409895
KIAA1468	0.238040949	0.778601631	0.396206245	0.609539757	0.727549936	0.739616925	0.976409895
KLF16	0.487232882	0.588725828	0.152020521	0.991177186	0.749714562	0.738690992	0.976409895
LOC100336013	0.669004903	0.759487596	0.540241178	0.215072708	0.551689553	0.739644678	0.976409895
LOC100848011	0.262206627	0.465106123	0.391782819	0.803339226	0.842474712	0.738302946	0.976409895
LOC112442408	0.261458716	0.517965086	0.301909258	0.817857809	0.965361519	0.737980632	0.976409895
LOC112443001	0.497075806	0.997835097	0.313538697	0.348631638	0.59889366	0.739073632	0.976409895
LOC112449358	0.402153339	0.686376577	0.370257617	0.660838404	0.478336423	0.738126512	0.976409895
LOC507930	0.796917362	0.432739296	0.292088886	0.514018735	0.625804599	0.738679473	0.976409895
LSM5	0.8694419	0.186410362	0.765465609	0.577078506	0.454744061	0.739567548	0.976409895
NUDT12	0.761980131	0.991295278	0.409105529	0.18887767	0.557758808	0.739555023	0.976409895
OXSR1	0.744903715	0.778344885	0.242184706	0.649519474	0.355116617	0.738597652	0.976409895
PTGDR2	0.658108429	0.4210375	0.723591319	0.249000804	0.647265352	0.738172906	0.976409895
PTGR2	0.77689919	0.77245777	0.387704848	0.29034706	0.481401963	0.73936458	0.976409895
RPS2	0.811168314	0.701884342	0.385222918	0.343364803	0.432037519	0.739450513	0.976409895
SFT2D2	0.472285788	0.507586892	0.210764151	0.965942381	0.665072892	0.739006319	0.976409895
SNX27	0.644913575	0.86061282	0.677791361	0.432465278	0.199008586	0.738531554	0.976409895
SOX18	0.593079213	0.721909877	0.419228939	0.25548784	0.70443515	0.738114178	0.976409895
THAP2	0.491998978	0.161147826	0.837807234	0.602558661	0.808180328	0.738364639	0.976409895
TMEM233	0.60694446	0.29510992	0.262114098	0.927391257	0.748124418	0.739662825	0.976409895
TRAPPC4	0.666269695	0.382734156	0.425985819	0.529126429	0.56492904	0.739076519	0.976409895
TSPYL5	0.877135748	0.869749177	0.269376238	0.348952717	0.454191696	0.739648185	0.976409895
TTC28	0.732523654	0.426851101	0.830897029	0.607219416	0.206482887	0.739669275	0.976409895
LOC112446667	0.182597157	0.668004643	0.583146502	0.481285124	0.952177915	0.739796763	0.976424994
LOC112447443	0.603167388	0.260153054	0.437955527	0.862234869	0.550121189	0.739800645	0.976424994
C28H1orf198	0.784429697	0.250401914	0.304848295	0.820921957	0.663441886	0.73988633	0.976459555
STAM2	0.307104391	0.880579722	0.543206377	0.336754236	0.659803003	0.740043856	0.976588913
LOC112443853	0.390067795	0.267955699	0.515986739	0.815410716	0.742737587	0.740175509	0.976684111
ACOX2	0.487245453	0.98937006	0.519134008	0.234645891	0.557110332	0.74047041	0.976692234
FBXW9	0.868054493	0.181069255	0.580463572	0.60929325	0.588643007	0.740515417	0.976692234
LOC101903682	0.247361939	0.839427637	0.327379131	0.565840427	0.850821153	0.740538753	0.976692234
MACO1	0.951790859	0.498088922	0.175057239	0.909560707	0.433476214	0.74050701	0.976692234
MAGOHB	0.357720482	0.653369649	0.553851307	0.744656976	0.339321948	0.74043776	0.976692234
SRP9	0.577515384	0.378872155	0.426774003	0.639363629	0.547752489	0.74040441	0.976692234
USP25	0.100535948	0.59694303	0.998946116	0.844450029	0.646667094	0.740603863	0.976699614
SMIM5	0.626234155	0.979327491	0.63187508	0.235727057	0.358514724	0.740673111	0.976712449
GPAT4	0.51002365	0.967330233	0.139162298	0.72824389	0.655510716	0.740815176	0.976813603
LOC104972830	0.283129421	0.910778334	0.277536796	0.623656567	0.735460617	0.741106951	0.976813603
LOC104973145	0.562615717	0.584365611	0.229883086	0.774175866	0.5608687	0.741055963	0.976813603
LOC112449111	0.522463327	0.865512115	0.390246138	0.288418408	0.644786548	0.741056888	0.976813603
LOC788634	0.72790257	0.78703983	0.749454402	0.393722169	0.194137283	0.741060096	0.976813603
ZFHX4	0.53038238	0.519707661	0.457558944	0.283731842	0.916624888	0.740966107	0.976813603

DHX32	0.410197029	0.887356354	0.605375531	0.161240819	0.92465846	0.741256526	0.976846153
EPG5	0.116978481	0.843083703	0.691623026	0.575975648	0.836997555	0.741429266	0.976846153
LOC112443214	0.922188427	0.882737885	0.274315854	0.191288896	0.769637121	0.741387947	0.976846153
LOC112449560	0.190418028	0.453908964	0.577720798	0.896598398	0.733689479	0.741227906	0.976846153
RFESD	0.466841498	0.90325056	0.387820003	0.35577137	0.564863861	0.741321269	0.976846153
CCDC190	0.448972846	0.566285529	0.678450447	0.391691248	0.487510043	0.741740952	0.977038963
LOC112447322	0.818168786	0.217468399	0.808648046	0.666120659	0.343700788	0.741754216	0.977038963
VAV3	0.860499971	0.656520185	0.307593263	0.89264639	0.212311222	0.741709671	0.977038963
HBA1	0.945326388	0.473791033	0.42540842	0.490236093	0.352814051	0.741837332	0.97707002
RARB	0.375814267	0.787689829	0.434814342	0.556709412	0.46037255	0.742027921	0.977242614
FAM229A	0.88926004	0.832259673	0.628762875	0.286406633	0.248187464	0.742528184	0.977432355
LGALS9	0.16854728	0.868394595	0.713473644	0.669052789	0.473266097	0.742461819	0.977432355
LOC104971030	0.883174571	0.187678063	0.65372037	0.658938578	0.463347878	0.742556796	0.977432355
MAP1LC3B	0.791941038	0.384558005	0.194556211	0.673643469	0.827785272	0.742318251	0.977432355
NEDD1	0.077689082	0.744339197	0.651877291	0.891892201	0.984169809	0.74258891	0.977432355
RNF216	0.663048316	0.303768457	0.325115929	0.867698843	0.581497882	0.742316388	0.977432355
TMEM237	0.704672275	0.43580243	0.963233159	0.450112751	0.248269566	0.742406311	0.977432355
ACADM	0.836062435	0.645598527	0.167685535	0.541608521	0.678406221	0.743528244	0.977435337
ANGEL1	0.30705948	0.308523017	0.553755638	0.688584795	0.91776164	0.742947276	0.977435337
ANKRD33	0.249442648	0.502965568	0.813512687	0.847913795	0.383815344	0.743303521	0.977435337
ANKRD49	0.446097207	0.811887553	0.445254274	0.274374582	0.749036424	0.742889328	0.977435337
BBS4	0.448293578	0.810233586	0.325627573	0.339695729	0.830502303	0.744150026	0.977435337
BMP4	0.731385586	0.238374576	0.361747807	0.992300585	0.53146078	0.743551601	0.977435337
BTBD1	0.397807851	0.468528842	0.544197643	0.618939511	0.530990459	0.743968757	0.977435337
CHD6	0.65487283	0.569134515	0.219989999	0.95856565	0.424160569	0.743979768	0.977435337
EXOC5	0.470795137	0.572377592	0.436648014	0.679207686	0.415880232	0.743418976	0.977435337
KIN	0.426164695	0.361412428	0.983257962	0.276628408	0.791134863	0.742895764	0.977435337
LOC101903056	0.818615771	0.157260003	0.367207313	0.832652298	0.847606006	0.744125447	0.977435337
LOC101908111	0.633680642	0.112101319	0.886696067	0.903190963	0.584905292	0.743634815	0.977435337
LOC112441807	0.72465591	0.809722508	0.391138428	0.309989158	0.467676041	0.743621579	0.977435337
LOC615223	0.779958595	0.235666189	0.884709847	0.946586391	0.216774361	0.744155588	0.977435337
MDN1	0.808750016	0.665511412	0.841834748	0.16724186	0.439466703	0.743783159	0.977435337
MYSM1	0.782824567	0.250427868	0.716616103	0.973397103	0.243934666	0.744095417	0.977435337
PAPOLG	0.188199103	0.628937473	0.887972196	0.316680027	0.999689078	0.743630076	0.977435337
PRIMA1	0.587186142	0.700235535	0.212358276	0.8886307	0.428080071	0.743298439	0.977435337
RIPOR1	0.671080811	0.358097357	0.333079698	0.885377561	0.469874087	0.743768812	0.977435337
SMCR8	0.931027872	0.860020922	0.655681937	0.069226069	0.917698221	0.744068562	0.977435337
SMPD1	0.349114794	0.261933871	0.777034199	0.601225498	0.774833935	0.742660441	0.977435337
STAG1	0.115727089	0.97987818	0.880526545	0.380371985	0.875641186	0.743533433	0.977435337
TFPI2	0.628488791	0.424000805	0.467062282	0.521191974	0.511076323	0.742950243	0.977435337
TTC39A	0.298003	0.70334009	0.585337744	0.626276584	0.431224499	0.742838707	0.977435337
TUBA8	0.59904082	0.201275813	0.979335119	0.491889111	0.572053432	0.743361678	0.977435337
ZKSCAN7	0.407016738	0.562365276	0.305086962	0.922621511	0.518038458	0.744199289	0.977435337
ZNF404	0.324594032	0.750964993	0.282998273	0.962274382	0.499651384	0.743030573	0.977435337

SPAG16	0.655908036	0.688017516	0.691646833	0.128529956	0.832304634	0.744273529	0.977454617
LOC515333	0.680696684	0.717919477	0.172937053	0.438537087	0.901634232	0.744419256	0.977489551
SAMM50	0.940358725	0.927278895	0.133557938	0.568980244	0.504159462	0.744369891	0.977489551
AJM1	0.526388334	0.783294688	0.240213933	0.468038577	0.726088854	0.745764574	0.977533842
AKAP8	0.326868249	0.589828362	0.934836528	0.268853024	0.693991397	0.745594533	0.977533842
AP5M1	0.296262089	0.882583894	0.751486281	0.194533678	0.884935667	0.746686369	0.977533842
ARL16	0.123953675	0.919216681	0.447319108	0.696978695	0.954685282	0.747162128	0.977533842
BAZ2A	0.77437158	0.663359707	0.800072629	0.122827134	0.668393277	0.746214453	0.977533842
BCL10	0.865686173	0.458952577	0.52695947	0.175138691	0.930811753	0.748345373	0.977533842
BET1L	0.277799875	0.344722975	0.741653588	0.604260327	0.793313843	0.747886318	0.977533842
BIRC6	0.249060986	0.963371964	0.831859431	0.179674408	0.946750745	0.747374168	0.977533842
C1QB	0.932489093	0.878269818	0.471693448	0.212851754	0.408671406	0.745457778	0.977533842
C20H5orf51	0.886462725	0.41086367	0.138286866	0.92006212	0.735796263	0.748159734	0.977533842
C23H6orf106	0.43114944	0.960637144	0.752926761	0.12630302	0.865463661	0.748112261	0.977533842
CASC1	0.783222811	0.455794883	0.670099137	0.496236646	0.282226009	0.744900635	0.977533842
CHAF1A	0.963681152	0.897332111	0.21075039	0.473814126	0.394733022	0.748096881	0.977533842
CHD7	0.342233149	0.947350255	0.698340947	0.491520439	0.301869481	0.745405595	0.977533842
CLCN3	0.715428879	0.346753955	0.410152188	0.500951932	0.67005448	0.74846879	0.977533842
CMIP	0.607695366	0.799872067	0.613265494	0.315965727	0.360573909	0.747424386	0.977533842
DENND1B	0.633484093	0.526260763	0.953724791	0.385964841	0.277364445	0.747837638	0.977533842
DOCK1	0.47011565	0.936795196	0.681661778	0.22401938	0.503255854	0.746785861	0.977533842
EXOSC10	0.673433878	0.876021825	0.221879913	0.503020957	0.509145206	0.745018604	0.977533842
FAM102A	0.648840162	0.635339167	0.140477319	0.805416146	0.728082676	0.747407893	0.977533842
FAM110A	0.30397722	0.57681381	0.820212009	0.293397234	0.809227138	0.748421991	0.977533842
FCER1G	0.239031978	0.926485433	0.839137145	0.359761946	0.500981051	0.744851193	0.977533842
FRZB	0.928413144	0.868813804	0.233075258	0.593965429	0.305660219	0.748351675	0.977533842
GMNN	0.536466377	0.83197586	0.878975214	0.621042014	0.138247229	0.745895394	0.977533842
GSK3A	0.729221651	0.859396997	0.337995307	0.576333059	0.276269437	0.746135932	0.977533842
HES1	0.24190351	0.435356625	0.786955728	0.47054291	0.858296642	0.744727269	0.977533842
HEXB	0.700995085	0.39106693	0.738786106	0.213654835	0.787179364	0.747971036	0.977533842
KAT6B	0.468783109	0.904506851	0.83597178	0.228115236	0.416059522	0.745672349	0.977533842
KCNJ11	0.70826808	0.360600532	0.445782695	0.611514822	0.489241174	0.747973601	0.977533842
LMO7	0.313100573	0.77369282	0.344100228	0.618831527	0.662146528	0.748479371	0.977533842
LOC100336644	0.923268874	0.48477282	0.209413148	0.911740403	0.393966844	0.745807392	0.977533842
LOC101903586	0.564376777	0.908272423	0.140260445	0.520710004	0.902891152	0.746554961	0.977533842
LOC101905188	0.733255877	0.258627665	0.488919858	0.488189333	0.751800924	0.747795443	0.977533842
LOC101907132	0.398870052	0.191013402	0.565029634	0.875013111	0.901752495	0.747458145	0.977533842
LOC107132942	0.876611725	0.595103774	0.739881049	0.759326205	0.115522404	0.746855752	0.977533842
LOC112441493	0.777049211	0.78939958	0.583352493	0.134128053	0.707455262	0.747384211	0.977533842
LOC112441655	0.428075086	0.413460169	0.328471919	0.916027375	0.630409999	0.745286982	0.977533842
LOC112442636	0.865516654	0.816828122	0.126585268	0.471073447	0.804235661	0.747113806	0.977533842
LOC112446406	0.771328795	0.917170144	0.988273985	0.094280298	0.507419495	0.744590683	0.977533842
LOC512175	0.676434704	0.600376397	0.314302725	0.365336072	0.725564959	0.74673198	0.977533842
LOC615112	0.473803981	0.818437241	0.762585876	0.835492183	0.135955081	0.745384112	0.977533842

LOC782673	0.89411991	0.120348606	0.912925795	0.766340211	0.452379178	0.747939199	0.977533842
MTMR11	0.762865967	0.364070731	0.977957172	0.631026186	0.198398406	0.747659015	0.977533842
MYBPHL	0.892508279	0.675816318	0.443959856	0.477603351	0.264207557	0.746488975	0.977533842
NDUFV3	0.729300286	0.890740202	0.42359685	0.136508572	0.899162719	0.746408344	0.977533842
NECTIN3	0.471243917	0.832576802	0.831069279	0.244861232	0.424945315	0.747241431	0.977533842
NVL	0.589524072	0.908884757	0.135176122	0.551632454	0.848161067	0.747018766	0.977533842
OTOP1	0.764234425	0.514354769	0.981086164	0.228246604	0.382970116	0.746048505	0.977533842
PAFAH1B1	0.870215692	0.295858151	0.240530912	0.766705454	0.705134657	0.74477332	0.977533842
PANK4	0.270090985	0.931358142	0.763385149	0.597125354	0.292381635	0.745031645	0.977533842
PDLIM4	0.888787784	0.784362721	0.077660913	0.769788973	0.805799853	0.745343811	0.977533842
PLAC9	0.785709919	0.8273358	0.147960113	0.534392818	0.65632932	0.746178934	0.977533842
PNPLA6	0.920043075	0.315550786	0.38526427	0.685511231	0.445522583	0.748503459	0.977533842
PYGO1	0.820027337	0.591869413	0.118675236	0.836444109	0.706232835	0.74776978	0.977533842
RAB3D	0.785903816	0.963118985	0.283463788	0.445091055	0.353247826	0.746180714	0.977533842
RASA2	0.376803956	0.958708211	0.8479825	0.23973163	0.462760208	0.747544183	0.977533842
RAVER1	0.669579817	0.633248971	0.240567372	0.379540976	0.87849006	0.747688734	0.977533842
SARNP	0.91043824	0.147117275	0.585418573	0.965874535	0.448684105	0.747532208	0.977533842
SHISA6	0.6954985	0.157020609	0.388746506	0.911495107	0.869291414	0.745652649	0.977533842
SHQ1	0.398511372	0.510438354	0.660067617	0.608565391	0.410796358	0.745254008	0.977533842
SPEF2	0.495672281	0.85554282	0.58316399	0.267906807	0.513730923	0.747832618	0.977533842
SVIP	0.143215339	0.707454403	0.701443181	0.711430449	0.670459703	0.747081544	0.977533842
TICAM1	0.38190791	0.716939576	0.708847199	0.331855213	0.521687566	0.745444922	0.977533842
TUBGCP4	0.859267837	0.48296569	0.879440662	0.145176379	0.64208438	0.747744315	0.977533842
USP8	0.504092658	0.527114745	0.691261828	0.807449824	0.228546373	0.747065491	0.977533842
ZFYVE16	0.544256669	0.630797607	0.74633823	0.173756019	0.753662608	0.745185629	0.977533842
ZNF283	0.983110478	0.279471658	0.796257641	0.936872743	0.164519579	0.746102393	0.977533842
ZNF445	0.835739551	0.452507874	0.560696181	0.305515957	0.524119272	0.747381502	0.977533842
LETMD1	0.480012512	0.604731234	0.186869123	0.705353401	0.893468852	0.74863904	0.977555322
ZBED1	0.764168362	0.614680617	0.657813142	0.149114349	0.741764323	0.748591243	0.977555322
SOD3	0.638616703	0.24575913	0.825056655	0.278531689	0.948445899	0.748759254	0.977634507
ZNF746	0.567439388	0.879152478	0.106106888	0.764379303	0.846124988	0.748908257	0.977751265
CNOT9	0.350890025	0.623108291	0.415722276	0.689850225	0.546616402	0.749123154	0.977809832
FBXO22	0.552656578	0.457408422	0.627916394	0.935263991	0.230899011	0.749141161	0.977809832
LOC104976078	0.650272032	0.261711368	0.55396936	0.5236969	0.694915792	0.749310612	0.977809832
LOC509184	0.761589123	0.835857369	0.22929322	0.720007298	0.326454081	0.749305303	0.977809832
RAPGEF2	0.377162866	0.568220377	0.445524352	0.453690679	0.791895669	0.74928056	0.977809832
SLC36A1	0.332778877	0.688776125	0.525195547	0.390996676	0.728709447	0.749253879	0.977809832
CRACR2A	0.473705423	0.709714546	0.30508391	0.554877674	0.603098661	0.749387833	0.977832848
ACBD5	0.371470832	0.798480981	0.165719523	0.848204312	0.839699844	0.753038037	0.977865023
ADAR	0.739614746	0.231954501	0.303919838	0.716144659	0.95409303	0.756243657	0.977865023
ARF6	0.652516871	0.603974832	0.919639853	0.651097014	0.147266159	0.751675476	0.977865023
ARHGAP4	0.254196063	0.482656166	0.997249528	0.715849209	0.402936699	0.754513366	0.977865023
ARL6IP6	0.292851251	0.777355508	0.982139952	0.18183299	0.849772013	0.750586849	0.977865023
ASB3	0.55937773	0.56466375	0.459762251	0.956168509	0.2516132	0.752659967	0.977865023

ASH2L	0.295953631	0.564883759	0.710422503	0.307956467	0.943102912	0.750303055	0.977865023
B3GALT6	0.858969888	0.315975939	0.983133353	0.811282641	0.160763556	0.75194179	0.977865023
BBIP1	0.217693514	0.461457173	0.899756749	0.599523928	0.646114928	0.753051092	0.977865023
BMP7	0.996792377	0.974892188	0.352509808	0.60840508	0.166700898	0.75162716	0.977865023
BRD7	0.953199845	0.667929036	0.493610677	0.122320273	0.900008772	0.750854156	0.977865023
C1QA	0.837413325	0.867741511	0.529355888	0.273528566	0.340424269	0.757233114	0.977865023
C29H11orf54	0.775977468	0.316393795	0.29809834	0.761471243	0.645323071	0.757978472	0.977865023
C5H12orf56	0.633780233	0.115822528	0.696509007	0.922494654	0.745701802	0.753885875	0.977865023
CABYR	0.859788954	0.539956623	0.415026254	0.744765799	0.250251196	0.757706279	0.977865023
CAMK2N2	0.194667267	0.469247788	0.483634136	0.919199852	0.879630354	0.756735234	0.977865023
CAPN5	0.269735918	0.819875074	0.375585547	0.661309232	0.633197795	0.751829328	0.977865023
CCDC151	0.525294664	0.646460032	0.275462376	0.38961495	0.979824304	0.756679687	0.977865023
CCDC96	0.602671762	0.206426501	0.918434652	0.414710297	0.748034177	0.755314152	0.977865023
CDK5RAP3	0.787017724	0.813929432	0.429964215	0.26515157	0.477592548	0.752345559	0.977865023
CGNL1	0.509219286	0.471463978	0.177330103	0.891298856	0.927710777	0.754048682	0.977865023
CLDN5	0.386894022	0.517743828	0.767995033	0.621267031	0.37627683	0.757971647	0.977865023
CLIC3	0.371937805	0.412549456	0.964381906	0.339971577	0.708971011	0.756458169	0.977865023
CNOT6	0.752114324	0.485919583	0.205900874	0.503207556	0.909409022	0.749990395	0.977865023
CSNK1E	0.853602803	0.99300111	0.964630843	0.966730604	0.044320233	0.753159617	0.977865023
CTR9	0.400532301	0.689364609	0.991675671	0.260138581	0.503781433	0.757571624	0.977865023
DBNDD2	0.808293492	0.489682918	0.50820014	0.401297344	0.443995234	0.757343817	0.977865023
DNAJB5	0.474529439	0.377778259	0.960945062	0.926052978	0.224219025	0.7569822	0.977865023
ELMOD3	0.661022134	0.326116495	0.682241624	0.826514305	0.292867862	0.756112698	0.977865023
FAM32A	0.357268522	0.985209914	0.671423472	0.309129601	0.486516661	0.755820674	0.977865023
FAM83H	0.63435496	0.746527388	0.532483652	0.228478353	0.610335442	0.753847948	0.977865023
FBXO5	0.499515405	0.387903897	0.316118977	0.95011645	0.606172706	0.754440497	0.977865023
FGD1	0.611978053	0.221056337	0.770347999	0.447183213	0.765856534	0.756581635	0.977865023
FRMPD4	0.231121812	0.515890826	0.700039303	0.544628024	0.79144649	0.758052454	0.977865023
FUCA2	0.738896667	0.722357859	0.369890116	0.220159498	0.813561912	0.754880837	0.977865023
GATB	0.349778846	0.394725583	0.574052838	0.714480588	0.623711676	0.754659909	0.977865023
GBX1	0.714997689	0.32808903	0.275490014	0.688968533	0.79131306	0.754208875	0.977865023
GPAM	0.538175251	0.741868091	0.971214412	0.378822783	0.243810881	0.757213487	0.977865023
GPKOW	0.462167107	0.642963143	0.238522321	0.56705864	0.885116047	0.755983022	0.977865023
GPRC5B	0.485436273	0.661868917	0.287466483	0.876282601	0.438985912	0.755747873	0.977865023
GSPT2	0.789865979	0.761439297	0.574949143	0.345268554	0.288308136	0.749913466	0.977865023
GYG2	0.337365724	0.776197913	0.553664969	0.405887589	0.584155195	0.749667696	0.977865023
HARS2	0.854794009	0.400026585	0.226632155	0.499869506	0.902713079	0.75282202	0.977865023
HDGF	0.442628319	0.95999624	0.605176893	0.502084631	0.277997762	0.757616435	0.977865023
HIST1H1E	0.531143302	0.932088455	0.68826606	0.877052042	0.115202874	0.749948277	0.977865023
HTRA4	0.986775373	0.816305848	0.640728793	0.126622947	0.53460927	0.752659163	0.977865023
ISG20L2	0.620705661	0.824539297	0.662485939	0.107747553	0.949936085	0.751419978	0.977865023
IST1	0.472353623	0.886210004	0.206069971	0.746368516	0.557851691	0.757735177	0.977865023
JUP	0.668763427	0.614875744	0.818242956	0.214990741	0.47880491	0.751056674	0.977865023
KLHL26	0.853274675	0.865118338	0.437660099	0.159699476	0.667362378	0.749970686	0.977865023

LDAH	0.381364345	0.972684567	0.214203028	0.562059863	0.787446296	0.753866264	0.977865023
LIMA1	0.864544688	0.565556594	0.341530327	0.536422946	0.390917547	0.753079507	0.977865023
LOC100847120	0.119632263	0.956758997	0.664275406	0.474293089	0.959504584	0.750874934	0.977865023
LOC100847613	0.313198427	0.989645054	0.698048564	0.952806036	0.171816288	0.755184123	0.977865023
LOC100848407	0.675760642	0.79463129	0.092884505	0.827829324	0.852947412	0.754130491	0.977865023
LOC100848995	0.463709048	0.431308509	0.419178378	0.620615981	0.680488129	0.755109003	0.977865023
LOC101902430	0.835427957	0.801082324	0.389786485	0.209686155	0.628733478	0.749750589	0.977865023
LOC101903126	0.228520077	0.285727257	0.950596185	0.728915561	0.783225791	0.755261471	0.977865023
LOC101904270	0.595054948	0.398215164	0.785961108	0.690042037	0.273883595	0.754025682	0.977865023
LOC101904573	0.448671302	0.260193545	0.4938305	0.721785042	0.854889201	0.755973764	0.977865023
LOC101904757	0.299454472	0.479301831	0.756351818	0.454871249	0.70334144	0.751565643	0.977865023
LOC101906477	0.634038097	0.6226108	0.812946486	0.287274446	0.38546541	0.755786005	0.977865023
LOC101906730	0.429713723	0.549723187	0.729407354	0.893339246	0.22575202	0.751660374	0.977865023
LOC101906855	0.830891022	0.402562763	0.933355856	0.408547403	0.275435989	0.753674217	0.977865023
LOC101907658	0.953364262	0.416009157	0.349644536	0.623443791	0.402808913	0.752060971	0.977865023
LOC104968634	0.338769389	0.452467752	0.611773181	0.473153136	0.805579843	0.756849399	0.977865023
LOC104969238	0.495948851	0.388794525	0.47516644	0.441363314	0.854921807	0.75071872	0.977865023
LOC104973760	0.585449536	0.284190816	0.690868831	0.533378731	0.568241823	0.752137008	0.977865023
LOC107132486	0.620208983	0.622889787	0.37097306	0.794437224	0.306360465	0.752357704	0.977865023
LOC112441602	0.233725525	0.922268931	0.25435548	0.969215508	0.672157786	0.756724312	0.977865023
LOC112441603	0.5232535	0.725426053	0.187100863	0.643141171	0.784544955	0.757319254	0.977865023
LOC112442245	0.500640747	0.729299351	0.15739589	0.942512272	0.654384145	0.755307542	0.977865023
LOC112444770	0.307789427	0.933322153	0.599342919	0.624302567	0.3282018	0.754440462	0.977865023
LOC112445001	0.779494166	0.506180268	0.16100198	0.745913005	0.734339415	0.75191213	0.977865023
LOC112445194	0.628387821	0.571655606	0.669802005	0.569665314	0.252563156	0.750962298	0.977865023
LOC112446390	0.363886028	0.754529592	0.58585269	0.826141339	0.262082998	0.752077277	0.977865023
LOC574091	0.1586147	0.973069821	0.772585584	0.510620293	0.570702804	0.751660645	0.977865023
LOC614402	0.353381057	0.298135571	0.577524564	0.797671792	0.722483491	0.753331544	0.977865023
LOC617224	0.983818852	0.993261694	0.858082898	0.172756176	0.245711139	0.756077794	0.977865023
LOC781108	0.686744526	0.358879127	0.329438362	0.510852317	0.856918985	0.755818285	0.977865023
LOC783920	0.477758256	0.766792532	0.590017223	0.176405959	0.933276472	0.75603883	0.977865023
LOC783988	0.684578674	0.562154055	0.676612808	0.471258408	0.289193843	0.755528321	0.977865023
LOC790098	0.68648252	0.746285548	0.159459213	0.645457617	0.658447141	0.751503769	0.977865023
LRP12	0.837524648	0.361650717	0.721495487	0.241026664	0.671037334	0.75479425	0.977865023
LRRC27	0.464645022	0.388630894	0.342801354	0.909513015	0.612838956	0.750349216	0.977865023
MAPRE3	0.864687844	0.25819351	0.273641745	0.688654541	0.829097838	0.752362069	0.977865023
METTL24	0.998334486	0.408991371	0.232724795	0.378979642	0.95691062	0.750121111	0.977865023
MIOS	0.791871933	0.314187124	0.635516853	0.907198623	0.248864113	0.756613901	0.977865023
MTUS1	0.62513573	0.65510082	0.262590273	0.882891031	0.370000391	0.753666725	0.977865023
NARS2	0.358671353	0.784120338	0.826127473	0.809721982	0.185850357	0.752799779	0.977865023
NCK2	0.54249009	0.657980443	0.569729642	0.338280989	0.512480579	0.754326914	0.977865023
NTSR2	0.209235257	0.430312839	0.878431798	0.858589927	0.51185364	0.751710204	0.977865023
ONECUT2	0.194660936	0.923329739	0.457247582	0.623056172	0.673206499	0.750181693	0.977865023
P3H2	0.996136029	0.869665744	0.601866816	0.848482234	0.079213812	0.75321935	0.977865023

PCSK5	0.382050866	0.680423986	0.566699868	0.555712292	0.422699255	0.750893	0.977865023
PHF12	0.568253647	0.904494181	0.414288573	0.213809896	0.788077992	0.757548349	0.977865023
PHF24	0.572877156	0.714431758	0.168681857	0.751203092	0.686096128	0.756021341	0.977865023
PHTF2	0.824295931	0.576027815	0.478777869	0.211736046	0.747073038	0.757957892	0.977865023
PIP4P2	0.625559937	0.335629406	0.361636349	0.719235585	0.65636422	0.75736647	0.977865023
PLIN3	0.42057977	0.849783114	0.282989264	0.662759128	0.53316924	0.756830265	0.977865023
PODXL	0.385140239	0.549398637	0.238189152	0.790803185	0.885046714	0.754426202	0.977865023
PPM1D	0.498698533	0.783495249	0.681442523	0.152226084	0.884933015	0.757487232	0.977865023
PPP1R1B	0.844113724	0.546729161	0.111982021	0.749253493	0.926144447	0.75745593	0.977865023
PRKDC	0.969237386	0.294327786	0.351813437	0.662874384	0.540595716	0.757982841	0.977865023
PSMG4	0.883861234	0.748207535	0.124354566	0.456781414	0.933605875	0.753357075	0.977865023
QRSL1	0.556834986	0.863941429	0.976622887	0.21374309	0.356038767	0.756906226	0.977865023
RAB27A	0.227521397	0.870303327	0.601092272	0.553459893	0.535853112	0.754554462	0.977865023
RAB30	0.905700658	0.903309953	0.604576149	0.099683364	0.701135948	0.750706812	0.977865023
RAMMET	0.547373879	0.66695803	0.582130312	0.659773905	0.252875108	0.755374731	0.977865023
RN7SL1	0.395021434	0.524221606	0.505633399	0.727738494	0.453391346	0.75058883	0.977865023
RNF111	0.556151574	0.489319836	0.678981644	0.196828055	0.989057204	0.758014376	0.977865023
RNF157	0.94896895	0.595715762	0.157213049	0.80149215	0.490219502	0.752563776	0.977865023
RNF215	0.571429845	0.471457121	0.686583239	0.214736558	0.903360816	0.757555772	0.977865023
RPS25	0.703611845	0.303309785	0.198824074	0.927408718	0.893630464	0.753856278	0.977865023
RUNX2	0.41937755	0.661530372	0.99312224	0.866020724	0.144921868	0.750758879	0.977865023
SCAP	0.706786301	0.935987099	0.86010304	0.169492947	0.371523435	0.757294382	0.977865023
SETD6	0.388353977	0.694360118	0.587284899	0.543549945	0.406019465	0.75272421	0.977865023
SGTB	0.321864944	0.628815287	0.369167163	0.64992109	0.711956695	0.750722387	0.977865023
SLC38A10	0.683658795	0.705690604	0.194530353	0.396058495	0.955283787	0.755640732	0.977865023
SLC9A7	0.646252889	0.86649381	0.223103873	0.350225874	0.82066039	0.757691333	0.977865023
SNRPB2	0.868291678	0.09221489	0.736808966	0.677962272	0.881065532	0.754245048	0.977865023
SPATA6L	0.597086296	0.900780961	0.935116278	0.500366482	0.139680018	0.753782858	0.977865023
SPN	0.46005477	0.475275792	0.410663617	0.508571466	0.754939868	0.750200094	0.977865023
SRD5A3	0.593017104	0.417362703	0.320431471	0.567581982	0.780623146	0.753715743	0.977865023
SRMS	0.613591548	0.159115661	0.53286938	0.828777725	0.824764205	0.755914787	0.977865023
STK32A	0.421857356	0.567542086	0.674129088	0.240725701	0.906048275	0.754052405	0.977865023
TGS1	0.834027622	0.27390239	0.480494321	0.461141566	0.698886859	0.754952279	0.977865023
THOC2	0.644717159	0.467604019	0.896994402	0.156798835	0.838324449	0.755834967	0.977865023
TMEM167A	0.639966282	0.373421467	0.521174591	0.728602698	0.391000587	0.755503242	0.977865023
TMEM243	0.713943733	0.671884533	0.107753853	0.701919673	0.98893029	0.757546899	0.977865023
TRIM32	0.337315018	0.563962707	0.889949907	0.207898201	0.998980564	0.753831584	0.977865023
TRIP6	0.396388573	0.782722722	0.22933374	0.893690208	0.549441803	0.752663985	0.977865023
TRPM3	0.760608727	0.591555388	0.135458229	0.686008922	0.833669981	0.752231264	0.977865023
TSPAN15	0.545365389	0.463815305	0.707162439	0.242980158	0.799795037	0.751728272	0.977865023
TUBB3	0.941037065	0.421807177	0.513115456	0.325643427	0.53488865	0.755475195	0.977865023
TXLNB	0.061548664	0.757307041	0.984228733	0.923362465	0.818843041	0.751328217	0.977865023
TXNRD1	0.379501905	0.214996957	0.830679951	0.839415504	0.617297163	0.753616238	0.977865023
UBE3D	0.075756378	0.727271951	0.847724207	0.860629512	0.887933948	0.756585366	0.977865023

WNT10B	0.774586942	0.612233986	0.311703756	0.239383058	0.970894298	0.749557257	0.977865023
XKRX	0.39320799	0.734748379	0.342597916	0.787847029	0.459213036	0.757191149	0.977865023
ZCCHC2	0.970325167	0.754283542	0.451334925	0.932554203	0.113476831	0.75276005	0.977865023
ZDHHC15	0.745194916	0.953744341	0.1855638	0.448710582	0.599252176	0.755403093	0.977865023
ZNF629	0.455453683	0.551360746	0.271825334	0.974027722	0.527199025	0.753262105	0.977865023
ZNF783	0.820098006	0.876792896	0.609594576	0.139851394	0.582525667	0.756677558	0.977865023
ZZEF1	0.446048749	0.888224308	0.750277164	0.133267056	0.898122759	0.756001154	0.977865023
NKAIN2	0.773956951	0.163890354	0.88549634	0.572951145	0.559818123	0.758296575	0.978025526
STXBP6	0.708942107	0.413233309	0.55569448	0.616318451	0.359248428	0.758389644	0.978025526
TTC36	0.407622914	0.952591569	0.110274426	0.929022572	0.905834786	0.758336161	0.978025526
UPF1	0.772477905	0.680419741	0.200899782	0.711285532	0.47997273	0.758415261	0.978025526
KIF24	0.858052518	0.230366405	0.882956317	0.467055519	0.442425467	0.758490263	0.978045392
MCCD1	0.316791595	0.523712811	0.903672506	0.301480596	0.798239742	0.758570269	0.978071707
PDSS2	0.502569297	0.398031714	0.334831881	0.695778184	0.774879345	0.758728218	0.978121664
TXNDC11	0.404900733	0.918294024	0.299026828	0.493082756	0.65857139	0.75869358	0.978121664
DLL1	0.296668357	0.388377938	0.670594952	0.477722381	0.979518183	0.758951091	0.97825529
ZEB1	0.647375854	0.583530324	0.331533701	0.589655417	0.489570965	0.758945784	0.97825529
B3GALNT2	0.483507496	0.987700539	0.370099795	0.268335743	0.76439936	0.759444849	0.978268
C15H11orf94	0.601389433	0.938881745	0.406152576	0.233237994	0.681436784	0.760427548	0.978268
CDC42BPG	0.973402617	0.36637778	0.442825346	0.50541866	0.456190792	0.760247103	0.978268
DDX28	0.38817535	0.491466724	0.485099445	0.533605339	0.733509433	0.759290071	0.978268
FAM83F	0.160982017	0.972394313	0.959184227	0.57419504	0.422523985	0.760323139	0.978268
FKBP8	0.753717588	0.641011159	0.325196673	0.245865084	0.942940675	0.760310077	0.978268
FOXL1	0.96112008	0.948898483	0.377295553	0.129427275	0.815180983	0.75970288	0.978268
GLA	0.717231489	0.417473405	0.260387378	0.701443859	0.663830787	0.759702653	0.978268
GRK6	0.345045475	0.924348982	0.180997166	0.868591622	0.725667751	0.760114817	0.978268
KLHL30	0.905236148	0.319194578	0.833283366	0.680954372	0.22115297	0.759476313	0.978268
LMOD3	0.885661228	0.726489982	0.284221582	0.300427697	0.664369602	0.760689715	0.978268
LOC101908204	0.144219267	0.925778156	0.706191436	0.443312474	0.866287537	0.75922518	0.978268
LOC104976281	0.131289192	0.990979572	0.827702133	0.422586939	0.799356629	0.760067203	0.978268
LOC112442246	0.59214371	0.887430444	0.30656212	0.606494658	0.372738372	0.76027198	0.978268
LOC112442721	0.918831917	0.286354239	0.85242187	0.364178669	0.447081551	0.760770245	0.978268
LOC112443422	0.898629917	0.839643463	0.531386751	0.557345457	0.162915294	0.760213832	0.978268
LOC112443452	0.353276433	0.740178038	0.707428359	0.392839857	0.501623808	0.760447001	0.978268
LOC112446793	0.783197451	0.471767453	0.817317773	0.305317094	0.392643831	0.759189543	0.978268
LOC112447845	0.310393644	0.75072482	0.331431503	0.95526951	0.493221764	0.760122388	0.978268
LOC782954	0.134753845	0.85276416	0.49597617	0.809049	0.785652052	0.759314195	0.978268
MCTP1	0.130005933	0.542067466	0.626672186	0.966571135	0.852890614	0.760218571	0.978268
MPEG1	0.732660644	0.947874112	0.996602443	0.279462688	0.1886881	0.760664387	0.978268
NALCN	0.963276026	0.175030933	0.771111785	0.300327461	0.93541396	0.760806522	0.978268
OGDH	0.908206191	0.863992273	0.614356233	0.255805865	0.296281182	0.760868487	0.978268
PCDHB11	0.572934307	0.545925816	0.395773287	0.877898238	0.336012568	0.760765667	0.978268
S100A4	0.963707324	0.261527118	0.579250159	0.30308475	0.824577586	0.76061385	0.978268
SDHAF3	0.711001062	0.944244946	0.841010405	0.855476532	0.075624014	0.76082432	0.978268

SPCS2	0.99896676	0.158024888	0.796021398	0.833812649	0.348569067	0.760796768	0.978268
SSFA2	0.40354503	0.953958265	0.281680364	0.899134347	0.372651038	0.759848523	0.978268
TOR3A	0.446449412	0.703954822	0.15272042	0.887317446	0.856538169	0.760578617	0.978268
TST	0.420590533	0.717595537	0.969837147	0.125347155	0.986286703	0.759111859	0.978268
UBE2I	0.47208866	0.162724336	0.966280605	0.619565017	0.788366356	0.759465116	0.978268
AIG1	0.643639811	0.777009151	0.171915292	0.700593451	0.607714547	0.761213258	0.978492448
L1CAM	0.512735553	0.445224222	0.497402798	0.776185092	0.415359495	0.76122193	0.978492448
LOC112442040	0.735922695	0.518278063	0.573691814	0.699315573	0.239097038	0.761117346	0.978492448
AGL	0.580393532	0.347436361	0.857848528	0.418233819	0.506539027	0.761419717	0.978650854
RNF39	0.868822909	0.140939024	0.536634483	0.627982596	0.888297011	0.761464429	0.978650854
ABTB2	0.699876916	0.769279075	0.378356387	0.195801285	0.921637983	0.761983494	0.978769031
ADCY8	0.583471706	0.696218748	0.373768461	0.748980423	0.323946629	0.762373762	0.978769031
B3GALT2	0.659348382	0.567381395	0.167327225	0.648625906	0.905051013	0.761916901	0.978769031
B3GNT9	0.559775473	0.546850667	0.587010609	0.370389413	0.551376746	0.761669741	0.978769031
B9D1	0.362186473	0.554747599	0.463812126	0.802162332	0.492553812	0.762278727	0.978769031
C16H1orf116	0.480617556	0.789286286	0.30481907	0.64379964	0.49677928	0.763078003	0.978769031
CBFA2T3	0.806360759	0.476004405	0.945409409	0.751887279	0.135950152	0.763623982	0.978769031
CRHR2	0.343363868	0.756826509	0.55536967	0.45895571	0.560636125	0.763830272	0.978769031
CTSL	0.120751084	0.950140963	0.88412773	0.640108112	0.567693184	0.762478276	0.978769031
EXOG	0.417895799	0.87869448	0.928420242	0.638670821	0.168762656	0.761908475	0.978769031
FUOM	0.801960813	0.491513868	0.755183244	0.401239111	0.307991453	0.762109734	0.978769031
GALT	0.218650672	0.581116378	0.607265741	0.608244689	0.790447492	0.763645506	0.978769031
HCST	0.282237509	0.783623603	0.366416974	0.926809084	0.490165193	0.762255581	0.978769031
HNRNPUL1	0.548446143	0.529060121	0.197899165	0.686098684	0.937685119	0.762883399	0.978769031
HOXC6	0.676819393	0.897427427	0.718107431	0.953899108	0.088891597	0.763092339	0.978769031
INO80C	0.383138078	0.294779909	0.902481897	0.530026506	0.682896121	0.762638936	0.978769031
KLHL36	0.225100415	0.951381188	0.589901213	0.338353608	0.865791714	0.763205435	0.978769031
LINGO1	0.690348358	0.62837993	0.177144291	0.702786223	0.687779	0.763874259	0.978769031
LOC100847876	0.425296615	0.876048879	0.16755991	0.726807642	0.813484467	0.762729236	0.978769031
LOC101903988	0.963765731	0.750220016	0.260431566	0.662447931	0.29705502	0.763435213	0.978769031
LOC101907140	0.170197308	0.456233137	0.521983464	0.960656165	0.943122762	0.761794454	0.978769031
LOC101907514	0.269960361	0.321195324	0.856114249	0.507501605	0.981355037	0.763025153	0.978769031
LOC112442278	0.244582688	0.742528867	0.283017669	0.757803478	0.94258099	0.761750524	0.978769031
LOC112447140	0.534891427	0.700373196	0.256014923	0.954029991	0.405430853	0.763643492	0.978769031
LRIG2	0.362741376	0.743530973	0.7098287	0.993515756	0.195125319	0.763726829	0.978769031
LZTFL1	0.264886297	0.748815564	0.908020534	0.561818636	0.36347066	0.762072923	0.978769031
MAEA	0.713594471	0.991674892	0.750404808	0.078990882	0.8855558	0.763882381	0.978769031
NME6	0.581556872	0.719715419	0.937619038	0.236683659	0.398967928	0.763453359	0.978769031
NUDC	0.665562789	0.304344828	0.247677775	0.747330809	0.983538006	0.762555888	0.978769031
OTUB2	0.837039411	0.445023646	0.118667966	0.89297794	0.935445184	0.762797388	0.978769031
PAX8	0.082389476	0.894655917	0.83251233	0.955643811	0.627741183	0.7622407	0.978769031
RASGEF1A	0.461489627	0.646734762	0.443505747	0.944049834	0.297097438	0.763786811	0.978769031
SCFD2	0.738221608	0.943044558	0.734266243	0.464525478	0.155171299	0.762407914	0.978769031
SFPQ	0.607945298	0.48274417	0.889148286	0.183433747	0.772200467	0.762983425	0.978769031

SNX8	0.964620702	0.674478806	0.118417081	0.558557487	0.857174853	0.762610655	0.978769031
TNKS1BP1	0.780217409	0.53649425	0.297918879	0.776770056	0.382166333	0.763259636	0.978769031
TRMT61B	0.730374476	0.332804673	0.837114715	0.596017967	0.305107173	0.76317917	0.978769031
TXNL4B	0.553584087	0.968207589	0.442068362	0.590116162	0.26557118	0.763819807	0.978769031
ZCWPW1	0.925948132	0.839179806	0.105544871	0.610674927	0.736517135	0.762608653	0.978769031
LOC101903928	0.458001057	0.934552973	0.304141472	0.694892369	0.41083477	0.763975182	0.978811516
COL23A1	0.552234733	0.569652205	0.374153569	0.558036393	0.566951433	0.764335835	0.979120707
LOC100336448	0.715725186	0.588849876	0.986012745	0.116908844	0.766401727	0.764313003	0.979120707
FRMD8	0.843802574	0.773330012	0.854418327	0.308670319	0.216463716	0.764404712	0.979132511
SFRP4	0.387529609	0.305768593	0.667584778	0.580990959	0.811010937	0.764506895	0.97918697
LTBR	0.598792181	0.969438528	0.671094895	0.599624404	0.159683025	0.764640077	0.979234726
POP7	0.83816642	0.418669411	0.223236078	0.507592921	0.938194642	0.764663519	0.979234726
HMGN3	0.313299864	0.787637401	0.705743808	0.22650367	0.94717077	0.764940926	0.979284307
KLHL5	0.523637676	0.745722502	0.695984992	0.239087337	0.574675849	0.764836459	0.979284307
VPS72	0.831042553	0.531751641	0.23026411	0.644000206	0.570138385	0.764935693	0.979284307
ZNF132	0.207922367	0.278466425	0.835541683	0.861518027	0.895741143	0.764795244	0.979284307
BAIAP2	0.615745871	0.752589918	0.783834263	0.302205753	0.341090769	0.765325722	0.979439378
LOC614882	0.849238617	0.605309325	0.602988731	0.181452674	0.665539057	0.765282374	0.979439378
RCN2	0.980494062	0.669925406	0.194211419	0.841285768	0.348758167	0.765266014	0.979439378
STK10	0.92833816	0.741090644	0.779167803	0.143109281	0.488035495	0.765313549	0.979439378
ZNF333	0.838372512	0.501786606	0.375756768	0.926705554	0.255643778	0.765360464	0.979439378
RPH3AL	0.357414481	0.645193692	0.604519545	0.483926318	0.555354677	0.765436747	0.97946062
BEX2	0.809857	0.334858582	0.360296881	0.712056665	0.539865251	0.765899999	0.979650742
C5H12orf57	0.646260764	0.571853385	0.733514942	0.782468663	0.177199552	0.766025735	0.979650742
CENPV	0.288793874	0.97894503	0.624175175	0.775400742	0.274564431	0.765938896	0.979650742
CRY2	0.752360076	0.743083054	0.958530434	0.210360648	0.33360246	0.766122578	0.979650742
OSBP	0.992640194	0.602656663	0.674968439	0.207013158	0.44908245	0.765790939	0.979650742
PTPN23	0.761873807	0.320657309	0.319332104	0.988361267	0.487363596	0.76598584	0.979650742
RAB11FIP1	0.480538449	0.776227606	0.645650265	0.314428182	0.496155405	0.765951641	0.979650742
SLC22A5	0.763491407	0.808360671	0.373484853	0.445328682	0.365474684	0.765686822	0.979650742
TLDC1	0.82146261	0.132540877	0.876479342	0.471835869	0.835084044	0.766096548	0.979650742
LOC101906676	0.895947134	0.855007964	0.152554842	0.434169546	0.741700705	0.766249541	0.979736753
CALY	0.551705475	0.304656746	0.576435973	0.86629068	0.449331231	0.766639215	0.980029483
LOC100847554	0.392266219	0.883820448	0.359544082	0.328317074	0.921622275	0.766657638	0.980029483
WDR82	0.810676028	0.406035804	0.859581999	0.333430104	0.399558226	0.766549186	0.980029483
LIMK1	0.389098957	0.840314121	0.794279107	0.42733285	0.340118119	0.766794351	0.980081283
LRCH2	0.285298629	0.414804767	0.568790186	0.810090013	0.692305533	0.766817602	0.980081283
ARHGAP42	0.790051018	0.714290463	0.493456484	0.309591135	0.440271218	0.767802858	0.980305107
AS3MT	0.638625072	0.939127607	0.167846305	0.558047595	0.675729764	0.767819116	0.980305107
CD300LF	0.877177456	0.278471171	0.889178565	0.576278375	0.303365403	0.767872668	0.980305107
CHKA	0.476262984	0.768996986	0.857945242	0.500338885	0.242630969	0.768700612	0.980305107
COG7	0.963372057	0.381110944	0.486141805	0.415160274	0.512503887	0.767900326	0.980305107
DDX50	0.915090019	0.571801906	0.72459741	0.115918052	0.864943367	0.768076924	0.980305107
DOK2	0.331194514	0.337404512	0.806993721	0.523967351	0.80590157	0.76838765	0.980305107

DRD2	0.802130751	0.338375966	0.972050494	0.177972441	0.811902522	0.768595887	0.980305107
EPHB6	0.296001506	0.480266027	0.866246318	0.585982787	0.525669511	0.767689071	0.980305107
GAS7	0.501437831	0.706904804	0.309567422	0.451494988	0.767167417	0.768048335	0.980305107
HOXA3	0.197456217	0.925559209	0.816705086	0.347472554	0.734123445	0.768362426	0.980305107
INTS7	0.815416688	0.132071244	0.969918574	0.392988335	0.929388591	0.768725028	0.980305107
ITGAE	0.576843404	0.515608526	0.446109115	0.296742929	0.967712171	0.768494035	0.980305107
LOC100847236	0.955597425	0.117706365	0.991291028	0.417774269	0.816250813	0.768117141	0.980305107
LOC104969353	0.976657496	0.74334287	0.925354057	0.148625384	0.379727941	0.767601521	0.980305107
LOC112442243	0.116461995	0.743417813	0.909077559	0.815409392	0.589707034	0.767278251	0.980305107
LOC112447080	0.687345319	0.60344497	0.444923059	0.224476029	0.918944917	0.76833206	0.980305107
LOC112447340	0.685419325	0.388238452	0.769640968	0.240300926	0.774933119	0.768667789	0.980305107
LOC512684	0.866291773	0.118469764	0.981446203	0.815010478	0.464650495	0.768694899	0.980305107
MOCS1	0.865836785	0.760986017	0.120203353	0.512385875	0.932625118	0.767279674	0.980305107
PLA2G2D4	0.733378556	0.290149812	0.298185463	0.809954092	0.739721209	0.768085471	0.980305107
PLEKHO1	0.443280378	0.637751631	0.613192432	0.582323882	0.375131555	0.767380455	0.980305107
PLP2	0.895768543	0.385950749	0.211451606	0.962309674	0.539295333	0.767716332	0.980305107
RGP1	0.498330599	0.68041793	0.767205326	0.192338993	0.758359444	0.767744341	0.980305107
S1PR2	0.936202972	0.47714197	0.155965778	0.675251376	0.803845601	0.767134237	0.980305107
SUGP2	0.775225073	0.083138912	0.834851321	0.962713493	0.731750187	0.767558237	0.980305107
TTF2	0.37510555	0.366068371	0.830505618	0.703243954	0.47392469	0.768047188	0.980305107
UNC5B	0.587529092	0.449416019	0.931395815	0.384583375	0.402282523	0.768239057	0.980305107
YRDC	0.733280543	0.32514964	0.451812347	0.414587327	0.852305452	0.768317808	0.980305107
CLEC14A	0.344013626	0.589018787	0.740352729	0.510163346	0.498783887	0.768835412	0.980369693
PICK1	0.317218569	0.525431344	0.876283259	0.355372101	0.736093824	0.768989696	0.980447697
RHOB	0.943633545	0.580335523	0.279620306	0.550662054	0.453171463	0.769016072	0.980447697
AMACR	0.622628265	0.813653354	0.171198318	0.556887051	0.793181528	0.769477545	0.980465336
COL5A3	0.609380022	0.462757557	0.189235336	0.87109058	0.823811105	0.769405739	0.980465336
ETV6	0.242051601	0.990410324	0.275310376	0.786226798	0.736842204	0.76912812	0.980465336
HSCB	0.74036474	0.772845421	0.652451683	0.178162863	0.575384818	0.769292261	0.980465336
LOC112443849	0.858809986	0.334694536	0.278612682	0.669816279	0.713134862	0.769213853	0.980465336
LOC784966	0.400108772	0.506902781	0.228523136	0.843314627	0.979171787	0.76930023	0.980465336
TRMT112	0.566471947	0.387927595	0.578365927	0.415686396	0.725241612	0.769507863	0.980465336
WDR5	0.546031243	0.494101693	0.333902749	0.859892067	0.494595102	0.769494244	0.980465336
ATG16L1	0.62500036	0.126187367	0.726224079	0.999960016	0.669924255	0.769755364	0.980575805
DUSP18	0.866256152	0.790554285	0.65956064	0.180172709	0.471517387	0.769773816	0.980575805
MED13L	0.926766764	0.723177688	0.31898987	0.317147433	0.565583023	0.769661668	0.980575805
ATP23	0.688053092	0.284726626	0.323427331	0.644229168	0.942013446	0.770150125	0.980607372
LOC112444752	0.592601645	0.129151761	0.939101085	0.932982579	0.573494996	0.77017277	0.980607372
LOC616094	0.558463437	0.428337367	0.598449068	0.574099374	0.467763988	0.770107512	0.980607372
NUPL2	0.992857591	0.896384887	0.442431651	0.110670977	0.88130366	0.769926695	0.980607372
PPP2R5B	0.310992481	0.712607305	0.545768429	0.661063882	0.481415638	0.770336375	0.980607372
PRDX6	0.909640835	0.722224169	0.091389642	0.759252115	0.844296719	0.770314699	0.980607372
SLIRP	0.975542059	0.45553214	0.258005051	0.392272586	0.855663414	0.770299773	0.980607372
SNX1	0.536439807	0.384882396	0.572432549	0.35912569	0.906754883	0.770310413	0.980607372

UCK1	0.325442393	0.747256297	0.764114523	0.523544318	0.395526998	0.770277985	0.980607372
LOC616304	0.225764936	0.383158679	0.906140454	0.972777748	0.505332209	0.770523053	0.980642805
PHF2	0.542135536	0.954085179	0.63477608	0.20977205	0.559296384	0.770474807	0.980642805
PLD1	0.534044141	0.394926465	0.360958054	0.965528487	0.524270234	0.770543475	0.980642805
COQ3	0.687806653	0.648644479	0.526314184	0.578399502	0.283901674	0.770644719	0.980695603
CERS5	0.867934094	0.410733468	0.222322315	0.971487885	0.501183078	0.770790008	0.980735499
KL	0.955954392	0.275621772	0.754590423	0.274236089	0.707847599	0.770816045	0.980735499
RIC3	0.845662358	0.208112495	0.270531611	0.871768112	0.930051106	0.770855354	0.980735499
CCDC167	0.424961949	0.540864623	0.63667619	0.424127028	0.623621758	0.771334422	0.980780209
DHRS12	0.51460788	0.746022593	0.244542226	0.739669292	0.55650187	0.771048734	0.980780209
FANCI	0.720676756	0.64151275	0.195692517	0.810737882	0.529948897	0.772106333	0.980780209
FGF9	0.888879722	0.190768929	0.323292789	0.896856094	0.792278456	0.772484854	0.980780209
FIZ1	0.873135691	0.271037487	0.707566063	0.713175396	0.326114302	0.772442161	0.980780209
FSD1L	0.966528156	0.42118205	0.163437358	0.618774397	0.939892283	0.771281141	0.980780209
FYB2	0.477670025	0.899277612	0.525559276	0.173793239	0.989262631	0.771838113	0.980780209
GPM6B	0.354288292	0.905563278	0.527811495	0.591797225	0.388287805	0.772291572	0.980780209
IFNAR1	0.812460087	0.921807556	0.573640408	0.106786766	0.84833698	0.772327543	0.980780209
LOC100847802	0.369077074	0.32488233	0.617917958	0.949247926	0.550343973	0.77133999	0.980780209
LOC100847861	0.534180751	0.238254599	0.325787474	0.946488193	0.98727035	0.771517407	0.980780209
LOC101904916	0.904174925	0.231028695	0.419864112	0.622316788	0.713764697	0.772504112	0.980780209
LOC101905254	0.78391495	0.770696345	0.35907091	0.974392775	0.183804022	0.772017998	0.980780209
LOC107132490	0.45695831	0.254040062	0.734331384	0.940616438	0.484115111	0.771856422	0.980780209
MLH3	0.32371892	0.634148494	0.377687968	0.986639718	0.509009686	0.772414193	0.980780209
MYO10	0.390076624	0.421417491	0.49099267	0.761410045	0.632909767	0.772215583	0.980780209
PCNA	0.27570928	0.780138215	0.543604164	0.435477539	0.760706795	0.77146492	0.980780209
PLA1A	0.328021189	0.842847529	0.829975868	0.418558888	0.403495836	0.771557589	0.980780209
PLEKHA3	0.311669783	0.260950405	0.70664055	0.92683333	0.728761088	0.771858897	0.980780209
RORB	0.424484488	0.232145913	0.576810779	0.685621099	0.996469539	0.771927932	0.980780209
RPLP0	0.880044648	0.244677963	0.549445894	0.560986478	0.584619635	0.771780986	0.980780209
SLC10A6	0.683887957	0.798582074	0.215859785	0.488951193	0.671047266	0.771218338	0.980780209
SLC43A3	0.857356444	0.605857243	0.27795321	0.560968519	0.479947856	0.772106945	0.980780209
TMEM87B	0.921158675	0.179569885	0.836982319	0.809537169	0.347267663	0.772334803	0.980780209
TNPO2	0.738314383	0.472956184	0.8953724	0.504488927	0.245047253	0.771082345	0.980780209
VKORC1	0.998167784	0.381461419	0.182466596	0.722181937	0.773793287	0.771888386	0.980780209
ZNF646	0.76169138	0.418440996	0.466244185	0.536567067	0.484663579	0.771050646	0.980780209
ARHGAP12	0.864382767	0.335820865	0.751936419	0.33855241	0.527903011	0.772745037	0.980878603
LOC104975686	0.62959953	0.464789897	0.471300289	0.316004827	0.89516021	0.77276092	0.980878603
UGDH	0.956139455	0.518917586	0.340637415	0.244918938	0.941908665	0.772648959	0.980878603
CELSR2	0.273908817	0.652921254	0.850095874	0.348199651	0.737925676	0.77299397	0.981022662
SIGLECL1	0.503772911	0.719573776	0.604080931	0.293063911	0.608611641	0.772965872	0.981022662
NCDN	0.405979336	0.79822247	0.435122774	0.826613992	0.335392008	0.773126323	0.981058032
ZNF777	0.927085984	0.383971788	0.689479788	0.206812439	0.770218642	0.773141401	0.981058032
AAR2	0.975532212	0.282122538	0.693380593	0.307867105	0.66773528	0.773757178	0.981126135
ADSS	0.779126068	0.654161072	0.903849728	0.104808672	0.811962383	0.77363428	0.981126135

CTNNBL1	0.781479228	0.615178238	0.343702894	0.406008435	0.584213785	0.773587358	0.981126135
DOCK11	0.567763147	0.415913396	0.232262553	0.935305152	0.764111633	0.773608469	0.981126135
EZR	0.694761113	0.798574082	0.235814202	0.7097867	0.421997914	0.773566817	0.981126135
FAXC	0.95872868	0.293869065	0.168250039	0.987366708	0.84022678	0.774196232	0.981126135
FRAT1	0.815636467	0.442678672	0.36349809	0.517612702	0.576103152	0.773332335	0.981126135
GEN1	0.582922994	0.430496174	0.337445071	0.760132856	0.611281394	0.774293226	0.981126135
ITGB4	0.39495933	0.470937643	0.506158571	0.733101765	0.570215328	0.774330979	0.981126135
KDM1B	0.555886836	0.70797852	0.594320196	0.318337159	0.527246286	0.773885119	0.981126135
LOC100847357	0.447347881	0.915782185	0.37153933	0.994849966	0.259219913	0.773860493	0.981126135
LOC100849023	0.448219309	0.556985795	0.52651601	0.340077146	0.880394531	0.774329124	0.981126135
LOC101907322	0.173499443	0.856994002	0.406941359	0.866418042	0.749262305	0.773984947	0.981126135
LOC107131573	0.4597818	0.26461292	0.711170321	0.664950075	0.683014822	0.774062131	0.981126135
LOC107132697	0.743331139	0.495273301	0.684404788	0.864961624	0.180371967	0.774124416	0.981126135
RPL31	0.668985213	0.413982301	0.242377721	0.710522312	0.822721793	0.773799817	0.981126135
SAP30	0.122605311	0.981754328	0.734630621	0.910814702	0.487551953	0.773928256	0.981126135
SLC25A41	0.550662669	0.614140131	0.492124906	0.309540982	0.762760769	0.774053587	0.981126135
TGM2	0.357970645	0.967101943	0.246022296	0.515066738	0.893968555	0.773699724	0.981126135
ATP2B4	0.423871944	0.474207122	0.449168403	0.438603975	0.994293389	0.774411015	0.981151793
CEND1	0.674952992	0.726199772	0.154141524	0.671132989	0.777543732	0.774652339	0.981230282
PTBP1	0.920728756	0.882398567	0.913229475	0.069607839	0.763040906	0.774569526	0.981230282
SPATA22	0.50361638	0.559775742	0.408804493	0.39036976	0.876230869	0.774628197	0.981230282
RAB33B	0.550582218	0.367954388	0.443081435	0.947027019	0.464050587	0.774753504	0.981282686
DHX33	0.792434952	0.774055535	0.848005673	0.590148845	0.128551168	0.774813303	0.981282692
AIDA	0.837904046	0.53128144	0.287157394	0.378993124	0.814820356	0.774879453	0.98129074
CACNA1C	0.408458315	0.901124044	0.204057689	0.710336383	0.740884423	0.775114029	0.981361644
LOC112446388	0.893457191	0.428289593	0.794867707	0.969752796	0.134098554	0.775234437	0.981361644
MGC127055	0.341420276	0.855983011	0.318522889	0.48653399	0.873246149	0.775215313	0.981361644
PAFAH1B3	0.847713644	0.592007207	0.147876635	0.700685449	0.760170899	0.775118268	0.981361644
RASA1	0.423415561	0.581369419	0.233870848	0.929207788	0.739307305	0.775210341	0.981361644
AADACL3	0.591103592	0.309006179	0.927144926	0.60963184	0.387433136	0.77723591	0.981402076
ABCA4	0.16382173	0.794624696	0.568063401	0.648250346	0.836535339	0.777694984	0.981402076
AGTPBP1	0.337677161	0.612566444	0.620912119	0.417591109	0.748992748	0.778006376	0.981402076
AKIRIN2	0.743929639	0.176806383	0.472096503	0.840588237	0.760652402	0.775911148	0.981402076
ALYREF	0.661438465	0.16733172	0.849268538	0.803039318	0.538985032	0.7802715	0.981402076
ANKRD16	0.361076489	0.51924185	0.633674141	0.391353672	0.879680935	0.781215809	0.981402076
ARAP1	0.518343723	0.287625988	0.336352661	0.897852691	0.904178921	0.780383917	0.981402076
C5H12orf45	0.412301094	0.884536269	0.472793683	0.699712347	0.339479265	0.781463696	0.981402076
CD247	0.268069869	0.938323003	0.772779982	0.762748061	0.275410737	0.780924325	0.981402076
CDC123	0.220528138	0.978128831	0.646630041	0.754831314	0.37993396	0.777248755	0.981402076
CDH3	0.76107818	0.883259344	0.289405644	0.323855055	0.633936035	0.776979541	0.981402076
CHRNA3	0.382810727	0.47397405	0.33792545	0.710469222	0.914889859	0.776589158	0.981402076
CLIP4	0.959679409	0.768529214	0.224520447	0.613051245	0.395359903	0.777849133	0.981402076
CNKSR1	0.898226513	0.40409517	0.274503624	0.417285906	0.952582947	0.775466923	0.981402076
CNRIP1	0.82453298	0.897047261	0.502978203	0.309050345	0.355593114	0.781144358	0.981402076

COL4A2	0.427355095	0.877820903	0.410533733	0.393995472	0.657122902	0.776674855	0.981402076
COX16	0.361149656	0.721048616	0.799017274	0.340735315	0.564718516	0.77740658	0.981402076
DCUN1D3	0.875521586	0.296737485	0.529454809	0.594895364	0.492112317	0.778442127	0.981402076
DCUN1D5	0.743965226	0.191209129	0.759294909	0.858870446	0.441559245	0.781485731	0.981402076
DGKZ	0.981443113	0.3029972	0.546479582	0.672659294	0.364203905	0.776401014	0.981402076
DIAPH2	0.781521428	0.49069231	0.34004141	0.397779574	0.781694245	0.779670232	0.981402076
E2F4	0.911779225	0.238889077	0.507388278	0.858256221	0.422983633	0.777781242	0.981402076
EMG1	0.777547737	0.506511659	0.650088736	0.757128044	0.208237928	0.778871956	0.981402076
ERI2	0.164751109	0.759303055	0.810400705	0.894443187	0.444286911	0.778518872	0.981402076
FAAP100	0.660470119	0.853921446	0.310297993	0.930016971	0.250393147	0.780573847	0.981402076
FAM57A	0.524757643	0.143148266	0.870270936	0.676786328	0.924665338	0.781259248	0.981402076
FUZ	0.741456746	0.124951264	0.957077307	0.466800174	0.965665695	0.77710757	0.981402076
GAS2L2	0.472427948	0.747126024	0.223105216	0.657059194	0.766215289	0.775648751	0.981402076
GPD1	0.59788874	0.78015851	0.984857288	0.257575934	0.334525531	0.775366544	0.981402076
GTF3C2	0.807011881	0.375739767	0.393724363	0.343210085	0.997845974	0.781155763	0.981402076
HBS1L	0.87041851	0.877848069	0.261037622	0.740801619	0.273336439	0.778967202	0.981402076
HIP1	0.137932912	0.602802815	0.816619281	0.700879557	0.840461837	0.777228249	0.981402076
HMBOX1	0.624068969	0.591766491	0.837054117	0.261057815	0.505838112	0.780869615	0.981402076
INTS5	0.537631358	0.62552755	0.73442902	0.710092977	0.228688072	0.777728814	0.981402076
ITGA11	0.772970739	0.573961546	0.538361168	0.465510618	0.356480865	0.775603711	0.981402076
KCNN1	0.525924838	0.149871976	0.854822509	0.591764688	0.995579336	0.775876003	0.981402076
KIAA1324L	0.396434684	0.6466916	0.586492897	0.322277146	0.844326385	0.781273994	0.981402076
KLHDC10	0.789611164	0.173883063	0.687418111	0.733239194	0.585113185	0.779430288	0.981402076
LOC100299025	0.354573861	0.781140603	0.975214976	0.332934407	0.445106776	0.777366246	0.981402076
LOC100336909	0.274406644	0.410253154	0.419522975	0.905139621	0.958127601	0.781466415	0.981402076
LOC100848339	0.559979276	0.922126277	0.285886809	0.341024381	0.804829424	0.779540631	0.981402076
LOC101903281	0.911883567	0.181850081	0.500468309	0.989492703	0.490589696	0.778519244	0.981402076
LOC101907577	0.572797434	0.947772752	0.694545871	0.402984688	0.262911523	0.777014058	0.981402076
LOC104969024	0.994139088	0.324561806	0.651725466	0.505329721	0.374911845	0.776523017	0.981402076
LOC104969299	0.838814986	0.380903519	0.432316508	0.877929539	0.332306464	0.778568985	0.981402076
LOC104976062	0.367480075	0.5758426	0.527213207	0.385451648	0.924245134	0.776097186	0.981402076
LOC107131649	0.894729051	0.859324278	0.326969297	0.985108878	0.163980904	0.779946012	0.981402076
LOC107132189	0.627434208	0.48689837	0.351484097	0.421628489	0.902452179	0.781026025	0.981402076
LOC112442312	0.454034699	0.473677883	0.803621624	0.53225265	0.432708815	0.776367979	0.981402076
LOC112442382	0.721152839	0.555331876	0.194540393	0.744563567	0.695530663	0.778785403	0.981402076
LOC112442649	0.321812245	0.58429617	0.724284199	0.312623151	0.956926273	0.780524985	0.981402076
LOC112444278	0.99982307	0.197252238	0.615377211	0.842610436	0.394065058	0.778568914	0.981402076
LOC112445944	0.806931564	0.703500878	0.99742398	0.124443765	0.577179221	0.780205325	0.981402076
LOC112445989	0.294960348	0.92694922	0.819078047	0.510392538	0.355587712	0.780094921	0.981402076
LOC112447342	0.285622116	0.949295908	0.295594566	0.900378074	0.559437198	0.778891613	0.981402076
LOC512149	0.536629993	0.269419522	0.964169537	0.401654516	0.713678732	0.777058745	0.981402076
LOC518980	0.602919309	0.700788498	0.613021344	0.760658571	0.206268695	0.780073954	0.981402076
LOC522174	0.278007318	0.484241627	0.82069429	0.974426487	0.378969029	0.780774432	0.981402076
LOC534630	0.641170593	0.154454479	0.722818008	0.614251096	0.90971534	0.777240744	0.981402076

LOC619026	0.952138499	0.953748571	0.174021242	0.378434226	0.676399778	0.779246687	0.981402076
LOC782024	0.77051344	0.321746905	0.282404078	0.893761544	0.640971991	0.777723331	0.981402076
LOC782305	0.734509007	0.924985456	0.381997789	0.327701254	0.481289091	0.781358184	0.981402076
LOC784914	0.621793211	0.332243161	0.410885034	0.520531702	0.905080152	0.777200389	0.981402076
LOC785216	0.594774811	0.809856439	0.417568195	0.512641219	0.388084177	0.777311996	0.981402076
LOC790101	0.987032598	0.62888102	0.292413401	0.494007586	0.452146803	0.779649288	0.981402076
MAP6D1	0.981164178	0.709224677	0.305437288	0.816512996	0.229747097	0.776666289	0.981402076
MTERF3	0.611565917	0.800698547	0.251087126	0.504287008	0.659786554	0.781252079	0.981402076
PC	0.672487751	0.654679166	0.448698935	0.261234873	0.779988648	0.778365777	0.981402076
PFKFB2	0.437374242	0.61297528	0.750943569	0.796340798	0.253031033	0.779758458	0.981402076
PGAP2	0.89336743	0.417296991	0.316844213	0.666482083	0.515048936	0.779668908	0.981402076
PIGT	0.235985586	0.57085586	0.730953858	0.913277804	0.445080388	0.777359691	0.981402076
PLGRKT	0.368355741	0.527414565	0.850101312	0.3261503	0.754070381	0.779981643	0.981402076
PPP1R35	0.320060775	0.987432386	0.705507422	0.203689995	0.882101043	0.777518748	0.981402076
PROSER1	0.376900112	0.804713777	0.969779953	0.444779098	0.311195213	0.780391252	0.981402076
PSMG1	0.762397142	0.330751801	0.401198239	0.607080356	0.645902818	0.775756657	0.981402076
RAB40C	0.961451421	0.265714135	0.929993983	0.281089722	0.606430134	0.779459367	0.981402076
RAPGEF3	0.478877753	0.663940949	0.184829997	0.922913854	0.753441123	0.781055007	0.981402076
REM1	0.924365377	0.928523291	0.134565366	0.957988224	0.369967402	0.781364902	0.981402076
RORC	0.932743354	0.16837364	0.840259497	0.57500639	0.53143386	0.778688296	0.981402076
RTN3	0.991173051	0.442052007	0.227832118	0.556968765	0.726165571	0.778907298	0.981402076
RYK	0.825711675	0.137007356	0.992211072	0.716159672	0.505367166	0.780011749	0.981402076
SAMD12	0.62598589	0.724101495	0.290707442	0.68946711	0.444277646	0.778859557	0.981402076
SGSM1	0.325717135	0.640087125	0.839005896	0.977829503	0.237494571	0.779998726	0.981402076
SH3BP2	0.195602576	0.809340081	0.626939055	0.870984807	0.465632843	0.778364855	0.981402076
SLC25A21	0.938671094	0.477304359	0.143000314	0.921789579	0.67177365	0.775775126	0.981402076
SLC25A6	0.400776031	0.54739164	0.94614873	0.393843945	0.500839682	0.781401284	0.981402076
SMIM15	0.516779999	0.18816546	0.625368794	0.80090645	0.815733019	0.776027324	0.981402076
SNRPA1	0.620458002	0.498679651	0.178747199	0.758635877	0.963340991	0.779105562	0.981402076
ST7L	0.345700571	0.549799504	0.68666427	0.491276426	0.623816833	0.777231425	0.981402076
STAT6	0.617633096	0.627783284	0.974321215	0.122025298	0.884851107	0.780737526	0.981402076
STRADB	0.798149867	0.404218934	0.155544073	0.974529772	0.835691435	0.78107901	0.981402076
TCF4	0.529297033	0.487370021	0.33470758	0.536919459	0.881111064	0.780983924	0.981402076
TCTN3	0.656609864	0.830750972	0.476518229	0.337649224	0.463445536	0.780228817	0.981402076
TEDC1	0.994648842	0.928950979	0.153535161	0.322436164	0.880818425	0.778535726	0.981402076
TJP1	0.365026658	0.929471113	0.740940441	0.587337913	0.26817725	0.775425465	0.981402076
TMEM121	0.384726154	0.830410483	0.439714603	0.346869616	0.824075726	0.777938433	0.981402076
TP53I11	0.889138491	0.71375978	0.270660687	0.281782761	0.824580898	0.776845061	0.981402076
TUBA4A	0.30290314	0.787238902	0.838586979	0.998975794	0.198429197	0.775617578	0.981402076
UBA52	0.922874702	0.67285695	0.183959412	0.462047142	0.756129037	0.7768349	0.981402076
VPS35L	0.336316329	0.85145131	0.315214624	0.800850507	0.554412005	0.77758825	0.981402076
VRK1	0.755540525	0.297347719	0.58743008	0.333748184	0.92156174	0.779859319	0.981402076
VWA5A	0.671775787	0.431913962	0.629253111	0.34765399	0.64240274	0.780671521	0.981402076
ZNF322	0.272128864	0.810801029	0.788222352	0.296667043	0.792841002	0.781241856	0.981402076

ZNF467	0.949581967	0.306802609	0.256095753	0.920359071	0.592529709	0.780285876	0.981402076
LOC523963	0.666164178	0.314643891	0.271028778	0.914473712	0.788949029	0.781587202	0.981454401
ADGRB3	0.147925336	0.799117453	0.813639786	0.559826093	0.763438883	0.782109794	0.981489945
ALG1	0.601653724	0.473291105	0.216964519	0.897900683	0.744690416	0.782991064	0.981489945
CD74	0.158101747	0.841006301	0.48034853	0.78535582	0.823525808	0.78297812	0.981489945
HESX1	0.615549961	0.547740369	0.231746084	0.558322825	0.946034698	0.782817421	0.981489945
HIPK1	0.490175841	0.693926584	0.960690713	0.130512743	0.967216351	0.782728566	0.981489945
ILKAP	0.663479872	0.743361458	0.38598587	0.719164618	0.300412307	0.782204912	0.981489945
KLF3	0.29858779	0.977613559	0.560046064	0.298128173	0.846422216	0.782739816	0.981489945
LAT2	0.766880113	0.765633903	0.225269257	0.938396809	0.332407326	0.782762132	0.981489945
LOC101903098	0.739650919	0.899506984	0.36316666	0.454400104	0.375885744	0.782812793	0.981489945
LOC112447411	0.88668367	0.591144253	0.574010936	0.67324507	0.203831697	0.782892773	0.981489945
LOC540707	0.496164691	0.928449872	0.460488203	0.478340071	0.406054313	0.782523357	0.981489945
NPAS2	0.991427822	0.424640913	0.374993029	0.904268854	0.288782673	0.782626049	0.981489945
PARP4	0.45871871	0.832059374	0.363774226	0.391181582	0.758165257	0.782421624	0.981489945
PGRMC2	0.803467263	0.503186778	0.13670059	0.82193214	0.909355733	0.782978735	0.981489945
PPP2R1A	0.560047733	0.564351688	0.765164165	0.252993239	0.674614037	0.782838216	0.981489945
SLC25A38	0.577099097	0.203898674	0.524645041	0.947619932	0.704869875	0.782666394	0.981489945
SPIN2B	0.28627066	0.73625201	0.514994733	0.745370101	0.509196747	0.782498816	0.981489945
SYNE3	0.48699653	0.281622733	0.606579557	0.51469724	0.962234923	0.782519507	0.981489945
TEKT3	0.396148533	0.708777871	0.307747497	0.738799033	0.644516886	0.782277786	0.981489945
TRAF3IP1	0.837318664	0.389785099	0.821940025	0.598643472	0.256718759	0.782628974	0.981489945
TRUB1	0.800983513	0.470174478	0.323887374	0.414478608	0.812957454	0.78208271	0.981489945
ZDHHC8	0.806606857	0.816735595	0.266161469	0.395682768	0.594788429	0.782799154	0.981489945
ZFAND2B	0.817448219	0.467358179	0.652695907	0.75460291	0.218957143	0.782513079	0.981489945
LOC101906135	0.986918616	0.304028568	0.742509499	0.213489362	0.869564129	0.783198594	0.981600132
MAP3K3	0.775506798	0.844380512	0.594744656	0.201952489	0.525747277	0.783159778	0.981600132
FNDC4	0.178373673	0.419461513	0.771676081	0.982595337	0.729568178	0.783330505	0.981690486
ADH5	0.394546067	0.370887315	0.947503357	0.318194639	0.939588697	0.783597035	0.981724631
GFRA3	0.659031486	0.348314617	0.906376416	0.820496559	0.242618976	0.78344875	0.981724631
IGBP1	0.868917032	0.86506485	0.20283445	0.344860419	0.788153601	0.78354469	0.981724631
S1PR1	0.504050962	0.500884186	0.52114729	0.32323703	0.974018461	0.783478384	0.981724631
ARL4A	0.152369814	0.989549174	0.790674057	0.706493764	0.493373035	0.784032366	0.981759008
CUX1	0.885056621	0.771836882	0.309779035	0.308523004	0.636976564	0.784171938	0.981759008
EMCN	0.433019386	0.869831901	0.653218458	0.440443663	0.383118351	0.783871928	0.981759008
GFRA4	0.769351144	0.512346378	0.164945016	0.879131006	0.727035033	0.784040061	0.981759008
LOC101903567	0.319331905	0.867347675	0.756340878	0.261497986	0.759642479	0.78428253	0.981759008
LOC101908205	0.633730321	0.435334905	0.428800325	0.989985149	0.354884224	0.784065381	0.981759008
MCPH1	0.195289554	0.555251829	0.886581612	0.628730818	0.687866557	0.78412994	0.981759008
MGAT5	0.47795776	0.45162252	0.815049936	0.815677632	0.289873007	0.78421928	0.981759008
NR3C1	0.820677125	0.85573082	0.086015747	0.986080927	0.697317558	0.783956083	0.981759008
SLC41A1	0.880911605	0.471853621	0.609144945	0.303750688	0.539500281	0.783767745	0.981759008
ZNF845	0.919803603	0.897818704	0.136541519	0.455872717	0.809366302	0.784244391	0.981759008
ABRACL	0.547035063	0.593877422	0.48914135	0.428936929	0.629203537	0.789605405	0.981985096

ADGRG1	0.459675492	0.660014294	0.316476695	0.698539635	0.632934402	0.787808234	0.981985096
ANKRD10	0.854410633	0.265955797	0.598313616	0.931874021	0.340545786	0.790659477	0.981985096
AP1S2	0.656695902	0.573459472	0.573851594	0.246261148	0.785100838	0.784998994	0.981985096
ARHGAP6	0.290572723	0.529320219	0.556566298	0.71346251	0.697904312	0.788522497	0.981985096
ARMH4	0.216049424	0.767116679	0.79105131	0.980784025	0.335147799	0.790453889	0.981985096
ASB14	0.620968749	0.599755095	0.649172512	0.605687782	0.294835809	0.790778714	0.981985096
AURKC	0.902006228	0.176806439	0.980383484	0.306413865	0.889403446	0.788463129	0.981985096
BEX5	0.68220812	0.408759904	0.415527671	0.37858782	0.961241093	0.786625458	0.981985096
BRPF1	0.8638359	0.624847175	0.809996129	0.260200526	0.373848883	0.788131204	0.981985096
C16H1orf159	0.837721234	0.64560471	0.260333764	0.495698964	0.612283295	0.788972238	0.981985096
C18H19orf12	0.904324095	0.223415303	0.563141117	0.662527478	0.566564247	0.788866982	0.981985096
C19H17orf100	0.585556229	0.494626802	0.73446258	0.526681945	0.378177652	0.787468914	0.981985096
C7H1orf35	0.425661835	0.725914636	0.989145778	0.141421743	0.998995215	0.790801863	0.981985096
CCDC126	0.911848511	0.903845504	0.657096715	0.275340699	0.285853471	0.788523633	0.981985096
CD46	0.847417875	0.17068026	0.548857867	0.714659625	0.762585399	0.79114043	0.981985096
CD47	0.289966927	0.51548718	0.91328803	0.416216086	0.752003232	0.788950175	0.981985096
CHD8	0.736993946	0.882607925	0.269876657	0.253408676	0.947145766	0.786483681	0.981985096
CHSY3	0.27589809	0.764352621	0.563510798	0.553890229	0.646106539	0.788123204	0.981985096
CPSF6	0.972548935	0.747817653	0.853863581	0.071135705	0.952691531	0.786280447	0.981985096
CRAMP1	0.844759002	0.153134147	0.594638043	0.7032249	0.77649395	0.785936374	0.981985096
CSF3	0.203057984	0.693749476	0.638281968	0.743930663	0.641806922	0.789783939	0.981985096
DBT	0.93625043	0.859532883	0.449773437	0.268923618	0.437639293	0.7884156	0.981985096
DCK	0.713015934	0.649645207	0.416412645	0.643587293	0.347668374	0.790713745	0.981985096
DDB2	0.665332669	0.580313083	0.552114604	0.362557523	0.544055911	0.786123955	0.981985096
DLGAP4	0.575625568	0.961614956	0.198523731	0.754085814	0.52240425	0.791242422	0.981985096
DOK4	0.934576011	0.437002303	0.452678269	0.272716459	0.842500678	0.787919182	0.981985096
DPAGT1	0.56790367	0.826389288	0.414068068	0.858798385	0.251868829	0.786061235	0.981985096
DYNLL1	0.678337227	0.117071234	0.862013181	0.856725155	0.731154741	0.789576925	0.981985096
DZIP3	0.760655676	0.544686343	0.273139325	0.960641636	0.388029563	0.786690889	0.981985096
E2F5	0.232733167	0.651196991	0.86103697	0.579351494	0.565518969	0.789059179	0.981985096
EMID1	0.939516471	0.313809658	0.294011576	0.885918998	0.561487496	0.790550997	0.981985096
EML1	0.734379705	0.306591039	0.33238309	0.779160934	0.723231493	0.786641497	0.981985096
ERG	0.219171055	0.494611349	0.976093672	0.681952478	0.589513088	0.78816936	0.981985096
FAM214A	0.788196079	0.88274669	0.628200873	0.745600002	0.131781621	0.789847649	0.981985096
FMO4	0.526408536	0.208565474	0.950352641	0.524758143	0.768632013	0.786277262	0.981985096
FZD6	0.424489593	0.308350439	0.532824708	0.947583013	0.646410955	0.788913423	0.981985096
GLRX2	0.587873679	0.343717458	0.418063874	0.640967813	0.796669061	0.790620977	0.981985096
GRASP	0.28466281	0.802900603	0.459364572	0.941842939	0.422399788	0.784943663	0.981985096
GRK5	0.460433917	0.843731103	0.574309441	0.651353955	0.294189032	0.789050706	0.981985096
HACL1	0.503612194	0.91435843	0.427369436	0.323322812	0.680507726	0.79128456	0.981985096
HOMER2	0.696432195	0.735638124	0.918932817	0.883370975	0.102476316	0.788496629	0.981985096
HOMEZ	0.479080308	0.39255814	0.461539302	0.498658782	0.969144649	0.785701609	0.981985096
ICE1	0.447364171	0.40864037	0.989441044	0.285717695	0.835355003	0.790765686	0.981985096
IRS1	0.985264661	0.620034488	0.233733304	0.535228944	0.56624674	0.791183271	0.981985096

KIAA0754	0.803313111	0.894804886	0.274069529	0.717291393	0.305836407	0.790950737	0.981985096
LDB2	0.998410931	0.508348441	0.322422954	0.709909332	0.367908012	0.789001311	0.981985096
LOC100300896	0.616997382	0.475625361	0.552647725	0.321843339	0.825297298	0.790382232	0.981985096
LOC100848439	0.284859225	0.380957316	0.543053726	0.911910641	0.795206171	0.788978028	0.981985096
LOC100849587	0.903286622	0.307380631	0.585839782	0.540472005	0.489076168	0.790050286	0.981985096
LOC101904592	0.805780521	0.522874363	0.502344741	0.780769172	0.253210891	0.78525691	0.981985096
LOC101905453	0.417393294	0.861680652	0.375568437	0.415378178	0.759679717	0.788521619	0.981985096
LOC101907320	0.368753279	0.995464752	0.675349475	0.236969635	0.720434615	0.787272946	0.981985096
LOC101910094	0.922062267	0.286951368	0.852958551	0.293058324	0.631761396	0.785005373	0.981985096
LOC104970913	0.423586351	0.302736556	0.450801259	0.991311363	0.748608541	0.789656336	0.981985096
LOC104974812	0.95408003	0.787007742	0.149271662	0.716176366	0.534525764	0.789685876	0.981985096
LOC107131643	0.547560336	0.391575919	0.466658931	0.812390482	0.520684271	0.787276686	0.981985096
LOC112441511	0.817993575	0.876870944	0.176891588	0.534267156	0.630397975	0.788971794	0.981985096
LOC112442083	0.765028754	0.683589347	0.603414938	0.322252722	0.42177871	0.78962135	0.981985096
LOC112442254	0.453730325	0.446525453	0.428407836	0.68245162	0.730014235	0.791050024	0.981985096
LOC112442384	0.464764095	0.739317596	0.663467361	0.606706328	0.306748838	0.787706146	0.981985096
LOC112443767	0.737989802	0.762203917	0.349835795	0.626540821	0.345310319	0.788314692	0.981985096
LOC112444531	0.521530045	0.425218572	0.797310331	0.752615228	0.322520788	0.789734373	0.981985096
LOC112445190	0.85375397	0.615555774	0.986753104	0.39457943	0.205682342	0.786282271	0.981985096
LOC112446407	0.287482285	0.800967164	0.880867616	0.273250679	0.761976647	0.786891785	0.981985096
LOC112446753	0.241404234	0.99402631	0.61728266	0.712946803	0.403575792	0.78850296	0.981985096
LOC112447350	0.188439958	0.668249666	0.843514114	0.786409258	0.50739671	0.787525814	0.981985096
LOC112448056	0.480471058	0.584002102	0.337864168	0.605169294	0.749291137	0.790018182	0.981985096
LOC112448075	0.64834755	0.363432511	0.554640107	0.686151282	0.474404635	0.788179656	0.981985096
LOC112448103	0.31598837	0.494068966	0.665084445	0.904582781	0.444477874	0.784854707	0.981985096
LOC509006	0.35834471	0.578978665	0.981526281	0.543989294	0.389343136	0.790599135	0.981985096
LOC511936	0.940048308	0.741404859	0.540141193	0.44355648	0.256876018	0.789627436	0.981985096
LOC518768	0.775296283	0.361402669	0.982703847	0.243759867	0.627967178	0.786542838	0.981985096
LOC784488	0.977797675	0.317714894	0.922549348	0.90148327	0.16468622	0.788211854	0.981985096
LYSMD4	0.918065086	0.337103337	0.676616813	0.218165099	0.93676611	0.789226831	0.981985096
MCF2	0.401801737	0.445411549	0.438747276	0.937437036	0.585370013	0.790426141	0.981985096
MRPL39	0.82073106	0.83849408	0.343021162	0.191101225	0.955622883	0.79051162	0.981985096
NFKBIB	0.732139431	0.727999923	0.728333656	0.985228077	0.112937517	0.790858559	0.981985096
NMRK1	0.942248752	0.298248154	0.668089959	0.595631112	0.380188502	0.788075379	0.981985096
OGFOD3	0.637013249	0.803346768	0.93034299	0.156509746	0.575517915	0.789590791	0.981985096
PCYOX1L	0.56437537	0.563519468	0.214891446	0.874159179	0.705154194	0.786457395	0.981985096
PDCD7	0.96723871	0.222315832	0.546684564	0.520604526	0.701139286	0.789695307	0.981985096
PIGG	0.905953871	0.089780749	0.898097693	0.874228308	0.660047472	0.786555875	0.981985096
PLPPR1	0.68148784	0.72105822	0.847166519	0.946882512	0.105934942	0.784894906	0.981985096
POPDC3	0.782969502	0.680229913	0.323709255	0.683225312	0.358410807	0.786835452	0.981985096
RASGRF2	0.658494497	0.494229345	0.460908077	0.797161187	0.360878884	0.7906857	0.981985096
RERG	0.831125641	0.478434071	0.479452938	0.251219208	0.88143641	0.786826454	0.981985096
RMDN2	0.180725415	0.559965161	0.591917955	0.733575295	0.983506701	0.790952762	0.981985096
RNGTT	0.611257652	0.578149666	0.959993539	0.691249643	0.182887583	0.78961394	0.981985096

RPS13	0.632426045	0.550179	0.202519852	0.755107565	0.806237597	0.789654888	0.981985096
SASH1	0.647782319	0.860189834	0.195868434	0.653512479	0.58760262	0.785543801	0.981985096
SLC22A3	0.716494626	0.521662475	0.82433863	0.396165332	0.343076762	0.78540148	0.981985096
SNX31	0.499037923	0.835990005	0.850211713	0.152763666	0.787687307	0.788756903	0.981985096
SRM	0.554537144	0.819143219	0.62018311	0.349590385	0.436625476	0.790069831	0.981985096
TAF10	0.67387351	0.695334192	0.375452404	0.409200713	0.57956719	0.784745916	0.981985096
TM4SF1	0.511182029	0.652539805	0.482793307	0.462963676	0.559196052	0.784618639	0.981985096
TMEM116	0.636309601	0.993306464	0.265862066	0.382757129	0.671326746	0.790791841	0.981985096
TMEM141	0.880009347	0.724942328	0.719317669	0.186021083	0.501107891	0.789149537	0.981985096
TNFRSF1B	0.589516764	0.546947423	0.689143677	0.766963992	0.250048821	0.78847954	0.981985096
TPO	0.536817021	0.81819293	0.881443364	0.825556531	0.132933263	0.787954596	0.981985096
TSPAN33	0.607564598	0.339773267	0.365986135	0.568577528	0.984100694	0.787069004	0.981985096
TUT1	0.574201607	0.652024903	0.878934855	0.977416873	0.132592426	0.788614327	0.981985096
UBAP2L	0.935523709	0.577176053	0.586522246	0.162480378	0.841175291	0.791223786	0.981985096
UBB	0.484239545	0.3746585	0.948746334	0.808092721	0.30270993	0.786361057	0.981985096
UBE2E3	0.732112032	0.881601919	0.159121111	0.635032975	0.655919451	0.789157548	0.981985096
VPS11	0.817292371	0.714066919	0.227005116	0.807805696	0.39475748	0.786952706	0.981985096
VPS39	0.432335243	0.596860672	0.572433227	0.696190366	0.42052482	0.791063531	0.981985096
WDR26	0.547645771	0.49135524	0.686483291	0.340964735	0.683140237	0.790177256	0.981985096
XPO1	0.450464693	0.708201337	0.80521467	0.485122656	0.34374149	0.789395637	0.981985096
ZFP69	0.44154329	0.750586047	0.544731418	0.241617892	0.967905579	0.786842585	0.981985096
ZNF350	0.793916417	0.87431401	0.106878497	0.942097778	0.606511466	0.787553088	0.981985096
ZNF7	0.584715751	0.148614004	0.919613552	0.87604535	0.616984374	0.79085053	0.981985096
ZNF853	0.195236975	0.950287531	0.911449016	0.551857287	0.462477654	0.790711719	0.981985096
ZSCAN4	0.814263314	0.357989156	0.313717536	0.538949618	0.860058104	0.787545758	0.981985096
PDE4A	0.640559082	0.428606985	0.892030208	0.646835014	0.273537517	0.791414858	0.981998279
UROC1	0.392436101	0.918860454	0.417229128	0.324580594	0.887063445	0.791359143	0.981998279
DEPDC5	0.888571712	0.201105785	0.453180943	0.812125699	0.659266442	0.791521585	0.982003684
LOC104972346	0.698459516	0.435052883	0.755382261	0.567567288	0.33294873	0.791590933	0.982003684
POLI	0.392553927	0.795958893	0.618998225	0.73166963	0.3066324	0.791658567	0.982003684
SNRPN	0.914725371	0.322311234	0.664823927	0.467983153	0.473028041	0.791649479	0.982003684
LOC101902106	0.57777693	0.348631082	0.745702774	0.717302914	0.403291319	0.791901001	0.982097999
LOC112448889	0.916837458	0.980271008	0.551525096	0.19919516	0.439936497	0.791844152	0.982097999
UBE2A	0.431886043	0.421433139	0.567972461	0.515128015	0.816029376	0.791914132	0.982097999
CREG2	0.517498607	0.318919056	0.510800648	0.685817923	0.753367476	0.792321555	0.982157945
DNAJB12	0.808713666	0.634354571	0.629558909	0.848931482	0.158767754	0.792217327	0.982157945
FMC1	0.65285705	0.6338585	0.553610883	0.294313171	0.644946042	0.792035907	0.982157945
LOC101903413	0.297534125	0.798721602	0.389141533	0.509127372	0.923846913	0.792083536	0.982157945
LOC615792	0.451640491	0.521554629	0.964723672	0.2724625	0.703351894	0.792288597	0.982157945
SH3BGRL2	0.439554043	0.92568262	0.602269883	0.329695429	0.538862931	0.792240945	0.982157945
PRDM8	0.594226393	0.289011553	0.63674479	0.908331666	0.438664382	0.792383037	0.982159971
NTN5	0.481049172	0.348992446	0.500638744	0.622873316	0.833225444	0.792577562	0.982252708
SCAMP4	0.713796466	0.492433059	0.254534993	0.787479605	0.618991909	0.792537752	0.982252708
GTF2H2	0.284372701	0.441986233	0.913379972	0.446106357	0.852706794	0.792776547	0.982340676

HIBCH	0.567967014	0.934803503	0.955528104	0.090921414	0.947026043	0.79282839	0.982340676
RBP4	0.207082859	0.647623541	0.51280649	0.752537612	0.844334433	0.792887977	0.982340676
USP40	0.620555703	0.482076643	0.437292547	0.958820877	0.348231034	0.792811832	0.982340676
SULT1C4	0.676537236	0.603926151	0.964445916	0.220753374	0.502784281	0.793040735	0.982455763
BRD3	0.924934592	0.720298415	0.204828692	0.592748096	0.541354692	0.793251574	0.982593854
TMEM268	0.969625788	0.859745562	0.379427324	0.406378359	0.340709081	0.79327195	0.982593854
LOC101909083	0.523631854	0.183609755	0.931324543	0.672811203	0.727193339	0.793331893	0.982593939
LCA5	0.398020941	0.770640015	0.862895515	0.352679608	0.469588328	0.793431409	0.982643035
ARHGEF11	0.757644008	0.240030363	0.473275826	0.717429172	0.710489323	0.793579811	0.982678509
RFX2	0.872863576	0.818301225	0.221909176	0.346894029	0.797753228	0.793546808	0.982678509
HYLS1	0.523132648	0.785008031	0.143925677	0.751889329	0.987932843	0.793709807	0.982765326
CERK	0.505131772	0.510134149	0.392917809	0.502190413	0.864260668	0.793869985	0.982815353
LOC100848912	0.659181475	0.39242637	0.314553471	0.746182526	0.723650279	0.793840841	0.982815353
LOC112448078	0.346402689	0.348227209	0.837179962	0.503447002	0.865239903	0.794050499	0.982964678
LOC107131489	0.388541891	0.780325827	0.923896256	0.238389207	0.659323331	0.794198768	0.98300809
LOC783185	0.624128835	0.465627215	0.225750373	0.754155759	0.889890074	0.794205367	0.98300809
ACSS3	0.451999949	0.74072432	0.564539004	0.415927501	0.58396563	0.801456159	0.983060722
ADSSL1	0.378380717	0.73280719	0.870913541	0.38136521	0.501028507	0.802330111	0.983060722
AK5	0.608930715	0.6848536	0.850936344	0.693851954	0.180475234	0.795813826	0.983060722
ANKRD6	0.770139387	0.766097099	0.162863805	0.892838018	0.522039235	0.797177335	0.983060722
ARHGAP39	0.404472641	0.570532797	0.484500449	0.642578171	0.624509878	0.797487392	0.983060722
ATP9B	0.87661456	0.911810779	0.279559144	0.760648751	0.267595356	0.799848704	0.983060722
BMP3	0.365111779	0.400783481	0.9363586	0.658305363	0.50837661	0.801256443	0.983060722
C5AR2	0.991162955	0.17263478	0.700825597	0.645707292	0.585576782	0.799312315	0.983060722
C5H12orf4	0.701526691	0.536957456	0.848888943	0.541404831	0.259634634	0.797803199	0.983060722
CCDC71	0.83917175	0.72814311	0.508508997	0.328128586	0.449982428	0.801341827	0.983060722
CCDC90B	0.745878276	0.15931149	0.711601293	0.866976154	0.619690504	0.799643261	0.983060722
CCNT2	0.635163751	0.919125797	0.958671933	0.104956871	0.775454744	0.800106644	0.983060722
CCPG1	0.654950607	0.485521636	0.321457312	0.486186328	0.928936933	0.802422113	0.983060722
CD44	0.23633576	0.676147371	0.384476438	0.867892689	0.844693894	0.798157057	0.983060722
CDK4	0.408418664	0.215267963	0.717335275	0.927838771	0.772207922	0.798718836	0.983060722
CHERP	0.584974945	0.801651996	0.285387952	0.370113271	0.929556572	0.801962191	0.983060722
CHODL	0.571131427	0.266710967	0.543001826	0.980186066	0.544423496	0.79464118	0.983060722
COL4A4	0.904173163	0.153960315	0.762366132	0.895693804	0.467124881	0.795681349	0.983060722
COMMD7	0.45235965	0.395151468	0.838634696	0.554956993	0.547327591	0.800038585	0.983060722
COX15	0.68993681	0.722294506	0.971699198	0.239055877	0.381598753	0.794776964	0.983060722
DACH1	0.284855198	0.991004342	0.93641026	0.649138119	0.265861273	0.800369195	0.983060722
DENND2C	0.200970977	0.932167782	0.86662845	0.768509888	0.357166787	0.79630803	0.983060722
DLST	0.965556407	0.98625602	0.728378036	0.112488287	0.586886105	0.801015955	0.983060722
EP400	0.725204637	0.832724812	0.534040147	0.145388862	0.944472903	0.795216254	0.983060722
FAM110D	0.34649506	0.65717563	0.546008544	0.459429667	0.781710845	0.79665316	0.983060722
FAM135A	0.270844582	0.325656992	0.823243721	0.997763751	0.622836824	0.798478333	0.983060722
FAM92A	0.875989508	0.353006548	0.176434458	0.929899603	0.909690679	0.802368797	0.983060722
FAR2	0.323340132	0.818391645	0.290085857	0.919048255	0.631849383	0.796355409	0.983060722

FAXDC2	0.323691084	0.333213632	0.974680691	0.874552442	0.484006588	0.796056562	0.983060722
G6PC	0.394091577	0.861098539	0.28248324	0.596837578	0.783617696	0.797357044	0.983060722
GAB2	0.357506694	0.757756466	0.745057885	0.677370255	0.335182112	0.801145246	0.983060722
GARNL3	0.718955386	0.776870253	0.419274047	0.308578407	0.610841242	0.794648685	0.983060722
GPR62	0.901412238	0.723427473	0.119829901	0.662566353	0.890914759	0.802271415	0.983060722
GUSB	0.190756723	0.527428137	0.915851908	0.538981102	0.928410359	0.802205112	0.983060722
H3F3A	0.707414623	0.150769866	0.913346203	0.492716989	0.957648354	0.801667984	0.983060722
HACD1	0.360161047	0.331734821	0.603725129	0.695778371	0.916465146	0.801781582	0.983060722
HERC1	0.312740113	0.915024077	0.950705154	0.20743755	0.817865655	0.802383232	0.983060722
HMCN2	0.208085675	0.512819393	0.587908438	0.808582439	0.896973931	0.79991661	0.983060722
HNRNPC	0.710835821	0.33619275	0.686560746	0.772103006	0.361976519	0.801257017	0.983060722
IFITM5	0.429933423	0.54318114	0.356783247	0.813214216	0.681759417	0.802523959	0.983060722
IFT22	0.482927914	0.374267979	0.602913011	0.749132757	0.549770657	0.797539642	0.983060722
IFT81	0.813122732	0.967984137	0.560495661	0.208846449	0.481321982	0.795457985	0.983060722
IGF2R	0.941229521	0.966362414	0.168652334	0.847098649	0.345700774	0.797700367	0.983060722
INF2	0.555837106	0.302979137	0.824514448	0.400519815	0.804877498	0.797080879	0.983060722
JKAMP	0.994699122	0.206369399	0.671639286	0.464632272	0.694012027	0.795894915	0.983060722
KHK	0.901810514	0.714098466	0.70123096	0.162756273	0.627510292	0.802249172	0.983060722
KIAA1522	0.986424335	0.556158492	0.46389419	0.20660123	0.878295342	0.802471783	0.983060722
KMT5A	0.70110727	0.596740447	0.949265257	0.516976169	0.221815603	0.800075666	0.983060722
LARP6	0.400593759	0.876618471	0.787011411	0.885077199	0.182801486	0.796898653	0.983060722
LGI4	0.662482822	0.937611553	0.515443249	0.575216403	0.250329495	0.802181245	0.983060722
LOC100335340	0.235605444	0.887420564	0.262835023	0.901079134	0.90732115	0.797724233	0.983060722
LOC100848405	0.805358144	0.857923254	0.688217553	0.742376624	0.126214251	0.796274004	0.983060722
LOC101902681	0.49325855	0.623125619	0.641666162	0.290340116	0.781628558	0.797062687	0.983060722
LOC101905293	0.984645484	0.789031148	0.901168935	0.28178556	0.229123113	0.798780228	0.983060722
LOC101906177	0.522052247	0.161981894	0.632990193	0.907001297	0.920862005	0.796867925	0.983060722
LOC101907523	0.843827174	0.242567369	0.599065285	0.77715491	0.464085104	0.794979004	0.983060722
LOC104969916	0.739853839	0.97517155	0.301537162	0.573703933	0.363587871	0.79945658	0.983060722
LOC104972400	0.769688953	0.815320356	0.601480379	0.215083705	0.544593462	0.794930719	0.983060722
LOC104974034	0.90996125	0.703408857	0.746123848	0.399578992	0.23591959	0.798078169	0.983060722
LOC107132537	0.244596521	0.644955708	0.970257883	0.905766229	0.329183844	0.80043438	0.983060722
LOC107132799	0.997964586	0.577632506	0.339076671	0.67785735	0.340020479	0.798197824	0.983060722
LOC112441682	0.860417499	0.282946324	0.838639225	0.963076827	0.227417166	0.796905028	0.983060722
LOC112442865	0.286418009	0.761832294	0.857159828	0.56283653	0.432899477	0.800183362	0.983060722
LOC112443503	0.60297365	0.517811978	0.477096998	0.998715465	0.30596284	0.79998312	0.983060722
LOC112444967	0.945154178	0.181963258	0.650443437	0.752223293	0.536034434	0.798408262	0.983060722
LOC112445996	0.652563261	0.608348939	0.815523719	0.17365022	0.79492713	0.796801814	0.983060722
LOC112447290	0.782528012	0.347604959	0.281578513	0.712068194	0.823268032	0.797614849	0.983060722
LOC112447495	0.827374803	0.496023504	0.172096286	0.654720454	0.975182544	0.79836138	0.983060722
LOC112447811	0.107583961	0.739594734	0.746155172	0.910704442	0.83796345	0.799180897	0.983060722
LOC112448021	0.780821649	0.240037482	0.614393741	0.447256126	0.87780557	0.79880592	0.983060722
LOC509155	0.132582508	0.976730699	0.935831308	0.437649813	0.8624147	0.800823755	0.983060722
LOC510185	0.848713771	0.393219948	0.573753845	0.71276614	0.335483664	0.800998296	0.983060722

LOC511409	0.635721796	0.64008878	0.677110785	0.979000647	0.168416824	0.799643946	0.983060722
LOC528802	0.960317075	0.521573458	0.586872732	0.337263877	0.463525185	0.801624183	0.983060722
LOC782202	0.534050463	0.777926077	0.364543065	0.746537136	0.40768735	0.802152069	0.983060722
LOC782525	0.847209067	0.338896142	0.89555901	0.688185166	0.257725336	0.800311841	0.983060722
LOC787554	0.984572904	0.436121549	0.387471731	0.724490256	0.365641484	0.794384115	0.983060722
LRR1	0.475533151	0.203035176	0.826077879	0.818788134	0.703130654	0.801490605	0.983060722
MAPK8IP3	0.802872358	0.608967302	0.262704523	0.596668614	0.591020408	0.799129362	0.983060722
MAPKAP1	0.840195844	0.656812898	0.364371204	0.32022333	0.711681322	0.801142857	0.983060722
MARVELD2	0.386922771	0.843982995	0.333454153	0.571279257	0.727850479	0.79906601	0.983060722
MCF2L	0.768151022	0.753259738	0.648027715	0.588900563	0.205096008	0.799105618	0.983060722
MDP1	0.515485585	0.4682192	0.608995977	0.448965494	0.696986568	0.801783014	0.983060722
MED7	0.866445879	0.871401092	0.511238699	0.164237023	0.721710053	0.800869669	0.983060722
MGLL	0.863024646	0.982255408	0.235357971	0.348141467	0.655401987	0.800004003	0.983060722
MME	0.832404594	0.332008616	0.939268915	0.365628961	0.480373211	0.800263831	0.983060722
NINJ1	0.399143569	0.411691424	0.779819113	0.375026642	0.924271409	0.795737422	0.983060722
NKAPL	0.593988691	0.225089878	0.909910373	0.675394713	0.540680763	0.795767423	0.983060722
NOL3	0.950925456	0.535709785	0.168433668	0.594776498	0.871712258	0.796008061	0.983060722
NR4A1	0.564382852	0.227048186	0.70122128	0.637684599	0.796959411	0.800540572	0.983060722
NR6A1	0.272210072	0.76709751	0.853738712	0.275578437	0.903076921	0.795534292	0.983060722
OSMR	0.438713195	0.848237528	0.924137782	0.357499255	0.361494011	0.795839483	0.983060722
P2RX6	0.453476261	0.796577756	0.478679597	0.300866674	0.871861798	0.799370689	0.983060722
PF4	0.222318902	0.978575725	0.801962073	0.298332301	0.857946982	0.796670077	0.983060722
PIBF1	0.613867956	0.981019204	0.825124532	0.109942597	0.833946597	0.800137755	0.983060722
PIGF	0.93115847	0.717922519	0.652488874	0.92772888	0.108903968	0.794366284	0.983060722
PIGP	0.950021734	0.576576724	0.495905266	0.219228044	0.75335363	0.797469462	0.983060722
PLEKHM1	0.769312266	0.926852634	0.087033971	0.86254872	0.852324734	0.800382241	0.983060722
PLSCR2	0.425555045	0.965662515	0.934184973	0.413632058	0.29015072	0.802074246	0.983060722
PMAIP1	0.449269018	0.722954338	0.531578013	0.466068397	0.553856964	0.796328339	0.983060722
POLB	0.684980549	0.764532785	0.350457695	0.563434105	0.441050185	0.800322677	0.983060722
PPP1R18	0.292437632	0.862446468	0.338658684	0.894205907	0.588974317	0.797939706	0.983060722
PRELP	0.873780425	0.605736013	0.632742718	0.141567937	0.942615768	0.796800949	0.983060722
PSMB8	0.552683281	0.849563216	0.724685133	0.340961724	0.384491989	0.796481539	0.983060722
RALB	0.561878421	0.604939036	0.779771039	0.728454292	0.23841275	0.80191602	0.983060722
RALGPS1	0.170472941	0.522882334	0.756488271	0.878141975	0.745930613	0.794762091	0.983060722
RAP1GDS1	0.51313119	0.637566129	0.631086938	0.718509846	0.309899215	0.801695136	0.983060722
RBM26	0.410301533	0.908014459	0.814300024	0.166487643	0.895640138	0.798911012	0.983060722
RC3H1	0.425962576	0.321484658	0.582778641	0.610773312	0.915471336	0.796539908	0.983060722
RCC1L	0.640837111	0.776559315	0.248151458	0.853188187	0.431360687	0.799719202	0.983060722
RING1	0.707665152	0.823469674	0.422541388	0.231214387	0.810904507	0.802422043	0.983060722
RND3	0.433361226	0.652872422	0.883340706	0.785032713	0.227564802	0.796635694	0.983060722
RNF26	0.958597283	0.438486705	0.424559966	0.372970644	0.690870985	0.801737679	0.983060722
RPL26L1	0.498381108	0.427124465	0.777169382	0.541966842	0.50564179	0.799290213	0.983060722
SCUBE3	0.672969823	0.517297828	0.635044337	0.228319202	0.903281643	0.800268911	0.983060722
SDF4	0.51822818	0.586180058	0.433047077	0.566603543	0.619439946	0.802436479	0.983060722

SERPINB9	0.337337522	0.3556965	0.957960229	0.49909216	0.780605205	0.797157489	0.983060722
SLC22A4	0.813129278	0.532657807	0.376259059	0.533532736	0.507948213	0.794742252	0.983060722
SLC25A42	0.921093129	0.989168821	0.804997741	0.06508925	0.948890493	0.799149401	0.983060722
SPRY4	0.501090174	0.226986566	0.550822402	0.797609102	0.887240869	0.795417842	0.983060722
SUMO2	0.771689578	0.331467227	0.76212272	0.433502931	0.546781041	0.802574343	0.983060722
TANGO2	0.459789323	0.646732737	0.436904659	0.369453231	0.934605446	0.797458999	0.983060722
TBC1D17	0.972862689	0.831696995	0.304822463	0.236439055	0.780417102	0.799952108	0.983060722
TCTN1	0.353098334	0.680168629	0.565619194	0.585833136	0.555467778	0.794900329	0.983060722
TEX261	0.559988759	0.722367356	0.263181562	0.427146871	0.987035391	0.797556765	0.983060722
TGFBR3	0.737227649	0.870750397	0.770121202	0.156367962	0.585715001	0.799067506	0.983060722
TIAM1	0.317478324	0.328992561	0.929092727	0.738074423	0.634008009	0.79957172	0.983060722
TM9SF1	0.866461651	0.730974277	0.324146667	0.416909503	0.522228656	0.796834165	0.983060722
TOE1	0.897691252	0.387068234	0.625677131	0.267425631	0.759540488	0.794719529	0.983060722
TPST2	0.551958969	0.77639495	0.420189778	0.674059273	0.371764794	0.798474479	0.983060722
UBXN1	0.868496484	0.465087543	0.167436746	0.891201053	0.742261829	0.796990515	0.983060722
USP31	0.989734592	0.971346075	0.734252692	0.071855905	0.906202791	0.801667401	0.983060722
VPS37B	0.613912635	0.897380522	0.671752484	0.301499197	0.410372364	0.80100462	0.983060722
WNT2B	0.168232562	0.914958051	0.539004525	0.658140045	0.812627185	0.79555979	0.983060722
WSCD1	0.637771278	0.935086725	0.676925043	0.192899209	0.587589996	0.800887624	0.983060722
YY1	0.768248097	0.837752304	0.462913272	0.194670918	0.76520106	0.795591893	0.983060722
ZNF174	0.262948419	0.9120976	0.839653537	0.22704007	0.975368564	0.796429084	0.983060722
ZNF268	0.928943017	0.42341547	0.93075441	0.293922046	0.427345374	0.801737203	0.983060722
ZNF655	0.513409593	0.489900609	0.418781016	0.579411454	0.740151415	0.798660074	0.983060722
ABHD16A	0.859330627	0.658454592	0.850119145	0.239305025	0.404045465	0.803696113	0.983105849
ALOX5	0.322847589	0.738870681	0.589674712	0.417705701	0.791618172	0.803702704	0.983105849
BRWD1	0.418582906	0.674103442	0.383199347	0.566145203	0.760223535	0.803796518	0.983105849
EFCAB7	0.981234131	0.301797227	0.351584013	0.531584295	0.840901443	0.803809291	0.983105849
FAM212B	0.583740984	0.333925988	0.467002336	0.759402905	0.67090396	0.8032112	0.983105849
GTPBP10	0.224155652	0.932529494	0.701492479	0.321034985	0.987119919	0.803541732	0.983105849
HES2	0.408776858	0.938530273	0.148857698	0.856964436	0.950917089	0.803800234	0.983105849
KBTBD3	0.612718081	0.794474611	0.461573828	0.44614879	0.463308994	0.803452651	0.983105849
LOC101907518	0.598220763	0.3319319	0.315579711	0.975145239	0.759558725	0.803341039	0.983105849
LOC112442227	0.781719142	0.226484766	0.819289897	0.420238568	0.759947381	0.803007248	0.983105849
LOC112444520	0.920481244	0.928453561	0.173021924	0.806886616	0.387663972	0.802744906	0.983105849
LOC112447462	0.941044449	0.488223435	0.422139608	0.250638865	0.954940792	0.803364583	0.983105849
LOC783504	0.463843774	0.571583911	0.703059175	0.323114439	0.769940875	0.803185588	0.983105849
MVB12B	0.956262634	0.857429762	0.209850892	0.380907915	0.709195244	0.803587134	0.983105849
OTOGL	0.740151074	0.997740949	0.492357855	0.420821063	0.303107245	0.803207543	0.983105849
POLD3	0.257055276	0.767850202	0.702443046	0.451829889	0.739366278	0.802984274	0.983105849
RAB23	0.646226739	0.499393468	0.538918756	0.344234171	0.77291003	0.802820064	0.983105849
SCARB1	0.588095505	0.483337926	0.666440069	0.490299846	0.499348746	0.80321258	0.983105849
ZFAND2A	0.706308154	0.598766187	0.773427288	0.454716087	0.311251002	0.802894527	0.983105849
ZNF154	0.280602695	0.865175915	0.780788194	0.730210777	0.336233021	0.803802907	0.983105849
GDAP2	0.573938458	0.243537112	0.38521345	0.960627352	0.900123433	0.803870437	0.983107366

PBX3	0.542156692	0.596793163	0.797468007	0.492950045	0.36635756	0.804021038	0.983218275
ATP6V0A2	0.844816142	0.829476697	0.743250194	0.919963427	0.097747482	0.8048927	0.983508564
C25H16orf58	0.873065773	0.140593565	0.837805912	0.729722703	0.623817517	0.804811294	0.983508564
ELMSAN1	0.914412056	0.651374308	0.783144455	0.327113819	0.306160277	0.804452457	0.983508564
ITGA7	0.898728444	0.560649059	0.254792181	0.477132395	0.763211938	0.804581781	0.983508564
LOC101905179	0.94551954	0.32074383	0.788200078	0.639001845	0.306538016	0.804843057	0.983508564
LOC512541	0.947059883	0.436535897	0.837492677	0.274028025	0.493542422	0.804861263	0.983508564
LOC785843	0.841612529	0.561956458	0.250731015	0.648510292	0.609117404	0.804917648	0.983508564
RHBDL3	0.980722615	0.673184871	0.484568237	0.477787852	0.30558768	0.804430441	0.983508564
TF	0.947527831	0.467466427	0.783330026	0.237832991	0.567605792	0.804903954	0.983508564
TTC30A	0.517417147	0.516434372	0.710650956	0.568562363	0.433555202	0.804796914	0.983508564
ZNF317	0.40215588	0.407083285	0.70752294	0.805281165	0.501907047	0.804818319	0.983508564
ACOT11	0.283985956	0.694881063	0.985955985	0.53045517	0.456417527	0.805877522	0.983557795
ATG10	0.597231597	0.469497398	0.305348569	0.674669365	0.814886879	0.805753172	0.983557795
CACNA1F	0.621033087	0.216219916	0.801902491	0.755430317	0.577329563	0.805355166	0.983557795
CLMN	0.785201436	0.814125524	0.735870847	0.611234877	0.163360457	0.805386277	0.983557795
CNTN1	0.208106807	0.951544208	0.877625498	0.552266715	0.490872558	0.805903611	0.983557795
DHX16	0.869546339	0.968812612	0.49663628	0.206569138	0.545561569	0.80603673	0.983557795
FAM122B	0.65137861	0.725845194	0.622262419	0.933638648	0.171345987	0.805730976	0.983557795
FER	0.870460202	0.761256567	0.603332844	0.214613107	0.549406299	0.805999975	0.983557795
FGFR1OP2	0.924156864	0.892410471	0.993375451	0.058532822	0.983123286	0.806017457	0.983557795
GTF2IRD1	0.665552959	0.342447436	0.611380905	0.425535511	0.791527028	0.805252567	0.983557795
LOC100848177	0.394802713	0.857032966	0.376495245	0.835114784	0.44136252	0.805327007	0.983557795
LOC101904268	0.381082348	0.526466668	0.640266563	0.431983259	0.844857441	0.805059694	0.983557795
LOC101905723	0.388792511	0.978633111	0.98613352	0.133323249	0.941759779	0.805894724	0.983557795
LOC107131498	0.789500409	0.944087563	0.549884527	0.214690002	0.535757862	0.806011666	0.983557795
LOC507443	0.344035271	0.54405938	0.774041187	0.357672218	0.909758075	0.806014801	0.983557795
MADD	0.449118076	0.785765133	0.826795167	0.275055556	0.586661363	0.805792153	0.983557795
PALMD	0.862466882	0.530954211	0.670023102	0.404245208	0.37980267	0.805883942	0.983557795
RHOBTB1	0.928156003	0.915325559	0.501801162	0.216794669	0.509261872	0.805736556	0.983557795
ADGRA2	0.86301851	0.778034079	0.848484814	0.40460687	0.208379325	0.809205163	0.983589637
ANGPT1	0.520277568	0.758104242	0.698494797	0.512210256	0.335491298	0.806735781	0.983589637
ANO10	0.784224638	0.575981889	0.133153178	0.808807503	0.978189929	0.807605109	0.983589637
ANXA3	0.719439916	0.604298589	0.86297726	0.142060444	0.891174693	0.807295751	0.983589637
ATP6AP1L	0.487680462	0.548728412	0.970133392	0.890255247	0.204292948	0.806277647	0.983589637
ATP6V0D2	0.63209985	0.375503692	0.901344155	0.223117274	0.997278956	0.807672315	0.983589637
BEAN1	0.722697666	0.550732546	0.19670547	0.765325154	0.788608432	0.806407137	0.983589637
BTC	0.253806198	0.91427578	0.388314833	0.558006036	0.957008257	0.809505442	0.983589637
C11H9orf78	0.744997598	0.964597993	0.243167247	0.406152796	0.678876551	0.80972783	0.983589637
C6H4orf19	0.706894265	0.47353858	0.627702026	0.283010281	0.799280531	0.80740704	0.983589637
COPB2	0.817937432	0.46976655	0.909591814	0.601948263	0.227630076	0.808690886	0.983589637
CYB5R1	0.669239316	0.462362558	0.367875929	0.820575932	0.514427222	0.809266816	0.983589637
DOC2B	0.540163371	0.681616652	0.756228102	0.389939719	0.441000307	0.808658354	0.983589637
DTWD1	0.246685625	0.852410077	0.707742286	0.535575248	0.593487283	0.806595279	0.983589637

EEF1G	0.755267952	0.558343989	0.35257921	0.525123936	0.612567117	0.80847047	0.983589637
FAM180B	0.295730447	0.530012124	0.58169486	0.766697132	0.690196979	0.809957492	0.983589637
FAM199X	0.480748159	0.472582983	0.537520042	0.410137701	0.964864646	0.810235929	0.983589637
FAM98B	0.490702627	0.496019251	0.605744525	0.995783103	0.32461557	0.807868606	0.983589637
FBXO10	0.717621632	0.320712677	0.689639285	0.741164282	0.404629851	0.80765883	0.983589637
FOXP4	0.967424015	0.905420979	0.081505714	0.761443746	0.880546695	0.808615716	0.983589637
GLE1	0.460383508	0.646355197	0.649598263	0.959594835	0.260563589	0.810255865	0.983589637
HPCAL1	0.624485563	0.617523131	0.315513195	0.634066976	0.620114636	0.808519535	0.983589637
INAFM1	0.454656116	0.536833274	0.730883751	0.903854909	0.299599281	0.810167086	0.983589637
KDELR1	0.833982211	0.818249391	0.649795744	0.145544352	0.738234029	0.8078169	0.983589637
KRT80	0.670988837	0.813582072	0.549669174	0.308327975	0.511085959	0.806526617	0.983589637
LOC100139360	0.264796488	0.943697487	0.437464534	0.617633221	0.708572418	0.808520509	0.983589637
LOC100297725	0.981926893	0.143478826	0.921358321	0.394833609	0.934271171	0.808669361	0.983589637
LOC101903375	0.477066755	0.872128875	0.25323135	0.888017044	0.514954013	0.809720196	0.983589637
LOC101903976	0.449802752	0.311442862	0.533559136	0.660893476	0.972842783	0.809285988	0.983589637
LOC101905686	0.688957591	0.700541361	0.182230679	0.574316127	0.93449091	0.80623108	0.983589637
LOC101906472	0.964476517	0.253061674	0.502817031	0.861749068	0.447886348	0.806822257	0.983589637
LOC101907503	0.856335523	0.715670658	0.992687047	0.08671478	0.908951337	0.808912517	0.983589637
LOC101907581	0.479546661	0.435369796	0.607615532	0.513144863	0.742481433	0.81025826	0.983589637
LOC104968479	0.738998571	0.232180845	0.40785187	0.865802728	0.789059557	0.808402292	0.983589637
LOC104975559	0.314266977	0.262335447	0.864548563	0.821910963	0.821858092	0.809602741	0.983589637
LOC104975676	0.225625257	0.877443491	0.44682237	0.81371164	0.665347261	0.808699958	0.983589637
LOC112441619	0.820058238	0.554727846	0.743155118	0.510380238	0.279685737	0.809994369	0.983589637
LOC112442223	0.438713206	0.950044456	0.324050105	0.363058847	0.964472522	0.806557599	0.983589637
LOC112442383	0.695589837	0.531869485	0.86096155	0.424422514	0.351548051	0.807391473	0.983589637
LOC112442544	0.819471668	0.845520266	0.213712032	0.368871683	0.882066325	0.809718811	0.983589637
LOC112444207	0.809502149	0.828359972	0.641328435	0.184161008	0.602633934	0.80811513	0.983589637
LOC112448335	0.794674254	0.957238556	0.515082007	0.500464902	0.245563653	0.809624915	0.983589637
LOC616254	0.78563034	0.888534869	0.733000329	0.135551466	0.696162176	0.810089431	0.983589637
LOC783421	0.732701191	0.219226813	0.377802213	0.90291847	0.868346226	0.807588351	0.983589637
LOC784208	0.414041394	0.926724452	0.32869243	0.437894222	0.870638537	0.809377568	0.983589637
LTBP4	0.254530177	0.640325516	0.858928186	0.67738109	0.505412247	0.808823402	0.983589637
MFAP3	0.695341737	0.536545549	0.415780883	0.961821479	0.322468171	0.809479126	0.983589637
NKG7	0.135417417	0.891041032	0.8331152	0.482694718	0.983048827	0.808019434	0.983589637
NUP88	0.829048956	0.832168542	0.965234881	0.118492259	0.609090157	0.809301197	0.983589637
OPA1	0.617720612	0.77474508	0.791248767	0.452201882	0.282025934	0.810118287	0.983589637
ORC3	0.795126379	0.934082802	0.541797603	0.533628752	0.223560193	0.809104015	0.983589637
PDCD10	0.823025134	0.505392299	0.234026566	0.509409955	0.956771104	0.807099647	0.983589637
PDK3	0.59163614	0.771685177	0.789962049	0.448723033	0.293737908	0.807436354	0.983589637
PEPD	0.298865856	0.391509911	0.859095945	0.995421147	0.478169341	0.808538758	0.983589637
PHACTR1	0.76486	0.608271369	0.36668703	0.555723308	0.503331864	0.808083372	0.983589637
PIK3IP1	0.497096758	0.692384788	0.597024769	0.269282988	0.862960489	0.808198181	0.983589637
PILRA	0.825258746	0.902198503	0.236291244	0.422693097	0.644392268	0.80879973	0.983589637
PPP1R26	0.592970003	0.482516753	0.780221953	0.238641616	0.905661668	0.809958075	0.983589637

PRRC2B	0.771474308	0.629110285	0.833174144	0.508846051	0.233839398	0.809493755	0.983589637
RAD9A	0.379479692	0.653435985	0.605652551	0.352596857	0.902585635	0.808355566	0.983589637
RNF169	0.433396586	0.713560006	0.869566729	0.221704981	0.799937556	0.807990212	0.983589637
RUBCN	0.270736045	0.25107176	0.865885102	0.916329325	0.884319887	0.807996001	0.983589637
SEMA6D	0.932892549	0.468986921	0.34598209	0.402362446	0.775383733	0.806312249	0.983589637
SETBP1	0.622412457	0.674778955	0.550240266	0.379174356	0.540767283	0.806887079	0.983589637
SLC30A6	0.978276564	0.445337387	0.769696697	0.898182645	0.157454906	0.807024556	0.983589637
SPATS2L	0.652420158	0.840463632	0.676469882	0.555500085	0.233658468	0.809600272	0.983589637
TLR2	0.598732215	0.845611507	0.709281345	0.466518414	0.284715661	0.808010518	0.983589637
TWF2	0.521344335	0.89933778	0.168909349	0.646064735	0.94084437	0.809574688	0.983589637
WDR45	0.745676747	0.551770649	0.205058517	0.80254416	0.710930009	0.809569909	0.983589637
YIPF1	0.196977876	0.400443957	0.733310812	0.929062774	0.888466409	0.808179642	0.983589637
ACOT13	0.418309796	0.660810517	0.29414034	0.795365162	0.74867345	0.810549212	0.983636347
ATG7	0.804720326	0.926167346	0.709419548	0.204349921	0.448017088	0.810517221	0.983636347
GATA2	0.42385739	0.605873364	0.38983777	0.734657157	0.658008647	0.810476301	0.983636347
LOC107132386	0.605597649	0.975170086	0.439051646	0.352347468	0.530100458	0.810596427	0.983636347
NTNG2	0.997258978	0.804334182	0.251862581	0.571084659	0.419525069	0.810501564	0.983636347
EDEM2	0.783378312	0.814037848	0.1302847	0.929997086	0.627394102	0.810761192	0.98364777
LMLN	0.982390134	0.853492932	0.807999794	0.109679083	0.652492127	0.810785655	0.98364777
LOC112444635	0.32457525	0.99939333	0.860373171	0.188760111	0.919894982	0.81070483	0.98364777
CINP	0.989302494	0.635936681	0.878990308	0.330967615	0.265199418	0.8109772	0.983807424
CERS1	0.781838203	0.348241161	0.211192971	0.870512307	0.970630205	0.811141047	0.983860733
KCNB1	0.898091774	0.895668897	0.749711896	0.168101333	0.479182282	0.811113494	0.983860733
LOC100297097	0.755304893	0.625614172	0.277624585	0.848426531	0.436880578	0.811280894	0.983884921
LOC101905203	0.438250707	0.308891777	0.597712976	0.684244496	0.878177433	0.811261968	0.983884921
LOC104975890	0.760915801	0.560780003	0.319576206	0.938865829	0.379943048	0.811343984	0.983888725
ZNF185	0.071516463	0.994442649	0.82807758	0.950677902	0.869249667	0.811425511	0.983914886
PPP2R2C	0.932466184	0.573665661	0.396439958	0.339613617	0.676039915	0.811500379	0.983932968
UXT	0.734875033	0.83610708	0.96908498	0.219759006	0.372461219	0.811671139	0.984067307
LOC101903806	0.804987792	0.46246104	0.278135954	0.854005816	0.552244808	0.812001845	0.984395529
CPQ	0.782857434	0.994088629	0.224957039	0.386179944	0.7231361	0.812197697	0.98448751
LOC112443215	0.635237617	0.971446313	0.520423306	0.953913642	0.159551087	0.812160114	0.98448751
LOC617875	0.56830446	0.854399025	0.455982745	0.569469701	0.388228885	0.812404553	0.984665518
AAK1	0.679205496	0.931423569	0.447489946	0.192463919	0.934872361	0.819085402	0.984681879
ACACB	0.902775124	0.693575965	0.579198957	0.578975298	0.247150589	0.822181278	0.984681879
ADAMTS4	0.537181077	0.916122999	0.46581188	0.318778643	0.710762324	0.822324526	0.984681879
ADGRA3	0.318684017	0.615453253	0.572046785	0.762035268	0.580452355	0.814725932	0.984681879
AGO4	0.928260177	0.943086229	0.551631241	0.189304213	0.53951856	0.813682238	0.984681879
AGTR1	0.614641236	0.223346475	0.751880114	0.924489812	0.535697733	0.81967552	0.984681879
AKR1C4	0.875110599	0.361579095	0.32868213	0.62411156	0.77127861	0.816190696	0.984681879
ALG6	0.948876588	0.843949472	0.757224974	0.683285412	0.126805877	0.824225017	0.984681879
ANXA2	0.416003291	0.775855638	0.573252477	0.630539686	0.425331786	0.814700887	0.984681879
APBB2	0.560595079	0.545570357	0.257332589	0.751872785	0.850318288	0.817041467	0.984681879
AQP7	0.575246212	0.758629781	0.261008814	0.57046528	0.788863612	0.82013493	0.984681879

ASB16	0.329509302	0.536194282	0.585210498	0.643355632	0.73773233	0.812833605	0.984681879
ASRGL1	0.982272788	0.336395938	0.956317162	0.875114205	0.184380185	0.819250632	0.984681879
B3GNT2	0.89247744	0.381333656	0.746773581	0.265987707	0.771352407	0.822975035	0.984681879
BBS1	0.389089473	0.309172691	0.755451824	0.870830411	0.642753411	0.818855765	0.984681879
BLM	0.485702798	0.221128192	0.64316402	0.724167853	0.98499742	0.813516934	0.984681879
BLMH	0.941433401	0.460419268	0.556114238	0.574512268	0.365695473	0.818121809	0.984681879
BRSK1	0.618786928	0.799619891	0.345698644	0.892490407	0.346627882	0.825399618	0.984681879
C10H15orf41	0.506847068	0.5461237	0.573962653	0.809420209	0.402703685	0.821833334	0.984681879
C10H15orf61	0.8037226	0.884690345	0.617109471	0.194583511	0.61419169	0.823912359	0.984681879
C25H7orf26	0.586351814	0.348653241	0.302055173	0.851010069	0.937571034	0.813503866	0.984681879
CAMSAP1	0.749339029	0.522251827	0.379776421	0.614129586	0.563028152	0.820558628	0.984681879
CASC4	0.751469888	0.946222051	0.390764155	0.1941167	0.946020343	0.819373031	0.984681879
CCNC	0.950899372	0.474101412	0.34123111	0.407179281	0.810130174	0.818455953	0.984681879
CD99L2	0.678543461	0.653874363	0.486640404	0.458373777	0.522818185	0.821695642	0.984681879
CDC14B	0.559821029	0.798902375	0.561571238	0.557767147	0.376733763	0.824962191	0.984681879
CDH20	0.285541009	0.706408901	0.550513739	0.5449352	0.837364629	0.818208852	0.984681879
CDO1	0.861213405	0.492800092	0.414527585	0.360661508	0.826306013	0.823876654	0.984681879
CEP290	0.750921104	0.159156807	0.758162257	0.761038017	0.749530436	0.821514889	0.984681879
CEP83	0.780354774	0.644806865	0.457157166	0.278529911	0.78353121	0.816653739	0.984681879
CGN	0.373244502	0.185087679	0.952935024	0.848300068	0.915790824	0.819755531	0.984681879
CLDN12	0.856834276	0.615040228	0.810584132	0.751456319	0.155403352	0.815590469	0.984681879
CLIC1	0.417471883	0.924881956	0.267422288	0.990404336	0.492639972	0.817246758	0.984681879
CLOCK	0.779042525	0.349010254	0.451353167	0.482830903	0.891872996	0.825181552	0.984681879
CLUAP1	0.977974896	0.822017589	0.505532971	0.53889573	0.227030099	0.815042632	0.984681879
CNST	0.713173699	0.538529071	0.17902449	0.946209675	0.775488896	0.817488161	0.984681879
COL18A1	0.964499131	0.679348306	0.15051179	0.823655707	0.610194054	0.814512858	0.984681879
COMTD1	0.818792114	0.43051967	0.715451218	0.202967529	0.996626655	0.819343485	0.984681879
COPS4	0.418582641	0.403952436	0.518108889	0.918587145	0.65297966	0.824247754	0.984681879
CREBZF	0.39977705	0.389018608	0.96047757	0.64840862	0.534347766	0.821732926	0.984681879
CST6	0.813736898	0.970994804	0.33191836	0.289819141	0.685269374	0.82279025	0.984681879
CUL2	0.218698886	0.481717659	0.604007027	0.797902642	0.96783442	0.813059113	0.984681879
DDX42	0.265215011	0.370656855	0.827122996	0.936405842	0.682615039	0.822431871	0.984681879
DICER1	0.888775642	0.763462287	0.64889317	0.235384545	0.493730495	0.819847854	0.984681879
DIS3L	0.153425913	0.384743115	0.971528628	0.982623329	0.920531931	0.822115676	0.984681879
DTX3L	0.648346112	0.665014507	0.735727198	0.381893073	0.42173825	0.819586656	0.984681879
EBF3	0.976387181	0.625597434	0.173250553	0.618405299	0.806171137	0.82490791	0.984681879
EEFSEC	0.924958091	0.776691505	0.823030127	0.348360105	0.239830694	0.813947201	0.984681879
EFHD1	0.750498043	0.56146631	0.181861866	0.812355935	0.802728967	0.815887188	0.984681879
EHBP1L1	0.575964847	0.596046631	0.858755458	0.785059386	0.21647458	0.816321706	0.984681879
ELFN2	0.245143735	0.65116486	0.47816325	0.785515408	0.871477737	0.823315053	0.984681879
ELMO1	0.344177538	0.790857713	0.873383721	0.639569982	0.3464394	0.82464588	0.984681879
ESYT1	0.600136783	0.972604313	0.229044159	0.443143449	0.891672398	0.825121438	0.984681879
EXOSC7	0.778215156	0.144609058	0.984425032	0.492955949	0.924739399	0.817651998	0.984681879
FAM110C	0.381193957	0.176190507	0.899426792	0.940629805	0.921480676	0.823657532	0.984681879

FBXO25	0.275077788	0.977674104	0.451884662	0.968139417	0.435742935	0.820163861	0.984681879
FGF12	0.427950094	0.438611131	0.710046934	0.527133733	0.721991063	0.818385811	0.984681879
FIGNL1	0.916921676	0.321666941	0.178052448	0.951012478	0.996865911	0.815260545	0.984681879
FXYD7	0.18248917	0.975972809	0.433578546	0.695623749	0.95987978	0.821116129	0.984681879
GALK1	0.830745401	0.617447325	0.622682512	0.17492729	0.943873245	0.824837245	0.984681879
GMEB1	0.533880901	0.651346451	0.405563146	0.381408074	0.937582237	0.817424327	0.984681879
GPR182	0.742772308	0.703324318	0.176936301	0.734792537	0.734786457	0.815664541	0.984681879
GRIN2D	0.862603123	0.353451099	0.405249435	0.410829235	0.995865742	0.817813545	0.984681879
GRIP2	0.90776888	0.890255685	0.971130698	0.232375366	0.279301436	0.819084496	0.984681879
GTF3C4	0.832838997	0.221230412	0.591657963	0.571725868	0.79625648	0.814721619	0.984681879
HDDC3	0.594026434	0.847651645	0.515776109	0.390738576	0.522133561	0.825612781	0.984681879
HECTD2	0.87988168	0.590112326	0.488888152	0.475429539	0.435425633	0.824253877	0.984681879
HIVEP2	0.751858355	0.862194564	0.118171431	0.777317946	0.833123552	0.814661384	0.984681879
HPS1	0.978514226	0.933698696	0.171316979	0.386241394	0.831838523	0.816945626	0.984681879
HS3ST4	0.623132786	0.781489783	0.46318657	0.395785643	0.578199871	0.821293506	0.984681879
HSD17B1	0.87306838	0.914720309	0.646117043	0.766919045	0.131528987	0.822675263	0.984681879
IARS2	0.800261761	0.472005013	0.329564094	0.473470216	0.869291472	0.820060865	0.984681879
IDS	0.909322357	0.174266565	0.950433954	0.736976103	0.459748965	0.819390415	0.984681879
IGF2	0.633729083	0.85439051	0.471007762	0.402373353	0.496689139	0.819188533	0.984681879
IGSF1	0.712471044	0.513982553	0.178959949	0.888272351	0.896091323	0.823037531	0.984681879
IKBIP	0.754365075	0.489643476	0.716520071	0.191728556	0.984132156	0.815771219	0.984681879
INTS9	0.258467416	0.949144278	0.913750335	0.682180868	0.321633792	0.813212462	0.984681879
IRAK1BP1	0.822984172	0.378567541	0.647953341	0.298783138	0.874720361	0.824912388	0.984681879
ISL2	0.868930699	0.816969381	0.30852097	0.933132171	0.250221566	0.819741538	0.984681879
KAT8	0.49853274	0.39855885	0.485093298	0.752277148	0.687480082	0.815469366	0.984681879
KCNMB4	0.664995631	0.312519717	0.372204831	0.853194152	0.764926763	0.817590632	0.984681879
KHNYN	0.795554562	0.717079553	0.508230642	0.256812313	0.706446061	0.824415008	0.984681879
KIAA0556	0.695334156	0.364889707	0.339675556	0.959079506	0.62949411	0.82261695	0.984681879
KIAA1841	0.259193385	0.917118205	0.796600034	0.43517369	0.638245629	0.824394896	0.984681879
KIF9	0.269127019	0.919205537	0.799982726	0.313830848	0.793312371	0.813509015	0.984681879
KRT18	0.408123509	0.349980576	0.837879102	0.598925727	0.696857468	0.815811303	0.984681879
KTN1	0.723304841	0.518970041	0.191867148	0.957284398	0.75146015	0.821909662	0.984681879
LETM2	0.259472932	0.383569261	0.69789967	0.770839462	0.925425355	0.814456918	0.984681879
LNP1	0.7957825	0.702523624	0.175944185	0.834483573	0.619271809	0.818738204	0.984681879
LOC100336369	0.734670976	0.409740087	0.66962717	0.459616027	0.53873588	0.815684377	0.984681879
LOC100847941	0.781841821	0.7885742	0.628771489	0.138482895	0.954841301	0.820139398	0.984681879
LOC100848478	0.971946047	0.945564232	0.232378511	0.233407715	0.986784133	0.813227897	0.984681879
LOC100850437	0.639900389	0.647242028	0.222407546	0.879225868	0.652484669	0.825176038	0.984681879
LOC101902124	0.622472551	0.504961366	0.291106056	0.573648565	0.999365896	0.823961907	0.984681879
LOC101904084	0.389829313	0.320630588	0.603017946	0.747030369	0.901921151	0.81857957	0.984681879
LOC101906522	0.958033768	0.580519009	0.135721668	0.760226086	0.914190256	0.8239717	0.984681879
LOC101907641	0.531699524	0.613312252	0.354687273	0.600180015	0.7410547	0.820730403	0.984681879
LOC101907920	0.984408617	0.259311291	0.512295971	0.564279607	0.695135506	0.820254299	0.984681879
LOC101909432	0.36463442	0.981377624	0.785539991	0.20627692	0.854904637	0.814532961	0.984681879

LOC104968820	0.566686666	0.752479499	0.379404311	0.49089382	0.647380848	0.820639341	0.984681879
LOC104972409	0.684473383	0.52144328	0.444206329	0.341626764	0.916462635	0.814759426	0.984681879
LOC104974473	0.788174368	0.284018162	0.643048799	0.932571693	0.393815156	0.825247145	0.984681879
LOC104975788	0.499295317	0.345462795	0.873481911	0.743414954	0.472700634	0.825490796	0.984681879
LOC107132398	0.523993356	0.334147131	0.955299873	0.929002376	0.316844037	0.813382898	0.984681879
LOC107132897	0.558645513	0.687357349	0.899665527	0.278755426	0.523339146	0.817305797	0.984681879
LOC112441455	0.587536496	0.181452244	0.955207865	0.564369003	0.908491351	0.823193298	0.984681879
LOC112441543	0.843489429	0.608490398	0.57757289	0.176753029	0.935230421	0.812590101	0.984681879
LOC112441611	0.971325046	0.328096411	0.755017566	0.548690425	0.386277207	0.819283562	0.984681879
LOC112442048	0.845043406	0.813945162	0.80467761	0.42642859	0.224231911	0.825418697	0.984681879
LOC112443170	0.627724415	0.481931673	0.745351097	0.839110256	0.275712424	0.823045485	0.984681879
LOC112443859	0.516344022	0.700426118	0.576572172	0.304853005	0.827941422	0.824510757	0.984681879
LOC112445985	0.723832569	0.258592472	0.995009929	0.342177047	0.815311671	0.822384498	0.984681879
LOC112446029	0.861940334	0.708485139	0.484363305	0.54352741	0.326938488	0.824291112	0.984681879
LOC112447370	0.561037935	0.481863978	0.595531097	0.823795055	0.398954067	0.825389679	0.984681879
LOC112447506	0.997644282	0.752640792	0.831440222	0.164381008	0.504724139	0.821867296	0.984681879
LOC112447842	0.674351525	0.459957749	0.876926727	0.488423443	0.394758782	0.823922331	0.984681879
LOC112448531	0.442359627	0.757185867	0.629539035	0.301968754	0.82226438	0.823647961	0.984681879
LOC112448753	0.502692869	0.410403959	0.858126458	0.459256676	0.647013304	0.824430664	0.984681879
LOC523461	0.803964551	0.327239454	0.764847259	0.248432283	0.999270284	0.815824612	0.984681879
LOC539009	0.929482208	0.781747565	0.14241492	0.571373946	0.871668018	0.821039971	0.984681879
LOC540014	0.110407813	0.971919686	0.708098585	0.736814456	0.941831058	0.82481801	0.984681879
LOC613570	0.771398783	0.87437326	0.153524364	0.927736138	0.543690512	0.823250511	0.984681879
LOC781421	0.947592496	0.708818691	0.134256982	0.809061676	0.70691217	0.821156995	0.984681879
LOC782385	0.531994805	0.921859312	0.421648288	0.509693509	0.502010631	0.825382552	0.984681879
LOC783294	0.879297703	0.573598347	0.874345973	0.191648778	0.624050411	0.824855108	0.984681879
LOC783533	0.936541848	0.3208835	0.57531787	0.326673163	0.900593297	0.818851634	0.984681879
LOC788142	0.247310117	0.460568956	0.831283751	0.888520182	0.621232166	0.823356191	0.984681879
LOC789715	0.644838997	0.781699044	0.819745807	0.566315866	0.215726396	0.817585299	0.984681879
LRP3	0.57182803	0.736958491	0.414955199	0.345760305	0.867834643	0.824008172	0.984681879
LSM3	0.559464572	0.576805452	0.217096998	0.795971016	0.947174402	0.825094017	0.984681879
MAL2	0.970224114	0.63095947	0.171420706	0.717451318	0.668993285	0.817207328	0.984681879
MAMDC2	0.852296382	0.838452379	0.314260238	0.836009528	0.263836825	0.814406901	0.984681879
MCCC2	0.943131311	0.920780472	0.294365016	0.664939882	0.308605324	0.82396199	0.984681879
METTL15	0.377811414	0.944090928	0.491875202	0.997868827	0.284674779	0.815437214	0.984681879
MIDN	0.24082923	0.788422976	0.415194738	0.749923057	0.884942896	0.823525554	0.984681879
MMD	0.89741952	0.977106232	0.490383553	0.319624178	0.357995584	0.813276048	0.984681879
MMS19	0.953482368	0.384155823	0.248192912	0.846298629	0.646061329	0.814988579	0.984681879
MOB1B	0.713328764	0.355046902	0.56996096	0.433350919	0.821357361	0.820526476	0.984681879
MON2	0.661337134	0.57415763	0.944321435	0.442893698	0.324391782	0.820966775	0.984681879
MRM2	0.76580182	0.832807558	0.554943685	0.192745795	0.726690842	0.814538441	0.984681879
MRPS28	0.634600663	0.702084687	0.919823431	0.254641903	0.507664676	0.825593474	0.984681879
MTMR2	0.40025165	0.573250841	0.730350364	0.580155311	0.533969384	0.822237209	0.984681879
MXD1	0.947526052	0.555043559	0.500388154	0.334340849	0.597286308	0.824264745	0.984681879

MYOF	0.674343189	0.712617786	0.221823261	0.558120389	0.829905731	0.813862571	0.984681879
NCBP2	0.411274745	0.966092883	0.820690226	0.477581369	0.330215859	0.820673074	0.984681879
NFATC2IP	0.4027319	0.292670257	0.85813855	0.545706092	0.92352023	0.819209742	0.984681879
NFS1	0.999532552	0.839316502	0.685292692	0.123259504	0.742416487	0.824443032	0.984681879
NOM1	0.279886945	0.702292883	0.389426927	0.901513408	0.717997159	0.814452121	0.984681879
OMP	0.998551967	0.53822582	0.139494163	0.883495376	0.791261317	0.823815744	0.984681879
OXCT1	0.994363504	0.798715582	0.281792546	0.660485338	0.346026472	0.819778278	0.984681879
P2RX3	0.585040831	0.43612388	0.491420531	0.78138682	0.531860052	0.822863469	0.984681879
PACS1	0.348206295	0.844950356	0.832800051	0.456335996	0.473556857	0.825504143	0.984681879
PCGF2	0.755074588	0.680671313	0.673565051	0.576222179	0.257256795	0.820324027	0.984681879
PDHB	0.707459323	0.949712256	0.781005095	0.192075386	0.499611517	0.817168993	0.984681879
PDZD9	0.881976237	0.920885854	0.286460001	0.559685466	0.384302063	0.816124096	0.984681879
PITPNM1	0.484614304	0.98046884	0.216319832	0.516119547	0.979969594	0.822473452	0.984681879
PKN1	0.724942277	0.519811147	0.299186907	0.61715033	0.751306714	0.823391487	0.984681879
PLPP5	0.494576804	0.963051048	0.934095988	0.179780744	0.625660054	0.816129017	0.984681879
PMF1	0.407283248	0.378455833	0.770777321	0.476130106	0.92033588	0.822711041	0.984681879
PNRC1	0.803009681	0.976827941	0.539662966	0.356455213	0.338345437	0.819466654	0.984681879
PPA2	0.979933272	0.691519647	0.138774402	0.825901158	0.667082938	0.821912366	0.984681879
PPARGC1B	0.585456629	0.655584693	0.175729132	0.774184333	0.96238572	0.816825571	0.984681879
PPP1R12C	0.638627251	0.493844394	0.52105708	0.701153561	0.442944599	0.819412432	0.984681879
PPP2R3B	0.91503515	0.346163959	0.575669832	0.31911118	0.874108271	0.818842315	0.984681879
PPP3CB	0.648616462	0.2670935	0.934483506	0.938315352	0.325254712	0.813976614	0.984681879
PTGER4	0.688271958	0.351455204	0.862917067	0.490346199	0.517684388	0.825618345	0.984681879
PTK2	0.430927923	0.558819967	0.342455807	0.856511556	0.725036236	0.819983221	0.984681879
PUF60	0.732401144	0.639312878	0.677170533	0.975068506	0.166225048	0.820564625	0.984681879
RAD51C	0.118707473	0.707501097	0.921218441	0.700591435	0.945749464	0.820149778	0.984681879
RCAN3	0.376033316	0.66109845	0.521888586	0.782411395	0.511814506	0.822370037	0.984681879
RGL1	0.645696112	0.947521663	0.368452979	0.61243942	0.377732417	0.822990667	0.984681879
RILP	0.950720117	0.534674943	0.677718626	0.536565926	0.265751085	0.813003923	0.984681879
RMI2	0.9527309	0.461436415	0.510627581	0.228793947	0.955810433	0.812892223	0.984681879
RPL17	0.460777775	0.584308694	0.27463931	0.935424071	0.736279384	0.81905254	0.984681879
RSPO1	0.121536974	0.65664554	0.776056327	0.920640818	0.893980913	0.819207394	0.984681879
RTKN	0.768927685	0.465302766	0.500674547	0.667844294	0.41069118	0.813033911	0.984681879
RXFP3	0.601369856	0.276881752	0.376275325	0.863525383	0.957666654	0.821917291	0.984681879
RXRB	0.480578171	0.462435376	0.439428489	0.706670031	0.740030798	0.81952151	0.984681879
SAO	0.873662167	0.888365819	0.766444563	0.180799375	0.479538464	0.82115537	0.984681879
SCFD1	0.983025627	0.488910273	0.836750613	0.507587024	0.25577721	0.823186967	0.984681879
SEC24C	0.934441989	0.470702891	0.971760348	0.862084304	0.140669729	0.821985059	0.984681879
SEPT8	0.926926848	0.237771011	0.461309811	0.595773191	0.811446131	0.813100151	0.984681879
SERPINC1	0.26882061	0.537080748	0.865349031	0.602189929	0.668202699	0.816892804	0.984681879
SESN3	0.496973467	0.697247579	0.347798672	0.979504464	0.444886997	0.824153474	0.984681879
SF3B4	0.71364914	0.163579098	0.740361538	0.664981104	0.866893768	0.815385119	0.984681879
SHC1	0.646234594	0.81001723	0.907688969	0.137177693	0.791266162	0.821151975	0.984681879
SHE	0.191499353	0.605431129	0.605783265	0.839069032	0.8357369	0.813441534	0.984681879

SIGLEC5	0.410911929	0.976975886	0.722088834	0.858617613	0.203327517	0.818003763	0.984681879
SLC29A3	0.589570808	0.296838638	0.629673992	0.689394244	0.652730286	0.814588424	0.984681879
SLC37A2	0.389451313	0.633995608	0.458121253	0.460280391	0.946925807	0.81361301	0.984681879
SMIM10	0.531204702	0.635913187	0.302647713	0.598857218	0.80562595	0.813688924	0.984681879
SNX14	0.291743558	0.568924861	0.828405767	0.628957148	0.603490994	0.823121781	0.984681879
SPTBN1	0.805896009	0.960101855	0.72450053	0.747840067	0.119585282	0.816424527	0.984681879
SPTY2D1	0.867966881	0.267757894	0.435151415	0.512308317	0.98692127	0.819724761	0.984681879
STX3	0.963462172	0.709077395	0.73194787	0.139357299	0.745111641	0.822271465	0.984681879
TEX14	0.508037401	0.83630073	0.786818977	0.644914338	0.245501538	0.825437418	0.984681879
TJP2	0.331179111	0.753387968	0.330946453	0.994567389	0.629377669	0.821517784	0.984681879
TLE1	0.967329151	0.401582308	0.736653317	0.574886618	0.317009831	0.822998336	0.984681879
TMCO4	0.93316552	0.658696226	0.680430759	0.127731083	0.931327661	0.81515116	0.984681879
TMEM86B	0.487713901	0.411360023	0.528365753	0.696137783	0.708497405	0.823412607	0.984681879
TNRC6A	0.696126379	0.60278527	0.889610655	0.823599617	0.160715586	0.81398805	0.984681879
TNRC6C	0.538490093	0.465331968	0.408228208	0.810535924	0.61881759	0.820291424	0.984681879
TOP3A	0.778521802	0.219276232	0.361562141	0.871193773	0.980030194	0.824721072	0.984681879
TOX2	0.585246794	0.780237533	0.367145649	0.932909128	0.335183407	0.823857809	0.984681879
TRAK2	0.927494722	0.349532709	0.860958106	0.28331264	0.650747022	0.820781716	0.984681879
TRIM3	0.729254483	0.627245771	0.847067843	0.522829522	0.249127236	0.817540725	0.984681879
TRIM45	0.529021253	0.41783918	0.887664892	0.535700005	0.501422562	0.824742824	0.984681879
TRNT1	0.84002848	0.625994291	0.551850062	0.26820314	0.649932386	0.817926163	0.984681879
TTLL9	0.29449533	0.683249994	0.543597077	0.519657277	0.927830051	0.824842873	0.984681879
UBTF	0.808892086	0.320359206	0.365533804	0.748047347	0.692378111	0.812785347	0.984681879
UFSP2	0.902195804	0.310403145	0.808561229	0.282612431	0.804461183	0.820849332	0.984681879
USF1	0.727375498	0.420499964	0.886172446	0.300075217	0.650604271	0.825399794	0.984681879
USP36	0.878152048	0.63021451	0.301328109	0.417260593	0.705580558	0.812911292	0.984681879
VPS37D	0.878686288	0.678098233	0.265451945	0.332215948	0.953239975	0.816275356	0.984681879
WISP2	0.724236221	0.887313607	0.143815584	0.88518951	0.640311074	0.823730268	0.984681879
ZBTB25	0.939894325	0.356297951	0.612057918	0.665212734	0.383114337	0.823267438	0.984681879
ZBTB42	0.488629648	0.673957497	0.979641515	0.359294016	0.434250662	0.817099008	0.984681879
ZC3H4	0.369298323	0.885696328	0.372822161	0.607952201	0.703520455	0.823014763	0.984681879
ZNF300	0.548675262	0.756416157	0.582882652	0.302078559	0.679243673	0.814754692	0.984681879
ZNF389	0.658178339	0.789719209	0.256058301	0.453753635	0.874348713	0.825047378	0.984681879
LRSAM1	0.758835746	0.752118985	0.978959333	0.134165599	0.707719794	0.825820676	0.984851618
LDHAL6B	0.557017376	0.302675668	0.865731091	0.412648372	0.881595826	0.825963495	0.984950365
ANAPC4	0.96713253	0.693210073	0.600823699	0.553193463	0.242418042	0.828777418	0.985066755
ANAPC7	0.963260562	0.667383007	0.555199643	0.659833261	0.233677616	0.831804487	0.985066755
BIN3	0.37608873	0.451344859	0.699611921	0.692805444	0.661369291	0.829968146	0.985066755
C18H19orf33	0.119245178	0.780707477	0.980241665	0.657939355	0.903034151	0.82938323	0.985066755
CA10	0.202660904	0.850335901	0.945509387	0.781563923	0.422644015	0.828185134	0.985066755
CA8	0.354284909	0.820523045	0.765773015	0.565397661	0.430445842	0.829256933	0.985066755
CAT	0.536204276	0.846375675	0.147671816	0.813196321	0.987369411	0.828147701	0.985066755
CCDC88A	0.733777149	0.892947075	0.850537829	0.70623255	0.140010084	0.832017677	0.985066755
CDC42BPB	0.951558616	0.431063855	0.633960579	0.617504091	0.337226155	0.829175986	0.985066755

CSAD	0.502576691	0.724700016	0.956905216	0.190164008	0.805706461	0.826891653	0.985066755
DEAF1	0.865191134	0.209804559	0.920040413	0.835793627	0.392437575	0.831051194	0.985066755
DLEC1	0.95447491	0.58809443	0.263527527	0.824613194	0.447750355	0.830572301	0.985066755
DSP	0.646035922	0.429731796	0.244795873	0.888578968	0.907575285	0.831138483	0.985066755
EFCAB5	0.713878721	0.163112768	0.678574945	0.928205557	0.731938023	0.827756931	0.985066755
ENOX2	0.203909287	0.743391657	0.97629932	0.835188044	0.436459908	0.828561718	0.985066755
ERC2	0.096237706	0.968091007	0.904748875	0.95989471	0.672168933	0.829885724	0.985066755
EXOSC6	0.452026343	0.713376976	0.97906843	0.381156087	0.450316709	0.829294781	0.985066755
FAM89A	0.806287386	0.416436637	0.369297111	0.68777233	0.627074876	0.827134773	0.985066755
FERMT3	0.258406219	0.574268858	0.751894738	0.736860719	0.668395999	0.831571118	0.985066755
FLT1	0.932810678	0.227135694	0.551869146	0.638722513	0.713692694	0.826590932	0.985066755
FLT3LG	0.575525137	0.24075713	0.597354346	0.673541304	0.986633061	0.831720834	0.985066755
FRK	0.778373422	0.773877403	0.230930897	0.801714638	0.485640215	0.829204595	0.985066755
GATM	0.283354682	0.873645768	0.72356244	0.961524255	0.312701488	0.828285418	0.985066755
GNAQ	0.639443014	0.527414798	0.743046813	0.271789148	0.799994581	0.830184866	0.985066755
HEXIM2	0.599640981	0.963012454	0.228376818	0.698876573	0.589062082	0.82960259	0.985066755
HIST2H2AA4	0.954251433	0.254489439	0.292077315	0.803681106	0.947747238	0.828802535	0.985066755
ICK	0.838348949	0.81654439	0.294189454	0.662957199	0.410108159	0.830980913	0.985066755
IKBKG	0.694078365	0.969240391	0.676839807	0.227523986	0.529479704	0.831275121	0.985066755
LMBRD2	0.567595582	0.75136508	0.377618381	0.701756782	0.486167358	0.83154162	0.985066755
LOC100335190	0.653877498	0.332754053	0.890228586	0.348895238	0.804013275	0.829731196	0.985066755
LOC100337390	0.370718401	0.846930206	0.463229353	0.93096117	0.403525505	0.830634676	0.985066755
LOC100848469	0.595603306	0.544423833	0.416027331	0.509553985	0.785431379	0.828692688	0.985066755
LOC100848472	0.532708573	0.460050764	0.487333323	0.837399602	0.538858068	0.828397082	0.985066755
LOC101904492	0.986119333	0.789264242	0.141884478	0.488671543	0.999285676	0.828496715	0.985066755
LOC101906451	0.333944636	0.875491193	0.770094001	0.449204933	0.538484855	0.830109351	0.985066755
LOC104968671	0.708739558	0.528655395	0.761074251	0.387264682	0.496837259	0.831314747	0.985066755
LOC104972724	0.972639363	0.464317288	0.692701154	0.818723109	0.213693799	0.830915802	0.985066755
LOC107132577	0.203306894	0.940167136	0.452288194	0.807568386	0.764135386	0.826735822	0.985066755
LOC112441638	0.609819486	0.881541148	0.355426868	0.668969287	0.42418945	0.829386166	0.985066755
LOC112443419	0.371756401	0.731837295	0.596252651	0.360347683	0.933200876	0.83037621	0.985066755
LOC112444190	0.468351923	0.654508391	0.876574105	0.306360229	0.656393717	0.828827446	0.985066755
LOC112445938	0.318669395	0.619473318	0.893300173	0.928695317	0.335628945	0.83160792	0.985066755
LOC112445965	0.853664948	0.89218536	0.294032553	0.603230186	0.397387968	0.827759587	0.985066755
LOC112447438	0.988621403	0.633153382	0.116536711	0.795566227	0.948439598	0.831829984	0.985066755
LOC112448057	0.451591608	0.701687611	0.689727468	0.404686241	0.622141588	0.831787853	0.985066755
LOC112448304	0.929040571	0.231254037	0.804436576	0.314451137	0.987379491	0.827690754	0.985066755
LOC112448364	0.977471732	0.595880907	0.972986769	0.120481083	0.785975222	0.827707806	0.985066755
LOC534520	0.823503362	0.310047457	0.537309698	0.750501311	0.520499419	0.82747846	0.985066755
LOC616051	0.943887414	0.476576354	0.436216024	0.507180722	0.544342794	0.82924698	0.985066755
LOC616957	0.743479988	0.827997414	0.113441694	0.998490128	0.762322265	0.82614295	0.985066755
LOC617648	0.290499488	0.760975396	0.418289048	0.641589203	0.920676881	0.830584647	0.985066755
LOC618409	0.561922802	0.91110333	0.4102349	0.520147503	0.502268041	0.831326161	0.985066755
LOC619156	0.69274481	0.376259284	0.40099687	0.95492955	0.548362212	0.83091534	0.985066755

LOC783376	0.9336533	0.217782458	0.384602814	0.898839431	0.770844145	0.829277086	0.985066755
LOC786435	0.224796792	0.969660391	0.505050019	0.638301273	0.760303599	0.826975542	0.985066755
LOC786930	0.781100548	0.751405716	0.87848447	0.278487742	0.383876596	0.832063448	0.985066755
LOC787714	0.469198114	0.23959444	0.772773608	0.739147404	0.85114947	0.830684004	0.985066755
LOC789733	0.230995984	0.832573275	0.85690708	0.781412523	0.42346984	0.830324997	0.985066755
MAP4	0.560521372	0.764546022	0.694055839	0.238630431	0.7511137	0.826622382	0.985066755
NAGS	0.179711682	0.949220813	0.67295087	0.979467216	0.482047953	0.829328676	0.985066755
NFAM1	0.736477776	0.457556478	0.546080818	0.514243953	0.564500082	0.826951973	0.985066755
NXN	0.194346387	0.959526855	0.376096289	0.946499835	0.815528938	0.829135445	0.985066755
PCK2	0.520724949	0.944923532	0.413488756	0.302205106	0.888950219	0.830693673	0.985066755
PEX26	0.587485902	0.982404901	0.353316204	0.367393911	0.725278266	0.829734422	0.985066755
PLCG1	0.563334714	0.737932102	0.868250632	0.457956518	0.3261309	0.828440552	0.985066755
PLEKHD1	0.695418793	0.868369548	0.626686555	0.203735583	0.689811032	0.826235619	0.985066755
PPM1N	0.403151475	0.556550518	0.93536193	0.548404049	0.474503009	0.830560457	0.985066755
PRDM11	0.99984644	0.907565175	0.178775914	0.496388056	0.662188049	0.826660561	0.985066755
PRR18	0.386309636	0.740560038	0.269416179	0.912986551	0.778495647	0.831064856	0.985066755
PTPN1	0.633986028	0.882539138	0.775721603	0.435883138	0.291356006	0.832063577	0.985066755
RAD17	0.549357685	0.640363399	0.217012488	0.876392303	0.813294101	0.829968623	0.985066755
RAMP2	0.809215211	0.692903859	0.549329636	0.429968341	0.403844979	0.827150777	0.985066755
RHEX	0.809839448	0.411065026	0.859778766	0.693846418	0.272278068	0.828939914	0.985066755
RNF141	0.938045311	0.33380791	0.337069564	0.513331328	0.994831487	0.828419124	0.985066755
RNF217	0.587349373	0.735502978	0.880008219	0.399398625	0.362924504	0.832017657	0.985066755
RXRG	0.649179818	0.53328284	0.297607633	0.567790742	0.927477125	0.829498039	0.985066755
SCG5	0.490631897	0.571758498	0.598311307	0.706228221	0.459913747	0.830270146	0.985066755
SHISAL1	0.137295958	0.956906443	0.999523426	0.779491095	0.531771503	0.830022543	0.985066755
SLC39A13	0.873456078	0.228245301	0.807206681	0.407703178	0.832176757	0.830521209	0.985066755
SMOX	0.582227347	0.973912237	0.736748376	0.132857107	0.96585553	0.827530733	0.985066755
STAT4	0.566573448	0.865575502	0.990159518	0.111042719	0.993731924	0.827454227	0.985066755
SV2A	0.512203829	0.695966929	0.398746826	0.554199604	0.679389084	0.827261031	0.985066755
TAF6	0.880043567	0.195467693	0.87443715	0.486896725	0.736395062	0.828520031	0.985066755
TBC1D7	0.777328982	0.878930556	0.176213597	0.464064156	0.961141459	0.827807984	0.985066755
TM6SF1	0.705762908	0.25517951	0.385453	0.830613513	0.94272749	0.829799347	0.985066755
TM7SF3	0.479922957	0.511096911	0.374985399	0.699554501	0.829759824	0.826864616	0.985066755
TMEM179B	0.888163184	0.282202721	0.781005852	0.421408105	0.665228332	0.831342202	0.985066755
TPCN2	0.795654936	0.350254356	0.546903698	0.718663629	0.501725011	0.831577119	0.985066755
TRAFD1	0.906697982	0.90866504	0.083055223	0.900113112	0.887134362	0.830645725	0.985066755
TRMT11	0.925314389	0.365959276	0.663176558	0.370927239	0.646295056	0.828224751	0.985066755
TSEN2	0.560381295	0.960545527	0.566311368	0.270775083	0.64715428	0.826944011	0.985066755
TUSC3	0.894474208	0.989139403	0.149267821	0.956116452	0.436180597	0.831934853	0.985066755
UBE2S	0.566360711	0.344009641	0.471650295	0.638345015	0.938553431	0.831871273	0.985066755
YES1	0.599500099	0.568620244	0.331283693	0.783819132	0.617116178	0.830598748	0.985066755
YPEL2	0.264280454	0.635509366	0.884887781	0.506412124	0.710882855	0.827209682	0.985066755
ZBTB4	0.537997578	0.850871151	0.667669799	0.842021379	0.212365111	0.830680251	0.985066755
ZEB2	0.697404804	0.769689115	0.37618916	0.580006948	0.460271195	0.828444062	0.985066755

ZNF496	0.464305135	0.51952792	0.470725667	0.751778132	0.638733086	0.830297229	0.985066755
ZNF557	0.56419942	0.835571792	0.724473606	0.637025214	0.247394671	0.828192804	0.985066755
ZNF598	0.686394498	0.222350938	0.702306169	0.501796069	0.996155604	0.827442165	0.985066755
ELMO2	0.714062986	0.645822544	0.468424638	0.988304967	0.258468723	0.832240712	0.985082458
NOL8	0.487136557	0.312517087	0.979253137	0.709547949	0.521948888	0.832330498	0.985082458
RAB42	0.42886109	0.943638269	0.229335186	0.714184798	0.833202063	0.832376969	0.985082458
THUMPD2	0.399107986	0.217195097	0.74684838	0.890034892	0.958019314	0.832302526	0.985082458
ZNF408	0.881144927	0.483000117	0.891866343	0.886429992	0.163907628	0.832147286	0.985082458
KCNK6	0.611573161	0.680729562	0.632021811	0.991282021	0.21190552	0.832504007	0.985148885
NIT2	0.726868444	0.560787146	0.483298927	0.295750074	0.948980348	0.832562591	0.985148885
RC3H2	0.478550741	0.35341306	0.83452431	0.673858069	0.581526536	0.832613188	0.985148885
ALDH2	0.251953824	0.909335919	0.302852685	0.848479346	0.953493287	0.835009166	0.985152527
ANKH	0.960183601	0.300928104	0.333199745	0.847878556	0.689755487	0.835496056	0.985152527
ANKRD52	0.84865805	0.564173432	0.612120257	0.194394074	0.993601819	0.836356327	0.985152527
APCDD1L	0.79597793	0.765018401	0.198344825	0.755395552	0.620853075	0.836459542	0.985152527
APH1A	0.683217893	0.755633583	0.362372413	0.562063166	0.534748946	0.835277713	0.985152527
C14H8orf76	0.347427932	0.358802604	0.600116399	0.949656903	0.796263759	0.836246251	0.985152527
C21H14orf132	0.461480472	0.702930587	0.821898138	0.852619253	0.245929794	0.83434705	0.985152527
CA2	0.961870058	0.423280983	0.744222276	0.202093138	0.910492118	0.833910972	0.985152527
CAMK2D	0.678964088	0.752346532	0.726656224	0.26320757	0.581237888	0.836861403	0.985152527
CCND2	0.868295828	0.553617549	0.559169411	0.252822964	0.824351063	0.834680778	0.985152527
CCS	0.242688406	0.526454288	0.882080468	0.6106085	0.823997084	0.836625424	0.985152527
CLIP1	0.492451572	0.828025412	0.943647826	0.582139948	0.247115511	0.832746198	0.985152527
DCLRE1B	0.762338999	0.749465439	0.222867384	0.626348342	0.697566116	0.83356608	0.985152527
DGKH	0.353283384	0.797529831	0.901031102	0.840616306	0.264316251	0.835785021	0.985152527
DIO2	0.734945925	0.927178602	0.512304929	0.304732142	0.522246344	0.833340493	0.985152527
DNPH1	0.384786438	0.677184735	0.7013382	0.374644945	0.830596523	0.837089424	0.985152527
EEF2K	0.550581109	0.280731693	0.597537721	0.973823771	0.624003742	0.834975819	0.985152527
EGLN3	0.801652242	0.313147619	0.46369288	0.536478301	0.898326409	0.834904015	0.985152527
ERGIC3	0.447833895	0.740332255	0.750673663	0.390840977	0.583497558	0.836783173	0.985152527
FAM184B	0.517924236	0.248945128	0.878619411	0.689906778	0.728332061	0.837246471	0.985152527
FAT4	0.534208039	0.964944615	0.335380049	0.868772913	0.370526459	0.833613651	0.985152527
FGR	0.81813395	0.717399797	0.721160933	0.622564455	0.212502317	0.834612602	0.985152527
FRMPD1	0.750209328	0.949354017	0.899692789	0.090060661	0.969149938	0.834414194	0.985152527
G2E3	0.475887732	0.847325538	0.903291856	0.159536056	0.952354161	0.832707338	0.985152527
HOXA4	0.552997626	0.663575383	0.539984855	0.471697675	0.594742073	0.833432188	0.985152527
IPMK	0.880243891	0.476334005	0.495266099	0.590770119	0.461109973	0.836245051	0.985152527
ITM2B	0.899264174	0.867600362	0.47557735	0.362793062	0.415945439	0.834596904	0.985152527
JADE1	0.754947565	0.304103458	0.616630739	0.411688352	0.974429085	0.836874725	0.985152527
JMJD8	0.393891549	0.623179354	0.856497943	0.441305826	0.612103013	0.836874019	0.985152527
KAZALD1	0.936465614	0.131248557	0.596196558	0.774786866	0.992043716	0.835547266	0.985152527
KCTD18	0.509581195	0.610659169	0.708829934	0.335027136	0.761995412	0.835509436	0.985152527
KDM5A	0.259526604	0.980740923	0.514556796	0.525833398	0.805212593	0.833038036	0.985152527
KLHL20	0.375501728	0.640078703	0.592655136	0.472457336	0.82579204	0.833392446	0.985152527

KLRK1	0.144354375	0.78513512	0.748124989	0.686792126	0.973963442	0.836666039	0.985152527
LNPEP	0.938934362	0.853711279	0.250673923	0.568404939	0.4875056	0.833694438	0.985152527
LOC101905687	0.592486657	0.935275435	0.453552228	0.437165267	0.507792353	0.83402301	0.985152527
LOC104973431	0.896151275	0.658742392	0.270374974	0.509320962	0.691125471	0.835148544	0.985152527
LOC107131357	0.265971638	0.767880598	0.392497991	0.966337671	0.718311588	0.833588636	0.985152527
LOC107132556	0.815872273	0.635660608	0.582588503	0.726421345	0.257268559	0.835952546	0.985152527
LOC112442715	0.952910486	0.984351465	0.813868079	0.088839728	0.820041142	0.833510901	0.985152527
LOC112444194	0.76536569	0.854567651	0.851122794	0.179959504	0.566708305	0.836823044	0.985152527
LOC112448155	0.85991691	0.993866411	0.255148984	0.554894303	0.466448017	0.835881256	0.985152527
LOC614522	0.141390797	0.757937465	0.671689803	0.786305504	0.996129289	0.835710567	0.985152527
LOC785605	0.511086685	0.645622108	0.243507261	0.96191368	0.725503485	0.834833074	0.985152527
MAP3K21	0.507177379	0.718435903	0.486608763	0.865827324	0.370813089	0.837255193	0.985152527
MCAM	0.802960064	0.597483797	0.326723686	0.93563957	0.379995843	0.833841326	0.985152527
MED14	0.590906862	0.441902934	0.882072212	0.266318778	0.920350655	0.835923149	0.985152527
MINDY4	0.598077875	0.529067577	0.189400263	0.964725551	0.983235189	0.837032746	0.985152527
MTMR3	0.602838963	0.630611829	0.492823064	0.84785543	0.349308852	0.83313393	0.985152527
NCF2	0.316396319	0.683737379	0.621002227	0.489521429	0.865857422	0.837298608	0.985152527
NDRG2	0.639711017	0.62643393	0.242701829	0.819653628	0.706900783	0.835633136	0.985152527
OCIAD2	0.948475853	0.611210232	0.969035863	0.571928697	0.174683177	0.834977328	0.985152527
OSCP1	0.905432231	0.505585805	0.404116909	0.34259616	0.885508428	0.834971147	0.985152527
PCNT	0.82676055	0.590550765	0.870692004	0.744543161	0.176781824	0.834487667	0.985152527
PHLDA1	0.908936195	0.387606085	0.274691422	0.673586977	0.872262997	0.837069449	0.985152527
POGK	0.626497618	0.723594914	0.248719435	0.842684562	0.583494074	0.833000031	0.985152527
PRKN	0.50862876	0.465125593	0.434636791	0.572058592	0.94873156	0.834061738	0.985152527
PRKRA	0.858137043	0.847440925	0.206474163	0.461436592	0.811671204	0.835301148	0.985152527
PURA	0.833592784	0.815197103	0.25247806	0.730315244	0.453592672	0.836998072	0.985152527
RAB38	0.168794625	0.625239927	0.698794036	0.829549066	0.924464373	0.836211996	0.985152527
RN18S1	0.281265735	0.857295141	0.983549853	0.768633177	0.306228715	0.834108939	0.985152527
RTL9	0.808232431	0.988524042	0.526968192	0.448086993	0.299296469	0.835947795	0.985152527
SMPDL3A	0.489735376	0.677376545	0.545543115	0.931903468	0.334035715	0.835582657	0.985152527
SPEF1	0.819581068	0.578254118	0.819644664	0.692906499	0.210350061	0.836384404	0.985152527
STIM2	0.330692068	0.534062324	0.648781263	0.617044005	0.786088881	0.833400313	0.985152527
TAF9	0.900641587	0.754216209	0.448488189	0.997644893	0.183875664	0.834291031	0.985152527
TARS2	0.812550804	0.600740521	0.891693629	0.133109991	0.964029779	0.83420013	0.985152527
TBX21	0.530939341	0.922465342	0.768702099	0.901290173	0.167368669	0.8368783	0.985152527
TMEM44	0.759074508	0.427690484	0.505245668	0.749093945	0.463257553	0.837240902	0.985152527
TMX1	0.91905411	0.996576083	0.616066206	0.157079014	0.628206986	0.833697039	0.985152527
TRNAU1AP	0.597787516	0.820342954	0.708730559	0.989051988	0.164833993	0.836508389	0.985152527
TRPC3	0.377602223	0.873781898	0.443133229	0.440216186	0.86767982	0.834179039	0.985152527
UACA	0.750692869	0.780557837	0.800123972	0.262262737	0.452687823	0.833645076	0.985152527
WDR5B	0.580669012	0.727310584	0.321221378	0.482523109	0.849497061	0.833486293	0.985152527
ZBTB8A	0.260560805	0.845792668	0.604158661	0.844531253	0.503900893	0.836506732	0.985152527
ZNF319	0.333236528	0.803482949	0.537861282	0.810595439	0.484428063	0.836192132	0.985152527
ZNF473	0.684084654	0.2470827	0.921520409	0.480181022	0.74329437	0.833445379	0.985152527

ZNF597	0.763534008	0.599777261	0.311082118	0.481861594	0.819818821	0.835415785	0.985152527
ADAL	0.724665448	0.82847551	0.527472789	0.241779309	0.749644568	0.838571352	0.985317629
ALG2	0.974187281	0.337930592	0.989920281	0.892492205	0.197184589	0.838444902	0.985317629
ANKRD28	0.860622318	0.429931506	0.399673641	0.612487364	0.637626333	0.839557722	0.985317629
APC	0.552272387	0.704392291	0.184501494	0.980866027	0.819017958	0.839297576	0.985317629
AUNIP	0.81529606	0.842795252	0.832009666	0.146942025	0.688018433	0.839679019	0.985317629
C23H6orf226	0.796517291	0.523821816	0.786817488	0.361401145	0.48720951	0.839695934	0.985317629
CCDC121	0.736116454	0.849032209	0.433922098	0.269148771	0.791400106	0.839590768	0.985317629
CFAP70	0.883332286	0.554468276	0.585462706	0.23064871	0.87429361	0.839750978	0.985317629
DET1	0.812780066	0.789383107	0.484320292	0.223912743	0.831526141	0.839838808	0.985317629
DHX8	0.707601901	0.810714081	0.781001593	0.159845979	0.80655793	0.839581589	0.985317629
EIF4ENIF1	0.879005818	0.746840681	0.959160343	0.152963461	0.597266248	0.83892921	0.985317629
FAM187A	0.43303169	0.651338462	0.821245656	0.379079641	0.656416511	0.839238371	0.985317629
GFOD2	0.965211085	0.661509955	0.473197963	0.212290074	0.889359506	0.83758464	0.985317629
IL20RB	0.94314737	0.380674983	0.49624444	0.386080998	0.831457291	0.838003906	0.985317629
IWS1	0.793877148	0.677762834	0.645217266	0.38916571	0.426165394	0.839069791	0.985317629
LOC101907005	0.792618084	0.530713856	0.53072733	0.403349294	0.639049709	0.838982485	0.985317629
LOC104973224	0.626837786	0.924179702	0.362693625	0.651083978	0.422926057	0.839840534	0.985317629
LOC107132288	0.310265205	0.653750684	0.556705031	0.730501083	0.695566719	0.838511927	0.985317629
LOC112443512	0.473552527	0.736446391	0.977815204	0.316855732	0.530762406	0.838437634	0.985317629
LOC112448032	0.505576336	0.689456806	0.312879092	0.620611068	0.845772247	0.838149297	0.985317629
LOC112449282	0.787215875	0.602421669	0.855689972	0.147578081	0.962585414	0.839261309	0.985317629
NLGN2	0.715912275	0.509638198	0.344734927	0.599842931	0.764039484	0.839257058	0.985317629
NRAP	0.61030576	0.902215941	0.320128584	0.361550816	0.898029893	0.838112259	0.985317629
NSUN4	0.360683514	0.466038569	0.613247035	0.714236431	0.779797643	0.838613846	0.985317629
PEA15	0.912237725	0.401285083	0.674831475	0.879038274	0.266270568	0.839743268	0.985317629
PEX7	0.827880877	0.542468869	0.937650956	0.290287263	0.471697183	0.839299119	0.985317629
PLEKHG7	0.814437443	0.353019971	0.219471333	0.936200793	0.969856927	0.838284647	0.985317629
RAB15	0.560924194	0.923739715	0.671734156	0.809033347	0.205433877	0.839817373	0.985317629
SLC9A3R2	0.725965304	0.634438921	0.925864427	0.234999444	0.570770966	0.838016362	0.985317629
SRSF10	0.662332577	0.372926433	0.514574145	0.962547753	0.467265242	0.837924852	0.985317629
ST3GAL2	0.871809201	0.494017875	0.920951021	0.195839079	0.737410084	0.838247005	0.985317629
TANK	0.451852695	0.881999677	0.215623511	0.717788701	0.926902192	0.837946532	0.985317629
TCF25	0.982945682	0.791024674	0.303819826	0.350632875	0.689830189	0.837850345	0.985317629
TMEM255A	0.872527818	0.777643441	0.833282637	0.924423857	0.109816662	0.838571239	0.985317629
TNFSF14	0.815097489	0.523217437	0.310842096	0.72057965	0.603820906	0.839352858	0.985317629
TP53RK	0.778684062	0.554824072	0.590133571	0.531510644	0.423798903	0.838662014	0.985317629
ZCCHC4	0.329918772	0.638820192	0.898197014	0.857317266	0.351841949	0.837745823	0.985317629
ZFHX2	0.397672001	0.490831827	0.440225166	0.752094272	0.883114595	0.837663184	0.985317629
ZMIZ2	0.934095026	0.41573484	0.4025713	0.392304006	0.936214114	0.838628789	0.985317629
ZNF239	0.502566689	0.617392335	0.865019737	0.244006111	0.879277959	0.839091089	0.985317629
BCAR1	0.328822152	0.9358815	0.840167899	0.248454461	0.90578253	0.840742237	0.985318921
CCDC127	0.789457274	0.357355415	0.537512618	0.469761709	0.816333302	0.840648732	0.985318921
CYSRT1	0.624660479	0.733364836	0.322457544	0.657110139	0.596904612	0.840069938	0.985318921

EDEM3	0.388063636	0.569960763	0.951390612	0.552057801	0.500415673	0.840597372	0.985318921
IGFBP3	0.445961649	0.98771949	0.712624826	0.325382299	0.568733951	0.840477846	0.985318921
KCTD9	0.994752438	0.19773039	0.44255497	0.974359152	0.684445502	0.840374917	0.985318921
LOC101905648	0.788913323	0.485438159	0.950088462	0.4004987	0.399038614	0.840641604	0.985318921
LOC104969177	0.66329699	0.977088601	0.363598463	0.776539623	0.316845333	0.840177932	0.985318921
LOC104970173	0.961490938	0.266321423	0.516993154	0.647496261	0.678514373	0.840674166	0.985318921
LOC614922	0.778553986	0.345835163	0.730742641	0.331590455	0.889860466	0.84038686	0.985318921
RTCA	0.823206449	0.669983142	0.339552582	0.485314533	0.640046797	0.840704229	0.985318921
THAP1	0.574830227	0.471683179	0.433119618	0.62697248	0.789948904	0.840678883	0.985318921
TM6SF2	0.662724525	0.888806921	0.876425453	0.131472393	0.854608963	0.840244689	0.985318921
TMEM184B	0.886133915	0.939620767	0.327248825	0.265168825	0.802869942	0.840259413	0.985318921
ZBTB39	0.793445302	0.995490219	0.572864057	0.638566863	0.200777866	0.840270404	0.985318921
SFXN4	0.65117911	0.527042417	0.538665416	0.50947758	0.618834828	0.841014702	0.985427122
TMEM183A	0.470632315	0.976182757	0.57651076	0.818167313	0.268968644	0.841014382	0.985427122
TMEM98	0.901085563	0.69437447	0.415672552	0.2986059	0.750371352	0.840985794	0.985427122
DLG3	0.63703547	0.686717216	0.225454145	0.915152567	0.646741374	0.841255159	0.985454301
FAM229B	0.902498901	0.892512498	0.092995447	0.848283485	0.917839972	0.841111675	0.985454301
LOC107132610	0.962683209	0.809218205	0.45592301	0.411767487	0.399203355	0.841278092	0.985454301
MTF2	0.567385352	0.85363845	0.300426629	0.698008603	0.574450703	0.841174947	0.985454301
LOC112447846	0.69691731	0.483060947	0.324401275	0.839942107	0.63794522	0.841646942	0.985675299
RNF138	0.545633543	0.799216158	0.841578406	0.182340909	0.874132059	0.841582286	0.985675299
VPS4B	0.915205915	0.651175507	0.158512661	0.849821984	0.728729032	0.841601895	0.985675299
SHF	0.325378258	0.74636266	0.613556928	0.596508212	0.658702622	0.841720053	0.98569058
LOC112446360	0.602726667	0.687129486	0.595885424	0.385705282	0.615553004	0.84184525	0.985705798
LOC782755	0.818049406	0.591979269	0.422831862	0.368148749	0.777297888	0.841853176	0.985705798
AIFM3	0.729543896	0.677248427	0.997017257	0.252802077	0.471941242	0.8423286	0.985827525
APOM	0.900124084	0.868938058	0.194158158	0.519651065	0.745206224	0.842424792	0.985827525
BBS10	0.753103845	0.824776259	0.827340285	0.366467069	0.311582545	0.842078457	0.985827525
C7H5orf15	0.74794664	0.304884233	0.427016547	0.698096157	0.863305428	0.84209547	0.985827525
HOXB2	0.66702599	0.723125525	0.842181623	0.23954575	0.604450124	0.842452435	0.985827525
LOC112442625	0.399787569	0.517658929	0.543292148	0.724313369	0.721192548	0.842224358	0.985827525
LOC112446696	0.97949622	0.800931888	0.733117404	0.19186128	0.533183414	0.842497778	0.985827525
SGSM2	0.728966882	0.420432668	0.451518458	0.721059612	0.589539859	0.842471691	0.985827525
TRIM31	0.134847846	0.755142315	0.831650783	0.852641823	0.813381307	0.842220895	0.985827525
TC2N	0.304628128	0.73285059	0.975546793	0.362202944	0.746185398	0.842569194	0.985840799
LOC100196898	0.338766817	0.771588989	0.695539128	0.906307028	0.357368998	0.842630411	0.985842138
GSC2	0.896221988	0.83328753	0.973818032	0.942989819	0.086029331	0.84293604	0.98612941
AHCYL1	0.939136757	0.751307302	0.189189663	0.471220262	0.944138866	0.843974031	0.986338052
CACTIN	0.937107217	0.296647756	0.379737257	0.645442724	0.86911002	0.843518228	0.986338052
CKS1B	0.92091001	0.334255201	0.422890113	0.64955764	0.699975713	0.843437547	0.986338052
EEF1AKMT1	0.477976682	0.217910678	0.952380411	0.94693808	0.631653911	0.843826499	0.986338052
LOC101903165	0.910045072	0.225298279	0.982529511	0.322517647	0.913942846	0.843950565	0.986338052
LOC101905239	0.444393927	0.767228524	0.302534771	0.916212791	0.625230852	0.843176786	0.986338052
LOC104972578	0.090923251	0.844563523	0.91010189	0.859689654	0.987559629	0.843828627	0.986338052

LOC104973551	0.499155994	0.964635735	0.625772041	0.699591517	0.281842454	0.844033085	0.986338052
NAF1	0.313450828	0.646106335	0.345278984	0.959577426	0.885649173	0.844076023	0.986338052
NUP62CL	0.584172704	0.370153664	0.3535583	0.837188759	0.927895664	0.84397519	0.986338052
ORC6	0.866910607	0.914084275	0.759342833	0.914002687	0.10782194	0.843738301	0.986338052
PLCD1	0.625207353	0.37034142	0.56902201	0.553287944	0.813994656	0.843838001	0.986338052
POLR3E	0.908816626	0.860277104	0.364514509	0.539061317	0.386002896	0.843740154	0.986338052
RPL23	0.614796461	0.641997558	0.190580393	0.835459459	0.942470651	0.843550916	0.986338052
TEN1	0.860468355	0.750205602	0.379965709	0.244079688	0.990408136	0.843721043	0.986338052
ZHX1	0.709743531	0.673752946	0.68556117	0.731515372	0.246944674	0.843527448	0.986338052
LOC104972542	0.969875603	0.856662882	0.156382322	0.981073024	0.466498137	0.844177247	0.986341401
LOC112447735	0.549105006	0.158083701	0.929463577	0.804309362	0.916644344	0.844225742	0.986341401
TTC7B	0.929838783	0.795894883	0.535215437	0.307592267	0.488341773	0.844259196	0.986341401
BCL2L11	0.978900002	0.808509315	0.968955989	0.126994314	0.614218959	0.845106462	0.986378216
CD24	0.863881223	0.910034126	0.354773	0.855950032	0.25036513	0.844979916	0.986378216
CDK11B	0.187110381	0.869268646	0.916693115	0.69935195	0.573505724	0.845061145	0.986378216
GPC6	0.392563333	0.860810809	0.676572013	0.440570277	0.592188394	0.844662629	0.986378216
LOC101902360	0.54418666	0.486518828	0.45803994	0.699812019	0.701920071	0.844450486	0.986378216
LOC112441827	0.489320833	0.918987563	0.338388738	0.544392674	0.722505336	0.845192278	0.986378216
LOC617698	0.507157368	0.756373358	0.986229698	0.217086846	0.72514379	0.844412402	0.986378216
LSM7	0.367701966	0.873546578	0.242143772	0.803739145	0.953255497	0.844508292	0.986378216
MTFMT	0.694053126	0.635914772	0.343353832	0.408949418	0.964712221	0.845021952	0.986378216
PAQR4	0.317301035	0.599974854	0.497531929	0.951402855	0.662165534	0.844716841	0.986378216
SDSL	0.532197518	0.911383589	0.688467487	0.395816566	0.451533123	0.844747403	0.986378216
SLC37A1	0.451638977	0.670965403	0.230179649	0.946872878	0.904538296	0.844904979	0.986378216
SPC25	0.600362281	0.715004142	0.913339125	0.66859302	0.228272347	0.845154942	0.986378216
SPRY1	0.375377214	0.50326148	0.484809467	0.685901655	0.952696803	0.845183857	0.986378216
ST6GALNAC6	0.91871568	0.62562103	0.196107845	0.908091955	0.583517036	0.84486657	0.986378216
CHST1	0.47441912	0.415318101	0.729456562	0.596790714	0.698830614	0.845431883	0.986453393
KIAA0895	0.811441421	0.982595231	0.568072346	0.77861935	0.169927603	0.845391522	0.986453393
MIS12	0.889310084	0.441258865	0.285574049	0.733649628	0.729115952	0.845437022	0.986453393
XPC	0.675398979	0.314550712	0.805808875	0.982864653	0.356546233	0.845560388	0.986527195
C17H5orf52	0.721194946	0.920616434	0.482022991	0.674980118	0.278244614	0.845857539	0.986733585
LOC104972843	0.84151734	0.30820434	0.57077513	0.662562692	0.612601026	0.845805157	0.986733585
CNN3	0.890607203	0.878510344	0.941292469	0.204733366	0.398813788	0.845929449	0.986747331
EXD2	0.20363803	0.496824178	0.941498936	0.665220473	0.951341269	0.846313679	0.986775468
GBP6	0.544120461	0.968612612	0.73541529	0.395882809	0.392465818	0.846156326	0.986775468
GGT7	0.30109549	0.62742214	0.932367891	0.656400035	0.521218451	0.846261984	0.986775468
PPM1H	0.999567212	0.304605005	0.8574548	0.254414576	0.906618419	0.846149299	0.986775468
URGCP	0.171784722	0.571496725	0.958423661	0.751784973	0.85218873	0.846314345	0.986775468
VAMP1	0.709617136	0.268559106	0.508985852	0.903147736	0.687497949	0.846175618	0.986775468
EMILIN2	0.101187385	0.945428099	0.98632707	0.662304292	0.965944439	0.846530356	0.986793352
LOC100462699	0.692850798	0.833417086	0.332665184	1	0.314426997	0.84661803	0.986793352
RBBP8	0.730453509	0.612836748	0.853588626	0.530648556	0.297900015	0.846630333	0.986793352
UBE2D4	0.395304266	0.964496097	0.841881165	0.55943175	0.33598223	0.846444242	0.986793352

ZNF248	0.757711971	0.421030761	0.379249894	0.945793763	0.527619338	0.846557351	0.986793352
ZNF41	0.447756874	0.631636588	0.429978265	0.729319954	0.681675157	0.846769878	0.986885907
DDX19B	0.455208243	0.861264796	0.51264325	0.404285827	0.745982616	0.847175044	0.98704687
GBP4	0.749131042	0.91941299	0.457410671	0.231298639	0.83213024	0.847232431	0.98704687
INO80D	0.215419507	0.948088319	0.732686517	0.816665701	0.49629995	0.84726886	0.98704687
PEX1	0.957392737	0.950828433	0.547013366	0.871543093	0.139666367	0.847172045	0.98704687
PPHLN1	0.785390012	0.955653935	0.186510068	0.46129139	0.939129952	0.84725047	0.98704687
SLU7	0.759215073	0.770077916	0.236561683	0.562279709	0.779019536	0.84709078	0.98704687
AFG1L	0.625929323	0.494111065	0.565538222	0.824398302	0.423006741	0.848149377	0.987060052
GFAP	0.614371931	0.809267363	0.463294731	0.642981058	0.411916712	0.848182369	0.987060052
GRTP1	0.315259888	0.405364553	0.683068829	0.772575291	0.90056001	0.847481275	0.987060052
HIC2	0.498714933	0.763708109	0.441801701	0.611163794	0.591522554	0.847733656	0.987060052
LOC101906218	0.839665179	0.415424041	0.515585794	0.531830441	0.634950734	0.847473923	0.987060052
LOC510913	0.286437125	0.460596987	0.987608072	0.581222372	0.801938503	0.84747625	0.987060052
OSBPL9	0.300284762	0.671082265	0.713011289	0.814985809	0.519910039	0.847858275	0.987060052
RABGAP1L	0.430532768	0.880429573	0.845926508	0.35311664	0.538438256	0.848074642	0.987060052
RPL28	0.613998271	0.91538923	0.408518362	0.41004703	0.645685685	0.84762725	0.987060052
SNAPC3	0.86682073	0.475735426	0.271815061	0.871229757	0.622213022	0.847555778	0.987060052
TBC1D2	0.432885621	0.316854721	0.644697255	0.849877372	0.810297686	0.847896378	0.987060052
ZCCHC7	0.466282186	0.784047491	0.421009759	0.572305389	0.69214721	0.84808256	0.987060052
ZFP36L2	0.732047778	0.91416182	0.615574131	0.201564837	0.733158556	0.847848179	0.987060052
ZNF200	0.560711625	0.547895688	0.437989459	0.545980317	0.829907042	0.848081798	0.987060052
ZNF774	0.344552703	0.34168854	0.818487344	0.697591248	0.906894695	0.848063311	0.987060052
HCK	0.96663468	0.881982282	0.932325002	0.325393119	0.236501088	0.848591349	0.987064182
LOC100848122	0.600534184	0.399623428	0.751772142	0.640050997	0.529471668	0.848520209	0.987064182
LOC784464	0.742628651	0.898556456	0.912890522	0.296665925	0.337980079	0.848363592	0.987064182
PHKG2	0.521896608	0.985633998	0.978062727	0.142367013	0.853262691	0.848458232	0.987064182
SRA1	0.90496321	0.360982981	0.620947656	0.822529455	0.366649052	0.848606943	0.987064182
TAP2	0.888644675	0.49514261	0.610298559	0.588600433	0.386526167	0.848401433	0.987064182
ZNF713	0.620495931	0.852741501	0.960072028	0.196262932	0.613296307	0.848533296	0.987064182
BBS2	0.78886908	0.897753767	0.714991859	0.283749748	0.427178779	0.849119401	0.987065525
GABPA	0.945245152	0.682708101	0.396015489	0.850822367	0.28176533	0.848837026	0.987065525
ITPR1	0.375581998	0.716041102	0.828558402	0.810539697	0.339034192	0.848753192	0.987065525
LOC101906914	0.922822261	0.325963073	0.964383321	0.331219079	0.638727726	0.849104704	0.987065525
LOC104976448	0.432404882	0.837287244	0.721084233	0.5457782	0.430376185	0.848979027	0.987065525
LOC112446004	0.713430748	0.842859332	0.533131619	0.196027486	0.976864051	0.849149417	0.987065525
PAG1	0.913463735	0.72823314	0.851390986	0.266361101	0.406164913	0.848853419	0.987065525
SNRNP200	0.798124656	0.619483678	0.445037888	0.673955056	0.413882158	0.849118259	0.987065525
ZBTB43	0.910501037	0.976405708	0.093491985	0.906175747	0.815061704	0.849147843	0.987065525
ADORA2A	0.833484869	0.563747797	0.337602734	0.477733995	0.811794343	0.849480773	0.98715536
ALKBH2	0.31863695	0.903455671	0.313109321	0.84091088	0.811567945	0.849464052	0.98715536
AP3D1	0.222497684	0.953836224	0.580421559	0.948501326	0.535428464	0.852062299	0.98715536
ATG101	0.916316013	0.688194301	0.36161494	0.292521357	0.927768235	0.850399825	0.98715536
BCAS4	0.873976981	0.949830448	0.573141424	0.695235801	0.189556127	0.852414759	0.98715536

BCKDHA	0.85747516	0.916915643	0.258468057	0.434363623	0.710039669	0.852349658	0.98715536
CCL26	0.288318422	0.815992163	0.519044917	0.585925174	0.865474742	0.850492374	0.98715536
CENPS	0.602288938	0.695782351	0.813911098	0.232295631	0.789877708	0.852123898	0.98715536
CTDSPL2	0.741335423	0.247529695	0.950863359	0.536951062	0.669082889	0.852378299	0.98715536
CTGF	0.174069023	0.929036668	0.439128403	0.997773337	0.870318646	0.849850006	0.98715536
DAB1	0.700448894	0.377938986	0.460007372	0.960475341	0.534497308	0.851960431	0.98715536
DOCK5	0.911579689	0.799910226	0.410574977	0.396840993	0.526310962	0.851993248	0.98715536
DPY19L4	0.980896498	0.912464612	0.562977191	0.329956105	0.375482271	0.851741025	0.98715536
EXOSC1	0.652027054	0.750654764	0.385781544	0.736954444	0.449740207	0.852121596	0.98715536
FAM208A	0.780064466	0.55923805	0.979877726	0.350096277	0.417775589	0.851972819	0.98715536
FAM45A	0.593776016	0.826411924	0.199555	0.766112043	0.825919442	0.850582001	0.98715536
GNG10	0.891089604	0.213334325	0.532920672	0.624455425	0.972291305	0.849452879	0.98715536
GPR153	0.877650774	0.295063673	0.465152798	0.755468956	0.683155552	0.851100065	0.98715536
GSTCD	0.642193734	0.310431783	0.495725335	0.938478032	0.674764067	0.852121508	0.98715536
HAUS5	0.945666181	0.343355271	0.342253979	0.869663542	0.644844333	0.851478795	0.98715536
HNRNPA1	0.684966603	0.791779731	0.531884364	0.296739473	0.725258426	0.850883253	0.98715536
HS1BP3	0.487096014	0.753429156	0.224027209	0.934276734	0.810307132	0.851282442	0.98715536
INPP5E	0.831399932	0.193734892	0.953550462	0.535715985	0.757624825	0.851519267	0.98715536
IRF2BP1	0.910458077	0.367509931	0.745678273	0.723279362	0.347105649	0.852262731	0.98715536
ITFG1	0.903954868	0.27614576	0.445862751	0.635595939	0.882794264	0.851794667	0.98715536
JAZF1	0.203373459	0.981560192	0.537488986	0.647894987	0.891046595	0.850537179	0.98715536
LIAS	0.937932095	0.797799463	0.46320058	0.19812747	0.903842369	0.85085216	0.98715536
LOC101904248	0.676606143	0.702368983	0.137897644	0.967466425	0.974029121	0.850067329	0.98715536
LOC104975849	0.974657908	0.862473982	0.494664473	0.150437619	0.991898875	0.85080281	0.98715536
LOC107132070	0.781593202	0.8258689	0.932305299	0.324796614	0.319265175	0.851683396	0.98715536
LOC112446855	0.754851278	0.409833364	0.286809632	0.866845018	0.805410527	0.850549766	0.98715536
LOC112447030	0.460596418	0.356170895	0.94981821	0.616401246	0.642537721	0.849966027	0.98715536
MAST2	0.25533399	0.493449637	0.607047834	0.845052254	0.962927298	0.851271253	0.98715536
MCUB	0.790524772	0.566798671	0.432878626	0.327724926	0.978891131	0.851236771	0.98715536
METTL7A	0.15695409	0.773403103	0.976070582	0.532294049	0.974608488	0.849345471	0.98715536
MISP3	0.622463384	0.300647097	0.856715526	0.388558526	0.990320045	0.849915377	0.98715536
NFKBIE	0.319815382	0.628428813	0.533624737	0.859827149	0.676865489	0.851715958	0.98715536
ORMDL2	0.819058074	0.682950685	0.499953047	0.646392996	0.346020602	0.852044453	0.98715536
PDZRN3	0.931556418	0.763083915	0.168766544	0.901930266	0.578752457	0.852222143	0.98715536
PHOSPHO2	0.316250723	0.901729242	0.552072215	0.844423165	0.469307683	0.851650877	0.98715536
POMC	0.632836761	0.840559365	0.372654149	0.691915492	0.448982901	0.849633529	0.98715536
PTAR1	0.566674129	0.953071467	0.475207788	0.683815478	0.353331492	0.850707136	0.98715536
PTGDS	0.810600016	0.9683395	0.125281494	0.995879388	0.639045953	0.852125261	0.98715536
PYCR3	0.754881033	0.641757795	0.909346505	0.441332674	0.319870363	0.851153269	0.98715536
RAB3C	0.346414731	0.507908877	0.44275875	0.869608089	0.911316148	0.850023152	0.98715536
SEC14L2	0.874720439	0.576210404	0.750868639	0.231760403	0.708954698	0.851136544	0.98715536
SNUPN	0.432144755	0.597590374	0.577065987	0.532093684	0.777020779	0.849715602	0.98715536
SRRM5	0.951940263	0.429224421	0.700545676	0.765726192	0.282662892	0.850568149	0.98715536
TMEM61	0.339275724	0.989510064	0.283298716	0.687190051	0.949793108	0.850870594	0.98715536

TNFRSF1A	0.97796359	0.675610267	0.435709301	0.594637913	0.360547682	0.849983646	0.98715536
TXNDC12	0.502067595	0.571250615	0.328911817	0.906918099	0.728711622	0.851534096	0.98715536
WDR37	0.645162137	0.752936585	0.275064442	0.59283817	0.781201046	0.850385912	0.98715536
ZFPL1	0.99895134	0.962949248	0.783611274	0.14968233	0.553232384	0.851723624	0.98715536
GPR146	0.565921386	0.440611971	0.770346344	0.518929317	0.63011663	0.852679944	0.987240981
IZUMO1	0.895022932	0.990193722	0.403083991	0.193859088	0.906904697	0.852669381	0.987240981
MDH1B	0.641439093	0.55996004	0.528928105	0.580810681	0.569407967	0.852729322	0.987240981
NPTN	0.407173283	0.285667638	0.583070022	0.989201763	0.935487676	0.85255844	0.987240981
LOC101903205	0.22402698	0.87769427	0.942248218	0.635877747	0.533782388	0.85286347	0.987292265
RPRD1A	0.796984742	0.583280349	0.440532347	0.622295454	0.493550076	0.85289394	0.987292265
BSCL2	0.323716034	0.851172519	0.978365636	0.486120217	0.480416259	0.853039265	0.987321206
TRAF1	0.771583657	0.913507563	0.533035646	0.16847185	0.994509525	0.853019251	0.987321206
FAM13A	0.76422394	0.490543088	0.385079835	0.723220166	0.603486995	0.853160265	0.987321988
UBC	0.747156647	0.777173241	0.579439859	0.696311117	0.268841744	0.853107612	0.987321988
PON2	0.837758882	0.468787312	0.211179917	0.984692796	0.773174505	0.853491561	0.987635736
BTD	0.592666839	0.469887997	0.925391729	0.714528649	0.343860426	0.85391692	0.987692623
CIB4	0.280435815	0.584446739	0.820590536	0.471824445	0.997169536	0.853818942	0.987692623
LOC107131341	0.78660705	0.88226437	0.45231583	0.439618755	0.458466086	0.853793367	0.987692623
MBD2	0.921659838	0.829812465	0.500178454	0.47572898	0.347399112	0.853681321	0.987692623
PPM1J	0.976297046	0.51669621	0.589389597	0.239259198	0.89037322	0.853962015	0.987692623
TRIM52	0.550156877	0.687448545	0.720570803	0.816255059	0.284607186	0.853896738	0.987692623
ZNF677	0.454396064	0.176654899	0.95890253	0.984080591	0.835437988	0.853827835	0.987692623
ACAD11	0.268956095	0.817673507	0.364334436	0.846452953	0.952298248	0.856934813	0.98779429
AOC3	0.49231299	0.265444073	0.792268885	0.688648634	0.907144207	0.857152461	0.98779429
APOL3	0.953989357	0.93949824	0.767066804	0.400379649	0.234888636	0.857098635	0.98779429
AREL1	0.933345882	0.420430551	0.495574943	0.658876643	0.500929494	0.85598716	0.98779429
ASAH1	0.710949624	0.832180395	0.300474149	0.364603823	0.992118151	0.856274531	0.98779429
ATP5MC2	0.930675323	0.613178222	0.427662082	0.380493263	0.698991662	0.857690615	0.98779429
BUD31	0.469986713	0.305492306	0.672685785	0.783888949	0.858196098	0.857841948	0.98779429
C1QTNF5	0.394760723	0.729530829	0.487836139	0.523569923	0.881736773	0.857572358	0.98779429
CCDC197	0.625428869	0.970196217	0.553367671	0.217635755	0.886968224	0.857476307	0.98779429
CDKAL1	0.231952933	0.776572701	0.653506848	0.929041482	0.582683841	0.854890591	0.98779429
CFP	0.413014312	0.876023554	0.84187793	0.599478526	0.35537618	0.857651364	0.98779429
CILP	0.651665841	0.696738523	0.410656263	0.44190259	0.783028042	0.856774083	0.98779429
CYP27B1	0.419779865	0.5619885	0.49010799	0.945696043	0.593560492	0.857674153	0.98779429
CYP2U1	0.50951042	0.80335875	0.387658629	0.508788113	0.792362699	0.85547771	0.98779429
ECI1	0.713853458	0.946444551	0.462410174	0.226118901	0.897959167	0.854196048	0.98779429
FIBIN	0.610862833	0.47893224	0.617144196	0.456057067	0.782419837	0.856559671	0.98779429
GNG3	0.621590799	0.492709216	0.541143369	0.394192693	0.974372304	0.854729276	0.98779429
HELQ	0.341734179	0.694672064	0.777765225	0.414896936	0.843947921	0.857086756	0.98779429
HSD17B8	0.891215321	0.79471912	0.731593201	0.153168678	0.816072286	0.857362995	0.98779429
JMJD4	0.755839871	0.179993181	0.767532429	0.838252722	0.739651459	0.857300267	0.98779429
KBTBD11	0.273545725	0.927940604	0.852733416	0.919117812	0.321361257	0.855391807	0.98779429
LOC100848721	0.819177513	0.80546467	0.613259484	0.177983255	0.88647104	0.855175809	0.98779429

LOC101905010	0.794661959	0.923718311	0.608583623	0.706471295	0.202849733	0.855596273	0.98779429
LOC101907747	0.994293994	0.468108274	0.842812509	0.619136792	0.266149606	0.857062989	0.98779429
LOC104971296	0.502745249	0.985834282	0.319472962	0.560485514	0.724796592	0.856315983	0.98779429
LOC107131941	0.378690512	0.734770065	0.87384106	0.48352298	0.551208148	0.857445452	0.98779429
LOC107132546	0.568481382	0.908612405	0.556195557	0.237331987	0.947403217	0.856961417	0.98779429
LOC107132967	0.523885174	0.638271952	0.36607369	0.908125013	0.583761035	0.857651076	0.98779429
LOC112443213	0.840515855	0.848477081	0.265255512	0.652409883	0.526290694	0.857792323	0.98779429
LOC112447418	0.957543021	0.584510269	0.239087075	0.829454118	0.57249136	0.854457405	0.98779429
LOC112448430	0.87918167	0.30597279	0.463868468	0.985024789	0.522370328	0.856041429	0.98779429
LOC112448540	0.929325445	0.706322367	0.389860999	0.982425219	0.252709125	0.854433894	0.98779429
LOC112449558	0.777858519	0.631782388	0.817758108	0.295426321	0.540989863	0.856093711	0.98779429
LOC785503	0.833273827	0.21839168	0.824574645	0.544798145	0.779688753	0.8549294	0.98779429
LPAR5	0.518834147	0.599137609	0.434519988	0.938093876	0.500445925	0.854140875	0.98779429
LRRFIP1	0.45453837	0.554141782	0.375681946	0.934573829	0.725084532	0.855842555	0.98779429
MORC3	0.298811457	0.828231708	0.81963839	0.750549458	0.422938548	0.85647735	0.98779429
MRPS26	0.857545879	0.251627555	0.929394327	0.481573777	0.659713033	0.854867147	0.98779429
MYH7B	0.396987481	0.868163554	0.697775524	0.89128833	0.298568571	0.855542006	0.98779429
NTN3	0.995226168	0.844654167	0.185689122	0.832111103	0.497254825	0.856938357	0.98779429
PARVG	0.413835048	0.84920532	0.934024333	0.329042979	0.58960084	0.854788238	0.98779429
PRKAR2B	0.847245523	0.36650083	0.312728135	0.830233624	0.791772079	0.855154472	0.98779429
PSPC1	0.523871798	0.952405723	0.854771923	0.829951313	0.179735449	0.854638772	0.98779429
RABIF	0.355393429	0.726716634	0.783897384	0.791546526	0.401381199	0.856316209	0.98779429
RITA1	0.487847077	0.411846367	0.93901362	0.597837099	0.568297007	0.855784768	0.98779429
RNASEH2C	0.378652528	0.826038179	0.482829367	0.890823781	0.482754487	0.857777041	0.98779429
RPA4	0.376278001	0.835577722	0.737342476	0.40539522	0.688516797	0.857221239	0.98779429
SAMD10	0.96816778	0.61629717	0.763853182	0.775450637	0.182111187	0.856412139	0.98779429
SEPT3	0.815067016	0.365027751	0.848095012	0.678615199	0.377555677	0.857085237	0.98779429
SMC6	0.289167612	0.927006923	0.501834233	0.547048942	0.86672181	0.855030585	0.98779429
SOCS2	0.221121352	0.872664044	0.777837533	0.508904927	0.832881677	0.854639827	0.98779429
SPATA21	0.801885626	0.830895796	0.889323491	0.337058178	0.321144928	0.855881317	0.98779429
SPSB3	0.812658571	0.523615093	0.819746851	0.186084654	0.979751836	0.854583595	0.98779429
TBC1D5	0.298578905	0.600697877	0.646350295	0.748409939	0.748340828	0.85773155	0.98779429
TBC1D8	0.513101552	0.864053771	0.298226887	0.531459566	0.908114591	0.855101844	0.98779429
THOC3	0.674560748	0.231233546	0.577236845	0.907521461	0.783567661	0.855613155	0.98779429
TRIM16	0.878257945	0.384117716	0.518952499	0.549932354	0.66611404	0.855862824	0.98779429
TSPAN32	0.915127717	0.861621928	0.433593664	0.939031149	0.202194985	0.857700063	0.98779429
USP18	0.858575365	0.80569461	0.794254107	0.230895646	0.508250174	0.856677849	0.98779429
ZNF142	0.894850967	0.283576528	0.371484448	0.792002253	0.86948162	0.857705879	0.98779429
ZNF35	0.725047118	0.22739769	0.774939212	0.820021077	0.616944369	0.857058239	0.98779429
ZNF574	0.520785493	0.713343646	0.651774663	0.328888071	0.800614095	0.854970073	0.98779429
ZNF683	0.696527576	0.933381018	0.686052548	0.371116055	0.391137502	0.857303471	0.98779429
MIPEP	0.655697275	0.221876871	0.639175488	0.719054836	0.972742571	0.85800077	0.987907853
AAED1	0.667441723	0.339469006	0.457285868	0.668337796	0.953811299	0.860311663	0.987961777
ABCA10	0.770589131	0.668056915	0.412823951	0.677990536	0.461106433	0.861197107	0.987961777

ACKR2	0.408591651	0.64365891	0.908326896	0.549029376	0.505980447	0.861021589	0.987961777
ARHGAP17	0.722947506	0.515523557	0.489163447	0.458769932	0.78434366	0.859291075	0.987961777
ATP11B	0.811952818	0.751007656	0.750595841	0.264268465	0.541020981	0.858920034	0.987961777
C25H16orf91	0.221485522	0.350286012	0.927471591	0.972441651	0.939849106	0.859665141	0.987961777
CAMK4	0.571018146	0.808610525	0.623746625	0.888963456	0.256557864	0.859483782	0.987961777
CENPM	0.985174522	0.36785285	0.880044421	0.468080346	0.439988655	0.859479288	0.987961777
EPB41L4B	0.370477979	0.947444684	0.271386564	0.909783328	0.765622482	0.861001074	0.987961777
F8	0.559349199	0.983422886	0.369311922	0.40785729	0.79162024	0.859267215	0.987961777
FBRSL1	0.723334648	0.719868972	0.251267714	0.766927249	0.658025257	0.860265498	0.987961777
FEZ2	0.705139097	0.175405307	0.725582188	0.864777421	0.854527219	0.860924226	0.987961777
FOLH1B	0.547996295	0.916632821	0.877681131	0.20289717	0.740084568	0.860659532	0.987961777
GCNT1	0.697934896	0.610508663	0.423862655	0.955391047	0.377944493	0.858399205	0.987961777
HERC5	0.803390349	0.552761912	0.853569572	0.369069302	0.468130633	0.859037258	0.987961777
HMCES	0.441828154	0.666577846	0.872277617	0.488911024	0.526635426	0.860532417	0.987961777
KAT2A	0.484292434	0.929936533	0.76987669	0.740281399	0.25918125	0.861388801	0.987961777
KLHL12	0.577205178	0.672761523	0.22316295	0.774309912	0.972038508	0.858423764	0.987961777
LCN6	0.733931767	0.628368455	0.952302758	0.759623103	0.195141503	0.858139055	0.987961777
LOC100848419	0.83476006	0.915781778	0.820967045	0.136104554	0.766298335	0.858957913	0.987961777
LOC101905029	0.682553424	0.72707754	0.908734547	0.183011532	0.794039669	0.859140075	0.987961777
LOC104968656	0.520670826	0.367701989	0.676015365	0.746215759	0.687324927	0.861064863	0.987961777
LOC104975162	0.482551383	0.546366728	0.62864859	0.477594164	0.832475213	0.859968154	0.987961777
LOC112443877	0.840490037	0.88778463	0.48123651	0.983800176	0.187236881	0.860531783	0.987961777
LOC112448488	0.647142279	0.41165691	0.333182291	0.996980659	0.744636926	0.859962039	0.987961777
LOC514680	0.54886633	0.9464742	0.20270427	0.75197561	0.839050869	0.861198578	0.987961777
LOC535280	0.626001509	0.414402095	0.946289171	0.3897829	0.687349132	0.859675848	0.987961777
LOC615271	0.961180282	0.155402688	0.926142947	0.507002129	0.941833913	0.860334384	0.987961777
LOC781982	0.498522373	0.422104117	0.505305405	0.725973642	0.858804418	0.860868676	0.987961777
LOC784297	0.341897711	0.388167792	0.759236088	0.821237188	0.801442379	0.860923902	0.987961777
LOC784735	0.742445869	0.657931126	0.345600609	0.395190816	0.99012915	0.860331934	0.987961777
LOC788334	0.608853962	0.98464483	0.731636677	0.234219239	0.644660493	0.860719823	0.987961777
LRRCC1	0.859964947	0.236094918	0.429963026	0.772090119	0.974710335	0.859509872	0.987961777
MANEA	0.593161801	0.955136932	0.284823633	0.46425005	0.875852756	0.859321327	0.987961777
NTM	0.482068176	0.737216247	0.222475974	0.936014554	0.898222364	0.861275275	0.987961777
OTULIN	0.898753791	0.245454726	0.986254691	0.445569203	0.684609265	0.861036202	0.987961777
POGZ	0.352234381	0.812330254	0.909724983	0.630658481	0.401018896	0.859818522	0.987961777
PPT1	0.882175118	0.600814556	0.340989759	0.732948057	0.498528967	0.860291082	0.987961777
RBM41	0.671471618	0.531353341	0.931648778	0.814150976	0.241318399	0.858613793	0.987961777
RIMS3	0.802165744	0.323296706	0.870974499	0.3163558	0.93053019	0.861318039	0.987961777
RUNDC3B	0.638930704	0.852422056	0.832725697	0.345128674	0.418935161	0.859231329	0.987961777
SLC1A1	0.576668634	0.324312161	0.426885814	0.844468468	0.98655597	0.861362587	0.987961777
SLC26A1	0.781955337	0.175487611	0.88126891	0.69060415	0.795983035	0.8612811	0.987961777
SPNS1	0.437258313	0.481177118	0.610928195	0.675906363	0.752938275	0.858863937	0.987961777
TADA2B	0.708499655	0.429020299	0.360622878	0.968614917	0.619670709	0.85973176	0.987961777
TAF3	0.651096081	0.970458792	0.961201537	0.14830234	0.726124044	0.858834899	0.987961777

TMEM38A	0.791015335	0.944816346	0.338803455	0.344967172	0.761239481	0.861318764	0.987961777
TMEM74	0.277757798	0.517173419	0.671644377	0.911467584	0.742485747	0.858583402	0.987961777
TNIP1	0.768133642	0.791018272	0.144610283	0.758769921	0.980434095	0.858750031	0.987961777
TRIM14	0.39465609	0.66744488	0.458473872	0.823133056	0.657082688	0.858641647	0.987961777
TTL	0.646299165	0.783993048	0.194314645	0.944054077	0.707297509	0.859616634	0.987961777
TWSG1	0.619949664	0.698831148	0.529296722	0.303469403	0.953417407	0.860990254	0.987961777
VWA8	0.893236584	0.640242613	0.22754854	0.804268589	0.634502718	0.861126014	0.987961777
YAE1D1	0.533926228	0.414753049	0.682142883	0.665577076	0.647761205	0.858196992	0.987961777
ZNF524	0.792803287	0.88549977	0.887109097	0.173044722	0.607811587	0.859064881	0.987961777
ZNF575	0.529231494	0.291436809	0.665609392	0.81447527	0.795760687	0.86141887	0.987961777
TAPBPL	0.605771696	0.396772389	0.340654221	0.8429947	0.96529127	0.861617644	0.988120696
ARHGEF4	0.527722809	0.877807257	0.228452953	0.955154436	0.659638906	0.861732634	0.988172096
CSNK1G3	0.471527006	0.59518465	0.314273484	0.828890983	0.912356032	0.861782892	0.988172096
CCDC162P	0.675427794	0.648502094	0.787490883	0.24783412	0.781049034	0.861936731	0.988279445
LOC112448390	0.26915779	0.689886038	0.82299699	0.467091747	0.936586679	0.862128299	0.988360986
WDR70	0.701805395	0.770109538	0.582994769	0.924461544	0.229508653	0.862124537	0.988360986
ADAMTS20	0.513195514	0.807414153	0.294626272	0.972817928	0.563391544	0.862251888	0.988382133
SFT2D3	0.646551199	0.86363659	0.727084246	0.258167202	0.638488925	0.862279549	0.988382133
ZNF512	0.684961938	0.56950197	0.999726213	0.363277915	0.472531567	0.862327425	0.988382133
RBM27	0.763695017	0.80678032	0.724198314	0.210975303	0.712303447	0.862573426	0.988526013
RNF6	0.207983916	0.687119217	0.855242823	0.61390818	0.893648338	0.862570657	0.988526013
SMIM15	0.476868262	0.757007134	0.387764899	0.585095686	0.819088255	0.862640328	0.988533651
ARHGAP35	0.93202228	0.970861771	0.909011023	0.105361786	0.782780334	0.864299671	0.98860466
CLPB	0.767710788	0.933867547	0.597456963	0.717826609	0.218552562	0.862894194	0.98860466
CNR1	0.491499051	0.702906935	0.287089944	0.822497356	0.830748152	0.864151769	0.98860466
CTDSP2	0.744484022	0.354784305	0.657312356	0.741322016	0.522420118	0.862981001	0.98860466
DGCR2	0.899836878	0.305852691	0.767793672	0.426632211	0.752423782	0.864286335	0.98860466
DPY19L3	0.834412783	0.910519497	0.256692728	0.781202917	0.442109579	0.86324097	0.98860466
FBXW4	0.461907109	0.89014826	0.229648514	0.841804934	0.849850798	0.863671012	0.98860466
GALNT16	0.458882173	0.831435138	0.393917498	0.570802603	0.790934361	0.864328783	0.98860466
GLB1L3	0.432077509	0.674702473	0.356574763	0.9292459	0.7002847	0.863874347	0.98860466
HS3ST3A1	0.144358332	0.794315035	0.739580881	0.946538454	0.840407694	0.86347058	0.98860466
KIAA2013	0.905751634	0.279711982	0.650709858	0.901331382	0.454628173	0.863675673	0.98860466
LOC112442264	0.289328907	0.796697641	0.944043036	0.542638934	0.573880178	0.864140486	0.98860466
LOC512248	0.826086474	0.973432241	0.213163069	0.703223132	0.562707279	0.864280231	0.98860466
LOC786256	0.464321777	0.652360058	0.947967085	0.77714198	0.303997395	0.864297635	0.98860466
LRRC32	0.340194181	0.74579463	0.447908834	0.957623452	0.622856859	0.864177841	0.98860466
MAP2K2	0.246455841	0.975963525	0.955877258	0.519192118	0.566983938	0.863957622	0.98860466
NRDE2	0.598048885	0.706494059	0.879303476	0.513640322	0.353910609	0.863637476	0.98860466
ORAI3	0.665415428	0.404546435	0.500691262	0.959708494	0.522561751	0.863764459	0.98860466
OTUD7A	0.940987849	0.888301653	0.960596313	0.181929565	0.461075139	0.863235098	0.98860466
PARP8	0.853301979	0.392998507	0.394862406	0.990109838	0.516773031	0.864110906	0.98860466
PFKM	0.975012442	0.7644905	0.336707776	0.386436243	0.697575442	0.863899819	0.98860466
PRSS33	0.900425164	0.61254111	0.205630887	0.951821417	0.628536024	0.864327296	0.98860466

RGS19	0.798214703	0.474633217	0.55977366	0.71933259	0.443913008	0.864041016	0.98860466
RPP14	0.938321785	0.266918227	0.392508316	0.736870825	0.9328765	0.863725568	0.98860466
TMEM178A	0.789522996	0.823393892	0.760172424	0.148428939	0.923453506	0.864075235	0.98860466
VEGFB	0.791996563	0.425400127	0.640226032	0.726786999	0.428426847	0.862816417	0.98860466
ZNF169	0.429737961	0.918262759	0.408715971	0.7545533	0.556447925	0.864037203	0.98860466
RESP18	0.209640558	0.665784618	0.893275296	0.5576665	0.977549413	0.864583271	0.988757914
ZNF263	0.408468796	0.689734846	0.873623627	0.309426088	0.892180175	0.864538029	0.988757914
C1D	0.795089817	0.341389554	0.370251282	0.72919411	0.930788138	0.865110806	0.988947718
HECW2	0.638938985	0.760712638	0.650296425	0.579990487	0.371924046	0.865046002	0.988947718
LOC104973746	0.468231741	0.937475268	0.396104404	0.51497998	0.761759751	0.865104589	0.988947718
NTS	0.312579301	0.831946571	0.369754544	0.92126774	0.768954597	0.864906193	0.988947718
PEX14	0.759869052	0.895378914	0.145714284	0.96568779	0.711846331	0.864979377	0.988947718
ZNF444	0.484889238	0.340212578	0.622454857	0.954009575	0.695013148	0.864834964	0.988947718
FBXO34	0.670416208	0.371980928	0.861791335	0.460255359	0.690020146	0.865202967	0.988969796
KPNA5	0.676178364	0.913951155	0.136937735	0.895689276	0.900899351	0.865275438	0.988969796
SNRPC	0.668203394	0.367022218	0.992402293	0.683647777	0.410510408	0.865310908	0.988969796
CD9	0.98777562	0.324397704	0.347032343	0.878396209	0.70002404	0.865468635	0.989012309
MALSU1	0.744923959	0.925084681	0.826431557	0.120443145	0.996692872	0.86544632	0.989012309
USP45	0.475427769	0.818807395	0.754546515	0.929048717	0.250691998	0.865544006	0.98902957
PPP3CC	0.868201103	0.661420862	0.293280467	0.496166294	0.819096117	0.865616087	0.989043069
GPR107	0.901628476	0.753810885	0.433762368	0.447019598	0.519586804	0.865678261	0.989045248
CGAS	0.87153712	0.418171921	0.759380254	0.397726207	0.622695163	0.865825316	0.989144397
JTB	0.590254026	0.728789657	0.465395694	0.386445608	0.886625713	0.865938128	0.989204415
C1H3orf33	0.918441056	0.684912964	0.530055566	0.351758613	0.588033256	0.866738554	0.989437693
CAPRIN2	0.932942908	0.921854799	0.096078954	0.905746006	0.920506105	0.866577541	0.989437693
LOC100849067	0.741443649	0.733403819	0.148701262	0.949767036	0.896193335	0.866433906	0.989437693
LOC101907857	0.194835852	0.871837018	0.828115753	0.724860374	0.67643318	0.866745248	0.989437693
MAP3K4	0.86573667	0.932545984	0.230082158	0.568265704	0.653255613	0.866711115	0.989437693
NUP43	0.829648696	0.840673171	0.359786191	0.285031004	0.963111457	0.866562513	0.989437693
PAF1	0.586116239	0.687678393	0.656295367	0.398132617	0.654323176	0.866614635	0.989437693
PI4KA	0.572222436	0.848458747	0.778753342	0.242820525	0.748507764	0.866204337	0.989437693
RP9	0.84428144	0.780102325	0.83535574	0.488629251	0.255808539	0.866315339	0.989437693
SMPD2	0.828978466	0.324454285	0.415712953	0.979926583	0.627970631	0.866389336	0.989437693
NRP1	0.558809605	0.472729692	0.785661633	0.4665579	0.712740074	0.866838118	0.989445259
SLC29A1	0.90795655	0.359306838	0.511531511	0.531107822	0.778869884	0.866872459	0.989445259
CRYBG1	0.697352066	0.560111469	0.226122097	0.860247291	0.911420521	0.867332431	0.989558254
KEAP1	0.931703415	0.798968962	0.857776479	0.124487665	0.871148763	0.867328614	0.989558254
LOC101902742	0.863509068	0.609602501	0.733008554	0.620079515	0.289085235	0.867158244	0.989558254
LOC101904667	0.802680335	0.476936385	0.934437839	0.66588793	0.290527223	0.867241176	0.989558254
PCMTD1	0.90854577	0.454529284	0.673256493	0.480763495	0.517925684	0.867290721	0.989558254
SHROOM4	0.662521076	0.976305351	0.644841557	0.940122936	0.176600147	0.867333248	0.989558254
LOC783539	0.422996371	0.716125511	0.430328077	0.645735146	0.823469546	0.867472788	0.989648657
BMPR1A	0.963436804	0.261827425	0.602920138	0.54987978	0.82987059	0.86765797	0.989653527
IL27RA	0.525774018	0.607703315	0.909142196	0.466572748	0.511965803	0.867626459	0.989653527

PDCD2L	0.778487504	0.914207764	0.333162366	0.553551198	0.528370756	0.867547578	0.989653527
LOC529930	0.43230581	0.249588048	0.985570231	0.867299255	0.752824943	0.867721755	0.989657497
ACADL	0.567939224	0.838495709	0.291933941	0.59257573	0.866396078	0.871734445	0.989686308
AGAP1	0.762900557	0.814577701	0.834041344	0.297697997	0.460791605	0.871175515	0.989686308
ARHGAP15	0.33129478	0.545467176	0.581856122	0.672657345	0.990540496	0.869033819	0.989686308
C1GALT1	0.400280188	0.92338813	0.487857758	0.400339767	0.987581588	0.871566689	0.989686308
C1H3orf58	0.637633093	0.695574877	0.532946484	0.810913361	0.364103079	0.868473384	0.989686308
C6H4orf3	0.679979864	0.603110266	0.248179497	0.907212771	0.773314852	0.871793146	0.989686308
CCDC107	0.593547596	0.381904729	0.956387068	0.499930001	0.651069206	0.870077882	0.989686308
DDB1	0.6093715	0.764965406	0.872005741	0.233110036	0.744698174	0.870079188	0.989686308
DIS3	0.730819922	0.952177399	0.56638373	0.247785828	0.720231042	0.869611452	0.989686308
DNAJC6	0.374796595	0.362065504	0.660334412	0.845004841	0.918434546	0.867953915	0.989686308
FCHSD2	0.446470967	0.605320839	0.338091756	0.798018004	0.964787797	0.869635106	0.989686308
FLOT2	0.747466332	0.850655378	0.244342593	0.761556253	0.601369373	0.871281873	0.989686308
FN3KRP	0.340496475	0.956728165	0.356825849	0.831030307	0.72820402	0.869624888	0.989686308
GCGR	0.357249544	0.375533721	0.634081285	0.85233251	0.975117361	0.870362346	0.989686308
GIT2	0.970542498	0.712541618	0.917640762	0.151763601	0.727752513	0.869095542	0.989686308
GPR157	0.480001924	0.872816392	0.676958545	0.318681463	0.779660063	0.869880265	0.989686308
GRIK5	0.946363888	0.713667801	0.218652796	0.750119685	0.628746791	0.868176733	0.989686308
GTF2F1	0.833032638	0.390955882	0.877445109	0.53471411	0.459089486	0.869223019	0.989686308
HADHA	0.81462075	0.874804299	0.34388429	0.339892329	0.839955965	0.868836237	0.989686308
HILPDA	0.662353697	0.668350536	0.218304364	0.935259138	0.772451023	0.86852802	0.989686308
HNRNPA0	0.830324619	0.681087325	0.428254042	0.529337649	0.544050823	0.868381643	0.989686308
HOOK2	0.403029895	0.749493916	0.971063791	0.650695016	0.367152909	0.869071631	0.989686308
IFT27	0.743147025	0.568193721	0.311207514	0.981595035	0.545024724	0.869538569	0.989686308
IRF5	0.612637737	0.964955103	0.643934318	0.427900249	0.433169658	0.870068635	0.989686308
ITIH5	0.739048924	0.672280622	0.721904409	0.736730301	0.268420984	0.870828768	0.989686308
KCNQ5	0.224569453	0.913999864	0.851081026	0.978357765	0.417041671	0.871533932	0.989686308
LOC100139990	0.922848445	0.695418727	0.408400277	0.561576418	0.48344238	0.871291403	0.989686308
LOC100847374	0.235820516	0.943028287	0.85366145	0.641521768	0.573563965	0.86860389	0.989686308
LOC101902542	0.757908506	0.686450884	0.758949742	0.556882693	0.323714889	0.871341121	0.989686308
LOC101907688	0.808891728	0.229419912	0.868550421	0.60426233	0.730436629	0.871260835	0.989686308
LOC104968807	0.937040495	0.968933615	0.609019965	0.710408644	0.181660327	0.871702721	0.989686308
LOC104973285	0.878636483	0.937830587	0.21448554	0.664620069	0.595006897	0.868685194	0.989686308
LOC107131660	0.656207595	0.757765113	0.437294593	0.842398941	0.387097827	0.870782072	0.989686308
LOC112442367	0.85893242	0.564670979	0.664274277	0.227776972	0.96018201	0.869871978	0.989686308
LOC112442987	0.671879353	0.684653079	0.315611128	0.705918623	0.679610197	0.868181128	0.989686308
LOC112448760	0.711717602	0.18154899	0.704917074	0.916029613	0.836872846	0.868544487	0.989686308
LOC781004	0.925173956	0.20524606	0.861944331	0.82724714	0.514259461	0.868136113	0.989686308
LOC781813	0.732123853	0.930385068	0.391318118	0.682728558	0.392343505	0.871782374	0.989686308
LOC783461	0.538449577	0.338514459	0.62694835	0.719030253	0.858476663	0.870027386	0.989686308
LSM2	0.4557508	0.42113888	0.491240198	0.945343704	0.795214923	0.870726152	0.989686308
MLPH	0.310941403	0.66981167	0.512207488	0.931945907	0.71589154	0.87132429	0.989686308
MOCOS	0.754007192	0.682587956	0.735604874	0.514566201	0.357933615	0.868347325	0.989686308

MOCS3	0.465280532	0.691609327	0.309686542	0.956977943	0.748042237	0.871660768	0.989686308
NGEF	0.390311315	0.738610754	0.958304905	0.704166566	0.365881914	0.871334846	0.989686308
PDE4C	0.802094918	0.452242985	0.291639529	0.688640757	0.980920828	0.871908319	0.989686308
PITRM1	0.570419639	0.47544892	0.653724039	0.465940917	0.862268108	0.871441104	0.989686308
PLEKHA7	0.555722919	0.330684865	0.706075386	0.554138467	0.992011574	0.871638359	0.989686308
PLPPR5	0.896250695	0.324986432	0.312220448	0.881213702	0.891222321	0.871826123	0.989686308
PPP5C	0.87315636	0.999845675	0.183871973	0.579258921	0.752317716	0.868815394	0.989686308
PRCC	0.953396148	0.602779677	0.452321786	0.370669532	0.722182182	0.868041186	0.989686308
PRR36	0.184220051	0.869320997	0.968338699	0.475322501	0.959595335	0.870425613	0.989686308
PTPN6	0.446110353	0.840263374	0.829551692	0.37425851	0.613033067	0.871670969	0.989686308
RIPPLY3	0.599428229	0.982042418	0.670609734	0.2072905	0.863818615	0.870331517	0.989686308
RNASEL	0.329186092	0.995531783	0.68423485	0.452128437	0.691603958	0.869153355	0.989686308
RPL36A	0.774583286	0.932899058	0.264294803	0.47520567	0.771368856	0.868923071	0.989686308
RPLP2	0.862598646	0.850919745	0.380753911	0.280419536	0.905422683	0.870886446	0.989686308
RWDD4	0.343931421	0.32449732	0.64269863	0.997580269	0.999287024	0.871993971	0.989686308
SFMBT1	0.539200761	0.67979197	0.580268531	0.87695123	0.383101731	0.871899821	0.989686308
SLC37A4	0.368934723	0.522906061	0.904092463	0.761390187	0.536150873	0.871379309	0.989686308
SMIM8	0.597309401	0.979812588	0.858302016	0.409061977	0.342907144	0.869866242	0.989686308
SSRP1	0.44039302	0.741416429	0.597096008	0.663552097	0.549228706	0.871077723	0.989686308
ST6GALNAC2	0.922545978	0.5137973	0.578744706	0.371756833	0.69812004	0.87137118	0.989686308
STAT3	0.84992398	0.666985106	0.953070267	0.365748033	0.354366593	0.868963636	0.989686308
TEX30	0.554678417	0.545873201	0.659453183	0.355407546	0.98542994	0.868766627	0.989686308
TSTD3	0.505299115	0.344357168	0.830002986	0.602358445	0.804779977	0.868934614	0.989686308
TTC26	0.90068274	0.818090412	0.315886389	0.417525079	0.733057024	0.87146055	0.989686308
UHRF1BP1	0.784068879	0.406373994	0.643375466	0.374383488	0.922576845	0.870573572	0.989686308
VWA5B2	0.458482723	0.577223816	0.875217099	0.959817223	0.320493618	0.871482702	0.989686308
WASHC3	0.553521231	0.944434552	0.321592654	0.730250738	0.582571041	0.872028762	0.989686308
ZRANB3	0.309696646	0.797244889	0.408770019	0.988787406	0.7141312	0.871515078	0.989686308
ZSWIM7	0.427355762	0.746893572	0.332529103	0.812378316	0.818729441	0.870143448	0.989686308
LOC112449548	0.655736154	0.943817043	0.964987094	0.455242317	0.263206827	0.872110154	0.989710238
CD302	0.940304184	0.968641676	0.939043513	0.155321216	0.539076973	0.872216594	0.989762586
ORC5	0.581474605	0.610376982	0.794733653	0.518852931	0.489691475	0.872322311	0.989814108
CSTF2	0.708502327	0.858389358	0.976419705	0.135121747	0.894206662	0.872490824	0.989902676
FECH	0.907555744	0.716623245	0.755066301	0.176585124	0.827585552	0.872521005	0.989902676
BTBD19	0.899195772	0.821079174	0.874206	0.576867824	0.192981157	0.872696033	0.989989326
LOC781728	0.702125979	0.536947961	0.367846282	0.737415576	0.703554864	0.872888037	0.989989326
PLB1	0.202677246	0.909021693	0.979034142	0.559049175	0.713559041	0.872899003	0.989989326
SLC4A1AP	0.305766281	0.988735579	0.765628339	0.924935671	0.335892209	0.872813623	0.989989326
ZNF664	0.701027285	0.507207643	0.308689378	0.820747746	0.798629244	0.872879233	0.989989326
CELF6	0.750129094	0.394325119	0.40244945	0.868095797	0.697227176	0.873093235	0.990006492
LOC107131356	0.693327382	0.548011338	0.682395342	0.54880201	0.506797512	0.873215768	0.990006492
LOC787074	0.78290183	0.779574839	0.737051305	0.826580476	0.193840042	0.873142117	0.990006492
SDR42E1	0.98002627	0.888856484	0.297126971	0.620154081	0.448815314	0.873071711	0.990006492
ZNF23	0.908430257	0.388737762	0.628486595	0.5897824	0.550748807	0.873174616	0.990006492

CLSTN2	0.709956769	0.381309232	0.75512887	0.735098922	0.480834796	0.873499374	0.990015797
LOC101907255	0.367313035	0.846890429	0.681424567	0.929080956	0.366533357	0.873359383	0.990015797
LOC104974937	0.421716924	0.648235351	0.438881057	0.603384085	0.997696133	0.873440136	0.990015797
LOC504858	0.643878072	0.890381655	0.493656527	0.714088287	0.357748109	0.873585934	0.990015797
LOC510193	0.85815642	0.697794112	0.767016452	0.750202127	0.209531528	0.873385066	0.990015797
WNK1	0.608699401	0.653911736	0.377709181	0.524950509	0.916072817	0.873583928	0.990015797
CAB39L	0.99069973	0.980053735	0.178287826	0.499853337	0.838664961	0.874117483	0.990110248
CFD	0.585437917	0.710128732	0.752340613	0.42722797	0.542702924	0.874021713	0.990110248
LOC107132994	0.510090358	0.845257982	0.869540914	0.235735679	0.822134622	0.874298734	0.990110248
LOC112444346	0.860134004	0.656269181	0.541135951	0.976215087	0.24316963	0.8740069	0.990110248
ME3	0.770249119	0.937778748	0.528559222	0.192100389	0.989138383	0.874073247	0.990110248
MED4	0.729600664	0.844647485	0.899378969	0.260459968	0.502421906	0.874041077	0.990110248
OLR1	0.541757693	0.815088721	0.478146712	0.481027096	0.715406286	0.874298326	0.990110248
PCCA	0.871441955	0.89300471	0.987936282	0.270072906	0.349195754	0.873994287	0.990110248
PDE10A	0.650247674	0.429298681	0.755491531	0.348229308	0.986250828	0.873845671	0.990110248
PLBD1	0.902490419	0.159503785	0.813235265	0.999909364	0.620167734	0.87416854	0.990110248
THRAP3	0.916716096	0.746435487	0.194179035	0.651008158	0.840199791	0.87433293	0.990110248
ACSF2	0.792923514	0.565909309	0.334609638	0.553160235	0.875912352	0.87447445	0.990182028
GTF2F2	0.651910479	0.74649354	0.777200191	0.66262441	0.290364188	0.87451699	0.990182028
CBLN3	0.682654699	0.659790059	0.36216438	0.974849085	0.45864418	0.87483562	0.99026951
RNF145	0.191675724	0.864560708	0.835990001	0.547960851	0.960679466	0.874824396	0.99026951
TBC1D25	0.395205522	0.425475133	0.949659076	0.855992926	0.533281465	0.874758573	0.99026951
ZNF687	0.481219654	0.829267248	0.372811032	0.719170492	0.681435505	0.874788721	0.99026951
SMAP1	0.821594184	0.22029231	0.858770784	0.764397061	0.614157997	0.874903745	0.99027832
LOC107132735	0.862008404	0.665998507	0.577529868	0.372626429	0.590924471	0.874979921	0.990296241
SHISA3	0.963356458	0.245611226	0.531220014	0.939728827	0.619370173	0.875274763	0.990561626
AFDN	0.76357633	0.565463111	0.378459025	0.474542623	0.947223165	0.875845572	0.990672715
KCNK5	0.790091851	0.896905425	0.528519756	0.634342103	0.309088346	0.875809041	0.990672715
LOC100847180	0.442203896	0.978842607	0.953448897	0.619648536	0.286831134	0.875649421	0.990672715
LOC101907800	0.992770512	0.668662967	0.603141845	0.688870605	0.266765713	0.876087913	0.990672715
LOC112448764	0.430281859	0.90058794	0.28526465	0.889528663	0.746869495	0.875822198	0.990672715
LRCH3	0.416242955	0.659911879	0.85714606	0.80027384	0.38981662	0.87584017	0.990672715
MRPL50	0.521042113	0.55126207	0.634938186	0.768600634	0.523083496	0.875593545	0.990672715
NAALAD2	0.546265311	0.975378104	0.656706262	0.237721653	0.883318175	0.875888335	0.990672715
OSGEP	0.715232734	0.526974019	0.805779336	0.634618213	0.382101959	0.876219201	0.990672715
PHC1	0.427833479	0.920725456	0.855161456	0.220048682	0.993323964	0.876192445	0.990672715
PPM1M	0.836879589	0.421117346	0.792452284	0.406000047	0.649049046	0.876120496	0.990672715
PPWD1	0.884181152	0.454086659	0.700939076	0.314187223	0.831592035	0.875994897	0.990672715
PTPRZ1	0.691924095	0.92703213	0.557580854	0.471561604	0.434522415	0.875520604	0.990672715
RAB8B	0.998539442	0.812518033	0.906584577	0.106302225	0.940669627	0.876036449	0.990672715
SLC25A44	0.57193611	0.659701956	0.483093077	0.779867202	0.51829712	0.876278419	0.990672715
LOC782966	0.9592784	0.214886554	0.818655437	0.882622576	0.495169397	0.876428273	0.990773879
GIPR	0.917386404	0.892747605	0.299220028	0.553989493	0.544628426	0.876784186	0.990903194
LOC112448847	0.96588159	0.387645167	0.620282661	0.663290725	0.479671137	0.876693401	0.990903194

RHBDD2	0.358997018	0.591987094	0.727769456	0.783874207	0.609152698	0.876619605	0.990903194
SIN3A	0.466368364	0.706687213	0.611406995	0.410849936	0.893035327	0.876772793	0.990903194
LOC112442547	0.963222576	0.821959936	0.71698807	0.183546065	0.710438987	0.876943308	0.991014779
APLP2	0.929083893	0.786683594	0.656536434	0.414476263	0.37764946	0.879003903	0.991101017
ARID3B	0.411990204	0.937584074	0.929852867	0.239635777	0.868307473	0.878301945	0.991101017
CAMKK2	0.400610393	0.346397109	0.994314998	0.943188308	0.572529422	0.87787324	0.991101017
CCDC138	0.423101553	0.873587839	0.590009799	0.792431976	0.432674498	0.878365927	0.991101017
CEP19	0.523606236	0.572811934	0.444223193	0.772084454	0.722541287	0.877524816	0.991101017
CRELD1	0.303967882	0.801592025	0.611543343	0.93367362	0.534836889	0.877680753	0.991101017
CTDSP1	0.430724047	0.793092075	0.871527312	0.531825604	0.474176184	0.878942505	0.991101017
CYB5D1	0.63500312	0.881802649	0.247701678	0.770459805	0.696312997	0.87768268	0.991101017
ECSCR	0.277350029	0.988477414	0.336908956	0.829717848	0.977244977	0.878595444	0.991101017
ENTPD3	0.640644975	0.815757359	0.708021904	0.406575453	0.496550621	0.87823484	0.991101017
IL15RA	0.911117993	0.839593262	0.462572647	0.925633932	0.227496549	0.877880039	0.991101017
INSL3	0.55251146	0.646304541	0.728762251	0.451644618	0.631172563	0.877252465	0.991101017
IQCG	0.752715091	0.495462954	0.717688644	0.89197297	0.3145518	0.878977804	0.991101017
LOC100847284	0.505044165	0.787280627	0.962157514	0.738751431	0.265836957	0.879041576	0.991101017
LOC100848581	0.755457108	0.871852145	0.541169027	0.513160775	0.409957945	0.87876945	0.991101017
LOC107132045	0.642091094	0.793215627	0.900918087	0.173550068	0.942788798	0.878942409	0.991101017
LOC112442713	0.941067092	0.367555764	0.384089654	0.961431573	0.581335968	0.877386905	0.991101017
LOC112448511	0.891334409	0.423519501	0.453188536	0.600707532	0.726835741	0.878222682	0.991101017
LSM14A	0.383752079	0.971801806	0.761666175	0.264156923	0.987870486	0.877137025	0.991101017
LZTS3	0.419392845	0.738348557	0.452482342	0.764168387	0.701847302	0.879072963	0.991101017
NNAT	0.338633578	0.523715421	0.97549378	0.925021637	0.468668661	0.878798393	0.991101017
PCYOX1	0.811012135	0.661862311	0.250491351	0.982627862	0.566895682	0.878607944	0.991101017
PHYKPL	0.842515224	0.614474436	0.79535041	0.384819788	0.472445839	0.878533231	0.991101017
PPFIA3	0.617586557	0.929399003	0.973570056	0.760562416	0.17661546	0.878915993	0.991101017
PRSS23	0.371410855	0.713201484	0.765068301	0.762407013	0.482546168	0.877962983	0.991101017
REXO5	0.703745	0.932593716	0.233136521	0.792499551	0.618856617	0.878875958	0.991101017
RLIM	0.712401378	0.981979885	0.726400783	0.501438258	0.294836753	0.879036379	0.991101017
RUFY1	0.872459017	0.964405389	0.35451498	0.28040028	0.888829377	0.877554281	0.991101017
STYXL1	0.29710394	0.762342049	0.795867996	0.588725342	0.699964623	0.877441008	0.991101017
TBC1D22A	0.592405941	0.880920876	0.777642967	0.670978737	0.273165287	0.877630666	0.991101017
TPRKB	0.319891613	0.63089478	0.638613686	0.82774863	0.695863954	0.877353948	0.991101017
UBAC2	0.387665459	0.804339995	0.427606069	0.873049863	0.641960631	0.878286215	0.991101017
WASHC5	0.671524087	0.599918435	0.367423977	0.645756333	0.781819557	0.878288949	0.991101017
YAP1	0.683296379	0.457967129	0.7193385	0.991378061	0.333761394	0.877819745	0.991101017
SLC12A7	0.726030675	0.816069372	0.954764146	0.251994277	0.527596365	0.879187755	0.991162344
LOC107132296	0.627517165	0.475958288	0.741073327	0.652782568	0.521306528	0.879396859	0.99132998
FAM213A	0.672141762	0.64118023	0.409070883	0.684735314	0.625577251	0.879760441	0.991331269
H2AFJ	0.85053631	0.747709542	0.245957994	0.670581547	0.719560007	0.879683655	0.991331269
LOC101903097	0.696049655	0.54069711	0.841117149	0.40739793	0.584686023	0.879550283	0.991331269
LOC101905365	0.887039268	0.460828949	0.724662534	0.268609343	0.947506962	0.879527559	0.991331269
LOC112445952	0.925728348	0.29259668	0.982683029	0.427661095	0.663273662	0.879733223	0.991331269

PYGL	0.962065006	0.734711108	0.368401555	0.339112801	0.855008785	0.879732685	0.991331269
ABHD17C	0.564719166	0.336240565	0.64534549	0.686274073	0.89900143	0.879918406	0.991359022
EHD4	0.521659302	0.850197208	0.925116424	0.289962306	0.635951779	0.880026703	0.991359022
LOC505918	0.187014793	0.936722161	0.889158314	0.900773936	0.538933645	0.879945518	0.991359022
LOC789384	0.340590155	0.775827115	0.859492026	0.93790124	0.355066739	0.87997423	0.991359022
LOC112449245	0.459717308	0.841130737	0.737356409	0.538093632	0.49341222	0.880101103	0.991374782
LOC101904133	0.482510127	0.596222409	0.964836386	0.562198857	0.485497027	0.880212382	0.99143208
MAN2A1	0.585081506	0.908285703	0.980583476	0.17033389	0.854330828	0.880343018	0.99151117
SLC39A9	0.656203764	0.549540194	0.575595617	0.868184294	0.421122064	0.880447644	0.991560958
GPR137C	0.978003524	0.788239394	0.837966199	0.125042289	0.940808526	0.880643131	0.991576976
LOC101905232	0.351310529	0.620272779	0.474856919	0.760276938	0.965160578	0.880522452	0.991576976
LOC101907613	0.896341851	0.542647507	0.669178257	0.49691347	0.469844953	0.880638444	0.991576976
AKR1A1	0.514572712	0.974817637	0.990353358	0.186394267	0.823078802	0.881045143	0.991621414
FMR1	0.693957459	0.580423111	0.306955114	0.76724524	0.802768987	0.880930469	0.991621414
GPRC5C	0.194127686	0.76731804	0.910602411	0.695010374	0.807065292	0.880806584	0.991621414
LOC112442740	0.673407252	0.678880692	0.298238244	0.906188856	0.615948996	0.88084078	0.991621414
NCR3LG1	0.578180737	0.297042024	0.854295212	0.94812887	0.547552405	0.880964306	0.991621414
RPL3	0.587064422	0.833133279	0.264011558	0.941871641	0.626489013	0.881011135	0.991621414
BMP5	0.957927366	0.969776727	0.563447968	0.25926453	0.562684193	0.881312045	0.991807762
TM2D2	0.440597709	0.302862983	0.726770872	0.847242115	0.92946579	0.881331582	0.991807762
LOC101902067	0.599048265	0.522593744	0.392522947	0.638655567	0.973773728	0.881424232	0.991844012
GPR31	0.990992493	0.658514349	0.691677654	0.956563611	0.17728217	0.881649331	0.991893273
LOC112446034	0.723442984	0.903988944	0.666086924	0.217368861	0.80800328	0.881582224	0.991893273
RFC2	0.882792291	0.53303517	0.253480758	0.697139562	0.920399322	0.881629198	0.991893273
PIAS3	0.960950835	0.891828004	0.238660252	0.747406799	0.501167877	0.881772419	0.991963749
NDC1	0.715755194	0.724635681	0.762393764	0.291823466	0.664586773	0.88191095	0.992034344
ZNF366	0.604137323	0.688415176	0.763517642	0.262997001	0.918587466	0.88195607	0.992034344
PLTP	0.454599336	0.561290416	0.691204502	0.829098145	0.524931144	0.882037564	0.992058012
ACAD8	0.623364491	0.359090806	0.740077391	0.491589335	0.945972491	0.882540174	0.992126504
ACSF3	0.883036061	0.784758481	0.763103632	0.156862784	0.939635108	0.884153883	0.992126504
ADGRF5	0.604040809	0.557529928	0.4757084	0.863152269	0.558421658	0.882865671	0.992126504
ASPH	0.72353707	0.591598283	0.31765933	0.634514025	0.909765061	0.885116692	0.992126504
ATM	0.505170031	0.92540207	0.401862307	0.498571086	0.837706793	0.885067629	0.992126504
BRSK2	0.847264603	0.549235506	0.612485718	0.419667437	0.647977844	0.883379619	0.992126504
C9H6orf163	0.681627738	0.548734701	0.582182525	0.385699575	0.926472458	0.883923388	0.992126504
CDK14	0.308423391	0.929827258	0.817066443	0.529293744	0.629014897	0.884276721	0.992126504
CDK19	0.561058019	0.853587039	0.964796648	0.443897156	0.375128189	0.88236425	0.992126504
CEP97	0.653707068	0.56044017	0.644015375	0.571614323	0.578895923	0.884386858	0.992126504
CHRM3	0.505864595	0.973961027	0.556090504	0.73281435	0.391077324	0.885166634	0.992126504
CHST12	0.96531254	0.374459413	0.528548228	0.495500016	0.813160241	0.882435686	0.992126504
CPED1	0.97574985	0.51162612	0.245511055	0.822066416	0.777944979	0.884926583	0.992126504
CSK	0.481276574	0.846571051	0.873361917	0.235985246	0.922973234	0.883375573	0.992126504
DCLRE1A	0.336053328	0.590169358	0.72832102	0.880671449	0.611107205	0.883792757	0.992126504
DOC2G	0.827965995	0.469157829	0.295680634	0.93884041	0.728089387	0.885151756	0.992126504

FBXO4	0.928926913	0.66218877	0.392848765	0.364617702	0.885147189	0.884237848	0.992126504
FBXO47	0.970304336	0.83121936	0.283815942	0.706189136	0.483063476	0.884410101	0.992126504
FSTL3	0.390738926	0.975017447	0.37014146	0.994734215	0.555648214	0.884152819	0.992126504
FXYD6	0.864041543	0.940489041	0.184524766	0.634121832	0.81990596	0.884186426	0.992126504
GAT	0.747939276	0.995604011	0.320134183	0.504314691	0.652140567	0.88496129	0.992126504
GLMN	0.855854872	0.651639304	0.899068664	0.629263929	0.248187112	0.884798319	0.992126504
HYAL1	0.859015377	0.522025945	0.427533333	0.438543633	0.914517311	0.882272987	0.992126504
KBTBD4	0.767878087	0.919939861	0.869995815	0.634391196	0.2010527	0.884932237	0.992126504
KLK10	0.710525162	0.155630714	0.93227631	0.924865447	0.812820439	0.883364837	0.992126504
LOC100296121	0.986993169	0.116082571	0.832159015	0.978056711	0.841368673	0.885059233	0.992126504
LOC100847934	0.649198876	0.328179357	0.916079955	0.828623288	0.479750575	0.883524611	0.992126504
LOC101902926	0.976680081	0.132378854	0.902351519	0.94006889	0.710931363	0.884203634	0.992126504
LOC101907353	0.497458092	0.434663279	0.695600535	0.930734004	0.558461207	0.884569146	0.992126504
LOC104970698	0.181993128	0.84825218	0.803176082	0.982549806	0.64418838	0.885097243	0.992126504
LOC112442208	0.796513556	0.410181129	0.67007541	0.639605412	0.559133597	0.884769201	0.992126504
LOC112448030	0.627182314	0.469868369	0.690574785	0.79611255	0.484801066	0.885211093	0.992126504
LOC780963	0.452065126	0.846815455	0.392759353	0.614168864	0.843812109	0.884113528	0.992126504
LRP5	0.386632251	0.814191753	0.44871993	0.66821822	0.815183047	0.882370687	0.992126504
MAFA	0.280201909	0.734789595	0.986319203	0.775814487	0.492387984	0.883499773	0.992126504
MAN1B1	0.723729627	0.87231009	0.600005109	0.375167685	0.54407111	0.88304357	0.992126504
NSMCE3	0.945132157	0.902100584	0.783570247	0.19607882	0.586708488	0.882212513	0.992126504
PAQR5	0.408018478	0.678614122	0.459601873	0.826774947	0.745236784	0.884973374	0.992126504
PNP	0.83134153	0.437668445	0.934965889	0.312419202	0.733955365	0.884265058	0.992126504
RELA	0.790548998	0.459879079	0.618131668	0.43015826	0.812709518	0.885242118	0.992126504
SLC16A10	0.769948016	0.855142552	0.880661456	0.185401334	0.726334816	0.884401544	0.992126504
SLC52A2	0.333367938	0.899580083	0.426032705	0.770132927	0.791450179	0.884032968	0.992126504
SNIP1	0.845477606	0.351358195	0.984144083	0.882814084	0.301589273	0.883969563	0.992126504
TAF7	0.325415731	0.401196888	0.876178095	0.936602918	0.725435017	0.883762109	0.992126504
TMEM117	0.742963117	0.554683944	0.773510322	0.649169588	0.379260561	0.885102488	0.992126504
TRABD2B	0.664746083	0.594426585	0.994064669	0.26003364	0.765331988	0.884556517	0.992126504
TRIM37	0.651558445	0.532972114	0.890267464	0.909683077	0.275951316	0.883558277	0.992126504
TSHZ1	0.634820298	0.311150189	0.628121492	0.746817575	0.843527711	0.884534472	0.992126504
TYW5	0.914646818	0.752568396	0.212241406	0.833547716	0.63478163	0.88301205	0.992126504
ZC3H12A	0.54178589	0.921597475	0.862957655	0.444305702	0.402660806	0.88262801	0.992126504
ZNF205	0.679615803	0.284054505	0.578981986	0.830650807	0.838061869	0.883915217	0.992126504
ZNF70	0.458986543	0.703838523	0.719642356	0.662639418	0.509049752	0.884992679	0.992126504
LOC107132237	0.34605972	0.9201599	0.518118379	0.514247571	0.928060395	0.885549602	0.992363196
LOC112442657	0.500326662	0.873415875	0.997232782	0.230446722	0.7842025	0.885574249	0.992363196
LOC785760	0.677198344	0.687477826	0.608130971	0.665500334	0.418952346	0.885893831	0.992653535
ELOA	0.895064566	0.66239143	0.490824334	0.641124833	0.423987227	0.886178824	0.992905079
CCDC194	0.875166484	0.915003236	0.928902286	0.449725728	0.237320575	0.886673782	0.993000424
DOCK3	0.486704562	0.746172336	0.444340611	0.935397867	0.526242689	0.886747987	0.993000424
ECE1	0.634151921	0.933866544	0.73588851	0.28577616	0.637302958	0.88664103	0.993000424
FAM181B	0.602478134	0.934894489	0.425849054	0.785077255	0.421003694	0.886482765	0.993000424

GULP1	0.948968861	0.450227523	0.470268771	0.422392159	0.935494947	0.886680361	0.993000424
IPP	0.375625755	0.479792226	0.890449731	0.713220717	0.693880036	0.886723929	0.993000424
LIN7B	0.593842413	0.566864829	0.312736601	0.791711803	0.951266381	0.886495584	0.993000424
RHOG	0.646315282	0.682208499	0.295544375	0.688762086	0.883595431	0.886529566	0.993000424
NUP37	0.719458036	0.625789916	0.919815528	0.433937704	0.442262323	0.88682296	0.993016621
ANK3	0.364941949	0.964213584	0.304476597	0.949907089	0.782664997	0.887125615	0.993287744
PTPN14	0.417244924	0.963135795	0.70130932	0.339979144	0.83187374	0.887216375	0.993321595
EDC4	0.350706372	0.497454309	0.853048762	0.70485084	0.760240988	0.887285644	0.99333138
ARHGAP26	0.963509243	0.31907112	0.932974197	0.288327736	0.966901029	0.887649056	0.99339939
CDH23	0.43962125	0.948289022	0.771382214	0.792681998	0.313552835	0.887591963	0.99339939
CH25H	0.790969858	0.473005552	0.907009515	0.998684879	0.235776653	0.887550877	0.99339939
KLF2	0.508955677	0.68207326	0.920506312	0.625302515	0.399794253	0.88751874	0.99339939
LOC101904258	0.746327841	0.405315885	0.615169824	0.586515159	0.732087266	0.887548719	0.99339939
ADGRF1	0.365583079	0.667244731	0.81423281	0.917791079	0.440247807	0.888142	0.993418047
ALPK2	0.59710985	0.822262475	0.980048652	0.594692857	0.281317855	0.888559863	0.993418047
KIF3B	0.891948111	0.49623262	0.383902288	0.818253713	0.578574702	0.888463574	0.993418047
LOC100847695	0.53046394	0.636827018	0.539635295	0.989453926	0.445537707	0.888328012	0.993418047
LOC101905141	0.515011048	0.432448942	0.916282632	0.945575578	0.415093897	0.887880761	0.993418047
LOC112442262	0.447754616	0.463844678	0.533244221	0.890574421	0.814786004	0.888326407	0.993418047
LOC783195	0.860697565	0.871485814	0.55840878	0.436409831	0.438423801	0.887952024	0.993418047
PLEKHA2	0.902437989	0.817026456	0.44707689	0.475041454	0.513173835	0.888319908	0.993418047
POMT2	0.61354713	0.38168256	0.715434798	0.848097259	0.56660573	0.888573732	0.993418047
SCN3B	0.192292542	0.944157218	0.937038071	0.571935684	0.827427753	0.888571971	0.993418047
SCX	0.893604458	0.314218546	0.864098902	0.449463615	0.73763019	0.888457594	0.993418047
SNX29	0.93539374	0.630274471	0.629047395	0.636917848	0.340719017	0.888524063	0.993418047
SYN1	0.483189909	0.891871091	0.808482332	0.629494236	0.365503374	0.887990039	0.993418047
TRDN	0.293583841	0.964105121	0.736953912	0.708965286	0.542651991	0.888136502	0.993418047
ZNF316	0.735459687	0.538895383	0.664802844	0.404238058	0.751634678	0.887811077	0.993418047
CFAP298	0.259312465	0.876836723	0.718528451	0.532309453	0.926229189	0.888642745	0.993427527
ATRX	0.295569602	0.94451416	0.505198816	0.632894393	0.909991968	0.889769446	0.993676337
AUH	0.96703121	0.84340363	0.51719492	0.226872157	0.850840545	0.890097414	0.993676337
BCL9L	0.917702967	0.653152387	0.264304446	0.680325262	0.753549989	0.889754097	0.993676337
CHPF	0.357877948	0.595751768	0.833591806	0.719482166	0.631915403	0.889066676	0.993676337
CIB2	0.553324165	0.861103911	0.650995713	0.496570784	0.52841085	0.890037633	0.993676337
COMMD9	0.998999492	0.892653923	0.203279981	0.631021985	0.708024501	0.889377944	0.993676337
DCUN1D2	0.68002837	0.5444802	0.782365129	0.414840799	0.676609235	0.889905187	0.993676337
DGAT1	0.252323133	0.743245335	0.87846311	0.717813874	0.683506828	0.889108532	0.993676337
DHRS7B	0.407899048	0.961520881	0.448946869	0.714632312	0.64681951	0.890039457	0.993676337
EIF3D	0.65146426	0.779712354	0.732363722	0.233978439	0.928674504	0.88911589	0.993676337
FAM196A	0.405155708	0.255522025	0.933292927	0.947696558	0.884836555	0.889429061	0.993676337
GPR108	0.669229893	0.623065261	0.85595855	0.475347313	0.476764092	0.88920435	0.993676337
HEXA	0.932434583	0.541975915	0.557370332	0.621288234	0.46376346	0.889655651	0.993676337
INTS14	0.431226646	0.547144693	0.795057573	0.524812769	0.822333681	0.889323067	0.993676337
KSR1	0.77566661	0.495264565	0.743126773	0.338816101	0.838227724	0.889522264	0.993676337

LOC112448772	0.46180607	0.494571888	0.9994634	0.604671149	0.586626734	0.88934733	0.993676337
LOC784473	0.776702276	0.294085859	0.705799421	0.621285485	0.812772381	0.89006977	0.993676337
PLPP3	0.734530178	0.427834531	0.870076771	0.39771811	0.749455125	0.890222406	0.993676337
RAP2A	0.936387746	0.479149975	0.476892867	0.589348677	0.646473615	0.890255487	0.993676337
SEPT4	0.305219623	0.842052663	0.966261511	0.487264495	0.672167335	0.889951764	0.993676337
SPATA2L	0.36813822	0.664515929	0.880395251	0.624870327	0.605752488	0.890257948	0.993676337
TMC8	0.580117763	0.806191141	0.83308415	0.478437428	0.436098032	0.889878684	0.993676337
UBALD2	0.527169214	0.973358268	0.372742586	0.647683497	0.653260977	0.889268003	0.993676337
RARG	0.676183259	0.916456703	0.616762971	0.477216587	0.447433016	0.890399721	0.99376699
UVSSA	0.894025717	0.154290325	0.799263887	0.901615903	0.821728625	0.890520286	0.993833963
CCDC36	0.585363331	0.404677468	0.507572962	0.833141387	0.817949791	0.890936268	0.993892672
HDAC8	0.4806168	0.666135185	0.971566837	0.332642392	0.791237265	0.890825361	0.993892672
LOC101906276	0.728259437	0.195456904	0.661682913	0.940129545	0.925239706	0.890921069	0.993892672
MLH1	0.704188187	0.901547463	0.638585387	0.229451351	0.880787362	0.890929995	0.993892672
MRNIP	0.772608575	0.715964883	0.89134364	0.674926127	0.245876427	0.890748695	0.993892672
TRPM6	0.59385859	0.941750337	0.667093281	0.436830475	0.502236773	0.890797166	0.993892672
LOC107132243	0.872808026	0.655201755	0.55616688	0.305907053	0.842998071	0.891070686	0.993975057
RAB33A	0.971981457	0.70213177	0.300787557	0.675097101	0.592366035	0.891186961	0.994037193
LOC101902141	0.430747091	0.333263834	0.979165971	0.632139968	0.925862161	0.891473959	0.994083222
LOC513573	0.242225016	0.99945309	0.674127265	0.573646298	0.87911351	0.891531099	0.994083222
RSRC1	0.7619542	0.700826731	0.521122573	0.38502549	0.768033156	0.891511568	0.994083222
UBAP1L	0.7895256	0.843745738	0.583136215	0.95656825	0.221430034	0.891496654	0.994083222
WASL	0.288205973	0.986182317	0.878704674	0.390798304	0.841924344	0.891321041	0.994083222
PRICKLE3	0.430657088	0.457588101	0.428663319	0.993242268	0.982748952	0.891779123	0.994224673
TM4SF5	0.418417299	0.711378475	0.980434719	0.310827954	0.908679524	0.891729851	0.994224673
AMN1	0.972440148	0.915657886	0.169398756	0.696989532	0.791742219	0.893032178	0.99429481
ATP8A1	0.592596021	0.865126473	0.875884818	0.616439533	0.300005343	0.892723373	0.99429481
C3H1orf50	0.891025735	0.540771877	0.313922216	0.965155405	0.568227232	0.892583077	0.99429481
EXOSC8	0.703208626	0.245344159	0.603919361	0.982881332	0.806319541	0.891971312	0.99429481
IFI44L	0.600537905	0.570049033	0.672922887	0.380441272	0.947394341	0.89270246	0.99429481
LOC101902043	0.405372803	0.461831018	0.497925047	0.988202592	0.902015517	0.892801829	0.99429481
LOC107132262	0.800801899	0.895881411	0.404993704	0.324329053	0.88318139	0.893014868	0.99429481
LOC112441879	0.53482112	0.771633838	0.413307911	0.59907583	0.814398433	0.893000408	0.99429481
PPP1R3G	0.376486254	0.899239624	0.757967158	0.972639242	0.333549631	0.893053775	0.99429481
RAB4B	0.501271643	0.718154049	0.71671299	0.358127373	0.897849686	0.892592703	0.99429481
RRAS2	0.439370353	0.99297935	0.381058869	0.95528083	0.520860729	0.892206779	0.99429481
RRP1B	0.967749317	0.502505702	0.84760256	0.222541953	0.905268653	0.892717478	0.99429481
SEL1L	0.944548521	0.272564914	0.65106544	0.558194684	0.88715643	0.892662257	0.99429481
SPIN4	0.552975016	0.708699925	0.223488493	0.994128742	0.951998983	0.892478048	0.99429481
SULF1	0.477692942	0.76726446	0.813751346	0.558579508	0.498866328	0.89283027	0.99429481
TEX11	0.597535415	0.899486919	0.6770796	0.52052122	0.436898145	0.892268365	0.99429481
TMEM115	0.876596666	0.789744893	0.942104726	0.91835167	0.138290156	0.892381365	0.99429481
TMEM123	0.478902799	0.965767985	0.532849748	0.475783582	0.705781946	0.892264713	0.99429481
UTP23	0.938434084	0.351912196	0.950074919	0.306509956	0.862110919	0.892509087	0.99429481

ZNF526	0.329864269	0.719259161	0.65955131	0.887879707	0.594048169	0.89190936	0.99429481
RPRML	0.484420142	0.403881206	0.91917966	0.740719949	0.626202011	0.893315184	0.994518383
ACSM2B	0.277284652	0.631257005	0.984571246	0.872276482	0.558813116	0.894243956	0.994560569
ADRB1	0.531985303	0.434936732	0.885483746	0.753361607	0.54336508	0.894031991	0.994560569
ARHGEF10	0.548878705	0.83497593	0.245878701	0.775753336	0.960053889	0.894119722	0.994560569
ARL15	0.535614169	0.522863861	0.590885449	0.574751075	0.879750351	0.89372257	0.994560569
ATP2B1	0.525460138	0.364340966	0.778286143	0.58857199	0.969145795	0.895778127	0.994560569
BLCAP	0.632481026	0.487974097	0.618635191	0.529240236	0.835810274	0.89495245	0.994560569
C16H1orf112	0.74961453	0.269357722	0.442894746	0.992826949	0.952894771	0.895178175	0.994560569
CCDC97	0.925453233	0.524761926	0.349079061	0.596558116	0.843456089	0.896253427	0.994560569
CHAMP1	0.489078888	0.627051025	0.545446284	0.53238644	0.955716034	0.895962913	0.994560569
DCHS1	0.942426944	0.305016054	0.66897846	0.453758192	0.977044738	0.896182992	0.994560569
DRAM1	0.937615826	0.613889904	0.751624082	0.497931355	0.396371496	0.896383242	0.994560569
ERMAP	0.828955537	0.868296574	0.295590947	0.62501095	0.637064322	0.895351734	0.994560569
FAS	0.371273088	0.97519154	0.881111165	0.667036273	0.393596453	0.893853742	0.994560569
FOXF2	0.889865702	0.401119231	0.675603806	0.583957019	0.60088092	0.895200185	0.994560569
GNB5	0.679858813	0.337322891	0.658707133	0.920280161	0.613267721	0.896184683	0.994560569
GPN2	0.374007326	0.643350892	0.80695135	0.496462588	0.876107395	0.894946339	0.994560569
GSTA4	0.820689749	0.604762884	0.677195332	0.249417228	0.997883244	0.89369248	0.994560569
IFT46	0.978750584	0.68356177	0.767393543	0.233463362	0.702210141	0.894502801	0.994560569
LOC100299845	0.537238341	0.479772981	0.483088523	0.906153921	0.755412366	0.896151163	0.994560569
LOC100336777	0.751381481	0.951314631	0.319970716	0.725756986	0.511122609	0.895547746	0.994560569
LOC100848105	0.720617518	0.799586074	0.971577516	0.380201748	0.396263769	0.894772942	0.994560569
LOC101902036	0.963337916	0.805045343	0.868951942	0.234832743	0.536698703	0.895690221	0.994560569
LOC101902907	0.433168051	0.482027291	0.538164732	0.895942545	0.847700733	0.896316567	0.994560569
LOC104970162	0.794956951	0.569429461	0.895706354	0.360581153	0.574901492	0.894318478	0.994560569
LOC104973390	0.331596075	0.969749353	0.664932336	0.484559776	0.815957625	0.895079951	0.994560569
LOC104975324	0.546152072	0.727237864	0.814463358	0.387896555	0.672155857	0.894773415	0.994560569
LOC112441843	0.946925764	0.478178955	0.417380143	0.960620186	0.463013589	0.89432999	0.994560569
LOC112442221	0.893330674	0.33662934	0.562585987	0.679711837	0.732457063	0.894595599	0.994560569
LOC112443225	0.987641682	0.85464641	0.778046373	0.282768053	0.454749034	0.894938554	0.994560569
LOC112448088	0.593744416	0.298884882	0.870049171	0.723978973	0.756179696	0.895060899	0.994560569
LOC112448770	0.651188718	0.308116305	0.967948634	0.926994331	0.470702261	0.895392728	0.994560569
LOC112449367	0.955727031	0.733608576	0.416925927	0.510031355	0.569804687	0.895719177	0.994560569
LOC786303	0.80476105	0.285774326	0.677890194	0.651490564	0.827929413	0.894380722	0.994560569
LRRC25	0.630061085	0.28422639	0.925578178	0.735676633	0.695976989	0.895587174	0.994560569
PATJ	0.348450567	0.576533608	0.836612359	0.54647355	0.922273786	0.895338977	0.994560569
PCDH9	0.684607385	0.874647038	0.602673226	0.498516415	0.472164601	0.895703852	0.994560569
PLOD3	0.960116194	0.410937006	0.488665233	0.742898622	0.59270165	0.895627613	0.994560569
RBFOX3	0.264075151	0.68282078	0.719936467	0.72392221	0.908503843	0.896370963	0.994560569
RFC4	0.697954068	0.350386654	0.854884441	0.64848974	0.628122496	0.896035001	0.994560569
SART3	0.849819125	0.707580517	0.867664261	0.218390408	0.744895178	0.89559947	0.994560569
SHROOM1	0.549536081	0.984626431	0.830457986	0.57845439	0.321714196	0.893643949	0.994560569
SLC26A9	0.743040983	0.298130027	0.563739388	0.914484065	0.744871255	0.895892065	0.994560569

STAG3	0.577189457	0.418725005	0.667805889	0.865652577	0.597643996	0.893448877	0.994560569
TCIRG1	0.450664837	0.741788673	0.452791074	0.635372841	0.873674963	0.894277418	0.994560569
TJP3	0.685930684	0.274837336	0.731022172	0.751555083	0.813234375	0.894596485	0.994560569
TMEM169	0.753044736	0.925977938	0.827175157	0.308855568	0.475465791	0.895331287	0.994560569
TMEM51	0.791856084	0.410250687	0.783411497	0.626481725	0.528374034	0.89461839	0.994560569
TRAM1L1	0.310271451	0.825020656	0.765353297	0.451675136	0.959203301	0.895606296	0.994560569
ZBTB3	0.989442251	0.738757032	0.434313001	0.894046631	0.298492878	0.89536009	0.994560569
ZNF579	0.800873926	0.655440491	0.695088889	0.566456927	0.411984849	0.896021957	0.994560569
ATR	0.316519684	0.967020291	0.95194151	0.308424244	0.953179393	0.896797743	0.99462265
H2B	0.70062767	0.449184583	0.869471527	0.769324705	0.406733838	0.896743117	0.99462265
HGS	0.67941599	0.617059517	0.532284031	0.818090517	0.469351044	0.896838399	0.99462265
LOC112444867	0.756671041	0.395467493	0.844842599	0.602179973	0.562642972	0.896792704	0.99462265
RAB11FIP2	0.384705491	0.737140365	0.688010364	0.794948028	0.552556504	0.896863443	0.99462265
SLC16A2	0.929772294	0.841382904	0.254767082	0.964552433	0.445644361	0.896815162	0.99462265
UBR4	0.666001669	0.287310735	0.714562004	0.695532497	0.900478476	0.896764213	0.99462265
PPOX	0.871076592	0.520864151	0.80660218	0.492338527	0.475932754	0.896941511	0.994642012
ALCAM	0.6199717	0.919927333	0.622349318	0.498545875	0.486388503	0.897419651	0.994788199
HHLA2	0.184628666	0.885103931	0.876913839	0.698345454	0.858431127	0.897173919	0.994788199
LOC100138633	0.611209551	0.401388437	0.968749412	0.539623668	0.670373523	0.897278052	0.994788199
LOC100295687	0.785324925	0.506435803	0.553911935	0.473382256	0.82500001	0.897369568	0.994788199
LOC101903831	0.278916442	0.610447626	0.908842821	0.954115426	0.583306668	0.897497658	0.994788199
LOC112444281	0.730729519	0.60951168	0.693218636	0.52543573	0.529636138	0.897197859	0.994788199
TRMT2B	0.357278303	0.880709392	0.64740967	0.962803434	0.438937621	0.89745277	0.994788199
BAIAP2L1	0.46074519	0.416825815	0.67549379	0.742730105	0.894280158	0.897567009	0.99479473
LOC112442867	0.394574493	0.60068265	0.866928832	0.475063243	0.883132704	0.897624786	0.99479473
LEKR1	0.250106006	0.918702493	0.468895542	0.922017725	0.869485181	0.897875851	0.994871419
LOC101906457	0.441775178	0.709900172	0.602754919	0.722658354	0.632150796	0.897850956	0.994871419
LOC112444198	0.336055147	0.900298059	0.917677362	0.325095638	0.956390029	0.897803243	0.994871419
LOC100336869	0.778262525	0.58100807	0.933455408	0.216807606	0.944321572	0.897941341	0.994876813
ACKR3	0.630877971	0.571650896	0.345290258	0.943823538	0.75040501	0.90056205	0.994886058
AGK	0.766114031	0.709783292	0.969878072	0.253220562	0.666722224	0.901774112	0.994886058
AJAP1	0.520187787	0.330511273	0.867890917	0.635500029	0.932976101	0.900958934	0.994886058
ALDOC	0.511845804	0.889538422	0.516819121	0.496857533	0.75497365	0.900667626	0.994886058
ARPC1B	0.574785687	0.907280921	0.959896962	0.534169442	0.328784149	0.900154257	0.994886058
BBOF1	0.861285107	0.76744144	0.902953119	0.922697758	0.161380617	0.901536579	0.994886058
BCL2A1	0.776097711	0.564122127	0.663116699	0.7247792	0.421160213	0.901174559	0.994886058
CAPN1	0.545041494	0.932404065	0.294362779	0.905142762	0.652507481	0.90078904	0.994886058
CASKIN1	0.841551276	0.32279243	0.525814829	0.644803205	0.9577941	0.900588227	0.994886058
CCDC43	0.808910451	0.232118932	0.683824309	0.893951778	0.761503946	0.899409914	0.994886058
CD7	0.511159447	0.709677554	0.735952057	0.641724765	0.505375092	0.898189546	0.994886058
CEACAM19	0.777158605	0.723790025	0.307979939	0.733792123	0.696614225	0.901079966	0.994886058
CLDN20	0.724509142	0.944075064	0.458025589	0.64866587	0.437773628	0.901666074	0.994886058
DZIP1	0.24504944	0.650356958	0.668671964	0.992265008	0.841180548	0.901644058	0.994886058
ECM2	0.883236686	0.423939791	0.525368061	0.713103012	0.616812628	0.898106439	0.994886058

FAM189B	0.591464438	0.659482887	0.832441979	0.624055276	0.42996664	0.898995907	0.994886058
FOXD2	0.520435131	0.779628675	0.904715563	0.276190121	0.870497292	0.900648788	0.994886058
GOPC	0.963407627	0.962182219	0.393734676	0.257191595	0.93482732	0.899917412	0.994886058
GPANK1	0.579617521	0.700924014	0.610537799	0.73153344	0.478979009	0.898678927	0.994886058
GTF2I	0.950378075	0.914720013	0.62081289	0.44906341	0.359339269	0.898940666	0.994886058
HAVCR2	0.222586573	0.833128745	0.898326365	0.816776023	0.653604402	0.901622894	0.994886058
HOXB8	0.914500158	0.64809477	0.551630597	0.555140537	0.483599091	0.899946871	0.994886058
IFI47	0.740315862	0.653680402	0.712190883	0.309332504	0.814874067	0.898625267	0.994886058
ITGB5	0.836365714	0.801279942	0.832538867	0.698541102	0.225152716	0.899915915	0.994886058
LOC100297676	0.950356845	0.900473577	0.677993053	0.7305697	0.208629306	0.900906739	0.994886058
LOC101902768	0.52450698	0.323446834	0.59445271	0.923826639	0.950805536	0.901122093	0.994886058
LOC101903793	0.862539546	0.440670538	0.833999008	0.948905069	0.295943706	0.90174786	0.994886058
LOC101906398	0.841387824	0.821736792	0.439140607	0.905139924	0.316929019	0.898956269	0.994886058
LOC101906966	0.974505567	0.773421132	0.629441942	0.192443593	0.956175773	0.899248727	0.994886058
LOC104969340	0.876864117	0.551314613	0.394604249	0.772493047	0.601643374	0.901231651	0.994886058
LOC104971926	0.923437001	0.608150313	0.42365097	0.74941899	0.499595865	0.901829565	0.994886058
LOC104974749	0.736099885	0.398039507	0.699057731	0.467246196	0.908892005	0.898785315	0.994886058
LOC107131906	0.929494819	0.925994256	0.544038157	0.503663656	0.372024184	0.89989887	0.994886058
LOC107132877	0.393500724	0.632113165	0.915910231	0.580623023	0.661694516	0.899588326	0.994886058
LOC112441568	0.910921511	0.78316274	0.426416173	0.608862105	0.480264712	0.901653053	0.994886058
LOC112446779	0.734099723	0.670766803	0.797015846	0.319002991	0.6956553	0.89894801	0.994886058
LOC112447469	0.786638872	0.347399935	0.767767214	0.918068596	0.451751485	0.898837362	0.994886058
LOC112448045	0.979038466	0.516132334	0.28373703	0.642124397	0.953659568	0.899985321	0.994886058
LOC112448169	0.621094055	0.406308531	0.47429038	0.744480933	0.974291101	0.898537613	0.994886058
LOC112449619	0.956747148	0.525413686	0.891690116	0.217584503	0.895416066	0.899298504	0.994886058
LOC507787	0.657832826	0.965055419	0.32239254	0.752620082	0.571633793	0.900355843	0.994886058
LOC614695	0.89600916	0.982389058	0.327027721	0.863347917	0.357056024	0.901341693	0.994886058
LOC788183	0.884387463	0.901492808	0.458553756	0.916866742	0.263521848	0.900759287	0.994886058
LOC790266	0.758530805	0.469542099	0.857887375	0.324671084	0.878618894	0.89904874	0.994886058
LRRC28	0.879975597	0.466566672	0.31223929	0.987831553	0.684244311	0.898289646	0.994886058
MARS2	0.901807184	0.659200137	0.480352362	0.336512603	0.916656466	0.900400708	0.994886058
MSH3	0.828040698	0.733142507	0.575465859	0.640921669	0.386225955	0.898033641	0.994886058
NDUFAF2	0.581594188	0.44894997	0.770441348	0.77584716	0.564770876	0.900491129	0.994886058
OVGP1	0.941376265	0.427122434	0.964514282	0.245234396	0.9308632	0.901045186	0.994886058
OXA1L	0.949192839	0.610424051	0.964700106	0.351625636	0.450728334	0.901128	0.994886058
PRKCG	0.507238155	0.970945038	0.790801459	0.416589506	0.543178775	0.900467317	0.994886058
RAB6B	0.355736331	0.847793614	0.60577612	0.584164327	0.823518937	0.900117962	0.994886058
RPL34	0.64740998	0.523607504	0.565600342	0.683870855	0.666073003	0.899306049	0.994886058
SLC13A5	0.448309454	0.730327737	0.478924077	0.706504524	0.798284238	0.900911135	0.994886058
SLC39A11	0.642706227	0.750783307	0.744593957	0.701407287	0.347523238	0.899663892	0.994886058
SMIM1	0.754342426	0.768744354	0.376342933	0.676298537	0.592371128	0.899446827	0.994886058
SOCS4	0.681238076	0.851407473	0.517332436	0.619662708	0.467191623	0.898613832	0.994886058
SUSD6	0.87775929	0.731001424	0.605145144	0.736144634	0.308317	0.900463989	0.994886058
TMEM102	0.512058449	0.973283281	0.359576638	0.761962192	0.645494273	0.900482309	0.994886058

USP30	0.827488789	0.874521023	0.702523485	0.509537775	0.338727071	0.899905688	0.994886058
WDR91	0.779284789	0.974029328	0.340658939	0.884143276	0.384223653	0.900045904	0.994886058
ZC3H7A	0.250933244	0.721754979	0.622291621	0.911736722	0.863192648	0.901287885	0.994886058
ZNF581	0.27910827	0.716244135	0.961263586	0.604477516	0.765608632	0.901622915	0.994886058
ZSWIM3	0.420709466	0.624536396	0.761249663	0.762649882	0.567827891	0.898242958	0.994886058
LOC112446381	0.675142542	0.295211825	0.734567703	0.754255917	0.80783309	0.902014035	0.995022675
C10H15orf59	0.866700246	0.17667823	0.991594326	0.629054363	0.938771501	0.902664839	0.995030296
CDR2L	0.970597004	0.377645456	0.407532327	0.91687914	0.654810957	0.902687895	0.995030296
FAM185A	0.266544856	0.61694292	0.92158019	0.991887664	0.596533303	0.902668196	0.995030296
HHAT	0.814347802	0.717689101	0.262656415	0.747627215	0.7785768	0.902223986	0.995030296
LOC101902959	0.683580985	0.974081582	0.235903462	0.959760024	0.593560655	0.902406639	0.995030296
LOC101906688	0.765451501	0.915626385	0.679810467	0.705696244	0.266640393	0.902645487	0.995030296
LOC104975415	0.928296213	0.528144574	0.281729135	0.844425263	0.768275839	0.902582028	0.995030296
LOC782258	0.874725531	0.691794302	0.629709822	0.842591887	0.278219764	0.90218713	0.995030296
PHF5A	0.91156108	0.391925517	0.4619478	0.795606491	0.682947657	0.902674699	0.995030296
TRPS1	0.780670853	0.396749423	0.510728562	0.63269251	0.895024774	0.902539113	0.995030296
ZNF513	0.260728935	0.926523459	0.957749957	0.826639094	0.468534148	0.902583607	0.995030296
ADPRHL2	0.731755765	0.972516764	0.211122519	0.642513508	0.954911212	0.906127539	0.995059176
AK7	0.917897345	0.625920208	0.237867037	0.969023137	0.694654439	0.905872326	0.995059176
APBB1	0.767825481	0.663845469	0.790835559	0.864090095	0.267320342	0.907369878	0.995059176
APEH	0.774346832	0.838797981	0.606721803	0.368830704	0.624145024	0.904131941	0.995059176
ARF4	0.734518577	0.494654933	0.515869526	0.747501984	0.653842602	0.905351011	0.995059176
ARMC8	0.463878006	0.98837085	0.434655018	0.544228638	0.848170359	0.905867342	0.995059176
ATL1	0.378497751	0.950547137	0.662709615	0.747812108	0.521841496	0.907280368	0.995059176
CASTOR1	0.655669666	0.972923332	0.590604786	0.422217895	0.584022903	0.90709174	0.995059176
CCDC85B	0.463195495	0.765338124	0.527577523	0.858657653	0.5698243	0.905217391	0.995059176
CEBPA	0.72569535	0.691523507	0.661247084	0.347894611	0.786612905	0.904258591	0.995059176
CPEB3	0.561086923	0.828865222	0.3703956	0.941993839	0.56623865	0.905722691	0.995059176
DLC1	0.432899646	0.650783195	0.723622931	0.453418295	0.984248209	0.904490955	0.995059176
DRD1	0.404360004	0.953888723	0.700592082	0.887496808	0.386116203	0.906690276	0.995059176
EIF2B4	0.83067295	0.487212477	0.55734393	0.499612279	0.805846426	0.904265316	0.995059176
ENTPD7	0.41442958	0.415975535	0.576096644	0.974208147	0.939951829	0.904442437	0.995059176
FAM120C	0.256406739	0.727970353	0.617219711	0.923091167	0.867928137	0.906289791	0.995059176
FHOD1	0.822537823	0.926535857	0.349540775	0.430072266	0.806765271	0.906459055	0.995059176
GSTZ1	0.325264203	0.625204794	0.60091249	0.935288777	0.797538575	0.904729345	0.995059176
HRH1	0.876678532	0.866423592	0.242216913	0.807631737	0.626706374	0.907382887	0.995059176
IFITM2	0.642506087	0.607990083	0.577546332	0.476882125	0.842931451	0.904094049	0.995059176
INTS3	0.627925357	0.630823271	0.907345095	0.682463477	0.37649524	0.906351389	0.995059176
KLHL9	0.712460242	0.719858797	0.394492739	0.752385514	0.609971342	0.907026266	0.995059176
LACTB	0.857266877	0.607821511	0.979134725	0.977296889	0.182268405	0.904356288	0.995059176
LCOR	0.572445772	0.94619006	0.825992623	0.599923773	0.344854668	0.906634768	0.995059176
LOC100297513	0.582076669	0.840125827	0.506668478	0.553398917	0.678556191	0.907275031	0.995059176
LOC100847363	0.697247616	0.791677911	0.503768697	0.369354051	0.878421472	0.903442697	0.995059176
LOC100848495	0.454806485	0.332516772	0.859074327	0.695812307	0.99789366	0.903423789	0.995059176

LOC101904377	0.71138532	0.428666237	0.867351789	0.472344929	0.739176331	0.906351286	0.995059176
LOC101904622	0.773039336	0.687019071	0.812422552	0.681775316	0.313872872	0.906328643	0.995059176
LOC101907697	0.387670441	0.588816737	0.907829023	0.685987606	0.639058636	0.904307655	0.995059176
LOC104972526	0.602846287	0.38862482	0.725491919	0.874467268	0.618284553	0.905743934	0.995059176
LOC107132664	0.659441429	0.481774972	0.860980873	0.640067391	0.527872779	0.906448889	0.995059176
LOC112444842	0.929310449	0.175155988	0.702114355	0.83537818	0.959188295	0.905308	0.995059176
LOC112445052	0.932125676	0.620420606	0.649336006	0.490034793	0.4955103	0.904770373	0.995059176
LOC112446701	0.676799116	0.613733548	0.434701782	0.951670947	0.52929293	0.904454757	0.995059176
LOC112447433	0.650725081	0.265002945	0.696827681	0.783343613	0.986236835	0.907000779	0.995059176
LOC112447838	0.791240543	0.665069761	0.809400385	0.915078634	0.23531333	0.905498272	0.995059176
LOC112449052	0.621855929	0.77111331	0.738846717	0.595327526	0.432797044	0.904912138	0.995059176
LOC112449300	0.74915762	0.732757274	0.517564297	0.562644495	0.569682487	0.904613415	0.995059176
LOC783163	0.940932372	0.886737032	0.314744282	0.768774103	0.447589543	0.903638869	0.995059176
LOC786332	0.81006573	0.511782919	0.503133359	0.730558261	0.598710342	0.904842593	0.995059176
LOC787057	0.768057457	0.400607316	0.551892354	0.860761014	0.61651125	0.903291354	0.995059176
LOC789388	0.570511474	0.421344986	0.767768858	0.620470951	0.794338598	0.904467975	0.995059176
LRPAP1	0.516318912	0.685637228	0.665568841	0.58338572	0.669292112	0.905879934	0.995059176
MARCH7	0.618958289	0.624758811	0.934290614	0.301136332	0.837690312	0.904710688	0.995059176
MAST3	0.763085811	0.488498337	0.271696281	0.922796567	0.971570566	0.904248968	0.995059176
MBLAC1	0.44810309	0.892631472	0.571128126	0.403197249	0.989948474	0.904771541	0.995059176
MECP2	0.520860521	0.83613389	0.909764233	0.731686419	0.319290309	0.906639262	0.995059176
MKLN1	0.868802971	0.701952819	0.573780422	0.842637823	0.314080018	0.906701736	0.995059176
MRPL38	0.813580842	0.806369978	0.832410315	0.181087948	0.922374302	0.904816265	0.995059176
NAPEPLD	0.811204714	0.356580421	0.959688586	0.680204179	0.481005225	0.904279815	0.995059176
NT5C2	0.617443535	0.715206158	0.780440179	0.862281212	0.302352346	0.902925489	0.995059176
PTPN18	0.383039228	0.980442119	0.680543327	0.359412887	0.997840677	0.905421363	0.995059176
RAB11FIP4	0.852551036	0.924379868	0.186267456	0.904058807	0.684483377	0.904297142	0.995059176
RAD1	0.41539631	0.50536044	0.887688942	0.833591412	0.593856918	0.906217983	0.995059176
RPS6	0.603923193	0.822631297	0.604857623	0.385599705	0.777634339	0.903280233	0.995059176
SEPT6	0.806883257	0.488645747	0.880449654	0.53053187	0.502181077	0.906537909	0.995059176
SHB	0.884686693	0.766329552	0.314464244	0.502115937	0.839495993	0.902945422	0.995059176
SLC25A40	0.444184932	0.581191351	0.948919113	0.583915361	0.650937933	0.907368431	0.995059176
SLC45A2	0.669069605	0.89765672	0.643193999	0.448743886	0.535890166	0.907083418	0.995059176
SPAM1	0.949298178	0.837557008	0.777927196	0.202103914	0.72340825	0.903732759	0.995059176
STEAP1	0.378090815	0.710922546	0.996429068	0.747014603	0.459446612	0.90578037	0.995059176
SYNRG	0.664526569	0.647645267	0.345678053	0.812344508	0.768721548	0.907092726	0.995059176
TATDN2	0.735988895	0.450976897	0.584518848	0.637261754	0.728131888	0.903164102	0.995059176
TBCCD1	0.768172497	0.766772351	0.561683717	0.479684149	0.572819696	0.904390281	0.995059176
TCEA3	0.596751465	0.86893911	0.573683854	0.442352316	0.704435463	0.90681811	0.995059176
TCF7	0.967044761	0.830180159	0.226100826	0.860307746	0.590524465	0.906176023	0.995059176
TMEM205	0.54957384	0.965258024	0.257314779	0.707727567	0.963903156	0.907377532	0.995059176
TMEM65	0.655008262	0.752669157	0.277536181	0.963735674	0.699591582	0.906221774	0.995059176
TSPAN5	0.489931986	0.604725716	0.451199318	0.779086618	0.883134617	0.905850871	0.995059176
UNC13D	0.690580391	0.914978	0.903259671	0.224445811	0.70305522	0.903218217	0.995059176

UNC93B1	0.345199276	0.871009564	0.91164862	0.695945773	0.48734838	0.907179247	0.995059176
UTP3	0.966666316	0.63113913	0.95393831	0.76243926	0.202882235	0.903169696	0.995059176
WIZ	0.612016627	0.623479788	0.841033156	0.657313825	0.440973123	0.907249784	0.995059176
ZNF281	0.759315868	0.981087074	0.618590277	0.300972773	0.665625892	0.906312412	0.995059176
ZNF580	0.650831013	0.799470376	0.409165651	0.759327041	0.557382784	0.903280228	0.995059176
ZNFX1	0.543359932	0.986165504	0.965770581	0.349582933	0.513871756	0.907173893	0.995059176
STUM	0.624732202	0.761698836	0.830734305	0.380321014	0.620162337	0.907536715	0.995161368
FAF2	0.793215063	0.681866897	0.782372616	0.687918694	0.320458639	0.907598572	0.995162703
AIFM2	0.436263054	0.530604215	0.483268012	0.98258563	0.864200906	0.90981727	0.995268046
FEZ1	0.700098458	0.758604292	0.507312754	0.471789774	0.738707182	0.908406524	0.995268046
GLB1	0.275498151	0.485502594	0.98400052	0.879098544	0.819055034	0.909527105	0.995268046
HOXC8	0.98666196	0.313890737	0.830761708	0.60643012	0.607615554	0.909576649	0.995268046
ILF3	0.981773155	0.560664788	0.586672495	0.827493712	0.353913666	0.909279146	0.995268046
KNSTRN	0.849115164	0.172538208	0.857249674	0.988664337	0.764280027	0.909696423	0.995268046
LOC100847890	0.180318137	0.762751172	0.759462353	0.939890979	0.962498828	0.909176395	0.995268046
LOC100848353	0.73172936	0.374245699	0.762004159	0.464697726	0.969701021	0.908576256	0.995268046
LOC101902048	0.820372582	0.718283563	0.66959067	0.685921709	0.346554954	0.90826319	0.995268046
LOC101903795	0.568667743	0.540050951	0.789333168	0.480272572	0.805481743	0.90824457	0.995268046
LOC101904963	0.644654361	0.66740441	0.533115813	0.951846734	0.429174874	0.908143381	0.995268046
LOC101905498	0.474476382	0.575015882	0.880625974	0.806286253	0.48976629	0.909669396	0.995268046
LOC107132327	0.889406299	0.427636933	0.454409746	0.710657248	0.766527856	0.908727844	0.995268046
LOC112442949	0.564733296	0.933647914	0.6194553	0.366227383	0.788353377	0.908924009	0.995268046
LOC112444521	0.627856522	0.700899901	0.641377591	0.681144404	0.492202609	0.909346807	0.995268046
LOC510798	0.84854999	0.989198693	0.376962381	0.707889927	0.423205581	0.909560703	0.995268046
LOC782159	0.456461175	0.30594023	0.934281543	0.946677926	0.756524632	0.907805374	0.995268046
MAP3K8	0.219746791	0.559930506	0.884842738	0.875580491	0.987660257	0.908732136	0.995268046
METTL23	0.713487709	0.871334081	0.198700787	0.792721374	0.965693152	0.909267443	0.995268046
NECAP2	0.322485022	0.655286435	0.69094933	0.847647627	0.762266432	0.908980484	0.995268046
PHF6	0.236647306	0.979598706	0.809827236	0.645922238	0.771489582	0.90794936	0.995268046
RBM15B	0.64519539	0.62415755	0.839519018	0.299245109	0.932005433	0.908910869	0.995268046
RIOK1	0.989095637	0.405520159	0.524493545	0.966906454	0.460545812	0.908117757	0.995268046
RTL1	0.869366214	0.901008036	0.780600286	0.159250287	0.969376154	0.909043346	0.995268046
SCIMP	0.601784009	0.380427991	0.493200547	0.971100642	0.865970331	0.909764545	0.995268046
SLC1A4	0.606296541	0.366983824	0.643549399	0.988896503	0.664500871	0.90865719	0.995268046
TDRD9	0.889244267	0.970589699	0.187431955	0.792474741	0.739277608	0.909537518	0.995268046
TLN2	0.813359042	0.547186174	0.445513958	0.979615142	0.482821185	0.908251168	0.995268046
TMPO	0.57676748	0.722561346	0.495507663	0.802772319	0.571979326	0.909595026	0.995268046
TNNI3	0.422862141	0.742285041	0.872320779	0.346979576	0.999775096	0.909805979	0.995268046
UFD1	0.925541057	0.804134785	0.448111175	0.297376918	0.956027302	0.909592022	0.995268046
ZDHHC19	0.174972261	0.886362164	0.825256035	0.936248312	0.780101296	0.907852816	0.995268046
ZFAND1	0.980095852	0.334515156	0.432887197	0.989362911	0.669954415	0.908628927	0.995268046
ZKSCAN4	0.615104926	0.914526098	0.343973861	0.985791832	0.491023414	0.908092324	0.995268046
ZNF891	0.878507486	0.563582212	0.626829271	0.777569435	0.389622384	0.908565561	0.995268046
LOC100337293	0.267699656	0.840461313	0.95415853	0.474964156	0.932567894	0.90993897	0.995280579

TUFT1	0.915256311	0.217595626	0.902868836	0.773898163	0.683385133	0.909950022	0.995280579
ORAI1	0.894803032	0.6515874	0.284432475	0.7602173	0.755545491	0.910148648	0.995380341
RBMS3	0.674646353	0.9157313	0.732641538	0.288631981	0.729200873	0.910162537	0.995380341
LOC101904098	0.873162155	0.685204341	0.704625727	0.502850153	0.449729177	0.910256277	0.995416523
BPGM	0.470074356	0.92350778	0.713570092	0.601552029	0.513053779	0.910595541	0.995486527
R3HDM4	0.949666294	0.615822242	0.460619119	0.473364624	0.74957598	0.910567673	0.995486527
RPL21	0.715902888	0.421223811	0.499410818	0.779132548	0.814512336	0.910554905	0.995486527
STX10	0.469560923	0.901621029	0.9745955	0.241518949	0.95959716	0.910623591	0.995486527
TTI2	0.591551584	0.827448887	0.448005629	0.88566671	0.491437384	0.910393721	0.995486527
MTHFR	0.730845119	0.994174005	0.690786866	0.62675646	0.304202681	0.910710784	0.995509387
PPP1R21	0.711788245	0.502710926	0.700210796	0.50908193	0.750595245	0.910765824	0.995509387
LOC112444593	0.576470739	0.858611726	0.699092878	0.954379614	0.290163802	0.910872076	0.995559216
PPP1R9A	0.379890322	0.739150265	0.863122685	0.581400457	0.680799065	0.911006762	0.995573815
RBM7	0.866864619	0.424395437	0.410908441	0.672511061	0.943208553	0.91095573	0.995573815
FNDC3B	0.805347706	0.920467125	0.368977899	0.424782714	0.82700208	0.911203314	0.995656008
LARGE1	0.825523024	0.449487439	0.938914962	0.352540148	0.782071212	0.911165112	0.995656008
AKAP1	0.285392002	0.962351089	0.549103368	0.716581053	0.892151391	0.911610265	0.995759723
ATAD5	0.780862854	0.963348586	0.20649011	0.84063072	0.738856323	0.911690388	0.995759723
CCDC125	0.958457666	0.670554585	0.807595415	0.229284521	0.810102537	0.911605369	0.995759723
GPATCH11	0.907124418	0.865836955	0.228526395	0.708658842	0.758689185	0.911722967	0.995759723
KLHDC1	0.625243262	0.330281041	0.946053641	0.981011232	0.502521479	0.911483864	0.995759723
MSX2	0.920245513	0.521553591	0.597021818	0.476546212	0.705272199	0.911477657	0.995759723
PRSS42	0.986408463	0.684523369	0.239481278	0.830808715	0.718027209	0.91167263	0.995759723
ACTR8	0.952723068	0.401953156	0.840355349	0.724577875	0.428209101	0.915770327	0.99580676
ADAMTS1	0.733381726	0.291506194	0.73533364	0.826973977	0.803625639	0.920990034	0.99580676
AES	0.989055992	0.378936297	0.409312089	0.940571906	0.674784513	0.91278731	0.99580676
AMPH	0.974658682	0.127838534	0.998491983	0.861868146	0.956616293	0.918895564	0.99580676
ANK2	0.679697378	0.680238776	0.671032971	0.504043303	0.634840244	0.915095842	0.99580676
ANKRD29	0.918868462	0.51190364	0.764863582	0.425358063	0.658903287	0.916915216	0.99580676
ANP32A	0.576257546	0.641298198	0.992154641	0.506927029	0.533187658	0.914886505	0.99580676
ANTXR1	0.511607509	0.728683099	0.47889323	0.614917924	0.925490394	0.917797645	0.99580676
APBB1IP	0.459512075	0.961036601	0.774063812	0.308629035	0.927269425	0.913352143	0.99580676
APOBEC3Z2	0.66813986	0.275313426	0.901630499	0.87267713	0.678309694	0.913775997	0.99580676
ARID5B	0.720270944	0.57464456	0.294728979	0.875808257	0.948981301	0.91755261	0.99580676
ATAD2	0.648459054	0.866709944	0.479729407	0.670410991	0.546642473	0.914537443	0.99580676
BAP1	0.849165425	0.405503542	0.446885193	0.69464087	0.949058683	0.917619757	0.99580676
BAX	0.540986857	0.461755775	0.588836092	0.883821673	0.781823253	0.91784117	0.99580676
BCAS3	0.701941221	0.781692094	0.267810613	0.868766068	0.780095822	0.915464675	0.99580676
C7H19orf38	0.761102126	0.888165577	0.310659188	0.587530551	0.790389529	0.912978021	0.99580676
CABLES2	0.623053138	0.992992352	0.3787932	0.514971321	0.826066083	0.915587965	0.99580676
CACNA2D1	0.672522461	0.860161522	0.232629331	0.82410331	0.919986904	0.918279621	0.99580676
CARD11	0.465439147	0.689491983	0.738115327	0.628071327	0.656010523	0.91307232	0.99580676
CCDC106	0.846701982	0.632390335	0.529123941	0.411435347	0.838559884	0.913256898	0.99580676
CCNG2	0.873613863	0.624863643	0.625879671	0.556166595	0.523703893	0.915375726	0.99580676

CCP110	0.662405088	0.76518401	0.856254413	0.262978413	0.889312243	0.917681228	0.99580676
CEP44	0.479873907	0.381075248	0.917917273	0.890765162	0.688887246	0.91937516	0.99580676
CIRBP	0.596388332	0.662021335	0.685873861	0.885020555	0.406690469	0.912914841	0.99580676
CPNE5	0.695469656	0.768026519	0.437810808	0.750656948	0.587076191	0.919434255	0.99580676
CRISPLD1	0.500388711	0.358729176	0.711670467	0.886360722	0.891939319	0.917101947	0.99580676
CROT	0.292490083	0.880184811	0.878464564	0.60128263	0.768270597	0.920988521	0.99580676
CST3	0.530266413	0.287989614	0.931121974	0.776943296	0.889731902	0.913918352	0.99580676
CTNNA1	0.74958243	0.450194783	0.431236276	0.978043541	0.684502824	0.912861486	0.99580676
DCAF16	0.391286197	0.40377363	0.892198529	0.727437445	0.977835117	0.916258049	0.99580676
DCLK3	0.537158408	0.942846341	0.577394477	0.455048578	0.782360004	0.920590883	0.99580676
DDX59	0.978338713	0.510755691	0.713329494	0.344892381	0.811760808	0.915705046	0.99580676
DENND4A	0.542625828	0.652157137	0.855025921	0.941029002	0.367115408	0.921049954	0.99580676
DIO1	0.588427473	0.468294759	0.621859787	0.765320689	0.754448432	0.914695316	0.99580676
EHD2	0.929380338	0.999801612	0.285612053	0.4296908	0.899653655	0.91891613	0.99580676
ENPP6	0.616848255	0.688670241	0.746017147	0.504509826	0.632344285	0.917225782	0.99580676
ERCC2	0.878351603	0.850866416	0.739565413	0.295409838	0.596368946	0.912800393	0.99580676
FAM200A	0.351303306	0.673008486	0.449462694	0.982994715	0.974040975	0.917963192	0.99580676
FAM57B	0.34991267	0.84339091	0.943832967	0.501677749	0.717323878	0.916222674	0.99580676
FBF1	0.911358578	0.219640384	0.865702201	0.617461225	0.952023998	0.918096985	0.99580676
FRA10AC1	0.804280978	0.863095132	0.826329689	0.376854739	0.474951061	0.919002734	0.99580676
GAA	0.355365431	0.892991566	0.471379417	0.862421819	0.78810828	0.917876357	0.99580676
GJC3	0.704663548	0.567267809	0.445897922	0.675877119	0.848227341	0.91845775	0.99580676
GPR143	0.959059508	0.726643768	0.288360227	0.529468575	0.92850047	0.914517746	0.99580676
GPR27	0.94266985	0.442516687	0.578017532	0.710046266	0.588812682	0.916886317	0.99580676
GPR52	0.361265382	0.432318512	0.87618738	0.979325296	0.737373593	0.914549563	0.99580676
GRID2IP	0.384535375	0.795162686	0.618665521	0.645014778	0.810378664	0.914621406	0.99580676
HAND1	0.948438996	0.808040822	0.493807114	0.312966561	0.847143372	0.916338679	0.99580676
HFE	0.889415206	0.222217579	0.907367603	0.637389896	0.888699613	0.917777128	0.99580676
HIST2H2AC	0.602437804	0.965413339	0.59528671	0.829233877	0.340226996	0.913171492	0.99580676
IQCE	0.452406118	0.896375616	0.576180183	0.549219535	0.801588092	0.919222423	0.99580676
IRX3	0.71347543	0.304211684	0.898018191	0.884774791	0.576830694	0.915331014	0.99580676
ITPR2	0.56656688	0.593155042	0.508875584	0.817808681	0.743451689	0.920448262	0.99580676
JMJD7	0.917086848	0.7152912	0.483448196	0.456690903	0.672950866	0.912911072	0.99580676
KLHL18	0.987172912	0.187358567	0.718412668	0.953293862	0.794301744	0.91666069	0.99580676
KLHL28	0.572285113	0.65395178	0.494634251	0.952081912	0.579429183	0.918386514	0.99580676
L3HYPDH	0.432588969	0.728816213	0.867692561	0.692542108	0.520076595	0.914203996	0.99580676
LOC100847759	0.546689006	0.888935194	0.494849627	0.484654739	0.85601156	0.915675729	0.99580676
LOC100848642	0.134603612	0.910022556	0.943995262	0.861566494	0.997404136	0.915200333	0.99580676
LOC100848906	0.983412143	0.970076368	0.601137499	0.674253906	0.258443385	0.915866995	0.99580676
LOC101902207	0.725649656	0.289529833	0.669547701	0.760166618	0.927770587	0.915013965	0.99580676
LOC101902668	0.289509853	0.939670786	0.869931957	0.592556425	0.735239876	0.919488633	0.99580676
LOC101902994	0.516465817	0.795838215	0.535719839	0.544493125	0.861100866	0.919638118	0.99580676
LOC101903397	0.336156501	0.802987273	0.638024175	0.880807786	0.651667816	0.914591182	0.99580676
LOC101903853	0.841250748	0.558361358	0.325095862	0.825686759	0.819464698	0.919729543	0.99580676

LOC101904822	0.59665838	0.948917148	0.2788889	0.903561082	0.696624697	0.915228239	0.99580676
LOC101905033	0.218004848	0.909585611	0.945892346	0.770592195	0.709021922	0.918789199	0.99580676
LOC101905668	0.921170191	0.764129858	0.620920913	0.294145363	0.791319969	0.917945253	0.99580676
LOC101905951	0.95898409	0.923558465	0.501044052	0.287045082	0.781625268	0.915433659	0.99580676
LOC101907886	0.570168575	0.750539535	0.874727474	0.308669425	0.883167057	0.91829862	0.99580676
LOC101908535	0.673275897	0.604877335	0.357363688	0.861717081	0.822008996	0.919469904	0.99580676
LOC104971501	0.77898743	0.357841411	0.891420836	0.461224856	0.905611761	0.920244652	0.99580676
LOC104971613	0.677536784	0.610050797	0.874803491	0.56477	0.497914833	0.917886291	0.99580676
LOC104975911	0.372989635	0.4543004	0.851777077	0.96779369	0.692721572	0.91204557	0.99580676
LOC107131494	0.530776864	0.62955327	0.88512871	0.684809064	0.497258382	0.916781304	0.99580676
LOC107132093	0.849997326	0.429331077	0.661696911	0.574279502	0.740229854	0.918980584	0.99580676
LOC112442619	0.631335932	0.782962967	0.877318476	0.486894023	0.489036511	0.919660289	0.99580676
LOC112443728	0.820592401	0.502371479	0.894674548	0.490145162	0.571205717	0.919660311	0.99580676
LOC112444289	0.530825847	0.90572874	0.85919192	0.376185152	0.668119744	0.920280777	0.99580676
LOC112446039	0.903581928	0.567660557	0.399329576	0.849562764	0.572884039	0.915582268	0.99580676
LOC112446709	0.698126435	0.663082319	0.92066221	0.882301173	0.275814912	0.920160145	0.99580676
LOC112447031	0.638861087	0.89937538	0.866708127	0.514756581	0.397436483	0.91811357	0.99580676
LOC112448062	0.843400469	0.565939344	0.898819166	0.42910811	0.55041887	0.917486222	0.99580676
LOC112448387	0.365574014	0.745295007	0.484449895	0.963128536	0.773835453	0.914016439	0.99580676
LOC112449363	0.486495383	0.892602394	0.880041717	0.422152964	0.629685519	0.917779042	0.99580676
LOC508455	0.362229503	0.805421933	0.861104685	0.643796423	0.624376053	0.917090987	0.99580676
LOC526769	0.797606216	0.801048761	0.609127618	0.261434461	0.969477603	0.914335772	0.99580676
LOC613519	0.758853836	0.893330469	0.435713201	0.565412652	0.601328449	0.91644447	0.99580676
LOC614643	0.92436751	0.631167307	0.749942171	0.394870407	0.570746224	0.914297138	0.99580676
LOC615258	0.854602503	0.944732317	0.145454595	0.868374333	0.982780532	0.916206532	0.99580676
LOC616295	0.434271309	0.532049774	0.940710208	0.527151714	0.884935263	0.91756106	0.99580676
LOC782437	0.747277124	0.557511471	0.569084601	0.937153592	0.440552443	0.913424578	0.99580676
MAD2L1BP	0.38505596	0.652040785	0.572071001	0.912002309	0.797000263	0.92091043	0.99580676
MAP3K20	0.47841453	0.303697181	0.835194381	0.942491895	0.910898677	0.92066937	0.99580676
MEGF9	0.765968209	0.679794991	0.345338871	0.750156681	0.736404183	0.91516341	0.99580676
METTL25	0.943719228	0.892076739	0.96971986	0.908629492	0.130661877	0.912244236	0.99580676
MTF1	0.871397993	0.856604017	0.500994761	0.315617601	0.874767847	0.919646898	0.99580676
NDFIP1	0.847980904	0.161507233	0.80166917	0.994094201	0.910915231	0.915265371	0.99580676
NME7	0.855195504	0.829805716	0.858629419	0.226329485	0.743338128	0.918826116	0.99580676
NOVA2	0.449667945	0.606616107	0.92452152	0.886238694	0.464288655	0.920219234	0.99580676
P2RX7	0.851501209	0.267395486	0.552305673	0.806733436	0.982504159	0.915564258	0.99580676
P2RY12	0.657108454	0.957859524	0.693855796	0.345144682	0.674705081	0.917909516	0.99580676
PCGF3	0.680848745	0.830747143	0.693554311	0.906100246	0.273289298	0.912512123	0.99580676
PHF20L1	0.740855415	0.459281882	0.895863283	0.441303131	0.755832706	0.917881265	0.99580676
PLEKHG3	0.481799438	0.369175076	0.88922883	0.75233835	0.872297559	0.920252905	0.99580676
PMP22	0.288575032	0.989081307	0.864143354	0.746796455	0.564373899	0.920424746	0.99580676
PNMA2	0.723124883	0.658719393	0.749074452	0.483843123	0.590419923	0.918170579	0.99580676
PPFIA1	0.358247964	0.759158133	0.852028024	0.660936536	0.643135413	0.914165608	0.99580676
PRIM1	0.637884853	0.51026872	0.474798457	0.782356398	0.845352968	0.918486369	0.99580676

PTER	0.920562976	0.735436761	0.98130813	0.172470373	0.911291402	0.920929504	0.99580676
QARS	0.840102067	0.220830387	0.588751989	0.969105289	0.958544035	0.917638431	0.99580676
QRICH1	0.91196321	0.990651404	0.24114611	0.668811182	0.697700798	0.917863679	0.99580676
RAI2	0.442291245	0.687535712	0.700074023	0.554296069	0.835275886	0.914243924	0.99580676
RBAK	0.727951644	0.817959092	0.894358653	0.335338631	0.571114686	0.918236617	0.99580676
RBM11	0.939178324	0.919990945	0.191241835	0.975593875	0.647585079	0.920904547	0.99580676
REST	0.410638112	0.939875133	0.924530706	0.298138956	0.924406662	0.913974749	0.99580676
RGS11	0.790879752	0.757547464	0.858260723	0.846412972	0.233780257	0.917964483	0.99580676
RHPN2	0.649059366	0.888317739	0.601151061	0.28843949	0.978664014	0.913370878	0.99580676
RNF130	0.686629936	0.915364357	0.336314426	0.949229033	0.484469679	0.912595007	0.99580676
RNF8	0.599259776	0.929535751	0.557926726	0.642096915	0.493725036	0.914196168	0.99580676
RPL27A	0.613393797	0.58815869	0.572612974	0.652051334	0.737005999	0.91509442	0.99580676
RPL36AL	0.779654971	0.288988212	0.693106845	0.932194979	0.709612282	0.919706599	0.99580676
RPL5	0.455200119	0.456351604	0.589200104	0.84848287	0.946702337	0.913944373	0.99580676
RPS11	0.671669836	0.667716383	0.285965759	0.995919356	0.790828552	0.917120544	0.99580676
RPS28	0.715836413	0.724407631	0.301914605	0.692984938	0.919922453	0.91571899	0.99580676
RPUSD3	0.552860904	0.651789019	0.938774095	0.556249744	0.519075567	0.913167904	0.99580676
RRH	0.652940555	0.827693802	0.655121785	0.436943908	0.672333406	0.920484995	0.99580676
SCYL3	0.737259656	0.631154932	0.445329623	0.562261019	0.842476359	0.91375652	0.99580676
SEMA4B	0.444561933	0.386823733	0.833847849	0.898428281	0.806980498	0.920433374	0.99580676
SERP2	0.944584846	0.544734424	0.660067803	0.393588635	0.73094689	0.913211325	0.99580676
SGTA	0.758256781	0.560445428	0.626427378	0.94576713	0.398517648	0.916337935	0.99580676
SIPA1L2	0.947784374	0.556798786	0.344879306	0.820646788	0.69123547	0.919639888	0.99580676
SIRT6	0.918227049	0.834469589	0.655704466	0.529896007	0.38882907	0.919945255	0.99580676
SLC27A1	0.933731597	0.336889214	0.990349662	0.762906391	0.437351578	0.920412686	0.99580676
SLX4IP	0.611493487	0.791971438	0.495564536	0.588963018	0.707833971	0.91600638	0.99580676
SMOC1	0.571623308	0.413205404	0.95711136	0.447345193	0.975241026	0.91431854	0.99580676
SSBP2	0.733318388	0.729298464	0.702116031	0.581487742	0.443809119	0.912221204	0.99580676
TCF12	0.897274246	0.940563696	0.285856817	0.642250727	0.62350289	0.911851485	0.99580676
TGDS	0.230408806	0.885142831	0.795909682	0.980971682	0.64130805	0.918381825	0.99580676
TMEM201	0.515016946	0.725410419	0.547564944	0.852636922	0.558701246	0.912893405	0.99580676
TPGS1	0.802531118	0.803297371	0.657840366	0.240776685	0.967032486	0.914460447	0.99580676
TSTD2	0.930927996	0.578049279	0.833189125	0.379284677	0.599199712	0.918132587	0.99580676
TTC21B	0.300497397	0.841734863	0.784271669	0.84327814	0.607875101	0.917894731	0.99580676
UBALD1	0.749241957	0.254054994	0.806231099	0.901104851	0.71447126	0.914529903	0.99580676
USP49	0.745409566	0.807164999	0.386698889	0.971172727	0.461405523	0.920755405	0.99580676
UTP11	0.418843186	0.559467027	0.939660987	0.991329924	0.453889278	0.914854938	0.99580676
VWA2	0.722329504	0.397013242	0.662298135	0.547885996	0.999216996	0.920450461	0.99580676
WDR93	0.728920108	0.918375388	0.252091877	0.639493605	0.908406235	0.913603796	0.99580676
WDYHV1	0.758913879	0.524107191	0.977645716	0.468300234	0.545061943	0.915071767	0.99580676
XKR4	0.790185518	0.526059003	0.591797725	0.668066288	0.633154592	0.92053483	0.99580676
ZC3HC1	0.816809648	0.380902281	0.613717479	0.980239591	0.550862127	0.919487304	0.99580676
ZNF212	0.68253211	0.642709854	0.550366861	0.836744148	0.509250545	0.919233104	0.99580676
ZNF674	0.724869395	0.365158781	0.700360922	0.577652656	0.970989889	0.920450725	0.99580676

LOC787530	0.945401004	0.85773947	0.789258077	0.226610604	0.721943445	0.921241874	0.995948642
C28H1orf131	0.858847584	0.280085457	0.824494213	0.85888595	0.616430291	0.921564485	0.995975746
CMTM8	0.779490479	0.968637493	0.747014462	0.734445991	0.253474542	0.921559695	0.995975746
CYGB	0.732912598	0.716143005	0.288688959	0.890249751	0.778742336	0.921609709	0.995975746
EGFL8	0.405590163	0.992562278	0.728421653	0.759280894	0.472142588	0.921691771	0.995975746
KLF10	0.495259526	0.933348091	0.870566994	0.824541023	0.316778658	0.921677017	0.995975746
MRPL45	0.884507558	0.61831623	0.350347192	0.835911903	0.654679741	0.921404942	0.995975746
VN1R1	0.523746591	0.424748586	0.970820396	0.714750769	0.680521047	0.921609832	0.995975746
LOC104971220	0.428429997	0.990610529	0.569983997	0.606484119	0.717407736	0.921828637	0.996049318
TOR1B	0.827966579	0.911919332	0.408591632	0.98184646	0.347641399	0.921881244	0.996049318
ADAP2	0.703199571	0.58849405	0.701182001	0.412451056	0.900150793	0.92442739	0.99606619
ADIPOQ	0.996792791	0.533520485	0.289594199	0.733500672	0.95314454	0.924367769	0.99606619
CAMKK1	0.419782606	0.531136854	0.843063111	0.810826963	0.700128546	0.923368205	0.99606619
CBWD2	0.669951163	0.570934823	0.498172576	0.650325469	0.877621658	0.925470046	0.99606619
CD180	0.625849515	0.51280763	0.79498385	0.990676222	0.417173361	0.922036057	0.99606619
CELF2	0.844010007	0.96503939	0.317628469	0.89895433	0.455972435	0.922671264	0.99606619
CHST15	0.599915856	0.734319821	0.379512751	0.836691077	0.763741182	0.923500682	0.99606619
CLDN15	0.880552508	0.467818953	0.917392009	0.418915193	0.672565754	0.92312466	0.99606619
CMC1	0.529716398	0.373836779	0.868259536	0.788166043	0.798258578	0.924884456	0.99606619
ECPAS	0.994710844	0.695189731	0.205861076	0.848712587	0.89716084	0.925104939	0.99606619
ENOPH1	0.318321814	0.662021095	0.688018207	0.904736599	0.808734442	0.922717164	0.99606619
FAM155B	0.926358071	0.515271317	0.565736344	0.475537634	0.82778515	0.922940081	0.99606619
FGD4	0.451808878	0.777111856	0.640091718	0.890208507	0.541475895	0.925040531	0.99606619
FZR1	0.654656415	0.688983333	0.533540472	0.558146056	0.798071282	0.923876045	0.99606619
GDA	0.942498744	0.288625242	0.972811379	0.709892901	0.56365759	0.922507035	0.99606619
GMPR2	0.633281847	0.873027031	0.581013609	0.612181957	0.553174478	0.925496869	0.99606619
GPR180	0.704296556	0.371364152	0.505943992	0.848969143	0.956087526	0.924097983	0.99606619
HEG1	0.502429059	0.6353044	0.53594879	0.836870971	0.748353841	0.923816276	0.99606619
HIRIP3	0.9344086	0.899865162	0.430062061	0.58099654	0.510376014	0.923909325	0.99606619
HNRNPDL	0.944768171	0.830914273	0.404915622	0.380098895	0.898809669	0.925308842	0.99606619
LOC100848538	0.688485968	0.865293701	0.631072417	0.500097989	0.560795553	0.922027375	0.99606619
LOC101901960	0.960657981	0.535130003	0.5923902	0.47378789	0.745133649	0.924201754	0.99606619
LOC101904902	0.853078578	0.569352843	0.374579862	0.639018195	0.935250191	0.925446884	0.99606619
LOC101905493	0.847259698	0.618273643	0.723413105	0.664724149	0.428735127	0.924700981	0.99606619
LOC101905821	0.737387268	0.648466197	0.247256206	0.974558194	0.91662064	0.922216408	0.99606619
LOC101907661	0.464200534	0.459853194	0.967147375	0.918148985	0.557737722	0.922328426	0.99606619
LOC101908046	0.468841522	0.749794472	0.658438135	0.911634757	0.506857182	0.923622805	0.99606619
LOC104970809	0.572843049	0.436867383	0.558385579	0.962764964	0.809059046	0.925564157	0.99606619
LOC107131323	0.796460491	0.935267878	0.515184676	0.703389832	0.403366311	0.925599256	0.99606619
LOC112442754	0.92721283	0.467904334	0.609444989	0.482262449	0.828757892	0.922282257	0.99606619
LOC112444287	0.397729763	0.726433621	0.846650117	0.912951544	0.474696401	0.922636024	0.99606619
LOC112446462	0.419674344	0.985401106	0.995309231	0.652689535	0.396881243	0.923279315	0.99606619
LOC512440	0.325340543	0.631657568	0.628013116	0.856170345	0.964844014	0.923267801	0.99606619
LOC613444	0.559899363	0.472288671	0.9431595	0.696335621	0.618477281	0.924097335	0.99606619

LOC781381	0.730864696	0.567957444	0.855247243	0.418654238	0.72842257	0.92497254	0.99606619
LOC786489	0.836416807	0.410272547	0.605185303	0.556732329	0.914455018	0.92233662	0.99606619
MAP7D1	0.978081367	0.641766865	0.704852979	0.26585267	0.903588354	0.922922318	0.99606619
MKRN2	0.707531706	0.453492805	0.404822496	0.969451145	0.84043138	0.922444326	0.99606619
NIPAL1	0.937667845	0.501637251	0.681654042	0.607255108	0.558502093	0.92545771	0.99606619
NNT	0.891420301	0.951206637	0.773308794	0.739217054	0.221173769	0.923885477	0.99606619
NOTCH2	0.824718931	0.946431301	0.55488389	0.406302749	0.603108916	0.922762648	0.99606619
P2RY2	0.885020396	0.748772373	0.254049891	0.883490966	0.712158707	0.922545527	0.99606619
PECAM1	0.35925855	0.794598809	0.947297716	0.828975215	0.480085186	0.92431583	0.99606619
PGBD1	0.566733762	0.976802994	0.250308861	0.804652008	0.972234873	0.925114115	0.99606619
PLK2	0.356306657	0.685673416	0.900309496	0.562732233	0.859937578	0.923086837	0.99606619
PLPPR3	0.661194094	0.555314702	0.808026085	0.585576177	0.626464426	0.925552377	0.99606619
PRAF2	0.947565553	0.911109953	0.306487939	0.755743137	0.539846097	0.924656086	0.99606619
PTPRU	0.592348599	0.899641297	0.392169563	0.958055653	0.543596441	0.925556077	0.99606619
RCE1	0.552104839	0.946622117	0.625458497	0.724555495	0.451712862	0.92365894	0.99606619
RELN	0.77989351	0.984357242	0.233230618	0.719890786	0.825324609	0.923025929	0.99606619
RNF170	0.619655994	0.993265544	0.332546346	0.995424798	0.528877021	0.924450885	0.99606619
SF3A2	0.483225376	0.515010561	0.95695587	0.710460637	0.630823566	0.923396122	0.99606619
TBCEL	0.937040008	0.250486037	0.716737373	0.886235506	0.725627024	0.924891561	0.99606619
TMC7	0.799727082	0.986824559	0.545067042	0.932322969	0.266241582	0.923439288	0.99606619
UBP1	0.928902855	0.460422577	0.968544836	0.412614641	0.632619836	0.924833097	0.99606619
USP33	0.650979616	0.828190601	0.778131484	0.884346624	0.288998638	0.923899044	0.99606619
WNT11	0.79315697	0.998139436	0.22493863	0.942686387	0.640686903	0.924245989	0.99606619
ZNF514	0.30478134	0.833018083	0.971627208	0.707953766	0.622849686	0.925491036	0.99606619
ZNF699	0.731621595	0.531696595	0.496437643	0.642470943	0.871910443	0.92488551	0.99606619
ZRANB2	0.953754165	0.480589303	0.99760078	0.925268334	0.252714204	0.923590756	0.99606619
ZXDB	0.670662623	0.978766333	0.401624348	0.469897822	0.876668425	0.925316842	0.99606619
CATSPER2	0.651935182	0.608164377	0.365312184	0.763803972	0.99419104	0.926701689	0.996082706
CCDC120	0.932129438	0.87169523	0.255085956	0.872927571	0.610619578	0.927185609	0.996082706
CD209	0.811019094	0.604980947	0.961050887	0.364913594	0.642044487	0.927185097	0.996082706
CHD9	0.6622187	0.820854732	0.999736921	0.227350826	0.893336376	0.927082888	0.996082706
DHTKD1	0.626206459	0.391074994	0.974822355	0.647363364	0.708800253	0.926258453	0.996082706
DMTF1	0.97265616	0.329088129	0.983006884	0.872903377	0.400974187	0.926844266	0.996082706
FRAT2	0.415919796	0.950870552	0.806066946	0.766182446	0.450629936	0.92678009	0.996082706
FTSJ1	0.839027755	0.755744714	0.720687504	0.251957419	0.948011066	0.925871797	0.996082706
GNAS	0.814623329	0.358881641	0.893606998	0.477752256	0.88596346	0.927284631	0.996082706
HIST1H2AC	0.944024698	0.919024522	0.929711238	0.452427087	0.303298378	0.927385371	0.996082706
HS3ST2	0.535118052	0.874572539	0.938483054	0.316873807	0.796866176	0.927601142	0.996082706
LGALS4	0.594493211	0.990343282	0.357903415	0.811669656	0.647981876	0.927525736	0.996082706
LOC101902154	0.791288168	0.236429658	0.870335386	0.980197044	0.685663074	0.92615092	0.996082706
LOC101907404	0.571644394	0.602171242	0.635353208	0.820352582	0.613448546	0.926776469	0.996082706
LOC104974758	0.881881065	0.429851364	0.507776808	0.752161152	0.755538698	0.92610598	0.996082706
LOC107133302	0.94228266	0.505469653	0.666807218	0.567378473	0.60615803	0.925946468	0.996082706
LOC112441530	0.815249553	0.568542323	0.29385719	0.975685144	0.83440174	0.927583964	0.996082706

LOC112447347	0.809935874	0.300592033	0.674547134	0.906130255	0.744327662	0.927464601	0.996082706
LOC506181	0.756621212	0.97897443	0.353146129	0.475360792	0.88667992	0.926965299	0.996082706
LOC789494	0.527622258	0.896905333	0.460942855	0.535948771	0.948788827	0.927617573	0.996082706
PCSK6	0.33578807	0.829634522	0.989824468	0.565173565	0.710760769	0.927469672	0.996082706
PYGB	0.766496657	0.833277779	0.388859978	0.940899405	0.467462502	0.925958559	0.996082706
RABL2B	0.487522914	0.451169712	0.869515838	0.791778218	0.727764553	0.926918376	0.996082706
RALGPS2	0.890537204	0.447127961	0.509434816	0.935875877	0.577277373	0.92630878	0.996082706
RPAIN	0.694908559	0.704710139	0.614435814	0.832734802	0.439398272	0.926811491	0.996082706
RRM1	0.804182739	0.496726375	0.898481273	0.697058775	0.440726435	0.926971538	0.996082706
SAMD13	0.434744093	0.867552147	0.676037242	0.482855983	0.895072233	0.926910543	0.996082706
TBC1D20	0.72162035	0.364230276	0.68525626	0.850942849	0.715696237	0.926407151	0.996082706
TBC1D2B	0.576069904	0.471770308	0.999181148	0.405748458	0.99655742	0.926518153	0.996082706
TMEM161A	0.451110364	0.952411489	0.547408525	0.635554695	0.730644919	0.925932105	0.996082706
TSGA10	0.423466246	0.536255158	0.943547116	0.839469698	0.607898453	0.926060962	0.996082706
ZNF24	0.903699823	0.949816607	0.258092424	0.997220438	0.501712007	0.927536742	0.996082706
ZNF621	0.602850244	0.359365905	0.806796147	0.820187472	0.772699838	0.927474288	0.996082706
CETN4	0.73066649	0.735015651	0.346432259	0.869365624	0.689676938	0.928231359	0.996210322
ISLR	0.596923926	0.467971066	0.770627169	0.991461563	0.523211059	0.928343456	0.996210322
KIF1C	0.593893165	0.762667444	0.771852828	0.321561664	0.991905663	0.928189351	0.996210322
LOC104971307	0.773083157	0.974823308	0.639511731	0.279135415	0.82825992	0.928107462	0.996210322
LOC515150	0.878124337	0.904231031	0.663563433	0.869696249	0.243579429	0.928291723	0.996210322
OLA1	0.866312165	0.710278819	0.998177875	0.408582055	0.444166814	0.928145728	0.996210322
RNASE13	0.857908243	0.636655054	0.787526227	0.35978557	0.719854203	0.928086669	0.996210322
RPS10	0.746353537	0.688791339	0.720062246	0.393688068	0.763273267	0.92792201	0.996210322
TNFSF13	0.408080481	0.974188914	0.695045064	0.70206534	0.573456531	0.927933277	0.996210322
TSPAN17	0.675826129	0.953265276	0.275652022	0.725004801	0.866860098	0.928285227	0.996210322
CACNG7	0.66453997	0.951909179	0.363904564	0.92196998	0.527428865	0.928602933	0.996242236
ELMOD2	0.728829047	0.830964803	0.355289556	0.770639228	0.6765465	0.92883848	0.996242236
GIT1	0.574602039	0.668203132	0.54661017	0.926952729	0.575931295	0.928700895	0.996242236
LOC781059	0.840401052	0.424386409	0.358250933	0.928913923	0.944967283	0.9288109	0.996242236
MICAL1	0.895221427	0.573431595	0.849261849	0.723578424	0.355705006	0.928860609	0.996242236
RTN4RL2	0.793668836	0.977428538	0.328616007	0.660835128	0.663536106	0.928451205	0.996242236
SIN3B	0.991455044	0.937420642	0.643717595	0.843402885	0.221992042	0.928674678	0.996242236
SRI	0.775265838	0.746002287	0.253111272	0.889791238	0.860369675	0.928724056	0.996242236
UBOX5	0.497357348	0.792521908	0.506949015	0.838439208	0.670120359	0.928919548	0.996242236
LOC112449266	0.27920309	0.807173231	0.999665907	0.755918206	0.659757896	0.929001496	0.996265017
LOC104970503	0.869414785	0.550504318	0.916363683	0.626885854	0.410104786	0.929379493	0.99655492
LOC107132796	0.349698128	0.838470051	0.773283218	0.59069224	0.842004411	0.929393276	0.99655492
KAT2B	0.802575155	0.898769982	0.223714776	0.818659217	0.855954494	0.929684446	0.996645867
LOC789192	0.638822124	0.981513357	0.363464309	0.794903589	0.624021573	0.929652418	0.996645867
NTHL1	0.690276769	0.235496407	0.859976924	0.919224442	0.880162789	0.929707985	0.996645867
TRMT1L	0.307997834	0.845912257	0.566546889	0.838071741	0.914412376	0.929721015	0.996645867
C1H3orf70	0.853971594	0.48509837	0.5848795	0.484185921	0.965621152	0.929874278	0.996679955
PLEKHO2	0.736549094	0.821534018	0.666179502	0.343538752	0.817925468	0.929861909	0.996679955

LOC112446357	0.678383921	0.725983537	0.694400659	0.752230919	0.440864308	0.929998917	0.996709291
RAI14	0.995525823	0.335435859	0.687522165	0.721231265	0.685477362	0.930083851	0.996709291
ZNF821	0.589475847	0.49782957	0.656790619	0.712735236	0.825889737	0.930036867	0.996709291
AHDC1	0.874076488	0.565128584	0.48378938	0.613974616	0.774425174	0.930197685	0.996717263
LOC616942	0.674339566	0.724369876	0.677215952	0.879304714	0.390696149	0.93021276	0.996717263
CTDP1	0.768164835	0.517493452	0.679198282	0.582230346	0.723774165	0.930336832	0.99675082
LOC100848325	0.371606673	0.680993204	0.906909053	0.962939871	0.51526651	0.930426288	0.99675082
SMCO3	0.929029507	0.804234901	0.664669446	0.429994699	0.53304939	0.930384308	0.99675082
ARL5A	0.984833189	0.375536568	0.793329213	0.502659019	0.773966712	0.930679949	0.996762294
NAP1L3	0.64238228	0.704619483	0.754831742	0.68340034	0.488411467	0.930581582	0.996762294
PPFIA4	0.431107213	0.946059713	0.328001153	0.943495968	0.903072733	0.930528709	0.996762294
SSH3	0.457725653	0.941673585	0.923188135	0.732283	0.391629953	0.930652392	0.996762294
GABRG3	0.892684837	0.535631975	0.880424841	0.640931417	0.423682931	0.930836641	0.996828813
JPH1	0.961534145	0.771870775	0.583648338	0.819497443	0.322301714	0.930924281	0.996828813
KLHL24	0.453947322	0.687599064	0.502005164	0.929832916	0.785049374	0.930895188	0.996828813
ARMC5	0.68097154	0.823733031	0.401666173	0.615398802	0.827342871	0.931203327	0.996865657
JARID2	0.932670128	0.426080461	0.344606554	0.987901589	0.847976781	0.931207679	0.996865657
LOC112442228	0.521234838	0.998588928	0.54041488	0.82281054	0.495117342	0.931090192	0.996865657
MYO9B	0.96573965	0.90527307	0.649176592	0.437994296	0.461186989	0.931136811	0.996865657
PNPLA8	0.316109321	0.463379017	0.963313514	0.844820241	0.962856376	0.931262409	0.996865657
ABCG4	0.469174536	0.490324539	0.90523626	0.951124247	0.585663937	0.932369446	0.996982683
AP4S1	0.849028031	0.261517879	0.648093557	0.998430608	0.815321112	0.93338176	0.996982683
ARL6	0.450105523	0.686284767	0.662495334	0.586661578	0.975308987	0.933339858	0.996982683
CALML6	0.691106818	0.469810648	0.48581845	0.998022169	0.739752973	0.932776927	0.996982683
CETN3	0.461324513	0.97790996	0.584413712	0.725738254	0.606978866	0.932492476	0.996982683
CLCN6	0.470247129	0.753367662	0.394816125	0.98076705	0.851357805	0.933072025	0.996982683
CLEC16A	0.906725799	0.834286104	0.945372248	0.594523897	0.275568673	0.933402029	0.996982683
CMTM3	0.349325124	0.889130767	0.425556732	0.939574129	0.94509669	0.933584182	0.996982683
CRLS1	0.937286237	0.971127356	0.329965836	0.39836384	0.974515858	0.932900412	0.996982683
DHH	0.865187004	0.932777432	0.357537383	0.574452811	0.707815041	0.933542279	0.996982683
FAM133A	0.682028363	0.439921227	0.599317894	0.654494345	0.993556407	0.933197526	0.996982683
GGCT	0.892042515	0.239546456	0.661011235	0.90629788	0.908827866	0.932673656	0.996982683
KIAA1671	0.862749071	0.556693122	0.931298044	0.377389383	0.687554233	0.932422143	0.996982683
LGALS7	0.733579808	0.549798153	0.966990387	0.87491364	0.338334077	0.931869474	0.996982683
LOC100294994	0.339142693	0.604239829	0.984566441	0.715539271	0.814238125	0.933741017	0.996982683
LOC100848699	0.504053939	0.624620405	0.722454602	0.56206559	0.919326952	0.93372656	0.996982683
LOC101907126	0.50474266	0.616342695	0.668396143	0.999296757	0.559135884	0.932530454	0.996982683
LOC107131225	0.512743751	0.506666314	0.68331721	0.886973802	0.734359696	0.932033153	0.996982683
LOC112442802	0.97409706	0.91541847	0.431796063	0.547459393	0.546971273	0.931733028	0.996982683
LOC112449360	0.285149136	0.605574074	0.891092762	0.877314632	0.85775624	0.932181362	0.996982683
LOC519208	0.867497189	0.555634734	0.87228628	0.719833587	0.386199598	0.93315644	0.996982683
LOC520104	0.609654793	0.91626182	0.463060324	0.835705095	0.533610962	0.931781665	0.996982683
LOC533093	0.986880491	0.981408463	0.382190328	0.629670132	0.497875536	0.932408309	0.996982683
LOC786417	0.431689573	0.834933122	0.958342734	0.396760459	0.845234835	0.932221766	0.996982683

LRRC69	0.840314898	0.744451309	0.59141489	0.772515749	0.409666024	0.933334273	0.996982683
LRRC72	0.364991593	0.99664613	0.954896258	0.946634037	0.357068703	0.933620644	0.996982683
MLKL	0.6044909	0.709619474	0.731139744	0.677025588	0.550424472	0.933146458	0.996982683
MYO1D	0.617593183	0.610006975	0.900603103	0.41726246	0.814093431	0.931693824	0.996982683
NEBL	0.551180259	0.518428912	0.906975515	0.877472148	0.515939132	0.933548596	0.996982683
PCDHGB4	0.921348825	0.545814017	0.57644661	0.53154613	0.74735503	0.931607401	0.996982683
PPIB	0.662810566	0.764021583	0.598631047	0.549385643	0.702519911	0.933258727	0.996982683
RNF144A	0.923751994	0.689140169	0.264044165	0.883409398	0.777223802	0.931836721	0.996982683
RPL39	0.602191814	0.466240086	0.729159172	0.627792319	0.910465961	0.933272115	0.996982683
SYT4	0.89233338	0.278432465	0.569409991	0.999447734	0.823122108	0.932711827	0.996982683
TMEM47	0.273339764	0.62212263	0.824976937	0.96802152	0.864937803	0.933662514	0.996982683
UROS	0.864258738	0.800211713	0.904726432	0.243359527	0.765289766	0.932841702	0.996982683
USE1	0.913455183	0.781481556	0.431308386	0.447393604	0.83901666	0.93198264	0.996982683
ZNF2	0.775207289	0.998301263	0.672641968	0.420689643	0.527727717	0.931978052	0.996982683
ZPBP	0.95668535	0.727928729	0.596513846	0.680543289	0.41445514	0.933406497	0.996982683
ABCA13	0.725281641	0.668979815	0.533535588	0.766510768	0.625829852	0.939253396	0.996999903
ACTR6	0.449605907	0.864564785	0.715677717	0.792239699	0.558022276	0.938301143	0.996999903
AP5B1	0.279578678	0.581583198	0.968433771	0.925503062	0.835393168	0.93729498	0.996999903
ARL13A	0.864631884	0.866607027	0.619571962	0.942735376	0.271628652	0.93489363	0.996999903
ASB6	0.518580219	0.402096589	0.972826699	0.714772341	0.867306686	0.940479062	0.996999903
BACH2	0.944942189	0.390461507	0.691501723	0.73964337	0.641253687	0.936690259	0.996999903
C1H3orf38	0.497299499	0.969880409	0.660461389	0.62868015	0.624662777	0.939973167	0.996999903
CALD1	0.718795416	0.810405271	0.381569662	0.931034221	0.579857965	0.935841766	0.996999903
CAPN2	0.883703404	0.594329163	0.325202825	0.884814049	0.822355949	0.939330543	0.996999903
CASKIN2	0.438990341	0.687179339	0.773058672	0.78448062	0.680744189	0.939534127	0.996999903
CCDC85A	0.604682995	0.790815286	0.443531828	0.624389734	0.939271706	0.939415017	0.996999903
CCDC92	0.970867712	0.878999205	0.70786967	0.259707492	0.801467644	0.940467927	0.996999903
CLCA3	0.509704006	0.856616015	0.798212294	0.830260441	0.421346789	0.937437774	0.996999903
CMTM4	0.817796823	0.559089415	0.782227597	0.715493444	0.482071108	0.938601473	0.996999903
CMTM7	0.949990808	0.817430097	0.172428237	0.963424052	0.936592173	0.93653252	0.996999903
CPLANE2	0.925272014	0.413189636	0.561763092	0.938632986	0.609004803	0.938126527	0.996999903
CPN2	0.689379731	0.984660807	0.405575497	0.596687812	0.763702888	0.940248287	0.996999903
CYTH3	0.849881095	0.665382673	0.789006821	0.591284065	0.459276032	0.936817525	0.996999903
DLG2	0.914123331	0.714193891	0.492828532	0.50039196	0.748742641	0.936304086	0.996999903
DUSP7	0.548741383	0.907614587	0.546934545	0.582260327	0.7499248	0.93494731	0.996999903
EEF2	0.313192052	0.91516866	0.557237932	0.958369878	0.813555809	0.939527235	0.996999903
EIF2AK1	0.653988677	0.92789876	0.519538059	0.470305442	0.840197639	0.939566966	0.996999903
EVI5L	0.614217826	0.997314195	0.648126113	0.388066062	0.79334974	0.937691559	0.996999903
FBXO42	0.981570475	0.363212957	0.883745573	0.724982035	0.538349653	0.938288982	0.996999903
FHIT	0.475779114	0.419900992	0.871818301	0.971592309	0.719863087	0.937354138	0.996999903
FOXN3	0.921063203	0.772648093	0.652356317	0.860039758	0.314324935	0.94028514	0.996999903
GULO	0.768386982	0.672267695	0.509441222	0.595308302	0.767777071	0.936079951	0.996999903
H2AFX	0.491218319	0.92538523	0.916223211	0.919467088	0.327870933	0.940326262	0.996999903
HIST1H2AK	0.965166283	0.353308327	0.480230383	0.855544378	0.891416603	0.939809446	0.996999903

HS2ST1	0.801176409	0.819687696	0.91957487	0.235929449	0.841233256	0.935723873	0.996999903
HYKK	0.932559587	0.70065009	0.383756773	0.761736154	0.62430659	0.935204234	0.996999903
ING5	0.730290913	0.681336032	0.56113403	0.792789765	0.566252444	0.940159642	0.996999903
KCTD5	0.834149562	0.281540712	0.739860814	0.922196142	0.746834151	0.935565467	0.996999903
KLRG1	0.473060614	0.957991957	0.747384262	0.539257154	0.656791099	0.935813169	0.996999903
LMO2	0.291407703	0.694737567	0.800435192	0.998469149	0.764469067	0.938865785	0.996999903
LOC100138078	0.639514764	0.753016603	0.640060515	0.44166309	0.867743856	0.934246255	0.996999903
LOC100848007	0.510499505	0.923139542	0.94285189	0.378226167	0.717062655	0.93627052	0.996999903
LOC101902831	0.768641376	0.926486958	0.24903983	0.939314609	0.751116273	0.93999358	0.996999903
LOC101904290	0.891751125	0.528749452	0.771780373	0.947360915	0.36274553	0.939938619	0.996999903
LOC101904314	0.619749571	0.801060225	0.937773245	0.584827918	0.458135042	0.939690874	0.996999903
LOC101904796	0.670042449	0.92671355	0.940125333	0.246213778	0.827027	0.934883081	0.996999903
LOC101904871	0.932355147	0.909647987	0.942484174	0.231159035	0.640184435	0.934384409	0.996999903
LOC101907250	0.950813074	0.600041776	0.270306434	0.978762755	0.827546641	0.939826056	0.996999903
LOC101907327	0.822372073	0.88431072	0.535574081	0.848695657	0.368545023	0.93735944	0.996999903
LOC107131749	0.958248441	0.640617328	0.352729667	0.620598084	0.928222941	0.939686532	0.996999903
LOC107131834	0.880382168	0.484976083	0.331831674	0.908304471	0.920866654	0.934571072	0.996999903
LOC107132255	0.379393875	0.881559666	0.844670015	0.470128441	0.904492887	0.93595389	0.996999903
LOC112441629	0.929353525	0.66367344	0.966096878	0.399118464	0.505726944	0.936075441	0.996999903
LOC112441659	0.386545272	0.965287181	0.711264294	0.956201046	0.484932867	0.938361113	0.996999903
LOC112442039	0.996150012	0.91189825	0.15356209	0.922779762	0.952905283	0.938038581	0.996999903
LOC112442708	0.719953153	0.796873893	0.468606561	0.497109529	0.88845034	0.934770833	0.996999903
LOC112443783	0.349184208	0.659435825	0.936403925	0.835539899	0.68171345	0.938165464	0.996999903
LOC112446427	0.845278903	0.74778739	0.647514547	0.307713098	0.957439941	0.936333179	0.996999903
LOC112447420	0.570686739	0.580888837	0.820678352	0.707834316	0.63900926	0.938357429	0.996999903
LOC112447510	0.482129132	0.9447618	0.36155199	0.830706467	0.860854435	0.933933246	0.996999903
LOC112448776	0.565708617	0.550728283	0.861375474	0.995560581	0.455636641	0.937284507	0.996999903
LOC112448832	0.494317671	0.693908416	0.996878526	0.364453224	0.97487213	0.937084701	0.996999903
LOC112449247	0.376901476	0.485948282	0.935650384	0.96426215	0.757248781	0.939996954	0.996999903
LOC548613	0.586907346	0.966900848	0.602639079	0.459115972	0.800637584	0.940444471	0.996999903
LOC787250	0.813980299	0.995561303	0.184755953	0.907816038	0.924516212	0.940405556	0.996999903
LOC787497	0.753582012	0.954537235	0.774868306	0.378436889	0.564058366	0.934977638	0.996999903
LOC788541	0.639245904	0.69551102	0.507314111	0.725744512	0.738189482	0.936545031	0.996999903
LOC789997	0.593573987	0.675621746	0.558506535	0.940022419	0.577811864	0.937220719	0.996999903
MED22	0.377226162	0.623356621	0.995074749	0.779647282	0.668613445	0.937481506	0.996999903
MTX2	0.986874484	0.688896957	0.636398388	0.651946182	0.432820007	0.937572495	0.996999903
MUS81	0.962441511	0.281288063	0.632341201	0.741225721	0.960536672	0.937406773	0.996999903
NECTIN2	0.351194412	0.952240581	0.827325561	0.462962252	0.964766082	0.938774226	0.996999903
NHS	0.535434476	0.825584437	0.537233288	0.5705177	0.92836557	0.940500609	0.996999903
NOBOX	0.685551301	0.444108429	0.789598629	0.81615928	0.621526699	0.937459195	0.996999903
NR1D1	0.855092587	0.377760659	0.561577695	0.962710941	0.69047191	0.93633235	0.996999903
NUDT1	0.349399388	0.876102479	0.632816811	0.649286528	0.98272796	0.938793809	0.996999903
OGFR	0.896691391	0.558411235	0.967179769	0.814418088	0.314939056	0.939280612	0.996999903
PCCB	0.717763206	0.822194265	0.938615753	0.24949839	0.861130281	0.935003723	0.996999903

PCIF1	0.614790756	0.701026333	0.90842259	0.793505497	0.395867162	0.938299737	0.996999903
PIEZO1	0.595338131	0.526095938	0.747345329	0.680510745	0.770580579	0.938107021	0.996999903
PIP4K2C	0.194308686	0.978556562	0.861571476	0.851992588	0.875221415	0.937631792	0.996999903
PLA2G7	0.56367448	0.312258543	0.946863098	0.857938875	0.863828379	0.938723313	0.996999903
PLAGL2	0.973180512	0.754795854	0.799543073	0.21874971	0.916053688	0.933862127	0.996999903
PM20D2	0.913288369	0.41947573	0.571371147	0.683544636	0.834308089	0.939763991	0.996999903
PPP1R16B	0.924948301	0.994039438	0.448801768	0.565935557	0.536327931	0.940088277	0.996999903
PRDM6	0.494881021	0.642814158	0.534303526	0.865763349	0.852200957	0.940209621	0.996999903
PRR29	0.750688813	0.730532613	0.364258783	0.665583907	0.894810045	0.934971647	0.996999903
RPL35A	0.690523365	0.615362218	0.307499681	0.981022925	0.973344674	0.939713078	0.996999903
RPS17	0.968164473	0.559155083	0.546732675	0.464968347	0.880825117	0.936861151	0.996999903
RPS29	0.689930594	0.827363148	0.339161965	0.637068846	0.978754762	0.936445196	0.996999903
SHANK3	0.90506053	0.665622496	0.51109795	0.536351109	0.740546231	0.937743461	0.996999903
SLC1A3	0.536253482	0.81503462	0.486138611	0.7039331	0.813225961	0.937201759	0.996999903
SLC35E4	0.563086377	0.98698621	0.834693922	0.364122443	0.742208871	0.94018088	0.996999903
SLC4A7	0.774174203	0.449296712	0.706014876	0.58756901	0.830093525	0.935656178	0.996999903
SLCO2A1	0.953169798	0.591864652	0.817279614	0.380060848	0.68991998	0.936594661	0.996999903
SNURF	0.904802602	0.975800689	0.737574765	0.310911655	0.601801294	0.937376841	0.996999903
SYNGR2	0.920329889	0.555905065	0.478813916	0.875852169	0.572356967	0.938153908	0.996999903
SYNPO	0.687979174	0.509892731	0.837510391	0.717790303	0.567142046	0.935507403	0.996999903
TASP1	0.493046505	0.73489518	0.676858643	0.66646536	0.745939471	0.937441386	0.996999903
TBX2	0.846318137	0.958020971	0.902287442	0.174217534	0.975993964	0.939419066	0.996999903
TCN2	0.614256398	0.843442873	0.584786538	0.862822724	0.469333296	0.938062015	0.996999903
TEF	0.662796423	0.976548738	0.627901591	0.354722566	0.862843056	0.939417651	0.996999903
TEK	0.416590988	0.501660749	0.920956312	0.924337613	0.671281849	0.935358143	0.996999903
TM9SF4	0.583934392	0.825289982	0.661924512	0.780025135	0.488944421	0.937224346	0.996999903
TMED9	0.89433426	0.506844376	0.464098704	0.71763562	0.782354932	0.934230648	0.996999903
TMEM9	0.947329678	0.93742861	0.514199139	0.374980152	0.688666835	0.934064304	0.996999903
TSPAN12	0.423210212	0.958984296	0.649336917	0.506592989	0.895036076	0.935414884	0.996999903
TYW3	0.893824321	0.390554745	0.390221735	0.93122526	0.938945165	0.935088172	0.996999903
XYLT1	0.877188622	0.881380608	0.841238179	0.458540891	0.408365802	0.937329035	0.996999903
ZC3H10	0.649287649	0.834160988	0.296831143	0.966939961	0.804713801	0.939968331	0.996999903
ZDHHC14	0.421164537	0.718927805	0.795228324	0.56467082	0.880883821	0.935649465	0.996999903
ZFP91	0.709144461	0.412659872	0.650398711	0.883702614	0.735703465	0.93890495	0.996999903
ZMYM6	0.604021616	0.69516661	0.50765926	0.998156171	0.586325603	0.939702153	0.996999903
ZNF394	0.834309238	0.613521583	0.839967491	0.90901227	0.321794225	0.940489122	0.996999903
ZNF584	0.412982398	0.865904526	0.974313088	0.36801713	0.96823877	0.939229246	0.996999903
ZNRF3	0.869479737	0.74855255	0.91647424	0.765773523	0.271341781	0.939064048	0.996999903
PDGFC	0.592661382	0.95273182	0.307089105	0.836674192	0.867577307	0.940564658	0.997003397
CDH22	0.998538554	0.819192705	0.504236814	0.496029313	0.61791227	0.94099009	0.997082295
CNIH4	0.652204978	0.97669598	0.663210965	0.811154027	0.369244221	0.941077172	0.997082295
DDX5	0.87935124	0.400178244	0.873049477	0.619898488	0.664031422	0.941022102	0.997082295
FBXL20	0.702689978	0.425333164	0.565333791	0.771201094	0.972159094	0.941185902	0.997082295
LOC512286	0.908079292	0.505988924	0.64867914	0.48991681	0.866652172	0.941088177	0.997082295

MKNK2	0.512347322	0.620345491	0.850550456	0.69565135	0.673531808	0.941173719	0.997082295
PIGK	0.666588579	0.720577707	0.837122804	0.369008299	0.850068862	0.940767621	0.997082295
SCARA3	0.580710489	0.491507548	0.910481249	0.734188754	0.66363426	0.941140371	0.997082295
SF3A1	0.743247556	0.812684779	0.594098544	0.36998794	0.95296392	0.941069608	0.997082295
LOC112444484	0.731512643	0.873178581	0.22111927	0.997985492	0.899294282	0.941246706	0.997082345
CENPP	0.988446732	0.89291385	0.54452376	0.311065331	0.850294763	0.941517095	0.997175672
HSF1	0.654878105	0.789683163	0.359661369	0.983517129	0.694507649	0.941465276	0.997175672
PIH1D1	0.759266295	0.89431728	0.94041588	0.507884193	0.391572777	0.941424801	0.997175672
AKT1	0.887491238	0.7517074	0.594827914	0.567298521	0.568603431	0.942182604	0.997205222
ARHGDIB	0.282905289	0.932925875	0.771965027	0.893022959	0.704197719	0.942274169	0.997205222
C1H21orf58	0.586317185	0.756919089	0.56269959	0.713418256	0.715568322	0.941793376	0.997205222
C22H3orf18	0.999660873	0.569344056	0.67636553	0.50768544	0.655532455	0.942264262	0.997205222
CASS4	0.421498139	0.606636882	0.530404582	0.99054139	0.953223191	0.942220669	0.997205222
CNPY4	0.82611156	0.758595647	0.799853872	0.375832389	0.679596845	0.942200123	0.997205222
GINS3	0.977914433	0.387108729	0.509834928	0.679076465	0.97578293	0.942097022	0.997205222
HIST1H2BL	0.50488376	0.945311719	0.690060495	0.399635781	0.973181297	0.942245386	0.997205222
HNMT	0.817331639	0.76269219	0.658407534	0.370055735	0.840691867	0.941945694	0.997205222
PTPRA	0.594620591	0.811052495	0.763029741	0.448270151	0.775709052	0.942148317	0.997205222
RNFT2	0.504264717	0.775102355	0.66407123	0.68403382	0.721446897	0.942245939	0.997205222
RPL36	0.695932568	0.909011452	0.849766498	0.315212997	0.753961873	0.941999149	0.997205222
CHFR	0.348197635	0.969261416	0.968099966	0.446580404	0.879719692	0.942446648	0.997212942
LOC100847143	0.51330126	0.434752373	0.921724049	0.940793384	0.663589282	0.942485555	0.997212942
LOC112445951	0.511024735	0.905572853	0.668129325	0.415836716	0.99767675	0.942382672	0.997212942
SLC35E2	0.859443936	0.694486638	0.848785387	0.605006716	0.419397321	0.942585288	0.997212942
ZNF814	0.247764152	0.966872864	0.690167817	0.781085266	0.994986428	0.942544424	0.997212942
CITED4	0.887177547	0.922879946	0.355358248	0.618093211	0.71594948	0.942736615	0.997308747
LOC101906221	0.675267255	0.970317252	0.815380363	0.975688968	0.247400657	0.942889916	0.997342341
TMEM131L	0.889560541	0.682080032	0.241294151	0.998019755	0.882561604	0.942885533	0.997342341
UBE2D1	0.793965133	0.525710808	0.771455121	0.61390319	0.653710874	0.943081944	0.997481166
SLC35B4	0.343826695	0.772898983	0.781668692	0.790315985	0.789322532	0.943341481	0.997691374
PHRF1	0.839474961	0.552535741	0.927027874	0.576787071	0.523009282	0.943438009	0.997729164
ABCA9	0.612628528	0.737558797	0.621582832	0.517782291	0.91511762	0.945818938	0.997740751
ADAMTSL1	0.81703616	0.651879635	0.68530126	0.421400918	0.871936405	0.946524635	0.997740751
ARFGEF2	0.981422921	0.819110483	0.301558284	0.676976121	0.803043681	0.944920166	0.997740751
ARHGAP32	0.74350174	0.81617136	0.378235942	0.611987942	0.952460365	0.946303291	0.997740751
BFSP1	0.733190409	0.802056187	0.400302782	0.682316963	0.814652805	0.944253845	0.997740751
CBX1	0.581840574	0.407746283	0.6024305	0.943118907	0.994913951	0.946520951	0.997740751
CC2D2B	0.536448858	0.494742737	0.912024793	0.857648598	0.645407547	0.946438081	0.997740751
CLEC3B	0.721867171	0.727711826	0.837148618	0.809729591	0.373928067	0.94586759	0.997740751
CNNM2	0.922337055	0.43521962	0.862734334	0.926104858	0.40872188	0.944424281	0.997740751
CUTC	0.830393397	0.978321201	0.519717505	0.651872682	0.478797689	0.944912714	0.997740751
CYP26B1	0.826658257	0.952495107	0.962334038	0.627614793	0.280436407	0.946014167	0.997740751
DDAH2	0.876459394	0.884334072	0.550470834	0.736204341	0.423098365	0.945693212	0.997740751
DENND3	0.969125853	0.202925239	0.807978032	0.918123421	0.905553736	0.945143534	0.997740751

DNAL1	0.919079891	0.468054086	0.939872518	0.366481565	0.899179568	0.945924462	0.997740751
DR1	0.897078735	0.886490033	0.665078361	0.452008565	0.5435109	0.9435995	0.997740751
FAM111B	0.811079625	0.478924038	0.939477739	0.673321478	0.54598495	0.946555917	0.997740751
FAM168A	0.552553856	0.667151463	0.614699092	0.594281387	0.986873384	0.945691731	0.997740751
GFRA2	0.705461096	0.396932635	0.984813591	0.65895916	0.722641445	0.944587497	0.997740751
HCAR1	0.458160529	0.956840428	0.415065063	0.950204704	0.770512462	0.945914627	0.997740751
HLTF	0.412297939	0.93480408	0.426227476	0.980750874	0.807672147	0.943736661	0.997740751
HOXB7	0.759716173	0.80578576	0.348258199	0.702983898	0.895699221	0.946610413	0.997740751
INTS2	0.771264202	0.789163674	0.986365115	0.914590835	0.244390415	0.946576707	0.997740751
IRF2BP2	0.70391731	0.980383382	0.510730678	0.592563784	0.623848556	0.943856798	0.997740751
LIPC	0.930392278	0.604996868	0.354283567	0.85704636	0.768850288	0.944649764	0.997740751
LOC100847773	0.897981095	0.457469141	0.820823282	0.867306307	0.455022448	0.945811968	0.997740751
LOC104975612	0.664529076	0.874387359	0.491526445	0.587807376	0.788264205	0.945300951	0.997740751
LOC112446053	0.757838557	0.87544697	0.95724343	0.330571989	0.625896262	0.944645704	0.997740751
LOC112446676	0.818956254	0.751216152	0.306751241	0.920906938	0.771910812	0.94655069	0.997740751
LOC112446775	0.893464509	0.933023352	0.398440565	0.709522372	0.564006869	0.945706254	0.997740751
LOC521656	0.709639881	0.356646065	0.720165437	0.998071361	0.720364189	0.944393875	0.997740751
LOC781741	0.596999233	0.676289882	0.80124796	0.954093982	0.433082781	0.946223354	0.997740751
LOC784522	0.990340359	0.258524992	0.649983923	0.878338728	0.902941054	0.945054126	0.997740751
LOC786783	0.435084942	0.716657275	0.595882377	0.832837772	0.862709929	0.946104584	0.997740751
LOC790886	0.588742169	0.935668559	0.514330833	0.670948153	0.693508895	0.944951781	0.997740751
LYPLAL1	0.784987341	0.785307228	0.792809087	0.287105052	0.932810054	0.944283051	0.997740751
MORC2	0.568595304	0.737115921	0.831502507	0.431510204	0.86838497	0.944068357	0.997740751
NEK3	0.670504398	0.74386932	0.698775846	0.458440047	0.836942475	0.94626092	0.997740751
ORC2	0.982345404	0.379710695	0.44407696	0.895083766	0.88963761	0.944998963	0.997740751
OSGEPL1	0.811353938	0.64166197	0.845559476	0.34685655	0.875603551	0.946240715	0.997740751
PKP2	0.721216385	0.772495568	0.399231216	0.68911805	0.853476851	0.944232914	0.997740751
PPM1B	0.497495472	0.79135879	0.476196653	0.704292985	0.999181684	0.945019124	0.997740751
RANBP17	0.65535422	0.794727745	0.565994592	0.771276918	0.581537454	0.945220882	0.997740751
RASEF	0.40615013	0.45060324	0.920248094	0.908008825	0.853052666	0.943970571	0.997740751
REL	0.770309249	0.8557086	0.746038236	0.312767461	0.855535162	0.944776972	0.997740751
RPL27	0.911090669	0.8102785	0.406200779	0.600432264	0.735327639	0.945345544	0.997740751
RPS27	0.781373013	0.613004684	0.529887446	0.537036197	0.966880343	0.944920092	0.997740751
RPS3A	0.774881391	0.406679562	0.512721701	0.826597666	0.984522456	0.944708079	0.997740751
SLC7A6OS	0.758809336	0.906499518	0.636646174	0.449944167	0.673793878	0.945600492	0.997740751
SMPD5	0.864255198	0.585916398	0.806190345	0.573039066	0.563647036	0.944967975	0.997740751
SOX17	0.345930603	0.931446141	0.914032473	0.637000822	0.715286499	0.946578676	0.997740751
TDG	0.854961912	0.872007163	0.658351965	0.824706613	0.321184705	0.943652915	0.997740751
ZC3H12C	0.743502884	0.971886656	0.96688517	0.967684968	0.196313103	0.945573471	0.997740751
CCDC3	0.688635396	0.453811942	0.801062512	0.560417778	0.972528169	0.948078324	0.997770075
CLTRN	0.69210128	0.66620701	0.886181174	0.832762522	0.397664077	0.947331996	0.997770075
CORO1B	0.773961755	0.725128907	0.964493127	0.343836789	0.733250188	0.948097407	0.997770075
GRIN3A	0.880725489	0.973504136	0.406281076	0.393847617	0.984489703	0.947166753	0.997770075
HERC2	0.798055932	0.830266362	0.836523237	0.274044578	0.890869623	0.947337241	0.997770075

HIST1H2BB	0.449008705	0.980528125	0.4399942	0.740098038	0.945812636	0.947522378	0.997770075
KLHL22	0.845597684	0.681177327	0.580598549	0.636614047	0.636513616	0.947465901	0.997770075
LOC100847573	0.749912853	0.997180556	0.52283165	0.453851201	0.768223086	0.947996183	0.997770075
LOC112441888	0.553825105	0.890361079	0.809854216	0.546497669	0.623994509	0.947906921	0.997770075
LOC112448022	0.883899145	0.97346684	0.250151368	0.867312947	0.720064321	0.946734733	0.997770075
MYDGF	0.451958914	0.729682739	0.765145166	0.612036474	0.875256275	0.94723855	0.997770075
NPDC1	0.85622232	0.593792659	0.62925364	0.95195784	0.445413482	0.947558044	0.997770075
PDE9A	0.792791481	0.734400685	0.629020872	0.771627653	0.481953322	0.947918302	0.997770075
POLE2	0.337260733	0.999868045	0.755464002	0.95368869	0.55776942	0.947465848	0.997770075
POLR2H	0.905678797	0.709880118	0.79104962	0.428070857	0.624979727	0.947830458	0.997770075
RASAL1	0.946699689	0.905831739	0.183159682	0.959714058	0.899444791	0.947511987	0.997770075
RPL19	0.599757363	0.760516239	0.45866482	0.895122905	0.718232698	0.946787732	0.997770075
SEPT11	0.865386642	0.981832991	0.738431292	0.310451221	0.698875117	0.947873079	0.997770075
SLC4A4	0.96488822	0.887019136	0.764122146	0.810150819	0.256685928	0.947788333	0.997770075
SMARCD1	0.430595284	0.638877305	0.759654191	0.824161862	0.788571347	0.94766741	0.997770075
STAT1	0.734048024	0.697110972	0.687564195	0.729615105	0.530981969	0.947989154	0.997770075
TFEB	0.875683781	0.793779921	0.872742753	0.345832132	0.644469879	0.94726189	0.997770075
WASHC1	0.910000235	0.590433074	0.467860146	0.822285706	0.652657714	0.947060964	0.997770075
ZNF614	0.760951113	0.766809437	0.486764766	0.585077503	0.817238446	0.947661066	0.997770075
DDX23	0.921936237	0.868683132	0.996717569	0.786005653	0.218022092	0.948307738	0.997927431
UNC45B	0.485586681	0.639105015	0.769247913	0.664506098	0.864278251	0.948512472	0.998078877
LIMD1	0.725219138	0.748403638	0.817367727	0.466026546	0.663903014	0.94861085	0.998101595
MGARP	0.927998395	0.808570899	0.878531496	0.640246751	0.325377041	0.948655699	0.998101595
ALKAL2	0.885794813	0.548380904	0.384905855	0.913784775	0.80633946	0.948936695	0.998248978
LOC112441810	0.789398573	0.765695515	0.925506898	0.393804028	0.626009882	0.949030843	0.998248978
LOC783301	0.610685824	0.776899651	0.843304054	0.42114197	0.817205764	0.948894873	0.998248978
LOC784088	0.658933754	0.71593695	0.667307858	0.452524815	0.968829381	0.949099921	0.998248978
SLC6A3	0.35091783	0.749848964	0.900839056	0.741459957	0.785020174	0.949071909	0.998248978
AKAP17A	0.943166146	0.874027472	0.647744423	0.373347299	0.706312124	0.950852602	0.998438289
ANGPTL1	0.69717591	0.408491174	0.867620515	0.743044435	0.755332389	0.949521567	0.998438289
BMT2	0.882518319	0.789151462	0.373478237	0.807582887	0.684381818	0.952630503	0.998438289
C21H15orf40	0.830877419	0.844356777	0.339103973	0.599071279	0.976307208	0.949815568	0.998438289
CEP41	0.639766991	0.909450071	0.90339873	0.880892886	0.306643645	0.951569852	0.998438289
CRIPT	0.970887176	0.409723284	0.580813125	0.965788623	0.635186984	0.951419844	0.998438289
EPB41L1	0.641772784	0.699242446	0.925737306	0.669613587	0.499923392	0.949767833	0.998438289
FKBP15	0.267696438	0.72285646	0.946515356	0.994392446	0.76238748	0.949632591	0.998438289
FOXQ1	0.367732342	0.883823224	0.940198419	0.737609451	0.634241938	0.952153399	0.998438289
H2AFV	0.90119767	0.471211415	0.920724457	0.459634828	0.78179037	0.950661276	0.998438289
IAH1	0.525953204	0.615819528	0.661425823	0.995380456	0.655573219	0.950224851	0.998438289
JUN	0.266810562	0.92262025	0.777524752	0.787131301	0.944119369	0.951723404	0.998438289
KCNJ13	0.565475639	0.581779094	0.994313341	0.462728843	0.928067631	0.950647814	0.998438289
KLHL2	0.540229288	0.885768891	0.926233773	0.448677601	0.722441087	0.952576025	0.998438289
LOC101902930	0.454667884	0.459246955	0.90458794	0.772265311	0.962442417	0.950594029	0.998438289
LOC101907653	0.853937313	0.975205641	0.684324529	0.910789379	0.272702002	0.951303066	0.998438289

LOC104973050	0.958601652	0.464166705	0.924196432	0.535580649	0.642023252	0.951216006	0.998438289
LOC107131296	0.316569302	0.541166046	0.94144801	0.98706543	0.87915693	0.950328511	0.998438289
LOC112444152	0.688127541	0.66551532	0.531095462	0.948752358	0.605120062	0.950124685	0.998438289
LOC112444215	0.705617707	0.687931509	0.699552835	0.94340197	0.446017111	0.952111229	0.998438289
LOC112444285	0.661533741	0.589431015	0.783624424	0.70779389	0.648404656	0.950496556	0.998438289
LOC112444339	0.786555098	0.663010402	0.79851443	0.380321954	0.910197977	0.952861406	0.998438289
LOC112446022	0.789931414	0.791244401	0.706560029	0.609869256	0.516742733	0.949835642	0.998438289
LOC112449056	0.551787984	0.606779966	0.900420889	0.734579614	0.647827546	0.952457036	0.998438289
LOC789996	0.860043909	0.294817816	0.936383607	0.835704363	0.703967136	0.950152694	0.998438289
MBD1	0.981702964	0.617009893	0.338344113	0.989312091	0.685794368	0.949754398	0.998438289
MDFIC	0.551130705	0.563182687	0.978333384	0.657939201	0.721344188	0.952841852	0.998438289
MELK	0.710289367	0.684426829	0.379273816	0.88240163	0.876702198	0.951963757	0.998438289
MELTF	0.853646903	0.785079072	0.817228404	0.572929878	0.452963888	0.951661364	0.998438289
MFF	0.78667793	0.905967761	0.463602373	0.492812286	0.875302855	0.951897128	0.998438289
MTERF1	0.832243849	0.773321179	0.900243288	0.876954055	0.282782089	0.952584452	0.998438289
MVB12A	0.865083906	0.474402436	0.768560866	0.522706321	0.873912025	0.952820395	0.998438289
MYOZ3	0.959347437	0.93868538	0.344403395	0.940586411	0.484911208	0.951250213	0.998438289
NME3	0.703962752	0.821305533	0.675001224	0.666056306	0.546466332	0.95160863	0.998438289
NR2C1	0.339855624	0.844652149	0.908159481	0.962658231	0.561735516	0.950954659	0.998438289
NUCB1	0.647042076	0.860059166	0.879259052	0.329502047	0.879080142	0.951416828	0.998438289
OMA1	0.952968079	0.623616706	0.454841265	0.639976373	0.833848488	0.95291735	0.998438289
PARG	0.985993964	0.761854455	0.955567854	0.779667603	0.249032991	0.949959532	0.998438289
PIAS4	0.483139435	0.915921746	0.751470198	0.812638664	0.531795204	0.952601128	0.998438289
PPARG	0.609797817	0.710424284	0.833327782	0.472669183	0.826849891	0.95102736	0.998438289
PRDM16	0.964324249	0.920297698	0.590204625	0.969588935	0.274225257	0.949893965	0.998438289
PRRG2	0.344252377	0.890248744	0.902927213	0.544780807	0.948734013	0.95219446	0.998438289
PTK2B	0.709169045	0.596024952	0.858827869	0.467439405	0.846785706	0.952588368	0.998438289
RAB11FIP3	0.910845915	0.683144819	0.820427922	0.43310058	0.637338993	0.950918563	0.998438289
RANBP10	0.313693196	0.767748057	0.870869864	0.740510738	0.917918272	0.951920219	0.998438289
RCAN2	0.910437186	0.902543309	0.688018005	0.878367691	0.287757409	0.952118541	0.998438289
RCOR3	0.577725041	0.752599868	0.811208511	0.939246968	0.424415622	0.950725284	0.998438289
RREB1	0.926260229	0.829355245	0.699662198	0.546791355	0.474856637	0.950074793	0.998438289
SEMA5A	0.793886806	0.717096471	0.362095692	0.802753469	0.842730401	0.950011178	0.998438289
SETD1B	0.848642717	0.626688222	0.727355206	0.375770784	0.968839928	0.95086684	0.998438289
SNX33	0.614665524	0.683126361	0.682711725	0.721480798	0.694306405	0.952536404	0.998438289
SSR2	0.682571469	0.491364142	0.874191083	0.506004351	0.960996614	0.951924474	0.998438289
STARD3NL	0.672151002	0.91819536	0.819312692	0.512713925	0.556877626	0.952991126	0.998438289
SUN1	0.617943767	0.517077292	0.823208168	0.858741713	0.636060846	0.9525798	0.998438289
SWAP70	0.533990399	0.829738482	0.563512038	0.716212167	0.793782371	0.951546863	0.998438289
SWT1	0.391510183	0.948755497	0.957536954	0.907104435	0.447401708	0.952975837	0.998438289
TRAPPC11	0.811541653	0.600378597	0.890122389	0.59371805	0.553168818	0.951843616	0.998438289
TRMT9B	0.88531179	0.365347903	0.78679324	0.725275011	0.77605098	0.952321766	0.998438289
VPS36	0.606052525	0.68922281	0.652997806	0.759367247	0.696568786	0.952934943	0.998438289
WDR78	0.688396389	0.890309797	0.652239013	0.563347174	0.630246334	0.951537352	0.998438289

ZFYVE26	0.695767192	0.612812434	0.756899218	0.975489933	0.440511947	0.949522033	0.998438289
AATK	0.801355305	0.463172451	0.763183548	0.549968574	0.927860671	0.95309424	0.998482577
PRKD3	0.915941726	0.964219035	0.210475178	0.808893787	0.963060959	0.953243967	0.998511951
PTPN3	0.636422621	0.859066785	0.362527224	0.815139378	0.895715178	0.95319073	0.998511951
ACVRL1	0.947014367	0.76594394	0.369587773	0.575293597	0.964294763	0.955455238	0.998598299
C14H8orf89	0.346786067	0.860253638	0.53159147	0.987155038	0.944076746	0.954943196	0.998598299
C16H1orf21	0.747921584	0.466366162	0.983541165	0.442419344	0.975971543	0.955130179	0.998598299
CD109	0.573972594	0.785234685	0.59972062	0.746290354	0.726769757	0.954274334	0.998598299
CFAP126	0.998497524	0.631307695	0.279316825	0.883116089	0.934971511	0.95357482	0.998598299
CSDC2	0.694446042	0.565067995	0.520480634	0.857891589	0.848787582	0.955456127	0.998598299
DCAF7	0.931915856	0.975561835	0.52043188	0.350885002	0.884189717	0.954381566	0.998598299
DPH2	0.89231364	0.951902846	0.587164824	0.34139203	0.869146391	0.955048786	0.998598299
EIF2AK4	0.929330124	0.86512954	0.514896954	0.804129123	0.437422041	0.953709067	0.998598299
FRMD3	0.936288232	0.867408824	0.578740179	0.405669458	0.767916604	0.954170874	0.998598299
FRMD4A	0.683568572	0.777345172	0.33572669	0.926440823	0.899229785	0.955398711	0.998598299
IL1RAPL2	0.726497474	0.500112273	0.639310709	0.767694523	0.816002649	0.953650447	0.998598299
LOC100337081	0.917302999	0.700660189	0.909041468	0.925242445	0.273688037	0.955029639	0.998598299
LOC100848171	0.864177515	0.511377314	0.832224473	0.444309805	0.897448764	0.95430036	0.998598299
LOC101904526	0.526423416	0.589837377	0.929675549	0.739683206	0.692419832	0.95497262	0.998598299
LOC112444843	0.759364329	0.642973304	0.502546626	0.694943905	0.860101947	0.954307757	0.998598299
LOC112447066	0.861310191	0.733161948	0.524427382	0.456703649	0.960739886	0.95353311	0.998598299
LOC112447832	0.768886697	0.979900815	0.871088097	0.537149437	0.418391954	0.954777444	0.998598299
LOC785386	0.845360423	0.836438543	0.575526145	0.629365427	0.580254687	0.955397264	0.998598299
LOC785761	0.739001861	0.691003091	0.952591294	0.350865288	0.870744139	0.955397368	0.998598299
MAPK7	0.915058822	0.685574146	0.378205887	0.862833785	0.708638093	0.953397551	0.998598299
MRPL10	0.801782429	0.743529613	0.383843982	0.65012273	0.989504342	0.954613505	0.998598299
MS4A13	0.748020882	0.426836794	0.992462102	0.679581002	0.687092812	0.955035472	0.998598299
MYO6	0.565095022	0.821731278	0.458039728	0.753869042	0.923751662	0.95512237	0.998598299
NOS3	0.498128219	0.964049339	0.522271888	0.846747068	0.688121792	0.954008516	0.998598299
PIK3R3	0.608384426	0.787851454	0.783359641	0.860380852	0.457434671	0.9549327	0.998598299
PIP4P1	0.895875396	0.589998596	0.381837896	0.786762784	0.930924031	0.954957753	0.998598299
PLXNA2	0.912139356	0.467806653	0.805887287	0.717698068	0.591059616	0.953858544	0.998598299
PTPRH	0.967542281	0.923299226	0.886440017	0.436543818	0.422590276	0.953980324	0.998598299
SNRNP40	0.675216585	0.975469244	0.353306797	0.655802135	0.972518962	0.955287393	0.998598299
SYN3	0.777594501	0.814987993	0.385876366	0.656485651	0.907211442	0.953726087	0.998598299
TDRD6	0.72394998	0.701624319	0.48013405	0.629441671	0.958879096	0.954608261	0.998598299
TRIT1	0.922169146	0.848712137	0.656067337	0.935312488	0.304917461	0.954181409	0.998598299
TYW1	0.815378902	0.861839686	0.912365787	0.495940499	0.459294764	0.95395478	0.998598299
ZNF235	0.839583481	0.607080936	0.814827584	0.643143825	0.552622986	0.954839752	0.998598299
ZFYVE27	0.67227371	0.410615196	0.884406387	0.848964949	0.718975638	0.955618112	0.998703995
AIF1L	0.723193639	0.577017502	0.474864733	0.955511949	0.794434639	0.956378554	0.998842876
DND1	0.636472426	0.957323349	0.703550281	0.650621487	0.53995946	0.956474223	0.998842876
FAM13B	0.784418596	0.452700188	0.953246608	0.912542431	0.485421873	0.956123225	0.998842876
FAM149A	0.392022751	0.751084267	0.754843344	0.766894389	0.877527806	0.955920977	0.998842876

GAB1	0.574596298	0.862258435	0.738256629	0.563082033	0.730898174	0.956439553	0.998842876
GPSM2	0.444107966	0.773321492	0.458730911	0.988292407	0.964724866	0.956264695	0.998842876
LOC100337328	0.932668329	0.897534924	0.563693507	0.386888381	0.824928131	0.956474312	0.998842876
LOC104974883	0.492625569	0.69446423	0.605042478	0.806891747	0.897649533	0.956111763	0.998842876
LOC107132849	0.849562004	0.383382898	0.934426505	0.795329193	0.622216573	0.956481372	0.998842876
LOC112441859	0.866889374	0.559372217	0.824895224	0.591009378	0.636385736	0.956391631	0.998842876
MTOR	0.530196524	0.920713753	0.833931065	0.728709243	0.506530893	0.956293779	0.998842876
SLC39A1	0.741227524	0.83756671	0.627180716	0.801426601	0.480537268	0.956126625	0.998842876
NCAM2	0.720715296	0.674841394	0.920584617	0.588263317	0.57238232	0.956560323	0.998861762
INO80E	0.857766538	0.514981384	0.570767675	0.802120798	0.747434004	0.956772347	0.998987617
LOC112447461	0.385758997	0.681536101	0.839480108	0.773635924	0.885613751	0.956802594	0.998987617
CRKL	0.786120292	0.702335563	0.816542376	0.581976444	0.587305141	0.958298876	0.999218952
GAS2L3	0.674337704	0.448451177	0.747880728	0.744612736	0.907971745	0.957689633	0.999218952
GNAO1	0.673353352	0.925276618	0.566618193	0.452192509	0.961288297	0.95797266	0.999218952
GPR151	0.459850742	0.950356419	0.73502259	0.521621594	0.91943663	0.958280761	0.999218952
HSDL2	0.805748095	0.771812641	0.304291295	0.820255984	0.987715155	0.957899879	0.999218952
IL10RB	0.925051005	0.925618793	0.849196988	0.432487876	0.488373967	0.958036337	0.999218952
KLF8	0.659235334	0.630591134	0.96833431	0.764593212	0.495867431	0.957540217	0.999218952
LOC100335177	0.898392933	0.878355598	0.817679776	0.718153872	0.332429958	0.95827283	0.999218952
LOC104969192	0.34116873	0.816541748	0.668377003	0.988535003	0.83275819	0.957881368	0.999218952
LOC112448507	0.874125549	0.789678746	0.436277259	0.745512381	0.676146868	0.957113477	0.999218952
LOC112449565	0.667301675	0.411751192	0.895092759	0.785089454	0.799342529	0.958424564	0.999218952
LOC781576	0.906886275	0.385737854	0.780460239	0.984781698	0.572946822	0.958275098	0.999218952
LOC789626	0.682409406	0.901993396	0.700184338	0.539448752	0.662461546	0.958260888	0.999218952
MXRA8	0.937153568	0.603876277	0.440819478	0.949559662	0.651505882	0.95842148	0.999218952
MYLIP	0.462057274	0.628518034	0.819052311	0.73622418	0.880376804	0.958339062	0.999218952
PARP2	0.832215406	0.48321749	0.485294386	0.795662737	0.985887817	0.957783065	0.999218952
RPL35	0.786292834	0.707595235	0.400343143	0.76098443	0.898844406	0.957403362	0.999218952
SHISA5	0.876962098	0.933632547	0.710470391	0.558302071	0.470688269	0.957667308	0.999218952
SKA1	0.596790769	0.768570488	0.54145881	0.793489822	0.777738558	0.957875207	0.999218952
STX11	0.87627595	0.499534335	0.80602937	0.654734898	0.666573998	0.958242671	0.999218952
TENM1	0.890940493	0.441864711	0.453182089	0.877188449	0.980624497	0.957977067	0.999218952
ZC3HAV1	0.847299116	0.965210708	0.309581711	0.826810136	0.736490665	0.958339669	0.999218952
ZNF570	0.94229805	0.706428229	0.748920309	0.500820673	0.617483727	0.958338311	0.999218952
GPHN	0.920190569	0.95729869	0.236832513	0.890824568	0.832551095	0.958621502	0.999304672
RPP25	0.625775545	0.901896501	0.953143941	0.8076324	0.356451012	0.958689462	0.999304672
SLC35B3	0.995971471	0.549280485	0.782650743	0.586686284	0.616332688	0.95866839	0.999304672
CHIC2	0.754641626	0.854152061	0.750120106	0.871982936	0.36998339	0.959253512	0.999362691
FBXO9	0.458770059	0.789406984	0.50638193	0.870498667	0.977265212	0.959263821	0.999362691
IKZF4	0.949927346	0.298671056	0.983389793	0.937287107	0.594646664	0.959011227	0.999362691
MEX3D	0.998298619	0.677442583	0.598920253	0.648142588	0.59356666	0.959172327	0.999362691
PPP4C	0.303868538	0.796381843	0.892346055	0.791928636	0.912626454	0.959293186	0.999362691
RNF126	0.529948471	0.621274495	0.659861437	0.787722826	0.909651007	0.959096601	0.999362691
SEPT2	0.975116783	0.552253358	0.391658277	0.881670451	0.839245685	0.959289302	0.999362691

SORCS1	0.77381747	0.95778564	0.70208696	0.405641932	0.736247283	0.958961711	0.999362691
UNC50	0.778695505	0.584065801	0.653242431	0.682543214	0.768173281	0.959145746	0.999362691
ANKRD24	0.703189259	0.851234931	0.950230299	0.359245745	0.764658124	0.959380461	0.999390171
ATP2A3	0.905194676	0.759814849	0.417773385	0.575496157	0.947224308	0.959571775	0.99939915
FCGRT	0.360982824	0.801327454	0.852069855	0.958641601	0.662785707	0.959556752	0.99939915
RUVBL2	0.529244387	0.893433464	0.928573145	0.932566019	0.382089442	0.9594818	0.99939915
A2M	0.768452739	0.879422843	0.777941747	0.497987941	0.602916779	0.960164144	0.999459585
ANKRD54	0.940649714	0.638393403	0.648933274	0.544370913	0.742548038	0.96000522	0.999459585
AVPR1A	0.975935388	0.779774221	0.708057266	0.29467302	0.996005862	0.960308654	0.999459585
FPGT	0.628222558	0.409414088	0.958590439	0.756377683	0.844384964	0.959979254	0.999459585
FYCO1	0.760471906	0.92837274	0.254659608	0.885843878	0.99365128	0.960360624	0.999459585
LOC104973767	0.521266984	0.822153146	0.600859369	0.937545203	0.652061329	0.959957701	0.999459585
LOC619131	0.657866716	0.454252414	0.796672424	0.987973197	0.671527221	0.960214355	0.999459585
LOC783060	0.758748882	0.619135956	0.959965552	0.733189261	0.477323194	0.960151863	0.999459585
MARC2	0.767160522	0.616116334	0.710810362	0.676412007	0.695312059	0.960244241	0.999459585
MRRF	0.886930903	0.830116508	0.347402789	0.880161279	0.696951645	0.959703212	0.999459585
MTRF1L	0.906231322	0.728098308	0.533772993	0.603368442	0.741343808	0.960014409	0.999459585
RACK1	0.547033296	0.539370958	0.781727389	0.92216674	0.741184536	0.960068076	0.999459585
LOC784659	0.791935593	0.93307569	0.408691184	0.912438089	0.575708652	0.960544752	0.999461065
RPS3	0.533709051	0.886855681	0.455803038	0.887405394	0.828457038	0.960530518	0.999461065
SLC27A4	0.604334331	0.504971049	0.656684427	0.972907736	0.812485894	0.960436195	0.999461065
CD2AP	0.901297405	0.483435276	0.987124859	0.830492484	0.452614622	0.961968866	0.999592796
CEBPB	0.796273977	0.531803548	0.435183545	0.897391151	0.988647239	0.962795481	0.999592796
CTU2	0.674332284	0.426345424	0.935166949	0.645352815	0.930827908	0.961891607	0.999592796
ECI2	0.488264062	0.535597532	0.992721046	0.74743279	0.837796355	0.962375934	0.999592796
ENTPD5	0.615370758	0.723802185	0.644402555	0.640790947	0.878541557	0.961926074	0.999592796
FZD3	0.800268148	0.842544532	0.845233947	0.477666892	0.596428288	0.962283711	0.999592796
HSPA12A	0.680896867	0.928341944	0.315529991	0.874714732	0.935849036	0.962692957	0.999592796
IMPDH1	0.831285448	0.920418552	0.491457676	0.800750573	0.539646421	0.962341448	0.999592796
KLRD1	0.92808411	0.757158534	0.611466835	0.507715488	0.749775707	0.962827441	0.999592796
LENG1	0.896912084	0.722009214	0.938577388	0.368123546	0.731118149	0.962835033	0.999592796
LOC100138131	0.473270108	0.679032635	0.896243064	0.873185679	0.646131343	0.96234565	0.999592796
LOC100294792	0.564688603	0.484747654	0.955698271	0.909903205	0.685572493	0.962657783	0.999592796
LOC100849069	0.992902323	0.337504425	0.749913261	0.871822629	0.749445161	0.96310775	0.999592796
LOC101906315	0.740391408	0.502629622	0.864855461	0.94910063	0.532093576	0.962362697	0.999592796
LOC104971057	0.660681355	0.910275626	0.935826285	0.668280606	0.430306023	0.962046393	0.999592796
LOC104972417	0.758513554	0.903639518	0.981949929	0.383735098	0.633677227	0.962869361	0.999592796
LOC104974542	0.620031631	0.992283922	0.702695767	0.64534193	0.570498615	0.960798436	0.999592796
LOC104975290	0.651271739	0.664426251	0.600508201	0.807040517	0.774034764	0.962265855	0.999592796
LOC112445988	0.593700692	0.734841663	0.960624117	0.609873466	0.624703717	0.961036128	0.999592796
LOC618220	0.830667179	0.871722237	0.267430156	0.859616437	0.983439301	0.962889629	0.999592796
LOC782776	0.346204399	0.82969877	0.706358656	0.940969949	0.859167191	0.963034981	0.999592796
LOC783926	0.944316206	0.570793075	0.687635949	0.721205559	0.595266019	0.960774611	0.999592796
LRRN4CL	0.894003094	0.963405454	0.406286596	0.578885108	0.786972204	0.960915177	0.999592796

MANSC4	0.58039658	0.620304141	0.927816101	0.94020061	0.512765669	0.961675433	0.999592796
MARCH9	0.705224891	0.992261854	0.419813479	0.972203027	0.560483434	0.961226995	0.999592796
MCM10	0.641065739	0.65515873	0.837499657	0.721900537	0.638734694	0.962205895	0.999592796
MR1	0.966730047	0.686626661	0.505082565	0.676954524	0.70963099	0.961683924	0.999592796
NCKIPSD	0.929666873	0.522027071	0.741608797	0.573296493	0.794318537	0.962974034	0.999592796
NRCAM	0.519308741	0.798765881	0.61526545	0.934919441	0.675724623	0.96176483	0.999592796
PCID2	0.420085131	0.515986922	0.860953497	0.889944609	0.973657532	0.961982899	0.999592796
PLPP4	0.80466127	0.865745599	0.882236856	0.268065023	0.98074158	0.961924474	0.999592796
RABGGTB	0.912402656	0.932560887	0.381009762	0.953564254	0.517153044	0.96112982	0.999592796
RAD18	0.779860622	0.398088986	0.765973689	0.6862904	0.988629541	0.961816434	0.999592796
RPL13	0.844422689	0.939053842	0.690463657	0.714027325	0.41902577	0.962936601	0.999592796
RPUSD4	0.793979771	0.918246975	0.759032099	0.490069466	0.602026658	0.962693001	0.999592796
SCAF1	0.970344625	0.847195218	0.431216726	0.648248986	0.714320302	0.963086751	0.999592796
SLC35B2	0.69592176	0.433777045	0.906804634	0.690464787	0.864492586	0.962750614	0.999592796
TERF1	0.515132796	0.88842736	0.990061701	0.856690595	0.412500365	0.961248044	0.999592796
TMCO3	0.994392153	0.398472664	0.797660582	0.849589511	0.61003874	0.962935613	0.999592796
ZNF548	0.495212142	0.708823907	0.506128056	0.997450611	0.925969762	0.963059599	0.999592796
GJB2	0.625987252	0.761251532	0.520990907	0.83897159	0.79086524	0.963343696	0.999634776
LOC112444653	0.899350451	0.604674089	0.780282418	0.79764819	0.486773238	0.963354839	0.999634776
LOC112447817	0.872820534	0.937429002	0.74917525	0.383125454	0.701013843	0.963300348	0.999634776
RPL8	0.891012265	0.686639642	0.483717202	0.79976217	0.69646091	0.963391848	0.999634776
MOB3B	0.870659942	0.846063879	0.546321634	0.433643867	0.945434594	0.96345915	0.999641406
CNTNAP3	0.629968648	0.975163277	0.868157757	0.627378355	0.493762245	0.963555436	0.999678105
LOC112443431	0.942975027	0.539087307	0.389927563	0.989510323	0.843081858	0.963620531	0.999682441
ABCB7	0.8000346	0.684011762	0.791034014	0.484049407	0.883664584	0.971233079	0.999863206
ABCG1	0.726900044	0.961125584	0.70441516	0.618595998	0.704415412	0.979493536	0.999863206
ACBD6	0.896106453	0.650034558	0.815252484	0.655954997	0.823696851	0.987256595	0.999863206
ACMSD	0.566341435	0.757793403	0.792204964	0.961201441	0.847804224	0.989862523	0.999863206
ACSBG1	0.610682219	0.994756246	0.896509216	0.854771628	0.417505235	0.974159078	0.999863206
ACTR1B	0.989238838	0.874521704	0.856397803	0.402193064	0.840158989	0.986334209	0.999863206
ADAM23	0.948603883	0.799072078	0.723102966	0.79381268	0.891082809	0.997083402	0.999863206
ADAMTS18	0.978284302	0.98862753	0.67444589	0.760696185	0.420202404	0.978050478	0.999863206
ADRA1B	0.735085695	0.95941902	0.86785654	0.809893346	0.402016046	0.975591744	0.999863206
AGA	0.951769437	0.901120486	0.961943969	0.625393512	0.853000642	0.998423127	0.999863206
AK1	0.356856121	0.978675215	0.797919641	0.761115559	0.94096224	0.975675555	0.999863206
AK8	0.90090896	0.999716478	0.620102262	0.621062542	0.483683071	0.964656974	0.999863206
AK9	0.769677476	0.817861945	0.585646331	0.963552367	0.584338609	0.977813915	0.999863206
AKNAD1	0.819014951	0.774757279	0.86691263	0.896585174	0.707547323	0.995465054	0.999863206
ANG2	0.824915246	0.829937079	0.610427212	0.76975338	0.849191039	0.989413563	0.999863206
ANKFY1	0.814808972	0.856675815	0.925021327	0.631129376	0.628278312	0.987177611	0.999863206
ANKRD13A	0.971728877	0.789862895	0.407992861	0.776809295	0.935552472	0.982338007	0.999863206
ANKRD9	0.773629244	0.926852746	0.636878954	0.550358062	0.866143899	0.980238005	0.999863206
APPBP2	0.948911127	0.804512411	0.54287419	0.975295407	0.584666207	0.983999438	0.999863206
ARHGAP18	0.840341528	0.732375821	0.755377673	0.478178695	0.981635604	0.980357249	0.999863206

ARHGEF15	0.984291816	0.450801462	0.720606086	0.928740664	0.973538555	0.991136368	0.999863206
ARHGEF38	0.85707846	0.960782114	0.627617208	0.926296104	0.534801522	0.987176619	0.999863206
ARMCX5	0.67890331	0.826810443	0.821980514	0.592010298	0.890912901	0.985217858	0.999863206
ARSG	0.598995569	0.860289822	0.669524644	0.872816695	0.888030759	0.988710107	0.999863206
ARV1	0.649580831	0.976794743	0.891665167	0.683099332	0.709044228	0.989513568	0.999863206
ASAP1	0.439821888	0.998248783	0.955936239	0.862381973	0.538629773	0.974338022	0.999863206
ASB5	0.912917257	0.898463103	0.610104829	0.495815984	0.761585866	0.972484651	0.999863206
ASB9	0.540621862	0.905604162	0.645562405	0.983302801	0.960275677	0.992012363	0.999863206
ASPN	0.533843944	0.899272608	0.817556794	0.84649803	0.689511684	0.982636066	0.999863206
ATP10A	0.57225192	0.943796623	0.537416357	0.906883683	0.692757742	0.970269429	0.999863206
ATP6V0E2	0.841801687	0.907879194	0.490730238	0.659515686	0.687216862	0.965578303	0.999863206
ATXN1	0.641398913	0.95664676	0.955737792	0.807036261	0.584052352	0.989789242	0.999863206
ATXN3	0.905255017	0.736407317	0.399866486	0.782151156	0.926111446	0.973775899	0.999863206
ATXN7L1	0.659178696	0.882840835	0.536885782	0.871894362	0.637341126	0.967038714	0.999863206
AVEN	0.772194919	0.619661861	0.891788975	0.868170089	0.991110717	0.996310607	0.999863206
AVPR2	0.896995242	0.658121832	0.819929358	0.882236024	0.889785815	0.996812018	0.999863206
BACE1	0.987136748	0.758376702	0.716710699	0.772247956	0.680651054	0.990408397	0.999863206
BARD1	0.97353664	0.720993063	0.668291624	0.386262167	0.937051021	0.965497064	0.999863206
BBX	0.954468868	0.903949866	0.991674521	0.823723974	0.639077193	0.998607069	0.999863206
BCAT1	0.632242198	0.608277023	0.932232719	0.93733465	0.657086666	0.980928661	0.999863206
BCL7C	0.569023912	0.992352128	0.47075973	0.808650768	0.866804006	0.971624175	0.999863206
BICD1	0.699387147	0.579595694	0.858010133	0.964947987	0.67007047	0.981790279	0.999863206
BICRAL	0.342783447	0.921789965	0.908639572	0.990930557	0.75779884	0.979760778	0.999863206
BLOC1S3	0.689834644	0.969239686	0.575325233	0.721726832	0.723948049	0.976068201	0.999863206
BORCS8	0.482455726	0.879169088	0.814862585	0.849290724	0.946762072	0.989958503	0.999863206
C11H2orf49	0.934011873	0.872474994	0.702764675	0.869446402	0.927407381	0.99878699	0.999863206
C11H2orf68	0.64062419	0.803563138	0.95230868	0.718817795	0.979621653	0.995268736	0.999863206
C14H8orf82	0.723997472	0.844357137	0.98331958	0.568975716	0.810284503	0.989870635	0.999863206
C19H17orf75	0.461565205	0.898809327	0.88847245	0.798103718	0.592555774	0.96730882	0.999863206
C2CD2	0.858853293	0.491647291	0.552004776	0.923810036	0.894153005	0.973606421	0.999863206
C3H1orf216	0.531743399	0.928287154	0.836387821	0.790385208	0.881436596	0.990988658	0.999863206
C5H12orf10	0.868727191	0.735004244	0.618678568	0.897657581	0.673614565	0.984452532	0.999863206
C7H19orf66	0.5946512	0.521385407	0.918552703	0.991665753	0.822513801	0.983254977	0.999863206
CALHM2	0.716151277	0.960431184	0.547204858	0.812702154	0.778605911	0.9843279	0.999863206
CAMK1D	0.866621439	0.977967539	0.82292635	0.60214968	0.476504431	0.975826686	0.999863206
CASC3	0.921824624	0.868689217	0.908784182	0.560212564	0.433460665	0.968226215	0.999863206
CASP6	0.709479932	0.822532414	0.971547204	0.655939506	0.569972378	0.978904788	0.999863206
CASP7	0.880887146	0.613355579	0.850338218	0.889803798	0.966962429	0.997328069	0.999863206
CASP9	0.995489429	0.764666572	0.607611146	0.799721092	0.628678011	0.983302247	0.999863206
CAST	0.926749394	0.934500721	0.655493088	0.556944451	0.861005848	0.989300397	0.999863206
CBY1	0.558909588	0.80273318	0.7741172	0.628319238	0.957048498	0.97813728	0.999863206
CC2D1A	0.991214389	0.523143694	0.904153337	0.515982989	0.880500555	0.979153645	0.999863206
CCDC130	0.882922917	0.907655899	0.548167845	0.949284004	0.640398852	0.988664878	0.999863206
CCDC170	0.525876791	0.942280402	0.857119898	0.989357184	0.997112411	0.997973336	0.999863206

CCDC50	0.932705996	0.699711231	0.714541268	0.81365538	0.504699235	0.973285959	0.999863206
CCL28	0.731467628	0.547023738	0.741165117	0.744387467	0.930044532	0.977231553	0.999863206
CCNK	0.991470521	0.453523293	0.552022475	0.866170939	0.997852434	0.979515106	0.999863206
CCR8	0.990992602	0.953778432	0.354636734	0.677333738	0.76455134	0.967024263	0.999863206
CD274	0.917010836	0.860109085	0.997495555	0.410620449	0.692730025	0.981563012	0.999863206
CDCA7L	0.562363498	0.804033879	0.663259172	0.676391382	0.862074376	0.967523981	0.999863206
CDH1	0.907135081	0.76800633	0.576155968	0.818250824	0.595110133	0.974488245	0.999863206
CECR2	0.9285625	0.738163939	0.807171085	0.517003093	0.858062868	0.985559941	0.999863206
CES2	0.791039199	0.898181917	0.889566127	0.867531152	0.674842186	0.996428405	0.999863206
CHML	0.870592443	0.83055047	0.764096613	0.493096196	0.952547377	0.987666573	0.999863206
CHRDL1	0.997883398	0.662366226	0.439196081	0.986402214	0.753439088	0.979794922	0.999863206
CHRNA5	0.777836188	0.637866195	0.944050535	0.43290324	0.959335377	0.974209464	0.999863206
CHST14	0.912801564	0.803877457	0.534170771	0.801459449	0.7646657	0.984686289	0.999863206
CITED1	0.734775454	0.940712149	0.9996418	0.425033577	0.58754052	0.966616503	0.999863206
CLDN12	0.775764698	0.991045607	0.8111847	0.997624448	0.289103207	0.969388777	0.999863206
CLEC1A	0.386548004	0.961146979	0.744685688	0.837408306	0.896728157	0.977862586	0.999863206
CLSTN1	0.900464513	0.893968708	0.710160726	0.386612338	0.781533494	0.966687777	0.999863206
CMTR1	0.963823599	0.398225226	0.992652753	0.809905886	0.607271528	0.971973833	0.999863206
CNKSR2	0.757648695	0.51349032	0.71442758	0.673370487	0.982634388	0.970808523	0.999863206
CNOT8	0.965597404	0.495226873	0.900210427	0.853957864	0.953822251	0.995549558	0.999863206
CNPY3	0.887753278	0.863048289	0.576180119	0.821702133	0.919127865	0.994597364	0.999863206
CNTF	0.574373902	0.945394908	0.87881207	0.678681233	0.840499655	0.989298847	0.999863206
CNTNAP4	0.482586693	0.409434832	0.990040071	0.948380092	0.914478238	0.965444519	0.999863206
COCH	0.47235749	0.666433089	0.934946247	0.797316904	0.838605143	0.974878054	0.999863206
COG5	0.970473632	0.957802751	0.774001986	0.959215776	0.347290672	0.984591865	0.999863206
COMP	0.813212229	0.621508727	0.919342978	0.915754849	0.52756506	0.981707188	0.999863206
CPSF4	0.651106982	0.933915413	0.956443154	0.609906057	0.760284403	0.988992777	0.999863206
CRADD	0.989060191	0.777521462	0.937035028	0.826838502	0.827446495	0.999177902	0.999863206
CRY1	0.72726021	0.571879627	0.880882644	0.802023367	0.972338386	0.990793937	0.999863206
CSNK1G1	0.687388049	0.788942142	0.98316186	0.878360222	0.583172491	0.989405867	0.999863206
CTIF	0.956483446	0.635707079	0.757252878	0.707509263	0.553259648	0.969519111	0.999863206
CTNND1	0.828385071	0.96522639	0.630746606	0.793373801	0.78326645	0.993241063	0.999863206
CTSW	0.951982423	0.698241666	0.889298137	0.739055342	0.608317256	0.988499389	0.999863206
CTTNBP2NL	0.375449158	0.720834169	0.76702202	0.872366099	0.969534776	0.967792999	0.999863206
CYBA	0.670991873	0.591711163	0.744684077	0.968596152	0.830142211	0.984252657	0.999863206
CYS1	0.787620112	0.558244161	0.681373188	0.983212475	0.802142944	0.983991928	0.999863206
DAAM2	0.793701794	0.98268421	0.824915409	0.737826409	0.395048619	0.972022488	0.999863206
DAB2IP	0.841540068	0.98504012	0.687602129	0.721943351	0.610580836	0.986472353	0.999863206
DAPK1	0.496610543	0.903015298	0.984563423	0.930696521	0.741771361	0.992560666	0.999863206
DCLRE1C	0.959100859	0.623232909	0.691909285	0.942106852	0.79109622	0.99284011	0.999863206
DDHD2	0.812772714	0.951726331	0.668308026	0.707548301	0.758818235	0.989918329	0.999863206
DEF8	0.911336852	0.549809531	0.923509601	0.882295145	0.785720782	0.993776488	0.999863206
DENND4C	0.673011377	0.888796443	0.808399468	0.496630197	0.804249761	0.973791726	0.999863206
DEPP1	0.815291362	0.962241493	0.523626766	0.728622505	0.919880428	0.989664704	0.999863206

DHX38	0.871872823	0.843815662	0.938503276	0.621585154	0.704840233	0.992366366	0.999863206
DIXDC1	0.743854454	0.429369016	0.831368927	0.880939567	0.940845235	0.980768359	0.999863206
DMXL2	0.438477921	0.692124413	0.948037645	0.716591077	0.840064504	0.96687224	0.999863206
DPM3	0.530858434	0.758345516	0.866866715	0.949711913	0.976308969	0.993967903	0.999863206
DPP8	0.630125185	0.618677377	0.933134668	0.786283837	0.99611882	0.990713272	0.999863206
DPYSL2	0.682684351	0.548050586	0.903473575	0.573051255	0.871151329	0.965068369	0.999863206
DSN1	0.963781921	0.891074847	0.620376687	0.518965813	0.820499649	0.982194387	0.999863206
DTD1	0.822220454	0.814014879	0.623520339	0.660515279	0.643644513	0.968489322	0.999863206
DYNC1H1	0.772703024	0.755944134	0.818178786	0.564432242	0.850441661	0.982699808	0.999863206
EDEM1	0.438799615	0.98106874	0.966810067	0.503487938	0.848500019	0.968633341	0.999863206
EDN1	0.921262059	0.952132946	0.501388662	0.433496547	0.995245154	0.972734811	0.999863206
EEF1A1	0.56832632	0.719586246	0.842427457	0.75386444	0.742207029	0.973677321	0.999863206
EFCAB6	0.909799565	0.502846009	0.687975115	0.864210972	0.681686319	0.971321123	0.999863206
EFNA5	0.942521947	0.986908047	0.977296039	0.702872773	0.744426421	0.99897789	0.999863206
EIF3F	0.829285648	0.80008541	0.38499694	0.914882586	0.807734766	0.972422621	0.999863206
EIF5A2	0.696659028	0.796997252	0.921686735	0.904328877	0.701960834	0.99405413	0.999863206
ELOVL4	0.873537681	0.994841826	0.880328818	0.262323256	0.986609068	0.975227209	0.999863206
ENO3	0.706546983	0.976269578	0.721313606	0.661691064	0.994492641	0.994221406	0.999863206
EPCAM	0.647608283	0.734226187	0.778135153	0.969727867	0.902901907	0.993993546	0.999863206
ESRP2	0.629582114	0.44194637	0.790673781	0.991496836	0.82501081	0.969419686	0.999863206
EXT1	0.684856655	0.508303095	0.851288439	0.938544661	0.610813308	0.965540175	0.999863206
FA2H	0.555079945	0.842029556	0.765770184	0.847617888	0.58094125	0.968048061	0.999863206
FABP9	0.714206162	0.932843751	0.679268726	0.827057421	0.888911053	0.994554976	0.999863206
FAM20C	0.33591166	0.927770367	0.809644223	0.979509775	0.813601231	0.976095069	0.999863206
FAM71F2	0.820898294	0.849774535	0.842527705	0.429389226	0.768918619	0.974066343	0.999863206
FAM83G	0.814788189	0.683011413	0.744451685	0.872907205	0.639945493	0.983091169	0.999863206
FAR1	0.57750322	0.825424891	0.999561001	0.881926103	0.530490469	0.981379897	0.999863206
FAU	0.62750409	0.89141197	0.896415603	0.920236027	0.855712649	0.99731407	0.999863206
FBXL17	0.781871623	0.803957312	0.914698106	0.951903914	0.742625058	0.99765265	0.999863206
FBXL4	0.647634007	0.637441775	0.895725176	0.979925559	0.950558592	0.995227864	0.999863206
FGFRL1	0.571148567	0.631739697	0.995384337	0.612263895	0.788597985	0.966955538	0.999863206
FGL2	0.761047472	0.901782754	0.923609035	0.410368646	0.760856767	0.975203214	0.999863206
FMO5	0.778264154	0.827488275	0.999292725	0.860934019	0.794036913	0.998419772	0.999863206
FOXK1	0.750250825	0.744867762	0.66135042	0.998215738	0.888457475	0.994245126	0.999863206
FSD2	0.982930952	0.767145582	0.600401076	0.902782008	0.664636445	0.989231475	0.999863206
FXR2	0.899497018	0.969366972	0.626073296	0.746523426	0.772055969	0.993333343	0.999863206
GABPB2	0.683543754	0.647358224	0.80610417	0.981697572	0.614513823	0.979665584	0.999863206
GALNT7	0.819292304	0.722715676	0.941626781	0.746367882	0.496884654	0.977610016	0.999863206
GAS6	0.546475651	0.891415701	0.863703669	0.843538139	0.905933713	0.993827887	0.999863206
GJA4	0.862148065	0.908679571	0.54778002	0.732725625	0.618447881	0.974191898	0.999863206
GJB6	0.879513669	0.909279038	0.537459219	0.970892315	0.504853918	0.978590861	0.999863206
GLTP	0.759386689	0.873546438	0.554708519	0.946046316	0.693206986	0.984875231	0.999863206
GNAT2	0.842240127	0.824480005	0.837901477	0.894008028	0.948551931	0.999182154	0.999863206
GOLGA3	0.552234887	0.428162522	0.894047333	0.960598124	0.994783397	0.97634747	0.999863206

GPR135	0.820550889	0.849295759	0.615300292	0.552973292	0.712818142	0.965180459	0.999863206
GRB14	0.980157682	0.76629607	0.680388024	0.998370115	0.398264751	0.976669415	0.999863206
GRK2	0.996783418	0.814838191	0.662998295	0.715671328	0.948610914	0.996243276	0.999863206
HACD4	0.963072207	0.882919813	0.840358348	0.380048179	0.650068012	0.968159778	0.999863206
HAUS6	0.929432022	0.784718601	0.905356314	0.792956618	0.952940815	0.999237908	0.999863206
HIST1H1C	0.673360505	0.807213408	0.4871354	0.987278637	0.958894159	0.986382989	0.999863206
HIST1H2BI	0.814267507	0.927986236	0.765346178	0.884613419	0.770980471	0.997300823	0.999863206
HOXA5	0.924254605	0.629806923	0.81581885	0.547290287	0.860431751	0.981528849	0.999863206
HOXC4	0.951034357	0.697240927	0.576872286	0.67258072	0.906418392	0.983426236	0.999863206
HPS3	0.831547122	0.732752994	0.996543285	0.737228615	0.656631825	0.991601948	0.999863206
IFI16	0.4685219	0.528082391	0.975331671	0.919002686	0.999554159	0.981113367	0.999863206
IFIT5	0.427278394	0.763340963	0.680328958	0.964005502	0.777959169	0.964076615	0.999863206
IFT52	0.961696589	0.587121674	0.970193217	0.962453252	0.554782089	0.991465681	0.999863206
IFT74	0.574641096	0.87325059	0.919125814	0.68005162	0.615620331	0.973776899	0.999863206
IL17RC	0.988437462	0.778110193	0.526926944	0.812228393	0.872872158	0.990958515	0.999863206
IL2RB	0.672418311	0.802169138	0.939019446	0.787030492	0.732328054	0.991411141	0.999863206
ILDR2	0.989829222	0.895550382	0.318748283	0.903421623	0.919824941	0.983722166	0.999863206
INPPL1	0.795356709	0.742494996	0.708006037	0.766948459	0.79923139	0.987214532	0.999863206
INVS	0.920067379	0.757727799	0.726477598	0.583001476	0.756093929	0.981450301	0.999863206
ITGAL	0.943388663	0.971357349	0.801056997	0.849775848	0.36471708	0.982323499	0.999863206
KAT5	0.652620668	0.959974866	0.901418326	0.8438854	0.539733275	0.987347417	0.999863206
KCTD13	0.889152196	0.678368322	0.576804889	0.977654238	0.802418072	0.989384644	0.999863206
KDM8	0.975746762	0.59734485	0.482566653	0.905015214	0.84098253	0.979405875	0.999863206
KIFC3	0.779193065	0.925748841	0.83571493	0.708871763	0.4423495	0.97250618	0.999863206
KLHDC3	0.872380624	0.782551105	0.565883764	0.585936192	0.809579807	0.970583356	0.999863206
KLRF2	0.954698993	0.69758037	0.577117021	0.946553221	0.887526351	0.993921161	0.999863206
LASP1	0.835983465	0.853308221	0.821269659	0.678772835	0.986857787	0.997238079	0.999863206
LAYN	0.999372372	0.996013079	0.627002831	0.501083595	0.819467143	0.987212206	0.999863206
LDHD	0.698452002	0.951330643	0.901149565	0.81821538	0.976481185	0.999012284	0.999863206
LEMD2	0.665928146	0.784881234	0.488420121	0.936573659	0.719984926	0.966454265	0.999863206
LFNG	0.694851607	0.605663856	0.832713561	0.924267646	0.709922059	0.982805163	0.999863206
LIN37	0.922619119	0.928833357	0.433750858	0.613084204	0.947099722	0.979815278	0.999863206
LOC100138449	0.64494003	0.895018859	0.944417053	0.928329605	0.703997969	0.995824943	0.999863206
LOC100139732	0.545117847	0.694698663	0.714703177	0.855197552	0.853489036	0.975098213	0.999863206
LOC100140873	0.971608486	0.511378491	0.744012621	0.721362132	0.782325061	0.978079469	0.999863206
LOC100140915	0.762649398	0.916646335	0.742263157	0.554632436	0.686034281	0.975066571	0.999863206
LOC100297616	0.736240606	0.88907724	0.899671544	0.790614823	0.732739285	0.995048143	0.999863206
LOC100298453	0.814212037	0.967978431	0.693911601	0.455677728	0.80218376	0.975769114	0.999863206
LOC100299281	0.679623016	0.932947703	0.692201645	0.497578366	0.824281344	0.96943831	0.999863206
LOC100300095	0.799793223	0.595854522	0.796222564	0.852487239	0.526021973	0.965649756	0.999863206
LOC100335205	0.926163341	0.747341215	0.965964541	0.585539905	0.982140237	0.996973739	0.999863206
LOC100335404	0.792332835	0.431337718	0.671637587	0.925006813	0.974279021	0.977633843	0.999863206
LOC100335635	0.921394935	0.960377065	0.58881259	0.843839752	0.780861038	0.995167099	0.999863206
LOC100336532	0.674031205	0.905951181	0.679039076	0.504425203	0.816662301	0.965918064	0.999863206

LOC100847156	0.861697651	0.782447667	0.389910335	0.698289428	0.954451915	0.967655177	0.999863206
LOC100847453	0.446848601	0.781400987	0.627064421	0.822768449	0.989915261	0.968826382	0.999863206
LOC100847745	0.879125913	0.738609471	0.740150657	0.813078684	0.641857431	0.986405992	0.999863206
LOC100847780	0.462629377	0.60247402	0.757901532	0.882005088	0.971542152	0.969798617	0.999863206
LOC100847841	0.769218888	0.738631679	0.748165838	0.720677319	0.718646745	0.980785572	0.999863206
LOC100848138	0.758979152	0.673894781	0.97986839	0.792998881	0.900818963	0.995906226	0.999863206
LOC100848357	0.86410236	0.493452139	0.983085618	0.847489926	0.541068481	0.973508105	0.999863206
LOC100848369	0.890663489	0.683482661	0.752859243	0.829458781	0.500843357	0.972940016	0.999863206
LOC100848492	0.875928901	0.861739002	0.33380292	0.964175112	0.740877685	0.969429679	0.999863206
LOC100848815	0.643107302	0.806614972	0.7187155	0.7031378	0.747685303	0.974648429	0.999863206
LOC100848869	0.90764021	0.633942925	0.754337604	0.965005216	0.898951222	0.996684024	0.999863206
LOC100848985	0.70493096	0.642590873	0.706677562	0.933708611	0.669338008	0.975810352	0.999863206
LOC100850436	0.66153739	0.909428155	0.508395963	0.777717943	0.883525468	0.978465043	0.999863206
LOC101902290	0.891832638	0.835882275	0.701905172	0.884462	0.587503372	0.989260555	0.999863206
LOC101902345	0.853640055	0.730803986	0.761556903	0.710993412	0.7184797	0.985107521	0.999863206
LOC101902663	0.971369397	0.748409039	0.638027052	0.99245911	0.434364903	0.975780819	0.999863206
LOC101902838	0.682060116	0.972756704	0.809691134	0.499552767	0.833926427	0.981564287	0.999863206
LOC101902841	0.542432022	0.575257225	0.937821107	0.945984948	0.73874576	0.977018985	0.999863206
LOC101903356	0.90770902	0.969744107	0.483626657	0.801854831	0.935257969	0.993669199	0.999863206
LOC101903572	0.773273295	0.824176939	0.874706915	0.555165094	0.706151067	0.980429258	0.999863206
LOC101904156	0.360114604	0.756648738	0.897998222	0.965975336	0.798721393	0.972428212	0.999863206
LOC101904173	0.958623063	0.748065855	0.374756845	0.782800552	0.944829936	0.975446057	0.999863206
LOC101904393	0.597144598	0.953048684	0.715117013	0.52633079	0.954684657	0.977016967	0.999863206
LOC101904413	0.767702892	0.664437007	0.553950377	0.962062965	0.686601561	0.971730713	0.999863206
LOC101904947	0.698580019	0.99698178	0.591708161	0.770146999	0.855008017	0.989197264	0.999863206
LOC101905049	0.864032396	0.876330072	0.472784523	0.814260839	0.693006076	0.976347257	0.999863206
LOC101905265	0.992076645	0.997213285	0.251961562	0.82977978	0.980919429	0.976587741	0.999863206
LOC101905513	0.58806973	0.791729325	0.810858828	0.874467732	0.561668899	0.971323584	0.999863206
LOC101905771	0.509652915	0.961896349	0.753491064	0.665846946	0.994458172	0.985421977	0.999863206
LOC101906110	0.922992897	0.616841385	0.659813019	0.859828905	0.955656511	0.992874845	0.999863206
LOC101906460	0.872416778	0.37356403	0.772801771	0.994490213	0.836237507	0.978288052	0.999863206
LOC101906836	0.82647215	0.879710781	0.701149124	0.717519983	0.702914631	0.987331085	0.999863206
LOC101907544	0.998280721	0.811829894	0.824847334	0.992696529	0.367165264	0.985267114	0.999863206
LOC101907883	0.539272711	0.694791158	0.907193305	0.847522392	0.732433959	0.978669269	0.999863206
LOC101907941	0.887960625	0.872278284	0.545831197	0.743809862	0.914418691	0.990981478	0.999863206
LOC101908577	0.767349356	0.531506456	0.881452397	0.848899951	0.929341873	0.990577187	0.999863206
LOC104969067	0.929928687	0.719972425	0.745061708	0.612866733	0.964320218	0.991682818	0.999863206
LOC104969259	0.797417965	0.968413067	0.870257704	0.931162935	0.725435284	0.998665745	0.999863206
LOC104970815	0.696317794	0.640377689	0.700235203	0.804305543	0.771067903	0.973944051	0.999863206
LOC104972026	0.908491211	0.97962832	0.943208026	0.395022152	0.798171667	0.988358706	0.999863206
LOC104974516	0.581735832	0.671451723	0.789396905	0.99844486	0.687868763	0.978856587	0.999863206
LOC104975283	0.652937505	0.597073337	0.788475475	0.883643934	0.950876465	0.987496063	0.999863206
LOC104975286	0.52181971	0.703076163	0.692982558	0.833177831	0.941439934	0.97563185	0.999863206
LOC104975610	0.771740214	0.570887723	0.870644595	0.974846284	0.977349804	0.996238332	0.999863206

LOC104975782	0.92255358	0.434081643	0.828792575	0.930742505	0.84353504	0.98781389	0.999863206
LOC104975979	0.703853263	0.760944821	0.63468134	0.958722968	0.77656772	0.986746986	0.999863206
LOC104976573	0.845438772	0.843172606	0.649090721	0.869248339	0.641075762	0.987435082	0.999863206
LOC104976575	0.824654113	0.752431165	0.945717261	0.94141068	0.562715916	0.993046479	0.999863206
LOC104976614	0.568979464	0.806245534	0.741719079	0.672191828	0.988295021	0.982027101	0.999863206
LOC107131452	0.911660871	0.374099657	0.843408695	0.62054545	0.966985931	0.966637893	0.999863206
LOC107131455	0.852958145	0.981447926	0.739509113	0.896670404	0.667560873	0.996450358	0.999863206
LOC107131525	0.963403791	0.854043139	0.846185485	0.483782659	0.657159824	0.981044041	0.999863206
LOC107131651	0.823126839	0.82107306	0.475906561	0.82981198	0.859349504	0.982690801	0.999863206
LOC107131652	0.487681064	0.919791543	0.917131575	0.52937603	0.976483743	0.979070621	0.999863206
LOC107132251	0.933786581	0.93303643	0.553147159	0.731926248	0.480650104	0.965398489	0.999863206
LOC107132374	0.783454739	0.556392592	0.881067897	0.651402887	0.675256793	0.965146237	0.999863206
LOC107132672	0.962658272	0.886163589	0.679795956	0.93119058	0.924686225	0.999241604	0.999863206
LOC107132793	0.759123943	0.666280977	0.732714329	0.594620131	0.908817993	0.975869762	0.999863206
LOC107132851	0.759698646	0.867141522	0.599820897	0.621157196	0.684967924	0.964805108	0.999863206
LOC107132987	0.687703696	0.872369666	0.714802378	0.848367724	0.920232726	0.994680423	0.999863206
LOC112441491	0.59478272	0.994760989	0.813943483	0.776156414	0.491979891	0.970803236	0.999863206
LOC112441846	0.846540906	0.772309778	0.782460417	0.600834002	0.91793778	0.990421041	0.999863206
LOC112442038	0.583063862	0.907639348	0.886983479	0.586513527	0.815998645	0.981742408	0.999863206
LOC112442244	0.890477904	0.628172512	0.910052491	0.469204727	0.978036975	0.983501935	0.999863206
LOC112442349	0.85114588	0.943803105	0.510917256	0.799419527	0.585389215	0.97346267	0.999863206
LOC112442602	0.472507211	0.848210246	0.576558775	0.845507246	0.902836941	0.968104614	0.999863206
LOC112442656	0.42991416	0.684646745	0.892832789	0.928965703	0.893636229	0.980344248	0.999863206
LOC112442676	0.816239929	0.351942491	0.907589962	0.93394665	0.691927002	0.964957655	0.999863206
LOC112442702	0.701126673	0.800771599	0.970940899	0.713577267	0.617287534	0.984669781	0.999863206
LOC112443007	0.863113861	0.560287031	0.997126123	0.851258952	0.620710232	0.986997735	0.999863206
LOC112443177	0.853999896	0.495007957	0.90999121	0.493369859	0.904739786	0.966282005	0.999863206
LOC112443178	0.996662619	0.670907825	0.645636751	0.966474202	0.615778967	0.98730582	0.999863206
LOC112443199	0.748110463	0.762667397	0.989029152	0.718253922	0.579309849	0.983722673	0.999863206
LOC112443437	0.868207334	0.716786594	0.832345238	0.682389013	0.512022012	0.969786823	0.999863206
LOC112443864	0.92662635	0.930273674	0.79868187	0.90726654	0.724853312	0.998646247	0.999863206
LOC112444464	0.527022805	0.902016814	0.903444748	0.9859593	0.666552429	0.990432787	0.999863206
LOC112444473	0.421014068	0.917491043	0.949332653	0.691838325	0.783183925	0.975425678	0.999863206
LOC112445088	0.941799976	0.468311263	0.688613977	0.893831255	0.883029399	0.984600336	0.999863206
LOC112445915	0.908507197	0.696197673	0.326158855	0.844383627	0.998179725	0.967138083	0.999863206
LOC112445971	0.732057935	0.717987064	0.58323542	0.702281207	0.817281621	0.967936642	0.999863206
LOC112446417	0.867724994	0.386972554	0.811988497	0.734448867	0.969919803	0.974120235	0.999863206
LOC112446452	0.673654694	0.706696722	0.987593253	0.744759796	0.929312053	0.994090245	0.999863206
LOC112446642	0.890970333	0.695874916	0.935242116	0.562163023	0.550381365	0.96922125	0.999863206
LOC112446725	0.868370603	0.988772751	0.885384773	0.586523842	0.7574783	0.994854289	0.999863206
LOC112446822	0.606677203	0.925264759	0.968318121	0.935143724	0.625270644	0.993566965	0.999863206
LOC112447026	0.611455933	0.973097819	0.411081748	0.955642995	0.754046517	0.968052671	0.999863206
LOC112447029	0.299336319	0.989876302	0.875661207	0.940258803	0.917670488	0.981581023	0.999863206
LOC112447291	0.699187709	0.856721981	0.885932037	0.757268089	0.949761506	0.996874075	0.999863206

LOC112447323	0.791237058	0.967346822	0.709905774	0.866392796	0.617742868	0.991303496	0.999863206
LOC112447346	0.854418553	0.779557712	0.841315016	0.597045935	0.535090031	0.969081227	0.999863206
LOC112447399	0.46846444	0.666234846	0.983110307	0.981348748	0.764391913	0.982848166	0.999863206
LOC112447435	0.851615133	0.79897812	0.912938883	0.750149572	0.902109081	0.998005921	0.999863206
LOC112447499	0.561911627	0.528784529	0.80650031	0.799246441	0.962377967	0.970949496	0.999863206
LOC112447508	0.544443393	0.711783539	0.746148581	0.887527227	0.656478376	0.964952212	0.999863206
LOC112447769	0.982039158	0.716314712	0.759500334	0.898968074	0.471318066	0.98209444	0.999863206
LOC112447797	0.610752085	0.679010943	0.977236532	0.481980592	0.891899246	0.967270432	0.999863206
LOC112449053	0.588513522	0.898220509	0.849597324	0.810506243	0.855554355	0.993090198	0.999863206
LOC112449092	0.931489603	0.886469621	0.523826467	0.781666776	0.581816209	0.974856197	0.999863206
LOC112449275	0.685164463	0.891164685	0.795857207	0.448670252	0.829397206	0.969733473	0.999863206
LOC112449338	0.638148908	0.88783424	0.842905538	0.633136504	0.691171579	0.978171288	0.999863206
LOC112449346	0.570390209	0.892922527	0.583910861	0.921655522	0.861980516	0.983989305	0.999863206
LOC508628	0.594574171	0.749765524	0.824006658	0.973400595	0.545269392	0.974342082	0.999863206
LOC515089	0.59793109	0.898653826	0.692501287	0.820993594	0.59771046	0.970355232	0.999863206
LOC516742	0.777273886	0.769012811	0.693903848	0.902686108	0.861052192	0.993886808	0.999863206
LOC519145	0.741249624	0.661873066	0.785283594	0.936121516	0.601967807	0.980106264	0.999863206
LOC530348	0.712746877	0.880427513	0.541733712	0.89532773	0.667767388	0.976683164	0.999863206
LOC534391	0.618084945	0.689773034	0.815225232	0.628384424	0.820226641	0.969123185	0.999863206
LOC534913	0.889224579	0.467008648	0.700981818	0.75553245	0.770057551	0.965323605	0.999863206
LOC614914	0.699401366	0.986293289	0.650974982	0.802290786	0.611141988	0.980789862	0.999863206
LOC615521	0.852863131	0.816242694	0.585692772	0.607948233	0.977435463	0.985038628	0.999863206
LOC616538	0.452089381	0.799761224	0.991190615	0.997607793	0.946642659	0.994894582	0.999863206
LOC618633	0.59925305	0.93078164	0.876715246	0.793446358	0.704911367	0.989452403	0.999863206
LOC782032	0.42131985	0.738206001	0.715380333	0.930651315	0.835102601	0.966764061	0.999863206
LOC782057	0.906900847	0.95982998	0.674673022	0.919749148	0.37658961	0.976728777	0.999863206
LOC782470	0.617277738	0.912830541	0.881135216	0.550254887	0.912609521	0.98617623	0.999863206
LOC782812	0.989208388	0.511161724	0.848878645	0.837561536	0.677146666	0.985231942	0.999863206
LOC783022	0.755563056	0.661389584	0.913514457	0.460692181	0.994406558	0.978207668	0.999863206
LOC783396	0.917335233	0.750901777	0.480673747	0.725535539	0.856279203	0.977332476	0.999863206
LOC785445	0.483901184	0.725613675	0.920481568	0.815320357	0.68471864	0.969590775	0.999863206
LOC787102	0.906659293	0.935695559	0.671176037	0.488535336	0.657352083	0.97044514	0.999863206
LOC787875	0.895034041	0.6990306	0.956831974	0.772389252	0.557359897	0.987417213	0.999863206
LOC788205	0.892070863	0.577015075	0.453670081	0.864522522	0.975037828	0.974894128	0.999863206
LOC788293	0.704416835	0.825216599	0.814451268	0.740383355	0.854974667	0.992123186	0.999863206
LOC790037	0.403772339	0.672852858	0.858802729	0.94579323	0.8795175	0.974077375	0.999863206
LOC790312	0.599700577	0.851758207	0.939746893	0.870058571	0.565074861	0.983941883	0.999863206
LOC790871	0.983865495	0.699551608	0.644305739	0.421783484	0.954996783	0.968934631	0.999863206
LRRC75A	0.671287736	0.726076546	0.597618212	0.755025208	0.968824209	0.97916833	0.999863206
LST1	0.572067605	0.937487755	0.866400778	0.951215974	0.681632379	0.992261072	0.999863206
LYVE1	0.988513371	0.887597617	0.699567892	0.585767917	0.90758079	0.994150196	0.999863206
LZTR1	0.973902071	0.889693583	0.567183741	0.36714695	0.976766206	0.968047511	0.999863206
MALL	0.798042649	0.674637531	0.711774925	0.878508091	0.783008328	0.988218936	0.999863206
MAP10	0.849457493	0.677172218	0.901914756	0.952910655	0.862532293	0.998143514	0.999863206

MAP3K7CL	0.983925345	0.527337101	0.944468795	0.815073315	0.791419748	0.993442612	0.999863206
MAPK12	0.91171716	0.425900729	0.951508174	0.816054195	0.637100704	0.973469863	0.999863206
MAVS	0.704774231	0.700055053	0.985135622	0.934195516	0.907097932	0.997796901	0.999863206
MBD4	0.909328979	0.95203955	0.601608648	0.575265264	0.636101258	0.972999817	0.999863206
MBTPS1	0.472190124	0.684722982	0.827391872	0.971368744	0.806435393	0.978313176	0.999863206
MCM7	0.934550693	0.541091371	0.484711716	0.897599913	0.861353044	0.972658332	0.999863206
MED15	0.756305102	0.680660358	0.880131118	0.728698017	0.816005746	0.988958979	0.999863206
MED25	0.674874674	0.482418676	0.920574505	0.840959017	0.793939669	0.975824406	0.999863206
MED26	0.940647672	0.952985306	0.599811129	0.75131859	0.729737985	0.991681163	0.999863206
MEN1	0.993754269	0.899521901	0.819455925	0.287997381	0.952274785	0.976042287	0.999863206
METRN	0.631823152	0.687584859	0.759354966	0.760453953	0.787698424	0.975114284	0.999863206
METTL2A	0.616084386	0.634571617	0.927423703	0.998590335	0.979579871	0.995748337	0.999863206
MFAP4	0.854434078	0.89032253	0.958751118	0.632338526	0.956181101	0.998439434	0.999863206
MICAL3	0.899140102	0.85604941	0.931131526	0.445147831	0.535292326	0.965904263	0.999863206
MICU2	0.848158238	0.891493493	0.930344511	0.292834744	0.867455102	0.968960195	0.999863206
MIGA1	0.81373564	0.46686966	0.643699407	0.863452291	0.839117298	0.968401414	0.999863206
MKRN3	0.908570197	0.399448576	0.742124664	0.992069946	0.691367366	0.971089523	0.999863206
MLLT6	0.827605483	0.903137945	0.90114839	0.501654422	0.79187919	0.988729609	0.999863206
MMP17	0.869675607	0.389204833	0.888407494	0.79579741	0.799185753	0.973208259	0.999863206
MNT	0.920148807	0.932123298	0.735127566	0.724747833	0.529783914	0.985006367	0.999863206
MOGAT1	0.934148397	0.979130009	0.87673084	0.607853745	0.971673902	0.998951903	0.999863206
MORF4L2	0.717358933	0.65825783	0.659873027	0.58166442	0.987340956	0.969054204	0.999863206
MORN4	0.713166093	0.813414795	0.947094436	0.840771436	0.93833555	0.998292209	0.999863206
MRE11	0.354309547	0.760115393	0.886656264	0.992875949	0.711144479	0.965007866	0.999863206
MRGPRF	0.536879404	0.697366006	0.787374179	0.714820212	0.974389124	0.977235128	0.999863206
MRPL3	0.948612504	0.786346549	0.508221471	0.911138492	0.71267823	0.985678064	0.999863206
MSH2	0.865411517	0.988481506	0.711874001	0.720869472	0.950066893	0.997926964	0.999863206
MSL3	0.480687142	0.92234453	0.721403361	0.91656599	0.94709097	0.989928211	0.999863206
MT3	0.747299362	0.993940162	0.706944318	0.882510845	0.891182221	0.997825059	0.999863206
MUC20	0.978040496	0.996870771	0.533391228	0.737210563	0.712893979	0.989429359	0.999863206
MYO1H	0.834332426	0.898808941	0.937396972	0.729565732	0.976151238	0.999253942	0.999863206
NAALADL2	0.90152534	0.640086141	0.775705292	0.915871149	0.899745687	0.996380993	0.999863206
NADK2	0.88111176	0.833175591	0.754088126	0.93645151	0.803009193	0.997907952	0.999863206
NCR3	0.629261	0.75241185	0.643330875	0.785676155	0.985678228	0.983920557	0.999863206
NEDD4L	0.573139296	0.621664988	0.983171534	0.935228802	0.83246663	0.989360355	0.999863206
NELFCD	0.64599843	0.601763061	0.882616511	0.968471541	0.705801015	0.983672893	0.999863206
NEU1	0.725165305	0.969161539	0.594883111	0.788892869	0.614584615	0.976537401	0.999863206
NOS1AP	0.834774737	0.912237932	0.965325177	0.985387861	0.358219316	0.987663261	0.999863206
NOTCH1	0.905338816	0.950756764	0.83524347	0.814299945	0.779962711	0.998708153	0.999863206
NOVA1	0.887067935	0.973561844	0.780529385	0.711078867	0.470285671	0.981900189	0.999863206
NPHP1	0.930207315	0.957489414	0.996467907	0.955543783	0.416472062	0.9956767	0.999863206
NRBP1	0.72886751	0.846512036	0.541870137	0.643819447	0.919815667	0.97522451	0.999863206
NRTN	0.627801995	0.878873554	0.892741169	0.43853827	0.815900487	0.968048935	0.999863206
NSL1	0.846473548	0.732836182	0.895662913	0.737982428	0.872265571	0.995889371	0.999863206

NSMAF	0.983380417	0.884706976	0.609859856	0.631532	0.92474872	0.992968465	0.999863206
NUDT2	0.557804557	0.754937233	0.938736231	0.813847675	0.973862767	0.993234174	0.999863206
NUFIP1	0.905573209	0.840034223	0.720602391	0.601755739	0.840372592	0.989879226	0.999863206
NXPE4	0.77999369	0.765320805	0.6227167	0.848297934	0.617092506	0.974229509	0.999863206
OCIAD1	0.769099905	0.531680354	0.852694188	0.810498934	0.926523819	0.987984331	0.999863206
ODF2L	0.729394005	0.852120042	0.920625209	0.911638506	0.717238681	0.996592264	0.999863206
ORMDL1	0.786208528	0.667613352	0.89846922	0.869109313	0.442375584	0.969904388	0.999863206
OTUD3	0.964426226	0.718766915	0.82288473	0.464573093	0.889655157	0.983898113	0.999863206
OTUD7B	0.533033016	0.891573496	0.758372784	0.736104244	0.987979154	0.988020372	0.999863206
P2RX4	0.696733881	0.664022832	0.838594416	0.766352266	0.934928405	0.98996547	0.999863206
PABPC4L	0.403387331	0.859881565	0.87591574	0.941322198	0.942236515	0.988967067	0.999863206
PACSIN1	0.740832282	0.988342315	0.994836514	0.63485819	0.676767992	0.993207863	0.999863206
PARP1	0.904861959	0.962752335	0.573807696	0.828588648	0.454097982	0.972200953	0.999863206
PARVB	0.872854639	0.817492067	0.51972114	0.759179375	0.806378249	0.982227405	0.999863206
PATZ1	0.742044579	0.715654831	0.794882553	0.74382794	0.78882651	0.985918944	0.999863206
PBX4	0.516223788	0.938042764	0.749862963	0.880767974	0.611580987	0.974527829	0.999863206
PDE4DIP	0.909235338	0.811378205	0.780879347	0.995119132	0.804253366	0.998776262	0.999863206
PDE6A	0.622524457	0.974924657	0.952961361	0.85334018	0.680247911	0.99473651	0.999863206
PDGFRL	0.954242898	0.571173046	0.662032598	0.563357948	0.957462353	0.974241348	0.999863206
PDZD2	0.975303113	0.864669943	0.687453787	0.648099541	0.73820243	0.989896676	0.999863206
PEG10	0.960310137	0.855089663	0.872833446	0.745667055	0.718425703	0.99695472	0.999863206
PEX12	0.755900093	0.54090797	0.649908367	0.857608097	0.791326845	0.969556082	0.999863206
PEX3	0.708222778	0.988466429	0.985859493	0.438550557	0.736822214	0.981399228	0.999863206
PFKL	0.863836378	0.739592067	0.647495033	0.762553085	0.657004063	0.97773279	0.999863206
PGAP1	0.676492873	0.9717407	0.983079799	0.79982134	0.910342711	0.998911046	0.999863206
PI4K2B	0.818478699	0.575992302	0.87409463	0.694403298	0.814525767	0.983402831	0.999863206
PIGL	0.996184593	0.674891144	0.789227818	0.407850276	0.800960101	0.966926556	0.999863206
PIGV	0.976109694	0.65328056	0.760112939	0.772832215	0.924416085	0.99532575	0.999863206
PIM2	0.955886641	0.22203187	0.970797193	0.876780852	0.963054541	0.967177677	0.999863206
PIP4K2B	0.885424601	0.648720759	0.553602907	0.732302618	0.926202596	0.97977954	0.999863206
PKD2	0.753971069	0.573024986	0.820822759	0.70248081	0.723902626	0.969557209	0.999863206
PLCB2	0.436158795	0.811012292	0.783955673	0.760617038	0.942393355	0.975448629	0.999863206
PLCD4	0.556171494	0.752269308	0.725385165	0.967169784	0.660387448	0.974004869	0.999863206
PNLDC1	0.515854047	0.929485808	0.897494822	0.900516575	0.698629468	0.989120635	0.999863206
PPP2R2B	0.94641581	0.588541369	0.577858682	0.937293485	0.905797676	0.989424114	0.999863206
PPP4R1	0.618997246	0.676953113	0.823744314	0.982533692	0.82939848	0.990329201	0.999863206
PPYR1	0.664797834	0.478371971	0.955643426	0.775762092	0.830216919	0.974569482	0.999863206
PRIM2	0.755069126	0.648133569	0.44644827	0.930510171	0.849907545	0.966710827	0.999863206
PRKCD	0.706710069	0.76576313	0.66553841	0.629071525	0.83159251	0.972308782	0.999863206
PRL	0.761257421	0.670786837	0.921822961	0.935622767	0.59918691	0.988256879	0.999863206
PROB1	0.810467726	0.554760224	0.472718067	0.929536491	0.867191441	0.966126376	0.999863206
PROX2	0.625044091	0.991624862	0.680600845	0.98460798	0.552053497	0.982677896	0.999863206
PRPF6	0.633329688	0.841950738	0.820826249	0.755456002	0.972654101	0.993833937	0.999863206
PTGIS	0.781643576	0.605979236	0.960822613	0.973778604	0.975286762	0.998267094	0.999863206

PTGS1	0.819242601	0.627619094	0.706428137	0.701424302	0.762578882	0.974138064	0.999863206
PTMA	0.778976921	0.521605722	0.909242483	0.968710571	0.615397597	0.980803786	0.999863206
RAB32	0.921116284	0.995455124	0.602914521	0.845952766	0.577880868	0.9890627	0.999863206
RAP1GAP	0.7939575	0.538103514	0.540448144	0.957241935	0.864660169	0.97316514	0.999863206
RASA3	0.966914237	0.960114338	0.966246809	0.333099553	0.699871833	0.978204449	0.999863206
RASSF7	0.745778225	0.768497542	0.852636542	0.473470521	0.743869835	0.966440939	0.999863206
RCC2	0.477851304	0.859716156	0.985068721	0.736606769	0.706291574	0.978556599	0.999863206
RDH14	0.719607118	0.872420799	0.826755955	0.91973927	0.422750247	0.976294682	0.999863206
RFTN2	0.702662701	0.990606623	0.415959792	0.872582035	0.732772391	0.971223046	0.999863206
RGS13	0.717055979	0.589690951	0.598804622	0.8429029	0.865583275	0.971089765	0.999863206
RHBDF2	0.986990733	0.919494059	0.540645372	0.968789136	0.526397703	0.986314265	0.999863206
RHNO1	0.947081689	0.982095874	0.681461754	0.662965848	0.56295851	0.984043454	0.999863206
RIN2	0.995439224	0.627335836	0.672935735	0.604075607	0.873629496	0.981135272	0.999863206
RNF149	0.8391073	0.906535359	0.460035319	0.996154574	0.735576836	0.987232859	0.999863206
RPL10	0.873653063	0.835940405	0.868089982	0.772962412	0.797724998	0.997189414	0.999863206
RPL10A	0.71949347	0.98038698	0.704798237	0.846223168	0.845565931	0.995799007	0.999863206
RPL11	0.986040674	0.862235595	0.698280987	0.8058558	0.923953364	0.998459054	0.999863206
RPL13A	0.588948269	0.940771479	0.860534811	0.906721424	0.70307646	0.992488955	0.999863206
RPL14	0.487449723	0.86747741	0.957704554	0.685834021	0.727852102	0.976387841	0.999863206
RPL18	0.928966101	0.646377209	0.490642102	0.720235293	0.829841771	0.967987649	0.999863206
RPL18A	0.912279757	0.908055411	0.617338382	0.804341156	0.934933544	0.996976416	0.999863206
RPL24	0.501367678	0.873871754	0.907101117	0.995798794	0.966806518	0.996907746	0.999863206
RPL29	0.709714235	0.976943409	0.713312333	0.712532571	0.743851526	0.98802381	0.999863206
RPL32	0.88421842	0.953799546	0.584247458	0.893999705	0.952312512	0.99798545	0.999863206
RPL36A	0.614634601	0.88135754	0.598670184	0.84699066	0.643421257	0.968234591	0.999863206
RPL37	0.816935137	0.887159855	0.618763746	0.899708277	0.950168847	0.996934117	0.999863206
RPL4	0.679655886	0.726381398	0.552937038	0.792860816	0.839649184	0.970050973	0.999863206
RPL6	0.560537231	0.691326147	0.635559258	0.735155869	0.985127961	0.9688405	0.999863206
RPL7	0.66458545	0.750457384	0.778492118	0.570267978	0.906171014	0.975972272	0.999863206
RPP25L	0.367601049	0.974964063	0.94404804	0.661586632	0.835014816	0.971817937	0.999863206
RPS12	0.767389242	0.747544598	0.540370092	0.991920051	0.97210707	0.992054	0.999863206
RPS14	0.313194742	0.760534311	0.839066417	0.936495892	0.981952755	0.970768134	0.999863206
RPS15	0.860068335	0.784048902	0.888458888	0.407545051	0.844538555	0.977464622	0.999863206
RPS15A	0.554568175	0.544920002	0.96209592	0.889177567	0.92011209	0.984276055	0.999863206
RPS16	0.864488022	0.859349566	0.678486738	0.511526728	0.955244888	0.985698072	0.999863206
RPS18	0.86777613	0.841997474	0.874755384	0.79319767	0.918473439	0.998843237	0.999863206
RPS19	0.386507263	0.942854623	0.924592219	0.95102662	0.839324464	0.988902252	0.999863206
RPS20	0.774417417	0.946976096	0.714965455	0.56850554	0.62870412	0.971979353	0.999863206
RPS21	0.880543725	0.831424528	0.828619558	0.872666679	0.939301012	0.999221172	0.999863206
RPS24	0.677233567	0.804921403	0.866403534	0.723768281	0.682480641	0.983443335	0.999863206
RPS27A	0.799466342	0.864614551	0.958428136	0.768795281	0.890510145	0.998659176	0.999863206
RPS27P	0.996065846	0.83197453	0.427963552	0.903718514	0.597951256	0.973332523	0.999863206
RPS5	0.63384742	0.866399676	0.635329241	0.842284611	0.631674763	0.971392279	0.999863206
RPS7	0.803865731	0.764292262	0.513479504	0.835612638	0.81901934	0.979832304	0.999863206

RPS9	0.961858747	0.784681017	0.985243372	0.537748431	0.819177117	0.994231806	0.999863206
RSPH3	0.629405294	0.576876585	0.691148688	0.986741218	0.785390332	0.974195965	0.999863206
SAMD4A	0.9005194	0.771056289	0.83949772	0.550022747	0.940690756	0.992288887	0.999863206
SART1	0.547184189	0.749221536	0.625586362	0.996932832	0.947372363	0.985028718	0.999863206
SBK1	0.662646203	0.418721832	0.958351366	0.868807463	0.991507433	0.982631961	0.999863206
SCD5	0.975369011	0.955843295	0.39979587	0.657349574	0.997948972	0.985408765	0.999863206
SCLT1	0.951745678	0.873307399	0.900902916	0.934124043	0.613535043	0.998202751	0.999863206
SCNM1	0.757342289	0.965588762	0.930782454	0.647484149	0.463126957	0.976913643	0.999863206
SEC61A2	0.706512851	0.95916289	0.719021958	0.808958954	0.680526181	0.988813816	0.999863206
SEMA3A	0.993294921	0.600234315	0.775670986	0.55777758	0.828982268	0.979350224	0.999863206
SEMA3C	0.619703204	0.922267895	0.441568096	0.742673464	0.971540247	0.970179489	0.999863206
SERF1A	0.953089805	0.521771648	0.957742929	0.783368787	0.854551315	0.993639295	0.999863206
SERGEF	0.958526382	0.976540076	0.430132764	0.788234044	0.629161679	0.975701431	0.999863206
SF3B2	0.927515718	0.910797592	0.304713335	0.973216974	0.738604598	0.971191617	0.999863206
SH2D7	0.909307232	0.608043846	0.642483764	0.997890947	0.58934562	0.978152591	0.999863206
SH3BP4	0.797217412	0.575009858	0.529311121	0.984996151	0.72426619	0.966833645	0.999863206
SH3RF3	0.970087445	0.968611659	0.80724341	0.71747728	0.636577538	0.995333954	0.999863206
SIKE1	0.818917059	0.47926286	0.864353923	0.886796716	0.649568593	0.974474098	0.999863206
SKI	0.872540116	0.962127849	0.57222382	0.948473559	0.650299944	0.991819145	0.999863206
SLC25A29	0.809763813	0.881083488	0.75754835	0.628491268	0.779318531	0.988366056	0.999863206
SLC26A10	0.551705023	0.866635572	0.890876724	0.876224008	0.559457488	0.978126369	0.999863206
SLC27A6	0.927251035	0.883970825	0.476864569	0.653323344	0.897044829	0.982634142	0.999863206
SLC35A2	0.792314187	0.896992062	0.893113473	0.68279193	0.999425052	0.99828609	0.999863206
SLC35D2	0.842554455	0.643099819	0.853279446	0.807528054	0.8397782	0.993251199	0.999863206
SLC38A7	0.464393397	0.683193954	0.913783562	0.791254643	0.768865137	0.968098116	0.999863206
SLC6A9	0.900339702	0.909240921	0.84353886	0.763274523	0.359808452	0.972703068	0.999863206
SNRPE	0.984707241	0.947422612	0.645076333	0.658203036	0.545648835	0.979885997	0.999863206
SNRPG	0.852132069	0.682880758	0.941580153	0.965967759	0.834346618	0.998450747	0.999863206
SPIN2	0.670890222	0.65817289	0.617543639	0.766575356	0.849950368	0.968581677	0.999863206
SPSB2	0.622484673	0.833819435	0.694770655	0.767974833	0.998433579	0.989799743	0.999863206
SREBF2	0.553918696	0.960828983	0.73479612	0.956721384	0.901821958	0.994835179	0.999863206
SSX2IP	0.993133809	0.876063568	0.675684045	0.790823779	0.583197548	0.989169188	0.999863206
ST6GALNAC5	0.894679617	0.683953547	0.558033452	0.991718629	0.679048685	0.982806809	0.999863206
STK11	0.845584837	0.933141173	0.953888296	0.496098235	0.868880688	0.994025699	0.999863206
STK11IP	0.703524806	0.688318264	0.658375047	0.893044808	0.74121799	0.978678646	0.999863206
STN1	0.999379462	0.714608107	0.970237379	0.538846821	0.829345652	0.992952044	0.999863206
STOML1	0.736651348	0.738095236	0.805095615	0.708820165	0.771635707	0.984549592	0.999863206
STX16	0.59975332	0.737829634	0.916031722	0.915823158	0.814016716	0.992340021	0.999863206
STXBP4	0.872000569	0.732585326	0.735517366	0.884119371	0.625834719	0.987731674	0.999863206
SULT1B1	0.919320517	0.943582847	0.752965103	0.792805468	0.727134217	0.996684275	0.999863206
SUMF1	0.872484086	0.68820261	0.403512195	0.99171479	0.870966057	0.978243762	0.999863206
SUPT5H	0.533004229	0.949626548	0.895043821	0.916273466	0.855754764	0.995774834	0.999863206
SYCE1L	0.638870532	0.764384143	0.982026589	0.651096021	0.873714734	0.989370185	0.999863206
SYDE1	0.828877901	0.929719512	0.783857994	0.992574269	0.695252803	0.997921823	0.999863206

SYT12	0.721026443	0.978522767	0.833817378	0.469064702	0.996741779	0.989632194	0.999863206
TADA2A	0.900238258	0.978857373	0.83817961	0.494144523	0.55638854	0.976635892	0.999863206
TAF12	0.60032892	0.666363521	0.975905281	0.994849978	0.604207006	0.983698089	0.999863206
TAF9B	0.531160713	0.927239751	0.775191472	0.543299931	0.810474976	0.9648021	0.999863206
TANC2	0.751868736	0.662807491	0.613121175	0.744971796	0.986645382	0.981727856	0.999863206
TAPBP	0.511269779	0.819316999	0.972544432	0.708612732	0.919345552	0.988452944	0.999863206
TBC1D12	0.848813498	0.843611795	0.773895032	0.730970244	0.489215727	0.975276127	0.999863206
TBCK	0.79397669	0.99380487	0.673255202	0.847521299	0.621931728	0.990191035	0.999863206
TBXAS1	0.709038837	0.877542329	0.733955546	0.515319107	0.82611815	0.974176196	0.999863206
TCEA2	0.943318617	0.736229622	0.517354232	0.931547462	0.534340787	0.969016634	0.999863206
TFPT	0.916800241	0.74197854	0.49974165	0.967081312	0.914888524	0.99221797	0.999863206
TG	0.905590936	0.949361589	0.67051736	0.78423798	0.797090347	0.9960134	0.999863206
TGFBR2	0.993060773	0.968700303	0.426816332	0.55607813	0.7886606	0.969458804	0.999863206
THAP11	0.924532178	0.892370795	0.759489156	0.543695512	0.801049262	0.989380723	0.999863206
THEM4	0.669878891	0.704518219	0.919032975	0.488710178	0.917527598	0.974198181	0.999863206
TLR3	0.86934802	0.784875164	0.630748296	0.733522426	0.686774032	0.980038899	0.999863206
TLR8	0.838745532	0.77996443	0.492543776	0.937154308	0.825711005	0.986178615	0.999863206
TMEM163	0.908235111	0.990872497	0.443989681	0.907639697	0.51808826	0.972138229	0.999863206
TMEM240	0.918275217	0.572558389	0.930340167	0.748603923	0.634176063	0.983241069	0.999863206
TMEM241	0.938932773	0.43919615	0.919889203	0.874188899	0.97965643	0.994054524	0.999863206
TMEM246	0.783169899	0.761689645	0.853787329	0.747804308	0.700706259	0.988641771	0.999863206
TMEM260	0.736921992	0.896337913	0.91220225	0.564324246	0.708093844	0.98478187	0.999863206
TMEM63B	0.904402316	0.937233976	0.696764755	0.670892718	0.704940396	0.990114617	0.999863206
TMEM74B	0.66723169	0.76633243	0.744662181	0.836676796	0.840893864	0.98876948	0.999863206
TNFAIP8	0.847224847	0.640025484	0.838504356	0.844047341	0.439832523	0.965086591	0.999863206
TRIM2	0.810508918	0.79259383	0.653445361	0.835148215	0.58843122	0.977485329	0.999863206
TRIM47	0.807899537	0.701188137	0.853568781	0.967460566	0.43043249	0.976170163	0.999863206
TRIQK	0.730539134	0.645598099	0.959851446	0.989333413	0.784871321	0.995594147	0.999863206
TRMO	0.971626359	0.595030539	0.978688277	0.936373242	0.581120117	0.992811965	0.999863206
TSEN15	0.841352741	0.364799853	0.812914549	0.874579815	0.821726579	0.969185238	0.999863206
TULP3	0.918714276	0.819377308	0.775178179	0.404246645	0.835856883	0.974988887	0.999863206
TULP4	0.866524477	0.976437834	0.967766351	0.587472067	0.753876695	0.996115791	0.999863206
TYRO3	0.817357049	0.525255408	0.555524494	0.897185212	0.980571502	0.978379086	0.999863206
U2SURP	0.734198635	0.723033558	0.675774483	0.670783321	0.893448372	0.979621165	0.999863206
UBE2L6	0.662979285	0.962855808	0.969419005	0.542708036	0.935294965	0.993294526	0.999863206
UBXN2B	0.962616722	0.45934155	0.536091232	0.989675369	0.938327466	0.980779251	0.999863206
UBXN6	0.906865289	0.953042893	0.254197307	0.896359383	0.900891403	0.968486369	0.999863206
ULBP21	0.802099453	0.63301625	0.714163404	0.81939568	0.577525331	0.966234653	0.999863206
UNC119	0.576387481	0.981763787	0.991392774	0.754325795	0.493655484	0.978151312	0.999863206
USP27X	0.855016607	0.429351631	0.890891858	0.862614924	0.836295988	0.983929427	0.999863206
VPS54	0.83256385	0.635306136	0.879736344	0.821620963	0.906964069	0.99535031	0.999863206
VWA1	0.27407097	0.827890789	0.870393295	0.951862328	0.95239181	0.969085623	0.999863206
WDR25	0.747035454	0.748644909	0.580085948	0.877141322	0.975599644	0.989925435	0.999863206
WDR41	0.857315086	0.954824377	0.837623989	0.416496224	0.779942375	0.981340316	0.999863206

WDR60	0.963177956	0.718796543	0.819389133	0.693052285	0.638391634	0.98643227	0.999863206
WHAMM	0.757304124	0.809483902	0.840998075	0.810188992	0.691633157	0.991115364	0.999863206
WNT7B	0.907159499	0.936066477	0.607116474	0.864883585	0.524142287	0.98352027	0.999863206
WNT8B	0.93549823	0.649454098	0.591394275	0.890151961	0.977837882	0.993191666	0.999863206
WRNIP1	0.985268127	0.580943336	0.897554809	0.616648894	0.781180526	0.985887188	0.999863206
WWTR1	0.988137463	0.966640734	0.488658828	0.786753307	0.902758299	0.994481132	0.999863206
XAB2	0.703941172	0.83752266	0.635992706	0.982471407	0.777057516	0.990850616	0.999863206
XKR8	0.641304245	0.394301891	0.930451296	0.995934378	0.923575078	0.979949237	0.999863206
XPNPEP3	0.95197618	0.756890573	0.886102818	0.589877029	0.597221878	0.98179893	0.999863206
XRCC2	0.656794451	0.8392378	0.836542375	0.626064372	0.584491294	0.965061289	0.999863206
YIPF3	0.955199314	0.741171244	0.911102165	0.687469371	0.912827022	0.997606534	0.999863206
ZBTB24	0.803147512	0.777395158	0.7896426	0.590018661	0.791634749	0.98287002	0.999863206
ZBTB41	0.974464412	0.838177124	0.716704629	0.51276751	0.835422295	0.986398183	0.999863206
ZC3HAV1L	0.78889084	0.955879704	0.755227596	0.931264237	0.575386793	0.992589637	0.999863206
ZDHHC24	0.807453784	0.719859488	0.718088365	0.60904297	0.936243489	0.984299796	0.999863206
ZDHHC5	0.881570694	0.617334235	0.675730781	0.719804349	0.653736819	0.966813651	0.999863206
ZER1	0.996696142	0.966111339	0.98783156	0.602664314	0.461776264	0.988364463	0.999863206
ZFP37	0.72472175	0.612662184	0.742141951	0.777974954	0.902994895	0.98310237	0.999863206
ZNF202	0.943999624	0.726194255	0.881359509	0.978151128	0.309224052	0.970408254	0.999863206
ZNF3	0.851081732	0.827629186	0.800112872	0.486868585	0.945065563	0.987640549	0.999863206
ZNF331	0.809281802	0.590819976	0.570463283	0.954034525	0.881018439	0.982671341	0.999863206
ZNF397	0.779249631	0.568160043	0.991784196	0.967779049	0.802312299	0.995036158	0.999863206
ZNF449	0.995357634	0.611591738	0.816118962	0.928406161	0.870957191	0.997519874	0.999863206
ZNF461	0.810004278	0.876335459	0.745043788	0.714371531	0.970300789	0.99628567	0.999863206
ZNF554	0.580973232	0.848264674	0.966862281	0.582619668	0.911339991	0.986734364	0.999863206
ZNF596	0.930038253	0.859622097	0.766712943	0.468078978	0.833380603	0.984495184	0.999863206
ZNF606	0.451508208	0.899270853	0.654210557	0.828532011	0.84647282	0.971612558	0.999863206
ZNF691	0.689882662	0.433334279	0.86133825	0.945664706	0.998686304	0.985189748	0.999863206
ZNF75A	0.492637625	0.892813175	0.802218326	0.937208611	0.523571055	0.966848858	0.999863206
ZNF787	0.675161589	0.567995611	0.746000746	0.913670597	0.811460214	0.978937068	0.999863206
ZNF8	0.587837347	0.974448934	0.905492136	0.95874383	0.338465106	0.964885389	0.999863206
ZNF865	0.992866855	0.978653494	0.809013385	0.787266563	0.47977297	0.991875655	0.999863206
ZSWIM9	0.860359395	0.684122045	0.890535248	0.931801486	0.67126333	0.994250118	0.999863206
ZXDC	0.687285266	0.408341891	0.8759861	0.806027092	0.882754386	0.967544167	0.999863206
CSNK1A1	0.965254473	0.739914772	0.957838218	0.917295945	0.81934463	0.999373503	0.99990517
HIST1H1D	0.954837026	0.997240553	0.658529294	0.869985352	0.985149513	0.999539596	0.99990517
PIGS	0.991186032	0.915741134	0.925459631	0.80554489	0.783125371	0.999490915	0.99990517
SMURF1	0.989955332	0.945555233	0.962847347	0.917392888	0.63976802	0.999484531	0.99990517
LOC112447731	0.927024681	0.765962226	0.996083999	0.944766895	0.861507941	0.99972908	0.999956775
MRI1	0.962884169	0.851490078	0.817296199	0.910989147	0.945479604	0.999734786	0.999956775
RPS8	0.960408017	0.907180759	0.810457856	0.881573719	0.944914891	0.999773979	0.999956775
FBXO48	0.97391698	0.961087088	0.889459212	0.845781912	0.925114117	0.999915522	0.999974781
MRPL48	0.941738	0.94266754	0.949479057	0.860327005	0.990226643	0.999974781	0.999974781
TMLHE	0.80788324	0.940793723	0.936392931	0.996115015	0.994284341	0.99996739	0.999974781

A2ML1	0.151324831	NaN	0.674930698	0.308936045	0.773740788	NaN	NaN
ABAT	0.517441962	0.92449532	NaN	0.048600517	0.120375983	NaN	NaN
ACBD7	0.044510188	0.368038305	0.164606353	NaN	0.031369219	NaN	NaN
ACOT2	0.87578184	0.73014153	NaN	0.018273784	0.870379002	NaN	NaN
ACP5	0.307986743	0.951584245	0.542966988	0.750044873	NaN	NaN	NaN
ACTA1	0.568779371	NaN	0.682120375	0.119392694	0.303076537	NaN	NaN
ACTN2	0.515979898	NaN	0.417101008	NaN	0.941622201	NaN	NaN
ADAM8	0.009862063	0.804228526	0.369319898	NaN	0.069396208	NaN	NaN
ADAMTS19	0.001321677	0.25054137	0.163269547	NaN	0.370991032	NaN	NaN
ADRA2C	0.799717143	NaN	0.110498306	0.340876226	0.890047326	NaN	NaN
ALB	0.285661596	0.352300994	NaN	0.750356355	0.448186895	NaN	NaN
ALDOB	NaN	0.886503838	0.222492832	0.318211017	0.284185516	NaN	NaN
AMZ1	0.345892833	0.04191924	0.027642257	0.543196834	NaN	NaN	NaN
APOA4	NaN	0.14214815	0.543990994	0.048566055	0.542211496	NaN	NaN
ARHGAP11A	0.342218692	0.004793307	NaN	0.388146077	0.96495514	NaN	NaN
ART3	0.670545829	0.467883873	0.143406175	NaN	0.305287431	NaN	NaN
ATP10B	0.212128512	0.939150419	NaN	0.710792529	0.488317791	NaN	NaN
BAIAP2L2	NaN	0.191897219	0.766006234	0.638453364	0.282702563	NaN	NaN
BCL2L15	NaN	0.226956453	0.761938486	0.240180024	0.063695017	NaN	NaN
BIRC5	0.472385175	0.020460235	NaN	0.157242278	0.58447482	NaN	NaN
BOLA	0.418893321	0.4317999	0.899589805	NaN	0.374908323	NaN	NaN
BOLA3	0.619318509	0.947767707	NaN	0.011683987	0.490863963	NaN	NaN
BSG	0.386388954	0.763054775	NaN	0.082548832	0.443538455	NaN	NaN
BUB1B	0.260275829	0.092929942	NaN	0.466787274	0.826891146	NaN	NaN
C10H15orf62	0.855171478	NaN	0.282237149	0.497296871	0.828157327	NaN	NaN
C19H17orf58	0.709418596	0.801813395	0.046861039	NaN	0.307831322	NaN	NaN
C4BPA	NaN	0.16336406	NaN	0.428060486	0.847229589	NaN	NaN
CA12	NaN	0.123380851	0.166839724	0.039199163	0.681693094	NaN	NaN
CA13	NaN	0.346506114	0.94478711	0.144425383	NaN	NaN	NaN
CADM4	0.263298654	0.68734169	0.467195091	NaN	0.770539833	NaN	NaN
CATHL5	0.713152128	0.837079694	NaN	0.335546458	0.689892152	NaN	NaN
CCDC73	0.71863191	0.961220211	NaN	0.365213493	0.650735277	NaN	NaN
CCL17	0.151142343	0.81337126	0.176984029	NaN	0.929165214	NaN	NaN
CCL5	NaN	0.924180627	0.384771094	0.946655902	0.452120901	NaN	NaN
CCR9	0.952293662	0.93022357	0.649739889	0.608746369	NaN	NaN	NaN
CD5L	0.859971882	0.154871634	0.758847543	0.042295333	NaN	NaN	NaN
CDC6	0.156788908	0.043929072	NaN	0.906027893	0.79284746	NaN	NaN
CDCA8	0.566926448	0.064030658	NaN	0.176775031	0.917016707	NaN	NaN
CDH17	NaN	0.440927521	0.612625343	0.016119574	0.338892566	NaN	NaN
CDH19	0.978588146	0.827535323	0.129089079	NaN	0.654386689	NaN	NaN
CDKN2A	0.371848429	0.265827568	NaN	0.855961584	0.976260117	NaN	NaN
CERS3	0.165782257	0.272725517	NaN	0.49744862	0.600358544	NaN	NaN
CFHR5	0.210983295	NaN	NaN	0.937031859	0.669526655	NaN	NaN
CHAD	NaN	0.096224766	0.579607046	0.127058768	0.257276921	NaN	NaN

CHGA	NaN	0.02033224	0.823153827	0.180847949	0.79845903	NaN	NaN
CKB	0.97854896	0.638353503	NaN	0.112444177	0.77385944	NaN	NaN
CKMT1A	NaN	0.476584543	0.68895914	0.004115176	0.890853811	NaN	NaN
CLDN3	NaN	0.682697213	0.789078215	0.724673424	0.989028139	NaN	NaN
COBL	0.922233068	0.546714564	NaN	0.949126661	0.798297388	NaN	NaN
COL24A1	0.243496348	NaN	0.007019589	0.646476568	0.820744466	NaN	NaN
COL2A1	0.255766238	0.757641864	0.089405912	NaN	0.095760664	NaN	NaN
CR2	0.222630296	0.676558118	0.932013808	0.254246817	NaN	NaN	NaN
CRB2	NaN	0.066432458	0.474008521	0.612574441	0.469128049	NaN	NaN
CXCL11	0.598227189	0.000813327	0.726578036	0.148824806	NaN	NaN	NaN
CXCL8	0.404347446	0.059779362	0.298096238	NaN	0.002688366	NaN	NaN
CXHXorf57	0.35470446	0.231703347	0.824070842	NaN	0.374220307	NaN	NaN
CYP21	0.590054089	0.334804467	NaN	0.723145432	0.604940506	NaN	NaN
CYP46A1	0.949179123	0.66387237	NaN	0.615378265	0.673929184	NaN	NaN
DDX58	0.941177981	0.304415529	0.373954803	NaN	0.77367404	NaN	NaN
DEFB7	0.235331801	0.743067425	0.185298319	NaN	0.20630825	NaN	NaN
DMRT3	0.360125793	NaN	0.386518616	0.176301688	0.751035526	NaN	NaN
DNAH9	0.839925388	0.413343279	0.52922249	NaN	0.640829214	NaN	NaN
DNAJB13	NaN	0.724660754	0.676535931	0.25612699	0.30081476	NaN	NaN
DPP10	0.01196677	0.45513653	0.543499831	NaN	0.688402569	NaN	NaN
DPP4	0.735566499	0.132569336	NaN	0.253820169	0.583427087	NaN	NaN
DSG2	NaN	0.624332718	0.801695683	0.160767756	0.835642196	NaN	NaN
EEF1A2	0.518683269	NaN	0.180094969	0.599960267	0.001479466	NaN	NaN
ELOVL6	0.599772313	0.801607937	NaN	0.072493463	0.11557845	NaN	NaN
FAM3B	NaN	0.888681206	0.841640558	0.048757623	0.704818401	NaN	NaN
FAM49B	0.974425266	0.627673126	NaN	0.210253572	0.130266195	NaN	NaN
FAM83D	0.015396018	NaN	0.352447279	0.017783443	0.021685205	NaN	NaN
FCGBP	NaN	0.418693741	0.155752749	NaN	0.440814753	NaN	NaN
FCGR2A	0.573874912	0.569268191	NaN	0.625661379	0.011474015	NaN	NaN
FGB	0.614832143	0.871032722	NaN	0.882006144	0.265954779	NaN	NaN
FOSL1	0.198672255	0.769497757	0.841830241	0.295620359	NaN	NaN	NaN
FOXD1	0.492925786	0.46034139	0.003461996	NaN	0.80247787	NaN	NaN
FOXD3	0.926438173	0.468036595	0.489499434	NaN	0.303281875	NaN	NaN
FREM2	0.430671712	0.355752865	0.238721856	NaN	0.997875667	NaN	NaN
FZD5	NaN	0.176987968	0.72951825	0.01432146	0.878923644	NaN	NaN
G6PD	0.876659324	0.959728689	NaN	0.007132816	0.567992527	NaN	NaN
GABRB3	0.85422766	0.744430642	0.304912565	NaN	0.495246317	NaN	NaN
GOLGA7B	0.105376275	0.142433923	0.576873099	0.274533493	NaN	NaN	NaN
GPT2	0.297810813	0.73482762	NaN	0.442052015	0.254729788	NaN	NaN
GRIA2	NaN	0.227273257	0.317971212	0.287282317	0.638927587	NaN	NaN
GRIK4	0.471943417	0.769018673	0.296201748	NaN	0.182057372	NaN	NaN
GSS	0.444121299	0.923854755	NaN	0.034339458	0.169043115	NaN	NaN
GSTA3	NaN	0.230451567	NaN	0.70063498	0.554074003	NaN	NaN
GSTM1	0.384918407	0.727384319	NaN	0.32952458	0.410430859	NaN	NaN

GUCY2C	NaN	0.726762496	0.935156467	0.318985967	0.592494096	NaN	NaN
GYS2	0.867631667	0.930023704	0.273336359	NaN	0.315676192	NaN	NaN
HAND2	0.721212251	NaN	0.848820185	0.451252889	0.648461097	NaN	NaN
HMOX1	0.973045565	0.632963867	NaN	0.010362934	0.799590817	NaN	NaN
HNF4G	NaN	0.609384628	0.072406073	0.148935129	0.353931068	NaN	NaN
HP	0.455667697	NaN	0.126106879	0.142549098	0.031732935	NaN	NaN
HPSE2	0.065776331	NaN	0.999671998	0.552016486	0.156629152	NaN	NaN
IFIH1	0.700308605	0.089163527	0.571643761	NaN	0.566456927	NaN	NaN
IGLL1	0.02874786	NaN	NaN	0.55469448	0.578815067	NaN	NaN
IGSF11	0.651266514	0.87747526	0.624111466	NaN	0.778275392	NaN	NaN
IGSF5	NaN	0.169793166	NaN	0.822741895	0.950923047	NaN	NaN
IGSF9B	0.204205724	0.15533945	0.231966118	NaN	0.217165548	NaN	NaN
IL1B	0.175961313	0.216947622	0.000951487	NaN	0.14553094	NaN	NaN
IRX1	0.983332802	0.490090064	NaN	0.439078957	0.2394986	NaN	NaN
ISG15	0.483853847	0.415134019	0.15280581	NaN	0.970448603	NaN	NaN
JAKMIP2	0.329131006	0.990053846	0.71285682	NaN	0.344057148	NaN	NaN
KCNA4	0.981023508	0.850454583	NaN	0.640779049	0.609309596	NaN	NaN
KCNK2	0.27983639	0.225656147	NaN	0.171939283	0.87721559	NaN	NaN
KCNN2	0.605770144	0.843584741	NaN	0.148467135	0.82458934	NaN	NaN
KIAA1211L	NaN	0.109354174	0.993094899	0.365873657	0.604968616	NaN	NaN
KIF11	0.883705987	0.04825394	NaN	0.383610235	0.744674271	NaN	NaN
KIF20A	0.351741603	0.046531656	NaN	0.148594402	0.994501896	NaN	NaN
KIF2C	0.749560483	0.008841222	NaN	0.063046771	0.840343482	NaN	NaN
KLHL31	0.48345665	0.566392568	NaN	0.108453039	0.144279969	NaN	NaN
LAMC3	0.024739918	0.896273044	NaN	0.083618865	0.195896934	NaN	NaN
LCN2	0.440854135	0.144294725	0.71261892	0.257576782	NaN	NaN	NaN
LDHB	0.92091096	0.913926561	NaN	0.15663571	0.894945694	NaN	NaN
LGI1	0.55565257	0.825863766	0.097336934	NaN	0.802104289	NaN	NaN
LGR5	0.568061115	0.683888979	0.586843144	0.75128526	NaN	NaN	NaN
LIN7A	0.530780103	0.673517697	NaN	0.870302624	0.557744255	NaN	NaN
LOC100138641	0.858970595	0.815983979	NaN	0.175296019	0.029556256	NaN	NaN
LOC100139670	NaN	0.612933007	0.717487866	NaN	0.948137568	NaN	NaN
LOC100139885	NaN	0.878208553	0.448784672	0.137070477	0.346796349	NaN	NaN
LOC100140226	NaN	0.706135908	0.218079389	0.286341886	0.998185675	NaN	NaN
LOC100297192	0.004233012	NaN	0.001167883	0.986698335	0.730549959	NaN	NaN
LOC100297779	NaN	0.60770781	0.054259101	0.322855651	NaN	NaN	NaN
LOC100298356	0.797534136	0.098454923	0.39083509	NaN	0.209893918	NaN	NaN
LOC100300115	0.011914019	0.922330858	0.386607968	0.581458891	NaN	NaN	NaN
LOC100301224	0.834324838	0.273859926	NaN	0.649756816	0.600370773	NaN	NaN
LOC100847119	0.024118088	NaN	2.75E-06	0.704292734	0.700862902	NaN	NaN
LOC100847415	0.427801304	NaN	0.932741609	0.226522981	0.471332425	NaN	NaN
LOC100847724	0.96583207	NaN	0.003802687	0.061536041	0.232688539	NaN	NaN
LOC100847981	0.430383419	0.444779775	0.997542941	NaN	0.608207514	NaN	NaN
LOC100848536	0.688354585	0.012905663	0.912971323	NaN	0.800946834	NaN	NaN

LOC100851369	0.607285387	0.740172782	NaN	0.249776433	0.91574013	NaN	NaN
LOC101901948	0.728933946	0.295808445	0.321433917	0.228194419	NaN	NaN	NaN
LOC101902787	0.611955232	0.331582467	0.893929269	NaN	0.184700235	NaN	NaN
LOC101903284	0.856673974	0.168655322	NaN	NaN	NaN	NaN	NaN
LOC101903734	NaN	0.136887655	0.146797722	0.815986128	0.468393014	NaN	NaN
LOC101905242	0.218736239	0.12190939	NaN	0.580070501	0.026707467	NaN	NaN
LOC101905509	0.179060394	0.533476005	0.458452958	NaN	0.087301064	NaN	NaN
LOC101906743	NaN	0.964339051	0.076494556	0.077678916	0.024027856	NaN	NaN
LOC101907335	0.683189149	0.867892535	NaN	0.361246982	0.513187777	NaN	NaN
LOC104968484	0.039921699	0.015334683	NaN	0.167854198	0.61514673	NaN	NaN
LOC104973965	0.607705772	0.490575147	NaN	0.030197701	0.754230697	NaN	NaN
LOC104974214	NaN	0.717029193	0.867349411	0.07413664	0.131159003	NaN	NaN
LOC104974444	0.730552584	0.849826152	NaN	0.170654524	0.554659207	NaN	NaN
LOC104974455	0.084732213	NaN	0.270996993	0.066973598	0.808746401	NaN	NaN
LOC104976942	0.894894025	NaN	0.90186717	0.1505157	0.070389004	NaN	NaN
LOC107131864	0.785881849	0.974879797	NaN	NaN	0.451478984	NaN	NaN
LOC107131942	0.000379771	NaN	NaN	0.851757191	NaN	NaN	NaN
LOC112441507	0.664083362	0.106899796	0.270731983	NaN	0.37222805	NaN	NaN
LOC112441557	0.589499054	0.790365722	0.866895996	NaN	0.739316882	NaN	NaN
LOC112441777	0.554974323	NaN	0.533910898	0.119105713	0.145363995	NaN	NaN
LOC112442062	0.026263282	NaN	0.001320508	0.709033292	0.435947423	NaN	NaN
LOC112443013	0.475992913	0.890355104	NaN	0.235066647	0.894012686	NaN	NaN
LOC112443862	0.2271784	0.929550461	0.066202086	0.772722834	NaN	NaN	NaN
LOC112444652	0.768807766	0.272630211	0.397835589	0.113224937	NaN	NaN	NaN
LOC112445090	0.81603425	0.940593853	NaN	0.483503969	0.268548051	NaN	NaN
LOC112446680	0.654633355	0.992207367	0.830190401	NaN	NaN	NaN	NaN
LOC112446726	0.703660337	NaN	0.778830603	0.958978675	0.167734764	NaN	NaN
LOC112447079	NaN	0.648886529	0.551785319	0.732340642	0.118076869	NaN	NaN
LOC112447816	0.866579784	NaN	0.141279587	0.406232505	0.248064103	NaN	NaN
LOC112448034	0.709716134	0.340383095	NaN	0.667041135	0.419197786	NaN	NaN
LOC505033	0.833448159	0.059650547	0.48835912	NaN	0.954240464	NaN	NaN
LOC507055	0.537998398	0.609134307	0.824113233	NaN	0.376255737	NaN	NaN
LOC508459	0.062260507	0.020017849	0.046086905	0.082833369	NaN	NaN	NaN
LOC509283	0.975258929	0.153069275	0.623973225	NaN	0.655954911	NaN	NaN
LOC509911	0.133467754	NaN	0.207962085	0.760226108	0.231534574	NaN	NaN
LOC511683	0.544763326	NaN	0.414333331	0.891842462	0.665485578	NaN	NaN
LOC513210	0.709959996	0.178362622	NaN	0.886028475	0.466853715	NaN	NaN
LOC514978	NaN	0.527405264	0.51558901	0.060464872	1.44E-06	NaN	NaN
LOC515676	0.136113628	0.302085949	0.520307236	NaN	NaN	NaN	NaN
LOC516421	0.501384404	NaN	0.358629923	0.717409538	0.743411465	NaN	NaN
LOC519274	0.01822856	NaN	0.028831818	0.655438918	NaN	NaN	NaN
LOC530653	NaN	0.179949309	0.907073642	0.744890376	0.461720216	NaN	NaN
LOC615051	0.25799346	0.631837083	NaN	NaN	NaN	NaN	NaN
LOC616782	NaN	0.496827519	0.973203341	0.173508812	0.215600112	NaN	NaN

LOC616830	0.002127202	0.323443727	NaN	0.250909057	0.039919187	NaN	NaN
LOC618297	0.981855557	0.782911191	NaN	0.010493851	0.94358996	NaN	NaN
LOC781736	NaN	0.390834042	0.812760655	0.715279607	0.863320111	NaN	NaN
LOC781796	NaN	0.258064896	0.884943968	0.024133418	0.025114505	NaN	NaN
LOC783106	0.203108723	0.908851536	NaN	0.041553191	0.533207875	NaN	NaN
LOC784266	0.574636314	0.825056446	0.309994438	0.235399082	NaN	NaN	NaN
LOC785161	0.920157717	NaN	0.345298078	0.965279984	0.72204744	NaN	NaN
LOC789829	NaN	0.206581356	0.676182791	0.060917225	0.069733668	NaN	NaN
LTF	NaN	0.990053843	0.857185807	0.183920988	0.231852257	NaN	NaN
LUZP2	0.612682512	0.746168562	0.067427602	NaN	0.833431985	NaN	NaN
MAPK4	0.130222761	NaN	0.519958493	0.007112596	0.674280923	NaN	NaN
MARCO	0.032441382	0.861369409	NaN	0.039942876	0.580821517	NaN	NaN
MEF2B	NaN	0.402313047	0.421811488	NaN	0.023601217	NaN	NaN
MEGF11	NaN	0.972671246	NaN	0.149138839	0.747405317	NaN	NaN
MID1IP1	0.416171614	0.444779992	NaN	0.069562824	0.007680241	NaN	NaN
MISP	NaN	0.848386795	0.43270564	0.086483741	0.874830328	NaN	NaN
MLXIPL	0.56601876	0.693820648	NaN	0.04437512	0.109967866	NaN	NaN
MMP12	0.455657932	0.580363589	0.245227631	NaN	0.057230452	NaN	NaN
MOXD1	0.889073348	NaN	0.15198882	NaN	0.749541973	NaN	NaN
MPC1	0.685050308	0.497136076	NaN	0.08906162	0.81533615	NaN	NaN
MPZ	0.936198954	0.586116016	0.05504531	NaN	0.508077156	NaN	NaN
MT1A	0.965731164	0.664524526	0.226748362	0.088862281	NaN	NaN	NaN
MUSK	0.111344188	NaN	0.734406818	0.014273479	0.2416411	NaN	NaN
MX1	0.977927638	0.025387408	0.306582532	NaN	0.647525476	NaN	NaN
MX2	NaN	0.182360865	0.714045445	NaN	0.514418262	NaN	NaN
MYO1A	NaN	0.707546276	0.292629075	0.159507273	0.032418449	NaN	NaN
NAALADL1	NaN	NaN	0.340506428	0.936403895	0.583107215	NaN	NaN
NPFFR2	0.498014944	NaN	0.342008816	0.298936619	0.488178809	NaN	NaN
OAS1Y	0.969480489	0.045537435	0.416944266	NaN	0.854129435	NaN	NaN
OAS1Z	NaN	0.381652068	0.464185236	0.219984357	0.786518555	NaN	NaN
OPCML	0.6132837	NaN	0.760099969	0.35180572	0.072730498	NaN	NaN
OSM	0.474146767	0.051207778	0.05436817	NaN	0.028797978	NaN	NaN
P2RX1	0.074898357	NaN	0.660850733	0.399781048	0.093387971	NaN	NaN
PARD6B	0.441303993	0.073200315	NaN	0.754696903	0.881887547	NaN	NaN
PBLD	0.46797645	0.514653903	NaN	0.391913116	0.107355853	NaN	NaN
PCDH10	0.993015992	0.715001234	0.411200881	NaN	0.768640159	NaN	NaN
PCLAF	0.41746197	0.081490944	NaN	0.055847189	0.785379146	NaN	NaN
PDZK1	NaN	0.367320531	0.209740221	0.401342717	0.267118887	NaN	NaN
PDZRN4	0.120422108	0.332171578	0.983061794	NaN	0.100545709	NaN	NaN
PEBP4	0.005084865	NaN	0.680206361	NaN	0.575332428	NaN	NaN
PHF21B	0.451946808	NaN	0.496198973	0.691843813	0.18522382	NaN	NaN
PIR	0.868092301	0.982881082	NaN	0.099826569	0.769611039	NaN	NaN
PKD2L1	0.770057417	0.572511532	NaN	NaN	0.573683956	NaN	NaN
PLA2G2D1	NaN	0.690228102	0.178644775	0.117012889	0.001842468	NaN	NaN

PLEK2	NaN	0.119142764	0.426042067	0.170832399	0.458478601	NaN	NaN
PLP1	0.622510839	0.693749934	0.058485746	NaN	0.663004743	NaN	NaN
PPDPFL	0.971936388	NaN	0.954916709	0.12160899	0.9423585	NaN	NaN
PPM1E	0.018187237	0.120120947	NaN	0.270209958	0.387811309	NaN	NaN
PRSS35	0.430545355	NaN	0.002046181	0.363676108	0.940710555	NaN	NaN
PTCH2	0.623175821	0.810616747	NaN	0.450049815	0.76428581	NaN	NaN
PTGIR	0.887430285	NaN	0.00038954	0.3277263	0.77059224	NaN	NaN
PTPRQ	0.928126155	0.659074068	NaN	0.172699866	0.870414703	NaN	NaN
RASL12	0.130297495	NaN	0.765682663	0.592493587	0.119747749	NaN	NaN
REC8	0.655894124	0.016059384	0.118140163	0.044166998	NaN	NaN	NaN
RHOD	0.652136538	0.194566581	0.001915792	0.906434731	NaN	NaN	NaN
RIPK4	NaN	0.677543296	0.894315542	0.003331101	0.797113341	NaN	NaN
RRM2	0.360735702	0.159085808	NaN	0.35243067	0.886036859	NaN	NaN
RSAD2	0.927161034	0.328646014	0.480353703	NaN	0.450507382	NaN	NaN
SAMD9	0.605533998	0.063986354	0.322944327	NaN	0.003748949	NaN	NaN
SCP2	0.916762896	0.862472608	NaN	0.208893679	0.432889925	NaN	NaN
SCUBE1	0.871768656	0.728314364	0.141236036	NaN	0.40349581	NaN	NaN
SECTM1A	0.480127858	0.409956032	0.993138042	0.52710762	NaN	NaN	NaN
SERHL2	0.308742699	0.126460774	NaN	0.209138521	0.603516488	NaN	NaN
SGCG	0.920043496	0.632040193	0.715236151	NaN	0.732333443	NaN	NaN
SH3RF2	0.147478942	0.460330153	NaN	0.742972754	0.873444536	NaN	NaN
SIX2	0.556551881	NaN	0.082053823	0.109964747	0.32170207	NaN	NaN
SLC13A3	0.939240178	0.675440552	NaN	0.36527242	0.442541378	NaN	NaN
SLC15A1	NaN	0.855791174	0.932644454	0.594294159	0.130290663	NaN	NaN
SLC16A1	0.067955649	0.401302969	NaN	0.128510021	0.046831798	NaN	NaN
SLC16A7	0.723729057	0.878047832	NaN	0.370164158	0.127825899	NaN	NaN
SLC25A4	0.697167626	0.979142226	NaN	0.030623158	0.666783244	NaN	NaN
SLC35F1	0.461514496	0.784131154	0.090095539	NaN	0.583169208	NaN	NaN
SLC51B	NaN	0.225911255	0.844187192	0.661406439	0.202622322	NaN	NaN
SLC5A9	0.88248853	0.582933786	NaN	0.450951722	0.795494428	NaN	NaN
SLC6A8	NaN	0.42210562	0.86357138	0.127357727	0.884768415	NaN	NaN
SLC9A2	0.09581167	0.728975265	0.672744904	0.29629208	NaN	NaN	NaN
SLC9A3	NaN	0.853750036	0.285579029	0.613552884	0.384232545	NaN	NaN
SNAP25	0.799674683	0.961938993	0.151632558	NaN	0.458798475	NaN	NaN
SNCA	0.866879636	0.468186223	0.903217711	NaN	0.802697842	NaN	NaN
SNRNP25	0.290528124	0.804035421	NaN	0.026242766	0.087333199	NaN	NaN
SOCS3	0.428049344	0.217686871	0.675246107	0.081974531	NaN	NaN	NaN
SOX10	0.545921302	0.457064429	0.330623816	NaN	0.873928002	NaN	NaN
SPCS3	0.726494446	0.034433232	0.658325737	0.291424636	NaN	NaN	NaN
SPHAR	0.041755335	0.314057018	0.04972047	NaN	NaN	NaN	NaN
SPOCK1	0.609757349	0.96368337	0.845595136	NaN	0.758813396	NaN	NaN
SPRY3	0.510374623	0.429455086	NaN	0.017979755	0.019267576	NaN	NaN
SPTB	0.769247934	0.304859583	0.282870764	0.412083017	NaN	NaN	NaN
SRXN1	0.027010909	0.264609457	NaN	0.283118974	0.34212069	NaN	NaN

ST14	NaN	0.429222242	0.059621153	0.151069593	0.252325039	NaN	NaN
ST8SIA2	0.813842003	0.070832474	0.028841164	0.864446496	NaN	NaN	NaN
SVOP	0.870682278	0.762878573	0.000724674	0.14638678	NaN	NaN	NaN
TDH	0.536565069	0.215306538	NaN	0.353919165	0.647308881	NaN	NaN
TECTB	0.377163158	0.458759698	NaN	0.414298029	0.090939014	NaN	NaN
TGM5	0.248286258	0.555450575	NaN	0.000592761	0.224920975	NaN	NaN
TIMD4	0.240596527	0.902250072	0.123786227	0.37393195	NaN	NaN	NaN
TKT	0.720842809	0.69803535	NaN	0.156985601	0.204149691	NaN	NaN
TMEM179	0.96715722	0.371526167	NaN	0.682138386	0.966562316	NaN	NaN
TMEM216	0.167602719	0.41924805	0.086020528	0.052294713	NaN	NaN	NaN
TMEM45B	NaN	0.912131259	0.824697435	0.395033863	0.626434487	NaN	NaN
TMPRSS2	NaN	0.402061388	0.971696597	0.004132356	0.03847643	NaN	NaN
TMPRSS5	0.912642914	0.78016479	0.254025335	NaN	0.739736343	NaN	NaN
TNFRSF11B	0.592287395	0.438684574	0.069514315	NaN	0.839123909	NaN	NaN
TRIB3	0.806291472	0.304308743	0.099067087	NaN	0.058323135	NaN	NaN
TRIM44	0.046380825	0.656315445	NaN	0.358533167	0.33779586	NaN	NaN
TTYH1	0.549465222	0.242941073	0.101558735	NaN	0.603513287	NaN	NaN
TUBA1D	NaN	0.845812333	0.192921307	0.665431207	0.674863489	NaN	NaN
UBE2C	0.276069533	0.030769296	NaN	0.219862116	0.982974088	NaN	NaN
UCHL3	0.406639024	0.400630554	NaN	0.146054138	0.277393118	NaN	NaN
UCMA	0.908746957	0.311484138	NaN	0.633609031	0.953197235	NaN	NaN
UCP1	NaN	0.121661283	0.303196977	0.268561089	0.365771228	NaN	NaN
UHRF1	0.533055369	0.087719503	NaN	0.484881082	0.864242888	NaN	NaN
VIL1	NaN	0.698076912	0.438244373	0.001734582	0.65813738	NaN	NaN
WNT6	0.98850129	0.639661875	0.233248598	NaN	0.203710822	NaN	NaN
YDJC	0.825505265	0.83376212	NaN	0.003837765	0.44740918	NaN	NaN
ZBTB37	NaN	0.004290168	NaN	0.623697488	0.20099663	NaN	NaN
ZDHHC23	0.506825465	0.840022635	0.382544188	NaN	NaN	NaN	NaN
