# Supplementary material for: Genes associated with body weight gain and feed intake identified by meta-analysis of the mesenteric fat from crossbred beef steers
Source: PLoS One. 2020 Jan 7;15(1):e0227154. doi: 10.1371/journal.pone.0227154 (PMC6946124; doi:10.1371/journal.pone.0227154)
Supplement: S4 Table — Genes with all gray cell indicate those that were excluded because they were also significant for the gain by intake interaction term. Jackknife 1 P-value gives the adjusted P-value for the meta-analysis with Cohort 1 removed, Jackknife 2 P-value gives the adjusted P-value for the meta-analysis with Cohort 2 removed, and so on. Yellow cells indicate jackknife analyses where the P-value was insignificant, i.e. the gene failed to pass the jackknife analysis. (PDF) [file pone.0227154.s004.pdf]

Supplemental Table 4. Jackknife sensitivity analysis results for the DEGs associated with the gain main effect. Genes with all gray cell indicate those that were excluded because they were also significant for the gain by intake interaction term.
Yellow cells indicate jackknife analyses where the P-value was insignificant, i.e. the gene failed to pass the jackknife analysis.

Gene	Original P-value	Jackknife 1 P-value	Jackknife 2 P-value	Jackknife 3 P-value	Jackknife 4 P-value	Jackknife 5 P-value
OLFML3	0.000109523	0.030462202	0.000144154	0.000537233	0.011147612	8.30E-05
MRPL14	0.00079315	0.025449072	0.001005427	0.000537233	0.169231007	0.000397807
HSPH1	0.00079315	0.001029316	0.000482766	0.007150564	0.239804724	0.002007083
GCAT	0.00079315	0.001029316	0.003064014	0.000537233	0.371487913	0.002890233
LOC100300716	0.000867323	0.023785131	0.008086698	0.051654146	0.001245533	0.000397807
EBPL	0.001335857	0.004399379	0.219428799	0.008407796	0.002955945	0.000799287
AACS	0.001335857	0.001029316	0.001558122	0.003383168	0.389234007	0.006495088
JCHAIN	0.001377188	0.020537795	0.006035246	0.144920501	0.002955945	0.000581505
PDLIM5	0.004323139	0.004399379	0.896821247	0.003776236	0.008410659	0.002007083
MGC152281	0.004323139	0.005011728	0.014317715	0.284249009	0.011623357	0.002890233
ADGRL4	0.004323139	0.006230779	0.003064014	0.003383168	0.959657413	0.002890233
PTGS2	0.004323139	0.028017292	0.006203401	0.101532419	0.005653708	0.00582196
ACSL3	0.004323139	0.009186373	0.003984942	0.026622838	0.093565007	0.016042047
NKAIN1	0.004323139	0.005833756	0.005195895	0.223420961	0.008410659	0.019762026
MOSPD1	0.004323139	0.014061127	0.004435091	0.005798246	0.072580055	0.027435762
FST	0.004323139	0.095597759	0.010251484	0.005798246	0.005932689	0.033395047
PTAFR	0.004323139	0.004399379	0.003064014	0.189843685	0.011007321	0.037938803
SFRP1	0.004323139	0.013515027	0.005195895	0.005798246	0.003494328	0.481287134
PUS10	0.005413007	0.004399379	0.319666529	0.142020376	0.005932689	0.003327788
LOC100848025	0.005413007	0.004399379	0.003064014	0.999981514	0.005932689	0.003327788
GALR3	0.005413007	0.140268596	0.007708813	0.01049822	0.016924709	0.012492098
RHOH	0.005618886	0.004399379	0.457051074	0.009294159	0.011007321	0.016042047
H19	0.006323489	0.046376349	0.040855823	0.013551762	0.028331951	0.008058916
GSTT2	0.006323489	0.023785131	0.004435091	0.005798246	0.259605072	0.02989897
HAS3	0.006945926	0.005143738	0.003984942	0.980532429	0.005932689	0.006609468
HPS6	0.006976907	1.19E-05	1.19E-05	1.19E-05	1.19E-05	1.19E-05
LOC782367	0.007758796	0.140628476	0.041270129	0.008407796	0.011623357	0.017465067
RUFY2	0.009510624	0.005011728	0.006176253	0.999981514	0.00622898	0.004634901
SRPX	0.009510624	0.023785131	0.016405609	0.013583458	0.410579416	0.004634901
LIPG	0.009510624	0.007854385	0.006035246	0.005798246	0.429580883	0.082833206
SMUG1	0.009510624	0.007854385	0.020279903	0.007150564	0.005932689	0.786936221
CCDC117	0.011850891	0.009186373	0.006035246	0.030347973	0.659373888	0.012492098
CDKN2B	0.012989121	0.771162191	0.006176253	0.013535312	0.021448293	0.006495088
ARL6IP1	0.013096166	0.006656726	0.008482949	0.792140715	0.011007321	0.030222631

GIMAP7	0.013096166	0.015602435	0.07493451	0.090665733	0.013418636	0.051612345
CCL1	0.013096166	0.028017292	0.010978012	0.010066023	0.071077861	0.236488033
MAP2K6	0.015082676	0.009186373	0.006176253	0.983397724	0.011147612	0.013224633
NRG2	0.015377584	0.193198829	0.056915186	0.017628974	0.050783194	0.009750867
AASDH	0.015377584	0.023785131	0.014317715	0.264971068	0.07934306	0.015096869
SLC30A2	0.015377584	0.101607236	0.008086698	0.317775964	0.012728453	0.019762026
NTNG1	0.015377584	0.531880429	0.006677879	0.014457074	0.011007321	0.067636557
PLN	0.015377584	0.407164443	0.010978012	0.008407796	0.011007321	0.12602847
RCOR2	0.016340519	0.019788047	0.010978012	0.016388096	0.459901956	0.050045876
STT3B	0.016340519	0.007854385	0.011282993	0.455750731	0.038641801	0.065527435
DLX3	0.017483197	0.013515027	0.011282993	0.493932071	0.104739407	0.013939876
RAB7B	0.019175109	0.020432119	0.010978012	0.013535312	0.833173678	0.02135416
ANXA9	0.019175109	0.02739935	0.070347905	0.195133816	0.035585427	0.024297337
LOC100848799	0.019473611	0.629520176	0.010978012	0.016918662	0.012660927	0.049043162
GLCE	0.021303906	0.013515027	0.010464615	0.899047543	0.050089145	0.010682683
KCNMA1	0.021429024	0.581251143	0.010978012	0.017628974	0.012660927	0.066453419
LOC100138922	0.022570293	0.01271104	0.016405609	0.733411985	0.091929235	0.011911095
ARHGEF26	0.022570293	0.261005811	0.134705643	0.017327625	0.016924709	0.026549236
CHAC1	0.022570293	0.38381979	0.019463809	0.013535312	0.07934306	0.030038419
CCDC18	0.024628316	0.014061127	0.016405609	0.142897139	0.012728453	0.607070178
SH2B2	0.02622284	0.028017292	0.02289723	0.421668661	0.088549922	0.023841394
TARDBP	0.02622284	0.013515027	0.010978012	0.017628974	0.817472827	0.078604224
ELP3	0.02622284	0.022183135	0.295198472	0.015929013	0.02350412	0.231591662
MAPT	0.02622284	0.028017292	0.023145393	0.017628974	0.078019742	0.462837324
CAB39	0.026807218	0.018074023	0.253411983	0.246894799	0.038641801	0.021279033
NINL	0.026807218	0.015111522	0.155962342	0.320552723	0.050089145	0.02218159
ANTXRL	0.026807218	0.018566029	0.627295843	0.03592926	0.016924709	0.059028456
IL18BP	0.026807218	0.018074023	0.02289723	0.158268938	0.0140837	0.529445217
AKR1B1	0.027025454	0.041698715	0.032579962	0.164561018	0.218907883	0.019216099
LOC789607	0.028024529	0.056200849	0.085055732	0.017628974	0.429015291	0.015096869
LOC101906455	0.028024529	0.014061127	0.928731949	0.017628974	0.019257861	0.038821918
TMED6	0.028024529	0.018074023	0.014317715	0.041662861	0.220650999	0.336632335
SLC6A16	0.028214073	0.710362373	0.02289723	0.089519983	0.016557913	0.016042047
UNC5A	0.028214073	0.03028188	0.028304215	0.527867968	0.088434446	0.019762026
VEGFC	0.028214073	0.018074023	0.011457996	0.676566861	0.114220493	0.030222631
BAG3	0.028214073	0.048373476	0.019460305	0.087007175	0.19372157	0.091509537
LPIN1	0.028214073	0.028017292	0.012606581	0.171973551	0.160299875	0.123340166
PAK5	0.029169028	0.112592492	0.16427018	0.051654146	0.054012024	0.032779511

CD5	0.029169028	0.101162337	0.068022262	0.017628974	0.115996316	0.096654902
CNTN4	0.030075892	0.471255215	0.056242327	0.017628974	0.050089145	0.036669109
LOC783540	0.030278483	0.053539426	0.040903847	0.021980166	0.059087931	0.41219904
NEIL1	0.030890334	0.034301242	0.013794871	0.265322159	0.405207591	0.017465067
ACLY	0.033461735	0.028017292	0.020279903	0.041662861	0.301814666	0.236892591
ACKR4	0.033678273	0.41466518	0.081070817	0.090191097	0.019010316	0.026549236
SHROOM3	0.033678273	0.019788047	0.057453544	0.244473266	0.145017318	0.077359386
SLIT1	0.033678273	0.021246977	0.024985638	0.021980166	0.67482152	0.116296903
PPTC7	0.033678273	0.023785131	0.015429751	0.149152044	0.31726277	0.123340166
CHMP1B	0.033678273	0.018566029	0.023145393	0.055922809	0.039275295	0.841312694
ACSM3	0.033716717	0.084171958	0.167895602	0.052265112	0.100663646	0.042109537
TIMM13	0.034450928	0.095597759	0.039244562	0.079487894	0.158789265	0.083443362
PRMT7	0.035007583	0.018566029	0.031572675	0.021980166	0.959565914	0.036669109
DENND2D	0.035007583	0.028017292	0.051303387	0.406380182	0.050089145	0.096181444
MYLK	0.035737836	0.775262502	0.041530229	0.031933946	0.021448293	0.041509544
EMB	0.037369194	0.102046985	0.071918694	0.25577133	0.023540359	0.082510165
PDE4B	0.038117298	0.028017292	0.031558913	0.852170016	0.048864729	0.033395047
GID8	0.039698306	0.046664108	0.027961657	0.026622838	0.788995893	0.051612345
PKHD1L1	0.040767539	0.173292591	0.123834939	0.061984512	0.035585427	0.131927377
TNNT3	0.040767539	0.029339947	0.070646249	0.20767138	0.103546734	0.131927377
HSPA1A	0.040767539	0.027624174	0.031558913	0.056999429	0.480281203	0.150393674
FAM210B	0.043702346	0.023785131	0.043637718	0.854623597	0.047731818	0.049043162
LDB3	0.044355989	0.899494273	0.042383342	0.033067695	0.024528975	0.038121044
LOC513659	0.045091712	0.034301242	0.05944237	0.028000912	0.900927045	0.026549236
RAP1A	0.045091712	0.028285269	0.07493451	0.348123948	0.048864729	0.171346752
CCL8	0.049621576	0.072299457	0.042383342	0.030347973	0.421697748	0.148288516
SGK2	0.050325967	0.084761484	0.030216025	0.197274509	0.375347002	0.037938803
SCG3	0.051321965	0.073625625	0.41604117	0.190916959	0.053948734	0.026549236
HSPB3	0.052095949	0.088301139	0.030216025	0.328845482	0.184917114	0.057923797
JMJD6	0.052386923	0.028017292	0.032104539	0.105673986	0.817472827	0.049043162
CIDEA	0.052386923	0.114482838	0.101251367	0.043122786	0.285914544	0.066453419
SLF1	0.052386923	0.028017292	0.07493451	0.574117341	0.053396783	0.106829802
CBARP	0.054265007	0.037830747	0.041270129	0.435037147	0.243749249	0.050890427
CDCA4	0.054265007	0.075283568	0.111760238	0.145175946	0.050089145	0.263947229
LOC107132475	0.054265007	0.025449072	0.039442167	0.03592926	0.410579416	0.416841281
TMEM126B	0.054265007	0.02739935	0.052129589	0.030347973	0.07934306	0.891088733
MGC139164	0.054506754	0.029339947	0.492173361	0.434462583	0.039275295	0.02989897
CYP2B6	0.054506754	0.196508483	0.046726841	0.14003237	0.243749249	0.041509544

KCTD10	0.054506754	0.03028188	0.824121854	0.142020376	0.039275295	0.045329159
INTS6	0.054506754	0.0406769	0.036675991	0.033062342	0.970980375	0.049043162
LOC100336414	0.054506754	0.028017292	0.029501066	0.244473266	0.523715832	0.082510165
DNAJA1	0.054506754	0.035143651	0.030216025	0.237294485	0.403447621	0.092525987
COL6A5	0.054506754	0.084171958	0.203043741	0.114865871	0.07934306	0.117143837
INSIG1	0.054506754	0.084171958	0.069255302	0.051654146	0.222363228	0.231591662
SBSPON	0.054506754	0.11643928	0.161747977	0.095245089	0.035585427	0.254708908
RIOX1	0.054506754	0.03028188	0.031558913	0.049304694	0.194489535	0.669558041
LOC618076	0.057101617	0.034301242	0.033383258	0.03592926	0.972160227	0.061498972
PGF	0.057101617	0.124515901	0.203043741	0.114341618	0.101887329	0.075812851
LOC107133024	0.057101617	0.028017292	0.036675991	0.144920501	0.361095252	0.261631306
CRYAB	0.057422091	0.0668104	0.059696172	0.146093585	0.092287824	0.345042155
ATP10D	0.058363091	0.029226373	0.062651398	0.094393659	0.809129327	0.051612345
TLL2	0.061652083	0.095597759	0.057453544	0.630535638	0.092287824	0.037938803
LOC107131807	0.062276278	0.03028188	0.033383258	0.999981514	0.039275295	0.051612345
FARSB	0.063066009	0.029339947	0.057453544	0.051654146	0.053396783	0.94844234
CHI3L1	0.064011732	0.051061474	0.620613545	0.087007175	0.107338764	0.058985585
KPNA1	0.064840771	0.049089448	0.466192774	0.223420961	0.07934306	0.05532141
HOXA6	0.064840771	0.089019844	0.042136051	0.493932071	0.07934306	0.148288516
RNF125	0.064840771	0.084171958	0.063610956	0.110392228	0.156696702	0.32341956
SYS1	0.064981832	0.03042085	0.999993442	0.080951073	0.046449093	0.037938803
HOGA1	0.066197685	0.102046985	0.070347905	0.175674204	0.383253602	0.049043162
PLEKHJ1	0.072183598	0.041182004	0.220089268	0.371526594	0.050089145	0.172403722
LOC784052	0.073975166	0.114482838	0.159249606	0.090191097	0.429580883	0.044343574
SOX4	0.073975166	0.071823075	0.056915186	0.852170016	0.078019742	0.050890427
LOC100140586	0.073975166	0.916448625	0.069255302	0.057717721	0.050089145	0.056323686
TUBB2A	0.073975166	0.04199274	0.507675433	0.330930008	0.053584861	0.065963161
MIEF2	0.073975166	0.298866179	0.071338954	0.061655563	0.304332925	0.074241587
CDH2	0.073975166	0.109201636	0.042383342	0.828736799	0.048864729	0.075931969
TCAF1	0.073975166	0.051061474	0.046726841	0.5222819	0.059897953	0.310534477
CCDC188	0.074092399	0.034507719	0.332000222	0.24308376	0.19372157	0.070847488
LOC782706	0.074092399	0.087980091	0.07493451	0.30124858	0.131147122	0.164000918
GPT	0.074253197	0.041698715	0.07493451	0.817230639	0.077405936	0.078604224
FKBP4	0.074253197	0.041182004	0.063610956	0.328845482	0.169231007	0.261631306
B4GALT6	0.074253197	0.109201636	0.293267587	0.070261735	0.050089145	0.345042155
TMEM165	0.074755011	0.095271091	0.060113608	0.069186027	0.113107212	0.645427231
WDR86	0.074979715	0.096288167	0.101251367	0.14003237	0.276583162	0.136024479
NCR1	0.075477982	0.046664108	0.042383342	0.834401906	0.092287824	0.105038472

KCNK12	0.075477982	0.047399453	0.380765282	0.271515526	0.070596012	0.131685293
RRAD	0.075477982	0.430562237	0.085583362	0.066639954	0.050101776	0.275185976
PAN2	0.075477982	0.034301242	0.608218151	0.095245089	0.050089145	0.316375257
ACSL6	0.075477982	0.079339867	0.071338954	0.075956851	0.268084837	0.365572588
CLN5	0.07673746	0.043840906	0.069812425	0.918256198	0.093548946	0.049043162
STOX2	0.07673746	0.041698715	0.055256725	0.33257772	0.243749249	0.226443947
EPM2AIP1	0.077178986	0.0406769	0.044604987	0.695681179	0.183682164	0.116776579
CASQ1	0.077178986	0.244044595	0.131579357	0.087007175	0.107338764	0.217784724
ZAR1L	0.077178986	0.048373476	0.123834939	0.265078026	0.071077861	0.401953772
KBTBD8	0.077178986	0.0406769	0.044125514	0.447647758	0.07934306	0.472996792
PDE4D	0.077356327	0.086295667	0.045774964	0.101532419	0.837915327	0.063093006
MZF1	0.077807206	0.048319212	0.407806986	0.613568161	0.050089145	0.049043162
PKNOX1	0.077807206	0.071925466	0.041530229	0.142897139	0.83704813	0.065963161
SPRYD7	0.077807206	0.098935364	0.40651486	0.092951282	0.220257143	0.070847488
CREB3L1	0.077807206	0.053539426	0.092939405	0.230408294	0.39364787	0.111965604
DEDD2	0.077807206	0.05948897	0.059696172	0.146093585	0.615387632	0.12602847
ITPKC	0.077807206	0.077261213	0.044125514	0.243580087	0.189029065	0.348834279
NOD2	0.079126056	0.065947845	0.057453544	0.094471326	0.15109727	0.710630057
SLC18B1	0.079226371	0.0406769	0.190906767	0.608306231	0.088549922	0.103007606
TRIP12	0.08243884	0.047487822	0.059696172	0.673160239	0.301814666	0.051612345
TNFAIP8L3	0.08243884	0.254378588	0.135792529	0.265078026	0.088549922	0.087682759
SLC7A8	0.08243884	0.099782223	0.07524322	0.328845482	0.175487094	0.165471616
LOC104970711	0.08243884	0.0406769	0.071338954	0.608689518	0.156585636	0.17082925
SPP1	0.08243884	0.044028744	0.057453544	0.237294485	0.06903251	0.774118367
RASSF9	0.083213149	0.04603057	0.069812425	0.588166711	0.309861372	0.067624612
PIK3R6	0.083370466	0.046376349	0.049891205	0.561120526	0.461764223	0.058081496
ATP6V1C2	0.083370466	0.171035293	0.057453544	0.307895098	0.308741687	0.067636557
SH3TC1	0.083370466	0.048373476	0.123834939	0.348123948	0.386670972	0.070847488
LOC530077	0.083370466	0.051061474	0.407806986	0.306245142	0.113350592	0.096181444
LSMEM2	0.083370466	0.041182004	0.157824336	0.318413172	0.261642081	0.137916361
LAT	0.083370466	0.058742114	0.247516611	0.208271387	0.210411804	0.143192822
ZMYND8	0.083370466	0.059022131	0.071338954	0.617759491	0.071077861	0.259961044
FABP7	0.083370466	0.428129561	0.063610956	0.076778815	0.072580055	0.352283712
FLNC	0.083997212	0.718258172	0.059696172	0.110392228	0.05501254	0.162010252
IFI6	0.084253852	0.090927734	0.266796445	0.156913424	0.232246616	0.117564974
RASAL2	0.084253852	0.054750513	0.044125514	0.087007175	0.613652691	0.348834279
BTAF1	0.084689538	0.074364469	0.045774964	0.060203812	0.999985752	0.050890427
LOC100297240	0.084689538	0.041698715	0.834683675	0.281172702	0.058191157	0.059044379

WDR90	0.084689538	0.041698715	0.627791512	0.31713801	0.059575681	0.111965604
KCTD12	0.084689538	0.231981784	0.123834939	0.297613285	0.078019742	0.146911658
AOX1	0.084689538	0.248786678	0.045110023	0.087007175	0.280065531	0.279773719
LSAMP	0.085426491	0.331055243	0.062651398	0.095720411	0.385312392	0.087368021
JAML	0.086605111	0.147717181	0.391019766	0.124274496	0.154086472	0.105038472
ADAM19	0.087650197	0.202942544	0.07493451	0.586290626	0.058191157	0.121775786
BBS9	0.08967177	0.439484206	0.059757896	0.092951282	0.150689744	0.243648755
TTC38	0.0900445	0.083089827	0.124162687	0.812453944	0.059575681	0.097150556
DNAJB9	0.09096423	0.147717181	0.118296051	0.418062303	0.106002215	0.150393674
TNC	0.091151194	0.154664113	0.106999866	0.486381297	0.160299875	0.075812851
PADI1	0.092539981	0.267299905	0.089282735	0.256428354	0.252969091	0.066453419
LOC112445144	0.092539981	0.047487822	0.066378107	0.272286871	0.797227609	0.067624612
PRG4	0.092539981	0.175761069	0.49874267	0.137390097	0.11856409	0.078604224
RNF165	0.092539981	0.10330052	0.063610956	0.233609578	0.553835124	0.101551068
BoLA	0.092539981	0.04603057	0.161747977	0.466614794	0.190389953	0.164000918
RBM47	0.093359431	0.60594617	0.07493451	0.144920501	0.060608263	0.188365181
GIMAP4	0.093359431	0.071823075	0.380765282	0.157512683	0.11856409	0.268031409
BCL11B	0.093359431	0.085117029	0.376259378	0.079487894	0.162368647	0.330239393
TFAM	0.095620491	0.046564385	0.457350984	0.095245089	0.321162042	0.17082925
FBXL6	0.096698346	0.061318677	0.131579357	0.819571278	0.108431149	0.083443362
FCER1A	0.096698346	0.10330052	0.18477696	0.158268938	0.181356269	0.307772114
ALPK1	0.098810265	0.061318677	0.123834939	0.759397892	0.07934306	0.171346752
PTGES3	0.098992399	0.095271091	0.07493451	0.285121972	0.373086408	0.165471616
NIF3L1	0.098992399	0.065947845	0.101251367	0.081045512	0.608253358	0.310738754
LOC101906101	0.099124739	0.213876395	0.092082677	0.468177997	0.162368647	0.082510165
MXRA5	0.100199813	0.0668104	0.204743396	0.840700255	0.07934306	0.067580837
LOC112449613	0.101363704	0.065278114	0.072857804	0.242468577	0.092287824	0.768168974
PTK7	0.104189428	0.096288167	0.224019804	0.434367671	0.214920985	0.075931969
VWA3B	0.105861015	0.051061474	0.07493451	0.25577133	0.349814923	0.35938146
WASF1	0.106099929	0.628096433	0.167253332	0.11098035	0.071077861	0.148288516
MAP3K1	0.106149397	0.150152701	0.608754919	0.17610268	0.104739407	0.094614164
RIMS1	0.106149397	0.48520817	0.221817649	0.144920501	0.100663646	0.096181444
FAIM2	0.106149397	0.382320412	0.057453544	0.271515526	0.126170987	0.210552471
SLC10A1	0.106149397	0.306854565	0.057453544	0.143339586	0.125352042	0.462837324
ANGPTL4	0.11057794	0.153770044	0.069812425	0.095245089	0.644194633	0.216520461
KYNU	0.11057794	0.126226436	0.159466813	0.258539834	0.107338764	0.370481724
KLHL25	0.11144605	0.124515901	0.069812425	0.142897139	0.160619085	0.710234013
FAM124B	0.11147891	0.092959139	0.064872197	0.690807874	0.300638192	0.087543443

EPB42	0.111554193	0.149848835	0.774191554	0.138399299	0.092287824	0.095486552
LOC101906565	0.111896155	0.055024792	0.996224523	0.108121458	0.092287824	0.088817057
PIK3C3	0.113671266	0.194981771	0.17678075	0.537984757	0.115565977	0.077802264
ST6GAL2	0.113889533	0.833713501	0.143292593	0.14003237	0.077405936	0.084547949
FGF10	0.116288919	0.082368783	0.096774872	0.087007175	0.478408771	0.485795983
SMAD7	0.117012141	0.05948897	0.95322206	0.090191097	0.15109727	0.108634721
LHX6	0.117012141	0.099818333	0.161460258	0.272012865	0.324735158	0.17082925
SHOX2	0.117477307	0.1900602	0.119508634	0.243421078	0.093565007	0.440866698
PDZD3	0.118397611	0.828704412	0.084844093	0.093617975	0.184917114	0.094614164
GSE1	0.118397611	0.05948897	0.123834939	0.944929997	0.099458616	0.116296903
TMEM266	0.118397611	0.244044595	0.072772621	0.268990576	0.337606409	0.133604581
GPR183	0.118397611	0.065947845	0.405548748	0.142020376	0.333691681	0.219804421
TRAT1	0.118397611	0.073625625	0.371177512	0.178713329	0.256619651	0.235278475
ATL2	0.118397611	0.095271091	0.131579357	0.100046148	0.588923286	0.278300597
FAM120B	0.118397611	0.071823075	0.069812425	0.373269014	0.362632321	0.29820838
ARL14EP	0.118397611	0.095271091	0.159336409	0.318413172	0.091589724	0.504596604
LOC782951	0.118518863	0.087980091	0.069812425	0.233609578	0.788007619	0.133604581
KCP	0.119496472	0.250992446	0.092939405	0.518542038	0.189880485	0.091509537
EMC9	0.119496472	0.142205008	0.147502403	0.095245089	0.690628811	0.148308142
LOC112444897	0.119496472	0.064780795	0.092082677	0.138399299	0.86010579	0.188365181
NDE1	0.122505827	0.114482838	0.069812425	0.421668661	0.088434446	0.613401904
HGH1	0.122780102	0.132320138	0.16748684	0.095245089	0.228803825	0.521756022
PER3	0.12329909	0.070471887	0.16187826	0.812453944	0.088549922	0.190336795
LENG8	0.123494822	0.065278114	0.289119143	0.596654953	0.11355332	0.188365181
ALPK3	0.123494822	0.129394194	0.135598368	0.105929082	0.107338764	0.81956221
TUBB	0.123580203	0.10330052	0.147502403	0.687568149	0.113350592	0.17082925
C4H7orf25	0.124179535	0.083089827	0.137232888	0.145175946	0.74028971	0.17082925
GSDME	0.124179535	0.096288167	0.071338954	0.495395077	0.140287234	0.444913711
MVD	0.124336542	0.072299457	0.142262294	0.095245089	0.510264075	0.440363475
LSM12	0.124336542	0.075915504	0.43322325	0.114951036	0.092287824	0.640434848
LOC112442023	0.124352906	0.095271091	0.421396526	0.281172702	0.127817537	0.236892591
TUBB4B	0.124352906	0.092340538	0.288302068	0.332251447	0.138308561	0.297599027
LOC104975559	0.124352906	0.222998185	0.083098714	0.246894799	0.175487094	0.380806889
ADCK5	0.124445023	0.079339867	0.622387278	0.472729963	0.107338764	0.082833206
IL7R	0.124445023	0.10330052	0.42111624	0.158268938	0.19915908	0.263947229
CELSR1	0.125033644	0.352921816	0.372748001	0.166782138	0.092287824	0.167287581
IPO7	0.125632598	0.082368783	0.276215914	0.287569241	0.333474676	0.165309689
ESPN	0.125632598	0.515680499	0.07493451	0.110106042	0.232246616	0.253576008

ATP1A2	0.12582926	0.984089343	0.077940002	0.105929082	0.092287824	0.117655874
CATSPERG	0.12582926	0.071823075	0.124789881	0.619232063	0.243749249	0.1947175
PPP1CB	0.12582926	0.082368783	0.247516611	0.258539834	0.338817987	0.217841795
LOC112444309	0.12582926	0.47375776	0.07493451	0.178713329	0.103463546	0.368852585
TMEM176A	0.12582926	0.171625678	0.227605298	0.258376333	0.088396315	0.404708082
TCEA1	0.127098958	0.067542799	0.237224665	0.144920501	0.169231007	0.692922947
IMPA2	0.127163964	0.085492713	0.121169568	0.46351377	0.114667091	0.497110172
IFI27	0.127227532	0.095271091	0.741683135	0.126739958	0.240993303	0.12602847
TMEM35A	0.127227532	0.779025263	0.07493451	0.158268938	0.093565007	0.219657046
LOC107131769	0.127227532	0.100821701	0.100305308	0.105929082	0.52068239	0.44566684
CFB	0.129074086	0.173610482	0.290289905	0.288575311	0.242691323	0.119273971
ZNF182	0.129074086	0.070471887	0.132044739	0.704815275	0.149911117	0.269043001
PLD2	0.129389275	0.072299457	0.131579357	0.939604997	0.093565007	0.162010252
LOC112445943	0.130121153	0.190906176	0.081281362	0.276955855	0.340652621	0.243648755
CD4	0.130121153	0.083089827	0.532764011	0.144191063	0.127464139	0.426772334
CABIN1	0.130495903	0.140268596	0.101251367	0.657738814	0.301402937	0.088817057
ESRP1	0.131046598	0.175122514	0.121169568	0.17425459	0.797227609	0.082833206
CLASRP	0.131046598	0.071823075	0.552362414	0.615029548	0.093285358	0.113858254
PLEKHA6	0.131046598	0.105621605	0.457767372	0.322717781	0.222363228	0.118789666
LOC785630	0.131046598	0.095271091	0.128747939	0.890704007	0.134809437	0.123427522
FLT4	0.131046598	0.291635793	0.311710447	0.333677631	0.107338764	0.12602847
ARMCX4	0.131046598	0.098935364	0.084844093	0.544789716	0.346226314	0.188365181
LOC112447770	0.131046598	0.480755771	0.143538098	0.138399299	0.149967974	0.290733382
SYNDIG1	0.131046598	0.406494847	0.080652773	0.146093585	0.123505091	0.495253191
COA7	0.131046598	0.133607584	0.096418492	0.158268938	0.175487094	0.768168974
TACC1	0.131733982	0.140268596	0.203043741	0.206482381	0.535694492	0.133604581
PYGM	0.131733982	0.843278276	0.143201225	0.126739958	0.104786396	0.148288516
NEGR1	0.131733982	0.692305416	0.108958286	0.100046148	0.169231007	0.238276647
CA3	0.131733982	0.152396903	0.123834939	0.383271918	0.188785007	0.338075577
BDH1	0.133187457	0.095271091	0.10976106	0.143339586	0.720896795	0.303619975
LOC104970537	0.133187457	0.071823075	0.315377577	0.328845482	0.093565007	0.513034959
SYTL3	0.134690392	0.10330052	0.100346545	0.612761692	0.321291154	0.162010252
DOK6	0.135427679	0.173614475	0.073337608	0.421668661	0.346226314	0.21818809
MAPRE1	0.136558755	0.079050419	0.781767828	0.406051248	0.11856409	0.087465777
SNX16	0.136558755	0.114698248	0.092077049	0.7694401	0.206311705	0.17082925
SPTSSA	0.136558755	0.10330052	0.220089268	0.124267267	0.242691323	0.614265476
CD93	0.138395001	0.102046985	0.07493451	0.586033659	0.52068239	0.116651559
FNDC1	0.138395001	0.140268596	0.332000222	0.509147405	0.169359047	0.131685293

POSTN	0.140007388	0.148578053	0.18102241	0.594220399	0.184917114	0.165471616
SLC32A1	0.140659411	0.079849806	0.305569839	0.1187671	0.655786713	0.23081184
RGS4	0.140771413	0.692305416	0.130692355	0.345539016	0.107338764	0.094614164
SIAE	0.140771413	0.142205008	0.100305308	0.631326261	0.206311705	0.236488033
HSP90AA1	0.140771413	0.095597759	0.100346545	0.418062303	0.445529822	0.243648755
GNAT1	0.140771413	0.105621605	0.137232888	0.17425459	0.508367859	0.348834279
GLUL	0.141128502	0.079998371	0.081281362	0.648660726	0.220650999	0.348834279
DCLRE1B	0.142959592	0.077354796	0.085055732	0.890704007	0.228803825	0.17082925
USPL1	0.1431229	0.098935364	0.089629633	0.178713329	0.951021192	0.133604581
PI4K2A	0.143493058	0.096288167	0.203043741	0.691025178	0.259960413	0.105038472
TYSND1	0.144948888	0.088310944	0.123834939	0.142045176	0.976292433	0.12602847
LOC781001	0.145243882	0.109201636	0.563372946	0.519487474	0.130221132	0.105038472
LOC101903647	0.145243882	0.148578053	0.333951286	0.298705716	0.364333362	0.112770103
SFRP2	0.145243882	0.202942544	0.137232888	0.424465138	0.317509486	0.153641783
IGFBP2	0.145243882	0.104799613	0.532764011	0.151348118	0.272369183	0.256625608
GPATCH2L	0.145243882	0.213604544	0.149945659	0.144920501	0.487063655	0.268031409
LOC512867	0.145243882	0.140628476	0.289119143	0.282975368	0.148854672	0.396383896
HK2	0.145243882	0.370464508	0.162384839	0.14003237	0.114220493	0.529083282
TATDN3	0.145243882	0.083089827	0.096774872	0.230749309	0.155786687	0.903500283
SLC30A6	0.145790914	0.709542765	0.131579357	0.149152044	0.220257143	0.148288516
TIMM17A	0.145790914	0.095271091	0.16427018	0.158268938	0.220650999	0.759003017
PPP1R3B	0.146734239	0.414881121	0.381375604	0.149152044	0.217512322	0.12602847
ABCD4	0.147260563	0.493052487	0.174138273	0.183802665	0.236546924	0.162010252
SATB1	0.147424657	0.085492713	0.333951286	0.153342782	0.523124197	0.259961044
NIP7	0.14969101	0.095271091	0.488784977	0.230749309	0.226813717	0.304857167
CNTNAP1	0.14969101	0.181364066	0.15750213	0.181930769	0.152869405	0.694197796
SVEP1	0.153948168	0.23567727	0.227605298	0.213302437	0.472139664	0.131685293
NUP210	0.154058002	0.095271091	0.631783694	0.200813545	0.236546924	0.239120492
LOC526966	0.154058002	0.085492713	0.204743396	0.149152044	0.110958057	0.930710695
NUDT8	0.154096358	0.095271091	0.108958286	0.20767138	0.976769047	0.107311997
TMEM242	0.154096358	0.74733285	0.213808553	0.124274496	0.11856409	0.219804421
ELOVL3	0.154096358	0.113674564	0.134734739	0.541206056	0.321162042	0.236892591
TTC25	0.154096358	0.231791372	0.084377673	0.230749309	0.543569691	0.247033418
DNAJC3	0.154096358	0.095343004	0.092939405	0.328845482	0.403447621	0.455556158
MMRN1	0.156053461	0.804542244	0.134705643	0.220997714	0.127073096	0.161784494
MOB3A	0.156137023	0.131255776	0.225417227	0.157295596	0.149967974	0.778711943
LMO7	0.157193601	0.098935364	0.086269132	0.358606834	0.801692604	0.164000918
GPM6A	0.157267581	0.554555702	0.264746077	0.244473266	0.158789265	0.12602847

CCDC171	0.157267581	0.195864427	0.130823623	0.71113156	0.15109727	0.21818809
MAT2A	0.157990336	0.134454067	0.196817785	0.764257755	0.243238607	0.103007606
HHIP	0.157990336	0.724028362	0.376259378	0.138399299	0.107338764	0.137718554
GIMAP7	0.157990336	0.152396903	0.488101319	0.263148753	0.19372157	0.243648755
SHROOM2	0.158864627	0.08605105	0.289119143	0.612227019	0.226813717	0.215682742
SYNM	0.159715375	0.809330769	0.160944555	0.159382087	0.115231146	0.215552418
KLF15	0.161170855	0.191143436	0.137232888	0.6331124	0.162368647	0.256650336
MON1A	0.162731095	0.225201246	0.376259378	0.153342782	0.152910322	0.453456176
NR2F1	0.163024688	0.621279921	0.140368605	0.371984821	0.162368647	0.121775786
WNT4	0.163024688	0.225929217	0.315377577	0.489523244	0.149967974	0.171346752
DNAJB4	0.163024688	0.262279886	0.10976106	0.218154192	0.324735158	0.414167655
TTR	0.163024688	0.158370343	0.454895041	0.243580087	0.11856409	0.420532744
NID2	0.163581037	0.175458312	0.131579357	0.183802665	0.187532394	0.780585188
CAPZA2	0.163587526	0.114698248	0.221817649	0.582177282	0.278967777	0.192427355
SMIM33	0.164447407	0.248967644	0.203043741	0.333677631	0.252553732	0.233861113
GLULP	0.165096417	0.092959139	0.121169568	0.831280166	0.228803825	0.243648755
KLHL13	0.166403166	0.683972894	0.194803525	0.162170298	0.250017312	0.131927377
TOP1	0.166403166	0.150152701	0.134531292	0.138399299	0.887514169	0.221822899
TMEM151A	0.166403166	0.10330052	0.147502403	0.610848628	0.350342841	0.227773298
DNER	0.166403166	0.096288167	0.182852532	0.695681179	0.226813717	0.243648755
CCDC141	0.166403166	0.194603254	0.52262636	0.230749309	0.138677713	0.318988997
LOC617565	0.166465688	0.11260585	0.263693478	0.893220599	0.115996316	0.148288516
RPGRIP1L	0.166465688	0.158370343	0.101251367	0.882057818	0.206311705	0.16305052
CATSPERD	0.166465688	0.095271091	0.28201526	0.760670924	0.130221132	0.25537536
HSPD1	0.166465688	0.098935364	0.128747939	0.509147405	0.19372157	0.587132866
LOC782779	0.169112528	0.126270088	0.16427018	0.328845482	0.508689889	0.259961044
LOC112441594	0.170152339	0.10330052	0.905023942	0.200813545	0.190962164	0.162010252
LOC107131704	0.170152339	0.130747353	0.485200576	0.379699598	0.115996316	0.345042155
PSMA7	0.170289943	0.140628476	0.232158906	0.178713329	0.206311705	0.717538808
KHDC4	0.17115477	0.118943126	0.12193059	0.1640952	0.962513981	0.175327295
HSP90AB1	0.17115477	0.10330052	0.101251367	0.327763847	0.696466814	0.290733382
GPX4	0.17115477	0.135098827	0.15750213	0.140657695	0.774940205	0.33731134
ADA	0.173595011	0.124515901	0.101251367	0.204008197	0.977167046	0.143192822
LYZ	0.173595011	0.1803923	0.177263486	0.519487474	0.321162042	0.188365181
DNAJB1	0.174616963	0.253117639	0.159249606	0.320552723	0.332977669	0.269261309
C6H4orf19	0.176032369	0.364362847	0.177744973	0.230749309	0.507950493	0.129676662
EVPL	0.176032369	0.095271091	0.455195587	0.466614794	0.321162042	0.164000918
LOC525426	0.176032369	0.107612874	0.123834939	0.929388614	0.192076892	0.216520461

SLC25A1	0.176032369	0.131083549	0.150395977	0.16362091	0.69149571	0.384886074
OGT	0.176032369	0.121751021	0.228879875	0.271515526	0.145017318	0.748921056
AASDHPPT	0.176032369	0.095271091	0.221817649	0.223420961	0.236546924	0.75640676
LOC101906178	0.179174672	0.126083867	0.357663999	0.149152044	0.810436875	0.148288516
BAHCC1	0.179174672	0.096288167	0.16427018	0.694666231	0.290115856	0.261631306
CYP4V2	0.179369724	0.582127335	0.221817649	0.259195167	0.202799203	0.164000918
LIMK2	0.182182352	0.244115226	0.225891302	0.510388001	0.307459189	0.131685293
LOC101907985	0.182182352	0.118943126	0.10976106	0.961898258	0.244188382	0.137601015
L3MBTL1	0.182301832	0.095343004	0.170027415	0.537984757	0.429015291	0.265629576
STIP1	0.182301832	0.114482838	0.126437518	0.459073822	0.492937023	0.310738754
NINJ2	0.183639019	0.156568523	0.641535145	0.23893604	0.338817987	0.148288516
LAMB3	0.183639019	0.212795409	0.128747939	0.459073822	0.529281738	0.162010252
PRPF40B	0.183639019	0.095597759	0.173043426	0.769701093	0.192076892	0.338075577
NUMA1	0.183639019	0.102846291	0.222506307	0.362359041	0.183729013	0.67453786
MTFP1	0.184589968	0.216060513	0.201838748	0.244473266	0.392114522	0.336632335
RUVBL1	0.184589968	0.213995435	0.155509506	0.230749309	0.183682164	0.748921056
ZMYM3	0.184707463	0.140891746	0.128531767	0.604750897	0.220257143	0.442234422
RASD2	0.184707463	0.280564227	0.121169568	0.243421078	0.140287234	0.774299863
FAM207A	0.184974846	0.181773962	0.134531292	0.16511126	0.870042072	0.227773298
SGPL1	0.186352825	0.34639555	0.12744446	0.521237523	0.321162042	0.157877061
LOC104975034	0.18670239	0.26001291	0.124357907	0.178713329	0.308741687	0.628917359
TXNL4A	0.186757636	0.132552473	0.154694246	0.466614794	0.43787689	0.306319401
GPR173	0.189424356	0.234569178	0.164539256	0.518542038	0.450726215	0.129837773
STEAP2	0.189424356	0.68903984	0.134705643	0.395781309	0.180323878	0.162010252
MYBL2	0.189424356	0.245371433	0.660365588	0.178713329	0.27339973	0.165471616
LOC112441884	0.189424356	0.54845263	0.156589345	0.220997714	0.195939907	0.352283712
LOC101902293	0.189424356	0.102046985	0.224019804	0.248055601	0.158789265	0.867907362
ADAMTS5	0.190008909	0.13290717	0.261891147	0.318413172	0.658667583	0.17082925
GALNT5	0.194515848	0.10330052	0.759357794	0.321113304	0.228803825	0.188365181
ELF3	0.194558536	0.250888294	0.21079102	0.244473266	0.501311839	0.238402701
LYPD6	0.194627749	0.474946062	0.487875367	0.189843685	0.158789265	0.226443947
EAF2	0.194627749	0.170707665	0.267633028	0.630951216	0.195625074	0.259409228
HINT3	0.194627749	0.283879605	0.156589345	0.230408294	0.245239639	0.587387684
FBN2	0.19596137	0.14028689	0.168423854	0.521723045	0.141622833	0.655022943
NAIP	0.196138148	0.152425796	0.855847948	0.158268938	0.216883868	0.243648755
SREBF1	0.196296866	0.830930037	0.18477696	0.158268938	0.189029065	0.232669707
LOC101906828	0.197184847	0.335464703	0.492173361	0.256688924	0.169231007	0.256625608
NR3C2	0.199265391	0.520216632	0.21772312	0.233609578	0.228803825	0.275185976

GZMB	0.199532677	0.189001172	0.27063412	0.762598894	0.222643686	0.137601015
TMEM120A	0.199532677	0.173292591	0.196361429	0.534853543	0.405540421	0.21292943
SPRN	0.199532677	0.143451808	0.659919107	0.320552723	0.158789265	0.331473604
ERGIC2	0.199532677	0.105621605	0.159627256	0.206524144	0.206311705	0.946112759
MYH3	0.201029339	0.10330052	0.201045475	0.930987593	0.265743557	0.136024479
CREM	0.201029339	0.194272101	0.12346755	0.190916959	0.973219333	0.155273283
KBTBD11	0.201029339	0.970934548	0.123834939	0.188754824	0.195939907	0.17082925
HEXIM1	0.201029339	0.178888094	0.203043741	0.657738814	0.30731562	0.1947175
RCAN1	0.201029339	0.10330052	0.140451505	0.93048026	0.210411804	0.268031409
NPPC	0.201029339	0.230102595	0.232158906	0.335976936	0.353844241	0.289777696
CHORDC1	0.201029339	0.11755015	0.135792529	0.328845482	0.766655597	0.293609234
WDR19	0.201029339	0.156568523	0.457350984	0.487829118	0.184917114	0.294807972
RGS7	0.201029339	0.549085151	0.264285773	0.258376333	0.141622833	0.321909778
LOC104975686	0.201029339	0.194981771	0.147502403	0.701248463	0.203098181	0.330375391
ZNF341	0.201029339	0.109201636	0.376259378	0.451094074	0.234246049	0.387697543
LMOD1	0.201029339	0.321117458	0.173043426	0.172761773	0.219689266	0.684994779
KCNA3	0.201029339	0.177235342	0.121169568	0.233609578	0.178930793	0.927426708
ATAD3A	0.201667579	0.141254813	0.290289905	0.158268938	0.285241994	0.751270885
LOC533307	0.202722299	0.166388584	0.263037513	0.739727518	0.194489535	0.236892236
FAM136A	0.202722299	0.156762843	0.289119143	0.190321073	0.206311705	0.785562356
HHEX	0.202863794	0.207927874	0.170027415	0.519487474	0.523124197	0.150393674
BEND6	0.202863794	0.162070877	0.135792529	0.42341558	0.568764846	0.300134855
DDX3X	0.203072241	0.167316382	0.23807348	0.415349648	0.60903578	0.162010252
ASZ1	0.204605489	0.154664113	0.148577691	0.230408294	0.602648954	0.495197104
LOC112444164	0.204735443	0.260376907	0.339614385	0.379605284	0.372759621	0.153641783
CCNJ	0.205353995	0.332382274	0.123834939	0.789139053	0.221363351	0.17082925
GBP5	0.205353995	0.244044595	0.224019804	0.276781355	0.642796813	0.17082925
ADAM12	0.205353995	0.126270088	0.513255922	0.614863577	0.220650999	0.184830482
LOC101904573	0.205353995	0.126226436	0.159627256	0.220997714	0.970980375	0.190336795
NKRF	0.205353995	0.164497297	0.541610391	0.359644843	0.290976138	0.219023879
KLK7	0.205353995	0.150231325	0.314465547	0.570760743	0.240988155	0.300899175
C2CD2L	0.205353995	0.426582179	0.209157652	0.260791097	0.271454139	0.327096775
HPD	0.205513929	0.339799881	0.134305012	0.302038497	0.692764366	0.153906753
RNF146	0.206266345	0.198150337	0.14445118	0.405144561	0.35025312	0.441709492
TMED10	0.206325211	0.164497297	0.872088608	0.271515526	0.213885249	0.157877061
KCNH8	0.206325211	0.36444467	0.161747977	0.551641524	0.206311705	0.269043001
LOC112441505	0.206938756	0.188051682	0.164085252	0.882395013	0.189029065	0.23081184
LOC516494	0.207415528	0.204261001	0.219097224	0.340792236	0.532662842	0.243648755

SPOCK2	0.207415528	0.171219494	0.747666034	0.258539834	0.220257143	0.247033418
BHLHE40	0.20774885	0.143723481	0.1660097	0.201596841	0.988881329	0.154015391
TBC1D4	0.20774885	0.140268596	0.162384839	0.756459079	0.371057408	0.226443947
NRM	0.207791007	0.156936741	0.29723319	0.24308376	0.803705802	0.17082925
MSTO1	0.207791007	0.131255776	0.332000222	0.183854397	0.391810273	0.609692015
LIMS1	0.210671732	0.22299815	0.213951827	0.379699598	0.224819698	0.51212265
NUS1	0.211843674	0.171219494	0.310480699	0.541206056	0.264393955	0.293609234
SEMA3F	0.21274127	0.474946062	0.15750213	0.181930769	0.596137752	0.224217031
DNAJA4	0.21274127	0.167091326	0.312920161	0.498444775	0.337806259	0.262707378
LOC112449258	0.21274127	0.114698248	0.174138273	0.901834772	0.160299875	0.348834279
JPH2	0.214835999	0.906658524	0.161747977	0.244473266	0.158789265	0.231591662
ENO2	0.214835999	0.417405254	0.16427018	0.206524144	0.189029065	0.679479899
DTL	0.21504812	0.324238107	0.161747977	0.27614882	0.346226314	0.427891722
MRPL16	0.21701469	0.149848835	0.238925543	0.177756738	0.307022464	0.811759324
PROX1	0.21713846	0.470862073	0.425565437	0.332251447	0.169231007	0.214807033
PARM1	0.21713846	0.140390197	0.203043741	0.216652631	0.526646233	0.610832246
SLC16A14	0.217229819	0.178293122	0.156589345	0.37939721	0.731607614	0.233861113
PLD4	0.217424818	0.454880625	0.43314299	0.268990576	0.220650999	0.219023879
CDC42EP3	0.217424818	0.40117451	0.332000222	0.181930769	0.410579416	0.246535972
TP53INP1	0.217424818	0.189453886	0.219428799	0.731537809	0.192076892	0.324230326
LOC112449100	0.218198571	0.191143436	0.419867844	0.183854397	0.75602825	0.169263829
LOC104975663	0.218198571	0.185779689	0.542055154	0.190916959	0.486387163	0.243648755
TPBG	0.218938548	0.330823549	0.462922708	0.394882194	0.206311705	0.221214729
TM9SF2	0.219609182	0.227667869	0.224019804	0.675426128	0.338817987	0.165471616
CFI	0.219609182	0.479687945	0.238925543	0.561120526	0.169231007	0.192013713
PYCR2	0.219609182	0.129960287	0.156954299	0.193597155	0.999985752	0.201641577
SIGLEC11	0.219609182	0.489987041	0.285893798	0.200813545	0.365608028	0.243648755
MKX	0.219609182	0.728504282	0.161747977	0.272012865	0.169231007	0.339003717
SLC4A11	0.219609182	0.250888294	0.551149334	0.230408294	0.206366562	0.387697543
BAG2	0.219609182	0.21952118	0.265489131	0.259195167	0.184917114	0.748921056
NAPSA	0.219749224	0.146143926	0.631008412	0.233609578	0.324735158	0.345042155
LOC100196897	0.219823385	0.550226079	0.157205474	0.412833005	0.317509486	0.184830482
PLD5	0.222382786	0.148578053	0.756426507	0.519487474	0.169231007	0.188365181
GATAD2A	0.222382786	0.173614475	0.161747977	0.876616876	0.206311705	0.296944628
LOC101907219	0.222382786	0.139013489	0.238925543	0.484080728	0.308741687	0.487514327
NOA1	0.222382786	0.132722662	0.203043741	0.200813545	0.375347002	0.820395402
LY75	0.222431387	0.155758461	0.485200576	0.291662687	0.274979387	0.44035446
SLC30A4	0.223387326	0.129394194	0.16748684	0.37131062	0.873366037	0.219260715

HSPA4L	0.224075432	0.15310767	0.16427018	0.284249009	0.713359648	0.381602573
DTD2	0.224481852	0.151666206	0.382828821	0.281172702	0.736942681	0.178122091
DNAJC16	0.22577837	0.221919611	0.16427018	0.233609578	0.230407118	0.846210629
DUOXA2	0.225939242	0.148279096	0.804171507	0.472732632	0.206311705	0.165471616
ITGA2B	0.225939242	0.140268596	0.599631267	0.414302555	0.236546924	0.329740076
RRS1	0.225939242	0.150231325	0.457350984	0.487965302	0.253232037	0.329740076
CXCL10	0.225939242	0.319654425	0.310213331	0.522681653	0.162368647	0.336145569
LOC112446690	0.225939242	0.213995435	0.135384648	0.418062303	0.383253602	0.497388408
LTB	0.226598845	0.152425796	0.222288059	0.373269014	0.510001595	0.382635416
TAF4B	0.227882813	0.144987161	0.147502403	0.891139257	0.383253602	0.19620601
UBIAD1	0.227882813	0.171219494	0.213808553	0.499858161	0.350342841	0.418173662
AHSA1	0.228558381	0.1423435	0.167253332	0.541206056	0.437165526	0.403290419
ARSI	0.228935447	0.147717181	0.372748001	0.549287144	0.460664408	0.188365181
PDE3A	0.228935447	0.975984829	0.177263486	0.203515264	0.183682164	0.219804421
NUDT9	0.228935447	0.158370343	0.312920161	0.76992117	0.246967945	0.233431327
ANGPTL8	0.228935447	0.185779689	0.161747977	0.325529737	0.760007802	0.296205915
MYH11	0.228935447	0.860600608	0.16427018	0.24308376	0.172554707	0.321713134
LOC100139916	0.228935447	0.35609607	0.170027415	0.356392963	0.403447621	0.325918022
HSPA8	0.228935447	0.158370343	0.186126264	0.467531709	0.545946638	0.336632335
PDLIM3	0.228935447	0.700130683	0.222288059	0.244473266	0.190962164	0.338075577
NTRK3	0.228935447	0.89417747	0.156589345	0.181930769	0.19372157	0.339003717
KCNAB1	0.228935447	0.130747353	0.186957689	0.230749309	0.242824673	0.947801358
GAL	0.230383316	0.150152701	0.194803525	0.486381297	0.638815482	0.275185976
WAPL	0.231376486	0.144447068	0.380765282	0.244473266	0.858495696	0.171346752
ARL8B	0.231376486	0.158370343	0.227605298	0.686487857	0.197146469	0.46459395
LOC101908206	0.234571257	0.23567727	0.196354908	0.958588196	0.183682164	0.171346752
HEATR4	0.234571257	0.154664113	0.367642473	0.645168798	0.206311705	0.35938146
PTI	0.234571257	0.311196652	0.332000222	0.421668661	0.206311705	0.379371038
LOC513508	0.234571257	0.171851848	0.596931535	0.404005354	0.169231007	0.419810556
DNAJA3	0.234571257	0.205410466	0.219428799	0.266681464	0.300638192	0.724693895
TRAPPC8	0.235339417	0.144447068	0.203043741	0.882057818	0.37499039	0.182142532
DAPK2	0.235339417	0.132533975	0.255262051	0.271515526	0.930118855	0.215168007
AMIGO2	0.235339417	0.532115072	0.299097274	0.421668661	0.195625074	0.236488033
RRP15	0.235339417	0.173610482	0.376259378	0.794386089	0.187532394	0.254861352
STBD1	0.235339417	0.45629714	0.388358382	0.206524144	0.353664429	0.261631306
VWCE	0.235339417	0.158370343	0.314465547	0.626698454	0.241159148	0.377503548
PTPRE	0.235339417	0.151666206	0.397871714	0.345572957	0.308741687	0.500317514
OLFML2A	0.236270019	0.307033248	0.25118033	0.518542038	0.351955331	0.236892591

PCDH19	0.236270019	0.158196326	0.156589345	0.245235814	0.455838272	0.779201808
COX19	0.236565746	0.910560869	0.263693478	0.228571182	0.183682164	0.22339476
WWP1	0.237504965	0.140268596	0.985171227	0.194234315	0.221116317	0.256650336
TMEM82	0.237504965	0.346007726	0.1550505	0.229616558	0.677185022	0.345042155
LOC783797	0.237504965	0.236790771	0.156026348	0.418062303	0.210411804	0.749116532
PPA1	0.238108408	0.158370343	0.265489131	0.195133816	0.402965379	0.764260585
LOC101902413	0.238902268	0.171851848	0.289119143	0.609054155	0.240993303	0.425037007
PPIF	0.238902268	0.140268596	0.225417227	0.223420961	0.389965946	0.823013262
LOC404103	0.238941109	0.283879605	0.41604117	0.328845482	0.294404294	0.334892097
TFCP2	0.239690123	0.185779689	0.18707947	0.461172896	0.695436567	0.243648755
TEP1	0.239690123	0.140268596	0.361384628	0.822387291	0.19372157	0.310534477
COL5A2	0.239690123	0.178293122	0.427152347	0.616503998	0.215355583	0.32617053
TRAF3	0.240501393	0.194603254	0.156589345	0.358674574	0.603965612	0.440512821
ZGRF1	0.243841541	0.144316373	0.288302068	0.518542038	0.194273748	0.695765939
CEP131	0.244677745	0.141254813	0.180986443	0.999981514	0.228803825	0.182087456
ATP2B2	0.244677745	0.386477216	0.680262176	0.244473266	0.221116317	0.236488033
MCMBP	0.244677745	0.234275431	0.18477696	0.543346467	0.530344927	0.247033418
SRGAP3	0.244677745	0.821767596	0.161747977	0.204008197	0.280961806	0.329740076
LOC100300510	0.244677745	0.389789684	0.517857593	0.237685681	0.232246616	0.337984312
PPID	0.244677745	0.139515631	0.18477696	0.543403516	0.540227484	0.382635416
RGMA	0.244677745	0.144316373	0.165995755	0.250894247	0.285241994	0.940583352
GUF1	0.24473968	0.150125022	0.201045475	0.258376333	0.695436567	0.524890122
TMCC1	0.24622266	0.167316382	0.161747977	0.954946234	0.285030586	0.23081184
ZNF684	0.246870483	0.140268596	0.22372518	0.466614794	0.797489438	0.235278475
PCBP1	0.246870483	0.146156841	0.312920161	0.328845482	0.596079788	0.391911239
RGS7BP	0.246870483	0.184794695	0.376259378	0.302038497	0.447765878	0.408480726
ECM1	0.246870483	0.144987161	0.397871714	0.549287144	0.218955741	0.500317514
HOXA9	0.247510058	0.176619828	0.693546993	0.359963043	0.413909508	0.17082925
PUM2	0.248259655	0.202942544	0.225248779	0.761359847	0.413792445	0.187866698
LOC505099	0.250504491	0.230102595	0.929691578	0.198862931	0.244337693	0.219804421
RGS12	0.250879657	0.175122514	0.263693478	0.95901497	0.240378459	0.17082925
SMARCA5	0.250879657	0.155758461	0.181166619	0.510246043	0.823508064	0.215552418
ADAMTS17	0.250879657	0.180248051	0.511417994	0.732262457	0.19372157	0.235278475
CACNA1G	0.250879657	0.142205008	0.264746077	0.96444269	0.221116317	0.236044227
SMPD3	0.250879657	0.207356717	0.21772312	0.900875874	0.215355583	0.262707378
PRDM1	0.250879657	0.241463151	0.193713592	0.324273469	0.20654875	0.836111377
EN1	0.252191761	0.164497297	0.982041666	0.230408294	0.184917114	0.289777696
TEC	0.252926953	0.141254813	0.402832408	0.230408294	0.946935016	0.171346752

CX3CR1	0.252926953	0.154699098	0.416802987	0.820971736	0.206311705	0.268031409
APBA1	0.252926953	0.834615638	0.261891147	0.223420961	0.222363228	0.289777696
LOC112441499	0.25322184	0.242912313	0.533570754	0.518542038	0.309861372	0.182142532
TMEM203	0.254080141	0.149848835	0.509514515	0.356392963	0.578333624	0.243648755
LOC112447832	0.254080141	0.737941984	0.156589345	0.204008197	0.19372157	0.620459631
NOX5	0.255310027	0.260850309	0.159249606	0.518542038	0.333691681	0.500317514
WDR13	0.255442106	0.171431224	0.873496239	0.230749309	0.403447621	0.212908803
POLR2K	0.25576994	0.171851848	0.95322206	0.271515526	0.19372157	0.268031409
GLIS3	0.25576994	0.357368153	0.293310696	0.328845482	0.425533882	0.316375257
GREB1L	0.25576994	0.236790771	0.238925543	0.232532422	0.459901956	0.628649974
LOC518526	0.25576994	0.154664113	0.229001428	0.335214405	0.189029065	0.927426708
FAM174A	0.255816879	0.486093343	0.225248779	0.24308376	0.228803825	0.609206121
IL7	0.255896705	0.191143436	0.774015998	0.518542038	0.194489535	0.222183376
H1F0	0.255896705	0.150152701	0.221817649	0.995565324	0.221694953	0.23081184
PRLR	0.255896705	0.793300166	0.211515827	0.415734949	0.189029065	0.243648755
PIEZO2	0.255896705	0.183787179	0.298472088	0.767092077	0.259377402	0.310534477
LOC107131817	0.255896705	0.202942544	0.17788487	0.47137884	0.236546924	0.751389857
MYRF	0.256221922	0.164497297	0.466192774	0.459073822	0.422271061	0.307772114
YBX3	0.256221922	0.202942544	0.199199432	0.5222819	0.295929564	0.601371091
UBB	0.256221922	0.259484869	0.196819665	0.383208814	0.206311705	0.779201808
LOC534967	0.257377824	0.228936145	0.162854421	0.220997714	0.939208227	0.310534477
PDE11A	0.257593401	0.303250787	0.810839887	0.318413172	0.22637806	0.19620601
GIMAP6	0.257593401	0.173614475	0.744735369	0.281172702	0.245844782	0.394427049
CD2	0.257593401	0.268224849	0.552362414	0.258539834	0.236546924	0.481925208
LOC101904177	0.257593401	0.173610482	0.279067921	0.223754377	0.645748057	0.564822443
C2H2orf72	0.258943116	0.52614209	0.289119143	0.535163392	0.239804724	0.215552418
VANGL2	0.260531685	0.193692992	0.226774312	0.528783909	0.364333362	0.476029406
LOC104975091	0.261333043	0.289052192	0.224019804	0.921950515	0.220257143	0.187866698
SMTNL2	0.261333043	0.178293122	0.161747977	0.934925429	0.332977669	0.243648755
BMPER	0.261333043	0.250888294	0.172578717	0.486381297	0.257473233	0.667995834
HBB	0.261822125	0.194981771	0.312920161	0.400190066	0.510264075	0.35938146
PIGZ	0.261822125	0.157536653	0.275234017	0.328845482	0.313855706	0.779201808
DCAF1	0.26215719	0.177235342	0.162854421	0.230749309	0.999985752	0.187719348
TSR1	0.26215719	0.235168132	0.33920471	0.429892744	0.222363228	0.603083588
POLR3A	0.26215719	0.279649429	0.196292496	0.306245142	0.308741687	0.733767439
PNPLA3	0.26215719	0.154664113	0.204988458	0.223420961	0.617915169	0.748750383
LRRC24	0.262990007	0.439484206	0.227605298	0.457902257	0.415457256	0.237475258
CACNA1A	0.263336155	0.171826296	0.611647147	0.28672141	0.605982335	0.232306921

TMEM100	0.263342308	0.148578053	0.716468083	0.229385586	0.5863408	0.284327136
DARS	0.263342308	0.154664113	0.355757388	0.268990576	0.307022464	0.794932301
POLR1B	0.264514086	0.148578053	0.487875367	0.479863522	0.220257143	0.590086394
LCMT2	0.266593976	0.153400043	0.415690997	0.318413172	0.487063655	0.47629249
ZSWIM6	0.266731907	0.155758461	0.17319299	0.235959082	0.999985752	0.206639162
RTEL1	0.266731907	0.198150337	0.636543516	0.565578589	0.264437467	0.23081184
C18H19orf18	0.266731907	0.172088931	0.220089268	0.356392963	0.799272579	0.345042155
IMP3	0.266731907	0.189001172	0.71967891	0.22813316	0.251454635	0.535620846
C5H12orf29	0.266731907	0.150231325	0.424739024	0.314962476	0.32137054	0.68185373
CACYBP	0.267154753	0.155758461	0.213808553	0.577737722	0.480104202	0.444913711
ZFAND2A	0.269719866	0.306780965	0.225248779	0.362359041	0.28155751	0.656746469
IFT20	0.269991041	0.38719103	0.636223522	0.244473266	0.338419262	0.243648755
LOC101903832	0.27040181	0.230072979	0.265656966	0.281619951	0.760136746	0.336632335
PACS2	0.270677931	0.262672636	0.369565002	0.321113304	0.502432219	0.339003717
ANO8	0.271388344	0.483305224	0.290289905	0.480047213	0.338817987	0.224217031
ARFGAP1	0.271388344	0.424965921	0.366279622	0.233609578	0.342493459	0.440866698
PLCL2	0.272482518	0.152425796	0.491965793	0.418062303	0.474619036	0.345042155
DUSP16	0.272482518	0.166260029	0.203043741	0.230749309	0.863970911	0.494195557
C18H16orf86	0.272636068	0.151666206	0.685018711	0.457902257	0.243238607	0.389420093
LOC782177	0.272763961	0.240559554	0.466207947	0.627280162	0.206311705	0.345042155
NDUFA3	0.272763961	0.17972533	0.237231427	0.233609578	0.87329316	0.392988847
TBL2	0.273230457	0.203864876	0.654924919	0.275712238	0.515453824	0.251531355
XK	0.273230457	0.180248051	0.534905861	0.760251847	0.206311705	0.270471051
IFIT3	0.275477445	0.750379179	0.201518545	0.263148753	0.47157751	0.224443735
CHP2	0.275477445	0.283879605	0.290289905	0.574117341	0.376447594	0.307772114
MAX	0.275477445	0.197707056	0.228879875	0.519487474	0.570107746	0.348834279
PNPLA7	0.275477445	0.173292591	0.395501027	0.600166011	0.206311705	0.575190599
ZBTB40	0.275477445	0.164497297	0.499640977	0.332251447	0.290976138	0.62884029
PHAX	0.276442902	0.164497297	0.409955029	0.349464413	0.216883868	0.808979368
HSD11B2	0.276543046	0.360897508	0.16748684	0.535570772	0.618159249	0.224217031
TSC22D3	0.276543046	0.213876395	0.273691978	0.768290504	0.383253602	0.253576008
ARHGAP28	0.276543046	0.216652731	0.204743396	0.819968583	0.383931047	0.268031409
CCDC68	0.276543046	0.160683347	0.219428799	0.984423083	0.240531754	0.292295101
GPC3	0.276543046	0.303250787	0.571415357	0.275712238	0.386673096	0.316375257
PROCR	0.276543046	0.204309696	0.794676674	0.256688924	0.304147038	0.348834279
PCYT2	0.276543046	0.180248051	0.29721858	0.230408294	0.855939488	0.380806889
AHSA2	0.276543046	0.167316382	0.195028494	0.46682385	0.701502867	0.412040609
SCN4B	0.276543046	0.516115205	0.18102241	0.351097779	0.240531754	0.593122637

RASSF10	0.277394113	0.699192406	0.289119143	0.410993929	0.220257143	0.284327136
FCRL5	0.277394113	0.306854565	0.487875367	0.346470306	0.301814666	0.396383896
RNASE1	0.277394113	0.173292591	0.48640588	0.630535638	0.215355583	0.440866698
FKBP5	0.277394113	0.185060987	0.201045475	0.468663128	0.429015291	0.632510367
LOC100300806	0.278015179	0.294802047	0.266796445	0.77872142	0.340652621	0.219023879
ERH	0.278015179	0.160520752	0.629779224	0.302038497	0.450288791	0.380806889
LOC107131530	0.278015179	0.42633922	0.18477696	0.229616558	0.353083291	0.72571141
FUBP1	0.279769977	0.158370343	0.177263486	0.258539834	0.999985752	0.206639162
COX8B	0.279769977	0.382853453	0.382449111	0.230408294	0.738811608	0.215682742
KIF14	0.279769977	0.649340041	0.376344992	0.276439276	0.346226314	0.233861113
CALM	0.279769977	0.156936741	0.804171507	0.571690689	0.210314633	0.277052677
TFRC	0.279769977	0.442980755	0.622890829	0.307108989	0.243749249	0.284327136
ABT1	0.279769977	0.213876395	0.735715719	0.44771463	0.240531754	0.310534477
FERMT1	0.279769977	0.249175931	0.457503431	0.577737722	0.248597119	0.348834279
KMT5C	0.279769977	0.156936741	0.497992443	0.528783909	0.361598024	0.369942484
RBM20	0.279769977	0.340788135	0.315377577	0.275712238	0.226813717	0.749726404
ERO1B	0.279919192	0.537166876	0.620801326	0.250705544	0.228803825	0.290733382
CBX2	0.280154421	0.164497297	0.174332119	0.580099141	0.867456282	0.236892236
CYSLTR1	0.280154421	0.528669672	0.305156052	0.513533242	0.228803825	0.310534477
GEMIN6	0.280154421	0.222843742	0.332000222	0.246894799	0.567426339	0.559845075
ZBTB26	0.280154421	0.17544367	0.203043741	0.612761692	0.258559309	0.730527465
SHC2	0.280188981	0.412100649	0.174138273	0.695125147	0.346226314	0.268031409
CADM2	0.280188981	0.830243527	0.273009815	0.328845482	0.206311705	0.30198063
ACTR3	0.280648666	0.188051682	0.799265115	0.383271918	0.362632321	0.23023862
EHF	0.280648666	0.37175727	0.219428799	0.230749309	0.872713065	0.261624235
LOC101905514	0.280648666	0.352921816	0.320772195	0.379510457	0.321162042	0.472996792
MARC1	0.281157318	0.173610482	0.376259378	0.907794002	0.276583162	0.227773298
AEBP2	0.281157318	0.255474579	0.225417227	0.230408294	0.923910588	0.310738754
CRB3	0.281157318	0.403328812	0.466192774	0.415734949	0.253527686	0.338075577
PGS1	0.281157318	0.460244277	0.424739024	0.271515526	0.28067829	0.427891722
LOC107131896	0.281622782	0.171851848	0.630012726	0.493932071	0.423015995	0.256625608
RRAGD	0.281622782	0.244044595	0.342240037	0.691025178	0.240993303	0.380806889
UQCRQ	0.281622782	0.222998185	0.320591795	0.259195167	0.755512904	0.383090164
PKIB	0.281622782	0.1900602	0.555917581	0.235617277	0.403447621	0.587387684
CEBPZ	0.283345737	0.274672834	0.199577642	0.594220399	0.520724297	0.329035696
SOST	0.283345737	0.269110524	0.28201526	0.362359041	0.422271061	0.537186568
SPOUT1	0.287191254	0.241463151	0.21772312	0.253726121	0.501311839	0.749726404
NME2	0.287191254	0.250888294	0.255362729	0.259195167	0.264393955	0.89209761

CXCR6	0.287670262	0.213876395	0.608218151	0.265078026	0.220257143	0.713707269
ACAP1	0.288060967	0.190906176	0.611647147	0.612227019	0.285241994	0.290853569
TRAF3IP3	0.288060967	0.186279351	0.528387114	0.358674574	0.309564851	0.571961045
HDLBP	0.288060967	0.266852211	0.361922003	0.324423853	0.217479948	0.783241453
SDS	0.28815548	0.189438866	0.353891991	0.549287144	0.502432219	0.338075577
ACTR2	0.289810192	0.198516766	0.532743798	0.570760743	0.261642081	0.385223859
LMBR1L	0.290523864	0.171219494	0.834683675	0.580099141	0.257179584	0.219804421
HSF4	0.290523864	0.175351046	0.480070708	0.901168085	0.226813717	0.236892591
WT1	0.290523864	0.194417397	0.755135544	0.344143836	0.392114522	0.290733382
LOC112441605	0.290523864	0.194981771	0.192012094	0.991955963	0.243749249	0.306319401
RELN	0.290523864	0.78357023	0.289119143	0.281172702	0.222643686	0.37954335
NDOR1	0.290851039	0.544401883	0.342601111	0.342830359	0.453790338	0.232306921
ABHD6	0.291003546	0.250888294	0.870765519	0.373269014	0.215164825	0.288674669
APH1B	0.29121061	0.169802082	0.197051222	0.271515526	0.244987575	0.999948228
SLC4A8	0.292453601	0.202942544	0.250811036	0.868530316	0.462744229	0.218566338
BGLAP	0.292853274	0.173292591	0.301630207	0.717645144	0.252969091	0.564822443
EFNB2	0.292907029	0.253228516	0.295198472	0.918693944	0.284494642	0.216087837
RNF182	0.292907029	0.64596808	0.386538031	0.375351105	0.321291154	0.219804421
OXTR	0.292907029	0.26040329	0.376259378	0.586290626	0.403447621	0.306319401
STK38L	0.292907029	0.311196652	0.264746077	0.76992117	0.285030586	0.324132086
LOC112441476	0.292907029	0.181364066	0.449596237	0.486767679	0.375347002	0.468732955
MRPS7	0.292907029	0.219308294	0.289119143	0.281172702	0.423015995	0.758175289
LOC112447762	0.293060122	0.239130734	0.228879875	0.48500625	0.409827576	0.587387684
CCSAP	0.293099094	0.279475276	0.263693478	0.270999523	0.257179584	0.878118151
CEP57	0.293326927	0.697643204	0.195976355	0.258376333	0.290976138	0.546170552
COG2	0.293900648	0.177235342	0.372748001	0.346904474	0.823508064	0.300599914
GRHL2	0.294307391	0.335464703	0.289119143	0.825308462	0.228803825	0.297599027
LOC101905866	0.294307391	0.188051682	0.194791658	0.908126016	0.368946575	0.329035696
MKS1	0.294307391	0.173610482	0.227605298	0.886831994	0.290976138	0.421566456
ATP5MD	0.294307391	0.198150337	0.315377577	0.258376333	0.808047125	0.438685707
TP53BP1	0.294307391	0.217274427	0.238925543	0.472729963	0.403447621	0.640434848
LOC526488	0.294307391	0.195864427	0.517382044	0.244473266	0.404092468	0.645719071
COL5A1	0.295095974	0.213876395	0.559304698	0.773694377	0.243749249	0.243648755
GPX3	0.296075915	0.171851848	0.478168172	0.460818045	0.395278001	0.490415687
XRCC3	0.296153272	0.487617269	0.332000222	0.271515526	0.591306131	0.274476706
OSCAR	0.296153272	0.285872549	0.700963878	0.335214405	0.362632321	0.289777696
FZD10	0.296153272	0.311196652	0.608218151	0.340792236	0.307022464	0.382635416
LOC112442189	0.296153272	0.303250787	0.397871714	0.627280162	0.218907883	0.437363114

ARFGAP3	0.296153272	0.173610482	0.397871714	0.259004169	0.362019552	0.833150766
XPOT	0.296153272	0.171851848	0.3167578	0.24289098	0.231325815	0.990924439
ABCA6	0.29636814	0.275926874	0.495423363	0.371678509	0.631537325	0.224217031
SLFN11	0.296405968	0.465004933	0.345120469	0.409803563	0.455516234	0.261631306
NDUFA4L2	0.296405968	0.173610482	0.381272753	0.320552723	0.863291666	0.309878132
SAP30L	0.296405968	0.869428129	0.220178433	0.269672536	0.272065317	0.35938146
PPP1R3D	0.296405968	0.181609043	0.582898646	0.258376333	0.553835124	0.457840591
FGD6	0.296405968	0.283879605	0.203043741	0.54857232	0.300638192	0.666292389
DNAJC2	0.296405968	0.175761069	0.505553867	0.362359041	0.221421896	0.783241453
IGF2BP2	0.29724877	0.370464508	0.391019766	0.314962476	0.385979159	0.45725625
LOC112449261	0.298548738	0.227667869	0.222338517	0.613744304	0.74028971	0.246535972
ASAH2	0.298548738	0.212550981	0.230578582	0.87522058	0.436968101	0.256650336
STK32C	0.298833335	0.209208844	0.237224665	0.999981514	0.228803825	0.221822899
MZT2B	0.298833335	0.29496073	0.495423363	0.244473266	0.797127163	0.232538153
SMU1	0.298833335	0.262279886	0.30455465	0.82163384	0.357092245	0.243648755
KIAA1217	0.298833335	0.230072979	0.29721858	0.505857338	0.752587077	0.256650336
DMPK	0.298833335	0.231981784	0.421777252	0.268990576	0.801692604	0.320661248
EPHB3	0.298833335	0.185060987	0.326609961	0.819213899	0.368182147	0.321909778
CCT4	0.298833335	0.182634887	0.246286355	0.500788064	0.547818665	0.543272495
IMPA1	0.298833335	0.185415374	0.245566442	0.259985535	0.324735158	0.960023723
LOC101905979	0.299334178	0.173610482	0.886829776	0.345539016	0.301814666	0.33731134
CBL	0.299531118	0.173610482	0.34510985	0.555557333	0.781724458	0.236447986
THBS4	0.299703311	0.192328514	0.199577642	0.419947065	0.622751397	0.63094682
RFXANK	0.300776412	0.184890524	0.584877063	0.745577087	0.304147038	0.25537536
TMSB4X	0.300776412	0.172363024	0.999993442	0.270046532	0.243749249	0.261631306
C1QTNF6	0.300776412	0.301414099	0.361988752	0.705422335	0.285241994	0.337754585
LARP4	0.301059502	0.173614475	0.361922003	0.707217807	0.267378467	0.539240716
GPR68	0.302231972	0.192902022	0.460184825	0.552451497	0.548274329	0.289777696
DES	0.302231972	0.816127864	0.255362729	0.284249009	0.220650999	0.452900632
CNOT7	0.302348401	0.312120962	0.509514515	0.599544809	0.338340057	0.247033418
LOC112442664	0.302390835	0.213995435	0.19660791	0.28524144	0.748925155	0.643003473
TPST1	0.302946329	0.213903854	0.220089268	0.263148753	0.999985752	0.236892236
ACAT2	0.302946329	0.213995435	0.285090082	0.284249009	0.590544812	0.682479856
ZNF605	0.302946329	0.184794695	0.291173692	0.59258231	0.221363351	0.799816679
CSNK1B	0.302946329	0.173610482	0.424739024	0.359013436	0.252553732	0.846210629
ZC3H12D	0.303447647	0.177941785	0.749453648	0.472217546	0.28067829	0.380806889
IFFO1	0.30412604	0.538774329	0.320164496	0.434462583	0.409022457	0.269043001
GJA1	0.305699278	0.445080617	0.224019804	0.676566861	0.257404016	0.392126702

IMMP1L	0.305699278	0.185779689	0.266796445	0.659427594	0.474869023	0.442730925
GLRB	0.305699278	0.414670902	0.543921757	0.27614882	0.238463867	0.545900472
CNOT1	0.305786888	0.346007726	0.255362729	0.267884784	0.944584705	0.224443735
CLPX	0.306063728	0.234819797	0.224019804	0.731537809	0.605982335	0.267753388
LOC112441484	0.306063728	0.370475687	0.70011973	0.302038497	0.290976138	0.348834279
PPP1CC	0.306063728	0.332081544	0.203043741	0.462657114	0.648267928	0.373692297
RIPOR3	0.306063728	0.582560314	0.284776553	0.291662687	0.376447594	0.440866698
SLC7A6	0.306202235	0.210892767	0.66149866	0.410993929	0.40786268	0.348834279
EIF2B2	0.306202235	0.303250787	0.390611004	0.306600881	0.472699899	0.519240641
HAS1	0.306503348	0.198150337	0.712435509	0.774214837	0.228803825	0.23081184
COL28A1	0.306503348	0.943754915	0.217488007	0.359644843	0.238463867	0.290733382
PRKD2	0.306503348	0.380409291	0.299097274	0.418062303	0.513223069	0.350409498
OBSCN	0.307518965	0.198436666	0.996224523	0.259195167	0.324735158	0.236892591
ARMCX3	0.307518965	0.311196652	0.238484886	0.424465138	0.852132157	0.243648755
HCN4	0.307518965	0.528669672	0.328810186	0.284249009	0.63648166	0.259961044
MZB1	0.307518965	0.282521927	0.492173361	0.694189359	0.307379953	0.268031409
IGFBP5	0.307518965	0.321010792	0.692461158	0.271515526	0.487063655	0.279393335
YTHDF2	0.307518965	0.205410466	0.28201526	0.522617886	0.804274107	0.284327136
NAV1	0.307518965	0.292474682	0.652724437	0.599807615	0.237547414	0.307772114
CKS2	0.307518965	0.213876395	0.724940313	0.574371412	0.244987575	0.339003717
LNX2	0.307518965	0.225665268	0.457350984	0.359013436	0.6274106	0.378410553
MICU3	0.307518965	0.465004933	0.227605298	0.456103613	0.409827576	0.420532744
TMEM132E	0.307518965	0.213876395	0.524780246	0.527867968	0.321162042	0.460497664
SLC31A2	0.307518965	0.234383429	0.301630207	0.799139231	0.253527686	0.470494326
CNTFR	0.307518965	0.693703257	0.203043741	0.259107184	0.384290341	0.504596604
ZNF438	0.307518965	0.194981771	0.225248779	0.845055741	0.255576901	0.564822443
HSPB1	0.307518965	0.367446738	0.244671934	0.486381297	0.290976138	0.645719071
IDI1	0.307518965	0.188051682	0.289119143	0.365822386	0.532662842	0.699447977
LOC104976020	0.307518965	0.350851666	0.203043741	0.331269626	0.396504336	0.751270885
PTCD2	0.307518965	0.254897553	0.329305578	0.30124858	0.383253602	0.769865166
ADAM11	0.307518965	0.205410466	0.21772312	0.271515526	0.468542094	0.891088733
NUDT18	0.308191582	0.177941785	0.485200576	0.284249009	0.819342514	0.345042155
RPF2	0.308191582	0.1900602	0.492173361	0.637708629	0.266640624	0.490415687
NF2	0.308618718	0.316933702	0.29721858	0.302038497	0.891726429	0.256625608
GIMAP8	0.308618718	0.360897508	0.269785804	0.500022989	0.612403767	0.302479349
SCYL2	0.308618718	0.231791372	0.79497298	0.481018469	0.269698277	0.319536891
PTPRS	0.308618718	0.23567727	0.487875367	0.356430136	0.487063655	0.462837324
HSD17B12	0.308618718	0.225929217	0.289119143	0.258376333	0.585254432	0.754218406

DHX58	0.309273128	0.304671591	0.678042414	0.291662687	0.527202266	0.277144273
RXFP3	0.309273128	0.195864427	0.510235798	0.512559037	0.53689463	0.327202343
NDUFA1	0.309273128	0.184794695	0.27649516	0.325529737	0.878451408	0.440866698
FBXO32	0.309273128	0.469272895	0.260752668	0.263114746	0.468542094	0.588384898
IDE	0.309273128	0.180248051	0.232158906	0.421668661	0.276583162	0.940017687
SMARCAL1	0.310369374	0.355271557	0.366681691	0.46682385	0.333691681	0.494676026
SH3BGR	0.310369374	0.579367653	0.323430342	0.281172702	0.252553732	0.628379498
RSPO2	0.310369374	0.302235128	0.3571052	0.30124858	0.240531754	0.862925606
TMEM30A	0.310796432	0.282826079	0.883950091	0.284249009	0.362632321	0.260904103
HIGD1B	0.310796432	0.526023059	0.264746077	0.275712238	0.228803825	0.78087541
CUBN	0.310881155	0.234383429	0.731960288	0.451094074	0.385800515	0.290497904
CYCS	0.310881155	0.244767648	0.43314299	0.265322159	0.462744229	0.67453786
EXD3	0.311257295	0.1803923	0.45863002	0.761359847	0.334605421	0.35938146
SLC5A6	0.311257295	0.370475687	0.332000222	0.279658152	0.445159316	0.616715863
SELENOK	0.311294847	0.198150337	0.481165053	0.886312303	0.309564851	0.243648755
CROCC2	0.311294847	0.23567727	0.525415185	0.415920568	0.658667583	0.268031409
ACAP3	0.311294847	0.210348867	0.390642802	0.896116841	0.238463867	0.348834279
TPPP	0.311294847	0.242912313	0.310213331	0.267416955	0.887514169	0.377627541
ENPP6	0.311294847	0.530945639	0.224019804	0.259195167	0.52068239	0.512846657
RWDD1	0.311294847	0.195717367	0.372748001	0.383208814	0.324909457	0.811296955
ERBB2	0.311294847	0.250888294	0.221817649	0.265078026	0.392114522	0.919124527
SURF4	0.311619936	0.309937145	0.294593768	0.888687722	0.228803825	0.348834279
SLC2A9	0.311655506	0.253046295	0.860877703	0.328845482	0.309564851	0.330239393
TXK	0.313615567	0.210977965	0.298472088	0.54857232	0.352530635	0.690728863
PDHX	0.314267328	0.244044595	0.279067921	0.348361463	0.547116225	0.673985072
HERPUD1	0.315389662	0.185060987	0.221817649	0.632683273	0.833864947	0.29173988
GNAI3	0.315836263	0.272971706	0.745084982	0.575014007	0.302323003	0.236488033
C14H8orf37	0.315836263	0.582560314	0.222288059	0.689227896	0.261642081	0.329035696
SCEL	0.315836263	0.360897508	0.513648728	0.493932071	0.339325818	0.338075577
LOC518495	0.315836263	0.194603254	0.384796164	0.432621514	0.520750873	0.57229939
TMEM97	0.315836263	0.236872105	0.497091612	0.281172702	0.472139664	0.620459631
CPM	0.315836263	0.231791372	0.320164496	0.707331521	0.243749249	0.621946029
GNB1L	0.3163122	0.416680499	0.668824581	0.435037147	0.264393955	0.310738754
LOC782264	0.3163122	0.21859524	0.211515827	0.786943075	0.611733649	0.324132086
MRPS21	0.3163122	0.250888294	0.397871714	0.277921392	0.85185197	0.33731134
IRF4	0.3163122	0.254909017	0.605687128	0.685601134	0.237094681	0.342620721
HSPA4	0.3163122	0.186677274	0.289119143	0.686487857	0.501311839	0.440866698
ERICH3	0.3163122	0.224822033	0.798119213	0.265322159	0.285914544	0.589397695

ITGAD	0.3163122	0.190906176	0.462410139	0.48880446	0.370917594	0.614425574
CCR2	0.3163122	0.242654059	0.372748001	0.329491543	0.521036349	0.627691291
SBF1	0.31696478	0.244220572	0.758146087	0.331269626	0.450288791	0.321909778
LOC112448105	0.318054496	0.238862972	0.289119143	0.557634977	0.332977669	0.699160709
ALDH18A1	0.318202821	0.22327969	0.289119143	0.384581248	0.627206089	0.609692015
OTUD6B	0.318356149	0.18508888	0.663513753	0.677374927	0.339983944	0.30198063
CASZ1	0.318356149	0.195864427	0.3167578	0.739727518	0.450288791	0.407114014
RRP7A	0.318743004	0.436471501	0.487875367	0.362867589	0.265743557	0.515726083
BYSL	0.318745194	0.213876395	0.579487023	0.459073822	0.342493459	0.520859926
PTX3	0.318745194	0.265713346	0.55950158	0.263114746	0.469323472	0.554291688
MCRIP2	0.319905365	0.319475864	0.397871714	0.263148753	0.736942681	0.398656825
SLC16A11	0.32094457	0.244044595	0.23978124	0.899552507	0.351411609	0.345042155
LSMEM1	0.32094457	0.25871338	0.293310696	0.911874416	0.236546924	0.392885476
TMEM236	0.32094457	0.18820162	0.266676602	0.825279787	0.403447621	0.442234422
AK2	0.32094457	0.234383429	0.289119143	0.798687063	0.246714398	0.587132866
LOC618456	0.321665312	0.199406542	0.34502002	0.867235478	0.346226314	0.352283712
ITGB8	0.321665312	0.454880625	0.22372518	0.680403382	0.278401607	0.45725625
FAM234A	0.321859734	0.340788135	0.266796445	0.291662687	0.935286646	0.281550325
PALB2	0.321859734	0.243922694	0.947651663	0.318413172	0.257404016	0.339003717
FLT3	0.321859734	0.193198829	0.64236045	0.408838245	0.361598024	0.544955425
ECSIT	0.322084685	0.259930339	0.361922003	0.269025298	0.879395665	0.35938146
PPP1R14B	0.322084685	0.358391448	0.413814922	0.302038497	0.467557398	0.539240716
AARSD1	0.322084685	0.213995435	0.555640272	0.317712944	0.269698277	0.806714845
LOC104970180	0.322084685	0.306854565	0.233921638	0.271515526	0.294404294	0.958790968
OSBPL11	0.322224222	0.198150337	0.225275137	0.941935077	0.535275659	0.242286545
FFAR3	0.322238875	0.213876395	0.29721858	0.362359041	0.802592922	0.481287134
CXCL16	0.32241625	0.230072979	0.237224665	0.808073654	0.605982335	0.290733382
TMEM64	0.32241625	0.214597731	0.371177512	0.364040151	0.900893337	0.297599027
GPBP1	0.32241625	0.250888294	0.225248779	0.318413172	0.936921784	0.377503548
ICAM3	0.32241625	0.210348867	0.332000222	0.632683273	0.476090036	0.472073165
ZNF555	0.322951977	0.262861959	0.361922003	0.322717781	0.472808893	0.700459582
FTSJ3	0.323724704	0.53848189	0.388358382	0.384581248	0.403447621	0.346280441
NOG	0.324002739	0.18820162	0.810647419	0.352535951	0.547116225	0.294865582
LOC112446791	0.324002739	0.651269643	0.364716867	0.462409524	0.240531754	0.396383896
ALKBH1	0.324425264	0.309937145	0.242685181	0.271515526	0.988238252	0.262707378
CCT5	0.32509361	0.21859524	0.420557205	0.588166711	0.332977669	0.605408237
REP15	0.325125762	0.213995435	0.289119143	0.618518556	0.372309454	0.669558041
ST3GAL5	0.325299016	0.194603254	0.289119143	0.808075336	0.583449841	0.321909778

LOC101908359	0.325299016	0.441931708	0.225248779	0.606611279	0.487063655	0.345042155
ARL4C	0.325299016	0.235205618	0.289119143	0.948668643	0.290976138	0.348834279
PARD6G	0.325299016	0.382320412	0.588125041	0.410993929	0.338817987	0.380806889
CRISPLD2	0.325299016	0.26595575	0.275234017	0.362359041	0.823508064	0.405793019
FHL5	0.325299016	0.563985547	0.332000222	0.324425579	0.404092468	0.458773762
LRRC59	0.325299016	0.261646566	0.46838416	0.451094074	0.252295227	0.739579536
CDK2AP1	0.325299016	0.213876395	0.372748001	0.451094074	0.35445418	0.762133815
TAGLN	0.325299016	0.321010792	0.332290029	0.327763847	0.274138218	0.864572584
ABCF2	0.325299016	0.252029632	0.33824575	0.383208814	0.269698277	0.874000314
LRRC8D	0.325299016	0.220882672	0.289119143	0.287569241	0.240993303	0.997862553
BOP1	0.325966328	0.319372498	0.40651486	0.297613285	0.383253602	0.734177434
MAP1LC3C	0.327969425	0.42120516	0.344420176	0.852170016	0.271454139	0.256650336
POMT1	0.32836495	0.319372498	0.545412419	0.554743688	0.375347002	0.332111976
ILVBL	0.32898842	0.213876395	0.345787032	0.318413172	0.844434232	0.458773762
CNN1	0.32898842	0.718258172	0.295198472	0.31780038	0.243749249	0.60624915
UQCR10	0.32898842	0.234275431	0.315377577	0.287569241	0.727256509	0.636866913
SIRPA	0.32898842	0.709247882	0.225417227	0.271515526	0.290976138	0.684524441
CIITA	0.329185761	0.250888294	0.273691978	0.469897262	0.795839201	0.380806889
C8H9orf131	0.329464515	0.265621994	0.372748001	0.368185783	0.517239659	0.616715863
ARHGAP20	0.329900021	0.358391448	0.532415258	0.504242357	0.413792445	0.325689296
EXTL1	0.329900021	0.337247845	0.286602304	0.707217807	0.380405851	0.417777723
CXCL13	0.329900021	0.343951817	0.238925543	0.795917151	0.30731562	0.458100647
SELL	0.329900021	0.258519684	0.628904157	0.393287802	0.403447621	0.458773762
LPCAT3	0.329900021	0.314377732	0.315377577	0.328845482	0.27339973	0.893838339
LOC107131975	0.330850363	0.21859524	0.939948201	0.460818045	0.257179584	0.309878132
DHRS1	0.331266653	0.350288067	0.69621934	0.301391172	0.294404294	0.525913971
TRIB2	0.332165892	0.307822412	0.22372518	0.885789665	0.450726215	0.30198063
ANGPTL5	0.333170529	0.355271557	0.234199204	0.546397256	0.288124662	0.748921056
SLC16A3	0.333298394	0.301414099	0.289119143	0.636027046	0.585398487	0.345042155
TMEM182	0.333832452	0.303936393	0.369656108	0.271515526	0.602648954	0.645719071
TYRP1	0.333832452	0.241463151	0.224350773	0.382057193	0.697778495	0.668191006
PLCB4	0.333890544	0.474946062	0.507083871	0.271515526	0.269698277	0.645719071
TLR7	0.334390297	0.262279886	0.290716943	0.777942892	0.593931025	0.279393335
RBM43	0.334390297	0.388315576	0.34510985	0.409020797	0.727256509	0.300599914
PEX6	0.334390297	0.200858277	0.759332589	0.518542038	0.429015291	0.310534477
LOC506828	0.334390297	0.242912313	0.497091612	0.876355536	0.253527686	0.327157687
VASN	0.334390297	0.228936145	0.266676602	0.930904695	0.396504336	0.332111976
HSPA5	0.334390297	0.239130734	0.255362729	0.780703865	0.560554936	0.348834279

SERINC5	0.334390297	0.321010792	0.345465441	0.561120526	0.472139664	0.43348847
MPV17L2	0.334390297	0.296106068	0.457051074	0.486381297	0.389234007	0.519240641
EIF4E	0.334390297	0.216060513	0.732274253	0.447005055	0.257404016	0.589397695
PANK1	0.334390297	0.267118782	0.409955029	0.275712238	0.524981289	0.702623424
CHST7	0.334897874	0.216060513	0.224350773	0.594931919	0.939343714	0.25090893
ANKRD17	0.334897874	0.244044595	0.230877992	0.284249009	0.999985752	0.261631306
ENPP1	0.334897874	0.258714703	0.307792941	0.340792236	0.9683846	0.270183276
GRIA1	0.334897874	0.196453729	0.911103174	0.519487474	0.317603468	0.286227386
LOC112445031	0.334897874	0.270970676	0.653833671	0.611379189	0.338817987	0.324230326
TIMM8B	0.334897874	0.25413552	0.503655463	0.30124858	0.738738532	0.394759278
ZSWIM4	0.334897874	0.213995435	0.286963816	0.775617102	0.332977669	0.622811146
LOC100847365	0.334897874	0.250992446	0.305210577	0.377558079	0.457135312	0.783241453
DIRAS2	0.334897874	0.213876395	0.332000222	0.58293921	0.294404294	0.79187813
ARL11	0.335744708	0.213876395	0.744735369	0.720787315	0.301814666	0.275185976
PLEKHG2	0.335744708	0.213876395	0.34510985	0.819968583	0.56180439	0.290733382
CCL21	0.335744708	0.709247882	0.252125559	0.672328815	0.258559309	0.318988997
LOC100139881	0.335744708	0.284832431	0.492173361	0.534082333	0.558285349	0.319622661
CAP2	0.335744708	0.694400411	0.384796164	0.47137884	0.300638192	0.332111976
LOC784289	0.335744708	0.227667869	0.29721858	0.699481317	0.629049686	0.352283712
NUAK1	0.335744708	0.204511711	0.340470457	0.454022278	0.854966579	0.370594065
ZNF771	0.335744708	0.207320457	0.333951286	0.908467146	0.314668689	0.409448021
AMT	0.335744708	0.202942544	0.565148263	0.561398298	0.294404294	0.621946029
RAD51D	0.335744708	0.202942544	0.266676602	0.58293921	0.533903668	0.65014676
LOC104974460	0.335744708	0.54845263	0.424739024	0.302038497	0.259605072	0.658600906
FARSA	0.335744708	0.332081544	0.455195587	0.406608123	0.257179584	0.752070741
SNAP23	0.335744708	0.202942544	0.472521949	0.383208814	0.403447621	0.75702988
LOC104973154	0.335744708	0.225201246	0.255362729	0.611320534	0.265743557	0.865767172
PCDH1	0.33609689	0.228114725	0.284113209	0.845867148	0.553835124	0.316375257
PLEKHG4	0.33609689	0.265514731	0.501988763	0.729705452	0.374313394	0.321909778
VASP	0.33609689	0.234266964	0.444582039	0.328845482	0.658667583	0.560879865
GUCD1	0.33623318	0.355271557	0.73091642	0.527867968	0.294404294	0.310534477
CD200	0.336318834	0.213876395	0.958410013	0.421401825	0.275770932	0.329035696
CKAP2L	0.337929749	0.23567727	0.755135544	0.460818045	0.352171754	0.402562285
PIK3R5	0.337929749	0.202942544	0.41426165	0.486381297	0.649653657	0.481287134
TRAM1	0.337929749	0.250992446	0.532415258	0.447895467	0.4182393	0.542061576
NDUFA5	0.337929749	0.258714703	0.369656108	0.326279159	0.571784062	0.690728863
ECD	0.338798949	0.202942544	0.371177512	0.284249009	0.848550619	0.552257767
MADCAM1	0.339314487	0.231791372	0.227605298	0.999981514	0.280065531	0.348834279

PRPS2	0.339314487	0.355271557	0.376259378	0.537956207	0.476961495	0.418772572
PPP1R10	0.339966732	0.298195068	0.255362729	0.904018489	0.457358633	0.283505942
ZBED5	0.343478649	0.260376907	0.315872098	0.744278279	0.560554936	0.338075577
NKTR	0.343478649	0.222998185	0.385796308	0.799480811	0.365608028	0.442234422
TBX1	0.343498125	0.589431944	0.43865289	0.561120526	0.256619651	0.348834279
LOC112441452	0.344010632	0.242912313	0.234199204	0.999981514	0.280788411	0.268031409
GALNT10	0.344010632	0.667258304	0.518829767	0.302572327	0.429015291	0.294660282
MTERF2	0.344010632	0.393018763	0.397871714	0.306245142	0.792867703	0.336632335
C2CD4B	0.344010632	0.203905324	0.498347424	0.806573529	0.403447621	0.338075577
HDC	0.344010632	0.386477216	0.466192774	0.654131191	0.301814666	0.380194245
INPP5F	0.344010632	0.256868883	0.596826854	0.324423853	0.457770074	0.613401904
MUSTN1	0.344010632	0.553132278	0.40835816	0.302618861	0.28418483	0.669558041
FOXO6	0.344010632	0.213876395	0.301630207	0.375351105	0.677185022	0.702623424
IQGAP2	0.344010632	0.242912313	0.332000222	0.328845482	0.626973048	0.721364949
TOMM70	0.344010632	0.283879605	0.56863752	0.351407996	0.260791746	0.775725294
SP100	0.344010632	0.319654425	0.261891147	0.351221202	0.338817987	0.90969547
SPCS1	0.344715387	0.245106054	0.580869674	0.282975368	0.385312392	0.754218406
OAS2	0.346266786	0.299590998	0.507787875	0.462657114	0.472808893	0.45725625
TAS1R3	0.34650191	0.434898742	0.611647147	0.613568161	0.313855706	0.269435809
MEG3	0.34650191	0.29496073	0.489549483	0.645168798	0.40786268	0.358352261
SYCP3	0.34650191	0.415447733	0.425565437	0.700026235	0.28418483	0.37954335
USP10	0.34650191	0.21952118	0.596931535	0.653503626	0.294404294	0.500317514
CXXC5	0.34654614	0.283879605	0.65062204	0.459828217	0.321162042	0.521791376
PRPF19	0.34654614	0.227300713	0.596931535	0.503087647	0.321162042	0.631731716
CRIM1	0.346887977	0.383384374	0.345787032	0.314962476	0.879776073	0.310738754
CYP2J2	0.346887977	0.324238107	0.71967891	0.309234137	0.555907302	0.339184707
SEMA4F	0.346887977	0.382853453	0.382449111	0.809871372	0.27339973	0.35938146
ARHGDIA	0.346887977	0.51352654	0.414110931	0.541206056	0.346226314	0.373692297
LOC101902570	0.346887977	0.311196652	0.41604117	0.627280162	0.445529822	0.41219904
ZFP36L1	0.346887977	0.442990825	0.457051074	0.519254756	0.338419262	0.442730925
MRPL11	0.346887977	0.250888294	0.560847675	0.302038497	0.493437841	0.645427231
PHACTR4	0.346887977	0.244044595	0.339614385	0.41814733	0.280065531	0.923807852
WDFY2	0.346976542	0.261979523	0.367733479	0.400088147	0.917407853	0.30198063
HMGCLL1	0.346976542	0.331055243	0.421777252	0.561120526	0.576970572	0.338075577
CDC25B	0.346976542	0.389385843	0.650613864	0.40243606	0.38204968	0.383090164
LOC514181	0.346976542	0.542918274	0.369656108	0.521237523	0.362257015	0.392988847
NECAB3	0.346976542	0.219469609	0.382449111	0.551641524	0.714597718	0.399528543
EIF3A	0.346976542	0.244044595	0.33920471	0.521237523	0.642796813	0.504596604

LOC789374	0.346976542	0.236790771	0.700963878	0.495562454	0.277542999	0.584339668
ITK	0.346976542	0.234275431	0.528387114	0.324273469	0.484413225	0.690728863
MTRF1	0.346976542	0.238862972	0.489268443	0.322881726	0.344401523	0.852459193
SELENOF	0.346976542	0.227667869	0.397871714	0.282975368	0.282111398	0.981216824
TUBB2B	0.347756942	0.292636255	0.487875367	0.828736799	0.362632321	0.279897622
CLDN10	0.347756942	0.382853453	0.402972276	0.640315346	0.509805204	0.289036125
CARMIL2	0.347756942	0.232696386	0.89478142	0.317775964	0.437165526	0.360629136
RND2	0.347756942	0.227667869	0.478270125	0.339974682	0.626503214	0.62493158
ASCL2	0.347756942	0.267118782	0.492173361	0.332251447	0.383253602	0.764310896
HTR2B	0.347980489	0.610379943	0.485824122	0.284249009	0.276583162	0.608200832
UCK2	0.34899899	0.311196652	0.351350646	0.309234137	0.337606409	0.908768184
BMF	0.349361229	0.303250787	0.457350984	0.724431807	0.426006781	0.338075577
TRADD	0.349361229	0.980289052	0.273691978	0.318413172	0.300638192	0.345042155
PHOSPHO2	0.35003361	0.262279886	0.328810186	0.348123948	0.301814666	0.952571698
PLCG2	0.350527789	0.984179983	0.388358382	0.302038497	0.262502754	0.289777696
GUCY1A1	0.35084641	0.230072979	0.759332589	0.387086654	0.392368763	0.495197104
ADM	0.352015823	0.228114725	0.28201526	0.438595551	0.946935016	0.357416359
COL21A1	0.352715117	0.238810433	0.458004431	0.606102383	0.61565225	0.360100385
CENPU	0.352715117	0.293898381	0.43164239	0.627280162	0.515394956	0.369797989
UGGT2	0.352715117	0.212575402	0.384210252	0.594220399	0.583449841	0.520859926
SLAMF1	0.353182725	0.285585459	0.710025682	0.30124858	0.705288263	0.318838278
AGPAT2	0.353604296	0.416680499	0.342240037	0.328845482	0.596079788	0.564822443
TESPA1	0.353604296	0.234383429	0.566069604	0.552451497	0.308741687	0.63863812
FBXO16	0.354075149	0.238862972	0.319745024	0.834401906	0.591306131	0.310534477
ADM5	0.354207497	0.382853453	0.355474568	0.758601772	0.463102881	0.297599027
CARD9	0.354207497	0.447485891	0.854567571	0.371678509	0.280065531	0.322425896
CCDC84	0.354207497	0.259930339	0.876414692	0.460818045	0.32659787	0.346280441
CCL22	0.355141554	0.321117458	0.411289123	0.886312303	0.365608028	0.275185976
INAFM2	0.355141554	0.238862972	0.754913805	0.348123948	0.571639895	0.380806889
LOC100847700	0.355652212	0.319220716	0.501808639	0.309234137	0.473692248	0.670089951
SERPINE2	0.355710178	0.465004933	0.427325038	0.535163392	0.503487214	0.310534477
TNFRSF11A	0.355710178	0.276295569	0.821683829	0.575014007	0.271454139	0.348834279
LOC101907302	0.355710178	0.222843742	0.263693478	0.964521432	0.391810273	0.385223859
SCN8A	0.355710178	0.275831305	0.335979698	0.487829118	0.707939546	0.470494326
DDX21	0.356589546	0.222998185	0.361922003	0.527867968	0.838137243	0.35938146
ICOSLG	0.356589546	0.644366672	0.320591795	0.342952467	0.583449841	0.36598082
TIMM10	0.356589546	0.213929789	0.289119143	0.528783909	0.844434232	0.442234422
CYHR1	0.35666582	0.594126609	0.533570754	0.317775964	0.484413225	0.332111976

NFASC	0.35666582	0.635138243	0.56802023	0.379699598	0.294404294	0.380806889
PSMD12	0.35666582	0.244044595	0.632141336	0.345572957	0.609470399	0.477997005
LOC112446426	0.357316668	0.395925974	0.301630207	0.588166711	0.655556663	0.338075577
RET	0.357316668	0.244044595	0.312920161	0.58293921	0.810436875	0.355246637
PDGFA	0.357316668	0.23567727	0.608218151	0.387086654	0.625219099	0.448702315
TMEM176B	0.357316668	0.34639555	0.361922003	0.764257755	0.268785569	0.550386028
INHA	0.357316668	0.225929217	0.507675433	0.355914547	0.292383704	0.903773776
LOC513779	0.357803254	0.811186016	0.365633542	0.486381297	0.361598024	0.275185976
SFI1	0.357803254	0.250888294	0.694535506	0.730712781	0.332977669	0.321909778
RYBP	0.357803254	0.258714703	0.475049246	0.438595551	0.823508064	0.329035696
GBP6	0.357803254	0.227667869	0.29885897	0.347765608	0.998287238	0.337984312
RCOR1	0.357803254	0.219308294	0.264746077	0.440192972	0.985250243	0.338075577
ATP13A2	0.357803254	0.347147986	0.48405583	0.521280234	0.578333624	0.346280441
LRRC51	0.357803254	0.244669582	0.665599776	0.777956162	0.285241994	0.352283712
CRELD2	0.357803254	0.533735495	0.290289905	0.659998592	0.323388588	0.440866698
ZNF184	0.357803254	0.382744251	0.409955029	0.640315346	0.301814666	0.524899551
SEPT1	0.357803254	0.275387717	0.663513753	0.356392963	0.422942299	0.59373266
RDH13	0.357803254	0.294802047	0.596931535	0.30124858	0.472139664	0.6467931
LOC782598	0.357803254	0.250888294	0.385007812	0.640315346	0.301402937	0.733767439
TM9SF3	0.357803254	0.283879605	0.332000222	0.362359041	0.502432219	0.793197037
S1PR5	0.358618004	0.306854565	0.513255922	0.718713712	0.405256527	0.338075577
MT1E	0.358663	0.412566059	0.456681692	0.317775964	0.463102881	0.640128675
LOC531747	0.359465428	0.228114725	0.369656108	0.705801894	0.309564851	0.723555931
LOC100847509	0.359512154	0.293520391	0.466192774	0.299601069	0.943702351	0.302949937
PDPK1	0.359512154	0.314389928	0.388358382	0.839725231	0.427233204	0.318838278
CENPB	0.359512154	0.231981784	0.81412252	0.335976936	0.463102881	0.470494326
TSSC4	0.359512154	0.36901158	0.397871714	0.297696608	0.571639895	0.669558041
LOC100848703	0.359838363	0.234275431	0.352295049	0.391160166	0.964301327	0.331473604
CCL2	0.359838363	0.242654059	0.289119143	0.999981514	0.302255232	0.345042155
MPP7	0.359838363	0.940500178	0.323430342	0.317775964	0.294404294	0.415053221
EFNB1	0.359838363	0.254897553	0.345465441	0.66637376	0.376990283	0.666011114
LOC100297044	0.360374055	0.244220572	0.562973926	0.811200887	0.428442971	0.290733382
LOC112445995	0.360374055	0.40372842	0.507675433	0.56187044	0.530344927	0.290733382
PTP4A3	0.360374055	0.314240268	0.29721858	0.819968583	0.533903668	0.336632335
AMD1	0.360374055	0.492794915	0.310480699	0.686250223	0.365608028	0.404708082
PFKFB4	0.360374055	0.733318711	0.332000222	0.416593832	0.376447594	0.428606899
FAP	0.360374055	0.232599148	0.584710697	0.424465138	0.652333594	0.440866698
GIMAP7	0.360374055	0.360723284	0.69881939	0.349464413	0.389657332	0.49105688

TMEM61	0.360374055	0.539106348	0.497711491	0.36230762	0.355490976	0.51212265
LOC100848246	0.360374055	0.461008609	0.315377577	0.3067656	0.659596244	0.551945488
PGP	0.360374055	0.320494959	0.457051074	0.416353908	0.541564331	0.574870258
PPARD	0.360374055	0.391697369	0.365095767	0.356392963	0.487063655	0.684994779
RAB5IF	0.360911704	0.319661234	0.457350984	0.356392963	0.459765599	0.72026427
CLINT1	0.361234551	0.826102395	0.303361099	0.349464413	0.332977669	0.491178906
KCNH2	0.361742059	0.808823388	0.315377577	0.365925543	0.370463024	0.427891722
SERP1	0.361817813	0.243922694	0.55950158	0.562572875	0.313855706	0.668851588
LOC100848699	0.362037928	0.943859632	0.296009287	0.480970947	0.308741687	0.289777696
JAK1	0.362037928	0.225201246	0.317443337	0.893051204	0.612403767	0.306319401
TUBA1A	0.362037928	0.38025903	0.552362414	0.729705452	0.307379953	0.339003717
KIAA1147	0.362037928	0.564100429	0.397871714	0.381249085	0.605738596	0.345042155
NR2F2	0.362037928	0.550226079	0.324612516	0.774800098	0.297903462	0.348834279
PSRC1	0.362037928	0.22327969	0.298472088	0.812453944	0.677185022	0.348834279
LUZP1	0.362037928	0.398295321	0.301630207	0.318413172	0.895067787	0.380806889
CD79A	0.362037928	0.334867943	0.398176384	0.861804954	0.282111398	0.427891722
RASGRP3	0.362037928	0.225929217	0.279067921	0.695125147	0.715786729	0.448443129
LOC112443499	0.362037928	0.235596866	0.612049436	0.472217546	0.523715832	0.487514327
SLC14A1	0.362037928	0.360897508	0.549193746	0.359013436	0.523124197	0.495197104
ZSWIM2	0.362037928	0.358391448	0.29721858	0.563228658	0.583449841	0.503701037
GTF2A2	0.362037928	0.227667869	0.411289123	0.381373394	0.32659787	0.93363377
RBM5	0.362037928	0.225201246	0.326181902	0.400190066	0.291544159	0.988796537
LOC101902444	0.363592158	0.309224335	0.332000222	0.424465138	0.455516234	0.795936781
LOC112441663	0.364352552	0.231981784	0.267818833	0.954946234	0.570107746	0.303619975
DCAF13	0.365051747	0.22327969	0.608218151	0.819571278	0.301238606	0.418173662
TRPC1	0.365051747	0.29496073	0.305569839	0.326345349	0.593931025	0.823031765
PDLIM7	0.365433126	0.29744634	0.363556211	0.820971736	0.428442971	0.396383896
LOC104976804	0.365433126	0.228114725	0.431636322	0.821450642	0.292356966	0.601500908
LIX1	0.365434148	0.295888679	0.268771009	0.622980329	0.803705802	0.36598082
LOC511847	0.366457086	0.273174055	0.341972274	0.318413172	0.986003146	0.337984312
LOC112448523	0.366643984	0.373964414	0.481165053	0.857837616	0.290907475	0.332111976
CCL11	0.366643984	0.323460072	0.556512199	0.76992117	0.337153403	0.345042155
CCNG1	0.366643984	0.232696386	0.372748001	0.410993929	0.357970049	0.925273613
GCHFR	0.367391659	0.242912313	0.405262458	0.598316303	0.716994185	0.404910071
IFRD1	0.367391659	0.292905378	0.411289123	0.47562064	0.735091896	0.43348847
RAPGEFL1	0.367447997	0.346488221	0.56802023	0.680884552	0.353179334	0.369942484
P4HB	0.367447997	0.387607688	0.45863002	0.586033659	0.307379953	0.601371091
ATP5MC1	0.367447997	0.324304652	0.443690681	0.306600881	0.560554936	0.72493952

LOC782675	0.3677093	0.753090681	0.280525198	0.534082333	0.300638192	0.482980366
GSC	0.367745128	0.579367653	0.693546993	0.34981443	0.364333362	0.345042155
FCHO1	0.367916896	0.225929217	0.71601562	0.486381297	0.527202266	0.419810556
CYTIP	0.367916896	0.228936145	0.814495418	0.46351377	0.35856792	0.503701037
DUSP12	0.368659566	0.36901158	0.705746499	0.328845482	0.678703442	0.297599027
HOXD3	0.368659566	0.26595575	0.33920471	0.808075336	0.516525043	0.396383896
ACER2	0.368778159	0.317357075	0.401354374	0.888687722	0.341397863	0.363651539
ZNF215	0.368778159	0.244044595	0.333951286	0.457902257	0.674089143	0.673210328
CCNC	0.368778159	0.227441371	0.542266375	0.438595551	0.321162042	0.841312694
PAK3	0.369086388	0.54021232	0.266796445	0.708604007	0.489930391	0.321909778
SNRPA	0.369086388	0.314389928	0.478270125	0.784595865	0.429015291	0.338075577
MMP28	0.369312656	0.358391448	0.312920161	0.424465138	0.860495437	0.380806889
EGFLAM	0.370792384	0.460244277	0.289119143	0.356392963	0.929896768	0.305566786
IL11RA	0.370792384	0.796253817	0.419761137	0.43260638	0.301814666	0.396383896
FAM118A	0.370792384	0.276679556	0.286602304	0.424465138	0.533903668	0.835231445
NUP98	0.371128673	0.265621994	0.369656108	0.34981443	0.996781978	0.290733382
NHSL2	0.371128673	0.589782216	0.603530331	0.457715968	0.407114819	0.300134855
MEOX2	0.371128673	0.262279886	0.309575181	0.794738001	0.723467697	0.310738754
LPP	0.371128673	0.846724749	0.332000222	0.549287144	0.294404294	0.343317756
MTR	0.371128673	0.293442873	0.332000222	0.913946495	0.421697748	0.348834279
ARVCF	0.371128673	0.764456895	0.312920161	0.399790374	0.487063655	0.369942484
LOC101907041	0.371128673	0.23567727	0.332000222	0.553146279	0.776537165	0.495999863
UBL3	0.371128673	0.230102595	0.312920161	0.626698454	0.719783218	0.513034959
CLDN11	0.371128673	0.347147986	0.677197181	0.379613587	0.311008295	0.669090959
C1R	0.371305055	0.506509735	0.328810186	0.379699598	0.823508064	0.321909778
LOC112445197	0.371305055	0.267980827	0.552362414	0.627280162	0.560554936	0.348834279
STK38	0.371305055	0.337583001	0.492173361	0.362359041	0.812997556	0.349527203
P2RY13	0.371305055	0.709247882	0.298472088	0.505857338	0.332977669	0.504596604
KLHL42	0.371921402	0.821674572	0.339614385	0.505499977	0.357092245	0.338075577
PARS2	0.371921402	0.247784456	0.308651338	0.457902257	0.961465747	0.348834279
SLA2	0.371921402	0.625469932	0.40651486	0.43080152	0.362257015	0.503701037
MEG8	0.371921402	0.258714703	0.681918771	0.598316303	0.308741687	0.557166513
LOC107133071	0.371921402	0.25796707	0.410561869	0.730052184	0.365608028	0.614511437
AKIRIN1	0.371921402	0.445080617	0.622387278	0.400088147	0.300638192	0.616114905
CCDC77	0.371921402	0.228936145	0.507083871	0.691020007	0.337606409	0.637719217
TMEM230	0.372781332	0.234383429	0.531780431	0.324423853	0.383253602	0.887722423
MRPL55	0.373369962	0.412566059	0.392757065	0.346904474	0.603965612	0.616975059
LOC100848598	0.373565193	0.370464508	0.476775388	0.456103613	0.395278001	0.668851588

C7H19orf70	0.373638929	0.322451563	0.458004431	0.317775964	0.846232586	0.440866698
MTAP	0.374394566	0.232599148	0.353392001	0.71886505	0.735485629	0.36598082
EOMES	0.376028552	0.319475864	0.621702102	0.550189288	0.386673096	0.490415687
MOK	0.376210371	0.250888294	0.745477682	0.600166011	0.301402937	0.51212265
KALRN	0.376508202	0.416680499	0.363342023	0.627280162	0.656015118	0.318838278
CHST13	0.376856043	0.311196652	0.342240037	0.859320133	0.508493183	0.339003717
LOC101908166	0.376856043	0.985997726	0.27649516	0.361976563	0.308741687	0.380806889
SPDYA	0.376856043	0.52001926	0.36648605	0.322717781	0.723054375	0.440866698
ULK3	0.376856043	0.23567727	0.598652788	0.654205968	0.307325498	0.632381609
AK6	0.376856043	0.386477216	0.407806986	0.383208814	0.338419262	0.832964812
RTP4	0.377085177	0.411108591	0.545866468	0.358687597	0.788007619	0.309878132
CD248	0.377102824	0.340143691	0.291173692	0.98673849	0.383253602	0.302218528
IL17RD	0.379206676	0.315723479	0.416802987	0.356220406	0.817950097	0.491178906
TWIST2	0.38059558	0.950986475	0.369656108	0.40243606	0.333691681	0.316375257
LPGAT1	0.38059558	0.303250787	0.507675433	0.510246043	0.788995893	0.327157687
WNT2	0.38059558	0.762563464	0.627295843	0.359644843	0.303418372	0.348834279
MIS18BP1	0.38059558	0.340788135	0.641535145	0.547780857	0.463377166	0.383090164
LOC527186	0.38059558	0.344296647	0.424739024	0.81112438	0.308741687	0.495999863
LOC100196901	0.38059558	0.479687945	0.621702102	0.331269626	0.429580883	0.497388408
B4GALT7	0.38059558	0.333827203	0.518894161	0.335214405	0.479364681	0.721767685
L3MBTL2	0.38059558	0.231981784	0.435041359	0.383271918	0.571639895	0.79187813
IGFALS	0.38059558	0.262929823	0.382449111	0.328845482	0.504746184	0.877014986
LOC783577	0.381131255	0.370475687	0.376259378	0.504242357	0.840108119	0.313078883
PCP4L1	0.382345183	0.549410627	0.457350984	0.575014007	0.489930391	0.310534477
LOC112442284	0.382345183	0.337247845	0.722770256	0.383271918	0.583789489	0.377139284
ZNF432	0.382345183	0.919540627	0.289119143	0.328845482	0.356507789	0.494195557
ZNF512B	0.382345183	0.238810433	0.372748001	0.939604997	0.328111581	0.494666285
NSUN6	0.382345183	0.240559554	0.289119143	0.746075358	0.527222445	0.632510367
ADAM33	0.382345183	0.275387717	0.485339975	0.685953876	0.308741687	0.669558041
GRIK2	0.383705422	0.893164557	0.291173692	0.630951216	0.301402937	0.310534477
LOC112444310	0.383705422	0.430996205	0.615161884	0.451094074	0.618977366	0.310738754
INPP5J	0.383705422	0.489987041	0.607319392	0.691025178	0.307379953	0.313477414
SLC23A2	0.383705422	0.247784456	0.579487023	0.849834721	0.428442971	0.324132086
ZNF420	0.383705422	0.366070217	0.955845583	0.356430136	0.346226314	0.331790617
FAM160A2	0.383705422	0.338196075	0.327860062	0.653503626	0.74028971	0.348834279
LOC531090	0.383705422	0.658496391	0.627295843	0.383208814	0.333474676	0.396383896
ALDH1A2	0.383705422	0.264702075	0.694535506	0.596654953	0.453983763	0.415609752
CKAP4	0.383705422	0.283879605	0.332000222	0.959805738	0.346226314	0.420532744

CCDC78	0.383705422	0.481256695	0.55950158	0.575014007	0.332753067	0.431260036
ATG4B	0.383705422	0.265514731	0.376259378	0.873302281	0.361598024	0.508039178
MFSD9	0.383705422	0.36901158	0.439568256	0.424465138	0.553835124	0.621946029
PGLYRP2	0.383705422	0.321010792	0.376259378	0.638269781	0.437165526	0.622811146
MOG	0.383705422	0.415447733	0.499164335	0.418062303	0.309564851	0.751270885
GP9	0.383996794	0.251173336	0.400888251	0.797312233	0.657647754	0.336632335
STX12	0.383996794	0.360897508	0.369656108	0.521723045	0.786661228	0.380806889
KIF4A	0.383996794	0.285046056	0.570681722	0.612761692	0.413792445	0.521756022
MRPL33	0.384131481	0.279475276	0.80412682	0.34981443	0.501932218	0.48181553
RFLNB	0.384154025	0.254909017	0.466192774	0.541206056	0.881061976	0.309878132
LTN1	0.384154025	0.358391448	0.481165053	0.678657085	0.580738755	0.325934599
RAB24	0.384154025	0.471949819	0.332000222	0.893220599	0.351007552	0.329035696
MBNL2	0.384154025	0.265514731	0.286831175	0.899855526	0.473692248	0.462837324
RASAL3	0.384154025	0.521188472	0.509514515	0.365822386	0.499400667	0.468732955
MAP3K6	0.384411599	0.485778913	0.70011973	0.348361463	0.567274296	0.321909778
ROM1	0.384411599	0.364362847	0.40835816	0.764257755	0.500876883	0.348834279
ACAN	0.384411599	0.369900105	0.418121393	0.701248463	0.450726215	0.440866698
DIAPH3	0.384634566	0.326411558	0.744735369	0.708604007	0.361598024	0.306319401
NADSYN1	0.384634566	0.262279886	0.607319392	0.852170016	0.409444837	0.310738754
FAM189A2	0.384634566	0.369899828	0.328810186	0.588166711	0.823508064	0.329740076
PEX11B	0.384634566	0.245371433	0.317433455	0.371678509	0.999985752	0.333656027
STAC2	0.384634566	0.536598701	0.608218151	0.43066905	0.483224704	0.340708111
PABPN1	0.384634566	0.250270282	0.638444487	0.576955372	0.627206089	0.357801765
ZBTB22	0.384634566	0.317357075	0.32119214	0.799712183	0.603965612	0.380806889
RSPO1	0.384634566	0.338196075	0.290289905	0.565933683	0.810436875	0.424653761
SLC30A5	0.384634566	0.541287559	0.470895554	0.481277757	0.434714683	0.448702315
DNAH12	0.384634566	0.253046295	0.289119143	0.962350885	0.302323003	0.594058991
FLNA	0.384634566	0.560109317	0.361988752	0.421401825	0.332977669	0.735392644
GRHPR	0.384634566	0.313975784	0.535077764	0.328845482	0.508689889	0.742309541
LOC783686	0.384839774	0.267007856	0.472823059	0.482216802	0.719783218	0.507632407
EPRS	0.38713823	0.242654059	0.565148263	0.49605271	0.338419262	0.812774915
CACNB2	0.387411684	0.916484772	0.481165053	0.379699598	0.333691681	0.321909778
B3GNT6	0.387411684	0.418848099	0.45863002	0.627280162	0.596680559	0.329740076
LOC107131684	0.387411684	0.3323766	0.345465441	0.670507175	0.775582766	0.336632335
DERL1	0.387411684	0.321010792	0.929661805	0.424465138	0.334500692	0.380806889
CCR4	0.387411684	0.460387616	0.382449111	0.349464413	0.821351993	0.396383896
TM7SF2	0.387411684	0.337247845	0.366279622	0.421668661	0.852746309	0.451485662
IKZF1	0.387411684	0.355271557	0.71601562	0.398962161	0.362632321	0.619388281

TBC1D13	0.387411684	0.244044595	0.347279336	0.646556987	0.463102881	0.745988271
DHX57	0.387411684	0.244841775	0.376259378	0.652644879	0.30731562	0.85532952
SOBP	0.387766204	0.461008609	0.374446792	0.37131062	0.887514169	0.328157876
SLC22A3	0.387766204	0.275387717	0.849389234	0.537984757	0.429015291	0.345042155
CCER2	0.387766204	0.580473137	0.376259378	0.77872142	0.321291154	0.351731967
SELENOP	0.387766204	0.242912313	0.440056291	0.813323291	0.578296318	0.380349323
MFSD8	0.387766204	0.283879605	0.298472088	0.716077462	0.712053021	0.442234422
STARD9	0.387766204	0.250270282	0.541610391	0.627280162	0.338419262	0.701800445
EIF2S1	0.388278168	0.25080363	0.813441395	0.55931035	0.333691681	0.503406995
COX7C	0.388960045	0.307914859	0.369656108	0.346904474	0.949311988	0.440512821
CNTROB	0.389019409	0.264702075	0.70638908	0.82868625	0.337806259	0.335662282
CPSF1	0.389019409	0.314377732	0.829751489	0.627280162	0.346226314	0.343317756
CELF1	0.389019409	0.29496073	0.988390948	0.362359041	0.309564851	0.40223864
KCNG1	0.389019409	0.443435932	0.307792941	0.869208308	0.362632321	0.428431098
SERINC2	0.389019409	0.469660184	0.674950575	0.359013436	0.491185174	0.428431098
NDUFS3	0.389019409	0.311196652	0.388358382	0.356392963	0.878451408	0.512846657
DENR	0.389019409	0.367446738	0.69454186	0.387140485	0.405207591	0.587132866
FABP1	0.389019409	0.580905654	0.361922003	0.333677631	0.541176598	0.621946029
DSTN	0.389019409	0.641707362	0.384796164	0.375351105	0.321162042	0.724693895
GNB3	0.389019409	0.249084198	0.442622095	0.731537809	0.321162042	0.737726964
IFRD2	0.389019409	0.311196652	0.33920471	0.441489462	0.533807401	0.819366282
SIT1	0.389019409	0.292332168	0.654545673	0.362867589	0.317509486	0.837789732
LOC101906664	0.389019409	0.262672636	0.346006247	0.451094074	0.484413225	0.872238439
UFM1	0.389019409	0.258714703	0.606660869	0.348361463	0.338817987	0.887722423
GABBR1	0.389358735	0.244115226	0.466192774	0.78116296	0.308741687	0.684994779
MANF	0.390070003	0.371049704	0.342240037	0.784595865	0.563212168	0.367606057
UFSP1	0.390592769	0.358391448	0.369656108	0.406608123	0.669123866	0.667758333
ZNF436	0.390906088	0.553132278	0.332000222	0.910575108	0.308741687	0.310738754
SLC25A51	0.390906088	0.528669672	0.552162548	0.418062303	0.56690435	0.362138574
MCEMP1	0.390906088	0.411108591	0.648593816	0.513533242	0.389922765	0.481287134
DHX29	0.390906088	0.354693769	0.498347424	0.362359041	0.402603236	0.821257366
ASB3	0.390906088	0.279649429	0.372748001	0.484080728	0.457135312	0.849885981
ELOF1	0.390920991	0.576461978	0.29721858	0.410993929	0.823508064	0.345042155
PICALM	0.391826315	0.250270282	0.411289123	0.965050971	0.414596876	0.348834279
ZMYND15	0.391826315	0.263251289	0.369656108	0.882057818	0.487063655	0.43348847
MITF	0.392608023	0.254897553	0.538447707	0.627280162	0.407452621	0.645427231
PTHLH	0.392608023	0.410883853	0.29666985	0.72187681	0.365608028	0.665695543
CD96	0.394266373	0.709247882	0.312920161	0.626698454	0.321162042	0.495197104

BICDL1	0.394675722	0.35609607	0.367733479	0.910854395	0.339270907	0.440866698
GMPS	0.394712449	0.785057176	0.471376314	0.515539653	0.383253602	0.325525685
KLHDC7A	0.394712449	0.808696335	0.366403539	0.400088147	0.551302181	0.341769356
CEP95	0.394712449	0.244044595	0.727870684	0.853058361	0.330336268	0.345042155
MIIP	0.394712449	0.318023067	0.716635829	0.627280162	0.385312392	0.419810556
TLCD2	0.394712449	0.346910196	0.501988763	0.456084509	0.61565225	0.546170552
LOC751811	0.395263057	0.587916027	0.421777252	0.513533242	0.308741687	0.632510367
P3H1	0.39527679	0.28021445	0.665599776	0.80717883	0.414742567	0.333656027
CLASP1	0.39527679	0.411108591	0.89478142	0.36448233	0.436968101	0.33731134
HSPA13	0.39527679	0.252029632	0.832065705	0.639639909	0.403447621	0.348834279
MINK1	0.39527679	0.25665513	0.495423363	0.918693944	0.380153307	0.380806889
CROCC	0.39527679	0.269843382	0.552362414	0.812999186	0.426006781	0.396383896
LRRC8B	0.39527679	0.317357075	0.454835278	0.812453944	0.483106459	0.396383896
LOC112446699	0.39527679	0.265446819	0.612793995	0.773429607	0.407452621	0.418173662
COL3A1	0.39527679	0.29496073	0.611794073	0.77580333	0.367214432	0.424489082
ATP6V0A1	0.39527679	0.244220572	0.513648728	0.853058361	0.321162042	0.560224188
GLDN	0.39527679	0.282521927	0.478168172	0.780539372	0.321291154	0.640128675
MRPL15	0.39527679	0.380041028	0.624829541	0.421668661	0.318448968	0.745988271
SAE1	0.39527679	0.311196652	0.680262176	0.391114534	0.367214432	0.748921056
SIGLEC1	0.39527679	0.262279886	0.315377577	0.336188007	0.664519875	0.885207772
TMEM167A	0.39527679	0.26595575	0.465496265	0.350939341	0.338817987	0.966659342
KIFC2	0.397160373	0.252029632	0.509302195	0.992930508	0.324735158	0.328550082
STAP2	0.397250634	0.321010792	0.429027801	0.868255052	0.487063655	0.347184142
LOC101904449	0.397250634	0.258714703	0.916294854	0.379699598	0.421697748	0.481287134
ZNF451	0.398642981	0.311196652	0.312920161	0.543346467	0.964301327	0.321909778
PRRX1	0.398642981	0.686033606	0.552362414	0.484080728	0.362019552	0.384409602
ZBED6CL	0.398642981	0.479687945	0.749417651	0.481018469	0.350342841	0.402563223
IL2RA	0.398642981	0.29496073	0.541610391	0.381424679	0.788007619	0.503701037
SOX9	0.398642981	0.321010792	0.312920161	0.819968583	0.487063655	0.526690149
ARHGAP40	0.398642981	0.306854565	0.395501027	0.598316303	0.656396948	0.545900472
WDR87	0.398642981	0.337583001	0.471438077	0.703663629	0.349906717	0.629427844
LOC112448070	0.398642981	0.367931057	0.725264968	0.373269014	0.332977669	0.700913346
MRVI1	0.400011122	0.517719875	0.384796164	0.394882194	0.33583181	0.83150957
CAPN10	0.401007274	0.558327847	0.623431824	0.58293921	0.385578499	0.333645233
APC2	0.401007274	0.750379179	0.497091612	0.546397256	0.361598024	0.336632335
PMS2	0.401007274	0.28021445	0.314465547	0.467730172	0.989334846	0.345042155
ZCCHC13	0.401166825	0.497687725	0.376853169	0.716077462	0.502432219	0.348834279
CTHRC1	0.401166825	0.268224849	0.649622134	0.718713712	0.429015291	0.432863285

CDK3	0.401166825	0.28021445	0.867222363	0.466614794	0.362646608	0.513034959
LOC788175	0.401493037	0.839128192	0.424739024	0.458535018	0.389670901	0.35938146
C5H12orf71	0.402298886	0.311738139	0.298472088	0.9745615	0.509805204	0.336632335
CCNJL	0.402298886	0.54021232	0.534905861	0.627280162	0.429015291	0.338075577
TAB1	0.402298886	0.254400782	0.417624775	0.418062303	0.983941532	0.345042155
SEC16B	0.402298886	0.328677561	0.605532453	0.792140715	0.38204968	0.386621411
UPK3B	0.402298886	0.247784456	0.577846633	0.689227896	0.550227273	0.442970192
LOC112447082	0.402298886	0.838155432	0.332000222	0.371678509	0.426006781	0.512846657
ATP2A2	0.402298886	0.324406814	0.517165421	0.541206056	0.47946353	0.636674289
MYBPH	0.402298886	0.669770548	0.341972274	0.342952467	0.376447594	0.761858515
C3	0.402320553	0.33848164	0.571415357	0.632683273	0.583956928	0.362710613
LOC101902537	0.402320553	0.340788135	0.662272069	0.362359041	0.468776461	0.68185373
PIGO	0.402430562	0.279475276	0.73826834	0.805499732	0.352171754	0.345299578
GAS2L2	0.402430562	0.265621994	0.310480699	0.486767679	0.499818667	0.902134012
CAPN8	0.402550995	0.439760003	0.789166592	0.617502141	0.332977669	0.336145569
FSBP	0.403343679	0.254958881	0.457350984	0.85005716	0.437810559	0.496801433
TAOK3	0.40336296	0.26040329	0.452644067	0.486381297	0.804274107	0.532354747
CFAP36	0.404133403	0.311196652	0.442078677	0.362359041	0.907094634	0.474572339
LOC788801	0.404934142	0.314389928	0.424739024	0.777956162	0.555526527	0.43348847
CCDC168	0.404934142	0.259699534	0.761415057	0.657738814	0.338340057	0.50354757
CD83	0.404934142	0.471255215	0.735388121	0.362930205	0.403447621	0.51212265
CD3D	0.404934142	0.447450909	0.800295805	0.346904474	0.353664429	0.560502405
RXFP1	0.404934142	0.373964414	0.333951286	0.528783909	0.443267567	0.801888363
EPOR	0.405143858	0.567198791	0.391019766	0.646965108	0.483224704	0.380806889
ZNF628	0.405143858	0.326285235	0.332000222	0.918693944	0.491185174	0.382635416
CEBPG	0.405143858	0.258714703	0.582638793	0.889415593	0.367944466	0.401953772
C23H6orf201	0.405143858	0.614976364	0.427016139	0.707217807	0.324735158	0.420370672
ZNRD1	0.405143858	0.346007726	0.952319163	0.404005354	0.332977669	0.427891722
SCRG1	0.405143858	0.398295321	0.361922003	0.379699598	0.911028086	0.431941338
LOC615733	0.405143858	0.445080617	0.546224399	0.451094074	0.474844367	0.584391974
MYO1E	0.405143858	0.258218955	0.369656108	0.601220553	0.714642011	0.621256155
UBE2D3	0.405143858	0.264032853	0.658345716	0.486381297	0.429015291	0.706176041
CCNB3	0.405143858	0.387656579	0.385271104	0.486381297	0.486113888	0.749726404
RPL22L1	0.405143858	0.262279886	0.502079089	0.531399091	0.418020354	0.7948807
DPT	0.405145124	0.414670902	0.305569839	0.479863522	0.906893576	0.381520146
APOC3	0.405209919	0.314377732	0.943836178	0.362359041	0.356507789	0.500317514
HAGH	0.405209919	0.314240268	0.400888251	0.365798962	0.870189808	0.587387684
DOPEY2	0.405209919	0.27418758	0.411289123	0.466614794	0.688889159	0.703769019

BCL7A	0.405350763	0.539073921	0.454835278	0.37131062	0.837399927	0.325934599
LOC781412	0.405350763	0.376775473	0.531780431	0.486381297	0.621029742	0.495197104
LOC615278	0.405350763	0.391482838	0.308651338	0.654797105	0.371487913	0.769865166
G0S2	0.406332154	0.51862711	0.546224399	0.37131062	0.429015291	0.638990067
STK19	0.407197197	0.259510013	0.582902669	0.9063051	0.337606409	0.422632938
SLC1A2	0.407390149	0.347575344	0.34374426	0.9745615	0.386673096	0.371881847
CDC45	0.408600218	0.324304652	0.608218151	0.808075336	0.324735158	0.469504329
LOC104968422	0.409385651	0.262929823	0.817582037	0.58293921	0.423015995	0.446618196
SDCBP	0.409517072	0.268224849	0.775552434	0.708466343	0.429015291	0.348834279
FFAR4	0.409669666	0.640172572	0.478168172	0.53435735	0.365608028	0.490495335
LOC104969545	0.410760292	0.402113442	0.380765282	0.356430136	0.970988226	0.343317756
RAB4A	0.410760292	0.292332168	0.966293373	0.37131062	0.495333297	0.345042155
OIT3	0.410760292	0.426582179	0.319666529	0.964521432	0.383253602	0.348834279
CBR4	0.410760292	0.541287559	0.431523929	0.46351377	0.712053021	0.378250649
ELAVL1	0.410760292	0.317357075	0.758146087	0.751643991	0.338340057	0.380806889
TRANK1	0.410760292	0.382853453	0.647867544	0.627280162	0.48361505	0.380806889
SCARB2	0.410760292	0.362371483	0.415543542	0.950643928	0.350342841	0.382635416
IGSF10	0.410760292	0.887455957	0.332000222	0.543403516	0.35463881	0.39318689
TGM1	0.410760292	0.833713501	0.371177512	0.373269014	0.553835124	0.394759278
PKDCC	0.410760292	0.319470703	0.454835278	0.891988949	0.4182393	0.428606899
TRAPPC3	0.410760292	0.355173924	0.831933739	0.410993929	0.500876883	0.431260036
CYP1A1	0.410760292	0.434208808	0.400888251	0.630951216	0.593860686	0.452024212
RGS18	0.410760292	0.321010792	0.532764011	0.797338497	0.368946575	0.503701037
EBP	0.410760292	0.341285659	0.502079089	0.418062303	0.780871086	0.512846657
HSPA6	0.410760292	0.378985237	0.579487023	0.509147405	0.48367155	0.580973226
ZNF12	0.410760292	0.262279886	0.424739024	0.701248463	0.533903668	0.629427844
COX7ALP1	0.410760292	0.314377732	0.344420176	0.433615397	0.831573375	0.640128675
ARHGAP29	0.410760292	0.383450541	0.310480699	0.356430136	0.847092933	0.657833313
SZT2	0.410760292	0.253228516	0.353392001	0.824230049	0.385312392	0.728993082
EIF2AK2	0.410760292	0.454880625	0.310213331	0.349464413	0.542799757	0.853056636
ENTPD2	0.411099017	0.36901158	0.332000222	0.999981514	0.333691681	0.345042155
UNC5C	0.411127863	0.687020192	0.497091612	0.587186699	0.404092468	0.348834279
CEP68	0.412125133	0.365524019	0.673165093	0.69459333	0.426231977	0.373692297
FN1	0.412265903	0.425336061	0.332000222	0.962899291	0.361598024	0.375310732
AKAP5	0.412265903	0.311196652	0.895801611	0.451094074	0.487063655	0.380806889
H2AFY2	0.412265903	0.375367521	0.478270125	0.362359041	0.87329316	0.442970192
LSS	0.412265903	0.279649429	0.418121393	0.384581248	0.849766114	0.643550981
LOC112442610	0.412265903	0.258714703	0.647477401	0.632683273	0.368946575	0.669558041

SLC35B1	0.412265903	0.436471501	0.547220001	0.469851076	0.337806259	0.749726404
AVPI1	0.412265903	0.262279886	0.376259378	0.55233084	0.596680559	0.77803403
LOC112444461	0.412265903	0.358391448	0.332000222	0.400088147	0.674294711	0.785636406
CAPZB	0.41325993	0.347147986	0.863543401	0.602157124	0.339892622	0.387697543
SMIM3	0.413436501	0.367446738	0.400888251	0.598316303	0.365608028	0.799889245
LOC101904103	0.414085404	0.872175143	0.492173361	0.420332527	0.405207591	0.345042155
PODXL2	0.414085404	0.283952337	0.363556211	0.952247001	0.443267567	0.444913711
RNF185	0.414275774	0.32719831	0.620613545	0.792140715	0.487063655	0.338075577
RAP2B	0.414275774	0.266852211	0.992937407	0.429892744	0.415305092	0.349240873
HDAC1	0.414275774	0.36901158	0.407806986	0.700026235	0.699191343	0.370949487
ARNTL	0.414275774	0.271208213	0.406919123	0.58734717	0.875687454	0.427722203
KCNH1	0.414275774	0.446686539	0.324612516	0.399246368	0.885786025	0.487514327
BID	0.414275774	0.313975784	0.342240037	0.908467146	0.429131373	0.512941039
PGM3	0.414275774	0.344296647	0.53939242	0.764257755	0.361598024	0.534954937
NAMPT	0.414275774	0.324794366	0.555428156	0.630951216	0.482155822	0.554751652
SNX19	0.414275774	0.331694641	0.476465349	0.418062303	0.658667583	0.700123604
LOC112444907	0.41476304	0.28021445	0.457350984	0.695125147	0.720896795	0.427722203
DISP1	0.415216805	0.457693965	0.4551661	0.695125147	0.407114819	0.51212265
KANK2	0.415801062	0.367446738	0.315377577	0.99542943	0.440694046	0.331788812
CA11	0.415801062	0.533735495	0.485200576	0.764257755	0.403447621	0.346280441
OLFML2B	0.415801062	0.630897425	0.388792304	0.507434672	0.635890386	0.382635416
HMCN2	0.41591827	0.692305416	0.810647419	0.379699598	0.330336268	0.35145363
FAM160B2	0.41591827	0.261979523	0.56802023	0.493678909	0.714642011	0.568308927
C5AR2	0.41591827	0.292474682	0.466192774	0.418062303	0.79453727	0.643003473
DOLK	0.41591827	0.359215228	0.70011973	0.375374604	0.484413225	0.655022943
GTSE1	0.41591827	0.437924391	0.369656108	0.527867968	0.512833021	0.700746926
KCNN1	0.416411367	0.361363832	0.995377403	0.438595551	0.338419262	0.335985533
SALL2	0.417114242	0.849513595	0.40855681	0.633006168	0.330336268	0.343317756
KIAA1024	0.417114242	0.313975784	0.45863002	0.464653418	0.954403133	0.345042155
NOXA1	0.417219024	0.311196652	0.533251947	0.898423855	0.419007823	0.373344999
LAMA2	0.417219024	0.391671365	0.532415258	0.384581248	0.797489438	0.45725625
APLP1	0.41785684	0.694834718	0.345465441	0.456261969	0.773874829	0.333656027
LOC518134	0.41785684	0.316728805	0.695084268	0.529579445	0.721096295	0.345042155
CALM3	0.41785684	0.528669672	0.350589195	0.418062303	0.881680572	0.369317789
SLC7A4	0.41785684	0.293520391	0.369656108	0.853058361	0.510264075	0.526690149
LOC514457	0.41785684	0.289811454	0.810647419	0.523261601	0.419827409	0.53440379
NDUFC2	0.41785684	0.285762422	0.456401352	0.381424679	0.658667583	0.808345278
LTV1	0.418334789	0.36159739	0.705746499	0.555557333	0.376990283	0.578851812

JSRP1	0.418732099	0.446395731	0.486000495	0.365925543	0.737633927	0.537186568
SLMAP	0.419519399	0.833323491	0.410561869	0.457902257	0.332977669	0.540876781
LOC100850659	0.420920388	0.332081544	0.486000495	0.486381297	0.847068842	0.440866698
ACD	0.421294423	0.262279886	0.846523249	0.799712183	0.362019552	0.338075577
CASTOR2	0.421294423	0.315690655	0.881070741	0.577737722	0.462507236	0.345042155
DFFB	0.421294423	0.28021445	0.846372547	0.736392704	0.33431614	0.394759278
RSRP1	0.421294423	0.331055243	0.41604117	0.486381297	0.864524107	0.487514327
ELL2	0.421294423	0.316933702	0.330749942	0.957493716	0.405207591	0.508671371
TOMM20	0.421294423	0.303059098	0.730661807	0.627280162	0.383253602	0.563801887
FFAR2	0.421294423	0.35609607	0.482939711	0.400627468	0.764213741	0.619532648
MRPL19	0.421294423	0.282521927	0.460075169	0.400190066	0.493545527	0.908768184
TRMT1	0.421568559	0.460453876	0.393857105	0.528783909	0.483224704	0.699162043
DGKQ	0.422808314	0.533324576	0.668824581	0.552451497	0.487063655	0.336632335
RGS1	0.422808314	0.292971214	0.612165542	0.428014152	0.917407853	0.345042155
ERO1A	0.422808314	0.294477622	0.764467593	0.670969025	0.521007676	0.346280441
MDGA2	0.422808314	0.710362373	0.635840248	0.369493567	0.521036349	0.348834279
TARBP1	0.422808314	0.27381867	0.685620658	0.894086736	0.360786315	0.366741209
PRKAR1A	0.422808314	0.283217657	0.653631615	0.610790896	0.682784145	0.396383896
ACTG2	0.422808314	0.91287376	0.395501027	0.410993929	0.338817987	0.514512902
CDC20	0.422808314	0.309937145	0.56802023	0.677545541	0.473071776	0.568004229
SGF29	0.422808314	0.583140563	0.380522912	0.500063521	0.474869023	0.632880775
TIGIT	0.422808314	0.423816704	0.784788059	0.362359041	0.362632321	0.665255561
FLVCR2	0.422808314	0.471255215	0.443690681	0.531399091	0.443267567	0.68185373
CCDC57	0.422808314	0.262929823	0.63135074	0.531399091	0.502432219	0.68185373
NDRG4	0.422808314	0.610421396	0.419761137	0.358687597	0.429015291	0.750967073
SLC31A1	0.422808314	0.3805254	0.424739024	0.684965277	0.333691681	0.75640676
SGIP1	0.422978912	0.358391448	0.546717582	0.518542038	0.664569452	0.504596604
C7H19orf25	0.423671163	0.324804737	0.332000222	0.576621615	0.964301327	0.346280441
MAGEL2	0.423702468	0.363346483	0.424739024	0.855232617	0.429015291	0.487407082
EFHB	0.423702468	0.274892607	0.471376314	0.498444775	0.737149805	0.667047801
TTC4	0.423702468	0.306854565	0.551149334	0.528783909	0.484413225	0.740861782
FCN1	0.423823586	0.337247845	0.487875367	0.379699598	0.804625939	0.621946029
RAMP3	0.423898433	0.872175143	0.499164335	0.39398546	0.349906717	0.466220233
NDST1	0.424807484	0.411819594	0.376259378	0.731459497	0.403447621	0.668851588
PODN	0.42561933	0.47375776	0.369656108	0.365925543	0.887514169	0.471903579
ZNF428	0.427394311	0.341285659	0.372748001	0.989707094	0.461764223	0.338075577
PTN	0.427755813	0.398295321	0.563473889	0.69193752	0.60903578	0.342620721
LOC100849865	0.427755813	0.384733167	0.679415149	0.440192972	0.788995893	0.345042155

MDM4	0.427755813	0.693513972	0.328810186	0.870210705	0.350342841	0.348834279
TONSL	0.427755813	0.321010792	0.965407935	0.544789716	0.362019552	0.348834279
ZNF335	0.427755813	0.445080617	0.862253703	0.447895467	0.486387163	0.348834279
CCBE1	0.427755813	0.439075262	0.735532321	0.416593832	0.687203978	0.348834279
LOC100849050	0.427755813	0.512513718	0.428870051	0.575014007	0.735091896	0.356332498
DNAJC27	0.427755813	0.373964414	0.424739024	0.543403516	0.884356471	0.35938146
BMP6	0.427755813	0.29744634	0.532743798	0.613568161	0.805615411	0.37954335
C18H16orf87	0.427755813	0.335464703	0.372748001	0.63966902	0.864524107	0.396383896
POLD4	0.427755813	0.454880625	0.609398456	0.731537809	0.38204968	0.414167655
MRPS24	0.427755813	0.309937145	0.473560084	0.395781309	0.965163023	0.418173662
MLYCD	0.427755813	0.398295321	0.465496265	0.407916519	0.872096228	0.45324563
REC114	0.427755813	0.587215076	0.611794073	0.411572762	0.512833021	0.462837324
LAMA3	0.427755813	0.664911321	0.501853676	0.518542038	0.429015291	0.476740315
UTP4	0.427755813	0.28288971	0.381816332	0.518542038	0.935286646	0.481287134
LOC101903540	0.427755813	0.311196652	0.369656108	0.849834721	0.590122535	0.487514327
ATG3	0.427755813	0.460200512	0.532415258	0.484080728	0.62911749	0.490594625
FUT1	0.427755813	0.279787852	0.40835816	0.896116841	0.385312392	0.631731716
GMPPB	0.427755813	0.52243101	0.485705164	0.535163392	0.403447621	0.65014676
CRYBG2	0.427755813	0.390997127	0.452747813	0.615029548	0.453983763	0.696550852
NPHP4	0.427755813	0.324237485	0.444417407	0.782567822	0.346226314	0.734177434
GSPT1	0.427755813	0.283879605	0.550289803	0.686250223	0.365608028	0.742309541
SH3BGRL	0.427755813	0.331055243	0.485148529	0.510562659	0.362019552	0.893706948
EIF5A	0.427755813	0.364362847	0.485339975	0.418062303	0.403447621	0.894694231
LOC100295797	0.428220723	0.345059299	0.472521949	0.527867968	0.429015291	0.832964812
EDA	0.428672454	0.525901055	0.332000222	0.698952489	0.383253602	0.663182265
LOC785408	0.429038677	0.499204258	0.673592902	0.641118121	0.409827576	0.363564487
LAMP3	0.429038677	0.303250787	0.735532321	0.391160166	0.383253602	0.819366282
LMNB1	0.42949175	0.382853453	0.518439818	0.609774743	0.64727886	0.454844961
SMARCC1	0.42949175	0.282521927	0.485200576	0.746075358	0.659596244	0.465577771
CAPS	0.42949175	0.610520254	0.457051074	0.649763076	0.361598024	0.514507804
AQP9	0.429834663	0.333891631	0.332000222	0.486381297	0.849434441	0.645719071
PPP2R1B	0.430848207	0.367095209	0.398176384	0.627280162	0.593931025	0.645719071
SCIN	0.431384263	0.346007726	0.534918375	0.866566721	0.349906717	0.508039178
POLR2F	0.431540595	0.479687945	0.384796164	0.441405457	0.798683736	0.512846657
LOC511617	0.431540595	0.319372498	0.681700278	0.407506997	0.569025406	0.687639605
HDAC10	0.43191091	0.524309619	0.521546054	0.433615397	0.80599066	0.343820447
CEP250	0.43191091	0.295503322	0.826913617	0.804434747	0.348941188	0.369839301
ALOX15	0.43191091	0.283217657	0.457051074	0.870210705	0.656015118	0.370530199

CCDC158	0.43191091	0.699211784	0.332000222	0.717652404	0.410579416	0.442970192
LOC101903604	0.43191091	0.314389928	0.828806956	0.632683273	0.382708431	0.470494326
GLI3	0.43191091	0.376659499	0.582638793	0.58293921	0.580738755	0.501733747
TPM2	0.43191091	0.790885918	0.389030278	0.457902257	0.352171754	0.647932306
CALR	0.431915403	0.314377732	0.411289123	0.882057818	0.618012018	0.396383896
PRKCQ	0.432504092	0.330796886	0.551149334	0.588863891	0.385312392	0.776811893
OPTC	0.433293013	0.296602679	0.450940071	0.418062303	0.38204968	0.980009318
PLPPR2	0.433490746	0.318023067	0.359728198	0.973832508	0.486387163	0.415609752
PLEKHA6	0.434230233	0.383545311	0.685018711	0.594220399	0.597314504	0.380806889
LRRC46	0.434230233	0.316933702	0.443690681	0.486381297	0.954814306	0.39890666
DPM1	0.434230233	0.309518408	0.454835278	0.498444775	0.487063655	0.869437527
CDAN1	0.434230233	0.303754531	0.604354543	0.460818045	0.339598774	0.911219114
SCN7A	0.43576439	0.886565095	0.342240037	0.689227896	0.341397863	0.35938146
GATA5	0.436063678	0.328677561	0.511116295	0.632683273	0.705288263	0.46459395
ADAP1	0.436752489	0.969051025	0.34502002	0.549573927	0.383253602	0.345042155
HID1	0.436752489	0.358391448	0.487875367	0.939604997	0.415898104	0.378250649
AZGP1	0.436752489	0.750379179	0.395501027	0.534082333	0.472699899	0.476740315
HOXA7	0.437245599	0.400941187	0.585284463	0.51403442	0.723054375	0.433152599
DYNC2H1	0.437355808	0.880565292	0.400888251	0.442001623	0.541176598	0.369925474
ZNF618	0.437355808	0.314240268	0.376259378	0.626698454	0.912617855	0.411693675
TPSB2	0.437355808	0.331910244	0.628489483	0.672328815	0.607561642	0.425800889
DIP2A	0.437355808	0.279475276	0.434811105	0.999981514	0.362257015	0.428431098
SHKBP1	0.437355808	0.447137916	0.473560084	0.418062303	0.870450746	0.436939175
SLC38A8	0.437355808	0.406494847	0.938011449	0.459073822	0.359078835	0.438685707
PRKAG2	0.437355808	0.980521483	0.390629973	0.406051248	0.352171754	0.452024212
DBF4B	0.437355808	0.284898988	0.345489021	0.500788064	0.976292433	0.46459395
ZNF311	0.437355808	0.332382274	0.482939711	0.891988949	0.429580883	0.481980128
LOC101906739	0.437355808	0.356015749	0.4551661	0.840541751	0.487063655	0.49105688
PGGHG	0.437355808	0.291635793	0.625861236	0.76992117	0.466059186	0.503701037
PCNX3	0.437355808	0.279649429	0.799265115	0.680418978	0.380153307	0.518095746
SETD3	0.437355808	0.283879605	0.817582037	0.505857338	0.483224704	0.587387684
LCK	0.437355808	0.518799911	0.728670337	0.418062303	0.368946575	0.61614241
SNCG	0.437355808	0.314377732	0.45863002	0.519684186	0.785840865	0.621946029
GCC1	0.437355808	0.456204284	0.333951286	0.69158245	0.507950493	0.640128675
LOC100300881	0.437355808	0.382320412	0.495423363	0.731537809	0.344401523	0.684994779
CCT7	0.437355808	0.303250787	0.478168172	0.824230049	0.361598024	0.694197796
RBM3	0.437562704	0.345527875	0.591348881	0.929491385	0.383253602	0.352283712
APEX1	0.438894496	0.375367521	0.49874267	0.415349648	0.35445418	0.935468188

GABARAPL1	0.440501418	0.285762422	0.517382044	0.882395013	0.457135312	0.501207489
SUSD3	0.440501418	0.406488087	0.605532453	0.514995	0.5863408	0.559845075
NDUFA6	0.440501418	0.347147986	0.491956841	0.391160166	0.79453727	0.669558041
NAA10	0.440501418	0.358391448	0.48405583	0.396676779	0.626973048	0.801888363
ME2	0.440674413	0.331977658	0.390076146	0.871338923	0.591306131	0.462837324
LOC112444326	0.441374683	0.285762422	0.487875367	0.379699598	0.40786268	0.980501512
CDH3	0.441970745	0.667258304	0.351965977	0.764257755	0.437029744	0.42204128
KCNMB1	0.441970745	0.77844414	0.550289803	0.379699598	0.463102881	0.485795983
GSDMB	0.441970745	0.368366921	0.332000222	0.443182693	0.795839201	0.748921056
COPS2	0.441970745	0.356628231	0.620613545	0.522681653	0.389234007	0.776734587
SIRPB1	0.442140137	0.454880625	0.341972274	0.43804734	0.70314895	0.74212863
SNU13	0.443057065	0.291635793	0.755135544	0.812453944	0.436502736	0.37147651
ZNF131	0.443449409	0.340788135	0.33920471	0.381424679	0.999985752	0.375377905
CCDC114	0.443620347	0.317273	0.641535145	0.929388614	0.395278001	0.348834279
SELENOI	0.444253313	0.356418931	0.716468083	0.882057818	0.364333362	0.345042155
TGFBI	0.444253313	0.363208684	0.458004431	0.760230321	0.738738532	0.359632759
CENPF	0.444253313	0.311196652	0.819734395	0.731537809	0.403447621	0.408480726
COX7A2	0.444253313	0.337247845	0.485705164	0.406608123	0.852234727	0.621946029
MAP3K12	0.444528709	0.311196652	0.579487023	0.910495312	0.403447621	0.43348847
MARCKSL1	0.445964992	0.332081544	0.333951286	0.689227896	0.893716526	0.410922175
SLC9A1	0.445970449	0.317357075	0.734143952	0.528798255	0.733278347	0.392988847
CHID1	0.445970449	0.728267541	0.454835278	0.451094074	0.560390886	0.490495335
HJURP	0.445970449	0.467147493	0.643516006	0.633703984	0.376447594	0.533968062
LOC101903193	0.445970449	0.40372842	0.429027801	0.418062303	0.687203978	0.748921056
TET3	0.446124906	0.32719831	0.455297509	0.646965108	0.687203978	0.587387684
LOC100335514	0.446124906	0.360897508	0.518894161	0.4908681	0.563212168	0.748921056
PLIN2	0.447578285	0.482217107	0.668824581	0.627280162	0.370917594	0.512846657
MED11	0.448570043	0.551003129	0.455797803	0.613744304	0.659373888	0.387697543
DUSP14	0.448881188	0.40372842	0.840055694	0.606611279	0.470098343	0.349240873
ASPSCR1	0.448881188	0.396058445	0.745348234	0.535570772	0.646707861	0.35938146
FANCF	0.448881188	0.285872549	0.355474568	0.646556987	0.827356651	0.596578269
CUL3	0.448932218	0.407339844	0.609398456	0.450758291	0.53689463	0.688619301
DCXR	0.449883046	0.367446738	0.478168172	0.418062303	0.944839728	0.440866698
ZNF624	0.449883046	0.60754196	0.376259378	0.598663987	0.468874683	0.627258867
LENG9	0.449883046	0.29496073	0.342240037	0.889415593	0.509805204	0.641401171
LOC101909003	0.449883046	0.314389928	0.503989485	0.65294576	0.476090036	0.742588975
TNFSF10	0.451082965	0.434208808	0.33920471	0.72169974	0.844384115	0.348834279
ZDHHC2	0.451082965	0.314377732	0.538447707	0.876703811	0.468776461	0.472881591

CDKN1A	0.452017836	0.346007726	0.487875367	0.813945621	0.491185174	0.531055466
FBXO36	0.452017836	0.531880429	0.36648605	0.661894736	0.457358633	0.659120631
USP4	0.45211211	0.306854565	0.598849326	0.982220679	0.383253602	0.348834279
LOC112449547	0.45211211	0.594199197	0.34510985	0.852958102	0.467076905	0.389619719
LAPTM4A	0.45211211	0.757078435	0.606660869	0.48500625	0.35856792	0.495197104
SAMD10	0.452154116	0.317357075	0.975126903	0.480047213	0.383253602	0.462837324
INO80B	0.453287911	0.553132278	0.376259378	0.527867968	0.799752471	0.429504806
SLC6A17	0.453287911	0.775033799	0.424739024	0.515539653	0.357092245	0.640128675
SNTA1	0.453287911	0.382320412	0.400888251	0.596654953	0.461235361	0.835338212
TOMM40	0.454051296	0.400145858	0.566409195	0.429892744	0.605738596	0.705204221
UBN1	0.454703246	0.323460072	0.752659039	0.396676779	0.887514169	0.348834279
LOC512486	0.454703246	0.479687945	0.533634902	0.499858161	0.821351993	0.35145363
LYAR	0.454703246	0.323983996	0.43314299	0.493609824	0.989447906	0.35145363
LOXL4	0.454703246	0.479687945	0.441807263	0.535659494	0.678703442	0.564221938
HEXIM2	0.454703246	0.53061969	0.648593816	0.527867968	0.38204968	0.621089184
CD3E	0.454703246	0.480755771	0.71633602	0.404005354	0.426006781	0.67453786
NDUFB4	0.454703246	0.40372842	0.566069604	0.468112509	0.577102225	0.690728863
COPG2	0.45516871	0.887455957	0.344420176	0.626698454	0.452330766	0.378410553
KIAA1143	0.45549012	0.321010792	0.524780246	0.53954651	0.651585054	0.695065923
TSPAN15	0.455493219	0.791046586	0.550289803	0.498444775	0.501311839	0.349850003
ACTA2	0.455534584	0.718258172	0.407806986	0.41814733	0.389670901	0.764041382
PREX1	0.455534584	0.489987041	0.372748001	0.391160166	0.386673096	0.952571698
ASNS	0.456733116	0.333891631	0.507675433	0.504242357	0.564695829	0.799889245
CADM1	0.456916836	0.382853453	0.653833671	0.493932071	0.404264313	0.770490368
SLC16A9	0.456921573	0.341904379	0.744735369	0.515539653	0.670245065	0.456099659
VDAC2	0.457294356	0.332382274	0.478168172	0.43080152	0.623804687	0.847193872
ZNF484	0.457294356	0.303250787	0.418121393	0.423067881	0.680301694	0.877048993
GCSH	0.457594705	0.452206305	0.454835278	0.434367671	0.789999838	0.609692015
ATG9B	0.459798269	0.791582069	0.363556211	0.451094074	0.704783515	0.421566456
RAB21	0.459891896	0.303059098	0.978014482	0.549287144	0.418020354	0.38897044
CALM2	0.459891896	0.355173924	0.89478142	0.64770518	0.373086408	0.419810556
GMPPA	0.459891896	0.364362847	0.764467593	0.771320656	0.383253602	0.42126634
BTBD11	0.459891896	0.952863279	0.422206667	0.40816348	0.362632321	0.529445217
FAM124A	0.459891896	0.506962444	0.759110916	0.466234452	0.427233204	0.533823331
CPA3	0.459891896	0.355271557	0.774924967	0.457902257	0.545645258	0.60624915
DDX54	0.459891896	0.58531281	0.382449111	0.553146279	0.560554936	0.627258867
SEMA6B	0.460093017	0.343951817	0.498347424	0.964521432	0.469611067	0.35806796
CHRNA1	0.460288704	0.641897615	0.42111624	0.457715968	0.677185022	0.522289438

LOC786139	0.460288704	0.29496073	0.351815148	0.543403516	0.916605572	0.621946029
MCUR1	0.460430451	0.589044534	0.819359511	0.418324989	0.492937023	0.372209768
SMDT1	0.460555296	0.38719103	0.525934706	0.457902257	0.635449581	0.725967807
PSMF1	0.460812327	0.303250787	0.869932384	0.598316303	0.569025406	0.394759278
ARHGAP27	0.460812327	0.343951817	0.913134155	0.555047431	0.424448433	0.453219699
CEP128	0.460812327	0.382853453	0.480037287	0.447647758	0.804274107	0.632510367
SLC25A5	0.460812327	0.439142493	0.499598421	0.486381297	0.376990283	0.873185607
RPP40	0.460852905	0.355271557	0.570681722	0.582177282	0.361598024	0.844759721
AIMP2	0.460858373	0.422787251	0.877824879	0.391160166	0.362632321	0.66881641
RALGAPB	0.461065755	0.317357075	0.606390596	0.912777307	0.501311839	0.352283712
LOC508933	0.461065755	0.522280519	0.844093851	0.617287092	0.364791996	0.357401263
TUBG2	0.461065755	0.363346483	0.582898646	0.647342496	0.733818161	0.411969094
LOC104976281	0.461065755	0.445080617	0.372748001	0.973075232	0.389670901	0.440866698
LIF	0.461065755	0.350510345	0.444805139	0.874170259	0.608486847	0.440866698
STAB1	0.461065755	0.647890563	0.492173361	0.733411985	0.375347002	0.452024212
IL16	0.461065755	0.321117458	0.397871714	0.912777307	0.403447621	0.665736967
TMED2	0.461065755	0.358391448	0.849636922	0.433615397	0.365608028	0.729319161
TTC19	0.461065755	0.319661234	0.497091612	0.636814098	0.52068239	0.749918186
CMSS1	0.461065755	0.346488221	0.644677344	0.491405184	0.436968101	0.811759324
ZBTB9	0.461710528	0.920978623	0.41426165	0.395781309	0.474619036	0.495197104
ZMAT5	0.46180201	0.885674643	0.40651486	0.402952198	0.677185022	0.35938146
RERE	0.46180201	0.314389928	0.400888251	0.39238643	0.37499039	0.999948228
NFYB	0.462287609	0.418930992	0.368024229	0.429892744	0.98865576	0.411969094
NDUFAF6	0.463053813	0.301414099	0.472521949	0.598316303	0.523124197	0.839906041
MEOX1	0.463473424	0.361164768	0.36648605	0.659998592	0.855761574	0.487514327
MSX1	0.463533513	0.451236244	0.397871714	0.852005554	0.482155822	0.515726083
RPS6KA6	0.464194063	0.452206305	0.507083871	0.804116763	0.367214432	0.597568377
ERC2	0.465082641	0.941958569	0.6506579	0.395781309	0.363195127	0.365718889
PDE5A	0.465082641	0.418930992	0.495423363	0.499858161	0.906893576	0.38581698
CPZ	0.465082641	0.355173924	0.649378275	0.670507175	0.642796813	0.440866698
XPO5	0.465274021	0.382320412	0.382449111	0.999981514	0.413792445	0.382635416
C15H11orf87	0.466888303	0.367446738	0.421396526	0.518542038	0.943702351	0.444913711
SENP1	0.466931488	0.966057895	0.481165053	0.429892744	0.434714683	0.380806889
FOXK2	0.466931488	0.340788135	0.552362414	0.658194793	0.788995893	0.420532744
HAPLN1	0.466931488	0.382320412	0.507675433	0.887642126	0.498546902	0.424146598
CXCL5	0.466931488	0.3805254	0.427152347	0.861301134	0.610145147	0.442970192
IL6	0.466931488	0.580473137	0.36648605	0.788323571	0.426006781	0.569429928
STK32B	0.466931488	0.37643767	0.612793995	0.627280162	0.553835124	0.587387684

NCAPD3	0.466931488	0.353273682	0.682166552	0.627280162	0.486387163	0.622811146
LOC530973	0.466931488	0.299590998	0.611216367	0.561398298	0.638815482	0.67453786
TYK2	0.466931488	0.309937145	0.513648728	0.541352894	0.682784145	0.729499398
PFN2	0.466993083	0.423751729	0.70638908	0.482915217	0.738738532	0.416841281
AP5Z1	0.466993083	0.38381979	0.829455601	0.626698454	0.460388293	0.436939175
SYT9	0.466993083	0.686033606	0.43314299	0.63494856	0.487063655	0.481980128
TATDN1	0.466993083	0.479687945	0.37614981	0.852005554	0.510264075	0.486586811
LOC787891	0.466993083	0.460605417	0.479690622	0.680219003	0.389234007	0.729319161
HMGB3	0.467678144	0.320189468	0.680262176	0.41814733	0.915775776	0.423241805
RRAGC	0.467678144	0.32719831	0.551149334	0.928404714	0.463102881	0.432652444
LOC101904526	0.467678144	0.332081544	0.436168983	0.999981514	0.375347002	0.447212551
STK36	0.467678144	0.392477353	0.596826854	0.742465161	0.517239659	0.487514327
ANKRD22	0.467678144	0.410883853	0.73765068	0.622980329	0.438638841	0.531055466
LOC112448014	0.467678144	0.309937145	0.466192774	0.866566721	0.555125415	0.563801887
DYNLL1	0.467678144	0.316933702	0.671231078	0.762598894	0.459901956	0.566196709
EIF3J	0.467678144	0.367146912	0.632469403	0.627280162	0.382497531	0.749726404
NAT14	0.467678144	0.333827203	0.454835278	0.456261969	0.429015291	0.973413452
LXN	0.467779452	0.373964414	0.487875367	0.959652624	0.402497797	0.447212551
ATF6	0.467779452	0.374036856	0.808493605	0.630951216	0.474619036	0.448403672
LOC782456	0.467779452	0.438643862	0.439933643	0.733411985	0.664519875	0.470494326
GPR35	0.467779452	0.376619022	0.810647419	0.426957178	0.670221463	0.479152098
LOC100298890	0.467779452	0.474946062	0.8042974	0.486381297	0.431226303	0.535971177
ATP5MC3	0.467779452	0.375367521	0.545866468	0.46351377	0.738333933	0.656746469
LOC100298530	0.467779452	0.64563665	0.501853676	0.518533249	0.392368763	0.690728863
NHP2	0.467779452	0.48559314	0.486000495	0.46682385	0.437425346	0.839565788
TMBIM7	0.468436794	0.589431944	0.480970735	0.42341558	0.832407979	0.426336374
ARID3A	0.469099402	0.337247845	0.485200576	0.551641524	0.836444721	0.571961045
MMP19	0.469189734	0.360897508	0.481165053	0.652519777	0.386670972	0.848298915
NFE2L2	0.469256109	0.357787901	0.415690997	0.812453944	0.671669027	0.501207489
HSF2	0.469459011	0.386477216	0.376259378	0.626698454	0.942786245	0.383090164
LOC784769	0.469459011	0.338196075	0.745477682	0.654131191	0.627206089	0.401953772
PET117	0.469459011	0.318023067	0.476775388	0.997239348	0.42782681	0.429408353
COX5A	0.469459011	0.376819185	0.495423363	0.451094074	0.877712931	0.563814955
COX6B1	0.469459011	0.347014064	0.528387114	0.40833214	0.889217664	0.601987815
ETF1	0.469459011	0.333827203	0.70011973	0.718713712	0.407114819	0.622811146
BMI1	0.469809211	0.321117458	0.754557912	0.534082333	0.380153307	0.78096075
TSN	0.470717493	0.358391448	0.668824581	0.531399091	0.365825688	0.839565788
PDGFRA	0.471226668	0.68903984	0.405470741	0.498444775	0.626973048	0.557469092

PIM3	0.471690122	0.318023067	0.49874267	0.44307697	0.986861597	0.426772334
PNPO	0.471712698	0.332681229	0.372748001	0.466234452	0.696466814	0.881947456
LPCAT2	0.472494339	0.373964414	0.481165053	0.674469953	0.465861906	0.766218451
LY6G5B	0.472494339	0.309937145	0.528511058	0.764257755	0.385670299	0.783901526
ALDH9A1	0.472494339	0.317357075	0.473560084	0.464653418	0.450726215	0.96885995
NFYC	0.473653946	0.382320412	0.628904157	0.508967067	0.607031816	0.669883077
COL23A1	0.473898003	0.31951646	0.999993442	0.459073822	0.395278001	0.415053221
SLC5A3	0.473898003	0.412566059	0.42111624	0.486381297	0.958654817	0.438685707
SMIM37	0.473898003	0.879185178	0.502079089	0.406051248	0.531351877	0.440866698
MOSPD3	0.473898003	0.447111556	0.495423363	0.442052083	0.916057607	0.440866698
FAM91A1	0.474058505	0.346007726	0.862602899	0.534082333	0.631537325	0.40223864
NKAPD1	0.474058505	0.341285659	0.608218151	0.768290504	0.626973048	0.441715562
RGS22	0.474058505	0.347732257	0.701625985	0.764257755	0.428442971	0.532230992
ZNF469	0.474104279	0.38381979	0.68898955	0.771412309	0.483224704	0.440866698
TNFAIP6	0.474104279	0.314325225	0.36648605	0.528783909	0.970988226	0.531055466
ZNF652	0.474771894	0.311196652	0.741228435	0.613568161	0.533807401	0.614200722
ATP5PD	0.474771894	0.343870533	0.566638914	0.464879331	0.81725718	0.621946029
TRA2A	0.474771894	0.329951891	0.445922811	0.481018469	0.883951804	0.667047801
KCTD17	0.475385767	0.439075262	0.433304678	0.959652624	0.4182393	0.440866698
PRAG1	0.476279291	0.528669672	0.380423932	0.638269781	0.773384033	0.448702315
ATP6V0E1	0.476279291	0.321010792	0.727703335	0.598316303	0.429015291	0.734177434
ARMCX2	0.476460362	0.54021232	0.405974447	0.727950912	0.626796691	0.466256919
GPR162	0.476680565	0.313144577	0.511417994	0.964521432	0.494000973	0.422632938
LOC100297399	0.476749839	0.434898742	0.397871714	0.575112315	0.864524107	0.503701037
LOC112448166	0.476749839	0.344296647	0.485445908	0.519487474	0.887514169	0.558375365
ROBO2	0.476749839	0.499204258	0.376217254	0.442878665	0.882206878	0.574870258
LONRF3	0.476837324	0.32719831	0.524780246	0.794386089	0.777428223	0.383090164
NRBP2	0.477107904	0.36444467	0.672696223	0.504242357	0.58186871	0.690728863
ATE1	0.477693813	0.589754585	0.538114316	0.481018469	0.686042858	0.489274479
SLAIN2	0.477693813	0.345527875	0.726746856	0.531399091	0.710478485	0.500317514
LOC112443143	0.477693813	0.324238107	0.466192774	0.88097449	0.507738123	0.608554064
MRPS30	0.478132654	0.321117458	0.489549483	0.531399091	0.560554936	0.867063767
PARP10	0.478272264	0.464892289	0.872088608	0.41814733	0.607958542	0.41219904
TRAM2	0.478272264	0.331055243	0.411289123	0.973075232	0.483106459	0.505016274
TPPP3	0.478653039	0.439075262	0.40855681	0.522681653	0.935093621	0.442970192
TMEM45A	0.478653039	0.457693965	0.409955029	0.812999186	0.443535384	0.645719071
TIMM23	0.478653039	0.358391448	0.592159701	0.441089439	0.617915169	0.798871707
SIX5	0.478827948	0.358391448	0.435579952	0.932914414	0.52068239	0.486586811

LOC112444603	0.478899921	0.356418931	0.487875367	0.420332527	0.992990026	0.423834625
TECPR1	0.479989789	0.343951817	0.879939804	0.705801894	0.461764223	0.383090164
ROGDI	0.479989789	0.375367521	0.813441395	0.484080728	0.695436567	0.440866698
LOC112441566	0.479989789	0.416798678	0.566638914	0.627280162	0.659943626	0.521756022
CLDN15	0.479989789	0.457605323	0.397871714	0.747199287	0.656015118	0.525913971
GIMAP5	0.479989789	0.407339844	0.752113836	0.604036098	0.410579416	0.628379498
ADRM1	0.479989789	0.398474333	0.648593816	0.472406569	0.509805204	0.764737727
LOC509118	0.480166772	0.345239927	0.574826765	0.916858019	0.547042845	0.371020753
CNTN2	0.480166772	0.346007726	0.464182805	0.751386915	0.659373888	0.605408237
THOP1	0.480166772	0.36901158	0.550289803	0.464653418	0.533903668	0.853009713
GPR88	0.480739194	0.327101033	0.376259378	0.664273743	0.97352263	0.378250649
LOC112442634	0.480739194	0.46147411	0.415629851	0.7817105	0.721096295	0.418173662
PNO1	0.480739194	0.326285235	0.734143952	0.738460631	0.562163729	0.448702315
TSR3	0.480739194	0.587740565	0.532764011	0.452423129	0.758424886	0.471233805
CLCN5	0.480739194	0.358391448	0.501853676	0.85981779	0.532662842	0.523933932
CYFIP2	0.480739194	0.447137916	0.903431343	0.418062303	0.450726215	0.551434111
MSL2	0.480739194	0.51352654	0.648593816	0.454517255	0.610004585	0.597568377
TOMM5	0.480739194	0.372319879	0.532764011	0.426957178	0.79152988	0.700123604
F2RL2	0.480739194	0.442374595	0.492173361	0.592708053	0.558285349	0.723644207
C2	0.481220066	0.477507105	0.700349135	0.552451497	0.600776371	0.466085264
VSIG10	0.481220066	0.337537989	0.492173361	0.612761692	0.415898104	0.903990996
NDUFB5	0.481636027	0.335464703	0.542266375	0.451094074	0.592313179	0.870490204
ITPKA	0.481762433	0.808478344	0.457350984	0.421668661	0.44198836	0.68185373
LOC112448373	0.481826601	0.382853453	0.369656108	0.999981514	0.480110166	0.380806889
SIX4	0.481826601	0.331694641	0.607986114	0.559525332	0.91563394	0.383090164
BLK	0.481826601	0.321117458	0.903431343	0.636814098	0.510264075	0.402562285
CEPT1	0.481826601	0.759814955	0.503989485	0.653503626	0.457770074	0.418173662
TENT5C	0.481826601	0.331055243	0.382449111	0.632683273	0.968585482	0.422632938
ADCY5	0.481826601	0.764799096	0.500533485	0.570760743	0.53689463	0.426772334
MTCL1	0.481826601	0.335464703	0.551149334	0.904417923	0.557011358	0.428431098
TROAP	0.481826601	0.436471501	0.794846789	0.600166011	0.539185944	0.429504806
TUBGCP5	0.481826601	0.314389928	0.560847675	0.939604997	0.410579416	0.537094861
LOC786474	0.481826601	0.530969645	0.419761137	0.464653418	0.690943093	0.704351773
TRPC4	0.481826601	0.547676372	0.603530331	0.487740758	0.429015291	0.737726964
YPEL1	0.481826601	0.347147986	0.409955029	0.86220603	0.421697748	0.75640676
TMEM126A	0.481826601	0.364606615	0.55004286	0.441405457	0.714642011	0.76139014
ANXA8L1	0.481873561	0.479682241	0.611794073	0.678056392	0.605738596	0.428606899
CDK1	0.481873561	0.468048766	0.429275047	0.631746507	0.78021215	0.501207489

SH2D1A	0.481873561	0.413330726	0.711148626	0.451748594	0.670221463	0.596578269
HAX1	0.481905143	0.728267541	0.682220784	0.500063521	0.403447621	0.503701037
LOC100847495	0.482460577	0.367446738	0.557524506	0.552451497	0.902907077	0.412040609
CENPX	0.482594421	0.350220269	0.457051074	0.457131305	0.996781978	0.440866698
CCDC86	0.482594421	0.649949716	0.507083871	0.527867968	0.403447621	0.724693895
KCNQ4	0.482594421	0.340788135	0.372748001	0.517860657	0.810436875	0.793197037
RASSF5	0.483472068	0.360897508	0.739106589	0.599544809	0.510264075	0.621946029
CD8B	0.483530875	0.697043768	0.523628873	0.472439714	0.687203978	0.440866698
RTCB	0.48373683	0.319220716	0.546068496	0.932656054	0.428442971	0.548381804
TCF15	0.483922975	0.346488221	0.552362414	0.528783909	0.958018667	0.383090164
LOC510382	0.483922975	0.423816704	0.511028724	0.41814733	0.976292433	0.389619719
LOC617785	0.483922975	0.481136135	0.587139265	0.680418978	0.657331824	0.406828769
TSR2	0.483922975	0.351317355	0.951392011	0.637708629	0.403447621	0.412040609
PREB	0.483922975	0.95462892	0.534918375	0.456103613	0.424445576	0.41219904
IL33	0.483922975	0.482081164	0.465496265	0.925353907	0.457358633	0.420370672
LOC104976276	0.483922975	0.969051025	0.376259378	0.593594064	0.396193633	0.431260036
ATP1A1	0.483922975	0.386477216	0.499640977	0.64770518	0.830835063	0.440866698
LOC104975162	0.483922975	0.414912961	0.400888251	0.627280162	0.909379774	0.440866698
MDM2	0.483922975	0.320192503	0.415690997	0.964864514	0.61647911	0.442234422
BTG2	0.483922975	0.454880625	0.513255922	0.864831081	0.476961495	0.468732955
PRR19	0.483922975	0.382320412	0.376259378	0.840247324	0.727256509	0.486586811
LOC516599	0.483922975	0.316933702	0.494944201	0.978404889	0.429015291	0.513034959
PKLR	0.483922975	0.512513718	0.492173361	0.764257755	0.4884215	0.529445217
CXCR4	0.483922975	0.373964414	0.663513753	0.586290626	0.690058599	0.529445217
AP1G2	0.483922975	0.345527875	0.684282335	0.836610893	0.383253602	0.582795441
ARPC5	0.483922975	0.370464508	0.867661896	0.58293921	0.404092468	0.597300308
LOC112441502	0.483922975	0.341285659	0.897322115	0.457131305	0.517239659	0.608200832
TMEM70	0.483922975	0.321010792	0.454835278	0.498444775	0.55863621	0.940017687
MRPL12	0.483969734	0.375367521	0.630408979	0.451094074	0.74307807	0.666011114
PAPOLA	0.486148373	0.383639708	0.397871714	0.654131191	0.788007619	0.625915593
LOC104970249	0.486578055	0.658174484	0.864847127	0.529049853	0.385312392	0.380806889
RABGEF1	0.486578055	0.327902524	0.630408979	0.727236849	0.788995893	0.396383896
STAC	0.486578055	0.335476613	0.40651486	0.626698454	0.976292433	0.41219904
ZFX	0.486578055	0.551459649	0.518439818	0.522681653	0.811276938	0.42204128
COL1A1	0.486578055	0.382320412	0.716468083	0.809886338	0.498657967	0.424146598
ALOX12	0.486578055	0.596368398	0.855268213	0.504242357	0.409827576	0.442970192
PLEKHH2	0.486578055	0.321010792	0.410261216	0.999981514	0.495333297	0.444913711
GPR171	0.486578055	0.496890044	0.538447707	0.688505637	0.610761857	0.48181553

FBF1	0.486578055	0.336861124	0.466192774	0.999981514	0.395278001	0.487514327
LIG1	0.486578055	0.318023067	0.906019013	0.612761692	0.496066793	0.489274479
P4HA2	0.486578055	0.484688573	0.441807263	0.76992117	0.593931025	0.521756022
RIOK2	0.486578055	0.421022405	0.425565437	0.791081996	0.666917776	0.528196382
LOC516108	0.486578055	0.65172183	0.478270125	0.457902257	0.70853015	0.563814955
LOC112442677	0.486578055	0.406606236	0.874063521	0.530577799	0.429015291	0.581350944
MVB12B	0.486578055	0.480755771	0.389030278	0.899308956	0.429015291	0.588422002
DYNLRB2	0.486578055	0.433279727	0.473560084	0.840247324	0.459901956	0.620459631
SMTN	0.486578055	0.891547502	0.444417407	0.47533304	0.383253602	0.640434848
LOC112448269	0.486578055	0.317357075	0.411289123	0.462446186	0.956041014	0.667047801
CDADC1	0.486578055	0.324304652	0.455297509	0.689370133	0.727879947	0.671759429
IKZF5	0.486578055	0.672505775	0.409955029	0.528783909	0.563212168	0.672418631
GDF10	0.486578055	0.423816704	0.566638914	0.670194455	0.481518205	0.68781527
ACYP2	0.486578055	0.38381979	0.476775388	0.588863891	0.695846971	0.703769019
ANKRA2	0.486578055	0.429673816	0.422397986	0.73982969	0.510264075	0.721192619
BCCIP	0.486578055	0.367446738	0.694535506	0.598316303	0.437165526	0.748921056
MS4A1	0.486578055	0.449821474	0.731847616	0.493932071	0.385979159	0.769865166
RFTN2	0.486578055	0.351248765	0.660365588	0.459073822	0.591515687	0.799104608
DDR1	0.486578055	0.331055243	0.45863002	0.611379189	0.584477791	0.846210629
LOC101902742	0.486578055	0.355173924	0.401200107	0.518542038	0.688556938	0.871200929
HSPB11	0.486578055	0.462496513	0.473560084	0.519684186	0.453983763	0.877014986
BMP1	0.486927591	0.397956302	0.644501365	0.953697985	0.386670972	0.385223859
PLIN5	0.487127175	0.483496628	0.384796164	0.693458387	0.695436567	0.582795441
MON1B	0.487127175	0.355173924	0.72058898	0.588863891	0.626973048	0.588600411
NHLH1	0.487127175	0.470133653	0.381356074	0.707217807	0.626796691	0.659134809
NMD3	0.487127175	0.382853453	0.649622134	0.486381297	0.555125415	0.776277826
PMM1	0.487316584	0.366629388	0.627084316	0.459073822	0.936921784	0.433152599
UBE4B	0.487345105	0.321117458	0.460075169	0.999981514	0.457770074	0.442970192
NDUFS6	0.487345105	0.476346865	0.511417994	0.43804734	0.823508064	0.601190935
EVC2	0.487345105	0.343951817	0.56863752	0.654131191	0.487063655	0.779201808
LMAN1	0.487551323	0.337247845	0.565148263	0.519487474	0.429015291	0.928820381
DOCK10	0.487724571	0.333827203	0.915120516	0.500788064	0.651951691	0.440866698
NYNRIN	0.487724571	0.447485891	0.487875367	0.694815488	0.65298501	0.564822443
LOC107133276	0.48877608	0.376466654	0.916323169	0.647855934	0.404092468	0.444913711
SMAD1	0.48877608	0.421022405	0.551149334	0.932914414	0.424445576	0.451434552
POLG2	0.48877608	0.363208684	0.53400059	0.681573779	0.472699899	0.78288746
HTR1E	0.490081625	0.428129561	0.89496118	0.488122553	0.56086474	0.448403672
MYO18A	0.490081625	0.445034796	0.457350984	0.934180032	0.45951987	0.494666285

ELAC2	0.490081625	0.83844057	0.497091612	0.457131305	0.492937023	0.560879865
API5	0.490091249	0.360723284	0.702094756	0.685261155	0.489920278	0.623319115
LOC100295712	0.490091249	0.321010792	0.855793459	0.539889587	0.455741172	0.694197796
SSBP1	0.490205595	0.355090648	0.681977537	0.443004068	0.82488413	0.591154283
NDUFAF5	0.490205595	0.42354307	0.647291859	0.441089439	0.733818161	0.645719071
LRRN1	0.490321477	0.649949716	0.752840524	0.596654953	0.457358633	0.39318689
ANP32E	0.490321477	0.355271557	0.999993442	0.462657114	0.429015291	0.412040609
LOC112442745	0.490321477	0.542567583	0.486538048	0.674194099	0.721096295	0.429504806
LOC101903248	0.490321477	0.439484206	0.527368302	0.889415593	0.510264075	0.441709492
CD22	0.490321477	0.622818484	0.623431824	0.707217807	0.404092468	0.482980366
SYNC	0.490321477	0.971455677	0.411495032	0.486381297	0.429015291	0.495197104
LOC101903289	0.490321477	0.355591197	0.421396526	0.913382681	0.467891996	0.668851588
TBC1D22B	0.490321477	0.321010792	0.427960468	0.959591127	0.419827409	0.67453786
ASIC2	0.490321477	0.383545311	0.849389234	0.424465138	0.429015291	0.762133815
NDUFB9	0.490321477	0.35816511	0.454835278	0.424465138	0.591306131	0.958790968
RRP12	0.49043146	0.507210923	0.487875367	0.459828217	0.583449841	0.806714845
PSMB6	0.490470119	0.415447733	0.603530331	0.507254322	0.512833021	0.814218101
DALRD3	0.490527622	0.579367653	0.810647419	0.451094074	0.461764223	0.544955425
TBL1XR1	0.490877751	0.360897508	0.726119774	0.931383184	0.405207591	0.387873306
SEMA4A	0.490877751	0.356418931	0.793399684	0.577939388	0.479364681	0.669558041
ABHD5	0.491736791	0.43720588	0.420822773	0.731537809	0.803705802	0.470494326
FAT3	0.491736791	0.919540627	0.443690681	0.58557509	0.412721376	0.481925208
CDC73	0.491845238	0.358391448	0.429481013	0.866566721	0.705202026	0.497110172
TMEM200A	0.49196327	0.43720588	0.454835278	0.429892744	0.999985752	0.394427049
LOC783776	0.49196327	0.792755412	0.693546993	0.466614794	0.473071776	0.440866698
ZCCHC11	0.493149438	0.447111556	0.427325038	0.808075336	0.632055507	0.540876781
LOC100337457	0.493149438	0.340788135	0.561599458	0.544789716	0.544248798	0.856833445
PTPRT	0.493912789	0.548622699	0.421396526	0.438595551	0.578296318	0.852504043
OLFML1	0.493970727	0.400145858	0.560469326	0.626698454	0.81911207	0.466750685
ARHGEF19	0.493990113	0.454880625	0.470538126	0.633053624	0.854289643	0.44159009
PPM1F	0.493990113	0.324304652	0.498512048	0.5116854	0.789999838	0.77694371
ADTRP	0.494353792	0.865101561	0.501988763	0.630951216	0.459901956	0.391411375
LURAP1	0.494353792	0.722195304	0.388358382	0.649446726	0.583789489	0.517000508
NGF	0.494353792	0.345527875	0.492173361	0.582333557	0.901853599	0.539240716
DHRS3	0.494353792	0.393018763	0.485445908	0.481018469	0.912199478	0.590055009
SYNCRIP	0.494353792	0.333827203	0.754913805	0.636814098	0.519899007	0.632880775
HENMT1	0.494353792	0.345239927	0.731960288	0.440192972	0.502432219	0.844759721
AKAP6	0.494516848	0.866862152	0.457350984	0.434462583	0.429015291	0.690728863

RAB29	0.49452552	0.358391448	0.611647147	0.882395013	0.548274329	0.45725625
CSPG5	0.49452552	0.362371483	0.407806986	0.549573927	0.953300742	0.55869127
PPARGC1A	0.494854443	0.457693965	0.799265115	0.577939388	0.459240337	0.578077816
ST6GAL1	0.495033351	0.480755771	0.429027801	0.819968583	0.627206089	0.49105688
LSP1	0.495033351	0.364362847	0.767559288	0.594220399	0.670245065	0.491178906
LOC101907574	0.495033351	0.355271557	0.427325038	0.896116841	0.405207591	0.759003017
LOC107132820	0.495033351	0.466588762	0.395501027	0.462657114	0.627206089	0.880355149
C15H11orf94	0.49504576	0.744774335	0.427152347	0.565578589	0.521036349	0.622811146
COL1A2	0.495141595	0.410855444	0.695416854	0.734460659	0.578296318	0.458837523
THAP3	0.495141595	0.490111519	0.843955813	0.575086938	0.457770074	0.487514327
CA9	0.495141595	0.335259566	0.497091612	0.88099367	0.577102225	0.593981666
NDUFAB1	0.495296894	0.362371483	0.560469326	0.451094074	0.87329316	0.645719071
PGM5	0.495419804	0.744671791	0.517859609	0.464653418	0.403447621	0.749918186
LOC100848208	0.495419804	0.375367521	0.507083871	0.543245865	0.631319169	0.81853698
DRG1	0.495419804	0.344296647	0.605191943	0.486381297	0.428442971	0.947838082
CHST6	0.495435867	0.32719831	0.731143569	0.731537809	0.593931025	0.51212265
IMP4	0.495435867	0.746712661	0.473560084	0.438595551	0.571639895	0.672418631
LOC101903758	0.495639504	0.553132278	0.555640272	0.525230287	0.605738596	0.641401171
COL8A1	0.495759067	0.492794915	0.605532453	0.712481274	0.656015118	0.408921503
SEMA3D	0.496459213	0.504727219	0.713233032	0.518542038	0.472808893	0.678166983
RAB5C	0.496807434	0.822956796	0.489549483	0.459073822	0.690450229	0.43348847
DAZAP2	0.496889227	0.360897508	0.98258069	0.528783909	0.487063655	0.420611221
PAQR7	0.496889227	0.333827203	0.481165053	0.730052184	0.858495696	0.49158696
LOC101902083	0.496889227	0.831153015	0.607986114	0.440192972	0.407114819	0.619388281
LOC112443469	0.496889227	0.355173924	0.521645647	0.586033659	0.574180707	0.844929051
OSBP2	0.497413457	0.612070685	0.502079089	0.596654953	0.786314903	0.400277507
LOC785804	0.497413457	0.576446091	0.676501557	0.551641524	0.670221463	0.418173662
ZBP1	0.497413457	0.690635606	0.565148263	0.486381297	0.738738532	0.426772334
ADAMTS2	0.497413457	0.449821474	0.754193103	0.636814098	0.554711391	0.481287134
COX5B	0.497413457	0.358391448	0.566638914	0.459073822	0.916605572	0.587132866
LOC100848469	0.497413457	0.413330726	0.531780431	0.806573529	0.498467324	0.629427844
IGF1	0.497413457	0.445080617	0.648593816	0.704647823	0.458370193	0.640128675
FADS6	0.497413457	0.479687945	0.397871714	0.777956162	0.533807401	0.68185373
TARS	0.497413457	0.350220269	0.658489624	0.724431807	0.439547962	0.737726964
LOC100848883	0.497413457	0.45450823	0.47471765	0.441405457	0.405207591	0.9790177
DAGLA	0.497808539	0.758609923	0.492173361	0.630951216	0.610004585	0.396383896
RAD50	0.497808539	0.412100649	0.628904157	0.907621547	0.445175487	0.452024212
ELFN1	0.497808539	0.435344594	0.488784977	0.930987593	0.495629679	0.470494326

SEC31A	0.497808539	0.398295321	0.489549483	0.765499767	0.737149805	0.503701037
DNAJC21	0.497808539	0.361164768	0.942357321	0.46682385	0.458370193	0.628379498
ATP5MF	0.497808539	0.370464508	0.542055154	0.451094074	0.901144467	0.628379498
C11H2orf40	0.497808539	0.490326582	0.395501027	0.466614794	0.886944613	0.640825046
DGKB	0.497808539	0.479073963	0.711148626	0.462303912	0.499400667	0.741459783
ADGRV1	0.497808539	0.479687945	0.397871714	0.797338497	0.403447621	0.784782855
DNAL4	0.497863914	0.469660184	0.630408979	0.609774743	0.682784145	0.506109202
DCTD	0.498325027	0.382320412	0.839760004	0.435097625	0.49499356	0.742687461
MTMR12	0.498325027	0.428728874	0.457422774	0.518542038	0.681367696	0.814218101
EGLN1	0.498325027	0.373964414	0.427325038	0.501491101	0.691186726	0.876861428
ZNF391	0.498376343	0.333891631	0.679415149	0.759397892	0.626973048	0.522289438
GFI1	0.498620354	0.52001926	0.874983674	0.515539653	0.467076905	0.491178906
CUL7	0.498620354	0.355271557	0.845525761	0.731537809	0.401768956	0.568308927
LOC787858	0.498885868	0.383639708	0.605191943	0.568299686	0.651951691	0.71289069
FAM167A	0.49906565	0.360723284	0.419761137	0.675426128	0.413792445	0.944973383
DLGAP5	0.499327388	0.383639708	0.818612484	0.728264598	0.487063655	0.458837523
LOC100848689	0.49937089	0.414302593	0.43314299	0.599807615	0.47341511	0.917024468
NDUFV2	0.499588816	0.340322836	0.605532453	0.466234452	0.81911207	0.709205243
ATP6AP1	0.499588816	0.428191139	0.651356851	0.695125147	0.407783132	0.720922938
CYC1	0.500468721	0.390262333	0.532764011	0.438595551	0.940870936	0.566407295
PLAA	0.500468721	0.364362847	0.513648728	0.528783909	0.487063655	0.930710695
S100PBP	0.500506399	0.583598898	0.429389644	0.83795554	0.403447621	0.626045106
ABCC5	0.500506399	0.333891631	0.552162548	0.645168798	0.486387163	0.857457228
SMYD5	0.502309438	0.445034796	0.720612298	0.466614794	0.428442971	0.835231445
CCDC189	0.503123628	0.587350756	0.797005537	0.554743688	0.533903668	0.414167655
FAM210A	0.503123628	0.355271557	0.739982136	0.486381297	0.624785761	0.736866513
EIF4EBP1	0.503172857	0.343951817	0.499640977	0.999981514	0.426006781	0.43348847
GPLD1	0.503172857	0.642929022	0.620613545	0.498444775	0.516804874	0.640128675
DYM	0.503172857	0.412566059	0.611794073	0.587839433	0.472808893	0.81853698
MAP4K1	0.503384291	0.457605323	0.774924967	0.528783909	0.492863282	0.645427231
AKIP1	0.503654618	0.423816704	0.754913805	0.495562454	0.823508064	0.42204128
STAM	0.503654618	0.364926892	0.606660869	0.838528071	0.6274106	0.470494326
CTSB	0.503654618	0.406494847	0.507675433	0.965724803	0.405207591	0.529931934
TMEM263	0.503654618	0.340788135	0.744735369	0.688019411	0.585856348	0.578851812
NDUFB3	0.503654618	0.375367521	0.487875367	0.466234452	0.956761712	0.587387684
CRLF1	0.503654618	0.383545311	0.528387114	0.558176707	0.849554534	0.601371091
CDC37L1	0.503661984	0.361145127	0.492173361	0.675565794	0.652333594	0.748323122
PHF11	0.503682066	0.332081544	0.727619107	0.707217807	0.429015291	0.741500497

PRNP	0.503682066	0.439142493	0.507675433	0.515352026	0.656015118	0.793197037
METTL21A	0.50481191	0.686033606	0.682700098	0.541206056	0.527685234	0.48181553
SHANK2	0.50481191	0.621713316	0.457350984	0.466614794	0.845802744	0.521756022
SPATA46	0.504946517	0.354693769	0.999993442	0.530577799	0.424448433	0.437877648
ANKRD23	0.504946517	0.367446738	0.916038418	0.691025178	0.429580883	0.465840174
SNCAIP	0.504946517	0.382320412	0.81412252	0.75404116	0.486387163	0.466700097
FANCE	0.504946517	0.469660184	0.665599776	0.648660726	0.599090915	0.529445217
DNMT3A	0.504946517	0.36444467	0.608218151	0.805302469	0.607459418	0.543766428
FASLG	0.504946517	0.571373541	0.51081359	0.637708629	0.590122535	0.601371091
LOC512005	0.504946517	0.382853453	0.819359511	0.554743688	0.506535763	0.645719071
TEFM	0.505180434	0.382853453	0.764467593	0.473606343	0.457770074	0.83150957
SQSTM1	0.505445202	0.377729751	0.509514515	0.748424012	0.419682467	0.83821684
ZFP41	0.505547128	0.337247845	0.48405583	0.677545541	0.766650514	0.695352244
CCNDBP1	0.505644705	0.382853453	0.797180963	0.627280162	0.491185174	0.622811146
CDV3	0.505644705	0.445034796	0.606660869	0.498444775	0.45951987	0.877014986
NOL4L	0.505981344	0.363208684	0.397871714	0.630535638	0.885118272	0.65014676
SYAP1	0.505981344	0.358391448	0.734143952	0.598316303	0.487063655	0.754218406
TMED3	0.506201519	0.672505775	0.623431824	0.66651301	0.469611067	0.487514327
GMPR	0.506201519	0.494454594	0.749854422	0.717652404	0.447765878	0.490013062
RNPEP	0.506201519	0.362371483	0.532764011	0.518542038	0.959565914	0.494666285
SMARCA1	0.506328982	0.722779653	0.41426165	0.480047213	0.814655215	0.50354757
LOC101904976	0.507278851	0.472312407	0.665599776	0.518542038	0.738738532	0.552868236
PIGQ	0.507278851	0.337426986	0.406919123	0.462657114	0.984386856	0.655022943
CPAMD8	0.507664548	0.376269829	0.608809506	0.482915217	0.86876405	0.616975059
LOC112447819	0.507664548	0.741310853	0.427325038	0.484737404	0.487063655	0.78096075
NPTX1	0.507832711	0.374472361	0.56802023	0.880851997	0.470098343	0.623290811
KERA	0.507832711	0.671536686	0.582294342	0.451094074	0.578333624	0.669558041
AKTIP	0.507897053	0.434208808	0.71601562	0.71389957	0.631537325	0.440866698
TMOD1	0.507897053	0.386477216	0.923578245	0.457902257	0.657647754	0.469313855
KLK8	0.507897053	0.530724395	0.642023154	0.827786827	0.427233204	0.475399219
NPNT	0.507897053	0.796253817	0.659919107	0.55931035	0.423015995	0.495999863
MAST4	0.507897053	0.473734192	0.478270125	0.514698825	0.934450558	0.496415296
NPTXR	0.507897053	0.40372842	0.415690997	0.889472032	0.681080458	0.518765111
MFGE8	0.507897053	0.441536334	0.457350984	0.686250223	0.821351993	0.521756022
EML3	0.507897053	0.341285659	0.592586313	0.910495312	0.517239659	0.529445217
SHISA4	0.507897053	0.404705887	0.552362414	0.71113156	0.684528053	0.594772227
RAD23A	0.507897053	0.450686302	0.752192353	0.631620874	0.472699899	0.621946029
WBP2	0.507897053	0.45627172	0.517382044	0.731537809	0.605738596	0.622811146

NUDT16L1	0.507897053	0.637453144	0.658489624	0.499579373	0.473071776	0.675688253
ZSCAN29	0.507897053	0.341285659	0.487875367	0.882395013	0.461235361	0.748921056
TSHZ3	0.507995122	0.364606615	0.489602416	0.848131881	0.764945406	0.470494326
DIRAS3	0.507995122	0.792967492	0.66149866	0.501491101	0.419682467	0.580973226
VPS18	0.507995122	0.364606615	0.428815497	0.699546881	0.470098343	0.911808092
CHD1	0.508091619	0.37787072	0.400888251	0.456103613	0.999985752	0.426707367
KDM7A	0.508091619	0.474946062	0.470895554	0.537984757	0.927086179	0.4899194
JPT2	0.508091619	0.355173924	0.71967891	0.707217807	0.495273374	0.672418631
ACOT2	0.508091619	0.361527232	0.46534436	0.484737404	0.925918507	0.696420214
GNGT2	0.509238835	0.647999098	0.625861236	0.598316303	0.618211409	0.458773762
GPR37	0.509238835	0.3805254	0.886829776	0.598316303	0.543569691	0.51212265
SPOPL	0.509238835	0.364362847	0.532743798	0.632177478	0.867529283	0.537186568
PAM	0.509238835	0.660882273	0.518829767	0.554743688	0.634894348	0.572514995
CLECL1	0.509238835	0.442990825	0.513255922	0.789139053	0.591306131	0.609692015
PRICKLE2	0.509238835	0.582051417	0.407806986	0.609204337	0.696466814	0.660603184
IL17B	0.509238835	0.582127335	0.64236045	0.481018469	0.57991354	0.669558041
CABCOCO1	0.509238835	0.446395731	0.465246113	0.454517255	0.686042858	0.872239777
PROS1	0.509358576	0.674524738	0.596931535	0.677545541	0.5863408	0.409664289
RORC	0.509358576	0.342824969	0.579487023	0.865076519	0.502432219	0.645719071
LOC618169	0.50961825	0.366629388	0.59950726	0.784595865	0.75602825	0.453456176
EIF5	0.50961825	0.355271557	0.533145072	0.534082333	0.437165526	0.96870908
ATPAF2	0.509690485	0.594126609	0.487694723	0.466614794	0.726283157	0.68185373
EIF4G2	0.510024176	0.348585097	0.855847948	0.80459801	0.487063655	0.437877648
BPHL	0.510024176	0.652446942	0.566638914	0.519487474	0.568764846	0.645719071
LOC104969384	0.510024176	0.39388102	0.569818343	0.790631706	0.535275659	0.652300161
ABCB8	0.510024176	0.434898742	0.485200576	0.451094074	0.909379774	0.667726353
KIAA1958	0.510024176	0.356628231	0.411289123	0.517860657	0.887688917	0.773306611
NDUFAF4	0.510024176	0.382853453	0.759332589	0.451094074	0.450726215	0.870018677
ATP6V0A2	0.510024176	0.38381979	0.685018711	0.464082401	0.456868044	0.911336121
LOC112446351	0.510477704	0.3805254	0.547053998	0.949605617	0.555125415	0.442970192
LOC107132469	0.510477704	0.489987041	0.499640977	0.62496659	0.487063655	0.814218101
CAND2	0.510477704	0.451236244	0.40651486	0.645168798	0.468776461	0.909056553
CARNS1	0.510900117	0.512513718	0.450940071	0.54857232	0.864524107	0.580973226
TCP11L2	0.510956141	0.376269829	0.71967891	0.715394268	0.738738532	0.418173662
LOC524810	0.510956141	0.41466518	0.774025101	0.676566861	0.670221463	0.420532744
OMA1	0.510956141	0.865455711	0.427325038	0.729912904	0.487063655	0.426336374
THBS3	0.510956141	0.477035321	0.55950158	0.764257755	0.715674124	0.431260036
LOC783604	0.510956141	0.51525676	0.684282335	0.705801894	0.607314284	0.440866698

ZNF688	0.510956141	0.425196086	0.886208004	0.695681179	0.437165526	0.470494326
IQANK1	0.510956141	0.358391448	0.917651622	0.654131191	0.491185174	0.471903579
NDUFS8	0.510956141	0.469272895	0.524780246	0.456103613	0.892446951	0.605297754
TMEM169	0.510956141	0.419392688	0.640084371	0.576242366	0.602648954	0.737726964
LOC787269	0.510956141	0.532096752	0.509514515	0.67024921	0.461235361	0.754218406
LOC101902665	0.510956141	0.367446738	0.522478083	0.698952489	0.517239659	0.82345874
MTBP	0.510956141	0.589754585	0.473560084	0.46351377	0.584450219	0.83150957
LOC112446012	0.510956141	0.48520817	0.497091612	0.59866737	0.487063655	0.840291265
EPHA4	0.510961514	0.377081072	0.405441139	0.998667933	0.514458714	0.515726083
TMEM223	0.510961514	0.412566059	0.627084316	0.519487474	0.811991102	0.614511437
PROKR1	0.510961514	0.45629714	0.57816803	0.680418978	0.477053031	0.749726404
IL6ST	0.510961514	0.376819185	0.487875367	0.53954651	0.450726215	0.973649216
BTG3	0.511382391	0.364362847	0.716635829	0.493932071	0.479782826	0.875650601
NELL1	0.511982576	0.690635606	0.457051074	0.609774743	0.788007619	0.440866698
APOBEC3H	0.511982576	0.45438297	0.487875367	0.886312303	0.443703224	0.666011114
ZNF318	0.511982576	0.343951817	0.68898955	0.712481274	0.410921459	0.806476213
HIPK3	0.512189156	0.358391448	0.58300263	0.659427594	0.90673121	0.433908714
PSPH	0.512189156	0.429850159	0.583883256	0.475888955	0.865075514	0.621946029
KPNA2	0.512189156	0.382853453	0.611647147	0.611379189	0.457770074	0.867063767
KIF27	0.512189156	0.42633922	0.478168172	0.695125147	0.433889339	0.876162019
IFI44	0.51325926	0.499388045	0.671469816	0.543245865	0.801692604	0.448702315
AGBL5	0.51325926	0.474946062	0.607986114	0.630804516	0.60702401	0.645719071
USP53	0.513409151	0.360897508	0.490749653	0.734472882	0.887514169	0.475170024
TXNL1	0.513409151	0.361363832	0.689992074	0.623608478	0.449903641	0.839489406
ATG13	0.514055248	0.471255215	0.731143569	0.613744304	0.489930391	0.655296683
BATF2	0.514299673	0.479687945	0.596826854	0.457902257	0.913013159	0.491178906
CERS4	0.514689649	0.480755771	0.422397986	0.733411985	0.788995893	0.531378095
ZNF554	0.515468502	0.796253817	0.460075169	0.796128007	0.483018928	0.426054677
IL1RAP	0.515692868	0.414912961	0.56863752	0.457715968	0.947112796	0.534954937
NTS	0.515847568	0.383639708	0.791198027	0.823183126	0.523124197	0.420611221
AKAP7	0.515847568	0.463158835	0.45863002	0.600433884	0.958797242	0.423791075
PERP	0.515847568	0.724633427	0.654545673	0.549287144	0.570517621	0.481473069
LOC112447371	0.516425113	0.378476235	0.684274697	0.95901497	0.468542094	0.41766438
HBEGF	0.516425113	0.482976744	0.818612484	0.466614794	0.738738532	0.457840591
HIST1H3G	0.517298609	0.41595634	0.489549483	0.673160239	0.463102881	0.877014986
HSPBP1	0.517437445	0.386477216	0.607293453	0.990975625	0.423319935	0.442520879
PNCK	0.518742856	0.904875256	0.499640977	0.627280162	0.473623717	0.442234422
GZMA	0.518742856	0.367446738	0.777154688	0.812453944	0.553835124	0.45324563

LETM1	0.518742856	0.693513972	0.525415185	0.493932071	0.776537165	0.503701037
MFSD13A	0.518742856	0.648503747	0.443690681	0.613744304	0.696466814	0.593981666
SHANK1	0.518742856	0.368470917	0.532415258	0.486381297	0.587200465	0.934112488
SLC23A1	0.519405006	0.558327847	0.499164335	0.759755663	0.457254874	0.684994779
WNT5A	0.519486704	0.457693965	0.505553867	0.868435169	0.418020354	0.699160709
TMEM87A	0.520122468	0.634059237	0.538528639	0.832489049	0.508689889	0.423834625
PBXIP1	0.520122468	0.460605417	0.533570754	0.466614794	0.429015291	0.963456717
PMVK	0.520517182	0.532096752	0.518439818	0.722996827	0.721096295	0.476101066
TRIM21	0.520517182	0.806326521	0.627084316	0.541053342	0.50652393	0.495999863
MBNL3	0.520517182	0.589651566	0.479024795	0.636814098	0.721096295	0.552868236
C29H11orf24	0.520517182	0.466901018	0.725264968	0.598316303	0.446373961	0.748921056
MAP3K13	0.520624275	0.674524738	0.487875367	0.748424012	0.527202266	0.529445217
LOC781304	0.520624275	0.483305224	0.66149866	0.609774743	0.695436567	0.540972908
ARHGEF5	0.520931289	0.387719774	0.854444064	0.705448435	0.553835124	0.462837324
SH3GLB1	0.520931289	0.3495324	0.897322115	0.565933683	0.597314504	0.566196709
GMFB	0.521615345	0.42744802	0.567712191	0.484390589	0.455516234	0.958281344
CAPS2	0.521640495	0.517719875	0.418121393	0.907621547	0.658667583	0.434454711
YTHDF3	0.521640495	0.463660565	0.658489624	0.863356729	0.533903668	0.440866698
ZNF862	0.521640495	0.489987041	0.824657779	0.518542038	0.696466814	0.447212551
TENM2	0.521640495	0.728267541	0.566638914	0.570760743	0.64727886	0.486586811
ZMYND19	0.521640495	0.583275815	0.871782109	0.541206056	0.469471718	0.491178906
MFNG	0.521640495	0.773939845	0.487875367	0.577737722	0.670221463	0.491178906
FAM71E1	0.521640495	0.582394134	0.772600931	0.610848628	0.484413225	0.519240641
GPC4	0.521640495	0.502437895	0.4551661	0.89478385	0.571639895	0.521756022
LOC616199	0.521640495	0.464957075	0.426474434	0.812354594	0.705202026	0.587387684
LOC104971374	0.521640495	0.355173924	0.648593816	0.874424802	0.472139664	0.641401171
FAM229A	0.521640495	0.412100649	0.534905861	0.493932071	0.883951804	0.668851588
C5H12orf75	0.521640495	0.710815877	0.481165053	0.622980329	0.502705054	0.672587014
CITED2	0.521640495	0.454880625	0.526976806	0.739954987	0.498467324	0.748921056
MRPS34	0.521640495	0.454880625	0.611794073	0.46351377	0.705202026	0.762133815
ITGA3	0.521640495	0.450686302	0.427489789	0.636814098	0.658667583	0.796402766
SEM1	0.521640495	0.412566059	0.52262636	0.528783909	0.429015291	0.968741623
CDC27	0.521848152	0.452650248	0.799265115	0.531399091	0.429015291	0.765070525
LOC101902656	0.522563604	0.415447733	0.476781476	0.9880671	0.44198836	0.564826731
CSNK2B	0.522761963	0.429710219	0.759332589	0.664953033	0.453983763	0.68185373
TMEM94	0.522761963	0.376659499	0.55950158	0.527867968	0.810436875	0.741319465
TSGA10	0.52293886	0.375367521	0.487875367	0.980564765	0.626733381	0.438685707
MPHOSPH9	0.52293886	0.378985237	0.971053208	0.498444775	0.623804687	0.447212551

TMEM119	0.52293886	0.383639708	0.538402463	0.962174761	0.52068239	0.493918515
TTI1	0.52293886	0.355173924	0.443690681	0.964521432	0.48367155	0.683495103
SPICE1	0.52293886	0.469660184	0.444417407	0.792386115	0.442033318	0.81956221
ARHGEF12	0.524082825	0.369330799	0.565148263	0.543346467	0.584450219	0.893838339
SYNE2	0.524340962	0.480060264	0.675096377	0.652519777	0.443519966	0.741017467
CAND1	0.524489806	0.452080332	0.604354543	0.598316303	0.834569503	0.514507804
SUN2	0.524489806	0.355591197	0.528387114	0.780184803	0.671669027	0.68185373
DCUN1D2	0.524489806	0.50468844	0.70011973	0.46351377	0.488757973	0.81956221
BEND3	0.525660187	0.360897508	0.495739729	0.912777307	0.764945406	0.440866698
NCAPD2	0.52568225	0.515680499	0.77843611	0.633006168	0.455838272	0.60936417
DIRC2	0.52602051	0.373964414	0.424739024	0.876703811	0.665098448	0.659761491
TAL1	0.526547798	0.471255215	0.817582037	0.612761692	0.439663672	0.659134809
LOC101906226	0.526547798	0.386477216	0.441071049	0.878291315	0.627206089	0.666292389
LOC112445999	0.526547798	0.362371483	0.492173361	0.90413565	0.502432219	0.700746926
NEK4	0.526547798	0.716749236	0.481165053	0.54137792	0.487063655	0.768168974
GPI	0.526547798	0.370475687	0.481165053	0.466234452	0.847173934	0.836705424
GLRX3	0.526547798	0.36901158	0.660344165	0.582177282	0.425159106	0.921843102
OGFOD2	0.527897789	0.495857219	0.560847675	0.552451497	0.883951804	0.489274479
SDC1	0.528042399	0.382853453	0.487875367	0.98981682	0.596709577	0.440512821
LOC112443141	0.528356376	0.355173924	0.708018623	0.833973634	0.656015118	0.458773762
LRRC17	0.529629512	0.618921732	0.507675433	0.941796131	0.433889339	0.431260036
ARF1	0.529629512	0.360897508	0.866467804	0.886662305	0.429015291	0.438685707
LRP6	0.529629512	0.668852983	0.43164239	0.486381297	0.936921784	0.444913711
NKD1	0.529629512	0.415447733	0.456936866	0.939604997	0.626973048	0.522828605
FAM219B	0.529629512	0.469660184	0.569273084	0.486381297	0.923910588	0.529445217
DUSP2	0.529629512	0.386477216	0.811480663	0.493932071	0.788007619	0.560309811
FSCN1	0.529629512	0.35609607	0.462160789	0.999981514	0.429015291	0.583681742
GPX7	0.529629512	0.36159739	0.711148626	0.903242359	0.427233204	0.587387684
UNC13B	0.529629512	0.45450823	0.788126358	0.504242357	0.678243175	0.609692015
SPIB	0.529629512	0.43124616	0.648593816	0.843593618	0.450288791	0.633344697
SAP130	0.529629512	0.382853453	0.421396526	0.983738237	0.472139664	0.663182265
LOC112442593	0.529629512	0.360897508	0.548262961	0.695125147	0.701686512	0.723555931
MPV17L	0.529629512	0.440549685	0.487578581	0.519254756	0.789915129	0.789030652
LOC101906546	0.529629512	0.367446738	0.427325038	0.493932071	0.903866359	0.801888363
LOC107131367	0.529629512	0.360897508	0.668727476	0.702327474	0.455838272	0.829202617
CBX5	0.529629512	0.395925974	0.615030265	0.472217546	0.591515687	0.900500341
POLA2	0.529629512	0.355271557	0.620613545	0.499858161	0.476090036	0.967308566
SLC19A2	0.53020887	0.366070217	0.509854589	0.999981514	0.491185174	0.440866698

PPP6R2	0.531084329	0.428324786	0.689000346	0.914778844	0.506010332	0.428431098
RGN	0.531130706	0.664859217	0.478270125	0.612761692	0.789915129	0.49105688
GOLT1B	0.531352275	0.386477216	0.885896294	0.727797158	0.450884601	0.537094861
EDNRA	0.531352275	0.831153015	0.545866468	0.550331985	0.463102881	0.628917359
RABEP2	0.531471541	0.411803396	0.57588937	0.960937718	0.507992942	0.476507871
MROH6	0.531542045	0.54021232	0.867222363	0.640315346	0.486387163	0.442970192
MTDH	0.531542045	0.382744251	0.92091815	0.586089426	0.483106459	0.621946029
KCNMB2	0.531542045	0.355591197	0.759332589	0.564748167	0.596709577	0.757776634
ZNF704	0.531542045	0.456224933	0.622754083	0.587186699	0.429580883	0.877014986
SEC61A1	0.531542045	0.531880429	0.555428156	0.551641524	0.443267567	0.877048993
AFF4	0.531614393	0.372319879	0.605656687	0.498444775	0.996781978	0.433152599
GTF2H1	0.531614393	0.42132992	0.45863002	0.554743688	0.98865576	0.481287134
TMEM177	0.531614393	0.430996205	0.533570754	0.466234452	0.986003146	0.490558738
TEX22	0.531614393	0.356628231	0.909173866	0.639281534	0.600125756	0.495197104
POLR3K	0.531614393	0.452134986	0.652681076	0.486381297	0.821557309	0.632381609
SARAF	0.531614393	0.375367521	0.517859609	0.92891806	0.499818667	0.645719071
SDHAF4	0.53175717	0.411778074	0.524780246	0.630535638	0.955623649	0.440866698
HECTD3	0.531926821	0.469660184	0.883500436	0.572764879	0.642796813	0.440866698
ENKD1	0.531935746	0.382853453	0.809840329	0.812453944	0.455838272	0.554751652
BLVRB	0.531935746	0.446062855	0.526325915	0.578735575	0.696466814	0.780283623
LOC112443479	0.531985104	0.623984886	0.648593816	0.833973634	0.433645579	0.456099659
UBR3	0.531985104	0.413330726	0.509514515	0.769286764	0.836444721	0.490415687
TCTN2	0.531985104	0.469660184	0.612165542	0.915013589	0.472808893	0.495197104
PRMT5	0.531985104	0.3805254	0.485705164	0.799480811	0.804037558	0.580125529
TFDP1	0.531985104	0.579825729	0.649378275	0.613568161	0.578333624	0.623996305
CYR61	0.531985104	0.386477216	0.627295843	0.898658215	0.457135312	0.632510367
NEXN	0.531985104	0.532096752	0.509514515	0.622980329	0.466153859	0.852504043
ALDOA	0.532766715	0.522698706	0.517859609	0.506045489	0.613652691	0.842654067
MYCN	0.532792462	0.460244277	0.700349135	0.888687722	0.457770074	0.490013062
F5	0.532792462	0.399167149	0.727619107	0.518542038	0.85231994	0.551701946
ADM2	0.532792462	0.360897508	0.693546993	0.609774743	0.802592922	0.613547915
CYP2S1	0.532885587	0.449821474	0.648593816	0.686250223	0.491185174	0.748921056
NDUFC1	0.532885587	0.454880625	0.582638793	0.467679691	0.792867703	0.75062693
STX18	0.532894004	0.414670902	0.794225743	0.731537809	0.652333594	0.440866698
AP2B1	0.53341876	0.386477216	0.64428623	0.65294576	0.882831953	0.452024212
LOC101907803	0.53341876	0.383450541	0.466192774	0.999981514	0.472139664	0.496415296
SORCS1	0.533807582	0.684972622	0.481165053	0.855893886	0.469471718	0.519240641
LOC112443176	0.533846904	0.47375776	0.587609898	0.867235478	0.495333297	0.587132866

GOT1L1	0.534025178	0.531880429	0.507675433	0.486381297	0.814253177	0.695765939
FAM20A	0.534730767	0.471255215	0.625861236	0.640315346	0.563212168	0.736468943
SSB	0.534940672	0.370464508	0.494944201	0.896116841	0.635733222	0.622811146
SPATA7	0.534940672	0.358391448	0.455195587	0.951936643	0.515394956	0.700326694
DOK5	0.535355324	0.709247882	0.492173361	0.621219513	0.781724458	0.452024212
EFCAB7	0.535355324	0.41398295	0.507675433	0.99542943	0.455838272	0.533823331
KCTD3	0.535355324	0.364926892	0.470222822	0.759591583	0.626973048	0.81956221
FKBP10	0.535988029	0.40372842	0.680262176	0.941467375	0.510001595	0.442970192
MYH10	0.535988029	0.452080332	0.454835278	0.734472882	0.91073527	0.455806019
PAK2	0.535988029	0.38719103	0.754913805	0.714751696	0.721826916	0.462837324
KLRG2	0.535988029	0.454880625	0.999993442	0.484737404	0.429015291	0.466256919
ENDOV	0.535988029	0.614976364	0.538402463	0.885789665	0.467557398	0.494666285
IRAK2	0.535988029	0.469544329	0.437954478	0.810557898	0.803705802	0.517000508
SLC43A2	0.535988029	0.454880625	0.791843351	0.594220399	0.582706032	0.631731716
HYPK	0.535988029	0.587539403	0.711148626	0.613744304	0.487063655	0.637500569
SLC16A6	0.535988029	0.559306577	0.48405583	0.691025178	0.488357336	0.783241453
CANX	0.535988029	0.366629388	0.574056986	0.565933683	0.431226303	0.973649216
TPM1	0.536499911	0.750379179	0.502079089	0.522681653	0.437165526	0.816246746
MYH9	0.536734064	0.415447733	0.476465349	0.493932071	0.999985752	0.47629249
NT5E	0.536756748	0.571373541	0.599676268	0.479863522	0.870042072	0.521756022
LLPH	0.536926706	0.38381979	0.905574702	0.531399091	0.461764223	0.736468943
MRPS18C	0.537471003	0.363208684	0.684382692	0.626698454	0.908528514	0.460497664
LOC100336476	0.537471003	0.471255215	0.855713041	0.64853083	0.530499378	0.507632407
FGD2	0.537471003	0.45629714	0.770497031	0.619201546	0.613652691	0.601767799
PNPT1	0.537471003	0.454880625	0.608218151	0.513533242	0.879395665	0.608554064
MSL1	0.537471003	0.358391448	0.507675433	0.641165011	0.902907077	0.625066198
C16H1orf159	0.537471003	0.725495584	0.450940071	0.730052184	0.511621842	0.631731716
KAZN	0.537471003	0.67048768	0.436168983	0.718713712	0.577855141	0.655022943
DBR1	0.537471003	0.362371483	0.549830614	0.570760743	0.900893337	0.668851588
NDUFA13	0.537471003	0.418480843	0.534947183	0.519684186	0.887514169	0.68185373
DNAJC28	0.537471003	0.368301394	0.546238984	0.549287144	0.89052284	0.690728863
FIG4	0.537471003	0.469544329	0.454835278	0.777956162	0.651585054	0.705204221
RARRES2	0.537471003	0.447485891	0.54405026	0.499858161	0.836444721	0.733694301
SQOR	0.537471003	0.386477216	0.608218151	0.723302519	0.535275659	0.783241453
CD164	0.537471003	0.372826863	0.532743798	0.682383061	0.483224704	0.91373422
PTPRCAP	0.537832934	0.582051417	0.550289803	0.69158245	0.531159884	0.68185373
MYL9	0.537963828	0.693373829	0.534905861	0.598316303	0.499400667	0.727229543
KLHL3	0.539882612	0.659622605	0.669511234	0.714451311	0.571784062	0.440866698

P4HA1	0.539914972	0.441007379	0.455195587	0.698605165	0.678783788	0.783005026
YKT6	0.539914972	0.440549685	0.745477682	0.546397256	0.487063655	0.818370468
MGC157082	0.539921275	0.360897508	0.742539165	0.708466343	0.487063655	0.754218406
C8G	0.54063484	0.434898742	0.915191964	0.692226	0.512833021	0.448403672
LRRC66	0.54063484	0.568485668	0.480312073	0.932914414	0.445175487	0.568770289
LOC100299242	0.541526589	0.363208684	0.481165053	0.687291299	0.573188535	0.911219114
SCX	0.542347321	0.822956796	0.81412252	0.478709485	0.489920278	0.442234422
LOC101907566	0.542798213	0.531880429	0.66149866	0.493932071	0.663923284	0.729319161
LOC100847304	0.542943077	0.469660184	0.711148626	0.499858161	0.765965392	0.645719071
DEXI	0.54324123	0.428728874	0.744735369	0.529579445	0.728185525	0.656746469
REEP1	0.543486875	0.40117451	0.999993442	0.543346467	0.498546902	0.440866698
MAOB	0.543486875	0.364749437	0.625439953	0.598316303	0.965676623	0.454893533
TMEM245	0.543486875	0.790429889	0.649622134	0.65294576	0.502957335	0.462837324
LSM14B	0.543486875	0.49771159	0.606230025	0.630951216	0.852234727	0.469601694
LOC100141253	0.543486875	0.947350735	0.643516006	0.493932071	0.461235361	0.495197104
CENPA	0.543486875	0.452206305	0.752113836	0.831577385	0.495333297	0.495999863
HSP90B1	0.543486875	0.425196086	0.450940071	0.939604997	0.705202026	0.500317514
TET2	0.543486875	0.428129561	0.541510288	0.868378265	0.716994185	0.501207489
CEMIP	0.543486875	0.470862073	0.50425164	0.941467375	0.58186871	0.501733747
GTF3C1	0.543486875	0.370464508	0.849389234	0.819213899	0.483224704	0.524890122
TAPT1	0.543486875	0.45329653	0.487875367	0.893220599	0.668182282	0.535620846
CLEC11A	0.543486875	0.584209799	0.546717582	0.559525332	0.788995893	0.609692015
CCL25	0.543486875	0.660882273	0.561599458	0.518542038	0.738738532	0.621946029
MTHFD2	0.543486875	0.505171225	0.560847675	0.793876439	0.57367759	0.632510367
ELP6	0.543486875	0.415447733	0.583120434	0.559525332	0.877712931	0.641823119
ADRB3	0.543486875	0.608823572	0.632469403	0.553396512	0.638815482	0.645719071
COL26A1	0.543486875	0.581251143	0.732274253	0.552451497	0.52589341	0.669558041
MRPL51	0.543486875	0.365882129	0.622890829	0.513533242	0.895067787	0.68185373
SMARCC2	0.543486875	0.428728874	0.567712191	0.622024344	0.772501291	0.702623424
MDH2	0.543486875	0.375979337	0.546717582	0.504242357	0.902170532	0.729499398
LOC509513	0.543486875	0.532115072	0.759332589	0.518542038	0.510264075	0.735122635
TMEM229A	0.543486875	0.660404537	0.552362414	0.613744304	0.501311839	0.737362929
BAG1	0.543486875	0.373964414	0.77566312	0.666541609	0.489920278	0.75062693
ADO	0.543486875	0.449965992	0.71967891	0.677374927	0.457358633	0.759003017
RASGRP1	0.543486875	0.54021232	0.596826854	0.51481969	0.664519875	0.762133815
LOC768255	0.543486875	0.438643862	0.639410099	0.658587613	0.571639895	0.766435538
LOC101903501	0.543486875	0.553132278	0.443690681	0.630535638	0.651951691	0.779201808
LAMB1	0.543486875	0.411108591	0.481165053	0.553146279	0.788995893	0.839906041

ANKRD26	0.543486875	0.367446738	0.566638914	0.786099236	0.483789259	0.841451122
CFAP300	0.543486875	0.4149983	0.625105036	0.686250223	0.460237064	0.852608759
ALG13	0.543486875	0.414881121	0.497992443	0.632177478	0.603965612	0.875297569
FAM98A	0.543486875	0.382744251	0.735532321	0.498626862	0.481583803	0.919124527
LOC100848684	0.54381997	0.479687945	0.671003058	0.705405905	0.721826916	0.500317514
CACNA1D	0.543912191	0.78357023	0.774015998	0.626698454	0.450726215	0.440866698
IGSF3	0.544195893	0.412566059	0.538402463	0.916858019	0.650485683	0.51212265
SNRPD3	0.544195893	0.412100649	0.733850691	0.656101697	0.533903668	0.74201596
PTPRO	0.5446716	0.54021232	0.744735369	0.619231292	0.71597087	0.458773762
C17H22orf39	0.5446716	0.428324786	0.458539546	0.518542038	0.999985752	0.503701037
NUDT13	0.544844659	0.38381979	0.526976806	0.518542038	0.811991102	0.831652079
DIP2B	0.544991198	0.378622993	0.478270125	0.973075232	0.626634623	0.551180671
IMPG2	0.545080536	0.43474227	0.481165053	0.541206056	0.887514169	0.735392644
RBFOX2	0.545080536	0.553638694	0.534905861	0.618288053	0.487063655	0.839565788
LOC104973322	0.545080536	0.40425517	0.58300263	0.637708629	0.489920278	0.899183258
HIGD1A	0.545270618	0.428324786	0.69621934	0.557947326	0.677185022	0.742568903
TIRAP	0.545688133	0.654696606	0.497992443	0.609054155	0.818179113	0.501733747
ALPL	0.545688133	0.393018763	0.81412252	0.582333557	0.585398487	0.71635798
LOXL1	0.54630308	0.411108591	0.561599458	0.999981514	0.457135312	0.442234422
PLAU	0.54630308	0.409470885	0.446879237	0.999981514	0.626033107	0.458100647
LOC101905956	0.54630308	0.471255215	0.779034209	0.817144975	0.522415863	0.465577771
KNTC1	0.54630308	0.376269829	0.794676674	0.627280162	0.823508064	0.466085264
HELB	0.54630308	0.483305224	0.513255922	0.982220679	0.487063655	0.494747873
LOC104971683	0.54630308	0.388315576	0.627295843	0.898188142	0.632055507	0.512100901
ATRNL1	0.54630308	0.418930992	0.742336488	0.736045965	0.674942708	0.512846657
ARCN1	0.54630308	0.382744251	0.849389234	0.773694377	0.526268673	0.53697518
RSRC2	0.54630308	0.38381979	0.586766766	0.682944876	0.87329316	0.560309811
USP54	0.54630308	0.498166344	0.658489624	0.559525332	0.804037558	0.587132866
USP43	0.54630308	0.791281103	0.455797803	0.654131191	0.612403767	0.587353805
LOC101905510	0.54630308	0.36997696	0.705746499	0.885789665	0.533903668	0.588384898
LOC112442049	0.54630308	0.372029782	0.526325915	0.594220399	0.950958352	0.61411085
CGN	0.54630308	0.412566059	0.532415258	0.868378265	0.64727886	0.627258867
KIF20B	0.54630308	0.422464369	0.70011973	0.821450642	0.487063655	0.645719071
ABCE1	0.54630308	0.366818918	0.705746499	0.686647893	0.714597718	0.645719071
RERGL	0.54630308	0.402160415	0.507675433	0.690807874	0.832407979	0.660603184
RBM39	0.54630308	0.382744251	0.489163551	0.518542038	0.970980375	0.667726353
KCNIP3	0.54630308	0.402062086	0.880163755	0.630535638	0.487063655	0.669558041
TIMM44	0.54630308	0.469660184	0.532764011	0.499858161	0.893716526	0.673985072

SNN	0.54630308	0.416674804	0.458004431	0.855893886	0.626634623	0.727876258
PPIA	0.54630308	0.379156	0.598995111	0.544789716	0.828142439	0.748921056
KBTBD2	0.54630308	0.61939332	0.56863752	0.531399091	0.576970572	0.78087541
POLR3H	0.54630308	0.470133653	0.630408979	0.498444775	0.688556938	0.806476213
PCBD2	0.54630308	0.534573879	0.502079089	0.527867968	0.697778495	0.812491979
TBCA	0.54630308	0.453089563	0.620613545	0.609054155	0.487063655	0.872456059
LOC100335822	0.54630308	0.390582142	0.520959511	0.703717381	0.494730686	0.909056553
PLEKHA1	0.546680248	0.465004933	0.676501557	0.931509349	0.483224704	0.475073702
ACTB	0.547644725	0.395134649	0.986511454	0.633086085	0.503487214	0.444913711
ARHGAP10	0.547644725	0.445034796	0.653631615	0.736045965	0.652961597	0.627109183
RAB12	0.547946315	0.36901158	0.927508464	0.541206056	0.652333594	0.584339668
LOC506495	0.548023121	0.414881121	0.734143952	0.839725231	0.64727886	0.451485662
DTX3	0.548023121	0.660977079	0.456401352	0.95901497	0.478500917	0.462837324
NCAPG	0.548023121	0.409897025	0.836691254	0.758601772	0.603965612	0.487514327
LOC101902468	0.548023121	0.522173354	0.533251947	0.812453944	0.721096295	0.494666285
CDH24	0.548023121	0.375367521	0.660365588	0.952247001	0.501311839	0.537186568
VAT1	0.548023121	0.380409291	0.630408979	0.911311261	0.571784254	0.574171361
LOC112443006	0.548023121	0.449821474	0.507675433	0.541206056	0.809129327	0.78288746
C19H17orf113	0.548023121	0.605916765	0.611647147	0.53954651	0.472139664	0.842214812
LOC101903026	0.548421384	0.416674804	0.860179257	0.865746543	0.460237064	0.457840591
LOC107131398	0.548452952	0.45450823	0.921434768	0.543403516	0.626973048	0.504596604
ATF4	0.549191462	0.530969645	0.565148263	0.766675276	0.652333594	0.587246156
PGBD5	0.549269552	0.741310853	0.620613545	0.486381297	0.60903578	0.672950726
FANCG	0.549269552	0.369330799	0.904274378	0.638739139	0.470098343	0.68781527
ENDOG	0.549269552	0.37175727	0.497992443	0.518542038	0.953300742	0.724693895
FZR1	0.549269552	0.383545311	0.457051074	0.518542038	0.666917776	0.970555603
PIH1D1	0.550266751	0.414881121	0.457051074	0.999981514	0.487063655	0.501207489
ALG11	0.550266751	0.434596717	0.694535506	0.707217807	0.727873981	0.559845075
TRPM2	0.550266751	0.423816704	0.914866994	0.518542038	0.619521862	0.621946029
MFSD6	0.550266751	0.660882273	0.683693005	0.511608297	0.501311839	0.751705208
LOC100847791	0.550388214	0.481934113	0.782699836	0.803690355	0.454877643	0.564822443
DDX18	0.550415201	0.434898742	0.700900214	0.808073654	0.695846971	0.471903579
DUSP26	0.550449481	0.45329653	0.555945698	0.777956162	0.801692604	0.513535906
LOC112443226	0.550449481	0.382853453	0.470762646	0.996033721	0.560390886	0.587387684
SLC39A7	0.550449481	0.58531281	0.891009505	0.519487474	0.486387163	0.601371091
SLC33A1	0.550449481	0.382853453	0.834683675	0.736520443	0.494730686	0.643615576
HOXD4	0.550449481	0.543645094	0.545866468	0.723043513	0.645748057	0.645719071
SEC61B	0.550449481	0.556813446	0.777154688	0.580133741	0.483106459	0.698733341

ZNF19	0.550449481	0.452080332	0.470895554	0.576844035	0.893716526	0.701906535
SEMA5B	0.550449481	0.614976364	0.58300263	0.610790896	0.553835124	0.748867818
CECR2	0.550449481	0.38719103	0.721489943	0.677374927	0.472808893	0.831652079
LOC112441500	0.550637711	0.530931154	0.466192774	0.528783909	0.926972948	0.629427844
LOC112441554	0.5507828	0.828516628	0.457051074	0.512906846	0.846745071	0.47629249
TMC4	0.5507828	0.842391145	0.466192774	0.627280162	0.670221463	0.503701037
CD52	0.5507828	0.469660184	0.757235604	0.641118121	0.591306131	0.645427231
IL12RB1	0.551351564	0.382853453	0.971053208	0.649763076	0.47785732	0.526690149
ATP5ME	0.551353771	0.499204258	0.579487023	0.519487474	0.882252274	0.623996305
TP53BP2	0.551353771	0.483580176	0.45863002	0.952247001	0.489930391	0.643003473
LOC112445176	0.55156333	0.517719875	0.473560084	0.874662174	0.695436567	0.528561091
LOC524576	0.551641555	0.422866317	0.457051074	0.706180917	0.957793213	0.503701037
RGS16	0.551690538	0.644366672	0.454835278	0.940438437	0.548400565	0.462837324
NMRAL1	0.551690538	0.559141865	0.529409119	0.528783909	0.790136352	0.711923954
PDE1B	0.551856352	0.454875439	0.466192774	0.513791921	0.999985752	0.468732955
SPRYD3	0.551856352	0.616467638	0.582638793	0.519487474	0.890348531	0.487514327
CCAR1	0.551856352	0.383450541	0.682974314	0.518542038	0.970988226	0.487514327
MEGF8	0.551856352	0.412566059	0.606230025	0.870210705	0.726564622	0.495197104
MCEE	0.553261933	0.587350756	0.497992443	0.608306231	0.688845988	0.742309541
SLC2A3	0.55384401	0.463158835	0.569818343	0.972467586	0.533903668	0.481287134
HHIPL1	0.55384401	0.674801662	0.45863002	0.630535638	0.864524107	0.491178906
EMP1	0.55384401	0.573719533	0.608008194	0.922002553	0.457358633	0.496415296
ABL1	0.55384401	0.376619022	0.487875367	0.519487474	0.999985752	0.501733747
NT5DC3	0.55384401	0.386477216	0.636814454	0.961516541	0.535275659	0.518765111
PHYHD1	0.55384401	0.449965992	0.487875367	0.984423083	0.570517621	0.521778441
SAV1	0.55384401	0.45629714	0.485200576	0.960016921	0.60903578	0.529445217
C18H19orf48	0.55384401	0.580473137	0.492173361	0.65483771	0.832119756	0.546062761
FAAH	0.55384401	0.517719875	0.781109205	0.618607324	0.635733222	0.571961045
USP2	0.55384401	0.386477216	0.497091612	0.808075336	0.853250878	0.578851812
MCHR1	0.55384401	0.424061706	0.526787889	0.939604997	0.601490796	0.587132866
ACOT4	0.55384401	0.381943695	0.613840553	0.751194677	0.80599066	0.596578269
ATG2A	0.55384401	0.461860331	0.730571184	0.850762358	0.465432916	0.601371091
PTGES2	0.55384401	0.476257657	0.570681722	0.518542038	0.931775933	0.605572924
MTFR1L	0.55384401	0.45450823	0.778802525	0.519487474	0.723054375	0.661029645
PIF1	0.55384401	0.452206305	0.635738982	0.801848398	0.553835124	0.682483304
AKAP4	0.55384401	0.479687945	0.476465349	0.773429607	0.70977288	0.690728863
LOC107131471	0.55384401	0.563489799	0.591414717	0.664953033	0.575928213	0.727876258
UBE2D2	0.55384401	0.37175727	0.77566312	0.686250223	0.510264075	0.765138165

PRKG1	0.55384401	0.746712661	0.533570754	0.549573927	0.487063655	0.783241453
ROMO1	0.55384401	0.446993842	0.611522181	0.527867968	0.738738532	0.806476213
ZNF831	0.55384401	0.562940544	0.523681249	0.519254756	0.618211409	0.862925606
NUDT17	0.55384401	0.389789684	0.53791136	0.546814352	0.663923284	0.922539606
ITGB7	0.553990014	0.47375776	0.752192353	0.554743688	0.605738596	0.742309541
PLPPR4	0.554198216	0.483305224	0.594921032	0.823935462	0.738738532	0.469313855
PLEKHM2	0.554198216	0.658496391	0.533570754	0.627280162	0.74028971	0.578077816
NAA38	0.554659861	0.45453159	0.542873405	0.802276874	0.804274107	0.513535906
SYNJ2	0.554866203	0.483305224	0.591348881	0.541206056	0.944839728	0.506515041
LOC617475	0.555089045	0.38719103	0.550289803	0.552451497	0.999985752	0.456325916
CBX6	0.555293332	0.414912961	0.52262636	0.837820405	0.848696349	0.486586811
NDUFA8	0.555293332	0.453089563	0.685018711	0.518542038	0.809913252	0.684994779
MBLAC2	0.555293332	0.392477353	0.606660869	0.576242366	0.801692604	0.770035496
RPS27L	0.555293332	0.446686539	0.594331059	0.58293921	0.737149805	0.78096075
LOC101906754	0.555293332	0.519450301	0.564048951	0.636814098	0.541176598	0.832540656
NOXO1	0.555293332	0.412100649	0.476465349	0.498444775	0.723467697	0.950798794
M6PR	0.556029682	0.382853453	0.774015998	0.903584224	0.556100527	0.471903579
PTPRN2	0.556029682	0.968166143	0.570681722	0.577737722	0.486387163	0.472421683
EFCAB11	0.556029682	0.884634125	0.528387114	0.636814098	0.558285349	0.503701037
COQ7	0.556029682	0.621713316	0.734143952	0.559525332	0.696466814	0.521756022
MPZL3	0.556029682	0.538848393	0.526976806	0.841788013	0.638815482	0.566196709
CEP170	0.556029682	0.393018763	0.732274253	0.577737722	0.870450746	0.568308927
SH3GL3	0.556029682	0.690635606	0.69687851	0.555559546	0.510920861	0.683230524
BVES	0.556029682	0.71189217	0.749417651	0.509797804	0.479364681	0.690728863
LOC101907369	0.556029682	0.623984886	0.478270125	0.823935462	0.486387163	0.699160709
BRD3OS	0.556029682	0.594199197	0.640121554	0.627280162	0.474619036	0.78104892
PSMD8	0.556029682	0.428324786	0.604354543	0.531399091	0.545604339	0.944330889
LOC107132317	0.556660327	0.38381979	0.523242525	0.731537809	0.789679352	0.721192619
EZH2	0.556770473	0.45629714	0.809673076	0.685601134	0.695436567	0.487514327
C3H1orf54	0.556770473	0.850640641	0.476781476	0.500788064	0.826634148	0.487514327
ADAMTS10	0.556770473	0.416674804	0.826913617	0.829298091	0.51036843	0.514975796
C25H16orf72	0.556770473	0.451236244	0.499725791	0.731537809	0.865076851	0.582812525
DECR1	0.556770473	0.414881121	0.507083871	0.555047431	0.86010579	0.783404933
LOC100140431	0.557014358	0.428129561	0.815587305	0.874170259	0.457770074	0.529931934
LOC100336282	0.557014358	0.550226079	0.745479541	0.657738814	0.481583803	0.684524441
UQCRC1	0.557209906	0.439142493	0.552368676	0.518542038	0.930451229	0.681479061
MIC1	0.55721214	0.439142493	0.492173361	0.98981682	0.60903578	0.495197104
FZD2	0.557224793	0.428324786	0.76174577	0.887642126	0.571639895	0.465404276

CFAP46	0.557224793	0.65159362	0.492173361	0.682383061	0.811359687	0.495197104
IFITM3	0.557224793	0.600246997	0.791843351	0.581559341	0.631537325	0.529931934
GYG1	0.557224793	0.589782216	0.693546993	0.707217807	0.553835124	0.601371091
LOC100141258	0.557224793	0.729565423	0.533251947	0.514100969	0.794743818	0.607349313
SPATA21	0.557224793	0.531880429	0.759332589	0.522681653	0.720896795	0.621946029
JMY	0.557224793	0.586798812	0.497091612	0.648660726	0.765961853	0.648376028
MANBAP1	0.557224793	0.489231082	0.727870684	0.69158245	0.555526527	0.672418631
PELI2	0.557224793	0.449965992	0.570681722	0.527867968	0.887514169	0.700746926
LOC100849652	0.557224793	0.528669672	0.630408979	0.780279943	0.479782826	0.711809436
LGALS3	0.557224793	0.522698706	0.637234901	0.777956162	0.458370193	0.741319465
LOC100847546	0.557224793	0.426582179	0.492173361	0.932914414	0.487063655	0.742309541
COX10	0.557224793	0.441012638	0.660365588	0.731537809	0.54683696	0.762657772
COX17	0.557224793	0.477618743	0.608218151	0.551641524	0.723054375	0.782330566
TEKT3	0.557224793	0.436471501	0.726119774	0.600567045	0.512497102	0.846210629
ZFP1	0.557224793	0.412566059	0.489549483	0.627280162	0.461764223	0.988866378
PIPOX	0.557740636	0.48520817	0.458004431	0.711667568	0.952264073	0.470494326
EIF1	0.557740636	0.382744251	0.774015998	0.8705821	0.545946638	0.552868236
SLC25A15	0.557740636	0.391697369	0.546238984	0.504242357	0.96532664	0.669883077
RAC2	0.558126745	0.469660184	0.759332589	0.553146279	0.705288263	0.668851588
TMEM109	0.558159342	0.378985237	0.943528149	0.688019411	0.60903578	0.476029406
SLC38A4	0.558159342	0.489987041	0.745477682	0.689227896	0.621029742	0.601987815
SMIM26	0.558159342	0.382320412	0.56802023	0.664953033	0.612403767	0.87754233
F11R	0.558603056	0.517719875	0.478270125	0.62191878	0.852959789	0.68185373
WIPF1	0.558818891	0.412566059	0.879939804	0.534768891	0.800547823	0.514507804
TTN	0.558863812	0.38641445	0.754913805	0.630535638	0.618159249	0.759003017
SPP2	0.558884915	0.553132278	0.534905861	0.564748167	0.834569503	0.656183348
ZKSCAN5	0.558901607	0.382853453	0.525415185	0.730926769	0.473881967	0.940583352
RNF208	0.559670605	0.440549685	0.492173361	0.819549243	0.863970911	0.513535906
PTPRU	0.559967282	0.599021986	0.708019215	0.534082333	0.555907302	0.752954485
KRT7	0.560045852	0.531798727	0.608068845	0.776578422	0.658667583	0.585359885
SSU72	0.560055419	0.50468844	0.657462506	0.500788064	0.687203978	0.806714845
SLC6A1	0.560271481	0.463660565	0.726119774	0.71113156	0.527991521	0.719460348
PSTPIP1	0.560647286	0.420442142	0.638444487	0.549573927	0.870246061	0.684994779
RGS14	0.561433953	0.579367653	0.679506848	0.519487474	0.707739077	0.690728863
ELAVL3	0.561668297	0.564100429	0.885535367	0.613744304	0.489920278	0.569398665
SIGIRR	0.562166932	0.411803396	0.518439818	0.948776099	0.707939546	0.51212265
IL18R1	0.562166932	0.406494847	0.679506848	0.53435735	0.789915129	0.772618249
FAM129A	0.562166932	0.609864167	0.628904157	0.627280162	0.496066793	0.775725294

NSDHL	0.563241952	0.465004933	0.56802023	0.575014007	0.792867703	0.768168974
ESM1	0.563470195	0.41169188	0.895031346	0.632654918	0.674942708	0.51840034
IL10RA	0.563470195	0.445080617	0.761504341	0.565578589	0.811991102	0.589917482
LOC112444279	0.563470195	0.767113995	0.518017118	0.630535638	0.652206871	0.609692015
PCED1B	0.563770524	0.846724749	0.533128651	0.565751353	0.627206089	0.598297881
DNAJC14	0.564077745	0.451925292	0.630408979	0.613744304	0.948421303	0.474696021
CNTRL	0.564077745	0.401222162	0.754557912	0.703663629	0.818179113	0.490495335
RAB43	0.564077745	0.614976364	0.561914716	0.764257755	0.712435151	0.503701037
OSBPL6	0.564077745	0.612637146	0.669511234	0.817146543	0.50652393	0.535620846
ING1	0.564077745	0.553489554	0.555640272	0.852170016	0.622751397	0.560502405
TRPM4	0.564077745	0.573865937	0.499687881	0.94469136	0.487063655	0.587387684
IL17RB	0.564077745	0.442990825	0.670070271	0.87522058	0.584450219	0.596578269
TCAF2	0.564077745	0.412566059	0.681918771	0.908467146	0.533903668	0.601371091
PAK6	0.564077745	0.512513718	0.69454186	0.636769069	0.735687381	0.601500908
OSBPL7	0.564077745	0.456208448	0.755135544	0.764054702	0.56959555	0.622811146
PPP1R37	0.564077745	0.766943819	0.744735369	0.5222819	0.498467324	0.640128675
PAPPA2	0.564077745	0.390629518	0.818951412	0.574117341	0.717677975	0.68185373
DNAJC19	0.564077745	0.479687945	0.627888616	0.531399091	0.825549715	0.699162043
NDUFAF8	0.564077745	0.54845263	0.665599776	0.535570772	0.666917776	0.754218406
TMEM160	0.564077745	0.45629714	0.627295843	0.528783909	0.795865165	0.769865166
COL7A1	0.564077745	0.398295321	0.608218151	0.64770518	0.73254131	0.78096075
LBP	0.564077745	0.48696531	0.694535506	0.528783909	0.652333594	0.799581725
ADRA1D	0.564077745	0.470862073	0.476781476	0.784595865	0.541176598	0.8537109
CD6	0.564725195	0.783112117	0.70011973	0.514995	0.608043638	0.600092797
FEM1A	0.564725195	0.54021232	0.630408979	0.748424012	0.593931025	0.659120631
CP	0.564725195	0.528292592	0.846960897	0.541053342	0.545973193	0.697356941
NACC1	0.564725195	0.582127335	0.526266326	0.5116854	0.854289643	0.697356941
SNAP29	0.564725195	0.714747526	0.740372388	0.543245865	0.47341511	0.700746926
ETFBKMT	0.564725195	0.596426784	0.700963878	0.654275895	0.487063655	0.713707269
OSGIN2	0.565884837	0.445080617	0.625439953	0.824230049	0.472808893	0.78087541
BLOC1S6	0.565937961	0.54671968	0.936203646	0.636814098	0.501311839	0.468732955
YWHAH	0.565937961	0.61939332	0.627295843	0.656831463	0.695436567	0.585719297
SAMD15	0.565937961	0.461047248	0.652724437	0.714751696	0.763229358	0.608200832
ARFGEF1	0.565973968	0.479687945	0.466192774	0.848592907	0.864524107	0.490013062
LOC104975814	0.565973968	0.762081499	0.606230025	0.819213899	0.483224704	0.494676026
NLRP12	0.565973968	0.516314841	0.498347424	0.855835231	0.780940257	0.507584347
DUOX2	0.565973968	0.434208808	0.916038418	0.784595865	0.480921321	0.512941039
PHOSPHO1	0.565973968	0.582127335	0.609398456	0.615029548	0.800943124	0.588600411

GEMIN4	0.565973968	0.479687945	0.495423363	0.944351122	0.56959555	0.627258867
GDF6	0.565973968	0.382853453	0.89478142	0.718713712	0.514458714	0.631731716
GALNT1	0.565973968	0.831653609	0.623785533	0.575014007	0.501311839	0.6467931
TMEM59L	0.565973968	0.51352654	0.517859609	0.741889739	0.736914075	0.669558041
NT5DC2	0.565973968	0.796253817	0.67171984	0.552451497	0.491199833	0.669883077
TMEM62	0.565973968	0.532427681	0.518439818	0.610848628	0.788007619	0.736866513
ATP5PB	0.565973968	0.42354307	0.641535145	0.535163392	0.716994185	0.854563457
MTHFD1L	0.565973968	0.518799911	0.489163551	0.762159685	0.484391124	0.867063767
IDH3A	0.565973968	0.452080332	0.630408979	0.554743688	0.6274106	0.876162019
MRPS5	0.565973968	0.479687945	0.579487023	0.509929823	0.696466814	0.877014986
DENND1A	0.565973968	0.439484206	0.472972947	0.519487474	0.473623717	0.999948228
DNLZ	0.566198287	0.572077017	0.659779853	0.59258231	0.67482152	0.699162043
HSD17B11	0.56632464	0.410855444	0.663738759	0.935282232	0.605738596	0.523933932
APLF	0.56632464	0.636038252	0.679415149	0.815919419	0.487063655	0.543446012
DNAJC5	0.56632464	0.596685917	0.63135074	0.812453944	0.580738755	0.54677303
DYNC2LI1	0.56632464	0.977196455	0.507083871	0.554743688	0.478662178	0.607070178
HMGN2	0.56632464	0.728565135	0.570681722	0.586033659	0.696466814	0.622811146
COX7A1	0.56632464	0.428728874	0.485339975	0.641118121	0.953300742	0.628379498
SETD7	0.56632464	0.389385843	0.691850804	0.828736799	0.607083502	0.668851588
ME1	0.56632464	0.400145858	0.504626782	0.587186699	0.960573822	0.669883077
CHMP6	0.56632464	0.553132278	0.604354543	0.64770518	0.543569691	0.806714845
CCT2	0.56632464	0.382853453	0.677197181	0.68918122	0.591306131	0.835231445
PRELID3B	0.56632464	0.445080617	0.652653727	0.549287144	0.480104202	0.958790968
GPR174	0.56732832	0.408570394	0.69621934	0.887642126	0.487063655	0.683779751
RALBP1	0.56742774	0.425196086	0.849389234	0.695125147	0.593931025	0.625066198
GRB7	0.56742774	0.382853453	0.492173361	0.82868625	0.788007619	0.699160709
PGD	0.56742774	0.487617269	0.546224399	0.518542038	0.837915327	0.780963557
LOC785693	0.56742774	0.445080617	0.64236045	0.677545541	0.545946638	0.847619123
PRR5L	0.567883822	0.38719103	0.945995021	0.764257755	0.486113888	0.526690149
TCEAL8	0.567883822	0.423816704	0.711636925	0.627280162	0.474619036	0.895446935
PRELID2	0.568735797	0.398330774	0.492173361	0.683393974	0.960048824	0.594348476
LIFR	0.568735797	0.424061706	0.614355922	0.764257755	0.804625939	0.601371091
GID4	0.568735797	0.595982761	0.586766766	0.664104969	0.650027114	0.690728863
IRF8	0.568735797	0.461008609	0.466192774	0.659998592	0.655786713	0.892171608
DCAF11	0.568744115	0.43720588	0.608218151	0.984289938	0.562163729	0.497137301
CAV3	0.568985019	0.806821185	0.473560084	0.543403516	0.837915327	0.529445217
MRM2	0.568985019	0.531880429	0.694535506	0.799712183	0.582706032	0.587132866
CNNM3	0.568985019	0.402113442	0.580555896	0.543403516	0.553835124	0.9790177

DDX6	0.569748831	0.448806308	0.955427429	0.757403199	0.474619036	0.473546947
DTX4	0.569748831	0.428728874	0.996291804	0.627280162	0.501311839	0.490641282
EXOC6	0.569748831	0.565421383	0.566638914	0.610790896	0.923910588	0.491178906
HEXDC	0.569748831	0.478699501	0.704088819	0.845867148	0.619385713	0.523933932
APBB1	0.569748831	0.513825592	0.478168172	0.999981514	0.472139664	0.558213767
DDX56	0.569748831	0.483305224	0.75756349	0.652644879	0.658667583	0.631731716
HPSE	0.569748831	0.454880625	0.713072075	0.576844035	0.797489438	0.675688253
MRPL57	0.569748831	0.48811343	0.546238984	0.519487474	0.870142361	0.751884708
MYOCD	0.569748831	0.738007931	0.500993788	0.561120526	0.622751397	0.772618249
LOC512863	0.569748831	0.392477353	0.466192774	0.636814098	0.891726429	0.775725294
TRIM25	0.570723949	0.493805901	0.854571175	0.841637378	0.484413225	0.472189988
LOC112446470	0.570723949	0.553132278	0.507675433	0.799139231	0.821557309	0.494676026
CMAS	0.570723949	0.650612374	0.865512879	0.598316303	0.473828582	0.601500908
BCAR3	0.570723949	0.454880625	0.48640588	0.617759491	0.964301327	0.613547915
LOC112442296	0.570723949	0.446062855	0.819734395	0.550678728	0.776537165	0.628379498
ABLIM1	0.570723949	0.439142493	0.480037287	0.940436902	0.626973048	0.669558041
FKBP1B	0.570723949	0.430353256	0.612793995	0.892071982	0.508269211	0.720865143
CD28	0.570723949	0.428728874	0.616480891	0.891988949	0.487063655	0.745952648
C2CD5	0.570723949	0.691730737	0.507675433	0.549287144	0.629421719	0.812091218
MSS51	0.570723949	0.38719103	0.517857593	0.727236849	0.583449841	0.916377759
OVOS2	0.570742591	0.584209799	0.489549483	0.996195248	0.502432219	0.470494326
ZBTB17	0.570742591	0.414881121	0.734143952	0.930987593	0.548532659	0.512846657
LOC101906024	0.570742591	0.396058445	0.555640272	0.999981514	0.472699899	0.521756022
HNRNPD	0.570742591	0.43720588	0.551149334	0.999981514	0.503635067	0.531378095
DNAJB11	0.570742591	0.425196086	0.513648728	0.823935462	0.791848145	0.645719071
WAS	0.570742591	0.513491832	0.731739801	0.521237523	0.760007802	0.695691829
PSD	0.570742591	0.616227995	0.587296907	0.528783909	0.78021215	0.708195638
LOC781339	0.570742591	0.750379179	0.612793995	0.609774743	0.487063655	0.748921056
PHLPP2	0.570742591	0.48811343	0.754913805	0.588863891	0.553835124	0.793197037
NRDE2	0.570742591	0.403985891	0.569658472	0.782811812	0.535275659	0.865889509
DHODH	0.570742591	0.436471501	0.630638844	0.594220399	0.593931025	0.907124813
EIF2S2	0.570742591	0.4069136	0.680262176	0.616503998	0.494730686	0.930710695
PTPN12	0.570779764	0.487617269	0.481777242	0.519487474	0.978453925	0.631731716
NUBP2	0.57114087	0.715546288	0.754193103	0.537667649	0.593931025	0.621946029
MRPL41	0.57114087	0.589351784	0.552362414	0.526429493	0.78646867	0.758422012
RAI14	0.57114087	0.51452013	0.552362414	0.519487474	0.695436567	0.87754233
TSPAN31	0.571311055	0.863064327	0.54159252	0.648660726	0.605738596	0.532354747
CLIC4	0.571537008	0.398295321	0.572514198	0.999981514	0.521450866	0.500317514

ACSS2	0.571537008	0.445080617	0.545084153	0.513533242	0.950723924	0.710234013
MMP15	0.572039657	0.808696335	0.497711491	0.534082333	0.811991102	0.563814955
MKL2	0.572325056	0.743602194	0.494944201	0.914026346	0.487063655	0.505866223
LOC101905099	0.572325056	0.473243816	0.894338686	0.527867968	0.58186871	0.706176041
LOC789148	0.572444823	0.569554583	0.70011973	0.812453944	0.613093988	0.500317514
VIT	0.572444823	0.774979074	0.494778641	0.518542038	0.890124729	0.519240641
LRRC3	0.572444823	0.41922051	0.711148626	0.695125147	0.848550619	0.529445217
CCDC88C	0.572444823	0.46147411	0.587296907	0.901353354	0.677185022	0.539240716
TBC1D10C	0.572444823	0.504542625	0.78930905	0.554743688	0.72350412	0.643003473
NEPRO	0.572444823	0.413266514	0.810647419	0.527867968	0.627206089	0.823824518
MSLN	0.572923263	0.514675796	0.685018711	0.742903483	0.656015118	0.622811146
THAP8	0.573026403	0.398295321	0.774015998	0.908467146	0.610761857	0.476029406
TSC1	0.573026403	0.454073827	0.579487023	0.872590202	0.501311839	0.767613107
CD79B	0.573026403	0.477035321	0.802277976	0.596654953	0.498206036	0.812774915
PHKG1	0.573026403	0.444900634	0.67984895	0.529049853	0.723467697	0.838913379
SIGMAR1	0.573026403	0.519450301	0.653026936	0.518542038	0.648899379	0.856833445
RSL24D1	0.573186348	0.425196086	0.641535145	0.777956162	0.498467324	0.841879197
PSMC5	0.573422874	0.483305224	0.666696467	0.609204337	0.605738596	0.839906041
SMAP2	0.573422874	0.446062855	0.500533485	0.554743688	0.788850323	0.886743984
PSMD14	0.573422874	0.436471501	0.641535145	0.528783909	0.527222445	0.966850975
FBXL22	0.573590526	0.927853707	0.532743798	0.549573927	0.595423977	0.601987815
PCF11	0.573855021	0.491313047	0.509514515	0.518542038	0.939343714	0.705204221
MEGF6	0.573914826	0.51452013	0.813054822	0.627280162	0.776537165	0.487514327
LOC614741	0.573914826	0.790429889	0.69454186	0.676566861	0.573116505	0.490013062
ADAMTS9	0.573914826	0.515892935	0.485200576	0.598316303	0.989334846	0.501207489
CEP295	0.573914826	0.430996205	0.806470927	0.820971736	0.578333624	0.574870258
AGFG2	0.573914826	0.536598701	0.595088127	0.610790896	0.862852956	0.621946029
KCNJ8	0.573914826	0.45922652	0.605532453	0.907794002	0.593006249	0.622187701
SPRYD4	0.573914826	0.45629714	0.77566312	0.534853543	0.823508064	0.628917359
WDR77	0.573914826	0.442980755	0.728269425	0.748837705	0.591306131	0.720865143
LOC112447381	0.573914826	0.442980755	0.728269425	0.598663987	0.658108379	0.793197037
BZW2	0.573914826	0.442980755	0.497091612	0.742465161	0.715654748	0.812491979
DGKI	0.574107653	0.445080617	0.57816803	0.965724803	0.643560251	0.503701037
PCDH17	0.574107653	0.465004933	0.830135741	0.806573529	0.533903668	0.564826731
SUPT4H1	0.574107653	0.4069136	0.914770879	0.598316303	0.656015118	0.632510367
ZDHHC21	0.574107653	0.483305224	0.688156363	0.670507175	0.750169765	0.640825046
NDUFB1	0.574107653	0.477035321	0.598652788	0.518533249	0.889301074	0.733767439
LOC100337355	0.574107653	0.479687945	0.689888669	0.549287144	0.660637622	0.83150957

SLITRK2	0.574107653	0.395925974	0.555640272	0.636027046	0.766655597	0.849731479
TBXA2R	0.575409454	0.416674804	0.641535145	0.519487474	0.998008754	0.513034959
TNFAIP3	0.575409454	0.412566059	0.787846685	0.586089426	0.871558258	0.571576949
DAPK3	0.575409454	0.531880429	0.489549483	0.627280162	0.819342514	0.748921056
URI1	0.575409454	0.709247882	0.497091612	0.613744304	0.626973048	0.77593589
LOC107132092	0.575409454	0.43720588	0.517382044	0.534082333	0.535275659	0.99828741
CILP	0.575634493	0.519450301	0.498563826	0.636769069	0.859754169	0.690728863
SCAPER	0.575754418	0.598286294	0.566959933	0.648660726	0.711090681	0.696461811
ABTB1	0.575823781	0.429850159	0.89478142	0.734460659	0.641551148	0.494666285
COX8A	0.575977661	0.416674804	0.605532453	0.54137792	0.909316444	0.742456493
LOC533597	0.576092326	0.438643862	0.728269425	0.777956162	0.800097282	0.494666285
CNTLN	0.576092326	0.45629714	0.693546993	0.695125147	0.762015885	0.640434848
DNAH10	0.576092326	0.469660184	0.829751489	0.632177478	0.622240553	0.671759429
UBA3	0.576092326	0.446395731	0.874983674	0.549573927	0.658667583	0.690728863
NSMCE4A	0.576211591	0.571983204	0.532743798	0.940436902	0.629421719	0.485795983
HABP4	0.576469872	0.624142952	0.514783121	0.652644879	0.882252274	0.519240641
ADAMTS15	0.576469872	0.467147493	0.525791261	0.587186699	0.721826916	0.885162483
CARD19	0.57660202	0.464892289	0.831312019	0.703717381	0.735091896	0.49105688
SLC45A3	0.57660202	0.453089563	0.734143952	0.822691957	0.656015118	0.552868236
CHUK	0.57660202	0.573498846	0.594921032	0.716147042	0.663173293	0.68392363
MIF	0.57660202	0.553132278	0.507675433	0.519487474	0.891304411	0.733366979
CYSTM1	0.57660202	0.48520817	0.555945698	0.764140634	0.626852413	0.779201808
UTP15	0.576766747	0.399167149	0.826465082	0.817146543	0.593006249	0.596578269
MDGA1	0.577003239	0.804185018	0.560699356	0.792140715	0.521036349	0.532354747
MAPK8IP2	0.577117931	0.624142952	0.754913805	0.518542038	0.832407979	0.495197104
C1H21orf91	0.577888787	0.542918274	0.828132549	0.636525	0.593216299	0.62532656
LOC101906221	0.577888787	0.609864167	0.668824581	0.627280162	0.658667583	0.68185373
TRIM34	0.578045951	0.560752029	0.532743798	0.643452505	0.918497533	0.521299803
LOC112441493	0.578104752	0.951949541	0.485339975	0.577737722	0.692827651	0.500495711
BCL6	0.578104752	0.861480696	0.517382044	0.630535638	0.705202026	0.513034959
NLRC3	0.578104752	0.550226079	0.659779853	0.899296336	0.533903668	0.537186568
DPYSL5	0.578432712	0.659462323	0.682700098	0.618477111	0.738738532	0.554675319
MARCKS	0.57864368	0.553132278	0.492173361	0.874257643	0.804625939	0.48181553
PRAM1	0.57864368	0.503404007	0.716468083	0.704815275	0.821557309	0.482980366
AFTPH	0.57864368	0.43720588	0.587296907	0.813323291	0.882252274	0.495197104
TOMT	0.57864368	0.446062855	0.612793995	0.604036098	0.977167046	0.49553442
KIF1B	0.57864368	0.40117451	0.527368302	0.999981514	0.508269211	0.507632407
LOC101902435	0.57864368	0.450686302	0.77566312	0.889416444	0.570107746	0.513034959

MORF4L1	0.57864368	0.434462153	0.684555229	0.870210705	0.714642011	0.529445217
PLPBP	0.57864368	0.51453344	0.614228787	0.636814098	0.823508064	0.645719071
MRPL43	0.57864368	0.579275375	0.736435559	0.541206056	0.659661953	0.727876258
PAQR8	0.57864368	0.470862073	0.524161037	0.695681179	0.521724147	0.927517509
SRSF3	0.578741857	0.426894049	0.838909114	0.81631926	0.530344927	0.614511437
PTGES	0.578741857	0.645611345	0.638444487	0.739098396	0.555544932	0.668191006
LOC112441545	0.579113474	0.825171788	0.620613545	0.724431807	0.571639895	0.496415296
SOX8	0.579113474	0.426894049	0.48405583	0.997792939	0.714642011	0.497110172
TMEM145	0.579113474	0.446993842	0.670239985	0.731537809	0.879395665	0.500691455
RCN3	0.579113474	0.441931708	0.746661392	0.887420349	0.569944182	0.584339668
KIAA0895	0.579113474	0.40751035	0.487694723	0.960016921	0.527202266	0.772618249
F13A1	0.579209832	0.713956321	0.57816803	0.636814098	0.578296318	0.738287596
USP42	0.579374483	0.445516027	0.487875367	0.544789716	0.999985752	0.482980366
CNKSR3	0.579451501	0.669828549	0.532195319	0.682944876	0.7744799	0.607349313
LOC112446402	0.579644516	0.515680499	0.571415357	0.760279212	0.541176598	0.8255975
ATP6AP2	0.579644516	0.430996205	0.555917581	0.718713712	0.519899007	0.92789871
LOC101904794	0.579900364	0.399053034	0.720612298	0.916858019	0.586110509	0.588384898
PIN4	0.580372529	0.502634382	0.762561451	0.571569169	0.808047125	0.608554064
FCHO2	0.580827743	0.61939332	0.533570754	0.878513543	0.642662413	0.539240716
SMAD6	0.581396649	0.434208808	0.754913805	0.549287144	0.891726429	0.609692015
TBC1D32	0.581493182	0.895777642	0.669511234	0.57824119	0.581445124	0.521756022
KLC1	0.581493182	0.829067393	0.567712191	0.537667649	0.797489438	0.521756022
SELENOO	0.581493182	0.593498372	0.532764011	0.637708629	0.78021215	0.702623424
RANBP6	0.581557927	0.444114888	0.676501557	0.589593123	0.878451408	0.673985072
RTRAF	0.581557927	0.442990825	0.680262176	0.577737722	0.833360418	0.734177434
TBRG4	0.581557927	0.607281278	0.719862199	0.519487474	0.517239659	0.850475013
ENKUR	0.581668654	0.450686302	0.533522641	0.82868625	0.846856925	0.572754665
RAN	0.581668654	0.423816704	0.711148626	0.627280162	0.487063655	0.927426708
ATP7A	0.58169066	0.553132278	0.682670221	0.727950912	0.738738532	0.550806936
EIF4H	0.581931804	0.413266514	0.749417651	0.822691957	0.503360023	0.742309541
GAK	0.581989146	0.791281103	0.777618267	0.600166011	0.596709577	0.487514327
CDK15	0.581989146	0.441012638	0.999993442	0.546397256	0.547042845	0.489184277
LOC101907944	0.581989146	0.741263262	0.518829767	0.900044333	0.558285349	0.490415687
RAET1L	0.581989146	0.423816704	0.487875367	0.999981514	0.61744201	0.503701037
LOC100141266	0.581989146	0.52001926	0.716120406	0.888687722	0.575928213	0.51840034
ZNF414	0.581989146	0.479687945	0.608218151	0.914850247	0.658667583	0.531936221
SRCIN1	0.581989146	0.575770651	0.716986439	0.686647893	0.707739077	0.571961045
LOC100335990	0.581989146	0.77844414	0.545842534	0.630951216	0.728185525	0.587246156

LOC785629	0.581989146	0.428728874	0.497091612	0.674469953	0.943160494	0.669558041
SCMH1	0.581989146	0.825243522	0.485200576	0.58293921	0.533903668	0.811759324
DPH3	0.582102304	0.414881121	0.729115949	0.994331516	0.502432219	0.487514327
WDR43	0.582102304	0.406647141	0.56802023	0.810665442	0.887514169	0.564826731
LOC787122	0.582102304	0.470243637	0.604354543	0.552451497	0.973219333	0.570076124
IPCEF1	0.582102304	0.469660184	0.820146472	0.522681653	0.858187492	0.582422147
MTX1	0.582102304	0.459379688	0.685018711	0.534082333	0.925171841	0.63512796
RMND5A	0.582102304	0.580473137	0.82155377	0.610790896	0.576744878	0.669558041
LMO3	0.582102304	0.816129287	0.537185735	0.565933683	0.607561642	0.736468943
KLHL22	0.582102304	0.402391801	0.487875367	0.957132838	0.560390886	0.772618249
FKRP	0.582102304	0.399994413	0.831933739	0.527867968	0.723467697	0.779201808
SFRP4	0.582102304	0.474018124	0.55950158	0.739098396	0.61565225	0.838729965
NUDT16	0.582102304	0.6385246	0.532415258	0.596654953	0.578296318	0.872721634
LOC107131567	0.582164259	0.709542765	0.63840857	0.627280162	0.782298976	0.521756022
CACNA1B	0.582164259	0.953345357	0.485339975	0.594220399	0.517239659	0.672100865
GLRX5	0.58217842	0.532096752	0.604354543	0.527867968	0.891726429	0.688206116
MAOA	0.58230376	0.412100649	0.755135544	0.554743688	0.87389153	0.665736967
KCNJ2	0.582596999	0.885313051	0.533570754	0.695125147	0.626973048	0.496415296
OTUB2	0.582596999	0.40372842	0.507675433	0.65483771	0.600394912	0.967792782
BCL7B	0.582814969	0.460244277	0.958307418	0.666598607	0.487063655	0.602791137
TRIM36	0.582814969	0.480935933	0.53939242	0.55713914	0.893376996	0.752070741
LOC101908113	0.582814969	0.65172183	0.608218151	0.731537809	0.488494916	0.761858515
LOC101903478	0.582814969	0.45627172	0.641535145	0.601475996	0.788995893	0.78288746
ARMH4	0.582814969	0.52001926	0.566607969	0.541206056	0.487063655	0.982422737
CUL9	0.582971623	0.412100649	0.736160515	0.940438437	0.533807401	0.588600411
TTYH3	0.582971623	0.434898742	0.92380537	0.575014007	0.631537325	0.669090959
ADAM9	0.583838474	0.449305022	0.509514515	0.780703865	0.6274106	0.855963573
MRPL34	0.583852739	0.517719875	0.717242466	0.537735901	0.797227609	0.709160118
CRABP2	0.583915973	0.425196086	0.615161884	0.870591999	0.593931025	0.748921056
CD69	0.584358012	0.40543681	0.653631615	0.682383061	0.891726429	0.628917359
NLRC5	0.584869904	0.445080617	0.95217331	0.665999974	0.554989446	0.583681742
GTPBP3	0.584990259	0.446395731	0.759332589	0.894086736	0.596079788	0.526690149
SLCO3A1	0.584990259	0.51352654	0.879939804	0.614125713	0.698346212	0.542524968
PRCP	0.584990259	0.586194382	0.538825602	0.969609355	0.494000973	0.569429928
RUM1	0.584990259	0.450686302	0.63135074	0.815919419	0.755431982	0.627258867
C11H9orf116	0.584990259	0.414881121	0.630408979	0.891988949	0.670961786	0.645719071
TMEM206	0.584990259	0.491135151	0.515497954	0.561120526	0.939516498	0.702623424
WDR89	0.584990259	0.412543461	0.557137242	0.731537809	0.823508064	0.742588975

LTBP2	0.584990259	0.745791496	0.622025849	0.58293921	0.52068239	0.788699374
EGLN3	0.584990259	0.407484019	0.861121921	0.551641524	0.608169547	0.823406796
ACOD1	0.584990259	0.594126609	0.616480891	0.703717381	0.487063655	0.831652079
ACBD4	0.585122186	0.548622699	0.822300339	0.64770518	0.613652691	0.627258867
NCALD	0.585206615	0.452206305	0.603530331	0.822708607	0.789915129	0.616715863
ECM2	0.58520836	0.981920173	0.517857593	0.626698454	0.553835124	0.513780222
COLEC12	0.58520836	0.489058914	0.596931535	0.647342496	0.887514169	0.632316453
LOC112447599	0.58520836	0.45450823	0.743532054	0.802267873	0.507738123	0.748921056
SYBU	0.585830721	0.549135888	0.487875367	0.626698454	0.964301327	0.564822443
GNA14	0.585830721	0.711197981	0.542873405	0.688505637	0.728185525	0.614511437
ZNF827	0.585830721	0.709348874	0.726119774	0.695681179	0.52068239	0.622811146
SERPINH1	0.585830721	0.445080617	0.622890829	0.764257755	0.821351993	0.631731716
NARF	0.586074666	0.449612614	0.651356851	0.560256928	0.987439758	0.508039178
PPDPF	0.586264635	0.412100649	0.611216367	0.995565324	0.612403767	0.522289438
C13H20orf27	0.586264635	0.710903449	0.671469816	0.531399091	0.833173678	0.532354747
PHF7	0.586264635	0.598069728	0.490752494	0.946503916	0.612403767	0.559845075
SIRPB2	0.586264635	0.426894049	0.652724437	0.549287144	0.970988226	0.608554064
CTC1	0.586264635	0.445080617	0.727870684	0.817903737	0.521036349	0.748921056
SIMC1	0.586264635	0.45629714	0.612793995	0.630535638	0.682784145	0.86324237
MSI2	0.586591643	0.461047248	0.579487023	0.555047431	0.917407853	0.733767439
TTLL11	0.586953963	0.452206305	0.911401689	0.745806541	0.545973193	0.587839909
P3H4	0.587014558	0.471255215	0.579487023	0.999981514	0.488757973	0.491178906
CDK16	0.587014558	0.496890044	0.624296996	0.823167001	0.820264258	0.494190902
GPALPP1	0.587014558	0.919173546	0.660365588	0.618288053	0.570517621	0.501207489
MYNN	0.587014558	0.991018684	0.55950158	0.613744304	0.503487214	0.508039178
SLC49A3	0.587014558	0.445080617	0.943120912	0.71656639	0.602648954	0.513034959
HOXB4	0.587014558	0.465004933	0.605532453	0.727236849	0.926972948	0.519240641
CMTM6	0.587014558	0.441012638	0.873674244	0.793916687	0.626973048	0.521756022
CCDC61	0.587014558	0.423816704	0.594331059	0.717645144	0.956041014	0.525913971
CDT1	0.587014558	0.434898742	0.693546993	0.672828315	0.925918507	0.529445217
AVIL	0.587014558	0.429816591	0.70011973	0.952662898	0.576970572	0.560309811
TRPC3	0.587014558	0.711503165	0.75407054	0.623608478	0.567274296	0.636674289
LOC112445927	0.587014558	0.628205932	0.550289803	0.852005554	0.5863408	0.645719071
ZC3H6	0.587014558	0.792661811	0.492173361	0.813323291	0.489930391	0.669558041
CCDC186	0.587014558	0.772336368	0.570681722	0.627280162	0.640551195	0.677365619
DMRT2	0.587014558	0.693703257	0.627477288	0.724431807	0.533807401	0.701800445
P2RY10	0.587014558	0.415447733	0.726119774	0.823167001	0.601145588	0.713351736
RSPRY1	0.587014558	0.429811586	0.541610391	0.69158245	0.872734644	0.737362929

GPR4	0.587014558	0.621713316	0.534905861	0.64770518	0.726283157	0.743842064
MYH7	0.587014558	0.416674804	0.495755857	0.659908345	0.89548293	0.774932208
KCNK1	0.587014558	0.575241982	0.487875367	0.610848628	0.696466814	0.874557852
EFHD1	0.587014558	0.423816704	0.507675433	0.654797105	0.69975451	0.923807852
CLCN3	0.587014558	0.414881121	0.611794073	0.716147042	0.519850807	0.93363377
INSL3	0.587014558	0.534573879	0.608218151	0.533011704	0.571639895	0.940017687
FAM120A	0.587014558	0.597345821	0.552162548	0.562498406	0.487063655	0.959025331
CEBPZOS	0.58790633	0.522698706	0.64236045	0.574117341	0.711809801	0.83150957
CYREN	0.58866192	0.532381384	0.560847675	0.598663987	0.979052133	0.495197104
STPG4	0.588916428	0.796666816	0.743624166	0.659998592	0.532662842	0.539240716
MPC2	0.588916428	0.463660565	0.625861236	0.590326511	0.821351993	0.78288746
EPB41L3	0.589107008	0.425813852	0.668824581	0.865222811	0.803705802	0.521756022
SOCS6	0.589107008	0.420543145	0.78790711	0.903584224	0.522415863	0.614511437
LOC101906779	0.589107008	0.415447733	0.977455115	0.619548588	0.571784254	0.622913023
MCM8	0.589107008	0.938817997	0.530370658	0.565578589	0.56959555	0.668851588
NAA50	0.589107008	0.461860331	0.768932648	0.771412309	0.576828974	0.700948005
LOC101907405	0.589107008	0.409897025	0.663738759	0.677581064	0.817472827	0.742309541
SFRS18	0.589107008	0.441931708	0.527368302	0.808073654	0.488805918	0.927897089
ZCCHC9	0.589107008	0.416674804	0.518017118	0.627280162	0.62911749	0.970105102
PGK1	0.589153456	0.460857983	0.608218151	0.646556987	0.730921558	0.831652079
LOC104975007	0.589297396	0.694788864	0.53791136	0.729912904	0.541176598	0.769865166
BEND5	0.589297396	0.530592604	0.549830614	0.731537809	0.634894348	0.819366282
ADGRF2	0.589326425	0.790429889	0.509514515	0.825279787	0.629494988	0.512846657
QPRT	0.589326425	0.560109317	0.509514515	0.586290626	0.804625939	0.811410743
LOC112448737	0.589475869	0.454880625	0.608218151	0.705801894	0.692827651	0.81956221
LOC112441603	0.589561502	0.441931708	0.497711491	0.930987593	0.797227609	0.588384898
FOXP3	0.589561502	0.722968232	0.569273084	0.577737722	0.837915327	0.601371091
HYOU1	0.589561502	0.482551032	0.502079089	0.972142185	0.613652691	0.629427844
BAZ2B	0.589561502	0.53061969	0.684274697	0.866566721	0.571421132	0.629881817
GADD45A	0.589561502	0.490326582	0.665599776	0.890422845	0.574180707	0.645719071
NELFB	0.589561502	0.479687945	0.644256366	0.764257755	0.772103109	0.65498499
LAIR1	0.589561502	0.541664445	0.607986114	0.85005716	0.598927903	0.682479856
TAB3	0.589561502	0.429850159	0.799533361	0.794299784	0.533903668	0.728699286
PRR33	0.589561502	0.429710219	0.768932648	0.643033645	0.721183131	0.737726964
ARRDC3	0.589561502	0.430612964	0.723132042	0.551587083	0.744695566	0.839906041
USP15	0.590164388	0.561143981	0.77699301	0.691025178	0.631537325	0.627258867
UBAP2	0.590164388	0.542771309	0.489549483	0.831709307	0.795839201	0.627258867
TEAD2	0.590667658	0.648119635	0.668721054	0.638269781	0.585398487	0.75640676

MYB	0.590929266	0.589754585	0.511417994	0.839725231	0.809317773	0.512846657
LOC112442053	0.590929266	0.439142493	0.745477682	0.890362631	0.560554936	0.640434848
TMOD4	0.590929266	0.440218555	0.66484453	0.768290504	0.52589341	0.86324237
CCR5	0.590929266	0.696525042	0.64236045	0.54857232	0.502432219	0.867482845
NPC2	0.591120219	0.439142493	0.892139418	0.886312303	0.498546902	0.520534974
FABP4	0.591120219	0.673096434	0.535625285	0.784595865	0.698406173	0.614511437
XBP1	0.591120219	0.444140556	0.663513753	0.78584336	0.805277279	0.622302832
PTRH2	0.591120219	0.565802716	0.835397351	0.630535638	0.567831747	0.68781527
ASGR1	0.591120219	0.551459649	0.492173361	0.669039225	0.87329316	0.690728863
GIMAP7	0.591120219	0.717684248	0.643576065	0.574117341	0.649653657	0.74201596
HRAS	0.591609593	0.491135151	0.705695072	0.649763076	0.821557309	0.628917359
PDXK	0.591609593	0.542567583	0.489549483	0.793916687	0.776537165	0.700746926
TIMP1	0.591618738	0.422321502	0.668192825	0.784838526	0.86930615	0.563814955
PHPT1	0.59161948	0.52243101	0.630408979	0.576822871	0.900893337	0.656859522
EPB41L5	0.59161948	0.460605417	0.594331059	0.64770518	0.66960879	0.880870288
ZNF75D	0.591626414	0.418480843	0.966232793	0.680293142	0.638815482	0.51212265
WFIKKN2	0.591931392	0.447485891	0.758146087	0.687437608	0.758424886	0.666292389
INCENP	0.592146381	0.464178788	0.892139418	0.554743688	0.644263455	0.733767439
C7H5orf63	0.592146381	0.437710144	0.69454186	0.531399091	0.618211409	0.944973383
ARIH1	0.592358659	0.479687945	0.58300263	0.633006168	0.97130055	0.526690149
PAIP1	0.592358659	0.445080617	0.679506848	0.652519777	0.642796813	0.862925606
ERP29	0.593140412	0.461008609	0.69687851	0.618607324	0.50652393	0.93363377
MMP23	0.593686201	0.582127335	0.726119774	0.828736799	0.640551195	0.507584347
TXN2	0.593686201	0.43720588	0.668824581	0.559525332	0.904206501	0.732191464
CD53	0.593755793	0.550226079	0.606660869	0.923220263	0.579443999	0.603083588
ZNF711	0.593755793	0.488101355	0.758146087	0.745577087	0.653399081	0.657689714
UBR5	0.59460996	0.486739486	0.497091612	0.614863577	0.980695939	0.621089184
LOC781663	0.59477197	0.690884835	0.709285034	0.757463039	0.656396948	0.503701037
DNAJB6	0.59477197	0.783634367	0.754913805	0.743443667	0.501311839	0.51212265
POLR2I	0.59477197	0.563316131	0.947176871	0.594220399	0.626973048	0.512846657
BBS12	0.59477197	0.821674572	0.668192825	0.784595865	0.507738123	0.51840034
LOC784007	0.59477197	0.823830365	0.629726901	0.812453944	0.501311839	0.521067427
LOC515697	0.59477197	0.579367653	0.532195319	0.541206056	0.986003146	0.544955425
HSPB7	0.59477197	0.954187594	0.605532453	0.613568161	0.498546902	0.614425574
SHOC2	0.59477197	0.486739486	0.612793995	0.687291299	0.887514169	0.621946029
LOC112449324	0.59477197	0.442374595	0.576113677	0.541206056	0.998196897	0.628379498
LOC107132308	0.59477197	0.414881121	0.669511234	0.887983383	0.714642011	0.62884029
TUFM	0.59477197	0.471949819	0.615251035	0.561120526	0.858741373	0.783404933

TPI1	0.59477197	0.519510456	0.628567322	0.580099141	0.738738532	0.838468539
RIPK2	0.59477197	0.450113576	0.649427086	0.627280162	0.658667583	0.888930405
YPEL3	0.59477197	0.416674804	0.622025849	0.656831463	0.547002755	0.960023723
PDE12	0.59477197	0.421022405	0.623785533	0.555557333	0.583449841	0.985165897
MDH1	0.595035719	0.454880625	0.734143952	0.587741387	0.821351993	0.729100237
FNIP1	0.595636193	0.564100429	0.664740692	0.748837705	0.800097282	0.540876781
PDCD1	0.595636193	0.51452013	0.735051644	0.690807874	0.804264617	0.577181134
ISCA1	0.595636193	0.445034796	0.717242466	0.728264598	0.702888149	0.741319465
LOC100848895	0.595636193	0.449612614	0.700963878	0.661894736	0.537834106	0.902722881
NLN	0.595636193	0.550226079	0.544077194	0.581479634	0.650027114	0.922361909
CGGBP1	0.595636193	0.471593143	0.499640977	0.601220553	0.496066793	0.999948228
C22H3orf67	0.596038946	0.588515985	0.731708587	0.81451387	0.626973048	0.537094861
LOC101904357	0.596038946	0.453467886	0.69417649	0.630535638	0.940870936	0.561829852
ITGA8	0.596038946	0.985428851	0.569601375	0.549573927	0.502432219	0.62884029
ELMO2	0.596038946	0.674801662	0.504126126	0.575014007	0.877712931	0.683500374
NCAPH2	0.59624087	0.51525676	0.531780431	0.96968491	0.612209898	0.609692015
CDR2	0.59624087	0.513491832	0.826785415	0.541206056	0.832119756	0.609692015
LOC112444498	0.59624087	0.672531785	0.561599458	0.769701093	0.642796813	0.677365619
LOC107132531	0.59624087	0.483305224	0.735532321	0.728264598	0.644194633	0.727876258
NSD2	0.596600833	0.474946062	0.957621643	0.626698454	0.596304942	0.598372273
CYP20A1	0.596882218	0.846724749	0.508079608	0.81451387	0.620007054	0.508039178
ZMPSTE24	0.597020831	0.521162219	0.853036433	0.574117341	0.501311839	0.83150957
LOC112444896	0.59748487	0.686984735	0.841294863	0.630535638	0.502957335	0.6467931
C5AR1	0.597959591	0.553132278	0.619994227	0.577939388	0.926972948	0.621946029
ANAPC10	0.598055191	0.445080617	0.89478142	0.660278677	0.784259667	0.515756244
HIP1R	0.599257155	0.428163797	0.51081359	0.999981514	0.634894348	0.501733747
CORO1C	0.599452143	0.424061706	0.834683675	0.894086736	0.619521862	0.51212265
RABL6	0.599452143	0.727473229	0.518265754	0.629215541	0.784972475	0.684994779
MRPL18	0.59946056	0.603542632	0.693546993	0.586089426	0.723054375	0.74212863
CORO2B	0.599498314	0.799590997	0.528387114	0.821450642	0.612403767	0.551807435
DCTN4	0.599498314	0.811059858	0.622920504	0.677545541	0.652333594	0.582422147
GPC1	0.599515853	0.787802226	0.507675433	0.706586091	0.766338619	0.575524376
PKNOX2	0.599515853	0.535202811	0.495423363	0.925353907	0.730921558	0.593981666
SLX4	0.599515853	0.566842413	0.835108464	0.649446726	0.674089143	0.596578269
LOC529196	0.599515853	0.469541075	0.911973589	0.598316303	0.735091896	0.604469389
CAMK2G	0.599515853	0.584209799	0.587609898	0.812453944	0.679048977	0.666011114
FASTKD2	0.600156343	0.477992327	0.557137242	0.573142003	0.870450746	0.8255975
MZT1	0.600383512	0.477035321	0.892343042	0.630535638	0.558285349	0.742455792

FRMD4B	0.600538441	0.528669672	0.727703335	0.814857183	0.58592304	0.668851588
RIC1	0.600618577	0.453089563	0.532703372	0.627280162	0.999985752	0.524890122
AIFM1	0.600618577	0.469272895	0.613634374	0.541206056	0.938316699	0.734269931
LOC788425	0.600618577	0.519450301	0.76555928	0.549287144	0.735091896	0.759003017
SCRN2	0.600948968	0.855067335	0.726177329	0.594220399	0.563212168	0.614511437
CAGE1	0.602010735	0.786691027	0.606660869	0.701248463	0.652333594	0.609692015
PHTF1	0.602116548	0.457488813	0.711148626	0.955466436	0.622751397	0.515756244
CDK8	0.602116548	0.532422913	0.528387114	0.87165456	0.847092933	0.51840034
CASK	0.602116548	0.605916765	0.734143952	0.695681179	0.776537165	0.522828605
LRRC45	0.602116548	0.727473229	0.745084982	0.806573529	0.50555325	0.531378095
PIMREG	0.602116548	0.573498846	0.779581461	0.736045965	0.687203978	0.542061576
LOC101904923	0.602116548	0.48559314	0.562914224	0.766675276	0.913064455	0.564826731
KRT19	0.602116548	0.526654774	0.805702274	0.63494856	0.766507736	0.609692015
LOC100296324	0.602116548	0.573711724	0.612793995	0.574981059	0.91563394	0.640128675
ADHFE1	0.602116548	0.674524738	0.55004286	0.573883521	0.834470237	0.706869246
OSR2	0.602116548	0.435344594	0.590666151	0.961898258	0.556361481	0.723555931
AMOTL1	0.602116548	0.482081164	0.535077764	0.606267291	0.935286646	0.733767439
HSPB6	0.602116548	0.479687945	0.679415149	0.808586366	0.638815482	0.735045772
PKP1	0.602116548	0.441273161	0.647741723	0.791549902	0.74372042	0.735122635
SGSM1	0.602116548	0.471255215	0.604354543	0.792140715	0.738738532	0.741319465
KLHL38	0.602116548	0.584923513	0.527925698	0.549287144	0.8992849	0.748323122
RAB10	0.602116548	0.423816704	0.773165287	0.842434279	0.514024062	0.750967073
GSTM3	0.602116548	0.434898742	0.635427585	0.645168798	0.791848145	0.83150957
TFB2M	0.602116548	0.474946062	0.726044482	0.681573779	0.585452075	0.842654067
WDR27	0.602116548	0.471255215	0.622025849	0.783197692	0.58186871	0.849595612
DMD	0.602116548	0.525344889	0.605532453	0.68362796	0.614663901	0.867907362
PLXNA3	0.60221404	0.528669672	0.729115949	0.854220165	0.555710752	0.652143749
MED12	0.60221404	0.51352654	0.651356851	0.636814098	0.815913413	0.728795223
SELENOW	0.602410509	0.571983204	0.513255922	0.677800818	0.847173934	0.715545192
ZWILCH	0.602410509	0.420543145	0.55950158	0.586089426	0.804037558	0.919475967
PLAUR	0.602411778	0.442980755	0.625861236	0.716348437	0.881521011	0.673985072
LOC515578	0.602519971	0.589431944	0.818612484	0.764257755	0.60702401	0.544955425
FICD	0.602519971	0.673096434	0.834683675	0.713866995	0.502432219	0.607349313
HIF3A	0.602519971	0.446686539	0.532764011	0.99045711	0.653434609	0.614511437
LOC101906006	0.602552567	0.439142493	0.532415258	0.603509689	0.987723553	0.670372456
GVQW3	0.602552567	0.471255215	0.591348881	0.763544094	0.658667583	0.837559792
GARS	0.602552567	0.50468844	0.682152967	0.654131191	0.52068239	0.90816713
CENPC	0.60262546	0.493407919	0.628489483	0.543403516	0.976292433	0.607349313

FKBP2	0.603556376	0.503492022	0.947176871	0.613744304	0.620169849	0.606878524
HSPA1L	0.603556376	0.53764322	0.497992443	0.581479634	0.932024143	0.749726404
CPNE5	0.603568914	0.539469592	0.525791261	0.784838526	0.687203978	0.784754767
PRKCI	0.604130244	0.517719875	0.499932053	0.609774743	0.999985752	0.524890122
TNRC18	0.604328685	0.425196086	0.67171984	0.828736799	0.612403767	0.789870764
STXBP5	0.604378823	0.742905463	0.560847675	0.561120526	0.931775933	0.512846657
RHEB	0.604378823	0.474946062	0.894316021	0.546397256	0.736158637	0.681450813
LOC112443144	0.604466965	0.439760003	0.611647147	0.617282578	0.691315145	0.927900405
LOC112442713	0.6047985	0.950336973	0.620613545	0.612761692	0.614078821	0.521756022
PITX3	0.6047985	0.720305841	0.529409119	0.672328815	0.823508064	0.613401904
SLC6A6	0.6047985	0.625182931	0.507675433	0.730052184	0.847173934	0.629427844
BHLHA15	0.6047985	0.482453139	0.742539165	0.898818046	0.510001595	0.666292389
SEPHS2	0.6047985	0.442980755	0.585370837	0.649763076	0.926172968	0.716096025
CHST8	0.6047985	0.872175143	0.55950158	0.543403516	0.585452075	0.776734587
TSPAN5	0.6047985	0.517719875	0.717242466	0.594220399	0.52589341	0.923033364
TRAF3IP1	0.6047985	0.425196086	0.608218151	0.544789716	0.714597718	0.963456717
SHMT1	0.604821276	0.47375776	0.636479697	0.853058361	0.658667583	0.71289069
DBI	0.604821276	0.454880625	0.616500251	0.544860672	0.937946177	0.754218406
EIF4A2	0.604821276	0.756473821	0.531780431	0.571690689	0.64566985	0.839489406
CRYZL1	0.60493702	0.747604001	0.675096377	0.843284421	0.571639895	0.504854636
CEMIP2	0.60493702	0.425196086	0.754917897	0.868530316	0.782990999	0.51212265
TCF19	0.60493702	0.51452013	0.883737851	0.768290504	0.633781036	0.51840034
KDELR3	0.60493702	0.434596717	0.788106994	0.882057818	0.680594277	0.551945488
LOC787057	0.60493702	0.438643862	0.819322085	0.654131191	0.852746309	0.587387684
NBEA	0.60493702	0.602500622	0.684443377	0.682944876	0.797227609	0.606025716
GBE1	0.60493702	0.55180706	0.500422254	0.768635572	0.89052284	0.614511437
TRA2B	0.60493702	0.439484206	0.503990806	0.680418978	0.976292433	0.655296683
EDIL3	0.60493702	0.517719875	0.591348881	0.865076519	0.69975451	0.669883077
LOC508666	0.60493702	0.479687945	0.667552729	0.606267291	0.877478619	0.729100237
FBXL14	0.60493702	0.844170799	0.55004286	0.626698454	0.571619794	0.761858515
LOC112443175	0.60493702	0.544106496	0.507675433	0.910575108	0.503360023	0.799889245
TREML2	0.60493702	0.517719875	0.768120148	0.656831463	0.554711391	0.82345874
C3H1orf226	0.60493702	0.626284422	0.566638914	0.730052184	0.547247419	0.842507193
EMC4	0.60493702	0.423816704	0.640878185	0.691025178	0.631537325	0.91373422
ORAI2	0.60493702	0.581849076	0.630408979	0.599807615	0.540985448	0.925726857
LOC100848263	0.605101343	0.423816704	0.744735369	0.876174657	0.575201044	0.728699286
GGPS1	0.605814902	0.707494835	0.813441395	0.678068768	0.578296318	0.587353805
STEAP4	0.605814902	0.444140556	0.550289803	0.9880671	0.657146412	0.622811146

SUPV3L1	0.605814902	0.573498846	0.793399684	0.553071371	0.696466814	0.742309541
LOC107132724	0.606938036	0.46147411	0.571415357	0.962350885	0.769529419	0.513780222
DNM2	0.606938036	0.648119635	0.644256366	0.598316303	0.714597718	0.769865166
UBR7	0.607136944	0.445080617	0.962797326	0.745577087	0.605738596	0.515756244
TK2	0.607136944	0.630477275	0.531780431	0.763544094	0.883951804	0.524890122
LOC101904601	0.607136944	0.516115205	0.604354543	0.803690355	0.887514169	0.529445217
CDC42EP2	0.607136944	0.531880429	0.648593816	0.941796131	0.639611119	0.535971177
LOC101902854	0.607136944	0.553132278	0.758146087	0.558176707	0.906827881	0.542061576
LCN1	0.607136944	0.47375776	0.507675433	0.999981514	0.613093988	0.552257767
ARHGEF1	0.607136944	0.457256223	0.79382647	0.866566721	0.670010317	0.552257767
NDUFB7	0.607136944	0.508613061	0.711148626	0.575014007	0.953300742	0.564826731
LOC616944	0.607136944	0.439484206	0.529409119	0.999981514	0.533779151	0.582422147
HOXB3	0.607136944	0.426249621	0.818612484	0.839725231	0.660390139	0.609692015
DOC2A	0.607136944	0.717848892	0.786126716	0.657497543	0.585327515	0.621946029
MYH15	0.607136944	0.520957387	0.849389234	0.71113156	0.625590452	0.645719071
BREH1	0.607136944	0.579367653	0.653833671	0.892047793	0.543569691	0.667995834
CD27	0.607136944	0.449612614	0.81412252	0.830187312	0.585472469	0.669558041
LOC112448354	0.607136944	0.651269643	0.63135074	0.839725231	0.54424469	0.68185373
KDELR2	0.607136944	0.452504201	0.879939804	0.731047585	0.585452075	0.690728863
GTF2IRD1	0.607136944	0.573865937	0.650009937	0.707386562	0.738738532	0.692269123
F3	0.607136944	0.53280405	0.570681722	0.829838753	0.647980002	0.755679512
GZMK	0.607136944	0.522400504	0.623611563	0.552451497	0.823508064	0.824780045
CHRAC1	0.607136944	0.550226079	0.517859609	0.792386115	0.628256006	0.839489406
LOC100141070	0.607136944	0.479687945	0.599489021	0.630535638	0.764945406	0.865767172
LYPLA1	0.607136944	0.479687945	0.69621934	0.630951216	0.621029742	0.883829611
LOC112447844	0.607136944	0.49316884	0.652724437	0.593594064	0.626973048	0.927633316
LRRC55	0.607136944	0.426582179	0.724540928	0.618518556	0.586507835	0.936951685
PSMA1	0.607184163	0.430996205	0.67796952	0.627280162	0.618211409	0.940583352
LOC101903831	0.607741728	0.622449596	0.758146087	0.786067589	0.510264075	0.669558041
LOC100139891	0.607819541	0.447587224	0.947567836	0.574371412	0.75602825	0.607986347
ARHGEF2	0.607819541	0.453089563	0.565148263	0.636814098	0.965676623	0.666292389
NUMB	0.607819541	0.428728874	0.526266326	0.610848628	0.958797242	0.77545502
ISCA2	0.607980163	0.482551032	0.969404007	0.698952489	0.576764113	0.532354747
SPNS2	0.60814011	0.519533906	0.586392082	0.827443542	0.811601139	0.616715863
CDK5RAP2	0.608215186	0.517404163	0.78676714	0.598316303	0.788739452	0.690728863
SLC22A18	0.608254512	0.709518358	0.744735369	0.599544809	0.741179466	0.601371091
PARP4	0.608254512	0.477992327	0.509514515	0.940436902	0.602168189	0.766534677
HUNK	0.608507167	0.741263262	0.700963878	0.863808152	0.529281738	0.513034959

FBXW11	0.608507167	0.452980749	0.705746499	0.854450063	0.804252817	0.539240716
TREX1	0.608507167	0.51452013	0.671231078	0.749535965	0.877712931	0.548381804
IDO1	0.608507167	0.528669672	0.658489624	0.673160239	0.917407853	0.564822443
KCND1	0.608507167	0.449821474	0.55950158	0.999981514	0.57991354	0.57934594
MTG1	0.608507167	0.482453139	0.69687851	0.813323291	0.750542783	0.629881817
ADAMTS6	0.608507167	0.452206305	0.793169467	0.903584224	0.54258013	0.640128675
FAM186B	0.608507167	0.763249441	0.700486014	0.675426128	0.571421132	0.676604463
LY6E	0.608507167	0.501718584	0.582902669	0.626698454	0.900927045	0.740672585
CTNS	0.608507167	0.573498846	0.552362414	0.784595865	0.651951691	0.784857648
TFG	0.608507167	0.565953284	0.822553407	0.610848628	0.52068239	0.81956221
COX11	0.608507167	0.581074652	0.605532453	0.636814098	0.686117762	0.839906041
CNIH1	0.608507167	0.488001171	0.745477682	0.618288053	0.533807401	0.913221224
CAPZA1	0.608507167	0.450686302	0.775552434	0.599544809	0.562981968	0.919124527
OFD1	0.609328549	0.559306577	0.69358179	0.869208308	0.721096295	0.512846657
KIF22	0.609328549	0.542391346	0.789663873	0.752840326	0.727873981	0.559845075
FGF11	0.609328549	0.991673407	0.58300263	0.565751353	0.546299503	0.607349313
PANK3	0.609804996	0.650600033	0.64222503	0.633006168	0.644945176	0.795936781
ORM1	0.609847113	0.491135151	0.62031191	0.838961268	0.710664679	0.70377729
LOC101907483	0.60987304	0.525901055	0.726119774	0.612227019	0.657388898	0.839906041
LOC782348	0.610510788	0.435818607	0.855971055	0.553146279	0.578333624	0.893585844
SLC26A7	0.610602196	0.452080332	0.57816803	0.736392704	0.605738596	0.927520621
VAMP5	0.610604147	0.499204258	0.566959933	0.999981514	0.568764846	0.563801887
TMBIM4	0.610604147	0.617216601	0.849389234	0.780279943	0.541176598	0.568308927
CPXM1	0.610604147	0.782783231	0.625105036	0.723043513	0.696466814	0.571961045
GTF2E1	0.610604147	0.649340041	0.892139418	0.63494856	0.578296318	0.607349313
HSD11B1	0.610604147	0.509765303	0.570681722	0.883236823	0.734324024	0.669558041
C15H11orf58	0.610604147	0.446062855	0.872088608	0.674469953	0.540227484	0.811195375
CARD6	0.610604147	0.449965992	0.761415057	0.631620874	0.71077874	0.812391447
ZBTB8OS	0.610977157	0.708137441	0.560847675	0.581297447	0.953300742	0.523933932
ZNF775	0.610977157	0.553132278	0.541610391	0.758499128	0.696466814	0.807285519
SET	0.610977157	0.474946062	0.637234901	0.736045965	0.521036349	0.923547711
ACOT9	0.611395717	0.519450301	0.71156186	0.78584336	0.829853428	0.520534974
LOC112447626	0.611395717	0.985997726	0.651735357	0.553146279	0.585398487	0.529445217
FMO3	0.611395717	0.469660184	0.689992074	0.81472008	0.655556663	0.749726404
PDSS1	0.611395717	0.493407919	0.744735369	0.643190603	0.571639895	0.873130182
MED13	0.611702328	0.548448956	0.552162548	0.587186699	0.999985752	0.521299803
CYP4B1	0.611702328	0.442990825	0.637234901	0.925457774	0.809317773	0.526690149
MED29	0.611702328	0.442374595	0.743442322	0.586033659	0.980016241	0.532841807

PET100	0.611702328	0.752229543	0.740207705	0.637708629	0.723054375	0.539240716
GLI4	0.611702328	0.447450909	0.531780431	0.839245804	0.94971901	0.53935279
FAM46B	0.611702328	0.855752352	0.561914716	0.626698454	0.805138244	0.542061576
KCNK17	0.611702328	0.553489554	0.58646427	0.587186699	0.983941532	0.562667338
IL9R	0.611702328	0.553132278	0.654732007	0.839690643	0.776537165	0.564822443
GRAMD1C	0.611702328	0.445080617	0.534905861	0.999981514	0.696466814	0.568308927
LOC101903038	0.611702328	0.48811343	0.532743798	0.852005554	0.895067787	0.574900933
BMP5	0.611702328	0.446395731	0.507675433	0.627280162	0.999985752	0.577181134
AZIN1	0.611702328	0.589351784	0.884172102	0.727236849	0.578333624	0.587132866
DLX5	0.611702328	0.975852983	0.620613545	0.581942629	0.585472469	0.587941456
MAGED1	0.611702328	0.445034796	0.594331059	0.998529748	0.644945176	0.597568377
EWSR1	0.611702328	0.454880625	0.603097231	0.999981514	0.567274296	0.625066198
USP35	0.611702328	0.45627172	0.69881939	0.964521432	0.527202266	0.657833313
LOC101906511	0.611702328	0.54671968	0.656335298	0.589571496	0.911028086	0.669548645
FRRS1	0.611702328	0.479687945	0.744735369	0.797338497	0.66960879	0.694197796
GDF11	0.611702328	0.482453139	0.586106764	0.949502614	0.60583938	0.699160709
LOC112445078	0.611702328	0.447485891	0.64236045	0.783796091	0.82114467	0.700746926
PPP1R12B	0.611702328	0.659408339	0.641535145	0.678604808	0.714642011	0.710078411
GRID2	0.611702328	0.82089312	0.537185735	0.677545541	0.642796813	0.711116516
MRTO4	0.611702328	0.54671968	0.730578564	0.852170016	0.516812553	0.711922168
SASH3	0.611702328	0.499204258	0.777712574	0.612761692	0.789999838	0.716532042
UQCRB	0.611702328	0.457693965	0.718374618	0.610790896	0.881680572	0.720141691
SERPINF1	0.611702328	0.45450823	0.685018711	0.80459801	0.735687381	0.720398359
MLF1	0.611702328	0.54671968	0.587296907	0.580133741	0.926071555	0.721192619
PRKAA2	0.611702328	0.445080617	0.886629485	0.58293921	0.674089143	0.779201808
FAM193B	0.611702328	0.512513718	0.708227907	0.686250223	0.626858142	0.836705424
TRDMT1	0.611702328	0.461047248	0.647065939	0.815888375	0.55605466	0.864514638
LOC112449614	0.611702328	0.461860331	0.596835991	0.762687521	0.52068239	0.944973383
GDF9	0.612274177	0.754676989	0.53400059	0.741401062	0.650485683	0.716863155
FAM96A	0.61366911	0.687651905	0.621702102	0.612761692	0.555544932	0.880870288
ACSL1	0.61372789	0.483976773	0.734143952	0.575289975	0.716950801	0.858321732
NDUFB11	0.614099942	0.493407919	0.694535506	0.630535638	0.872096228	0.695065923
TRIM31	0.614099942	0.871924797	0.631699427	0.554743688	0.647949888	0.699162043
SEPT6	0.614099942	0.833429351	0.534905861	0.627280162	0.634894348	0.762133815
EXOSC5	0.614099942	0.541763982	0.509302195	0.691442543	0.766655597	0.852504043
LOC100847995	0.614103675	0.445080617	0.779209064	0.682944876	0.900893337	0.563814955
DEPDC1	0.614103675	0.479687945	0.908072171	0.7694401	0.600776371	0.60936417
MAML2	0.614103675	0.634059237	0.582638793	0.628724992	0.91563394	0.614511437

DPM2	0.614103675	0.589651566	0.758146087	0.609774743	0.821351993	0.623290811
FBLIM1	0.614103675	0.647890563	0.524780246	0.955981799	0.571639895	0.631731716
CENPO	0.614103675	0.453089563	0.978014482	0.630535638	0.612403767	0.63202365
NDUFS5	0.614103675	0.51862711	0.693546993	0.594220399	0.926813827	0.645719071
TOP3B	0.614103675	0.479687945	0.798782742	0.80459801	0.643560251	0.669883077
GRPEL1	0.614103675	0.608823572	0.754913805	0.796934171	0.544248798	0.68185373
AFF1	0.614103675	0.460244277	0.582638793	0.716077462	0.879268517	0.748921056
EDF1	0.614103675	0.590368852	0.630408979	0.618607324	0.787177225	0.779201808
LOC112448454	0.614103675	0.826488263	0.645276799	0.594220399	0.545946638	0.783241453
ZAP70	0.614103675	0.450686302	0.570681722	0.912777307	0.578296318	0.823701731
MED16	0.614103675	0.495857219	0.608218151	0.630951216	0.765646002	0.866726566
LOC104969050	0.614103675	0.482217107	0.63135074	0.777326875	0.593931025	0.867063767
TIMM8A	0.614103675	0.460244277	0.717242466	0.659427594	0.626973048	0.884001927
TIMM9	0.614103675	0.447485891	0.723132042	0.577737722	0.651951691	0.933903265
CADPS2	0.614175917	0.720304917	0.847776765	0.564694471	0.652333594	0.628640603
BCO2	0.614175917	0.517719875	0.833583844	0.581052873	0.655786713	0.798336045
HMBS	0.614196646	0.687651905	0.752113836	0.570760743	0.750542783	0.668191006
ZNF572	0.61423383	0.750379179	0.726119774	0.765499767	0.567274296	0.609692015
CAVIN3	0.614803878	0.494454594	0.942357321	0.608306231	0.750369638	0.560987471
MPP2	0.615308512	0.718258172	0.532743798	0.832401065	0.567274296	0.740774964
RIPK1	0.615628207	0.48811343	0.550289803	0.613091606	0.999985752	0.52479594
C16H1orf115	0.615628207	0.449965992	0.654545673	0.844863645	0.891726429	0.524899551
SCN1B	0.615628207	0.544401883	0.813942299	0.703717381	0.631537325	0.699160709
HES6	0.615628207	0.728455575	0.551149334	0.677545541	0.756363818	0.702623424
SMPDL3B	0.615628207	0.534089022	0.685018711	0.590326511	0.769686429	0.829441883
OVCA2	0.615628207	0.479687945	0.544667159	0.780279943	0.696466814	0.860966325
TUBA1B	0.615813179	0.584209799	0.774924967	0.728264598	0.597314504	0.710036675
XPO1	0.61583265	0.532096752	0.52262636	0.844863645	0.591306131	0.85532952
LOC112442226	0.616130136	0.535710688	0.649063946	0.710172832	0.832407979	0.677355984
VCPIP1	0.616405661	0.454880625	0.728670337	0.999981514	0.53709747	0.546062761
DENND6B	0.616405661	0.454880625	0.768050411	0.898818046	0.579443999	0.669558041
FGG	0.616405661	0.558704912	0.845113191	0.728264598	0.527129253	0.727876258
COX7B	0.616405661	0.464892289	0.660861667	0.580099141	0.935286646	0.742309541
SLC25A25	0.61703968	0.648958657	0.846424224	0.69158245	0.567831747	0.645719071
UCKL1	0.617050458	0.829653291	0.689888669	0.792140715	0.572464745	0.521756022
LOC112449523	0.617050458	0.499204258	0.745348234	0.939604997	0.534012164	0.623319115
SEC23B	0.617050458	0.50850907	0.972162594	0.627280162	0.571639895	0.640128675
MFSD6L	0.617143098	0.49587005	0.565148263	0.973906748	0.738738532	0.533968062

HINT1	0.617143098	0.48559314	0.711148626	0.612761692	0.777218526	0.8255975
STEAP3	0.617238305	0.550942014	0.524780246	0.999981514	0.549773723	0.632510367
ZNF277	0.617238305	0.692305416	0.563473889	0.838528071	0.651951691	0.667047801
NLRP3	0.617496827	0.482217107	0.685018711	0.594220399	0.887514169	0.751884708
FBXW12	0.617533376	0.47375776	0.906019013	0.636027046	0.583721163	0.765948605
NEDD4	0.617585582	0.544401883	0.532764011	0.619686917	0.999985752	0.540314488
LOC101905779	0.617585582	0.448447766	0.53400059	0.902696094	0.736942681	0.751705208
LOC100140403	0.61774574	0.571339777	0.625861236	0.684149461	0.801692604	0.741017467
KIF21B	0.618214419	0.479687945	0.69687851	0.685601134	0.687203978	0.841312694
STK17B	0.618487769	0.470243637	0.784833275	0.652519777	0.626796691	0.845747012
TSPAN9	0.619288121	0.571983204	0.647741723	0.821865782	0.824072221	0.526690149
NUDT22	0.619288121	0.761907799	0.8042974	0.60232178	0.630794687	0.63202365
CENPJ	0.619288121	0.609864167	0.681918771	0.812313105	0.596079788	0.718444845
STOM	0.619288121	0.449612614	0.700963878	0.703663629	0.696466814	0.845783187
TBC1D15	0.619427164	0.479687945	0.5846414	0.849313593	0.674048869	0.801888363
RPRD2	0.619863796	0.722968232	0.641535145	0.717238192	0.809129327	0.531055466
B3GNTL1	0.619863796	0.452206305	0.75029951	0.627280162	0.929896768	0.631731716
MRPL4	0.619863796	0.687651905	0.624052322	0.630535638	0.820477096	0.669883077
NDUFA2	0.619863796	0.454880625	0.682166552	0.573142003	0.953300742	0.716074186
DRAP1	0.619863796	0.542391346	0.755940516	0.731537809	0.645616811	0.733767439
TH	0.619863796	0.45629714	0.70675041	0.830940997	0.642662413	0.764310896
CHCHD2	0.619863796	0.482453139	0.694535506	0.630951216	0.815913413	0.781629683
P2RY6	0.620196031	0.454880625	0.731143569	0.64770518	0.70977288	0.852504043
ZMYND11	0.620319961	0.512513718	0.65062204	0.780279943	0.612403767	0.840768468
CDS1	0.620343825	0.62215202	0.64428623	0.898423855	0.638815482	0.597128696
KCNK3	0.620343825	0.746341488	0.702094756	0.577737722	0.804625939	0.621089184
GADD45G	0.620343825	0.580905654	0.647065939	0.812453944	0.769529419	0.62884029
COMMD5	0.620343825	0.531880429	0.681700278	0.892071982	0.658667583	0.632510367
NDUFA11	0.620343825	0.545258886	0.637928356	0.58293921	0.956904152	0.645427231
NFE2	0.620343825	0.729001857	0.80412682	0.627280162	0.607958542	0.668851588
ADPRH	0.620343825	0.613273559	0.719862199	0.763544094	0.652333594	0.68185373
LOC515736	0.620343825	0.88044109	0.565148263	0.736045965	0.531351877	0.690728863
S100A10	0.620343825	0.51726061	0.620613545	0.627280162	0.935286646	0.690728863
DBP	0.620343825	0.516972202	0.590686872	0.794386089	0.804274107	0.718149424
LOC100140121	0.620343825	0.514675796	0.951392011	0.565933683	0.598907641	0.729499398
WDR12	0.620343825	0.447450909	0.79497298	0.792161305	0.607314284	0.769865166
ALS2CL	0.620343825	0.472523617	0.594331059	0.890362631	0.614663901	0.811759324
BET1L	0.620343825	0.600886305	0.749453648	0.565933683	0.631319169	0.845593437

ZPBP2	0.620343825	0.495378253	0.807254925	0.627280162	0.60903578	0.847386898
GRAMD1B	0.620343825	0.445080617	0.586392082	0.887187234	0.560554936	0.877014986
GIGYF1	0.620343825	0.469660184	0.668727476	0.717645144	0.527685234	0.941413984
SNPH	0.620356028	0.48811343	0.684382692	0.896116841	0.56180439	0.751100989
BTG1	0.620858733	0.449612614	0.879939804	0.821450642	0.659373888	0.593122637
TOX2	0.620858733	0.473243816	0.999993442	0.616373358	0.552121564	0.596578269
SH3PXD2A	0.620858733	0.51452013	0.605532453	0.940436902	0.672729318	0.645719071
PLCB1	0.620858733	0.571983204	0.700963878	0.623608478	0.879395665	0.645719071
MRPL21	0.620858733	0.499388045	0.694535506	0.561120526	0.929802526	0.706869246
ATP5IF1	0.620858733	0.471255215	0.677197181	0.613744304	0.928630535	0.713238512
EML6	0.620858733	0.865570309	0.546452802	0.691655428	0.60443186	0.713707269
PMF1	0.620858733	0.48811343	0.684443377	0.724431807	0.788007619	0.749918186
THY1	0.62176264	0.596540379	0.76555928	0.812453944	0.696466814	0.535620846
ZDHHC18	0.62176264	0.452134986	0.588125041	0.736045965	0.976259416	0.582927878
ANGPTL7	0.62176264	0.565421383	0.645276799	0.901353354	0.576970572	0.700459582
CNBP	0.62176264	0.442990825	0.784788059	0.792386115	0.61565225	0.78087541
PRUNE2	0.62176264	0.496890044	0.660861667	0.739273899	0.680934071	0.835231445
KIF17	0.62176264	0.509765303	0.693546993	0.56187044	0.541010839	0.980167535
PLEKHF1	0.622102111	0.526654774	0.622025849	0.901353354	0.546299503	0.778302445
CCDC85C	0.622226931	0.517719875	0.612793995	0.960916501	0.735091896	0.526690149
RSPH10B	0.622226931	0.460605417	0.979584814	0.609774743	0.716994185	0.584339668
PAPD5	0.622226931	0.469660184	0.550289803	0.952247001	0.716994185	0.68185373
AREL1	0.622226931	0.584228062	0.582638793	0.627280162	0.923666555	0.68185373
SRGN	0.622226931	0.648503747	0.630408979	0.587186699	0.826634148	0.748750383
PFDN6	0.622226931	0.741263262	0.720612298	0.596654953	0.60903578	0.779201808
LOC788745	0.622226931	0.454880625	0.708019215	0.627280162	0.638815482	0.931410194
DHX35	0.622794613	0.510547475	0.871491704	0.606267291	0.823508064	0.614511437
WFDC3	0.622958722	0.522698706	0.957052938	0.719770646	0.61565225	0.529445217
TSPAN4	0.622958722	0.465004933	0.764467593	0.887983383	0.666619775	0.628379498
RUNDC3B	0.62314951	0.485778913	0.836459233	0.819213899	0.652996371	0.62532656
GRAP2	0.62314951	0.515680499	0.928927123	0.577939388	0.718810344	0.660603184
RNF7	0.62314951	0.52701263	0.874983674	0.627280162	0.735091896	0.666011114
NOS1	0.62314951	0.472312407	0.533570754	0.817903737	0.902907077	0.668851588
SLBP	0.62314951	0.461047248	0.71967891	0.784838526	0.807575053	0.669883077
CAMK1G	0.62314951	0.62215202	0.627295843	0.596654953	0.799272579	0.79187813
MUC16	0.623365302	0.54845263	0.740372388	0.830940997	0.642796813	0.661029645
MYOM1	0.623365302	0.550442307	0.607986114	0.565578589	0.626033107	0.96870908
LOC112443504	0.623375988	0.474946062	0.667814218	0.682383061	0.891304411	0.702623424

NMNAT1	0.623375988	0.564100429	0.634391045	0.777956162	0.640551195	0.803098894
PCYT1A	0.623864098	0.518261575	0.641535145	0.886312303	0.797227609	0.589397695
MAPK11	0.624507827	0.450686302	0.734143952	0.893051204	0.753088486	0.597568377
DPH5	0.624520076	0.598069728	0.632469403	0.609204337	0.691315145	0.875650601
KPNA4	0.624923908	0.532096752	0.759357794	0.852170016	0.694783366	0.587353805
SUGT1	0.624923908	0.521662141	0.668824581	0.731537809	0.883152894	0.627258867
FAM131A	0.624923908	0.492794915	0.55004286	0.575014007	0.585452075	0.999948228
KIT	0.625225286	0.583140563	0.818951412	0.646058642	0.769529419	0.628649974
ATP6V1G1	0.625225286	0.500811762	0.854567571	0.730705148	0.530499378	0.78288746
SPEN	0.625356763	0.585763624	0.643576065	0.716077462	0.891304411	0.587387684
LOC785842	0.625356763	0.471593143	0.535557143	0.985435217	0.698346212	0.645719071
ANOS1	0.62542569	0.496890044	0.75669748	0.739199454	0.795865165	0.652116614
PTPN2	0.626144989	0.465158408	0.69687851	0.78584336	0.906803277	0.560502405
N6AMT1	0.626144989	0.482801836	0.546068496	0.768635572	0.958797242	0.614511437
LOC104969028	0.62630479	0.692305416	0.791001445	0.765499767	0.580561884	0.622811146
NFKBID	0.626507541	0.465275901	0.874983674	0.864771297	0.656015118	0.545900472
ASB8	0.626507541	0.473243816	0.642566325	0.600433884	0.999985752	0.566196709
METTL8	0.626749181	0.587350756	0.876414692	0.751437238	0.58186871	0.62884029
PPP2CB	0.627685782	0.484688573	0.658489624	0.972942834	0.632055507	0.621946029
FAM13C	0.627790372	0.517268685	0.526976806	0.969088992	0.696466814	0.657174611
LOC104974923	0.627860312	0.565257607	0.617730771	0.887983383	0.801692604	0.554751652
LLGL2	0.627860312	0.821674572	0.570681722	0.659998592	0.770343524	0.640128675
JRKL	0.628577709	0.659408339	0.552362414	0.822708607	0.738738532	0.671759429
PFKFB1	0.628577709	0.722779653	0.62439117	0.580099141	0.85545716	0.68185373
TMCO1	0.628577709	0.481934113	0.667782878	0.586033659	0.613652691	0.974962631
LOC101905312	0.629050124	0.460857983	0.823108128	0.576242366	0.962513981	0.546170552
SLC39A2	0.629050124	0.446395731	0.63135074	0.932914414	0.647980002	0.748750383
SRSF5	0.629050124	0.446395731	0.532764011	0.843593618	0.847173934	0.754218406
STYK1	0.629668559	0.589782216	0.694535506	0.896116841	0.543569691	0.684994779
VIRMA	0.629696422	0.895311167	0.552362414	0.636814098	0.804037558	0.552257767
STARD7	0.629716947	0.543554867	0.755135544	0.832489049	0.691315145	0.61614241
CDH1	0.629716947	0.479687945	0.627295843	0.765499767	0.930451229	0.61614241
NIPSNAP1	0.629716947	0.553132278	0.679415149	0.63494856	0.930451229	0.621946029
TRERF1	0.629738456	0.469016365	0.668824581	0.854966424	0.819124214	0.639142898
TPD52L2	0.629986432	0.622818484	0.668824581	0.66651301	0.809129327	0.690728863
SP140L	0.629986432	0.522698706	0.809114624	0.767092077	0.654168701	0.692528599
LOC101903877	0.629986432	0.489541299	0.680262176	0.742465161	0.607314284	0.88313584
THBD	0.63036019	0.750087757	0.788126358	0.681730579	0.618012018	0.629427844

PIGC	0.63036019	0.471255215	0.854567571	0.717645144	0.78021215	0.638990067
CCDC14	0.63036019	0.470133653	0.609398456	0.959616431	0.642796813	0.702623424
FASN	0.63036019	0.470923083	0.594331059	0.598316303	0.928363269	0.821573137
DRAM2	0.63036019	0.621713316	0.649063946	0.685261155	0.650027114	0.836111377
CBLN2	0.63036019	0.65172183	0.677197181	0.636027046	0.605738596	0.859032412
ALDH5A1	0.63067924	0.697043768	0.543652077	0.715394268	0.811276938	0.690728863
BBS5	0.63067924	0.491829344	0.55950158	0.896116841	0.655556663	0.797662039
MB	0.63067924	0.474946062	0.630408979	0.630535638	0.805615411	0.87501921
MALT1	0.630743038	0.471255215	0.817582037	0.594220399	0.541564331	0.938894901
THAP5	0.630809669	0.592003056	0.883500436	0.693458387	0.692827651	0.574870258
SIRT4	0.630809669	0.462981381	0.69931173	0.784838526	0.890273522	0.609692015
HNRNPAB	0.630809669	0.479687945	0.849389234	0.853058361	0.612403767	0.640670691
FBXL5	0.630809669	0.657066596	0.685018711	0.769701093	0.687203978	0.675176731
MRPL37	0.630809669	0.528504292	0.751079565	0.586290626	0.799272579	0.786849547
SLC38A2	0.630809669	0.683744977	0.566638914	0.672328815	0.697778495	0.83150957
KBTBD7	0.630809669	0.449821474	0.716635829	0.793841296	0.638815482	0.842214812
FKBP3	0.630809669	0.556813446	0.70011973	0.626698454	0.64727886	0.88457156
PHLDB2	0.630809669	0.50468844	0.531780431	0.777956162	0.628507266	0.927520621
C13H20orf202	0.630830863	0.580444201	0.557137242	0.999981514	0.543569691	0.613401904
TCP1	0.630830863	0.465004933	0.80412682	0.710070489	0.583449841	0.856640874
GUCY1B1	0.630830863	0.531880429	0.629143322	0.675565794	0.595153566	0.938894901
ANAPC15	0.63106907	0.609772211	0.874797723	0.656831463	0.734324024	0.572514995
SPOP	0.63106907	0.449612614	0.587296907	0.766675276	0.901853599	0.734983106
HBQ1	0.6312452	0.486372821	0.589828096	0.641696066	0.998196897	0.600092797
IDH3B	0.6312452	0.479687945	0.671579333	0.586290626	0.991182251	0.621946029
PHLDA2	0.6312452	0.753090681	0.603953115	0.666446733	0.821557309	0.632510367
LMLN	0.631292596	0.459370989	0.570681722	0.610790896	0.999985752	0.552257767
SNX22	0.631292596	0.584209799	0.943836178	0.628601595	0.688556938	0.564822443
ANKRD13B	0.631292596	0.54021232	0.855892553	0.812453944	0.652333594	0.587132866
MAGED4B	0.631292596	0.614976364	0.651356851	0.947799524	0.57836033	0.622811146
POLRMT	0.631292596	0.718477366	0.758146087	0.682944876	0.687203978	0.62884029
EMILIN3	0.631292596	0.965482645	0.596931535	0.610848628	0.627206089	0.640128675
SNURF	0.631292596	0.449965992	0.533570754	0.586033659	0.999985752	0.645719071
OCIAD2	0.631292596	0.657122966	0.91882781	0.574371412	0.634184199	0.652116614
DHX40	0.631292596	0.710645465	0.627295843	0.764257755	0.695436567	0.682479856
CHTF18	0.631292596	0.45450823	0.916323169	0.78877021	0.561655662	0.699160709
COG1	0.631292596	0.54021232	0.641535145	0.949565453	0.565792249	0.702623424
A1CF	0.631292596	0.759245424	0.694535506	0.731537809	0.572813055	0.72026427

TMEM128	0.631292596	0.598069728	0.81412252	0.724431807	0.570431346	0.74880923
RPS19BP1	0.631292596	0.722195304	0.670239985	0.623608478	0.723054375	0.749918186
GCFC2	0.631292596	0.470133653	0.637504121	0.918105555	0.596680559	0.783241453
OLFM2	0.631292596	0.840785555	0.546224399	0.654797105	0.572523505	0.828287671
ASNA1	0.631292596	0.527574316	0.688151858	0.627280162	0.78773451	0.835231445
KANK1	0.631292596	0.718228672	0.561599458	0.648660726	0.66960879	0.841312694
NMT1	0.631292596	0.582127335	0.754913805	0.604036098	0.652333594	0.847443063
KCND3	0.631292596	0.487617269	0.652724437	0.796128007	0.576970572	0.893838339
LOC100336669	0.631469399	0.587387416	0.603097231	0.688942799	0.943702351	0.603083588
ADAM1A	0.631469399	0.454880625	0.735532321	0.816226107	0.846464841	0.61411085
KIAA1551	0.631469399	0.517683932	0.711148626	0.74577892	0.769686429	0.741319465
MED6	0.631542441	0.614976364	0.53791136	0.601220553	0.891304411	0.783969061
ACACA	0.631591171	0.477618743	0.585284463	0.596654953	0.887514169	0.871998318
LOC112446456	0.631698243	0.699192406	0.845113191	0.748837705	0.560554936	0.620459631
PQBP1	0.631698243	0.561716964	0.838341783	0.586290626	0.735091896	0.748921056
TOMM22	0.631698243	0.54845263	0.768120148	0.664953033	0.63648166	0.824780045
MGST1	0.631698243	0.483948377	0.660344165	0.701248463	0.663923284	0.903500283
CASC4	0.631698243	0.550226079	0.533251947	0.615089094	0.696466814	0.960023723
ETFA	0.631871529	0.539483465	0.614355922	0.581479634	0.965163023	0.690728863
SYT11	0.631871529	0.727534248	0.608265631	0.622980329	0.58186871	0.891423889
MAP2K3	0.632106537	0.527777396	0.901982947	0.688704343	0.61033296	0.702623424
NIFK	0.632106537	0.469660184	0.802277976	0.645168798	0.789915129	0.762133815
GSTO1	0.632106537	0.480755771	0.754913805	0.641118121	0.677185022	0.867063767
C23H6orf62	0.632106537	0.460200512	0.651458903	0.598316303	0.56690435	0.999948228
LOC112446757	0.632153976	0.692305416	0.606146644	0.627280162	0.940870936	0.585646285
EPDR1	0.632867024	0.610421396	0.627371121	0.606267291	0.972160227	0.578851812
LOC782057	0.632867024	0.450686302	0.993024884	0.682944876	0.642796813	0.598118579
C1S	0.632867024	0.71366091	0.625861236	0.632683273	0.893716526	0.601371091
ZNF276	0.632867024	0.474946062	0.97773026	0.652134587	0.662671151	0.613401904
NIN	0.632867024	0.489193793	0.605532453	0.893220599	0.804274107	0.656859522
ZNF470	0.632867024	0.532096752	0.65933691	0.932764647	0.635127008	0.669883077
MRPL2	0.632867024	0.630230756	0.749453648	0.604750897	0.817472827	0.68185373
QRICH2	0.632867024	0.499204258	0.700963878	0.836610893	0.741086403	0.683722496
LOC112445193	0.632867024	0.45627172	0.696535103	0.598316303	0.964301327	0.690728863
IL1RL1	0.632867024	0.465419715	0.749417651	0.698952489	0.85545716	0.694197796
TOE1	0.632867024	0.460244277	0.685018711	0.769631679	0.832394138	0.733200009
EDN1	0.632867024	0.46019627	0.608218151	0.908467146	0.69975451	0.755679512
LOC101906966	0.633279904	0.452080332	0.566638914	0.989707094	0.660390139	0.699160709

RNASEK	0.633388898	0.542873579	0.759357794	0.610848628	0.56690435	0.923092436
POMGNT2	0.633619997	0.520957387	0.924855847	0.695125147	0.56180439	0.719460348
CHCHD4	0.633997296	0.54021232	0.831933739	0.632177478	0.626973048	0.818370468
ABCB9	0.63405933	0.573865937	0.968204727	0.641927161	0.570517621	0.63094682
NKD2	0.634513311	0.474946062	0.597046406	0.799712183	0.925918507	0.651781292
PLSCR3	0.634679075	0.581059045	0.651502209	0.977711979	0.593931025	0.594285548
FGF7	0.634679075	0.681192035	0.674950575	0.882057818	0.593931025	0.645719071
USP12	0.634679075	0.476247505	0.735532321	0.948668643	0.585398487	0.673244165
PER2	0.634679075	0.516632812	0.630408979	0.690807874	0.887514169	0.736866513
RTL6	0.634679075	0.474946062	0.600357181	0.961898258	0.553835124	0.793197037
CCT8	0.635212289	0.468668438	0.684934371	0.748837705	0.58186871	0.927426708
WDR73	0.635345571	0.615769899	0.572476783	0.695125147	0.817472827	0.766218451
RAD51	0.636559394	0.469660184	0.949855095	0.654131191	0.738738532	0.618303855
DNAJC25	0.636731036	0.484688573	0.734143952	0.712481274	0.918512595	0.601371091
SELENOT	0.636731036	0.473734192	0.596931535	0.587839433	0.562981968	0.999948228
APOL3	0.637077133	0.882242226	0.634857444	0.598316303	0.600776371	0.769865166
CCL19	0.63730687	0.482453139	0.8735515	0.751045386	0.788007619	0.587941456
LOC786586	0.637375764	0.469660184	0.726746856	0.999981514	0.553835124	0.559845075
TVP23B	0.637375764	0.471778712	0.804902355	0.89684883	0.717315449	0.560987471
PGAP3	0.637375764	0.452206305	0.570836276	0.705448435	0.965163023	0.704950309
TPMT	0.637375764	0.587350756	0.66149866	0.630535638	0.691186726	0.877014986
RBMX2	0.637854537	0.465004933	0.749417651	0.731537809	0.695890051	0.83150957
POLR2E	0.637905664	0.584209799	0.749417651	0.599807615	0.819679279	0.739579536
LOC101903574	0.638350251	0.474946062	0.744735369	0.994689556	0.605738596	0.542061576
SLC12A2	0.638608409	0.49771159	0.590381768	0.645168798	0.999985752	0.551807435
RRBP1	0.638608409	0.553489554	0.679415149	0.937615893	0.693337723	0.584339668
TUBB6	0.638608409	0.734546826	0.655886028	0.780703865	0.738738532	0.605297754
PTPRF	0.638608409	0.834615638	0.71967891	0.630951216	0.696466814	0.622811146
DLK1	0.638608409	0.599021986	0.677197181	0.652644879	0.826736355	0.733767439
TECR	0.638608409	0.522698706	0.716724771	0.627280162	0.848550619	0.765644158
YARS	0.638608409	0.474946062	0.69621934	0.724431807	0.619521862	0.91373422
CBX7	0.638608409	0.582127335	0.585284463	0.615079168	0.630569631	0.963456717
MTMR7	0.638938476	0.950986475	0.572476783	0.608301577	0.593931025	0.742309541
CORO1A	0.639059137	0.535967468	0.855811972	0.601220553	0.690633636	0.785562356
NAA40	0.639059137	0.567198791	0.69687851	0.599807615	0.809317773	0.814218101
RFC1	0.639563811	0.470243637	0.597922877	0.95901497	0.626973048	0.765070525
METAP1	0.639669174	0.454880625	0.668824581	0.980532429	0.738738532	0.571765275
H2AFY	0.639746067	0.639920656	0.541610391	0.712481274	0.954071324	0.587132866

GNAL	0.639758451	0.45629714	0.627295843	0.819968583	0.652333594	0.883307547
TIMM21	0.640535066	0.470862073	0.734143952	0.624159166	0.952796169	0.669883077
OXNAD1	0.64059443	0.750379179	0.79497298	0.653466517	0.750277829	0.553389239
PHETA1	0.640654655	0.855067335	0.622890829	0.60125663	0.667763384	0.758175289
GNL1	0.640746679	0.532096752	0.546717582	0.707217807	0.976292433	0.625245189
TMEM250	0.640746679	0.469541075	0.670070271	0.586089426	0.936108073	0.79187813
COQ9	0.640829272	0.524051946	0.641535145	0.609081266	0.976259416	0.666011114
LOC101904265	0.641307504	0.77139884	0.720612298	0.706180917	0.702888149	0.621946029
XYLT2	0.641307504	0.553132278	0.56863752	0.838528071	0.84620147	0.669558041
SNX17	0.641307504	0.469660184	0.693546993	0.641118121	0.954014484	0.684615686
MOB4	0.641307504	0.51452013	0.927635169	0.691655428	0.580707198	0.730208866
LOC112448736	0.641307504	0.47375776	0.695084268	0.858330221	0.725950216	0.742309541
DNAJC7	0.641307504	0.694445189	0.726119774	0.685261155	0.637102655	0.761939071
REEP2	0.641307504	0.547676372	0.679415149	0.677545541	0.818179113	0.769865166
LOC101903615	0.641383499	0.472315604	0.561623293	0.999981514	0.591515687	0.622811146
ZNF582	0.641383499	0.522173354	0.587609898	0.627280162	0.996781978	0.631731716
SELENOH	0.641383499	0.658075243	0.628904157	0.687330897	0.693671558	0.828287671
NDUFB6	0.641383499	0.479687945	0.755135544	0.613568161	0.795839201	0.839906041
PLEKHM3	0.641383499	0.466912164	0.701512486	0.818166381	0.584450219	0.87501921
UGP2	0.641383499	0.5641165	0.670239985	0.596333047	0.655786713	0.940017687
LOC112448078	0.641672285	0.49771159	0.637928356	0.999981514	0.626033107	0.566196709
TMF1	0.641672285	0.534062577	0.538132476	0.634552521	0.999985752	0.566196709
MED12L	0.641672285	0.863286109	0.846622323	0.632177478	0.575928213	0.581350944
VANGL1	0.641672285	0.658075243	0.574826765	0.677545541	0.944839728	0.60417388
MEG9	0.641672285	0.456203151	0.881778842	0.85721441	0.648899379	0.629427844
TEAD1	0.641672285	0.79358003	0.585204095	0.827786827	0.66101983	0.632510367
GTPBP6	0.641672285	0.580905654	0.632141336	0.598316303	0.956041014	0.68185373
SLC2A6	0.641672285	0.461610272	0.542873405	0.999981514	0.58186871	0.738287596
DCP1B	0.641672285	0.479687945	0.897725902	0.731537809	0.569025406	0.772618249
ERI3	0.642031963	0.455869075	0.874843419	0.657497543	0.887514169	0.593981666
PEMT	0.642535613	0.861395263	0.668824581	0.632683273	0.793993073	0.569398665
LOC781022	0.642535613	0.624142952	0.867661896	0.792071212	0.618977366	0.588600411
LOC101905262	0.642535613	0.492943907	0.685018711	0.882057818	0.817950097	0.618592325
C29H11orf95	0.642535613	0.474946062	0.774015998	0.859799195	0.707739077	0.67453786
FBXO8	0.642535613	0.46147411	0.736531189	0.794386089	0.815913413	0.698787824
LOC100295347	0.642535613	0.461860331	0.623308784	0.630951216	0.944839728	0.781473646
YIPF5	0.642535613	0.479687945	0.86347788	0.596654953	0.58186871	0.912531667
CXCL9	0.64255453	0.778487216	0.779052868	0.682944876	0.567274296	0.700326694

LOC112447032	0.642651522	0.512872801	0.76555928	0.813323291	0.658667583	0.742455792
AHNAK2	0.643196953	0.524576105	0.679506848	0.97731816	0.60903578	0.632880775
EPHA5	0.643209442	0.754676989	0.552362414	0.804862068	0.8282759	0.564826731
FABP5	0.644183763	0.763758778	0.778210036	0.596654953	0.823508064	0.558375365
LOC112447526	0.644341475	0.624142952	0.821457385	0.866566721	0.579443999	0.601371091
TRIM7	0.644341475	0.720304917	0.734143952	0.762598894	0.716406563	0.601371091
LOC112447316	0.644341475	0.846724749	0.758146087	0.661894736	0.626973048	0.619388281
LOC100848639	0.644341475	0.550442307	0.830420708	0.791081996	0.691315145	0.643003473
MATN4	0.644341475	0.482453139	0.559053784	0.633006168	0.998287238	0.68185373
UBE2E1	0.644341475	0.479687945	0.855892553	0.642073478	0.571784062	0.88542704
FNBP1	0.644341475	0.481653805	0.663738759	0.623608478	0.578333624	0.995379819
LOC101906916	0.64436006	0.534529958	0.606660869	0.999981514	0.626973048	0.563814955
LOC112448524	0.64436006	0.622818484	0.590381768	0.978404889	0.5863408	0.643003473
SH3GLB2	0.64436006	0.85988793	0.726119774	0.602157124	0.696466814	0.648195749
LOC112449284	0.64436006	0.534529958	0.800716826	0.63966902	0.695436567	0.818370468
CSTB	0.644966348	0.546899736	0.607986114	0.845055741	0.610376834	0.855286622
HPN	0.645103784	0.474946062	0.566638914	0.729705452	0.970988226	0.68185373
RRP8	0.645183168	0.531880429	0.754913805	0.617282578	0.908820136	0.672418631
PRIMPOL	0.646026207	0.460387616	0.640121554	0.970868622	0.804037558	0.566196709
ADAMTS12	0.646026207	0.530020372	0.56802023	0.999981514	0.586511866	0.596578269
CWF19L1	0.646026207	0.659195551	0.775552434	0.632683273	0.788007619	0.673985072
RNF219	0.647299311	0.571983204	0.892343042	0.672181572	0.649653657	0.706842017
DTNB	0.647344465	0.482217107	0.791198027	0.724271417	0.909379774	0.57856676
PHLDB1	0.647344465	0.901275501	0.671717793	0.676566861	0.642662413	0.634096451
C1QTNF3	0.647344465	0.50468844	0.869932384	0.646556987	0.823508064	0.645719071
AKAP8L	0.647344465	0.553132278	0.774015998	0.674469953	0.801692604	0.71016497
LOC107131715	0.647344465	0.630038239	0.679415149	0.701248463	0.801692604	0.718444845
AOAH	0.647344465	0.457605323	0.876414692	0.627280162	0.688845988	0.835231445
LOC107131209	0.647376709	0.653179887	0.719475516	0.896508582	0.656015118	0.568308927
ANPEP	0.647376709	0.565421383	0.679506848	0.600946	0.854289643	0.809567434
DYRK1A	0.647589203	0.730581055	0.69687851	0.672328815	0.858179728	0.568308927
ADSL	0.647672133	0.469660184	0.728269425	0.618518556	0.87329316	0.814745299
THNSL2	0.648504603	0.476257657	0.604753359	0.96951401	0.787177225	0.627258867
GPR137B	0.648504603	0.524929534	0.726119774	0.932832695	0.567274296	0.716925595
ZNF800	0.648637506	0.527777396	0.56745809	0.610699285	0.999985752	0.593981666
LOC787074	0.648637506	0.463158835	0.879939804	0.619231292	0.593931025	0.893838339
GFPT2	0.64872048	0.477992327	0.810647419	0.648660726	0.833864947	0.739579536
ZNF566	0.648900497	0.553132278	0.810647419	0.703717381	0.627065919	0.799581725

ALDH3B1	0.649055248	0.7889129	0.68898955	0.649446726	0.612403767	0.78288746
LOC100847818	0.649092933	0.460200512	0.668824581	0.900044333	0.65298501	0.799254373
SLC29A2	0.649213857	0.718258172	0.552362414	0.952247001	0.626973048	0.61614241
CFAP161	0.649213857	0.824188473	0.754913805	0.619201546	0.73254131	0.61614241
EIF4A3	0.649213857	0.469660184	0.957295966	0.812999186	0.560701594	0.640128675
ACP2	0.649213857	0.926852499	0.672696223	0.652644879	0.6274106	0.640128675
LOC618737	0.649213857	0.630038239	0.947952648	0.631620874	0.5863408	0.667758333
GPR50	0.649213857	0.573498846	0.659831992	0.827443542	0.726295891	0.739095574
LOC506989	0.649213857	0.474946062	0.731143569	0.614863577	0.61565225	0.969564816
SIVA1	0.649239251	0.649221583	0.608218151	0.84478631	0.848717914	0.561792744
MTMR1	0.649239251	0.48157544	0.559304698	0.993416552	0.788007619	0.601987815
SLC16A13	0.649239251	0.672505775	0.67171984	0.628378059	0.927973846	0.611960528
CCDC32	0.649239251	0.875932472	0.606146644	0.837820405	0.575928213	0.628379498
LARS	0.649239251	0.573865937	0.743278326	0.755893058	0.788007619	0.681807713
NOP10	0.649239251	0.583140563	0.823979873	0.664953033	0.652333594	0.78096075
GPR141	0.649239251	0.474946062	0.764467593	0.636027046	0.82688838	0.793643414
DNAJC24	0.649239251	0.51352654	0.740372388	0.62527333	0.797127163	0.842178536
RPS4Y1	0.649239251	0.48811343	0.774025101	0.695125147	0.569025406	0.919124527
SLCO1C1	0.649344385	0.496355097	0.784833275	0.652644879	0.882206878	0.684615686
AARS	0.649344385	0.522698706	0.774015998	0.661390091	0.699191343	0.839565788
CRTAC1	0.649643016	0.791176621	0.73826834	0.839245804	0.593931025	0.560502405
CNOT4	0.649643016	0.508210155	0.608008194	0.676566861	0.998060725	0.607349313
JAG1	0.649643016	0.595156245	0.705746499	0.630535638	0.926813827	0.643003473
SLC35C2	0.649643016	0.628660167	0.729359017	0.682944876	0.823508064	0.668851588
LOC511386	0.649643016	0.48811343	0.949855095	0.634717693	0.721096295	0.669883077
MRPL54	0.649643016	0.612070685	0.695127588	0.630951216	0.887514169	0.688206116
PRPS1	0.649643016	0.466912164	0.883737851	0.852170016	0.585398487	0.704351773
TMEM225B	0.649643016	0.614976364	0.552362414	0.904271346	0.655556663	0.754218406
POU2AF1	0.649643016	0.577757207	0.69454186	0.835194309	0.623804687	0.77803403
LOC101905499	0.649643016	0.553132278	0.612941017	0.871134824	0.631537325	0.821910421
LOC505479	0.649697091	0.496355097	0.598655764	0.944351122	0.626973048	0.789870764
GLUD1	0.650066657	0.483305224	0.555917581	0.719816078	0.693337723	0.960023723
CACNG4	0.650084791	0.647365653	0.949855095	0.598663987	0.657171354	0.631731716
PRR7	0.650084791	0.591296685	0.662879727	0.610062671	0.90654624	0.746853748
VPS37A	0.650141978	0.461008609	0.914770879	0.728264598	0.656015118	0.742309541
ATP5MG	0.650208354	0.499204258	0.684274697	0.627280162	0.882494878	0.814218101
METTL23	0.65272353	0.613632195	0.694535506	0.676612863	0.567831747	0.919124527
BNIP2	0.652785734	0.511936523	0.845525761	0.812453944	0.799272579	0.56419933

BDP1	0.653244865	0.479687945	0.608218151	0.598316303	0.999985752	0.589397695
PPP4R4	0.653244865	0.471255215	0.660344165	0.999981514	0.653434609	0.63202365
NFE2L1	0.653481424	0.472312407	0.64236045	0.96444269	0.578333624	0.786002175
SLC1A1	0.65360266	0.477035321	0.608218151	0.999981514	0.681182883	0.563801887
STRIP2	0.65360266	0.487617269	0.55345323	0.868378265	0.93723117	0.627258867
SHISA7	0.65360266	0.609209091	0.77566312	0.810132355	0.69314679	0.645719071
MAF1	0.65360266	0.474946062	0.732274253	0.701248463	0.926972948	0.669558041
MVK	0.65360266	0.527574316	0.704680319	0.659998592	0.890124729	0.735122635
MCM3	0.65360266	0.624142952	0.693546993	0.736045965	0.735091896	0.755679512
NDUFA4	0.65360266	0.483305224	0.649427086	0.612761692	0.957588298	0.769865166
LOC112443444	0.65360266	0.593498372	0.679415149	0.64770518	0.803705802	0.806476213
ANKRD11	0.654642102	0.54845263	0.826785415	0.664953033	0.877712931	0.609692015
MKI67	0.654642102	0.51452013	0.880476435	0.81472008	0.637102655	0.669558041
TAX1BP1	0.654642102	0.527553585	0.874063521	0.736045965	0.659373888	0.720865143
ACO2	0.654642102	0.54021232	0.675096377	0.627280162	0.908820136	0.762133815
TMEM88	0.654696715	0.495202621	0.693546993	0.728264598	0.958797242	0.609287712
LOC112442386	0.654696715	0.51352654	0.644256366	0.918971786	0.612403767	0.793197037
SPAG5	0.65487851	0.479687945	0.872088608	0.898818046	0.591306131	0.645719071
SMIM12	0.655224443	0.609209091	0.817051566	0.632683273	0.886785729	0.581214144
FAHD1	0.655224443	0.569645599	0.776076303	0.707217807	0.795839201	0.695765939
LEP	0.655224443	0.835486075	0.7293348	0.707217807	0.569004261	0.706165228
ZNF410	0.655224443	0.642598915	0.569601375	0.878513543	0.714642011	0.729499398
DBT	0.655224443	0.531880429	0.663513753	0.62496659	0.85545716	0.845747012
GPR83	0.655224443	0.514966729	0.84685641	0.606611279	0.681182883	0.862925606
DLAT	0.655584242	0.483305224	0.7293348	0.615079168	0.85545716	0.837173937
RAB27B	0.65577724	0.527777396	0.668824581	0.630535638	0.847092933	0.845747012
EBD	0.656102768	0.630038239	0.696535103	0.598316303	0.744684207	0.860414702
MINOS1	0.656270867	0.614976364	0.694535506	0.615089094	0.943702351	0.640434848
CYB5R4	0.656470216	0.519533906	0.979494108	0.764257755	0.583449841	0.601987815
SYT17	0.656470216	0.798589766	0.572476783	0.627280162	0.879776073	0.668851588
SPARCL1	0.656470216	0.861395263	0.700900214	0.649446726	0.593931025	0.745988271
TMEM14A	0.656556094	0.600246997	0.78057665	0.941467375	0.578296318	0.587387684
RHBDD1	0.656556094	0.823175902	0.652724437	0.856195359	0.579443999	0.632510367
HNRNPK	0.656556094	0.469660184	0.761415057	0.882395013	0.655556663	0.754218406
LOC107132921	0.656556094	0.471949819	0.819173863	0.777956162	0.670221463	0.784381044
FDPS	0.656556094	0.572077017	0.759110916	0.604750897	0.735091896	0.8537109
SLC25A33	0.656659831	0.469660184	0.583883256	0.999981514	0.584450219	0.601500908
LPIN3	0.656659831	0.495378253	0.916323169	0.84478631	0.582154532	0.645719071

WSB2	0.656659831	0.522698706	0.829455601	0.820971736	0.696466814	0.668851588
FGF2	0.656659831	0.502964446	0.579487023	0.942883863	0.738738532	0.730527465
LOC101906607	0.656659831	0.506509735	0.606660869	0.733411985	0.605982335	0.970071797
LOC112442253	0.657273514	0.479687945	0.740372388	0.831577385	0.870189808	0.611142532
ATP5F1D	0.657273514	0.49771159	0.687462433	0.613744304	0.954403133	0.743842064
FBXL7	0.657759769	0.740343181	0.653924522	0.66637376	0.847173934	0.66459017
PRELID1	0.657759769	0.660882273	0.684952951	0.759397892	0.655786713	0.789870764
SETMAR	0.657952355	0.901028838	0.557137242	0.741170082	0.753088486	0.608554064
DLX4	0.657952355	0.622818484	0.909762947	0.676566861	0.705202026	0.620459631
TRPT1	0.657952355	0.610421396	0.743624166	0.641696066	0.902907077	0.628917359
GRAMD4	0.657952355	0.493317252	0.8735515	0.899552507	0.596304942	0.632880775
FBXO33	0.657952355	0.489987041	0.608008194	0.999981514	0.5863408	0.640128675
WRAP73	0.657952355	0.496111927	0.987610559	0.645168798	0.656015118	0.659761491
FAIM	0.657952355	0.542918274	0.942357321	0.634717693	0.700612826	0.675895249
LOC107131699	0.657952355	0.659837453	0.834683675	0.645168798	0.656015118	0.749726404
MAIP1	0.657952355	0.492794915	0.72058898	0.641118121	0.89743683	0.763582226
DNAJC11	0.657952355	0.516972202	0.744735369	0.614863577	0.82488413	0.832964812
LOC104975460	0.657952355	0.522079637	0.653026936	0.828736799	0.60941238	0.877048993
LOC101906280	0.658105503	0.549121527	0.815661698	0.896116841	0.618211409	0.638990067
AMPD3	0.658105503	0.479687945	0.715158198	0.676566861	0.958852623	0.667726353
RELT	0.658105503	0.524051946	0.596080143	0.76992117	0.879776073	0.763466477
TMEM35B	0.6584086	0.769416761	0.796783363	0.7817105	0.596680559	0.621946029
TBC1D2	0.6584086	0.594199197	0.709649545	0.705448435	0.677185022	0.844759721
RTCA	0.658573674	0.609209091	0.673165093	0.645168798	0.716994185	0.876162019
RND1	0.658604244	0.493407919	0.902215582	0.839632707	0.69149571	0.587865358
LOC786332	0.658604244	0.709198375	0.915713148	0.631746507	0.690450229	0.596578269
STXBP2	0.658604244	0.587740565	0.604354543	0.681899077	0.960419102	0.660200814
SEC63	0.658604244	0.476955291	0.885896294	0.751451145	0.747400879	0.68185373
CSTF1	0.658604244	0.560109317	0.778802525	0.739750555	0.793993073	0.683495103
NIPA2	0.658604244	0.469660184	0.720612298	0.926970666	0.652333594	0.748750383
PINX1	0.658653964	0.760623317	0.648593816	0.771699334	0.603965612	0.779201808
CLU	0.659117796	0.744241031	0.583006927	0.613744304	0.964301327	0.601371091
LARS2	0.659117796	0.624292003	0.633096422	0.60068965	0.92549567	0.748921056
LOC536097	0.659117796	0.882858125	0.560847675	0.631746507	0.626973048	0.83437817
RPAP1	0.659117796	0.501598223	0.894497585	0.669514212	0.570107746	0.85643253
LOC107132949	0.659389156	0.558327847	0.682974314	0.979644238	0.655556663	0.603040282
C16H1orf105	0.659389156	0.580322769	0.885896294	0.608306231	0.603143049	0.842473495
LRP11	0.659761799	0.824188473	0.814495418	0.627280162	0.696466814	0.613401904

LYPLA2	0.65997349	0.630230756	0.771066142	0.598316303	0.681182883	0.85532952
B3GALT1	0.660328091	0.779025263	0.809673076	0.610790896	0.735133749	0.657174611
TPK1	0.660337141	0.745860095	0.779209064	0.682944876	0.66960879	0.690728863
BCL11A	0.660460026	0.479687945	0.70011973	0.780279943	0.804037558	0.785562356
IL2RG	0.660804267	0.580530872	0.736160515	0.703717381	0.932036998	0.571961045
SLC25A26	0.660804267	0.526654774	0.698275826	0.627280162	0.990937627	0.608554064
LOC107132431	0.660804267	0.531880429	0.761415057	0.690807874	0.912691379	0.629427844
SPIRE2	0.660804267	0.474946062	0.816117673	0.710626372	0.865076851	0.682263171
UQCRH	0.660804267	0.506509735	0.711148626	0.639948763	0.906893576	0.762451586
CPT2	0.660804267	0.584209799	0.627295843	0.636814098	0.887514169	0.79187813
ASB10	0.660804267	0.568485668	0.700963878	0.623608478	0.864524107	0.792412747
LOC101906317	0.660804267	0.479687945	0.612793995	0.915039335	0.655556663	0.835231445
LRRC42	0.660804267	0.559306577	0.605532453	0.912777307	0.573188535	0.841312694
BBS1	0.660863152	0.757251331	0.627084316	0.876703811	0.715674124	0.601371091
MED9	0.660863152	0.568856941	0.833583844	0.85721441	0.665098448	0.622811146
CENPW	0.661160221	0.551459649	0.879939804	0.751045386	0.71767272	0.65014676
ADCY4	0.661360141	0.476257657	0.614355922	0.999981514	0.612403767	0.643003473
FBXO24	0.661462247	0.483305224	0.854571175	0.914026346	0.679186011	0.582422147
CLIP2	0.661462247	0.580322769	0.681700278	0.94857292	0.674719204	0.636674289
LOC104973054	0.661680808	0.479667805	0.831933739	0.830427818	0.782443723	0.640128675
TSEN54	0.661680808	0.632321074	0.668824581	0.742465161	0.847092933	0.683592201
GORASP2	0.661680808	0.48811343	0.989494193	0.632683273	0.605982335	0.734983106
HS6ST1	0.661680808	0.477035321	0.70011973	0.610480724	0.670245065	0.980009318
LOC101901950	0.661680808	0.493723355	0.623611563	0.656831463	0.571639895	0.999948228
GIPC3	0.661835557	0.541287559	0.907626082	0.705422335	0.810436875	0.572514995
C4A	0.661835557	0.802045751	0.69621934	0.808073654	0.720059315	0.580973226
COX14	0.661835557	0.673435317	0.752659039	0.613744304	0.929896768	0.587387684
TRIM11	0.661835557	0.54845263	0.681652094	0.760270895	0.954403133	0.587387684
PPL	0.661835557	0.482801836	0.95219206	0.746075358	0.738738532	0.588384898
TLDC1	0.661835557	0.479687945	0.933884582	0.868378265	0.64727886	0.590055009
SV2B	0.661835557	0.927973597	0.571415357	0.836387732	0.632055507	0.591919512
THAP9	0.661835557	0.482453139	0.757565308	0.89684883	0.811991102	0.596578269
TOP2A	0.661835557	0.616020041	0.847776765	0.853058361	0.638815482	0.601371091
SLITRK6	0.661835557	0.750379179	0.593098683	0.705448435	0.92549567	0.605408237
LOC101902760	0.661835557	0.674801662	0.923364099	0.652519777	0.696466814	0.607349313
HELLS	0.661835557	0.573498846	0.625439953	0.976125636	0.721096295	0.609692015
KCNS2	0.661835557	0.882858125	0.849389234	0.623632909	0.617915169	0.612307446
DCAF10	0.661835557	0.775033799	0.649427086	0.687568149	0.870450746	0.614233457

LOC104974057	0.661835557	0.69959307	0.830410896	0.610848628	0.847173934	0.614511437
EME2	0.661835557	0.530470574	0.863546668	0.863269783	0.674048869	0.621946029
CABP7	0.661835557	0.54845263	0.641535145	0.898818046	0.84620147	0.621946029
KY	0.661835557	0.694092819	0.668824581	0.888687722	0.696466814	0.622811146
SLC29A3	0.661835557	0.484607868	0.717242466	0.627280162	0.998287238	0.622811146
FOPNL	0.661835557	0.474946062	0.669864986	0.812453944	0.948421303	0.622913023
KCTD1	0.661835557	0.749370809	0.605532453	0.888198716	0.721826916	0.628379498
ADCY6	0.661835557	0.81232489	0.680262176	0.627280162	0.849766114	0.631731716
TMEM173	0.661835557	0.560109317	0.89478142	0.852287362	0.593216299	0.640128675
NEO1	0.661835557	0.78357023	0.612793995	0.891732037	0.633781036	0.640128675
PKN2	0.661835557	0.492855313	0.716468083	0.956685049	0.726283157	0.640128675
EFCAB5	0.661835557	0.521967606	0.679415149	0.982220679	0.67654501	0.643615576
HDAC9	0.661835557	0.913116251	0.700963878	0.627280162	0.699191343	0.645719071
NADK	0.661835557	0.811059858	0.620613545	0.720787315	0.789915129	0.669558041
REPS1	0.661835557	0.532096752	0.865512879	0.865076519	0.611555745	0.669883077
TET1	0.661835557	0.614976364	0.585370837	0.896116841	0.79453727	0.672587014
RGS5	0.661835557	0.54671968	0.944126714	0.627280162	0.738738532	0.673985072
BLOC1S1	0.661835557	0.679199291	0.694535506	0.627280162	0.901144467	0.675247254
PRKX	0.661835557	0.549410627	0.855847948	0.707217807	0.789999838	0.675688253
RINL	0.661835557	0.553132278	0.915858924	0.636814098	0.762812927	0.677355984
CCR7	0.661835557	0.49771159	0.969404007	0.627280162	0.729180196	0.679437565
LGALS3BP	0.661835557	0.760778257	0.69454186	0.674891586	0.799272579	0.679803128
STXBP6	0.661835557	0.596649723	0.925953504	0.636027046	0.677889535	0.684994779
ZFAND5	0.661835557	0.54845263	0.596931535	0.952247001	0.738738532	0.684994779
CD164L2	0.661835557	0.879093989	0.718840994	0.666598607	0.638815482	0.68781527
MROH1	0.661835557	0.486739486	0.879939804	0.889055166	0.582706032	0.688206116
CLSTN3	0.661835557	0.616227995	0.648593816	0.630951216	0.942786245	0.68969695
FASTKD5	0.661835557	0.550442307	0.886829776	0.809356235	0.605738596	0.690728863
JAKMIP3	0.661835557	0.641897615	0.82155377	0.76992117	0.655556663	0.699162043
C26H10orf82	0.661835557	0.781222833	0.665648059	0.799712183	0.650057649	0.700123604
MLX	0.661835557	0.560109317	0.744735369	0.648660726	0.89052284	0.703969699
LRRC14	0.661835557	0.482551032	0.971053208	0.602157124	0.727356071	0.710630057
FAM219A	0.661835557	0.818489019	0.694535506	0.763544094	0.612403767	0.714321361
LOC518961	0.661835557	0.755789173	0.649378275	0.819213899	0.642796813	0.729499398
ATP6V0C	0.661835557	0.639909661	0.700128649	0.893220599	0.598364653	0.730527465
NCOR2	0.661835557	0.718228672	0.680262176	0.839245804	0.626033107	0.732191464
LOC104974666	0.661835557	0.624142952	0.63135074	0.931509349	0.610761857	0.733767439
TMEM156	0.661835557	0.560109317	0.871285807	0.613744304	0.784972475	0.742568903

RPP30	0.661835557	0.47375776	0.805479206	0.659427594	0.879776073	0.745952648
KLF9	0.661835557	0.52001926	0.691971775	0.867235478	0.738811608	0.745988271
SLC39A14	0.661835557	0.528167223	0.563292548	0.916858019	0.792867703	0.745988271
TAZ	0.661835557	0.479687945	0.899106435	0.659998592	0.760007802	0.752213117
LOC101903752	0.661835557	0.482081164	0.66149866	0.898818046	0.764945406	0.752213117
RNF152	0.661835557	0.696358351	0.668824581	0.87522058	0.58186871	0.759003017
MGRN1	0.661835557	0.626284422	0.77283514	0.809886338	0.591306131	0.769865166
WDR74	0.661835557	0.554555702	0.736160515	0.799147532	0.71767272	0.769865166
NDUFB8	0.661835557	0.4971494	0.671469816	0.68362796	0.91563394	0.770035496
NAXD	0.661835557	0.750466772	0.571415357	0.633006168	0.823508064	0.784754767
C14H8orf59	0.661835557	0.480755771	0.943836178	0.627280162	0.682784145	0.785562356
EXOSC4	0.661835557	0.641897615	0.794676674	0.609774743	0.740929839	0.786849547
LOC112449531	0.661835557	0.54021232	0.611794073	0.803690355	0.810436875	0.789337092
PLEKHB2	0.661835557	0.522678607	0.731143569	0.890362631	0.592840293	0.797662039
SLC25A13	0.661835557	0.609772211	0.667552729	0.659427594	0.817472827	0.810508177
APBB3	0.661835557	0.493805901	0.761415057	0.837820405	0.619787296	0.824025963
SUSD2	0.661835557	0.474946062	0.630408979	0.773429607	0.854966579	0.827474082
PSAT1	0.661835557	0.701348278	0.69621934	0.633006168	0.726564622	0.83150957
ABCD2	0.661835557	0.678023292	0.809673119	0.633006168	0.605738596	0.839231958
PAN3	0.661835557	0.561355727	0.640065634	0.715394268	0.797227609	0.846210629
LHFPL4	0.661835557	0.645547325	0.660344165	0.613744304	0.801692604	0.851325852
ACSS1	0.661835557	0.697306013	0.570836276	0.618518556	0.804274107	0.858245612
SMC3	0.661835557	0.692305416	0.608218151	0.695681179	0.698563589	0.858507498
EIF1AX	0.661835557	0.52001926	0.774924967	0.733411985	0.652333594	0.860966325
MRPL53	0.661835557	0.474946062	0.791070941	0.665999974	0.721672235	0.874557852
TCTEX1D1	0.661835557	0.664859217	0.71659461	0.685261155	0.596304942	0.877014986
CCT3	0.661835557	0.498324072	0.701777234	0.769631679	0.670221463	0.877048993
PGR	0.661835557	0.495202621	0.89385865	0.612761692	0.627206089	0.87754233
FAM166B	0.661835557	0.551459649	0.604753359	0.883236823	0.582706032	0.881939613
SRP19	0.661835557	0.563489799	0.805702274	0.627280162	0.652206871	0.891088733
GSTM1	0.661835557	0.541943971	0.605532453	0.748424012	0.736233814	0.905034785
KLF11	0.661835557	0.624142952	0.630638844	0.627280162	0.738738532	0.909056553
FILIP1L	0.661835557	0.550226079	0.632469403	0.731537809	0.682784145	0.923092436
C7H19orf71	0.661835557	0.584209799	0.588533868	0.708803752	0.707939546	0.923547711
GCNA	0.661835557	0.474946062	0.75197571	0.764140634	0.597314504	0.923807852
EPHA3	0.661835557	0.575241982	0.564522177	0.641118121	0.804274107	0.923807852
HSPA9	0.661835557	0.482453139	0.694535506	0.674469953	0.737297505	0.928416436
FAM43B	0.661835557	0.522596388	0.579487023	0.866566721	0.582544518	0.932049622

TCP11L1	0.661835557	0.48161576	0.611647147	0.82163384	0.629421719	0.93531275
LOC112444773	0.661835557	0.616467638	0.609398456	0.654797105	0.647602397	0.954465286
VARS	0.661835557	0.532096752	0.749453648	0.640315346	0.583449841	0.958707771
LRRC4	0.661835557	0.492855313	0.644256366	0.615029548	0.713877228	0.979505709
SLC2A1	0.662015874	0.615769899	0.630408979	0.988408846	0.656105076	0.605408237
CLEC4D	0.662015874	0.684946765	0.748230839	0.774800098	0.750542783	0.654416032
LOC104969981	0.662015874	0.580473137	0.698320032	0.797338497	0.631537325	0.853548296
LOC512684	0.662015874	0.571839975	0.711636925	0.731537809	0.661305864	0.872238439
RFFL	0.662409696	0.652446942	0.802150905	0.618288053	0.796497513	0.742309541
BBIP1	0.662409696	0.617042679	0.719815462	0.609774743	0.832486638	0.806714845
GPR18	0.662459672	0.51452013	0.838341783	0.896116841	0.651585054	0.660576629
LOC101905293	0.662971154	0.507024057	0.56802023	0.978404889	0.732302754	0.721192619
PPIL3	0.663028982	0.54021232	0.668727476	0.627280162	0.999985752	0.608554064
ZNF304	0.663179756	0.54911059	0.817582037	0.812453944	0.607314284	0.78096075
ROBO3	0.663183247	0.607454605	0.689992074	0.780184803	0.670221463	0.833150766
MICALL1	0.663459264	0.821674572	0.630408979	0.741170082	0.735091896	0.683495103
CHMP2B	0.663459264	0.534671863	0.916968887	0.731537809	0.603965612	0.751100989
KDM4A	0.663459264	0.477992327	0.833583844	0.626698454	0.593931025	0.958474163
KCNS3	0.663459264	0.553132278	0.596826854	0.613744304	0.737633927	0.973218068
FASTK	0.663763064	0.614976364	0.848278925	0.72187681	0.823508064	0.584339668
POGLUT1	0.663763064	0.840646333	0.622920504	0.612227019	0.820264258	0.718567063
AKNA	0.66386295	0.479687945	0.915858924	0.731876542	0.788995893	0.655022943
CFDP1	0.66386295	0.591296685	0.942357321	0.653503626	0.658108379	0.690728863
BUB1	0.66386295	0.535967468	0.810647419	0.80921192	0.714642011	0.724693895
MMADHC	0.66386295	0.477992327	0.789663873	0.636814098	0.790136352	0.865767172
CILP2	0.664085349	0.530592604	0.729290628	0.811831345	0.607314284	0.874037262
KRT79	0.664281511	0.579451739	0.637234901	0.707217807	0.970988226	0.621946029
FGD3	0.664281511	0.609864167	0.761415057	0.61731688	0.870450746	0.733767439
ABI1	0.664292802	0.498155476	0.728269425	0.730052184	0.954071324	0.631731716
SAMD3	0.664292802	0.494789709	0.686711082	0.610848628	0.737633927	0.965783646
PDZRN3	0.665085506	0.594126609	0.591348881	0.76992117	0.797489438	0.832964812
CENPE	0.665311038	0.517719875	0.851633941	0.900875874	0.637102655	0.64753123
TXNDC15	0.665311038	0.493407919	0.972482052	0.627280162	0.648267928	0.762133815
ZNF550	0.665354877	0.480755771	0.957295966	0.868435169	0.595423977	0.609692015
DAPP1	0.665354877	0.474946062	0.971255098	0.752081152	0.679186011	0.640128675
POLQ	0.665354877	0.522698706	0.855971055	0.692226	0.855740473	0.653830641
RALA	0.665354877	0.519787678	0.808037655	0.853058361	0.723990421	0.68185373
RRP9	0.665354877	0.622600605	0.846523249	0.630951216	0.687203978	0.793197037

LOC782101	0.665354877	0.531880429	0.744735369	0.673875708	0.741891558	0.872238439
LOC540403	0.665627279	0.51862711	0.971556427	0.640315346	0.680301694	0.699720332
LOC529399	0.665748452	0.524051946	0.635797358	0.889415593	0.900747635	0.613270659
EPPK1	0.665748452	0.648503747	0.761415057	0.731537809	0.696466814	0.758175289
IFNGR1	0.665748452	0.524929534	0.732480837	0.690798131	0.788007619	0.848566057
AGGF1	0.665822744	0.48811343	0.632469403	0.999981514	0.764945406	0.601371091
SEMA4D	0.666005478	0.565257607	0.935266153	0.645092912	0.723054375	0.684615686
ATP5PO	0.666005478	0.50468844	0.747884618	0.632683273	0.932024143	0.743720206
BATF3	0.666005478	0.833713501	0.793169467	0.627280162	0.583449841	0.765948605
TOMM6	0.666005478	0.608823572	0.69621934	0.627280162	0.71077874	0.91373422
SUGP1	0.666208251	0.477035321	0.669511234	0.898818046	0.864524107	0.667995834
ZNF326	0.666208251	0.479073963	0.611647147	0.707217807	0.903410824	0.847193872
CCDC134	0.666239269	0.819747113	0.608008194	0.777956162	0.695436567	0.724693895
LOC112448366	0.66633248	0.693194821	0.717242466	0.796843021	0.821777864	0.593981666
CNTN3	0.666335155	0.600886305	0.831933739	0.630535638	0.911568867	0.601371091
DNASE1	0.666335155	0.493052487	0.676501557	0.659908345	0.996089557	0.651781292
ZNF692	0.666335155	0.519533906	0.8644528	0.902776185	0.596304942	0.669883077
MRPL36	0.666335155	0.692305416	0.736435559	0.627280162	0.773384033	0.788618009
GPRC5B	0.666335155	0.522698706	0.620613545	0.659988757	0.810263299	0.923092436
SERPINB5	0.666531855	0.728267541	0.854571175	0.619201546	0.612403767	0.78288746
LOC112446002	0.666547041	0.51862711	0.627295843	0.83431635	0.738738532	0.850190854
SLA	0.666694943	0.51352654	0.854567571	0.610848628	0.903404534	0.695765939
TTC14	0.66681513	0.499858047	0.685620658	0.972142185	0.793993073	0.591919512
EFHC1	0.66681513	0.515892935	0.587296907	0.972142185	0.849915122	0.60417388
KCNE4	0.66681513	0.643640444	0.735532321	0.736045965	0.868722067	0.626045106
SUOX	0.66681513	0.489058914	0.758146087	0.636814098	0.970988226	0.659120631
LTA4H	0.66681513	0.758609923	0.879939804	0.707217807	0.596680559	0.665736967
ELN	0.66681513	0.584209799	0.610032576	0.962369147	0.714642011	0.671526037
PRRC2C	0.66681513	0.50230849	0.668824581	0.654131191	0.988049878	0.681450813
GTF2IRD2	0.66681513	0.524576105	0.66149866	0.892047793	0.823508064	0.684524441
PPP1R2	0.66681513	0.493983544	0.949855095	0.731537809	0.679048977	0.684994779
TARBP2	0.66681513	0.517719875	0.809673119	0.734423422	0.849786155	0.687128561
INPP5B	0.66681513	0.50468844	0.579487023	0.999981514	0.656015118	0.699977246
DNASE1L3	0.66681513	0.584209799	0.583883256	0.733411985	0.927086179	0.742309541
LAMP2	0.66681513	0.534573879	0.608218151	0.969088992	0.655610947	0.75702988
PITPNM2	0.66681513	0.68903984	0.603530331	0.888948777	0.596137752	0.809783905
PFDN4	0.66681513	0.479687945	0.731143569	0.652484448	0.851351885	0.85532952
IFIT5	0.66681513	0.748660215	0.608218151	0.71656639	0.652333594	0.865767172

BLOC1S2	0.66681513	0.622818484	0.751957722	0.695125147	0.618977366	0.874000314
VMA21	0.66681513	0.485755959	0.743624166	0.673160239	0.671225322	0.944330889
RDH16	0.66681513	0.50598388	0.637234901	0.684402602	0.589215866	0.99828741
STX11	0.666867158	0.855141737	0.620613545	0.617759491	0.776537165	0.763760487
VPS4A	0.666867158	0.560109317	0.796499368	0.719816078	0.680301694	0.831652079
SAPCD1	0.666910437	0.60754196	0.950971816	0.621508012	0.738738532	0.645427231
TMED4	0.666957883	0.498608227	0.93190909	0.764257755	0.735091896	0.640128675
C22H3orf22	0.666957883	0.587482651	0.897322115	0.71656639	0.669123866	0.706869246
HMGCS1	0.666957883	0.528669672	0.768247414	0.764257755	0.669761703	0.846210629
CCDC153	0.667093867	0.584209799	0.885896294	0.611379189	0.887514169	0.61614241
TRMT10C	0.667093867	0.499388045	0.872088608	0.655088715	0.607958542	0.897522188
PARP6	0.667167224	0.518261575	0.880476435	0.828337624	0.695436567	0.66881641
C8H9orf152	0.6678827	0.52614209	0.842694695	0.684402602	0.784972475	0.773093509
PLA2G5	0.668182824	0.486739486	0.816553355	0.898818046	0.723481165	0.655022943
RBM28	0.668182824	0.495378253	0.749453648	0.808073654	0.864524107	0.683230524
LOC100298923	0.668689398	0.479687945	0.829455601	0.636027046	0.83704813	0.806714845
AMPD2	0.668689398	0.584209799	0.77566312	0.651507079	0.679048977	0.87501921
RNFT1	0.668689398	0.524576105	0.860877703	0.627280162	0.669123866	0.87754233
LIME1	0.668890925	0.518261575	0.791843351	0.687568149	0.932024143	0.645719071
LOC537848	0.668890925	0.621713316	0.651356851	0.845954103	0.83704813	0.657174611
MPPE1	0.668890925	0.709247882	0.726119774	0.686647893	0.834311833	0.67453786
MEIS2	0.668890925	0.549135888	0.666696467	0.989601002	0.637102655	0.675688253
LAX1	0.668890925	0.518799911	0.570681722	0.978559648	0.788007619	0.68185373
LOC100138864	0.668890925	0.51862711	0.751510697	0.912777307	0.68748005	0.704123433
FAM47E	0.668890925	0.741263262	0.869932384	0.659427594	0.636058041	0.704950309
LOC101902869	0.668890925	0.790837224	0.684282335	0.784595865	0.592388443	0.772618249
HEBP2	0.668913217	0.813759037	0.579487023	0.810132355	0.832407979	0.59373266
KIAA1324	0.668913217	0.989303356	0.731470249	0.627280162	0.612209898	0.596578269
DEPTOR	0.668913217	0.757078435	0.596931535	0.821450642	0.834035648	0.621453491
ZNF503	0.668913217	0.99488888	0.628904157	0.630951216	0.590122535	0.677365619
LOC614732	0.668913217	0.490326582	0.946791586	0.720787315	0.592537033	0.785636406
TRIM66	0.668913217	0.54845263	0.734143952	0.641414122	0.80599066	0.853824859
EMC3	0.668913217	0.583140563	0.731143569	0.705489419	0.695436567	0.869696744
CCAR2	0.66949816	0.528669672	0.883032292	0.80966716	0.612403767	0.754218406
FGFR1OP	0.669818002	0.884128797	0.707488381	0.680418978	0.738738532	0.628379498
SPTLC1	0.669818002	0.543708557	0.849704325	0.886312303	0.653968366	0.666292389
TMEM254	0.669818002	0.621279921	0.612793995	0.948668643	0.682784145	0.702623424
BCL2L1	0.669818002	0.514675796	0.700963878	0.907513131	0.612403767	0.831652079

TMEM136	0.669818002	0.614976364	0.671003058	0.660261452	0.610761857	0.960023723
UBR2	0.669999022	0.580129905	0.631008412	0.892047793	0.87329316	0.61644051
SLC25A45	0.669999022	0.488703353	0.679506848	0.853058361	0.886785729	0.690728863
SEC24A	0.669999022	0.63270619	0.700900214	0.808586366	0.789915129	0.702623424
PGAM5	0.669999022	0.600839448	0.69687851	0.698952489	0.847173934	0.768572658
SMIM10L1	0.670114422	0.54615571	0.711148626	0.96951401	0.735091896	0.596578269
CREBRF	0.670114422	0.724496927	0.70011973	0.88097449	0.707551381	0.621946029
GTF2H4	0.670114422	0.492881625	0.749417651	0.631620874	0.956041014	0.728795223
ZNF34	0.670114422	0.51862711	0.620613545	0.953263369	0.593931025	0.845747012
SLC28A1	0.670165785	0.483496628	0.653793242	0.887983383	0.943610727	0.593122637
ACP4	0.670165785	0.560109317	0.871929137	0.665999974	0.854289643	0.648195749
MYL6	0.670165785	0.54021232	0.796499368	0.777956162	0.838628427	0.668851588
MYCBP2	0.670165785	0.600886305	0.775013999	0.640315346	0.881680572	0.702623424
LOC786948	0.670165785	0.481035337	0.792493806	0.83559345	0.789915129	0.720455716
WDR76	0.670165785	0.522698706	0.652681076	0.984423083	0.593931025	0.766534677
ARMC8	0.670165785	0.517404163	0.579487023	0.636814098	0.889301074	0.91373422
PRSS53	0.670165785	0.489193793	0.71967891	0.775617102	0.645589595	0.925233445
SEC24B	0.670261503	0.654079939	0.625105036	0.614863577	0.999453276	0.605408237
SUSD4	0.670261503	0.496890044	0.942357321	0.705448435	0.682784145	0.743301322
NEK2	0.670819749	0.49771159	0.961437412	0.822691957	0.637102655	0.621946029
IRF7	0.670819749	0.584228062	0.849389234	0.637708629	0.891726429	0.63584104
TELO2	0.670819749	0.509425844	0.916323169	0.7817105	0.733613832	0.65866127
DNAJC6	0.670819749	0.527777396	0.999993442	0.627280162	0.632545657	0.659761491
PRR11	0.670819749	0.772618617	0.700349135	0.632177478	0.870042072	0.668263315
CNPPD1	0.670819749	0.587215076	0.590381768	0.852481062	0.900891209	0.66881641
EIF6	0.670819749	0.516972202	0.885896294	0.891988949	0.620939114	0.669883077
FOXM1	0.670819749	0.542302787	0.854571175	0.808073654	0.744684207	0.672015032
PDRG1	0.670819749	0.692305416	0.883950091	0.724175944	0.650743327	0.674960401
TNFRSF21	0.670819749	0.983594252	0.630012726	0.646556987	0.625219099	0.68185373
FRRS1L	0.670819749	0.744774335	0.733850691	0.863275598	0.60443186	0.68781527
WHRN	0.670819749	0.513801187	0.842567034	0.670507175	0.878451408	0.706556814
SLC39A8	0.670819749	0.823133291	0.573433996	0.76154751	0.769529419	0.727632739
CEP41	0.670819749	0.510907649	0.693546993	0.630951216	0.970885781	0.748223279
PADI2	0.670819749	0.58531281	0.708019215	0.799712183	0.784972475	0.761143135
ADRB1	0.670819749	0.532115072	0.813441395	0.626248961	0.857988103	0.782330566
MAGI3	0.670819749	0.51352654	0.829751489	0.6956751	0.771893039	0.813775367
LOC112444931	0.670819749	0.543103725	0.711148626	0.652644879	0.871558258	0.824780045
NR1H3	0.670819749	0.658075243	0.630638844	0.809277776	0.688845988	0.835231445

RRP36	0.670819749	0.573498846	0.916294854	0.6331124	0.593237225	0.842214812
EMC7	0.670819749	0.482217107	0.770497031	0.730052184	0.696466814	0.892605403
RBM48	0.671802962	0.62215202	0.67821881	0.888687722	0.804588949	0.632510367
TTC13	0.671802962	0.802638906	0.69687851	0.627280162	0.86930615	0.647275211
RPN1	0.672197812	0.48811343	0.754557912	0.839632707	0.613652691	0.874000314
EIF3B	0.672197812	0.52001926	0.745477682	0.813323291	0.605738596	0.888475311
NUP50	0.672444525	0.58217015	0.798782742	0.717645144	0.832407979	0.697356941
FOXO3	0.672444525	0.531071725	0.651356851	0.774214837	0.9096389	0.743842064
PNMA8B	0.672521784	0.528644841	0.731739801	0.797312233	0.910027101	0.643003473
LOC104975244	0.672752211	0.519533906	0.744735369	0.82868625	0.847092933	0.681450813
COX7B2	0.672917134	0.68903984	0.805212947	0.782217967	0.696466814	0.675688253
ARRB2	0.672967011	0.590038452	0.833583844	0.823972839	0.744684207	0.631731716
PPP1R16A	0.672967011	0.509765303	0.818612484	0.882057818	0.750277829	0.668851588
COX6A1	0.672967011	0.544401883	0.667782878	0.661894736	0.964301327	0.727632739
UQCR11	0.672967011	0.534573879	0.71967891	0.627280162	0.960048824	0.733767439
UBLCP1	0.672999676	0.542567583	0.620613545	0.716843352	0.78547726	0.920481994
RBFOX1	0.673079856	0.856868887	0.627295843	0.822708607	0.738738532	0.601371091
CNN2	0.673162533	0.524929534	0.606660869	0.9063051	0.923910588	0.616642648
POLR2D	0.673508513	0.589120792	0.885896294	0.682685243	0.677185022	0.772618249
ATXN2L	0.673546538	0.572077017	0.772600931	0.627280162	0.896589592	0.748750383
PEG3	0.673546538	0.663309441	0.660365588	0.659908345	0.84620147	0.806476213
RNF214	0.673546538	0.519327423	0.620613545	0.645168798	0.804625939	0.952571698
FBXO11	0.673584147	0.750379179	0.586766766	0.809277776	0.848550619	0.655022943
MAP1LC3A	0.673601103	0.703189238	0.652571125	0.641118121	0.792867703	0.845783187
KIAA1109	0.673602391	0.569554583	0.608218151	0.839632707	0.893935775	0.690977997
RAC1	0.673602391	0.485755959	0.867661896	0.885336966	0.64727886	0.722536153
LOC112445030	0.673892223	0.558327847	0.735388121	0.653491823	0.956906331	0.669883077
STX7	0.673892223	0.49771159	0.89385865	0.840541751	0.615050367	0.748921056
LOC101904013	0.674025657	0.929409572	0.582898646	0.839245804	0.652333594	0.622811146
ZFYVE21	0.674025657	0.669124554	0.605191943	0.922503563	0.794813456	0.632510367
TBX3	0.674025657	0.590368852	0.80412682	0.731537809	0.844250202	0.669558041
LOC101908760	0.674025657	0.512866562	0.847357383	0.874424802	0.687109055	0.702623424
RRAGA	0.674025657	0.483305224	0.990037865	0.631746507	0.687203978	0.741459783
ABCC2	0.674025657	0.760778257	0.730571184	0.701248463	0.731607614	0.742588975
PTGFR	0.674025657	0.54021232	0.614111118	0.843593618	0.859351739	0.75640676
FAM107A	0.674025657	0.517719875	0.74792215	0.796843021	0.762352103	0.812774915
SF3B1	0.674206687	0.524309619	0.744735369	0.808073654	0.799752471	0.765138165
FAM222A	0.674237196	0.533735495	0.603864365	0.97731816	0.622751397	0.806476213

MAP3K5	0.674621941	0.658075243	0.819734395	0.680884552	0.862205587	0.623290811
TUBG1	0.674621941	0.614976364	0.935266153	0.669493024	0.682871954	0.694496432
AAGAB	0.674621941	0.745791496	0.742539165	0.691025178	0.690058599	0.78087541
ODC1	0.674859461	0.52243101	0.794676674	0.853058361	0.821351993	0.639142898
ZBTB11	0.674859461	0.55180706	0.607319392	0.637708629	0.999985752	0.640128675
DUSP4	0.674859461	0.513491832	0.926834962	0.699414374	0.769686429	0.700247288
RNF14	0.674859461	0.4971494	0.76555928	0.659872042	0.852926251	0.835231445
BZW1	0.674979879	0.486739486	0.894338686	0.727236849	0.613652691	0.860414702
OAZ1	0.67504759	0.584209799	0.736923472	0.900044333	0.664742123	0.727429267
TMEM170A	0.67504759	0.544401883	0.733319943	0.806573529	0.738738532	0.810508177
TIFA	0.67504759	0.77844414	0.716986439	0.685601134	0.655556663	0.816415069
COL6A3	0.675351608	0.544401883	0.735388121	0.845954103	0.750277829	0.759003017
THRSP	0.675351608	0.528669672	0.71967891	0.686250223	0.911837627	0.765298372
CISD2	0.675351608	0.519450301	0.809673119	0.764257755	0.612403767	0.888475311
FANCA	0.675766013	0.663620961	0.674777778	0.808073654	0.603567103	0.872237727
SLIT2	0.675799675	0.542391346	0.761415057	0.78584336	0.908820136	0.621089184
EMX2	0.675799675	0.877893862	0.725264968	0.812453944	0.621787839	0.622811146
LOC104976061	0.675992915	0.58531281	0.738483855	0.910575108	0.625219099	0.748750383
CYP1B1	0.675992915	0.650612374	0.584710697	0.657497543	0.928951321	0.782330566
MRPL32	0.675992915	0.573711724	0.894338686	0.636027046	0.706856083	0.798057364
CCT6A	0.675992915	0.485778913	0.829745504	0.751437238	0.625219099	0.895633366
FAM214B	0.67611339	0.62117273	0.758146087	0.7817105	0.642796813	0.827832928
SCNN1D	0.676191043	0.489541299	0.997300234	0.765499767	0.626973048	0.631731716
SLC35G2	0.676191043	0.621279921	0.749417651	0.874170259	0.78021215	0.631731716
CBX4	0.676191043	0.48811343	0.764467593	0.757345832	0.935286646	0.66881641
CDKL1	0.676191043	0.861395263	0.700963878	0.661894736	0.784259667	0.669558041
RAB6A	0.676191043	0.48811343	0.93475587	0.812453944	0.655556663	0.701700929
TMEM8A	0.676191043	0.722779653	0.794225743	0.763258191	0.66871441	0.716804193
BCS1L	0.676191043	0.589782216	0.774015998	0.627280162	0.826634148	0.812091218
USP28	0.676191043	0.532096752	0.730571184	0.817230639	0.631537325	0.881939613
ST8SIA4	0.676627158	0.506509735	0.73091642	0.821450642	0.943540106	0.607349313
LOC510913	0.676627158	0.783112117	0.930817886	0.665999974	0.632055507	0.611994375
PCK1	0.676627158	0.997471998	0.707407957	0.636814098	0.596680559	0.61411085
BANF1	0.676627158	0.548448956	0.726119774	0.920867825	0.793993073	0.639063682
HEBP1	0.676627158	0.724496927	0.694535506	0.876703811	0.732388993	0.640128675
RBM12	0.676627158	0.664985713	0.899725007	0.69158245	0.710664679	0.669883077
RBM25	0.676627158	0.487617269	0.637844154	0.852005554	0.947299702	0.669883077
BNC1	0.676627158	0.519533906	0.867719797	0.853058361	0.6855631	0.700459582

POLR2L	0.676627158	0.696914218	0.715158198	0.724431807	0.823508064	0.702623424
ACVR1C	0.676627158	0.679199291	0.606660869	0.912777307	0.721096295	0.711923954
SH3BP1	0.676627158	0.526654774	0.813441395	0.638739139	0.911568867	0.72571141
ATP5PF	0.676627158	0.529619156	0.851822885	0.652519777	0.864521109	0.742309541
ADGRE3	0.676627158	0.766943819	0.605532453	0.869208308	0.626973048	0.78087541
PNISR	0.676627158	0.485755959	0.711148626	0.928404714	0.648267928	0.818021392
NDUFV1	0.676627158	0.535202811	0.673327876	0.627280162	0.937932469	0.82709156
CPT1A	0.676627158	0.543554867	0.754913805	0.703717381	0.804037558	0.83150957
PDK1	0.676627158	0.52509947	0.70011973	0.848131881	0.723467697	0.835231445
YBX1	0.676627158	0.49561318	0.860877703	0.80459801	0.612403767	0.839906041
STK40	0.676627158	0.610379943	0.79497298	0.695681179	0.682550281	0.844759721
ALG5	0.676627158	0.550226079	0.79497298	0.683393974	0.735091896	0.858451225
LOC100848307	0.676627158	0.549135888	0.665599776	0.812453944	0.696466814	0.884203066
LOC101905390	0.676884797	0.759814955	0.681700278	0.912777307	0.656015118	0.629427844
SLC37A3	0.676884797	0.548622699	0.679506848	0.832489049	0.803705802	0.779201808
KIF23	0.677096767	0.531880429	0.605532453	0.819213899	0.885217562	0.783241453
HNRNPF	0.677834794	0.522051351	0.608218151	0.761649762	0.996781978	0.640128675
CDCA3	0.677834794	0.634059237	0.791557166	0.796934171	0.781724458	0.67357674
ATXN1L	0.677834794	0.572313942	0.616620774	0.999981514	0.616552625	0.710630057
PAICS	0.677834794	0.573498846	0.865794303	0.731537809	0.741891558	0.739151902
AHR	0.677834794	0.520957387	0.620613545	0.99987014	0.623574736	0.764737727
TTC33	0.677834794	0.582324384	0.599489021	0.965050971	0.655786713	0.77545502
SYNPO2	0.677834794	0.913120359	0.701777234	0.626698454	0.596709577	0.810156973
CXHXorf36	0.678247845	0.530644127	0.596931535	0.968436292	0.85185197	0.634208749
PPIE	0.678474041	0.518799911	0.813441395	0.66637376	0.949927999	0.658600906
ZNF608	0.678474041	0.513491832	0.8644528	0.806838917	0.804037558	0.669558041
GRAMD2A	0.678474041	0.487617269	0.788209682	0.810665442	0.802151151	0.762657772
PLK4	0.678474041	0.687020192	0.806651923	0.72169974	0.667589489	0.779201808
LOC112444926	0.678567799	0.56685163	0.736160515	0.744617133	0.788850323	0.822427019
CCDC58	0.67858613	0.514675796	0.851822885	0.651507079	0.792867703	0.833150766
UGT8	0.67858613	0.697043768	0.608218151	0.655447257	0.735091896	0.918081882
DPH7	0.678759149	0.622818484	0.730571184	0.641210745	0.887514169	0.757357628
LPAR6	0.679026666	0.52509947	0.605191943	0.999981514	0.781724458	0.616715863
ZFPM2	0.679617939	0.718477366	0.754913805	0.773429607	0.821351993	0.609692015
PDIA6	0.679617939	0.622818484	0.647547719	0.989601002	0.692764366	0.622811146
LOC101902968	0.679617939	0.598407024	0.727364203	0.882057818	0.655786713	0.779284546
FHDC1	0.679729063	0.687651905	0.754913805	0.958588196	0.608043638	0.608554064
IL6R	0.679729063	0.528167223	0.697914586	0.806139084	0.943734737	0.645427231

PRICKLE1	0.679729063	0.609772211	0.809931129	0.717510559	0.850566017	0.675688253
METTL14	0.679729063	0.927973597	0.739344407	0.670229436	0.642796813	0.68185373
TRAPPC6A	0.679729063	0.929409572	0.638444487	0.688942799	0.730921558	0.68185373
ARL14EPL	0.679729063	0.499204258	0.652979882	0.664953033	0.999985752	0.695765939
TRIP10	0.679729063	0.621279921	0.810647419	0.630535638	0.882831953	0.708090768
WDR6	0.679729063	0.490326582	0.608008194	0.881449261	0.920318083	0.718946833
CFL2	0.679729063	0.511445452	0.894338686	0.825279787	0.678703442	0.727429267
C10H15orf59	0.679729063	0.741310853	0.705746499	0.828736799	0.669123866	0.729499398
PHF21A	0.679729063	0.490326582	0.841294863	0.891988949	0.685561272	0.729499398
CD226	0.679729063	0.584228062	0.714622432	0.71656639	0.887514169	0.748921056
TRMT61A	0.679729063	0.678023292	0.758146087	0.627280162	0.721826916	0.860966325
TRMT10B	0.679729063	0.535967468	0.653631615	0.627280162	0.891304411	0.889662488
TMEM138	0.679729063	0.489659522	0.76555928	0.648660726	0.66101983	0.968076143
ADGRB1	0.679955579	0.499745396	0.781179133	0.676566861	0.687203978	0.936263602
C1QBP	0.680335363	0.535710688	0.705913177	0.677374927	0.788739452	0.91373422
TIPARP	0.680658662	0.522698706	0.685620658	0.739416956	0.993780193	0.612307446
FKBP7	0.680658662	0.522173354	0.754193103	0.9880671	0.696466814	0.621089184
SCRIB	0.680658662	0.689824411	0.915858924	0.777956162	0.652333594	0.622811146
FAM96B	0.680658662	0.692305416	0.935266153	0.652644879	0.735091896	0.628379498
RPS6KB2	0.680658662	0.52001926	0.794225743	0.711837881	0.943610727	0.656746469
PNPLA6	0.680658662	0.637566275	0.789166592	0.918693944	0.612403767	0.669883077
EFR3A	0.680658662	0.582127335	0.861585977	0.834562052	0.687203978	0.684615686
ZNHIT3	0.680658662	0.648119635	0.681700278	0.764257755	0.886785729	0.690728863
SEMA3E	0.680658662	0.878551917	0.594331059	0.796125318	0.714642011	0.702623424
LOC112442693	0.680658662	0.856115337	0.73826834	0.753267542	0.623804687	0.709662163
LOC112447030	0.680658662	0.536692773	0.694535506	0.796128007	0.902907077	0.716804193
B3GNT7	0.680658662	0.52001926	0.700963878	0.935282232	0.727268681	0.746853748
LOC101907276	0.680658662	0.538568518	0.809673119	0.828202581	0.735091896	0.748921056
SIGLECL1	0.680658662	0.691759664	0.627084316	0.891916807	0.686905158	0.764260585
DHPS	0.680658662	0.580322769	0.728269425	0.729481641	0.847173934	0.779201808
MAN1A1	0.680658662	0.624142952	0.714173992	0.632654918	0.887514169	0.783361008
SLC25A39	0.680658662	0.591296685	0.611647147	0.670969025	0.936215792	0.806714845
BTBD3	0.680658662	0.789260516	0.744735369	0.652519777	0.645511731	0.836111377
PXMP4	0.680658662	0.538848393	0.754913805	0.630535638	0.804625939	0.889238459
LOC112447492	0.680658662	0.583140563	0.677197181	0.832489049	0.622751397	0.895426195
CSTF2T	0.680658662	0.521967606	0.700128649	0.692226	0.788995893	0.91618909
ZC4H2	0.680658662	0.531880429	0.668824581	0.74577892	0.626973048	0.973649216
LOC530348	0.680658662	0.565421383	0.612793995	0.689554882	0.655665696	0.988866378

IER3IP1	0.680863897	0.517719875	0.872088608	0.689227896	0.630794687	0.892394114
SLC25A12	0.680893448	0.541611621	0.606660869	0.999981514	0.642662413	0.620459631
ELOVL7	0.680893448	0.531880429	0.732073575	0.904374668	0.756378178	0.72571141
CAVIN4	0.681180466	0.530656147	0.682602079	0.870591999	0.937932469	0.607349313
LOC101904595	0.681523769	0.51862711	0.99341711	0.690807874	0.724334049	0.632510367
NR4A2	0.681793492	0.681192035	0.736435559	0.728264598	0.786314903	0.765948605
HOXB5	0.681793492	0.689589681	0.594331059	0.764257755	0.695436567	0.898470276
SLC22A31	0.681883743	0.983483207	0.630408979	0.687568149	0.696466814	0.628379498
LOC104971030	0.681883743	0.531880429	0.734143952	0.999981514	0.626973048	0.63202365
IQGAP3	0.681883743	0.750466772	0.817582037	0.815919419	0.656015118	0.643392483
NOV	0.681883743	0.795955721	0.682700098	0.646270278	0.902258286	0.645427231
ATP6V1E1	0.681883743	0.692305416	0.758146087	0.736045965	0.656015118	0.82345874
GNPNAT1	0.681883743	0.573498846	0.789166592	0.840507942	0.603965612	0.835231445
NOB1	0.681883743	0.589754585	0.689000346	0.822069434	0.721672235	0.835331508
MAGED2	0.681883743	0.609864167	0.806470927	0.76311249	0.609894299	0.856295543
UBE2G1	0.681883743	0.493052487	0.876414692	0.765499767	0.626033107	0.860966325
SMIM20	0.682220725	0.531880429	0.744735369	0.664953033	0.834510805	0.864139695
CYP2R1	0.682320663	0.558327847	0.750313078	0.869068788	0.799272579	0.690728863
SUSD5	0.682320663	0.664104209	0.677350667	0.640315346	0.906893576	0.768168974
MACF1	0.682320663	0.622818484	0.605532453	0.762472696	0.891726429	0.773506763
KBTBD6	0.682374805	0.524051946	0.774015998	0.710946563	0.943055455	0.681450813
LOC100848226	0.682374805	0.661223524	0.648593816	0.812453944	0.817472827	0.7502369
IGF2BP3	0.682374805	0.60754196	0.648593816	0.731537809	0.890124729	0.779284546
BTBD9	0.682374805	0.535710688	0.711148626	0.67119815	0.626634623	0.986042926
KLHL17	0.682445249	0.549085151	0.916323169	0.896116841	0.613652691	0.631731716
ALS2	0.682917865	0.614976364	0.641535145	0.985437486	0.738738532	0.621946029
MAGEF1	0.682917865	0.63850019	0.628904157	0.9880671	0.723054375	0.622811146
ANG	0.682917865	0.801585146	0.755135544	0.813472855	0.667763384	0.645719071
CDH22	0.682917865	0.524051946	0.89478142	0.641118121	0.658667583	0.878642
MT2A	0.682917865	0.512513718	0.696778723	0.669802063	0.83365499	0.913722377
TRIOBP	0.682971063	0.796253817	0.596080143	0.777956162	0.766101355	0.766900958
LOC100335828	0.683023611	0.975997485	0.71156186	0.703717381	0.627206089	0.625066198
MAFF	0.683023611	0.718258172	0.834140212	0.808586366	0.656015118	0.672466112
GXYLT2	0.683209324	0.582127335	0.69621934	0.907794002	0.776537165	0.700123604
ANXA7	0.683249957	0.633062614	0.916081147	0.812453944	0.613093988	0.675176731
EPHB6	0.683375861	0.573498846	0.693546993	0.982220679	0.677001223	0.679437565
POLR3D	0.684054343	0.630897425	0.96327718	0.627280162	0.618211409	0.762451586
NUSAP1	0.684281255	0.524051946	0.907477369	0.907794002	0.635127008	0.643003473

COPA	0.684281255	0.659408339	0.789166592	0.654131191	0.91509147	0.643003473
LOC615989	0.684281255	0.805283017	0.840107596	0.754355205	0.64727886	0.651781292
TLR6	0.684281255	0.839660873	0.876213859	0.630535638	0.649309225	0.684994779
EI24	0.684281255	0.552362863	0.78930905	0.827203798	0.750277829	0.754218406
UBAC1	0.684281255	0.652509786	0.717242466	0.677545541	0.870450746	0.763582226
TMEM106A	0.684281255	0.673435317	0.770991724	0.843613441	0.60903578	0.773183944
GNS	0.684388711	0.544401883	0.829751489	0.896116841	0.626634623	0.750967073
B4GALT2	0.684388711	0.622818484	0.607986114	0.932083159	0.634184199	0.83150957
SOX6	0.684522927	0.816540275	0.818612484	0.75404116	0.655556663	0.657950857
SDC2	0.684559	0.687020192	0.829455601	0.703717381	0.809317773	0.671352522
RNF41	0.684559	0.570437403	0.668824581	0.905772309	0.833173678	0.68185373
NINJ1	0.684559	0.535967468	0.708274076	0.636814098	0.962166847	0.762133815
SARS	0.684559	0.621713316	0.829455601	0.74577892	0.653653376	0.823013262
SNRK	0.684593549	0.687020192	0.627084316	0.654131191	0.893716526	0.801888363
EPSTI1	0.684721399	0.757078435	0.880163755	0.68362796	0.772501291	0.610832246
CFAP100	0.684721399	0.589782216	0.836482	0.85721441	0.716994185	0.67453786
ERBIN	0.684721399	0.571839975	0.608265631	0.676566861	0.977193348	0.749726404
ATP5F1E	0.684721399	0.51525676	0.682700098	0.645168798	0.955370715	0.820182674
TMEM229B	0.684721399	0.516972202	0.927635169	0.698991211	0.651585054	0.832425518
VPS16	0.684741105	0.539935734	0.90226609	0.865076519	0.664727575	0.675688253
LOC100847454	0.684741105	0.672819246	0.711148626	0.751643991	0.878668281	0.68185373
DNAJB14	0.684741105	0.553489554	0.736435559	0.687291299	0.935396488	0.734177434
MDK	0.684856552	0.742851755	0.921411252	0.767092077	0.610761857	0.622811146
RABEP1	0.684856552	0.517719875	0.671469816	0.752454758	0.989334846	0.645719071
SAP25	0.685123265	0.834615638	0.79382647	0.636814098	0.622751397	0.791689746
DDA1	0.685289748	0.718258172	0.942357321	0.682944876	0.678035103	0.638990067
PDE6C	0.685289748	0.895777642	0.680052524	0.674194099	0.804625939	0.657833313
TMEM72	0.685289748	0.973189491	0.620467805	0.627280162	0.787234817	0.668851588
MYBPHL	0.685289748	0.51352654	0.668824581	0.849834721	0.942786245	0.674223046
CCR10	0.685289748	0.519327423	0.916294854	0.630951216	0.887514169	0.683230524
TMEM248	0.685289748	0.517719875	0.750313078	0.959616431	0.707939546	0.690728863
SLC2A10	0.685289748	0.573711724	0.675096377	0.999981514	0.617915169	0.704351773
CSF1	0.685289748	0.531880429	0.622387278	0.923663924	0.823508064	0.748750383
MTUS2	0.685289748	0.543708557	0.680262176	0.748424012	0.935286646	0.749918186
STON2	0.685289748	0.691301661	0.754913805	0.795909483	0.627206089	0.818021392
ORC3	0.685289748	0.516115205	0.79497298	0.883040163	0.642662413	0.823701731
CCL24	0.685289748	0.515892935	0.623611563	0.830427818	0.859351739	0.831652079
NAT1	0.685289748	0.513825592	0.79497298	0.712481274	0.804037558	0.845747012

XYLB	0.685289748	0.573498846	0.620355108	0.87592304	0.670031265	0.89209761
AP1M1	0.685289748	0.538568518	0.736160515	0.755493626	0.680934071	0.921843102
LOC112448084	0.685289748	0.49771159	0.669511234	0.735464965	0.652333594	0.98804672
BMP2	0.685397748	0.625011831	0.829455601	0.817146543	0.705202026	0.711118977
RALGAPA1	0.685415911	0.603542632	0.644256366	0.753267542	0.965676623	0.658042301
FDX2	0.685415911	0.767113995	0.686711082	0.682944876	0.821557309	0.751884708
HEATR3	0.685467249	0.588515985	0.641535145	0.952247001	0.788007619	0.682483304
SAR1B	0.685467249	0.528669672	0.81412252	0.876703811	0.665973931	0.779201808
TDRD12	0.685532533	0.648503747	0.756426507	0.932406746	0.610145147	0.700123604
HNF4A	0.685613329	0.640172572	0.605532453	0.961831293	0.823508064	0.61411085
NOSIP	0.685613329	0.542918274	0.628904157	0.999981514	0.677185022	0.620344327
ZC3H4	0.685613329	0.640335813	0.749417651	0.975678715	0.642516521	0.621946029
PPP2R3A	0.685613329	0.499745396	0.62401908	0.96599541	0.882206878	0.639142898
EVA1A	0.685613329	0.572077017	0.651356851	0.97731816	0.779385814	0.658684706
LOC787287	0.685613329	0.54845263	0.608218151	0.868378265	0.939208227	0.68185373
THSD1	0.685613329	0.716005622	0.834683675	0.821450642	0.63648166	0.684994779
CXXC1	0.685613329	0.506509735	0.893427844	0.907258317	0.626973048	0.702623424
SLC9B2	0.685613329	0.666009746	0.618684669	0.905103731	0.788995893	0.702623424
TTLL3	0.685613329	0.622449596	0.754913805	0.891139257	0.680696229	0.729319161
APOE	0.685613329	0.670494813	0.599475136	0.881493849	0.797227609	0.754218406
KLHL8	0.685613329	0.517408153	0.695084268	0.771980793	0.904084756	0.779201808
LOC781064	0.685613329	0.553132278	0.736435559	0.730052184	0.875895345	0.780283623
CLK1	0.685613329	0.517719875	0.731960288	0.777956162	0.795839201	0.85643253
SOD2	0.685613329	0.515892935	0.627295843	0.682944876	0.799752471	0.966850975
PIP5K1C	0.685961867	0.672505775	0.679506848	0.786099236	0.781724458	0.797546932
SPG11	0.686249911	0.663152438	0.67171984	0.961898258	0.670961786	0.675688253
LOC515169	0.686249911	0.640995899	0.655190845	0.973075232	0.61033296	0.748921056
SEC11C	0.686249911	0.681192035	0.810647419	0.632683273	0.738738532	0.83150957
FAM171A2	0.686293861	0.512513718	0.70011973	0.948668643	0.86876405	0.621946029
KRTCAP3	0.686543257	0.580530872	0.854571175	0.809886338	0.657647754	0.779201808
PPCS	0.686543257	0.655974568	0.724797892	0.674194099	0.820477096	0.827413316
PPARGC1B	0.68698792	0.649340041	0.684282335	0.919688166	0.81247611	0.613547915
GBA2	0.68698792	0.571839975	0.70011973	0.914850247	0.863970911	0.618303855
LYST	0.68698792	0.609864167	0.709285034	0.823935462	0.910508458	0.621946029
CXCR1	0.68698792	0.519450301	0.871285807	0.656831463	0.912199478	0.695691829
PCDH12	0.68698792	0.510204295	0.663738759	0.952247001	0.764945406	0.762133815
COX7A2L	0.68698792	0.553638694	0.76555928	0.673875708	0.784785055	0.883688554
MRPS16	0.687091207	0.648958657	0.817582037	0.630951216	0.871558258	0.733467771

LITAF	0.687091207	0.573498846	0.73826834	0.802977147	0.811991102	0.770496306
DEF6	0.687091207	0.571983204	0.731960288	0.630535638	0.878451408	0.855082018
LOC100336941	0.687465406	0.585547344	0.908072171	0.757403199	0.687203978	0.742687461
INSR	0.687465406	0.543645094	0.651855625	0.952247001	0.720896795	0.776734587
SLC27A3	0.687465406	0.823478017	0.668333235	0.629713796	0.776537165	0.828124017
FADS3	0.68753148	0.511445452	0.874983674	0.815432466	0.849766114	0.631731716
ZSCAN16	0.687676329	0.572342135	0.73442593	0.715394268	0.687203978	0.927520621
COMMD2	0.688026026	0.927973597	0.746818005	0.674469953	0.683310213	0.671759429
DDT	0.688131079	0.589044534	0.744735369	0.63494856	0.928951321	0.766534677
LIPA	0.688831147	0.582560314	0.745942803	0.896116841	0.618728497	0.816461945
THEM6	0.688983293	0.703571355	0.627295843	0.64770518	0.970988226	0.684615686
C25H16orf45	0.689256953	0.595838503	0.915120516	0.808075336	0.687203978	0.675176731
KCNT1	0.689256953	0.522400504	0.791557166	0.865746543	0.811991102	0.702623424
WEE1	0.689256953	0.610379943	0.648767122	0.688704343	0.964833865	0.726852722
LOC509155	0.689256953	0.696797615	0.847776765	0.632683273	0.660633095	0.845747012
PWWP2A	0.689256953	0.616227995	0.623431824	0.815919419	0.766655597	0.86324237
LOC100299303	0.689256953	0.808478344	0.630012726	0.676566861	0.62146056	0.923092436
DMTN	0.68932146	0.919245978	0.630408979	0.684149461	0.78646867	0.700746926
FGFR3	0.68932146	0.674801662	0.751661482	0.812453944	0.759888741	0.729319161
H2AFZ	0.68932146	0.591296685	0.883629347	0.751194677	0.612403767	0.832770678
GTPBP4	0.689531188	0.703500334	0.916871067	0.695693564	0.674089143	0.689625555
NXPE3	0.689531188	0.51862711	0.808493605	0.809277776	0.804625939	0.763466477
IL1A	0.689531188	0.640172572	0.629132356	0.839632707	0.804049674	0.784754767
RANBP9	0.689531188	0.621279921	0.852916849	0.76992117	0.64727886	0.801888363
PITPNM3	0.690100877	0.888900446	0.676501557	0.815888375	0.659373888	0.677964856
PARD3	0.690100877	0.553132278	0.700963878	0.630951216	0.987381787	0.723555931
VAV1	0.690100877	0.580530872	0.76555928	0.689370133	0.917407853	0.727632739
ZMAT2	0.690100877	0.572077017	0.971255098	0.665999974	0.690687154	0.733767439
MAATS1	0.690100877	0.591547993	0.797675887	0.830877557	0.705202026	0.765948605
FTH1	0.690214621	0.611197509	0.852014608	0.764257755	0.851827871	0.629427844
LOC100847122	0.690214621	0.53280405	0.999993442	0.680977089	0.680301694	0.649823379
MAML3	0.690367862	0.52001926	0.755940516	0.866566721	0.81066793	0.745952648
THUMPD1	0.690367862	0.599059532	0.707407957	0.882024184	0.781724458	0.748750383
CCDC47	0.690367862	0.528669672	0.749453648	0.812453944	0.648209263	0.912894278
DTX1	0.690560055	0.615769899	0.68346497	0.964521432	0.638949662	0.745988271
LOC104968476	0.690811212	0.527553585	0.791843351	0.675565794	0.631537325	0.966850975
LOC783854	0.69082152	0.507726133	0.693546993	0.707217807	0.721669351	0.971545917
CHL1	0.691044979	0.741310853	0.608218151	0.949502614	0.765866216	0.628379498

TLR4	0.691044979	0.866901725	0.689888669	0.630951216	0.814120141	0.734269931
LOC112447302	0.691044979	0.745791496	0.813441395	0.636814098	0.801692604	0.736404047
VAMP8	0.691044979	0.804542244	0.691986709	0.637708629	0.842759044	0.750967073
LOC107131418	0.691044979	0.524929534	0.677197181	0.792140715	0.811991102	0.875297569
TOMM7	0.691052949	0.572836284	0.949772	0.729705452	0.788995893	0.632316453
FAM160B1	0.691052949	0.90323787	0.680262176	0.734472882	0.788007619	0.632510367
VAMP1	0.691334274	0.614976364	0.681652094	0.819223189	0.881680572	0.70951022
CLPP	0.691334274	0.6385246	0.77566312	0.722996827	0.789915129	0.786849547
CYB5B	0.691334274	0.52001926	0.736435559	0.751451145	0.810436875	0.866494771
AP1S2	0.691334274	0.622818484	0.689000346	0.655015747	0.825740937	0.877048993
TINF2	0.691446466	0.582560314	0.620613545	0.999981514	0.69314679	0.65014676
RHBDF1	0.691472811	0.519450301	0.954249102	0.763194165	0.723467697	0.700123604
LOC512672	0.691472811	0.782721907	0.755135544	0.734472882	0.723054375	0.741459783
INPP5D	0.691472811	0.51862711	0.886629485	0.718713712	0.788315735	0.783241453
OOEP	0.691472811	0.56685163	0.744735369	0.707217807	0.87329316	0.803594141
PCNX4	0.691472811	0.589754585	0.744069765	0.652519777	0.811991102	0.876861428
PLXDC1	0.69153492	0.899490486	0.637234901	0.710070489	0.838461406	0.640128675
RBM4	0.69153492	0.573498846	0.774015998	0.812453944	0.86864151	0.68185373
GALNT9	0.69153492	0.938087741	0.728670337	0.630951216	0.721096295	0.700123604
GDF1	0.69153492	0.611297444	0.77566312	0.896116841	0.61647911	0.777374794
LOC516442	0.69153492	0.660882273	0.744735369	0.688019411	0.658667583	0.91373422
SNAPC5	0.691598572	0.540077548	0.6249319	0.736392386	0.940870936	0.81956221
ABCC3	0.691876475	0.586798812	0.79497298	0.979385838	0.644945176	0.628917359
GPRC5A	0.691922556	0.65172183	0.71601562	0.803690355	0.846464841	0.716925595
GDI1	0.691957748	0.648119635	0.829992055	0.884493789	0.720059315	0.639142898
MYCT1	0.692269264	0.651269643	0.608008194	0.948668643	0.847173934	0.623319115
STRN3	0.692269264	0.520957387	0.689000346	0.792140715	0.983941532	0.635271082
TNNT2	0.692269264	0.627997081	0.804002279	0.832489049	0.799752471	0.658684706
DESI2	0.692269264	0.811354298	0.653631615	0.727236849	0.847092933	0.700746926
NANOS1	0.692269264	0.528669672	0.654545673	0.999981514	0.631537325	0.71289069
RAPGEF1	0.692269264	0.51352654	0.894338686	0.717652404	0.815061105	0.75544128
USP13	0.692269264	0.51862711	0.713310017	0.636814098	0.938879379	0.846210629
LSM1	0.692269264	0.559306577	0.73826834	0.664953033	0.674089143	0.966850975
DOK7	0.692423753	0.573498846	0.693546993	0.704815275	0.983010476	0.669883077
NOL11	0.692423753	0.62215202	0.693546993	0.654020714	0.935286646	0.777197325
ZNF296	0.692600548	0.681192035	0.848071679	0.764257755	0.801692604	0.645719071
LOC104970976	0.692600548	0.529372009	0.660344165	0.860937578	0.901853599	0.742456493
TPGS2	0.692600548	0.554555702	0.894338686	0.64770518	0.704783515	0.862925606

FBXO32	0.692600548	0.60754196	0.645276799	0.645168798	0.885217562	0.88313584
PEX11G	0.692720732	0.821415604	0.683693005	0.705448435	0.855939488	0.674521823
NAT9	0.692931506	0.565948458	0.698295081	0.808886078	0.964301327	0.621453491
SLC25A27	0.692931506	0.678885463	0.701928205	0.859320133	0.85317858	0.636365162
POT1	0.692931506	0.919245978	0.622025849	0.693120183	0.847173934	0.643392483
TMEM132C	0.692931506	0.813849014	0.647867969	0.695681179	0.900747635	0.667995834
STAG2	0.692931506	0.863613561	0.717242466	0.680418978	0.820411915	0.668191006
ENTPD6	0.692931506	0.660882273	0.864477161	0.640315346	0.879776073	0.668851588
CC2D2A	0.692931506	0.694159627	0.726119774	0.923738645	0.684537031	0.677365619
NUMBL	0.692931506	0.645160587	0.749417651	0.682383061	0.937932469	0.681807713
CFH	0.692931506	0.524929534	0.739982136	0.9063051	0.819329746	0.700123604
NDUFS7	0.692931506	0.648503747	0.731143569	0.691655428	0.920622084	0.715560211
MAMDC4	0.692931506	0.544401883	0.916038418	0.80966716	0.695436567	0.733366979
SCML4	0.692931506	0.66711334	0.669511234	0.674503998	0.939343714	0.748921056
MS4A2	0.692931506	0.626568786	0.795595114	0.812453944	0.688556938	0.789870764
KATNA1	0.692931506	0.553305933	0.698295081	0.837820405	0.801692604	0.81853698
CENPI	0.692931506	0.624142952	0.771066142	0.631620874	0.723481165	0.915486568
SH2D2A	0.692931506	0.624804325	0.768120148	0.682383061	0.658667583	0.923547711
LOC508131	0.692931506	0.697306013	0.724076353	0.702508869	0.61565225	0.927520621
AGER	0.692931506	0.515680499	0.740372388	0.677596835	0.759720089	0.951493662
KDM5C	0.69314325	0.664859217	0.755321685	0.707217807	0.657276571	0.893838339
MRPL23	0.693263305	0.704181021	0.736435559	0.753267542	0.765514485	0.783241453
JPT1	0.693682854	0.799590997	0.669511234	0.782811812	0.63941345	0.838468539
ADAMTSL2	0.693913627	0.601485212	0.791557166	0.682944876	0.942786245	0.669883077
TSSK2	0.694545284	0.54021232	0.784833275	0.685953876	0.964301327	0.677365619
CSE1L	0.694545284	0.861395263	0.758472515	0.705801894	0.664519875	0.748323122
LOC101903400	0.695764137	0.590038452	0.630408979	0.71113156	0.906827881	0.842537695
RPS6KA3	0.695778819	0.741310853	0.685620658	0.947574232	0.665088846	0.666292389
RBL1	0.695938609	0.722968232	0.664740692	0.731537809	0.809317773	0.814218101
SMG9	0.696122664	0.916484772	0.700963878	0.790145374	0.663467384	0.669558041
TPC3	0.696122664	0.95530536	0.716120406	0.636814098	0.705288263	0.700703362
LOC101909196	0.696568097	0.598069728	0.988390948	0.69158245	0.696466814	0.660166206
UBA7	0.696733941	0.760778257	0.745942803	0.684965277	0.906893576	0.628917359
B3GALT2	0.696733941	0.830243527	0.803342698	0.759413746	0.665973931	0.690728863
MRPL20	0.696733941	0.593498372	0.933884582	0.695125147	0.695436567	0.765948605
ACIN1	0.696733941	0.52509947	0.879608555	0.777956162	0.710664679	0.81673655
CCNI	0.696733941	0.589754585	0.698166631	0.683393974	0.881680572	0.847386898
ZC3H11A	0.696733941	0.549834049	0.695084268	0.808073654	0.795839201	0.865767172

ABHD17B	0.696803353	0.54671968	0.648593816	0.728264598	0.618211409	0.99976973
TAB2	0.696902672	0.567198791	0.652979882	0.63942692	0.999985752	0.629427844
LOC104970821	0.697146324	0.671536686	0.774015998	0.925294859	0.622751397	0.706704597
LOC112443849	0.697146324	0.514220836	0.69454186	0.952247001	0.737149805	0.780585188
MAPK9	0.697146324	0.756712161	0.680262176	0.695681179	0.683296297	0.893838339
GNPTAB	0.697387584	0.608342753	0.830887859	0.712481274	0.906893576	0.652143749
NDUFB10	0.697388489	0.582560314	0.859328338	0.677545541	0.886944613	0.706704597
FHOD3	0.69771359	0.894014677	0.641535145	0.886312303	0.669761703	0.643003473
ICE2	0.69771359	0.790429889	0.668824581	0.92139105	0.624785761	0.714553008
UBE2F	0.69771359	0.598069728	0.908072171	0.760372943	0.626796691	0.806476213
RXRA	0.697841005	0.763758778	0.634391045	0.752796989	0.935286646	0.640128675
CDC42	0.697841005	0.51352654	0.913609237	0.927802946	0.638815482	0.67453786
ZNF521	0.697841005	0.722968232	0.80412682	0.840700255	0.69314679	0.68185373
ZFP30	0.697841005	0.74733285	0.816144767	0.830187312	0.66101983	0.68392363
LOC104973739	0.697841005	0.521162219	0.843776796	0.87522058	0.697641832	0.774932208
DPYSL3	0.697921702	0.978857858	0.627295843	0.757434902	0.716994185	0.630637814
LOC615559	0.698080255	0.648503747	0.706064882	0.999981514	0.626796691	0.632510367
HSD17B14	0.698080255	0.624373062	0.924855847	0.695125147	0.794877876	0.672418631
EDN3	0.698080255	0.819210468	0.625439953	0.92743463	0.65298501	0.688619301
HIF1A	0.698080255	0.519450301	0.641535145	0.819213899	0.91563394	0.801888363
MLLT1	0.698080255	0.541253945	0.751957722	0.838273964	0.622751397	0.91373422
CLTB	0.698293418	0.607281278	0.823731372	0.733411985	0.801692604	0.770035496
ECHDC2	0.698293418	0.694960349	0.69687851	0.760614955	0.714597718	0.865889509
LOC104975590	0.698686222	0.744774335	0.753409204	0.75404116	0.626973048	0.850475013
S100G	0.698788852	0.750379179	0.711072455	0.822305562	0.810436875	0.668851588
FLRT1	0.698954929	0.58531281	0.727870684	0.653491823	0.998196897	0.643532185
POLR3B	0.698954929	0.641897615	0.652724437	0.901171792	0.804625939	0.728795223
GPD1L	0.699024222	0.660882273	0.734527982	0.907997414	0.796861564	0.629881817
CHRNB1	0.699024222	0.739236562	0.69621934	0.852481062	0.706302033	0.759003017
UBASH3A	0.699942863	0.58531281	0.714173992	0.659908345	0.658667583	0.982937529
VSTM2L	0.700053004	0.517719875	0.877334778	0.738460631	0.907455286	0.668815635
SOWAHC	0.700053004	0.703571355	0.866467804	0.805568606	0.695104667	0.684524441
SP7	0.700053004	0.594199197	0.749417651	0.891988949	0.803705802	0.690728863
REX1BD	0.700053004	0.849936168	0.701512486	0.680418978	0.788007619	0.750967073
IFI44L	0.700053004	0.867885278	0.668824581	0.645168798	0.821351993	0.754218406
AUP1	0.700053004	0.697486102	0.855811972	0.698187821	0.721826916	0.772618249
POP4	0.700053004	0.565421383	0.887132069	0.819213899	0.658108379	0.779284546
UBFD1	0.700053004	0.801709626	0.796499368	0.659427594	0.714642011	0.78087541

SYNE1	0.700053004	0.588902702	0.77566312	0.789086796	0.797227609	0.786849547
RIPOR2	0.700053004	0.813759037	0.774924967	0.670229436	0.692827651	0.797991983
MAF	0.700053004	0.627743951	0.774015998	0.641118121	0.871906908	0.806476213
CAPG	0.700053004	0.673930726	0.717242466	0.790252503	0.696466814	0.858245612
ZMAT1	0.700053004	0.572836284	0.608809506	0.7817105	0.730641268	0.960023723
SLC1A7	0.700053004	0.633062614	0.611647147	0.741401062	0.658667583	0.97521287
SPIRE1	0.700218559	0.837945129	0.795595114	0.820024304	0.655556663	0.643392483
ATP5F1C	0.700218559	0.548850389	0.754979443	0.680418978	0.943702351	0.762133815
FRS3	0.700316395	0.683744977	0.847357383	0.809277776	0.801692604	0.627258867
POLG	0.700316395	0.720320927	0.915713148	0.765499767	0.705202026	0.632510367
FHOD1	0.700316395	0.722195304	0.693546993	0.698952489	0.887514169	0.749726404
BRI3BP	0.700316395	0.759245424	0.685018711	0.684402602	0.804037558	0.83150957
RPLP2	0.700316395	0.662013091	0.731960288	0.730052184	0.705202026	0.888475311
DDX17	0.700316395	0.571955649	0.66149866	0.640315346	0.887514169	0.917930485
COL22A1	0.70038318	0.551459649	0.612793995	0.654131191	0.999985752	0.639142898
LOC100848940	0.70038318	0.884168437	0.67171984	0.794533639	0.788007619	0.643003473
ANKRD39	0.70038318	0.586798812	0.841294863	0.748424012	0.905825822	0.643003473
CEP72	0.70038318	0.750379179	0.617721225	0.925294859	0.738738532	0.699162043
SLTM	0.70038318	0.553132278	0.705746499	0.84844684	0.902907077	0.702623424
DLG1	0.70038318	0.915013697	0.6506579	0.637708629	0.809921436	0.745988271
PLAGL1	0.70038318	0.703571355	0.69454186	0.754719584	0.804625939	0.806476213
MEIS3	0.70038318	0.624142952	0.734143952	0.888687722	0.665013921	0.811410743
UBE2H	0.70038318	0.52001926	0.755135544	0.853058361	0.741086403	0.844759721
ARMCX1	0.70038318	0.596590801	0.660861667	0.823935462	0.735091896	0.891088733
FCMR	0.70038318	0.746712661	0.755135544	0.652519777	0.642796813	0.909056553
WDR18	0.70038318	0.551459649	0.697093367	0.638739139	0.644945176	0.999948228
LOC101902527	0.700428875	0.596368398	0.794225743	0.656875071	0.936388637	0.727876258
SLC41A3	0.700428875	0.561716964	0.854571175	0.808586366	0.788995893	0.742309541
NTMT1	0.700428875	0.59136789	0.81412252	0.742989529	0.756115173	0.835231445
LOC112447010	0.700428875	0.54021232	0.64236045	0.682383061	0.797227609	0.973649216
LOC104969496	0.700762609	0.91475257	0.817582037	0.688096109	0.653653376	0.681479061
C10H14orf93	0.701340179	0.692305416	0.70011973	0.907794002	0.66960879	0.762133815
COPZ1	0.701340179	0.559306577	0.874983674	0.815919419	0.66960879	0.798229124
MMACHC	0.701340179	0.582127335	0.8042974	0.645168798	0.87389153	0.81956221
LOC101906134	0.701610321	0.69959307	0.755940516	0.728264598	0.908551994	0.645719071
OXSM	0.701667124	0.524051946	0.70011973	0.676274746	0.999985752	0.645719071
RIOK3	0.701667124	0.555690642	0.777712574	0.753267542	0.899042139	0.742588975
PNMA1	0.701667124	0.724496927	0.772600931	0.742989529	0.665088846	0.839906041

RLF	0.702437649	0.635148479	0.693546993	0.670969025	0.983941532	0.680644329
ATP5MPL	0.702437649	0.580322769	0.730839225	0.724431807	0.943702351	0.742309541
NDUFB2	0.702437649	0.643640444	0.733850691	0.722611168	0.881680572	0.769865166
PPP4R3B	0.702437649	0.559306577	0.874983674	0.787102027	0.699191343	0.808200663
C8H9orf40	0.702437649	0.644366672	0.662272069	0.809277776	0.629421719	0.940017687
LOC112444352	0.702824493	0.621592704	0.735532321	0.78584336	0.902907077	0.690728863
FCHSD1	0.702912158	0.526959926	0.819527307	0.77580333	0.695846971	0.890967932
PTPN22	0.703156598	0.573498846	0.849389234	0.648660726	0.767678283	0.877048993
NAV3	0.703560341	0.789494428	0.791557166	0.819549243	0.670221463	0.698362131
LOC112444341	0.703937592	0.600004269	0.943836178	0.777956162	0.758618121	0.640434848
MAP4K2	0.703937592	0.526903667	0.69687851	0.999981514	0.642796813	0.645719071
ASTN2	0.703937592	0.544848881	0.77566312	0.890362631	0.834470237	0.684994779
CRHR2	0.703937592	0.712132686	0.623007852	0.816169012	0.844384115	0.768572658
CD40	0.703937592	0.811354298	0.630408979	0.823935462	0.64727886	0.836111377
ACKR2	0.703937592	0.580003885	0.73826834	0.64045509	0.853985138	0.892605403
LOC614625	0.704102193	0.583275815	0.70011973	0.999981514	0.655556663	0.68781527
ANP32A	0.704102193	0.954731859	0.627477288	0.640315346	0.823508064	0.68781527
YME1L1	0.704102193	0.54021232	0.813441395	0.64770518	0.797227609	0.90969547
ADAMTS7	0.704209071	0.627997081	0.810839887	0.923220263	0.710664679	0.655022943
HLX	0.704209071	0.544401883	0.679415149	0.891988949	0.887514169	0.728699286
CDYL2	0.704209071	0.692305416	0.804171507	0.82868625	0.627206089	0.792412747
MND1	0.704209071	0.52243101	0.971594714	0.694471701	0.674089143	0.801888363
UBE2A	0.704209071	0.577487692	0.754913805	0.668134034	0.670221463	0.970105102
PRAP1	0.704209415	0.570844841	0.660365588	0.921950515	0.900927045	0.655022943
LOC511229	0.704209415	0.689826137	0.693546993	0.984423083	0.656015118	0.670180759
SACM1L	0.704209415	0.591296685	0.63071957	0.856836633	0.934067848	0.701906535
MAB21L2	0.704209415	0.95687851	0.730695973	0.694666231	0.652333594	0.706869246
GDPD1	0.704209415	0.710645465	0.677197181	0.906880358	0.697129248	0.754218406
LAMTOR4	0.704209415	0.679897494	0.761415057	0.677545541	0.872096228	0.768168974
DGKG	0.704209415	0.928658222	0.694535506	0.698952489	0.631537325	0.795129319
VDAC1	0.704209415	0.584209799	0.743624166	0.738460631	0.80599066	0.86324237
LOC519309	0.704209415	0.552361981	0.836459233	0.673160239	0.804037558	0.864572584
GART	0.704209415	0.528669672	0.788126358	0.730052184	0.696466814	0.939662884
UBA2	0.704382059	0.58531281	0.685620658	0.811200887	0.943610727	0.699162043
BIRC2	0.704382059	0.54845263	0.865444995	0.733411985	0.677185022	0.882122026
AGFG1	0.704382059	0.54021232	0.805772321	0.724431807	0.658667583	0.945480515
PRRT2	0.704586409	0.553489554	0.759332589	0.988408846	0.706856083	0.661029645
SYT1	0.704586409	0.52001926	0.627295843	0.734472882	0.999985752	0.675895249

PEX2	0.704586409	0.544401883	0.973237403	0.687291299	0.710664679	0.761858515
HM13	0.704586409	0.614976364	0.867661896	0.777956162	0.626973048	0.844334911
NUP153	0.704708461	0.644366672	0.671704122	0.695125147	0.998060725	0.637719217
PAMR1	0.704708461	0.692305416	0.928514467	0.677800818	0.799051801	0.6467931
AARS2	0.704708461	0.543645094	0.906787307	0.874170259	0.727268681	0.680780892
RAB9A	0.704708461	0.573865937	0.705695072	0.9063051	0.857562998	0.689309487
ZNF217	0.704708461	0.532096752	0.950055019	0.689554882	0.823508064	0.703669004
SAFB	0.704708461	0.579840876	0.732274253	0.698952489	0.966822961	0.71289069
KIF18A	0.704708461	0.626185042	0.88462169	0.731537809	0.66101983	0.83150957
MEMO1	0.704708461	0.54845263	0.830420708	0.675426128	0.762020956	0.905509632
LMAN2L	0.704743893	0.929409572	0.759137214	0.712481274	0.737149805	0.629427844
LOC614129	0.704940088	0.688775944	0.637928356	0.799139231	0.856951734	0.785562356
LOC112449602	0.704940088	0.70880006	0.754913805	0.745577087	0.669123866	0.867063767
IFI30	0.704942376	0.602773648	0.660861667	0.751451145	0.947299702	0.761991719
P4HA3	0.705171429	0.565953284	0.69687851	0.946503916	0.769935313	0.751100989
CD63	0.70547183	0.543708557	0.858032788	0.941935077	0.705202026	0.66881641
BRAT1	0.70547183	0.571839975	0.881070741	0.843335547	0.725081247	0.731082028
FLCN	0.70547183	0.615602441	0.69621934	0.676566861	0.781850172	0.940017687
ASTE1	0.705561196	0.74241072	0.711148626	0.652644879	0.948711141	0.683230524
CFAP97	0.705636431	0.824188473	0.652653727	0.890704007	0.665973931	0.735045772
SUSD1	0.70572131	0.624142952	0.77566312	0.80459801	0.90654624	0.639142898
ADGRG5	0.706197464	0.929204871	0.684282335	0.819968583	0.695528035	0.643392483
TSPO	0.706197464	0.559306577	0.66149866	0.685893052	0.999985752	0.68781527
MMAB	0.706197464	0.553132278	0.798782742	0.690807874	0.943160494	0.741319465
BANP	0.706197464	0.630477275	0.693546993	0.866566721	0.727357021	0.839906041
FBXO41	0.706197464	0.622423182	0.768438632	0.712481274	0.798683736	0.852383414
RAD54L2	0.706197464	0.608781165	0.659919107	0.676566861	0.879701773	0.896529476
KRT23	0.706254195	0.582560314	0.627295843	0.96444269	0.88127953	0.640434848
TMEM184A	0.706368412	0.522400504	0.755135544	0.925294859	0.789915129	0.743720206
NFATC1	0.706729974	0.595982761	0.867661896	0.935985829	0.640259949	0.675247254
FNBP4	0.706729974	0.54845263	0.880612713	0.885789665	0.736942681	0.696550852
NUAK2	0.70676198	0.615769899	0.807254925	0.896116841	0.796633553	0.643003473
PCDHGA2	0.70697838	0.928427136	0.647867969	0.777956162	0.796633553	0.640434848
KIAA0754	0.70697838	0.550226079	0.999993442	0.71656639	0.682856038	0.682483304
LOC101905897	0.70697838	0.709348874	0.711148626	0.764257755	0.779549045	0.831652079
PARP12	0.707156665	0.589754585	0.95219206	0.65294576	0.81725718	0.700123604
MANEAL	0.707156665	0.614976364	0.947176871	0.680418978	0.788850323	0.704950309
LOC101906837	0.707156665	0.564092381	0.802277976	0.922967576	0.695890051	0.748921056

LOC107131728	0.707156665	0.870334242	0.668824581	0.714751696	0.799272579	0.748921056
AGPS	0.707156665	0.549778156	0.761415057	0.958978724	0.664519875	0.765948605
NNAT	0.707156665	0.572077017	0.731143569	0.978503042	0.638815482	0.769865166
LIG3	0.707156665	0.554555702	0.71338011	0.962437357	0.642796813	0.81956221
C29H11orf80	0.707156665	0.646834837	0.706064882	0.892047793	0.664519875	0.835231445
LOC112444314	0.707156665	0.573498846	0.799533361	0.707217807	0.632545657	0.960023723
PNRC1	0.707156665	0.526654774	0.682220784	0.762472696	0.745891168	0.963701289
LOC100336897	0.707239046	0.582127335	0.625105036	0.946938434	0.856136977	0.716096025
ARHGEF39	0.707239046	0.527777396	0.805212947	0.901353354	0.691315145	0.806714845
C11H9orf50	0.707266705	0.906927139	0.727520588	0.792140715	0.749752717	0.631731716
GRP	0.707266705	0.977196455	0.731739801	0.672828315	0.738738532	0.632510367
CUEDC1	0.707266705	0.614976364	0.726119774	0.995565324	0.707939546	0.640128675
DACT3	0.707266705	0.722348907	0.72304829	0.868435169	0.832486638	0.640434848
SOSTDC1	0.707266705	0.786392346	0.779052868	0.810132355	0.795839201	0.643003473
LOC112447360	0.707266705	0.788544942	0.672696223	0.839245804	0.850435498	0.643003473
SEPT5	0.707266705	0.697996754	0.916038418	0.789139053	0.699191343	0.666292389
THSD4	0.707266705	0.94422395	0.744735369	0.731537809	0.687203978	0.668851588
PLS3	0.707266705	0.7095693	0.945995021	0.650818142	0.782990999	0.668851588
WDR17	0.707266705	0.559306577	0.957052938	0.731910397	0.788739452	0.687128561
GJD3	0.707266705	0.597345821	0.668824581	0.910575108	0.879268517	0.690728863
ANKRD40	0.707266705	0.536598701	0.794225743	0.904018489	0.819329746	0.695065923
ANO6	0.707266705	0.532096752	0.758146087	0.916858019	0.823508064	0.702623424
ZNF839	0.707266705	0.615769899	0.687462433	0.815919419	0.929896768	0.702623424
LOC112447005	0.707266705	0.855752352	0.701512486	0.796934171	0.74028971	0.706704597
ZDHHC13	0.707266705	0.981207384	0.642661551	0.718713712	0.687203978	0.706869246
HSPA2	0.707266705	0.609772211	0.668824581	0.915779841	0.846464841	0.70951022
IL17REL	0.707266705	0.764456895	0.851633941	0.731537809	0.736914075	0.718413142
CFAP54	0.707266705	0.727534248	0.730571184	0.865248466	0.75602825	0.728699286
CRYL1	0.707266705	0.542391346	0.723132042	0.998529748	0.670221463	0.733767439
ZCCHC18	0.707266705	0.764456895	0.889098668	0.724431807	0.66960879	0.734509981
LOC101908535	0.707266705	0.580530872	0.855268213	0.925353907	0.641212999	0.735122635
KRBA2	0.707266705	0.597711676	0.828806956	0.925210648	0.642796813	0.748323122
TNFSF9	0.707266705	0.854961334	0.713233032	0.799148505	0.675071981	0.75702988
TFAP4	0.707266705	0.551459649	0.71967891	0.728264598	0.959658682	0.758175289
PTS	0.707266705	0.580473137	0.85894223	0.830187312	0.735091896	0.762133815
LRAT	0.707266705	0.660882273	0.803342698	0.864831081	0.687203978	0.765948605
COPE	0.707266705	0.584209799	0.810647419	0.731537809	0.868722067	0.772618249
HEATR5B	0.707266705	0.692305416	0.685018711	0.919510399	0.658667583	0.783579115

KIFC1	0.707266705	0.550442307	0.879939804	0.786099236	0.74416708	0.796158754
EPHB2	0.707266705	0.753131028	0.652724437	0.82868625	0.736942681	0.812091218
BAALC	0.707266705	0.553638694	0.888596622	0.81631926	0.665098448	0.81853698
LZTS1	0.707266705	0.532096752	0.627295843	0.794386089	0.950852294	0.81956221
MNF1	0.707266705	0.727781617	0.779581461	0.683232054	0.781724458	0.820395402
ABCF3	0.707266705	0.584209799	0.857372289	0.684402602	0.809129327	0.829441883
RANGRF	0.707266705	0.744774335	0.747666034	0.66651301	0.803705802	0.83150957
CHD2	0.707266705	0.555931044	0.802787375	0.808073654	0.769529419	0.837596789
AKR7A2	0.707266705	0.529619156	0.743624166	0.694141249	0.926813827	0.840768468
ZKSCAN2	0.707266705	0.71096688	0.805542815	0.757345832	0.637102685	0.857911901
MARS	0.707266705	0.572077017	0.895801611	0.754782628	0.629421719	0.867063767
RGS9	0.707266705	0.595982761	0.744735369	0.827443542	0.702094252	0.867482845
ARRDC5	0.707266705	0.627743951	0.848278925	0.718713712	0.658667583	0.882690335
LOC787234	0.707266705	0.525504435	0.817582037	0.688019411	0.814120141	0.887542005
ACSL5	0.707266705	0.582051417	0.739106589	0.777956162	0.726283157	0.909056553
RORB	0.707266705	0.600084034	0.84099486	0.680418978	0.691315145	0.912894278
ADGRA2	0.707266705	0.522940516	0.68005166	0.64770518	0.789915129	0.993890045
LOC101907084	0.707587269	0.600626027	0.833583844	0.686487857	0.809129327	0.838468539
RNF122	0.707824974	0.582324384	0.638444487	0.765499767	0.999453276	0.667047801
LOC101906818	0.707824974	0.533735495	0.746818005	0.988408846	0.759888741	0.670249703
LARP4B	0.707910686	0.728504282	0.629946682	0.949565453	0.759888741	0.690728863
RTTN	0.707910686	0.589782216	0.682166552	0.748424012	0.738738532	0.948705062
TIE1	0.7082076	0.559796438	0.836719427	0.888198716	0.690058599	0.78096075
SLX1A	0.70838594	0.587350756	0.961437412	0.777956162	0.701123622	0.690728863
EPN1	0.70838594	0.942088099	0.681700278	0.65483771	0.753088486	0.753016904
MAN1B1	0.70838594	0.60754196	0.660816053	0.980564765	0.699693139	0.761143135
MARK2	0.70838594	0.772342166	0.776353067	0.731537809	0.75564854	0.768168974
FPGT	0.70838594	0.572077017	0.734869284	0.914026346	0.760007802	0.774178526
TGM3	0.70838594	0.567744993	0.749417651	0.74577892	0.906893576	0.783241453
FREM1	0.70838594	0.744774335	0.81412252	0.646556987	0.704783515	0.861855564
DIRAS1	0.7088105	0.535967468	0.720612298	0.902137829	0.913910082	0.667758333
SMARCA2	0.7088105	0.692305416	0.702147813	0.959652624	0.725081247	0.669883077
SLC38A1	0.7088105	0.734546826	0.796168865	0.701248463	0.870246061	0.699160709
CHP1	0.7088105	0.616227995	0.628904157	0.997792939	0.735091896	0.706704597
STXBP3	0.7088105	0.708218805	0.69881939	0.882057818	0.781159411	0.743842064
LOC101902937	0.7088105	0.547676372	0.755022914	0.664953033	0.964301327	0.770800978
PDE6G	0.7088105	0.716415149	0.860742804	0.652644879	0.788007619	0.779201808
BUD23	0.7088105	0.625011831	0.682677321	0.688019411	0.80531082	0.92789871

LOC786512	0.709624454	0.880565292	0.633096422	0.946503916	0.651951691	0.643059978
UFL1	0.709624454	0.813658213	0.682974314	0.751045386	0.87329316	0.688206116
MCOLN2	0.709624454	0.548622699	0.834683675	0.889055166	0.795035487	0.711923954
HSH2D	0.709624454	0.61939332	0.867222363	0.819213899	0.69149571	0.77803403
LOC104974345	0.709873791	0.70454105	0.680262176	0.680884552	0.982538884	0.668191006
ZNF207	0.710344986	0.532096752	0.754913805	0.661362364	0.999985752	0.684994779
C26H10orf62	0.710344986	0.546072402	0.77566312	0.9745615	0.710664679	0.706704597
WDR35	0.710344986	0.829067393	0.637234901	0.907794002	0.695846971	0.70951022
MLH1	0.710344986	0.636523083	0.670239985	0.822691957	0.924222719	0.718444845
C25H7orf50	0.710344986	0.65172183	0.807254925	0.812453944	0.800097282	0.737726964
GLS	0.710344986	0.664859217	0.652653727	0.803690355	0.907678664	0.754218406
WDTC1	0.710344986	0.553132278	0.911973589	0.653503626	0.650485683	0.925697832
CFD	0.710617013	0.65172183	0.821695426	0.731537809	0.85185197	0.742309541
COL15A1	0.710784303	0.597345821	0.89478142	0.930987593	0.670221463	0.63512796
SAR1A	0.710784303	0.565257607	0.946791586	0.854450063	0.696466814	0.669883077
SH3BP5	0.710784303	0.579451739	0.731739801	0.707217807	0.992500414	0.671759429
TSTA3	0.710784303	0.829067393	0.734143952	0.700026235	0.862716067	0.683230524
ADIG	0.710784303	0.693513972	0.732480837	0.764257755	0.864524107	0.754218406
SPTBN5	0.710784303	0.528669672	0.866467804	0.904374668	0.691315145	0.758175289
AMZ2	0.710784303	0.621713316	0.809673119	0.823167001	0.735091896	0.796158754
SNX6	0.710784303	0.962068181	0.640084371	0.664273743	0.697778495	0.796402766
SDHA	0.710784303	0.600886305	0.69417649	0.687568149	0.953300742	0.803098894
MCRS1	0.710784303	0.623984886	0.869932384	0.676566861	0.807468091	0.805925428
MBNL1	0.710784303	0.55180706	0.641535145	0.994395954	0.655556663	0.831652079
UBE2L3	0.710784303	0.644366672	0.754913805	0.682944876	0.832407979	0.858245612
EXD1	0.710784303	0.587350756	0.726119774	0.896116841	0.658667583	0.867635709
SCD	0.710784303	0.550226079	0.650009937	0.685601134	0.889301074	0.932688327
LOC789231	0.710890041	0.615769899	0.694535506	0.965050971	0.66101983	0.783423501
LOC101902106	0.711327497	0.655096367	0.693546993	0.731537809	0.658667583	0.963701289
STX17	0.711584492	0.532096752	0.830420708	0.759055324	0.72341123	0.90607344
LOC112446716	0.711737872	0.831564725	0.63135074	0.96951401	0.645411168	0.668851588
ANKHD1	0.711737872	0.772618617	0.652724437	0.730052184	0.953300742	0.668851588
LOC104972545	0.711737872	0.789957129	0.913609237	0.774214837	0.636203556	0.677355984
SRP72	0.711737872	0.832556888	0.668824581	0.733933516	0.883951804	0.690728863
HAP1	0.711737872	0.550226079	0.81336501	0.916858019	0.769686429	0.720865143
NREP	0.711737872	0.767570093	0.692461158	0.886831994	0.718810344	0.749726404
PDE7A	0.711737872	0.621856619	0.731143569	0.695125147	0.759808196	0.933897626
CC2D1B	0.711994916	0.82177045	0.736160515	0.822691957	0.773264077	0.670180759

PURG	0.711994916	0.683744977	0.668824581	0.796128007	0.857738578	0.80383591
GLYR1	0.711994916	0.649949716	0.946924479	0.680884552	0.651951691	0.815129379
TRIM37	0.712140594	0.673096434	0.700486014	0.9880671	0.65298501	0.706869246
UBQLN1	0.712140594	0.542302787	0.846372547	0.887983383	0.705202026	0.788462021
BTF3L4	0.712140594	0.54021232	0.886829776	0.698859815	0.696466814	0.906637848
A2M	0.712350054	0.531880429	0.693546993	0.680884552	0.999985752	0.648195749
TMEM265	0.712350054	0.550117048	0.774015998	0.813472855	0.809129327	0.835231445
SLC8A1	0.712390889	0.91763262	0.817582037	0.731537809	0.653434609	0.684994779
SPTAN1	0.712390889	0.531880429	0.70011973	0.892071982	0.934751389	0.690728863
VHL	0.712390889	0.551281365	0.73519629	0.657774122	0.999985752	0.706704597
FANCI	0.712390889	0.54021232	0.818612484	0.830289573	0.858495696	0.739579536
RAB11FIP4	0.712390889	0.553132278	0.955221146	0.749104032	0.638815482	0.83150957
ANKRD13C	0.712390889	0.534529958	0.958410013	0.658194793	0.716406563	0.844334911
LEF1	0.712390889	0.580111602	0.813441395	0.69158245	0.84620147	0.846210629
AOX2	0.712545315	0.606559706	0.879820377	0.890704007	0.763224862	0.645719071
ANLN	0.712550205	0.615769899	0.826534865	0.868378265	0.732405592	0.755679512
ZNF350	0.712550205	0.610580181	0.754557912	0.812453944	0.79453727	0.831652079
STARD3	0.712914877	0.60754196	0.980761647	0.796128007	0.696466814	0.640128675
PAXBP1	0.712914877	0.53098199	0.739106589	0.999981514	0.721096295	0.677365619
ZNF609	0.712914877	0.541918349	0.810647419	0.692590198	0.976769047	0.68185373
CAV2	0.712914877	0.66603796	0.709649545	0.824323293	0.900927045	0.689625555
RAB2A	0.712914877	0.587800209	0.999993442	0.700943067	0.659373888	0.708508856
KDELC2	0.712914877	0.927034887	0.747884618	0.728264598	0.652206871	0.752213117
STT3A	0.712914877	0.653225995	0.80412682	0.819968583	0.788007619	0.754218406
ARMC12	0.712914877	0.589754585	0.660816053	0.701248463	0.960573822	0.81956221
CPNE9	0.712914877	0.594199197	0.81319557	0.731537809	0.773264077	0.867635709
ZC3H15	0.712914877	0.54615571	0.850624788	0.690807874	0.687203978	0.943638116
KIAA0319L	0.713028088	0.553132278	0.885896294	0.840247324	0.706876268	0.782330566
LPAR3	0.71307679	0.90323787	0.693546993	0.69459333	0.87329316	0.645719071
SIPA1L1	0.71307679	0.702211146	0.744735369	0.882057818	0.716406563	0.766218451
PLCXD2	0.71307679	0.844170799	0.660365588	0.808075336	0.750277829	0.768572658
MED19	0.71307679	0.599021986	0.652681076	0.695125147	0.982087479	0.772618249
ATP5F1B	0.71307679	0.550226079	0.734143952	0.76561391	0.91383048	0.806476213
TSPYL6	0.71307679	0.568485668	0.634391045	0.69158245	0.976292433	0.83150957
ANKRD55	0.713319016	0.550226079	0.888989166	0.907794002	0.776537165	0.645719071
STX1A	0.713319016	0.790429889	0.628658187	0.856764312	0.858488348	0.68185373
LOC112448395	0.713319016	0.533735495	0.785516539	0.760230321	0.879435126	0.828124017
SOX5	0.713319016	0.61939332	0.744735369	0.724431807	0.738738532	0.925233445

CHMP2A	0.713779305	0.610421396	0.813441395	0.775528043	0.859672963	0.748921056
EPYC	0.713969126	0.573498846	0.744735369	0.999981514	0.652333594	0.684615686
HDAC7	0.713969126	0.531880429	0.865697455	0.892047793	0.691315145	0.785562356
LOC617905	0.714010649	0.614976364	0.863546668	0.688019411	0.817472827	0.811487478
CDK6	0.714081158	0.620485532	0.727870684	0.705422335	0.799272579	0.917930485
USP47	0.714244255	0.565421383	0.904919322	0.792140715	0.872229135	0.645719071
TSPAN7	0.714244255	0.692305416	0.945995021	0.677374927	0.738738532	0.728699286
SYTL1	0.714599632	0.56685163	0.801754158	0.872590202	0.753508098	0.802390255
ACVRL1	0.714599632	0.594199197	0.677197181	0.734423422	0.788007619	0.948913998
KIF2A	0.714704992	0.724380878	0.896821247	0.731971176	0.797489438	0.663570907
LOC112444622	0.714704992	0.750379179	0.729359017	0.822262805	0.665973931	0.839565788
DNAJA2	0.714881686	0.532427681	0.892343042	0.827786827	0.720896795	0.80383591
COL6A1	0.71495154	0.774934769	0.749417651	0.871338923	0.795021983	0.644451469
ID1	0.71495154	0.634059237	0.807254925	0.827786827	0.75602825	0.78288746
B3GAT3	0.71495154	0.742703566	0.734143952	0.691020007	0.859005924	0.786849547
VBP1	0.71495154	0.54021232	0.881778842	0.74577892	0.642796813	0.923092436
DSE	0.715136201	0.790370995	0.750313078	0.819968583	0.823508064	0.640825046
ARHGAP26	0.715136201	0.989303356	0.745477682	0.654131191	0.72350412	0.642725087
C7H19orf38	0.715136201	0.766967329	0.89478142	0.715394268	0.799272579	0.645427231
SASS6	0.715136201	0.533735495	0.999993442	0.731537809	0.670245065	0.645719071
NFKB1	0.715136201	0.589754585	0.723110374	0.672328815	0.999985752	0.645719071
PCOLCE	0.715136201	0.693373829	0.774015998	0.874170259	0.825284223	0.650195003
LOC101907540	0.715136201	0.531880429	0.901736458	0.69459333	0.953391502	0.655022943
LOC515551	0.715136201	0.584209799	0.655439346	0.999981514	0.771915356	0.669558041
PARP14	0.715136201	0.823478017	0.824121854	0.714065591	0.801692604	0.677355984
KRCC1	0.715136201	0.811114624	0.837787117	0.799712183	0.69975451	0.683230524
LOC613660	0.715136201	0.60754196	0.916294854	0.69705012	0.854289643	0.704351773
YIF1B	0.715136201	0.725061573	0.670239985	0.85981779	0.848038632	0.728686831
LOC112443510	0.715136201	0.64563665	0.744735369	0.759591583	0.916605572	0.74212863
CYP4A11	0.715136201	0.579367653	0.832065705	0.686647893	0.916284118	0.768572658
RHOF	0.715136201	0.696757704	0.875900843	0.794386089	0.665973931	0.776734587
E2F2	0.715136201	0.649949716	0.894316021	0.7694401	0.714597718	0.777197325
CMC2	0.715136201	0.616292283	0.749417651	0.657100373	0.913732532	0.835231445
KIF7	0.715136201	0.549085151	0.759110916	0.907993229	0.696466814	0.841312694
RRAGB	0.715136201	0.555931044	0.668192825	0.882057818	0.833864947	0.844869475
SOD1	0.715136201	0.624142952	0.73091642	0.765499767	0.786286008	0.888309103
TLE4	0.715136201	0.636057435	0.671231078	0.687568149	0.833864947	0.927743378
SLC25A28	0.715136201	0.634059237	0.677197181	0.792140715	0.64727886	0.969014913

LOC101907503	0.715192043	0.543103725	0.860877703	0.715394268	0.964301327	0.650195003
LOC112446689	0.715192043	0.615769899	0.685620658	0.896116841	0.902907077	0.677355984
LY86	0.715364376	0.918234392	0.684282335	0.686250223	0.847173934	0.690728863
LOC107132783	0.715364376	0.614976364	0.70011973	0.861243324	0.87329316	0.754218406
SAC3D1	0.715364376	0.70880006	0.755940516	0.75842437	0.80653881	0.795226088
GJA5	0.715526274	0.591412538	0.884121008	0.745806541	0.823508064	0.752213117
MGAT4A	0.715526274	0.693513972	0.739888939	0.898890982	0.656396948	0.803924112
FAM84A	0.715606938	0.823561191	0.751957722	0.772860262	0.705202026	0.778344889
DYNLRB1	0.71561775	0.60754196	0.843955813	0.715394268	0.870189808	0.770035496
LOC100848941	0.715984396	0.664859217	0.774015998	0.680418978	0.887514169	0.799581725
SOGA3	0.716008322	0.618921732	0.637234901	0.78584336	0.849786155	0.888475311
VWA5A	0.716092473	0.750379179	0.689888669	0.706180917	0.92915293	0.742309541
EHMT2	0.716618872	0.563234314	0.874983674	0.912777307	0.769686429	0.668851588
SLC22A17	0.716618872	0.601808502	0.682700098	0.865746543	0.914280652	0.733767439
TAOK2	0.71673116	0.649949716	0.835574833	0.819213899	0.792867703	0.739579536
UHRF1BP1L	0.716836272	0.665053816	0.696778723	0.765558953	0.970988226	0.660603184
LOC101902922	0.716836272	0.792109226	0.755135544	0.812453944	0.809317773	0.672411349
LOC112444726	0.716836272	0.657192178	0.668824581	0.686250223	0.998008754	0.699160709
C6H4orf48	0.716836272	0.732885933	0.704088819	0.961898258	0.677965402	0.700123604
WDR83	0.716836272	0.981520642	0.735532321	0.678068768	0.648467848	0.741319465
ATG14	0.716836272	0.7889129	0.855892553	0.727236849	0.723467697	0.748739294
C3H1orf109	0.716836272	0.850640641	0.717242466	0.729705452	0.723467697	0.816246746
GALNT6	0.716836272	0.642929022	0.726741709	0.874424802	0.741891558	0.83150957
TMEM200C	0.716836272	0.761754776	0.749417651	0.739059155	0.714597718	0.85532952
LOC112441457	0.716836272	0.582127335	0.915120516	0.681573779	0.738738532	0.85643253
LUZP6	0.716836272	0.593498372	0.853546772	0.784595865	0.66101983	0.879884117
CXCL3	0.716836272	0.54845263	0.680262176	0.932406746	0.658667583	0.911219114
TMEM50B	0.716836272	0.582127335	0.794350709	0.724431807	0.690633636	0.950798794
MAGEH1	0.717425454	0.808279036	0.874983674	0.669493024	0.821351993	0.661029645
GNAZ	0.717425454	0.532427681	0.999993442	0.710070489	0.656015118	0.670249703
CHDH	0.717425454	0.62215202	0.75407054	0.92532303	0.797227609	0.699160709
LOC101905887	0.717425454	0.722195304	0.691850804	0.664953033	0.976259416	0.702104086
BRIX1	0.717425454	0.573711724	0.727870684	0.680884552	0.97464569	0.779201808
CCHCR1	0.717425454	0.768359345	0.8042974	0.76992117	0.659943626	0.8255975
ARC	0.717669394	0.822956796	0.815932548	0.677545541	0.807922723	0.727632739
LSG1	0.717669394	0.757251331	0.680262176	0.777956162	0.879268517	0.75062693
CRLF3	0.717669394	0.946820717	0.719188447	0.758601772	0.64566985	0.750967073
CHCHD3	0.717669394	0.598833902	0.797180963	0.687568149	0.810436875	0.889406298

KIAA1755	0.717669394	0.605710874	0.67821881	0.707217807	0.831903251	0.936951685
LOC112444936	0.717669394	0.54021232	0.749417651	0.764257755	0.738274405	0.951081819
CCDC191	0.717705373	0.622818484	0.818612484	0.839725231	0.735091896	0.798057364
C7H19orf57	0.717808816	0.595982761	0.669827112	0.999981514	0.75602825	0.682479856
CNDP2	0.718213134	0.740847451	0.758146087	0.944351122	0.688740147	0.669883077
ACTG1	0.718213134	0.664859217	0.951392011	0.758499128	0.74965664	0.669883077
NFYA	0.718213134	0.553132278	0.685165356	0.972467586	0.796861564	0.75640676
NDUFA10	0.718213134	0.609534144	0.685620658	0.687568149	0.976292433	0.778711943
SDK1	0.718213134	0.553132278	0.872088608	0.89684883	0.678959674	0.780283623
MYOZ1	0.718213134	0.688775944	0.684282335	0.954946234	0.666917776	0.784826714
LOC101906086	0.718213134	0.54671968	0.71967891	0.874424802	0.86864151	0.795936781
SLC8A3	0.718213134	0.941251179	0.668824581	0.667385661	0.738811608	0.80383591
PVR	0.718213134	0.593498372	0.67171984	0.688704343	0.972160227	0.811410743
ZBTB7C	0.718213134	0.545932635	0.794501834	0.813495071	0.821351993	0.835231445
PSMB7	0.718213134	0.627743951	0.808037655	0.698952489	0.795865165	0.874490166
LOC112441542	0.718213134	0.609864167	0.668721054	0.82868625	0.805126365	0.878118151
SLCO4A1	0.718213134	0.54845263	0.652724437	0.789139053	0.893716526	0.883688554
ATIC	0.718213134	0.620485532	0.819359511	0.689227896	0.738853921	0.912531667
MAPKBP1	0.71843535	0.580530872	0.75029951	0.828337624	0.779205591	0.867063767
LOC101907487	0.718703676	0.741263262	0.744735369	0.673875708	0.960182847	0.671759429
LOC112444920	0.718703676	0.614805655	0.749417651	0.96444269	0.705202026	0.742309541
ESRRA	0.718703676	0.696757704	0.813441395	0.659998592	0.770132758	0.87501921
ZBTB7B	0.718711774	0.833429351	0.677197181	0.915039335	0.754995923	0.645719071
TNFSF8	0.718774053	0.664837103	0.766722666	0.707217807	0.954403133	0.690728863
VPS53	0.718774053	0.54021232	0.781148064	0.819968583	0.938316699	0.702623424
ME3	0.718774053	0.54021232	0.682974314	0.996090365	0.75602825	0.754218406
PLAC8	0.718774053	0.580111602	0.832192335	0.748837705	0.832407979	0.829441883
IL17D	0.718904775	0.550226079	0.893988897	0.751451145	0.738738532	0.85532952
IKZF3	0.718904775	0.790702675	0.744791594	0.777956162	0.653399081	0.858245612
LOC112444355	0.71930628	0.546398474	0.671469816	0.687568149	0.999985752	0.655022943
HIPK1	0.71930628	0.572077017	0.681700278	0.999981514	0.788995893	0.657833313
IL34	0.71930628	0.605710874	0.819359511	0.901171792	0.81247611	0.669883077
SCN3A	0.71930628	0.787904651	0.70011973	0.96951401	0.656015118	0.67453786
APPBP2	0.71930628	0.539255096	0.774924967	0.843593618	0.936108073	0.695065923
GMCL1	0.71930628	0.687662016	0.716635829	0.929325365	0.791848145	0.700019951
PSIP1	0.71930628	0.826102395	0.685620658	0.908467146	0.69975451	0.702623424
PLRG1	0.71930628	0.839128192	0.731960288	0.887642126	0.661674859	0.717538808
KLHL24	0.71930628	0.936319037	0.693546993	0.687809326	0.799272579	0.733767439

LRRTM2	0.71930628	0.709624468	0.77566312	0.762598894	0.868528934	0.734983106
HUS1	0.71930628	0.791281103	0.652653727	0.822035058	0.832486638	0.750967073
NSA2	0.71930628	0.648958657	0.77566312	0.828736799	0.816070658	0.761991719
ADAMTS16	0.71930628	0.64939288	0.649622134	0.854450063	0.883043829	0.781629683
TOMM40L	0.71930628	0.60689126	0.879939804	0.767989359	0.788850323	0.783241453
TMED7	0.71930628	0.589651566	0.826785415	0.853058361	0.69906048	0.839906041
UNKL	0.71930628	0.615769899	0.804171507	0.821450642	0.721096295	0.851718562
GFM1	0.71930628	0.579451739	0.739888939	0.678604808	0.895757462	0.881875045
RUNX3	0.71930628	0.546072402	0.732130173	0.777956162	0.818179113	0.912531667
DIAPH1	0.71930628	0.739786945	0.774015998	0.703717381	0.651951691	0.923092436
MYL5	0.719316433	0.884128797	0.754979443	0.784838526	0.70314895	0.727632739
DACT1	0.719978934	0.943712473	0.652724437	0.886386107	0.678703442	0.666011114
MNAT1	0.719978934	0.712357273	0.915858924	0.813375274	0.658667583	0.72026427
SAMSN1	0.719978934	0.627743951	0.80412682	0.765499767	0.887514169	0.748223279
WDR3	0.719978934	0.543554867	0.80412682	0.819213899	0.833173678	0.81956221
UBE3B	0.719978934	0.554864655	0.670239985	0.799712183	0.936108073	0.83150957
TMEM71	0.719978934	0.553132278	0.707407957	0.912229943	0.677622204	0.903990996
EEF1E1	0.719978934	0.554321821	0.764872024	0.790377592	0.661712531	0.960023723
MUTYH	0.720231101	0.678023292	0.831933739	0.876703811	0.74028971	0.718413142
CDC37	0.720231101	0.622818484	0.792493806	0.774214837	0.879776073	0.762133815
GDE1	0.720231101	0.708218805	0.726353513	0.81451387	0.735091896	0.85513515
THUMPD3	0.720231101	0.551459649	0.677225445	0.764555331	0.842704431	0.93531275
TMED5	0.72044301	0.54021232	0.810831922	0.865746543	0.78130551	0.831652079
LOC101905743	0.720571644	0.54845263	0.668824581	0.92532303	0.959151723	0.645719071
VDR	0.720571644	0.554321821	0.829455601	0.681899077	0.989334846	0.669558041
ANO1	0.720571644	0.715664279	0.875900843	0.703717381	0.856814762	0.689729828
TEPSIN	0.720571644	0.614976364	0.802277976	0.736045965	0.950855218	0.69516836
LOC783202	0.720571644	0.550226079	0.721294664	0.892047793	0.930970429	0.698787824
TDP1	0.720571644	0.573865937	0.999993442	0.753267542	0.680301694	0.699160709
GOLGB1	0.720571644	0.596426784	0.745942803	0.940436902	0.799272579	0.730527465
ZBTB16	0.720571644	0.582127335	0.646946768	0.97828838	0.821557309	0.744315306
FAM198A	0.720571644	0.583140563	0.846960897	0.87522058	0.771469454	0.76139014
MED27	0.720571644	0.550226079	0.855268213	0.812453944	0.823508064	0.782330566
PABPC4	0.720571644	0.581074652	0.693546993	0.754782628	0.962513981	0.78288746
TRAF4	0.720571644	0.60754196	0.77566312	0.896116841	0.723054375	0.812091218
TMEM69	0.720571644	0.622818484	0.70011973	0.878513543	0.797489438	0.831652079
ACE	0.720571644	0.687020192	0.845113191	0.676566861	0.804037558	0.835338212
RMRP	0.720571644	0.592003056	0.698295081	0.952298386	0.695436567	0.836111377

MYSM1	0.720571644	0.599075208	0.765365298	0.902218883	0.688556938	0.839573661
RIMKLB	0.720571644	0.624142952	0.671704122	0.952247001	0.657647754	0.862925606
POLR1A	0.720571644	0.612800724	0.740372388	0.717645144	0.86010579	0.875297569
CDC42EP5	0.720571644	0.553132278	0.782415108	0.887877413	0.658667583	0.891423889
LOC107131416	0.720571644	0.558233871	0.663513753	0.695125147	0.916605572	0.923092436
POC5	0.720793994	0.550226079	0.834683675	0.98544125	0.725105178	0.666881531
SYNGAP1	0.720793994	0.666266365	0.731143569	0.946503916	0.788007619	0.696461811
FBLN7	0.720793994	0.594199197	0.76555928	0.995565324	0.685561272	0.697356941
MYL12A	0.720793994	0.614976364	0.841294863	0.903584224	0.756483092	0.708090768
RECQL4	0.720793994	0.715705323	0.892343042	0.854966424	0.655786713	0.712844937
PSMB10	0.720793994	0.55180706	0.734527982	0.979433033	0.790339877	0.722073353
LOC112443178	0.720793994	0.999987083	0.649378275	0.680418978	0.652333594	0.72569511
LDLRAP1	0.720793994	0.716415149	0.849704325	0.74577892	0.823508064	0.727876258
ADGRA1	0.720793994	0.678023292	0.968204727	0.673160239	0.738738532	0.7353049
SECISBP2	0.720793994	0.60754196	0.681700278	0.729912904	0.985238194	0.748323122
GPAT2	0.720793994	0.802570554	0.855847948	0.688942799	0.776537165	0.748739294
TRMU	0.720793994	0.826102395	0.784788059	0.733933516	0.754089854	0.765138165
LOC533921	0.720793994	0.542391346	0.755940516	0.695125147	0.953300742	0.83150957
SERPINA5	0.720793994	0.630897425	0.749453648	0.701200081	0.900893337	0.832964812
MAT2B	0.720793994	0.572077017	0.916323169	0.74577892	0.733613832	0.835231445
MKRN1	0.720793994	0.584209799	0.6813705	0.825279787	0.834431851	0.876861428
HDAC5	0.720793994	0.618921732	0.645276799	0.72169974	0.886041168	0.917024468
LMO2	0.721085975	0.649949716	0.735916998	0.896116841	0.885217562	0.660837985
TMEM202	0.721085975	0.822956796	0.652724437	0.942145064	0.677185022	0.733467771
LOC112444864	0.721085975	0.54845263	0.707407957	0.808073654	0.960419102	0.761858515
UTP18	0.721085975	0.602500622	0.731960288	0.882057818	0.851927006	0.766900958
RBBP6	0.721085975	0.614976364	0.727619107	0.764257755	0.911664199	0.807219446
CRYZ	0.721085975	0.675274606	0.731960288	0.731537809	0.87329316	0.83150957
CERS1	0.721085975	0.740642434	0.77566312	0.760230321	0.744684207	0.836111377
TRHDE	0.721085975	0.694842866	0.777106205	0.707217807	0.823508064	0.838131346
LOC104972821	0.721085975	0.800193122	0.66149866	0.822871493	0.714597718	0.846210629
FAM173B	0.721085975	0.559306577	0.694535506	0.888687722	0.817472827	0.853735179
RNF170	0.721085975	0.551275739	0.908072171	0.730052184	0.729058286	0.873130182
TRAPPC9	0.721085975	0.613590941	0.666670866	0.762598894	0.797227609	0.94844234
FARS2	0.721085975	0.557264832	0.694535506	0.707217807	0.83704813	0.959198304
LOC101906240	0.721374583	0.662013091	0.713072075	0.874170259	0.670221463	0.888475311
BTBD10	0.721543459	0.696797615	0.70011973	0.812453944	0.939813331	0.681807713
MPP5	0.721543459	0.634752242	0.669511234	0.999981514	0.702850222	0.700326694

WISP1	0.721543459	0.588939818	0.945471374	0.825308462	0.696466814	0.74201596
WNK2	0.721543459	0.878926117	0.732480837	0.723043513	0.706265272	0.81956221
COQ8A	0.721543459	0.60754196	0.668824581	0.685953876	0.855920132	0.952563377
LOC112441473	0.721543459	0.606343396	0.670239985	0.703717381	0.817950097	0.963015949
POLD1	0.72176039	0.614976364	0.978014482	0.754782628	0.738738532	0.690728863
DCAF15	0.721988047	0.582127335	0.805479206	0.852170016	0.682784145	0.881947456
EIF2AK3	0.721988485	0.589782216	0.652724437	0.947313672	0.935286646	0.648195749
NR2F6	0.722059586	0.847323359	0.841294863	0.692661174	0.820078109	0.668851588
LOC781710	0.722662706	0.878462725	0.669511234	0.668134034	0.946749073	0.669558041
C19H17orf53	0.722662706	0.790722217	0.843955813	0.738460631	0.66960879	0.816415069
LOC616903	0.722662706	0.573689402	0.791198027	0.904018489	0.6855631	0.848423144
AFAP1	0.722662706	0.60594617	0.641535145	0.895877524	0.801692604	0.867063767
ATP6V1A	0.722779092	0.582560314	0.776353067	0.881493849	0.658667583	0.891423889
XPC	0.723859639	0.565948458	0.716437951	0.999981514	0.684537031	0.727876258
PAQR3	0.723859639	0.568765648	0.677225445	0.899855526	0.895084325	0.76666104
NPEPL1	0.724005759	0.584209799	0.776418599	0.748424012	0.976259416	0.68185373
LOC112448518	0.724735467	0.636781917	0.677225445	0.916858019	0.911837627	0.669558041
IGSF9	0.724735467	0.676138557	0.854567571	0.808073654	0.738738532	0.783241453
RASGRP4	0.724735467	0.644366672	0.822300339	0.764257755	0.804625939	0.821372302
CCM2	0.725147133	0.817310492	0.69881939	0.949565453	0.680301694	0.684615686
LOC616948	0.725147133	0.58217015	0.862253703	0.719816078	0.935286646	0.722754623
PLSCR4	0.725147133	0.804185018	0.731708587	0.707217807	0.862665065	0.766218451
LOC112446691	0.725185825	0.637453144	0.881402575	0.826028128	0.721096295	0.774715551
LOC104974348	0.72551342	0.573498846	0.93190909	0.812933188	0.73021076	0.766218451
TSPYL1	0.72553073	0.590210355	0.943211357	0.808586366	0.784972475	0.700746926
GDI2	0.725680898	0.547676372	0.846372547	0.74577892	0.669123866	0.958281344
LOC781256	0.726256493	0.844279755	0.846099482	0.712481274	0.75602825	0.72886149
NDUFAF7	0.726256493	0.571983204	0.69621934	0.741475688	0.976259416	0.783241453
LOC789694	0.726264228	0.630038239	0.76555928	0.761799332	0.965676623	0.671759429
GET4	0.726264228	0.757515036	0.817582037	0.742465161	0.823508064	0.742309541
POLR2J	0.726264228	0.615769899	0.819359511	0.703663629	0.943610727	0.742309541
B4GALNT3	0.726264228	0.621713316	0.717242466	0.973075232	0.749858579	0.747009564
LRRN4	0.726264228	0.583275815	0.894338686	0.828736799	0.781159411	0.754218406
SIX1	0.726264228	0.833429351	0.717134809	0.876703811	0.659373888	0.774299863
KDM6A	0.726264228	0.692305416	0.693546993	0.708398401	0.947299702	0.783241453
SIGLEC15	0.726330528	0.582560314	0.892139418	0.845867148	0.849704586	0.669558041
RENBP	0.726330528	0.582127335	0.833583844	0.886312303	0.870042072	0.67453786
COA5	0.726330528	0.747553137	0.822300339	0.798687063	0.776537165	0.749726404

LOC112442613	0.726450627	0.573865937	0.80412682	0.729705452	0.970988226	0.702623424
MLIP	0.726506258	0.811354298	0.8644528	0.777956162	0.756284717	0.677125043
VMAC	0.726506258	0.62117273	0.79497298	0.893220599	0.799272579	0.748323122
LOC107131939	0.726506258	0.543708557	0.681700278	0.806573529	0.976292433	0.764310896
ATXN7L2	0.726506258	0.622818484	0.75029951	0.949565453	0.656015118	0.822427019
ALG14	0.726506258	0.592027146	0.839046961	0.777790625	0.796861564	0.846210629
TMEM209	0.726506258	0.54845263	0.737314964	0.688019411	0.801692604	0.973649216
GNA15	0.726527892	0.864668925	0.720612298	0.730052184	0.864524107	0.701800445
ZNF622	0.726527892	0.556778765	0.766724881	0.853058361	0.847519063	0.814218101
PTPRZ1	0.726597868	0.941817736	0.663738759	0.780184803	0.707939546	0.769865166
ST6GALNAC4	0.726597868	0.551109685	0.951392011	0.780184803	0.741086403	0.779201808
PTP4A1	0.726828777	0.54671968	0.96102707	0.764257755	0.670221463	0.836157024
RAD52	0.726828777	0.710815877	0.694535506	0.777956162	0.686224451	0.940583352
EMP2	0.726829475	0.714027042	0.768247414	0.760270895	0.942786245	0.666292389
GMEB2	0.726829475	0.764775882	0.744182994	0.755893058	0.901662258	0.704123433
ARID3B	0.726829475	0.635269571	0.648593816	0.728755498	0.999985752	0.706842017
MPHOSPH10	0.726829475	0.573498846	0.728269425	0.792140715	0.953885477	0.769865166
CYB5R2	0.726829475	0.872175143	0.66149866	0.799480811	0.777218526	0.78096075
LOC100335744	0.726829475	0.543645094	0.735532321	0.707217807	0.970988226	0.815387748
LOC101905593	0.726829475	0.571839975	0.685018711	0.789139053	0.935286646	0.839906041
CA4	0.726829475	0.660882273	0.700110354	0.674194099	0.855740473	0.925233445
NARS2	0.726829475	0.679897494	0.689992074	0.80459801	0.683310213	0.948685461
SEC13	0.727473642	0.835999607	0.817582037	0.794299784	0.795839201	0.657833313
ENY2	0.727474793	0.571839975	0.855268213	0.767989359	0.954403133	0.668851588
DYNC1I2	0.727531689	0.610421396	0.717242466	0.777956162	0.996781978	0.659761491
ASAP1	0.727531689	0.985997726	0.708274076	0.710070489	0.738738532	0.683495103
TTC22	0.727531689	0.608454581	0.744735369	0.873157737	0.896589592	0.725522874
LOC112443015	0.727531689	0.697306013	0.693546993	0.959480252	0.764945406	0.730477834
DLGAP1	0.727531689	0.62215202	0.771066142	0.907621547	0.814984338	0.733767439
TMEM131	0.727531689	0.553132278	0.657305905	0.892047793	0.952796169	0.748921056
SLC41A1	0.727531689	0.609457398	0.679415149	0.852170016	0.917407853	0.78087541
CALM1	0.727531689	0.606343396	0.858820898	0.838528071	0.760136746	0.79187813
TMEM251	0.727531689	0.584209799	0.784260981	0.948050281	0.690687154	0.805925428
CLK4	0.727531689	0.664859217	0.676501557	0.724175944	0.93630105	0.835461633
DPY19L1	0.727531689	0.662013091	0.76555928	0.75404116	0.762015885	0.899428912
GPNMB	0.727531689	0.583140563	0.731847616	0.892910741	0.657171354	0.923092436
TRMT6	0.727531689	0.550442307	0.793169467	0.751451145	0.68576536	0.972695131
NEU3	0.727531689	0.555277331	0.71967891	0.731537809	0.784972475	0.973413452

RNASE12	0.727570107	0.698963184	0.860179257	0.821450642	0.788007619	0.717538808
ACER1	0.72785409	0.830230945	0.867222363	0.673160239	0.721096295	0.78288746
AUTS2	0.727879069	0.587215076	0.705695072	0.764257755	0.995870928	0.702623424
COL6A2	0.727922819	0.704646482	0.778210036	0.898818046	0.814253177	0.669558041
PHACTR1	0.727922819	0.696525042	0.685018711	0.999981514	0.695436567	0.681807713
BAZ1A	0.727922819	0.582127335	0.845113191	0.716077462	0.965676623	0.689309487
ENPP4	0.727922819	0.767113995	0.886829776	0.792140715	0.738738532	0.700123604
HSD17B10	0.727922819	0.684972622	0.73826834	0.738687937	0.954403133	0.727632739
CHD1L	0.727922819	0.625469932	0.774015998	0.949502614	0.723387409	0.754218406
SAP18	0.727922819	0.62720176	0.916294854	0.731537809	0.797227609	0.774575102
ADA2	0.727922819	0.636781917	0.794846789	0.764257755	0.879776073	0.786849547
MCFD2	0.727922819	0.551281365	0.894338686	0.789086796	0.793430687	0.8255975
INSL6	0.727922819	0.602019792	0.749453648	0.68362796	0.949311988	0.83150957
GNPTG	0.727922819	0.649949716	0.845525761	0.676566861	0.848038632	0.839906041
ALMS1	0.727922819	0.616390046	0.758146087	0.843593618	0.759393281	0.865767172
CCRL2	0.727922819	0.691638455	0.711148626	0.808075336	0.788995893	0.872238439
COMMD6	0.727922819	0.602981016	0.720806436	0.722483146	0.891726429	0.882690335
LOC112449590	0.727922819	0.573498846	0.668824581	0.689227896	0.906893576	0.940017687
EFNA4	0.727922819	0.573498846	0.796135494	0.768290504	0.656015118	0.967759699
LOC784058	0.728008741	0.54845263	0.884710729	0.887983383	0.763224862	0.764985164
IRGQ	0.728745658	0.562520928	0.716635829	0.984423083	0.66960879	0.839906041
CWC15	0.729571143	0.550226079	0.916294854	0.764257755	0.659373888	0.903773776
LOC112443512	0.730407818	0.623984886	0.749764665	0.67498747	0.927049941	0.852504043
GIPC1	0.730886849	0.653683126	0.659919107	0.928404714	0.801692604	0.806476213
LOXL2	0.731232904	0.549085151	0.818612484	0.964521432	0.795839201	0.690728863
SUGCT	0.731232904	0.639920656	0.694535506	0.879344676	0.938565449	0.690728863
ZNF467	0.731232904	0.566864873	0.78057665	0.75404116	0.970988226	0.732191464
CARD10	0.731232904	0.584209799	0.992511421	0.727236849	0.696466814	0.769865166
PTPRC	0.731232904	0.582560314	0.882643407	0.692258797	0.883940132	0.801018631
POLA1	0.731267051	0.898550443	0.800422157	0.759591583	0.744684207	0.697619856
YWHAQ	0.731394373	0.663620961	0.731143569	0.764257755	0.972160227	0.683495103
SUMF2	0.731937068	0.846721805	0.855847948	0.716147042	0.812127322	0.669558041
SIRT1	0.731937068	0.709409381	0.753188445	0.761359847	0.950958352	0.68185373
DUSP15	0.731937068	0.638044641	0.775013999	0.840247324	0.911028086	0.690728863
CDCP1	0.731937068	0.553132278	0.771066142	0.972142185	0.809129327	0.702623424
LOC100337213	0.731937068	0.833429351	0.695127588	0.677545541	0.940870936	0.718567063
LOC101907648	0.731937068	0.857674524	0.726119774	0.833667906	0.770343524	0.721192619
CEBPB	0.731937068	0.553132278	0.671231078	0.712312845	0.999985752	0.742588975

RPGR	0.731937068	0.872175143	0.744735369	0.68362796	0.848550619	0.748750383
CD1D	0.731937068	0.657192178	0.694535506	0.925294859	0.832437297	0.749726404
DLG4	0.731937068	0.610520254	0.843955813	0.845114238	0.821557309	0.751100989
OTUD7A	0.731937068	0.553489554	0.861580887	0.839725231	0.84620147	0.759010664
CYSLTR2	0.731937068	0.758609923	0.663513753	0.95925588	0.707551381	0.761858515
UCP2	0.731937068	0.586798812	0.916294854	0.812453944	0.78021215	0.766534677
TRPM1	0.731937068	0.65172183	0.73091642	0.984331039	0.661674859	0.772618249
LOC787497	0.731937068	0.553132278	0.999993442	0.68918122	0.711891526	0.779201808
PTGDR2	0.731937068	0.560109317	0.740372388	0.907794002	0.847092933	0.783241453
LOC112445980	0.731937068	0.551459649	0.970427737	0.748711846	0.727256509	0.80383591
EGR1	0.731937068	0.722172907	0.80412682	0.799912445	0.764945406	0.813140661
PRPSAP1	0.731937068	0.579451739	0.671871883	0.961898258	0.795839201	0.81853698
CIP2A	0.731937068	0.692305416	0.709285034	0.941467375	0.658667583	0.835231445
GALNT15	0.731937068	0.558704912	0.79281326	0.825032424	0.810436875	0.860966325
FCGRT	0.731937068	0.596590801	0.772276679	0.891988949	0.705202026	0.867073248
MECR	0.731937068	0.707290221	0.700963878	0.731537809	0.855920132	0.872238439
FBXO46	0.731937068	0.571011361	0.829751489	0.808073654	0.735485629	0.902100676
PPEF1	0.731955076	0.708218805	0.826785415	0.677545541	0.823508064	0.842259463
MRPL44	0.732086996	0.61939332	0.731960288	0.819213899	0.886785729	0.811759324
CXCL14	0.732086996	0.648119635	0.695250624	0.764257755	0.674942708	0.982937529
UBE2V2	0.732167814	0.586194382	0.830420708	0.822035058	0.805226714	0.8255975
GBGT1	0.732428956	0.629418148	0.718840994	0.9063051	0.823508064	0.778344889
SYDE2	0.732428956	0.870298381	0.708019215	0.731537809	0.75602825	0.835231445
CTSO	0.732428956	0.775033799	0.70011973	0.819571278	0.765961853	0.839906041
SLC19A1	0.732428956	0.664859217	0.69687851	0.745577087	0.887514169	0.864197513
SLC6A2	0.732990644	0.759245424	0.78930905	0.690807874	0.921710688	0.721444677
RCBTB1	0.733323358	0.549834049	0.749417651	0.97828838	0.788007619	0.754218406
ZNF181	0.733330101	0.782783231	0.735064172	0.962046395	0.680696229	0.687639605
MTCP1	0.733330101	0.621279921	0.890697966	0.852170016	0.789492208	0.733767439
TMEM102	0.733330101	0.605920804	0.79497298	0.934466438	0.733218722	0.78087541
AQP11	0.733330101	0.686033606	0.930759114	0.727236849	0.733613832	0.783969061
LOC112447402	0.733330101	0.731024992	0.668824581	0.936631044	0.691315145	0.832201748
ZSCAN26	0.733330101	0.7070747	0.836494447	0.819213899	0.659596244	0.848298915
FBXO28	0.733711387	0.625168319	0.92271578	0.866566721	0.788007619	0.666011114
PDK3	0.733711387	0.768359345	0.860877703	0.751451145	0.725663367	0.789870764
ATAT1	0.733711387	0.598603907	0.68898955	0.896116841	0.854289643	0.81956221
NXT2	0.733711387	0.711166977	0.734143952	0.918693944	0.66101983	0.831176431
RBL2	0.733832968	0.598407024	0.681669526	0.912777307	0.954403133	0.669090959

CCNB2	0.733832968	0.61939332	0.777373522	0.902204742	0.823508064	0.742309541
LOC112446481	0.733832968	0.620036149	0.749453648	0.753267542	0.666917776	0.9790177
FTL	0.73434444	0.621713316	0.810167226	0.93048026	0.795839201	0.706176041
LOC112444904	0.73434444	0.572313942	0.728269425	0.701248463	0.741332772	0.995906915
FOXP2	0.734408098	0.796253817	0.704680319	0.738460631	0.845387518	0.81956221
NUDT14	0.734815963	0.929807858	0.696778723	0.705448435	0.87329316	0.684615686
LOC112447459	0.73497769	0.865570309	0.744735369	0.805568606	0.823508064	0.67453786
KPNA6	0.73497769	0.550226079	0.77566312	0.999981514	0.711090681	0.704351773
ADGRG6	0.73497769	0.897183343	0.805479206	0.748837705	0.72350412	0.739151902
RANBP1	0.73497769	0.708031655	0.874983674	0.723302519	0.705202026	0.863020449
HSPE1	0.735109712	0.628205932	0.751876894	0.758601772	0.80587765	0.911219114
REEP3	0.735341244	0.664911321	0.894671468	0.782811812	0.81911207	0.730527465
SHCBP1L	0.735341244	0.816129287	0.903150496	0.712481274	0.687203978	0.765948605
RTL5	0.735353655	0.691301661	0.68518078	0.969088992	0.804625939	0.700746926
CLASP2	0.735353655	0.606343396	0.717242466	0.817230639	0.953300742	0.754218406
FXR1	0.73561949	0.664104209	0.669511234	0.799147532	0.864524107	0.876162019
LOC781218	0.735843465	0.573512091	0.976484937	0.852365348	0.696466814	0.706704597
LOC101903678	0.736135497	0.679199291	0.972897558	0.731537809	0.756274174	0.700123604
PIK3C2A	0.736135497	0.610379943	0.660807263	0.877652482	0.943160494	0.759003017
PACSIN2	0.736135497	0.573498846	0.743624166	0.935958061	0.781724458	0.827870367
OAS1X	0.736135497	0.62542886	0.660365588	0.751045386	0.936215792	0.867063767
VPS35	0.736135497	0.632321074	0.779581461	0.839632707	0.668508052	0.914655402
NOC4L	0.736135497	0.692305416	0.761415057	0.764257755	0.696466814	0.928976746
PAPD4	0.736462764	0.899490486	0.736435559	0.69158245	0.894364008	0.669090959
CHKB	0.736462764	0.731850993	0.717242466	0.934959901	0.817472827	0.68185373
TUBB4A	0.736682196	0.638842915	0.685018711	0.731537809	0.935737262	0.858415238
USP34	0.736774184	0.675765841	0.717242466	0.715394268	0.991671024	0.700123604
LOC112441525	0.736774184	0.571559213	0.71601562	0.97828838	0.789915129	0.77803403
PDIA5	0.736948863	0.573865937	0.829751489	0.953401589	0.823508064	0.666292389
CMTM8	0.736948863	0.692463931	0.66355596	0.736520443	0.987516261	0.733767439
LOC531038	0.736948863	0.792624847	0.773165287	0.833011444	0.696466814	0.802390255
SETDB1	0.736948863	0.57439391	0.808037655	0.899855526	0.690633636	0.867073248
OST4	0.737653989	0.614805655	0.884121008	0.809277776	0.900891209	0.669558041
GNA13	0.737653989	0.551850362	0.885896294	0.994965893	0.665098448	0.68185373
GJC2	0.737653989	0.716596673	0.716635829	0.761251529	0.967116582	0.690728863
LOC104974667	0.737653989	0.722968232	0.684443377	0.999981514	0.687203978	0.695065923
RPS6KL1	0.737653989	0.710645465	0.730571184	0.687291299	0.982087479	0.712495864
LOC112441887	0.737653989	0.580003885	0.700963878	0.822691957	0.971117951	0.743301322

KIAA0825	0.737653989	0.792483444	0.770538618	0.886312303	0.710664679	0.750967073
OPA3	0.737653989	0.610421396	0.700349135	0.806898312	0.960190226	0.765948605
LCTL	0.737653989	0.582127335	0.754193103	0.853058361	0.900891209	0.77803403
LOC107131843	0.737653989	0.565257607	0.763521949	0.731537809	0.970988226	0.779201808
NLK	0.737653989	0.574215245	0.91469924	0.834401906	0.753088486	0.785999833
WASF2	0.737653989	0.791281103	0.684282335	0.894625439	0.734324024	0.79363949
CCND2	0.737653989	0.594199197	0.69621934	0.876703811	0.879395665	0.830162774
EPS8L2	0.737653989	0.559306577	0.860877703	0.848592907	0.695104667	0.882690335
C7H19orf24	0.737653989	0.806240543	0.693546993	0.687291299	0.72527924	0.940256832
RAB3A	0.738199082	0.600246997	0.827062418	0.88904348	0.821148296	0.75062693
HECTD1	0.738255918	0.648503747	0.749417651	0.982220679	0.797489438	0.668851588
KDELC1	0.738255918	0.610128989	0.776353067	0.98981682	0.771915356	0.675697431
PTPMT1	0.738255918	0.589754585	0.997300234	0.700026235	0.797227609	0.702623424
TCTEX1D2	0.738255918	0.688237052	0.959725886	0.681899077	0.806034958	0.727876258
LAMTOR1	0.738255918	0.659408339	0.771066142	0.784012709	0.927086179	0.748867818
PMM2	0.738255918	0.767113995	0.822898819	0.718071196	0.86010579	0.749918186
NDUFAF3	0.738255918	0.571528531	0.860877703	0.716147042	0.950855218	0.752070741
ANKMY2	0.738255918	0.683744977	0.749417651	0.799147532	0.881680572	0.789870764
YWHAB	0.738255918	0.582127335	0.781106602	0.931203167	0.711090681	0.840271658
ENAH	0.738255918	0.60689126	0.658489624	0.8418384	0.893716526	0.858321732
PAX5	0.738255918	0.624142952	0.855268213	0.853058361	0.669123866	0.862925606
NPR2	0.738255918	0.573711724	0.794676674	0.769305101	0.837915327	0.887722423
NR2C2AP	0.738255918	0.738007931	0.774015998	0.769701093	0.678703442	0.91373422
LRPPRC	0.738255918	0.589754585	0.751957722	0.698882151	0.804037558	0.963456717
STC1	0.738269864	0.937448751	0.8644528	0.712025145	0.670221463	0.710630057
DBN1	0.738309527	0.720673049	0.668824581	0.988408846	0.784259667	0.690728863
LOC107132853	0.738309527	0.599075208	0.684366528	0.717878052	0.855939488	0.960023723
RBPMS	0.738738501	0.624373062	0.775013999	0.764140634	0.776537165	0.923944552
LOC100847870	0.738740733	0.777309385	0.829421247	0.764140634	0.862219803	0.699160709
LOC112446127	0.739038593	0.61939332	0.796499368	0.882057818	0.887514169	0.702623424
MANSC1	0.739038593	0.676397431	0.775552434	0.947574232	0.776537165	0.710630057
PDCL3	0.739038593	0.61939332	0.937334712	0.901353354	0.670221463	0.711010694
NDUFA9	0.739038593	0.59263018	0.754913805	0.711837881	0.977167046	0.768168974
UPK1B	0.739038593	0.716415149	0.77566312	0.887877413	0.764945406	0.769865166
LOC784148	0.739038593	0.571983204	0.682947788	0.985701713	0.78773451	0.806476213
TRIP13	0.739038593	0.610733542	0.916968376	0.738687937	0.763170536	0.839565788
TBC1D8	0.739038593	0.766943819	0.774015998	0.774214837	0.715674124	0.871998318
LIN52	0.739038593	0.568485668	0.922552585	0.739273899	0.712053021	0.891088733

PPP2R3C	0.739038593	0.561716964	0.726119774	0.764257755	0.768005485	0.9803583
RAB2B	0.739322521	0.824188473	0.95322206	0.695681179	0.699191343	0.69516836
SLC8A2	0.739322521	0.811354298	0.759332589	0.712606346	0.855920132	0.77593589
SNX11	0.739322521	0.600886305	0.806783121	0.707217807	0.887514169	0.866494771
LRIG3	0.739621874	0.839128192	0.668824581	0.896116841	0.753088486	0.75807902
YPEL5	0.739621874	0.582247952	0.831933739	0.762598894	0.782443723	0.915303189
LOC100847708	0.739643784	0.605401983	0.753895435	0.892071982	0.757419334	0.864139695
CGREF1	0.73987819	0.934045162	0.741334388	0.865076519	0.682109555	0.684994779
PHF14	0.73987819	0.614976364	0.702807255	0.999981514	0.687203978	0.716925595
LOC104972216	0.73987819	0.624744876	0.862093582	0.889415593	0.798443817	0.729499398
GSTT1	0.73987819	0.958639992	0.669663317	0.696501426	0.847092933	0.729499398
PARD3B	0.73987819	0.658075243	0.879939804	0.728264598	0.858741373	0.77803403
PRR16	0.73987819	0.798589766	0.77566312	0.779773412	0.792867703	0.783241453
NLRC4	0.73987819	0.555688032	0.839046961	0.805302469	0.887514169	0.797679046
NOL6	0.73987819	0.587350756	0.916294854	0.731537809	0.680301694	0.91373422
GAPDH	0.740001533	0.644132227	0.804171507	0.753267542	0.879776073	0.824780045
STARD5	0.74004451	0.74241072	0.711148626	0.843335547	0.933183898	0.669558041
RUNDC3A	0.74022237	0.781543413	0.745348234	0.788323571	0.901662258	0.700123604
GTPBP10	0.740341159	0.81518533	0.83252813	0.709098739	0.87329316	0.690728863
ABHD17A	0.740341159	0.606343396	0.749140224	0.815888375	0.764945406	0.927426708
DPY30	0.740951511	0.581251143	0.999993442	0.771412309	0.671669027	0.684615686
MYBPC2	0.741195128	0.72742557	0.799533361	0.886312303	0.741541989	0.764395168
EPC2	0.741205795	0.883458323	0.744735369	0.689227896	0.872096228	0.740843731
DESI1	0.741205795	0.750466772	0.774015998	0.799480811	0.788007619	0.828124017
VDAC3	0.741205795	0.582051417	0.829455601	0.710070489	0.841307824	0.90645885
SAMM50	0.741205795	0.705008978	0.764467593	0.805568606	0.691315145	0.91373422
MPI	0.741330165	0.589754585	0.955427429	0.745806541	0.833186643	0.737726964
TAF8	0.741330165	0.696797615	0.715158198	0.972142185	0.707939546	0.769865166
LPXN	0.741330165	0.630928576	0.80412682	0.717645144	0.939516498	0.782330566
BCKDHB	0.741330165	0.621713316	0.745477682	0.723642816	0.857562998	0.917146354
PPP4R2	0.741541179	0.573498846	0.671469816	0.853058361	0.973219333	0.754218406
SELENBP1	0.742610801	0.837244723	0.722770256	0.705448435	0.954071324	0.672418631
SPHK2	0.742610801	0.77844414	0.755940516	0.75404116	0.911930323	0.717538808
GCSAML	0.742610801	0.576461978	0.826785415	0.96951401	0.7744799	0.720022925
GPR146	0.742610801	0.926049158	0.822109641	0.731537809	0.695436567	0.749726404
PLPP7	0.742610801	0.7889129	0.700128649	0.815919419	0.79669563	0.835231445
ABCG2	0.742610801	0.580322769	0.915407439	0.828736799	0.69314679	0.847193872
ROR1	0.742610801	0.846724749	0.682166552	0.806573529	0.738738532	0.851995112

SUMO1	0.743565925	0.582127335	0.893427844	0.727236849	0.829280764	0.851325852
CD1B	0.744033929	0.684946765	0.99615564	0.752081152	0.71077874	0.688206116
FYB1	0.744033929	0.600886305	0.841294863	0.730052184	0.82114467	0.888475311
ZFC3H1	0.74431679	0.604324442	0.885896294	0.764140634	0.903888186	0.737726964
HTRA3	0.744507306	0.681192035	0.796135494	0.907353815	0.735361993	0.783241453
APOO	0.744507306	0.580111602	0.772276679	0.695561237	0.879776073	0.923807852
TBC1D14	0.744599191	0.658496391	0.663513753	0.801848398	0.85185197	0.91373422
EHD1	0.745374792	0.636781917	0.920681987	0.82868625	0.765965392	0.748750383
VPS29	0.745374792	0.573498846	0.885896294	0.78584336	0.674089143	0.926480924
LOC100336368	0.745494485	0.699211784	0.667782878	0.999981514	0.693984901	0.684524441
RADIL	0.745494485	0.676397431	0.899793024	0.848086379	0.776537165	0.726512102
BMS1	0.745494485	0.750466772	0.682700098	0.825279787	0.930451229	0.727876258
LOC511936	0.745494485	0.976596938	0.676501557	0.727236849	0.797127163	0.741319465
ACSM5	0.745494485	0.598069728	0.817582037	0.910575108	0.823508064	0.742456493
METTL13	0.745494485	0.757078435	0.736435559	0.819213899	0.870042072	0.754218406
GINS1	0.745494485	0.573711724	0.727520588	0.727236849	0.999453276	0.764260585
ZNF280B	0.745494485	0.748625319	0.694535506	0.731537809	0.916877832	0.81956221
TRMT5	0.745494485	0.692305416	0.854571175	0.730052184	0.772569315	0.867063767
LOC100848324	0.745494485	0.615769899	0.732480837	0.839725231	0.796861564	0.900648457
PLOD3	0.745494485	0.836427846	0.705746499	0.759902002	0.695436567	0.907152267
FAM229B	0.746346227	0.744671791	0.679506848	0.82868625	0.80911802	0.860966325
UPF2	0.746690382	0.589754585	0.741510374	0.780599386	0.976769047	0.748921056
TMEM25	0.746972489	0.692305416	0.842387629	0.887983383	0.826634148	0.680741064
C2H2orf69	0.746972489	0.70880006	0.818612484	0.887642126	0.821351993	0.699160709
WWC1	0.746972489	0.608465508	0.938011449	0.764257755	0.847173934	0.737726964
NOS2	0.746972489	0.735827981	0.673165093	0.78584336	0.959251971	0.748323122
LOC101902768	0.746972489	0.598069728	0.744735369	0.999981514	0.67113613	0.762133815
ASL	0.746972489	0.865101561	0.730248597	0.818504791	0.766100834	0.772618249
GFER	0.746972489	0.790429889	0.810647419	0.745806541	0.789915129	0.815129379
ADNP	0.746972489	0.692305416	0.668824581	0.812453944	0.90300429	0.835231445
TMEM33	0.746972489	0.602981016	0.895801611	0.76871084	0.774289699	0.855349987
GYPC	0.746972489	0.647890563	0.746661392	0.812453944	0.791220688	0.906637848
LRRN3	0.746972489	0.592365586	0.879608555	0.799147532	0.696466814	0.907124813
PRRG3	0.746972489	0.565421383	0.679415149	0.746434518	0.919166785	0.930710695
AK8	0.746972489	0.609209091	0.819359511	0.728264598	0.711809801	0.965456017
HERC6	0.747034835	0.822956796	0.872088608	0.689227896	0.858179728	0.690728863
PACRGL	0.747166578	0.60754196	0.746661392	0.777956162	0.787234817	0.94844234
GRK3	0.747336617	0.622449596	0.71967891	0.910575108	0.741332772	0.876162019

ACTRT3	0.747413609	0.973712113	0.67171984	0.703717381	0.878451408	0.675140237
HOMEZ	0.747413609	0.622818484	0.995377403	0.812453944	0.721824417	0.677125043
QPCT	0.747413609	0.687651905	0.973562292	0.830187312	0.687203978	0.68392363
LOC530102	0.747413609	0.693513972	0.830135741	0.724431807	0.958797242	0.686077455
TMEM258	0.747413609	0.768359345	0.966911424	0.746253306	0.716805303	0.690728863
TIMM22	0.747413609	0.630928576	0.79497298	0.819213899	0.953300742	0.690728863
STK24	0.747413609	0.658520696	0.761738351	0.943808491	0.823508064	0.702623424
LRIG1	0.747413609	0.590368852	0.926161087	0.874170259	0.797227609	0.706869246
KLHL15	0.747413609	0.643417752	0.716437951	0.729724066	0.999723274	0.724693895
JAM2	0.747413609	0.75769517	0.822300339	0.884452623	0.763224862	0.733366979
SEMA6A	0.747413609	0.653179887	0.77566312	0.87522058	0.883117333	0.737726964
S100A16	0.747413609	0.594126609	0.791843351	0.876703811	0.903410824	0.739270623
APOBR	0.747413609	0.973321075	0.791843351	0.708604007	0.688556938	0.748323122
SMPDL3A	0.747413609	0.605377203	0.999993442	0.731537809	0.677965402	0.748921056
PPP2CA	0.747413609	0.579659644	0.981301073	0.799147532	0.735361993	0.75640676
LOC100335608	0.747413609	0.742703566	0.797180963	0.812453944	0.84367638	0.75640676
TP63	0.747413609	0.71189217	0.749417651	0.784595865	0.910290884	0.766218451
RAVER2	0.747413609	0.717684248	0.758146087	0.769701093	0.906766758	0.769865166
DCTN6	0.747413609	0.586194382	0.921266625	0.889472032	0.701123622	0.77803403
APOLD1	0.747413609	0.571011361	0.730571184	0.999981514	0.735091896	0.778344889
LOC101902449	0.747413609	0.573498846	0.734143952	0.892071982	0.89052284	0.801888363
MLLT3	0.747413609	0.727791766	0.819359511	0.730553988	0.848038632	0.811759324
ASMTL	0.747413609	0.671536686	0.759332589	0.745806541	0.907455286	0.827832928
LOC112442218	0.747413609	0.709247882	0.744735369	0.816337428	0.832407979	0.83150957
CSPP1	0.747413609	0.589754585	0.8644528	0.80459801	0.772501291	0.876162019
EMC8	0.747413609	0.687020192	0.874063521	0.724431807	0.729180196	0.889406298
LMX1B	0.747413609	0.628205932	0.867222363	0.786914837	0.691696983	0.911888512
MYF6	0.747413609	0.863286109	0.679415149	0.688019411	0.75602825	0.923064357
ERAS	0.747413609	0.575351685	0.730571184	0.716147042	0.900927045	0.927520621
GNG10	0.747413609	0.565421383	0.852214402	0.822691957	0.691315145	0.927900405
SMKR1	0.747413609	0.584209799	0.885896294	0.700026235	0.695436567	0.960023723
LOC101902851	0.747413609	0.60594617	0.77699301	0.693458387	0.804625939	0.960023723
SLC25A43	0.747418106	0.697043768	0.828935179	0.940438437	0.735091896	0.711922168
PEX16	0.747418106	0.644366672	0.809673119	0.741170082	0.916605572	0.798336045
FGF1	0.747457373	0.936521326	0.830420708	0.817230639	0.67482152	0.673985072
ITIH4	0.747457373	0.919201618	0.75669748	0.842084202	0.680301694	0.737726964
TRIM38	0.747490072	0.814177827	0.668824581	0.921950515	0.721096295	0.792374188
HRC	0.747517792	0.573498846	0.732480837	0.999981514	0.735091896	0.754218406

NAP1L1	0.747517792	0.895777642	0.754557912	0.747502044	0.804037558	0.754218406
MB21D2	0.747517792	0.696358351	0.682700098	0.828736799	0.912033904	0.793197037
NAV2	0.747517792	0.614805655	0.668824581	0.984421126	0.74965664	0.839906041
FANCC	0.747517792	0.671442847	0.711148626	0.852170016	0.759888741	0.91348389
CNNM4	0.747755236	0.788980101	0.716120406	0.896508582	0.864524107	0.674519254
MAP3K10	0.747755236	0.901984573	0.736435559	0.762598894	0.873957915	0.675688253
HAPLN3	0.747755236	0.650567453	0.680262176	0.999981514	0.738738532	0.684994779
CIT	0.747755236	0.657192178	0.867222363	0.959511917	0.695436567	0.706869246
JARID2	0.747755236	0.565257607	0.949939862	0.757577698	0.887514169	0.721192619
EIPR1	0.747755236	0.725636614	0.851822885	0.695125147	0.896490594	0.759003017
UBXN11	0.747755236	0.76003522	0.679415149	0.943447418	0.788007619	0.762657772
TSPEAR	0.747755236	0.744774335	0.942357321	0.711415619	0.741086403	0.770035496
ZNF446	0.747755236	0.614976364	0.747666034	0.819968583	0.938316699	0.774575102
TDRD10	0.747755236	0.800193122	0.777504633	0.882057818	0.682784145	0.79187813
RPS6KA5	0.747755236	0.741263262	0.749417651	0.746075358	0.891726429	0.804551322
SLC7A11	0.747755236	0.644366672	0.843514405	0.911258863	0.677185022	0.827870367
MGC148714	0.747755236	0.565953284	0.754557912	0.695108693	0.979759416	0.832964812
LRRFIP2	0.747755236	0.781543413	0.8644528	0.724431807	0.721826916	0.847193872
CCDC136	0.747755236	0.580322769	0.796738707	0.929663263	0.723467697	0.850475013
ZC3H14	0.747755236	0.587350756	0.744735369	0.812453944	0.799272579	0.93363377
PSMD2	0.747755236	0.582127335	0.759332589	0.763977006	0.707939546	0.988639786
SPATA24	0.748454112	0.672596838	0.943028511	0.877455139	0.720896795	0.690728863
SYT2	0.748551594	0.573865937	0.806651923	0.999981514	0.75925	0.700123604
ZCCHC6	0.748551594	0.620171743	0.831933739	0.830289573	0.900747635	0.739270623
LOC112447433	0.748551594	0.902525869	0.754557912	0.856195359	0.687203978	0.748323122
NUCB2	0.748551594	0.573711724	0.73027251	0.980564765	0.834569503	0.748921056
ECHDC1	0.748551594	0.786442687	0.820959147	0.760372943	0.810436875	0.78288746
FOXN2	0.748551594	0.654971251	0.892343042	0.760230321	0.804264617	0.824976535
HACD4	0.748761002	0.741310853	0.885896294	0.695681179	0.887514169	0.724693895
ATP13A4	0.748761002	0.722779653	0.711148626	0.952247001	0.721096295	0.799889245
NAA15	0.748775722	0.615769899	0.870734949	0.847997885	0.753088486	0.837559792
TANC1	0.748921232	0.610421396	0.768247414	0.695125147	0.999985752	0.695065923
BOLA1	0.748921232	0.846724749	0.855268213	0.734472882	0.795839201	0.737726964
DDX46	0.748921232	0.722690977	0.711148626	0.815919419	0.924795709	0.759003017
WFDC1	0.748953735	0.750379179	0.731143569	0.882057818	0.738738532	0.840768468
KCNB1	0.748957958	0.709137178	0.671003058	0.955981799	0.826634148	0.748921056
HPGD	0.749086052	0.579451739	0.754979443	0.812453944	0.958797242	0.775725294
SPSB1	0.749327491	0.7889129	0.884710729	0.806573529	0.710664679	0.764310896

PEX5	0.749327491	0.704801601	0.853546772	0.812313105	0.687203978	0.87100843
PHF24	0.749658741	0.786691027	0.736160515	0.804862068	0.687109055	0.911638347
ATAD2B	0.749934617	0.60594617	0.744735369	0.777956162	0.999453276	0.68781527
LOC104971162	0.749934617	0.624373062	0.954020369	0.80966716	0.781724458	0.742455792
ADGRL1	0.749934617	0.855067335	0.685620658	0.916858019	0.735091896	0.748867818
UPF3B	0.749934617	0.582127335	0.832192335	0.95837151	0.721096295	0.791354446
SMPX	0.749934617	0.788544942	0.829137153	0.757544248	0.723054375	0.85532952
LOC100847345	0.749934617	0.839128192	0.688925042	0.707217807	0.804625939	0.890862406
PHB	0.749934617	0.628736003	0.808785521	0.765499767	0.784972475	0.923092436
IFIT2	0.750276924	0.94651182	0.69621934	0.707217807	0.864524107	0.729319161
LOC104969670	0.750276924	0.938235006	0.770295064	0.775617102	0.738738532	0.734177434
C17H12orf65	0.750276924	0.741310853	0.832268748	0.703717381	0.894555985	0.768572658
SDF2L1	0.750283063	0.781543413	0.793169467	0.786099236	0.816045604	0.788462021
KLHL23	0.750283063	0.980521483	0.696535103	0.731537809	0.680594277	0.816415069
HPCAL1	0.75040829	0.589455549	0.774015998	0.80459801	0.887514169	0.855545794
NRP2	0.750605415	0.60594617	0.984886031	0.815919419	0.721096295	0.739579536
MRPL17	0.750605415	0.659408339	0.922552585	0.820971736	0.69149571	0.818021392
RETREG3	0.750605415	0.714088981	0.683693005	0.699414374	0.771532192	0.98140846
SAG	0.750670052	0.686805478	0.670239985	0.796125318	0.999985752	0.67453786
ARHGEF10L	0.750670052	0.822956796	0.805270634	0.805138513	0.856136977	0.68185373
LOC107131331	0.750670052	0.781836924	0.849389234	0.889415593	0.73254131	0.707127136
RPUSD2	0.750670052	0.634752242	0.735916998	0.88097449	0.942786245	0.720865143
LOC101902366	0.750670052	0.589754585	0.849704325	0.964521432	0.772501291	0.721192619
MEST	0.750670052	0.781543413	0.754913805	0.796128007	0.887514169	0.748660929
ZFR	0.750670052	0.575241982	0.892343042	0.863508746	0.804037558	0.788462021
ARHGAP30	0.750670052	0.661031998	0.866497255	0.710190773	0.880672837	0.816246746
ZFYVE27	0.750785201	0.856868887	0.716635829	0.862651416	0.764945406	0.770035496
SMIM11A	0.751095797	0.767737619	0.879939804	0.76992117	0.86587625	0.679803128
XAF1	0.751095797	0.713956321	0.916323169	0.707217807	0.901144467	0.681450813
CBLL1	0.751095797	0.99960614	0.737528643	0.734472882	0.6855631	0.68185373
TSPYL4	0.751095797	0.985594367	0.711148626	0.808073654	0.735091896	0.68185373
ANKRD33B	0.751095797	0.65159362	0.89385865	0.937654716	0.739593742	0.688206116
COL16A1	0.751095797	0.663152438	0.913400043	0.887983383	0.782769176	0.690728863
CBR1	0.751095797	0.759245424	0.690093381	0.886312303	0.911837627	0.697356941
SELENON	0.751095797	0.647999098	0.93475587	0.903564774	0.716994185	0.699720332
S100A2	0.751095797	0.880129816	0.849389234	0.80459801	0.738811608	0.700123604
TLL1	0.751095797	0.865101561	0.929661805	0.710190773	0.721096295	0.706842017
DTX3L	0.751095797	0.824188473	0.841294863	0.731537809	0.84620147	0.731474118

PIK3CA	0.751095797	0.704512234	0.764467593	0.796128007	0.934813966	0.742588975
TRIM24	0.751095797	0.744774335	0.685620658	0.857420777	0.910231534	0.748921056
LOC100295687	0.751095797	0.895395302	0.820959147	0.712481274	0.788007619	0.75640676
EIF3E	0.751095797	0.961625046	0.696778723	0.751451145	0.766507736	0.7765939
LVRN	0.751095797	0.694400411	0.79497298	0.741170082	0.927086179	0.778302445
AFF2	0.751095797	0.806436265	0.89399247	0.736045965	0.741086403	0.778344889
VPS9D1	0.751095797	0.572077017	0.791843351	0.947574232	0.811991102	0.780585188
AIF1	0.751095797	0.583275815	0.810647419	0.764257755	0.944839728	0.803177592
PDE10A	0.751095797	0.621713316	0.860877703	0.873956209	0.762447693	0.82345874
LOC509810	0.751095797	0.599075208	0.860877703	0.767989359	0.871558258	0.835231445
LOC112441478	0.751095797	0.602019792	0.901736458	0.825279787	0.741086403	0.841312694
PSMB5	0.751095797	0.694148907	0.813441395	0.722996827	0.859672963	0.853548296
ANXA1	0.751095797	0.657192178	0.734143952	0.898818046	0.788007619	0.8537109
MCF2L	0.751095797	0.60754196	0.804996461	0.9063051	0.738333933	0.853735179
LOC112443431	0.751095797	0.885313051	0.689992074	0.797338497	0.725950216	0.858507498
SSH1	0.751095797	0.65172183	0.923521499	0.710070489	0.764945406	0.862925606
SRRM3	0.751095797	0.643640444	0.70011973	0.934445963	0.771893039	0.862925606
TSC22D4	0.751095797	0.796882729	0.702256337	0.710190773	0.872096228	0.862925606
SFR1	0.751095797	0.664439271	0.754913805	0.896116841	0.705202026	0.88313584
CAMSAP3	0.751095797	0.785346067	0.705746499	0.782238761	0.773264077	0.902100676
LOC112442967	0.751095797	0.571839975	0.732092376	0.888123511	0.809317773	0.903278153
ZNF154	0.751095797	0.792109226	0.755940516	0.757403199	0.687691369	0.923547711
ZNF214	0.751095797	0.692305416	0.754913805	0.777956162	0.772501291	0.923944552
TMEM51	0.751095797	0.796253817	0.704088819	0.757403199	0.695436567	0.948705062
STMN1	0.751095797	0.649340041	0.692020726	0.804472003	0.723467697	0.9802309
CSNK1E	0.751095797	0.572077017	0.670070271	0.695125147	0.764945406	0.999948228
APLNR	0.751508569	0.62720176	0.744735369	0.893220599	0.954403133	0.677355984
MCAT	0.751508569	0.741310853	0.731143569	0.925294859	0.869108362	0.677365619
ERAP1	0.751508569	0.94043655	0.826269935	0.733411985	0.788850323	0.68185373
RGCC	0.751508569	0.671536686	0.805479206	0.994395954	0.723467697	0.683230524
FBXO31	0.751508569	0.908364002	0.75197571	0.815919419	0.804037558	0.690728863
HINT2	0.751508569	0.687020192	0.903150496	0.820971736	0.844384115	0.695352244
NUFIP2	0.751508569	0.634059237	0.698295081	0.728264598	0.999985752	0.699160709
MESD	0.751508569	0.691301661	0.895428817	0.930164453	0.696466814	0.700746926
ATF1	0.751508569	0.595982761	0.696802779	0.729724066	0.999985752	0.703769019
ZNF585A	0.751508569	0.629312112	0.774015998	0.999981514	0.706936135	0.727876258
LOC101906588	0.751508569	0.767882792	0.69621934	0.964521432	0.776857944	0.732191464
GPR75	0.751508569	0.581074652	0.917155067	0.731537809	0.927086179	0.743720206

L1CAM	0.751508569	0.89465829	0.745479541	0.876949026	0.696466814	0.748921056
PXMP2	0.751508569	0.624804325	0.728670337	0.801848398	0.935286646	0.835231445
LOC100295750	0.751508569	0.580530872	0.923578245	0.695125147	0.864524107	0.839573661
SPART	0.751508569	0.657192178	0.731960288	0.952247001	0.723054375	0.842980036
PLEKHF2	0.751508569	0.64661775	0.794676674	0.927802946	0.695528035	0.847193872
ANG2	0.751508569	0.599021986	0.990974506	0.708604007	0.690058599	0.850475013
LOC112446756	0.751508569	0.600004269	0.713592297	0.813323291	0.895757462	0.879576441
LOC509415	0.751508569	0.687020192	0.744735369	0.896116841	0.706302033	0.883688554
MYBBP1A	0.751508569	0.589782216	0.776160956	0.886312303	0.764945406	0.893838339
LOC522610	0.751508569	0.720426741	0.719862199	0.816337428	0.683310213	0.951493662
COPS6	0.751600634	0.60754196	0.916294854	0.756729528	0.821148296	0.828124017
CCSER1	0.751763516	0.819439092	0.871285807	0.777956162	0.810436875	0.700123604
HMGB2	0.751763516	0.753090681	0.749417651	0.907794002	0.755086175	0.784754767
TIPRL	0.751763516	0.572077017	0.946791586	0.805499732	0.71767272	0.85532952
LOC107133095	0.751984216	0.796253817	0.8735515	0.767989359	0.821148296	0.720865143
COL13A1	0.751984216	0.648503747	0.855793459	0.840616458	0.863651062	0.748921056
ANKRD12	0.751984216	0.635148479	0.794676674	0.703717381	0.965676623	0.785562356
RARRES1	0.751984216	0.613273559	0.78057665	0.912777307	0.707939546	0.87754233
SOX7	0.752039265	0.842884359	0.700310857	0.744153611	0.959443572	0.682478463
SLC9A5	0.752039265	0.573498846	0.866497255	0.943669023	0.721096295	0.803177592
ETNK2	0.752039265	0.636937576	0.731708587	0.940438437	0.794743818	0.83150957
LOC104969611	0.752039265	0.766595431	0.688864545	0.854966424	0.804274107	0.850475013
PABPC1	0.752039265	0.618921732	0.791198027	0.822691957	0.816306826	0.877048993
HSPB8	0.752039265	0.846724749	0.768247414	0.717645144	0.701502867	0.900219465
FAM92A	0.75254264	0.575241982	0.937812682	0.798687063	0.714642011	0.872238439
GMDS	0.752699029	0.847093186	0.688565918	0.727950912	0.84620147	0.857911901
UBASH3B	0.753283055	0.679189456	0.871491704	0.764257755	0.84620147	0.806476213
EGR2	0.753534207	0.844170799	0.819359511	0.748837705	0.853250878	0.721192619
CNOT11	0.753534207	0.594126609	0.955427429	0.853058361	0.75602825	0.749726404
WAC	0.753534207	0.607084892	0.735916998	0.727236849	0.999985752	0.749918186
UBE2M	0.754677831	0.770126217	0.893761108	0.774800098	0.75602825	0.781016584
SRPX2	0.754677831	0.692305416	0.69687851	0.96968491	0.750542783	0.807200313
ID2	0.754677831	0.655096367	0.68255592	0.97731816	0.786314903	0.815129379
ARL8A	0.754748413	0.767113995	0.865512879	0.916858019	0.716994185	0.684994779
COPB1	0.754748413	0.72828303	0.97773026	0.742989856	0.727256509	0.739579536
LOC104971345	0.754748413	0.630477275	0.957052938	0.812160271	0.714597718	0.795752855
MERTK	0.754748413	0.582127335	0.854571175	0.767989359	0.89052284	0.839906041
ETV2	0.754748413	0.664104209	0.736435559	0.840247324	0.865076851	0.844759721

LOC107132121	0.754876364	0.682216234	0.745477682	0.731537809	0.87329316	0.900219465
RPRD1B	0.754877617	0.649678141	0.846372547	0.876121618	0.872734644	0.721192619
MRPL24	0.754877617	0.691301661	0.754193103	0.790889911	0.852959789	0.867635709
ZMYM4	0.754882889	0.919171954	0.694535506	0.719816078	0.934825476	0.684994779
RBPJ	0.754882889	0.901631302	0.862602899	0.715225048	0.804625939	0.698787824
RACGAP1	0.754882889	0.794937711	0.761415057	0.733411985	0.738738532	0.912894278
ADAMTS3	0.755016916	0.651269643	0.723132042	0.780279943	0.999985752	0.68185373
GOLGA7	0.75526669	0.587350756	0.76555928	0.818060287	0.964301327	0.75640676
LAMA5	0.75526669	0.587350756	0.732073575	0.963132425	0.784972475	0.839906041
KANSL3	0.755368636	0.961368616	0.681918771	0.728264598	0.797227609	0.79187813
PSMA6	0.755740818	0.582127335	0.832971131	0.839745443	0.72350412	0.926189934
BCL6B	0.75594784	0.886924072	0.711148626	0.799135547	0.834311833	0.758917718
HECW1	0.755978151	0.749370809	0.943237386	0.74577892	0.82488413	0.684994779
DTYMK	0.756195094	0.775033799	0.679415149	0.888687722	0.930451229	0.687639605
FOXRED1	0.756195094	0.647102837	0.761415057	0.708466343	0.999985752	0.702623424
ZNF148	0.756195094	0.600246997	0.971053208	0.700026235	0.891726429	0.723555931
GATA6	0.756195094	0.779343136	0.867661896	0.731537809	0.867529283	0.742687461
ZYG11B	0.756195094	0.661302208	0.865512879	0.765499767	0.918052462	0.748323122
NCKAP5L	0.756195094	0.589351784	0.768932648	0.984423083	0.804625939	0.748921056
TGFBR1	0.756195094	0.614805655	0.955427429	0.820971736	0.776537165	0.758917718
ZNF641	0.756195094	0.61939332	0.99647733	0.707568004	0.783491263	0.762451586
LOC782343	0.756195094	0.676397431	0.831933739	0.842434279	0.852234727	0.769865166
LOC101904258	0.756195094	0.794015013	0.709834758	0.717645144	0.952264073	0.774932208
SP1	0.756195094	0.86285541	0.798782742	0.796128007	0.769529419	0.779201808
LOC112449505	0.756195094	0.708137441	0.732274253	0.886312303	0.86864151	0.78087541
SELP	0.756195094	0.628205932	0.845113191	0.948668643	0.696466814	0.804551322
LOC101907642	0.756195094	0.924125044	0.694535506	0.821450642	0.697778495	0.831652079
MCM5	0.756195094	0.609772211	0.69621934	0.935239391	0.847405031	0.836111377
NUPR2	0.756195094	0.664439271	0.810647419	0.887983383	0.765961853	0.839906041
C17H4orf46	0.756195094	0.726740894	0.879939804	0.813945621	0.693337723	0.845747012
BTF3	0.756195094	0.587249397	0.71967891	0.960916501	0.747103777	0.874870038
ANO9	0.756195094	0.589754585	0.682677321	0.753267542	0.935286646	0.927253029
STS	0.756195094	0.612937827	0.724543483	0.718713712	0.886785729	0.950631909
ELP1	0.756250281	0.692305416	0.976484937	0.82868625	0.712689649	0.699160709
ZNF638	0.756250281	0.621713316	0.814495418	0.71113156	0.993831963	0.728699286
LOC785568	0.756250281	0.77434858	0.867661896	0.771980793	0.828207883	0.748921056
C29H11orf68	0.756250281	0.649340041	0.860963813	0.819213899	0.882418284	0.754218406
PPP1R1A	0.756250281	0.656833203	0.679506848	0.823167001	0.977167046	0.754218406

NDUFS4	0.756250281	0.666228153	0.845113191	0.718713712	0.951918183	0.758394846
CLEC10A	0.756250281	0.686805478	0.76746129	0.890362631	0.864524107	0.761991719
PBK	0.756250281	0.693905757	0.824121854	0.938303773	0.721096295	0.772618249
MGAT5B	0.756250281	0.806821185	0.785806653	0.698952489	0.887514169	0.794932301
FAM214A	0.756250281	0.62215202	0.731315913	0.715394268	0.984494338	0.81956221
KCNQ1	0.756250281	0.579367653	0.749417651	0.866543173	0.920024826	0.823410836
PAPSS1	0.756250281	0.628736003	0.784260981	0.865076519	0.854289643	0.83150957
NRN1	0.756250281	0.852526348	0.779581461	0.707217807	0.81066793	0.831652079
SLC1A5	0.756250281	0.65159362	0.882346431	0.777956162	0.793993073	0.856548815
PIK3CD	0.756250281	0.61939332	0.845113191	0.796128007	0.831673407	0.865767172
CCDC150	0.756250281	0.580322769	0.749453648	0.850395689	0.804625939	0.927633316
ATP8B4	0.756292725	0.731024992	0.89478142	0.731537809	0.900893337	0.699160709
LOC786372	0.756297303	0.585242829	0.999993442	0.819213899	0.707939546	0.718865013
NR2C1	0.756297303	0.844170799	0.684382692	0.805499732	0.701123622	0.917930485
LOC112442598	0.756297303	0.659408339	0.688656264	0.840541751	0.795839201	0.940079339
RBBP5	0.756309053	0.71366091	0.951297932	0.81472008	0.781724458	0.690728863
SPEF2	0.756309053	0.981614672	0.679415149	0.822832329	0.766608741	0.702623424
LOC112443415	0.756309053	0.609209091	0.855971055	0.760230321	0.970988226	0.704351773
RBX1	0.756309053	0.588248603	0.879939804	0.780279943	0.956041014	0.713351736
LOC100847951	0.756309053	0.940961445	0.705695072	0.80459801	0.780871086	0.765644158
TIMP3	0.756309053	0.63734659	0.886829776	0.823167001	0.844566722	0.769865166
WDR36	0.756309053	0.580905654	0.755135544	0.766675276	0.986019537	0.779201808
TAGAP	0.756309053	0.636038252	0.894338686	0.728264598	0.78021215	0.901618453
SSSCA1	0.756441935	0.741263262	0.726119774	0.745577087	0.808650572	0.927520621
POLR2C	0.756768388	0.65172183	0.964807927	0.885789665	0.726283157	0.68839953
NDNF	0.756768388	0.930293015	0.846960897	0.765499767	0.765961853	0.690728863
GRHL1	0.756768388	0.95687851	0.686366599	0.812453944	0.823508064	0.695765939
RAB3GAP2	0.756768388	0.641897615	0.717242466	0.999981514	0.738738532	0.700123604
ZNF567	0.756768388	0.993002684	0.756014804	0.717645144	0.738811608	0.71016497
NAA25	0.756768388	0.718258172	0.804002279	0.764257755	0.953300742	0.721192619
LOC104973100	0.756768388	0.616467638	0.831933739	0.970812491	0.788739452	0.727632739
LOC100847410	0.756768388	0.786825403	0.732639736	0.808073654	0.929896768	0.733366979
KLHL4	0.756768388	0.826153046	0.890761634	0.828736799	0.703397779	0.737726964
ZCRB1	0.756768388	0.580322769	0.999993442	0.736392386	0.773264077	0.751705208
ZDHHC20	0.756768388	0.696797615	0.899106435	0.894086736	0.710478485	0.754731949
LYN	0.756768388	0.660882273	0.759332589	0.808075336	0.953300742	0.762133815
TK1	0.756768388	0.772618617	0.679506848	0.729705452	0.974738415	0.769865166
LOC786352	0.756768388	0.590802323	0.700963878	0.730052184	0.999985752	0.78096075

NCEH1	0.756768388	0.585786551	0.849389234	0.93745545	0.786314903	0.784754767
SAPCD2	0.756768388	0.915570087	0.690291254	0.739098396	0.835959418	0.806714845
C7	0.756768388	0.644795013	0.879939804	0.819968583	0.812092965	0.817529182
MRPS23	0.756768388	0.589754585	0.849389234	0.739098396	0.914280652	0.845747012
TMX2	0.756768388	0.65159362	0.843955813	0.753267542	0.867558388	0.8537109
LOC104974891	0.756768388	0.617216601	0.693546993	0.984423083	0.738738532	0.85532952
LOC101906923	0.756768388	0.589782216	0.754557912	0.940438437	0.781724458	0.859993218
EIF3CL	0.756768388	0.6465114	0.751957722	0.854220165	0.804274107	0.891151691
SURF6	0.756768388	0.718258172	0.695084268	0.823935462	0.823508064	0.89209761
PSMB4	0.756768388	0.663309441	0.834683675	0.775405413	0.796633553	0.893578168
MKKS	0.756768388	0.594199197	0.855793459	0.777956162	0.776537165	0.927426708
LOC784087	0.756768388	0.661467856	0.805479206	0.731537809	0.799867929	0.93363377
LOC618541	0.756768388	0.678023292	0.711148626	0.731537809	0.72341123	0.997206681
HAS2	0.75686444	0.750352025	0.688226981	0.984548238	0.78021215	0.742687461
CGRRF1	0.756881352	0.649340041	0.845113191	0.822691957	0.713822796	0.912894278
LOC101904796	0.756881352	0.594199197	0.679506848	0.921950515	0.799272579	0.917849087
ZNF502	0.757379648	0.614976364	0.68005166	0.731876542	0.999985752	0.684994779
GFRA1	0.757379648	0.692305416	0.916294854	0.832489049	0.810436875	0.716096025
GPR155	0.757379648	0.636937576	0.751130079	0.972142185	0.823508064	0.745988271
MSRB1	0.757379648	0.77139884	0.892139418	0.716077462	0.847092933	0.764260585
RFX3	0.757379648	0.649340041	0.840107596	0.81451387	0.787108353	0.87754233
RBM18	0.757514073	0.648958657	0.916323169	0.774214837	0.871558258	0.750967073
EFS	0.757598675	0.697643204	0.749854422	0.988408846	0.733613832	0.749726404
PLXDC2	0.757614739	0.783112117	0.771367402	0.764257755	0.956904152	0.683500374
THUMPD2	0.757614739	0.77844414	0.830135741	0.939604997	0.691696983	0.7353049
LOC100141145	0.757614739	0.737083529	0.939353663	0.839725231	0.716406563	0.738551306
LOC112444633	0.757614739	0.968602591	0.749417651	0.786096053	0.695436567	0.765948605
ZFP62	0.757614739	0.74241072	0.701928205	0.874562224	0.750439419	0.893838339
POLR1D	0.757614739	0.613062249	0.723132042	0.899855526	0.735091896	0.93531275
PSMC4	0.757946422	0.657900042	0.849389234	0.731537809	0.81900808	0.899428912
FNDC10	0.75817103	0.669828549	0.736435559	0.884482389	0.95102225	0.708090768
LOC101907843	0.75817103	0.644132227	0.776353067	0.998529748	0.729058286	0.748323122
CDC42BPG	0.758476235	0.614976364	0.76555928	0.999981514	0.717315449	0.718413142
UQCRC2	0.758476235	0.58531281	0.817582037	0.724431807	0.938731934	0.863020449
PGM2L1	0.758619503	0.750379179	0.944501125	0.753858518	0.772569315	0.75640676
DHX36	0.758632414	0.750379179	0.727870684	0.736005865	0.943702351	0.806714845
PIGA	0.758745161	0.614976364	0.997400824	0.731537809	0.845802744	0.684994779
TP53I13	0.758745161	0.848383949	0.754913805	0.866566721	0.84620147	0.688206116

TRABD	0.758745161	0.709137178	0.945995021	0.893051204	0.691708563	0.702623424
FOSB	0.758745161	0.660882273	0.997400824	0.780279943	0.738738532	0.703969699
MAP4K3	0.758745161	0.669828549	0.79497298	0.973075232	0.784972475	0.733366979
XRCC5	0.758745161	0.808785257	0.757565308	0.853058361	0.850031541	0.737726964
KIAA1257	0.758745161	0.621856619	0.962797326	0.890362631	0.70314895	0.742309541
DHRS11	0.758745161	0.822956796	0.831933739	0.756459079	0.84620147	0.759003017
CPLANE1	0.758745161	0.586628625	0.834683675	0.98981682	0.720896795	0.768168974
INPP1	0.758745161	0.804542244	0.775552434	0.892047793	0.749740098	0.773506763
PML	0.758745161	0.717848892	0.816032984	0.777956162	0.862852956	0.82909875
HNRNPH3	0.758745161	0.60754196	0.700497565	0.973832508	0.804725002	0.831908977
TNFRSF8	0.758745161	0.609772211	0.751957722	0.943808491	0.796633553	0.841451122
LOC107131792	0.758745161	0.602762504	0.798782742	0.904715959	0.804274107	0.844334911
ACYP1	0.758745161	0.660882273	0.828449846	0.753267542	0.87329316	0.860966325
ZFP2	0.758745161	0.634059237	0.788106994	0.891988949	0.738811608	0.888475311
FDFT1	0.758745161	0.644366672	0.841294863	0.736045965	0.819679279	0.912531667
EPOP	0.758745161	0.687651905	0.731960288	0.764257755	0.774289699	0.967056707
NFKB2	0.759429479	0.667035851	0.684282335	0.819213899	0.999985752	0.690728863
UBE2G2	0.759429479	0.600246997	0.980761647	0.892047793	0.716406563	0.704950309
RSAD1	0.759429479	0.65172183	0.98426364	0.863350574	0.705202026	0.706176041
RABGAP1L	0.759429479	0.692305416	0.874983674	0.766675276	0.927086179	0.716804193
CHRNA3	0.759429479	0.600886305	0.943836178	0.817146543	0.766655597	0.821257366
FAT2	0.759429479	0.649949716	0.688925042	0.996033721	0.728185525	0.835231445
LOC112441885	0.759429479	0.607707577	0.686755664	0.841299504	0.957588298	0.835231445
ST13	0.759429479	0.583275815	0.731960288	0.865746543	0.895067787	0.867063767
AP3S1	0.759429479	0.716270512	0.881778842	0.757301622	0.728185525	0.883688554
LOC112446717	0.759686791	0.606559706	0.826409457	0.995312373	0.738333933	0.742687461
LOC112449302	0.760115683	0.582324384	0.779581461	0.716077462	0.732388993	0.999948228
ERCC6L	0.760193615	0.705088508	0.879939804	0.852315557	0.805615411	0.754218406
TXNRD3	0.760193615	0.738007931	0.800428397	0.780279943	0.913732532	0.762133815
WDR75	0.760193615	0.589754585	0.749417651	0.912777307	0.924277487	0.762133815
KIF1BP	0.760193615	0.886565095	0.761415057	0.809277776	0.716994185	0.83150957
MRPS36	0.760193615	0.663152438	0.819527307	0.730052184	0.92337151	0.835231445
S100A1	0.760193615	0.688237052	0.759332589	0.929325365	0.705660117	0.865745718
PRMT3	0.760193615	0.686033606	0.755135544	0.727236849	0.764945406	0.97521287
PTGFRN	0.760263301	0.939956757	0.795458525	0.813323291	0.763477194	0.695765939
ELF1	0.760263301	0.582394134	0.741228435	0.733411985	0.999985752	0.733767439
LOC112441506	0.760270305	0.786516395	0.695084268	0.802977147	0.796861564	0.911808092
POMP	0.760270305	0.584209799	0.894316021	0.812313105	0.711422807	0.925233445

RIN1	0.760406779	0.582127335	0.853036433	0.838551707	0.870450746	0.83150957
NEMF	0.760668878	0.683704605	0.791985797	0.985437486	0.775771176	0.711118977
VCL	0.760668878	0.609864167	0.852916849	0.952247001	0.782443723	0.762133815
INTS6L	0.760668878	0.589651566	0.745348234	0.950643928	0.847173934	0.809830825
PTPRR	0.760694147	0.76071489	0.897322115	0.773429607	0.834569503	0.741319465
SLC22A16	0.760694147	0.918854612	0.819359511	0.812453944	0.723054375	0.742588975
ASB11	0.760694147	0.590038452	0.937993336	0.920690301	0.723467697	0.752070741
TTC7A	0.760694147	0.70496036	0.805479206	0.910495312	0.810436875	0.75640676
CCNQ	0.760694147	0.786392346	0.777540303	0.941467375	0.705202026	0.765948605
ZNF697	0.760694147	0.652246332	0.708274076	0.843593618	0.953300742	0.79187813
RSU1	0.760694147	0.643640444	0.971594714	0.799912445	0.723054375	0.795129319
SLC7A2	0.760694147	0.760451944	0.76555928	0.815919419	0.864524107	0.798452828
RPF1	0.760694147	0.816129287	0.761415057	0.822691957	0.796861564	0.818370468
LYZ2	0.760694147	0.748690795	0.708274076	0.76992117	0.935286646	0.822744521
LIMD2	0.760694147	0.716415149	0.819359511	0.742989529	0.887514169	0.830697497
SERPINE1	0.760694147	0.685486361	0.838341783	0.819968583	0.814120141	0.841451122
CMPK1	0.760694147	0.648958657	0.776353067	0.731537809	0.823508064	0.947838082
EIF2S3	0.760694147	0.709542765	0.705695072	0.757921478	0.823508064	0.94844234
LOC112445051	0.760694147	0.644366672	0.827969228	0.765499767	0.74426152	0.952563377
C22H3orf62	0.760780691	0.709542765	0.805924802	0.952247001	0.750277829	0.754218406
AJM1	0.761024955	0.996728887	0.725264968	0.782811812	0.727256509	0.700459582
RECQL	0.761024955	0.599276559	0.920779692	0.865187072	0.854966579	0.728795223
NDUFA7	0.761024955	0.709247882	0.80412682	0.710070489	0.974403222	0.745988271
PPM1G	0.761024955	0.621279921	0.999993442	0.80459801	0.695436567	0.754218406
VAPA	0.761024955	0.660404537	0.89478142	0.889415593	0.788007619	0.76139014
THBS1	0.761024955	0.684946765	0.77566312	0.769286764	0.960573822	0.766218451
ADH6	0.761024955	0.659408339	0.785411061	0.965729104	0.738738532	0.792412747
RAB9B	0.761024955	0.72742557	0.694535506	0.866566721	0.872022568	0.839906041
KIAA1324L	0.761024955	0.624373062	0.950004587	0.731537809	0.752560181	0.87100843
MINPP1	0.761024955	0.736846903	0.89385865	0.707217807	0.763477194	0.878754045
PDHB	0.761024955	0.589868638	0.831933739	0.80459801	0.846285074	0.89209761
TMEM132A	0.761024955	0.584228062	0.810647419	0.786067589	0.788007619	0.960023723
PPP5C	0.761058783	0.692305416	0.718323041	0.95901497	0.847092933	0.754218406
RPP38	0.761213503	0.7889129	0.731960288	0.870210705	0.849434441	0.777592695
LOC104971817	0.761213503	0.622818484	0.894338686	0.856085962	0.710661387	0.870522865
LOC112442047	0.761213503	0.659195551	0.805479206	0.768629299	0.858495696	0.885162483
PENK	0.761798756	0.594199197	0.711148626	0.915013589	0.976292433	0.690728863
ABCG1	0.761798756	0.628547317	0.724908605	0.72742295	0.738738532	0.999948228

SYVN1	0.761819571	0.870334242	0.734143952	0.892047793	0.801692604	0.720865143
MID1	0.761819571	0.7022048	0.700349135	0.893220599	0.788739452	0.891088733
SF3B5	0.761941884	0.870334242	0.819359511	0.717645144	0.738738532	0.858245612
USP25	0.762115601	0.846798773	0.70011973	0.707217807	0.814984338	0.913250128
TERC	0.762857068	0.587740565	0.749417651	0.729705452	0.97234838	0.864243158
DTWD1	0.762881821	0.791281103	0.689000346	0.999981514	0.721826916	0.713351736
KCTD15	0.762881821	0.583894729	0.744735369	0.940436902	0.927086179	0.748921056
ANKS6	0.762881821	0.691301661	0.79281326	0.97382992	0.735091896	0.765948605
DUSP19	0.762881821	0.648119635	0.741510374	0.95901497	0.832407979	0.78087541
SLC35G1	0.762881821	0.692305416	0.754913805	0.87522058	0.882145303	0.793197037
IDUA	0.762881821	0.893164557	0.732073575	0.853058361	0.738738532	0.79363949
MCM2	0.762881821	0.647020437	0.77566312	0.868378265	0.847092933	0.851325852
AADAT	0.762881821	0.690635606	0.737930899	0.759591583	0.932024143	0.858245612
GRIP1	0.762881821	0.709542765	0.871491704	0.809277776	0.727873981	0.873130182
LOC112442559	0.762881821	0.634059237	0.94955109	0.764257755	0.705202026	0.87754233
IGSF1	0.763330465	0.692305416	0.848426432	0.808262813	0.900927045	0.756591895
TRMT2A	0.763330465	0.674524738	0.973562292	0.739750555	0.804274107	0.756871392
BIRC3	0.763330465	0.66246801	0.726119774	0.973373329	0.823508064	0.768168974
CCNA2	0.763330465	0.627743951	0.870734949	0.919643615	0.755441633	0.797662039
MSRA	0.763330465	0.759245424	0.717242466	0.812453944	0.911568867	0.80383591
LOC100848077	0.763330465	0.77139884	0.877965851	0.729912904	0.797489438	0.839906041
KIF24	0.763644217	0.635148479	0.998096104	0.715394268	0.734324024	0.807084664
LOC104972888	0.76370768	0.605958163	0.706064882	0.999981514	0.856297415	0.737726964
GGA1	0.763885608	0.624292003	0.853546772	0.996054502	0.72350412	0.713351736
NCAM1	0.7639302	0.786392346	0.71601562	0.982220679	0.723054375	0.756606016
PDF	0.7639302	0.682216234	0.826913617	0.71113156	0.816306826	0.930710695
HTR4	0.764118541	0.613397345	0.911973589	0.799480811	0.789999838	0.865933526
RAPH1	0.764436786	0.942546802	0.693546993	0.724431807	0.879395665	0.768572658
SPDL1	0.764436786	0.790837224	0.689000346	0.906528	0.818179113	0.801888363
LOC782159	0.764436786	0.912670574	0.741510374	0.715394268	0.811991102	0.836111377
ABCB6	0.764436786	0.667258304	0.768438632	0.777956162	0.872096228	0.893550479
S1PR2	0.764445264	0.83844057	0.747666034	0.885789665	0.810436875	0.748832955
GTF3C6	0.764445264	0.809057302	0.69687851	0.82868625	0.810436875	0.86324237
PSMB3	0.764445264	0.659837453	0.870734949	0.728264598	0.804037558	0.91373422
ZNF385D	0.764505804	0.591722409	0.77566312	0.901353354	0.956761712	0.704351773
LOC107133032	0.764631628	0.62117273	0.949855095	0.754782628	0.807468091	0.832928063
PPP1R3F	0.764673993	0.844170799	0.788563419	0.726676396	0.918426817	0.740861782
SARM1	0.764673993	0.750379179	0.874983674	0.889384035	0.764945406	0.748223279

HEPH	0.764673993	0.768359345	0.774015998	0.817146543	0.902907077	0.749726404
SLC25A22	0.764673993	0.777309385	0.871782109	0.870210705	0.696466814	0.795129319
LOC112443526	0.764809996	0.636038252	0.916294854	0.889415593	0.726141965	0.806476213
LOC101904849	0.765382404	0.77022554	0.758146087	0.900875874	0.736914075	0.835231445
RNASE6	0.765382404	0.651269643	0.834683675	0.77800934	0.887514169	0.842214812
ATF3	0.765646332	0.830715727	0.818612484	0.9063051	0.736046437	0.727876258
OSER1	0.765646332	0.600246997	0.894316021	0.897371376	0.8568751	0.733673343
ALKBH3	0.765646332	0.622818484	0.86347788	0.96444269	0.78021215	0.742309541
GALNT12	0.765646332	0.847718043	0.805479206	0.853058361	0.788995893	0.748921056
KIAA0556	0.765646332	0.608342753	0.813441395	0.999981514	0.702888149	0.75640676
LOC614785	0.765646332	0.865101561	0.77566312	0.764140634	0.788007619	0.841312694
LOC101902059	0.765646332	0.649949716	0.768120148	0.830289573	0.709066987	0.963456717
VAT1L	0.765889103	0.765866962	0.745477682	0.907794002	0.887514169	0.690728863
C20H5orf22	0.766209447	0.670209048	0.93190909	0.825279787	0.738738532	0.815033455
RFT1	0.766312469	0.643640444	0.731739801	0.828082731	0.858187492	0.91373422
FAM168B	0.76644905	0.895777642	0.813441395	0.813798865	0.804264617	0.710467724
LOC515358	0.766960978	0.639909661	0.819359511	0.950173531	0.797489438	0.77593589
DHX15	0.766960978	0.646834837	0.751876894	0.80459801	0.958852623	0.806714845
LOC101907813	0.767141356	0.868390063	0.693546993	0.931509349	0.789915129	0.742309541
VLDLR	0.767141356	0.602500622	0.890961496	0.754719584	0.959658682	0.748921056
BAP1	0.767141356	0.930293015	0.834683675	0.774800098	0.735984709	0.754218406
ADAM22	0.767141356	0.660882273	0.8644528	0.828736799	0.891726429	0.754218406
ANXA11	0.767141356	0.738561239	0.88370567	0.82868625	0.797227609	0.779201808
ZFP57	0.767141356	0.811345153	0.781767828	0.912777307	0.707939546	0.78711973
UQCRFS1	0.767141356	0.624142952	0.79497298	0.736301189	0.964301327	0.83150957
NFRKB	0.767141356	0.66246801	0.916294854	0.793916687	0.788007619	0.842214812
SIRT5	0.767141356	0.622797538	0.754913805	0.901168085	0.828207883	0.86359986
RIDA	0.767141356	0.649949716	0.728670337	0.852170016	0.797489438	0.93983168
POR	0.767141356	0.663620961	0.728269425	0.716077462	0.818179113	0.983850813
S100A3	0.767141356	0.615769899	0.731739801	0.733411985	0.750277829	0.999948228
UBL7	0.767204848	0.675765841	0.791557166	0.784595865	0.988049878	0.693216036
COA6	0.767204848	0.719629484	0.826785415	0.800283296	0.881680572	0.797991983
LOC787554	0.767447642	0.588939818	0.848278925	0.910495312	0.741086403	0.872239777
CD244	0.76753751	0.793382089	0.777154688	0.808073654	0.915934652	0.733467771
LARP1B	0.76753751	0.713726826	0.689000346	0.854220165	0.938879379	0.796929234
KHK	0.76753751	0.870222905	0.747666034	0.764257755	0.806034958	0.842473495
ATP6V1C1	0.767796221	0.614805655	0.745281157	0.999981514	0.755431982	0.78087541
ACOX3	0.768387986	0.872175143	0.812446906	0.827786827	0.716994185	0.801888363

LDHA	0.768656099	0.759245424	0.758146087	0.97828838	0.720896795	0.75640676
EPHX2	0.768667509	0.819353713	0.706064882	0.82868625	0.930451229	0.733767439
PABPC1L2A	0.768667509	0.829067393	0.731143569	0.935958061	0.74416708	0.769585703
MYRIP	0.768667509	0.766595431	0.955221146	0.738460631	0.720896795	0.808590736
NUDCD1	0.768669794	0.648503747	0.749417651	0.929663263	0.89052284	0.762133815
LOC101904942	0.768669794	0.660882273	0.867625133	0.761359847	0.802592922	0.905509632
RHOA	0.7687068	0.624142952	0.881849916	0.962369147	0.737633927	0.754218406
GLT8D2	0.7687068	0.888057447	0.734143952	0.896116841	0.755086175	0.75640676
PPP1R15A	0.7687068	0.981614672	0.697667813	0.827786827	0.721826916	0.759003017
SLC25A46	0.7687068	0.600886305	0.893761108	0.886312303	0.760489317	0.850138656
ABCA3	0.768879155	0.774644712	0.71967891	0.863350574	0.721096295	0.922361909
TXNDC17	0.768910539	0.823133291	0.706064882	0.739967325	0.735091896	0.960023723
CHAC2	0.768910539	0.638895543	0.810647419	0.776284562	0.770626806	0.960023723
TMEM144	0.770209902	0.796253817	0.858376167	0.852365348	0.788007619	0.754731949
TALDO1	0.770257074	0.782783231	0.761415057	0.799497281	0.926831826	0.755679512
MFAP1	0.77080525	0.60754196	0.941507802	0.889384035	0.795839201	0.754218406
FAM3C	0.77080525	0.731024992	0.70011973	0.758601772	0.900233561	0.905034785
APRT	0.77080525	0.661806153	0.726119774	0.777956162	0.854966579	0.947801358
RRAS	0.770818591	0.642862769	0.892343042	0.839299113	0.717677975	0.891088733
NAXE	0.77115194	0.704346844	0.817582037	0.728264598	0.953300742	0.790348826
HAUS5	0.771956035	0.62215202	0.736435559	0.999981514	0.707939546	0.770035496
METAP2	0.771956035	0.64939288	0.799122572	0.812453944	0.955623649	0.770035496
YWHAG	0.771956035	0.745791496	0.772600931	0.948668643	0.731874279	0.806892223
RSL1D1	0.771956035	0.60594617	0.80412682	0.846996336	0.924277487	0.81853698
LOC112443475	0.771956035	0.681192035	0.759332589	0.961898258	0.730829032	0.839906041
NET1	0.771956035	0.741263262	0.755135544	0.791081996	0.874672682	0.861855564
LOC112444300	0.771956035	0.615769899	0.749417651	0.764257755	0.958797242	0.867063767
ABHD16B	0.772022383	0.693501292	0.727703335	0.982220679	0.834569503	0.742309541
MMP14	0.772148421	0.786691027	0.744735369	0.763977006	0.976769047	0.700123604
LOC104968522	0.772663366	0.61939332	0.879939804	0.828736799	0.883853714	0.789870764
TFB1M	0.772941787	0.728513666	0.730571184	0.780703865	0.91563394	0.857911901
ELOVL5	0.772941787	0.690635606	0.804888406	0.726524321	0.911837627	0.868748913
PLAT	0.773003365	0.589754585	0.883200248	0.729912904	0.955623649	0.806714845
LMTK3	0.773003365	0.60754196	0.723132042	0.821450642	0.891726429	0.923092436
ZNF365	0.773152821	0.634059237	0.69454186	0.999981514	0.815175669	0.697356941
LOC615899	0.773516004	0.765910578	0.852214402	0.830187312	0.804625939	0.786849547
VPS41	0.773723135	0.660882273	0.747666034	0.988408846	0.721096295	0.835231445
SLC4A2	0.773871748	0.614976364	0.754913805	0.83795554	0.996781978	0.71016497

SP110	0.773871748	0.915269318	0.868479534	0.758499128	0.789999838	0.729499398
G3BP1	0.773871748	0.622449596	0.865697455	0.899296336	0.879395665	0.742309541
EEF2KMT	0.773871748	0.757078435	0.726741709	0.843613441	0.948711141	0.743350163
LOC783920	0.773871748	0.95687851	0.711148626	0.786776958	0.832407979	0.748921056
AMOTL2	0.773871748	0.837244723	0.928731949	0.729912904	0.788739452	0.749918186
LOC101904121	0.773871748	0.667258304	0.779581461	0.836610893	0.943160494	0.770490368
SMURF2	0.773871748	0.75517176	0.711148626	0.780279943	0.967563858	0.772618249
TRIM23	0.773871748	0.746457441	0.708274076	0.972142185	0.790136352	0.78096075
COA3	0.773871748	0.644366672	0.808037655	0.757463039	0.964301327	0.800936414
C15H11orf49	0.773871748	0.834615638	0.700963878	0.820034368	0.871558258	0.815129379
PPP3R1	0.773871748	0.686805478	0.879939804	0.890362631	0.738738532	0.83150957
PCGF5	0.773871748	0.829067393	0.883950091	0.761171025	0.735091896	0.835231445
RXYLT1	0.773871748	0.671759506	0.794353972	0.866566721	0.865075514	0.835231445
GNG11	0.773871748	0.869160875	0.789663873	0.830877557	0.714642011	0.835338212
MTA3	0.773871748	0.916448625	0.720612298	0.780184803	0.741086403	0.869867401
ITPR3	0.773871748	0.625011831	0.829137153	0.834401906	0.801692604	0.906637848
ATF5	0.773871748	0.760623317	0.7786153	0.751194677	0.817472827	0.911219114
PDPR	0.773871748	0.658075243	0.788126358	0.825279787	0.788995893	0.930710695
LOC104974850	0.773871748	0.644366672	0.770497031	0.733411985	0.723467697	0.999463809
CAPN11	0.774135521	0.973712113	0.711148626	0.871946375	0.766655597	0.699720332
PRDM5	0.774135521	0.629418148	0.929661805	0.853058361	0.866324844	0.724693895
AP3B1	0.774135521	0.68903984	0.889098668	0.792140715	0.889087404	0.769865166
LOC101903851	0.774135521	0.719964051	0.950055019	0.741475688	0.817472827	0.776734587
NDEL1	0.774135521	0.637453144	0.728269425	0.817146543	0.983941532	0.779653589
HPS1	0.774135521	0.609209091	0.731739801	0.996033721	0.753508098	0.84413042
GRB10	0.774135521	0.665400209	0.754913805	0.910575108	0.818179113	0.852504043
RUFY1	0.774135521	0.594199197	0.789166592	0.954783411	0.784972475	0.858289382
CARF	0.774135521	0.802570554	0.71338011	0.874170259	0.789915129	0.863020449
PEBP1	0.774135521	0.619072791	0.722252778	0.764257755	0.939442711	0.919124527
POU6F1	0.774135521	0.764456895	0.735916998	0.801848398	0.727268681	0.958790968
PUSL1	0.774135521	0.692305416	0.744735369	0.748424012	0.737633927	0.994788615
POLR3F	0.774142909	0.76003522	0.855892553	0.844174859	0.847092933	0.748275566
TMEM38B	0.774142909	0.594199197	0.891662311	0.854966424	0.90730011	0.749918186
LYPD3	0.774142909	0.638895543	0.860877703	0.78584336	0.820411915	0.899460485
PTH1R	0.774206459	0.686805478	0.836482	0.922002553	0.847092933	0.737726964
TRAPPC6B	0.774206459	0.6385246	0.95322206	0.817146543	0.804625939	0.779201808
XIAP	0.774206459	0.722968232	0.899106435	0.74577892	0.852026472	0.812091218
ADORA1	0.774206459	0.82707084	0.824121854	0.766675276	0.823508064	0.8150125

DUSP23	0.774206459	0.788544942	0.70011973	0.82868625	0.85317858	0.865745718
LDLRAD3	0.774253706	0.659408339	0.749417651	0.940436902	0.856297415	0.797953744
PIK3CG	0.774292118	0.664513512	0.951392011	0.830187312	0.847092933	0.700913346
FAM126A	0.774292118	0.600246997	0.69454186	0.765499767	0.760007802	0.999948228
CD1A	0.774425358	0.596368398	0.982232295	0.887983383	0.779385814	0.715732654
TSPAN6	0.774449992	0.991673407	0.733319943	0.813495071	0.720896795	0.736237609
STAT5A	0.774449992	0.60754196	0.819322085	0.731537809	0.87329316	0.936778056
HMGXB3	0.774685346	0.674524738	0.927508464	0.81451387	0.89052284	0.700123604
PARP9	0.774685346	0.808510536	0.910957946	0.728264598	0.847092933	0.754061883
LOC112446760	0.774685346	0.614976364	0.69687851	0.999981514	0.738738532	0.76139014
IRF2BPL	0.774685346	0.614976364	0.842692778	0.852170016	0.935093621	0.766900958
GTF3C5	0.774685346	0.783112117	0.830887859	0.852170016	0.804037558	0.788462021
RNF168	0.774685346	0.872175143	0.80412682	0.764257755	0.788007619	0.838251328
PIAS1	0.77484549	0.898279284	0.862253703	0.725213594	0.870450746	0.699160709
TJP3	0.77484549	0.70880006	0.833583844	0.925294859	0.721826916	0.831652079
ANGPT4	0.77484549	0.848979728	0.791038191	0.798687063	0.721826916	0.877014986
AP5M1	0.77484549	0.872244611	0.704088819	0.727236849	0.849766114	0.877014986
FBXO44	0.77484549	0.615769899	0.723132042	0.932677501	0.735485629	0.940256832
CCDC62	0.774921094	0.614976364	0.80412682	0.812453944	0.980695939	0.747871787
SMAD2	0.774999495	0.7022048	0.93190909	0.914653338	0.753088486	0.699160709
CCDC157	0.775329538	0.682825402	0.73826834	0.999981514	0.804274107	0.711923954
MPND	0.775329538	0.886976958	0.853546772	0.865076519	0.716994185	0.742309541
SCLY	0.775329538	0.766943819	0.869932384	0.904018489	0.721096295	0.772959565
LOC107132278	0.775329538	0.709247882	0.93475587	0.792715129	0.745325398	0.838530462
ARMC6	0.775329538	0.775614137	0.796783363	0.799147532	0.819549575	0.861855564
RCSD1	0.775329538	0.730372512	0.776418599	0.898818046	0.733613832	0.87501921
AIMP1	0.775329538	0.636038252	0.700963878	0.882395013	0.742554126	0.973649216
NAPA	0.775387803	0.662498852	0.794225743	0.757403199	0.930451229	0.865889509
PXK	0.775567315	0.60754196	0.916294854	0.874424802	0.814984338	0.798057364
TACO1	0.775685572	0.699211784	0.770595941	0.751437238	0.944839728	0.841312694
LOC112447838	0.775692824	0.915269318	0.71967891	0.859320133	0.714642011	0.839906041
MTPN	0.775808749	0.599945835	0.807254925	0.999981514	0.716994185	0.766435538
TMEM18	0.775808749	0.790429889	0.763368729	0.809356235	0.821148296	0.864139695
ASB2	0.775808749	0.718228672	0.788665027	0.724175944	0.772614149	0.97061907
LOC107131542	0.775929224	0.61939332	0.753334016	0.990955345	0.883951804	0.704351773
ADAMTS18	0.775929224	0.815436463	0.694535506	0.892047793	0.879776073	0.765948605
DNMBP	0.775929224	0.679668575	0.720612298	0.745806541	0.960573822	0.873130182
GUCA1A	0.776480109	0.764775882	0.735532321	0.896116841	0.832119756	0.816409111

GNB1	0.776551661	0.722779653	0.789166592	0.973075232	0.771532192	0.75640676
LOC107132301	0.776751053	0.997425219	0.728670337	0.786099236	0.759888741	0.716074186
SMG5	0.776751053	0.882102283	0.819359511	0.730052184	0.870042072	0.762133815
USP11	0.776751053	0.635339433	0.758146087	0.94222181	0.857562998	0.809961137
TFE3	0.776751053	0.855067335	0.759332589	0.799480811	0.750277829	0.877014986
RBKS	0.776809315	0.697043768	0.825687859	0.901168085	0.883951804	0.731778552
ACOT7	0.776904822	0.649340041	0.70011973	0.969088992	0.912199478	0.749726404
FAM19A5	0.776904822	0.674524738	0.877334778	0.770660417	0.909316444	0.795539146
LOC112444290	0.776904822	0.638044641	0.744735369	0.821450642	0.844434232	0.93988265
TXNRD2	0.777079237	0.715664279	0.822898819	0.757463039	0.954403133	0.769865166
CCDC15	0.777118485	0.644366672	0.78406673	0.926970666	0.936962513	0.700123604
KATNB1	0.777118485	0.88008538	0.851822885	0.731459497	0.865505927	0.742456493
LOC100299503	0.777173161	0.811354298	0.784788059	0.886312303	0.872734644	0.703391424
TMEM161B	0.777173161	0.643640444	0.737043937	0.96951401	0.848550619	0.789998967
GALNT3	0.77723984	0.737396716	0.888275071	0.88904348	0.721096295	0.810156973
LOC112443850	0.777294958	0.595995663	0.700963878	0.887983383	0.958797242	0.835231445
PPP2R2C	0.777461216	0.985428851	0.788813378	0.764257755	0.7744799	0.718149424
LOC101906120	0.777461216	0.692305416	0.711148626	0.999981514	0.821351993	0.722497558
GNG5	0.777461216	0.610580181	0.949939862	0.730052184	0.910070259	0.779201808
LOC515823	0.777461216	0.674524738	0.83068481	0.812999186	0.911568867	0.806892223
COPS9	0.777461216	0.728565135	0.759357794	0.815919419	0.884872176	0.849595612
METRN	0.777524388	0.991673407	0.717242466	0.848592907	0.738738532	0.702623424
ASIP	0.777524388	0.763614683	0.999993442	0.724271417	0.714642011	0.70951022
NUTF2	0.777524388	0.621279921	0.849389234	0.962437357	0.810144664	0.756871392
LOC112443837	0.777524388	0.83844057	0.810647419	0.731537809	0.906766758	0.766218451
PMPCA	0.777524388	0.732885933	0.754913805	0.747198125	0.960573822	0.8150125
CDC16	0.777524388	0.602500622	0.838909114	0.728264598	0.962513981	0.845747012
LOC107132883	0.777524388	0.631580394	0.76555928	0.885789665	0.881680572	0.85532952
FSTL4	0.777524388	0.602500622	0.947155158	0.804862068	0.784972475	0.864243158
LOC101902475	0.777524388	0.665819884	0.81412252	0.937654716	0.716406563	0.867635709
IGDCC4	0.777614506	0.988824757	0.717242466	0.874170259	0.728185525	0.702623424
ZBTB14	0.777614506	0.648503747	0.836914718	0.968114032	0.854289643	0.702623424
PPP1R36	0.777614506	0.864945924	0.700963878	0.990955345	0.737149805	0.704351773
ATP2C2	0.777614506	0.83866979	0.930736996	0.820971736	0.75925	0.704950309
PLOD2	0.777614506	0.890045072	0.786799553	0.813323291	0.870450746	0.711750433
CDK10	0.777614506	0.7889129	0.891662311	0.82868625	0.845802744	0.715808305
XKR5	0.777614506	0.68858196	0.711148626	0.999981514	0.774232717	0.718149424
GPR21	0.777614506	0.919540627	0.76555928	0.915013589	0.721096295	0.727876258

VCAN	0.777614506	0.681192035	0.836482	0.96444269	0.819679279	0.728993082
CCDC80	0.777614506	0.816412341	0.927508464	0.85981779	0.717315449	0.729499398
LGALS8	0.777614506	0.621279921	0.780096377	0.999981514	0.73254131	0.729499398
TSHZ2	0.777614506	0.702520574	0.890354549	0.889415593	0.84836347	0.729499398
SLC26A2	0.777614506	0.62215202	0.700963878	0.999981514	0.799272579	0.732900489
MYCL	0.777614506	0.7889129	0.86427915	0.82163384	0.864524107	0.741971799
LCP1	0.777614506	0.72828303	0.883737851	0.885789665	0.823508064	0.742687461
RINT1	0.777614506	0.738007931	0.824121854	0.839245804	0.915279373	0.744016261
USP20	0.777614506	0.819910026	0.727870684	0.915039335	0.847173934	0.748323122
KPNA3	0.777614506	0.632321074	0.999993442	0.732130385	0.795839201	0.755679512
GHDC	0.777614506	0.737941984	0.744735369	0.874023052	0.936108073	0.75640676
MGAT4B	0.777614506	0.683368775	0.829745504	0.989485949	0.736914075	0.758917718
TMEM107	0.777614506	0.819623256	0.708227907	0.934629728	0.823508064	0.759003017
ATP6V0D1	0.777614506	0.767113995	0.777504633	0.964521432	0.738738532	0.765948605
FOXJ3	0.777614506	0.692305416	0.747666034	0.828736799	0.962513981	0.769865166
YAF2	0.777614506	0.693373829	0.916323169	0.890704007	0.753088486	0.774562005
ZPR1	0.777614506	0.610580181	0.758146087	0.93660503	0.91563394	0.779201808
AMMECR1	0.777614506	0.697306013	0.755265458	0.925457774	0.872096228	0.780585188
LASP1	0.777614506	0.60754196	0.70011973	0.729481641	0.999985752	0.780933489
LOC101903649	0.777614506	0.618921732	0.914770879	0.904271346	0.804037558	0.78096075
EMC6	0.777614506	0.764456895	0.874063521	0.780279943	0.867650653	0.78096075
THBS2	0.777614506	0.675635323	0.872088608	0.893220599	0.822424846	0.782168676
STAT2	0.777614506	0.72108575	0.802277976	0.752454758	0.961465747	0.782330566
BPTF	0.777614506	0.675285188	0.774015998	0.769893371	0.977193348	0.783901526
GPR137	0.777614506	0.753090681	0.916038418	0.777956162	0.819329746	0.785562356
LOC785745	0.777614506	0.614976364	0.951028911	0.891988949	0.741463451	0.785636406
CD48	0.777614506	0.722968232	0.8735515	0.764257755	0.892446951	0.800364528
FBXO3	0.777614506	0.692305416	0.85453084	0.872916849	0.845802744	0.80383591
N4BP2L2	0.777614506	0.901071863	0.789166592	0.840700255	0.735091896	0.807084664
HIST1H1C	0.777614506	0.609506522	0.764467593	0.824507073	0.965163023	0.812774915
ALAS1	0.777614506	0.672237251	0.879939804	0.744617133	0.927086179	0.814218101
DKKL1	0.777614506	0.946859049	0.69687851	0.812999186	0.788007619	0.814745299
DOLPP1	0.777614506	0.834615638	0.860877703	0.74577892	0.817472827	0.816246746
RMI1	0.777614506	0.783112117	0.724908605	0.925353907	0.800097282	0.81956221
CDC34	0.777614506	0.833323491	0.867661896	0.752616152	0.804274107	0.820286425
NPC1	0.777614506	0.636038252	0.737124809	0.983233117	0.81066793	0.821910421
POLDIP2	0.777614506	0.614976364	0.93190909	0.799712183	0.848550619	0.83150957
POLB	0.777614506	0.616227995	0.964807927	0.840700255	0.737149805	0.831652079

GEM	0.777614506	0.733509477	0.7292782	0.917892197	0.823508064	0.832770678
RABGGTA	0.777614506	0.636038252	0.732480837	0.748424012	0.996310017	0.833150766
LOC112442805	0.777614506	0.622818484	0.943836178	0.812453944	0.802395087	0.835231445
SERF2	0.777614506	0.697043768	0.777540303	0.780279943	0.938316699	0.835231445
ELMO3	0.777614506	0.687288091	0.91469924	0.822691957	0.790339877	0.837596789
GPBP1L1	0.777614506	0.645547325	0.902549865	0.828736799	0.817472827	0.839565788
TP53INP2	0.777614506	0.642929022	0.862253703	0.762199111	0.917407853	0.846210629
CRYM	0.777614506	0.762081499	0.758146087	0.909765627	0.766655597	0.847619123
CMTR2	0.777614506	0.741310853	0.705695072	0.768290504	0.954403133	0.849595612
DYRK1B	0.777614506	0.632459972	0.951237862	0.812453944	0.75602825	0.850475013
ANAPC11	0.777614506	0.781222833	0.813942299	0.731459497	0.879268517	0.851995112
CCDC89	0.777614506	0.811059858	0.69687851	0.727236849	0.943610727	0.851995112
C1QTNF9	0.777614506	0.816202069	0.801754158	0.764257755	0.826452966	0.8537109
GNL3	0.777614506	0.709247882	0.89478142	0.757463039	0.820477096	0.865432393
TMEM129	0.777614506	0.772669268	0.921434639	0.762159685	0.714642011	0.865745718
SORBS2	0.777614506	0.823830365	0.805702274	0.764257755	0.801692604	0.871273798
LOC112443463	0.777614506	0.753090681	0.719188447	0.799497281	0.883951804	0.884925084
DOHH	0.777614506	0.750432618	0.871929137	0.773429607	0.760634495	0.891154545
PRF1	0.777614506	0.650612374	0.885896294	0.85647796	0.723054375	0.893838339
GATA3	0.777614506	0.6385246	0.759332589	0.839245804	0.883612892	0.893838339
CORO7	0.777614506	0.855067335	0.846960897	0.731537809	0.716994185	0.895517058
EPHA2	0.777614506	0.742575051	0.705695072	0.819968583	0.857562998	0.908099327
OPRL1	0.777614506	0.831549062	0.70011973	0.806573529	0.804625939	0.911219114
CEBPA	0.777614506	0.61939332	0.799533361	0.738687937	0.931871771	0.911219114
PSMD7	0.777614506	0.605799206	0.913400043	0.813798865	0.755431982	0.917024468
SNRNP48	0.777614506	0.602019792	0.890355277	0.731459497	0.847173934	0.923092436
ENTPD1	0.777614506	0.790149523	0.744735369	0.734488268	0.834268541	0.927426708
DMBT1	0.777614506	0.624142952	0.709704032	0.919688166	0.796633553	0.928416436
ASB12	0.777614506	0.692305416	0.702256337	0.803690355	0.865638205	0.944973383
LOC101902128	0.777614506	0.660882273	0.800848425	0.865210554	0.721096295	0.947801358
ZNF584	0.777614506	0.644366672	0.898179681	0.72670074	0.771805357	0.948705062
SERPIND1	0.777614506	0.644366672	0.774015998	0.801954011	0.811567827	0.960023723
KLK13	0.777614506	0.649340041	0.836459233	0.724431807	0.815175669	0.960023723
CYTH2	0.777614506	0.689824411	0.726119774	0.724431807	0.750277829	0.999948228
HMCN1	0.778146814	0.892252816	0.701512486	0.900044333	0.820546747	0.749726404
LOC614923	0.778146814	0.687651905	0.700963878	0.838528071	0.979759416	0.777374794
PFDN2	0.778347529	0.716974822	0.962034224	0.839632707	0.788007619	0.729499398
LOC507696	0.778347529	0.615562349	0.791198027	0.999981514	0.804625939	0.754366249

CACUL1	0.778548867	0.60305629	0.939981892	0.896116841	0.801692604	0.770035496
XPR1	0.778597662	0.64014286	0.817582037	0.751451145	0.983884995	0.783856067
RAB31	0.779164789	0.664859217	0.817582037	0.777956162	0.788995893	0.960023723
LOC104969140	0.779348387	0.614976364	0.999993442	0.792161305	0.811706964	0.706869246
EFR3B	0.779348387	0.796253817	0.791843351	0.892047793	0.837915327	0.756035665
COQ10B	0.779348387	0.744774335	0.784792499	0.821450642	0.911568867	0.793197037
LOC112442952	0.779348387	0.741312096	0.723132042	0.856195359	0.903410824	0.835231445
HBP1	0.779348387	0.709542765	0.854231171	0.830187312	0.772501291	0.885162483
LOC616063	0.779348387	0.717684248	0.707407957	0.748424012	0.928951321	0.917849087
GPC5	0.779348387	0.644132227	0.736377019	0.882057818	0.723467697	0.975788372
SLC46A2	0.77936171	0.700353734	0.817582037	0.916858019	0.848550619	0.768168974
TAF7	0.779489649	0.999987083	0.740372388	0.731537809	0.716406563	0.711923954
MYH6	0.779643078	0.962416786	0.721255681	0.818481906	0.804625939	0.754731949
SNTB1	0.780059163	0.869754045	0.895801611	0.733397157	0.847173934	0.733366979
SETD2	0.780248929	0.681192035	0.805212947	0.748837705	0.999985752	0.728993082
LOC104974812	0.780248929	0.952863279	0.822300339	0.827786827	0.723054375	0.742309541
CLTC	0.780248929	0.657066596	0.912020408	0.960937718	0.723467697	0.754218406
UNC13C	0.780248929	0.614976364	0.80412682	0.876703811	0.959658682	0.75640676
SBF2	0.780248929	0.648503747	0.740372388	0.95057248	0.916605572	0.758917718
LOC112446454	0.780248929	0.749236881	0.77566312	0.840552589	0.91509147	0.779201808
LOC101904590	0.780248929	0.634720956	0.754913805	0.887642126	0.91563394	0.839906041
INPP5E	0.780248929	0.609457398	0.989494193	0.751451145	0.738340365	0.874000314
LOC101903820	0.780248929	0.740083874	0.732274253	0.857073952	0.738738532	0.948705062
TXN	0.780248929	0.729001857	0.755135544	0.757301622	0.80599066	0.960023723
TAF6L	0.780608437	0.622818484	0.755940516	0.900875874	0.967205491	0.739270623
LOC514507	0.780608437	0.899682309	0.848490402	0.7817105	0.816045604	0.750623484
IARS	0.780753352	0.660977079	0.849389234	0.729705452	0.738738532	0.984046914
TCTA	0.780780632	0.775508074	0.819359511	0.769701093	0.963790274	0.711118977
HES2	0.780780632	0.649340041	0.700349135	0.999981514	0.735091896	0.737726964
PPP1R3C	0.780780632	0.833323491	0.871285807	0.866566721	0.78021215	0.749726404
HARBI1	0.780780632	0.67093609	0.745477682	0.797338497	0.997422012	0.759003017
SINHCAF	0.780780632	0.694148907	0.817582037	0.884452623	0.896589592	0.769865166
LOC112447353	0.780780632	0.755252885	0.744735369	0.930346498	0.850461911	0.778302445
PITPNB	0.780780632	0.781543413	0.764467593	0.905209994	0.833864947	0.78087541
IKBKE	0.780780632	0.812449121	0.874797723	0.821083913	0.78021215	0.804385539
DUSP11	0.780780632	0.722968232	0.856952806	0.797338497	0.843396588	0.853548296
BCAS1	0.780780632	0.740343181	0.75029951	0.764257755	0.904643542	0.883688554
LOC101906366	0.780780632	0.610580181	0.731143569	0.818166381	0.917407853	0.923807852

SLC25A40	0.780784164	0.754676989	0.812446906	0.87165456	0.907455286	0.730048817
UBE2W	0.780839917	0.704181021	0.913134155	0.853344701	0.784972475	0.81853698
FAM76A	0.780852875	0.710645465	0.771066142	0.88858839	0.815913413	0.871200929
ADD2	0.780882387	0.991673407	0.726119774	0.792140715	0.771171962	0.76047709
LRFN4	0.780882387	0.652509786	0.876414692	0.882057818	0.837028469	0.814745299
LOC112443235	0.7809225	0.624142952	0.777154688	0.812453944	0.798683736	0.971545917
EYA3	0.781115984	0.609864167	0.899106435	0.774800098	0.977167046	0.71289069
KIF18B	0.781115984	0.634752242	0.906019013	0.889415593	0.887514169	0.730498873
TMEM183A	0.781115984	0.653683126	0.80412682	0.796128007	0.766507736	0.973649216
TRMT12	0.781440451	0.622423182	0.745477682	0.999981514	0.753088486	0.83150957
LOC104973099	0.781496421	0.974846249	0.812446906	0.7694401	0.766655597	0.748750383
SLC38A9	0.781496421	0.927973597	0.700963878	0.78252493	0.881680572	0.783241453
UBD	0.782019961	0.70880006	0.879608555	0.830187312	0.883951804	0.769735861
SHC4	0.782338286	0.847718043	0.71967891	0.964521432	0.769529419	0.754218406
KIZ	0.782338286	0.728285587	0.851633941	0.731537809	0.964301327	0.754218406
ARID1A	0.782338286	0.658496391	0.8267276	0.817230639	0.933426897	0.81956221
BNIP3	0.782338286	0.718382634	0.717242466	0.976125636	0.737633927	0.863216707
NCLN	0.782338286	0.843278276	0.77566312	0.760279212	0.823644365	0.868748913
CEP126	0.782338286	0.687651905	0.730126401	0.903584224	0.82775883	0.888930405
LOC107131368	0.782460559	0.907054337	0.874063521	0.761359847	0.738738532	0.806714845
DST	0.782503244	0.683368775	0.764467593	0.999981514	0.781724458	0.75916457
HEPACAM	0.782503244	0.693373829	0.906019013	0.780279943	0.821777864	0.856640874
ALDOC	0.782503244	0.636781917	0.903431343	0.81451387	0.752382345	0.917930485
CACNB3	0.782644418	0.691301661	0.719862199	0.748424012	0.970988226	0.871273798
CA8	0.782644418	0.766943819	0.827969228	0.752616152	0.776857944	0.93363377
CNGA3	0.782649064	0.933100216	0.849389234	0.768644469	0.788995893	0.759144917
MTMR4	0.782649064	0.652246332	0.740372388	0.945300947	0.810436875	0.87501921
SCYL1	0.782661281	0.98098403	0.855713041	0.777956162	0.727256509	0.71289069
BARX1	0.782661281	0.737941984	0.832268748	0.876121618	0.916605572	0.714321361
FCER2	0.782661281	0.688310668	0.877334778	0.957008595	0.799272579	0.733767439
TXNIP	0.782661281	0.628660167	0.855892553	0.930987593	0.834431851	0.784754767
LOC107132382	0.782661281	0.643417752	0.774924967	0.982220679	0.821351993	0.785562356
LOC782560	0.782661281	0.643640444	0.97773026	0.813323291	0.788007619	0.799104608
C11H2orf50	0.782661281	0.629418148	0.916294854	0.892071982	0.727246643	0.860376457
ARMC1	0.782661281	0.651269643	0.860877703	0.805302469	0.766655597	0.945480515
LYPLAL1	0.783088213	0.7889129	0.705746499	0.751643991	0.796861564	0.972695131
LOC786614	0.78316585	0.657122966	0.906019013	0.786099236	0.961465747	0.713707269
LOC512869	0.78316585	0.630477275	0.739415743	0.889415593	0.988881329	0.729499398

TCF25	0.78316585	0.659462323	0.768247414	0.999981514	0.77889993	0.733767439
CRACR2A	0.78316585	0.848383949	0.945732557	0.808075336	0.738738532	0.736468943
DYRK3	0.78316585	0.899293191	0.775552434	0.896116841	0.78021215	0.748921056
ATP6V0B	0.78316585	0.753385865	0.936898293	0.888687722	0.721096295	0.760780768
TXNDC5	0.78316585	0.758609923	0.771697162	0.989707094	0.742450542	0.761143135
CUX2	0.78316585	0.816912013	0.71967891	0.991955963	0.735091896	0.76139014
ZNF782	0.78316585	0.794494758	0.957052938	0.777956162	0.776857944	0.76139014
PTEN	0.78316585	0.738007931	0.718840994	0.922002553	0.911568867	0.766534677
PASK	0.78316585	0.616227995	0.955427429	0.861243324	0.823508064	0.769865166
SDK2	0.78316585	0.61939332	0.973562292	0.815919419	0.833360418	0.769865166
NEMP1	0.78316585	0.863286109	0.915858924	0.80459801	0.735091896	0.772618249
LOC107132465	0.78316585	0.678023292	0.708227907	0.993481918	0.85545716	0.772618249
EBF4	0.78316585	0.980462136	0.727364203	0.828736799	0.738738532	0.78096075
VTI1B	0.78316585	0.876304005	0.852916849	0.852170016	0.723054375	0.793197037
LOC112442243	0.78316585	0.836640468	0.727619107	0.949468104	0.764945406	0.793197037
LOC615112	0.78316585	0.955652503	0.707407957	0.762598894	0.837915327	0.818370468
LOC112443428	0.78316585	0.64563665	0.872088608	0.854220165	0.877712931	0.821257366
STX8	0.78316585	0.687020192	0.8644528	0.758499128	0.926972948	0.835231445
MRPL40	0.78316585	0.846724749	0.791843351	0.782811812	0.845095928	0.83821684
CCDC88B	0.78316585	0.634059237	0.924855847	0.755247535	0.887514169	0.839906041
UBL5	0.78316585	0.651269643	0.916038418	0.805217242	0.847092933	0.841312694
PRRT4	0.78316585	0.906064108	0.731708587	0.780279943	0.827452098	0.844759721
METTL9	0.78316585	0.664859217	0.799265115	0.874554652	0.880890828	0.846210629
STX6	0.78316585	0.788544942	0.735532321	0.799480811	0.887514169	0.867063767
MSMO1	0.78316585	0.634059237	0.836719427	0.928404714	0.769529419	0.872239777
SSR1	0.78316585	0.609864167	0.924855847	0.78584336	0.826634148	0.877014986
LMO4	0.78316585	0.718258172	0.809673119	0.887983383	0.771469454	0.883688554
C3H1orf52	0.78316585	0.610421396	0.707407957	0.999180255	0.750169765	0.888475311
LOC112444206	0.78316585	0.746341488	0.749854422	0.853058361	0.823508064	0.890967932
GPS1	0.78316585	0.697885203	0.804002279	0.784158627	0.882145303	0.891088733
ZBTB34	0.78316585	0.67048768	0.720612298	0.809277776	0.924277487	0.913221224
MAU2	0.78316585	0.643417752	0.786126716	0.890362631	0.735091896	0.952817435
PSMD10	0.78316585	0.659622605	0.838341783	0.792161305	0.788007619	0.957318253
LOC112448744	0.78316585	0.615602441	0.731143569	0.731537809	0.859351739	0.996813547
GOT2	0.783246293	0.628547317	0.779581461	0.764780853	0.936108073	0.910085997
CHAF1B	0.783274585	0.681222457	0.817582037	0.755247535	0.999859158	0.729499398
ZNF768	0.783274585	0.658075243	0.836482	0.891988949	0.931438412	0.745952648
CHCHD6	0.783274585	0.792266336	0.91882781	0.825530631	0.767678283	0.777374794

VPS37C	0.783274585	0.709247882	0.77566312	0.87522058	0.738738532	0.93531275
MRPS10	0.783376539	0.626321326	0.846302631	0.812453944	0.797227609	0.945870804
CUEDC2	0.783593771	0.737127482	0.754913805	0.773429607	0.998287238	0.733767439
LOC504548	0.783593771	0.972007528	0.715271956	0.817144975	0.833864947	0.74201596
LOC100139549	0.783593771	0.709348874	0.96030829	0.823434028	0.794743818	0.763466477
LOC112442719	0.783593771	0.840785555	0.920462513	0.808075336	0.749131936	0.768572658
IQCA1	0.783593771	0.872175143	0.716120406	0.925294859	0.801692604	0.77112874
FRZB	0.783593771	0.774979074	0.707407957	0.987591144	0.788007619	0.785562356
PUF60	0.783593771	0.648904409	0.916294854	0.896116841	0.75925	0.832964812
LOC100848331	0.783593771	0.718220849	0.709649545	0.873302281	0.891726429	0.870490204
LOC513329	0.783593771	0.74906219	0.75197571	0.828658981	0.847092933	0.89148147
NLRX1	0.783593771	0.68903984	0.770595941	0.777956162	0.900893337	0.911638347
FAM204A	0.784197966	0.697306013	0.855268213	0.822871493	0.905825822	0.789870764
LOC112441472	0.784197966	0.614976364	0.774015998	0.965050971	0.810436875	0.8537109
LOC510520	0.784197966	0.692305416	0.7786153	0.954832177	0.738811608	0.867063767
SSR3	0.784371769	0.638084745	0.777540303	0.97731816	0.832486638	0.795129319
DEGS1	0.784371769	0.62215202	0.804002279	0.846996336	0.891304411	0.879576441
TOR2A	0.784401904	0.622818484	0.813441395	0.999981514	0.819329746	0.742588975
SOCS7	0.784401904	0.709247882	0.841294863	0.786943075	0.962513981	0.754218406
LOC504773	0.784401904	0.766595431	0.720612298	0.906709343	0.87389153	0.814218101
DERL3	0.784401904	0.682216234	0.924855847	0.812453944	0.804274107	0.844759721
ELOB	0.784401904	0.683744977	0.803342698	0.762598894	0.943160494	0.865767172
OLA1	0.784401904	0.690635606	0.869932384	0.731537809	0.810436875	0.938894901
NUPR1	0.784489575	0.612070685	0.744735369	0.993481918	0.84620147	0.805925428
FAM92B	0.78480792	0.707638393	0.874983674	0.758601772	0.953391502	0.766435538
CTH	0.784930624	0.687020192	0.955427429	0.904018489	0.781724458	0.727876258
BASP1	0.785011743	0.833323491	0.774015998	0.868378265	0.83704813	0.791275236
CREB1	0.785053879	0.674048073	0.794225743	0.832489049	0.984386856	0.72571141
LOC112447474	0.785053879	0.737941984	0.746818005	0.819968583	0.788739452	0.955210102
CHRM1	0.785178872	0.77844414	0.879608555	0.914643464	0.776537165	0.748921056
RNF121	0.785369178	0.725876283	0.766709403	0.885789665	0.891205587	0.818370468
TRAPPC13	0.785417421	0.636523083	0.858032788	0.764140634	0.833186643	0.940583352
LOC783224	0.785528903	0.93738232	0.833583844	0.78252493	0.788739452	0.772589817
PPP1R9B	0.785597827	0.621713316	0.845113191	0.868255052	0.964301327	0.740843731
DUS1L	0.785597827	0.740319373	0.916323169	0.792140715	0.86010579	0.779201808
SKP1	0.785597827	0.617216601	0.78930905	0.860166343	0.818179113	0.943285784
PAPSS2	0.78567918	0.947350735	0.788106994	0.866566721	0.735091896	0.75702988
CDON	0.78567918	0.811354298	0.880357899	0.796843021	0.804625939	0.81853698

LGI2	0.78567918	0.681192035	0.750313078	0.974286747	0.789999838	0.845075821
LOC101907577	0.78567918	0.709303377	0.720925701	0.94778564	0.804274107	0.872238439
SLC25A30	0.78567918	0.72742557	0.774924967	0.852481062	0.823508064	0.893838339
TROVE2	0.786102645	0.689723517	0.873674244	0.889415593	0.900927045	0.729499398
COLQ	0.786211047	0.8857401	0.75029951	0.944351122	0.781724458	0.733767439
LTC4S	0.786211047	0.715801466	0.925320071	0.916858019	0.75602825	0.748921056
TOR1AIP2	0.786211047	0.676397431	0.850941364	0.959616431	0.823508064	0.749918186
SLC48A1	0.786211047	0.629235398	0.896821247	0.925294859	0.847173934	0.751714113
PIK3R3	0.786211047	0.659408339	0.724436373	0.78584336	0.999985752	0.754218406
ANKRD45	0.786211047	0.868390063	0.773165287	0.855651954	0.832486638	0.780933489
RP2	0.786211047	0.712535219	0.744735369	0.990955345	0.782990999	0.803177592
MRPL27	0.786211047	0.692305416	0.852214402	0.792140715	0.916605572	0.835231445
ITIH1	0.786211047	0.84238625	0.758146087	0.812453944	0.833186643	0.854935725
REPIN1	0.786211047	0.753090681	0.727870684	0.780279943	0.91563394	0.891151691
LOC107131619	0.786211047	0.716712413	0.810647419	0.852170016	0.723467697	0.943972369
LOC101907545	0.786220459	0.709247882	0.711148626	0.869868859	0.943702351	0.835231445
AURKAIP1	0.786220459	0.658496391	0.856952806	0.777956162	0.935286646	0.840531072
RNF2	0.786464213	0.813759037	0.716120406	0.886831994	0.870450746	0.821586021
PM20D1	0.786549385	0.668634225	0.717242466	0.962437357	0.926813827	0.761858515
LRP4	0.786549385	0.760515997	0.91882781	0.886312303	0.754089854	0.769735861
CD247	0.786674107	0.893615781	0.862577505	0.837820405	0.738738532	0.77593589
DENND1B	0.786674107	0.811567701	0.832065705	0.862823588	0.81911207	0.788462021
CNKSR1	0.786674107	0.621279921	0.751957722	0.984423083	0.823508064	0.835231445
NT5C	0.786674107	0.757515036	0.809673119	0.88099367	0.818328184	0.841451122
STK17A	0.786779731	0.718382634	0.950055019	0.91285419	0.727879947	0.74201596
LOC104974937	0.786779731	0.663834635	0.732073575	0.785246104	0.999985752	0.742161724
SNF8	0.786779731	0.699144965	0.916294854	0.890499731	0.801692604	0.774932208
PEX11A	0.786779731	0.791194847	0.797180963	0.936882654	0.789915129	0.78096075
MTFR1	0.786779731	0.652446942	0.852916849	0.883695093	0.907323851	0.780963557
JUNB	0.786779731	0.622818484	0.855847948	0.958588196	0.810436875	0.799322384
PDZD11	0.786779731	0.699192406	0.899325654	0.812313105	0.877712931	0.801888363
DDX39A	0.786779731	0.687020192	0.810167226	0.906186004	0.851927006	0.832770678
MMP16	0.786779731	0.750087757	0.77566312	0.870591999	0.870450746	0.836111377
HSD17B7	0.786779731	0.634059237	0.860731747	0.765499767	0.950729461	0.840421342
BRB	0.786779731	0.766595431	0.774015998	0.774800098	0.882013597	0.888475311
SERBP1	0.786779731	0.622818484	0.855811972	0.854220165	0.774169346	0.93640423
KANSL1	0.786941195	0.775033799	0.754913805	0.763968157	0.996781978	0.729499398
MSN	0.786941195	0.65159362	0.828806956	0.832489049	0.976292433	0.741319465

FANCM	0.786941195	0.693703257	0.79497298	0.993481918	0.811276938	0.742687461
LOC107133049	0.786941195	0.866901725	0.750313078	0.889415593	0.84620147	0.762133815
LOC112446423	0.786941195	0.877893862	0.754913805	0.764257755	0.929896768	0.770748374
ARFRP1	0.786941195	0.871924797	0.79497298	0.77872142	0.870450746	0.802390255
LOC104976232	0.786941195	0.673096434	0.971594714	0.734472882	0.850085238	0.812036123
OPHN1	0.786941195	0.727966344	0.893761108	0.870210705	0.794743818	0.824025963
AP4B1	0.786941195	0.622818484	0.779209064	0.96582208	0.838461406	0.83150957
LOC112444521	0.786941195	0.823589054	0.942357321	0.749510262	0.735091896	0.834911683
GLRX	0.786941195	0.81745062	0.868360233	0.764257755	0.818977667	0.842473495
LOC104975324	0.786941195	0.650600033	0.869212413	0.748837705	0.943055455	0.847193872
LOC101903232	0.786941195	0.664837103	0.723132042	0.977002843	0.788007619	0.87778492
STIM1	0.786941195	0.614976364	0.871782109	0.757403199	0.881680572	0.923547711
C5H12orf73	0.786941195	0.659408339	0.754913805	0.777956162	0.860594228	0.966850975
KCTD20	0.787001485	0.915269318	0.854571175	0.819968583	0.79453727	0.747871787
VPS13C	0.787446107	0.766595431	0.87706952	0.793333588	0.917407853	0.749726404
HTR1B	0.787680961	0.863064327	0.806470927	0.812999186	0.852746309	0.785562356
SPCS2	0.787958688	0.649340041	0.923327823	0.898818046	0.787248506	0.814218101
BCL2	0.787993179	0.659195551	0.768247414	0.999981514	0.728185525	0.742309541
RPAP2	0.787993179	0.675765841	0.849389234	0.999981514	0.735287397	0.748921056
PLXND1	0.787993179	0.824188473	0.747666034	0.900497667	0.86864151	0.762133815
IFNLR1	0.787993179	0.823478017	0.77566312	0.890362631	0.841758501	0.78087541
MMP25	0.787993179	0.752383445	0.933884582	0.805138513	0.799272579	0.811296955
NPAS3	0.787993179	0.692775132	0.721294664	0.898818046	0.939203547	0.8150125
CLEC5A	0.788034918	0.757917107	0.880665752	0.898818046	0.844384115	0.727876258
MICAL2	0.788063589	0.761754776	0.822300339	0.889881783	0.847092933	0.789870764
MRPS31	0.788153407	0.679199291	0.867222363	0.753267542	0.788995893	0.960023723
METRNL	0.788270275	0.692305416	0.79497298	0.874170259	0.926972948	0.801888363
PKIG	0.788270275	0.901326757	0.77566312	0.762472696	0.847092933	0.835338212
IL20RA	0.788442021	0.759773597	0.99341711	0.777956162	0.781724458	0.742309541
FHIT	0.788442021	0.846724749	0.743624166	0.944351122	0.817950097	0.748867818
C5H22orf23	0.788442021	0.616227995	0.819322085	0.942955143	0.887514169	0.785562356
PALMD	0.788454935	0.77022554	0.945575068	0.853058361	0.776537165	0.754218406
LOC112442547	0.788454935	0.709247882	0.71967891	0.765499767	0.999985752	0.775725294
HLF	0.788454935	0.624744876	0.871285807	0.899855526	0.891726429	0.778344889
PRODH	0.788564784	0.719152669	0.80412682	0.990955345	0.801692604	0.739270623
USP24	0.788564784	0.678023292	0.819359511	0.852875175	0.963550093	0.748921056
DHX30	0.788564784	0.693513972	0.864153038	0.855232617	0.926972948	0.754218406
MAFK	0.788564784	0.758119468	0.879939804	0.912922827	0.760007802	0.784529314

BPI	0.788564784	0.791281103	0.947651663	0.820971736	0.735091896	0.785562356
ORC4	0.788564784	0.774644712	0.804002279	0.925353907	0.809913252	0.785562356
EVA1B	0.788564784	0.673435317	0.929661805	0.815919419	0.870450746	0.786849547
PIGW	0.788564784	0.705532659	0.957052938	0.799147532	0.808945365	0.806476213
CLBA1	0.788564784	0.709247882	0.916294854	0.830427818	0.823508064	0.812476232
LOC614208	0.788564784	0.808478344	0.844022087	0.777956162	0.796861564	0.885202945
C1QTNF7	0.788564784	0.790885918	0.720612298	0.805217242	0.872096228	0.911186983
PPFIA4	0.788564784	0.712535219	0.711148626	0.952247001	0.760007802	0.91373422
RNF144B	0.788564784	0.642929022	0.744735369	0.896116841	0.769529419	0.966196012
DNAJC30	0.788814682	0.662013091	0.731143569	0.812999186	0.999985752	0.765070525
ADAMTSL1	0.788863623	0.708204618	0.998335361	0.812453944	0.788007619	0.724693895
TRUB2	0.789341042	0.716415149	0.799265115	0.774214837	0.999453276	0.723555931
ALKBH8	0.789341042	0.939956757	0.754913805	0.771412309	0.893376996	0.748750383
UTP6	0.789341042	0.68903984	0.852916849	0.904018489	0.891304411	0.754218406
LOC112445044	0.789341042	0.763249441	0.76555928	0.977689659	0.801692604	0.765644158
ZNF644	0.789341042	0.769416761	0.770295064	0.745577087	0.976769047	0.78288746
ALKBH5	0.789341042	0.614976364	0.852624484	0.98471945	0.799272579	0.785562356
FAM216A	0.789341042	0.869754045	0.809673119	0.828736799	0.80599066	0.814745299
EIF3K	0.789341042	0.713792742	0.851633941	0.884452623	0.796861564	0.865745718
PHB2	0.789341042	0.709247882	0.915858924	0.759755663	0.834510805	0.867447114
MRPS2	0.789341042	0.766595431	0.746818005	0.827786827	0.887514169	0.867482845
PPAN	0.789341042	0.678023292	0.778210036	0.831460524	0.875469001	0.911219114
ARSA	0.789591607	0.855310926	0.777154688	0.908467146	0.847092933	0.726043679
PKD1L2	0.789591607	0.748933195	0.739894258	0.89684883	0.954403133	0.739682579
PPP1R14A	0.789591607	0.714027042	0.838909114	0.77580333	0.795839201	0.94844234
GHSR	0.789591607	0.628558955	0.79497298	0.827461395	0.837915327	0.950798794
CSTF3	0.789591607	0.658075243	0.802277976	0.746624292	0.753088486	0.999948228
PGAM1	0.789620316	0.648830734	0.79497298	0.823682103	0.879395665	0.921772742
BAK1	0.789691236	0.718382634	0.77566312	0.784838526	0.89490694	0.904076478
LOC112443250	0.789791119	0.652509786	0.818612484	0.859320133	0.864524107	0.888887063
GRTP1	0.789970447	0.767113995	0.996267659	0.751194677	0.737149805	0.78087541
IFFO2	0.789991875	0.644121862	0.744735369	0.819571278	0.871558258	0.960023723
LOC539166	0.790133547	0.767570093	0.924855847	0.854623597	0.737149805	0.816245398
ASS1	0.79054308	0.757251331	0.832192335	0.757403199	0.964301327	0.768168974
FMNL3	0.79054308	0.636683453	0.826465082	0.896116841	0.932024143	0.782330566
POLE3	0.79054308	0.687020192	0.836691254	0.990955345	0.738738532	0.785562356
PRR22	0.790997525	0.632321074	0.798535967	0.962369147	0.823508064	0.839906041
LOC112443243	0.791458121	0.72828303	0.775552434	0.989485949	0.804037558	0.762133815

C1QTNF2	0.791458121	0.768359345	0.745477682	0.979385838	0.784972475	0.803177592
KNL1	0.791458121	0.674524738	0.872088608	0.918971786	0.820411915	0.806476213
LOC781494	0.791458121	0.835883963	0.71601562	0.929388614	0.751239872	0.862925606
MBOAT2	0.791458121	0.781965607	0.71633602	0.843593618	0.864524107	0.895426195
CPXM2	0.791458121	0.658520696	0.874983674	0.898818046	0.737633927	0.895855654
ZNF667	0.791458121	0.659837453	0.731708587	0.893220599	0.877712931	0.909056553
SLC46A1	0.791458121	0.659408339	0.885896294	0.779773412	0.795865165	0.943972369
TMEM154	0.791969843	0.685572308	0.89478142	0.805568606	0.930451229	0.779201808
EML4	0.792501451	0.792109226	0.774015998	0.813323291	0.916877832	0.81956221
DNAJC4	0.792532049	0.690635606	0.964382669	0.866566721	0.810436875	0.748921056
EXOC3L2	0.792532049	0.741310853	0.713592297	0.912777307	0.943702351	0.765948605
COQ6	0.792532049	0.687020192	0.882643407	0.753267542	0.956041014	0.798336045
LOC107131356	0.792777203	0.781543413	0.842891367	0.973075232	0.753088486	0.745988271
IFT172	0.792777203	0.622818484	0.955427429	0.894086736	0.750755368	0.83150957
CHCHD7	0.792777203	0.688618972	0.969404007	0.812453944	0.750277829	0.84413042
OMP	0.792777203	0.664859217	0.86986745	0.952247001	0.735091896	0.849595612
LOC107132032	0.792777203	0.704512234	0.8735515	0.76992117	0.887514169	0.865767172
NT5DC1	0.792777203	0.707837135	0.749453648	0.884819185	0.862205587	0.89148147
MGAT3	0.792777203	0.741263262	0.728269425	0.901115098	0.763959048	0.937689834
ARFIP1	0.793146739	0.814177827	0.788106994	0.817230639	0.849766114	0.85634226
RSF1	0.793380141	0.764456895	0.846372547	0.827228897	0.911664199	0.769865166
ATP7B	0.793380141	0.931588197	0.81412252	0.813323291	0.799752471	0.778711943
IL17RA	0.793380141	0.715129484	0.777540303	0.934466438	0.821148296	0.842654067
TMEFF2	0.793396704	0.790429889	0.835664645	0.786099236	0.958384396	0.737362929
CRIP1	0.793396704	0.775033799	0.731143569	0.782811812	0.971475922	0.814218101
HDAC2	0.793585106	0.645547325	0.830135741	0.799480811	0.89052284	0.915414597
SPIDR	0.793585106	0.669828549	0.855892553	0.80084173	0.74434995	0.970071797
NKIRAS1	0.793994657	0.731024992	0.805479206	0.783908833	0.97352263	0.775725294
LOC100337108	0.794010325	0.938087741	0.724908605	0.961898258	0.735091896	0.737726964
KCNG3	0.794010325	0.741310853	0.730571184	0.999981514	0.799752471	0.748323122
LMBR1	0.794010325	0.621713316	0.833583844	0.979644238	0.867558388	0.755679512
MNS1	0.794010325	0.620171743	0.891662311	0.902793721	0.886041168	0.781629683
OBSL1	0.794010325	0.740343181	0.745942803	0.985717249	0.802592922	0.796929234
STK16	0.794010325	0.744671791	0.90226609	0.790377592	0.883143938	0.801888363
LEPR	0.794010325	0.929019822	0.724981308	0.837820405	0.829853428	0.809961137
SNX5	0.794010325	0.761907799	0.759332589	0.964521432	0.772501291	0.833150766
LOC786726	0.794010325	0.640335813	0.875382795	0.942955143	0.780744244	0.845747012
ABCA4	0.794010325	0.802570554	0.727870684	0.777956162	0.934481683	0.865767172

STIL	0.794010325	0.773508874	0.744735369	0.912777307	0.753088486	0.908667118
XCL2	0.794010325	0.697306013	0.720612298	0.827652764	0.887514169	0.93363377
HDAC6	0.794010325	0.649949716	0.814495418	0.836610893	0.772614149	0.969754487
DNAJC1	0.794041542	0.768359345	0.753710115	0.769321578	0.965933092	0.820286425
OTUD1	0.794062754	0.664104209	0.869932384	0.95901497	0.790136352	0.805925428
GLO1	0.794097928	0.683368775	0.755135544	0.85647796	0.953300742	0.837559792
CD55	0.794168944	0.750379179	0.953470857	0.7694401	0.864524107	0.762133815
WDHD1	0.794330781	0.684972622	0.784788059	0.894086736	0.958797242	0.754218406
DDX24	0.794330781	0.692463931	0.964382669	0.793333588	0.750277829	0.867063767
COBLL1	0.794330781	0.648503747	0.744735369	0.876616876	0.917407853	0.888475311
JAGN1	0.79439934	0.781836924	0.893761108	0.889415593	0.823508064	0.748323122
LOC100336208	0.79439934	0.702392068	0.761415057	0.839245804	0.988881329	0.748323122
GARNL3	0.79439934	0.880565292	0.888566052	0.780279943	0.818977667	0.772589817
FBXO6	0.79439934	0.690635606	0.911535369	0.887642126	0.845802744	0.782330566
CEP55	0.79439934	0.824156489	0.813441395	0.940136897	0.753139644	0.783241453
PDAP1	0.79439934	0.907585165	0.894316021	0.777956162	0.766444631	0.791101465
SNAI2	0.79439934	0.802570554	0.727619107	0.769701093	0.964301327	0.830504438
LOC782021	0.79439934	0.752229543	0.77566312	0.841411503	0.845387518	0.89438401
VPS8	0.794537708	0.69591721	0.854231171	0.903564774	0.837919392	0.824025963
FCRLA	0.794537708	0.758733912	0.781109205	0.924425698	0.774289699	0.867063767
ACADM	0.794537708	0.622423182	0.745208017	0.886312303	0.837028469	0.958707771
LGR4	0.79471778	0.722968232	0.863546668	0.915039335	0.784972475	0.835231445
MFSD10	0.794958289	0.879093989	0.916323169	0.771699334	0.814984338	0.749726404
CDC40	0.794958289	0.920382325	0.822300339	0.819968583	0.776537165	0.809961137
COL4A6	0.794959907	0.995373357	0.723132042	0.777956162	0.823508064	0.748921056
PSMG2	0.794959907	0.648958657	0.899952655	0.792140715	0.753088486	0.958790968
OIP5	0.794969249	0.751470284	0.987700803	0.853058361	0.75925	0.728699286
LOC112449072	0.794969249	0.877893862	0.917155067	0.853058361	0.735091896	0.745694174
GPR45	0.794969249	0.914514746	0.756014804	0.764257755	0.939203547	0.748223279
ESPL1	0.794969249	0.687651905	0.916323169	0.908467146	0.833173678	0.749918186
LOC101905875	0.794969249	0.69349295	0.810167226	0.949565453	0.888656048	0.754218406
SLC19A3	0.794969249	0.648503747	0.93190909	0.949565453	0.789492208	0.75640676
LOC107131516	0.794969249	0.669828549	0.912183977	0.748327767	0.964301327	0.769865166
DCAF6	0.794969249	0.882242226	0.743278326	0.941796131	0.788007619	0.772618249
RNF19B	0.794969249	0.634059237	0.732480837	0.98981682	0.892446951	0.783969061
SEC31B	0.794969249	0.65833629	0.774015998	0.945113651	0.906893576	0.792412747
LOC787714	0.794969249	0.687651905	0.999993442	0.757463039	0.767160171	0.796158754
VMP1	0.794969249	0.718382634	0.97773026	0.815919419	0.752560181	0.812036123

NUP43	0.794969249	0.807742849	0.73826834	0.948668643	0.797489438	0.830187739
MMAA	0.794969249	0.624373062	0.779581461	0.885789665	0.953300742	0.832964812
ANKRD42	0.794969249	0.660882273	0.754557912	0.933718942	0.893716526	0.839565788
TMEM86A	0.794969249	0.666011367	0.776098141	0.809277776	0.958384396	0.864139695
LOC112442081	0.794969249	0.69820785	0.867661896	0.774800098	0.891304411	0.873130182
CSRP1	0.794969249	0.800861368	0.816553355	0.764257755	0.834569503	0.905509632
CCDC6	0.794969249	0.65159362	0.817582037	0.892558706	0.792867703	0.927215832
PLEKHH3	0.79513934	0.732885933	0.867222363	0.898818046	0.804602594	0.824780045
HSPG2	0.795473587	0.664859217	0.874063521	0.998529748	0.789679352	0.727876258
GPR20	0.795473587	0.730581055	0.852225514	0.796934171	0.948421303	0.783241453
ANKZF1	0.795616653	0.636937576	0.95077817	0.774214837	0.844384115	0.867907362
SCN2B	0.795735674	0.906064108	0.75624592	0.777956162	0.804037558	0.879576441
LOC112446759	0.795745667	0.626740757	0.849389234	0.962369147	0.823508064	0.816246746
LOC782938	0.796354611	0.672505775	0.948653258	0.907621547	0.73609618	0.814218101
RANBP10	0.796354611	0.742703566	0.76746129	0.762159685	0.769529419	0.993808733
SNAPC4	0.796527297	0.649949716	0.964807927	0.896116841	0.776537165	0.782168676
LOC101906870	0.796578907	0.941655994	0.830718919	0.749104032	0.784972475	0.839906041
NUP160	0.796659033	0.811059858	0.8042974	0.831577385	0.943702351	0.734177434
LOC107131293	0.796659033	0.625469932	0.744735369	0.822069434	0.999985752	0.748223279
LOC104969409	0.796659033	0.997471998	0.71967891	0.782217967	0.820264258	0.750241232
PSMC1	0.796659033	0.694400411	0.865717285	0.861243324	0.760007802	0.923092436
MEIOB	0.796659033	0.712357273	0.745803187	0.76992117	0.863694174	0.968138839
PLXNA1	0.796659033	0.709247882	0.761415057	0.782217967	0.788007619	0.991421487
CEP112	0.796849566	0.783112117	0.810647419	0.848592907	0.852968331	0.848282393
CDH6	0.796904298	0.872175143	0.740393806	0.868530316	0.887514169	0.77333513
LPAR2	0.796904298	0.739236562	0.845113191	0.928404714	0.838628427	0.776734587
RAD54B	0.796904298	0.649340041	0.999993442	0.810557898	0.755735635	0.78087541
GRAMD1A	0.796904298	0.782783231	0.849912291	0.745806541	0.954913166	0.78087541
INTS12	0.796904298	0.748669855	0.899106435	0.777956162	0.765961853	0.917930485
PHYH	0.796904298	0.672048448	0.749417651	0.929342214	0.804274107	0.9221477
CX3CL1	0.797383623	0.88349845	0.77797137	0.959159289	0.777218526	0.729499398
RANBP2	0.797383623	0.685297756	0.810647419	0.836387732	0.990192394	0.730265513
IFT80	0.797383623	0.855067335	0.896847099	0.803690724	0.828756107	0.762133815
RANGAP1	0.797383623	0.741310853	0.780367031	0.918693944	0.738738532	0.911219114
MYL4	0.797383623	0.901984573	0.774924967	0.796128007	0.737297505	0.91373422
EIF2A	0.797383623	0.644366672	0.872204943	0.757751836	0.855920132	0.944973383
NMI	0.797525647	0.997678999	0.769830851	0.777956162	0.774289699	0.754218406
LOC789569	0.797525647	0.72742557	0.731143569	0.764257755	0.93315562	0.928416436

TESK2	0.797689711	0.702655356	0.734143952	0.870210705	0.766655597	0.9790177
LOC101906484	0.797753016	0.898706315	0.731143569	0.949502614	0.793993073	0.759003017
LOC101903383	0.797753016	0.696797615	0.754913805	0.956685049	0.773874829	0.899183258
IL23A	0.798231607	0.981670901	0.749453648	0.796843021	0.862852956	0.732191464
LOC101904227	0.798231607	0.792109226	0.809673119	0.941467375	0.810263299	0.779201808
EMILIN1	0.798231607	0.77766577	0.727703335	0.943808491	0.854893996	0.82345874
LOC104976082	0.798231607	0.710835581	0.806470927	0.952247001	0.769529419	0.86324237
MIGA2	0.798494533	0.808785257	0.835725901	0.751643991	0.962513981	0.752213117
CALU	0.798634878	0.728513666	0.838909114	0.97828838	0.82114467	0.739151902
RHPN1	0.798662521	0.630230756	0.805674392	0.999981514	0.865505927	0.742400088
CSGALNACT2	0.798662521	0.648503747	0.744735369	0.802977147	0.999985752	0.762133815
PHLDA3	0.798662521	0.915269318	0.744735369	0.844621856	0.864524107	0.78087541
TMEM26	0.798677946	0.651269643	0.867257372	0.955954309	0.887514169	0.737726964
NKAP	0.798677946	0.822956796	0.916323169	0.900875874	0.737633927	0.748223279
NTN4	0.798677946	0.880565292	0.798782742	0.812453944	0.821351993	0.842214812
ZNF76	0.798677946	0.649949716	0.947462602	0.872590202	0.788850323	0.842473495
HIST1H1E	0.798677946	0.912602201	0.758146087	0.766750054	0.864524107	0.844304528
LAMC2	0.798677946	0.690635606	0.95875611	0.808075336	0.790319629	0.852504043
CISD1	0.79903121	0.669828549	0.78502605	0.771412309	0.959251971	0.888475311
CTXN1	0.79956782	0.684946765	0.916294854	0.880401606	0.865234947	0.780283623
NSFL1C	0.79956782	0.722172907	0.895047296	0.795909483	0.864524107	0.855049294
AIFM3	0.79956782	0.64688053	0.75029951	0.866566721	0.924222719	0.903990996
FOXJ2	0.799734052	0.866269779	0.916294854	0.777956162	0.791463181	0.800967059
FKTN	0.799734052	0.933100216	0.731143569	0.754719584	0.738738532	0.940308142
LOC784914	0.800182437	0.741310853	0.764467593	0.896116841	0.833864947	0.879576441
ANXA6	0.800342788	0.694960349	0.862253703	0.777956162	0.823508064	0.940017687
POLR2A	0.800404716	0.684919506	0.77699301	0.799480811	0.999985752	0.747934012
SLC2A4	0.800404716	0.658075243	0.855892553	0.773429607	0.996781978	0.758917718
ARF5	0.800404716	0.81232489	0.727870684	0.819968583	0.970988226	0.769865166
TACC3	0.800404716	0.722779653	0.915120516	0.876355536	0.810436875	0.814745299
OSBPL5	0.800404716	0.7973731	0.791843351	0.961898258	0.747947334	0.81853698
MAGT1	0.800404716	0.821674572	0.91416437	0.812453944	0.772103109	0.835231445
MANBA	0.800404716	0.70496036	0.777540303	0.855886739	0.935093621	0.844759721
MAFG	0.800404716	0.697015989	0.79497298	0.956685049	0.764945406	0.881189314
LOC101904614	0.800404716	0.696797615	0.834683675	0.775276339	0.891304411	0.91373422
LOC101906676	0.800404716	0.641142913	0.824121854	0.892910741	0.804049674	0.92789871
RAD23B	0.800566365	0.657900042	0.899106435	0.880851997	0.738738532	0.91618909
LOC101907133	0.800566365	0.801469106	0.796499368	0.78584336	0.799272579	0.93839036

LOC100336564	0.801340987	0.796253817	0.885896294	0.901115098	0.789915129	0.77593589
RASGEF1B	0.80136877	0.66922042	0.893988897	0.930882136	0.826634148	0.786936221
SULT1A1	0.80136877	0.663309441	0.739894258	0.952247001	0.882252274	0.855963573
DUSP8	0.801449433	0.693905757	0.731143569	0.757984796	0.788007619	0.999948228
C18H16orf46	0.801502717	0.658496391	0.847776765	0.765499767	0.917407853	0.912531667
C3H1orf43	0.801502717	0.693513972	0.847835111	0.830187312	0.81954052	0.923092436
TEAD3	0.802038027	0.99488888	0.754913805	0.853058361	0.740514915	0.742588975
TNK1	0.802038027	0.805580108	0.813441395	0.907794002	0.816540569	0.806476213
TMEM168	0.802038027	0.768359345	0.827969228	0.774800098	0.900927045	0.867063767
TICAM1	0.802038027	0.831506983	0.730571184	0.843613441	0.858869582	0.876861428
SORT1	0.802042142	0.705008978	0.820248241	0.908467146	0.942462162	0.740672585
RPRM	0.802099394	0.731596902	0.860877703	0.949502614	0.849786155	0.742456493
GABRE	0.802099394	0.661138839	0.979494108	0.799480811	0.882252274	0.762133815
GAS8	0.802099394	0.928658222	0.771066142	0.792161305	0.879776073	0.783241453
RIMKLA	0.802099394	0.913116251	0.816005434	0.751643991	0.891304411	0.783241453
NECTIN4	0.802099394	0.883890743	0.730571184	0.817146543	0.924795709	0.793197037
ZNF706	0.802099394	0.704346844	0.84685641	0.951936643	0.823508064	0.795936781
HECA	0.802099394	0.675765841	0.805702274	0.876703811	0.936108073	0.83150957
LOC618367	0.802099394	0.646834837	0.808037655	0.959805738	0.751764383	0.912486829
SWT1	0.802099394	0.796253817	0.834683675	0.809277776	0.738738532	0.940017687
CDCA7	0.802278862	0.878155433	0.744735369	0.929663263	0.784972475	0.816246746
GPR17	0.80232647	0.850337973	0.768932648	0.852481062	0.764554616	0.907145443
DCAF4	0.802543533	0.660882273	0.746818005	0.990955345	0.876359217	0.792412747
TMEM14C	0.802543533	0.783112117	0.805212947	0.822069434	0.878451408	0.86324237
LOC514189	0.802543533	0.737740845	0.828449846	0.827443542	0.882206878	0.868117674
BAIAP2	0.802543533	0.829067393	0.828449846	0.756729528	0.799272579	0.925995537
GABRG3	0.802779824	0.63850019	0.749140224	0.787435087	0.999453276	0.849493381
SYT7	0.802886221	0.6385246	0.959725886	0.768290504	0.887514169	0.835231445
DMAC2	0.802950605	0.713792742	0.831933739	0.852170016	0.956906331	0.765948605
LOC104974272	0.802950605	0.683735904	0.754913805	0.908467146	0.891726429	0.867063767
LOC786039	0.802950605	0.697306013	0.745477682	0.915013589	0.82114467	0.919124527
LOC789748	0.802950605	0.710645465	0.855793459	0.763977006	0.81725718	0.955990509
CD3EAP	0.802950605	0.649360352	0.788126358	0.885789665	0.804274107	0.958790968
HR	0.803523289	0.731024992	0.955221146	0.882057818	0.799272579	0.762133815
LOC101908185	0.803523289	0.813305033	0.779034209	0.819968583	0.755089345	0.945854375
PFKFB3	0.803552415	0.89417747	0.920682633	0.812453944	0.801692604	0.734177434
LOC112447066	0.803552415	0.649340041	0.980032241	0.870210705	0.854966579	0.735998098
KIF5C	0.803552415	0.708204618	0.731708587	0.999981514	0.881273907	0.737726964

PDS5B	0.803552415	0.923232089	0.735532321	0.870591999	0.89052284	0.742309541
RUSC1	0.803552415	0.760778257	0.797180963	0.946605077	0.891304411	0.742309541
FERMT2	0.803552415	0.739786945	0.972073392	0.855569741	0.804625939	0.745988271
MMS22L	0.803552415	0.744774335	0.966232793	0.784595865	0.870450746	0.753391198
NUDCD3	0.803552415	0.734509989	0.916323169	0.892047793	0.837915327	0.755647138
ARF2	0.803552415	0.664985713	0.905752358	0.979644238	0.744548748	0.783241453
ZNRF2	0.803552415	0.699144965	0.886829776	0.952247001	0.769529419	0.816246746
SEC61G	0.803552415	0.644132227	0.999993442	0.792161305	0.750542783	0.821257366
CDK5	0.803552415	0.692305416	0.806753023	0.792140715	0.97352263	0.832964812
GPATCH3	0.803552415	0.855141737	0.728269425	0.764257755	0.943160494	0.847012179
SLC25A36	0.803552415	0.708137441	0.810647419	0.858030541	0.823508064	0.923547711
BEX2	0.803552415	0.72828303	0.77447994	0.797338497	0.789679352	0.982871032
ZER1	0.803552415	0.630038239	0.75029951	0.892047793	0.765961853	0.988866378
C1QL3	0.803552415	0.737941984	0.736160515	0.76992117	0.789999838	0.99828741
LOC100140958	0.803583763	0.697043768	0.936898293	0.918693944	0.823508064	0.737726964
KCNG2	0.803583763	0.744241031	0.818131588	0.9880671	0.816306826	0.748750383
ASCC1	0.803583763	0.699192406	0.745348234	0.789139053	0.999985752	0.748921056
RECQL5	0.803583763	0.709247882	0.879608555	0.917892197	0.784972475	0.847012179
GYG2	0.803583763	0.863064327	0.86347788	0.812453944	0.787248506	0.8537109
MED17	0.803583763	0.697306013	0.866497255	0.764257755	0.89052284	0.903990996
AVL9	0.804004477	0.649340041	0.860877703	0.999981514	0.750277829	0.737726964
CEP290	0.804004477	0.648503747	0.842694695	0.999981514	0.750542783	0.742588975
LOC100126544	0.804004477	0.88008538	0.753188445	0.852170016	0.935286646	0.745694174
MINDY1	0.804004477	0.855079912	0.749417651	0.812453944	0.964963602	0.745952648
AHI1	0.804004477	0.651269643	0.728441914	0.763544094	0.999985752	0.746853748
KIAA0930	0.804004477	0.713646993	0.916294854	0.951936643	0.797227609	0.748323122
GDF5	0.804004477	0.704346844	0.990037865	0.774800098	0.884872176	0.748323122
RNF139	0.804004477	0.939956757	0.916294854	0.771699334	0.795839201	0.748750383
MAP3K8	0.804004477	0.878462725	0.851633941	0.93660503	0.741086403	0.748921056
LOC101904087	0.804004477	0.687651905	0.808037655	0.999981514	0.83704813	0.748921056
IQCA1L	0.804004477	0.905550751	0.930963858	0.812453944	0.759375734	0.749726404
EVL	0.804004477	0.76533726	0.798782742	0.99461374	0.801692604	0.751884708
DYNC1LI1	0.804004477	0.649340041	0.832632542	0.829793071	0.992984551	0.752213117
PPP3CA	0.804004477	0.766943819	0.949855095	0.759591583	0.896490594	0.754218406
ELF2	0.804004477	0.920702629	0.813441395	0.764257755	0.906893576	0.75640676
CKAP2	0.804004477	0.651269643	0.929661805	0.948668643	0.818179113	0.758917718
PTGER1	0.804004477	0.731024992	0.749417651	0.999981514	0.788995893	0.759003017
LOC112446753	0.804004477	0.848383949	0.972205442	0.782217967	0.760278238	0.762133815

IFITM3	0.804004477	0.916448625	0.804627059	0.817146543	0.879395665	0.765298372
BGN	0.804004477	0.768359345	0.867661896	0.898818046	0.864524107	0.765948605
PIK3R2	0.804004477	0.753090681	0.791557166	0.980532429	0.823508064	0.768572658
SLC43A1	0.804004477	0.718258172	0.946791586	0.904950341	0.790136352	0.769865166
NAB2	0.804004477	0.753022598	0.755135544	0.891988949	0.956904152	0.769865166
NEMP2	0.804004477	0.686805478	0.992511421	0.819835879	0.823508064	0.770035496
AKAP11	0.804004477	0.792109226	0.914816911	0.763977006	0.912033904	0.772618249
ZNRF1	0.804004477	0.708218805	0.731143569	0.989485949	0.89052284	0.77301049
FAN1	0.804004477	0.814246237	0.916294854	0.795430313	0.870450746	0.77593589
ZNF354C	0.804004477	0.791614315	0.872088608	0.932677501	0.788007619	0.779201808
PPP1R14C	0.804004477	0.644366672	0.998899782	0.852481062	0.788007619	0.78087541
PRKAG3	0.804004477	0.786691027	0.764872024	0.9745615	0.815913413	0.78096075
RAB22A	0.804004477	0.637855406	0.931080531	0.965724803	0.771532192	0.782330566
PPIP5K2	0.804004477	0.752229543	0.92323046	0.892047793	0.797227609	0.783241453
SMYD3	0.804004477	0.848383949	0.794676674	0.874424802	0.871558258	0.783241453
AKAP9	0.804004477	0.660882273	0.843776796	0.940025301	0.893376996	0.783241453
KIF3A	0.804004477	0.684972622	0.852916849	0.928075638	0.884872176	0.79187813
FAM221A	0.804004477	0.884168437	0.928417566	0.780184803	0.774940205	0.793197037
LSM10	0.804004477	0.648119635	0.966700703	0.896116841	0.789915129	0.793197037
FAM135A	0.804004477	0.788230732	0.775552434	0.866566721	0.922834705	0.811759324
BCL2L14	0.804004477	0.821674572	0.894447947	0.837820405	0.804049674	0.812476232
ASF1A	0.804004477	0.649052162	0.979494108	0.758601772	0.887514169	0.815129379
LOC101906526	0.804004477	0.947350735	0.755940516	0.765499767	0.87329316	0.81853698
MFSD4A	0.804004477	0.658503182	0.749764665	0.999981514	0.837915327	0.81956221
LOC100847765	0.804004477	0.898550443	0.826785415	0.784595865	0.854289643	0.81956221
AASS	0.804004477	0.791281103	0.818612484	0.808073654	0.928951321	0.81956221
INTS13	0.804004477	0.812449121	0.798782742	0.868435169	0.870356079	0.827126869
NDC80	0.804004477	0.647871625	0.957052938	0.898818046	0.776537165	0.829809521
SPSB4	0.804004477	0.819210468	0.784788059	0.817146543	0.916877832	0.829955257
TNFSF13B	0.804004477	0.652717399	0.999841067	0.815570184	0.758424886	0.83150957
TDP2	0.804004477	0.741263262	0.914057049	0.887187234	0.789915129	0.83150957
LOC104975111	0.804004477	0.72828303	0.729115949	0.999981514	0.789915129	0.831652079
ZNF219	0.804004477	0.704801601	0.877924496	0.852170016	0.889301074	0.831652079
USP21	0.804004477	0.632321074	0.852916849	0.973075232	0.804274107	0.836705424
THEMIS2	0.804004477	0.716758461	0.922552585	0.819213899	0.849786155	0.836705424
CDK5R1	0.804004477	0.749236881	0.832268748	0.882057818	0.864524107	0.839906041
SLC9A3R1	0.804004477	0.798434093	0.736435559	0.893220599	0.886944613	0.841312694
RB1	0.804004477	0.774934769	0.849389234	0.800283296	0.901853599	0.841312694

SLAMF8	0.804004477	0.663309441	0.837414887	0.942303202	0.826898407	0.850475013
RGL3	0.804004477	0.664859217	0.964382669	0.850762358	0.766655597	0.859032412
SLC25A37	0.804004477	0.708218805	0.867661896	0.907794002	0.801692604	0.862925606
TRIM13	0.804004477	0.647890563	0.831933739	0.786099236	0.967116582	0.86324237
LOC112443614	0.804004477	0.766943819	0.903431343	0.836248024	0.788995893	0.865767172
IRS2	0.804004477	0.672596838	0.802277976	0.812453944	0.956994968	0.867063767
NDUFS1	0.804004477	0.659195551	0.799533361	0.764257755	0.977193348	0.872238439
LOC788599	0.804004477	0.750432618	0.756302945	0.762472696	0.959565914	0.877014986
APOD	0.804004477	0.878551917	0.744735369	0.888687722	0.756033063	0.887542005
ZNF135	0.804004477	0.870296749	0.764467593	0.784595865	0.847092933	0.892605403
BOLA	0.804004477	0.709542765	0.91882781	0.815919419	0.788007619	0.903500283
SKP2	0.804004477	0.691301661	0.754913805	0.90413565	0.86930615	0.905509632
ELAC1	0.804004477	0.696797615	0.781992943	0.764257755	0.954403133	0.90607344
NLGN3	0.804004477	0.880565292	0.759357794	0.801629663	0.796861564	0.91373422
PRRT1	0.804004477	0.805283017	0.814765112	0.828736799	0.781850172	0.923092436
TAC3	0.804004477	0.685572308	0.838341783	0.904018489	0.772103109	0.923573245
EIF2B1	0.804004477	0.684946765	0.892343042	0.812453944	0.810436875	0.92789871
NLGN4X	0.804004477	0.781543413	0.779581461	0.85005716	0.796861564	0.93129461
WDR97	0.804004477	0.697306013	0.807254925	0.8418384	0.832486638	0.936263602
AHCY	0.804004477	0.704181021	0.77699301	0.869208308	0.801692604	0.958288244
METTL1	0.804004477	0.697015989	0.819527307	0.870408722	0.753088486	0.960023723
CALCOCO1	0.804004477	0.690635606	0.752695804	0.916858019	0.759375734	0.960023723
SIAH1	0.804004477	0.872175143	0.734527982	0.764257755	0.74028971	0.973649216
LANCL1	0.804004477	0.686805478	0.796517614	0.766020069	0.812127322	0.994347099
ZNF319	0.804004477	0.692305416	0.730695973	0.806898312	0.823508064	0.996453303
ANAPC5	0.80416085	0.64532731	0.813441395	0.967883482	0.823508064	0.854481531
C18H19orf84	0.804998004	0.64532731	0.731739801	0.792386115	0.893716526	0.986042926
BAMBI	0.805019712	0.777309385	0.768583827	0.903004767	0.803705802	0.893585844
VPS26B	0.805239915	0.687020192	0.879608555	0.999981514	0.788007619	0.742568903
KITLG	0.805239915	0.681192035	0.880476435	0.866566721	0.959658682	0.742568903
LOC104972567	0.805239915	0.749369443	0.979494108	0.885789665	0.753508098	0.754218406
TMEM140	0.805239915	0.865582541	0.86347788	0.839245804	0.844873801	0.77803403
LOC101908014	0.805239915	0.679199291	0.846372547	0.898818046	0.913732532	0.806714845
GGA2	0.805239915	0.758609923	0.904170228	0.853058361	0.802592922	0.849348058
REXO2	0.805239915	0.855752352	0.872088608	0.775617102	0.796861564	0.876103791
ICOS	0.805239915	0.654319826	0.971114007	0.818473754	0.776537165	0.876861428
PRMT6	0.805239915	0.673793679	0.906019013	0.797336494	0.789915129	0.947801358
B4GALT5	0.805239915	0.690635606	0.753188445	0.764257755	0.935286646	0.950798794

KCMF1	0.805239915	0.669828549	0.752695804	0.914770746	0.788007619	0.961265889
BST1	0.805239915	0.664439271	0.828449846	0.773694377	0.864524107	0.966914618
FYN	0.805995665	0.766491149	0.78057665	0.896116841	0.951996251	0.749726404
ALDH1B1	0.805995665	0.658174484	0.991936822	0.852005554	0.825799491	0.764467077
LOC104975593	0.806552782	0.865582541	0.800716826	0.984423083	0.748655165	0.742309541
MAZ	0.806552782	0.669124554	0.885896294	0.995565324	0.784972475	0.766218451
PIK3R1	0.806552782	0.65172183	0.744735369	0.999981514	0.87329316	0.770035496
LOC112442295	0.806552782	0.690635606	0.970427737	0.887877413	0.759137056	0.819366282
FAM21A	0.806552782	0.672531785	0.892343042	0.960937718	0.771532192	0.835231445
LOC782688	0.806552782	0.808478344	0.759332589	0.890362631	0.864524107	0.846210629
SESTD1	0.806552782	0.77139884	0.831933739	0.777956162	0.929802526	0.85519889
GLP1R	0.806552782	0.879093989	0.755135544	0.840247324	0.845387518	0.85643253
LOC112446129	0.806552782	0.750379179	0.908072171	0.829793071	0.795839201	0.875297569
SPPL2A	0.806552782	0.750379179	0.812051167	0.881493849	0.823508064	0.893585844
LOC107132589	0.806552782	0.652246332	0.916294854	0.843593618	0.769686429	0.930146713
LOC616957	0.806552782	0.812449121	0.731847616	0.887983383	0.755086175	0.945532151
ZNF275	0.806775763	0.669828549	0.776883713	0.812453944	0.999985752	0.769865166
EGFR	0.80693741	0.72742557	0.774924967	0.935278793	0.943160494	0.751705208
PMS1	0.807151461	0.688775944	0.771646484	0.793216078	0.91563394	0.94456864
PPP2R5E	0.807207563	0.800916955	0.929085764	0.823935462	0.87329316	0.742309541
ITSN1	0.807207563	0.729001857	0.749417651	0.784838526	0.999985752	0.748750383
GSDMD	0.807207563	0.783112117	0.939353663	0.882395013	0.80281845	0.762133815
E2F6	0.807207563	0.884798729	0.849389234	0.861243324	0.788007619	0.824025963
NICN1	0.807207563	0.652504746	0.947651663	0.827786827	0.848369675	0.848566057
HACD2	0.807207563	0.741310853	0.881070741	0.822691957	0.797227609	0.91373422
PITPNM1	0.807280038	0.811059858	0.895826828	0.828658981	0.892446951	0.746567999
MDFI	0.807280038	0.936822825	0.862253703	0.800283296	0.845095928	0.751100989
MYZAP	0.807280038	0.941400719	0.855793459	0.812453944	0.816306826	0.762133815
LOC101903868	0.807280038	0.718382634	0.731143569	0.999981514	0.790339877	0.779201808
C13H10orf113	0.807280038	0.656991364	0.751133563	0.999981514	0.754127588	0.783241453
CYB5RL	0.807280038	0.764456895	0.749417651	0.999981514	0.786362341	0.789870764
TJAP1	0.807280038	0.692305416	0.836691254	0.870210705	0.894364008	0.862925606
SUCLG1	0.807280038	0.693062595	0.845113191	0.796934171	0.943160494	0.867073248
SLC15A4	0.807343156	0.652869504	0.741510374	0.908467146	0.998196897	0.748921056
GRIN3A	0.807343156	0.639528357	0.999993442	0.796125318	0.792574144	0.815129379
EPAS1	0.807624353	0.687020192	0.920544372	0.821450642	0.929896768	0.78087541
LMO1	0.807624353	0.647102837	0.944126714	0.812313105	0.89052284	0.839906041
LOC101905367	0.807624353	0.831153015	0.746158282	0.866566721	0.883951804	0.851995112

SNRPD1	0.807624353	0.7889129	0.886829776	0.887642126	0.765866216	0.8537109
IFI16	0.808283195	0.772355877	0.991936822	0.777956162	0.845085806	0.742456493
IRAK1	0.808283195	0.878551917	0.89478142	0.792386115	0.786362341	0.842462038
ALDH4A1	0.808283195	0.766595431	0.755135544	0.780184803	0.964768333	0.858245612
LOC527388	0.808307506	0.696757704	0.892343042	0.887877413	0.775771176	0.904383301
NIPA1	0.808318946	0.691730737	0.818612484	0.999981514	0.823508064	0.742309541
DBF4	0.808318946	0.694134212	0.951392011	0.952247001	0.769686429	0.751705208
PKP2	0.808318946	0.99960614	0.735532321	0.812453944	0.796861564	0.751884708
VEGFD	0.808318946	0.952760839	0.855793459	0.778239004	0.838461406	0.75640676
CCND1	0.808318946	0.737721966	0.890716604	0.892047793	0.88885475	0.762133815
PSMA3	0.808318946	0.649340041	0.869932384	0.809886338	0.896570023	0.911234149
RFX2	0.808318946	0.744774335	0.735388121	0.834935925	0.900927045	0.923092436
C16H1orf74	0.808318946	0.692305416	0.867661896	0.777956162	0.871558258	0.930710695
NES	0.808347185	0.745791496	0.808785521	0.774800098	0.999453276	0.75640676
TMEM256	0.808347185	0.792661811	0.854571175	0.889415593	0.888234373	0.761143135
LOC787904	0.808347185	0.750379179	0.851822885	0.912922827	0.890153743	0.762133815
SENP6	0.808347185	0.660224009	0.734143952	0.82868625	0.999985752	0.776734587
NPEPPS	0.808347185	0.718477366	0.795299823	0.927802946	0.925171841	0.78096075
MARCH2	0.808347185	0.804185018	0.77566312	0.820971736	0.911568867	0.85532952
TNFRSF25	0.808347185	0.67048768	0.829745504	0.947313672	0.823508064	0.867063767
FOXC2	0.808347185	0.941817736	0.735532321	0.792071212	0.823508064	0.87501921
LOC112441639	0.808347185	0.759245424	0.759332589	0.865076519	0.864524107	0.912531667
CYP51A1	0.808469308	0.70880006	0.827969228	0.999981514	0.750277829	0.748323122
RCHY1	0.808469831	0.65159362	0.818612484	0.886312303	0.947346268	0.83821684
PLCH1	0.808537942	0.664439271	0.916038418	0.956685862	0.823508064	0.768168974
BOD1L1	0.808668276	0.709247882	0.884454208	0.921950515	0.862205587	0.781984152
MET	0.808671605	0.943244588	0.893761108	0.771699334	0.832407979	0.748323122
AKT1S1	0.808671605	0.882007869	0.734143952	0.784595865	0.870450746	0.903773776
LOC112441456	0.808671605	0.651269643	0.894338686	0.854450063	0.753978076	0.954853954
CAPN5	0.808695986	0.851948902	0.842694695	0.840700255	0.906893576	0.748921056
GPR65	0.809015981	0.710061038	0.81412252	0.796128007	0.991673538	0.784754767
PBX2	0.809242944	0.742303692	0.786411737	0.896116841	0.925950159	0.816246746
PLCH2	0.809520365	0.961663204	0.749417651	0.794299784	0.879776073	0.786936221
PRR15L	0.809636757	0.879574076	0.749453648	0.947799524	0.80599066	0.785562356
CAMTA1	0.809636757	0.687651905	0.78502605	0.769701093	0.864524107	0.982937529
HNRNPA2B1	0.809649133	0.76003522	0.745477682	0.965050971	0.808945365	0.860966325
LOC101907491	0.809739809	0.697032395	0.740372388	0.777956162	0.999985752	0.749918186
LOC790271	0.809739809	0.824201296	0.817582037	0.819835879	0.823508064	0.893585844

NUDT5	0.809807205	0.750466772	0.807258702	0.777956162	0.998287238	0.762133815
ENTPD7	0.809931762	0.687020192	0.898179681	0.836610893	0.964301327	0.748223279
LOC112445242	0.809931762	0.929737582	0.768247414	0.924160679	0.81066793	0.748323122
CDK2AP2	0.809931762	0.869754045	0.849218971	0.821450642	0.907678664	0.749726404
RAB7A	0.809931762	0.869953468	0.819527307	0.821440208	0.928951321	0.753373279
LOC104973519	0.809931762	0.710788298	0.927508464	0.965729104	0.772501291	0.754218406
KRIT1	0.809931762	0.934225172	0.811480663	0.777956162	0.907455286	0.754218406
ZC3H8	0.809931762	0.696914218	0.972410614	0.796002239	0.879776073	0.786849547
NLRP1	0.809931762	0.673793679	0.935266153	0.821450642	0.923910588	0.788462021
AGMO	0.809931762	0.68903984	0.789166592	0.999981514	0.769529419	0.814218101
RNASE4	0.809931762	0.96365708	0.81319557	0.801848398	0.801692604	0.814218101
PDCD11	0.809931762	0.659837453	0.744735369	0.96591587	0.924222719	0.829716215
TBL3	0.809931762	0.791281103	0.89399247	0.808073654	0.875880101	0.83150957
GNAI1	0.809931762	0.885620498	0.74088638	0.943808491	0.784972475	0.831652079
ZNF185	0.809931762	0.840785555	0.855811972	0.882395013	0.798683736	0.831652079
CBFB	0.809931762	0.69820785	0.916294854	0.843593618	0.87329316	0.835231445
LOC104970645	0.809931762	0.692305416	0.732073575	0.995565324	0.848696349	0.839565788
RPS6KB1	0.809931762	0.757251331	0.892343042	0.87165456	0.823508064	0.841312694
PDZD7	0.809931762	0.936822825	0.791843351	0.812453944	0.797227609	0.856770593
CHEK2	0.809931762	0.741310853	0.924405743	0.763841036	0.870189808	0.865767172
MDC1	0.809931762	0.709247882	0.754913805	0.892071982	0.926734976	0.865889509
RCCD1	0.809931762	0.710903449	0.860877703	0.819213899	0.906557107	0.865933526
VIPR2	0.809931762	0.696688354	0.850624788	0.92139105	0.821351993	0.867063767
LOC112449102	0.809931762	0.814162633	0.853455703	0.839725231	0.773264077	0.905509632
ZNF683	0.809931762	0.65097235	0.754557912	0.969088992	0.801692604	0.925125664
SERPINC1	0.809931762	0.813759037	0.756735411	0.764257755	0.849444776	0.958281344
CTTN	0.809931762	0.758609923	0.776353067	0.766675276	0.857014385	0.965705577
HS3ST1	0.810249697	0.66246801	0.95558643	0.805302469	0.789915129	0.91373422
TAF5	0.810429053	0.994185904	0.755135544	0.822691957	0.818179113	0.742687461
LOC101903126	0.810429053	0.915576843	0.867661896	0.815919419	0.862205587	0.748921056
SRGAP1	0.810429053	0.684972622	0.913100432	0.819213899	0.965676623	0.748921056
LOC112445007	0.810429053	0.674566493	0.862602899	0.999981514	0.763224862	0.759003017
NEK9	0.810429053	0.648119635	0.977455115	0.889415593	0.847092933	0.762133815
LOC112444681	0.810429053	0.95462892	0.838290164	0.819968583	0.809913252	0.769865166
RHOT1	0.810429053	0.762184568	0.805479206	0.816169012	0.976088926	0.780283623
THPO	0.810429053	0.695672965	0.916323169	0.856085962	0.90300429	0.783241453
ZCCHC17	0.810429053	0.77844414	0.863680222	0.834401906	0.89743683	0.81853698
PISD	0.810429053	0.762081499	0.798782742	0.812999186	0.961465747	0.823824518

FNTB	0.810429053	0.868390063	0.847776765	0.780703865	0.886785729	0.83150957
CBR3	0.810429053	0.893213198	0.791843351	0.812999186	0.809051283	0.881189314
LURAP1L	0.810688899	0.702818587	0.810647419	0.948668643	0.815913413	0.874490166
EFEMP2	0.810799503	0.678950251	0.855811972	0.938303773	0.935286646	0.748323122
PMEPA1	0.810799503	0.757251331	0.999993442	0.786914837	0.781724458	0.764310896
AAMP	0.810799503	0.769416761	0.911973589	0.931383184	0.78021215	0.783241453
TADA3	0.810799503	0.686033606	0.916038418	0.937654716	0.823508064	0.789870764
SGSM3	0.810799503	0.662013091	0.885896294	0.851965812	0.944839728	0.810156973
TIMM10B	0.810799503	0.676349839	0.925244197	0.899792456	0.823508064	0.83150957
C1H21orf62	0.810799503	0.821709833	0.804888406	0.940436902	0.788007619	0.839565788
SPAG7	0.810799503	0.709542765	0.955427429	0.768290504	0.880672837	0.839906041
LOC616840	0.810799503	0.888057447	0.744735369	0.922002553	0.788995893	0.844092922
LOC101902721	0.810799503	0.709247882	0.778210036	0.963601374	0.849766114	0.844759721
PRMT1	0.810799503	0.671947397	0.961437412	0.888687722	0.772501291	0.845747012
TCIM	0.810799503	0.770459279	0.744735369	0.911470775	0.890348531	0.8537109
DHRS13	0.810799503	0.901710313	0.745477682	0.784595865	0.900747635	0.85643253
TUBD1	0.810799503	0.672819246	0.788813378	0.982220679	0.821148296	0.860966325
TSC22D1	0.810799503	0.724171476	0.809673119	0.941467375	0.816045604	0.872238439
MLF2	0.810799503	0.77844414	0.873674244	0.798687063	0.872096228	0.872239777
DIDO1	0.810799503	0.758609923	0.849389234	0.86458365	0.848550619	0.873130182
LOC101904447	0.810799503	0.658496391	0.857446271	0.85647796	0.91090967	0.873130182
LOC112442257	0.810799503	0.644366672	0.751957722	0.999981514	0.802349909	0.877750901
ZNF518B	0.810799503	0.777249749	0.765365298	0.896116841	0.833173678	0.89567043
FIBP	0.810799503	0.704346844	0.838341783	0.803690355	0.911837627	0.907145443
HMG20B	0.810799503	0.669898746	0.788512873	0.804434747	0.936921784	0.930710695
AIG1	0.810799503	0.660882273	0.749453648	0.855893886	0.78021215	0.999948228
AMHR2	0.810905318	0.649340041	0.927508464	0.805302469	0.917736327	0.846210629
LOC100847171	0.810987939	0.737120625	0.873219549	0.899091941	0.799752471	0.871241787
SERTAD1	0.811067028	0.750379179	0.81412252	0.999981514	0.756033063	0.748750383
LOC781977	0.811067028	0.941676269	0.879939804	0.82868625	0.771893039	0.78087541
BCNT2	0.811067028	0.737941984	0.77566312	0.961898258	0.891726429	0.781984152
MEAF6	0.811067028	0.712357273	0.874983674	0.910744545	0.887514169	0.783241453
CCDC174	0.811067028	0.839128192	0.817582037	0.798687063	0.925918507	0.824025963
G3BP2	0.811067028	0.692305416	0.879939804	0.949864417	0.789915129	0.849731479
DNAJC10	0.811067028	0.929409572	0.736531189	0.828736799	0.823508064	0.866494771
WNT9B	0.811067028	0.792109226	0.916294854	0.812453944	0.788007619	0.877014986
CRK	0.811099049	0.699042243	0.850624788	0.999981514	0.810436875	0.747871787
DNAH2	0.811099049	0.938701207	0.747025306	0.839725231	0.911837627	0.75062693

MEX3B	0.811099049	0.670209048	0.905686078	0.989601002	0.805615411	0.750967073
GPAT4	0.811099049	0.68204808	0.819359511	0.999981514	0.804274107	0.754218406
BLM	0.811099049	0.77844414	0.93784999	0.893051204	0.804625939	0.766900958
TUB	0.811099049	0.752460325	0.744735369	0.92139105	0.958797242	0.769865166
SFXN2	0.811099049	0.661627228	0.747025306	0.908467146	0.991531798	0.770490368
QPCTL	0.811099049	0.783178342	0.944126714	0.80459801	0.877712931	0.779201808
LOC112443193	0.811099049	0.7889129	0.754557912	0.815681983	0.987723553	0.779201808
FYTTD1	0.811099049	0.744774335	0.916294854	0.903584224	0.819449204	0.790348826
DPF3	0.811099049	0.792109226	0.884121008	0.871338923	0.860072627	0.799389365
RPA3	0.811099049	0.708066593	0.824121854	0.948668643	0.887514169	0.804967091
LOC112444889	0.811099049	0.829345514	0.855268213	0.815919419	0.891726429	0.812774915
TPCN1	0.811099049	0.950675653	0.810647419	0.768290504	0.847092933	0.829441883
NUP133	0.811099049	0.94422395	0.791198027	0.780279943	0.858914398	0.83150957
DCAKD	0.811099049	0.766595431	0.867222363	0.797338497	0.927086179	0.837596789
FAAP20	0.811099049	0.814995661	0.916294854	0.765558953	0.859351739	0.838468539
METTL22	0.811099049	0.684972622	0.87118003	0.799480811	0.956904152	0.847012179
SLC50A1	0.811099049	0.829345514	0.761692603	0.928404714	0.810436875	0.847193872
P4HTM	0.811099049	0.669124554	0.885896294	0.908467146	0.836515016	0.860414702
GLYAT	0.811099049	0.683704605	0.779034209	0.815888375	0.976259416	0.864703284
LOC101906195	0.811099049	0.71815098	0.916294854	0.854966424	0.795839201	0.883688554
DKK2	0.811099049	0.887455957	0.754913805	0.825279787	0.833173678	0.884426509
SH3GL2	0.811099049	0.648119635	0.873674244	0.891988949	0.823508064	0.91373422
PTP4A2	0.811099049	0.718382634	0.822300339	0.827786827	0.758424886	0.981530701
SLC10A5	0.811114676	0.663309441	0.78057665	0.942363973	0.926831826	0.835231445
ABCA2	0.811224325	0.688775944	0.920462513	0.9819628	0.795839201	0.749726404
CCDC93	0.811224325	0.744774335	0.810647419	0.777956162	0.943047881	0.891151691
PTPRG	0.811224325	0.648503747	0.927635169	0.867533077	0.776537165	0.923092436
COX20	0.811225682	0.800193122	0.924855847	0.800283296	0.911568867	0.749918186
PRPF8	0.811225682	0.686805478	0.794676674	0.812453944	0.999985752	0.756871392
DEK	0.811225682	0.92565465	0.875900843	0.786099236	0.855920132	0.769865166
LOC100295848	0.811225682	0.739786945	0.754557912	0.989601002	0.864524107	0.792412747
LOC783466	0.811225682	0.652717399	0.821026363	0.960937718	0.884872176	0.831652079
ZNF677	0.811225682	0.730634526	0.839760004	0.815888375	0.953300742	0.839489406
SAXO2	0.811225682	0.738832446	0.77566312	0.812453944	0.887514169	0.943638116
TMEM167B	0.811444649	0.674524738	0.990802991	0.823935462	0.781724458	0.853576299
SERTM1	0.811444649	0.891384768	0.794846789	0.817146543	0.801692604	0.894875736
GFM2	0.811444649	0.71799389	0.788106994	0.765499767	0.935286646	0.93363377
PALM2	0.811554076	0.696757704	0.869739827	0.908467146	0.939203547	0.749726404

SIPA1L2	0.811554076	0.679897494	0.774015998	0.941796131	0.970491451	0.754218406
MTMR14	0.811554076	0.686984735	0.744735369	0.97828838	0.891726429	0.836111377
VGF	0.811554076	0.696757704	0.76555928	0.898890982	0.928245056	0.866680565
FBXL19	0.811567943	0.738007931	0.916323169	0.925294859	0.781724458	0.81956221
WDR53	0.811567943	0.666269218	0.838909114	0.999981514	0.773264077	0.820286425
IFT74	0.811567943	0.742673431	0.735532321	0.974286747	0.8568751	0.844759721
LRMP	0.812049066	0.672505775	0.96608402	0.816169012	0.817472827	0.867063767
RER1	0.812049066	0.697469149	0.91882781	0.823935462	0.815792965	0.902883152
MOB3B	0.812049066	0.649340041	0.847776765	0.885789665	0.877712931	0.911336121
NT5C3B	0.812071752	0.689108253	0.870734949	0.898818046	0.901853599	0.820374589
DSB	0.812071752	0.781836924	0.77283514	0.777956162	0.865075514	0.960023723
FAM161B	0.812075593	0.692305416	0.99647733	0.808075336	0.803705802	0.823701731
APOPT1	0.812266349	0.839128192	0.76555928	0.896116841	0.91090967	0.77977421
CFLAR	0.812266349	0.648119635	0.877334778	0.959616431	0.887514169	0.78087541
RGS2	0.812666783	0.687020192	0.893761108	0.925294859	0.847092933	0.83150957
MICALL2	0.813032028	0.659221677	0.943237386	0.812313105	0.894364008	0.849074285
LHFPL2	0.813148007	0.750379179	0.761725783	0.961898258	0.795839201	0.88542704
TOGARAM1	0.814119142	0.920786925	0.79497298	0.812453944	0.91563394	0.765179756
FAM122A	0.814119142	0.796558979	0.835443208	0.806838917	0.962513981	0.779284546
MMP2	0.814119142	0.669007504	0.811480663	0.912777307	0.879776073	0.888475311
LOC104974473	0.814119142	0.728504282	0.916323169	0.82868625	0.760278238	0.924740534
UPRT	0.814119142	0.663663903	0.955427429	0.768247745	0.811991102	0.927426708
S100A5	0.814119142	0.673793679	0.832065705	0.804862068	0.799272579	0.994347099
DDX11	0.814167303	0.746712661	0.874983674	0.891732037	0.81725718	0.867063767
NUDT4	0.814620472	0.70496036	0.744735369	0.995434268	0.887514169	0.791291767
BANK1	0.814620472	0.648958657	0.89478142	0.949864417	0.823508064	0.839565788
LOC112447290	0.81463596	0.669124554	0.847776765	0.999981514	0.775582766	0.770490368
TMA7	0.815054033	0.808696335	0.789166592	0.777956162	0.99971266	0.748323122
WWP2	0.815094866	0.817329649	0.831933739	0.913048575	0.85545716	0.785636406
LOC101902346	0.815094866	0.813305033	0.899106435	0.819968583	0.845802744	0.836111377
SPATA5L1	0.815465003	0.704503729	0.981383697	0.904018489	0.794877876	0.759003017
DOCK9	0.815465003	0.756568666	0.89385865	0.907794002	0.853973085	0.784958125
LAG3	0.815465003	0.855310926	0.864847127	0.853271157	0.78021215	0.867063767
CORO2A	0.815465003	0.892108342	0.788106994	0.886312303	0.788007619	0.867063767
GFRA4	0.815465003	0.722195304	0.757565308	0.960937718	0.837915327	0.87501921
TOP2B	0.815668435	0.70880006	0.841294863	0.806573529	0.979290191	0.814218101
KCTD8	0.815760791	0.664859217	0.745477682	0.891988949	0.795839201	0.992934504
ZNF593	0.815784411	0.77844414	0.867222363	0.852481062	0.769529419	0.927215832

PHLDA1	0.815903036	0.776670017	0.933884582	0.944351122	0.78021215	0.751884708
TRAK1	0.815903036	0.741263262	0.969404007	0.890704007	0.781159411	0.786936221
SRRD	0.815903036	0.684972622	0.847776765	0.866566721	0.962513981	0.810414874
LOC112448791	0.815903036	0.758609923	0.835610539	0.866566721	0.932024143	0.816246746
DENND4B	0.815903036	0.664985713	0.927508464	0.872590202	0.886785729	0.83150957
PRPF40A	0.815903036	0.726224601	0.852214402	0.777956162	0.962682426	0.847791663
USP5	0.815903036	0.830930037	0.854571175	0.76992117	0.902258286	0.850475013
TRAPPC2	0.815903036	0.724089233	0.943836178	0.78584336	0.834035648	0.882690335
CD300LB	0.815903036	0.802045751	0.79497298	0.935239391	0.766655597	0.888309103
EMILIN2	0.815903036	0.820534347	0.76555928	0.887420349	0.789915129	0.927426708
LOC112449367	0.815903036	0.649360352	0.894030462	0.807214761	0.810436875	0.96870908
CTNNAL1	0.816130114	0.76043092	0.818612484	0.942363973	0.807575053	0.861014665
UBE2N	0.816130114	0.687651905	0.954020369	0.865746543	0.796861564	0.867063767
RHOJ	0.816164528	0.792624847	0.84137172	0.9819628	0.7744799	0.789030652
SND1	0.816278132	0.796253817	0.86347788	0.792161305	0.787108353	0.948852198
EFCAB14	0.81644743	0.758609923	0.9965509	0.81451387	0.82114467	0.748921056
ATP6V1B2	0.81644743	0.657965823	0.813054822	0.999981514	0.769529419	0.783241453
TRNP1	0.81644743	0.683744977	0.894316021	0.931488699	0.804274107	0.862925606
EGR3	0.816472994	0.924173915	0.879939804	0.889415593	0.791141016	0.748921056
LOC100336868	0.816472994	0.775033799	0.916323169	0.814600427	0.935286646	0.749726404
LOC112446645	0.816472994	0.853946295	0.828935179	0.891988949	0.878451408	0.774636709
MRPS33	0.816472994	0.674524738	0.924855847	0.863269783	0.883143938	0.839906041
RDH10	0.816472994	0.771208827	0.806651923	0.794386089	0.962513981	0.845783187
LOC100616098	0.816472994	0.738007931	0.819359511	0.80882845	0.943540106	0.876162019
RBPMS2	0.816472994	0.664966859	0.935266153	0.821450642	0.851927006	0.888475311
TMEM267	0.816549454	0.659408339	0.92422067	0.823935462	0.795839201	0.940256832
CYGB	0.816688944	0.694842866	0.744735369	0.89684883	0.999985752	0.749726404
DPEP3	0.816688944	0.884773193	0.836676892	0.954343461	0.776857944	0.759003017
ADGRF3	0.816688944	0.659622605	0.999993442	0.839245804	0.799272579	0.768168974
CIAO1	0.816688944	0.663620961	0.886829776	0.823167001	0.979988991	0.774575102
LANCL2	0.816688944	0.731850993	0.886829776	0.98981682	0.775771176	0.77803403
PAWR	0.816688944	0.927034887	0.817582037	0.893051204	0.765866216	0.8255975
PPP1R15B	0.816688944	0.741310853	0.802277976	0.912777307	0.907094634	0.831652079
PEAK1	0.816688944	0.686033606	0.838341783	0.854966424	0.960419102	0.835231445
LOC112448627	0.816688944	0.753090681	0.927635169	0.845719581	0.797489438	0.867907362
SKA2	0.816688944	0.7889129	0.739888939	0.899792456	0.857562998	0.905069789
LOC112442377	0.816688944	0.741671849	0.745477682	0.830289573	0.925114091	0.925458723
RBBP4	0.816688944	0.672505775	0.906019013	0.859320133	0.792867703	0.933903265

TFIP11	0.816688944	0.788980101	0.805212947	0.79380551	0.809051283	0.96870908
EPHA1	0.816688944	0.660882273	0.748392726	0.942433199	0.788995893	0.970186217
RAC3	0.817081815	0.694400411	0.89385865	0.787640064	0.996781978	0.751705208
LOC782566	0.817081815	0.864945924	0.894671468	0.916858019	0.781724458	0.759531886
ZFAND4	0.817081815	0.839128192	0.800716826	0.914026346	0.889217664	0.768572658
PARVA	0.817081815	0.661031998	0.943120912	0.933718942	0.852959789	0.774562005
SRRM1	0.817081815	0.658075243	0.873674244	0.910575108	0.947112796	0.77593589
ATP1B1	0.817081815	0.710788298	0.830717895	0.824230049	0.98865576	0.78096075
ZKSCAN4	0.817081815	0.973189491	0.818612484	0.832401065	0.799867929	0.783241453
RAP1B	0.817081815	0.737127482	0.852214402	0.977689659	0.822410248	0.79187813
ARRB1	0.817081815	0.816919386	0.927508464	0.844838486	0.81911207	0.806476213
LOC101905818	0.817081815	0.79280401	0.982708154	0.780184803	0.809051283	0.810156973
LIPT1	0.817081815	0.691301661	0.855268213	0.889415593	0.943702351	0.811410743
PPP1CA	0.817081815	0.709542765	0.852916849	0.893051204	0.929896768	0.813112397
HOXB9	0.817081815	0.870296749	0.75029951	0.916892022	0.851174659	0.826557348
ACTR5	0.817081815	0.764775882	0.879939804	0.773429607	0.953300742	0.830572943
CDK7	0.817081815	0.782783231	0.817582037	0.76992117	0.964301327	0.842654067
LSM4	0.817081815	0.777249749	0.819359511	0.792051803	0.958797242	0.847193872
GJC1	0.817081815	0.696525042	0.77566312	0.891988949	0.954403133	0.850475013
RUNX1	0.817081815	0.667519609	0.862253703	0.940436902	0.853985138	0.859032412
ATP1B3	0.817081815	0.823133291	0.76113663	0.961898258	0.784259667	0.860966325
PSMD3	0.817081815	0.811445496	0.894338686	0.821450642	0.804037558	0.878754045
LOC101905876	0.817081815	0.669124554	0.800716826	0.821450642	0.967116582	0.879135421
FAHD2A	0.817081815	0.777309385	0.78502605	0.808073654	0.930451229	0.89438401
SRP14	0.817081815	0.685572308	0.903150496	0.843613441	0.847092933	0.908405626
LOC112443339	0.817081815	0.752314536	0.879939804	0.825279787	0.804274107	0.927021567
AP1S3	0.817081815	0.659408339	0.865512879	0.784595865	0.926972948	0.927520621
RAB37	0.817081815	0.709863077	0.741510374	0.959616431	0.804264617	0.928976746
VSIG2	0.817081815	0.717684248	0.830664444	0.784595865	0.891726429	0.944973383
CCNE2	0.817081815	0.795559378	0.804002279	0.806573529	0.823508064	0.954465286
TOB2	0.817081815	0.657066596	0.750567094	0.798687063	0.905990244	0.988252822
LOC112447340	0.81725582	0.792770008	0.964807927	0.799139231	0.884679971	0.749918186
TIMM17B	0.81725582	0.699192406	0.879939804	0.792071212	0.998287238	0.754218406
LOC101907247	0.817292399	0.745846221	0.97773026	0.783796091	0.853985138	0.816246746
RNF103	0.817496924	0.816841439	0.817582037	0.904018489	0.828190913	0.849595612
HSF2BP	0.81759337	0.709247882	0.759332589	0.896116841	0.996781978	0.754218406
PPP1R18	0.81759337	0.85415953	0.754913805	0.832489049	0.959565914	0.783969061
DNPEP	0.81759337	0.674524738	0.758146087	0.890704007	0.976292433	0.839565788

NAA20	0.81759337	0.682964967	0.897322115	0.84229953	0.927086179	0.839906041
TCEA2	0.81759337	0.734546826	0.774924967	0.863269783	0.831607792	0.960877268
PDXP	0.817768648	0.783896922	0.828449846	0.786943075	0.976259416	0.805925428
SCO1	0.817768648	0.72445188	0.77566312	0.98981682	0.818977667	0.845714049
ABCC9	0.817768648	0.756360309	0.774015998	0.912922827	0.885217562	0.867063767
SNX4	0.817857183	0.692305416	0.779625724	0.999981514	0.870042072	0.754218406
PMPCB	0.817910883	0.693373829	0.800428397	0.889472032	0.940881754	0.861631711
MKLN1	0.817910883	0.672552593	0.853694398	0.799912445	0.853599134	0.970216353
MANBAL	0.818086092	0.824188473	0.916323169	0.900044333	0.814694993	0.754731949
MRC2	0.818086092	0.722195304	0.907477369	0.907621547	0.900092357	0.758175289
M1AP	0.818086092	0.850894788	0.754913805	0.952247001	0.864524107	0.78096075
LOC783033	0.818086092	0.689826137	0.854567571	0.999981514	0.781159411	0.799901412
VSIG4	0.818086092	0.775033799	0.818612484	0.947574232	0.848087203	0.827870367
ZDBF2	0.818086092	0.863286109	0.766722666	0.80459801	0.935286646	0.847193872
HRH4	0.818086092	0.664691175	0.955427429	0.891988949	0.804264617	0.853440904
YTHDC2	0.818086092	0.916141416	0.744735369	0.821450642	0.886785729	0.855349987
ASB13	0.818086092	0.66246801	0.743195871	0.812453944	0.930451229	0.972695131
SECISBP2L	0.818363284	0.709247882	0.871285807	0.925294859	0.869537678	0.832964812
SMIM4	0.818363284	0.77844414	0.871929137	0.828337624	0.904206501	0.839489406
EIF4A1	0.818363284	0.710645465	0.9377363	0.85721441	0.81954052	0.866494771
LOC101904332	0.818468398	0.690620255	0.747666034	0.869068788	0.999985752	0.768168974
CDC42EP1	0.818468398	0.768359345	0.772600931	0.861531477	0.976259416	0.789870764
PIK3IP1	0.818468398	0.861395263	0.899106435	0.823935462	0.821148296	0.829118045
LOC613460	0.818563595	0.83844057	0.861447545	0.922189411	0.776537165	0.83150957
CDH15	0.81871473	0.659408339	0.93190909	0.799912445	0.935286646	0.847193872
CYP27B1	0.81892366	0.999987083	0.761738351	0.815919419	0.78021215	0.761991719
PCDH7	0.819211647	0.712357273	0.894316021	0.891988949	0.880617822	0.835231445
SEC24D	0.81947381	0.772909657	0.755355191	0.929663263	0.956041014	0.763466477
CREB3	0.81947381	0.785057176	0.869932384	0.886312303	0.920505592	0.768168974
ANK3	0.81947381	0.997332173	0.763021995	0.823434028	0.773384033	0.806476213
DENND1C	0.81947381	0.744774335	0.929661805	0.819549243	0.896541204	0.812491979
PSAP	0.819560446	0.681110826	0.833583844	0.999981514	0.768297035	0.768168974
TMEM11	0.819560446	0.788524937	0.93475587	0.812999186	0.840108119	0.841312694
KMT2E	0.819560446	0.7889129	0.809673119	0.82183466	0.942786245	0.850475013
RNH1	0.819679385	0.758609923	0.854571175	0.789139053	0.977158496	0.790348826
AGBL2	0.820261302	0.69591721	0.992511421	0.899552507	0.789915129	0.770490368
PSPN	0.820261302	0.697306013	0.8644528	0.972142185	0.789915129	0.856833445
PTCD1	0.820567849	0.694630014	0.951392011	0.885789665	0.78021215	0.87501921

CCDC8	0.820890348	0.806415971	0.75029951	0.913048575	0.87329316	0.865767172
LOC104975027	0.820989155	0.808478344	0.77566312	0.777956162	0.916605572	0.921843102
CLGN	0.820989155	0.802840415	0.846965262	0.801228158	0.847092933	0.923547711
LOC101902029	0.821029521	0.846724749	0.789166592	0.892047793	0.878451408	0.832964812
HMGN1	0.821090147	0.751470284	0.91882781	0.796128007	0.823508064	0.90969547
DNM1	0.821131997	0.728285587	0.912020408	0.871338923	0.89743683	0.806476213
STOML2	0.821257524	0.708137441	0.930736996	0.799480811	0.891304411	0.865767172
FLOT1	0.821257524	0.773508874	0.885469405	0.886727784	0.787083646	0.893838339
IP6K1	0.821324699	0.745488999	0.768438632	0.786914837	0.999985752	0.754218406
MIER1	0.821333198	0.824828477	0.755135544	0.821450642	0.926813827	0.877014986
KLHDC4	0.821369019	0.918625585	0.943836178	0.799480811	0.784972475	0.783241453
GPD2	0.821369019	0.821674572	0.774015998	0.848253453	0.914322944	0.864572584
COPS8	0.821482113	0.671759506	0.990615319	0.896116841	0.84367638	0.751270885
RBM38	0.821482113	0.855067335	0.886064678	0.797312233	0.930451229	0.765948605
ADRB2	0.821482113	0.892108342	0.824434404	0.925210648	0.804274107	0.784754767
CFAP44	0.821482113	0.674524738	0.874983674	0.884452623	0.803705802	0.944973383
CD300LF	0.821482113	0.69959307	0.871285807	0.777956162	0.793993073	0.991416486
FAM155B	0.821926403	0.74501367	0.999993442	0.799912445	0.795839201	0.752070741
MECOM	0.821926403	0.750379179	0.755355191	0.9914926	0.890841443	0.773680578
LOC101905498	0.821926403	0.979651412	0.817582037	0.854966424	0.784259667	0.788697467
MRPL46	0.821926403	0.820235509	0.870922751	0.816169012	0.926972948	0.801888363
LOC104973105	0.821926403	0.867460706	0.826409457	0.892047793	0.85545716	0.80889835
RFWD3	0.821926403	0.81847736	0.759332589	0.907794002	0.91563394	0.814218101
KCNK7	0.821926403	0.741263262	0.958454497	0.874170259	0.811706964	0.81956221
DZIP1L	0.821926403	0.696504109	0.763521949	0.999981514	0.816249936	0.835231445
PHC3	0.821926403	0.957328149	0.872088608	0.784595865	0.781724458	0.840768468
MTCH2	0.821926403	0.686984735	0.862577505	0.861301134	0.943702351	0.850432365
HDGFL3	0.821926403	0.796253817	0.841294863	0.852170016	0.893716526	0.852504043
NSD1	0.821926403	0.659622605	0.853455703	0.992930508	0.797127163	0.855082018
KIFAP3	0.821926403	0.855067335	0.866497255	0.876703811	0.78547726	0.865767172
COX4I1	0.821926403	0.709247882	0.831933739	0.822691957	0.936108073	0.893585844
LOC101904377	0.821926403	0.774979074	0.774015998	0.887983383	0.795865165	0.959723327
SOAT1	0.821926403	0.692305416	0.800716826	0.907794002	0.772103109	0.973579501
SMYD2	0.821926403	0.663620961	0.796499368	0.896116841	0.771532192	0.989457616
LOC112442032	0.822312427	0.773708461	0.817582037	0.995312373	0.836444721	0.759003017
NMT2	0.822312427	0.889387648	0.845113191	0.884493789	0.796861564	0.842654067
SH2D4A	0.822417037	0.757812284	0.791557166	0.808075336	0.956994968	0.879576441
UBL4A	0.82242597	0.7889129	0.89478142	0.812453944	0.958384396	0.756591895

CCDC30	0.82242597	0.669828549	0.916294854	0.852170016	0.964833865	0.765948605
RNF13	0.82242597	0.918491096	0.802277976	0.845114238	0.906893576	0.766900958
DNAJC18	0.82242597	0.880129816	0.937993336	0.812453944	0.821351993	0.781629683
LOC101902551	0.82242597	0.710788298	0.995489533	0.777956162	0.887514169	0.782330566
KIF15	0.82242597	0.681192035	0.94422142	0.96900924	0.794877876	0.783241453
HIGD2A	0.82242597	0.7889129	0.820353079	0.833118758	0.968585482	0.783241453
LOC100848315	0.82242597	0.697306013	0.942134405	0.908467146	0.852234727	0.793308041
PPP1R7	0.82242597	0.731300596	0.858277296	0.945300947	0.85545716	0.83150957
PNPLA4	0.82242597	0.929514642	0.76555928	0.848592907	0.86876405	0.832770678
TMC6	0.82242597	0.708218805	0.833233621	0.853058361	0.964301327	0.835231445
MRFAP1L1	0.82242597	0.692305416	0.897827774	0.938303773	0.828207883	0.841312694
PTPDC1	0.82242597	0.703887794	0.849389234	0.885789665	0.926972948	0.85634055
PPM1K	0.82242597	0.829710539	0.770497031	0.839245804	0.927086179	0.857457228
MIEF1	0.82242597	0.68204808	0.889098668	0.821450642	0.93630105	0.867063767
AP1S1	0.82242597	0.712357273	0.855793459	0.812453944	0.943160494	0.877014986
FAM69C	0.82242597	0.740343181	0.95219206	0.792140715	0.833173678	0.877048993
LOC518623	0.82242597	0.741332329	0.754913805	0.882057818	0.935286646	0.888309103
L2HGDH	0.82242597	0.707837135	0.871482445	0.792140715	0.935286646	0.894737145
PCBP2	0.82242597	0.68858196	0.838341783	0.868981727	0.909316444	0.89950552
AP3B2	0.82242597	0.72742557	0.798782742	0.874023052	0.889301074	0.919124527
LOC100337053	0.82242597	0.842391145	0.836482	0.777956162	0.823508064	0.936263602
ST6GALNAC3	0.82242597	0.685572308	0.852916849	0.852170016	0.776537165	0.985717525
ACCS	0.822783559	0.774934769	0.874797723	0.898818046	0.891726429	0.786849547
LOC112445063	0.822783559	0.843278276	0.911850355	0.899855526	0.788007619	0.797991983
LRIG2	0.822783559	0.858384828	0.784260981	0.815681983	0.78021215	0.96248183
SNAP91	0.822818751	0.663620961	0.921266625	0.843613441	0.970988226	0.75640676
LRP3	0.822818751	0.90116376	0.749417651	0.876703811	0.943702351	0.75702988
DOCK5	0.822818751	0.678812333	0.869932384	0.961898258	0.86587625	0.826426663
SLC39A3	0.822818751	0.718258172	0.875053718	0.886312303	0.891726429	0.853548296
ALDH7A1	0.822818751	0.681192035	0.855713041	0.840247324	0.944839728	0.874000314
FAM177A1	0.822818751	0.679199291	0.942357321	0.888687722	0.788739452	0.896027932
MRPS11	0.822818751	0.767113995	0.813250419	0.812453944	0.911568867	0.912531667
ALKBH2	0.82323879	0.994185904	0.771697162	0.823913856	0.821148296	0.772618249
LOC104968820	0.823526932	0.999278945	0.749417651	0.843613441	0.801692604	0.766534677
LOC101904768	0.823526932	0.664859217	0.911535369	0.792071212	0.788995893	0.984509067
RSPO3	0.823587226	0.764079757	0.77699301	0.868435169	0.905571216	0.900219465
RAB14	0.823710263	0.687020192	0.949855095	0.88106101	0.923910588	0.759342754
ASPHD2	0.824205004	0.677370365	0.935266153	0.876703811	0.887514169	0.833150766

SH3PXD2B	0.824603545	0.710645465	0.908072171	0.910495312	0.921710688	0.754731949
ZNF385B	0.824603545	0.732885933	0.833583844	0.820971736	0.916057607	0.911219114
STYX	0.824723954	0.664104209	0.749417651	0.849834721	0.987906024	0.879576441
LOC100139996	0.82473825	0.857871704	0.854567571	0.908428501	0.871558258	0.75702988
LOC112445912	0.82473825	0.709247882	0.867222363	0.937726848	0.906803277	0.788462021
PATL2	0.82473825	0.687020192	0.879939804	0.99004392	0.815913413	0.806476213
CDK18	0.82473825	0.786691027	0.85942296	0.832489049	0.943702351	0.809961137
SLC16A5	0.82473825	0.718258172	0.872088608	0.796843021	0.981261122	0.810156973
TERF2IP	0.82473825	0.753090681	0.938011449	0.872590202	0.834470237	0.832770678
FAM169A	0.82473825	0.863286109	0.853497226	0.792140715	0.907678664	0.839489406
UBE2K	0.82473825	0.699192406	0.87118003	0.891988949	0.906893576	0.849731479
LOC112448038	0.82473825	0.866113361	0.852214402	0.857139436	0.816070658	0.865745718
ENO4	0.82473825	0.669007504	0.774924967	0.891329697	0.89052284	0.947801358
RPL36A	0.82473825	0.670480954	0.770595941	0.894086736	0.797227609	0.993414951
LOC522763	0.82473825	0.741263262	0.774025101	0.838412155	0.788007619	0.996813547
TMEM37	0.824963926	0.823602247	0.885896294	0.947382877	0.811991102	0.763965283
DDB2	0.824963926	0.869789153	0.901736458	0.812453944	0.84620147	0.83150957
LOC101906717	0.824963926	0.753090681	0.834683675	0.98614952	0.788850323	0.841312694
LOC112441650	0.824963926	0.686805478	0.948128856	0.874257643	0.816306826	0.876162019
TSPAN13	0.824963926	0.748690795	0.843955813	0.944563237	0.801692604	0.879759038
FAM198B	0.824963926	0.664985713	0.959725886	0.796128007	0.877991959	0.880846873
LOC786733	0.824963926	0.748700806	0.759332589	0.970039043	0.810263299	0.895803603
PIGY	0.825079657	0.722779653	0.958197827	0.866566721	0.86876405	0.793197037
TSLP	0.825138122	0.693373829	0.804002279	0.799147532	0.980189546	0.88313584
LOC101908104	0.825179936	0.741263262	0.990118874	0.828736799	0.81911207	0.806476213
USP32	0.825179936	0.745791496	0.781767828	0.893220599	0.959367717	0.824780045
SRSF6	0.825400243	0.684946765	0.871491704	0.918693944	0.903866359	0.835231445
COMMD3	0.825400243	0.672045313	0.889098668	0.822387291	0.964301327	0.839565788
GNL3L	0.825400243	0.718382634	0.915858924	0.832401065	0.887514169	0.867063767
LOC782293	0.825400243	0.783896922	0.78502605	0.863269783	0.880672837	0.918580372
FUBP3	0.82540611	0.912764028	0.810647419	0.881355084	0.879435126	0.78096075
LOC613677	0.82540611	0.701348278	0.817582037	0.827786827	0.964301327	0.873130182
ANKRD37	0.82540611	0.673435317	0.880163755	0.952247001	0.799272579	0.888930405
PTPN23	0.82540611	0.696504109	0.86427915	0.949864417	0.801692604	0.891423889
MAP10	0.82540611	0.910651459	0.754913805	0.887983383	0.781724458	0.909056553
THRAP3	0.82540611	0.661806153	0.814495418	0.94469136	0.848717914	0.918461982
LOC618787	0.825468293	0.681192035	0.991454341	0.937565894	0.788995893	0.764260585
RBM33	0.825468293	0.898791459	0.916294854	0.806573529	0.804625939	0.831652079

CAMK4	0.825468293	0.758609923	0.8644528	0.948226346	0.788995893	0.867907362
KIAA1522	0.825468293	0.757251331	0.810647419	0.888687722	0.864166961	0.91373422
USP14	0.825576357	0.687651905	0.873438881	0.887877413	0.804625939	0.945480515
TNNT1	0.825631018	0.932394105	0.810647419	0.780279943	0.844434232	0.87754233
ASPM	0.825731773	0.787802226	0.899106435	0.952247001	0.82114467	0.765948605
LOC104970966	0.825731773	0.716415149	0.960695288	0.843613441	0.872096228	0.818370468
RASSF8	0.825731773	0.704346844	0.8735515	0.805499732	0.976769047	0.829809521
TSTD1	0.825731773	0.69591721	0.988307089	0.792140715	0.879776073	0.83150957
CD72	0.825731773	0.797999637	0.899106435	0.892047793	0.81725718	0.839489406
UFSP2	0.825731773	0.663309441	0.997850143	0.791615737	0.836515016	0.85532952
HERC4	0.825731773	0.733689113	0.77566312	0.973373329	0.857738578	0.858964118
TIGD5	0.825731773	0.721685298	0.829751489	0.812453944	0.970988226	0.859032412
CCSER2	0.825731773	0.717684248	0.907585583	0.896116841	0.838788415	0.864139695
WDR44	0.825731773	0.833429351	0.77566312	0.948668643	0.784972475	0.88313584
SIRT3	0.825731773	0.835486075	0.781767828	0.886312303	0.833789832	0.903500283
LBH	0.825731773	0.934225172	0.759332589	0.812453944	0.788995893	0.930146713
SLC4A3	0.825731773	0.709247882	0.776353067	0.935563591	0.795839201	0.959075802
ATXN2	0.825731773	0.691785155	0.749854422	0.786943075	0.915775776	0.988639786
SS18L2	0.825758549	0.767737619	0.924855847	0.828736799	0.789915129	0.911219114
GCH1	0.826249436	0.796253817	0.841294863	0.943808491	0.891304411	0.766059933
LOC101904239	0.826249436	0.901984573	0.923521499	0.853271157	0.804274107	0.768572658
LOC101902407	0.826249436	0.740343181	0.829751489	0.995565324	0.846464841	0.782515535
MYH7B	0.826249436	0.746712661	0.76555928	0.965416137	0.892654422	0.835231445
PSMC6	0.826249436	0.700130683	0.890945134	0.870591999	0.823508064	0.925472172
ZNF385A	0.8263763	0.964134676	0.811480663	0.874023052	0.847173934	0.754218406
LCA5	0.8263763	0.711166977	0.997300234	0.812453944	0.834431851	0.811759324
SFSWAP	0.8263763	0.686805478	0.951028911	0.899552507	0.844384115	0.83150957
LOC783541	0.8263763	0.758609923	0.759357794	0.960937718	0.860032529	0.867063767
NRK	0.8263763	0.724380878	0.784788059	0.812453944	0.939203547	0.933861738
HSDL1	0.826971427	0.672531785	0.997400824	0.876703811	0.820458937	0.786588477
LRRC61	0.826971427	0.889387648	0.927635169	0.808586366	0.836444721	0.788686554
NUF2	0.826971427	0.710645465	0.942357321	0.941467375	0.790136352	0.83150957
NDUFS2	0.826971427	0.692305416	0.841294863	0.797312233	0.970988226	0.884925084
DNTTIP2	0.826971427	0.678023292	0.945995021	0.840247324	0.805277279	0.923092436
LOC101907152	0.826971427	0.882858125	0.774015998	0.798687063	0.823508064	0.945480515
DIS3L2	0.826971427	0.679897494	0.885896294	0.812453944	0.811991102	0.977503497
NUCKS1	0.827217023	0.952760839	0.945995021	0.808262813	0.78021215	0.755679512
IER5L	0.827217023	0.709247882	0.78561065	0.941467375	0.96870902	0.774932208

MEFV	0.827217023	0.867277242	0.961437412	0.814600427	0.810436875	0.779201808
TGFBR3	0.827217023	0.767113995	0.929661805	0.79267243	0.948421303	0.78096075
USP49	0.827217023	0.887479443	0.751957722	0.960016921	0.823508064	0.810414874
RAB18	0.827217023	0.725876283	0.916294854	0.941467375	0.821851334	0.817529182
ALOX5AP	0.827217023	0.806881517	0.944949386	0.866566721	0.809129327	0.821257366
NUP62CL	0.827217023	0.878551917	0.818612484	0.904018489	0.826634148	0.833150766
LOC788634	0.827217023	0.675765841	0.75407054	0.999981514	0.80587765	0.839906041
S100B	0.827217023	0.816412341	0.80412682	0.812999186	0.956761712	0.844759721
LOC101903616	0.827217023	0.714027042	0.764398531	0.960016921	0.911664199	0.846210629
NRIP2	0.827217023	0.880152719	0.794676674	0.890362631	0.846464841	0.8537109
TMOD2	0.827217023	0.831694163	0.924855847	0.780279943	0.847092933	0.860966325
PANX1	0.827217023	0.728267541	0.815587305	0.912777307	0.89992403	0.867063767
TES	0.827217023	0.891547502	0.783944582	0.865746543	0.818179113	0.893838339
MEDAG	0.827217023	0.766943819	0.827969228	0.804862068	0.935286646	0.902100676
TMEM147	0.827217023	0.69959307	0.794676674	0.812453944	0.9683846	0.908049203
SFN	0.827217023	0.712357273	0.874797723	0.801166782	0.890124729	0.933897626
STUB1	0.827447132	0.845338797	0.826785415	0.822691957	0.960177245	0.77926303
RGS20	0.827498026	0.764456895	0.912183977	0.897371376	0.878451408	0.79187813
LRRN2	0.827549007	0.738007931	0.955427429	0.870210705	0.78021215	0.876861428
LRRC4C	0.827579619	0.705088508	0.810647419	0.905886285	0.788739452	0.970105102
LOC514257	0.828280343	0.866113361	0.916038418	0.812453944	0.862205587	0.814745299
AP2S1	0.828280343	0.741310853	0.830420708	0.793914945	0.947299702	0.911219114
RABL3	0.828529906	0.703500334	0.894338686	0.867533077	0.89743683	0.867482845
CS	0.828534457	0.696504109	0.839406764	0.82868625	0.961465747	0.874386228
MAMLD1	0.828572853	0.702362909	0.91882781	0.82906342	0.962513981	0.779201808
TTC1	0.828710501	0.699192406	0.922552585	0.893220599	0.891726429	0.816246746
UBE3A	0.828901831	0.772618617	0.892343042	0.863288407	0.870450746	0.862023846
LOC615271	0.828958144	0.664859217	0.927508464	0.874170259	0.797489438	0.93363377
CINP	0.829001504	0.717684248	0.94565648	0.78584336	0.837915327	0.921843102
WRB	0.82901711	0.69722211	0.999993442	0.889343851	0.80599066	0.766534677
PRKCZ	0.82901711	0.768359345	0.926234868	0.949502614	0.797576462	0.783969061
CENPBD1	0.82901711	0.833323491	0.829455601	0.886312303	0.892446951	0.83150957
PATL1	0.82901711	0.77194497	0.916294854	0.782811812	0.937932469	0.83150957
PIGN	0.82901711	0.823478017	0.813441395	0.901168085	0.82835186	0.877014986
RBM10	0.829126688	0.713792742	0.975774524	0.914653338	0.798443817	0.790348826
ESPNL	0.829158916	0.669934428	0.972806636	0.925294859	0.84044793	0.778447279
FAM110A	0.829158916	0.879150176	0.935219877	0.796934171	0.864524107	0.784754767
TRIM4	0.829158916	0.716415149	0.966847507	0.85005716	0.864524107	0.821257366

SHTN1	0.829158916	0.792661811	0.845113191	0.887877413	0.872734644	0.867063767
MARCH8	0.829158916	0.682216234	0.940712253	0.824323293	0.887514169	0.876162019
TMEM208	0.829158916	0.709348874	0.885469405	0.844621856	0.895067787	0.891423889
NAT10	0.829158916	0.799590997	0.830104583	0.878074356	0.804625939	0.930146713
CD99	0.829158916	0.674524738	0.901579001	0.808073654	0.816306826	0.973649216
RIC8B	0.82937449	0.761280186	0.971556427	0.848592907	0.885217562	0.75640676
ACBD3	0.82937449	0.673793679	0.842776542	0.825279787	0.999985752	0.765644158
SPON1	0.82937449	0.744049882	0.915858924	0.881493849	0.936215792	0.768168974
FMOD	0.82937449	0.77139884	0.927327441	0.9063051	0.870450746	0.769410472
PARP3	0.82937449	0.704870978	0.963627763	0.949565453	0.802592922	0.779201808
MTG2	0.82937449	0.671536686	0.778210036	0.830289573	0.999985752	0.779201808
BCKDK	0.82937449	0.771718176	0.822300339	0.786099236	0.999985752	0.783241453
TSSK3	0.82937449	0.843278276	0.982232295	0.822691957	0.786887726	0.790348826
CBX8	0.82937449	0.692420359	0.77566312	0.812453944	0.999985752	0.79187813
ALG9	0.82937449	0.722968232	0.897322115	0.822708607	0.959658682	0.814218101
C18H19orf54	0.82937449	0.704646482	0.829137153	0.978503042	0.884872176	0.820182674
GPATCH8	0.82937449	0.943754915	0.903431343	0.796934171	0.803705802	0.824780045
FES	0.82937449	0.744774335	0.803428123	0.827443542	0.980695939	0.840768468
SCPEP1	0.82937449	0.722779653	0.89478142	0.910854395	0.862302249	0.846210629
TMEM205	0.82937449	0.831564725	0.894316021	0.887877413	0.794877876	0.862925606
OGG1	0.82937449	0.824188473	0.92305034	0.813323291	0.815913413	0.872064287
FUNDC2	0.82937449	0.692305416	0.81946399	0.865861024	0.959658682	0.872239777
CDIPT	0.82937449	0.748933195	0.873674244	0.80459801	0.935286646	0.87754233
BTLA	0.82937449	0.944261937	0.810647419	0.812453944	0.804049674	0.88542704
FKBP14	0.82937449	0.716712413	0.916294854	0.852170016	0.858091718	0.890967932
CTTNBP2	0.82937449	0.687651905	0.854567571	0.929288192	0.864524107	0.891088733
SIRT2	0.82937449	0.7973731	0.838341783	0.882395013	0.847092933	0.895446935
ENO3	0.82937449	0.755089315	0.961769207	0.794880581	0.798701806	0.907384701
LOC101905706	0.82937449	0.696797615	0.79497298	0.990955345	0.788995893	0.909968586
FBL	0.82937449	0.727763882	0.758146087	0.97828838	0.817472827	0.912531667
LOC537017	0.82937449	0.694842866	0.758146087	0.839725231	0.970980375	0.91373422
NOC2L	0.82937449	0.872175143	0.834683675	0.806573529	0.80599066	0.930710695
FAAP100	0.82937449	0.836488699	0.811480663	0.854450063	0.786314903	0.950631909
OGFOD1	0.82937449	0.796253817	0.791843351	0.790984179	0.872734644	0.964058484
MDFIC2	0.82937449	0.720673049	0.758146087	0.784595865	0.901144467	0.987785166
LIG4	0.829385451	0.81556898	0.957052938	0.896116841	0.791848145	0.780283623
DGKA	0.829407002	0.731238567	0.845113191	0.896116841	0.958797242	0.795936781
DCUN1D1	0.829407002	0.690635606	0.867760342	0.829793071	0.890841443	0.933903265

ESRP2	0.829538625	0.715334857	0.829745504	0.962437357	0.883951804	0.838530462
LOC112448863	0.830080302	0.805283017	0.880163755	0.832401065	0.947299702	0.78096075
LOC112442280	0.830496117	0.988444882	0.832717775	0.853058361	0.796861564	0.774932208
HHLA2	0.830496117	0.805580108	0.999993442	0.812999186	0.789078975	0.779201808
CARS	0.830496117	0.760623317	0.868962356	0.812453944	0.976292433	0.794301059
MED24	0.830496117	0.792109226	0.809673119	0.979644238	0.848038632	0.806476213
NOTCH3	0.830496117	0.722348907	0.754913805	0.96591587	0.902223188	0.859032412
IPO5	0.830496117	0.759245424	0.761537172	0.94864068	0.873488171	0.882690335
PCYT1B	0.830496117	0.741310853	0.860306567	0.875836286	0.797489438	0.953509765
CPSF3	0.830496117	0.679199291	0.927508464	0.800826586	0.811991102	0.964898868
PDE8A	0.830877721	0.716270512	0.826789985	0.976196758	0.869580831	0.839489406
KRT8	0.830877721	0.758609923	0.867625133	0.876703811	0.912199478	0.846210629
KCTD2	0.830877721	0.764027588	0.810647419	0.961898258	0.823508064	0.872238439
FNDC3A	0.830877721	0.786392346	0.89478142	0.889415593	0.80587765	0.877048993
ECI2	0.830877721	0.696797615	0.8644528	0.838528071	0.789915129	0.987840151
PNKP	0.831306414	0.71189217	0.926656614	0.973373329	0.83704813	0.75986955
SNX10	0.831306414	0.846215781	0.927508464	0.885789665	0.848550619	0.763760487
LOC787102	0.831306414	0.998847958	0.75197571	0.865746543	0.810436875	0.766900958
EPM2A	0.831306414	0.687020192	0.915858924	0.830427818	0.981652321	0.769865166
TMEM50A	0.831306414	0.697043768	0.999993442	0.797338497	0.887514169	0.770035496
AP4E1	0.831306414	0.737721966	0.858820898	0.999981514	0.827356651	0.775725294
NDP	0.831306414	0.7973731	0.926161087	0.815919419	0.930513915	0.779201808
PDIA3	0.831306414	0.744774335	0.7786153	0.999981514	0.857562998	0.784754767
LYSMD3	0.831306414	0.759245424	0.791843351	0.945113651	0.943160494	0.793308041
MARCH6	0.831306414	0.750379179	0.905017412	0.874023052	0.929896768	0.797991983
LOC101902221	0.831306414	0.678512921	0.95322206	0.963981839	0.789915129	0.818370468
TOLLIP	0.831306414	0.865582541	0.754913805	0.95876517	0.849766114	0.827707374
ACADS	0.831306414	0.7889129	0.784764046	0.827600292	0.976292433	0.839489406
COPG1	0.831306414	0.796254453	0.894316021	0.883236823	0.847092933	0.85365082
LOC515828	0.831306414	0.694400411	0.995746693	0.797312233	0.847173934	0.8537109
NFIC	0.831306414	0.693513972	0.849704325	0.977689659	0.837915327	0.858507498
ADCY3	0.831306414	0.777309385	0.841294863	0.89684883	0.871085053	0.876810151
CLCC1	0.831306414	0.848470752	0.831933739	0.871338923	0.847173934	0.876861428
PCGF6	0.831306414	0.882242226	0.803342698	0.853762905	0.847173934	0.885089102
BCAT2	0.831306414	0.804185018	0.848278925	0.796934171	0.917407853	0.893585844
PREP	0.831306414	0.674524738	0.830887859	0.975653996	0.788007619	0.927520621
GAMT	0.831306414	0.709247882	0.847061113	0.817230639	0.886944613	0.959723327
LOC112448153	0.831306414	0.733030814	0.824121854	0.887642126	0.796861564	0.97061907

LOC112445029	0.831550764	0.722779653	0.972410614	0.937654716	0.803840921	0.772618249
TRAPPC1	0.831719993	0.796253817	0.927635169	0.817230639	0.927087035	0.78096075
ISOC1	0.831719993	0.755089315	0.854567571	0.843613441	0.976769047	0.783241453
CDKN2AIPNL	0.831719993	0.821674572	0.895114336	0.905630971	0.823508064	0.823610158
ITGA4	0.831719993	0.71532732	0.981566174	0.792140715	0.886785729	0.839906041
LOC101909718	0.831719993	0.89465829	0.810647419	0.813375274	0.911568867	0.844759721
EIF3G	0.831719993	0.727225517	0.8588642	0.988408846	0.78547726	0.860966325
PCSK7	0.831719993	0.940956417	0.805479206	0.792140715	0.872178532	0.865767172
KPNB1	0.831719993	0.688775944	0.855160666	0.808075336	0.976292433	0.872238439
TOB1	0.831719993	0.861395263	0.794846789	0.914850247	0.816070658	0.875297569
FDXR	0.831719993	0.940546131	0.770826735	0.848131881	0.817472827	0.886743984
TMEM104	0.831770286	0.826316517	0.871782109	0.792161305	0.936215792	0.841451122
LOC789337	0.832500764	0.76533726	0.828035534	0.812354594	0.989334846	0.817522068
TTYH2	0.832595814	0.957385532	0.774924967	0.885789665	0.847092933	0.809830825
BTBD6	0.832672796	0.889457989	0.819359511	0.886312303	0.786314903	0.899557238
PURB	0.83287671	0.694400411	0.865375115	0.9819628	0.865075514	0.81956221
MRPS12	0.832877392	0.738007931	0.853455703	0.890362631	0.848550619	0.923092436
C19H17orf80	0.833021667	0.694400411	0.916323169	0.917158741	0.846285074	0.858507498
CD9	0.833162124	0.816129287	0.854571175	0.959616431	0.788995893	0.844334911
NPW	0.833162124	0.69820785	0.77566312	0.95901497	0.886025454	0.897315753
SMIM17	0.833476758	0.811445496	0.975774524	0.809277776	0.799752471	0.846210629
LOC539009	0.833898379	0.96643574	0.755135544	0.888687722	0.859755271	0.789870764
VNN2	0.833923662	0.708204618	0.810167226	0.882057818	0.970988226	0.847386898
CDKN2AIP	0.834041924	0.805580108	0.847014764	0.901353354	0.943160494	0.765948605
EIF3M	0.834041924	0.816129287	0.754913805	0.916858019	0.886944613	0.876162019
LOC786065	0.834514515	0.709247882	0.89478142	0.985118339	0.821351993	0.809350773
H2AFX	0.834514515	0.806029227	0.768438632	0.999981514	0.787177225	0.845241321
CDKN2C	0.834514515	0.811059858	0.913712666	0.809886338	0.893716526	0.846210629
TAF9	0.834514515	0.681192035	0.983582266	0.812999186	0.804274107	0.912531667
FABP3	0.834518022	0.805580108	0.832192335	0.896116841	0.821351993	0.909635302
PIGF	0.834518022	0.722779653	0.886829776	0.839245804	0.852234727	0.935468188
LOC783045	0.834526386	0.786392346	0.935266153	0.953331149	0.788995893	0.78288746
JOSD2	0.834526386	0.875506677	0.806470927	0.791081996	0.926972948	0.874490166
SPATA6L	0.834526386	0.691301661	0.774924967	0.820971736	0.943702351	0.960023723
CEP89	0.834531647	0.741263262	0.935266153	0.887877413	0.823508064	0.865889509
LOC112449318	0.834531647	0.833323491	0.754913805	0.874170259	0.870042072	0.926122394
TAF1	0.834816367	0.750466772	0.949892469	0.843613441	0.912597537	0.783241453
SDHAF2	0.834816367	0.812449121	0.885469405	0.839245804	0.925874018	0.819366282

FBP2	0.834816367	0.745846221	0.964807927	0.876854671	0.797227609	0.85532952
HIST1H2AC	0.834816367	0.819210468	0.79497298	0.853058361	0.805277279	0.96248183
LOC101906756	0.834911528	0.737396716	0.851633941	0.808073654	0.998287238	0.804385539
ANKRD34A	0.834993028	0.902241567	0.897432125	0.899855526	0.81911207	0.763151085
RAMP1	0.834993028	0.83359488	0.826269935	0.80459801	0.989339508	0.769865166
CEP350	0.834993028	0.741263262	0.810647419	0.808073654	0.999985752	0.769865166
CSMD2	0.834993028	0.956406487	0.927508464	0.796128007	0.815913413	0.77545502
SPATA20	0.834993028	0.891111357	0.880612713	0.885789665	0.864524107	0.779201808
LOC101903424	0.834993028	0.717037143	0.917651622	0.886312303	0.943702351	0.779201808
SORL1	0.834993028	0.69959307	0.78790711	0.999981514	0.818179113	0.783241453
SELENOS	0.834993028	0.704503729	0.999993442	0.87522058	0.804274107	0.788462021
DENND2A	0.834993028	0.805283017	0.901955007	0.870591999	0.904912019	0.79187813
GRWD1	0.834993028	0.686805478	0.894338686	0.999981514	0.804037558	0.799889245
VNN1	0.834993028	0.757251331	0.810167226	0.97731816	0.893716526	0.801888363
FAM173A	0.834993028	0.863286109	0.8042974	0.812453944	0.970988226	0.802390255
SSR4	0.834993028	0.687651905	0.85942296	0.913048575	0.959658682	0.806476213
ARID2	0.834993028	0.825171788	0.81550927	0.821450642	0.97234838	0.806714845
PDCD4	0.834993028	0.781543413	0.964382669	0.809356235	0.887514169	0.810508177
CDH4	0.834993028	0.697043768	0.799077215	0.956685049	0.954071324	0.818370468
LOC614531	0.834993028	0.992795654	0.7560439	0.855232617	0.810436875	0.81956221
CTSZ	0.834993028	0.850502007	0.805212947	0.908467146	0.892616401	0.820645649
LOC781989	0.834993028	0.686033606	0.862602899	0.9880671	0.847092933	0.841312694
USF3	0.834993028	0.766943819	0.865288669	0.825410839	0.954403133	0.845747012
LOC101905997	0.834993028	0.674524738	0.86885176	0.812453944	0.989334846	0.847193872
ACAD10	0.834993028	0.817524348	0.780486258	0.972142185	0.821351993	0.8537109
NUDC	0.834993028	0.724380878	0.889098668	0.940136897	0.829853428	0.867063767
MRPL50	0.834993028	0.697043768	0.996224523	0.809277776	0.817472827	0.870640897
USO1	0.834993028	0.72108575	0.996624589	0.804472003	0.790136352	0.877048993
ITGA10	0.834993028	0.781163738	0.928514467	0.830427818	0.836444721	0.877750901
PA2G4	0.834993028	0.691584935	0.803183436	0.908467146	0.935286646	0.891088733
TRAP1	0.834993028	0.779343136	0.819527307	0.812453944	0.947020167	0.896529476
ADAMTS20	0.834993028	0.69959307	0.759332589	0.999981514	0.799272579	0.902100676
WDR46	0.834993028	0.687651905	0.87708434	0.929388614	0.836444721	0.911219114
POU2F2	0.834993028	0.738561239	0.883906525	0.928404714	0.788007619	0.91373422
TMEM204	0.834993028	0.700629302	0.882360707	0.888687722	0.867456282	0.91373422
TMCO4	0.834993028	0.691301661	0.802277976	0.914026346	0.906893576	0.915414597
SRP68	0.834993028	0.710645465	0.93475587	0.830427818	0.801692604	0.940050644
LOC786015	0.834993028	0.742019939	0.892343042	0.815919419	0.838090622	0.947937638

LOC107132486	0.834993028	0.691301661	0.822300339	0.956685049	0.789679352	0.950540248
HOMER1	0.834993028	0.775033799	0.87391133	0.80459801	0.810436875	0.969014913
LOC101909754	0.835060473	0.826032283	0.810185568	0.999981514	0.795839201	0.785562356
LOC112446761	0.835182567	0.683972894	0.791557166	0.934445963	0.847092933	0.958281344
SELENOV	0.835225242	0.94592331	0.862253703	0.803690355	0.90533486	0.770035496
ENDOD1	0.835269897	0.72321922	0.972482052	0.957796224	0.796497513	0.766887422
ABRAXAS2	0.835269897	0.783896922	0.929661805	0.920843019	0.86010579	0.768659551
NIPAL2	0.835269897	0.839128192	0.783854721	0.999981514	0.799051801	0.770035496
TIA1	0.835269897	0.704181021	0.880540893	0.89684883	0.959658682	0.79187813
TCF21	0.835269897	0.952760839	0.788941145	0.812399046	0.923666555	0.806714845
LOC112444847	0.835269897	0.955652503	0.837162788	0.825308462	0.833173678	0.835231445
LOC101903557	0.835269897	0.889679752	0.764467593	0.949605617	0.834569503	0.835231445
LUC7L3	0.835269897	0.691301661	0.834683675	0.916858019	0.956041014	0.836705424
LOC101905179	0.835269897	0.683368775	0.810647419	0.999981514	0.795839201	0.838530462
CNRIP1	0.835269897	0.792109226	0.908072171	0.813323291	0.911568867	0.847786883
GYS1	0.835269897	0.7583338	0.788106994	0.806573529	0.997789733	0.85102506
NMB	0.835269897	0.696525042	0.8735515	0.948668643	0.88127953	0.853576299
STPG1	0.835269897	0.71311249	0.879939804	0.952247001	0.84620147	0.86078454
DLK2	0.835269897	0.690635606	0.774015998	0.941467375	0.937932469	0.877048993
KRI1	0.835269897	0.731024992	0.955427429	0.860937578	0.811359687	0.882690335
C2H2orf88	0.835269897	0.921382572	0.802277976	0.87522058	0.797227609	0.893252687
SPRED2	0.835269897	0.699192406	0.8588642	0.90413565	0.864524107	0.919124527
PLEKHA8	0.835269897	0.741310853	0.858032788	0.892047793	0.837399927	0.927426708
TGDS	0.835269897	0.870298381	0.777712574	0.88099367	0.788995893	0.945532151
EEF1D	0.835269897	0.722779653	0.895801611	0.812453944	0.857826764	0.948705062
PTGDR	0.835269897	0.741310853	0.772600931	0.925210648	0.809051283	0.963456717
TUBE1	0.835269897	0.721685298	0.84808383	0.812999186	0.802592922	0.99666605
ADGRD1	0.835380109	0.779325129	0.855971055	0.976069331	0.85185197	0.793197037
U2SURP	0.835380109	0.697306013	0.76555928	0.999981514	0.851054476	0.793873776
TRARG1	0.835380109	0.962423197	0.777712574	0.915039335	0.80917607	0.799581725
LRCH4	0.835708442	0.893615781	0.915698649	0.792071212	0.917407853	0.769865166
TCP11	0.835708442	0.847323359	0.944126714	0.896116841	0.801692604	0.783241453
PELI1	0.835708442	0.737120625	0.947462602	0.875471208	0.87329316	0.835231445
LOC112443484	0.835979282	0.738031602	0.964661607	0.951479029	0.80281845	0.77508144
TTC21A	0.835979282	0.816984836	0.972410614	0.82868625	0.826634148	0.808345278
LOC101904698	0.835979282	0.874981732	0.871491704	0.803627275	0.906893576	0.842473495
LOC107131239	0.835979282	0.704346844	0.802277976	0.949502614	0.935286646	0.852504043
NFKBIZ	0.835979282	0.76003522	0.879939804	0.952247001	0.807325011	0.86078454

RHOBTB2	0.835979282	0.753976609	0.825463618	0.961898258	0.846745071	0.867635709
BRD8	0.835979282	0.687020192	0.93190909	0.918693944	0.797227609	0.898470276
TMUB2	0.835979282	0.678705386	0.894030462	0.856195359	0.865075514	0.944330889
SH3TC2	0.835979282	0.737396716	0.847301381	0.843613441	0.80972094	0.9803583
MFSD14B	0.83608917	0.898550443	0.89385865	0.860937578	0.882252274	0.769865166
PRR12	0.836499344	0.748702456	0.892139418	0.992930508	0.828207883	0.768572658
CD59	0.836499344	0.7095693	0.983534298	0.889415593	0.87329316	0.770035496
TNFRSF6B	0.836499344	0.717848892	0.876414692	0.989601002	0.789492208	0.858245612
ADAM17	0.836499344	0.788980101	0.804579956	0.978006499	0.823508064	0.858507498
CKMT2	0.836499344	0.802045751	0.797180963	0.805568606	0.788995893	0.99828741
MAP2K4	0.836722913	0.727534248	0.999993442	0.802276874	0.823508064	0.769865166
ARID4B	0.836722913	0.726454526	0.849389234	0.940025301	0.95721434	0.772618249
RORA	0.836722913	0.694842866	0.896821247	0.845521561	0.984328593	0.783241453
TBC1D8B	0.836722913	0.809600412	0.853546772	0.944913633	0.882013597	0.793873776
RPLP1	0.836722913	0.740642434	0.798396577	0.999981514	0.819549575	0.813140661
FBXO30	0.836722913	0.840089521	0.916294854	0.817146543	0.891726429	0.820723094
LUC7L	0.836722913	0.696797615	0.951028911	0.892047793	0.887514169	0.829181492
SH2D1B	0.836722913	0.681192035	0.992245264	0.815919419	0.887514169	0.838983503
CCR6	0.836722913	0.745846221	0.841294863	0.960937718	0.864524107	0.852504043
KIAA0232	0.836722913	0.68204808	0.761415057	0.998529748	0.890348531	0.860966325
MSRB3	0.836722913	0.872921026	0.886064678	0.870591999	0.801692604	0.867073248
SLF2	0.836722913	0.816369086	0.798782742	0.891988949	0.891304411	0.877750901
SEMA4G	0.836722913	0.687651905	0.849389234	0.889236731	0.844585092	0.963456717
IFT43	0.836722913	0.718258172	0.854571175	0.830427818	0.833864947	0.976020793
LIPJ	0.836722913	0.686805478	0.77566312	0.885789665	0.823508064	0.997383134
TEX9	0.836904209	0.938235006	0.876414692	0.914850247	0.791848145	0.770035496
YIF1A	0.837021758	0.718258172	0.996224523	0.806838917	0.900891209	0.783241453
PXYLP1	0.837286511	0.753090681	0.850624788	0.958978724	0.902907077	0.800967059
EMC2	0.837286511	0.740343181	0.923189411	0.96444269	0.811991102	0.810508177
ZNF383	0.837286511	0.95687851	0.772739244	0.894086736	0.795839201	0.861071121
C17H12orf49	0.837569987	0.838775377	0.911535369	0.915605519	0.826634148	0.789030652
CXCR2	0.837569987	0.819210468	0.849704325	0.804472003	0.930451229	0.882572902
C11H2orf42	0.837569987	0.811354298	0.770037479	0.934466438	0.804274107	0.930710695
TSFM	0.837572781	0.780985952	0.88846032	0.808075336	0.904206501	0.895803603
CCDC9B	0.837914069	0.838154275	0.791557166	0.817146543	0.989334846	0.791354446
PID1	0.837914069	0.931043812	0.855268213	0.887983383	0.83439397	0.80105726
NOL7	0.837914069	0.759814955	0.957052938	0.852170016	0.893376996	0.801888363
LOC112446795	0.837914069	0.789494428	0.901983585	0.911470775	0.868722067	0.8170478

SRP54	0.837914069	0.764371601	0.964110173	0.813323291	0.883951804	0.838729965
LOC529792	0.837914069	0.750466772	0.927635169	0.918693944	0.799272579	0.865212385
SCARF1	0.837914069	0.904754143	0.855892553	0.859320133	0.804037558	0.877048993
COPS5	0.837914069	0.718258172	0.916038418	0.836387732	0.89743683	0.890967932
IFITM1	0.837914069	0.915269318	0.759357794	0.80459801	0.844434232	0.944973383
TTC30B	0.837914069	0.686805478	0.784115813	0.799480811	0.970980375	0.954496768
ASB16	0.837914069	0.804498656	0.879939804	0.812453944	0.799051801	0.960023723
POLR3G	0.837914069	0.691638455	0.855892553	0.904018489	0.809051283	0.963255451
NABP2	0.838180545	0.833323491	0.867222363	0.819213899	0.846464841	0.923092436
ADIPOR1	0.838196629	0.783896922	0.8735515	0.899855526	0.879395665	0.85513515
CCDC102B	0.838273838	0.686033606	0.871782109	0.821450642	0.821148296	0.992568593
HIF1AN	0.838356451	0.702392068	0.925509132	0.887877413	0.925950159	0.829441883
HDHD5	0.838617076	0.816369086	0.875873031	0.805499732	0.976292433	0.780283623
DOCK6	0.838617076	0.772669268	0.91469924	0.952247001	0.816070658	0.81956221
LOC112445972	0.838617076	0.95360445	0.798782742	0.88858839	0.820546747	0.839565788
ATP6V0A4	0.838617076	0.709329006	0.775552434	0.999981514	0.823508064	0.839906041
ANGPTL1	0.838617076	0.912776769	0.841294863	0.823935462	0.821351993	0.899428912
LOC787309	0.838617076	0.817022405	0.849835502	0.888412547	0.797227609	0.931410194
CCDC102A	0.838665519	0.683704605	0.871285807	0.999981514	0.801692604	0.770035496
SUPT16H	0.838868046	0.792109226	0.894030462	0.874688075	0.954071324	0.766218451
TST	0.838868046	0.941676269	0.849389234	0.812999186	0.928951321	0.768168974
PTOV1	0.838868046	0.811059858	0.955427429	0.799147532	0.904643542	0.806714845
LOC112442298	0.838868046	0.902525869	0.828449846	0.870210705	0.887514169	0.83150957
SNHG12	0.838868046	0.812449121	0.81412252	0.98981682	0.804625939	0.835231445
UIMC1	0.838868046	0.734509989	0.907477369	0.812453944	0.957793213	0.847619123
LOC101905151	0.838868046	0.906658524	0.881849916	0.819968583	0.828832304	0.865767172
FARP2	0.838868046	0.790149523	0.862253703	0.866566721	0.852234727	0.9221477
IGIP	0.838868046	0.706007408	0.809673119	0.888687722	0.887514169	0.9557845
ZNF532	0.83897453	0.690635606	0.807254925	0.949864417	0.832437297	0.947838082
KIAA0895L	0.839080194	0.694834718	0.809673119	0.96951401	0.964301327	0.779201808
CC2D2B	0.839080194	0.729001857	0.791557166	0.965462948	0.794877876	0.945480515
UMPS	0.839310857	0.68903984	0.860306567	0.853058361	0.797227609	0.996310803
CDK12	0.839327035	0.907836324	0.870965946	0.82163384	0.92549567	0.78096075
LRRC34	0.839327035	0.744774335	0.871929137	0.828736799	0.989334846	0.785562356
S1PR3	0.839327035	0.826488263	0.794676674	0.85721441	0.958797242	0.841312694
LOC101907965	0.839327035	0.863286109	0.893988897	0.799712183	0.903866359	0.845714049
KMT5B	0.839327035	0.951172741	0.805479206	0.812453944	0.875392598	0.85532952
KIAA0040	0.839327035	0.71930931	0.869932384	0.970039043	0.804625939	0.882705842

LOC112442851	0.8393651	0.799204728	0.961800035	0.859320133	0.791942644	0.860966325
RCN1	0.839662926	0.691301661	0.922552585	0.960016921	0.864524107	0.816246746
EPHB1	0.839662926	0.915269318	0.784788059	0.823913856	0.946454221	0.821372302
LANCL3	0.839662926	0.822956796	0.76538687	0.940693919	0.907323851	0.841312694
STK4	0.839662926	0.722195304	0.817051566	0.809886338	0.972806049	0.911219114
WDR11	0.839662926	0.709247882	0.810647419	0.916294903	0.804264617	0.973779337
IGHMBP2	0.839767821	0.737941984	0.9377363	0.964521432	0.799272579	0.81956221
PDIA4	0.839767821	0.757251331	0.761415057	0.995565324	0.884872176	0.836111377
ZFYVE26	0.839767821	0.722184539	0.999993442	0.827786827	0.804037558	0.839906041
CEP164	0.839767821	0.72828303	0.879939804	0.967883482	0.828832304	0.8537109
ZBTB1	0.839767821	0.733509477	0.818612484	0.979644238	0.80296868	0.911219114
NFKBIA	0.839876054	0.705532659	0.824121854	0.979644238	0.936962513	0.789870764
ODF2	0.839876054	0.692305416	0.855713041	0.885336966	0.909379774	0.923547711
TAF10	0.839876054	0.716415149	0.89478142	0.828736799	0.870450746	0.94095689
HDGF	0.839876054	0.792109226	0.819761308	0.851164437	0.808945365	0.973649216
APCDD1	0.839982234	0.696797615	0.999993442	0.828736799	0.887514169	0.770490368
AMDHD1	0.839982234	0.868390063	0.89193017	0.915039335	0.847173934	0.782001574
SNX15	0.839982234	0.709348874	0.990118874	0.805499732	0.923910588	0.806476213
DCTN3	0.839982234	0.776428476	0.916323169	0.82868625	0.944839728	0.809961137
PRPF4B	0.839982234	0.793300166	0.816553355	0.952247001	0.905571216	0.816246746
PDK4	0.839982234	0.761137241	0.879939804	0.904715959	0.926972948	0.816785597
SLCO2B1	0.839982234	0.805934634	0.818612484	0.893051204	0.948711141	0.823701731
LOC614141	0.839982234	0.796253817	0.958197827	0.852481062	0.852968331	0.8255975
AEN	0.839982234	0.712357273	0.8267276	0.956685049	0.91563394	0.851325852
EXT2	0.839982234	0.708031655	0.896821247	0.952247001	0.852968331	0.8537109
LOC104973229	0.839982234	0.814246237	0.885896294	0.874023052	0.85545716	0.875650601
GATM	0.839982234	0.761570067	0.902867554	0.806573529	0.836444721	0.954496768
MID2	0.840021666	0.74733285	0.804002279	0.888123511	0.998835841	0.779201808
FRMPD1	0.840021666	0.689824411	0.774924967	0.943808491	0.983941532	0.814218101
ZNHIT2	0.840021666	0.708278125	0.884121008	0.812453944	0.964301327	0.877014986
TIMM50	0.840021666	0.713726826	0.915858924	0.828736799	0.86876405	0.931410194
SDHD	0.840102888	0.684972622	0.911535369	0.825032424	0.976292433	0.835231445
THAP4	0.840277476	0.938087741	0.836482	0.80459801	0.870450746	0.867063767
EIF3I	0.840277476	0.744774335	0.867326337	0.884452623	0.809129327	0.959723327
NFATC3	0.840620869	0.7889129	0.916323169	0.866566721	0.943540106	0.772618249
LOC101902030	0.840620869	0.716005622	0.925320071	0.922967576	0.91563394	0.78087541
LOC790037	0.840620869	0.722348907	0.927508464	0.987281997	0.789999838	0.818021392
UEVLD	0.840620869	0.766943819	0.907477369	0.908467146	0.818179113	0.877048993

MYOM3	0.840620869	0.894014677	0.838341783	0.874023052	0.826634148	0.877048993
BRINP1	0.840620869	0.808495385	0.771066142	0.92139105	0.804037558	0.951081819
SCD5	0.840620869	0.778518806	0.810647419	0.824230049	0.799272579	0.99666605
ADI1	0.840640386	0.870716141	0.774924967	0.870591999	0.897352056	0.88313584
RCAN3	0.840640386	0.727473229	0.921266625	0.852170016	0.852746309	0.918249293
MOSPD2	0.840722324	0.727534248	0.852225514	0.973373329	0.887514169	0.83150957
RPH3AL	0.84082238	0.749370809	0.848180652	0.817146543	0.999985752	0.801888363
ERP44	0.84082238	0.899494273	0.804171507	0.820024304	0.916605572	0.865745718
IQCH	0.84082238	0.718228672	0.878390815	0.886312303	0.902258286	0.895666013
ZNF423	0.841205288	0.886565095	0.802277976	0.97676218	0.854365297	0.769865166
SAFB2	0.841205288	0.768359345	0.885896294	0.808075336	0.997264208	0.770035496
ENC1	0.841205288	0.914902719	0.802277976	0.812453944	0.972722722	0.775854424
ZDHHC4	0.841205288	0.739786945	0.964807927	0.813375274	0.953300742	0.77803403
ANKRD24	0.841205288	0.696797615	0.948904459	0.919689139	0.903513738	0.78096075
ZNF507	0.841205288	0.727534248	0.820146472	0.876703811	0.999985752	0.78096075
LOC101904477	0.841205288	0.709348874	0.816114918	0.999981514	0.837915327	0.782775967
SGCA	0.841205288	0.91537041	0.971053208	0.806838917	0.810436875	0.783241453
ZFYVE9	0.841205288	0.84312966	0.963164828	0.891329697	0.801692604	0.784782855
GPR132	0.841205288	0.880565292	0.863546668	0.837820405	0.931775933	0.801888363
RNF115	0.841205288	0.759033998	0.879939804	0.959652624	0.885790728	0.803177592
LOC511531	0.841205288	0.938235006	0.766722666	0.819213899	0.956653075	0.806714845
DAZAP1	0.841205288	0.697306013	0.991108777	0.939604997	0.795839201	0.806892223
FPGS	0.841205288	0.712357273	0.79497298	0.903584224	0.996089557	0.810156973
MPG	0.841205288	0.757251331	0.85942296	0.853058361	0.977167046	0.810159134
NR2C2	0.841205288	0.68903984	0.850908982	0.809886338	0.999985752	0.820971043
COASY	0.841205288	0.77844414	0.836482	0.873302281	0.970491451	0.823031765
OAZ2	0.841205288	0.764799096	0.8267276	0.888198716	0.966665737	0.83150957
CDH11	0.841205288	0.737396716	0.874983674	0.959616431	0.871593517	0.839906041
ABI3	0.841205288	0.692305416	0.895826828	0.918693944	0.919194647	0.839906041
BRD2	0.841205288	0.71189217	0.855268213	0.888687722	0.964833865	0.839906041
CEP152	0.841205288	0.785057176	0.8644528	0.950746405	0.856725527	0.84048788
TXLNA	0.841205288	0.778666381	0.886829776	0.821450642	0.953300742	0.846210629
KCNC4	0.841205288	0.709348874	0.886829776	0.874424802	0.952796169	0.8537109
TAF4	0.841205288	0.718258172	0.915858924	0.799497281	0.964301327	0.856640874
ZSCAN30	0.841205288	0.687651905	0.965819476	0.875836286	0.867993134	0.86324237
USF2	0.841205288	0.881942703	0.806470927	0.939604997	0.807468091	0.867482845
LOC784357	0.841205288	0.68903984	0.86684572	0.999793541	0.79876313	0.872238439
PIGX	0.841205288	0.704512234	0.798782742	0.932914414	0.939442711	0.877547163

METTL6	0.841205288	0.877009583	0.883737851	0.806573529	0.854289643	0.89438401
INPP5A	0.841205288	0.792661811	0.828935179	0.812453944	0.949404261	0.904026143
TMCC3	0.841205288	0.714920127	0.836447778	0.896116841	0.901853599	0.920605619
FASTKD1	0.841205288	0.709247882	0.854567571	0.838528071	0.894364008	0.955140534
GSKIP	0.841205288	0.692305416	0.899106435	0.892047793	0.804264617	0.958281344
EFCAB2	0.841205288	0.749369443	0.916294854	0.819968583	0.799272579	0.965109715
HILPDA	0.841205288	0.740362216	0.774015998	0.868435169	0.81725718	0.99828741
GRIN1	0.841205288	0.740362216	0.77283514	0.852161263	0.821351993	0.999948228
REEP4	0.841392122	0.904847484	0.810647419	0.898818046	0.91563394	0.78096075
LOC107131944	0.841392122	0.839128192	0.809673119	0.921482057	0.906803277	0.83150957
ZSWIM5	0.841392122	0.697306013	0.824121854	0.80459801	0.895404843	0.989184798
KRR1	0.841449958	0.808711476	0.797180963	0.819571278	0.999985752	0.770035496
NRARP	0.841449958	0.748656757	0.823731372	0.85647796	0.999985752	0.772618249
EMC10	0.841449958	0.792109226	0.774025101	0.886831994	0.998008754	0.78096075
IQCC	0.841449958	0.772336368	0.8042974	0.999981514	0.81725718	0.783241453
DVL1	0.841449958	0.955652503	0.819527307	0.843515965	0.901662258	0.783241453
ACTL6A	0.841449958	0.731024992	0.999993442	0.828337624	0.819329746	0.793197037
LSM11	0.841449958	0.940994886	0.784260981	0.898188142	0.89052284	0.793308041
NSF	0.841449958	0.870334242	0.8644528	0.881493849	0.907956183	0.806476213
LRIF1	0.841449958	0.81848543	0.774015998	0.808073654	0.999985752	0.819366282
HAUS4	0.841449958	0.744241031	0.818131588	0.952247001	0.943702351	0.81956221
MCL1	0.841449958	0.709542765	0.867625133	0.999981514	0.804049674	0.83150957
ACHE	0.841449958	0.741310853	0.871482445	0.829793071	0.979840175	0.83150957
B3GALNT2	0.841449958	0.870334242	0.764398531	0.940436902	0.89548293	0.831652079
LOC107131660	0.841449958	0.961055437	0.784260981	0.916480703	0.803705802	0.835231445
EPS15	0.841449958	0.7889129	0.864832853	0.843613441	0.960048824	0.835231445
IFT122	0.841449958	0.710788298	0.916038418	0.810580554	0.974222572	0.839906041
DEFB4A	0.841449958	0.782783231	0.922552585	0.940438437	0.799272579	0.846792135
LRRC57	0.841449958	0.846721805	0.893988897	0.908467146	0.801692604	0.85861062
UNC79	0.841449958	0.848886451	0.813810146	0.824507073	0.935286646	0.877014986
NCOA6	0.841449958	0.827824094	0.81319557	0.812999186	0.943160494	0.895517058
NBEAL2	0.841449958	0.818838644	0.935823375	0.839725231	0.799867929	0.898470276
GHITM	0.841449958	0.72828303	0.805479206	0.896116841	0.936921784	0.90645885
ABCC10	0.841449958	0.691950381	0.932477853	0.928404714	0.795839201	0.912531667
LONP1	0.841449958	0.804185018	0.80412682	0.831477463	0.931357161	0.917930485
C18H19orf81	0.841449958	0.759397946	0.874983674	0.812999186	0.821351993	0.977525788
UBE2B	0.841449958	0.700204995	0.831933739	0.882057818	0.804625939	0.994887496
DGCR8	0.841451072	0.774644712	0.997400824	0.874424802	0.804625939	0.79187813

LEKR1	0.841499587	0.955652503	0.939948201	0.819835879	0.801692604	0.78096075
FBXO17	0.841499587	0.861395263	0.854567571	0.894086736	0.926972948	0.782330566
TNFAIP8L1	0.841499587	0.958814007	0.879939804	0.861301134	0.823508064	0.792412747
ELP5	0.841499587	0.848383949	0.805212947	0.980564765	0.846464841	0.812774915
NAPEPLD	0.841499587	0.919171954	0.880476435	0.886312303	0.810436875	0.835231445
SLITRK5	0.841877213	0.876831363	0.820146472	0.892047793	0.86010579	0.872238439
NFIL3	0.842323652	0.692305416	0.943836178	0.823935462	0.977167046	0.783241453
MAP2K7	0.842323652	0.984089343	0.853546772	0.843613441	0.810436875	0.810156973
TDRD3	0.842323652	0.816369086	0.860306567	0.927300606	0.855920132	0.853548296
NIPSNAP2	0.842323652	0.709247882	0.905023942	0.852005554	0.956653075	0.853576299
CD3G	0.842323652	0.886669497	0.916323169	0.812453944	0.82488413	0.872237727
GUK1	0.842323652	0.764775882	0.798846681	0.802462225	0.989334846	0.879576441
ADPGK	0.842323652	0.703887794	0.89478142	0.918693944	0.83704813	0.915414597
PIAS3	0.842464983	0.722968232	0.999993442	0.863269783	0.811991102	0.821910421
LBR	0.842954342	0.804185018	0.916294854	0.903242359	0.854289643	0.835231445
LOC104975196	0.843162966	0.781543413	0.968249948	0.839725231	0.879395665	0.820395402
ANKRD63	0.843162966	0.760778257	0.829751489	0.956685049	0.858545936	0.881246845
TMEM17	0.843600777	0.895777642	0.871929137	0.870210705	0.91563394	0.776734587
MBP	0.843600777	0.767113995	0.869932384	0.885789665	0.973219333	0.782330566
ARMC10	0.843600777	0.836427846	0.972180683	0.854220165	0.823508064	0.801398889
DOCK3	0.843600777	0.933545282	0.827969228	0.822691957	0.935146118	0.802342358
DCAF5	0.843600777	0.709247882	0.810647419	0.806838917	0.976259416	0.930710695
ZNF654	0.84361389	0.739786945	0.817582037	0.849989809	0.980016241	0.867063767
PUDP	0.843686713	0.708652332	0.895801611	0.938303773	0.953300742	0.77508144
LOC112446033	0.843686713	0.933886329	0.807254925	0.822262805	0.947299702	0.797953744
MPST	0.843686713	0.88008538	0.851822885	0.84844684	0.943610727	0.801888363
KAT14	0.843686713	0.709542765	0.867661896	0.915013589	0.958797242	0.823031765
SLC44A1	0.843686713	0.829067393	0.859987058	0.913048575	0.89052284	0.828655205
ARHGAP45	0.843686713	0.849513595	0.916294854	0.801848398	0.891726429	0.857457228
AFG3L2	0.843686713	0.705532659	0.860306567	0.867380977	0.9683846	0.865767172
MCM6	0.843686713	0.7889129	0.76555928	0.999981514	0.799272579	0.87501921
CFL1	0.843686713	0.788980101	0.897322115	0.882057818	0.851927006	0.891423889
KLF7	0.843686713	0.722690977	0.810167226	0.815919419	0.970491451	0.926927742
LOC100848939	0.843688764	0.823478017	0.818131588	0.915039335	0.887514169	0.867063767
ZNF346	0.843688764	0.693513972	0.77566312	0.990955345	0.900747635	0.872238439
LOC101902385	0.843691768	0.722968232	0.893761108	0.899792456	0.961465747	0.792374188
LOC104973139	0.843691768	0.746341488	0.987610559	0.872590202	0.852968331	0.816246746
NRAS	0.843691768	0.722195304	0.957052938	0.932764647	0.845773773	0.81956221

GNE	0.843691768	0.973321075	0.799533361	0.812453944	0.89743683	0.83150957
ANGPT2	0.843691768	0.969051025	0.808037655	0.868435169	0.844384115	0.835231445
KDM2B	0.843691768	0.824188473	0.819359511	0.97828838	0.82488413	0.842473495
MRPL13	0.843691768	0.718258172	0.915858924	0.819968583	0.956041014	0.865745718
MYADM	0.843691768	0.713956321	0.97918842	0.852005554	0.85545716	0.865767172
LOC785403	0.843691768	0.887281124	0.903431343	0.843515965	0.797156657	0.891088733
UBXN4	0.843691768	0.709247882	0.957052938	0.824507073	0.881680572	0.893838339
NPLOC4	0.843691768	0.7889129	0.921266625	0.870210705	0.802592922	0.918869728
CLSPN	0.843691768	0.796253817	0.880163755	0.88904348	0.797227609	0.936263602
AATF	0.843691768	0.713792742	0.916323169	0.853058361	0.833173678	0.946347861
DNM1L	0.843691768	0.692305416	0.859584617	0.805499732	0.857203077	0.997383134
FAM89B	0.843695921	0.970950021	0.841827714	0.829793071	0.820079097	0.851995112
IGFLR1	0.84390501	0.690635606	0.99341711	0.817802131	0.911568867	0.83150957
SCAF8	0.843928159	0.77194497	0.854571175	0.830187312	0.999453276	0.779201808
MPPED2	0.844103916	0.834615638	0.829455601	0.887877413	0.904912019	0.867063767
RAB36	0.844119247	0.750379179	0.845525761	0.948668643	0.893716526	0.860410905
RHOU	0.844190445	0.842884359	0.854087255	0.980564765	0.847092933	0.779201808
PRRG1	0.844190445	0.758389761	0.879939804	0.890362631	0.970988226	0.78087541
SSNA1	0.844190445	0.829408576	0.971053208	0.821865782	0.887514169	0.78096075
TMEM80	0.844190445	0.741310853	0.915858924	0.865210554	0.970988226	0.781984152
SUZ12	0.844190445	0.823133291	0.871491704	0.950643928	0.886779248	0.783241453
LOC104970450	0.844190445	0.744685673	0.906019013	0.898818046	0.953885477	0.783241453
SERF1A	0.844190445	0.868390063	0.984886031	0.803690355	0.847173934	0.785869233
FAM161A	0.844190445	0.792109226	0.83804464	0.999981514	0.819549575	0.789870764
ARHGAP17	0.844190445	0.724171476	0.833583844	0.996054502	0.904202474	0.790705747
U2AF1	0.844190445	0.692305416	0.890697966	0.999981514	0.801692604	0.814218101
CMBL	0.844190445	0.753090681	0.855793459	0.815919419	0.982538884	0.8537109
ERCC6	0.844190445	0.766595431	0.832065705	0.865746543	0.942462162	0.896434668
PPIL6	0.844190445	0.781543413	0.783856677	0.904018489	0.901149683	0.919993751
PRDX1	0.844190445	0.77139884	0.886829776	0.87522058	0.847092933	0.92725789
LOC101906512	0.844190445	0.77844414	0.882643407	0.868530316	0.823508064	0.940583352
NOP14	0.844296977	0.718228672	0.849218971	0.833241946	0.838461406	0.991847494
NCAPG2	0.844739086	0.766943819	0.997300234	0.854220165	0.806321189	0.831652079
ARIH2OS	0.844739086	0.774644712	0.850499069	0.882057818	0.960190226	0.839573661
PRDM15	0.844739086	0.728504282	0.967696262	0.832401065	0.887514169	0.85532952
DDHD1	0.844739086	0.713956321	0.954249102	0.853058361	0.857183568	0.89950552
SETDB2	0.844744534	0.782387091	0.8655261	0.999981514	0.809129327	0.776734587
IDNK	0.844744534	0.757251331	0.978014482	0.876703811	0.887514169	0.782330566

TUBA1C	0.844744534	0.796253817	0.916323169	0.965578567	0.82488413	0.78288746
LOC101906235	0.844744534	0.829345514	0.992377532	0.876703811	0.799867929	0.783241453
IER2	0.844744534	0.761391528	0.879939804	0.965109987	0.893716526	0.788462021
DCBLD1	0.844744534	0.791281103	0.846523249	0.828337624	0.996781978	0.795129319
ROR2	0.844744534	0.696914218	0.854567571	0.999981514	0.81725718	0.796402766
LOC615610	0.844744534	0.710645465	0.915858924	0.9063051	0.948421303	0.802390255
RAP1GAP2	0.844744534	0.775737793	0.819173863	0.999981514	0.848550619	0.806714845
WDR47	0.844744534	0.740343181	0.89496118	0.882395013	0.961465747	0.814218101
LOC548613	0.844744534	0.709247882	0.770595941	0.912922827	0.99971266	0.81956221
DSC1	0.844744534	0.717037143	0.804002279	0.999981514	0.887514169	0.821257366
LRRC41	0.844744534	0.946258461	0.849389234	0.882057818	0.842810018	0.83150957
LOC783022	0.844744534	0.817553533	0.926161087	0.860301856	0.887514169	0.833150766
LOC112443216	0.844744534	0.82177045	0.903844825	0.868378265	0.900927045	0.837559792
ATP12A	0.844744534	0.821674572	0.921266625	0.863269783	0.878269439	0.845714049
ACAT1	0.844744534	0.731493547	0.809673119	0.848592907	0.998008754	0.853548296
UNG	0.844744534	0.704646482	0.916038418	0.839725231	0.957596412	0.859993218
LOC112446024	0.844744534	0.807919676	0.925192628	0.899308956	0.817472827	0.860414702
LRRC8C	0.844744534	0.835486075	0.810831922	0.932764647	0.878668281	0.860966325
CD68	0.844744534	0.774237994	0.860877703	0.959591127	0.817472827	0.887239327
CXHXorf21	0.844744534	0.718258172	0.95219206	0.865114984	0.864524107	0.887722423
LOC104974144	0.844744534	0.889387648	0.879939804	0.828736799	0.81900808	0.909056553
ABO	0.844744534	0.855291433	0.834683675	0.852170016	0.858091718	0.923807852
SEC62	0.844744534	0.702392068	0.853546772	0.962008027	0.823508064	0.924333
DEFB13	0.844744534	0.710788298	0.79382647	0.817802131	0.981460283	0.926480924
PTPN7	0.844744534	0.812449121	0.894316021	0.839725231	0.829853428	0.92789871
LATS2	0.844744534	0.71815098	0.911850355	0.866566721	0.804725002	0.965117554
LUM	0.844744534	0.69959307	0.810647419	0.951290705	0.811359687	0.966914618
RAB1A	0.844744534	0.738007931	0.774015998	0.908467146	0.837399927	0.981216824
VEZF1	0.84484232	0.836427846	0.800428397	0.908467146	0.953300742	0.811759324
PI16	0.84484232	0.696757704	0.94565648	0.929663263	0.893935775	0.814218101
ARMC2	0.84484232	0.781543413	0.943836178	0.9063051	0.867706849	0.817529182
OGFRL1	0.84484232	0.693513972	0.949855095	0.916858019	0.900747635	0.81956221
LRWD1	0.84484232	0.697306013	0.979584814	0.940436902	0.810144664	0.83150957
LOC104970930	0.84484232	0.849119707	0.810167226	0.935956158	0.887514169	0.841312694
C3H1orf162	0.84484232	0.715334857	0.944403177	0.896116841	0.887514169	0.847193872
SEC22B	0.84484232	0.816412341	0.869129976	0.888687722	0.878408909	0.87754233
DUSP6	0.84484232	0.882858125	0.819359511	0.854220165	0.891304411	0.881054489
PDCD6	0.84484232	0.788414159	0.892343042	0.812160271	0.911568867	0.912888794

P2RY14	0.84484232	0.750466772	0.77566312	0.910575108	0.935093621	0.912888794
LOC112444909	0.844859803	0.918455779	0.893761108	0.815919419	0.870450746	0.846210629
POLL	0.844859803	0.711474183	0.996224523	0.860937578	0.823508064	0.857673662
CBFA2T2	0.845439737	0.733437549	0.935672252	0.904417923	0.872229135	0.85513515
FURIN	0.845586952	0.992144201	0.818612484	0.827786827	0.879776073	0.77803403
ZNF705A	0.845586952	0.713792742	0.817582037	0.989601002	0.891726429	0.853056636
RICTOR	0.845586952	0.947247457	0.865512879	0.82868625	0.83704813	0.855963573
KCNJ15	0.845586952	0.872175143	0.943237386	0.808075336	0.837915327	0.865889509
FAM107B	0.845586952	0.704181021	0.780400817	0.961898258	0.929896768	0.888475311
EIF4EBP3	0.845586952	0.772669268	0.779209064	0.890704007	0.823508064	0.982937529
MYLIP	0.845586952	0.727657689	0.78406673	0.812453944	0.801692604	0.999948228
UTRN	0.845685874	0.741012865	0.849389234	0.876703811	0.982087479	0.832956306
FGL2	0.845685874	0.951172741	0.860877703	0.808586366	0.86864151	0.852504043
SIPA1	0.845877083	0.805580108	0.996224523	0.885789665	0.801692604	0.783241453
BICC1	0.846002125	0.697043768	0.89478142	0.904018489	0.961497644	0.81853698
RNF128	0.8460124	0.910438009	0.788106994	0.9745615	0.837915327	0.802390255
KLHDC2	0.846015515	0.711368546	0.916038418	0.899308956	0.857772698	0.911638347
ABR	0.846076645	0.694842866	0.803342698	0.891988949	0.996781978	0.851995112
EP300	0.846107689	0.753613004	0.89478142	0.82868625	0.984386856	0.801109854
MRPL30	0.846107689	0.720304917	0.896574746	0.843335547	0.931357161	0.904642337
LOC101905925	0.846291404	0.845750059	0.958410013	0.839725231	0.817472827	0.851995112
SERPING1	0.846454055	0.968509005	0.8644528	0.853058361	0.853985138	0.796929234
ARMH3	0.846454055	0.696797615	0.891662311	0.888687722	0.976259416	0.82150922
SAMD4B	0.846454055	0.694842866	0.872088608	0.816402285	0.999985752	0.824780045
LOC785087	0.846454055	0.863286109	0.784260981	0.925294859	0.823508064	0.914132808
PLD3	0.846513176	0.779216815	0.803342698	0.999981514	0.818179113	0.851995112
SLC25A42	0.846681125	0.883756544	0.829751489	0.853058361	0.960573822	0.793214332
OTULIN	0.846681125	0.696914218	0.999993442	0.836387732	0.864524107	0.839573661
MSANTD3	0.846681125	0.918854612	0.802277976	0.896116841	0.877712931	0.849595612
GSTP1	0.846681125	0.783112117	0.858715613	0.828082731	0.964301327	0.864128961
LMNB2	0.846681125	0.845750059	0.854087255	0.93003185	0.834569503	0.867063767
TTC8	0.846681125	0.758609923	0.831933739	0.888198716	0.955623649	0.873166373
PAGR1	0.846681125	0.823478017	0.901955007	0.867197605	0.834569503	0.901618453
LTBP1	0.846961148	0.87976124	0.8042974	0.992339478	0.843023657	0.78087541
SEPT7	0.847235425	0.842461837	0.964807927	0.887983383	0.821557309	0.795703661
DLGAP3	0.847265563	0.888057447	0.833583844	0.922967576	0.833173678	0.859949878
ANKRD2	0.847332397	0.796253817	0.928927123	0.882057818	0.936215792	0.776811893
DNAJC17	0.847332397	0.709303377	0.874797723	0.999981514	0.803705802	0.847193872

CDIP1	0.847332397	0.796253817	0.905023942	0.947574232	0.821351993	0.850475013
LOC613401	0.847332397	0.75098267	0.916294854	0.865210554	0.924222719	0.863216707
MEIS1	0.847332397	0.802570554	0.783845551	0.998987812	0.816045604	0.868748913
SVIL	0.847332397	0.926330259	0.827400186	0.892047793	0.818549341	0.876861428
GALE	0.847332397	0.866862152	0.89478142	0.822691957	0.872096228	0.878754045
PER1	0.847332397	0.821674572	0.814495418	0.827652764	0.95221242	0.895426195
DDRGK1	0.847332397	0.767580432	0.874983674	0.925457774	0.801692604	0.930710695
ARF6	0.847332397	0.704850212	0.845113191	0.85981779	0.918426817	0.95578593
AKNAD1	0.847332397	0.712357273	0.788106994	0.898818046	0.87329316	0.978979999
SNRNP70	0.847343447	0.738832446	0.917772116	0.995565324	0.823508064	0.785562356
RAB3IL1	0.847343447	0.927853707	0.842405438	0.939604997	0.846232586	0.786936221
TPRG1	0.847343447	0.99964543	0.786126716	0.853058361	0.804625939	0.807084664
LOC112443328	0.847343447	0.880129816	0.850624788	0.870210705	0.880617822	0.872238439
UNC80	0.847343447	0.728504282	0.845525761	0.853271157	0.916353643	0.947801358
TXLNB	0.847503713	0.899223474	0.855892553	0.808073654	0.964768333	0.791275236
GOLGA4	0.847503713	0.703571355	0.810647419	0.925353907	0.996781978	0.799636331
SLC38A3	0.847503713	0.821674572	0.845113191	0.992442163	0.845085806	0.799816679
ADARB1	0.847503713	0.783896922	0.820956248	0.999981514	0.811991102	0.812036123
UMAD1	0.847503713	0.808696335	0.816032984	0.999981514	0.808047125	0.819366282
TLR3	0.847503713	0.760778257	0.845113191	0.995312373	0.859793828	0.832964812
CAMK2N2	0.847503713	0.821674572	0.8735515	0.935985829	0.858187492	0.846210629
LOC101902172	0.847503713	0.708137441	0.915858924	0.925294859	0.90730011	0.846210629
TERF2	0.847503713	0.783896922	0.817582037	0.819213899	0.995870928	0.8537109
IPPK	0.847503713	0.940077933	0.871929137	0.828736799	0.804625939	0.888475311
LRR1	0.847503713	0.733509477	0.857316023	0.828736799	0.955623649	0.916193353
SHMT2	0.847503713	0.802638906	0.896821247	0.812453944	0.887514169	0.919124527
RETREG2	0.847503713	0.707163364	0.809673119	0.990955345	0.805615411	0.933897626
SYTL2	0.847503713	0.796253817	0.79648765	0.836248024	0.927087035	0.942982678
LOC100847376	0.847503713	0.754676989	0.833583844	0.886312303	0.888368075	0.944973383
LOC101905757	0.847503713	0.760778257	0.794501834	0.928416242	0.801692604	0.976567668
MTMR2	0.8476494	0.744049882	0.791557166	0.825279787	0.999985752	0.795703661
TSPAN18	0.847699737	0.696797615	0.901816431	0.990955345	0.887514169	0.786936221
SNAPC2	0.847744671	0.90323787	0.946791586	0.809356235	0.889301074	0.783241453
ARPP19	0.84780012	0.802570554	0.885896294	0.948668643	0.844384115	0.850475013
ETFB	0.84780012	0.740343181	0.896023027	0.887877413	0.927109614	0.867907362
RIC8A	0.848185967	0.765910578	0.906019013	0.899855526	0.941645866	0.806476213
COLGALT1	0.848185967	0.730581055	0.779209064	0.999981514	0.887514169	0.83150957
FBH1	0.848185967	0.764456895	0.991936822	0.821450642	0.877712931	0.835231445

ZFAT	0.848185967	0.741263262	0.887132069	0.819213899	0.98865576	0.835331508
IP6K2	0.848185967	0.722779653	0.941507802	0.809356235	0.95978143	0.8537109
DUS4L	0.848185967	0.697043768	0.874983674	0.995434268	0.801692604	0.89209761
VAPB	0.848185967	0.7095693	0.905023942	0.952247001	0.801692604	0.919580629
RASL11A	0.848185967	0.796253817	0.899952655	0.870210705	0.814253177	0.93531275
SMARCAD1	0.848185967	0.808478344	0.843955813	0.906590982	0.801692604	0.950415692
LOC104973252	0.848185967	0.741263262	0.831933739	0.910575108	0.817472827	0.971982373
SUCLA2	0.848207158	0.740040635	0.910284791	0.925294859	0.852026472	0.887989933
SMPD4	0.848264789	0.795715992	0.915858924	0.947574232	0.887514169	0.78096075
PCDH18	0.848264789	0.936693847	0.775552434	0.886312303	0.94542482	0.785562356
LOC100335467	0.848264789	0.734546826	0.852916849	0.959616431	0.950085378	0.805925428
ALDH16A1	0.848264789	0.927853707	0.892343042	0.828736799	0.867558388	0.839906041
LOC101904435	0.848264789	0.836131298	0.810647419	0.898658215	0.900927045	0.881697198
C26H10orf143	0.848264789	0.808586422	0.829455601	0.82868625	0.799752471	0.995525297
N4BP2	0.848270439	0.766943819	0.838840947	0.827443542	0.999985752	0.784754767
LOC101905343	0.848270439	0.902525869	0.879939804	0.887983383	0.849562516	0.842214812
LOC101902754	0.848310951	0.7973731	0.835318542	0.999981514	0.875687454	0.78087541
ALG10	0.848404184	0.899490486	0.916294854	0.904277308	0.819329746	0.806476213
SCARA5	0.848404184	0.77139884	0.93190909	0.816169012	0.879268517	0.917150486
TADA1	0.848404184	0.741671849	0.79497298	0.817146543	0.943160494	0.969754487
MYMK	0.848445476	0.792690308	0.861585977	0.999981514	0.802592922	0.801888363
MED31	0.848445476	0.758169869	0.919214273	0.94778564	0.880239832	0.816245398
CD1E	0.848445476	0.711880957	0.957102261	0.886727784	0.864524107	0.877529959
CCDC124	0.848445476	0.76003522	0.858376167	0.876703811	0.943540106	0.88313584
RTL1	0.848445476	0.829067393	0.81412252	0.812313105	0.959739966	0.898470276
LOC101906989	0.848445476	0.731238567	0.95322206	0.843260607	0.87329316	0.899428912
GINS4	0.848445476	0.709247882	0.841294863	0.868435169	0.964301327	0.904383301
LOC104975222	0.848487128	0.697643204	0.796499368	0.982220679	0.804037558	0.960877268
ZXDB	0.848579625	0.798327403	0.980061232	0.912777307	0.821351993	0.779201808
THTPA	0.848579625	0.811354298	0.881778842	0.876703811	0.963550093	0.784754767
RDH8	0.848579625	0.759814955	0.92358634	0.9745615	0.810436875	0.832964812
XRRA1	0.848579625	0.758609923	0.841294863	0.9819628	0.877712931	0.85102506
EPHX3	0.848579625	0.782783231	0.922552585	0.907794002	0.847092933	0.865933526
MAN2A2	0.848782993	0.765724506	0.872088608	0.948668643	0.907455286	0.835231445
CTXND1	0.848782993	0.965482645	0.78930905	0.812453944	0.887514169	0.877014986
NAGK	0.848820803	0.804185018	0.867222363	0.961898258	0.86930615	0.834624227
NAPB	0.848820803	0.750379179	0.929661805	0.912777307	0.871558258	0.8537109
WBP11	0.848820803	0.709247882	0.915120516	0.955981799	0.84367638	0.877048993

TCN1	0.848900411	0.937723596	0.924855847	0.887983383	0.80599066	0.785869233
CREB3L4	0.849039627	0.84927532	0.942357321	0.889924182	0.856136977	0.806714845
TGFB1I1	0.849039627	0.833323491	0.832971131	0.839725231	0.817677764	0.973413452
SF1	0.849106973	0.704181021	0.854396475	0.988408846	0.917736327	0.824025963
ACSL4	0.849106973	0.781543413	0.879939804	0.908467146	0.8568751	0.905509632
TOX4	0.849404509	0.740343181	0.972806636	0.813472855	0.964301327	0.780283623
USP6NL	0.849404509	0.788980101	0.86641857	0.819968583	0.999985752	0.782330566
APOBEC2	0.849404509	0.726638987	0.991936822	0.932406746	0.836182483	0.783241453
LOC104976321	0.849404509	0.850640641	0.79497298	0.817146543	0.999985752	0.783241453
TEX264	0.849404509	0.991883165	0.833583844	0.865076519	0.833173678	0.795936781
IQGAP1	0.849404509	0.713792742	0.927635169	0.926970666	0.911028086	0.83150957
LYZ1	0.849404509	0.807742849	0.785478462	0.999981514	0.834278521	0.831652079
ERFE	0.849404509	0.855079912	0.889098668	0.812453944	0.950852294	0.839565788
STRA6	0.849404509	0.740343181	0.955427429	0.910575108	0.821148296	0.874490166
MTMR11	0.849404509	0.710645465	0.830420708	0.91185657	0.954071324	0.887722423
RAF1	0.849404509	0.828583132	0.865717285	0.922967576	0.804625939	0.911219114
JAM3	0.849404509	0.742303692	0.885896294	0.940436902	0.833173678	0.91373422
LOC100336976	0.849404509	0.709247882	0.809931129	0.876121618	0.951461287	0.941781555
ENPP2	0.849408934	0.799590997	0.849704325	0.848253453	0.804274107	0.984046914
CNR1	0.849718382	0.727473229	0.982367155	0.886312303	0.902907077	0.79187813
ASPN	0.849718382	0.718382634	0.801754158	0.81472008	0.999985752	0.855049294
TXNDC9	0.849718382	0.726447332	0.957052938	0.852170016	0.851054476	0.915735519
MCU	0.850108685	0.874981732	0.805479206	0.908467146	0.950958352	0.793197037
LOC101905167	0.850108685	0.755789173	0.964807927	0.886831994	0.886944613	0.83150957
P2RY1	0.850108685	0.901028838	0.847357383	0.902490239	0.809129327	0.891088733
KLK12	0.850108685	0.770652022	0.834377194	0.865076519	0.872178532	0.966850975
LOC101904275	0.850225568	0.722779653	0.794225743	0.89684883	0.887514169	0.971545917
ERBB4	0.850270708	0.803408292	0.98258069	0.892047793	0.825920661	0.801888363
CYP2D14	0.85043628	0.872331637	0.883681256	0.932914414	0.879435126	0.78288746
TTF1	0.85043628	0.737564781	0.8644528	0.999981514	0.864524107	0.811709507
VAV2	0.85043628	0.710835581	0.947651663	0.944949439	0.874462038	0.83150957
ZKSCAN8	0.85043628	0.800861368	0.829455601	0.999981514	0.801692604	0.858577001
HIVEP3	0.85043628	0.702818587	0.838341804	0.874662174	0.95845082	0.923092436
LOC101906021	0.85043628	0.697885203	0.955427429	0.865746543	0.809129327	0.950415692
IL4I1	0.850449005	0.855079912	0.978530068	0.827203798	0.806321189	0.847619123
TPM4	0.850479272	0.822956796	0.810167226	0.859883031	0.996781978	0.79363949
LOC112447362	0.850479272	0.714027042	0.860877703	0.950162237	0.953300742	0.831652079
MED10	0.850479272	0.716596673	0.953572282	0.952247001	0.819679279	0.8537109

SMAD4	0.850479272	0.69959307	0.957052938	0.922967576	0.857394434	0.859949878
SLC25A3	0.850479272	0.717447227	0.886829776	0.815919419	0.960573822	0.913221224
LOC101906408	0.850479272	0.733509477	0.959863581	0.848131881	0.828832304	0.923807852
CAAP1	0.850565989	0.709303377	0.90676547	0.999981514	0.858040731	0.801888363
NTHL1	0.850565989	0.727534248	0.871285807	0.973075232	0.875469001	0.865767172
KLHL11	0.850567274	0.884804337	0.813441395	0.950746405	0.893368406	0.801888363
CCDC96	0.850567274	0.879093989	0.883604967	0.810557898	0.958797242	0.816785597
FAM171A1	0.850567274	0.768359345	0.916294854	0.908467146	0.872229135	0.866494771
LOC101907622	0.850567274	0.882858125	0.784115813	0.821450642	0.956761712	0.877750901
PCMT1	0.850567274	0.697306013	0.953176526	0.916858019	0.837915327	0.885202945
ARFGAP2	0.850632401	0.710645465	0.916294854	0.946992713	0.838461406	0.893838339
CCDC162P	0.85066899	0.749236881	0.791843351	0.822708607	0.999985752	0.84882411
S1PR4	0.85066899	0.708218805	0.894316021	0.822691957	0.935286646	0.936778056
SRD5A1	0.8508018	0.931233175	0.779581461	0.875836286	0.953300742	0.801888363
MIB2	0.851008188	0.78945719	0.87118003	0.992560663	0.823508064	0.835231445
LTB4R	0.851151	0.913120359	0.77566312	0.872590202	0.970980375	0.788462021
FAM69A	0.851187864	0.993481224	0.79497298	0.812453944	0.918426817	0.785562356
TRMT13	0.851365224	0.724380878	0.915120516	0.999981514	0.810436875	0.78096075
TRAF5	0.851365224	0.833323491	0.975774524	0.821450642	0.879395665	0.819366282
CACHD1	0.851365224	0.819210468	0.895801611	0.868435169	0.930451229	0.839906041
ZNF792	0.851365224	0.811114624	0.89478142	0.911874416	0.87329316	0.854935725
ATP6V1H	0.851365224	0.759629362	0.819359511	0.948668643	0.832754891	0.944973383
UBE2J2	0.851365224	0.802638906	0.89478142	0.868435169	0.804274107	0.952571698
POPDC2	0.851365224	0.828516628	0.829751489	0.82868625	0.877712931	0.960023723
SAMD14	0.851413522	0.877893862	0.897322115	0.948776099	0.832486638	0.789870764
GEMIN5	0.85169907	0.819210468	0.985156077	0.81313549	0.854289643	0.844759721
ZNF202	0.851736931	0.819439092	0.886829776	0.907794002	0.804037558	0.919124527
SRPK3	0.851852094	0.89417747	0.809673076	0.892047793	0.823508064	0.925233445
ATPAF1	0.851886903	0.742673431	0.854567571	0.876703811	0.970980375	0.867063767
DDIT4L	0.851886903	0.796666816	0.813441395	0.910575108	0.891726429	0.915546224
RFX5	0.851957271	0.7889129	0.885896294	0.999981514	0.816249936	0.793197037
DOC2B	0.851957271	0.738007931	0.999993442	0.840862991	0.856136977	0.811759324
PLCD3	0.851957271	0.741310853	0.829751489	0.999981514	0.86876405	0.814334714
C27H4orf47	0.851957271	0.81518533	0.816032984	0.964521432	0.924795709	0.815129379
LOC104968434	0.851957271	0.910651459	0.914866994	0.870408722	0.859672963	0.82345874
FAM181B	0.851957271	0.985428851	0.879939804	0.836387732	0.804625939	0.835231445
LOC100297097	0.851957271	0.807350682	0.983504244	0.886386107	0.804274107	0.839565788
LRFN3	0.851957271	0.741310853	0.807254925	0.918693944	0.976292433	0.845075821

TTLL5	0.851957271	0.737941984	0.951049907	0.890704007	0.893716526	0.849595612
SPRY4	0.851957271	0.746562816	0.996224523	0.884452623	0.804625939	0.856833445
NAE1	0.851957271	0.709247882	0.831933739	0.879425058	0.984386856	0.875297569
AAED1	0.851957271	0.761907799	0.964382669	0.830187312	0.810436875	0.92789871
SCHIP1	0.851957271	0.827824094	0.848278925	0.882057818	0.804625939	0.963255451
CHN2	0.852001271	0.81518533	0.841294863	0.985701713	0.871558258	0.81956221
RTKN2	0.852119559	0.899490486	0.789166592	0.990955345	0.842818179	0.802390255
PITHD1	0.852119559	0.699211784	0.957052938	0.982220679	0.823508064	0.816415069
NEURL4	0.852119559	0.718382634	0.976484937	0.898818046	0.81954052	0.876861428
PRRT1B	0.852119559	0.805331198	0.802277976	0.853058361	0.917407853	0.945854375
AGAP2	0.852666651	0.758119468	0.818951412	0.999981514	0.887514169	0.783241453
GNPDA1	0.852666651	0.790429889	0.943836178	0.970039043	0.821351993	0.790639219
FAT1	0.852666651	0.741671849	0.798782742	0.892047793	0.999985752	0.79187813
CHCHD5	0.852666651	0.929737582	0.949939862	0.812453944	0.847173934	0.81956221
SRPRA	0.852666651	0.919315138	0.947155158	0.868378265	0.804037558	0.824025963
LOC112447802	0.852666651	0.994993704	0.846704346	0.815919419	0.821148296	0.839906041
PLXNA4	0.852666651	0.915576843	0.89193017	0.884819185	0.843396588	0.846210629
CCR3	0.852666651	0.994185904	0.789663873	0.812999186	0.869580831	0.846210629
E2F1	0.852666651	0.760145173	0.917155067	0.907794002	0.894306143	0.852504043
PHKA1	0.852666651	0.893680105	0.846276242	0.949565453	0.804274107	0.864572584
PRDX2	0.852666651	0.808478344	0.871782109	0.822691957	0.959658682	0.867907362
DOCK2	0.852666651	0.754676989	0.93475587	0.821450642	0.935286646	0.876315929
ZNF143	0.852666651	0.758733912	0.900828975	0.820971736	0.954071324	0.883829611
LOC101904891	0.852666651	0.798290851	0.797180963	0.98981682	0.810436875	0.903990996
ESAM	0.852666651	0.793820847	0.847357383	0.85721441	0.935286646	0.911984937
RNASE10	0.852666651	0.878462725	0.865444995	0.869208308	0.804625939	0.933897626
KCNB2	0.852666651	0.874564847	0.820146472	0.859320133	0.855920132	0.937715964
TDRD7	0.852691208	0.779025263	0.871782109	0.928453481	0.931402923	0.838279398
OSR1	0.852691208	0.846721805	0.786126716	0.93048026	0.940870936	0.839565788
LOC101903015	0.852691208	0.843278276	0.879939804	0.928404714	0.804625939	0.891423889
BAIAP2L1	0.852698061	0.976596938	0.908072171	0.834401906	0.832407979	0.794932301
SH2D5	0.852698061	0.838308162	0.852916849	0.893014287	0.833173678	0.929215023
ABHD1	0.852780156	0.939140452	0.789166592	0.915013589	0.923910588	0.783241453
LOC104975022	0.852780156	0.735768592	0.830135741	0.999038184	0.930565685	0.79391909
LPAR1	0.852780156	0.970084348	0.784260981	0.861594988	0.912149124	0.823824518
LOC101906392	0.852780156	0.728565135	0.869573453	0.943808491	0.952264073	0.832964812
WDSUB1	0.852780156	0.811486218	0.939981892	0.898818046	0.862852956	0.839906041
LOC112447756	0.852780156	0.774644712	0.879939804	0.975118969	0.847092933	0.85532952

ASAP2	0.852780156	0.737396716	0.93190909	0.913946495	0.868722067	0.876162019
BORCS7	0.852780156	0.814995661	0.908072171	0.876703811	0.865075514	0.893838339
HCLS1	0.852780156	0.725495584	0.923578245	0.824230049	0.924795709	0.91832348
INF2	0.852780156	0.726171565	0.834683675	0.949468104	0.887514169	0.923092436
ACTN1	0.852780156	0.741310853	0.911535369	0.896116841	0.857562998	0.924241027
LOC104968435	0.852780156	0.791281103	0.874983674	0.830427818	0.864524107	0.960023723
LOC786974	0.852780156	0.708137441	0.819322085	0.898818046	0.891726429	0.969197073
SGPP1	0.852780156	0.727846054	0.794676674	0.893051204	0.816045604	0.999948228
SCN2A	0.852984197	0.819210468	0.875900843	0.9880671	0.8282759	0.81956221
CAMLG	0.852984197	0.746712661	0.980496473	0.929388614	0.810436875	0.837559792
CRBN	0.852984197	0.737120625	0.803342698	0.997467114	0.91563394	0.841312694
MMP24	0.852984197	0.713792742	0.996224523	0.840247324	0.887514169	0.845747012
SDAD1	0.852984197	0.742905463	0.779581461	0.987084426	0.891304411	0.89209761
LOC782054	0.852984197	0.76003522	0.871782109	0.904271346	0.847173934	0.94328669
TEF	0.852984197	0.741263262	0.799923795	0.82868625	0.902907077	0.993013409
XG	0.853211386	0.702101563	0.855811972	0.885789665	0.976259416	0.877014986
LOC619159	0.853497763	0.965482645	0.838909114	0.892071982	0.821557309	0.844334911
KDM4B	0.853497763	0.743602194	0.857250535	0.928510139	0.936108073	0.867063767
RNPEPL1	0.853497763	0.938087741	0.80412682	0.840700255	0.902258286	0.874459658
FCF1	0.853604358	0.728267541	0.997400824	0.862790635	0.906893576	0.78288746
LOC112442347	0.853782654	0.744774335	0.805610288	0.92726712	0.832119756	0.975788372
CNTN1	0.853794172	0.905271052	0.780367031	0.935251689	0.917289552	0.81956221
ARHGEF18	0.853794172	0.833713501	0.965229237	0.828736799	0.877926588	0.836111377
DEAF1	0.853794172	0.915269318	0.916323169	0.821450642	0.823508064	0.876861428
FUS	0.853794172	0.759814955	0.94422142	0.860166343	0.846285074	0.923092436
LRP2BP	0.85444459	0.774979074	0.949855095	0.890704007	0.935093621	0.785562356
XPO4	0.854471959	0.866955566	0.784196515	0.911874416	0.890909775	0.895446935
SSTR1	0.854479882	0.753090681	0.879939804	0.908467146	0.955148901	0.839565788
RNF44	0.854479882	0.792690308	0.978014482	0.839725231	0.879776073	0.839906041
LOC112448743	0.854479882	0.749011813	0.970873534	0.840700255	0.873818479	0.88313584
GPR34	0.854479882	0.811463186	0.899106435	0.852170016	0.90645589	0.887722423
ADD1	0.854479882	0.709542765	0.836241961	0.929388614	0.936921784	0.907124813
NPM1	0.854479882	0.744049882	0.79497298	0.854220165	0.951996251	0.959075802
INO80	0.855027994	0.832398588	0.819359511	0.812453944	0.999985752	0.788462021
RBP4	0.855062197	0.980521483	0.79497298	0.952247001	0.80917607	0.796158754
CLTA	0.855062197	0.7973731	0.921266625	0.978503042	0.810436875	0.81956221
AMMECR1L	0.855215204	0.744774335	0.810839887	0.906084437	0.999985752	0.783438303
LOC101907189	0.855215204	0.971413741	0.819359511	0.898818046	0.884872176	0.784754767

USP38	0.855215204	0.740642434	0.957052938	0.961841509	0.868722067	0.785562356
CMKLR1	0.855215204	0.709198375	0.929661805	0.90413565	0.965676623	0.785562356
MKL1	0.855215204	0.788544942	0.992511421	0.887983383	0.865505927	0.786849547
ALDH1A3	0.855215204	0.77844414	0.927635169	0.974272706	0.851124834	0.796158754
HOMER3	0.855215204	0.859034448	0.881402575	0.92532303	0.900891209	0.801888363
SYK	0.855215204	0.742703566	0.897322115	0.940438437	0.943876051	0.806476213
SCML2	0.855215204	0.846724749	0.81047208	0.845954103	0.997422012	0.806476213
BTBD8	0.855215204	0.755089315	0.894316021	0.999981514	0.817472827	0.806714845
EIF4E3	0.855215204	0.808478344	0.899106435	0.874257643	0.956041014	0.818370468
QTRT1	0.855215204	0.750379179	0.962142631	0.921482057	0.877712931	0.81956221
STAR	0.855215204	0.808478344	0.864847127	0.959652624	0.899505018	0.821910421
NEDD8	0.855215204	0.768359345	0.933884582	0.828736799	0.965163023	0.82345874
NCKAP1	0.855215204	0.871924797	0.904919322	0.891988949	0.887514169	0.826875426
GATD1	0.855215204	0.883756544	0.79497298	0.871338923	0.970988226	0.829441883
NAA60	0.855215204	0.845750059	0.829137153	0.817146543	0.993461941	0.83186501
TNFSF4	0.855215204	0.713956321	0.957052938	0.912922827	0.895757462	0.835231445
PSD4	0.855215204	0.832398588	0.829455601	0.988637603	0.84367638	0.845714049
LOC784697	0.855215204	0.772618617	0.846906704	0.93775206	0.933426897	0.862023846
C15H11orf74	0.855215204	0.823133291	0.916294854	0.882057818	0.883951804	0.864703284
VASH1	0.855215204	0.741263262	0.892343042	0.988408846	0.823508064	0.870018677
NOP2	0.855215204	0.804185018	0.791985797	0.853058361	0.984386856	0.872721634
PDCD1LG2	0.855215204	0.911290584	0.879939804	0.821450642	0.887514169	0.877014986
PAM16	0.855215204	0.83359488	0.908072171	0.819213899	0.895067787	0.90071654
KCNA5	0.855215204	0.745846221	0.916323169	0.900875874	0.87329316	0.903795325
SLC26A11	0.855215204	0.809693907	0.854567571	0.899047543	0.859005924	0.927520621
TMEM186	0.855215204	0.728267541	0.800422157	0.812999186	0.995018375	0.927520621
DNAAF2	0.855215204	0.742575051	0.789663873	0.832136826	0.958797242	0.963255451
ANKRD27	0.855337112	0.758609923	0.854571175	0.838528071	0.930451229	0.947937638
BOLA2B	0.855423765	0.731024992	0.946791586	0.980564765	0.828190913	0.824025963
PRDM2	0.855423765	0.807919676	0.849389234	0.892047793	0.943540106	0.867063767
ESD	0.855848308	0.829067393	0.99647733	0.825654835	0.872096228	0.784754767
NRBF2	0.855848308	0.855310926	0.871888627	0.950643928	0.838090622	0.852112206
GCA	0.855848308	0.72742557	0.891035201	0.943447418	0.829853428	0.931410194
PLEKHB1	0.855850519	0.853476078	0.853546772	0.952247001	0.900927045	0.804551322
DDX19A	0.855850519	0.768359345	0.916038418	0.882395013	0.959658682	0.81956221
UNK	0.856031002	0.756568666	0.96030829	0.892047793	0.826634148	0.889662488
LOC101907255	0.856031002	0.826395702	0.849389234	0.904642425	0.804625939	0.953509765
LOC112448833	0.856111015	0.941400719	0.895801611	0.896116841	0.821148296	0.8255975

RAD9B	0.856111015	0.718258172	0.925081962	0.972142185	0.846464841	0.858507498
NDST2	0.856111015	0.730539687	0.964661607	0.907353815	0.818977667	0.89209761
MCTS1	0.856111015	0.709247882	0.923521499	0.865076519	0.91563394	0.917930485
ARHGAP15	0.856211337	0.802045751	0.999993442	0.823708225	0.868722067	0.789870764
BEND7	0.856211337	0.804185018	0.796499368	0.93048026	0.964301327	0.838530462
MLC1	0.856211337	0.847255873	0.834683675	0.876703811	0.960573822	0.841312694
ATP6V1F	0.856211337	0.77899313	0.855892553	0.887877413	0.952310018	0.877750901
RBMS2	0.856211337	0.748138542	0.846960897	0.915129182	0.924222719	0.911808092
PRKAA1	0.856230069	0.910651459	0.793169467	0.866566721	0.976259416	0.799889245
LOC781726	0.856230069	0.74241072	0.999993442	0.839245804	0.823508064	0.806236908
INTS5	0.856230069	0.760778257	0.829455601	0.830427818	0.959565914	0.93531275
ENOSF1	0.856465038	0.712357273	0.849218971	0.953697985	0.817472827	0.965486169
ARHGEF17	0.856489916	0.980289052	0.819527307	0.876703811	0.886785729	0.810156973
APLN	0.856489916	0.764456895	0.943237386	0.881493849	0.899042139	0.867063767
APAF1	0.856489916	0.819910026	0.802707828	0.851398061	0.961465747	0.906637848
ANKLE2	0.85662975	0.744671791	0.971053208	0.933240019	0.886785729	0.786849547
PIAS2	0.85662975	0.839800081	0.802277976	0.899308956	0.977193348	0.806476213
TRIM65	0.85662975	0.710645465	0.886829776	0.96951401	0.931357161	0.831652079
PP2D1	0.85662975	0.946148902	0.818951412	0.892071982	0.867966092	0.85532952
LRRC3B	0.85662975	0.821790484	0.980761647	0.822691957	0.853599134	0.857457228
KDM3A	0.85662975	0.741805593	0.828806956	0.892047793	0.970988226	0.88542704
MGAT4C	0.85662975	0.758609923	0.885896294	0.870591999	0.909657705	0.925233445
LOC112442843	0.85662975	0.912609424	0.817582037	0.832401065	0.834470237	0.9557845
RTN2	0.85662975	0.718382634	0.813441395	0.912777307	0.871558258	0.978087957
FADS1	0.85662975	0.775289791	0.789166592	0.834401906	0.864524107	0.999795635
ARMCX6	0.856730871	0.7889129	0.79497298	0.960190213	0.970988226	0.799816679
PALM3	0.856730871	0.894089177	0.911535369	0.92139105	0.843023657	0.815129379
IER5	0.856730871	0.718228672	0.871782109	0.999981514	0.855939488	0.822427019
DOCK7	0.856730871	0.90715891	0.804002279	0.867235478	0.926972948	0.876162019
TCAP	0.85709044	0.840402825	0.886829776	0.821450642	0.81725718	0.967759699
LOC112444479	0.857131514	0.766595431	0.886829776	0.844621856	0.87389153	0.960877268
CTSC	0.857224398	0.775033799	0.949939862	0.852170016	0.964301327	0.784754767
TLR10	0.857224398	0.816442476	0.8588642	0.820971736	0.999453276	0.806476213
TMEM150C	0.857224398	0.766943819	0.860742804	0.974793558	0.929802526	0.813775367
MAMSTR	0.857224398	0.888057447	0.827400186	0.998529748	0.809317773	0.816246746
ATP9B	0.857224398	0.709247882	0.871285807	0.916858019	0.844585092	0.967056564
HAUS8	0.857307628	0.709348874	0.852214402	0.998529748	0.928951321	0.818370468
CCDC51	0.857307628	0.776553131	0.898179681	0.853058361	0.972160227	0.835231445

GK	0.857307628	0.800861368	0.841294863	0.999981514	0.810436875	0.846608532
NBDY	0.857307628	0.94622198	0.884121008	0.819968583	0.864524107	0.867063767
CARNMT1	0.857307628	0.710645465	0.966232793	0.945300635	0.814120141	0.872239777
MS4A8	0.857500901	0.772618617	0.852214402	0.940438437	0.877879264	0.915303189
LOC785873	0.857596242	0.790819434	0.801754158	0.982220679	0.823508064	0.923807852
MYL12B	0.85760948	0.784217552	0.915858924	0.828736799	0.989334846	0.785562356
TMEM59	0.857631005	0.840402825	0.849389234	0.972142185	0.858179728	0.845714049
NPAT	0.857631005	0.715705323	0.927635169	0.898818046	0.943702351	0.846210629
LOC107131703	0.857652219	0.779372555	0.853546772	0.908467146	0.984692578	0.785562356
LOC101906008	0.857829845	0.872684104	0.875900843	0.984548238	0.820477096	0.801888363
TNFSF15	0.857829845	0.786825403	0.860877703	0.938094344	0.940870936	0.838729965
SREK1	0.857829845	0.709247882	0.874632512	0.959591127	0.950958352	0.838913379
LRFN1	0.857829845	0.749370809	0.879939804	0.852170016	0.977167046	0.865767172
SPC24	0.857829845	0.785303212	0.889098668	0.949565453	0.823508064	0.905509632
LOC112446659	0.857829845	0.778487216	0.856466821	0.853058361	0.954071324	0.911336121
GCNT1	0.857829845	0.718382634	0.855811972	0.892047793	0.826634148	0.990943966
LOC112444505	0.858022689	0.756473821	0.810647419	0.836610893	0.999985752	0.83821684
CPEB4	0.858498448	0.839660873	0.92380537	0.821450642	0.955076828	0.81956221
KIF26B	0.858498448	0.76003522	0.959725886	0.922002553	0.871558258	0.832964812
ASMT	0.858498448	0.781222833	0.971053208	0.868435169	0.891726429	0.839565788
SREK1IP1	0.858498448	0.804185018	0.858032788	0.9880671	0.847173934	0.851995112
TYRO3	0.858498448	0.728513666	0.829745504	0.828736799	0.934729126	0.9790177
USP19	0.858765286	0.853678591	0.922552585	0.921811299	0.872178532	0.806476213
LOC104972400	0.858765286	0.829067393	0.930736996	0.825530631	0.877712931	0.90182838
LOC101905801	0.858765286	0.816149664	0.831933739	0.843613441	0.940870936	0.925233445
ERI1	0.858787345	0.823133291	0.886829776	0.941935077	0.909379774	0.811759324
GPN3	0.858787345	0.796253817	0.894338686	0.91185657	0.943702351	0.81853698
TRPV4	0.858787345	0.870195838	0.912183977	0.900044333	0.833864947	0.866494771
KATNAL1	0.85907605	0.790429889	0.978014482	0.819835879	0.82250626	0.917849087
TMUB1	0.859233529	0.750432618	0.837770432	0.993656085	0.935286646	0.806476213
MRPS18A	0.859233529	0.750379179	0.842222423	0.907621547	0.954814306	0.888475311
LMCD1	0.859610914	0.787500872	0.794225743	0.849313593	0.951461287	0.953140004
KLRK1	0.859610914	0.823830365	0.889098668	0.828337624	0.844384115	0.96248183
FAM241A	0.85962247	0.728565135	0.802277976	0.994711026	0.823508064	0.940017687
LOC788205	0.85963824	0.709542765	0.880476435	0.839725231	0.977827804	0.893838339
MBTPS2	0.859647915	0.77844414	0.916323169	0.888687722	0.958797242	0.816246746
SLAMF6	0.859647915	0.744241031	0.867222363	0.9880671	0.851927006	0.881189314
KIF16B	0.859647915	0.741310853	0.906019013	0.892047793	0.929896768	0.887542005

FLYWCH1	0.859666512	0.821767596	0.845525761	0.907997414	0.864521109	0.92789871
WTIP	0.859694418	0.787092649	0.99341711	0.927560164	0.814984338	0.796402766
DGAT2	0.860063579	0.7889129	0.87118003	0.821450642	0.970980375	0.888309103
AP3M2	0.860308671	0.727534248	0.986920775	0.852481062	0.937932469	0.819366282
FGD5	0.860308671	0.718258172	0.957295966	0.98673849	0.823508064	0.821910421
FOXP1	0.860683671	0.792967492	0.97316848	0.955954309	0.817472827	0.789870764
TNXB	0.860683671	0.80085193	0.879939804	0.827455669	0.996781978	0.818021392
LOC112441611	0.860683671	0.713792742	0.911103174	0.999981514	0.843396588	0.83150957
JDP2	0.860683671	0.722779653	0.865697455	0.941467375	0.936448903	0.876861428
LOC104971464	0.860683671	0.78945719	0.937812682	0.881493849	0.887514169	0.877014986
BBS2	0.860890345	0.959396625	0.864593507	0.868378265	0.877012056	0.832964812
PTPRD	0.860890345	0.866901725	0.855713041	0.952247001	0.871558258	0.841312694
LOC107132283	0.860890345	0.826316517	0.906019013	0.943808491	0.82114467	0.873130182
NAA35	0.860890345	0.872175143	0.903431343	0.827443542	0.832407979	0.93363377
TAF13	0.860890345	0.797906794	0.896821247	0.820971736	0.87329316	0.958790968
XCR1	0.861169554	0.811354298	0.999993442	0.821865782	0.853985138	0.818370468
XPO6	0.861169554	0.725636614	0.978530068	0.896116841	0.905571216	0.824025963
UROD	0.861169554	0.762081499	0.899106435	0.852170016	0.982796995	0.835231445
ATF7IP	0.861169554	0.848456869	0.822300339	0.984548238	0.872178532	0.836705424
VGLL4	0.861169554	0.816369086	0.957052938	0.903584224	0.852746309	0.837596789
LOC101904308	0.861169554	0.722195304	0.883398046	0.988889838	0.877712931	0.862925606
LOC112443485	0.861169554	0.777309385	0.865787276	0.904018489	0.868722067	0.943287933
KIAA0100	0.861169554	0.744774335	0.813441395	0.82868625	0.902907077	0.996310803
TMEM214	0.8612541	0.728504282	0.849389234	0.888687722	0.998661549	0.839565788
FAM110B	0.861577973	0.895338171	0.794465534	0.819968583	0.982087479	0.850475013
GGA3	0.861727603	0.96365708	0.884710729	0.868435169	0.887514169	0.786849547
ZNF527	0.861727603	0.717684248	0.954020369	0.899855526	0.889087404	0.876162019
LOC515042	0.861937175	0.713716824	0.999993442	0.889472032	0.864524107	0.829955257
BCAS2	0.861937175	0.754676989	0.89478142	0.819549243	0.84868194	0.988639786
DCP1A	0.862329431	0.779343136	0.916038418	0.852170016	0.959151723	0.85532952
RHBG	0.862444905	0.963573631	0.894030462	0.898658215	0.842279027	0.788462021
GRIPAP1	0.862444905	0.973780375	0.918247946	0.825279787	0.859351739	0.793873776
LOC101904492	0.862444905	0.713792742	0.803428123	0.999981514	0.92535945	0.803177592
FAM171B	0.862444905	0.946820717	0.822300339	0.909500328	0.88127953	0.835231445
LOC529125	0.862444905	0.750432618	0.793169467	0.839245804	0.999985752	0.858451225
ACOT8	0.862444905	0.822956796	0.860333557	0.859466553	0.960573822	0.867063767
REV1	0.862444905	0.790149523	0.883816089	0.832136826	0.970988226	0.869437527
DNMT1	0.862444905	0.780799374	0.915858924	0.952247001	0.847173934	0.872238439

ERCC1	0.862444905	0.829067393	0.879939804	0.893051204	0.897352056	0.881951302
SNX13	0.862444905	0.718258172	0.955963987	0.915039335	0.82114467	0.917930485
ATRIP	0.862444905	0.744774335	0.896821247	0.861243324	0.912199478	0.935770262
CHTF8	0.862444905	0.775532414	0.838341783	0.82868625	0.927086179	0.966914618
CD160	0.862444905	0.737956652	0.891972402	0.892047793	0.823645587	0.97521287
CYB561D2	0.862444905	0.717848892	0.834683675	0.865076519	0.920318083	0.979480605
RAB11FIP2	0.862444905	0.758609923	0.798782742	0.950643928	0.816540569	0.980741977
BSN	0.86265537	0.745791496	0.810167226	0.889415593	0.969396685	0.919124527
RYR1	0.862840927	0.891514707	0.836691254	0.977691426	0.820264258	0.849595612
LOC788648	0.863061273	0.727781617	0.916294854	0.999981514	0.810436875	0.822317091
LOC112442191	0.863061273	0.732885933	0.852916849	0.999981514	0.875687454	0.830187739
LOC104969097	0.863061273	0.822956796	0.91882781	0.925210648	0.887514169	0.831652079
FAM53B	0.863061273	0.951112641	0.818951412	0.843613441	0.938731934	0.837596789
PAICSP	0.863061273	0.826488263	0.818612484	0.95901497	0.933426897	0.838913379
KCTD14	0.863061273	0.991673407	0.791557166	0.903597775	0.823508064	0.839906041
NDFIP2	0.863061273	0.839507565	0.830135741	0.882057818	0.970988226	0.846210629
TARSL2	0.863061273	0.759245424	0.908072171	0.979644238	0.848717914	0.856640874
SAMHD1	0.863061273	0.826316517	0.855268213	0.928404714	0.906893576	0.866680565
WDR62	0.863061273	0.728814417	0.999993442	0.852170016	0.847279395	0.871503289
C3AR1	0.863061273	0.930647265	0.873496239	0.862094125	0.867925537	0.873130182
SMIM30	0.863061273	0.738007931	0.955427429	0.890704007	0.870847438	0.899183258
PPIL1	0.863061273	0.749365295	0.915858924	0.825279787	0.955623649	0.904592039
PSME2	0.863061273	0.794510324	0.912183977	0.833973634	0.848550619	0.960023723
SNED1	0.863075778	0.718382634	0.979494108	0.949565453	0.847092933	0.835231445
LAPTM5	0.863119788	0.83777309	0.893988897	0.970785492	0.879776073	0.79391909
TTPA	0.863119788	0.739799306	0.810647419	0.999981514	0.865075514	0.798229124
S100A14	0.863119788	0.760623317	0.855847948	0.935985829	0.976769047	0.801022176
MAGEE2	0.863119788	0.873814011	0.830420708	0.925210648	0.936921784	0.827870367
GTF2H5	0.863119788	0.72742557	0.924588495	0.844881916	0.987723553	0.832770678
SLC5A2	0.863119788	0.837399973	0.91469924	0.89684883	0.879776073	0.865767172
ZSWIM1	0.863119788	0.834615638	0.89478142	0.839245804	0.943702351	0.867063767
LOC514011	0.863119788	0.778487216	0.974656086	0.819213899	0.900927045	0.873130182
ATOX1	0.863119788	0.815362549	0.879939804	0.886312303	0.903606765	0.907384701
LOC107131289	0.863119788	0.862299245	0.899106435	0.854220165	0.864524107	0.91373422
SPON2	0.863209218	0.982515431	0.822300339	0.904018489	0.819679279	0.842654067
MRS2	0.863525135	0.802996442	0.886829776	0.88904348	0.891304411	0.91373422
LOC104975635	0.863525135	0.76003522	0.853036433	0.854623597	0.964301327	0.917849087
SLC39A6	0.863535598	0.797421707	0.865787276	0.891732037	0.969746326	0.839906041

S100A12	0.863960426	0.77844414	0.809673119	0.975150022	0.960573822	0.81853698
FAM101A	0.864123916	0.764775882	0.849389234	0.819549243	0.999985752	0.803920886
RCL1	0.864123916	0.77844414	0.832268748	0.999981514	0.823508064	0.874490166
IRF1	0.864123916	0.727065913	0.811480663	0.870442442	0.998040182	0.893838339
URGCP	0.864123916	0.96643574	0.842580963	0.82163384	0.81725718	0.927520621
TOMM34	0.864123916	0.722195304	0.844195628	0.962899291	0.816306826	0.968697704
PPP1R11	0.864175176	0.716415149	0.99341711	0.908467146	0.901853599	0.790348826
ADGRB2	0.864175176	0.7583338	0.855268213	0.999981514	0.858741373	0.806476213
NFATC4	0.864175176	0.847093186	0.947651663	0.874170259	0.886893907	0.839906041
LOC781280	0.864175176	0.755089315	0.878390815	0.999981514	0.832407979	0.852504043
CRAT	0.864243271	0.878551917	0.841294863	0.888198716	0.967682066	0.806892223
RAB3B	0.864338578	0.890045072	0.836691254	0.998529748	0.81725718	0.81956221
NRF1	0.864338578	0.823478017	0.831933739	0.822691957	0.999985752	0.83150957
TNIP2	0.864338578	0.737956652	0.965407935	0.824836902	0.964528248	0.832964812
BRD9	0.864338578	0.988540349	0.813441395	0.854220165	0.867558388	0.853009713
MTERF3	0.864338578	0.818303808	0.849389234	0.914263396	0.890841443	0.911808092
SLC44A3	0.864338578	0.802570554	0.798782742	0.955466436	0.891304411	0.91618909
CXHXorf38	0.864338578	0.869754045	0.870965946	0.878513543	0.823708771	0.943621425
LOC101903261	0.864338578	0.722195304	0.800428397	0.975346638	0.84620147	0.966212838
RNASEL	0.864420833	0.864668925	0.99341711	0.859320133	0.841549705	0.799901412
FAM114A2	0.864420833	0.808013061	0.892343042	0.961898258	0.907455286	0.810156973
CLEC4A	0.864636967	0.842884359	0.901982947	0.89684883	0.878451408	0.87501921
RGL2	0.864815502	0.734546826	0.996509242	0.952247001	0.823508064	0.801888363
BAAT	0.864908053	0.718228672	0.998364815	0.874023052	0.878451408	0.849595612
LOC112448335	0.864925085	0.872175143	0.884753588	0.865017704	0.87329316	0.912894278
LOC107132450	0.864949057	0.750954205	0.96030829	0.929663263	0.882409565	0.835231445
AXL	0.865025087	0.829345514	0.797180963	0.839725231	0.936108073	0.955210102
TMEM273	0.865121718	0.796253817	0.89385865	0.890362631	0.825284223	0.958790968
COPS3	0.86560009	0.750379179	0.955427429	0.887983383	0.855922702	0.914132808
LOC112446383	0.865772053	0.746341488	0.945995021	0.965724803	0.837915327	0.854935725
RAB19	0.865772053	0.758062181	0.888566052	0.86458365	0.832486638	0.987516427
METTL3	0.866010859	0.819210468	0.834683675	0.854220165	0.999453276	0.827530095
LOC104970589	0.866010859	0.744241031	0.982603155	0.885789665	0.887514169	0.853576299
MRM3	0.866010859	0.775033799	0.889098668	0.867235478	0.929896768	0.923092436
PRDM11	0.866015066	0.737941984	0.797409012	0.900875874	0.846530377	0.999701868
CHN1	0.866176824	0.734509989	0.818612484	0.999981514	0.881680572	0.821234952
LOC112442214	0.866188303	0.872244611	0.916294854	0.926970666	0.847173934	0.837557183
AZI2	0.866188303	0.741310853	0.827062418	0.999981514	0.826634148	0.891088733

WDR59	0.866188303	0.875506677	0.89478142	0.853058361	0.817472827	0.944227672
CEP192	0.866404528	0.718382634	0.933012945	0.982220679	0.906766758	0.793197037
MINDY2	0.866404528	0.760623317	0.957052938	0.907794002	0.891726429	0.845714049
KDM2A	0.866404528	0.78039095	0.865697455	0.908467146	0.963253256	0.849885981
LOC112444585	0.866404528	0.988008216	0.834683675	0.837449473	0.870061356	0.8537109
RILPL1	0.866404528	0.792109226	0.957295966	0.843284421	0.907094634	0.867482845
NARS	0.866404528	0.745791496	0.883906525	0.943447418	0.899042139	0.898085102
CREG1	0.866404528	0.808711476	0.849389234	0.941467375	0.826634148	0.940583352
KMT5A	0.866404528	0.766943819	0.944126714	0.865187072	0.833173678	0.948219411
KCTD6	0.866404528	0.737941984	0.814495418	0.901834772	0.871558258	0.989184798
RMDN2	0.866492254	0.813759037	0.948810088	0.886312303	0.909379774	0.835231445
LOC782799	0.866492254	0.941817736	0.831933739	0.865076519	0.911052879	0.862871483
CPSF2	0.866666555	0.814177827	0.93573893	0.868431455	0.883951804	0.890862406
TLDC2	0.866856655	0.752129142	0.957295966	0.888687722	0.916877832	0.853029499
CHIC1	0.866856655	0.918854612	0.852225514	0.832489049	0.899388069	0.901981092
RPRML	0.866856655	0.840402825	0.963335792	0.828736799	0.819342514	0.91373422
ZNF473	0.867024664	0.788980101	0.999993442	0.821450642	0.85185197	0.807084664
RNF5	0.867024664	0.728370183	0.999993442	0.852005554	0.887514169	0.816511068
LOC534578	0.867024664	0.862468468	0.881957212	0.927721166	0.916057607	0.81956221
GTPBP2	0.867024664	0.775033799	0.894545897	0.91185657	0.964301327	0.81956221
ID4	0.867024664	0.992144201	0.908072171	0.831709307	0.817472827	0.821257366
RASL10B	0.867024664	0.753597772	0.947567836	0.887877413	0.954403133	0.829955257
RAP2A	0.867024664	0.805580108	0.961800035	0.946503916	0.82488413	0.83150957
LCORL	0.867024664	0.779025263	0.854571175	0.944913633	0.956041014	0.841312694
ABHD2	0.867024664	0.929007264	0.862253703	0.839632707	0.935286646	0.845747012
LOC112446369	0.867024664	0.813759037	0.874063521	0.922967576	0.932024143	0.852504043
RILP	0.867024664	0.781836924	0.923364099	0.956685049	0.864524107	0.8537109
LOC101906545	0.867024664	0.923776511	0.834683675	0.923221248	0.859005924	0.867063767
CPOX	0.867024664	0.758609923	0.894338686	0.931361128	0.868722067	0.923092436
CWC25	0.867024664	0.761907799	0.849389234	0.850825191	0.967205491	0.923807852
SDR16C5	0.867024664	0.745846221	0.79497298	0.896116841	0.961670582	0.937715964
BUB3	0.867024664	0.741310853	0.91469924	0.865187072	0.895065229	0.947801358
TMEM219	0.867250021	0.829067393	0.893988897	0.895877524	0.91563394	0.867063767
DYNLL2	0.867388402	0.807350682	0.976589023	0.914026346	0.870061356	0.800967059
VAMP2	0.867388402	0.842884359	0.937334712	0.916858019	0.881680572	0.821257366
WFS1	0.867388402	0.889457989	0.935219877	0.882057818	0.882252274	0.83150957
UBE2QL1	0.867388402	0.775262502	0.879939804	0.999981514	0.847173934	0.846210629
LOC781499	0.867388402	0.727207722	0.851070786	0.98981682	0.928951321	0.852504043

LRRC1	0.867388402	0.722779653	0.899106435	0.893051204	0.969887518	0.863592771
FBXO10	0.867388402	0.724633427	0.874983674	0.999981514	0.845773773	0.867063767
MRPS18B	0.867388402	0.77766577	0.95077817	0.854220165	0.929896768	0.867063767
CHD6	0.867388402	0.72828303	0.964807927	0.937615893	0.818179113	0.893838339
PLOD1	0.867565282	0.752229543	0.866497255	0.999981514	0.909379774	0.81956221
GALNT11	0.867565282	0.774979074	0.893761108	0.907794002	0.964301327	0.834172836
SEC23A	0.867565282	0.913899522	0.916294854	0.922967576	0.823508064	0.835231445
PCBD1	0.867565282	0.90715891	0.865512879	0.886312303	0.929802526	0.839906041
LOC112448808	0.867565282	0.722779653	0.945995021	0.903584224	0.935286646	0.853735179
CLYBL	0.867565282	0.807919676	0.858376167	0.979644238	0.879395665	0.858165602
LOC104975974	0.867565282	0.786691027	0.972806636	0.908467146	0.823508064	0.867635709
NKAIN2	0.867565282	0.915269318	0.883737851	0.82163384	0.881680572	0.911219114
FITM1	0.867565282	0.760623317	0.830420708	0.868530316	0.955741702	0.944973383
PDIK1L	0.867565282	0.760672527	0.86885176	0.871338923	0.821351993	0.99666605
PPFIA2	0.867594031	0.867518836	0.885559767	0.874424802	0.964301327	0.806476213
GLYCTK	0.867594031	0.872175143	0.8644528	0.840700255	0.983941532	0.807084664
NHSL1	0.867594031	0.970084348	0.849389234	0.912922827	0.826634148	0.839906041
ISY1	0.867594031	0.823478017	0.997400824	0.870591999	0.818179113	0.841312694
TRIM59	0.867594031	0.871091574	0.809673119	0.965050971	0.906893576	0.841312694
PIDD1	0.867594031	0.796253817	0.949855095	0.952247001	0.840108119	0.842214812
MYO5B	0.867594031	0.789957129	0.879939804	0.97828838	0.890841443	0.842654067
KL	0.867594031	0.743752921	0.863803757	0.907794002	0.982208423	0.847020266
LOC107132262	0.867594031	0.95028107	0.809673119	0.898658215	0.900927045	0.847386898
SPATS2	0.867594031	0.722195304	0.89478142	0.892047793	0.976292433	0.856833445
BICRA	0.867594031	0.885940782	0.891972402	0.825279787	0.943540106	0.861779975
TMEM39B	0.867594031	0.947911876	0.842694695	0.844615499	0.91563394	0.862925606
EBAG9	0.867594031	0.760404518	0.999993442	0.874023052	0.81725718	0.873130182
RFC5	0.867594031	0.739236562	0.916294854	0.97828838	0.854289643	0.874490166
PSMA5	0.867594031	0.738007931	0.961800035	0.825279787	0.948711141	0.874490166
NDUFV3	0.867594031	0.808478344	0.867661896	0.86458365	0.961465747	0.880846873
C18H16orf74	0.867594031	0.785057176	0.883216179	0.888687722	0.943734737	0.89016973
SH3D21	0.867594031	0.782783231	0.894316021	0.952247001	0.858495696	0.898021919
LYRM7	0.867594031	0.750379179	0.928514467	0.887642126	0.870450746	0.935770262
PECR	0.867594031	0.750379179	0.930718777	0.828736799	0.896589592	0.945854375
LOC112446013	0.867594031	0.83359488	0.92091815	0.848086379	0.823508064	0.950798794
CNP	0.867594031	0.777309385	0.831933739	0.951193444	0.847173934	0.958699521
LOC112447305	0.867594031	0.740343181	0.807254925	0.886312303	0.849434441	0.999948228
FARP1	0.867595267	0.760778257	0.951028911	0.957419137	0.891726429	0.806476213

MFAP5	0.867595267	0.796748952	0.895801611	0.949468104	0.900747635	0.849731479
LOC104975626	0.867595267	0.741263262	0.807254925	0.999981514	0.887514169	0.859219598
CLEC12A	0.867595267	0.805283017	0.879939804	0.853058361	0.901144467	0.944973383
GRIK3	0.867624665	0.878746339	0.818612484	0.893220599	0.870042072	0.938894901
C29H11orf98	0.867624665	0.758609923	0.958410013	0.822691957	0.823508064	0.966136679
GRIA3	0.867693329	0.872175143	0.824121854	0.908467146	0.962513981	0.821910421
PPP1R8	0.867807352	0.720627712	0.915858924	0.865076519	0.891726429	0.96053762
ST3GAL6	0.86818488	0.776670017	0.943836178	0.85981779	0.878269439	0.927426708
PRC1	0.868454432	0.756160885	0.985172237	0.935239391	0.847173934	0.835231445
IL18	0.868505933	0.76627546	0.820007835	0.999981514	0.847034404	0.795936781
CCDC12	0.868505933	0.940500178	0.881778842	0.919084646	0.86864151	0.811759324
XIRP1	0.868505933	0.866399172	0.854571175	0.840862991	0.983941532	0.83150957
CLCN7	0.868505933	0.864685893	0.855971055	0.992930508	0.82488413	0.839906041
SLC17A9	0.868505933	0.808478344	0.893988897	0.976125636	0.855920132	0.854935725
FAM98C	0.868505933	0.863286109	0.894316021	0.922967576	0.86864151	0.867063767
MLLT11	0.868505933	0.758733912	0.892343042	0.911004319	0.901853599	0.918461982
ABCD1	0.868505933	0.722779653	0.954020369	0.859320133	0.903888186	0.921557022
LOC101906266	0.868838381	0.88008538	0.89385865	0.960016921	0.82114467	0.852112206
LOC510613	0.868936237	0.828365124	0.89478142	0.994689556	0.848550619	0.806476213
RNF38	0.869003213	0.722779653	0.999993442	0.940438437	0.823508064	0.804967091
ICA1	0.869003213	0.879150176	0.968548891	0.903584224	0.834035648	0.806476213
PSMD6	0.869003213	0.757349588	0.929661805	0.839725231	0.878451408	0.959198304
MGAT2	0.869003213	0.740362216	0.892139418	0.89684883	0.820546747	0.985165897
LOC101905228	0.869070344	0.759629362	0.874983674	0.883184408	0.936215792	0.930710695
RCC1	0.869071821	0.762299274	0.916323169	0.82906342	0.82488413	0.988320707
ZNF322	0.869152214	0.880565292	0.831933739	0.897371376	0.947020167	0.853440904
LOC104975861	0.869417355	0.7973731	0.850941364	0.982220679	0.821148296	0.918461982
CTSH	0.869423044	0.829345514	0.831933739	0.999981514	0.87329316	0.836705424
NCS1	0.869423044	0.749632153	0.971556427	0.950893883	0.857014385	0.836744339
YWHAE	0.869643901	0.729001857	0.895534805	0.960016921	0.877578545	0.911219114
ZSWIM3	0.86966745	0.943754915	0.842694695	0.82868625	0.849554534	0.936263602
LOC788201	0.869806457	0.933545282	0.818612484	0.896116841	0.953885477	0.806892223
HOXD9	0.869806457	0.773411104	0.865717285	0.907353815	0.976292433	0.84234728
SCAF11	0.869963987	0.866901725	0.849389234	0.825279787	0.999453276	0.813571897
ACO1	0.869963987	0.783112117	0.906019013	0.999981514	0.818179113	0.817185907
EGFL8	0.869963987	0.943213236	0.832268748	0.973673712	0.828207883	0.824780045
CARD14	0.869963987	0.968509005	0.864832853	0.898658215	0.864524107	0.827038823
LOC107131330	0.869963987	0.730634526	0.920681987	0.951193444	0.943702351	0.828655205

LOC100337323	0.869963987	0.758609923	0.831933739	0.889415593	0.999985752	0.83150957
FITM2	0.869963987	0.929204871	0.849389234	0.912777307	0.896589592	0.835231445
ZFYVE19	0.869963987	0.806029227	0.798782742	0.999981514	0.909737016	0.836705424
ARHGAP39	0.869963987	0.993481224	0.860877703	0.853058361	0.823508064	0.847386898
ATP13A3	0.869963987	0.831549062	0.835443208	0.943808491	0.939442711	0.853029499
ABHD10	0.869963987	0.796866016	0.891662311	0.868255052	0.964301327	0.87100843
FAM43A	0.869963987	0.934225172	0.86885176	0.907794002	0.832437297	0.875118526
LOC112443151	0.869963987	0.722779653	0.853036433	0.984423083	0.885163738	0.91373422
ARL1	0.869963987	0.744774335	0.954020369	0.904374668	0.85231994	0.915546224
GPATCH4	0.869963987	0.847493166	0.940954497	0.872004186	0.823508064	0.918461982
TRAF6	0.869963987	0.867595051	0.845113191	0.928404714	0.844585092	0.923807852
SDHAF3	0.869963987	0.755252885	0.968679299	0.853058361	0.820477096	0.950631909
TPX2	0.869963987	0.739236562	0.813441395	0.907997414	0.943055455	0.953580778
CDK2	0.869963987	0.727781617	0.879939804	0.88106101	0.906803277	0.966850975
SF3B3	0.870042807	0.727791766	0.826660577	0.935239391	0.996781978	0.841312694
ARL6IP4	0.870042807	0.901028838	0.949855095	0.852170016	0.858741373	0.857853613
PGLS	0.870042807	0.838196095	0.845113191	0.832489049	0.943963542	0.930710695
NOX4	0.870042807	0.818303808	0.822300339	0.828736799	0.931775933	0.966850975
RTN4RL1	0.870450133	0.985428851	0.844251803	0.872590202	0.902907077	0.801109854
TMEM199	0.870450133	0.728504282	0.999993442	0.868435169	0.916877832	0.810156973
MED23	0.870450133	0.992539037	0.843955813	0.852170016	0.887514169	0.821910421
MAP9	0.870450133	0.722348907	0.958554319	0.921482057	0.935286646	0.83150957
PLEKHG6	0.870450133	0.792661811	0.89478142	0.996740986	0.852234727	0.835231445
SCAMP2	0.870450133	0.744049882	0.867719797	0.999981514	0.823508064	0.839565788
RPS6KA4	0.870450133	0.887455957	0.830789082	0.949502614	0.900747635	0.851995112
NHLRC1	0.870450133	0.816912013	0.858521535	0.999981514	0.834035648	0.858391424
MATR3	0.870450133	0.916484772	0.855811972	0.865076519	0.932024143	0.864207753
LOC101907744	0.870450133	0.943859632	0.819527307	0.889415593	0.890909775	0.876162019
KLF12	0.870450133	0.737396716	0.885535367	0.870210705	0.984386856	0.876162019
BEX5	0.870450133	0.895249755	0.850624788	0.855232617	0.940870936	0.877014986
MRPS25	0.870450133	0.837399973	0.848818476	0.827786827	0.976292433	0.884402686
IL15	0.870450133	0.741263262	0.802277976	0.959591127	0.95182754	0.913221224
LOC112444171	0.870450133	0.738832446	0.865512879	0.990955345	0.850582836	0.91373422
LOC101906206	0.870450133	0.728370183	0.95322206	0.840247324	0.926972948	0.919124527
LOC539973	0.870450133	0.760778257	0.916294854	0.941467375	0.847092933	0.919195656
TAMM41	0.870450133	0.938087741	0.810647419	0.853058361	0.882418284	0.927520621
ZNF613	0.870450133	0.797765031	0.903526927	0.82868625	0.916057607	0.938894901
PI15	0.870450133	0.757251331	0.836719427	0.852170016	0.970988226	0.940017687

AGPAT1	0.870450133	0.741263262	0.800428397	0.866769648	0.983941532	0.940583352
RAB11B	0.870450133	0.722779653	0.818951412	0.929663263	0.887514169	0.978087957
TMEM200B	0.870450133	0.753597772	0.819527307	0.853058361	0.935088219	0.982168135
DHRS9	0.870588024	0.878551917	0.915858924	0.886312303	0.939326536	0.810508177
SYTL4	0.870588024	0.866767436	0.810647419	0.887983383	0.935286646	0.91373422
DGKD	0.870612822	0.738007931	0.854567571	0.882057818	0.999985752	0.811759324
TUBA8	0.870612822	0.918455779	0.894338686	0.896116841	0.902258286	0.822427019
DDR2	0.870612822	0.973321075	0.867625133	0.886312303	0.837915327	0.859629526
SNX30	0.870612822	0.738054694	0.916294854	0.852958493	0.984386856	0.860414702
IVD	0.870612822	0.802605511	0.834683675	0.825279787	0.999985752	0.862925606
BNC2	0.870612822	0.85265333	0.894316021	0.891988949	0.896540092	0.883688554
RNF39	0.870612822	0.722968232	0.904170228	0.972423308	0.880672837	0.891088733
HK3	0.870612822	0.782783231	0.894316021	0.975453864	0.829853428	0.900500341
LOC787397	0.870612822	0.753131028	0.858376167	0.98981682	0.848550619	0.91373422
POP1	0.870612822	0.781543413	0.874983674	0.853058361	0.887514169	0.973413452
ALG12	0.870752935	0.895777642	0.846372547	0.894099772	0.954403133	0.832964812
SCOC	0.870752935	0.808785257	0.927274303	0.887983383	0.864524107	0.919124527
ALG1	0.870840366	0.753090681	0.838341783	0.999981514	0.907762824	0.806714845
FAM126B	0.870840366	0.959263706	0.829751489	0.868378265	0.937096422	0.832201748
PAX8	0.870840366	0.901631302	0.810647419	0.974286747	0.832437297	0.879135421
RNF34	0.870840366	0.750379179	0.920682633	0.870210705	0.823508064	0.98140846
LOC104974890	0.870898079	0.99528564	0.8042974	0.91285419	0.852234727	0.809466925
ADIRF	0.870898079	0.944354781	0.880163755	0.894086736	0.896490594	0.814218101
SH3D19	0.870898079	0.875447594	0.896023027	0.949502614	0.887514169	0.816246746
SLIT3	0.870898079	0.877822131	0.836482	0.999981514	0.823508064	0.831652079
LOC100848148	0.870898079	0.878551917	0.879939804	0.912777307	0.911568867	0.844759721
CAPN15	0.870898079	0.968509005	0.916294854	0.82868625	0.821148296	0.872238439
SHCBP1	0.871007054	0.722779653	0.916323169	0.965050971	0.868722067	0.896529476
ZDHHC14	0.871486681	0.999987083	0.83068481	0.82868625	0.87329316	0.806476213
SHPK	0.871486681	0.77139884	0.858032788	0.999981514	0.891205587	0.806476213
ABCC1	0.871486681	0.782783231	0.885896294	0.827786827	0.999985752	0.808832219
LOC534627	0.871486681	0.78945719	0.831933739	0.946938434	0.980189546	0.821257366
CHSY3	0.871486681	0.796253817	0.986894787	0.926098747	0.846285074	0.8255975
GPRIN3	0.871486681	0.779407705	0.878164336	0.973373329	0.936388637	0.83150957
TTC32	0.871486681	0.758609923	0.874983674	0.944351122	0.970988226	0.83150957
CRY1	0.871486681	0.841902289	0.819359511	0.996033721	0.893854857	0.831652079
NPR1	0.871486681	0.945598957	0.828449846	0.836610893	0.964301327	0.83186501
LOC112444350	0.871486681	0.946241678	0.927635169	0.852481062	0.871558258	0.835231445

MS4A7	0.871486681	0.863286109	0.949855095	0.896116841	0.882252274	0.835231445
FUT11	0.871486681	0.989303356	0.805212947	0.878513543	0.895067787	0.835231445
TMEM67	0.871486681	0.823602247	0.813652211	0.9880671	0.928951321	0.838468539
ASCC2	0.871486681	0.969811486	0.879608555	0.903584224	0.832407979	0.838530462
MGME1	0.871486681	0.758609923	0.987789469	0.896116841	0.887514169	0.846210629
PNPLA2	0.871486681	0.944346754	0.851822885	0.887642126	0.905571216	0.847791663
GLIS2	0.871486681	0.766943819	0.894316021	0.981156002	0.890124729	0.855349987
HS1BP3	0.871486681	0.929409572	0.845113191	0.92532303	0.87329316	0.860966325
ZNF500	0.871486681	0.734509989	0.955189235	0.962610411	0.85231994	0.867063767
ZDHHC7	0.871486681	0.748690795	0.894338686	0.998529748	0.846464841	0.873185607
RBM8A	0.871486681	0.796253817	0.924855847	0.885789665	0.931357161	0.875118526
LOC112444288	0.871486681	0.730581055	0.957052938	0.928416242	0.858741373	0.896529476
SPTY2D1OS	0.871486681	0.794959033	0.958410013	0.886312303	0.826634148	0.923092436
RFX1	0.871486681	0.753090681	0.946791586	0.887983383	0.879776073	0.923807852
BLZF1	0.871486681	0.797421707	0.927508464	0.886662305	0.847092933	0.939948644
LOC101906632	0.871486681	0.774644712	0.836482	0.961898258	0.836515016	0.959487749
POP5	0.871486681	0.786817529	0.897322115	0.853271157	0.881680572	0.965117554
LOC107131224	0.871486681	0.750379179	0.834683675	0.8443913	0.929896768	0.987840151
NEDD9	0.871691902	0.785374969	0.894316021	0.886312303	0.977167046	0.84172106
FUK	0.871726645	0.750379179	0.938624264	0.952247001	0.911028086	0.839906041
PDP1	0.871726645	0.894014677	0.857372289	0.866566721	0.946970109	0.865971618
NXPH3	0.871726645	0.74241072	0.999993442	0.865076519	0.847092933	0.884993859
AP3D1	0.871835881	0.997810586	0.850624788	0.853058361	0.844434232	0.841312694
PPCDC	0.871835881	0.77766577	0.862253703	0.922189411	0.959007232	0.872239777
TOR1A	0.871835881	0.77139884	0.841294863	0.938681987	0.947313295	0.895446935
NGLY1	0.872028316	0.737396716	0.8042974	0.956685049	0.984692578	0.859219598
FAM151B	0.872028316	0.789202232	0.886829776	0.952247001	0.865638205	0.91373422
SYNGR1	0.872080906	0.942088099	0.846704346	0.880872925	0.943702351	0.828124017
LOC112441839	0.872104866	0.760778257	0.914876997	0.962610411	0.891304411	0.865212385
LOC101906363	0.872514307	0.750379179	0.89478142	0.874023052	0.968973598	0.89148147
LOC104972390	0.872530433	0.796253817	0.915858924	0.893220599	0.938879379	0.867635709
TLCD1	0.872879419	0.842884359	0.996224523	0.85647796	0.887514169	0.803767714
CMIP	0.872879419	0.811089435	0.984886031	0.874662174	0.870042072	0.858165602
LARP1	0.872879419	0.780830698	0.831933739	0.994395954	0.895757462	0.873130182
NSMF	0.872879419	0.796253817	0.824121854	0.905772309	0.960573822	0.90388238
TM4SF19	0.872879419	0.849119707	0.858820898	0.952247001	0.844434232	0.91373422
PLK1	0.872879419	0.845347956	0.869932384	0.907794002	0.852746309	0.938616551
MCCC1	0.872879419	0.769009241	0.869932384	0.896116841	0.921625807	0.940050644

COX18	0.872914013	0.734193706	0.916323169	0.965724803	0.870061356	0.893838339
GOSR2	0.87298557	0.757251331	0.852388625	0.852170016	0.987198672	0.91373422
ATOH8	0.873040963	0.798434093	0.849389234	0.892577971	0.864524107	0.975558533
ITPA	0.873086337	0.863613561	0.895801611	0.839245804	0.967430558	0.839906041
LOC101902174	0.873086337	0.816540275	0.806470927	0.894086736	0.887514169	0.973649216
CCNY	0.87325791	0.757201997	0.944126714	0.959652624	0.821148296	0.90182838
TM6SF1	0.873631636	0.748933195	0.999993442	0.872590202	0.854966579	0.805424719
SNX24	0.873631636	0.916484772	0.994035957	0.840700255	0.823508064	0.813528842
CLIC3	0.873631636	0.94622198	0.878390815	0.915039335	0.887514169	0.813528842
LOC107132228	0.873631636	0.731945941	0.996224523	0.896116841	0.923910588	0.814218101
LOC112441654	0.873631636	0.792109226	0.867661896	0.999981514	0.823508064	0.818374372
LOC101902211	0.873631636	0.918455779	0.937093664	0.910854395	0.849704586	0.81956221
ACKR1	0.873631636	0.893164557	0.85942296	0.995565324	0.832437297	0.821257366
DBNDD1	0.873631636	0.807623624	0.875900843	0.892047793	0.987723553	0.821257366
SPECC1	0.873631636	0.802840415	0.86347788	0.97828838	0.939132857	0.821910421
NEXMIF	0.873631636	0.995373357	0.817728993	0.896116841	0.858179728	0.824780045
LOC101906451	0.873631636	0.783896922	0.910135571	0.999981514	0.863651062	0.828184367
CEP170B	0.873631636	0.904266915	0.885896294	0.95901497	0.864524107	0.83150957
FRMPD4	0.873631636	0.826124039	0.879939804	0.997485079	0.864524107	0.83150957
SCLT1	0.873631636	0.760623317	0.806470927	0.999981514	0.915840352	0.83150957
CEBPD	0.873631636	0.798175865	0.893427844	0.961898258	0.929896768	0.831652079
PHF6	0.873631636	0.890669166	0.813441395	0.984423083	0.891726429	0.836111377
PGM2	0.873631636	0.816984836	0.852916849	0.89684883	0.984386856	0.837559792
CLCN4	0.873631636	0.763758778	0.817582037	0.999981514	0.935286646	0.839906041
LOC104969027	0.873631636	0.855067335	0.882281083	0.882057818	0.960573822	0.845747012
HOOK2	0.873631636	0.999987083	0.83068481	0.832401065	0.832407979	0.846210629
NPRL3	0.873631636	0.872175143	0.841294863	0.9880671	0.86864151	0.849595612
SCAMP3	0.873631636	0.814177827	0.963335792	0.830427818	0.940870936	0.849885981
GNG2	0.873631636	0.872175143	0.916038418	0.952247001	0.832407979	0.852504043
L3MBTL3	0.873631636	0.758609923	0.895698572	0.9063051	0.970988226	0.852504043
ZBTB32	0.873631636	0.801273171	0.933884582	0.979584502	0.823508064	0.85634055
ZNF362	0.873631636	0.744671791	0.863546668	0.885962241	0.999985752	0.85634055
MRAS	0.873631636	0.928835826	0.916294854	0.887314831	0.858495696	0.858321732
LOC104972031	0.873631636	0.971823688	0.894497585	0.852137114	0.854966579	0.861765471
LOC112447408	0.873631636	0.785346067	0.933884582	0.979385838	0.833173678	0.86324237
RTF1	0.873631636	0.742673431	0.908072171	0.843613441	0.996781978	0.86324237
LOC107132757	0.873631636	0.837945129	0.879939804	0.9819628	0.847519063	0.864197513
TMEFF1	0.873631636	0.823647199	0.845113191	0.912777307	0.960419102	0.867063767

SIPA1L3	0.873631636	0.787904651	0.999993442	0.85721441	0.834569503	0.867907362
LOC104970812	0.873631636	0.835452905	0.842694695	0.843834406	0.989334846	0.870018677
LOC112447473	0.873631636	0.835319802	0.849389234	0.941467375	0.929896768	0.870490204
FUNDC1	0.873631636	0.929409572	0.903526927	0.840932024	0.89052284	0.872238439
MYO1F	0.873631636	0.792690308	0.914002845	0.860448498	0.963533116	0.873130182
CYTH4	0.873631636	0.795020265	0.95322206	0.900875874	0.88127953	0.876784504
NOC3L	0.873631636	0.741310853	0.810647419	0.988408846	0.944839728	0.880622346
GABRA1	0.873631636	0.847093186	0.831933739	0.993215785	0.823508064	0.891088733
ZRSR2	0.873631636	0.894702415	0.86404457	0.876616876	0.91563394	0.891088733
RAB3GAP1	0.873631636	0.753090681	0.916323169	0.955466436	0.877712931	0.892560108
ISPD	0.873631636	0.741263262	0.916038418	0.873182682	0.956041014	0.9136642
SEPT10	0.873631636	0.796666816	0.954020369	0.887642126	0.854966579	0.915414597
MRPL52	0.873631636	0.813759037	0.920784786	0.853058361	0.907678664	0.919124527
ZC3H7B	0.873631636	0.865945453	0.855811972	0.8705821	0.92549567	0.919124527
ZNF770	0.873631636	0.744774335	0.880357899	0.868378265	0.964301327	0.926480924
PXDC1	0.873631636	0.748625319	0.826913617	0.985701713	0.879395665	0.932688327
TRPM6	0.873631636	0.744774335	0.960685112	0.839632707	0.894177708	0.933638318
AP4M1	0.873631636	0.737941984	0.987610559	0.837820405	0.854966579	0.939948644
CDKN1B	0.873631636	0.894702415	0.848468312	0.839725231	0.89052284	0.948705062
TMEM9B	0.873631636	0.740040635	0.937812682	0.908467146	0.828142439	0.959075802
HIST2H2BE	0.873631636	0.779372555	0.846372547	0.885789665	0.91506312	0.96870908
NEURL1	0.873631636	0.929204871	0.809673119	0.85797315	0.826634148	0.970105102
LOC101905254	0.873631636	0.766203241	0.819527307	0.876703811	0.927973846	0.9803583
CGNL1	0.873631636	0.800861368	0.833583844	0.902776185	0.847092933	0.986856275
TMEM8B	0.873631636	0.814177827	0.809673119	0.901171792	0.82775883	0.996310803
LOC101905141	0.873631636	0.750466772	0.836482	0.926970666	0.821351993	0.996453303
TMEM92	0.873968549	0.88008538	0.89478142	0.974941201	0.82488413	0.84391477
WBP4	0.873968549	0.7889129	0.871285807	0.872590202	0.989379051	0.860414702
OSBPL8	0.873968549	0.729703804	0.89478142	0.985701713	0.892336551	0.875650601
CUL4A	0.873968549	0.938087741	0.838852919	0.905209994	0.879656633	0.878754045
NFX1	0.874294664	0.821674572	0.843955813	0.85981779	0.977167046	0.891088733
FAM13B	0.874357292	0.734075141	0.860306567	0.944913633	0.988010662	0.836157024
LOC101902361	0.874357292	0.792483444	0.842694695	0.879344676	0.917407853	0.966850975
EML5	0.874398023	0.840431179	0.87118003	0.951936643	0.882206878	0.883688554
CATSPERE	0.874434348	0.734546826	0.991138474	0.97828838	0.838054502	0.815597809
IRAK3	0.874434348	0.857276429	0.912183977	0.896116841	0.949311988	0.820182674
SLC25A11	0.874434348	0.918234392	0.915713148	0.840210569	0.943702351	0.83150957
ZDHHC9	0.874434348	0.796666816	0.954020369	0.863269783	0.961465747	0.83150957

HDAC11	0.874434348	0.831564725	0.886829776	0.952247001	0.929896768	0.831652079
LOC107131498	0.874434348	0.737127482	0.877334778	0.960016921	0.972160227	0.831652079
CAD	0.874434348	0.888057447	0.93475587	0.915013589	0.872022568	0.832964812
MAPKAPK5	0.874434348	0.910651459	0.881849916	0.889415593	0.935286646	0.838530462
NR3C1	0.874434348	0.771654685	0.820959147	0.962437357	0.976292433	0.840271658
ARL13B	0.874434348	0.913116251	0.915858924	0.908467146	0.87329316	0.840768468
CDS2	0.874434348	0.833884514	0.947155158	0.959652624	0.823508064	0.845747012
HNRNPA3	0.874434348	0.905599077	0.916038418	0.904018489	0.877712931	0.846210629
HOPX	0.874434348	0.764456895	0.896821247	0.999981514	0.862205587	0.848282393
CLN3	0.874434348	0.87976124	0.978014482	0.866566721	0.852234727	0.849348058
CBWD2	0.874434348	0.741310853	0.999993442	0.889415593	0.878451408	0.853576299
FKBP1A	0.874434348	0.760404518	0.916294854	0.9063051	0.956041014	0.864243158
LOC100297099	0.874434348	0.804893203	0.831933739	0.925294859	0.964833865	0.872239777
C16H1orf53	0.874434348	0.848480716	0.881070741	0.920843549	0.893716526	0.883688554
CCND3	0.874434348	0.737120625	0.867661896	0.982220679	0.91506312	0.888930405
PSMD13	0.874434348	0.786691027	0.908072171	0.874170259	0.956904152	0.889662488
ARFGEF3	0.874434348	0.808478344	0.948594902	0.92386951	0.837399927	0.891423889
LOC101902288	0.874434348	0.886909552	0.916323169	0.890362631	0.849444776	0.898085102
CSRNP1	0.874434348	0.796253817	0.847014764	0.990955345	0.858716235	0.903795325
KLHL30	0.874434348	0.883756544	0.955427429	0.840247324	0.829853428	0.911219114
ATG12	0.874434348	0.737721966	0.94422142	0.899792456	0.828207883	0.960877268
PREPL	0.874696315	0.779372555	0.860877703	0.955954309	0.976292433	0.811296955
CCDC66	0.874696315	0.740343181	0.95217331	0.959616431	0.929896768	0.812774915
HK1	0.874696315	0.791281103	0.867222363	0.979217817	0.950958352	0.81853698
BRF1	0.874696315	0.808478344	0.924855847	0.955466436	0.89541085	0.835231445
HNRNPLL	0.874696315	0.775532414	0.849804464	0.925294859	0.983941532	0.846210629
LAP3	0.874696315	0.760778257	0.833042299	0.999981514	0.879776073	0.872238439
LOC107131948	0.874696315	0.741332329	0.957052938	0.940438437	0.85407843	0.899428912
MUC1	0.874696315	0.787904651	0.861447545	0.891717819	0.895757462	0.964898868
CPNE2	0.874712964	0.882242226	0.854567571	0.986562897	0.887514169	0.81956221
LOC781533	0.874712964	0.846798773	0.953572282	0.959141938	0.837915327	0.821910421
ZCWPW2	0.874712964	0.796253817	0.958197827	0.863269783	0.958852623	0.83150957
LOC614522	0.874712964	0.961663204	0.810647419	0.929388614	0.887514169	0.842473495
MOB3C	0.874712964	0.793300166	0.831933739	0.932406746	0.981652321	0.847012179
LOC104970387	0.874712964	0.863286109	0.833583844	0.999981514	0.851174659	0.851995112
LOC104975006	0.874712964	0.811059858	0.89478142	0.959591127	0.894364008	0.860966325
UGCG	0.874712964	0.808478344	0.888730544	0.912152327	0.953300742	0.860966325
C18H19orf12	0.874712964	0.946820717	0.903844825	0.890704007	0.833864947	0.86977568

LOC531462	0.874712964	0.7889129	0.835798494	0.999981514	0.848550619	0.876784504
COQ4	0.874712964	0.823478017	0.885896294	0.830427818	0.976259416	0.877048993
IZUMO4	0.874712964	0.766595431	0.8735515	0.989601002	0.828207883	0.923807852
DHDDS	0.874712964	0.817310492	0.944949386	0.865248466	0.854168394	0.935770262
LYRM1	0.874712964	0.843211838	0.880163755	0.896116841	0.864524107	0.944330889
MVP	0.874712964	0.795955721	0.853739258	0.925353907	0.880876632	0.955210102
SLC22A23	0.874778588	0.786691027	0.949939862	0.989601002	0.854966579	0.814218101
SMC4	0.874778588	0.786392346	0.95219206	0.969837124	0.87329316	0.831176431
FIBIN	0.874778588	0.871176243	0.849389234	0.896116841	0.97464569	0.832964812
RHOV	0.874778588	0.7889129	0.958197827	0.954343717	0.864524107	0.844759721
UGDH	0.874778588	0.801273171	0.833042299	0.95901497	0.956761712	0.860966325
ETFRF1	0.874778588	0.760778257	0.955427429	0.932406746	0.881680572	0.87501921
CLPB	0.874778588	0.750379179	0.860179257	0.999981514	0.887514169	0.875650601
SLU7	0.874778588	0.741263262	0.95219206	0.919628651	0.871558258	0.91373422
TNFRSF4	0.874778588	0.792690308	0.913609237	0.952247001	0.846167107	0.914132808
TGM2	0.874778588	0.848383949	0.894545897	0.892071982	0.865638205	0.931410194
LOC101905267	0.874778588	0.813442974	0.96102707	0.841796491	0.834431851	0.944927301
TMEM222	0.874778588	0.822956796	0.8644528	0.832489049	0.944584705	0.944973383
QSER1	0.874778588	0.879093989	0.828035534	0.837820405	0.886944613	0.975954071
PIP5K1A	0.874806876	0.927369042	0.874983674	0.830940997	0.932024143	0.87754233
LOC618939	0.874869904	0.771236128	0.999993442	0.892047793	0.853985138	0.822427019
LOC107132617	0.874897917	0.929409572	0.879608555	0.928404714	0.902258286	0.814218101
VPS52	0.874897917	0.762100436	0.97763883	0.914850247	0.926972948	0.816245398
DYSF	0.874897917	0.826102395	0.948904459	0.869208308	0.958797242	0.81956221
RMND1	0.874897917	0.748937365	0.884172102	0.865076519	0.999985752	0.838954188
LOC101905786	0.874897917	0.835486075	0.838909114	0.990955345	0.86864151	0.87501921
SNHG3	0.874897917	0.758609923	0.86347788	0.929388614	0.893716526	0.954496768
TGFB3	0.875285077	0.847718043	0.850624788	0.901353354	0.986861597	0.814218101
TNFSF18	0.875285077	0.752085497	0.913400043	0.972467586	0.943540106	0.8255975
EFCC1	0.875285077	0.863414694	0.830664444	0.965728173	0.907094634	0.864139695
RARS	0.875285077	0.785300379	0.91882781	0.866566721	0.953300742	0.89438401
CYP27A1	0.875747669	0.94502659	0.830135741	0.940257809	0.854966579	0.87501921
LOC112447309	0.875747669	0.873814011	0.818925429	0.859320133	0.847092933	0.991416486
CXHXorf56	0.875851256	0.750379179	0.899106435	0.934445963	0.976727507	0.824602886
KIF1A	0.875851256	0.740343181	0.966199203	0.941467375	0.858495696	0.888309103
BBS7	0.875851256	0.865570309	0.871285807	0.896116841	0.926831826	0.891088733
ZNF383	0.875851256	0.856560491	0.829092326	0.949502614	0.906893576	0.893706948
CRIP2	0.875851256	0.796253817	0.819527307	0.853535289	0.98094737	0.927520621

TPD52	0.875851256	0.741310853	0.974656086	0.868378265	0.838788415	0.955140534
ROBO1	0.875932505	0.738802109	0.849389234	0.948050281	0.984386856	0.85643253
PSMB2	0.875932505	0.808478344	0.94422142	0.873302281	0.87329316	0.927517509
SERTAD2	0.875968055	0.8332127	0.870922751	0.916480703	0.962558935	0.841312694
TYW3	0.875968055	0.932394105	0.908237602	0.870591999	0.891304411	0.857673662
TCF3	0.876038572	0.775262502	0.879608555	0.84908329	0.999985752	0.847193872
BHLHE22	0.8762255	0.760778257	0.828806956	0.999981514	0.844384115	0.858289382
ADGB	0.876329168	0.807285501	0.976967187	0.933718942	0.879395665	0.81956221
RSBN1	0.876329168	0.887139959	0.929661805	0.840700255	0.953300742	0.831652079
PTGER4	0.876329168	0.77139884	0.853546772	0.960016921	0.976259416	0.832964812
HERPUD2	0.876329168	0.822956796	0.8644528	0.999981514	0.855920132	0.836111377
KIAA1211	0.876329168	0.821674572	0.935266153	0.870210705	0.960048824	0.839906041
SEMA6C	0.876329168	0.781836924	0.862253703	0.999981514	0.852746309	0.853056636
CACNG7	0.876329168	0.800861368	0.996224523	0.907621547	0.824009924	0.85365082
MREG	0.876329168	0.845347956	0.916323169	0.948776099	0.877712931	0.8537109
LOC100847609	0.876329168	0.952863279	0.842694695	0.910575108	0.871558258	0.873130182
FXN	0.876329168	0.756706633	0.916294854	0.915779841	0.944839728	0.877014986
ARFIP2	0.876329168	0.7973731	0.947155158	0.868255052	0.929896768	0.883530991
CLEC16A	0.876329168	0.969051025	0.865617673	0.853058361	0.87329316	0.887542005
PODNL1	0.876329168	0.778032596	0.845113191	0.949565453	0.937932469	0.912531667
PCNT	0.876329168	0.821709833	0.823731372	0.952247001	0.852234727	0.959275214
RNF10	0.876329168	0.816202069	0.855892553	0.853058361	0.906803277	0.972314037
C17H5orf52	0.876329168	0.741310853	0.815587305	0.859466553	0.959565914	0.98140846
LOC101902122	0.876329168	0.741263262	0.816005434	0.911735195	0.87329316	0.99828741
CHCHD10	0.876411382	0.828516628	0.875873031	0.86458365	0.989379051	0.845241321
EPS8	0.876411382	0.737120625	0.978014482	0.853058361	0.954403133	0.865767172
P2RY8	0.876411382	0.780799374	0.951756885	0.830427818	0.924222719	0.923527309
EXOC8	0.876802172	0.762081499	0.916294854	0.934425211	0.922834705	0.884382952
KDM4C	0.876802172	0.827824094	0.841294863	0.889415593	0.961465747	0.908099327
LOC104974678	0.876802172	0.95310464	0.855847948	0.870393589	0.846285074	0.925233445
HPGDS	0.876802172	0.782783231	0.948926379	0.889415593	0.865075514	0.933224627
LOC112444924	0.876802172	0.823133291	0.942134405	0.886312303	0.837121276	0.939024684
NQO1	0.876802172	0.77657783	0.883816089	0.874424802	0.944839728	0.940017687
MFHAS1	0.876802172	0.834615638	0.810647419	0.965578567	0.847092933	0.950415692
LOC101903545	0.876802172	0.811354298	0.869932384	0.843335547	0.917407853	0.967792782
OCLN	0.877110066	0.792690308	0.859987058	0.982220679	0.931871771	0.852383414
FAM50A	0.87711149	0.980521483	0.858032788	0.856471902	0.906893576	0.844759721
PPME1	0.87711149	0.827590337	0.966199203	0.839245804	0.926972948	0.864243158

PTMA	0.87711149	0.918854612	0.87118003	0.907794002	0.857909058	0.903210685
ARHGEF37	0.877177296	0.891306676	0.854571175	0.840247324	0.989334846	0.836705424
C25H16orf54	0.877177296	0.770652022	0.95322206	0.873782043	0.947299702	0.872239777
LOC100294792	0.877177296	0.782571577	0.855811972	0.85981779	0.915775776	0.981216824
ZNFX1	0.877245785	0.835883963	0.855730513	0.865210554	0.999985752	0.832964812
PSMC2	0.877245785	0.753090681	0.955221146	0.852170016	0.854289643	0.970105102
CCL16	0.877303118	0.772248352	0.853497226	0.999981514	0.914322944	0.83150957
RNASEH1	0.877303118	0.892252816	0.854567571	0.921811299	0.898420043	0.88542704
LOC615454	0.877303118	0.936319037	0.893988897	0.888418302	0.858495696	0.888887063
SH3KBP1	0.877303118	0.741310853	0.97251482	0.854450063	0.926813827	0.908768184
FADS2	0.877303118	0.899490486	0.883216179	0.923768152	0.832486638	0.912894278
LACTB2	0.877303118	0.745791496	0.871285807	0.940436902	0.940870936	0.919124527
NCOA2	0.877303118	0.806975285	0.862879463	0.882057818	0.949128489	0.930777372
TUBB1	0.877303118	0.768359345	0.885896294	0.855232617	0.958797242	0.938013912
C1RL	0.877303118	0.796253817	0.854231171	0.85981779	0.867529283	0.997862553
MAPK14	0.877421323	0.750379179	0.828935179	0.883236823	0.940509808	0.98140846
RNF215	0.877436037	0.781543413	0.878604976	0.999981514	0.895757462	0.81956221
BAZ1B	0.877436037	0.823133291	0.858521535	0.886312303	0.996781978	0.840187224
IQSEC2	0.877436037	0.743260404	0.881778842	0.999981514	0.87329316	0.841312694
PCSK1N	0.877436037	0.902525869	0.824743946	0.903021188	0.958720483	0.855878683
OLFM1	0.877436037	0.778585145	0.888566052	0.999981514	0.848003552	0.874490166
ZDHHC3	0.877436037	0.90154488	0.838909114	0.888687722	0.936921784	0.892171608
VPS26A	0.877436037	0.800916955	0.964661607	0.912777307	0.837915327	0.89721271
LOC101906367	0.877436037	0.802638906	0.841294863	0.934445963	0.901853599	0.940922833
FOXS1	0.877436037	0.779025263	0.870734949	0.870591999	0.953300742	0.946112759
LOC101908154	0.877436037	0.816202069	0.8869541	0.887983383	0.878451408	0.960023723
ARHGEF3	0.87750879	0.823830365	0.990118874	0.848067505	0.911664199	0.841312694
NACAD	0.87750879	0.76533726	0.884222215	0.999981514	0.846464841	0.89438401
FOXO4	0.87750879	0.888002573	0.816697148	0.944351122	0.882252274	0.917930485
MGC126945	0.878024257	0.820171846	0.850899991	0.912229943	0.996781978	0.815545636
BRD4	0.878024257	0.84312966	0.865717285	0.853052441	0.999985752	0.81956221
SPHK1	0.878024257	0.886909552	0.891662311	0.962046395	0.887514169	0.83150957
LOC101904642	0.878024257	0.759857785	0.97773026	0.982220679	0.832119756	0.835231445
LOC112443139	0.878024257	0.741310853	0.822300339	0.899792456	0.999985752	0.860414702
FAM212A	0.878024257	0.846724749	0.819359511	0.962046395	0.941800786	0.862925606
LOC101907916	0.878024257	0.857370953	0.860877703	0.967883482	0.834569503	0.91373422
PPP2R3B	0.878024854	0.794565957	0.957052938	0.870652413	0.943540106	0.860966325
SNX25	0.878186139	0.757251331	0.870127948	0.961898258	0.972806049	0.836111377

LOC101904062	0.878186139	0.750379179	0.927508464	0.892071982	0.924795709	0.925040815
SHC1	0.878345021	0.750379179	0.846965262	0.864696894	0.999985752	0.861779975
LOC112444348	0.878345021	0.796253817	0.849389234	0.97828838	0.863970911	0.930710695
LOC112448368	0.878384248	0.819353713	0.971944422	0.839725231	0.961465747	0.817529182
RAPGEF6	0.878384248	0.797999637	0.899106435	0.833973634	0.999985752	0.81956221
TOPBP1	0.878384248	0.792661811	0.836719427	0.849313593	0.999985752	0.839906041
HTR6	0.878384248	0.756473821	0.879939804	0.874023052	0.999985752	0.839906041
SARS2	0.878384248	0.750466772	0.908237602	0.955466436	0.956884203	0.844759721
ZDHHC17	0.878384248	0.916448625	0.826785415	0.898981048	0.954403133	0.856833445
XRN1	0.878384248	0.837244723	0.849389234	0.85647796	0.996781978	0.860966325
CPT1C	0.878384248	0.804185018	0.894338686	0.965050971	0.877712931	0.89148147
PSMC3	0.878384248	0.865101561	0.942134405	0.862273791	0.887514169	0.899557238
WDR55	0.878384248	0.7889129	0.916323169	0.843515965	0.958654817	0.91373422
LOC112445939	0.878384248	0.7973731	0.817582037	0.852170016	0.982796995	0.938013912
POU3F1	0.878384248	0.855752352	0.879939804	0.892047793	0.86930615	0.950540248
LOC101905403	0.878384248	0.768359345	0.940843312	0.882057818	0.865075514	0.960023723
SLC35C1	0.878384248	0.796253817	0.865450223	0.856195359	0.878269439	0.99442139
TMEM185A	0.878384248	0.745846221	0.871285807	0.85498137	0.891726429	0.997603451
NKPD1	0.878731448	0.863286109	0.999993442	0.865517249	0.853985138	0.81956221
VSIG10L	0.878731448	0.782034776	0.998096104	0.867235478	0.916605572	0.833150766
TAGLN2	0.878731448	0.824188473	0.834683675	0.953968239	0.970988226	0.835231445
BRCA2	0.878731448	0.741310853	0.945995021	0.940436902	0.893935775	0.892042538
PLA2G12A	0.878731448	0.839128192	0.927508464	0.887877413	0.856297415	0.932688327
LOC615514	0.878758135	0.804074698	0.863546668	0.995434268	0.887514169	0.865767172
LOC101907613	0.878900947	0.928835826	0.831933739	0.89684883	0.872096228	0.927520621
TCF20	0.878938207	0.764456895	0.966700703	0.976756543	0.849786155	0.844759721
REEP5	0.878938207	0.83866979	0.835574833	0.999981514	0.872229135	0.8537109
IKBIP	0.879270403	0.918455779	0.851822885	0.899855526	0.935286646	0.86324237
TCEANC2	0.879270403	0.786691027	0.849389234	0.965724803	0.841307824	0.963112102
PTPA	0.879298754	0.855067335	0.916323169	0.892155958	0.911568867	0.876861428
LOC104971852	0.879326458	0.870298381	0.959725886	0.892047793	0.887514169	0.839906041
IL32	0.879547801	0.845125482	0.884148516	0.896116841	0.970988226	0.83821684
GNA12	0.879547801	0.767741317	0.855847948	0.916858019	0.976259416	0.883530991
DZANK1	0.879547801	0.823133291	0.937869586	0.905477428	0.858187492	0.91773106
TRIP4	0.879726642	0.879552187	0.85526474	0.950643928	0.901662258	0.872237727
MOB1A	0.879848497	0.842884359	0.881656689	0.898818046	0.948711141	0.876162019
ARMC9	0.879848497	0.807350682	0.916323169	0.898818046	0.929896768	0.891088733
TNFSF12	0.879848497	0.849513595	0.860551134	0.888058665	0.896589592	0.950631909

PCSK4	0.879848497	0.744774335	0.945995021	0.891473194	0.864524107	0.960023723
LOC101905666	0.879848497	0.741310853	0.8735515	0.889415593	0.918512595	0.975247881
SPTY2D1	0.879958091	0.741310853	0.870439924	0.999981514	0.854966579	0.835231445
DKK3	0.879958091	0.960185644	0.855268213	0.906428647	0.902258286	0.839906041
BCL2L13	0.879958091	0.910560869	0.845113191	0.925294859	0.940302215	0.846210629
MAP7D1	0.879958091	0.924125044	0.865787276	0.890704007	0.943540106	0.848298915
STK26	0.879958091	0.74501367	0.942357321	0.87522058	0.966121925	0.877048993
DNAJC12	0.879958091	0.879243746	0.93190909	0.852170016	0.901853599	0.89148147
JADE2	0.879958091	0.829067393	0.881778842	0.871946375	0.963550093	0.892560108
LOC788736	0.879958091	0.779281985	0.845113191	0.999981514	0.877712931	0.897071936
LOC104971814	0.879958091	0.76043092	0.850624788	0.999981514	0.859351739	0.923033364
CBS	0.879958091	0.792690308	0.91882781	0.86458365	0.924222719	0.93531275
COMMD8	0.879958091	0.753090681	0.885896294	0.949502614	0.871558258	0.958281344
FXYD1	0.879978383	0.872175143	0.955427429	0.892047793	0.858495696	0.87501921
CLEC6A	0.879996289	0.821674572	0.997400824	0.853058361	0.895067787	0.839565788
STAT6	0.879997603	0.880565292	0.866956563	0.853058361	0.976259416	0.86324237
UBXN7	0.880005037	0.802570554	0.916294854	0.89684883	0.972128973	0.836111377
DIP2C	0.880005037	0.98098403	0.89385865	0.868378265	0.858741373	0.860414702
H1FX	0.880013314	0.796253817	0.879939804	0.996054502	0.87329316	0.872237727
LOC112442392	0.880116421	0.760778257	0.975774524	0.887983383	0.870450746	0.923199665
C16H1orf21	0.880241673	0.750379179	0.916323169	0.882057818	0.910716004	0.958790968
LRRC71	0.880302864	0.856560491	0.823731372	0.908467146	0.940531552	0.918869728
LRRC10B	0.880375492	0.813669639	0.894338686	0.868255052	0.998008754	0.835231445
DHX34	0.880762989	0.885449385	0.834683675	0.942955143	0.943055455	0.85519889
C24H18orf25	0.881111645	0.825171788	0.917155067	0.922002553	0.909379774	0.872238439
ZBTB47	0.881111645	0.779216815	0.897322115	0.868378265	0.988049878	0.872238439
CTNNBIP1	0.881111645	0.813240774	0.841294863	0.999981514	0.87329316	0.872239777
EARS2	0.881111645	0.846798773	0.852916849	0.916858019	0.887514169	0.943972369
NUDT7	0.881111645	0.782783231	0.834683675	0.848592907	0.872745471	0.999948228
HNRNPDL	0.881233828	0.741310853	0.828035534	0.999981514	0.887514169	0.81956221
KRTCAP2	0.881233828	0.741263262	0.999993442	0.865076519	0.926972948	0.81956221
RAD21	0.881233828	0.781543413	0.916294854	0.892047793	0.976259416	0.856640874
TBCB	0.881233828	0.846215781	0.944126714	0.885789665	0.914280652	0.873130182
EBI3	0.881233828	0.755252885	0.820146472	0.984423083	0.960573822	0.874490166
FOLR2	0.881233828	0.814245795	0.916294854	0.959652624	0.855920132	0.895446935
CHM	0.881233828	0.786691027	0.817582037	0.853058361	0.997165254	0.927520621
FAM131B	0.881233828	0.827824094	0.841294863	0.929663263	0.912617855	0.931410194
HIVEP1	0.881233828	0.782783231	0.93190909	0.894086736	0.85185197	0.960023723

DPP3	0.881233828	0.864668925	0.8735515	0.861645817	0.887514169	0.960877268
DOCK8	0.881233828	0.792767257	0.83472756	0.91285419	0.910070259	0.965117554
LOC107131846	0.881233828	0.840431179	0.881778842	0.868435169	0.877926588	0.969754487
C25H16orf89	0.881456655	0.788544942	0.891972402	0.891988949	0.999985752	0.820395402
NSUN5	0.881752558	0.815362549	0.971053208	0.929342214	0.856136977	0.864572584
FAM160A1	0.882219329	0.954827347	0.924855847	0.886312303	0.87389153	0.836705424
LRRC49	0.882219329	0.912609424	0.85942296	0.914643464	0.933940422	0.85532952
ZMYM2	0.882219329	0.755312035	0.855793459	0.876703811	0.99971266	0.89438401
MCTP2	0.882219329	0.846724749	0.885896294	0.898818046	0.904206501	0.923199665
JUP	0.882219329	0.751996186	0.89478142	0.876703811	0.900893337	0.980167535
LOC112449363	0.882219329	0.768359345	0.819359511	0.891732037	0.87329316	0.999948228
CLIC2	0.882228786	0.773508874	0.891972402	0.935251689	0.86864151	0.960023723
LMOD3	0.8822394	0.750379179	0.999993442	0.896116841	0.893716526	0.83150957
LOC112442652	0.8822394	0.845347956	0.825144372	0.959652624	0.962066414	0.844759721
ALKBH7	0.8822394	0.901028838	0.916294854	0.878291315	0.935737262	0.845747012
AKAP13	0.8822394	0.752344321	0.966293373	0.843613441	0.970988226	0.862925606
HASPIN	0.8822394	0.7583338	0.935266153	0.960016921	0.893376996	0.877014986
LOC101904344	0.8822394	0.869794572	0.947952648	0.888465847	0.878451408	0.882250973
IQCB1	0.8822394	0.898515323	0.834683675	0.8418384	0.963790274	0.911808092
SUV39H2	0.8822394	0.753090681	0.858820898	0.915039335	0.891726429	0.979480605
LAMTOR2	0.882299598	0.869909596	0.893778284	0.869113625	0.961827673	0.867063767
CLDN5	0.882411268	0.861395263	0.955427429	0.849822298	0.906803277	0.8826027
CERS2	0.882620244	0.781836924	0.97773026	0.918693944	0.83683285	0.904762432
RIBC1	0.882633217	0.805580108	0.82022448	0.965050971	0.870450746	0.960023723
ALAD	0.882734534	0.838308162	0.948637575	0.863288407	0.887514169	0.917930485
PRKAB2	0.882762963	0.816412341	0.884121008	0.980632768	0.846285074	0.913221224
LOC786303	0.882903083	0.764456895	0.895801611	0.855651954	0.999985752	0.82400432
CENPT	0.882961851	0.759245424	0.947651663	0.890704007	0.964301327	0.860414702
PHLPP1	0.883004829	0.802840415	0.831933739	0.952247001	0.943702351	0.911219114
B4GALT3	0.883099565	0.764607241	0.819359511	0.866566721	0.935286646	0.996335668
LRRC8A	0.883122356	0.981952108	0.817582037	0.887877413	0.943540106	0.829983945
TNFRSF12A	0.883122356	0.822956796	0.834683675	0.892071982	0.991481886	0.871804449
NTM	0.883235606	0.767741317	0.849389234	0.950643928	0.847092933	0.984046914
DNAJC8	0.8832822	0.7973731	0.999993442	0.853058361	0.845802744	0.845747012
TSKU	0.8832822	0.879088966	0.832574011	0.925294859	0.961465747	0.855471132
DKK1	0.8832822	0.91537041	0.850624788	0.892047793	0.887514169	0.927520621
RNF223	0.8832822	0.843278276	0.832971131	0.866566721	0.837915327	0.999948228
LOC112445925	0.883302728	0.750379179	0.846622323	0.999981514	0.891726429	0.83150957

LPCAT1	0.883302728	0.927701869	0.879608555	0.907794002	0.873957915	0.891195244
EHMT1	0.883302728	0.796253817	0.982232295	0.877132866	0.849786155	0.923033364
PRPSAP2	0.883602053	0.962416786	0.892343042	0.941467375	0.850435498	0.83150957
LOC101902839	0.883602053	0.81848543	0.93475587	0.843593618	0.972301893	0.860414702
LOC101907140	0.883602053	0.83844057	0.969026634	0.929672739	0.833173678	0.867635709
DPH1	0.883602053	0.766943819	0.911973589	0.915039335	0.957793213	0.881452113
COG6	0.883602053	0.951949541	0.8644528	0.840117405	0.929896768	0.884867749
EXOC6B	0.883602053	0.796253817	0.870164776	0.868378265	0.960573822	0.938894901
NPY5R	0.883602053	0.901631302	0.879939804	0.867281023	0.854966579	0.960023723
GPR161	0.883602053	0.796253817	0.843955813	0.95901497	0.848369675	0.970216353
UTP23	0.883635262	0.994405848	0.855971055	0.840247324	0.927943699	0.819391396
HTRA2	0.883635262	0.836217146	0.957246663	0.882057818	0.956041014	0.81956221
MPHOSPH8	0.883635262	0.753090681	0.927635169	0.961898258	0.956041014	0.824780045
TSC2	0.883635262	0.812449121	0.978014482	0.970039043	0.833789832	0.83150957
PIP4K2A	0.883635262	0.760623317	0.916323169	0.886312303	0.996781978	0.844092922
OSBPL1A	0.883635262	0.81232489	0.818951412	0.999981514	0.879776073	0.844759721
LOC100337044	0.883635262	0.753090681	0.999993442	0.854220165	0.89052284	0.844759721
RABGAP1	0.883635262	0.88817075	0.945995021	0.852170016	0.939208227	0.845747012
CWC22	0.883635262	0.887018937	0.908072171	0.843613441	0.965163023	0.853056636
RAD51AP1	0.883635262	0.745860095	0.945995021	0.984423083	0.887514169	0.8537109
MDH1B	0.883635262	0.77194497	0.8644528	0.969837124	0.958797242	0.865767172
SKA3	0.883635262	0.748969774	0.911973589	0.878116099	0.996781978	0.867063767
PPIL2	0.883635262	0.796253817	0.972741989	0.908467146	0.887514169	0.870018677
LOC112444351	0.883635262	0.802638906	0.879939804	0.896116841	0.965163023	0.89148147
ZMAT3	0.883635262	0.766595431	0.831933739	0.9880671	0.92400341	0.914132808
PLEKHH1	0.883635262	0.782783231	0.833583844	0.999981514	0.878729931	0.919124527
PIGV	0.883635262	0.74400458	0.851822885	0.932406746	0.935286646	0.960023723
PLEKHG5	0.883635262	0.861395263	0.908072171	0.861096909	0.864524107	0.960877268
GAPDHS	0.883635262	0.778219881	0.956655464	0.845521561	0.877712931	0.96274079
LOC617692	0.883934064	0.95297524	0.894338686	0.853271157	0.94754975	0.829955257
CD84	0.883934064	0.833429351	0.926161087	0.96444269	0.887514169	0.84413042
SPTBN4	0.884128007	0.851161648	0.842694695	0.916858019	0.971475922	0.860414702
SYP	0.884128007	0.872175143	0.840107596	0.93660503	0.953300742	0.865767172
LOC789764	0.884128007	0.808328971	0.879939804	0.965416137	0.864524107	0.927520621
ADRA1B	0.884128755	0.831153015	0.939981892	0.886312303	0.943160494	0.865767172
DMAC1	0.884128755	0.811059858	0.902649664	0.941935077	0.900927045	0.902100676
PPT1	0.884128755	0.942546802	0.871888627	0.899047543	0.844384115	0.919475967
TMEM252	0.884128755	0.78945719	0.982051025	0.889472032	0.847173934	0.922361909

TRIM62	0.884128755	0.946820717	0.834521849	0.887983383	0.858545936	0.940647189
MCM4	0.884254344	0.811114624	0.867661896	0.921950515	0.956041014	0.893585844
IGSF6	0.884403958	0.788197838	0.92422067	0.886312303	0.953300742	0.903500283
CUL1	0.8844417	0.855067335	0.915858924	0.951101818	0.930451229	0.824025963
PPP6C	0.8844417	0.78945719	0.98191733	0.961898258	0.863694174	0.831652079
LOC100849587	0.8844417	0.916448625	0.872088608	0.856435757	0.974738415	0.841312694
UBA6	0.8844417	0.983816461	0.836482	0.863269783	0.936921784	0.844759721
LOC112444588	0.8844417	0.851864064	0.841294863	0.999981514	0.855740473	0.853029499
CRYBG3	0.8844417	0.773708461	0.883500436	0.886831994	0.999985752	0.858245612
PDE1A	0.8844417	0.878551917	0.89478142	0.965729104	0.872734644	0.858289382
JAK2	0.8844417	0.865455711	0.849704325	0.890362631	0.980695939	0.860966325
FAM213B	0.8844417	0.798327403	0.892343042	0.852481062	0.998338718	0.865889509
LLGL1	0.8844417	0.873718123	0.935824202	0.939604997	0.847092933	0.87501921
LOC100124497	0.8844417	0.929514642	0.916038418	0.865746543	0.894177708	0.87754233
YJU2	0.8844417	0.758609923	0.879939804	0.854450063	0.999985752	0.87754233
LOC112448082	0.8844417	0.751957659	0.858032788	0.999981514	0.887514169	0.896777161
MBTD1	0.8844417	0.930293015	0.83598882	0.899091941	0.892654422	0.917831216
CLCN2	0.8844417	0.790659235	0.946791586	0.896116841	0.847173934	0.953140004
SERTAD4	0.8844417	0.764775882	0.871285807	0.898818046	0.887514169	0.986042926
LOC101905908	0.884553374	0.971413741	0.841294863	0.887983383	0.932024143	0.847193872
DENND6A	0.884560876	0.805283017	0.91882781	0.989601002	0.889301074	0.836111377
ENOX1	0.884560876	0.847255873	0.923353904	0.891988949	0.943702351	0.860966325
WWOX	0.884630576	0.764456895	0.841294863	0.999981514	0.847173934	0.912531667
DCTPP1	0.88483467	0.807350682	0.855268213	0.95901497	0.944839728	0.883688554
LOC104976274	0.885331192	0.758609923	0.972162594	0.980564765	0.864524107	0.845241321
NCOA3	0.885331192	0.781222833	0.909588452	0.990955345	0.847092933	0.903990996
C10H15orf48	0.885331192	0.804185018	0.855847948	0.899792456	0.966967496	0.91348389
IER3	0.885331192	0.758609923	0.855160666	0.995565324	0.858187492	0.940583352
ZNF354A	0.885410862	0.792109226	0.964807927	0.992930508	0.844384115	0.826557348
TLE3	0.885410862	0.749369443	0.842891367	0.92532303	0.999985752	0.8537109
LAPTM4B	0.885410862	0.781543413	0.944126714	0.9880671	0.864524107	0.860966325
ROCK1	0.885410862	0.872346752	0.859328338	0.928510139	0.884872176	0.927520621
RYK	0.885410862	0.758609923	0.955427429	0.855651954	0.89052284	0.960023723
CNOT10	0.885410862	0.883420505	0.838341783	0.889867419	0.856855389	0.9790177
KIAA2012	0.885472383	0.745791496	0.87706952	0.988077819	0.873267294	0.936263602
PSENEN	0.885517657	0.759906124	0.955427429	0.904374668	0.958384396	0.8537109
AURKB	0.885517657	0.97304892	0.829751489	0.893051204	0.871558258	0.909635302
BRCC3	0.885517657	0.750379179	0.867661896	0.876703811	0.935093621	0.98469083

AP1AR	0.885661608	0.786392346	0.911502207	0.999981514	0.885844206	0.849595612
CHST10	0.885661608	0.948801019	0.89478142	0.871946375	0.893716526	0.87501921
GIPR	0.885661608	0.750379179	0.908072171	0.952247001	0.935286646	0.898470276
MFN1	0.885661608	0.792690308	0.89385865	0.862286925	0.982796995	0.899183258
RCBTB2	0.885661608	0.817580794	0.843955813	0.868378265	0.947299702	0.965117554
TRIM56	0.885661608	0.757251331	0.8735515	0.938303773	0.887514169	0.96870908
LOC104974269	0.885661608	0.745791496	0.866360056	0.908467146	0.858545936	0.99828741
SPTLC3	0.885727525	0.778219881	0.865697455	0.888687722	0.940870936	0.966212838
MUL1	0.885934854	0.779407705	0.935266153	0.96951401	0.895757462	0.864572584
ULK2	0.885934854	0.817524348	0.849943652	0.893051204	0.917407853	0.966779561
LAGE3	0.885948759	0.786442687	0.944126714	0.922931949	0.955076828	0.840582689
ZNF366	0.886039457	0.99960614	0.85942296	0.852170016	0.900927045	0.820920598
ST8SIA5	0.886039457	0.927853707	0.883950091	0.984548238	0.852234727	0.83150957
LOC783641	0.886039457	0.833429351	0.886829776	0.906260102	0.983010476	0.832770678
HIPK2	0.886039457	0.822956796	0.831933739	0.999981514	0.875687454	0.833718346
SNRNP27	0.886039457	0.757251331	0.855002692	0.896116841	0.999985752	0.838729965
LOC112442619	0.886039457	0.758609923	0.999993442	0.870210705	0.887514169	0.839906041
LOC101903992	0.886039457	0.802638906	0.836719427	0.959652624	0.982087479	0.842214812
CCDC71L	0.886039457	0.943754915	0.958197827	0.852170016	0.88333383	0.842473495
LOC112446375	0.886039457	0.855319025	0.999993442	0.87522058	0.84002678	0.846210629
F2RL1	0.886039457	0.750379179	0.931080531	0.894086736	0.988010662	0.847193872
LOC107131675	0.886039457	0.942546802	0.951392011	0.850825191	0.887514169	0.848282393
TRAF7	0.886039457	0.865570309	0.922552585	0.882057818	0.958797242	0.849595612
LOC112441645	0.886039457	0.840431179	0.913100432	0.874424802	0.972160227	0.859032412
NISCH	0.886039457	0.833713501	0.879939804	0.936586091	0.953300742	0.864572584
LOC615663	0.886039457	0.850514112	0.961437412	0.937726848	0.846232586	0.865971618
ADIPOR2	0.886039457	0.885494685	0.830104583	0.999981514	0.847092933	0.874870038
LOC107132852	0.886039457	0.816369086	0.858032788	0.975351263	0.926972948	0.87754233
LOC107131643	0.886039457	0.81848543	0.951229848	0.952247001	0.851102673	0.882250973
LOC112443170	0.886039457	0.761907799	0.976484937	0.942955143	0.867558388	0.88313584
VTA1	0.886039457	0.842884359	0.937869586	0.870591999	0.938731934	0.88313584
SGO2	0.886039457	0.855310926	0.918641217	0.907794002	0.900511685	0.888683725
LOC101902469	0.886039457	0.872175143	0.860462374	0.964521432	0.878451408	0.899428912
CETN2	0.886039457	0.916448625	0.924855847	0.879425058	0.865075514	0.909056553
CSRNP3	0.886039457	0.80442913	0.913100432	0.887983383	0.953300742	0.911781676
LOC101906018	0.886039457	0.807285501	0.910845937	0.952247001	0.868473464	0.926341424
LOC104976247	0.886039457	0.835486075	0.93190909	0.87522058	0.887514169	0.935770262
PCNP	0.886039457	0.748099858	0.916294854	0.899047543	0.917407853	0.952571698

TTC37	0.886039457	0.749199931	0.871491704	0.882057818	0.964301327	0.959198304
TNFAIP2	0.886039457	0.822484848	0.868360233	0.941935077	0.859351739	0.960023723
PFKP	0.886039457	0.764456895	0.893988897	0.928404714	0.870450746	0.972695131
FBXO40	0.886039457	0.750379179	0.836719427	0.852170016	0.850582836	0.999948228
NEK6	0.88611318	0.856560491	0.944126714	0.868255052	0.887514169	0.923064357
SHANK3	0.886168153	0.83844057	0.917155067	0.91673213	0.950897336	0.844759721
RPN2	0.88619862	0.790429889	0.915858924	0.999981514	0.846464841	0.835231445
LOC515570	0.88625462	0.768359345	0.853546772	0.940436902	0.852026472	0.993890045
CHPF2	0.886427418	0.767113995	0.898179681	0.931361128	0.858716235	0.971009015
CSNK2A1	0.886500095	0.799590997	0.980761647	0.888198716	0.846745071	0.926480924
RMDN1	0.886597227	0.757917107	0.912396874	0.886312303	0.989442936	0.874490166
CCNT1	0.886778313	0.927163124	0.894338686	0.8986831	0.946454221	0.831176431
SMIM14	0.886778313	0.796666816	0.916323169	0.921950515	0.864524107	0.950631909
FXR2	0.886795385	0.997678999	0.856237958	0.856836633	0.852234727	0.877014986
SCUBE2	0.887052858	0.920978623	0.836719427	0.889415593	0.95721434	0.876861428
ETNPPL	0.887060167	0.953101202	0.870734949	0.910575108	0.884872176	0.872238439
ARMT1	0.887076607	0.753090681	0.999993442	0.854450063	0.863651062	0.883688554
JADE3	0.887171843	0.852243254	0.894316021	0.932247787	0.960573822	0.833065678
GLI1	0.887171843	0.941676269	0.834683675	0.952247001	0.913732532	0.842214812
DHX37	0.887171843	0.977196455	0.849804464	0.888198716	0.892446951	0.876162019
NAGA	0.887171843	0.79181577	0.833583844	0.867281023	0.911568867	0.99828741
AGMAT	0.887544125	0.749236881	0.943836178	0.999981514	0.892654422	0.83150957
CCDC183	0.887544125	0.796253817	0.946791586	0.970039043	0.891726429	0.847020266
ABHD3	0.887544125	0.764775882	0.903431343	0.999981514	0.847173934	0.852112206
LOC789035	0.887544125	0.764456895	0.918351026	0.896116841	0.983941532	0.860414702
WIF1	0.887544125	0.793300166	0.858032788	0.978559648	0.954071324	0.864197513
MGC137036	0.887544125	0.896067917	0.8644528	0.979217817	0.87329316	0.867063767
TTC7B	0.887544125	0.997471998	0.8267276	0.874170259	0.884679971	0.867537626
BTC	0.887544125	0.919245978	0.893988897	0.930164453	0.880672837	0.874000314
LOC789352	0.887544125	0.759391821	0.957052938	0.959652624	0.887514169	0.877014986
LOC100141185	0.887544125	0.764775882	0.880163755	0.963601374	0.954403133	0.877048993
HEYL	0.887544125	0.916484772	0.95875611	0.877132866	0.848550619	0.880139883
CLDN1	0.887544125	0.826153046	0.906019013	0.868435169	0.964301327	0.89438401
DANCR	0.887544125	0.779407705	0.929661805	0.914850247	0.934067848	0.902238606
NEURL2	0.887544125	0.796253817	0.874063521	0.967883482	0.872734644	0.940583352
MACO1	0.887544125	0.781543413	0.957052938	0.915779841	0.838788415	0.944927301
ZNF41	0.887544125	0.818303808	0.861481426	0.965729104	0.854966579	0.950631909
INTS10	0.887544125	0.797999637	0.883906525	0.889416444	0.935125041	0.954550352

ADAMTSL5	0.887544125	0.822613497	0.880357899	0.94222181	0.844566722	0.963456717
ITGAV	0.887544125	0.762274611	0.833334792	0.88990469	0.938879379	0.988639786
FAM196A	0.887783166	0.829067393	0.978014482	0.852170016	0.953300742	0.842259463
MAP1B	0.887856118	0.872244611	0.8392324	0.999981514	0.858495696	0.841312694
LOC107133166	0.887856118	0.750379179	0.846960897	0.999981514	0.900927045	0.847193872
FBLN1	0.887937016	0.7973731	0.97773026	0.878513543	0.946935016	0.852048593
GSTA1	0.887937016	0.75769517	0.899651559	0.912777307	0.959658682	0.91373422
FBXO45	0.887947038	0.832902156	0.924855847	0.980632768	0.891726429	0.833500833
SF3B6	0.887947038	0.766943819	0.999993442	0.940946275	0.854966579	0.839906041
ITGA11	0.888208708	0.893680105	0.855847948	0.876703811	0.970980375	0.876861428
RELB	0.888231209	0.855310926	0.845297459	0.99542943	0.929802526	0.838983503
IPO11	0.888231209	0.889457989	0.988390948	0.852170016	0.900893337	0.839565788
TMOD3	0.888231209	0.762081499	0.999993442	0.880566335	0.917407853	0.844759721
TGIF1	0.888231209	0.876831363	0.911973589	0.874662174	0.959443572	0.865889509
PIH1D2	0.888231209	0.875621295	0.955189235	0.919601062	0.854966579	0.873130182
LOC112441616	0.888231209	0.927103901	0.83252813	0.976005921	0.865505927	0.877750901
C20H5orf51	0.888231209	0.753090681	0.874063521	0.929663263	0.962513981	0.922361909
RPL7L1	0.888231209	0.753287083	0.958410013	0.930164453	0.874462038	0.924333
FMN1	0.888449901	0.940500178	0.84404544	0.886312303	0.977167046	0.826963407
ARHGEF40	0.888449901	0.818303808	0.834683675	0.995152316	0.936108073	0.864139695
TMEM192	0.888449901	0.849513595	0.894316021	0.948668643	0.893716526	0.895446935
YEATS2	0.888516613	0.762563464	0.849389234	0.868378265	0.987723553	0.947801358
KLF6	0.888630437	0.766911473	0.881221246	0.999981514	0.954403133	0.8255975
PPP2R5C	0.888630437	0.77139884	0.971053208	0.982815793	0.884872176	0.83150957
STAT4	0.888630437	0.767113995	0.89478142	0.976069331	0.970118548	0.83150957
SCARF2	0.888630437	0.804185018	0.838840947	0.999981514	0.877578545	0.832964812
SRGAP2	0.888630437	0.882102283	0.879939804	0.992930508	0.886944613	0.835231445
RHOT2	0.888630437	0.950126059	0.963821408	0.866566721	0.872096228	0.838983503
ABHD13	0.888630437	0.846721805	0.951028911	0.948668643	0.893716526	0.839565788
IRF9	0.888630437	0.804973066	0.997300234	0.850496025	0.943055455	0.839906041
SON	0.888630437	0.809693907	0.845113191	0.886312303	0.999985752	0.839906041
NAGS	0.888630437	0.952760839	0.868360233	0.955466436	0.87329316	0.845747012
LOC107131311	0.888630437	0.948822318	0.87118003	0.914026346	0.917617128	0.845747012
NOL8	0.888630437	0.802570554	0.89478142	0.986051493	0.927086179	0.849885981
FCGR3A	0.888630437	0.813904552	0.968317646	0.928404714	0.895757462	0.8537109
NCKAP1L	0.888630437	0.866767436	0.941230744	0.891988949	0.938879379	0.8537109
TCOF1	0.888630437	0.77844414	0.981332474	0.958103924	0.871906908	0.85643253
MILR1	0.888630437	0.796253817	0.960922789	0.853058361	0.962843288	0.872239777

DNAJB2	0.888630437	0.805283017	0.927635169	0.952247001	0.903888186	0.876162019
C21H14orf132	0.888630437	0.780799374	0.999993442	0.888687722	0.847173934	0.881875045
LOC104971021	0.888630437	0.904875256	0.951392011	0.868435169	0.870450746	0.898470276
SCRN1	0.888630437	0.883420505	0.940632769	0.89684883	0.847092933	0.919124527
LOC101904701	0.888630437	0.768359345	0.898179681	0.984548238	0.858187492	0.928976746
LOC112449615	0.888630437	0.796253817	0.849389234	0.999981514	0.8568751	0.930146713
ITGB3	0.888630437	0.912602201	0.895428817	0.89684883	0.854966579	0.930710695
HEMK1	0.888630437	0.813994669	0.916323169	0.898890982	0.906766758	0.930946379
POGZ	0.888630437	0.867518836	0.856082558	0.906186004	0.855920132	0.973649216
PSMD1	0.888630437	0.750379179	0.916323169	0.916858019	0.85185197	0.98140846
LOC112447811	0.888699398	0.884272556	0.982195333	0.935282232	0.844434232	0.828736538
MFAP2	0.888699398	0.846724749	0.942357321	0.972142185	0.871558258	0.844759721
LOC101902991	0.888699398	0.750466772	0.8735515	0.999981514	0.887514169	0.876861428
TMEM198	0.888699398	0.901642841	0.881070741	0.972142185	0.852234727	0.87754233
ATP2A3	0.888699398	0.750379179	0.994887288	0.90554873	0.883951804	0.891088733
LTB4R2	0.888829709	0.910560869	0.891972402	0.908467146	0.932024143	0.862925606
KIAA0586	0.888829709	0.913116251	0.939983859	0.899091941	0.882252274	0.866280163
SLC6A4	0.888829709	0.779343136	0.845113191	0.912229943	0.996781978	0.890967932
ENO1	0.888829709	0.786819777	0.858032788	0.884819185	0.900927045	0.996310803
UCHL1	0.888940689	0.769009241	0.836691254	0.884482389	0.999453276	0.923092436
HEY2	0.888940689	0.788230732	0.894671468	0.893220599	0.948711141	0.937715964
ITGB1BP2	0.888946394	0.823647199	0.8644528	0.9063051	0.95594186	0.922539606
FN3K	0.888947387	0.759629362	0.899106435	0.922967576	0.970988226	0.885207772
MEX3A	0.88897012	0.7973731	0.846906704	0.995565324	0.917407853	0.888930405
CEP76	0.88897012	0.757078435	0.867222363	0.950643928	0.958797242	0.917930485
NAA30	0.889075355	0.772077493	0.910979747	0.916858019	0.86930615	0.973413452
KHNYN	0.889227244	0.855079912	0.912396874	0.908124518	0.891726429	0.921557022
TLR2	0.890066195	0.832105548	0.855104573	0.934740386	0.942786245	0.917930485
HAUS2	0.890234233	0.786817529	0.999993442	0.852365348	0.863082535	0.863020449
LOC112444616	0.890234233	0.818933054	0.867222363	0.9063051	0.976292433	0.883688554
SDHB	0.890234233	0.7889129	0.908072171	0.92386951	0.932024143	0.923092436
GPSM2	0.890397429	0.999987083	0.849389234	0.87522058	0.845802744	0.835231445
OMD	0.890397429	0.793678771	0.862253703	0.937277106	0.852234727	0.987843572
NANS	0.890936719	0.839128192	0.89478142	0.98981682	0.855939488	0.880622346
LOC112444775	0.891364414	0.792109226	0.856082558	0.941467375	0.887514169	0.972695131
JAG2	0.891485256	0.789957129	0.830104583	0.999981514	0.958797242	0.83150957
LRMDA	0.891485256	0.989303356	0.836241961	0.952247001	0.858545936	0.83821684
PUS7L	0.891485256	0.855067335	0.935266153	0.892071982	0.926813827	0.885202945

ACVR2A	0.891485256	0.875144563	0.867222363	0.979644238	0.87329316	0.891088733
ABCB4	0.891485256	0.807919676	0.830135741	0.887983383	0.973219333	0.947937638
CUL5	0.891485256	0.787731652	0.943836178	0.907621547	0.871558258	0.952663521
TMEM106C	0.891485256	0.922610146	0.852916849	0.869703378	0.872096228	0.969014913
PROX2	0.891746239	0.961663204	0.830135741	0.876703811	0.902258286	0.923944552
SH2B3	0.891802985	0.790879122	0.879306811	0.911591687	0.998287238	0.849731479
FAM184B	0.891856239	0.968509005	0.955427429	0.852170016	0.865075514	0.850571158
FLI1	0.892113491	0.814177827	0.845113191	0.913828818	0.999985752	0.83821684
IGFBP3	0.892113491	0.813759037	0.983504244	0.944563237	0.884872176	0.838983503
FAM20B	0.892113491	0.767741317	0.917117076	0.96951401	0.938316699	0.865889509
RCN2	0.892113491	0.783896922	0.858934339	0.96900924	0.879495058	0.960023723
C3H1orf210	0.892185095	0.943213236	0.867222363	0.888687722	0.935286646	0.876162019
LIMS2	0.892202461	0.835452905	0.89478142	0.892071982	0.891726429	0.960023723
KCNJ10	0.892614986	0.883756544	0.906019013	0.934466438	0.918426817	0.862925606
EXOC5	0.892682929	0.788980101	0.844338127	0.892047793	0.999985752	0.855349987
RUNX2	0.892682929	0.807285501	0.873438881	0.999180255	0.864521109	0.912531667
MYBL1	0.892682929	0.866862152	0.896821247	0.907993229	0.904643542	0.919124527
PHTF2	0.892715955	0.759391821	0.966232793	0.886386107	0.977167046	0.841312694
CHRNA3	0.892855948	0.894014677	0.990118874	0.904018489	0.855428064	0.835231445
CCNL2	0.892855948	0.775033799	0.958454497	0.960016921	0.855740473	0.909056553
COL4A5	0.892953964	0.99960614	0.831933739	0.888687722	0.879268517	0.846210629
PLAG1	0.892953964	0.895777642	0.893988897	0.880401606	0.943160494	0.898436105
TRPA1	0.893129296	0.994185904	0.841294863	0.893220599	0.860495437	0.877014986
RPS23	0.893553335	0.8332127	0.86347788	0.999981514	0.891304411	0.856833445
LOC101906410	0.893679501	0.797356381	0.834683675	0.999981514	0.937253749	0.852504043
KIAA0753	0.893679501	0.860553498	0.951028911	0.91294441	0.906766758	0.860966325
LOC101908339	0.893679501	0.846724749	0.896821247	0.974717682	0.892962265	0.870640897
SELENOM	0.893760726	0.790702675	0.854231171	0.999981514	0.900747635	0.839565788
KREMEN1	0.893760726	0.808510536	0.853176216	0.984423083	0.870083369	0.944973383
CLEC2B	0.893792577	0.833429351	0.883032292	0.999981514	0.847173934	0.888475311
LOC614226	0.894030732	0.823133291	0.846960897	0.995312373	0.935286646	0.864243158
SARDH	0.894030732	0.889457989	0.929661805	0.880401606	0.885217562	0.923092436
TMEM63A	0.894120613	0.757014314	0.965819476	0.896116841	0.976259416	0.839565788
TRPV1	0.894206473	0.821674572	0.865697455	0.932677501	0.916353643	0.944927301
NBR1	0.894365826	0.792483444	0.916294854	0.999981514	0.858488348	0.855963573
CRTAM	0.894365826	0.849119707	0.99341711	0.893051204	0.85317858	0.876162019
ISLR2	0.894365826	0.87078416	0.884121008	0.991606593	0.854289643	0.876861428
ATP8B1	0.894365826	0.835486075	0.943836178	0.85708308	0.958797242	0.883688554

CFAP20	0.894436111	0.7889129	0.998899782	0.9063051	0.909379774	0.835231445
OSTC	0.894436111	0.867120886	0.916323169	0.96951401	0.871558258	0.867482845
LDLRAD4	0.894436111	0.837945129	0.851633941	0.853058361	0.999985752	0.877014986
LOC101906001	0.894440732	0.820534347	0.944949386	0.91761928	0.956761712	0.839906041
LOC112444846	0.894440732	0.852335319	0.870965946	0.999981514	0.868722067	0.858415238
LOC101906656	0.894440732	0.923007853	0.891035201	0.908467146	0.926813827	0.867063767
BCORL1	0.894440732	0.767570093	0.894338686	0.976196758	0.952796169	0.872239777
PDGFB	0.894440732	0.820902954	0.884148516	0.882057818	0.984649787	0.891088733
SRSF12	0.894440732	0.901631302	0.860531176	0.94714048	0.902125794	0.895803603
EHHADH	0.894440732	0.814937616	0.867222363	0.87522058	0.977158496	0.925995537
SP140	0.894518054	0.898515323	0.982195333	0.874023052	0.87329316	0.865862969
RHBDL3	0.894518054	0.768359345	0.997300234	0.875836286	0.932024143	0.867073248
COL25A1	0.894518054	0.855752352	0.900102512	0.891916807	0.887514169	0.958217292
GOLM1	0.894671075	0.807623624	0.895801611	0.991955963	0.943610727	0.83150957
LOC101902084	0.894671075	0.981952108	0.865512879	0.944929997	0.878451408	0.835231445
GTF3A	0.894671075	0.926905849	0.889098668	0.980564765	0.872734644	0.838530462
PBRM1	0.894671075	0.99960614	0.858376167	0.866566721	0.893376996	0.838729965
EML1	0.894671075	0.88008538	0.990037865	0.896116841	0.882206878	0.839906041
FANCL	0.894671075	0.79181577	0.927635169	0.965724803	0.943734737	0.840768468
LOC101907322	0.894671075	0.932778537	0.833583844	0.980564765	0.903410824	0.846210629
TSNARE1	0.894671075	0.768359345	0.982195333	0.873302281	0.969470443	0.850138656
BDKRB2	0.894671075	0.960747241	0.91173001	0.892071982	0.893716526	0.855082018
NFU1	0.894671075	0.796253817	0.927635169	0.989601002	0.89052284	0.862925606
ANKRD6	0.894671075	0.924099465	0.849389234	0.972142185	0.887514169	0.874870038
BRK1	0.894671075	0.829067393	0.926234868	0.891988949	0.960573822	0.87501921
MARK4	0.894671075	0.859861862	0.894338686	0.903584224	0.959103409	0.876861428
MRPL49	0.894671075	0.779407705	0.929661805	0.9063051	0.965163023	0.877048993
LOC101902561	0.894671075	0.790837224	0.91882781	0.989484139	0.887514169	0.87754233
KIAA2026	0.894671075	0.890669166	0.875900843	0.887983383	0.961670582	0.885957057
C23H6orf141	0.894671075	0.796253817	0.940413	0.941467375	0.917407853	0.888475311
DCTN5	0.894671075	0.791194847	0.955427429	0.896116841	0.943540106	0.891088733
IGFBP4	0.894671075	0.926330259	0.845297459	0.955466436	0.887514169	0.898470276
AHNAK	0.894671075	0.755089315	0.957052938	0.970665366	0.852746309	0.917150486
ZBTB49	0.894671075	0.809188108	0.951392011	0.919643615	0.882418284	0.919124527
MUT	0.894671075	0.779025263	0.881790227	0.887515247	0.984386856	0.919124527
ATP5F1A	0.894671075	0.773508874	0.881778842	0.886312303	0.987723553	0.920153823
RBBP9	0.894671075	0.796253817	0.950185097	0.891931114	0.916605572	0.923547711
PLPPR1	0.894671075	0.838155432	0.980761647	0.886312303	0.848038632	0.923807852

URM1	0.894671075	0.859204465	0.916569251	0.882057818	0.916605572	0.927253029
C23H6orf132	0.894671075	0.760778257	0.871285807	0.892047793	0.984386856	0.929461379
TMEM220	0.894671075	0.813639295	0.864847127	0.99045711	0.857909058	0.93363377
LOC107132296	0.894671075	0.77139884	0.91416437	0.868435169	0.970988226	0.93363377
FAAP24	0.894671075	0.786392346	0.972940212	0.870591999	0.900893337	0.933897626
LOC509006	0.894671075	0.91078662	0.849389234	0.941935077	0.864524107	0.938894901
FCGR2B	0.894671075	0.951172741	0.8588642	0.896116841	0.854966579	0.940017687
RWDD4	0.894671075	0.813904552	0.894523258	0.889415593	0.943055455	0.944927301
DDX47	0.894671075	0.757078435	0.951028911	0.909765627	0.89052284	0.947801358
LOC112442080	0.894671075	0.787305351	0.843439964	0.995565324	0.872734644	0.948705062
ZNF345	0.894671075	0.938087741	0.830420708	0.867533077	0.904643542	0.958790968
PRIMA1	0.894671075	0.799352476	0.849636922	0.925294859	0.935286646	0.960023723
DNHD1	0.894671075	0.759245424	0.899600099	0.889415593	0.935286646	0.970115855
LSM8	0.894671075	0.77139884	0.894316021	0.882057818	0.927973846	0.980189436
RPL27	0.894671075	0.768359345	0.883737851	0.907794002	0.864524107	0.997206681
ADGRG2	0.894843832	0.786819777	0.856362287	0.882395013	0.999985752	0.835231445
IDH2	0.894843832	0.826316517	0.877334778	0.916858019	0.986861597	0.8537109
KYAT3	0.894843832	0.759397946	0.971053208	0.865210554	0.977167046	0.864572584
EHD3	0.894956089	0.77766577	0.909440935	0.941467375	0.988881329	0.835231445
EBF3	0.895178049	0.872244611	0.910224442	0.979644238	0.859023122	0.875118526
PUS7	0.895178049	0.811354298	0.912252673	0.940436902	0.870061356	0.952571698
THOC5	0.895310625	0.932191409	0.865401218	0.999981514	0.85545716	0.835231445
PDS5A	0.895310625	0.770190457	0.944309407	0.879344676	0.992620733	0.857673662
LOC101902531	0.895315838	0.926852499	0.856466821	0.984423083	0.893716526	0.839906041
LOC101904378	0.895423173	0.998847958	0.838392315	0.892047793	0.887514169	0.847193872
SEPHS1	0.895423173	0.775289791	0.871313178	0.999981514	0.929802526	0.873130182
BECN1	0.895423173	0.760778257	0.895314389	0.938094344	0.891726429	0.967759699
NRIP3	0.895461675	0.929409572	0.880163755	0.893051204	0.965676623	0.835231445
PIK3C2B	0.895461675	0.792109226	0.879939804	0.999981514	0.936215792	0.840531072
ZBED8	0.895461675	0.844170799	0.966733809	0.95901497	0.873488171	0.842259463
UBE3C	0.895461675	0.786255694	0.855268213	0.98981682	0.969746326	0.849885981
LOC512464	0.895461675	0.828365124	0.922552585	0.994395954	0.870042072	0.860966325
AXIN2	0.895461675	0.955002097	0.930759114	0.870210705	0.891726429	0.866494771
NRXN2	0.895461675	0.885585919	0.881778842	0.990955345	0.865076851	0.867482845
ANK1	0.895461675	0.930411132	0.832268748	0.973832508	0.879776073	0.88542704
QDPR	0.895461675	0.883756544	0.881070741	0.867683265	0.971133034	0.890967932
RAB8B	0.895461675	0.774237994	0.917155067	0.890362631	0.970980375	0.91373422
ZNF672	0.895461675	0.861395263	0.901955007	0.941467375	0.887514169	0.916193353

NR1H2	0.895461675	0.7973731	0.851633941	0.999981514	0.850461911	0.921843102
PALD1	0.895461675	0.846724749	0.912183977	0.876703811	0.938316699	0.930206677
HDAC3	0.895509031	0.882858125	0.860212681	0.984423083	0.87329316	0.898021919
KRBA1	0.895832138	0.828516628	0.899106435	0.98061629	0.89992403	0.876162019
LOC107131225	0.895895466	0.998847958	0.899106435	0.869113625	0.852959789	0.847020266
RAB26	0.895926103	0.85988793	0.899600099	0.967883482	0.943160494	0.831652079
TNFAIP8L2	0.895926103	0.846724749	0.895826828	0.860309157	0.98865576	0.874557852
HACE1	0.896010324	0.810010174	0.852225514	0.890362631	0.917407853	0.988635999
USP7	0.89601125	0.773411104	0.881070741	0.998472013	0.956906331	0.841312694
LOC101903564	0.89601125	0.77844414	0.979494108	0.972467586	0.879656633	0.853029499
ZBTB18	0.89601125	0.814245795	0.924898651	0.959616431	0.937932469	0.8537109
LOC787257	0.89601125	0.786420102	0.901955007	0.959616431	0.964301327	0.860966325
TAF1D	0.89601125	0.767741317	0.983504244	0.908467146	0.926831826	0.875991349
STX5	0.89601125	0.791281103	0.924855847	0.955466436	0.92549567	0.891088733
KMT2A	0.89601125	0.790249697	0.89478142	0.910495312	0.970988226	0.90182838
CETN3	0.89601125	0.983983727	0.836459233	0.902137829	0.854289643	0.918629092
LOC107132225	0.89601125	0.816202069	0.916038418	0.978503042	0.85545716	0.919124527
NUDT21	0.89601125	0.782783231	0.885896294	0.883236823	0.980695939	0.927520621
ZC3H3	0.89601125	0.791281103	0.93891936	0.932914414	0.870450746	0.945480515
WARS	0.896279332	0.980833065	0.849218971	0.862506252	0.950958352	0.865889509
PRR14	0.896341193	0.775033799	0.997400824	0.950160436	0.8568751	0.86324237
RHBDD3	0.896341193	0.954187594	0.841294863	0.882057818	0.964301327	0.865745718
FJX1	0.896341193	0.767095476	0.894316021	0.999981514	0.879361555	0.865767172
COTL1	0.896415978	0.816540275	0.958888096	0.9063051	0.95978143	0.836705424
RBM27	0.896415978	0.763758778	0.999993442	0.870591999	0.918512595	0.842473495
LOC101904574	0.896415978	0.816202069	0.927084242	0.897601295	0.964768333	0.874459658
TLNRD1	0.896429218	0.768359345	0.915858924	0.915039335	0.902907077	0.963456717
EOGT	0.896439293	0.910560869	0.937993336	0.918131532	0.898833204	0.8537109
KDM5B	0.896558167	0.790885918	0.855847948	0.965050971	0.976292433	0.867063767
F12	0.896558167	0.779025263	0.926161087	0.961898258	0.900927045	0.91373422
DDX55	0.896653903	0.798290851	0.979584814	0.892047793	0.911930323	0.891423889
LOC112443437	0.896693009	0.775262502	0.898179681	0.887983383	0.992245178	0.899183258
WFIKKN1	0.896693009	0.855444194	0.912187636	0.944949439	0.887514169	0.91373422
PDGFC	0.896795738	0.807919676	0.999993442	0.89684883	0.888197631	0.844759721
COL8A2	0.896795738	0.792109226	0.875900843	0.999981514	0.895404843	0.844759721
TMEM158	0.896795738	0.790646828	0.901736458	0.953949097	0.964301327	0.865767172
PROCA1	0.896795738	0.867277242	0.838940013	0.951193444	0.954814306	0.889662488
DPP9	0.896795738	0.845942715	0.895826828	0.962437357	0.854966579	0.931410194

FAM180B	0.896795738	0.867579696	0.853546772	0.886312303	0.900891209	0.980167535
TBX18	0.896858474	0.826316517	0.945471374	0.986337023	0.883951804	0.844759721
SQLE	0.896858474	0.811980171	0.939237164	0.985701713	0.887514169	0.860966325
CIB1	0.897069731	0.941053277	0.921266625	0.929663263	0.89992403	0.831652079
TNIP1	0.897239837	0.884804337	0.879939804	0.999981514	0.877578545	0.846210629
PDE2A	0.897402036	0.918234392	0.916038418	0.889924182	0.93630105	0.868004108
MRPS22	0.897402036	0.76071489	0.943836178	0.888687722	0.959658682	0.923092436
LOC100296205	0.897402036	0.870334242	0.886064678	0.890704007	0.891726429	0.960877268
LOC100139345	0.897402036	0.768359345	0.927508464	0.910854395	0.893935775	0.96274079
DNASE2	0.897651818	0.889387648	0.876414692	0.949502614	0.879776073	0.923807852
LOC112447103	0.897651818	0.7889129	0.842694695	0.956685862	0.854365297	0.996310803
PALLD	0.897868325	0.869754045	0.860877703	0.999981514	0.886944613	0.850475013
FGL1	0.897868325	0.907054337	0.867801577	0.988408846	0.891726429	0.852112206
SNAPC1	0.897868325	0.953841555	0.89478142	0.911874416	0.902907077	0.85643253
ZFP90	0.897868325	0.808478344	0.851822885	0.907794002	0.999985752	0.867063767
PHLDB3	0.897868325	0.790722217	0.874983674	0.999981514	0.858545936	0.877014986
BAHD1	0.897868325	0.863613561	0.955221146	0.896116841	0.89052284	0.903990996
LOC107131941	0.897868325	0.912693158	0.950223118	0.869068788	0.871558258	0.919124527
ARF4	0.897868325	0.771162191	0.996224523	0.890362631	0.871558258	0.925553067
LOC112445150	0.897868325	0.816912013	0.849389234	0.999981514	0.854289643	0.930710695
ELL3	0.897868325	0.856606693	0.903431343	0.916858019	0.894364008	0.93129461
PCDHGA8	0.897868325	0.850640641	0.871285807	0.886312303	0.956906331	0.938013912
WRN	0.897868325	0.962037123	0.853546772	0.887420349	0.870042072	0.944973383
PEX10	0.897868325	0.759629362	0.943836178	0.915013589	0.860495437	0.973649216
PLEKHO1	0.897894115	0.879093989	0.892343042	0.882057818	0.983941532	0.858507498
UFC1	0.897894115	0.799665594	0.95322206	0.889415593	0.943734737	0.90645885
POLR2B	0.898009961	0.870334242	0.895801611	0.910575108	0.976259416	0.840271658
AQP1	0.898009961	0.834795173	0.891555464	0.890362631	0.999453276	0.846210629
LYSMD1	0.898009961	0.791281103	0.991447919	0.926970666	0.894825378	0.865695113
ADORA3	0.898009961	0.901275501	0.896821247	0.910575108	0.892360667	0.917930485
ATXN7L3	0.898009961	0.796748952	0.893988897	0.932764647	0.891726429	0.963456717
LOC112445177	0.898141516	0.842884359	0.871036575	0.931203167	0.987685932	0.845747012
METTL16	0.898141516	0.805632148	0.894523258	0.934878139	0.979290191	0.858577001
INPP5K	0.898141516	0.969051025	0.885896294	0.888687722	0.895404843	0.881875045
LOC101907348	0.898302077	0.820155944	0.894338686	0.986051493	0.918424492	0.872239777
SYNJ1	0.898302077	0.8013363	0.997400824	0.9063051	0.879776073	0.873130182
EXTL2	0.898302077	0.895777642	0.894338686	0.950643928	0.894364008	0.884382952
PFDN1	0.898302077	0.78766817	0.94422142	0.889415593	0.947766231	0.923199665

MAP4K4	0.898453526	0.792661811	0.841294863	0.896116841	0.999985752	0.836705424
LOC100848443	0.898453526	0.941676269	0.851822885	0.921950515	0.960573822	0.839906041
ROCK2	0.898453526	0.855141737	0.908072171	0.916858019	0.972432486	0.841451122
ZNF35	0.898453526	0.777309385	0.899106435	0.988637603	0.958797242	0.845747012
GAS2	0.898453526	0.880129816	0.997400824	0.898658215	0.854966579	0.847386898
POFUT2	0.898453526	0.760778257	0.8644528	0.999981514	0.906893576	0.869437527
MN1	0.898453526	0.806326521	0.954020369	0.896116841	0.960048824	0.871998318
PRRX2	0.898453526	0.808713975	0.881778842	0.999981514	0.854966579	0.91373422
UBXN1	0.898453526	0.952863279	0.865717285	0.933718942	0.852746309	0.917930485
C23H6orf136	0.898453526	0.76533726	0.916294854	0.903242359	0.964301327	0.923547711
PAFAH1B3	0.898453526	0.7889129	0.874983674	0.935239391	0.955370715	0.931410194
FAM71F2	0.898453526	0.764456895	0.858032788	0.867281023	0.906893576	0.999948228
CHD4	0.898594823	0.768359345	0.838341783	0.999981514	0.976769047	0.845747012
CMPK2	0.898594823	0.913120359	0.856952806	0.865665372	0.996781978	0.8537109
SSC5D	0.898607157	0.852526348	0.926234868	0.992930508	0.887514169	0.83821684
SLC45A4	0.898607157	0.8292724	0.95219206	0.935563591	0.947020167	0.839906041
LMNA	0.898607157	0.782783231	0.838341783	0.999981514	0.953300742	0.841312694
DNMT3B	0.898607157	0.790095871	0.969404007	0.994965893	0.865075514	0.844759721
TIMP2	0.898607157	0.804185018	0.868360233	0.999981514	0.87329316	0.845647064
ATAD1	0.898607157	0.821674572	0.997400824	0.912777307	0.890348531	0.845747012
LOC104972290	0.898607157	0.880129816	0.857358031	0.941467375	0.976259416	0.845747012
SYN2	0.898607157	0.993481224	0.916038418	0.865076519	0.886785729	0.847193872
RAB3IP	0.898607157	0.760778257	0.891662311	0.999981514	0.85545716	0.850432365
CAVIN2	0.898607157	0.775033799	0.99647733	0.911553824	0.927086179	0.850700833
ZEB2	0.898607157	0.847255873	0.945995021	0.899855526	0.958797242	0.853029499
PTGER3	0.898607157	0.796253817	0.841294863	0.999981514	0.925503344	0.8537109
KRT24	0.898607157	0.90265862	0.851822885	0.941935077	0.961465747	0.857038672
THAP7	0.898607157	0.855067335	0.972021503	0.870210705	0.950085378	0.857673662
OARD1	0.898607157	0.946820717	0.854567571	0.876703811	0.973603253	0.858507498
LOC107131992	0.898607157	0.801585146	0.91882781	0.999347752	0.891726429	0.859949878
AP3S2	0.898607157	0.823801613	0.949939862	0.915013589	0.953300742	0.859949878
LOC112442949	0.898607157	0.78357023	0.836691254	0.999981514	0.926813827	0.859993218
GOLPH3	0.898607157	0.774644712	0.900890831	0.978404889	0.957793213	0.866280163
DOK4	0.898607157	0.871543546	0.896821247	0.959616431	0.923430168	0.872238439
ZNF829	0.898607157	0.764775882	0.895801611	0.887515247	0.999985752	0.872239777
VEPH1	0.898607157	0.76556609	0.912183977	0.999981514	0.901853599	0.87501921
LOC104974020	0.898607157	0.946008705	0.879939804	0.890362631	0.939208227	0.875297569
NR1D1	0.898607157	0.851161648	0.922488509	0.990084447	0.854289643	0.876162019

TSSK1B	0.898607157	0.931706707	0.895801611	0.941467375	0.870450746	0.892605403
OTUD5	0.898607157	0.839660873	0.93872685	0.907794002	0.930451229	0.895803603
BCL9L	0.898607157	0.978405231	0.855847948	0.906186004	0.882831953	0.899428912
DDX27	0.898607157	0.924173915	0.855713041	0.866566721	0.967563029	0.90071654
MAP6	0.898607157	0.906064108	0.834683675	0.898658215	0.964833865	0.903626415
CD163	0.898607157	0.796253817	0.915858924	0.925457774	0.956653075	0.905188617
WDR83OS	0.898607157	0.827992008	0.917155067	0.874424802	0.964301327	0.912531667
AMER1	0.898607157	0.830930037	0.949855095	0.93048026	0.877712931	0.917930485
ACAA1	0.898607157	0.830243527	0.896821247	0.984423083	0.864524107	0.919124527
CAPN7	0.898607157	0.796253817	0.89478142	0.896116841	0.97420903	0.919124527
TUBGCP6	0.898607157	0.763758778	0.965407935	0.952247001	0.872096228	0.921843102
TXLNG	0.898607157	0.822659311	0.867661896	0.9880671	0.889301074	0.923092436
LOC781770	0.898607157	0.807623624	0.943836178	0.93745545	0.891726429	0.923092436
FIGNL2	0.898607157	0.78357023	0.864847127	0.975346638	0.940870936	0.923092436
LOC101906200	0.898607157	0.796253817	0.916323169	0.899047543	0.950085378	0.930830535
LOC617654	0.898607157	0.761280186	0.893988897	0.950643928	0.938316699	0.940017687
LOC112442271	0.898607157	0.884151397	0.849218971	0.949502614	0.891304411	0.943285784
CACNB1	0.898607157	0.791281103	0.866467804	0.990955345	0.878269439	0.945480515
AVPR1A	0.898607157	0.949902864	0.855268213	0.870591999	0.896589592	0.94763673
COQ10A	0.898607157	0.792410373	0.892343042	0.922503563	0.935286646	0.951081819
LOC527796	0.898607157	0.811354298	0.872088608	0.914850247	0.930306915	0.963336022
ZNF407	0.898607157	0.795559378	0.881221246	0.916858019	0.922953685	0.968697704
CLCF1	0.898607157	0.767124704	0.855847948	0.905039693	0.959658682	0.970105102
SEC16A	0.898607157	0.816129287	0.871938867	0.944672402	0.879495058	0.972415704
SLC25A6	0.898607157	0.818303808	0.858376167	0.949252011	0.882409565	0.973413452
LOC100849237	0.898607157	0.761391528	0.841294863	0.991955963	0.857562998	0.980167535
MIPOL1	0.898607157	0.807919676	0.852916849	0.896116841	0.939203547	0.980167535
KIF5B	0.898607157	0.821133604	0.854567571	0.928404714	0.879268517	0.988273129
LOC781688	0.898607157	0.764456895	0.935219877	0.870210705	0.862773747	0.999463809
AFDN	0.898713399	0.847323359	0.86347788	0.999981514	0.87329316	0.836111377
ECI1	0.898713399	0.913116251	0.912396874	0.888687722	0.960048824	0.856548815
PTPN9	0.898791037	0.767737619	0.87384462	0.957251577	0.999985752	0.835231445
USP39	0.898791037	0.835942551	0.855847948	0.98981682	0.962513981	0.839489406
XKRX	0.898791037	0.999278945	0.854567571	0.912777307	0.855433483	0.8537109
FH	0.898791037	0.78757544	0.899952655	0.893220599	0.976769047	0.917930485
LPIN2	0.898791037	0.89417747	0.853546772	0.942883863	0.857014385	0.962412275
GEMIN8	0.89879198	0.904570743	0.945995021	0.920163802	0.87329316	0.881875045
CALHM5	0.89879198	0.973321075	0.845113191	0.861499607	0.891726429	0.944330889

SUFU	0.89879198	0.823478017	0.836482	0.948668643	0.926972948	0.960023723
TTC39B	0.89879198	0.767113995	0.894316021	0.900044333	0.933898805	0.975639156
LOXL3	0.898878282	0.786691027	0.905023942	0.989485949	0.960048824	0.839906041
ARSK	0.898878282	0.94502659	0.855811972	0.97828838	0.900927045	0.842214812
LOC784127	0.898878282	0.821674572	0.999993442	0.925294859	0.854289643	0.842650666
LOC104970145	0.898878282	0.789740412	0.992991376	0.893051204	0.943239508	0.860966325
LOC101905586	0.898878282	0.910044299	0.964702402	0.916858019	0.861348302	0.867635709
GNG7	0.898878282	0.779025263	0.871929137	0.999981514	0.943540106	0.872238439
XDH	0.898878282	0.806326521	0.896023027	0.95901497	0.960573822	0.87229131
CTNND2	0.898878282	0.866862152	0.955221146	0.889415593	0.932024143	0.881189314
POLE2	0.898878282	0.993481224	0.841294863	0.918216988	0.856136977	0.886662989
LOC112447506	0.898878282	0.764631524	0.86347788	0.999981514	0.937932469	0.887542005
KIF13A	0.898878282	0.855141737	0.867661896	0.995434268	0.891304411	0.888887063
NOL10	0.898878282	0.853678591	0.963164828	0.901308803	0.896589592	0.892171608
IPO13	0.898878282	0.817763894	0.99341711	0.882057818	0.887514169	0.910085997
ADGRF1	0.898878282	0.872175143	0.979494108	0.868435169	0.880876632	0.912531667
RPS6KA1	0.898878282	0.880565292	0.916294854	0.891988949	0.935286646	0.912894278
PVALB	0.898878282	0.831153015	0.845113191	0.999981514	0.86930615	0.927520621
KIAA1549	0.898878282	0.767113995	0.93190909	0.97390792	0.883143938	0.927520621
LOC107133289	0.898878282	0.842884359	0.924855847	0.936586091	0.879776073	0.930146713
LOC112446734	0.898878282	0.816129287	0.91882781	0.906186004	0.910705824	0.947937638
ZNF696	0.898878282	0.785689574	0.912666347	0.896116841	0.901908484	0.980167535
HSBP1L1	0.898996321	0.893164557	0.857250535	0.999981514	0.853973085	0.864572584
FAM98B	0.898996321	0.83844057	0.962797326	0.889924182	0.926972948	0.894808664
GPR108	0.898996321	0.942298781	0.866360056	0.930882136	0.887514169	0.909056553
LOC112441886	0.898996321	0.993804376	0.84201467	0.870591999	0.886785729	0.91373422
LAMTOR3	0.899119557	0.831549062	0.968204727	0.965050971	0.860594228	0.870280355
LRRC74B	0.899119557	0.926852499	0.980061232	0.870591999	0.864524107	0.877014986
PIK3AP1	0.89921088	0.809666542	0.964661607	0.899091941	0.943702351	0.877014986
LOC781261	0.899242642	0.952344105	0.885483585	0.96951401	0.857203077	0.864139695
NCR3LG1	0.899242642	0.884804337	0.894316021	0.980564765	0.893716526	0.867063767
ARHGAP21	0.899242642	0.830041449	0.894338686	0.870591999	0.979988991	0.91618909
NRXN1	0.899242642	0.826316517	0.893988897	0.940436902	0.88127953	0.963107047
LOC112441778	0.89928819	0.764206109	0.864832853	0.922993716	0.970885781	0.948091259
TRAIP	0.899478061	0.819439092	0.880163755	0.978503042	0.956761712	0.865507429
CTGF	0.899478061	0.895777642	0.870965946	0.891988949	0.9683846	0.892171608
CPEB1	0.899478061	0.905550751	0.871285807	0.939604997	0.887514169	0.92789871
LOC112449346	0.899478061	0.808478344	0.89478142	0.9880671	0.857562998	0.93983168

PDE3B	0.899750951	0.87134357	0.883816089	0.999981514	0.878708123	0.851325852
SFXN5	0.899791388	0.973712113	0.843955813	0.932083159	0.943610727	0.836111377
ACTR3B	0.899791388	0.912627552	0.894316021	0.901353354	0.970988226	0.842178536
COMMD4	0.899791388	0.946148902	0.886829776	0.949502614	0.916204944	0.842473495
LOC112447080	0.899791388	0.955652503	0.916968887	0.889415593	0.930451229	0.847193872
SMTNL1	0.899791388	0.805580108	0.883950091	0.875836286	0.999985752	0.8537109
LOC101902786	0.899791388	0.872331637	0.871491704	0.904018489	0.989442936	0.855963573
DHRSX	0.899791388	0.839660873	0.916294854	0.89684883	0.982538884	0.857457228
MFSD14A	0.899791388	0.805283017	0.962553873	0.934445963	0.943540106	0.858245612
LOC104968411	0.899791388	0.779343136	0.955427429	0.92139105	0.962513981	0.870018677
LOC101906606	0.899791388	0.792109226	0.996068003	0.886727784	0.935286646	0.873699988
LOC101905845	0.899791388	0.939027328	0.927635169	0.92139105	0.875924242	0.875297569
CSNK2A2	0.899791388	0.795955721	0.855713041	0.940436902	0.996781978	0.877014986
B3GNT8	0.899791388	0.783178342	0.875900843	0.997660427	0.943047881	0.88050125
SRBD1	0.899791388	0.8353133	0.843955813	0.999981514	0.929896768	0.882690335
SEC11A	0.899791388	0.819210468	0.888566052	0.999981514	0.887514169	0.885162483
SIDT2	0.899791388	0.785261204	0.855811972	0.964521432	0.976769047	0.891088733
UNC13D	0.899791388	0.785374969	0.959903168	0.887877413	0.960715767	0.899061149
GTF2H3	0.899791388	0.821767596	0.927635169	0.878074356	0.970988226	0.903500283
METTL5	0.899791388	0.766595431	0.944126714	0.98981682	0.876359217	0.905794324
GAPT	0.899791388	0.775262502	0.999993442	0.882057818	0.873198769	0.907124813
RFXAP	0.899791388	0.870296749	0.845113191	0.892047793	0.984494338	0.910785883
FXYD5	0.899791388	0.878624872	0.892343042	0.918693944	0.935286646	0.911781676
DLD	0.899791388	0.806821185	0.914256466	0.893051204	0.970988226	0.91373422
TRRAP	0.899791388	0.837244723	0.926161087	0.866566721	0.962513981	0.915735519
UHRF2	0.899791388	0.820534347	0.971053208	0.878728467	0.924795709	0.919124527
LOC112446053	0.899791388	0.773939845	0.921266625	0.890362631	0.976259416	0.923092436
ADGRL3	0.899791388	0.91763262	0.92355285	0.893220599	0.881521011	0.923807852
MRPS35	0.899791388	0.781222833	0.951392011	0.8995158	0.938316699	0.92725789
FAM46A	0.899791388	0.863286109	0.880357899	0.948478716	0.909379774	0.927471252
CDH20	0.899791388	0.82707084	0.856082558	0.929663263	0.958852623	0.929214184
PIN1	0.899791388	0.83844057	0.929661805	0.904768656	0.906893576	0.93363377
RBM24	0.899791388	0.932471268	0.935117456	0.865517249	0.854966579	0.943972369
RAD54L	0.899791388	0.796253817	0.858032788	0.998177865	0.879268517	0.945480515
LOC101908205	0.899791388	0.77899313	0.874983674	0.999981514	0.864524107	0.950540248
RBM4B	0.899791388	0.863414694	0.89478142	0.903021188	0.895757462	0.959740646
MSANTD2	0.899791388	0.837797314	0.854087103	0.890362631	0.956906331	0.960023723
ZNF10	0.899791388	0.781543413	0.906019013	0.883236823	0.956906331	0.963015949

EEF1AKMT3	0.899791388	0.806726634	0.879939804	0.925294859	0.926813827	0.963107047
GGTA1	0.899791388	0.783112117	0.890716604	0.952247001	0.887514169	0.971545917
DSCC1	0.899791388	0.822956796	0.91469924	0.885789665	0.911664199	0.971545917
MRPL22	0.899791388	0.799590997	0.849389234	0.894086736	0.959565914	0.973649216
TSNAX	0.899791388	0.844170799	0.890499307	0.889415593	0.891726429	0.981216824
HDDC2	0.899791388	0.870298381	0.916294854	0.867235478	0.855740473	0.987516427
LOC112446357	0.899791388	0.870264196	0.845525761	0.887983383	0.882418284	0.997383134
TWF1	0.899791388	0.791858901	0.916294854	0.882057818	0.872096228	0.997862553
KIDINS220	0.900139019	0.811059858	0.865444995	0.870210705	0.999985752	0.919124527
NOP16	0.900139019	0.83866979	0.891662311	0.956685049	0.910717783	0.925432855
GTF2E2	0.900139019	0.77139884	0.957052938	0.896116841	0.902907077	0.958790968
PFN1	0.900289674	0.94422395	0.933884582	0.910575108	0.888368075	0.860966325
LOC790009	0.900361593	0.955652503	0.923364099	0.871946375	0.930451229	0.862925606
TMPRSS6	0.900592196	0.991673407	0.916323169	0.870591999	0.900809233	0.839565788
MTRF1L	0.9006947	0.795559378	0.999993442	0.882057818	0.895757462	0.88313584
NUBPL	0.900971206	0.811059858	0.883737851	0.999981514	0.858914398	0.917930485
NYAP1	0.901004659	0.805958674	0.992263335	0.947574232	0.87329316	0.874490166
CEP85	0.901004659	0.81232489	0.912018831	0.960937718	0.904643542	0.925040815
SFRP5	0.901216996	0.867740903	0.89478142	0.964521432	0.901853599	0.899428912
TP53I3	0.901260961	0.911562695	0.895826828	0.97828838	0.883294083	0.867063767
SLC25A17	0.901260961	0.807285501	0.891096535	0.898818046	0.996089557	0.888930405
TBKBP1	0.901260961	0.818303808	0.945732557	0.888687722	0.938879379	0.927517509
AOC1	0.901260961	0.872175143	0.855268213	0.891988949	0.964963602	0.930710695
MYO5C	0.901402113	0.833323491	0.913609237	0.999981514	0.871558258	0.865767172
PLA2G4B	0.901520289	0.790370995	0.912020408	0.999981514	0.887514169	0.891423889
GRID1	0.901606587	0.930092771	0.924855847	0.910575108	0.942786245	0.837559792
LOC783730	0.901606587	0.893615781	0.914866994	0.901171792	0.972160227	0.842214812
SLC7A5	0.901606587	0.928733588	0.942357321	0.957132838	0.870042072	0.846210629
CEP104	0.901606587	0.886565095	0.846372547	0.999981514	0.906893576	0.846210629
SNAPIN	0.901606587	0.7889129	0.935266153	0.945300947	0.976259416	0.847012179
LOC100300896	0.901606587	0.796253817	0.874983674	0.993481918	0.964301327	0.850700833
ATF7	0.901606587	0.935001231	0.946760569	0.874424802	0.931775933	0.862871483
ZGPAT	0.901606587	0.888057447	0.869932384	0.949468104	0.957104426	0.871503289
ANAPC16	0.901606587	0.796253817	0.916323169	0.932677501	0.960573822	0.895633366
LCP2	0.901606587	0.878551917	0.91882781	0.873302281	0.958832188	0.902445346
TREM2	0.901606587	0.849896139	0.935266153	0.929388614	0.909379774	0.908475983
EVI2B	0.901606587	0.796748952	0.959725886	0.884482389	0.955623649	0.911808092
DDX1	0.901606587	0.77844414	0.979494108	0.874023052	0.903606765	0.951081819

AGPAT5	0.901606587	0.78571923	0.93190909	0.952247001	0.869580831	0.959075802
LOC112447438	0.901606587	0.802570554	0.850624788	0.995312373	0.858869582	0.966136679
MAK16	0.901606587	0.7889129	0.949383783	0.908467146	0.864524107	0.972314037
LOC104973826	0.901686196	0.789957129	0.886829776	0.868255052	0.962513981	0.973649216
PRADC1	0.901887912	0.819210468	0.94789262	0.886312303	0.960573822	0.900988212
SHARPIN	0.902598033	0.822956796	0.979494108	0.890422845	0.964301327	0.839906041
TTC28	0.902598033	0.780233411	0.920682633	0.997374305	0.893716526	0.891423889
ERBB3	0.902598033	0.925232913	0.856943813	0.939142733	0.895067787	0.927215832
ECHDC3	0.902627428	0.903396355	0.858032788	0.898818046	0.959658682	0.91373422
ZNF516	0.902652489	0.77844414	0.931161015	0.901171792	0.999985752	0.845747012
LOC101904498	0.902652489	0.816202069	0.90780986	0.973075232	0.958797242	0.85643253
NAP1L5	0.902652489	0.823478017	0.939913423	0.882057818	0.982090215	0.87326437
LOC512953	0.902652489	0.770934591	0.903431343	0.877455139	0.999985752	0.874000314
NELFE	0.902652489	0.778279435	0.994887288	0.93048026	0.901853599	0.876861428
CAMSAP2	0.902652489	0.7889129	0.947155158	0.922993716	0.951996251	0.900219465
CUL4B	0.902652489	0.856536345	0.849389234	0.999981514	0.884872176	0.905188617
CISD3	0.902652489	0.77766577	0.972073392	0.910575108	0.935286646	0.906632034
LOC104972827	0.902652489	0.932191409	0.872088608	0.870442442	0.953300742	0.919557825
SUPT3H	0.902652489	0.848383949	0.844022087	0.885789665	0.989334846	0.92789871
CARMIL1	0.902652489	0.870716141	0.855268213	0.88099367	0.974931134	0.933897626
NNT	0.902652489	0.783788501	0.872088608	0.964521432	0.916057607	0.960023723
PCED1A	0.902676043	0.797906794	0.999993442	0.916858019	0.881680572	0.839489406
LOC112442657	0.902676043	0.997425219	0.860179257	0.896116841	0.911568867	0.845647064
CPD	0.902676043	0.982011436	0.927635169	0.889415593	0.89052284	0.845783187
FRYL	0.902676043	0.808279036	0.873219549	0.999981514	0.891726429	0.851995112
TOM1L2	0.902676043	0.846724749	0.862253703	0.902696094	0.999985752	0.851995112
FBXL15	0.902676043	0.871834641	0.881778842	0.907794002	0.989334846	0.8537109
ANKRD31	0.902676043	0.809056302	0.916323169	0.952247001	0.964301327	0.86324237
LOC782479	0.902676043	0.893056502	0.872088608	0.961353555	0.94542482	0.867063767
RAB11FIP1	0.902676043	0.910651459	0.965961035	0.893220599	0.893716526	0.872238439
SLC35A5	0.902676043	0.825171788	0.935672252	0.992339478	0.878451408	0.874490166
TRPM7	0.902676043	0.83844057	0.874797723	0.868649772	0.999985752	0.888887063
GCNT3	0.902676043	0.921940584	0.945995021	0.919643615	0.86864151	0.890014046
DUSP28	0.902676043	0.884457875	0.853546772	0.940136897	0.961465747	0.891423889
LOC100850276	0.902676043	0.807350682	0.889098668	0.999981514	0.870042072	0.927426708
GIT1	0.902676043	0.801273171	0.867625133	0.894086736	0.987723553	0.932688327
BATF	0.902676043	0.811059858	0.871285807	0.995312373	0.887514169	0.93529968
RASIP1	0.902676043	0.884499778	0.865717285	0.876703811	0.959565914	0.943285784

ATG4A	0.902676043	0.832498507	0.943836178	0.899047543	0.884872176	0.958790968
BORCS6	0.902676043	0.81232489	0.848278925	0.876703811	0.983941532	0.960023723
YTHDF1	0.902745962	0.788544942	0.911973589	0.960016921	0.983941532	0.845747012
C9H6orf203	0.902745962	0.787305351	0.999993442	0.870591999	0.887514169	0.8537109
EXOSC2	0.902745962	0.774644712	0.956655464	0.897371376	0.983941532	0.865767172
ARL10	0.902807367	0.793660624	0.8644528	0.932254825	0.864524107	0.999948228
ATL3	0.90282245	0.954237721	0.935672252	0.908467146	0.9096389	0.839906041
STK32A	0.90282245	0.817323509	0.964807927	0.976196758	0.911052879	0.839906041
HCFC1R1	0.90282245	0.813759037	0.915713148	0.888687722	0.999985752	0.839906041
MSH6	0.90282245	0.993035141	0.916294854	0.867281023	0.910747101	0.841312694
LOC512149	0.90282245	0.820882294	0.999993442	0.870210705	0.887514169	0.845747012
LOC112448057	0.90282245	0.818720168	0.885104909	0.980564765	0.970988226	0.845747012
LOC104970628	0.90282245	0.806326521	0.849389234	0.999981514	0.936962513	0.846210629
MESP2	0.90282245	0.915269318	0.874983674	0.917653833	0.974931134	0.847012179
ITGB3BP	0.90282245	0.846721805	0.886829776	0.999981514	0.86979089	0.853009713
PHIP	0.90282245	0.870334242	0.915858924	0.889415593	0.987723553	0.853576299
SLC7A10	0.90282245	0.847255873	0.916323169	0.940438437	0.964301327	0.853882876
TMEM60	0.90282245	0.782783231	0.992719412	0.870210705	0.973603253	0.856833445
LOC107132490	0.90282245	0.796666816	0.999993442	0.898890982	0.865505927	0.857165842
ALCAM	0.90282245	0.999987083	0.846578721	0.886312303	0.880066194	0.858321732
LOC112442265	0.90282245	0.999987083	0.867661896	0.892047793	0.864524107	0.862925606
CAP1	0.90282245	0.872331637	0.937281477	0.961898258	0.900809233	0.865767172
TSC22D2	0.90282245	0.833429351	0.877193584	0.882057818	0.999985752	0.871273798
TTLL12	0.90282245	0.8013363	0.852225514	0.999981514	0.883951804	0.875991349
SRRM5	0.90282245	0.798589766	0.916294854	0.999981514	0.864524107	0.876861428
LOC101907697	0.90282245	0.84927532	0.855730513	0.905477428	0.997422012	0.880981659
SLC15A3	0.90282245	0.851775118	0.943836178	0.887983383	0.954403133	0.898470276
GFRA2	0.90282245	0.875506677	0.880476435	0.872590202	0.986019537	0.900244228
HECW2	0.90282245	0.800193122	0.849704325	0.913048575	0.999453276	0.909056553
UBE2V1	0.90282245	0.824188473	0.932786033	0.887983383	0.950958352	0.930710695
POLH	0.90282245	0.816308985	0.93190909	0.899792456	0.936797477	0.933638318
PDP2	0.90282245	0.810010174	0.924855847	0.869510763	0.964301327	0.939024684
LOC101907000	0.90282245	0.782721907	0.881778842	0.87522058	0.979290191	0.960023723
CD86	0.90282245	0.855752352	0.855892553	0.889415593	0.932024143	0.979480605
ARPC3	0.90282245	0.783835122	0.924855847	0.925294859	0.862302249	0.98469083
IBA57	0.903274555	0.885494685	0.892343042	0.870210705	0.999985752	0.839565788
EIF1AD	0.903274555	0.819210468	0.972741989	0.970812491	0.900891209	0.846210629
BRAP	0.903274555	0.804185018	0.957295966	0.917933896	0.970988226	0.846210629

NCAPH	0.903274555	0.880565292	0.929661805	0.925210648	0.955049621	0.849973192
ARHGAP5	0.903274555	0.89465829	0.91882781	0.886312303	0.976322141	0.852504043
ERLEC1	0.903274555	0.857742576	0.953572282	0.95901497	0.911028086	0.854734677
RUFY3	0.903274555	0.7889129	0.981655709	0.885789665	0.976292433	0.85532952
PRKCG	0.903274555	0.869953468	0.992511421	0.907621547	0.892446951	0.85643253
MTPAP	0.903274555	0.801802651	0.845113191	0.973075232	0.993831963	0.866680565
TMEM108	0.903274555	0.969051025	0.891662311	0.921950515	0.895757462	0.871998318
KIAA1191	0.903274555	0.77844414	0.921266625	0.941796131	0.976727507	0.874490166
MATK	0.903274555	0.844170799	0.947155158	0.96060703	0.878451408	0.903210685
LOC100139764	0.903274555	0.808478344	0.921266625	0.900875874	0.970988226	0.90596786
HAGHL	0.903274555	0.904754143	0.854571175	0.899792456	0.964301327	0.91373422
STK35	0.903274555	0.77844414	0.939353663	0.868435169	0.970988226	0.938013912
DDIAS	0.903274555	0.772618617	0.966232793	0.904018489	0.909737016	0.944973383
CAMK2B	0.903274555	0.910651459	0.895801611	0.868378265	0.887514169	0.970105102
SLAMF9	0.903274555	0.811059858	0.849389234	0.889415593	0.886785729	0.999948228
ZWINT	0.903329582	0.77139884	0.923993948	0.929663263	0.976292433	0.891088733
LOC616868	0.903435054	0.986900642	0.895801611	0.905772309	0.887514169	0.860436712
MYPOP	0.903435054	0.81745062	0.879939804	0.936625532	0.976259416	0.903500283
LOC790218	0.903435054	0.786691027	0.944613162	0.974873127	0.873366037	0.92789871
SNHG4	0.903435054	0.805283017	0.858376167	0.918693944	0.979290191	0.930710695
CYP3A4	0.903676751	0.821767596	0.893988897	0.91558617	0.999985752	0.841312694
HOMER2	0.903676751	0.887455957	0.927635169	0.935985829	0.860032529	0.930710695
NDUFAF1	0.903676751	0.777245655	0.968043317	0.893220599	0.935286646	0.930814839
LYPD1	0.903676751	0.796666816	0.849389234	0.918693944	0.972160227	0.955140534
METTL25	0.90370007	0.782783231	0.893988897	0.899296336	0.886893907	0.999948228
LIMCH1	0.903733585	0.805283017	0.945732557	0.941467375	0.972160227	0.842650666
MIB1	0.903733585	0.772618617	0.885896294	0.999981514	0.880890828	0.853440904
WBP1L	0.903733585	0.90420761	0.957052938	0.905886285	0.911664199	0.867907362
MRPS14	0.903733585	0.844170799	0.931161015	0.894870298	0.964833865	0.888309103
LOC527744	0.903733585	0.77844414	0.990974506	0.885789665	0.931400945	0.921772742
ECHS1	0.903733585	0.779025263	0.883629347	0.896116841	0.993571905	0.929027441
RPE	0.903733585	0.791393612	0.986920775	0.890704007	0.878451408	0.952563377
LYRM4	0.903733585	0.78945719	0.879939804	0.912922827	0.926972948	0.984415161
URB2	0.903849826	0.77844414	0.911103174	0.96900924	0.948421303	0.9136642
PLPPR3	0.903849826	0.782783231	0.865697455	0.907794002	0.980695939	0.94844234
MPZL2	0.903906148	0.77844414	0.937993336	0.949863561	0.962757238	0.877048993
NCK1	0.903955544	0.772618617	0.845113191	0.999981514	0.944839728	0.878754045
NDRG1	0.904145609	0.779025263	0.932933417	0.990955345	0.891726429	0.907384701

ETS1	0.904191193	0.816129287	0.930720084	0.919114762	0.976292433	0.863020449
SMG1	0.904881484	0.821674572	0.91469924	0.916858019	0.991531798	0.857853613
RASSF2	0.905075717	0.7973731	0.999993442	0.882057818	0.954071324	0.842650666
CD274	0.905075717	0.848114285	0.979354564	0.870210705	0.970491451	0.844759721
PSMB9	0.905075717	0.927853707	0.899484481	0.957579201	0.884872176	0.889406298
ELP2	0.905075717	0.811304457	0.949855095	0.935563591	0.924222719	0.91373422
LOC782609	0.905101929	0.950336973	0.917155067	0.931383184	0.911028086	0.845747012
TMEM189	0.905101929	0.815090615	0.899106435	0.999981514	0.943702351	0.845747012
PCDHB14	0.905101929	0.824637127	0.872088608	0.984421126	0.975181971	0.847193872
POLE	0.905101929	0.826986239	0.924855847	0.930882136	0.934156546	0.923033364
PPP1R12A	0.905101929	0.903341833	0.912183977	0.892047793	0.92337151	0.930206677
SH3YL1	0.905165457	0.779025263	0.990278947	0.994408995	0.864524107	0.8537109
IFNAR1	0.905229148	0.80014899	0.916038418	0.918921815	0.999985752	0.845241321
STRIP1	0.905260749	0.811354298	0.885896294	0.990955345	0.960048824	0.859032412
FAM162A	0.905260749	0.802605511	0.963164828	0.886312303	0.972722722	0.878642
NSUN2	0.905260749	0.870444737	0.94422142	0.872783968	0.893716526	0.958707771
LOC101907682	0.905260749	0.832556888	0.93475587	0.919601062	0.865505927	0.966212838
POLDIP3	0.90548374	0.836131298	0.987324693	0.955466436	0.887514169	0.853075722
CHST11	0.905528572	0.969193477	0.852214402	0.893220599	0.976259416	0.841312694
DDX52	0.905528572	0.895395302	0.964807927	0.869868859	0.948856947	0.865971618
LOC107132360	0.905528572	0.842948165	0.869573453	0.896116841	0.999453276	0.888475311
LOC112448893	0.905538252	0.834615638	0.994339207	0.886312303	0.953300742	0.845747012
GABARAP	0.905538252	0.791281103	0.996224523	0.973075232	0.886944613	0.846210629
ACTN3	0.905538252	0.800193122	0.926299163	0.978559648	0.959393538	0.847012179
LOC107132270	0.905538252	0.907054337	0.915858924	0.994395954	0.878451408	0.847193872
REEP6	0.905538252	0.811354298	0.892343042	0.973075232	0.947020167	0.90645885
MED25	0.905538252	0.796253817	0.984886031	0.925294859	0.887514169	0.914375652
LOC515227	0.905538252	0.941483684	0.872088608	0.8856246	0.935286646	0.931410194
SNX3	0.905538252	0.7973731	0.95219206	0.912777307	0.906033379	0.945854375
LOC100848575	0.905591468	0.794494758	0.89478142	0.999981514	0.891726429	0.848298915
RALGDS	0.905591468	0.918455779	0.916323169	0.952247001	0.900747635	0.867907362
PCTP	0.905949441	0.822484848	0.971053208	0.893220599	0.972128973	0.849885981
BCL2L11	0.905949441	0.778219881	0.964382669	0.935958061	0.970988226	0.850475013
GALNS	0.905949441	0.850894788	0.958200097	0.908467146	0.956904152	0.855878683
PNN	0.905949441	0.786691027	0.945732557	0.949565453	0.963550093	0.871998318
CRELD1	0.905949441	0.997425219	0.852916849	0.93048026	0.864518064	0.876162019
OXLD1	0.905949441	0.777246451	0.848278925	0.960177245	0.999985752	0.876162019
CAPN6	0.905949441	0.991673407	0.855811972	0.940726907	0.86930615	0.877048993

NUP62	0.905949441	0.94422395	0.88462169	0.891988949	0.953840825	0.885957057
LOC112443751	0.905949441	0.808478344	0.978014482	0.940438437	0.86864151	0.923807852
RAP1GAP	0.905949441	0.781582351	0.862602899	0.959159289	0.961465747	0.94337298
ADORA2B	0.905949441	0.81848543	0.871482445	0.887642126	0.980189546	0.948886628
RBM45	0.905949441	0.794494758	0.949892469	0.87522058	0.943702351	0.955210102
C17H12orf43	0.905949441	0.877893862	0.848278925	0.884819185	0.894364008	0.998240456
ZC3H18	0.906203292	0.943244588	0.880163755	0.891988949	0.977193348	0.845714049
TESC	0.906203292	0.819210468	0.896821247	0.999981514	0.902258286	0.846210629
SS18L1	0.906203292	0.872684104	0.94565648	0.925294859	0.954403133	0.849074285
PDLIM4	0.906203292	0.899655864	0.912187636	0.999981514	0.87329316	0.849595612
LOC100299757	0.906203292	0.870444737	0.979494108	0.916858019	0.914280652	0.855471132
FRA10AC1	0.906203292	0.899186915	0.895570861	0.923738645	0.96755608	0.858507498
DENND5A	0.906203292	0.78945719	0.903431343	0.980564765	0.97234838	0.858507498
CRCP	0.906203292	0.779809577	0.999993442	0.89684883	0.881680572	0.868748913
MRPS6	0.906203292	0.7889129	0.966567676	0.985878137	0.887514169	0.876861428
RIPK3	0.906203292	0.863064327	0.957052938	0.870591999	0.964301327	0.87699793
EMP3	0.906203292	0.919936324	0.855847948	0.999981514	0.865076851	0.877014986
SMO	0.906203292	0.939857309	0.914816911	0.959616431	0.865505927	0.883688554
SCFD1	0.906203292	0.798327403	0.999993442	0.87522058	0.882252274	0.888309103
UQCC1	0.906203292	0.848383949	0.915120516	0.887983383	0.982087479	0.893585844
LOC512627	0.906203292	0.7973731	0.943836178	0.936631044	0.951996251	0.903990996
TWNK	0.906203292	0.796253817	0.894523258	0.898818046	0.993181315	0.916193353
TTK	0.906203292	0.781836924	0.919082405	0.981295935	0.917407853	0.919124527
SETD5	0.906203292	0.895777642	0.893761108	0.887325381	0.943702351	0.940017687
FAM69B	0.906203292	0.7889129	0.912396874	0.982220679	0.887514169	0.945480515
PATJ	0.906203292	0.823133291	0.96102707	0.902776185	0.887133502	0.948913998
CALHM6	0.906203292	0.843422472	0.949939862	0.898890982	0.887514169	0.952663521
PACS1	0.906203292	0.839128192	0.899106435	0.899855526	0.939208227	0.958281344
LOC112442079	0.906451854	0.823133291	0.972806636	0.896116841	0.943610727	0.891088733
LOC112441602	0.90663311	0.846721805	0.854567571	0.999981514	0.891726429	0.864572584
LOC531557	0.90663311	0.87976124	0.911535369	0.95901497	0.942786245	0.867701833
CORO6	0.906822612	0.899490486	0.928927123	0.96900924	0.912617855	0.846210629
LOC522540	0.906822612	0.837263562	0.855793459	0.913048575	0.999985752	0.850475013
TCF24	0.906822612	0.823133291	0.906019013	0.989485949	0.953300742	0.855963573
NFS1	0.906822612	0.869953468	0.913609237	0.925294859	0.9683846	0.867907362
TPM3	0.906822612	0.788980101	0.94565648	0.916858019	0.980016241	0.871998318
MYO10	0.906822612	0.985428851	0.874063521	0.930987593	0.889119631	0.872238439
MOV10	0.906822612	0.889345904	0.990630783	0.916858019	0.868722067	0.87229131

LOC101906077	0.906822612	0.787305351	0.904858534	0.999981514	0.913732532	0.879884117
SERINC4	0.906822612	0.964418016	0.88955259	0.875836286	0.947299702	0.888309103
TMEM121B	0.906822612	0.911063463	0.895801611	0.896116841	0.959393538	0.895517058
ACAD9	0.906822612	0.848456869	0.894316021	0.932914414	0.956906331	0.914769128
STARD10	0.906822612	0.845338797	0.961800035	0.937654716	0.882145303	0.917849087
KDM6B	0.906822612	0.791281103	0.975774524	0.929663263	0.903888186	0.918869728
TMEM259	0.906822612	0.925026329	0.93190909	0.90413565	0.877712931	0.926341424
PAK4	0.906822612	0.77844414	0.875873031	0.999981514	0.881680572	0.930710695
DDX39B	0.906822612	0.781543413	0.927635169	0.98673849	0.87329316	0.940583352
P3H2	0.906822612	0.867518836	0.958676768	0.889415593	0.882206878	0.945532151
USP46	0.906822612	0.788197838	0.889098668	0.892047793	0.981652321	0.950631909
PSME3	0.906822612	0.83552723	0.961437412	0.894086736	0.87329316	0.960023723
PVRIG	0.906822612	0.918854612	0.870965946	0.892047793	0.900893337	0.966800477
USP16	0.906822612	0.779407705	0.941360985	0.901988458	0.918424492	0.970105102
RNLS	0.906822612	0.783896922	0.868015424	0.874170259	0.960419102	0.995525297
PLXNB2	0.906837247	0.786493332	0.916294854	0.999981514	0.87329316	0.878118151
GDPD5	0.906907547	0.792690308	0.886829776	0.992073429	0.953885477	0.887722423
CAPRIN1	0.906907547	0.828012731	0.907669615	0.990955345	0.903606765	0.898470276
RGL1	0.906907547	0.822956796	0.862577505	0.908932731	0.983010476	0.931410194
LOC616254	0.906950748	0.783112117	0.849704325	0.999981514	0.976292433	0.846210629
ZNF543	0.906950748	0.788544942	0.89478142	0.882057818	0.999985752	0.857911901
VIPR1	0.906950748	0.936592735	0.916294854	0.890362631	0.959565914	0.860966325
THAP2	0.906950748	0.887413559	0.996769243	0.873956209	0.909657705	0.86324237
CLCA3	0.906950748	0.799590997	0.874983674	0.97828838	0.983941532	0.865767172
SHISAL1	0.906950748	0.999987083	0.848966671	0.874170259	0.867993134	0.870522865
ZBTB43	0.906950748	0.826316517	0.972073392	0.972142185	0.887514169	0.871503289
LOC101908214	0.906950748	0.855444194	0.871782109	0.89684883	0.999985752	0.87501921
FBXL2	0.906950748	0.796253817	0.883950091	0.999981514	0.897352056	0.876278015
STAMBPL1	0.906950748	0.893615781	0.872088608	0.995565324	0.899042139	0.877014986
ZSCAN2	0.906950748	0.811059858	0.916294854	0.999981514	0.87329316	0.885089102
GON4L	0.906950748	0.894702415	0.916038418	0.960016921	0.895757462	0.892605403
RABAC1	0.906950748	0.878551917	0.949855095	0.885789665	0.953300742	0.893282904
CCDC127	0.906950748	0.880565292	0.929661805	0.898818046	0.935286646	0.917849087
PPP1R9A	0.906950748	0.863948462	0.852916849	0.900875874	0.986019537	0.919124527
LHPP	0.906950748	0.912627552	0.876414692	0.907794002	0.943160494	0.926480924
LOC101902232	0.906950748	0.7889129	0.979494108	0.936066665	0.865075514	0.943285784
PSMA2	0.906950748	0.782720431	0.968679299	0.899792456	0.923931669	0.944973383
MAP1A	0.906950748	0.872175143	0.867222363	0.982220679	0.864524107	0.951340373

FRMD8	0.906950748	0.779372555	0.888254866	0.887420349	0.948006937	0.994347099
LOC112448304	0.907276667	0.7889129	0.983534298	0.959616431	0.943160494	0.841312694
ZNF395	0.907276667	0.856560491	0.999993442	0.935985829	0.879776073	0.842537695
ARHGEF9	0.907276667	0.824188473	0.916038418	0.999981514	0.911028086	0.851718562
LOC107132767	0.907276667	0.7973731	0.916294854	0.997332186	0.950958352	0.8537109
UBE2J1	0.907276667	0.838775377	0.999993442	0.942955143	0.87389153	0.85519889
IL17RE	0.907276667	0.929007264	0.871782109	0.993215785	0.902258286	0.85532952
PAAF1	0.907276667	0.783248473	0.990615319	0.898818046	0.965676623	0.860966325
FAXDC2	0.907276667	0.958897845	0.922552585	0.892047793	0.935286646	0.862925606
SLC25A44	0.907276667	0.813759037	0.879939804	0.999981514	0.902907077	0.865745718
EEPD1	0.907276667	0.7973731	0.883032292	0.980532429	0.976259416	0.872238439
LOC104970779	0.907276667	0.851287385	0.897322115	0.999981514	0.867993134	0.873130182
WWC2	0.907276667	0.807919676	0.885896294	0.999981514	0.944839728	0.874490166
SH3RF1	0.907276667	0.957328149	0.878164336	0.950643928	0.90240862	0.876162019
LOC112448773	0.907276667	0.855752352	0.965819476	0.926098747	0.91563394	0.879576441
ZBTB5	0.907276667	0.983594252	0.858376167	0.944351122	0.873957915	0.893838339
RBM14	0.907276667	0.817329649	0.854571175	0.972142185	0.976292433	0.893838339
NODAL	0.907276667	0.880565292	0.874983674	0.949502614	0.956041014	0.898470276
DHTKD1	0.907276667	0.791281103	0.880163755	0.908467146	0.99971266	0.911219114
EID1	0.907276667	0.823133291	0.99042659	0.908467146	0.887514169	0.91373422
PQLC1	0.907276667	0.915269318	0.852916849	0.982220679	0.894364008	0.91373422
LOC112448847	0.907276667	0.829067393	0.879939804	0.958103924	0.944839728	0.931496018
ST3GAL1	0.907276667	0.928427136	0.894316021	0.892047793	0.92535945	0.933897626
KIAA1614	0.907276667	0.782783231	0.947155158	0.944351122	0.908820136	0.944927301
LOC112446044	0.907276667	0.796253817	0.852860621	0.892047793	0.999453276	0.944973383
LIN9	0.907276667	0.83866979	0.91882781	0.940436902	0.900891209	0.945480515
BNIP3L	0.907276667	0.792596927	0.916323169	0.975346638	0.877712931	0.958790968
C17H4orf33	0.907276667	0.846721805	0.916294854	0.907794002	0.904643542	0.963107047
WASF3	0.907408508	0.902468141	0.862577505	0.908467146	0.939553343	0.945480515
MGA	0.907524264	0.864727593	0.963164828	0.882057818	0.977167046	0.842537695
ABCB1	0.907524264	0.91950087	0.928731949	0.941467375	0.935286646	0.846210629
ZNF783	0.907524264	0.786691027	0.912808445	0.994965893	0.946935016	0.877048993
RFC3	0.907524264	0.792109226	0.938624264	0.912229943	0.982203967	0.885089102
SLC1A4	0.907524264	0.927973597	0.912396874	0.969088992	0.865505927	0.893838339
HIST3H2A	0.907524264	0.848383949	0.966911424	0.889415593	0.926972948	0.919557825
MRE11	0.907524264	0.943244588	0.915124089	0.87522058	0.916877832	0.927021567
FADD	0.907524264	0.918854612	0.885469405	0.874170259	0.944839728	0.943972369
PTBP2	0.907524264	0.863948462	0.872088608	0.886518579	0.887514169	0.99841577

PDE1C	0.907575326	0.971413741	0.911535369	0.918693944	0.921710688	0.848830027
LOC101907729	0.907575326	0.81745062	0.89478142	0.896116841	0.999985752	0.864139695
RETREG1	0.907575326	0.927649637	0.879939804	0.995565324	0.878451408	0.872238439
TRIT1	0.907575326	0.786283227	0.874983674	0.999981514	0.902907077	0.89438401
C1H21orf2	0.907605978	0.870298381	0.908072171	0.956685049	0.894177708	0.930206677
STRADB	0.907605978	0.7889129	0.888989166	0.950162237	0.865075514	0.997383134
EID2	0.907940718	0.837987203	0.906019013	0.988408846	0.906827881	0.905011296
ZFHX3	0.907990031	0.833429351	0.892343042	0.999981514	0.906264219	0.853735179
RNF138	0.907990031	0.811354298	0.854571175	0.929413746	0.999985752	0.858507498
SLC26A10	0.907990031	0.817329649	0.854567571	0.932764647	0.999985752	0.86324237
SENP8	0.907990031	0.811059858	0.886829776	0.998831122	0.956761712	0.865767172
NSD3	0.907990031	0.857370953	0.89385865	0.952247001	0.976259416	0.865889509
VSTM1	0.907990031	0.90116376	0.957052938	0.950643928	0.887514169	0.870018677
TPRG1L	0.907990031	0.863414694	0.881778842	0.999981514	0.900927045	0.874870038
LOC107132911	0.907990031	0.875396087	0.892343042	0.905630971	0.987723553	0.87501921
NUDT3	0.907990031	0.796253817	0.95322206	0.998529748	0.890124729	0.876162019
COPRS	0.907990031	0.860419291	0.903431343	0.955466436	0.958797242	0.876162019
SBK1	0.907990031	0.92132606	0.972410614	0.889415593	0.90300429	0.877014986
CENPN	0.907990031	0.895395302	0.915858924	0.944351122	0.929896768	0.893838339
LOC783163	0.907990031	0.805283017	0.980761647	0.965050971	0.87329316	0.901618453
PPP6R1	0.907990031	0.861395263	0.942357321	0.950160436	0.909379774	0.903990996
KIAA2013	0.907990031	0.872175143	0.906019013	0.961898258	0.926972948	0.904428259
MAN1C1	0.907990031	0.857748111	0.917678884	0.989485949	0.878452585	0.904744538
COQ5	0.907990031	0.792483444	0.920682633	0.955000045	0.959565914	0.90645885
MASTL	0.907990031	0.792248775	0.964807927	0.963601374	0.900233561	0.910004719
CD300A	0.907990031	0.84312966	0.924855847	0.876703811	0.976292433	0.917024468
SPEG	0.907990031	0.936622887	0.852214402	0.967883482	0.891726429	0.919124527
LOC101903713	0.907990031	0.826316517	0.951521942	0.899792456	0.943540106	0.923807852
SMIM15	0.907990031	0.889581399	0.934401467	0.92139105	0.893376996	0.926480924
ZSCAN25	0.907990031	0.87976124	0.971053208	0.907794002	0.867529283	0.927520621
COQ3	0.907990031	0.818303808	0.996769243	0.892071982	0.878451408	0.927520621
DPF2	0.907990031	0.882858125	0.914770879	0.908467146	0.934067848	0.931410194
CCNF	0.907990031	0.792109226	0.964661607	0.960016921	0.877712931	0.932816123
SEMA4B	0.907990031	0.832398588	0.910851361	0.979385838	0.877712931	0.942982678
PARK7	0.907990031	0.853678591	0.939376972	0.893220599	0.926831826	0.944973383
FLRT3	0.907990031	0.81848543	0.850624788	0.896179114	0.993831963	0.945480515
MARCH5	0.907990031	0.786825403	0.935266153	0.947313672	0.91563394	0.952571698
OGA	0.907990031	0.804542244	0.942357321	0.95925588	0.872229135	0.958790968

CYB561A3	0.907990031	0.95187005	0.89478142	0.885789665	0.875924242	0.960023723
LOC101906477	0.907990031	0.791281103	0.964762618	0.887983383	0.926972948	0.960023723
AKR1C4	0.907990031	0.849513595	0.921266625	0.907794002	0.890124729	0.96870908
MTERF4	0.907990031	0.786691027	0.891035201	0.901353354	0.96087844	0.971589829
RYR2	0.907990031	0.8857401	0.877911619	0.892047793	0.923910588	0.972695131
COIL	0.907990031	0.796253817	0.916595776	0.914026346	0.907762824	0.980009318
ARHGAP23	0.907990031	0.791281103	0.871491704	0.881493849	0.965676623	0.988273129
KIAA1841	0.907990031	0.918234392	0.851633941	0.876616876	0.887514169	0.996335668
AGT	0.907990031	0.792109226	0.943836178	0.874170259	0.891726429	0.996335668
SOX15	0.907990031	0.814177827	0.871285807	0.888687722	0.935286646	0.997603451
FBP1	0.907990031	0.798489117	0.853455703	0.881493849	0.943540106	0.999948228
LOC101905668	0.908068539	0.867460706	0.996224523	0.896116841	0.935286646	0.846210629
SPATA9	0.908068539	0.899490486	0.988307089	0.922967576	0.892446951	0.850379754
EMID1	0.908068539	0.870298381	0.905023942	0.910575108	0.9902393	0.857853613
CHMP1A	0.908068539	0.789957129	0.921266625	0.922967576	0.999985752	0.859032412
SCN11A	0.908068539	0.951172741	0.895801611	0.919688166	0.943702351	0.862925606
TEN1	0.908068539	0.978017713	0.867222363	0.934466438	0.925503344	0.870490204
SCAI	0.908068539	0.834615638	0.885896294	0.973075232	0.970988226	0.871998318
GDPD4	0.908068539	0.913116251	0.893427844	0.961898258	0.936342434	0.872239777
PAG1	0.908068539	0.885494685	0.995377403	0.878513543	0.912617855	0.87501921
STEAP1	0.908068539	0.819210468	0.975774524	0.908467146	0.943160494	0.891423889
LOC112445002	0.908068539	0.916448625	0.944734215	0.918693944	0.89052284	0.905509632
CDPF1	0.908068539	0.94592331	0.927635169	0.886312303	0.910234468	0.9136642
UBN2	0.908068539	0.806326521	0.904534602	0.984423083	0.929896768	0.919124527
FAM172A	0.908068539	0.882858125	0.955845583	0.894086736	0.900927045	0.927520621
ASB4	0.908068539	0.811354298	0.855811972	0.911735195	0.997422012	0.927520621
SYPL1	0.908068539	0.783112117	0.883950091	0.999981514	0.89052284	0.941867365
MED18	0.908068539	0.821674572	0.894316021	0.87522058	0.983941532	0.947218832
PPP3CC	0.908068539	0.783112117	0.8644528	0.957008595	0.965676623	0.94844234
MAP3K14	0.908068539	0.799665594	0.949855095	0.920397967	0.904643542	0.959075802
LOC101904691	0.908068539	0.78838783	0.874797723	0.98981682	0.867558388	0.980501512
SIDT1	0.908435554	0.902525869	0.957052938	0.890704007	0.906803277	0.914769128
ABCA5	0.908463605	0.816412341	0.916323169	0.999981514	0.900927045	0.849595612
NFE2L3	0.908518118	0.916141416	0.995746693	0.899047543	0.869108362	0.871200929
PHF20	0.908518118	0.807350682	0.996624589	0.912777307	0.917407853	0.881054489
LOC112442683	0.908761797	0.930293015	0.881221246	0.924425698	0.970988226	0.855286622
LOC505600	0.908959229	0.939482337	0.883629347	0.923220263	0.926972948	0.91373422
PWP1	0.909005321	0.79096947	0.948904459	0.910495312	0.963790274	0.919124527

CTF1	0.909005321	0.893164557	0.869932384	0.925294859	0.935286646	0.947143537
IQSEC1	0.909016612	0.834615638	0.871313178	0.999981514	0.954403133	0.845647064
IL1RN	0.909016612	0.993481224	0.916539526	0.890499731	0.887514169	0.862925606
B3GAT1	0.909016612	0.980833065	0.91882781	0.927721166	0.879352099	0.865767172
OAT	0.909016612	0.876304005	0.896821247	0.999981514	0.883951804	0.872238439
PAPPA	0.909016612	0.804707639	0.894338686	0.999981514	0.892446951	0.872238439
SNRPD2	0.909016612	0.870222905	0.992263335	0.928332965	0.886785729	0.874000314
LY6D	0.909016612	0.905271052	0.880163755	0.925210648	0.953885477	0.91373422
LOC101903793	0.909016612	0.811445496	0.916294854	0.995717805	0.886785729	0.923092436
SRRM2	0.909016612	0.796253817	0.935491635	0.940438437	0.947299702	0.926530353
CLIP4	0.909016612	0.818303808	0.949855095	0.928404714	0.910508458	0.940256832
HSPBAP1	0.909016612	0.788414159	0.971053208	0.908932731	0.891304411	0.960023723
CLPTM1	0.909016612	0.796253817	0.899294652	0.925353907	0.87329316	0.99828741
GALNT16	0.909041248	0.998847958	0.879939804	0.899792456	0.914280652	0.847193872
LOC104969159	0.909041248	0.786691027	0.999993442	0.916858019	0.943540106	0.8537109
SMC2	0.909041248	0.94372165	0.95322206	0.949565453	0.875924242	0.85643253
LOC101904871	0.909041248	0.906064108	0.908072171	0.877132866	0.998060725	0.85643253
SH2B1	0.909041248	0.855067335	0.999993442	0.889415593	0.900927045	0.859993218
GGN	0.909041248	0.837875963	0.906019013	0.892047793	0.999985752	0.86324237
STRBP	0.909041248	0.936628011	0.918351026	0.891732037	0.961465747	0.867063767
TPRN	0.909041248	0.843278276	0.899106435	0.960016921	0.971117951	0.867907362
RTN4R	0.909041248	0.78945719	0.935266153	0.929388614	0.991481886	0.867907362
LOC100848011	0.909041248	0.949902864	0.911973589	0.972142185	0.873488171	0.872239777
TBPL1	0.909041248	0.988540349	0.852916849	0.907353815	0.936921784	0.87501921
LOC112448856	0.909041248	0.796748952	0.992511421	0.969837124	0.887514169	0.876162019
SUCNR1	0.909041248	0.906428409	0.899106435	0.919543876	0.964301327	0.876162019
LOC614614	0.909041248	0.841902289	0.996224523	0.923220263	0.891726429	0.877014986
PRRC1	0.909041248	0.805958674	0.854571175	0.999981514	0.961465747	0.882690335
CASC1	0.909041248	0.973321075	0.895801611	0.904018489	0.91563394	0.885089102
DUSP5	0.909041248	0.861975125	0.999350606	0.898818046	0.872734644	0.901981092
GTF3C3	0.909041248	0.941990146	0.899106435	0.921950515	0.92120858	0.903500283
CEP63	0.909041248	0.927973597	0.916294854	0.959652624	0.87329316	0.907145443
CIDEB	0.909041248	0.832142291	0.964661607	0.96951401	0.87329316	0.907145443
SHF	0.909041248	0.92565465	0.860877703	0.907258317	0.965676623	0.911219114
FAM53C	0.909041248	0.833713501	0.929661805	0.907794002	0.964301327	0.911781676
NQO2	0.909041248	0.791263947	0.93190909	0.978503042	0.932024143	0.912531667
LOC104969299	0.909041248	0.943754915	0.883950091	0.954935279	0.889301074	0.91348389
PCDH11X	0.909041248	0.876304005	0.93190909	0.961898258	0.887514169	0.913529327

NRIP1	0.909041248	0.812449121	0.946791586	0.889415593	0.975270955	0.91373422
WDR54	0.909041248	0.7889129	0.943836178	0.948050281	0.954023497	0.917024468
SLC38A6	0.909041248	0.966032124	0.867222363	0.952247001	0.86864151	0.923033364
PHF10	0.909041248	0.847255873	0.894030462	0.92386951	0.964301327	0.923807852
DAP3	0.909041248	0.890669166	0.930736996	0.907621547	0.924135649	0.926487302
IPO4	0.909041248	0.924917071	0.943836178	0.898818046	0.883998248	0.930710695
AUH	0.909041248	0.872175143	0.885896294	0.915779841	0.953300742	0.936263602
TUFT1	0.909041248	0.801351194	0.957052938	0.943808491	0.902907077	0.936778056
LOC510860	0.909041248	0.875506677	0.885469405	0.908467146	0.955623649	0.938894901
NOL12	0.909041248	0.857499283	0.880180025	0.992339478	0.87329316	0.940583352
SLC25A34	0.909041248	0.853678591	0.927635169	0.935282232	0.901688672	0.944330889
MPEG1	0.909041248	0.81848543	0.911973589	0.899047543	0.964833865	0.946729593
MANSC4	0.909041248	0.922610146	0.864832853	0.898818046	0.937932469	0.950631909
LOC614091	0.909041248	0.828365124	0.923713948	0.929663263	0.909379774	0.959198304
GABBR2	0.909041248	0.796253817	0.955189235	0.907794002	0.911664199	0.963255451
CHPT1	0.909041248	0.824149383	0.885896294	0.972142185	0.887514169	0.966850975
IL5RA	0.909041248	0.7889129	0.927635169	0.9063051	0.926813827	0.9790177
CERS5	0.909041248	0.792109226	0.91882781	0.935563591	0.882252274	0.987516427
LOC112445938	0.909041248	0.833323491	0.872088608	0.882057818	0.937096422	0.996679516
ACTR1B	0.909041248	0.790429889	0.89385865	0.905103731	0.89743683	0.999948228
MIEN1	0.909106783	0.918455779	0.945995021	0.949502614	0.926972948	0.846210629
CBLB	0.909106783	0.821767596	0.913609237	0.889415593	0.992104882	0.917150486
SLC41A2	0.909263369	0.950336973	0.855847948	0.890704007	0.986861597	0.867635709
PLCB3	0.90933558	0.929843695	0.916294854	0.966348631	0.914280652	0.85532952
DEF8	0.90933558	0.842856444	0.96030829	0.928416242	0.943160494	0.883688554
MICU1	0.90933558	0.827824094	0.910845937	0.951936643	0.953300742	0.923092436
PALM	0.909463391	0.824188473	0.957052938	0.990955345	0.895067787	0.867482845
LOC101903200	0.909539198	0.813759037	0.90780986	0.967596803	0.944017777	0.923092436
SPSB3	0.909545866	0.977196455	0.916294854	0.881225924	0.954071324	0.854563457
C26H10orf88	0.909545866	0.981952108	0.95322206	0.889415593	0.887514169	0.860414702
TM4SF18	0.909545866	0.785346067	0.904919322	0.999981514	0.917407853	0.890249222
LOC112447070	0.909567768	0.889387648	0.91882781	0.950643928	0.953300742	0.865767172
ADD3	0.909567768	0.817329649	0.999993442	0.886386107	0.93630105	0.877014986
MST1R	0.909655725	0.796253817	0.920462513	0.924425698	0.996089557	0.877048993
CSTF2	0.909964947	0.824188473	0.860877703	0.998529748	0.976292433	0.8537109
TRIM35	0.910047623	0.796253817	0.892343042	0.954946234	0.91563394	0.973731616
NTAN1	0.91038974	0.787034124	0.921266625	0.910575108	0.999985752	0.873130182
EPB41	0.91038974	0.811567701	0.911535369	0.95901497	0.906893576	0.960227605

LOC107131134	0.910757467	0.811059858	0.855793459	0.999981514	0.887514169	0.872238439
MLH3	0.910796992	0.848456869	0.865697455	0.999981514	0.891726429	0.870018677
GPN1	0.910796992	0.875506677	0.89478142	0.999981514	0.893854857	0.872238439
HMGA1	0.910898694	0.882007869	0.947155158	0.989601002	0.891304411	0.853101578
LOC786978	0.910898694	0.786392346	0.880540893	0.999981514	0.870450746	0.945854375
CHRNA7	0.910898694	0.822956796	0.949855095	0.946503916	0.872734644	0.960023723
WDFY4	0.91106548	0.846724749	0.916038418	0.999981514	0.926972948	0.849885981
PRIM1	0.91106548	0.927853707	0.879939804	0.975653996	0.887514169	0.91373422
ARPC4	0.91106548	0.840402825	0.96030829	0.926740082	0.901853599	0.927520621
RPS6KC1	0.91106548	0.816129287	0.892139418	0.96968491	0.946454221	0.930710695
DFFA	0.911202227	0.938087741	0.916294854	0.989601002	0.873198769	0.86078454
TBC1D9	0.911202227	0.855141737	0.999993442	0.934878139	0.879435126	0.860966325
ATXN7L3B	0.911202227	0.942088099	0.923578245	0.899855526	0.917407853	0.907124813
PPAT	0.911202227	0.846724749	0.964661607	0.896116841	0.871558258	0.966850975
ZNF689	0.911202227	0.786825403	0.957052938	0.942759064	0.872096228	0.971545917
AP2A2	0.911202227	0.861975125	0.865697455	0.888687722	0.924277487	0.996679516
ARL5B	0.91127087	0.955138222	0.949939862	0.940438437	0.891726429	0.847012179
ZNHIT1	0.91127087	0.993481224	0.879608555	0.915779841	0.923666555	0.85532952
SNX12	0.91127087	0.89417747	0.974656086	0.952247001	0.891726429	0.858245612
SPAG9	0.91127087	0.808711476	0.944714965	0.995152316	0.935286646	0.859032412
NOX1	0.91127087	0.7973731	0.982080536	0.999981514	0.87329316	0.859219598
ZNF32	0.91127087	0.808478344	0.867719797	0.999981514	0.923931669	0.862925606
LOC101904810	0.91127087	0.826124039	0.999993442	0.894086736	0.93630105	0.865745718
C8H8orf58	0.91127087	0.799832125	0.944126714	0.952247001	0.976259416	0.865767172
CHMP4A	0.91127087	0.849119707	0.935266153	0.980564765	0.935093621	0.867063767
LARP7	0.91127087	0.898951086	0.865512879	0.949565453	0.977167046	0.869668409
GUSB	0.91127087	0.875974117	0.873674244	0.999981514	0.887514169	0.872237727
KPTN	0.91127087	0.80321632	0.916038418	0.944617608	0.991481886	0.87501921
SLITRK4	0.91127087	0.852526348	0.880163755	0.992560663	0.954071324	0.876162019
NME4	0.91127087	0.899098089	0.927635169	0.973396104	0.896589592	0.877048993
STUM	0.91127087	0.924125044	0.911636686	0.972467586	0.900233561	0.881697198
LOC100847363	0.91127087	0.968166143	0.921266625	0.893220599	0.918052462	0.883530991
LOC787851	0.91127087	0.874981732	0.996224523	0.896116841	0.900233561	0.883688554
TRMT61B	0.91127087	0.802570554	0.916323169	0.949502614	0.979290191	0.887542005
TAF1C	0.91127087	0.804973066	0.91469924	0.999981514	0.901144467	0.891088733
VPS25	0.91127087	0.798718768	0.955427429	0.891988949	0.988049878	0.891088733
FKBP9	0.91127087	0.847093186	0.855268213	0.888198716	0.999985752	0.891088733
LOC101907383	0.91127087	0.834615638	0.940445817	0.96223045	0.930451229	0.903500283

TNFSF13	0.91127087	0.927973597	0.885896294	0.898818046	0.964301327	0.905188617
DDX31	0.91127087	0.790429889	0.999993442	0.94218374	0.870450746	0.91373422
FBN1	0.91127087	0.808823388	0.906019013	0.973373329	0.953107977	0.917930485
LOC782418	0.91127087	0.799856566	0.937118288	0.954946234	0.934481683	0.930710695
NR4A3	0.91127087	0.904875256	0.910845937	0.916858019	0.926813827	0.932688327
SRPK1	0.91127087	0.79341825	0.937869586	0.907512576	0.965676623	0.933897626
SIK1	0.91127087	0.821767596	0.899106435	0.955466436	0.944839728	0.936263602
SLIRP	0.91127087	0.786691027	0.982504315	0.925074676	0.894364008	0.943972369
GDPD3	0.91127087	0.833713501	0.9377363	0.929158185	0.913732532	0.94844234
ZFYVE1	0.91127087	0.808478344	0.940467884	0.917875952	0.924222719	0.960877268
RAB44	0.91127087	0.7973731	0.916294854	0.94565162	0.917407853	0.966914618
WDR92	0.91127087	0.846724749	0.944949386	0.902776185	0.887514169	0.971982373
PTCD3	0.91127087	0.804542244	0.893988897	0.925353907	0.935286646	0.980167535
ESS2	0.91127087	0.884128797	0.874797723	0.88904348	0.89743683	0.99642553
DCAF17	0.91127087	0.828151573	0.891572374	0.886312303	0.891726429	0.999948228
LOC107133190	0.911345211	0.907694305	0.863543401	0.98981682	0.879435126	0.930710695
GCLM	0.911495189	0.831506983	0.916294854	0.996621929	0.930451229	0.876162019
RPP21	0.911495189	0.853678591	0.896821247	0.957008595	0.902907077	0.957558074
SMCO4	0.911661363	0.871091574	0.957052938	0.925353907	0.948006937	0.873130182
LOC112442082	0.911661363	0.786817529	0.880357899	0.995565324	0.937932469	0.933897626
CFAP69	0.911774297	0.851948403	0.957295966	0.973075232	0.926972948	0.858507498
XRN2	0.91182912	0.87976124	0.906019013	0.940438437	0.940870936	0.923199665
FMO4	0.91184891	0.831153015	0.916038418	0.999981514	0.887514169	0.90645885
ARV1	0.91229862	0.861395263	0.996224523	0.885789665	0.926831826	0.891088733
LOC784322	0.912339441	0.794494758	0.999993442	0.886312303	0.893716526	0.923547711
CSDC2	0.912339441	0.913120359	0.891972402	0.908467146	0.877712931	0.9790177
LOC101905219	0.912579504	0.792483444	0.996068003	0.925210648	0.953300742	0.867063767
PLA2G7	0.912579504	0.846724749	0.962034224	0.959616431	0.920318083	0.87754233
LYRM9	0.912579504	0.791046586	0.903122466	0.908467146	0.999985752	0.888930405
UQCC3	0.912579504	0.823478017	0.905023942	0.976679676	0.953300742	0.909056553
SNRPB	0.912579504	0.796666816	0.990118874	0.952247001	0.882206878	0.921557022
DCLK2	0.912579504	0.787802226	0.855847948	0.932914414	0.900927045	0.999948228
NPRL2	0.912606517	0.839507565	0.929176945	0.950643928	0.976292433	0.85532952
LOC100139548	0.912606517	0.91876635	0.916323169	0.882086275	0.976259416	0.883530991
SLC25A35	0.912606517	0.809330769	0.98915348	0.912777307	0.948302449	0.884993859
XRCC4	0.912606517	0.986900642	0.872705694	0.887983383	0.923910588	0.917150486
ZNF729	0.912606517	0.792690308	0.971053208	0.959159289	0.893716526	0.927520621
PSMA4	0.912606517	0.796253817	0.972482052	0.886312303	0.960573822	0.927897089

LOC539893	0.912606517	0.790429889	0.992937407	0.91691351	0.887514169	0.942105787
ARMC4	0.912842149	0.984410135	0.871491704	0.966343269	0.887514169	0.866494771
GLP2R	0.912860331	0.796253817	0.999993442	0.903584224	0.890124729	0.8537109
PGGT1B	0.912860331	0.901905553	0.982708154	0.940438437	0.884033936	0.868538865
IMMP2L	0.912860331	0.789445628	0.943836178	0.965724803	0.961465747	0.884925084
LOC781197	0.912860331	0.906658524	0.897702337	0.890704007	0.970988226	0.91373422
AP5B1	0.912860331	0.861395263	0.891972402	0.916858019	0.881680572	0.994788615
RTN4IP1	0.912900751	0.829067393	0.967696262	0.885789665	0.964301327	0.911336121
TTC39C	0.912900751	0.821767596	0.97524026	0.930987593	0.890909775	0.934360589
CERCAM	0.913033068	0.861395263	0.915858924	0.999981514	0.882252274	0.905034785
ARHGEF11	0.913033068	0.867518836	0.935266153	0.907997414	0.926972948	0.944973383
CCL26	0.913033068	0.889387648	0.916323169	0.89684883	0.926972948	0.95508469
DMXL1	0.913036989	0.912012478	0.881522424	0.999981514	0.87329316	0.854935725
SAT2	0.913036989	0.892252816	0.925244197	0.982220679	0.891726429	0.88542704
PLIN3	0.913137153	0.961663204	0.860877703	0.922947889	0.887514169	0.950631909
BMP2K	0.913249165	0.829067393	0.964661607	0.891916807	0.964301327	0.911219114
HGF	0.913400594	0.912764028	0.922552585	0.948668643	0.887514169	0.92725789
LOC787905	0.913400594	0.930266314	0.916294854	0.916858019	0.905184827	0.92725789
ATP5S	0.913744313	0.796748952	0.89478142	0.973428299	0.895084325	0.973218068
NCF4	0.913987723	0.855067335	0.989494193	0.892047793	0.917407853	0.91373422
SOCS5	0.913987723	0.814995661	0.955427429	0.925294859	0.955566302	0.91373422
ATG101	0.914035382	0.78945719	0.864133419	0.999981514	0.953300742	0.867063767
LOC617875	0.914042827	0.805580108	0.856952806	0.999981514	0.885844206	0.966212838
SLC12A6	0.914042827	0.846724749	0.955427429	0.894086736	0.891304411	0.969197073
MTUS1	0.914042827	0.839128192	0.899106435	0.923623116	0.908528514	0.980464176
CD81	0.914050419	0.846724749	0.957052938	0.943447418	0.942572522	0.889662488
NF1	0.914062324	0.850508324	0.916323169	0.889415593	0.999985752	0.852504043
ZNF518A	0.914062324	0.866614438	0.916294854	0.935958061	0.982037317	0.865767172
HEATR5A	0.914062324	0.836427846	0.880163755	0.960937718	0.979290191	0.89148147
RTN1	0.914062324	0.7889129	0.92271578	0.961898258	0.964833865	0.911219114
CLK2	0.914062324	0.791281103	0.951392011	0.954343717	0.943702351	0.919124527
CEP85L	0.914124315	0.938454625	0.93475587	0.890704007	0.953300742	0.880981659
TCF4	0.914144353	0.822956796	0.877334778	0.973045775	0.996781978	0.866494771
AKT2	0.914144353	0.884773193	0.97998887	0.899855526	0.929802526	0.88313584
FBXL3	0.914144353	0.989303356	0.93891936	0.890362631	0.876359217	0.888309103
LOC104972830	0.914144353	0.884164817	0.894742182	0.929663263	0.972432486	0.893838339
JPH4	0.914144353	0.892252816	0.922552585	0.914850247	0.943540106	0.919993751
RASGRP2	0.914454845	0.984331753	0.937162781	0.898818046	0.916605572	0.850475013

INTS4	0.914454845	0.991673407	0.879939804	0.891988949	0.958797242	0.852048593
TMEM150B	0.914454845	0.842948165	0.93891936	0.999981514	0.926972948	0.853056636
ZBTB2	0.914454845	0.913120359	0.888989166	0.9063051	0.996781978	0.858451225
CHRNB4	0.914454845	0.847135393	0.995377403	0.963132425	0.887514169	0.859032412
PAK1IP1	0.914454845	0.882242226	0.881070741	0.999981514	0.911568867	0.860966325
LOC101906358	0.914454845	0.846724749	0.933884582	0.96951401	0.960419102	0.862925606
WBP1	0.914454845	0.952760839	0.940411348	0.891988949	0.953885477	0.864514638
CD101	0.914454845	0.948801019	0.877193584	0.898890982	0.982526545	0.867063767
APEX2	0.914454845	0.8332127	0.915191964	0.92743463	0.998287238	0.867482845
IRX3	0.914454845	0.892108342	0.972349147	0.962864391	0.884872176	0.870490204
PTBP3	0.914454845	0.840785555	0.891972402	0.930987593	0.999985752	0.872132138
CALY	0.914454845	0.814995661	0.916294854	0.999981514	0.926813827	0.873130182
FBXO21	0.914454845	0.89489192	0.948594902	0.887983383	0.974738415	0.874490166
BHLHE41	0.914454845	0.846242937	0.95940506	0.892047793	0.979840175	0.87501921
L3HYPDH	0.914454845	0.901028838	0.987610559	0.933240019	0.883951804	0.876861428
TMX4	0.914454845	0.791281103	0.943028511	0.999981514	0.906033379	0.876861428
TMBIM1	0.914454845	0.793300166	0.935266153	0.999981514	0.929896768	0.876861428
FAM162B	0.914454845	0.874783523	0.983504244	0.925294859	0.911664199	0.87754233
LOC101902458	0.914454845	0.808478344	0.94422142	0.999981514	0.902258286	0.884402686
SNTB2	0.914454845	0.80640506	0.955427429	0.898818046	0.988049878	0.886350023
LIN54	0.914454845	0.808711476	0.872088608	0.886312303	0.999985752	0.887722423
R3HCC1L	0.914454845	0.871924797	0.894338686	0.890362631	0.999985752	0.89243935
ERGIC1	0.914454845	0.817524348	0.983504244	0.973812087	0.879268517	0.898470276
GSAP	0.914454845	0.856047169	0.924855847	0.984423083	0.911930323	0.903210685
DOCK4	0.914454845	0.985428851	0.872088608	0.888687722	0.938316699	0.909056553
AGO2	0.914454845	0.804185018	0.911973589	0.908467146	0.998835841	0.909579961
PRPF18	0.914454845	0.799339314	0.893988897	0.999981514	0.917407853	0.910841645
CYYR1	0.914454845	0.918455779	0.95219206	0.920412583	0.892446951	0.913250128
LOC783396	0.914454845	0.934402043	0.886829776	0.98061629	0.878542125	0.91373422
NFAT5	0.914454845	0.872244611	0.937869586	0.90413565	0.956041014	0.91832348
RAD9A	0.914454845	0.826316517	0.867661896	0.984423083	0.959658682	0.922543509
SNRPB2	0.914454845	0.790429889	0.957295966	0.988951957	0.883143938	0.925233445
P2RY12	0.914454845	0.961663204	0.906019013	0.893220599	0.909379774	0.92789871
TIMM29	0.914454845	0.826316517	0.976484937	0.952298386	0.877712931	0.928708614
MLXIP	0.914454845	0.818303808	0.942357321	0.910575108	0.960573822	0.930710695
TCEAL9	0.914454845	0.840402825	0.877863957	0.992930508	0.923910588	0.931410194
CPT1B	0.914454845	0.888057447	0.858376167	0.999981514	0.882252274	0.933897626
MAST3	0.914454845	0.796253817	0.979354564	0.95901497	0.882206878	0.935967823

OTOR	0.914454845	0.826316517	0.980761647	0.887983383	0.917407853	0.947801358
ZW10	0.914454845	0.818933054	0.955427429	0.925353907	0.919660109	0.947801358
DMKN	0.914454845	0.938235006	0.888566052	0.900875874	0.909379774	0.958790968
GADD45GIP1	0.914454845	0.840886089	0.916453343	0.916858019	0.935286646	0.960023723
LOC100849681	0.914454845	0.792624847	0.929661805	0.901353354	0.960573822	0.960023723
PHACTR3	0.914454845	0.852526348	0.867222363	0.915681158	0.954403133	0.971545917
MEF2D	0.914454845	0.845338797	0.896821247	0.896116841	0.943702351	0.978087957
CABLES2	0.914454845	0.801802651	0.916294854	0.912777307	0.935286646	0.981216824
VOPP1	0.914479735	0.952760839	0.972741989	0.898818046	0.893716526	0.870490204
EMG1	0.914479735	0.804185018	0.955427429	0.943447418	0.900233561	0.959198304
SPX	0.915111626	0.796253817	0.90676547	0.975118969	0.976259416	0.891423889
SUB1	0.915368998	0.861641368	0.999993442	0.916858019	0.87329316	0.862925606
INTS1	0.915368998	0.866901725	0.916294854	0.92532303	0.983941532	0.874490166
SOX12	0.915368998	0.886565095	0.93475587	0.99739416	0.881680572	0.876162019
ZBTB6	0.915368998	0.804185018	0.916323169	0.987605107	0.964301327	0.876162019
LOC100847604	0.915368998	0.909789853	0.997300234	0.891732037	0.889249582	0.883688554
LDB2	0.915368998	0.795514291	0.999993442	0.943447418	0.892654422	0.890754919
LOC101904536	0.915368998	0.831153015	0.921266625	0.938303773	0.970988226	0.907145443
E2F7	0.915368998	0.849153241	0.964134334	0.952247001	0.907678664	0.908099327
NPY1R	0.915368998	0.997613842	0.867661896	0.888687722	0.887514169	0.923807852
SLC15A2	0.915368998	0.85265333	0.883950091	0.912922827	0.982481582	0.927520621
ASRGL1	0.915368998	0.7973731	0.92355285	0.999981514	0.882013597	0.929814744
NT5M	0.915368998	0.872175143	0.881070741	0.898658215	0.982203967	0.931500173
MAPK15	0.915368998	0.926551255	0.923521499	0.935563591	0.883951804	0.934671838
RPA1	0.915368998	0.831564725	0.971594714	0.901653135	0.902907077	0.954550352
AP1B1	0.915368998	0.950336973	0.885896294	0.885789665	0.917407853	0.960023723
CTPS1	0.915368998	0.849513595	0.899106435	0.896116841	0.887514169	0.999463809
DEPDC7	0.915469901	0.848383949	0.948653258	0.93660503	0.964301327	0.877014986
LDHC	0.915596452	0.926551255	0.937841596	0.896116841	0.964301327	0.873179954
SEMA3G	0.915596452	0.813639295	0.885896294	0.999981514	0.886451706	0.882690335
LOC107132870	0.915596452	0.986900642	0.859987058	0.959652624	0.882206878	0.899183258
ZNF835	0.915596452	0.938235006	0.875900843	0.8986831	0.943160494	0.945854375
OSMR	0.915822014	0.923232089	0.944126714	0.886312303	0.935286646	0.923807852
LOC101910153	0.915852428	0.895777642	0.959725886	0.964521432	0.902907077	0.865889509
MORN2	0.915855834	0.835486075	0.949855095	0.996033721	0.890124729	0.888475311
CARS2	0.915948438	0.826395702	0.949855095	0.892047793	0.984386856	0.901216608
ACTR1A	0.915996644	0.846724749	0.99341711	0.910575108	0.906557107	0.907124813
ABCF1	0.916038866	0.834615638	0.957052938	0.892047793	0.906893576	0.970105102

LOC101906167	0.916039287	0.824188473	0.91882781	0.985701713	0.919660109	0.923092436
SPATA5	0.916039287	0.845750059	0.865444995	0.898818046	0.878451408	0.999948228
VRK2	0.916220932	0.872175143	0.947176871	0.913048575	0.967470055	0.877014986
LOC512248	0.916613472	0.806695542	0.879939804	0.999981514	0.954403133	0.870047002
TFPI	0.916613472	0.861480696	0.901955007	0.961898258	0.943160494	0.92451208
KIF13B	0.916613472	0.83359488	0.971255098	0.903584224	0.935286646	0.930710695
BEX3	0.916613472	0.811059858	0.892343042	0.960016921	0.933426897	0.969014913
LOC781254	0.916784249	0.799648942	0.976011371	0.973373329	0.950958352	0.858245612
ITPRIP	0.916784249	0.968186752	0.880612713	0.940436902	0.953300742	0.86324237
PRKACA	0.916784249	0.81232489	0.978014482	0.982220679	0.926813827	0.864139695
TSKS	0.916784249	0.811354298	0.978014482	0.892047793	0.989379051	0.864207753
LOC100298868	0.916784249	0.823478017	0.984886031	0.967577396	0.913180847	0.874459658
FBXW8	0.916784249	0.929204871	0.876414692	0.960016921	0.947003843	0.893838339
STARD13	0.916784249	0.855389438	0.872088608	0.901353354	0.999985752	0.911219114
KIF3B	0.916784249	0.826316517	0.896821247	0.999981514	0.893716526	0.91373422
HIKESHI	0.916784249	0.823647199	0.978014482	0.935282232	0.92549567	0.91373422
CCNB1IP1	0.916784249	0.811354298	0.990037865	0.961898258	0.881680572	0.914207441
STRN4	0.916784249	0.941506707	0.922552585	0.899792456	0.927086179	0.9221477
CDC26	0.916784249	0.826316517	0.957052938	0.949565453	0.917617128	0.928708614
LOC112446793	0.916784249	0.845347956	0.890499307	0.910495312	0.983010476	0.933638318
IL20RB	0.916784249	0.823844235	0.891662311	0.979644238	0.929802526	0.950798794
ILK	0.916784249	0.802570554	0.95219206	0.943808491	0.918426817	0.952571698
LPL	0.916784249	0.880146316	0.916323169	0.901970399	0.929802526	0.960487142
FBRS	0.916784249	0.815362549	0.938624264	0.889472032	0.959026737	0.963112102
MAPRE3	0.916784249	0.861395263	0.918641217	0.93745545	0.887514169	0.971545917
MED30	0.916859293	0.829067393	0.930736996	0.952247001	0.943610727	0.927520621
INTS8	0.916905488	0.847093186	0.911973589	0.890362631	0.972160227	0.951123163
FAM118B	0.91691226	0.792967492	0.925320071	0.914026346	0.999985752	0.859949878
PI4KB	0.91691226	0.796253817	0.989144553	0.886312303	0.977167046	0.890967932
ASCC3	0.91691226	0.833323491	0.867326337	0.961898258	0.980695939	0.917849087
AKAIN1	0.91691226	0.904875256	0.886829776	0.912777307	0.966328884	0.923092436
MFSD3	0.91691226	0.895777642	0.951028911	0.912777307	0.911930323	0.927426708
FKBP11	0.91691226	0.871245137	0.874797723	0.969088992	0.902907077	0.963456717
SMAD5	0.916933198	0.90715891	0.867964007	0.961898258	0.964833865	0.888475311
LOC511161	0.91701745	0.842391145	0.871285807	0.929388614	0.999453276	0.907152267
GRN	0.91701745	0.880146316	0.901736458	0.96951401	0.926972948	0.923807852
ATP11A	0.91701745	0.908364002	0.894338686	0.934466438	0.943160494	0.929814744
GPR39	0.917161617	0.876873679	0.895826828	0.975653996	0.887514169	0.951081819

LOC509972	0.917217965	0.855067335	0.964807927	0.973075232	0.93630105	0.853496009
SASH1	0.917217965	0.868376989	0.906395507	0.999981514	0.892058675	0.864139695
C8H9orf72	0.917217965	0.908504395	0.881070741	0.911606774	0.996781978	0.874000314
C24H18orf32	0.917217965	0.7973731	0.999993442	0.91294441	0.905825822	0.879759038
USP48	0.917217965	0.792109226	0.864832853	0.907794002	0.999985752	0.927520621
METTL24	0.917363695	0.981520642	0.892343042	0.970367101	0.903149543	0.8537109
WNT5B	0.917363695	0.988444882	0.923521499	0.950643928	0.882252274	0.853994103
CEP78	0.917363695	0.877822131	0.971053208	0.969214881	0.917410944	0.857673662
CCR1	0.917363695	0.912602201	0.927635169	0.980564765	0.932024143	0.857911901
SNRPF	0.917363695	0.829067393	0.999993442	0.952247001	0.9096389	0.858321732
LOC107133343	0.917363695	0.867277242	0.999993442	0.9063051	0.924662947	0.859032412
SOS1	0.917363695	0.870298381	0.915858924	0.893220599	0.999985752	0.859949878
LOC101903645	0.917363695	0.885951248	0.97316848	0.969837124	0.901853599	0.861631711
PAFAH1B2	0.917363695	0.929267209	0.94955109	0.969256019	0.900747635	0.862871483
DISC1	0.917363695	0.855141737	0.89478142	0.999981514	0.943610727	0.862925606
TBC1D5	0.917363695	0.879035304	0.99341711	0.961898258	0.883951804	0.864139695
CRACR2B	0.917363695	0.796748952	0.949855095	0.999981514	0.925918507	0.865745718
BCL2L12	0.917363695	0.872331637	0.89478142	0.999981514	0.936215792	0.865767172
HYI	0.917363695	0.952760839	0.955427429	0.944351122	0.889217664	0.865889509
BACE2	0.917363695	0.885951248	0.89478142	0.999981514	0.8992849	0.867063767
LOC107132524	0.917363695	0.989303356	0.879939804	0.958582027	0.900927045	0.867063767
LOC104975676	0.917363695	0.931540327	0.945995021	0.949468104	0.925918507	0.867063767
LOC101903301	0.917363695	0.843278276	0.997850143	0.896116841	0.95388482	0.867063767
SPAG8	0.917363695	0.844170799	0.946791586	0.949565453	0.970988226	0.867063767
SCAF4	0.917363695	0.811059858	0.881790227	0.918693944	0.999985752	0.867063767
NPL	0.917363695	0.946241678	0.924855847	0.949565453	0.926972948	0.868117674
LINGO1	0.917363695	0.897910327	0.939912874	0.965050971	0.935286646	0.871503289
CBLN4	0.917363695	0.895777642	0.916294854	0.958588196	0.959658682	0.872238439
RBMS1	0.917363695	0.805580108	0.879608555	0.999981514	0.972160227	0.875650601
DDX41	0.917363695	0.829067393	0.89478142	0.947574232	0.999453276	0.876162019
CD34	0.917363695	0.872175143	0.93190909	0.935563591	0.970988226	0.87754233
PRIM2	0.917363695	0.846724749	0.894338686	0.999981514	0.878451408	0.879576441
LOC112447857	0.917363695	0.822057273	0.8735515	0.999981514	0.942786245	0.882690335
CSPG4	0.917363695	0.842273005	0.997400824	0.915039335	0.91563394	0.891088733
EPN3	0.917363695	0.821383763	0.894545897	0.999981514	0.925171841	0.891088733
FBXW7	0.917363695	0.822956796	0.957052938	0.912573312	0.976769047	0.891088733
FILIP1	0.917363695	0.956095821	0.911401689	0.906186004	0.943702351	0.894808664
ONECUT2	0.917363695	0.950336973	0.966293373	0.904018489	0.887514169	0.896027932

HMOX2	0.917363695	0.890669166	0.916294854	0.974286747	0.929896768	0.898352859
PIGU	0.917363695	0.877501251	0.955427429	0.979385838	0.886944613	0.899428912
CDKL2	0.917363695	0.855389438	0.891745553	0.990955345	0.943702351	0.905509632
ARHGEF25	0.917363695	0.951772495	0.8644528	0.983233117	0.900879375	0.90607344
TSG101	0.917363695	0.883756544	0.981655709	0.942955143	0.883095372	0.907678449
TMEM110	0.917363695	0.887752955	0.916294854	0.9880671	0.900809233	0.911219114
LOC616720	0.917363695	0.846721805	0.899600099	0.891988949	0.999985752	0.911638347
MINDY3	0.917363695	0.813442974	0.991138474	0.949565453	0.902258286	0.912486829
ADNP2	0.917363695	0.905550751	0.982504315	0.912777307	0.879776073	0.914132808
SEH1L	0.917363695	0.832556888	0.898179681	0.992560663	0.938316699	0.915735519
EIF4E2	0.917363695	0.828583132	0.999993442	0.902490239	0.893376996	0.917930485
LOC786258	0.917363695	0.849119707	0.940467884	0.95901497	0.933344708	0.919124527
AGAP3	0.917363695	0.867518836	0.922552585	0.985701713	0.893716526	0.923092436
TNFRSF18	0.917363695	0.984089343	0.868479534	0.908467146	0.916605572	0.923092436
AJUBA	0.917363695	0.816248177	0.916294854	0.889415593	0.999985752	0.923092436
LOC783497	0.917363695	0.811486218	0.981383697	0.938303773	0.91563394	0.923807852
MRPL35	0.917363695	0.821790484	0.948653258	0.891732037	0.977167046	0.923807852
ING2	0.917363695	0.871176243	0.874315179	0.985701713	0.940870936	0.923944552
FAM117A	0.917363695	0.938087741	0.916323169	0.934466438	0.901853599	0.925057945
KCNMB3	0.917363695	0.938235006	0.946791586	0.910575108	0.882418284	0.932534796
NABP1	0.917363695	0.921881538	0.942357321	0.93660503	0.878451408	0.93531275
PTGER2	0.917363695	0.814995661	0.968679299	0.898818046	0.95221242	0.938038311
RNF187	0.917363695	0.796253817	0.944538814	0.889415593	0.983941532	0.940017687
DEPDC5	0.917363695	0.829067393	0.966847507	0.916858019	0.92337151	0.944895331
EFNA2	0.917363695	0.899494273	0.899952655	0.962437357	0.89052284	0.945624606
GKAP1	0.917363695	0.826955807	0.943836178	0.961898258	0.89052284	0.958281344
LOC107131642	0.917363695	0.873915965	0.899634571	0.975346638	0.882418284	0.958790968
PQLC2	0.917363695	0.882858125	0.865697455	0.901115098	0.960573822	0.970105102
CSF2RB	0.917363695	0.810212541	0.895801611	0.984421126	0.887514169	0.975558533
LOC100847490	0.917363695	0.883391071	0.889098668	0.924425698	0.879776073	0.993477308
H4	0.917363695	0.813305033	0.903431343	0.952247001	0.879776073	0.99666605
MBD2	0.917363695	0.812449121	0.867661896	0.898964391	0.895067787	0.999948228
LOC112447118	0.917363695	0.856868887	0.879939804	0.893051204	0.906676706	0.999948228
TBC1D2B	0.917363695	0.812449121	0.883737851	0.893031155	0.936108073	0.999948228
SLC35A3	0.917379262	0.890669166	0.96030829	0.972142185	0.884872176	0.891088733
RCE1	0.917439478	0.818303808	0.999993442	0.915039335	0.882766929	0.928416436
CREBBP	0.917766142	0.846724749	0.944538814	0.891732037	0.996781978	0.881236815
LOC100848665	0.917766142	0.820800377	0.957052938	0.92532303	0.944839728	0.934360589

CST7	0.917766142	0.804185018	0.892343042	0.999981514	0.893716526	0.950631909
CPNE1	0.917766142	0.853678591	0.96123049	0.887983383	0.935286646	0.955210102
ADRA2B	0.917766142	0.814995661	0.898179681	0.955981799	0.891726429	0.991140568
LOC101905525	0.917920886	0.893615781	0.916323169	0.961898258	0.889301074	0.940017687
PHF13	0.918030595	0.878488273	0.867661896	0.896116841	0.999985752	0.908099327
LOC107131429	0.918063967	0.870195838	0.892139418	0.888687722	0.91383048	0.99828741
PAPOLB	0.918218009	0.813658213	0.91882781	0.904018489	0.999985752	0.877014986
PTPN13	0.918524659	0.796253817	0.961554145	0.904277308	0.996781978	0.879576441
PLXNB3	0.918524659	0.994185904	0.920681987	0.893220599	0.886785729	0.893838339
MRPL28	0.918524659	0.855141737	0.956656968	0.898188142	0.964301327	0.915735519
LMBRD1	0.918524659	0.823995537	0.93475587	0.95901497	0.927037956	0.945854375
C14H8orf88	0.918611392	0.7973731	0.927508464	0.912777307	0.97352263	0.947838082
THOC1	0.918631735	0.83844057	0.946924479	0.960016921	0.932024143	0.921557022
SLC3A2	0.918727264	0.840402825	0.924855847	0.992930508	0.953300742	0.867907362
LOC613822	0.918727264	0.870222905	0.946791586	0.992475566	0.891726429	0.88542704
LOC101907797	0.918727264	0.832398588	0.924855847	0.888687722	0.983941532	0.938894901
EIF4G1	0.918944564	0.925291049	0.894338686	0.898818046	0.943702351	0.952571698
SERPINB8	0.918944564	0.808586422	0.883984752	0.972142185	0.905825822	0.985863239
DNAH5	0.918962943	0.955827589	0.894338686	0.912152327	0.894364008	0.951081819
SUPT6H	0.918978896	0.821674572	0.903431343	0.947313672	0.999985752	0.876162019
POLR3C	0.918991334	0.918455779	0.911850355	0.947574232	0.950958352	0.899183258
LOC101909173	0.918991334	0.823133291	0.939981892	0.984423083	0.893376996	0.937942868
CCDC125	0.919251101	0.829345514	0.964807927	0.989485949	0.934419113	0.860966325
NUP155	0.919251101	0.968509005	0.867625133	0.891988949	0.976292433	0.89209761
KCNC3	0.919251101	0.942546802	0.877334778	0.902696094	0.962513981	0.923807852
QKI	0.919251101	0.847323359	0.924855847	0.990955345	0.887514169	0.930710695
JMJD7	0.919259806	0.886168102	0.887132069	0.999981514	0.90645589	0.904206635
LOC107132487	0.919259806	0.800861368	0.917155067	0.982220679	0.926972948	0.94844234
POLR1E	0.919259806	0.821790484	0.879939804	0.949864417	0.953300742	0.971938133
SGK1	0.919267435	0.846885681	0.89478142	0.96900924	0.984386856	0.87501921
HCN2	0.919267435	0.892108342	0.942195449	0.948668643	0.954071324	0.877048993
LRBA	0.919267435	0.82177045	0.870410781	0.903004767	0.999985752	0.901216608
RPA2	0.919267435	0.938087741	0.886829776	0.972142185	0.925171841	0.903500283
RBFA	0.919267435	0.894702415	0.915858924	0.930854558	0.953300742	0.923807852
TANGO6	0.919813832	0.873218028	0.896109455	0.907353815	0.951996251	0.966779561
GON7	0.919861857	0.892252816	0.943836178	0.999981514	0.891726429	0.857911901
EXOC1L	0.919861857	0.826316517	0.982963534	0.916858019	0.964301327	0.876162019
ARHGAP22	0.919861857	0.912693158	0.870734949	0.963132425	0.946384846	0.923807852

SLC7A7	0.919861857	0.845367708	0.905023942	0.964521432	0.956653075	0.925472172
RUSC2	0.919861857	0.946148902	0.916038418	0.892047793	0.893032264	0.963107047
CD37	0.919937108	0.846724749	0.949855095	0.908467146	0.97352263	0.906637848
PPIH	0.919942393	0.827824094	0.99341711	0.889415593	0.953300742	0.915546224
ASXL3	0.919943826	0.872175143	0.871929137	0.999981514	0.891304411	0.877014986
CIDEC	0.919943826	0.940500178	0.893848993	0.989601002	0.906893576	0.878754045
GHR	0.919943826	0.819210468	0.872088608	0.999981514	0.915279373	0.891088733
KCTD5	0.919943826	0.888057447	0.915858924	0.989601002	0.880315832	0.930710695
HAND1	0.919943826	0.7973731	0.988585686	0.901353354	0.926813827	0.954496768
CYB5A	0.919943826	0.797765031	0.883737851	0.943808491	0.970988226	0.965117554
CHMP7	0.919973515	0.822956796	0.999993442	0.925294859	0.891726429	0.859032412
METAP1D	0.919973515	0.833429351	0.999993442	0.900044333	0.925918507	0.872239777
TF	0.919973515	0.836427846	0.975774524	0.918739801	0.969746326	0.873185607
LOC112444841	0.919973515	0.836198562	0.983504244	0.938303773	0.953300742	0.87501921
PEX13	0.919973515	0.814995661	0.999993442	0.951936643	0.895067787	0.89438401
CABP1	0.919973515	0.960519707	0.871285807	0.983233117	0.887514169	0.911234149
CCS	0.919973515	0.911250516	0.897322115	0.960937718	0.940870936	0.91373422
RELL1	0.919973515	0.831153015	0.885896294	0.941467375	0.996089557	0.917930485
LRRC47	0.919973515	0.811354298	0.916323169	0.999981514	0.917407853	0.925472172
LOC100336104	0.919973515	0.950675653	0.874894845	0.896116841	0.928951321	0.963255451
ENSA	0.920004085	0.812449121	0.955427429	0.959652624	0.883951804	0.964898868
DNASE1L1	0.920235199	0.878328949	0.964807927	0.927560164	0.935286646	0.910062576
C1GALT1C1	0.920235199	0.863286109	0.935266153	0.890499731	0.97234838	0.932534796
SLC25A16	0.920369417	0.835486075	0.982823002	0.957725993	0.902366469	0.911219114
TM2D1	0.920505522	0.915269318	0.877193584	0.921950515	0.996781978	0.874870038
DAGLB	0.920765859	0.880819975	0.916294854	0.959616431	0.958797242	0.895517058
GPRC5C	0.920894543	0.904754143	0.916294854	0.996621929	0.927086179	0.860966325
LOC100847780	0.920894543	0.846724749	0.929661805	0.932914414	0.931871771	0.960023723
LRRC40	0.92097324	0.991673407	0.879939804	0.896116841	0.926972948	0.91373422
TBC1D31	0.92097324	0.823478017	0.951678465	0.952247001	0.891304411	0.963456717
SRF	0.921077176	0.986900642	0.937281477	0.892071982	0.920952484	0.875118526
RNF167	0.921077176	0.846721805	0.989252436	0.959616431	0.909750936	0.883688554
LOC112442288	0.921077176	0.823589054	0.879939804	0.905772309	0.999985752	0.911808092
RBP1	0.921433364	0.92522948	0.974470955	0.900875874	0.954827657	0.856833445
LOC614423	0.921433364	0.813442974	0.992511421	0.943808491	0.960784627	0.860414702
EXT1	0.921433364	0.822956796	0.999993442	0.899296336	0.947299702	0.866280163
HS2ST1	0.921433364	0.912622732	0.971255098	0.900044333	0.956906331	0.869620106
NCOA5	0.921433364	0.915269318	0.944113072	0.890362631	0.983941532	0.869668409

SEMA6D	0.921433364	0.932778537	0.978014482	0.925210648	0.905825822	0.87229131
LOC101903997	0.921433364	0.970084348	0.885896294	0.925353907	0.957104426	0.877014986
RBM19	0.921433364	0.999987083	0.874983674	0.888687722	0.907762824	0.878118151
WDR91	0.921433364	0.826316517	0.885896294	0.96444269	0.998196897	0.88313584
LOC100847946	0.921433364	0.808478344	0.984886031	0.951479029	0.936388637	0.90388238
SLC25A21	0.921433364	0.811354298	0.989986643	0.960016921	0.884679971	0.930710695
FNBP1L	0.921590092	0.973712113	0.893427844	0.96444269	0.900747635	0.888475311
ARHGAP9	0.921590092	0.96643574	0.916038418	0.908467146	0.935286646	0.906251135
LOC786616	0.921590092	0.863286109	0.887453166	0.938553091	0.974738415	0.93363377
CD19	0.921590092	0.813916354	0.99341711	0.896116841	0.895067787	0.963015949
MRPS9	0.921600439	0.826316517	0.95875611	0.899792456	0.987723553	0.891423889
SCAMP1	0.921703632	0.99960614	0.882643407	0.904018489	0.926972948	0.858451225
ETFDH	0.921703632	0.862299245	0.911636686	0.904018489	0.999985752	0.891088733
ABCD3	0.921703632	0.822879294	0.984886031	0.948668643	0.929802526	0.910004719
SERPINA5	0.921703632	0.90323787	0.915407439	0.902776185	0.958852623	0.938161998
DCPS	0.921703632	0.835452905	0.867661896	0.980632768	0.913732532	0.977166378
C6H4orf3	0.921703632	0.816129287	0.879939804	0.949502614	0.931775933	0.993890045
SLC2A13	0.921896607	0.812308035	0.971053208	0.961898258	0.964553547	0.865767172
DPP6	0.922288529	0.857815763	0.93475587	0.90413565	0.932024143	0.970105102
SLC52A2	0.922320842	0.927853707	0.978014482	0.925353907	0.926972948	0.86324237
SCAND1	0.922320842	0.912776769	0.935117456	0.952247001	0.936108073	0.894808664
MRPL47	0.922320842	0.868390063	0.942357321	0.940436902	0.935286646	0.933897626
TBL1X	0.92234124	0.892108342	0.94422142	0.889415593	0.993010827	0.873130182
TTL	0.922418547	0.821790484	0.871929137	0.999981514	0.960419102	0.864197513
ERCC4	0.922418547	0.808823388	0.971996404	0.948965036	0.968290818	0.877048993
PJA1	0.922418547	0.855141737	0.955427429	0.890704007	0.939813331	0.960023723
ALG3	0.922418547	0.913116251	0.91882781	0.896116841	0.89050859	0.981216824
MLST8	0.922506374	0.940381381	0.911535369	0.901970399	0.98865576	0.860966325
VSTM4	0.922506374	0.890315896	0.910851361	0.996033721	0.943160494	0.867482845
ZFP3	0.922506374	0.884850838	0.999993442	0.922503563	0.893376996	0.872239777
LOC100336589	0.922506374	0.968509005	0.897322115	0.916858019	0.960573822	0.873130182
RASSF3	0.922506374	0.816412341	0.962832886	0.997332186	0.89112005	0.909056553
HDHD3	0.922506374	0.805283017	0.951392011	0.952247001	0.964301327	0.91373422
PHGDH	0.922506374	0.840402825	0.912252673	0.999981514	0.911028086	0.919124527
EEF1AKMT2	0.922506374	0.969051025	0.883737851	0.892071982	0.953300742	0.926480924
LOC112444194	0.922506374	0.804185018	0.874983674	0.963132425	0.972160227	0.955990509
RXFP4	0.922565077	0.918854612	0.914256466	0.961898258	0.958852623	0.871503289
MORC4	0.922565077	0.942088099	0.868546176	0.916858019	0.988010662	0.880622346

TMEM30B	0.922565077	0.819439092	0.997400824	0.899855526	0.907678664	0.940079339
SMG7	0.922565077	0.813317206	0.988120522	0.955466436	0.882252274	0.940956155
LOC112442997	0.922582841	0.882483488	0.872088608	0.889415593	0.999985752	0.877750901
FAM8A1	0.922602869	0.808279036	0.99647733	0.928404714	0.959658682	0.872239777
PARL	0.922602869	0.802570554	0.992324476	0.973075232	0.887514169	0.922539606
ATP6V1D	0.922602869	0.816369086	0.927635169	0.940438437	0.961670582	0.943972369
FAM149B1	0.922602869	0.865101561	0.910845937	0.985701713	0.890348531	0.953423944
SFXN4	0.922602869	0.817329649	0.944613162	0.908467146	0.956906331	0.960023723
DECR2	0.922603751	0.981520642	0.928514467	0.932406746	0.89052284	0.888475311
RABGAP1L	0.922648368	0.846724749	0.951392011	0.908467146	0.996781978	0.871998318
D2HGDH	0.922648368	0.913116251	0.95219206	0.904374668	0.970980375	0.872239777
PUS3	0.922648368	0.826395702	0.997400824	0.916858019	0.918426817	0.91373422
KCTD11	0.922648368	0.912670574	0.905023942	0.951936643	0.887514169	0.964898868
SPRY2	0.922648368	0.845750059	0.927508464	0.906186004	0.943540106	0.971545917
EXOG	0.922648368	0.845131166	0.867661896	0.891988949	0.986003146	0.973649216
MYLK3	0.922730617	0.946904846	0.89478142	0.940438437	0.953300742	0.898470276
KLHL28	0.922749069	0.980833065	0.969370452	0.901495718	0.887514169	0.872238439
TTC9C	0.922780303	0.816592355	0.927635169	0.908467146	0.999985752	0.865767172
HDGFL2	0.922780303	0.927853707	0.871285807	0.975118969	0.970491451	0.868866029
LOC101904705	0.922780303	0.848383949	0.916081147	0.930987593	0.999985752	0.871998318
LOC112446457	0.922780303	0.893164557	0.999833345	0.896116841	0.900891209	0.899428912
RAB38	0.922780303	0.991186561	0.917651622	0.898818046	0.886617004	0.919124527
FXYD3	0.922863038	0.906064108	0.972806636	0.940436902	0.935286646	0.869904046
PPIL4	0.922863038	0.94043655	0.916038418	0.899792456	0.980016241	0.874870038
KCNE3	0.922863038	0.893615781	0.935491635	0.943447418	0.964833865	0.877014986
SORCS2	0.922863038	0.829438596	0.945995021	0.910575108	0.991747435	0.895803603
NPHP3	0.922863038	0.889345904	0.892343042	0.982220679	0.935286646	0.923547711
NIPAL3	0.922863038	0.835452905	0.879820377	0.899855526	0.984263497	0.96870908
LOC616051	0.922928011	0.804185018	0.937869586	0.907621547	0.976292433	0.948705062
ZMYM5	0.922940839	0.853678591	0.892343042	0.916858019	0.999985752	0.891088733
LNPEP	0.923168225	0.976299197	0.872088608	0.9880671	0.900927045	0.876861428
LOC104974070	0.923308765	0.811354298	0.894594614	0.978503042	0.998287238	0.874490166
SLC16A12	0.923308765	0.89915699	0.957052938	0.928404714	0.886944613	0.94844234
DPH6	0.923412897	0.823478017	0.978014482	0.894345525	0.907455286	0.971730151
U2AF2	0.923432833	0.869754045	0.911973589	0.991479413	0.964768333	0.861049553
DDOST	0.923493154	0.875326701	0.922722926	0.9880671	0.953300742	0.874000314
INHBB	0.923493154	0.881942703	0.967696262	0.985118339	0.887514169	0.885202945
SLC2A12	0.923624325	0.87976124	0.916323169	0.960016921	0.966328884	0.887989933

ARPC5L	0.923669001	0.811059858	0.886829776	0.948668643	0.887514169	0.999948228
ALDH3A2	0.923855622	0.802570554	0.920487566	0.905351625	0.999985752	0.905034785
SORD	0.923855622	0.884457875	0.920462513	0.948050281	0.958797242	0.91373422
FAM78B	0.923855622	0.889387648	0.943211357	0.942955143	0.906766758	0.944927301
SOD3	0.923855622	0.863948462	0.93894628	0.892071982	0.964301327	0.945854375
HMGCL	0.924078681	0.860419291	0.903431343	0.999981514	0.956653075	0.860966325
SOGA1	0.924078681	0.846724749	0.916038418	0.999981514	0.889978339	0.927426708
SULT1B1	0.924078681	0.808696335	0.876414692	0.935985829	0.999985752	0.930146713
NEK3	0.924078681	0.819210468	0.872088608	0.907794002	0.999985752	0.933903265
CD209	0.924078681	0.955827589	0.904919322	0.893220599	0.913013159	0.960023723
P2RX4	0.924078681	0.808785257	0.916323169	0.896116841	0.953331803	0.993175779
FAF1	0.924078681	0.813759037	0.928731949	0.892047793	0.935286646	0.99666605
FBXW5	0.92416743	0.94043655	0.888254866	0.893105703	0.999985752	0.860966325
CNPY2	0.92416743	0.888322912	0.89478142	0.95901497	0.976292433	0.890862406
SLC12A9	0.92416743	0.812449121	0.999145937	0.922967576	0.935286646	0.904383301
STX2	0.92416743	0.827824094	0.914770879	0.999981514	0.933940422	0.915414597
LOC782527	0.92416743	0.811354298	0.916038418	0.940946275	0.965163023	0.958790968
NSUN3	0.924225467	0.802840415	0.957052938	0.960016921	0.954814306	0.922613891
CTPS2	0.924404062	0.869754045	0.964807927	0.9880671	0.904643542	0.877014986
PEX19	0.924404062	0.905512356	0.980061232	0.914026346	0.906893576	0.91373422
UBXN2A	0.924404062	0.808478344	0.988142154	0.981156002	0.89052284	0.914132808
CDC14B	0.924430422	0.950986475	0.883950091	0.901168085	0.892446951	0.980167535
C5H12orf45	0.924449324	0.878551917	0.874983674	0.906362302	0.964301327	0.971982373
EAF1	0.924492353	0.819210468	0.945995021	0.955954309	0.993946355	0.86324237
LOC104975612	0.924492353	0.855310926	0.999993442	0.952247001	0.899157497	0.867447114
LOC100335340	0.924492353	0.872175143	0.873674244	0.973075232	0.996781978	0.867635709
GALNT17	0.924492353	0.822484848	0.93891936	0.999981514	0.935286646	0.868748913
GABPB1	0.924492353	0.99488888	0.895801611	0.890704007	0.947299702	0.876162019
WIPF3	0.924492353	0.904875256	0.968492346	0.948668643	0.918166705	0.891088733
LOC112445011	0.924492353	0.95530536	0.915124089	0.900044333	0.960573822	0.902339039
RNF4	0.924492353	0.878809061	0.947952648	0.922002553	0.965392454	0.903500283
NME6	0.924492353	0.869794572	0.989494193	0.89668616	0.945062246	0.911888512
VEGFA	0.924492353	0.876304005	0.886829776	0.999981514	0.911417136	0.912531667
SYNGR3	0.924492353	0.850894788	0.889080797	0.999981514	0.933940422	0.91373422
PDPN	0.924492353	0.808478344	0.971053208	0.9880671	0.906766758	0.91618909
NUB1	0.924492353	0.833025853	0.928927123	0.999981514	0.891726429	0.919124527
LOC101906230	0.924492353	0.88856027	0.912183977	0.90595069	0.983941532	0.919124527
SC5D	0.924492353	0.927853707	0.893427844	0.907794002	0.976259416	0.92097743

PCCA	0.924492353	0.872175143	0.916294854	0.916858019	0.977193348	0.921843102
LOC112449106	0.924492353	0.993343185	0.905023942	0.907353815	0.887514169	0.923092436
ARL2	0.924492353	0.875391655	0.921266625	0.901353354	0.977158496	0.925553067
POLR2M	0.924492353	0.833323491	0.911535369	0.997792939	0.930451229	0.927520621
SERPINB1	0.924492353	0.889387648	0.924855847	0.950662233	0.937932469	0.927520621
ITGA7	0.924492353	0.850640641	0.894338686	0.982220679	0.953300742	0.929027441
PLA2G6	0.924492353	0.892108342	0.894338686	0.901168085	0.970980375	0.953140004
DACT2	0.924492353	0.817329649	0.916294854	0.949605617	0.923910588	0.98377195
LOC112446740	0.924492353	0.842195544	0.879939804	0.896116841	0.9683846	0.9884289
AK7	0.924492353	0.822484848	0.879608555	0.949565453	0.935286646	0.994347099
PCGF1	0.924492353	0.811059858	0.879939804	0.941467375	0.938101148	0.997054363
CLMN	0.924492353	0.85317418	0.91469924	0.904904807	0.892446951	0.999948228
IRAK4	0.924562932	0.83778644	0.99647733	0.952247001	0.926813827	0.876861428
GGT1	0.924562932	0.938087741	0.880163755	0.969256019	0.953300742	0.895517058
MCOLN3	0.924562932	0.880565292	0.895801611	0.989601002	0.888656048	0.953140004
TPT1	0.924591981	0.816411963	0.878604976	0.982220679	0.900927045	0.991416486
NAGLU	0.924635615	0.905108627	0.984886031	0.898818046	0.954403133	0.874490166
RNF31	0.924635615	0.833713501	0.99647733	0.929663263	0.952007172	0.875297569
LOC615959	0.924635615	0.927973597	0.886829776	0.99570283	0.935286646	0.876861428
DUSP3	0.924635615	0.964925317	0.984886031	0.892071982	0.887514169	0.891423889
SYF2	0.924635615	0.807285501	0.941228678	0.995565324	0.953300742	0.893838339
LOC101905588	0.924635615	0.826316517	0.999159888	0.94857292	0.904643542	0.903990996
LOC112443816	0.924635615	0.85265333	0.935491635	0.960937718	0.96080502	0.90607344
KRAS	0.924635615	0.879552187	0.8735515	0.94957823	0.979988991	0.925233445
SMIM13	0.924635615	0.955827589	0.926161087	0.892047793	0.935286646	0.927520621
CTSA	0.924635615	0.918455779	0.874983674	0.995565324	0.891726429	0.934671838
MIR3064	0.924635615	0.806029227	0.936203646	0.98981682	0.887514169	0.963255451
TMEM232	0.924635615	0.837244723	0.967590756	0.900504516	0.887514169	0.985165897
PEA15	0.924635615	0.829067393	0.897322115	0.978404889	0.886944613	0.988320707
SRP9	0.924635615	0.82955476	0.928731949	0.908467146	0.89052284	0.999655834
GTF2H2	0.924734909	0.861641368	0.999993442	0.891988949	0.964301327	0.86324237
ARHGAP18	0.924734909	0.855079912	0.874797723	0.959652624	0.912617855	0.988866378
PEX26	0.924757163	0.950336973	0.89478142	0.9880671	0.916605572	0.878118151
AAMDC	0.924757163	0.860419291	0.971053208	0.943808491	0.956041014	0.885471103
CLDN4	0.924757163	0.989799017	0.929661805	0.90318376	0.910119841	0.888475311
WDR7	0.924757163	0.821505027	0.91882781	0.999981514	0.953300742	0.896529476
MPP6	0.924757163	0.929204871	0.914002845	0.896116841	0.976292433	0.911219114
GMIP	0.924757163	0.901071863	0.94422142	0.908467146	0.961465747	0.91373422

NXF1	0.924757163	0.833713501	0.947875514	0.932914414	0.969801592	0.922361909
CDCA2	0.924757163	0.863613561	0.927508464	0.995434268	0.89050859	0.930710695
AGPAT4	0.924757163	0.842391145	0.927635169	0.893105703	0.976292433	0.952563377
PARP11	0.924757163	0.809693907	0.923521499	0.919153842	0.954403133	0.978087957
DCLK1	0.924777007	0.962037123	0.97773026	0.935563591	0.887514169	0.867063767
AMY2B	0.924777007	0.811114624	0.961437412	0.989601002	0.948421303	0.877750901
LOC107131510	0.924777007	0.857815763	0.939912874	0.976855823	0.899332763	0.943621425
BNIP1	0.925100032	0.880129816	0.999993442	0.89684883	0.895067787	0.884402686
KCNJ13	0.925100032	0.82089312	0.895826828	0.996033721	0.961465747	0.91373422
SBNO2	0.925100032	0.898667357	0.956655464	0.93048026	0.933940422	0.9221477
KCNMB4	0.925100032	0.813759037	0.934401467	0.941467375	0.976259416	0.930710695
UTP14A	0.925100032	0.941817736	0.895826828	0.918693944	0.942557024	0.940017687
VIM	0.925100032	0.926905849	0.879939804	0.978503042	0.896589592	0.94844234
FZD1	0.925100032	0.873915965	0.879939804	0.959591127	0.954071324	0.95578593
PIKFYVE	0.925100032	0.831153015	0.95322206	0.919711717	0.929896768	0.968245362
POLK	0.925100032	0.955981683	0.879939804	0.934466438	0.887514169	0.96944627
C1GALT1	0.925189964	0.843758099	0.911636686	0.999981514	0.934450558	0.873130182
FNTA	0.925189964	0.816202069	0.94422142	0.965724803	0.969087007	0.905188617
SLC7A1	0.925189964	0.87976124	0.93190909	0.932914414	0.965676623	0.91373422
CCDC28A	0.925189964	0.811059858	0.939912874	0.952247001	0.976292433	0.919124527
IL3RA	0.925189964	0.849896139	0.955427429	0.978503042	0.900893337	0.927520621
TBX21	0.925189964	0.826102395	0.931623453	0.989601002	0.930513915	0.927520621
ADRA2A	0.925189964	0.858400865	0.958197827	0.95925588	0.887514169	0.952571698
PAIP2	0.925189964	0.836384702	0.937993336	0.959805738	0.900893337	0.969014913
GPR37L1	0.925189964	0.808478344	0.899106435	0.972142185	0.930709114	0.9790177
TULP2	0.925270693	0.821591763	0.999993442	0.892047793	0.953300742	0.903210685
TC2N	0.925328994	0.995373357	0.911103174	0.929388614	0.91563394	0.865889509
SOWAHD	0.925328994	0.817329649	0.92380537	0.9063051	0.999985752	0.895803603
MAGI1	0.925328994	0.973321075	0.879939804	0.960016921	0.897723763	0.923807852
ERCC5	0.925370495	0.869754045	0.911535369	0.999981514	0.926831826	0.909953142
LOC101907998	0.925370495	0.884773193	0.898254437	0.979644238	0.892446951	0.960023723
ADGRB3	0.925405906	0.906064108	0.916323169	0.999981514	0.895067787	0.872238439
ELOVL1	0.925405906	0.850514112	0.916038418	0.999981514	0.909379774	0.888475311
IMPDH1	0.925405906	0.987887518	0.894208525	0.924052222	0.926972948	0.905152236
ABHD17C	0.925405906	0.871091574	0.911973589	0.979330173	0.916605572	0.947974638
RIN2	0.925405906	0.831549062	0.945995021	0.960016921	0.891726429	0.969754487
TCEAL4	0.925405906	0.830715727	0.926161087	0.902315549	0.889301074	0.999948228
ZBTB24	0.925434354	0.816202069	0.999993442	0.921950515	0.900092357	0.867635709

NRGN	0.925434354	0.872175143	0.945732557	0.934466438	0.986003146	0.867635709
GPATCH1	0.925434354	0.913120359	0.938624264	0.957008595	0.960154383	0.869437527
SLC25A20	0.925434354	0.927973597	0.966232793	0.908467146	0.953300742	0.876861428
ERAL1	0.925434354	0.847093186	0.889098668	0.941467375	0.999985752	0.879576441
LOC100847320	0.925434354	0.927853707	0.979494108	0.932674949	0.89743683	0.895426195
SWSAP1	0.925434354	0.837244723	0.918351026	0.999981514	0.915775776	0.895446935
RBM34	0.925434354	0.861395263	0.960922789	0.963132425	0.926972948	0.915735519
LOC101905630	0.925434354	0.829067393	0.908412457	0.960016921	0.979290191	0.923092436
FOXF1	0.925434354	0.824226631	0.939912874	0.984421126	0.930970429	0.932129053
ZUP1	0.925434354	0.863286109	0.916294854	0.936631044	0.9683846	0.935603672
SLC22A15	0.925434354	0.928483112	0.951392011	0.9063051	0.910350852	0.940583352
TCEAL1	0.925434354	0.969051025	0.894316021	0.915039335	0.891726429	0.960023723
NANP	0.925434354	0.940543846	0.91882781	0.914850247	0.893854857	0.960877268
HNRNPH2	0.925434354	0.855141737	0.964661607	0.929388614	0.887514169	0.969197073
ADSS	0.925443925	0.846215781	0.972741989	0.896116841	0.989334846	0.879884117
RRAS2	0.925827564	0.943244588	0.913134155	0.93048026	0.887514169	0.963145894
DAB1	0.925927938	0.895777642	0.910224442	0.9819628	0.953300742	0.895683298
ATN1	0.925927938	0.816770158	0.92442058	0.999981514	0.943055455	0.905586088
SLCO5A1	0.926013419	0.833713501	0.999993442	0.930987593	0.902258286	0.911808092
PLK3	0.926013419	0.950942764	0.885896294	0.950643928	0.935286646	0.927426708
DLL1	0.926013419	0.861395263	0.943836178	0.912777307	0.944417042	0.960023723
PLA2G4A	0.9260817	0.949902864	0.882643407	0.999981514	0.900927045	0.867635709
ATRN	0.9260817	0.834615638	0.999993442	0.898818046	0.964301327	0.872238439
VRK1	0.9260817	0.983305314	0.927635169	0.901308803	0.937932469	0.88313584
ADGRE5	0.9260817	0.883960313	0.983504244	0.973179867	0.89052284	0.886430939
HGFAC	0.9260817	0.852526348	0.999993442	0.91231772	0.891726429	0.911808092
DYRK2	0.9260817	0.863064327	0.894338686	0.999981514	0.91563394	0.93363377
BMX	0.9260817	0.942546802	0.927635169	0.892071982	0.907455286	0.963456717
COL12A1	0.926097889	0.863948462	0.915858924	0.995434268	0.964301327	0.872239777
ITFG1	0.926097889	0.878551917	0.924855847	0.999981514	0.935093621	0.877048993
MAPK6	0.926097889	0.846724749	0.999993442	0.918216988	0.895757462	0.898085102
LOC104975673	0.926097889	0.85265333	0.988120522	0.892047793	0.970988226	0.898085102
MBD3	0.926097889	0.924128126	0.959192525	0.952247001	0.900747635	0.909318728
ITGA9	0.926097889	0.945574389	0.908072171	0.98544125	0.891726429	0.91228365
NPAS2	0.926097889	0.918460462	0.959725886	0.958103924	0.887514169	0.918461982
LZTS2	0.926097889	0.824149383	0.880612713	0.989707094	0.976259416	0.922361909
TRPV2	0.926097889	0.887265261	0.929572921	0.98981682	0.890841443	0.926418736
SPATA16	0.926097889	0.863286109	0.964661607	0.915039335	0.935286646	0.946760037

KIF3C	0.926097889	0.873915965	0.879939804	0.997792939	0.889301074	0.96248183
COL4A2	0.926097889	0.935661607	0.905023942	0.922002553	0.911664199	0.963701289
SMOC2	0.926097889	0.834615638	0.916294854	0.975118969	0.907094634	0.970728712
ARID1B	0.926152299	0.872341235	0.979494108	0.912777307	0.974958543	0.866428791
SP3	0.926152299	0.985428851	0.924855847	0.905772309	0.951021192	0.867063767
ARL2BP	0.926152299	0.873915965	0.916323169	0.952247001	0.989334846	0.873130182
CNTNAP2	0.926152299	0.811354298	0.99341711	0.961898258	0.906893576	0.919124527
MUM1	0.926152299	0.891379558	0.874983674	0.959159289	0.945908526	0.960023723
LOC104975749	0.926180166	0.833323491	0.978014482	0.914850247	0.977158496	0.885089102
DCN	0.926180166	0.926905849	0.916294854	0.902776185	0.983941532	0.895559724
COL5A3	0.926224366	0.998847958	0.897432125	0.952247001	0.889251503	0.865933526
USE1	0.926224366	0.927853707	0.997400824	0.912777307	0.906766758	0.871503289
ZNF784	0.926224366	0.808328971	0.957052938	0.999981514	0.936215792	0.888475311
RNF20	0.926224366	0.91763262	0.916038418	0.898818046	0.996781978	0.888475311
TBRG1	0.926224366	0.811567701	0.999993442	0.907794002	0.932024143	0.896777161
HIC1	0.926224366	0.80907783	0.964382669	0.957132838	0.964833865	0.907922835
LY96	0.926224366	0.846724749	0.944126714	0.980532429	0.942527603	0.91373422
LOC104974443	0.926224366	0.808916694	0.999993442	0.952247001	0.887514169	0.929027441
GRK6	0.926224366	0.850521787	0.956397579	0.96951401	0.911930323	0.93363377
KMT2C	0.926224366	0.826339887	0.966232793	0.939604997	0.936921784	0.945480515
LOC100848439	0.926224366	0.864668925	0.912020408	0.970725878	0.928951321	0.958790968
KIAA0513	0.926469127	0.905550751	0.893761108	0.996684642	0.954071324	0.876861428
HEY1	0.926469127	0.81848543	0.881656689	0.999981514	0.928951321	0.93363377
SEMA4C	0.926469127	0.840402825	0.885896294	0.993215785	0.928951321	0.960023723
LOC513767	0.92659994	0.863286109	0.894316021	0.999981514	0.89052284	0.871503289
ACE2	0.92659994	0.98098403	0.895801611	0.972467586	0.901144467	0.88542704
USP1	0.926603314	0.993481224	0.885896294	0.896116841	0.965676623	0.871273798
LOC101905041	0.926603314	0.907585165	0.97773026	0.966838467	0.907956321	0.873130182
LOC112444333	0.926603314	0.846724749	0.924855847	0.9880671	0.972160227	0.873130182
GCDH	0.926603314	0.855067335	0.915713148	0.896116841	0.999985752	0.874490166
TDRKH	0.926603314	0.893164557	0.916294854	0.995565324	0.943702351	0.877048993
PRELID3A	0.926603314	0.82707084	0.886829776	0.908982438	0.999985752	0.88457156
THYN1	0.926603314	0.888808283	0.944501125	0.955466436	0.958852623	0.891423889
PKMYT1	0.926603314	0.922923898	0.949855095	0.939604997	0.943702351	0.896529476
SUCO	0.926603314	0.880565292	0.950336741	0.914653338	0.976292433	0.896529476
PRKDC	0.926603314	0.849513595	0.957052938	0.962437357	0.954403133	0.899428912
ABRACL	0.926603314	0.826395702	0.920462513	0.938303773	0.998287238	0.907384701
LOC104969833	0.926603314	0.918854612	0.957052938	0.933718942	0.935093621	0.911219114

LOC104973224	0.926603314	0.91318565	0.887132069	0.999981514	0.891726429	0.917024468
B3GALT4	0.926603314	0.834615638	0.914002845	0.904018489	0.999985752	0.919124527
GAL3ST4	0.926603314	0.938087741	0.872945921	0.999981514	0.887514169	0.919195656
TLR8	0.926603314	0.814246237	0.896109455	0.999981514	0.943702351	0.923807852
AADACL3	0.926603314	0.837244723	0.946791586	0.99313054	0.907455286	0.926530353
MAFB	0.926603314	0.901928494	0.924096511	0.898818046	0.970988226	0.931500173
HSPB2	0.926603314	0.853194713	0.991855429	0.899792456	0.935286646	0.934114816
NGFR	0.926603314	0.960355725	0.903844825	0.918131532	0.932024143	0.93531275
ICA1L	0.926603314	0.959732252	0.916294854	0.928075638	0.904643542	0.93839036
SACS	0.926603314	0.878504776	0.894338686	0.893220599	0.988049878	0.953140004
NUP188	0.926603314	0.833713501	0.995377403	0.925353907	0.887514169	0.953359824
MKNK1	0.926603314	0.871176243	0.957052938	0.899855526	0.944839728	0.955990509
ACP1	0.926603314	0.855067335	0.914479281	0.899855526	0.973219333	0.966659342
TUSC1	0.926603314	0.95856975	0.879939804	0.919688166	0.909548748	0.967056707
NOP56	0.926603314	0.863948462	0.947651663	0.916858019	0.926831826	0.970105102
EFCAB8	0.926603314	0.863064327	0.93190909	0.942883863	0.898833204	0.980741977
IFI27L2	0.926677991	0.955333923	0.879939804	0.930987593	0.976088926	0.892885933
PCM1	0.926677991	0.955652503	0.893988897	0.919643615	0.970988226	0.895800655
CISH	0.926709151	0.861395263	0.999993442	0.918693944	0.889081761	0.876162019
DACH1	0.926718314	0.904754143	0.8735515	0.896116841	0.999985752	0.91373422
PCIF1	0.926718314	0.823602247	0.879939804	0.924425698	0.925481162	0.999948228
STAP1	0.926778508	0.923927608	0.927635169	0.903004767	0.923931669	0.965783646
CRYBB1	0.926786746	0.865518737	0.949855095	0.999981514	0.901853599	0.891088733
PPIB	0.926792944	0.905145212	0.933884582	0.959644972	0.956906331	0.891088733
NOP9	0.926863927	0.902525869	0.95322206	0.947574232	0.964301327	0.872238439
MINDY4	0.926863927	0.982515431	0.915191964	0.965271935	0.901662258	0.876162019
FAM81A	0.926863927	0.992040539	0.916294854	0.904018489	0.942786245	0.877048993
AIDA	0.926863927	0.882858125	0.916038418	0.964521432	0.964357593	0.90969547
PFKFB2	0.926863927	0.943244588	0.935266153	0.96444269	0.891486132	0.916924937
LOC112443214	0.926863927	0.882471806	0.874983674	0.999981514	0.94542482	0.920222784
PTPN4	0.926863927	0.967034954	0.927635169	0.940436902	0.893716526	0.923092436
SCAP	0.926863927	0.882242226	0.907795285	0.896116841	0.996781978	0.930710695
ZBTB10	0.926884717	0.952760839	0.873674244	0.972142185	0.950958352	0.903500283
SMNDC1	0.926884717	0.830930037	0.913134155	0.901988458	0.999985752	0.925125664
RBM22	0.92707906	0.824188473	0.894316021	0.959652624	0.999985752	0.87229131
LEPROTL1	0.927199503	0.843278276	0.999993442	0.928416242	0.935093621	0.87754233
KIRREL1	0.927199503	0.901631302	0.911535369	0.947574232	0.974896634	0.910431588
FAM57B	0.927199503	0.927701869	0.912187636	0.973812087	0.904643542	0.93363377

RTL8C	0.927268613	0.878551917	0.928514467	0.921482057	0.998196897	0.877048993
LOC112443012	0.9274517	0.938087741	0.927508464	0.95901497	0.943702351	0.891088733
ARRDC2	0.9274517	0.829067393	0.945995021	0.985118339	0.953300742	0.911219114
LACTB	0.927641748	0.820874681	0.886829776	0.925353907	0.906766758	0.999948228
FAM206A	0.927661567	0.885494685	0.999993442	0.914026346	0.936921784	0.869620106
ACAA2	0.927785245	0.869754045	0.91882781	0.975346638	0.983928296	0.867063767
UST	0.927785245	0.894014677	0.916294854	0.896116841	0.999985752	0.870018677
LOC107132192	0.927785245	0.999987083	0.899106435	0.906186004	0.916877832	0.872239777
AMOT	0.927785245	0.83844057	0.927635169	0.976069331	0.985250243	0.873130182
ANGPTL2	0.927785245	0.920702629	0.996224523	0.913946495	0.901853599	0.895446935
LOC100336909	0.927785245	0.981952108	0.89740351	0.952247001	0.91563394	0.89950552
HES7	0.927785245	0.866955566	0.875900843	0.907794002	0.999985752	0.904642337
SLAMF7	0.927785245	0.868493314	0.950962607	0.973373329	0.924277487	0.925125664
TACR2	0.927785245	0.870296749	0.997400824	0.899047543	0.909379774	0.933224627
GOT1	0.927785245	0.814937616	0.952341787	0.933718942	0.970988226	0.935497122
ADCK2	0.927785245	0.820235509	0.906019013	0.969214881	0.971075533	0.940583352
PITPNC1	0.927785245	0.850998314	0.897322115	0.922292236	0.988589472	0.944973383
SCCPDH	0.927785245	0.819439092	0.916038418	0.952247001	0.964301327	0.960023723
ZNF227	0.927785245	0.837263562	0.879939804	0.948965036	0.943160494	0.994347099
HMGB1	0.927884575	0.846724749	0.999993442	0.949565453	0.917407853	0.879576441
LOC112444600	0.927884575	0.912670574	0.957052938	0.916858019	0.91563394	0.945955807
LOC104973058	0.927884575	0.815511484	0.916294854	0.912777307	0.932024143	0.999948228
BTBD7	0.928074731	0.888936768	0.949855095	0.915039335	0.988238252	0.87501921
UROC1	0.928074731	0.977196455	0.916323169	0.950643928	0.932024143	0.877014986
ERF	0.928074731	0.921940584	0.895801611	0.917892197	0.996089557	0.893252687
PLEKHG1	0.928074731	0.958814007	0.916038418	0.908467146	0.943055455	0.930710695
LOC112447342	0.928074731	0.948801019	0.89478142	0.973075232	0.887514169	0.940583352
PRKCA	0.928074731	0.835883963	0.879939804	0.965729104	0.96870902	0.960023723
GPRASP1	0.928097882	0.899494273	0.884172102	0.999981514	0.93146031	0.881947456
FIS1	0.928113317	0.954222802	0.948653258	0.935985829	0.945062246	0.872238439
NAP1L4	0.928113317	0.898726299	0.935824202	0.910575108	0.996781978	0.876861428
KIF26A	0.928122339	0.811354298	0.942357321	0.999981514	0.960754497	0.865897019
LOC100298453	0.928122339	0.816248177	0.999993442	0.898981048	0.908528514	0.867063767
ABCA1	0.928122339	0.825950451	0.999993442	0.980564765	0.918052462	0.867063767
NIPBL	0.928122339	0.905271052	0.935491635	0.898818046	0.999985752	0.867063767
PCDHB8	0.928122339	0.994185904	0.937869586	0.925353907	0.905571216	0.867907362
LOC101906850	0.928122339	0.816202069	0.999993442	0.896116841	0.936108073	0.875650601
ACVR2B	0.928122339	0.946806157	0.92323046	0.942145064	0.960573822	0.87754233

FHL2	0.928122339	0.946148902	0.892343042	0.999981514	0.921710688	0.881992627
LOC112446668	0.928122339	0.930266314	0.947462602	0.972142185	0.92592492	0.883688554
LOC100337495	0.928122339	0.883391071	0.975774524	0.97731816	0.909859639	0.888475311
LOC100847782	0.928122339	0.927992601	0.916323169	0.912922827	0.983941532	0.888475311
HUWE1	0.928122339	0.837244723	0.916081147	0.941467375	0.999985752	0.888475311
TTC9B	0.928122339	0.985997726	0.879939804	0.900044333	0.973502409	0.889406298
LOC104973517	0.928122339	0.846724749	0.943836178	0.999981514	0.911568867	0.891195244
DLST	0.928122339	0.855079912	0.935266153	0.918693944	0.999453276	0.893838339
MAP4K5	0.928122339	0.995373357	0.894523258	0.940436902	0.901853599	0.899428912
PLXNC1	0.928122339	0.898446328	0.951521942	0.938303773	0.958797242	0.905509632
ZBED4	0.928122339	0.95310464	0.911535369	0.95754063	0.935286646	0.912531667
KCNK13	0.928122339	0.830930037	0.975718134	0.903584224	0.982090215	0.913152907
LOC112449280	0.928122339	0.859034448	0.894338686	0.998177865	0.958654817	0.91373422
HSCB	0.928122339	0.900674317	0.892343042	0.905209994	0.999985752	0.91373422
GREM1	0.928122339	0.906064108	0.979885406	0.943808491	0.900809233	0.915414597
FGFR2	0.928122339	0.942159543	0.945995021	0.929342214	0.927086179	0.921843102
POMK	0.928122339	0.855079912	0.945732557	0.957796224	0.954071324	0.927520621
PAQR4	0.928122339	0.880565292	0.945732557	0.981327579	0.896589592	0.93363377
SERPINB6	0.928122339	0.857815763	0.966199203	0.898818046	0.958797242	0.945480515
COL4A3BP	0.928122339	0.842391145	0.927508464	0.9880671	0.924662947	0.946760037
ZBTB7A	0.928122339	0.846724749	0.916294854	0.909925598	0.977167046	0.960023723
CXCL2	0.928122339	0.879093989	0.885896294	0.968447717	0.935146118	0.96870908
LOC100139363	0.928122339	0.835883963	0.897322115	0.949565453	0.964235922	0.96870908
LYSMD4	0.928122339	0.856868887	0.913155467	0.937654716	0.954412818	0.970071797
LOC112446042	0.928122339	0.902525869	0.899600099	0.908467146	0.954071324	0.971545917
LOC112449080	0.928122339	0.839128192	0.914256466	0.980532429	0.906893576	0.975170737
SLC6A20	0.928122339	0.852526348	0.883500436	0.985437486	0.918512595	0.975558533
LOC100295130	0.928122339	0.869754045	0.885896294	0.984423083	0.900927045	0.977503497
SLC35E3	0.928122339	0.84238625	0.948129084	0.952247001	0.893376996	0.978979999
ZNF653	0.928122339	0.833126007	0.923521499	0.92532303	0.93630105	0.989184798
UBP1	0.928122339	0.819210468	0.896023027	0.961898258	0.91563394	0.99666605
LOC112448777	0.928122339	0.822956796	0.946791586	0.922967576	0.89052284	0.99828741
PDXDC1	0.928122339	0.837244723	0.891972402	0.956822441	0.891726429	0.999948228
ENDOU	0.928122339	0.82700126	0.916294854	0.926970666	0.893716526	0.999948228
VTI1A	0.928301426	0.928658222	0.990615319	0.916858019	0.926972948	0.883688554
CSRP2	0.928301426	0.969051025	0.937281477	0.907794002	0.927086179	0.922695415
LOC101903913	0.928301426	0.846724749	0.995377403	0.959652624	0.891304411	0.923547711
NPHP1	0.928301426	0.814245795	0.876719527	0.931383184	0.892446951	0.999948228

SPATA6	0.928450334	0.962037123	0.901955007	0.982220679	0.935286646	0.872239777
PGM1	0.928450334	0.843278276	0.971053208	0.961898258	0.924135649	0.931410194
TMEM74	0.928450334	0.882858125	0.939981892	0.915013589	0.893716526	0.988796537
SMARCB1	0.928454172	0.928427136	0.947875514	0.974286747	0.935286646	0.873166373
ARID4A	0.928454172	0.855067335	0.946791586	0.92139105	0.999453276	0.87754233
LOC112441481	0.928454172	0.81848543	0.943836178	0.948478716	0.997165254	0.893838339
MRM1	0.928454172	0.833713501	0.899600099	0.932406746	0.938316699	0.99642553
GMNN	0.928454172	0.886669497	0.89478142	0.896116841	0.935286646	0.998240456
WDR34	0.92851904	0.918455779	0.89478142	0.995565324	0.960573822	0.867063767
PTGR1	0.92851904	0.821709833	0.999993442	0.945261452	0.932024143	0.87501921
LOC112444635	0.92851904	0.96643574	0.876414692	0.898818046	0.990192394	0.895602373
HHAT	0.92851904	0.864945924	0.942357321	0.999981514	0.891726429	0.909635302
CABLES1	0.92851904	0.879185178	0.916038418	0.917117252	0.933426897	0.985165897
LYNX1	0.928596121	0.837263562	0.880163755	0.904018489	0.999985752	0.890967932
ZNF81	0.928855165	0.870444737	0.916294854	0.949502614	0.976088926	0.926480924
ZNF282	0.928956574	0.846724749	0.957295966	0.941935077	0.910747101	0.969014913
TRPC6	0.928967974	0.837945129	0.894316021	0.928332965	0.999985752	0.934360589
HOXC5	0.928982565	0.899490486	0.951392011	0.914026346	0.946454221	0.940017687
LOC100847190	0.929063585	0.956095821	0.903844825	0.96951401	0.956904152	0.867907362
ORAOV1	0.929063585	0.988008216	0.899106435	0.97828838	0.906893576	0.87100843
LOC112448253	0.929063585	0.931610273	0.97251482	0.948668643	0.930451229	0.876784504
PDZD4	0.929063585	0.904847484	0.943237386	0.98981682	0.911028086	0.90388238
LOC112447031	0.929063585	0.839800081	0.879608555	0.929663263	0.999985752	0.944330889
ING4	0.929063585	0.972007528	0.877824879	0.934180032	0.891726429	0.96870908
EDNRB	0.929079469	0.849513595	0.905023942	0.934466438	0.999985752	0.867907362
LOC100335635	0.929079469	0.856560491	0.999993442	0.905772309	0.891304411	0.87100843
SLC20A2	0.929079469	0.853678591	0.965407935	0.935563591	0.988049878	0.871998318
RHOBTB3	0.929079469	0.927103901	0.879939804	0.999981514	0.943702351	0.872238439
MPV17	0.929079469	0.833323491	0.927635169	0.93048026	0.999985752	0.872238439
SNX7	0.929079469	0.877893862	0.975774524	0.992442163	0.906766758	0.876162019
LOC104976344	0.929079469	0.954505363	0.911103174	0.948668643	0.967116582	0.876810151
LOC783378	0.929079469	0.916141416	0.988660944	0.941467375	0.931357161	0.876861428
MYD88	0.929079469	0.827824094	0.900890831	0.999981514	0.967116582	0.876861428
HCRTR1	0.929079469	0.912602201	0.894338686	0.999981514	0.899373824	0.877014986
SSX2IP	0.929079469	0.968495451	0.91882781	0.973812087	0.915775776	0.880238246
LOC101905188	0.929079469	0.840402825	0.999993442	0.93745545	0.935286646	0.880981659
PDE9A	0.929079469	0.911469424	0.945732557	0.974588495	0.944839728	0.88313584
ZC3H13	0.929079469	0.877893862	0.919202798	0.947574232	0.996781978	0.88313584

TEX2	0.929079469	0.835486075	0.945995021	0.999981514	0.896490594	0.883688554
HGS	0.929079469	0.977196455	0.91493299	0.978503042	0.897352056	0.887722423
PLA2G2C	0.929079469	0.824188473	0.92669814	0.999981514	0.917407853	0.890249222
NECAP1	0.929079469	0.91078662	0.962797326	0.952247001	0.944839728	0.890967932
G6PC3	0.929079469	0.892108342	0.942357321	0.898658215	0.998287238	0.89148147
AMACR	0.929079469	0.969051025	0.936203646	0.959652624	0.903606765	0.893585844
PTPRJ	0.929079469	0.913116251	0.92323046	0.943808491	0.976259416	0.89438401
HYAL2	0.929079469	0.835883963	0.964807927	0.998529748	0.917407853	0.900219465
RUNDC1	0.929079469	0.914629393	0.958454497	0.965724803	0.916605572	0.903500283
LYL1	0.929079469	0.950986475	0.935824202	0.907794002	0.961465747	0.90645885
PITPNA	0.929079469	0.839128192	0.93891936	0.999981514	0.936342434	0.909953142
DMTF1	0.929079469	0.814995661	0.990615319	0.995565324	0.893160105	0.911336121
EME1	0.929079469	0.830230945	0.923993948	0.999981514	0.891304411	0.912531667
LOC100848504	0.929079469	0.816129287	0.891662311	0.999981514	0.962513981	0.91291467
LOC104975684	0.929079469	0.95687851	0.929661805	0.934629728	0.937932469	0.913210801
SLC17A5	0.929079469	0.895311167	0.94422142	0.999180255	0.89052284	0.913221224
TRIM68	0.929079469	0.854961334	0.92442058	0.903584224	0.999985752	0.91373422
LOC616200	0.929079469	0.855141737	0.92271578	0.908467146	0.999985752	0.91373422
SKIDA1	0.929079469	0.870296749	0.883950091	0.932914414	0.999985752	0.91373422
LOC112448540	0.929079469	0.815147563	0.959903168	0.941135794	0.983941532	0.914132808
PRR13	0.929079469	0.881316277	0.93475587	0.964521432	0.957588298	0.915414597
MAPK1	0.929079469	0.822956796	0.964807927	0.998529748	0.906893576	0.917930485
PNLDC1	0.929079469	0.853678591	0.877911619	0.941467375	0.999985752	0.918461982
EPC1	0.929079469	0.879035304	0.921266625	0.907794002	0.996089557	0.923092436
EXOC3L1	0.929079469	0.819910026	0.97998887	0.985437486	0.908770755	0.924333
FAM25A	0.929079469	0.975612288	0.894338686	0.908467146	0.954071324	0.927426708
LOC112442307	0.929079469	0.814177827	0.968233314	0.988408846	0.923666555	0.928416436
NUP37	0.929079469	0.836148393	0.983504244	0.962369147	0.913732532	0.930146713
PRR14L	0.929079469	0.837945129	0.971053208	0.972142185	0.917407853	0.930710695
PTMS	0.929079469	0.962037123	0.915858924	0.91002948	0.947112796	0.931410194
CLDND1	0.929079469	0.847493166	0.91882781	0.908467146	0.996781978	0.934701435
LOC107132098	0.929079469	0.941817736	0.929661805	0.947313672	0.912597537	0.937396169
KLHL21	0.929079469	0.955652503	0.89478142	0.973075232	0.891304411	0.939662884
LOC100847284	0.929079469	0.848383949	0.972806636	0.902108759	0.962513981	0.940079339
IFT57	0.929079469	0.886924072	0.910284791	0.972142185	0.944909181	0.941585207
SLC35A4	0.929079469	0.918854612	0.917651622	0.915039335	0.959026737	0.943285784
SH2D3C	0.929079469	0.948801019	0.915120516	0.916858019	0.936108073	0.950631909
LOC783504	0.929079469	0.853946295	0.905023942	0.900044333	0.997422012	0.953140004

LOC616427	0.929079469	0.924099465	0.894316021	0.952662898	0.935286646	0.959344811
SEPT9	0.929079469	0.890964982	0.894338686	0.907512022	0.972160227	0.965783646
NTF3	0.929079469	0.855067335	0.883216179	0.984423083	0.936921784	0.969014913
NXPE2	0.929079469	0.850337973	0.894671468	0.916858019	0.977158496	0.969197073
LOC112442048	0.929079469	0.833713501	0.892373046	0.972276398	0.954643373	0.970115855
SHLD1	0.929079469	0.888900446	0.892343042	0.896116841	0.976259416	0.97061907
LOC112445989	0.929079469	0.892108342	0.911535369	0.95901497	0.90654624	0.974500551
LOC112449087	0.929079469	0.903254657	0.880163755	0.934466438	0.934154779	0.988252822
COMMD7	0.929079469	0.898279284	0.929661805	0.898818046	0.91563394	0.989700755
CALD1	0.929079469	0.829067393	0.899106435	0.921950515	0.894364008	0.999948228
E2F3	0.929079469	0.872175143	0.889405623	0.919601062	0.924795709	0.999948228
LOC100847841	0.929108806	0.866901725	0.999993442	0.935239391	0.897454688	0.872239777
ATP2C1	0.929108806	0.823589054	0.971053208	0.999981514	0.926813827	0.873130182
ERC1	0.929108806	0.915570087	0.886829776	0.910854395	0.999985752	0.876162019
ST6GALNAC6	0.929108806	0.950986475	0.921266625	0.957008595	0.954071324	0.88313584
LOC104975607	0.929108806	0.913120359	0.992410032	0.910575108	0.939442711	0.893561294
NT5C2	0.929108806	0.830004338	0.999993442	0.932406746	0.891304411	0.911219114
LOC101908111	0.929108806	0.829067393	0.999993442	0.916858019	0.912136413	0.927517509
DNAAF3	0.929108806	0.851721601	0.917155067	0.912152327	0.996781978	0.935497122
QTRT2	0.929108806	0.90265862	0.915858924	0.921950515	0.970988226	0.940017687
LOC112448378	0.929108806	0.842391145	0.899106435	0.98981682	0.948711141	0.948705062
LOC104974050	0.929108806	0.83844057	0.924820085	0.916858019	0.955730694	0.982868841
ANGEL1	0.929436043	0.869754045	0.944457577	0.9819628	0.930513915	0.927520621
CENPK	0.929436043	0.817329649	0.979584814	0.98981682	0.890909775	0.93363377
ITGB1	0.929466765	0.872175143	0.978710459	0.951936643	0.943963542	0.903500283
RNF180	0.929504782	0.952863279	0.939948201	0.903242359	0.94542482	0.927426708
CAV1	0.929507595	0.816412341	0.943836178	0.973075232	0.992500414	0.879576441
PSMD9	0.929507595	0.890562099	0.93784999	0.957008595	0.976259416	0.888475311
ZNF548	0.929507595	0.913116251	0.968679299	0.912152327	0.943702351	0.923807852
CLNS1A	0.929507595	0.869754045	0.993073581	0.945326519	0.909379774	0.924740534
ZNF213	0.929559384	0.822956796	0.93190909	0.925294859	0.999985752	0.871200929
DEDD	0.929559384	0.882340482	0.905023942	0.972142185	0.976292433	0.908768184
TMEM218	0.929559384	0.853946295	0.996224523	0.9063051	0.956041014	0.915414597
RNF43	0.929559384	0.955652503	0.883032292	0.925353907	0.953300742	0.945955807
ZNF106	0.929578825	0.872175143	0.913609237	0.965416137	0.911490334	0.978087957
HIBADH	0.929769945	0.822766393	0.923578245	0.952247001	0.999453276	0.908099327
LOC101905014	0.92979326	0.984410135	0.895801611	0.947574232	0.930451229	0.911219114
FLAD1	0.92979326	0.889387648	0.949939862	0.965050971	0.944839728	0.912531667

LOC107131710	0.92979326	0.869754045	0.879939804	0.965050971	0.903583337	0.99666605
MRPL39	0.929806601	0.833323491	0.937281477	0.935985829	0.979290191	0.939662884
RIMBP2	0.929835636	0.850640641	0.943836178	0.977888476	0.915775776	0.958540151
LOC100299201	0.929877177	0.847255873	0.916294854	0.944929997	0.992984551	0.923199665
PDK2	0.929884184	0.819210468	0.916323169	0.933116136	0.999985752	0.871200929
PRDX4	0.929884184	0.816202069	0.949855095	0.999981514	0.931775933	0.874490166
HPF1	0.929884184	0.868390063	0.994887288	0.929663263	0.961465747	0.87501921
REXO1	0.929884184	0.852526348	0.999993442	0.913048575	0.944839728	0.877014986
SYPL2	0.929884184	0.943244588	0.924855847	0.973075232	0.946675189	0.878642
LOC100847118	0.929884184	0.863414694	0.999993442	0.91185657	0.91563394	0.88313584
FGF9	0.929884184	0.874981732	0.949709881	0.999981514	0.893716526	0.891423889
LOC112449596	0.929884184	0.846724749	0.969404007	0.959616431	0.964301327	0.893706948
CCL14	0.929884184	0.823133291	0.998899782	0.908982438	0.970988226	0.893838339
TAPBPL	0.929884184	0.884457875	0.944538814	0.999981514	0.895067787	0.905509632
ANAPC13	0.929884184	0.884168437	0.941312902	0.920843019	0.988881329	0.905509632
LOC101904355	0.929884184	0.830715727	0.91882781	0.897601295	0.999985752	0.912894278
RNF40	0.929884184	0.916484772	0.975774524	0.952247001	0.904206501	0.91373422
GALNT2	0.929884184	0.831153015	0.885896294	0.951936643	0.999985752	0.91373422
ZRANB1	0.929884184	0.829067393	0.971255098	0.961898258	0.956904152	0.914629989
LOC112446406	0.929884184	0.833429351	0.947651663	0.931509349	0.986003146	0.923807852
CCDC194	0.929884184	0.887383587	0.911502207	0.999038184	0.929896768	0.925125664
RABEPK	0.929884184	0.832398588	0.947155158	0.999981514	0.90300429	0.93363377
LPCAT4	0.929884184	0.878551917	0.937281477	0.984423083	0.89437032	0.954496768
SLC40A1	0.929884184	0.866505227	0.924855847	0.961898258	0.937946177	0.960023723
CDK20	0.929884184	0.83866979	0.911103174	0.899855526	0.995308621	0.966212838
ZNF511	0.929884184	0.924316541	0.915858924	0.912777307	0.943160494	0.96870908
CCDC34	0.929884184	0.905550751	0.958410013	0.899047543	0.912033904	0.971545917
SYMPK	0.929913822	0.888322912	0.975774524	0.907621547	0.987723553	0.870490204
GPER1	0.929913822	0.848383949	0.91469924	0.946503916	0.999985752	0.873130182
CD14	0.929913822	0.888057447	0.943836178	0.97828838	0.965676623	0.875118526
LAYN	0.929913822	0.997763501	0.935266153	0.905772309	0.919352575	0.877750901
LONRF1	0.929913822	0.993481224	0.916294854	0.906186004	0.944839728	0.890967932
FAM3A	0.929913822	0.898726299	0.971053208	0.96951401	0.931357161	0.891088733
RGS3	0.929913822	0.859034448	0.970873534	0.984423083	0.939813331	0.891088733
TRIR	0.929913822	0.899490486	0.961800035	0.973075232	0.935286646	0.893706948
HELZ	0.929913822	0.818303808	0.911973589	0.941514611	0.999985752	0.898470276
LOC112448520	0.929913822	0.994088914	0.894545897	0.961898258	0.891726429	0.902883152
ISCU	0.929913822	0.871924797	0.896821247	0.972142185	0.985250243	0.911219114

PRDX6	0.929913822	0.816984836	0.942357321	0.988637603	0.95978143	0.923807852
COG4	0.929913822	0.833429351	0.932786033	0.950643928	0.983941532	0.927520621
TTLL1	0.929913822	0.993481224	0.886829776	0.915039335	0.927087035	0.928820381
UBA5	0.929913822	0.846215781	0.983504244	0.950643928	0.929896768	0.933897626
RNF181	0.929913822	0.910651459	0.957052938	0.929388614	0.936921784	0.934360589
RBP2	0.929913822	0.931453307	0.957052938	0.9063051	0.932006841	0.942938877
C8H9orf3	0.929913822	0.902392633	0.883950091	0.999793541	0.906766758	0.952571698
B9D2	0.929913822	0.882007869	0.912252673	0.944913633	0.943702351	0.972784753
LOC782987	0.929913822	0.829345514	0.898179681	0.97828838	0.892336551	0.997862553
IST1	0.930097778	0.940500178	0.957052938	0.960016921	0.913732532	0.903500283
LOC100336602	0.930524528	0.890669166	0.964807927	0.985717249	0.895067787	0.915006717
CLNK	0.930610722	0.893164557	0.980761647	0.9063051	0.964833865	0.900219465
C7H1orf35	0.930883967	0.99528564	0.894545897	0.902733965	0.969470443	0.870018677
CDC14A	0.930883967	0.894555763	0.919202798	0.984590227	0.970988226	0.875650601
VPS28	0.930883967	0.873915965	0.945995021	0.949565453	0.989334846	0.877014986
C23H6orf106	0.930883967	0.903618936	0.896128396	0.901171792	0.976292433	0.96274079
PLEKHD1	0.931318153	0.865613843	0.944949386	0.967450445	0.953300742	0.930146713
ZCCHC14	0.931452709	0.846724749	0.955427429	0.999981514	0.956761712	0.87100843
GULP1	0.931452709	0.969051025	0.90780986	0.95901497	0.961465747	0.872238439
MEF2C	0.931452709	0.93172121	0.945995021	0.98981682	0.927086179	0.873130182
LOC104974883	0.931452709	0.85265333	0.892343042	0.907794002	0.999985752	0.873130182
LCLAT1	0.931452709	0.987782772	0.949939862	0.948668643	0.905825822	0.874000314
SNW1	0.931452709	0.873915965	0.92323046	0.903551489	0.999985752	0.874459658
USB1	0.931452709	0.999987083	0.885896294	0.901171792	0.936388637	0.876861428
IFNAR2	0.931452709	0.929204871	0.911973589	0.999981514	0.926972948	0.877048993
ACTR10	0.931452709	0.833323491	0.999993442	0.925353907	0.953407241	0.883688554
DDO	0.931452709	0.845131166	0.916323169	0.999981514	0.946414267	0.888930405
KLF4	0.931452709	0.876873679	0.938074902	0.999981514	0.903888186	0.895446935
LOC104968518	0.931452709	0.823844235	0.928927123	0.999981514	0.954403133	0.895446935
RAB11A	0.931452709	0.822956796	0.963821408	0.998529748	0.943702351	0.897219825
RNF19A	0.931452709	0.865570309	0.951237862	0.978404889	0.959658682	0.899428912
LOC112447011	0.931452709	0.94422395	0.891972402	0.999981514	0.905825822	0.903210685
CIART	0.931452709	0.871924797	0.899106435	0.996667757	0.964301327	0.908099327
ZNF852	0.931452709	0.909096476	0.911535369	0.988761333	0.954403133	0.909056553
ISOC2	0.931452709	0.855067335	0.942357321	0.912777307	0.999985752	0.911219114
CFP	0.931452709	0.866573673	0.922552585	0.925294859	0.999985752	0.911808092
LOC104968964	0.931452709	0.961663204	0.95219206	0.907794002	0.942527603	0.91373422
TLE2	0.931452709	0.818337049	0.916294854	0.999981514	0.954403133	0.916662891

PGAP2	0.931452709	0.91318565	0.91882781	0.992560663	0.926813827	0.917150486
GAS1	0.931452709	0.836627269	0.955427429	0.962437357	0.960557169	0.92789871
UTP20	0.931452709	0.829067393	0.952862977	0.976069331	0.953300742	0.93363377
SPG7	0.931452709	0.927973597	0.894338686	0.912777307	0.976292433	0.940583352
ZNF524	0.931452709	0.918854612	0.947875514	0.898818046	0.955623649	0.946760037
LOC100140372	0.931452709	0.836148393	0.96744958	0.972142185	0.900747635	0.960023723
NBAS	0.931452709	0.90116376	0.916323169	0.927284668	0.943702351	0.970983408
RRNAD1	0.931452709	0.918455779	0.894316021	0.934466438	0.919360896	0.988639786
LOC107131424	0.931452709	0.882858125	0.907585583	0.941467375	0.900927045	0.997383134
DSN1	0.931452709	0.831549062	0.884148516	0.969214881	0.911930323	0.999948228
NPM3	0.931452709	0.829067393	0.89478142	0.914026346	0.917407853	0.999948228
NAPG	0.931545345	0.956232956	0.949855095	0.961898258	0.938879379	0.872237727
LOC615258	0.931545345	0.853946295	0.999993442	0.972142185	0.929896768	0.87229131
PPP1R35	0.931545345	0.918234392	0.998848708	0.904374668	0.951996251	0.874459658
LOC104971510	0.931545345	0.913756356	0.949855095	0.994395954	0.931775933	0.876162019
ASF1B	0.931545345	0.84238625	0.884710729	0.999981514	0.976292433	0.876162019
LOC104973073	0.931545345	0.999987083	0.894338686	0.907794002	0.903410824	0.877750901
ARGLU1	0.931545345	0.845750059	0.964807927	0.999981514	0.937932469	0.879576441
IMPAD1	0.931545345	0.884850838	0.935266153	0.999981514	0.943702351	0.888475311
CCDC166	0.931545345	0.949902864	0.925192628	0.901171792	0.986003146	0.891423889
NAGPA	0.931545345	0.839128192	0.999145937	0.919643615	0.961465747	0.896458128
CUTA	0.931545345	0.916484772	0.971053208	0.899792456	0.970988226	0.899183258
EFEMP1	0.931545345	0.95856975	0.927635169	0.979311704	0.907678664	0.899428912
TOPORS	0.931545345	0.90715891	0.927508464	0.900875874	0.99971266	0.902395868
PTDSS2	0.931545345	0.915269318	0.927635169	0.948668643	0.976088926	0.903500283
GLIPR2	0.931545345	0.89201381	0.891972402	0.965729104	0.992245178	0.905034785
TMEM132B	0.931545345	0.863948462	0.888951326	0.999981514	0.943734737	0.911219114
LOC101907174	0.931545345	0.95387031	0.892343042	0.955954309	0.964301327	0.911219114
RIT1	0.931545345	0.946820717	0.908072171	0.91833103	0.977193348	0.911336121
C11H9orf16	0.931545345	0.883756544	0.92335454	0.973075232	0.970491451	0.911611715
FNIP2	0.931545345	0.839128192	0.992511421	0.979330173	0.911417136	0.914207441
MIER2	0.931545345	0.913899522	0.988142154	0.935282232	0.91563394	0.919124527
STAMBP	0.931545345	0.824889863	0.943836178	0.995434268	0.949311988	0.925995537
PARP16	0.931545345	0.863286109	0.945995021	0.941935077	0.976259416	0.927426708
RMC1	0.931545345	0.918234392	0.893988897	0.97828838	0.954643373	0.927900405
ARIH2	0.931545345	0.927853707	0.916294854	0.980564765	0.911837627	0.938616551
LOC112441834	0.931545345	0.870334242	0.937812682	0.959853833	0.943702351	0.95147983
LDB1	0.931545345	0.88856027	0.894316021	0.995434268	0.926972948	0.953977276

HTT	0.931545345	0.893164557	0.894098617	0.941935077	0.96637945	0.960120928
NOTCH4	0.931545345	0.955536844	0.886829776	0.91285419	0.947112796	0.963701289
PRKCH	0.931545345	0.826102395	0.943836178	0.980532429	0.92549567	0.965628973
SLC35A1	0.931545345	0.927853707	0.89478142	0.935563591	0.948006937	0.966136679
RNPC3	0.931545345	0.843278276	0.91882781	0.952247001	0.953300742	0.973649216
IFI35	0.931545345	0.882242226	0.951392011	0.916480703	0.927086179	0.975126734
NSMAF	0.931545345	0.893615781	0.946791586	0.907794002	0.933426897	0.975200512
SKAP1	0.931545345	0.823478017	0.947651663	0.929388614	0.934751389	0.990564043
DNAJB5	0.931545345	0.847493166	0.916294854	0.958582027	0.901853599	0.997383134
HOXB6	0.931705226	0.865570309	0.951392011	0.999981514	0.902258286	0.91373422
RNF112	0.931727763	0.848383949	0.99341711	0.96900924	0.943702351	0.885089102
TINAGL1	0.931727763	0.955652503	0.928731949	0.995565324	0.893376996	0.887710732
ESF1	0.931727763	0.860553498	0.916453343	0.937657986	0.999985752	0.894808664
MPDU1	0.931727763	0.877822131	0.942134405	0.973673712	0.962513981	0.911219114
NCKAP5	0.931727763	0.833323491	0.907705835	0.964521432	0.918424492	0.99666605
ADAT1	0.93200538	0.95462892	0.899600099	0.970039043	0.924222719	0.931904429
ZNF286A	0.932074363	0.991673407	0.913429569	0.900875874	0.935093621	0.927633316
CHMP5	0.932130361	0.822956796	0.939948201	0.973396104	0.935286646	0.969197073
PRND	0.932552762	0.973780375	0.894171177	0.991955963	0.934450558	0.876162019
ZNF529	0.932552762	0.940381381	0.972741989	0.903584224	0.954071324	0.90645885
CDC42BPB	0.932552762	0.984913707	0.944949386	0.915039335	0.923137951	0.911219114
COP1	0.932552762	0.873156844	0.89478142	0.999981514	0.944839728	0.923772625
TMEM249	0.932552762	0.867518836	0.955427429	0.913946495	0.982796995	0.925233445
ATG4C	0.932552762	0.857815763	0.968548891	0.96951401	0.936108073	0.927426708
LOC112442091	0.932552762	0.891547502	0.974586624	0.907794002	0.953300742	0.936951685
LOC100294994	0.932552762	0.870296749	0.894316021	0.941467375	0.951128877	0.98926637
CNPY3	0.93260625	0.831153015	0.892343042	0.935825185	0.999985752	0.872239777
LOC107133459	0.93260625	0.890669166	0.901736458	0.999981514	0.958797242	0.878364726
IL4R	0.93260625	0.822956796	0.89478142	0.91185657	0.999985752	0.93363377
LOC104975782	0.93260625	0.959396625	0.911103174	0.907353815	0.893716526	0.985223183
C18H19orf47	0.932614367	0.872175143	0.964807927	0.904768656	0.91563394	0.982422737
MED1	0.932796818	0.875391655	0.928927123	0.916858019	0.999985752	0.874490166
LIPT2	0.932796818	0.939795204	0.937841596	0.947313672	0.943702351	0.923807852
ASAP3	0.932815346	0.83359488	0.999993442	0.921950515	0.911568867	0.906637848
MCRIP1	0.932815346	0.902559762	0.916038418	0.940436902	0.989379051	0.912531667
C21H14orf28	0.932815346	0.927794672	0.93475587	0.913946495	0.929896768	0.970071797
MLPH	0.93292714	0.926852499	0.922552585	0.999981514	0.921710688	0.88755642
DHX9	0.933062565	0.926551255	0.949855095	0.929663263	0.976292433	0.882690335

TMEM41B	0.933062565	0.822956796	0.999993442	0.980564765	0.893716526	0.89209761
KIAA0141	0.933062565	0.823589054	0.966232793	0.995434268	0.911568867	0.93531275
THOC3	0.933359565	0.829067393	0.949855095	0.999981514	0.895757462	0.877014986
C7H5orf24	0.933359565	0.927973597	0.999993442	0.908467146	0.906572071	0.877048993
TRIP11	0.933359565	0.872175143	0.906019013	0.950643928	0.999985752	0.888887063
PARVG	0.933359565	0.868390063	0.927635169	0.940726907	0.99971266	0.900219465
MS4A13	0.933359565	0.82440829	0.999993442	0.962899291	0.901662258	0.903773776
ABHD11	0.933359565	0.941817736	0.916294854	0.973075232	0.953300742	0.904215473
TMEM47	0.933359565	0.998847958	0.916294854	0.928404714	0.894364008	0.90969547
TNFRSF17	0.933359565	0.957588005	0.947432432	0.96951401	0.893716526	0.912531667
ADCK1	0.933359565	0.833429351	0.980761647	0.928404714	0.9683846	0.927520621
ANAPC1	0.933359565	0.890176137	0.895801611	0.930987593	0.989965404	0.940017687
HECTD4	0.933359565	0.938235006	0.923521499	0.952247001	0.907762824	0.960023723
SH3GL1	0.933359565	0.960845618	0.916323169	0.912922827	0.917407853	0.963456717
RBM23	0.933359565	0.846721805	0.916294854	0.992702099	0.906766758	0.973649216
LOC407171	0.933359565	0.87063977	0.935194852	0.908467146	0.960419102	0.973649216
ZNF384	0.933359565	0.869754045	0.935219877	0.940438437	0.936921784	0.9790177
SYNE3	0.933359565	0.846724749	0.924820085	0.918693944	0.929896768	0.999463809
RNF123	0.933412373	0.885494685	0.957052938	0.923623116	0.98094737	0.909488315
CD36	0.933639458	0.950986475	0.972741989	0.975653996	0.900893337	0.873130182
HTR2A	0.933639458	0.916448625	0.935266153	0.952247001	0.982538884	0.877014986
OAF	0.933639458	0.99960614	0.916294854	0.934445963	0.901853599	0.888475311
RAB34	0.933639458	0.967034954	0.899952655	0.97828838	0.943734737	0.89148147
LOC524181	0.933639458	0.842884359	0.943028511	0.992339478	0.970980375	0.893838339
NCBP1	0.933639458	0.955333923	0.893988897	0.961516541	0.967205491	0.899428912
LOC508153	0.933639458	0.855067335	0.885896294	0.961898258	0.999985752	0.905509632
SZRD1	0.933639458	0.915269318	0.906019013	0.918693944	0.999985752	0.911219114
ZNF512	0.933639458	0.994185904	0.89478142	0.93660503	0.929896768	0.91373422
LRRC8E	0.933639458	0.835486075	0.924855847	0.929342214	0.999985752	0.914132808
KIAA1328	0.933639458	0.895777642	0.921266625	0.980605008	0.958852623	0.915546224
PEF1	0.933639458	0.893164557	0.942134405	0.94751394	0.976259416	0.916193353
SGMS2	0.933639458	0.937448751	0.925244197	0.965155716	0.943540106	0.919124527
RPS6KA2	0.933639458	0.946859049	0.886829776	0.940436902	0.977167046	0.921792022
CADM3	0.933639458	0.950986475	0.955221146	0.9063051	0.952264073	0.923092436
NR1D2	0.933639458	0.939956757	0.955221146	0.916858019	0.953300742	0.923092436
ACOT6	0.933639458	0.927853707	0.903431343	0.940436902	0.979290191	0.923092436
LOC112446696	0.933639458	0.833713501	0.949855095	0.944351122	0.970980375	0.948705062
SLCO4C1	0.933639458	0.908364002	0.927508464	0.903565968	0.954071324	0.973218068

APMAP	0.933639458	0.919245978	0.908072171	0.907794002	0.935286646	0.989184798
ZFPL1	0.933648494	0.823589054	0.999993442	0.912777307	0.976259416	0.890014046
LOC100336644	0.933819564	0.890875255	0.908072171	0.990955345	0.911930323	0.960023723
LOC112448582	0.933819564	0.878797095	0.894316021	0.916858019	0.952264073	0.997206681
LOC112442384	0.933985524	0.911461743	0.916323169	0.928075638	0.906893576	0.992680267
B4GALT1	0.934109924	0.97359232	0.899106435	0.964521432	0.927086179	0.923092436
MRO	0.934189178	0.857523251	0.895801611	0.986051493	0.926831826	0.980556133
LOC101907893	0.934274606	0.867885278	0.970873534	0.929342214	0.953300742	0.945854375
C5H12orf56	0.93433355	0.833323491	0.989494193	0.94222181	0.985360041	0.876162019
PAFAH1B1	0.93433355	0.823478017	0.999993442	0.964521432	0.900747635	0.886912202
IKBKB	0.93433355	0.870195838	0.957295966	0.999981514	0.926813827	0.893585844
CDKN1C	0.93433355	0.856560491	0.916294854	0.972467586	0.996089557	0.904106475
LOC104975004	0.93433355	0.870195838	0.899600099	0.985878137	0.980189546	0.91373422
RAPGEF4	0.93433355	0.880565292	0.946791586	0.919153842	0.986861597	0.923092436
FMNL2	0.93433355	0.823133291	0.948949412	0.908467146	0.999985752	0.923807852
ITPRIPL2	0.93433355	0.835883963	0.915858924	0.999981514	0.935286646	0.952663521
DYNLT3	0.93433355	0.850514112	0.958454497	0.958103924	0.926831826	0.966659342
NTN1	0.934357161	0.83359488	0.946791586	0.908467146	0.999985752	0.873456456
VPS72	0.934357161	0.913116251	0.903431343	0.96444269	0.993831963	0.884068792
MEF2A	0.934357161	0.870222905	0.979354564	0.961898258	0.961465747	0.887812286
SERGEF	0.934357161	0.850640641	0.944126714	0.995565324	0.958797242	0.905034785
FANCD2	0.934357161	0.863286109	0.916294854	0.959616431	0.998914459	0.90645885
SARNP	0.934357161	0.831359179	0.999993442	0.941754203	0.896589592	0.909056553
WDR1	0.934357161	0.848383949	0.949855095	0.999981514	0.923961062	0.923807852
NOCT	0.934357161	0.941400719	0.894316021	0.907794002	0.996089557	0.923807852
GPR55	0.934357161	0.926852499	0.894338686	0.943808491	0.982538884	0.926487302
CYP8B1	0.934357161	0.869754045	0.975185526	0.917273503	0.958852623	0.940079339
VAMP7	0.934357161	0.826316517	0.955427429	0.99313054	0.931357161	0.940423784
FAM217B	0.934357161	0.863286109	0.88846032	0.978559648	0.948711141	0.975558533
PARD6A	0.934375185	0.945942733	0.940413	0.901353354	0.989334846	0.883688554
SLC46A3	0.934421242	0.823647199	0.906279973	0.999981514	0.908820136	0.960877268
LOC107131649	0.934578356	0.831549062	0.97773026	0.940436902	0.951996251	0.955140534
SLC4A5	0.934705479	0.863486436	0.89385865	0.999981514	0.970988226	0.917930485
CCNT2	0.934750698	0.916484772	0.906019013	0.910575108	0.989379051	0.936263602
MAP2K1	0.934805189	0.825171788	0.921266625	0.960566165	0.991481886	0.93363377
LOC781379	0.934923919	0.823478017	0.999993442	0.925210648	0.942786245	0.874000314
PF4	0.934937412	0.89833876	0.999993442	0.918693944	0.953300742	0.876162019
RGP1	0.934937412	0.932394105	0.91469924	0.960016921	0.986003146	0.876861428

MYL6B	0.934937412	0.834615638	0.892139418	0.999981514	0.938879379	0.891088733
LAMTOR5	0.934937412	0.869754045	0.929661805	0.990036762	0.964301327	0.90969547
EPHB4	0.934937412	0.868390063	0.999993442	0.924040493	0.921710688	0.925125664
LOC786987	0.934937412	0.889345904	0.941507802	0.959652624	0.964301327	0.925273613
ULK1	0.934937412	0.838355508	0.981655709	0.982220679	0.917667911	0.930710695
EIF4B	0.934937412	0.934225172	0.916294854	0.928404714	0.964301327	0.940583352
LOC112442215	0.934937412	0.872175143	0.970873534	0.922002553	0.942786245	0.960023723
INTS2	0.934937412	0.832142291	0.962314622	0.959159289	0.926813827	0.971982373
XYLT1	0.934937412	0.95462892	0.889098668	0.9063051	0.942786245	0.9803583
ZSWIM8	0.934937412	0.930293015	0.924855847	0.910575108	0.913388714	0.986441687
RAB4B	0.935297416	0.893164557	0.991936822	0.976196758	0.935286646	0.876162019
DDAH1	0.935297416	0.901710313	0.916294854	0.9880671	0.977158496	0.876162019
KLC4	0.935297416	0.870222905	0.889859292	0.96968491	0.999985752	0.876162019
SH3BP5L	0.935297416	0.872175143	0.990037865	0.921950515	0.983941532	0.876861428
LOC112442408	0.935297416	0.980521483	0.958197827	0.944913633	0.926998996	0.877014986
AURKC	0.935297416	0.843278276	0.964807927	0.998529748	0.956041014	0.881992627
DENND5B	0.935297416	0.829067393	0.927635169	0.999981514	0.958797242	0.883688554
PABPC5	0.935297416	0.999987083	0.894338686	0.933718942	0.900927045	0.893838339
LOC100847835	0.935297416	0.864945924	0.975774524	0.929388614	0.984386856	0.893838339
LOC112444967	0.935297416	0.833429351	0.980032241	0.999981514	0.904833739	0.89438401
ZBTB44	0.935297416	0.915269318	0.949855095	0.955466436	0.9683846	0.89438401
LOC112447523	0.935297416	0.885940782	0.980032241	0.953577223	0.957596412	0.896434668
SFT2D1	0.935297416	0.971000021	0.963164828	0.916858019	0.935286646	0.907124813
GPX8	0.935297416	0.846724749	0.999993442	0.964521432	0.913355547	0.907729038
LOC404051	0.935297416	0.916484772	0.911973589	0.999981514	0.906893576	0.909318728
COA1	0.935297416	0.904875256	0.966199203	0.910495312	0.976292433	0.912486829
IGSF8	0.935297416	0.901631302	0.967590756	0.984331039	0.91563394	0.91373422
PRDX5	0.935297416	0.927973597	0.966232793	0.93048026	0.951996251	0.917849087
SLC22A4	0.935297416	0.88008538	0.901982947	0.932764647	0.999985752	0.922361909
MXD4	0.935297416	0.880565292	0.96030829	0.978861394	0.936108073	0.923807852
ADK	0.935297416	0.91763262	0.969000554	0.929388614	0.947299702	0.925233445
KLHL41	0.935297416	0.991673407	0.89478142	0.916858019	0.947020167	0.925432855
USP18	0.935297416	0.971931061	0.911973589	0.915039335	0.959026737	0.930710695
RASD1	0.935297416	0.844170799	0.91882781	0.999981514	0.900893337	0.931410194
LOC100336161	0.935297416	0.942088099	0.927508464	0.917892197	0.963662524	0.93363377
SRSF9	0.935297416	0.938235006	0.918606509	0.922418106	0.964301327	0.940256832
LOC100337507	0.935297416	0.930293015	0.905017412	0.989601002	0.91563394	0.944973383
AK1	0.935297416	0.885620498	0.99647733	0.907794002	0.924795709	0.944973383

PYCARD	0.935297416	0.895777642	0.916294854	0.95901497	0.961465747	0.947937638
DUS2	0.935297416	0.823478017	0.976883418	0.92139105	0.970885781	0.948705062
CAVIN1	0.935297416	0.839128192	0.973034514	0.977689659	0.914280652	0.951081819
RARA	0.935297416	0.824188473	0.886829776	0.972142185	0.996089557	0.951081819
TMCO6	0.935297416	0.863613561	0.961437412	0.955466436	0.939203547	0.952663521
YBX2	0.935297416	0.880129816	0.948904459	0.948050281	0.947299702	0.958790968
LOC101907883	0.935297416	0.983887536	0.916294854	0.916858019	0.900927045	0.960023723
SRSF1	0.935297416	0.889581399	0.975774524	0.91231772	0.926813827	0.966212838
FYB2	0.935297416	0.938087741	0.8869541	0.932914414	0.958654817	0.966850975
HMGXB4	0.935297416	0.893229467	0.932217079	0.96951401	0.904996355	0.970105102
PNRC2	0.935297416	0.865455711	0.916323169	0.970039043	0.936108073	0.973649216
HSF5	0.935297416	0.829067393	0.897432125	0.98981682	0.943160494	0.978923235
FGFR1	0.935308767	0.877468389	0.893988897	0.999981514	0.906893576	0.919124527
CCDC90B	0.935348541	0.831694163	0.999993442	0.907794002	0.900893337	0.91373422
PRDM4	0.935582765	0.988444882	0.895801611	0.934180032	0.956906331	0.909056553
APOOL	0.935582765	0.827594227	0.965819476	0.905772309	0.998287238	0.927520621
UGGT1	0.936500299	0.855141737	0.926161087	0.970039043	0.943702351	0.969754487
SOX11	0.936504036	0.916484772	0.922552585	0.960016921	0.932024143	0.960023723
PELO	0.936725393	0.825171788	0.999993442	0.946503916	0.89992403	0.904744538
CCNB1	0.936995339	0.857370953	0.974470955	0.999981514	0.939553343	0.876162019
PLCL1	0.936995339	0.960845618	0.978014482	0.930987593	0.93630105	0.882690335
RETN	0.936995339	0.888057447	0.944485826	0.934466438	0.998040182	0.893585844
USP50	0.936995339	0.905727888	0.972897558	0.937033554	0.952264073	0.923807852
NFIA	0.936995339	0.889387648	0.996224523	0.922967576	0.938011667	0.923878545
NKG7	0.936995339	0.943403405	0.900964393	0.932914414	0.976292433	0.927520621
UNC119B	0.936995339	0.866113361	0.91882781	0.97828838	0.972160227	0.928820381
SFT2D2	0.936995339	0.919171954	0.915124089	0.973075232	0.957588298	0.930146713
MYO9A	0.936995339	0.933100216	0.935266153	0.913946495	0.964301327	0.940583352
MRPL42	0.936995339	0.85265333	0.989494193	0.935884171	0.937932469	0.950631909
DOK3	0.936995339	0.946859049	0.916323169	0.924992287	0.927086179	0.971545917
MFSD2B	0.936995339	0.88008538	0.927635169	0.959277542	0.926998996	0.980167535
LOC112442227	0.937328852	0.886669497	0.963821408	0.964524528	0.927618579	0.940583352
CNOT3	0.937427951	0.882242226	0.970873534	0.976125636	0.961465747	0.880302064
PCID2	0.937427951	0.861395263	0.916294854	0.960937718	0.999985752	0.896529476
MTMR6	0.937427951	0.855752352	0.910845937	0.999981514	0.939326536	0.912531667
CSF2RA	0.937427951	0.872175143	0.971780659	0.980532429	0.932024143	0.923199665
LOC519208	0.937427951	0.860419291	0.908603738	0.974286747	0.936108073	0.982937529
TMEM238	0.937429335	0.943244588	0.959725886	0.941467375	0.965163023	0.876162019

PSEN2	0.937429335	0.899490486	0.91882781	0.958917811	0.998287238	0.885202945
ATP6V1E2	0.937429335	0.991673407	0.905023942	0.939604997	0.958797242	0.890653851
C16H1orf116	0.937429335	0.933100216	0.957268178	0.990955345	0.910717783	0.89209761
NOSTRIN	0.937429335	0.927853707	0.895826828	0.973075232	0.97077135	0.922361909
LOC101905053	0.937429335	0.942088099	0.929661805	0.989601002	0.906766758	0.923807852
TAF1A	0.937429335	0.871003459	0.999993442	0.914778844	0.900891209	0.93363377
TMEM178A	0.937429335	0.898706315	0.891292772	0.907794002	0.999985752	0.936263602
RALB	0.937429335	0.845411477	0.916323169	0.938303773	0.996781978	0.950798794
CLHC1	0.937429335	0.863613561	0.89478142	0.997660427	0.933344708	0.969470835
TWISTNB	0.937429335	0.848383949	0.903431343	0.987680286	0.948421303	0.971545917
PLEK	0.938032277	0.897000209	0.990037865	0.989601002	0.916605572	0.876861428
IRF3	0.938032277	0.915269318	0.913100432	0.999981514	0.964301327	0.877014986
LOC100847876	0.938032277	0.872175143	0.992377532	0.978503042	0.935286646	0.893706948
THAP12	0.938032277	0.872175143	0.986511454	0.988347114	0.911028086	0.91373422
UBA1	0.938089529	0.88008538	0.976295638	0.952247001	0.91563394	0.958707771
RERG	0.938100152	0.829067393	0.906019013	0.999981514	0.960048824	0.879576441
ELP4	0.938100152	0.845338797	0.89478142	0.961898258	0.947020167	0.995702108
DRG2	0.938123521	0.855067335	0.927635169	0.991955963	0.933426897	0.960023723
IPO8	0.938438773	0.998847958	0.966232793	0.907621547	0.900927045	0.877014986
CTNNB1	0.938438773	0.863286109	0.916294854	0.999981514	0.947020167	0.877014986
TIGD7	0.938438773	0.856808434	0.993297549	0.979584502	0.954071324	0.877014986
KLC2	0.938438773	0.988824757	0.898179681	0.991054578	0.915775776	0.877048993
GTSF1	0.938438773	0.840909162	0.937334712	0.999981514	0.972160227	0.879576441
HTR7	0.938438773	0.968509005	0.964807927	0.951645599	0.925918507	0.891088733
AHCTF1	0.938438773	0.918381186	0.960713825	0.929342214	0.977158496	0.893585844
LRRK1	0.938438773	0.882858125	0.999993442	0.907794002	0.959393538	0.895446935
DIABLO	0.938438773	0.833429351	0.999993442	0.919604295	0.961465747	0.897071936
FMNL1	0.938438773	0.890669166	0.955427429	0.915039335	0.998141915	0.902100676
ART4	0.938438773	0.997425219	0.908072171	0.935956158	0.936215792	0.903990996
NUDCD2	0.938438773	0.862299245	0.990118874	0.933718942	0.970988226	0.908667118
ERMAP	0.938438773	0.84471528	0.914256466	0.999981514	0.950958352	0.911638347
MBD6	0.938438773	0.840402825	0.99341711	0.990955345	0.91563394	0.912531667
LONP2	0.938438773	0.831816777	0.999993442	0.949502614	0.946384846	0.914132808
DTNA	0.938438773	0.980289052	0.944126714	0.925294859	0.936215792	0.920121131
ECE1	0.938438773	0.889387648	0.987610559	0.910575108	0.965163023	0.9221477
GXYLT1	0.938438773	0.932394105	0.94422142	0.97828838	0.931357161	0.922539606
NSG1	0.938438773	0.982011436	0.936354212	0.93048026	0.931775933	0.923092436
LOC112441868	0.938438773	0.864945924	0.95940506	0.921950515	0.988881329	0.923944552

COL27A1	0.938438773	0.847735457	0.945995021	0.999981514	0.901853599	0.925273613
CHKA	0.938438773	0.944324631	0.89478142	0.994231354	0.920393744	0.93531275
PICK1	0.938438773	0.90715891	0.907477369	0.997660427	0.936108073	0.938894901
BPNT1	0.938438773	0.859469124	0.943836178	0.940438437	0.983941532	0.940256832
LOC112443528	0.938438773	0.842884359	0.964807927	0.955466436	0.956904152	0.950798794
KLHL33	0.938438773	0.855067335	0.907128613	0.999981514	0.941364574	0.960023723
LOC112446663	0.938438773	0.893213198	0.900847693	0.965729104	0.961465747	0.960023723
GSTM2	0.938438773	0.96643574	0.899106435	0.949864417	0.91509147	0.964898868
MED14	0.938438773	0.84312966	0.949939862	0.907794002	0.972432486	0.973649216
GLTP	0.938438773	0.833429351	0.94422142	0.935884171	0.962513981	0.9792065
LOC112445968	0.938438773	0.846724749	0.937281477	0.908467146	0.976259416	0.9803583
MXI1	0.938438773	0.928427136	0.916294854	0.931383184	0.926813827	0.98469083
ZNF331	0.938438773	0.910044299	0.932786033	0.946264276	0.907678664	0.985223183
LOC510798	0.938438773	0.932429983	0.88955259	0.933718942	0.936921784	0.988273129
MAP3K21	0.938438773	0.851721601	0.89478142	0.960016921	0.903410824	0.999948228
LOC509034	0.938438773	0.839958186	0.914002845	0.941467375	0.911568867	0.999948228
FYCO1	0.938623516	0.88008538	0.894778002	0.999981514	0.911568867	0.877048993
EIF1B	0.938623516	0.863286109	0.905023942	0.992470001	0.999453276	0.877048993
CHMP3	0.938623516	0.846721805	0.995377403	0.993215785	0.935286646	0.882690335
PHACTR2	0.938623516	0.829345514	0.916294854	0.97731816	0.999985752	0.889238459
APBA3	0.938623516	0.935001231	0.963821408	0.980564765	0.927087035	0.89209761
ZMYM6	0.938623516	0.898726299	0.894523258	0.999981514	0.900927045	0.902100676
LOC788414	0.938623516	0.841427489	0.893988897	0.918693944	0.999985752	0.91373422
CRNKL1	0.938623516	0.829067393	0.996224523	0.918693944	0.976292433	0.919124527
CITED1	0.938623516	0.934225172	0.95322206	0.908467146	0.970988226	0.923807852
MAPK8	0.938623516	0.96130201	0.927635169	0.910575108	0.935286646	0.96274079
CRY2	0.938623516	0.846724749	0.897827774	0.907966636	0.984386856	0.990817897
SELPLG	0.938797869	0.939140452	0.980032241	0.907794002	0.965676623	0.891088733
KMT2B	0.938797869	0.853678591	0.972410614	0.960937718	0.970988226	0.912531667
LOC784522	0.938797869	0.855752352	0.933884582	0.999981514	0.926972948	0.925125664
TMC7	0.938797869	0.939442799	0.955427429	0.925294859	0.956041014	0.927520621
LOC100139325	0.938797869	0.847255873	0.945336172	0.955466436	0.982087479	0.933903265
MAPK3	0.938797869	0.873915965	0.943836178	0.960190213	0.961465747	0.945870804
ZNF557	0.938797869	0.938235006	0.906019013	0.959652624	0.929896768	0.967047449
PKD1	0.938799767	0.851775118	0.955427429	0.995312373	0.970980375	0.885247403
PAOX	0.938879038	0.838308162	0.999993442	0.959616431	0.927943699	0.908768184
ACOX2	0.938879038	0.90116376	0.978014482	0.920867825	0.964235922	0.923092436
AFAP1L1	0.938879038	0.890045072	0.989977673	0.952247001	0.906766758	0.944973383

SNRNP35	0.938879038	0.844546559	0.940653871	0.955340531	0.901853599	0.99841577
C5H12orf4	0.938883703	0.837263562	0.975154765	0.964485876	0.959565914	0.930710695
BTD	0.939063367	0.888322912	0.95322206	0.999981514	0.906893576	0.925273613
MPRIP	0.939187223	0.927853707	0.897322115	0.989601002	0.970491451	0.90388238
CHSY1	0.939187223	0.867277242	0.894338686	0.97828838	0.999985752	0.912531667
COQ8B	0.939190665	0.927853707	0.951028911	0.989701998	0.950980504	0.877014986
GJC3	0.939190665	0.99528564	0.899106435	0.959652624	0.943702351	0.87754233
MRPS15	0.939190665	0.895202184	0.969404007	0.929663263	0.988238252	0.887722423
SFMBT2	0.939190665	0.869953468	0.899106435	0.999981514	0.904643542	0.891088733
DCAF8	0.939190665	0.833713501	0.92091815	0.999981514	0.916353643	0.891088733
ZNF200	0.939190665	0.879209957	0.984886031	0.972142185	0.953885477	0.893706948
NEURL3	0.939190665	0.833323491	0.89478142	0.999981514	0.954403133	0.896529476
TGIF2	0.939190665	0.91078662	0.916294854	0.950643928	0.998141915	0.902445346
FBXL20	0.939190665	0.927853707	0.968204727	0.980564765	0.909737016	0.91373422
KLKB1	0.939190665	0.928658222	0.943120912	0.949468104	0.964301327	0.922665469
MRPL58	0.939190665	0.872331637	0.973562292	0.937615893	0.970988226	0.923092436
LOC100847802	0.939190665	0.941400719	0.993049856	0.922002553	0.901853599	0.930710695
BRF2	0.939190665	0.836053314	0.974656086	0.916858019	0.984692578	0.933897626
GPAT3	0.939190665	0.871543546	0.949855095	0.959652624	0.961432343	0.944973383
CTNNA3	0.939190665	0.97225074	0.894316021	0.941467375	0.953300742	0.945532151
C7H19orf44	0.939190665	0.882858125	0.941149858	0.952247001	0.964301327	0.950631909
LOC785477	0.939190665	0.829345514	0.975774524	0.978503042	0.918512595	0.960023723
SMIM7	0.939190665	0.839128192	0.999993442	0.927721166	0.909737016	0.960877268
KIAA0408	0.939190665	0.878551917	0.903150496	0.976125636	0.953300742	0.971608881
INPP4B	0.939190665	0.87976124	0.942357321	0.910575108	0.943540106	0.993890045
TECPR2	0.939190665	0.844170799	0.948129084	0.924494167	0.929802526	0.998458498
RSBN1L	0.93926768	0.997471998	0.935266153	0.943669023	0.915775776	0.888475311
PLCE1	0.939306903	0.99306529	0.89478142	0.96861531	0.902258286	0.926418736
EIF2B3	0.939392415	0.851793828	0.965327631	0.94469136	0.950958352	0.964898868
LOC104972346	0.93968389	0.837263562	0.915713148	0.912777307	0.999985752	0.923547711
LOC101905708	0.93992054	0.954131334	0.951028911	0.982220679	0.935286646	0.881029291
MTSS1	0.939940957	0.955827589	0.894030462	0.908467146	0.999985752	0.887722423
TCEANC	0.940103371	0.861767941	0.964085416	0.973812087	0.964301327	0.917024468
ANKLE1	0.940103371	0.837244723	0.917973089	0.913048575	0.973208134	0.994788615
PCYOX1L	0.94017706	0.859469124	0.901575815	0.959853833	0.983941532	0.960023723
PRPF39	0.940202232	0.855141737	0.951028911	0.999981514	0.956952436	0.891088733
NKAPL	0.940202232	0.991673407	0.972940212	0.91185657	0.911568867	0.895633366
CALCRL	0.940202232	0.872175143	0.916323169	0.989485949	0.927086179	0.971271151

CYB5R3	0.940243734	0.930293015	0.908072171	0.999981514	0.942786245	0.911960508
LOC787812	0.940272291	0.998847958	0.965229237	0.907794002	0.912199478	0.877822428
EXD2	0.940272291	0.913116251	0.999993442	0.914850247	0.943055455	0.880981659
PANX2	0.940272291	0.919525428	0.900541227	0.999981514	0.950085378	0.885089102
SPECC1L	0.940272291	0.833429351	0.999993442	0.949502614	0.943540106	0.888887063
MED28	0.940272291	0.900239737	0.975774524	0.999981514	0.904084756	0.891088733
SSPN	0.940272291	0.995373357	0.925320071	0.952247001	0.924222719	0.891088733
LOC574091	0.940272291	0.996728887	0.911973589	0.929251053	0.956041014	0.891088733
BRPF3	0.940272291	0.934225172	0.916323169	0.926740082	0.997422012	0.907145443
HSD3B7	0.940272291	0.886565095	0.937993336	0.999981514	0.936108073	0.911234149
LOC104974749	0.940272291	0.842391145	0.999993442	0.949565453	0.930451229	0.919124527
GRIP2	0.940272291	0.965456931	0.916038418	0.949565453	0.959338941	0.923547711
THAP11	0.940272291	0.831153015	0.911973589	0.999981514	0.927149659	0.940583352
KIF21A	0.940272291	0.842884359	0.938212761	0.995565324	0.953300742	0.944927301
ARAP3	0.940272291	0.883756544	0.975774524	0.952247001	0.92337151	0.959075802
GM2A	0.940272291	0.941309293	0.92422067	0.96444269	0.916800726	0.960023723
CDKL4	0.940272291	0.913120359	0.930148372	0.982868459	0.904084756	0.963107047
CEP295NL	0.940272291	0.880129816	0.947651663	0.979644238	0.916353643	0.963701289
WDR4	0.940272291	0.920882164	0.892139418	0.942955143	0.935286646	0.995379819
ANO3	0.940348556	0.833429351	0.915858924	0.999981514	0.986019537	0.87754233
SEMA5A	0.940348556	0.994405848	0.948949412	0.945300947	0.918426817	0.880981659
FXYD6	0.940348556	0.957667701	0.912396874	0.965729104	0.976292433	0.881875045
AES	0.940348556	0.83777309	0.996224523	0.95901497	0.972160227	0.885471103
LOC783838	0.940348556	0.99950853	0.899725007	0.923220263	0.954071324	0.891088733
ARNT	0.940348556	0.931588197	0.927508464	0.949502614	0.987723553	0.894808664
MLKL	0.940348556	0.87976124	0.942357321	0.999981514	0.929802526	0.898470276
DNAAF5	0.940348556	0.922285873	0.927327441	0.988408846	0.960573822	0.903500283
CYBRD1	0.940348556	0.967469801	0.89478142	0.911606774	0.997422012	0.90763123
ZNF639	0.940348556	0.863286109	0.997400824	0.948668643	0.955741702	0.911219114
VAMP3	0.940348556	0.831153015	0.992511421	0.991955963	0.935286646	0.913221224
LOC112448474	0.940348556	0.930591251	0.92422067	0.9745615	0.954403133	0.926480924
CEP135	0.940348556	0.872175143	0.99647733	0.923060035	0.954814306	0.926487302
S100A8	0.940348556	0.881706092	0.915713148	0.944929997	0.999453276	0.929457383
ATRAID	0.940348556	0.882102283	0.915120516	0.999981514	0.94542482	0.93363377
ZNF112	0.940348556	0.93796343	0.95322206	0.932914414	0.951918183	0.938894901
RPUSD3	0.940348556	0.864945924	0.896023027	0.999981514	0.917007149	0.953509765
TMEM135	0.940348556	0.831694163	0.958197827	0.992930508	0.912386282	0.963456717
GAS7	0.940348556	0.875506677	0.899106435	0.948050281	0.929059391	0.999948228

LOC112449358	0.940441569	0.869754045	0.896821247	0.999981514	0.91506312	0.923092436
LOC112447027	0.940441569	0.83844057	0.942357321	0.952247001	0.961465747	0.973779337
ZNF18	0.940462409	0.892108342	0.899725007	0.982220679	0.99971266	0.885202945
DCP2	0.940462409	0.902525869	0.91882781	0.995565324	0.953300742	0.926433572
PAXX	0.940462409	0.960747241	0.894030462	0.989601002	0.932024143	0.930710695
LOC782114	0.940462409	0.872175143	0.965961035	0.978404889	0.940870936	0.933897626
DIO3	0.940462409	0.955652503	0.924797209	0.940438437	0.953300742	0.940583352
LOC112443004	0.940462409	0.913120359	0.899106435	0.95901497	0.975921255	0.948705062
COG8	0.940462409	0.839660873	0.980032241	0.952247001	0.910508458	0.979480605
ICE1	0.941075146	0.883945911	0.999993442	0.911258863	0.959367717	0.887722423
HDAC4	0.941075146	0.833323491	0.999993442	0.919628651	0.964301327	0.888475311
C23H6orf226	0.941075146	0.834736959	0.95322206	0.999981514	0.935286646	0.923807852
PSMB1	0.941075146	0.927369042	0.99341711	0.927284668	0.91563394	0.93531275
SPTLC2	0.941075146	0.913120359	0.916038418	0.932677501	0.989334846	0.938894901
PDE4A	0.941075146	0.961663204	0.915858924	0.95901497	0.935286646	0.947801358
HBS1L	0.941075146	0.849119707	0.958410013	0.959616431	0.935286646	0.973964249
LOC112442849	0.941075146	0.90715891	0.95558643	0.934995498	0.911664199	0.981216824
NATD1	0.941189523	0.995275863	0.89478142	0.95901497	0.956041014	0.878118151
NSRP1	0.941189523	0.990045341	0.899190814	0.960016921	0.960419102	0.881939613
PDCD5	0.941189523	0.878504776	0.996224523	0.918105555	0.979290191	0.888475311
VCPKMT	0.941189523	0.836131298	0.999993442	0.972142185	0.909379774	0.912531667
LOC104976574	0.941189523	0.867518836	0.916038418	0.999981514	0.935286646	0.914132808
ETAA1	0.941189523	0.955536844	0.942357321	0.984423083	0.914280652	0.919124527
GIN1	0.941189523	0.83844057	0.972806636	0.973396104	0.965930435	0.922361909
LOC101905114	0.941189523	0.872175143	0.916038418	0.999981514	0.964301327	0.926319563
LOC112442039	0.941189523	0.839800081	0.990615319	0.977002843	0.935286646	0.933224627
RUNX1T1	0.941189523	0.893615781	0.98915348	0.914026346	0.955315195	0.940017687
LOC100847819	0.941189523	0.939442799	0.903431343	0.989601002	0.915775776	0.958707771
LOC101902390	0.941189523	0.920382325	0.911103174	0.939960459	0.950958352	0.981216824
CRIPT	0.941189523	0.833126007	0.937603618	0.925353907	0.906893576	0.999948228
LOC101907920	0.941189523	0.851775118	0.937559174	0.934466438	0.917407853	0.999948228
ADGRL2	0.941320873	0.872175143	0.89478142	0.983738237	0.999985752	0.888475311
TM6SF2	0.941320873	0.905335407	0.985172237	0.912777307	0.983941532	0.891088733
LYVE1	0.941320873	0.999987083	0.901983585	0.913025089	0.936215792	0.893706948
LOC101906513	0.941320873	0.875142087	0.967590756	0.979385838	0.964301327	0.901981092
LOC112446779	0.941320873	0.861395263	0.897432125	0.999981514	0.955049621	0.911336121
GLIPR1	0.941320873	0.906658524	0.944501125	0.972142185	0.970980375	0.911984937
HMG20A	0.941320873	0.918996897	0.976295638	0.940438437	0.909316444	0.960023723

SCN9A	0.941447523	0.846721805	0.991138474	0.9880671	0.960573822	0.880981659
CPEB2	0.941447523	0.83359488	0.980199146	0.999981514	0.935286646	0.883688554
LOC100847861	0.941447523	0.947347707	0.965819476	0.990955345	0.911490334	0.883829611
N4BP1	0.941447523	0.870222905	0.927508464	0.999981514	0.973219333	0.88542704
LOC101904069	0.941447523	0.950465218	0.905023942	0.961898258	0.986003146	0.891088733
AKAP17A	0.941447523	0.847093186	0.916294854	0.999981514	0.9683846	0.895426195
AOC2	0.941447523	0.887139959	0.945995021	0.98051571	0.976292433	0.898021919
LOC112443502	0.941447523	0.842883767	0.944606182	0.959616431	0.999985752	0.898933037
C15H11orf71	0.941447523	0.941676269	0.943836178	0.959644972	0.968973598	0.899183258
LOC100297170	0.941447523	0.943213236	0.937367686	0.941796131	0.976292433	0.911638347
CHURC1	0.941447523	0.872175143	0.998899782	0.952247001	0.938622071	0.918580372
MTX3	0.941447523	0.834615638	0.935266153	0.999981514	0.953300742	0.940583352
LOC104969378	0.941447523	0.872175143	0.942134405	0.983012396	0.954403133	0.945532151
ELL	0.941447523	0.941801945	0.916294854	0.916858019	0.976292433	0.945532151
CLEC4G	0.941447523	0.918854612	0.951392011	0.941796131	0.944839728	0.958707771
C5H12orf66	0.941447523	0.925232913	0.927635169	0.977689659	0.926972948	0.959075802
LOC104972065	0.941447523	0.884128797	0.93891936	0.969426656	0.953300742	0.959198304
FBXL4	0.941447523	0.853129493	0.942134405	0.999981514	0.908528514	0.960023723
PSMD11	0.941447523	0.839128192	0.964382669	0.974272706	0.921710688	0.973649216
HPDL	0.941447523	0.876304005	0.932217079	0.955954309	0.929896768	0.989493848
MORN1	0.941447523	0.835486075	0.950223118	0.941467375	0.937946177	0.996335668
MTA1	0.941487618	0.966069053	0.996224523	0.941796131	0.908820136	0.881939613
LFNG	0.941487618	0.936822825	0.957295966	0.924193829	0.986642926	0.887989933
MYEF2	0.941487618	0.913899522	0.964807927	0.9880671	0.935286646	0.910004719
LOC101905813	0.941487618	0.865101561	0.997400824	0.973075232	0.924795709	0.9221477
NCK2	0.941487618	0.861395263	0.899106435	0.989601002	0.996217975	0.922361909
ZNF668	0.941487618	0.846215781	0.895801611	0.999981514	0.961465747	0.923199665
RGS6	0.941487618	0.939566131	0.90780986	0.929663263	0.989379051	0.929814744
ZNF133	0.941487618	0.839958186	0.999993442	0.930882136	0.932036998	0.958790968
NFKBIB	0.941487618	0.994088914	0.899106435	0.92139105	0.913732532	0.960023723
LOC107131749	0.941487618	0.888057447	0.904274378	0.982220679	0.935286646	0.980464176
CCDC184	0.941487618	0.921940584	0.905023942	0.921950515	0.943702351	0.995702108
LOC107132606	0.941640677	0.867245111	0.980061232	0.973075232	0.976088926	0.881875045
LOC509941	0.941640677	0.895777642	0.913609237	0.999981514	0.982724663	0.882690335
DLG5	0.941640677	0.969051025	0.938624264	0.930987593	0.973219333	0.893838339
SLC45A2	0.941640677	0.965950444	0.916323169	0.995434268	0.935286646	0.895446935
SLC6A14	0.941640677	0.955827589	0.987610559	0.922967576	0.943540106	0.900219465
OR51E1	0.941640677	0.98098403	0.91493299	0.931383184	0.970988226	0.912486829

LOC101905239	0.941640677	0.872175143	0.89478142	0.999981514	0.966121925	0.923092436
NAPRT	0.941640677	0.867277242	0.972482052	0.933718942	0.976292433	0.933897626
TBX6	0.941640677	0.889457989	0.894338686	0.988580812	0.958797242	0.96053762
RPTOR	0.941640677	0.853476078	0.924855847	0.959652624	0.976259416	0.964898868
GEMIN7	0.941640677	0.927794672	0.916038418	0.949502614	0.953300742	0.970105102
BFAR	0.941640677	0.833429351	0.926161087	0.932914414	0.980695939	0.981216824
RN7SL1	0.941802139	0.883391071	0.941102978	0.997660427	0.910747101	0.960000414
DGUOK	0.942080124	0.855067335	0.978079673	0.929388614	0.960573822	0.960023723
VPS33B	0.942144044	0.919540627	0.916038418	0.999981514	0.954403133	0.894808664
KCTD21	0.942144044	0.89807935	0.997833363	0.930174753	0.958797242	0.903500283
LOC100848484	0.942144044	0.846724749	0.916038418	0.916858019	0.999985752	0.922361909
MACROD1	0.942144044	0.973321075	0.924855847	0.952247001	0.940870936	0.931410194
FUT8	0.942144044	0.862299245	0.905023942	0.999981514	0.956904152	0.935490012
C23H6orf47	0.942144044	0.901984573	0.925320071	0.960016921	0.960419102	0.959198304
ATP9A	0.942144044	0.911290584	0.899294652	0.923060035	0.984386856	0.966850975
PPT2	0.942144044	0.860782858	0.966232793	0.941467375	0.953300742	0.969014913
TMSB10	0.942269293	0.98978863	0.94422142	0.973812087	0.91506312	0.881939613
CFDP2	0.942269293	0.835452142	0.959903168	0.964521432	0.996089557	0.911219114
PYCR1	0.942269293	0.863286109	0.996224523	0.960566165	0.956906331	0.911336121
ZC2HC1A	0.942269293	0.892108342	0.949939862	0.999981514	0.91563394	0.91373422
FBLL1	0.942269293	0.882483488	0.999280799	0.950643928	0.941800786	0.913945794
DPYD	0.942269293	0.843278276	0.922552585	0.999981514	0.970980375	0.915546224
LOC782258	0.942269293	0.939956757	0.997400824	0.921950515	0.907762824	0.92789871
MRRF	0.942269293	0.924099465	0.929661805	0.948668643	0.973799161	0.933897626
LOC112442414	0.942269293	0.894014677	0.935824202	0.941467375	0.984533042	0.938013912
SPINT2	0.942269293	0.869406822	0.975774524	0.952247001	0.949437495	0.952663521
HLCS	0.942518538	0.885951248	0.969000554	0.946503916	0.956904152	0.947838082
INTS11	0.942785661	0.915269318	0.946791586	0.943464106	0.98485685	0.914132808
ZFP64	0.942785661	0.981952108	0.916294854	0.930174753	0.940302215	0.94844234
TNS2	0.942785661	0.899682309	0.957986379	0.935563591	0.939343714	0.971545917
LOC112448805	0.942871574	0.886761349	0.957295966	0.999981514	0.906893576	0.887239327
COL14A1	0.942871574	0.878488273	0.943836178	0.961898258	0.99971266	0.891423889
AAAS	0.942871574	0.893857042	0.998899782	0.949473031	0.935286646	0.917906589
ZNF576	0.942871574	0.846721805	0.973555428	0.912229943	0.996781978	0.93363377
WDR48	0.942871574	0.855067335	0.912183977	0.999981514	0.920622084	0.945480515
EPB41L4B	0.942871574	0.935001231	0.89478142	0.953697985	0.954023497	0.972088812
CYTH1	0.943031607	0.87976124	0.971053208	0.959616431	0.964301327	0.927426708
DDIT3	0.943031607	0.901631302	0.947438677	0.935190952	0.983941532	0.931410194

LOC101902301	0.943031607	0.907054337	0.973562292	0.972142185	0.91563394	0.940583352
IARS2	0.943031607	0.865570309	0.955427429	0.947313672	0.964301327	0.96248183
AGRN	0.943031607	0.896603391	0.925876488	0.929388614	0.962513981	0.979505709
CDC5L	0.943096909	0.948038876	0.939981892	0.914263396	0.99971266	0.88313584
LOC104975610	0.943096909	0.84238625	0.999993442	0.982220679	0.953300742	0.885957057
COPS7A	0.943096909	0.889387648	0.979584814	0.959616431	0.97352263	0.891154545
HIBCH	0.943096909	0.856560491	0.983504244	0.910575108	0.999985752	0.894694231
SLC25A10	0.943096909	0.981670901	0.907585583	0.990955345	0.935286646	0.898470276
LOC100847941	0.943096909	0.855067335	0.895801611	0.999981514	0.970988226	0.906517494
BABAM2	0.943096909	0.869160875	0.967590756	0.99570283	0.953300742	0.911808092
CDO1	0.943096909	0.909789853	0.957052938	0.941467375	0.979840175	0.919124527
MYO1G	0.943096909	0.877893862	0.957681559	0.965050971	0.976259416	0.922361909
GPR182	0.943096909	0.991673407	0.897322115	0.970813955	0.926813827	0.925273613
SLC35F5	0.943096909	0.888322912	0.94354642	0.996621929	0.952796169	0.925458723
LOC511713	0.943096909	0.932530199	0.972741989	0.929388614	0.950085378	0.933903265
LOC112448816	0.943096909	0.857815763	0.992991376	0.961898258	0.943160494	0.935770262
LRRC2	0.943096909	0.918234392	0.916294854	0.940436902	0.976292433	0.958707771
PUM3	0.943096909	0.91763262	0.97773026	0.932764647	0.926172968	0.96053762
LOC104973848	0.943096909	0.885823207	0.910284791	0.995565324	0.943047881	0.964361107
FDXACB1	0.943096909	0.880565292	0.946924479	0.962610411	0.947346268	0.966212838
C5H12orf57	0.943096909	0.870222905	0.908072171	0.916858019	0.999453276	0.96870908
LOC112442979	0.943096909	0.944261937	0.905023942	0.940726907	0.943702351	0.978112039
ZSCAN31	0.943096909	0.847093186	0.89478142	0.960016921	0.977193348	0.981216824
DIMT1	0.943096909	0.877501251	0.947875514	0.919643615	0.930306915	0.99828741
LOC618289	0.943096909	0.869754045	0.935219877	0.933718942	0.927086179	0.999948228
PRSS23	0.943105693	0.8857401	0.916294854	0.999981514	0.930451229	0.921557022
GANAB	0.943130142	0.847093186	0.949855095	0.973373329	0.943160494	0.973649216
LOC101903413	0.943508363	0.920217996	0.999993442	0.941935077	0.929896768	0.897812
FAM32A	0.943508363	0.927973597	0.947462602	0.932914414	0.947045868	0.964898868
HSPA14	0.943600273	0.845750059	0.916294854	0.964521432	0.987723553	0.960023723
SYNJ2BP	0.943638609	0.96025412	0.964762618	0.912777307	0.916800726	0.960023723
PDLIM2	0.943748666	0.890176137	0.927635169	0.995312373	0.983941532	0.887722423
APIP	0.943748666	0.941400719	0.969026634	0.965416137	0.911930323	0.93129461
RBSN	0.943869832	0.966069053	0.915120516	0.978127727	0.960419102	0.906637848
MPZL1	0.944113574	0.906064108	0.915742138	0.995565324	0.955623649	0.93839036
CLIP3	0.944238351	0.942088099	0.912020408	0.999981514	0.935286646	0.905188617
ADGRA3	0.944238351	0.96989035	0.957295966	0.943808491	0.950958352	0.908049203
CALN1	0.944238351	0.988444882	0.921266625	0.925294859	0.956906331	0.923460925

LOC534155	0.944238351	0.94422395	0.927635169	0.961898258	0.961465747	0.926480924
R3HDM2	0.944287703	0.913116251	0.968679299	0.96357554	0.958797242	0.914132808
MTMR3	0.944287703	0.861395263	0.911502207	0.943808491	0.947299702	0.999948228
IRF2	0.944326404	0.980289052	0.990118874	0.934445963	0.923194531	0.889662488
HERC1	0.944326404	0.901984573	0.90780986	0.947574232	0.999985752	0.893838339
LOC100335751	0.944326404	0.851622183	0.999993442	0.948668643	0.927951765	0.923547711
SDHAF1	0.944326404	0.869953468	0.998899782	0.926970666	0.943540106	0.945532151
SLC36A4	0.944326404	0.946859049	0.936203646	0.957008595	0.912900863	0.967759699
MKRN3	0.944326404	0.855141737	0.917070334	0.976196758	0.908820136	0.99976973
LEMD3	0.944340576	0.904754143	0.915713148	0.941670722	0.999985752	0.89062462
FAM83G	0.944340576	0.943244588	0.970873534	0.96900924	0.946454221	0.893838339
ZNF263	0.944340576	0.882242226	0.920462513	0.999981514	0.946384846	0.90182838
STX4	0.944340576	0.840402825	0.933884582	0.984423083	0.998800466	0.911219114
OGN	0.944340576	0.948801019	0.943237386	0.935563591	0.976292433	0.91373422
HBA	0.944340576	0.901984573	0.89478142	0.95901497	0.999985752	0.919124527
LOC112448848	0.944340576	0.969051025	0.908072171	0.982220679	0.936388637	0.930301995
LOC100141168	0.944609931	0.870296749	0.963164828	0.941796131	0.999985752	0.888475311
TIMP4	0.94469009	0.870195838	0.972297	0.960016921	0.960252528	0.940079339
SRSF7	0.944705731	0.863286109	0.960685112	0.99739416	0.970988226	0.888475311
LOC100297420	0.944705731	0.83844057	0.993024884	0.975678715	0.970988226	0.89438401
TRIB1	0.944705731	0.874981732	0.915858924	0.992339478	0.987723553	0.917906589
LOC104975890	0.944705731	0.845750059	0.910224442	0.999981514	0.936108073	0.937469898
CTSV	0.944740379	0.964925317	0.899106435	0.973373329	0.980189546	0.887722423
HSBP1	0.944908147	0.931043812	0.93190909	0.952247001	0.996781978	0.888309103
OSBPL10	0.944908147	0.869754045	0.999993442	0.923220263	0.964301327	0.901618453
PPRC1	0.944908147	0.849217933	0.916294854	0.932914414	0.999985752	0.903626415
NEK7	0.944908147	0.846721805	0.999993442	0.973075232	0.926972948	0.915303189
LOC100296121	0.944908147	0.913116251	0.972741989	0.948720795	0.933426897	0.958790968
ZNF333	0.944916208	0.928835826	0.979494108	0.944351122	0.938316699	0.933897626
BORCS5	0.944916208	0.908364002	0.931319315	0.949605617	0.976292433	0.944973383
CCM2L	0.944977454	0.922245127	0.996224523	0.97828838	0.926813827	0.884898241
DHH	0.944977454	0.97628064	0.895811483	0.947574232	0.9902393	0.891423889
LOC100299712	0.944977454	0.927853707	0.915858924	0.997467114	0.936921784	0.940910488
UNC93B1	0.94513445	0.889345904	0.99341711	0.912777307	0.964301327	0.933897626
MPLKIP	0.945168708	0.856560491	0.927635169	0.999981514	0.929802526	0.900219465
FUCA1	0.945310153	0.980833065	0.93190909	0.960016921	0.947346268	0.911219114
ALDH1A1	0.945325668	0.855067335	0.896109455	0.999981514	0.961465747	0.903990996
PPIC	0.945325668	0.840431179	0.93891936	0.999981514	0.936108073	0.909056553

MBOAT1	0.945325668	0.870298381	0.959903168	0.952247001	0.996781978	0.911638347
YIPF2	0.945857071	0.884804337	0.987789469	0.915039335	0.998287238	0.887722423
IDH3G	0.945857732	0.853946295	0.982303092	0.917892197	0.974738415	0.954961112
SPRED3	0.94593477	0.887064545	0.94422142	0.999981514	0.964301327	0.891088733
LOC100300938	0.94593477	0.855752352	0.917155067	0.998529748	0.989334846	0.911960508
CD300E	0.94593477	0.918455779	0.964807927	0.973075232	0.927086179	0.940639747
RNF11	0.94593477	0.846724749	0.985156077	0.995565324	0.911568867	0.943972369
PAK1	0.946088245	0.899805713	0.957295966	0.985748796	0.943160494	0.932534796
CCDC190	0.946201643	0.982515431	0.916294854	0.922002553	0.964235922	0.93363377
LOC112442401	0.946234792	0.961368616	0.927508464	0.944351122	0.954403133	0.944973383
AR	0.946234792	0.861395263	0.992263335	0.941467375	0.943702351	0.960023723
FBXW4	0.946250891	0.993481224	0.912666347	0.98981682	0.916932722	0.891088733
PELI3	0.946506072	0.888057447	0.9377363	0.96951401	0.975270955	0.940017687
MYCBPAP	0.94666907	0.880990186	0.948653258	0.999981514	0.964768333	0.887239327
SESN1	0.94666907	0.846721805	0.916323169	0.999981514	0.961670582	0.890754919
CCL3	0.94666907	0.936296621	0.963164828	0.98981682	0.943610727	0.891088733
LOC112441880	0.94666907	0.904875256	0.962838954	0.998177865	0.952264073	0.893838339
ADAT2	0.94666907	0.985428851	0.921311126	0.914850247	0.984386856	0.894430697
FBXW2	0.94666907	0.941817736	0.937993336	0.999981514	0.91563394	0.895446935
R3HCC1	0.94666907	0.892108342	0.963814739	0.999981514	0.950958352	0.899428912
NR5A2	0.94666907	0.863064327	0.895801611	0.999981514	0.977167046	0.905017065
LOC112443142	0.94666907	0.893164557	0.999993442	0.915013589	0.936921784	0.905509632
PJVK	0.94666907	0.997678999	0.93190909	0.953697985	0.917407853	0.909056553
LOC616304	0.94666907	0.985428851	0.967590756	0.922002553	0.939650698	0.909056553
MAGOH	0.94666907	0.842884359	0.95875611	0.999981514	0.967116582	0.909056553
ATP6V0D2	0.94666907	0.923874332	0.957052938	0.954343717	0.976292433	0.911336121
LOC107132971	0.94666907	0.862845348	0.907585583	0.995434268	0.998287238	0.91373422
SSFA2	0.94666907	0.975984829	0.927508464	0.992339478	0.913732532	0.915735519
RHBDL2	0.94666907	0.946258461	0.916323169	0.996667757	0.94629095	0.917277552
PORCN	0.94666907	0.89417747	0.99341711	0.958588196	0.951996251	0.917930485
SPR	0.94666907	0.964925317	0.945995021	0.978404889	0.929896768	0.919124527
MAN2A1	0.94666907	0.901631302	0.908072171	0.979644238	0.996781978	0.919124527
CAMK2N1	0.94666907	0.927034887	0.946791586	0.94469136	0.982203967	0.923092436
CIAPIN1	0.94666907	0.938390602	0.970873534	0.960016921	0.937932469	0.927426708
CPPED1	0.94666907	0.882858125	0.915120516	0.999981514	0.918426817	0.927517509
CD7	0.94666907	0.938087741	0.903431343	0.999981514	0.925226185	0.927520621
ARHGAP33	0.94666907	0.853678591	0.943028511	0.973993549	0.987723553	0.931410194
KDM3B	0.94666907	0.901710313	0.957052938	0.98981682	0.938879379	0.93363377

VSIR	0.94666907	0.840909162	0.916294854	0.96444269	0.999985752	0.936263602
GALNT14	0.94666907	0.998847958	0.908701801	0.941467375	0.911417136	0.940079339
GPD1	0.94666907	0.927853707	0.942357321	0.950643928	0.965676623	0.940639747
KCNK5	0.94666907	0.987709638	0.899106435	0.965050971	0.924242108	0.945984601
TMEM178B	0.94666907	0.966052556	0.928739281	0.928416242	0.956906331	0.950631909
MOCS2	0.94666907	0.957667701	0.959725886	0.925283079	0.935286646	0.952007067
NFATC2	0.94666907	0.843278276	0.990037865	0.989601002	0.911028086	0.952914371
GFPT1	0.94666907	0.937448751	0.926161087	0.960190213	0.956041014	0.953140004
MFSD12	0.94666907	0.929007264	0.916294854	0.994965893	0.927109614	0.955990509
VARS2	0.94666907	0.901028838	0.939912874	0.990955345	0.926972948	0.960023723
RUBCNL	0.94666907	0.930266314	0.916323169	0.925294859	0.964301327	0.975200512
DDX10	0.94666907	0.922923898	0.927635169	0.949468104	0.943702351	0.980167535
ENTR1	0.94666907	0.882102283	0.924855847	0.946503916	0.964501754	0.98140846
HACD1	0.94666907	0.882858125	0.943836178	0.961898258	0.916605572	0.994347099
MSANTD4	0.94666907	0.84312966	0.949855095	0.952247001	0.943702351	0.996335668
SPATS2L	0.94666907	0.870296749	0.955189235	0.937615893	0.933426897	0.996679516
ZNF664	0.94666907	0.847135393	0.915858924	0.959227327	0.931357161	0.999948228
ANKRD44	0.946888193	0.899494273	0.898179681	0.938094344	0.989379051	0.969197073
CST3	0.946903696	0.873915965	0.97773026	0.962610411	0.929896768	0.965117554
AGO1	0.946999962	0.863064327	0.911535369	0.999981514	0.980016241	0.932816123
DHRS4	0.947066866	0.942088099	0.916294854	0.952247001	0.976292433	0.933224627
LGALS1	0.947069917	0.916484772	0.979494108	0.968436292	0.964301327	0.888475311
LOC100847695	0.947089125	0.89417747	0.955427429	0.980564765	0.943702351	0.947838082
LOC100847453	0.947226622	0.902616821	0.907669615	0.999981514	0.913180847	0.905204647
MIF4GD	0.947339152	0.979272895	0.938074902	0.975453864	0.951996251	0.891088733
RFC4	0.947339152	0.985579976	0.971053208	0.915039335	0.943702351	0.908099327
ZNF174	0.947339152	0.992795654	0.935266153	0.916858019	0.959658682	0.91348389
LOC101908149	0.947339152	0.927369042	0.916294854	0.972142185	0.982203967	0.923547711
SRFBP1	0.947339152	0.887064545	0.927635169	0.948668643	0.999985752	0.927900405
FIGN	0.947339152	0.863064327	0.949939862	0.982220679	0.976259416	0.930814839
RPL28	0.947339152	0.91110501	0.908412457	0.978404889	0.959393538	0.965652257
CCDC137	0.947339152	0.843278276	0.916294854	0.999981514	0.946935016	0.974962631
ZNF33B	0.947605393	0.973321075	0.946791586	0.932764647	0.912617855	0.963456717
CDK9	0.947615869	0.867083204	0.999993442	0.972845645	0.915279373	0.917146354
PRDX3	0.947615869	0.846724749	0.963044414	0.919153842	0.999985752	0.923460925
HSD11B1L	0.94771561	0.860218913	0.896573058	0.999981514	0.91563394	0.923092436
STAG3	0.94771561	0.872175143	0.948105765	0.995565324	0.964301327	0.923547711
PROZ	0.947949302	0.935001231	0.999993442	0.925353907	0.944417042	0.888887063

AQP4	0.947949302	0.96299056	0.899764763	0.973075232	0.956041014	0.940079339
PI3	0.947968569	0.95689647	0.979584814	0.955466436	0.922834705	0.923092436
BLCAP	0.947968569	0.942088099	0.99647733	0.927721166	0.928384972	0.927517509
DCDC2B	0.948167482	0.875852152	0.967590756	0.999981514	0.913064455	0.944927301
ERMP1	0.948167482	0.928427136	0.897322115	0.991088092	0.953300742	0.95508469
HTATIP2	0.948167482	0.847255873	0.972897558	0.952247001	0.926972948	0.989084358
ZMIZ1	0.948192188	0.843278276	0.94565648	0.999981514	0.924222719	0.894808664
MMP11	0.948444808	0.893164557	0.964807927	0.999981514	0.91563394	0.89438401
TTC26	0.948444808	0.980521483	0.899106435	0.957132838	0.972528668	0.917146354
SNX2	0.948480768	0.898951086	0.938011449	0.995565324	0.979874385	0.891088733
LRRK2	0.948480768	0.988384541	0.925320071	0.93048026	0.976292433	0.891423889
C28H1orf198	0.948480768	0.864668925	0.95219206	0.999981514	0.926813827	0.897315753
JRK	0.948480768	0.886669497	0.908072171	0.96900924	0.999859158	0.930710695
LOC112446798	0.948480768	0.844170799	0.980761647	0.959591127	0.944839728	0.969014913
CSDE1	0.948480768	0.855752352	0.946791586	0.999981514	0.914280652	0.973649216
UBE2Q1	0.948480768	0.849491537	0.935219877	0.96951401	0.966967496	0.973649216
REPS2	0.948480768	0.864685893	0.946791586	0.980632768	0.936108073	0.9792065
DDX42	0.948480768	0.924125044	0.927635169	0.960016921	0.927086179	0.984046914
C10H14orf1	0.948480768	0.906927139	0.918247946	0.925353907	0.964301327	0.988639786
LOC101903114	0.948480768	0.849513595	0.916294854	0.999981514	0.916605572	0.991847494
PPP2R2A	0.948493519	0.928427136	0.906019013	0.928075638	0.999985752	0.899428912
KDR	0.948567911	0.873562762	0.959903168	0.980632768	0.987723553	0.893706948
NEFH	0.948567911	0.846724749	0.964807927	0.944351122	0.999985752	0.895426195
LOC101907641	0.948567911	0.925557011	0.924855847	0.989485949	0.958797242	0.932674432
ITSN2	0.948635485	0.883646769	0.942134405	0.96444269	0.999985752	0.891088733
FOXC1	0.948635485	0.995373357	0.958019774	0.928841374	0.932339125	0.902351886
LOC788334	0.948635485	0.884272556	0.916038418	0.920690301	0.999985752	0.903990996
SF3A3	0.948635485	0.907294328	0.927635169	0.999981514	0.964301327	0.914132808
RB1CC1	0.948635485	0.880565292	0.924855847	0.999981514	0.974931134	0.919124527
LOC101904520	0.948635485	0.963297767	0.946791586	0.919628651	0.976259416	0.923092436
IGFBP6	0.948635485	0.901028838	0.942357321	0.95901497	0.989771098	0.923807852
NUP35	0.948635485	0.941817736	0.916294854	0.922967576	0.988238252	0.9465036
NCF1	0.948635485	0.918234392	0.933884582	0.916858019	0.965676623	0.979480605
CIRBP	0.948635485	0.897183343	0.916294854	0.982220679	0.919719454	0.991416486
TMEM181	0.948677188	0.908364002	0.942357321	0.918693944	0.958384396	0.987785166
PRRG2	0.948768376	0.98364041	0.902867554	0.999981514	0.943610727	0.888475311
CALCOCO2	0.948768376	0.870222905	0.897827774	0.999981514	0.935093621	0.888930405
OSCP1	0.948768376	0.866862152	0.991936822	0.999981514	0.943702351	0.889479413

GTF2B	0.948768376	0.852526348	0.999993442	0.94857292	0.979500822	0.895426195
LOC104970173	0.948768376	0.882594165	0.943836178	0.999981514	0.960048824	0.895446935
LOC112448090	0.948768376	0.92546629	0.916294854	0.962437357	0.999985752	0.896529476
LOC112449406	0.948768376	0.886565095	0.950415303	0.999981514	0.938879379	0.905509632
LIN7C	0.948768376	0.895637433	0.999993442	0.95901497	0.943160494	0.912937481
N4BP2L1	0.948768376	0.964418016	0.912396874	0.961898258	0.932024143	0.966850975
LOC618071	0.948768376	0.908975049	0.949855095	0.950643928	0.956041014	0.966850975
SLC9A8	0.948768376	0.954187594	0.915858924	0.971264934	0.929802526	0.969014913
MARVELD2	0.948768376	0.913899522	0.899952655	0.973075232	0.959367717	0.973649216
LOC781646	0.948768376	0.849119707	0.935266153	0.980564765	0.917964923	0.997862553
KBTBD3	0.948777268	0.864668925	0.939118199	0.999981514	0.943540106	0.935497122
LOC100847182	0.948803277	0.872175143	0.999993442	0.952247001	0.921710688	0.923547711
HFE	0.948803277	0.908364002	0.957052938	0.958103924	0.927086179	0.978087957
PTRH1	0.948806588	0.904875256	0.923578245	0.941467375	0.977167046	0.966850975
LOC786173	0.948818346	0.893164557	0.945575068	0.940438437	0.995590117	0.940017687
LOC112447351	0.948825382	0.955652503	0.990037865	0.928404714	0.940870936	0.923092436
GSN	0.94895532	0.893806048	0.989494193	0.952247001	0.962513981	0.923547711
TNFAIP1	0.94895532	0.861395263	0.913182371	0.962610411	0.957793213	0.997054363
LOC112442374	0.948971523	0.845167221	0.999993442	0.999981514	0.91563394	0.891088733
ELFN2	0.948971523	0.98978863	0.913100432	0.95925588	0.970988226	0.891423889
SPARC	0.948971523	0.95462892	0.966232793	0.988537917	0.932024143	0.893585844
SERPINI1	0.948971523	0.993481224	0.915858924	0.919084646	0.980106629	0.898021919
PDGFRB	0.948971523	0.856560491	0.925320071	0.999981514	0.934729126	0.899428912
PTPN13	0.948971523	0.918854612	0.949939862	0.935812075	0.999985752	0.9010458
SEC14L1	0.948971523	0.912602201	0.92091815	0.961898258	0.999985752	0.911219114
ELK4	0.948971523	0.929204871	0.955427429	0.921811299	0.998287238	0.91145312
CMC4	0.948971523	0.851026498	0.999993442	0.960937718	0.935286646	0.91373422
RBM17	0.948971523	0.927853707	0.993024884	0.959141938	0.943160494	0.91373422
GDA	0.948971523	0.953042388	0.97998887	0.916858019	0.963550093	0.917930485
LOC512978	0.948971523	0.870222905	0.999993442	0.94857292	0.943702351	0.923092436
SDC4	0.948971523	0.849513595	0.999993442	0.947574232	0.97028586	0.923092436
LOC783926	0.948971523	0.890669166	0.916294854	0.925294859	0.999985752	0.923807852
FIGNL1	0.948971523	0.977769595	0.93190909	0.982220679	0.924222719	0.925233445
SSBP2	0.948971523	0.955652503	0.935219877	0.988408846	0.93568841	0.927520621
BRI3	0.948971523	0.876873679	0.916323169	0.999981514	0.929802526	0.932688327
GLB1L	0.948971523	0.893164557	0.92323046	0.999981514	0.961465747	0.935497122
VCAM1	0.948971523	0.870195838	0.999993442	0.952247001	0.942786245	0.935770262
SLC11A1	0.948971523	0.846724749	0.916323169	0.999981514	0.949528657	0.936263602

OSBPL3	0.948971523	0.938235006	0.950971816	0.946938434	0.963634663	0.940583352
CHIC2	0.948971523	0.928427136	0.927635169	0.952247001	0.964301327	0.960023723
ZSCAN21	0.948971523	0.849632699	0.965407935	0.979385838	0.953300742	0.962419223
GCNT2	0.948971523	0.87976124	0.959903168	0.949864417	0.96419154	0.963456717
LOC101904963	0.948971523	0.899494273	0.916323169	0.999981514	0.91563394	0.96870908
DQX1	0.948971523	0.918460462	0.916294854	0.952247001	0.965676623	0.970105102
CXADR	0.948971523	0.85265333	0.916323169	0.999981514	0.942786245	0.971545917
ZDHHC1	0.948971523	0.902525869	0.951392011	0.951936643	0.934450558	0.985156335
FUT7	0.949263469	0.846724749	0.951756885	0.999981514	0.936388637	0.905509632
GORASP1	0.949457824	0.929409572	0.962734642	0.947313672	0.983941532	0.906637848
SRPK2	0.949457824	0.861589503	0.929661805	0.969088992	0.996781978	0.940583352
SCUBE3	0.949464411	0.981670901	0.916294854	0.982220679	0.959565914	0.89364769
KCNAB2	0.949464411	0.948801019	0.992377532	0.923060035	0.943702351	0.926641512
PYGL	0.949510977	0.845750059	0.999993442	0.96968491	0.943702351	0.891088733
RALGAPA2	0.949510977	0.867740903	0.999993442	0.921950515	0.949508117	0.899428912
CCNG2	0.949510977	0.846724749	0.999993442	0.935985829	0.935286646	0.913221224
ASPA	0.949510977	0.991883165	0.927635169	0.941796131	0.937932469	0.93363377
KCNH4	0.949625223	0.955652503	0.95219206	0.997792939	0.931357161	0.896328583
VWA2	0.949803748	0.971458291	0.93190909	0.996033721	0.935286646	0.903500283
HINFP	0.949803748	0.893366963	0.957052938	0.999981514	0.953300742	0.914132808
LOC112443177	0.949803748	0.846724749	0.948949412	0.999981514	0.943540106	0.919124527
KCNJ12	0.949803748	0.976527871	0.955427429	0.953401589	0.943702351	0.919124527
LOC107133075	0.949803748	0.895777642	0.909048387	0.999981514	0.928363269	0.945480515
LOC100847120	0.949812458	0.949224146	0.926161087	0.999981514	0.935286646	0.895426195
TMEM53	0.949812458	0.949323635	0.957052938	0.973373329	0.959443572	0.903500283
LOC112444276	0.949812458	0.855067335	0.917155067	0.995152316	0.999985752	0.903500283
XKR8	0.949812458	0.999987083	0.926161087	0.921482057	0.930451229	0.908768184
HAUS3	0.949812458	0.919218013	0.916294854	0.952247001	0.999985752	0.912404912
GLT8D1	0.949812458	0.914629393	0.945995021	0.999981514	0.953300742	0.923092436
ULK4	0.949812458	0.976624004	0.912396874	0.995565324	0.926831826	0.927520621
SAMD5	0.949812458	0.934225172	0.916323169	0.995565324	0.955623649	0.931018395
TCF23	0.949812458	0.985097978	0.923521499	0.918693944	0.965163023	0.934071377
ITGB4	0.949812458	0.918234392	0.999993442	0.939180916	0.924222719	0.935770262
TIGD4	0.949812458	0.894014677	0.909654415	0.999981514	0.926831826	0.943972369
LGI4	0.949812458	0.997678999	0.905023942	0.934466438	0.938879379	0.944416747
TMX1	0.949812458	0.929007264	0.908192841	0.929943858	0.976292433	0.975200512
LOC112447087	0.949963402	0.869734727	0.95322206	0.999981514	0.954116896	0.923092436
ITGA2	0.94998991	0.882858125	0.955845583	0.999981514	0.960573822	0.891088733

LOC783963	0.950028174	0.929409572	0.967590756	0.928075638	0.989334846	0.910231415
CACNA1H	0.950073358	0.993427125	0.930963858	0.9589395	0.927049941	0.927426708
PELP1	0.950139868	0.980521483	0.955427429	0.964521432	0.943160494	0.905509632
PTPN3	0.950139868	0.916721068	0.961437412	0.949502614	0.991481886	0.905509632
SGSH	0.950139868	0.906927139	0.93475587	0.999981514	0.935286646	0.91373422
TMED1	0.950139868	0.967034954	0.916294854	0.959119039	0.976259416	0.923092436
FSTL1	0.950139868	0.892252816	0.926161087	0.999981514	0.958797242	0.923772625
LOC104970105	0.950139868	0.887018937	0.916539526	0.999981514	0.965930435	0.930710695
WNK4	0.950139868	0.866901725	0.955427429	0.943808491	0.996781978	0.945092617
LOC112441683	0.950139868	0.870195838	0.999993442	0.918921815	0.930856225	0.946760037
LOC784488	0.950139868	0.928512049	0.939353663	0.989601002	0.935286646	0.951081819
MARF1	0.950139868	0.878551917	0.971255098	0.967883482	0.957396931	0.951493662
VPS37D	0.950139868	0.863173005	0.916294854	0.960016921	0.999985752	0.9557845
GSK3B	0.950139868	0.878311791	0.982708154	0.945326519	0.949927999	0.966850975
LOC107132944	0.950139868	0.924426163	0.944126714	0.930987593	0.952264073	0.983208118
SLC30A9	0.950139868	0.871924797	0.961800035	0.950643928	0.946806164	0.984172542
DBNL	0.950139868	0.878311791	0.966293373	0.929388614	0.943702351	0.991421487
TPCN2	0.950139868	0.853678591	0.957052938	0.966213678	0.926813827	0.996310803
LOC101902895	0.950237982	0.884804337	0.972741989	0.999981514	0.93624434	0.919124527
LOC112444531	0.950237982	0.869406822	0.951392011	0.921811299	0.996361295	0.963456717
UBQLN2	0.950237982	0.870716141	0.935824202	0.952247001	0.929802526	0.999948228
LOC112449560	0.950290333	0.988444882	0.983534298	0.942759064	0.918166705	0.899428912
TRIO	0.950290333	0.883524089	0.997400824	0.990955345	0.926972948	0.91373422
METTL4	0.950290333	0.980764484	0.916294854	0.955070008	0.973219333	0.91373422
EDC3	0.950290333	0.920217996	0.997833363	0.973832508	0.91563394	0.91618909
KCNN4	0.950290333	0.870334242	0.946791586	0.999981514	0.924222719	0.960023723
ID3	0.950365316	0.952760839	0.925320071	0.961898258	0.942557024	0.963456717
TNRC6B	0.950409018	0.870222905	0.999993442	0.979644238	0.935286646	0.915414597
DHCR7	0.950409018	0.878624872	0.993024884	0.948668643	0.970988226	0.927520621
FCER1G	0.950409018	0.916484772	0.955221146	0.919689139	0.996781978	0.931410194
CHST12	0.950409018	0.90323787	0.933884582	0.952247001	0.983884995	0.955210102
LOC101901983	0.950409018	0.872175143	0.949855095	0.955466436	0.982538884	0.958790968
LOC101904757	0.950409018	0.955652503	0.916323169	0.937895404	0.952796169	0.975558533
FUT10	0.95042618	0.915576843	0.964661607	0.999981514	0.947531863	0.900219465
GPR31	0.95042618	0.849119707	0.977455101	0.96900924	0.916204944	0.988363574
B2M	0.950815457	0.947152616	0.99869493	0.944913633	0.935286646	0.908099327
SLC38A5	0.951029221	0.901331238	0.991138474	0.999981514	0.929896768	0.895374575
PCBP3	0.951029221	0.973868672	0.943836178	0.960016921	0.965869975	0.900219465

CDC42SE1	0.951029221	0.878488273	0.949855095	0.990955345	0.976292433	0.9221477
RAD18	0.951029221	0.99528564	0.944949386	0.922967576	0.944839728	0.923064357
LOC112442787	0.951029221	0.916418368	0.916294854	0.997660427	0.955623649	0.947478418
PRPF38B	0.951190017	0.880565292	0.914816911	0.965050971	0.999985752	0.91373422
ETHE1	0.951190017	0.988444882	0.930817886	0.927284668	0.970988226	0.917146354
LOC112447731	0.951428287	0.940342068	0.971053208	0.999981514	0.929896768	0.89209761
C7H5orf30	0.951428287	0.855067335	0.957052938	0.974793558	0.983941532	0.938894901
RPUSD1	0.951428287	0.889387648	0.911103174	0.989601002	0.977193348	0.950798794
LOC520336	0.951428287	0.863286109	0.980032241	0.960016921	0.943610727	0.970994034
LOC789587	0.951514413	0.99528564	0.938624264	0.974286747	0.92337151	0.902254268
ABCC11	0.951514413	0.847440829	0.955221146	0.999981514	0.943702351	0.906798203
TSACC	0.951514413	0.989303356	0.929779163	0.929388614	0.972160227	0.912531667
PPM1L	0.951514413	0.885951248	0.916294854	0.990955345	0.986003146	0.933034848
NXPH2	0.951514413	0.981952108	0.916323169	0.973075232	0.936388637	0.937689834
LOC107131566	0.951514413	0.899963841	0.910845937	0.999981514	0.936108073	0.944973383
FLNB	0.951514413	0.920781392	0.923364099	0.978404889	0.954403133	0.965456017
BMP4	0.951514413	0.869754045	0.948637575	0.995312373	0.926813827	0.978087957
LOC104974516	0.951514413	0.864597377	0.964771309	0.951193444	0.955741702	0.981216824
PAXIP1	0.951514413	0.871107291	0.971556427	0.940946275	0.943540106	0.988639786
SIKE1	0.951514413	0.850593568	0.944949386	0.921950515	0.970988226	0.997383134
MCAM	0.951514413	0.855752352	0.915858924	0.995565324	0.924222719	0.998284411
TYROBP	0.951962748	0.930293015	0.971053208	0.995565324	0.943540106	0.898470276
LOC101907294	0.951962748	0.872175143	0.996224523	0.949565453	0.964301327	0.933897626
STK25	0.951962748	0.88008538	0.927635169	0.961898258	0.982208423	0.963898271
SDHC	0.951962748	0.858967509	0.937812682	0.949468104	0.970988226	0.989493848
CACNA2D1	0.951962748	0.865582541	0.904919322	0.973432601	0.943702351	0.999948228
LOC112442278	0.95213589	0.950512146	0.983504244	0.965462948	0.954071324	0.893550479
NRAP	0.95213589	0.992040539	0.908072171	0.990955345	0.946679482	0.893838339
FMR1	0.95213589	0.867579696	0.916038418	0.999981514	0.976259416	0.893838339
NAAA	0.95213589	0.929204871	0.927127212	0.98981682	0.984386856	0.899460485
CDC7	0.95213589	0.941817736	0.926821911	0.969088992	0.996217975	0.900219465
RAB28	0.95213589	0.905599077	0.990037865	0.984423083	0.956761712	0.901404231
MYLK2	0.95213589	0.892108342	0.999993442	0.965271935	0.953300742	0.903500283
KLF3	0.95213589	0.946806157	0.908072171	0.944351122	0.999985752	0.903500283
SGCD	0.95213589	0.977817481	0.916294854	0.999981514	0.929896768	0.905509632
SLC9A7	0.95213589	0.861395263	0.982232295	0.969505895	0.989334846	0.90596786
PRSS42	0.95213589	0.946148902	0.920682633	0.999981514	0.926831826	0.906637848
LOC104976614	0.95213589	0.969051025	0.997400824	0.929388614	0.934067848	0.910004719

WLS	0.95213589	0.968932899	0.927635169	0.979644238	0.964301327	0.911219114
TRAPPC5	0.95213589	0.898951086	0.95219206	0.937277106	0.999985752	0.91373422
LOC506408	0.95213589	0.861395263	0.999993442	0.97828838	0.925171841	0.919993751
CDC42EP4	0.95213589	0.941817736	0.929572921	0.999732027	0.953300742	0.9221477
TMEM269	0.95213589	0.968186752	0.968679299	0.938303773	0.958797242	0.923092436
IDH1	0.95213589	0.866113361	0.99341711	0.980564765	0.956041014	0.923807852
CEP120	0.95213589	0.902392633	0.972741989	0.952247001	0.981449294	0.923807852
SCP2D1	0.95213589	0.912768407	0.969000554	0.982220679	0.956906331	0.924617356
GAS2L1	0.95213589	0.98098403	0.907626082	0.94222181	0.982087479	0.926530208
ZMYM1	0.95213589	0.912693158	0.927635169	0.999981514	0.936921784	0.92725789
OTULINL	0.95213589	0.855310926	0.91882781	0.997374305	0.996361295	0.927520621
LOC101903326	0.95213589	0.850640641	0.946791586	0.979385838	0.998287238	0.92789871
LOC781100	0.95213589	0.894014677	0.963164828	0.960016921	0.979804647	0.928965038
BCOR	0.95213589	0.976043292	0.911535369	0.928510139	0.987723553	0.933638318
SLK	0.95213589	0.929204871	0.955427429	0.996054502	0.929630092	0.93531275
LOC540014	0.95213589	0.994185904	0.927635169	0.949864417	0.932024143	0.93531275
MTX2	0.95213589	0.882340482	0.997400824	0.930987593	0.961465747	0.944330889
FAM102B	0.95213589	0.951172741	0.916294854	0.995565324	0.93630105	0.944973383
CHD5	0.95213589	0.947350735	0.917155067	0.961898258	0.972160227	0.944973383
LOC101906730	0.95213589	0.899490486	0.916294854	0.96951401	0.996089557	0.944973383
RFTN1	0.95213589	0.874078599	0.999993442	0.927657435	0.959367717	0.945480515
FLII	0.95213589	0.867885278	0.916294854	0.999981514	0.923931669	0.945918286
GCNT4	0.95213589	0.948278108	0.916294854	0.99313054	0.943702351	0.946347861
HERC3	0.95213589	0.927973597	0.930759114	0.999981514	0.924222719	0.946760037
PSMC3IP	0.95213589	0.910651459	0.980032241	0.98061629	0.923910588	0.947801358
LOC104972797	0.95213589	0.849513595	0.949939862	0.952377278	0.998287238	0.94844234
PARN	0.95213589	0.880565292	0.970873534	0.973179867	0.952796169	0.960023723
LOC100848339	0.95213589	0.86846037	0.967770797	0.973075232	0.959658682	0.960023723
MAP1S	0.95213589	0.863948462	0.939981892	0.956685049	0.996089557	0.960023723
DVL2	0.95213589	0.913120359	0.945995021	0.961898258	0.960419102	0.963255451
POLD3	0.95213589	0.940500178	0.930736996	0.958588196	0.954071324	0.967759699
ADPRHL2	0.95213589	0.857370953	0.959903168	0.988408846	0.942786245	0.970138435
PSMD4	0.95213589	0.870298381	0.980657691	0.941935077	0.958797242	0.971537655
LOC107131458	0.95213589	0.980521483	0.922552585	0.937615893	0.934637853	0.973649216
PYM1	0.95213589	0.918455779	0.924855847	0.989601002	0.928951321	0.974962631
CREB5	0.95213589	0.884773193	0.957052938	0.923583128	0.976259416	0.977503497
TBCC	0.95213589	0.866269779	0.946924479	0.950918623	0.970988226	0.980464176
LOC112447817	0.95213589	0.859204465	0.916294854	0.950173531	0.983941532	0.988639786

ELF4	0.95213589	0.870298381	0.972940212	0.934466438	0.936921784	0.994991252
PRKACB	0.95213589	0.926905849	0.942134405	0.925294859	0.927037956	0.999655834
IQCN	0.95213589	0.853129493	0.94796441	0.943447418	0.929896768	0.999948228
UBC	0.95213589	0.938087741	0.912183977	0.932914414	0.938316699	0.999948228
YJEFN3	0.952354863	0.912693158	0.971255098	0.943447418	0.983941532	0.925458723
KLK10	0.952354863	0.955333923	0.964661607	0.954229828	0.955623649	0.927471252
TRIM63	0.952569403	0.882242226	0.944126714	0.988408846	0.924222719	0.987785166
RAB23	0.952692736	0.973780375	0.927635169	0.941467375	0.993946355	0.899183258
CSNK1G2	0.952692736	0.997236605	0.94565648	0.929388614	0.957104426	0.906714945
LOC101907514	0.952692736	0.966661805	0.941507802	0.922993716	0.996781978	0.911219114
TDRP	0.952692736	0.939956757	0.988585686	0.944351122	0.955972645	0.925553067
LOC100848995	0.952692736	0.878551917	0.924588495	0.999981514	0.931277282	0.929461379
TBP	0.952909622	0.87976124	0.997300234	0.959616431	0.976292433	0.895803603
WDR20	0.952909622	0.870716141	0.917155067	0.925491377	0.999985752	0.896892269
MGST3	0.952909622	0.942088099	0.971053208	0.95901497	0.976259416	0.899428912
QSOX2	0.952909622	0.991673407	0.920779692	0.965050971	0.958797242	0.911219114
NMUR1	0.952909622	0.902392633	0.924855847	0.980564765	0.999453276	0.91373422
PWWP2B	0.952909622	0.882471806	0.96327718	0.9880671	0.973219333	0.923092436
BLOC1S4	0.952909622	0.905727888	0.930736996	0.9880671	0.983941532	0.923807852
LOC104975849	0.952909622	0.863286109	0.983066857	0.952247001	0.986287964	0.930131506
EED	0.952909622	0.941400719	0.996224523	0.943808491	0.935286646	0.930206677
FLYWCH2	0.952909622	0.899490486	0.924855847	0.984423083	0.980016241	0.940583352
LRG1	0.952909622	0.863286109	0.957295966	0.985701713	0.964301327	0.954925542
MMP9	0.952909622	0.865570309	0.976563428	0.984548238	0.935286646	0.966390476
ZBTB3	0.952909622	0.901028838	0.927635169	0.961898258	0.929896768	0.99828741
LOC107132798	0.952946178	0.993481224	0.924855847	0.944351122	0.943540106	0.940079339
CENPV	0.953174822	0.910438009	0.957052938	0.97731816	0.969244211	0.932145031
PDE7B	0.953269536	0.999987083	0.907626082	0.929388614	0.936108073	0.899428912
ZFP36L2	0.953269536	0.861395263	0.999993442	0.941467375	0.964301327	0.89950552
MYH14	0.953269536	0.98338119	0.91882781	0.982220679	0.960695315	0.90071654
PIK3R4	0.953269536	0.863286109	0.906019013	0.992339478	0.999985752	0.901618453
KCTD9	0.953269536	0.878462725	0.959725886	0.968996947	0.999859158	0.908099327
ZNF316	0.953269536	0.861395263	0.999993442	0.95901497	0.943702351	0.908405626
RILPL2	0.953269536	0.858384828	0.914816911	0.999981514	0.958852623	0.909056553
ATAD5	0.953269536	0.967251014	0.983504244	0.973075232	0.923430168	0.911219114
EPS15L1	0.953269536	0.916484772	0.93475587	0.964007727	0.999985752	0.911219114
COPZ2	0.953269536	0.967192911	0.964807927	0.952247001	0.961465747	0.911234149
MFSD2A	0.953269536	0.910438009	0.957295966	0.997467114	0.962682426	0.91373422

LOC112442867	0.953269536	0.946859049	0.927508464	0.9880671	0.972594401	0.91373422
TRIM41	0.953269536	0.905108627	0.94422142	0.9880671	0.986003146	0.91373422
MMS19	0.953269536	0.872175143	0.93190909	0.999981514	0.970988226	0.923092436
RGS10	0.953269536	0.915908807	0.946791586	0.984423083	0.976292433	0.923092436
ZFPM1	0.953269536	0.866901725	0.937281477	0.982131178	0.999985752	0.924939539
N4BP3	0.953269536	0.901331238	0.915858924	0.987187885	0.996781978	0.927520621
SIGLEC8	0.953269536	0.997471998	0.949855095	0.935884171	0.930451229	0.92789871
CCDC106	0.953269536	0.864945924	0.975774524	0.972142185	0.981652321	0.92789871
CHAF1A	0.953269536	0.971289048	0.93190909	0.988408846	0.936962513	0.930206677
RAB5A	0.953269536	0.901631302	0.97316848	0.995312373	0.936215792	0.936263602
GK5	0.953269536	0.918854612	0.917075851	0.999981514	0.953300742	0.938013912
RDH11	0.953269536	0.89812791	0.97773026	0.979385838	0.953300742	0.93850525
ZNF287	0.953269536	0.895777642	0.982195333	0.995565324	0.925576986	0.938894901
SAA3	0.953269536	0.991585358	0.939981892	0.960016921	0.921710688	0.940583352
CYLD	0.953269536	0.902488025	0.937869586	0.98981682	0.9683846	0.943972369
TMBIM6	0.953269536	0.961663204	0.944126714	0.965050971	0.943702351	0.948705062
BUD31	0.953269536	0.905359761	0.996068003	0.95901497	0.926813827	0.958790968
RAD51B	0.953269536	0.872175143	0.950004587	0.999981514	0.925918507	0.960023723
RDX	0.953269536	0.851948403	0.996224523	0.978503042	0.931357161	0.960023723
TGFBR3L	0.953269536	0.878311791	0.948949412	0.987659016	0.961465747	0.960023723
PSMD5	0.953269536	0.872175143	0.999993442	0.940436902	0.926831826	0.963456717
LONRF2	0.953269536	0.889387648	0.939912874	0.973075232	0.970988226	0.963456717
MED21	0.953269536	0.957328149	0.951521942	0.957132838	0.927086179	0.966850975
GPCPD1	0.953269536	0.869953468	0.916294854	0.999981514	0.938316699	0.973287151
MAPK8IP1	0.953269536	0.862468468	0.951237862	0.99313054	0.943540106	0.97439943
SRSF10	0.953269536	0.884773193	0.979494108	0.941467375	0.953300742	0.976124527
MRPS27	0.953269536	0.859833245	0.962797326	0.948050281	0.970980375	0.980009318
TMX3	0.953269536	0.901028838	0.935266153	0.943447418	0.970988226	0.981216824
CCNL1	0.953269536	0.886565095	0.949855095	0.925353907	0.970491451	0.988866378
RNF130	0.953269536	0.868447954	0.962797326	0.964521432	0.926972948	0.995525297
LOC112444278	0.95348522	0.884457875	0.99869493	0.98981682	0.929896768	0.923064357
TANGO2	0.953578	0.985997726	0.949855095	0.961898258	0.953300742	0.911219114
LOC528767	0.953578	0.988008216	0.927508464	0.960016921	0.960573822	0.917906589
RCC1L	0.953578	0.955333923	0.979354564	0.967450445	0.934092515	0.9267033
CCDC115	0.953578	0.918854612	0.99042659	0.939353813	0.968472466	0.929027441
CORIN	0.953578	0.890045072	0.942357321	0.932764647	0.999985752	0.930146713
LOC101907749	0.953578	0.926905849	0.916323169	0.995565324	0.963790274	0.943621425
FBXO25	0.953578	0.989303356	0.916294854	0.972142185	0.931357161	0.945480515

LYSMD2	0.953578	0.869754045	0.920501366	0.964007727	0.999985752	0.952563377
EPB41L4A	0.953578	0.924917071	0.947155158	0.953697985	0.973208134	0.954496768
CASP4	0.953578	0.906970018	0.949855095	0.961898258	0.935286646	0.988796537
STK39	0.953615636	0.884773193	0.968249948	0.929342214	0.923910588	0.999948228
WRAP53	0.953704504	0.871091574	0.957052938	0.999981514	0.939813331	0.919303252
LOC112443163	0.953704504	0.936310161	0.912183977	0.995565324	0.954071324	0.958699521
HVCN1	0.953895744	0.923232089	0.997300234	0.932562244	0.976292433	0.898933037
CD8A	0.953895744	0.964925317	0.916294854	0.952298386	0.944839728	0.97521287
KAT6A	0.953895744	0.864586691	0.942357321	0.99570283	0.927086179	0.989055333
CLUAP1	0.954062623	0.946820717	0.927508464	0.951101818	0.935286646	0.989022544
C8H9orf85	0.954147224	0.855067335	0.996224523	0.964521432	0.989379051	0.895446935
ZZEF1	0.954147224	0.92042855	0.996624589	0.935239391	0.976769047	0.898470276
LOC107131652	0.954147224	0.98978863	0.955189235	0.96968491	0.937932469	0.90645885
TSPYL2	0.954147224	0.869514182	0.997400824	0.984423083	0.954023497	0.922361909
PBX1	0.954147224	0.943119518	0.912396874	0.999981514	0.943540106	0.930814839
LEO1	0.954147224	0.890669166	0.946791586	0.960016921	0.976292433	0.963701289
LOC100140533	0.954147224	0.858384828	0.957052938	0.965724803	0.972722722	0.969014913
RTL8C	0.954215012	0.958043794	0.950055019	0.949605617	0.9902393	0.895855654
HMMR	0.954215012	0.878624872	0.999993442	0.932914414	0.943702351	0.903990996
LOC104971220	0.954215012	0.971931061	0.927635169	0.991955963	0.956041014	0.911234149
FTCDNL1	0.954215012	0.865582541	0.975774524	0.999981514	0.935286646	0.911638347
RNASEH2B	0.954215012	0.864886058	0.968317646	0.960016921	0.999985752	0.9136642
BTBD2	0.954215012	0.872175143	0.924855847	0.999981514	0.96870902	0.914132808
LOC512323	0.954215012	0.917822211	0.999993442	0.952247001	0.927086179	0.917024468
PHF8	0.954215012	0.878311791	0.929661805	0.949605617	0.999985752	0.919124527
C13H20orf96	0.954215012	0.885951248	0.983504244	0.999981514	0.926972948	0.920793507
PRUNE1	0.954215012	0.919245978	0.999993442	0.938434021	0.948421303	0.923092436
CTNND1	0.954215012	0.878551917	0.915858924	0.974717682	0.999985752	0.923092436
EZR	0.954215012	0.944505732	0.972641134	0.96951401	0.954071324	0.923573245
EFL1	0.954215012	0.875293698	0.912020408	0.999981514	0.998040182	0.925472172
FRAT1	0.954215012	0.886565095	0.999993442	0.952247001	0.936921784	0.93363377
LOC101904290	0.954215012	0.957961435	0.927635169	0.938303773	0.987723553	0.933897626
ZCCHC10	0.954215012	0.949902864	0.949892509	0.960016921	0.961465747	0.943285784
ARAP2	0.954215012	0.865101561	0.916038418	0.985748796	0.996781978	0.955990509
MFSD4B	0.954215012	0.902407744	0.927381195	0.936882654	0.999497678	0.960023723
RAB40B	0.954215012	0.984410135	0.916294854	0.92532303	0.956906331	0.966408025
TRMT44	0.954215012	0.884440241	0.995746693	0.941467375	0.938316699	0.973218068
SYT3	0.954238488	0.992316823	0.932112545	0.929388614	0.929802526	0.965428409

FCAR	0.954304393	0.887018937	0.924855847	0.999981514	0.943702351	0.917930485
MCC	0.954444494	0.998847958	0.916323169	0.965050971	0.955623649	0.898470276
NSL1	0.954444494	0.863613561	0.999993442	0.934466438	0.931357161	0.899428912
ZNF300	0.954444494	0.98098403	0.963164828	0.948705762	0.964301327	0.905509632
CD320	0.954444494	0.92132606	0.983504244	0.999981514	0.926972948	0.909953142
ABHD18	0.954444494	0.94651182	0.966700703	0.990955345	0.943540106	0.91373422
PPIP5K1	0.954444494	0.927853707	0.916294854	0.952247001	0.999985752	0.917024468
GPX1	0.954444494	0.897910327	0.959903168	0.988408846	0.972128973	0.925995537
LOC112446639	0.954444494	0.912890368	0.944818235	0.999981514	0.947003843	0.938894901
METTL18	0.954444494	0.99960614	0.916323169	0.94222181	0.925918507	0.940256832
DAB2	0.954444494	0.872175143	0.913100432	0.940438437	0.999985752	0.940606882
UNC45A	0.954444494	0.927034887	0.946791586	0.929388614	0.996781978	0.946347861
SHPRH	0.954444494	0.963481074	0.94798401	0.973673712	0.936797477	0.947527219
MED4	0.954444494	0.872175143	0.985171227	0.973075232	0.958797242	0.951340373
ARPC2	0.954444494	0.884359909	0.988585686	0.980532429	0.937946177	0.954925542
IRS1	0.954444494	0.969051025	0.918606509	0.969609355	0.942786245	0.963456717
ARHGAP19	0.954444494	0.918234392	0.912183977	0.990955345	0.964301327	0.963456717
CLSTN1	0.954444494	0.953449552	0.930736996	0.93048026	0.9683846	0.969014913
LOC101906855	0.954444494	0.889387648	0.99341711	0.934466438	0.943702351	0.972695131
LOC534181	0.954444494	0.855079912	0.922552585	0.999981514	0.943702351	0.972695131
ABLIM2	0.954444494	0.889748855	0.996224523	0.936540846	0.936530177	0.973218068
STOML1	0.954444494	0.927853707	0.91469924	0.964521432	0.958797242	0.985564959
GTDC1	0.954444494	0.904754143	0.946791586	0.965728173	0.940870936	0.988451994
IQCE	0.954444494	0.884457875	0.916294854	0.970785492	0.943047881	0.999948228
TMEM134	0.954450317	0.898706315	0.949855095	0.995565324	0.960573822	0.940256832
FGF18	0.954450317	0.943702928	0.950223118	0.98981682	0.937932469	0.940942561
JADE1	0.954549644	0.8857401	0.944126714	0.999981514	0.969746326	0.898470276
COCH	0.954549644	0.925713742	0.999993442	0.925353907	0.965676623	0.899428912
LOC101907017	0.954549644	0.917822211	0.916323169	0.999981514	0.964235922	0.902100676
TMSB15B	0.954549644	0.988540349	0.913155467	0.997467114	0.951996251	0.904052262
LOC614882	0.954549644	0.862468468	0.999993442	0.941467375	0.970980375	0.907729038
EP400	0.954549644	0.876884737	0.954020369	0.989601002	0.996781978	0.912531667
LOC112448364	0.954549644	0.857489449	0.957052938	0.995565324	0.992500414	0.91373422
C7H19orf53	0.954549644	0.882102283	0.955845583	0.986214799	0.996089557	0.91373422
LOC112446381	0.954549644	0.930293015	0.999993442	0.929703482	0.935286646	0.914132808
C25H16orf91	0.954549644	0.946148902	0.996224523	0.959616431	0.943160494	0.91618909
GINS3	0.954549644	0.998847958	0.93190909	0.950662233	0.947313295	0.916193353
PCOLCE2	0.954549644	0.980521483	0.916038418	0.938729416	0.996781978	0.917930485

RWDD3	0.954549644	0.912484369	0.997400824	0.961898258	0.953300742	0.923807852
WDR82	0.954549644	0.946148902	0.955427429	0.986051493	0.953885477	0.926433572
SH3BGRL2	0.954549644	0.96967304	0.971594714	0.937615893	0.960048824	0.927426708
NUBP1	0.954549644	0.969051025	0.975774524	0.927393867	0.96079081	0.927426708
IMPACT	0.954549644	0.969051025	0.926161087	0.992930508	0.943610727	0.930657508
EEA1	0.954549644	0.878488273	0.916038418	0.999981514	0.970988226	0.931410194
PLP2	0.954549644	0.855752352	0.946791586	0.999981514	0.926972948	0.933903265
HEATR1	0.954549644	0.918854612	0.948594902	0.949468104	0.99253365	0.940017687
LOC101906508	0.954549644	0.893174452	0.926821911	0.999981514	0.938316699	0.944973383
ITFG2	0.954549644	0.876831363	0.957052938	0.999981514	0.954403133	0.944973383
JOSD1	0.954549644	0.891220169	0.93141958	0.982220679	0.984386856	0.946760037
S100A13	0.954549644	0.910651459	0.933291874	0.999981514	0.944839728	0.947838082
CCDC146	0.954549644	0.918447234	0.915858924	0.999981514	0.953300742	0.947838082
KTN1	0.954549644	0.865570309	0.948904459	0.995565324	0.964301327	0.959198304
SMURF1	0.954549644	0.901130244	0.911535369	0.950643928	0.999985752	0.960023723
RPS26	0.954549644	0.883756544	0.916323169	0.946503916	0.999453276	0.971545917
FGR	0.954549644	0.918854612	0.916323169	0.930987593	0.989334846	0.975200512
BSDC1	0.954549644	0.882242226	0.912020408	0.98279984	0.943702351	0.999463809
ZNF740	0.954664428	0.871924797	0.99341711	0.999981514	0.937681236	0.923807852
SPTSSB	0.954698381	0.94422395	0.916323169	0.940436902	0.999985752	0.907145443
LOC618256	0.954769549	0.877501251	0.959725886	0.940438437	0.950958352	0.998240456
CRAMP1	0.954770209	0.863286109	0.999993442	0.973373329	0.930451229	0.935497122
CUTC	0.954770209	0.863286109	0.999993442	0.965050971	0.932024143	0.958707771
LOC101902154	0.954801674	0.872244611	0.978530068	0.999981514	0.953300742	0.921772742
WSB1	0.954801674	0.865570309	0.938624264	0.973075232	0.998141915	0.950798794
ATF6B	0.954801674	0.863286109	0.959903168	0.979330173	0.944839728	0.98469083
ZCCHC2	0.954801674	0.894702415	0.916323169	0.965050971	0.926047649	0.999948228
GMEB1	0.954801674	0.891435594	0.927635169	0.951936643	0.947112796	0.999948228
SUPT7L	0.954803077	0.989303356	0.95322206	0.928332965	0.952007172	0.940382532
TAF6	0.95482849	0.96643574	0.996624589	0.948668643	0.942455745	0.908475983
ZC3H7A	0.95482849	0.936822825	0.915858924	0.999981514	0.976292433	0.916939552
LOC112449282	0.95482849	0.863286109	0.98915348	0.972142185	0.981190294	0.925233445
MLEC	0.95482849	0.913116251	0.921411252	0.932914414	0.999985752	0.949475996
LOC104976293	0.954842225	0.893938241	0.943028511	0.976221833	0.99971266	0.923064357
TUBGCP2	0.954927211	0.912316725	0.934354308	0.960016921	0.996781978	0.944973383
ABCA7	0.954960076	0.913116251	0.95875611	0.999981514	0.929037565	0.90816713
RXRB	0.954960076	0.949902864	0.979584814	0.961898258	0.964301327	0.910879598
MYO7A	0.954960076	0.946806157	0.957052938	0.949913449	0.989502048	0.912894278

PLEKHA2	0.954960076	0.861191187	0.965407935	0.999981514	0.970988226	0.917024468
LOC101908048	0.954960076	0.902468141	0.96608402	0.999981514	0.933344708	0.918461982
YARS2	0.954960076	0.89489192	0.913609237	0.947574232	0.999985752	0.919124527
NUPL2	0.954960076	0.973321075	0.916038418	0.949468104	0.989379051	0.927520621
LOC112442757	0.954960076	0.878551917	0.988307089	0.996394267	0.94677113	0.931410194
MACROD2	0.954960076	0.950336973	0.937093664	0.995565324	0.947020167	0.933897626
LOC112448511	0.954960076	0.904728572	0.93573893	0.98981682	0.978453925	0.935770262
CARHSP1	0.954960076	0.933100216	0.982195333	0.929663263	0.973219333	0.936951685
WNT9A	0.954960076	0.872331637	0.980061232	0.999981514	0.929896768	0.953140004
LOC101903385	0.954960076	0.941483684	0.956381241	0.948776099	0.964301327	0.958790968
LOC101905770	0.954960076	0.91196842	0.949939862	0.990955345	0.943610727	0.96274079
EHBP1L1	0.954960076	0.889387648	0.924855847	0.998801621	0.961465747	0.964898868
NACA	0.954960076	0.872175143	0.929661805	0.989601002	0.976259416	0.966071454
GTPBP1	0.954960076	0.861395263	0.928731949	0.961241178	0.998892262	0.966850975
PEPD	0.954960076	0.977196455	0.961437412	0.932254825	0.926972948	0.967759699
ALPK2	0.954960076	0.912484369	0.996840573	0.928339235	0.938879379	0.96870908
WDCP	0.954960076	0.897327772	0.942296737	0.999981514	0.928951321	0.969014913
ETV5	0.954960076	0.942159543	0.911973589	0.943808491	0.972499705	0.98140846
ZBED3	0.954960076	0.861589503	0.93190909	0.9745615	0.976292433	0.98140846
NUDT6	0.954960076	0.884128797	0.924855847	0.995565324	0.935286646	0.98926637
GLRX2	0.955004544	0.893164557	0.992714073	0.95901497	0.959658682	0.947937638
TMEM74B	0.955050344	0.870489012	0.916038418	0.953708932	0.999985752	0.903990996
GIPC2	0.955050344	0.896232903	0.972806636	0.999981514	0.964301327	0.905509632
TNNI2	0.955050344	0.865101561	0.999993442	0.997332186	0.943734737	0.911219114
LOC112449618	0.955050344	0.859986967	0.929661805	0.999981514	0.999251855	0.911219114
NCAM2	0.955050344	0.955652503	0.992511421	0.928075638	0.9683846	0.91373422
RBCK1	0.955050344	0.893164557	0.969370452	0.961898258	0.996781978	0.918461982
CDC25A	0.955050344	0.90265862	0.935266153	0.999981514	0.977167046	0.922361909
PRKAR2B	0.955050344	0.914902719	0.990037865	0.999981514	0.929896768	0.923092436
TMEM184C	0.955050344	0.930411132	0.958410013	0.999981514	0.935286646	0.923199665
LHCGR	0.955050344	0.876954722	0.916323169	0.999981514	0.987723553	0.923298221
CCZ1	0.955050344	0.935661607	0.939912874	0.999981514	0.953885477	0.923807852
RALGPS2	0.955050344	0.997678999	0.946791586	0.94565162	0.940870936	0.925233445
PLA2G15	0.955050344	0.870444737	0.999993442	0.984423083	0.934813966	0.925704954
APBB1IP	0.955050344	0.936319037	0.972741989	0.96900924	0.960573822	0.930710695
SIRT7	0.955050344	0.930293015	0.988307089	0.98673849	0.928951321	0.932534796
ACADVL	0.955050344	0.931610273	0.964807927	0.973075232	0.964301327	0.93363377
DAD1	0.955050344	0.893229467	0.996224523	0.943808491	0.971075533	0.933903265

AQP3	0.955050344	0.865887622	0.966199203	0.989601002	0.976292541	0.934671838
FAM49A	0.955050344	0.888057447	0.972482052	0.999793541	0.948421303	0.940079339
RAB20	0.955050344	0.918491096	0.96412644	0.999793541	0.927086179	0.94844234
IQCD	0.955050344	0.895777642	0.999280799	0.944913633	0.954071324	0.950540248
ZNF358	0.955050344	0.942273776	0.916294854	0.999981514	0.937932469	0.951678343
CLUH	0.955050344	0.929409572	0.979494108	0.949565453	0.957104426	0.954961112
ICMT	0.955050344	0.872175143	0.964382669	0.96968491	0.976292433	0.95883396
CUX1	0.955050344	0.9574162	0.929661805	0.964521432	0.955076828	0.963107047
ABHD12	0.955050344	0.965405402	0.916294854	0.928026192	0.979290191	0.965117554
KANSL1L	0.955050344	0.943244588	0.913100432	0.955954309	0.977167046	0.968168677
NCBP2	0.955050344	0.894702415	0.99341711	0.949864417	0.943055455	0.969564816
EXOC1	0.955050344	0.861395263	0.972806636	0.973179867	0.961465747	0.970071797
ZDHHC6	0.955050344	0.861975125	0.997300234	0.95901497	0.935286646	0.977166378
PIM1	0.955050344	0.961663204	0.916038418	0.928332965	0.9683846	0.9803583
LOC101905533	0.955050344	0.918455779	0.957295966	0.948050281	0.930451229	0.996335668
LOC100847612	0.955050344	0.880565292	0.935824202	0.943808491	0.956041014	0.999948228
ITM2B	0.955050344	0.872175143	0.915191964	0.948668643	0.956041014	0.999948228
SPG21	0.955165459	0.867460706	0.975774524	0.999981514	0.937932469	0.911219114
TERF1	0.955165459	0.942088099	0.935266153	0.957008595	0.956041014	0.978087957
SSBP3	0.955188713	0.968509005	0.924855847	0.935956158	0.999985752	0.905188617
ZADH2	0.955188713	0.882007869	0.922551937	0.949502614	0.999985752	0.960023723
DTX2	0.955280956	0.927853707	0.997400824	0.940436902	0.960573822	0.930146713
MFSD11	0.955280956	0.975612288	0.916453343	0.940946275	0.944436335	0.982908937
CMTM3	0.955402443	0.982664716	0.934354308	0.961898258	0.977193348	0.90240926
LOC107133150	0.955402443	0.946881447	0.91416437	0.999981514	0.965163023	0.90607344
TRIM28	0.955402443	0.863414694	0.91882781	0.999981514	0.932024143	0.911219114
LOC100297498	0.955402443	0.861395263	0.915713148	0.97828838	0.999985752	0.911234149
MAP7	0.955402443	0.877221102	0.943836178	0.999981514	0.953300742	0.919124527
ZNF2	0.955402443	0.998847958	0.926107649	0.940438437	0.964301327	0.919124527
TYW1	0.955402443	0.863414694	0.999993442	0.941467375	0.940870936	0.927426708
CEP162	0.955402443	0.863613561	0.916294854	0.999981514	0.964301327	0.940017687
SOWAHA	0.955402443	0.947247457	0.957052938	0.977689659	0.953300742	0.943972369
STK3	0.955402443	0.910560869	0.917155067	0.948050281	0.999985752	0.947245743
VPS39	0.955402443	0.916141416	0.927635169	0.999981514	0.931357161	0.948705062
SEC22C	0.955402443	0.872684104	0.993024884	0.956685049	0.970988226	0.948913998
LOC100297056	0.955402443	0.875506677	0.93891936	0.999981514	0.964301327	0.951081819
NFXL1	0.955402443	0.89969745	0.992447094	0.940946275	0.964301327	0.958790968
NDFIP1	0.955402443	0.863613561	0.999993442	0.952247001	0.926972948	0.963456717

ZNF16	0.955402443	0.873915965	0.927635169	0.986363498	0.983941532	0.963456717
NR4A1	0.955402443	0.910651459	0.957052938	0.989601002	0.942786245	0.966212838
LOC614617	0.955402443	0.916484772	0.916038418	0.946162866	0.989379051	0.978073306
RPS2	0.955402443	0.870296749	0.949939862	0.984423083	0.954403133	0.982937529
OCIAD1	0.955402443	0.910651459	0.963335792	0.940136897	0.939343714	0.996335668
LOC101907327	0.955402443	0.863286109	0.916294854	0.955466436	0.935286646	0.999948228
SHISA6	0.95573846	0.936822825	0.971003926	0.981295935	0.927087035	0.960023723
LOC107132410	0.955751428	0.880129816	0.943836178	0.999981514	0.93315562	0.912531667
WDR93	0.955821392	0.933886329	0.997400824	0.969837124	0.955623649	0.902445346
CHMP4B	0.955821392	0.90715891	0.999993442	0.982220679	0.943702351	0.912894278
MAP3K20	0.955821392	0.96365708	0.999993442	0.932764647	0.939208227	0.923092436
ACTN4	0.955821392	0.889763131	0.959725886	0.992470001	0.970988226	0.936263602
MCM3AP	0.955821392	0.872175143	0.999993442	0.942955143	0.935286646	0.945480515
LOC101906312	0.955821392	0.913116251	0.957052938	0.997374305	0.953300742	0.945480515
HACD3	0.955821392	0.927973597	0.957052938	0.999981514	0.928260428	0.948705062
MORC3	0.955821392	0.955652503	0.979494108	0.930987593	0.954071324	0.952571698
RBM6	0.955821392	0.866901725	0.973562292	0.995565324	0.941800786	0.966212838
MAP2	0.955821392	0.913899522	0.951028911	0.980564765	0.930080843	0.98469083
NR6A1	0.955821392	0.916484772	0.916038418	0.945261452	0.983941532	0.98469083
TP53I11	0.956105734	0.936582263	0.959725886	0.999981514	0.959007232	0.904240925
TRPC2	0.956105734	0.912627552	0.99647733	0.9880671	0.943540106	0.919124527
ATG5	0.956105734	0.953203609	0.984886031	0.972142185	0.930451229	0.935497122
SLC25A23	0.956105734	0.920217996	0.947506672	0.979311704	0.976292433	0.940910488
ATG2B	0.956109125	0.968509005	0.950727498	0.961898258	0.979290191	0.906517494
LOC100848721	0.956109125	0.872175143	0.999145937	0.940438437	0.985360041	0.927253029
SETX	0.956109125	0.922610146	0.996224523	0.961898258	0.938879379	0.945480515
CCDC25	0.956109125	0.91078662	0.939936316	0.978503042	0.976292433	0.95883396
ZNF260	0.956495299	0.931588197	0.957052938	0.976196758	0.984386856	0.91373422
CDK5RAP3	0.956495299	0.906331397	0.99341711	0.975653996	0.964301327	0.919124527
EZH1	0.956495299	0.894014677	0.922552585	0.989601002	0.930451229	0.998284411
RECK	0.95670556	0.970965452	0.93190909	0.941467375	0.998287238	0.915303189
KIAA0391	0.956997991	0.932191409	0.916294854	0.949565453	0.932024143	0.999948228
MDM1	0.957014237	0.872175143	0.986238163	0.950643928	0.933324246	0.995895432
LOC101904173	0.957014237	0.86285541	0.916323169	0.997660427	0.930513915	0.999948228
GLA	0.957035183	0.947350735	0.93475587	0.999981514	0.946935016	0.911219114
LOC784738	0.957292097	0.890669166	0.942357321	0.999981514	0.972890315	0.932507146
LOC100848212	0.957479078	0.947911876	0.975774524	0.984423083	0.953885477	0.917930485
TMEM150A	0.957479078	0.988384541	0.957052938	0.957008595	0.956653075	0.919124527

BET1	0.957479078	0.894702415	0.996224523	0.940438437	0.972160227	0.945532151
SIN3B	0.957479078	0.875391655	0.953300281	0.973075232	0.930451229	0.999655834
HAT1	0.957673684	0.99960614	0.946791586	0.93660503	0.959565914	0.903990996
LOC780968	0.957673684	0.871924797	0.99341711	0.974286747	0.989334846	0.903990996
MAPK1IP1L	0.957673684	0.89417747	0.999993442	0.961831293	0.954071324	0.907145443
NRROS	0.957673684	0.933100216	0.930248841	0.996884833	0.986257323	0.90969547
GPR27	0.957673684	0.991673407	0.973034514	0.959616431	0.935093621	0.91373422
LYRM2	0.957673684	0.880565292	0.996224523	0.999981514	0.937932469	0.915414597
STRADA	0.957673684	0.939956757	0.999734445	0.97731816	0.931402923	0.917024468
STARD8	0.957673684	0.950336973	0.957295966	0.97731816	0.970988226	0.919124527
LOC513573	0.957673684	0.925557011	0.999993442	0.937657986	0.936921784	0.920974152
LOC112444921	0.957673684	0.878854854	0.999993442	0.982220679	0.929802526	0.940017687
LOC104971307	0.957673684	0.883756544	0.999993442	0.941467375	0.964301327	0.940174044
LOC104973551	0.957673684	0.919245978	0.981044281	0.984423083	0.938316699	0.94844234
MFF	0.957673684	0.868447954	0.97773026	0.95901497	0.989334846	0.948666557
SYCE2	0.957673684	0.887139959	0.962832886	0.934466438	0.99971266	0.952663521
DAPK1	0.957673684	0.93172121	0.999993442	0.936587163	0.928951321	0.960023723
LOC100335553	0.957673684	0.930293015	0.969370452	0.959591127	0.953300742	0.966212838
STXBP4	0.957673684	0.95038008	0.916294854	0.943447418	0.943610727	0.99828741
DOK2	0.957830144	0.921940584	0.982823002	0.96405425	0.983010476	0.911638347
C27H8orf48	0.957830144	0.980764484	0.945995021	0.999981514	0.934450558	0.911808092
CDRT4	0.957830144	0.915269318	0.959725886	0.99045711	0.976292433	0.919195656
ABL2	0.957830144	0.863948462	0.99341711	0.964521432	0.988881329	0.922361909
OSBP	0.957830144	0.966507289	0.933884582	0.980632768	0.970980375	0.925472172
LOC101905127	0.957830144	0.875506677	0.975774524	0.989601002	0.956761712	0.960000414
UCHL5	0.957830144	0.871924797	0.992511421	0.979644238	0.947020167	0.96166694
LOC107132958	0.957830144	0.913120359	0.972641134	0.930987593	0.976292433	0.964307876
PARP8	0.957830144	0.89417747	0.964807927	0.960937718	0.946935016	0.988320707
SFXN3	0.95796667	0.981520642	0.935266153	0.993215785	0.958797242	0.907124813
RGMB	0.95796667	0.871924797	0.915713148	0.97731816	0.937932469	0.999948228
RIOX2	0.958088207	0.869953468	0.934541067	0.999981514	0.976259416	0.935728367
LOC112447359	0.958112019	0.889302699	0.999993442	0.931383184	0.962513981	0.907384701
SPAST	0.958112019	0.889387648	0.916038418	0.969214881	0.999985752	0.908405626
TAF9B	0.958112019	0.925281429	0.926161087	0.999981514	0.939208227	0.923092436
CCNH	0.958112019	0.968932899	0.99341711	0.952247001	0.930451229	0.931410194
LOC112444340	0.958112019	0.895768247	0.99647733	0.948668643	0.976259416	0.93363377
FAM234B	0.958112019	0.962494531	0.93475587	0.992470001	0.945908526	0.944927301
ANKRD28	0.958112019	0.915269318	0.985156077	0.961241178	0.960573822	0.948915539

MRC1	0.958112019	0.935350625	0.971053208	0.940726907	0.970988226	0.958790968
ZNF283	0.958112019	0.899490486	0.949855095	0.96223045	0.982519862	0.963456717
LOC107132335	0.958596822	0.959732252	0.955427429	0.990955345	0.960573822	0.911336121
RAD51C	0.958596822	0.99950853	0.967590756	0.941467375	0.935093621	0.91373422
LOC101905156	0.958596822	0.994185904	0.97960335	0.944351122	0.929802526	0.922361909
LSM2	0.958596822	0.90265862	0.944818235	0.972142185	0.998016692	0.937715964
SLC30A7	0.958596822	0.911290584	0.928514467	0.976714093	0.970988226	0.974962631
UPF1	0.958596822	0.868390063	0.948594902	0.9880671	0.941800786	0.996335668
LRRC25	0.958920634	0.903618936	0.971053208	0.99000498	0.976292433	0.916193353
PDZD8	0.959034705	0.919245978	0.99647733	0.967512088	0.956904152	0.926487302
ZBTB25	0.959034705	0.870296749	0.999993442	0.986051493	0.936388637	0.938894901
CAMKMT	0.959078521	0.871107291	0.958454497	0.979357217	0.999985752	0.910085997
SETD1A	0.959078521	0.933545282	0.996224523	0.955466436	0.970988226	0.911638347
IFNGR2	0.959078521	0.913116251	0.917155067	0.96900924	0.999985752	0.917930485
PROSER3	0.959078521	0.899490486	0.999993442	0.985118339	0.937932469	0.923092436
RXRG	0.959078521	0.927488825	0.999993442	0.973373329	0.940531552	0.923092436
NCL	0.959078521	0.879552187	0.964807927	0.963132425	0.998016692	0.938812939
LOC112442221	0.959078521	0.871176243	0.945471374	0.9880671	0.996781978	0.940583352
GRSF1	0.959078521	0.949323635	0.987786638	0.960016921	0.935286646	0.952571698
WSCD2	0.959078521	0.872331637	0.944714965	0.999981514	0.963790274	0.961107601
RNF24	0.959078521	0.869754045	0.918247946	0.999981514	0.976259416	0.965117554
PWP2	0.959078521	0.94592331	0.939981892	0.973373329	0.956041014	0.967759699
LOC104970809	0.959078521	0.880650029	0.951392011	0.973075232	0.932024143	0.999872573
HYAL3	0.959078521	0.884272556	0.939912874	0.95901497	0.941800786	0.999948228
TMED8	0.959191365	0.868390063	0.959725886	0.98981682	0.940870936	0.987785166
UBE2S	0.95922304	0.941817736	0.957052938	0.984533143	0.948711141	0.953509765
XPNPEP1	0.95922304	0.871176243	0.99341711	0.934466438	0.977167046	0.963456717
TRMT9B	0.95922304	0.94592331	0.958410013	0.952247001	0.953300742	0.971742751
LOC104969648	0.95922304	0.865582541	0.916294854	0.941467375	0.998287238	0.996335668
SMC6	0.959334618	0.954827347	0.943585644	0.960016921	0.957293377	0.969014913
LOC100847613	0.95947791	0.991498596	0.916038418	0.982220679	0.930451229	0.960023723
SBDS	0.95947791	0.888057447	0.99341711	0.97828838	0.943540106	0.960153446
DYNLT1	0.959594897	0.969051025	0.995746693	0.957419137	0.943610727	0.915546224
NAB1	0.959594897	0.999987083	0.916294854	0.952247001	0.935286646	0.925472172
HEATR6	0.959594897	0.888057447	0.939912874	0.999981514	0.976259416	0.938013912
FBXO9	0.959594897	0.927973597	0.949855095	0.948668643	0.961465747	0.98469083
TMEM106B	0.959778153	0.924255599	0.935266153	0.999981514	0.948711141	0.927426708
DCUN1D4	0.959821691	0.912627552	0.987610559	0.9880671	0.935286646	0.956827114

SLC27A1	0.959863894	0.91287376	0.951392011	0.941467375	0.996781978	0.960023723
SP4	0.960089161	0.975612288	0.949939862	0.982815793	0.964301327	0.911219114
PMP2	0.960089161	0.887479443	0.916294854	0.999981514	0.969253072	0.911219114
SLC8B1	0.960245527	0.930293015	0.945732557	0.999981514	0.929896768	0.950540248
MAP3K7	0.960245527	0.869406822	0.951237862	0.979644238	0.931775933	0.999948228
CLEC4E	0.960326277	0.882102283	0.975774524	0.98471945	0.996781978	0.911219114
AGTR2	0.960326277	0.918854612	0.999993442	0.952247001	0.965676623	0.91373422
PLCG1	0.960326277	0.915570087	0.999993442	0.954724745	0.948421303	0.939662884
LOC104974137	0.960326277	0.916721068	0.920194747	0.973812087	0.936921784	0.999948228
ALYREF	0.960338354	0.895777642	0.999993442	0.994965893	0.939442711	0.917930485
SLC2A8	0.960438721	0.882858125	0.990615319	0.999981514	0.935286646	0.919124527
OXCT1	0.960438721	0.870195838	0.960998979	0.969256019	0.976292433	0.972332728
RNF135	0.960577337	0.867518836	0.917772116	0.988580812	0.999985752	0.918461982
IQCK	0.960577337	0.870026439	0.933884582	0.999981514	0.938316699	0.986042926
LNPK	0.960577337	0.893213198	0.929661805	0.969214881	0.960048824	0.997054363
RAPGEF5	0.96060552	0.974846249	0.924855847	0.999981514	0.935286646	0.91373422
MTRR	0.960630299	0.99488888	0.928927123	0.976714093	0.937946177	0.93363377
EGLN2	0.960630299	0.894702415	0.964661607	0.99045711	0.961465747	0.958281344
LOC101903526	0.960630299	0.889345904	0.957052938	0.949565453	0.976769047	0.981216824
TMEM38A	0.960630299	0.870298381	0.944126714	0.99045711	0.956653075	0.988639786
SPIN1	0.960667147	0.919525428	0.961437412	0.955466436	0.987723553	0.945480515
LOC101902861	0.960667147	0.89417747	0.929661805	0.995565324	0.970491451	0.967759699
POC1A	0.960782104	0.870222905	0.977641717	0.941796131	0.999985752	0.933897626
LY9	0.960782104	0.883945911	0.947306164	0.955466436	0.999985752	0.939662884
DERL2	0.960782104	0.955652503	0.978530068	0.978503042	0.932036998	0.943972369
LOC101902290	0.960782104	0.882858125	0.990974506	0.940136897	0.942786245	0.995525297
LOC104975911	0.960782723	0.904875256	0.996224523	0.972142185	0.976259416	0.912531667
AEBP1	0.960782723	0.941817736	0.93475587	0.99542943	0.982538884	0.919124527
LOC112447140	0.960782723	0.903341833	0.918641217	0.999981514	0.964768333	0.945477886
IL17RC	0.960794926	0.999987083	0.923364099	0.950643928	0.943702351	0.919124527
COMMD10	0.960794926	0.872175143	0.960945531	0.959616431	0.962513981	0.993890045
LOC783421	0.961125238	0.888322912	0.96043275	0.960016921	0.998287238	0.943972369
CD58	0.961356529	0.872175143	0.971255098	0.982220679	0.987723553	0.93988265
AOC3	0.961356529	0.960747241	0.955221146	0.964733972	0.956904152	0.956820511
SLC38A7	0.961356529	0.980289052	0.92157194	0.973373329	0.947512899	0.964898868
GAREM1	0.961356529	0.933100216	0.926234868	0.949565453	0.938316699	0.999948228
REV3L	0.961424737	0.910651459	0.999145937	0.942955143	0.976292433	0.926480924
BRIP1	0.961424737	0.880129816	0.992377532	0.948720795	0.964301327	0.973218068

ENHO	0.961454044	0.950336973	0.964807927	0.952247001	0.989771098	0.917930485
IL1R1	0.961454044	0.97304892	0.99341711	0.950643928	0.950958352	0.923092436
MOCS1	0.961454044	0.951172741	0.916323169	0.999981514	0.964235922	0.923807852
OPLAH	0.961454044	0.898667357	0.966232793	0.973373329	0.988049878	0.935770262
CIC	0.961454044	0.882858125	0.988120522	0.988408846	0.964301327	0.940079339
LOC101903586	0.961454044	0.914089415	0.957295966	0.999981514	0.950897336	0.945480515
RPP25	0.961454044	0.919686429	0.999993442	0.939604997	0.935286646	0.959198304
TMA16	0.961454044	0.882858125	0.95940506	0.955466436	0.976292433	0.980501512
PPHLN1	0.961571228	0.882102283	0.916294854	0.999981514	0.958797242	0.913221224
PPOX	0.961625235	0.905019377	0.996624589	0.995565324	0.943702351	0.923092436
YOD1	0.961625235	0.942088099	0.916323169	0.964864514	0.999985752	0.929461379
ZNF74	0.961625235	0.879243786	0.999993442	0.992339478	0.935286646	0.931315757
LOC101904097	0.961625235	0.880129816	0.933884582	0.999981514	0.935286646	0.984122543
LOC112447858	0.961625235	0.903350914	0.959076299	0.934466438	0.958797242	0.998240456
PKM	0.961634577	0.952323707	0.926161087	0.999981514	0.97352263	0.91373422
SLC35B3	0.961634577	0.938087741	0.955427429	0.999981514	0.942527603	0.916662891
SIL1	0.961634577	0.972007528	0.957052938	0.987281997	0.952343558	0.927426708
LOC100337328	0.961943268	0.998847958	0.917848891	0.984331039	0.953300742	0.912531667
CLP1	0.961943268	0.895777642	0.95219206	0.979752657	0.999985752	0.91348389
ATG16L2	0.961943268	0.955536844	0.996224523	0.939604997	0.970988226	0.915988786
LOC101907126	0.961943268	0.898699441	0.942357321	0.999981514	0.964301327	0.919124527
LOC100848527	0.961943268	0.93431906	0.920544372	0.999981514	0.977167046	0.925057945
TXNRD1	0.961943268	0.902241567	0.979354564	0.985118339	0.973219333	0.930710695
MIA3	0.961943268	0.894702415	0.927635169	0.949468104	0.999985752	0.931410194
NDUFAF2	0.961943268	0.887265261	0.99647733	0.939604997	0.983941532	0.945532151
TIMELESS	0.961943268	0.870195838	0.999993442	0.950643928	0.939650698	0.9790177
TEX14	0.961943268	0.926852499	0.916294854	0.960190213	0.938879379	0.999948228
RPUSD4	0.961943268	0.938087741	0.916323169	0.938303773	0.955076828	0.999948228
TM4SF5	0.962314624	0.897183343	0.91882781	0.9880671	0.999985752	0.929814744
ERCC6L2	0.962314624	0.936738131	0.93475587	0.999981514	0.958797242	0.943621425
LOC101904042	0.962314624	0.899490486	0.957052938	0.95901497	0.999251855	0.945480515
MUM1L1	0.962314624	0.87976124	0.955427429	0.949468104	0.944909181	0.999948228
LOC112446733	0.962372001	0.872175143	0.959076299	0.999981514	0.960754497	0.957165156
LOC100848869	0.962372001	0.889748855	0.997400824	0.984423083	0.934825476	0.959792861
SMIM1	0.962372001	0.890669166	0.966232793	0.959616431	0.980016241	0.966408025
SPAG1	0.962372001	0.977929455	0.946791586	0.952298386	0.944839728	0.969197073
LOC112444752	0.962372001	0.886924072	0.993073581	0.979385838	0.932024143	0.973649216
RWDD2A	0.962746385	0.974402411	0.94796441	0.995434268	0.958702487	0.913977389

ANKRD46	0.962746385	0.969051025	0.957052938	0.973812087	0.947299702	0.951081819
ECH1	0.962746385	0.899494273	0.96030829	0.969426656	0.976292433	0.964128552
LOC112444190	0.962746385	0.918234392	0.937812682	0.948668643	0.9683846	0.996310803
CNTNAP4	0.962792371	0.910651459	0.944126714	0.999981514	0.935286646	0.955281097
SAAL1	0.962971323	0.978518618	0.929176945	0.993481918	0.958797242	0.927633316
F8A1	0.963188116	0.89915699	0.948904459	0.961898258	0.999985752	0.911219114
LOC101906426	0.963188116	0.90116376	0.929176945	0.989601002	0.999985752	0.911219114
HNRNPL	0.963188116	0.999987083	0.9377363	0.962899291	0.935286646	0.911336121
EXO5	0.963188116	0.930551178	0.959725886	0.999981514	0.954071324	0.91373422
LOC101902204	0.963188116	0.936058126	0.990615319	0.954343717	0.984386856	0.91373422
LOC112443147	0.963188116	0.951172741	0.999993442	0.952443827	0.943702351	0.914132808
SOS2	0.963188116	0.972007528	0.980032241	0.961898258	0.962513981	0.915414597
CWF19L2	0.963188116	0.991673407	0.971053208	0.952247001	0.954814306	0.918461982
LACC1	0.963188116	0.993709317	0.923578245	0.960016921	0.976259416	0.921843102
DHDH	0.963188116	0.993481224	0.924588495	0.999793541	0.935286646	0.922469878
SEC23IP	0.963188116	0.898744477	0.979494108	0.99045711	0.976292433	0.923092436
TMEM143	0.963188116	0.889345904	0.946791586	0.961898258	0.999985752	0.923092436
NMNAT2	0.963188116	0.993203157	0.961227258	0.949502614	0.960177245	0.923199665
KCNJ11	0.963188116	0.901631302	0.999993442	0.961160238	0.943540106	0.924939539
CPE	0.963188116	0.878551917	0.964661607	0.999981514	0.947313295	0.926480924
ENGASE	0.963188116	0.938087741	0.927508464	0.999793541	0.981652321	0.927426708
PLAC8	0.963188116	0.90715891	0.999993442	0.948668643	0.976259416	0.928416436
PRPF31	0.963188116	0.99960614	0.924855847	0.948668643	0.959658682	0.928708614
BABAM1	0.963188116	0.91667357	0.947155158	0.951193444	0.999985752	0.929505673
CHRM3	0.963188116	0.943244588	0.996624589	0.948668643	0.963790274	0.930146713
ZNF236	0.963188116	0.905271052	0.997400824	0.944351122	0.983941532	0.930710695
DCSTAMP	0.963188116	0.928483112	0.953572282	0.999981514	0.960576267	0.93363377
FKBPL	0.963188116	0.975997485	0.955845583	0.969088992	0.961465747	0.93363377
CDYL	0.963188116	0.906658524	0.999993442	0.96951401	0.948856947	0.933897626
STRAP	0.963188116	0.882242226	0.999993442	0.959159289	0.961465747	0.933897626
SLC39A10	0.963188116	0.909954806	0.948810088	0.972142185	0.999985752	0.933897626
TRMT11	0.963188116	0.882858125	0.985156077	0.999981514	0.954412818	0.934148044
TWIST1	0.963188116	0.915269318	0.916294854	0.989707094	0.999480355	0.937184729
GSTA2	0.963188116	0.997425219	0.922552585	0.977888476	0.943055455	0.938013912
GMFG	0.963188116	0.930304997	0.996224523	0.944351122	0.969739417	0.938013912
ZNF317	0.963188116	0.989303356	0.972940212	0.956928634	0.935286646	0.938894901
BCL2L2	0.963188116	0.946241678	0.982232295	0.959652624	0.962558935	0.940017687
ACBD5	0.963188116	0.985997726	0.927635169	0.995565324	0.935286646	0.940256832

PTPN6	0.963188116	0.899490486	0.962034224	0.939604997	0.999985752	0.941835384
MR1	0.963188116	0.983594252	0.933884582	0.952247001	0.970988226	0.947801358
MT3	0.963188116	0.99960614	0.923327823	0.941796131	0.944839728	0.950540248
MYO1C	0.963188116	0.948915926	0.966700703	0.960016921	0.965163023	0.950631909
ARL13A	0.963188116	0.882858125	0.918641217	0.999981514	0.977158496	0.950631909
NMRK1	0.963188116	0.917655233	0.999993442	0.943808491	0.943047881	0.950798794
CEACAM19	0.963188116	0.96643574	0.923521499	0.990955345	0.953300742	0.960023723
RND3	0.963188116	0.975612288	0.922552585	0.938303773	0.983941532	0.960023723
LOC785605	0.963188116	0.944324631	0.943211357	0.975453864	0.965676623	0.963107047
DTNBP1	0.963188116	0.928213372	0.91882781	0.999981514	0.93723117	0.971545917
RHPN2	0.963188116	0.879093989	0.927635169	0.9880671	0.98865576	0.971545917
LOC100296832	0.963188116	0.882858125	0.963723107	0.989485949	0.959565914	0.973579501
LOC112441770	0.963188116	0.934225172	0.91882781	0.970202518	0.976259416	0.977503497
LOC617224	0.963188116	0.893164557	0.922552585	0.941796131	0.976259416	0.999948228
TTC12	0.963213703	0.938087741	0.96030829	0.999981514	0.947299702	0.927520621
DNPH1	0.963213703	0.958764172	0.947176871	0.944949439	0.993571905	0.940017687
RPL37A	0.963244206	0.915269318	0.969026634	0.999981514	0.943702351	0.930710695
LOC112442248	0.963244206	0.930293015	0.940521631	0.990955345	0.972128973	0.952563377
LOC100297725	0.963260686	0.888322912	0.991936822	0.996033721	0.954071324	0.940079339
LOC788672	0.963260686	0.892108342	0.925615238	0.999981514	0.946935016	0.964885036
LOC112444653	0.963286342	0.906331397	0.955427429	0.999981514	0.943540106	0.923807852
PSMG1	0.963286342	0.883945911	0.999993442	0.980564765	0.956041014	0.937478275
DNM3	0.963286342	0.927853707	0.976484937	0.95901497	0.940870936	0.982979905
LOC101905399	0.963287233	0.924099465	0.955427429	0.99542943	0.989334846	0.912894278
FAM83H	0.963287233	0.948801019	0.927635169	0.999981514	0.970988226	0.919124527
MYC	0.963287233	0.941676269	0.950736796	0.998529748	0.970491451	0.925995537
ODR4	0.963287233	0.933100216	0.937869586	0.946938434	0.999985752	0.932688327
DKC1	0.963287233	0.990052293	0.971053208	0.951019428	0.943702351	0.937478275
LOC112447392	0.963287233	0.927973597	0.99341711	0.948668643	0.972128973	0.940583352
USP8	0.963287233	0.91287376	0.946791586	0.999981514	0.956906331	0.963456717
POLE4	0.963287233	0.927973597	0.947176871	0.960435829	0.960573822	0.987785166
PLIN4	0.963287233	0.89201381	0.943836178	0.955466436	0.976259416	0.995099097
LOC107133284	0.963311022	0.927973597	0.946791586	0.967450445	0.999985752	0.925057945
PILRA	0.963376529	0.90116376	0.946791586	0.995565324	0.996781978	0.925057945
ZNF329	0.96372269	0.928519855	0.961437412	0.94857292	0.977356551	0.966779561
ZNF292	0.963847807	0.975612288	0.964807927	0.979433033	0.961670582	0.914207441
LOC100296952	0.96387619	0.969051025	0.941312902	0.999981514	0.943702351	0.914132808
KIF1C	0.96387619	0.983126365	0.951449985	0.960016921	0.970988226	0.927520621

LOC533308	0.96387619	0.978518618	0.921266625	0.997332186	0.935286646	0.960023723
LOC786914	0.96387619	0.88008538	0.97773026	0.978503042	0.953300742	0.982871032
MSC	0.96387619	0.886611588	0.945995021	0.999981514	0.93568841	0.982908937
YIPF4	0.96387619	0.889387648	0.943028511	0.998529748	0.946384846	0.988796537
OXSR1	0.96387619	0.891220169	0.923189411	0.982220679	0.957396931	0.999463809
GLS2	0.964290529	0.911290584	0.978530068	0.999981514	0.961569801	0.917849087
PLA2R1	0.964290529	0.892559835	0.943836178	0.999981514	0.979516371	0.923807852
TMEM39A	0.964290529	0.91078662	0.966199203	0.988408846	0.977945109	0.934360589
ZBTB21	0.964467703	0.892108342	0.950727498	0.989585692	0.998287238	0.93363377
LOC100848570	0.964514483	0.890669166	0.999993442	0.950154952	0.986003146	0.923547711
E2F8	0.964514483	0.872175143	0.957052938	0.999981514	0.963550093	0.952571698
FBXO38	0.964542955	0.913116251	0.951392011	0.999981514	0.969619627	0.923092436
LOC107132577	0.964542955	0.938235006	0.957052938	0.999981514	0.944839728	0.930710695
ARSB	0.964542955	0.887064545	0.978014482	0.999981514	0.958797242	0.932688327
LOC112445194	0.964542955	0.891435594	0.975774524	0.950643928	0.947112796	0.998284411
LOC112444520	0.964587328	0.950675653	0.916323169	0.988408846	0.936921784	0.98926637
SETD4	0.964617927	0.96476359	0.961437412	0.963132425	0.984386856	0.915414597
SDC3	0.964617927	0.88869789	0.949855095	0.999981514	0.953391502	0.917930485
LOC107132748	0.964617927	0.993481224	0.916453343	0.999981514	0.938879379	0.923092436
SH3BP2	0.964617927	0.946581569	0.996224523	0.952247001	0.965676623	0.927426708
ACAP2	0.964617927	0.907585165	0.985350221	0.990955345	0.966328884	0.927520621
MTFR2	0.964617927	0.969288757	0.952862977	0.985118339	0.958797242	0.935770262
EIF2D	0.964617927	0.946258461	0.961437412	0.955466436	0.987789919	0.9374034
LOC107133302	0.964617927	0.893164557	0.999993442	0.950643928	0.944839728	0.948913998
HORMAD2	0.964617927	0.915073021	0.921266625	0.979644238	0.999985752	0.952663521
DYNC1I1	0.964617927	0.927853707	0.949855095	0.998529748	0.958852623	0.959075802
BCDIN3D	0.964617927	0.895777642	0.965327631	0.961898258	0.976292433	0.972695131
LOC112446001	0.964617927	0.968509005	0.948988112	0.952247001	0.954814306	0.975056853
LOC112449552	0.964617927	0.943213236	0.937869586	0.989601002	0.947299702	0.976020793
FGF16	0.964617927	0.902525869	0.91882781	0.989601002	0.964713591	0.989084358
MBD1	0.964617927	0.872175143	0.925320071	0.99739416	0.953300742	0.99828741
LOC107132327	0.964617927	0.872175143	0.947155158	0.97731816	0.943702351	0.999948228
ANKS1A	0.964644874	0.942546802	0.984886031	0.965050971	0.954403133	0.952571698
CLIP1	0.964644874	0.895311167	0.917973089	0.985347282	0.983941532	0.980501512
LOC112444763	0.964644874	0.877079008	0.939081737	0.959591127	0.959658682	0.999948228
TRAPPC2L	0.964730969	0.938235006	0.928731949	0.9914926	0.966648836	0.963107047
UHMK1	0.964802717	0.872331637	0.943857983	0.999981514	0.958797242	0.960023723
LOC101905319	0.964903479	0.902646689	0.924855847	0.951193444	0.999985752	0.919124527

NACC2	0.964903479	0.916484772	0.944818235	0.999981514	0.983941532	0.927520621
MAMDC2	0.964903479	0.872331637	0.997213619	0.973812087	0.970988226	0.945480515
RAP2C	0.964903479	0.882242226	0.988120522	0.989485949	0.945372504	0.97061907
TRAF2	0.964907788	0.915269318	0.942128842	0.949863561	0.999985752	0.927517509
CTSS	0.964926393	0.942088099	0.99341711	0.988408846	0.954071324	0.917930485
PPP2R5B	0.964988537	0.984410135	0.931161015	0.999981514	0.946970109	0.927426708
TTC9	0.965007374	0.902525869	0.955427429	0.999981514	0.967470055	0.931410194
LOC104972578	0.965007374	0.985195756	0.933884582	0.952247001	0.935286646	0.98469083
FCHSD2	0.965030912	0.940956417	0.937363244	0.999981514	0.936108073	0.917024468
TOM1	0.965030912	0.879552187	0.999993442	0.955466436	0.987723553	0.919124527
UBR1	0.965030912	0.994185904	0.93921542	0.94714048	0.982538884	0.919993751
MADD	0.965030912	0.988008216	0.963164828	0.940726907	0.977167046	0.920098946
APPL1	0.965030912	0.938087741	0.978014482	0.961433319	0.989379051	0.921772742
LOC615183	0.965030912	0.999987083	0.930148372	0.940436902	0.957396931	0.923064357
NUP210L	0.965030912	0.941400719	0.999829453	0.9880671	0.936108073	0.923092436
GNB2	0.965030912	0.899494273	0.964807927	0.992560663	0.989334846	0.925233445
FRK	0.965030912	0.889457989	0.999993442	0.980564765	0.960573822	0.925432855
TRIM5	0.965030912	0.902468141	0.999993442	0.939604997	0.970099629	0.927703633
EMSY	0.965030912	0.96643574	0.929661805	0.944929997	0.999985752	0.930168877
TBC1D10B	0.965030912	0.878311791	0.92323046	0.999981514	0.999985752	0.930710695
ARSH	0.965030912	0.946148902	0.949855095	0.992885501	0.972128973	0.932507146
LOC112442263	0.965030912	0.950675653	0.945732557	0.997696233	0.965676623	0.932688327
ADAMTS14	0.965030912	0.915269318	0.944538814	0.999981514	0.9457833	0.936263602
NTRK2	0.965030912	0.99386673	0.949855095	0.961898258	0.948711141	0.940256832
ZCCHC3	0.965030912	0.991177966	0.935266153	0.940973093	0.970988226	0.955990509
LHFPL1	0.965030912	0.886746033	0.925320071	0.999981514	0.964301327	0.959275214
LOC112444285	0.965030912	0.884548529	0.929661805	0.999981514	0.94677113	0.966850975
LOC112445035	0.965030912	0.873915965	0.96043275	0.984423083	0.9683846	0.980293741
DSTYK	0.965030912	0.929204871	0.955344563	0.95901497	0.958797242	0.989084358
DCLRE1C	0.965030912	0.875506677	0.929661805	0.94415318	0.936342434	0.999948228
LOC104974260	0.965453921	0.901984573	0.960009003	0.994965893	0.996089557	0.922361909
LOC521224	0.965453921	0.987731372	0.944126714	0.995434268	0.943055455	0.930146713
SSBP4	0.965512831	0.908364002	0.990974506	0.999981514	0.943702351	0.927520621
BORA	0.965551883	0.893615781	0.999993442	0.991955963	0.94671521	0.920222784
GRAP	0.965551883	0.93738232	0.959725886	0.952247001	0.999985752	0.930131506
TNPO3	0.965551883	0.877893862	0.999993442	0.961898258	0.953885477	0.944973383
LRRC20	0.965551883	0.933100216	0.97918842	0.988408846	0.952007172	0.951649115
PYROXD1	0.965551883	0.88869789	0.964807927	0.999981514	0.961465747	0.960023723

PRKCB	0.965551883	0.889387648	0.964807927	0.999981514	0.938879379	0.976124527
IVNS1ABP	0.965551883	0.967668839	0.961987531	0.948776099	0.943963542	0.9790177
GOLIM4	0.965586137	0.969051025	0.924797209	0.98981682	0.97464569	0.936263602
PHKB	0.965586137	0.902525869	0.975774524	0.973075232	0.970988226	0.963456717
SAYSD1	0.965597992	0.890669166	0.95583423	0.957132838	0.999453276	0.963898271
FCRL1	0.96570182	0.916721068	0.999993442	0.96444269	0.958654817	0.923298221
LOC507581	0.965726055	0.906064108	0.966232793	0.999981514	0.960573822	0.914207441
LOC112443419	0.965726055	0.997613842	0.927635169	0.950154952	0.958018667	0.953140004
TLR9	0.965726055	0.894014677	0.995377403	0.967450445	0.954403133	0.970105102
MRPL38	0.965726055	0.879093989	0.99341711	0.942243773	0.976292433	0.973218068
UBE2Z	0.965890017	0.892252816	0.945995021	0.999981514	0.986287964	0.91373422
CD200R1L	0.965890017	0.985428851	0.955427429	0.969214881	0.974738415	0.915117421
LOC781612	0.965890017	0.965648998	0.946791586	0.999981514	0.965676623	0.917930485
LOC100848569	0.965890017	0.915269318	0.959725886	0.949502614	0.999985752	0.925233445
LOC101904468	0.965890017	0.941817736	0.999993442	0.949863561	0.961465747	0.925873491
AQR	0.965890017	0.965405402	0.91882781	0.976125636	0.998060725	0.927520621
OSTM1	0.965890017	0.893857042	0.958454497	0.999981514	0.942786245	0.944330889
RAE1	0.965890017	0.886669497	0.957052938	0.999981514	0.960600393	0.945854375
TCAM1	0.965890017	0.919540627	0.927677875	0.945113651	0.999985752	0.945984601
ZDHHC16	0.965890017	0.927853707	0.971053208	0.996033721	0.953300742	0.953140004
LOC781224	0.965890017	0.88008538	0.983504244	0.989601002	0.970031315	0.956154589
PRRG4	0.965890017	0.964132382	0.963044414	0.984423083	0.937932469	0.960023723
LOC789715	0.965890017	0.884168437	0.946759056	0.999981514	0.964833865	0.962127558
KNOP1	0.965890017	0.90180894	0.93573893	0.984423083	0.960419102	0.994887496
BIN2	0.966082606	0.894014677	0.990037865	0.961898258	0.983010476	0.950798794
ARSJ	0.96618814	0.997661867	0.955427429	0.976196758	0.944584705	0.917930485
DCBLD2	0.96618814	0.890669166	0.999993442	0.97828838	0.953300742	0.9221477
BCLAF3	0.96618814	0.895777642	0.939912874	0.999981514	0.990937627	0.932688327
YTHDC1	0.96618814	0.937448751	0.943836178	0.940438437	0.999985752	0.933571471
BCL9	0.96618814	0.901437685	0.943836178	0.999981514	0.953300742	0.94844234
LOC783612	0.96618814	0.890819105	0.99341711	0.950643928	0.970916104	0.970105102
IFT22	0.966297223	0.965250433	0.949772	0.950162237	0.999985752	0.923807852
GATAD2B	0.966297223	0.915269318	0.95940506	0.961898258	0.999985752	0.927897089
CCL4	0.966297223	0.927973597	0.944113072	0.950352843	0.943702351	0.999948228
PIP4P2	0.966446445	0.927853707	0.999993442	0.9745615	0.952393745	0.919124527
ASGR2	0.966446445	0.916260474	0.927508464	0.999981514	0.954814306	0.919124527
MSR1	0.966446445	0.922587381	0.945995021	0.999981514	0.940870936	0.922361909
DGCR6L	0.966446445	0.950512146	0.935266153	0.965462948	0.999985752	0.923092436

HMGN5	0.966446445	0.881942703	0.978014482	0.999981514	0.977016274	0.92789871
LOC112447303	0.966446445	0.999987083	0.925250835	0.947574232	0.962513981	0.929461379
RBBP7	0.966446445	0.954827347	0.959609498	0.999981514	0.944839728	0.938894901
ENOX2	0.966446445	0.949323635	0.946791586	0.986214799	0.977167046	0.938894901
INSIG2	0.966446445	0.90154488	0.98258069	0.963132425	0.992730609	0.940017687
CTBP1	0.966446445	0.955149104	0.927635169	0.943447418	0.999985752	0.940017687
CD24	0.966446445	0.93988991	0.955427429	0.979217817	0.976292433	0.950631909
MAP2K2	0.966446445	0.984498838	0.961437412	0.95901497	0.953300742	0.952563377
BDKRB1	0.966446445	0.883945911	0.927508464	0.999981514	0.986257323	0.960023723
KLHDC3	0.966446445	0.882858125	0.942357321	0.999981514	0.944054333	0.962872722
CHD7	0.966446445	0.925557011	0.944734215	0.946503916	0.999985752	0.966196012
LOC518980	0.966446445	0.969051025	0.962034224	0.95901497	0.940870936	0.975170737
SNAI1	0.966446445	0.969051025	0.936126875	0.961898258	0.956041014	0.9803583
EIF3H	0.966446445	0.902791592	0.927978312	0.999981514	0.956041014	0.981216824
PRORSD1	0.966446445	0.903350914	0.922552585	0.990955345	0.965676623	0.989365627
TFEC	0.966460188	0.933737961	0.992263335	0.976196758	0.965457884	0.93129461
ADAR	0.966460188	0.931411766	0.997850143	0.972142185	0.959393538	0.931500173
BCL10	0.966460188	0.883945911	0.990615319	0.984421126	0.983941532	0.933762041
ZNF140	0.966460188	0.93738232	0.964382669	0.999981514	0.943702351	0.939662884
ZNF568	0.966460188	0.957667701	0.924855847	0.999981514	0.944839728	0.968697704
LOC782950	0.966460188	0.96643574	0.959725886	0.960016921	0.954071324	0.969197073
CUL2	0.966460188	0.950986475	0.990974506	0.952247001	0.939208227	0.970206971
TREML1	0.966460188	0.882858125	0.942899366	0.999981514	0.940870936	0.99666605
PSTPIP2	0.966560772	0.984126877	0.93475587	0.959227327	0.976292433	0.946760037
PPP1R13B	0.966560772	0.967668839	0.965819476	0.942955143	0.976292433	0.951649115
CRTC2	0.966560772	0.950203225	0.975774524	0.943447418	0.976292433	0.95652195
ZBTB8A	0.966560772	0.982011436	0.947155158	0.949864417	0.939208227	0.983430934
LOC112449172	0.966560772	0.889387648	0.965407935	0.952247001	0.954814306	0.999948228
DNAI1	0.96668156	0.983212485	0.924855847	0.999981514	0.954403133	0.921557022
BLOC1S3	0.96668156	0.901028838	0.984886031	0.999981514	0.959565914	0.923092436
LOC112442245	0.96668156	0.929737582	0.962734642	0.999981514	0.936215792	0.927517509
MESP1	0.96668156	0.997678999	0.948949412	0.960937718	0.956653075	0.929027441
TLR5	0.96668156	0.899490486	0.980930024	0.999981514	0.943160494	0.930146713
POMGNT1	0.96668156	0.935148144	0.99341711	0.948965036	0.983999763	0.930814839
LOC112446882	0.96668156	0.957667701	0.999993442	0.945113651	0.954071324	0.933897626
BEGAIN	0.96668156	0.906064108	0.979494108	0.999981514	0.953300742	0.93983168
LOC112443011	0.96668156	0.958764172	0.974586624	0.975886364	0.958852623	0.943972369
ZNF175	0.96668156	0.876873679	0.995377403	0.997332186	0.958797242	0.948705062

LOC112447313	0.96668156	0.927279391	0.931002363	0.978404889	0.996781978	0.960023723
C15H11orf96	0.96668156	0.941817736	0.96638833	0.988408846	0.943702351	0.966779561
ATP2A1	0.96668156	0.883945911	0.968679299	0.999981514	0.94754975	0.969754487
ANP32B	0.96668156	0.905550751	0.927508464	0.973075232	0.988049878	0.982908937
MCF2	0.96668156	0.940956417	0.946791586	0.961898258	0.940870936	0.99828741
SMARCA4	0.966682925	0.925557011	0.966700703	0.999981514	0.943702351	0.945480515
LOC101907138	0.967289672	0.915269318	0.976883418	0.962350885	0.95721434	0.982908937
SMARCD3	0.967300448	0.895777642	0.966293373	0.950643928	0.984866432	0.979888141
PAFAH2	0.967387597	0.946859049	0.953572282	0.999981514	0.943734737	0.9221477
EXOSC7	0.967620694	0.89968376	0.992324476	0.999021176	0.954071324	0.944973383
LOC784841	0.967657126	0.940711772	0.965819476	0.999981514	0.965676623	0.919993751
PPP2R5A	0.967657126	0.92924712	0.97998887	0.999981514	0.937463101	0.920222784
FZD8	0.967657126	0.916484772	0.923521499	0.999981514	0.996781978	0.933762041
UBALD2	0.967657126	0.941483684	0.943836178	0.999981514	0.943702351	0.93531275
XPA	0.967657126	0.912670574	0.946791586	0.999981514	0.960419102	0.938894901
S1PR1	0.967657126	0.898726299	0.95940506	0.999981514	0.97077135	0.950037116
MYO19	0.967657126	0.918455779	0.989977673	0.980564765	0.960573822	0.95524009
UBE2R2	0.967657126	0.882102283	0.94565648	0.999981514	0.972160227	0.958699521
CYP7B1	0.967657126	0.880565292	0.93190909	0.999981514	0.956653075	0.988796537
LOC100337293	0.967657126	0.930266314	0.935266153	0.98471945	0.958797242	0.988796537
LPAR5	0.967657126	0.922825452	0.955427429	0.97731816	0.936921784	0.997383134
LOC112441659	0.967657126	0.962037123	0.936203646	0.94851252	0.946935016	0.99841577
ASB9	0.967657126	0.896281821	0.922552585	0.979385838	0.939208227	0.999948228
MSI1	0.967888295	0.886924072	0.955427429	0.999981514	0.964833865	0.944927301
GOSR1	0.967916774	0.953042388	0.927635169	0.999981514	0.964301327	0.917930485
LOC100462699	0.968040271	0.883756544	0.943237386	0.96951401	0.999985752	0.957428727
RPAP3	0.968229503	0.936822825	0.993049856	0.962046395	0.976292433	0.929640982
KIF9	0.968760109	0.95297524	0.930736996	0.963684916	0.989334846	0.961107601
NOTCH2	0.968760109	0.98098403	0.921266625	0.959652624	0.968101305	0.972980807
CDK13	0.968863645	0.887479443	0.958410013	0.955466436	0.999985752	0.923807852
ADRA1A	0.968863645	0.941817736	0.999993442	0.948050281	0.958797242	0.950074649
LOC112447727	0.968931931	0.889387648	0.939948201	0.984548238	0.972432486	0.992190617
CBLN3	0.969022093	0.999091529	0.949855095	0.966950568	0.943963542	0.930710695
SYNPR	0.969128542	0.991673407	0.985444929	0.944563237	0.964963602	0.91373422
ZSCAN20	0.969128542	0.912670574	0.999993442	0.96444269	0.977016274	0.916909662
ENG	0.969128542	0.885519885	0.982195333	0.978503042	0.999985752	0.917930485
CLEC7A	0.969128542	0.912693158	0.951521942	0.999981514	0.970988226	0.923092436
TAF11	0.969128542	0.879150176	0.999993442	0.97731816	0.959565914	0.923547711

LOC112449086	0.969128542	0.912627552	0.942357321	0.978503042	0.999985752	0.924939539
FOXO1	0.969128542	0.906064108	0.925320071	0.999981514	0.996781978	0.927426708
MRPL9	0.969128542	0.925557011	0.979494108	0.975346638	0.986003146	0.933224627
PRKN	0.969128542	0.992144201	0.944296942	0.984548238	0.954071324	0.93363377
PMEL	0.969128542	0.972948584	0.990037865	0.961898258	0.956904152	0.93363377
ALKAL2	0.969128542	0.88008538	0.971053208	0.999981514	0.944583036	0.933897626
LOC112445888	0.969128542	0.958424951	0.941230744	0.999981514	0.965676623	0.940017687
IBTK	0.969128542	0.933100216	0.989720033	0.995312373	0.948856947	0.942208298
LOC101906067	0.969128542	0.919936324	0.999993442	0.948668643	0.954412818	0.944973383
ABRAXAS1	0.969128542	0.905550751	0.999993442	0.973373329	0.946849735	0.947838082
EPG5	0.969128542	0.972316453	0.990615319	0.949468104	0.956041014	0.947838082
MARCH9	0.969128542	0.915034433	0.935266153	0.999981514	0.967116582	0.952663521
MATN2	0.969128542	0.878809061	0.981376717	0.999981514	0.965163023	0.953140004
LOC112443223	0.969128542	0.91287376	0.948129084	0.95901497	0.999985752	0.960023723
SEMA3B	0.969128542	0.969051025	0.97923564	0.961831293	0.941800786	0.965428409
FAM76B	0.969128542	0.973321075	0.937869586	0.952247001	0.964301327	0.98140846
TRPC4AP	0.969128542	0.901984573	0.996224523	0.948668643	0.953300742	0.988639786
EXOSC3	0.969128542	0.886746033	0.95322206	0.990955345	0.953300742	0.996335668
RNMT	0.969128542	0.906658524	0.935117456	0.98981682	0.956906331	0.99666605
CCDC85A	0.969128542	0.936673875	0.924855847	0.969837124	0.956906331	0.999463809
TESK1	0.969128542	0.896812213	0.92323046	0.984423083	0.941800786	0.999948228
TPRKB	0.969128542	0.92546629	0.943836178	0.957579201	0.942786245	0.999948228
LOC107132534	0.969128542	0.905550751	0.942357321	0.947574232	0.953300742	0.999948228
DR1	0.969128542	0.913120359	0.923578245	0.965724803	0.956041014	0.999948228
LOC112448075	0.969296939	0.888322912	0.999993442	0.964521432	0.943610727	0.928976746
LOC104971845	0.969306958	0.915269318	0.999993442	0.957132838	0.993010827	0.914207441
LOC617648	0.969306958	0.967583778	0.972897558	0.9819628	0.975921255	0.915303189
GPR160	0.969306958	0.904754143	0.93475587	0.999981514	0.979759416	0.919124527
H2AFJ	0.969306958	0.881706092	0.935266153	0.999981514	0.999985752	0.923092436
CLSTN2	0.969306958	0.885906727	0.999993442	0.95901497	0.943160494	0.92789871
NPR3	0.969306958	0.953841555	0.996769243	0.978404889	0.943702351	0.933897626
POLI	0.969306958	0.951112641	0.964661607	0.988408846	0.964301327	0.944973383
PIP4K2C	0.969306958	0.955204917	0.924855847	0.999981514	0.943540106	0.951081819
VCP	0.969306958	0.893615781	0.997400824	0.98981682	0.952264073	0.957428727
LOC112442292	0.969306958	0.954237721	0.957052938	0.965050971	0.976292433	0.958281344
SKIL	0.969306958	0.968509005	0.938212761	0.984423083	0.964301327	0.96248183
HPS4	0.969306958	0.933545282	0.935266153	0.999981514	0.956041014	0.963456717
ZNF189	0.969306958	0.953042388	0.955427429	0.949565453	0.982538884	0.966850975

MBD4	0.969306958	0.932739256	0.927635169	0.985118339	0.97234838	0.9803583
MYOC	0.969306958	0.964925317	0.928927123	0.961648576	0.964301327	0.98469083
NUP205	0.969306958	0.928519855	0.942357321	0.980181029	0.959658682	0.992568593
FBRSL1	0.969306958	0.921825868	0.93891936	0.997383273	0.943055455	0.994788615
LOC100848766	0.969306958	0.884128797	0.93475587	0.955466436	0.986003146	0.999729224
LOC112443509	0.969363346	0.968509005	0.951392011	0.998767332	0.965676623	0.923807852
FEZ1	0.969509827	0.993481224	0.92669814	0.999981514	0.954071324	0.920222784
GATA2	0.969554722	0.94502659	0.999993442	0.964521432	0.954403133	0.93531275
GRO1	0.969554722	0.916448625	0.942357321	0.999981514	0.976259416	0.944973383
C22H3orf49	0.969554722	0.912670574	0.945958356	0.999981514	0.977167046	0.946760037
AFF3	0.969554722	0.909954806	0.947155158	0.950643928	0.965405395	0.999948228
LOC100850437	0.96955636	0.968590259	0.94422142	0.999981514	0.939208227	0.923092436
ABCG4	0.96955636	0.912627552	0.999993442	0.955954309	0.956904152	0.966850975
GPS2	0.969562887	0.91078662	0.979494108	0.955466436	0.999985752	0.924248476
PKN3	0.969573698	0.890176137	0.99520354	0.999981514	0.939442711	0.934071377
HNRNPU	0.969621893	0.938087741	0.97251482	0.961499817	0.988881329	0.944973383
TEDC2	0.969623127	0.894702415	0.999353029	0.987659016	0.972047845	0.927633316
TEX30	0.969726187	0.895395302	0.937281477	0.952247001	0.999985752	0.917024468
LGMN	0.969726187	0.951172741	0.964807927	0.999981514	0.950085378	0.919124527
DDX20	0.969726187	0.988444882	0.955427429	0.947313672	0.995054863	0.919124527
RUBCN	0.969726187	0.999987083	0.962232862	0.947574232	0.944839728	0.923092436
CFAP126	0.969726187	0.962037123	0.972806636	0.990955345	0.964301327	0.923092436
ACBD6	0.969726187	0.893615781	0.937993336	0.995565324	0.999985752	0.923807852
SCO2	0.969726187	0.948801019	0.952862977	0.961898258	0.999985752	0.927268011
HDHD2	0.969726187	0.927853707	0.964807927	0.973812087	0.998287238	0.93363377
LOC785503	0.969726187	0.890669166	0.978014482	0.994231354	0.986003146	0.936983348
PHETA2	0.969726187	0.959478257	0.935266153	0.999981514	0.956761712	0.940942561
FBXO7	0.969726187	0.993481224	0.927635169	0.972699051	0.965676623	0.944927301
ABHD14A	0.969726187	0.96365708	0.976295638	0.955466436	0.974738415	0.945854375
THRB	0.969726187	0.918854612	0.951028911	0.9880671	0.991481886	0.951342758
PRTFDC1	0.969726187	0.890669166	0.937334712	0.98981682	0.999859158	0.960023723
LOC104972724	0.969726187	0.896512504	0.99341711	0.951193444	0.981652321	0.966850975
B4GALNT1	0.969726187	0.938701207	0.925509132	0.990955345	0.956761712	0.988796537
LOC100847326	0.969726187	0.905550751	0.941360985	0.998529748	0.958797242	0.989184798
SRL	0.969726187	0.955536844	0.955427429	0.94714048	0.954814306	0.995895432
TCHP	0.969726187	0.91475257	0.927635169	0.958103924	0.983884995	0.997862553
LOC112443859	0.969732458	0.899293191	0.966232793	0.999981514	0.976259416	0.933897626
NTNG2	0.969732458	0.984410135	0.943836178	0.980532429	0.967116582	0.940079339

ZHX3	0.969732458	0.917024872	0.999993442	0.948590079	0.970988226	0.945918286
LOC510193	0.969732458	0.923193439	0.971594714	0.949565453	0.996781978	0.960023723
MAP1LC3B	0.969732458	0.89465829	0.948653258	0.994395954	0.976259416	0.975558533
TMEM234	0.969732458	0.885494685	0.926234868	0.982220679	0.97352263	0.999463809
CDKN3	0.969885115	0.896281821	0.999993442	0.985435217	0.9683846	0.928976746
SHB	0.969928962	0.944354781	0.989069245	0.98981682	0.970385983	0.91832348
ATP11C	0.969928962	0.933886329	0.937334712	0.979385838	0.999985752	0.923807852
TIGAR	0.970011519	0.912693158	0.999993442	0.953736955	0.965392454	0.933897626
PRR15	0.970011519	0.907054337	0.947651663	0.97731816	0.983941532	0.980148159
TNFRSF19	0.970011519	0.904754143	0.947155158	0.951580624	0.943702351	0.999948228
PXN	0.970428813	0.895777642	0.99341711	0.980564765	0.976259416	0.951123163
REL	0.970563502	0.919540627	0.996747575	0.952247001	0.989334846	0.932534796
RPL22	0.970563502	0.92641214	0.978014482	0.98981682	0.939813331	0.976452727
HMGCR	0.970743218	0.881174371	0.99301092	0.999981514	0.958797242	0.920222784
DPP7	0.970743218	0.986900642	0.929572921	0.999981514	0.953300742	0.923807852
TBC1D20	0.970743218	0.952344105	0.999993442	0.949913449	0.941800786	0.926487302
TTC30A	0.970743218	0.977901258	0.977455101	0.949565453	0.965676623	0.950631909
MELTF	0.970743218	0.924173915	0.996224523	0.959591127	0.964301327	0.960977144
LOC112442262	0.970743218	0.985432041	0.959725886	0.96488029	0.943160494	0.966379194
AMH	0.970743218	0.92522948	0.949939862	0.958103924	0.996781978	0.971700843
CWC27	0.970743218	0.895777642	0.946791586	0.950643928	0.995054863	0.994788615
ZFAND3	0.970763806	0.884272556	0.949855095	0.999981514	0.947003843	0.925057945
CD82	0.970823982	0.927173411	0.955427429	0.999981514	0.953300742	0.934071377
DSEL	0.970871814	0.901028838	0.996224523	0.9745615	0.977167046	0.940583352
NLGN1	0.970871814	0.91078662	0.977455115	0.982220679	0.964301327	0.972695131
AMBRA1	0.97102714	0.928658222	0.991138474	0.958588196	0.984692578	0.944330889
DGCR2	0.971217463	0.967959965	0.957295966	0.999981514	0.956761712	0.923092436
AAR2	0.971217463	0.916484772	0.996224523	0.955466436	0.997422012	0.927426708
LOC505918	0.971217463	0.999987083	0.926161087	0.952247001	0.965869975	0.929831752
SEC22A	0.971217463	0.967486811	0.964807927	0.961898258	0.987723553	0.930710695
LOC112445033	0.971217463	0.929204871	0.94955109	0.953309243	0.999985752	0.960023723
RPL36	0.971217463	0.97969515	0.927508464	0.959652624	0.976292433	0.970071797
THRA	0.971217463	0.933100216	0.971594714	0.960190213	0.976259416	0.970105102
TBC1D23	0.971217463	0.923389147	0.946791586	0.997467114	0.953300742	0.985863239
LOC112446010	0.971217463	0.916484772	0.939912874	0.989485949	0.964301327	0.991416486
BSCL2	0.9712617	0.969051025	0.942357321	0.999981514	0.954071324	0.930946379
SHROOM1	0.9712617	0.96734064	0.997400824	0.948668643	0.953300742	0.951081819
SH2D3A	0.9712617	0.956095821	0.959609498	0.964733972	0.977167046	0.958281344

EVI2A	0.9712617	0.946820717	0.951392011	0.955466436	0.987723553	0.969014913
LOC112442383	0.9712617	0.939442799	0.947155158	0.979644238	0.964301327	0.982937529
SIAH2	0.971343226	0.925801778	0.983504244	0.999981514	0.970988226	0.919124527
TRAPPC10	0.971343226	0.972829016	0.927635169	0.999981514	0.975611541	0.919124527
MMP24OS	0.971343226	0.911593575	0.943836178	0.988437392	0.999985752	0.922361909
KCNK4	0.971343226	0.955522526	0.949939862	0.964521432	0.999985752	0.923064357
GOLGA2	0.971343226	0.928742608	0.99341711	0.999981514	0.945908526	0.924617356
IKBKG	0.971343226	0.942088099	0.927508464	0.959616431	0.999985752	0.927520621
SRC	0.971343226	0.895777642	0.943836178	0.999981514	0.995356208	0.929814744
TRIM45	0.971343226	0.934301396	0.999993442	0.957008595	0.95978143	0.930814839
TRAPPC4	0.971343226	0.983594252	0.982303092	0.964521432	0.959367717	0.931410194
GRB2	0.971343226	0.906064108	0.963821408	0.999981514	0.956906331	0.93983168
PRR36	0.971343226	0.99488888	0.955427429	0.947313672	0.972160227	0.940017687
CMYA5	0.971343226	0.94502659	0.959609498	0.973812087	0.98865576	0.944973383
FGF14	0.971343226	0.968166143	0.989720033	0.95901497	0.963550093	0.946760037
MXRA7	0.971343226	0.988540349	0.945995021	0.978503042	0.959723959	0.950798794
FER1L5	0.971343226	0.910651459	0.996840573	0.987659016	0.96009858	0.951081819
VEGFB	0.971343226	0.943754915	0.99341711	0.982220679	0.948421303	0.952571698
LOC107132924	0.971343226	0.955652503	0.944403177	0.999981514	0.961465747	0.953140004
SGCB	0.971343226	0.911461743	0.999993442	0.982220679	0.946749073	0.960023723
LAS1L	0.971343226	0.952344105	0.953572282	0.973075232	0.979290191	0.960023723
PPM1A	0.971343226	0.887765804	0.93475587	0.999981514	0.943702351	0.960877268
MAP2K5	0.971343226	0.913116251	0.999993442	0.973812087	0.940870936	0.963112102
LOC112442704	0.971343226	0.886565095	0.99341711	0.980564765	0.970988226	0.963456717
CCDC91	0.971343226	0.889345904	0.997850143	0.989601002	0.952264073	0.966071454
RMI2	0.971343226	0.924099465	0.957052938	0.961898258	0.996781978	0.966850975
RNF217	0.971343226	0.96476359	0.957295966	0.948776099	0.976292433	0.969197073
KAT7	0.971343226	0.984089343	0.939353663	0.968176314	0.954403133	0.975056853
SLAIN1	0.971343226	0.904728572	0.96412644	0.976714093	0.970988226	0.985223183
ATXN3	0.971343226	0.916721068	0.990974506	0.965050971	0.947512899	0.987516427
CD80	0.971343226	0.934225172	0.971053208	0.954967866	0.962513981	0.987843572
EIF4G3	0.971343226	0.952344105	0.959725886	0.969837124	0.946935016	0.988639786
SMIM8	0.971343226	0.895777642	0.982041666	0.948668643	0.976259416	0.988910972
SEPT4	0.971343226	0.927853707	0.95875611	0.961898258	0.964301327	0.993890045
LOC788183	0.971343226	0.887598271	0.97251482	0.962437357	0.964301327	0.996813547
LSR	0.971343226	0.890045072	0.943836178	0.969256019	0.965163023	0.999948228
RNF145	0.971353769	0.997678999	0.933884582	0.948720795	0.996781978	0.919124527
POLN	0.971353769	0.893615781	0.946760569	0.97676218	0.999985752	0.919124527

RNASET2	0.971353769	0.914977101	0.978188041	0.999981514	0.962513981	0.922361909
GZF1	0.971353769	0.91287376	0.944126714	0.96951401	0.999985752	0.922543509
RNF114	0.971353769	0.906658524	0.974975876	0.999981514	0.956904152	0.923092436
CPLANE2	0.971353769	0.888322912	0.975774524	0.999981514	0.987723553	0.927520621
LOC100335268	0.971353769	0.998847958	0.945995021	0.976069331	0.958797242	0.927633316
ZNF180	0.971353769	0.987639395	0.95219206	0.999981514	0.943702351	0.930710695
INPP4A	0.971353769	0.980462136	0.982194426	0.982220679	0.954403133	0.930710695
DPCD	0.971353769	0.913116251	0.980761647	0.980564765	0.996781978	0.930710695
KLHL7	0.971353769	0.998847958	0.943836178	0.961898258	0.963253256	0.933897626
CLMP	0.971353769	0.961368616	0.967696262	0.99004392	0.964301327	0.93988265
LOC112443853	0.971353769	0.947350735	0.96183803	0.999981514	0.943702351	0.940017687
VPS11	0.971353769	0.8857401	0.965819476	0.999981514	0.953300742	0.950561417
PRKAG1	0.971353769	0.93659581	0.985156077	0.976196758	0.963550093	0.960023723
NOP58	0.971353769	0.883756544	0.979494108	0.969214881	0.997165254	0.960877268
NCOA7	0.971353769	0.888322912	0.988307089	0.9745615	0.979759416	0.966382321
ARG2	0.971353769	0.927973597	0.95322206	0.999981514	0.95151881	0.969014913
LOC101903988	0.971353769	0.885494685	0.995746693	0.9819628	0.959658682	0.973649216
RAMMET	0.971353769	0.902525869	0.997400824	0.95901497	0.956625221	0.981216824
GPR176	0.971353769	0.899490486	0.941312902	0.989601002	0.982266918	0.982422737
SEC24C	0.971353769	0.885313051	0.999993442	0.948104294	0.943160494	0.985156335
RTKN	0.971353769	0.968186752	0.962734642	0.959616431	0.94542482	0.986042926
VPS50	0.971353769	0.985432041	0.945995021	0.952247001	0.942786245	0.991299214
LOC104975299	0.971353769	0.889387648	0.938011449	0.987187885	0.98227455	0.991847494
NBN	0.971353769	0.920407654	0.957295966	0.973673712	0.953300742	0.997862553
LOC107132532	0.971353769	0.939989548	0.944949386	0.95901497	0.956041014	0.999948228
PRRC2B	0.971353769	0.89465829	0.957295966	0.953404888	0.963253256	0.999948228
NID1	0.971410475	0.994185904	0.939981892	0.948226346	0.989334846	0.93363377
VASH2	0.971410475	0.887265261	0.99647733	0.991955963	0.976292433	0.935497122
LOC104974934	0.971410475	0.927973597	0.966700703	0.966786897	0.947020167	0.997383134
C24H18orf21	0.971466612	0.952863279	0.999993442	0.948776099	0.962166847	0.926341424
LOC522174	0.971568449	0.955652503	0.943836178	0.964521432	0.996217975	0.958790968
PCSK6	0.97160935	0.945598957	0.927635169	0.959277542	0.999985752	0.966136679
LOC100126043	0.971621076	0.957385532	0.95322206	0.999981514	0.950855218	0.959075802
TTPAL	0.971621076	0.934225172	0.996624589	0.95925588	0.946384846	0.976020793
RPSA	0.971621076	0.903618936	0.97998887	0.976399741	0.950958352	0.994788615
NDST3	0.971637632	0.926852499	0.974656086	0.951193444	0.999985752	0.925472172
LOC100299281	0.971637632	0.974846249	0.955427429	0.98981682	0.972128973	0.93148901
PCNX1	0.971637632	0.951172741	0.955427429	0.948478716	0.999985752	0.940583352

CCDC112	0.971637632	0.938087741	0.929176945	0.999981514	0.960252528	0.981216824
FTO	0.971637632	0.916484772	0.991936822	0.964521432	0.947299702	0.988796537
USP37	0.97173176	0.950336973	0.974470955	0.954935279	0.999985752	0.9221477
JMJD4	0.97173176	0.88809849	0.981332474	0.999981514	0.943702351	0.923092436
FAM19A3	0.97173176	0.980462136	0.99568697	0.956176521	0.960419102	0.933224627
FUT4	0.97173176	0.916484772	0.999993442	0.964521432	0.964301327	0.943285784
CLEC18C	0.97173176	0.940618635	0.971053208	0.985118339	0.976292433	0.947937638
ZC2HC1C	0.97173176	0.920882164	0.966232793	0.991955963	0.982087479	0.950631909
ESR1	0.97173176	0.915269318	0.973254083	0.960016921	0.999985752	0.951081819
LOC529930	0.97173176	0.99488888	0.964807927	0.952247001	0.953300742	0.954925542
LOC101906012	0.97173176	0.895777642	0.936203646	0.986051493	0.999985752	0.958645249
ETNK1	0.97173176	0.907836324	0.982195333	0.96968491	0.986003146	0.960251802
LOC101905711	0.97173176	0.905550751	0.95940506	0.989485949	0.988881329	0.96063559
TMC8	0.97173176	0.915269318	0.93190909	0.957008595	0.999985752	0.960877268
MARCH7	0.97173176	0.92565465	0.944126714	0.999981514	0.966967496	0.963015949
FAM166A	0.97173176	0.941400719	0.951028911	0.960937718	0.976259416	0.985564959
E2F5	0.97173176	0.979272895	0.947651663	0.952247001	0.954023497	0.989493848
INIP	0.97173176	0.927973597	0.961437412	0.952247001	0.961465747	0.99828741
LOC789192	0.971780166	0.92259022	0.935649184	0.972142185	0.953885477	0.999948228
LOC112446777	0.972116283	0.893615781	0.95160432	0.999981514	0.961465747	0.95645237
PRX	0.97212741	0.927973597	0.993024884	0.949565453	0.974931134	0.970105102
SGTB	0.972290124	0.943712473	0.944126714	0.999981514	0.990904356	0.923807852
ATL1	0.972290124	0.928427136	0.951237862	0.952247001	0.999985752	0.969014913
PSCA	0.972467621	0.886565095	0.990630783	0.999981514	0.94682471	0.973649216
DARS2	0.972473327	0.895768247	0.964807927	0.999981514	0.998800466	0.923547711
LOC112445889	0.972473327	0.885585919	0.994715737	0.990955345	0.959443572	0.96870908
SYT15	0.972473327	0.896890955	0.947567836	0.952247001	0.986642926	0.998458498
LOC786489	0.972480268	0.984410135	0.957052938	0.955466436	0.956653075	0.973643118
LOC100294723	0.972480268	0.931883544	0.958200097	0.978503042	0.960048824	0.988796537
PROSER2	0.972503566	0.938235006	0.999993442	0.97828838	0.956041014	0.9234914
HTRA1	0.972503566	0.958764172	0.953572282	0.999793541	0.976292433	0.930710695
MOB1B	0.972503566	0.939442799	0.999993442	0.958588196	0.959393538	0.93363377
LOC100848177	0.972503566	0.91763262	0.999993442	0.969837124	0.954403133	0.937942868
MUC20	0.972503566	0.953345357	0.987789469	0.996033721	0.944839728	0.944973383
MAPRE2	0.972503566	0.980521483	0.950223118	0.949502614	0.991130873	0.951493662
COL4A4	0.972503566	0.899490486	0.999993442	0.954724745	0.943540106	0.954465286
HCK	0.972503566	0.904728572	0.964807927	0.949864417	0.999985752	0.965705577
SRA1	0.972503566	0.904875256	0.997400824	0.958588196	0.966967496	0.973218068

RPL38	0.972503566	0.916484772	0.94422142	0.993684984	0.976292433	0.978026735
LOC107132300	0.972503566	0.905550751	0.942357321	0.984423083	0.977356551	0.989840954
LOC781059	0.97269896	0.895777642	0.999993442	0.972467586	0.947020167	0.923944552
TMEM42	0.97269896	0.922610146	0.990615319	0.999981514	0.961465747	0.927520621
KEAP1	0.97269896	0.948801019	0.976484937	0.960663647	0.996089557	0.938034451
TBC1D19	0.97269896	0.935001231	0.978014482	0.999981514	0.947020167	0.940079339
BLMH	0.97269896	0.90715891	0.999993442	0.959591127	0.958797242	0.971236348
WIPI1	0.972938175	0.991673407	0.978014482	0.979385838	0.958797242	0.919993751
FEM1B	0.972938175	0.99528564	0.943028511	0.969837124	0.983941532	0.922361909
LOC101904840	0.972938175	0.886565095	0.999993442	0.999981514	0.944839728	0.923092436
CDC23	0.972938175	0.928427136	0.944126714	0.999981514	0.97464569	0.925057945
ZBTB12	0.972938175	0.885887367	0.971780659	0.999981514	0.976292433	0.927021567
TUSC2	0.972938175	0.930266314	0.999993442	0.972142185	0.953300742	0.930710695
LOC101907606	0.972938175	0.899490486	0.949939862	0.999981514	0.999985752	0.930710695
TGFBR2	0.972938175	0.905550751	0.997400824	0.965050971	0.996781978	0.931410194
MME	0.972938175	0.946558826	0.975774524	0.999981514	0.961465747	0.933897626
ASXL1	0.972938175	0.916141416	0.999993442	0.988493925	0.958797242	0.936951685
LOC107133209	0.972938175	0.924917071	0.986967706	0.979385838	0.987723553	0.940079339
CLEC9A	0.972938175	0.927973597	0.971053208	0.999981514	0.965405395	0.940583352
NCDN	0.972938175	0.972287585	0.957052938	0.999981514	0.943702351	0.945480515
RALY	0.972938175	0.922213189	0.94422142	0.9880671	0.999985752	0.950561417
MAP3K11	0.972938175	0.90116376	0.946760569	0.999981514	0.976292433	0.952571698
KLHL6	0.972938175	0.943213236	0.989494193	0.976756543	0.9683846	0.952663521
RBM12B	0.972938175	0.961663204	0.939912874	0.9745615	0.992326084	0.958790968
SNX20	0.972938175	0.906658524	0.999993442	0.953309243	0.967470055	0.960023723
AP1G1	0.972938175	0.918854612	0.99341711	0.973075232	0.970988226	0.960023723
LAT2	0.972938175	0.890669166	0.990974506	0.9880671	0.976259416	0.963112102
HARS	0.972938175	0.957494685	0.99647733	0.959616431	0.943702351	0.966800477
LOC104968873	0.972938175	0.973189491	0.941507802	0.995565324	0.953300742	0.970071797
LOC101902428	0.972938175	0.942546802	0.937281477	0.952247001	0.999985752	0.970105102
PHC2	0.972938175	0.899494273	0.96412644	0.972142185	0.996781978	0.970555603
ARHGAP6	0.972938175	0.988444882	0.957013563	0.959616431	0.950958352	0.973649216
RNF166	0.972938175	0.920217996	0.975774524	0.990036762	0.956041014	0.978445076
SLC11A2	0.972938175	0.950336973	0.99341711	0.952247001	0.947020167	0.980009318
LOC107132697	0.972938175	0.893056502	0.991936822	0.96951401	0.97130055	0.980167535
PCDH20	0.972938175	0.913899522	0.959183993	0.96951401	0.984386856	0.98140846
EXTL3	0.972938175	0.89417747	0.961437412	0.972142185	0.983941532	0.988273129
LOC112444281	0.972938175	0.901130244	0.964762618	0.992930508	0.956906331	0.991140568

LOC534630	0.972938175	0.915269318	0.983504244	0.954343717	0.964235922	0.993890045
COLEC10	0.972938175	0.886623149	0.937993336	0.999981514	0.964301327	0.995525297
CLEC3B	0.972938175	0.894014677	0.997400824	0.950643928	0.947313295	0.997383134
ZBTB42	0.972938175	0.941817736	0.955427429	0.949864417	0.964301327	0.99828741
GNB5	0.972938175	0.89489192	0.962232862	0.96582208	0.943702351	0.999948228
LOC101905951	0.973110684	0.918455779	0.999993442	0.972142185	0.970491451	0.923092436
MTHFSD	0.973110684	0.997471998	0.954020369	0.960937718	0.974738415	0.927517509
IGF2	0.973110684	0.966057895	0.974967552	0.961898258	0.988010662	0.933903265
MPP1	0.973110684	0.887694214	0.963821408	0.999981514	0.959565914	0.940017687
LOC112446018	0.973110684	0.936319037	0.939913423	0.950643928	0.999985752	0.946347861
CRABP1	0.973110684	0.888322912	0.959725886	0.999981514	0.953300742	0.951081819
CPSF7	0.973110684	0.891384768	0.942357321	0.980564765	0.999985752	0.960023723
SLC37A2	0.973110684	0.942159543	0.970873534	0.969214881	0.982203967	0.961968919
SIK3	0.973110684	0.913120359	0.949939862	0.999981514	0.97077135	0.966850975
TMEM43	0.973110684	0.940190923	0.964807927	0.961898258	0.97058792	0.985624998
TUBA4A	0.973110684	0.967034954	0.95219206	0.957132838	0.956653075	0.994788615
NUP93	0.973110684	0.921973207	0.957052938	0.961898258	0.943702351	0.999948228
MLLT10	0.973143563	0.931883544	0.980032241	0.999981514	0.950958352	0.962872722
LOC101903056	0.973157434	0.957352756	0.984886031	0.999981514	0.943702351	0.923092436
ATRX	0.973157434	0.931610273	0.93475587	0.972142185	0.999985752	0.923092436
SPN	0.973157434	0.91287376	0.999993442	0.984548238	0.959251971	0.923547711
LOC101905357	0.973157434	0.950336973	0.960945531	0.995565324	0.987723553	0.926487302
MED15	0.973157434	0.910651459	0.999993442	0.973075232	0.961465747	0.927426708
MAPKAPK2	0.973157434	0.891111357	0.992511421	0.999981514	0.976292433	0.92789871
CRB1	0.973157434	0.929409572	0.999993442	0.950643928	0.962558935	0.930146713
CKS1B	0.973157434	0.887455957	0.975774524	0.999981514	0.996781978	0.932688327
ASH1L	0.973157434	0.95128993	0.971996404	0.950643928	0.999985752	0.932688327
DCTN2	0.973157434	0.922923898	0.971053208	0.999981514	0.953885477	0.934071377
XKR6	0.973157434	0.999987083	0.939912874	0.959652624	0.95721434	0.934671838
LOC112442623	0.973157434	0.913116251	0.929572921	0.999981514	0.996781978	0.935497122
BOD1	0.973157434	0.905108627	0.954020369	0.999981514	0.995590117	0.93881025
LOC784251	0.973157434	0.968509005	0.975774524	0.984421126	0.9683846	0.940017687
GPAA1	0.973157434	0.981952108	0.950185097	0.961353555	0.98865576	0.942208298
AURKA	0.973157434	0.952209524	0.971053208	0.999981514	0.943702351	0.943194375
TMTC1	0.973157434	0.927369042	0.979494108	0.999981514	0.943702351	0.944927301
COMTD1	0.973157434	0.890669166	0.999993442	0.968103864	0.986861597	0.945480515
ABCB10	0.973157434	0.954187594	0.947155158	0.995152316	0.983884995	0.947801358
LMF2	0.973157434	0.915269318	0.997850143	0.999981514	0.943702351	0.952571698

BCR	0.973157434	0.926931466	0.998848708	0.979644238	0.956041014	0.960023723
WDR45B	0.973157434	0.898951086	0.980761647	0.999981514	0.958384396	0.960023723
SPC25	0.973157434	0.96299056	0.99341711	0.961898258	0.954071324	0.963107047
ZCWPW1	0.973157434	0.950986475	0.930736996	0.999981514	0.958797242	0.963456717
ESRRG	0.973157434	0.936296621	0.96434562	0.960016921	0.983941532	0.976601373
LOC112449516	0.973157434	0.940500178	0.983504244	0.970039043	0.955741702	0.9803583
SLC27A5	0.973157434	0.959263706	0.966232793	0.963132425	0.960190226	0.981216824
CNKSR2	0.973157434	0.892252816	0.96030829	0.999981514	0.956041014	0.987565013
COLGALT2	0.973157434	0.940618635	0.949855095	0.980564765	0.960419102	0.993890045
FAM222B	0.973157434	0.941817736	0.937128793	0.969214881	0.965676623	0.99828741
LOC100296627	0.973278815	0.944261937	0.961800035	0.955466436	0.964833865	0.994347099
CNIH4	0.973366826	0.901130244	0.929661805	0.955466436	0.999985752	0.994991252
NOL9	0.973425872	0.937448751	0.999993442	0.952247001	0.947512899	0.971742751
RMND5B	0.973464707	0.983594252	0.964661607	0.991955963	0.965163023	0.925125664
LOC107131772	0.973464707	0.974792832	0.999993442	0.962437357	0.948421303	0.925233445
LOC112442538	0.973464707	0.991673407	0.978014482	0.984423083	0.953300742	0.927426708
MXRA8	0.973464707	0.993481224	0.941312902	0.999981514	0.943702351	0.927897089
OSGIN1	0.973464707	0.952344105	0.957052938	0.999981514	0.958852623	0.927900405
LOC107133268	0.973464707	0.938087741	0.94565648	0.999981514	0.959658682	0.930710695
OTUD3	0.973464707	0.930293015	0.964807927	0.999981514	0.964301327	0.938894901
ATXN7	0.973464707	0.994185904	0.972806636	0.952247001	0.965392454	0.938894901
TGOLN2	0.973464707	0.918854612	0.998364815	0.984548238	0.970988226	0.940017687
PSMG3	0.973464707	0.973712113	0.983504244	0.961241178	0.976259416	0.940583352
SGCE	0.973464707	0.991673407	0.947155158	0.961898258	0.982538884	0.942105787
GNL2	0.973464707	0.918854612	0.981849526	0.999981514	0.97352263	0.944973383
EML2	0.973464707	0.940956417	0.968233314	0.972142185	0.996781978	0.951081819
LOC783533	0.973464707	0.896067917	0.968679299	0.999981514	0.964833865	0.960023723
DAXX	0.973464707	0.92546629	0.955427429	0.999981514	0.969087007	0.960464023
COL4A1	0.973464707	0.991673407	0.949855095	0.973812087	0.954403133	0.967056564
AK3	0.973464707	0.899490486	0.964661607	0.995565324	0.977193348	0.970105102
EGFL7	0.973464707	0.938087741	0.964382669	0.961898258	0.987789919	0.970555603
FAM133B	0.973464707	0.908364002	0.939913423	0.998529748	0.991481886	0.970555603
FAM174B	0.973464707	0.888769932	0.97251482	0.999981514	0.965392454	0.978087957
SCNN1A	0.973464707	0.893164557	0.978185847	0.999981514	0.954071324	0.9790177
LOC112441638	0.973464707	0.940956417	0.93190909	0.975346638	0.991481886	0.980009318
RASA3	0.973464707	0.938235006	0.944734215	0.963132425	0.984386856	0.988273129
ETV3	0.973464707	0.921940584	0.939676808	0.974873127	0.987723553	0.989840954
CDH8	0.973464707	0.91763262	0.99341711	0.95901497	0.953300742	0.994347099

NHEJ1	0.973464707	0.930293015	0.971053208	0.950452807	0.963550093	0.99828741
RTL9	0.973464707	0.895777642	0.942357321	0.976196758	0.961465747	0.999948228
HCAR1	0.973516204	0.951763113	0.943836178	0.970725878	0.953300742	0.99976973
TBK1	0.973591695	0.893164557	0.947875514	0.9819628	0.996781978	0.983208118
LOC101903397	0.973690044	0.95310464	0.978014482	0.982220679	0.988049878	0.927426708
CCDC28B	0.973690044	0.927853707	0.999993442	0.965724803	0.959367717	0.930710695
TAF15	0.974102276	0.899490486	0.980761647	0.957132838	0.9683846	0.997862553
SYCP2	0.974102276	0.931842602	0.948129084	0.984548238	0.956904152	0.998240456
LOC107132045	0.974210165	0.905297247	0.999993442	0.959652624	0.983941532	0.953509765
LOC100847831	0.974210165	0.918854612	0.93190909	0.961898258	0.999985752	0.984509067
CROT	0.974453565	0.953449552	0.942357321	0.999981514	0.989334846	0.923807852
CARM1	0.974453565	0.928427136	0.944949386	0.999981514	0.984386856	0.927426708
LOC100139144	0.974453565	0.934225172	0.988120522	0.989707094	0.983941532	0.930710695
KHDRBS3	0.974453565	0.889387648	0.949855095	0.991955963	0.999985752	0.930710695
DAG1	0.974453565	0.912622732	0.968043317	0.950643928	0.999985752	0.931414945
WSCD1	0.974453565	0.916484772	0.949939862	0.995565324	0.999985752	0.935421237
LOC104969340	0.974453565	0.898208229	0.944126714	0.999981514	0.955049621	0.935497122
AGO3	0.974453565	0.937448751	0.937869586	0.952247001	0.999985752	0.940423784
MAGI2	0.974453565	0.949323635	0.956423184	0.999981514	0.979290191	0.943621425
LOC516849	0.974453565	0.96643574	0.984886031	0.996195248	0.947045868	0.944973383
SULF2	0.974453565	0.95687851	0.93475587	0.999981514	0.964301327	0.946760037
FBXO34	0.974453565	0.912670574	0.999993442	0.952247001	0.959367717	0.959792861
ELK3	0.974520039	0.893164557	0.950994492	0.999981514	0.962682426	0.931410194
FRG1	0.974520039	0.937243624	0.969404007	0.997792939	0.970988226	0.958790968
KLF14	0.974780123	0.913116251	0.957052938	0.97731816	0.976292433	0.991847494
SP2	0.974941407	0.910560869	0.955427429	0.999981514	0.952264073	0.996335668
COX4I2	0.974982669	0.988444882	0.964661607	0.999981514	0.953300742	0.92789871
SIK2	0.975013999	0.965482645	0.944126714	0.999981514	0.945710873	0.935770262
ITM2C	0.975021177	0.951949541	0.964807927	0.999981514	0.965676623	0.93363377
PNMA2	0.975021177	0.927853707	0.947155158	0.999981514	0.983941532	0.948705062
ELMSAN1	0.975021177	0.978518618	0.954020369	0.952247001	0.970988226	0.979480605
BAG4	0.975080348	0.904754143	0.999993442	0.999981514	0.960345165	0.927426708
LOC616094	0.975080348	0.913120359	0.95219206	0.999981514	0.976292433	0.9374034
IRX5	0.975080348	0.915269318	0.943836178	0.999981514	0.976292433	0.946112759
LOC788724	0.975080348	0.896890955	0.988120522	0.970039043	0.99971266	0.954550352
FABP2	0.975080348	0.967340733	0.964807927	0.956685049	0.976292433	0.970105102
URB1	0.97521315	0.895777642	0.955427429	0.999981514	0.953300742	0.971545917
ARPC1A	0.975260898	0.918854612	0.982875453	0.974941201	0.961465747	0.987785166

MFSD5	0.975310164	0.89915699	0.957052938	0.999981514	0.964301327	0.973218068
ABHD8	0.975330034	0.960747241	0.94796441	0.999981514	0.996089557	0.923199665
TIAL1	0.975330034	0.95577784	0.999993442	0.960016921	0.946935016	0.930206677
LOC107132952	0.975330034	0.934225172	0.988660944	0.979385838	0.972160227	0.960781671
PTDSS1	0.975330034	0.926051993	0.949855095	0.999981514	0.947346268	0.966779561
DCLRE1A	0.975359039	0.99960614	0.955189235	0.969837124	0.961465747	0.930131506
ITPKB	0.975359039	0.949323635	0.949855095	0.999981514	0.989379051	0.93531275
LOC101903853	0.975359039	0.892148187	0.995746693	0.982220679	0.998040182	0.936983348
AGL	0.975359039	0.978454122	0.984886031	0.965050971	0.964301327	0.951081819
USHBP1	0.975362625	0.929204871	0.962034224	0.999981514	0.976259416	0.925432855
TNKS	0.975362625	0.992144201	0.950223118	0.95901497	0.996781978	0.92789871
XPNPEP3	0.975362625	0.933886329	0.949855095	0.952247001	0.999985752	0.933596357
ADAM15	0.975362625	0.89417747	0.950223118	0.999981514	0.999985752	0.935770262
RHBDD2	0.975362625	0.938087741	0.999993442	0.976125636	0.952796169	0.936263602
ISLR	0.975362625	0.999987083	0.948904459	0.965050971	0.954023497	0.936263602
CAMTA2	0.975362625	0.929204871	0.996224523	0.999981514	0.953300742	0.945480515
LOC101902809	0.975362625	0.901984573	0.974656086	0.999981514	0.959565914	0.946112759
ARHGAP12	0.975362625	0.988444882	0.95940506	0.965578567	0.972432486	0.951951972
LGALSL	0.975362625	0.913445628	0.986511454	0.999981514	0.959565914	0.953140004
NFIX	0.975362625	0.927973597	0.957295966	0.999981514	0.977167046	0.958790968
LOC112446708	0.975362625	0.915269318	0.955427429	0.973075232	0.999985752	0.959275214
PLEKHG3	0.975362625	0.913120359	0.957052938	0.989601002	0.996089557	0.966659342
LOC107132247	0.975362625	0.95054234	0.996224523	0.952443827	0.963550093	0.969197073
TNRC6A	0.975362625	0.89833876	0.94796441	0.999981514	0.960048824	0.972334437
STX10	0.975362625	0.922610146	0.93891936	0.995312373	0.976292433	0.987785166
LOC112449115	0.975362625	0.921382572	0.943836178	0.973373329	0.976769047	0.997862553
RCOR3	0.975362625	0.938087741	0.949855095	0.986642278	0.953300742	0.99828741
LEPROT	0.975415174	0.973712113	0.992511421	0.973993549	0.976292433	0.923807852
MGMT	0.975415174	0.926905849	0.944126714	0.999981514	0.999985752	0.923807852
LOC112447462	0.975415174	0.906684077	0.948594902	0.999981514	0.986642926	0.923878545
LOC781913	0.975415174	0.993203157	0.979494108	0.959616431	0.976292433	0.923944552
GPIHBP1	0.975415174	0.980833065	0.951392011	0.980564765	0.996781978	0.925704954
TMEM246	0.975415174	0.999987083	0.955484273	0.952247001	0.949998733	0.927426708
DMWD	0.975415174	0.933100216	0.972073392	0.952247001	0.999985752	0.927426708
KIAA1468	0.975415174	0.972496661	0.937397154	0.975653996	0.999985752	0.927426708
LOC112446796	0.975415174	0.919783389	0.95219206	0.999981514	0.965676623	0.927517509
LIX1L	0.975415174	0.999987083	0.947155158	0.965729104	0.953300742	0.927520621
OSGEPL1	0.975415174	0.89417747	0.999993442	0.959159289	0.977167046	0.927520621

PUM1	0.975415174	0.928427136	0.958410013	0.952247001	0.999985752	0.927520621
COPS7B	0.975415174	0.901631302	0.999993442	0.961898258	0.982203967	0.930146713
TMEM170B	0.975415174	0.910044299	0.999993442	0.999981514	0.953300742	0.930494708
CD44	0.975415174	0.986098932	0.944126714	0.999981514	0.947299702	0.930659059
ISG20	0.975415174	0.902525869	0.999993442	0.959591127	0.999985752	0.930710695
PLEC	0.975415174	0.906064108	0.999993442	0.989707094	0.971133034	0.93129461
FAM120C	0.975415174	0.947350735	0.999993442	0.977888476	0.953300742	0.933903265
LOC112443130	0.975415174	0.930092771	0.999993442	0.976756543	0.970988226	0.936468627
ASCL4	0.975415174	0.901028838	0.972741989	0.952247001	0.999985752	0.937715964
LOC506181	0.975415174	0.942159543	0.972806636	0.999981514	0.959443572	0.938013912
LOC107132796	0.975415174	0.946258461	0.993024884	0.999981514	0.956653075	0.938894901
RNF25	0.975415174	0.968509005	0.992263335	0.952247001	0.983941532	0.939127532
ANKRD29	0.975415174	0.943244588	0.972806636	0.990364645	0.987723553	0.940017687
LOC112445041	0.975415174	0.941895237	0.972806636	0.980564765	0.997165254	0.940583352
CNOT6L	0.975415174	0.939442799	0.958554319	0.962369147	0.999985752	0.940583352
DNAH17	0.975415174	0.904754143	0.94955109	0.999981514	0.970980375	0.946760037
LOC528802	0.975415174	0.892108342	0.959725886	0.999981514	0.983941532	0.948705062
CD180	0.975415174	0.969051025	0.997850143	0.97828838	0.947003843	0.950074649
A4GALT	0.975415174	0.986900642	0.943028511	0.988408846	0.97352263	0.951340373
TUSC3	0.975415174	0.895395302	0.984886031	0.999981514	0.965163023	0.951342758
CASP1	0.975415174	0.99831878	0.942357321	0.987389559	0.955623649	0.952563377
HRH2	0.975415174	0.912670574	0.983582726	0.999981514	0.956906331	0.952563377
MTHFR	0.975415174	0.931254913	0.971053208	0.999981514	0.976259416	0.952663521
CANT1	0.975415174	0.952664179	0.997400824	0.973075232	0.960573822	0.954925542
SMOX	0.975415174	0.906133257	0.964771309	0.971356031	0.999985752	0.955990509
FZD9	0.975415174	0.941895237	0.999993442	0.965724803	0.960419102	0.95883396
RWDD2B	0.975415174	0.89915699	0.999993442	0.984548238	0.965676623	0.95883396
LOC107131848	0.975415174	0.950336973	0.997300234	0.982220679	0.954071324	0.959198304
LOC112443225	0.975415174	0.901275501	0.943028511	0.999981514	0.970988226	0.960023723
FSD1L	0.975415174	0.911461743	0.991936822	0.999981514	0.959658682	0.963456717
AFMID	0.975415174	0.969051025	0.949855095	0.986116445	0.972499705	0.966196012
TPST2	0.975415174	0.918854612	0.999993442	0.970725878	0.962513981	0.969014913
SUPT20H	0.975415174	0.918447234	0.996224523	0.989485949	0.954403133	0.970105102
WDR81	0.975415174	0.893164557	0.99647733	0.965729104	0.983941532	0.970105102
KRT18	0.975415174	0.921940584	0.963552292	0.976855823	0.996781978	0.970105102
TNPO2	0.975415174	0.929204871	0.999993442	0.959591127	0.958384396	0.9729325
LOC112445952	0.975415174	0.934225172	0.964382669	0.999981514	0.960573822	0.973413452
ZNF226	0.975415174	0.938087741	0.939981892	0.990036762	0.983941532	0.976124527

PIGB	0.975415174	0.933100216	0.97773026	0.989601002	0.960573822	0.977503497
CYBC1	0.975415174	0.989144081	0.940956064	0.96444269	0.964301327	0.9790177
MTREX	0.975415174	0.977769595	0.950223118	0.952247001	0.976292433	0.979480605
LOC104971613	0.975415174	0.913120359	0.943836178	0.999981514	0.969396685	0.980167535
ITIH3	0.975415174	0.96643574	0.939912874	0.962046395	0.984386856	0.980167535
STAT3	0.975415174	0.912627552	0.958197827	0.952247001	0.999985752	0.9803583
SMAGP	0.975415174	0.957961435	0.971255098	0.972142185	0.958797242	0.98140846
DTWD2	0.975415174	0.941817736	0.955427429	0.95901497	0.976292433	0.996335668
LMF1	0.975415174	0.91196842	0.989494193	0.955466436	0.954403133	0.999948228
INTS7	0.975436163	0.956672713	0.992324476	0.976679676	0.987198672	0.924156542
CENPQ	0.975436163	0.90494821	0.997400824	0.966786897	0.989334846	0.95662324
LOC784866	0.975436163	0.989303356	0.937281477	0.998529748	0.957104426	0.960023723
COG3	0.975436163	0.89417747	0.938074902	0.996033721	0.999985752	0.967759699
SPPL3	0.975436163	0.905550751	0.996224523	0.952247001	0.970988226	0.989309315
ZNF419	0.975925675	0.977196455	0.97998887	0.999981514	0.953300742	0.925233445
NSMCE1	0.975925675	0.988008216	0.954020369	0.999981514	0.953300742	0.940079339
GFOD2	0.975925675	0.942088099	0.948594902	0.979644238	0.999985752	0.941413984
LOC100138933	0.975925675	0.894452577	0.957295966	0.999981514	0.961465747	0.943285784
BBC3	0.975925675	0.941817736	0.94422142	0.999981514	0.9683846	0.944973383
ALG8	0.975925675	0.977196455	0.963291208	0.999981514	0.953300742	0.958790968
EMC1	0.975925675	0.936582263	0.955427429	0.992560663	0.991481886	0.959723327
YPEL4	0.975942306	0.918742331	0.968233314	0.973373329	0.999985752	0.927517509
BMPR2	0.975942306	0.901631302	0.978014482	0.999981514	0.970980375	0.927520621
LOC515333	0.975942306	0.957494685	0.989494193	0.997660427	0.970988226	0.927520621
SMYD4	0.975942306	0.938087741	0.992378548	0.989601002	0.987969079	0.927520621
HOOK3	0.975942306	0.906658524	0.971556427	0.967883482	0.999985752	0.927520621
PDLIM1	0.975942306	0.930293015	0.992511421	0.985437486	0.995515529	0.92789871
MDP1	0.975942306	0.918854612	0.95940506	0.999981514	0.963790274	0.927900405
USP3	0.975942306	0.927794672	0.979494108	0.983233117	0.999985752	0.930710695
GPATCH2	0.975942306	0.934225172	0.95940506	0.98656293	0.999985752	0.930710695
TIMMDC1	0.975942306	0.900196378	0.978014482	0.999981514	0.996781978	0.930777372
CIZ1	0.975942306	0.93431906	0.994035957	0.999981514	0.964301327	0.931459545
LOC101906177	0.975942306	0.99488888	0.988307089	0.960016921	0.960573822	0.93363377
POLR3GL	0.975942306	0.945598957	0.987610559	0.999981514	0.970988226	0.93363377
SETBP1	0.975942306	0.991673407	0.949855095	0.988408846	0.976259416	0.933897626
MTURN	0.975942306	0.919245978	0.991447919	0.999981514	0.960419102	0.935967823
LOC107131919	0.975942306	0.927973597	0.999993442	0.961898258	0.989334846	0.937469898
PHRF1	0.975942306	0.981952108	0.999993442	0.961898258	0.956653075	0.937715964

ARRDC4	0.975942306	0.926330259	0.942357321	0.984423083	0.999985752	0.937787793
HTRA4	0.975942306	0.904754143	0.937993336	0.999981514	0.999985752	0.938013912
CLIC1	0.975942306	0.991673407	0.958197827	0.98981682	0.964301327	0.938161998
ZFP36	0.975942306	0.915576843	0.996224523	0.999981514	0.957793213	0.938894901
HERC5	0.975942306	0.997678999	0.959725886	0.969088992	0.965676623	0.939024684
LGALS9	0.975942306	0.993035141	0.99615564	0.959616431	0.954403133	0.940017687
LOC101904769	0.975942306	0.99960614	0.950185097	0.957611026	0.97352263	0.940079339
SPTA1	0.975942306	0.915269318	0.99615564	0.992560663	0.983941532	0.940079339
ACOT11	0.975942306	0.998847958	0.979354564	0.954724745	0.958797242	0.940256832
PTPN21	0.975942306	0.977196455	0.946791586	0.999981514	0.962513981	0.943621425
CACFD1	0.975942306	0.915269318	0.964807927	0.999981514	0.969396685	0.943972369
GAPVD1	0.975942306	0.955652503	0.942357321	0.995434268	0.998008754	0.944973383
SAT1	0.975942306	0.969051025	0.987610559	0.99739416	0.953885477	0.945480515
LOC101907603	0.975942306	0.915269318	0.957052938	0.999981514	0.977167046	0.947838082
TTBK2	0.975942306	0.955981683	0.945995021	0.999981514	0.983941532	0.950631909
IGF2R	0.975942306	0.910651459	0.955427429	0.999981514	0.952796169	0.951734631
BRCA1	0.975942306	0.922004059	0.999993442	0.979385838	0.953300742	0.954496768
SLC35A2	0.975942306	0.927973597	0.999993442	0.972142185	0.954403133	0.957876917
BUD13	0.975942306	0.904754143	0.999993442	0.960016921	0.970988226	0.958707771
LOC518775	0.975942306	0.902241567	0.999993442	0.991955963	0.949858142	0.958790968
LOC112441718	0.975942306	0.991673407	0.960544729	0.986642278	0.950958352	0.960023723
RNF216	0.975942306	0.930092771	0.971053208	0.999981514	0.958797242	0.960023723
HP1BP3	0.975942306	0.954704613	0.997400824	0.958588196	0.970491451	0.960023723
INVS	0.975942306	0.984145514	0.939912874	0.967883482	0.988010662	0.960023723
BRD1	0.975942306	0.899490486	0.998848708	0.963601374	0.990192394	0.960023723
LOC107131403	0.975942306	0.913120359	0.939912874	0.994331516	0.999985752	0.960023723
LSM3	0.975942306	0.924173915	0.997400824	0.997467114	0.952264073	0.96248183
DNAJC15	0.975942306	0.952053757	0.984886031	0.995152316	0.956041014	0.96248183
TMEM98	0.975942306	0.918854612	0.942357321	0.999981514	0.996089557	0.96274079
ORMDL2	0.975942306	0.944354781	0.943237386	0.999005991	0.985238194	0.963145894
FZD6	0.975942306	0.920702629	0.999993442	0.965724803	0.948421303	0.963456717
CAPNS1	0.975942306	0.906658524	0.991138474	0.99313054	0.976259416	0.963456717
LOC112442585	0.975942306	0.94422395	0.970873534	0.982220679	0.977356551	0.965705577
HIST2H2AA4	0.975942306	0.931933841	0.971053208	0.983012396	0.986019537	0.966850975
LOC101907653	0.975942306	0.895777642	0.957052938	0.999981514	0.955741702	0.968138839
CYB561	0.975942306	0.91763262	0.955221146	0.999981514	0.97028586	0.969197073
DGKZ	0.975942306	0.906927139	0.999993442	0.974286747	0.954023497	0.969754487
BCL3	0.975942306	0.937723596	0.957052938	0.999981514	0.953300742	0.970105102

C9H6orf120	0.975942306	0.912670574	0.947952648	0.999981514	0.990192394	0.970492307
C1QC	0.975942306	0.929409572	0.964382669	0.97676218	0.996089557	0.971545917
LOC617141	0.975942306	0.95856975	0.966232793	0.982220679	0.965163023	0.973649216
MARCH3	0.975942306	0.927853707	0.972806636	0.961898258	0.996089557	0.973649216
PPP2R2B	0.975942306	0.971455677	0.961800035	0.98981682	0.948856947	0.975170737
PGPEP1	0.975942306	0.92342846	0.996224523	0.96951401	0.965163023	0.975558533
LOC534913	0.975942306	0.895777642	0.958410013	0.999981514	0.956041014	0.978087957
LOC101904442	0.975942306	0.901326757	0.959903168	0.999981514	0.956904152	0.978087957
ARNT2	0.975942306	0.955652503	0.939948201	0.999981514	0.951889995	0.9790177
TBC1D16	0.975942306	0.940500178	0.943836178	0.996033721	0.976259416	0.981216824
GCN1	0.975942306	0.910520898	0.99869493	0.96951401	0.956672344	0.988866378
ZBTB39	0.975942306	0.957328149	0.975774524	0.961898258	0.955623649	0.991299214
SOX13	0.975942306	0.89465829	0.99341711	0.967883482	0.955623649	0.99828741
ATG9A	0.975942306	0.918234392	0.947155158	0.982220679	0.961465747	0.999948228
SMG8	0.975974431	0.940994886	0.949855095	0.999981514	0.999985752	0.927520621
NYX	0.976073704	0.912764028	0.943237386	0.999981514	0.976292433	0.93531275
SLC35E1	0.976073704	0.895777642	0.988585686	0.999981514	0.962513981	0.970105102
FBXL21	0.976094381	0.959732252	0.944501125	0.999981514	0.988049878	0.940017687
CCDC151	0.976094381	0.923007853	0.999703935	0.98981682	0.966967496	0.9512199
LOC100336734	0.976094381	0.90116376	0.999993442	0.977477147	0.964833865	0.967056564
LOC512165	0.976094381	0.915269318	0.996624589	0.984423083	0.969253072	0.967759699
HNRNPR	0.976094381	0.915511799	0.999993442	0.989601002	0.955623649	0.970071797
CELA1	0.976094381	0.912622732	0.939912874	0.991024671	0.999985752	0.973649216
PPARA	0.976094381	0.955827589	0.974656086	0.969088992	0.968207779	0.980167535
SORBS3	0.976094381	0.905599077	0.957295966	0.959616431	0.988049878	0.999463809
UBQLN4	0.97613139	0.924099465	0.939981892	0.99542943	0.977193348	0.991416486
LOC101902840	0.97621379	0.895777642	0.972741989	0.999981514	0.954403133	0.982908937
TAF5L	0.97621379	0.919245978	0.955221146	0.97828838	0.98865576	0.989084358
MGAT1	0.976317429	0.947911876	0.95219206	0.99045711	0.999985752	0.930946379
JCAD	0.976328121	0.938395301	0.963335792	0.982332902	0.977167046	0.979480605
ZFHX2	0.976356742	0.929409572	0.999993442	0.976196758	0.986019537	0.926487302
NIT1	0.976356742	0.946411824	0.970618773	0.988347114	0.999985752	0.930657508
TMEM52	0.976356742	0.941895237	0.999993442	0.974286747	0.953300742	0.935770262
INO80C	0.976356742	0.973321075	0.999993442	0.967883482	0.959251971	0.940639747
LOC100847236	0.976356742	0.97225074	0.996224523	0.972142185	0.964235922	0.946760037
LOC782202	0.976356742	0.916141416	0.962734642	0.999981514	0.970988226	0.951081819
ISM1	0.976356742	0.904728572	0.992511421	0.973075232	0.988010662	0.970463092
ST3GAL4	0.976356742	0.917462806	0.999993442	0.967883482	0.959658682	0.980840077

LOC112443140	0.976397178	0.915389218	0.945995021	0.989601002	0.999985752	0.926487302
TLN1	0.976397178	0.919540627	0.976484937	0.999981514	0.970988226	0.927268011
CNNM2	0.976397178	0.895777642	0.984886031	0.991955963	0.998939043	0.947245743
GFAP	0.976397178	0.984089343	0.943028511	0.999981514	0.959565914	0.950798794
MTIF3	0.976397178	0.936319037	0.996224523	0.960864406	0.983941532	0.963456717
TSPAN2	0.976397178	0.96989035	0.952862977	0.976221833	0.984386856	0.966379194
WNT11	0.976397178	0.899490486	0.937281477	0.999981514	0.999453276	0.972695131
CNOT2	0.97673799	0.941676269	0.943237386	0.96900924	0.999985752	0.930657508
NENF	0.97673799	0.90656885	0.980032241	0.986051493	0.964301327	0.994788615
LOC100847269	0.976754475	0.924173915	0.93891936	0.999981514	0.953300742	0.93363377
USP31	0.976754475	0.901130244	0.991936822	0.976005921	0.999985752	0.93363377
LOC104974330	0.976754475	0.906658524	0.992410032	0.972142185	0.999985752	0.951081819
EPHX1	0.976754475	0.948801019	0.95322206	0.999981514	0.959151723	0.98140846
CYP39A1	0.976754475	0.902488025	0.949939862	0.999981514	0.96080502	0.999729224
LOC100174924	0.976767143	0.90042817	0.999993442	0.97828838	0.991481886	0.940423784
YAE1D1	0.976767143	0.938674217	0.999993442	0.960016921	0.97352263	0.94844234
CALR3	0.976767143	0.940342068	0.93891936	0.999981514	0.958797242	0.975117753
GGACT	0.97676908	0.918234392	0.955427429	0.995434268	0.999985752	0.927520621
C3H1orf123	0.97676908	0.916484772	0.990118874	0.965462948	0.999985752	0.92789871
PAPLN	0.97676908	0.937448751	0.957052938	0.979644238	0.999985752	0.932534796
LOC789895	0.97676908	0.912693158	0.946791586	0.989601002	0.999985752	0.932688327
CNIH3	0.97676908	0.94592331	0.946791586	0.999981514	0.956653075	0.93363377
FOXRED2	0.97676908	0.901631302	0.982195333	0.989601002	0.999985752	0.941835384
ZNF513	0.97676908	0.994185904	0.979494108	0.955466436	0.970988226	0.943972369
MEA1	0.97676908	0.969051025	0.996224523	0.984240987	0.958797242	0.946347861
AHSP	0.97676908	0.981952108	0.962924758	0.961516541	0.989334846	0.952415156
PRKAR2A	0.97676908	0.930633315	0.975774524	0.999981514	0.95721434	0.960023723
TAP1	0.97676908	0.952344105	0.957295966	0.959652624	0.999985752	0.96248183
LOC107131940	0.97676908	0.943244588	0.938624264	0.989485949	0.999985752	0.963107047
CTSK	0.97676908	0.96025412	0.96327718	0.985437486	0.974738415	0.969197073
PPP1R3E	0.976774116	0.958814007	0.996624589	0.984548238	0.97464569	0.930710695
ACADL	0.976774116	0.981952108	0.972741989	0.985701713	0.977193348	0.931402465
RIC3	0.976774116	0.991882821	0.980851214	0.98981682	0.953300742	0.933897626
LOC508455	0.976774116	0.999987083	0.958200097	0.961898258	0.964301327	0.935770262
TICAM2	0.976774116	0.940342068	0.955427429	0.991955963	0.999985752	0.935770262
ACY1	0.976774116	0.98412146	0.964661607	0.990955345	0.972128973	0.940583352
LOC104976078	0.976774116	0.94592331	0.999993442	0.985748796	0.961465747	0.944973383
EBF2	0.976774116	0.980793805	0.982765283	0.987659016	0.964301327	0.944973383

SUMO3	0.976774116	0.902525869	0.997400824	0.974717682	0.999453276	0.944973383
TOR4A	0.976774116	0.905271052	0.943836178	0.982220679	0.999985752	0.950540248
AGPAT3	0.976774116	0.969051025	0.957052938	0.985118339	0.988049878	0.953359824
CA5B	0.976774116	0.908271963	0.949855095	0.989601002	0.999985752	0.959976849
TRMT1L	0.976774116	0.966661805	0.970873534	0.999981514	0.953300742	0.960023723
LOC101904529	0.976774116	0.95530536	0.968679299	0.96582208	0.996781978	0.960023723
LOC101906364	0.976774116	0.932191409	0.951392011	0.999981514	0.961465747	0.963107047
GSTZ1	0.976774116	0.937549597	0.996224523	0.976714093	0.976259416	0.963456717
LOC789384	0.976774116	0.969051025	0.999993442	0.957365387	0.955623649	0.965645137
LOC107131341	0.976774116	0.92905835	0.939936316	0.999981514	0.976769047	0.969014913
CTSL	0.976774116	0.980521483	0.937993336	0.999981514	0.958832188	0.971545917
ZNF655	0.976774116	0.958632969	0.991138474	0.975366569	0.958797242	0.972695131
ADCY7	0.976774116	0.913899522	0.960945531	0.959616431	0.999985752	0.977503497
LOC104972622	0.976774116	0.927853707	0.955221146	0.999981514	0.970988226	0.9790177
XRCC1	0.976774116	0.916260474	0.999993442	0.961898258	0.953300742	0.979505709
CNOT6	0.976774116	0.926330259	0.954020369	0.999981514	0.968739995	0.988866378
LOC784521	0.976774116	0.94622198	0.97773026	0.985437486	0.953300742	0.990943966
ANKRD61	0.976774116	0.918234392	0.982195333	0.960016921	0.962513981	0.999948228
ZNF132	0.977083128	0.99116226	0.998899782	0.959375021	0.960048824	0.934360589
LOC788467	0.977083128	0.948038876	0.944126714	0.999981514	0.957104426	0.973649216
LOC112444778	0.977083128	0.913120359	0.978014482	0.972142185	0.959565914	0.999948228
TTC23	0.977148984	0.906658524	0.982823002	0.985701713	0.999985752	0.93983168
CHRD	0.977148984	0.916484772	0.999993442	0.975653996	0.984386856	0.940583352
TOP3A	0.977384823	0.938087741	0.986511454	0.979385838	0.954403133	0.992680267
LRSAM1	0.977411466	0.919540627	0.957052938	0.999981514	0.997790653	0.930710695
C28H10orf71	0.977411466	0.94592331	0.941616329	0.999981514	0.970491451	0.937715964
LOC104970103	0.977411466	0.968509005	0.967549444	0.999981514	0.96080502	0.940017687
LMX1A	0.977411466	0.918491096	0.949855095	0.999981514	0.956906331	0.940647189
LOC510362	0.977411466	0.981952108	0.971053208	0.989601002	0.970988226	0.944927301
DMAP1	0.977411466	0.930293015	0.99647733	0.963981839	0.997422012	0.94621529
ERCC8	0.977411466	0.997425219	0.95322206	0.980532429	0.964727486	0.951757326
HNRNPUL2	0.977411466	0.899490486	0.968317646	0.998046286	0.997264208	0.963456717
BTRC	0.977411466	0.90715891	0.944126714	0.999981514	0.956906331	0.971545917
DGAT1	0.977411466	0.980462136	0.973237403	0.95901497	0.970988226	0.973649216
ALDH6A1	0.977411466	0.918854612	0.955427429	0.999981514	0.965163023	0.97521287
LOC101905734	0.977411466	0.976124961	0.942030889	0.95901497	0.988010662	0.98469083
HPRT1	0.977411466	0.966069053	0.964382669	0.985118339	0.953885477	0.988639786
PBDC1	0.977411466	0.950439405	0.955427429	0.973075232	0.976769047	0.989184798

LOC101910094	0.977601342	0.899490486	0.969404007	0.983012396	0.999985752	0.93363377
KLHL26	0.977601342	0.907054337	0.944126714	0.999981514	0.998287238	0.942874931
SMG6	0.977601342	0.929204871	0.943237386	0.984331039	0.954403133	0.999948228
LOC530929	0.977611985	0.942088099	0.973562292	0.999981514	0.953300742	0.952819903
GATC	0.977617181	0.934225172	0.983504244	0.969837124	0.991481886	0.967047449
UBE3D	0.977624943	0.992144201	0.96030829	0.999981514	0.964301327	0.928416436
SMCHD1	0.977624943	0.997678999	0.949855095	0.967883482	0.989379051	0.930146713
CFAP70	0.977624943	0.915269318	0.999993442	0.96900924	0.981652321	0.930710695
SMC1A	0.977624943	0.913116251	0.954020369	0.980605008	0.999985752	0.930710695
KLF16	0.977624943	0.926330259	0.969000554	0.999981514	0.956041014	0.933097499
FAM104A	0.977624943	0.943244588	0.94951784	0.984423083	0.999985752	0.934967882
S100A11	0.977624943	0.914629393	0.990118874	0.96951401	0.999985752	0.935770262
NOD1	0.977624943	0.957494685	0.989969382	0.978503042	0.988238252	0.939662884
SLC9A9	0.977624943	0.950675653	0.947155158	0.964521432	0.999985752	0.940583352
ZNF280D	0.977624943	0.982515431	0.951678465	0.999981514	0.964768333	0.943316981
SGMS1	0.977624943	0.904754143	0.972741989	0.999981514	0.979966065	0.944973383
CCDC9	0.977624943	0.980833065	0.943836178	0.961898258	0.999985752	0.946112759
ACSS3	0.977624943	0.94592331	0.966367125	0.996033721	0.98865576	0.948705062
MTM1	0.977624943	0.905145212	0.996844843	0.999981514	0.962513981	0.950631909
GABRB1	0.977624943	0.998389057	0.94955109	0.988408846	0.958797242	0.958250054
GATAD1	0.977624943	0.952863279	0.946791586	0.960016921	0.999985752	0.958790968
CASP3	0.977624943	0.973868672	0.962034224	0.98981682	0.972722722	0.960023723
PRR5	0.977624943	0.904728572	0.979584814	0.999981514	0.977167046	0.960133749
RN18S1	0.977624943	0.980521483	0.979584814	0.982220679	0.960419102	0.963112102
KMT2D	0.977624943	0.927794672	0.997300234	0.9880671	0.965676623	0.966850975
KCNK6	0.977624943	0.95298741	0.953470857	0.995434268	0.982087479	0.967056707
BROX	0.977624943	0.927853707	0.957052938	0.999981514	0.962513981	0.971545917
CD38	0.977624943	0.95577784	0.971053208	0.980532429	0.976292433	0.972695131
PLVAP	0.977624943	0.971775102	0.978014482	0.961898258	0.970988226	0.973649216
LOC101903438	0.977624943	0.981670901	0.943732649	0.984423083	0.976259416	0.973649216
ERLIN2	0.977624943	0.912602201	0.947952648	0.999981514	0.984386856	0.973649216
MPHOSPH6	0.977624943	0.935426633	0.981383697	0.967883482	0.982796995	0.9803583
RPL7A	0.977624943	0.927853707	0.946099836	0.999981514	0.972722722	0.98140846
WDR60	0.977624943	0.899490486	0.949855095	0.978503042	0.999985752	0.98469083
LOC510536	0.977624943	0.972829016	0.974044536	0.965050971	0.959658682	0.986042926
LOC787550	0.977624943	0.904754143	0.958197827	0.999981514	0.958852623	0.988273129
GNB4	0.977624943	0.910309058	0.999993442	0.965728173	0.953300742	0.988796537
BORCS8	0.977624943	0.98572429	0.963821408	0.959159289	0.953300742	0.993175779

LOC112441607	0.977624943	0.950658813	0.944126714	0.974286747	0.982538884	0.994887496
LOC104970503	0.977624943	0.913892615	0.950727498	0.9589395	0.976292433	0.999948228
LOC107132302	0.977624943	0.918234392	0.939981892	0.987084426	0.976292433	0.999948228
DBNDD2	0.977646359	0.991673407	0.95219206	0.97731816	0.964768333	0.970105102
ORC1	0.977669505	0.912012478	0.943836178	0.999981514	0.965163023	0.998458498
DLL4	0.977672596	0.995373357	0.982195333	0.9880671	0.954403133	0.931410194
PXDN	0.977672596	0.989303356	0.980761647	0.973373329	0.976769047	0.93145966
CNST	0.977672596	0.937723596	0.978014482	0.999981514	0.953300742	0.93363377
CMTM4	0.977672596	0.912764028	0.951392011	0.999981514	0.961465747	0.944973383
RITA1	0.977672596	0.941895237	0.999993442	0.974793558	0.959658682	0.948705062
TMEM88B	0.977672596	0.927973597	0.987610559	0.980564765	0.998196897	0.954496768
RAI1	0.977672596	0.913120359	0.99341711	0.999981514	0.956906331	0.958061107
GREB1	0.977672596	0.927853707	0.988307089	0.999981514	0.96419154	0.960023723
GLG1	0.977672596	0.906658524	0.991138474	0.999981514	0.976769047	0.960023723
ZNF8	0.977672596	0.918234392	0.942134405	0.999981514	0.976259416	0.975558533
UBE2Q2	0.977672596	0.910309058	0.976484937	0.999981514	0.954071324	0.980009318
CSF1R	0.977787389	0.975984829	0.995746693	0.95901497	0.964833865	0.964898868
YBEY	0.977816873	0.963243963	0.958410013	0.965050971	0.985250243	0.980189436
E2F4	0.977858598	0.901984573	0.999280799	0.999981514	0.956041014	0.950798794
RPGRIP1	0.977874498	0.998847958	0.978014482	0.960016921	0.965676623	0.940017687
PLAC9	0.977874498	0.952053757	0.95558643	0.999981514	0.963790274	0.963456717
TMTC3	0.977943717	0.918234392	0.996224523	0.9880671	0.976292433	0.967056707
LOC112444871	0.977943717	0.927794672	0.990615319	0.965050971	0.972160227	0.990755602
RABGGTB	0.977943717	0.902525869	0.976883418	0.980564765	0.976259416	0.99666605
RPL23A	0.978005735	0.965456931	0.971053208	0.98673849	0.958797242	0.981716775
FAM129C	0.978117419	0.977196455	0.996224523	0.979357217	0.976292433	0.92789871
ZNF879	0.978117419	0.98098403	0.942195449	0.999981514	0.970988226	0.963098281
BFSP1	0.978117419	0.994013976	0.95219206	0.978503042	0.956041014	0.973649216
LOC104974113	0.97812022	0.947736156	0.955427429	0.996195248	0.996781978	0.955990509
LOC112443001	0.978200971	0.959732252	0.958454497	0.999981514	0.976259416	0.940583352
LOC101902757	0.978200971	0.98098403	0.97316848	0.999981514	0.958654817	0.944973383
STK10	0.978200971	0.903618936	0.955427429	0.976196758	0.999985752	0.950415692
CD74	0.978200971	0.977769595	0.997400824	0.982220679	0.954814306	0.950631909
PLCXD3	0.978200971	0.98098403	0.965407935	0.984423083	0.980016241	0.951081819
ZBTB33	0.978200971	0.997471998	0.955845583	0.95901497	0.983884995	0.952571698
EXOC7	0.978200971	0.927853707	0.999993442	0.979385838	0.960048824	0.956820511
MCM9	0.978200971	0.936319037	0.959903168	0.999981514	0.965676623	0.963456717
ARHGAP1	0.978200971	0.942546802	0.957052938	0.999981514	0.970988226	0.97521287

CXHXorf58	0.978200971	0.915269318	0.981655709	0.982611153	0.979759416	0.983430934
LOC101903629	0.978200971	0.920217996	0.955427429	0.999981514	0.961465747	0.988639786
ALG6	0.978200971	0.912627552	0.96021005	0.982557317	0.958797242	0.999948228
LOC104975054	0.978200971	0.930293015	0.947590988	0.96900924	0.979290191	0.999948228
ALKBH6	0.978235891	0.948801019	0.976484937	0.972142185	0.999985752	0.950631909
LOC781692	0.978235891	0.982515431	0.978923213	0.967512088	0.980695939	0.951081819
LOC112444888	0.978235891	0.918234392	0.978014482	0.959652624	0.998287238	0.98377195
LOC100847999	0.978235891	0.905727888	0.943836178	0.999981514	0.961465747	0.99666605
EIF2B5	0.97824248	0.96365708	0.988390948	0.995434268	0.976259416	0.93988265
MYOF	0.97824248	0.952863279	0.99647733	0.994331516	0.960048824	0.955210102
LOC107131531	0.97824248	0.940383554	0.955427429	0.999981514	0.976292433	0.959198304
LOC100850875	0.97824248	0.968186752	0.942357321	0.999981514	0.97352263	0.966914618
UBR4	0.97837938	0.981952108	0.978014482	0.989601002	0.979988991	0.929027441
GPR156	0.978445732	0.915269318	0.951276289	0.999981514	0.960182847	0.945480515
RAB11FIP5	0.978445732	0.932778537	0.971255098	0.999981514	0.970988226	0.960023723
ADPRM	0.978445732	0.969051025	0.966232793	0.9880671	0.970988226	0.97061907
HOXD8	0.978523509	0.938087741	0.964807927	0.98981682	0.996781978	0.963112102
LOC112447324	0.978655554	0.930293015	0.97316848	0.991955963	0.999985752	0.940256832
DDIT4	0.978838572	0.926852499	0.961437412	0.999981514	0.964301327	0.930710695
LOC112442715	0.978838572	0.985923446	0.963164828	0.962899291	0.999985752	0.931410194
LOC104968634	0.978838572	0.993481224	0.957052938	0.999981514	0.9683846	0.932688327
LOC112446676	0.978838572	0.904728572	0.996224523	0.999981514	0.958797242	0.933402442
MIS12	0.978838572	0.927005854	0.957052938	0.999981514	0.970041959	0.93363377
PTRHD1	0.978838572	0.977817481	0.961437412	0.970725878	0.999985752	0.93363377
LSM7	0.978838572	0.952760839	0.969404007	0.999981514	0.969470443	0.934071377
RIN3	0.978838572	0.929409572	0.950055019	0.999981514	0.999985752	0.934071377
LOC104974912	0.978838572	0.927853707	0.975774524	0.998529748	0.999985752	0.934671838
TMTC2	0.978838572	0.991673407	0.987789469	0.995434268	0.954403133	0.935770262
PTPN11	0.978838572	0.927973597	0.965819476	0.999981514	0.956906331	0.935770262
FAXC	0.978838572	0.902525869	0.983504244	0.999981514	0.974931134	0.935770262
CST6	0.978838572	0.910651459	0.976484937	0.999981514	0.996781978	0.935770262
SVBP	0.978838572	0.99960614	0.945995021	0.982220679	0.976292433	0.936468627
FLT3LG	0.978838572	0.981670901	0.999993442	0.978404889	0.960573822	0.937025487
LOC783680	0.978838572	0.981952108	0.999993442	0.977691426	0.959658682	0.937478275
FLT1	0.978838572	0.991217881	0.996224523	0.972142185	0.961670582	0.940017687
MTMR10	0.978838572	0.946241678	0.958200097	0.999981514	0.962166847	0.940017687
CHODL	0.978838572	0.946148902	0.999993442	0.973812087	0.954071324	0.940079339
PPP4R3A	0.978838572	0.948801019	0.969026634	0.960566165	0.999985752	0.940583352

PTBP1	0.978838572	0.904326175	0.943836178	0.992930508	0.999985752	0.941413984
LOC783255	0.978838572	0.92132606	0.972897558	0.999981514	0.981652321	0.944973383
ITGB2	0.978838572	0.95462892	0.980851214	0.999981514	0.964120325	0.948705062
NECTIN1	0.978838572	0.918491096	0.943028511	0.999981514	0.989334846	0.951340373
GCGR	0.978838572	0.969477436	0.966232793	0.999981514	0.96405787	0.952571698
CCNI2	0.978838572	0.95187005	0.957052938	0.989485949	0.999985752	0.953509765
PYGO2	0.978838572	0.916141416	0.983534298	0.999981514	0.958797242	0.954496768
CNEP1R1	0.978838572	0.915269318	0.999993442	0.97828838	0.976769047	0.958707771
EDRF1	0.978838572	0.981952108	0.949855095	0.959591127	0.999985752	0.959198304
LCAT	0.978838572	0.973712113	0.955484273	0.965050971	0.999985752	0.959198304
RHOQ	0.978838572	0.955652503	0.972897558	0.999981514	0.955741702	0.960000414
PHYKPL	0.978838572	0.981546364	0.957621643	0.997660427	0.96870902	0.960023723
JAK3	0.978838572	0.904754143	0.999993442	0.980564765	0.980189546	0.960023723
LOC112443417	0.978838572	0.96643574	0.960937375	0.988408846	0.988010662	0.960023723
NAALAD2	0.978838572	0.991673407	0.947875514	0.973075232	0.989334846	0.960023723
LOC617698	0.978838572	0.937448751	0.988585686	0.960016921	0.999985752	0.960023723
GCNT7	0.978838572	0.929409572	0.95875611	0.999981514	0.976259416	0.960877268
TMEM185B	0.978838572	0.939956757	0.982195333	0.999981514	0.962922212	0.963015949
FGGY	0.978838572	0.955652503	0.94714672	0.999981514	0.954071324	0.963255451
C6	0.978838572	0.970084348	0.943237386	0.999981514	0.983304479	0.963456717
ENPP5	0.978838572	0.941483684	0.975774524	0.964521432	0.999985752	0.963456717
C20H5orf34	0.978838572	0.931043812	0.989494193	0.997792939	0.976259416	0.964361107
SBNO1	0.978838572	0.930293015	0.955427429	0.995434268	0.999859158	0.964898868
APPL2	0.978838572	0.916466865	0.949939862	0.999981514	0.999985752	0.964898868
ZNF710	0.978838572	0.944324631	0.987610559	0.993481918	0.965676623	0.96870908
LOC101906283	0.978838572	0.951628925	0.998777236	0.961898258	0.970988226	0.969564816
LOC101908075	0.978838572	0.954827347	0.951237862	0.999981514	0.965676623	0.970105102
ADAM20	0.978838572	0.933545282	0.951028911	0.988761333	0.999985752	0.971545917
PIGM	0.978838572	0.923007853	0.951392011	0.991955963	0.999985752	0.972314037
FNDC3B	0.978838572	0.919245978	0.966232793	0.999981514	0.967116582	0.972695131
BTBD19	0.978838572	0.996075126	0.964807927	0.960190213	0.960182847	0.973218068
LOC112449053	0.978838572	0.927103901	0.957295966	0.999981514	0.976769047	0.973218068
TCF7L1	0.978838572	0.941676269	0.958197827	0.98981682	0.991747435	0.973413452
CSRNP2	0.978838572	0.954237721	0.96412644	0.973075232	0.991588867	0.974962631
IREB2	0.978838572	0.909246615	0.996224523	0.999981514	0.954412818	0.97528579
LOC104972045	0.978838572	0.943244588	0.945995021	0.999981514	0.973173346	0.976831292
LOC100297152	0.978838572	0.946008705	0.968317646	0.98673849	0.97938347	0.978519031
RETSAT	0.978838572	0.95462892	0.943836178	0.998529748	0.976292433	0.980148159

DCX	0.978838572	0.933100216	0.99341711	0.994965893	0.956904152	0.980167535
TP53	0.978838572	0.942088099	0.947952648	0.999981514	0.974738415	0.980464176
B3GALNT1	0.978838572	0.981952108	0.944126714	0.997467114	0.956906331	0.981216824
RPS17	0.978838572	0.925557011	0.967590756	0.999981514	0.964301327	0.98140846
PCGF3	0.978838572	0.976776018	0.945995021	0.9880671	0.970988226	0.98140846
HCST	0.978838572	0.947124432	0.992263335	0.9819628	0.960177245	0.981716775
MAFA	0.978838572	0.945574389	0.951392011	0.999981514	0.956041014	0.983208118
LOC112447041	0.978838572	0.910651459	0.966700703	0.98981682	0.988238252	0.985649472
LOC104970913	0.978838572	0.930293015	0.971053208	0.999981514	0.954403133	0.986042926
MIA	0.978838572	0.919540627	0.998899782	0.97828838	0.955623649	0.992190617
IGF1R	0.978838572	0.93431906	0.968233314	0.960016921	0.983941532	0.997206681
DIXDC1	0.978838572	0.9419174	0.966232793	0.984548238	0.960048824	0.998284411
DHRS7B	0.978838572	0.938668827	0.945995021	0.979752657	0.960048824	0.999948228
LOC781439	0.978851368	0.952760839	0.982041666	0.9880671	0.998835841	0.934671838
BCAP29	0.978851368	0.988008216	0.99341711	0.976221833	0.961465747	0.948705062
TRIM8	0.978851368	0.919171954	0.985081637	0.959687593	0.999985752	0.951081819
TCTN3	0.978922	0.967042629	0.979584814	0.990036762	0.976292433	0.958281344
CENPM	0.978922	0.91078662	0.999993442	0.981295935	0.970988226	0.959198304
ENTPD3	0.978922	0.930293015	0.979494108	0.97828838	0.983941532	0.983564069
C22H3orf18	0.979004336	0.916448625	0.999993442	0.967512088	0.970212692	0.940017687
ARL6IP5	0.979014227	0.912693158	0.996224523	0.999981514	0.976292433	0.955308873
UBXN8	0.979040806	0.918854612	0.999993442	0.986337023	0.959026737	0.93145966
GINM1	0.979040806	0.937448751	0.988585686	0.990955345	0.999453276	0.934114816
VPS13A	0.979040806	0.932457887	0.996224523	0.973396104	0.999985752	0.937675392
GBF1	0.979040806	0.977196455	0.97773026	0.965724803	0.99971266	0.940017687
LOC112449563	0.979040806	0.918854612	0.951392011	0.999981514	0.966121925	0.940583352
SYNGR2	0.979040806	0.906550273	0.999993442	0.982220679	0.957588298	0.944330889
FAM57A	0.979040806	0.938087741	0.999993442	0.995565324	0.958797242	0.946760037
PIK3CB	0.979040806	0.912693158	0.991936822	0.995809088	0.998287238	0.946760037
PTK2B	0.979040806	0.977196455	0.99341711	0.985701713	0.970988226	0.947563293
LOC618409	0.979040806	0.919245978	0.978530068	0.979644238	0.999985752	0.947669118
H3F3B	0.979040806	0.959478257	0.999993442	0.969837124	0.958852623	0.947838082
AKIRIN2	0.979040806	0.911063463	0.996224523	0.982815793	0.999985752	0.94844234
SERAC1	0.979040806	0.928427136	0.95322206	0.999981514	0.959367717	0.949740463
PSKH1	0.979040806	0.942478777	0.955427429	0.999981514	0.996781978	0.951081819
LOC112446036	0.979040806	0.964418016	0.978530068	0.972467586	0.998287238	0.952563377
CCDC82	0.979040806	0.931411766	0.955845583	0.999981514	0.999985752	0.953140004
TMEM201	0.979040806	0.955652503	0.979584814	0.999981514	0.961465747	0.954496768

FAM192A	0.979040806	0.91475257	0.974586624	0.990955345	0.999985752	0.955541887
ZDHHC19	0.979040806	0.98098403	0.949855095	0.961898258	0.999985752	0.955990509
TSEN15	0.979040806	0.910560869	0.999993442	0.991085695	0.958797242	0.958707771
C10H14orf119	0.979040806	0.985997726	0.943836178	0.9880671	0.989334846	0.959198304
USP9X	0.979040806	0.961368616	0.996224523	0.9880671	0.964301327	0.960023723
AMFR	0.979040806	0.927369042	0.992991376	0.997792939	0.980016241	0.960023723
PRKRIP1	0.979040806	0.968166143	0.957052938	0.99045711	0.988238252	0.960023723
PQLC3	0.979040806	0.984179983	0.972741989	0.972142185	0.976292433	0.963456717
NUDT15	0.979040806	0.930266314	0.99341711	0.999981514	0.955623649	0.970105102
FBXL8	0.979040806	0.904875256	0.984886031	0.998529748	0.984386856	0.970105102
IMMT	0.979040806	0.918460462	0.972806636	0.985701713	0.998287238	0.973413452
LOC112445190	0.979040806	0.94622198	0.959903168	0.999981514	0.976292433	0.973649216
SRR	0.979040806	0.941400719	0.963821408	0.972467586	0.999344768	0.978445076
LOC783185	0.979040806	0.931842602	0.957148447	0.995312373	0.991481886	0.978923235
LOC112444215	0.979040806	0.981952108	0.962734642	0.989707094	0.955623649	0.981216824
LOC112442312	0.979040806	0.930293015	0.957052938	0.995434268	0.984328593	0.986042926
OCEL1	0.979040806	0.948801019	0.949939862	0.991955963	0.976292433	0.988639786
LOC515547	0.979040806	0.910651459	0.94565648	0.999981514	0.983941532	0.996335668
ANKRD13A	0.979040806	0.927853707	0.951292549	0.999858551	0.964713591	0.998458498
RPL39	0.979040806	0.919171954	0.957052938	0.973075232	0.959565914	0.999948228
FEM1C	0.979403507	0.951862885	0.989494193	0.98981682	0.966967496	0.971608881
DHFR	0.979459498	0.918455779	0.969026634	0.973373329	0.988049878	0.99666605
HNRNPM	0.979503113	0.909246615	0.958410013	0.999981514	0.987702131	0.963145894
SDF2	0.979503113	0.913435208	0.979494108	0.96951401	0.999251855	0.983430934
PRMT9	0.979692982	0.946148902	0.989969382	0.976756543	0.999453276	0.94844234
VPS33A	0.979768873	0.916448625	0.996224523	0.999981514	0.974931134	0.939948644
LOC531152	0.979768873	0.927746665	0.968679299	0.999981514	0.996089557	0.954496768
TTC27	0.979768873	0.94592331	0.951392011	0.999981514	0.996781978	0.960877268
POLR3E	0.979768873	0.96643574	0.964762618	0.999981514	0.964301327	0.96248183
LOC531679	0.979768873	0.948801019	0.94422142	0.999981514	0.955623649	0.964307876
WDR33	0.979768873	0.955652503	0.947155158	0.997660427	0.991481886	0.96885995
TWSG1	0.979813602	0.995373357	0.947282719	0.972142185	0.976292433	0.967056564
HCFC1	0.979871942	0.946820717	0.999993442	0.980532429	0.984386856	0.940256832
PLCB2	0.979871942	0.935426633	0.991046292	0.962899291	0.999985752	0.960023723
SEPT11	0.979871942	0.929204871	0.944126714	0.999981514	0.999985752	0.960023723
HOXC8	0.980000471	0.969051025	0.974470955	0.999981514	0.970988226	0.93988265
H3F3C	0.980000471	0.946148902	0.976188884	0.965724803	0.999985752	0.944144347
EIF3L	0.980000471	0.930293015	0.957295966	0.984423083	0.964301327	0.999948228

LOC100848886	0.980017144	0.919171954	0.949855095	0.998529748	0.999985752	0.93363377
COMT	0.980017144	0.933100216	0.996224523	0.985878137	0.99971266	0.933897626
GIGYF2	0.980017144	0.913116251	0.958200097	0.999981514	0.999985752	0.93531275
CD46	0.980017144	0.972007528	0.998848708	0.999669429	0.958797242	0.935770262
LNX1	0.980017144	0.98098403	0.948904459	0.999981514	0.988238252	0.935770262
JKAMP	0.980017144	0.954005878	0.991138474	0.995434268	0.987723553	0.936263602
ZNF614	0.980017144	0.988008216	0.978837953	0.983079972	0.982087479	0.938894901
ZNF462	0.980017144	0.934225172	0.975774524	0.999043216	0.999985752	0.940423784
LOC782966	0.980017144	0.911461743	0.999993442	0.995565324	0.956906331	0.945480515
HES1	0.980017144	0.973712113	0.961437412	0.999981514	0.9683846	0.958790968
LOC104973746	0.980017144	0.980521483	0.954020369	0.984423083	0.996781978	0.959075802
LDLR	0.980017144	0.912693158	0.99341711	0.986051493	0.984386856	0.975247881
GOLGA5	0.980017144	0.916141416	0.947155158	0.999981514	0.982538884	0.97529557
PES1	0.980017144	0.990697047	0.947155158	0.982220679	0.964301327	0.98469083
SUMO2	0.980017144	0.937448751	0.972741989	0.989032224	0.966024339	0.996335668
CHCHD8	0.980017144	0.915269318	0.981504131	0.978503042	0.962513981	0.999948228
UBE2E3	0.980190721	0.94422395	0.997400824	0.999981514	0.961465747	0.940256832
LGR6	0.980190721	0.980833065	0.957052938	0.999981514	0.959658682	0.97061907
SNRPA1	0.980270958	0.939116609	0.996224523	0.999981514	0.958852623	0.970105102
CEP57L1	0.980270958	0.968369135	0.966232793	0.995565324	0.958797242	0.984046914
CAMSAP1	0.980366298	0.99960614	0.955427429	0.989707094	0.964301327	0.944330889
DUS3L	0.980430965	0.950336973	0.999993442	0.969689331	0.970988226	0.960023723
COPB2	0.980454491	0.927853707	0.999993442	0.979385838	0.962513981	0.969014913
SMCR8	0.980505214	0.920882164	0.987610559	0.9880671	0.999985752	0.932688327
MIER3	0.980505214	0.972287585	0.993073581	0.995434268	0.976259416	0.935770262
SNX31	0.980505214	0.955652503	0.957052938	0.99739416	0.999985752	0.936468627
LOC784451	0.980505214	0.92132606	0.95322206	0.999981514	0.962513981	0.94844234
FAM117B	0.980505214	0.963146208	0.984886031	0.980605008	0.989334846	0.955457361
LOC112447460	0.980505214	0.981952108	0.987610559	0.961898258	0.986003146	0.958281344
YPEL2	0.980505214	0.977196455	0.966232793	0.982220679	0.989906659	0.959075802
EID3	0.980505214	0.952760839	0.966232793	0.984331039	0.99971266	0.96053762
EXOC3	0.980505214	0.994185904	0.975774524	0.973075232	0.964301327	0.960877268
FGD4	0.980525158	0.956095821	0.956423184	0.999981514	0.970041959	0.946858406
LOC513580	0.980525158	0.915269318	0.971556427	0.989601002	0.971133034	0.999463809
GLMN	0.980609107	0.913116251	0.999993442	0.968436292	0.960419102	0.967047449
PPP1R21	0.980659466	0.926905849	0.957052938	0.999981514	0.970212692	0.956218347
LOC104969024	0.980659466	0.913116251	0.991936822	0.999981514	0.976292433	0.957558074
GALC	0.980675793	0.943860167	0.945732557	0.990955345	0.999859158	0.975200512

PSEN1	0.98072957	0.910651459	0.965327631	0.999981514	0.958797242	0.936331898
CYB5D2	0.980886919	0.952344105	0.96030829	0.97439926	0.999985752	0.958707771
TSPOAP1	0.980886919	0.988008216	0.958454497	0.999981514	0.961465747	0.963112102
IFT52	0.980886919	0.930293015	0.95217331	0.978503042	0.999985752	0.96885995
KHDRBS1	0.980886919	0.968509005	0.980061232	0.982220679	0.976259416	0.970105102
TBC1D24	0.980886919	0.924316541	0.996224523	0.999981514	0.964833865	0.971545917
LHFPL3	0.980886919	0.91078662	0.990118874	0.97731816	0.996781978	0.981216824
PLPP2	0.980886919	0.930266314	0.976563428	0.973075232	0.997422012	0.981672932
MAN1A2	0.981058817	0.959478257	0.958410013	0.979644238	0.999985752	0.93363377
FAM167B	0.981058817	0.939956757	0.949855095	0.999981514	0.970980375	0.93526981
METTL26	0.981058817	0.983594252	0.990118874	0.97828838	0.986003146	0.936263602
EFHD2	0.981058817	0.944324631	0.996224523	0.980564765	0.99971266	0.936263602
NECAB2	0.981058817	0.925026329	0.996224523	0.975150022	0.999985752	0.936263602
CSNK1A1	0.981058817	0.997840295	0.98385084	0.961898258	0.977167046	0.937469898
SEMA3A	0.981058817	0.966661805	0.955427429	0.999981514	0.962513981	0.943972369
UBXN10	0.981058817	0.915871693	0.957770624	0.999981514	0.976292433	0.943972369
LOC112443244	0.981058817	0.947350735	0.95558643	0.999981514	0.983941532	0.944330889
KCNA2	0.981058817	0.935661607	0.955427429	0.995312373	0.999985752	0.944927301
LOC614424	0.981058817	0.939457142	0.997400824	0.999981514	0.964301327	0.944973383
HELQ	0.981058817	0.981670901	0.961437412	0.999981514	0.973219333	0.947838082
FRMD6	0.981058817	0.948801019	0.99647733	0.976679676	0.996089557	0.949740463
LSM6	0.981058817	0.912627552	0.999993442	0.964521432	0.977167046	0.951081819
KCTD18	0.981058817	0.997471998	0.96043275	0.96591587	0.984386856	0.952663521
LOC104969425	0.981058817	0.981952108	0.996509242	0.97731816	0.964833865	0.956820511
TRAK2	0.981058817	0.910651459	0.972741989	0.999981514	0.983941532	0.959198304
DDX50	0.981058817	0.928427136	0.97316848	0.99570283	0.999985752	0.959723327
ELMO1	0.981058817	0.944449061	0.999993442	0.963132425	0.964301327	0.960023723
ST7	0.981058817	0.926852499	0.999993442	0.9880671	0.976259416	0.960023723
REXO4	0.981058817	0.977196455	0.983504244	0.996090365	0.961465747	0.960877268
EEF2K	0.981058817	0.927103901	0.989977673	0.994395954	0.989334846	0.963456717
ELMOD3	0.981058817	0.994013976	0.975774524	0.980564765	0.956906331	0.968138839
EEF1B2	0.981058817	0.939956757	0.987610559	0.999981514	0.960419102	0.968138839
ATP8B2	0.981058817	0.940500178	0.997300234	0.984423083	0.976259416	0.96870908
FBXL12	0.981058817	0.966069053	0.99341711	0.984423083	0.959658682	0.974500551
SDCCAG8	0.981058817	0.91763262	0.999993442	0.978225003	0.970988226	0.98140846
IL15RA	0.981058817	0.927973597	0.996894724	0.982220679	0.970988226	0.98140846
PPP1R1B	0.981058817	0.915269318	0.959192525	0.999981514	0.972160227	0.987516427
ZNF703	0.981058817	0.961663204	0.979494108	0.976221833	0.970980375	0.988796537

SHISA5	0.981058817	0.969811486	0.96030829	0.965050971	0.979290191	0.991847494
CDKN2D	0.981058817	0.924472315	0.974975876	0.969214881	0.995870928	0.994991252
HIP1	0.981058817	0.980833065	0.971053208	0.96444269	0.959658682	0.996813547
DERA	0.981058817	0.930266314	0.996509242	0.972142185	0.956653075	0.99841577
PCSK5	0.981058817	0.951172741	0.957052938	0.972467586	0.963550093	0.999948228
LOC101902918	0.981058817	0.929204871	0.945995021	0.973373329	0.964301327	0.999948228
KDSR	0.981062371	0.95687851	0.990615319	0.988408846	0.989334846	0.948091259
ANKRD35	0.981067074	0.933545282	0.945995021	0.999981514	0.999985752	0.95662324
MST1	0.981070536	0.994492827	0.962553873	0.98693839	0.982087479	0.940583352
CEND1	0.981070536	0.94019273	0.999993442	0.999981514	0.960177245	0.950540248
PLEKHM1	0.981070536	0.980266807	0.949855095	0.999981514	0.962513981	0.953140004
SURF2	0.981070536	0.943244588	0.990118874	0.999981514	0.961465747	0.958790968
LOC112449300	0.981070536	0.938087741	0.983504244	0.974272706	0.999985752	0.963107047
DCAF16	0.981075944	0.993481224	0.996859483	0.983512531	0.960573822	0.933903265
GAREM2	0.981075944	0.98098403	0.999993442	0.966786897	0.970988226	0.93531275
NEDD1	0.981075944	0.999987083	0.958197827	0.972142185	0.960048824	0.935497122
ANKRD54	0.981075944	0.994185904	0.99341711	0.96900924	0.970988226	0.938894901
XRCC6	0.981075944	0.918447234	0.995377403	0.999981514	0.973219333	0.940017687
LOC112445985	0.981075944	0.936947145	0.999993442	0.961898258	0.999985752	0.940017687
LOC104970852	0.981075944	0.941483684	0.947462602	0.999981514	0.983941532	0.940050644
EVA1C	0.981075944	0.962037123	0.980761647	0.98981682	0.998141915	0.940256832
GRID2IP	0.981075944	0.942088099	0.951028911	0.999981514	0.9683846	0.940583352
TADA2B	0.981075944	0.933886329	0.996224523	0.999981514	0.956904152	0.940639747
RBM42	0.981075944	0.942088099	0.999993442	0.972142185	0.987723553	0.942982678
LOC101907132	0.981075944	0.938087741	0.99341711	0.999981514	0.958852623	0.943972369
LHFPL6	0.981075944	0.961663204	0.954020369	0.999981514	0.999985752	0.944927301
LOC112441644	0.981075944	0.927853707	0.996224523	0.999981514	0.971133034	0.945480515
IFT140	0.981075944	0.939442799	0.999993442	0.999981514	0.961465747	0.950798794
LOC101910045	0.981075944	0.953345357	0.999993442	0.979644238	0.970988226	0.951123163
CACNB4	0.981075944	0.927973597	0.95219206	0.999981514	0.976419607	0.954496768
LOC100335642	0.981075944	0.913116251	0.957052938	0.999981514	0.984386856	0.955210102
ZMYND12	0.981075944	0.98978863	0.957052938	0.98981682	0.983941532	0.958707771
NEURL1B	0.981075944	0.937448751	0.991138474	0.999981514	0.970980375	0.958790968
LOC614402	0.981075944	0.989303356	0.975774524	0.984423083	0.976259416	0.958790968
SLC35F2	0.981075944	0.963193873	0.987610559	0.988408846	0.983941532	0.959075802
LOC104969353	0.981075944	0.933886329	0.999993442	0.964521432	0.998060725	0.960023723
EVI5	0.981075944	0.913116251	0.974656086	0.999981514	0.986019537	0.960877268
CDK5RAP1	0.981075944	0.976596938	0.948129084	0.973673712	0.999985752	0.960877268

RASGEF1A	0.981075944	0.934225172	0.966199203	0.999981514	0.970988226	0.963107047
ADAMTSL3	0.981075944	0.955137018	0.999993442	0.969088992	0.97130055	0.963112102
ARHGAP32	0.981075944	0.944505732	0.949772	0.991418569	0.999985752	0.965486169
BRAF	0.981075944	0.971775102	0.957052938	0.961898258	0.999985752	0.966212838
EIF2B4	0.981075944	0.942855072	0.993073581	0.998177865	0.972594401	0.966850975
TOX	0.981075944	0.966303622	0.990037865	0.99542943	0.963037022	0.967792782
KLHDC9	0.981075944	0.942088099	0.95219206	0.97828838	0.999985752	0.968697704
BARD1	0.981075944	0.965405402	0.966199203	0.972521866	0.999453276	0.96870908
LOC614376	0.981075944	0.918447234	0.995746693	0.995565324	0.983941532	0.968832802
LOC790312	0.981075944	0.929204871	0.978014482	0.999981514	0.970988226	0.973649216
KCNJ16	0.981075944	0.941895237	0.951028911	0.97828838	0.999985752	0.973649216
KLHDC8B	0.981075944	0.950986475	0.959725886	0.999981514	0.970988226	0.975558533
MTHFD1	0.981075944	0.969051025	0.988585686	0.964521432	0.977369136	0.977503497
HEXA	0.981075944	0.9574162	0.997400824	0.9745615	0.962513981	0.980501512
LOC112447328	0.981075944	0.915269318	0.971053208	0.988408846	0.999251855	0.98140846
EBNA1BP2	0.981075944	0.941400719	0.969404007	0.998529748	0.976259416	0.98469083
S100A9	0.981075944	0.968932899	0.958554319	0.982220679	0.977167046	0.987785166
CCDC149	0.981075944	0.936082042	0.960945531	0.9880671	0.992984551	0.988866378
FAR2	0.981075944	0.955652503	0.972741989	0.993849725	0.959658682	0.991847494
FOCAD	0.981075944	0.911593575	0.988307089	0.964521432	0.970988226	0.999948228
PTER	0.981110977	0.915034433	0.999993442	0.96444269	0.999985752	0.934671838
LOC107131296	0.981110977	0.991177966	0.978014482	0.999981514	0.961465747	0.93531275
SLC35B2	0.981110977	0.997810586	0.962734642	0.999981514	0.960784627	0.935421237
RTF2	0.981110977	0.918854612	0.999993442	0.980532429	0.999985752	0.936951685
LOC100139115	0.981110977	0.945574389	0.96327718	0.999981514	0.997422012	0.938894901
MAEA	0.981110977	0.953841555	0.95219206	0.97731816	0.999985752	0.940017687
TKFC	0.981110977	0.984089343	0.978014482	0.976399741	0.997422012	0.940256832
SHBG	0.981110977	0.918854612	0.95322206	0.999981514	0.998016692	0.944973383
LOC107131974	0.981110977	0.930058688	0.999993442	0.995565324	0.971075533	0.947245743
NAT8L	0.981110977	0.927853707	0.999993442	0.999981514	0.977158496	0.94844234
LOC101904579	0.981110977	0.928835826	0.99461027	0.972142185	0.999985752	0.951493662
EHBP1	0.981110977	0.920978623	0.952702375	0.999981514	0.998016692	0.952663521
CRTAP	0.981110977	0.938017087	0.988307089	0.999981514	0.988238252	0.953423944
KANK4	0.981110977	0.918854612	0.95322206	0.988437392	0.999985752	0.960251802
SLC37A1	0.981110977	0.933100216	0.965819476	0.999981514	0.964553547	0.963701289
OSGEP	0.981110977	0.975612288	0.957295966	0.995312373	0.979874385	0.971545917
MARCH1	0.981110977	0.946881447	0.966232793	0.995434268	0.977167046	0.984046914
LOC511409	0.981110977	0.941817736	0.966199203	0.995565324	0.979988991	0.985027129

FIZ1	0.981110977	0.946859049	0.999993442	0.969837124	0.960048824	0.989084358
GRASP	0.981110977	0.952344105	0.957052938	0.999981514	0.964301327	0.993890045
INCA1	0.981110977	0.943244588	0.957052938	0.999793541	0.964301327	0.998284411
CAMK2D	0.981110977	0.928213372	0.95219206	0.969088992	0.98572619	0.999948228
POLM	0.981114677	0.979651412	0.975774524	0.989601002	0.988049878	0.951342758
NUP107	0.981269277	0.960355725	0.999993442	0.969837124	0.991481886	0.938161998
TMED9	0.981269277	0.918854612	0.999993442	0.999981514	0.960048824	0.940017687
PDGFD	0.981269277	0.945755356	0.957052938	0.999981514	0.996781978	0.943621425
LOC616400	0.981269277	0.927746665	0.960977611	0.999981514	0.976292433	0.960023723
EDEM3	0.981269277	0.977196455	0.975121416	0.96951401	0.998016692	0.960023723
ELK1	0.981269277	0.991673407	0.96102707	0.96444269	0.979290191	0.976020793
KCNIP2	0.981269277	0.941400719	0.997300234	0.970386534	0.970988226	0.989184798
CHEK1	0.981282136	0.942594366	0.988430001	0.990955345	0.961465747	0.991847494
GBP4	0.981465746	0.997678999	0.954789609	0.989485949	0.98865576	0.937469898
ARRDC1	0.981465746	0.980521483	0.979494108	0.994408995	0.987723553	0.940017687
SVIP	0.981465746	0.995204627	0.958410013	0.996033721	0.976769047	0.940256832
CSNK1D	0.981465746	0.91763262	0.984886031	0.973075232	0.999985752	0.940583352
SMARCD2	0.981465746	0.980616436	0.966700703	0.995312373	0.996217975	0.940942561
SETD9	0.981465746	0.97304892	0.984358443	0.996884833	0.983941532	0.944330889
MED13L	0.981465746	0.91287376	0.999993442	0.995152316	0.983941532	0.944973383
LOC112442365	0.981465746	0.947350735	0.978014482	0.999981514	0.991481886	0.944973383
LOC101907943	0.981465746	0.915570087	0.970873534	0.978503042	0.999985752	0.947838082
FRMD5	0.981465746	0.991673407	0.957052938	0.997792939	0.977167046	0.951566529
UBOX5	0.981465746	0.947911876	0.949855095	0.999981514	0.999985752	0.953140004
LOC104973485	0.981465746	0.916484772	0.998848708	0.980564765	0.999985752	0.958707771
LOC112446034	0.981465746	0.927034887	0.96030829	0.997536986	0.999985752	0.958707771
MAPK13	0.981465746	0.925557011	0.999993442	0.999981514	0.958797242	0.958790968
TNK2	0.981465746	0.994185904	0.954020369	0.98981682	0.976292433	0.960023723
ITGAM	0.981465746	0.957328149	0.97773026	0.999981514	0.976292433	0.960023723
CDNF	0.981465746	0.985428851	0.980761647	0.972142185	0.976419607	0.967759699
IZUMO1	0.981465746	0.933545282	0.959725886	0.999981514	0.991481886	0.972695131
LGALS12	0.981465746	0.927853707	0.999993442	0.988408846	0.960573822	0.973218068
TMEM86B	0.981465746	0.972460638	0.975774524	0.988408846	0.976259416	0.973649216
CPSF6	0.981465746	0.913120359	0.96412644	0.978503042	0.999985752	0.973649216
LOC516742	0.981465746	0.980521483	0.959725886	0.972142185	0.990937627	0.978923235
LOC104973382	0.981465746	0.985428851	0.966199203	0.99542943	0.958797242	0.980148159
TCF7L2	0.981465746	0.918854612	0.97918842	0.975653996	0.999985752	0.9803583
THNSL1	0.981465746	0.991673407	0.965684927	0.96968491	0.964301327	0.985717525

EIF2AK4	0.981465746	0.917822211	0.972741989	0.999981514	0.961465747	0.986042926
R3HDM1	0.981465746	0.92259022	0.964382669	0.999981514	0.964301327	0.986042926
HNRNPUL1	0.981465746	0.952863279	0.967590756	0.999981514	0.961670582	0.988320707
ZC3H12C	0.981465746	0.985428851	0.962034224	0.964521432	0.958654817	0.999701868
NELFA	0.98148782	0.919245978	0.999993442	0.988252869	0.988881329	0.964898868
NTN3	0.98148782	0.980521483	0.959863581	0.999981514	0.958852623	0.970105102
TMEM175	0.981573936	0.941817736	0.957295966	0.999981514	0.999985752	0.941585207
GSTK1	0.981577457	0.927369042	0.979494108	0.999981514	0.996781978	0.935497122
LIN7B	0.981577457	0.976596938	0.99341711	0.999981514	0.97464569	0.938894901
IK	0.981577457	0.929007264	0.997850143	0.995565324	0.998196897	0.939024684
NCOA1	0.981577457	0.946148902	0.949939862	0.982220679	0.999985752	0.960023723
LOC112442866	0.981577457	0.99960614	0.972741989	0.96900924	0.963550093	0.960227605
CAPN1	0.981577457	0.956095821	0.980761647	0.992930508	0.98924357	0.960533245
CLPTM1L	0.981577457	0.938087741	0.991936822	0.9880671	0.996781978	0.963456717
PREX2	0.981577457	0.961663204	0.957052938	0.969837124	0.999985752	0.966610103
SLC12A7	0.981577457	0.991673407	0.968679299	0.965416137	0.983941532	0.970105102
SMPD2	0.981577457	0.915269318	0.976484937	0.999981514	0.958797242	0.977525788
HIC2	0.981577457	0.929737582	0.979494108	0.986610305	0.970988226	0.99828741
PLPP1	0.981578249	0.925557011	0.985444929	0.999981514	0.982208423	0.95883396
LOC101906469	0.981585711	0.943213236	0.992714073	0.999981514	0.970885781	0.936468627
PYGO1	0.981585711	0.918234392	0.966724799	0.999981514	0.998287238	0.944973383
PPP1R42	0.981585711	0.919540627	0.999993442	0.999981514	0.964301327	0.960023723
ALDH1L2	0.981775007	0.949902864	0.964011761	0.999981514	0.970980375	0.940017687
ARHGEF4	0.981775007	0.975589223	0.94955109	0.999981514	0.964768333	0.944330889
LOC789157	0.981775007	0.948801019	0.984886031	0.999981514	0.964301327	0.947801358
KCNT2	0.981775007	0.91475257	0.99341711	0.999981514	0.992326084	0.951081819
IL21R	0.981775007	0.998847958	0.987610559	0.96582208	0.964963602	0.953140004
LOC512617	0.981775007	0.978857858	0.957052938	0.995565324	0.996781978	0.955140534
ZNF197	0.981775007	0.956527556	0.983504244	0.98471945	0.999344768	0.9557845
TCEA3	0.981775007	0.94422395	0.973562292	0.999981514	0.996781978	0.958790968
CTDSP2	0.981775007	0.955827589	0.99647733	0.97082713	0.993190318	0.960023723
DCAF12	0.981775007	0.920382325	0.97763883	0.999981514	0.982796995	0.970044042
APP	0.981775007	0.915073021	0.99341711	0.991955963	0.987723553	0.9790177
MAPK8IP3	0.981775007	0.918199716	0.955427429	0.999981514	0.972499705	0.98140846
LOC112443215	0.981775007	0.924625436	0.964807927	0.980632768	0.999985752	0.986646873
LOC112446360	0.981876178	0.932394105	0.95219206	0.999981514	0.97234838	0.962412275
CASTOR1	0.981986948	0.988824757	0.955427429	0.989909951	0.976259416	0.973413452
C25H7orf43	0.982018412	0.927973597	0.997400824	0.999981514	0.963550093	0.940942561

ZNF93	0.982018412	0.916448625	0.990974506	0.999981514	0.970980375	0.946858406
BCL2L10	0.982018412	0.976527871	0.978014482	0.999981514	0.970988226	0.949280707
ATMIN	0.982018412	0.963957751	0.997850143	0.992930508	0.970988226	0.957628177
GSTT4	0.982018412	0.973712113	0.987610559	0.999981514	0.965676623	0.958790968
ENOPH1	0.982018412	0.950336973	0.999993442	0.980532429	0.958797242	0.977053057
RAP1GDS1	0.982018412	0.941400719	0.972806636	0.973075232	0.989334846	0.996335668
KCNC2	0.982032821	0.975612288	0.996624589	0.994965893	0.976259416	0.940079339
ZFAND1	0.982032821	0.938817997	0.978014482	0.999981514	0.959007232	0.946112759
PTPN14	0.982032821	0.946859049	0.99341711	0.982220679	0.996781978	0.960023723
MAML1	0.982035793	0.941655994	0.999993442	0.976196758	0.989334846	0.938894901
TRIP6	0.982035793	0.993481224	0.953176526	0.999793541	0.961670582	0.972695131
MEPCE	0.982035793	0.935001231	0.966199203	0.999981514	0.972160227	0.9790177
TMEM187	0.982035793	0.955652503	0.989494193	0.980564765	0.963790274	0.995379819
LOC104972584	0.982201585	0.941400719	0.999993442	0.96582208	0.999985752	0.940256832
OSTF1	0.982201585	0.946806157	0.998335361	0.999981514	0.959026737	0.941762089
TCF7	0.982201585	0.927853707	0.979584814	0.999981514	0.969396685	0.985564959
PDCD6IP	0.982211049	0.932191409	0.987346948	0.999981514	0.976259416	0.973970782
TGFBRAP1	0.982254298	0.918455779	0.996224523	0.996033721	0.999985752	0.944927301
SAMD8	0.982254298	0.984126877	0.973034514	0.97731816	0.99971266	0.946760037
GAB3	0.982254298	0.92259022	0.999993442	0.999981514	0.964301327	0.948705062
RAPGEF2	0.982254298	0.983594252	0.957052938	0.982220679	0.999985752	0.954465286
ARL3	0.982254298	0.933545282	0.999993442	0.967883482	0.976292433	0.966212838
CIPC	0.982254298	0.966507289	0.979494108	0.978503042	0.986003146	0.978087957
ADCY8	0.982254298	0.99528564	0.955845583	0.98673849	0.960419102	0.98140846
FAM212B	0.982254298	0.933545282	0.979494108	0.999981514	0.960784627	0.989840954
TTLL7	0.982254298	0.954222802	0.95940506	0.98981682	0.976292433	0.996453303
ANGPTL6	0.982254298	0.929737582	0.997300234	0.96900924	0.966328884	0.99828741
TAX1BP3	0.982420845	0.95462892	0.99341711	0.969837124	0.999985752	0.953140004
PDCD10	0.982420845	0.93738232	0.961800035	0.999981514	0.976292433	0.98679538
MSANTD1	0.982481497	0.930293015	0.973254083	0.999981514	0.976259416	0.958474163
ABCD4	0.982481497	0.957328149	0.999993442	0.990955345	0.959565914	0.960023723
THSD7A	0.98255946	0.980521483	0.962034224	0.995565324	0.999985752	0.937715964
TTC36	0.98255946	0.964925317	0.99647733	0.999981514	0.959658682	0.940017687
CIB2	0.98255946	0.955516767	0.964661607	0.999981514	0.974222572	0.971545917
CDKL5	0.98255946	0.953449552	0.972806636	0.990955345	0.995515529	0.971589829
SOCS1	0.98255946	0.929409572	0.957052938	0.973075232	0.999985752	0.98140846
ZBTB48	0.982657241	0.927992601	0.982041666	0.973075232	0.970980375	0.999948228
LOC101902807	0.982699983	0.930293015	0.95875611	0.999981514	0.976292433	0.945870804

VGLL3	0.982699983	0.945942733	0.958595987	0.999981514	0.999985752	0.960023723
CIR1	0.982699983	0.933100216	0.957052938	0.999981514	0.976586416	0.96870908
TUG1	0.982828665	0.975509159	0.961437412	0.985118339	0.999985752	0.93839036
TRPM3	0.982828665	0.969051025	0.973034514	0.999981514	0.970988226	0.940017687
ZNF266	0.982828665	0.936822825	0.99341711	0.999981514	0.970980375	0.958790968
BBS10	0.982828665	0.920217996	0.996224523	0.980605008	0.989334846	0.981672932
CCDC88A	0.982828665	0.94592331	0.99341711	0.970725878	0.977167046	0.989840954
ZBTB4	0.982828665	0.950986475	0.951392011	0.999981514	0.969087007	0.996813547
DHCR24	0.982828665	0.918491096	0.953572282	0.999981514	0.977158496	0.999948228
AIF1L	0.982841856	0.966684938	0.99341711	0.986214799	0.996089557	0.946347861
GGNBP2	0.982841856	0.94019273	0.999993442	0.999038184	0.964301327	0.954550352
NRTN	0.982863254	0.940546131	0.999993442	0.989485949	0.973219333	0.952571698
EMCN	0.983032191	0.939857309	0.97773026	0.999981514	0.996781978	0.960023723
SEL1L3	0.983032191	0.937598531	0.999993442	0.9880671	0.967205491	0.9790177
ST7L	0.983082536	0.95038008	0.957681559	0.999981514	0.970988226	0.945480515
SLC25A32	0.983082536	0.930293015	0.975774524	0.999981514	0.999453276	0.959198304
CD2BP2	0.983139552	0.951159584	0.977811584	0.998529748	0.998287238	0.958040819
KLB	0.983161603	0.942088099	0.965961035	0.999981514	0.970980375	0.938894901
LOC104975666	0.983161603	0.959263706	0.992377532	0.990955345	0.983941532	0.962412275
ACER3	0.983161603	0.923232089	0.975718134	0.989032224	0.999985752	0.963456717
MEX3C	0.983203346	0.919540627	0.99341711	0.999981514	0.996089557	0.950037116
PLA2G16	0.983203346	0.938087741	0.996224523	0.999981514	0.976292433	0.963456717
SAP30BP	0.983203346	0.933839487	0.971053208	0.999981514	0.959565914	0.98140846
ZNF691	0.98335679	0.973712113	0.99869493	0.999981514	0.960573822	0.938894901
KXD1	0.98335679	0.981670901	0.991046292	0.995565324	0.983941532	0.939662884
GTPBP8	0.98335679	0.988008216	0.958454497	0.999981514	0.964301327	0.940079339
TIPIN	0.98335679	0.928763536	0.999993442	0.999981514	0.964833865	0.945480515
CGAS	0.98335679	0.991673407	0.972073392	0.973432601	0.999453276	0.945480515
AUNIP	0.98335679	0.94622198	0.999993442	0.972467586	0.999453276	0.947478418
NKAIN3	0.98335679	0.988540349	0.972897558	0.96951401	0.999985752	0.947801358
TPR	0.98335679	0.923007853	0.995377403	0.999005991	0.999985752	0.947937638
MARVELD1	0.98335679	0.938235006	0.997400824	0.999981514	0.976259416	0.948705062
LOC507443	0.98335679	0.997430868	0.982232295	0.978503042	0.976259416	0.948913998
LRRC27	0.98335679	0.950336973	0.978014482	0.999981514	0.974931134	0.950540248
FBXO2	0.98335679	0.942546802	0.959903168	0.999981514	0.986003146	0.950631909
PDZD2	0.98335679	0.969051025	0.964382669	0.999981514	0.964301327	0.960023723
CHCHD1	0.98335679	0.933367097	0.990974506	0.98981682	0.999985752	0.960023723
MYO5A	0.98335679	0.999987083	0.95322206	0.974873127	0.974931134	0.961449284

SRSF2	0.98335679	0.927005854	0.997400824	0.988889838	0.997422012	0.963456717
LOC112447346	0.98335679	0.957328149	0.958410013	0.999981514	0.989442936	0.964898868
TGFB2	0.98335679	0.927742019	0.961437412	0.999981514	0.998287238	0.964898868
WNT2B	0.98335679	0.994185904	0.951028911	0.990955345	0.979290191	0.965117554
SNX18	0.98335679	0.918234392	0.972806636	0.99542943	0.999985752	0.966850975
PGRMC1	0.98335679	0.922610146	0.961800035	0.993171895	0.999985752	0.969014913
ESYT2	0.98335679	0.938087741	0.998674796	0.972562401	0.996781978	0.969197073
LOC101904592	0.98335679	0.99431777	0.959725886	0.996033721	0.961465747	0.971545917
PNKD	0.98335679	0.94422395	0.978014482	0.999981514	0.9683846	0.971545917
MALSU1	0.98335679	0.922610146	0.96327718	0.975351263	0.999985752	0.971608881
ABHD14B	0.98335679	0.982011436	0.950185097	0.990660526	0.989334846	0.972695131
GABARAPL2	0.98335679	0.936319037	0.972410614	0.999981514	0.979290191	0.974527074
ATP5MC2	0.98335679	0.933100216	0.97773026	0.996033721	0.988049878	0.983669172
LOC104972407	0.98335679	0.924316541	0.972741989	0.999981514	0.976292433	0.984509067
TSPAN11	0.98335679	0.942088099	0.950971816	0.984423083	0.999985752	0.984509067
CHTOP	0.98335679	0.929490454	0.97251482	0.975118969	0.999985752	0.988866378
MOSMO	0.98335679	0.954827347	0.990671805	0.978503042	0.973219333	0.990924439
CYSRT1	0.98335679	0.969066375	0.957052938	0.994641979	0.976259416	0.991421487
LOC509184	0.98335679	0.943244588	0.967590756	0.998529748	0.976292433	0.993890045
LOC112443864	0.98335679	0.953042388	0.976484937	0.998667933	0.963790274	0.994788615
MSRB2	0.983457556	0.949323635	0.980657691	0.999981514	0.976292433	0.973218068
CALML4	0.983528013	0.938087741	0.999993442	0.999981514	0.960573822	0.940079339
STXBP1	0.983528013	0.967668839	0.954249102	0.999981514	0.998914459	0.958281344
LOC112448853	0.983528013	0.927853707	0.983504244	0.985717249	0.999985752	0.958699521
LOC107131623	0.983528013	0.927794672	0.972410614	0.999981514	0.979840175	0.970071797
TFDP2	0.983528013	0.965649989	0.950971816	0.976196758	0.999985752	0.971545917
LOC112441682	0.983528013	0.955652503	0.991936822	0.979644238	0.984100479	0.979480605
NCR3	0.983550423	0.952760839	0.96327718	0.999981514	0.999985752	0.947801358
RFX7	0.983615892	0.956232956	0.993123964	0.999981514	0.970988226	0.948705062
ORC6	0.983615892	0.971775102	0.962034224	0.978503042	0.964301327	0.999948228
ENPEP	0.983684606	0.99960614	0.972897558	0.979433033	0.98094737	0.940256832
LOC781565	0.983684606	0.955652503	0.99647733	0.974717682	0.999985752	0.945480515
LOC535280	0.983684606	0.928658222	0.99341711	0.973075232	0.999985752	0.946112759
MAST2	0.983684606	0.998847958	0.993024884	0.976196758	0.960573822	0.958707771
LOC112442865	0.983684606	0.99960614	0.984886031	0.969837124	0.965676623	0.958790968
THOC7	0.983684606	0.938235006	0.996291804	0.996054502	0.988881329	0.960023723
FOS	0.983684606	0.993417214	0.959725886	0.974941201	0.996781978	0.960877268
TAOK1	0.983684606	0.988540349	0.955221146	0.969256019	0.999985752	0.963107047

ZNF853	0.983684606	0.998847958	0.982232295	0.96900924	0.966328884	0.965783646
NTPCR	0.983684606	0.936058126	0.997300234	0.99313054	0.977167046	0.973649216
TSSK4	0.983684606	0.960355725	0.974975876	0.975753165	0.973219333	0.99828741
HSF1	0.983778584	0.992040539	0.957295966	0.989601002	0.960419102	0.98905196
LOC101907570	0.983796768	0.973847382	0.996224523	0.997660427	0.976259416	0.947838082
RIF1	0.983874367	0.973712113	0.98915348	0.991955963	0.996781978	0.940017687
APTX	0.983900507	0.947341093	0.960945531	0.989432811	0.999985752	0.952563377
STAT1	0.983900507	0.999987083	0.955427429	0.979647979	0.976259416	0.958790968
LOC540707	0.983900507	0.982515431	0.957052938	0.984423083	0.999985752	0.960000414
SMIM10	0.983900507	0.955652503	0.99341711	0.999981514	0.964301327	0.960023723
TM9SF1	0.983900507	0.993481224	0.971053208	0.990955345	0.976259416	0.960023723
LOC101902838	0.983900507	0.96365708	0.955427429	0.999981514	0.987198672	0.963158362
MIDN	0.983900507	0.969051025	0.957052938	0.999981514	0.972160227	0.963456717
GAB1	0.983900507	0.980521483	0.961800035	0.980564765	0.999985752	0.963456717
MOGS	0.983900507	0.934225172	0.968548891	0.999981514	0.987439758	0.972536788
KLHL29	0.983900507	0.918455779	0.999993442	0.97828838	0.962513981	0.972986817
C1QB	0.983900507	0.943754915	0.978530068	0.982220679	0.999453276	0.979480605
TMEM123	0.983900507	0.955333923	0.993024884	0.980564765	0.970988226	0.99218589
KCTD7	0.983900507	0.95530536	0.96327718	0.985878137	0.964301327	0.999948228
ZMIZ2	0.983923577	0.955051921	0.975774524	0.999981514	0.989334846	0.940496128
LOC112442656	0.983923577	0.955333923	0.980061232	0.999981514	0.976292433	0.943621425
ZBTB45	0.983923577	0.952863279	0.971053208	0.999981514	0.984692578	0.967759699
NDN	0.983936294	0.955137018	0.993049856	0.975118969	0.960419102	0.999655834
CSTB	0.983967013	0.961663204	0.972741989	0.999981514	0.970988226	0.950631909
HNRNPH1	0.984169479	0.918854612	0.999993442	0.985701713	0.996781978	0.955990509
LMTK2	0.984169479	0.922285873	0.958410013	0.997374305	0.999985752	0.966212838
WDFY1	0.984169479	0.918854612	0.962734642	0.999981514	0.999985752	0.971545917
GJA10	0.984722059	0.924099465	0.989494193	0.988408846	0.979840175	0.997054363
MICAL1	0.984914824	0.935661607	0.99341711	0.999981514	0.965676623	0.976568885
CFAP53	0.984914824	0.923232089	0.973237403	0.972467586	0.999985752	0.98140846
SLC25A19	0.984914824	0.939442799	0.964807927	0.984421126	0.999985752	0.988796537
CHST9	0.984914824	0.920786925	0.964807927	0.973373329	0.999985752	0.999463809
EIF5B	0.984914824	0.930293015	0.972073392	0.985701713	0.976769047	0.999948228
ZNF581	0.985088452	0.976310661	0.961800035	0.999981514	0.965676623	0.943285784
ZNF774	0.985099366	0.982062841	0.99341711	0.973075232	0.999453276	0.940256832
GAS2L3	0.985099366	0.99960614	0.966199203	0.999981514	0.967116582	0.940647189
LOC790871	0.985099366	0.941708558	0.999993442	0.97731816	0.987756516	0.944927301
PUS1	0.985099366	0.985594367	0.999993442	0.973075232	0.964301327	0.947937638

ZNF445	0.985099366	0.939956757	0.99647733	0.996033721	0.998141915	0.952532102
SLC2A4RG	0.985099366	0.991673407	0.96638833	0.96951401	0.999985752	0.957165156
LOC100296211	0.985099366	0.989303356	0.992263335	0.970785492	0.988049878	0.958790968
HAUS1	0.985099366	0.964418016	0.976484937	0.999981514	0.966121925	0.959723327
LOC112442071	0.985099366	0.961368616	0.999993442	0.999981514	0.965163023	0.960023723
VKORC1L1	0.985099366	0.922285873	0.999993442	0.999981514	0.96080502	0.963015949
PSME4	0.985099366	0.933100216	0.954020369	0.999981514	0.999985752	0.969197073
TRAPPC12	0.985099366	0.98098403	0.99341711	0.973075232	0.971075533	0.9803583
VPS26C	0.985099366	0.939956757	0.959725886	0.9880671	0.999985752	0.988635999
FBXO15	0.985099366	0.954237721	0.961354574	0.999981514	0.973219333	0.988796537
KYAT1	0.985110386	0.919525428	0.963219965	0.999981514	0.977167046	0.999948228
AGTRAP	0.985200826	0.948626145	0.966232793	0.999981514	0.972432486	0.943972369
CD300LG	0.985200826	0.99528564	0.983582726	0.996621929	0.968739995	0.945480515
ETV1	0.985200826	0.94019273	0.969404007	0.999981514	0.98865576	0.965117554
PRKCE	0.985200826	0.99960614	0.967590756	0.974823341	0.970491451	0.969197073
BAG6	0.985200826	0.930293015	0.99341711	0.999981514	0.964301327	0.983430934
TMEM19	0.985200826	0.920978623	0.986920775	0.989485949	0.970988226	0.999948228
ANKRD16	0.985277538	0.951172741	0.999993442	0.976196758	0.976292433	0.940910488
TUBGCP4	0.985277538	0.922285873	0.999993442	0.970176086	0.999985752	0.948705062
F2R	0.985277538	0.949902864	0.989069245	0.999981514	0.976292433	0.958707771
ACSBG1	0.985277538	0.941817736	0.969404007	0.999981514	0.999985752	0.960023723
FBLN5	0.985277538	0.988444882	0.97773026	0.97828838	0.989334846	0.964898868
CPTP	0.985277538	0.934301396	0.955427429	0.999981514	0.999985752	0.973218068
CCDC120	0.985401029	0.955204917	0.962832886	0.999981514	0.987723553	0.957165156
OTOGL	0.985401029	0.971458291	0.957681124	0.999981514	0.988049878	0.967693922
RFNG	0.985401029	0.96365708	0.975774524	0.996195248	0.979874385	0.9802309
CSNK1G3	0.985545194	0.99431777	0.963164828	0.999981514	0.966967496	0.944330889
CCDC69	0.985545194	0.959396625	0.968679299	0.999981514	0.999985752	0.945532151
FNDC4	0.985545194	0.976596938	0.983534298	0.978503042	0.999985752	0.946760037
FAM129B	0.985545194	0.921940584	0.956423184	0.999981514	0.999985752	0.950798794
MTMR9	0.985545194	0.938087741	0.999993442	0.995565324	0.991747435	0.95662324
BACH1	0.985545194	0.994993704	0.980199146	0.974286747	0.983884995	0.960023723
ESCO1	0.985545194	0.947911876	0.97773026	0.978503042	0.999985752	0.960877268
PCMTD2	0.985545194	0.993481224	0.981655709	0.984423083	0.965392454	0.971545917
E4F1	0.985545194	0.927973597	0.9965509	0.995565324	0.984866432	0.975200512
ARMC7	0.985545194	0.977769595	0.962797326	0.999981514	0.961465747	0.9790177
LOC112447846	0.985545194	0.957328149	0.964661607	0.999981514	0.962166847	0.982587107
LOC104971266	0.985545194	0.943712473	0.989494193	0.980605008	0.976292433	0.997486942

LOC112444147	0.985582357	0.936822825	0.99341711	0.984423083	0.996781978	0.979480605
EIF4EBP2	0.985583093	0.926905849	0.974967552	0.999981514	0.965405395	0.952663521
HPS5	0.985583093	0.953042388	0.957295966	0.986051493	0.999985752	0.986042926
LOC101907615	0.985738598	0.926905849	0.978014482	0.999981514	0.965405395	0.967308566
BLOC1S5	0.985864769	0.941838807	0.999993442	0.988408846	0.965163023	0.985165897
AKAP2	0.98598265	0.961663204	0.989494193	0.996033721	0.999985752	0.941867365
GSTP1	0.98598265	0.936319037	0.992511421	0.981863335	0.999985752	0.945532151
KDM1A	0.98598265	0.931411766	0.968028851	0.999981514	0.999985752	0.947937638
GPR19	0.98598265	0.942088099	0.989640118	0.999981514	0.972432486	0.950540248
LOC104975044	0.98598265	0.934225172	0.999993442	0.982611153	0.999985752	0.958281344
PRKAR1B	0.98598265	0.930293015	0.991138474	0.999981514	0.964301327	0.96248183
LOC101905571	0.98598265	0.929514642	0.979584814	0.999981514	0.983941532	0.970105102
ZFHX4	0.98598265	0.936244328	0.99647733	0.989601002	0.996494051	0.970216353
GNPDA2	0.98598265	0.959263706	0.965961035	0.999981514	0.977158496	0.971545917
PPP3CB	0.98598265	0.949902864	0.999993442	0.973576494	0.964768333	0.975558533
TRIAP1	0.98598265	0.94422395	0.989069245	0.995565324	0.986003146	0.979505709
OGDH	0.98598265	0.920217996	0.981655709	0.9880671	0.999985752	0.988252822
LOC107132251	0.98598265	0.971823688	0.972806636	0.979644238	0.972160227	0.998284411
LOC783803	0.98598265	0.950675653	0.964382669	0.99074899	0.98094737	0.99828741
ELMOD2	0.98598265	0.926905849	0.956842825	0.992930508	0.964301327	0.999948228
WHAMM	0.985993103	0.930633315	0.996624589	0.999981514	0.974738415	0.947838082
SNX8	0.985999289	0.962037123	0.972641134	0.999981514	0.995515529	0.945282233
RIMS3	0.985999289	0.976596938	0.999993442	0.972142185	0.986003146	0.945480515
RNF144A	0.985999289	0.933100216	0.999993442	0.999021176	0.972160227	0.946112759
RBM11	0.985999289	0.981920173	0.996224523	0.999981514	0.961465747	0.954925542
VKORC1	0.985999289	0.922587381	0.999993442	0.999981514	0.964301327	0.958707771
LOC112441494	0.985999289	0.955652503	0.973562292	0.990955345	0.999985752	0.958790968
ADAMTSL4	0.985999289	0.964761409	0.964382669	0.999981514	0.982087479	0.960023723
MAGOHB	0.985999289	0.950126059	0.999993442	0.985435217	0.979290191	0.970071797
UBAP1	0.985999289	0.947247457	0.979674024	0.996054502	0.998196897	0.970105102
ICK	0.985999289	0.952760839	0.957052938	0.999981514	0.983941532	0.973413452
PPFIBP2	0.985999289	0.927973597	0.965819476	0.996033721	0.999985752	0.9803583
MRPS17	0.985999289	0.927163124	0.997400824	0.999981514	0.970988226	0.981216824
SLC35F6	0.985999289	0.951949541	0.981383697	0.999180255	0.969887518	0.994347099
TMEM164	0.985999289	0.932191409	0.961437412	0.997536986	0.968585482	0.999948228
KIFC3	0.985999289	0.931588197	0.973562292	0.972142185	0.982526545	0.999948228
TRIL	0.985999289	0.927853707	0.965229237	0.972142185	0.996781978	0.999948228
BFSP2	0.986010222	0.958814007	0.976484937	0.999981514	0.964301327	0.988190394

NUDT1	0.98602291	0.985997726	0.982823002	0.979385838	0.970980375	0.985951746
IKZF2	0.98602291	0.938087741	0.996224523	0.992930508	0.976292433	0.987565013
LOC101905265	0.986094009	0.928213372	0.999993442	0.999981514	0.962513981	0.965117554
PHF5A	0.986094009	0.926905849	0.999993442	0.999981514	0.965676623	0.973649216
INPPL1	0.986094009	0.948801019	0.973562292	0.9819628	0.996809631	0.991585102
RBFOX3	0.986202427	0.990546209	0.999993442	0.98061629	0.964301327	0.951057971
RAB33B	0.986299361	0.947350735	0.979494108	0.987680286	0.970988226	0.999948228
LARGE1	0.986312578	0.95856975	0.992728681	0.999981514	0.989379051	0.945480515
RSPH3	0.986312578	0.936082042	0.996224523	0.979644238	0.999985752	0.945480515
CXCL12	0.986312578	0.946148902	0.964382669	0.999981514	0.999985752	0.945480515
TWF2	0.986312578	0.938087741	0.96102707	0.999981514	0.999985752	0.946842302
ERCC3	0.986312578	0.935661607	0.999993442	0.990955345	0.998287238	0.947801358
MMP17	0.986312578	0.998847958	0.97251482	0.98981682	0.982087479	0.948493473
HLTF	0.986312578	0.997332173	0.99341711	0.989601002	0.968472466	0.950631909
ACKR3	0.986312578	0.947350735	0.999993442	0.990955345	0.962513981	0.950798794
LOC534742	0.986312578	0.93988991	0.979584814	0.999981514	0.996089557	0.954279607
PPM1H	0.986312578	0.938701207	0.996844843	0.975346638	0.999985752	0.954496768
ACOX1	0.986312578	0.991673407	0.975774524	0.999981514	0.964301327	0.957876917
TCHH	0.986312578	0.96643574	0.978014482	0.999981514	0.976292433	0.958790968
GOLPH3L	0.986312578	0.934301396	0.96030829	0.999981514	0.997354523	0.958790968
ISG20L2	0.986312578	0.946148902	0.961227258	0.999981514	0.999985752	0.95883396
HYLS1	0.986312578	0.973712113	0.992377532	0.999981514	0.964301327	0.960023723
ETV6	0.986312578	0.97523862	0.996224523	0.999981514	0.968726264	0.960023723
RING1	0.986312578	0.982011436	0.987610559	0.9880671	0.991481886	0.960023723
GULO	0.986312578	0.938817997	0.957295966	0.996033721	0.999985752	0.960023723
UAP1L1	0.986312578	0.935661607	0.976484937	0.999981514	0.985360041	0.962419223
GNG12	0.986312578	0.924173915	0.999993442	0.999981514	0.967116582	0.962872722
C19H17orf49	0.986312578	0.99386673	0.963164828	0.97731816	0.998287238	0.963145894
NCOA4	0.986312578	0.92546629	0.959903168	0.999981514	0.970988226	0.963456717
GRPEL2	0.986312578	0.926551255	0.991138474	0.991955963	0.999985752	0.965428409
TOM1L1	0.986312578	0.940543846	0.996624589	0.999981514	0.976292433	0.966212838
NDUFA12	0.986312578	0.98098403	0.990118874	0.978503042	0.991531798	0.966850975
CTSD	0.986312578	0.98098403	0.977642672	0.999981514	0.964301327	0.96870908
OXR1	0.986312578	0.927853707	0.976443473	0.999981514	0.983941532	0.96870908
GNPAT	0.986312578	0.926051993	0.999993442	0.997660427	0.987723553	0.969014913
CHST3	0.986312578	0.930293015	0.999993442	0.992560663	0.972499705	0.970105102
TBCD	0.986312578	0.968509005	0.99341711	0.9880671	0.988049878	0.970105102
ELOC	0.986312578	0.976520191	0.980496473	0.982220679	0.996781978	0.970105102

NIM1K	0.986312578	0.930293015	0.984886031	0.98981682	0.999985752	0.970105102
ARHGAP31	0.986312578	0.98098403	0.989720033	0.99542943	0.97234838	0.971742751
CRYBA4	0.986312578	0.931411766	0.999993442	0.992930508	0.97352263	0.972314037
UBE2E2	0.986312578	0.943820266	0.964762618	0.999981514	0.995870928	0.972695131
CASP2	0.986312578	0.981520642	0.971556427	0.999981514	0.967563029	0.97701
LOC101904039	0.986312578	0.991673407	0.975774524	0.97676218	0.983620937	0.978087957
LRRC32	0.986312578	0.953449552	0.999993442	0.994395954	0.966967496	0.979480605
NOP53	0.986312578	0.976823261	0.97773026	0.996054502	0.976292433	0.979480605
UAP1	0.986312578	0.950986475	0.97763883	0.990955345	0.996781978	0.980840077
JMJD8	0.986312578	0.947350735	0.976295638	0.995565324	0.996089557	0.98140846
LOC101909384	0.986312578	0.94592331	0.990118874	0.999981514	0.974635634	0.983430934
LOC112444502	0.986312578	0.950986475	0.980761647	0.982220679	0.998016692	0.984829839
ZRANB3	0.986312578	0.991673407	0.96043275	0.989601002	0.976259416	0.985027129
ING5	0.986312578	0.964418016	0.998777236	0.988408846	0.964301327	0.98575911
ATP6AP1L	0.986312578	0.950675653	0.964382669	0.98981682	0.999985752	0.986042926
NIPAL1	0.986312578	0.936822825	0.999993442	0.980564765	0.984386856	0.986638746
MFAP3L	0.986312578	0.947350735	0.959903168	0.999981514	0.968726264	0.988639786
LOC100139990	0.986312578	0.921973207	0.976589023	0.999981514	0.989334846	0.988639786
MAP6D1	0.986312578	0.923232089	0.957052938	0.999981514	0.976259416	0.989084358
LOC101907581	0.986312578	0.948801019	0.99341711	0.984421126	0.976419607	0.994887496
LOC101902705	0.986312578	0.931411766	0.992511421	0.985118339	0.987723553	0.994887496
PLBD2	0.986312578	0.972007528	0.957052938	0.999981514	0.965163023	0.996335668
C22H3orf14	0.986312578	0.948801019	0.982823002	0.980564765	0.984386856	0.997383134
TRDN	0.986312578	0.95687851	0.964807927	0.989601002	0.964768333	0.999948228
LOC784297	0.986312578	0.95310464	0.972741989	0.988408846	0.976259416	0.999948228
PRSS48	0.986312578	0.940956417	0.957052938	0.979644238	0.976292433	0.999948228
EIF3F	0.986312578	0.928658222	0.95558643	0.999981514	0.976769047	0.999948228
INHBA	0.986348243	0.99385517	0.991138474	0.993215785	0.976259416	0.948081656
LOC104975925	0.986348243	0.966400376	0.969026634	0.996195248	0.999985752	0.966850975
SERINC1	0.986348243	0.929409572	0.980851214	0.999981514	0.96755608	0.993890045
NEK8	0.986387224	0.946258461	0.999993442	0.999981514	0.986861597	0.944973383
SERINC3	0.986387224	0.969193477	0.978014482	0.999981514	0.989334846	0.951081819
MXD1	0.986387224	0.939442799	0.999993442	0.9880671	0.983941532	0.953140004
ORAI3	0.986387224	0.999987083	0.973562292	0.9880671	0.964301327	0.960023723
HOXB2	0.986387224	0.969051025	0.990615319	0.973812087	0.999985752	0.960023723
IL27RA	0.986387224	0.945755356	0.999993442	0.999981514	0.977193348	0.963456717
IRAK1BP1	0.986387224	0.981952108	0.976484937	0.992212416	0.984386856	0.971545917
PGA5	0.986387224	0.939956757	0.988390948	0.995434268	0.996781978	0.9790177

LOC526769	0.986387224	0.927853707	0.976484937	0.983960202	0.999985752	0.988273129
ISYNA1	0.986387224	0.934102864	0.975774524	0.999981514	0.971133034	0.989184798
LOC100300483	0.986416026	0.998847958	0.972806636	0.985878137	0.97028586	0.970105102
LOC107133048	0.986587028	0.980609227	0.957052938	0.99313054	0.990937627	0.98140846
TMEM115	0.986623795	0.927973597	0.999993442	0.973744943	0.970988226	0.996335668
BOC	0.986826143	0.938235006	0.992714073	0.992212416	0.999985752	0.955210102
PDHA1	0.986826143	0.925047999	0.976563428	0.999981514	0.999985752	0.959198304
FASTKD3	0.986826143	0.926905849	0.99647733	0.974272706	0.969253072	0.999948228
SPRY1	0.986919143	0.95298741	0.972741989	0.999981514	0.999985752	0.947838082
ANXA5	0.987001521	0.953042388	0.975774524	0.999981514	0.986003146	0.946165841
NOM1	0.987001521	0.999987083	0.964807927	0.98981682	0.964301327	0.950415692
AHCYL1	0.987001521	0.927853707	0.957052938	0.999981514	0.999985752	0.954496768
ANKRD49	0.987001521	0.978998282	0.981655709	0.992339478	0.999453276	0.955140534
ATP13A1	0.987001521	0.991760961	0.968028851	0.989144862	0.998287238	0.957588281
LOC112443007	0.987001521	0.99960614	0.967590756	0.995565324	0.970212692	0.958281344
ERLIN1	0.987001521	0.937448751	0.999993442	0.999981514	0.979290191	0.960023723
RAB32	0.987001521	0.982559387	0.958200097	0.998529748	0.999859158	0.960023723
ABI3BP	0.987001521	0.932394105	0.992511421	0.996033721	0.999985752	0.963456717
LOC100140207	0.987001521	0.954336228	0.955427429	0.999981514	0.97234838	0.966382321
ZSCAN12	0.987001521	0.985195756	0.957052938	0.999981514	0.993190318	0.966850975
RASAL1	0.987001521	0.948038876	0.984886031	0.999981514	0.991481886	0.967056707
MXD3	0.987001521	0.926330259	0.999993442	0.999981514	0.981652321	0.971545917
CFAP298	0.987001521	0.96672074	0.957052938	0.991955963	0.999985752	0.9790177
TRIM2	0.987001521	0.989303356	0.957052938	0.991955963	0.9683846	0.996310803
LOC100848205	0.987001521	0.954005878	0.973562292	0.9880671	0.97077135	0.999948228
C10H15orf65	0.987001521	0.938454625	0.959725886	0.992930508	0.973219333	0.999948228
DXO	0.98704626	0.943213236	0.991936822	0.989485949	0.976292433	0.99666605
KLF5	0.987163425	0.971455677	0.962832886	0.998667933	0.999985752	0.95883396
NGEF	0.987233818	0.948801019	0.971594714	0.973075232	0.999985752	0.984957404
GPR1	0.987257806	0.932778537	0.999993442	0.992930508	0.997264208	0.945532151
RMDN3	0.987257806	0.950336973	0.999993442	0.999981514	0.984386856	0.950540248
WDR5	0.987257806	0.950336973	0.968028851	0.999981514	0.976292433	0.978073306
TUBA3E	0.987257806	0.953694236	0.958410013	0.999981514	0.984152256	0.99666605
COG7	0.98734377	0.927853707	0.999993442	0.988637603	0.976292433	0.960023723
TMEM101	0.98743963	0.979118997	0.997400824	0.997792939	0.984386856	0.944973383
LOC515418	0.98743963	0.991673407	0.999993442	0.990036762	0.964301327	0.945480515
MFSD1	0.98743963	0.991673407	0.984886031	0.999981514	0.970988226	0.945532151
GBX1	0.98743963	0.977196455	0.996224523	0.999981514	0.984386856	0.946760037

PLEKHA7	0.98743963	0.940500178	0.999993442	0.992339478	0.974026015	0.947801358
IFITM2	0.98743963	0.991673407	0.995377403	0.982748016	0.989379051	0.948219411
TMCC2	0.98743963	0.993275893	0.978014482	0.999981514	0.970988226	0.94844234
AQP7	0.98743963	0.993481224	0.979494108	0.999981514	0.972432486	0.948705062
SERPINB9	0.98743963	0.99950853	0.980761647	0.995434268	0.973502409	0.950631909
LOC100848419	0.98743963	0.929204871	0.980032241	0.999981514	0.999985752	0.951081819
ZNF597	0.98743963	0.955652503	0.962797326	0.999981514	0.999985752	0.951493662
KLF13	0.98743963	0.981520642	0.979494108	0.999981514	0.976292433	0.953095171
PRKAB1	0.98743963	0.927853707	0.999993442	0.999981514	0.996781978	0.953509765
ALDH2	0.98743963	0.999621646	0.961437412	0.999981514	0.964449703	0.955140534
ALDH1L1	0.98743963	0.993481224	0.988307089	0.999981514	0.970980375	0.955210102
GLMP	0.98743963	0.985997726	0.984886031	0.999981514	0.983010476	0.955210102
BIVM	0.98743963	0.991673407	0.964398608	0.982815793	0.999985752	0.955990509
LOC112447301	0.98743963	0.977196455	0.968233314	0.9880671	0.999985752	0.955990509
LOC101907006	0.98743963	0.948801019	0.996224523	0.999981514	0.976259416	0.958790968
PDCL	0.98743963	0.955652503	0.999841067	0.986879372	0.999301912	0.958790968
ZNF830	0.98743963	0.998847958	0.96102707	0.999981514	0.968585482	0.960023723
ZNF569	0.98743963	0.993028532	0.991936822	0.989601002	0.979290191	0.960023723
LRP12	0.98743963	0.950336973	0.978014482	0.999981514	0.995018375	0.960023723
SNX21	0.98743963	0.94270411	0.958454497	0.999981514	0.999985752	0.960023723
LOC112449099	0.98743963	0.953841555	0.963821408	0.999981514	0.999985752	0.960023723
TTLL4	0.98743963	0.939442799	0.999993442	0.979644238	0.999985752	0.96053762
AP3M1	0.98743963	0.956406487	0.999993442	0.999981514	0.969887518	0.961265889
LOC112444463	0.98743963	0.988444882	0.999993442	0.980564765	0.976259416	0.96248183
GCLC	0.98743963	0.965482645	0.957295966	0.999981514	0.999985752	0.96274079
LOC618733	0.98743963	0.965405402	0.971053208	0.999981514	0.976259416	0.963107047
TXNDC16	0.98743963	0.961663204	0.96030829	0.999981514	0.964301327	0.965117554
METTL17	0.98743963	0.961055437	0.962553873	0.999669429	0.999985752	0.965117554
RASA1	0.98743963	0.965648998	0.964807927	0.999981514	0.966328884	0.965705577
TBC1D1	0.98743963	0.936822825	0.998848708	0.999981514	0.964501754	0.966850975
LOC782024	0.98743963	0.953345357	0.997850143	0.998529748	0.985360041	0.967047449
RNPS1	0.98743963	0.983100982	0.957052938	0.999981514	0.979759416	0.967056707
PIGP	0.98743963	0.955652503	0.997850143	0.989601002	0.995318217	0.96870908
RRP1	0.98743963	0.988008216	0.958197827	0.998529748	0.992245178	0.969014913
LOC790098	0.98743963	0.930633315	0.958185071	0.999981514	0.982087479	0.969197073
PKP4	0.98743963	0.964206062	0.999993442	0.985118339	0.987723553	0.970105102
MMD	0.98743963	0.952344105	0.99615564	0.986051493	0.999453276	0.970464944
NUDT19	0.98743963	0.927746665	0.999993442	0.995312373	0.99581977	0.971545917

FBXW9	0.98743963	0.988008216	0.996224523	0.985701713	0.971133034	0.973294257
GAB2	0.98743963	0.95310464	0.999993442	0.97731816	0.970988226	0.973450579
NLE1	0.98743963	0.946859049	0.96608402	0.999981514	0.984386856	0.973480626
ZNF169	0.98743963	0.983305314	0.964807927	0.999981514	0.965163023	0.973649216
ANKRD13D	0.98743963	0.929409572	0.999993442	0.999981514	0.967205491	0.974962631
LZTFL1	0.98743963	0.96643574	0.966232793	0.999981514	0.976292433	0.975170737
ELOA	0.98743963	0.939956757	0.999993442	0.9880671	0.984386856	0.976246852
LOC513969	0.98743963	0.932191409	0.984886031	0.993481918	0.999985752	0.978087957
RAB27A	0.98743963	0.970965452	0.999993442	0.982220679	0.976292433	0.9790177
DOT1L	0.98743963	0.942088099	0.999993442	0.978654231	0.987723553	0.9790177
POP7	0.98743963	0.933100216	0.974431297	0.999981514	0.976259416	0.980167535
NME9	0.98743963	0.964185302	0.978014482	0.9880671	0.998338718	0.9803583
MAD1L1	0.98743963	0.938087741	0.999993442	0.981295935	0.976292433	0.980556133
LOC101903402	0.98743963	0.954237721	0.99615564	0.995565324	0.978453925	0.981216824
PTGS1	0.98743963	0.977196455	0.963821408	0.999981514	0.976292433	0.98140846
LOC613664	0.98743963	0.957667701	0.989494193	0.995434268	0.984386856	0.98140846
RANBP17	0.98743963	0.944261937	0.957963367	0.999981514	0.986019537	0.98140846
EPHA7	0.98743963	0.943213236	0.955845583	0.990364645	0.999985752	0.985165897
KDELR1	0.98743963	0.927853707	0.959725886	0.99739416	0.999985752	0.987840151
KRT10	0.98743963	0.954237721	0.958410013	0.999981514	0.998287238	0.98829088
C22H3orf20	0.98743963	0.930293015	0.999145937	0.999981514	0.970988226	0.988320707
LOC781158	0.98743963	0.938390602	0.99341711	0.995565324	0.985250243	0.988639786
ITGB1BP1	0.98743963	0.929204871	0.968679299	0.994331516	0.999985752	0.988796537
TSPAN32	0.98743963	0.950986475	0.964807927	0.989485949	0.999985752	0.988866378
MAP3K9	0.98743963	0.99386673	0.975774524	0.974793558	0.964301327	0.996453303
LOC101906347	0.98743963	0.930293015	0.996224523	0.989601002	0.980189546	0.996679516
LOC112446004	0.98743963	0.932245603	0.961437412	0.992930508	0.999985752	0.997206681
HPCAL4	0.98743963	0.928483112	0.992991376	0.992930508	0.977167046	0.998175127
RPS21	0.98743963	0.933100216	0.957052938	0.986116445	0.999985752	0.998284411
LOC100847759	0.98743963	0.942273776	0.959725886	0.999981514	0.976259416	0.998458498
RNGTT	0.98743963	0.960185644	0.977455115	0.995434268	0.967116582	0.999729224
LOC112441827	0.98743963	0.9640374	0.95940506	0.999981514	0.970988226	0.999729224
FBXL17	0.98743963	0.946503637	0.95940506	0.997792939	0.971133034	0.999948228
TTC21B	0.987555645	0.991673407	0.957295966	0.999981514	0.976088926	0.959597988
PCK2	0.987696141	0.948801019	0.992511421	0.990955345	0.999985752	0.965456017
LOC101908034	0.987696141	0.932530199	0.980813082	0.98981682	0.986642926	0.999948228
CERK	0.987732445	0.942088099	0.999993442	0.992560663	0.97464569	0.947801358
GAN	0.987732445	0.990421548	0.975774524	0.999981514	0.983941532	0.952563377

DIS3L	0.987732445	0.985997726	0.975774524	0.999981514	0.964727486	0.960023723
MOCS3	0.987732445	0.948801019	0.998899782	0.999981514	0.976292433	0.966850975
LOC112443452	0.987732445	0.955652503	0.996224523	0.983233117	0.997422012	0.975170737
LOC614207	0.987732445	0.98098403	0.958410013	0.984423083	0.999985752	0.983590245
PRMT2	0.987732445	0.927973597	0.957148447	0.999981514	0.972160227	0.997206681
DHX32	0.987767831	0.966069053	0.959903168	0.999981514	0.999985752	0.959275214
LOC515150	0.987767831	0.98572429	0.990615319	0.98240262	0.970491451	0.988866378
PANK2	0.987812103	0.942902732	0.97773026	0.982220679	0.999985752	0.978087957
TXNDC11	0.987840182	0.98098403	0.987786638	0.999981514	0.989334846	0.960023723
TTF2	0.987848424	0.953841555	0.991109288	0.979385838	0.967116582	0.999948228
KIAA0355	0.988014622	0.932778537	0.97998887	0.999981514	0.991534656	0.958707771
LCMT1	0.988113	0.973321075	0.999993442	0.999981514	0.969470443	0.947838082
UBE2T	0.988113	0.95689647	0.996224523	0.975346638	0.998060725	0.9803583
CRLS1	0.988128193	0.934102864	0.998364815	0.999981514	0.996781978	0.960023723
LOC112448169	0.988139492	0.938087741	0.999993442	0.9880671	0.984386856	0.947478418
SLC26A9	0.988139492	0.953449552	0.994035957	0.999981514	0.976259416	0.967056707
LOC112446029	0.988139492	0.969051025	0.959725886	0.999981514	0.999985752	0.969014913
NIPSNAP3A	0.988414099	0.950336973	0.985172237	0.999981514	0.998287238	0.972314037
TTC17	0.988557836	0.964984046	0.994979324	0.984423083	0.999985752	0.960023723
MYORG	0.98858654	0.973712113	0.999993442	0.983233117	0.975762765	0.969564816
LOC505199	0.988665044	0.980833065	0.980657691	0.999981514	0.976292433	0.951081819
ST8SIA1	0.988665044	0.99960614	0.964807927	0.997792939	0.979290191	0.95524009
ZNF274	0.988665044	0.977196455	0.999993442	0.990955345	0.964501754	0.970105102
LOC101908204	0.988665044	0.99386673	0.987789469	0.979433033	0.976292433	0.978087957
AGO4	0.988694293	0.927973597	0.999280799	0.984423083	0.999985752	0.970723729
RAB8A	0.988961407	0.953177704	0.990615319	0.989601002	0.97077135	0.999948228
XPO7	0.98896747	0.929460134	0.978530068	0.999981514	0.981652321	0.986435724
PPP1R12C	0.98896747	0.944532792	0.99341711	0.998529748	0.977193348	0.989840954
LOC100848478	0.989040567	0.938817997	0.966232793	0.999981514	0.999985752	0.947937638
LOC112441469	0.989040567	0.999987083	0.95940506	0.988408846	0.983397176	0.94844234
ICAM1	0.989040567	0.943859632	0.999993442	0.980632768	0.991531798	0.948713925
LAMC1	0.989040567	0.974143317	0.999993442	0.9880671	0.976259416	0.953140004
PEX14	0.989040567	0.95462892	0.995746693	0.999981514	0.984386856	0.956208083
PROM1	0.989040567	0.981520642	0.999993442	0.98981682	0.967003003	0.960023723
VPS13B	0.989040567	0.966057895	0.968233314	0.988408846	0.999985752	0.966212838
LOC112444626	0.989040567	0.960295155	0.957052938	0.97860339	0.999985752	0.967759699
THOC6	0.989040567	0.954237721	0.999993442	0.982220679	0.992104882	0.971545917
TBC1D7	0.989040567	0.969051025	0.980061232	0.999981514	0.977193348	0.971608881

ARFGEF2	0.989040567	0.95028107	0.963164828	0.997660427	0.999985752	0.972314037
NFKBIL1	0.989040567	0.994185904	0.984886031	0.995565324	0.966967496	0.973218068
LOC514680	0.989040567	0.942088099	0.966232793	0.999981514	0.999985752	0.978922587
DND1	0.989040567	0.936319037	0.996224523	0.984423083	0.999985752	0.9790177
NME1	0.989040567	0.943244588	0.975774524	0.99739416	0.999985752	0.986598172
GEN1	0.989040567	0.942088099	0.973237403	0.995565324	0.999985752	0.988639786
ABI2	0.989040567	0.989303356	0.971053208	0.991955963	0.972432486	0.989642028
LMBRD2	0.989040567	0.940618635	0.99341711	0.996033721	0.980985349	0.994347099
LOC112447291	0.989040567	0.933100216	0.980061232	0.989707094	0.976259416	0.999948228
MCCD1	0.989086032	0.967037247	0.975185526	0.999981514	0.996781978	0.950631909
CNMD	0.989086032	0.967034954	0.999993442	0.989601002	0.970988226	0.954925542
GDPGP1	0.989086032	0.999987083	0.968679299	0.998177865	0.969739417	0.958474163
LOC112446021	0.989086032	0.92905835	0.966232793	0.999981514	0.997422012	0.958707771
MASP1	0.989086032	0.988444882	0.999993442	0.990955345	0.970988226	0.958790968
LOC616295	0.989086032	0.947911876	0.991363218	0.996033721	0.999985752	0.959275214
LOC100847825	0.989086032	0.936891528	0.999993442	0.999981514	0.972128973	0.960023723
MTFMT	0.989086032	0.940956417	0.999993442	0.999981514	0.977167046	0.960023723
LOC112448260	0.989086032	0.98098403	0.985206685	0.999981514	0.987723553	0.960023723
MLLT6	0.989086032	0.94592331	0.958200097	0.98981682	0.999985752	0.960023723
BTK	0.989086032	0.954237721	0.997400824	0.991955963	0.999985752	0.960120928
UPP1	0.989086032	0.997678999	0.963723107	0.999981514	0.970980375	0.960251802
PPM1D	0.989086032	0.943754915	0.999993442	0.983233117	0.999985752	0.961873176
NCOR1	0.989086032	0.966661805	0.999993442	0.98981682	0.985250243	0.96248183
LOC101906411	0.989086032	0.938087741	0.999993442	0.988761333	0.999985752	0.96274079
PLEKHA4	0.989086032	0.96365708	0.999993442	0.995434268	0.976292433	0.966850975
TAP2	0.989086032	0.95530536	0.990615319	0.999981514	0.974931134	0.967759699
LAMP1	0.989086032	0.950126059	0.990118874	0.999981514	0.9683846	0.970071797
LOC100852077	0.989086032	0.98098403	0.963164828	0.995312373	0.999985752	0.970071797
IRF2BP1	0.989086032	0.946148902	0.99341711	0.999981514	0.9683846	0.972695131
DOK1	0.989086032	0.98098403	0.993024884	0.996667757	0.976292433	0.973218068
MARK1	0.989086032	0.993249767	0.972806636	0.995565324	0.979290191	0.973649216
LOC101906472	0.989086032	0.969811486	0.993024884	0.999981514	0.972476988	0.973675921
AOX4	0.989086032	0.949902864	0.999993442	0.999981514	0.967470055	0.97521287
LOC112444474	0.989086032	0.937598531	0.981655709	0.999981514	0.982208423	0.9790177
LOC101905894	0.989086032	0.998847958	0.968679299	0.98981682	0.970491451	0.982422737
SNX27	0.989086032	0.950986475	0.994035957	0.9880671	0.996781978	0.982937529
LOC100848872	0.989086032	0.939457142	0.979494108	0.97828838	0.999985752	0.982937529
OSBPL9	0.989086032	0.991673407	0.972482052	0.998529748	0.970988226	0.984172542

CHMP4C	0.989086032	0.966052556	0.976011371	0.979217817	0.999985752	0.98469083
MFN2	0.989086032	0.941112519	0.99341711	0.995434268	0.989334846	0.988796537
MRPL45	0.989086032	0.931610273	0.997833363	0.995565324	0.976292433	0.99666605
LOC107132942	0.989086032	0.929737582	0.995746693	0.982998681	0.996781978	0.99666605
CETN4	0.989086032	0.957385532	0.972806636	0.998831122	0.965163023	0.999948228
LOC104975290	0.989086032	0.975612288	0.964807927	0.985701713	0.965676623	0.999948228
LOC101902856	0.989246259	0.953449552	0.964661607	0.999981514	0.977167046	0.995895432
TNIK	0.989413705	0.95462892	0.971594714	0.986340119	0.999985752	0.989309315
FOXK1	0.989477255	0.932739256	0.999993442	0.984289938	0.983941532	0.963107047
LOC112441455	0.989477255	0.939956757	0.99647733	0.979644238	0.999985752	0.963107047
BICRAL	0.989538455	0.993481224	0.999993442	0.979584502	0.970988226	0.951081819
DCHS2	0.989541818	0.98098403	0.97316848	0.999981514	0.972806049	0.955990509
HOXC9	0.989541818	0.9640374	0.999993442	0.98981682	0.988881329	0.969197073
SLC6A3	0.98963428	0.999987083	0.961800035	0.980564765	0.989379051	0.951081819
LOC112447347	0.98963428	0.977196455	0.992167185	0.999981514	0.965676623	0.953667689
MCPH1	0.98963428	0.980462136	0.999993442	0.988408846	0.976259416	0.973218068
ATG4D	0.989642953	0.968828874	0.999703589	0.999981514	0.983941532	0.953509765
SHISAL2A	0.989642953	0.964185302	0.999993442	0.979584502	0.976259416	0.954496768
LOC101902067	0.989642953	0.946148902	0.999993442	0.999981514	0.976292433	0.971545917
TGS1	0.989642953	0.942088099	0.999993442	0.988637603	0.976292433	0.975170737
SLC4A7	0.989642953	0.941400719	0.99341711	0.984423083	0.999985752	0.977503497
RPL12	0.989670433	0.942546802	0.985081637	0.999981514	0.984864146	0.992190617
MON2	0.989670433	0.94019273	0.984886031	0.995565324	0.976292433	0.999948228
LOC112448894	0.989688358	0.99960614	0.992324476	0.992560663	0.972160227	0.949201688
ZNHIT6	0.989688358	0.974143317	0.997400824	0.991207957	0.990937627	0.963112102
LIAS	0.989688358	0.936822825	0.996224523	0.995312373	0.999985752	0.966850975
FEN1	0.989688358	0.957328149	0.961437412	0.999981514	0.979251887	0.99841577
WIPI2	0.989829672	0.991217881	0.964661607	0.999981514	0.988049878	0.951081819
LOC780963	0.989829672	0.952760839	0.999993442	0.995565324	0.999985752	0.952571698
LOC112448032	0.989829672	0.94422395	0.970873534	0.999981514	0.996089557	0.952642975
FZD7	0.989829672	0.930293015	0.971053208	0.999981514	0.999985752	0.952921716
BLNK	0.989829672	0.983594252	0.990118874	0.999981514	0.972160227	0.954465286
ZNF146	0.989829672	0.985428851	0.959725886	0.986617251	0.999985752	0.955210102
WWC3	0.989829672	0.968186752	0.978837953	0.999981514	0.976292433	0.958281344
S100A4	0.989829672	0.946148902	0.999993442	0.999981514	0.996781978	0.958707771
SFT2D3	0.989829672	0.999987083	0.961108613	0.980564765	0.996781978	0.958790968
ZNF621	0.989829672	0.973718725	0.999993442	0.980564765	0.998008754	0.958790968
LOC786363	0.989829672	0.95462892	0.999993442	0.999981514	0.970988226	0.960023723

ADCYAP1R1	0.989829672	0.999987083	0.972482052	0.979584502	0.987723553	0.960023723
LOC112448753	0.989829672	0.948801019	0.999993442	0.979644238	0.998196897	0.960023723
AXIN1	0.989829672	0.974410978	0.985171227	0.986642278	0.999985752	0.960023723
LOC781298	0.989829672	0.971446403	0.99647733	0.989783183	0.999985752	0.960023723
SMAD9	0.989829672	0.985428851	0.972741989	0.999981514	0.969739417	0.962412275
PLXNA2	0.989829672	0.938087741	0.999993442	0.993215785	0.989334846	0.965117554
PRKCSH	0.989829672	0.942088099	0.999993442	0.998529748	0.971893495	0.965705577
NAIF1	0.989829672	0.985428851	0.965407935	0.999981514	0.976259416	0.965826151
MYDGF	0.989829672	0.968509005	0.979494108	0.999981514	0.996397016	0.966850975
BTN3A3	0.989829672	0.946859049	0.998447145	0.999981514	0.993461941	0.967759699
ZNF646	0.989829672	0.933545282	0.999993442	0.989701998	0.988010662	0.96870908
PTAR1	0.989829672	0.995373357	0.962980373	0.999981514	0.976259416	0.969754487
TRAF3IP2	0.989829672	0.940190923	0.98258069	0.999981514	0.998661549	0.970105102
GLDC	0.989829672	0.954877525	0.989977673	0.999981514	0.986019537	0.971545917
LOC786417	0.989829672	0.999987083	0.961037916	0.980605008	0.979759416	0.972695131
LRCH2	0.989829672	0.988633241	0.976883418	0.999981514	0.983941532	0.972695131
LOC104969916	0.989829672	0.935001231	0.959903168	0.999981514	0.988049878	0.972695131
TSPYL5	0.989829672	0.962494531	0.972741989	0.999981514	0.989334846	0.972695131
SLC9C2	0.989829672	0.96643574	0.963164828	0.999981514	0.966967496	0.973218068
C2H2orf76	0.989829672	0.981952108	0.97251482	0.995565324	0.996781978	0.973649216
C16H1orf174	0.989829672	0.956527556	0.999993442	0.997025804	0.9683846	0.975639156
NAF1	0.989829672	0.969051025	0.976295638	0.999981514	0.995318217	0.977053057
SLC10A6	0.989829672	0.938087741	0.960998979	0.999981514	0.998922204	0.977166378
PRDM8	0.989829672	0.985997726	0.99341711	0.995434268	0.971117951	0.979480605
SLC10A3	0.989829672	0.961368616	0.987610559	0.999180255	0.994821374	0.980464176
SATB2	0.989829672	0.988008216	0.970873534	0.982220679	0.998287238	0.98140846
WNT7B	0.989829672	0.954622533	0.972741989	0.999981514	0.998800466	0.982422737
APOL3	0.989829672	0.995343428	0.975774524	0.983079972	0.979966065	0.982908937
TENM4	0.989829672	0.949902864	0.991138474	0.98051571	0.999985752	0.982937529
LOC101903375	0.989829672	0.972658336	0.959844128	0.999981514	0.987723553	0.984129452
MIOS	0.989829672	0.939956757	0.997400824	0.995312373	0.994756807	0.984957404
LOC101902664	0.989829672	0.94422395	0.978014482	0.999981514	0.999985752	0.986042926
LOC101901960	0.989829672	0.936319037	0.990118874	0.999981514	0.989379051	0.986638746
OLR1	0.989829672	0.981952108	0.962797326	0.999981514	0.984386856	0.988639786
GNAO1	0.989829672	0.966221925	0.986511454	0.985118339	0.996781978	0.988639786
P2RY2	0.989829672	0.933100216	0.964416364	0.999981514	0.999985752	0.989084358
MRPL1	0.989829672	0.942159543	0.996624589	0.985748796	0.996781978	0.989309315
B3GALT6	0.989829672	0.933100216	0.995377403	0.999981514	0.972128973	0.991847494

TSTD3	0.989829672	0.952344105	0.999993442	0.982220679	0.976769047	0.994887496
LOC104974669	0.989829672	0.937260971	0.972482052	0.999981514	0.988881329	0.995604307
DNAJC13	0.989829672	0.972496661	0.99647733	0.982220679	0.976259416	0.995895432
KLF8	0.989829672	0.99306529	0.980851214	0.97828838	0.970988226	0.996367479
IQCG	0.989829672	0.937723596	0.972741989	0.999981514	0.996781978	0.997206681
B3GNT2	0.989829672	0.946820717	0.97918842	0.990084447	0.989442936	0.999655834
DDAH2	0.989829672	0.965359774	0.95875611	0.986214799	0.996781978	0.999701868
ZNF280C	0.989829672	0.955652503	0.97998887	0.9880671	0.970980375	0.999948228
CTCFL	0.98991441	0.950336973	0.987786638	0.984423083	0.989334846	0.999463809
RNF150	0.990274854	0.944112289	0.982232295	0.999981514	0.970980375	0.962419223
NDC1	0.990333969	0.985997726	0.979584814	0.999981514	0.990937627	0.957876917
RNF26	0.990333969	0.931233175	0.999993442	0.999981514	0.999985752	0.958790968
FAM114A1	0.990333969	0.997471998	0.984886031	0.996033721	0.977158496	0.961107601
ZNF70	0.990333969	0.981670901	0.965819476	0.999981514	0.976292433	0.963456717
LOC786930	0.990333969	0.968596537	0.991447919	0.998529748	0.996781978	0.970994034
PEX7	0.990333969	0.934225172	0.999993442	0.995434268	0.989334846	0.979505709
LOC789258	0.990369843	0.976063495	0.99341711	0.979385838	0.999985752	0.959723327
ITGA1	0.990369843	0.932394105	0.999993442	0.995312373	0.976292433	0.960023723
PPIG	0.990369843	0.968080358	0.975774524	0.995565324	0.999985752	0.960023723
GLOD4	0.990369843	0.942088099	0.989494193	0.999981514	0.996781978	0.963112102
DCUN1D5	0.990369843	0.944354781	0.996224523	0.999981514	0.990937627	0.968697704
HOXC10	0.990369843	0.950336973	0.978530068	0.999981514	0.983941532	0.996335668
LOC112445065	0.990369843	0.952863279	0.97773026	0.998630959	0.989334846	0.996813547
TRIM33	0.990418133	0.99386673	0.981044281	0.9880671	0.999985752	0.953140004
APCDD1L	0.990599773	0.988540349	0.972806636	0.999981514	0.977158496	0.963456717
WIZ	0.99069917	0.95462892	0.999993442	0.999981514	0.976292433	0.970071797
RRM2B	0.990719572	0.932394105	0.998899782	0.999981514	0.98998448	0.960877268
KANK3	0.990719572	0.983594252	0.996224523	0.999981514	0.976292433	0.966657572
HIST1H1E	0.990719572	0.948801019	0.980851214	0.984423083	0.999859158	0.997260961
B9D1	0.990988062	0.961138122	0.981044281	0.999981514	0.976769047	0.96870908
GTF3C4	0.990988062	0.939956757	0.999993442	0.991955963	0.998835841	0.970105102
SEC14L2	0.990988062	0.946241678	0.971780659	0.989601002	0.999985752	0.993890045
MAP3K2	0.991101551	0.961663204	0.97773026	0.999981514	0.999985752	0.966212838
CSK	0.99116073	0.948801019	0.999993442	0.995565324	0.999985752	0.960000414
ADAMTS4	0.99116073	0.952760839	0.991936822	0.999981514	0.988010662	0.9790177
LOC101903905	0.99116073	0.939922389	0.99341711	0.999981514	0.984386856	0.991847494
MBIP	0.991290521	0.965456931	0.999841067	0.999981514	0.976769047	0.955140534
NUP54	0.991290521	0.948810556	0.963723107	0.999981514	0.999985752	0.959792861

SRSF11	0.991371763	0.960848894	0.997400824	0.999981514	0.9683846	0.981216824
LOC783942	0.99162188	0.952344105	0.999993442	0.999981514	0.970988226	0.978087957
CELF6	0.99162188	0.978189471	0.980851214	0.993215785	0.976292433	0.99666605
ACADSB	0.991621995	0.970084348	0.971053208	0.999981514	0.999985752	0.967047449
LOC101905648	0.991873076	0.964251568	0.974586624	0.995565324	0.999985752	0.97721867
GDAP2	0.992057258	0.943860167	0.999993442	0.999981514	0.970988226	0.958790968
PCDHA13	0.992057258	0.999987083	0.966232793	0.988408846	0.9683846	0.961265889
C7H19orf66	0.992057258	0.947350735	0.999993442	0.988408846	0.9683846	0.980167535
PHC1	0.992057258	0.953200124	0.985156077	0.982220679	0.999985752	0.9848451
LOC104971296	0.992093102	0.973712113	0.999993442	0.998529748	0.977158496	0.960023723
CSKMT	0.992093102	0.947350735	0.97773026	0.999981514	0.989246103	0.96053762
CCDC17	0.992093102	0.941676269	0.980761647	0.999981514	0.976292433	0.963107047
ZNF212	0.992093102	0.943244588	0.999993442	0.988408846	0.970988226	0.998009873
LOC618268	0.992093102	0.933100216	0.980032241	0.982220679	0.976259416	0.999948228
CSNK1G1	0.992189779	0.939956757	0.999993442	0.9880671	0.970988226	0.960023723
COPS4	0.992189779	0.962423197	0.999993442	0.989601002	0.990192394	0.964898868
UBXN6	0.992189779	0.998847958	0.972897558	0.999981514	0.970980375	0.967602061
CRYBG1	0.992214703	0.951172741	0.972806636	0.999981514	0.989334846	0.98140846
LOC104968751	0.992240143	0.993275893	0.96327718	0.999981514	0.970988226	0.986042926
PM20D2	0.99226258	0.934225172	0.99341711	0.995565324	0.976769047	0.999948228
ERN1	0.99230959	0.936319037	0.972410614	0.985118339	0.999985752	0.952571698
RRN3	0.99230959	0.95462892	0.993073581	0.999981514	0.999985752	0.952571698
CDC42SE2	0.99230959	0.98098403	0.973562292	0.999981514	0.976292433	0.953140004
LOC786252	0.99230959	0.967251014	0.964807927	0.999981514	0.999985752	0.954925542
ECT2	0.99230959	0.981670901	0.979494108	0.999981514	0.977167046	0.9557845
LOC104969719	0.99230959	0.940500178	0.999993442	0.984548238	0.972432486	0.960023723
USP22	0.99230959	0.941400719	0.999993442	0.999981514	0.991588867	0.960023723
SMAP1	0.99230959	0.985940669	0.999993442	0.997332186	0.971475922	0.96053762
CPNE8	0.99230959	0.995373357	0.972806636	0.9880671	0.999985752	0.960877268
ECSCR	0.99230959	0.988008216	0.998364815	0.999981514	0.970988226	0.966196012
SAMD11	0.99230959	0.938701207	0.984886031	0.999981514	0.983941532	0.966850975
SYT5	0.99230959	0.936319037	0.999993442	0.992930508	0.999985752	0.968697704
LOC101906131	0.99230959	0.933545282	0.999993442	0.999981514	0.98227455	0.96870908
LIPE	0.99230959	0.962269569	0.975774524	0.999981514	0.976292433	0.969197073
C10H15orf61	0.99230959	0.980833065	0.99341711	0.9880671	0.99971266	0.970105102
LOC101902440	0.99230959	0.955652503	0.99341711	0.996033721	0.999985752	0.970105102
LOC101902668	0.99230959	0.988444882	0.999993442	0.9880671	0.974931134	0.972695131
GPR89A	0.99230959	0.981670901	0.969404007	0.999981514	0.976292433	0.974527074

PTPN18	0.99230959	0.9574162	0.978014482	0.990955345	0.999985752	0.982401177
AK9	0.99230959	0.938235006	0.962797326	0.999981514	0.972160227	0.984509067
ARHGAP25	0.99230959	0.981670901	0.972482052	0.999981514	0.979290191	0.984509067
CYB561D1	0.99230959	0.963297767	0.970427737	0.999981514	0.983941532	0.990924439
SGK3	0.99230959	0.969811486	0.963814739	0.999981514	0.989379051	0.993890045
LOC100299025	0.99230959	0.980806952	0.977285557	0.989485949	0.987723553	0.99666605
LOC112443199	0.99230959	0.941817736	0.989494193	0.984548238	0.976292433	0.999948228
ANKAR	0.992403338	0.980521483	0.999993442	0.985701713	0.972160227	0.955541887
TAAR1	0.992403338	0.946148902	0.982232295	0.984423083	0.999985752	0.958707771
STMP1	0.992403338	0.934225172	0.999993442	0.999981514	0.996781978	0.973649216
LRRCC1	0.992403338	0.936822825	0.999993442	0.999038184	0.976259416	0.978087957
LSM14A	0.992403338	0.959570234	0.983377931	0.982220679	0.996781978	0.99841577
LOC112448889	0.992412383	0.936521326	0.965327631	0.994395954	0.999985752	0.967759699
PDE6D	0.992460098	0.984631976	0.99341711	0.984423083	0.999985752	0.960023723
C18H19orf33	0.992503333	0.99960614	0.99341711	0.984445628	0.986861597	0.955990509
PHF3	0.992503333	0.95687851	0.996624589	0.980632768	0.999985752	0.957588281
LOC100336448	0.992503333	0.965482645	0.991046292	0.99313054	0.999985752	0.958281344
RBP7	0.992503333	0.984631976	0.983735552	0.999981514	0.981652321	0.958790968
ARL6IP6	0.992503333	0.998847958	0.964807927	0.988437392	0.999985752	0.958790968
LOC100848007	0.992503333	0.963052738	0.999993442	0.9880671	0.984692578	0.959075802
SV2A	0.992503333	0.976615289	0.999993442	0.996033721	0.985250243	0.959075802
VAC14	0.992503333	0.989407995	0.971053208	0.999981514	0.983768748	0.960023723
ADAM23	0.992503333	0.999987083	0.964661607	0.98544125	0.996781978	0.960023723
PKDREJ	0.992503333	0.980521483	0.996224523	0.989432811	0.999985752	0.96179157
MGLL	0.992503333	0.973712113	0.985156077	0.999981514	0.986003146	0.963112102
SPI1	0.992503333	0.983594252	0.996291804	0.992442163	0.996089557	0.963456717
LOC101903806	0.992503333	0.938087741	0.999993442	0.999981514	0.970988226	0.966212838
LOC534520	0.992503333	0.947911876	0.990118874	0.999981514	0.991673538	0.966850975
LOC101906460	0.992503333	0.992116558	0.982232295	0.985118339	0.999985752	0.96870908
KCNQ5	0.992503333	0.99960614	0.978014482	0.993656085	0.977158496	0.970105102
SIRT6	0.992503333	0.988444882	0.99341711	0.999981514	0.977167046	0.970680228
STON1	0.992503333	0.977196455	0.996224523	0.999981514	0.986050453	0.971545917
KLHL18	0.992503333	0.948801019	0.999993442	0.999981514	0.972043489	0.972088812
PRR3	0.992503333	0.997471998	0.978014482	0.999981514	0.976769047	0.972695131
MAD2L2	0.992503333	0.972007528	0.989761927	0.999981514	0.970980375	0.973413452
CDH7	0.992503333	0.953449552	0.979584814	0.999981514	0.987789919	0.973649216
LOC100848264	0.992503333	0.981952108	0.978014482	0.999981514	0.995054863	0.973649216
ACVR1B	0.992503333	0.95462892	0.984142978	0.982220679	0.999985752	0.973649216

PLBD1	0.992503333	0.969051025	0.999993442	0.999981514	0.975611541	0.97439943
SCAMP4	0.992503333	0.962037123	0.982195333	0.999981514	0.996089557	0.980840077
TTC3	0.992503333	0.942509482	0.99341711	0.999981514	0.97234838	0.98140846
HMGN4	0.992503333	0.989303356	0.992511421	0.98981682	0.978453925	0.98140846
SSH2	0.992503333	0.974060899	0.978735356	0.984423083	0.999985752	0.983430934
ZFAND2B	0.992503333	0.993481224	0.988307089	0.990955345	0.976292433	0.984046914
NRCAM	0.992503333	0.999987083	0.965407935	0.987659016	0.97077135	0.986042926
LTBP4	0.992503333	0.977070024	0.972741989	0.982220679	0.999985752	0.98665664
MRPS28	0.992503333	0.944261937	0.999993442	0.980564765	0.996089557	0.988620333
CERS6	0.992503333	0.968509005	0.971053208	0.999981514	0.984386856	0.988796537
FBXL18	0.992503333	0.945755356	0.999993442	0.995152316	0.984494338	0.98926637
LOC107133473	0.992503333	0.950336973	0.984886031	0.98981682	0.999985752	0.992466226
MMRN2	0.992503333	0.969051025	0.999993442	0.989485949	0.970885781	0.992680267
BBOF1	0.992503333	0.939857309	0.97424602	0.999981514	0.977167046	0.993890045
PBX4	0.992503333	0.951172741	0.983504244	0.999981514	0.970980375	0.996335668
PIGK	0.992503333	0.942088099	0.99341711	0.989320128	0.999453276	0.996453303
ZNF268	0.992503333	0.938235006	0.979494108	0.981327579	0.999985752	0.996453303
ARHGEF6	0.992503333	0.950512146	0.996224523	0.989601002	0.990937627	0.99666605
SLC10A7	0.992503333	0.947350735	0.99341711	0.991955963	0.983941532	0.99976973
GSC2	0.992503333	0.937448751	0.979494108	0.991955963	0.970988226	0.999948228
BCAS4	0.992503333	0.985428851	0.968204727	0.98981682	0.973799161	0.999948228
TEX12	0.992503333	0.980521483	0.976484937	0.984548238	0.976322141	0.999948228
SLC25A41	0.992503333	0.969051025	0.971594714	0.98981682	0.989334846	0.999948228
SNX1	0.992503333	0.947350735	0.987610559	0.98981682	0.989379051	0.999948228
TSTD2	0.992503333	0.938017087	0.971053208	0.9880671	0.999985752	0.999948228
CMTR1	0.992716294	0.991673407	0.990037865	0.982220679	0.996781978	0.97521287
AMPH	0.992884254	0.9604921	0.999993442	0.984423083	0.999985752	0.958707771
NUP58	0.992884254	0.98667582	0.990974506	0.999981514	0.986003146	0.958790968
GPR63	0.992884254	0.959478257	0.982195333	0.999981514	0.998279993	0.960023723
LOC112448770	0.992884254	0.942088099	0.990974506	0.999981514	0.972160227	0.965211535
P2RX5	0.992884254	0.95530536	0.974470955	0.999981514	0.970988226	0.966212838
LOC112449052	0.992884254	0.943754915	0.979494108	0.9880671	0.999985752	0.971545917
PRDM10	0.992884254	0.942088099	0.999993442	0.999981514	0.970988226	0.98140846
LOC100297676	0.992884254	0.974000935	0.997850143	0.999981514	0.972128973	0.986366353
MOB2	0.992884254	0.952863279	0.99341711	0.984062548	0.996089557	0.99828741
LOC107133294	0.992909234	0.965565049	0.987610559	0.986340119	0.976419607	0.999948228
CREG2	0.993056139	0.957130277	0.990037865	0.999981514	0.977158496	0.958707771
TANC2	0.993056738	0.939956757	0.970427737	0.999981514	0.999985752	0.960023723

APOA1	0.993056738	0.948801019	0.999993442	0.984423083	0.995515529	0.988273129
LOC100851323	0.993056738	0.962483234	0.999993442	0.999981514	0.976088926	0.989084358
RAB35	0.993104817	0.952760839	0.999993442	0.999981514	0.974738415	0.954997829
CLN8	0.993104817	0.962494531	0.99341711	0.982220679	0.999985752	0.955140534
SKA1	0.993104817	0.95297524	0.996224523	0.995565324	0.999985752	0.958707771
LOC523963	0.993104817	0.953200124	0.999993442	0.999981514	0.976088926	0.958790968
ARAF	0.993104817	0.972496661	0.994035957	0.999981514	0.997499364	0.959075802
DNAAF4	0.993104817	0.964418016	0.999993442	0.989701998	0.999985752	0.959198304
RCC2	0.993104817	0.95724185	0.998899782	0.999981514	0.976292433	0.959275214
LOC112447385	0.993104817	0.94422395	0.964661607	0.999981514	0.999985752	0.960023723
LOC100849067	0.993104817	0.94622198	0.971996404	0.999981514	0.999985752	0.960023723
NSUN4	0.993104817	0.969101539	0.986311618	0.9880671	0.999985752	0.963107047
TBCE	0.993104817	0.972496661	0.982232295	0.996033721	0.999985752	0.963456717
GATD3A	0.993104817	0.981952108	0.987610559	0.999981514	0.990066164	0.963701289
LOC112444869	0.993104817	0.946148902	0.999993442	0.999981514	0.999251855	0.965428409
CCT6B	0.993104817	0.956095821	0.974656086	0.999981514	0.999453276	0.966071454
LZIC	0.993104817	0.953954642	0.999993442	0.999669429	0.976259416	0.966212838
C5H12orf10	0.993104817	0.940546131	0.999993442	0.989701998	0.999985752	0.967308566
EAPP	0.993104817	0.987731372	0.99615564	0.999981514	0.982087479	0.968697704
B3GNT9	0.993104817	0.99528564	0.975774524	0.998630959	0.98998448	0.970105102
UNC119	0.993104817	0.949902864	0.999993442	0.98981682	0.983941532	0.971742751
DOC2G	0.993104817	0.939442799	0.985538977	0.999981514	0.996781978	0.971982373
TNPO1	0.993104817	0.951172741	0.996224523	0.999981514	0.976259416	0.972314037
EEF1AKMT1	0.993104817	0.980806952	0.999993442	0.999981514	0.976259416	0.972695131
AKT3	0.993104817	0.961663204	0.979494108	0.990955345	0.999985752	0.97439943
LOC112446855	0.993104817	0.941817736	0.99341711	0.999981514	0.999985752	0.974527074
LOC101904622	0.993104817	0.941817736	0.999993442	0.984423083	0.976292433	0.975558533
NECTIN3	0.993104817	0.998847958	0.971053208	0.984423083	0.996781978	0.975558533
LOC100847934	0.993104817	0.942594366	0.990037865	0.999981514	0.995600485	0.976338092
ATXN10	0.993104817	0.974846249	0.994887288	0.999981514	0.989334846	0.977503497
LOC524650	0.993104817	0.969051025	0.964807927	0.999793541	0.999985752	0.979505709
DUSP10	0.993104817	0.970084348	0.970873534	0.982220679	0.999985752	0.982871032
AKAP10	0.993104817	0.974846249	0.999993442	0.982220679	0.983941532	0.982908937
PIP5K1B	0.993104817	0.981952108	0.998364815	0.988437392	0.976292433	0.988796537
PTPRK	0.993104817	0.991673407	0.971053208	0.998529748	0.984386856	0.989184798
LOC101904339	0.993104817	0.982515431	0.966232793	0.985701713	0.999985752	0.991847494
RAB5B	0.993104817	0.942159543	0.983534298	0.98981682	0.999985752	0.994788615
LOC104973285	0.993104817	0.938087741	0.964661607	0.999981514	0.980016241	0.995099097

ABHD15	0.993104817	0.942088099	0.999993442	0.98981682	0.987723553	0.99666605
LOC104972542	0.993104817	0.943859632	0.967549444	0.999981514	0.973799161	0.997383134
SLC23A3	0.993104817	0.9419174	0.983504244	0.995434268	0.999985752	0.997383134
RAD1	0.993104817	0.962037123	0.983504244	0.990955345	0.970988226	0.999948228
KCNH3	0.993104817	0.946820717	0.971053208	0.990955345	0.972594401	0.999948228
CCDC197	0.993144642	0.959263706	0.968679299	0.999981514	0.998287238	0.971742751
AIP	0.993144642	0.939857309	0.989069245	0.999981514	0.976292433	0.990755602
UBTD1	0.993408424	0.952863279	0.977455101	0.999981514	0.996089557	0.971545917
BCAP31	0.993468355	0.960185644	0.981655709	0.999981514	0.996781978	0.987677304
LOC112444484	0.993491249	0.954827347	0.998061638	0.999981514	0.998060725	0.960023723
NIPAL4	0.993491249	0.957328149	0.969000554	0.999981514	0.996781978	0.976020793
JMJD1C	0.993600832	0.98098403	0.986311618	0.999981514	0.999859158	0.958790968
FGFRL1	0.993600832	0.999987083	0.984823134	0.992212416	0.976292433	0.95883396
KLK4	0.993600832	0.981952108	0.971053208	0.999981514	0.998060725	0.960023723
NDRG3	0.993600832	0.988444882	0.993073581	0.990955345	0.999985752	0.960023723
LOC104972595	0.993600832	0.972007528	0.981332474	0.999981514	0.986861597	0.961492377
RNF227	0.993600832	0.969748836	0.971053208	0.999981514	0.999985752	0.963112102
ABLIM3	0.993600832	0.985097978	0.972806636	0.996033721	0.999985752	0.963255451
DNAH1	0.993600832	0.94019273	0.999993442	0.988889838	0.999453276	0.963456717
KPNA5	0.993600832	0.943244588	0.978014482	0.999981514	0.976769047	0.966850975
THAP6	0.993600832	0.980521483	0.991936822	0.999981514	0.989334846	0.970105102
ITGBL1	0.993600832	0.9640374	0.999993442	0.984423083	0.999985752	0.970105102
PRPF4	0.993600832	0.941483684	0.965407935	0.999981514	0.999985752	0.971545917
CRKL	0.993600832	0.964415116	0.971255098	0.999981514	0.989334846	0.975558533
SLFNL1	0.993600832	0.994185904	0.991108777	0.99313054	0.977193348	0.980148159
NRDC	0.993600832	0.967668839	0.999993442	0.999981514	0.976769047	0.980177119
TTC31	0.993600832	0.941817736	0.996224523	0.999981514	0.999985752	0.985156335
LOC101902812	0.993600832	0.95530536	0.972410614	0.999981514	0.977193348	0.985717525
RNF113A	0.993600832	0.949850381	0.999993442	0.999180255	0.983129429	0.989184798
SUCLG2	0.993600832	0.947911876	0.988585686	0.999981514	0.982087479	0.989840954
DCTN1	0.993600832	0.9533882	0.997300234	0.999981514	0.976259416	0.996310803
LOC107131323	0.993600832	0.954187594	0.982232295	0.999981514	0.980189546	0.996335668
LOC510454	0.993600832	0.964418016	0.964807927	0.999981514	0.970988226	0.999948228
LOC100138633	0.993600832	0.946859049	0.997300234	0.982220679	0.976769047	0.999948228
ERMARD	0.993701784	0.990052293	0.984886031	0.999981514	0.97352263	0.958790968
STYXL1	0.993701784	0.981511914	0.999993442	0.984423083	0.980682226	0.972695131
C1D	0.993728929	0.975612288	0.999993442	0.999981514	0.983941532	0.958281344
RFK	0.993728929	0.955652503	0.978014482	0.99313054	0.999985752	0.958790968

LOC100848991	0.993728929	0.99960614	0.99341711	0.990036762	0.986861597	0.960000414
GORAB	0.993728929	0.977929455	0.968679299	0.999981514	0.999985752	0.960023723
COQ2	0.993728929	0.947247457	0.997400824	0.999981514	0.999985752	0.960023723
THAP1	0.993728929	0.964418016	0.999993442	0.995434268	0.976292433	0.961873176
COL11A2	0.993728929	0.991177966	0.97998887	0.989485949	0.999985752	0.972784535
GNRH2	0.99373445	0.955051921	0.999993442	0.984423083	0.999453276	0.971545917
ZNF565	0.993739931	0.942159543	0.975774524	0.989485949	0.998287238	0.999948228
ETS2	0.993782862	0.942088099	0.999993442	0.999981514	0.999985752	0.958789052
ARMCX5	0.993782862	0.943924846	0.995377403	0.999981514	0.989379051	0.958790968
LOC100335936	0.993782862	0.973321075	0.975774524	0.999981514	0.97442835	0.959730261
LOC783657	0.993782862	0.985432041	0.999460457	0.984421126	0.999985752	0.959976849
UFD1	0.993782862	0.962423197	0.999993442	0.995565324	0.996089557	0.960000414
IGFBP7	0.993782862	0.959263706	0.981044281	0.999981514	0.991481886	0.960023723
LOC783261	0.993782862	0.943213236	0.967138739	0.999981514	0.998287238	0.960023723
MED7	0.993782862	0.941483684	0.99615564	0.992560663	0.999985752	0.960023723
EHD4	0.993782862	0.961055437	0.999993442	0.983960202	0.999985752	0.96063559
TYMS	0.993782862	0.971455677	0.999993442	0.999981514	0.983941532	0.960877268
LOC101905046	0.993782862	0.952760839	0.999993442	0.999981514	0.976769047	0.96248183
ZNF713	0.993782862	0.968509005	0.971053208	0.999981514	0.999985752	0.962872722
TUBGCP3	0.993782862	0.955026114	0.999993442	0.999981514	0.995870928	0.963112102
ST3GAL3	0.993782862	0.973712113	0.982367155	0.999981514	0.982519862	0.963456717
BICD2	0.993782862	0.943244588	0.978014482	0.999981514	0.986050453	0.964898868
TMEM116	0.993782862	0.946806157	0.969000554	0.999981514	0.999985752	0.966136679
POFUT1	0.993782862	0.978518618	0.991138474	0.999981514	0.998287238	0.967759699
LOC112446879	0.993782862	0.969051025	0.999993442	0.98981682	0.976292433	0.969197073
DIAPH2	0.993782862	0.946258461	0.979494108	0.999981514	0.999985752	0.972415704
LOC112448760	0.993782862	0.946411824	0.999993442	0.999981514	0.972722722	0.97456385
LRP5	0.993782862	0.981920173	0.967590756	0.999981514	0.988283637	0.977167015
PRKD1	0.993782862	0.980833065	0.999993442	0.996033721	0.983620937	0.980009318
INTS9	0.993782862	0.980289052	0.975774524	0.984423083	0.999985752	0.9803583
LOC101904270	0.993782862	0.948801019	0.999993442	0.9880671	0.996781978	0.981216824
ARHGEF10	0.993782862	0.951172741	0.999993442	0.999981514	0.976292433	0.98140846
SULT1C4	0.993782862	0.94422395	0.980061232	0.997374305	0.999985752	0.98140846
LOC100848507	0.993782862	0.969051025	0.990615319	0.999981514	0.976259416	0.990755602
RAB40C	0.993782862	0.955652503	0.999280799	0.98981682	0.996089557	0.992190617
ATP1B2	0.993782862	0.973712113	0.964863184	0.999981514	0.987723553	0.993890045
SLC39A13	0.993782862	0.941817736	0.996224523	0.991955963	0.999985752	0.994576386
MFAP3	0.993782862	0.955333923	0.985156077	0.996033721	0.999859158	0.995895432

ABCA13	0.993782862	0.976624004	0.987324693	0.999981514	0.977193348	0.996453303
LOC107132793	0.993782862	0.945209003	0.972806636	0.999981514	0.976292433	0.999463809
LOC101902542	0.993782862	0.991673407	0.971053208	0.989485949	0.977167046	0.999730148
RBM15	0.993782862	0.956095821	0.976484937	0.997792939	0.985238194	0.999948228
ABHD4	0.993797794	0.95530536	0.968924534	0.999981514	0.982538884	0.999948228
ORC2	0.993798181	0.995373357	0.999280799	0.999981514	0.972128973	0.958790968
LOC101909140	0.993798181	0.972496661	0.976677948	0.999981514	0.983941532	0.969014913
SMC5	0.993811444	0.962838725	0.992991376	0.999981514	0.999985752	0.958790968
C14H8orf33	0.993811444	0.981952108	0.97773026	0.999981514	0.998287238	0.983430934
LOC101903165	0.99383711	0.981952108	0.999993442	0.988437392	0.999985752	0.958790968
ASB7	0.99383711	0.973780375	0.999993442	0.999981514	0.977193348	0.960000414
NHLRC2	0.99383711	0.994749993	0.999993442	0.989601002	0.98865576	0.960023723
PTTG1	0.99383711	0.969051025	0.992511421	0.999981514	0.979052133	0.966212838
P2RX7	0.99383711	0.942088099	0.996224523	0.999981514	0.976292433	0.967759699
RAB1B	0.99383711	0.97304892	0.991936822	0.999981514	0.9902393	0.97529557
HDDC3	0.99383711	0.949224146	0.969404007	0.999981514	0.999985752	0.979480605
MGARP	0.99383711	0.946820717	0.999993442	0.984423083	0.999985752	0.980009318
WDR24	0.99383711	0.973712113	0.999993442	0.984423083	0.984386856	0.994887496
RAMP2	0.99383711	0.94502659	0.999993442	0.990955345	0.986003146	0.995165705
SIGLEC5	0.99383711	0.975984829	0.992377532	0.999981514	0.979759416	0.996310803
LOC112444770	0.99383711	0.992144201	0.966232793	0.989601002	0.993010827	0.996367479
PATZ1	0.99383711	0.960708527	0.987610559	0.994395954	0.980189546	0.999948228
RPS10	0.99383711	0.947350735	0.983504244	0.999981514	0.988049878	0.999948228
TMEM159	0.993840567	0.95687851	0.970427737	0.992930508	0.999985752	0.959075802
PPARG	0.993840567	0.949726	0.999993442	0.985118339	0.999985752	0.959198304
YIPF1	0.993840567	0.994185904	0.999993442	0.992560663	0.973123933	0.960023723
ZCCHC24	0.993840567	0.989303356	0.972940212	0.999981514	0.996781978	0.960023723
AP2M1	0.993840567	0.973712113	0.999993442	0.999981514	0.998800466	0.960023723
LOC112441543	0.993840567	0.983594252	0.972897558	0.992560663	0.999985752	0.960023723
CDR2L	0.993840567	0.940273185	0.985156077	0.999981514	0.999985752	0.96053762
TMEM9	0.993840567	0.941483684	0.999993442	0.999981514	0.989334846	0.963112102
LOC100847567	0.993840567	0.948801019	0.999993442	0.999981514	0.998287238	0.969014913
ZYX	0.993840567	0.955652503	0.999993442	0.999981514	0.986861597	0.970105102
FAM208B	0.993840567	0.946859049	0.992991376	0.992930508	0.999985752	0.970105102
LOC789018	0.993840567	0.969051025	0.997400824	0.999981514	0.985854456	0.971742751
RHBDL1	0.993840567	0.974846249	0.99647733	0.999981514	0.976292433	0.978087957
FCGR1A	0.993840567	0.985432041	0.999993442	0.998177865	0.976292433	0.9790177
CRTC1	0.993840567	0.963007593	0.983066857	0.999981514	0.976259416	0.980464176

IRF5	0.993840567	0.993481224	0.979523323	0.989485949	0.998287238	0.980501512
LOC101905821	0.993840567	0.975612288	0.998899782	0.999981514	0.972594401	0.981216824
LOC112442708	0.993840567	0.959263706	0.972410614	0.999981514	0.995318217	0.982868841
LAMB2	0.993840567	0.946148902	0.992377532	0.999981514	0.98865576	0.982937529
PSME1	0.993840567	0.997471998	0.975774524	0.999981514	0.976292433	0.986042926
C1QA	0.993840567	0.975984829	0.989523691	0.990955345	0.998196897	0.991416486
CKAP5	0.993840567	0.959396625	0.971053208	0.997374305	0.999985752	0.993890045
ACVR1	0.993840567	0.980289052	0.988307089	0.994331516	0.989379051	0.995099097
PHKA2	0.993840567	0.96365708	0.982195333	0.988408846	0.999985752	0.998175127
LIMD1	0.993840567	0.943244588	0.999993442	0.989601002	0.984386856	0.99828741
PROKR2	0.993840567	0.95530536	0.998899782	0.999981514	0.974026015	0.999463809
GPR62	0.993840567	0.941817736	0.972806636	0.999981514	0.976292433	0.999701868
LOC101902490	0.993840567	0.959478257	0.984886031	0.999981514	0.974403222	0.999948228
LOC107132537	0.993840567	0.986210679	0.979584814	0.988408846	0.976259416	0.999948228
LOC100196898	0.993878933	0.969051025	0.999993442	0.998996992	0.98865576	0.973649216
B4GALT4	0.994053545	0.973633627	0.978014482	0.999981514	0.988010662	0.973779337
LOC101905595	0.994100122	0.990421548	0.999993442	0.9880671	0.976259416	0.960023723
ACMSD	0.994100122	0.953852086	0.999993442	0.999981514	0.976259416	0.960023723
ANGEL2	0.994100122	0.99293293	0.978014482	0.999981514	0.976292433	0.960023723
WNT8B	0.994100122	0.991673407	0.999993442	0.999981514	0.976292433	0.960023723
LOC786553	0.994100122	0.980462136	0.996224523	0.999981514	0.983941532	0.960023723
SPATA22	0.994100122	0.962311281	0.999993442	0.999981514	0.998287238	0.960023723
LOC112449619	0.994100122	0.980521483	0.99341711	0.999981514	0.999888682	0.960023723
FOXD2	0.994100122	0.991883165	0.990037865	0.988408846	0.999985752	0.960023723
LOC788405	0.994100122	0.955652503	0.988307089	0.999981514	0.999985752	0.960023723
STAG1	0.994100122	0.999987083	0.971053208	0.986386218	0.999453276	0.960781671
ITGAE	0.994100122	0.956527556	0.999993442	0.999981514	0.996781978	0.961599731
LRRC58	0.994100122	0.960848894	0.978014482	0.999981514	0.999985752	0.96248183
PRPF38A	0.994100122	0.981952108	0.999993442	0.989484139	0.99810638	0.96274079
FUZ	0.994100122	0.94422395	0.999993442	0.985701713	0.999985752	0.963015949
RALGPS1	0.994100122	0.988540349	0.999993442	0.999981514	0.974931134	0.963112102
SLC35B4	0.994100122	0.999987083	0.975185526	0.988408846	0.981652321	0.963112102
LOC100848407	0.994100122	0.948801019	0.984886031	0.999981514	0.977158496	0.963456717
ARHGEF28	0.994100122	0.997471998	0.976484937	0.999981514	0.989334846	0.963456717
TAF2	0.994100122	0.955333923	0.969356117	0.999981514	0.999859158	0.963456717
NUP214	0.994100122	0.969811486	0.991138474	0.997792939	0.999985752	0.963456717
APELA	0.994100122	0.984410135	0.999993442	0.98981682	0.976292433	0.964898868
EIF4ENIF1	0.994100122	0.993481224	0.972940212	0.996054502	0.999985752	0.965117554

ECE2	0.994100122	0.96365708	0.999993442	0.999981514	0.995018375	0.966212838
METTL27	0.994100122	0.977769595	0.999993442	0.999981514	0.976292433	0.966773564
OGFOD3	0.994100122	0.991217881	0.988073662	0.98544125	0.999985752	0.966779561
IL12RB2	0.994100122	0.993028532	0.99647733	0.989601002	0.999453276	0.966850975
LOC107132515	0.994100122	0.962037123	0.996224523	0.999981514	0.999985752	0.967694794
RPIA	0.994100122	0.941309293	0.999993442	0.991955963	0.999985752	0.967759699
GALNT7	0.994100122	0.942088099	0.9965509	0.999981514	0.998287238	0.969197073
CENPH	0.994100122	0.992795654	0.99341711	0.98981682	0.996781978	0.971545917
FRAT2	0.994100122	0.985997726	0.999993442	0.995434268	0.976259416	0.972334437
CCNYL1	0.994100122	0.941817736	0.988390948	0.994395954	0.999985752	0.972536788
VAMP4	0.994100122	0.973321075	0.999841067	0.999981514	0.975921255	0.972695131
SMPD1	0.994100122	0.997471998	0.996224523	0.994965893	0.977807915	0.972695131
ST5	0.994100122	0.980521483	0.987324693	0.999981514	0.986003146	0.972695131
LOC784054	0.994100122	0.993028532	0.982195333	0.998529748	0.998835841	0.972695131
NOL3	0.994100122	0.953200124	0.979494108	0.999981514	0.999985752	0.972784753
FHL3	0.994100122	0.974846249	0.982195333	0.999981514	0.999985752	0.973218068
SPATA2L	0.994100122	0.969051025	0.97998887	0.999981514	0.991481886	0.973413452
GALK1	0.994100122	0.942273776	0.988390948	0.995565324	0.999985752	0.973649216
LRRC28	0.994100122	0.95856975	0.999993442	0.999981514	0.976259416	0.975126734
DLGAP4	0.994100122	0.980521483	0.971053208	0.999981514	0.998648991	0.975126734
ZNF382	0.994100122	0.988553076	0.997300234	0.989485949	0.996089557	0.975170737
LOC101902043	0.994100122	0.981670901	0.990037865	0.999981514	0.97464569	0.975200512
HEXB	0.994100122	0.95577784	0.996224523	0.990955345	0.999985752	0.97521287
KRT80	0.994100122	0.960355725	0.970873534	0.992930508	0.999985752	0.975558533
C25H16orf71	0.994100122	0.973189491	0.986649629	0.997467114	0.999985752	0.977167015
TMEM121	0.994100122	0.981952108	0.992263335	0.999981514	0.989334846	0.978087957
LOC112444328	0.994100122	0.96643574	0.997400824	0.999981514	0.977158496	0.978530896
GLB1L3	0.994100122	0.97304892	0.999993442	0.999981514	0.974738415	0.979480605
CCNK	0.994100122	0.969162328	0.999993442	0.991955963	0.976292433	0.980189436
WDPCP	0.994100122	0.945942733	0.999145937	0.999981514	0.979840175	0.9803583
PPA2	0.994100122	0.956095821	0.996224523	0.999981514	0.983941532	0.980464176
CAMK2A	0.994100122	0.960185644	0.978837953	0.999981514	0.982037317	0.980501512
METTL15	0.994100122	0.972287585	0.984886031	0.999981514	0.998196897	0.98140846
LOC100848492	0.994100122	0.952344105	0.983881246	0.999981514	0.999453276	0.98140846
B3GNT5	0.994100122	0.963957751	0.993486876	0.999981514	0.979290191	0.982422737
TNFSF14	0.994100122	0.965648998	0.99869493	0.999981514	0.987381787	0.982717982
OGFR	0.994100122	0.943119518	0.998364815	0.988408846	0.999985752	0.98304899
NGDN	0.994100122	0.96643574	0.999993442	0.984548238	0.984692578	0.985156335

ACRBP	0.994100122	0.983594252	0.990118874	0.999981514	0.980325376	0.985223183
CCDC126	0.994100122	0.943213236	0.999993442	0.999981514	0.996781978	0.98679538
ABCB7	0.994100122	0.988444882	0.988660944	0.999981514	0.984386856	0.987207163
LOC112445982	0.994100122	0.952863279	0.984886031	0.999981514	0.999985752	0.987281476
LOC112441629	0.994100122	0.943244588	0.975774524	0.999981514	0.996781978	0.987843572
ACP6	0.994100122	0.980833065	0.991138474	0.989485949	0.999985752	0.988225178
TNFRSF1B	0.994100122	0.95187005	0.999993442	0.992930508	0.996781978	0.988252822
SEPT2	0.994100122	0.969051025	0.979494108	0.999981514	0.974738415	0.988320707
ARL4D	0.994100122	0.985428851	0.978014482	0.999981514	0.98865576	0.988320707
RFESD	0.994100122	0.977196455	0.975774524	0.999981514	0.989334846	0.988639786
LOC101902808	0.994100122	0.957328149	0.974470955	0.994395954	0.999985752	0.989184798
CTSW	0.994100122	0.966052556	0.972482052	0.999981514	0.998287238	0.991299214
GINS2	0.994100122	0.954827347	0.99341711	0.999981514	0.973799161	0.995099097
RAB15	0.994100122	0.955652503	0.984886031	0.999981514	0.996781978	0.995099097
OXA1L	0.994100122	0.952760839	0.990615319	0.995565324	0.999985752	0.995895432
DZIP3	0.994100122	0.946904846	0.976484937	0.999981514	0.972499705	0.996335668
CASP8AP2	0.994100122	0.943244588	0.984886031	0.999981514	0.999985752	0.996335668
PKIA	0.994100122	0.991673407	0.972806636	0.999981514	0.977193348	0.997383134
TUT1	0.994100122	0.95038008	0.996224523	0.999981514	0.975762765	0.998284411
WDFY3	0.994100122	0.959263706	0.992410032	0.990660526	0.996781978	0.999701868
LOC100336381	0.994100122	0.961663204	0.97251482	0.999981514	0.983941532	0.999729224
TMEM255A	0.994100122	0.981520642	0.978014482	0.995565324	0.986003146	0.999729224
UVSSA	0.994100122	0.944261937	0.999993442	0.989601002	0.976088926	0.999948228
LOC112446388	0.994100122	0.969051025	0.992377532	0.999981514	0.976088926	0.999948228
CDH23	0.994100122	0.961663204	0.987789469	0.9880671	0.982519862	0.999948228
ARF3	0.994100122	0.968509005	0.971053208	0.999981514	0.983941532	0.999948228
TBCCD1	0.994100122	0.947678531	0.978014482	0.999981514	0.983941532	0.999948228
HSD17B1	0.994100122	0.94422395	0.994734716	0.98981682	0.996781978	0.999948228
RHOC	0.994100122	0.964925317	0.978923213	0.995565324	0.996781978	0.999948228
RDH5	0.994399261	0.966661805	0.999993442	0.999981514	0.984386856	0.963701289
ZKSCAN1	0.994463429	0.986664684	0.99341711	0.989485949	0.999985752	0.960023723
GALNT4	0.994463429	0.993481224	0.999993442	0.995565324	0.980695939	0.963015949
SLC30A1	0.994463429	0.973321075	0.979494108	0.999981514	0.999985752	0.963456717
LOC107131273	0.994463429	0.997425219	0.99341711	0.999981514	0.98865576	0.965486169
HSD17B4	0.994463429	0.974846249	0.999993442	0.999981514	0.998287238	0.966212838
LOC104973145	0.994463429	0.954827347	0.992511421	0.999981514	0.998835841	0.966212838
TM4SF1	0.994463429	0.984089343	0.996624589	0.99570283	0.999985752	0.966850975
SRMS	0.994463429	0.9640374	0.999993442	0.995434268	0.986861597	0.978087957

LOC101904749	0.994463429	0.993055804	0.99341711	0.999981514	0.976769047	0.984122543
CEACAM1	0.994463429	0.98098403	0.984886031	0.999981514	0.994606412	0.992190617
PLK2	0.994463429	0.969051025	0.991138474	0.9880671	0.987723553	0.999948228
LOC112442633	0.994517822	0.943244588	0.984886031	0.989485949	0.999453276	0.999948228
GPKOW	0.994592882	0.97523862	0.999993442	0.999981514	0.980325376	0.963015949
GRIK5	0.994592882	0.99960614	0.975774524	0.999981514	0.976292433	0.966408025
ZHX2	0.994592882	0.9640374	0.996224523	0.999981514	0.979988991	0.986366353
LOC101904753	0.994652198	0.973321075	0.972180683	0.999981514	0.999985752	0.97721867
TMPPE	0.994661241	0.954237721	0.990278947	0.987659016	0.999985752	0.969754487
OCRL	0.994691805	0.966221925	0.975774524	0.999981514	0.999985752	0.987516427
TLE6	0.994699244	0.99293293	0.991936822	0.999981514	0.996781978	0.960023723
LOC112447461	0.994699244	0.96643574	0.999993442	0.98981682	0.999985752	0.960877268
LOC112446407	0.994699244	0.986900642	0.999829453	0.990036762	0.999985752	0.963456717
LOC112443783	0.994699244	0.998847958	0.981242297	0.989601002	0.999985752	0.969754487
B4GAT1	0.994699244	0.973712113	0.996224523	0.994965893	0.999985752	0.970105102
C14H8orf76	0.994699244	0.973321075	0.999993442	0.999981514	0.976292433	0.971545917
LOC101903795	0.994699244	0.976527871	0.978530068	0.998667933	0.999985752	0.971545917
CNN3	0.994699244	0.95387031	0.988307089	0.999981514	0.999985752	0.97345098
ITGAX	0.994699244	0.966791666	0.999993442	0.999981514	0.982796995	0.974500551
NUP85	0.994699244	0.968509005	0.999993442	0.999981514	0.976292433	0.9790177
TPP2	0.994699244	0.954237721	0.978014482	0.999981514	0.999985752	0.980189436
LOC112447495	0.994699244	0.944354781	0.990615319	0.999981514	0.998196897	0.981852178
ROBO4	0.994699244	0.985997726	0.978014482	0.999981514	0.999985752	0.982937529
TCIRG1	0.994699244	0.980833065	0.99341711	0.997322684	0.998287238	0.984086745
ZNF22	0.994699244	0.96025412	0.999993442	0.997467114	0.987723553	0.986042926
LOC112448488	0.994699244	0.955652503	0.996224523	0.999981514	0.976259416	0.98905196
RAB30	0.994699244	0.948801019	0.971053208	0.999981514	0.999985752	0.989084358
LOC787875	0.994699244	0.961368616	0.993024884	0.988493925	0.999985752	0.990951824
LOC784980	0.994699244	0.950465218	0.975774524	0.999981514	0.983941532	0.998240456
C11H2orf49	0.994699244	0.955652503	0.971255098	0.999981514	0.983941532	0.999948228
PLPP4	0.994728594	0.955333923	0.990037865	0.999981514	0.998040182	0.960533245
LOC101904855	0.994728594	0.95687851	0.999993442	0.993684984	0.999985752	0.967759699
POLD2	0.994728594	0.946859049	0.989494193	0.999981514	0.999985752	0.978112039
TSGA10IP	0.994728594	0.945598957	0.999993442	0.999981514	0.976259416	0.997383134
FBXO47	0.994728594	0.961663204	0.979584814	0.999981514	0.980016241	0.999948228
RPS15	0.994801297	0.945942733	0.974967552	0.999981514	0.999985752	0.963701289
WDR37	0.994928234	0.970029875	0.972741989	0.999981514	0.979290191	0.993890045
LOC104976573	0.995019659	0.960845618	0.999993442	0.997332186	0.983941532	0.973579501

LOC112446462	0.995019659	0.964134676	0.999993442	0.989485949	0.980695939	0.975788372
UBTF	0.995019659	0.952760839	0.997400824	0.999981514	0.981652321	0.99828741
WDR45	0.995138997	0.983594252	0.979494108	0.999981514	0.984386856	0.965705577
CDCA5	0.995138997	0.952344105	0.999993442	0.999981514	0.987723553	0.96870908
BIN1	0.995138997	0.974229033	0.999841067	0.998529748	0.999985752	0.969197073
LOC100848369	0.995138997	0.944449061	0.996224523	0.999981514	0.976419607	0.970216353
FAM83F	0.995138997	0.993481224	0.972741989	0.999981514	0.983010476	0.973649216
UBA52	0.995138997	0.968016981	0.99341711	0.999981514	0.987723553	0.986042926
ADSSL1	0.995138997	0.981950382	0.987324693	0.993481918	0.999985752	0.986042926
SCFD2	0.995138997	0.953449552	0.99615564	0.990955345	0.999985752	0.996679516
EXOC2	0.995318903	0.960185644	0.984886031	0.999981514	0.999985752	0.96053762
OTUD4	0.995318903	0.965648998	0.992044485	0.999981514	0.999985752	0.966212838
EPCAM	0.995318903	0.999987083	0.980761647	0.999981514	0.976259416	0.971545917
LDAH	0.995334775	0.967668839	0.996224523	0.999981514	0.986019537	0.982422737
C2CD3	0.995348279	0.991673407	0.999993442	0.988408846	0.998287238	0.960023723
LOC112441810	0.995348279	0.946258461	0.989069245	0.999981514	0.999985752	0.965117554
MCOLN1	0.995348279	0.98978863	0.988227304	0.999981514	0.983941532	0.9790177
DZIP1	0.995348279	0.999987083	0.981566174	0.990036762	0.976259416	0.984590164
NASP	0.995348279	0.956627744	0.974967552	0.999981514	0.999985752	0.989840954
GABPA	0.995348279	0.956963688	0.990615319	0.999981514	0.977167046	0.999948228
LOC112449059	0.995348279	0.949902864	0.990615319	0.999981514	0.984328593	0.999948228
ALKBH4	0.995421857	0.984126877	0.982041666	0.999981514	0.999985752	0.960877268
LOC101904057	0.995421857	0.951172741	0.999993442	0.998529748	0.996781978	0.969197073
MANEA	0.995421857	0.994993704	0.975774524	0.999981514	0.988049878	0.977034298
LOC789867	0.995421857	0.952760839	0.975774524	0.999981514	0.999985752	0.994347099
LTBP3	0.995475423	0.973712113	0.997400824	0.999981514	0.999453276	0.960023723
ANTXR1	0.995475423	0.999987083	0.974470955	0.996181341	0.984328593	0.960877268
DENND4C	0.995475423	0.969811486	0.999993442	0.999981514	0.984386856	0.960877268
TMEM184B	0.995475423	0.947350735	0.986649629	0.999981514	0.999985752	0.960877268
OPTN	0.995475423	0.962037123	0.99615564	0.999981514	0.989334846	0.963098281
PKN1	0.995475423	0.992144201	0.994715737	0.999981514	0.980189546	0.963255451
EXOSC9	0.995475423	0.946411824	0.999993442	0.98981682	0.988238252	0.964898868
GNA11	0.995475423	0.955509162	0.999993442	0.999981514	0.976292433	0.965117554
WDR26	0.995475423	0.998847958	0.995898294	0.994395954	0.996781978	0.965117554
TPRA1	0.995475423	0.991177966	0.990742065	0.999981514	0.996781978	0.965117554
AP5S1	0.995475423	0.962037123	0.990118874	0.995434268	0.999985752	0.965652257
HSPA12B	0.995475423	0.959367958	0.999993442	0.989601002	0.999985752	0.966196012
GATB	0.995475423	0.981952108	0.999993442	0.999981514	0.998016692	0.966212838

PAPOLG	0.995475423	0.993481224	0.997850143	0.990955345	0.999453276	0.966659342
MARK3	0.995475423	0.970084348	0.975067671	0.995565324	0.999985752	0.966850975
SFXN1	0.995475423	0.976310661	0.983504244	0.999981514	0.999985752	0.967056707
UPF3A	0.995475423	0.974846249	0.983504244	0.999981514	0.980016241	0.967759699
DPP8	0.995475423	0.981614672	0.977811584	0.999981514	0.984386856	0.967759699
C1H3orf38	0.995475423	0.999987083	0.973562292	0.997792939	0.985293323	0.967759699
WISP2	0.995475423	0.973712113	0.972741989	0.999981514	0.996089557	0.967759699
CTCF	0.995475423	0.990546209	0.974470955	0.999981514	0.999985752	0.967759699
GANC	0.995475423	0.945709513	0.984886031	0.999981514	0.999985752	0.970105102
GTF3C2	0.995475423	0.954237721	0.997400824	0.999981514	0.999985752	0.971608881
HS3ST3B1	0.995475423	0.9533882	0.979584814	0.999981514	0.976292433	0.972314037
RNASEH2A	0.995475423	0.995373357	0.979494108	0.999981514	0.988010662	0.973649216
NTSR2	0.995475423	0.999987083	0.993073581	0.98981682	0.976769047	0.973731616
UBE2O	0.995475423	0.985097978	0.992263335	0.999981514	0.999859158	0.976915135
TEX10	0.995475423	0.968166143	0.99341711	0.988408846	0.999985752	0.978087957
QRSL1	0.995475423	0.969051025	0.992511421	0.999981514	0.999985752	0.978445076
ARSE	0.995475423	0.99831878	0.972741989	0.999981514	0.995870928	0.9790177
DUSP22	0.995475423	0.977196455	0.992410032	0.999981514	0.984692578	0.980464176
COX16	0.995475423	0.969051025	0.999993442	0.989601002	0.993010827	0.981216824
LOC101902926	0.995475423	0.965648998	0.999993442	0.999793541	0.993010827	0.981216824
SLC26A6	0.995475423	0.972007528	0.999993442	0.98981682	0.976292433	0.988639786
SETD6	0.995475423	0.973189491	0.979584814	0.999981514	0.987723553	0.989084358
FAM110C	0.995475423	0.969051025	0.999993442	0.999981514	0.976292433	0.989309315
LOC615768	0.995475423	0.977070024	0.996224523	0.999981514	0.977167046	0.993890045
TMEM68	0.995475423	0.960185644	0.992263335	0.999981514	0.99581977	0.99642553
PYROXD2	0.995475423	0.980833065	0.99647733	0.998529748	0.983941532	0.996679516
RREB1	0.995475423	0.994013976	0.97251482	0.990955345	0.996781978	0.997054363
PIM2	0.995475423	0.946148902	0.999993442	0.990036762	0.976292433	0.998284411
SUV39H1	0.995475423	0.973712113	0.990037865	0.999981514	0.989334846	0.998284411
YEATS4	0.995475423	0.970084348	0.99341711	0.999981514	0.984386856	0.99828741
GAR1	0.995475423	0.955137018	0.997850143	0.999981514	0.983941532	0.999795635
FAM205C	0.995475423	0.989869972	0.975774524	0.997660427	0.976292433	0.999948228
ANXA4	0.995475423	0.954222802	0.997400824	0.999981514	0.976535793	0.999948228
EFNA1	0.995873272	0.99488888	0.996224523	0.999981514	0.996781978	0.960877268
GGH	0.995873272	0.966069053	0.987789469	0.999981514	0.999985752	0.963107047
ERAP2	0.995873272	0.961055437	0.991138474	0.999981514	0.999985752	0.970105102
RAB13	0.995873272	0.958400846	0.97773026	0.999981514	0.976292433	0.972314037
CCDC85B	0.995873272	0.973712113	0.974470955	0.999793541	0.999985752	0.972695131

LOC521580	0.995873272	0.985997726	0.996509242	0.990955345	0.999985752	0.973413452
CAMK1	0.995873272	0.980833065	0.997400824	0.999981514	0.986861597	0.981216824
ACSM1	0.995873272	0.968509005	0.99341711	0.999981514	0.999985752	0.981216824
CCDC43	0.995873272	0.988444882	0.999993442	0.998529748	0.976292433	0.986441687
RPL26	0.995873272	0.983887536	0.974656086	0.999981514	0.984386856	0.987785166
LOC112443476	0.995873272	0.96643574	0.972641134	0.999981514	0.991481886	0.9884289
CRTC3	0.995950917	0.95462892	0.99615564	0.989485949	0.983941532	0.999948228
EHD2	0.995966547	0.99960614	0.979354564	0.999981514	0.989334846	0.964898868
APOM	0.995966547	0.994185904	0.988430001	0.999981514	0.987723553	0.965117554
ATXN1	0.995966547	0.952863279	0.997300234	0.989485949	0.999985752	0.969996755
ARHGAP44	0.995966547	0.95687851	0.99341711	0.999981514	0.999985752	0.972986817
RTN3	0.995966547	0.968509005	0.999993442	0.999981514	0.983941532	0.973649216
LOC789996	0.995966547	0.981952108	0.99647733	0.999981514	0.976292433	0.98140846
FUOM	0.995966547	0.952344105	0.999993442	0.990955345	0.995318217	0.981672932
EDEM2	0.995966547	0.952760839	0.972897558	0.999981514	0.977158496	0.997054363
SPAAR	0.996217523	0.962924498	0.996224523	0.999981514	0.984386856	0.963145894
MITD1	0.996217523	0.95577784	0.999993442	0.999981514	0.987702131	0.981216824
LOC100296900	0.996217523	0.969051025	0.997400824	0.999981514	0.996781978	0.991416486
SCIMP	0.996221791	0.985997726	0.996224523	0.999981514	0.983941532	0.966779561
LOC511937	0.996221791	0.998847958	0.973034514	0.999981514	0.996781978	0.979480605
TPP1	0.996221791	0.948801019	0.985172237	0.999981514	0.976292433	0.989082925
KCNN3	0.996221791	0.947350735	0.988142154	0.999981514	0.989379051	0.991847494
ZXDC	0.996221791	0.955652503	0.999993442	0.989601002	0.987723553	0.99841577
MRNIP	0.996221791	0.960845618	0.999993442	0.998529748	0.980016241	0.999948228
XPNPEP2	0.996261215	0.981952108	0.997400824	0.999981514	0.998661549	0.969579195
PAIP2B	0.996333965	0.954877525	0.999993442	0.999981514	0.983941532	0.965456017
LOC533093	0.996344487	0.948801019	0.997300234	0.999981514	0.982087479	0.981216824
ZNF575	0.996445331	0.965125462	0.999993442	0.999981514	0.999985752	0.965705577
FAM213A	0.996445331	0.952863279	0.999993442	0.999981514	0.979290191	0.966850975
WTAP	0.996445331	0.95687851	0.977455115	0.999981514	0.999985752	0.973218068
GFRA3	0.996445331	0.95687851	0.999993442	0.989601002	0.977167046	0.994788615
GMPR2	0.996445331	0.989303356	0.996224523	0.995565324	0.983941532	0.996335668
USP30	0.996446603	0.981670901	0.995377403	0.999981514	0.987198672	0.996679516
ANKEF1	0.996446603	0.948801019	0.988000015	0.999981514	0.996217975	0.997383134
LOC104976062	0.996464704	0.991673407	0.982195333	0.999981514	0.999985752	0.967047449
PLEKHO2	0.996635796	0.970084348	0.999993442	0.999981514	0.996781978	0.963255451
PHF19	0.996635796	0.994185904	0.999993442	0.999981514	0.982276824	0.966212838
LOC112447824	0.996635796	0.988008216	0.983504244	0.999981514	0.997165254	0.973649216

RAVER1	0.99666343	0.955652503	0.999993442	0.999981514	0.991360611	0.972088812
LOC789551	0.99666343	0.955536844	0.978014482	0.999981514	0.998008754	0.973649216
LOC784966	0.996688531	0.969051025	0.999993442	0.999981514	0.996781978	0.971545917
RYR3	0.996738681	0.957667701	0.999703589	0.999981514	0.977167046	0.969014913
ZNF789	0.996767921	0.988008216	0.985172237	0.997792939	0.999985752	0.985950607
DSP	0.996786771	0.961663204	0.990974506	0.999981514	0.984692578	0.988639786
SEMA7A	0.996859175	0.952760839	0.990974506	0.999981514	0.999985752	0.979505709
FAM241B	0.996924262	0.985940669	0.979494108	0.999981514	0.982087479	0.994788615
SUDS3	0.99712618	0.964418016	0.99341711	0.999981514	0.99351564	0.963015949
FOSL2	0.99712618	0.972167256	0.992263335	0.999981514	0.999453276	0.96870908
LOC101907886	0.99718068	0.988444882	0.995377403	0.999981514	0.999985752	0.963336022
SCRN3	0.99718068	0.981952108	0.992728681	0.999981514	0.988881329	0.963456717
ASH2L	0.99718068	0.981952108	0.997833363	0.992930508	0.999985752	0.963456717
TMEM231	0.99718068	0.975612288	0.978014482	0.999981514	0.999985752	0.963456717
SDR39U1	0.99718068	0.993481224	0.987610559	0.999981514	0.999985752	0.963456717
THOC2	0.99718068	0.972496661	0.988307089	0.98981682	0.999985752	0.964898868
ASB1	0.99718068	0.95856975	0.982041666	0.999981514	0.999985752	0.965783646
DHRS7	0.99718068	0.960708527	0.99341711	0.999981514	0.989334846	0.966017321
RARS2	0.99718068	0.950169081	0.999993442	0.999981514	0.999985752	0.966196012
LOC784808	0.99718068	0.988444882	0.999993442	0.999981514	0.989379051	0.966212838
CDK17	0.99718068	0.993481224	0.996224523	0.995565324	0.999985752	0.966850975
LRRC7	0.99718068	0.96299056	0.999993442	0.999981514	0.999985752	0.966850975
PRKG2	0.99718068	0.983887536	0.997400824	0.999981514	0.999859158	0.966914618
MGST2	0.99718068	0.961663204	0.978014482	0.999981514	0.984386856	0.967602061
KLHL32	0.99718068	0.993481224	0.99647733	0.999981514	0.977158496	0.968002076
GDAP1	0.99718068	0.977341627	0.996224523	0.999981514	0.989334846	0.968697704
LOC518768	0.99718068	0.954131334	0.99341711	0.999981514	0.999985752	0.96870908
LOC101906836	0.99718068	0.982515431	0.979494108	0.999981514	0.996089557	0.969014913
LOC112445944	0.99718068	0.96643574	0.990037865	0.999981514	0.999985752	0.969197073
CCDC142	0.99718068	0.968509005	0.999993442	0.990955345	0.996781978	0.969754487
LOC104972526	0.99718068	0.983594252	0.999993442	0.999981514	0.981652321	0.970105102
LOC112448062	0.99718068	0.969051025	0.997850143	0.999981514	0.989334846	0.970105102
ALOX5	0.99718068	0.977196455	0.999993442	0.999981514	0.996217975	0.970105102
PRPF3	0.99718068	0.955137018	0.999993442	0.994395954	0.998287238	0.970105102
FGFR1OP2	0.99718068	0.950126059	0.990974506	0.989707094	0.999985752	0.971545917
VPS13D	0.99718068	0.985579976	0.989494193	0.999981514	0.999985752	0.971545917
CSF3	0.99718068	0.989303356	0.979494108	0.999981514	0.999985752	0.973218068
ACOT13	0.99718068	0.969051025	0.999703935	0.999981514	0.999453276	0.973919852

RDM1	0.99718068	0.958764172	0.999993442	0.992930508	0.983941532	0.975200512
POGK	0.99718068	0.9574162	0.999993442	0.999981514	0.986287964	0.975200512
PRSS33	0.99718068	0.980521483	0.992511421	0.999981514	0.989334846	0.97521287
ACTR6	0.99718068	0.988444882	0.999993442	0.992930508	0.982538884	0.97529557
RNF6	0.99718068	0.99385517	0.97918842	0.98981682	0.999985752	0.977702717
MTSS1L	0.99718068	0.955201601	0.976677948	0.999981514	0.999985752	0.978445076
ORMDL3	0.99718068	0.981670901	0.998174982	0.999981514	0.987723553	0.9790177
GSTCD	0.99718068	0.997425219	0.997850143	0.999981514	0.976769047	0.980167535
NXT1	0.99718068	0.95687851	0.997850143	0.999981514	0.999453276	0.980167535
RNASE13	0.99718068	0.98978863	0.996224523	0.999981514	0.996781978	0.981216824
LOC112442544	0.99718068	0.964134676	0.990615319	0.999981514	0.998287238	0.981216824
CCDC36	0.99718068	0.96643574	0.999993442	0.999981514	0.984386856	0.98140846
SH3BGRL3	0.99718068	0.991673407	0.981655709	0.995312373	0.999985752	0.98140846
GEMIN2	0.99718068	0.955630894	0.999993442	0.995565324	0.999985752	0.982937529
CCDC160	0.99718068	0.969051025	0.998899782	0.999981514	0.984152256	0.984086745
ANKRD10	0.99718068	0.994405848	0.997850143	0.996621929	0.984328593	0.98469083
FKBP8	0.99718068	0.98098403	0.990118874	0.999981514	0.999859158	0.985564959
NBEAL1	0.99718068	0.969051025	0.972897558	0.999981514	0.999985752	0.986042926
ZNF330	0.99718068	0.96643574	0.988257612	0.999981514	0.999985752	0.987785166
C21H15orf40	0.99718068	0.952863279	0.992511421	0.999981514	0.999985752	0.988273129
LOC112448453	0.99718068	0.99386673	0.975774524	0.999981514	0.977193348	0.988796537
PJA2	0.99718068	0.95310464	0.999993442	0.996621929	0.997165254	0.991416486
BIN3	0.99718068	0.980521483	0.999993442	0.999981514	0.979988991	0.991847494
NFKBIE	0.99718068	0.979651412	0.993049856	0.999981514	0.976769047	0.992941653
CNTNAP3	0.99718068	0.963218312	0.999993442	0.999981514	0.987723553	0.993890045
TLE1	0.99718068	0.949396195	0.999993442	0.999981514	0.988049878	0.993890045
CHML	0.99718068	0.973712113	0.999993442	0.993215785	0.993190318	0.994788615
MAD2L1BP	0.99718068	0.977341627	0.999993442	0.997660427	0.983884995	0.994887496
AFG1L	0.99718068	0.969051025	0.999993442	0.995565324	0.977193348	0.995604307
WASHC4	0.99718068	0.978518618	0.979584814	0.999981514	0.988010662	0.99562654
ASB14	0.99718068	0.965250433	0.982296455	0.999981514	0.999985752	0.996310803
NCSTN	0.99718068	0.978265699	0.98385084	0.995565324	0.999985752	0.99666605
UBE4A	0.99718068	0.962269569	0.974975876	0.999981514	0.989334846	0.997862553
SPATA13	0.99718068	0.950336973	0.982195333	0.999981514	0.999985752	0.997862553
UTP3	0.99718068	0.988008216	0.989494193	0.999981514	0.977167046	0.998284411
HRH1	0.99718068	0.949902864	0.996699281	0.999981514	0.977167046	0.99828741
FAM45A	0.99718068	0.957336947	0.977455115	0.999981514	0.996781978	0.999872573
LOC112441655	0.99718068	0.969811486	0.989977673	0.999981514	0.977167046	0.999948228

P2RX3	0.99718068	0.957385532	0.990037865	0.999981514	0.977167046	0.999948228
SPA17	0.99718068	0.95530536	0.999993442	0.998529748	0.980695939	0.999948228
IL1RAPL2	0.99718068	0.953203609	0.989494193	0.999981514	0.987723553	0.999948228
TBC1D10A	0.99718068	0.962416786	0.999993442	0.98981682	0.988049878	0.999948228
SPPL2B	0.99718068	0.968495451	0.99647733	0.999981514	0.989334846	0.999948228
CLN6	0.99718068	0.952344105	0.987789469	0.995434268	0.999985752	0.999948228
PEX1	0.99718068	0.949902864	0.988585686	0.999981514	0.999985752	0.999948228
ATF7IP2	0.997210325	0.966661805	0.999993442	0.995565324	0.996781978	0.994148575
CREBL2	0.997210325	0.955981683	0.999993442	0.999981514	0.977167046	0.997206681
PPP6R3	0.99735813	0.969051025	0.982232295	0.999981514	0.999985752	0.963861337
LOC783142	0.99735813	0.99960614	0.978014482	0.999981514	0.979840175	0.965428409
RHEX	0.99735813	0.962037123	0.995688679	0.999981514	0.999985752	0.9884289
SRD5A3	0.997365676	0.958764172	0.999993442	0.999981514	0.996781978	0.966872814
CHD3	0.997365676	0.983887536	0.997850143	0.999981514	0.996217975	0.981216824
LOC112446771	0.997387233	0.980521483	0.995377403	0.999981514	0.979759416	0.983430934
TMEM120B	0.997482798	0.970965452	0.994035957	0.999981514	0.999985752	0.963701289
SF3B4	0.997482798	0.954237721	0.999993442	0.999981514	0.986019537	0.964128552
FGD1	0.997482798	0.966752969	0.999993442	0.999981514	0.999985752	0.966136679
SNUPN	0.997482798	0.969101539	0.998592799	0.999981514	0.988049878	0.966850975
CPQ	0.997482798	0.99431777	0.989494193	0.999981514	0.997264208	0.967308566
POU2F1	0.997482798	0.99950853	0.994339207	0.992930508	0.998287238	0.971545917
KIN	0.997482798	0.972007528	0.999993442	0.995565324	0.999985752	0.971545917
INTU	0.997482798	0.953042388	0.999993442	0.992930508	0.999985752	0.973413452
PI4K2B	0.997482798	0.959478257	0.999993442	0.999981514	0.999985752	0.97345098
RARB	0.997482798	0.980616436	0.999993442	0.999981514	0.986003146	0.973649216
C1H21orf58	0.997482798	0.99528564	0.985156077	0.999981514	0.996781978	0.98304899
CENPL	0.997482798	0.981952108	0.999993442	0.996195248	0.982087479	0.988796537
LOC788142	0.997482798	0.989769855	0.990615319	0.990955345	0.999453276	0.996335668
TNKS1BP1	0.997482798	0.960848894	0.984886031	0.999981514	0.996822897	0.998284411
MGP	0.997892333	0.988444882	0.980851214	0.995565324	0.999985752	0.969197073
GPSM1	0.997892333	0.96367188	0.997400824	0.999981514	0.999985752	0.973450579
BBX	0.997923712	0.95175343	0.97773026	0.999981514	0.999985752	0.96870908
AKAP12	0.997923712	0.984410135	0.990118874	0.999981514	0.999985752	0.972695131
TOR1B	0.997976164	0.954237721	0.984886031	0.999981514	0.999985752	0.98140846
SOX17	0.998061624	0.98098403	0.978530068	0.990955345	0.999985752	0.99828741
TTC5	0.998061624	0.964418016	0.996624589	0.998529748	0.980016241	0.999948228
KATNBL1	0.99814244	0.965405402	0.982303092	0.999981514	0.999985752	0.984509067
LOC101907518	0.99814244	0.960080899	0.996224523	0.999981514	0.983941532	0.99828741

AS3MT	0.998184605	0.956232956	0.999280799	0.999981514	0.985989554	0.980425178
FAM110D	0.998238551	0.978405231	0.999993442	0.999981514	0.989334846	0.981216824
STN1	0.998266317	0.991177966	0.995377403	0.999981514	0.986003146	0.966659342
MIA2	0.998294125	0.980521483	0.999993442	0.999981514	0.989334846	0.987840151
LOC104975977	0.998562404	0.955827589	0.999993442	0.999981514	0.981382267	0.973649216
ST3GAL2	0.998562404	0.988008216	0.997400824	0.996033721	0.999985752	0.975558533
STAU1	0.998639149	0.983594252	0.992714073	0.999981514	0.999985752	0.966212838
NAA16	0.998639149	0.988008216	0.99341711	0.999981514	0.977193348	0.970105102
DDX59	0.998639149	0.993481224	0.999993442	0.996033721	0.998287238	0.971545917
HAVCR2	0.998693781	0.988008216	0.999993442	0.994408995	0.997422012	0.984509067
KHSRP	0.998844067	0.963508389	0.988307089	0.999981514	0.999985752	0.98140846
RAB3C	0.998910316	0.991673407	0.999993442	0.999981514	0.999985752	0.965117554
ZDHHC12	0.998910316	0.99528564	0.986955604	0.999981514	0.984386856	0.966800477
UNC45B	0.998910316	0.98098403	0.996224523	0.999981514	0.991097291	0.966850975
MRGBP	0.998910316	0.993481224	0.99647733	0.999981514	0.996781978	0.967643148
MAN2B1	0.998910316	0.971413741	0.997850143	0.999981514	0.999985752	0.967759699
ZNF7	0.998910316	0.99431777	0.999993442	0.991955963	0.983941532	0.970105102
FAM84B	0.998910316	0.998847958	0.999107548	0.994331516	0.996781978	0.971545917
TSPAN33	0.998910316	0.981670901	0.997400824	0.999981514	0.999985752	0.971545917
LOC100847719	0.998910316	0.99488888	0.99341711	0.999981514	0.997422012	0.972986817
SPAG16	0.998910316	0.961368616	0.997440573	0.999981514	0.999985752	0.973649216
ZNF536	0.998910316	0.973321075	0.99341711	0.999981514	0.991531798	0.973970782
LOC782385	0.998910316	0.99528564	0.985877047	0.999981514	0.999985752	0.976124527
LOC782673	0.998910316	0.972496661	0.999993442	0.997467114	0.98865576	0.980009318
ACAD11	0.998910316	0.984410135	0.999993442	0.999981514	0.984386856	0.98140846
ZNF25	0.998910316	0.981614672	0.984886031	0.999981514	0.990422796	0.985156335
ZNF793	0.998910316	0.960708527	0.999993442	0.997374305	0.999985752	0.98679538
SLC4A10	0.998910316	0.96643574	0.999993442	0.995565324	0.999859158	0.988273129
POLR2G	0.998910316	0.984410135	0.999993442	0.992560663	0.996781978	0.988796537
UBAP1L	0.998910316	0.989303356	0.978014482	0.999981514	0.998196897	0.999463809
NECAP2	0.998910316	0.984307512	0.981655709	0.995312373	0.980016241	0.999948228
TBC1D22A	0.998910316	0.985428851	0.990615319	0.995565324	0.983941532	0.999948228
GRM8	0.998910316	0.95187005	0.99341711	0.999981514	0.984386856	0.999948228
SLC35D1	0.998921321	0.968509005	0.989494193	0.994408995	0.982208423	0.999948228
PHKG2	0.998985578	0.985997726	0.991071739	0.999981514	0.999985752	0.971545917
LOC101904187	0.998985578	0.95462892	0.999993442	0.999981514	0.988050858	0.971742751
ARHGEF16	0.999225799	0.994185904	0.991187638	0.998472013	0.999453276	0.986042926
CDRT1	0.999231695	0.952760839	0.982232295	0.997025804	0.999985752	0.985165897

GPBAR1	0.99954921	0.998847958	0.990037865	0.999981514	0.991603388	0.973020901
ANXA3	0.999594065	0.955652503	0.992378548	0.999981514	0.999985752	0.966850975
CRISPLD1	0.999594065	0.969101539	0.999993442	0.999981514	0.979290191	0.967759699
LOC107132672	0.999594065	0.99960614	0.992410032	0.999981514	0.979290191	0.969197073
PON2	0.999594065	0.976299197	0.990118874	0.999981514	0.98094737	0.970105102
ORAI1	0.999594065	0.96643574	0.999993442	0.999981514	0.999985752	0.973649216
LOC112441807	0.999594065	0.99528564	0.988307089	0.999981514	0.999985752	0.976436822
NEU1	0.999594065	0.998847958	0.977641717	0.999981514	0.987439758	0.977927398
BLVRA	0.999594065	0.988444882	0.979354564	0.999981514	0.983941532	0.978112039
ZNF814	0.999594065	0.998847958	0.97773026	0.999981514	0.996781978	0.980464176
IKZF4	0.999594065	0.975984829	0.99647733	0.999981514	0.979290191	0.988866378
NDRG2	0.999594065	0.968186752	0.99341711	0.999981514	0.980189546	0.996335668
GADD45B	0.999667263	0.965405402	0.99341711	0.999981514	0.999985752	0.98150605
EEF2	0.999667263	0.98098403	0.978014482	0.998529748	0.996781978	0.999948228
TANK	0.999987489	0.974229033	0.999993442	0.999981514	0.993190318	0.973218068
VPS51	0.999987489	0.974060899	0.980851214	0.999981514	0.999985752	0.973413452
LOC112446427	0.999987489	0.977901258	0.982195333	0.999981514	0.999985752	0.973649216
CD99L2	0.999987489	0.999987083	0.987789469	0.999981514	0.995870928	0.976124527
NR1I3	0.999987489	0.997425219	0.999430375	0.992339478	0.998287238	0.978519031
PDSS2	0.999987489	0.998847958	0.994715737	0.999981514	0.984386856	0.9790177
HYKK	0.999987489	0.95297524	0.984886031	0.999981514	0.989334846	0.980464176
LOC101905033	0.999987489	0.990421548	0.978530068	0.999981514	0.98500191	0.98140846
COX15	0.999987489	0.95687851	0.999993442	0.995312373	0.999985752	0.982422737
NOLC1	0.999987489	0.95687851	0.990278947	0.999981514	0.999985752	0.986042926
ARHGAP24	0.999987489	0.972948584	0.980061232	0.999981514	0.999985752	0.98926637
BAZ2A	0.999987489	0.956406487	0.99341711	0.995565324	0.999985752	0.992841537
KAT2A	0.999987489	0.972658336	0.999993442	0.994395954	0.998008754	0.993523243
LOC520104	0.999987489	0.980833065	0.980761647	0.999981514	0.981449294	0.994788615
GSTA4	0.999987489	0.955652503	0.999993442	0.999793541	0.999985752	0.996065581
LRP10	0.999987489	0.96130201	0.996224523	0.999981514	0.982538884	0.997862553
SWI5	0.999987489	0.962494531	0.982296455	0.999981514	0.999985752	0.99841577
DOCK11	0.999987489	0.962423197	0.992714073	0.999981514	0.980189546	0.999948228
EFNA5	0.999987489	0.957494685	0.999993442	0.999981514	0.996089557	0.999948228
CLK3	0.999987489	0.96643574	0.982195333	0.999981514	0.998835841	0.999948228
HRASLS	0.999987489	0.953954642	0.999993442	0.992560663	0.999985752	0.999948228
GRIN2D	0.999995916	0.966057895	0.999993442	0.999981514	0.998141915	0.966659342
UVRAG	0.999995916	0.957494685	0.991936822	0.999981514	0.999985752	0.966850975
FAM133A	0.999995916	0.988444882	0.993083875	0.999981514	0.999985752	0.967308566

TSPAN3	0.999995916	0.999987083	0.999993442	0.998529748	0.983941532	0.96870908
HADH	0.999995916	0.993249767	0.988120522	0.999981514	0.98865576	0.96870908
PFDN5	0.999995916	0.971894866	0.999993442	0.999981514	0.996781978	0.96870908
HES4	0.999995916	0.973321075	0.992263335	0.999981514	0.999985752	0.96870908
FAM78A	0.999995916	0.988444882	0.999993442	0.999981514	0.983941532	0.969014913
GOPC	0.999995916	0.997471998	0.996685706	0.999981514	0.999985752	0.969014913
YY1	0.999995916	0.959396625	0.979494108	0.999981514	0.999985752	0.969032994
FLRT2	0.999995916	0.972287585	0.999993442	0.999981514	0.984386856	0.969197073
BBS4	0.999995916	0.977196455	0.99341711	0.999981514	0.999985752	0.969754487
RBM7	0.999995916	0.997332173	0.99341711	0.999981514	0.987723553	0.969884023
FABP9	0.999995916	0.980521483	0.990615319	0.999981514	0.999985752	0.970044042
SUGP2	0.999995916	0.972007528	0.999993442	0.999981514	0.980325376	0.970105102
FAM200A	0.999995916	0.996738167	0.999353029	0.999981514	0.982538884	0.970105102
TBCEL	0.999995916	0.977196455	0.999993442	0.999981514	0.987723553	0.970105102
TMEM260	0.999995916	0.963957751	0.999993442	0.999981514	0.996217975	0.970105102
GPR153	0.999995916	0.997471998	0.997300234	0.999981514	0.996781978	0.970105102
KANTR	0.999995916	0.969051025	0.979716436	0.999981514	0.999985752	0.970105102
G2E3	0.999995916	0.991673407	0.982195333	0.999981514	0.999985752	0.970105102
ZBED1	0.999995916	0.991673407	0.991108777	0.999981514	0.999985752	0.970105102
BCLAF1	0.999995916	0.98978863	0.996224523	0.999981514	0.999985752	0.970105102
BRWD3	0.999995916	0.979651412	0.996224523	0.999981514	0.999985752	0.970115855
GRAMD2B	0.999995916	0.976297362	0.999993442	0.999981514	0.999985752	0.970216353
ITPK1	0.999995916	0.985997726	0.99615564	0.999981514	0.999985752	0.970555603
RPP14	0.999995916	0.97225074	0.999993442	0.999981514	0.999985752	0.970555603
ARL5A	0.999995916	0.969101539	0.997850143	0.999981514	0.992245178	0.970680228
LETMD1	0.999995916	0.969162328	0.999993442	0.999981514	0.988881329	0.971545917
AKR1A1	0.999995916	0.988444882	0.999993442	0.999038184	0.999985752	0.971545917
CTDNEP1	0.999995916	0.981952108	0.979494108	0.999981514	0.999985752	0.971545917
PPP1R26	0.999995916	0.969457788	0.999993442	0.999981514	0.999985752	0.971545917
RASSF4	0.999995916	0.99960614	0.999993442	0.999180255	0.995870928	0.971742751
LOC101902430	0.999995916	0.955652503	0.991936822	0.999981514	0.999985752	0.971742751
SRRT	0.999995916	0.972007528	0.99647733	0.999981514	0.999985752	0.971982373
HCFC2	0.999995916	0.99960614	0.988585686	0.999981514	0.999985752	0.972314037
FOLH1B	0.999995916	0.981952108	0.983582726	0.996195248	0.999985752	0.972332728
LOC101902998	0.999995916	0.998847958	0.986069541	0.997660427	0.999985752	0.972332728
FAM13A	0.999995916	0.959478257	0.999993442	0.999981514	0.999985752	0.972334437
FAM193A	0.999995916	0.982664716	0.999993442	0.999981514	0.999985752	0.972334437
TRUB1	0.999995916	0.974580085	0.996224523	0.999981514	0.999985752	0.972415704

STRN	0.999995916	0.99528564	0.999280799	0.999981514	0.999985752	0.972493748
LOC112449510	0.999995916	0.959263706	0.999993442	0.999981514	0.983941532	0.972695131
PRRC2A	0.999995916	0.966069053	0.996224523	0.999981514	0.999985752	0.972980807
ZZZ3	0.999995916	0.998847958	0.992511421	0.999981514	0.999985752	0.972986817
HADHB	0.999995916	0.968509005	0.998899782	0.999981514	0.999453276	0.973020901
SYN3	0.999995916	0.963052738	0.981040333	0.999981514	0.999985752	0.973020901
FAM180A	0.999995916	0.994185904	0.984886031	0.999981514	0.987723553	0.973218068
ZNF583	0.999995916	0.991673407	0.999993442	0.999981514	0.988283637	0.973218068
CYBB	0.999995916	0.99528564	0.99341711	0.999981514	0.998196897	0.973218068
CDK4	0.999995916	0.993481224	0.999993442	0.999981514	0.995907022	0.973413452
MBD5	0.999995916	0.973321075	0.99341711	0.999981514	0.999985752	0.973413452
LOC532875	0.999995916	0.98098403	0.99341711	0.999981514	0.999985752	0.973413452
TMTC4	0.999995916	0.96734064	0.984886031	0.999981514	0.986766713	0.97345098
NEDD4L	0.999995916	0.96643574	0.999993442	0.99542943	0.983941532	0.973649216
SCAMP5	0.999995916	0.999987083	0.999993442	0.999793541	0.98865576	0.973649216
FDX1	0.999995916	0.973321075	0.999993442	0.999981514	0.991673538	0.973649216
PLXNB1	0.999995916	0.999987083	0.982232295	0.999981514	0.996781978	0.973649216
LOC112443240	0.999995916	0.965648998	0.999993442	0.999981514	0.996781978	0.973649216
CTU2	0.999995916	0.971894866	0.999993442	0.999981514	0.996781978	0.973649216
ZNF75D	0.999995916	0.980289052	0.999993442	0.999981514	0.999453276	0.973649216
OTUB1	0.999995916	0.988540349	0.999993442	0.996195248	0.999985752	0.973649216
PRKD3	0.999995916	0.98643346	0.984886031	0.999981514	0.999985752	0.973649216
LOC507930	0.999995916	0.961663204	0.996224523	0.999981514	0.999985752	0.973649216
SCN3B	0.999995916	0.995373357	0.99647733	0.999981514	0.999985752	0.973649216
PEX3	0.999995916	0.968186752	0.999993442	0.999981514	0.999985752	0.973649216
ELOVL4	0.999995916	0.969051025	0.999993442	0.999981514	0.999985752	0.973649216
YLPM1	0.999995916	0.980521483	0.999993442	0.999981514	0.999985752	0.973649216
HDAC8	0.999995916	0.980833065	0.999993442	0.999981514	0.999985752	0.973649216
FMC1	0.999995916	0.988008216	0.999993442	0.999981514	0.999985752	0.973649216
ATM	0.999995916	0.997425219	0.999993442	0.999981514	0.999985752	0.973649216
DET1	0.999995916	0.977196455	0.997400824	0.999981514	0.999985752	0.973970782
NGRN	0.999995916	0.992040539	0.999993442	0.999981514	0.984328593	0.974527074
GIT2	0.999995916	0.965405402	0.99341711	0.999981514	0.999985752	0.975089707
TENM3	0.999995916	0.976310661	0.997400824	0.999981514	0.999985752	0.975089707
LOC112449254	0.999995916	0.981670901	0.99341711	0.999981514	0.999985752	0.975124497
LOC101903600	0.999995916	0.965456931	0.999993442	0.999981514	0.996781978	0.975170737
TIAM1	0.999995916	0.985432041	0.999993442	0.999981514	0.999985752	0.975170737
SESN2	0.999995916	0.993203157	0.998592799	0.999981514	0.999985752	0.975200512

GRK2	0.999995916	0.968186752	0.999993442	0.999981514	0.999985752	0.975200512
SLC25A38	0.999995916	0.972068759	0.999993442	0.999981514	0.996781978	0.97521287
SLC44A2	0.999995916	0.981614672	0.999993442	0.999981514	0.996781978	0.97521287
PSMG4	0.999995916	0.962311281	0.996224523	0.999981514	0.998008754	0.97521287
LMAN2	0.999995916	0.997678999	0.99341711	0.999981514	0.999985752	0.97521287
LOC614922	0.999995916	0.968509005	0.999993442	0.999981514	0.999985752	0.97521287
XXYLT1	0.999995916	0.98098403	0.990962474	0.999981514	0.999985752	0.975247881
MYCBP	0.999995916	0.981520642	0.999993442	0.999981514	0.996089557	0.975558533
FLVCR1	0.999995916	0.985697207	0.997213619	0.999981514	0.999985752	0.975558533
ATP2B1	0.999995916	0.991177966	0.997400824	0.999981514	0.999985752	0.975558533
FAM199X	0.999995916	0.999278945	0.999884681	0.999981514	0.999985752	0.975558533
ARHGAP35	0.999995916	0.98098403	0.999993442	0.999981514	0.999985752	0.975558533
TBC1D17	0.999995916	0.994993704	0.991440116	0.999981514	0.999985752	0.975788372
LOC101904248	0.999995916	0.966661805	0.989494193	0.999981514	0.985360041	0.975983804
ITCH	0.999995916	0.981952108	0.99341711	0.999981514	0.999985752	0.976124527
LOC101903976	0.999995916	0.984089343	0.999993442	0.999981514	0.999859158	0.976246852
CDK14	0.999995916	0.981952108	0.999993442	0.996033721	0.998835841	0.976376506
NFAM1	0.999995916	0.975612288	0.999993442	0.999981514	0.992652493	0.976452727
CSAD	0.999995916	0.993481224	0.992511421	0.996667757	0.999985752	0.977034298
BAG5	0.999995916	0.980793805	0.993024884	0.999981514	0.982087479	0.977166378
UTP25	0.999995916	0.971289048	0.999993442	0.999981514	0.998287238	0.977166378
ANKS3	0.999995916	0.992795654	0.999993442	0.999981514	0.999985752	0.977166378
WNT10B	0.999995916	0.99488888	0.987610559	0.999981514	0.999985752	0.97721867
LOC101905049	0.999995916	0.965456931	0.986511454	0.999981514	0.999985752	0.97805715
SNRPG	0.999995916	0.969051025	0.999993442	0.999981514	0.984386856	0.978087957
SEL1L	0.999995916	0.957328149	0.999993442	0.999981514	0.992984551	0.978087957
LOC112445915	0.999995916	0.965482645	0.992511421	0.999981514	0.993461941	0.978087957
CYFIP1	0.999995916	0.990429195	0.99341711	0.999981514	0.997429556	0.978087957
SDE2	0.999995916	0.999987083	0.99341711	0.999981514	0.999453276	0.978087957
RFC2	0.999995916	0.955652503	0.991447919	0.999981514	0.999985752	0.978087957
EVC	0.999995916	0.985428851	0.996224523	0.999981514	0.999985752	0.978087957
TMEM233	0.999995916	0.971413741	0.998364815	0.999981514	0.999985752	0.978087957
AKAP8	0.999995916	0.992795654	0.999993442	0.999981514	0.999985752	0.978087957
WDR31	0.999995916	0.992144201	0.999993442	0.999981514	0.989379051	0.978112039
TGFB1	0.999995916	0.96734064	0.999993442	0.999981514	0.999985752	0.978112039
SENP3	0.999995916	0.980521483	0.999993442	0.999981514	0.999985752	0.978112039
CD151	0.999995916	0.989303356	0.980496473	0.999981514	0.999453276	0.978134946
LOC781421	0.999995916	0.960185644	0.999993442	0.999981514	0.983941532	0.978445076

KLHL12	0.999995916	0.99960614	0.991138474	0.999981514	0.991481886	0.978445076
PLPPR5	0.999995916	0.973633627	0.999993442	0.999981514	0.996089557	0.978445076
SLC25A37	0.999995916	0.96365708	0.999993442	0.999981514	0.996781978	0.978445076
LOC101905977	0.999995916	0.976527871	0.990118874	0.999981514	0.999985752	0.978445076
FZD3	0.999995916	0.961368616	0.99341711	0.999981514	0.999985752	0.978445076
ANKRD52	0.999995916	0.993481224	0.99341711	0.999981514	0.999985752	0.978445076
PEAR1	0.999995916	0.969051025	0.997400824	0.999981514	0.999985752	0.978445076
UXS1	0.999995916	0.961663204	0.983582726	0.999981514	0.999985752	0.978519031
FUCA2	0.999995916	0.973927752	0.99341711	0.999981514	0.999985752	0.978923235
IFT88	0.999995916	0.96643574	0.982232295	0.999981514	0.983941532	0.9790177
FAM149A	0.999995916	0.998847958	0.999993442	0.999981514	0.994505441	0.9790177
CCDC107	0.999995916	0.98098403	0.999993442	0.999981514	0.996781978	0.9790177
LOC104973050	0.999995916	0.979046404	0.999993442	0.996033721	0.997165254	0.9790177
CIB4	0.999995916	0.985997726	0.989069245	0.999981514	0.997429556	0.9790177
LOC781108	0.999995916	0.980833065	0.999993442	0.999981514	0.998279993	0.9790177
LOC112441888	0.999995916	0.981950382	0.979584814	0.995312373	0.999985752	0.9790177
TRMT10A	0.999995916	0.973712113	0.999993442	0.998529748	0.999985752	0.9790177
PLGRKT	0.999995916	0.981952108	0.999993442	0.998529748	0.999985752	0.9790177
PAPD7	0.999995916	0.969811486	0.992263335	0.999981514	0.999985752	0.9790177
TEX35	0.999995916	0.974000935	0.99341711	0.999981514	0.999985752	0.9790177
TRIM32	0.999995916	0.993481224	0.997941789	0.999981514	0.999985752	0.9790177
LOC112444842	0.999995916	0.955652503	0.999993442	0.999981514	0.999985752	0.9790177
IFT46	0.999995916	0.96643574	0.999993442	0.999981514	0.999985752	0.9790177
WARS2	0.999995916	0.955652503	0.999993442	0.999981514	0.999985752	0.97907351
KAT8	0.999995916	0.981520642	0.997400824	0.999981514	0.995054863	0.979480605
DSC2	0.999995916	0.975612288	0.989494193	0.999981514	0.996781978	0.979480605
LOC787679	0.999995916	0.995373357	0.997400824	0.999981514	0.998279174	0.979480605
LOC782470	0.999995916	0.980289052	0.992378548	0.999981514	0.999985752	0.979480605
ZNF596	0.999995916	0.981670901	0.996224523	0.999981514	0.999985752	0.979480605
LOC104970698	0.999995916	0.996608947	0.996224523	0.999981514	0.999985752	0.979480605
TCFL5	0.999995916	0.963957751	0.997300234	0.999981514	0.999985752	0.979480605
LOC107131607	0.999995916	0.968460927	0.998899782	0.999981514	0.999985752	0.979480605
USP36	0.999995916	0.976043292	0.999993442	0.999981514	0.999985752	0.979480605
H3F3A	0.999995916	0.980121799	0.999993442	0.999981514	0.996781978	0.979505709
LOC104971057	0.999995916	0.968509005	0.99341711	0.999981514	0.986003146	0.979838305
ARL4A	0.999995916	0.995373357	0.999993442	0.999981514	0.995515529	0.979838305
MGC127055	0.999995916	0.991883165	0.998096104	0.999981514	0.999985752	0.979838305
NRADD	0.999995916	0.984179983	0.999993442	0.999981514	0.996781978	0.980009318

LOC104968656	0.999995916	0.980833065	0.998899782	0.999981514	0.999453276	0.980009318
LOC100847156	0.999995916	0.992539037	0.99341711	0.999981514	0.999985752	0.980073023
LOC101902959	0.999995916	0.980833065	0.990615319	0.999981514	0.988881329	0.980167535
TNKS2	0.999995916	0.961663204	0.999993442	0.999981514	0.989334846	0.980167535
CEP70	0.999995916	0.964134676	0.983504244	0.999981514	0.999985752	0.980167535
PDCD2	0.999995916	0.999987083	0.99341711	0.999981514	0.999985752	0.980167535
PDCD7	0.999995916	0.967251014	0.999993442	0.999981514	0.999985752	0.980167535
LOC112448531	0.999995916	0.981952108	0.999993442	0.999981514	0.999985752	0.980167535
HMGN3	0.999995916	0.989303356	0.999993442	0.999981514	0.999985752	0.980167535
IDS	0.999995916	0.980521483	0.999993442	0.995434268	0.991481886	0.9802309
CTDP1	0.999995916	0.980833065	0.999993442	0.999981514	0.99581977	0.9802309
ADH5	0.999995916	0.981920173	0.999993442	0.998529748	0.999985752	0.9802309
UACA	0.999995916	0.95687851	0.983504244	0.999981514	0.999985752	0.9803583
LOC787530	0.999995916	0.984410135	0.992377532	0.999981514	0.999985752	0.9803583
C11H2orf68	0.999995916	0.985428851	0.992511421	0.999981514	0.999985752	0.9803583
CD2AP	0.999995916	0.971431535	0.999993442	0.999981514	0.983941532	0.980464176
LOC101909083	0.999995916	0.971636622	0.999993442	0.999981514	0.998287238	0.980464176
NCF2	0.999995916	0.98978863	0.996224523	0.999981514	0.999453276	0.980464176
LOC112447399	0.999995916	0.993203157	0.999993442	0.999981514	0.999453276	0.980464176
ZFP28	0.999995916	0.973712113	0.999993442	0.999981514	0.999985752	0.980464176
TRIM17	0.999995916	0.994013976	0.999993442	0.999981514	0.999985752	0.980556133
TENM1	0.999995916	0.999987083	0.999993442	0.999981514	0.996781978	0.980840077
C8H9orf64	0.999995916	0.997678999	0.985350221	0.999981514	0.999985752	0.980840077
KLHL9	0.999995916	0.999987083	0.987610559	0.999981514	0.983941532	0.981216824
LOC112447469	0.999995916	0.980238585	0.999993442	0.999981514	0.987723553	0.981216824
LOC107133180	0.999995916	0.998191056	0.991138474	0.999981514	0.988049878	0.981216824
ARHGEF38	0.999995916	0.973712113	0.986511454	0.999981514	0.995870928	0.981216824
TMEM127	0.999995916	0.976124961	0.999993442	0.999981514	0.996089557	0.981216824
LOC104971926	0.999995916	0.96643574	0.999993442	0.999981514	0.996781978	0.981216824
FAM228B	0.999995916	0.981520642	0.999993442	0.999981514	0.996781978	0.981216824
METTL7A	0.999995916	0.999987083	0.993024884	0.999981514	0.997165254	0.981216824
KDM8	0.999995916	0.99960614	0.996224523	0.999981514	0.998661549	0.981216824
NVL	0.999995916	0.975852983	0.984886031	0.999981514	0.999985752	0.981216824
ARID5A	0.999995916	0.988444882	0.987786638	0.999981514	0.999985752	0.981216824
VRK3	0.999995916	0.983594252	0.988142154	0.999981514	0.999985752	0.981216824
LOC112442223	0.999995916	0.991673407	0.989977673	0.999981514	0.999985752	0.981216824
ERCC2	0.999995916	0.985682995	0.991936822	0.999981514	0.999985752	0.981216824
LOC100139360	0.999995916	0.991673407	0.992511421	0.999981514	0.999985752	0.981216824

C1QTNF5	0.999995916	0.994993704	0.992511421	0.999981514	0.999985752	0.981216824
LOC104974459	0.999995916	0.98572429	0.992719412	0.999981514	0.999985752	0.981216824
ASB5	0.999995916	0.971458291	0.99341711	0.999981514	0.999985752	0.981216824
FIP1L1	0.999995916	0.988008216	0.99341711	0.999981514	0.999985752	0.981216824
LOC101905365	0.999995916	0.976596383	0.994035957	0.999981514	0.999985752	0.981216824
PTPRM	0.999995916	0.995716364	0.996224523	0.999981514	0.999985752	0.981216824
BDH2	0.999995916	0.998847958	0.996224523	0.999981514	0.999985752	0.981216824
CTBP2	0.999995916	0.961663204	0.997300234	0.999981514	0.999985752	0.981216824
GTF2A1	0.999995916	0.968468454	0.997300234	0.999981514	0.999985752	0.981216824
LRCH1	0.999995916	0.981952108	0.997400824	0.999981514	0.999985752	0.981216824
CA2	0.999995916	0.973321075	0.999993442	0.999981514	0.999985752	0.981216824
ARL15	0.999995916	0.982515431	0.999993442	0.999981514	0.999985752	0.981216824
DYNC1LI2	0.999995916	0.988444882	0.999993442	0.999981514	0.999985752	0.981216824
LDHAL6B	0.999995916	0.991882821	0.999993442	0.999981514	0.999985752	0.981216824
MBLAC1	0.999995916	0.997154508	0.999993442	0.999981514	0.999985752	0.981216824
DVL3	0.999995916	0.973712113	0.996224523	0.999981514	0.999985752	0.98139581
PCBP4	0.999995916	0.968166143	0.999993442	0.999981514	0.996089557	0.98140846
CLIC5	0.999995916	0.980521483	0.999993442	0.999981514	0.996217975	0.98140846
ZKSCAN7	0.999995916	0.981952108	0.999993442	0.999981514	0.996781978	0.98140846
LOC112449561	0.999995916	0.980521483	0.991936822	0.999981514	0.997264208	0.98140846
FERMT3	0.999995916	0.99554111	0.999993442	0.999981514	0.997422012	0.98140846
LOC785843	0.999995916	0.979046404	0.999993442	0.999981514	0.998287238	0.98140846
DUSP1	0.999995916	0.977196455	0.991936822	0.999981514	0.998800466	0.98140846
C23H6orf89	0.999995916	0.971455677	0.998899782	0.995565324	0.999985752	0.98140846
ZNF580	0.999995916	0.991673407	0.984886031	0.999981514	0.999985752	0.98140846
ZNF404	0.999995916	0.98978863	0.987346948	0.999981514	0.999985752	0.98140846
MYOZ3	0.999995916	0.99960614	0.987610559	0.999981514	0.999985752	0.98140846
ABCA10	0.999995916	0.969051025	0.988307089	0.999981514	0.999985752	0.98140846
METTL2A	0.999995916	0.963007593	0.990118874	0.999981514	0.999985752	0.98140846
ADAL	0.999995916	0.980833065	0.99341711	0.999981514	0.999985752	0.98140846
TRAM1L1	0.999995916	0.997425219	0.99341711	0.999981514	0.999985752	0.98140846
AGTPBP1	0.999995916	0.999987083	0.99341711	0.999981514	0.999985752	0.98140846
IPO9	0.999995916	0.98978863	0.996224523	0.999981514	0.999985752	0.98140846
TRABD2B	0.999995916	0.99960614	0.996224523	0.999981514	0.999985752	0.98140846
GJB2	0.999995916	0.999987083	0.996224523	0.999981514	0.999985752	0.98140846
ZNF239	0.999995916	0.999987083	0.996224523	0.999981514	0.999985752	0.98140846
PNP	0.999995916	0.967665316	0.99647733	0.999981514	0.999985752	0.98140846
TNS3	0.999995916	0.96643574	0.997300234	0.999981514	0.999985752	0.98140846

WNK1	0.999995916	0.99386673	0.997400824	0.999981514	0.999985752	0.98140846
GNG3	0.999995916	0.981952108	0.997850143	0.999981514	0.999985752	0.98140846
LOC101902994	0.999995916	0.998847958	0.997850143	0.999981514	0.999985752	0.98140846
SLC9A3R2	0.999995916	0.969051025	0.999993442	0.999981514	0.999985752	0.98140846
RHNO1	0.999995916	0.970010586	0.999993442	0.999981514	0.999985752	0.98140846
PHF23	0.999995916	0.971413741	0.999993442	0.999981514	0.999985752	0.98140846
PLEKHA5	0.999995916	0.974846249	0.999993442	0.999981514	0.999985752	0.98140846
DEPDC1B	0.999995916	0.981614672	0.999993442	0.999981514	0.999985752	0.98140846
ITPRIPL1	0.999995916	0.981952108	0.999993442	0.999981514	0.999985752	0.98140846
BIRC6	0.999995916	0.997678999	0.999993442	0.999981514	0.999985752	0.98140846
NKIRAS2	0.999995916	0.98098403	0.999993442	0.999981514	0.999985752	0.981467471
SETD1B	0.999995916	0.980289052	0.999993442	0.999981514	0.999985752	0.981672932
HIVEP2	0.999995916	0.98098403	0.99341711	0.999981514	0.998008754	0.981716775
LOC782120	0.999995916	0.993804376	0.997300234	0.999981514	0.999985752	0.98182783
ZBTB38	0.999995916	0.99960614	0.991046292	0.999981514	0.996781978	0.981989887
SPATA2	0.999995916	0.978518618	0.999993442	0.998529748	0.999985752	0.981989887
MARS2	0.999995916	0.988540349	0.99341711	0.999981514	0.999985752	0.981989887
CAST	0.999995916	0.98098403	0.996224523	0.999981514	0.999985752	0.981989887
ASAH1	0.999995916	0.997471998	0.999993442	0.999981514	0.999985752	0.981989887
CYP3A5	0.999995916	0.976596383	0.991936822	0.999981514	0.999985752	0.982401177
AK4	0.999995916	0.984410135	0.9965509	0.999981514	0.999985752	0.982422737
MAP3K3	0.999995916	0.96643574	0.999993442	0.999981514	0.999985752	0.982422737
AGK	0.999995916	0.982011436	0.999993442	0.999981514	0.999985752	0.982422737
TSPAN14	0.999995916	0.986900642	0.999993442	0.999981514	0.999985752	0.982422737
TLK1	0.999995916	0.991673407	0.996685706	0.999981514	0.999985752	0.982441918
PIGH	0.999995916	0.988008216	0.999993442	0.999981514	0.999985752	0.982587107
ZSWIM9	0.999995916	0.999987083	0.99341711	0.999981514	0.991983087	0.982868841
TRAF1	0.999995916	0.98553119	0.999993442	0.999981514	0.999985752	0.982868841
LOC107132395	0.999995916	0.991673407	0.999993442	0.999981514	0.999985752	0.982868841
TOR3A	0.999995916	0.985432041	0.999993442	0.999981514	0.996781978	0.982871032
GPR107	0.999995916	0.966069053	0.999993442	0.999981514	0.999985752	0.982871032
FRY	0.999995916	0.994185904	0.999993442	0.999981514	0.989771098	0.982908937
PRR18	0.999995916	0.98978863	0.99341711	0.999981514	0.993190318	0.982908937
MEGF10	0.999995916	0.985997726	0.980061232	0.999981514	0.984386856	0.982937529
ERG	0.999995916	0.998847958	0.999993442	0.999981514	0.989334846	0.982937529
PLEKHA3	0.999995916	0.999987083	0.999993442	0.999981514	0.996781978	0.982937529
INO80E	0.999995916	0.999278945	0.999993442	0.999981514	0.997264208	0.982937529
HESX1	0.999995916	0.982318163	0.999993442	0.999981514	0.998287238	0.982937529

CBX3	0.999995916	0.971950465	0.99341711	0.999981514	0.999985752	0.982937529
GPR151	0.999995916	0.987621888	0.997942715	0.999981514	0.999985752	0.982937529
ST6GALNAC2	0.999995916	0.969811486	0.999993442	0.999981514	0.999985752	0.982937529
EMD	0.999995916	0.981952108	0.999993442	0.999981514	0.999985752	0.982937529
RAB6B	0.999995916	0.991673407	0.999993442	0.999981514	0.999985752	0.982937529
PIGG	0.999995916	0.988008216	0.999993442	0.999981514	0.996781978	0.98304899
SNRPE	0.999995916	0.988444882	0.999993442	0.999981514	0.996781978	0.98304899
TMEM240	0.999995916	0.960747241	0.999993442	0.999981514	0.989334846	0.983430934
CDK11B	0.999995916	0.999987083	0.991936822	0.999981514	0.996781978	0.983430934
LOC101903682	0.999995916	0.991882821	0.984886031	0.999981514	0.998196897	0.983430934
AAK1	0.999995916	0.99385517	0.99647733	0.999981514	0.999985752	0.983430934
SEPT8	0.999995916	0.980289052	0.999993442	0.999981514	0.999985752	0.983564069
TTLL9	0.999995916	0.997154508	0.999993442	0.999981514	0.999985752	0.983590245
MNT	0.999995916	0.998847958	0.997300234	0.999981514	0.999279201	0.98377195
LOC101902831	0.999995916	0.998558572	0.995468549	0.999981514	0.999985752	0.98377195
LOC783195	0.999995916	0.988444882	0.996224523	0.999981514	0.999985752	0.98377195
U2AF1L4	0.999995916	0.967668839	0.999993442	0.999981514	0.999985752	0.98377195
DONSON	0.999995916	0.976527871	0.999993442	0.999981514	0.999985752	0.98377195
LOC786783	0.999995916	0.980833065	0.999993442	0.999981514	0.998287238	0.984046914
ABTB2	0.999995916	0.993481224	0.996224523	0.999981514	0.999985752	0.984046914
NALCN	0.999995916	0.982515431	0.999993442	0.999981514	0.999985752	0.984046914
SLC38A10	0.999995916	0.988444882	0.999993442	0.999981514	0.999985752	0.984046914
MICU2	0.999995916	0.993035141	0.999993442	0.999981514	0.999985752	0.984046914
DHX8	0.999995916	0.981546364	0.999993442	0.999981514	0.999985752	0.984070454
MAL	0.999995916	0.969051025	0.999993442	0.999981514	0.985250243	0.984086745
GFOD1	0.999995916	0.993481224	0.99869493	0.999981514	0.996781978	0.984086745
MTF1	0.999995916	0.979075658	0.999993442	0.999981514	0.999985752	0.984122543
WIPF2	0.999995916	0.970007713	0.999993442	0.999981514	0.999985752	0.984129452
LOC100847839	0.999995916	0.96643574	0.996068003	0.999981514	0.999985752	0.984172542
TBC1D12	0.999995916	0.98098403	0.999993442	0.999981514	0.999985752	0.984377504
DENND3	0.999995916	0.987731372	0.999993442	0.999981514	0.999985752	0.984397021
EXOSC8	0.999995916	0.977769595	0.999993442	0.999981514	0.98865576	0.984498359
C1QTNF1	0.999995916	0.976596938	0.999993442	0.999981514	0.996781978	0.984509067
CDH5	0.999995916	0.989303356	0.991447919	0.999981514	0.999985752	0.984509067
ZFYVE16	0.999995916	0.998847958	0.99647733	0.999981514	0.999985752	0.984509067
LOC783294	0.999995916	0.969811486	0.999993442	0.999981514	0.999985752	0.984509067
LOC100299705	0.999995916	0.997471998	0.999993442	0.999981514	0.996781978	0.984516291
ORC5	0.999995916	0.998847958	0.99341711	0.999981514	0.999985752	0.984546618

STX3	0.999995916	0.965405402	0.994734716	0.999981514	0.999985752	0.984669451
ADCY9	0.999995916	0.986900642	0.997850143	0.999981514	0.996089557	0.98469083
MTHFS	0.999995916	0.992539037	0.999993442	0.999981514	0.996089557	0.98469083
RASL11B	0.999995916	0.991786545	0.999993442	0.999981514	0.998338718	0.98469083
LOC101907213	0.999995916	0.96643574	0.996224523	0.997792939	0.999985752	0.98469083
ERRFI1	0.999995916	0.976271642	0.989977673	0.999981514	0.999985752	0.98469083
ZNF177	0.999995916	0.982664716	0.991326941	0.999981514	0.999985752	0.98469083
GALNT18	0.999995916	0.954222802	0.996224523	0.999981514	0.999985752	0.98469083
CLCN6	0.999995916	0.989303356	0.997400824	0.999981514	0.999985752	0.98469083
TRPS1	0.999995916	0.961663204	0.997850143	0.999981514	0.999985752	0.98469083
LOC101905723	0.999995916	0.993481224	0.999280799	0.999981514	0.999985752	0.98469083
LOC112446007	0.999995916	0.983594252	0.999841067	0.999981514	0.999985752	0.98469083
POU2F3	0.999995916	0.96130201	0.999993442	0.999981514	0.999985752	0.98469083
LOC781381	0.999995916	0.97694487	0.999993442	0.999981514	0.999985752	0.98469083
LOC112442625	0.999995916	0.991673407	0.999993442	0.999981514	0.999985752	0.98469083
CASP8	0.999995916	0.994185904	0.999993442	0.999981514	0.999985752	0.98469083
ZNF674	0.999995916	0.995373357	0.999993442	0.999981514	0.999985752	0.98469083
LOC616281	0.999995916	0.980462136	0.999993442	0.999981514	0.999985752	0.984829839
LOC107132967	0.999995916	0.993275893	0.99341711	0.999981514	0.987723553	0.98483967
SHQ1	0.999995916	0.985997726	0.989494193	0.999981514	0.998008754	0.98483967
FOXL1	0.999995916	0.982011436	0.996224523	0.999981514	0.999985752	0.985156335
ADGRF5	0.999995916	0.99960614	0.995947704	0.999981514	0.999985752	0.985165897
ZSWIM7	0.999995916	0.992539037	0.980761647	0.999981514	0.989442936	0.985223183
VWF	0.999995916	0.999278945	0.992937407	0.995565324	0.997741313	0.985223183
WASHC3	0.999995916	0.974846249	0.999993442	0.999981514	0.998835841	0.985223183
HAUS7	0.999995916	0.985097978	0.983735552	0.999981514	0.999985752	0.985223183
LOC101907320	0.999995916	0.991318226	0.996224523	0.999981514	0.999985752	0.985223183
LOC789494	0.999995916	0.987530688	0.999993442	0.999981514	0.999985752	0.985223183
TSHZ1	0.999995916	0.985682995	0.999993442	0.999981514	0.998287238	0.985551221
CSGALNACT1	0.999995916	0.998847958	0.996224523	0.999981514	0.987723553	0.985564959
LOC789960	0.999995916	0.989303356	0.999993442	0.999981514	0.98865576	0.985564959
SAP30	0.999995916	0.99960614	0.997400824	0.999981514	0.989442936	0.985564959
REXO5	0.999995916	0.997471998	0.987610559	0.999981514	0.990937627	0.985564959
BRD7	0.999995916	0.988444882	0.99647733	0.999981514	0.999985752	0.985564959
TFPI2	0.999995916	0.989303356	0.996224523	0.999981514	0.998196897	0.985633224
FAM151A	0.999995916	0.983594252	0.997400824	0.999981514	0.996781978	0.985649472
LOC100847573	0.999995916	0.999987083	0.997400824	0.999981514	0.999985752	0.985649472
GPN2	0.999995916	0.993481224	0.997400824	0.999981514	0.999985752	0.985687436

PINK1	0.999995916	0.997604725	0.99647733	0.999981514	0.999985752	0.985717525
PGRMC2	0.999995916	0.981614672	0.999993442	0.999981514	0.998287238	0.985863239
LOC521656	0.999995916	0.980462136	0.999993442	0.999981514	0.999985752	0.985863239
LOC100300095	0.999995916	0.976615289	0.987789469	0.999981514	0.983941532	0.985951746
LOC112448772	0.999995916	0.996656754	0.996224523	0.999981514	0.996781978	0.986042926
C23H6orf52	0.999995916	0.98181237	0.998777236	0.999981514	0.998338718	0.986042926
ITGA5	0.999995916	0.981511914	0.997833363	0.999981514	0.999888682	0.986042926
LOC786055	0.999995916	0.99528564	0.989069245	0.999981514	0.999985752	0.986042926
TOR1AIP1	0.999995916	0.971413741	0.99341711	0.999981514	0.999985752	0.986042926
NAP1L3	0.999995916	0.995373357	0.99341711	0.999981514	0.999985752	0.986042926
HIST1H2AK	0.999995916	0.98275486	0.996224523	0.999981514	0.999985752	0.986042926
SKAP2	0.999995916	0.98098403	0.997400824	0.999981514	0.999985752	0.986042926
GNAI2	0.999995916	0.961663204	0.999993442	0.999981514	0.999985752	0.986042926
LOC534391	0.999995916	0.984089343	0.999993442	0.999981514	0.999985752	0.986042926
RNASEH2C	0.999995916	0.984179983	0.999993442	0.999981514	0.999985752	0.986042926
ZDHHC5	0.999995916	0.985559976	0.999993442	0.999981514	0.999985752	0.986042926
FAS	0.999995916	0.99960614	0.988632381	0.999981514	0.996781978	0.986441687
ATP10A	0.999995916	0.984179983	0.999993442	0.999981514	0.999985752	0.986441687
PWWP2B	0.999995916	0.985997726	0.999993442	0.999981514	0.999985752	0.986585145
ZNF845	0.999995916	0.980833065	0.999993442	0.999981514	0.999985752	0.986638746
LOC112448271	0.999995916	0.97304892	0.999993442	0.999981514	0.98865576	0.986646873
SNRPN	0.999995916	0.98164714	0.999993442	0.999981514	0.999985752	0.986646873
CDH13	0.999995916	0.985428851	0.998096104	0.999981514	0.999859158	0.98679538
LOC104976664	0.999995916	0.999987083	0.982504315	0.996621929	0.996781978	0.987207163
ETV4	0.999995916	0.98098403	0.999993442	0.999981514	0.996781978	0.987516427
EXOSC10	0.999995916	0.977196455	0.99869493	0.999981514	0.998287238	0.987516427
C25H16orf58	0.999995916	0.979651412	0.999993442	0.999981514	0.998287238	0.987516427
LOC104973431	0.999995916	0.985428851	0.999993442	0.999981514	0.999301912	0.987516427
LOC107132288	0.999995916	0.998847958	0.999993442	0.999981514	0.999888682	0.987516427
EXOC4	0.999995916	0.98098403	0.999993442	0.999981514	0.999985752	0.987516427
CASD1	0.999995916	0.985997726	0.99341711	0.999981514	0.996781978	0.987785166
LOC104969177	0.999995916	0.981520642	0.999993442	0.999981514	0.996781978	0.987785166
ANTXR2	0.999995916	0.996656754	0.999993442	0.999981514	0.996781978	0.987785166
TEX11	0.999995916	0.973321075	0.999993442	0.999981514	0.998287238	0.987785166
DLC1	0.999995916	0.981952108	0.996068003	0.999981514	0.999344768	0.987785166
SHLD2	0.999995916	0.973189491	0.981383697	0.999981514	0.999888682	0.987785166
LOC782954	0.999995916	0.999987083	0.99341711	0.999981514	0.999985752	0.987785166
CDK19	0.999995916	0.976596938	0.999993442	0.999981514	0.999985752	0.987785166

KIF25	0.999995916	0.991673407	0.999993442	0.999981514	0.999985752	0.987785166
MECP2	0.999995916	0.973321075	0.999993442	0.995565324	0.999985752	0.987823302
RBM15B	0.999995916	0.985428851	0.999993442	0.999981514	0.999985752	0.987840151
LOC101903281	0.999995916	0.980521483	0.999993442	0.999981514	0.989379051	0.987843572
ANXA2	0.999995916	0.984179983	0.99341711	0.999981514	0.999985752	0.987843572
CNTF	0.999995916	0.997430868	0.999993442	0.999981514	0.998835841	0.988134267
TJP2	0.999995916	0.999987083	0.99341711	0.999981514	0.996089557	0.988252822
ARAP1	0.999995916	0.981952108	0.999993442	0.999981514	0.996781978	0.988252822
CHFR	0.999995916	0.999278945	0.995377403	0.999981514	0.999251855	0.988252822
LOC107132784	0.999995916	0.969051025	0.996224523	0.999981514	0.999985752	0.988252822
MCUB	0.999995916	0.981952108	0.999993442	0.999981514	0.999985752	0.988252822
APBB2	0.999995916	0.991673407	0.999993442	0.999981514	0.999985752	0.988252822
RPL36AL	0.999995916	0.974846249	0.999993442	0.999981514	0.99605513	0.988273129
SLC39A1	0.999995916	0.997471998	0.990118874	0.999981514	0.998287238	0.988273129
LOC112445060	0.999995916	0.980289052	0.988585686	0.999981514	0.999985752	0.988273129
HACL1	0.999995916	0.98667582	0.999993442	0.999981514	0.999985752	0.988273129
PBX3	0.999995916	0.999987083	0.992511421	0.999185251	0.996781978	0.988320707
ZNF623	0.999995916	0.981952108	0.999993442	0.999981514	0.999985752	0.988320707
IGBP1	0.999995916	0.994185904	0.999993442	0.999981514	0.999985752	0.988320707
SPAM1	0.999995916	0.995373357	0.999993442	0.999981514	0.999985752	0.988320707
FAH	0.999995916	0.997041849	0.99341711	0.999981514	0.997165254	0.9884289
LAMA4	0.999995916	0.98978863	0.991138474	0.999981514	0.999985752	0.9884289
LOC112445178	0.999995916	0.991673407	0.997300234	0.999981514	0.999985752	0.9884289
TM2D3	0.999995916	0.967668839	0.997833363	0.999981514	0.999985752	0.9884289
ZNF235	0.999995916	0.980521483	0.999993442	0.999981514	0.999985752	0.9884289
LOC100138645	0.999995916	0.993481224	0.996224523	0.999981514	0.999985752	0.988565413
LOC112443159	0.999995916	0.966052556	0.989494193	0.999981514	0.999985752	0.988620333
LOC784088	0.999995916	0.984410135	0.999993442	0.999793541	0.996089557	0.988635999
LOC104973803	0.999995916	0.99960614	0.998364815	0.999981514	0.999453276	0.988635999
UROS	0.999995916	0.998847958	0.999993442	0.999981514	0.999985752	0.988635999
CMTM7	0.999995916	0.955827589	0.999993442	0.999981514	0.983941532	0.988639786
TBC1D9B	0.999995916	0.983594252	0.982195333	0.999981514	0.988050858	0.988639786
AFAP1L2	0.999995916	0.993035141	0.999993442	0.999981514	0.994700298	0.988639786
ZNF75A	0.999995916	0.99960614	0.999993442	0.999981514	0.998196897	0.988639786
APC	0.999995916	0.99960614	0.999280799	0.999981514	0.998287238	0.988639786
TNRC6C	0.999995916	0.982515431	0.999993442	0.999981514	0.999453276	0.988639786
SLC12A4	0.999995916	0.97225074	0.988390948	0.999981514	0.999985752	0.988639786
FRMPD3	0.999995916	0.981520642	0.996224523	0.999981514	0.999985752	0.988639786

NHLRC3	0.999995916	0.999987083	0.996291804	0.999981514	0.999985752	0.988639786
PLPP6	0.999995916	0.985997726	0.997400824	0.999981514	0.999985752	0.988639786
SPSB2	0.999995916	0.99337291	0.997400824	0.999981514	0.999985752	0.988639786
STAM2	0.999995916	0.99960614	0.997400824	0.999981514	0.999985752	0.988639786
PTCH1	0.999995916	0.981207384	0.99869493	0.999981514	0.999985752	0.988639786
LOC101903253	0.999995916	0.987616583	0.999841067	0.999981514	0.999985752	0.988639786
PNMA8A	0.999995916	0.975612288	0.999993442	0.999981514	0.999985752	0.988639786
DDX19B	0.999995916	0.979272895	0.999993442	0.999981514	0.999985752	0.988639786
BRD3	0.999995916	0.980521483	0.999993442	0.999981514	0.999985752	0.988639786
DCAF7	0.999995916	0.982515431	0.999993442	0.999981514	0.999985752	0.988639786
LOC101907195	0.999995916	0.984482951	0.999993442	0.999981514	0.999985752	0.988639786
LOC101902124	0.999995916	0.985682995	0.999993442	0.999981514	0.999985752	0.988639786
EXOSC6	0.999995916	0.985997726	0.999993442	0.999981514	0.999985752	0.988639786
DDX28	0.999995916	0.99488888	0.999993442	0.999981514	0.999985752	0.988639786
ZNF444	0.999995916	0.997471998	0.999993442	0.999981514	0.999985752	0.988639786
HSPA12A	0.999995916	0.99960614	0.999993442	0.999981514	0.999985752	0.988639786
LOC101906273	0.999995916	0.999987083	0.999993442	0.999981514	0.999985752	0.988639786
LOC112449056	0.999995916	0.969051025	0.987789469	0.999981514	0.989334846	0.988796537
KLHL36	0.999995916	0.991673407	0.982232295	0.999981514	0.999985752	0.988796537
FMO5	0.999995916	0.976596938	0.995746693	0.999981514	0.999985752	0.988796537
COMMD9	0.999995916	0.985428851	0.999993442	0.999981514	0.996217975	0.988823507
HOXA3	0.999995916	0.999987083	0.996224523	0.999981514	0.999985752	0.988823507
FAM168A	0.999995916	0.981520642	0.999993442	0.999981514	0.996781978	0.988866378
LOC107132175	0.999995916	0.95687851	0.991936822	0.999981514	0.999985752	0.988866378
PCNA	0.999995916	0.999987083	0.997400824	0.999981514	0.999985752	0.988866378
SLC20A1	0.999995916	0.962037123	0.999993442	0.999981514	0.999985752	0.988866378
DRD2	0.999995916	0.984469201	0.999993442	0.999981514	0.999985752	0.988866378
DAP	0.999995916	0.988444882	0.999993442	0.999981514	0.999985752	0.988866378
AIFM2	0.999995916	0.997425219	0.999993442	0.999981514	0.999985752	0.988866378
SHE	0.999995916	0.99960614	0.999993442	0.999981514	0.999985752	0.988866378
DAAM1	0.999995916	0.999987083	0.999993442	0.999981514	0.999985752	0.988866378
GPHN	0.999995916	0.985428851	0.999993442	0.999981514	0.999985752	0.988941608
TLK2	0.999995916	0.975984829	0.998899782	0.999981514	0.999985752	0.989022544
RIOK1	0.999995916	0.972007528	0.999993442	0.999981514	0.998914459	0.989084358
SOCS4	0.999995916	0.98098403	0.992378548	0.999981514	0.999985752	0.989084358
LOC781741	0.999995916	0.985428851	0.99341711	0.999981514	0.999985752	0.989084358
TCERG1	0.999995916	0.974846249	0.999993442	0.999981514	0.999985752	0.989084358
ATP2B4	0.999995916	0.99960614	0.999993442	0.999981514	0.999985752	0.989084358

LOC112444593	0.999995916	0.994185904	0.998447145	0.999981514	0.983941532	0.989184798
LOC786256	0.999995916	0.99960614	0.996224523	0.99615053	0.989442936	0.989184798
TBC1D30	0.999995916	0.976596938	0.998174982	0.999981514	0.992633314	0.989184798
LOC112447435	0.999995916	0.982011436	0.999993442	0.999981514	0.99971266	0.989184798
JUND	0.999995916	0.968509005	0.995377403	0.999981514	0.999985752	0.989184798
TRIM27	0.999995916	0.993481224	0.997850143	0.999981514	0.999985752	0.989184798
LOC104974837	0.999995916	0.982839621	0.999993442	0.999981514	0.999985752	0.989184798
CDCA7L	0.999995916	0.984179983	0.999993442	0.999981514	0.999985752	0.989184798
RIPPLY3	0.999995916	0.995373357	0.999993442	0.999981514	0.999985752	0.989184798
PTTG1IP	0.999995916	0.991673407	0.999993442	0.999981514	0.99932493	0.98926637
CTU1	0.999995916	0.964589091	0.998899782	0.999981514	0.999985752	0.98926637
SLC26A1	0.999995916	0.98098403	0.999993442	0.999981514	0.999985752	0.98926637
TARS2	0.999995916	0.991673407	0.999993442	0.999981514	0.999985752	0.98926637
ZBTB46	0.999995916	0.99960614	0.999993442	0.999981514	0.999985752	0.98926637
ZDHHC24	0.999995916	0.999987083	0.99341711	0.999981514	0.999453276	0.989309315
CD276	0.999995916	0.99960614	0.999829453	0.999981514	0.996809631	0.989312665
LOC100850436	0.999995916	0.999987083	0.999993442	0.999981514	0.998287238	0.989312665
LOC518080	0.999995916	0.991177966	0.999993442	0.999981514	0.999985752	0.989365627
CCDC59	0.999995916	0.966052556	0.999993442	0.999981514	0.986003146	0.989383207
SIGLEC10	0.999995916	0.973633627	0.992263335	0.995434268	0.999985752	0.989457616
SNAPC3	0.999995916	0.984631976	0.999993442	0.999981514	0.997429556	0.989493848
CACNA1F	0.999995916	0.981952108	0.999993442	0.999981514	0.998287238	0.989493848
ATG7	0.999995916	0.988444882	0.996224523	0.999981514	0.999985752	0.989493848
NUDT12	0.999995916	0.983594252	0.999993442	0.999981514	0.999985752	0.989493848
C16H1orf112	0.999995916	0.988008216	0.999993442	0.999981514	0.999985752	0.989493848
BCL7C	0.999995916	0.991673407	0.997942715	0.999981514	0.999985752	0.989642028
LOC613444	0.999995916	0.990421548	0.999993442	0.999981514	0.999985752	0.989642028
ARPIN	0.999995916	0.990421548	0.995746693	0.999981514	0.999453276	0.989700755
CHD8	0.999995916	0.976299197	0.999993442	0.999981514	0.999985752	0.989700755
ANGPT1	0.999995916	0.981670901	0.999993442	0.999981514	0.999985752	0.989700755
PPFIBP1	0.999995916	0.981952108	0.999993442	0.999981514	0.999985752	0.989700755
THG1L	0.999995916	0.975509159	0.988307089	0.999981514	0.999985752	0.989742709
PPP2R5D	0.999995916	0.982515431	0.999993442	0.999981514	0.999985752	0.989840954
LOC101907688	0.999995916	0.985428851	0.999993442	0.999981514	0.999985752	0.989840954
LOC782922	0.999995916	0.985559976	0.999993442	0.999981514	0.999985752	0.989840954
LOC112446799	0.999995916	0.99528564	0.999993442	0.999981514	0.999985752	0.989840954
KLHL20	0.999995916	0.998847958	0.999993442	0.999981514	0.999985752	0.989840954
CNOT8	0.999995916	0.985997726	0.999993442	0.999981514	0.999985752	0.990017477

IFT27	0.999995916	0.968166143	0.985270134	0.999981514	0.999985752	0.990035056
PSMB8	0.999995916	0.993427125	0.999993442	0.999981514	0.999985752	0.990076968
YWHAZ	0.999995916	0.974798116	0.999993442	0.999981514	0.999859158	0.990466545
LOC101906522	0.999995916	0.994185904	0.999993442	0.999981514	0.999985752	0.990544734
FGF12	0.999995916	0.99528564	0.999993442	0.999981514	0.999985752	0.990564508
CLEC1A	0.999995916	0.991673407	0.988307089	0.999981514	0.98865576	0.990755602
GGT7	0.999995916	0.998847958	0.999993442	0.999981514	0.998835841	0.990755602
MEX3D	0.999995916	0.975250907	0.999993442	0.999981514	0.999985752	0.990755602
SGO1	0.999995916	0.994185904	0.999993442	0.999981514	0.999985752	0.990755602
UBTD2	0.999995916	0.997425219	0.999993442	0.999981514	0.999985752	0.990755602
CYBA	0.999995916	0.989303356	0.99341711	0.999981514	0.999985752	0.990924439
ZNF598	0.999995916	0.993481224	0.999993442	0.999981514	0.999985752	0.990924439
POMT2	0.999995916	0.99960614	0.999993442	0.999981514	0.998287238	0.990951824
LOC112446667	0.999995916	0.999657245	0.999993442	0.999981514	0.999985752	0.990951824
TEAD4	0.999995916	0.99488888	0.999993442	0.999981514	0.997165254	0.991140568
GABPB2	0.999995916	0.999987083	0.999993442	0.999981514	0.998008754	0.991140568
WWTR1	0.999995916	0.985997726	0.999993442	0.999981514	0.999985752	0.991140568
CALML6	0.999995916	0.98572429	0.999993442	0.999981514	0.999453276	0.991141055
GALM	0.999995916	0.969051025	0.984886031	0.999981514	0.983941532	0.991416486
C19H17orf75	0.999995916	0.993481224	0.980032241	0.999981514	0.988049878	0.991416486
PECAM1	0.999995916	0.989303356	0.999993442	0.999981514	0.996781978	0.991416486
LOC104970908	0.999995916	0.990697047	0.999993442	0.999981514	0.998287238	0.991416486
HS3ST6	0.999995916	0.981614672	0.99341711	0.999981514	0.999453276	0.991416486
ZNF592	0.999995916	0.969051025	0.999993442	0.997660427	0.999985752	0.991416486
PDE8B	0.999995916	0.978518618	0.99341711	0.999981514	0.999985752	0.991416486
RNF157	0.999995916	0.981952108	0.996224523	0.999981514	0.999985752	0.991416486
LOC100848912	0.999995916	0.981670901	0.999993442	0.999981514	0.999985752	0.991416486
ACSF3	0.999995916	0.988824757	0.999993442	0.999981514	0.999985752	0.991416486
MRPS26	0.999995916	0.994185904	0.999993442	0.999981514	0.999985752	0.991416486
BACE1	0.999995916	0.999987083	0.999993442	0.999981514	0.999985752	0.991416486
MED20	0.999995916	0.999987083	0.999993442	0.999981514	0.999985752	0.991416486
LOC783376	0.999995916	0.970084348	0.999993442	0.999981514	0.989334846	0.991421487
RESP18	0.999995916	0.99528564	0.991936822	0.999981514	0.996089557	0.991421487
ANO10	0.999995916	0.986900642	0.999993442	0.999981514	0.999859158	0.991421487
ZCCHC4	0.999995916	0.987731372	0.99341711	0.999981514	0.999985752	0.991421487
BRPF1	0.999995916	0.998847958	0.996224523	0.999981514	0.999985752	0.991421487
LOC101902345	0.999995916	0.984179983	0.999993442	0.999981514	0.999985752	0.991421487
LOC112442367	0.999995916	0.993481224	0.999993442	0.999981514	0.999985752	0.991421487

LOC107132189	0.999995916	0.995373357	0.999993442	0.999981514	0.999985752	0.991421487
INTS14	0.999995916	0.998847958	0.999993442	0.999981514	0.999985752	0.991527539
PTPRB	0.999995916	0.997471998	0.999993442	0.999981514	0.996781978	0.991847494
GGT5	0.999995916	0.987887518	0.99341711	0.999981514	0.998892262	0.991847494
LOC112448579	0.999995916	0.992144201	0.99341711	0.997696233	0.999985752	0.991847494
LOC516355	0.999995916	0.984089343	0.997400824	0.999981514	0.999985752	0.991847494
AKAP1	0.999995916	0.999987083	0.997400824	0.999981514	0.999985752	0.991847494
ZCCHC7	0.999995916	0.993481224	0.998848708	0.999981514	0.999985752	0.991847494
UBAP2L	0.999995916	0.986849931	0.999993442	0.999981514	0.999985752	0.991847494
P2RX6	0.999995916	0.993481224	0.999993442	0.999981514	0.999985752	0.991847494
SENP7	0.999995916	0.99488888	0.999993442	0.999981514	0.999985752	0.991847494
ZNF746	0.999995916	0.99488888	0.999993442	0.999981514	0.999985752	0.991847494
PTGIS	0.999995916	0.998847958	0.999993442	0.999981514	0.999985752	0.991847494
AHCYL2	0.999995916	0.981952108	0.999993442	0.999981514	0.999859158	0.992085109
AP4S1	0.999995916	0.981952108	0.999993442	0.999981514	0.999985752	0.992085109
PLB1	0.999995916	0.998847958	0.999993442	0.999981514	0.999985752	0.992085109
FAM102A	0.999995916	0.988444882	0.999993442	0.999981514	0.999985752	0.99218589
LOC101904902	0.999995916	0.994185904	0.999993442	0.999981514	0.999985752	0.99218589
LOC112442740	0.999995916	0.998847958	0.999993442	0.999981514	0.996781978	0.992190617
BCKDHA	0.999995916	0.995373357	0.996291804	0.999981514	0.999985752	0.992190617
HERC2	0.999995916	0.99431777	0.999993442	0.999981514	0.999985752	0.992190617
DUT	0.999995916	0.998847958	0.999993442	0.999981514	0.999985752	0.992190617
LATS1	0.999995916	0.99960614	0.999993442	0.999981514	0.999985752	0.992190617
TUBB3	0.999995916	0.995373357	0.999993442	0.999981514	0.999985752	0.992209621
UTP11	0.999995916	0.988444882	0.999993442	0.999981514	0.991481886	0.992389954
YIPF6	0.999995916	0.964761409	0.999993442	0.999981514	0.996781978	0.992466226
PRKCD	0.999995916	0.98098403	0.987610559	0.999981514	0.999985752	0.992466226
LOC101903356	0.999995916	0.991673407	0.999993442	0.999981514	0.999985752	0.992568593
RAI2	0.999995916	0.999987083	0.998899782	0.999981514	0.999985752	0.992651393
LOC112441530	0.999995916	0.985428851	0.998899782	0.999981514	0.996781978	0.992680267
NXPE4	0.999995916	0.977769595	0.999993442	0.999981514	0.996781978	0.992680267
KAZALD1	0.999995916	0.980521483	0.999993442	0.999981514	0.996781978	0.992680267
AJAP1	0.999995916	0.997678999	0.999993442	0.999981514	0.999985752	0.992680267
PLIN1	0.999995916	0.985997726	0.997300234	0.999981514	0.997264208	0.992934504
LOC112442611	0.999995916	0.998847958	0.990037865	0.999981514	0.988881329	0.992941653
ANKRD50	0.999995916	0.988444882	0.988307089	0.999981514	0.999985752	0.993175779
LOC112447443	0.999995916	0.978998282	0.999993442	0.999981514	0.992104882	0.993477308
LNP1	0.999995916	0.993481224	0.999993442	0.999981514	0.999453276	0.993477308

LOC541276	0.999995916	0.99960614	0.999993442	0.999981514	0.999985752	0.993477308
CAPRIN2	0.999995916	0.989251449	0.999993442	0.999981514	0.999985752	0.993523243
LOC104975415	0.999995916	0.992539037	0.999993442	0.999981514	0.999985752	0.993729926
LOC784659	0.999995916	0.985997726	0.982823002	0.999981514	0.984386856	0.993890045
LOC101907661	0.999995916	0.991673407	0.99647733	0.999981514	0.984386856	0.993890045
HGSNAT	0.999995916	0.987530425	0.99647733	0.999981514	0.987702131	0.993890045
CCDC50	0.999995916	0.988444882	0.999993442	0.995565324	0.988049878	0.993890045
PFAS	0.999995916	0.962506437	0.999993442	0.999981514	0.988049878	0.993890045
C3H1orf50	0.999995916	0.990429195	0.999993442	0.999981514	0.996781978	0.993890045
SLC22A5	0.999995916	0.967665316	0.992378548	0.999981514	0.998141915	0.993890045
LOC112446775	0.999995916	0.975256555	0.99615564	0.999981514	0.998287238	0.993890045
SCNM1	0.999995916	0.974042599	0.999993442	0.999981514	0.999453276	0.993890045
VPS36	0.999995916	0.99960614	0.999993442	0.999981514	0.999453276	0.993890045
PYURF	0.999995916	0.993481224	0.989494193	0.995565324	0.999985752	0.993890045
C21H15orf39	0.999995916	0.984469201	0.999993442	0.998529748	0.999985752	0.993890045
BOK	0.999995916	0.990546209	0.988790673	0.999981514	0.999985752	0.993890045
ZC3HAV1	0.999995916	0.985668939	0.991936822	0.999981514	0.999985752	0.993890045
MAP3K4	0.999995916	0.99578865	0.997400824	0.999981514	0.999985752	0.993890045
FOXQ1	0.999995916	0.999987083	0.997400824	0.999981514	0.999985752	0.993890045
PAQR6	0.999995916	0.99960614	0.998899782	0.999981514	0.999985752	0.993890045
PTGR2	0.999995916	0.970010586	0.999993442	0.999981514	0.999985752	0.993890045
STAT5B	0.999995916	0.974658129	0.999993442	0.999981514	0.999985752	0.993890045
ZDHHC15	0.999995916	0.981920173	0.999993442	0.999981514	0.999985752	0.993890045
LOC112448030	0.999995916	0.98338119	0.999993442	0.999981514	0.999985752	0.993890045
TCAIM	0.999995916	0.983900149	0.999993442	0.999981514	0.999985752	0.993890045
LOC101902644	0.999995916	0.985997726	0.999993442	0.999981514	0.999985752	0.993890045
LOC100138078	0.999995916	0.988008216	0.999993442	0.999981514	0.999985752	0.993890045
HOXA5	0.999995916	0.989501533	0.999993442	0.999981514	0.999985752	0.993890045
ATG16L1	0.999995916	0.993481224	0.999993442	0.999981514	0.999985752	0.993890045
HOXA2	0.999995916	0.993481224	0.999993442	0.999981514	0.999985752	0.993890045
RAPGEF3	0.999995916	0.99386673	0.999993442	0.999981514	0.999985752	0.993890045
NONO	0.999995916	0.997471998	0.999993442	0.999981514	0.999985752	0.993890045
SAMD1	0.999995916	0.96085254	0.980032241	0.999981514	0.999985752	0.993916825
LIMK1	0.999995916	0.985997726	0.991326941	0.999981514	0.999985752	0.994059388
LOC112445076	0.999995916	0.993249767	0.999993442	0.999981514	0.999985752	0.994059388
DLG3	0.999995916	0.995373357	0.993297549	0.999981514	0.986003146	0.994347099
LOC112448776	0.999995916	0.981952108	0.997300234	0.999981514	0.996781978	0.994347099
MIPEP	0.999995916	0.993481224	0.999993442	0.999981514	0.998287238	0.994347099

PLA1A	0.999995916	0.990421548	0.992511421	0.999981514	0.999985752	0.994347099
HS3ST4	0.999995916	0.984410135	0.996685706	0.999981514	0.999985752	0.994347099
LOC112445001	0.999995916	0.981952108	0.999993442	0.999981514	0.999985752	0.994347099
PPM1J	0.999995916	0.998847958	0.999993442	0.999981514	0.999985752	0.994347099
LINS1	0.999995916	0.99960614	0.999993442	0.999981514	0.999985752	0.994347099
STK11	0.999995916	0.99960614	0.999993442	0.999981514	0.999985752	0.994347099
CHST1	0.999995916	0.999987083	0.999993442	0.999981514	0.999985752	0.994347099
LPAR4	0.999995916	0.980521483	0.999993442	0.999981514	0.999985752	0.99442139
LOC101902983	0.999995916	0.982515431	0.999993442	0.999981514	0.999985752	0.99475582
MTCH1	0.999995916	0.976596938	0.999993442	0.999981514	0.997789733	0.994776918
SMIM5	0.999995916	0.998847958	0.991936822	0.999981514	0.999985752	0.994776918
PRELP	0.999995916	0.991673407	0.999993442	0.999981514	0.999985752	0.994776918
HS3ST2	0.999995916	0.992795654	0.999993442	0.999981514	0.999985752	0.994776918
MCTP1	0.999995916	0.998847958	0.99424874	0.999981514	0.983941532	0.994788615
ISL2	0.999995916	0.980833065	0.997400824	0.999981514	0.986861597	0.994788615
GLE1	0.999995916	0.976063495	0.999993442	0.999981514	0.98865576	0.994788615
HRCT1	0.999995916	0.991673407	0.99341711	0.999981514	0.989334846	0.994788615
UBE2I	0.999995916	0.980289052	0.999993442	0.999981514	0.998141915	0.994788615
LRP8	0.999995916	0.993481224	0.997300234	0.999981514	0.998196897	0.994788615
ITGA6	0.999995916	0.969051025	0.99869493	0.999981514	0.998196897	0.994788615
LOC101906009	0.999995916	0.991673407	0.999993442	0.999981514	0.999453276	0.994788615
PSTK	0.999995916	0.99488888	0.999993442	0.999981514	0.999453276	0.994788615
LOC112442597	0.999995916	0.967042629	0.999993442	0.999981514	0.999859158	0.994788615
CD302	0.999995916	0.980521483	0.979459751	0.999981514	0.999985752	0.994788615
ARHGEF7	0.999995916	0.973321075	0.989969382	0.999981514	0.999985752	0.994788615
LOC112442352	0.999995916	0.996866547	0.990627783	0.999981514	0.999985752	0.994788615
ING3	0.999995916	0.991673407	0.99647733	0.999981514	0.999985752	0.994788615
RPS4X	0.999995916	0.998847958	0.99647733	0.999981514	0.999985752	0.994788615
RAB42	0.999995916	0.993481224	0.997300234	0.999981514	0.999985752	0.994788615
SORBS1	0.999995916	0.99358456	0.997400824	0.999981514	0.999985752	0.994788615
CLEC14A	0.999995916	0.99960614	0.997400824	0.999981514	0.999985752	0.994788615
HS3ST3A1	0.999995916	0.999987083	0.997833363	0.999981514	0.999985752	0.994788615
SART3	0.999995916	0.981952108	0.999993442	0.999981514	0.999985752	0.994788615
SNAP47	0.999995916	0.981952108	0.999993442	0.999981514	0.999985752	0.994788615
FOXP4	0.999995916	0.984126877	0.999993442	0.999981514	0.999985752	0.994788615
LOC112446452	0.999995916	0.984179983	0.999993442	0.999981514	0.999985752	0.994788615
LOC104973760	0.999995916	0.985997726	0.999993442	0.999981514	0.999985752	0.994788615
ZSCAN4	0.999995916	0.988444882	0.999993442	0.999981514	0.999985752	0.994788615

VPS35L	0.999995916	0.993804376	0.999993442	0.999981514	0.999985752	0.994788615
NSMCE2	0.999995916	0.994185904	0.999993442	0.999981514	0.999985752	0.994788615
LOC615792	0.999995916	0.99528564	0.999993442	0.999981514	0.999985752	0.994788615
TEX261	0.999995916	0.995373357	0.999993442	0.999981514	0.999985752	0.994788615
MTIF2	0.999995916	0.998847958	0.999993442	0.999981514	0.999985752	0.994788615
LOC512440	0.999995916	0.999987083	0.999993442	0.999981514	0.999985752	0.994788615
ESCO2	0.999995916	0.974846249	0.999993442	0.999981514	0.998287238	0.994826505
OSBPL2	0.999995916	0.980764484	0.999993442	0.999981514	0.987930594	0.994887496
LOC100335177	0.999995916	0.981952108	0.999993442	0.999981514	0.996089557	0.994887496
ARHGAP4	0.999995916	0.999987083	0.997300234	0.999981514	0.996781978	0.994887496
LOC107132374	0.999995916	0.993804376	0.999993442	0.999981514	0.998287238	0.994887496
NFIB	0.999995916	0.985432041	0.99341711	0.999981514	0.999251855	0.994887496
LOC104969238	0.999995916	0.981670901	0.99952024	0.999981514	0.99971266	0.994887496
WDR78	0.999995916	0.988444882	0.998899782	0.999981514	0.999985752	0.994887496
LOC100848642	0.999995916	0.999987083	0.999703589	0.999981514	0.999985752	0.994887496
ZNF48	0.999995916	0.985997726	0.999993442	0.999981514	0.999985752	0.994887496
SENP5	0.999995916	0.990052293	0.999993442	0.999981514	0.999985752	0.994887496
BEAN1	0.999995916	0.991673407	0.999993442	0.999981514	0.999985752	0.994887496
MAN1B1	0.999995916	0.991673407	0.999993442	0.999981514	0.999985752	0.994887496
LOC112442323	0.999995916	0.99386673	0.999993442	0.999981514	0.999985752	0.994887496
CCNE1	0.999995916	0.99528564	0.999993442	0.999981514	0.999985752	0.994887496
KLHL2	0.999995916	0.999987083	0.999993442	0.999981514	0.999985752	0.994887496
LZTR1	0.999995916	0.999987083	0.999993442	0.999981514	0.999985752	0.994887496
LOC100847947	0.999995916	0.969051025	0.997400824	0.999981514	0.999985752	0.994991252
PPFIA1	0.999995916	0.999987083	0.998364815	0.999981514	0.999985752	0.994991252
C19H17orf100	0.999995916	0.977196455	0.999993442	0.999981514	0.999985752	0.994991252
RNF111	0.999995916	0.984410135	0.999993442	0.999981514	0.999985752	0.994991252
RNF169	0.999995916	0.99831878	0.999993442	0.999981514	0.999985752	0.994991252
LOC101905453	0.999995916	0.993028532	0.997300234	0.999981514	0.999985752	0.995099097
AMN1	0.999995916	0.97969515	0.999993442	0.999981514	0.998661549	0.99511113
LRPAP1	0.999995916	0.99960614	0.99341711	0.999981514	0.999985752	0.99511113
WDR61	0.999995916	0.956095821	0.999993442	0.999981514	0.996781978	0.995165705
IPMK	0.999995916	0.985097978	0.999993442	0.999981514	0.996781978	0.995165705
SPIN4	0.999995916	0.995373357	0.998364815	0.999981514	0.999985752	0.995165705
CMC1	0.999995916	0.993481224	0.999993442	0.999981514	0.999985752	0.995165705
CCDC167	0.999995916	0.99960614	0.999993442	0.999981514	0.999985752	0.995165705
FN3KRP	0.999995916	0.99960614	0.999993442	0.999981514	0.999985752	0.995165705
LOC112449073	0.999995916	0.999987083	0.999993442	0.999981514	0.989379051	0.995379819

RPP25L	0.999995916	0.998847958	0.998899782	0.999981514	0.999985752	0.995379819
FRS2	0.999995916	0.990697047	0.999993442	0.999981514	0.999985752	0.995379819
GNAS	0.999995916	0.98098403	0.999993442	0.999981514	0.999985752	0.995421961
GALT	0.999995916	0.998847958	0.999993442	0.999981514	0.996781978	0.995525297
STARD3NL	0.999995916	0.981952108	0.999993442	0.996033721	0.998287238	0.995525297
ANKIB1	0.999995916	0.981520642	0.996291804	0.999981514	0.999985752	0.995525297
TRAFD1	0.999995916	0.991177966	0.999993442	0.999981514	0.999985752	0.995525297
MPDZ	0.999995916	0.99528564	0.999993442	0.999981514	0.999985752	0.995525297
BRMS1	0.999995916	0.998847958	0.999993442	0.999981514	0.999985752	0.995525297
DHRS12	0.999995916	0.984089343	0.99341711	0.999981514	0.988049878	0.995565214
KLRG1	0.999995916	0.993481224	0.999993442	0.999981514	0.999985752	0.995604307
LOC100847890	0.999995916	0.999987083	0.999993442	0.999981514	0.999985752	0.995604307
SIN3A	0.999995916	0.999987083	0.999993442	0.999981514	0.999985752	0.995653107
PDE4C	0.999995916	0.99528564	0.999993442	0.999981514	0.999985752	0.995702108
LIN37	0.999995916	0.99831878	0.999993442	0.999981514	0.999985752	0.995752939
CHERP	0.999995916	0.96456272	0.999145937	0.999981514	0.996781978	0.995895432
ARID5B	0.999995916	0.991882821	0.999993442	0.999981514	0.997499364	0.995895432
SSH3	0.999995916	0.984289531	0.996224523	0.999981514	0.998196897	0.995895432
RGS19	0.999995916	0.993427125	0.999993442	0.999981514	0.999453276	0.995895432
FER	0.999995916	0.988540349	0.993073581	0.999981514	0.999985752	0.995895432
LOC101904962	0.999995916	0.9640374	0.99341711	0.999981514	0.999985752	0.995895432
PGBD2	0.999995916	0.988633241	0.99615564	0.999981514	0.999985752	0.995895432
CREBZF	0.999995916	0.988008216	0.997400824	0.999981514	0.999985752	0.995895432
OVGP1	0.999995916	0.99386673	0.999993442	0.999981514	0.999985752	0.995895432
CHST2	0.999995916	0.995301874	0.999993442	0.999981514	0.999985752	0.995895432
LOC101909432	0.999995916	0.997471998	0.999993442	0.999981514	0.999985752	0.995895432
MAN2C1	0.999995916	0.980521483	0.999993442	0.999981514	0.996781978	0.995906915
LOC112447735	0.999995916	0.991673407	0.999993442	0.999981514	0.996781978	0.995906915
ATR	0.999995916	0.999987083	0.997850143	0.999981514	0.999985752	0.995906915
MAL2	0.999995916	0.985997726	0.999993442	0.999981514	0.999985752	0.995906915
PPM1M	0.999995916	0.987621888	0.999993442	0.999981514	0.999985752	0.995906915
FECH	0.999995916	0.988444882	0.999993442	0.999981514	0.999985752	0.995906915
TIGD2	0.999995916	0.980521483	0.99341711	0.999981514	0.999985752	0.995950237
ZNF574	0.999995916	0.982515431	0.999993442	0.999981514	0.999985752	0.995950237
ZNF526	0.999995916	0.999987083	0.999993442	0.999981514	0.999985752	0.995950237
LGALS4	0.999995916	0.99528564	0.999993442	0.999981514	0.999985752	0.995999503
TICRR	0.999995916	0.981520642	0.999993442	0.999981514	0.998287238	0.996065581
DLEC1	0.999995916	0.983594252	0.990615319	0.999981514	0.984236549	0.996310803

TMEM243	0.999995916	0.957328149	0.984886031	0.999981514	0.987723553	0.996310803
LOC101905232	0.999995916	0.998847958	0.997400824	0.999981514	0.998008754	0.996310803
WASL	0.999995916	0.994185904	0.994035957	0.999793541	0.998060725	0.996310803
DCUN1D3	0.999995916	0.971413741	0.999993442	0.999981514	0.999888682	0.996310803
SMAD3	0.999995916	0.976063495	0.99341711	0.999981514	0.999985752	0.996310803
ABHD16A	0.999995916	0.980521483	0.998364815	0.999981514	0.999985752	0.996310803
KTI12	0.999995916	0.985997726	0.999993442	0.999981514	0.999985752	0.996310803
CPED1	0.999995916	0.988008216	0.999993442	0.999981514	0.999985752	0.996310803
KDM1B	0.999995916	0.990421548	0.999993442	0.999981514	0.999985752	0.996310803
LOC107132994	0.999995916	0.991673407	0.999993442	0.999981514	0.999985752	0.996310803
KDM5A	0.999995916	0.994185904	0.999993442	0.999981514	0.999985752	0.996310803
STIM2	0.999995916	0.998847958	0.999993442	0.999981514	0.999985752	0.996310803
PODXL	0.999995916	0.99960614	0.999993442	0.999981514	0.999985752	0.996310803
ERICH1	0.999995916	0.98978863	0.997400824	0.995545226	0.997422012	0.996325012
ANKH	0.999995916	0.991883165	0.999993442	0.999981514	0.994154822	0.996335668
ZNF599	0.999995916	0.962068181	0.990615319	0.999981514	0.996217975	0.996335668
HIST2H2AC	0.999995916	0.99960614	0.992714073	0.999981514	0.996781978	0.996335668
SLC16A2	0.999995916	0.991673407	0.993073581	0.999981514	0.996781978	0.996335668
RIPOR1	0.999995916	0.960845618	0.999993442	0.999981514	0.996781978	0.996335668
BMT2	0.999995916	0.988540349	0.996224523	0.999981514	0.998287238	0.996335668
LOC112448021	0.999995916	0.966069053	0.999993442	0.999981514	0.999251855	0.996335668
NEBL	0.999995916	0.999987083	0.999993442	0.999981514	0.99971266	0.996335668
TP53RK	0.999995916	0.977769595	0.999993442	0.999981514	0.999859158	0.996335668
SLC25A53	0.999995916	0.977341627	0.990037865	0.999981514	0.999985752	0.996335668
C13H20orf194	0.999995916	0.982515431	0.997300234	0.999981514	0.999985752	0.996335668
AP2A1	0.999995916	0.990429195	0.998364815	0.999981514	0.999985752	0.996335668
CCDC92	0.999995916	0.981614672	0.99869493	0.999981514	0.999985752	0.996335668
TPBGL	0.999995916	0.981952108	0.998899782	0.999981514	0.999985752	0.996335668
ZNF398	0.999995916	0.980289052	0.999993442	0.999981514	0.999985752	0.996335668
ZNF389	0.999995916	0.985997726	0.999993442	0.999981514	0.999985752	0.996335668
LOC107131525	0.999995916	0.98978863	0.999993442	0.999981514	0.999985752	0.996335668
RGS2	0.999995916	0.991177966	0.999993442	0.999981514	0.999985752	0.996335668
LOC112442636	0.999995916	0.991673407	0.999993442	0.999981514	0.999985752	0.996335668
CTDSPL2	0.999995916	0.992040539	0.999993442	0.999981514	0.999985752	0.996335668
PRCC	0.999995916	0.992512743	0.999993442	0.999981514	0.999985752	0.996335668
ZNF777	0.999995916	0.993028532	0.999993442	0.999981514	0.999985752	0.996335668
EIF2AK1	0.999995916	0.993481224	0.999993442	0.999981514	0.999985752	0.996335668
ESYT1	0.999995916	0.993481224	0.999993442	0.999981514	0.999985752	0.996335668

MAP4	0.999995916	0.993481224	0.999993442	0.999981514	0.999985752	0.996335668
PIGL	0.999995916	0.993481224	0.999993442	0.999981514	0.999985752	0.996335668
NTN5	0.999995916	0.994993704	0.999993442	0.999981514	0.999985752	0.996335668
TRIM16	0.999995916	0.995253163	0.999993442	0.999981514	0.999985752	0.996335668
LOC512286	0.999995916	0.997236605	0.999993442	0.999981514	0.999985752	0.996335668
RNF141	0.999995916	0.997471998	0.999993442	0.999981514	0.999985752	0.996335668
FAM189B	0.999995916	0.998847958	0.999993442	0.999981514	0.999985752	0.996335668
NFATC2IP	0.999995916	0.998847958	0.999993442	0.999981514	0.999985752	0.996335668
PIGT	0.999995916	0.994749993	0.99341711	0.999981514	0.998800466	0.996387874
LOC100297513	0.999995916	0.999987083	0.999993442	0.999981514	0.999985752	0.996387874
ANK2	0.999995916	0.985594367	0.999993442	0.999981514	0.999985752	0.996414919
GPM6B	0.999995916	0.99528564	0.996224523	0.999981514	0.999985752	0.99642553
WDR5B	0.999995916	0.994185904	0.999993442	0.999981514	0.999985752	0.99642553
SMS	0.999995916	0.997425219	0.999993442	0.999981514	0.999985752	0.99642553
LOC104973767	0.999995916	0.99960614	0.999993442	0.999981514	0.999985752	0.99642553
SLC6A9	0.999995916	0.999987083	0.999993442	0.999981514	0.998287238	0.996453303
PNPLA8	0.999995916	0.998847958	0.997850143	0.999981514	0.999985752	0.996453303
PLSCR2	0.999995916	0.998847958	0.999145937	0.999981514	0.999985752	0.996453303
STAU2	0.999995916	0.979143479	0.999993442	0.999981514	0.999985752	0.996453303
FAM208A	0.999995916	0.984179983	0.999993442	0.999981514	0.999985752	0.996453303
GOLGA3	0.999995916	0.999278945	0.999993442	0.999981514	0.999985752	0.996453303
SMARCD1	0.999995916	0.976596938	0.999993442	0.999981514	0.983941532	0.99666605
TDG	0.999995916	0.981952108	0.989494193	0.999981514	0.988881329	0.99666605
LOC101902930	0.999995916	0.993481224	0.999993442	0.999981514	0.989334846	0.99666605
LOC787250	0.999995916	0.988444882	0.996224523	0.999981514	0.995515529	0.99666605
UBE2D4	0.999995916	0.99960614	0.984886031	0.999981514	0.996781978	0.99666605
REM1	0.999995916	0.985682995	0.999993442	0.999981514	0.996781978	0.99666605
TAF1B	0.999995916	0.981952108	0.999993442	0.999981514	0.997374918	0.99666605
LOC104972409	0.999995916	0.980833065	0.989720033	0.999981514	0.998196897	0.99666605
EXOSC1	0.999995916	0.98338119	0.999993442	0.999981514	0.998287238	0.99666605
PFKL	0.999995916	0.991673407	0.999993442	0.999981514	0.998287238	0.99666605
RPL9	0.999995916	0.996484544	0.999993442	0.999981514	0.998338718	0.99666605
GCK	0.999995916	0.96672074	0.99341711	0.999981514	0.999985752	0.99666605
LOC112442630	0.999995916	0.989407995	0.993517607	0.999981514	0.999985752	0.99666605
CC2D1A	0.999995916	0.971413741	0.996224523	0.999981514	0.999985752	0.99666605
DPM3	0.999995916	0.991673407	0.996224523	0.999981514	0.999985752	0.99666605
SAMD13	0.999995916	0.991673407	0.996224523	0.999981514	0.999985752	0.99666605
LOC112442687	0.999995916	0.991673407	0.997400824	0.999981514	0.999985752	0.99666605

LOC112447845	0.999995916	0.995373357	0.997400824	0.999981514	0.999985752	0.99666605
LOC101908759	0.999995916	0.999987083	0.997400824	0.999981514	0.999985752	0.99666605
LOC781004	0.999995916	0.985997726	0.999993442	0.999981514	0.999985752	0.99666605
LOC782305	0.999995916	0.986664684	0.999993442	0.999981514	0.999985752	0.99666605
ZNF23	0.999995916	0.988444882	0.999993442	0.999981514	0.999985752	0.99666605
PHF2	0.999995916	0.991673407	0.999993442	0.999981514	0.999985752	0.99666605
ATF2	0.999995916	0.993481224	0.999993442	0.999981514	0.999985752	0.99666605
PIBF1	0.999995916	0.993481224	0.999993442	0.999981514	0.999985752	0.99666605
ZNF205	0.999995916	0.993804376	0.999993442	0.999981514	0.999985752	0.99666605
TMEM131L	0.999995916	0.99386673	0.999993442	0.999981514	0.999985752	0.99666605
ENTPD4	0.999995916	0.994185904	0.999993442	0.999981514	0.999985752	0.99666605
LOC101902360	0.999995916	0.995204627	0.999993442	0.999981514	0.999985752	0.99666605
SLC16A10	0.999995916	0.997471998	0.999993442	0.999981514	0.999985752	0.99666605
MTF2	0.999995916	0.99831878	0.999993442	0.999981514	0.999985752	0.99666605
SDF4	0.999995916	0.99831878	0.999993442	0.999981514	0.999985752	0.99666605
GPR157	0.999995916	0.998847958	0.999993442	0.999981514	0.999985752	0.99666605
SPNS1	0.999995916	0.998847958	0.999993442	0.999981514	0.999985752	0.99666605
C14H8orf89	0.999995916	0.99960614	0.999993442	0.999981514	0.999985752	0.99666605
CACNG5	0.999995916	0.99960614	0.999993442	0.999981514	0.999985752	0.99666605
DOPEY1	0.999995916	0.99960614	0.999993442	0.999981514	0.999985752	0.99666605
KCTD13	0.999995916	0.99960614	0.999993442	0.999981514	0.999985752	0.99666605
MTMR9	0.999995916	0.99960614	0.999993442	0.999981514	0.999985752	0.99666605
LOC100335190	0.999995916	0.999987083	0.999993442	0.999981514	0.999985752	0.99666605
TMEM63B	0.999995916	0.999987083	0.999993442	0.999981514	0.999985752	0.99666605
TMPO	0.999995916	0.999987083	0.999993442	0.999981514	0.999985752	0.99666605
OCSTAMP	0.999995916	0.991673407	0.999993442	0.999981514	0.996781978	0.996679516
LOC112442246	0.999995916	0.98098403	0.9965509	0.999981514	0.998060725	0.996679516
NT5C3A	0.999995916	0.978493738	0.990037865	0.999981514	0.999453276	0.996679516
IFITM5	0.999995916	0.980521483	0.991936822	0.999981514	0.999859158	0.996679516
LOC784208	0.999995916	0.99960614	0.999280799	0.999981514	0.999985752	0.996679516
TACC2	0.999995916	0.978405231	0.999993442	0.999981514	0.999985752	0.996679516
CCDC3	0.999995916	0.992144201	0.999993442	0.999981514	0.999985752	0.996679516
GAT	0.999995916	0.99358456	0.999993442	0.999981514	0.999985752	0.996679516
PLAGL2	0.999995916	0.99528564	0.999993442	0.999981514	0.999985752	0.996679516
SOCS2	0.999995916	0.999987083	0.999993442	0.999981514	0.999985752	0.996679516
SLC39A9	0.999995916	0.99488888	0.999993442	0.999981514	0.996614711	0.996813547
OTOP1	0.999995916	0.99386673	0.996509242	0.999981514	0.999985752	0.996813547
CTR9	0.999995916	0.985428851	0.997300234	0.999981514	0.999985752	0.996813547

RNF220	0.999995916	0.988444882	0.99869493	0.999981514	0.999985752	0.996813547
CACNA1C	0.999995916	0.99960614	0.99869493	0.999981514	0.999985752	0.996813547
LIMA1	0.999995916	0.981920173	0.999993442	0.999981514	0.999985752	0.996813547
PPP1R16B	0.999995916	0.985428851	0.999993442	0.999981514	0.999985752	0.996813547
HECTD2	0.999995916	0.98572429	0.999993442	0.999981514	0.999985752	0.996813547
MALL	0.999995916	0.991673407	0.999993442	0.999981514	0.999985752	0.996813547
BCAM	0.999995916	0.993203157	0.999993442	0.999981514	0.999985752	0.996813547
NOS3	0.999995916	0.993481224	0.999993442	0.999981514	0.999985752	0.996813547
LOC112447350	0.999995916	0.999987083	0.999993442	0.999981514	0.999985752	0.996813547
LOC104975099	0.999995916	0.984923589	0.999993442	0.999981514	0.999985752	0.996933933
TEDC1	0.999995916	0.991883165	0.999993442	0.999981514	0.999985752	0.996933933
RASSF7	0.999995916	0.99960614	0.999993442	0.999981514	0.999985752	0.996933933
MIS18A	0.999995916	0.999621646	0.999993442	0.999981514	0.999985752	0.996933933
B3GNT3	0.999995916	0.977070024	0.99341711	0.999981514	0.990556568	0.997054363
LOC781799	0.999995916	0.985428851	0.999993442	0.999981514	0.995054863	0.997054363
CYS1	0.999995916	0.999987083	0.999993442	0.999981514	0.998800466	0.997054363
RGS11	0.999995916	0.981952108	0.987346948	0.999981514	0.999985752	0.997054363
C18H16orf70	0.999995916	0.96734064	0.990118874	0.999981514	0.999985752	0.997054363
DICER1	0.999995916	0.985428851	0.99341711	0.999981514	0.999985752	0.997054363
ZSCAN23	0.999995916	0.99960614	0.99647733	0.999981514	0.999985752	0.997054363
CBX1	0.999995916	0.988444882	0.998364815	0.999981514	0.999985752	0.997054363
ZNF165	0.999995916	0.988008216	0.999993442	0.999981514	0.999985752	0.997054363
CASP9	0.999995916	0.988540349	0.999993442	0.999981514	0.999985752	0.997054363
LOC107132664	0.999995916	0.988540349	0.999993442	0.999981514	0.999985752	0.997054363
GLCCI1	0.999995916	0.988824757	0.999993442	0.999981514	0.999985752	0.997054363
FAM187A	0.999995916	0.993481224	0.999993442	0.999981514	0.999985752	0.997054363
IAH1	0.999995916	0.994185904	0.999993442	0.999981514	0.999985752	0.997054363
LOC101903900	0.999995916	0.998847958	0.999993442	0.999981514	0.999985752	0.997054363
ARL16	0.999995916	0.999987083	0.999993442	0.999981514	0.999985752	0.997054363
LEMD2	0.999995916	0.999987083	0.999993442	0.999981514	0.999985752	0.997054363
NCBP3	0.999995916	0.975781839	0.996224523	0.999981514	0.999985752	0.997064418
LOC100336369	0.999995916	0.957588005	0.999993442	0.995565324	0.99040634	0.997206681
CACTIN	0.999995916	0.98519101	0.999993442	0.999981514	0.998060725	0.997206681
LOC101902841	0.999995916	0.980616436	0.999993442	0.999981514	0.999453276	0.997206681
MTHFD2L	0.999995916	0.980833065	0.99869493	0.999981514	0.999985752	0.997206681
PACSIN1	0.999995916	0.991673407	0.999993442	0.999981514	0.999985752	0.997206681
LOC107132713	0.999995916	0.993481224	0.999993442	0.999981514	0.999985752	0.997206681
LOC112449360	0.999995916	0.993481224	0.999993442	0.999981514	0.999985752	0.997206681

GPR158	0.999995916	0.997471998	0.999993442	0.999981514	0.999985752	0.997206681
LOC101907523	0.999995916	0.99960614	0.999993442	0.999981514	0.999985752	0.997206681
PITRM1	0.999995916	0.999987083	0.999993442	0.999981514	0.999985752	0.997206681
C25H7orf26	0.999995916	0.985195756	0.999993442	0.999981514	0.996781978	0.997260961
SNX33	0.999995916	0.993374813	0.999993442	0.999981514	0.999985752	0.997260961
C11H9orf78	0.999995916	0.994185904	0.999993442	0.999981514	0.999985752	0.997260961
C1H3orf70	0.999995916	0.994185904	0.999993442	0.999981514	0.999985752	0.997260961
RBM26	0.999995916	0.998191056	0.999993442	0.999981514	0.999985752	0.997355127
HIRIP3	0.999995916	0.985428851	0.999993442	0.999981514	0.999985752	0.997355881
DOCK1	0.999995916	0.99528564	0.999993442	0.999981514	0.999985752	0.997355881
MAS1	0.999995916	0.998847958	0.999993442	0.999981514	0.999985752	0.997355881
ZNF865	0.999995916	0.98098403	0.999993442	0.999981514	0.99932493	0.997383134
TJP1	0.999995916	0.993804376	0.996224523	0.999981514	0.999497678	0.997383134
MTA2	0.999995916	0.991673407	0.99647733	0.999981514	0.999985752	0.997383134
PARPBP	0.999995916	0.98098403	0.9965509	0.999981514	0.999985752	0.997383134
LOC112449245	0.999995916	0.987050697	0.999993442	0.999981514	0.999985752	0.997383134
GALK2	0.999995916	0.993481224	0.999993442	0.999981514	0.999985752	0.997383134
PMAIP1	0.999995916	0.994185904	0.999993442	0.999981514	0.999985752	0.997383134
LOC107131834	0.999995916	0.99488888	0.999993442	0.999981514	0.999985752	0.997383134
LOC112447797	0.999995916	0.99528564	0.999993442	0.999981514	0.999985752	0.997383134
PRKRA	0.999995916	0.998847958	0.999993442	0.999981514	0.999985752	0.997383134
VN1R1	0.999995916	0.998847958	0.999993442	0.999981514	0.999985752	0.997383134
ZNF699	0.999995916	0.998847958	0.999993442	0.999981514	0.999985752	0.997383134
CDC123	0.999995916	0.99960614	0.999993442	0.999981514	0.999453276	0.997538592
G6PC	0.999995916	0.99960614	0.999993442	0.999981514	0.999985752	0.997538592
NOS1AP	0.999995916	0.987731372	0.999993442	0.999981514	0.988881329	0.997603451
COMMD1	0.999995916	0.998847958	0.999993442	0.999981514	0.999985752	0.997629692
LOC107132196	0.999995916	0.981952108	0.999993442	0.999981514	0.992298407	0.997797826
ILDR2	0.999995916	0.988540349	0.989494193	0.999981514	0.990937627	0.997862553
DNTTIP1	0.999995916	0.97225074	0.999993442	0.999981514	0.996089557	0.997862553
ANKRD33	0.999995916	0.993481224	0.996926242	0.999981514	0.996781978	0.997862553
SUMF1	0.999995916	0.99306529	0.999993442	0.999981514	0.997794658	0.997862553
VPS45	0.999995916	0.98098403	0.994035957	0.999981514	0.999985752	0.997862553
CTSF	0.999995916	0.981952108	0.99615564	0.999981514	0.999985752	0.997862553
LOC101906569	0.999995916	0.981670901	0.999993442	0.999981514	0.999985752	0.997862553
LOC112447370	0.999995916	0.990396408	0.999993442	0.999981514	0.999985752	0.997862553
GPR143	0.999995916	0.993481224	0.999993442	0.999981514	0.999985752	0.997862553
LOC112443728	0.999995916	0.993481224	0.999993442	0.999981514	0.999985752	0.997862553

TBXAS1	0.999995916	0.993481224	0.999993442	0.999981514	0.999985752	0.997862553
LOC112448381	0.999995916	0.99386673	0.999993442	0.999981514	0.999985752	0.997862553
LOC112445996	0.999995916	0.997678999	0.999993442	0.999981514	0.999985752	0.997862553
RNFT2	0.999995916	0.997810586	0.999993442	0.999981514	0.999985752	0.997862553
EVI5L	0.999995916	0.99960614	0.999993442	0.999981514	0.999985752	0.997862553
DNAJC9	0.999995916	0.99964543	0.999993442	0.999981514	0.999985752	0.997862553
PAF1	0.999995916	0.999987083	0.999993442	0.999981514	0.999985752	0.997862553
SLC27A4	0.999995916	0.999987083	0.999993442	0.999981514	0.999985752	0.997862553
MAPKAP1	0.999995916	0.992539037	0.999993442	0.999981514	0.999985752	0.997881914
JTB	0.999995916	0.99960614	0.999993442	0.999981514	0.999985752	0.997895076
ATXN7L1	0.999995916	0.988444882	0.999993442	0.999981514	0.998060725	0.997938444
LOC112441619	0.999995916	0.981520642	0.999993442	0.999981514	0.999985752	0.998009873
SPINDOC	0.999995916	0.988008216	0.99869493	0.999981514	0.997165254	0.998175127
LOC512175	0.999995916	0.993481224	0.999993442	0.999981514	0.999985752	0.998175127
TIAM2	0.999995916	0.993481224	0.999993442	0.999981514	0.999985752	0.998175127
SRSF4	0.999995916	0.955652503	0.999993442	0.999981514	0.996781978	0.998240456
FZD4	0.999995916	0.964925317	0.984886031	0.999981514	0.999251855	0.998240456
SLC4A1AP	0.999995916	0.999987083	0.997400824	0.999981514	0.999453276	0.998240456
NIT2	0.999995916	0.977196455	0.998899782	0.999981514	0.999985752	0.998240456
LOC112441879	0.999995916	0.991673407	0.999993442	0.999981514	0.999985752	0.998240456
RHOG	0.999995916	0.992040539	0.999993442	0.999981514	0.999985752	0.998240456
ZDHHC8	0.999995916	0.994185904	0.999993442	0.999981514	0.999985752	0.998240456
BAD	0.999995916	0.999987083	0.999993442	0.999981514	0.999985752	0.998240456
TMEM44	0.999995916	0.969288757	0.999993442	0.999981514	0.992902239	0.998284411
INO80D	0.999995916	0.998847958	0.999993442	0.999981514	0.997898563	0.998284411
LOC112445965	0.999995916	0.988540349	0.999993442	0.999981514	0.999251855	0.998284411
GRK4	0.999995916	0.985997726	0.999993442	0.999981514	0.999453276	0.998284411
GSPT2	0.999995916	0.991673407	0.985350221	0.999981514	0.999497678	0.998284411
PTPRH	0.999995916	0.993481224	0.99341711	0.998039602	0.999985752	0.998284411
SAO	0.999995916	0.962423197	0.983066857	0.999981514	0.999985752	0.998284411
LOC112443425	0.999995916	0.998847958	0.99341711	0.999981514	0.999985752	0.998284411
ACACB	0.999995916	0.984631976	0.99647733	0.999981514	0.999985752	0.998284411
GGCX	0.999995916	0.968435705	0.997400824	0.999981514	0.999985752	0.998284411
C10H15orf41	0.999995916	0.990759058	0.997400824	0.999981514	0.999985752	0.998284411
LOC100336013	0.999995916	0.987908386	0.999993442	0.999981514	0.999985752	0.998284411
SEPT3	0.999995916	0.988008216	0.999993442	0.999981514	0.999985752	0.998284411
GOLGA1	0.999995916	0.988444882	0.999993442	0.999981514	0.999985752	0.998284411
RPS25	0.999995916	0.98978863	0.999993442	0.999981514	0.999985752	0.998284411

CEP19	0.999995916	0.991673407	0.999993442	0.999981514	0.999985752	0.998284411
CEP83	0.999995916	0.991673407	0.999993442	0.999981514	0.999985752	0.998284411
HSDL2	0.999995916	0.991673407	0.999993442	0.999981514	0.999985752	0.998284411
LOC101904396	0.999995916	0.991882821	0.999993442	0.999981514	0.999985752	0.998284411
LOC619026	0.999995916	0.991883165	0.999993442	0.999981514	0.999985752	0.998284411
LOC101904947	0.999995916	0.992795654	0.999993442	0.999981514	0.999985752	0.998284411
CLOCK	0.999995916	0.993481224	0.999993442	0.999981514	0.999985752	0.998284411
CRADD	0.999995916	0.993481224	0.999993442	0.999981514	0.999985752	0.998284411
GPR137C	0.999995916	0.993481224	0.999993442	0.999981514	0.999985752	0.998284411
RPL26L1	0.999995916	0.993481224	0.999993442	0.999981514	0.999985752	0.998284411
ZNF367	0.999995916	0.993481224	0.999993442	0.999981514	0.999985752	0.998284411
CHST15	0.999995916	0.994185904	0.999993442	0.999981514	0.999985752	0.998284411
LRRC69	0.999995916	0.994185904	0.999993442	0.999981514	0.999985752	0.998284411
ADCY2	0.999995916	0.99431777	0.999993442	0.999981514	0.999985752	0.998284411
LOC101904822	0.999995916	0.997471998	0.999993442	0.999981514	0.999985752	0.998284411
CHST14	0.999995916	0.997604725	0.999993442	0.999981514	0.999985752	0.998284411
LETM2	0.999995916	0.998847958	0.999993442	0.999981514	0.999985752	0.998284411
RC3H1	0.999995916	0.998847958	0.999993442	0.999981514	0.999985752	0.998284411
UNC5B	0.999995916	0.99950853	0.999993442	0.999981514	0.999985752	0.998284411
C24H18orf54	0.999995916	0.999680685	0.999993442	0.999981514	0.999985752	0.998284411
LOC100848815	0.999995916	0.999987083	0.999993442	0.999981514	0.999985752	0.998284411
EFTUD2	0.999995916	0.969051025	0.999993442	0.999981514	0.984386856	0.99828741
LOC107132987	0.999995916	0.980521483	0.999993442	0.999981514	0.986003146	0.99828741
AMIGO1	0.999995916	0.969342656	0.999993442	0.999981514	0.988049878	0.99828741
NXN	0.999995916	0.99528564	0.99647733	0.999981514	0.989334846	0.99828741
ZNF142	0.999995916	0.973321075	0.999993442	0.999981514	0.995870928	0.99828741
RTN4	0.999995916	0.981952108	0.999993442	0.999981514	0.996089557	0.99828741
LOC513894	0.999995916	0.993481224	0.99647733	0.999981514	0.997422012	0.99828741
C1H3orf58	0.999995916	0.997678999	0.999993442	0.999981514	0.998008754	0.99828741
SNRPC	0.999995916	0.969288757	0.999993442	0.999981514	0.998016692	0.99828741
LOC112442602	0.999995916	0.989303356	0.99341711	0.999981514	0.998196897	0.99828741
LOC112448103	0.999995916	0.99488888	0.999993442	0.999981514	0.999453276	0.99828741
APEH	0.999995916	0.985997726	0.999841067	0.999981514	0.99971266	0.99828741
LOC101904098	0.999995916	0.964418016	0.999993442	0.999981514	0.999859158	0.99828741
LOC790886	0.999995916	0.969051025	0.982823002	0.999981514	0.999985752	0.99828741
LOC112442649	0.999995916	0.981670901	0.989494193	0.999981514	0.999985752	0.99828741
RSRC1	0.999995916	0.981952108	0.991936822	0.999981514	0.999985752	0.99828741
LOC112446039	0.999995916	0.967668839	0.99341711	0.999981514	0.999985752	0.99828741

KLHL5	0.999995916	0.990697047	0.99341711	0.999981514	0.999985752	0.99828741
LOC101906688	0.999995916	0.994993704	0.99341711	0.999981514	0.999985752	0.99828741
LOC112444198	0.999995916	0.998426018	0.99341711	0.999981514	0.999985752	0.99828741
STMN3	0.999995916	0.993481224	0.99615564	0.999981514	0.999985752	0.99828741
SPESP1	0.999995916	0.973321075	0.996224523	0.999981514	0.999985752	0.99828741
SOX18	0.999995916	0.984179983	0.997400824	0.999981514	0.999985752	0.99828741
DROSHA	0.999995916	0.985428851	0.997400824	0.999981514	0.999985752	0.99828741
RHBDF2	0.999995916	0.991958541	0.997400824	0.999981514	0.999985752	0.99828741
SEPSECS	0.999995916	0.981670901	0.999993442	0.999981514	0.999985752	0.99828741
LOC104975788	0.999995916	0.981952108	0.999993442	0.999981514	0.999985752	0.99828741
NUP88	0.999995916	0.985940087	0.999993442	0.999981514	0.999985752	0.99828741
USP40	0.999995916	0.985997726	0.999993442	0.999981514	0.999985752	0.99828741
HSD17B8	0.999995916	0.988008216	0.999993442	0.999981514	0.999985752	0.99828741
LOC107132735	0.999995916	0.988008216	0.999993442	0.999981514	0.999985752	0.99828741
LOC101907835	0.999995916	0.988444882	0.999993442	0.999981514	0.999985752	0.99828741
KAT5	0.999995916	0.989303356	0.999993442	0.999981514	0.999985752	0.99828741
PTGDS	0.999995916	0.989303356	0.999993442	0.999981514	0.999985752	0.99828741
SNX9	0.999995916	0.989407995	0.999993442	0.999981514	0.999985752	0.99828741
NSMCE3	0.999995916	0.98978863	0.999993442	0.999981514	0.999985752	0.99828741
PPM1B	0.999995916	0.98978863	0.999993442	0.999981514	0.999985752	0.99828741
BRSK1	0.999995916	0.990205953	0.999993442	0.999981514	0.999985752	0.99828741
C29H11orf54	0.999995916	0.990447956	0.999993442	0.999981514	0.999985752	0.99828741
ATP23	0.999995916	0.990697047	0.999993442	0.999981514	0.999985752	0.99828741
CTNNBL1	0.999995916	0.991558617	0.999993442	0.999981514	0.999985752	0.99828741
IRF2BP2	0.999995916	0.991673407	0.999993442	0.999981514	0.999985752	0.99828741
LOC101907549	0.999995916	0.991673407	0.999993442	0.999981514	0.999985752	0.99828741
LOC112444207	0.999995916	0.991673407	0.999993442	0.999981514	0.999985752	0.99828741
LOC519145	0.999995916	0.991673407	0.999993442	0.999981514	0.999985752	0.99828741
RPL31	0.999995916	0.991673407	0.999993442	0.999981514	0.999985752	0.99828741
LOC101906135	0.999995916	0.991992136	0.999993442	0.999981514	0.999985752	0.99828741
CCDC121	0.999995916	0.992539037	0.999993442	0.999981514	0.999985752	0.99828741
LOC104972026	0.999995916	0.992795654	0.999993442	0.999981514	0.999985752	0.99828741
AGTR1	0.999995916	0.993481224	0.999993442	0.999981514	0.999985752	0.99828741
CBY1	0.999995916	0.993481224	0.999993442	0.999981514	0.999985752	0.99828741
IFT81	0.999995916	0.993481224	0.999993442	0.999981514	0.999985752	0.99828741
LOC101906276	0.999995916	0.993481224	0.999993442	0.999981514	0.999985752	0.99828741
SMIM19	0.999995916	0.993481224	0.999993442	0.999981514	0.999985752	0.99828741
F8	0.999995916	0.993804376	0.999993442	0.999981514	0.999985752	0.99828741

PARP2	0.999995916	0.99386673	0.999993442	0.999981514	0.999985752	0.99828741
LOC787237	0.999995916	0.994013976	0.999993442	0.999981514	0.999985752	0.99828741
HPS3	0.999995916	0.994185904	0.999993442	0.999981514	0.999985752	0.99828741
NAALADL2	0.999995916	0.994185904	0.999993442	0.999981514	0.999985752	0.99828741
IP6K3	0.999995916	0.99488888	0.999993442	0.999981514	0.999985752	0.99828741
LOC112448056	0.999995916	0.994993704	0.999993442	0.999981514	0.999985752	0.99828741
CHAMP1	0.999995916	0.995373357	0.999993442	0.999981514	0.999985752	0.99828741
DDB1	0.999995916	0.995373357	0.999993442	0.999981514	0.999985752	0.99828741
LOC789388	0.999995916	0.997332173	0.999993442	0.999981514	0.999985752	0.99828741
ARHGEF12	0.999995916	0.997471998	0.999993442	0.999981514	0.999985752	0.99828741
LOC112444289	0.999995916	0.997471998	0.999993442	0.999981514	0.999985752	0.99828741
LOC100848325	0.999995916	0.998218353	0.999993442	0.999981514	0.999985752	0.99828741
ACSF2	0.999995916	0.998847958	0.999993442	0.999981514	0.999985752	0.99828741
BRMS1L	0.999995916	0.998847958	0.999993442	0.999981514	0.999985752	0.99828741
FEZ2	0.999995916	0.998847958	0.999993442	0.999981514	0.999985752	0.99828741
INTS3	0.999995916	0.998847958	0.999993442	0.999981514	0.999985752	0.99828741
LOC112442208	0.999995916	0.998847958	0.999993442	0.999981514	0.999985752	0.99828741
LRRFIP1	0.999995916	0.998847958	0.999993442	0.999981514	0.999985752	0.99828741
NUDT2	0.999995916	0.998847958	0.999993442	0.999981514	0.999985752	0.99828741
RAB33A	0.999995916	0.998847958	0.999993442	0.999981514	0.999985752	0.99828741
TRMT112	0.999995916	0.998847958	0.999993442	0.999981514	0.999985752	0.99828741
ZFP69	0.999995916	0.998847958	0.999993442	0.999981514	0.999985752	0.99828741
LOC100847554	0.999995916	0.99960614	0.999993442	0.999981514	0.999985752	0.99828741
LOC112447510	0.999995916	0.99960614	0.999993442	0.999981514	0.999985752	0.99828741
ACAD8	0.999995916	0.999987083	0.999993442	0.999981514	0.999985752	0.99828741
LOC107131573	0.999995916	0.999987083	0.999993442	0.999981514	0.999985752	0.99828741
LOC112448088	0.999995916	0.999987083	0.999993442	0.999981514	0.999985752	0.99828741
PGAP1	0.999995916	0.999987083	0.999993442	0.999981514	0.999985752	0.99828741
LOC112449565	0.999995916	0.99960614	0.999993442	0.999981514	0.998287238	0.99841577
PFKM	0.999995916	0.965648998	0.989969382	0.999981514	0.99971266	0.99841577
ANAPC4	0.999995916	0.983816461	0.996224523	0.999981514	0.999985752	0.99841577
LOC785216	0.999995916	0.984410135	0.999993442	0.999981514	0.999985752	0.99841577
LSM5	0.999995916	0.988444882	0.999993442	0.999981514	0.999985752	0.99841577
INAFM1	0.999995916	0.990421548	0.999993442	0.999981514	0.999985752	0.99841577
LOC101906218	0.999995916	0.992795654	0.999993442	0.999981514	0.999985752	0.99841577
CDC42BPA	0.999995916	0.993709317	0.999993442	0.999981514	0.999985752	0.99841577
EDEM1	0.999995916	0.99386673	0.999993442	0.999981514	0.999985752	0.99841577
HNRNPA0	0.999995916	0.998847958	0.999993442	0.999981514	0.999985752	0.99841577

TMCO3	0.999995916	0.998847958	0.999993442	0.999981514	0.999985752	0.99841577
PLCD1	0.999995916	0.993481224	0.999993442	0.999981514	0.999985752	0.998458498
RPLP0	0.999995916	0.969051025	0.999993442	0.999981514	0.999888682	0.99867548
ATG10	0.999995916	0.998191056	0.999993442	0.999981514	0.999985752	0.99887027
CABYR	0.999995916	0.984410135	0.996224523	0.999981514	0.998784447	0.999038835
RPL18A	0.999995916	0.982011436	0.996224523	0.999981514	0.999985752	0.999075292
TAF3	0.999995916	0.993028532	0.999993442	0.999981514	0.999985752	0.999075292
PLEKHG7	0.999995916	0.993481224	0.999993442	0.999981514	0.999985752	0.999075292
LOC785445	0.999995916	0.998847958	0.999993442	0.999981514	0.999985752	0.999075292
CREB3L2	0.999995916	0.991673407	0.999993442	0.999981514	0.999985752	0.999208871
LOC101902207	0.999995916	0.995373357	0.999993442	0.999981514	0.999985752	0.999208871
LOC100298774	0.999995916	0.983594252	0.999993442	0.999981514	0.997210259	0.999350873
HIST1H1D	0.999995916	0.991673407	0.99341711	0.999981514	0.994903228	0.999463809
PHF1	0.999995916	0.99528564	0.99341711	0.999981514	0.998287238	0.999463809
LOC784735	0.999995916	0.981952108	0.992324476	0.999981514	0.999859158	0.999463809
OPA1	0.999995916	0.966221925	0.996323098	0.998987812	0.999985752	0.999463809
LOC104969067	0.999995916	0.968369135	0.990037865	0.999981514	0.999985752	0.999463809
LOC107132546	0.999995916	0.980289052	0.996224523	0.999981514	0.999985752	0.999463809
SMCO3	0.999995916	0.99960614	0.996224523	0.999981514	0.999985752	0.999463809
LOC101906457	0.999995916	0.99960614	0.999280799	0.999981514	0.999985752	0.999463809
LST1	0.999995916	0.97970612	0.999993442	0.999981514	0.999985752	0.999463809
LOC112444346	0.999995916	0.980764484	0.999993442	0.999981514	0.999985752	0.999463809
DLG2	0.999995916	0.980833065	0.999993442	0.999981514	0.999985752	0.999463809
GPAM	0.999995916	0.988540349	0.999993442	0.999981514	0.999985752	0.999463809
TULP3	0.999995916	0.988553076	0.999993442	0.999981514	0.999985752	0.999463809
LOC101906315	0.999995916	0.98978863	0.999993442	0.999981514	0.999985752	0.999463809
KANSL2	0.999995916	0.991673407	0.999993442	0.999981514	0.999985752	0.999463809
PRSS2	0.999995916	0.991673407	0.999993442	0.999981514	0.999985752	0.999463809
LOC107133226	0.999995916	0.991883165	0.999993442	0.999981514	0.999985752	0.999463809
CNPY4	0.999995916	0.993481224	0.999993442	0.999981514	0.999985752	0.999463809
RPL30	0.999995916	0.995253163	0.999993442	0.999981514	0.999985752	0.999463809
HOXA4	0.999995916	0.995373357	0.999993442	0.999981514	0.999985752	0.999463809
RPL15	0.999995916	0.997471998	0.999993442	0.999981514	0.999985752	0.999463809
TMEM141	0.999995916	0.997471998	0.999993442	0.999981514	0.999985752	0.999463809
LOC783461	0.999995916	0.997678999	0.999993442	0.999981514	0.999985752	0.999463809
MOGAT1	0.999995916	0.99960614	0.999993442	0.999981514	0.999985752	0.999463809
LOC107132237	0.999995916	0.99964543	0.999993442	0.999981514	0.999985752	0.999463809
IFI47	0.999995916	0.999987083	0.999993442	0.999981514	0.999985752	0.999463809

SEMA3C	0.999995916	0.999987083	0.999993442	0.999981514	0.999985752	0.999463809
DTD1	0.999995916	0.993481224	0.999993442	0.999981514	0.999985752	0.999530242
TRIM14	0.999995916	0.988008216	0.999993442	0.999981514	0.988881329	0.999655834
ATP8A1	0.999995916	0.971413741	0.991138474	0.999981514	0.994974235	0.999655834
ZRANB2	0.999995916	0.973321075	0.999993442	0.999981514	0.996781978	0.999655834
KLF2	0.999995916	0.988008216	0.999993442	0.999981514	0.997967575	0.999655834
DENND2C	0.999995916	0.999987083	0.997850143	0.999981514	0.998835841	0.999655834
R3HDM4	0.999995916	0.981670901	0.99647733	0.999981514	0.999453276	0.999655834
CAB39L	0.999995916	0.98098403	0.996685706	0.999981514	0.999859158	0.999655834
LOC510185	0.999995916	0.96734064	0.996224523	0.999981514	0.999985752	0.999655834
WASHC1	0.999995916	0.990697047	0.996624589	0.999981514	0.999985752	0.999655834
SLC25A29	0.999995916	0.998847958	0.997440573	0.999981514	0.999985752	0.999655834
SLC29A1	0.999995916	0.977830531	0.999993442	0.999981514	0.999985752	0.999655834
LOC101906397	0.999995916	0.981952108	0.999993442	0.999981514	0.999985752	0.999655834
LOC112446701	0.999995916	0.981952108	0.999993442	0.999981514	0.999985752	0.999655834
SFPQ	0.999995916	0.991673407	0.999993442	0.999981514	0.999985752	0.999655834
RELA	0.999995916	0.993481224	0.999993442	0.999981514	0.999985752	0.999655834
LOC101904314	0.999995916	0.994185904	0.999993442	0.999981514	0.999985752	0.999655834
LRP1	0.999995916	0.994185904	0.999993442	0.999981514	0.999985752	0.999655834
AVEN	0.999995916	0.998847958	0.999993442	0.999981514	0.999985752	0.999655834
ENTPD5	0.999995916	0.998847958	0.999993442	0.999981514	0.999985752	0.999655834
SNX14	0.999995916	0.998847958	0.999993442	0.999981514	0.999985752	0.999655834
LOC783539	0.999995916	0.99960614	0.999993442	0.999981514	0.999985752	0.999655834
MKRN2	0.999995916	0.99960614	0.999993442	0.999981514	0.999985752	0.999655834
PACSIN3	0.999995916	0.99960614	0.999993442	0.999981514	0.999985752	0.999655834
CAMKK1	0.999995916	0.999987083	0.999993442	0.999981514	0.999985752	0.999655834
CELF2	0.999995916	0.982515431	0.999993442	0.999981514	0.999888682	0.999701868
DUSP18	0.999995916	0.982515431	0.990118874	0.999981514	0.999985752	0.999701868
RASA2	0.999995916	0.99960614	0.999833345	0.999981514	0.999985752	0.999701868
HNRNPC	0.999995916	0.98338119	0.999993442	0.999981514	0.999985752	0.999701868
TMEM87B	0.999995916	0.988008216	0.999993442	0.999981514	0.999985752	0.999701868
KCNA6	0.999995916	0.993481224	0.999993442	0.999981514	0.999985752	0.999701868
TMEM81	0.999995916	0.99386673	0.999993442	0.999981514	0.999985752	0.999701868
PHF12	0.999995916	0.994185904	0.999993442	0.999981514	0.999985752	0.999701868
YRDC	0.999995916	0.994993704	0.999993442	0.999981514	0.999985752	0.999701868
TBX2	0.999995916	0.99528564	0.999993442	0.999981514	0.999985752	0.999701868
SPRED1	0.999995916	0.995292516	0.999993442	0.999981514	0.999985752	0.999701868
LOC100138449	0.999995916	0.998847958	0.999993442	0.999981514	0.999985752	0.999701868

NARFL	0.999995916	0.998847958	0.999993442	0.999981514	0.999985752	0.999701868
LOC101903097	0.999995916	0.99960614	0.999993442	0.999981514	0.999985752	0.999701868
LOC101903567	0.999995916	0.99960614	0.999993442	0.999981514	0.999985752	0.999701868
CALHM2	0.999995916	0.999987083	0.999993442	0.999981514	0.999985752	0.999701868
STX16	0.999995916	0.999987083	0.999993442	0.999981514	0.999985752	0.999701868
KAT6B	0.999995916	0.992794014	0.999993442	0.999981514	0.999985752	0.999729224
BICD1	0.999995916	0.99528564	0.999993442	0.999981514	0.999985752	0.999729224
TRIM9	0.999995916	0.997471998	0.999993442	0.999981514	0.999985752	0.999729224
TXNDC12	0.999995916	0.997810586	0.999993442	0.999981514	0.999985752	0.999729224
FXYD7	0.999995916	0.999987083	0.999993442	0.999981514	0.999985752	0.999729224
LOC112442987	0.999995916	0.981952108	0.995746693	0.999981514	0.999985752	0.999767722
TRIM47	0.999995916	0.988444882	0.999993442	0.999981514	0.999985752	0.99976973
LOC507787	0.999995916	0.995373357	0.999993442	0.999981514	0.999985752	0.99976973
CAT	0.999995916	0.99960614	0.999993442	0.999981514	0.999985752	0.99976973
DIO1	0.999995916	0.99960614	0.999993442	0.999981514	0.999985752	0.99976973
CEP44	0.999995916	0.99960614	0.999993442	0.999981514	0.999985752	0.999795635
UBALD1	0.999995916	0.99488888	0.999993442	0.999981514	0.999985752	0.999872573
SESN3	0.999995916	0.975984829	0.990118874	0.999981514	0.983941532	0.999948228
SLC24A3	0.999995916	0.981511914	0.991108777	0.999981514	0.983941532	0.999948228
LOC100139638	0.999995916	0.980999707	0.982195333	0.995565324	0.984386856	0.999948228
PRDM16	0.999995916	0.983594252	0.987789469	0.999981514	0.984386856	0.999948228
LOC112448515	0.999995916	0.969051025	0.998416047	0.999981514	0.984386856	0.999948228
SERTAD3	0.999995916	0.99386673	0.989430795	0.999981514	0.984494338	0.999948228
CCR8	0.999995916	0.955652503	0.99341711	0.999981514	0.986003146	0.999948228
KNSTRN	0.999995916	0.973712113	0.999993442	0.999981514	0.986003146	0.999948228
DGKE	0.999995916	0.979456887	0.99647733	0.999981514	0.987106594	0.999948228
LTBR	0.999995916	0.977213776	0.99341711	0.99739416	0.987723553	0.999948228
LOC112447769	0.999995916	0.96402095	0.989430795	0.999981514	0.987723553	0.999948228
LOC112447508	0.999995916	0.969288757	0.999993442	0.999981514	0.987723553	0.999948228
HOXC6	0.999995916	0.977196455	0.999993442	0.999981514	0.987723553	0.999948228
LOC781813	0.999995916	0.970084348	0.995377403	0.999981514	0.987789919	0.999948228
PPP1R13L	0.999995916	0.981520642	0.994035957	0.999981514	0.988010662	0.999948228
LCN6	0.999995916	0.96643574	0.988390948	0.99542943	0.988049878	0.999948228
LOC104975811	0.999995916	0.98098403	0.991447919	0.999981514	0.988049878	0.999948228
LOC112445088	0.999995916	0.994117102	0.99341711	0.999981514	0.988049878	0.999948228
SPTBN1	0.999995916	0.991673407	0.996224523	0.999981514	0.988049878	0.999948228
GRB14	0.999995916	0.961663204	0.996291804	0.999981514	0.988049878	0.999948228
ZNF687	0.999995916	0.978808875	0.997833363	0.999981514	0.988050858	0.999948228

GSR	0.999995916	0.981511914	0.991108777	0.999981514	0.98865576	0.999948228
LOC112447085	0.999995916	0.99386673	0.99341711	0.999981514	0.988881329	0.999948228
ITIH5	0.999995916	0.994993704	0.990118874	0.999981514	0.989334846	0.999948228
MT1E	0.999995916	0.991673407	0.996224523	0.999981514	0.989334846	0.999948228
ADIPOQ	0.999995916	0.96643574	0.997400824	0.999981514	0.989334846	0.999948228
FRMD4A	0.999995916	0.997471998	0.993024884	0.999981514	0.989379051	0.999948228
QRICH1	0.999995916	0.96643574	0.979584814	0.999981514	0.989442936	0.999948228
ADAMTS1	0.999995916	0.969452363	0.999993442	0.999981514	0.990937627	0.999948228
PGBD1	0.999995916	0.98098403	0.983547826	0.999981514	0.992104882	0.999948228
IL2RB	0.999995916	0.96643574	0.988307089	0.999981514	0.993190318	0.999948228
RASSF1	0.999995916	0.975315616	0.99341711	0.999981514	0.99351564	0.999948228
SLC1A3	0.999995916	0.977196455	0.999993442	0.999981514	0.993831963	0.999948228
ZNF629	0.999995916	0.980521483	0.990037865	0.999981514	0.994700298	0.999948228
PSPC1	0.999995916	0.981952108	0.999993442	0.999981514	0.994747842	0.999948228
LOC112448387	0.999995916	0.990546209	0.99341711	0.999981514	0.995870928	0.999948228
IMPDH2	0.999995916	0.961663204	0.980761647	0.999981514	0.996089557	0.999948228
TYW5	0.999995916	0.97304892	0.999993442	0.999981514	0.996089557	0.999948228
FAM122B	0.999995916	0.991883165	0.99647733	0.999981514	0.996217975	0.999948228
SLC18A2	0.999995916	0.968509005	0.999993442	0.995565324	0.996781978	0.999948228
LOC112446022	0.999995916	0.977425528	0.99647733	0.997374305	0.996781978	0.999948228
DPF1	0.999995916	0.993028532	0.986920775	0.999981514	0.996781978	0.999948228
LOC104975073	0.999995916	0.968186752	0.990118874	0.999981514	0.996781978	0.999948228
ZNF3	0.999995916	0.964185302	0.991936822	0.999981514	0.996781978	0.999948228
SCYL3	0.999995916	0.962993211	0.99341711	0.999981514	0.996781978	0.999948228
CLDN12	0.999995916	0.975550225	0.996224523	0.999981514	0.996781978	0.999948228
LOC107131494	0.999995916	0.981520642	0.996224523	0.999981514	0.996781978	0.999948228
DRD1	0.999995916	0.988444882	0.996224523	0.999981514	0.996781978	0.999948228
RNF126	0.999995916	0.992316823	0.996224523	0.999981514	0.996781978	0.999948228
CBFA2T3	0.999995916	0.981511914	0.998899782	0.999981514	0.996781978	0.999948228
GLB1	0.999995916	0.99431777	0.999280799	0.999981514	0.996781978	0.999948228
LARP6	0.999995916	0.991673407	0.999703589	0.999981514	0.996781978	0.999948228
VIPAS39	0.999995916	0.981920173	0.999993442	0.999981514	0.996781978	0.999948228
CTDSPL	0.999995916	0.983594252	0.999993442	0.999981514	0.996781978	0.999948228
MKNK2	0.999995916	0.993481224	0.999280799	0.999981514	0.997165254	0.999948228
LOC100848472	0.999995916	0.988444882	0.999993442	0.999981514	0.997363093	0.999948228
SH3BP4	0.999995916	0.977196455	0.999993442	0.999981514	0.997422012	0.999948228
LOC112443503	0.999995916	0.987731372	0.997850143	0.999981514	0.997741313	0.999948228
ZHX1	0.999995916	0.980521483	0.999993442	0.999981514	0.998040182	0.999948228

LOC112442382	0.999995916	0.983970583	0.997300234	0.999981514	0.998060725	0.999948228
PIAS4	0.999995916	0.984631976	0.99341711	0.999981514	0.998141915	0.999948228
KLHDC10	0.999995916	0.980289052	0.999993442	0.999981514	0.998196897	0.999948228
SHROOM4	0.999995916	0.972948584	0.980496473	0.999981514	0.998287238	0.999948228
ZNF45	0.999995916	0.991673407	0.996224523	0.999981514	0.998287238	0.999948228
FAR1	0.999995916	0.999278945	0.996224523	0.999981514	0.998287238	0.999948228
LOC112446709	0.999995916	0.98098403	0.9965509	0.999981514	0.998287238	0.999948228
RPS7	0.999995916	0.982011436	0.996624589	0.999981514	0.998287238	0.999948228
RPL24	0.999995916	0.988444882	0.997400824	0.999981514	0.998287238	0.999948228
RPS27A	0.999995916	0.980616436	0.997833363	0.999981514	0.998287238	0.999948228
SGTA	0.999995916	0.983594252	0.99869493	0.999981514	0.998287238	0.999948228
MORF4L2	0.999995916	0.98098403	0.999280799	0.999981514	0.998287238	0.999948228
ZCCHC8	0.999995916	0.972007528	0.999993442	0.999981514	0.998287238	0.999948228
BMP7	0.999995916	0.97304892	0.999993442	0.999981514	0.998287238	0.999948228
ASNSD1	0.999995916	0.973712113	0.999993442	0.999981514	0.998287238	0.999948228
LOC101907941	0.999995916	0.980289052	0.999993442	0.999981514	0.998287238	0.999948228
TMEM41A	0.999995916	0.980521483	0.999993442	0.999981514	0.998287238	0.999948228
ILF2	0.999995916	0.980833065	0.999993442	0.999981514	0.998287238	0.999948228
SPEF1	0.999995916	0.988071578	0.999993442	0.999981514	0.998287238	0.999948228
GTF2F2	0.999995916	0.991217881	0.999993442	0.999981514	0.998287238	0.999948228
LOC112449075	0.999995916	0.997471998	0.999993442	0.999981514	0.998287238	0.999948228
RASEF	0.999995916	0.997471998	0.999993442	0.999981514	0.998287238	0.999948228
PIEZO1	0.999995916	0.99960614	0.999993442	0.999981514	0.998287238	0.999948228
SLC25A14	0.999995916	0.992795654	0.999993442	0.999981514	0.998338718	0.999948228
RASGRF2	0.999995916	0.991673407	0.999993442	0.999981514	0.998661549	0.999948228
LOC786435	0.999995916	0.997906448	0.998899782	0.999981514	0.998800466	0.999948228
DGKH	0.999995916	0.998847958	0.999993442	0.999981514	0.998800466	0.999948228
LOC619156	0.999995916	0.989303356	0.999993442	0.999981514	0.998835841	0.999948228
LOC101905199	0.999995916	0.977196455	0.99647733	0.999981514	0.999251855	0.999948228
LOC112449092	0.999995916	0.993481224	0.999993442	0.999981514	0.999251855	0.999948228
C11H2orf92	0.999995916	0.988008216	0.9965509	0.999981514	0.99932493	0.999948228
IL13RA1	0.999995916	0.972829016	0.99341711	0.998529748	0.999453276	0.999948228
LOC112448022	0.999995916	0.968186752	0.993073581	0.999981514	0.999453276	0.999948228
RANBP3	0.999995916	0.977830531	0.99341711	0.999981514	0.999453276	0.999948228
TMEM65	0.999995916	0.982515431	0.99341711	0.999981514	0.999453276	0.999948228
THADA	0.999995916	0.991177966	0.996224523	0.999981514	0.999453276	0.999948228
RPS29	0.999995916	0.981614672	0.998364815	0.999981514	0.999453276	0.999948228
USP45	0.999995916	0.984089343	0.99869493	0.999981514	0.999453276	0.999948228

ZFAND6	0.999995916	0.981614672	0.998899782	0.999981514	0.999453276	0.999948228
LOC112443416	0.999995916	0.978462031	0.999993442	0.999981514	0.999453276	0.999948228
BMPR1A	0.999995916	0.980367301	0.999993442	0.999981514	0.999453276	0.999948228
LOC112441491	0.999995916	0.982011436	0.999993442	0.999981514	0.999453276	0.999948228
KIRREL3	0.999995916	0.993481224	0.999993442	0.999981514	0.999453276	0.999948228
TRNAU1AP	0.999995916	0.994185904	0.999993442	0.999981514	0.99971266	0.999948228
LOC104971503	0.999995916	0.996075126	0.999993442	0.999981514	0.999859158	0.999948228
POC1B	0.999995916	0.985428851	0.988660944	0.995565324	0.999985752	0.999948228
SPRTN	0.999995916	0.96365708	0.99341711	0.997025804	0.999985752	0.999948228
POLR1C	0.999995916	0.963297767	0.999993442	0.998529748	0.999985752	0.999948228
RABL2B	0.999995916	0.969051025	0.990974506	0.999240374	0.999985752	0.999948228
EEFSEC	0.999995916	0.971967233	0.989517957	0.999981514	0.999985752	0.999948228
LOC112449558	0.999995916	0.963146208	0.989720033	0.999981514	0.999985752	0.999948228
LOC107132897	0.999995916	0.973868672	0.990615319	0.999981514	0.999985752	0.999948228
RBMS3	0.999995916	0.991673407	0.992511421	0.999981514	0.999985752	0.999948228
DHX33	0.999995916	0.959263706	0.992714073	0.999981514	0.999985752	0.999948228
RPS6	0.999995916	0.980121799	0.992728681	0.999981514	0.999985752	0.999948228
SENP2	0.999995916	0.968590259	0.99341711	0.999981514	0.999985752	0.999948228
RPL8	0.999995916	0.972829016	0.99341711	0.999981514	0.999985752	0.999948228
LOC107132610	0.999995916	0.973712113	0.99341711	0.999981514	0.999985752	0.999948228
RLIM	0.999995916	0.987530688	0.99341711	0.999981514	0.999985752	0.999948228
LOC101907857	0.999995916	0.997613842	0.994035957	0.999981514	0.999985752	0.999948228
LOC539069	0.999995916	0.975612288	0.996224523	0.999981514	0.999985752	0.999948228
GNAQ	0.999995916	0.980121799	0.996224523	0.999981514	0.999985752	0.999948228
RPS20	0.999995916	0.980521483	0.996224523	0.999981514	0.999985752	0.999948228
RPL13	0.999995916	0.988008216	0.996224523	0.999981514	0.999985752	0.999948228
RAB39A	0.999995916	0.989769855	0.996224523	0.999981514	0.999985752	0.999948228
H2B	0.999995916	0.974580085	0.99647733	0.999981514	0.999985752	0.999948228
VAV3	0.999995916	0.984410135	0.99647733	0.999981514	0.999985752	0.999948228
NEK1	0.999995916	0.988008216	0.99647733	0.999981514	0.999985752	0.999948228
KARS	0.999995916	0.991673407	0.996685706	0.999981514	0.999985752	0.999948228
HIST1H2BL	0.999995916	0.993481224	0.997213619	0.999981514	0.999985752	0.999948228
EPB41L2	0.999995916	0.968166143	0.997300234	0.999981514	0.999985752	0.999948228
PPFIA3	0.999995916	0.980521483	0.997300234	0.999981514	0.999985752	0.999948228
LOC101907713	0.999995916	0.981952108	0.997300234	0.999981514	0.999985752	0.999948228
ZNRF3	0.999995916	0.98098403	0.997400824	0.999981514	0.999985752	0.999948228
LOC104976575	0.999995916	0.981952108	0.997400824	0.999981514	0.999985752	0.999948228
CCDC170	0.999995916	0.993275893	0.997400824	0.999981514	0.999985752	0.999948228

LOC104972417	0.999995916	0.995994413	0.997400824	0.999981514	0.999985752	0.999948228
ZNF606	0.999995916	0.99960614	0.997400824	0.999981514	0.999985752	0.999948228
LOC104968807	0.999995916	0.988444882	0.997700289	0.999981514	0.999985752	0.999948228
IL10RB	0.999995916	0.993481224	0.997833363	0.999981514	0.999985752	0.999948228
KSR1	0.999995916	0.977196455	0.997850143	0.999981514	0.999985752	0.999948228
RPS9	0.999995916	0.981670901	0.997850143	0.999981514	0.999985752	0.999948228
BPGM	0.999995916	0.992144201	0.997850143	0.999981514	0.999985752	0.999948228
MBOAT7	0.999995916	0.993481224	0.997850143	0.999981514	0.999985752	0.999948228
TMEM237	0.999995916	0.980521483	0.998096104	0.999981514	0.999985752	0.999948228
RCAN2	0.999995916	0.99960614	0.998335361	0.999981514	0.999985752	0.999948228
NHS	0.999995916	0.99960614	0.99869493	0.999981514	0.999985752	0.999948228
SMN2	0.999995916	0.98098403	0.998899782	0.999981514	0.999985752	0.999948228
NEIL2	0.999995916	0.981952108	0.998899782	0.999981514	0.999985752	0.999948228
BCHE	0.999995916	0.985997726	0.998899782	0.999981514	0.999985752	0.999948228
LOC100848538	0.999995916	0.993481224	0.998899782	0.999981514	0.999985752	0.999948228
LOC101905687	0.999995916	0.997471998	0.998899782	0.999981514	0.999985752	0.999948228
USF1	0.999995916	0.98098403	0.999280799	0.999981514	0.999985752	0.999948228
LOC782437	0.999995916	0.981952108	0.999280799	0.999981514	0.999985752	0.999948228
LOC100848906	0.999995916	0.997678999	0.999280799	0.999981514	0.999985752	0.999948228
GRINA	0.999995916	0.989303356	0.999350606	0.999981514	0.999985752	0.999948228
RPS28	0.999995916	0.993203157	0.999475981	0.999981514	0.999985752	0.999948228
DPAGT1	0.999995916	0.985299169	0.99948073	0.999981514	0.999985752	0.999948228
PCMTD1	0.999995916	0.972496661	0.999993442	0.999981514	0.999985752	0.999948228
HARS2	0.999995916	0.974846249	0.999993442	0.999981514	0.999985752	0.999948228
MRPL3	0.999995916	0.976271642	0.999993442	0.999981514	0.999985752	0.999948228
DDX5	0.999995916	0.977196455	0.999993442	0.999981514	0.999985752	0.999948228
RPL21	0.999995916	0.980289052	0.999993442	0.999981514	0.999985752	0.999948228
LOC100848405	0.999995916	0.981520642	0.999993442	0.999981514	0.999985752	0.999948228
SCML1	0.999995916	0.981614672	0.999993442	0.999981514	0.999985752	0.999948228
EFCAB6	0.999995916	0.981670901	0.999993442	0.999981514	0.999985752	0.999948228
LOC781576	0.999995916	0.981670901	0.999993442	0.999981514	0.999985752	0.999948228
CYB5R1	0.999995916	0.981950382	0.999993442	0.999981514	0.999985752	0.999948228
CCDC97	0.999995916	0.981952108	0.999993442	0.999981514	0.999985752	0.999948228
EEF1G	0.999995916	0.981952108	0.999993442	0.999981514	0.999985752	0.999948228
LOC104975960	0.999995916	0.981952108	0.999993442	0.999981514	0.999985752	0.999948228
APOBEC3Z2	0.999995916	0.982839621	0.999993442	0.999981514	0.999985752	0.999948228
ARHGEF15	0.999995916	0.982910911	0.999993442	0.999981514	0.999985752	0.999948228
LOC101907800	0.999995916	0.983100982	0.999993442	0.999981514	0.999985752	0.999948228

TMEM268	0.999995916	0.983212485	0.999993442	0.999981514	0.999985752	0.999948228
LIPC	0.999995916	0.983305314	0.999993442	0.999981514	0.999985752	0.999948228
GTF2I	0.999995916	0.983483207	0.999993442	0.999981514	0.999985752	0.999948228
EBF1	0.999995916	0.983594252	0.999993442	0.999981514	0.999985752	0.999948228
MCCC2	0.999995916	0.983594252	0.999993442	0.999981514	0.999985752	0.999948228
ANAPC7	0.999995916	0.984179983	0.999993442	0.999981514	0.999985752	0.999948228
ANKFY1	0.999995916	0.984179983	0.999993442	0.999981514	0.999985752	0.999948228
LOC101907747	0.999995916	0.984631976	0.999993442	0.999981514	0.999985752	0.999948228
LOC101902141	0.999995916	0.984913707	0.999993442	0.999981514	0.999985752	0.999948228
PARG	0.999995916	0.984931795	0.999993442	0.999981514	0.999985752	0.999948228
AHDC1	0.999995916	0.985432041	0.999993442	0.999981514	0.999985752	0.999948228
LOC107131651	0.999995916	0.985697207	0.999993442	0.999981514	0.999985752	0.999948228
LOC100335404	0.999995916	0.98572429	0.999993442	0.999981514	0.999985752	0.999948228
POPDC3	0.999995916	0.985997726	0.999993442	0.999981514	0.999985752	0.999948228
ZC3HC1	0.999995916	0.985997726	0.999993442	0.999981514	0.999985752	0.999948228
MELK	0.999995916	0.986441438	0.999993442	0.999981514	0.999985752	0.999948228
ASXL2	0.999995916	0.986900642	0.999993442	0.999981514	0.999985752	0.999948228
CASC3	0.999995916	0.987616583	0.999993442	0.999981514	0.999985752	0.999948228
LOC112447411	0.999995916	0.988008216	0.999993442	0.999981514	0.999985752	0.999948228
LOC101904133	0.999995916	0.988444882	0.999993442	0.999981514	0.999985752	0.999948228
PCDHGC3	0.999995916	0.988444882	0.999993442	0.999981514	0.999985752	0.999948228
DNAJB12	0.999995916	0.988540349	0.999993442	0.999981514	0.999985752	0.999948228
ITGB5	0.999995916	0.988540349	0.999993442	0.999981514	0.999985752	0.999948228
LOC112441511	0.999995916	0.988553076	0.999993442	0.999981514	0.999985752	0.999948228
FBXO4	0.999995916	0.989303356	0.999993442	0.999981514	0.999985752	0.999948228
LOC101902048	0.999995916	0.989303356	0.999993442	0.999981514	0.999985752	0.999948228
LOC101907005	0.999995916	0.989303356	0.999993442	0.999981514	0.999985752	0.999948228
LOC112447322	0.999995916	0.989303356	0.999993442	0.999981514	0.999985752	0.999948228
NELFCD	0.999995916	0.989303356	0.999993442	0.999981514	0.999985752	0.999948228
TRIM3	0.999995916	0.989303356	0.999993442	0.999981514	0.999985752	0.999948228
TXNL4B	0.999995916	0.989303356	0.999993442	0.999981514	0.999985752	0.999948228
CYP2U1	0.999995916	0.989407995	0.999993442	0.999981514	0.999985752	0.999948228
RGS13	0.999995916	0.989491693	0.999993442	0.999981514	0.999985752	0.999948228
LOC112448155	0.999995916	0.98978863	0.999993442	0.999981514	0.999985752	0.999948228
MTERF1	0.999995916	0.98978863	0.999993442	0.999981514	0.999985752	0.999948228
EPN2	0.999995916	0.990052293	0.999993442	0.999981514	0.999985752	0.999948228
LOC112442721	0.999995916	0.990081924	0.999993442	0.999981514	0.999985752	0.999948228
FBXO42	0.999995916	0.990421548	0.999993442	0.999981514	0.999985752	0.999948228

PGAM2	0.999995916	0.990429195	0.999993442	0.999981514	0.999985752	0.999948228
CCDC22	0.999995916	0.990546209	0.999993442	0.999981514	0.999985752	0.999948228
CAPN3	0.999995916	0.991177966	0.999993442	0.999981514	0.999985752	0.999948228
LOC101902036	0.999995916	0.991177966	0.999993442	0.999981514	0.999985752	0.999948228
PTPRA	0.999995916	0.991177966	0.999993442	0.999981514	0.999985752	0.999948228
LOC618633	0.999995916	0.991186561	0.999993442	0.999981514	0.999985752	0.999948228
CITED4	0.999995916	0.991673407	0.999993442	0.999981514	0.999985752	0.999948228
COMP	0.999995916	0.991673407	0.999993442	0.999981514	0.999985752	0.999948228
CORO1B	0.999995916	0.991673407	0.999993442	0.999981514	0.999985752	0.999948228
HNMT	0.999995916	0.991673407	0.999993442	0.999981514	0.999985752	0.999948228
KBTBD4	0.999995916	0.991673407	0.999993442	0.999981514	0.999985752	0.999948228
LOC100848568	0.999995916	0.991673407	0.999993442	0.999981514	0.999985752	0.999948228
LOC101902663	0.999995916	0.991673407	0.999993442	0.999981514	0.999985752	0.999948228
LOC101908123	0.999995916	0.991673407	0.999993442	0.999981514	0.999985752	0.999948228
LOC112442254	0.999995916	0.991673407	0.999993442	0.999981514	0.999985752	0.999948228
LOC112444843	0.999995916	0.991673407	0.999993442	0.999981514	0.999985752	0.999948228
LOC112448430	0.999995916	0.991673407	0.999993442	0.999981514	0.999985752	0.999948228
LOC504858	0.999995916	0.991673407	0.999993442	0.999981514	0.999985752	0.999948228
LOC615223	0.999995916	0.991673407	0.999993442	0.999981514	0.999985752	0.999948228
LOC784354	0.999995916	0.991673407	0.999993442	0.999981514	0.999985752	0.999948228
NPDC1	0.999995916	0.991673407	0.999993442	0.999981514	0.999985752	0.999948228
PURA	0.999995916	0.991673407	0.999993442	0.999981514	0.999985752	0.999948228
QARS	0.999995916	0.991673407	0.999993442	0.999981514	0.999985752	0.999948228
RBM41	0.999995916	0.991673407	0.999993442	0.999981514	0.999985752	0.999948228
RPL4	0.999995916	0.991673407	0.999993442	0.999981514	0.999985752	0.999948228
SNIP1	0.999995916	0.991673407	0.999993442	0.999981514	0.999985752	0.999948228
TNS1	0.999995916	0.991673407	0.999993442	0.999981514	0.999985752	0.999948228
ZNF24	0.999995916	0.991882821	0.999993442	0.999981514	0.999985752	0.999948228
SS18	0.999995916	0.991883165	0.999993442	0.999981514	0.999985752	0.999948228
GRK5	0.999995916	0.991992136	0.999993442	0.999981514	0.999985752	0.999948228
SAMD4A	0.999995916	0.992066998	0.999993442	0.999981514	0.999985752	0.999948228
EIF3D	0.999995916	0.992144201	0.999993442	0.999981514	0.999985752	0.999948228
FBXO22	0.999995916	0.992144201	0.999993442	0.999981514	0.999985752	0.999948228
LOC112445988	0.999995916	0.992144201	0.999993442	0.999981514	0.999985752	0.999948228
ZNF397	0.999995916	0.992144201	0.999993442	0.999981514	0.999985752	0.999948228
ZPBP	0.999995916	0.992144201	0.999993442	0.999981514	0.999985752	0.999948228
LOC112442083	0.999995916	0.992293464	0.999993442	0.999981514	0.999985752	0.999948228
GSK3A	0.999995916	0.992539037	0.999993442	0.999981514	0.999985752	0.999948228

SLC43A3	0.999995916	0.992539037	0.999993442	0.999981514	0.999985752	0.999948228
LOC100337081	0.999995916	0.993028532	0.999993442	0.999981514	0.999985752	0.999948228
DHX16	0.999995916	0.993035141	0.999993442	0.999981514	0.999985752	0.999948228
MOCOS	0.999995916	0.993035141	0.999993442	0.999981514	0.999985752	0.999948228
CES2	0.999995916	0.993203157	0.999993442	0.999981514	0.999985752	0.999948228
SGSM2	0.999995916	0.993249767	0.999993442	0.999981514	0.999985752	0.999948228
ARL6	0.999995916	0.993309933	0.999993442	0.999981514	0.999985752	0.999948228
IWS1	0.999995916	0.993427125	0.999993442	0.999981514	0.999985752	0.999948228
ADAM10	0.999995916	0.993481224	0.999993442	0.999981514	0.999985752	0.999948228
CCDC71	0.999995916	0.993481224	0.999993442	0.999981514	0.999985752	0.999948228
CCPG1	0.999995916	0.993481224	0.999993442	0.999981514	0.999985752	0.999948228
DDX49	0.999995916	0.993481224	0.999993442	0.999981514	0.999985752	0.999948228
GTF2F1	0.999995916	0.993481224	0.999993442	0.999981514	0.999985752	0.999948228
PPYR1	0.999995916	0.993481224	0.999993442	0.999981514	0.999985752	0.999948228
RRH	0.999995916	0.993481224	0.999993442	0.999981514	0.999985752	0.999948228
SLC39A11	0.999995916	0.993481224	0.999993442	0.999981514	0.999985752	0.999948228
USP27X	0.999995916	0.993481224	0.999993442	0.999981514	0.999985752	0.999948228
AGAP1	0.999995916	0.993804376	0.999993442	0.999981514	0.999985752	0.999948228
MEN1	0.999995916	0.993804376	0.999993442	0.999981514	0.999985752	0.999948228
MGAT5	0.999995916	0.993804376	0.999993442	0.999981514	0.999985752	0.999948228
C7H5orf15	0.999995916	0.99380699	0.999993442	0.999981514	0.999985752	0.999948228
FAM89A	0.999995916	0.994013976	0.999993442	0.999981514	0.999985752	0.999948228
LOC614695	0.999995916	0.994013976	0.999993442	0.999981514	0.999985752	0.999948228
PIGS	0.999995916	0.994013976	0.999993442	0.999981514	0.999985752	0.999948228
ALG2	0.999995916	0.994185904	0.999993442	0.999981514	0.999985752	0.999948228
AMDHD2	0.999995916	0.994185904	0.999993442	0.999981514	0.999985752	0.999948228
C28H1orf131	0.999995916	0.994185904	0.999993442	0.999981514	0.999985752	0.999948228
CTDSP1	0.999995916	0.994185904	0.999993442	0.999981514	0.999985752	0.999948228
FRMD3	0.999995916	0.994185904	0.999993442	0.999981514	0.999985752	0.999948228
LOC112441843	0.999995916	0.994185904	0.999993442	0.999981514	0.999985752	0.999948228
MDN1	0.999995916	0.994185904	0.999993442	0.999981514	0.999985752	0.999948228
PCDHB11	0.999995916	0.994185904	0.999993442	0.999981514	0.999985752	0.999948228
PPP2R1A	0.999995916	0.994185904	0.999993442	0.999981514	0.999985752	0.999948228
RBBP8	0.999995916	0.994185904	0.999993442	0.999981514	0.999985752	0.999948228
SYNRG	0.999995916	0.994185904	0.999993442	0.999981514	0.999985752	0.999948228
THEM4	0.999995916	0.994185904	0.999993442	0.999981514	0.999985752	0.999948228
DDX51	0.999995916	0.99431777	0.999993442	0.999981514	0.999985752	0.999948228
FBLN2	0.999995916	0.994486346	0.999993442	0.999981514	0.999985752	0.999948228

LGALS7	0.999995916	0.994749993	0.999993442	0.999981514	0.999985752	0.999948228
LOC790101	0.999995916	0.99488888	0.999993442	0.999981514	0.999985752	0.999948228
PON3	0.999995916	0.99488888	0.999993442	0.999981514	0.999985752	0.999948228
RHOBTB1	0.999995916	0.99488888	0.999993442	0.999981514	0.999985752	0.999948228
RPL3	0.999995916	0.99488888	0.999993442	0.999981514	0.999985752	0.999948228
FLOT2	0.999995916	0.994993704	0.999993442	0.999981514	0.999985752	0.999948228
GLI2	0.999995916	0.994993704	0.999993442	0.999981514	0.999985752	0.999948228
LOC101905686	0.999995916	0.994993704	0.999993442	0.999981514	0.999985752	0.999948228
LOC101906398	0.999995916	0.994993704	0.999993442	0.999981514	0.999985752	0.999948228
LOC104974758	0.999995916	0.994993704	0.999993442	0.999981514	0.999985752	0.999948228
LOC112444152	0.999995916	0.994993704	0.999993442	0.999981514	0.999985752	0.999948228
LOC614914	0.999995916	0.994993704	0.999993442	0.999981514	0.999985752	0.999948228
PI4KA	0.999995916	0.994993704	0.999993442	0.999981514	0.999985752	0.999948228
PLTP	0.999995916	0.994993704	0.999993442	0.999981514	0.999985752	0.999948228
RPL36A	0.999995916	0.994993704	0.999993442	0.999981514	0.999985752	0.999948228
SUSD6	0.999995916	0.994993704	0.999993442	0.999981514	0.999985752	0.999948228
TAF12	0.999995916	0.994993704	0.999993442	0.999981514	0.999985752	0.999948228
TFEB	0.999995916	0.994993704	0.999993442	0.999981514	0.999985752	0.999948228
LOC784243	0.999995916	0.995048226	0.999993442	0.999981514	0.999985752	0.999948228
PDGFRL	0.999995916	0.995128956	0.999993442	0.999981514	0.999985752	0.999948228
HIST1H2BD	0.999995916	0.995204627	0.999993442	0.999981514	0.999985752	0.999948228
LOC100847180	0.999995916	0.995204627	0.999993442	0.999981514	0.999985752	0.999948228
ARPC1B	0.999995916	0.99528564	0.999993442	0.999981514	0.999985752	0.999948228
LOC107132556	0.999995916	0.99528564	0.999993442	0.999981514	0.999985752	0.999948228
PPM1N	0.999995916	0.99528564	0.999993442	0.999981514	0.999985752	0.999948228
PRR29	0.999995916	0.99528564	0.999993442	0.999981514	0.999985752	0.999948228
RPL35A	0.999995916	0.99528564	0.999993442	0.999981514	0.999985752	0.999948228
ASPH	0.999995916	0.995343428	0.999993442	0.999981514	0.999985752	0.999948228
CNOT9	0.999995916	0.995373357	0.999993442	0.999981514	0.999985752	0.999948228
LOC782525	0.999995916	0.995373357	0.999993442	0.999981514	0.999985752	0.999948228
PLPP5	0.999995916	0.995373357	0.999993442	0.999981514	0.999985752	0.999948228
PTMA	0.999995916	0.995373357	0.999993442	0.999981514	0.999985752	0.999948228
SKI	0.999995916	0.995373357	0.999993442	0.999981514	0.999985752	0.999948228
SYT4	0.999995916	0.995373357	0.999993442	0.999981514	0.999985752	0.999948228
LOC112448045	0.999995916	0.995510633	0.999993442	0.999981514	0.999985752	0.999948228
LOC100847357	0.999995916	0.995994413	0.999993442	0.999981514	0.999985752	0.999948228
OS9	0.999995916	0.995994413	0.999993442	0.999981514	0.999985752	0.999948228
RPL7	0.999995916	0.995994413	0.999993442	0.999981514	0.999985752	0.999948228

SSUH2	0.999995916	0.995994413	0.999993442	0.999981514	0.999985752	0.999948228
ZNF891	0.999995916	0.995994413	0.999993442	0.999981514	0.999985752	0.999948228
LOC512541	0.999995916	0.996000962	0.999993442	0.999981514	0.999985752	0.999948228
TDRD9	0.999995916	0.996075126	0.999993442	0.999981514	0.999985752	0.999948228
RHOB	0.999995916	0.996335745	0.999993442	0.999981514	0.999985752	0.999948228
TPO	0.999995916	0.99635564	0.999993442	0.999981514	0.999985752	0.999948228
HAUS6	0.999995916	0.996728887	0.999993442	0.999981514	0.999985752	0.999948228
LOC101905010	0.999995916	0.996738167	0.999993442	0.999981514	0.999985752	0.999948228
ATAD2	0.999995916	0.996867386	0.999993442	0.999981514	0.999985752	0.999948228
LOC101907658	0.999995916	0.996867386	0.999993442	0.999981514	0.999985752	0.999948228
DIS3	0.999995916	0.996915629	0.999993442	0.999981514	0.999985752	0.999948228
ZNF248	0.999995916	0.996915629	0.999993442	0.999981514	0.999985752	0.999948228
ZNF408	0.999995916	0.997154508	0.999993442	0.999981514	0.999985752	0.999948228
LOC515089	0.999995916	0.997199115	0.999993442	0.999981514	0.999985752	0.999948228
RPS13	0.999995916	0.997236605	0.999993442	0.999981514	0.999985752	0.999948228
LOC112442754	0.999995916	0.997332173	0.999993442	0.999981514	0.999985752	0.999948228
MYO6	0.999995916	0.997332173	0.999993442	0.999981514	0.999985752	0.999948228
VPS4B	0.999995916	0.997332173	0.999993442	0.999981514	0.999985752	0.999948228
CCDC130	0.999995916	0.997425219	0.999993442	0.999981514	0.999985752	0.999948228
NOBOX	0.999995916	0.997425219	0.999993442	0.999981514	0.999985752	0.999948228
PYCR3	0.999995916	0.997425219	0.999993442	0.999981514	0.999985752	0.999948228
RPAIN	0.999995916	0.997425219	0.999993442	0.999981514	0.999985752	0.999948228
SMARCE1	0.999995916	0.997425219	0.999993442	0.999981514	0.999985752	0.999948228
FAF2	0.999995916	0.997430868	0.999993442	0.999981514	0.999985752	0.999948228
ADAP2	0.999995916	0.997471998	0.999993442	0.999981514	0.999985752	0.999948228
CAMK1D	0.999995916	0.997471998	0.999993442	0.999981514	0.999985752	0.999948228
DCLK3	0.999995916	0.997471998	0.999993442	0.999981514	0.999985752	0.999948228
FBXO5	0.999995916	0.997471998	0.999993442	0.999981514	0.999985752	0.999948228
GCC2	0.999995916	0.997471998	0.999993442	0.999981514	0.999985752	0.999948228
GPR180	0.999995916	0.997471998	0.999993442	0.999981514	0.999985752	0.999948228
HBA1	0.999995916	0.997471998	0.999993442	0.999981514	0.999985752	0.999948228
LOC104970162	0.999995916	0.997471998	0.999993442	0.999981514	0.999985752	0.999948228
LOC107132799	0.999995916	0.997471998	0.999993442	0.999981514	0.999985752	0.999948228
LOC112442349	0.999995916	0.997471998	0.999993442	0.999981514	0.999985752	0.999948228
LOX	0.999995916	0.997471998	0.999993442	0.999981514	0.999985752	0.999948228
PPWD1	0.999995916	0.997471998	0.999993442	0.999981514	0.999985752	0.999948228
RPS27	0.999995916	0.997471998	0.999993442	0.999981514	0.999985752	0.999948228
SAMD12	0.999995916	0.997471998	0.999993442	0.999981514	0.999985752	0.999948228

STK11IP	0.999995916	0.997471998	0.999993442	0.999981514	0.999985752	0.999948228
TMEM163	0.999995916	0.997471998	0.999993442	0.999981514	0.999985752	0.999948228
UXT	0.999995916	0.997471998	0.999993442	0.999981514	0.999985752	0.999948228
ZEB1	0.999995916	0.997471998	0.999993442	0.999981514	0.999985752	0.999948228
ZNF496	0.999995916	0.997471998	0.999993442	0.999981514	0.999985752	0.999948228
TADA2A	0.999995916	0.997557921	0.999993442	0.999981514	0.999985752	0.999948228
MISP3	0.999995916	0.997604725	0.999993442	0.999981514	0.999985752	0.999948228
CH25H	0.999995916	0.997637352	0.999993442	0.999981514	0.999985752	0.999948228
ADORA2A	0.999995916	0.997678999	0.999993442	0.999981514	0.999985752	0.999948228
CPNE3	0.999995916	0.997678999	0.999993442	0.999981514	0.999985752	0.999948228
CTIF	0.999995916	0.997678999	0.999993442	0.999981514	0.999985752	0.999948228
LOC100848581	0.999995916	0.997678999	0.999993442	0.999981514	0.999985752	0.999948228
LOC104975979	0.999995916	0.997678999	0.999993442	0.999981514	0.999985752	0.999948228
LOC112443422	0.999995916	0.997678999	0.999993442	0.999981514	0.999985752	0.999948228
LOC112444287	0.999995916	0.997678999	0.999993442	0.999981514	0.999985752	0.999948228
LOC112444613	0.999995916	0.997678999	0.999993442	0.999981514	0.999985752	0.999948228
LOC112447418	0.999995916	0.997678999	0.999993442	0.999981514	0.999985752	0.999948228
MEGF9	0.999995916	0.997678999	0.999993442	0.999981514	0.999985752	0.999948228
MYO9B	0.999995916	0.997678999	0.999993442	0.999981514	0.999985752	0.999948228
RPL23	0.999995916	0.997678999	0.999993442	0.999981514	0.999985752	0.999948228
RUVBL2	0.999995916	0.997678999	0.999993442	0.999981514	0.999985752	0.999948228
ANKRD9	0.999995916	0.997840295	0.999993442	0.999981514	0.999985752	0.999948228
LCOR	0.999995916	0.997951673	0.999993442	0.999981514	0.999985752	0.999948228
RTN4RL2	0.999995916	0.998094116	0.999993442	0.999981514	0.999985752	0.999948228
ZFP91	0.999995916	0.998094116	0.999993442	0.999981514	0.999985752	0.999948228
GDNF	0.999995916	0.99831878	0.999993442	0.999981514	0.999985752	0.999948228
LOC101904916	0.999995916	0.99831878	0.999993442	0.999981514	0.999985752	0.999948228
LOC614643	0.999995916	0.99831878	0.999993442	0.999981514	0.999985752	0.999948228
POLR2H	0.999995916	0.998558572	0.999993442	0.999981514	0.999985752	0.999948228
TMEM241	0.999995916	0.998819285	0.999993442	0.999981514	0.999985752	0.999948228
AGA	0.999995916	0.998847958	0.999993442	0.999981514	0.999985752	0.999948228
AK5	0.999995916	0.998847958	0.999993442	0.999981514	0.999985752	0.999948228
ARMC5	0.999995916	0.998847958	0.999993442	0.999981514	0.999985752	0.999948228
BOLA	0.999995916	0.998847958	0.999993442	0.999981514	0.999985752	0.999948228
BRSK2	0.999995916	0.998847958	0.999993442	0.999981514	0.999985752	0.999948228
C2CD2	0.999995916	0.998847958	0.999993442	0.999981514	0.999985752	0.999948228
CAPN2	0.999995916	0.998847958	0.999993442	0.999981514	0.999985752	0.999948228
CASKIN2	0.999995916	0.998847958	0.999993442	0.999981514	0.999985752	0.999948228

CATSPER2	0.999995916	0.998847958	0.999993442	0.999981514	0.999985752	0.999948228
CENPS	0.999995916	0.998847958	0.999993442	0.999981514	0.999985752	0.999948228
CEP97	0.999995916	0.998847958	0.999993442	0.999981514	0.999985752	0.999948228
CHRNA5	0.999995916	0.998847958	0.999993442	0.999981514	0.999985752	0.999948228
CYP26B1	0.999995916	0.998847958	0.999993442	0.999981514	0.999985752	0.999948228
DCHS1	0.999995916	0.998847958	0.999993442	0.999981514	0.999985752	0.999948228
DIO2	0.999995916	0.998847958	0.999993442	0.999981514	0.999985752	0.999948228
DRAM1	0.999995916	0.998847958	0.999993442	0.999981514	0.999985752	0.999948228
ECPAS	0.999995916	0.998847958	0.999993442	0.999981514	0.999985752	0.999948228
EIF5A2	0.999995916	0.998847958	0.999993442	0.999981514	0.999985752	0.999948228
FAM111B	0.999995916	0.998847958	0.999993442	0.999981514	0.999985752	0.999948228
FAT4	0.999995916	0.998847958	0.999993442	0.999981514	0.999985752	0.999948228
FTSJ1	0.999995916	0.998847958	0.999993442	0.999981514	0.999985752	0.999948228
GGCT	0.999995916	0.998847958	0.999993442	0.999981514	0.999985752	0.999948228
GJA4	0.999995916	0.998847958	0.999993442	0.999981514	0.999985752	0.999948228
GNAT2	0.999995916	0.998847958	0.999993442	0.999981514	0.999985752	0.999948228
HMBOX1	0.999995916	0.998847958	0.999993442	0.999981514	0.999985752	0.999948228
HNRNPA1	0.999995916	0.998847958	0.999993442	0.999981514	0.999985752	0.999948228
ILKAP	0.999995916	0.998847958	0.999993442	0.999981514	0.999985752	0.999948228
KLHDC1	0.999995916	0.998847958	0.999993442	0.999981514	0.999985752	0.999948228
KLRD1	0.999995916	0.998847958	0.999993442	0.999981514	0.999985752	0.999948228
LOC100337390	0.999995916	0.998847958	0.999993442	0.999981514	0.999985752	0.999948228
LOC100847745	0.999995916	0.998847958	0.999993442	0.999981514	0.999985752	0.999948228
LOC100848122	0.999995916	0.998847958	0.999993442	0.999981514	0.999985752	0.999948228
LOC100849046	0.999995916	0.998847958	0.999993442	0.999981514	0.999985752	0.999948228
LOC100849069	0.999995916	0.998847958	0.999993442	0.999981514	0.999985752	0.999948228
LOC101903928	0.999995916	0.998847958	0.999993442	0.999981514	0.999985752	0.999948228
LOC101904667	0.999995916	0.998847958	0.999993442	0.999981514	0.999985752	0.999948228
LOC101905029	0.999995916	0.998847958	0.999993442	0.999981514	0.999985752	0.999948228
LOC104968671	0.999995916	0.998847958	0.999993442	0.999981514	0.999985752	0.999948228
LOC104975283	0.999995916	0.998847958	0.999993442	0.999981514	0.999985752	0.999948228
LOC104976448	0.999995916	0.998847958	0.999993442	0.999981514	0.999985752	0.999948228
LOC107132851	0.999995916	0.998847958	0.999993442	0.999981514	0.999985752	0.999948228
LOC112442040	0.999995916	0.998847958	0.999993442	0.999981514	0.999985752	0.999948228
LOC112442228	0.999995916	0.998847958	0.999993442	0.999981514	0.999985752	0.999948228
LOC112443877	0.999995916	0.998847958	0.999993442	0.999981514	0.999985752	0.999948228
LOC112444339	0.999995916	0.998847958	0.999993442	0.999981514	0.999985752	0.999948228
LOC112445052	0.999995916	0.998847958	0.999993442	0.999981514	0.999985752	0.999948228

LOC112445971	0.999995916	0.998847958	0.999993442	0.999981514	0.999985752	0.999948228
LOC112447026	0.999995916	0.998847958	0.999993442	0.999981514	0.999985752	0.999948228
LOC112447842	0.999995916	0.998847958	0.999993442	0.999981514	0.999985752	0.999948228
LOC112448762	0.999995916	0.998847958	0.999993442	0.999981514	0.999985752	0.999948228
LOC112449111	0.999995916	0.998847958	0.999993442	0.999981514	0.999985752	0.999948228
LOC112449338	0.999995916	0.998847958	0.999993442	0.999981514	0.999985752	0.999948228
LOC523461	0.999995916	0.998847958	0.999993442	0.999981514	0.999985752	0.999948228
LOC613519	0.999995916	0.998847958	0.999993442	0.999981514	0.999985752	0.999948228
LOC613570	0.999995916	0.998847958	0.999993442	0.999981514	0.999985752	0.999948228
LOC782755	0.999995916	0.998847958	0.999993442	0.999981514	0.999985752	0.999948228
LOC783060	0.999995916	0.998847958	0.999993442	0.999981514	0.999985752	0.999948228
LOC788541	0.999995916	0.998847958	0.999993442	0.999981514	0.999985752	0.999948228
LOC790266	0.999995916	0.998847958	0.999993442	0.999981514	0.999985752	0.999948228
LRRN4CL	0.999995916	0.998847958	0.999993442	0.999981514	0.999985752	0.999948228
MAPK12	0.999995916	0.998847958	0.999993442	0.999981514	0.999985752	0.999948228
MUS81	0.999995916	0.998847958	0.999993442	0.999981514	0.999985752	0.999948228
MYO1D	0.999995916	0.998847958	0.999993442	0.999981514	0.999985752	0.999948228
NCKIPSD	0.999995916	0.998847958	0.999993442	0.999981514	0.999985752	0.999948228
NME3	0.999995916	0.998847958	0.999993442	0.999981514	0.999985752	0.999948228
PARVB	0.999995916	0.998847958	0.999993442	0.999981514	0.999985752	0.999948228
PC	0.999995916	0.998847958	0.999993442	0.999981514	0.999985752	0.999948228
PCDHGB4	0.999995916	0.998847958	0.999993442	0.999981514	0.999985752	0.999948228
PCGF2	0.999995916	0.998847958	0.999993442	0.999981514	0.999985752	0.999948228
PDCD2L	0.999995916	0.998847958	0.999993442	0.999981514	0.999985752	0.999948228
PDZD9	0.999995916	0.998847958	0.999993442	0.999981514	0.999985752	0.999948228
PEX12	0.999995916	0.998847958	0.999993442	0.999981514	0.999985752	0.999948228
PLA2G2D4	0.999995916	0.998847958	0.999993442	0.999981514	0.999985752	0.999948228
PLD1	0.999995916	0.998847958	0.999993442	0.999981514	0.999985752	0.999948228
PRAF2	0.999995916	0.998847958	0.999993442	0.999981514	0.999985752	0.999948228
RAB11FIP3	0.999995916	0.998847958	0.999993442	0.999981514	0.999985752	0.999948228
RAB3D	0.999995916	0.998847958	0.999993442	0.999981514	0.999985752	0.999948228
RABIF	0.999995916	0.998847958	0.999993442	0.999981514	0.999985752	0.999948228
RACK1	0.999995916	0.998847958	0.999993442	0.999981514	0.999985752	0.999948228
RAD17	0.999995916	0.998847958	0.999993442	0.999981514	0.999985752	0.999948228
RP9	0.999995916	0.998847958	0.999993442	0.999981514	0.999985752	0.999948228
RPS18	0.999995916	0.998847958	0.999993442	0.999981514	0.999985752	0.999948228
RPS8	0.999995916	0.998847958	0.999993442	0.999981514	0.999985752	0.999948228
SDR42E1	0.999995916	0.998847958	0.999993442	0.999981514	0.999985752	0.999948228

SEC61A2	0.999995916	0.998847958	0.999993442	0.999981514	0.999985752	0.999948228
SF3A2	0.999995916	0.998847958	0.999993442	0.999981514	0.999985752	0.999948228
SNRNP40	0.999995916	0.998847958	0.999993442	0.999981514	0.999985752	0.999948228
SNX29	0.999995916	0.998847958	0.999993442	0.999981514	0.999985752	0.999948228
SYNPO	0.999995916	0.998847958	0.999993442	0.999981514	0.999985752	0.999948228
TDRD6	0.999995916	0.998847958	0.999993442	0.999981514	0.999985752	0.999948228
TMEM117	0.999995916	0.998847958	0.999993442	0.999981514	0.999985752	0.999948228
TMEM179B	0.999995916	0.998847958	0.999993442	0.999981514	0.999985752	0.999948228
TRNT1	0.999995916	0.998847958	0.999993442	0.999981514	0.999985752	0.999948228
UBXN2B	0.999995916	0.998847958	0.999993442	0.999981514	0.999985752	0.999948228
UCK1	0.999995916	0.998847958	0.999993442	0.999981514	0.999985752	0.999948228
VPS37B	0.999995916	0.998847958	0.999993442	0.999981514	0.999985752	0.999948228
VWA5B2	0.999995916	0.998847958	0.999993442	0.999981514	0.999985752	0.999948228
VWA8	0.999995916	0.998847958	0.999993442	0.999981514	0.999985752	0.999948228
XAB2	0.999995916	0.998847958	0.999993442	0.999981514	0.999985752	0.999948228
YAP1	0.999995916	0.998847958	0.999993442	0.999981514	0.999985752	0.999948228
ZNF449	0.999995916	0.998847958	0.999993442	0.999981514	0.999985752	0.999948228
TSEN34	0.999995916	0.999043477	0.999993442	0.999981514	0.999985752	0.999948228
MVB12A	0.999995916	0.999167135	0.999993442	0.999981514	0.999985752	0.999948228
GAS6	0.999995916	0.999170961	0.999993442	0.999981514	0.999985752	0.999948228
LOC101906914	0.999995916	0.999278945	0.999993442	0.999981514	0.999985752	0.999948228
LOC107132386	0.999995916	0.999278945	0.999993442	0.999981514	0.999985752	0.999948228
LOC112441859	0.999995916	0.999278945	0.999993442	0.999981514	0.999985752	0.999948228
LOC784464	0.999995916	0.999311624	0.999993442	0.999981514	0.999985752	0.999948228
LOC101907250	0.999995916	0.99950853	0.999993442	0.999981514	0.999985752	0.999948228
LOC781982	0.999995916	0.99950853	0.999993442	0.999981514	0.999985752	0.999948228
PCYOX1	0.999995916	0.99950853	0.999993442	0.999981514	0.999985752	0.999948228
RPS3A	0.999995916	0.99950853	0.999993442	0.999981514	0.999985752	0.999948228
LOC101902681	0.999995916	0.999522358	0.999993442	0.999981514	0.999985752	0.999948228
AKT1	0.999995916	0.99960614	0.999993442	0.999981514	0.999985752	0.999948228
APH1A	0.999995916	0.99960614	0.999993442	0.999981514	0.999985752	0.999948228
ARHGAP42	0.999995916	0.99960614	0.999993442	0.999981514	0.999985752	0.999948228
BCAT1	0.999995916	0.99960614	0.999993442	0.999981514	0.999985752	0.999948228
BMP3	0.999995916	0.99960614	0.999993442	0.999981514	0.999985752	0.999948228
BTBD1	0.999995916	0.99960614	0.999993442	0.999981514	0.999985752	0.999948228
BTNL9	0.999995916	0.99960614	0.999993442	0.999981514	0.999985752	0.999948228
C1H3orf33	0.999995916	0.99960614	0.999993442	0.999981514	0.999985752	0.999948228
CAMKK2	0.999995916	0.99960614	0.999993442	0.999981514	0.999985752	0.999948228

CCL28	0.999995916	0.99960614	0.999993442	0.999981514	0.999985752	0.999948228
CCP110	0.999995916	0.99960614	0.999993442	0.999981514	0.999985752	0.999948228
CD47	0.999995916	0.99960614	0.999993442	0.999981514	0.999985752	0.999948228
CDKAL1	0.999995916	0.99960614	0.999993442	0.999981514	0.999985752	0.999948228
CELSR2	0.999995916	0.99960614	0.999993442	0.999981514	0.999985752	0.999948228
CENPP	0.999995916	0.99960614	0.999993442	0.999981514	0.999985752	0.999948228
CHRNE	0.999995916	0.99960614	0.999993442	0.999981514	0.999985752	0.999948228
CLDN20	0.999995916	0.99960614	0.999993442	0.999981514	0.999985752	0.999948228
COG5	0.999995916	0.99960614	0.999993442	0.999981514	0.999985752	0.999948228
COL18A1	0.999995916	0.99960614	0.999993442	0.999981514	0.999985752	0.999948228
CTNNA1	0.999995916	0.99960614	0.999993442	0.999981514	0.999985752	0.999948228
CTTNBP2NL	0.999995916	0.99960614	0.999993442	0.999981514	0.999985752	0.999948228
DCK	0.999995916	0.99960614	0.999993442	0.999981514	0.999985752	0.999948228
DDHD2	0.999995916	0.99960614	0.999993442	0.999981514	0.999985752	0.999948228
DDX23	0.999995916	0.99960614	0.999993442	0.999981514	0.999985752	0.999948228
DIAPH2	0.999995916	0.99960614	0.999993442	0.999981514	0.999985752	0.999948228
DNAL1	0.999995916	0.99960614	0.999993442	0.999981514	0.999985752	0.999948228
DPY19L3	0.999995916	0.99960614	0.999993442	0.999981514	0.999985752	0.999948228
DPY19L4	0.999995916	0.99960614	0.999993442	0.999981514	0.999985752	0.999948228
DUSP7	0.999995916	0.99960614	0.999993442	0.999981514	0.999985752	0.999948228
EPB41L1	0.999995916	0.99960614	0.999993442	0.999981514	0.999985752	0.999948228
FAM185A	0.999995916	0.99960614	0.999993442	0.999981514	0.999985752	0.999948228
FAU	0.999995916	0.99960614	0.999993442	0.999981514	0.999985752	0.999948228
FOXF2	0.999995916	0.99960614	0.999993442	0.999981514	0.999985752	0.999948228
GJB6	0.999995916	0.99960614	0.999993442	0.999981514	0.999985752	0.999948228
GPATCH11	0.999995916	0.99960614	0.999993442	0.999981514	0.999985752	0.999948228
HADHA	0.999995916	0.99960614	0.999993442	0.999981514	0.999985752	0.999948228
HEG1	0.999995916	0.99960614	0.999993442	0.999981514	0.999985752	0.999948228
HIST1H2BI	0.999995916	0.99960614	0.999993442	0.999981514	0.999985752	0.999948228
HOXB7	0.999995916	0.99960614	0.999993442	0.999981514	0.999985752	0.999948228
ITPR1	0.999995916	0.99960614	0.999993442	0.999981514	0.999985752	0.999948228
LOC100138131	0.999995916	0.99960614	0.999993442	0.999981514	0.999985752	0.999948228
LOC100140873	0.999995916	0.99960614	0.999993442	0.999981514	0.999985752	0.999948228
LOC100140915	0.999995916	0.99960614	0.999993442	0.999981514	0.999985752	0.999948228
LOC100336532	0.999995916	0.99960614	0.999993442	0.999981514	0.999985752	0.999948228
LOC100336777	0.999995916	0.99960614	0.999993442	0.999981514	0.999985752	0.999948228
LOC100336869	0.999995916	0.99960614	0.999993442	0.999981514	0.999985752	0.999948228
LOC100847773	0.999995916	0.99960614	0.999993442	0.999981514	0.999985752	0.999948228

LOC100848105	0.999995916	0.99960614	0.999993442	0.999981514	0.999985752	0.999948228
LOC100848353	0.999995916	0.99960614	0.999993442	0.999981514	0.999985752	0.999948228
LOC101903098	0.999995916	0.99960614	0.999993442	0.999981514	0.999985752	0.999948228
LOC101906110	0.999995916	0.99960614	0.999993442	0.999981514	0.999985752	0.999948228
LOC101907544	0.999995916	0.99960614	0.999993442	0.999981514	0.999985752	0.999948228
LOC104968479	0.999995916	0.99960614	0.999993442	0.999981514	0.999985752	0.999948228
LOC104970815	0.999995916	0.99960614	0.999993442	0.999981514	0.999985752	0.999948228
LOC104972843	0.999995916	0.99960614	0.999993442	0.999981514	0.999985752	0.999948228
LOC104974034	0.999995916	0.99960614	0.999993442	0.999981514	0.999985752	0.999948228
LOC107131489	0.999995916	0.99960614	0.999993442	0.999981514	0.999985752	0.999948228
LOC107131906	0.999995916	0.99960614	0.999993442	0.999981514	0.999985752	0.999948228
LOC107132070	0.999995916	0.99960614	0.999993442	0.999981514	0.999985752	0.999948228
LOC107132093	0.999995916	0.99960614	0.999993442	0.999981514	0.999985752	0.999948228
LOC107132243	0.999995916	0.99960614	0.999993442	0.999981514	0.999985752	0.999948228
LOC107132398	0.999995916	0.99960614	0.999993442	0.999981514	0.999985752	0.999948228
LOC112442244	0.999995916	0.99960614	0.999993442	0.999981514	0.999985752	0.999948228
LOC112442676	0.999995916	0.99960614	0.999993442	0.999981514	0.999985752	0.999948228
LOC112442802	0.999995916	0.99960614	0.999993442	0.999981514	0.999985752	0.999948228
LOC112443213	0.999995916	0.99960614	0.999993442	0.999981514	0.999985752	0.999948228
LOC112443767	0.999995916	0.99960614	0.999993442	0.999981514	0.999985752	0.999948228
LOC112444464	0.999995916	0.99960614	0.999993442	0.999981514	0.999985752	0.999948228
LOC112444867	0.999995916	0.99960614	0.999993442	0.999981514	0.999985752	0.999948228
LOC112446390	0.999995916	0.99960614	0.999993442	0.999981514	0.999985752	0.999948228
LOC112446725	0.999995916	0.99960614	0.999993442	0.999981514	0.999985752	0.999948228
LOC112447420	0.999995916	0.99960614	0.999993442	0.999981514	0.999985752	0.999948228
LOC112448507	0.999995916	0.99960614	0.999993442	0.999981514	0.999985752	0.999948228
LOC112448832	0.999995916	0.99960614	0.999993442	0.999981514	0.999985752	0.999948228
LOC112449275	0.999995916	0.99960614	0.999993442	0.999981514	0.999985752	0.999948228
LOC112449548	0.999995916	0.99960614	0.999993442	0.999981514	0.999985752	0.999948228
LOC616942	0.999995916	0.99960614	0.999993442	0.999981514	0.999985752	0.999948228
LOC619131	0.999995916	0.99960614	0.999993442	0.999981514	0.999985752	0.999948228
LOC782776	0.999995916	0.99960614	0.999993442	0.999981514	0.999985752	0.999948228
LOC783988	0.999995916	0.99960614	0.999993442	0.999981514	0.999985752	0.999948228
LOC784473	0.999995916	0.99960614	0.999993442	0.999981514	0.999985752	0.999948228
LOC785760	0.999995916	0.99960614	0.999993442	0.999981514	0.999985752	0.999948228
LZTS3	0.999995916	0.99960614	0.999993442	0.999981514	0.999985752	0.999948228
MAP3K7CL	0.999995916	0.99960614	0.999993442	0.999981514	0.999985752	0.999948228
MAVS	0.999995916	0.99960614	0.999993442	0.999981514	0.999985752	0.999948228

MICAL3	0.999995916	0.99960614	0.999993442	0.999981514	0.999985752	0.999948228
MRPL10	0.999995916	0.99960614	0.999993442	0.999981514	0.999985752	0.999948228
MSH2	0.999995916	0.99960614	0.999993442	0.999981514	0.999985752	0.999948228
MSH3	0.999995916	0.99960614	0.999993442	0.999981514	0.999985752	0.999948228
MSX2	0.999995916	0.99960614	0.999993442	0.999981514	0.999985752	0.999948228
NLGN2	0.999995916	0.99960614	0.999993442	0.999981514	0.999985752	0.999948228
NOVA1	0.999995916	0.99960614	0.999993442	0.999981514	0.999985752	0.999948228
NPTN	0.999995916	0.99960614	0.999993442	0.999981514	0.999985752	0.999948228
NRP1	0.999995916	0.99960614	0.999993442	0.999981514	0.999985752	0.999948228
PANK4	0.999995916	0.99960614	0.999993442	0.999981514	0.999985752	0.999948228
PHF20L1	0.999995916	0.99960614	0.999993442	0.999981514	0.999985752	0.999948228
PIP4P1	0.999995916	0.99960614	0.999993442	0.999981514	0.999985752	0.999948228
POMC	0.999995916	0.99960614	0.999993442	0.999981514	0.999985752	0.999948228
PPP4R1	0.999995916	0.99960614	0.999993442	0.999981514	0.999985752	0.999948228
PRICKLE3	0.999995916	0.99960614	0.999993442	0.999981514	0.999985752	0.999948228
PTK2	0.999995916	0.99960614	0.999993442	0.999981514	0.999985752	0.999948228
PTPN1	0.999995916	0.99960614	0.999993442	0.999981514	0.999985752	0.999948228
QSOX1	0.999995916	0.99960614	0.999993442	0.999981514	0.999985752	0.999948228
RARG	0.999995916	0.99960614	0.999993442	0.999981514	0.999985752	0.999948228
RNF149	0.999995916	0.99960614	0.999993442	0.999981514	0.999985752	0.999948228
RPL13A	0.999995916	0.99960614	0.999993442	0.999981514	0.999985752	0.999948228
RPL19	0.999995916	0.99960614	0.999993442	0.999981514	0.999985752	0.999948228
RPS15A	0.999995916	0.99960614	0.999993442	0.999981514	0.999985752	0.999948228
RPS19	0.999995916	0.99960614	0.999993442	0.999981514	0.999985752	0.999948228
RPS3	0.999995916	0.99960614	0.999993442	0.999981514	0.999985752	0.999948228
RRM1	0.999995916	0.99960614	0.999993442	0.999981514	0.999985752	0.999948228
RRP1B	0.999995916	0.99960614	0.999993442	0.999981514	0.999985752	0.999948228
SDSL	0.999995916	0.99960614	0.999993442	0.999981514	0.999985752	0.999948228
SERP2	0.999995916	0.99960614	0.999993442	0.999981514	0.999985752	0.999948228
SF3A1	0.999995916	0.99960614	0.999993442	0.999981514	0.999985752	0.999948228
SFMBT1	0.999995916	0.99960614	0.999993442	0.999981514	0.999985752	0.999948228
SHISA3	0.999995916	0.99960614	0.999993442	0.999981514	0.999985752	0.999948228
SLC13A5	0.999995916	0.99960614	0.999993442	0.999981514	0.999985752	0.999948228
SLC27A6	0.999995916	0.99960614	0.999993442	0.999981514	0.999985752	0.999948228
SLCO2A1	0.999995916	0.99960614	0.999993442	0.999981514	0.999985752	0.999948228
SNRNP200	0.999995916	0.99960614	0.999993442	0.999981514	0.999985752	0.999948228
SULF1	0.999995916	0.99960614	0.999993442	0.999981514	0.999985752	0.999948228
SYDE1	0.999995916	0.99960614	0.999993442	0.999981514	0.999985752	0.999948228

SYN1	0.999995916	0.99960614	0.999993442	0.999981514	0.999985752	0.999948228
TATDN2	0.999995916	0.99960614	0.999993442	0.999981514	0.999985752	0.999948228
TLN2	0.999995916	0.99960614	0.999993442	0.999981514	0.999985752	0.999948228
TM2D2	0.999995916	0.99960614	0.999993442	0.999981514	0.999985752	0.999948228
TMEM161A	0.999995916	0.99960614	0.999993442	0.999981514	0.999985752	0.999948228
TNFAIP8	0.999995916	0.99960614	0.999993442	0.999981514	0.999985752	0.999948228
TNFRSF1A	0.999995916	0.99960614	0.999993442	0.999981514	0.999985752	0.999948228
TPGS1	0.999995916	0.99960614	0.999993442	0.999981514	0.999985752	0.999948228
TRAPPC11	0.999995916	0.99960614	0.999993442	0.999981514	0.999985752	0.999948228
TRIM52	0.999995916	0.99960614	0.999993442	0.999981514	0.999985752	0.999948228
TRMO	0.999995916	0.99960614	0.999993442	0.999981514	0.999985752	0.999948228
TTC39A	0.999995916	0.99960614	0.999993442	0.999981514	0.999985752	0.999948228
TTI2	0.999995916	0.99960614	0.999993442	0.999981514	0.999985752	0.999948228
UHRF1BP1	0.999995916	0.99960614	0.999993442	0.999981514	0.999985752	0.999948228
ULBP21	0.999995916	0.99960614	0.999993442	0.999981514	0.999985752	0.999948228
UNC50	0.999995916	0.99960614	0.999993442	0.999981514	0.999985752	0.999948228
USP33	0.999995916	0.99960614	0.999993442	0.999981514	0.999985752	0.999948228
WDR41	0.999995916	0.99960614	0.999993442	0.999981514	0.999985752	0.999948228
WNT16	0.999995916	0.99960614	0.999993442	0.999981514	0.999985752	0.999948228
YES1	0.999995916	0.99960614	0.999993442	0.999981514	0.999985752	0.999948228
ZC3H10	0.999995916	0.99960614	0.999993442	0.999981514	0.999985752	0.999948228
ZNF394	0.999995916	0.99960614	0.999993442	0.999981514	0.999985752	0.999948228
ZNF514	0.999995916	0.99960614	0.999993442	0.999981514	0.999985752	0.999948228
ZNF570	0.999995916	0.99960614	0.999993442	0.999981514	0.999985752	0.999948228
ZNF579	0.999995916	0.99960614	0.999993442	0.999981514	0.999985752	0.999948228
GPANK1	0.999995916	0.999621646	0.999993442	0.999981514	0.999985752	0.999948228
WDR70	0.999995916	0.999621646	0.999993442	0.999981514	0.999985752	0.999948228
IPP	0.999995916	0.99964543	0.999993442	0.999981514	0.999985752	0.999948228
RPS12	0.999995916	0.99964543	0.999993442	0.999981514	0.999985752	0.999948228
MCM10	0.999995916	0.999657245	0.999993442	0.999981514	0.999985752	0.999948228
LOC101904268	0.999995916	0.999794908	0.999993442	0.999981514	0.999985752	0.999948228
AATK	0.999995916	0.999987083	0.999993442	0.999981514	0.999985752	0.999948228
ABCA9	0.999995916	0.999987083	0.999993442	0.999981514	0.999985752	0.999948228
ACSM2B	0.999995916	0.999987083	0.999993442	0.999981514	0.999985752	0.999948228
ACTR8	0.999995916	0.999987083	0.999993442	0.999981514	0.999985752	0.999948228
ADGRG1	0.999995916	0.999987083	0.999993442	0.999981514	0.999985752	0.999948228
APLP2	0.999995916	0.999987083	0.999993442	0.999981514	0.999985752	0.999948228
ARHGDIB	0.999995916	0.999987083	0.999993442	0.999981514	0.999985752	0.999948228

ARSG	0.999995916	0.999987083	0.999993442	0.999981514	0.999985752	0.999948228
ASB6	0.999995916	0.999987083	0.999993442	0.999981514	0.999985752	0.999948228
ATP11B	0.999995916	0.999987083	0.999993442	0.999981514	0.999985752	0.999948228
ATP6V0E2	0.999995916	0.999987083	0.999993442	0.999981514	0.999985752	0.999948228
AVPR2	0.999995916	0.999987083	0.999993442	0.999981514	0.999985752	0.999948228
BACH2	0.999995916	0.999987083	0.999993442	0.999981514	0.999985752	0.999948228
BAX	0.999995916	0.999987083	0.999993442	0.999981514	0.999985752	0.999948228
BCAR1	0.999995916	0.999987083	0.999993442	0.999981514	0.999985752	0.999948228
BCAS3	0.999995916	0.999987083	0.999993442	0.999981514	0.999985752	0.999948228
BCL2A1	0.999995916	0.999987083	0.999993442	0.999981514	0.999985752	0.999948228
BRWD1	0.999995916	0.999987083	0.999993442	0.999981514	0.999985752	0.999948228
C14H8orf82	0.999995916	0.999987083	0.999993442	0.999981514	0.999985752	0.999948228
C3H1orf216	0.999995916	0.999987083	0.999993442	0.999981514	0.999985752	0.999948228
C9H6orf163	0.999995916	0.999987083	0.999993442	0.999981514	0.999985752	0.999948228
CA10	0.999995916	0.999987083	0.999993442	0.999981514	0.999985752	0.999948228
CARD11	0.999995916	0.999987083	0.999993442	0.999981514	0.999985752	0.999948228
CASKIN1	0.999995916	0.999987083	0.999993442	0.999981514	0.999985752	0.999948228
CASP6	0.999995916	0.999987083	0.999993442	0.999981514	0.999985752	0.999948228
CASP7	0.999995916	0.999987083	0.999993442	0.999981514	0.999985752	0.999948228
CASS4	0.999995916	0.999987083	0.999993442	0.999981514	0.999985752	0.999948228
CCDC138	0.999995916	0.999987083	0.999993442	0.999981514	0.999985752	0.999948228
CD109	0.999995916	0.999987083	0.999993442	0.999981514	0.999985752	0.999948228
CHD9	0.999995916	0.999987083	0.999993442	0.999981514	0.999985752	0.999948228
CHPF	0.999995916	0.999987083	0.999993442	0.999981514	0.999985752	0.999948228
CHRDL1	0.999995916	0.999987083	0.999993442	0.999981514	0.999985752	0.999948228
CLDN12	0.999995916	0.999987083	0.999993442	0.999981514	0.999985752	0.999948228
CLTRN	0.999995916	0.999987083	0.999993442	0.999981514	0.999985752	0.999948228
CPEB3	0.999995916	0.999987083	0.999993442	0.999981514	0.999985752	0.999948228
CPN2	0.999995916	0.999987083	0.999993442	0.999981514	0.999985752	0.999948228
CPSF4	0.999995916	0.999987083	0.999993442	0.999981514	0.999985752	0.999948228
CYB5D1	0.999995916	0.999987083	0.999993442	0.999981514	0.999985752	0.999948228
CYTH3	0.999995916	0.999987083	0.999993442	0.999981514	0.999985752	0.999948228
DAAM2	0.999995916	0.999987083	0.999993442	0.999981514	0.999985752	0.999948228
DAB2IP	0.999995916	0.999987083	0.999993442	0.999981514	0.999985752	0.999948228
DENND4A	0.999995916	0.999987083	0.999993442	0.999981514	0.999985752	0.999948228
DEPP1	0.999995916	0.999987083	0.999993442	0.999981514	0.999985752	0.999948228
DMXL2	0.999995916	0.999987083	0.999993442	0.999981514	0.999985752	0.999948228
DPH2	0.999995916	0.999987083	0.999993442	0.999981514	0.999985752	0.999948228

DPYSL2	0.999995916	0.999987083	0.999993442	0.999981514	0.999985752	0.999948228
DYNC1H1	0.999995916	0.999987083	0.999993442	0.999981514	0.999985752	0.999948228
EDC4	0.999995916	0.999987083	0.999993442	0.999981514	0.999985752	0.999948228
EEF1A1	0.999995916	0.999987083	0.999993442	0.999981514	0.999985752	0.999948228
ERGIC3	0.999995916	0.999987083	0.999993442	0.999981514	0.999985752	0.999948228
ERI2	0.999995916	0.999987083	0.999993442	0.999981514	0.999985752	0.999948228
FA2H	0.999995916	0.999987083	0.999993442	0.999981514	0.999985752	0.999948228
FAM20C	0.999995916	0.999987083	0.999993442	0.999981514	0.999985752	0.999948228
FBXO48	0.999995916	0.999987083	0.999993442	0.999981514	0.999985752	0.999948228
FKBP15	0.999995916	0.999987083	0.999993442	0.999981514	0.999985752	0.999948228
FOXN3	0.999995916	0.999987083	0.999993442	0.999981514	0.999985752	0.999948228
FSD2	0.999995916	0.999987083	0.999993442	0.999981514	0.999985752	0.999948228
FSTL3	0.999995916	0.999987083	0.999993442	0.999981514	0.999985752	0.999948228
GAA	0.999995916	0.999987083	0.999993442	0.999981514	0.999985752	0.999948228
GBA	0.999995916	0.999987083	0.999993442	0.999981514	0.999985752	0.999948228
GPC6	0.999995916	0.999987083	0.999993442	0.999981514	0.999985752	0.999948228
GPR135	0.999995916	0.999987083	0.999993442	0.999981514	0.999985752	0.999948228
GPR52	0.999995916	0.999987083	0.999993442	0.999981514	0.999985752	0.999948228
H2AFV	0.999995916	0.999987083	0.999993442	0.999981514	0.999985752	0.999948228
HIST1H2BB	0.999995916	0.999987083	0.999993442	0.999981514	0.999985752	0.999948228
HMCES	0.999995916	0.999987083	0.999993442	0.999981514	0.999985752	0.999948228
HOXB8	0.999995916	0.999987083	0.999993442	0.999981514	0.999985752	0.999948228
HOXC4	0.999995916	0.999987083	0.999993442	0.999981514	0.999985752	0.999948228
HYAL1	0.999995916	0.999987083	0.999993442	0.999981514	0.999985752	0.999948228
ILF3	0.999995916	0.999987083	0.999993442	0.999981514	0.999985752	0.999948228
ITPR2	0.999995916	0.999987083	0.999993442	0.999981514	0.999985752	0.999948228
JAZF1	0.999995916	0.999987083	0.999993442	0.999981514	0.999985752	0.999948228
JPH1	0.999995916	0.999987083	0.999993442	0.999981514	0.999985752	0.999948228
JUN	0.999995916	0.999987083	0.999993442	0.999981514	0.999985752	0.999948228
KAT2B	0.999995916	0.999987083	0.999993442	0.999981514	0.999985752	0.999948228
KIAA1671	0.999995916	0.999987083	0.999993442	0.999981514	0.999985752	0.999948228
KLF10	0.999995916	0.999987083	0.999993442	0.999981514	0.999985752	0.999948228
KLRF2	0.999995916	0.999987083	0.999993442	0.999981514	0.999985752	0.999948228
LDHD	0.999995916	0.999987083	0.999993442	0.999981514	0.999985752	0.999948228
LENG1	0.999995916	0.999987083	0.999993442	0.999981514	0.999985752	0.999948228
LOC100139732	0.999995916	0.999987083	0.999993442	0.999981514	0.999985752	0.999948228
LOC100297616	0.999995916	0.999987083	0.999993442	0.999981514	0.999985752	0.999948228
LOC100299845	0.999995916	0.999987083	0.999993442	0.999981514	0.999985752	0.999948228

LOC100335205	0.999995916	0.999987083	0.999993442	0.999981514	0.999985752	0.999948228
LOC100847143	0.999995916	0.999987083	0.999993442	0.999981514	0.999985752	0.999948228
LOC100847374	0.999995916	0.999987083	0.999993442	0.999981514	0.999985752	0.999948228
LOC100848171	0.999995916	0.999987083	0.999993442	0.999981514	0.999985752	0.999948228
LOC100848357	0.999995916	0.999987083	0.999993442	0.999981514	0.999985752	0.999948228
LOC100848495	0.999995916	0.999987083	0.999993442	0.999981514	0.999985752	0.999948228
LOC100848985	0.999995916	0.999987083	0.999993442	0.999981514	0.999985752	0.999948228
LOC100849023	0.999995916	0.999987083	0.999993442	0.999981514	0.999985752	0.999948228
LOC101902907	0.999995916	0.999987083	0.999993442	0.999981514	0.999985752	0.999948228
LOC101903205	0.999995916	0.999987083	0.999993442	0.999981514	0.999985752	0.999948228
LOC101903572	0.999995916	0.999987083	0.999993442	0.999981514	0.999985752	0.999948228
LOC101904084	0.999995916	0.999987083	0.999993442	0.999981514	0.999985752	0.999948228
LOC101904156	0.999995916	0.999987083	0.999993442	0.999981514	0.999985752	0.999948228
LOC101904393	0.999995916	0.999987083	0.999993442	0.999981514	0.999985752	0.999948228
LOC101904413	0.999995916	0.999987083	0.999993442	0.999981514	0.999985752	0.999948228
LOC101905203	0.999995916	0.999987083	0.999993442	0.999981514	0.999985752	0.999948228
LOC101905493	0.999995916	0.999987083	0.999993442	0.999981514	0.999985752	0.999948228
LOC101905513	0.999995916	0.999987083	0.999993442	0.999981514	0.999985752	0.999948228
LOC101905771	0.999995916	0.999987083	0.999993442	0.999981514	0.999985752	0.999948228
LOC101907353	0.999995916	0.999987083	0.999993442	0.999981514	0.999985752	0.999948228
LOC101907404	0.999995916	0.999987083	0.999993442	0.999981514	0.999985752	0.999948228
LOC101908046	0.999995916	0.999987083	0.999993442	0.999981514	0.999985752	0.999948228
LOC101908577	0.999995916	0.999987083	0.999993442	0.999981514	0.999985752	0.999948228
LOC104969192	0.999995916	0.999987083	0.999993442	0.999981514	0.999985752	0.999948228
LOC104969259	0.999995916	0.999987083	0.999993442	0.999981514	0.999985752	0.999948228
LOC104971501	0.999995916	0.999987083	0.999993442	0.999981514	0.999985752	0.999948228
LOC104973390	0.999995916	0.999987083	0.999993442	0.999981514	0.999985752	0.999948228
LOC104974542	0.999995916	0.999987083	0.999993442	0.999981514	0.999985752	0.999948228
LOC104975286	0.999995916	0.999987083	0.999993442	0.999981514	0.999985752	0.999948228
LOC107131357	0.999995916	0.999987083	0.999993442	0.999981514	0.999985752	0.999948228
LOC107131452	0.999995916	0.999987083	0.999993442	0.999981514	0.999985752	0.999948228
LOC107131455	0.999995916	0.999987083	0.999993442	0.999981514	0.999985752	0.999948228
LOC107131615	0.999995916	0.999987083	0.999993442	0.999981514	0.999985752	0.999948228
LOC107132255	0.999995916	0.999987083	0.999993442	0.999981514	0.999985752	0.999948228
LOC107132849	0.999995916	0.999987083	0.999993442	0.999981514	0.999985752	0.999948228
LOC107132877	0.999995916	0.999987083	0.999993442	0.999981514	0.999985752	0.999948228
LOC112441568	0.999995916	0.999987083	0.999993442	0.999981514	0.999985752	0.999948228
LOC112441846	0.999995916	0.999987083	0.999993442	0.999981514	0.999985752	0.999948228

LOC112442038	0.999995916	0.999987083	0.999993442	0.999981514	0.999985752	0.999948228
LOC112442264	0.999995916	0.999987083	0.999993442	0.999981514	0.999985752	0.999948228
LOC112442702	0.999995916	0.999987083	0.999993442	0.999981514	0.999985752	0.999948228
LOC112444473	0.999995916	0.999987083	0.999993442	0.999981514	0.999985752	0.999948228
LOC112445951	0.999995916	0.999987083	0.999993442	0.999981514	0.999985752	0.999948228
LOC112446642	0.999995916	0.999987083	0.999993442	0.999981514	0.999985752	0.999948228
LOC112446822	0.999995916	0.999987083	0.999993442	0.999981514	0.999985752	0.999948228
LOC112447029	0.999995916	0.999987083	0.999993442	0.999981514	0.999985752	0.999948228
LOC112447323	0.999995916	0.999987083	0.999993442	0.999981514	0.999985752	0.999948228
LOC112447499	0.999995916	0.999987083	0.999993442	0.999981514	0.999985752	0.999948228
LOC112448390	0.999995916	0.999987083	0.999993442	0.999981514	0.999985752	0.999948228
LOC112448764	0.999995916	0.999987083	0.999993442	0.999981514	0.999985752	0.999948228
LOC112449247	0.999995916	0.999987083	0.999993442	0.999981514	0.999985752	0.999948228
LOC112449266	0.999995916	0.999987083	0.999993442	0.999981514	0.999985752	0.999948228
LOC508628	0.999995916	0.999987083	0.999993442	0.999981514	0.999985752	0.999948228
LOC615521	0.999995916	0.999987083	0.999993442	0.999981514	0.999985752	0.999948228
LOC616538	0.999995916	0.999987083	0.999993442	0.999981514	0.999985752	0.999948228
LOC618220	0.999995916	0.999987083	0.999993442	0.999981514	0.999985752	0.999948228
LOC781728	0.999995916	0.999987083	0.999993442	0.999981514	0.999985752	0.999948228
LOC782032	0.999995916	0.999987083	0.999993442	0.999981514	0.999985752	0.999948228
LOC782812	0.999995916	0.999987083	0.999993442	0.999981514	0.999985752	0.999948228
LOC783301	0.999995916	0.999987083	0.999993442	0.999981514	0.999985752	0.999948228
LOC785386	0.999995916	0.999987083	0.999993442	0.999981514	0.999985752	0.999948228
LOC785761	0.999995916	0.999987083	0.999993442	0.999981514	0.999985752	0.999948228
LOC788293	0.999995916	0.999987083	0.999993442	0.999981514	0.999985752	0.999948228
LOC789626	0.999995916	0.999987083	0.999993442	0.999981514	0.999985752	0.999948228
LOC789733	0.999995916	0.999987083	0.999993442	0.999981514	0.999985752	0.999948228
LOC789997	0.999995916	0.999987083	0.999993442	0.999981514	0.999985752	0.999948228
LRCH3	0.999995916	0.999987083	0.999993442	0.999981514	0.999985752	0.999948228
LRRC72	0.999995916	0.999987083	0.999993442	0.999981514	0.999985752	0.999948228
LRRC75A	0.999995916	0.999987083	0.999993442	0.999981514	0.999985752	0.999948228
MAPK7	0.999995916	0.999987083	0.999993442	0.999981514	0.999985752	0.999948228
MAPKAPK3	0.999995916	0.999987083	0.999993442	0.999981514	0.999985752	0.999948228
MARC2	0.999995916	0.999987083	0.999993442	0.999981514	0.999985752	0.999948228
MBTPS1	0.999995916	0.999987083	0.999993442	0.999981514	0.999985752	0.999948228
MCM7	0.999995916	0.999987083	0.999993442	0.999981514	0.999985752	0.999948228
MDFIC	0.999995916	0.999987083	0.999993442	0.999981514	0.999985752	0.999948228
MED22	0.999995916	0.999987083	0.999993442	0.999981514	0.999985752	0.999948228

MED26	0.999995916	0.999987083	0.999993442	0.999981514	0.999985752	0.999948228
MFAP4	0.999995916	0.999987083	0.999993442	0.999981514	0.999985752	0.999948228
MIGA1	0.999995916	0.999987083	0.999993442	0.999981514	0.999985752	0.999948228
MORC2	0.999995916	0.999987083	0.999993442	0.999981514	0.999985752	0.999948228
MORN4	0.999995916	0.999987083	0.999993442	0.999981514	0.999985752	0.999948228
MRGPRF	0.999995916	0.999987083	0.999993442	0.999981514	0.999985752	0.999948228
MRI1	0.999995916	0.999987083	0.999993442	0.999981514	0.999985752	0.999948228
MRPL48	0.999995916	0.999987083	0.999993442	0.999981514	0.999985752	0.999948228
MSL3	0.999995916	0.999987083	0.999993442	0.999981514	0.999985752	0.999948228
MTO1	0.999995916	0.999987083	0.999993442	0.999981514	0.999985752	0.999948228
MTOR	0.999995916	0.999987083	0.999993442	0.999981514	0.999985752	0.999948228
MYO1B	0.999995916	0.999987083	0.999993442	0.999981514	0.999985752	0.999948228
MYO1H	0.999995916	0.999987083	0.999993442	0.999981514	0.999985752	0.999948228
NADK2	0.999995916	0.999987083	0.999993442	0.999981514	0.999985752	0.999948228
NECTIN2	0.999995916	0.999987083	0.999993442	0.999981514	0.999985752	0.999948228
NME7	0.999995916	0.999987083	0.999993442	0.999981514	0.999985752	0.999948228
NOTCH1	0.999995916	0.999987083	0.999993442	0.999981514	0.999985752	0.999948228
NOVA2	0.999995916	0.999987083	0.999993442	0.999981514	0.999985752	0.999948228
NRBP1	0.999995916	0.999987083	0.999993442	0.999981514	0.999985752	0.999948228
NUCB1	0.999995916	0.999987083	0.999993442	0.999981514	0.999985752	0.999948228
NUFIP1	0.999995916	0.999987083	0.999993442	0.999981514	0.999985752	0.999948228
ODF2L	0.999995916	0.999987083	0.999993442	0.999981514	0.999985752	0.999948228
ORMDL1	0.999995916	0.999987083	0.999993442	0.999981514	0.999985752	0.999948228
OTUD7B	0.999995916	0.999987083	0.999993442	0.999981514	0.999985752	0.999948228
PABPC4L	0.999995916	0.999987083	0.999993442	0.999981514	0.999985752	0.999948228
PAQR5	0.999995916	0.999987083	0.999993442	0.999981514	0.999985752	0.999948228
PARP1	0.999995916	0.999987083	0.999993442	0.999981514	0.999985752	0.999948228
PCCB	0.999995916	0.999987083	0.999993442	0.999981514	0.999985752	0.999948228
PCDH9	0.999995916	0.999987083	0.999993442	0.999981514	0.999985752	0.999948228
PDE4DIP	0.999995916	0.999987083	0.999993442	0.999981514	0.999985752	0.999948228
PDE6A	0.999995916	0.999987083	0.999993442	0.999981514	0.999985752	0.999948228
PEG10	0.999995916	0.999987083	0.999993442	0.999981514	0.999985752	0.999948228
PIP4K2B	0.999995916	0.999987083	0.999993442	0.999981514	0.999985752	0.999948228
PKD2	0.999995916	0.999987083	0.999993442	0.999981514	0.999985752	0.999948228
PLCD4	0.999995916	0.999987083	0.999993442	0.999981514	0.999985752	0.999948228
PLPP3	0.999995916	0.999987083	0.999993442	0.999981514	0.999985752	0.999948228
PMP22	0.999995916	0.999987083	0.999993442	0.999981514	0.999985752	0.999948228
PPP1R3G	0.999995916	0.999987083	0.999993442	0.999981514	0.999985752	0.999948228

PPP4C	0.999995916	0.999987083	0.999993442	0.999981514	0.999985752	0.999948228
PRDM6	0.999995916	0.999987083	0.999993442	0.999981514	0.999985752	0.999948228
PRL	0.999995916	0.999987083	0.999993442	0.999981514	0.999985752	0.999948228
PROB1	0.999995916	0.999987083	0.999993442	0.999981514	0.999985752	0.999948228
PROSER1	0.999995916	0.999987083	0.999993442	0.999981514	0.999985752	0.999948228
PRPF6	0.999995916	0.999987083	0.999993442	0.999981514	0.999985752	0.999948228
PSD3	0.999995916	0.999987083	0.999993442	0.999981514	0.999985752	0.999948228
PYGB	0.999995916	0.999987083	0.999993442	0.999981514	0.999985752	0.999948228
RBAK	0.999995916	0.999987083	0.999993442	0.999981514	0.999985752	0.999948228
RC3H2	0.999995916	0.999987083	0.999993442	0.999981514	0.999985752	0.999948228
RDH14	0.999995916	0.999987083	0.999993442	0.999981514	0.999985752	0.999948228
REST	0.999995916	0.999987083	0.999993442	0.999981514	0.999985752	0.999948228
RNF8	0.999995916	0.999987083	0.999993442	0.999981514	0.999985752	0.999948228
RPA4	0.999995916	0.999987083	0.999993442	0.999981514	0.999985752	0.999948228
RPL10A	0.999995916	0.999987083	0.999993442	0.999981514	0.999985752	0.999948228
RPL11	0.999995916	0.999987083	0.999993442	0.999981514	0.999985752	0.999948228
RPL14	0.999995916	0.999987083	0.999993442	0.999981514	0.999985752	0.999948228
RPL17	0.999995916	0.999987083	0.999993442	0.999981514	0.999985752	0.999948228
RPL18	0.999995916	0.999987083	0.999993442	0.999981514	0.999985752	0.999948228
RPL27A	0.999995916	0.999987083	0.999993442	0.999981514	0.999985752	0.999948228
RPL29	0.999995916	0.999987083	0.999993442	0.999981514	0.999985752	0.999948228
RPL32	0.999995916	0.999987083	0.999993442	0.999981514	0.999985752	0.999948228
RPL34	0.999995916	0.999987083	0.999993442	0.999981514	0.999985752	0.999948228
RPL35	0.999995916	0.999987083	0.999993442	0.999981514	0.999985752	0.999948228
RPL37	0.999995916	0.999987083	0.999993442	0.999981514	0.999985752	0.999948228
RPL5	0.999995916	0.999987083	0.999993442	0.999981514	0.999985752	0.999948228
RPL6	0.999995916	0.999987083	0.999993442	0.999981514	0.999985752	0.999948228
RPRD1A	0.999995916	0.999987083	0.999993442	0.999981514	0.999985752	0.999948228
RPS11	0.999995916	0.999987083	0.999993442	0.999981514	0.999985752	0.999948228
RPS14	0.999995916	0.999987083	0.999993442	0.999981514	0.999985752	0.999948228
RPS16	0.999995916	0.999987083	0.999993442	0.999981514	0.999985752	0.999948228
RPS24	0.999995916	0.999987083	0.999993442	0.999981514	0.999985752	0.999948228
RPS27P	0.999995916	0.999987083	0.999993442	0.999981514	0.999985752	0.999948228
RPS5	0.999995916	0.999987083	0.999993442	0.999981514	0.999985752	0.999948228
SART1	0.999995916	0.999987083	0.999993442	0.999981514	0.999985752	0.999948228
SCAF1	0.999995916	0.999987083	0.999993442	0.999981514	0.999985752	0.999948228
SCARA3	0.999995916	0.999987083	0.999993442	0.999981514	0.999985752	0.999948228
SCARB1	0.999995916	0.999987083	0.999993442	0.999981514	0.999985752	0.999948228

SCG5	0.999995916	0.999987083	0.999993442	0.999981514	0.999985752	0.999948228
SF3B2	0.999995916	0.999987083	0.999993442	0.999981514	0.999985752	0.999948228
SH2D7	0.999995916	0.999987083	0.999993442	0.999981514	0.999985752	0.999948228
SH3RF3	0.999995916	0.999987083	0.999993442	0.999981514	0.999985752	0.999948228
SLC35D2	0.999995916	0.999987083	0.999993442	0.999981514	0.999985752	0.999948228
SLC35E2	0.999995916	0.999987083	0.999993442	0.999981514	0.999985752	0.999948228
SLC35E4	0.999995916	0.999987083	0.999993442	0.999981514	0.999985752	0.999948228
SLC36A1	0.999995916	0.999987083	0.999993442	0.999981514	0.999985752	0.999948228
SLC37A4	0.999995916	0.999987083	0.999993442	0.999981514	0.999985752	0.999948228
SLC7A6OS	0.999995916	0.999987083	0.999993442	0.999981514	0.999985752	0.999948228
SLX4IP	0.999995916	0.999987083	0.999993442	0.999981514	0.999985752	0.999948228
SMIM15	0.999995916	0.999987083	0.999993442	0.999981514	0.999985752	0.999948228
SMOC1	0.999995916	0.999987083	0.999993442	0.999981514	0.999985752	0.999948228
SMPD5	0.999995916	0.999987083	0.999993442	0.999981514	0.999985752	0.999948228
SPIN2	0.999995916	0.999987083	0.999993442	0.999981514	0.999985752	0.999948228
SPIN2B	0.999995916	0.999987083	0.999993442	0.999981514	0.999985752	0.999948228
SREBF2	0.999995916	0.999987083	0.999993442	0.999981514	0.999985752	0.999948228
SRI	0.999995916	0.999987083	0.999993442	0.999981514	0.999985752	0.999948228
SRM	0.999995916	0.999987083	0.999993442	0.999981514	0.999985752	0.999948228
SSR2	0.999995916	0.999987083	0.999993442	0.999981514	0.999985752	0.999948228
SSRP1	0.999995916	0.999987083	0.999993442	0.999981514	0.999985752	0.999948228
ST6GALNAC5	0.999995916	0.999987083	0.999993442	0.999981514	0.999985752	0.999948228
SUN1	0.999995916	0.999987083	0.999993442	0.999981514	0.999985752	0.999948228
SUPT5H	0.999995916	0.999987083	0.999993442	0.999981514	0.999985752	0.999948228
SWAP70	0.999995916	0.999987083	0.999993442	0.999981514	0.999985752	0.999948228
SYCE1L	0.999995916	0.999987083	0.999993442	0.999981514	0.999985752	0.999948228
SYT12	0.999995916	0.999987083	0.999993442	0.999981514	0.999985752	0.999948228
TAPBP	0.999995916	0.999987083	0.999993442	0.999981514	0.999985752	0.999948228
TASP1	0.999995916	0.999987083	0.999993442	0.999981514	0.999985752	0.999948228
TBC1D25	0.999995916	0.999987083	0.999993442	0.999981514	0.999985752	0.999948228
TBCK	0.999995916	0.999987083	0.999993442	0.999981514	0.999985752	0.999948228
TCF12	0.999995916	0.999987083	0.999993442	0.999981514	0.999985752	0.999948228
TCN2	0.999995916	0.999987083	0.999993442	0.999981514	0.999985752	0.999948228
TCTN1	0.999995916	0.999987083	0.999993442	0.999981514	0.999985752	0.999948228
TEK	0.999995916	0.999987083	0.999993442	0.999981514	0.999985752	0.999948228
TFPT	0.999995916	0.999987083	0.999993442	0.999981514	0.999985752	0.999948228
TG	0.999995916	0.999987083	0.999993442	0.999981514	0.999985752	0.999948228
TM7SF3	0.999995916	0.999987083	0.999993442	0.999981514	0.999985752	0.999948228

TM9SF4	0.999995916	0.999987083	0.999993442	0.999981514	0.999985752	0.999948228
TMLHE	0.999995916	0.999987083	0.999993442	0.999981514	0.999985752	0.999948228
TNNI3	0.999995916	0.999987083	0.999993442	0.999981514	0.999985752	0.999948228
TRIQK	0.999995916	0.999987083	0.999993442	0.999981514	0.999985752	0.999948228
TRMT2B	0.999995916	0.999987083	0.999993442	0.999981514	0.999985752	0.999948228
TSEN2	0.999995916	0.999987083	0.999993442	0.999981514	0.999985752	0.999948228
TSPAN12	0.999995916	0.999987083	0.999993442	0.999981514	0.999985752	0.999948228
TSPAN17	0.999995916	0.999987083	0.999993442	0.999981514	0.999985752	0.999948228
TULP4	0.999995916	0.999987083	0.999993442	0.999981514	0.999985752	0.999948228
UBAC2	0.999995916	0.999987083	0.999993442	0.999981514	0.999985752	0.999948228
UBE2D1	0.999995916	0.999987083	0.999993442	0.999981514	0.999985752	0.999948228
UBE2L6	0.999995916	0.999987083	0.999993442	0.999981514	0.999985752	0.999948228
VPS54	0.999995916	0.999987083	0.999993442	0.999981514	0.999985752	0.999948228
VWA1	0.999995916	0.999987083	0.999993442	0.999981514	0.999985752	0.999948228
WASHC5	0.999995916	0.999987083	0.999993442	0.999981514	0.999985752	0.999948228
WDR25	0.999995916	0.999987083	0.999993442	0.999981514	0.999985752	0.999948228
WDYHV1	0.999995916	0.999987083	0.999993442	0.999981514	0.999985752	0.999948228
WRNIP1	0.999995916	0.999987083	0.999993442	0.999981514	0.999985752	0.999948228
XKR4	0.999995916	0.999987083	0.999993442	0.999981514	0.999985752	0.999948228
XRCC2	0.999995916	0.999987083	0.999993442	0.999981514	0.999985752	0.999948228
YIPF3	0.999995916	0.999987083	0.999993442	0.999981514	0.999985752	0.999948228
ZBTB41	0.999995916	0.999987083	0.999993442	0.999981514	0.999985752	0.999948228
ZC3H12A	0.999995916	0.999987083	0.999993442	0.999981514	0.999985752	0.999948228
ZC3HAV1L	0.999995916	0.999987083	0.999993442	0.999981514	0.999985752	0.999948228
ZFP37	0.999995916	0.999987083	0.999993442	0.999981514	0.999985752	0.999948228
ZNF281	0.999995916	0.999987083	0.999993442	0.999981514	0.999985752	0.999948228
ZNF787	0.999995916	0.999987083	0.999993442	0.999981514	0.999985752	0.999948228
ZNF821	0.999995916	0.999987083	0.999993442	0.999981514	0.999985752	0.999948228
DHX38	0.999995916	0.99960614	0.99999468	0.999981514	0.999985752	0.999948228
LOC112446417	0.999995916	0.999987083	0.99999468	0.999981514	0.999985752	0.999948228
ITGAL	0.999995916	0.999987083	0.999993442	0.999981514	0.999985752	0.999984586
RPL10	0.999995916	0.999987083	0.999993442	0.999981514	0.999985752	0.999984586
SLC4A4	0.999995916	0.999987083	0.999993442	0.999981514	0.999985752	0.999984586
LOC100848138	0.999995916	0.999987083	0.99999468	0.999981514	0.999985752	0.999984586
ZNF461	0.999995916	0.999987083	0.99999468	0.999981514	0.999985752	0.999984586
REC8	NaN	NaN	NaN	NaN	NaN	0.050890427
LOC508459	NaN	NaN	NaN	NaN	NaN	0.289777696
CXCL11	NaN	NaN	NaN	NaN	NaN	0.300134855

SVOP	NaN	NaN	NaN	NaN	NaN	0.521756022
TMEM216	NaN	NaN	NaN	NaN	NaN	0.522289438
LOC101901948	NaN	NaN	NaN	NaN	NaN	0.539240716
SPCS3	NaN	NaN	NaN	NaN	NaN	0.575524376
AMZ1	NaN	NaN	NaN	NaN	NaN	0.595899153
CD5L	NaN	NaN	NaN	NaN	NaN	0.604469389
LCN2	NaN	NaN	NaN	NaN	NaN	0.623319115
RHOD	NaN	NaN	NaN	NaN	NaN	0.634079576
ST8SIA2	NaN	NaN	NaN	NaN	NaN	0.700123604
LOC784266	NaN	NaN	NaN	NaN	NaN	0.772959565
LOC100300115	NaN	NaN	NaN	NaN	NaN	0.784381044
SOCS3	NaN	NaN	NaN	NaN	NaN	0.85643253
LOC112444652	NaN	NaN	NaN	NaN	NaN	0.864572584
GOLGA7B	NaN	NaN	NaN	NaN	NaN	0.867063767
SPTB	NaN	NaN	NaN	NaN	NaN	0.893838339
LOC112443862	NaN	NaN	NaN	NaN	NaN	0.923199665
TIMD4	NaN	NaN	NaN	NaN	NaN	0.924740534
SLC9A2	NaN	NaN	NaN	NaN	NaN	0.927426708
FOSL1	NaN	NaN	NaN	NaN	NaN	0.93363377
CR2	NaN	NaN	NaN	NaN	NaN	0.936263602
MT1A	NaN	NaN	NaN	NaN	NaN	0.940017687
ACP5	NaN	NaN	NaN	NaN	NaN	0.944330889
LGR5	NaN	NaN	NaN	NaN	NaN	0.959723327
SECTM1A	NaN	NaN	NaN	NaN	NaN	0.995275602
CCR9	NaN	NaN	NaN	NaN	NaN	0.999948228
ADAMTS19	NaN	NaN	NaN	NaN	0.011007321	NaN
OSM	NaN	NaN	NaN	NaN	0.050089145	NaN
IL1B	NaN	NaN	NaN	NaN	0.19915908	NaN
LOC101905509	NaN	NaN	NaN	NaN	0.20654875	NaN
ADAM8	NaN	NaN	NaN	NaN	0.228803825	NaN
CXCL8	NaN	NaN	NaN	NaN	0.292046886	NaN
SAMD9	NaN	NaN	NaN	NaN	0.295929564	NaN
SLC35F1	NaN	NaN	NaN	NaN	0.309564851	NaN
DPP10	NaN	NaN	NaN	NaN	0.351955331	NaN
TRIB3	NaN	NaN	NaN	NaN	0.426006781	NaN
C19H17orf58	NaN	NaN	NaN	NaN	0.429015291	NaN
LOC505033	NaN	NaN	NaN	NaN	0.461235361	NaN
PDZRN4	NaN	NaN	NaN	NaN	0.47282425	NaN

ACBD7	NaN	NaN	NaN	NaN	0.510001595	NaN
GRIK4	NaN	NaN	NaN	NaN	0.568817116	NaN
PCDH10	NaN	NaN	NaN	NaN	0.56959555	NaN
LOC100848536	NaN	NaN	NaN	NaN	0.578333624	NaN
IGSF9B	NaN	NaN	NaN	NaN	0.610761857	NaN
PLP1	NaN	NaN	NaN	NaN	0.621029742	NaN
ART3	NaN	NaN	NaN	NaN	0.626796691	NaN
IGSF11	NaN	NaN	NaN	NaN	0.652333594	NaN
SOX10	NaN	NaN	NaN	NaN	0.658667583	NaN
IFIH1	NaN	NaN	NaN	NaN	0.665670485	NaN
MPZ	NaN	NaN	NaN	NaN	0.670221463	NaN
COL2A1	NaN	NaN	NaN	NaN	0.690687154	NaN
MX1	NaN	NaN	NaN	NaN	0.695436567	NaN
LOC101902787	NaN	NaN	NaN	NaN	0.69606091	NaN
TNFRSF11B	NaN	NaN	NaN	NaN	0.696466814	NaN
FOXD1	NaN	NaN	NaN	NaN	0.721826916	NaN
CADM4	NaN	NaN	NaN	NaN	0.743260392	NaN
OAS1Y	NaN	NaN	NaN	NaN	0.753088486	NaN
CDH19	NaN	NaN	NaN	NaN	0.788007619	NaN
LOC100298356	NaN	NaN	NaN	NaN	0.788007619	NaN
SNCA	NaN	NaN	NaN	NaN	0.788790565	NaN
LOC112441507	NaN	NaN	NaN	NaN	0.811991102	NaN
CXHXorf57	NaN	NaN	NaN	NaN	0.881680572	NaN
LOC112441557	NaN	NaN	NaN	NaN	0.886944613	NaN
DDX58	NaN	NaN	NaN	NaN	0.887514169	NaN
LOC509283	NaN	NaN	NaN	NaN	0.887514169	NaN
TTYH1	NaN	NaN	NaN	NaN	0.899042139	NaN
ISG15	NaN	NaN	NaN	NaN	0.902907077	NaN
FOXD3	NaN	NaN	NaN	NaN	0.907094634	NaN
CCL17	NaN	NaN	NaN	NaN	0.912691379	NaN
DEFB7	NaN	NaN	NaN	NaN	0.927086179	NaN
RSAD2	NaN	NaN	NaN	NaN	0.929630092	NaN
JAKMIP2	NaN	NaN	NaN	NaN	0.935286646	NaN
SGCG	NaN	NaN	NaN	NaN	0.951918183	NaN
MMP12	NaN	NaN	NaN	NaN	0.960573822	NaN
DNAH9	NaN	NaN	NaN	NaN	0.961465747	NaN
SPOCK1	NaN	NaN	NaN	NaN	0.962166847	NaN
WNT6	NaN	NaN	NaN	NaN	0.964301327	NaN

SNAP25	NaN	NaN	NaN	NaN	0.964833865	NaN
FREM2	NaN	NaN	NaN	NaN	0.972128973	NaN
LUZP2	NaN	NaN	NaN	NaN	0.973219333	NaN
SCUBE1	NaN	NaN	NaN	NaN	0.973430139	NaN
LGI1	NaN	NaN	NaN	NaN	0.974403222	NaN
BOLA	NaN	NaN	NaN	NaN	0.983941532	NaN
LOC100847981	NaN	NaN	NaN	NaN	0.98865576	NaN
LOC507055	NaN	NaN	NaN	NaN	0.993461941	NaN
GYS2	NaN	NaN	NaN	NaN	0.999251855	NaN
GABRB3	NaN	NaN	NaN	NaN	0.999985752	NaN
TMPRSS5	NaN	NaN	NaN	NaN	0.999985752	NaN
LOC101905242	NaN	NaN	NaN	0.014457074	NaN	NaN
G6PD	NaN	NaN	NaN	0.051654146	NaN	NaN
TGM5	NaN	NaN	NaN	0.168856518	NaN	NaN
CKB	NaN	NaN	NaN	0.223420961	NaN	NaN
LOC616830	NaN	NaN	NaN	0.284249009	NaN	NaN
PPM1E	NaN	NaN	NaN	0.297696608	NaN	NaN
MID1IP1	NaN	NaN	NaN	0.30124858	NaN	NaN
TDH	NaN	NaN	NaN	0.421668661	NaN	NaN
SNRNP25	NaN	NaN	NaN	0.451094074	NaN	NaN
SPRY3	NaN	NaN	NaN	0.451094074	NaN	NaN
YDJC	NaN	NaN	NaN	0.481277757	NaN	NaN
TKT	NaN	NaN	NaN	0.486381297	NaN	NaN
LOC104973965	NaN	NaN	NaN	0.50474615	NaN	NaN
ELOVL6	NaN	NaN	NaN	0.527327473	NaN	NaN
SLC16A1	NaN	NaN	NaN	0.527867968	NaN	NaN
BOLA3	NaN	NaN	NaN	0.561120526	NaN	NaN
LOC104968484	NaN	NaN	NaN	0.598663987	NaN	NaN
HMOX1	NaN	NaN	NaN	0.630535638	NaN	NaN
KIF2C	NaN	NaN	NaN	0.630951216	NaN	NaN
ARHGAP11A	NaN	NaN	NaN	0.653866049	NaN	NaN
FCGR2A	NaN	NaN	NaN	0.656158567	NaN	NaN
MLXIPL	NaN	NaN	NaN	0.68362796	NaN	NaN
DPP4	NaN	NaN	NaN	0.71113156	NaN	NaN
LAMC3	NaN	NaN	NaN	0.718713712	NaN	NaN
LOC101907335	NaN	NaN	NaN	0.721877283	NaN	NaN
MPC1	NaN	NaN	NaN	0.729724066	NaN	NaN
UCHL3	NaN	NaN	NaN	0.736045965	NaN	NaN

GSS	NaN	NaN	NaN	0.764257755	NaN	NaN
SLC25A4	NaN	NaN	NaN	0.765813436	NaN	NaN
TECTB	NaN	NaN	NaN	0.768644469	NaN	NaN
SLC16A7	NaN	NaN	NaN	0.76992117	NaN	NaN
SRXN1	NaN	NaN	NaN	0.773091002	NaN	NaN
LOC100138641	NaN	NaN	NaN	0.774800098	NaN	NaN
BSG	NaN	NaN	NaN	0.780279943	NaN	NaN
BIRC5	NaN	NaN	NaN	0.789139053	NaN	NaN
KIF20A	NaN	NaN	NaN	0.799147532	NaN	NaN
GSTM1	NaN	NaN	NaN	0.799480811	NaN	NaN
PIR	NaN	NaN	NaN	0.805499732	NaN	NaN
SERHL2	NaN	NaN	NaN	0.812354594	NaN	NaN
LIN7A	NaN	NaN	NaN	0.812453944	NaN	NaN
LOC783106	NaN	NaN	NaN	0.817511717	NaN	NaN
ABAT	NaN	NaN	NaN	0.819968583	NaN	NaN
CDCA8	NaN	NaN	NaN	0.820971736	NaN	NaN
GPT2	NaN	NaN	NaN	0.822691957	NaN	NaN
UBE2C	NaN	NaN	NaN	0.82388508	NaN	NaN
PARD6B	NaN	NaN	NaN	0.827600292	NaN	NaN
TRIM44	NaN	NaN	NaN	0.828736799	NaN	NaN
KCNN2	NaN	NaN	NaN	0.830187312	NaN	NaN
FAM49B	NaN	NaN	NaN	0.84312441	NaN	NaN
KLHL31	NaN	NaN	NaN	0.852170016	NaN	NaN
TMEM179	NaN	NaN	NaN	0.854220165	NaN	NaN
CDC6	NaN	NaN	NaN	0.854450063	NaN	NaN
MARCO	NaN	NaN	NaN	0.854966424	NaN	NaN
FGB	NaN	NaN	NaN	0.85981779	NaN	NaN
KCNK2	NaN	NaN	NaN	0.86458365	NaN	NaN
PCLAF	NaN	NaN	NaN	0.870210705	NaN	NaN
ACOT2	NaN	NaN	NaN	0.890362631	NaN	NaN
LOC112448034	NaN	NaN	NaN	0.893051204	NaN	NaN
CERS3	NaN	NaN	NaN	0.893220599	NaN	NaN
LOC618297	NaN	NaN	NaN	0.893220599	NaN	NaN
SLC13A3	NaN	NaN	NaN	0.913048575	NaN	NaN
SCP2	NaN	NaN	NaN	0.91569235	NaN	NaN
CYP46A1	NaN	NaN	NaN	0.918693944	NaN	NaN
ALB	NaN	NaN	NaN	0.928075638	NaN	NaN
KCNA4	NaN	NaN	NaN	0.93048026	NaN	NaN

CATHL5	NaN	NaN	NaN	0.930882136	NaN	NaN
RRM2	NaN	NaN	NaN	0.952247001	NaN	NaN
KIF11	NaN	NaN	NaN	0.958600841	NaN	NaN
CCDC73	NaN	NaN	NaN	0.958951038	NaN	NaN
LOC112443013	NaN	NaN	NaN	0.966055368	NaN	NaN
BUB1B	NaN	NaN	NaN	0.975351263	NaN	NaN
UCMA	NaN	NaN	NaN	0.978404889	NaN	NaN
LDHB	NaN	NaN	NaN	0.978503042	NaN	NaN
PTPRQ	NaN	NaN	NaN	0.982220679	NaN	NaN
UHRF1	NaN	NaN	NaN	0.982220679	NaN	NaN
PBLD	NaN	NaN	NaN	0.988951957	NaN	NaN
LOC100301224	NaN	NaN	NaN	0.98981682	NaN	NaN
LOC100851369	NaN	NaN	NaN	0.993481918	NaN	NaN
SH3RF2	NaN	NaN	NaN	0.997467114	NaN	NaN
ATP10B	NaN	NaN	NaN	0.999981514	NaN	NaN
CDKN2A	NaN	NaN	NaN	0.999981514	NaN	NaN
COBL	NaN	NaN	NaN	0.999981514	NaN	NaN
CYP21	NaN	NaN	NaN	0.999981514	NaN	NaN
IRX1	NaN	NaN	NaN	0.999981514	NaN	NaN
LOC104974444	NaN	NaN	NaN	0.999981514	NaN	NaN
LOC112445090	NaN	NaN	NaN	0.999981514	NaN	NaN
LOC513210	NaN	NaN	NaN	0.999981514	NaN	NaN
PTCH2	NaN	NaN	NaN	0.999981514	NaN	NaN
SLC5A9	NaN	NaN	NaN	0.999981514	NaN	NaN
LOC100847119	NaN	NaN	0.040855823	NaN	NaN	NaN
HP	NaN	NaN	0.071338954	NaN	NaN	NaN
LOC100297192	NaN	NaN	0.142262294	NaN	NaN	NaN
FAM83D	NaN	NaN	0.151095791	NaN	NaN	NaN
LOC112442062	NaN	NaN	0.17678075	NaN	NaN	NaN
HPSE2	NaN	NaN	0.315377577	NaN	NaN	NaN
PRSS35	NaN	NaN	0.333951286	NaN	NaN	NaN
PTGIR	NaN	NaN	0.457350984	NaN	NaN	NaN
MUSK	NaN	NaN	0.461305553	NaN	NaN	NaN
EEF1A2	NaN	NaN	0.561623293	NaN	NaN	NaN
LOC100847724	NaN	NaN	0.570681722	NaN	NaN	NaN
COL24A1	NaN	NaN	0.734143952	NaN	NaN	NaN
P2RX1	NaN	NaN	0.736435559	NaN	NaN	NaN
SIX2	NaN	NaN	0.736435559	NaN	NaN	NaN

MAPK4	NaN	NaN	0.744735369	NaN	NaN	NaN
LOC104976942	NaN	NaN	0.767540961	NaN	NaN	NaN
RASL12	NaN	NaN	0.791153417	NaN	NaN	NaN
A2ML1	NaN	NaN	0.871782109	NaN	NaN	NaN
LOC112447816	NaN	NaN	0.881522424	NaN	NaN	NaN
LOC104974455	NaN	NaN	0.881778842	NaN	NaN	NaN
DMRT3	NaN	NaN	0.892343042	NaN	NaN	NaN
ACTA1	NaN	NaN	0.89478142	NaN	NaN	NaN
HAND2	NaN	NaN	0.89478142	NaN	NaN	NaN
LOC509911	NaN	NaN	0.908072171	NaN	NaN	NaN
OPCML	NaN	NaN	0.914256466	NaN	NaN	NaN
NPFFR2	NaN	NaN	0.916038418	NaN	NaN	NaN
LOC511683	NaN	NaN	0.947176871	NaN	NaN	NaN
C10H15orf62	NaN	NaN	0.957052938	NaN	NaN	NaN
ADRA2C	NaN	NaN	0.964661607	NaN	NaN	NaN
LOC112441777	NaN	NaN	0.964807927	NaN	NaN	NaN
LOC785161	NaN	NaN	0.967696262	NaN	NaN	NaN
LOC100847415	NaN	NaN	0.984886031	NaN	NaN	NaN
PHF21B	NaN	NaN	0.988660944	NaN	NaN	NaN
LOC516421	NaN	NaN	0.996224523	NaN	NaN	NaN
LOC112446726	NaN	NaN	0.999993442	NaN	NaN	NaN
PPDPFL	NaN	NaN	0.999993442	NaN	NaN	NaN
DNAJB13	NaN	0.0053959	NaN	NaN	NaN	NaN
LOC514978	NaN	0.007854385	NaN	NaN	NaN	NaN
LOC781796	NaN	0.178293122	NaN	NaN	NaN	NaN
RIPK4	NaN	0.188051682	NaN	NaN	NaN	NaN
PLA2G2D1	NaN	0.225929217	NaN	NaN	NaN	NaN
LOC101906743	NaN	0.280306881	NaN	NaN	NaN	NaN
LOC789829	NaN	0.338196075	NaN	NaN	NaN	NaN
TMPRSS2	NaN	0.366629388	NaN	NaN	NaN	NaN
FZD5	NaN	0.447450909	NaN	NaN	NaN	NaN
CDH17	NaN	0.469660184	NaN	NaN	NaN	NaN
LOC112447079	NaN	0.474946062	NaN	NaN	NaN	NaN
PDZK1	NaN	0.506509735	NaN	NaN	NaN	NaN
CHGA	NaN	0.528882488	NaN	NaN	NaN	NaN
CLDN3	NaN	0.534301627	NaN	NaN	NaN	NaN
MISP	NaN	0.542391346	NaN	NaN	NaN	NaN
VIL1	NaN	0.54845263	NaN	NaN	NaN	NaN

CA12	NaN	0.589782216	NaN	NaN	NaN	NaN
LTF	NaN	0.60754196	NaN	NaN	NaN	NaN
SLC6A8	NaN	0.634752242	NaN	NaN	NaN	NaN
CRB2	NaN	0.642273994	NaN	NaN	NaN	NaN
OAS1Z	NaN	0.651269643	NaN	NaN	NaN	NaN
APOA4	NaN	0.664911321	NaN	NaN	NaN	NaN
CHAD	NaN	0.674524738	NaN	NaN	NaN	NaN
LOC104974214	NaN	0.674586986	NaN	NaN	NaN	NaN
BCL2L15	NaN	0.697306013	NaN	NaN	NaN	NaN
CKMT1A	NaN	0.697306013	NaN	NaN	NaN	NaN
GRIA2	NaN	0.722968232	NaN	NaN	NaN	NaN
PLEK2	NaN	0.760623317	NaN	NaN	NaN	NaN
MYO1A	NaN	0.775033799	NaN	NaN	NaN	NaN
ST14	NaN	0.7973731	NaN	NaN	NaN	NaN
CCL5	NaN	0.816369086	NaN	NaN	NaN	NaN
FAM3B	NaN	0.834086942	NaN	NaN	NaN	NaN
KIAA1211L	NaN	0.844170799	NaN	NaN	NaN	NaN
LOC616782	NaN	0.850337973	NaN	NaN	NaN	NaN
HNF4G	NaN	0.863613561	NaN	NaN	NaN	NaN
LOC100140226	NaN	0.910651459	NaN	NaN	NaN	NaN
ALDOB	NaN	0.91078662	NaN	NaN	NaN	NaN
LOC101903734	NaN	0.91287376	NaN	NaN	NaN	NaN
GUCY2C	NaN	0.916484772	NaN	NaN	NaN	NaN
UCP1	NaN	0.918234392	NaN	NaN	NaN	NaN
BAIAP2L2	NaN	0.922285873	NaN	NaN	NaN	NaN
SLC9A3	NaN	0.927853707	NaN	NaN	NaN	NaN
SLC15A1	NaN	0.936822825	NaN	NaN	NaN	NaN
LOC100139885	NaN	0.95038008	NaN	NaN	NaN	NaN
SLC51B	NaN	0.969051025	NaN	NaN	NaN	NaN
DSG2	NaN	0.980289052	NaN	NaN	NaN	NaN
TUBA1D	NaN	0.992144201	NaN	NaN	NaN	NaN
LOC530653	NaN	0.998847958	NaN	NaN	NaN	NaN
TMEM45B	NaN	0.99960614	NaN	NaN	NaN	NaN
LOC781736	NaN	0.999987083	NaN	NaN	NaN	NaN
ACTN2	NaN	NaN	NaN	NaN	NaN	NaN
C4BPA	NaN	NaN	NaN	NaN	NaN	NaN
CA13	NaN	NaN	NaN	NaN	NaN	NaN
CFHR5	NaN	NaN	NaN	NaN	NaN	NaN

FCGBP	NaN	NaN	NaN	NaN	NaN	NaN
GSTA3	NaN	NaN	NaN	NaN	NaN	NaN
IGLL1	NaN	NaN	NaN	NaN	NaN	NaN
IGSF5	NaN	NaN	NaN	NaN	NaN	NaN
LOC100139670	NaN	NaN	NaN	NaN	NaN	NaN
LOC100297779	NaN	NaN	NaN	NaN	NaN	NaN
LOC101903284	NaN	NaN	NaN	NaN	NaN	NaN
LOC107131864	NaN	NaN	NaN	NaN	NaN	NaN
LOC107131942	NaN	NaN	NaN	NaN	NaN	NaN
LOC112446680	NaN	NaN	NaN	NaN	NaN	NaN
LOC515676	NaN	NaN	NaN	NaN	NaN	NaN
LOC519274	NaN	NaN	NaN	NaN	NaN	NaN
LOC615051	NaN	NaN	NaN	NaN	NaN	NaN
MEF2B	NaN	NaN	NaN	NaN	NaN	NaN
MEGF11	NaN	NaN	NaN	NaN	NaN	NaN
MOXD1	NaN	NaN	NaN	NaN	NaN	NaN
MX2	NaN	NaN	NaN	NaN	NaN	NaN
NAALADL1	NaN	NaN	NaN	NaN	NaN	NaN
PEBP4	NaN	NaN	NaN	NaN	NaN	NaN
PKD2L1	NaN	NaN	NaN	NaN	NaN	NaN
SPHAR	NaN	NaN	NaN	NaN	NaN	NaN
ZBTB37	NaN	NaN	NaN	NaN	NaN	NaN
ZDHHC23	NaN	NaN	NaN	NaN	NaN	NaN
