# Supplementary material for: Genes associated with body weight gain and feed intake identified by meta-analysis of the mesenteric fat from crossbred beef steers
Source: PLoS One. 2020 Jan 7;15(1):e0227154. doi: 10.1371/journal.pone.0227154 (PMC6946124; doi:10.1371/journal.pone.0227154)
Supplement: S5 Table — Genes with all gray cell indicate those that were excluded because they were also significant for the gain by intake interaction term. Jackknife 1 P-value gives the adjusted P-value for the meta-analysis with Cohort 1 removed, Jackknife 2 P-value gives the adjusted P-value for the meta-analysis with Cohort 2 removed, and so on. Yellow cells indicate jackknife analyses where the P-value was insignificant, i.e. the gene failed to pass the jackknife analysis. (PDF) [file pone.0227154.s005.pdf]

Supplemental Table 5. Jackknife sensitivity analysis results for the DEGs associated with the intake main effect. Genes with all gray cell indicate those that were excluded because they were also significant for the gain by intake interaction term.
Yellow cells indicate jackknife analyses where the P-value was insignificant, i.e. the gene failed to pass the jackknife analysis.

Gene	Original P-value	Jackknife 1 P-value	Jackknife 2 P-value	Jackknife 3 P-value	Jackknife 4 P-value	Jackknife 5 P-value
IL18BP	8.71E-06	6.19E-06	9.17E-06	0.200374485	7.09E-06	0.004758767
LOC100300716	0.000205934	0.0121771	0.003197268	0.028927806	0.000269228	0.000130204
LOC112445144	0.000239889	0.041885134	0.000397415	0.002374853	0.059731166	0.000130204
NF2	0.001391992	0.000840006	0.000490689	0.011172292	0.961025202	0.000599597
ECE2	0.001743624	0.000881596	0.001234305	0.380156582	0.148879636	0.000928475
PRMT7	0.001743624	0.000840006	0.000775139	0.002374853	0.999985264	0.001554213
FST	0.001743624	0.184684932	0.002023609	0.002374853	0.002450456	0.020372329
JCHAIN	0.002484096	0.06204226	0.00533377	0.034171633	0.023101833	0.001560003
SPP1	0.002662926	0.002366327	0.001416493	0.385729122	0.001756541	0.110293052
SOBP	0.002809374	0.003692807	0.001738579	0.003535991	0.999985264	0.00136681
RIPK1	0.006251633	0.008218071	0.002271573	0.004207086	0.999985264	0.003027093
LY86	0.006579026	0.026637122	0.005537689	0.930183705	0.004176353	0.003027093
MRPL14	0.006579026	0.128444845	0.004079917	0.009961635	0.241792949	0.006171092
AACS	0.006579026	0.003692807	0.002023609	0.011172292	0.798938088	0.025327961
PTAFR	0.009117906	0.008763173	0.002830426	0.434318747	0.050009811	0.054460007
LOC782367	0.009403144	0.243601706	0.013894604	0.011172292	0.023101833	0.029645073
PTRH1	0.012202208	0.00600424	0.003701636	0.022306326	0.999985264	0.024390419
PDLIM5	0.01975543	0.018253496	0.720073352	0.011172292	0.083746014	0.007423908
GCAT	0.01975543	0.012922093	0.010770179	0.034171633	0.999985264	0.012261974
CCDC117	0.019841436	0.013902766	0.010173579	0.011172292	0.999985264	0.009067233
CPM	0.019841436	0.010039246	0.011558904	0.949602784	0.023101833	0.051425394
H19	0.020627354	0.092518197	0.145502901	0.035639866	0.089222316	0.012261974
HPS6	0.025659453	0.013902766	0.011603341	0.011172292	0.999985264	0.034049188
LOC101907219	0.025659453	0.012495605	0.012784634	0.560163102	0.114093013	0.062818891
CD5	0.025659453	0.134639957	0.021126817	0.019220526	0.199437132	0.062818891
DHRS9	0.027237602	0.015496274	0.010770179	0.953188062	0.072735116	0.033542566
LOC112441663	0.031216637	0.018253496	0.010770179	0.99999488	0.104838607	0.012577925
B3GAT1	0.032213091	0.041885134	0.010770179	0.99999488	0.050009811	0.012577925
GLCE	0.032213091	0.013902766	0.011558904	0.99999488	0.078042948	0.012577925
LOC100138922	0.033305234	0.014689652	0.010770179	0.99999488	0.173708236	0.012577925
LYZ	0.033305234	0.018253496	0.026124628	0.963012661	0.092922399	0.018173669
RCOR2	0.033305234	0.028625215	0.012784634	0.034171633	0.847202014	0.076677748
CD53	0.035678354	0.031665173	0.019253086	0.99999488	0.061691969	0.020692759
SRPX	0.040269792	0.070147783	0.062519886	0.056802676	0.568311949	0.020372329
NOG	0.050749425	0.022957953	0.733964676	0.111496006	0.089222316	0.025327961

HOXA9	0.052019736	0.018253496	0.405170393	0.102213782	0.323041702	0.02182385
ITGB8	0.052142675	0.035643893	0.401122009	0.139713122	0.072735116	0.083941866
PYCR2	0.052816256	0.05930047	0.021126817	0.034171633	0.999985264	0.020692759
GSTT2	0.052816256	0.042345617	0.045210516	0.035531913	0.999985264	0.029645073
EPM2AIP1	0.052816256	0.026637122	0.021126817	0.812057455	0.258000341	0.038781731
DLK1	0.062885086	0.040606228	0.291592084	0.035007216	0.677061328	0.024390419
RGS12	0.062885086	0.026637122	0.058278897	0.99999488	0.078042948	0.029281471
CLEC5A	0.067443872	0.041885134	0.03937657	0.99999488	0.089222316	0.026598
BTAF1	0.067443872	0.042345617	0.021126817	0.034171633	0.999985264	0.028695345
LOC107131807	0.067443872	0.065090349	0.023353916	0.99999488	0.072735116	0.03299652
OLFML3	0.067443872	0.041885134	0.045210516	0.214066263	0.901763837	0.03299652
ADGRL4	0.067443872	0.0557507	0.022712214	0.035007216	0.999985264	0.033542566
HSPH1	0.068783516	0.052061594	0.060940949	0.035007216	0.999985264	0.029645073
MZF1	0.079652178	0.062132643	0.284758321	0.589303553	0.089222316	0.029281471
FADS6	0.079652178	0.041885134	0.026124628	0.99999488	0.089222316	0.076612215
SCIN	0.080453228	0.041885134	0.058278897	0.99999488	0.078042948	0.063965159
PDE4B	0.088908499	0.040606228	0.035128751	0.99999488	0.089222316	0.063965159
KBTBD8	0.088908499	0.040606228	0.081247766	0.760971637	0.072735116	0.24556932
MXRA5	0.088991259	0.041885134	0.189411024	0.878491623	0.11927017	0.03299652
PLPPR2	0.097149509	0.041885134	0.035128751	0.99999488	0.11927017	0.040613955
CYP1B1	0.097149509	0.059548889	0.034407785	0.0667843	0.999985264	0.076612215
SBF1	0.097642973	0.041885134	0.259530141	0.0667843	0.974716308	0.035141823
ENTPD2	0.097642973	0.046250953	0.046945561	0.645998678	0.324790018	0.10375857
SLC6A16	0.09981439	0.950786203	0.035128751	0.246741625	0.089222316	0.037481659
TENM2	0.09981439	0.113020586	0.37511602	0.118049329	0.306037672	0.04196817
LIPA	0.09981439	0.042345617	0.067950176	0.99999488	0.089222316	0.09509626
CAMK1G	0.100532435	0.0557507	0.075607013	0.468727346	0.388290499	0.098996184
BOP1	0.100532435	0.057247246	0.03544227	0.137899533	0.478188626	0.419282739
ELN	0.103791728	0.041885134	0.063500233	0.99999488	0.119428945	0.04559089
SYS1	0.103791728	0.065090349	0.939147816	0.137899533	0.096488476	0.046502824
SLC7A8	0.103791728	0.041885134	0.195581875	0.398217024	0.535395397	0.046666552
PTK7	0.103791728	0.057247246	0.207637821	0.597946081	0.298584245	0.047315715
STAP2	0.103791728	0.111050808	0.063175645	0.322360579	0.741080831	0.051425394
CAB39	0.103791728	0.041885134	0.414030603	0.522793127	0.15274657	0.054460007
KCNK12	0.103791728	0.059063395	0.387533191	0.398217024	0.222343724	0.064359328
LGALS3	0.103791728	0.07507513	0.076443212	0.99999488	0.089222316	0.092064409
NUMB	0.103791728	0.041885134	0.045210516	0.088039905	0.999985264	0.10375857
RAB7B	0.103791728	0.057247246	0.057453282	0.286555733	0.707353372	0.141385306
ITGAD	0.103791728	0.041885134	0.106953788	0.825154704	0.11927017	0.178101465

BYSL	0.103791728	0.134639957	0.096857179	0.265823	0.230494679	0.178458141
LOC516494	0.103791728	0.041885134	0.09633452	0.386026888	0.263287549	0.266771051
KCNA3	0.103791728	0.077647692	0.03937657	0.181805405	0.172082634	0.738324902
LOC513659	0.105394749	0.041885134	0.091541869	0.111496006	0.999985264	0.076612215
VWA3B	0.106746651	0.042345617	0.058729402	0.391980658	0.787374898	0.10375857
IL1RN	0.106948279	0.128444845	0.055008323	0.985023854	0.146805081	0.064187795
LOC789607	0.111767696	0.104176007	0.200402587	0.088039905	0.937177132	0.046666552
NCR1	0.111767696	0.063992162	0.046945561	0.486936947	0.724248838	0.087292739
KYNU	0.111767696	0.109756302	0.080229764	0.468727346	0.096488476	0.378213674
LOC104969545	0.112243805	0.066359076	0.055105161	0.08478126	0.999985264	0.051425394
CNTFR	0.112243805	0.890565478	0.062519886	0.086296786	0.258000341	0.064359328
LRRC8B	0.112243805	0.063992162	0.081480217	0.884650797	0.309181682	0.079296161
FHL5	0.112243805	0.209652575	0.090377097	0.157445472	0.183591192	0.369978547
KCTD10	0.114111679	0.109756302	0.910426741	0.117444226	0.142684581	0.047315715
LOC782951	0.12244345	0.067530039	0.076443212	0.088039905	0.999985264	0.051425394
TNNT3	0.123495787	0.078260926	0.179774584	0.416149605	0.287977417	0.141385306
CCNJ	0.124639201	0.160609674	0.06274151	0.398230618	0.6707745	0.063965159
TBL1X	0.125655673	0.194701951	0.057453282	0.263650572	0.934591321	0.062818891
USP50	0.125655673	0.992977597	0.108050024	0.094182401	0.104068981	0.076612215
TREML1	0.125655673	0.0557507	0.062519886	0.99999488	0.096488476	0.087292739
MAP2K6	0.125655673	0.08767081	0.064350931	0.949825107	0.263287549	0.09584397
TIMM13	0.125655673	0.118229533	0.081247766	0.246741625	0.672072713	0.152626222
FBLN7	0.126870503	0.063992162	0.091541869	0.99999488	0.187659185	0.072071781
UNC5A	0.126870503	0.064243125	0.155253856	0.784700372	0.258000341	0.10375857
ARHGEF26	0.126870503	0.64113929	0.101842537	0.137899533	0.10158927	0.255783167
DRAM1	0.128489644	0.057247246	0.080229764	0.117444226	0.999985264	0.072071781
PDE4D	0.128813854	0.114611735	0.090454437	0.125014992	0.999985264	0.066461388
CDS1	0.128813854	0.099618844	0.081247766	0.878491623	0.359994407	0.076612215
ASAH2	0.128813854	0.099328055	0.081247766	0.785872741	0.124241694	0.294088757
SRGN	0.12956304	0.134639957	0.075607013	0.92106221	0.189748655	0.110293052
ADAM12	0.131592282	0.065090349	0.440030283	0.48240694	0.244065513	0.077265797
VEGFC	0.132210343	0.065090349	0.098106498	0.886224908	0.299421094	0.125634132
SHTN1	0.133447348	0.07507513	0.076443212	0.99999488	0.151707188	0.092064409
CDCA4	0.133447348	0.099983638	0.254322211	0.322360579	0.124241694	0.358046095
ECM1	0.133447348	0.065090349	0.160521013	0.468727346	0.11927017	0.50187271
HK3	0.135990858	0.065090349	0.090454437	0.99999488	0.11927017	0.098996184
PKNOX1	0.137882527	0.113885889	0.155253856	0.111496006	0.999985264	0.063965159
NDST1	0.137882527	0.089859412	0.067950176	0.74725201	0.404175423	0.160365035
MMP19	0.141446686	0.07507513	0.092927805	0.99999488	0.11927017	0.226723998

SNAP23	0.143623094	0.063992162	0.805319114	0.137899533	0.222343724	0.191384378
CA3	0.148151146	0.206473832	0.161020897	0.385729122	0.535395397	0.076234153
IGFBP2	0.148151146	0.105860591	0.440030283	0.398217024	0.250795474	0.101952388
PRLR	0.148151146	0.574311353	0.363631419	0.146523049	0.11927017	0.113557466
KLK7	0.148151146	0.105860591	0.166243488	0.481043972	0.411806973	0.128911494
PLIN5	0.148151146	0.148897667	0.071023339	0.573623074	0.426783288	0.158370687
LOC100139916	0.148334691	0.19552858	0.077160718	0.380156582	0.435567313	0.217879405
LOC101906455	0.150791704	0.065090349	0.973406453	0.181805405	0.124241694	0.122049454
LOC512486	0.151972255	0.065090349	0.165103343	0.868998353	0.388611323	0.09509626
SYNJ2	0.152078591	0.337920983	0.224307217	0.246741625	0.400840387	0.081688354
GSE1	0.152078591	0.067530039	0.092927805	0.99999488	0.16429036	0.114003333
PERP	0.152078591	0.533101609	0.229035721	0.127935521	0.250795474	0.128911494
TRIP12	0.152485938	0.078260926	0.09633452	0.855919388	0.669849392	0.076612215
KPNA1	0.152485938	0.110194992	0.568964092	0.434318747	0.244065513	0.076677748
CD83	0.152485938	0.10000994	0.091623784	0.99999488	0.149034773	0.09584397
RASSF9	0.152485938	0.071445998	0.096536381	0.904225644	0.535395397	0.101952388
FOSB	0.152485938	0.124355352	0.316237181	0.280349316	0.416265674	0.142732938
NTNG1	0.152485938	0.931488015	0.080862743	0.24289713	0.124241694	0.167283025
LOC107132475	0.152485938	0.067530039	0.106953788	0.144512373	0.741080831	0.465198839
ATAD3A	0.152485938	0.082046167	0.090480114	0.246741625	0.57461724	0.482266815
LOC783797	0.152485938	0.176619878	0.075607013	0.320311732	0.258000341	0.569013454
MAPRE1	0.158254401	0.099618844	0.819296429	0.322360579	0.221476705	0.09019732
ADGRB2	0.158270366	0.081762102	0.099984969	0.99999488	0.124241694	0.076677748
KIFC2	0.158270366	0.14979621	0.153245299	0.99999488	0.234281659	0.076677748
LOC101906101	0.158270366	0.222896492	0.114093188	0.672549466	0.365173383	0.097493148
CHI3L1	0.158270366	0.103545079	0.840781773	0.26302296	0.243946479	0.098996184
LOC112441499	0.158270366	0.128444845	0.272213652	0.543066914	0.343471486	0.098996184
BHLHA15	0.158270366	0.118229533	0.156084633	0.322988714	0.662093891	0.174766512
SLC31A2	0.158270366	0.102480232	0.113693972	0.989312306	0.18131345	0.210240612
ACSL6	0.158270366	0.08767081	0.106953788	0.283509667	0.546848192	0.462858576
PCDH19	0.158270366	0.071445998	0.362408227	0.207764263	0.299421094	0.468561589
TSPAN9	0.160210802	0.165637953	0.081247766	0.306653676	0.999985264	0.076677748
FAM210B	0.160210802	0.071445998	0.096857179	0.99999488	0.244065513	0.114003333
B3GNT6	0.160962807	0.221525208	0.334513987	0.30351281	0.414082187	0.097493148
LOC112442745	0.160962807	0.176619878	0.081480217	0.99999488	0.244065513	0.098996184
C16H1orf115	0.160962807	0.099618844	0.081247766	0.302375345	0.999985264	0.114211216
LOC100850659	0.161287167	0.103920029	0.199664704	0.434318747	0.474284035	0.19508181
GALNT5	0.161674787	0.092518197	0.591762752	0.448455803	0.357979213	0.079807231
CCDC188	0.161674787	0.265001767	0.476215563	0.157340173	0.417908713	0.098349811

GIMAP8	0.161674787	0.128444845	0.184360468	0.889823701	0.277004628	0.10375857
ETNK2	0.161674787	0.156483766	0.096187237	0.99999488	0.189748655	0.17061333
NR3C2	0.161674787	0.391489689	0.157494975	0.21906639	0.426783288	0.17061333
GTPBP4	0.161674787	0.124355352	0.151133731	0.246741625	0.688375802	0.280389643
TSR1	0.161674787	0.128444845	0.113693972	0.398230618	0.400840387	0.348752662
IMPA2	0.161674787	0.099618844	0.131449176	0.820583858	0.189748655	0.376881894
SVEP1	0.162036736	0.208474194	0.191953245	0.402703314	0.566515062	0.092064409
ACTR3	0.162924197	0.080755828	0.531763398	0.468727346	0.344374876	0.110293052
SLC25A51	0.164024289	0.191832936	0.18041586	0.92398295	0.228197054	0.101952388
SLAMF7	0.164024289	0.099618844	0.14248662	0.99999488	0.18131345	0.114211216
CREB3L1	0.164024289	0.128444845	0.171107906	0.413421898	0.656196025	0.142686991
ZNF775	0.164024289	0.105860591	0.113693972	0.566159233	0.250795474	0.505759802
MYLK	0.16492686	0.978089982	0.14248662	0.265823	0.146805081	0.101952388
LOC617565	0.16492686	0.095766253	0.113693972	0.99999488	0.156571782	0.128911494
LOC101906664	0.165028376	0.092518197	0.40793937	0.246741625	0.578351163	0.186644486
PLAC8	0.16585706	0.078260926	0.151133731	0.849357329	0.332378603	0.250540237
FAM124B	0.166844427	0.099618844	0.096857179	0.99999488	0.499785211	0.101952388
SCG3	0.170106297	0.370995618	0.159428204	0.77377244	0.227405518	0.097493148
CNOT6	0.170106297	0.867840435	0.156084633	0.251443857	0.244065513	0.10375857
CASQ1	0.170106297	0.540247468	0.106953788	0.385729122	0.326453474	0.168268435
CLINT1	0.170106297	0.461438762	0.219183317	0.229022096	0.332847247	0.192249392
IL7R	0.170106297	0.128444845	0.16515644	0.322360579	0.58909742	0.259161757
NLRC5	0.170896857	0.08767081	0.834068744	0.501376968	0.222343724	0.09509626
LOC107131896	0.170896857	0.095766253	0.271782816	0.306653676	0.400840387	0.392180614
SUSD5	0.170896857	0.187908701	0.188659578	0.169115437	0.397079609	0.521002923
NID2	0.170896857	0.207927892	0.144601247	0.30438873	0.242640259	0.615805309
REEP2	0.172875971	0.124355352	0.154599788	0.251443857	0.999985264	0.191384378
STYK1	0.175794981	0.222896492	0.282885049	0.400037191	0.189748655	0.298349735
NDOR1	0.179306265	0.337920983	0.225564375	0.263650572	0.608701929	0.10375857
SNX16	0.179306265	0.128444845	0.200402587	0.884650797	0.277004628	0.17061333
HSPB3	0.182463282	0.099618844	0.162872155	0.969770561	0.51883282	0.09584397
ITGB2	0.18467923	0.109756302	0.125684646	0.99999488	0.189748655	0.110293052
RRS1	0.18467923	0.113020586	0.220039139	0.540024507	0.496438607	0.217879405
CD93	0.187542314	0.108911243	0.140781463	0.617959671	0.961025202	0.099633867
FCER1A	0.18874595	0.181840329	0.192072163	0.351905173	0.357979213	0.410427392
LOC101903647	0.190140526	0.104508486	0.338611837	0.385729122	0.880252174	0.096780556
MARC1	0.190532573	0.206473832	0.154599788	0.949602784	0.345114626	0.10375857
LSAMP	0.190586142	0.561346167	0.149863504	0.590636502	0.278477149	0.10375857
HHIPL1	0.190586142	0.181331017	0.106953788	0.551603271	0.908980492	0.10375857

PLAU	0.195353331	0.100316702	0.14248662	0.830046103	0.707353372	0.128911494
TXNDC5	0.197710791	0.322966392	0.151133731	0.691124578	0.424723237	0.10375857
NOD2	0.199954156	0.099618844	0.106953788	0.246741625	0.357979213	0.960343814
IFRD1	0.201773842	0.124355352	0.268313268	0.297858309	0.864446669	0.182727362
WNT4	0.206274468	0.110194992	0.387533191	0.99999488	0.189748655	0.12376005
TARDBP	0.206274468	0.099983638	0.14750445	0.228966923	0.93150494	0.484575909
EN1	0.206552107	0.113441485	0.825497047	0.415184463	0.300576675	0.110293052
DUSP16	0.206552107	0.125812461	0.106731161	0.221633241	0.999985264	0.203471474
MRPL16	0.206552107	0.165545483	0.151133731	0.214963764	0.362364704	0.824559256
SMAD7	0.20881881	0.181269113	0.938203102	0.265823	0.241708471	0.110293052
CCDC84	0.20881881	0.302735571	0.590933668	0.251443857	0.359994407	0.122049454
NIP7	0.20881881	0.118953294	0.505866449	0.246741625	0.546848192	0.239342196
RXFP1	0.20881881	0.222896492	0.114681219	0.441185602	0.258000341	0.627277292
IKZF1	0.209704067	0.182635979	0.14248662	0.952783349	0.243946479	0.26631152
HOXA7	0.210016596	0.342974476	0.143908842	0.306653676	0.456843913	0.341188562
CFH	0.210197786	0.565729718	0.16515644	0.415184463	0.320722163	0.180497198
PLEK	0.21053069	0.113762716	0.151133731	0.99999488	0.243946479	0.10375857
CBX6	0.21053069	0.114611735	0.14248662	0.991596938	0.608701929	0.110293052
COL5A1	0.21053069	0.134422244	0.403466151	0.940265849	0.244225693	0.110300936
PTGES3	0.21053069	0.206560872	0.16201354	0.265823	0.999985264	0.110300936
WT1	0.21053069	0.114996889	0.837693962	0.325316306	0.357979213	0.128911494
PHF13	0.21053069	0.134639957	0.145502901	0.280349316	0.999985264	0.134776299
ACAN	0.21053069	0.218727412	0.218728455	0.747849903	0.403462825	0.141385306
CLECL1	0.21053069	0.156483766	0.114681219	0.687904478	0.727974484	0.186644486
DHODH	0.21053069	0.120585538	0.116500431	0.320311732	0.901104314	0.414123435
FARSB	0.21053069	0.108911243	0.126946906	0.297858309	0.359994407	0.927969856
LOC101903868	0.210993745	0.38625193	0.266523444	0.434318747	0.222343724	0.266771051
CABIN1	0.211124928	0.105860591	0.160521013	0.907700124	0.771578767	0.10375857
SV2B	0.211124928	0.119645186	0.411900922	0.450604811	0.529750417	0.191384378
PTCD2	0.211124928	0.16442218	0.171107906	0.26302296	0.841924245	0.347652735
ELOVL3	0.211124928	0.134422244	0.144601247	0.695630809	0.448668453	0.348752662
ELOF1	0.213107497	0.288565095	0.14248662	0.515120624	0.78585317	0.114211216
PSMB8	0.213714086	0.237931589	0.114093188	0.540024507	0.901104314	0.128911494
WDR13	0.214157315	0.120688164	0.780560307	0.246741625	0.633938808	0.117949896
LOC101907041	0.214157315	0.121234529	0.16515644	0.400037191	0.999985264	0.192283976
ELP3	0.214157315	0.176619878	0.353596276	0.229022096	0.299421094	0.644790352
STK17B	0.214597913	0.118229533	0.114093188	0.434318747	0.999985264	0.255783167
RNF165	0.214763451	0.10997488	0.14248662	0.48240694	0.999985264	0.195472806
PTN	0.214868048	0.150599738	0.271782816	0.773769459	0.552747179	0.110293052

LRRC3	0.214868048	0.128444845	0.204203579	0.99999488	0.343259247	0.128911494
CHAC1	0.214868048	0.674837172	0.151133731	0.246741625	0.546848192	0.189880281
GK5	0.214868048	0.189246757	0.133286581	0.99999488	0.189748655	0.234267766
DRAM2	0.215082092	0.165930787	0.282885049	0.554774022	0.332847247	0.312093451
ZNF684	0.218774097	0.113229511	0.179774584	0.563617963	0.931058866	0.178458141
CACNA1G	0.223911813	0.128444845	0.188659578	0.99999488	0.247620525	0.128911494
CLDN10	0.223911813	0.296898016	0.243811958	0.263650572	0.901763837	0.142732938
FEM1A	0.223911813	0.225280182	0.154264023	0.765740785	0.546848192	0.184013011
HSPA1A	0.223911813	0.134422244	0.206953721	0.246741625	0.999985264	0.186644486
LARP7	0.223911813	0.274904243	0.566721071	0.251443857	0.343471486	0.256039472
ERAP2	0.225117007	0.16442218	0.419946262	0.69049726	0.447414777	0.114211216
THOP1	0.225469788	0.126837996	0.151133731	0.385729122	0.499664211	0.73024728
CCDC158	0.225573547	0.350278804	0.14291978	0.540024507	0.741080831	0.128911494
LGALS9	0.225573547	0.209652575	0.125684646	0.400037191	0.999985264	0.128911494
H1F0	0.225573547	0.124355352	0.224798284	0.99999488	0.243946479	0.138778226
HOGA1	0.225573547	0.235772942	0.151133731	0.354841497	0.999985264	0.178458141
ACSM3	0.225573547	0.145201407	0.52966531	0.251443857	0.721547193	0.19535302
NFAM1	0.225573547	0.114148906	0.14248662	0.99999488	0.258000341	0.255783167
CRYM	0.225573547	0.206560872	0.385324267	0.475426312	0.250795474	0.367899858
TRIM36	0.225671772	0.113020586	0.23466285	0.263650572	0.999985264	0.255783167
ADCK5	0.22823402	0.19552858	0.746258329	0.473753805	0.332847247	0.114211216
ATG9B	0.22823402	0.322939396	0.140671722	0.265823	0.999985264	0.128911494
EMB	0.22823402	0.265001767	0.320369755	0.901707661	0.244065513	0.152296873
EMID1	0.22823402	0.113885889	0.224307217	0.563443606	0.982217275	0.152626222
GALNT6	0.22823402	0.145201407	0.16201354	0.99999488	0.258000341	0.224692647
ACKR3	0.22823402	0.149983587	0.143908842	0.265823	0.999985264	0.284190065
LOC112447770	0.22823402	0.416990571	0.254879532	0.283509667	0.393203728	0.319769466
TUBB4B	0.22823402	0.281172131	0.172738308	0.389258512	0.343259247	0.55740152
CYP2B6	0.23204055	0.525945198	0.179774584	0.525112056	0.546848192	0.117949896
PUS10	0.23204055	0.174034073	0.988922732	0.265823	0.258000341	0.128911494
ITGAL	0.23204055	0.118229533	0.159428204	0.99999488	0.244065513	0.142732938
TYSND1	0.23204055	0.118229533	0.151133731	0.251443857	0.999985264	0.163700172
LOC107131944	0.23204055	0.362289084	0.260150309	0.857917548	0.240033377	0.178458141
SIGLEC11	0.23204055	0.454975491	0.14248662	0.419318621	0.643950455	0.20453268
SYNM	0.23204055	0.958098081	0.16515644	0.33657035	0.228671709	0.204886275
ERI3	0.23204055	0.191832936	0.272519427	0.385729122	0.785107052	0.217879405
ANXA9	0.23204055	0.235772942	0.144601247	0.94169445	0.296713952	0.298349735
LOC112448523	0.232842407	0.25270365	0.459026472	0.687904478	0.244065513	0.191384378
WISP1	0.235944337	0.128444845	0.387533191	0.995196415	0.344642644	0.12376005

CASP1	0.236579692	0.367205885	0.155990192	0.99999488	0.230494679	0.148851957
ATP2A2	0.236579692	0.118229533	0.336578295	0.855453165	0.356028467	0.264713475
NIF3L1	0.245615791	0.128444845	0.151133731	0.26302296	0.999985264	0.496096399
ZBTB11	0.247677822	0.182635979	0.151133731	0.263650572	0.999985264	0.139251836
SOX4	0.247850488	0.160609674	0.151133731	0.99999488	0.241792949	0.141385306
TEC	0.247850488	0.122185751	0.156084633	0.529574194	0.999985264	0.141385306
HSD11B2	0.247850488	0.433007404	0.155253856	0.573623074	0.653566713	0.152296873
LOC101906565	0.247850488	0.128444845	0.998936876	0.322360579	0.244065513	0.166051777
KCP	0.247850488	0.530795065	0.154599788	0.794035382	0.357979213	0.168301715
COL6A5	0.247850488	0.33857298	0.172738308	0.568953816	0.403462825	0.306727331
MVD	0.247850488	0.127941907	0.156084633	0.318829028	0.983747207	0.482010691
HHEX	0.248112524	0.160609674	0.179774584	0.698527845	0.970485217	0.141385306
ADAMTS17	0.248112524	0.163284659	0.421076728	0.907045085	0.331821475	0.175305619
TMEM100	0.248112524	0.147976039	0.718226122	0.306653676	0.439247298	0.277985628
DDX54	0.248112524	0.206560872	0.151133731	0.468727346	0.884208058	0.298349735
GPR37	0.248954942	0.433007404	0.58523401	0.385729122	0.241708471	0.217879405
SDR16C5	0.248954942	0.131215855	0.219183317	0.354841497	0.999985264	0.219485161
INTS6	0.250601629	0.134639957	0.156380029	0.265823	0.999985264	0.129137193
HSPA6	0.250601629	0.176619878	0.316634322	0.280349316	0.999985264	0.152626222
CENPB	0.250601629	0.205058781	0.785177733	0.322360579	0.397079609	0.20453268
LOC104976082	0.250601629	0.149983587	0.171107906	0.99999488	0.241792949	0.222364321
LOC100848883	0.250601629	0.599298196	0.220039139	0.263650572	0.356802567	0.422940396
HACD4	0.251298241	0.128444845	0.151133731	0.99999488	0.474284035	0.135268227
CD300E	0.2533422	0.174034073	0.165103343	0.99999488	0.244065513	0.176812856
CBARP	0.2533422	0.176619878	0.155253856	0.915296749	0.719742223	0.190684434
TTC38	0.2533422	0.16442218	0.272519427	0.99999488	0.256954517	0.194050676
LTBP2	0.2533422	0.279898072	0.174902673	0.982713285	0.357979213	0.194050676
PADI2	0.2533422	0.128444845	0.270811008	0.978440951	0.381857971	0.234267766
PTPRZ1	0.2533422	0.128444845	0.268313268	0.281852858	0.52539338	0.779686358
OIT3	0.253593496	0.205636283	0.570175675	0.896748401	0.244225693	0.156958788
LOC513508	0.253593496	0.644729858	0.660036437	0.27154182	0.243946479	0.178458141
ATG4B	0.253593496	0.128444845	0.151133731	0.98846246	0.546848192	0.275375051
PCDH1	0.253593496	0.134422244	0.151133731	0.808495733	0.556807967	0.431130427
NEIL1	0.255430567	0.19242721	0.230150273	0.388779061	0.999985264	0.148851957
NCLN	0.257540458	0.207927892	0.16201354	0.347082552	0.901763837	0.430632753
LAPTM5	0.258444309	0.185495253	0.188659578	0.99999488	0.359994407	0.141385306
LOC100847719	0.258518752	0.310125056	0.254322211	0.628506385	0.357979213	0.31561418
LCP1	0.258955918	0.181840329	0.188659578	0.99999488	0.357979213	0.152626222
MYBBP1A	0.258955918	0.202479778	0.161020897	0.641348265	0.546848192	0.446372578

NAT14	0.258955918	0.19552858	0.224307217	0.845465538	0.244225693	0.52928764
PNO1	0.260082149	0.215793385	0.264940776	0.609127566	0.51883282	0.315396729
HMGCLL1	0.261225564	0.504825401	0.418438893	0.351905173	0.397079609	0.20453268
NRM	0.261659632	0.209652575	0.16201354	0.398230618	0.999985264	0.157813491
PGLYRP2	0.261659632	0.134639957	0.224307217	0.560163102	0.901104314	0.272594278
PAK5	0.261659632	0.502850182	0.156084633	0.468727346	0.456336784	0.333401168
ITGAX	0.262538873	0.161096146	0.210955602	0.99999488	0.265836378	0.189880281
TMEM156	0.262538873	0.19552858	0.220039139	0.707846893	0.6911547	0.252337751
CD68	0.263622985	0.169552676	0.204203579	0.99999488	0.250795474	0.25556725
GDF11	0.266844122	0.145201407	0.156084633	0.820920896	0.503338662	0.513288396
CD200R1L	0.268305966	0.209652575	0.16515644	0.99999488	0.298584245	0.192283976
SLAMF6	0.268305966	0.149983587	0.188659578	0.99999488	0.269746768	0.210477133
EMILIN3	0.271507112	0.741594487	0.189411024	0.428841505	0.51883282	0.182727362
CTGF	0.271523536	0.19552858	0.785248842	0.434318747	0.382521731	0.20453268
MGC157082	0.272246859	0.134422244	0.243371049	0.99999488	0.274546869	0.284190065
TRPC1	0.272644042	0.154845038	0.188659578	0.30438873	0.901763837	0.609986278
TAB1	0.273175616	0.181269113	0.240925949	0.388779061	0.999985264	0.153599643
NOC4L	0.273175616	0.19558086	0.173375488	0.483232843	0.657701189	0.544438709
ALPK3	0.273175616	0.174034073	0.154599788	0.284925278	0.344642644	0.999960414
SH2B2	0.277424468	0.202479778	0.154924488	0.99999488	0.52539338	0.205415468
TUBB	0.277424468	0.425252947	0.179774584	0.844450108	0.299421094	0.305331668
GZMB	0.278725486	0.335989493	0.172697446	0.99999488	0.366051534	0.194050676
LOC788801	0.278725486	0.225280182	0.172738308	0.907045085	0.663849161	0.211102683
ARL11	0.280831727	0.206398712	0.492557292	0.959611215	0.299421094	0.17255454
GTF2E1	0.280831727	0.790159199	0.437433425	0.322360579	0.284301644	0.196482518
KIF14	0.28390898	0.836515843	0.186990733	0.468727346	0.355674321	0.234267766
SLC30A4	0.285643294	0.14979621	0.188659578	0.445958983	0.999985264	0.194050676
NKD1	0.287908537	0.233399564	0.16515644	0.99999488	0.425292769	0.191384378
JMJD6	0.288714659	0.174034073	0.223107593	0.306418945	0.999985264	0.152626222
HMGA1	0.288714659	0.274904243	0.220039139	0.99999488	0.300576675	0.155966137
MYO1G	0.288714659	0.15456771	0.16515644	0.99999488	0.306037672	0.16367207
WAPL	0.288714659	0.176619878	0.387533191	0.322360579	0.999985264	0.17390792
LOC107131398	0.288714659	0.207927892	0.94634922	0.400037191	0.323980448	0.178458141
ATP6V1C2	0.288714659	0.610403345	0.162872155	0.92398295	0.332847247	0.178458141
TEPSIN	0.288714659	0.164598899	0.171188958	0.398217024	0.999985264	0.178458141
KCNJ10	0.288714659	0.166129063	0.418438893	0.643846037	0.671485036	0.211800949
PPL	0.288714659	0.176619878	0.459542294	0.35350473	0.873468654	0.264713475
BMP6	0.288714659	0.20961134	0.264940776	0.566159233	0.691827619	0.322855973
FARSA	0.288714659	0.235570183	0.176843978	0.401509068	0.575910187	0.676348472

MAT2A	0.289324648	0.212128906	0.260061205	0.99999488	0.463830955	0.169629953
MERTK	0.289324648	0.156483766	0.231158535	0.947995037	0.566515062	0.272302366
SLC41A3	0.289502188	0.16442218	0.401122009	0.30438873	0.999985264	0.16367207
CTSB	0.289502188	0.209652575	0.264541087	0.99999488	0.299421094	0.178101465
TUBA1A	0.289502188	0.748233963	0.224307217	0.622160052	0.300576675	0.226723998
MEOX2	0.291533838	0.170400779	0.673566693	0.601209712	0.546848192	0.176812856
CREM	0.294538587	0.267584154	0.165103343	0.322360579	0.999985264	0.163700172
PIEZO2	0.297702705	0.208474194	0.353596276	0.99999488	0.422500465	0.160365035
ATF3	0.297702705	0.286573624	0.848312163	0.388779061	0.425292769	0.169629953
CD48	0.297702705	0.225280182	0.16515644	0.99999488	0.442580702	0.217879405
KHDC4	0.297702705	0.209652575	0.188659578	0.306653676	0.999985264	0.226723998
TP53BP2	0.297702705	0.235772942	0.222457783	0.99999488	0.354915601	0.255783167
ZMYND8	0.297702705	0.237931589	0.285474251	0.869065101	0.357979213	0.34249435
AOX1	0.304644856	0.260620465	0.220039139	0.400037191	0.712429088	0.52928764
CXHXorf21	0.30551531	0.181269113	0.172738308	0.99999488	0.320722163	0.25655431
RRAGD	0.306533684	0.184637771	0.298069649	0.878491623	0.546848192	0.282693017
L3MBTL2	0.311524864	0.20961134	0.237294301	0.388779061	0.781939574	0.574610109
NDRG4	0.311721628	0.270621658	0.260150309	0.306653676	0.357979213	0.914825249
ZSWIM6	0.312473989	0.165207902	0.16515644	0.398230618	0.999985264	0.175305619
ARMCX3	0.312515869	0.222896492	0.493015519	0.354841497	0.999985264	0.181048498
MMP2	0.312515869	0.311493981	0.224307217	0.99999488	0.362185809	0.217879405
LOC506828	0.312515869	0.203760472	0.37511602	0.953917057	0.449266926	0.218049304
LOC788175	0.312515869	0.792047562	0.354577206	0.322360579	0.393203728	0.25655431
JAML	0.312515869	0.550447041	0.224307217	0.5736757	0.507683468	0.272302366
DNASE1L3	0.312515869	0.36365584	0.379593497	0.385729122	0.657836458	0.298460621
DUSP26	0.312515869	0.205375425	0.266523444	0.785363096	0.657472521	0.310467609
C4H7orf25	0.312515869	0.163957026	0.266523444	0.398230618	0.999985264	0.314555295
PRMT1	0.312515869	0.19552858	0.222808183	0.99999488	0.369061381	0.321563767
RIMS1	0.312515869	0.509768617	0.282219258	0.430247169	0.369061381	0.431733793
INSIG1	0.312515869	0.337318291	0.188659578	0.320311732	0.798938088	0.55740152
TBRG4	0.312515869	0.313964231	0.189411024	0.398230618	0.608470909	0.609823525
GRP	0.312732535	0.661182871	0.254322211	0.317840464	0.798938088	0.211012174
SLC25A27	0.313597993	0.225280182	0.243811958	0.398217024	0.999985264	0.210240612
PEX6	0.315710725	0.401842761	0.387533191	0.5736757	0.568311949	0.191384378
ARHGAP4	0.315710725	0.209652575	0.233790607	0.92106221	0.744678006	0.210240612
SCN4B	0.315710725	0.225280182	0.403466151	0.490137677	0.607711588	0.380378402
HGH1	0.315710725	0.198229544	0.189411024	0.354841497	0.847202014	0.72144082
RUVBL1	0.315710725	0.237931589	0.189411024	0.417809577	0.546848192	0.802788605
LOC112441594	0.316958424	0.19552858	0.970903823	0.401519611	0.320722163	0.228686236

TAS1R3	0.31697123	0.48968951	0.49177635	0.765740785	0.299421094	0.181048498
GMPR	0.31697123	0.237931589	0.592530632	0.925818215	0.326453474	0.181048498
TRIM25	0.31697123	0.225280182	0.62306879	0.931835237	0.306037672	0.192283976
EHF	0.31697123	0.487641245	0.179774584	0.322360579	0.999985264	0.240189056
CSPG5	0.31697123	0.20961134	0.189411024	0.400037191	0.999985264	0.298349735
ZBTB8OS	0.31697123	0.346080451	0.306075466	0.322360579	0.77926164	0.386443339
CTSS	0.317617015	0.209652575	0.230150273	0.99999488	0.51883282	0.210126646
MZB1	0.317617015	0.288905896	0.401122009	0.855453165	0.412249445	0.234267766
LOC618076	0.318660996	0.166967387	0.189753522	0.322360579	0.999985264	0.178101465
TFRC	0.31894088	0.406295444	0.609340165	0.529183231	0.343259247	0.254646668
TIRAP	0.31894088	0.208474194	0.272153329	0.566159233	0.708864153	0.434569255
TM4SF19	0.319278746	0.225280182	0.222808183	0.99999488	0.300576675	0.26631152
CTHRC1	0.319748503	0.189960072	0.539713639	0.855453165	0.546848192	0.193259398
LOC112447408	0.321795123	0.19552858	0.266523444	0.99999488	0.298584245	0.210240612
HOXA6	0.323697313	0.237931589	0.233790607	0.680109779	0.535395397	0.547278473
GRK3	0.324618715	0.222896492	0.189411024	0.99999488	0.299421094	0.370526188
LOC784052	0.325085747	0.25270365	0.394168076	0.388779061	0.999985264	0.186644486
EPHA5	0.325085747	0.311493981	0.771096787	0.465243948	0.359994407	0.25571884
SMPD3	0.325085747	0.174034073	0.266523444	0.99999488	0.356802567	0.298460621
SOST	0.325464759	0.189960072	0.267206344	0.398217024	0.999985264	0.298460621
DDX56	0.327215188	0.326705874	0.222457783	0.758838341	0.594728091	0.345652317
GIMAP7	0.32873721	0.225280182	0.236752223	0.551603271	0.625232521	0.596516103
SLC2A3	0.328807961	0.288565095	0.405170393	0.907039918	0.299421094	0.322855973
TMEM177	0.328869102	0.174034073	0.188659578	0.347082552	0.999985264	0.211800949
MPHOSPH9	0.329131565	0.225280182	0.946479014	0.388779061	0.487511192	0.184013011
MFN1	0.329131565	0.342195215	0.188659578	0.66895898	0.785107052	0.305488991
OXTR	0.329131565	0.274904243	0.36532409	0.725623067	0.397079609	0.428725468
LPAR2	0.32948208	0.225280182	0.237294301	0.99999488	0.364328619	0.211800949
MTFP1	0.32948208	0.307095857	0.222457783	0.476062614	0.849055371	0.407020776
IL2RG	0.329495788	0.336693283	0.189411024	0.941409556	0.707353372	0.196290235
EFNB2	0.329495788	0.189246757	0.405095497	0.99999488	0.361671779	0.200185176
SCYL2	0.329495788	0.284121857	0.75028318	0.589303553	0.397079609	0.211800949
LOC101904923	0.329495788	0.483069943	0.214552824	0.560163102	0.86390584	0.211800949
PRPSAP2	0.329495788	0.369069593	0.220039139	0.398217024	0.928683582	0.365993262
LOC100336414	0.330508303	0.19552858	0.214057387	0.322360579	0.999985264	0.192249392
LY9	0.333744905	0.19242721	0.188659578	0.99999488	0.478188626	0.205415468
LOC101905979	0.335693048	0.206560872	0.875823795	0.501376968	0.382860241	0.272302366
POU3F1	0.335693048	0.243691024	0.220039139	0.99999488	0.362364704	0.385430007
H2AFJ	0.33793374	0.301588167	0.214552824	0.468727346	0.999985264	0.191384378

CASTOR2	0.33793374	0.181840329	0.575199502	0.859289191	0.51883282	0.250279815
COL5A2	0.33793374	0.243691024	0.471348003	0.951237479	0.345114626	0.259750133
PMF1	0.33793374	0.216566806	0.214552824	0.322360579	0.999985264	0.347652735
LOC100848703	0.338566585	0.296717545	0.206984809	0.351905173	0.999985264	0.217770141
ACSL3	0.339273332	0.19552858	0.509151676	0.763371278	0.617894804	0.25745626
THBS4	0.339273332	0.19552858	0.189411024	0.794035382	0.719583646	0.514316109
DAPK3	0.339273332	0.342195215	0.240925949	0.417809577	0.679405937	0.551449383
MYH10	0.339926599	0.270621658	0.311651171	0.948570368	0.655307354	0.194050676
HAS3	0.342027897	0.19242721	0.252810282	0.99999488	0.326433843	0.209847159
ENDOV	0.342027897	0.230474554	0.224307217	0.511435398	0.999985264	0.211800949
C18H16orf86	0.342027897	0.296726456	0.746258329	0.608471943	0.357979213	0.256003861
LOC101904121	0.342027897	0.416445008	0.195581875	0.859289191	0.608701929	0.266771051
SEPT1	0.342027897	0.409220402	0.448027787	0.385729122	0.546848192	0.39743828
LOC101902413	0.342027897	0.220553436	0.411900922	0.896694735	0.326453474	0.462858576
TRAF3	0.342027897	0.219847651	0.214552824	0.69049726	0.741080831	0.521002923
SURF2	0.342027897	0.265001767	0.261325745	0.66085809	0.51883282	0.601639843
TYROBP	0.342221298	0.235570183	0.239916066	0.99999488	0.357979213	0.19535302
DNAJB1	0.34285922	0.235772942	0.271782816	0.384002022	0.999985264	0.199435573
XK	0.34285922	0.222896492	0.477167064	0.949602784	0.448994037	0.234267766
PHC3	0.34285922	0.921549	0.297626948	0.465243948	0.356028467	0.252444961
TF	0.343058803	0.406061927	0.214552824	0.35350473	0.999985264	0.200185176
LOC514457	0.343974208	0.423334704	0.494727878	0.415582646	0.439247298	0.404873878
DCAF1	0.344272311	0.225280182	0.189753522	0.378298905	0.999985264	0.193259398
GALNT10	0.344272311	0.736960298	0.408370911	0.354841497	0.608701929	0.211800949
CLASRP	0.344272311	0.332945137	0.785429296	0.594975772	0.359994407	0.212403014
TRPV2	0.344272311	0.211779848	0.243371049	0.99999488	0.345114626	0.239128267
BHLHE40	0.344272311	0.288905896	0.224307217	0.419734129	0.999985264	0.282693017
LOC112442851	0.344272311	0.915367594	0.314468393	0.385729122	0.332378603	0.347652735
VASP	0.344272311	0.281172131	0.353596276	0.425873414	0.847202014	0.403519537
MAOB	0.346593399	0.195254229	0.314468393	0.380156582	0.999985264	0.195472806
AHCY	0.34725505	0.207927892	0.26627627	0.99999488	0.411806973	0.298460621
DNAJB4	0.34725505	0.403109879	0.239916066	0.385729122	0.999985264	0.324935242
AFF4	0.347623547	0.202961747	0.399025922	0.380156582	0.999985264	0.19535302
CDK16	0.347623547	0.207927892	0.356595446	0.820583858	0.942175494	0.195472806
TPST1	0.347623547	0.207927892	0.247173592	0.388779061	0.999985264	0.204919664
POSTN	0.347623547	0.310125056	0.372681117	0.99999488	0.323041702	0.234267766
IFI27	0.347623547	0.225280182	0.807323012	0.687904478	0.359994407	0.272302366
ZNF391	0.347623547	0.19558086	0.40793937	0.99999488	0.357979213	0.275900023
LYAR	0.347623547	0.209652575	0.192357661	0.388779061	0.999985264	0.556159073

SMYD5	0.349227542	0.225280182	0.243811958	0.540024507	0.707353372	0.653862943
PPP2R2C	0.349355727	0.19552858	0.257934262	0.385729122	0.999985264	0.205995656
LOC784289	0.349355727	0.270621658	0.224798284	0.917553107	0.901104314	0.217879405
GK	0.349355727	0.237931589	0.233790607	0.99999488	0.323041702	0.231112863
DEFB4A	0.349355727	0.235772942	0.326842621	0.99999488	0.32443157	0.255783167
PKDCC	0.349355727	0.209652575	0.405095497	0.967551965	0.546848192	0.262776479
DKK2	0.349355727	0.304735985	0.254322211	0.426249931	0.999985264	0.392180614
LOC100847376	0.349355727	0.873113713	0.214552824	0.413421898	0.448994037	0.39743828
CXCL13	0.349355727	0.36365584	0.27139172	0.925818215	0.357979213	0.404873878
PTPRT	0.349355727	0.384132949	0.353923994	0.398230618	0.499848016	0.622335178
WDR86	0.350264368	0.38625193	0.405095497	0.802408477	0.535395397	0.231967092
GIMAP4	0.350742701	0.364955743	0.281243612	0.5736757	0.545924011	0.519407686
AQP9	0.351111897	0.206560872	0.211778638	0.725623067	0.939144222	0.437496223
POMT1	0.351111897	0.235570183	0.586654479	0.501376968	0.357979213	0.621229636
LOC529792	0.352072001	0.223655692	0.322027383	0.99999488	0.332847247	0.272302366
SMU1	0.353429009	0.237126854	0.385324267	0.99999488	0.546848192	0.203717451
CYP20A1	0.354948582	0.550265412	0.219972862	0.687904478	0.862059617	0.203717451
DNAH2	0.355032545	0.347981673	0.219183317	0.453449517	0.999985264	0.210477133
FAM160A2	0.355032545	0.402545802	0.266523444	0.71610479	0.830960616	0.238943791
SCIMP	0.355032545	0.211779848	0.225564375	0.99999488	0.362364704	0.25556725
GLULP	0.355032545	0.19558086	0.440030283	0.99999488	0.388611323	0.255783167
LAG3	0.355032545	0.225280182	0.324660229	0.590046473	0.999985264	0.292719589
SLC14A1	0.355032545	0.335989493	0.420270937	0.398217024	0.849822622	0.347652735
SYCP3	0.355032545	0.206560872	0.683285603	0.490137677	0.369372075	0.601032588
ZNF207	0.355374399	0.312714438	0.27122364	0.385729122	0.999985264	0.210240612
CYSLTR1	0.355374399	0.378106622	0.297626948	0.756513267	0.824490203	0.21049443
GBP5	0.355374399	0.222896492	0.485378752	0.717413645	0.856853826	0.217879405
ADAMTSL2	0.355374399	0.207927892	0.40793937	0.803037807	0.894715133	0.231967092
NLK	0.355374399	0.51620899	0.3544574	0.495803249	0.737989863	0.25655431
TPPP	0.355374399	0.281172131	0.297626948	0.365596939	0.999985264	0.26631152
SDS	0.355374399	0.225280182	0.322027383	0.896370331	0.729522487	0.293316846
CLEC12A	0.355374399	0.216989731	0.273235234	0.99999488	0.417722928	0.388523226
LOC112441505	0.355374399	0.225280182	0.214552824	0.99999488	0.357979213	0.410427392
ERBB3	0.355374399	0.243819453	0.485378752	0.501376968	0.546848192	0.538873169
FLVCR2	0.355374399	0.408874345	0.247143516	0.796555088	0.382860241	0.547278473
SFRP1	0.355374399	0.591124857	0.224307217	0.691124578	0.345114626	0.559024093
CLGN	0.355374399	0.220553436	0.295005899	0.437458118	0.864446669	0.603560332
BCL11B	0.355374399	0.419498004	0.273235234	0.398230618	0.58342251	0.621376973
WDR18	0.355374399	0.260828162	0.214057387	0.502809653	0.685269737	0.772769165

UCK2	0.355374399	0.225280182	0.214057387	0.398556815	0.856124022	0.812725657
HK2	0.356382813	0.322966392	0.271782816	0.400037191	0.58909742	0.770633383
TNFSF10	0.357582993	0.276761548	0.268681156	0.747849903	0.999985264	0.239510119
PTPN22	0.357582993	0.234337692	0.214552824	0.92106221	0.497727099	0.622335178
OSBPL8	0.359639851	0.222896492	0.255458748	0.99999488	0.856853826	0.210809471
ENC1	0.359639851	0.411165859	0.252810282	0.760007034	0.818885877	0.255783167
CAPG	0.36118378	0.228086543	0.233790607	0.99999488	0.365770365	0.412423814
LOC107131817	0.361409624	0.284606115	0.224798284	0.554774022	0.357979213	0.943860392
SEPT5	0.362555356	0.336693283	0.547354523	0.816792716	0.545924011	0.217879405
CAPZB	0.362555356	0.346080451	0.691847112	0.777342221	0.365040595	0.24556932
KLRG2	0.362555356	0.274904243	0.941118576	0.539894645	0.332847247	0.273011972
RNF182	0.362555356	0.370995618	0.268544695	0.794939965	0.535395397	0.445262677
CYP3A4	0.363467354	0.265001767	0.710429228	0.485154352	0.787383034	0.226455758
NOA1	0.366572349	0.2108387	0.358281509	0.385729122	0.813331114	0.734689625
CEP72	0.366643018	0.38625193	0.239916066	0.99999488	0.357979213	0.210477133
RPLP1	0.366643018	0.336029024	0.254322211	0.77377244	0.999985264	0.231967092
TPBG	0.366643018	0.520857951	0.453095481	0.763371278	0.458955101	0.239342196
GPBP1	0.366643018	0.286429495	0.222808183	0.385729122	0.999985264	0.259161757
SLC7A7	0.366643018	0.234337692	0.224307217	0.99999488	0.425292769	0.264713475
POLRMT	0.366643018	0.225280182	0.404711529	0.39972447	0.999985264	0.272302366
LOC100297044	0.366643018	0.217635436	0.541765557	0.94911704	0.442580702	0.306727331
LOC514181	0.366643018	0.591239507	0.271782816	0.589303553	0.609388545	0.335813741
LOC512867	0.366643018	0.345758663	0.277735273	0.77377244	0.448994037	0.574959923
LOC618541	0.366643018	0.259920507	0.239916066	0.384759887	0.77825208	0.856510591
CLN5	0.367401252	0.225280182	0.271782816	0.99999488	0.359994407	0.210477133
MATK	0.367401252	0.235772942	0.291592084	0.99999488	0.357979213	0.255783167
MED9	0.367401252	0.294756438	0.372719233	0.940265849	0.594728091	0.264715282
LOC784148	0.367401252	0.568300739	0.230150273	0.855453165	0.546848192	0.303654324
GNL3	0.367401252	0.281172131	0.272519427	0.400037191	0.999985264	0.4543016
PPP1R1A	0.368235535	0.225280182	0.322027143	0.491151532	0.999985264	0.255783167
CYP4V2	0.36844731	0.727539585	0.385888252	0.540024507	0.608701929	0.211800949
ELAC2	0.36844731	0.207927892	0.235361507	0.543496777	0.999985264	0.453090281
FTH1	0.369359454	0.282634514	0.414030603	0.904225644	0.707353372	0.220578095
LOC101902527	0.369359454	0.358086855	0.35879437	0.401519611	0.999985264	0.299897872
LOC101907985	0.369851701	0.209652575	0.224307217	0.99999488	0.552747179	0.219830535
LOC112444603	0.369851701	0.272711529	0.264940776	0.573623074	0.999985264	0.232996301
PPP1R26	0.369851701	0.279898072	0.250899182	0.99999488	0.546848192	0.254646668
GPT	0.369851701	0.225280182	0.237294301	0.99999488	0.456843913	0.256039472
C26H10orf82	0.369851701	0.493379784	0.387533191	0.465243948	0.801204085	0.284190065

CDK8	0.369851701	0.310125056	0.277735273	0.857323853	0.755207159	0.312093451
CCL2	0.369851701	0.223441943	0.266523444	0.99999488	0.367835214	0.316018978
BBS9	0.369851701	0.286429495	0.224307217	0.573623074	0.798938088	0.621376973
RAB7A	0.369977525	0.702802202	0.270811008	0.791188744	0.574203003	0.22611387
ABI1	0.369977525	0.212583021	0.291592084	0.495803249	0.999985264	0.231967092
AKAP7	0.369977525	0.274904243	0.51144778	0.490137677	0.998639844	0.255783167
LOC100140431	0.369977525	0.228678066	0.447056602	0.99999488	0.357979213	0.262776479
BGLAP	0.369977525	0.282634514	0.268544695	0.99999488	0.545924011	0.284190065
SLC16A3	0.369977525	0.341770652	0.224394157	0.894489259	0.847202014	0.284190065
HMGB3	0.369977525	0.226966556	0.349554411	0.419734129	0.999985264	0.284190065
LOC101904344	0.369977525	0.850692013	0.353596276	0.448455803	0.446470263	0.332569608
BCAT2	0.369977525	0.29825202	0.27517087	0.417809577	0.999985264	0.33960992
POMGNT2	0.369977525	0.356422038	0.348934016	0.812057455	0.596250513	0.347652735
NIPAL2	0.369977525	0.356422038	0.23466285	0.99999488	0.357979213	0.476152636
PAQR8	0.370017321	0.274904243	0.243811958	0.744871119	0.403462825	0.858593649
LOC782706	0.371205057	0.315490653	0.224307217	0.982713285	0.357979213	0.615326593
FAM136A	0.373376898	0.249242701	0.271782816	0.434318747	0.660803051	0.873463288
VASN	0.376723348	0.22047588	0.296427658	0.99999488	0.545924011	0.255783167
NFIC	0.376723348	0.286573624	0.278631552	0.99999488	0.57461724	0.298460621
CEBPZ	0.376723348	0.343863091	0.233790607	0.907045085	0.757173298	0.321563767
KCNG1	0.376723348	0.379157225	0.266914089	0.99999488	0.479528738	0.331540119
FGG	0.376723348	0.29825202	0.403466151	0.941409556	0.497727099	0.34879456
GID8	0.376723348	0.650665822	0.27517087	0.401519611	0.789479114	0.378213674
LOC515418	0.37732954	0.242843581	0.303323323	0.99999488	0.357979213	0.250279815
CARD9	0.377921117	0.370995618	0.678433983	0.786398759	0.397079609	0.268645572
IFI44	0.379572516	0.217422878	0.37511602	0.949602784	0.707353372	0.315141158
CKAP4	0.380861238	0.310125056	0.276807616	0.99999488	0.400840387	0.347652735
MAGEL2	0.382084949	0.311493981	0.358281509	0.99999488	0.400840387	0.383423168
GNA15	0.382189289	0.474122619	0.231158535	0.963012661	0.657836458	0.255783167
SLF1	0.382189289	0.330856412	0.465747691	0.493932625	0.685269737	0.453090281
PRAG1	0.384271676	0.55750909	0.23466285	0.644821001	0.671485036	0.419552755
MAFG	0.385803917	0.265001767	0.775848114	0.747076834	0.357979213	0.407861507
KNTC1	0.386218202	0.237931589	0.757137435	0.584503674	0.712429088	0.264713475
NUAK1	0.386218202	0.217422878	0.575199502	0.560163102	0.999985264	0.26631152
CACNB2	0.386218202	0.87010028	0.264940776	0.428841505	0.376764909	0.571518232
FBXO40	0.386218202	0.219260535	0.272519427	0.812057455	0.568311949	0.715526355
VARS	0.386218202	0.237931589	0.240925949	0.687904478	0.660075125	0.779686358
PLEKHG4	0.388372518	0.271328522	0.546845998	0.99999488	0.52539338	0.226723998
LOC104970249	0.388372518	0.568712199	0.858024919	0.401509068	0.442580702	0.234267766

LRFN4	0.388372518	0.237126854	0.475942277	0.859289191	0.529750417	0.453090281
CAP2	0.389785792	0.869999083	0.272519427	0.847714599	0.397079609	0.231967092
RGS10	0.389785792	0.338068267	0.230150273	0.99999488	0.47399696	0.255783167
SCNN1D	0.389785792	0.550447041	0.841717609	0.398230618	0.397079609	0.298460621
LOC782348	0.389960677	0.342195215	0.553217285	0.540024507	0.426783288	0.601032588
SLCO2B1	0.393265444	0.283340687	0.230150273	0.830949185	0.999985264	0.305331668
NT5C3A	0.393265444	0.243691024	0.230448325	0.963012661	0.91945292	0.321563767
NSUN2	0.393265444	0.288565095	0.276954794	0.622527961	0.999985264	0.387823865
COL21A1	0.393265444	0.237931589	0.405170393	0.757550318	0.78143481	0.412423814
INPP5F	0.393265444	0.237931589	0.372038256	0.71610479	0.741080831	0.523730974
LOC782456	0.393807947	0.336029024	0.577015792	0.680109779	0.688375802	0.275900023
GEMIN6	0.39387583	0.281499211	0.321136619	0.454736226	0.999985264	0.423244169
SPN	0.394165022	0.283340687	0.254322211	0.99999488	0.546848192	0.231967092
ZNF335	0.394165022	0.243691024	0.705567274	0.479547402	0.999985264	0.239128267
DNAJA1	0.394165022	0.281172131	0.297626948	0.398217024	0.999985264	0.240189056
PPTC7	0.394165022	0.286573624	0.408861677	0.617959671	0.999985264	0.25084741
LPAR3	0.394165022	0.270621658	0.344157642	0.687904478	0.999985264	0.264713475
SAMSN1	0.394165022	0.246019036	0.237294301	0.99999488	0.625232521	0.273011972
ASS1	0.394165022	0.421030287	0.353596276	0.798459589	0.662093891	0.33309039
CALM3	0.394165022	0.533394878	0.271782816	0.401519611	0.999985264	0.347652735
LRRC46	0.394165022	0.272850575	0.353797704	0.49753871	0.999985264	0.347652735
THBS2	0.394165022	0.281172131	0.398835626	0.966629392	0.546848192	0.349020434
ACOD1	0.394165022	0.294918944	0.291592084	0.99999488	0.397079609	0.407284616
LOC100848307	0.394165022	0.342195215	0.260061205	0.554774022	0.999985264	0.419894928
LOC101906024	0.394165022	0.225280182	0.278631552	0.99999488	0.357979213	0.621376973
MSL2	0.394165022	0.413107515	0.282885049	0.434318747	0.778765368	0.627549535
KLHL25	0.394165022	0.376301215	0.243811958	0.483232843	0.741080831	0.720732312
LOC615278	0.394165022	0.421030287	0.233790607	0.777342221	0.435141949	0.721134875
HAS1	0.396125048	0.225280182	0.595621168	0.99999488	0.361671779	0.259161757
MBLAC2	0.396385111	0.246019036	0.254322211	0.490980599	0.999985264	0.404873878
ACER2	0.396442119	0.530773348	0.243811958	0.989312306	0.424723237	0.385951766
P3H1	0.396617602	0.303193306	0.665391565	0.978440951	0.433794986	0.234267766
CCR1	0.396617602	0.302735571	0.272153329	0.99999488	0.499848016	0.234267766
NUP98	0.396617602	0.302235146	0.282219258	0.401509068	0.999985264	0.234267766
PENK	0.396617602	0.235772942	0.593672982	0.645998678	0.999985264	0.234267766
AKIP1	0.396617602	0.225280182	0.357303278	0.477591597	0.999985264	0.239128267
DDX3X	0.396617602	0.311493981	0.411900922	0.634541844	0.999985264	0.25556725
CD1B	0.396617602	0.286671197	0.683588966	0.99999488	0.359994407	0.255783167
APLP1	0.396617602	0.816518153	0.272519427	0.478118439	0.847202014	0.255783167

SLC5A3	0.396617602	0.336693283	0.295005899	0.502809653	0.999985264	0.255783167
APOC3	0.396617602	0.419498004	0.83711556	0.645998678	0.412249445	0.25655431
HBQ1	0.396617602	0.274904243	0.619592912	0.402703314	0.999985264	0.26631152
SPRN	0.396617602	0.561419114	0.541480881	0.794035382	0.439247298	0.275900023
SAP30L	0.396617602	0.991365955	0.305961682	0.490137677	0.419627723	0.284190065
DEDD2	0.396617602	0.288565095	0.266523444	0.398230618	0.999985264	0.29092101
RPTOR	0.396617602	0.288905896	0.237294301	0.77377244	0.999985264	0.298349735
KLHL6	0.396617602	0.288565095	0.328332837	0.925818215	0.813331114	0.298460621
LOC112442226	0.396617602	0.494978451	0.373363914	0.543066914	0.861807159	0.314555295
LOC112448378	0.396617602	0.816518153	0.244388028	0.554774022	0.671485036	0.347652735
TRA2A	0.396617602	0.274904243	0.296427658	0.468727346	0.999985264	0.349401965
GATA5	0.396617602	0.29443215	0.471348003	0.976631804	0.456843913	0.361520322
HTR2B	0.396617602	0.725393911	0.353596276	0.398230618	0.736346045	0.385430007
ACLY	0.396617602	0.235570183	0.268313268	0.451551297	0.999985264	0.412423814
C2CD2L	0.396617602	0.809135999	0.365811456	0.451993645	0.535395397	0.420434059
CIITA	0.396617602	0.237931589	0.268313268	0.647358816	0.999985264	0.445262677
TMED6	0.396617602	0.243819453	0.271782816	0.573623074	0.999985264	0.496096399
B4GALT7	0.396617602	0.337500891	0.237804547	0.794035382	0.60615126	0.654736169
SLC7A11	0.396617602	0.281172131	0.266914089	0.917553107	0.535395397	0.671843481
CCSAP	0.396617602	0.537303163	0.243371049	0.454736226	0.361265906	0.932608891
ADAMTS2	0.397316312	0.376089517	0.675208988	0.743631929	0.605218423	0.24556396
LOC616840	0.397536322	0.946274461	0.277735273	0.617959671	0.51883282	0.255783167
MCAT	0.397536322	0.309058687	0.322027143	0.99999488	0.683248455	0.255783167
SLFN11	0.397536322	0.270621658	0.305226713	0.99999488	0.644373506	0.332569608
TRAT1	0.39991997	0.302735571	0.27122364	0.573623074	0.999985264	0.404873878
ESRP1	0.399974038	0.286573624	0.421076728	0.554774022	0.999985264	0.240189056
CCL22	0.399974038	0.368543363	0.294677521	0.99999488	0.546848192	0.243107325
LOC107131975	0.399974038	0.302507633	0.954823586	0.434318747	0.487540676	0.314555295
TRIM7	0.401319932	0.356422038	0.271782816	0.851621164	0.546848192	0.596379492
KIF5C	0.401663067	0.302235146	0.448027787	0.978440951	0.608701929	0.272594278
EIF4EBP1	0.404326096	0.227607078	0.318608982	0.99999488	0.546848192	0.256812752
MKX	0.404913665	0.836515843	0.408370911	0.623907391	0.397079609	0.32073005
NPY1R	0.404913665	0.650665822	0.322027143	0.412075059	0.546848192	0.644629963
PCDHGA2	0.40650743	0.650665822	0.39008807	0.691124578	0.664366052	0.26631152
LRRC66	0.406758664	0.397499451	0.314468393	0.99999488	0.367376283	0.298349735
UGT8	0.406758664	0.406480185	0.272519427	0.501376968	0.657836458	0.770633383
AVPI1	0.406758664	0.237931589	0.277735273	0.566968003	0.588526968	0.931163114
MED13	0.407258129	0.284606115	0.27122364	0.401519611	0.999985264	0.24886255
OLFML1	0.407258129	0.270621658	0.405095497	0.794035382	0.999985264	0.25556725

FNDC1	0.407258129	0.240227152	0.493534355	0.99999488	0.568311949	0.266192567
CACNA1A	0.407258129	0.260828162	0.803677695	0.455709502	0.879141216	0.298349735
WDR90	0.407258129	0.307095857	0.95106368	0.554774022	0.382860241	0.32073005
FN1	0.407258129	0.601973618	0.267206344	0.99999488	0.425292769	0.321563767
LOC112445031	0.408125234	0.33857298	0.826595939	0.890282205	0.367835214	0.255783167
DAPK2	0.408400541	0.286573624	0.291592084	0.540024507	0.999985264	0.24556932
ABRACL	0.408400541	0.242843581	0.399025922	0.99999488	0.51883282	0.255783167
TLL2	0.408400541	0.375225978	0.401122009	0.99999488	0.474284035	0.308232485
SLX1A	0.408400541	0.286429495	0.515899039	0.689182046	0.6707745	0.489481105
PPP1CB	0.409786761	0.270621658	0.591030763	0.747849903	0.935138282	0.252337751
NLN	0.409786761	0.357465581	0.264940776	0.468727346	0.999985264	0.593347482
IL6	0.409786761	0.47266331	0.277735273	0.511435398	0.676931424	0.678677938
NTN1	0.410291917	0.310658204	0.365811456	0.857323853	0.999985264	0.25084741
APC2	0.410291917	0.870922303	0.412953991	0.707659473	0.433794986	0.255783167
CCDC68	0.410291917	0.237931589	0.700695194	0.74725201	0.873468654	0.255783167
BLNK	0.410291917	0.312714438	0.265938983	0.99999488	0.417908713	0.256039472
ST3GAL5	0.410291917	0.323872487	0.271782816	0.907700124	0.999985264	0.259161757
LOC518134	0.410291917	0.271737569	0.270187309	0.99999488	0.612322329	0.261104005
RRBP1	0.410291917	0.408874345	0.344157642	0.991596938	0.662093891	0.264713475
DLX5	0.410291917	0.985204209	0.373363914	0.430247169	0.546848192	0.272302366
CEP170B	0.410291917	0.591124857	0.260061205	0.999314272	0.597527476	0.272302366
FGFR1	0.410291917	0.310658204	0.254322211	0.99999488	0.566515062	0.284190065
DBF4B	0.410291917	0.25270365	0.401122009	0.589303553	0.999985264	0.284190065
ATP7A	0.410291917	0.341872638	0.401122009	0.675702198	0.999985264	0.29558415
LOC101905956	0.410291917	0.445237461	0.40365456	0.996471643	0.499664211	0.298349735
CPZ	0.410291917	0.366642903	0.471348003	0.99999488	0.474284035	0.298460621
RHEX	0.410291917	0.235772942	0.278631552	0.99999488	0.546848192	0.318477406
MREG	0.410291917	0.329919334	0.302770885	0.99999488	0.456843913	0.320369901
TOP1	0.410291917	0.281172131	0.322027143	0.419734129	0.999985264	0.320469762
SERPINE1	0.410291917	0.423334704	0.817700149	0.543066914	0.54072714	0.321563767
SNX10	0.410291917	0.27045622	0.322027143	0.963012661	0.83400817	0.376881894
ARMCX4	0.410291917	0.284121857	0.296427658	0.99999488	0.6707745	0.385430007
DHX58	0.410291917	0.243691024	0.517947068	0.855453165	0.68104332	0.427149545
EIF4G1	0.410291917	0.284121857	0.270726262	0.65216037	0.999985264	0.433588995
MCOLN3	0.410291917	0.282634514	0.294677521	0.99999488	0.422500465	0.436673268
DEXI	0.410291917	0.281172131	0.254322211	0.921764051	0.901104314	0.453090281
MRPL15	0.410291917	0.296726456	0.405170393	0.760605313	0.792861833	0.465198839
ANTXRL	0.410291917	0.270621658	0.926552506	0.434318747	0.434976541	0.519472498
ILVBL	0.410291917	0.303193306	0.297750614	0.569291976	0.999985264	0.522589003

DIP2B	0.410291917	0.240463317	0.288099709	0.978440951	0.676931424	0.533511539
BMPER	0.410291917	0.64037592	0.269444269	0.794035382	0.490281033	0.537810684
AFAP1	0.410291917	0.281172131	0.254322211	0.99999488	0.546848192	0.54675724
EAPP	0.410291917	0.385418738	0.282219258	0.468727346	0.939144222	0.60359273
RRP9	0.410291917	0.50481472	0.311651171	0.56961436	0.655307354	0.614862466
FGF10	0.410291917	0.288565095	0.509523994	0.434318747	0.741080831	0.654736169
RPL22L1	0.410291917	0.329514394	0.534748074	0.573623074	0.499864527	0.693616839
NOL6	0.410291917	0.271328522	0.425914657	0.560163102	0.68104332	0.714215429
CARD6	0.410291917	0.250841926	0.478716829	0.691124578	0.456843913	0.789976745
POLR1B	0.410291917	0.235772942	0.451511676	0.623431927	0.546843279	0.816226172
SNTA1	0.410291917	0.243819453	0.387533191	0.674479545	0.535395397	0.856224954
ABCF2	0.410291917	0.270621658	0.265938983	0.622527961	0.546848192	0.943220018
C1QL3	0.410291917	0.347981673	0.31513696	0.434318747	0.456843913	0.972919703
TBL1XR1	0.41131895	0.283340687	0.566721071	0.99999488	0.452997928	0.355934508
SLC23A2	0.412632889	0.239751512	0.465918382	0.99999488	0.546848192	0.25745626
LIG3	0.413234975	0.411165859	0.260061205	0.99999488	0.393203728	0.414123435
P2RX7	0.413303143	0.237931589	0.4844733	0.884809097	0.937177132	0.275900023
ELP1	0.413303143	0.286429495	0.926552506	0.59523567	0.515907379	0.321563767
GPR162	0.413412758	0.238800768	0.371662326	0.99999488	0.497727099	0.256812752
ALKBH1	0.413748131	0.397160637	0.268313268	0.428841505	0.999985264	0.256419143
C18H19orf48	0.413748131	0.56780353	0.271782816	0.566159233	0.980522362	0.374697138
PQBP1	0.413748131	0.343992179	0.322027143	0.490137677	0.999985264	0.410427392
KY	0.413748131	0.476083641	0.297626948	0.907700124	0.433794986	0.594584164
LOC783577	0.414241303	0.357465581	0.338611837	0.590046473	0.999985264	0.428217797
WDFY4	0.416277069	0.286429495	0.271782816	0.99999488	0.503338662	0.255783167
ANKRD17	0.416574814	0.303078947	0.266523444	0.439336917	0.999985264	0.255783167
PLEKHM2	0.416574814	0.266176294	0.260061205	0.502809653	0.999985264	0.298349735
CD226	0.416574814	0.378297385	0.346622368	0.613584928	0.999985264	0.397980881
GAL	0.416741901	0.281172131	0.310260582	0.92106221	0.934591321	0.378213674
CNOT1	0.418462742	0.336029024	0.272213652	0.448455803	0.999985264	0.255783167
SIX4	0.418472138	0.265001767	0.481599427	0.848389672	0.999985264	0.255783167
PTPN6	0.418472138	0.274904243	0.272213652	0.99999488	0.755784104	0.275117802
LOC782177	0.418472138	0.487467317	0.373234212	0.949602784	0.414082187	0.468561589
LOC107132537	0.419362905	0.578087256	0.580469773	0.854322524	0.400840387	0.314555295
LNX2	0.419445952	0.311493981	0.264201831	0.468727346	0.999985264	0.358684674
LOC112442023	0.419878337	0.413107515	0.805753285	0.506542466	0.584509232	0.34879456
BRI3BP	0.419878337	0.288565095	0.348012485	0.462799363	0.999985264	0.445262677
STPG4	0.421527099	0.674944018	0.441586616	0.813963687	0.54072714	0.284190065
NPTX1	0.421527099	0.281172131	0.405170393	0.99999488	0.435567313	0.321563767

TOB2	0.421527099	0.304735985	0.281243612	0.476062614	0.999985264	0.537810684
PBXIP1	0.421527099	0.503649548	0.372681117	0.554774022	0.403462825	0.850385256
ADM5	0.422208168	0.311493981	0.443036005	0.966694193	0.706185473	0.312093451
DAZAP2	0.422398945	0.344671752	0.917154286	0.540024507	0.655410555	0.255783167
ICAM3	0.422398945	0.283340687	0.278631552	0.949602784	0.691155429	0.582816513
C7H19orf25	0.424554269	0.343013097	0.272519427	0.691124578	0.999985264	0.267063051
ZNF404	0.424631619	0.999992444	0.266914089	0.490137677	0.546848192	0.298460621
ZFX	0.426488245	0.433354148	0.645166627	0.540024507	0.904842818	0.266771051
RAPGEFL1	0.426630292	0.421030287	0.608572985	0.71610479	0.497727099	0.475846099
LAMB3	0.427248867	0.487641245	0.311949976	0.925818215	0.813331114	0.275375051
IGF2BP3	0.427248867	0.322939396	0.306075466	0.99999488	0.397079609	0.424924923
ZMAT1	0.427248867	0.491830057	0.306075466	0.437458118	0.961025202	0.578094648
LOC101906870	0.428514125	0.310658204	0.368764929	0.71879265	0.999985264	0.385430007
WAS	0.428514125	0.369069593	0.318608982	0.99999488	0.594728091	0.415932896
RASSF4	0.429044245	0.329514394	0.314468393	0.99999488	0.546848192	0.264221141
LOC100336868	0.429044245	0.647062519	0.576741963	0.622527961	0.664079708	0.264713475
KLHL17	0.429044245	0.336693283	0.666104182	0.725623067	0.835076615	0.267604146
ALKBH8	0.429044245	0.999992444	0.270811008	0.465243948	0.546848192	0.312093451
USPL1	0.429209733	0.311493981	0.282219258	0.434318747	0.999985264	0.263057471
LPAR6	0.429209733	0.504913968	0.271632266	0.976631804	0.834754713	0.267411573
TMEM203	0.429209733	0.312714438	0.524275507	0.760007034	0.999985264	0.272302366
TMBIM7	0.429209733	0.312714438	0.458143546	0.543066914	0.999985264	0.275900023
POT1	0.429209733	0.634298188	0.272519427	0.830046103	0.856124022	0.284190065
CHORDC1	0.429209733	0.384769897	0.518971669	0.434318747	0.999985264	0.284190065
RYBP	0.429209733	0.281172131	0.477167064	0.573623074	0.999985264	0.284190065
SPRY2	0.429209733	0.337920983	0.922400936	0.468621806	0.707353372	0.288822775
FCGR1A	0.429209733	0.305501687	0.272519427	0.99999488	0.688375802	0.298349735
NUDT9	0.429209733	0.283340687	0.43661424	0.99999488	0.612198268	0.302587214
GRIK2	0.429209733	0.326936652	0.757137435	0.803037807	0.604847705	0.305331668
ITIH4	0.429209733	0.587721224	0.38143799	0.99999488	0.425292769	0.308232485
CLK4	0.429209733	0.459982696	0.308654643	0.48240694	0.999985264	0.396212881
ALG5	0.429209733	0.322939396	0.427421518	0.462879801	0.999985264	0.402739177
FABP1	0.429209733	0.819379885	0.387533191	0.447226346	0.707353372	0.407020776
TAOK3	0.429209733	0.271328522	0.270726262	0.896748401	0.999985264	0.478098211
SELL	0.429209733	0.305501687	0.403466151	0.945247973	0.566923246	0.535906285
LOC112445943	0.429209733	0.650780684	0.291592084	0.454736226	0.849864394	0.559024093
OAS2	0.429209733	0.274904243	0.408370911	0.794035382	0.612322329	0.697337654
SLC29A2	0.430278115	0.283340687	0.270811008	0.948570368	0.999985264	0.321151792
GDF6	0.430535193	0.275093864	0.632118699	0.99999488	0.458955101	0.387823865

MYH3	0.430987541	0.346961098	0.427421518	0.99999488	0.545924011	0.264713475
DGKI	0.430987541	0.387827411	0.353596276	0.99999488	0.771578767	0.266771051
PSAP	0.430987541	0.380987958	0.311949976	0.99999488	0.456843913	0.273011972
AKR1B1	0.430987541	0.548801965	0.474987636	0.927129324	0.546848192	0.284190065
DFFB	0.430987541	0.661182871	0.785248842	0.589303553	0.426783288	0.298460621
GPR137B	0.430987541	0.304735985	0.399025922	0.99999488	0.47399696	0.347652735
TREM2	0.430987541	0.337920983	0.343706531	0.99999488	0.478188626	0.347652735
TIMM44	0.430987541	0.288565095	0.296566679	0.545974633	0.999985264	0.378213674
RAC2	0.430987541	0.38625193	0.309703078	0.943090854	0.712429088	0.443181556
FRRS1L	0.430987541	0.281172131	0.418866598	0.99999488	0.433794986	0.453090281
TMEM236	0.430987541	0.281172131	0.308654643	0.99999488	0.608701929	0.513288396
NEU3	0.430987541	0.262424991	0.506949813	0.566159233	0.798938088	0.675571489
FAIM2	0.430987541	0.29443215	0.270780202	0.917553107	0.583644292	0.775144909
RGS4	0.435228889	0.509042229	0.285528161	0.99999488	0.546848192	0.272302366
ZNF19	0.435228889	0.323640247	0.296427658	0.672549466	0.999985264	0.436631145
BAALC	0.435228889	0.33049774	0.578947523	0.475426312	0.60615126	0.772479356
LOC781001	0.435336314	0.342195215	0.922400936	0.622527961	0.585367388	0.272594278
PGF	0.435336314	0.491830057	0.383280149	0.798549183	0.947692031	0.272594278
C25H16orf54	0.435336314	0.294756438	0.399025922	0.976631804	0.861287091	0.332569608
UNC5C	0.435336314	0.895540475	0.40793937	0.69049726	0.447414777	0.347652735
CFI	0.435336314	0.755100312	0.492460512	0.762590159	0.456843913	0.347652735
UBD	0.435336314	0.808097876	0.291592084	0.566159233	0.858096179	0.347652735
RABGEF1	0.435336314	0.286573624	0.592707491	0.794035382	0.81217611	0.388523226
SCML4	0.435336314	0.337436711	0.605479618	0.48240694	0.999985264	0.39743828
STXBP3	0.435336314	0.551538509	0.308654643	0.794035382	0.826782609	0.397980881
PTGS2	0.435336314	0.341872638	0.637673225	0.796555088	0.565236258	0.453973624
SIT1	0.435336314	0.567551894	0.38066575	0.518269294	0.448994037	0.847577522
ABCF3	0.435781927	0.329921024	0.295005899	0.5736757	0.999985264	0.442435723
LOC101908206	0.436086878	0.285893165	0.28669104	0.99999488	0.433794986	0.332348353
EVI2B	0.436454099	0.281172131	0.272519427	0.99999488	0.697132345	0.35354287
LMOD1	0.437225852	0.480300487	0.388697206	0.540024507	0.56049568	0.807187146
XRCC5	0.437283104	0.591239507	0.414091442	0.526131477	0.999985264	0.312093451
CYB5D2	0.437283104	0.661914444	0.372719233	0.5736757	0.741080831	0.451091614
NFE2L2	0.439920108	0.284773648	0.308654643	0.969770561	0.999985264	0.292243113
CAPN8	0.439920108	0.367205885	0.805753285	0.872834104	0.496438607	0.312093451
ARHGDIA	0.439920108	0.788635502	0.353596276	0.540024507	0.573890459	0.554947685
SURF6	0.439920108	0.329033389	0.412953991	0.791188744	0.616780785	0.67496387
LOC618737	0.440377479	0.345911791	0.372681117	0.991596938	0.712429088	0.386258495
CRABP1	0.440377479	0.270621658	0.374903616	0.794035382	0.921581207	0.580415308

PAK2	0.440488102	0.312137325	0.580738818	0.714531977	0.999985264	0.272594278
TNNT2	0.440488102	0.351000342	0.456961614	0.760007034	0.999985264	0.284190065
C2CD4B	0.440488102	0.270621658	0.582599433	0.99999488	0.662093891	0.298460621
SLC32A1	0.440488102	0.270416395	0.691847112	0.454736226	0.999985264	0.347652735
TENT5C	0.440488102	0.274904243	0.283399364	0.468727346	0.999985264	0.347652735
CTNND2	0.440488102	0.486333441	0.353596276	0.99999488	0.546848192	0.358046095
AQP4	0.440488102	0.282634514	0.708328647	0.757550318	0.732546652	0.407020776
LOC789035	0.440488102	0.830519875	0.430117243	0.449675422	0.676931424	0.414123435
GJC3	0.440488102	0.300555639	0.76260462	0.645998678	0.707353372	0.426595122
CDC16	0.440488102	0.288905896	0.272519427	0.468727346	0.999985264	0.54675724
CHST13	0.441443991	0.311493981	0.287578902	0.99999488	0.961025202	0.327455154
HCN4	0.441700392	0.742481138	0.294677521	0.483278747	0.981774771	0.397564689
CSTB	0.442301549	0.366827156	0.349491657	0.99999488	0.61901016	0.275900023
PSRC1	0.442842566	0.274904243	0.302770885	0.96412514	0.999985264	0.298349735
LOC617141	0.445660955	0.304768949	0.831723974	0.963659492	0.491621738	0.284190065
CKS2	0.445660955	0.274904243	0.865420641	0.847714599	0.474284035	0.415932896
POPDC2	0.445660955	0.685985172	0.398905253	0.454736226	0.77926164	0.530560642
EDIL3	0.445660955	0.345911791	0.430950682	0.645998678	0.771578767	0.632752232
CD4	0.445660955	0.341770652	0.518971669	0.566159233	0.546848192	0.827190118
HDAC10	0.446762905	0.376089517	0.476140374	0.493932625	0.999985264	0.279755664
ZNF428	0.446762905	0.343863091	0.314468393	0.99999488	0.497727099	0.28308236
LOC781022	0.446762905	0.357465581	0.592707491	0.99999488	0.51883282	0.292719589
SH3TC1	0.446762905	0.329514394	0.386210449	0.99999488	0.899432059	0.29558415
ACTB	0.446762905	0.530773348	0.932653752	0.540024507	0.478188626	0.298460621
MEGF8	0.446762905	0.288565095	0.403466151	0.791243098	0.999985264	0.305488991
PLEKHJ1	0.446762905	0.335617536	0.78255891	0.66085809	0.51883282	0.561314399
SMUG1	0.446762905	0.341770652	0.746258329	0.490137677	0.425292769	0.812825712
LIMK2	0.447596127	0.310658204	0.353596276	0.977598122	0.999985264	0.284190065
SMTNL2	0.447596127	0.286573624	0.282219258	0.99999488	0.655410555	0.312093451
LOC107132724	0.447596127	0.819937634	0.420369512	0.760007034	0.546848192	0.336226519
WASF1	0.447596127	0.87010028	0.336658841	0.566159233	0.52819768	0.560845144
LOC782675	0.449858612	0.870142804	0.380872288	0.540024507	0.546848192	0.499718183
LOC781977	0.450012465	0.560080598	0.38143799	0.99999488	0.452997928	0.284190065
ANPEP	0.451904707	0.517722585	0.372681117	0.590046473	0.969092995	0.44392264
BID	0.452150716	0.347067838	0.374816105	0.99999488	0.612198268	0.349401965
ZNF311	0.456086468	0.796531384	0.403466151	0.49753871	0.931118794	0.304252478
ALG11	0.456086468	0.308891887	0.43661424	0.99999488	0.625232521	0.355934508
PMP2	0.456097296	0.561419114	0.295005899	0.966054859	0.77825208	0.321563767
TNNI2	0.456097296	0.279898072	0.412953991	0.907045085	0.999985264	0.347652735

BMP1	0.456598782	0.341872638	0.689932449	0.99999488	0.487511192	0.284190065
PRPF19	0.456598782	0.348631542	0.398905253	0.901707661	0.546848192	0.699549917
NCF2	0.457053982	0.322939396	0.294848545	0.99999488	0.535395397	0.278565753
RBM39	0.458898454	0.405007903	0.324743601	0.49753871	0.999985264	0.465198839
M6PR	0.459578738	0.379157225	0.601420135	0.99999488	0.546848192	0.298349735
LOC100300806	0.459578738	0.310125056	0.40365456	0.99999488	0.656196025	0.305488991
NFYB	0.459578738	0.433007404	0.286580035	0.493932625	0.999985264	0.312093451
VSTM2L	0.459578738	0.317076178	0.599813846	0.917553107	0.7946823	0.316018978
PLEKHA6	0.459578738	0.311493981	0.888545123	0.571289919	0.736346045	0.358484708
ADAM19	0.459578738	0.286429495	0.358498433	0.99999488	0.433794986	0.493956701
VWCE	0.459578738	0.326705874	0.311949976	0.99999488	0.546848192	0.559024093
TMED10	0.460526418	0.303707231	0.96290532	0.589303553	0.625232521	0.301557927
PDZD3	0.460526418	0.647062519	0.37623012	0.479547402	0.934591321	0.50479158
ABT1	0.460526418	0.350278804	0.776282112	0.691124578	0.535395397	0.536719591
JAK1	0.461420621	0.281172131	0.332817482	0.99999488	0.901104314	0.281536736
GRN	0.463409384	0.336693283	0.311651171	0.99999488	0.546848192	0.321563835
RENBP	0.463561659	0.306875604	0.348334982	0.99999488	0.947449184	0.298460621
MCM8	0.463867585	0.900867274	0.383900825	0.853181717	0.51883282	0.302587214
POLG2	0.463867585	0.644729858	0.408370911	0.691124578	0.608701929	0.541323364
LOC112445011	0.465684558	0.548801965	0.755152442	0.468727346	0.672072713	0.433466574
CNDP2	0.467117001	0.445561167	0.405095497	0.99999488	0.453998366	0.298349735
TARBP1	0.467117001	0.341770652	0.628800812	0.99999488	0.546848192	0.321563767
LOC101907405	0.467117001	0.281172131	0.871925227	0.557668692	0.821945987	0.404873878
NAAA	0.46892638	0.308891887	0.360685245	0.99999488	0.964631108	0.306223626
FBXO28	0.469304274	0.338960015	0.926552506	0.760007034	0.586408232	0.284190065
PPP1R14B	0.469304274	0.549349774	0.344157642	0.56637458	0.83400817	0.645467494
CBL	0.469492616	0.285515319	0.427959961	0.917553107	0.999985264	0.314555295
BEND6	0.470391156	0.375633553	0.294677521	0.909784383	0.999985264	0.380378402
INHA	0.470391156	0.299096397	0.890096618	0.59523567	0.706931395	0.408694653
ZNF469	0.471049398	0.283340687	0.640903956	0.951237479	0.707353372	0.387585743
MED21	0.471049398	0.351000342	0.37511602	0.99999488	0.741080831	0.419282739
LOC107131704	0.471049398	0.356422038	0.825497047	0.554774022	0.443037875	0.692319327
ARC	0.471487942	0.659816181	0.566353811	0.48240694	0.608701929	0.583476019
DNAJC2	0.471739957	0.341770652	0.302770885	0.617959671	0.546848192	0.986787448
TXLNA	0.473597783	0.322939396	0.297626948	0.643826897	0.999985264	0.433466574
LARS2	0.474614188	0.3033493	0.313948449	0.594975772	0.999985264	0.404873878
LOC783680	0.47475947	0.398462449	0.380701737	0.978440951	0.941510628	0.298460621
MITF	0.475009664	0.286573624	0.295005899	0.99999488	0.478188626	0.513668906
CA4	0.475093533	0.4916534	0.305226713	0.484720117	0.999985264	0.687272719

HSD11B1	0.47536301	0.600396627	0.36065063	0.99999488	0.546848192	0.347652735
HBB	0.47536301	0.406480185	0.427421518	0.687904478	0.922384168	0.52928764
SIRPA	0.47536301	0.823801668	0.310260582	0.502809653	0.568311949	0.719032397
CD84	0.475831486	0.338068267	0.408267931	0.99999488	0.546848192	0.312093451
FBXL7	0.475831486	0.493550152	0.35879437	0.540024507	0.999985264	0.385513832
CADM1	0.475831486	0.322966392	0.405095497	0.949602784	0.546848192	0.721134875
PHACTR4	0.475831486	0.533435858	0.357047636	0.622611633	0.546848192	0.870708914
GPATCH4	0.477736057	0.411312632	0.305498587	0.927647996	0.797752972	0.545565041
INO80B	0.479146151	0.745276769	0.405095497	0.566159233	0.999985264	0.298460621
GNA12	0.479146151	0.416511972	0.36532409	0.952783349	0.789479114	0.445567073
NOP14	0.479146151	0.30132249	0.482693989	0.796555088	0.961025202	0.486472357
CX3CL1	0.479146151	0.494978451	0.405095497	0.917553107	0.546308973	0.599905787
TBC1D32	0.480182459	0.991488652	0.480714058	0.48240694	0.600951296	0.292719589
SRGAP3	0.480591262	0.999992444	0.340354802	0.48240694	0.657836458	0.349020434
USP4	0.480671441	0.303193306	0.519697319	0.99999488	0.474284035	0.52928764
MTPAP	0.481153383	0.589631719	0.360806372	0.973503136	0.764583416	0.314555295
SEC16B	0.481153383	0.368543363	0.567003593	0.99999488	0.546848192	0.388523226
TNPO2	0.481153383	0.296726456	0.322027143	0.917553107	0.999985264	0.402739177
STEAP2	0.481153383	0.510762296	0.387533191	0.99999488	0.546848192	0.404873878
LOC101902211	0.481153383	0.637749095	0.399025922	0.573623074	0.937177132	0.449721384
RGS14	0.481153383	0.4916534	0.386210449	0.945038047	0.672072713	0.475348039
KCNH1	0.481153383	0.459215288	0.316368724	0.687904478	0.999985264	0.52928764
RDH13	0.481153383	0.378106622	0.39008807	0.558959587	0.965672975	0.692319327
METTL1	0.481153383	0.337398416	0.362408227	0.795238168	0.63582413	0.863280322
IPO7	0.482030449	0.329919334	0.726885375	0.708414872	0.849864394	0.406585757
SCARB2	0.482137083	0.29825202	0.43661424	0.99999488	0.51883282	0.298349735
SLC6A6	0.482137083	0.510016527	0.405095497	0.99999488	0.561363177	0.298349735
KIAA1191	0.482137083	0.448821794	0.40793937	0.796555088	0.999985264	0.312093451
AP2B1	0.482137083	0.345758663	0.493015519	0.718002256	0.999985264	0.382799455
SH2D1B	0.482137083	0.301112494	0.373363914	0.99999488	0.600951296	0.387585743
LOC104969384	0.482137083	0.36365584	0.550189265	0.896370331	0.78143481	0.420434059
SLC38A4	0.482137083	0.551235148	0.706110318	0.744246818	0.535395397	0.451091614
HECTD3	0.482248839	0.503649548	0.774363763	0.490137677	0.91945292	0.312093451
VSTM1	0.482248839	0.395637933	0.432473682	0.99999488	0.546848192	0.321151792
LOC781710	0.482248839	0.416445008	0.405095497	0.813963687	0.792861833	0.578823016
SLC37A2	0.483798785	0.332945137	0.311651171	0.99999488	0.565236258	0.298460621
SFN	0.483811764	0.427983557	0.93198623	0.502809653	0.546848192	0.446372578
ARHGAP20	0.484274758	0.38156866	0.923604668	0.490137677	0.835076615	0.314555295
ACTR2	0.484274758	0.322966392	0.766712208	0.921764051	0.608701929	0.383423168

PIP4K2A	0.48504717	0.304768949	0.308654643	0.917553107	0.999985264	0.321563767
CD63	0.485758491	0.412965824	0.570175675	0.816792716	0.941811024	0.314555295
GCHFR	0.485758491	0.316033833	0.353596276	0.907700124	0.944638139	0.572723243
SIRPB2	0.486878441	0.307095857	0.344345603	0.99999488	0.922362358	0.383688194
MYCT1	0.487788882	0.332945137	0.387533191	0.937020992	0.999985264	0.298460621
LRRC47	0.489060858	0.311493981	0.468000957	0.99999488	0.6707745	0.513288396
OAZ2	0.490743314	0.310658204	0.362408227	0.699968468	0.999985264	0.358046095
LOC101905029	0.492539136	0.4916534	0.970945672	0.501376968	0.568311949	0.298884447
SGPL1	0.494510869	0.322966392	0.456961614	0.942391899	0.999985264	0.331540119
SGK2	0.494714369	0.366740223	0.379593497	0.859289191	0.999985264	0.314555295
MBNL1	0.494714369	0.551235148	0.309728905	0.917553107	0.921998583	0.401077962
TP53INP1	0.494714369	0.367205885	0.353596276	0.99999488	0.546848192	0.453090281
SERPINE2	0.494714369	0.408874345	0.459542294	0.876702931	0.777608317	0.513288396
ZNF182	0.494714369	0.336693283	0.506949813	0.99999488	0.51883282	0.528012162
TSSC4	0.494714369	0.411312632	0.344345603	0.580714846	0.999985264	0.681518797
SLITRK5	0.494714369	0.322939396	0.595339754	0.493932625	0.707353372	0.861670395
RAB43	0.494721127	0.356422038	0.306446706	0.99999488	0.894715133	0.347652735
PALB2	0.494721127	0.399245555	0.930678591	0.617959671	0.518388455	0.459569137
WARS	0.494721127	0.561419114	0.326842621	0.839050812	0.771578767	0.571769738
KCNE4	0.495220073	0.813113924	0.420270937	0.613584928	0.901104314	0.317426533
PRRT4	0.497222587	0.322966392	0.440026382	0.594975772	0.999985264	0.556965527
MARCKS	0.497257184	0.470599569	0.322027143	0.99999488	0.815550318	0.312093451
PIPOX	0.497257184	0.382043649	0.322027383	0.917553107	0.999985264	0.347652735
PKIB	0.497257184	0.344903278	0.485378752	0.884870518	0.741080831	0.580415308
DNAJC16	0.497257184	0.394487048	0.518123552	0.502809653	0.794349969	0.784749233
NUDT18	0.497448329	0.303193306	0.52966531	0.563443606	0.999985264	0.436631145
SERPINI1	0.497840797	0.523245437	0.43661424	0.502345596	0.999985264	0.312093451
MRPS21	0.497840797	0.306986282	0.405095497	0.554774022	0.999985264	0.376881894
PRG4	0.497840797	0.384769897	0.935136095	0.594975772	0.546848192	0.451091614
EIF2S1	0.497840797	0.390845983	0.771096787	0.794035382	0.596250513	0.468561589
LOC101906178	0.498544928	0.323872487	0.522266075	0.560163102	0.999985264	0.308232485
SHISA4	0.498544928	0.523245437	0.450532894	0.953188062	0.815550318	0.314555295
YJU2	0.498544928	0.345758663	0.638383601	0.540024507	0.999985264	0.316018978
PPDPF	0.498544928	0.368543363	0.536995142	0.99999488	0.657836458	0.331230897
MMP28	0.498544928	0.345758663	0.32009369	0.575416797	0.999985264	0.353918138
CDH2	0.498544928	0.408552266	0.36532409	0.99999488	0.546848192	0.402739177
CRIP1	0.498544928	0.647062519	0.355565796	0.573623074	0.999985264	0.41854243
KIAA1217	0.498544928	0.366278182	0.432491666	0.851621164	0.928683582	0.513288396
CORO1A	0.498544928	0.577792766	0.311651171	0.978440951	0.608701929	0.554947685

LOC112441884	0.498544928	0.764537649	0.401122009	0.695630809	0.608701929	0.560845144
C2H2orf72	0.498544928	0.406061927	0.578947523	0.937020992	0.528374331	0.586220428
LOC112449547	0.498544928	0.332945137	0.342443969	0.99999488	0.52539338	0.807566643
TMEM126B	0.498544928	0.384132949	0.374903616	0.515887724	0.565236258	0.993086089
EMILIN1	0.499266834	0.727377621	0.326842621	0.92106221	0.6707745	0.437919952
OSBPL11	0.500462264	0.302507633	0.534417481	0.992168706	0.887453777	0.363053792
DIAPH3	0.501503159	0.330856412	0.780158644	0.976631804	0.664686023	0.308960121
CCAR1	0.50169508	0.399819771	0.543896126	0.566159233	0.999985264	0.318527813
LOC782264	0.50169508	0.342195215	0.313136429	0.99999488	0.957235535	0.321563767
PPIF	0.50169508	0.322966392	0.403466151	0.543496777	0.999985264	0.728036947
GTPBP6	0.501743812	0.311493981	0.322027143	0.777342221	0.999985264	0.445262677
LENG9	0.502078546	0.370995618	0.477167064	0.99999488	0.798938088	0.34879456
TNFRSF18	0.502228928	0.598092257	0.322027143	0.99999488	0.529750417	0.419894928
PLA2G7	0.504459611	0.336693283	0.372719233	0.99999488	0.901104314	0.310228844
TELO2	0.504459611	0.326936652	0.788870461	0.691124578	0.996075618	0.321563767
AP4E1	0.504459611	0.864154704	0.405095497	0.917553107	0.546848192	0.335813387
SDK2	0.504459611	0.311493981	0.601128026	0.99999488	0.741080831	0.337549184
SERPINB6	0.504459611	0.502850182	0.353596276	0.950949206	0.956114729	0.360142945
CYTH4	0.504459611	0.348631542	0.322027383	0.99999488	0.880252174	0.413669144
EIF4A3	0.504459611	0.406954343	0.771154197	0.92106221	0.532489404	0.430466706
GAS2L3	0.504459611	0.369431296	0.63799633	0.876702931	0.607100736	0.58581641
ECD	0.504459611	0.306265072	0.387533191	0.540024507	0.999985264	0.732471021
CCR2	0.504459611	0.417495017	0.353596276	0.695630809	0.899432059	0.75441123
VAT1	0.505813991	0.491830057	0.405095497	0.98846246	0.656196025	0.504328062
DNAJC11	0.505813991	0.336029024	0.358281509	0.590046473	0.999985264	0.580415308
PEG3	0.505813991	0.408874345	0.595396914	0.554774022	0.901104314	0.610043612
DOLK	0.505813991	0.365512144	0.408267931	0.691124578	0.847202014	0.775755327
LOC101906312	0.506552375	0.339023773	0.373363914	0.99999488	0.588526968	0.371648492
LOC112447626	0.507675011	0.97671655	0.52619906	0.540024507	0.651633281	0.314555295
VSIG4	0.507675011	0.417495017	0.401122009	0.99999488	0.597486077	0.396426203
ICOSLG	0.507755829	0.725393911	0.372681117	0.573623074	0.999985264	0.358046095
SCRG1	0.507783449	0.33922431	0.380872288	0.554774022	0.999985264	0.312093451
SLC25A15	0.507783449	0.309138367	0.380872288	0.755002367	0.999985264	0.34249435
TMEM17	0.507783449	0.991488652	0.525702179	0.609742602	0.496438607	0.348752662
HEBP2	0.507783449	0.316597478	0.36065063	0.691124578	0.999985264	0.354233502
CLEC4A	0.507783449	0.423194756	0.504764189	0.99999488	0.546848192	0.39743828
C5AR1	0.507783449	0.425252947	0.384443607	0.896481111	0.999985264	0.404873878
SLC2A10	0.507783449	0.38625193	0.322027383	0.99999488	0.858096179	0.419282739
RGL3	0.507783449	0.893642384	0.609340165	0.573623074	0.497727099	0.459569137

HEY2	0.507783449	0.504825401	0.443945812	0.597506286	0.6707745	0.812831419
VPS18	0.507783449	0.548801965	0.353596276	0.687904478	0.57461724	0.895349622
APH1B	0.507783449	0.311493981	0.441586616	0.566159233	0.613524413	0.982250307
MSR1	0.50829293	0.338068267	0.374816105	0.99999488	0.515907379	0.329745654
BST1	0.50829293	0.312714438	0.570175675	0.99999488	0.535395397	0.412423814
FMO3	0.50829293	0.494849189	0.393874142	0.813963687	0.999985264	0.440179295
CD3EAP	0.50829293	0.509042229	0.623909299	0.805785692	0.546848192	0.601946079
TOMM40	0.50829293	0.377122341	0.36065063	0.554774022	0.999985264	0.601946079
LPIN1	0.508378193	0.308245864	0.634126063	0.99999488	0.546848192	0.541323364
ZNF711	0.509195541	0.674944018	0.592391392	0.81936725	0.6707745	0.355934508
GNAZ	0.509195541	0.874697957	0.486678526	0.770235811	0.608019099	0.361684438
FICD	0.509195541	0.705186914	0.626056936	0.851621164	0.546848192	0.391016005
SLC11A1	0.509195541	0.310125056	0.360541765	0.99999488	0.535395397	0.410197113
EPCAM	0.509195541	0.408030893	0.595396914	0.566159233	0.999985264	0.487965891
MPV17L2	0.509195541	0.450029284	0.417480833	0.924872024	0.729918988	0.559779949
MMP9	0.509683355	0.310658204	0.464239008	0.99999488	0.546848192	0.385513832
DOK7	0.510035638	0.574311353	0.40793937	0.543496777	0.999985264	0.385430007
SLC1A2	0.510164297	0.366642903	0.353596276	0.99999488	0.594728091	0.465198839
HDLBP	0.510164297	0.480871166	0.405170393	0.803237525	0.736651407	0.675571489
OTUD1	0.510450274	0.322966392	0.923953195	0.744246818	0.75447368	0.349401965
PAN2	0.510450274	0.411312632	0.998936876	0.568953816	0.497727099	0.378213674
LOC101904976	0.510450274	0.50499616	0.575199502	0.693790329	0.944638139	0.414123435
SLIT1	0.510450274	0.451314075	0.43661424	0.568953816	0.999985264	0.508556226
CD1E	0.510477892	0.322966392	0.593672982	0.99999488	0.515907379	0.32206159
LOC101908014	0.510477892	0.87010028	0.487628979	0.689182046	0.671485036	0.387823865
SMIM15	0.510477892	0.540247468	0.865420641	0.695630809	0.546848192	0.419282739
LOC101905876	0.510477892	0.859484941	0.378600654	0.586112202	0.771578767	0.520418463
GOLGA2	0.510500558	0.345911791	0.363631419	0.949602784	0.999985264	0.315141158
PUM2	0.510500558	0.450029284	0.40365456	0.977598122	0.969092995	0.347652735
AMPD3	0.510500558	0.324437981	0.448183673	0.949602784	0.999985264	0.358046095
EFCAB11	0.510500558	0.90213106	0.40365456	0.849357329	0.546848192	0.404873878
LOC101907813	0.510500558	0.491830057	0.337039502	0.92106221	0.999985264	0.407020776
TBL3	0.510500558	0.445237461	0.492557292	0.898357642	0.80986851	0.493273626
TAF4B	0.510500558	0.332945137	0.353596276	0.98846246	0.908980492	0.573142748
USP13	0.510500558	0.341770652	0.344157642	0.917553107	0.983747207	0.603050301
GNAI3	0.511442134	0.405435557	0.911018006	0.82674192	0.607100736	0.330617714
CLTB	0.513066729	0.64037592	0.520782676	0.812057455	0.677061328	0.479463211
LOC782779	0.513525918	0.380987958	0.459026472	0.545974633	0.999985264	0.35354287
TUBB2A	0.513525918	0.673847368	0.870186741	0.543066914	0.541873428	0.445567073

DMBT1	0.513525918	0.394487048	0.40793937	0.99999488	0.545924011	0.59171134
FAM118A	0.515148889	0.387494607	0.344522617	0.693790329	0.798938088	0.897965262
PACS2	0.519991539	0.316033833	0.336658841	0.661469607	0.999985264	0.631711796
CD3E	0.520337447	0.754374533	0.451511676	0.605202917	0.68458318	0.601946079
TBC1D22B	0.520642548	0.411312632	0.448977122	0.99999488	0.565236258	0.440179295
TRIM34	0.522278728	0.322939396	0.350879261	0.917553107	0.999985264	0.347652735
DLX3	0.522278728	0.384132949	0.432491666	0.99999488	0.519333426	0.370245109
STOX2	0.522278728	0.346956059	0.38351278	0.99999488	0.920863785	0.3814217
HDC	0.522278728	0.705186914	0.510346325	0.96968307	0.546848192	0.394404724
GUCD1	0.522278728	0.341770652	0.861497791	0.952783349	0.551732047	0.396212881
EPHB3	0.522278728	0.336693283	0.387533191	0.99999488	0.51883282	0.408694653
RFT1	0.522278728	0.434988507	0.39008807	0.540024507	0.999985264	0.422940396
LOC100849652	0.522278728	0.795255481	0.571817462	0.695630809	0.546848192	0.52928764
ASGR1	0.522278728	0.488021584	0.372719233	0.794035382	0.999985264	0.529436487
FYB1	0.522278728	0.359713072	0.408267931	0.937020992	0.831019096	0.617537824
KCNMA1	0.523945115	0.999992444	0.385324267	0.691124578	0.546848192	0.321563767
TMEM242	0.524021159	0.996680853	0.605719038	0.543496777	0.546848192	0.32206159
ZNF451	0.524021159	0.385418738	0.387533191	0.589303553	0.999985264	0.327545736
LOC617475	0.524021159	0.340166065	0.405170393	0.645998678	0.999985264	0.329042185
CHD1	0.524021159	0.356422038	0.344157642	0.540024507	0.999985264	0.349401965
CA8	0.524021159	0.377122341	0.341331708	0.99999488	0.849864394	0.358484708
FNDC10	0.524021159	0.406061927	0.394168076	0.813963687	0.999985264	0.387783704
CENPA	0.524021159	0.403109879	0.746258329	0.98846246	0.546848192	0.407020776
FGD2	0.524021159	0.448821794	0.459026472	0.99999488	0.568311949	0.419552755
POLR2K	0.524021159	0.569818891	0.921010865	0.648146722	0.529750417	0.422342133
IL10RA	0.524021159	0.384769897	0.349554411	0.99999488	0.983747207	0.433466574
CENPC	0.524021159	0.52907722	0.418438893	0.545974633	0.999985264	0.437919952
NKG7	0.524021159	0.432161395	0.347982086	0.99999488	0.664079708	0.440179295
AIF1	0.524021159	0.398462449	0.349491657	0.99999488	0.943686834	0.453090281
AARSD1	0.524021159	0.367205885	0.385324267	0.71610479	0.546848192	0.986408155
PIGZ	0.524021159	0.328441536	0.434111304	0.540024507	0.608701929	0.999960414
DPT	0.524271452	0.919191546	0.385112713	0.8398706	0.709947147	0.332569608
ZNF148	0.524961496	0.348631542	0.73604716	0.566159233	0.999985264	0.407020776
KIF1BP	0.525206177	0.710369326	0.606008934	0.757550318	0.546848192	0.544438709
TNFAIP8L3	0.525233881	0.705186914	0.363357217	0.99999488	0.529750417	0.396426203
LOC618268	0.525255909	0.342219984	0.360541765	0.99999488	0.52819768	0.586032676
CBX2	0.526163158	0.329514394	0.353923994	0.99999488	0.999985264	0.362026633
GPR20	0.526513205	0.517052652	0.554521346	0.708414872	0.86832979	0.540658715
GIMAP6	0.526513205	0.47266331	0.40793937	0.798459589	0.933605839	0.594984216

TRMT1	0.526513205	0.486333441	0.373234212	0.744246818	0.919929212	0.704679988
TROAP	0.528629039	0.445561167	0.80372615	0.851621164	0.729918988	0.358484708
COL3A1	0.528629039	0.366278182	0.667274767	0.99999488	0.614047054	0.397980881
SPIB	0.528629039	0.821194038	0.351888203	0.978440951	0.52539338	0.514316109
LOC100298890	0.528629039	0.491830057	0.824972187	0.712096118	0.58738912	0.525799302
WDR12	0.528629039	0.337920983	0.396792446	0.816792716	0.999985264	0.662497224
LOC112443504	0.530857975	0.47266331	0.433819606	0.695630809	0.999985264	0.493956701
HTR6	0.531076673	0.337318291	0.509523994	0.575074485	0.999985264	0.420434059
TMC4	0.531285939	0.805068817	0.40365456	0.949602784	0.708833788	0.346268185
MEG8	0.531285939	0.366642903	0.926552506	0.859289191	0.608701929	0.378213674
REPS1	0.531285939	0.332945137	0.566721071	0.99999488	0.546848192	0.385430007
SLC2A9	0.531285939	0.370995618	0.805753285	0.639929738	0.999985264	0.387823865
F5	0.531285939	0.38625193	0.355565796	0.98846246	0.999985264	0.453090281
NKD2	0.531285939	0.341872638	0.43661424	0.938185243	0.999985264	0.456040124
LOC616063	0.531285939	0.366642903	0.353596276	0.854395119	0.999985264	0.574432662
ZNF395	0.531285939	0.43425879	0.771154197	0.549818084	0.823340951	0.591266823
RHOH	0.531285939	0.390870678	0.372531846	0.966694193	0.707022204	0.743798578
RGS7	0.531285939	0.809104286	0.37511602	0.77377244	0.529750417	0.74674547
CXCL3	0.531285939	0.663430554	0.408370911	0.573623074	0.532489404	0.926673043
TIMP1	0.531337985	0.471965963	0.528575568	0.876343662	0.947449184	0.414123435
ZNF131	0.531644432	0.433007404	0.353246317	0.554774022	0.999985264	0.347652735
IFNGR2	0.531644432	0.50655961	0.473495008	0.566159233	0.999985264	0.347652735
FCHO2	0.531644432	0.596319881	0.520782676	0.991596938	0.662093891	0.390688306
SEMA6A	0.531644432	0.503649548	0.40365456	0.7019305	0.999985264	0.401077962
LCN1	0.531644432	0.365512144	0.362408227	0.99999488	0.68104332	0.402739177
TMCC1	0.531644432	0.336891938	0.411900922	0.99999488	0.715824378	0.418348746
RGS1	0.531644432	0.346080451	0.39008807	0.925818215	0.999985264	0.419552755
LOC112441494	0.531644432	0.372262879	0.380701737	0.99999488	0.712429088	0.419894928
SAC3D1	0.531644432	0.705186914	0.576058288	0.739782855	0.733867563	0.453647982
HTR7	0.531644432	0.694996311	0.360806372	0.99999488	0.546848192	0.489481105
PCYT2	0.531644432	0.348631542	0.372719233	0.58516979	0.999985264	0.52928764
SLC4A11	0.531644432	0.778248571	0.459026472	0.645998678	0.707353372	0.588733625
ATRIP	0.531644432	0.329514394	0.419376076	0.554774022	0.999985264	0.603050301
LCMT2	0.531644432	0.393482958	0.690770494	0.744246818	0.644373506	0.733745412
LOC100847365	0.531644432	0.406480185	0.43661424	0.716410729	0.85796104	0.785716654
LOC112443444	0.531644432	0.813113924	0.40365456	0.601209712	0.529750417	0.839572429
LOC783540	0.531644432	0.550265412	0.399025922	0.573623074	0.771578767	0.875840132
LOC518526	0.531644432	0.342219984	0.521866639	0.543066914	0.657836458	0.977753483
CEMIP	0.533646604	0.652895539	0.40365456	0.99999488	0.662093891	0.396426203

FAP	0.533674764	0.442411343	0.403466151	0.99999488	0.608701929	0.54675724
ITGAM	0.534139535	0.375633553	0.385324267	0.99999488	0.546848192	0.434569255
CCL3	0.534284515	0.336693283	0.445933661	0.99999488	0.675459166	0.347652735
CAGE1	0.534284515	0.859484941	0.485378752	0.813963687	0.708864153	0.361684438
MILR1	0.534284515	0.33818895	0.398905253	0.99999488	0.77926164	0.374697138
MAP3K6	0.534284515	0.33857298	0.833419544	0.589303553	0.999985264	0.384536388
ZBP1	0.534284515	0.337500891	0.546969041	0.884650797	0.999985264	0.451091614
MON1B	0.534284515	0.451314075	0.695533457	0.777023782	0.764583416	0.541323364
C11H9orf116	0.534284515	0.341053537	0.456961614	0.949602784	0.935138282	0.555648397
ZNF432	0.534284515	0.991365955	0.353596276	0.622527961	0.552747179	0.578823016
IRF1	0.534284515	0.540247468	0.609340165	0.747849903	0.662093891	0.644690751
RPP40	0.534284515	0.330508869	0.392709537	0.791188744	0.946408931	0.820927993
LAIR1	0.534341096	0.461438762	0.462647747	0.907700124	0.908980492	0.510140739
PIGO	0.534489398	0.459767182	0.945678616	0.825154704	0.546848192	0.347652735
TMEM64	0.534489398	0.39322293	0.585745323	0.738644224	0.999985264	0.347652735
PGGHG	0.534489398	0.341770652	0.704226721	0.960794858	0.864446669	0.380378402
PDCD1	0.534489398	0.674944018	0.535844878	0.786817493	0.873903927	0.404873878
CD180	0.534489398	0.366642903	0.380872288	0.99999488	0.574203003	0.419552755
ZNF75D	0.534489398	0.336693283	0.903175962	0.878491623	0.657836458	0.442784096
C25H16orf72	0.534489398	0.525458484	0.405095497	0.711602989	0.999985264	0.448052852
EARS2	0.534489398	0.391489689	0.387533191	0.66895898	0.999985264	0.496096399
PGP	0.534489398	0.395705956	0.383807223	0.855453165	0.999985264	0.58581641
GRIK3	0.534489398	0.425359784	0.773525557	0.568455312	0.757173298	0.654736169
PRICKLE2	0.534489398	0.341770652	0.592707491	0.794035382	0.785107052	0.738324902
NDUFAF4	0.534489398	0.358086855	0.524560092	0.608345795	0.804329357	0.885874544
MED30	0.534489398	0.345758663	0.502541452	0.760007034	0.708864153	0.888948455
LOC112449613	0.534489398	0.396628714	0.372327552	0.617959671	0.745595852	0.968772302
LOC101904573	0.534545538	0.663430554	0.411900922	0.672549466	0.999985264	0.349020434
EIF6	0.534545538	0.565729718	0.579891228	0.938185243	0.546848192	0.571889717
FOLR2	0.5355271	0.406954343	0.455857083	0.99999488	0.566515062	0.364726051
FABP3	0.5355271	0.452107085	0.414860121	0.99999488	0.546848192	0.450521621
POU2AF1	0.5355271	0.744638901	0.451511676	0.880028563	0.546848192	0.601032588
PREX1	0.5355271	0.551235148	0.408267931	0.66895898	0.535395397	0.960343814
LOC618169	0.535992679	0.338373464	0.393874142	0.99999488	0.919006503	0.397980881
ERO1A	0.537050271	0.456367947	0.815067929	0.813352686	0.847202014	0.347652735
CNTN2	0.537050271	0.36365584	0.416598617	0.99999488	0.535395397	0.378213674
HS6ST1	0.537050271	0.446508992	0.372531846	0.869065101	0.640946067	0.902590955
CLASP1	0.538105226	0.345758663	0.94634922	0.589303553	0.815874035	0.43457459
MUSTN1	0.538501753	0.705186914	0.592707491	0.71610479	0.546848192	0.64722711

LOC101902665	0.538874852	0.699199783	0.471348003	0.869065101	0.546848192	0.644094886
UBASH3A	0.538874852	0.754891312	0.403466151	0.573623074	0.565236258	0.884033654
C15H11orf87	0.540773004	0.337318291	0.381255077	0.693790329	0.999985264	0.347652735
ARNTL	0.541188922	0.401529469	0.414860121	0.925818215	0.999985264	0.428217797
LAX1	0.542224647	0.87010028	0.358281509	0.945038047	0.676931424	0.414123435
C3H1orf52	0.542224647	0.337920983	0.567599362	0.99999488	0.662093891	0.478345369
ZSWIM7	0.543053189	0.97015318	0.50846757	0.622611633	0.719583646	0.35195337
DLGAP5	0.543053189	0.364216588	0.809278649	0.9781951	0.688375802	0.391016005
ELF3	0.543053189	0.650665822	0.480714058	0.586112202	0.999985264	0.391016005
TNFAIP8L2	0.543053189	0.441450065	0.380872288	0.99999488	0.882312475	0.407020776
KDM7A	0.543053189	0.400553499	0.459542294	0.785363096	0.999985264	0.41854243
MLYCD	0.543053189	0.383289042	0.380872288	0.617959671	0.999985264	0.437919952
IL34	0.543053189	0.488021584	0.63799633	0.573623074	0.999985264	0.44392264
STBD1	0.543053189	0.667396315	0.450984432	0.60997889	0.999985264	0.456040124
SLC25A30	0.543053189	0.401454737	0.51144778	0.66085809	0.999985264	0.464676278
SPDYA	0.543053189	0.585950998	0.592707491	0.71879265	0.794349969	0.5747275
EIF3B	0.543053189	0.469045272	0.399025922	0.884809097	0.922362358	0.623057106
DCTN6	0.543053189	0.343013097	0.746258329	0.855453165	0.597486077	0.718350328
TTR	0.543053189	0.530773348	0.473127265	0.69049726	0.707353372	0.829811046
SPRED2	0.543053189	0.347762512	0.387104289	0.675179349	0.68458318	0.993606543
LOC104970180	0.543053189	0.53683564	0.36532409	0.5736757	0.578351163	0.99382486
ZFPM2	0.544349148	0.396050184	0.466002553	0.99999488	0.83400817	0.407020776
CCL11	0.544349148	0.729000253	0.455857083	0.977598122	0.657836458	0.422940396
DOC2A	0.544349148	0.337920983	0.939147816	0.794939965	0.697132345	0.428725468
CHRNA1	0.544349148	0.816518153	0.40793937	0.573623074	0.922362358	0.559779949
ABCC5	0.544349148	0.634336339	0.505866449	0.791188744	0.546843279	0.807665096
SCN1B	0.544370897	0.547053013	0.48156033	0.99999488	0.671485036	0.385112797
LOC785629	0.54474481	0.378297385	0.362408227	0.978440951	0.999985264	0.528012162
RASGRP1	0.54474481	0.486183065	0.40793937	0.803237525	0.742946425	0.819073042
HOXD3	0.545343496	0.565729718	0.42675714	0.978440951	0.906448097	0.404873878
TMEM263	0.545343496	0.403109879	0.498883267	0.781663566	0.999985264	0.475332036
LOC100139881	0.545343496	0.46094408	0.459026472	0.907700124	0.965672975	0.519472498
CCDC168	0.545343496	0.565729718	0.721605112	0.857323853	0.546848192	0.560845144
FASLG	0.545343496	0.827123416	0.360806372	0.657704227	0.915346056	0.56695901
LOC112447082	0.545343496	0.941495217	0.385324267	0.725623067	0.609009208	0.575428978
LOC789374	0.545343496	0.612694128	0.798553824	0.573623074	0.546848192	0.692319327
TRMT6	0.545773009	0.341770652	0.40365456	0.74725201	0.707353372	0.972825932
P2RY13	0.546787121	0.691364494	0.376044916	0.99999488	0.574203003	0.541323364
LOC112444310	0.547226733	0.423411884	0.76457744	0.825057511	0.957776687	0.358046095

CLIP2	0.547226733	0.343863091	0.628800812	0.99999488	0.566923246	0.367899858
GPX7	0.547226733	0.344903278	0.654174689	0.99999488	0.546848192	0.419282739
SMTN	0.547226733	0.979263532	0.420618887	0.750220059	0.546848192	0.511786447
KLHL24	0.547226733	0.999992444	0.374903616	0.589303553	0.55498633	0.582816513
S100A12	0.547534416	0.377122341	0.640617895	0.99999488	0.693376338	0.385430007
TLR6	0.547534416	0.634298188	0.398905253	0.99999488	0.566515062	0.422940396
SNX19	0.547534416	0.355142066	0.387533191	0.89716396	0.999985264	0.620694111
PIM3	0.547706637	0.503649548	0.430241014	0.614415882	0.999985264	0.420434059
ANKRD13B	0.547706637	0.461438762	0.70108275	0.96412514	0.677821933	0.445262677
PTPRC	0.547706637	0.38156866	0.418866598	0.99999488	0.798938088	0.504328062
HSPA4L	0.547765188	0.760975059	0.510180536	0.5736757	0.903345596	0.535906285
ESS2	0.549188041	0.649879097	0.408370911	0.707846893	0.864188653	0.679091734
TMEM165	0.549188041	0.350278804	0.48934817	0.645998678	0.777489275	0.940358165
SLAIN2	0.549488121	0.366642903	0.746258329	0.817200124	0.999985264	0.380378402
LOC100847509	0.550094678	0.466018853	0.405095497	0.566489656	0.999985264	0.387823865
SH3BGR	0.550094678	0.836065971	0.521866639	0.582040981	0.565236258	0.737269972
LAMP3	0.550094678	0.367205885	0.408267931	0.796555088	0.847202014	0.882867473
TRAPPC8	0.551245655	0.403840106	0.453676391	0.99999488	0.798938088	0.390913469
PSTPIP1	0.551245655	0.405435557	0.434111304	0.760007034	0.999985264	0.588186617
XAF1	0.551849154	0.370995618	0.524560092	0.99999488	0.937177132	0.414123435
LOC104976276	0.551849154	0.819379885	0.600307359	0.691124578	0.693190874	0.489481105
STEAP1	0.551849154	0.462935718	0.43661424	0.749661913	0.999985264	0.501823619
PHOSPHO1	0.551891298	0.486333441	0.921646762	0.744246818	0.671485036	0.420434059
BATF3	0.551891298	0.935274301	0.387533191	0.880028563	0.561363177	0.521002923
FSCN1	0.551891298	0.342195215	0.408370911	0.99999488	0.57461724	0.523730974
ANKRD63	0.552553797	0.378297385	0.408405973	0.99999488	0.707353372	0.513288396
SEMA4F	0.553272543	0.773422401	0.517947068	0.941409556	0.719583646	0.359301141
SCN8A	0.553272543	0.408552266	0.387533191	0.945038047	0.999985264	0.507948284
CYP4B1	0.553272543	0.359713072	0.46546631	0.99999488	0.861287091	0.541323364
LPP	0.555381132	0.634336339	0.411900922	0.949602784	0.608701929	0.67496387
LOC786586	0.556230687	0.650665822	0.566721071	0.99999488	0.554002562	0.370245109
FTL	0.556230687	0.487868804	0.568829521	0.99999488	0.794694113	0.414123435
BANK1	0.556230687	0.351000342	0.416598617	0.99999488	0.777489275	0.513288396
LENG8	0.556230687	0.4916534	0.815067929	0.904225644	0.546848192	0.541323364
TMEM129	0.556230687	0.401454737	0.43661424	0.907045085	0.999985264	0.559024093
PLA2G2C	0.556230687	0.408874345	0.589920437	0.938185243	0.657836458	0.695049955
RASAL2	0.556230687	0.38625193	0.43661424	0.687904478	0.999985264	0.738324902
FSTL4	0.556230687	0.356901832	0.553217285	0.791243098	0.671485036	0.914382882
LIPG	0.558004304	0.512755822	0.37511602	0.817200124	0.999985264	0.615805309

RAB21	0.558335416	0.378297385	0.988922732	0.675179349	0.736423482	0.374775759
LXN	0.558335416	0.421030287	0.385324267	0.99999488	0.729522487	0.418521645
PDE1B	0.558335416	0.387494607	0.432689426	0.622527961	0.999985264	0.451091614
GET4	0.558335416	0.553465911	0.386210449	0.816081719	0.999985264	0.456540289
PI4K2A	0.558335416	0.359713072	0.690770494	0.907045085	0.933861931	0.478861735
RAD23A	0.558335416	0.642323832	0.527035268	0.948570368	0.667906644	0.52928764
GCC1	0.558335416	0.533101609	0.38143799	0.989312306	0.876385027	0.547278473
GIMAP7	0.558335416	0.662093116	0.392709537	0.877873868	0.901763837	0.554947685
FASTKD2	0.558335416	0.410223322	0.374816105	0.594370904	0.999985264	0.770633383
DIRAS2	0.558335416	0.358086855	0.405095497	0.989312306	0.546848192	0.923360345
FAM167A	0.558335416	0.454578306	0.408267931	0.904225644	0.565236258	0.92658818
FILIP1L	0.558673282	0.384132949	0.451511676	0.760007034	0.908980492	0.838520085
F11R	0.559016288	0.530773348	0.374903616	0.609742602	0.999985264	0.513288396
DOK2	0.559316572	0.399245555	0.408370911	0.99999488	0.662093891	0.367899858
MMACHC	0.559316572	0.366740223	0.412953991	0.794035382	0.999985264	0.504328062
GRAMD4	0.559481605	0.348631542	0.716305079	0.99999488	0.546848192	0.644421332
CRHR2	0.559671728	0.45671495	0.575199502	0.948570368	0.766639843	0.581287271
RRP7A	0.559671728	0.594515957	0.380872288	0.794035382	0.901104314	0.733365024
SLC7A5	0.559992359	0.612694128	0.83126054	0.575416797	0.901763837	0.407020776
XCR1	0.560209357	0.991365955	0.51324314	0.632618228	0.657836458	0.424924923
C22H3orf67	0.560943291	0.471965963	0.759038925	0.991596938	0.707353372	0.37642397
CARNS1	0.560943291	0.543083933	0.468578328	0.691124578	0.999985264	0.514698521
CHST6	0.560943291	0.346228047	0.628496505	0.948570368	0.860350547	0.575719205
VSIG10	0.560943291	0.486333441	0.387533191	0.747849903	0.655410555	0.966921967
CENPF	0.561103132	0.358022547	0.865066354	0.99999488	0.656196025	0.3814217
TSR2	0.561103132	0.459867738	0.935768849	0.884809097	0.546848192	0.419552755
MIEF1	0.561103132	0.345758663	0.441586616	0.892000264	0.999985264	0.513288396
LIPJ	0.561103132	0.684021956	0.401621735	0.794035382	0.679852849	0.792819696
UTP4	0.561446264	0.356312653	0.401122009	0.691124578	0.999985264	0.620254304
CLIC4	0.562615248	0.378106622	0.497969977	0.99999488	0.594728091	0.385430007
SMARCA5	0.562615248	0.378297385	0.412953991	0.89716396	0.999985264	0.385513832
BIN2	0.562615248	0.370230677	0.399025922	0.99999488	0.718216606	0.422940396
TMEM106A	0.562615248	0.420660542	0.475942277	0.99999488	0.752497172	0.453090281
DTX4	0.562615248	0.399245555	0.956565728	0.760432761	0.608019099	0.493956701
KIF4A	0.562615248	0.384132949	0.691847112	0.978440951	0.736651407	0.525799302
PVR	0.562615248	0.705186914	0.421926617	0.622527961	0.964631108	0.631711796
GPNMB	0.562615248	0.366278182	0.494727878	0.99999488	0.546848192	0.692319327
RFXANK	0.563072157	0.445244414	0.831723974	0.949602784	0.722852917	0.378213674
GABARAP	0.563072157	0.476433748	0.606564873	0.803237525	0.999985264	0.385430007

PIK3R6	0.563072157	0.391938223	0.40365456	0.99999488	0.999985264	0.394404724
TFEC	0.563072157	0.421664755	0.411900922	0.99999488	0.671485036	0.404873878
CD28	0.563072157	0.384769897	0.566721071	0.99999488	0.650563192	0.555614471
TIGD5	0.563101798	0.380987958	0.387533191	0.99999488	0.999985264	0.402739177
LOC104969719	0.565554571	0.831782537	0.676439937	0.625191166	0.546848192	0.621376973
CUL3	0.565988084	0.650665822	0.738487246	0.812057455	0.794349969	0.402739177
CRLF1	0.566258258	0.442411343	0.539713639	0.993124804	0.983747207	0.396212881
POMK	0.566258258	0.410223322	0.387533191	0.99999488	0.999985264	0.40888949
FTSJ3	0.566258258	0.637749095	0.485378752	0.803237525	0.880252174	0.591266823
PEX11B	0.566488789	0.38625193	0.408370911	0.7019305	0.999985264	0.428725468
IGSF10	0.567014695	0.768934833	0.39008807	0.851621164	0.937177132	0.479463211
TGFBI	0.568011337	0.380987958	0.524324128	0.99999488	0.957776687	0.396212881
LOC784697	0.568162402	0.647579301	0.408405973	0.691124578	0.999985264	0.378213674
RGS5	0.568162402	0.673756899	0.592707491	0.606208572	0.999985264	0.39743828
SCAPER	0.568162402	0.610403345	0.575199502	0.590130006	0.999985264	0.520276531
NR2C2AP	0.568162402	0.574311353	0.437242628	0.870282231	0.664079708	0.812831419
CD248	0.568358682	0.413107515	0.405095497	0.99999488	0.660075125	0.39743828
C1QBP	0.568358682	0.384132949	0.437433425	0.623431927	0.999985264	0.76926818
RRP12	0.568358682	0.375633553	0.387104289	0.794035382	0.999985264	0.818895181
PSMF1	0.569675471	0.574311353	0.809486619	0.848389672	0.719583646	0.419552755
C13H20orf202	0.569675471	0.396628714	0.477167064	0.99999488	0.571673342	0.428725468
EIF1	0.569675471	0.449326704	0.600307359	0.99999488	0.684791634	0.533511539
DDX21	0.569675471	0.378297385	0.488164683	0.691124578	0.999985264	0.556366678
SLC16A14	0.569675471	0.433007404	0.385324267	0.722070872	0.999985264	0.573142748
LOC768255	0.569675471	0.509042229	0.464239008	0.744246818	0.999985264	0.661355141
COQ10B	0.571284397	0.509768617	0.746258329	0.760007034	0.935138282	0.456040124
MRPS26	0.571848668	0.480338277	0.569091034	0.99999488	0.761285588	0.388523226
ANP32E	0.571848668	0.380987958	0.999558179	0.66895898	0.660075125	0.396426203
TUBA1B	0.571848668	0.938597964	0.44808294	0.699968468	0.608701929	0.612764179
CD86	0.571848668	0.416445008	0.387533191	0.99999488	0.879141216	0.62260916
SURF4	0.571931325	0.732009317	0.48377808	0.973503136	0.703363142	0.464676278
B2M	0.572254136	0.469767836	0.484615563	0.989312306	0.999985264	0.3814217
BRIX1	0.572676225	0.486333441	0.506676728	0.708414872	0.999985264	0.599905787
NEDD4	0.572714963	0.459867738	0.405095497	0.687904478	0.999985264	0.410427392
IFIT3	0.573740043	0.366740223	0.535110445	0.98846246	0.999985264	0.487965891
TCAF1	0.573740043	0.413107515	0.43661424	0.99999488	0.655410555	0.578094648
STX18	0.573740043	0.422741902	0.633133298	0.917553107	0.853611376	0.584692851
PCF11	0.573740043	0.537303163	0.40365456	0.632945362	0.999985264	0.603560332
LOC100298530	0.574179388	0.408874345	0.525702179	0.99999488	0.655410555	0.691757815

NAT10	0.575143419	0.525945198	0.401122009	0.884809097	0.961025202	0.664012525
ADRA2B	0.57610038	0.703838914	0.628272393	0.718002256	0.6707745	0.661576605
UBR5	0.576169242	0.408874345	0.405095497	0.609127566	0.999985264	0.40763141
FADS1	0.576169242	0.445011294	0.393036301	0.672549466	0.649051802	0.999960414
LOC530929	0.576339026	0.367205885	0.890096618	0.935177636	0.599374017	0.543439563
ZNF354A	0.578161119	0.694134018	0.542792917	0.99999488	0.546848192	0.387585743
ROBO2	0.578161119	0.433007404	0.503266127	0.609127566	0.999985264	0.563215326
NEK4	0.578161119	0.471965963	0.539713639	0.691124578	0.901763837	0.809130895
SLIT2	0.580202537	0.441450065	0.576058288	0.99999488	0.983747207	0.385430007
LOC112446717	0.580202537	0.38625193	0.543052495	0.99999488	0.57461724	0.410427392
LOC787309	0.580202537	0.641537037	0.577568168	0.994573982	0.736466308	0.415932896
ZDHHC20	0.580345841	0.366642903	0.864441458	0.938185243	0.794349969	0.420434059
OSR1	0.581362607	0.610403345	0.408267931	0.826554135	0.999985264	0.407284616
ALDH1A2	0.582486718	0.4916534	0.886192181	0.798459589	0.657836458	0.537810684
C6H4orf19	0.582953992	0.411831249	0.575199502	0.644821001	0.999985264	0.419282739
KLF15	0.582953992	0.37011492	0.583273932	0.99999488	0.608701929	0.440179295
CCNJL	0.582953992	0.753449964	0.75659683	0.825154704	0.625232521	0.451635222
PARD3	0.582953992	0.439991972	0.477453582	0.913854136	0.999985264	0.48837418
MAFF	0.582953992	0.439991972	0.691575438	0.940265849	0.865815384	0.489481105
FBXW12	0.582953992	0.656315974	0.825497047	0.763371278	0.634541261	0.52928764
ARL4C	0.582953992	0.433007404	0.488036481	0.99999488	0.798938088	0.584872334
FAM174A	0.582953992	0.422357614	0.580363758	0.687904478	0.947902179	0.783345949
GOT1L1	0.583490366	0.723136577	0.467264558	0.622527961	0.999985264	0.505513788
KCNA5	0.583490366	0.406061927	0.785248842	0.851621164	0.860385366	0.537677429
NOX1	0.584894746	0.929230097	0.575977904	0.917553107	0.591834629	0.385430007
TBXA2R	0.584894746	0.445561167	0.391031875	0.896748401	0.999985264	0.392180614
DMRT2	0.586404622	0.76933827	0.579891228	0.758838341	0.712429088	0.603560332
CACFD1	0.587331947	0.485537897	0.393034075	0.99999488	0.77926164	0.402739177
ARRB2	0.587331947	0.672555467	0.408861677	0.99999488	0.696577739	0.456040124
FGF1	0.589797816	0.647062519	0.834173166	0.794035382	0.697132345	0.453090281
MED19	0.589797816	0.406480185	0.40365456	0.609127566	0.999985264	0.504328062
PLN	0.589797816	0.928925667	0.546479844	0.625191166	0.608701929	0.638709845
UBN1	0.589919733	0.368543363	0.776282112	0.617959671	0.999985264	0.407971604
ZNF536	0.589919733	0.38625193	0.644916374	0.791188744	0.706931395	0.888645453
TRABD2B	0.591599266	0.384769897	0.40365456	0.711602989	0.999985264	0.402739177
FAM91A1	0.591599266	0.366642903	0.897767405	0.794035382	0.964631108	0.419894928
BDH1	0.591599266	0.370230677	0.421087054	0.734738603	0.999985264	0.478345369
SLC18B1	0.591599266	0.480008967	0.774728133	0.952783349	0.672072713	0.520998854
GPRC5A	0.591599266	0.568712199	0.459542294	0.791799957	0.896630985	0.754852502

RNF185	0.592278132	0.38625193	0.756568208	0.99999488	0.707353372	0.419282739
LOXL4	0.592278132	0.656082241	0.522360985	0.809884527	0.999985264	0.462042323
CYBB	0.592278132	0.470599569	0.432473682	0.99999488	0.664686023	0.475846099
TMEM144	0.592278132	0.565729718	0.539791196	0.99999488	0.662093891	0.476152636
FAM47E	0.592278132	0.783973824	0.654174689	0.610363776	0.901763837	0.501267871
TMEM120A	0.592278132	0.458383763	0.427959961	0.913854136	0.999985264	0.52928764
RRAGB	0.592278132	0.439137203	0.874485384	0.679601533	0.815550318	0.609616735
LOC101903383	0.592278132	0.428546926	0.412953991	0.99999488	0.660075125	0.609823525
IL2RA	0.592278132	0.557992078	0.421076728	0.69049726	0.999985264	0.609823525
IFT43	0.592278132	0.554630136	0.590691361	0.803237525	0.77926164	0.72144082
WDR46	0.592278132	0.503649548	0.396289633	0.917553107	0.901763837	0.763713143
TMEM62	0.592619983	0.850364054	0.525391806	0.617959671	0.999985264	0.391016005
NAT9	0.592619983	0.43425879	0.44400488	0.796555088	0.999985264	0.391016005
CD14	0.592619983	0.462935718	0.412953991	0.99999488	0.798938088	0.404873878
ADAMTS7	0.592619983	0.503361866	0.601420135	0.99999488	0.707353372	0.408680438
ME2	0.592619983	0.368543363	0.480151299	0.99999488	0.901104314	0.514740511
CCDC125	0.592619983	0.55750909	0.477167064	0.939864142	0.908980492	0.580415308
SPATA7	0.592619983	0.510762296	0.437433425	0.744246818	0.999985264	0.594584164
TMEM132A	0.592619983	0.367205885	0.632118699	0.680109779	0.999985264	0.736708637
ATG13	0.592619983	0.379013183	0.575199502	0.98846246	0.691790458	0.769113651
DNAJA3	0.592619983	0.393482958	0.43661424	0.794035382	0.999985264	0.793763761
PTPRS	0.593806981	0.408874345	0.590933668	0.99999488	0.921581207	0.446372578
OBSL1	0.594744041	0.451314075	0.459542294	0.99999488	0.60615126	0.391016005
ATP7B	0.594744041	0.6725213	0.735869895	0.71879265	0.708864153	0.603050301
BTG2	0.595956941	0.442619337	0.99699426	0.660952609	0.684987032	0.445262677
LOC112443250	0.596346476	0.384132949	0.628800812	0.917553107	0.919929212	0.629039793
IL16	0.596346476	0.439762521	0.401122009	0.679601533	0.999985264	0.818895181
CNKSR1	0.597037859	0.553882269	0.433819606	0.99999488	0.649051802	0.398160568
CAPN11	0.597037859	0.829029204	0.726885375	0.876702931	0.657836458	0.404873878
BAHCC1	0.597037859	0.370230677	0.554127594	0.99999488	0.924009849	0.437381806
CDK3	0.597037859	0.591124857	0.926552506	0.764325384	0.656196025	0.465198839
RND2	0.597037859	0.450029284	0.491806042	0.803037807	0.829541927	0.883750044
TOMM70	0.597037859	0.634381235	0.566721071	0.760007034	0.569794269	0.89589047
DDX49	0.597127808	0.568712199	0.408370911	0.907700124	0.981774771	0.621229636
BTK	0.597288935	0.408874345	0.403466151	0.99999488	0.700650764	0.413669144
LOC107131542	0.597288935	0.494978451	0.575199502	0.977598122	0.942132162	0.475846099
MOB3A	0.597288935	0.530773348	0.574684386	0.728778565	0.664079708	0.918400465
SMCO4	0.597288935	0.647062519	0.467264558	0.77377244	0.608701929	0.919755394
ASTE1	0.597349621	0.667396315	0.458143546	0.747849903	0.999985264	0.45199452

CSMD2	0.597349621	0.661182871	0.675780972	0.89716396	0.794349969	0.462858576
ZNF550	0.597914933	0.793370989	0.851718619	0.647051562	0.759221661	0.407020776
PIKFYVE	0.597914933	0.400595502	0.546479844	0.99999488	0.607100736	0.408694653
DLX4	0.597914933	0.591586809	0.873452279	0.794035382	0.798043944	0.410197113
LOC100850436	0.597914933	0.999992444	0.403466151	0.687904478	0.608701929	0.414123435
LRIF1	0.597914933	0.511046592	0.399025922	0.71610479	0.999985264	0.419445795
VEGFD	0.598549357	0.779783885	0.580991263	0.848389672	0.901104314	0.419282739
SEC24B	0.598549357	0.533435858	0.408370911	0.641348265	0.999985264	0.44392264
SLBP	0.598549357	0.466435357	0.514032609	0.941409556	0.999985264	0.505513788
CEP128	0.598549357	0.519497908	0.405095497	0.622527961	0.999985264	0.578094648
ARIH1	0.599077702	0.445011294	0.44912319	0.705835699	0.999985264	0.407854888
MROH6	0.599211898	0.761984265	0.86178438	0.791188744	0.649051802	0.415932896
CD74	0.599221383	0.680103209	0.40365456	0.99999488	0.847202014	0.445262677
STX12	0.599732539	0.470599569	0.50519837	0.889823701	0.999985264	0.621376973
CLPX	0.60134027	0.420271086	0.578947523	0.99999488	0.95660507	0.402739177
EXOC6	0.60134027	0.568712199	0.662111034	0.978440951	0.834754713	0.424924923
LOC781304	0.60134027	0.564348044	0.451511676	0.69049726	0.999985264	0.484575909
FCN1	0.60134027	0.537303163	0.510576843	0.830046103	0.921581207	0.704966434
PRMT3	0.60134027	0.570683052	0.517812329	0.848389672	0.773111283	0.780629037
ZNF446	0.601363384	0.438805051	0.408370911	0.949602784	0.999985264	0.419282739
LOC112447011	0.601363384	0.75020046	0.40793937	0.907045085	0.999985264	0.445262677
DDX41	0.601363384	0.389941608	0.404295945	0.674372864	0.999985264	0.453090281
GMPPB	0.601363384	0.705186914	0.40365456	0.779163521	0.933861931	0.718350328
MSTO1	0.601363384	0.459867738	0.405095497	0.77377244	0.966075914	0.894723749
RDH10	0.602904963	0.588625967	0.72674783	0.69049726	0.874406643	0.620085999
DCAF13	0.602904963	0.423334704	0.562087244	0.99999488	0.65226281	0.649701943
INAFM2	0.603666008	0.450458388	0.870264123	0.779163521	0.999985264	0.408694653
GPR183	0.604159761	0.480941848	0.414249786	0.794035382	0.999985264	0.748495031
TMEM35B	0.604294763	0.650665822	0.592286584	0.99999488	0.737989863	0.414123435
RAB3A	0.604392551	0.406480185	0.50519837	0.99999488	0.798938088	0.537999986
LOC618733	0.604790538	0.867755878	0.412953991	0.833359511	0.999985264	0.404873878
LOC112445925	0.604790538	0.501891487	0.414249786	0.99999488	0.57461724	0.408694653
B3GALT4	0.604790538	0.659816181	0.632928415	0.794035382	0.999985264	0.419282739
CDKN1A	0.605111592	0.445011294	0.63799633	0.99999488	0.78585317	0.513288396
ADAMTS9	0.605304358	0.548801965	0.700674899	0.622527961	0.999985264	0.412423814
PTCD3	0.605462873	0.455528654	0.405095497	0.675179349	0.999985264	0.755905488
SPATA46	0.606709901	0.457973718	0.999558179	0.687904478	0.657836458	0.422342133
LRRC24	0.606709901	0.610403345	0.525272446	0.976631804	0.924009849	0.468803272
C3AR1	0.606709901	0.478562952	0.405095497	0.99999488	0.908980492	0.52928764

SLC25A22	0.607028319	0.525945198	0.558639627	0.99999488	0.629131525	0.559605167
ARHGEF5	0.607174546	0.537303163	0.973303821	0.780585407	0.58342251	0.468561589
COTL1	0.609318618	0.511239537	0.408267931	0.99999488	0.83400817	0.408694653
GSTP1	0.609318618	0.628133877	0.514775565	0.959611215	0.999985264	0.40888949
PLXNC1	0.609318618	0.491830057	0.43661424	0.99999488	0.704076141	0.414123435
ZNF436	0.609318618	0.647062519	0.503391083	0.99999488	0.594728091	0.41854243
LOC101905743	0.609318618	0.384132949	0.543495009	0.967551965	0.999985264	0.41854243
SPI1	0.609318618	0.471965963	0.427295264	0.99999488	0.708864153	0.419282739
LOC112441484	0.609318618	0.483069943	0.930127869	0.92106221	0.68104332	0.420434059
USP15	0.609318618	0.87010028	0.566721071	0.92106221	0.741080831	0.422940396
BLOC1S6	0.609318618	0.655648116	0.888692542	0.817200124	0.662093891	0.452017112
TTYH2	0.609318618	0.573366037	0.417480833	0.99999488	0.867495559	0.456040124
PIK3AP1	0.609318618	0.413107515	0.448183673	0.99999488	0.671485036	0.470820118
TFDP1	0.609318618	0.63744795	0.645275104	0.870282231	0.939144222	0.475846099
LOC101906916	0.609318618	0.416445008	0.43661424	0.99999488	0.769672519	0.482827846
PLD4	0.609318618	0.809104286	0.525702179	0.855453165	0.864446669	0.509082923
ODC1	0.609318618	0.530162509	0.654174689	0.953188062	0.885116932	0.509136455
MIS18BP1	0.609318618	0.421664755	0.830186385	0.952793359	0.741080831	0.52928764
TMEM176B	0.609318618	0.483251676	0.657690219	0.99999488	0.608701929	0.533511539
LOC784007	0.609318618	0.6598011	0.642046114	0.957636128	0.707353372	0.537999986
SLA	0.609318618	0.48968951	0.480714058	0.989312306	0.999985264	0.541323364
PIGN	0.609318618	0.565729718	0.566721071	0.925818215	0.943686834	0.54675724
KDELC2	0.609318618	0.993316598	0.580991263	0.644821001	0.597486077	0.547278473
CDC42BPB	0.609318618	0.408874345	0.457840076	0.966629392	0.999985264	0.559024093
ARHGAP30	0.609318618	0.476083641	0.414030603	0.99999488	0.824863432	0.57507561
GLI3	0.609318618	0.491830057	0.762340742	0.794035382	0.917539697	0.596516103
IGF1	0.609318618	0.414755075	0.683588966	0.99999488	0.637762548	0.612764179
ANOS1	0.609318618	0.588625967	0.539713639	0.889823701	0.901763837	0.621376973
SMIM10L1	0.609318618	0.453221457	0.521866639	0.969770561	0.901763837	0.651092183
CHPF2	0.609318618	0.608311055	0.40793937	0.99999488	0.714598747	0.692319327
LOC530973	0.609318618	0.710432617	0.678433983	0.691124578	0.707353372	0.718350328
PDGFA	0.609318618	0.442619337	0.481599427	0.940265849	0.942132162	0.728468552
SIAE	0.609318618	0.568380042	0.420270937	0.967551965	0.77926164	0.740386152
ELP5	0.609318618	0.561419114	0.437685431	0.989341529	0.707353372	0.766909088
FBN2	0.609318618	0.568712199	0.518971669	0.989312306	0.608470909	0.770030068
PRKCQ	0.609318618	0.445011294	0.437782735	0.925818215	0.946408931	0.773716179
GCNA	0.609318618	0.628133877	0.590691361	0.77032376	0.712429088	0.818484413
ACAT2	0.609318618	0.485537897	0.477167064	0.698527845	0.999985264	0.847577522
ZNF831	0.609318618	0.736960298	0.408370911	0.622527961	0.849864394	0.873463288

LOC104976020	0.609318618	0.540247468	0.43299062	0.755274145	0.766639843	0.943492944
ERP29	0.609318618	0.39350257	0.654174689	0.763371278	0.644467441	0.960343814
LOC514507	0.60962107	0.663430554	0.593672982	0.77377244	0.895905895	0.629039793
SLC28A1	0.611080092	0.53120828	0.443376928	0.904225644	0.999985264	0.471668047
GEMIN4	0.611102996	0.451314075	0.459026472	0.994265515	0.967727289	0.617537824
PRKX	0.611313317	0.480008967	0.613423863	0.99999488	0.58909742	0.519472498
LOC112444600	0.611313317	0.927072093	0.72136241	0.695630809	0.608701929	0.52928764
FGF2	0.611313317	0.601197167	0.513676108	0.99999488	0.755207159	0.588733625
ZNF12	0.611313317	0.38625193	0.408370911	0.855453165	0.928683582	0.929852609
ESM1	0.611325418	0.466901079	0.785248842	0.99999488	0.729522487	0.434903353
MBP	0.611325418	0.50499616	0.409286511	0.99999488	0.935138282	0.546034764
GPR34	0.611325418	0.502850182	0.417480833	0.99999488	0.708864153	0.580415308
LOC104973519	0.611863084	0.462935718	0.726885375	0.98846246	0.741080831	0.573142748
CCDC114	0.611924437	0.501732129	0.68741937	0.99999488	0.655307354	0.422940396
LOC112441545	0.611924437	0.826575339	0.412953991	0.66895898	0.999985264	0.427373524
ITGA2B	0.611924437	0.591586809	0.932657945	0.812057455	0.675459166	0.451091614
ALOX5AP	0.611924437	0.703838914	0.50725481	0.917553107	0.965227764	0.479463211
ARL14EP	0.611924437	0.64113929	0.683588966	0.99999488	0.604847705	0.509305644
SERTM1	0.611924437	0.977094262	0.409227828	0.687904478	0.729522487	0.646411189
SLC5A6	0.611924437	0.388111971	0.512130225	0.687904478	0.999985264	0.816226172
LOC509118	0.612402536	0.446386385	0.456961614	0.99999488	0.9300268	0.412423814
VNN2	0.612402536	0.481742711	0.427421518	0.99999488	0.974716308	0.488972518
HPD	0.612720451	0.471965963	0.405170393	0.959611215	0.999985264	0.415932896
TET2	0.612720451	0.417495017	0.418004577	0.99999488	0.999985264	0.516702363
SPATA5L1	0.613395679	0.755100312	0.800518725	0.917553107	0.63582413	0.428725468
PARP14	0.613395679	0.398462449	0.626056936	0.99999488	0.901763837	0.537810684
TNC	0.613395679	0.735221655	0.518971669	0.99999488	0.608701929	0.560067883
GPC3	0.613572432	0.656315974	0.707485652	0.761207079	0.999985264	0.408694653
MANBA	0.613572432	0.589631719	0.420270937	0.965065108	0.999985264	0.521422381
CILP2	0.613572432	0.825438977	0.605719038	0.691124578	0.630052244	0.773689802
PXDC1	0.613572432	0.561419114	0.419946262	0.977598122	0.68104332	0.847501251
FGF11	0.613739939	0.999992444	0.465918382	0.711602989	0.637762548	0.53438506
DNAAF5	0.613975188	0.564128591	0.516458375	0.905565531	0.999985264	0.436631145
TLR7	0.613975188	0.530162509	0.415004621	0.991596938	0.999985264	0.437919952
PSKH1	0.613975188	0.64546528	0.471348003	0.99999488	0.847202014	0.468561589
PTPRN2	0.613975188	0.439762521	0.506676728	0.99999488	0.999985264	0.489481105
ABCA6	0.613975188	0.790213802	0.474987636	0.691124578	0.999985264	0.513288396
ASPHD2	0.613975188	0.604615112	0.690864342	0.81936725	0.946408931	0.52928764
EVI2A	0.613975188	0.461438762	0.40793937	0.99999488	0.78046156	0.561314399

LOC533307	0.613975188	0.674944018	0.459026472	0.99999488	0.667311615	0.564361912
GPX3	0.613975188	0.439762521	0.485378752	0.925818215	0.849864394	0.844563769
TMUB1	0.614145331	0.473017249	0.453676391	0.99999488	0.907875746	0.560845144
ACAP1	0.614176128	0.565729718	0.86178438	0.952793359	0.708833788	0.419282739
DHRS1	0.614176128	0.699070323	0.673099635	0.695630809	0.707353372	0.770633383
SNCG	0.614176128	0.425932126	0.566721071	0.763371278	0.924089345	0.885874544
TCEA1	0.614233958	0.431952903	0.492460512	0.865261766	0.729522487	0.949718683
RSRP1	0.614988698	0.634381235	0.575199502	0.675179349	0.999985264	0.500888858
LYN	0.615557178	0.439991972	0.489644904	0.98846246	0.999985264	0.484575909
LOC100848940	0.615876802	0.487868804	0.923604668	0.855573807	0.847202014	0.419552755
RAD50	0.616351574	0.83698359	0.714371099	0.880829722	0.662093891	0.475348039
HECA	0.616351574	0.478562952	0.425914657	0.8398706	0.999985264	0.496364568
LOC515551	0.616410679	0.525945198	0.746258329	0.99999488	0.671485036	0.414123435
PCNX3	0.616410679	0.397499451	0.892943118	0.88041408	0.983747207	0.419552755
SRGAP2	0.616410679	0.63996058	0.49480606	0.907700124	0.999985264	0.422940396
MZT2B	0.616410679	0.509042229	0.414249786	0.644821001	0.999985264	0.433466574
PARVG	0.616410679	0.444580366	0.408370911	0.99999488	0.671485036	0.44392264
SIRT1	0.616410679	0.62408671	0.476611225	0.760007034	0.999985264	0.447084938
ACAP3	0.616410679	0.534959211	0.645275104	0.99999488	0.6707745	0.448374934
LOC534967	0.616410679	0.497162414	0.453676391	0.675179349	0.999985264	0.493956701
SLC9B2	0.616410679	0.91765877	0.439619012	0.925818215	0.729522487	0.52297188
SLC22A23	0.616410679	0.416445008	0.503391083	0.99999488	0.662093891	0.541777213
BCL7A	0.616410679	0.439953013	0.607189486	0.77377244	0.999985264	0.560067883
FLNC	0.616410679	0.981520287	0.524238325	0.794035382	0.625232521	0.569229015
GCN1	0.616410679	0.509042229	0.495842848	0.907700124	0.999985264	0.569563821
NCAPD2	0.616410679	0.661182871	0.644916374	0.964973086	0.655410555	0.615805309
LOC100300510	0.616410679	0.893743321	0.511472792	0.687904478	0.840528426	0.626804781
ELAVL3	0.616410679	0.612694128	0.691847112	0.917553107	0.697132345	0.628575862
LSMEM1	0.616410679	0.533435858	0.554234799	0.979945333	0.847202014	0.62904095
LOC101908359	0.616410679	0.402847296	0.534417481	0.99999488	0.864446669	0.699045591
LOC104972821	0.616410679	0.451314075	0.692872372	0.870282231	0.856124022	0.719097202
POLR1A	0.616410679	0.433007404	0.412953991	0.880028563	0.999985264	0.738983095
SOX5	0.616410679	0.656315974	0.606008934	0.744246818	0.801204085	0.772479356
PINX1	0.616410679	0.591586809	0.414030603	0.796555088	0.740020385	0.944457836
CD300LB	0.616410679	0.551052067	0.457913033	0.848077461	0.607100736	0.970849664
LOC101906818	0.61799649	0.544144809	0.724658338	0.913752239	0.916667939	0.516702363
TRMT61A	0.618181086	0.522689142	0.412953991	0.812057455	0.999985264	0.770633383
ITIH1	0.618654055	0.858885162	0.471356071	0.857323853	0.736346045	0.649957433
RNF112	0.619177828	0.434037958	0.589920437	0.911513036	0.999985264	0.434569255

B3GNT5	0.619454209	0.863793573	0.436665374	0.92106221	0.637029706	0.695049955
ZNF688	0.619939597	0.52907722	0.926552506	0.794939965	0.84872286	0.447871072
THAP9	0.619939597	0.450029284	0.825497047	0.691124578	0.999985264	0.453090281
DCXR	0.619939597	0.502850182	0.514775565	0.746076296	0.999985264	0.465198839
HSPA1L	0.619939597	0.444580366	0.566353811	0.704143314	0.999985264	0.500391313
ZDHHC13	0.620212125	0.938597964	0.446898302	0.741569405	0.999985264	0.420434059
PLEKHB2	0.620343937	0.473017249	0.670826239	0.99999488	0.612198268	0.545755335
RIPOR3	0.620343937	0.779783885	0.645275104	0.687237586	0.787383034	0.693397277
FREM1	0.620343937	0.59662083	0.655928749	0.830046103	0.787383034	0.726340954
LY75	0.620343937	0.486333441	0.431994576	0.949602784	0.878312529	0.832622862
SPTBN5	0.620820975	0.617969341	0.623372433	0.809884527	0.999985264	0.573142748
SP140	0.620863774	0.414755075	0.571877797	0.99999488	0.999985264	0.448607816
CHCHD6	0.622236268	0.842852797	0.670826239	0.779786063	0.684987032	0.612911427
LOC530077	0.622236268	0.445011294	0.43661424	0.99999488	0.97687524	0.638709845
TYRP1	0.622510608	0.480338277	0.416598617	0.794035382	0.901763837	0.948675165
CUL1	0.624729115	0.503649548	0.55966476	0.99999488	0.85717397	0.419282739
IFI6	0.624729115	0.461438762	0.575199502	0.99999488	0.787383034	0.438362302
PMM1	0.624729115	0.506284531	0.487591209	0.699561137	0.999985264	0.457194978
SPINT2	0.624729115	0.705186914	0.578947523	0.803037807	0.999985264	0.508556226
GTF2H4	0.624729115	0.446386385	0.562087244	0.691124578	0.999985264	0.588733625
LRRN3	0.624729115	0.50655961	0.941118576	0.796555088	0.63582413	0.609823525
RCC1	0.624729115	0.517204742	0.421926617	0.680109779	0.794694113	0.995391635
MCRIP2	0.624862482	0.467205779	0.667274767	0.661901668	0.999985264	0.478345369
PRR11	0.625435126	0.635853191	0.539474394	0.744246818	0.999985264	0.518142056
TOB1	0.625435126	0.478562952	0.695533457	0.794035382	0.999985264	0.588781291
SCN7A	0.625456945	0.48512827	0.78262245	0.931835237	0.757173298	0.617537824
C3	0.625565545	0.565729718	0.923953195	0.794035382	0.769672519	0.512260179
LPXN	0.625565545	0.561419114	0.437433425	0.99999488	0.684791634	0.52928764
PIK3CG	0.625565545	0.403238571	0.815602423	0.854322524	0.933917579	0.615805309
LOC513894	0.625565545	0.658402276	0.515996187	0.707659473	0.967727289	0.775675327
DLK2	0.625565545	0.482841882	0.43661424	0.937020992	0.965672975	0.792944216
LOC104970537	0.625612252	0.674837172	0.83396257	0.794035382	0.608701929	0.662512478
MRTO4	0.625939071	0.566397357	0.43661424	0.99999488	0.907875746	0.621376973
IMPG2	0.625939071	0.445237461	0.458143546	0.71879265	0.999985264	0.621376973
SYPL2	0.626515952	0.565729718	0.485378752	0.820583858	0.961025202	0.795293834
SIRPB1	0.626996522	0.647062519	0.43661424	0.889823701	0.703363142	0.904813143
FANCI	0.627633074	0.647062519	0.467264558	0.959611215	0.999985264	0.445262677
LOC112447302	0.627633074	0.87010028	0.663165825	0.673960257	0.895499865	0.537311716
NHP2	0.627633074	0.542800218	0.468000957	0.747849903	0.939144222	0.901809636

LOC784769	0.627672909	0.715899004	0.609340165	0.951237479	0.924089345	0.427373524
ZDHHC2	0.627672909	0.542314378	0.677011563	0.99999488	0.662093891	0.476724596
ARMC8	0.627672909	0.608311055	0.718226122	0.794939965	0.999985264	0.482266815
S1PR5	0.628099468	0.531215068	0.865066354	0.846458317	0.736651407	0.605340728
HMGN5	0.628216746	0.416445008	0.592620368	0.99999488	0.757173298	0.436351167
MAP6	0.628216746	0.548616419	0.489609531	0.777342221	0.825465701	0.931270478
RAP1A	0.629547623	0.469767836	0.654174689	0.99999488	0.740523538	0.433414418
PSPH	0.629547623	0.471965963	0.535900798	0.687904478	0.999985264	0.523380534
P4HB	0.629547623	0.670199175	0.485378752	0.889823701	0.781521342	0.804474221
C5H12orf29	0.629938292	0.406694709	0.471348003	0.878491623	0.965672975	0.89589047
SH3BGRL3	0.630452246	0.705186914	0.434850377	0.98846246	0.999985264	0.453090281
HIPK3	0.630817954	0.431925659	0.534417481	0.907700124	0.999985264	0.437381806
UCKL1	0.630971242	0.56780353	0.558137974	0.896748401	0.999985264	0.530798769
MYO1E	0.630971242	0.448821794	0.43661424	0.92106221	0.999985264	0.673027356
LOC104973154	0.630971242	0.674837172	0.442270997	0.671008739	0.662093891	0.987457361
YIPF2	0.631144001	0.681665332	0.609340165	0.99999488	0.880252174	0.422940396
LPCAT2	0.632249945	0.940269477	0.457840076	0.944661721	0.771578767	0.465198839
FXN	0.632249945	0.424736511	0.5662684	0.757550318	0.999985264	0.491451054
LOC112442218	0.632249945	0.87010028	0.505866449	0.794035382	0.964631108	0.52928764
NOC2L	0.632249945	0.566397357	0.421926617	0.794035382	0.99980018	0.865911659
HSH2D	0.633709702	0.850692013	0.52966531	0.979293963	0.664366052	0.561190412
INSL6	0.633709702	0.789663062	0.518971669	0.7019305	0.999985264	0.573142748
DNAJC3	0.633709702	0.42079669	0.570175675	0.820583858	0.999985264	0.593603038
SPAG5	0.633709702	0.411312632	0.803677695	0.99999488	0.662093891	0.618235079
KCNH3	0.633709702	0.445011294	0.511326755	0.855453165	0.999985264	0.631711796
LTV1	0.633709702	0.494978451	0.516458375	0.760007034	0.999985264	0.812825712
C1QTNF6	0.635464456	0.471965963	0.649927227	0.99999488	0.671485036	0.573142748
LOC107132524	0.635464456	0.515630499	0.495842848	0.784174371	0.999985264	0.721134875
SRSF6	0.6355797	0.667813705	0.569091034	0.749661913	0.999985264	0.451091614
ABL1	0.6355797	0.503649548	0.427421518	0.749661913	0.999985264	0.465198839
MAP3K12	0.6355797	0.591586809	0.683588966	0.99999488	0.671485036	0.508556226
CYTIP	0.6355797	0.408874345	0.592707491	0.99999488	0.773709332	0.716004541
GPIHBP1	0.636049161	0.850692013	0.456961614	0.687904478	0.999985264	0.436631145
PF4	0.636049161	0.599298196	0.474987636	0.99999488	0.707873066	0.496096399
ADGRB1	0.636049161	0.667396315	0.931146757	0.747849903	0.676931424	0.543439563
GIMAP7	0.636049161	0.644729858	0.539474394	0.925818215	0.901763837	0.652992721
ALG3	0.636049161	0.540247468	0.440030283	0.687904478	0.999985264	0.677379211
KLK13	0.636049161	0.453476133	0.72674783	0.978440951	0.676931424	0.758510984
HSPA2	0.637419598	0.461438762	0.445802474	0.959611215	0.999985264	0.449721384

FKBP10	0.637419598	0.461387587	0.708177705	0.99999488	0.657836458	0.453090281
MRPS24	0.637419598	0.465630893	0.460525656	0.684141313	0.999985264	0.530587857
PYGM	0.637419598	0.981067569	0.494727878	0.878491623	0.661721509	0.555614471
LARS	0.637419598	0.48512827	0.667228538	0.705835699	0.999985264	0.748495031
PUSL1	0.637419598	0.465343585	0.48891003	0.803237525	0.961025202	0.915476587
TNFSF8	0.638457113	0.509042229	0.448183673	0.99999488	0.998298811	0.508556226
DERL1	0.638457113	0.540247468	0.904140521	0.917553107	0.679405937	0.553445201
ADGRE3	0.640303369	0.711878823	0.455857083	0.99999488	0.664079708	0.446372578
YPEL1	0.640303369	0.727539585	0.459542294	0.99999488	0.785107052	0.465198839
H2AFY2	0.640303369	0.467243107	0.675115467	0.744043714	0.999985264	0.468561589
INPP5J	0.640303369	0.603322291	0.841739726	0.949602784	0.707022204	0.520418463
NINL	0.640657065	0.595636522	0.776282112	0.744246818	0.858733392	0.693397277
EMC9	0.640747012	0.487641245	0.569091034	0.687904478	0.999985264	0.427655417
PTPN12	0.640747012	0.537303163	0.460525656	0.707846893	0.999985264	0.573614149
SUSD4	0.640747012	0.417495017	0.862345036	0.98846246	0.715259968	0.593347482
MAGED1	0.640768672	0.498017932	0.581807838	0.99999488	0.773111283	0.4411687
FUBP1	0.640768672	0.471965963	0.43661424	0.687904478	0.999985264	0.448607816
CAPZA2	0.640768672	0.540247468	0.689170915	0.99999488	0.719583646	0.457194978
FZD2	0.640768672	0.441450065	0.690864342	0.99999488	0.84872286	0.470820118
ATP6AP1	0.640768672	0.658402276	0.68741937	0.99999488	0.707353372	0.509305644
STK38L	0.640768672	0.441450065	0.466884594	0.99999488	0.901763837	0.519407686
CD6	0.640768672	0.873289191	0.770213049	0.691124578	0.755207159	0.555648397
FFAR3	0.640768672	0.480300487	0.601231486	0.813963687	0.999985264	0.579273673
LRP4	0.640768672	0.591124857	0.494727878	0.99999488	0.729522487	0.627277292
FBXL3	0.640768672	0.826434903	0.620035975	0.884809097	0.66506033	0.661576605
CLK1	0.640768672	0.799180421	0.580469773	0.791706526	0.856853826	0.665217821
LOC783604	0.640768672	0.494978451	0.774363763	0.816792716	0.901104314	0.692319327
GPR4	0.640768672	0.796531384	0.524560092	0.796555088	0.792861833	0.769515026
SAT2	0.64133633	0.634336339	0.570175675	0.896370331	0.999985264	0.496096399
EME2	0.641437856	0.644729858	0.76260462	0.896748401	0.978668933	0.434660154
TMED4	0.641437856	0.511046592	0.870264123	0.978440951	0.798938088	0.449713844
COL1A2	0.641437856	0.425252947	0.774363763	0.99999488	0.908980492	0.451635222
ZC3H7B	0.641437856	0.425252947	0.516458375	0.99999488	0.999985264	0.456040124
CDKN2B	0.641437856	0.721370958	0.570175675	0.794035382	0.999985264	0.456990723
ARHGEF2	0.641437856	0.547417474	0.908572237	0.794035382	0.936175954	0.470820118
TMEM88	0.641437856	0.531215068	0.43661424	0.820583858	0.999985264	0.478345369
REEP4	0.641437856	0.753515888	0.853055742	0.785872741	0.777608317	0.512609659
KIF20B	0.641437856	0.561419114	0.645275104	0.99999488	0.841924245	0.51963883
WDR6	0.641437856	0.419200187	0.43661424	0.99999488	0.999985264	0.527456297

RABEP1	0.641437856	0.431925659	0.471348003	0.8398706	0.999985264	0.541323364
ATP6V0A1	0.641437856	0.445561167	0.677520363	0.99999488	0.657836458	0.551826127
GALNT12	0.641437856	0.974355032	0.566721071	0.744246818	0.773206807	0.568559353
AEBP2	0.641437856	0.607621421	0.43661424	0.7019305	0.999985264	0.571520557
ATL2	0.641437856	0.592672828	0.590933668	0.687904478	0.999985264	0.594584164
FKBP1B	0.641437856	0.486333441	0.683588966	0.99999488	0.684987032	0.614862466
SLC39A14	0.641437856	0.445561167	0.474987636	0.99999488	0.924009849	0.62904095
MINPP1	0.641437856	0.827873637	0.466516651	0.813963687	0.904842818	0.674504592
RASGRP3	0.641437856	0.421664755	0.451629734	0.99999488	0.961025202	0.715526355
LSS	0.641437856	0.422357614	0.43661424	0.77377244	0.999985264	0.72144082
GIMAP7	0.641437856	0.699986019	0.589920437	0.889823701	0.781939574	0.728264017
SRRM3	0.641437856	0.4916534	0.458143546	0.99999488	0.824424612	0.779686358
ANGPTL5	0.641437856	0.710289322	0.544909362	0.92106221	0.693376338	0.790569257
C23H6orf62	0.641437856	0.747155043	0.496742583	0.794035382	0.657836458	0.926596859
SAMD3	0.641437856	0.551235148	0.566721071	0.691124578	0.84872286	0.948213108
ATP13A2	0.641773686	0.554045796	0.783759914	0.848389672	0.999985264	0.499611057
SLC15A2	0.641773686	0.542800218	0.436108201	0.817200124	0.999985264	0.620947023
GJA1	0.642488852	0.729820154	0.459026472	0.99999488	0.719583646	0.475348039
USP42	0.642538713	0.4916534	0.440030283	0.697843564	0.999985264	0.475846099
P3H4	0.643546496	0.496760238	0.57992604	0.99999488	0.660429906	0.451635222
CLEC10A	0.643546496	0.487868804	0.492460512	0.99999488	0.858096179	0.565085989
PICALM	0.643740932	0.471965963	0.566721071	0.99999488	0.716196295	0.475348039
MAP4K1	0.644993609	0.476219255	0.665391565	0.99999488	0.757173298	0.656368128
HSPB11	0.645746227	0.533101609	0.455857083	0.896748401	0.708864153	0.966921967
CPSF1	0.647124802	0.473017249	0.915215321	0.989312306	0.77926164	0.440179295
LOC508153	0.647124802	0.431925659	0.437433425	0.99999488	0.999985264	0.54675724
LOC112447526	0.647614401	0.577792766	0.785248842	0.99999488	0.662093891	0.436673268
CHRD	0.647614401	0.471965963	0.644279997	0.99999488	0.998439184	0.452684071
DPH1	0.647614401	0.467243107	0.436108201	0.99999488	0.999985264	0.465198839
EPOR	0.647614401	0.445296003	0.55422436	0.99999488	0.999985264	0.513288396
MFSD9	0.647614401	0.661182871	0.44912319	0.747849903	0.999985264	0.807689737
ARFGEF1	0.647651011	0.596319881	0.43661424	0.99999488	0.999985264	0.452306012
TMEM245	0.647651011	0.883856603	0.673099635	0.907700124	0.769672519	0.464676278
KCNK17	0.647651011	0.466018853	0.638383601	0.755274145	0.999985264	0.475348039
LIF	0.647651011	0.474914864	0.488045766	0.99999488	0.999985264	0.478345369
TCF21	0.647651011	0.821033198	0.443376928	0.825434574	0.999985264	0.478861735
PLEKHA1	0.647651011	0.520409947	0.687781916	0.99999488	0.719742223	0.486426539
AFTPH	0.647651011	0.476083641	0.573644627	0.99999488	0.999985264	0.489481105
ARID4A	0.647651011	0.551235148	0.526888973	0.855453165	0.999985264	0.490483272

ADAMTS10	0.647651011	0.661332345	0.843674782	0.937020992	0.712429088	0.505513788
TRABD	0.647651011	0.658817102	0.925348822	0.823788475	0.729522487	0.509305644
LOC101905630	0.647651011	0.783314535	0.600305808	0.937020992	0.880459369	0.516702363
ZNF213	0.647651011	0.498898871	0.457028922	0.943090854	0.999985264	0.525799302
MYOM3	0.647651011	0.904961534	0.602936506	0.794035382	0.860350547	0.541323364
LOC112444931	0.647651011	0.487868804	0.686023128	0.984913325	0.986043731	0.541323364
TMSB10	0.647651011	0.985406717	0.545111954	0.817200124	0.676931424	0.578094648
ANKMY2	0.647651011	0.559186884	0.575199502	0.878491623	0.999985264	0.621376973
AXIN2	0.647651011	0.714111753	0.524238325	0.98846246	0.798938088	0.628644572
ITGB7	0.647651011	0.809599669	0.55422436	0.813352686	0.847202014	0.715526355
HHIP	0.647651011	0.683150214	0.78255891	0.791157558	0.664686023	0.772431293
TOMM34	0.647651011	0.462935718	0.455857083	0.947995037	0.999985264	0.819275036
FLT3	0.647651011	0.551764571	0.575199502	0.917553107	0.825465701	0.828248574
ARRDC5	0.647651011	0.647062519	0.63962071	0.772908752	0.715259968	0.900681286
NPC2	0.648827454	0.471965963	0.882515776	0.99999488	0.715259968	0.440179295
RALGAPB	0.648827454	0.457973718	0.726793848	0.99999488	0.829541927	0.443181556
LOC100297240	0.648827454	0.4529723	0.999558179	0.826149187	0.660075125	0.445567073
HMCN1	0.648827454	0.727209548	0.485378752	0.925818215	0.999985264	0.448147194
LAMA2	0.648827454	0.688798329	0.86638329	0.71610479	0.999985264	0.45199452
FAM180B	0.648827454	0.565729718	0.556511841	0.99999488	0.707353372	0.453090281
COL1A1	0.648827454	0.448821794	0.816532117	0.99999488	0.813331114	0.454910332
PTP4A3	0.648827454	0.445561167	0.571697442	0.99999488	0.999985264	0.459805827
GTF2H1	0.648827454	0.561419114	0.510576843	0.812057455	0.999985264	0.461948448
MEG9	0.648827454	0.439762521	0.93987882	0.989312306	0.742946425	0.462243264
ZNF800	0.648827454	0.663430554	0.480714058	0.698527845	0.999985264	0.471415595
CXCL16	0.648827454	0.664171921	0.525272446	0.99999488	0.719244798	0.475348039
TWIST2	0.648827454	0.941495217	0.451629734	0.911513036	0.928683582	0.481202569
GPR173	0.648827454	0.644729858	0.477167064	0.99999488	0.901104314	0.484575909
PIK3C3	0.648827454	0.997426798	0.485378752	0.825154704	0.798938088	0.486472357
PTOV1	0.648827454	0.565729718	0.497630672	0.896748401	0.999985264	0.503717194
TMEM109	0.648827454	0.548801965	0.935781943	0.937020992	0.684787787	0.508556226
PSMB9	0.648827454	0.816518153	0.451511676	0.99999488	0.901763837	0.513288396
LOC613822	0.648827454	0.525945198	0.592620368	0.99999488	0.676931424	0.515470846
MN1	0.648827454	0.593189944	0.623909299	0.935556917	0.999985264	0.525799302
LOC112445030	0.648827454	0.595177859	0.593672982	0.77032376	0.999985264	0.52928764
FAM160B2	0.648827454	0.445244414	0.443376928	0.98846246	0.999985264	0.530560642
ECSIT	0.648827454	0.486333441	0.509523994	0.702914991	0.999985264	0.53438506
PGM5	0.648827454	0.991488652	0.592565415	0.813963687	0.679405937	0.541777213
SMURF2	0.648827454	0.461281364	0.453676391	0.71610479	0.999985264	0.54675724

UBIAD1	0.648827454	0.483069943	0.743915133	0.955973055	0.999985264	0.54675724
LDB3	0.648827454	0.992977597	0.518971669	0.889928167	0.655410555	0.55740152
CCR4	0.648827454	0.832807683	0.440030283	0.695630809	0.999985264	0.559024093
SDK1	0.648827454	0.467243107	0.775962349	0.99999488	0.676931424	0.565085989
LOC101906397	0.648827454	0.978510121	0.456961614	0.892000264	0.741080831	0.578823016
TRANK1	0.648827454	0.431925659	0.774363763	0.982713285	0.937177132	0.579273673
GCFC2	0.648827454	0.882505302	0.545111954	0.82585253	0.901763837	0.581287271
FAM222A	0.648827454	0.50655961	0.809486619	0.99999488	0.688375802	0.588178204
RNASE6	0.648827454	0.482841882	0.46406527	0.99999488	0.999985264	0.594584164
CNTLN	0.648827454	0.591124857	0.675115467	0.791188744	0.999985264	0.598059647
NCF1	0.648827454	0.533733977	0.474987636	0.99999488	0.798938088	0.601946079
ENKD1	0.648827454	0.48968951	0.862345036	0.941409556	0.777489275	0.615805309
TAB2	0.648827454	0.491878713	0.455857083	0.691124578	0.999985264	0.615805309
CD52	0.648827454	0.925580392	0.456961614	0.785363096	0.901763837	0.631427808
SEPHS2	0.648827454	0.663430554	0.497969977	0.812057455	0.999985264	0.65137146
GPR155	0.648827454	0.537303163	0.595396914	0.99999488	0.677061328	0.662512478
ZNF385B	0.648827454	0.589631719	0.657239931	0.749661913	0.999985264	0.693397277
WFS1	0.648827454	0.608589691	0.605523279	0.77377244	0.999985264	0.693616839
LOC615733	0.648827454	0.72165336	0.68741937	0.794035382	0.83400817	0.723102808
LOC519309	0.648827454	0.470599569	0.821698192	0.953588494	0.68104332	0.74674547
RPS26	0.648827454	0.540247468	0.645166627	0.71610479	0.999985264	0.747408201
LCK	0.648827454	0.746555252	0.502827629	0.953588494	0.708864153	0.764052305
LOC789748	0.648827454	0.760975059	0.539713639	0.917553107	0.660075125	0.818895181
ITPKA	0.648827454	0.941793392	0.459026472	0.691124578	0.655410555	0.890433418
KCNS3	0.648827454	0.525856511	0.465747691	0.77032376	0.946408931	0.944906868
CXCR6	0.648827454	0.6725213	0.448133555	0.747849903	0.736466308	0.972662617
ITGA3	0.648827454	0.445237461	0.575199502	0.757550318	0.84872286	0.977753483
LRRC8D	0.648827454	0.454578306	0.441586616	0.691124578	0.965353772	0.996066257
SAA3	0.651116355	0.722542524	0.570175675	0.99999488	0.708833788	0.541777213
SFMBT2	0.652683349	0.485537897	0.590933668	0.99999488	0.659553789	0.493956701
ZBTB40	0.653638835	0.537303163	0.724584302	0.901855229	0.702777257	0.856860151
LYNX1	0.653734055	0.455528654	0.453676391	0.712722338	0.999985264	0.478345369
CHPF	0.654988697	0.753515888	0.462647747	0.99999488	0.864188653	0.457887711
ZNF470	0.654988697	0.670199175	0.580738818	0.99999488	0.727974484	0.586631499
KCTD15	0.654988697	0.435964613	0.517812329	0.99999488	0.983747207	0.675571489
KCNMB1	0.655252098	0.836126124	0.76260462	0.707659473	0.813331114	0.616845571
CAD	0.65594328	0.568300739	0.657808416	0.92398295	0.999985264	0.514316109
TUBG2	0.65594328	0.539697215	0.492460512	0.99999488	0.999985264	0.52928764
RANBP2	0.657039062	0.503649548	0.563808685	0.829458576	0.999985264	0.451091614

METTL3	0.657039062	0.745276769	0.667817366	0.701707998	0.999985264	0.456040124
KIF1A	0.657039062	0.446386385	0.746258329	0.99999488	0.678499214	0.486472357
RAI1	0.657039062	0.445237461	0.524324128	0.959611215	0.999985264	0.525799302
ABHD16B	0.657039062	0.533394878	0.489413188	0.99999488	0.999985264	0.525799302
CD37	0.657039062	0.505787722	0.477167064	0.99999488	0.965353772	0.52928764
PPP1R3B	0.657039062	0.870839685	0.586899194	0.814994678	0.961025202	0.541323364
PFN1	0.657039062	0.957700659	0.644916374	0.806055879	0.679852849	0.599905787
ITPKC	0.657039062	0.462326559	0.620035975	0.937020992	0.999985264	0.610043612
FBLIM1	0.657039062	0.471965963	0.459026472	0.99999488	0.671485036	0.639425605
AMT	0.657039062	0.652133366	0.825497047	0.907700124	0.672072713	0.665217821
EPPK1	0.657039062	0.566528058	0.658401387	0.716029091	0.708864153	0.966522116
RASGEF1A	0.658219744	0.47266331	0.464576285	0.99999488	0.657836458	0.523730974
LOC112445197	0.658777201	0.510117656	0.848312163	0.853556804	0.999985264	0.453647982
ZBTB7B	0.658868903	0.6725213	0.493015519	0.99999488	0.969703428	0.453090281
GLIS3	0.658868903	0.661182871	0.52966531	0.99999488	0.999985264	0.475846099
MYO1F	0.658868903	0.510762296	0.448027787	0.99999488	0.944638139	0.52928764
CACNB3	0.658868903	0.628680252	0.456961614	0.96268133	0.999985264	0.656368128
UCP2	0.658868903	0.650665822	0.595339754	0.907045085	0.767122015	0.839030006
LOC100848799	0.658868903	0.706295081	0.595396914	0.880829722	0.657836458	0.893150073
MRPL23	0.659091232	0.591586809	0.573450552	0.907045085	0.999985264	0.62260916
LOC104970976	0.659544277	0.477299609	0.522266075	0.99999488	0.915346056	0.464175901
TRAM2	0.659544277	0.462935718	0.502827629	0.99999488	0.777489275	0.514316109
SLC45A3	0.659544277	0.647579301	0.575908071	0.949825107	0.999985264	0.518142056
FCGR2B	0.659544277	0.709395	0.480714058	0.931835237	0.980522362	0.670092907
MAP1LC3C	0.659571327	0.808483763	0.464239008	0.99999488	0.719583646	0.520276531
CFAP46	0.659571327	0.674837172	0.494727878	0.876702931	0.999985264	0.52928764
CYSLTR2	0.659571327	0.877795869	0.509523994	0.99999488	0.708864153	0.530982983
TCF19	0.659596019	0.491830057	0.886962419	0.978440951	0.901104314	0.453090281
WIPF1	0.659778602	0.486333441	0.547354523	0.99999488	0.999985264	0.493956701
DTNBP1	0.659797206	0.741594487	0.640310147	0.99999488	0.849864394	0.508556226
SUPT3H	0.659797206	0.498708996	0.450984432	0.741071438	0.999985264	0.519407686
ABLIM1	0.661029082	0.5285062	0.493015519	0.92106221	0.999985264	0.656368128
DGKQ	0.661336752	0.626092982	0.825497047	0.794939965	0.999985264	0.456040124
C17H22orf39	0.661336752	0.486183065	0.455857083	0.813963687	0.999985264	0.468803272
MLLT1	0.661336752	0.466688669	0.468578328	0.99999488	0.904786242	0.69084747
TTC39C	0.66140117	0.466018853	0.605479618	0.825952127	0.6911547	0.992853834
TRUB2	0.661806223	0.515904022	0.563852379	0.760007034	0.999985264	0.465198839
GNGT2	0.661806223	0.892443429	0.529846128	0.889823701	0.978076141	0.509082923
CFB	0.661806223	0.673847368	0.877799478	0.880829722	0.777608317	0.545755335

FABP4	0.661806223	0.782417587	0.541633361	0.976631804	0.924095594	0.553445201
EBPL	0.661806223	0.466901079	0.567552006	0.99999488	0.961025202	0.612111034
MFSD8	0.661806223	0.510762296	0.522371341	0.948570368	0.999985264	0.621376973
DNER	0.661806223	0.735757186	0.596040322	0.99999488	0.68104332	0.677379211
RRP36	0.661806223	0.515630499	0.546969041	0.946427018	0.996075618	0.775511809
LOC104975814	0.662235997	0.769454808	0.547354523	0.99999488	0.707353372	0.475846099
NRIP1	0.662429112	0.486333441	0.821546364	0.921764051	0.801204085	0.721168613
ARHGAP25	0.662731396	0.548616419	0.481599427	0.99999488	0.901104314	0.613432706
RIOX1	0.663394179	0.510762296	0.481502186	0.907700124	0.999985264	0.559024093
NT5C3B	0.663871513	0.591586809	0.675121942	0.951237479	0.999985264	0.52928764
DUSP2	0.663871513	0.486333441	0.692027671	0.998140932	0.866103914	0.683998081
SH2D2A	0.663982063	0.76933827	0.526438568	0.77377244	0.837824474	0.873463288
WNK4	0.664146651	0.500919651	0.579260925	0.878491623	0.901104314	0.917028736
WDR19	0.666061205	0.634336339	0.886962419	0.949825107	0.754368292	0.508556226
SHOC2	0.666530973	0.551274104	0.577015792	0.855573807	0.999985264	0.464175901
LOC112448166	0.666530973	0.514847149	0.547354523	0.847714599	0.999985264	0.629039793
LOC112447819	0.66663841	0.913160799	0.862345036	0.747849903	0.761285588	0.465387405
COG6	0.66663841	0.565729718	0.85276575	0.707846893	0.999985264	0.468803272
SUSD1	0.66663841	0.551764571	0.467264558	0.99999488	0.781939574	0.469994927
TM9SF2	0.66663841	0.503649548	0.624757637	0.99999488	0.999985264	0.476152636
LOC101902043	0.66663841	0.4916534	0.576058288	0.99999488	0.662093891	0.479463211
PARM1	0.66663841	0.45539181	0.608011885	0.909784383	0.999985264	0.543439563
ABLIM2	0.66663841	0.520409947	0.925745065	0.949602784	0.708864153	0.584459217
IGSF6	0.66663841	0.493550152	0.488045766	0.99999488	0.91322793	0.593347482
AGPS	0.66663841	0.551235148	0.605479618	0.99999488	0.798938088	0.610600013
LOC781339	0.66663841	0.858808025	0.575199502	0.907045085	0.729522487	0.721134875
NDUFAF5	0.66736264	0.610403345	0.547354523	0.904832658	0.999985264	0.537810684
LOC112446690	0.66736264	0.563926315	0.456961614	0.880829722	0.999985264	0.675571489
RABGGTA	0.667542495	0.563854845	0.50519837	0.779922342	0.999985264	0.486472357
GSDME	0.667542495	0.733210627	0.517947068	0.99999488	0.787850759	0.50479158
CAMK2N2	0.667558706	0.612694128	0.493111169	0.917553107	0.999985264	0.580415308
ESCO1	0.667646801	0.826434903	0.543896126	0.794035382	0.999985264	0.502276558
XPR1	0.668038742	0.483069943	0.502541452	0.967551965	0.999985264	0.528993941
CDC20	0.669004507	0.485340159	0.768852397	0.99999488	0.901763837	0.53438506
PSD4	0.669203966	0.531215068	0.494727878	0.99999488	0.727974484	0.541323364
TTLL11	0.669203966	0.471965963	0.939147816	0.949602784	0.721208887	0.593352427
HOMER1	0.669203966	0.451712459	0.539713639	0.859289191	0.999985264	0.628644572
VWF	0.669487889	0.83698359	0.459026472	0.708414872	0.999985264	0.529736353
DUSP4	0.669487889	0.754509129	0.921379317	0.735045054	0.750008217	0.583476019

GSAP	0.669487889	0.530773348	0.536995142	0.99999488	0.830453488	0.594584164
PUS7	0.669487889	0.729000253	0.591762752	0.830046103	0.999985264	0.670092907
PPP1R9A	0.669995817	0.49887989	0.57285637	0.917553107	0.999985264	0.489481105
JPH2	0.669995817	0.9582784	0.525262038	0.935500683	0.671485036	0.612911427
SLC7A6	0.670495455	0.528475831	0.628496505	0.917553107	0.999985264	0.593352427
POR	0.670495455	0.488021584	0.519697319	0.917553107	0.980522362	0.893921142
TBC1D14	0.67055863	0.561419114	0.580469773	0.83856608	0.999985264	0.80980964
LOC112442677	0.67151618	0.774472123	0.923604668	0.755274145	0.860350547	0.462042323
LOC101902128	0.673090606	0.921934673	0.590933668	0.784700372	0.757159723	0.738193315
NSL1	0.673107825	0.999992444	0.495842848	0.749661913	0.708864153	0.468803272
SELENOK	0.673107825	0.568300739	0.769499495	0.99999488	0.798938088	0.496096399
MLLT3	0.673107825	0.626400323	0.55422436	0.813963687	0.999985264	0.583476019
GLUL	0.673872651	0.451314075	0.641573776	0.99999488	0.736346045	0.580415308
CCDC171	0.673872651	0.755100312	0.614864417	0.857249106	0.952554537	0.654736169
CCL19	0.674182215	0.745168963	0.534584077	0.99999488	0.999985264	0.476724596
IFI44L	0.67480065	0.4916534	0.485378752	0.99999488	0.999985264	0.556965527
C23H6orf141	0.675138244	0.462935718	0.509029335	0.99999488	0.702076457	0.786482582
MTMR2	0.675311373	0.518243898	0.492557292	0.794035382	0.999985264	0.505759802
LOC104970387	0.675963775	0.707737892	0.800518725	0.99999488	0.707353372	0.529355689
SAMHD1	0.676874846	0.509042229	0.518494004	0.99999488	0.729522487	0.542919974
COL6A2	0.676877264	0.789000771	0.610641809	0.99999488	0.865815384	0.50689567
AASDH	0.676877264	0.873289191	0.571817462	0.831276941	0.999985264	0.523006013
PLXDC1	0.676877264	0.859484941	0.519712749	0.813210232	0.999985264	0.54675724
TMEM119	0.676877264	0.461281364	0.595396914	0.99999488	0.676931424	0.588139353
AK5	0.676877264	0.474122619	0.506949813	0.77377244	0.999985264	0.588139353
LOC112447316	0.676877264	0.729000253	0.628800812	0.949602784	0.939144222	0.591266823
POLR3B	0.676877264	0.592672828	0.477167064	0.99999488	0.981860698	0.614040014
ITGB3	0.676877264	0.568712199	0.522323281	0.99999488	0.835076615	0.671843481
RASSF5	0.676877264	0.640417642	0.462089355	0.99999488	0.821095276	0.675571489
TMEM206	0.676877264	0.551235148	0.471348003	0.796555088	0.999985264	0.695049955
MAP3K1	0.676877264	0.589631719	0.833419544	0.921764051	0.729522487	0.711815036
TRIM5	0.676877264	0.559186884	0.76260462	0.777532895	0.999985264	0.743462219
AASDHPPT	0.676877264	0.592672828	0.609340165	0.907700124	0.946408931	0.784370223
SORBS2	0.676877264	0.607313379	0.595339754	0.794035382	0.999985264	0.828248574
ANK3	0.676877264	0.50481472	0.688865302	0.845465538	0.950839268	0.844563769
SMIM3	0.676877264	0.577106114	0.588780799	0.921764051	0.835076615	0.881211326
RAMP1	0.679360092	0.642323832	0.645275104	0.725623067	0.999985264	0.644094886
TIMM17A	0.679360092	0.471965963	0.542383341	0.938185243	0.999985264	0.681790665
PTPRO	0.679608765	0.650224401	0.657808416	0.907700124	0.998439184	0.649701943

RCN3	0.682246709	0.649541115	0.645495442	0.99999488	0.736423482	0.52386762
EVPL	0.682246709	0.461438762	0.994601277	0.896748401	0.721547193	0.55740152
HENMT1	0.682556796	0.482841882	0.932657945	0.744246818	0.793270571	0.807689737
PTI	0.682556796	0.59662083	0.59132149	0.99999488	0.719583646	0.819073042
MEG3	0.682853515	0.612694128	0.865837237	0.99999488	0.707353372	0.475846099
PIGA	0.68341753	0.978510121	0.708177705	0.750220059	0.868188283	0.471415595
IL11RA	0.68341753	0.999992444	0.477167064	0.816792716	0.77926164	0.489481105
SOS2	0.68341753	0.592962209	0.621354558	0.884870518	0.999985264	0.493273626
FLT4	0.68341753	0.694145861	0.516927579	0.99999488	0.740020385	0.494748648
GFRA1	0.68341753	0.752475942	0.774363763	0.948516678	0.858974474	0.513288396
PDE11A	0.68341753	0.579368072	0.870567942	0.99999488	0.676931424	0.52928764
CASP8	0.68341753	0.518580134	0.577409904	0.937020992	0.999985264	0.556337453
COL4A4	0.68341753	0.533733977	0.998936876	0.794035382	0.726622353	0.560845144
PAQR7	0.68341753	0.505588987	0.573644627	0.989312306	0.999985264	0.583476019
NFE2L1	0.68341753	0.489012471	0.539474394	0.99999488	0.999985264	0.588139353
GADD45G	0.68341753	0.492421508	0.600307359	0.978440951	0.999985264	0.594584164
MKNK1	0.68341753	0.642654038	0.622598236	0.99999488	0.773206807	0.615805309
DOK3	0.68341753	0.487868804	0.541480881	0.951237479	0.999985264	0.645467494
TACO1	0.68341753	0.565729718	0.573450552	0.982713285	0.999985264	0.650914382
RPF1	0.68341753	0.84528629	0.644916374	0.830046103	0.864188653	0.675571489
PSME2	0.68341753	0.667396315	0.605719038	0.907700124	0.999985264	0.675571489
ABCE1	0.68341753	0.50655961	0.575199502	0.813963687	0.999985264	0.699375122
USP10	0.68341753	0.551235148	0.690770494	0.978440951	0.819073469	0.772479356
GDF10	0.68341753	0.610403345	0.605479618	0.99999488	0.719583646	0.812831419
TRIM66	0.68341753	0.591124857	0.667228538	0.851621164	0.944638139	0.816959462
CNOT7	0.683833063	0.656082241	0.817177032	0.949602784	0.919506952	0.508556226
LOC618289	0.684054521	0.494849189	0.542428732	0.99999488	0.785107052	0.828248574
UGGT2	0.684178592	0.462326559	0.665391565	0.99999488	0.999985264	0.504939444
HSPA13	0.685023947	0.478396037	0.898815524	0.98846246	0.908750851	0.519407686
ST8SIA4	0.685023947	0.539697215	0.645275104	0.907700124	0.999985264	0.54675724
C3H1orf162	0.685023947	0.487641245	0.729977435	0.99999488	0.84872286	0.569013454
KDM6A	0.685023947	0.589631719	0.569091034	0.744246818	0.999985264	0.571533062
TIMM10	0.685023947	0.476083641	0.492557292	0.941409556	0.999985264	0.580118533
ZNF599	0.685023947	0.992977597	0.518971669	0.917553107	0.708864153	0.582372695
CSNK1B	0.685023947	0.616083911	0.770524164	0.907700124	0.745595852	0.802149108
TMEM132E	0.685023947	0.462935718	0.513676108	0.989312306	0.942132162	0.885874544
KCNN4	0.685470827	0.487035825	0.580738818	0.99999488	0.755902323	0.559024093
C3H1orf210	0.686210391	0.789654884	0.534417481	0.749661913	0.999985264	0.594984216
DNAH10	0.686210391	0.551235148	0.923604668	0.878491623	0.813331114	0.631711796

RHOF	0.686210391	0.778248571	0.712242615	0.889823701	0.847202014	0.650914382
ADAMTS5	0.686210391	0.471965963	0.556511841	0.896748401	0.999985264	0.845594867
TRAF5	0.686210391	0.473965378	0.645150516	0.77377244	0.771578767	0.999960414
ANKRD23	0.686662198	0.525410834	0.981039752	0.749661913	0.911651126	0.555648397
MAST4	0.686662198	0.588625967	0.474987636	0.760007034	0.999985264	0.584459217
LMNB1	0.686662198	0.554630136	0.800952304	0.998668948	0.879755684	0.584692851
MTX1	0.686662198	0.506284531	0.518494004	0.77377244	0.999985264	0.591189297
PLAG1	0.686662198	0.647062519	0.590691361	0.762590159	0.999985264	0.594984216
ADNP	0.686662198	0.647062519	0.503391083	0.917553107	0.999985264	0.599905787
XKR5	0.686662198	0.573366037	0.575199502	0.99999488	0.688375802	0.601946079
QTRT1	0.686662198	0.661182871	0.670826239	0.878491623	0.999985264	0.605046442
CATSPERD	0.686662198	0.794946953	0.817700149	0.922379064	0.688375802	0.608269365
BRIP1	0.686662198	0.565729718	0.834173166	0.851621164	0.999985264	0.614862466
CREBRF	0.686662198	0.935401063	0.494727878	0.99999488	0.697132345	0.615326593
PPP1R14C	0.686662198	0.504913968	0.922400936	0.92106221	0.793270571	0.645467494
LOC112446791	0.686662198	0.958098081	0.517947068	0.840951535	0.815550318	0.675571489
DTX1	0.686662198	0.503649548	0.59132149	0.99999488	0.815874035	0.738324902
FARS2	0.686662198	0.615586822	0.514775565	0.788951948	0.999985264	0.918400465
LOC781494	0.686828447	0.693386354	0.738189003	0.99999488	0.702777257	0.661149383
PIK3CD	0.687003565	0.52907722	0.474987636	0.99999488	0.973902302	0.569563821
AHSA2	0.687003565	0.534091773	0.543052495	0.760007034	0.999985264	0.626027519
LOC100848419	0.687306604	0.478562952	0.641573776	0.99999488	0.999985264	0.504328062
UBA7	0.687880403	0.494978451	0.654174689	0.976631804	0.999985264	0.52928764
PMS2	0.689053849	0.467243107	0.498883267	0.813963687	0.999985264	0.478098211
ATP10D	0.68984339	0.470599569	0.592707491	0.959611215	0.999985264	0.552527014
TSPYL1	0.68984339	0.606772993	0.876758498	0.917553107	0.880086683	0.580118533
BOLA	0.68984339	0.553465911	0.785248842	0.917553107	0.999985264	0.581399054
NLRP3	0.68984339	0.52907722	0.591210717	0.884809097	0.999985264	0.721134875
LOC511617	0.68984339	0.610403345	0.503391083	0.99999488	0.773111283	0.816959462
TAPBP	0.690133709	0.727539585	0.58523401	0.791188744	0.999985264	0.484575909
SOX8	0.690321738	0.477027103	0.78255891	0.99999488	0.861287091	0.509305644
HSD17B11	0.690324531	0.522689142	0.566721071	0.99999488	0.944638139	0.52928764
TLR2	0.690539912	0.489012471	0.485378752	0.99999488	0.740020385	0.588139353
ACKR4	0.690745394	0.935742738	0.488045766	0.99999488	0.787472587	0.513288396
RIC1	0.690863088	0.493550152	0.513676108	0.747849903	0.999985264	0.53438506
HRAS	0.690863088	0.693386354	0.553217285	0.959611215	0.999985264	0.555614471
LOC112446470	0.690885515	0.62408671	0.628800812	0.907700124	0.999985264	0.639767265
ACTG1	0.691238548	0.682316491	0.960133832	0.816792716	0.741080831	0.54675724
UHRF1BP1L	0.691238548	0.727539585	0.55730426	0.803237525	0.999985264	0.567763858

KCTD17	0.691238548	0.471965963	0.566721071	0.99999488	0.708864153	0.571533062
ELFN1	0.691238548	0.520857951	0.641573776	0.99999488	0.831019096	0.57593203
ZNF365	0.691238548	0.482841882	0.554521346	0.99999488	0.973714462	0.578094648
LOC100847861	0.691238548	0.700359766	0.575199502	0.927129324	0.999985264	0.603560332
GPATCH8	0.691238548	0.612694128	0.899576888	0.744246818	0.998439184	0.612764179
CCL1	0.691238548	0.862811597	0.63655368	0.884809097	0.905146204	0.615805309
KIF18B	0.691238548	0.487641245	0.763358761	0.989312306	0.906448097	0.691706801
EFR3B	0.691445037	0.658990072	0.821546364	0.927129324	0.983747207	0.498948797
ALOX15	0.691445037	0.520857951	0.592707491	0.982713285	0.901763837	0.844277572
WFIKKN2	0.692170139	0.511130754	0.771154197	0.976631804	0.999985264	0.607421354
VHL	0.692519906	0.510117656	0.730602626	0.791759362	0.999985264	0.543705218
LOC100847171	0.693364498	0.869999083	0.654174689	0.99999488	0.762071999	0.518142056
WNT2	0.695665171	0.9582784	0.535110445	0.813352686	0.829541927	0.697770772
CTSC	0.695841811	0.560520097	0.667274767	0.969770561	0.999985264	0.513288396
LOC112442610	0.695841811	0.525856511	0.785248842	0.986534694	0.796491725	0.743798578
TTC33	0.697558692	0.626400323	0.541765557	0.99999488	0.78774318	0.673916311
NLRC3	0.697584399	0.667396315	0.774363763	0.99999488	0.847202014	0.54675724
CX3CR1	0.697596336	0.49546171	0.615380158	0.99999488	0.721468892	0.53424523
LOC104975607	0.697596336	0.61100105	0.554273043	0.99999488	0.732426407	0.543705218
UTP15	0.697596336	0.509042229	0.873452279	0.99999488	0.767161377	0.584872334
VIPR1	0.697596336	0.919209432	0.70071733	0.896748401	0.729522487	0.615805309
PQLC2	0.697596336	0.551235148	0.485378752	0.907045085	0.999985264	0.654291391
OPTC	0.697596336	0.502850182	0.595396914	0.812057455	0.729858321	0.999960414
KCNB1	0.697621857	0.64037592	0.647431961	0.907039918	0.999985264	0.559024093
SUPT4H1	0.697621857	0.591586809	0.895626254	0.846458317	0.957776687	0.596379492
DMD	0.697621857	0.555412755	0.707964225	0.941409556	0.910490499	0.779686358
SPOUT1	0.697621857	0.509042229	0.52619906	0.817200124	0.999985264	0.873463288
FAM129A	0.697621857	0.715899004	0.673099635	0.77377244	0.719855511	0.939035437
NPTXR	0.698180721	0.56780353	0.510576843	0.99999488	0.999985264	0.50479158
APBA3	0.698180721	0.482318158	0.715757781	0.959611215	0.999985264	0.516702363
CHP2	0.698180721	0.551235148	0.606564873	0.99999488	0.999985264	0.599905787
EML6	0.698180721	0.80694956	0.831723974	0.8011796	0.835076615	0.627277292
ADAM33	0.698180721	0.772911907	0.736259816	0.907700124	0.852744493	0.645467494
TUBB6	0.698180721	0.91058693	0.606051701	0.816792716	0.907134049	0.654736169
PDF	0.698639712	0.55837549	0.558137974	0.889928167	0.999985264	0.767595486
AMIGO2	0.698648652	0.827661136	0.575199502	0.99999488	0.777489275	0.614040014
XPOT	0.698648652	0.610403345	0.489637147	0.839050812	0.733924156	0.999960414
DHX35	0.699243767	0.559186884	0.920629231	0.785363096	0.999985264	0.582816513
ADAMTS15	0.699243767	0.795255481	0.570175675	0.99999488	0.901104314	0.582828194

CAMK2G	0.699243767	0.62408671	0.592707491	0.99999488	0.917915833	0.721168613
CLDN11	0.699374181	0.999992444	0.494727878	0.878491623	0.77926164	0.495992985
AATK	0.699374181	0.49546171	0.994626276	0.859289191	0.890963305	0.499611057
DNAJB14	0.699374181	0.680103209	0.645275104	0.823788475	0.999985264	0.504328062
LOC100849050	0.699374181	0.634298188	0.645275104	0.907700124	0.999985264	0.505513788
EZH2	0.699374181	0.592962209	0.859430492	0.977598122	0.924009849	0.525799302
LOC112443484	0.699374181	0.849474629	0.803677695	0.951237479	0.707353372	0.55740152
ATF6	0.699374181	0.55750909	0.934694748	0.92106221	0.847202014	0.58105996
CALM	0.699374181	0.483069943	0.999558179	0.777342221	0.712429088	0.594584164
LCP2	0.699374181	0.656082241	0.558137974	0.99999488	0.754767672	0.594584164
LOC510520	0.699374181	0.56780353	0.581743487	0.99999488	0.864446669	0.608269365
USP2	0.699374181	0.528475831	0.592565415	0.99999488	0.999985264	0.612911427
ZNF608	0.699374181	0.479144523	0.86638329	0.930183705	0.911651126	0.692319327
JPT2	0.699374181	0.4916534	0.728362962	0.907700124	0.999985264	0.779686358
DUOXA2	0.699564604	0.478562952	0.922400936	0.99999488	0.798938088	0.494748648
POLR2F	0.699564604	0.699070323	0.590933668	0.869065101	0.999985264	0.52928764
TPP1	0.699564604	0.487641245	0.682888727	0.99999488	0.77926164	0.564360345
EBP	0.699564604	0.540247468	0.575199502	0.830046103	0.999985264	0.644421332
KMT5C	0.699564604	0.543654388	0.831863359	0.99999488	0.696407681	0.670092907
CDC42EP3	0.699564604	0.915367594	0.762327091	0.757550318	0.773206807	0.685090267
TMEM37	0.699810045	0.813113924	0.70108275	0.884650797	0.937177132	0.615805309
PTGER2	0.700393167	0.560520097	0.669698125	0.978440951	0.999985264	0.63161713
SPOPL	0.700720515	0.666616267	0.592707491	0.925818215	0.999985264	0.717437533
CISH	0.702870148	0.486686408	0.95230212	0.925818215	0.905874828	0.537810684
NOP16	0.703016872	0.517052652	0.541480881	0.976631804	0.999985264	0.766507584
LOC112448084	0.703016872	0.491830057	0.534044053	0.938185243	0.732426407	0.998331177
TSPAN18	0.703055552	0.530816655	0.628800812	0.99999488	0.921998583	0.521002923
ARMC10	0.703170595	0.753515888	0.862345036	0.820583858	0.952275018	0.537999986
SYP	0.703170595	0.639638256	0.862345036	0.924872024	0.944638139	0.547278473
GDI1	0.703170595	0.771984021	0.714371099	0.907700124	0.919006503	0.62904095
CP	0.703170595	0.673847368	0.785177733	0.796555088	0.901104314	0.780958584
LOC522763	0.703170595	0.619998703	0.708177705	0.917553107	0.707353372	0.919755394
CBX7	0.703170595	0.586824593	0.619337076	0.796555088	0.795272338	0.982250307
MAP2K4	0.70347819	0.537835999	0.940717701	0.851321364	0.999985264	0.494748648
SCRIB	0.70347819	0.650665822	0.904354738	0.963012661	0.864446669	0.497484228
CENPX	0.70347819	0.559186884	0.517812329	0.794035382	0.999985264	0.505759802
TTC25	0.70347819	0.733210627	0.55422436	0.917553107	0.999985264	0.508556226
PGM2L1	0.70347819	0.752475942	0.817177032	0.917553107	0.946408931	0.516702363
HJURP	0.70347819	0.622887279	0.825736269	0.99999488	0.707353372	0.599905787

KANK2	0.70347819	0.525945198	0.570175675	0.99999488	0.798938088	0.603050301
BARX1	0.70347819	0.500197446	0.895626254	0.823173273	0.999985264	0.641885255
RPS20	0.70347819	0.649879097	0.546479844	0.762590159	0.999985264	0.653527101
STX1A	0.70347819	0.754374533	0.494727878	0.931835237	0.873468654	0.859537053
IDH3A	0.70347819	0.551235148	0.583675883	0.894501136	0.999985264	0.873463288
HTR1B	0.70347819	0.688212161	0.738487246	0.760007034	0.798938088	0.911997747
LMAN1	0.70347819	0.510762296	0.489637147	0.945038047	0.868761851	0.972662617
SFI1	0.70351446	0.709149248	0.94634922	0.925818215	0.741080831	0.496096399
CENPW	0.703591007	0.582088443	0.933791093	0.951237479	0.894715133	0.496096399
TMEM200A	0.703591007	0.537303163	0.590933668	0.77377244	0.999985264	0.508556226
SELENBP1	0.703591007	0.727390627	0.569091034	0.794939965	0.999985264	0.583476019
TMEM30B	0.703591007	0.510938249	0.649927227	0.99999488	0.901763837	0.588781291
MTAP	0.703591007	0.505588987	0.599813846	0.959611215	0.999985264	0.594584164
LOC101909196	0.703591007	0.638045424	0.944932814	0.820583858	0.856124022	0.599905787
LOC100336476	0.703591007	0.805901612	0.837254326	0.794035382	0.864446669	0.629483566
FERMT2	0.703782465	0.705186914	0.780954783	0.896481111	0.961025202	0.612911427
TNFRSF21	0.705499636	0.975677199	0.518494004	0.823173273	0.935138282	0.621376973
FRRS1	0.705499636	0.561419114	0.497969977	0.851621164	0.999985264	0.710221641
TMEM26	0.707482088	0.53000554	0.890248427	0.917553107	0.999985264	0.513288396
PRKAR1A	0.707482088	0.573366037	0.803709097	0.922850423	0.999985264	0.514316109
MGC152281	0.707482088	0.564128591	0.803677695	0.855453165	0.999985264	0.560845144
GPR65	0.707482088	0.656589815	0.570175675	0.917553107	0.999985264	0.578094648
LOC104972031	0.707482088	0.661332345	0.95230212	0.859289191	0.712429088	0.617537824
KBTBD2	0.707482088	0.698915525	0.64882681	0.867138552	0.999985264	0.724432737
REP15	0.707482088	0.588625967	0.509151676	0.99999488	0.7946823	0.837143123
FADS2	0.707482088	0.796754942	0.588049069	0.948570368	0.707353372	0.862679714
RAET1L	0.707583332	0.509042229	0.573414999	0.99999488	0.773206807	0.578823016
CD151	0.707832446	0.816518153	0.525262038	0.989312306	0.983747207	0.603050301
LOC100140586	0.707971964	0.999992444	0.673566693	0.825154704	0.761285588	0.499611057
ZFC3H1	0.709117023	0.850095234	0.741183564	0.812057455	0.999985264	0.571053412
MRPL12	0.709117023	0.56780353	0.598765064	0.765740785	0.999985264	0.601946079
LOC512627	0.709243819	0.519740019	0.65017087	0.99999488	0.757173298	0.525139009
INPP5A	0.709243819	0.768079117	0.575132831	0.95442132	0.983747207	0.661576605
KIAA1024	0.711336061	0.587721224	0.502827629	0.859289191	0.999985264	0.52928764
STK32C	0.711664495	0.540247468	0.573414999	0.99999488	0.719855511	0.52928764
LOC100141145	0.711664495	0.706496014	0.750974849	0.99999488	0.877605164	0.55740152
MYRF	0.711664495	0.511239537	0.922400936	0.957896521	0.856632395	0.618171804
USP35	0.711664495	0.511739055	0.700695194	0.99999488	0.815550318	0.629039793
RAB12	0.711664495	0.4916534	0.973406453	0.905565531	0.769672519	0.654736169

PABPC1	0.711664495	0.525856511	0.52619906	0.99999488	0.999985264	0.695049955
RGS18	0.711664495	0.534091773	0.606564873	0.99999488	0.792861833	0.724432737
FGD6	0.711664495	0.69663723	0.643747653	0.876702931	0.999985264	0.770633383
EIF4EBP3	0.711664495	0.722585736	0.668594622	0.907700124	0.77926164	0.868103112
UFSP1	0.711735079	0.650770664	0.575199502	0.92106221	0.999985264	0.704679988
LRR1	0.712603002	0.994754253	0.632118699	0.937020992	0.741080831	0.513288396
MMP23	0.712603002	0.610403345	0.76260462	0.99999488	0.894715133	0.513288396
CKAP2L	0.712603002	0.491830057	0.95477544	0.988576086	0.852744493	0.520276531
IFFO1	0.712603002	0.978089982	0.673099635	0.851321364	0.901763837	0.523730974
DHX30	0.712603002	0.530795065	0.614864417	0.92106221	0.999985264	0.535906285
LOC101904794	0.712603002	0.516236607	0.842585804	0.99999488	0.847202014	0.537810684
LOC404051	0.712603002	0.705186914	0.551558801	0.99999488	0.732546652	0.539585315
C6H4orf48	0.712603002	0.72931078	0.591210717	0.99999488	0.715259968	0.541323364
RLF	0.712603002	0.661182871	0.632928415	0.791188744	0.999985264	0.55740152
TSHZ3	0.712603002	0.533354903	0.580469773	0.99999488	0.999985264	0.576884439
SMAD1	0.712603002	0.565729718	0.602936506	0.99999488	0.763423599	0.578094648
CNTNAP1	0.712603002	0.747155043	0.775848114	0.978440951	0.852744493	0.581287271
LOC104976232	0.712603002	0.88167071	0.825497047	0.794035382	0.864446669	0.599905787
LOC104968422	0.712603002	0.50481472	0.806978356	0.99999488	0.84401945	0.650740854
PARP9	0.712603002	0.525458484	0.773092345	0.982713285	0.998439184	0.653862943
LOC788425	0.712603002	0.525945198	0.888710954	0.848389672	0.999985264	0.661576605
CPAMD8	0.712603002	0.503649548	0.706110318	0.820154442	0.999985264	0.692319327
SIPA1L2	0.712603002	0.517052652	0.623909299	0.907045085	0.999985264	0.69378777
DALRD3	0.712603002	0.64037592	0.644916374	0.796555088	0.999985264	0.701003695
ABCB8	0.712603002	0.525692457	0.524238325	0.857249106	0.999985264	0.704966434
A1CF	0.712603002	0.991488652	0.592620368	0.794035382	0.741080831	0.721134875
ZC3H12D	0.712603002	0.502850182	0.821546364	0.99999488	0.761285588	0.721134875
RRP15	0.712603002	0.565729718	0.714371099	0.99999488	0.833887736	0.72144082
NAV2	0.712603002	0.533394878	0.645275104	0.99999488	0.757159723	0.728036947
NKTR	0.712603002	0.61414244	0.708177705	0.99999488	0.815550318	0.733745412
LRRC71	0.712603002	0.656589815	0.514338058	0.99999488	0.708864153	0.734175643
EPB42	0.712603002	0.946274461	0.524238325	0.859289191	0.847202014	0.754429032
ABHD17B	0.712603002	0.867777719	0.573644627	0.907700124	0.707353372	0.873463288
KIF21B	0.712603002	0.53063462	0.767682114	0.917553107	0.864446669	0.873463288
AFF3	0.712603002	0.78821881	0.604472666	0.796555088	0.749793356	0.944906868
PDE12	0.712603002	0.497678167	0.526438568	0.878491623	0.946408931	0.98341414
LAMP2	0.713200907	0.690700426	0.606564873	0.99999488	0.808410765	0.537677429
LOC787234	0.713200907	0.493550152	0.673842846	0.989312306	0.944638139	0.819073042
PROKR1	0.713200907	0.519740019	0.770213049	0.974583211	0.792861833	0.861030464

VPS37A	0.713344645	0.568712199	0.907484111	0.978440951	0.922384168	0.52928764
VLDLR	0.713344645	0.4916534	0.726885375	0.773769459	0.999985264	0.52928764
GSDMD	0.713344645	0.650973121	0.76260462	0.99999488	0.77825208	0.550777054
GTSE1	0.713399537	0.748233963	0.593672982	0.942391899	0.999985264	0.631711796
NUS1	0.71350732	0.657400432	0.898815524	0.803037807	0.999985264	0.599905787
ADAMTS16	0.71350732	0.542800218	0.517947068	0.830046103	0.999985264	0.620254304
MPPE1	0.713571533	0.819379885	0.778565768	0.907045085	0.908980492	0.588890004
TMEM59L	0.714426866	0.610403345	0.622846239	0.99999488	0.999985264	0.513288396
ZNF654	0.714426866	0.732202418	0.662111348	0.917553107	0.999985264	0.522578059
NDUFA1	0.714426866	0.4916534	0.534417481	0.904225644	0.999985264	0.525799302
RGS16	0.714426866	0.729820154	0.505866449	0.99999488	0.884208058	0.528012162
SLC49A3	0.714426866	0.56695737	0.903957821	0.99999488	0.852744493	0.537999986
NDUFS3	0.714426866	0.548616419	0.593672982	0.825154704	0.999985264	0.543705218
FMOD	0.714426866	0.634298188	0.65329932	0.99999488	0.942404196	0.556337453
TMSB4X	0.714426866	0.525458484	0.999558179	0.825154704	0.724196748	0.588178204
COL12A1	0.714426866	0.562433468	0.558137974	0.99999488	0.934529944	0.588733625
JMY	0.714426866	0.869428256	0.592620368	0.922850423	0.999985264	0.588781291
CNGA3	0.714426866	0.938597964	0.599813846	0.817200124	0.999985264	0.605976415
HBEGF	0.714426866	0.634336339	0.815067929	0.796555088	0.999985264	0.627277292
OSCAR	0.714426866	0.705186914	0.726258339	0.99999488	0.794349969	0.653862943
CDH15	0.714426866	0.647062519	0.673566693	0.946427018	0.999985264	0.654736169
IRF4	0.714426866	0.623767283	0.515996187	0.991596938	0.999985264	0.662624496
MRPL57	0.714426866	0.571254278	0.547354523	0.958273417	0.999985264	0.675571489
RANBP6	0.714426866	0.647062519	0.593672982	0.839050812	0.999985264	0.69311359
SNPH	0.714426866	0.52907722	0.638383601	0.99999488	0.798938088	0.761979307
MED6	0.714426866	0.663430554	0.534417481	0.803237525	0.999985264	0.770083907
LOC524810	0.714426866	0.586911842	0.898815524	0.907700124	0.719583646	0.80980964
PTPRK	0.714426866	0.634298188	0.610641809	0.796555088	0.999985264	0.827439931
TTC19	0.714426866	0.522974997	0.568829521	0.813963687	0.999985264	0.867843546
LOC101907857	0.714426866	0.634298188	0.541480881	0.993124804	0.873468654	0.883999232
LOC112446481	0.714426866	0.703007983	0.70108275	0.870282231	0.709947147	0.931163114
MFHAS1	0.714426866	0.517204742	0.506630409	0.953588494	0.715259968	0.999960414
CREB1	0.714515381	0.610403345	0.592620368	0.809884527	0.999985264	0.510140739
LOC112442284	0.714515381	0.568712199	0.76260462	0.859289191	0.999985264	0.588733625
DYNC1I2	0.714601281	0.499013692	0.566690404	0.917553107	0.999985264	0.535797307
FAM78A	0.714601281	0.607313379	0.577647688	0.99999488	0.801204085	0.554947685
TSPO	0.714601281	0.530301508	0.569091034	0.803037807	0.999985264	0.6128041
LOC782021	0.714601281	0.703838914	0.575199502	0.925818215	0.999985264	0.733745412
TTC4	0.714601281	0.533435858	0.663165825	0.949602784	0.982217275	0.846111851

TUBB2B	0.715005678	0.974812121	0.787474031	0.813963687	0.741080831	0.580415308
LPGAT1	0.716207321	0.506284531	0.628800812	0.99999488	0.999985264	0.541323364
AREL1	0.716207321	0.649541115	0.590691361	0.816792716	0.999985264	0.578094648
IDI1	0.716207321	0.505588987	0.533418803	0.99999488	0.999985264	0.769129136
FERMT1	0.716477395	0.644729858	0.640867126	0.959611215	0.835076615	0.882867473
ARF1	0.718116739	0.649879097	0.890248427	0.998140932	0.757173298	0.601032588
ARHGAP45	0.718116739	0.701847401	0.534417481	0.99999488	0.901104314	0.612462034
CHRNB1	0.718116739	0.732009317	0.593672982	0.99999488	0.913608545	0.702742967
ADA2	0.718781309	0.56101196	0.533418803	0.99999488	0.999985264	0.697878268
EPSTI1	0.718960974	0.551235148	0.744618167	0.99999488	0.999985264	0.580415308
LOC614625	0.718998961	0.574311353	0.58238704	0.99999488	0.964631108	0.560067883
PDLIM7	0.719491149	0.530816655	0.513676108	0.99999488	0.999985264	0.537311716
FCF1	0.719491149	0.650665822	0.939501959	0.825434574	0.858096179	0.649073257
CD79A	0.719491149	0.759500463	0.576058288	0.99999488	0.719742223	0.721134875
PDSS1	0.719491149	0.537303163	0.580738818	0.907039918	0.999985264	0.912262222
MFSD11	0.71978233	0.705186914	0.55422436	0.889823701	0.999985264	0.692319327
CHMP6	0.719843487	0.700359766	0.57285637	0.982713285	0.835844193	0.86871626
MYBL2	0.720714993	0.636891725	0.999558179	0.830046103	0.736651407	0.52928764
CYB5R2	0.721607257	0.785734341	0.645275104	0.88664838	0.999985264	0.693004154
SERPINF1	0.72199873	0.628133877	0.708177705	0.99999488	0.999985264	0.514740511
PLPBP	0.72199873	0.6217927	0.569091034	0.865047094	0.999985264	0.583476019
RPP38	0.72199873	0.730465779	0.554098831	0.907700124	0.999985264	0.596516103
RSPRY1	0.72199873	0.594515957	0.569091034	0.949602784	0.999985264	0.635743158
GNL3L	0.72199873	0.61469452	0.5662684	0.848731351	0.999985264	0.683998081
LOC527796	0.72199873	0.845554546	0.570175675	0.989312306	0.852744493	0.716598315
SYNGR1	0.72199873	0.87544156	0.706073772	0.791188744	0.921581207	0.736516695
MAN2A2	0.722478111	0.647579301	0.657808416	0.937020992	0.999985264	0.602867378
FBXO32	0.722563471	0.723805811	0.640310147	0.794035382	0.901104314	0.931163114
CBFB	0.723292939	0.574311353	0.692872372	0.99999488	0.999985264	0.52928764
MYB	0.723292939	0.705186914	0.54092971	0.99999488	0.999985264	0.530748083
MCMBP	0.723292939	0.586911842	0.541983115	0.99999488	0.999985264	0.547278473
BCORL1	0.723292939	0.599298196	0.72674783	0.945038047	0.999985264	0.591008297
EIF5A	0.723292939	0.650266299	0.588780799	0.794035382	0.946408931	0.957171025
ATP1A1	0.723757274	0.542602246	0.807497252	0.94911704	0.999985264	0.565085989
PLD2	0.724346124	0.705186914	0.683588966	0.99999488	0.721208887	0.536719591
LOC504548	0.724346124	0.978510121	0.518494004	0.823076443	0.999985264	0.552976392
AGMO	0.724346124	0.565729718	0.566706891	0.99999488	0.831424839	0.560067883
MYL9	0.724514983	0.901232581	0.675115467	0.859289191	0.821082556	0.750879856
OLFM1	0.726114564	0.503649548	0.640617895	0.99999488	0.813331114	0.580415308

KCNK1	0.726611744	0.873963285	0.516458375	0.907700124	0.999985264	0.712249777
NUDT8	0.726771593	0.56695737	0.606008934	0.798459589	0.999985264	0.541323364
PET100	0.727296466	0.971672461	0.654174689	0.859289191	0.961025202	0.533511539
NT5DC3	0.72729669	0.747155043	0.750974849	0.989312306	0.937177132	0.569518672
CD79B	0.727408064	0.727623521	0.767682114	0.884809097	0.773111283	0.861618934
MYBPC2	0.727695765	0.688316725	0.68741937	0.99999488	0.981812717	0.59171134
ADH6	0.727695765	0.6725213	0.666104182	0.99999488	0.798938088	0.681790665
HAPLN1	0.728306869	0.642323832	0.708177705	0.98846246	0.999985264	0.582816513
MYBPH	0.728306869	0.869407223	0.638383601	0.825154704	0.999985264	0.594584164
NR4A2	0.728306869	0.599298196	0.80757077	0.813210232	0.999985264	0.608004025
ATE1	0.728306869	0.86538354	0.700157555	0.832508373	0.999985264	0.617537824
TCAF2	0.728306869	0.723805811	0.707714287	0.99999488	0.77825208	0.628644572
THSD4	0.728306869	0.935742738	0.554098831	0.98846246	0.788218905	0.677379211
ADRA1D	0.728306869	0.560520097	0.665391565	0.99999488	0.864446669	0.770030068
RASD2	0.728306869	0.591586809	0.690969178	0.98846246	0.937177132	0.787517751
SMAP2	0.728306869	0.506145822	0.580189859	0.925818215	0.999985264	0.894723749
FAM107B	0.728480207	0.893642384	0.651092963	0.795238168	0.999985264	0.652992721
PRKCI	0.728773703	0.568300739	0.546479844	0.859289191	0.999985264	0.598059647
C7H19orf57	0.729861836	0.659816181	0.719683682	0.99999488	0.866288117	0.55740152
ZNHIT2	0.729861836	0.591586809	0.652966731	0.99999488	0.999985264	0.568559353
LOC508666	0.729861836	0.534877355	0.524238325	0.99999488	0.999985264	0.736686635
DENR	0.729861836	0.703838914	0.648583755	0.880916253	0.999985264	0.811283748
LOC112441839	0.730579761	0.530162509	0.578068042	0.99999488	0.999985264	0.571769738
DHRS11	0.730579761	0.805901612	0.63799633	0.924872024	0.999985264	0.688917793
LOC782598	0.730579761	0.699986019	0.688463551	0.99999488	0.824424612	0.691757815
TAF15	0.731458447	0.729000253	0.588685035	0.794939965	0.908980492	0.949764317
ARPC5	0.731852178	0.61100105	0.962346588	0.989312306	0.747771116	0.530748083
LOC101903248	0.732062096	0.821574372	0.717074345	0.993124804	0.924089345	0.547278473
ASB1	0.733058352	0.533394878	0.771154197	0.99999488	0.999985264	0.52928764
ZNF827	0.733058352	0.869999083	0.665391565	0.959611215	0.982916677	0.55740152
CYB5RL	0.733058352	0.805853817	0.526888973	0.99999488	0.970485217	0.599905787
LOC112441683	0.733058352	0.665320167	0.944085862	0.880772564	0.873903927	0.601946079
BCR	0.733058352	0.665320167	0.580469773	0.99999488	0.999985264	0.604532054
OXLD1	0.733058352	0.540247468	0.565442221	0.939864142	0.999985264	0.640391082
MYL12A	0.733058352	0.61414244	0.76260462	0.99999488	0.978076141	0.641183409
SUSD2	0.733058352	0.799180421	0.706110318	0.998140932	0.847202014	0.649701943
GABARAPL1	0.733058352	0.566118403	0.524238325	0.99999488	0.806038879	0.671843481
WDR43	0.733058352	0.571415248	0.606008934	0.941409556	0.999985264	0.687272719
ZNF770	0.733058352	0.738038006	0.544859334	0.859289191	0.999985264	0.736909525

TEAD2	0.733058352	0.670447167	0.52966531	0.925818215	0.999985264	0.809130895
LOC101903501	0.733058352	0.64849773	0.524238325	0.949459572	0.999985264	0.847577522
ATIC	0.733058352	0.610403345	0.652054096	0.881324867	0.999985264	0.894723749
ITK	0.733058352	0.568300739	0.590933668	0.823076443	0.999985264	0.939035437
F2RL1	0.733377773	0.727539585	0.690770494	0.855919388	0.999985264	0.531219161
CORO2B	0.733377773	0.709395	0.613270892	0.989312306	0.999985264	0.54675724
AGAP1	0.733377773	0.736264262	0.524238325	0.99999488	0.834754713	0.580415308
ZNF644	0.733377773	0.752475942	0.524238325	0.823788475	0.999985264	0.602271571
FKBP14	0.733377773	0.729000253	0.590691361	0.830046103	0.999985264	0.615805309
SYT2	0.733377773	0.525458484	0.791775061	0.99999488	0.758378717	0.662624496
LOC101902537	0.733377773	0.674837172	0.914001994	0.794035382	0.849864394	0.773689802
SH3GL1	0.733377773	0.519497908	0.546479844	0.901707661	0.999985264	0.815564853
CD2	0.733377773	0.78130286	0.576230569	0.876702931	0.884208058	0.918400465
RTP4	0.733941529	0.548801965	0.724584302	0.99999488	0.999985264	0.594584164
FABP7	0.733941529	0.634381235	0.552901215	0.794939965	0.784399954	0.999960414
PDXK	0.735183728	0.588625967	0.708177705	0.996471643	0.847202014	0.849138453
KRI1	0.736030732	0.540247468	0.849633432	0.820583858	0.999985264	0.691757815
CCDC18	0.737283566	0.673847368	0.691847112	0.939758802	0.901104314	0.839734604
LGI2	0.738117798	0.649879097	0.803677695	0.99999488	0.835837522	0.530748083
PPP1CC	0.738942339	0.800657503	0.544859334	0.816792716	0.999985264	0.528012162
AVL9	0.738942339	0.733210627	0.738454814	0.99999488	0.847202014	0.52928764
RRAGC	0.738942339	0.551538509	0.750765111	0.99999488	0.864446669	0.52928764
CORO1C	0.738942339	0.599818384	0.848312163	0.949602784	0.999985264	0.52928764
GDA	0.738942339	0.509768617	0.655928749	0.99999488	0.999985264	0.543439563
FMNL1	0.738942339	0.617347838	0.567003593	0.99999488	0.998639844	0.547278473
MAGEF1	0.738942339	0.619474811	0.539135601	0.99999488	0.894715133	0.621376973
AP4B1	0.738942339	0.656082241	0.593672982	0.99999488	0.847202014	0.656292237
MEGF10	0.738942339	0.592672828	0.864737879	0.959611215	0.922362358	0.675571489
KCNJ8	0.738942339	0.73051929	0.534417481	0.99999488	0.792861833	0.71363761
C1H21orf62	0.738942339	0.989547896	0.524560092	0.820583858	0.83400817	0.805249326
SPIDR	0.738942339	0.560520097	0.632118699	0.957896521	0.999985264	0.837143123
CAND2	0.738942339	0.565729718	0.568829521	0.991596938	0.792861833	0.967874696
RIMKLA	0.739364048	0.755100312	0.657408622	0.813963687	0.999985264	0.654736169
MLF1	0.739364048	0.729820154	0.590691361	0.851621164	0.999985264	0.706584473
TRMT9B	0.740424824	0.909577824	0.817700149	0.830046103	0.864446669	0.617537824
FOXK2	0.740424824	0.661182871	0.785173725	0.803237525	0.999985264	0.62260916
CALCOCO1	0.740424824	0.638012375	0.593672982	0.989312306	0.769672519	0.941265666
DOHH	0.740541587	0.694145861	0.609340165	0.989312306	0.999985264	0.653862943
COL8A1	0.741331196	0.821194038	0.647431961	0.99999488	0.815550318	0.547278473

LDLRAP1	0.741788767	0.896996319	0.724658338	0.794035382	0.999985264	0.570743097
FAM120B	0.741788767	0.517052652	0.770213049	0.92398295	0.999985264	0.593347482
ZSWIM2	0.741788767	0.905019423	0.562216435	0.881982604	0.952554537	0.773507109
CEMIP2	0.742303601	0.525945198	0.641573776	0.99999488	0.999985264	0.52928764
SEMA6B	0.742303601	0.570683052	0.640558462	0.99999488	0.864446669	0.533511539
SFRP2	0.742303601	0.677438555	0.718226122	0.99999488	0.901763837	0.541323364
FXYD3	0.742303601	0.56101196	0.912008683	0.976631804	0.999985264	0.543705218
ZMAT5	0.742303601	0.883624935	0.554098831	0.917553107	0.999985264	0.545755335
BNIP2	0.742303601	0.530816655	0.856449202	0.978440951	0.999985264	0.552865513
C8H9orf72	0.742303601	0.587162018	0.930678591	0.859289191	0.999985264	0.563215326
ADRA1B	0.742303601	0.533354903	0.595396914	0.848077461	0.999985264	0.571769738
CERS4	0.742303601	0.64037592	0.691847112	0.942391899	0.999985264	0.5747275
HSPBP1	0.742303601	0.744638901	0.577647688	0.99999488	0.864446669	0.580415308
EPC2	0.742303601	0.937626884	0.591210717	0.794035382	0.999985264	0.601946079
DPYSL5	0.742303601	0.729000253	0.675115467	0.989312306	0.999985264	0.609705
ZNF219	0.742303601	0.733210627	0.651313506	0.99999488	0.946408931	0.610043612
CCL8	0.742303601	0.847686989	0.689932449	0.796555088	0.999985264	0.610043612
CFAP69	0.742303601	0.592962209	0.746258329	0.878491623	0.999985264	0.615326593
ENDOG	0.742303601	0.531215068	0.606564873	0.896748401	0.999985264	0.654291391
PER3	0.742303601	0.76933827	0.801232877	0.951237479	0.781939574	0.738324902
NPPC	0.742303601	0.755100312	0.683588966	0.907045085	0.950839268	0.805442659
TMEM35A	0.742303601	0.90024974	0.570175675	0.951237479	0.77926164	0.819073042
LOC100848246	0.742303601	0.698927866	0.619790755	0.848389672	0.999985264	0.844563769
CDC27	0.742303601	0.755456121	0.811018607	0.880028563	0.741080831	0.856011656
CLHC1	0.743287809	0.755100312	0.573414999	0.880829722	0.798938088	0.964476713
NEK2	0.743400839	0.533733977	0.970700752	0.99999488	0.761285588	0.541323364
LOC101907540	0.743400839	0.659816181	0.877799478	0.839050812	0.999985264	0.555648397
ARHGAP9	0.743400839	0.755100312	0.577092785	0.99999488	0.741080831	0.588178204
GPR50	0.743400839	0.820605162	0.746258329	0.993124804	0.901104314	0.59171134
PARP10	0.743400839	0.551538509	0.866680636	0.907700124	0.999985264	0.617537824
LRRC4	0.743400839	0.525945198	0.825497047	0.98846246	0.879141216	0.812831419
NYNRIN	0.743400839	0.531215068	0.708177705	0.99999488	0.804315588	0.837143123
LOC104975663	0.743703267	0.518580134	0.68741937	0.875335495	0.999985264	0.547278473
LOC618367	0.745238877	0.589631719	0.63799633	0.99999488	0.921581207	0.868513869
PCOLCE	0.745504837	0.78130286	0.7536262	0.99999488	0.978076141	0.529436487
CCDC28B	0.745504837	0.573366037	0.923604668	0.798459589	0.816824542	0.885874544
RBM12	0.74562135	0.783973824	0.896055927	0.884650797	0.901436701	0.607538907
PNMA1	0.747230094	0.663430554	0.73245868	0.848389672	0.90115184	0.921771959
CD300LG	0.748628507	0.819937634	0.600307359	0.817200124	0.999985264	0.537810684

DNAJC5	0.748628507	0.647062519	0.667817366	0.99999488	0.999985264	0.628307831
ABCF1	0.74900983	0.573366037	0.569138905	0.855453165	0.999985264	0.914756067
PDCD1LG2	0.749281172	0.602393689	0.609340165	0.99999488	0.999985264	0.695049955
PHGDH	0.749589743	0.661182871	0.63799633	0.989312306	0.999985264	0.729827809
WDR77	0.749589743	0.661182871	0.583675883	0.989312306	0.933861931	0.873083064
ATG14	0.749852921	0.999347049	0.566721071	0.813963687	0.740523538	0.816959462
IDUA	0.749964572	0.918952092	0.572842879	0.99999488	0.961025202	0.551826127
OSBPL1A	0.749964572	0.553465911	0.539880753	0.99999488	0.944638139	0.575719205
FBXO11	0.749964572	0.661182871	0.536995142	0.949602784	0.999985264	0.580233963
LCORL	0.749964572	0.715614299	0.580363758	0.99999488	0.937177132	0.601946079
TERF2IP	0.749964572	0.677438555	0.72674783	0.99999488	0.864446669	0.617537824
DDX51	0.749964572	0.684910993	0.601420135	0.957896521	0.999985264	0.644112933
RASSF10	0.749964572	0.844174041	0.586972294	0.99999488	0.759221661	0.652992721
TSLP	0.749964572	0.530795065	0.843587792	0.907700124	0.999985264	0.655665506
TMEM176A	0.749964572	0.722585736	0.778280092	0.99999488	0.757173298	0.684089745
S100A1	0.749964572	0.588625967	0.632118699	0.99999488	0.776915111	0.751788817
RMRP	0.749964572	0.573366037	0.706073772	0.99999488	0.7946823	0.836831695
NEXN	0.749964572	0.691302101	0.70108275	0.978440951	0.815550318	0.859200033
EIF3CL	0.749964572	0.552756705	0.542792917	0.99999488	0.999985264	0.895349622
RERE	0.749964572	0.533435858	0.573414999	0.809884527	0.757173298	0.999960414
ID2	0.750393299	0.592838823	0.749648665	0.99999488	0.93150494	0.537810684
ELL2	0.750662129	0.568712199	0.55422436	0.99999488	0.856124022	0.571518232
MOSPD1	0.752101816	0.619449417	0.871925227	0.948570368	0.999985264	0.629039793
COX10	0.752726606	0.5736053	0.606008934	0.87159977	0.999985264	0.931163114
UTP18	0.752746585	0.610403345	0.767682114	0.99999488	0.915346056	0.74674547
FAM20A	0.753825325	0.682316491	0.572842879	0.99999488	0.867495559	0.614101486
NDEL1	0.754008436	0.596095252	0.595396914	0.896748401	0.999985264	0.684032381
ZNF362	0.755496792	0.557992078	0.615612025	0.917553107	0.999985264	0.661195029
TAP1	0.755526252	0.741594487	0.576058288	0.979293963	0.999985264	0.612911427
TIA1	0.757096439	0.777422141	0.739250184	0.932532289	0.999985264	0.588733625
ADCY5	0.757690727	0.659816181	0.774363763	0.98846246	0.999985264	0.571769738
PCDH7	0.757713293	0.587721224	0.586654479	0.898245017	0.999985264	0.678579877
FCER1G	0.758330355	0.62096594	0.590933668	0.99999488	0.956655472	0.569518672
PLEKHG2	0.758580412	0.592962209	0.698169084	0.99999488	0.901104314	0.580415308
TMEM248	0.758580412	0.537448684	0.645275104	0.99999488	0.755902323	0.596379492
NOM1	0.758580412	0.816518153	0.55730426	0.99999488	0.999985264	0.60011172
LMTK3	0.758580412	0.562433468	0.568829521	0.99999488	0.901763837	0.945794274
PRRT2	0.758580412	0.533733977	0.55422436	0.99999488	0.77825208	0.960343814
ISCA2	0.758917698	0.540247468	0.9808801	0.981779494	0.871609728	0.54675724

MTR	0.760341608	0.565729718	0.632928415	0.99999488	0.798938088	0.654291391
RASAL3	0.760372523	0.834860292	0.598765064	0.855453165	0.999985264	0.770633383
PABPC4	0.760372523	0.649866599	0.609340165	0.803037807	0.999985264	0.809513929
GPR88	0.760937467	0.548616419	0.564575598	0.99999488	0.999985264	0.541323364
SLC26A2	0.760937467	0.563039115	0.575199502	0.99999488	0.999985264	0.541777213
POC1A	0.760937467	0.540247468	0.670870221	0.91166192	0.999985264	0.54675724
HHLA2	0.760937467	0.830132999	0.55422436	0.99999488	0.928683582	0.55740152
PILRA	0.760937467	0.542800218	0.567599362	0.99999488	0.858096179	0.559024093
LOC112443428	0.760937467	0.540247468	0.907484111	0.99999488	0.999985264	0.580118533
C2	0.760937467	0.647062519	0.881938573	0.948570368	0.999985264	0.594584164
ANGPTL8	0.760937467	0.55837549	0.569091034	0.884809097	0.999985264	0.608269365
LOC784087	0.760937467	0.988850552	0.681730747	0.925818215	0.810386314	0.609616735
TTC22	0.760937467	0.664442591	0.849960855	0.998140932	0.933861931	0.614862466
PAMR1	0.760937467	0.674837172	0.88153193	0.99999488	0.787472587	0.622341636
LOC112441650	0.760937467	0.670600139	0.948420301	0.889823701	0.816099555	0.691757815
L1CAM	0.760937467	0.647062519	0.921010865	0.830046103	0.96948473	0.722837886
C14H8orf37	0.760937467	0.865926615	0.655928749	0.878491623	0.999985264	0.743462219
CCNDBP1	0.760937467	0.553465911	0.877799478	0.999314272	0.864446669	0.743798578
LOC100847495	0.760937467	0.577525057	0.72005175	0.943090854	0.999985264	0.812831419
CRABP2	0.760937467	0.550265412	0.655416187	0.99999488	0.864446669	0.831603744
MCUR1	0.760937467	0.551235148	0.938905004	0.889928167	0.821082556	0.83405895
LOC101905514	0.760937467	0.610403345	0.70090879	0.92106221	0.982217275	0.894420979
FERMT3	0.761742778	0.634336339	0.576230125	0.99999488	0.793270571	0.57860474
GPN3	0.76183433	0.865184773	0.825497047	0.921764051	0.916667939	0.591008297
GPX4	0.761956402	0.561419114	0.610641809	0.812057455	0.999985264	0.617132044
BEND3	0.762912167	0.884739596	0.620035975	0.959611215	0.999985264	0.55740152
OPA3	0.763058432	0.779783885	0.656285423	0.952783349	0.999985264	0.543705218
MANSC1	0.763309148	0.553465911	0.576058288	0.99999488	0.999985264	0.55740152
LOC112444346	0.763309148	0.610403345	0.566721071	0.99999488	0.999985264	0.615805309
SAMD15	0.763309148	0.741111932	0.784079944	0.989312306	0.873421342	0.703356006
TM7SF2	0.763309148	0.61100105	0.644916374	0.878491623	0.999985264	0.714633736
CDR2	0.763309148	0.711509889	0.743915133	0.813963687	0.999985264	0.718350328
LOC112447381	0.763309148	0.832584599	0.825497047	0.884809097	0.83400817	0.762686295
CFL1	0.763309148	0.917537324	0.708177705	0.907045085	0.829541927	0.763715224
LOC112448736	0.763436194	0.864945604	0.71183539	0.848077461	0.880459369	0.836520183
FAM43B	0.763995045	0.565729718	0.547354523	0.99999488	0.894715133	0.962703352
LOC112448893	0.764660224	0.870839685	0.825497047	0.816792716	0.999985264	0.609196585
TEX11	0.764881679	0.667396315	0.586568869	0.99999488	0.794349969	0.581941631
RETN	0.764881679	0.705186914	0.76260462	0.99999488	0.964616886	0.627549535

LOC101907682	0.764881679	0.582088443	0.941782902	0.921764051	0.798938088	0.777136982
EPDR1	0.764999815	0.718570773	0.647235003	0.825154704	0.999985264	0.593335761
PRDM5	0.764999815	0.549349774	0.908181052	0.954491441	0.999985264	0.594287724
RSRC2	0.764999815	0.612694128	0.691575438	0.901855229	0.999985264	0.602271571
STK40	0.765938535	0.649879097	0.667274767	0.948570368	0.999985264	0.839734604
LOC101906195	0.767287186	0.550447041	0.774363763	0.99999488	0.864446669	0.56695901
GMPPA	0.767287186	0.649879097	0.833419544	0.99999488	0.787383034	0.588781291
TRPA1	0.767287186	0.985406717	0.706073772	0.931835237	0.785107052	0.621376973
OIP5	0.767287186	0.694145861	0.957541703	0.896456511	0.785107052	0.684032381
ZAR1L	0.767287186	0.636415912	0.886901854	0.921764051	0.84872286	0.812831419
LOC509513	0.767287186	0.8756206	0.683763181	0.852513382	0.880459369	0.846334585
AIMP2	0.767287186	0.727623521	0.663165825	0.991596938	0.810386314	0.883507907
SAE1	0.767287186	0.6598011	0.778565768	0.865261766	0.864446669	0.931163114
TSKU	0.768545916	0.880163543	0.673148477	0.989312306	0.864446669	0.675571489
GRWD1	0.768944584	0.663430554	0.638602654	0.99999488	0.858963201	0.691757815
LOC539973	0.768944584	0.696893897	0.818784725	0.952783349	0.961025202	0.698422388
LOC101903289	0.768944584	0.595636522	0.594439052	0.99999488	0.77926164	0.748495031
KATNAL1	0.768944584	0.650266299	0.983077441	0.809884527	0.781939574	0.752465035
P2RX5	0.768944584	0.589510121	0.580469773	0.99999488	0.999985264	0.779686358
BRD3OS	0.768944584	0.850364054	0.690770494	0.982713285	0.785107052	0.789976745
LOC112448791	0.768944584	0.644291771	0.602936506	0.98846246	0.999985264	0.805462926
STT3B	0.769213245	0.591863434	0.785177733	0.99999488	0.942132162	0.568559353
HNMT	0.769213245	0.570683052	0.566353811	0.99999488	0.981774771	0.584692851
IL33	0.769213245	0.759500463	0.596040322	0.99999488	0.864446669	0.591711015
PLS3	0.769213245	0.589631719	0.914001994	0.825952127	0.999985264	0.612911427
NCOR2	0.769213245	0.544251731	0.671210214	0.99999488	0.999985264	0.626027519
NUCB2	0.769213245	0.582088443	0.729977435	0.99999488	0.937177132	0.658487395
SSTR1	0.769717605	0.59460811	0.683588966	0.949602784	0.999985264	0.583476019
POLD4	0.770671684	0.713094172	0.77602067	0.99999488	0.924009849	0.601946079
NRG2	0.770843612	0.867755878	0.707485652	0.859289191	0.999985264	0.603560332
ARG2	0.770892705	0.628680252	0.619986953	0.99999488	0.83400817	0.599905787
CKAP2	0.770892705	0.570683052	0.840760291	0.99999488	0.908980492	0.645467494
IFT57	0.770892705	0.612694128	0.746258329	0.943090854	0.999985264	0.675571489
FAM212A	0.770892705	0.537884107	0.560546656	0.969770561	0.999985264	0.953254073
PSMA7	0.770892705	0.663430554	0.615380158	0.865261766	0.983747207	0.953658739
BRD8	0.771170484	0.869999083	0.706110318	0.951237479	0.773709332	0.814496203
SKA3	0.771335001	0.76933827	0.798401881	0.907700124	0.999985264	0.559609585
OGFOD2	0.771335001	0.701623819	0.576058288	0.8398706	0.999985264	0.612764179
CACNB1	0.771335001	0.943802122	0.63799633	0.925818215	0.785107052	0.802688077

ABCA3	0.771335001	0.649879097	0.673099635	0.941409556	0.965227764	0.901809636
COL6A1	0.77161221	0.867755878	0.678433983	0.99999488	0.952330707	0.580415308
TRIM24	0.77161221	0.773422401	0.575199502	0.99999488	0.999985264	0.584459217
ZFPL1	0.77161221	0.557957838	0.556265776	0.917553107	0.999985264	0.617537824
IRX3	0.77161221	0.551235148	0.88153193	0.99999488	0.944638139	0.656561507
TNFSF13	0.77161221	0.591455943	0.55422436	0.99999488	0.942132162	0.693137826
LOC101905312	0.771825922	0.707737892	0.794324488	0.898472566	0.999985264	0.583476019
KCNE3	0.771825922	0.65897587	0.670870221	0.99999488	0.901763837	0.626804781
WBP2	0.772715522	0.816100073	0.654174689	0.99999488	0.981774771	0.594584164
PABPN1	0.772715522	0.721370958	0.916975216	0.963012661	0.899432059	0.609196585
LOC781726	0.772715522	0.565729718	0.956565728	0.99999488	0.798938088	0.616994145
ABCD4	0.772715522	0.661182871	0.767682114	0.948570368	0.999985264	0.617537824
SLITRK6	0.772715522	0.555377781	0.570175675	0.917553107	0.999985264	0.762686295
LOC100336669	0.772715522	0.557992078	0.594439052	0.907700124	0.999985264	0.858593649
LOC107133024	0.772747166	0.571415248	0.591210717	0.941409556	0.999985264	0.571651727
LOC783641	0.772747166	0.564128591	0.72628476	0.896748401	0.999985264	0.600769945
LOC540403	0.772747166	0.634298188	0.93198623	0.884809097	0.961025202	0.706474384
DPM1	0.772747166	0.864884124	0.586654479	0.917553107	0.999985264	0.787029868
HMGN1	0.772747166	0.650665822	0.691847112	0.851621164	0.999985264	0.931163114
RTEL1	0.773195546	0.650665822	0.971747951	0.947995037	0.903345596	0.554947685
NACC1	0.773195546	0.604983602	0.575199502	0.890282205	0.999985264	0.631711796
LOC101907152	0.773195546	0.754374533	0.68741937	0.98846246	0.849565759	0.847204221
RBM47	0.773195546	0.565729718	0.708177705	0.989312306	0.982624506	0.866439215
NPM3	0.773195546	0.553465911	0.591970585	0.831276941	0.999985264	0.995391635
MCRS1	0.77353012	0.76933827	0.62088615	0.979945333	0.999985264	0.684089745
KRBA2	0.773711756	0.732009317	0.799361358	0.87276246	0.77926164	0.929458454
HSF2	0.773738878	0.709904249	0.577092785	0.87276246	0.999985264	0.556965527
ZNF341	0.773738878	0.546324975	0.938203102	0.99999488	0.937177132	0.55740152
ZBTB14	0.773738878	0.649866599	0.911018006	0.99999488	0.995549184	0.55740152
EXD3	0.773738878	0.592962209	0.865377161	0.99999488	0.999985264	0.569507857
ASPA	0.773738878	0.821194038	0.654174689	0.99999488	0.829541927	0.569518672
AARS2	0.773738878	0.599818384	0.900351064	0.92106221	0.999985264	0.569518672
DGCR8	0.773738878	0.591586809	0.93955737	0.99999488	0.932859235	0.570630978
DMPK	0.773738878	0.631258266	0.692027671	0.820583858	0.999985264	0.571769738
ARID4B	0.773738878	0.605131338	0.656140087	0.973421737	0.999985264	0.572356362
LOC112448805	0.773738878	0.599298196	0.670826239	0.99999488	0.769672519	0.573384986
LOC506495	0.773738878	0.733210627	0.865420641	0.99999488	0.835076615	0.580118533
CEP95	0.773738878	0.710594949	0.944847074	0.999314272	0.815550318	0.583476019
ZNF638	0.773738878	0.826936949	0.673099635	0.844596239	0.999985264	0.583476019

TPPP3	0.773738878	0.705186914	0.586568869	0.924872024	0.999985264	0.586032676
NAV1	0.773738878	0.74820894	0.926552506	0.978440951	0.858974474	0.588890004
SLC25A33	0.773738878	0.573366037	0.663165825	0.99999488	0.858096179	0.58995269
ZDHHC18	0.773738878	0.555377781	0.576058288	0.99999488	0.999985264	0.593352427
KCTD14	0.773738878	0.747149262	0.728629222	0.99999488	0.999985264	0.594584164
DIP2C	0.773738878	0.963268227	0.63799633	0.99999488	0.861287091	0.595036301
IFI27L2	0.773738878	0.999992444	0.571697442	0.925818215	0.939144222	0.599905787
SENP6	0.773738878	0.741594487	0.595396914	0.907700124	0.999985264	0.599905787
ELAVL1	0.773738878	0.62408671	0.935572367	0.976631804	0.943670708	0.601946079
RAB9A	0.773738878	0.612694128	0.655928749	0.949602784	0.999985264	0.601946079
CTBP1	0.773738878	0.655648116	0.573414999	0.99999488	0.999985264	0.601946079
MGC139164	0.773738878	0.591586809	0.94634922	0.969770561	0.944638139	0.603050301
LOC100141266	0.773738878	0.999992444	0.639782943	0.949602784	0.798938088	0.609616735
FAN1	0.773738878	0.642323832	0.606051701	0.960794858	0.999985264	0.609616735
SPSB1	0.773738878	0.999992444	0.665391565	0.92106221	0.808842544	0.610600013
YTHDF2	0.773738878	0.568712199	0.683588966	0.926433531	0.999985264	0.612111034
DENND6A	0.773738878	0.732202418	0.724128712	0.99999488	0.970485217	0.614040014
LOC100337213	0.773738878	0.862707207	0.593672982	0.907045085	0.999985264	0.614040014
SLITRK2	0.773738878	0.636004657	0.684617023	0.99999488	0.995610149	0.617537824
ARPC3	0.773738878	0.560520097	0.671210214	0.99999488	0.999985264	0.619364593
TMEM108	0.773738878	0.850692013	0.884119589	0.885570604	0.922362358	0.623057106
NRCAM	0.773738878	0.592672828	0.566721071	0.99999488	0.911651126	0.628644572
CYP27A1	0.773738878	0.813113924	0.567542941	0.99999488	0.815550318	0.62904095
MYH11	0.773738878	0.999992444	0.62088615	0.907045085	0.785107052	0.654291391
ADORA3	0.773738878	0.673405033	0.576058288	0.99999488	0.835076615	0.654736169
SLCO4A1	0.773738878	0.591239507	0.571817462	0.917553107	0.999985264	0.658971109
DHX40	0.773738878	0.73157551	0.770213049	0.978440951	0.999985264	0.65937855
KLHDC7A	0.773738878	0.588325495	0.95106368	0.870282231	0.999985264	0.662512478
UTRN	0.773738878	0.606994513	0.575373244	0.840951535	0.999985264	0.664012525
ARSI	0.773738878	0.599298196	0.718226122	0.99999488	0.973714462	0.670235195
SNAP47	0.773738878	0.619474811	0.718951436	0.99999488	0.999985264	0.671843481
HMGCR	0.773738878	0.752475942	0.691847112	0.989312306	0.999985264	0.678756699
MYADM	0.773738878	0.873289191	0.872152937	0.830949185	0.901104314	0.692319327
LOC101908760	0.773738878	0.674944018	0.916975216	0.949459572	0.901104314	0.693397277
CXCR4	0.773738878	0.622790006	0.581214226	0.99999488	0.999985264	0.704895174
PLIN2	0.773738878	0.560029081	0.889744621	0.99999488	0.915346056	0.709726351
PKLR	0.773738878	0.935742738	0.688463551	0.917553107	0.901104314	0.72144082
SLC39A8	0.773738878	0.928525825	0.638912432	0.907045085	0.983747207	0.728036947
SFR1	0.773738878	0.653258736	0.748903111	0.99999488	0.999388501	0.729812118

LUZP1	0.773738878	0.84430803	0.620035975	0.820583858	0.999985264	0.734689625
PLCXD2	0.773738878	0.980867747	0.570175675	0.925818215	0.901104314	0.738324902
LOC100848684	0.773738878	0.6725213	0.716039143	0.952783349	0.999985264	0.747408201
LOC101902293	0.773738878	0.729820154	0.825497047	0.98846246	0.858096179	0.751946348
SLMAP	0.773738878	0.975462313	0.699187003	0.880916253	0.794694113	0.763785238
ARFGAP2	0.773738878	0.626400323	0.765522222	0.99999488	0.946408931	0.772431293
MSX1	0.773738878	0.645616236	0.654174689	0.99999488	0.935138282	0.772479356
LOC788599	0.773738878	0.678244127	0.645275104	0.820583858	0.999985264	0.772479356
SMARCC1	0.773738878	0.555377781	0.730171751	0.967551965	0.999985264	0.772479356
CCT5	0.773738878	0.729546716	0.678433983	0.973503136	0.999985264	0.787783489
FAM131A	0.773738878	0.668131227	0.666562848	0.859289191	0.999985264	0.790011024
LOC101905267	0.773738878	0.661182871	0.762986489	0.99999488	0.777489275	0.794870105
LRIG3	0.773738878	0.737307609	0.746258329	0.978440951	0.887261118	0.812316576
TH	0.773738878	0.752475942	0.68741937	0.907700124	0.999985264	0.812831419
PTPRG	0.773738878	0.655648116	0.855775419	0.978440951	0.800902502	0.829320798
CMSS1	0.773738878	0.560520097	0.66951603	0.92106221	0.999985264	0.87805762
FCMR	0.773738878	0.846845458	0.77618424	0.817200124	0.77926164	0.902893979
PNMA8A	0.773738878	0.793805955	0.683588966	0.907045085	0.901104314	0.905099683
EPB41L5	0.773738878	0.783973824	0.665391565	0.904225644	0.911651126	0.911997747
GZMK	0.773738878	0.674272153	0.598765064	0.820583858	0.999985264	0.918145254
GRB7	0.773738878	0.572327231	0.640558462	0.889823701	0.999985264	0.921771959
CYB561A3	0.773738878	0.814219866	0.573906533	0.966694193	0.847202014	0.926480606
SH2D4A	0.773738878	0.916036681	0.580189859	0.853181717	0.794349969	0.948213108
NTRK3	0.773738878	0.850692013	0.577647688	0.875836685	0.847202014	0.954101579
PLCB4	0.773738878	0.86538354	0.628800812	0.840951535	0.798938088	0.956550292
MOG	0.773738878	0.591454495	0.821698192	0.816792716	0.81058423	0.985811281
LOC511713	0.773738878	0.64243875	0.571817462	0.907700124	0.84872286	0.999960414
FKRP	0.773754008	0.703838914	0.882515776	0.989312306	0.952275018	0.601946079
TMEM128	0.774189446	0.921934673	0.851718619	0.961309253	0.805527405	0.580415308
CORO2A	0.775063245	0.935742738	0.590691361	0.99999488	0.943686834	0.582816513
LOC112446423	0.775063245	0.565729718	0.654174689	0.855453165	0.999985264	0.746326297
SLC9A1	0.775147155	0.638045424	0.82705847	0.959611215	0.999985264	0.598997211
LRRC61	0.775147155	0.882505302	0.58238704	0.99999488	0.901104314	0.746239631
SPATA5	0.775147155	0.802415056	0.591210717	0.99999488	0.840395005	0.795293834
LRRC59	0.775147155	0.709904249	0.594439052	0.948570368	0.799606341	0.982250307
ZKSCAN5	0.775271292	0.586911842	0.645275104	0.825154704	0.787472587	0.999960414
RPS12	0.775798607	0.612694128	0.670826239	0.869065101	0.999985264	0.609986278
CFAP44	0.775798607	0.759500463	0.724658338	0.876702931	0.787472587	0.958193903
SDHAF4	0.776838435	0.574311353	0.606564873	0.99999488	0.999985264	0.564361912

SYK	0.776838435	0.587361212	0.573450552	0.99999488	0.999985264	0.580906862
NELL1	0.776838435	0.565729718	0.70071733	0.953588494	0.999985264	0.644790352
MVK	0.776838435	0.760417583	0.701191215	0.878491623	0.999985264	0.704679988
TRAF3IP3	0.776838435	0.623767283	0.605719038	0.99999488	0.946408931	0.921416283
RBP7	0.777295496	0.893642384	0.833496026	0.907700124	0.96948473	0.588733625
NRP2	0.777295496	0.599298196	0.851718619	0.99999488	0.92336492	0.5902767
CPNE9	0.777295496	0.722585736	0.774363763	0.904225644	0.983747207	0.839859968
STK38	0.777479729	0.661182871	0.577647688	0.92106221	0.999985264	0.692319327
ANO1	0.777974495	0.741594487	0.807323012	0.939758802	0.999985264	0.588178204
PPP6R2	0.778335475	0.686708208	0.724584302	0.99999488	0.999985264	0.563215326
COG2	0.778335475	0.604615112	0.643747653	0.859289191	0.999985264	0.614040014
TAP2	0.778335475	0.926949474	0.63577421	0.885819492	0.999985264	0.627549535
PPP1R3F	0.778335475	0.703522187	0.595339754	0.869065101	0.999985264	0.816226172
PIK3R5	0.778335475	0.570683052	0.670826239	0.99999488	0.999985264	0.83405895
CD3D	0.778335475	0.819937634	0.778280092	0.840951535	0.944638139	0.838520085
LOC100847120	0.778348895	0.893743321	0.865066354	0.951539068	0.815874035	0.611153303
RERGL	0.778348895	0.685985172	0.691847112	0.884809097	0.999985264	0.736516695
DRG1	0.778348895	0.557992078	0.665391565	0.959611215	0.864446669	0.987011114
MAP4K4	0.778363571	0.647062519	0.577092785	0.924872024	0.999985264	0.641507019
LOC101905925	0.778363571	0.729820154	0.923604668	0.917553107	0.944638139	0.645467494
CCBE1	0.779011151	0.564128591	0.907484111	0.937509939	0.999985264	0.578094648
UBE3B	0.779011151	0.573366037	0.593672982	0.945038047	0.999985264	0.582816513
MDM4	0.779011151	0.992977597	0.57992604	0.99999488	0.794349969	0.588733625
PRAP1	0.779011151	0.565729718	0.623909299	0.960794858	0.999985264	0.661576605
LOC101906754	0.779011151	0.552756705	0.762661971	0.978440951	0.794349969	0.963903108
C2CD5	0.779011151	0.61100105	0.586568869	0.907700124	0.999985264	0.983542592
SOCS6	0.77912963	0.628177084	0.822003523	0.99999488	0.821945987	0.583922833
FLCN	0.779829775	0.674944018	0.593672982	0.901707661	0.999985264	0.885874544
HERPUD1	0.780546379	0.650665822	0.575528813	0.884650797	0.999985264	0.578094648
IRF5	0.780546379	0.573366037	0.570175675	0.99999488	0.901763837	0.597690769
PDHX	0.780546379	0.582088443	0.592620368	0.949602784	0.999985264	0.64722711
WDR76	0.780546379	0.567336107	0.620035975	0.99999488	0.983747207	0.798034534
SNRK	0.780546379	0.816518153	0.683588966	0.854395119	0.999985264	0.809513929
COL7A1	0.780546379	0.574751845	0.771154197	0.99999488	0.956025338	0.812825712
IL18R1	0.780546379	0.615586822	0.724584302	0.869065101	0.999985264	0.872307149
LOC101902059	0.780546379	0.605074845	0.610641809	0.949602784	0.999985264	0.909427764
CHTF18	0.780571812	0.563114067	0.954864482	0.989312306	0.942132162	0.609616735
S100A5	0.780824851	0.563039115	0.817223866	0.907700124	0.946408931	0.931163114
RXRA	0.781248267	0.634298188	0.606051701	0.907700124	0.999985264	0.588733625

PM20D1	0.781248267	0.574560576	0.666562848	0.99999488	0.965353772	0.85637643
GPR151	0.78134379	0.993777777	0.748903111	0.948570368	0.835076615	0.567763858
FAM234A	0.78134379	0.741139067	0.632928415	0.907700124	0.999985264	0.569013454
DMWD	0.78134379	0.670025008	0.744618167	0.99999488	0.999985264	0.578094648
FAM135A	0.78134379	0.647062519	0.670870221	0.949602784	0.999985264	0.580415308
CENPE	0.78134379	0.650665822	0.864737879	0.99999488	0.834754713	0.586349474
ZNF512B	0.78134379	0.580125964	0.691847112	0.99999488	0.841924245	0.588126998
CBX4	0.78134379	0.663430554	0.825497047	0.952783349	0.999985264	0.594584164
TNFAIP8L1	0.78134379	0.858808025	0.770484158	0.99999488	0.831019096	0.60042403
ADAT2	0.78134379	0.850692013	0.870264123	0.825154704	0.999985264	0.631726864
PABPC1L2A	0.78134379	0.832584599	0.605523279	0.99999488	0.821082556	0.650914382
RGS2	0.78134379	0.587721224	0.843587792	0.99999488	0.999985264	0.654291391
DES	0.78134379	0.999347049	0.690864342	0.917553107	0.815874035	0.654736169
LOC101903038	0.78134379	0.599298196	0.600902205	0.99999488	0.999985264	0.664012525
MESD	0.78134379	0.821194038	0.774363763	0.938185243	0.999388501	0.701119933
FAM198A	0.78134379	0.572327231	0.817816439	0.99999488	0.922384168	0.705710928
ST3GAL6	0.78134379	0.647062519	0.63799633	0.99999488	0.901763837	0.737158489
ALDH18A1	0.78134379	0.596319881	0.610361628	0.99999488	0.999985264	0.742551138
AKAP8L	0.78134379	0.736960298	0.794324488	0.92106221	0.999985264	0.75331823
RBM34	0.78134379	0.663928441	0.903957821	0.938185243	0.922362358	0.754852502
PLAUR	0.78134379	0.575110138	0.595396914	0.99999488	0.999985264	0.779686358
LOC404103	0.78134379	0.776219332	0.689932449	0.99999488	0.864446669	0.819073042
ACSL4	0.78134379	0.842852797	0.610641809	0.951237479	0.961025202	0.841443168
ANGPTL4	0.78134379	0.819937634	0.575908071	0.92106221	0.999985264	0.844292822
CXHXorf38	0.78134379	0.607621421	0.715414989	0.99999488	0.831424839	0.897467714
PTX3	0.78134379	0.626400323	0.701191215	0.853181717	0.999985264	0.918400465
ZNF704	0.78134379	0.667396315	0.662111348	0.836593623	0.944638139	0.985664957
C7H5orf63	0.78134379	0.591124857	0.593672982	0.92106221	0.965672975	0.993564907
PTGES2	0.782750706	0.6725213	0.609340165	0.907700124	0.999985264	0.614101486
ARHGAP19	0.782750706	0.579145828	0.696292819	0.99999488	0.999985264	0.617537824
GALR3	0.782750706	0.6725213	0.903957821	0.884809097	0.999985264	0.660540853
OTUD6B	0.782750706	0.584503543	0.691847112	0.99999488	0.907875746	0.692319327
AFG3L2	0.782750706	0.6598011	0.675121942	0.99999488	0.999985264	0.695049955
LOC614207	0.782750706	0.610403345	0.665391565	0.978440951	0.999985264	0.733745412
MYL12B	0.78297171	0.752475942	0.825736269	0.907700124	0.999985264	0.580415308
ANKRD33B	0.78297171	0.561419114	0.834068744	0.99999488	0.999985264	0.580415308
PLXNB3	0.78297171	0.850095234	0.962142195	0.846687492	0.864446669	0.601946079
LOC101903026	0.78297171	0.568712199	0.961024709	0.99999488	0.798938088	0.610600013
SWI5	0.78297171	0.568712199	0.591210717	0.99999488	0.999985264	0.615326593

DNASE2	0.78297171	0.735949796	0.63962071	0.99999488	0.907875746	0.621376973
IL12RB1	0.78297171	0.565729718	0.926552506	0.99999488	0.798043944	0.646816549
PSME1	0.78297171	0.824023254	0.594439052	0.99999488	0.999985264	0.653862943
DPH7	0.78297171	0.68530874	0.644916374	0.977598122	0.999985264	0.66542216
DAPP1	0.78297171	0.698915525	0.690864342	0.99999488	0.965672975	0.670235195
LOC100336869	0.78297171	0.74820894	0.592565415	0.99999488	0.981774771	0.670235195
CD164L2	0.78297171	0.974812121	0.591210717	0.989312306	0.901104314	0.67496387
LOC786352	0.78297171	0.589631719	0.644279997	0.92106221	0.999985264	0.67764684
DTX3L	0.78297171	0.599818384	0.580738818	0.99999488	0.999985264	0.73024728
NOL11	0.78297171	0.741111932	0.744618167	0.827995257	0.999985264	0.769515026
IFIT2	0.78297171	0.56780353	0.613720561	0.99999488	0.934601785	0.809513929
PCMTD2	0.78297171	0.73589068	0.70108275	0.924872024	0.999985264	0.818895181
LOC107131418	0.78297171	0.893642384	0.634080201	0.917553107	0.905874828	0.873463288
RBFOX2	0.78297171	0.693386354	0.728629222	0.996471643	0.856853826	0.894723749
TIGIT	0.78297171	0.819379885	0.714371099	0.904225644	0.84401945	0.928634547
CD244	0.78364074	0.620964055	0.640558462	0.99999488	0.999985264	0.604197867
IPCEF1	0.78364074	0.701786893	0.575199502	0.99999488	0.999985264	0.649701943
ARMCX1	0.78364074	0.59602732	0.816589116	0.978440951	0.996075618	0.818895181
LOC104975676	0.78364074	0.911731694	0.75028318	0.876702931	0.847202014	0.848830665
KRT79	0.783676762	0.628680252	0.6158151	0.99999488	0.999985264	0.58581641
ITGA9	0.78375428	0.998131745	0.594439052	0.99999488	0.84872286	0.578823016
SAV1	0.78375428	0.677224344	0.634080201	0.99999488	0.890963305	0.594584164
LOC112446734	0.78375428	0.926675185	0.770899781	0.914097104	0.999985264	0.601946079
LOC112449523	0.78375428	0.736701713	0.886962419	0.99999488	0.860941122	0.614862466
MGC126945	0.78375428	0.663430554	0.654174689	0.99999488	0.999985264	0.621376973
TNFRSF25	0.78375428	0.789654884	0.700157555	0.941409556	0.999985264	0.753766324
CD3G	0.78375428	0.816518153	0.707485652	0.878491623	0.999985264	0.755815194
PLAT	0.78375428	0.768079117	0.577647688	0.89716396	0.999985264	0.764052305
SOX11	0.78375428	0.661548817	0.746258329	0.99999488	0.969128502	0.819129695
DCTPP1	0.78375428	0.650973121	0.590691361	0.957896521	0.999985264	0.82386188
PRF1	0.78375428	0.699199783	0.696194157	0.989312306	0.968452969	0.850790144
CAPN7	0.78375428	0.753515888	0.667274767	0.917553107	0.999985264	0.863221317
OSCP1	0.78375428	0.705186914	0.595396914	0.977598122	0.920207885	0.94847852
LOC101904275	0.78375428	0.650665822	0.622970432	0.907700124	0.965353772	0.982250307
FAM219B	0.783820997	0.729820154	0.641573776	0.881324867	0.999985264	0.601946079
GAPT	0.784520621	0.599298196	0.746258329	0.99999488	0.944638139	0.661355141
SEPT9	0.784520621	0.746209367	0.634080201	0.952783349	0.999985264	0.76766284
SNAP29	0.784520621	0.729000253	0.70108275	0.99999488	0.901763837	0.824556283
NEFH	0.784520621	0.610403345	0.591210717	0.907045085	0.999985264	0.940358165

EVA1B	0.786162385	0.797577617	0.665391565	0.99999488	0.999985264	0.588781291
HMGN2	0.786162385	0.705186914	0.724381672	0.918070079	0.999985264	0.596516103
HELLS	0.786162385	0.656589815	0.654174689	0.99999488	0.965672975	0.607538907
NCKAP1L	0.786162385	0.6598011	0.614864417	0.99999488	0.964779023	0.608269365
LIG1	0.786162385	0.61414244	0.973406453	0.996471643	0.864446669	0.609196585
TK1	0.786162385	0.936657629	0.593672982	0.857323853	0.999985264	0.613432706
FBXL18	0.786162385	0.64546528	0.677011563	0.99999488	0.996075618	0.664012525
RGS20	0.786162385	0.656082241	0.713543621	0.99999488	0.981774771	0.683998081
SASH3	0.786162385	0.723870388	0.577382047	0.99999488	0.978076141	0.770633383
HEATR4	0.786162385	0.650973121	0.780672993	0.949602784	0.864446669	0.932608891
CAP1	0.786162385	0.674944018	0.746258329	0.917553107	0.840528426	0.967874696
GSTO1	0.786162385	0.584503543	0.654174689	0.869065101	0.924089345	0.999960414
SLC16A9	0.786554223	0.649879097	0.733455408	0.99999488	0.999985264	0.615805309
LOC781663	0.787049415	0.661332345	0.761533155	0.99999488	0.999985264	0.580233963
UBL4A	0.787049415	0.694145861	0.667817366	0.99999488	0.999985264	0.584459217
SIRT4	0.787049415	0.582088443	0.683588966	0.92106221	0.999985264	0.593352427
MTCL1	0.787049415	0.610403345	0.749648665	0.99999488	0.801204085	0.633084466
FBXO33	0.787049415	0.605827965	0.592707491	0.99999488	0.999985264	0.649073257
BAZ1A	0.787049415	0.595996951	0.714371099	0.92106221	0.999985264	0.685692039
LSM10	0.787049415	0.685985172	0.746258329	0.99999488	0.995085624	0.68755161
LOC112441476	0.787049415	0.649541115	0.81294064	0.839050812	0.93150494	0.952669273
INSR	0.787857537	0.58014251	0.640558462	0.99999488	0.983747207	0.715526355
NFASC	0.787857537	0.9582784	0.606564873	0.99999488	0.862059617	0.724218979
LHX6	0.787920591	0.6725213	0.807608869	0.99999488	0.813331114	0.695530652
CYP2J2	0.788013269	0.943802122	0.673566693	0.976631804	0.999985264	0.580118533
SRRD	0.788013269	0.610403345	0.716305079	0.959611215	0.999985264	0.594584164
TMOD3	0.788013269	0.583009741	0.590933668	0.931835237	0.999985264	0.596960727
MKI67	0.788013269	0.624463485	0.900890709	0.99999488	0.895905895	0.627549535
UIMC1	0.788013269	0.610403345	0.851718619	0.948570368	0.999985264	0.675571489
COMP	0.788013269	0.652628913	0.672194399	0.99999488	0.861628345	0.706653436
SH2D5	0.788013269	0.864945604	0.581743487	0.92106221	0.999985264	0.816226172
GFM2	0.788013269	0.732009317	0.606051701	0.851621164	0.999985264	0.831459089
PPAT	0.788013269	0.852035396	0.606564873	0.99999488	0.813331114	0.892187201
LSM5	0.7886147	0.87010028	0.785627328	0.973503136	0.985698852	0.615326593
NDUFAF6	0.7886147	0.634298188	0.667228538	0.989312306	0.999985264	0.885874544
NAIP	0.788891504	0.605074845	0.673482515	0.99999488	0.964631108	0.803939828
GPD1L	0.789435415	0.697485824	0.834173166	0.952783349	0.999985264	0.588139353
TRIOBP	0.789435415	0.599298196	0.889272129	0.99999488	0.821945987	0.601946079
EMG1	0.789435415	0.64343582	0.656285423	0.963583981	0.999985264	0.834573894

CDAN1	0.789435415	0.568300739	0.859566702	0.878491623	0.7946823	0.987365188
KCNH8	0.79122852	0.674837172	0.670826239	0.99999488	0.977475352	0.815564853
LOC101906739	0.791471002	0.6725213	0.706073772	0.99999488	0.924009849	0.588733625
SF3B1	0.791471002	0.826936949	0.751173926	0.907700124	0.999985264	0.742551138
STARD5	0.792139267	0.805110753	0.655928749	0.99999488	0.999985264	0.594584164
BDP1	0.793378366	0.64037592	0.593672982	0.851621164	0.999985264	0.604389904
SPP2	0.793378366	0.921759937	0.694790334	0.963012661	0.999985264	0.650158634
LOC112441807	0.793378366	0.852035396	0.842063863	0.931835237	0.946408931	0.67764684
TMCC2	0.793378366	0.734320293	0.648583755	0.99999488	0.83400817	0.927969856
SSC5D	0.793781328	0.745276769	0.762661971	0.99999488	0.981774771	0.580415308
TANC1	0.793781328	0.64113929	0.646284184	0.851621164	0.999985264	0.580415308
BROX	0.793781328	0.999992444	0.644073743	0.978440951	0.808139545	0.593603038
RNF138	0.793781328	0.76933827	0.63655368	0.869065101	0.999985264	0.607421354
KIF22	0.793781328	0.727539585	0.873452279	0.99999488	0.901104314	0.612111034
LOC618456	0.793781328	0.718044024	0.746258329	0.99999488	0.864446669	0.612462034
RASL10B	0.793781328	0.772379945	0.622893996	0.99999488	0.999985264	0.626804781
SDC4	0.793781328	0.792003681	0.930678591	0.859706585	0.999985264	0.632752232
LOC107131134	0.793781328	0.661182871	0.606564873	0.99999488	0.901763837	0.654291391
LOC104974516	0.793781328	0.598092257	0.670870221	0.99999488	0.829541927	0.656368128
AOX4	0.793781328	0.61414244	0.957669868	0.99999488	0.829541927	0.683998081
ARFGAP1	0.793781328	0.960125322	0.593886181	0.874380188	0.999985264	0.738324902
TMEM250	0.793781328	0.680103209	0.647235003	0.92106221	0.999985264	0.760015114
DDR1	0.793781328	0.587721224	0.738225468	0.998140932	0.999985264	0.765330803
TPD52L2	0.793781328	0.75238192	0.585345908	0.940265849	0.999985264	0.789800327
LOC104976247	0.793781328	0.834860292	0.789951578	0.855453165	0.999985264	0.804171005
LOC790009	0.793781328	0.967276215	0.632928415	0.855453165	0.957776687	0.819073042
HSPG2	0.794981868	0.647062519	0.654174689	0.99999488	0.894715133	0.58105996
STK19	0.795224104	0.830654932	0.919618001	0.957896521	0.849565759	0.654736169
DNAJC27	0.795224104	0.703838914	0.676439937	0.889823701	0.999985264	0.660138574
ZFP2	0.795224104	0.826936949	0.758498919	0.917553107	0.944638139	0.844923594
GPR83	0.795224104	0.647062519	0.595396914	0.92106221	0.999985264	0.923146498
PSAT1	0.795224104	0.738412248	0.673571108	0.908765361	0.941510628	0.952669273
PRKD2	0.79550151	0.92239337	0.592620368	0.859289191	0.999985264	0.6978527
UBFD1	0.796059641	0.834860292	0.70071733	0.941409556	0.999985264	0.757395858
LSMEM2	0.796133947	0.573366037	0.770213049	0.99999488	0.999985264	0.601946079
SNU13	0.796133947	0.732009317	0.815602423	0.99999488	0.921581207	0.692319327
FYB2	0.796133947	0.694996311	0.628800812	0.878491623	0.999985264	0.827439931
ELF1	0.796746106	0.569727345	0.586972294	0.914097104	0.999985264	0.610043612
MACF1	0.796805348	0.745276769	0.619358682	0.889823701	0.999985264	0.74674547

STUB1	0.796832508	0.698898696	0.590933668	0.948570368	0.999985264	0.606743017
GPR171	0.796832508	0.796000666	0.610361628	0.99999488	0.999985264	0.65137146
MAML3	0.796832508	0.638257273	0.775693389	0.917553107	0.999985264	0.770633383
CTSV	0.797554577	0.865099263	0.609340165	0.99999488	0.999985264	0.615947715
RBBP6	0.797554577	0.727539585	0.667228538	0.94911704	0.999985264	0.83405895
NIPA2	0.798388987	0.779783885	0.691855149	0.99999488	0.816789174	0.620254304
LOC112446756	0.798388987	0.589631719	0.593672982	0.99999488	0.999985264	0.89266134
TERF2	0.798515748	0.719377488	0.643747653	0.925818215	0.999985264	0.828248574
GAS2	0.798839337	0.616522574	0.91207077	0.938065999	0.999985264	0.588139353
LOC101904177	0.80041923	0.707737892	0.834068744	0.893717778	0.95245554	0.894723749
CFAP36	0.800710903	0.610999069	0.766128244	0.937020992	0.999985264	0.599905787
CRYBG2	0.801182978	0.852760886	0.777580059	0.917553107	0.969092995	0.781688852
PPP1R16A	0.80142579	0.607132821	0.86259773	0.99999488	0.999985264	0.614040014
MAT2B	0.80142579	0.585950998	0.923604668	0.99999488	0.908980492	0.770030068
COL16A1	0.801507849	0.675630177	0.877799478	0.99999488	0.935067743	0.601946079
CCNA2	0.803402317	0.591124857	0.785248842	0.99999488	0.911651126	0.814309336
YKT6	0.803402317	0.643710341	0.640310147	0.92106221	0.999985264	0.90109791
CDC25A	0.803646245	0.670447167	0.661326459	0.921764051	0.999985264	0.584872334
LMAN2	0.803646245	0.816518153	0.609340165	0.99999488	0.999985264	0.586487792
LOC107131209	0.803646245	0.677224344	0.76260462	0.99999488	0.967727289	0.594584164
TEAD1	0.803646245	0.851001595	0.640558462	0.99999488	0.904842818	0.598059647
DCAF11	0.803646245	0.676609157	0.640310147	0.99999488	0.880252174	0.602339943
PIK3CA	0.803646245	0.821194038	0.691847112	0.924872024	0.999985264	0.602867378
GLI4	0.803646245	0.574311353	0.601420135	0.99999488	0.999985264	0.608060743
CROCC2	0.803646245	0.809104286	0.94634922	0.948570368	0.880086683	0.621376973
CCDC61	0.803646245	0.61345881	0.691847112	0.896748401	0.999985264	0.621376973
GTF3C1	0.803646245	0.647062519	0.814724984	0.99999488	0.907875746	0.662512478
ANKRD44	0.803646245	0.719377488	0.599813846	0.99999488	0.999985264	0.670092907
GPR35	0.803646245	0.751438848	0.872386998	0.886605805	0.999985264	0.675571489
YBX1	0.803646245	0.825265917	0.711029036	0.98846246	0.999985264	0.692319327
NANOS1	0.803646245	0.589631719	0.746258329	0.99999488	0.999985264	0.695049955
DDX24	0.803646245	0.628133877	0.675208988	0.978440951	0.999985264	0.742551138
NAA10	0.803646245	0.646258196	0.643747653	0.99999488	0.999985264	0.754336759
ATXN1L	0.803646245	0.653451223	0.620035975	0.99999488	0.847202014	0.761516688
PES1	0.803646245	0.705186914	0.589715314	0.949602784	0.999985264	0.812831419
RSL1D1	0.803646245	0.700359766	0.720073352	0.855573807	0.999985264	0.83405895
ABCC2	0.803646245	0.789663062	0.671167173	0.978440951	0.969128502	0.870893258
TIMM9	0.803646245	0.698768403	0.673842846	0.878491623	0.999985264	0.873836462
AP3B2	0.803646245	0.661182871	0.673099635	0.957896521	0.999985264	0.910018813

ADSL	0.803646245	0.593189944	0.647235003	0.855453165	0.999985264	0.925260365
LOC112444505	0.804116457	0.592962209	0.834068744	0.889823701	0.999985264	0.621376973
NFKB1	0.804116457	0.738042027	0.618936949	0.884870518	0.999985264	0.628575862
FLNA	0.804334974	0.891793027	0.726885375	0.917553107	0.894348855	0.86871626
ZBTB16	0.804334974	0.617347838	0.644916374	0.99999488	0.999985264	0.884033654
CDC45	0.804344648	0.741111932	0.911018006	0.99999488	0.913670312	0.609986278
TRAM1	0.804344648	0.661182871	0.925520255	0.880829722	0.999985264	0.638709845
MCEMP1	0.804344648	0.754509129	0.683588966	0.976631804	0.999985264	0.812831419
MYCL	0.804569396	0.869999083	0.67612378	0.978440951	0.999985264	0.695049955
PTPN7	0.80489365	0.723870388	0.600305808	0.99999488	0.999985264	0.755815194
LOC783686	0.805126175	0.585950998	0.600307359	0.973503136	0.999985264	0.864430758
MOK	0.805126175	0.589631719	0.915117288	0.926788908	0.814027459	0.937393304
STXBP5	0.805247801	0.842852797	0.634570997	0.855453165	0.999985264	0.588781291
CPEB4	0.805247801	0.76933827	0.919745437	0.863532812	0.999985264	0.593352427
WRB	0.805247801	0.76933827	0.941336964	0.99999488	0.864446669	0.594287724
LTN1	0.805247801	0.816518153	0.822003523	0.959611215	0.999985264	0.595089487
RAPGEF6	0.805247801	0.699199783	0.70108275	0.907700124	0.999985264	0.596960727
LOC104969670	0.805247801	0.925580392	0.80757077	0.99999488	0.901104314	0.599905787
LOC112446351	0.805247801	0.661182871	0.640374252	0.99999488	0.946408931	0.599905787
NFKBID	0.805247801	0.816518153	0.891893039	0.99999488	0.907875746	0.601946079
NDUFA3	0.805247801	0.589631719	0.746258329	0.896748401	0.999985264	0.601946079
UPF1	0.805247801	0.589631719	0.687085396	0.99999488	0.999985264	0.611153303
TCHH	0.805247801	0.831782537	0.957541703	0.92106221	0.880252174	0.612462034
SH3YL1	0.805247801	0.73942687	0.970903823	0.978440951	0.847202014	0.612725931
DEPDC1	0.805247801	0.652673829	0.954823586	0.99999488	0.868761851	0.621376973
MRPS18C	0.805247801	0.591278815	0.595621168	0.967551965	0.999985264	0.621376973
TPRN	0.805247801	0.840946688	0.63799633	0.99999488	0.999985264	0.621376973
RPS6KA1	0.805247801	0.753515888	0.593672982	0.99999488	0.873593176	0.626337092
ZNF385D	0.805247801	0.610403345	0.62088615	0.99999488	0.999985264	0.629039793
SLC30A2	0.805247801	0.6725213	0.876758498	0.99999488	0.884139051	0.641885255
PDE4A	0.805247801	0.826936949	0.742557674	0.949602784	0.999985264	0.654736169
EIPR1	0.805247801	0.816518153	0.695533457	0.878491623	0.999985264	0.65702535
TNFRSF1B	0.805247801	0.606480397	0.638383601	0.99999488	0.957776687	0.660138574
LOC100847995	0.805247801	0.598092257	0.845368791	0.921764051	0.999985264	0.661149383
ARMCX2	0.805247801	0.699199783	0.592707491	0.99999488	0.999985264	0.662512478
LOC107132382	0.805247801	0.6725213	0.771154197	0.99999488	0.937177132	0.675571489
CD300A	0.805247801	0.647062519	0.655577179	0.99999488	0.969092995	0.695049955
TLR4	0.805247801	0.6725213	0.593672982	0.99999488	0.999985264	0.695049955
IER3	0.805247801	0.674944018	0.835038876	0.99999488	0.849565759	0.736516695

RBM28	0.805247801	0.610403345	0.662111034	0.99999488	0.999985264	0.743212015
SERPINB5	0.805247801	0.850692013	0.892053976	0.941409556	0.84872286	0.743462219
HSBP1L1	0.805247801	0.821617615	0.640310147	0.99999488	0.837824474	0.748830616
GALNT1	0.805247801	0.998131745	0.692027671	0.907700124	0.847202014	0.758510984
SYT17	0.805247801	0.732009317	0.636202317	0.99999488	0.884208058	0.772431293
TUFM	0.805247801	0.610403345	0.689800749	0.925818215	0.999985264	0.793369726
FAHD2A	0.805247801	0.721924826	0.648996122	0.989312306	0.999985264	0.79872286
LOC100336282	0.805247801	0.921759937	0.654174689	0.99999488	0.834621532	0.812831419
ITGB1BP2	0.805247801	0.883856603	0.644279997	0.930183705	0.999985264	0.820845891
MRM3	0.805247801	0.647062519	0.690864342	0.907045085	0.999985264	0.827439931
ZNF605	0.805247801	0.89880506	0.721605112	0.949602784	0.821082556	0.877940484
CCDC77	0.805247801	0.6725213	0.778565768	0.99999488	0.858096179	0.8876985
RANBP1	0.805247801	0.772071953	0.691847112	0.970769712	0.957235535	0.891439324
MTBP	0.805247801	0.957700659	0.692027671	0.876702931	0.829541927	0.897467714
P2RY6	0.805247801	0.666536383	0.72674783	0.938185243	0.999985264	0.926480606
PANK1	0.805247801	0.594008495	0.672945326	0.907700124	0.999985264	0.940358165
UMPS	0.805247801	0.591586809	0.665391565	0.907700124	0.999985264	0.977753483
TMEM138	0.805247801	0.591239507	0.805753285	0.901707661	0.901104314	0.987454496
CAVIN3	0.80532699	0.667813705	0.912511938	0.98846246	0.999985264	0.605304412
NLGN4X	0.80532699	0.592962209	0.89269406	0.931835237	0.999985264	0.778339877
TIMM8B	0.806022756	0.612694128	0.741183564	0.953188062	0.999985264	0.617537824
DTL	0.806022756	0.915313641	0.63799633	0.99999488	0.998439184	0.673027356
CDC25B	0.806022756	0.744558551	0.667228538	0.984913325	0.999985264	0.675571489
RNF167	0.806022756	0.650665822	0.639782943	0.925818215	0.999985264	0.691757815
LOC112445999	0.806022756	0.83698359	0.665391565	0.99999488	0.999985264	0.712249777
FAM217B	0.806022756	0.972298153	0.657808416	0.92106221	0.901104314	0.818895181
PISD	0.806022756	0.816518153	0.673482515	0.926788908	0.999985264	0.849046935
OLFM2	0.806022756	0.813746447	0.600902205	0.925818215	0.999985264	0.921771959
SP4	0.806252849	0.780728249	0.773092345	0.907700124	0.999985264	0.59171134
LIME1	0.80678499	0.715899004	0.859086279	0.865261766	0.999985264	0.694747436
NRF1	0.80692757	0.76933827	0.716305079	0.904225644	0.999985264	0.838520085
RND1	0.807578371	0.833935981	0.916975216	0.870282231	0.999985264	0.631726864
BORA	0.808206512	0.742976874	0.788870461	0.99999488	0.999985264	0.609705
CRISPLD2	0.808206512	0.594008495	0.785248842	0.938185243	0.999985264	0.671846838
TRHDE	0.808206512	0.813113924	0.700157555	0.99999488	0.999985264	0.712878507
LOC107132921	0.808206512	0.588325495	0.892574927	0.99999488	0.999985264	0.72144082
CEP57L1	0.808214199	0.999992444	0.654174689	0.907700124	0.852744493	0.748495031
CA11	0.808433244	0.668131227	0.728362962	0.99999488	0.847202014	0.612764179
HNRNPF	0.808530522	0.638045424	0.622893996	0.925818215	0.999985264	0.611893919

LOC507581	0.808609078	0.599818384	0.683588966	0.99999488	0.999985264	0.599905787
LOC515358	0.808609078	0.744147489	0.800518725	0.99999488	0.911651126	0.672055518
LOC100849865	0.808682337	0.764050973	0.770213049	0.896748401	0.999985264	0.594584164
ESPN	0.808682337	0.846742296	0.628827707	0.99999488	0.999985264	0.609196585
MADCAM1	0.808682337	0.647062519	0.714371099	0.99999488	0.894715133	0.627277292
PLRG1	0.808682337	0.882505302	0.743972127	0.99999488	0.901763837	0.671843481
REPS2	0.808682337	0.612694128	0.647235003	0.99999488	0.944638139	0.779686358
SSSCA1	0.808682337	0.638579158	0.695555702	0.99999488	0.999985264	0.781949871
FAM207A	0.808682337	0.798470892	0.746258329	0.976631804	0.999985264	0.80980964
TMBIM1	0.808682337	0.634336339	0.714371099	0.99999488	0.961025202	0.828248574
FANCC	0.808682337	0.611881327	0.685273962	0.92106221	0.999985264	0.849046935
IZUMO4	0.808682337	0.591586809	0.817410775	0.99999488	0.847202014	0.923360345
ARHGAP26	0.809444519	0.955128586	0.670819018	0.963012661	0.909266267	0.777939849
TK2	0.809787094	0.864811968	0.609340165	0.881982604	0.999985264	0.594584164
LOC537848	0.81037341	0.716547849	0.606564873	0.901707661	0.999985264	0.704551299
IL17B	0.81037341	0.955727918	0.738977782	0.880028563	0.999985264	0.750879856
NOC3L	0.810509415	0.610403345	0.600307359	0.99999488	0.999985264	0.736708637
HMMR	0.810550146	0.687373347	0.938203102	0.944661721	0.998639844	0.704966434
TSPYL4	0.810550146	0.915367594	0.610876626	0.948570368	0.983747207	0.85182524
CD99	0.810550146	0.668131227	0.837103057	0.889928167	0.864446669	0.967874696
LOC101904239	0.810563566	0.663430554	0.68201731	0.978440951	0.999985264	0.844563769
DACT3	0.810587024	0.661182871	0.730602626	0.99999488	0.999985264	0.608004025
IFITM5	0.810587024	0.636103412	0.811233159	0.953588494	0.999985264	0.617537824
RBM3	0.810587024	0.703838914	0.645275104	0.99999488	0.908750851	0.628644572
PARP6	0.810587024	0.827123416	0.917634327	0.945038047	0.968357741	0.641507019
PIGQ	0.810587024	0.826642757	0.606008934	0.917553107	0.999985264	0.653862943
SPATA9	0.810587024	0.731556816	0.954620171	0.954221644	0.894715133	0.692319327
SNX17	0.810587024	0.661182871	0.644916374	0.946427018	0.999985264	0.743189002
HERC6	0.810587024	0.591586809	0.762340742	0.99999488	0.999985264	0.849266568
NSDHL	0.810587024	0.705186914	0.663063376	0.937020992	0.999985264	0.856510591
HDAC2	0.810587024	0.594515957	0.691847112	0.99999488	0.999985264	0.901809636
TMEM252	0.810587024	0.6725213	0.835038876	0.926788908	0.864446669	0.948213108
PKD1	0.811082592	0.609588562	0.731527745	0.99999488	0.999985264	0.601946079
BUB1	0.811082592	0.674272153	0.863299295	0.99999488	0.999985264	0.621229636
FGFR3	0.811082592	0.991488652	0.726885375	0.973503136	0.876385027	0.653862943
CRELD2	0.811082592	0.891316528	0.644279997	0.99999488	0.944638139	0.795293834
NKAIN1	0.811170944	0.624309586	0.644916374	0.99999488	0.928683582	0.602271571
FAM19A3	0.811170944	0.747155043	0.82336109	0.952783349	0.999985264	0.609616735
FBLL1	0.811170944	0.647062519	0.825497047	0.99999488	0.901763837	0.63161713

PALLD	0.811170944	0.813113924	0.665391565	0.99999488	0.847202014	0.645467494
UCHL1	0.811170944	0.62408671	0.638602654	0.917553107	0.999985264	0.966921967
DENND1A	0.811170944	0.634298188	0.606564873	0.875335495	0.921998583	0.999960414
ALKBH5	0.811281264	0.747293429	0.770213049	0.99999488	0.833165768	0.599905787
RSPO3	0.811281264	0.664882391	0.694895009	0.976631804	0.999985264	0.601946079
SHARPIN	0.811281264	0.656315974	0.840781773	0.989312306	0.999985264	0.601946079
MARCKSL1	0.811281264	0.69663723	0.599813846	0.99999488	0.999985264	0.604197867
SCARF2	0.811281264	0.661182871	0.696292819	0.99999488	0.880459369	0.608269365
LONRF3	0.811281264	0.643710341	0.862345036	0.99999488	0.999985264	0.609196585
DBF4	0.811281264	0.747155043	0.907061478	0.99999488	0.866840018	0.609616735
NAT8L	0.811281264	0.780728249	0.63799633	0.99999488	0.999985264	0.609616735
PKN2	0.811281264	0.645842563	0.825497047	0.99999488	0.999985264	0.609616735
SPON1	0.811281264	0.642323832	0.802997196	0.99999488	0.999985264	0.609823525
CCDC12	0.811281264	0.850692013	0.783264179	0.99999488	0.91945292	0.610600013
ASPSCR1	0.811281264	0.706295081	0.864737879	0.925818215	0.999985264	0.613432706
ZNF276	0.811281264	0.629487817	0.983419007	0.947995037	0.999985264	0.614040014
SMAD5	0.811281264	0.869999083	0.640558462	0.949602784	0.999985264	0.615805309
PCYT1A	0.811281264	0.624463485	0.76260462	0.99999488	0.999985264	0.617537824
NACC2	0.811281264	0.640417642	0.628800812	0.99999488	0.999985264	0.627277292
CAPS	0.811281264	0.999992444	0.72274579	0.889823701	0.84872286	0.629039793
BAG3	0.811281264	0.912316394	0.688463551	0.907045085	0.999985264	0.631711796
XRCC3	0.811281264	0.753515888	0.817700149	0.869065101	0.999985264	0.639425605
LOC101907276	0.811281264	0.693386354	0.841717609	0.99999488	0.999985264	0.645467494
RITA1	0.811281264	0.816100073	0.780137056	0.99999488	0.999985264	0.652772144
RPL8	0.811281264	0.7302004	0.645275104	0.901707661	0.999985264	0.656368128
EPN1	0.811281264	0.887644042	0.644916374	0.99999488	0.999985264	0.656368128
CAV2	0.811281264	0.883856603	0.632928415	0.989312306	0.999985264	0.666382992
ZNF181	0.811281264	0.893642384	0.60319572	0.99999488	0.978076141	0.678579877
LOC112441478	0.811281264	0.674944018	0.884273683	0.99999488	0.961025202	0.679091734
CLEC7A	0.811281264	0.667396315	0.644279997	0.99999488	0.937177132	0.704679988
KMT5B	0.811281264	0.992977597	0.619337076	0.884809097	0.999985264	0.712878507
SEMA3E	0.811281264	0.821194038	0.638734792	0.99999488	0.977475352	0.715526355
DANCR	0.811281264	0.921549	0.729977435	0.99999488	0.861287091	0.721134875
PTGFR	0.811281264	0.710432617	0.670870221	0.99999488	0.999985264	0.731312503
SELENOO	0.811281264	0.747155043	0.708177705	0.907700124	0.999985264	0.737027647
GATAD2A	0.811281264	0.650266299	0.628800812	0.99999488	0.948597428	0.742551138
NLE1	0.811281264	0.649866599	0.769732676	0.99999488	0.999985264	0.74674547
GYG1	0.811281264	0.862811597	0.865837237	0.925818215	0.924009849	0.754336759
KIAA0040	0.811281264	0.729000253	0.76260462	0.99999488	0.999985264	0.764052305

LOC101906511	0.811281264	0.658817102	0.715414989	0.878491623	0.999985264	0.7655306
SMOC2	0.811281264	0.759310562	0.606564873	0.99999488	0.864446669	0.768934839
SPARCL1	0.811281264	0.985406717	0.706073772	0.925818215	0.901104314	0.769515026
MAP2	0.811281264	0.723136577	0.691847112	0.948570368	0.999985264	0.770030068
FFAR2	0.811281264	0.741264723	0.655928749	0.874380188	0.999985264	0.792944216
GCA	0.811281264	0.728501112	0.774632155	0.99999488	0.915346056	0.812831419
THBS1	0.811281264	0.729000253	0.684276131	0.889823701	0.999985264	0.83405895
ASTN2	0.811281264	0.626400323	0.941782902	0.941409556	0.911651126	0.845045999
CCDC186	0.811281264	0.938597964	0.666104182	0.917553107	0.965227764	0.847464988
CD96	0.811281264	0.778248571	0.644916374	0.99999488	0.983747207	0.865855226
RAVER2	0.811281264	0.732009317	0.622893996	0.945038047	0.999985264	0.869877865
LOC107131471	0.811281264	0.615826759	0.93036537	0.917553107	0.907875746	0.901809636
LOC100335514	0.811281264	0.61100105	0.809486619	0.907045085	0.999985264	0.905391122
KBTBD7	0.811281264	0.63261584	0.76260462	0.99999488	0.934591321	0.919755394
AKAP6	0.811281264	0.90213106	0.692872372	0.88041408	0.874006704	0.939035437
GNAT1	0.811281264	0.727539585	0.763201042	0.925818215	0.91945292	0.94847852
CCR5	0.811281264	0.78130286	0.60119157	0.949602784	0.978076141	0.955374205
MYOM1	0.811281264	0.751959025	0.666562848	0.884870518	0.927270318	0.994845239
PSMD12	0.811714539	0.615164422	0.852782377	0.92106221	0.999985264	0.713138353
TRPT1	0.811745758	0.650665822	0.606564873	0.970645122	0.999985264	0.698422388
LOC112443528	0.811745758	0.642323832	0.912511938	0.98846246	0.999985264	0.743798578
RABEP2	0.812316228	0.638045424	0.78255891	0.99999488	0.965672975	0.615805309
KIAA1257	0.812316228	0.727390627	0.945690614	0.99999488	0.919006503	0.618987715
MPRIP	0.812316228	0.597753534	0.628451118	0.99999488	0.999985264	0.64722711
YBX3	0.812316228	0.663430554	0.76260462	0.99999488	0.969092995	0.681790665
LOC616903	0.812316228	0.987516216	0.81294064	0.925818215	0.84872286	0.692319327
SHISA3	0.812316228	0.599298196	0.872364252	0.99999488	0.84872286	0.754852502
FASTK	0.81252289	0.703838914	0.746258329	0.99999488	0.999985264	0.60042403
LOC101904013	0.81252289	0.978089982	0.615380158	0.945038047	0.999985264	0.601946079
AP5Z1	0.81252289	0.838314316	0.862345036	0.957896521	0.999985264	0.601946079
FAM206A	0.81252289	0.703838914	0.774363763	0.991596938	0.999985264	0.604197867
MEGF6	0.81252289	0.752475942	0.865837237	0.99999488	0.999985264	0.604389904
LOC112446426	0.81252289	0.661182871	0.63799633	0.99999488	0.999985264	0.608269365
USP19	0.81252289	0.736053393	0.809401378	0.989312306	0.999985264	0.612911427
AKT1	0.81252289	0.864945604	0.685835676	0.99999488	0.981774771	0.615805309
PHYHD1	0.81252289	0.646275014	0.618274047	0.99999488	0.969092995	0.619451333
PADI1	0.81252289	0.755100312	0.628496505	0.99999488	0.999985264	0.621376973
RNF146	0.81252289	0.795255481	0.75868797	0.99999488	0.999985264	0.621376973
TRA2B	0.81252289	0.64037592	0.606008934	0.907700124	0.999985264	0.63161713

BACE2	0.81252289	0.760829911	0.778565768	0.99999488	0.901104314	0.631711796
HSF2BP	0.81252289	0.599298196	0.654174689	0.99999488	0.999985264	0.631711796
ANG2	0.81252289	0.729000253	0.851718619	0.907700124	0.999985264	0.645467494
E2F2	0.81252289	0.778248571	0.938203102	0.98846246	0.908980492	0.65937855
ATP6V1B2	0.81252289	0.59662083	0.714371099	0.99999488	0.849864394	0.666562345
SH2B1	0.81252289	0.650665822	0.91005986	0.99999488	0.999985264	0.673027356
TMEM189	0.81252289	0.839149526	0.749381997	0.99999488	0.981774771	0.675571489
CYB5R3	0.81252289	0.846198976	0.643457792	0.99999488	0.985698852	0.68827479
SHKBP1	0.81252289	0.644729858	0.724658338	0.882226211	0.999985264	0.68999472
SHANK2	0.81252289	0.921855042	0.622893996	0.941874456	0.999985264	0.68999472
FOXP3	0.81252289	0.727539585	0.623909299	0.907700124	0.999985264	0.693616839
PYCARD	0.81252289	0.723638326	0.641573776	0.99999488	0.96948473	0.72144082
TOMM5	0.81252289	0.693386354	0.714371099	0.917553107	0.999985264	0.734689625
ESRRG	0.81252289	0.815852027	0.609340165	0.99999488	0.920207885	0.738324902
LOC101905499	0.81252289	0.974812121	0.675121942	0.966629392	0.919552152	0.765696021
PTPMT1	0.81252289	0.732530744	0.83711556	0.989312306	0.999985264	0.770633383
GALC	0.81252289	0.615586822	0.8664043	0.937020992	0.999985264	0.798878799
AHR	0.81252289	0.64546528	0.644916374	0.99999488	0.901104314	0.839734604
DHRSX	0.81252289	0.781927288	0.822332225	0.943090854	0.961025202	0.851533524
TAGAP	0.81252289	0.650665822	0.645275104	0.99999488	0.999985264	0.895235998
LOC100295797	0.81252289	0.723870388	0.783497666	0.949602784	0.964906636	0.919758204
LOC112448070	0.81252289	0.745276769	0.903957821	0.87940915	0.84872286	0.931163114
LOC104973322	0.81252289	0.755100312	0.606564873	0.907700124	0.939144222	0.997713313
PLEKHH1	0.81252289	0.634336339	0.640558462	0.99999488	0.84872286	0.999960414
EXT2	0.813055135	0.830519875	0.781149314	0.99999488	0.920225927	0.619451333
KERA	0.813055135	0.693980599	0.766334745	0.931835237	0.934591321	0.960343814
TATDN1	0.813114291	0.809104286	0.641573776	0.99999488	0.911651126	0.635743158
LAT	0.813671268	0.705186914	0.775688011	0.98846246	0.999985264	0.818895181
RIOK3	0.814313205	0.715899004	0.794434202	0.962092791	0.999985264	0.603560332
BRAF	0.814439909	0.938476452	0.632118699	0.878491623	0.999985264	0.673027356
TTLL12	0.814439909	0.830519875	0.645275104	0.99999488	0.878957968	0.715337992
CEPT1	0.814586286	0.90024974	0.77819094	0.99999488	0.93150494	0.64568148
PPIL3	0.814651393	0.6725213	0.730602626	0.907700124	0.999985264	0.604389904
PDZRN3	0.814651393	0.599298196	0.673571108	0.99999488	0.999985264	0.652992721
LOC786987	0.814651393	0.779284948	0.76260462	0.99999488	0.999985264	0.665508161
CLDND1	0.814651393	0.634298188	0.670826239	0.928873093	0.999985264	0.696385367
LOC112444864	0.814651393	0.617347838	0.634080201	0.99999488	0.999985264	0.758091231
PPM1F	0.814651393	0.650665822	0.730374662	0.963012661	0.999985264	0.902893979
PCSK1N	0.814651393	0.655075375	0.70108275	0.907700124	0.999985264	0.954641022

ZNF318	0.814651393	0.610403345	0.800184523	0.99999488	0.84872286	0.960343814
NT5E	0.814875519	0.703838914	0.793478166	0.952783349	0.999985264	0.603050301
CNOT4	0.814875519	0.691302101	0.681365368	0.917553107	0.999985264	0.607621072
RGCC	0.814875519	0.67209328	0.755816517	0.99999488	0.996075618	0.608004025
CCAR2	0.814875519	0.687656817	0.97843676	0.969209391	0.995549184	0.609616735
ITSN2	0.814875519	0.678522413	0.717666539	0.983736128	0.999985264	0.61298375
TOP2A	0.814875519	0.655648116	0.878254532	0.99999488	0.999985264	0.614040014
C7H19orf70	0.814875519	0.676425921	0.732364232	0.880028563	0.999985264	0.621229636
HSP90AA1	0.814875519	0.673756899	0.751181541	0.92106221	0.999985264	0.62260916
CIDEA	0.814875519	0.687117225	0.708177705	0.907700124	0.999985264	0.627277292
CARF	0.814875519	0.978089982	0.834173166	0.907045085	0.972524588	0.63161713
RUFY3	0.814875519	0.73589068	0.639699071	0.99999488	0.999985264	0.63161713
LOC112449261	0.814875519	0.742976874	0.632928415	0.99999488	0.999985264	0.64722711
LGMN	0.814875519	0.717031803	0.673566693	0.99999488	0.901104314	0.654291391
AGMAT	0.814875519	0.610403345	0.771154197	0.99999488	0.918494274	0.655379707
ARHGEF19	0.814875519	0.826641183	0.670826239	0.917553107	0.999985264	0.656368128
COMMD5	0.814875519	0.727539585	0.673566693	0.99999488	0.983747207	0.656717415
FBXO3	0.814875519	0.69663723	0.745215355	0.976631804	0.999985264	0.661576605
CNPPD1	0.814875519	0.830654932	0.813142592	0.911513036	0.999985264	0.678579877
CSRNP1	0.814875519	0.873113713	0.818876578	0.99999488	0.853611376	0.691757815
LRRC57	0.814875519	0.808483763	0.962346588	0.949602784	0.847202014	0.693397277
TUBA4A	0.814875519	0.949285285	0.775693389	0.880772564	0.999985264	0.736516695
TIMMDC1	0.814875519	0.785562513	0.746258329	0.98846246	0.999985264	0.745702199
EPHA4	0.814875519	0.601197167	0.609340165	0.99999488	0.999985264	0.793525005
IFRD2	0.814875519	0.673405033	0.644279997	0.939864142	0.999985264	0.827439931
TMEM82	0.814875519	0.610403345	0.638383601	0.917553107	0.999985264	0.828547227
TIMM8A	0.814875519	0.687945116	0.770213049	0.924872024	0.999985264	0.862046449
RTCB	0.814875519	0.663430554	0.718312559	0.99999488	0.901763837	0.873463288
LOC536097	0.814875519	0.974812121	0.622893996	0.925818215	0.901104314	0.885874544
PMS1	0.814875519	0.885295091	0.665391565	0.925818215	0.952554537	0.919755394
LOC112443175	0.814875519	0.612694128	0.638383601	0.99999488	0.861287091	0.931163114
FOXS1	0.814875519	0.626834487	0.785248842	0.907700124	0.999985264	0.949718683
LOC101907574	0.814875519	0.638045424	0.620520867	0.99999488	0.847202014	0.962383033
CNOT11	0.81515729	0.692431693	0.970874203	0.99999488	0.84872286	0.675571489
CFAP100	0.81515729	0.6725213	0.866507741	0.99999488	0.981774771	0.693178721
GUF1	0.81580236	0.695496638	0.709285091	0.92398295	0.999985264	0.960343814
BMP2K	0.816008156	0.622521791	0.724658338	0.982713285	0.999985264	0.695049955
ARMC9	0.816008156	0.741111932	0.63799633	0.99999488	0.999985264	0.704679988
LOC100848895	0.816008156	0.781373573	0.920154807	0.976631804	0.864446669	0.787783489

STRIP2	0.816008156	0.650665822	0.63799633	0.978440951	0.999985264	0.796770444
ZMYM4	0.817228909	0.913204869	0.641573776	0.901707661	0.999985264	0.617537824
SRF	0.817228909	0.997794477	0.731527745	0.907700124	0.929139458	0.737158489
IL23A	0.817513001	0.999992444	0.636609282	0.878491623	0.999985264	0.606743017
LOC100196897	0.817513001	0.716267524	0.748903111	0.99999488	0.999985264	0.650154396
ZNRD1	0.817513001	0.610403345	0.993594894	0.938185243	0.922362358	0.754852502
BNIP3	0.817513001	0.729000253	0.609340165	0.99999488	0.937177132	0.846001048
TMED3	0.817823022	0.853296877	0.841773648	0.99999488	0.91945292	0.671843481
PRELID1	0.817823022	0.816518153	0.700157555	0.907700124	0.999985264	0.941265666
LOC524576	0.817960955	0.786382788	0.631033717	0.99999488	0.999985264	0.683998081
APLF	0.817960955	0.84060143	0.766334745	0.889823701	0.999985264	0.692319327
TLCD2	0.817960955	0.716267524	0.930678591	0.985023854	0.928683582	0.766713899
AQP3	0.817960955	0.647579301	0.93198623	0.969770561	0.999985264	0.770633383
SLC16A6	0.817960955	0.877795869	0.673099635	0.951237479	0.864446669	0.943220018
PRPF8	0.818814562	0.747155043	0.668594622	0.982713285	0.999985264	0.653862943
OGFOD1	0.819947952	0.821194038	0.666861957	0.932532289	0.999985264	0.937108046
SIX5	0.819950096	0.61469452	0.762007355	0.99999488	0.999985264	0.609705
LOC112449300	0.819950096	0.63996058	0.645275104	0.99999488	0.981774771	0.626027519
TIAM1	0.819950096	0.670199175	0.674629692	0.99999488	0.983747207	0.628644572
FHDC1	0.819950096	0.793370989	0.666104182	0.99999488	0.873421342	0.649701943
RALGAPA1	0.819950096	0.750919518	0.628800812	0.889767014	0.999985264	0.654736169
SH3BP5L	0.819950096	0.650973121	0.788332464	0.989312306	0.999985264	0.65702535
SLC9A5	0.819950096	0.808097876	0.821546364	0.99999488	0.890963305	0.662512478
KANK4	0.819950096	0.621010583	0.95230212	0.901707661	0.999985264	0.695049955
UHRF2	0.819950096	0.985406717	0.702366288	0.917553107	0.974716308	0.770030068
NUDT17	0.819950096	0.691302101	0.738977782	0.99999488	0.969092995	0.809513929
FAM71E1	0.819950096	0.650665822	0.920629231	0.996471643	0.937177132	0.819073042
F13A1	0.819950096	0.6725213	0.648996122	0.99999488	0.999985264	0.859200033
ZNF879	0.819950096	0.786382788	0.798401881	0.981779494	0.901763837	0.894723749
GRIN1	0.819950096	0.845554546	0.789951578	0.921764051	0.933861931	0.897965262
LOC104969050	0.819950096	0.772195648	0.778280092	0.880829722	0.999985264	0.927969856
MRPL19	0.819950096	0.647062519	0.687448391	0.931835237	0.999985264	0.95096765
XPO1	0.819950096	0.747293429	0.771714229	0.884650797	0.983747207	0.957984797
DHX29	0.819950096	0.819379885	0.683588966	0.917553107	0.964779023	0.960343814
PSMD8	0.819950096	0.744638901	0.708177705	0.944348064	0.952275018	0.966921967
SLC35B1	0.819950096	0.793373656	0.710429228	0.92106221	0.925091544	0.967874696
DARS	0.819950096	0.674944018	0.734011225	0.952783349	0.944638139	0.972391591
LOC101905151	0.819988495	0.999992444	0.645275104	0.924980169	0.943686834	0.609616735
NKAPD1	0.819988495	0.69663723	0.938905004	0.969770561	0.999985264	0.617537824

WSB1	0.819988495	0.661182871	0.721605112	0.943090854	0.999985264	0.692319327
BBS5	0.819988495	0.647062519	0.628800812	0.99999488	0.999985264	0.884143505
BCCIP	0.819988495	0.647062519	0.708177705	0.968672019	0.999985264	0.967045771
SP100	0.819988495	0.610403345	0.687781916	0.925818215	0.858096179	0.999960414
FAXC	0.820923805	0.661182871	0.774363763	0.99999488	0.999985264	0.616845571
LOC785842	0.820923805	0.727539585	0.673099635	0.99999488	0.924089345	0.622335178
ASZ1	0.820923805	0.72165336	0.744618167	0.967551965	0.999985264	0.628644572
LOC101904042	0.820923805	0.802939066	0.853166364	0.884168782	0.999985264	0.631711796
LRRC41	0.820923805	0.859757246	0.695555702	0.99999488	0.901104314	0.644094886
RAB5C	0.820923805	0.97671655	0.683588966	0.945038047	0.999985264	0.64722711
DNAJB9	0.820923805	0.9582784	0.726885375	0.884809097	0.999985264	0.649701943
CCDC3	0.820923805	0.647062519	0.746258329	0.896748401	0.999985264	0.662512478
C3H1orf54	0.820923805	0.936392293	0.691847112	0.901374316	0.999985264	0.662512478
METTL16	0.820923805	0.661182871	0.735524395	0.99999488	0.999985264	0.691757815
TMEM150C	0.820923805	0.865926615	0.645275104	0.99999488	0.946408931	0.692319327
CUEDC1	0.820923805	0.9582784	0.680040594	0.99999488	0.938391537	0.72144082
ERLIN1	0.820923805	0.673405033	0.734356561	0.99999488	0.864446669	0.732471021
TYK2	0.820923805	0.628133877	0.663165825	0.99999488	0.999985264	0.764393198
GRHL2	0.820923805	0.61981418	0.785248842	0.99999488	0.908980492	0.770030068
LOC101905403	0.820923805	0.645842563	0.964152013	0.978440951	0.905874828	0.781688852
ESAM	0.820923805	0.769626411	0.70090879	0.92106221	0.999985264	0.809513929
LOC112444633	0.820923805	0.989872618	0.747454661	0.884809097	0.866840018	0.847204221
LOC100139549	0.820923805	0.818448534	0.744618167	0.963012661	0.999985264	0.873463288
ZSCAN29	0.820923805	0.741594487	0.660231139	0.99999488	0.937177132	0.919755394
GRAMD1C	0.821640106	0.741111932	0.670826239	0.99999488	0.999985264	0.715526355
TSHZ2	0.822696358	0.656082241	0.837103057	0.99999488	0.999985264	0.662512478
SDC1	0.822841962	0.650665822	0.665391565	0.99999488	0.908980492	0.703356006
COL6A3	0.822880772	0.702248346	0.762007355	0.99999488	0.999985264	0.628307831
LOC100848025	0.82353484	0.686606529	0.673099635	0.99999488	0.999985264	0.644094886
ZNF862	0.82353484	0.830519875	0.822003523	0.92106221	0.999985264	0.650914382
GNS	0.82353484	0.634381235	0.822003523	0.99999488	0.919006503	0.744164643
IL1A	0.823622885	0.727539585	0.785248842	0.99999488	0.999985264	0.6155859
LOC107131948	0.823622885	0.937231686	0.831723974	0.99999488	0.907875746	0.640401607
LOC614732	0.823889735	0.991488652	0.805870442	0.989312306	0.85796104	0.627403213
FUT4	0.823992871	0.999992444	0.770899781	0.917553107	0.901104314	0.692319327
EPHB1	0.823992871	0.94984369	0.673508205	0.918070079	0.999985264	0.718350328
LOC112444290	0.823992871	0.85337372	0.70108275	0.927129324	0.981938571	0.925260365
LOXL1	0.82415582	0.753515888	0.664793956	0.99999488	0.872418804	0.617537824
POLR3F	0.82415582	0.999992444	0.655928749	0.99999488	0.922384168	0.651846161

CLDN4	0.82415582	0.752475942	0.998936876	0.918070079	0.901763837	0.657557645
ZNF624	0.82415582	0.83360496	0.817223866	0.96412514	0.999985264	0.776885453
GRAMD1A	0.82415582	0.752475942	0.706110318	0.896748401	0.999985264	0.827190118
NFE2L3	0.82415582	0.76933827	0.683588966	0.99999488	0.946408931	0.85182524
KIAA1147	0.82415582	0.6725213	0.638383601	0.989312306	0.999985264	0.873463288
S1PR4	0.82415582	0.661182871	0.805753285	0.884870518	0.999985264	0.921771959
GALNT17	0.824219486	0.740822434	0.822356437	0.99999488	0.994270709	0.617537824
USP12	0.824219486	0.619486097	0.834173166	0.99999488	0.999985264	0.698422388
CACNA1D	0.824423573	0.986637746	0.645166627	0.99999488	0.864446669	0.738324902
GSPT1	0.824423573	0.629487817	0.746258329	0.99999488	0.937177132	0.939035437
KBTBD6	0.824483352	0.650665822	0.692027671	0.938185243	0.999985264	0.901614166
COX7C	0.82476214	0.672731254	0.68741937	0.907700124	0.999985264	0.614040014
LOC101904357	0.82476214	0.745276769	0.645275104	0.99999488	0.999985264	0.614862466
CUL7	0.82476214	0.650665822	0.980558494	0.99999488	0.901104314	0.641885255
SEC14L1	0.82476214	0.825265917	0.827021762	0.947995037	0.999985264	0.661149383
SPOP	0.82476214	0.619486097	0.68741937	0.917553107	0.999985264	0.662624496
LOC104970930	0.82476214	0.76933827	0.645275104	0.99999488	0.999985264	0.67764684
MCM6	0.82476214	0.658805706	0.746258329	0.99999488	0.999985264	0.750879856
WWP1	0.82476214	0.634336339	0.993594894	0.904225644	0.999985264	0.751815267
MRPS27	0.82476214	0.752475942	0.647235003	0.961309253	0.999985264	0.868513869
STARD7	0.824785828	0.656589815	0.786738262	0.99999488	0.999985264	0.621376973
FAM53B	0.824785828	0.696376974	0.656285423	0.941409556	0.999985264	0.670803607
TMEM269	0.824785828	0.991488652	0.746258329	0.977598122	0.934591321	0.672602199
MTMR12	0.824785828	0.73157551	0.708177705	0.937020992	0.999985264	0.856510591
ZNF354C	0.825514506	0.938597964	0.72125893	0.99999488	0.901104314	0.653001371
CAND1	0.825553765	0.699785139	0.805753285	0.96412514	0.999985264	0.732471021
OXSM	0.8258629	0.619474811	0.683588966	0.940265849	0.999985264	0.622341636
PRKG2	0.8258629	0.863793573	0.746258329	0.938185243	0.999985264	0.627549535
STAM	0.8258629	0.650973121	0.837074149	0.99999488	0.999985264	0.639767265
ZBTB32	0.8258629	0.992977597	0.809486619	0.95762164	0.901104314	0.645467494
LOC781989	0.8258629	0.696893897	0.892943118	0.99999488	0.944638139	0.657296513
HIGD2A	0.8258629	0.674837172	0.645275104	0.985023854	0.999985264	0.675571489
ATP13A4	0.8258629	0.799180421	0.667228538	0.99999488	0.999985264	0.694747436
TMEM107	0.8258629	0.873289191	0.641573776	0.99999488	0.924089345	0.695049955
SYTL3	0.8258629	0.753515888	0.746258329	0.922379064	0.999985264	0.729240869
AVIL	0.8258629	0.650665822	0.794745005	0.99999488	0.901763837	0.737814085
RBL2	0.8258629	0.661182871	0.785248842	0.99999488	0.999985264	0.741213204
MIEF2	0.8258629	0.755056511	0.694895009	0.918070079	0.999985264	0.765696021
KPNA6	0.8258629	0.778758964	0.721396269	0.99999488	0.999985264	0.76762685

PPP1R2	0.8258629	0.64113929	0.956209414	0.99999488	0.874406643	0.792944216
HNRNPK	0.8258629	0.697485824	0.807825655	0.99999488	0.960543381	0.798638511
LOC104975054	0.8258629	0.658817102	0.95230212	0.917553107	0.999985264	0.815172397
PLEKHF1	0.8258629	0.813113924	0.730374662	0.99999488	0.932859235	0.861670395
C18H16orf46	0.8258629	0.723870388	0.803560874	0.99999488	0.921581207	0.862046449
GPBP1L1	0.8258629	0.659870071	0.859991705	0.971460441	0.999985264	0.863221317
PPARD	0.8258629	0.747155043	0.670870221	0.941409556	0.999985264	0.883999232
IKZF5	0.8258629	0.800657503	0.713543621	0.951237479	0.999985264	0.885874544
LOC786039	0.8258629	0.799180421	0.661431236	0.999314272	0.999985264	0.885874544
UBE2H	0.8258629	0.642146849	0.68741937	0.982713285	0.999985264	0.909427764
NOXO1	0.8258629	0.729000253	0.706110318	0.959611215	0.999985264	0.913527577
HMGCS1	0.8258629	0.755100312	0.683588966	0.99999488	0.961025202	0.928145377
PHLPP1	0.8258629	0.619486097	0.663165825	0.99999488	0.999985264	0.968772302
LSP1	0.826003165	0.685248684	0.9426263	0.99999488	0.988998158	0.617537824
YTHDF3	0.826003165	0.727623521	0.708177705	0.99999488	0.999985264	0.621376973
KCNJ2	0.826003165	0.992977597	0.642046114	0.99999488	0.999985264	0.626027519
LOC112441452	0.826003165	0.631298774	0.721033378	0.99999488	0.983747207	0.627549535
MFSD6L	0.826003165	0.72931078	0.746258329	0.99999488	0.921581207	0.628644572
OVOS2	0.826003165	0.755100312	0.63799633	0.99999488	0.872995322	0.645467494
CACNB4	0.826003165	0.869999083	0.746258329	0.978440951	0.999985264	0.653862943
ADGRV1	0.826003165	0.766227894	0.638383601	0.99999488	0.999985264	0.675571489
SNRPA	0.826003165	0.761984265	0.667817366	0.99999488	0.999985264	0.691757815
AMD1	0.826003165	0.993316598	0.710429228	0.921764051	0.981774771	0.737010604
KCND1	0.826003165	0.649541115	0.692027671	0.99999488	0.893695017	0.743798578
LOC514257	0.826003165	0.90213106	0.666562217	0.921764051	0.999985264	0.837143123
ZMAT2	0.826003165	0.747155043	0.938203102	0.917553107	0.937177132	0.84525008
SIVA1	0.826996358	0.827661136	0.641573776	0.99999488	0.999985264	0.646411189
LOC101906508	0.827139486	0.673756899	0.666104182	0.991596938	0.999985264	0.639425605
MICU3	0.827139486	0.9582784	0.69152129	0.99999488	0.960952394	0.649701943
EIF3M	0.827139486	0.757483795	0.631165609	0.99999488	0.999985264	0.679091734
PTPN2	0.827139486	0.654686148	0.746258329	0.99999488	0.999985264	0.704966434
CHUK	0.827139486	0.835868428	0.746258329	0.989312306	0.999985264	0.738983095
METTL13	0.827139486	0.800657503	0.70108275	0.99999488	0.999985264	0.772769165
BCL2A1	0.827139486	0.656315974	0.6485936	0.99999488	0.999985264	0.820574582
LOC526488	0.827139486	0.699986019	0.822003523	0.952793359	0.999985264	0.884143505
EIF4A1	0.827139486	0.815092066	0.774363763	0.893717778	0.964631108	0.954519353
IMPA1	0.827139486	0.6725213	0.662111348	0.917553107	0.946408931	0.999960414
GTSF1	0.827153048	0.650977403	0.732862402	0.99999488	0.999985264	0.738324902
SSR3	0.82746732	0.651292982	0.746258329	0.99999488	0.999985264	0.621376973

LOC100847818	0.827914263	0.786382788	0.841773648	0.99999488	0.981774771	0.72144082
PLD3	0.828036035	0.73942687	0.708177705	0.99999488	0.868761851	0.718350328
CNN2	0.828119176	0.74820894	0.645275104	0.99999488	0.999985264	0.660138574
WNK2	0.828119176	0.890682633	0.671210214	0.951237479	0.901763837	0.948213108
CPA3	0.828170964	0.655360237	0.683588966	0.907700124	0.999985264	0.989606676
RNASET2	0.828990777	0.748445257	0.706073772	0.99999488	0.884139051	0.622335178
PLIN3	0.828990777	0.813113924	0.684276131	0.99999488	0.999985264	0.644094886
C26H10orf62	0.828990777	0.863793573	0.724765586	0.99999488	0.943670708	0.661576605
ZCCHC13	0.828990777	0.721287499	0.688092486	0.99999488	0.999985264	0.736516695
FAM187A	0.828990777	0.663430554	0.922400936	0.925818215	0.999985264	0.885874544
PLXNA1	0.828990777	0.707737892	0.673099635	0.996471643	0.999985264	0.918400465
CCDC189	0.829487128	0.728980759	0.973303821	0.912267147	0.999985264	0.63161713
SVIL	0.829487128	0.919191546	0.691847112	0.99999488	0.901763837	0.638709845
CD1D	0.829487128	0.760068124	0.656285423	0.99999488	0.999985264	0.656368128
PLEKHA6	0.829487128	0.625694428	0.946479014	0.959611215	0.999985264	0.670092907
SUN2	0.829487128	0.650665822	0.76260462	0.99999488	0.938391537	0.695049955
FAM19A5	0.829487128	0.656082241	0.673571108	0.948570368	0.999985264	0.747408201
ZNF215	0.829487128	0.634381235	0.640558462	0.99999488	0.999985264	0.820845891
COPS3	0.829487128	0.633194506	0.6882732	0.99999488	0.944638139	0.972662617
TSR3	0.830430297	0.745276769	0.663165825	0.951237479	0.999985264	0.693004154
CYCS	0.830430297	0.73589068	0.729316615	0.917553107	0.999985264	0.772220282
TUBGCP5	0.830748675	0.895242264	0.835038876	0.99999488	0.873468654	0.657047929
TNRC18	0.830911975	0.672731254	0.644916374	0.99999488	0.928683582	0.949764317
BAK1	0.831240811	0.813113924	0.706110318	0.976631804	0.999985264	0.872365139
P4HA2	0.831355593	0.872023861	0.71274291	0.99999488	0.965672975	0.617537824
ATPAF2	0.832027563	0.755100312	0.677520363	0.917553107	0.999985264	0.818484413
DRG2	0.832027563	0.659870071	0.717074345	0.99999488	0.967727289	0.947433142
TESPA1	0.832634073	0.642323832	0.743915133	0.99999488	0.864446669	0.953495274
LOC112442634	0.833042501	0.827123416	0.739906937	0.99999488	0.944638139	0.666794437
LOC104969027	0.833042501	0.705186914	0.953704305	0.92106221	0.999985264	0.679854258
PRCP	0.833042501	0.656589815	0.640310147	0.99999488	0.999985264	0.701832753
DDX18	0.833042501	0.663430554	0.841739726	0.99999488	0.999985264	0.72831783
FXR1	0.833256334	0.922995823	0.667817366	0.967551965	0.999985264	0.644094886
GLT8D2	0.833256334	0.932142712	0.68741937	0.99999488	0.952554537	0.769515026
ADGRB3	0.833268612	0.756296711	0.852468219	0.99999488	0.999985264	0.628644572
ARSA	0.833268612	0.884739596	0.692027671	0.99999488	0.999985264	0.68999472
TMEM223	0.833268612	0.664442591	0.766334745	0.996471643	0.999985264	0.695049955
UBE2V2	0.833268612	0.892008332	0.746258329	0.99999488	0.999985264	0.74674547
IL7	0.833268612	0.647062519	0.746258329	0.99999488	0.999985264	0.76007433

AMOTL1	0.833268612	0.732212345	0.75028318	0.99999488	0.999985264	0.802788605
SMARCAD1	0.833268612	0.846198976	0.77618424	0.989312306	0.908980492	0.894723749
MAPT	0.833268612	0.864945604	0.644279997	0.945038047	0.864446669	0.991014754
UTP6	0.834303414	0.830654932	0.783264179	0.99999488	0.999985264	0.662512478
PSD	0.834303414	0.926955605	0.745264238	0.918070079	0.999985264	0.695049955
ANKRD13C	0.834838316	0.647062519	0.969880768	0.949459572	0.868188283	0.884143505
LOC512869	0.834866139	0.661182871	0.640558462	0.99999488	0.999985264	0.644690751
VPS13C	0.834866139	0.670447167	0.669782708	0.978440951	0.999985264	0.716470349
ERAP1	0.834866139	0.957700659	0.803560874	0.907039918	0.999985264	0.75331823
PAX8	0.83492616	0.87010028	0.638383601	0.99999488	0.981774771	0.839734604
MRPS12	0.835398728	0.721370958	0.706765598	0.963012661	0.999985264	0.819073042
HLX	0.835862743	0.674944018	0.769780734	0.99999488	0.999985264	0.819073042
CUBN	0.836126544	0.819937634	0.746258329	0.99999488	0.999985264	0.736909525
PELI1	0.836126544	0.6725213	0.825497047	0.951237479	0.999985264	0.753766324
DUSP11	0.836422317	0.723136577	0.714371099	0.989312306	0.999985264	0.818484413
TBX3	0.83644565	0.723805811	0.940815039	0.99999488	0.999985264	0.627549535
AMDHD1	0.83644565	0.974079639	0.907484111	0.949602784	0.901436701	0.628644572
C17H12orf65	0.83644565	0.816518153	0.716305079	0.911513036	0.999985264	0.703584472
CHID1	0.83644565	0.779783885	0.708177705	0.95025581	0.999985264	0.816924515
UFM1	0.83644565	0.744975757	0.770436657	0.91166192	0.981774771	0.976814573
HSF4	0.837605643	0.830519875	0.95106368	0.99579057	0.906448097	0.657047929
SRPK3	0.837631502	0.655648116	0.732364232	0.952783349	0.969092995	0.996066257
APOBEC3H	0.837698956	0.664490276	0.673099635	0.99999488	0.999985264	0.869157217
LOC104975559	0.837958601	0.754509129	0.717074345	0.99999488	0.944638139	0.868513869
LOC112443510	0.837996552	0.806647675	0.704226721	0.937020992	0.999985264	0.721134875
MIER1	0.83815031	0.813113924	0.654174689	0.921764051	0.999985264	0.77941434
NEMF	0.838838813	0.810079748	0.785248842	0.99999488	0.999985264	0.650154396
KIF18A	0.838838813	0.76933827	0.884320747	0.99999488	0.879141216	0.844859177
GPC5	0.839112249	0.675630177	0.76260462	0.99999488	0.901104314	0.967874696
LOC101907302	0.839436211	0.850095234	0.683588966	0.99999488	0.871609728	0.654736169
PLXNB2	0.840316094	0.650780684	0.697395563	0.99999488	0.964631108	0.622335178
ABHD5	0.840316094	0.637749095	0.793478166	0.99999488	0.999985264	0.728262915
HIST1H3G	0.840448072	0.699917998	0.822356437	0.99999488	0.880252174	0.95096765
P2RY12	0.840673592	0.650973121	0.656313811	0.99999488	0.999985264	0.644629963
TMEM186	0.840673592	0.642651175	0.708177705	0.925818215	0.999985264	0.847204221
NRROS	0.841019489	0.674837172	0.756568208	0.98846246	0.999985264	0.644094886
RAC1	0.841019489	0.663430554	0.916975216	0.99999488	0.999985264	0.656368128
TSC22D3	0.841019489	0.693753849	0.689071241	0.99999488	0.938391537	0.692319327
LOC101903540	0.841019489	0.867755878	0.673099635	0.947995037	0.999985264	0.826043635

FAM102B	0.841019489	0.650665822	0.683285603	0.99999488	0.961025202	0.972919703
MTURN	0.841408769	0.661182871	0.922400936	0.99999488	0.999985264	0.738324902
C29H11orf24	0.843147501	0.798975807	0.691847112	0.99999488	0.999985264	0.894723749
MORF4L1	0.843349466	0.721287499	0.80757077	0.99999488	0.999985264	0.656368128
GM2A	0.843938867	0.809518247	0.729977435	0.99999488	0.901689347	0.627277292
LOC789388	0.843938867	0.755100312	0.706110318	0.976631804	0.999985264	0.627277292
KEAP1	0.843938867	0.91407604	0.645275104	0.99999488	0.999985264	0.644629963
C18H16orf87	0.843938867	0.727623521	0.759038925	0.99999488	0.999985264	0.658487395
ZFP36L1	0.843938867	0.724480265	0.76260462	0.99999488	0.999985264	0.662089339
LOC104970711	0.843938867	0.883624935	0.803560874	0.99999488	0.873468654	0.693397277
TMED1	0.843938867	0.826485535	0.684617023	0.99999488	0.999985264	0.696385367
ACOT4	0.843938867	0.729000253	0.672577499	0.99999488	0.999985264	0.749618526
PRR12	0.843938867	0.689358518	0.776282112	0.99999488	0.982217275	0.789976745
GPR37L1	0.843938867	0.657779335	0.881942256	0.992168706	0.978668933	0.898917692
LOC507787	0.843938867	0.76933827	0.706110318	0.99999488	0.894715133	0.936885223
LOC112444461	0.843938867	0.686708208	0.661729852	0.907045085	0.999985264	0.95096765
TAGLN	0.844703596	0.741264723	0.840781773	0.917553107	0.952554537	0.960343814
LOC100126544	0.845158291	0.867755878	0.673099635	0.989312306	0.999985264	0.712561714
NCAPG	0.845315049	0.646277578	0.946479014	0.99999488	0.999985264	0.628917868
TIAL1	0.845315049	0.985406717	0.770213049	0.99999488	0.880252174	0.644112933
NUP210	0.845315049	0.6598011	0.86259773	0.99999488	0.999985264	0.832093857
DCTD	0.845318499	0.650665822	0.88153193	0.917553107	0.999985264	0.939035437
ZPBP2	0.845318499	0.885024111	0.655928749	0.907700124	0.999985264	0.946666773
LLPH	0.846366919	0.673756899	0.865066354	0.976631804	0.924009849	0.948213108
LMO7	0.847375648	0.745276769	0.645275104	0.940265849	0.999985264	0.645467494
USP7	0.847449035	0.661332345	0.671210214	0.99999488	0.999985264	0.686604302
DUSP3	0.847610116	0.991488652	0.874485384	0.941401688	0.894006166	0.68827479
TMEM94	0.848778779	0.710249945	0.865837237	0.907700124	0.999985264	0.680082272
CDK1	0.849791174	0.746079539	0.666562848	0.99999488	0.999985264	0.671846838
TMEM30A	0.849998454	0.755100312	0.914001994	0.941409556	0.999985264	0.631711796
CCDC82	0.849998454	0.856670857	0.663632169	0.99999488	0.999985264	0.681676783
SYT1	0.849998454	0.796531384	0.680995695	0.978440951	0.999985264	0.718350328
LSM14B	0.850284439	0.819937634	0.814724984	0.982713285	0.999985264	0.662512478
CYB5R4	0.850284439	0.727539585	0.907381775	0.99999488	0.999985264	0.675571489
TRAK2	0.850284439	0.661182871	0.698896024	0.978440951	0.999985264	0.704966434
IFT22	0.850284439	0.663430554	0.689932449	0.99999488	0.999985264	0.712878507
LOC112443417	0.850284439	0.661182871	0.890248427	0.907700124	0.999985264	0.715526355
GP9	0.850284439	0.667396315	0.726885375	0.99999488	0.999985264	0.718350328
C5H12orf57	0.850284439	0.665387007	0.685835676	0.907700124	0.999985264	0.764052305

RNF168	0.850284439	0.819937634	0.675121942	0.996471643	0.999985264	0.839734604
ADGRL1	0.850284439	0.816518153	0.645275104	0.99999488	0.911651126	0.874975722
TMEM97	0.850284439	0.73157551	0.724584302	0.957896521	0.999985264	0.920531105
POLR3A	0.850284439	0.649541115	0.726885375	0.99999488	0.999985264	0.932608891
AK2	0.850284439	0.741111932	0.822003523	0.977598122	0.90115184	0.958984414
SMAD6	0.850757996	0.754509129	0.864737879	0.99999488	0.999985264	0.639767265
STARD13	0.850757996	0.662093116	0.667817366	0.966694193	0.999985264	0.702428374
ADD2	0.850757996	0.999992444	0.670870221	0.949602784	0.931475332	0.703356006
PHLDB1	0.850757996	0.821194038	0.810767074	0.99999488	0.998296838	0.714356884
MUL1	0.850757996	0.719377488	0.708177705	0.99999488	0.999985264	0.715526355
OAS1X	0.850757996	0.799180421	0.683588966	0.99999488	0.999985264	0.746239631
LAMA3	0.850757996	0.927997582	0.851718619	0.979293963	0.933861931	0.765330803
GFRA2	0.850757996	0.655391029	0.68741937	0.99999488	0.999985264	0.873463288
ENAH	0.850758051	0.709904249	0.672194399	0.99999488	0.999985264	0.895349622
RPRD1B	0.850929916	0.700312175	0.809486619	0.99999488	0.999985264	0.644629963
NAP1L4	0.850929916	0.733210627	0.644916374	0.952783349	0.999985264	0.802880474
TMEM70	0.850929916	0.650780684	0.683588966	0.978440951	0.999985264	0.929130195
RPS6KL1	0.851023646	0.6725213	0.841773648	0.953588494	0.999985264	0.63161713
ID1	0.851023646	0.943802122	0.718295892	0.99999488	0.999985264	0.631711796
CDCA3	0.851023646	0.816518153	0.845368791	0.99999488	0.999985264	0.64170636
VPS8	0.851023646	0.727390627	0.834068744	0.99999488	0.999985264	0.766529049
LOC107132820	0.851023646	0.862811597	0.691847112	0.92106221	0.995549184	0.966921967
IL32	0.851204219	0.928525825	0.645275104	0.92106221	0.999985264	0.639013883
TMF1	0.851204219	0.729820154	0.655928749	0.907700124	0.999985264	0.653862943
RPS6KB2	0.85133072	0.741594487	0.762661971	0.907700124	0.999985264	0.695049955
VDR	0.851392829	0.941495217	0.886192181	0.931835237	0.999985264	0.656368128
SLC43A1	0.851392829	0.873289191	0.907484111	0.98846246	0.999985264	0.675571489
ZBTB47	0.851392829	0.732009317	0.692027671	0.99999488	0.999985264	0.715526355
RPL36A	0.851392829	0.6598011	0.735869895	0.959611215	0.999985264	0.780958584
VMA21	0.851392829	0.850095234	0.780560307	0.951237479	0.961025202	0.926326154
TMEM266	0.851421915	0.650780684	0.691847112	0.92106221	0.999985264	0.715526355
PPP2R1A	0.851709502	0.839504448	0.667817366	0.992168706	0.999985264	0.862046449
KIAA1958	0.851735745	0.661182871	0.645275104	0.99999488	0.999985264	0.691757815
HOXB3	0.851735745	0.755100312	0.771154197	0.99999488	0.929723444	0.82386188
LOC112447362	0.851735745	0.651292982	0.739525715	0.99999488	0.908980492	0.89935427
ZBTB7C	0.851830116	0.649541115	0.815067929	0.941409556	0.999985264	0.939035437
CD47	0.851890519	0.721370958	0.734356561	0.949602784	0.999985264	0.641183409
WFDC3	0.851890519	0.686708208	0.996348351	0.991596938	0.982217275	0.644094886
RAB30	0.851890519	0.87010028	0.746258329	0.941409556	0.999985264	0.644112933

FOXRED1	0.851890519	0.729546716	0.731527745	0.918070079	0.999985264	0.649073257
LOC112442253	0.851890519	0.76933827	0.774264043	0.99999488	0.999985264	0.654659468
ABTB1	0.851890519	0.658817102	0.95477544	0.99999488	0.999985264	0.65937855
LOC101903758	0.851890519	0.723870388	0.76260462	0.989312306	0.999985264	0.660138574
RIOK2	0.851890519	0.867755878	0.710479008	0.99999488	0.999985264	0.673232785
RSPH10B	0.851890519	0.6725213	0.988922732	0.951237479	0.999985264	0.67496387
ZNF414	0.851890519	0.655648116	0.770213049	0.99999488	0.999985264	0.675571489
PRKCSH	0.851890519	0.76933827	0.909070782	0.99999488	0.901763837	0.681790665
LGALS8	0.851890519	0.674364837	0.730602626	0.99999488	0.880459369	0.692319327
HABP4	0.851890519	0.768934833	0.667817366	0.993124804	0.999985264	0.69311359
NSMCE4A	0.851890519	0.722585736	0.683588966	0.99999488	0.999985264	0.693397277
PIMREG	0.851890519	0.647062519	0.914001994	0.99999488	0.999985264	0.707299561
LOC516108	0.851890519	0.852760886	0.691855149	0.949602784	0.999985264	0.712249777
SSBP1	0.851890519	0.687821048	0.738977782	0.917553107	0.999985264	0.72728191
H1FX	0.851890519	0.836111245	0.708177705	0.99999488	0.999985264	0.734689625
CDH20	0.851890519	0.727390627	0.75028318	0.92106221	0.999985264	0.746239631
DNAJC28	0.851890519	0.651606958	0.716305079	0.978440951	0.999985264	0.770633383
DNLZ	0.851890519	0.814676928	0.756747088	0.989312306	0.999985264	0.779686358
KCNG3	0.851890519	0.650973121	0.666562848	0.99999488	0.957235535	0.789800327
UPF3B	0.851890519	0.983850863	0.841957362	0.907700124	0.924803903	0.796010841
CLU	0.851890519	0.729000253	0.708177705	0.911513036	0.999985264	0.812831419
CD22	0.851890519	0.921550129	0.724381672	0.99999488	0.887453777	0.814735279
TPSB2	0.851890519	0.665320167	0.770213049	0.99999488	0.999985264	0.819129695
RHEB	0.851890519	0.661182871	0.955638134	0.945038047	0.999985264	0.838520085
FLOT1	0.851890519	0.89880506	0.691847112	0.99999488	0.999985264	0.841424601
FIP1L1	0.851890519	0.661182871	0.655220783	0.99999488	0.999985264	0.856510591
SHROOM3	0.851890519	0.794965819	0.881942256	0.99999488	0.901763837	0.870708914
GNG11	0.851890519	0.805110753	0.666562848	0.99999488	0.999985264	0.895349622
FOXRED2	0.851890519	0.652628913	0.852782377	0.979293963	0.999985264	0.922063125
ILK	0.851890519	0.821617615	0.762700105	0.992168706	0.946408931	0.92658818
CCT8	0.851890519	0.656315974	0.710429228	0.951237479	0.999985264	0.939035437
TNFRSF19	0.851890519	0.815489392	0.706073772	0.92106221	0.981774771	0.986338245
USHBP1	0.852305651	0.667396315	0.707964225	0.99999488	0.999985264	0.644629963
CLEC4D	0.852305651	0.859484941	0.747072631	0.99999488	0.999985264	0.645467494
ZMYM6	0.852305651	0.999992444	0.670826239	0.989312306	0.999985264	0.654291391
PIAS2	0.852305651	0.751282982	0.716305079	0.99999488	0.999985264	0.654291391
LARP4B	0.852305651	0.663430554	0.683285603	0.99999488	0.999985264	0.656368128
RCOR1	0.852305651	0.658990072	0.667274767	0.99999488	0.999985264	0.658487395
MAPK13	0.852305651	0.974812121	0.746258329	0.989312306	0.999985264	0.673232785

SETMAR	0.852305651	0.999992444	0.670340904	0.925818215	0.999985264	0.675571489
FRA10AC1	0.852305651	0.999992444	0.65017087	0.92106221	0.937177132	0.678579877
SLC19A2	0.852305651	0.674944018	0.841739726	0.99999488	0.999985264	0.715526355
S100B	0.852305651	0.729820154	0.715414989	0.922850423	0.999985264	0.73024728
PAOX	0.852305651	0.661182871	0.915215321	0.99999488	0.999985264	0.737027647
SLTM	0.852305651	0.727539585	0.756747088	0.976631804	0.999985264	0.772479356
RRP8	0.852305651	0.709395	0.68741937	0.941409556	0.999985264	0.843256981
F3	0.852305651	0.655446351	0.825497047	0.99999488	0.937177132	0.847577522
RPL36A	0.852305651	0.6725213	0.730171751	0.942613132	0.999985264	0.890433418
PPA1	0.852585556	0.656082241	0.799381787	0.917553107	0.999985264	0.987457361
DDX19A	0.853167931	0.691302101	0.834068744	0.989312306	0.999985264	0.753766324
IQGAP3	0.853828374	0.815700306	0.877799478	0.99999488	0.944638139	0.661576605
SEC13	0.853828374	0.999347049	0.706110318	0.99999488	0.950839268	0.664012525
LOC785408	0.853828374	0.671246003	0.851718619	0.99999488	0.999985264	0.680407972
NUPR1	0.853828374	0.650665822	0.68899489	0.99999488	0.999985264	0.765330803
C19H17orf53	0.854252947	0.830132999	0.898719232	0.99999488	0.999985264	0.639425605
KCNK4	0.854252947	0.935588259	0.710429228	0.941409556	0.999985264	0.645467494
UQCRQ	0.854252947	0.705186914	0.734356561	0.907700124	0.999985264	0.662512478
SEC22A	0.854252947	0.9527938	0.735595656	0.98846246	0.999985264	0.692319327
BATF2	0.854252947	0.81184075	0.822003523	0.99999488	0.999985264	0.695049955
ZC2HC1A	0.854252947	0.895813197	0.77392777	0.99999488	0.999985264	0.697878268
SNX20	0.854252947	0.690700426	0.684276131	0.99999488	0.981812717	0.729827809
APBB3	0.854252947	0.76933827	0.948420301	0.971910254	0.978076141	0.770083907
SFRP5	0.854252947	0.805577519	0.726258339	0.99999488	0.999985264	0.806724906
HCLS1	0.854252947	0.703838914	0.732868094	0.99999488	0.999985264	0.839734604
KCNT2	0.854252947	0.693980599	0.907484111	0.976631804	0.999985264	0.844563769
LOC781412	0.854252947	0.911418256	0.812769099	0.925818215	0.999985264	0.856224954
CTSO	0.854252947	0.694145861	0.672295687	0.966629392	0.999985264	0.856510591
SLC31A1	0.854252947	0.816100073	0.746258329	0.99999488	0.904795834	0.947452706
DNAJC14	0.854464976	0.779783885	0.86178438	0.921764051	0.999985264	0.657047929
TXNRD3	0.855532765	0.718044024	0.774363763	0.959611215	0.999985264	0.639425605
F8	0.855532765	0.709385406	0.773092345	0.942391899	0.999985264	0.645467494
TMEM231	0.855532765	0.66443742	0.691847112	0.99999488	0.999985264	0.645467494
TACC1	0.855532765	0.650665822	0.870567942	0.99999488	0.999985264	0.649073257
TBC1D15	0.855532765	0.84979258	0.826801319	0.99999488	0.999985264	0.649701943
IQCD	0.855532765	0.661182871	0.999558179	0.953588494	0.908980492	0.653862943
LOC112449100	0.855532765	0.9582784	0.923604668	0.92106221	0.999985264	0.655665506
NUFIP2	0.855532765	0.78130286	0.713543621	0.92106221	0.999985264	0.673027356
EMP3	0.855532765	0.985406717	0.658714534	0.99999488	0.998439184	0.677379211

USP53	0.855532765	0.671246003	0.759038925	0.99999488	0.999985264	0.680407972
KNL1	0.855532765	0.661182871	0.811233159	0.99999488	0.999985264	0.683998081
GPR75	0.855532765	0.650973121	0.734356561	0.99999488	0.999985264	0.692319327
RNF2	0.855532765	0.768934833	0.670826239	0.978440951	0.999985264	0.695049955
LOC784914	0.855532765	0.999992444	0.715414989	0.96412514	0.921581207	0.715526355
ARHGDIB	0.855532765	0.893642384	0.675121942	0.99999488	0.999985264	0.721134875
RAB3GAP2	0.855532765	0.696004643	0.673099635	0.99999488	0.999985264	0.72144082
LOC104972843	0.855532765	0.656352365	0.774363763	0.99999488	0.901763837	0.740009368
PUS1	0.855532765	0.869999083	0.834068744	0.925818215	0.999985264	0.740009368
LRBA	0.855532765	0.748445257	0.654174689	0.925818215	0.999985264	0.746239631
THUMPD1	0.855532765	0.850692013	0.811500923	0.99999488	0.999985264	0.760189361
VAV1	0.855532765	0.707737892	0.671491848	0.99999488	0.999985264	0.770030068
LST1	0.855532765	0.686708208	0.714371099	0.99999488	0.901104314	0.779686358
AKAP4	0.855532765	0.755100312	0.76260462	0.99999488	0.999985264	0.819112831
ZNF184	0.855532765	0.829029204	0.691847112	0.99999488	0.981774771	0.828248574
LOC531557	0.855532765	0.752475942	0.746258329	0.99999488	0.999985264	0.897965262
SLC9A3R1	0.855532765	0.89880506	0.710479008	0.948570368	0.999985264	0.89935427
IMP4	0.855532765	0.752475942	0.713674367	0.917553107	0.999985264	0.953254073
TMEM182	0.855532765	0.650665822	0.865420641	0.925818215	0.999985264	0.965048604
NEPRO	0.855532765	0.736053393	0.691847112	0.917553107	0.999985264	0.993564907
LOC101902851	0.855532765	0.738038006	0.691847112	0.917553107	0.999985264	0.993883944
MBTPS2	0.85559185	0.985406717	0.714371099	0.941409556	0.999985264	0.712878507
C4A	0.855814396	0.76933827	0.775034451	0.99999488	0.999985264	0.645467494
AKT1S1	0.855814396	0.675630177	0.726885375	0.940265849	0.999985264	0.688876212
KCNA2	0.855814396	0.883624935	0.746258329	0.959611215	0.999985264	0.738324902
PLCL2	0.855814396	0.650973121	0.831723974	0.978440951	0.999985264	0.812831419
TET1	0.855814396	0.661182871	0.691575438	0.99999488	0.999985264	0.828248574
WDR3	0.856131822	0.655648116	0.699021482	0.970742089	0.999985264	0.932886425
AGBL2	0.856195751	0.699785139	0.884407415	0.99999488	0.999985264	0.666794437
LOC511386	0.856459934	0.661182871	0.93198623	0.99999488	0.999985264	0.781688852
PPP2CB	0.856532125	0.717908913	0.770899781	0.99999488	0.981774771	0.729827809
COL25A1	0.856532125	0.76933827	0.690969178	0.95442132	0.999985264	0.982250307
NR4A3	0.8572372	0.74725619	0.985789565	0.917553107	0.999985264	0.679091734
RFXAP	0.857439314	0.99490847	0.671210214	0.937020992	0.999985264	0.661576605
SLC4A2	0.857972426	0.809518247	0.734780506	0.99999488	0.999985264	0.664787134
RNF19B	0.857972426	0.727539585	0.673099635	0.99999488	0.999985264	0.733745412
C25H16orf45	0.858020619	0.746079539	0.981218677	0.99999488	0.901104314	0.695049955
LOC511229	0.858020619	0.717031803	0.708177705	0.99999488	0.999985264	0.714356884
IRF8	0.858020619	0.732009317	0.657424505	0.99999488	0.995610149	0.991014754

ATP1B1	0.858463789	0.819937634	0.672295687	0.982713285	0.999985264	0.838101199
GSTA4	0.859139392	0.745276769	0.691847112	0.99999488	0.999985264	0.644094886
PLEKHF2	0.859139392	0.755100312	0.770524164	0.99999488	0.999985264	0.652947131
LOC616944	0.859139392	0.663430554	0.661326459	0.99999488	0.901763837	0.653527101
LOC785745	0.859139392	0.662839835	0.998936876	0.99999488	0.901104314	0.654736169
OLFML2B	0.859139392	0.826575339	0.724584302	0.99999488	0.999985264	0.695049955
PHKG1	0.859139392	0.699986019	0.713543621	0.99999488	0.999985264	0.716989032
ADM	0.859139392	0.661332345	0.691575438	0.99999488	0.999985264	0.931163114
TRIM21	0.859312034	0.703838914	0.927600835	0.99999488	0.999985264	0.667839158
CBX3	0.859312034	0.978510121	0.681075334	0.989312306	0.999985264	0.67496387
LOC614522	0.859312034	0.999992444	0.670826239	0.924872024	0.904842818	0.884143505
DOCK2	0.859362477	0.706611337	0.673571108	0.99999488	0.999985264	0.773507109
SLC35A5	0.85939628	0.998131745	0.75028318	0.99999488	0.901104314	0.661576605
LOC112441616	0.85939628	0.94984369	0.809486619	0.99999488	0.946408931	0.74498257
CNTRL	0.85949631	0.69621473	0.870567942	0.99999488	0.999985264	0.645467494
ESPL1	0.85949631	0.707496775	0.900890709	0.99999488	0.999985264	0.645467494
STK32A	0.85949631	0.819937634	0.859316601	0.99999488	0.999985264	0.659128316
LOC783045	0.85949631	0.699785139	0.991285432	0.99999488	0.908980492	0.661576605
BTG3	0.85949631	0.782368029	0.886962419	0.98846246	0.924126458	0.901809636
SHCBP1	0.85949631	0.658817102	0.802974969	0.99999488	0.972524588	0.921150136
PSMB6	0.860048421	0.811327744	0.734548843	0.978440951	0.999985264	0.931163114
MFGE8	0.860278141	0.662541554	0.747074913	0.994265515	0.999985264	0.760015114
FOPNL	0.860447157	0.795600408	0.779233624	0.978440951	0.999985264	0.656368128
SYT9	0.860589231	0.785562513	0.819296429	0.99999488	0.999985264	0.724432737
ASF1B	0.860589231	0.683818372	0.746258329	0.99999488	0.999985264	0.779686358
NSA2	0.860589231	0.656155148	0.785698168	0.99999488	0.999985264	0.816226172
PLA2R1	0.860589231	0.688212161	0.746258329	0.99999488	0.999985264	0.871960581
XYLB	0.860589231	0.721370958	0.705281717	0.99999488	0.999985264	0.931163114
OSGIN1	0.860934295	0.745276769	0.690770494	0.99999488	0.999985264	0.678756699
LOC107131403	0.860993852	0.6725213	0.75028318	0.978629275	0.999985264	0.727162787
MTSS1	0.861016582	0.815489392	0.66090256	0.99999488	0.983747207	0.796010841
FAM169A	0.861214874	0.832807683	0.865420641	0.92106221	0.999985264	0.765330803
NAP1L1	0.861231678	0.96712131	0.672194399	0.953188062	0.999985264	0.67764684
ALG13	0.861491186	0.864945604	0.744618167	0.952817651	0.999985264	0.885874544
SMC4	0.861497137	0.752475942	0.86763298	0.99999488	0.999985264	0.64722711
HAPLN3	0.861497137	0.68944077	0.665391565	0.99999488	0.999985264	0.666562345
CNTN4	0.861637134	0.992977597	0.706110318	0.945038047	0.999985264	0.656368128
ATP6V0C	0.861637134	0.872368602	0.825497047	0.99999488	0.92060323	0.674504592
DNAJC25	0.861637134	0.723771691	0.825497047	0.938185243	0.999985264	0.715526355

PNPLA7	0.861637134	0.729820154	0.970874203	0.99999488	0.901763837	0.770030068
POP1	0.861637134	0.711870077	0.815067929	0.982713285	0.999985264	0.939855019
AMOTL2	0.861875406	0.938597964	0.931533759	0.97688804	0.956445523	0.654736169
C7H19orf24	0.862003924	0.661182871	0.732033481	0.99999488	0.999985264	0.846639166
SLAMF8	0.862003924	0.752674825	0.730602626	0.99999488	0.974716308	0.847577522
HSP90AB1	0.86216923	0.732530744	0.766334745	0.925818215	0.999985264	0.646210786
MIIP	0.86216923	0.769979732	0.9808801	0.92398295	0.999985264	0.743462219
LOC112447010	0.862307822	0.730725094	0.686023128	0.937020992	0.999985264	0.996999374
DIRAS3	0.862631434	0.999992444	0.714371099	0.952783349	0.920863785	0.733745412
GPR141	0.862631434	0.667396315	0.692027671	0.942613132	0.999985264	0.894723749
ANO9	0.86273477	0.862707207	0.667817366	0.949602784	0.999985264	0.856510591
KIAA1551	0.862750845	0.826642757	0.778565768	0.989312306	0.999985264	0.816226172
LOC100294723	0.862902802	0.740652389	0.673571108	0.99999488	0.999985264	0.828222601
CBR4	0.862965445	0.786382788	0.746258329	0.999114211	0.999985264	0.695049955
NSUN5	0.862965445	0.752475942	0.92487482	0.99999488	0.999985264	0.715526355
IRGQ	0.862965445	0.681244807	0.762661971	0.99999488	0.904842818	0.773507109
KIAA0895L	0.863326354	0.750176907	0.663165825	0.99999488	0.999985264	0.654291391
SOAT1	0.863326354	0.673847368	0.759038925	0.99999488	0.919506952	0.692319327
ARFIP2	0.863326354	0.727623521	0.684276131	0.99999488	0.999985264	0.772479356
ANP32B	0.863326354	0.747155043	0.673482515	0.924981389	0.999985264	0.812825712
SNRPB	0.863326354	0.874563416	0.817700149	0.967551965	0.999985264	0.873463288
SLC19A1	0.863326354	0.83989094	0.744618167	0.99999488	0.999985264	0.881211326
CYC1	0.863402446	0.689041824	0.721033378	0.931835237	0.999985264	0.659515332
SLC4A8	0.863402446	0.745276769	0.667274767	0.99999488	0.999985264	0.675571489
LOC101902435	0.863402446	0.832584599	0.939147816	0.977598122	0.999985264	0.686272224
ADAM23	0.863402446	0.698898696	0.834173166	0.969770561	0.999985264	0.910018813
ARSH	0.863402446	0.741594487	0.736018006	0.917553107	0.999985264	0.999960414
EEF2K	0.863472498	0.675630177	0.785248842	0.949602784	0.929139458	0.999960414
ITGBL1	0.86364539	0.708249651	0.803560874	0.99999488	0.999985264	0.649701943
EDNRB	0.86364539	0.73589068	0.669782708	0.960794858	0.999985264	0.661576605
ETAA1	0.86364539	0.960125322	0.746258329	0.99999488	0.999985264	0.696385367
CASZ1	0.86364539	0.663430554	0.760766074	0.99999488	0.999985264	0.698216252
CTSZ	0.86364539	0.885948415	0.859991705	0.99999488	0.946408931	0.738324902
HPGDS	0.86364539	0.750176907	0.746258329	0.965065108	0.999985264	0.917028736
NMD3	0.86364539	0.73995988	0.781290064	0.960794858	0.999985264	0.972692909
CYP3A5	0.864463022	0.693386354	0.875823795	0.99999488	0.999985264	0.742551138
IPO13	0.864463022	0.76933827	0.770213049	0.99999488	0.999985264	0.779686358
SIGLEC1	0.864463022	0.689084791	0.670826239	0.978440951	0.999985264	0.856224954
LOC101902742	0.864463022	0.779783885	0.683588966	0.99999488	0.961025202	0.94921096

C10H15orf65	0.865390958	0.742143113	0.714371099	0.99999488	0.999985264	0.894723749
HTR4	0.865502545	0.999992444	0.672194399	0.989312306	0.999985264	0.652760582
ADGRF2	0.865502545	0.999347049	0.689932449	0.99999488	0.999985264	0.653862943
FAM166B	0.865502545	0.805123419	0.670826239	0.99999488	0.919506952	0.952669273
CDC37L1	0.866140398	0.93699217	0.765427745	0.99999488	0.999985264	0.656368128
EIF4A2	0.866140398	0.934871569	0.768324078	0.98846246	0.999985264	0.748495031
RPUSD1	0.866140398	0.656315974	0.673099635	0.99999488	0.999985264	0.770671825
MRPS7	0.866140398	0.674837172	0.684276131	0.984913325	0.999985264	0.895349622
DLG1	0.866140398	0.673847368	0.817700149	0.917553107	0.999985264	0.909427764
LOC107131530	0.866140398	0.815092066	0.692027671	0.925818215	0.999985264	0.939035437
CBX8	0.866157979	0.707737892	0.684617023	0.99999488	0.999985264	0.72144082
LOC512672	0.86620934	0.725016061	0.803942311	0.959611215	0.999985264	0.738324902
ROR2	0.866792387	0.661182871	0.785248842	0.99999488	0.999985264	0.683998081
SBSPON	0.866792387	0.935588259	0.743915133	0.99999488	0.963346365	0.812831419
CRYBB1	0.866792387	0.76933827	0.748903111	0.99999488	0.999985264	0.835553391
CFP	0.866961636	0.727377621	0.667228538	0.99999488	0.999985264	0.722837886
SOX9	0.867095989	0.718044024	0.809486619	0.99999488	0.999985264	0.657047929
SNX4	0.867095989	0.905019423	0.726885375	0.99999488	0.987067181	0.675571489
SLC16A13	0.867550836	0.815596111	0.72251505	0.917553107	0.999985264	0.704966434
EXD1	0.867550836	0.707496775	0.776282112	0.99999488	0.932933	0.887158291
ZBTB18	0.867550836	0.6725213	0.83711556	0.99999488	0.999985264	0.9160237
FBLN1	0.867742781	0.673847368	0.900890709	0.99999488	0.999985264	0.653862943
NDUFB3	0.867742781	0.698915525	0.726885375	0.948570368	0.999985264	0.656292237
STRN3	0.867742781	0.729000253	0.809278649	0.99999488	0.999985264	0.657047929
DYRK1A	0.867742781	0.744695215	0.785248842	0.92106221	0.999985264	0.662512478
ADA	0.867742781	0.720651109	0.785248842	0.989312306	0.999985264	0.662512478
CCDC66	0.867742781	0.850095234	0.852782377	0.959611215	0.999985264	0.674393308
LOC533921	0.867742781	0.673405033	0.778565768	0.99999488	0.999985264	0.674504592
CASS4	0.867742781	0.717580718	0.684276131	0.99999488	0.91945292	0.67496387
CACNA1F	0.867742781	0.671246003	0.759038925	0.99999488	0.907875746	0.675571489
PPFIA2	0.867742781	0.697256706	0.879253779	0.99999488	0.999985264	0.679091734
FLI1	0.867742781	0.743395398	0.667817366	0.971511747	0.999985264	0.683998081
BICC1	0.867742781	0.670199175	0.825497047	0.99999488	0.999985264	0.687854495
CNTN3	0.867742781	0.738445703	0.926552506	0.931835237	0.999985264	0.698890452
LOC112444164	0.867742781	0.721287499	0.908181052	0.979293963	0.999985264	0.709552096
MTMR1	0.867742781	0.661182871	0.724381672	0.99999488	0.999985264	0.714215429
ZBED6CL	0.867742781	0.998131745	0.881942256	0.92106221	0.942175494	0.716254676
TAPT1	0.867742781	0.839292361	0.746258329	0.99999488	0.999985264	0.73024728
FRZB	0.867742781	0.661182871	0.965714506	0.99999488	0.983747207	0.738324902

DPM2	0.867742781	0.811327744	0.781111723	0.969770561	0.999985264	0.743462219
FBXO7	0.867742781	0.817772879	0.830970408	0.98846246	0.999985264	0.754336759
LOC101906230	0.867742781	0.691364494	0.675121942	0.99999488	0.999985264	0.762686295
PNPT1	0.867742781	0.721370958	0.710429228	0.989312306	0.999985264	0.767631112
SYNPR	0.867742781	0.9639833	0.713178271	0.918070079	0.999985264	0.768785338
VCL	0.867742781	0.745276769	0.841739726	0.99999488	0.999985264	0.772479356
GNB1L	0.867742781	0.778579649	0.958627535	0.96412514	0.999458517	0.782973939
LOC100848263	0.867742781	0.747293429	0.876758498	0.99999488	0.967727289	0.793906942
APOLD1	0.867742781	0.673847368	0.787862242	0.99999488	0.946408931	0.809513929
SYNCRIP	0.867742781	0.771826499	0.920629231	0.966473049	0.999985264	0.831532663
LOC112448743	0.867742781	0.707496775	0.941336964	0.938185243	0.999985264	0.837143123
NLRC4	0.867742781	0.659816181	0.68741937	0.99999488	0.999985264	0.839734604
PELI2	0.867742781	0.708003771	0.722609258	0.963012661	0.999985264	0.873463288
METAP2	0.867742781	0.750176907	0.70108275	0.999114211	0.999985264	0.875113722
LOC515570	0.867742781	0.747155043	0.774363763	0.99999488	0.964631108	0.87805762
RGS9	0.867742781	0.891316528	0.800952304	0.99999488	0.927270318	0.887798625
TPGS2	0.867742781	0.76933827	0.903957821	0.925818215	0.999985264	0.925481882
C16H1orf105	0.867742781	0.859484941	0.862345036	0.917553107	0.957776687	0.939035437
EIF2B1	0.867742781	0.695496638	0.700695194	0.989312306	0.999985264	0.939035437
VMP1	0.867742781	0.722585736	0.766334745	0.978440951	0.921581207	0.999960414
IFITM3	0.86855038	0.755100312	0.916975216	0.99999488	0.999985264	0.654291391
ATP2B2	0.86855038	0.958967142	0.841739726	0.98846246	0.946875845	0.770633383
THAP3	0.868701073	0.747155043	0.994601277	0.932016193	0.999985264	0.759126917
LOC107133284	0.868701073	0.864945604	0.815067929	0.98846246	0.999985264	0.763713143
ANXA1	0.868701073	0.799180421	0.750765111	0.99999488	0.999985264	0.775511809
CD101	0.868701073	0.865926615	0.708177705	0.969770561	0.999985264	0.895349622
SLA2	0.868701073	0.808097876	0.78336402	0.99999488	0.999985264	0.901643249
SUPV3L1	0.868993747	0.816518153	0.770213049	0.949602784	0.999985264	0.883999232
RAD17	0.869006318	0.999992444	0.686023128	0.981636466	0.999985264	0.661576605
SRPRA	0.869260235	0.830519875	0.76260462	0.99999488	0.999985264	0.777367442
EIF4E	0.869260235	0.673405033	0.998936876	0.945038047	0.944638139	0.820388257
SPTSSB	0.869683998	0.709904249	0.670870221	0.92106221	0.999985264	0.738324902
STOML2	0.869683998	0.722585736	0.689932449	0.99999488	0.999985264	0.9271369
LMX1B	0.869830359	0.790213802	0.857072251	0.982713285	0.941510628	0.940358165
EVL	0.869945339	0.821033198	0.774363763	0.99999488	0.968452969	0.692319327
GSTT1	0.870133282	0.746209367	0.678677273	0.99999488	0.999985264	0.675571489
EPB41L4B	0.870133282	0.717915819	0.675208988	0.99999488	0.999985264	0.695049955
WWC1	0.870133282	0.755100312	0.999558179	0.966994712	0.907875746	0.712249777
MED11	0.870133282	0.829864864	0.714371099	0.99999488	0.999985264	0.728484954

RBFOX1	0.870133282	0.850692013	0.706110318	0.99999488	0.999985264	0.763312237
EOMES	0.870133282	0.74820894	0.815067929	0.99999488	0.999985264	0.888234257
ASPM	0.870174515	0.771984021	0.870567942	0.99999488	0.999985264	0.701003695
PLA2G4B	0.870454906	0.864154704	0.769466762	0.989312306	0.999985264	0.695049955
SELP	0.870454906	0.751959025	0.743946755	0.99999488	0.934591321	0.931163114
TACC3	0.87067529	0.817678561	0.898815524	0.99999488	0.998439184	0.718350328
SLC25A1	0.87067529	0.674944018	0.708177705	0.966629392	0.999985264	0.76762685
CXCL10	0.87067529	0.885011801	0.786953469	0.963012661	0.999985264	0.907296322
ERBB4	0.871131276	0.844433938	0.701191215	0.99999488	0.955767719	0.952851247
NUSAP1	0.871153787	0.661182871	0.935768849	0.99999488	0.946408931	0.681790665
IER5	0.871153787	0.862707207	0.880229444	0.99999488	0.999985264	0.715526355
DPP9	0.871230529	0.900867274	0.766334745	0.948570368	0.999985264	0.836520183
MTHFD2	0.871230529	0.951765021	0.70881811	0.996471643	0.999985264	0.856510591
DUSP14	0.871461653	0.87010028	0.916975216	0.978440951	0.999985264	0.661149383
BSN	0.871461653	0.700365247	0.96210311	0.98846246	0.999985264	0.757395858
KCTD5	0.871510328	0.87010028	0.785177733	0.99999488	0.999985264	0.661576605
MAPKAPK3	0.871510328	0.741594487	0.70108275	0.99999488	0.934529944	0.719097202
TMEM92	0.871510328	0.87010028	0.770213049	0.99999488	0.917539697	0.735738931
DDX27	0.871510328	0.667396315	0.715414989	0.99999488	0.999985264	0.746239631
WAC	0.871510328	0.703838914	0.691847112	0.949602784	0.999985264	0.818895181
TUBA1C	0.871510328	0.819379885	0.915215321	0.989312306	0.999985264	0.818895181
ARHGAP40	0.871510328	0.677224344	0.824972187	0.92106221	0.999985264	0.873463288
RPSA	0.871510328	0.821033198	0.809401378	0.989312306	0.999985264	0.873463288
LYPLA2	0.871510328	0.829363839	0.704758063	0.92106221	0.998296838	0.999960414
CDKN1B	0.872183107	0.991365955	0.746258329	0.945038047	0.999985264	0.700868523
PSMB10	0.872183107	0.754374533	0.914001994	0.99999488	0.999985264	0.73024728
SRPX2	0.872183107	0.802498185	0.785248842	0.99999488	0.999985264	0.818895181
C19H17orf113	0.872183107	0.684021956	0.984765193	0.953588494	0.934591321	0.874975722
KIF1B	0.87243084	0.674272153	0.732290342	0.99999488	0.999985264	0.692319327
LRRC34	0.872553472	0.863528984	0.82336109	0.977598122	0.999985264	0.661576605
SLCO1C1	0.872553472	0.938597964	0.715414989	0.959611215	0.999985264	0.677817124
DNAH17	0.872553472	0.694145861	0.730171751	0.99999488	0.969128502	0.691757815
RET	0.872553472	0.690700426	0.747072631	0.99999488	0.999985264	0.695049955
ZNF410	0.872553472	0.870839685	0.68899489	0.99999488	0.999985264	0.832197795
SLC22A18	0.872750757	0.893642384	0.834173166	0.965065108	0.999985264	0.67764684
GID4	0.872750757	0.727539585	0.731949795	0.99999488	0.999985264	0.809513929
C1R	0.873570867	0.721370958	0.766334745	0.941409556	0.999985264	0.675571489
N4BP1	0.873570867	0.786382788	0.724584302	0.99999488	0.999985264	0.691757815
FANCF	0.873570867	0.696574176	0.691847112	0.963012661	0.999985264	0.873463288

CD8A	0.873570867	0.747155043	0.824972187	0.99999488	0.999985264	0.899020749
WDR5B	0.873793289	0.999992444	0.724128712	0.963012661	0.941510628	0.675571489
POLR3K	0.873793289	0.72165336	0.76260462	0.969770561	0.999985264	0.688187637
PNRC2	0.873793289	0.874697957	0.746258329	0.99999488	0.999985264	0.688187637
NAMPT	0.873793289	0.769482402	0.86638329	0.99999488	0.909266267	0.702758631
FUT1	0.873793289	0.691302101	0.746258329	0.99999488	0.999985264	0.70329486
BCL7B	0.873793289	0.809104286	0.981039752	0.99999488	0.919006503	0.712561714
BMS1	0.873793289	0.757483795	0.73604716	0.99999488	0.999985264	0.777136982
LOC100847454	0.873793289	0.830519875	0.746258329	0.99579057	0.999985264	0.783345949
LOC107131843	0.873793289	0.689899438	0.691847112	0.98846246	0.999985264	0.803939828
CD69	0.873793289	0.667396315	0.72136241	0.989312306	0.999985264	0.80980964
LOC107131429	0.873793289	0.673756899	0.972853588	0.949602784	0.999985264	0.818895181
RPF2	0.873793289	0.696681089	0.871474302	0.99999488	0.999985264	0.870893258
BoLA	0.873793289	0.753515888	0.916975216	0.966629392	0.999985264	0.90109791
POLR2A	0.874125374	0.768934833	0.676439937	0.952783349	0.999985264	0.693397277
KLF5	0.874125374	0.865926615	0.825497047	0.968653855	0.999985264	0.873083064
LRP6	0.874446117	0.786382788	0.694895009	0.967551965	0.999985264	0.658971109
MRPL43	0.874446117	0.764537649	0.793478166	0.978440951	0.999985264	0.859537053
POC1B	0.874446117	0.962389936	0.726885375	0.937020992	0.999985264	0.885874544
ATG3	0.874446117	0.769482402	0.762661971	0.99999488	0.999985264	0.907070674
TSHZ1	0.874628931	0.721370958	0.746258329	0.99999488	0.999985264	0.773507109
TAX1BP1	0.874907551	0.763345458	0.969656316	0.99999488	0.941727981	0.748495031
SQOR	0.874907551	0.706611337	0.848786751	0.99999488	0.999985264	0.869877865
TRMT2A	0.875264742	0.698898696	0.746258329	0.99999488	0.999985264	0.799216519
FKBP7	0.875303059	0.694996311	0.825497047	0.99999488	0.999985264	0.675571489
LOC100295712	0.875303059	0.663928441	0.998816213	0.978440951	0.999985264	0.69084747
CCDC88B	0.875303059	0.698768403	0.746258329	0.99999488	0.999985264	0.753766324
NFE2	0.875303059	0.925580392	0.874752438	0.94962185	0.999985264	0.819073042
ATF1	0.875511868	0.692610187	0.706110318	0.941409556	0.999985264	0.675571489
NIFK	0.875864112	0.709395	0.76260462	0.94911704	0.999985264	0.939035437
JUND	0.876102385	0.833935981	0.921646762	0.977738069	0.999985264	0.693397277
PLXNA3	0.876102385	0.761362415	0.886472449	0.99999488	0.999985264	0.722837886
KIF23	0.877060522	0.755100312	0.6882732	0.99999488	0.999985264	0.748495031
METTL24	0.878032471	0.6725213	0.782338606	0.99999488	0.999985264	0.703242753
CNTROB	0.87824739	0.719377488	0.981039752	0.99999488	0.933861931	0.675571489
TIMM23	0.87824739	0.727377621	0.7536262	0.96412514	0.999985264	0.861670395
MAX	0.878370556	0.694145861	0.88687452	0.99999488	0.999985264	0.675571489
UBR3	0.878370556	0.752475942	0.807323012	0.99999488	0.999985264	0.677379211
INTS13	0.878370556	0.999992444	0.700192518	0.978440951	0.999985264	0.691757815

PLCB2	0.878370556	0.708003771	0.683588966	0.99999488	0.920207885	0.693397277
ST6GALNAC4	0.878370556	0.864945604	0.946479014	0.99999488	0.969092995	0.695049955
APBB1IP	0.878370556	0.705186914	0.677520363	0.99999488	0.998639844	0.695049955
LOC104974923	0.878370556	0.809135999	0.742557674	0.99999488	0.999985264	0.704679988
HERC5	0.878370556	0.6725213	0.70071733	0.99999488	0.999985264	0.729812118
BLM	0.878370556	0.718036056	0.767419831	0.99999488	0.999985264	0.736516695
PLSCR3	0.878370556	0.744415271	0.770213049	0.99999488	0.919006503	0.738324902
LOC107131516	0.878370556	0.80179593	0.857021454	0.945038047	0.999985264	0.738324902
CALM1	0.878370556	0.862811597	0.886192181	0.99999488	0.999985264	0.741213204
HOXD4	0.878370556	0.873289191	0.778565768	0.99999488	0.999985264	0.758510984
KLF4	0.878370556	0.993316598	0.76260462	0.99999488	0.924009849	0.783677757
PTPRF	0.878370556	0.714371951	0.864257597	0.982713285	0.999985264	0.790953407
GTF2F1	0.878370556	0.76933827	0.751173926	0.99999488	0.996950237	0.802788605
TNFRSF6B	0.878370556	0.76933827	0.825497047	0.99999488	0.999985264	0.802880474
FAM13C	0.878370556	0.74313037	0.775688011	0.99999488	0.999985264	0.814155301
SMIM5	0.878370556	0.697256706	0.899248502	0.965065108	0.999985264	0.814496203
PPM1G	0.878370556	0.745276769	0.953457715	0.99999488	0.928683582	0.820936624
TIMM50	0.878370556	0.764050973	0.70108275	0.978440951	0.999985264	0.873836462
SH3TC2	0.878370556	0.850095234	0.862345036	0.989312306	0.964906636	0.904915514
FNTB	0.878370556	0.819937634	0.681730747	0.98846246	0.999985264	0.939035437
PDZD7	0.878370556	0.850517908	0.752249769	0.971460441	0.999985264	0.945670698
HM13	0.878370556	0.799180421	0.680995695	0.99999488	0.999985264	0.947433142
ACTA2	0.878370556	0.863813934	0.781453389	0.949602784	0.977475352	0.966921967
PSMD6	0.878370556	0.703838914	0.718295892	0.985829712	0.999985264	0.968695729
TXK	0.878370556	0.73995988	0.720073352	0.99999488	0.999985264	0.972391591
AHI1	0.87882908	0.759500463	0.712513153	0.924872024	0.999985264	0.676283713
CLEC4E	0.879117559	0.722585736	0.708177705	0.99999488	0.999985264	0.715526355
TP63	0.879117559	0.766651965	0.689932449	0.99999488	0.999985264	0.754929012
STK32B	0.879117559	0.769482402	0.904323644	0.99999488	0.999985264	0.779686358
GTF3C6	0.879117559	0.927997582	0.691847112	0.99999488	0.965227764	0.897965262
UBLCP1	0.879972054	0.710594949	0.726885375	0.978440951	0.999985264	0.977569033
COL22A1	0.880688241	0.692611818	0.683588966	0.924872024	0.999985264	0.683946026
CUX2	0.880688241	0.840946688	0.708177705	0.99999488	0.999985264	0.691757815
SDCBP	0.880688241	0.700534564	0.822356437	0.99999488	0.999985264	0.695049955
ZNF326	0.880688241	0.896996319	0.710429228	0.98846246	0.999985264	0.72547134
MMP15	0.880688241	0.904961534	0.684276131	0.945038047	0.999985264	0.758510984
TSSK3	0.880688241	0.842782808	0.973406453	0.959611215	0.919006503	0.809513929
FBXO36	0.880688241	0.93531922	0.689170915	0.99999488	0.999985264	0.812831419
IL1RAPL2	0.880688241	0.745276769	0.857490704	0.99999488	0.999985264	0.868513869

PA2G4	0.880688241	0.798751099	0.746258329	0.99999488	0.999985264	0.884143505
ATP5MD	0.880935278	0.728913712	0.790350338	0.937020992	0.999985264	0.670524268
FAM189A2	0.880935278	0.869999083	0.785248842	0.99999488	0.999985264	0.684403983
CYREN	0.880935278	0.757483795	0.693417174	0.945038047	0.999985264	0.695049955
PTHLH	0.880935278	0.893743321	0.76260462	0.99999488	0.983747207	0.696738052
TEDC2	0.880935278	0.845554546	0.866582678	0.99999488	0.999985264	0.721134875
ACP4	0.880935278	0.915367594	0.767682114	0.984913325	0.999985264	0.737027647
NME9	0.880935278	0.999992444	0.746258329	0.949602784	0.999985264	0.747408201
P4HTM	0.880935278	0.920253543	0.746258329	0.99999488	0.999985264	0.80980964
QRICH2	0.880935278	0.751327936	0.930678591	0.949602784	0.999985264	0.850342872
BTBD11	0.880935278	0.999992444	0.718745637	0.933238662	0.922217699	0.905840072
ARMC12	0.880935278	0.717826799	0.762661971	0.949602784	0.964631108	0.999960414
PI16	0.881204837	0.795127474	0.905909805	0.99999488	0.999985264	0.665508161
LOC101905156	0.881204837	0.911418256	0.803677695	0.925818215	0.999985264	0.695049955
DENND1C	0.881204837	0.76933827	0.817700149	0.99999488	0.999985264	0.731312503
SLC25A13	0.881204837	0.741992039	0.702366288	0.978440951	0.999985264	0.901614166
PALD1	0.881892458	0.805123419	0.786568605	0.99999488	0.999985264	0.802880474
LOC107132897	0.881892458	0.974407708	0.778565768	0.951038127	0.999985264	0.847577522
TGM1	0.881940714	0.999992444	0.799361358	0.939271482	0.999985264	0.675571489
GPR45	0.881940714	0.978089982	0.766334745	0.984913325	0.999985264	0.698422388
LMCD1	0.881940714	0.753294829	0.926552506	0.951237479	0.999985264	0.897965262
LOC101904942	0.881940714	0.750176907	0.870995265	0.963012661	0.999985264	0.939035437
ACSM5	0.881958699	0.730465779	0.841957362	0.99999488	0.999985264	0.738324902
CDC42EP2	0.882119063	0.729546716	0.785248842	0.99999488	0.999985264	0.693178721
MNS1	0.882119063	0.744558551	0.881942256	0.99999488	0.999985264	0.807689737
PCED1B	0.882119063	0.970426668	0.76260462	0.967551965	0.999985264	0.812825712
EXTL1	0.882119063	0.781188626	0.746258329	0.99999488	0.999985264	0.812831419
NUDT4	0.882119063	0.736701713	0.851718619	0.99999488	0.982217275	0.856510591
YWHAQ	0.882394824	0.683818372	0.771714229	0.95312502	0.999985264	0.670235195
ANG	0.882394824	0.850692013	0.694350021	0.99999488	0.999985264	0.673027356
LOC112449072	0.882394824	0.936489465	0.882380454	0.99999488	0.937177132	0.67496387
LOC783224	0.882394824	0.994388319	0.756747088	0.959611215	0.969128502	0.847204221
LOXL2	0.882394824	0.705186914	0.803942311	0.99999488	0.97687524	0.858593649
WDR74	0.883060332	0.733210627	0.806978356	0.99999488	0.999985264	0.836887084
LOC787122	0.883423473	0.745276769	0.70108275	0.966629392	0.999985264	0.762686295
C1H21orf91	0.883423473	0.805863654	0.970517055	0.978440951	0.933861931	0.828248574
TRPM1	0.883427606	0.761716845	0.751173926	0.99999488	0.933861931	0.780958584
NHLRC1	0.883482159	0.835317461	0.774363763	0.99999488	0.999985264	0.738324902
NUP153	0.883893287	0.816409944	0.733989453	0.925818215	0.999985264	0.728036947

H2AFY	0.885146449	0.873289191	0.754993747	0.949602784	0.999985264	0.671273512
LOC100299712	0.885146449	0.80278938	0.714371099	0.99999488	0.999985264	0.712249777
ACTN1	0.885146449	0.957700659	0.802997196	0.99999488	0.996075618	0.736516695
CDC73	0.885146449	0.738042027	0.774363763	0.99999488	0.999985264	0.738324902
MAP4K5	0.885146449	0.999992444	0.708177705	0.991596938	0.957235535	0.772769165
BEND7	0.885146449	0.815092066	0.684617023	0.99999488	0.999985264	0.781688852
TUBG1	0.885146449	0.892206663	0.778565768	0.99999488	0.999985264	0.787783489
ANKRA2	0.885146449	0.708003771	0.848312163	0.99999488	0.999985264	0.819073042
FAM198B	0.885146449	0.916036681	0.821546364	0.978440951	0.999985264	0.849046935
SPHK1	0.885146449	0.87010028	0.742075034	0.99999488	0.999985264	0.856224954
NMT2	0.885146449	0.898789587	0.925745065	0.959611215	0.941510628	0.856510591
WEE1	0.885146449	0.746344971	0.68899489	0.976631804	0.999985264	0.858593649
ENG	0.885440875	0.757483795	0.759934666	0.99999488	0.999985264	0.717322461
BTBD10	0.885454816	0.808848805	0.778565768	0.99999488	0.999985264	0.697086772
TFAM	0.885454816	0.684790041	0.930678591	0.99999488	0.996075618	0.72144082
VGF	0.885454816	0.741111932	0.994601277	0.99999488	0.922362358	0.746239631
CORO7	0.885531968	0.918883775	0.710479008	0.99999488	0.93150494	0.764052305
CRYL1	0.885648886	0.777422141	0.789951578	0.99999488	0.928683582	0.738324902
MYOC	0.885752155	0.820161757	0.766334745	0.99999488	0.999985264	0.763713143
MDM2	0.885792655	0.729000253	0.692027671	0.99999488	0.999985264	0.721134875
CEP68	0.885792655	0.913160799	0.826510552	0.99999488	0.999985264	0.746239631
LOC101906569	0.885792655	0.693386354	0.770213049	0.99999488	0.999985264	0.769515026
USP32	0.885792655	0.76933827	0.774363763	0.98846246	0.999985264	0.770633383
MICALL1	0.885792655	0.896085343	0.752051085	0.99999488	0.999985264	0.793525005
C1H21orf2	0.885792655	0.994481342	0.714371099	0.978440951	0.999985264	0.818895181
LOC112444775	0.885792655	0.893642384	0.800952304	0.99999488	0.999985264	0.885874544
PNPO	0.885792655	0.703838914	0.6882732	0.989312306	0.999985264	0.897965262
HBP1	0.885792655	0.976819696	0.708177705	0.982713285	0.937177132	0.929458454
GNA13	0.885963817	0.724585536	0.870567942	0.99999488	0.964631108	0.704679988
HNRNPAB	0.885963817	0.830519875	0.935175447	0.989312306	0.999985264	0.80980964
ZNF705A	0.885963817	0.811801317	0.793478166	0.99999488	0.999985264	0.814496203
COL26A1	0.885963817	0.877142156	0.837139208	0.978440951	0.999985264	0.873463288
CDKL1	0.886030743	0.948421431	0.807323012	0.966629392	0.940842815	0.918343194
GPALPP1	0.88626882	0.985406717	0.886192181	0.99999488	0.961025202	0.673027356
ELOB	0.88626882	0.78130286	0.787474031	0.99999488	0.999985264	0.687854495
EIF3A	0.88626882	0.692683276	0.811233159	0.996471643	0.999985264	0.826043635
RAP2B	0.886300795	0.675265969	0.766334745	0.99999488	0.999985264	0.74674547
TJP3	0.886361715	0.867755878	0.761960605	0.99999488	0.944638139	0.941724934
PRUNE2	0.88677687	0.789663062	0.82413265	0.99999488	0.999985264	0.76926818

PNCK	0.887011181	0.999347049	0.691847112	0.99999488	0.949679366	0.697878268
LOC101902841	0.887070819	0.999992444	0.76260462	0.925939733	0.999985264	0.770030068
RFNG	0.887335498	0.850095234	0.746258329	0.99999488	0.999985264	0.816959462
IQSEC1	0.887375291	0.893642384	0.690864342	0.99999488	0.999985264	0.743241781
MRPL11	0.887579477	0.76933827	0.902552409	0.949602784	0.999985264	0.795293834
LOC618071	0.887822704	0.893642384	0.690969178	0.99999488	0.999985264	0.773507109
CCDC78	0.887822704	0.799180421	0.938215065	0.989312306	0.969128502	0.875200779
HDGFL2	0.888000702	0.748233963	0.691847112	0.99999488	0.999985264	0.67496387
ADAMTS12	0.888000702	0.770063367	0.770213049	0.99999488	0.965227764	0.678677938
LMBR1L	0.888000702	0.723136577	0.990780762	0.99999488	0.999985264	0.684032381
SUZ12	0.888000702	0.980496345	0.785248842	0.99999488	0.999985264	0.692319327
LOC104976274	0.888000702	0.999347049	0.907484111	0.937020992	0.964631108	0.699118435
CTNS	0.888000702	0.850692013	0.72674783	0.99999488	0.999985264	0.699118435
C2H2orf69	0.888000702	0.798975807	0.892574927	0.99999488	0.999985264	0.704966434
PAPD5	0.888000702	0.813746447	0.726885375	0.953588494	0.999985264	0.76161727
TMEM204	0.888000702	0.910384774	0.879253779	0.99999488	0.999985264	0.789800327
NAA40	0.888000702	0.796000666	0.83126054	0.99999488	0.999985264	0.809513929
CELF1	0.888000702	0.747155043	0.998936876	0.938065999	0.95385298	0.836520183
LOC104975590	0.888000702	0.845554546	0.848786751	0.959611215	0.948940193	0.960343814
FBXO41	0.888000702	0.73449175	0.841717609	0.949602784	0.999985264	0.986787448
SIGLEC15	0.888233083	0.727623521	0.771154197	0.99999488	0.999985264	0.769515026
PAPOLA	0.888351661	0.958098081	0.745961896	0.978629275	0.999985264	0.744164643
PSIP1	0.889025819	0.76933827	0.745717284	0.99999488	0.999985264	0.828248574
NRBP2	0.889153534	0.790159199	0.973406453	0.971460441	0.999985264	0.733921524
MCFD2	0.889153534	0.690700426	0.920629231	0.99999488	0.999985264	0.837019875
CACNA1B	0.889153534	0.999992444	0.714371099	0.98846246	0.961025202	0.841443168
AASS	0.889153534	0.915367594	0.837103057	0.949602784	0.99980018	0.923360345
ARHGEF16	0.889197361	0.912485935	0.956565728	0.949602784	0.999985264	0.681790665
SORT1	0.889197361	0.796869696	0.771154197	0.99999488	0.999985264	0.68999472
UBA6	0.889197361	0.991488652	0.707964225	0.99999488	0.999985264	0.721134875
MVB12B	0.889197361	0.805110753	0.774363763	0.99999488	0.999985264	0.864549074
LOC613660	0.889197361	0.813113924	0.938203102	0.937020992	0.999985264	0.872307149
MINDY2	0.889209681	0.796000666	0.907484111	0.99999488	0.999985264	0.704551299
MPEG1	0.889209681	0.694996311	0.690864342	0.99999488	0.999985264	0.781688852
CMTM6	0.889919388	0.693386354	0.981622418	0.99999488	0.999985264	0.67764684
NDUFB7	0.889919388	0.78130286	0.774363763	0.949602784	0.999985264	0.688187637
BRF1	0.889919388	0.776219332	0.897043265	0.99999488	0.999985264	0.692319327
CIT	0.889919388	0.778579649	0.876188482	0.99999488	0.97687524	0.703584472
COL14A1	0.889919388	0.864945604	0.76260462	0.99999488	0.999985264	0.704966434

CEP131	0.889919388	0.745276769	0.806978356	0.99999488	0.999985264	0.704966434
ZNRF2	0.889919388	0.744415271	0.886641616	0.99999488	0.944638139	0.721134875
SLAMF1	0.889919388	0.728913712	0.788870461	0.966629392	0.999985264	0.764052305
LUC7L	0.889919388	0.835868428	0.913211244	0.99999488	0.999985264	0.764052305
GALNT3	0.889919388	0.936273761	0.691847112	0.99999488	0.999985264	0.772431293
TMEM202	0.889919388	0.910719401	0.693776732	0.99999488	0.961025202	0.772479356
CYR61	0.889919388	0.836126124	0.876188482	0.99999488	0.937177132	0.773507109
SRSF11	0.889919388	0.741594487	0.785173725	0.989312306	0.999985264	0.778146437
PPIG	0.889919388	0.994753342	0.770213049	0.952783349	0.999985264	0.779686358
TWNK	0.889919388	0.739096585	0.888107357	0.99999488	0.999985264	0.787607112
LOC112442740	0.889919388	0.769482402	0.746258329	0.99999488	0.937177132	0.789800327
LOC107131239	0.889919388	0.698898696	0.826965593	0.99999488	0.999985264	0.800822576
PCDH20	0.889919388	0.745276769	0.708177705	0.98846246	0.999985264	0.805442659
MRPL4	0.889919388	0.858808025	0.726885375	0.976631804	0.999985264	0.809513929
LOC534181	0.889919388	0.76933827	0.692027671	0.99999488	0.999985264	0.814496203
MUC16	0.889919388	0.821194038	0.946479014	0.99999488	0.965672975	0.816959462
SLC1A4	0.889919388	0.978089982	0.785177733	0.967551965	0.999985264	0.819073042
EI24	0.889919388	0.801501688	0.848312163	0.99999488	0.999985264	0.827966127
SPRYD4	0.889919388	0.719377488	0.786053266	0.966629392	0.999985264	0.839734604
HCN2	0.889919388	0.816518153	0.825497047	0.99999488	0.969092995	0.847204221
SLC39A7	0.889919388	0.908693037	0.72002151	0.99999488	0.999985264	0.856510591
DKC1	0.889919388	0.747293429	0.778565768	0.99999488	0.999985264	0.856510591
NELFE	0.889919388	0.931488015	0.752746024	0.99999488	0.999985264	0.868256799
PPIE	0.889919388	0.683818372	0.857021454	0.948570368	0.999985264	0.882867473
B3GNT8	0.889919388	0.850692013	0.746258329	0.99999488	0.969703428	0.885874544
LOC107132851	0.889919388	0.883856603	0.794878847	0.989312306	0.999985264	0.907993548
MDFIC2	0.889919388	0.752135896	0.690770494	0.989312306	0.999985264	0.958434611
AMPD2	0.889919388	0.821194038	0.690864342	0.979498903	0.999985264	0.992644147
GZMA	0.88996466	0.802452784	0.944932814	0.99999488	0.999985264	0.731312503
KAZN	0.88996466	0.700365247	0.690969178	0.99999488	0.999985264	0.844563769
GPS1	0.88996466	0.738042027	0.715414989	0.99999488	0.999985264	0.919755394
LOC112442713	0.88996466	0.72165336	0.805753285	0.99999488	0.999985264	0.966921967
STAT4	0.890075644	0.850496315	0.695533457	0.99999488	0.999985264	0.765964057
ICE2	0.890075644	0.921661251	0.835038876	0.99999488	0.93150494	0.837482843
RBM5	0.890075644	0.729000253	0.774587294	0.96412514	0.983747207	0.999960414
LOC112448354	0.890164447	0.977354623	0.848312163	0.99999488	0.999985264	0.721168613
G2E3	0.890593638	0.938597964	0.811233159	0.991596938	0.999985264	0.680407972
FAM101A	0.890593638	0.869304939	0.692027671	0.96412514	0.999985264	0.69084747
TIMP2	0.890593638	0.850692013	0.754967534	0.99999488	0.965227764	0.691757815

MICALL2	0.890593638	0.704559141	0.973661483	0.99999488	0.933861931	0.692319327
CHMP2B	0.890593638	0.985406717	0.892053976	0.99999488	0.946408931	0.692319327
CCNB2	0.890593638	0.761716845	0.827021762	0.99999488	0.999985264	0.693004154
BCL2	0.890593638	0.738412248	0.74318117	0.99999488	0.999985264	0.693494816
C11H9orf16	0.890593638	0.859182763	0.81294064	0.99999488	0.999985264	0.698422388
CEP295	0.890593638	0.882863398	0.939799459	0.99999488	0.999985264	0.703354476
VCPIP1	0.890593638	0.723805811	0.908181052	0.99999488	0.964616886	0.703980684
APELA	0.890593638	0.790159199	0.938905004	0.979293963	0.999985264	0.715526355
PARP12	0.890593638	0.702198224	0.945678616	0.99999488	0.999985264	0.719097202
SCO2	0.890593638	0.793231404	0.813142592	0.982713285	0.999985264	0.72144082
RASD1	0.890593638	0.821194038	0.804494522	0.99999488	0.935138282	0.723102808
IFNLR1	0.890593638	0.90116492	0.796729927	0.99999488	0.999985264	0.726340954
LOC112449280	0.890593638	0.957700659	0.76260462	0.99999488	0.965672975	0.727162787
KCNK3	0.890593638	0.915367594	0.934694748	0.978440951	0.999985264	0.728942623
LOC100297099	0.890593638	0.921759937	0.791717601	0.99999488	0.999985264	0.731064684
HR	0.890593638	0.891316528	0.944871472	0.99999488	0.981812717	0.744164643
POFUT2	0.890593638	0.87010028	0.751985247	0.99999488	0.999985264	0.751815267
RSPO2	0.890593638	0.999992444	0.716305079	0.99999488	0.937177132	0.753212529
ATP6V1C1	0.890593638	0.68812008	0.84018083	0.99999488	0.978076141	0.754852502
TAGLN2	0.890593638	0.918911961	0.718226122	0.99999488	0.999985264	0.754852502
DACH1	0.890593638	0.755456121	0.746258329	0.952793359	0.999985264	0.763713143
USP34	0.890593638	0.768934833	0.693776732	0.959611215	0.999985264	0.763713143
FAM114A2	0.890593638	0.883856603	0.77618424	0.99999488	0.999985264	0.7655306
DYNLT3	0.890593638	0.999992444	0.771154197	0.99999488	0.942404196	0.767595486
TRPV1	0.890593638	0.745276769	0.734356561	0.99999488	0.999985264	0.772431293
RALBP1	0.890593638	0.778758964	0.940815039	0.99999488	0.999985264	0.773689802
PRMT5	0.890593638	0.707737892	0.746258329	0.989312306	0.999985264	0.777136982
CLPTM1L	0.890593638	0.809518247	0.724584302	0.99999488	0.999985264	0.784370223
TBC1D24	0.890593638	0.943802122	0.88153193	0.99999488	0.934591321	0.790569257
FKBP9	0.890593638	0.869999083	0.691847112	0.978440951	0.999985264	0.793763761
LOC100335608	0.890593638	0.801501688	0.834173166	0.99999488	0.999985264	0.809513929
NUBP2	0.890593638	0.901205367	0.70108275	0.98846246	0.999985264	0.819073042
SLC6A17	0.890593638	0.974812121	0.807608869	0.99999488	0.934018648	0.820927993
GORASP2	0.890593638	0.721740903	0.881942256	0.99999488	0.999985264	0.828248574
RMND5A	0.890593638	0.867755878	0.746258329	0.998140932	0.999985264	0.83405895
ZSCAN21	0.890593638	0.779783885	0.946479014	0.984913325	0.999985264	0.839734604
PALM3	0.890593638	0.887644042	0.875030741	0.99999488	0.961025202	0.850842307
CH25H	0.890593638	0.982289317	0.825497047	0.938185243	0.999985264	0.863192816
IMMP2L	0.890593638	0.766169816	0.830186385	0.99999488	0.999985264	0.865911659

CNN1	0.890593638	0.993316598	0.812450514	0.952783349	0.934591321	0.871960581
SLC43A2	0.890593638	0.821194038	0.812450514	0.960794858	0.999985264	0.897455868
LOC112446002	0.890593638	0.693980599	0.70108275	0.99999488	0.999985264	0.910018813
DHDDS	0.890593638	0.827700623	0.7547647	0.98846246	0.999985264	0.920531105
CIART	0.890593638	0.769022935	0.780954783	0.99999488	0.999985264	0.923360345
CNP	0.890593638	0.92462339	0.731527745	0.99999488	0.983747207	0.931163114
CSTB	0.890593638	0.768079117	0.76260462	0.99999488	0.944638139	0.955374205
BCNT2	0.89060107	0.961479426	0.788870461	0.968653855	0.999985264	0.772769165
KIAA1143	0.890765025	0.835868428	0.809486619	0.99999488	0.999985264	0.772908324
RFC5	0.890797771	0.998131745	0.799381787	0.99999488	0.967727289	0.767595486
ADRA2A	0.890850684	0.76933827	0.851718619	0.99999488	0.999985264	0.873463288
TET3	0.891002364	0.747155043	0.787426502	0.99999488	0.999985264	0.868256799
PACRGL	0.891002364	0.71953582	0.771154197	0.989312306	0.999985264	0.996066257
APAF1	0.891308437	0.727539585	0.721033378	0.976631804	0.999985264	0.721134875
SPRYD7	0.891308437	0.73942687	0.938905004	0.99999488	0.999985264	0.748495031
MYO5C	0.891389942	0.751813286	0.775962349	0.99999488	0.937177132	0.987454496
ATAD2B	0.891568091	0.792068341	0.724584302	0.99999488	0.999985264	0.692319327
VAT1L	0.891568091	0.89880506	0.754096594	0.99999488	0.999985264	0.692319327
LUC7L3	0.891568091	0.94095416	0.801163935	0.99999488	0.999985264	0.697024096
ALDH1B1	0.891568091	0.724993944	0.946479014	0.99579057	0.999985264	0.697878268
DDX6	0.891568091	0.703838914	0.999558179	0.979293963	0.999985264	0.702742967
CCDC146	0.891568091	0.925062074	0.809401378	0.99999488	0.999985264	0.704966434
ELOVL5	0.891568091	0.816518153	0.718312559	0.947235053	0.999985264	0.743462219
ZNF296	0.891568091	0.883856603	0.884320747	0.99999488	0.999985264	0.746239631
CTPS2	0.891568091	0.858753382	0.76260462	0.99999488	0.999985264	0.781688852
LARP4	0.891568091	0.779783885	0.841739726	0.99999488	0.999985264	0.787607112
CCL21	0.891568091	0.751282982	0.737519581	0.99999488	0.999985264	0.794068
CDNF	0.891568091	0.83698359	0.824988148	0.99999488	0.999985264	0.798878799
SETD7	0.891568091	0.703838914	0.807323012	0.99999488	0.941510628	0.812725657
LOC789867	0.891568091	0.783973824	0.70071733	0.99999488	0.999985264	0.815564853
HPN	0.891568091	0.84979258	0.771154197	0.991596938	0.999985264	0.818895181
LOC100847700	0.891568091	0.835868428	0.776282112	0.963012661	0.999985264	0.819073042
ALPK1	0.891568091	0.72165336	0.881040986	0.99999488	0.999985264	0.820936624
NOP10	0.891568091	0.778758964	0.787426502	0.998140932	0.999985264	0.828248574
OBSCN	0.891568091	0.885718559	0.841957362	0.949602784	0.999985264	0.82889587
PNISR	0.891568091	0.88167071	0.825497047	0.99999488	0.999985264	0.845804607
TMEM181	0.891568091	0.769482402	0.716039143	0.99999488	0.999985264	0.861670395
CHMP2A	0.891568091	0.832584599	0.76260462	0.99999488	0.999985264	0.862046449
RUNX1	0.891568091	0.7302004	0.922431991	0.99999488	0.999985264	0.873463288

CBR3	0.891568091	0.864945604	0.732033481	0.99999488	0.999985264	0.885698639
KNOP1	0.891568091	0.775336858	0.926552506	0.989312306	0.999985264	0.885874544
NLRX1	0.891568091	0.836065971	0.70108275	0.977880066	0.999985264	0.917028736
MPP7	0.891568091	0.938476452	0.759038925	0.99999488	0.935138282	0.927969856
RAB31	0.891568091	0.816518153	0.831239215	0.978440951	0.999985264	0.933698703
LHFPL4	0.891568091	0.867755878	0.771714229	0.951237479	0.999985264	0.952948574
TREML2	0.891568091	0.768934833	0.785248842	0.99999488	0.983747207	0.955374205
DEFB13	0.891568091	0.72165336	0.726885375	0.967551965	0.999985264	0.958083698
LOC515169	0.891568091	0.779783885	0.746258329	0.99999488	0.957776687	0.964476713
LOC107132469	0.891568091	0.909577824	0.785173725	0.949602784	0.974716308	0.967991416
ZNF484	0.891568091	0.72165336	0.707485652	0.948570368	0.999985264	0.969801844
CCDC190	0.891568091	0.90116492	0.774363763	0.948570368	0.983747207	0.977185271
APEX1	0.891568091	0.766942526	0.722255965	0.992168706	0.999985264	0.982568113
SELPLG	0.891806697	0.839149526	0.75028318	0.99999488	0.999985264	0.751788817
SPON2	0.891806697	0.999992444	0.734356561	0.99999488	0.937177132	0.758510984
PRKCZ	0.891806697	0.938597964	0.738225468	0.99999488	0.961025202	0.772431293
TCN1	0.891900547	0.978089982	0.908181052	0.99999488	0.996075618	0.69311359
DIRC2	0.891900547	0.694331004	0.771154197	0.99999488	0.999985264	0.722837886
CLN3	0.891900547	0.864945604	0.921010865	0.994265515	0.999985264	0.781688852
ARL14EPL	0.891900547	0.877795869	0.809486619	0.978440951	0.999985264	0.861618934
ETF1	0.891900547	0.745276769	0.856079665	0.99999488	0.999985264	0.901809636
THPO	0.892132139	0.821132416	0.910748743	0.991596938	0.999985264	0.769515026
IDO1	0.892132139	0.694996311	0.770213049	0.99999488	0.999985264	0.818895181
PIGM	0.892132139	0.995532499	0.698896024	0.981069985	0.999985264	0.873463288
MRPL21	0.892549359	0.745276769	0.778565768	0.989312306	0.999985264	0.738324902
SETDB2	0.892659921	0.885718559	0.724584302	0.99999488	0.959329174	0.693494816
SP110	0.892659921	0.749605793	0.851718619	0.99999488	0.999985264	0.818895181
MRPS18B	0.892659921	0.723805811	0.788097144	0.98846246	0.999985264	0.82386188
SH3GL3	0.892659921	0.936273761	0.875823795	0.994265515	0.999985264	0.828248574
HNRNPH3	0.892659921	0.706611337	0.72628476	0.99999488	0.999985264	0.829811046
ATP5MC1	0.893903289	0.703838914	0.826801319	0.948570368	0.999985264	0.731312503
CNTNAP2	0.893903289	0.753515888	0.830456514	0.99999488	0.999985264	0.746239631
SECISBP2	0.893903289	0.78821881	0.746258329	0.989312306	0.999985264	0.799216519
LMO3	0.894254857	0.87010028	0.785248842	0.977362925	0.999985264	0.95096765
LAMB2	0.894254857	0.705186914	0.778565768	0.99999488	0.981860698	0.977753483
GANC	0.89435747	0.847051507	0.726885375	0.99999488	0.999985264	0.868103112
TTC9C	0.89435747	0.813113924	0.747074913	0.951237479	0.999985264	0.918400465
HARBI1	0.894382906	0.754509129	0.756568208	0.989312306	0.999985264	0.820927993
EPRS	0.894382906	0.727209548	0.75028318	0.99999488	0.999985264	0.999960414

LOC101905099	0.894435687	0.69663723	0.993585211	0.959611215	0.944638139	0.918417922
CCDC15	0.894466223	0.858808025	0.841739726	0.99999488	0.999985264	0.691757815
NCOA7	0.894466223	0.703838914	0.886962419	0.949602784	0.999985264	0.743212015
LOC112445044	0.894466223	0.720253692	0.776282112	0.99999488	0.999985264	0.812825712
CENPI	0.894899109	0.905019423	0.76260462	0.99999488	0.999985264	0.929458454
WWOX	0.894932259	0.862787631	0.730602626	0.99999488	0.946408931	0.746239631
TFCP2	0.894932259	0.727390627	0.771154197	0.99999488	0.999985264	0.814496203
LOC112444479	0.894932259	0.753515888	0.724584302	0.99999488	0.999985264	0.934749052
LOC514189	0.894932259	0.755900817	0.718295892	0.99999488	0.999985264	0.977753483
IL1RAP	0.894981166	0.826642757	0.714371099	0.959611215	0.999985264	0.691757815
DYRK3	0.894981166	0.938597964	0.921010865	0.963012661	0.999985264	0.720372714
AEBP1	0.894981166	0.81184075	0.738454814	0.99999488	0.999985264	0.726340954
CWF19L1	0.894981166	0.969534305	0.872364252	0.99999488	0.999985264	0.729240869
TBC1D23	0.894981166	0.993316598	0.771096787	0.99999488	0.999985264	0.738324902
STAC	0.894981166	0.727390627	0.809278649	0.99999488	0.999985264	0.743462219
GNPDA1	0.894981166	0.821194038	0.875823795	0.99999488	0.965227764	0.750879856
VCPKMT	0.894981166	0.885718559	0.938911935	0.98846246	0.999985264	0.754852502
LOC100297399	0.894981166	0.88981187	0.715414989	0.99999488	0.999985264	0.763570777
GXYLT2	0.894981166	0.745276769	0.804991269	0.99999488	0.999985264	0.772431293
MAFK	0.894981166	0.822377918	0.843587792	0.99999488	0.999985264	0.779316087
SLC18A2	0.894981166	0.852472905	0.933046493	0.942391899	0.999985264	0.812831419
ARHGAP23	0.894981166	0.736701713	0.72274579	0.99999488	0.999985264	0.812831419
CDT1	0.894981166	0.729820154	0.748903111	0.99999488	0.999985264	0.814658726
PRPF40A	0.894981166	0.87010028	0.834068744	0.949602784	0.999985264	0.818484413
RAB5IF	0.894981166	0.778297572	0.787862242	0.99999488	0.999985264	0.870708914
PAPPA2	0.894981166	0.745276769	0.946479014	0.997623111	0.999985264	0.873463288
CLIC5	0.894981166	0.825265917	0.873092146	0.99999488	0.999985264	0.902893979
C17H12orf43	0.894981166	0.816518153	0.759038925	0.977880066	0.999985264	0.958984414
CKMT2	0.894981166	0.851001595	0.706110318	0.948516678	0.946408931	0.999960414
TLR10	0.89514792	0.816518153	0.731527745	0.99999488	0.999985264	0.703356006
GPRC5C	0.89514792	0.836126124	0.746258329	0.982713285	0.999985264	0.928814001
NRAS	0.895154941	0.732530744	0.934959203	0.99999488	0.999985264	0.69311359
ANKRD22	0.895417863	0.732530744	0.920629231	0.99999488	0.944638139	0.878070222
DENND6B	0.895525471	0.865926615	0.916975216	0.985023854	0.999985264	0.74674547
LOC112445888	0.895525471	0.81978796	0.875823795	0.99999488	0.999985264	0.763570777
HEXIM1	0.895525471	0.699854969	0.862345036	0.99999488	0.999985264	0.765330803
LOC515697	0.895525471	0.893642384	0.746258329	0.946427018	0.999985264	0.812831419
LOC100848598	0.895525471	0.862982	0.708177705	0.99999488	0.999985264	0.925260365
HACE1	0.895525471	0.87010028	0.834068744	0.957896521	0.999985264	0.944906868

LOC104974460	0.895525471	0.893642384	0.812450514	0.952783349	0.967727289	0.973330026
NMT1	0.895525471	0.729546716	0.746258329	0.976631804	0.999985264	0.989539011
LOC101906001	0.895532366	0.89064218	0.807323012	0.98846246	0.999985264	0.719097202
GADD45A	0.895725093	0.699917998	0.860461765	0.99999488	0.999985264	0.769515026
ANKRD12	0.895725093	0.869304939	0.738487246	0.951237479	0.999985264	0.826474547
KLK8	0.895725093	0.727539585	0.893044539	0.99999488	0.981774771	0.836520183
CCDC121	0.895725093	0.991365955	0.794878847	0.978440951	0.999985264	0.844861566
CCDC86	0.895725093	0.880001923	0.716305079	0.99999488	0.999985264	0.859537053
LOC104971345	0.895725093	0.809698987	0.965065403	0.99999488	0.942132162	0.863774505
FDXACB1	0.89613069	0.744638901	0.766334745	0.99999488	0.999985264	0.905557485
LOC534913	0.896193974	0.705186914	0.848832109	0.996471643	0.999985264	0.709552096
SOS1	0.896193974	0.850095234	0.781613915	0.945038047	0.999985264	0.721134875
LIMS1	0.896193974	0.827123416	0.834068744	0.99999488	0.999985264	0.786805278
TBL2	0.896193974	0.719377488	0.785248842	0.99999488	0.999985264	0.812831419
INCENP	0.896334871	0.716267524	0.985966356	0.99999488	0.999985264	0.786805278
LOC617785	0.896450177	0.859484941	0.713543621	0.99999488	0.992722375	0.758510984
LAS1L	0.896450177	0.949285285	0.751181541	0.99999488	0.999985264	0.758510984
BMP2	0.896593815	0.829688455	0.926552506	0.99999488	0.999985264	0.703584472
PPAN	0.896640052	0.815852027	0.707385221	0.998140932	0.999985264	0.978682689
ATG16L1	0.896824611	0.755100312	0.831723974	0.99999488	0.999985264	0.712249777
FGL1	0.896824611	0.824297013	0.746258329	0.99999488	0.999985264	0.919758204
LOC104973739	0.896953225	0.714123414	0.860189237	0.99999488	0.999985264	0.939035437
MYCN	0.897108541	0.757483795	0.857263009	0.99999488	0.999985264	0.816959462
TTN	0.897216095	0.834860292	0.926552506	0.99999488	0.965672975	0.693397277
HPSE	0.897216095	0.728501112	0.835038876	0.977598122	0.999985264	0.895370805
OSER1	0.897581274	0.727377621	0.938911935	0.99999488	0.999985264	0.754215263
PTBP3	0.897581274	0.769979732	0.75772402	0.99999488	0.999985264	0.780629037
ESYT2	0.897581274	0.830519875	0.849633432	0.99999488	0.999985264	0.819941939
LOC787904	0.897581274	0.768934833	0.800518725	0.99999488	0.999985264	0.868513869
CCDC141	0.897758136	0.752475942	0.746258329	0.99999488	0.999985264	0.955374205
GLP1R	0.89784115	0.915539284	0.785248842	0.99999488	0.999985264	0.819073042
PLAC8	0.89784115	0.839553386	0.713543621	0.99999488	0.999985264	0.837470362
JUNB	0.89784115	0.741264723	0.994601277	0.949602784	0.999985264	0.865855226
WDR44	0.89784115	0.805123419	0.770213049	0.99999488	0.999985264	0.915476587
LOC615610	0.89784115	0.699986019	0.745587382	0.99999488	0.999985264	0.923360345
SIAH1	0.89784115	0.852472905	0.832801768	0.949602784	0.999985264	0.929631053
LOC786065	0.898189589	0.927997582	0.809439813	0.99999488	0.999985264	0.76926818
MAP4K2	0.89839377	0.779783885	0.94634922	0.99999488	0.961025202	0.714215429
SEPHS1	0.89839377	0.723136577	0.76260462	0.99999488	0.999985264	0.779686358

AURKB	0.89839377	0.999992444	0.718226122	0.99999488	0.957235535	0.816959462
FANCG	0.89839377	0.728501112	0.993132892	0.99999488	0.948597428	0.828248574
KIAA1841	0.89839377	0.927997582	0.728484144	0.949602784	0.998639844	0.986787448
TEX22	0.898429583	0.701167969	0.999558179	0.977598122	0.969092995	0.816959462
POP4	0.898763881	0.81928857	0.906449732	0.99999488	0.999985264	0.871960581
FAM126B	0.899160055	0.999992444	0.742557674	0.959673894	0.999985264	0.703356006
EMP1	0.899160055	0.754891312	0.85773697	0.99999488	0.963400757	0.711923053
CDK2AP2	0.899160055	0.901232581	0.794878847	0.99999488	0.999985264	0.71363761
CHST7	0.899160055	0.779783885	0.814724984	0.99999488	0.999985264	0.721134875
PFN2	0.899160055	0.836435076	0.914001994	0.982713285	0.999985264	0.728262915
LAMTOR3	0.899160055	0.835868428	0.837095307	0.99999488	0.943804729	0.738324902
LOC101906240	0.899160055	0.816518153	0.968232195	0.99999488	0.999985264	0.743800772
CDK9	0.899160055	0.748160388	0.93198623	0.99999488	0.999985264	0.751788817
TREX1	0.899160055	0.746958874	0.754096594	0.99999488	0.999985264	0.769978848
DTYMK	0.899160055	0.881001912	0.706704201	0.99999488	0.999985264	0.770633383
LOC112444585	0.899160055	0.999992444	0.834173166	0.949602784	0.97210194	0.785716654
KCNH2	0.899160055	0.943802122	0.803677695	0.99999488	0.999985264	0.806724906
DDX31	0.899160055	0.748445257	0.852670802	0.99999488	0.999985264	0.809513929
ALDH5A1	0.899160055	0.938597964	0.713178271	0.99999488	0.999985264	0.836520183
ZGRF1	0.899160055	0.832807683	0.902527616	0.99999488	0.961025202	0.858593649
TMEM200C	0.899160055	0.769626411	0.841773648	0.99999488	0.999985264	0.860624144
MRPL55	0.899160055	0.865099263	0.743915133	0.976631804	0.999985264	0.868256799
RNF122	0.899160055	0.772554806	0.849633432	0.952783349	0.999985264	0.894723749
LOC112447005	0.899160055	0.962968114	0.774363763	0.978440951	0.999985264	0.904813143
SEC61A1	0.899160055	0.874192034	0.738225468	0.99999488	0.999985264	0.939035437
ZNF593	0.899160055	0.825855255	0.789951578	0.99999488	0.999985264	0.952124699
LOC107131416	0.899160055	0.76933827	0.731527745	0.953588494	0.999985264	0.970594306
TMEM209	0.899160055	0.703838914	0.768990269	0.951237479	0.999985264	0.999960414
GAK	0.899581967	0.764469173	0.962346588	0.99999488	0.999985264	0.702742967
PCTP	0.899581967	0.755100312	0.866582678	0.99999488	0.999985264	0.705703919
LOC101904378	0.899581967	0.935742738	0.785248842	0.99999488	0.999985264	0.714356884
VANGL1	0.899581967	0.747155043	0.821546364	0.998140932	0.999985264	0.715526355
ASCC1	0.899581967	0.806647675	0.834173166	0.977880066	0.999985264	0.742551138
SLC30A5	0.899581967	0.930955919	0.853195748	0.99999488	0.999985264	0.743212015
GMIP	0.899581967	0.820161757	0.746258329	0.99999488	0.999985264	0.757140928
LOC104971162	0.899581967	0.849474629	0.946479014	0.99999488	0.999985264	0.762686295
GALK2	0.899581967	0.745276769	0.819293705	0.99999488	0.999985264	0.765580778
NDUFS8	0.899581967	0.76933827	0.76260462	0.948570368	0.999985264	0.769129136
SREK1	0.899581967	0.885024111	0.807323012	0.99999488	0.999985264	0.770633383

PLOD1	0.899581967	0.759699299	0.862345036	0.99999488	0.999985264	0.772431293
KIAA0586	0.899581967	0.992977597	0.841957362	0.9998663	0.999985264	0.773507109
SHC4	0.899581967	0.865926615	0.856449202	0.99999488	0.981774771	0.779686358
TRIB2	0.899581967	0.790159199	0.715223887	0.99999488	0.999985264	0.779686358
S100A8	0.899581967	0.752769535	0.945678616	0.984913325	0.999985264	0.796010841
BTNL9	0.899581967	0.816168906	0.881469946	0.949602784	0.999985264	0.818895181
USP21	0.899581967	0.779783885	0.920629231	0.99999488	0.981774771	0.819073042
RAB44	0.899581967	0.931488015	0.766334745	0.98846246	0.999985264	0.901809636
IMP3	0.899581967	0.79397203	0.887673	0.989312306	0.999985264	0.927969856
ROBO3	0.899581967	0.813113924	0.762340742	0.99999488	0.983070991	0.931163114
SLC7A4	0.899581967	0.816518153	0.778565768	0.99999488	0.999985264	0.95096765
MTHFD1L	0.899581967	0.869999083	0.759038925	0.989312306	0.999985264	0.976814573
DUSP19	0.89959961	0.999347049	0.735869895	0.99999488	0.999985264	0.791277572
PRDM1	0.900182918	0.719377488	0.781111723	0.99999488	0.94767184	0.999960414
SLC16A11	0.900544081	0.754509129	0.831723974	0.99999488	0.999985264	0.747408201
IFI16	0.900544081	0.744638901	0.785248842	0.99999488	0.999985264	0.753766324
SLC16A5	0.900544081	0.944868298	0.765427745	0.959611215	0.999985264	0.76926818
FMO4	0.900544081	0.935588259	0.804471775	0.99999488	0.96948473	0.807566643
H4	0.900544081	0.705186914	0.964920281	0.99999488	0.946408931	0.816226172
SGTB	0.900544081	0.796869696	0.72674783	0.978440951	0.999985264	0.863331236
P2RY10	0.900544081	0.727377621	0.801828611	0.99999488	0.999985264	0.888645453
USP28	0.900544081	0.847015513	0.851718619	0.991596938	0.999985264	0.95096765
ANLN	0.901156707	0.709904249	0.884615251	0.99999488	0.999985264	0.832622862
ARHGEF10L	0.901770715	0.836111245	0.863098782	0.99999488	0.999985264	0.697878268
DIS3	0.901770715	0.999992444	0.731544721	0.959611215	0.999985264	0.704966434
STRBP	0.901770715	0.993316598	0.738225468	0.99999488	0.999985264	0.738983095
LOC518080	0.901909735	0.748445257	0.746258329	0.99999488	0.999985264	0.738324902
LOC112447359	0.902213739	0.799180421	0.946479014	0.978440951	0.999985264	0.698422388
SYPL1	0.902213739	0.790159199	0.938203102	0.99999488	0.999985264	0.703356006
PDGFB	0.902213739	0.886820818	0.734356561	0.99999488	0.999985264	0.714356884
LOC112443526	0.902213739	0.799180421	0.945766581	0.99999488	0.999985264	0.715526355
LOC101903752	0.902213739	0.813622817	0.791717601	0.99999488	0.999985264	0.719032397
UNC79	0.902213739	0.879007958	0.877742202	0.978440951	0.999985264	0.721134875
ZNF385A	0.902213739	0.999992444	0.745717284	0.99999488	0.999985264	0.73024728
SERTAD1	0.902213739	0.978510121	0.864737879	0.99999488	0.999985264	0.73024728
LOC615559	0.902213739	0.905019423	0.807323012	0.99999488	0.998639844	0.736516695
LOC107132301	0.902213739	0.999992444	0.787862242	0.992168706	0.969703428	0.737027647
XYLT2	0.902213739	0.769482402	0.762007355	0.99999488	0.999985264	0.742551138
LOC100848941	0.902213739	0.745276769	0.770213049	0.99999488	0.999985264	0.742551138

TMX3	0.902213739	0.962377515	0.862345036	0.99999488	0.999985264	0.748495031
CACUL1	0.902213739	0.842782808	0.956565728	0.99999488	0.964631108	0.754336759
RBM48	0.902213739	0.958967142	0.724584302	0.99999488	0.999985264	0.758091231
DYNLRB2	0.902213739	0.757079226	0.908181052	0.99999488	0.969128502	0.763715224
TDRD3	0.902213739	0.873963285	0.77618424	0.99999488	0.999985264	0.764052305
BET1	0.902213739	0.999992444	0.751173926	0.959611215	0.999985264	0.769515026
FNIP1	0.902213739	0.840946688	0.77618424	0.99999488	0.999985264	0.769515026
LOC101907642	0.902213739	0.974573659	0.82336109	0.99999488	0.999985264	0.769515026
SLX4	0.902213739	0.835868428	0.954823586	0.998140932	0.999985264	0.770633383
TULP2	0.902213739	0.898905584	0.944085862	0.99999488	0.999985264	0.770724447
GFPT2	0.902213739	0.741111932	0.938203102	0.994398173	0.999985264	0.772431293
ZNF652	0.902213739	0.730725094	0.9808801	0.99999488	0.998439184	0.773507109
UNC93B1	0.902213739	0.757758865	0.714371099	0.99999488	0.999985264	0.773507109
SLC15A3	0.902213739	0.783314535	0.738977782	0.99999488	0.999985264	0.781592893
LOC112448808	0.902213739	0.958224615	0.900890709	0.99999488	0.972524588	0.795293834
LOC783497	0.902213739	0.783973824	0.965214449	0.99999488	0.999985264	0.805442659
ITGA10	0.902213739	0.937231686	0.911922552	0.977598122	0.999985264	0.806724906
KIT	0.902213739	0.825265917	0.848786751	0.989312306	0.999985264	0.809513929
TIMM21	0.902213739	0.72165336	0.733934293	0.99999488	0.999985264	0.816959462
NRXN1	0.902213739	0.783314535	0.721605112	0.99999488	0.999985264	0.819073042
ZNF383	0.902213739	0.850095234	0.939147816	0.99999488	0.999985264	0.819073042
SLC38A8	0.902213739	0.754374533	0.998936876	0.978440951	0.988998158	0.826043635
BRCA2	0.902213739	0.827123416	0.938215065	0.99999488	0.999985264	0.861618934
ARFIP1	0.902213739	0.951760302	0.79490271	0.99999488	0.999985264	0.861875794
LOC112444896	0.902213739	0.752475942	0.985966356	0.991596938	0.978076141	0.868103112
COPG1	0.902213739	0.882505302	0.715414989	0.99999488	0.999985264	0.872823435
CENPBD1	0.902213739	0.938476452	0.774363763	0.991596938	0.999985264	0.892187201
FAT3	0.902213739	0.967520941	0.773092345	0.99999488	0.964616886	0.897467714
MED12	0.902213739	0.826642757	0.76260462	0.994398173	0.999985264	0.905391122
TWF2	0.902213739	0.806657225	0.716792416	0.99999488	0.999985264	0.911089313
LOC101907369	0.902213739	0.836094224	0.738484869	0.99999488	0.999985264	0.911089313
LOC112446013	0.902213739	0.885024111	0.875823795	0.99999488	0.965227764	0.923360345
SKP2	0.902213739	0.789663062	0.817223866	0.99999488	0.999985264	0.923360345
KANSL1L	0.902213739	0.915967228	0.767682114	0.958253703	0.999985264	0.925260365
LOC112443006	0.902213739	0.955128586	0.775743447	0.991596938	0.999985264	0.925260365
BCL11A	0.902213739	0.715899004	0.746258329	0.99999488	0.999985264	0.927969856
MRPL46	0.902213739	0.813746447	0.787862242	0.984394118	0.999985264	0.931163114
FDPS	0.902213739	0.853484779	0.774363763	0.989312306	0.999985264	0.931163114
ZBTB1	0.902213739	0.813113924	0.724658338	0.99999488	0.999985264	0.938885777

STAT5A	0.902213739	0.717352763	0.743915133	0.98846246	0.999985264	0.97101811
RUNX3	0.902213739	0.755100312	0.724381672	0.99999488	0.999985264	0.977067252
TEFM	0.902213739	0.750176907	0.879253779	0.976631804	0.949679366	0.999960414
LOC101905757	0.902213739	0.773422401	0.747072631	0.978440951	0.964526819	0.999960414
ANGPTL7	0.902213739	0.741594487	0.757793835	0.99999488	0.965353772	0.999960414
GGA3	0.902326146	0.880001923	0.825497047	0.949602784	0.999985264	0.715526355
CNOT6L	0.902548082	0.855219713	0.716039143	0.989312306	0.999985264	0.733505379
EMC10	0.902548082	0.847051507	0.746258329	0.99999488	0.999985264	0.756776717
ASB13	0.902548082	0.747155043	0.76260462	0.976631804	0.999985264	0.987454496
MAP4K3	0.902806879	0.836065971	0.851718619	0.99999488	0.999985264	0.754852502
IL1RL1	0.902806879	0.76933827	0.778565768	0.989312306	0.999985264	0.816226172
DOK5	0.903326499	0.809533197	0.750974849	0.99999488	0.999985264	0.817797256
NFATC1	0.903470463	0.783973824	0.780560307	0.99999488	0.972524588	0.738324902
CAPN10	0.903470463	0.882505302	0.946479014	0.99999488	0.998639844	0.747408201
TSSK2	0.903470463	0.813113924	0.746258329	0.99999488	0.999985264	0.760189361
CIDEB	0.903470463	0.957700659	0.886192181	0.99999488	0.969092995	0.76926818
BRD9	0.903470463	0.957700659	0.731527745	0.979293963	0.999985264	0.770633383
GRPEL2	0.903470463	0.921934673	0.738189003	0.960794858	0.999985264	0.772431293
CTSH	0.903470463	0.753515888	0.849633432	0.99999488	0.995610149	0.779686358
PPP2R5E	0.903470463	0.893748757	0.902552409	0.977598122	0.999985264	0.779686358
PPME1	0.903470463	0.785276332	0.76260462	0.966629392	0.999985264	0.785716654
EXTL2	0.903470463	0.862707207	0.76260462	0.99999488	0.999985264	0.812825712
CREB5	0.903470463	0.783973824	0.907381775	0.980460211	0.999985264	0.816226172
GABRE	0.903470463	0.736701713	0.987959756	0.99999488	0.999985264	0.82386188
LOC509415	0.903470463	0.827661136	0.877742202	0.99999488	0.999985264	0.831666995
MAN1C1	0.903470463	0.873289191	0.730171751	0.967551965	0.999985264	0.851533524
TMEM126A	0.903470463	0.73157551	0.778565768	0.978440951	0.999985264	0.872307149
PHF11	0.903470463	0.83961513	0.922569757	0.99999488	0.965227764	0.873463288
ADRM1	0.903470463	0.76933827	0.785177733	0.99999488	0.999985264	0.874975722
TERC	0.903470463	0.718036056	0.825860584	0.951237479	0.999985264	0.882867473
PRR15L	0.903470463	0.927147798	0.845368791	0.976631804	0.999985264	0.919755394
FCHO1	0.903470463	0.717031803	0.863098782	0.951237479	0.999985264	0.919758204
GALNT4	0.903470463	0.874807581	0.77618424	0.99999488	0.999985264	0.949764317
LOC524650	0.904181755	0.999992444	0.735595656	0.966508559	0.999985264	0.832833221
CDK15	0.904349336	0.764469173	0.999558179	0.95312502	0.999985264	0.780941577
ZPR1	0.904606379	0.795127474	0.815067929	0.99999488	0.999985264	0.787607112
CAPS2	0.90511736	0.876273882	0.743915133	0.99999488	0.999985264	0.704802849
SMIM10	0.90511736	0.820100152	0.884320747	0.991596938	0.999985264	0.738324902
SAPCD1	0.905207749	0.747155043	0.993594894	0.957896521	0.999985264	0.844292822

NTF3	0.905422415	0.871390547	0.76260462	0.99999488	0.961025202	0.856510591
LOC104975593	0.905738115	0.893642384	0.775693389	0.99999488	0.999985264	0.758091231
NCK1	0.905904533	0.941495217	0.745215355	0.99999488	0.999985264	0.769515026
LOC100849587	0.906650545	0.715899004	0.999558179	0.966629392	0.999985264	0.751815267
SERGEF	0.90683849	0.864154704	0.81983962	0.98846246	0.999985264	0.736516695
LSG1	0.90683849	0.877142156	0.734011225	0.99999488	0.999985264	0.751815267
RXYLT1	0.90683849	0.773422401	0.888700204	0.99999488	0.999985264	0.757280697
GLO1	0.90683849	0.91000013	0.76260462	0.99999488	0.999985264	0.788866553
LOC786726	0.90683849	0.751959025	0.917559681	0.99999488	0.998639844	0.841424601
PGAM5	0.90683849	0.752475942	0.759038925	0.99999488	0.999985264	0.84991826
CDIPT	0.90683849	0.850364054	0.775526567	0.985023854	0.999985264	0.927969856
ZMPSTE24	0.90683849	0.845554546	0.920629231	0.99999488	0.961025202	0.932608891
TMSB15B	0.90683849	0.917849199	0.751173926	0.99999488	0.981774771	0.972380059
IL17D	0.907198092	0.958967142	0.944932814	0.951237479	0.981774771	0.816349753
SPAG8	0.907713073	0.863793573	0.841957362	0.99999488	0.999985264	0.743462219
RECQL	0.907739746	0.729820154	0.930678591	0.99999488	0.999985264	0.722837886
NARFL	0.907739746	0.762086693	0.898125038	0.98846246	0.999985264	0.738324902
LOC112443012	0.907739746	0.999992444	0.81983962	0.961309253	0.999985264	0.751179678
BCL2L1	0.907739746	0.850692013	0.857021454	0.99999488	0.999985264	0.772479356
STEAP3	0.907739746	0.754509129	0.726885375	0.99999488	0.999985264	0.806933882
LOC787554	0.907739746	0.999992444	0.755816517	0.978629275	0.983747207	0.812831419
C11H2orf50	0.907739746	0.821033198	0.930678591	0.99999488	0.964631108	0.832833221
DRAP1	0.907739746	0.864811968	0.805753285	0.996471643	0.999985264	0.949764317
CEP57	0.907739746	0.910384774	0.731527745	0.959611215	0.999985264	0.962718528
RAP1GAP2	0.907769504	0.935588259	0.833747891	0.99999488	0.983747207	0.721134875
ESPNL	0.907769504	0.799180421	0.962653182	0.99999488	0.999985264	0.721134875
HELB	0.907769504	0.83989094	0.733964676	0.99999488	0.965672975	0.752017243
MDGA1	0.907769504	0.799180421	0.778565768	0.99999488	0.999985264	0.809513929
RNPC3	0.907769504	0.999992444	0.785248842	0.952783349	0.985745965	0.897170807
TOP3B	0.907930539	0.845554546	0.951822586	0.999114211	0.999985264	0.729827809
C5H12orf71	0.907930539	0.833511194	0.726885375	0.99999488	0.999985264	0.737027647
ZSWIM4	0.907930539	0.750176907	0.811233159	0.99999488	0.999985264	0.738324902
ANAPC10	0.907930539	0.744558551	0.962346588	0.959611215	0.999985264	0.743798578
UBE2D3	0.907930539	0.919209432	0.882380454	0.99999488	0.999985264	0.754852502
HIF1AN	0.907930539	0.783314535	0.787862242	0.99999488	0.999985264	0.763713143
ENY2	0.907930539	0.83698359	0.826965593	0.99999488	0.999985264	0.765962135
CRLS1	0.907930539	0.991488652	0.87398841	0.98846246	0.999985264	0.767595486
ARHGAP28	0.907930539	0.723870388	0.80757077	0.99999488	0.999985264	0.76762685
OSBPL7	0.907930539	0.865926615	0.954823586	0.989312306	0.999985264	0.771539286

FBLN5	0.907930539	0.993777777	0.750974849	0.99999488	0.999985264	0.772431293
SNAPC5	0.907930539	0.745276769	0.738911558	0.99999488	0.999985264	0.773689802
FBXO16	0.907930539	0.732009317	0.781111723	0.99999488	0.999985264	0.773689802
MANBAP1	0.907930539	0.727539585	0.898815524	0.99999488	0.999985264	0.822863905
USP54	0.907930539	0.867755878	0.83396257	0.99999488	0.999985264	0.827796589
LOC788334	0.907930539	0.992977597	0.72136241	0.99999488	0.999985264	0.834119413
CTSA	0.907930539	0.835868428	0.776401204	0.99999488	0.999985264	0.836520183
IQCB1	0.907930539	0.967463091	0.746258329	0.951237479	0.999985264	0.840513775
UEVLD	0.907930539	0.783973824	0.873452279	0.99999488	0.999985264	0.844277572
ADM2	0.907930539	0.779783885	0.923604668	0.99999488	0.999985264	0.845594867
MPND	0.907930539	0.869304939	0.841739726	0.978440951	0.999985264	0.845834943
TOR1A	0.907930539	0.936273761	0.803942311	0.959611215	0.999985264	0.872307149
PRPF40B	0.907930539	0.819127076	0.864441458	0.99999488	0.998639844	0.874975722
MED27	0.907930539	0.796869696	0.778565768	0.99999488	0.999985264	0.882867473
LOC101902869	0.907930539	0.970426668	0.84160836	0.994265515	0.983747207	0.894420979
LOC112443226	0.907930539	0.729000253	0.841739726	0.99999488	0.999985264	0.917028736
LOC100140403	0.907930539	0.747155043	0.759038925	0.99999488	0.999985264	0.922982646
MAGI3	0.907930539	0.741594487	0.795046332	0.989312306	0.999985264	0.939035437
NAPSA	0.907930539	0.778579649	0.803942311	0.99999488	0.999985264	0.952093196
AGRN	0.907930539	0.839292361	0.811233159	0.978026405	0.999985264	0.977569033
MS4A1	0.907930539	0.90024974	0.726885375	0.99999488	0.961025202	0.987457361
LBP	0.907930539	0.720651109	0.881942256	0.978440951	0.999985264	0.992727599
SLC25A5	0.907930539	0.84060143	0.788870461	0.99999488	0.982217275	0.995470031
KPNA2	0.907930539	0.775729202	0.776282112	0.99999488	0.999985264	0.999960414
SMIM12	0.908138957	0.816518153	0.862131495	0.997217712	0.999985264	0.743462219
KIF15	0.908138957	0.746079539	0.923089792	0.99999488	0.999985264	0.789800327
ZCCHC18	0.908138957	0.999347049	0.845368791	0.976631804	0.999985264	0.80980964
GABBR1	0.908138957	0.813113924	0.917410616	0.989312306	0.963400757	0.965920258
CYTH2	0.908138957	0.751282982	0.76260462	0.952783349	0.999985264	0.999960414
LOC107131330	0.908292345	0.729364371	0.871925227	0.99999488	0.999985264	0.816226172
RNF40	0.908292345	0.834860292	0.807323012	0.99999488	0.999985264	0.858593649
MYH15	0.908456935	0.985406717	0.785248842	0.99999488	0.998439184	0.836520183
SOWAHD	0.909413053	0.816518153	0.729189568	0.99999488	0.999985264	0.773507109
LOC511936	0.909413053	0.727539585	0.944085862	0.99999488	0.999985264	0.868513869
SPIRE2	0.909581119	0.848070173	0.774363763	0.99999488	0.999985264	0.84652292
EPHB6	0.909581119	0.783973824	0.77618424	0.99999488	0.999985264	0.999960414
SEC31A	0.909720538	0.744638901	0.774363763	0.99999488	0.999985264	0.812831419
SSR4	0.909720538	0.792003681	0.873092146	0.99999488	0.999985264	0.872364241
FIBP	0.909720538	0.786382788	0.778565768	0.978440951	0.999985264	0.969837958

ZFP90	0.909828369	0.932593501	0.745215355	0.991596938	0.999985264	0.779686358
ELK1	0.910259088	0.89875294	0.857021454	0.952783349	0.999985264	0.721134875
ZFYVE21	0.910259088	0.759500463	0.734356561	0.99999488	0.999985264	0.74498257
ZNF502	0.910259088	0.832584599	0.76260462	0.978440951	0.999985264	0.750879856
TP53BP1	0.910259088	0.794965819	0.773321573	0.99999488	0.999985264	0.779686358
KLHL13	0.910259088	0.740431054	0.791938217	0.99999488	0.999985264	0.82386188
NCOA2	0.910259088	0.795255481	0.733017121	0.99999488	0.999985264	0.885874544
MGST2	0.910350756	0.748160388	0.746258329	0.99999488	0.999985264	0.72144082
GNPTAB	0.910350756	0.728501112	0.791800799	0.99999488	0.999985264	0.754429032
LOC112447857	0.910350756	0.745276769	0.825497047	0.99999488	0.999985264	0.765580778
TMED7	0.910350756	0.767055852	0.841957362	0.99999488	0.999985264	0.89935427
TBXAS1	0.91049814	0.729000253	0.851718619	0.99999488	0.999985264	0.718350328
HMGXB3	0.91049814	0.862982	0.945678616	0.978440951	0.999985264	0.718906784
LOC107131715	0.91049814	0.754891312	0.935094475	0.99999488	0.999985264	0.738193315
RHOT2	0.91049814	0.949285285	0.916214824	0.963012661	0.999985264	0.738765733
CATSPERG	0.91049814	0.835868428	0.89038419	0.99999488	0.999985264	0.752387159
TIMM17B	0.91049814	0.819379885	0.775008152	0.969770561	0.999985264	0.768603857
FDX2	0.91049814	0.90465634	0.803677695	0.98846246	0.999985264	0.807566643
TIGD4	0.91049814	0.852963351	0.887626998	0.99999488	0.991867802	0.818895181
INPP5B	0.91049814	0.727539585	0.865420641	0.99999488	0.999985264	0.836520183
HEMK1	0.91049814	0.985406717	0.8664043	0.978440951	0.998298811	0.873463288
MRPL1	0.91049814	0.894140115	0.746854945	0.96412514	0.999985264	0.987454496
ELP6	0.910689803	0.742976874	0.726258339	0.99999488	0.999985264	0.885874544
TBX1	0.911185991	0.89942898	0.785177733	0.99999488	0.964631108	0.732351583
CEP350	0.911185991	0.766942526	0.724381672	0.977598122	0.999985264	0.738324902
APLP2	0.911185991	0.867755878	0.747072631	0.989312306	0.999985264	0.769113651
POLR2L	0.911185991	0.901232581	0.742557674	0.991596938	0.999985264	0.770724447
VANGL2	0.911185991	0.779783885	0.822003523	0.99999488	0.999985264	0.772479356
B3GNT7	0.911185991	0.850692013	0.746258329	0.99999488	0.999985264	0.787607112
MDK	0.911185991	0.9582784	0.825497047	0.99999488	0.999985264	0.79506749
MTMR14	0.911185991	0.76933827	0.738225468	0.99999488	0.999985264	0.806830222
GREB1L	0.911185991	0.864058283	0.858024919	0.956466388	0.999985264	0.888645453
ZNF516	0.911185991	0.816518153	0.825497047	0.961309253	0.999985264	0.900681286
CEP55	0.911209881	0.845554546	0.787862242	0.99999488	0.999985264	0.772431293
LOC539893	0.911857297	0.819937634	0.927022752	0.99999488	0.999985264	0.893392612
MANEAL	0.911936082	0.934142668	0.853055742	0.99999488	0.999985264	0.806724906
HCK	0.912160871	0.730725094	0.746258329	0.99999488	0.999985264	0.804787661
C23H6orf201	0.912240224	0.999992444	0.748903111	0.99999488	0.972524588	0.728036947
KDELR3	0.912240224	0.819937634	0.866445809	0.99999488	0.999985264	0.74308994

MARCH6	0.912240224	0.867755878	0.884320747	0.994265515	0.999985264	0.757395858
GMFG	0.912240224	0.816518153	0.726885375	0.99999488	0.999985264	0.772479356
NUMBL	0.912240224	0.766942526	0.861171188	0.982713285	0.999985264	0.847186604
CARMIL2	0.912240224	0.729546716	0.950948716	0.96412514	0.999985264	0.857135464
ST6GAL1	0.912240224	0.742976874	0.774363763	0.99999488	0.999985264	0.869877865
INSIG2	0.912240224	0.992977597	0.841773648	0.98846246	0.969092995	0.874975722
UNC13B	0.912240224	0.884739596	0.859316601	0.985023854	0.999985264	0.883999232
IRS2	0.912240224	0.813113924	0.94634922	0.99999488	0.999985264	0.888645453
GCSAML	0.912240224	0.729546716	0.815890139	0.99999488	0.999985264	0.894723749
RBPMS	0.912240224	0.813746447	0.844490512	0.99999488	0.999985264	0.912499952
CTCFL	0.912240224	0.884739596	0.778565768	0.99999488	0.999985264	0.925260365
FAT2	0.912240224	0.755247448	0.865066354	0.99999488	0.999985264	0.935349027
BRI3	0.912429049	0.827166566	0.841739726	0.99999488	0.999985264	0.735739204
LOC529196	0.912429049	0.822970473	0.806978356	0.99999488	0.999985264	0.738324902
RBBP5	0.912467715	0.816518153	0.958627535	0.99999488	0.999985264	0.743189002
LOC112442262	0.9133985	0.806647675	0.999558179	0.99999488	0.965672975	0.780941577
RCHY1	0.9133985	0.978510121	0.82413265	0.99999488	0.999985264	0.812870286
LOC112446456	0.913480125	0.943802122	0.95106368	0.99999488	0.967727289	0.760189361
FAAH	0.913480125	0.74820894	0.968232195	0.963012661	0.999985264	0.795293834
MTG1	0.913639073	0.816518153	0.872147381	0.99999488	0.999985264	0.772431293
EXOC3	0.913712806	0.837990885	0.746258329	0.99999488	0.999985264	0.773689802
ST13	0.914044568	0.737073459	0.793478166	0.99999488	0.999985264	0.734431285
LOC101903564	0.914044568	0.991488652	0.928642875	0.991596938	0.973714462	0.758510984
TSPYL6	0.914044568	0.850274237	0.726885375	0.978440951	0.999985264	0.758510984
CNKSR3	0.914044568	0.9527938	0.785429296	0.976631804	0.999985264	0.809513929
ELP4	0.914044568	0.821194038	0.726885375	0.99999488	0.999985264	0.935160451
ACSL1	0.914044568	0.755100312	0.776401204	0.953917057	0.999985264	0.982250307
HMGB2	0.914084304	0.789729989	0.752735548	0.99999488	0.999985264	0.746239631
ENPP5	0.914084304	0.754374533	0.77602067	0.99999488	0.999985264	0.809513929
UBAP2	0.914084304	0.729000253	0.734107387	0.99999488	0.999985264	0.812831419
BRF2	0.914084304	0.873289191	0.891893039	0.994398173	0.999985264	0.816226172
RPS4Y1	0.914084304	0.801588323	0.875823795	0.99999488	0.999985264	0.954519353
FAM98A	0.914084304	0.815700306	0.788870461	0.99999488	0.999985264	0.967874696
LOC513580	0.914084304	0.768079117	0.877786216	0.99999488	0.999985264	0.97025409
KCTD3	0.914084304	0.825265917	0.746258329	0.99999488	0.999985264	0.999960414
LOC112447492	0.914694834	0.999992444	0.803942311	0.982713285	0.969092995	0.743212015
ITGA4	0.914694834	0.742976874	0.785248842	0.99999488	0.999985264	0.832833221
APOBEC2	0.914734303	0.796531384	0.923953195	0.99999488	0.999985264	0.738324902
GPR68	0.914734303	0.755100312	0.949038056	0.99999488	0.981774771	0.738983095

CC2D1B	0.914734303	0.936657629	0.853055742	0.99999488	0.999985264	0.751815267
MRPL54	0.914734303	0.852760886	0.76260462	0.99999488	0.999985264	0.779686358
ZNF674	0.914734303	0.913160799	0.815035039	0.996471643	0.999985264	0.812143248
GPX1	0.914734303	0.87010028	0.803677695	0.99999488	0.999985264	0.816226172
CISD2	0.914734303	0.842852797	0.926552506	0.966054859	0.999985264	0.90109791
PPP1R12B	0.914734303	0.816100073	0.825881042	0.99999488	0.999985264	0.927969856
LOC104974669	0.914734303	0.844457578	0.739880392	0.99999488	0.983747207	0.996156089
RPL12	0.914734303	0.766621389	0.77618424	0.99999488	0.999985264	0.998331177
ZBTB22	0.915117112	0.796000666	0.861497791	0.99999488	0.999985264	0.743212015
RNASEH2C	0.915117112	0.779783885	0.82336109	0.99999488	0.999985264	0.7655306
SEMA4B	0.915117112	0.927147798	0.785248842	0.978440951	0.999985264	0.95096765
ELMOD2	0.915491485	0.999992444	0.76260462	0.992168706	0.999985264	0.738193315
ARHGEF9	0.915491485	0.734608537	0.774735207	0.99999488	0.999985264	0.750879856
IDH2	0.915491485	0.940269477	0.809486619	0.99999488	0.999985264	0.757085909
SOX7	0.915491485	0.883624935	0.757793835	0.99999488	0.999985264	0.764052305
LRRC10B	0.915491485	0.792068341	0.880309898	0.978440951	0.999985264	0.77941434
PCBP2	0.915491485	0.76933827	0.850804682	0.99999488	0.999985264	0.805869777
MGAT4A	0.915491485	0.870839685	0.857390841	0.99999488	0.999985264	0.809513929
LOC112448395	0.915491485	0.734662971	0.884119589	0.99999488	0.999985264	0.821634966
BFSP2	0.915491485	0.754374533	0.789951578	0.99999488	0.969128502	0.841443168
STAP1	0.915491485	0.88463751	0.755274116	0.99999488	0.999985264	0.846111851
GBGT1	0.915491485	0.855538599	0.743915133	0.99999488	0.999985264	0.856510591
SAR1B	0.915491485	0.754374533	0.781111723	0.99999488	0.999985264	0.8865725
ATP1A2	0.915491485	0.978510121	0.780954783	0.98846246	0.99980018	0.939657518
AP1M1	0.915491485	0.918952092	0.79490271	0.96412514	0.999985264	0.977753483
RNF214	0.915491485	0.883624935	0.746258329	0.979293963	0.999985264	0.993086089
LYSMD3	0.915646963	0.885621767	0.789951578	0.978440951	0.999985264	0.72144082
RECQL4	0.915646963	0.89880506	0.926552506	0.99999488	0.999985264	0.747408201
POLR2C	0.915646963	0.836094224	0.939478907	0.99999488	0.999985264	0.763312237
ATP5MC3	0.915646963	0.815092066	0.785248842	0.959611215	0.999985264	0.831477641
LRRN4	0.915646963	0.803699753	0.973364552	0.99999488	0.999985264	0.836520183
KLHL21	0.915646963	0.850095234	0.76260462	0.99999488	0.985177894	0.885874544
LOC787891	0.915646963	0.911418256	0.827259524	0.982713285	0.999985264	0.888645453
LOC786974	0.915646963	0.729820154	0.770213049	0.99999488	0.999985264	0.90109791
CXXC5	0.915646963	0.855341047	0.807323012	0.99999488	0.999985264	0.953908163
LOC101903604	0.915906841	0.76933827	0.998936876	0.99999488	0.978076141	0.780629037
PHETA1	0.915906841	0.984068885	0.788870461	0.99999488	0.999985264	0.812725657
TGFB2	0.915906841	0.747293429	0.744618167	0.99999488	0.999985264	0.815172397
MSI2	0.915906841	0.752475942	0.742075034	0.99999488	0.999985264	0.816226172

PRRC2C	0.915906841	0.751438848	0.746258329	0.976631804	0.999985264	0.819129695
SPRYD3	0.915974111	0.948421431	0.807323012	0.976631804	0.999985264	0.728262915
PDPK1	0.915994069	0.729820154	0.876721743	0.99999488	0.999985264	0.75331823
ATRNL1	0.915994069	0.821194038	0.923604668	0.982713285	0.999985264	0.84705359
LOC101907483	0.915994069	0.754891312	0.86259773	0.984913325	0.999985264	0.887231766
ERBB2	0.915994069	0.881001912	0.746258329	0.99999488	0.999985264	0.901614166
MVP	0.915994069	0.869999083	0.785248842	0.99999488	0.999985264	0.931163114
CFAP20	0.916065481	0.745276769	0.994626276	0.99999488	0.999985264	0.732024435
STAG2	0.916065481	0.867755878	0.770213049	0.99999488	0.999985264	0.738193315
USP6NL	0.916065481	0.869304939	0.734011225	0.98846246	0.999985264	0.738324902
RHPN1	0.916065481	0.729820154	0.825497047	0.99999488	0.999985264	0.738324902
RHOU	0.916065481	0.907069508	0.898815524	0.99999488	0.999985264	0.748495031
LOC100848484	0.916065481	0.72931078	0.774264043	0.978440951	0.999985264	0.754852502
LOC112448269	0.916065481	0.729546716	0.746258329	0.982713285	0.999985264	0.773507109
EIF3K	0.916065481	0.874192034	0.774363763	0.99999488	0.999985264	0.901809636
NOB1	0.916065481	0.836126124	0.76260462	0.985023854	0.999985264	0.939035437
CRYAB	0.916164437	0.821194038	0.781859509	0.99999488	0.999985264	0.724264917
EPYC	0.916164437	0.747293429	0.80757077	0.99999488	0.999985264	0.728262915
NPAS3	0.916164437	0.87789341	0.746258329	0.99999488	0.999985264	0.731312503
SLC35G2	0.916164437	0.836126124	0.879620211	0.99999488	0.999985264	0.736516695
CUEDC2	0.916164437	0.916036681	0.732364232	0.99999488	0.999985264	0.738324902
TMEM87A	0.916164437	0.835638623	0.861441442	0.99999488	0.999985264	0.738324902
LOC112449619	0.916164437	0.999992444	0.890248427	0.96412514	0.999985264	0.742361982
TCF15	0.916164437	0.754509129	0.770213049	0.99999488	0.999985264	0.742361982
NDUFAB1	0.916164437	0.747155043	0.788487235	0.966629392	0.999985264	0.742834762
PTPRJ	0.916164437	0.799180421	0.784501556	0.99999488	0.999985264	0.743462219
COLEC12	0.916164437	0.844174041	0.771714229	0.99999488	0.999985264	0.754215263
OST4	0.916164437	0.89695121	0.881938573	0.966629392	0.999985264	0.754852502
LOC534578	0.916164437	0.733210627	0.923604668	0.99999488	0.999985264	0.754852502
CCDC124	0.916164437	0.893642384	0.826965593	0.99999488	0.999985264	0.757395858
CXCR1	0.916164437	0.756730124	0.938203102	0.98846246	0.999985264	0.763312237
FNDC4	0.916164437	0.849474629	0.85109851	0.99999488	0.977475352	0.763570777
FILIP1	0.916164437	0.877142156	0.862345036	0.978440951	0.999985264	0.763713143
ANKS6	0.916164437	0.818854225	0.841957362	0.99999488	0.999985264	0.764052305
S1PR3	0.916164437	0.76933827	0.805870442	0.998140932	0.999985264	0.766593775
RP2	0.916164437	0.85337372	0.789951578	0.99999488	0.999985264	0.76801464
CCDC71L	0.916164437	0.985058069	0.90339136	0.99999488	0.999985264	0.769515026
NUDT19	0.916164437	0.78188084	0.822003523	0.99999488	0.999985264	0.770633383
SSFA2	0.916164437	0.806346891	0.929188473	0.99999488	0.999985264	0.772479356

UBL3	0.916164437	0.744415271	0.762007355	0.99999488	0.999985264	0.773507109
IK	0.916164437	0.794965819	0.933791093	0.99999488	0.999985264	0.773689802
CDS2	0.916164437	0.816518153	0.907484111	0.99999488	0.999985264	0.77941434
TUG1	0.916164437	0.9677397	0.882515776	0.99999488	0.999985264	0.779686358
DNAJC30	0.916164437	0.805123419	0.746258329	0.99999488	0.999985264	0.780958584
LOC781256	0.916164437	0.998131745	0.77602067	0.99999488	0.999985264	0.787263963
CAPN6	0.916164437	0.998131745	0.746167127	0.99999488	0.999985264	0.802688077
SPTLC1	0.916164437	0.742611159	0.927516734	0.99999488	0.999985264	0.802800269
LOC101907886	0.916164437	0.748308862	0.779146896	0.99999488	0.999985264	0.807689737
ANKZF1	0.916164437	0.779783885	0.956565728	0.991596938	0.999985264	0.812831419
NIN	0.916164437	0.755456121	0.763201042	0.99999488	0.999985264	0.816959462
NFKBIZ	0.916164437	0.958967142	0.890724268	0.99999488	0.981774771	0.818484413
SYNC	0.916164437	0.991488652	0.757137435	0.99999488	0.999985264	0.818895181
CRB3	0.916164437	0.938017364	0.886192181	0.99999488	0.999985264	0.818895181
DCLRE1B	0.916164437	0.736701713	0.890248427	0.99999488	0.999985264	0.818895181
TCP11	0.916164437	0.962968114	0.805753285	0.978440951	0.999985264	0.819073042
MTFR1L	0.916164437	0.755100312	0.845368791	0.993124804	0.999985264	0.820845891
CSRP2	0.916164437	0.87010028	0.902552409	0.959611215	0.999985264	0.831603744
SEC23B	0.916164437	0.916320187	0.939819448	0.984913325	0.999985264	0.83405895
ZCCHC17	0.916164437	0.893748757	0.805753285	0.99999488	0.999985264	0.838179582
STRA6	0.916164437	0.794965819	0.914001994	0.99999488	0.999985264	0.839734604
ARVCF	0.916164437	0.90116492	0.741830288	0.98846246	0.999985264	0.840513775
TINAGL1	0.916164437	0.940459751	0.885552508	0.99999488	0.999985264	0.851595584
DHX37	0.916164437	0.76933827	0.76260462	0.99999488	0.999985264	0.856224954
CD164	0.916164437	0.921759937	0.830398164	0.99999488	0.995549184	0.858593649
ARMC1	0.916164437	0.777376648	0.877742202	0.99999488	0.999985264	0.872365139
TM2D1	0.916164437	0.869999083	0.834068744	0.99999488	0.999985264	0.885874544
UBL5	0.916164437	0.830132999	0.863239204	0.99999488	0.999985264	0.885874544
SCMH1	0.916164437	0.998962836	0.759395765	0.99999488	0.999985264	0.88682376
TMEM52	0.916164437	0.813746447	0.862345036	0.99999488	0.983747207	0.888645453
ZNF185	0.916164437	0.904961534	0.88153193	0.964626155	0.999985264	0.897467714
SRP19	0.916164437	0.910133637	0.807825655	0.99999488	0.999985264	0.909412965
PDCL3	0.916164437	0.813771426	0.862345036	0.99999488	0.99980018	0.911089313
USP5	0.916164437	0.837990885	0.745939079	0.993124804	0.999985264	0.916081555
MYH7B	0.916164437	0.915367594	0.849633432	0.989312306	0.999985264	0.925260365
GIMAP5	0.916164437	0.816518153	0.751103874	0.99999488	0.999985264	0.927969856
IPO4	0.916164437	0.825265917	0.802997196	0.99999488	0.999985264	0.931163114
E2F6	0.91640285	0.964611012	0.930678591	0.99999488	0.999985264	0.737027647
LOC112447070	0.91640285	0.992977597	0.756747088	0.968653855	0.999985264	0.742551138

SCEL	0.91640285	0.85337372	0.973406453	0.988589291	0.999985264	0.754852502
ADCY6	0.91640285	0.755100312	0.759038925	0.99999488	0.999985264	0.754852502
AP1G2	0.91640285	0.873289191	0.944085862	0.99999488	0.978076141	0.773689802
LOC100847410	0.91640285	0.855538599	0.770213049	0.99999488	0.999985264	0.780941577
GALNT9	0.91640285	0.865184773	0.857454688	0.974583211	0.999985264	0.784749233
VSIG10L	0.91640285	0.883856603	0.968232195	0.99999488	0.999985264	0.785308385
STK36	0.91640285	0.877795869	0.884320747	0.99999488	0.999985264	0.788831815
TOX4	0.91640285	0.808848805	0.962346588	0.978440951	0.999985264	0.811424526
TBK1	0.91640285	0.867755878	0.746258329	0.99999488	0.999985264	0.827439931
SAP18	0.91640285	0.89942898	0.856449202	0.99999488	0.999985264	0.873463288
NSFL1C	0.91640285	0.834086888	0.76260462	0.99999488	0.999985264	0.920531105
RARRES2	0.916469107	0.835868428	0.821546364	0.99999488	0.999985264	0.84517471
NAT1	0.916549954	0.904518324	0.841717609	0.99999488	0.999985264	0.802788605
TRAPPC9	0.916549954	0.807905014	0.746258329	0.99999488	0.999985264	0.985332098
ANXA7	0.91676428	0.765748122	0.981622418	0.99999488	0.999985264	0.77941434
FAIM	0.91730897	0.747155043	0.999558179	0.99999488	0.999985264	0.787783489
CD8B	0.917341891	0.991488652	0.76260462	0.99999488	0.999985264	0.772908324
PRR36	0.917341891	0.870839685	0.935094475	0.966629392	0.999985264	0.901809636
RPS6	0.917370431	0.816518153	0.805753285	0.977598122	0.999985264	0.773689802
ERBIN	0.917370431	0.744638901	0.746258329	0.977598122	0.999985264	0.873083064
ALDH3A2	0.917763255	0.826485535	0.845368791	0.99999488	0.999985264	0.793369726
SUGT1	0.917826457	0.909633468	0.822003523	0.99999488	0.999985264	0.732471021
DVL1	0.917826457	0.805123419	0.75028318	0.99999488	0.999985264	0.738324902
GBA2	0.917826457	0.76933827	0.817177032	0.99999488	0.999985264	0.742361982
LAMB1	0.917826457	0.855474738	0.862345036	0.996471643	0.999985264	0.748830616
FAM69A	0.917826457	0.911236358	0.813142592	0.999314272	0.999985264	0.762686295
LOC101905897	0.917826457	0.87010028	0.874752438	0.99999488	0.999985264	0.787783489
VTI1B	0.917826457	0.992977597	0.841773648	0.99999488	0.981011898	0.819129695
LOC107131567	0.917826457	0.987559864	0.876758498	0.978440951	0.999985264	0.856510591
NPY5R	0.917826457	0.97286093	0.833419544	0.99999488	0.999985264	0.861030464
PURG	0.917826457	0.881261173	0.76457744	0.99999488	0.999985264	0.862046449
PTMS	0.917826457	0.999992444	0.781112489	0.977598122	0.999985264	0.894420979
CGREF1	0.917844814	0.906270195	0.817700149	0.99999488	0.999985264	0.787783489
FBXW11	0.917844814	0.768815704	0.837103057	0.99999488	0.999985264	0.879798493
LOC112449565	0.917928288	0.826936949	0.999558179	0.969770561	0.995549184	0.743462219
DUSP8	0.917928288	0.826434903	0.887626998	0.981779494	0.998296838	0.992644147
CNIH1	0.918137349	0.947212907	0.951735967	0.978440951	0.999782381	0.841424601
RSAD1	0.918286231	0.742658006	0.995045982	0.99999488	0.999985264	0.742361982
LOC112447371	0.918409193	0.907069508	0.9808801	0.99999488	0.999985264	0.732471021

RTL6	0.918409193	0.862811597	0.789951578	0.99999488	0.983747207	0.734363836
HECTD1	0.918409193	0.794351181	0.833419544	0.99999488	0.999985264	0.73565119
DCAF10	0.918409193	0.938597964	0.783497666	0.970742089	0.999985264	0.738324902
KLHL3	0.918409193	0.883624935	0.787426502	0.99999488	0.999985264	0.738324902
ZBED5	0.918409193	0.890682633	0.845165598	0.99999488	0.999985264	0.738324902
LOC100336208	0.918409193	0.76933827	0.843587792	0.99999488	0.999985264	0.742551138
RNF7	0.918409193	0.845554546	0.937874396	0.99999488	0.999985264	0.757395858
SAP130	0.918409193	0.766515706	0.756747088	0.99999488	0.999985264	0.760118245
LOC782966	0.918409193	0.986637746	0.83396257	0.989312306	0.999985264	0.762686295
C13H20orf27	0.918409193	0.893642384	0.778280092	0.976631804	0.999985264	0.772479356
VOPP1	0.918409193	0.95697765	0.920629231	0.99999488	0.999985264	0.806724906
LOC104970812	0.918409193	0.835868428	0.817700149	0.967551965	0.999985264	0.827971245
HK1	0.918409193	0.936657629	0.813142592	0.99999488	0.999985264	0.83360591
WNT9B	0.918409193	0.864945604	0.778565768	0.99999488	0.999985264	0.852196216
LOC107132531	0.918409193	0.807286491	0.938203102	0.985023854	0.999985264	0.863331236
LOC786474	0.918409193	0.961479426	0.746258329	0.982713285	0.999985264	0.871746584
EIF4E2	0.918409193	0.813113924	0.927600835	0.99999488	0.999985264	0.873463288
YEATS2	0.918409193	0.752475942	0.785248842	0.99999488	0.999985264	0.900914171
DDX47	0.918409193	0.830519875	0.864257597	0.99999488	0.988876383	0.939035437
MON2	0.918409193	0.754374533	0.941118576	0.981779494	0.999985264	0.945670698
FCRL5	0.918409193	0.870839685	0.770213049	0.99999488	0.999985264	0.960294693
CNTNAP4	0.918409193	0.845554546	0.821546364	0.989312306	0.999985264	0.964333944
METTL4	0.91895705	0.991365955	0.750974849	0.99999488	0.999985264	0.734175643
SNAPC2	0.91895705	0.873289191	0.805753285	0.99999488	0.999985264	0.743462219
FGF14	0.91895705	0.860182841	0.862345036	0.99999488	0.999985264	0.757750121
GEMIN2	0.91895705	0.893093235	0.781111723	0.969770561	0.999985264	0.779686358
NINJ2	0.91895705	0.88981187	0.968325332	0.977880066	0.999985264	0.779686358
MTCP1	0.91895705	0.985406717	0.946479014	0.966629392	0.999985264	0.780958584
CEP85L	0.91895705	0.910656676	0.75028318	0.969770561	0.999985264	0.806724906
BAZ2B	0.91895705	0.816100073	0.902527616	0.99999488	0.999985264	0.809513929
KRT19	0.91895705	0.901205367	0.956565728	0.976631804	0.999985264	0.850790144
PXMP4	0.919388549	0.808922621	0.774363763	0.976631804	0.999985264	0.977753483
MRPS16	0.919662708	0.873429424	0.817678425	0.99999488	0.999985264	0.76801464
ZNF135	0.919818719	0.967276215	0.834173166	0.991596938	0.999985264	0.74498257
PHPT1	0.919956147	0.864811968	0.831723974	0.98846246	0.999985264	0.740017592
INPP4B	0.919956147	0.78130286	0.83126054	0.99999488	0.999985264	0.986408155
NCF4	0.92069743	0.84313917	0.915401612	0.99999488	0.998639844	0.842880838
TBC1D4	0.920821479	0.850095234	0.895962167	0.99999488	0.999985264	0.785117739
OSR2	0.921055374	0.79258665	0.769780734	0.99999488	0.999985264	0.841443168

GRPEL1	0.921055374	0.935060764	0.826965593	0.99999488	0.999985264	0.885874544
PHLDB2	0.921121687	0.8143226	0.771154197	0.985306489	0.999985264	0.997713313
SELENOP	0.921143424	0.745276769	0.751173926	0.99999488	0.999985264	0.758510984
CSF1	0.921143424	0.751327936	0.920154807	0.99999488	0.999985264	0.769395336
FBH1	0.921143424	0.765254186	0.922400936	0.99999488	0.999985264	0.839734604
NCAM2	0.921227317	0.896575712	0.968232195	0.989312306	0.999985264	0.772431293
PLPP1	0.921227317	0.760068124	0.841957362	0.99999488	0.999985264	0.794870105
PLK4	0.921227317	0.793373656	0.921010865	0.99999488	0.999985264	0.807689737
LOC112444841	0.921227317	0.893743321	0.939414234	0.989312306	0.999985264	0.819073042
LOC112443751	0.921227317	0.915367594	0.944085862	0.99999488	0.983747207	0.847577522
ACTR1A	0.921227317	0.88167071	0.862345036	0.99999488	0.999985264	0.885874544
HEXIM2	0.921437541	0.752475942	0.95230212	0.99999488	0.999985264	0.754992305
SAFB	0.921488152	0.753515888	0.801163935	0.99999488	0.999985264	0.828257209
SPECC1	0.921488152	0.745276769	0.907381775	0.99999488	0.999985264	0.832622862
PTPRE	0.921488152	0.864154704	0.806778505	0.99999488	0.999985264	0.972919703
PCBP1	0.921504635	0.850095234	0.857072251	0.994265515	0.999985264	0.736516695
HCRTR1	0.921549356	0.813113924	0.901414204	0.99999488	0.999985264	0.780958584
TBRG1	0.921549356	0.753515888	0.972362683	0.979293963	0.999985264	0.80980964
PHLDA2	0.921549356	0.877795869	0.826965593	0.99999488	0.999985264	0.833388946
YTHDF1	0.921549356	0.862811597	0.801163935	0.99999488	0.999985264	0.839734604
ZNF566	0.921549356	0.867755878	0.89029356	0.99999488	0.999985264	0.861875794
SRSF12	0.921549356	0.907069508	0.80757077	0.99999488	0.999985264	0.918343194
KLHDC2	0.921549356	0.972764622	0.805753285	0.974583211	0.999985264	0.923087984
LOC100848405	0.921549356	0.76933827	0.746258329	0.99999488	0.999985264	0.927969856
TMEM45A	0.92155281	0.805110753	0.785248842	0.99999488	0.999985264	0.740087063
GAS8	0.92155281	0.941495217	0.877799478	0.99999488	0.999985264	0.772479356
CTSK	0.92155281	0.827123416	0.862911439	0.99999488	0.999985264	0.856224954
TERF1	0.92178675	0.984068885	0.901758346	0.993124804	0.999985264	0.779686358
FOXO6	0.921806013	0.741594487	0.869194969	0.99999488	0.999985264	0.880673048
LOC107132697	0.921938651	0.861747495	0.770899781	0.99999488	0.983747207	0.954519353
LOC101907566	0.922851519	0.875435394	0.80757077	0.99579057	0.999985264	0.738324902
NCS1	0.923795517	0.76933827	0.877799478	0.99999488	0.999985264	0.779686358
MAFB	0.924195659	0.773422401	0.746258329	0.99999488	0.999985264	0.82889587
PRKRIP1	0.924207592	0.847686989	0.980558494	0.982713285	0.999985264	0.747408201
MGAT3	0.924207592	0.935060764	0.892943118	0.99999488	0.999985264	0.779686358
ENPP2	0.924207592	0.999992444	0.837003989	0.99999488	0.999985264	0.783345949
LOC112447756	0.924207592	0.850692013	0.863239204	0.99999488	0.999985264	0.807665096
ZNF277	0.924207592	0.862811597	0.762327091	0.99999488	0.999985264	0.839734604
LOC787851	0.924207592	0.858808025	0.923902957	0.989312306	0.999985264	0.864806954

DCAF5	0.924207592	0.769979732	0.78255891	0.99999488	0.999985264	0.931163114
BCL3	0.924229915	0.991488652	0.770899781	0.99999488	0.999985264	0.869877865
GOLGA3	0.924753052	0.771984021	0.77602067	0.99999488	0.999985264	0.738324902
KLC2	0.924753052	0.850692013	0.779432747	0.99999488	0.999985264	0.738324902
PIK3R4	0.924753052	0.741594487	0.75028318	0.99999488	0.999985264	0.738983095
NR2F2	0.924753052	0.847051507	0.770213049	0.99999488	0.999985264	0.743241781
SAG	0.924753052	0.845262032	0.76260462	0.99999488	0.999985264	0.75331823
GTPBP3	0.924753052	0.942353856	0.946479014	0.984913325	0.999985264	0.753766324
SEC63	0.924753052	0.76933827	0.92760291	0.99999488	0.999985264	0.764825559
SERINC3	0.924753052	0.761716845	0.817177032	0.99999488	0.999985264	0.773689802
MAMDC4	0.924753052	0.893748757	0.95955996	0.99999488	0.999985264	0.781063387
CCNG2	0.924753052	0.978154072	0.948420301	0.978440951	0.999985264	0.787783489
NFATC3	0.924753052	0.815645917	0.782403833	0.99999488	0.999985264	0.802688077
MTERF2	0.924753052	0.862707207	0.75921229	0.99999488	0.999985264	0.809513929
GJA5	0.924753052	0.744613859	0.955141547	0.99999488	0.999985264	0.816226172
ERGIC1	0.924753052	0.935742738	0.77455741	0.99999488	0.999985264	0.818895181
PRC1	0.924753052	0.785917734	0.944031108	0.99999488	0.999985264	0.821675213
LOC100337053	0.924753052	0.999347049	0.851718619	0.99579057	0.999985264	0.837143123
UPK3B	0.924753052	0.754891312	0.999558179	0.978440951	0.999985264	0.894144329
SERPINA5	0.924753052	0.846070848	0.923604668	0.99999488	0.999985264	0.895349622
DESI1	0.924753052	0.855341047	0.793478166	0.99999488	0.999985264	0.896481747
SNX11	0.924753052	0.819937634	0.865837237	0.982713285	0.999985264	0.927969856
CYB561D2	0.924753052	0.803699753	0.756747088	0.99999488	0.999985264	0.960294693
KIFAP3	0.924753052	0.845554546	0.796735721	0.976631804	0.999985264	0.999960414
PAGR1	0.925015057	0.866462388	0.991285432	0.99999488	0.999985264	0.818895181
AEN	0.925015057	0.826936949	0.809401378	0.99999488	0.999985264	0.844472083
REC114	0.925015057	0.951038017	0.785248842	0.99999488	0.999985264	0.856510591
PTRH2	0.925015057	0.799180421	0.806978356	0.99999488	0.999985264	0.939035437
LOC513329	0.925015057	0.754374533	0.799381787	0.99999488	0.999985264	0.964675807
FAM84A	0.92521855	0.957700659	0.857072251	0.99999488	0.999985264	0.844472083
LOC100141258	0.925259987	0.917849199	0.769499495	0.99999488	0.999985264	0.926326154
PSMB2	0.925259987	0.864945604	0.825497047	0.99999488	0.999985264	0.972825932
SNAPC4	0.926061705	0.883624935	0.973406453	0.99999488	0.999985264	0.754852502
TAF1D	0.926061705	0.984366454	0.913259951	0.99999488	0.999985264	0.760189361
MLIP	0.926061705	0.999992444	0.77455741	0.99999488	0.999985264	0.773716179
VPS37C	0.926061705	0.976819696	0.825497047	0.99999488	0.999985264	0.80980964
ITGB3BP	0.926061705	0.838314316	0.807323012	0.99999488	0.999985264	0.816226172
MPP5	0.926061705	0.795255481	0.77602067	0.99999488	0.999985264	0.827190118
LOC101903193	0.926061705	0.806647675	0.757137435	0.99999488	0.999985264	0.863280322

KCTD11	0.926061705	0.883856603	0.746258329	0.99999488	0.998439184	0.908047942
COQ8A	0.926061705	0.9582784	0.75028318	0.978440951	0.999985264	0.942975882
FOXO3	0.926061705	0.751282982	0.807323012	0.99999488	0.999985264	0.986873741
SELENOT	0.926061705	0.938597964	0.785248842	0.978440951	0.999985264	0.99382486
ZCCHC6	0.92607217	0.900370677	0.865837237	0.99999488	0.999985264	0.79506749
AK6	0.92607217	0.941495217	0.751173926	0.99999488	0.999985264	0.885874544
LOC101903126	0.92607217	0.835868428	0.813142592	0.99999488	0.999985264	0.979046621
LOC104973099	0.926077958	0.991365955	0.746258329	0.99999488	0.999985264	0.773689802
CPT1C	0.926332316	0.968062432	0.843587792	0.99999488	0.999985264	0.753766324
PPP4R2	0.926636038	0.756730124	0.76260462	0.99999488	0.999985264	0.865195569
SLC25A26	0.92690022	0.768815704	0.76260462	0.99999488	0.999985264	0.740309811
JDP2	0.92690022	0.883624935	0.779335232	0.978440951	0.999985264	0.864275069
LOC104974348	0.92690022	0.747155043	0.964582964	0.99999488	0.999985264	0.866439215
NAA15	0.92690022	0.831782537	0.864257597	0.99999488	0.999985264	0.927969856
FCGR3A	0.927090016	0.809104286	0.76260462	0.99999488	0.999985264	0.785716654
CANT1	0.927325085	0.826052922	0.916975216	0.99999488	0.999985264	0.802450101
UBE2W	0.927799541	0.948421431	0.930678591	0.99999488	0.999985264	0.748495031
LOC783033	0.927799541	0.755100312	0.862968824	0.99999488	0.999985264	0.831532663
FBXL14	0.927824429	0.965422267	0.759038925	0.99999488	0.999985264	0.780958584
MYPOP	0.927891837	0.78188084	0.833722253	0.99999488	0.999985264	0.752465035
RTL1	0.927891837	0.783973824	0.930127869	0.99999488	0.999985264	0.763570777
COX11	0.927891837	0.935274301	0.766128244	0.99999488	0.999985264	0.812831419
EIF3E	0.927891837	0.987262615	0.761533155	0.99999488	0.999985264	0.819073042
LIX1	0.927891837	0.827661136	0.785248842	0.99999488	0.999985264	0.931163114
EEF1E1	0.927891837	0.806657225	0.804211069	0.99999488	0.999985264	0.956548594
DCLK2	0.927891837	0.757483795	0.825497047	0.99999488	0.999985264	0.999960414
COQ9	0.927894371	0.747155043	0.770899781	0.99999488	0.999985264	0.75331823
ABHD13	0.927894371	0.845554546	0.910426741	0.99999488	0.999985264	0.765330803
FAM46B	0.927894371	0.929230097	0.852468219	0.99999488	0.999985264	0.769515026
ZNF768	0.927894371	0.766651965	0.837623898	0.99999488	0.999985264	0.770083907
LOC100196901	0.927894371	0.992977597	0.756747088	0.98846246	0.999985264	0.771364381
THEM6	0.927894371	0.821194038	0.751181541	0.978440951	0.999985264	0.772479356
TMEM59	0.927894371	0.862811597	0.78185687	0.99999488	0.999985264	0.775144909
GNG7	0.927894371	0.755100312	0.770899781	0.99999488	0.999985264	0.805442659
LOC112448520	0.927894371	0.998962836	0.929207633	0.982713285	0.988998158	0.81375158
REXO4	0.927894371	0.939679212	0.755152442	0.99999488	0.999985264	0.819073042
SLC46A2	0.927894371	0.836126124	0.766334745	0.99999488	0.999985264	0.839405979
PLEKHH3	0.927894371	0.873113713	0.841773648	0.99999488	0.999985264	0.875113722
RNF103	0.927894371	0.918254521	0.778565768	0.98846246	0.999985264	0.885874544

WDR97	0.927894371	0.958098081	0.862345036	0.99999488	0.999985264	0.885874544
CACHD1	0.927894371	0.894727259	0.825497047	0.989312306	0.999985264	0.897965262
SEPT6	0.927894371	0.779422163	0.843587792	0.977598122	0.999985264	0.901809636
EIF2B2	0.927894371	0.847051507	0.842063863	0.99999488	0.999985264	0.912262222
BBC3	0.927894371	0.864154704	0.831723974	0.99999488	0.999985264	0.927969856
CCR6	0.927894371	0.76933827	0.82413265	0.99999488	0.999985264	0.937880392
TRPM3	0.927894371	0.909633468	0.771154197	0.99999488	0.999985264	0.948441365
TNFRSF12A	0.927894371	0.955128586	0.799891121	0.989312306	0.999985264	0.950336578
PSMA1	0.927894371	0.77876644	0.825497047	0.99999488	0.999985264	0.955561861
NME2	0.927894371	0.911418256	0.791411212	0.98846246	0.999985264	0.976119159
TCEA2	0.928037611	0.809135999	0.825497047	0.99999488	0.999985264	0.837143123
SUGP2	0.928163157	0.873963285	0.914449935	0.99999488	0.999985264	0.770083907
WSCD1	0.928163157	0.76933827	0.968232195	0.98846246	0.999985264	0.812725657
SLC8A2	0.92836935	0.967374613	0.831723974	0.982574928	0.999985264	0.854842173
DOCK8	0.929610424	0.834860292	0.785080434	0.99999488	0.999985264	0.758510984
C1QTNF3	0.929695843	0.768639517	0.958627535	0.99999488	0.999985264	0.752465035
AGTR1	0.929695843	0.826936949	0.83396257	0.99999488	0.999985264	0.769113651
RPRD2	0.929695843	0.830857826	0.843587792	0.99999488	0.999985264	0.779686358
LRRC45	0.929695843	0.989373299	0.921065686	0.99999488	0.999985264	0.779686358
TMEM106C	0.929695843	0.859484941	0.807323012	0.99999488	0.999985264	0.949764317
ERCC6L	0.929702032	0.821194038	0.942675769	0.99999488	0.999985264	0.743462219
PPM1B	0.929702032	0.999992444	0.757137435	0.989312306	0.999985264	0.807187146
SLF2	0.929702032	0.891922985	0.748903111	0.99999488	0.999985264	0.885874544
PIK3R1	0.929702032	0.747155043	0.922147082	0.99999488	0.998639844	0.904300404
POLR3G	0.929702032	0.813746447	0.81294064	0.99999488	0.999985264	0.934109451
IRF7	0.929706381	0.795255481	0.932653752	0.99999488	0.999985264	0.819112831
LAMC2	0.929706381	0.859182763	0.86259773	0.99999488	0.999985264	0.89935427
BNC1	0.929706381	0.785562513	0.992742113	0.991596938	0.999985264	0.911997747
LIMS2	0.929937411	0.91058693	0.862345036	0.99999488	0.999985264	0.919755394
TNFSF14	0.93019418	0.754509129	0.825497047	0.99999488	0.999985264	0.772479356
IKBKE	0.93019418	0.934905044	0.851523772	0.99999488	0.999985264	0.831114788
LTBP3	0.930534065	0.883624935	0.834068744	0.99999488	0.999985264	0.747408201
THY1	0.930534065	0.752475942	0.941118576	0.99999488	0.999985264	0.754929012
LOC101908113	0.930534065	0.985406717	0.759038925	0.982713285	0.999985264	0.886185283
LIMD2	0.930534065	0.940459751	0.802997196	0.989312306	0.999985264	0.928634547
NT5DC2	0.93059834	0.999992444	0.910686358	0.989312306	0.999985264	0.770633383
TCAP	0.93059834	0.826936949	0.93198623	0.989312306	0.999985264	0.951197866
PRKAG2	0.930767983	0.985406717	0.778565768	0.99999488	0.999985264	0.894723749
CALR	0.930967259	0.985406717	0.884320747	0.99999488	0.999985264	0.764052305

ANKRD45	0.930967259	0.946274461	0.822003523	0.99999488	0.999985264	0.809494002
TMED5	0.931041727	0.753515888	0.921065686	0.99999488	0.999985264	0.795302792
SYNGAP1	0.931434333	0.92239337	0.874559186	0.99999488	0.999985264	0.746239631
DENND5B	0.931434333	0.850692013	0.826965593	0.99999488	0.999985264	0.750224633
B4GALT2	0.931434333	0.90024974	0.773525557	0.99999488	0.999985264	0.770030068
CRIM1	0.931434333	0.859484941	0.862345036	0.994398173	0.999985264	0.779686358
ANKRD55	0.931434333	0.769482402	0.879481883	0.99999488	0.999985264	0.785716654
TMEM173	0.931434333	0.752475942	0.881942256	0.99999488	0.999985264	0.788831815
LOC112445078	0.931434333	0.769482402	0.86178438	0.99999488	0.999985264	0.802375178
RIT1	0.931434333	0.873289191	0.78278372	0.996471643	0.999985264	0.802880474
TTC21A	0.931434333	0.958967142	0.954620171	0.99999488	0.999985264	0.802880474
HOXD9	0.931434333	0.880001923	0.770213049	0.99999488	0.999985264	0.804506451
PAK3	0.931434333	0.916939994	0.774363763	0.99999488	0.999985264	0.839734604
SKIL	0.931434333	0.869304939	0.95230212	0.99999488	0.999985264	0.862679714
OSBPL9	0.931434333	0.819937634	0.892943118	0.98846246	0.999985264	0.872877052
AAGAB	0.931434333	0.932925766	0.816532117	0.99999488	0.999985264	0.922708624
CHCHD4	0.931434333	0.848070173	0.851743111	0.99999488	0.999985264	0.922982646
TNFAIP8	0.931434333	0.867840435	0.817177032	0.99999488	0.999985264	0.931163114
MED17	0.931434333	0.81184075	0.84018083	0.99999488	0.999985264	0.996066257
ADAMTS6	0.931444183	0.76933827	0.952207545	0.99999488	0.999985264	0.781939845
GNB3	0.931527533	0.89064218	0.900148803	0.99999488	0.999985264	0.766909088
AMMECR1	0.931527533	0.920253543	0.821546364	0.99999488	0.999985264	0.833388946
ENTPD1	0.931874011	0.883856603	0.781111723	0.99999488	0.999985264	0.962703352
LOC101904595	0.932330734	0.816518153	0.979462526	0.99999488	0.999985264	0.821675213
EFHB	0.932506405	0.750732633	0.817700149	0.99999488	0.999985264	0.772220282
PFKFB3	0.932506405	0.839292361	0.944993804	0.99999488	0.999985264	0.921771959
GFM1	0.932506405	0.74993087	0.790350338	0.99999488	0.999985264	0.95096765
DCAKD	0.932741955	0.864861694	0.756568208	0.99999488	0.999985264	0.918400465
SZRD1	0.932911148	0.893642384	0.871474302	0.99999488	0.999985264	0.751874426
SGCD	0.932911148	0.999992444	0.770213049	0.99999488	0.999985264	0.769515026
CSPG4	0.932911148	0.928759674	0.911063423	0.99999488	0.999985264	0.772431293
KAT14	0.932911148	0.869304939	0.788487235	0.99999488	0.999985264	0.779686358
MRPL2	0.932911148	0.847051507	0.806978356	0.99999488	0.999985264	0.792680582
NREP	0.932911148	0.756648131	0.766334745	0.99999488	0.999985264	0.819275036
UNC13D	0.932911148	0.811327744	0.75773367	0.99999488	0.999985264	0.819922188
CSF1R	0.932911148	0.867755878	0.76260462	0.99999488	0.999985264	0.827439931
RASGRP4	0.932911148	0.808405248	0.800952304	0.99999488	0.999985264	0.839734604
LOC112441542	0.932911148	0.82858414	0.778542909	0.99999488	0.999985264	0.962718528
CCL14	0.933046169	0.937626884	0.825497047	0.99999488	0.999985264	0.788976673

GCSH	0.933399704	0.862707207	0.785248842	0.996471643	0.999985264	0.813678203
CSPP1	0.934044603	0.850095234	0.929213642	0.983736128	0.999985264	0.939035437
DUOX2	0.934286623	0.78130286	0.95401762	0.99999488	0.999985264	0.758177212
FUCA1	0.934286623	0.878790155	0.809486619	0.99999488	0.999985264	0.779686358
GRB10	0.934286623	0.837543897	0.871385134	0.99999488	0.999985264	0.927969856
LOC101906006	0.934647096	0.755100312	0.778565768	0.98846246	0.999985264	0.764052305
CUL4A	0.934647096	0.816578975	0.773011937	0.99999488	0.999985264	0.901210136
MPHOSPH10	0.934766214	0.769022935	0.791938217	0.99999488	0.999985264	0.873463288
SLC36A4	0.935021026	0.991488652	0.811233159	0.99999488	0.999985264	0.802788605
ZNF461	0.935484754	0.991574265	0.778369	0.99999488	0.999985264	0.754852502
ATP8B4	0.935484754	0.942911665	0.910426741	0.99999488	0.999985264	0.754852502
PLA2G2D4	0.935484754	0.850692013	0.841739726	0.99999488	0.999985264	0.760015114
IDNK	0.935484754	0.799180421	0.841957362	0.99999488	0.999985264	0.765330803
ANKLE2	0.935484754	0.815852027	0.946479014	0.99999488	0.999985264	0.769515026
CLEC2B	0.935484754	0.883856603	0.774363763	0.99999488	0.999985264	0.770633383
WNK1	0.935484754	0.821194038	0.766334745	0.99999488	0.999985264	0.772431293
PHF14	0.935484754	0.893642384	0.794324488	0.99999488	0.999985264	0.772479356
LTA4H	0.935484754	0.999992444	0.771154197	0.99999488	0.999985264	0.772769165
LOC107133276	0.935484754	0.844433938	0.95106368	0.99999488	0.999985264	0.772769165
UBR7	0.935484754	0.752475942	0.995045982	0.99999488	0.999985264	0.773689802
LOC100335635	0.935484754	0.999347049	0.803677695	0.99999488	0.999985264	0.777136982
LOC789337	0.935484754	0.954790265	0.785177733	0.989312306	0.999985264	0.779686358
CHODL	0.935484754	0.935742738	0.981736235	0.989312306	0.999985264	0.780629037
SPARC	0.935484754	0.883624935	0.925520255	0.99999488	0.999985264	0.780629037
SOCS2	0.935484754	0.921934673	0.853195748	0.978440951	0.999985264	0.780958584
CHRM1	0.935484754	0.814252344	0.932653752	0.99999488	0.999985264	0.781688852
KRR1	0.935484754	0.978426416	0.834068744	0.99999488	0.999985264	0.785716654
PPP2R2A	0.935484754	0.835868428	0.833496026	0.98846246	0.999985264	0.787783489
UQCRC1	0.935484754	0.754509129	0.804770072	0.99999488	0.999985264	0.800219234
ZBTB10	0.935484754	0.830519875	0.837103057	0.99999488	0.999985264	0.807566643
RUM1	0.935484754	0.752475942	0.913211244	0.98846246	0.999985264	0.809024657
MSI1	0.935484754	0.81978796	0.788870461	0.99999488	0.999985264	0.809513929
SGSH	0.935484754	0.865926615	0.771154197	0.99999488	0.999985264	0.813723642
DOCK4	0.935484754	0.940762397	0.881942256	0.99999488	0.999985264	0.818895181
PRMT2	0.935484754	0.752475942	0.771154197	0.99999488	0.999985264	0.827439931
SYVN1	0.935484754	0.93699217	0.762661971	0.99999488	0.999985264	0.828248574
LOC112442189	0.935484754	0.978089982	0.958290185	0.978440951	0.999985264	0.837143123
MLX	0.935484754	0.755456121	0.785248842	0.99999488	0.999985264	0.844557292
API5	0.935484754	0.826936949	0.957251472	0.99999488	0.999985264	0.856510591

IFIT5	0.935484754	0.794946953	0.762340742	0.99999488	0.999985264	0.859246266
LOC515828	0.935484754	0.869999083	0.877554488	0.99999488	0.999985264	0.870708914
TSPAN7	0.935484754	0.89942898	0.939147816	0.990802377	0.999985264	0.873463288
CDH8	0.935484754	0.87010028	0.916975216	0.99999488	0.999985264	0.873463288
ERH	0.935484754	0.769454808	0.925520255	0.99999488	0.999985264	0.89069589
HIF1A	0.935484754	0.759500463	0.817177032	0.99999488	0.999985264	0.897965262
CRISPLD1	0.935484754	0.994135588	0.803677695	0.99999488	0.999985264	0.899020749
GFAP	0.935484754	0.805123419	0.946479014	0.99999488	0.999985264	0.910018813
IFT122	0.935484754	0.771984021	0.831723974	0.99999488	0.999985264	0.911997747
TAB3	0.935484754	0.753515888	0.891893039	0.99999488	0.999985264	0.911997747
MS4A2	0.935484754	0.943802122	0.815067929	0.99999488	0.999985264	0.919755394
ZNF217	0.935484754	0.816518153	0.905611306	0.98846246	0.999985264	0.919758204
ING2	0.935484754	0.835868428	0.785248842	0.99999488	0.999985264	0.920531105
RANGRF	0.935484754	0.967276215	0.770213049	0.991596938	0.999985264	0.923360345
SUFU	0.935484754	0.831782537	0.851718619	0.99999488	0.999985264	0.925260365
SOWAHA	0.935484754	0.991476708	0.771154197	0.99999488	0.999985264	0.926673043
SESN1	0.935484754	0.81753407	0.785429296	0.99999488	0.999985264	0.926955927
LOC101903261	0.935484754	0.806647675	0.778565768	0.99999488	0.999985264	0.960613782
SSH2	0.935484754	0.76933827	0.824984963	0.991596938	0.999985264	0.967874696
LOC107132853	0.935484754	0.76933827	0.766128244	0.99999488	0.999985264	0.99382486
GARS	0.935484754	0.896400219	0.794295711	0.989312306	0.999985264	0.996066257
MRPS30	0.935484754	0.752475942	0.83396257	0.996471643	0.999985264	0.999960414
NLGN3	0.935484754	0.826642757	0.805753285	0.99999488	0.999985264	0.999960414
LRRC17	0.935547743	0.757483795	0.853055742	0.99999488	0.999985264	0.754852502
BCL6B	0.935547743	0.993316598	0.890096618	0.99999488	0.999985264	0.754852502
ACTR3B	0.935547743	0.871627189	0.830970408	0.994529297	0.999985264	0.762686295
LOC107132796	0.935547743	0.861747495	0.778565768	0.99999488	0.999985264	0.764052305
AGGF1	0.935547743	0.958967142	0.791717601	0.99999488	0.999985264	0.766519031
UPF2	0.935547743	0.877795869	0.83711556	0.982713285	0.999985264	0.768291185
IL20RA	0.935547743	0.94984369	0.890248427	0.993124804	0.999985264	0.768603857
LOC512286	0.935547743	0.778248571	0.780954783	0.99999488	0.999985264	0.769515026
ROCK2	0.935547743	0.835868428	0.851197548	0.99999488	0.999985264	0.770633383
CBLL1	0.935547743	0.999992444	0.785173725	0.98846246	0.999985264	0.772479356
SON	0.935547743	0.819937634	0.785248842	0.99999488	0.999985264	0.773689802
C28H1orf198	0.935547743	0.838314316	0.865837237	0.99999488	0.999985264	0.777136982
TMEM170A	0.935547743	0.881001912	0.841773648	0.99999488	0.999985264	0.779686358
DPH3	0.935547743	0.754509129	0.832063317	0.99999488	0.999985264	0.781688852
SLC6A4	0.935547743	0.813113924	0.848312163	0.99999488	0.999985264	0.786482582
ADARB1	0.935547743	0.754891312	0.825497047	0.99999488	0.999985264	0.79716281

DYNC1LI1	0.935547743	0.76933827	0.855132242	0.99999488	0.999985264	0.806724906
SEMA6C	0.935547743	0.782204219	0.825497047	0.99999488	0.999985264	0.812725657
FGF7	0.935547743	0.825645694	0.874485384	0.99999488	0.999985264	0.812725657
LRP3	0.935547743	0.813113924	0.785248842	0.99999488	0.999985264	0.812825712
KHDRBS3	0.935547743	0.824013041	0.807323012	0.99999488	0.999985264	0.818484413
IL9R	0.935547743	0.874192034	0.881942256	0.99999488	0.999985264	0.819073042
ENPP4	0.935547743	0.916036681	0.960133832	0.99999488	0.999985264	0.819073042
LOC112442032	0.935547743	0.871695579	0.818784725	0.99999488	0.999985264	0.851595584
EBF4	0.935547743	0.999992444	0.799381482	0.99999488	0.999985264	0.856011656
NAGA	0.935547743	0.873289191	0.76260462	0.99999488	0.999985264	0.861670395
LOC781381	0.935547743	0.760347778	0.774363763	0.98846246	0.999985264	0.868513869
CDCA7	0.935547743	0.764050973	0.825497047	0.99999488	0.999985264	0.871746584
LOC104970821	0.935547743	0.830519875	0.920629231	0.99999488	0.999985264	0.872307149
LOC112443479	0.935547743	0.94984369	0.811233159	0.99999488	0.999985264	0.873463288
FGD3	0.935547743	0.793307242	0.825497047	0.99999488	0.999985264	0.873463288
LOC112448515	0.935547743	0.999992444	0.826965593	0.978440951	0.999985264	0.884033654
SLC38A2	0.935547743	0.978510121	0.785177733	0.99999488	0.999985264	0.885874544
CYBC1	0.935547743	0.896536444	0.774363763	0.99999488	0.999985264	0.888645453
ASMTL	0.935547743	0.830519875	0.853055742	0.99999488	0.999985264	0.890433418
PWP2	0.935547743	0.873113713	0.803942311	0.99999488	0.999985264	0.892908035
SAP30BP	0.935547743	0.861747495	0.785248842	0.99999488	0.999985264	0.899223071
FBXL5	0.935547743	0.819937634	0.803709097	0.99999488	0.999985264	0.900686992
GIPC1	0.935547743	0.869999083	0.88153193	0.99999488	0.999985264	0.90109791
PDCD11	0.935547743	0.92202441	0.770213049	0.99999488	0.999985264	0.901809636
PEX5	0.935547743	0.858808025	0.76260462	0.99999488	0.999985264	0.909427764
DEF6	0.935547743	0.775729202	0.771154197	0.99999488	0.999985264	0.910018813
TMEM222	0.935547743	0.87010028	0.769499495	0.99999488	0.999985264	0.91208425
MRVI1	0.935547743	0.979263532	0.821698192	0.99999488	0.999985264	0.917028736
LOC112441502	0.935547743	0.81978796	0.988537521	0.98846246	0.999985264	0.923360345
LOC574091	0.935547743	0.991365955	0.778280092	0.99999488	0.999985264	0.928928703
ENHO	0.935547743	0.830685465	0.912247957	0.99999488	0.999985264	0.928928703
MND1	0.935547743	0.769482402	0.938203102	0.99999488	0.999985264	0.934749052
SYNE2	0.935547743	0.957252213	0.844729395	0.99999488	0.999985264	0.940358165
EXOSC4	0.935547743	0.896536444	0.76260462	0.99999488	0.999985264	0.94847204
GSTT4	0.935547743	0.805110753	0.849221966	0.99999488	0.999985264	0.949718683
MAPK9	0.935547743	0.95697765	0.76260462	0.996471643	0.999985264	0.95096765
PIP5K1C	0.935547743	0.873289191	0.799197908	0.99999488	0.999985264	0.966921967
ZC3H14	0.935547743	0.81629099	0.788870461	0.99999488	0.999985264	0.978682689
ZWILCH	0.935547743	0.830132999	0.888710954	0.999114211	0.999985264	0.988817857

LOC506989	0.935547743	0.76933827	0.803677695	0.982475718	0.999985264	0.999960414
IQCN	0.935547743	0.76933827	0.870567942	0.999314272	0.999985264	0.999960414
TMEM71	0.935547743	0.760829911	0.761533155	0.99999488	0.999985264	0.999960414
SLAMF9	0.935547743	0.796531384	0.762007355	0.99999488	0.999985264	0.999960414
LOC112442271	0.935547743	0.795255481	0.787862242	0.99999488	0.999985264	0.999960414
CEP120	0.936100923	0.999992444	0.825497047	0.98846246	0.999985264	0.795293834
HSD17B14	0.936100923	0.870839685	0.95477544	0.99999488	0.999985264	0.826043635
MRPL36	0.936100923	0.931564202	0.822003523	0.99999488	0.999985264	0.832833221
ARHGAP27	0.936100923	0.911418256	0.922400936	0.99999488	0.999985264	0.866578338
LOC104969409	0.936100923	0.97015318	0.77618424	0.979293963	0.999985264	0.911089313
PDP1	0.93615201	0.991488652	0.837103057	0.994398173	0.999985264	0.815564853
GGA2	0.93615201	0.869999083	0.938911935	0.99999488	0.999985264	0.885874544
PIGW	0.93615201	0.769482402	0.942686702	0.99999488	0.999985264	0.885874544
DNAJC8	0.936283748	0.816518153	0.999558179	0.99999488	0.999985264	0.772479356
REEP1	0.936283748	0.816518153	0.998936876	0.99999488	0.999985264	0.802962946
SUOX	0.936283748	0.766942526	0.794895759	0.99999488	0.999985264	0.816226172
TIMM29	0.936283748	0.759310562	0.912470556	0.99999488	0.999985264	0.827971245
TMUB2	0.936283748	0.999992444	0.785248842	0.998140932	0.999985264	0.875113722
IL17RB	0.936283748	0.835638623	0.81184654	0.99999488	0.999985264	0.926326154
C8H9orf131	0.936283748	0.78130286	0.880229444	0.99999488	0.999985264	0.935834055
LOC112442047	0.936283748	0.943802122	0.849221966	0.99999488	0.999985264	0.939035437
YARS	0.936283748	0.816100073	0.824984963	0.99999488	0.999985264	0.999960414
LOC112445176	0.936456402	0.819379885	0.785248842	0.99999488	0.999985264	0.774022385
PHB2	0.936745575	0.847051507	0.804471775	0.99999488	0.999985264	0.911997747
LRRC25	0.936767192	0.769626411	0.840760291	0.99999488	0.999985264	0.766713899
CAVIN1	0.936767192	0.89064218	0.907484111	0.99999488	0.999985264	0.943492944
TROVE2	0.936883371	0.839292361	0.908181052	0.99999488	0.999985264	0.757395858
ATP5ME	0.936966053	0.848070173	0.876758498	0.984913325	0.999985264	0.758510984
SNX22	0.936966053	0.893642384	0.999558179	0.994398173	0.999985264	0.760189361
KCTD1	0.936966053	0.860833999	0.794745005	0.99999488	0.999985264	0.765330803
ABCA2	0.936966053	0.805863654	0.935094475	0.99999488	0.999985264	0.765330803
WRAP53	0.936966053	0.819937634	0.946479014	0.99999488	0.999985264	0.793560536
LOC112444355	0.936966053	0.816518153	0.780158644	0.99999488	0.999985264	0.819073042
PGA5	0.936966053	0.836126124	0.813142592	0.99999488	0.999985264	0.837143123
NCEH1	0.936966053	0.768079117	0.920154807	0.99999488	0.999985264	0.865911659
MYH7	0.936966053	0.863813934	0.872386998	0.99999488	0.999985264	0.923360345
PIF1	0.936966053	0.860833999	0.884119589	0.99999488	0.999985264	0.936885223
NUMA1	0.936966053	0.76933827	0.886962419	0.99999488	0.999985264	0.999960414
ATP11C	0.936986704	0.836435076	0.813596247	0.99999488	0.999985264	0.778339877

LOC783776	0.936986704	0.999992444	0.864441458	0.99999488	0.999985264	0.793763761
LOC101902561	0.936986704	0.873289191	0.770213049	0.99999488	0.999985264	0.819073042
LOC101906410	0.936986704	0.817847494	0.771714229	0.99999488	0.999985264	0.819073042
PRPS1	0.936986704	0.764050973	0.965714506	0.99999488	0.999985264	0.831603744
RAB13	0.936986704	0.782417587	0.76260462	0.99999488	0.999985264	0.837470362
C18H16orf74	0.936986704	0.813649909	0.811018607	0.99999488	0.999985264	0.837785413
NUDT16	0.936986704	0.999992444	0.774363763	0.99999488	0.999985264	0.856510591
LEKR1	0.936986704	0.83698359	0.865420641	0.99999488	0.999985264	0.870708914
LRPPRC	0.936986704	0.816518153	0.785248842	0.989312306	0.999985264	0.999960414
LOC107133268	0.937177259	0.836126124	0.941782902	0.99999488	0.999985264	0.816226172
IL21R	0.937177259	0.935742738	0.83711556	0.99999488	0.999985264	0.86871626
ZFP41	0.93813312	0.837713196	0.815035039	0.996471643	0.999985264	0.939035437
FAM241A	0.938284771	0.974079639	0.826965593	0.99999488	0.999985264	0.772431293
ACER3	0.938284771	0.978089982	0.785173725	0.99999488	0.999985264	0.812831419
SERPIND1	0.938284771	0.864945604	0.911018006	0.99999488	0.999985264	0.88310116
PSME3	0.938284771	0.832807683	0.811233159	0.99999488	0.999985264	0.977821653
B4GALT5	0.938411188	0.821033198	0.884615251	0.98846246	0.999985264	0.779686358
LURAP1	0.938411188	0.821194038	0.770213049	0.99999488	0.999985264	0.842880838
QDPR	0.938411188	0.943802122	0.781859509	0.99999488	0.999985264	0.882867473
FXYD5	0.938411188	0.913160799	0.770213049	0.99999488	0.999985264	0.885874544
PRSS53	0.938411188	0.941495217	0.887978771	0.99999488	0.999985264	0.919758204
SCX	0.938411188	0.765748122	0.851718619	0.984299362	0.999985264	0.999960414
LUM	0.938566944	0.994388319	0.771154197	0.99999488	0.999985264	0.772431293
IRAK2	0.938790669	0.790169865	0.920629231	0.99999488	0.999985264	0.763312237
ASAP3	0.938790669	0.873289191	0.993594894	0.99999488	0.999985264	0.809513929
RCL1	0.938790669	0.768079117	0.811233159	0.99999488	0.999985264	0.979046621
LOC526966	0.938790669	0.786382788	0.787006358	0.99999488	0.999985264	0.999960414
CLEC6A	0.939132996	0.850692013	0.862345036	0.99999488	0.999985264	0.812825712
MPZL3	0.939180192	0.873113713	0.813142592	0.99999488	0.999985264	0.812831419
TMA16	0.93963371	0.826434903	0.988922732	0.99999488	0.999985264	0.780707719
PTPN13	0.939811788	0.934886531	0.851718619	0.99999488	0.999985264	0.869877865
NENF	0.939811788	0.864945604	0.841957362	0.99999488	0.999985264	0.872307149
LOC107132534	0.939811788	0.926955605	0.822003523	0.996513698	0.999985264	0.973330026
NFXL1	0.939811788	0.832584599	0.856449202	0.989312306	0.999985264	0.994607516
TMEM72	0.939875426	0.999347049	0.80383368	0.989312306	0.999985264	0.761909911
FOXJ3	0.939875426	0.87010028	0.837948687	0.989312306	0.999985264	0.76762685
KRIT1	0.939875426	0.978089982	0.865066354	0.99999488	0.999985264	0.773689802
ACTG2	0.939875426	0.999347049	0.857006083	0.99999488	0.999985264	0.85653214
ARIH2OS	0.939875426	0.853296877	0.849152155	0.99999488	0.999985264	0.872307149

ARID5A	0.939875426	0.888208796	0.925604199	0.98846246	0.999985264	0.904477651
SYDE2	0.939875426	0.992977597	0.785248842	0.99999488	0.999985264	0.916081555
ELL3	0.939875426	0.827123416	0.862345036	0.99999488	0.999985264	0.921213122
MSL1	0.939875426	0.76933827	0.838909418	0.99999488	0.999985264	0.923593784
SLC23A1	0.939875426	0.991365955	0.817223866	0.99999488	0.999985264	0.931163114
PROSER2	0.939875426	0.850692013	0.902552409	0.99999488	0.999985264	0.936885223
LOC112441655	0.939875426	0.809104286	0.865066354	0.99999488	0.999985264	0.939035437
KITLG	0.940026481	0.76933827	0.926552506	0.99999488	0.999985264	0.769436293
FBXL6	0.940026481	0.76933827	0.946479014	0.99999488	0.999985264	0.781688852
DNMT3A	0.940026481	0.84060143	0.926552506	0.99999488	0.999985264	0.802788605
LOC104969097	0.940026481	0.999992444	0.86178438	0.99999488	0.999985264	0.812725657
GEMIN5	0.940026481	0.83698359	0.946479014	0.99999488	0.999985264	0.812825712
GMCL1	0.940026481	0.90116492	0.774363763	0.99999488	0.999985264	0.812831419
SLC17A5	0.940026481	0.883624935	0.803677695	0.99999488	0.999985264	0.949718683
LOC112443235	0.940026481	0.869716769	0.773092345	0.99999488	0.999985264	0.958193903
SERPINC1	0.940026481	0.9527938	0.809401378	0.99999488	0.999985264	0.973330026
LOC112448737	0.940026481	0.883624935	0.835553767	0.99999488	0.999985264	0.993740805
MRPL48	0.940026481	0.809599669	0.76457744	0.99999488	0.999985264	0.999960414
MBTD1	0.940071594	0.9582784	0.844036059	0.99999488	0.999985264	0.910813075
WIZ	0.940624005	0.862811597	0.778542909	0.99999488	0.999985264	0.79872286
SLC25A45	0.940624005	0.766621389	0.848312163	0.99999488	0.999985264	0.799216519
AZIN1	0.940624005	0.830519875	0.981666445	0.99999488	0.999985264	0.816226172
ARL8B	0.940624005	0.76451758	0.900890709	0.99999488	0.999985264	0.819073042
LOC112447402	0.940624005	0.815700306	0.769310317	0.99999488	0.999985264	0.861670395
LOC112442377	0.940624005	0.819937634	0.770213049	0.99999488	0.999985264	0.98564799
LOC101903795	0.940823617	0.796531384	0.871925227	0.989312306	0.999985264	0.769129136
TMEM38B	0.940823617	0.819155365	0.916975216	0.99999488	0.999985264	0.769515026
PCDH12	0.940823617	0.76933827	0.770532824	0.99999488	0.999985264	0.781688852
TLE3	0.940823617	0.835868428	0.776395793	0.99999488	0.999985264	0.866439215
LOC101902468	0.940823617	0.867541582	0.921065686	0.994265515	0.999985264	0.894420979
PPP1CA	0.940823617	0.934905044	0.851718619	0.989312306	0.999985264	0.895349622
GRID2	0.940823617	0.950729918	0.880399877	0.993124804	0.999985264	0.910472319
RRAD	0.940823617	0.999992444	0.779432747	0.99999488	0.999985264	0.918400465
TMEM168	0.940823617	0.830132999	0.877742202	0.99999488	0.999985264	0.923360345
GABRA1	0.940823617	0.870142804	0.77602067	0.99999488	0.999985264	0.926480606
LOC112442719	0.940823617	0.821033198	0.952414682	0.99999488	0.999985264	0.927969856
ZNF438	0.940823617	0.808097876	0.825497047	0.99999488	0.999985264	0.931163114
RIC8B	0.940939658	0.917849199	0.964152013	0.99999488	0.999985264	0.763715224
VPS26B	0.940939658	0.826936949	0.770213049	0.99999488	0.999985264	0.764747443

USP22	0.940939658	0.892008332	0.929207633	0.99999488	0.999985264	0.769515026
SPNS2	0.940939658	0.830654932	0.825497047	0.99999488	0.999985264	0.770633383
NAF1	0.940939658	0.999992444	0.785248842	0.99999488	0.999985264	0.798638511
GRTP1	0.940939658	0.958967142	0.841773648	0.99999488	0.999985264	0.809513929
MMP11	0.940939658	0.816518153	0.876758498	0.99999488	0.999985264	0.812831419
ARHGAP33	0.940939658	0.9582784	0.834068744	0.99999488	0.999985264	0.832966318
AADAT	0.940939658	0.769482402	0.785177733	0.99999488	0.999985264	0.836520183
NFKB2	0.940939658	0.862865667	0.817177032	0.99999488	0.999985264	0.837268226
AP3M2	0.940939658	0.921549	0.914001994	0.98846246	0.999985264	0.855712996
TM9SF3	0.940939658	0.898789587	0.785173725	0.99999488	0.999985264	0.896078284
RAD51AP1	0.940939658	0.793670583	0.86638329	0.99999488	0.999985264	0.918400465
GSTM3	0.940939658	0.941495217	0.864441458	0.99999488	0.999985264	0.919441558
CTXND1	0.940939658	0.883624935	0.963068909	0.992168706	0.999985264	0.934749052
MAIP1	0.940939658	0.816518153	0.821930959	0.99999488	0.999985264	0.939035437
DHX57	0.940939658	0.897308951	0.881942256	0.99999488	0.999985264	0.968059852
TMEM25	0.941075726	0.893642384	0.945678616	0.99999488	0.999985264	0.772431293
TOLLIP	0.941075726	0.837468586	0.771154197	0.99999488	0.999985264	0.79506749
SIPA1L3	0.941075726	0.883624935	0.964349087	0.99999488	0.999985264	0.901614166
MEOX1	0.941211526	0.916036681	0.862345036	0.99999488	0.999985264	0.770030068
LOC101902754	0.941211526	0.845554546	0.834068744	0.99999488	0.999985264	0.847186604
RDH16	0.941469894	0.782417587	0.815602423	0.99999488	0.999985264	0.999960414
ABHD10	0.941758941	0.994481342	0.803942311	0.99999488	0.999985264	0.836520183
U2AF2	0.941786163	0.819978014	0.826965593	0.99999488	0.999985264	0.764052305
LY6E	0.941786163	0.769454808	0.825497047	0.989312306	0.999985264	0.806724906
CCDC102A	0.941786163	0.773422401	0.865837237	0.99999488	0.999985264	0.807566643
POLE	0.942511088	0.76933827	0.911018006	0.99999488	0.999985264	0.869877865
CSGALNACT2	0.942570938	0.779944002	0.789951578	0.98846246	0.999985264	0.816226172
HDHD3	0.942642723	0.815092066	0.878344997	0.99999488	0.999985264	0.772479356
WDTC1	0.942642723	0.948421431	0.77618424	0.99999488	0.999985264	0.960343814
MARVELD1	0.942671082	0.971819361	0.88153193	0.99999488	0.999985264	0.839734604
POLR3H	0.942671082	0.840712878	0.882380454	0.99999488	0.999985264	0.872307149
LOC101904590	0.942692506	0.850095234	0.785248842	0.99999488	0.999985264	0.911997747
LOC100847708	0.942829351	0.879007958	0.848786751	0.99999488	0.999985264	0.960343814
RPL39	0.943331219	0.786382788	0.873452279	0.99999488	0.999985264	0.831532663
MTERF1	0.943331219	0.962665318	0.900351064	0.99999488	0.999985264	0.858593649
ZUP1	0.943331219	0.949285285	0.83126054	0.99999488	0.999985264	0.873463288
MAPK3	0.943337204	0.980496345	0.825497047	0.99999488	0.999985264	0.861670395
ARL5B	0.943345469	0.859484941	0.985966356	0.99999488	0.999985264	0.770633383
TMEM63A	0.943345469	0.832115014	0.923604668	0.99999488	0.999985264	0.845594867

GNAL	0.943345469	0.9582784	0.800460393	0.99999488	0.999985264	0.847204221
MCTP2	0.943345469	0.839292361	0.908181052	0.99999488	0.999985264	0.885874544
LOC100299242	0.943345469	0.883856603	0.802095986	0.99999488	0.999985264	0.911997747
KCNG2	0.943470926	0.816578975	0.879092985	0.99999488	0.999985264	0.773507109
CPE	0.943470926	0.778297572	0.825497047	0.99999488	0.999985264	0.80980964
KIF3A	0.943470926	0.915367594	0.771154197	0.99999488	0.999985264	0.83405895
SGMS1	0.943470926	0.77071675	0.893701265	0.99999488	0.999985264	0.836520183
MYCBP2	0.943470926	0.865184773	0.903957821	0.989312306	0.999985264	0.844861566
GALE	0.943470926	0.878186176	0.881938573	0.99999488	0.999985264	0.871960581
C29H11orf95	0.943470926	0.819937634	0.817177032	0.99999488	0.999985264	0.873463288
PDE7A	0.943470926	0.90116492	0.787807613	0.99999488	0.999985264	0.894723749
CEP126	0.943470926	0.90387438	0.809401378	0.99999488	0.999985264	0.89589047
TRMT5	0.943470926	0.835868428	0.778542909	0.99999488	0.999985264	0.968059852
AKR1C4	0.943470926	0.802939066	0.833496026	0.99999488	0.999985264	0.969741463
PTGR1	0.943842536	0.816518153	0.946479014	0.99999488	0.999985264	0.767595486
THBS3	0.943842536	0.809135999	0.921010865	0.99999488	0.999985264	0.770030068
MTA1	0.943842536	0.769626411	0.93088519	0.99999488	0.999985264	0.770030068
MED29	0.943842536	0.782368029	0.811233159	0.996471643	0.999985264	0.770633383
LOC100337328	0.943842536	0.852760886	0.837103057	0.99999488	0.999985264	0.772431293
RHBDF1	0.943842536	0.816518153	0.999558179	0.99999488	0.999985264	0.772431293
GNA11	0.943842536	0.976819696	0.886192181	0.99999488	0.999985264	0.772479356
PURB	0.943842536	0.796210279	0.915388802	0.99999488	0.999985264	0.772479356
ATP6V0E2	0.943842536	0.999992444	0.774363763	0.99999488	0.999985264	0.773689802
SLC8A1	0.943842536	0.999992444	0.848312163	0.99999488	0.999985264	0.773716179
NR1H3	0.943842536	0.873289191	0.96210311	0.99999488	0.999985264	0.775511809
COX6B1	0.943842536	0.798363588	0.864737879	0.989312306	0.999985264	0.779686358
ZDHHC14	0.943842536	0.985406717	0.778565768	0.99999488	0.999985264	0.779686358
NR2F6	0.943842536	0.991488652	0.806978356	0.99999488	0.999985264	0.779686358
SPATA20	0.943842536	0.998131745	0.902552409	0.99999488	0.999985264	0.779686358
LOC789148	0.943842536	0.896400219	0.939819448	0.99999488	0.999985264	0.779686358
LOC101905041	0.943842536	0.92239337	0.957541703	0.99999488	0.999985264	0.779686358
LOC785568	0.943842536	0.938017364	0.925745065	0.99999488	0.999985264	0.780698013
TOM1L2	0.943842536	0.826485535	0.774632155	0.99999488	0.999985264	0.787607112
NLRP1	0.943842536	0.813113924	0.862345036	0.99999488	0.999985264	0.787607112
CHST10	0.943842536	0.985406717	0.821546364	0.99999488	0.999985264	0.789976745
STX7	0.943842536	0.81928857	0.962777771	0.99999488	0.999985264	0.790569257
LYRM4	0.943842536	0.902683528	0.817816439	0.99999488	0.999985264	0.791582618
ABHD3	0.943842536	0.855971295	0.865420641	0.99999488	0.999985264	0.793906942
NDUFC2	0.943842536	0.819937634	0.845368791	0.99999488	0.999985264	0.794870105

SELENOS	0.943842536	0.78130286	0.993585211	0.99999488	0.999985264	0.79506749
PPP1R3C	0.943842536	0.941495217	0.961024709	0.99999488	0.999985264	0.796563177
MEF2A	0.943842536	0.9582784	0.880229444	0.99999488	0.999985264	0.796770444
FHL2	0.943842536	0.9582784	0.824972187	0.99999488	0.999985264	0.802345382
DDIAS	0.943842536	0.850692013	0.946479014	0.99999488	0.999985264	0.804128815
LOC101907603	0.943842536	0.885718559	0.803942311	0.99999488	0.999985264	0.805249326
RPS6KA3	0.943842536	0.835868428	0.843587792	0.99999488	0.999985264	0.805442659
IKBKG	0.943842536	0.78130286	0.771714229	0.991596938	0.999985264	0.807689737
NUDCD3	0.943842536	0.904961534	0.887626998	0.99999488	0.999985264	0.807689737
LOC112446796	0.943842536	0.909577824	0.882515776	0.99999488	0.999985264	0.812831419
LRRTM2	0.943842536	0.916036681	0.907381775	0.99999488	0.999985264	0.812991893
UCK1	0.943842536	0.835795712	0.778565768	0.99999488	0.999985264	0.813723642
GATD1	0.943842536	0.783973824	0.775526567	0.99999488	0.999985264	0.814496203
AAMP	0.943842536	0.89875294	0.817700149	0.99999488	0.999985264	0.816226172
CD82	0.943842536	0.935742738	0.83396257	0.99999488	0.999985264	0.816226172
LOC101902721	0.943842536	0.915367594	0.95230212	0.99999488	0.999985264	0.816959462
MAP3K10	0.943842536	0.837468586	0.791938217	0.99999488	0.999985264	0.818895181
EIF4H	0.943842536	0.926949474	0.939501959	0.99999488	0.999985264	0.819073042
LOC101904087	0.943842536	0.869999083	0.871385134	0.99999488	0.999985264	0.821089634
SLC13A5	0.943842536	0.819937634	0.809486619	0.99999488	0.999985264	0.82174652
UBA2	0.943842536	0.90024974	0.775848114	0.99999488	0.999985264	0.831666995
CD300LF	0.943842536	0.85337372	0.908572237	0.99999488	0.999985264	0.832833221
HSPB8	0.943842536	0.989547896	0.898815524	0.99999488	0.999985264	0.833359144
RUNDC3B	0.943842536	0.992977597	0.906524923	0.99999488	0.999985264	0.837172285
LOC107131675	0.943842536	0.958098081	0.95230212	0.989312306	0.999985264	0.8381256
AP2M1	0.943842536	0.993100184	0.913211244	0.99999488	0.999985264	0.839734604
LOC100139548	0.943842536	0.891316528	0.833496026	0.99999488	0.999985264	0.841443168
CLBA1	0.943842536	0.797034906	0.851718619	0.99999488	0.999985264	0.842880838
CSNK1G2	0.943842536	0.990248517	0.830626565	0.99999488	0.999985264	0.846111851
SPSB4	0.943842536	0.991488652	0.875030741	0.989312306	0.999985264	0.849046935
LOC112443485	0.943842536	0.929230097	0.876188482	0.99999488	0.999985264	0.849266568
NEMP2	0.943842536	0.847051507	0.998936876	0.98846246	0.999985264	0.850797738
HDAC11	0.943842536	0.794965819	0.925835926	0.99999488	0.999985264	0.85182524
ATP8B2	0.943842536	0.970426668	0.907381775	0.99999488	0.999985264	0.856510591
TEX264	0.943842536	0.991365955	0.785429296	0.99999488	0.999985264	0.856666693
PGBD5	0.943842536	0.999992444	0.907134138	0.98846246	0.999985264	0.858593649
INPP1	0.943842536	0.927545644	0.896570082	0.99999488	0.999985264	0.859200033
BAG2	0.943842536	0.944868298	0.89038419	0.99999488	0.999985264	0.861670395
LOC112446759	0.943842536	0.842852797	0.876758498	0.99999488	0.999985264	0.869017773

ZNF829	0.943842536	0.816518153	0.837254326	0.99999488	0.999985264	0.870708914
DHPS	0.943842536	0.76933827	0.81983962	0.99999488	0.999985264	0.873463288
EED	0.943842536	0.974079639	0.848832109	0.99999488	0.999985264	0.873463288
LOC112448105	0.943842536	0.943802122	0.826965593	0.99999488	0.999985264	0.874718379
NGF	0.943842536	0.81978796	0.878053482	0.99999488	0.999985264	0.877838449
ARFRP1	0.943842536	0.850692013	0.803942311	0.99999488	0.999985264	0.881211326
ABHD6	0.943842536	0.883856603	0.774363763	0.99999488	0.999985264	0.881781612
DYNC2LI1	0.943842536	0.919884594	0.828879745	0.99999488	0.999985264	0.882867473
MAGED4B	0.943842536	0.802204033	0.861480822	0.99999488	0.999985264	0.888855047
PDLIM3	0.943842536	0.999992444	0.825497047	0.99999488	0.999985264	0.894236694
LOC112444152	0.943842536	0.859920407	0.807608869	0.99999488	0.999985264	0.901809636
LOC112448153	0.943842536	0.836126124	0.964152013	0.99999488	0.999985264	0.904300404
SNX5	0.943842536	0.907069508	0.809561323	0.99999488	0.999985264	0.906118365
TSC22D1	0.943842536	0.850839746	0.863098782	0.99999488	0.999985264	0.909427764
MRO	0.943842536	0.819937634	0.841739726	0.99999488	0.999985264	0.911997747
FOXO4	0.943842536	0.928525825	0.793478166	0.99999488	0.999985264	0.915476587
LOC104975626	0.943842536	0.778248571	0.785177733	0.99999488	0.999985264	0.919441558
LOC104972797	0.943842536	0.911291478	0.863098782	0.99999488	0.999985264	0.919758204
LOC104972827	0.943842536	0.999992444	0.807323012	0.99999488	0.999985264	0.921771959
SYTL1	0.943842536	0.813113924	0.968232195	0.99999488	0.999985264	0.923360345
LRRC72	0.943842536	0.999992444	0.821546364	0.98846246	0.999985264	0.927969856
LRRN2	0.943842536	0.816518153	0.938203102	0.99999488	0.999985264	0.928634547
TPI1	0.943842536	0.867535124	0.826965593	0.99999488	0.999985264	0.928906685
TFG	0.943842536	0.915367594	0.861314069	0.99999488	0.999985264	0.929458454
KLF7	0.943842536	0.819379885	0.779432747	0.99999488	0.999985264	0.931377206
RGMA	0.943842536	0.796000666	0.815067929	0.99999488	0.999985264	0.932608891
WDHD1	0.943842536	0.811327744	0.864737879	0.99999488	0.999985264	0.935349027
GUCY1A1	0.943842536	0.813746447	0.906268775	0.99999488	0.999985264	0.939035437
LOC781197	0.943842536	0.935742738	0.775848114	0.99999488	0.999985264	0.940358165
RNASE1	0.943842536	0.816518153	0.950948716	0.99999488	0.999985264	0.942340826
ZNF557	0.943842536	0.998011664	0.771714229	0.99999488	0.999985264	0.945570512
PCNX4	0.943842536	0.813113924	0.785248842	0.991596938	0.999985264	0.95096765
KLF11	0.943842536	0.867840435	0.90339136	0.989312306	0.999985264	0.958193903
C1QTNF2	0.943842536	0.826434903	0.809401378	0.99999488	0.999985264	0.958193903
DENND2D	0.943842536	0.835868428	0.886192181	0.99999488	0.999985264	0.967991416
GAS2L2	0.943842536	0.896575712	0.833419544	0.99999488	0.999985264	0.976814573
LOC531747	0.943842536	0.821194038	0.911922552	0.996471643	0.999985264	0.993564907
LOC100849237	0.943842536	0.774642135	0.773794134	0.99999488	0.999985264	0.999960414
BTBD9	0.943842536	0.775729202	0.920629231	0.99999488	0.999985264	0.999960414

R3HCC1	0.943874021	0.808097876	0.988249967	0.99999488	0.999985264	0.850299382
NDUFA5	0.944009681	0.865926615	0.864737879	0.99999488	0.999985264	0.786482582
KLHDC4	0.944151521	0.96895079	0.841957362	0.99999488	0.999985264	0.772431293
EFEMP2	0.944151521	0.830654932	0.886962419	0.99999488	0.999985264	0.772431293
NPEPPS	0.944151521	0.819937634	0.913976361	0.99999488	0.999985264	0.773689802
NR4A1	0.944151521	0.805123419	0.999558179	0.989312306	0.999985264	0.773716179
LOC112443216	0.944151521	0.999992444	0.785248842	0.99999488	0.999985264	0.781063387
MTG2	0.944151521	0.863793573	0.785248842	0.99999488	0.999985264	0.796770444
ZBTB33	0.944151521	0.999992444	0.807323012	0.992168706	0.999985264	0.80980964
PODN	0.944151521	0.835868428	0.828038	0.99999488	0.999985264	0.812831419
SERINC2	0.944151521	0.834860292	0.997208738	0.99999488	0.999985264	0.812831419
MEAF6	0.944151521	0.827661136	0.934959203	0.99999488	0.999985264	0.816930408
SPOCK2	0.944151521	0.973912396	0.888545123	0.99999488	0.999985264	0.818895181
PRKCB	0.944151521	0.88981187	0.862131495	0.99999488	0.999985264	0.820845891
CHDH	0.944151521	0.842132999	0.841739726	0.99999488	0.999985264	0.831532663
RHOT1	0.944151521	0.883624935	0.830186385	0.996471643	0.999985264	0.834948594
WDR53	0.944151521	0.808097876	0.862345036	0.99999488	0.999985264	0.85653214
MARCH1	0.944151521	0.78130286	0.778816535	0.99999488	0.999985264	0.873463288
PLIN4	0.944151521	0.821194038	0.837139208	0.99999488	0.999985264	0.911997747
MSLN	0.944151521	0.893642384	0.94634922	0.99999488	0.999985264	0.911997747
RMI1	0.944151521	0.936273761	0.805753285	0.99999488	0.999985264	0.929458454
TRMT10C	0.944151521	0.835868428	0.807323012	0.99999488	0.999985264	0.999960414
SIRT7	0.944161449	0.849474629	0.999558179	0.99999488	0.999985264	0.796812106
LOC104975635	0.944161449	0.9582784	0.778565768	0.99999488	0.999985264	0.887158291
LOC100848315	0.944379581	0.868350171	0.946479014	0.99999488	0.999985264	0.795293834
SH3GLB1	0.944379581	0.779783885	0.973406453	0.99999488	0.999985264	0.820927993
TYW3	0.944379581	0.968062432	0.824093932	0.99999488	0.999985264	0.848830665
MECR	0.944379581	0.845554546	0.77602067	0.99999488	0.999985264	0.923360345
STARD9	0.944379581	0.810880325	0.933447488	0.99999488	0.999985264	0.960294693
LOC619131	0.944442987	0.795679871	0.851718619	0.991596938	0.999985264	0.828248574
POP5	0.944442987	0.816518153	0.809486619	0.99999488	0.999985264	0.934749052
TTPAL	0.944526621	0.877795869	0.930678591	0.996471643	0.999985264	0.957171025
ASB10	0.944754957	0.848246309	0.775688011	0.99999488	0.999985264	0.931163114
RNASE10	0.944754957	0.97505371	0.822003523	0.99999488	0.999985264	0.939855019
ROGDI	0.945120278	0.815700306	0.848786751	0.99999488	0.999985264	0.779686358
STPG1	0.945120278	0.816044556	0.95230212	0.99999488	0.999985264	0.806094408
GPI	0.945120278	0.773422401	0.81983962	0.99999488	0.999985264	0.806724906
IQCA1	0.945120278	0.957700659	0.825497047	0.99999488	0.999985264	0.809513929
DPP6	0.945120278	0.847015513	0.77455741	0.989312306	0.999985264	0.962283819

KIFC1	0.945195663	0.783193939	0.929188473	0.99999488	0.999985264	0.793211254
SPATA16	0.945195663	0.813113924	0.834068744	0.99999488	0.999985264	0.897965262
NIPA1	0.94524378	0.848246309	0.865420641	0.99999488	0.999985264	0.772431293
HTRA2	0.94524378	0.905019423	0.914001994	0.99999488	0.999985264	0.772479356
NDUFS6	0.94524378	0.91058693	0.822332225	0.99999488	0.999985264	0.790953407
MYD88	0.94524378	0.83989094	0.785177733	0.99999488	0.999985264	0.802714348
MPPED2	0.94524378	0.867755878	0.862345036	0.99999488	0.999985264	0.807665096
LOC101906526	0.94524378	0.999992444	0.791717601	0.99999488	0.999985264	0.825478387
LOC101902656	0.94524378	0.866708784	0.785248842	0.99999488	0.999985264	0.832197795
LOC112444921	0.94524378	0.926955605	0.95106368	0.99999488	0.999985264	0.836520183
LOC100299201	0.94524378	0.882022911	0.833419544	0.993124804	0.999985264	0.838101199
NEO1	0.94524378	0.922700337	0.825497047	0.99999488	0.999985264	0.839734604
ACSL5	0.94524378	0.885718559	0.83711556	0.99999488	0.999985264	0.987011114
EIF2AK2	0.94524378	0.783973824	0.77618424	0.99999488	0.999985264	0.989354156
NPEPL1	0.946468765	0.873289191	0.864441458	0.989312306	0.999985264	0.812831419
SASS6	0.946641313	0.918883775	0.923604668	0.99999488	0.999985264	0.779686358
ANXA3	0.947297366	0.821194038	0.805870442	0.99999488	0.999985264	0.772479356
LOC101906426	0.947297366	0.904961534	0.914001994	0.99999488	0.999985264	0.772479356
ZNF692	0.947297366	0.985406717	0.956565728	0.99999488	0.999985264	0.778339877
MIGA2	0.947297366	0.862707207	0.781111723	0.99999488	0.999985264	0.779686358
AQP1	0.947297366	0.859484941	0.851718619	0.99999488	0.999985264	0.779686358
ALS2	0.947297366	0.882505302	0.875823795	0.99999488	0.999985264	0.779686358
LAMTOR1	0.947297366	0.884739596	0.837931285	0.99999488	0.999985264	0.781688852
ATP6V0B	0.947297366	0.864945604	0.988922732	0.99999488	0.999985264	0.786805278
EMC6	0.947297366	0.916036681	0.861441442	0.99999488	0.999985264	0.796010841
SCO1	0.947297366	0.913160799	0.874559186	0.99999488	0.999985264	0.799216519
TONSL	0.947297366	0.836094224	0.999558179	0.99999488	0.999985264	0.799386524
EPB41L3	0.947297366	0.894140115	0.778565768	0.99999488	0.999985264	0.802149108
CYHR1	0.947297366	0.867755878	0.807608869	0.99999488	0.999985264	0.807566643
FUBP3	0.947297366	0.949285285	0.825736269	0.99999488	0.999985264	0.807689737
LOC112445242	0.947297366	0.994135588	0.830970408	0.99999488	0.999985264	0.807689737
BTBD7	0.947297366	0.869999083	0.848312163	0.99999488	0.999985264	0.807689737
CASP7	0.947297366	0.883624935	0.841957362	0.989312306	0.999985264	0.809513929
LMF2	0.947297366	0.836094224	0.888692542	0.99999488	0.999985264	0.80980964
SLC2A1	0.947297366	0.802112404	0.928478887	0.99999488	0.999985264	0.80980964
PEF1	0.947297366	0.835638623	0.803560874	0.99999488	0.999985264	0.810352947
TMEM140	0.947297366	0.884739596	0.840781773	0.99579057	0.999985264	0.812831419
EFNB1	0.947297366	0.9582784	0.8815145	0.99999488	0.999985264	0.812831419
LOC530102	0.947297366	0.847051507	0.916975216	0.99999488	0.999985264	0.812831419

KLHL15	0.947297366	0.8756206	0.815067929	0.989312306	0.999985264	0.814658726
MAF	0.947297366	0.89880506	0.851197548	0.99999488	0.999985264	0.816226172
CRTC1	0.947297366	0.816518153	0.874559186	0.99999488	0.999985264	0.816924515
C23H6orf106	0.947297366	0.819379885	0.785248842	0.99999488	0.999985264	0.818895181
ANKRD39	0.947297366	0.836126124	0.785177733	0.99999488	0.999985264	0.827439931
ELF2	0.947297366	0.998131745	0.825497047	0.994398173	0.999985264	0.832622862
LOC101904265	0.947297366	0.9582784	0.922400936	0.99999488	0.999985264	0.833244331
CEBPG	0.947297366	0.806647675	0.938203102	0.99999488	0.999985264	0.836520183
MIF	0.947297366	0.856066321	0.776282112	0.99999488	0.999985264	0.836741573
SP7	0.947297366	0.806647675	0.843587792	0.99999488	0.999985264	0.839734604
RAB40C	0.947297366	0.957700659	0.852782377	0.99999488	0.999985264	0.839734604
TDP1	0.947297366	0.821033198	0.991992782	0.99999488	0.999985264	0.839734604
LOC112442049	0.947297366	0.915367594	0.923604668	0.99999488	0.999985264	0.841443168
MRPL34	0.947297366	0.850496315	0.856449202	0.99999488	0.999985264	0.847577522
SMARCAL1	0.947297366	0.94431067	0.925520255	0.99999488	0.999985264	0.84833012
UBE2M	0.947297366	0.927997582	0.883109976	0.99999488	0.999985264	0.851595584
SH3BP1	0.947297366	0.891316528	0.821546364	0.99999488	0.999985264	0.858593649
SUSD3	0.947297366	0.935742738	0.95230212	0.99999488	0.999985264	0.860624144
LOC112449531	0.947297366	0.921934673	0.841739726	0.99999488	0.999985264	0.861670395
RPS15A	0.947297366	0.837468586	0.876188482	0.99999488	0.999985264	0.863774505
ADAM22	0.947297366	0.948421431	0.957541703	0.989312306	0.999985264	0.86871626
PAK6	0.947297366	0.895304124	0.780158644	0.99999488	0.999985264	0.86871626
RSL24D1	0.947297366	0.893748757	0.807825655	0.99999488	0.999985264	0.869877865
PKHD1L1	0.947297366	0.948421431	0.778565768	0.99999488	0.999985264	0.873463288
CRYBG3	0.947297366	0.816518153	0.778565768	0.99999488	0.999985264	0.880829713
LOC101902757	0.947297366	0.877142156	0.861497791	0.99999488	0.999985264	0.882867473
RPS18	0.947297366	0.858808025	0.865420641	0.989312306	0.999985264	0.883999232
KMT2E	0.947297366	0.865099263	0.817700149	0.99999488	0.999985264	0.888645453
LOC100335990	0.947297366	0.915367594	0.785248842	0.99999488	0.999985264	0.893313235
THEMIS2	0.947297366	0.79258665	0.796415125	0.99999488	0.999985264	0.894723749
RAB11B	0.947297366	0.955266582	0.831723974	0.99999488	0.999985264	0.894723749
CARM1	0.947297366	0.921934673	0.848786751	0.99999488	0.999985264	0.899223071
PPP1R7	0.947297366	0.921759937	0.824563098	0.99999488	0.999985264	0.90408677
DNAJB12	0.947297366	0.835868428	0.802997196	0.99999488	0.999985264	0.904300404
LOC112445002	0.947297366	0.862811597	0.921646762	0.99999488	0.999985264	0.904300404
ANKRD29	0.947297366	0.816518153	0.815879696	0.99999488	0.999985264	0.927969856
TLN1	0.947297366	0.870839685	0.83126054	0.99999488	0.999985264	0.929527103
LOC112448454	0.947297366	0.952345011	0.908380876	0.99999488	0.999985264	0.935349027
LOC101902490	0.947297366	0.816518153	0.88153193	0.99999488	0.999985264	0.939035437

ARF3	0.947297366	0.938597964	0.787006358	0.99999488	0.999985264	0.940358165
SLC44A3	0.947297366	0.835868428	0.787862242	0.99999488	0.999985264	0.943220018
TRIM13	0.947297366	0.789663062	0.886192181	0.99999488	0.999985264	0.945794274
LOC100337457	0.947297366	0.874807581	0.789951578	0.99999488	0.999985264	0.949718683
PAX5	0.947297366	0.90024974	0.861480822	0.99999488	0.999985264	0.95096765
NEGR1	0.947297366	0.927997582	0.864441458	0.999314272	0.999985264	0.951922741
RARRES1	0.947297366	0.845554546	0.907484111	0.99999488	0.999985264	0.955326589
RNF34	0.947297366	0.845554546	0.939147816	0.99999488	0.999985264	0.968695729
SEPT10	0.947297366	0.941495217	0.83711556	0.99999488	0.999985264	0.972391591
TAF9	0.947297366	0.846198976	0.812450514	0.99999488	0.999985264	0.973588942
LOC100141070	0.947297366	0.821617615	0.864257597	0.99999488	0.999985264	0.986873741
GSDMB	0.947297366	0.894140115	0.812450514	0.99999488	0.999985264	0.989348186
IRF2	0.947297366	0.799180421	0.925745065	0.99999488	0.999985264	0.999960414
BTBD6	0.94749623	0.884739596	0.864737879	0.99999488	0.999985264	0.849046935
REEP3	0.947607196	0.816518153	0.956565728	0.99999488	0.999985264	0.773689802
TEP1	0.947607196	0.840946688	0.970945672	0.99999488	0.999985264	0.775100948
LOC104969140	0.947607196	0.830519875	0.998936876	0.99999488	0.999985264	0.777136982
C28H1orf131	0.947607196	0.783973824	0.999697966	0.99999488	0.999985264	0.795293834
LOC101905319	0.947607196	0.825438977	0.785248842	0.99999488	0.999985264	0.805869777
SLC25A10	0.947607196	0.883856603	0.785248842	0.99999488	0.999985264	0.840547083
MON1A	0.947607196	0.891793027	0.80757077	0.99999488	0.999985264	0.875113722
NDUFAF8	0.947607196	0.874697957	0.858024919	0.99999488	0.999985264	0.887277447
LOC100848331	0.947607196	0.819937634	0.785248842	0.99999488	0.999985264	0.934749052
HIGD1B	0.947607196	0.935742738	0.947033142	0.997439039	0.999985264	0.939035437
SMARCC2	0.947607196	0.839292361	0.825497047	0.99999488	0.999985264	0.952669273
WDR35	0.947607196	0.85337372	0.803484516	0.99999488	0.999985264	0.976814573
CAPN15	0.947919225	0.816518153	0.933791093	0.99999488	0.999985264	0.824556283
GOLGA4	0.948453115	0.839292361	0.817700149	0.99999488	0.999985264	0.776440933
FARP1	0.948453115	0.920975242	0.962346588	0.99999488	0.999985264	0.777136982
EYA3	0.948453115	0.821194038	0.807015061	0.99999488	0.999985264	0.779686358
ATP5F1E	0.948453115	0.795255481	0.916975216	0.993124804	0.999985264	0.781688852
TRIM28	0.948453115	0.819937634	0.807323012	0.99999488	0.999985264	0.783345949
CGAS	0.948453115	0.796000666	0.875823795	0.99999488	0.999985264	0.790953407
ATP5MF	0.948453115	0.816518153	0.882515776	0.991596938	0.999985264	0.791922285
MRPL18	0.948453115	0.883624935	0.877799478	0.99999488	0.999985264	0.794870105
LOC615183	0.948453115	0.999992444	0.834068744	0.99999488	0.999985264	0.795293834
SRFBP1	0.948453115	0.881001912	0.938203102	0.99999488	0.999985264	0.806724906
PDCD7	0.948453115	0.956655737	0.993594894	0.99999488	0.999985264	0.806724906
SELENON	0.948453115	0.948421431	0.876758498	0.99999488	0.999985264	0.809513929

LRP11	0.948453115	0.999347049	0.895644023	0.99999488	0.999985264	0.809513929
SEMA3D	0.948453115	0.999992444	0.788487235	0.99999488	0.999985264	0.812870286
C7	0.948453115	0.999992444	0.851718619	0.99999488	0.999985264	0.818895181
IQGAP1	0.948453115	0.826642757	0.921010865	0.99999488	0.999985264	0.824556283
SNED1	0.948453115	0.795255481	0.920629231	0.99999488	0.999985264	0.827796589
B3GALT1	0.948453115	0.985406717	0.958627535	0.99999488	0.999985264	0.836520183
BEAN1	0.948453115	0.795255481	0.850804682	0.99999488	0.999985264	0.846001048
LOC107132852	0.948453115	0.992977597	0.814724984	0.99999488	0.999985264	0.856510591
OLR1	0.948453115	0.826052922	0.812450514	0.99999488	0.999985264	0.859200033
ZNF582	0.948453115	0.799180421	0.806978356	0.99999488	0.999985264	0.872307149
GNL1	0.948453115	0.991488652	0.794878847	0.99999488	0.999985264	0.873463288
IRAK4	0.948453115	0.872993581	0.95230212	0.99999488	0.999985264	0.880829713
KIF3C	0.948453115	0.885948415	0.788487235	0.99999488	0.999985264	0.884143505
SLC9A9	0.948453115	0.852035396	0.800518725	0.99999488	0.999985264	0.897965262
MBD3	0.948453115	0.893748757	0.862131495	0.99999488	0.999985264	0.911997747
TIMM10B	0.948453115	0.814676928	0.857072251	0.99999488	0.999985264	0.920531105
FBXL22	0.948453115	0.9527938	0.83711556	0.99999488	0.999985264	0.921760865
PER2	0.948453115	0.886820818	0.831723974	0.99999488	0.999985264	0.921771959
C18H19orf18	0.948453115	0.783314535	0.896570082	0.99999488	0.999985264	0.927969856
FOXJ2	0.948453115	0.915367594	0.94634922	0.99999488	0.999985264	0.932608891
RRAS	0.948453115	0.853777444	0.88687452	0.99999488	0.999985264	0.941205126
ATXN7L2	0.948453115	0.796000666	0.86638329	0.99999488	0.999985264	0.94182698
GPM6A	0.948453115	0.915367594	0.93198623	0.99999488	0.999985264	0.948213108
ZNF407	0.948453115	0.852760886	0.837103057	0.99999488	0.999985264	0.95096765
CCT7	0.948453115	0.873113619	0.848312163	0.99999488	0.999985264	0.965920258
PHF24	0.948453115	0.811327744	0.817700149	0.99999488	0.999985264	0.999960414
RGN	0.948592596	0.921759937	0.794895759	0.99999488	0.999985264	0.808608716
HSPA8	0.949047684	0.869999083	0.884320747	0.99999488	0.999985264	0.781063387
ANO8	0.949047684	0.918254521	0.920629231	0.99999488	0.999985264	0.785716654
GCNT2	0.949047684	0.853107715	0.884615251	0.99999488	0.999985264	0.832622862
NPW	0.949047684	0.87010028	0.780158644	0.99999488	0.999985264	0.933374538
TRDMT1	0.949047684	0.813113924	0.890248427	0.99999488	0.999985264	0.999960414
SNX24	0.949636238	0.992977597	0.870264123	0.99999488	0.999985264	0.779316087
SNRNP70	0.949865807	0.864945604	0.923604668	0.99999488	0.999985264	0.781688852
ARGLU1	0.949865807	0.896124237	0.907484111	0.99999488	0.999985264	0.787607112
NUF2	0.949865807	0.850095234	0.94634922	0.99999488	0.999985264	0.792819696
AUTS2	0.949865807	0.805123419	0.863209655	0.99999488	0.999985264	0.795293834
TMED9	0.949865807	0.820962752	0.830456514	0.99999488	0.999985264	0.802880474
PDIA6	0.949865807	0.970426668	0.848312163	0.99999488	0.999985264	0.806724906

CEP162	0.949865807	0.911731694	0.964152013	0.99999488	0.999985264	0.809513929
LOC100296952	0.949865807	0.90024974	0.833419544	0.99999488	0.999985264	0.816226172
SQSTM1	0.949865807	0.850692013	0.927600835	0.99999488	0.999985264	0.820845891
RIC8A	0.949865807	0.910319939	0.82336109	0.99999488	0.999985264	0.827190118
ELMO2	0.949865807	0.797577617	0.900148803	0.99999488	0.999985264	0.856860151
LZTS1	0.949865807	0.81753407	0.803677695	0.99999488	0.999985264	0.864806954
MAF1	0.949865807	0.826485535	0.900890709	0.99999488	0.999985264	0.873463288
IRAK1	0.949865807	0.859484941	0.946479014	0.99999488	0.999985264	0.873792335
PRRG4	0.949865807	0.95697765	0.856449202	0.99999488	0.999985264	0.885874544
MSRB3	0.949865807	0.867755878	0.862345036	0.99999488	0.999985264	0.892187201
SNRPD2	0.949865807	0.998011664	0.857021454	0.99999488	0.999985264	0.893921142
CDKL2	0.949865807	0.848246309	0.800952304	0.99999488	0.999985264	0.894236694
LOC112444498	0.949865807	0.943802122	0.834173166	0.991596938	0.999985264	0.89589047
ASNA1	0.949865807	0.909577824	0.848312163	0.99999488	0.999985264	0.89935427
LOC104974050	0.949865807	0.941876954	0.834068744	0.99999488	0.999985264	0.932608891
PPIL6	0.949865807	0.870839685	0.817700149	0.99999488	0.999985264	0.942195927
LOC617905	0.949865807	0.858808025	0.956565728	0.99999488	0.999985264	0.953254073
SEL1L3	0.949865807	0.826434903	0.912008683	0.99999488	0.999985264	0.955374205
SNRNP48	0.949865807	0.904961534	0.851718619	0.99999488	0.999985264	0.967316498
PSMD1	0.949865807	0.795127474	0.821698192	0.99999488	0.999985264	0.999960414
IDE	0.949865807	0.832807683	0.831723974	0.99999488	0.999985264	0.999960414
KIF27	0.950180532	0.846070848	0.83711556	0.99999488	0.999985264	0.87150355
OARD1	0.950565514	0.967520941	0.87762421	0.99999488	0.999985264	0.779686358
TGFB3	0.950565514	0.811327744	0.840781773	0.99999488	0.999985264	0.798878799
NES	0.950565514	0.9409629	0.917154286	0.99999488	0.999985264	0.809513929
SNX21	0.950565514	0.832807683	0.817700149	0.99999488	0.999985264	0.812825712
PPARGC1B	0.950565514	0.813113924	0.948420301	0.99999488	0.999985264	0.826043635
ASB11	0.950565514	0.978510121	0.946900905	0.99999488	0.999985264	0.841443168
DBN1	0.950565514	0.893470298	0.793478166	0.99999488	0.999985264	0.847577522
SLC38A9	0.950565514	0.958967142	0.785248842	0.99999488	0.999985264	0.86132915
LRRC51	0.950565514	0.81184075	0.831723974	0.99999488	0.999985264	0.894420979
ZNF667	0.950565514	0.91765877	0.787474031	0.99999488	0.999985264	0.905557485
NCAPH	0.950565514	0.922995823	0.841957362	0.99999488	0.999985264	0.919755394
LIMK1	0.950565514	0.885024111	0.870567942	0.99999488	0.999985264	0.931673132
TNIK	0.950565514	0.796000666	0.787862242	0.99999488	0.999985264	0.935335444
ENSA	0.950565514	0.893748757	0.865161179	0.99999488	0.999985264	0.946666773
IFI35	0.950565514	0.893642384	0.844729395	0.99999488	0.999985264	0.948213108
IRAK3	0.950741203	0.896996319	0.938203102	0.99999488	0.999985264	0.779686358
KDR	0.95135226	0.85337372	0.848312163	0.99999488	0.999985264	0.787607112

MYNN	0.95135226	0.999992444	0.785173725	0.99999488	0.999985264	0.791922285
GIPC2	0.95135226	0.873289191	0.890096618	0.99999488	0.999985264	0.792473222
CCR3	0.95135226	0.999992444	0.815389809	0.99999488	0.999985264	0.793763761
CBR1	0.95135226	0.942353856	0.878053482	0.99999488	0.999985264	0.805442659
GRHL1	0.95135226	0.999992444	0.825497047	0.99999488	0.999985264	0.809513929
TMBIM4	0.95135226	0.883624935	0.956565728	0.99999488	0.999985264	0.812725657
CLEC11A	0.95135226	0.928525825	0.886962419	0.99999488	0.999985264	0.812825712
GLRX5	0.95135226	0.87010028	0.86638329	0.998140932	0.999985264	0.816226172
CDC42BPG	0.95135226	0.999992444	0.843587792	0.99999488	0.999985264	0.816226172
LOC100126043	0.95135226	0.827123416	0.923604668	0.99999488	0.999985264	0.818895181
FAM189B	0.95135226	0.813622817	0.848312163	0.99999488	0.999985264	0.828248574
FES	0.95135226	0.836435076	0.833496026	0.99999488	0.999985264	0.83405895
LOC112447435	0.95135226	0.844174041	0.990752015	0.99999488	0.999985264	0.834119413
LOC112447353	0.95135226	0.933832654	0.785248842	0.99999488	0.999985264	0.870160734
SMARCA1	0.95135226	0.816518153	0.803677695	0.99999488	0.999985264	0.870160734
LOC100848226	0.95135226	0.921934673	0.838677332	0.99999488	0.999985264	0.893240103
HPRT1	0.95135226	0.821033198	0.873452279	0.99999488	0.999985264	0.897467714
RBKS	0.95135226	0.867755878	0.799381787	0.99999488	0.999985264	0.899223071
LOC100139891	0.95135226	0.87010028	0.862345036	0.997217712	0.999985264	0.90109791
TJP2	0.95135226	0.993777777	0.787862242	0.99999488	0.999985264	0.91208425
NR1H2	0.95135226	0.882505302	0.825497047	0.99999488	0.999985264	0.927969856
BORCS6	0.95135226	0.941495217	0.787006358	0.9998663	0.999985264	0.932351795
LOC510454	0.95135226	0.879402553	0.785248842	0.99999488	0.999985264	0.939035437
VSIG2	0.95135226	0.893642384	0.903957821	0.99999488	0.999985264	0.94921096
HTRA3	0.95135226	0.799180421	0.877799478	0.99999488	0.999985264	0.949718683
BAG6	0.95135226	0.985204209	0.818820085	0.99999488	0.999985264	0.957171025
KLHL8	0.95135226	0.796210279	0.809401378	0.99999488	0.999985264	0.9787163
WDR87	0.95135226	0.836126124	0.907381775	0.99999488	0.999985264	0.987454496
RUNX1T1	0.95135226	0.799180421	0.851718619	0.99999488	0.999985264	0.999960414
ZNF839	0.951361135	0.899394562	0.929188473	0.99999488	0.999985264	0.812831419
IL6ST	0.951361135	0.839292361	0.848786751	0.99999488	0.999985264	0.939035437
RBM10	0.951361348	0.826936949	0.981388276	0.99999488	0.999985264	0.807689737
LOC100848575	0.951361348	0.87010028	0.851718619	0.99999488	0.999985264	0.812825712
PFKFB4	0.951361348	0.985406717	0.877742202	0.99999488	0.999985264	0.812831419
DHCR7	0.951361348	0.998131745	0.835461206	0.99999488	0.999985264	0.821634966
ZC3H11A	0.951361348	0.981155176	0.833003624	0.99999488	0.999985264	0.85653214
LOC104971374	0.951361348	0.882505302	0.958627535	0.99999488	0.999985264	0.879798493
HIPK2	0.951361348	0.848070173	0.803677695	0.99999488	0.999985264	0.88807369
LOC101906226	0.951361348	0.935588259	0.803272674	0.99999488	0.999985264	0.948441365

XCL2	0.951361348	0.892206663	0.811233159	0.99999488	0.999985264	0.953908163
DNM2	0.951361348	0.816518153	0.852782377	0.99999488	0.999985264	0.978682689
PROS1	0.951520164	0.843635687	0.927600835	0.99999488	0.999985264	0.781688852
HOXC5	0.951520164	0.87010028	0.876758498	0.99999488	0.999985264	0.785716654
SCN2B	0.951520164	0.935742738	0.99035966	0.99999488	0.999985264	0.787783489
IL6R	0.951520164	0.830519875	0.785248842	0.99999488	0.999985264	0.790569257
CYP51A1	0.951520164	0.850692013	0.865066354	0.99999488	0.999985264	0.795293834
LOC101902207	0.951520164	0.985406717	0.851718619	0.99999488	0.999985264	0.818484413
CASD1	0.951520164	0.999992444	0.785248842	0.99999488	0.999985264	0.819073042
SNF8	0.951520164	0.831018329	0.956565728	0.99999488	0.999985264	0.827439931
RABL6	0.951520164	0.943802122	0.805319114	0.99999488	0.999985264	0.832622862
MBNL2	0.951520164	0.830519875	0.799266054	0.99999488	0.999985264	0.832833221
LOC782479	0.951520164	0.917537324	0.825497047	0.99999488	0.999985264	0.844292822
CAMK4	0.951520164	0.830519875	0.944085862	0.99999488	0.999985264	0.862838984
SENP7	0.951520164	0.967919743	0.864737879	0.99999488	0.999985264	0.872307149
RPS19BP1	0.951520164	0.943802122	0.846030883	0.99999488	0.999985264	0.885874544
RNF150	0.951520164	0.827661136	0.837103057	0.99999488	0.999985264	0.899020749
CDIP1	0.951520164	0.958098081	0.911922552	0.99999488	0.999985264	0.901809636
SNHG3	0.951520164	0.94984369	0.824972187	0.99999488	0.999985264	0.904161516
TRAF6	0.951520164	0.917537324	0.849633432	0.99999488	0.999985264	0.918400465
ULK4	0.951520164	0.925580392	0.879253779	0.99999488	0.999985264	0.921991933
RFFL	0.951520164	0.849474629	0.843674782	0.99999488	0.999985264	0.926480606
DISP1	0.951520164	0.819379885	0.901758346	0.99999488	0.999985264	0.931163114
MSANTD2	0.951520164	0.909633468	0.812450514	0.99999488	0.999985264	0.939035437
MYOZ1	0.951520164	0.859892902	0.785248842	0.99999488	0.999985264	0.943220018
NUDT16L1	0.951520164	0.911418256	0.848312163	0.99999488	0.999985264	0.945670698
LOC112442664	0.951520164	0.869999083	0.837103057	0.99999488	0.999985264	0.995564948
RETREG3	0.951520164	0.944701115	0.825497047	0.994632228	0.999985264	0.996949674
NOX5	0.951531461	0.91503472	0.803677695	0.99999488	0.999985264	0.818484413
TMEM86A	0.951531461	0.830519875	0.807608869	0.99999488	0.999985264	0.82386188
TTLL3	0.951557474	0.941495217	0.903175962	0.99999488	0.999985264	0.831603744
FOXM1	0.951666009	0.799180421	0.965059328	0.99999488	0.999985264	0.872307149
TVP23B	0.951891432	0.830132999	0.886192181	0.99999488	0.999985264	0.785716654
NUDT14	0.951891432	0.9582784	0.834068744	0.99999488	0.999985264	0.795293834
USP24	0.951891432	0.852760886	0.834173166	0.99999488	0.999985264	0.818895181
SREK1IP1	0.951891432	0.951765021	0.902527616	0.99999488	0.999985264	0.831532663
LOC615899	0.951891432	0.850095234	0.975538142	0.99999488	0.999985264	0.832017501
HAGH	0.951891432	0.815596111	0.824972187	0.99999488	0.999985264	0.83405895
SLC38A6	0.951891432	0.9582784	0.807608869	0.99999488	0.999985264	0.836520183

LOC112442295	0.951891432	0.921934673	0.978993134	0.99999488	0.999985264	0.84705359
CDC42EP5	0.951891432	0.874192034	0.841773648	0.99999488	0.999985264	0.858593649
SORCS2	0.951891432	0.862811597	0.88153193	0.99999488	0.999985264	0.858593649
APCDD1	0.951891432	0.821033198	0.862345036	0.99999488	0.999985264	0.884033654
RUSC1	0.951891432	0.90465634	0.848312163	0.99999488	0.999985264	0.894723749
C22H3orf22	0.951891432	0.90868193	0.992742113	0.99999488	0.999985264	0.897467714
LOC781261	0.951891432	0.975462313	0.817177032	0.99999488	0.999985264	0.900681286
NR2C2	0.951891432	0.820161757	0.807608869	0.99999488	0.999985264	0.910018813
PDGFRA	0.951891432	0.978089982	0.833496026	0.99999488	0.999985264	0.911997747
LOC786139	0.951891432	0.821617615	0.807323012	0.99999488	0.999985264	0.914088493
PODXL2	0.951891432	0.835868428	0.794530329	0.99999488	0.999985264	0.919755394
HEYL	0.951891432	0.915367594	0.941336964	0.99999488	0.999985264	0.919758204
TSFM	0.951891432	0.831782537	0.861441442	0.99999488	0.999985264	0.926596859
TGFB1I1	0.951891432	0.893906901	0.888545123	0.99999488	0.999985264	0.98341414
KRT23	0.951903985	0.861747495	0.789951578	0.99999488	0.999985264	0.790569257
ROM1	0.951903985	0.816518153	0.902527616	0.99999488	0.999985264	0.805869777
UQCR10	0.951903985	0.819379885	0.841739726	0.99999488	0.999985264	0.807187146
LOC112449406	0.951903985	0.825265917	0.985363931	0.99999488	0.999985264	0.80980964
C26H10orf88	0.951903985	0.999992444	0.926552506	0.99999488	0.999985264	0.815069494
RIPK3	0.951903985	0.87010028	0.920629231	0.99999488	0.999985264	0.839405979
SFXN1	0.951903985	0.87010028	0.851718619	0.99999488	0.999985264	0.856510591
SHC2	0.951903985	0.813113924	0.898815524	0.99999488	0.999985264	0.885874544
DLAT	0.951903985	0.821194038	0.834068744	0.99999488	0.999985264	0.89935427
HMBS	0.951903985	0.938017364	0.833419544	0.99999488	0.999985264	0.901614166
ARHGEF17	0.951903985	0.924095664	0.849221966	0.99999488	0.999985264	0.916064976
FAM219A	0.951903985	0.985406717	0.878344997	0.99999488	0.999985264	0.917028736
CD247	0.951903985	0.864811968	0.813142592	0.99999488	0.999985264	0.939035437
LOC112442979	0.951903985	0.83698359	0.946479014	0.99999488	0.999985264	0.94485591
CNKSR2	0.951903985	0.826485535	0.898125038	0.99999488	0.999985264	0.999960414
GALNT18	0.952045493	0.836435076	0.95230212	0.99999488	0.999985264	0.849138453
ZNF143	0.952045493	0.907069508	0.920629231	0.99999488	0.999985264	0.875840132
GOLGB1	0.952045493	0.862811597	0.880309898	0.99999488	0.999985264	0.886642513
EEF1G	0.952045493	0.869999083	0.898017577	0.99999488	0.999985264	0.89329705
EVA1A	0.952045493	0.805123419	0.837139208	0.99999488	0.999985264	0.897965262
GJC1	0.952045493	0.883624935	0.811233159	0.99999488	0.999985264	0.913766719
NDC80	0.952155323	0.831988573	0.992080972	0.99999488	0.999985264	0.805442659
LOC100848639	0.952155323	0.808483763	0.999558179	0.99999488	0.999985264	0.836520183
C2H2orf88	0.952155323	0.832584599	0.851718619	0.99999488	0.999985264	0.940358165
EGFR	0.952255447	0.805123419	0.86259773	0.99999488	0.999985264	0.873254557

MTCH2	0.952255447	0.816518153	0.873452279	0.99999488	0.999985264	0.927969856
LOC112442053	0.952362322	0.886198145	0.932653752	0.99999488	0.999985264	0.805869777
KIAA0930	0.952362322	0.882022911	0.92487482	0.99999488	0.999985264	0.816924515
LOC100848504	0.952362322	0.820100152	0.790826129	0.99999488	0.999985264	0.856510591
STX2	0.952362322	0.819937634	0.841957362	0.99999488	0.999985264	0.865195569
HNRNPD	0.952362322	0.819937634	0.854323176	0.99999488	0.999985264	0.892231098
CCDC34	0.95254638	0.867755878	0.948420301	0.99999488	0.999985264	0.939035437
MGRN1	0.952583588	0.809135999	0.937822865	0.99999488	0.999985264	0.888645453
TTL	0.952909485	0.984068885	0.851718619	0.99999488	0.999985264	0.809513929
STAC2	0.952909485	0.991488652	0.813142592	0.99999488	0.999985264	0.816930408
HAS2	0.953059472	0.832584599	0.787694703	0.99999488	0.999985264	0.799828183
PET117	0.953059472	0.873289191	0.788870461	0.99999488	0.999985264	0.812831419
FAM3C	0.953059472	0.919884594	0.80757077	0.99999488	0.999985264	0.833244331
LOC107133473	0.953059472	0.815489392	0.857490704	0.99999488	0.999985264	0.934749052
LOC112447385	0.953080906	0.904961534	0.830972393	0.99999488	0.999985264	0.83405895
ZNF628	0.953266583	0.88463751	0.815067929	0.99999488	0.999985264	0.795293834
LOC104971683	0.953266583	0.845698648	0.941118576	0.99999488	0.999985264	0.802880474
THNSL2	0.953266583	0.836111245	0.851718619	0.99999488	0.999985264	0.808608716
RASSF8	0.953266583	0.8324185	0.89483889	0.99999488	0.999985264	0.818484413
SPPL2B	0.953266583	0.90024974	0.862345036	0.99999488	0.999985264	0.923360345
IQSEC2	0.953266583	0.845262032	0.809401378	0.99999488	0.999985264	0.943633749
NBN	0.953266583	0.992977597	0.835038876	0.99999488	0.999985264	0.944906868
LOC104968476	0.953266583	0.926949474	0.938911935	0.99999488	0.999985264	0.948213108
SH3GL2	0.953266583	0.825645694	0.881942256	0.99999488	0.999985264	0.949315689
MT1E	0.953266583	0.826642757	0.905757315	0.99999488	0.999985264	0.976814573
LOC101908214	0.953266583	0.901205367	0.81983962	0.99999488	0.999985264	0.977753483
RNF4	0.953284346	0.957700659	0.831723974	0.99999488	0.999985264	0.790569257
RHBDD1	0.953284346	0.893470298	0.954864482	0.99999488	0.999985264	0.790569257
SPAST	0.953284346	0.831798801	0.834068744	0.99999488	0.999985264	0.793560536
POGLUT1	0.953284346	0.999992444	0.824972187	0.99999488	0.999985264	0.794870105
LOC112446689	0.953284346	0.936657629	0.807608869	0.99999488	0.999985264	0.795293834
NSMCE2	0.953284346	0.999992444	0.822003523	0.99999488	0.999985264	0.795293834
LOC781224	0.953284346	0.821194038	0.877799478	0.99999488	0.999985264	0.795293834
DOK6	0.953284346	0.89064218	0.895626254	0.99999488	0.999985264	0.796770444
PLEKHO2	0.953284346	0.870839685	0.817145109	0.99999488	0.999985264	0.796812106
FBXO30	0.953284346	0.999992444	0.803942311	0.99999488	0.999985264	0.802880474
ADCY2	0.953284346	0.821194038	0.8664043	0.99999488	0.999985264	0.806724906
TCIRG1	0.953284346	0.835868428	0.803677695	0.99999488	0.999985264	0.811240325
CCNI	0.953284346	0.915367594	0.873452279	0.99999488	0.999985264	0.812825712

CDK7	0.953284346	0.845554546	0.820679808	0.99999488	0.999985264	0.812831419
IER2	0.953284346	0.916036681	0.95106368	0.99999488	0.999985264	0.812831419
CCND1	0.953284346	0.91000013	0.923177807	0.99999488	0.999985264	0.818484413
APH1A	0.953284346	0.900867274	0.825497047	0.99999488	0.999985264	0.819073042
TMEM238	0.953284346	0.90213106	0.887626998	0.99999488	0.999985264	0.819073042
CREB3L4	0.953284346	0.917849199	0.95230212	0.99999488	0.999985264	0.819195992
SCN3A	0.953284346	0.920040168	0.797322227	0.99999488	0.999985264	0.82386188
PPP1R11	0.953284346	0.873289191	0.960095471	0.99999488	0.999985264	0.824632953
LOC104975034	0.953284346	0.935742738	0.825497047	0.99999488	0.999985264	0.827439931
C5H22orf23	0.953284346	0.869999083	0.920629231	0.99999488	0.999985264	0.828248574
RPUSD3	0.953284346	0.938476452	0.944993804	0.99999488	0.999985264	0.833388946
TM4SF18	0.953284346	0.832130119	0.931955158	0.99999488	0.999985264	0.836520183
PITPNA	0.953284346	0.943802122	0.892664791	0.99999488	0.999985264	0.837143123
AKIRIN1	0.953284346	0.916036681	0.922400936	0.99999488	0.999985264	0.839859968
LOC112445995	0.953284346	0.942353856	0.88754627	0.99999488	0.999985264	0.845834943
CSRNP2	0.953284346	0.915251309	0.877799478	0.99999488	0.999985264	0.847577522
ANAPC11	0.953284346	0.95697765	0.857072251	0.99999488	0.999985264	0.847964496
PIK3R2	0.953284346	0.862982	0.861140642	0.99999488	0.999985264	0.849046935
GYS1	0.953284346	0.890682633	0.817700149	0.99999488	0.999985264	0.849266568
POLR2E	0.953284346	0.85337372	0.822003523	0.99999488	0.999985264	0.850790144
EXTL3	0.953284346	0.9582784	0.849637051	0.99999488	0.999985264	0.856224954
ZFP37	0.953284346	0.999992444	0.866582678	0.99999488	0.999985264	0.856510591
P4HA1	0.953284346	0.864945604	0.945678616	0.99999488	0.999985264	0.856510591
GPLD1	0.953284346	0.999992444	0.805753285	0.99999488	0.999985264	0.861837269
CDCP1	0.953284346	0.845554546	0.998936876	0.99999488	0.999985264	0.862890646
CACYBP	0.953284346	0.821194038	0.900351064	0.99999488	0.999985264	0.863192816
CTSF	0.953284346	0.896507811	0.817700149	0.99999488	0.999985264	0.863331236
CCR10	0.953284346	0.867755878	0.886192181	0.99999488	0.999985264	0.871960581
CCDC174	0.953284346	0.94254418	0.790264407	0.99999488	0.999985264	0.872307149
NUP62CL	0.953284346	0.819937634	0.926552506	0.99999488	0.999985264	0.872307149
STAB1	0.953284346	0.866235664	0.865347217	0.99999488	0.999985264	0.874439646
BPTF	0.953284346	0.87010028	0.817700149	0.99999488	0.999985264	0.874975722
CENPK	0.953284346	0.829363839	0.92768724	0.99999488	0.999985264	0.877940484
FHOD3	0.953284346	0.919369362	0.813142592	0.99999488	0.999985264	0.878070222
BCAS4	0.953284346	0.928759674	0.881040986	0.99999488	0.999985264	0.880216491
MRPS23	0.953284346	0.816100073	0.871925227	0.99999488	0.999985264	0.88262005
HYPK	0.953284346	0.984068885	0.923604668	0.99999488	0.999985264	0.885874544
GARNL3	0.953284346	0.957700659	0.906268775	0.99999488	0.999985264	0.886185283
LOC783838	0.953284346	0.985406717	0.833419544	0.99999488	0.999985264	0.891439324

DNM1	0.953284346	0.836094224	0.938203102	0.99999488	0.999985264	0.89329705
STARD10	0.953284346	0.926955605	0.886962419	0.99999488	0.999985264	0.896480535
POLE3	0.953284346	0.91058693	0.914827591	0.99999488	0.999985264	0.89668604
PROZ	0.953284346	0.815852027	0.991992782	0.99999488	0.999985264	0.897965262
PAWR	0.953284346	0.989547896	0.862345036	0.99999488	0.999985264	0.901614166
STK4	0.953284346	0.850692013	0.825497047	0.99999488	0.999985264	0.904813143
LOC100296832	0.953284346	0.862982	0.816317002	0.99999488	0.999985264	0.911089313
NKRF	0.953284346	0.830654932	0.903389425	0.99999488	0.999985264	0.912889828
APBB1	0.953284346	0.830654932	0.934941286	0.99999488	0.999985264	0.915476587
MGAT5B	0.953284346	0.935060764	0.833496026	0.99999488	0.999985264	0.919755394
FANCE	0.953284346	0.850692013	0.826801319	0.99999488	0.999985264	0.919758204
KBTBD4	0.953284346	0.808097876	0.794745005	0.99999488	0.999985264	0.923146498
PMM2	0.953284346	0.896996319	0.825497047	0.99999488	0.999985264	0.927969856
WDR4	0.953284346	0.920639601	0.805319114	0.99999488	0.999985264	0.931480862
LOC112444778	0.953284346	0.817847494	0.825497047	0.99999488	0.999985264	0.948213108
IFFO2	0.953284346	0.858808025	0.930678591	0.99999488	0.999985264	0.948213108
ACVR2A	0.953284346	0.893642384	0.881040986	0.99999488	0.999985264	0.94847852
DERL3	0.953284346	0.893642384	0.954823586	0.99999488	0.999985264	0.949718683
TMEM198	0.953284346	0.938597964	0.841717609	0.99999488	0.999985264	0.949764317
NUDT21	0.953284346	0.867755878	0.859566702	0.99999488	0.999985264	0.949764317
SPATA2	0.953284346	0.893743321	0.815067929	0.99999488	0.999985264	0.95096765
MICAL2	0.953284346	0.90387438	0.851718619	0.99999488	0.999985264	0.95096765
TMEM232	0.953284346	0.840038535	0.926552506	0.99999488	0.999985264	0.953795193
PARD3B	0.953284346	0.81184075	0.923604668	0.99999488	0.999985264	0.955374205
NRIP2	0.953284346	0.864945604	0.922400936	0.99999488	0.999985264	0.956550292
RANGAP1	0.953284346	0.850692013	0.834173166	0.99999488	0.999985264	0.960613782
RYR2	0.953284346	0.957700659	0.832037703	0.99999488	0.999985264	0.972825932
EMC8	0.953284346	0.873963285	0.839661294	0.99999488	0.999985264	0.975051853
MAP3K9	0.953284346	0.892785043	0.831723974	0.99999488	0.999985264	0.982250307
BUB3	0.953284346	0.858808025	0.831723974	0.99999488	0.999985264	0.993564907
LOC112448582	0.953284346	0.936273761	0.800952304	0.99999488	0.999985264	0.993606543
TSN	0.953284346	0.859484941	0.922400936	0.99999488	0.999985264	0.996066257
FBL	0.953284346	0.907069508	0.803677695	0.99999488	0.999985264	0.998331177
YPEL3	0.953284346	0.813113924	0.805753285	0.99999488	0.999985264	0.999960414
ARMC2	0.953284346	0.849474629	0.892664791	0.99999488	0.999985264	0.999960414
RNF208	0.953772179	0.907069508	0.845165598	0.99999488	0.999985264	0.793105162
NUAK2	0.953772179	0.836126124	0.862345036	0.99999488	0.999985264	0.803939828
PLOD2	0.953772179	0.867840435	0.863098782	0.99999488	0.999985264	0.812825712
NDE1	0.953772179	0.999992444	0.816532117	0.99999488	0.999985264	0.812831419

GPR132	0.953772179	0.993316598	0.831723974	0.99999488	0.999985264	0.832093857
DEDD	0.953772179	0.938597964	0.851718619	0.99999488	0.999985264	0.840732705
NAA60	0.953772179	0.862811597	0.805753285	0.99999488	0.999985264	0.868103112
LOC100847304	0.953772179	0.867755878	0.880594299	0.99999488	0.999985264	0.894723749
CYP2S1	0.953772179	0.936657629	0.970874203	0.99999488	0.999985264	0.901809636
REXO2	0.953772179	0.935742738	0.837103057	0.99999488	0.999985264	0.969551076
CSTF2T	0.953772179	0.867755878	0.845368791	0.99999488	0.999985264	0.999960414
MRPS34	0.954089847	0.867755878	0.851718619	0.99999488	0.999985264	0.897965262
CXCL5	0.954426453	0.830519875	0.812450514	0.99999488	0.999985264	0.863331236
KAZALD1	0.954955212	0.991488652	0.917154286	0.99999488	0.999985264	0.819073042
MAZ	0.954955212	0.875829726	0.918170048	0.99999488	0.999985264	0.821080672
C22H3orf62	0.954955212	0.883624935	0.852670802	0.99999488	0.999985264	0.87805762
STK26	0.954955212	0.835868428	0.851718619	0.99999488	0.999985264	0.885874544
SLC26A11	0.954955212	0.885024111	0.852782377	0.99999488	0.999985264	0.95096765
HGF	0.955315887	0.927853996	0.86638329	0.99999488	0.999985264	0.901809636
TRAPPC3	0.95555421	0.8756206	0.970874203	0.99999488	0.999985264	0.812831419
MROH1	0.95555421	0.941495217	0.999558179	0.99999488	0.999985264	0.812831419
MEIS3	0.955615814	0.913160799	0.900890709	0.99999488	0.999985264	0.839734604
TEX9	0.95596351	0.999992444	0.830045657	0.99999488	0.999985264	0.807689737
TLK1	0.95596351	0.994434609	0.80757077	0.99999488	0.999985264	0.812825712
STK24	0.95596351	0.937231686	0.83711556	0.99999488	0.999985264	0.837019875
BBIP1	0.95596351	0.994135588	0.841957362	0.99999488	0.999985264	0.847577522
EXOSC2	0.95596351	0.811937459	0.982433474	0.99999488	0.999985264	0.847577522
PDRG1	0.95596351	0.830519875	0.908595488	0.99999488	0.999985264	0.873463288
LOC112442228	0.95596351	0.957700659	0.814724984	0.99999488	0.999985264	0.87805762
CCHCR1	0.95596351	0.958967142	0.922400936	0.99999488	0.999985264	0.894723749
TMEM258	0.95596351	0.999992444	0.860189237	0.99999488	0.999985264	0.897467714
RPP30	0.95596351	0.864945604	0.909070782	0.99999488	0.999985264	0.925481882
FAM3A	0.95596351	0.920253543	0.837693962	0.99999488	0.999985264	0.926885454
PSMA5	0.95596351	0.82555829	0.851718619	0.99999488	0.999985264	0.931163114
MBOAT2	0.95596351	0.850274237	0.825497047	0.99999488	0.999985264	0.932608891
LOC101905866	0.95596351	0.883624935	0.815864189	0.99999488	0.999985264	0.939035437
LOC112448744	0.95596351	0.826434903	0.837948687	0.99999488	0.999985264	0.999960414
DYNLL2	0.95608998	0.881505398	0.946479014	0.99999488	0.999985264	0.802788605
ZFYVE19	0.95608998	0.921661251	0.884320747	0.99999488	0.999985264	0.809513929
USP1	0.95608998	0.935742738	0.877799478	0.99999488	0.999985264	0.809729441
PAK4	0.95608998	0.825438977	0.829269849	0.99999488	0.999985264	0.820845891
CIAO1	0.95608998	0.827123416	0.851718619	0.99999488	0.999985264	0.827190118
LY6G5B	0.95608998	0.865926615	0.945678616	0.99999488	0.999985264	0.898510469

CAMSAP2	0.95608998	0.881001912	0.914630535	0.99999488	0.999985264	0.90408677
STS	0.95608998	0.813113924	0.877799478	0.99999488	0.999985264	0.927969856
NPNT	0.95608998	0.985406717	0.809561323	0.99999488	0.999985264	0.944457836
RIPOR2	0.95608998	0.958967142	0.825736269	0.99999488	0.999985264	0.949764317
ORAI2	0.95608998	0.905019423	0.815067929	0.99999488	0.999985264	0.999960414
TECPR1	0.956707172	0.867045149	0.981039752	0.99999488	0.999985264	0.812831419
ATP6V0E1	0.956707172	0.821617615	0.996715234	0.99999488	0.999985264	0.816959462
MRPL51	0.956707172	0.816518153	0.890248427	0.99999488	0.999985264	0.819090616
MASTL	0.956707172	0.819937634	0.938203102	0.99999488	0.999985264	0.904686193
WDR55	0.956860879	0.842782808	0.904959653	0.99999488	0.999985264	0.870893258
ALKBH4	0.956860879	0.836126124	0.930678591	0.99999488	0.999985264	0.912262222
ZNF852	0.956953366	0.97161271	0.825685674	0.99999488	0.999985264	0.911089313
LOC101904749	0.956979522	0.938597964	0.949038056	0.99999488	0.999985264	0.820927993
TTPA	0.957078674	0.826642757	0.825497047	0.99999488	0.999985264	0.807665096
HRC	0.957227569	0.835868428	0.877742202	0.99999488	0.999985264	0.837143123
AGO3	0.957487471	0.949544652	0.799266054	0.99999488	0.999985264	0.849046935
UFSP2	0.957553075	0.943802122	0.997208738	0.99999488	0.999985264	0.812831419
KDELC1	0.957553075	0.869999083	0.873771031	0.99999488	0.999985264	0.825478387
IFI30	0.957553075	0.889433819	0.817700149	0.99999488	0.999985264	0.861837269
LLGL2	0.957553075	0.948421431	0.857376578	0.99999488	0.999985264	0.873083064
PAICS	0.957553075	0.84979258	0.960804071	0.99999488	0.999985264	0.90109791
NABP1	0.957553075	0.863793573	0.881942256	0.99999488	0.999985264	0.982250307
SPTY2D1	0.957569435	0.896536444	0.881942256	0.99999488	0.999985264	0.849138453
CRTAC1	0.957569435	0.958967142	0.802997196	0.99999488	0.999985264	0.850790144
LOC104973139	0.957569435	0.869999083	0.982128584	0.99999488	0.999985264	0.885874544
RPL13A	0.957569435	0.872223076	0.851718619	0.99999488	0.999985264	0.892908035
LAGE3	0.957569435	0.821194038	0.86772394	0.99999488	0.999985264	0.894723749
NR1D1	0.957832794	0.864945604	0.83711556	0.99999488	0.999985264	0.999960414
COLGALT1	0.958154628	0.921934673	0.825497047	0.99999488	0.999985264	0.856510591
LOC104971817	0.958271488	0.862811597	0.957541703	0.99999488	0.999985264	0.890433418
LOC112442215	0.958295239	0.957700659	0.821546364	0.99999488	0.999985264	0.925260365
PPP1R15A	0.958528961	0.937231686	0.993585211	0.99999488	0.999985264	0.858593649
GTF2B	0.958796275	0.85337372	0.988922732	0.99999488	0.999985264	0.807689737
KCNA6	0.958796275	0.865184773	0.841957362	0.99999488	0.999985264	0.816959462
POLI	0.958796275	0.999992444	0.803942311	0.99999488	0.999985264	0.856510591
RNF13	0.958796275	0.936273761	0.821546364	0.99999488	0.999985264	0.882867473
IFITM1	0.958796275	0.867535124	0.80757077	0.99999488	0.999985264	0.927969856
FAM180A	0.958796275	0.90116492	0.803942311	0.99999488	0.999985264	0.956283599
PNPLA3	0.958796275	0.819937634	0.813282256	0.99999488	0.999985264	0.968772302

NDUFA11	0.959003455	0.863793573	0.861480822	0.99999488	0.999985264	0.812831419
COL15A1	0.959083025	0.866462388	0.803677695	0.99999488	0.999985264	0.807566643
LOC616199	0.959083025	0.93570298	0.825497047	0.99999488	0.999985264	0.834119413
POLR3D	0.959464012	0.880276234	0.811233159	0.99999488	0.999985264	0.948213108
SNHG12	0.959559655	0.927447588	0.862345036	0.99999488	0.999985264	0.819275036
RPS29	0.959559655	0.864024589	0.856079665	0.99999488	0.999985264	0.872364241
KIAA2012	0.959559655	0.938597964	0.803942311	0.99999488	0.999985264	0.936352893
LOC112442401	0.960650431	0.858808025	0.884320747	0.99999488	0.999985264	0.894723749
GLRX	0.960956494	0.935384315	0.888107357	0.99999488	0.999985264	0.866439215
ADTRP	0.960956494	0.907069508	0.886192181	0.99999488	0.999985264	0.893921142
SLC25A12	0.96099582	0.847686989	0.813142592	0.99999488	0.999985264	0.827439931
TMEM161B	0.961020136	0.904961534	0.900148803	0.99999488	0.999985264	0.837032263
VAV2	0.961020136	0.813755213	0.940815039	0.99999488	0.999985264	0.850307641
MRPL20	0.961020136	0.883624935	0.900351064	0.99999488	0.999985264	0.918400465
P2RY8	0.961020136	0.835868428	0.813142592	0.99999488	0.999985264	0.937108046
NTS	0.961020136	0.9677397	0.86638329	0.99999488	0.999985264	0.939035437
AOAH	0.961020136	0.821033198	0.837254326	0.99999488	0.999985264	0.989539011
CTPS1	0.961020136	0.863793573	0.864441458	0.99999488	0.999985264	0.992853834
TMOD4	0.961020136	0.836435076	0.83711556	0.99999488	0.999985264	0.997580124
TESK2	0.961020136	0.827661136	0.848473573	0.99999488	0.999985264	0.999960414
SLC2A8	0.961057875	0.824377616	0.953265249	0.99999488	0.999985264	0.822863905
PDK2	0.961057875	0.850095234	0.840781773	0.99999488	0.999985264	0.828248574
LOC617875	0.961057875	0.936273761	0.851718619	0.99999488	0.999985264	0.837517492
ASCL2	0.961057875	0.862982	0.949038056	0.99999488	0.999985264	0.849547326
PRDX3	0.961160491	0.905019423	0.848832109	0.99999488	0.999985264	0.8503922
SLC7A10	0.961160491	0.839292361	0.909070782	0.99999488	0.999985264	0.850842307
RNF19A	0.961160491	0.883856603	0.938203102	0.99999488	0.999985264	0.885266141
AHNAK2	0.961160491	0.850095234	0.890248427	0.99999488	0.999985264	0.969801844
STT3A	0.961266996	0.96728174	0.869864092	0.99999488	0.999985264	0.902508792
CSNK2B	0.961266996	0.928088831	0.867952378	0.99999488	0.999985264	0.923360345
GRHPR	0.961266996	0.816518153	0.831723974	0.99999488	0.999985264	0.952124699
LOC505479	0.961266996	0.925580392	0.821698192	0.99999488	0.999985264	0.982250307
MTRF1	0.961266996	0.862811597	0.826801319	0.99999488	0.999985264	0.996949674
NDUFA13	0.961447432	0.830519875	0.876188482	0.99999488	0.999985264	0.811240325
ACTR5	0.961602012	0.928113285	0.945678616	0.99999488	0.999985264	0.807566643
ANKHD1	0.961602012	0.935742738	0.818876578	0.99999488	0.999985264	0.809513929
IQANK1	0.961602012	0.821033198	0.999558179	0.99999488	0.999985264	0.809729441
SLC12A2	0.961602012	0.850692013	0.825497047	0.99999488	0.999985264	0.812831419
TLR8	0.961602012	0.816100073	0.900148803	0.99999488	0.999985264	0.812831419

CYP2R1	0.961602012	0.821033198	0.938203102	0.99999488	0.999985264	0.812831419
SP140L	0.961602012	0.827123416	0.964186888	0.99999488	0.999985264	0.816226172
RHOA	0.961602012	0.938597964	0.910426741	0.99999488	0.999985264	0.827439931
PKIG	0.961602012	0.999992444	0.871925227	0.99999488	0.999985264	0.841487109
LOC790101	0.961602012	0.83875524	0.837361321	0.99999488	0.999985264	0.856011656
ITIH3	0.961602012	0.963717626	0.831723974	0.99999488	0.999985264	0.881211326
PPP1R15B	0.961602012	0.883624935	0.857790793	0.99999488	0.999985264	0.887130847
ZRANB2	0.961602012	0.999992444	0.830970408	0.99999488	0.999985264	0.901545041
CDK5RAP2	0.961602012	0.874697957	0.877799478	0.99999488	0.999985264	0.906495263
RPLP0	0.961602012	0.874192034	0.88153193	0.99999488	0.999985264	0.919755394
CDK2	0.961602012	0.816518153	0.843587792	0.99999488	0.999985264	0.972391591
PHTF1	0.961815029	0.879773164	0.852468219	0.99999488	0.999985264	0.814309336
GATAD2B	0.961815029	0.998131745	0.862345036	0.99999488	0.999985264	0.818895181
PIAS1	0.961815029	0.992977597	0.825497047	0.99999488	0.999985264	0.828248574
RAD51C	0.961815029	0.999992444	0.833496026	0.99999488	0.999985264	0.861618934
SLC6A2	0.961815029	0.816518153	0.932139039	0.99999488	0.999985264	0.89329705
BTLA	0.961815029	0.999992444	0.873452279	0.99999488	0.999985264	0.919755394
DDX17	0.961888239	0.895540475	0.875823795	0.99999488	0.999985264	0.807689737
SCYL1	0.961888239	0.985406717	0.880229444	0.99999488	0.999985264	0.807689737
MEA1	0.961888239	0.907069508	0.998936876	0.99999488	0.999985264	0.812725657
PDE6G	0.961888239	0.891316528	0.999558179	0.99999488	0.999985264	0.815564853
PGM2	0.961888239	0.869999083	0.88153193	0.99999488	0.999985264	0.816226172
LOC112448082	0.961888239	0.918648578	0.881942256	0.99999488	0.999985264	0.816226172
LOC100847604	0.961888239	0.973092298	0.973406453	0.99999488	0.999985264	0.816226172
CDK2AP1	0.961888239	0.835868428	0.857021454	0.99999488	0.999985264	0.828248574
NARF	0.961888239	0.913204869	0.866240823	0.99999488	0.999985264	0.83405895
THAP7	0.961888239	0.914761777	0.817177032	0.99999488	0.999985264	0.839734604
SACM1L	0.961888239	0.925580392	0.887790797	0.99999488	0.999985264	0.841443168
CALM2	0.961888239	0.837678919	0.999558179	0.99999488	0.999985264	0.844277572
TRIP4	0.961888239	0.946274461	0.887626998	0.99999488	0.999985264	0.856224954
RBM25	0.961888239	0.912518351	0.849637051	0.99999488	0.999985264	0.859321224
ZC3H8	0.961888239	0.999992444	0.817700149	0.99999488	0.999985264	0.861030464
BRAT1	0.961888239	0.83698359	0.964186888	0.99999488	0.999985264	0.861837269
FGD4	0.961888239	0.974812121	0.80757077	0.99999488	0.999985264	0.866439215
RALB	0.961888239	0.852760886	0.847427167	0.99999488	0.999985264	0.873836462
ATP6V1A	0.961888239	0.832584599	0.926552506	0.99999488	0.999985264	0.881288523
LOC104974812	0.961888239	0.816518153	0.812450514	0.99999488	0.999985264	0.882867473
NCDN	0.961888239	0.847015513	0.885842815	0.99999488	0.999985264	0.895349622
CTTN	0.961888239	0.819127076	0.872364252	0.99999488	0.999985264	0.923360345

NFYC	0.961888239	0.987559864	0.915215321	0.99999488	0.999985264	0.931163114
WDR83	0.961888239	0.992977597	0.811233159	0.99999488	0.999985264	0.932425574
HTT	0.961888239	0.852035396	0.817700149	0.99999488	0.999985264	0.937108046
CPT1A	0.961888239	0.850692013	0.826826413	0.99999488	0.999985264	0.939035437
CSE1L	0.961888239	0.967276215	0.907381775	0.99999488	0.999985264	0.940731625
TPM1	0.961888239	0.977991484	0.887790797	0.99999488	0.999985264	0.946303774
LOC101909003	0.961888239	0.87010028	0.930678591	0.99999488	0.999985264	0.964333944
FSBP	0.961888239	0.840946688	0.824972187	0.99999488	0.999985264	0.977753483
EIF4G3	0.961910821	0.89695121	0.851718619	0.99999488	0.999985264	0.888234257
EFHC1	0.961940521	0.952346363	0.83711556	0.99999488	0.999985264	0.809513929
PLCL1	0.961940521	0.989958965	0.892053976	0.99999488	0.999985264	0.812825712
AJAP1	0.961940521	0.921934673	0.908181052	0.99999488	0.999985264	0.816226172
RGS7BP	0.961940521	0.821194038	0.833496026	0.99999488	0.999985264	0.921150136
AP1B1	0.961940521	0.926949474	0.825497047	0.99999488	0.999985264	0.949643184
MAK16	0.961940521	0.873289191	0.884407415	0.99999488	0.999985264	0.993086089
OFD1	0.962032542	0.999347049	0.938203102	0.99999488	0.999985264	0.819073042
LOC107132247	0.962032542	0.916036681	0.921010865	0.99999488	0.999985264	0.908596393
INPP5D	0.962032542	0.827661136	0.825497047	0.99999488	0.999985264	0.939035437
WSB2	0.962056602	0.832497353	0.939501959	0.99999488	0.999985264	0.818895181
RPAP2	0.962298218	0.991488652	0.93198623	0.99999488	0.999985264	0.826043635
HID1	0.962298218	0.90116492	0.9426263	0.99999488	0.999985264	0.828248574
COPS7B	0.962298218	0.858165479	0.945766581	0.99999488	0.999985264	0.839734604
PHLDA3	0.962298218	0.943802122	0.825497047	0.99999488	0.999985264	0.862046449
SPDL1	0.962298218	0.876631119	0.805753285	0.99999488	0.999985264	0.863331236
CCNE2	0.962400479	0.9409629	0.900148803	0.99999488	0.999985264	0.948347116
ANKRD52	0.962447471	0.848246309	0.840781773	0.99999488	0.999985264	0.814496203
PDE7B	0.962447471	0.999992444	0.825497047	0.99999488	0.999985264	0.818895181
MAPK8IP2	0.962447471	0.986637746	0.962653182	0.99999488	0.999985264	0.820927993
FAM149B1	0.962447471	0.998131745	0.865066354	0.99999488	0.999985264	0.821433361
HMGB1	0.962447471	0.917849199	0.973303821	0.99999488	0.999985264	0.837143123
BEGAIN	0.962447471	0.941495217	0.862257903	0.99999488	0.999985264	0.85182524
SOX6	0.962447471	0.991365955	0.920154807	0.99999488	0.999985264	0.85182524
TMEM160	0.962447471	0.915251309	0.862911439	0.99999488	0.999985264	0.855645922
BOD1	0.962447471	0.845554546	0.809561323	0.99999488	0.999985264	0.873836462
C7H19orf44	0.962447471	0.982918944	0.874485384	0.99999488	0.999985264	0.881350165
IP6K1	0.962447471	0.872223076	0.834800132	0.99999488	0.999985264	0.888645453
LOC783541	0.962447471	0.91407604	0.862345036	0.99999488	0.999985264	0.918400465
CD27	0.962447471	0.816518153	0.956472932	0.99999488	0.999985264	0.926326154
TATDN3	0.962447471	0.867755878	0.864257597	0.99999488	0.999985264	0.939035437

LOC112442374	0.962718498	0.816518153	0.964396605	0.99999488	0.999985264	0.831603744
BFAR	0.962835586	0.869304939	0.900148803	0.99999488	0.999985264	0.943220018
LOC112442652	0.962950198	0.93825132	0.807270151	0.99999488	0.999985264	0.812831419
LYPD3	0.962950198	0.832807683	0.993594894	0.99999488	0.999985264	0.818895181
WFIKKN1	0.962950198	0.819155365	0.95230212	0.99999488	0.999985264	0.827971245
SETD2	0.962950198	0.885718559	0.811233159	0.99999488	0.999985264	0.84393496
GEM	0.962950198	0.907069508	0.865420641	0.99999488	0.999985264	0.877838449
NPDC1	0.962950198	0.928759674	0.873452279	0.99999488	0.999985264	0.890433418
CCNY	0.962950198	0.84979258	0.938215065	0.99999488	0.999985264	0.907397567
EFNA4	0.962950198	0.840946688	0.915215321	0.99999488	0.999985264	0.964476713
CDC42EP1	0.963310517	0.936273761	0.834068744	0.99999488	0.999985264	0.839734604
SEC16A	0.963310517	0.834860292	0.84160836	0.99999488	0.999985264	0.960343814
ZDHHC17	0.963581363	0.999347049	0.865420641	0.99999488	0.999985264	0.840213271
ZFYVE16	0.963636227	0.902754188	0.958125965	0.99999488	0.999985264	0.812825712
LOC508933	0.963636227	0.922995823	0.935094475	0.99999488	0.999985264	0.819073042
MGAT2	0.963636227	0.893642384	0.852670802	0.99999488	0.999985264	0.991488481
PDXP	0.963745367	0.867755878	0.877901599	0.99999488	0.999985264	0.811060122
PRDX4	0.963745367	0.846198976	0.926552506	0.99999488	0.999985264	0.812831419
ATP5PD	0.963745367	0.841862449	0.93198623	0.99999488	0.999985264	0.812831419
NUP50	0.963745367	0.864157215	0.938203102	0.99999488	0.999985264	0.812831419
MRC2	0.963745367	0.864945604	0.95230212	0.99999488	0.999985264	0.812831419
UBE2G1	0.963745367	0.949285285	0.939799459	0.99999488	0.999985264	0.816959462
RPH3AL	0.963745367	0.952346363	0.837095307	0.99999488	0.999985264	0.821089634
MCM3	0.963745367	0.938597964	0.907381775	0.99999488	0.999985264	0.821634966
CHST3	0.963745367	0.991671026	0.913194357	0.99999488	0.999985264	0.824559256
NOXA1	0.963745367	0.896507811	0.95758013	0.99999488	0.999985264	0.827971245
CCL24	0.963745367	0.840946688	0.886962419	0.99999488	0.999985264	0.84525008
HSD17B10	0.963745367	0.827661136	0.841739726	0.99999488	0.999985264	0.850307641
NDUFA9	0.963745367	0.842852797	0.862345036	0.99999488	0.999985264	0.850790144
LOC101902407	0.963745367	0.840770466	0.921379317	0.99999488	0.999985264	0.85395895
MYL5	0.963745367	0.943802122	0.962346588	0.99999488	0.999985264	0.859008195
GFOD2	0.963745367	0.997426798	0.824984963	0.99999488	0.999985264	0.873463288
MAPK1	0.963745367	0.9582784	0.865066354	0.99999488	0.999985264	0.873463288
PDE3A	0.963745367	0.999992444	0.878254532	0.99999488	0.999985264	0.873463288
LOC101907255	0.963745367	0.999992444	0.917410615	0.99999488	0.999985264	0.885874544
BTF3	0.963745367	0.826642757	0.837103057	0.99999488	0.999985264	0.8865725
ZC3H12C	0.963745367	0.930069267	0.809486619	0.99999488	0.999985264	0.888645453
SHROOM2	0.963745367	0.840896809	0.939147816	0.99999488	0.999985264	0.888681581
CTTNBP2	0.963745367	0.943802122	0.890096618	0.99999488	0.999985264	0.889409839

LOC504773	0.963745367	0.929230097	0.817700149	0.99999488	0.999985264	0.892601101
PSMC3IP	0.963745367	0.891316528	0.930678591	0.99999488	0.999985264	0.894723749
SELENOI	0.963745367	0.832115014	0.999558179	0.99999488	0.999985264	0.895349622
CXCL14	0.963745367	0.943802122	0.886962419	0.99999488	0.999985264	0.89668604
RHOBTB2	0.963745367	0.882022911	0.851718619	0.99999488	0.999985264	0.899223071
NMRAL1	0.963745367	0.897406772	0.915215321	0.99999488	0.999985264	0.90109791
GPBAR1	0.963745367	0.946274461	0.956565728	0.99999488	0.999985264	0.90513586
DYNC2H1	0.963745367	0.941495217	0.825497047	0.99999488	0.999985264	0.910018813
PPRC1	0.963745367	0.845554546	0.83396257	0.99999488	0.999985264	0.913593134
CD24	0.963745367	0.850692013	0.812450514	0.99999488	0.999985264	0.919755394
PGD	0.963745367	0.842782808	0.825497047	0.99999488	0.999985264	0.921771959
KDM4C	0.963745367	0.905019423	0.825497047	0.99999488	0.999985264	0.927969856
DPAGT1	0.963745367	0.836126124	0.877786216	0.99999488	0.999985264	0.927969856
DNAJA2	0.963745367	0.830519875	0.944085862	0.99999488	0.999985264	0.927969856
PITPNM2	0.963745367	0.867755878	0.851718619	0.99999488	0.999985264	0.928145377
LRRC1	0.963745367	0.949285285	0.844036059	0.99999488	0.999985264	0.931163114
LOC101903526	0.963745367	0.955266582	0.916975216	0.99999488	0.999985264	0.937108046
LOC100847947	0.963745367	0.960125322	0.886962419	0.99999488	0.999985264	0.945670698
RER1	0.963745367	0.869333348	0.895644023	0.99999488	0.999985264	0.95096765
IL17RD	0.963745367	0.819379885	0.825497047	0.99999488	0.999985264	0.962703352
EIF3L	0.963745367	0.968780194	0.813142592	0.99999488	0.999985264	0.966921967
C16H1orf74	0.963745367	0.859484941	0.874752438	0.99999488	0.999985264	0.979961637
MED12L	0.963745367	0.928525825	0.809486619	0.99999488	0.999985264	0.999960414
PSMG2	0.963745367	0.836126124	0.851718619	0.99999488	0.999985264	0.999960414
LOC101902366	0.963893333	0.86639122	0.945678616	0.99999488	0.999985264	0.816226172
F2R	0.963893333	0.871627189	0.871385134	0.99999488	0.999985264	0.831666995
NKIRAS2	0.963893333	0.827123416	0.897767405	0.99999488	0.999985264	0.83405895
LOC101908104	0.963893333	0.946480858	0.94602826	0.99999488	0.999985264	0.863331236
LOC783963	0.963893333	0.905019423	0.946479014	0.99999488	0.999985264	0.866439215
LOC101906607	0.963893333	0.916939994	0.857021454	0.99999488	0.999985264	0.972391591
GSTK1	0.964104266	0.844106733	0.988922732	0.99999488	0.999985264	0.812831419
NRN1	0.964104266	0.944701115	0.825685674	0.99999488	0.999985264	0.838478213
PPIL2	0.964104266	0.867777719	0.958627535	0.99999488	0.999985264	0.838520085
ELOVL1	0.964104266	0.864154704	0.998816213	0.99999488	0.999985264	0.839734604
FHL3	0.964104266	0.909633468	0.873452279	0.99999488	0.999985264	0.872307149
DNMBP	0.964104266	0.884739596	0.849633432	0.99999488	0.999985264	0.927969856
MAPK15	0.964104266	0.9527938	0.919763144	0.99999488	0.999985264	0.948455284
LOC112449324	0.964276178	0.836111245	0.873452279	0.99999488	0.999985264	0.814658726
ADAM11	0.964276178	0.984068885	0.826801319	0.99999488	0.999985264	0.816959462

FBRSL1	0.964276178	0.829276743	0.848312163	0.99999488	0.999985264	0.818484413
RAB14	0.964276178	0.828207064	0.962346588	0.99999488	0.999985264	0.839734604
LOC107131772	0.964276178	0.865274556	0.999558179	0.99999488	0.999985264	0.851533524
ROCK1	0.964276178	0.976665444	0.890592965	0.99999488	0.999985264	0.863331236
KDELR2	0.964276178	0.830519875	0.981388276	0.99999488	0.999985264	0.865911659
IRF2BPL	0.964276178	0.835868428	0.902527616	0.99999488	0.999985264	0.894572878
LOC112442080	0.964276178	0.862707207	0.809943111	0.99999488	0.999985264	0.946666773
LOC104975460	0.964276178	0.826434903	0.85634783	0.99999488	0.999985264	0.967874696
ZNF280B	0.964276178	0.921855042	0.864737879	0.99999488	0.999985264	0.968772302
LOC101903326	0.964304275	0.867755878	0.86326697	0.99999488	0.999985264	0.953565168
TBX21	0.96440187	0.836126124	0.840760291	0.99999488	0.999985264	0.923360345
LOC512005	0.96440187	0.892008332	0.981218677	0.99999488	0.999985264	0.923360345
C17H4orf46	0.96440187	0.836787113	0.968663342	0.99999488	0.999985264	0.952669273
GDF9	0.96440187	0.993640257	0.83711556	0.99999488	0.999985264	0.955374205
LOC104974678	0.964674869	0.999992444	0.913976361	0.99999488	0.999985264	0.841443168
PTPN13	0.964674869	0.821194038	0.942675769	0.99999488	0.999985264	0.894420979
LOC112446383	0.964699404	0.827661136	0.947957719	0.99999488	0.999985264	0.85064213
SCAND1	0.964699404	0.948421431	0.821698192	0.99999488	0.999985264	0.863331236
LOC112447351	0.964699404	0.974812121	0.96210311	0.99999488	0.999985264	0.911089313
KCNK5	0.964699404	0.826642757	0.885552508	0.99999488	0.999985264	0.939035437
IKZF3	0.964699404	0.922700337	0.890248427	0.99999488	0.999985264	0.970849664
AGO1	0.964699404	0.846070848	0.822003523	0.99999488	0.999985264	0.999960414
CITED2	0.96479558	0.88463751	0.870995265	0.99999488	0.999985264	0.868513869
RAB22A	0.965079971	0.864945604	0.946479014	0.99999488	0.999985264	0.816226172
EDF1	0.965079971	0.893743321	0.844817525	0.99999488	0.999985264	0.849046935
RAD51	0.965079971	0.830519875	0.939799459	0.99999488	0.999985264	0.870160734
LOC100297676	0.96524714	0.821194038	0.841957362	0.99999488	0.999985264	0.885874544
ZNF383	0.965338475	0.999992444	0.83396257	0.99999488	0.999985264	0.831666995
LOC100297056	0.965338475	0.845554546	0.886192181	0.99999488	0.999985264	0.868103112
GORASP1	0.965341739	0.941495217	0.834173166	0.99999488	0.999985264	0.863280322
FRMPD1	0.965341739	0.82255131	0.824563098	0.99999488	0.999985264	0.912585098
CCNT1	0.965475861	0.973921957	0.923604668	0.99999488	0.999985264	0.812831419
LOC101904601	0.965475861	0.932723391	0.835038876	0.99999488	0.999985264	0.833388946
PDE8A	0.965873304	0.834860292	0.812450514	0.99999488	0.999985264	0.862890646
C13H20orf96	0.966046045	0.943802122	0.952875254	0.99999488	0.999985264	0.816226172
LOC101903713	0.966116755	0.942353856	0.948946702	0.99999488	0.999985264	0.922063125
LOC786948	0.966345855	0.850692013	0.879253779	0.99999488	0.999985264	0.957739231
VAMP5	0.966493671	0.89880506	0.862345036	0.99999488	0.999985264	0.819090616
DNAJC19	0.966493671	0.886242361	0.839056678	0.99999488	0.999985264	0.839572429

SAP25	0.966493671	0.859484941	0.94634922	0.99999488	0.999985264	0.968059852
CPT1B	0.966572312	0.998131745	0.821930959	0.99999488	0.999985264	0.884143505
ISM1	0.966572312	0.835868428	0.902552409	0.99999488	0.999985264	0.897965262
SLC22A16	0.966572312	0.948421431	0.923604668	0.99999488	0.999985264	0.95096765
LOC782922	0.966830547	0.948421431	0.907484111	0.99999488	0.999985264	0.839734604
SMARCA4	0.966830547	0.839292361	0.895626254	0.99999488	0.999985264	0.924295622
GLDN	0.967093118	0.864945604	0.938203102	0.99999488	0.999985264	0.949718683
KIAA0391	0.967157715	0.974812121	0.833003624	0.99999488	0.999985264	0.980032028
MINOS1	0.967274992	0.867755878	0.892943118	0.99999488	0.999985264	0.818895181
RNF115	0.967274992	0.958967142	0.886192181	0.99999488	0.999985264	0.827190118
SLC41A1	0.967274992	0.848246309	0.899248502	0.99999488	0.999985264	0.844277572
RFC1	0.967274992	0.982981183	0.898815524	0.99999488	0.999985264	0.845842942
SYAP1	0.967274992	0.821194038	0.956504686	0.99999488	0.999985264	0.847577522
NIPSNAP1	0.967274992	0.835868428	0.82336109	0.99999488	0.999985264	0.85182524
NFIA	0.967274992	0.821194038	0.957541703	0.99999488	0.999985264	0.861670395
RTN4RL1	0.967274992	0.999992444	0.862345036	0.99999488	0.999985264	0.873656025
CDC14A	0.967274992	0.883856603	0.824972187	0.99999488	0.999985264	0.90109791
JAKMIP3	0.967274992	0.999992444	0.869837069	0.99999488	0.999985264	0.910018813
NEURL1	0.967274992	0.999992444	0.841739726	0.99999488	0.999985264	0.927969856
LRRC2	0.967274992	0.896085343	0.821546364	0.99999488	0.999985264	0.985469637
LOC112445965	0.967274992	0.967276215	0.817177032	0.99999488	0.999985264	0.986757457
NOP56	0.967274992	0.830519875	0.894199923	0.99999488	0.999985264	0.986873741
AGFG1	0.967274992	0.864945604	0.873452279	0.99999488	0.999985264	0.999960414
ZFAND5	0.967554255	0.883624935	0.848312163	0.99999488	0.999985264	0.865911659
LOC112442288	0.967563715	0.864945604	0.86638329	0.99999488	0.999985264	0.894144329
PKNOX2	0.967679726	0.883856603	0.826965593	0.99999488	0.999985264	0.881211326
LEF1	0.967679726	0.866462388	0.877742202	0.99999488	0.999985264	0.966921967
DCP1A	0.967880645	0.869304939	0.95758013	0.99999488	0.999985264	0.826474547
LOC112442243	0.967880645	0.985406717	0.914001994	0.99999488	0.999985264	0.842566328
CD40	0.967880645	0.844457578	0.833419544	0.99999488	0.999985264	0.986408155
CSNK2A2	0.967918528	0.848246309	0.857021454	0.99999488	0.999985264	0.850842307
DHRS13	0.967918528	0.991365955	0.860189237	0.99999488	0.999985264	0.913527577
UNC5B	0.967918528	0.984068885	0.840781773	0.99999488	0.999985264	0.924295622
BVES	0.967918528	0.95697765	0.884119589	0.99999488	0.999985264	0.954519353
VWA5A	0.967928629	0.832807683	0.929213642	0.99999488	0.999985264	0.873463288
NDUFB1	0.967955603	0.873113713	0.916975216	0.99999488	0.999985264	0.815564853
UQCC3	0.967955603	0.870351626	0.821698192	0.99999488	0.999985264	0.816226172
LOC112446757	0.967955603	0.840770466	0.902527616	0.99999488	0.999985264	0.816226172
USP43	0.967955603	0.999992444	0.83126054	0.99999488	0.999985264	0.819073042

FZD10	0.967955603	0.991488652	0.895626254	0.99999488	0.999985264	0.819073042
CD1A	0.967955603	0.883624935	0.944871472	0.99999488	0.999985264	0.819073042
TMEM251	0.967955603	0.850095234	0.908181052	0.99999488	0.999985264	0.825478387
CPQ	0.967955603	0.991488652	0.900148803	0.99999488	0.999985264	0.828248574
PHF8	0.967955603	0.847051507	0.833846637	0.99999488	0.999985264	0.833388946
KATNB1	0.967955603	0.981520287	0.834068744	0.99999488	0.999985264	0.837517492
PARD6G	0.967955603	0.999347049	0.822356437	0.99999488	0.999985264	0.839734604
ETS1	0.967955603	0.90024974	0.916975216	0.99999488	0.999985264	0.839734604
NAA50	0.967955603	0.985406717	0.94634922	0.99999488	0.999985264	0.845834943
AP5B1	0.967955603	0.997975376	0.877805795	0.99999488	0.999985264	0.847553834
C1S	0.967955603	0.893748757	0.857021454	0.99999488	0.999985264	0.847577522
TOR1AIP2	0.967955603	0.938017364	0.882121777	0.99999488	0.999985264	0.848810537
ZGPAT	0.967955603	0.867755878	0.825881042	0.99999488	0.999985264	0.84917208
KPNA4	0.967955603	0.850095234	0.933046493	0.99999488	0.999985264	0.861030464
CBLB	0.967955603	0.90777161	0.858024919	0.99999488	0.999985264	0.864806954
KLF10	0.967955603	0.927447588	0.93955737	0.99999488	0.999985264	0.874975722
SNTB1	0.967955603	0.835868428	0.95230212	0.99999488	0.999985264	0.877760721
SIGLEC10	0.967955603	0.850095234	0.939147816	0.99999488	0.999985264	0.883999232
PCGF5	0.967955603	0.931336072	0.95106368	0.99999488	0.999985264	0.893849774
IQGAP2	0.967955603	0.829029204	0.851718619	0.99999488	0.999985264	0.897965262
LOC101903905	0.967955603	0.985406717	0.881942256	0.99999488	0.999985264	0.897965262
LOC101906779	0.967955603	0.844174041	0.999558179	0.99999488	0.999985264	0.911089313
FASTKD3	0.967955603	0.833676228	0.945673638	0.99999488	0.999985264	0.912262222
ABO	0.967955603	0.921549	0.938203102	0.99999488	0.999985264	0.922063125
LOC107131728	0.967955603	0.992977597	0.841739726	0.99999488	0.999985264	0.923360345
TMEM178B	0.967955603	0.874697957	0.859991705	0.99999488	0.999985264	0.924295622
AURKAIP1	0.967955603	0.867755878	0.908572237	0.99999488	0.999985264	0.925260365
POMT2	0.967955603	0.995073696	0.886192181	0.99999488	0.999985264	0.931163114
LOC107131699	0.967955603	0.845262032	0.961160234	0.99999488	0.999985264	0.931471274
NECAB3	0.967955603	0.873289191	0.870264123	0.99999488	0.999985264	0.943633749
JAM3	0.967955603	0.895242264	0.959314104	0.99999488	0.999985264	0.943633749
TMEM208	0.967955603	0.883856603	0.833419544	0.99999488	0.999985264	0.944457836
LOC786733	0.967955603	0.943802122	0.861480822	0.99999488	0.999985264	0.95096765
XG	0.967955603	0.821194038	0.873078755	0.99999488	0.999985264	0.955561861
ADGRA2	0.967955603	0.96566261	0.817700149	0.99999488	0.999985264	0.977753483
PDP2	0.967955603	0.850692013	0.898815524	0.99999488	0.999985264	0.986873741
SCD	0.96812373	0.821194038	0.822356437	0.99999488	0.999985264	0.967874696
DAG1	0.968176132	0.893642384	0.865347217	0.99999488	0.999985264	0.839734604
SCAF11	0.968448958	0.933208464	0.830045657	0.99999488	0.999985264	0.815564853

LOC101904449	0.968448958	0.883624935	0.991697739	0.99999488	0.999985264	0.816226172
NAXD	0.968448958	0.952346363	0.83711556	0.99999488	0.999985264	0.818484413
KANSL1	0.968448958	0.921549	0.877786216	0.99999488	0.999985264	0.819073042
USP38	0.968448958	0.852182782	0.941336964	0.99999488	0.999985264	0.819073042
LOC787397	0.968448958	0.991574265	0.844817525	0.99999488	0.999985264	0.819090616
PSMD9	0.968448958	0.904961534	0.938203102	0.99999488	0.999985264	0.826043635
AKAP11	0.968448958	0.90116492	0.933791093	0.99999488	0.999985264	0.831666995
AK8	0.968448958	0.9527938	0.865066354	0.99999488	0.999985264	0.839828214
TRIM59	0.968448958	0.864945604	0.881942256	0.99999488	0.999985264	0.840732705
CUTA	0.968448958	0.893748757	0.881942256	0.99999488	0.999985264	0.841424601
DONSON	0.968448958	0.992977597	0.938203102	0.99999488	0.999985264	0.872307149
LOC513767	0.968448958	0.864945604	0.825497047	0.99999488	0.999985264	0.872879549
LOC100847791	0.968448958	0.920253543	0.861360113	0.99999488	0.999985264	0.883750044
MCM5	0.968448958	0.863793573	0.824988148	0.99999488	0.999985264	0.890477991
GPRIN3	0.968448958	0.970085192	0.848786751	0.99999488	0.999985264	0.897467714
LOC788736	0.968448958	0.842852797	0.852670802	0.99999488	0.999985264	0.902893979
TMEM225B	0.968448958	0.938702715	0.9222878	0.99999488	0.999985264	0.932608891
HSPB2	0.968448958	0.869304939	0.948420301	0.99999488	0.999985264	0.939035437
DZANK1	0.968448958	0.9582784	0.834173166	0.99999488	0.999985264	0.943220018
MGAT4C	0.968448958	0.869999083	0.859597269	0.99999488	0.999985264	0.957171025
MARS	0.968448958	0.867755878	0.826965593	0.99999488	0.999985264	0.999960414
PITPNM1	0.968567705	0.999347049	0.821546364	0.99999488	0.999985264	0.816226172
AGAP2	0.968567705	0.900368858	0.852782377	0.99999488	0.999985264	0.816226172
COX5B	0.968567705	0.842852797	0.886730817	0.99999488	0.999985264	0.818895181
MANBAL	0.968567705	0.935742738	0.95230212	0.99999488	0.999985264	0.818895181
LOC107132335	0.968567705	0.963867256	0.887707591	0.99999488	0.999985264	0.819073042
PARD6A	0.968567705	0.918883775	0.956565728	0.99999488	0.999985264	0.819073042
TESC	0.968567705	0.830654932	0.86638329	0.99999488	0.999985264	0.820845891
TEAD3	0.968567705	0.991488652	0.869864092	0.99999488	0.999985264	0.827190118
TUBGCP6	0.968567705	0.873289191	0.973661483	0.99999488	0.999985264	0.827190118
LY6D	0.968567705	0.938597964	0.864330883	0.99999488	0.999985264	0.827948205
ZDHHC9	0.968567705	0.991488652	0.851718619	0.99999488	0.999985264	0.828248574
IDH3B	0.968567705	0.83698359	0.851718619	0.99999488	0.999985264	0.831603744
BCKDK	0.968567705	0.921525526	0.862345036	0.99999488	0.999985264	0.836520183
NCAM1	0.968567705	0.890682633	0.944085862	0.99999488	0.999985264	0.83652174
TRAK1	0.968567705	0.916036681	0.991285432	0.99999488	0.999985264	0.841424601
COPS2	0.968567705	0.986637746	0.933992957	0.99999488	0.999985264	0.845804607
CHST11	0.968567705	0.991488652	0.81983962	0.99999488	0.999985264	0.846001048
SLC35A3	0.968567705	0.948421431	0.914001994	0.99999488	0.999985264	0.847577522

LOC112448381	0.968567705	0.845554546	0.923604668	0.99999488	0.999985264	0.851533524
CFDP1	0.968567705	0.919505104	0.991285432	0.99999488	0.999985264	0.851595584
BRB	0.968567705	0.922995823	0.918170048	0.99999488	0.999985264	0.856510591
TMEM127	0.968567705	0.991365955	0.900148803	0.99999488	0.999985264	0.858593649
LOC101908048	0.968567705	0.921759937	0.930127869	0.99999488	0.999985264	0.859008195
STXBP2	0.968567705	0.9582784	0.871925227	0.99999488	0.999985264	0.859537053
TMEM132C	0.968567705	0.831988573	0.879092985	0.99999488	0.999985264	0.859537053
MYBL1	0.968567705	0.928759674	0.862345036	0.99999488	0.999985264	0.865855226
UBE2G2	0.968567705	0.84091959	0.998816213	0.99999488	0.999985264	0.865855226
MUC1	0.968567705	0.883856603	0.871925227	0.99999488	0.999985264	0.866439215
LIG4	0.968567705	0.916036681	0.980593046	0.99999488	0.999985264	0.870160734
IQCC	0.968567705	0.92976013	0.825497047	0.99999488	0.999985264	0.870708914
LOC101906012	0.968567705	0.880627967	0.848786751	0.99999488	0.999985264	0.870708914
LOC783466	0.968567705	0.879694242	0.862345036	0.99999488	0.999985264	0.870708914
ZRSR2	0.968567705	0.999992444	0.902552409	0.99999488	0.999985264	0.872365139
SBF2	0.968567705	0.89880506	0.845368791	0.99999488	0.999985264	0.873463288
CCNB1IP1	0.968567705	0.883624935	0.95477544	0.99999488	0.999985264	0.873463288
LOC107132398	0.968567705	0.999992444	0.898815524	0.99999488	0.999985264	0.877018187
ARMC4	0.968567705	0.998011664	0.851718619	0.99999488	0.999985264	0.882867473
HEATR3	0.968567705	0.893642384	0.81983962	0.99999488	0.999985264	0.884143505
LOC101903832	0.968567705	0.876631119	0.820249118	0.99999488	0.999985264	0.885874544
GPR18	0.968567705	0.877795869	0.849633432	0.99999488	0.999985264	0.885874544
ISLR2	0.968567705	0.869999083	0.922076383	0.99999488	0.999985264	0.885874544
NBEA	0.968567705	0.930955919	0.818784725	0.99999488	0.999985264	0.893030708
EXOSC9	0.968567705	0.898905584	0.935136095	0.99999488	0.999985264	0.900905186
ADIG	0.968567705	0.921934673	0.862345036	0.99999488	0.999985264	0.901614166
PDCD6	0.968567705	0.86538354	0.920629231	0.99999488	0.999985264	0.901809636
ZNF706	0.968567705	0.869999083	0.923089792	0.99999488	0.999985264	0.901809636
MFSD4A	0.968567705	0.869333348	0.862345036	0.99999488	0.999985264	0.904813143
AKAP5	0.968567705	0.893642384	0.862345036	0.99999488	0.999985264	0.910018813
YBEY	0.968567705	0.9582784	0.825497047	0.99999488	0.999985264	0.911089313
ALAS1	0.968567705	0.918648578	0.856449202	0.99999488	0.999985264	0.911997747
FAM193B	0.968567705	0.935742738	0.926552506	0.99999488	0.999985264	0.911997747
PYM1	0.968567705	0.920253543	0.841739726	0.99999488	0.999985264	0.912262222
NAV3	0.968567705	0.885024111	0.940815039	0.99999488	0.999985264	0.916081555
KCNQ1	0.968567705	0.831988573	0.920629231	0.99999488	0.999985264	0.917880648
DUS4L	0.968567705	0.893642384	0.853055742	0.99999488	0.999985264	0.925260365
WDR24	0.968567705	0.881261173	0.912008683	0.99999488	0.999985264	0.925260365
TMC8	0.968567705	0.991488652	0.862345036	0.99999488	0.999985264	0.926326154

RBM17	0.968567705	0.84091959	0.938203102	0.99999488	0.999985264	0.926326154
FKTN	0.968567705	0.999992444	0.876188482	0.99999488	0.999985264	0.927969856
BAG1	0.968567705	0.850496315	0.962346588	0.99999488	0.999985264	0.927969856
LOC107131367	0.968567705	0.859182763	0.968232195	0.99999488	0.999985264	0.927969856
PIBF1	0.968567705	0.981298804	0.850263886	0.99999488	0.999985264	0.929458454
CCL26	0.968567705	0.883856603	0.954823586	0.99999488	0.999985264	0.931163114
LOC112447031	0.968567705	0.985058069	0.821698192	0.99999488	0.999985264	0.932386503
LOC101903616	0.968567705	0.938777693	0.851718619	0.99999488	0.999985264	0.932608891
MKNK2	0.968567705	0.988850552	0.861140642	0.99999488	0.999985264	0.933698703
LOC101902204	0.968567705	0.941495217	0.848786751	0.99999488	0.999985264	0.93590083
KCNT1	0.968567705	0.845262032	0.963233239	0.99999488	0.999985264	0.936984097
TOMM20	0.968567705	0.87010028	0.965059328	0.99999488	0.999985264	0.943220018
TNFSF13B	0.968567705	0.864575754	0.884119589	0.99999488	0.999985264	0.94572902
NUP155	0.968567705	0.940762397	0.886192181	0.99999488	0.999985264	0.947452706
CCND3	0.968567705	0.919251805	0.825497047	0.99999488	0.999985264	0.949764317
SIGMAR1	0.968567705	0.913160799	0.85109851	0.99999488	0.999985264	0.952669273
SH2D1A	0.968567705	0.919446414	0.851718619	0.99999488	0.999985264	0.95905828
LOC527388	0.968567705	0.87010028	0.932653752	0.99999488	0.999985264	0.962703352
PSMC1	0.968567705	0.915367594	0.861790833	0.99999488	0.999985264	0.96512394
ERICH3	0.968567705	0.903377723	0.941118576	0.99999488	0.999985264	0.976814573
MALT1	0.968567705	0.893748757	0.900890709	0.99999488	0.999985264	0.98114037
LOC101903877	0.968567705	0.883856603	0.884320747	0.99999488	0.999985264	0.984869777
BZW1	0.968567705	0.90116492	0.914538737	0.99999488	0.999985264	0.987457361
EIF5B	0.968567705	0.870598408	0.830061817	0.99999488	0.999985264	0.999960414
GART	0.968567705	0.826936949	0.852670802	0.99999488	0.999985264	0.999960414
LOC107131224	0.968567705	0.835868428	0.888545123	0.99999488	0.999985264	0.999960414
LOC112444314	0.968567705	0.862811597	0.939147816	0.99999488	0.999985264	0.999960414
TMEM33	0.968649469	0.995029734	0.944085862	0.99999488	0.999985264	0.827190118
TXNDC11	0.968649469	0.999992444	0.9222878	0.99999488	0.999985264	0.841143656
TOX	0.968649469	0.904961534	0.935094475	0.99999488	0.999985264	0.851595584
HMOX2	0.968649469	0.983781707	0.849816872	0.99999488	0.999985264	0.866578338
EME1	0.968649469	0.90116492	0.851718619	0.99999488	0.999985264	0.868483865
PORCN	0.968649469	0.893642384	0.852782377	0.99999488	0.999985264	0.90109791
TOPBP1	0.968649469	0.862982	0.841957362	0.99999488	0.999985264	0.901614166
GPR158	0.968649469	0.882022911	0.862345036	0.99999488	0.999985264	0.924201993
KLF12	0.968649469	0.85337372	0.822003523	0.99999488	0.999985264	0.926596859
DSTN	0.968649469	0.979263532	0.926552506	0.99999488	0.999985264	0.927969856
CMTM8	0.968682467	0.869304939	0.862345036	0.99999488	0.999985264	0.88310116
LRRC49	0.968682467	0.864811968	0.872386998	0.99999488	0.999985264	0.896276108

OGT	0.968682467	0.926818306	0.971967312	0.99999488	0.999985264	0.901614166
AKR7A2	0.968682467	0.869999083	0.825497047	0.99999488	0.999985264	0.93845573
SCUBE2	0.968682467	0.9582784	0.834068744	0.99999488	0.999985264	0.95096765
RPL7L1	0.968682467	0.84091959	0.877799478	0.99999488	0.999985264	0.968240454
ASIC2	0.968682467	0.883856603	0.890096618	0.99999488	0.999985264	0.999960414
ROMO1	0.968685816	0.896400219	0.900890709	0.99999488	0.999985264	0.837143123
OCSTAMP	0.968685816	0.837468586	0.915307132	0.99999488	0.999985264	0.865855226
LOC112446663	0.968761263	0.934142668	0.862345036	0.99999488	0.999985264	0.926955927
SELENOM	0.968978426	0.835638623	0.862345036	0.99999488	0.999985264	0.819073042
LOC101907729	0.969279614	0.883856603	0.873452279	0.99999488	0.999985264	0.818895181
RGS22	0.969279614	0.881001912	0.831723974	0.99999488	0.999985264	0.924201993
SAMD11	0.969279614	0.830132999	0.843147286	0.99999488	0.999985264	0.931163114
LOC100336589	0.969419787	0.978004343	0.83711556	0.99999488	0.999985264	0.837143123
PITHD1	0.969419787	0.834579123	0.967926798	0.99999488	0.999985264	0.837143123
CARD14	0.969419787	0.989547896	0.922400936	0.99999488	0.999985264	0.839734604
CASK	0.969419787	0.994481342	0.880594299	0.99999488	0.999985264	0.858593649
GSR	0.969419787	0.869999083	0.862968824	0.99999488	0.999985264	0.871960581
LOC104974020	0.969419787	0.999992444	0.933791093	0.99999488	0.999985264	0.897965262
RPGRIP1L	0.969684653	0.974812121	0.834068744	0.99999488	0.999985264	0.919755394
C7H5orf30	0.969890822	0.831782537	0.94018971	0.99999488	0.999985264	0.82889587
VSTM4	0.970001548	0.961479426	0.938203102	0.99999488	0.999985264	0.83405895
LOC518775	0.970001548	0.84528629	0.938203102	0.99999488	0.999985264	0.949764317
ZADH2	0.970134115	0.865274556	0.926552506	0.99999488	0.999985264	0.987365188
MDH2	0.970335314	0.835868428	0.865420641	0.99999488	0.999985264	0.841424601
PITPNM3	0.970335314	0.830132999	0.841773648	0.99999488	0.999985264	0.986873741
PSENEN	0.970549544	0.924222289	0.917154286	0.99999488	0.999985264	0.873463288
ARPC1B	0.970549544	0.946274461	0.825497047	0.99999488	0.999985264	0.888645453
ATXN2L	0.970549544	0.847376288	0.95477544	0.99999488	0.999985264	0.894174543
RBM19	0.970549544	0.999992444	0.825497047	0.99999488	0.999985264	0.923360345
SARDH	0.970549544	0.873289191	0.883109976	0.99999488	0.999985264	0.927969856
HSD17B12	0.970549544	0.851001595	0.849221966	0.99999488	0.999985264	0.955374205
COPS8	0.970650749	0.893642384	0.938203102	0.99999488	0.999985264	0.858593649
NUDT3	0.970650749	0.974812121	0.938203102	0.99999488	0.999985264	0.88682376
PHF21A	0.970650749	0.84060143	0.932284584	0.99999488	0.999985264	0.926673043
PREPL	0.970944281	0.86538354	0.887626998	0.99999488	0.999985264	0.828248574
ANAPC4	0.970944281	0.999992444	0.849316006	0.99999488	0.999985264	0.919755394
TFB2M	0.970944281	0.894140115	0.872386998	0.99999488	0.999985264	0.927969856
COX7B2	0.971367829	0.9582784	0.83126054	0.99999488	0.999985264	0.900681286
FJX1	0.971650735	0.830519875	0.984631623	0.99999488	0.999985264	0.856510591

CMAS	0.971874047	0.985406717	0.939147816	0.99999488	0.999985264	0.902508792
LOC539166	0.971877582	0.957169634	0.973364552	0.99999488	0.999985264	0.841424601
PVALB	0.971877582	0.958215923	0.825497047	0.99999488	0.999985264	0.915574092
HIST1H2BI	0.971877582	0.992977597	0.884407415	0.99999488	0.999985264	0.967081351
LOC107132045	0.971986362	0.87010028	0.852670802	0.99999488	0.999985264	0.881768147
TMED2	0.971986362	0.919884594	0.9808801	0.99999488	0.999985264	0.901809636
CHMP1A	0.972117595	0.950500085	0.879092985	0.99999488	0.999985264	0.820845891
LOC783920	0.972117595	0.9093283	0.825497047	0.99999488	0.999985264	0.82209307
TSEN54	0.972117595	0.895632001	0.840760291	0.99999488	0.999985264	0.831532663
SLC48A1	0.972117595	0.883624935	0.916975216	0.99999488	0.999985264	0.844277572
MCPH1	0.972117595	0.999992444	0.890035877	0.99999488	0.999985264	0.846769116
ABCC3	0.972117595	0.865184773	0.927600835	0.99999488	0.999985264	0.850790144
FNBP1L	0.972117595	0.96493942	0.848312163	0.99999488	0.999985264	0.851595584
LOC101906469	0.972117595	0.940459751	0.903354205	0.99999488	0.999985264	0.856510591
NPR1	0.972117595	0.999992444	0.862345036	0.99999488	0.999985264	0.85775232
MKL2	0.972117595	0.999992444	0.825497047	0.99999488	0.999985264	0.858593649
SLC35F2	0.972117595	0.920639601	0.941118576	0.99999488	0.999985264	0.86295162
LBR	0.972117595	0.935588259	0.945678616	0.99999488	0.999985264	0.873463288
MCHR1	0.972117595	0.920040168	0.903175962	0.99999488	0.999985264	0.895349622
ATP6V0A4	0.972117595	0.869999083	0.82336109	0.99999488	0.999985264	0.896480535
KCNN3	0.972117595	0.878790155	0.890248427	0.99999488	0.999985264	0.901809636
LOC112447313	0.972117595	0.871627189	0.920154807	0.99999488	0.999985264	0.901809636
XRCC4	0.972117595	0.999992444	0.897043265	0.99999488	0.999985264	0.918400465
NHEJ1	0.972117595	0.938476452	0.933992957	0.99999488	0.999985264	0.921150136
ATP6V0D1	0.972117595	0.978089982	0.907484111	0.99999488	0.999985264	0.924201993
LGALS1	0.972117595	0.935060764	0.888645848	0.99999488	0.999985264	0.927969856
ARMC6	0.972117595	0.888660199	0.837095307	0.99999488	0.999985264	0.934749052
TFIP11	0.972117595	0.864945604	0.83396257	0.99999488	0.999985264	0.948213108
SEMA4A	0.972117595	0.938970117	0.868188614	0.99999488	0.999985264	0.957171025
ZBTB25	0.972117595	0.872368602	0.913211244	0.99999488	0.999985264	0.958869789
MTHFD1	0.972117595	0.907069508	0.847427167	0.99999488	0.999985264	0.960343814
CSKMT	0.972117595	0.850692013	0.865837237	0.99999488	0.999985264	0.977753483
URB2	0.972318897	0.957700659	0.841717609	0.99999488	0.999985264	0.966921967
PRR33	0.972379439	0.831988573	0.9808801	0.99999488	0.999985264	0.856510591
PIK3C2A	0.972382318	0.874697957	0.893044539	0.99999488	0.999985264	0.821992346
TMEM43	0.972382318	0.921934673	0.848312163	0.99999488	0.999985264	0.999960414
HOXC9	0.972710357	0.946274461	0.925520255	0.99999488	0.999985264	0.839405979
ARL8A	0.972710357	0.95697765	0.95230212	0.99999488	0.999985264	0.839734604
SLC10A1	0.972710357	0.962968114	0.863098782	0.99999488	0.999985264	0.862046449

ADCYAP1R1	0.972710357	0.976717114	0.83711556	0.99999488	0.999985264	0.873463288
GRO1	0.972710357	0.989627874	0.848312163	0.99999488	0.999985264	0.949764317
TTC39B	0.972710357	0.850692013	0.857006083	0.99999488	0.999985264	0.987454496
VSIR	0.972719953	0.999992444	0.843587792	0.99999488	0.999985264	0.848830665
ABL2	0.972719953	0.859484941	0.851718619	0.99999488	0.999985264	0.856510591
RPL7A	0.972719953	0.90024974	0.862345036	0.99999488	0.999985264	0.895370805
GNA14	0.972719953	0.958967142	0.922400936	0.99999488	0.999985264	0.918343194
MYO5B	0.972719953	0.991488652	0.925520255	0.99999488	0.999985264	0.919758204
KIAA2026	0.972719953	0.901126458	0.862345036	0.99999488	0.999985264	0.926326154
RAB40B	0.972719953	0.981298804	0.938905004	0.99999488	0.999985264	0.949315689
C24H18orf25	0.972719953	0.91058693	0.933307828	0.99999488	0.999985264	0.955374205
GABPB2	0.972719953	0.844174041	0.833696877	0.99999488	0.999985264	0.999960414
SDC3	0.9728811	0.943802122	0.841739726	0.99999488	0.999985264	0.832622862
LOC100847870	0.9728811	0.964246761	0.930678591	0.99999488	0.999985264	0.837143123
SMNDC1	0.9728811	0.997975376	0.841739726	0.99999488	0.999985264	0.839734604
LOC107132952	0.9728811	0.836126124	0.946479014	0.99999488	0.999985264	0.846769116
USP25	0.9728811	0.992977597	0.843587792	0.99999488	0.999985264	0.849046935
LIFR	0.9728811	0.859484941	0.917154286	0.99999488	0.999985264	0.850790144
ACIN1	0.9728811	0.893642384	0.9808801	0.99999488	0.999985264	0.86945248
LOC101906280	0.9728811	0.882426228	0.865420641	0.99999488	0.999985264	0.885874544
SMIM13	0.9728811	0.991488652	0.906406315	0.99999488	0.999985264	0.894236694
SGIP1	0.9728811	0.865926615	0.908181052	0.99999488	0.999985264	0.905391122
LOC784738	0.9728811	0.986445172	0.865066354	0.99999488	0.999985264	0.939035437
GRIP2	0.9728811	0.934515915	0.886962419	0.99999488	0.999985264	0.955374205
ZNF527	0.9728811	0.986637746	0.843587792	0.99999488	0.999985264	0.966921967
LOC516442	0.9728811	0.866462388	0.945678616	0.99999488	0.999985264	0.999960414
KLHL41	0.973418737	0.999992444	0.849633432	0.99999488	0.999985264	0.827190118
ASGR2	0.973418737	0.957700659	0.848312163	0.99999488	0.999985264	0.827971245
CRK	0.973418737	0.858808025	0.930678591	0.99999488	0.999985264	0.827971245
LOC614226	0.973418737	0.896507811	0.825497047	0.99999488	0.999985264	0.828248574
UBL7	0.973418737	0.881001912	0.826801319	0.99999488	0.999985264	0.831413041
HORMAD2	0.973418737	0.84197881	0.945766581	0.99999488	0.999985264	0.837482843
GAS1	0.973418737	0.836126124	0.925520255	0.99999488	0.999985264	0.844292822
YWHAH	0.973418737	0.885718559	0.874752438	0.99999488	0.999985264	0.85046698
LOC100139764	0.973418737	0.958098081	0.945678616	0.99999488	0.999985264	0.862519203
TAF1A	0.973418737	0.979263532	0.981593378	0.99999488	0.999985264	0.864806954
PDE1A	0.973418737	0.991335402	0.902527616	0.99999488	0.999985264	0.873463288
RHOG	0.973418737	0.941495217	0.835038876	0.99999488	0.999985264	0.874975722
JAM2	0.973418737	0.922102474	0.862345036	0.99999488	0.999985264	0.877838449

SCRN1	0.973418737	0.948421431	0.962346588	0.99999488	0.999985264	0.902958012
LOC100847951	0.973418737	0.939834725	0.938203102	0.99999488	0.999985264	0.905010417
TRPM7	0.973418737	0.978510121	0.841957362	0.99999488	0.999985264	0.917757534
LIPT2	0.973418737	0.943802122	0.922400936	0.99999488	0.999985264	0.931163114
MMAA	0.973418737	0.845554546	0.89029356	0.99999488	0.999985264	0.935106628
ARPC4	0.973418737	0.970426668	0.94634922	0.99999488	0.999985264	0.935349027
PTGDR	0.973418737	0.869999083	0.839056678	0.99999488	0.999985264	0.943220018
WDR36	0.973418737	0.837990885	0.917154286	0.99999488	0.999985264	0.948675165
POLR1E	0.973418737	0.883624935	0.841957362	0.99999488	0.999985264	0.955184323
TEKT3	0.973418737	0.869999083	0.929207633	0.99999488	0.999985264	0.972391591
LRRC4C	0.973418737	0.869180218	0.857021454	0.99999488	0.999985264	0.995391635
TNFAIP2	0.973418737	0.89880506	0.901414204	0.99999488	0.999985264	0.996929482
LOC112444936	0.973418737	0.867755878	0.871626027	0.99999488	0.999985264	0.999960414
LOC783854	0.973418737	0.834860292	0.908181052	0.99999488	0.999985264	0.999960414
COPA	0.973568813	0.878790155	0.860189237	0.99999488	0.999985264	0.827971245
ERAL1	0.973568813	0.850692013	0.825497047	0.99999488	0.999985264	0.846639166
FAM83F	0.973568813	0.93298315	0.841739726	0.99999488	0.999985264	0.873463288
KCNJ15	0.973568813	0.9582784	0.93198623	0.99999488	0.999985264	0.913174443
EFS	0.973616693	0.846198976	0.912008683	0.99999488	0.999985264	0.856510591
ARAP2	0.973616693	0.837468586	0.912008683	0.99999488	0.999985264	0.926673043
ZNF532	0.973620481	0.885718559	0.851718619	0.99999488	0.999985264	0.94485591
HIVEP3	0.973620481	0.961479426	0.890096618	0.99999488	0.999985264	0.957171025
WBP4	0.973646299	0.95697765	0.887626998	0.99999488	0.999985264	0.886185283
FEM1C	0.973646299	0.852760886	0.946479014	0.99999488	0.999985264	0.895370805
KPNB1	0.973646299	0.88167071	0.920629231	0.99999488	0.999985264	0.904813143
COX8A	0.973870978	0.845554546	0.887626998	0.99999488	0.999985264	0.827439931
LOC101908339	0.974111733	0.991488652	0.925745065	0.99999488	0.999985264	0.880216491
SERPINB8	0.974258781	0.862982	0.825497047	0.99999488	0.999985264	0.863280322
LOC101904468	0.974348072	0.869999083	0.999558179	0.99999488	0.999985264	0.828248574
NCOA5	0.974348072	0.9582784	0.914001994	0.99999488	0.999985264	0.868256799
RPS6KA4	0.974348072	0.991563824	0.848312163	0.99999488	0.999985264	0.87805762
B4GALT3	0.974348072	0.883624935	0.828875895	0.99999488	0.999985264	0.996929482
FGFR1OP	0.974377272	0.999992444	0.865129879	0.99999488	0.999985264	0.826043635
TMEM50A	0.974377272	0.858753382	0.999558179	0.99999488	0.999985264	0.83911834
HAVCR2	0.974377272	0.905019423	0.851718619	0.99999488	0.999985264	0.850307641
LITAF	0.974377272	0.907069508	0.893044539	0.99999488	0.999985264	0.88310116
PTCD1	0.974377272	0.867755878	0.964152013	0.99999488	0.999985264	0.926480606
ZBTB38	0.974377272	0.94254418	0.863239204	0.99999488	0.999985264	0.994845153
OSTF1	0.974601811	0.873113713	0.903957821	0.99999488	0.999985264	0.859200033

TIPARP	0.97465367	0.836787113	0.841717609	0.99999488	0.999985264	0.823935707
ACBD3	0.97465367	0.872223076	0.877799478	0.99999488	0.999985264	0.828547227
MAB21L2	0.97465367	0.999992444	0.834173166	0.99999488	0.999985264	0.829320798
SRGAP1	0.97465367	0.845554546	0.947957719	0.99999488	0.999985264	0.838101199
DUSP12	0.97465367	0.869999083	0.927600835	0.99999488	0.999985264	0.8381256
GLA	0.97465367	0.850511001	0.865066354	0.99999488	0.999985264	0.838520085
ZMYND15	0.97465367	0.87010028	0.921270971	0.99999488	0.999985264	0.868513869
LOC101902084	0.97465367	0.991488652	0.923604668	0.99999488	0.999985264	0.87805762
LIPT1	0.97465367	0.849474629	0.912915416	0.99999488	0.999985264	0.904915514
MYOCD	0.97465367	0.991476708	0.900148803	0.99999488	0.999985264	0.921771959
SLC39A3	0.97465367	0.920253543	0.881942256	0.99999488	0.999985264	0.925481882
LOC516599	0.97465367	0.844417133	0.840760291	0.99999488	0.999985264	0.929458454
BMF	0.97465367	0.836111245	0.848312163	0.99999488	0.999985264	0.929458454
MATN4	0.97465367	0.88167071	0.912014666	0.99999488	0.999985264	0.929527103
CHD2	0.97465367	0.892983059	0.892943118	0.99999488	0.999985264	0.95096765
SPTLC3	0.97465367	0.883856603	0.917154286	0.99999488	0.999985264	0.987454496
RNF139	0.974944464	0.948257806	0.997859293	0.99999488	0.999985264	0.828248574
PIH1D2	0.975048971	0.941495217	0.981039752	0.99999488	0.999985264	0.897965262
LOC107132225	0.975075653	0.911418256	0.951822586	0.99999488	0.999985264	0.85653214
VPS16	0.975278132	0.867755878	0.998936876	0.99999488	0.999985264	0.83405895
CEP89	0.975278132	0.912485935	0.946479014	0.99999488	0.999985264	0.948213108
SH3PXD2A	0.975342646	0.882505302	0.844476312	0.99999488	0.999985264	0.885254561
PRADC1	0.975397185	0.882505302	0.851523772	0.99999488	0.999985264	0.874975722
ZNF394	0.975397185	0.999992444	0.916214824	0.99999488	0.999985264	0.882774066
MFAP2	0.975766355	0.922102474	0.941118576	0.99999488	0.999985264	0.839734604
ACVR2B	0.975766355	0.991488652	0.866582678	0.99999488	0.999985264	0.873463288
C15H11orf58	0.975766355	0.924139925	0.973406453	0.99999488	0.999985264	0.873463288
FAM214B	0.975766355	0.922700337	0.966991646	0.99999488	0.999985264	0.874975722
TTC1	0.975766355	0.839292361	0.903957821	0.99999488	0.999985264	0.894333557
LOC100336369	0.975766355	0.999992444	0.862345036	0.99999488	0.999985264	0.895349622
GPATCH3	0.975766355	0.989627874	0.835038876	0.99999488	0.999985264	0.900681286
GSK3A	0.975766355	0.89880506	0.862345036	0.99999488	0.999985264	0.939035437
LOC100300483	0.975766355	0.992977597	0.831723974	0.99999488	0.999985264	0.986408155
LYZ2	0.975766355	0.938476452	0.851197548	0.99999488	0.999985264	0.992853834
LOC112447305	0.975766355	0.864811968	0.857021454	0.99999488	0.999985264	0.999960414
LOC112444309	0.975766355	0.943802122	0.862345036	0.99999488	0.999985264	0.999960414
LOC100337108	0.976389077	0.999992444	0.848312163	0.99999488	0.999985264	0.828248574
CFAP298	0.976389077	0.934886531	0.857021454	0.99999488	0.999985264	0.828248574
IRF3	0.976389077	0.921934673	0.857454688	0.99999488	0.999985264	0.837019875

QPCT	0.976389077	0.935742738	0.998816213	0.99999488	0.999985264	0.838520085
KIF9	0.976389077	0.936273761	0.999558179	0.99999488	0.999985264	0.839734604
COX5A	0.976389077	0.871627189	0.887319394	0.99999488	0.999985264	0.847204221
MEX3B	0.976389077	0.865274556	0.911018006	0.99999488	0.999985264	0.848951731
PDIA3	0.976389077	0.925062074	0.907484111	0.99999488	0.999985264	0.849138453
PBRM1	0.976389077	0.999992444	0.858024919	0.99999488	0.999985264	0.851533524
LOC112447080	0.976389077	0.904518324	0.886962419	0.99999488	0.999985264	0.856510591
PPT2	0.976389077	0.901232581	0.995045982	0.99999488	0.999985264	0.85653214
AHCYL2	0.976389077	0.867755878	0.959355629	0.99999488	0.999985264	0.861618934
FADS3	0.976389077	0.957199613	0.886192181	0.99999488	0.999985264	0.861837269
PRRC2A	0.976389077	0.926675185	0.870264123	0.99999488	0.999985264	0.863192816
LYRM9	0.976389077	0.882505302	0.851718619	0.99999488	0.999985264	0.863774505
LOC112441605	0.976389077	0.938597964	0.841957362	0.99999488	0.999985264	0.866439215
TXNRD1	0.976389077	0.873289191	0.927516734	0.99999488	0.999985264	0.866439215
SLC3A2	0.976389077	0.957700659	0.898815524	0.99999488	0.999985264	0.86871626
HTR2A	0.976389077	0.937626884	0.923604668	0.99999488	0.999985264	0.873463288
CLCN7	0.976389077	0.936489465	0.841773648	0.99999488	0.999985264	0.874849554
KCTD8	0.976389077	0.880276234	0.841773648	0.99999488	0.999985264	0.882867473
BIRC2	0.976389077	0.958098081	0.946479014	0.99999488	0.999985264	0.888645453
EPAS1	0.976389077	0.858808025	0.95230212	0.99999488	0.999985264	0.89589047
LURAP1L	0.976389077	0.922102474	0.870567942	0.99999488	0.999985264	0.900681286
LOC112444352	0.976389077	0.999992444	0.938905004	0.99999488	0.999985264	0.910018813
JAGN1	0.976389077	0.904961534	0.914021366	0.99999488	0.999985264	0.915574092
PDZD4	0.976389077	0.928525825	0.884615251	0.99999488	0.999985264	0.919755394
CDH4	0.976389077	0.850692013	0.939147816	0.99999488	0.999985264	0.923467533
F2RL2	0.976389077	0.95697765	0.920629231	0.99999488	0.999985264	0.925260365
KIF2A	0.976389077	0.839149526	0.852670802	0.99999488	0.999985264	0.926027375
TPM2	0.976389077	0.999347049	0.895626254	0.99999488	0.999985264	0.931163114
RCSD1	0.976389077	0.916036681	0.95230212	0.99999488	0.999985264	0.931163114
BCO2	0.976389077	0.881001912	0.862345036	0.99999488	0.999985264	0.937880392
STIL	0.976389077	0.978089982	0.855064966	0.99999488	0.999985264	0.939035437
CCL4	0.976389077	0.938597964	0.851718619	0.99999488	0.999985264	0.944906868
C10H15orf48	0.976389077	0.869999083	0.865066354	0.99999488	0.999985264	0.945670698
AKAP12	0.976389077	0.924216699	0.86638329	0.99999488	0.999985264	0.946666773
LOC112449172	0.976389077	0.91058693	0.923953195	0.99999488	0.999985264	0.955236184
PFAS	0.976389077	0.85337372	0.915266146	0.99999488	0.999985264	0.960343814
CHD5	0.976389077	0.978089982	0.834068744	0.99999488	0.999985264	0.966921967
LAMA5	0.976389077	0.845554546	0.859991705	0.99999488	0.999985264	0.981855491
LOC112447474	0.976389077	0.90284443	0.916975216	0.99999488	0.999985264	0.986408155

SREBF1	0.976389077	0.927997582	0.857021454	0.99999488	0.999985264	0.987457361
ANXA6	0.976389077	0.893642384	0.841957362	0.99999488	0.999985264	0.987872708
LOC112449302	0.976389077	0.859484941	0.871385134	0.99999488	0.999985264	0.999960414
AGFG2	0.976399529	0.850692013	0.834173166	0.99999488	0.999985264	0.856510591
NAE1	0.976490185	0.949971468	0.830398164	0.99999488	0.999985264	0.927933967
ZCCHC14	0.976700483	0.957700659	0.908181052	0.99999488	0.999985264	0.828248574
MS4A8	0.976700483	0.978510121	0.93198623	0.99999488	0.999985264	0.841443168
LOC790312	0.976700483	0.999992444	0.843674782	0.99999488	0.999985264	0.85182524
MCM2	0.976700483	0.991488652	0.902527616	0.99999488	0.999985264	0.862046449
CHAF1B	0.976700483	0.874697957	0.865837237	0.99999488	0.999985264	0.871960581
VNN1	0.976700483	0.87010028	0.830186385	0.99999488	0.999985264	0.952764661
LOC112447301	0.976756998	0.893642384	0.862345036	0.99999488	0.999985264	0.849046935
MEF2D	0.976756998	0.889390207	0.89029356	0.99999488	0.999985264	0.978896655
PGGT1B	0.97683422	0.974812121	0.902527616	0.99999488	0.999985264	0.850842307
DIRAS1	0.977194234	0.84313917	0.881040986	0.99999488	0.999985264	0.861670395
LOC104974850	0.977194234	0.877142156	0.853055742	0.99999488	0.999985264	0.999960414
PTP4A1	0.977434443	0.991488652	0.956565728	0.99999488	0.999985264	0.868103112
PRR16	0.977434443	0.874697957	0.948420301	0.99999488	0.999985264	0.926480606
DUSP15	0.977614555	0.859484941	0.841739726	0.99999488	0.999985264	0.837143123
EHD1	0.977614555	0.986637746	0.865129879	0.99999488	0.999985264	0.89668604
KDM5B	0.97777313	0.850095234	0.848312163	0.99999488	0.999985264	0.846769116
ALG9	0.97777313	0.871627189	0.938203102	0.99999488	0.999985264	0.873463288
GPX8	0.97777313	0.905019423	0.973406453	0.99999488	0.999985264	0.899223071
ACSS2	0.97777313	0.855390299	0.834068744	0.99999488	0.999985264	0.923360345
HES2	0.97777313	0.970653127	0.83126054	0.99999488	0.999985264	0.926480606
ZNF7	0.97777313	0.997083202	0.940724051	0.99999488	0.999985264	0.928634547
MAP2K3	0.97777313	0.928113285	0.938905004	0.99999488	0.999985264	0.939035437
TES	0.97777313	0.948421431	0.934941286	0.99999488	0.999985264	0.952948574
SLC6A20	0.97777313	0.862982	0.852782377	0.99999488	0.999985264	0.960343814
UBXN10	0.97777313	0.845554546	0.886962419	0.99999488	0.999985264	0.966921967
NET1	0.97777313	0.850095234	0.900890709	0.99999488	0.999985264	0.972391591
RORC	0.97777313	0.867535124	0.925745065	0.99999488	0.999985264	0.999960414
ZFP64	0.978053641	0.974812121	0.857021454	0.99999488	0.999985264	0.930540897
LOC104974749	0.978193909	0.847686989	0.863239204	0.99999488	0.999985264	0.832833221
CLIC2	0.978193909	0.865926615	0.978389389	0.99999488	0.999985264	0.836520183
MRPL41	0.978193909	0.93094333	0.926552506	0.99999488	0.999985264	0.84393496
COLQ	0.978193909	0.913605849	0.851718619	0.99999488	0.999985264	0.844563769
ICA1	0.978193909	0.949285285	0.988922732	0.99999488	0.999985264	0.849046935
PLEKHA4	0.978193909	0.893495573	0.944031108	0.99999488	0.999985264	0.856224954

LOC101907661	0.978193909	0.888468187	0.86178438	0.99999488	0.999985264	0.85637643
ZMYM5	0.978193909	0.973334182	0.854974646	0.99999488	0.999985264	0.884033654
PRKAG3	0.978193909	0.952346363	0.880008612	0.99999488	0.999985264	0.894723749
TRARG1	0.978193909	0.935060764	0.841957362	0.99999488	0.999985264	0.905099683
SINHCAF	0.978193909	0.89880506	0.849269107	0.99999488	0.999985264	0.920650439
LOC100847695	0.978193909	0.882022911	0.851718619	0.99999488	0.999985264	0.939035437
DNM1L	0.978193909	0.869999083	0.862345036	0.99999488	0.999985264	0.999960414
ACD	0.978228509	0.863813934	0.998936876	0.99999488	0.999985264	0.841424601
SLC45A2	0.978228509	0.999992444	0.834173166	0.99999488	0.999985264	0.858593649
LOC100848011	0.978392897	0.922995823	0.957541703	0.99999488	0.999985264	0.83405895
DSE	0.978392897	0.901205367	0.933046493	0.99999488	0.999985264	0.847577522
HOXD8	0.978392897	0.919884594	0.870264123	0.99999488	0.999985264	0.849046935
LOC782343	0.978392897	0.869999083	0.944932814	0.99999488	0.999985264	0.858593649
UMAD1	0.978392897	0.896507811	0.871925227	0.99999488	0.999985264	0.865855226
SAT1	0.978392897	0.991488652	0.864441458	0.99999488	0.999985264	0.86871626
RAD21	0.978392897	0.873963285	0.900148803	0.99999488	0.999985264	0.870708914
KIAA1109	0.978392897	0.913204869	0.848832109	0.99999488	0.999985264	0.882867473
METAP1	0.978392897	0.845262032	0.925520255	0.99999488	0.999985264	0.884124443
HOPX	0.978392897	0.87010028	0.922400936	0.99999488	0.999985264	0.89069589
MTDH	0.978392897	0.893748757	0.998936876	0.99999488	0.999985264	0.894723749
ZNHIT3	0.978392897	0.867777719	0.88825152	0.99999488	0.999985264	0.897965262
CACNG5	0.978392897	0.932593501	0.891893039	0.99999488	0.999985264	0.897965262
LOC104975974	0.978392897	0.885718559	0.996391386	0.99999488	0.999985264	0.921771959
CLPP	0.978392897	0.895813197	0.890096618	0.99999488	0.999985264	0.922403963
COPZ1	0.978392897	0.957700659	0.86638329	0.99999488	0.999985264	0.939035437
CYFIP2	0.978392897	0.994481342	0.856449202	0.99999488	0.999985264	0.948675165
TSSK4	0.978392897	0.883624935	0.935094475	0.99999488	0.999985264	0.949545831
LOC510536	0.978392897	0.986637746	0.886192181	0.99999488	0.999985264	0.954519353
NXPH3	0.978392897	0.915367594	0.849088328	0.99999488	0.999985264	0.960343814
C29H11orf98	0.978392897	0.867045149	0.886192181	0.99999488	0.999985264	0.999960414
FAM102A	0.978702561	0.935742738	0.861140642	0.99999488	0.999985264	0.846769116
CD320	0.978702561	0.870839685	0.964079616	0.99999488	0.999985264	0.885874544
NTHL1	0.979098365	0.869999083	0.964920281	0.99999488	0.999985264	0.832622862
SS18L2	0.979098365	0.890565478	0.922400936	0.99999488	0.999985264	0.844277572
TNFRSF17	0.979098365	0.958967142	0.920629231	0.99999488	0.999985264	0.858593649
TRAF4	0.979098365	0.918254521	0.892933333	0.99999488	0.999985264	0.883443672
ZCCHC11	0.979098365	0.922700337	0.902527616	0.99999488	0.999985264	0.897965262
ZSCAN26	0.979098365	0.944701115	0.926552506	0.99999488	0.999985264	0.944906868
VCAN	0.979112371	0.840712878	0.938203102	0.99999488	0.999985264	0.847204221

WNT5A	0.979112371	0.883856603	0.914001994	0.99999488	0.999985264	0.871960581
ADCK2	0.979224534	0.852472905	0.863098782	0.99999488	0.999985264	0.932608891
RNF223	0.979224534	0.882505302	0.925745065	0.99999488	0.999985264	0.997713313
FIBIN	0.979415303	0.935742738	0.995045982	0.99999488	0.999985264	0.839734604
UBE3A	0.979415303	0.891316528	0.910426741	0.99999488	0.999985264	0.931163114
LOC112441457	0.979415303	0.912518351	0.930127869	0.99999488	0.999985264	0.931163114
CLEC4G	0.979415303	0.883856603	0.889567735	0.99999488	0.999985264	0.95096765
CCDC93	0.979415303	0.912518351	0.862345036	0.99999488	0.999985264	0.960343814
SPAG7	0.979415303	0.893642384	0.8664043	0.99999488	0.999985264	0.999960414
STARD3	0.9794645	0.893642384	0.999558179	0.99999488	0.999985264	0.834119413
LOC104968411	0.9794645	0.87010028	0.946479014	0.99999488	0.999985264	0.840547083
PPID	0.9794645	0.849474629	0.886192181	0.99999488	0.999985264	0.841424601
ABHD18	0.9794645	0.9409629	0.946832224	0.99999488	0.999985264	0.847204221
STEAP4	0.9794645	0.883624935	0.892943118	0.99999488	0.999985264	0.849138453
ADCY3	0.9794645	0.884739596	0.887349218	0.99999488	0.999985264	0.868483865
TOE1	0.9794645	0.991365955	0.905254232	0.99999488	0.999985264	0.873463288
SNAI2	0.9794645	0.949285285	0.840781773	0.99999488	0.999985264	0.875113722
HDAC7	0.9794645	0.850692013	0.94634922	0.99999488	0.999985264	0.885874544
LOC508628	0.9794645	0.922700337	0.881942256	0.99999488	0.999985264	0.894236694
CDH11	0.9794645	0.883624935	0.93198623	0.99999488	0.999985264	0.896480535
PPIA	0.9794645	0.850692013	0.881942256	0.99999488	0.999985264	0.910018813
LOC112446018	0.9794645	0.911291478	0.881942256	0.99999488	0.999985264	0.918343194
LOC112446798	0.9794645	0.921934673	0.939147816	0.99999488	0.999985264	0.939035437
CEP112	0.9794645	0.938597964	0.892664791	0.99999488	0.999985264	0.949764317
ST5	0.9794645	0.935060764	0.842443565	0.99999488	0.999985264	0.977180137
COX7ALP1	0.979536102	0.868384898	0.945678616	0.99999488	0.999985264	0.839855875
SMAD9	0.979536102	0.943802122	0.840781773	0.99999488	0.999985264	0.883443672
ANXA11	0.979536102	0.953844371	0.925520255	0.99999488	0.999985264	0.91208425
FKBP5	0.979536102	0.846198976	0.915215321	0.99999488	0.999985264	0.925558146
DOPEY2	0.979536102	0.869999083	0.892574927	0.99999488	0.999985264	0.943220018
MRPL35	0.979899842	0.883624935	0.861480822	0.99999488	0.999985264	0.892908035
ACYP1	0.979954782	0.8756206	0.956565728	0.99999488	0.999985264	0.858593649
TMEM237	0.979954782	0.850095234	0.86638329	0.99999488	0.999985264	0.918400465
DNAAF3	0.979954782	0.893743321	0.891893039	0.99999488	0.999985264	0.920531105
FZD1	0.979954782	0.850095234	0.855642445	0.99999488	0.999985264	0.923360345
LIN9	0.979954782	0.899466275	0.86576143	0.99999488	0.999985264	0.949718683
COX6A1	0.980199122	0.845554546	0.894199923	0.99999488	0.999985264	0.836831695
FNIP2	0.980199122	0.848246309	0.944085862	0.99999488	0.999985264	0.85653214
ZMYM1	0.980199122	0.999992444	0.837103057	0.99999488	0.999985264	0.866578338

LOC782057	0.980199122	0.999992444	0.858024919	0.99999488	0.999985264	0.882867473
ZBTB21	0.980199122	0.882505302	0.962346588	0.99999488	0.999985264	0.883507907
CDC37	0.980199122	0.999347049	0.840760291	0.99999488	0.999985264	0.894723749
LOC112443837	0.980199122	0.919209432	0.86178438	0.99999488	0.999985264	0.921150136
LOC101907133	0.980199122	0.930710774	0.923604668	0.99999488	0.999985264	0.939657518
COASY	0.980397345	0.852760886	0.876765981	0.99999488	0.999985264	0.869423085
SKAP1	0.980397345	0.867755878	0.908181052	0.99999488	0.999985264	0.89935427
TMEM61	0.980493243	0.999992444	0.851718619	0.99999488	0.999985264	0.836520183
TNXB	0.980493243	0.931488015	0.912008683	0.99999488	0.999985264	0.836520183
SCAF8	0.980493243	0.918911961	0.861314069	0.99999488	0.999985264	0.837517492
DDX46	0.980493243	0.921934673	0.907381775	0.99999488	0.999985264	0.837517492
RSU1	0.980493243	0.932593501	0.999558179	0.99999488	0.999985264	0.844277572
LOC784451	0.980493243	0.847051507	0.945678616	0.99999488	0.999985264	0.84517471
CCER2	0.980493243	0.999992444	0.892943118	0.99999488	0.999985264	0.846769116
LOC101902786	0.980493243	0.935742738	0.886192181	0.99999488	0.999985264	0.849046935
LOC104970450	0.980493243	0.880691411	0.941730265	0.99999488	0.999985264	0.849547326
LOC100335744	0.980493243	0.873113713	0.89029356	0.99999488	0.999985264	0.850307641
PAFAH2	0.980493243	0.925580392	0.911131131	0.99999488	0.999985264	0.850790144
ADPRH	0.980493243	0.870839685	0.973406453	0.99999488	0.999985264	0.850797738
LOC100847374	0.980493243	0.999992444	0.850804682	0.99999488	0.999985264	0.851595584
LOC518961	0.980493243	0.920639601	0.925745065	0.99999488	0.999985264	0.85637643
SENP8	0.980493243	0.912485935	0.890248427	0.99999488	0.999985264	0.85653214
GPER1	0.980493243	0.883856603	0.844396077	0.99999488	0.999985264	0.859321224
POLDIP3	0.980493243	0.936273761	0.95935771	0.99999488	0.999985264	0.870160734
INTS1	0.980493243	0.864945604	0.914001994	0.99999488	0.999985264	0.870893258
AKAP9	0.980493243	0.87010028	0.921646762	0.99999488	0.999985264	0.870893258
PLEKHG6	0.980493243	0.999992444	0.855775419	0.99999488	0.999985264	0.872365139
IGIP	0.980493243	0.988170127	0.880399877	0.99999488	0.999985264	0.878070222
IRF9	0.980493243	0.894140115	0.96870883	0.99999488	0.999985264	0.881768147
EXOC7	0.980493243	0.874697957	0.881419724	0.99999488	0.999985264	0.885874544
SHMT1	0.980493243	0.873289191	0.880399877	0.99999488	0.999985264	0.892231098
NLRP12	0.980493243	0.872925496	0.871925227	0.99999488	0.999985264	0.892552781
LOC100847876	0.980493243	0.950729918	0.837074149	0.99999488	0.999985264	0.894723749
LOC112443415	0.980493243	0.884739596	0.946832224	0.99999488	0.999985264	0.894723749
FZD9	0.980493243	0.967535824	0.938203102	0.99999488	0.999985264	0.897014912
LOC112447392	0.980493243	0.999992444	0.945766581	0.99999488	0.999985264	0.897965262
PATL1	0.980493243	0.884842564	0.956565728	0.99999488	0.999985264	0.897965262
LOC785503	0.980493243	0.999992444	0.93198623	0.99999488	0.999985264	0.900681286
SF3B3	0.980493243	0.919884594	0.851718619	0.99999488	0.999985264	0.907789191

GATA3	0.980493243	0.930659282	0.849633432	0.99999488	0.999985264	0.910018813
ASCC2	0.980493243	0.999347049	0.851718619	0.99999488	0.999985264	0.918400465
SLC35A4	0.980493243	0.904518324	0.874559186	0.99999488	0.999985264	0.921771959
UBN2	0.980493243	0.999992444	0.884466521	0.99999488	0.999985264	0.921771959
SHANK1	0.980493243	0.850692013	0.911018006	0.99999488	0.999985264	0.924201993
C29H11orf80	0.980493243	0.991488652	0.926552506	0.99999488	0.999985264	0.929458454
LOC112449284	0.980493243	0.867755878	0.973406453	0.99999488	0.999985264	0.931163114
LGALS7	0.980493243	0.943802122	0.96210311	0.99999488	0.999985264	0.939035437
URM1	0.980493243	0.94984369	0.865837237	0.99999488	0.999985264	0.946666773
KLF14	0.980493243	0.94254418	0.899576888	0.99999488	0.999985264	0.947433142
PGLS	0.980493243	0.934515915	0.841739726	0.99999488	0.999985264	0.95096765
PPP2R1B	0.980493243	0.928095609	0.855064966	0.99999488	0.999985264	0.955184323
LOC107131642	0.980493243	0.922700337	0.876758498	0.99999488	0.999985264	0.960343814
POU2F2	0.980493243	0.883624935	0.908181052	0.99999488	0.999985264	0.972391591
COPS6	0.980493243	0.893470298	0.890248427	0.99999488	0.999985264	0.978249551
DPP3	0.980493243	0.9582784	0.851718619	0.99999488	0.999985264	0.986873741
TMEM229A	0.980493243	0.850095234	0.848312163	0.99999488	0.999985264	0.999960414
YME1L1	0.980493243	0.883856603	0.862345036	0.99999488	0.999985264	0.999960414
MT2A	0.980527682	0.84979258	0.857454688	0.99999488	0.999985264	0.968695729
MMP16	0.980621403	0.935742738	0.886962419	0.99999488	0.999985264	0.931163114
ST7L	0.981039619	0.999992444	0.865420641	0.99999488	0.999985264	0.849046935
MAML2	0.981039619	0.9582784	0.848312163	0.99999488	0.999985264	0.887277447
PUS3	0.981039619	0.881261173	0.895626254	0.99999488	0.999985264	0.926596859
LOC101902570	0.981481661	0.946480858	0.964908146	0.99999488	0.999985264	0.911997747
NTMT1	0.981596235	0.916320187	0.902271049	0.99999488	0.999985264	0.922063125
RAB18	0.981796772	0.943802122	0.930678591	0.99999488	0.999985264	0.875113722
MRPL49	0.981796772	0.87010028	0.892943118	0.99999488	0.999985264	0.888645453
BTF3L4	0.981796772	0.999992444	0.918170048	0.99999488	0.999985264	0.892056797
MMP25	0.981796772	0.999992444	0.894199923	0.99999488	0.999985264	0.90408677
DDX10	0.981796772	0.9582784	0.86178438	0.99999488	0.999985264	0.972391591
ADCY8	0.981796772	0.864945604	0.876758498	0.99999488	0.999985264	0.999960414
PAQR3	0.981918074	0.846198976	0.85773697	0.99999488	0.999985264	0.897467714
E2F4	0.981918074	0.927147798	0.933447488	0.99999488	0.999985264	0.931163114
WHRN	0.98202299	0.888660199	0.919763144	0.99999488	0.999985264	0.90408677
PRR22	0.982059138	0.89064218	0.945678616	0.99999488	0.999985264	0.862046449
KPTN	0.982059138	0.937231686	0.851718619	0.99999488	0.999985264	0.89668604
TGFBR1	0.982059138	0.877142156	0.998936876	0.99999488	0.999985264	0.901809636
ACO1	0.982059138	0.893642384	0.918351194	0.99999488	0.999985264	0.912262222
IL17RA	0.982059138	0.87010028	0.930678591	0.99999488	0.999985264	0.920531105

RHOC	0.982059138	0.998131745	0.86259773	0.99999488	0.999985264	0.95905828
VAPA	0.982303812	0.850692013	0.938911935	0.99999488	0.999985264	0.850552215
CFAP161	0.982303812	0.974812121	0.88153193	0.99999488	0.999985264	0.932608891
DSP	0.982303812	0.941779521	0.954823586	0.99999488	0.999985264	0.939035437
GAS2L1	0.983069595	0.941495217	0.83711556	0.99999488	0.999985264	0.888645453
GABBR2	0.983069595	0.958967142	0.837103057	0.99999488	0.999985264	0.953252635
CCDC136	0.983085906	0.858808025	0.864257597	0.99999488	0.999985264	0.992727599
BREH1	0.98309071	0.884690135	0.886500191	0.99999488	0.999985264	0.856510591
CBLN2	0.98309071	0.999992444	0.852468219	0.99999488	0.999985264	0.86871626
DDIT4	0.98309071	0.873963285	0.998936876	0.99999488	0.999985264	0.870708914
LOC100335553	0.98309071	0.9527938	0.95230212	0.99999488	0.999985264	0.911089313
RAB8A	0.98309071	0.999347049	0.898815524	0.99999488	0.999985264	0.940358165
MET	0.98309071	0.999992444	0.871925227	0.99999488	0.999985264	0.94487565
DNTTIP2	0.98309071	0.867755878	0.930127869	0.99999488	0.999985264	0.987454496
POLG	0.983225633	0.905019423	0.999558179	0.99999488	0.999985264	0.840513775
LOC101905887	0.983225633	0.90284443	0.865884303	0.99999488	0.999985264	0.85049547
ANKRD27	0.983225633	0.896536444	0.857021454	0.99999488	0.999985264	0.981216942
LEP	0.983344013	0.978089982	0.869906363	0.99999488	0.999985264	0.929852609
METTL21A	0.983686362	0.999992444	0.879253779	0.99999488	0.999985264	0.870160734
SGCA	0.983991834	0.992977597	0.973406453	0.99999488	0.999985264	0.86578423
TRAPPC6B	0.983991834	0.998962836	0.880229444	0.99999488	0.999985264	0.879832891
CERS6	0.983991834	0.92462339	0.849633432	0.99999488	0.999985264	0.929631053
UTP14A	0.983991834	0.965433188	0.864737879	0.99999488	0.999985264	0.946666773
GLRX3	0.983991834	0.869999083	0.944085862	0.99999488	0.999985264	0.982250307
SIDT1	0.984106161	0.901758989	0.926552506	0.99999488	0.999985264	0.9467439
ADO	0.984106161	0.887644042	0.921065686	0.99999488	0.999985264	0.999960414
MAPK11	0.984588747	0.882505302	0.973661483	0.99999488	0.999985264	0.838520085
PLXDC2	0.984685153	0.87010028	0.914630535	0.99999488	0.999985264	0.838510925
OXR1	0.984685153	0.936273761	0.862345036	0.99999488	0.999985264	0.872307149
C1GALT1C1	0.984685153	0.885024111	0.995045982	0.99999488	0.999985264	0.888234257
LOC101902390	0.984685153	0.961479426	0.860638608	0.99999488	0.999985264	0.89935427
ARL6IP1	0.984685153	0.862707207	0.999558179	0.99999488	0.999985264	0.931163114
MSMO1	0.98504407	0.859484941	0.914630535	0.99999488	0.999985264	0.939035437
TENM3	0.985046574	0.869999083	0.938203102	0.99999488	0.999985264	0.854325286
FUS	0.985198447	0.893743321	0.871925227	0.99999488	0.999985264	0.982250307
FYN	0.985300025	0.941495217	0.865837237	0.99999488	0.999985264	0.853892523
UXT	0.985300025	0.883856603	0.841739726	0.99999488	0.999985264	0.897467714
LOC100335751	0.985371173	0.883856603	0.986180419	0.99999488	0.999985264	0.858593649
ATF4	0.98551405	0.892206663	0.930678591	0.99999488	0.999985264	0.872307149

POLR2I	0.98554345	0.958967142	0.9808801	0.99999488	0.999985264	0.868483865
LOC107132911	0.98554345	0.867755878	0.884320747	0.99999488	0.999985264	0.90109791
TNFSF12	0.98554345	0.992977597	0.862345036	0.99999488	0.999985264	0.9467439
RICTOR	0.98554345	0.992977597	0.842881104	0.99999488	0.999985264	0.95956081
SRM	0.98554345	0.993316598	0.863098782	0.99999488	0.999985264	0.960343814
C29H11orf68	0.985765893	0.979263532	0.890248427	0.99999488	0.999985264	0.882867473
IFT140	0.985910991	0.898204214	0.94634922	0.99999488	0.999985264	0.872307149
KIAA1755	0.985910991	0.87010028	0.926552506	0.99999488	0.999985264	0.982250307
PLPPR4	0.986125578	0.883624935	0.864312509	0.99999488	0.999985264	0.882867473
SEMA4D	0.986125578	0.943802122	0.852782377	0.99999488	0.999985264	0.948213108
PDXDC1	0.986125578	0.941876954	0.863239204	0.99999488	0.999985264	0.966921967
PDIA4	0.986441525	0.978089982	0.900148803	0.99999488	0.999985264	0.895349622
TTC32	0.986580657	0.862982	0.907381775	0.99999488	0.999985264	0.848286219
SYT5	0.986580657	0.941495217	0.857021454	0.99999488	0.999985264	0.863774505
ADAMTS3	0.986580657	0.884739596	0.877742202	0.99999488	0.999985264	0.873463288
PRR19	0.986580657	0.864945604	0.857072251	0.99999488	0.999985264	0.873836462
LOC100848995	0.986580657	0.991488652	0.866582678	0.99999488	0.999985264	0.970849664
TIMP3	0.986604213	0.938597964	0.892053976	0.99999488	0.999985264	0.923146498
MSS51	0.986629585	0.952346363	0.876188482	0.99999488	0.999985264	0.982250307
LRRCC1	0.98667857	0.999992444	0.936914995	0.99999488	0.999985264	0.839734604
LRMDA	0.98667857	0.991488652	0.844394756	0.99999488	0.999985264	0.841424601
SERPINB9	0.98667857	0.999992444	0.886962419	0.99999488	0.999985264	0.844640551
TYMS	0.98667857	0.943802122	0.946479014	0.99999488	0.999985264	0.849046935
PARP3	0.98667857	0.924817361	0.994601277	0.99999488	0.999985264	0.849138453
IFT20	0.98667857	0.999992444	0.916975216	0.99999488	0.999985264	0.85653214
MARVELD2	0.98667857	0.927853996	0.915215321	0.99999488	0.999985264	0.859200033
DMAC2	0.98667857	0.890682633	0.877742202	0.99999488	0.999985264	0.866439215
THAP4	0.98667857	0.927997582	0.881942256	0.99999488	0.999985264	0.866439215
RCN1	0.98667857	0.863793573	0.993594894	0.99999488	0.999985264	0.868103112
LOC783504	0.98667857	0.893748757	0.998936876	0.99999488	0.999985264	0.872307149
NEDD9	0.98667857	0.961267851	0.886730817	0.99999488	0.999985264	0.885824404
GBE1	0.98667857	0.977007804	0.888076972	0.99999488	0.999985264	0.885874544
PNN	0.98667857	0.936562168	0.939799459	0.99999488	0.999985264	0.887231766
HES6	0.98667857	0.85337372	0.981388276	0.99999488	0.999985264	0.893921142
ABHD17A	0.98667857	0.958967142	0.930678591	0.99999488	0.999985264	0.894723749
SETD3	0.98667857	0.885718559	0.954620171	0.99999488	0.999985264	0.894749423
LOC112448833	0.98667857	0.999347049	0.935094475	0.99999488	0.999985264	0.895370805
COPS7A	0.98667857	0.977165015	0.877786216	0.99999488	0.999985264	0.89589047
EID1	0.98667857	0.9582784	0.865066354	0.99999488	0.999985264	0.900681286

PNPLA4	0.98667857	0.986445172	0.841739726	0.99999488	0.999985264	0.923360345
DMAC1	0.98667857	0.916036681	0.924604843	0.99999488	0.999985264	0.932008586
ACTRT3	0.98667857	0.999347049	0.848832109	0.99999488	0.999985264	0.944511198
SUV39H2	0.98667857	0.978510121	0.866377477	0.99999488	0.999985264	0.949764317
PRKACB	0.98667857	0.902714827	0.938911935	0.99999488	0.999985264	0.953254073
LOC112442298	0.98667857	0.981155176	0.848786751	0.99999488	0.999985264	0.955374205
PAN3	0.98667857	0.918911961	0.848786751	0.99999488	0.999985264	0.959920062
SEMA4C	0.98667857	0.933208464	0.886132368	0.99999488	0.999985264	0.977753483
TRIL	0.98667857	0.91058693	0.877799478	0.99999488	0.999985264	0.999960414
KLK12	0.98667857	0.862982	0.886192181	0.99999488	0.999985264	0.999960414
EIF4G2	0.986688437	0.864024589	0.999558179	0.99999488	0.999985264	0.870160734
ERMARD	0.986814658	0.999992444	0.904140521	0.99999488	0.999985264	0.84517471
SCPEP1	0.986814658	0.864811968	0.964582964	0.99999488	0.999985264	0.858593649
ZYG11B	0.986814658	0.941495217	0.962346588	0.99999488	0.999985264	0.868103112
B3GALNT2	0.986814658	0.883624935	0.980859619	0.99999488	0.999985264	0.868103112
WDR1	0.986814658	0.982313546	0.923604668	0.99999488	0.999985264	0.872307149
BLOC1S1	0.986814658	0.95830623	0.877786216	0.99999488	0.999985264	0.882867473
KIAA1324L	0.986814658	0.9582784	0.888545123	0.99999488	0.999985264	0.884033654
CHN2	0.986814658	0.965625946	0.886962419	0.99999488	0.999985264	0.887277447
LAT2	0.986814658	0.859484941	0.851718619	0.99999488	0.999985264	0.887607259
NFKBIA	0.986814658	0.869303784	0.856449202	0.99999488	0.999985264	0.892908035
LOC112448816	0.986814658	0.911291478	0.952386544	0.99999488	0.999985264	0.89935427
MEDAG	0.986814658	0.925739259	0.904140521	0.99999488	0.999985264	0.901545041
KIF13A	0.986814658	0.974407708	0.892943118	0.99999488	0.999985264	0.923360345
LOC104975673	0.986814658	0.908923824	0.980847826	0.99999488	0.999985264	0.924461747
CRB1	0.986814658	0.901205367	0.956565728	0.99999488	0.999985264	0.943220018
UBA3	0.986814658	0.921549	0.859430492	0.99999488	0.999985264	0.982250307
PSMD14	0.986814658	0.871695579	0.898815524	0.99999488	0.999985264	0.999960414
PRICKLE1	0.986858878	0.856670857	0.86638329	0.99999488	0.999985264	0.877838449
ZBTB5	0.986858878	0.999992444	0.863098782	0.99999488	0.999985264	0.906644037
CADPS2	0.986858878	0.985406717	0.93955737	0.99999488	0.999985264	0.907993548
WFDC1	0.986858878	0.952639631	0.94634922	0.99999488	0.999985264	0.939035437
GOT1	0.987059173	0.883856603	0.907484111	0.99999488	0.999985264	0.89668604
LOC112447032	0.987059173	0.895632001	0.938905004	0.99999488	0.999985264	0.960343814
CBX5	0.987137566	0.864154704	0.999558179	0.99999488	0.999985264	0.856510591
DCSTAMP	0.987137566	0.915367594	0.881942256	0.99999488	0.999985264	0.871960581
PHIP	0.987137566	0.949285285	0.86638329	0.99999488	0.999985264	0.881288523
TGFBR3L	0.987137566	0.869999083	0.944168373	0.99999488	0.999985264	0.919755394
LOC107133049	0.987847676	0.999347049	0.871832208	0.99999488	0.999985264	0.866439215

NEXMIF	0.987872466	0.998131745	0.877786216	0.99999488	0.999985264	0.856224954
LOC100140121	0.987905821	0.859484941	0.999558179	0.99999488	0.999985264	0.885874544
MTFMT	0.988002001	0.999992444	0.919618001	0.99999488	0.999985264	0.842457432
SLC2A4	0.988002001	0.864945604	0.865129879	0.99999488	0.999985264	0.866439215
FOXC1	0.988002001	0.935742738	0.981039752	0.99999488	0.999985264	0.873049694
NAA25	0.988002001	0.919505104	0.935726137	0.99999488	0.999985264	0.873463288
CCDC14	0.988002001	0.978089982	0.922400936	0.99999488	0.999985264	0.876287892
SLC25A25	0.988002001	0.938597964	0.991206238	0.99999488	0.999985264	0.884033654
RPS25	0.988002001	0.911418256	0.898815524	0.99999488	0.999985264	0.886185283
PLCH2	0.988002001	0.991488652	0.870995265	0.99999488	0.999985264	0.918400465
RUBCN	0.988002001	0.921661251	0.930127869	0.99999488	0.999985264	0.95096765
TFB1M	0.988002001	0.869999083	0.841957362	0.99999488	0.999985264	0.964476713
KIF16B	0.988002001	0.867755878	0.852782377	0.99999488	0.999985264	0.966921967
LYRM1	0.988002001	0.860752613	0.886730817	0.99999488	0.999985264	0.968059852
LOC101906828	0.988002001	0.933208464	0.921379317	0.99999488	0.999985264	0.975680198
LOC101904768	0.988002001	0.885718559	0.965714506	0.99999488	0.999985264	0.977753483
SHOX2	0.988115831	0.999992444	0.88153193	0.99999488	0.999985264	0.868103112
LOC112447459	0.988115831	0.924095664	0.866680636	0.99999488	0.999985264	0.931163114
TTLL5	0.988115831	0.916036681	0.890096618	0.99999488	0.999985264	0.972391591
LOC112448777	0.988115831	0.91058693	0.913976361	0.99999488	0.999985264	0.999960414
LOC101906317	0.988370514	0.974407708	0.881942256	0.99999488	0.999985264	0.954519353
MYLK3	0.988500062	0.999992444	0.881942256	0.99999488	0.999985264	0.895349622
ANKRD54	0.988980701	0.864154704	0.908572237	0.99999488	0.999985264	0.865911659
LOC786614	0.989014814	0.89064218	0.871925227	0.99999488	0.999985264	0.911997747
LOC104974057	0.989014814	0.943802122	0.869864092	0.99999488	0.999985264	0.915958083
HAUS8	0.989212641	0.862505333	0.898815524	0.99999488	0.999985264	0.892187201
MRPS5	0.989265428	0.866462388	0.91866621	0.99999488	0.999985264	0.89935427
CASP8AP2	0.989381875	0.9582784	0.865066354	0.99999488	0.999985264	0.945670698
CALML4	0.989492231	0.999347049	0.848786751	0.99999488	0.999985264	0.847204221
RAB1A	0.989492231	0.924139925	0.90339136	0.99999488	0.999985264	0.957310055
SLC20A1	0.989492231	0.899394562	0.95230212	0.99999488	0.999985264	0.968695729
MAP1A	0.989586945	0.884739596	0.849088328	0.99999488	0.999985264	0.899020749
ABCC1	0.989588016	0.931127395	0.865837237	0.99999488	0.999985264	0.849266568
FKBPL	0.989588016	0.975677199	0.851718619	0.99999488	0.999985264	0.865911659
TMPRSS6	0.989588016	0.999992444	0.926932508	0.99999488	0.999985264	0.866439215
TIMM22	0.989588016	0.883856603	0.880594299	0.99999488	0.999985264	0.867565269
NAB1	0.989588016	0.991345032	0.848312163	0.99999488	0.999985264	0.873463288
MAPKAPK2	0.989588016	0.967919743	0.933791093	0.99999488	0.999985264	0.876749994
RILPL2	0.989588016	0.877196482	0.862968824	0.99999488	0.999985264	0.8865725

KRTCAP3	0.989588016	0.928525825	0.988171751	0.99999488	0.999985264	0.927472114
LOC101907916	0.989588016	0.998131745	0.857021454	0.99999488	0.999985264	0.958193903
HEXDC	0.989695039	0.957252213	0.94212771	0.99999488	0.999985264	0.849138453
LOC782938	0.989695039	0.938597964	0.991242913	0.99999488	0.999985264	0.849266568
ACOT9	0.989695039	0.925580392	0.872386998	0.99999488	0.999985264	0.850790144
ADAM1A	0.989695039	0.869542732	0.975701948	0.99999488	0.999985264	0.868513869
EGFL8	0.989695039	0.883856603	0.993132892	0.99999488	0.999985264	0.882170916
LOC104975244	0.989695039	0.883624935	0.938203102	0.99999488	0.999985264	0.903461312
CC2D2A	0.989695039	0.916036681	0.907484111	0.99999488	0.999985264	0.923360345
PTPRR	0.989695039	0.964591213	0.925745065	0.99999488	0.999985264	0.927472114
KIAA0100	0.989695039	0.911418256	0.851718619	0.99999488	0.999985264	0.999960414
NELFA	0.989843553	0.885085629	0.923953195	0.99999488	0.999985264	0.856510591
MTMR7	0.989843553	0.999992444	0.865420641	0.99999488	0.999985264	0.901809636
RABL3	0.989977613	0.941793392	0.922400936	0.99999488	0.999985264	0.888948455
USP18	0.990273182	0.859182763	0.909070782	0.99999488	0.999985264	0.872236938
CAPNS1	0.990273182	0.954238252	0.902552409	0.99999488	0.999985264	0.895349622
LOC107132994	0.990273182	0.957700659	0.853574884	0.99999488	0.999985264	0.907070674
ZNF503	0.990273182	0.994434609	0.875823795	0.99999488	0.999985264	0.922063125
TRIP10	0.990273182	0.882022911	0.946479014	0.99999488	0.999985264	0.927969856
SRP68	0.990273182	0.926487359	0.923953195	0.99999488	0.999985264	0.931163114
SPART	0.990273182	0.921550129	0.908181052	0.99999488	0.999985264	0.944457836
GIPC3	0.991311503	0.938597964	0.964152013	0.99999488	0.999985264	0.847204221
LOC101907944	0.991311503	0.939104531	0.879092985	0.99999488	0.999985264	0.849046935
RFLNB	0.991311503	0.883624935	0.926552506	0.99999488	0.999985264	0.849046935
ATP5MPL	0.991311503	0.883624935	0.927600835	0.99999488	0.999985264	0.8503922
LOC101907843	0.991311503	0.864945604	0.922076383	0.99999488	0.999985264	0.85395895
GLYR1	0.991311503	0.970426668	0.999558179	0.99999488	0.999985264	0.856224954
DENND5A	0.991311503	0.929943407	0.892933333	0.99999488	0.999985264	0.856510591
NEK9	0.991311503	0.869999083	0.998936876	0.99999488	0.999985264	0.85653214
ME1	0.991311503	0.87010028	0.863209655	0.99999488	0.999985264	0.858315645
GOLM1	0.991311503	0.873113713	0.900148803	0.99999488	0.999985264	0.861837269
S100A10	0.991311503	0.917537324	0.895962167	0.99999488	0.999985264	0.866439215
LOC101902922	0.991311503	0.992977597	0.907125411	0.99999488	0.999985264	0.868513869
CCDC62	0.991311503	0.864945604	0.939478907	0.99999488	0.999985264	0.870102785
EFHD2	0.991311503	0.940269477	0.883623568	0.99999488	0.999985264	0.870160734
MSN	0.991311503	0.934142668	0.941336964	0.99999488	0.999985264	0.871960581
CMPK2	0.991311503	0.928575646	0.851718619	0.99999488	0.999985264	0.873463288
MFSD13A	0.991311503	0.941495217	0.879253779	0.99999488	0.999985264	0.873463288
MTRR	0.991311503	0.992977597	0.891893039	0.99999488	0.999985264	0.87805762

ANP32A	0.991311503	0.87010028	0.899248502	0.99999488	0.999985264	0.882867473
CARNMT1	0.991311503	0.999992444	0.926552506	0.99999488	0.999985264	0.882867473
ANKRD11	0.991311503	0.873289191	0.944871472	0.99999488	0.999985264	0.884033654
PKMYT1	0.991311503	0.988850552	0.930678591	0.99999488	0.999985264	0.885874544
ZNF729	0.991311503	0.912485935	0.979650244	0.99999488	0.999985264	0.885874544
ORM1	0.991311503	0.925580392	0.907015206	0.99999488	0.999985264	0.887277447
NIPSNAP2	0.991311503	0.867755878	0.915401612	0.99999488	0.999985264	0.887798625
TTYH3	0.991311503	0.945955989	0.970874203	0.99999488	0.999985264	0.888645453
FPGS	0.991311503	0.869999083	0.876280962	0.99999488	0.999985264	0.889409839
LOC104969159	0.991311503	0.935290542	0.981039752	0.99999488	0.999985264	0.892187201
DSC1	0.991311503	0.873289191	0.879620211	0.99999488	0.999985264	0.89329705
LOC112446454	0.991311503	0.999992444	0.926552506	0.99999488	0.999985264	0.89329705
CCDC80	0.991311503	0.934142668	0.98480229	0.99999488	0.999985264	0.894723749
DDA1	0.991311503	0.96566261	0.941336964	0.99999488	0.999985264	0.897467714
RPS11	0.991311503	0.928525825	0.886962419	0.99999488	0.999985264	0.89935427
ZFAND2A	0.991311503	0.867755878	0.996391386	0.99999488	0.999985264	0.900681286
COL8A2	0.991311503	0.883624935	0.900148803	0.99999488	0.999985264	0.90109791
FAHD1	0.991311503	0.893642384	0.891893039	0.99999488	0.999985264	0.901809636
XPA	0.991311503	0.945306277	0.971759453	0.99999488	0.999985264	0.90408677
FRYL	0.991311503	0.891316528	0.851718619	0.99999488	0.999985264	0.912889828
RPS6KA6	0.991311503	0.944181275	0.913211244	0.99999488	0.999985264	0.915476587
LOC786512	0.991311503	0.883624935	0.852782377	0.99999488	0.999985264	0.917028736
GLS	0.991311503	0.919884594	0.851718619	0.99999488	0.999985264	0.917322788
MOB3C	0.991311503	0.941495217	0.875030741	0.99999488	0.999985264	0.919758204
RPL30	0.991311503	0.91058693	0.879092985	0.99999488	0.999985264	0.919758204
LOC107132283	0.991311503	0.991488652	0.931367689	0.99999488	0.999985264	0.923693943
TDRD12	0.991311503	0.901906037	0.960133832	0.99999488	0.999985264	0.925260365
ARHGAP5	0.991311503	0.924543195	0.877742202	0.99999488	0.999985264	0.925558146
PRPF4B	0.991311503	0.944868298	0.886962419	0.99999488	0.999985264	0.927969856
RPLP2	0.991311503	0.925387304	0.899576888	0.99999488	0.999985264	0.929458454
CREB3	0.991311503	0.900370677	0.853055742	0.99999488	0.999985264	0.931163114
ZBTB45	0.991311503	0.9677397	0.870264123	0.99999488	0.999985264	0.931163114
SPATA13	0.991311503	0.921934673	0.926552506	0.99999488	0.999985264	0.931163114
ZNF74	0.991311503	0.919209432	0.985363931	0.99999488	0.999985264	0.932608891
GATA6	0.991311503	0.927465399	0.862345036	0.99999488	0.999985264	0.939035437
S100A11	0.991311503	0.925580392	0.914001994	0.99999488	0.999985264	0.943220018
ADPGK	0.991311503	0.867045149	0.9808801	0.99999488	0.999985264	0.948213108
LOC782950	0.991311503	0.956956287	0.962346588	0.99999488	0.999985264	0.951197866
TMC6	0.991311503	0.866462388	0.923604668	0.99999488	0.999985264	0.952669273

LOC518495	0.991311503	0.969534305	0.876758498	0.99999488	0.999985264	0.955184323
RUBCNL	0.991311503	0.979756265	0.886962419	0.99999488	0.999985264	0.957682692
ABCB4	0.991311503	0.927447588	0.849960855	0.99999488	0.999985264	0.963727152
ZFP57	0.991311503	0.992977597	0.923604668	0.99999488	0.999985264	0.96577301
APOA1	0.991311503	0.9582784	0.865837237	0.99999488	0.999985264	0.96860672
LOC101904574	0.991311503	0.869999083	0.881942256	0.99999488	0.999985264	0.972825932
LOC101906717	0.991311503	0.926949474	0.900148803	0.99999488	0.999985264	0.973330026
APRT	0.991311503	0.915367594	0.864441458	0.99999488	0.999985264	0.98534845
SACS	0.991311503	0.921549	0.879481883	0.99999488	0.999985264	0.986408155
DBP	0.991311503	0.880001923	0.884320747	0.99999488	0.999985264	0.987454496
POLR2D	0.991311503	0.863813934	0.871925227	0.99999488	0.999985264	0.991014754
ARHGEF25	0.991311503	0.864945604	0.861480822	0.99999488	0.999985264	0.99512563
EPHA3	0.991311503	0.881001912	0.900148803	0.99999488	0.999985264	0.999960414
ORC6	0.991311503	0.869999083	0.904140521	0.99999488	0.999985264	0.999960414
SELENOF	0.991311503	0.916036681	0.908181052	0.99999488	0.999985264	0.999960414
LOC112445051	0.991311503	0.90116492	0.912470556	0.99999488	0.999985264	0.999960414
CSTF3	0.991311503	0.911418256	0.930678591	0.99999488	0.999985264	0.999960414
GPC1	0.991352912	0.935588259	0.922400936	0.99999488	0.999985264	0.895349622
LOC784251	0.991352912	0.9582784	0.898815524	0.99999488	0.999985264	0.937880392
EIF3J	0.991352912	0.872223076	0.926552506	0.99999488	0.999985264	0.955561861
NGFR	0.991778852	0.862811597	0.95106368	0.99999488	0.999985264	0.943860392
DUS3L	0.99201327	0.877795869	0.941118576	0.99999488	0.999985264	0.939035437
ZNF835	0.992131844	0.943802122	0.95498522	0.99999488	0.999985264	0.952669273
CWF19L2	0.99214939	0.978510121	0.922400936	0.99999488	0.999985264	0.85653214
RGL2	0.992231526	0.867755878	0.999558179	0.99999488	0.999985264	0.858593649
LOC101905262	0.992231526	0.910133637	0.969880768	0.99999488	0.999985264	0.90109791
LOC112444348	0.992231526	0.943802122	0.907484111	0.99999488	0.999985264	0.94847204
ARSE	0.992231526	0.963737928	0.938203102	0.99999488	0.999985264	0.954519353
CLCN5	0.992231526	0.882863398	0.9808801	0.99999488	0.999985264	0.960343814
SERBP1	0.992231526	0.882505302	0.892574927	0.99999488	0.999985264	0.999960414
LOC107131273	0.992536699	0.958579042	0.884320747	0.99999488	0.999985264	0.856510591
PARP11	0.992895609	0.866462388	0.922147082	0.99999488	0.999985264	0.940731625
CPPED1	0.993276405	0.92202441	0.873452279	0.99999488	0.999985264	0.901809636
POLR1D	0.993276405	0.90213106	0.851718619	0.99999488	0.999985264	0.902893979
PTK2B	0.993276405	0.885948415	0.890248427	0.99999488	0.999985264	0.90408677
NPR2	0.993276405	0.883856603	0.933564465	0.99999488	0.999985264	0.941724934
LOC104973229	0.993276405	0.936657629	0.94212771	0.99999488	0.999985264	0.955561861
RTKN2	0.993590554	0.937511653	0.86638329	0.99999488	0.999985264	0.926480606
ANKRD31	0.993590554	0.948984325	0.861314069	0.99999488	0.999985264	0.966921967

MGAT4B	0.993741959	0.957700659	0.919618001	0.99999488	0.999985264	0.850790144
LOC112441602	0.993741959	0.931828419	0.912008683	0.99999488	0.999985264	0.922403963
PPP2R5D	0.993825293	0.9582784	0.903175962	0.99999488	0.999985264	0.858593649
HAUS3	0.993890341	0.96718649	0.862345036	0.99999488	0.999985264	0.85637643
DAP	0.993890341	0.957700659	0.930678591	0.99999488	0.999985264	0.871960581
DTD2	0.993904039	0.925580392	0.877742202	0.99999488	0.999985264	0.868513869
SPECC1L	0.994020709	0.87010028	0.95230212	0.99999488	0.999985264	0.863774505
TUB	0.994020709	0.877949935	0.865066354	0.99999488	0.999985264	0.895349622
FAM171A1	0.994020709	0.883856603	0.944993804	0.99999488	0.999985264	0.897965262
GREM1	0.994020709	0.949285285	0.930678591	0.99999488	0.999985264	0.912823155
SH2B3	0.994020709	0.909577824	0.902552409	0.99999488	0.999985264	0.919755394
TWISTNB	0.994020709	0.943802122	0.857021454	0.99999488	0.999985264	0.976082276
SH2D3A	0.994020709	0.991574265	0.862345036	0.99999488	0.999985264	0.998331177
LOC512248	0.994020709	0.927997582	0.898815524	0.99999488	0.999985264	0.999960414
LOC112447844	0.994020709	0.884739596	0.9222878	0.99999488	0.999985264	0.999960414
ARMCX6	0.994043908	0.883856603	0.862345036	0.99999488	0.999985264	0.95096765
NCKAP1	0.994675174	0.999347049	0.910426741	0.99999488	0.999985264	0.855349614
RNF114	0.994675174	0.909577824	0.909383349	0.99999488	0.999985264	0.85653214
LOC101904579	0.994675174	0.950715915	0.907484111	0.99999488	0.999985264	0.857136501
CDC7	0.994675174	0.89880506	0.884220809	0.99999488	0.999985264	0.859321224
GGA1	0.994675174	0.89064218	0.915388802	0.99999488	0.999985264	0.865195569
LOC112448753	0.994675174	0.882022911	0.894525066	0.99999488	0.999985264	0.866439215
VEPH1	0.994675174	0.869304939	0.930678591	0.99999488	0.999985264	0.883999232
TM9SF4	0.994675174	0.905019423	0.914001994	0.99999488	0.999985264	0.884033654
UBXN7	0.994675174	0.992977597	0.874752438	0.99999488	0.999985264	0.89589047
LOC104972888	0.994675174	0.978089982	0.876758498	0.99999488	0.999985264	0.896297062
CCDC30	0.994675174	0.935742738	0.961013192	0.99999488	0.999985264	0.89935427
TMEM69	0.994675174	0.883856603	0.904959653	0.99999488	0.999985264	0.906644037
CPNE2	0.994675174	0.97015318	0.886192181	0.99999488	0.999985264	0.927969856
MCM9	0.994675174	0.893748757	0.921065686	0.99999488	0.999985264	0.927969856
PRDM2	0.994675174	0.927997582	0.885737706	0.99999488	0.999985264	0.928814001
DDX11	0.994675174	0.925580392	0.946479014	0.99999488	0.999985264	0.931163114
NOLC1	0.994675174	0.938597964	0.871925227	0.99999488	0.999985264	0.940358165
SBNO2	0.994675174	0.910384774	0.946479014	0.99999488	0.999985264	0.941724934
BCL2L14	0.994675174	0.94984369	0.890248427	0.99999488	0.999985264	0.948213108
MIR3064	0.994675174	0.987516216	0.924604843	0.99999488	0.999985264	0.949718683
RNASEH1	0.994675174	0.87010028	0.874559186	0.99999488	0.999985264	0.958869789
THUMPD3	0.994675174	0.921550129	0.898815524	0.99999488	0.999985264	0.960343814
BICDL1	0.994675174	0.956531374	0.938203102	0.99999488	0.999985264	0.960343814

SMPDL3A	0.994675174	0.882505302	0.961734809	0.99999488	0.999985264	0.960343814
C17H5orf52	0.994675174	0.985406717	0.852670802	0.99999488	0.999985264	0.986408155
LOC112444926	0.994675174	0.985406717	0.916975216	0.99999488	0.999985264	0.986873741
FIGN	0.994675174	0.922700337	0.900351064	0.99999488	0.999985264	0.999960414
LOC107132949	0.994699359	0.938597964	0.930678591	0.99999488	0.999985264	0.910018813
CDC42SE1	0.994847277	0.9527938	0.898815524	0.99999488	0.999985264	0.870708914
EEF2KMT	0.994847277	0.985406717	0.879253779	0.99999488	0.999985264	0.872307149
ADRB3	0.994847277	0.978089982	0.876758498	0.99999488	0.999985264	0.892908035
IMMP1L	0.994847277	0.993777777	0.88153193	0.99999488	0.999985264	0.892908035
PRR5L	0.994847277	0.978089982	0.884320747	0.99999488	0.999985264	0.894723749
METTL15	0.994847277	0.999992444	0.857021454	0.99999488	0.999985264	0.901545871
CDC42	0.994847277	0.901232581	0.998816213	0.99999488	0.999985264	0.90408677
ADCK1	0.994847277	0.873289191	0.94634922	0.99999488	0.999985264	0.919530287
LOC100847345	0.994847277	0.999992444	0.884220809	0.99999488	0.999985264	0.95096765
FOXN2	0.994847277	0.941495217	0.91207077	0.99999488	0.999985264	0.957739231
LOC101910045	0.994847277	0.928575646	0.907381775	0.99999488	0.999985264	0.960294693
FCRL1	0.99513775	0.90024974	0.911018006	0.99999488	0.999985264	0.853135371
TTC14	0.99513775	0.999992444	0.930678591	0.99999488	0.999985264	0.855108375
LOC101902907	0.99513775	0.916036681	0.876721743	0.99999488	0.999985264	0.856073541
THAP2	0.99513775	0.999992444	0.926552506	0.99999488	0.999985264	0.85653214
ID3	0.99513775	0.954790265	0.969880768	0.99999488	0.999985264	0.856666693
CLDN15	0.99513775	0.871390547	0.875823795	0.99999488	0.999985264	0.861837269
CFLAR	0.99513775	0.882505302	0.862345036	0.99999488	0.999985264	0.865195569
ESD	0.99513775	0.92817225	0.995045982	0.99999488	0.999985264	0.865855226
AFF2	0.99513775	0.992977597	0.948946702	0.99999488	0.999985264	0.873463288
LOC112448014	0.99513775	0.938597964	0.95106368	0.99999488	0.999985264	0.873463288
RPL29	0.99513775	0.943737806	0.870567942	0.99999488	0.999985264	0.873836462
GTF2IRD2	0.99513775	0.871611007	0.881942256	0.99999488	0.999985264	0.894723749
GNAI2	0.99513775	0.999992444	0.917634327	0.99999488	0.999985264	0.910018813
GTF3C5	0.99513775	0.883856603	0.908572237	0.99999488	0.999985264	0.911997747
SLC25A39	0.99513775	0.873688559	0.863239204	0.99999488	0.999985264	0.919792225
TUBB4A	0.99513775	0.921934673	0.861166952	0.99999488	0.999985264	0.939035437
TMEM104	0.99513775	0.925580392	0.891552252	0.99999488	0.999985264	0.939035437
PPP1R3D	0.99513775	0.893642384	0.945280564	0.99999488	0.999985264	0.940731625
DNAJC21	0.99513775	0.88167071	0.999558179	0.99999488	0.999985264	0.945794274
CXCL2	0.99513775	0.885085629	0.926552506	0.99999488	0.999985264	0.9618678
COLGALT2	0.99513775	0.995073696	0.877799478	0.99999488	0.999985264	0.966921967
BZW2	0.99513775	0.864861694	0.903175962	0.99999488	0.999985264	0.982250307
PCDHB8	0.995443105	0.921934673	0.93955737	0.99999488	0.999985264	0.872307149

CIP2A	0.995443105	0.985406717	0.886192181	0.99999488	0.999985264	0.873463288
ERC1	0.99548256	0.915367594	0.85773697	0.99999488	0.999985264	0.86871626
GPATCH1	0.99554559	0.999992444	0.94634922	0.99999488	0.999985264	0.87312749
LOC782054	0.99554559	0.995073696	0.907381775	0.99999488	0.999985264	0.89935427
AIFM2	0.99577943	0.925580392	0.892664791	0.99999488	0.999985264	0.856510591
LOC101907803	0.99577943	0.885718559	0.856079665	0.99999488	0.999985264	0.873463288
AIFM3	0.99577943	0.872223076	0.908181052	0.99999488	0.999985264	0.87805762
TCF23	0.995841337	0.991488652	0.956565728	0.99999488	0.999985264	0.868103112
LOC788648	0.995864144	0.869910356	0.949038056	0.99999488	0.999985264	0.894333557
PKD1L2	0.995923644	0.978510121	0.863336545	0.99999488	0.999985264	0.856510591
COPG2	0.995923644	0.987516216	0.885737706	0.99999488	0.999985264	0.856510591
SUMO1	0.995923644	0.893642384	0.980169282	0.99999488	0.999985264	0.856510591
TSC2	0.995923644	0.874697957	0.986266701	0.99999488	0.999985264	0.856510591
KCNN1	0.995923644	0.926949474	0.999558179	0.99999488	0.999985264	0.856510591
ITPK1	0.995923644	0.999347049	0.862345036	0.99999488	0.999985264	0.858593649
AP1S2	0.995923644	0.992977597	0.874138224	0.99999488	0.999985264	0.858593649
LOC100139115	0.995923644	0.957252213	0.890248427	0.99999488	0.999985264	0.859537053
PELI3	0.995923644	0.940762397	0.877742202	0.99999488	0.999985264	0.86132915
LARGE1	0.995923644	0.87010028	0.95230212	0.99999488	0.999985264	0.863331236
ASIP	0.995923644	0.955128586	0.999558179	0.99999488	0.999985264	0.865855226
KRT24	0.995923644	0.941495217	0.877799478	0.99999488	0.999985264	0.866301544
ATP5IF1	0.995923644	0.875435394	0.898815524	0.99999488	0.999985264	0.866439215
CCDC157	0.995923644	0.913160799	0.923604668	0.99999488	0.999985264	0.866439215
GJD3	0.995923644	0.915539284	0.892664791	0.99999488	0.999985264	0.866578338
SVIP	0.995923644	0.964246761	0.884407415	0.99999488	0.999985264	0.868103112
TRPM4	0.995923644	0.922700337	0.920629231	0.99999488	0.999985264	0.868256799
TUBD1	0.995923644	0.963084698	0.853055742	0.99999488	0.999985264	0.868513869
MGAT1	0.995923644	0.916036681	0.887626998	0.99999488	0.999985264	0.868513869
NDUFB11	0.995923644	0.935274301	0.931367689	0.99999488	0.999985264	0.86871626
TTC9B	0.995923644	0.999992444	0.862345036	0.99999488	0.999985264	0.872307149
LIN54	0.995923644	0.938597964	0.898815524	0.99999488	0.999985264	0.872307149
TXN2	0.995923644	0.869999083	0.903516034	0.99999488	0.999985264	0.872307149
ISOC2	0.995923644	0.915367594	0.921646762	0.99999488	0.999985264	0.872307149
ABCC11	0.995923644	0.873289191	0.86638329	0.99999488	0.999985264	0.873083064
KLHL2	0.995923644	0.934142668	0.864737879	0.99999488	0.999985264	0.873463288
MPG	0.995923644	0.879745708	0.870567942	0.99999488	0.999985264	0.873463288
MCL1	0.995923644	0.912316394	0.939478907	0.99999488	0.999985264	0.873463288
GGTA1	0.995923644	0.973912396	0.955141547	0.99999488	0.999985264	0.873463288
LYSMD1	0.995923644	0.915367594	0.999558179	0.99999488	0.999985264	0.873463288

TSC22D2	0.995923644	0.912523334	0.887626998	0.99999488	0.999985264	0.873836462
B3GNTL1	0.995923644	0.895304124	0.949038056	0.99999488	0.999985264	0.873836462
LOC101904057	0.995923644	0.938777693	0.954620171	0.99999488	0.999985264	0.873836462
RNH1	0.995923644	0.926955605	0.879253779	0.99999488	0.999985264	0.875113722
DOCK5	0.995923644	0.883624935	0.999558179	0.99999488	0.999985264	0.875317728
LOC787102	0.995923644	0.992977597	0.890248427	0.99999488	0.999985264	0.875576073
ARRDC4	0.995923644	0.943802122	0.865129879	0.99999488	0.999985264	0.879798493
HDHD5	0.995923644	0.952346363	0.861480822	0.99999488	0.999985264	0.881288523
HSPA5	0.995923644	0.991488652	0.862345036	0.99999488	0.999985264	0.881288523
VIRMA	0.995923644	0.947212907	0.857021454	0.99999488	0.999985264	0.882867473
TMEM60	0.995923644	0.958967142	0.888215738	0.99999488	0.999985264	0.88310116
NDUFB8	0.995923644	0.873429424	0.902552409	0.99999488	0.999985264	0.884033654
SLC15A4	0.995923644	0.870839685	0.875823795	0.99999488	0.999985264	0.884143505
AKTIP	0.995923644	0.869999083	0.938215065	0.99999488	0.999985264	0.885874544
LOC107131331	0.995923644	0.999992444	0.939478907	0.99999488	0.999985264	0.885954735
ISY1	0.995923644	0.938597964	0.999558179	0.99999488	0.999985264	0.888645453
SPEN	0.995923644	0.941495217	0.877786216	0.99999488	0.999985264	0.890433418
YAF2	0.995923644	0.998011664	0.898815524	0.99999488	0.999985264	0.892187201
ANXA8L1	0.995923644	0.92202441	0.966991646	0.99999488	0.999985264	0.893392612
LOC522174	0.995923644	0.999992444	0.884220809	0.99999488	0.999985264	0.894236694
GGN	0.995923644	0.890682633	0.95230212	0.99999488	0.999985264	0.894236694
LRRK2	0.995923644	0.986445172	0.909070782	0.99999488	0.999985264	0.894420979
SFRP4	0.995923644	0.946274461	0.992709008	0.99999488	0.999985264	0.894723749
TOR2A	0.995923644	0.877142156	0.89038419	0.99999488	0.999985264	0.895349622
TMCC3	0.995923644	0.901232581	0.88650818	0.99999488	0.999985264	0.896480535
MRPL44	0.995923644	0.936562168	0.912247957	0.99999488	0.999985264	0.897467714
NUDCD1	0.995923644	0.872023861	0.908181052	0.99999488	0.999985264	0.897965262
SAMD4B	0.995923644	0.89880506	0.884320747	0.99999488	0.999985264	0.901614166
ARPP19	0.995923644	0.991488652	0.879092985	0.99999488	0.999985264	0.901809636
ARID3A	0.995923644	0.87010028	0.941336964	0.99999488	0.999985264	0.90408677
CEBPB	0.995923644	0.913581457	0.972420732	0.99999488	0.999985264	0.904813143
RLIM	0.995923644	0.992977597	0.861831449	0.99999488	0.999985264	0.904958457
LOC100139144	0.995923644	0.992977597	0.930678591	0.99999488	0.999985264	0.906644037
PEA15	0.995923644	0.995532499	0.929207633	0.99999488	0.999985264	0.907055373
PNKD	0.995923644	0.980496345	0.923604668	0.99999488	0.999985264	0.907923546
JOSD1	0.995923644	0.941495217	0.945280564	0.99999488	0.999985264	0.908047942
MYL6	0.995923644	0.936392293	0.938203102	0.99999488	0.999985264	0.909427764
LOC112444889	0.995923644	0.938597964	0.95230212	0.99999488	0.999985264	0.909427764
RAB26	0.995923644	0.931488015	0.964920281	0.99999488	0.999985264	0.910813075

NAXE	0.995923644	0.893748757	0.919763144	0.99999488	0.999985264	0.911997747
CWC22	0.995923644	0.969534305	0.944085862	0.99999488	0.999985264	0.911997747
SEMA3G	0.995923644	0.869999083	0.964152013	0.99999488	0.999985264	0.911997747
FABP2	0.995923644	0.999992444	0.915215321	0.99999488	0.999985264	0.918343194
LOC101904268	0.995923644	0.911418256	0.930678591	0.99999488	0.999985264	0.918400465
MRPS22	0.995923644	0.873289191	0.886962419	0.99999488	0.999985264	0.919755394
SCLY	0.995923644	0.92462339	0.935094475	0.99999488	0.999985264	0.919755394
PLCB1	0.995923644	0.946274461	0.970945672	0.99999488	0.999985264	0.919755394
LOC112444341	0.995923644	0.893642384	0.924866532	0.99999488	0.999985264	0.919758204
BRD2	0.995923644	0.935742738	0.900890709	0.99999488	0.999985264	0.920531105
LOC104972216	0.995923644	0.941495217	0.981039752	0.99999488	0.999985264	0.921771959
LOC112443850	0.995923644	0.895242264	0.908700926	0.99999488	0.999985264	0.923360345
SF3B5	0.995923644	0.938597964	0.945280564	0.99999488	0.999985264	0.923360345
TUBA8	0.995923644	0.883624935	0.938203102	0.99999488	0.999985264	0.924201993
LOC788467	0.995923644	0.928526556	0.933447488	0.99999488	0.999985264	0.924461747
EDA	0.995923644	0.999992444	0.884466521	0.99999488	0.999985264	0.925481882
LOC101902428	0.995923644	0.95697765	0.862345036	0.99999488	0.999985264	0.925558146
SNCAIP	0.995923644	0.991365955	0.859566702	0.99999488	0.999985264	0.927969856
LOC104972065	0.995923644	0.90116492	0.914001994	0.99999488	0.999985264	0.927969856
GABARAPL2	0.995923644	0.900867274	0.899576888	0.99999488	0.999985264	0.928634547
SOWAHC	0.995923644	0.870351626	0.970945672	0.99999488	0.999985264	0.929458454
LOC112446042	0.995923644	0.943802122	0.882380454	0.99999488	0.999985264	0.931163114
ITGB1BP1	0.995923644	0.948435978	0.900890709	0.99999488	0.999985264	0.931163114
AGO4	0.995923644	0.871627189	0.93198623	0.99999488	0.999985264	0.931163114
LOC101905127	0.995923644	0.947032486	0.938203102	0.99999488	0.999985264	0.932270046
ELMO3	0.995923644	0.92462339	0.998936876	0.99999488	0.999985264	0.932608891
GLP2R	0.995923644	0.865926615	0.999558179	0.99999488	0.999985264	0.935349027
SLC10A5	0.995923644	0.993316598	0.865837237	0.99999488	0.999985264	0.937108046
LOC101907581	0.995923644	0.916036681	0.998523328	0.99999488	0.999985264	0.937108046
LYPLA1	0.995923644	0.991365955	0.881942256	0.99999488	0.999985264	0.937880392
NOP2	0.995923644	0.893495573	0.864257597	0.99999488	0.999985264	0.939035437
PDK1	0.995923644	0.87010028	0.879253779	0.99999488	0.999985264	0.939035437
PFDN2	0.995923644	0.943802122	0.916975216	0.99999488	0.999985264	0.939035437
CXADR	0.995923644	0.938017364	0.920629231	0.99999488	0.999985264	0.939035437
ULK3	0.995923644	0.936273761	0.993594894	0.99999488	0.999985264	0.939035437
TUSC3	0.995923644	0.867755878	0.901414204	0.99999488	0.999985264	0.940358165
TMEM115	0.995923644	0.9582784	0.862345036	0.99999488	0.999985264	0.943220018
LOC531462	0.995923644	0.913160799	0.926552506	0.99999488	0.999985264	0.944457836
SLC35G1	0.995923644	0.885718559	0.857376578	0.99999488	0.999985264	0.944906868

GPD2	0.995923644	0.936273761	0.906584165	0.99999488	0.999985264	0.945794274
LOC112443614	0.995923644	0.936392293	0.914021366	0.99999488	0.999985264	0.949718683
EPOP	0.995923644	0.938597964	0.93198623	0.99999488	0.999985264	0.949718683
MCTS1	0.995923644	0.87010028	0.876758498	0.99999488	0.999985264	0.949764317
ZNF248	0.995923644	0.995532499	0.884220809	0.99999488	0.999985264	0.95096765
SLC25A43	0.995923644	0.869304939	0.907381775	0.99999488	0.999985264	0.95096765
QTRT2	0.995923644	0.893642384	0.948359914	0.99999488	0.999985264	0.952669273
ALCAM	0.995923644	0.90213106	0.930678591	0.99999488	0.999985264	0.954042188
CXCR2	0.995923644	0.941793392	0.922400936	0.99999488	0.999985264	0.955184323
UBASH3B	0.995923644	0.948200018	0.892053976	0.99999488	0.999985264	0.955374205
GOSR2	0.995923644	0.884837506	0.862345036	0.99999488	0.999985264	0.958984414
NOL12	0.995923644	0.936657629	0.889272129	0.99999488	0.999985264	0.960343814
SNRPD3	0.995923644	0.946274461	0.935243018	0.99999488	0.999985264	0.960343814
AFF1	0.995923644	0.904961534	0.88153193	0.99999488	0.999985264	0.960613782
PER1	0.995923644	0.927825701	0.863239204	0.99999488	0.999985264	0.969741463
TSACC	0.995923644	0.993316598	0.869906363	0.99999488	0.999985264	0.974234571
PSMB4	0.995923644	0.935742738	0.922400936	0.99999488	0.999985264	0.974376225
NOTCH3	0.995923644	0.867755878	0.882380454	0.99999488	0.999985264	0.976814573
MXD4	0.995923644	0.890682633	0.94634922	0.99999488	0.999985264	0.976814573
P2RY1	0.995923644	0.873289191	0.962777771	0.99999488	0.999985264	0.976814573
LOC112444622	0.995923644	0.893642384	0.922431991	0.99999488	0.999985264	0.977753483
LOC112444206	0.995923644	0.943802122	0.899576888	0.99999488	0.999985264	0.98341414
KCNIP3	0.995923644	0.890682633	0.909070782	0.99999488	0.999985264	0.983479593
BUD23	0.995923644	0.919884594	0.862345036	0.99999488	0.999985264	0.986338245
BHLHE22	0.995923644	0.925580392	0.865837237	0.99999488	0.999985264	0.987454496
SLC26A9	0.995923644	0.883856603	0.929952093	0.99999488	0.999985264	0.991014754
JADE2	0.995923644	0.943802122	0.862345036	0.99999488	0.999985264	0.999960414
LOC112442598	0.995923644	0.958967142	0.871925227	0.99999488	0.999985264	0.999960414
PSMD2	0.995923644	0.885295091	0.874752438	0.99999488	0.999985264	0.999960414
POU6F1	0.995923644	0.928525825	0.888545123	0.99999488	0.999985264	0.999960414
ALG8	0.995923644	0.885718559	0.899576888	0.99999488	0.999985264	0.999960414
RNF39	0.995923644	0.883856603	0.93198623	0.99999488	0.999985264	0.999960414
CARD19	0.995960728	0.926955605	0.927600835	0.99999488	0.999985264	0.873463288
PREP	0.996006604	0.882226341	0.921646762	0.99999488	0.999985264	0.987454496
HMGCL	0.996019692	0.873289191	0.895626254	0.99999488	0.999985264	0.945794274
ZNF345	0.996019692	0.981520287	0.861171188	0.99999488	0.999985264	0.98341414
PDS5A	0.996046327	0.978089982	0.919618001	0.99999488	0.999985264	0.89329705
GUK1	0.996046327	0.893748757	0.89937883	0.99999488	0.999985264	0.897467714
ALG12	0.996046327	0.9582784	0.862345036	0.99999488	0.999985264	0.904300404

WARS2	0.996046327	0.881001912	0.920629231	0.99999488	0.999985264	0.906644037
LOC112446659	0.996046327	0.91407604	0.93198623	0.99999488	0.999985264	0.923360345
ERMP1	0.996046327	0.947212907	0.865066354	0.99999488	0.999985264	0.938625326
COG8	0.996046327	0.905019423	0.944085862	0.99999488	0.999985264	0.939035437
ZBTB43	0.996046327	0.96718649	0.888545123	0.99999488	0.999985264	0.955374205
DGKB	0.996046327	0.9582784	0.86178438	0.99999488	0.999985264	0.998331177
VIPR2	0.996551427	0.916036681	0.945678616	0.99999488	0.999985264	0.883278571
KIZ	0.997030091	0.97587222	0.886730817	0.99999488	0.999985264	0.873463288
OSGIN2	0.997030091	0.87010028	0.945678616	0.99999488	0.999985264	0.89935427
PDCL	0.997030091	0.935060764	0.934941286	0.99999488	0.999985264	0.902893979
COX18	0.997030091	0.87010028	0.888863248	0.99999488	0.999985264	0.934749052
SIMC1	0.997030091	0.922700337	0.913616129	0.99999488	0.999985264	0.939035437
MRM1	0.997030091	0.883624935	0.914001994	0.99999488	0.999985264	0.978249551
LRWD1	0.997122522	0.90213106	0.997208738	0.99999488	0.999985264	0.868103112
TUBA3E	0.997122522	0.921549	0.973406453	0.99999488	0.999985264	0.903445296
LOC112441682	0.997122522	0.985406717	0.909070782	0.99999488	0.999985264	0.927969856
LOC101902122	0.997122522	0.883624935	0.881419724	0.99999488	0.999985264	0.999960414
ZRANB1	0.99713196	0.883856603	0.950948716	0.99999488	0.999985264	0.921771959
TOM1	0.997359712	0.929230097	0.998936876	0.99999488	0.999985264	0.861837269
LOC104976061	0.997359712	0.941495217	0.902552409	0.99999488	0.999985264	0.868103112
USP47	0.997359712	0.887644042	0.964920281	0.99999488	0.999985264	0.873463288
LOC107132360	0.997359712	0.92462339	0.870567942	0.99999488	0.999985264	0.879212286
FAXDC2	0.997359712	0.921759937	0.95106368	0.99999488	0.999985264	0.897965262
SAMD5	0.997359712	0.936273761	0.912008683	0.99999488	0.999985264	0.919755394
GTF2H3	0.997359712	0.9582784	0.914827591	0.99999488	0.999985264	0.945544446
ADGRG2	0.99741512	0.935742738	0.886962419	0.99999488	0.999985264	0.972391591
CEP192	0.997609322	0.893642384	0.973406453	0.99999488	0.999985264	0.863774505
LOC101904308	0.997609322	0.992887837	0.923604668	0.99999488	0.999985264	0.885874544
LOC788745	0.997609322	0.896507811	0.954620171	0.99999488	0.999985264	0.987454496
ZCCHC2	0.997641049	0.920639601	0.908572237	0.99999488	0.999985264	0.947012976
PML	0.997677173	0.894656415	0.902552409	0.99999488	0.999985264	0.925260365
NDUFS5	0.99774582	0.925580392	0.926552506	0.99999488	0.999985264	0.870708914
LOXL3	0.99774582	0.885718559	0.92768724	0.99999488	0.999985264	0.910022116
PRKCA	0.99774582	0.99241354	0.876758498	0.99999488	0.999985264	0.956550292
LOC781741	0.99774582	0.885024111	0.907381775	0.99999488	0.999985264	0.999960414
CDKN1C	0.997863854	0.967919743	0.898815524	0.99999488	0.999985264	0.861670395
LOC112444773	0.997938511	0.999347049	0.884320747	0.99999488	0.999985264	0.955420827
KDM4B	0.997945777	0.895540475	0.916975216	0.99999488	0.999985264	0.867896916
TAF11	0.997945777	0.942353856	0.956565728	0.99999488	0.999985264	0.884423852

LOC112443130	0.997945777	0.896507811	0.981039752	0.99999488	0.999985264	0.920531105
C23H6orf47	0.997945777	0.916036681	0.898719232	0.99999488	0.999985264	0.929458454
RPL3	0.997945777	0.900479208	0.862345036	0.99999488	0.999985264	0.977753483
CCDC183	0.998120193	0.994481342	0.969880768	0.99999488	0.999985264	0.907993548
PHF7	0.998120193	0.986445172	0.930678591	0.99999488	0.999985264	0.918400465
MLEC	0.998880023	0.885024111	0.895626254	0.99999488	0.999985264	0.914755003
LOC789231	0.998998658	0.972544855	0.895644023	0.99999488	0.999985264	0.862046449
SMC5	0.999258362	0.998131745	0.921065686	0.99999488	0.999985264	0.862046449
ATP6V1E2	0.999287746	0.999992444	0.877799478	0.99999488	0.999985264	0.897467714
RNF121	0.999287746	0.918911961	0.908181052	0.99999488	0.999985264	0.897467714
CACNA1H	0.999398181	0.999347049	0.867224246	0.99999488	0.999985264	0.949764317
FITM2	0.999405905	0.869999083	0.890248427	0.99999488	0.999985264	0.895370805
STAR	0.999405905	0.943802122	0.93198623	0.99999488	0.999985264	0.897965262
PLP2	0.999568635	0.957252213	0.891081265	0.99999488	0.999985264	0.902958012
SYT11	0.999568635	0.999992444	0.923089792	0.99999488	0.999985264	0.919758204
MYRIP	0.999568635	0.975677199	0.973406453	0.99999488	0.999985264	0.920531105
LOC100138864	0.999593577	0.90024974	0.956565728	0.99999488	0.999985264	0.864275069
RHBG	0.999593577	0.999992444	0.90339136	0.99999488	0.999985264	0.864806954
GGCT	0.999593577	0.941495217	0.926552506	0.99999488	0.999985264	0.865195569
LCMT1	0.999593577	0.913605849	0.930127869	0.99999488	0.999985264	0.865855226
MFAP4	0.999593577	0.970426668	0.907484111	0.99999488	0.999985264	0.865911659
LOC101904477	0.999593577	0.883624935	0.86391003	0.99999488	0.999985264	0.870708914
MPHOSPH8	0.999593577	0.986637746	0.922400936	0.99999488	0.999985264	0.871960581
RAB29	0.999593577	0.89942898	0.882515776	0.99999488	0.999985264	0.872307149
ANO6	0.999593577	0.882505302	0.946479014	0.99999488	0.999985264	0.872365139
IFNAR2	0.999593577	0.883856603	0.877742202	0.99999488	0.999985264	0.873463288
PGAP3	0.999593577	0.937231686	0.916975216	0.99999488	0.999985264	0.873463288
MEF2C	0.999593577	0.991488652	0.938203102	0.99999488	0.999985264	0.873463288
PPP3CA	0.999593577	0.946274461	0.973406453	0.99999488	0.999985264	0.873463288
LOC614424	0.999593577	0.95697765	0.9808801	0.99999488	0.999985264	0.873463288
LOC613460	0.999593577	0.987860004	0.94634922	0.99999488	0.999985264	0.877760721
CCDC153	0.999593577	0.943802122	0.873452279	0.99999488	0.999985264	0.883278571
H3F3A	0.999593577	0.915367594	0.935243018	0.99999488	0.999985264	0.883750044
LOC522540	0.999593577	0.921095578	0.873452279	0.99999488	0.999985264	0.885266141
BMT2	0.999593577	0.999992444	0.864467486	0.99999488	0.999985264	0.885874544
GFRA4	0.999593577	0.985406717	0.934694748	0.99999488	0.999985264	0.885874544
SLC22A31	0.999593577	0.999992444	0.86763298	0.99999488	0.999985264	0.890433418
CMC2	0.999593577	0.921661251	0.937004034	0.99999488	0.999985264	0.892187201
FNBP4	0.999593577	0.9527938	0.986948824	0.99999488	0.999985264	0.892231098

LOC101904258	0.999593577	0.905019423	0.966991646	0.99999488	0.999985264	0.894144329
LOC101905367	0.999593577	0.978004343	0.862345036	0.99999488	0.999985264	0.894723749
WTAP	0.999593577	0.874697957	0.870995265	0.99999488	0.999985264	0.894723749
FCGRT	0.999593577	0.999992444	0.927600835	0.99999488	0.999985264	0.894723749
ANGPT2	0.999593577	0.999992444	0.932653752	0.99999488	0.999985264	0.894723749
ZBTB17	0.999593577	0.917849199	0.946479014	0.99999488	0.999985264	0.894723749
RBM18	0.999593577	0.944481254	0.968232195	0.99999488	0.999985264	0.894723749
RXFP4	0.999593577	0.96895079	0.996715234	0.99999488	0.999985264	0.897965262
GAPDHS	0.999593577	0.949285285	0.999558179	0.99999488	0.999985264	0.897965262
LOC104969496	0.999593577	0.999992444	0.913211244	0.99999488	0.999985264	0.899223071
GRK4	0.999593577	0.893748757	0.985966356	0.99999488	0.999985264	0.900681286
GUCA1A	0.999593577	0.975677199	0.907381775	0.99999488	0.999985264	0.90109791
HSPB6	0.999593577	0.918883775	0.917154286	0.99999488	0.999985264	0.901809636
PODNL1	0.999593577	0.905019423	0.993594894	0.99999488	0.999985264	0.901809636
TPK1	0.999593577	0.999992444	0.890248427	0.99999488	0.999985264	0.902958012
LOC112448856	0.999593577	0.87010028	0.981715036	0.99999488	0.999985264	0.905099683
LOC104969833	0.999593577	0.97671655	0.944085862	0.99999488	0.999985264	0.906644037
NUB1	0.999593577	0.885718559	0.945280564	0.99999488	0.999985264	0.913593134
PP2D1	0.999593577	0.941495217	0.938905004	0.99999488	0.999985264	0.915476587
PEPD	0.999593577	0.921934673	0.908181052	0.99999488	0.999985264	0.916081555
HOXB9	0.999593577	0.958098081	0.875823795	0.99999488	0.999985264	0.916313772
TRIM37	0.999593577	0.911418256	0.877799478	0.99999488	0.999985264	0.918256134
RPS15	0.999593577	0.927147798	0.865347217	0.99999488	0.999985264	0.921771959
EVC2	0.999593577	0.893642384	0.968844378	0.99999488	0.999985264	0.922403963
ARL10	0.999593577	0.935742738	0.877799478	0.99999488	0.999985264	0.924461747
GCNT3	0.999593577	0.873963285	0.903175962	0.99999488	0.999985264	0.925260365
RPS28	0.999593577	0.967919743	0.874752438	0.99999488	0.999985264	0.925740594
CMYA5	0.999593577	0.904961534	0.920629231	0.99999488	0.999985264	0.926680635
BTRC	0.999593577	0.924808708	0.877742202	0.99999488	0.999985264	0.928634547
LOC616868	0.999593577	0.978089982	0.920629231	0.99999488	0.999985264	0.929458454
LOC100336381	0.999593577	0.9541326	0.872386998	0.99999488	0.999985264	0.931163114
LOC510860	0.999593577	0.967276215	0.862345036	0.99999488	0.999985264	0.934439243
MKRN1	0.999593577	0.87010028	0.88153193	0.99999488	0.999985264	0.934749052
MAATS1	0.999593577	0.92202441	0.998936876	0.99999488	0.999985264	0.935349027
SLC2A6	0.999593577	0.87010028	0.862345036	0.99999488	0.999985264	0.939035437
MAMDC2	0.999593577	0.938597964	0.864737879	0.99999488	0.999985264	0.939035437
SOGA3	0.999593577	0.874697957	0.920629231	0.99999488	0.999985264	0.939035437
MED31	0.999593577	0.921934673	0.929207633	0.99999488	0.999985264	0.939035437
RINL	0.999593577	0.921549	0.956504686	0.99999488	0.999985264	0.939035437

PRDM15	0.999593577	0.884739596	0.973406453	0.99999488	0.999985264	0.939087382
SORCS1	0.999593577	0.89880506	0.970945672	0.99999488	0.999985264	0.939855019
C23H6orf136	0.999593577	0.985406717	0.932446739	0.99999488	0.999985264	0.942340826
WASF2	0.999593577	0.920253543	0.879253779	0.99999488	0.999985264	0.944906868
DHX36	0.999593577	0.943802122	0.882380454	0.99999488	0.999985264	0.949764317
NCK2	0.999593577	0.930374078	0.870264123	0.99999488	0.999985264	0.95096765
LOC112443147	0.999593577	0.935401063	0.925745065	0.99999488	0.999985264	0.95096765
PPP4R3B	0.999593577	0.943802122	0.944871472	0.99999488	0.999985264	0.95096765
SERP1	0.999593577	0.900368858	0.998936876	0.99999488	0.999985264	0.95096765
LOC100850276	0.999593577	0.90116492	0.923604668	0.99999488	0.999985264	0.952948574
SOX12	0.999593577	0.883624935	0.95230212	0.99999488	0.999985264	0.958083698
RWDD1	0.999593577	0.884739596	0.914001994	0.99999488	0.999985264	0.958869789
ZFR	0.999593577	0.896536444	0.910748743	0.99999488	0.999985264	0.960613782
HSPA9	0.999593577	0.87010028	0.907484111	0.99999488	0.999985264	0.964333944
CCM2	0.999593577	0.992977597	0.93198623	0.99999488	0.999985264	0.973330026
WNT16	0.999593577	0.966014364	0.908181052	0.99999488	0.999985264	0.976814573
CLUH	0.999593577	0.881001912	0.873452279	0.99999488	0.999985264	0.9787163
FAM120A	0.999593577	0.928525825	0.945678616	0.99999488	0.999985264	0.986873741
GNPTG	0.999593577	0.937231686	0.927022752	0.99999488	0.999985264	0.987365188
GRAMD1B	0.999593577	0.916036681	0.925745065	0.99999488	0.999985264	0.987454496
ZNF34	0.999593577	0.941495217	0.954620171	0.99999488	0.999985264	0.994062777
TLE4	0.999593577	0.87010028	0.862345036	0.99999488	0.999985264	0.996949674
LOC100296205	0.999593577	0.975677199	0.871626027	0.99999488	0.999985264	0.996949674
CPEB3	0.999593577	0.964246761	0.863209655	0.99999488	0.999985264	0.999960414
DYRK2	0.999593577	0.873289191	0.884407415	0.99999488	0.999985264	0.999960414
DCAF17	0.999593577	0.927997582	0.907484111	0.99999488	0.999985264	0.999960414
ARHGAP1	0.999622572	0.958098081	0.899576888	0.99999488	0.999985264	0.919758204
CDK12	0.999839779	0.985406717	0.916975216	0.99999488	0.999985264	0.88310116
NDUFA4L2	0.999839779	0.878533356	0.862345036	0.99999488	0.999985264	0.939035437
ZNF555	0.999839779	0.911418256	0.883109976	0.99999488	0.999985264	0.957171025
ATP5F1D	0.999892635	0.880001923	0.91005986	0.99999488	0.999985264	0.882431247
CELSR1	0.999892635	0.999347049	0.88153193	0.99999488	0.999985264	0.884423852
LOC100848443	0.999892635	0.999992444	0.86772394	0.99999488	0.999985264	0.890433418
KALRN	0.999892635	0.991488652	0.880774328	0.99999488	0.999985264	0.892996981
CD36	0.999892635	0.967276215	0.925745065	0.99999488	0.999985264	0.895349622
P2RX3	0.999892635	0.999992444	0.910748743	0.99999488	0.999985264	0.89668604
UBQLN1	0.999892635	0.91507531	0.934299387	0.99999488	0.999985264	0.89935427
KCNS2	0.999892635	0.999992444	0.895626254	0.99999488	0.999985264	0.900681286
ITGB1	0.999892635	0.915367594	0.969880768	0.99999488	0.999985264	0.901809636

KANSL3	0.999892635	0.978089982	0.873452279	0.99999488	0.999985264	0.906118365
MPZL1	0.999892635	0.89064218	0.921065686	0.99999488	0.999985264	0.915219665
LOC780968	0.999892635	0.9582784	0.898125038	0.99999488	0.999985264	0.945705257
LOC101904592	0.999892635	0.992977597	0.922431991	0.99999488	0.999985264	0.95096765
NBDY	0.999892635	0.927447588	0.944085862	0.99999488	0.999985264	0.95096765
CCDC137	0.999892635	0.883856603	0.895626254	0.99999488	0.999985264	0.966921967
TMEM185A	0.999892635	0.967276215	0.926552506	0.99999488	0.999985264	0.973238011
RAF1	0.999892635	0.928525825	0.892574927	0.99999488	0.999985264	0.981855491
LOC100848205	0.999892635	0.946480858	0.891081265	0.99999488	0.999985264	0.992727599
TRIM63	0.999892635	0.90161039	0.924604843	0.99999488	0.999985264	0.994062777
THOC3	0.999892635	0.883624935	0.923604668	0.99999488	0.999985264	0.999960414
BRK1	0.999922091	0.930395237	0.930678591	0.99999488	0.999985264	0.90408677
KCTD9	0.999939598	0.870839685	0.9808801	0.99999488	0.999985264	0.882867473
ILDR2	0.999939598	0.999992444	0.934127018	0.99999488	0.999985264	0.885874544
ARHGAP42	0.999939598	0.999992444	0.892664791	0.99999488	0.999985264	0.894723749
ADAM10	0.999939598	0.90116492	0.892664791	0.99999488	0.999985264	0.896480535
CYP1A1	0.999939598	0.95697765	0.877799478	0.99999488	0.999985264	0.918356615
MAPK14	0.999939598	0.883624935	0.877799478	0.99999488	0.999985264	0.931163114
VIT	0.999964829	0.981644273	0.964920281	0.99999488	0.999985264	0.868103112
CLEC14A	0.999964829	0.938587041	0.914262201	0.99999488	0.999985264	0.885874544
IER3IP1	0.999964829	0.978426416	0.948359914	0.99999488	0.999985264	0.886185283
LOC112442757	0.999964829	0.998131745	0.926552506	0.99999488	0.999985264	0.905099683
STYX	0.999964829	0.938597964	0.885552508	0.99999488	0.999985264	0.907296322
LOC100141185	0.999964829	0.982981183	0.94634922	0.99999488	0.999985264	0.928213338
ACACA	0.999964829	0.873486784	0.865066354	0.99999488	0.999985264	0.945670698
DOK4	0.999996191	0.958098081	0.922400936	0.99999488	0.999985264	0.86871626
LOC107132228	0.999996191	0.881001912	0.983061067	0.99999488	0.999985264	0.869815046
RC3H1	0.999996191	0.974812121	0.921065686	0.99999488	0.999985264	0.869877865
HEBP1	0.999996191	0.89064218	0.941118576	0.99999488	0.999985264	0.869877865
LOC100848665	0.999996191	0.919361975	0.91207077	0.99999488	0.999985264	0.87150355
LOC112447360	0.999996191	0.916036681	0.884615251	0.99999488	0.999985264	0.871746584
ZNF529	0.999996191	0.998131745	0.946479014	0.99999488	0.999985264	0.871960581
HIST2H2AA4	0.999996191	0.927997582	0.978389389	0.99999488	0.999985264	0.872307149
ANKRD24	0.999996191	0.938597964	0.872482267	0.99999488	0.999985264	0.873463288
IQCE	0.999996191	0.90116492	0.888545123	0.99999488	0.999985264	0.873463288
LOC101907320	0.999996191	0.999992444	0.910426741	0.99999488	0.999985264	0.873463288
ZNF777	0.999996191	0.949285285	0.919618001	0.99999488	0.999985264	0.873463288
MMP14	0.999996191	0.927997582	0.922076383	0.99999488	0.999985264	0.873463288
RALGDS	0.999996191	0.991488652	0.933447488	0.99999488	0.999985264	0.873463288

PDIA5	0.999996191	0.883856603	0.939799459	0.99999488	0.999985264	0.873463288
LOC107132617	0.999996191	0.936273761	0.946479014	0.99999488	0.999985264	0.873463288
UTP25	0.999996191	0.941793392	0.973406453	0.99999488	0.999985264	0.873463288
TUSC2	0.999996191	0.92202441	0.981039752	0.99999488	0.999985264	0.873463288
CXXC1	0.999996191	0.896996319	0.994601277	0.99999488	0.999985264	0.873463288
LOC112444351	0.999996191	0.999992444	0.933447488	0.99999488	0.999985264	0.873836462
LOC100335828	0.999996191	0.985406717	0.900148803	0.99999488	0.999985264	0.87404666
DPYSL2	0.999996191	0.960180915	0.881516598	0.99999488	0.999985264	0.874439646
SFXN2	0.999996191	0.896536444	0.919004956	0.99999488	0.999985264	0.874975722
TMEM80	0.999996191	0.943802122	0.945766581	0.99999488	0.999985264	0.874975722
SMC2	0.999996191	0.883624935	0.949038056	0.99999488	0.999985264	0.874975722
COMT	0.999996191	0.946274461	0.971838819	0.99999488	0.999985264	0.874975722
CHAC2	0.999996191	0.918883775	0.985363931	0.99999488	0.999985264	0.874975722
CHCHD10	0.999996191	0.91407604	0.926552506	0.99999488	0.999985264	0.875113722
NEURL4	0.999996191	0.958967142	0.993585211	0.99999488	0.999985264	0.875113722
C20H5orf34	0.999996191	0.994481342	0.926552506	0.99999488	0.999985264	0.875200779
ACOT2	0.999996191	0.893748757	0.892574927	0.99999488	0.999985264	0.875212974
ETFA	0.999996191	0.916036681	0.907015206	0.99999488	0.999985264	0.875837549
ARMH3	0.999996191	0.881001912	0.926552506	0.99999488	0.999985264	0.87805762
GABPB1	0.999996191	0.999992444	0.927600835	0.99999488	0.999985264	0.87805762
WBP1L	0.999996191	0.910133637	0.999558179	0.99999488	0.999985264	0.878070222
RPA3	0.999996191	0.9093283	0.914630535	0.99999488	0.999985264	0.879798493
SPHK2	0.999996191	0.915971865	0.865129879	0.99999488	0.999985264	0.880673048
TNKS	0.999996191	0.989547896	0.870995265	0.99999488	0.999985264	0.882867473
EPS15	0.999996191	0.92202441	0.892053976	0.99999488	0.999985264	0.882867473
LOC513779	0.999996191	0.941793392	0.908181052	0.99999488	0.999985264	0.882867473
CYSTM1	0.999996191	0.917537324	0.917410616	0.99999488	0.999985264	0.882867473
STIP1	0.999996191	0.89880506	0.941961525	0.99999488	0.999985264	0.882867473
LOC783378	0.999996191	0.9582784	0.964920281	0.99999488	0.999985264	0.882867473
CUL9	0.999996191	0.948366017	0.985363931	0.99999488	0.999985264	0.882867473
HDAC3	0.999996191	0.986445172	0.911131131	0.99999488	0.999985264	0.88310116
TUBGCP3	0.999996191	0.90400337	0.932892118	0.99999488	0.999985264	0.88310116
UNC45A	0.999996191	0.985406717	0.922400936	0.99999488	0.999985264	0.883278571
HCFC1R1	0.999996191	0.96566261	0.907026738	0.99999488	0.999985264	0.883507907
GPR160	0.999996191	0.913160799	0.881942256	0.99999488	0.999985264	0.883999232
LOC104976293	0.999996191	0.883624935	0.907381775	0.99999488	0.999985264	0.883999232
COX7B	0.999996191	0.89880506	0.920629231	0.99999488	0.999985264	0.883999232
LOC782609	0.999996191	0.998131745	0.92487482	0.99999488	0.999985264	0.883999232
ZNF621	0.999996191	0.898789587	0.938911935	0.99999488	0.999985264	0.883999232

LOC100297725	0.999996191	0.993316598	0.946479014	0.99999488	0.999985264	0.883999232
SCN9A	0.999996191	0.957700659	0.969880768	0.99999488	0.999985264	0.883999232
MIER2	0.999996191	0.9582784	0.979650244	0.99999488	0.999985264	0.883999232
STX5	0.999996191	0.96493942	0.938203102	0.99999488	0.999985264	0.884033654
ZNF75D	0.999996191	0.973322746	0.938905004	0.99999488	0.999985264	0.884033654
PCBD2	0.999996191	0.946274461	0.902552409	0.99999488	0.999985264	0.885120898
PRRG2	0.999996191	0.963570838	0.941336964	0.99999488	0.999985264	0.885698639
SOCS7	0.999996191	0.958098081	0.884119589	0.99999488	0.999985264	0.885874544
LOC112442636	0.999996191	0.967276215	0.886730817	0.99999488	0.999985264	0.885874544
PPP1R35	0.999996191	0.935742738	0.88687452	0.99999488	0.999985264	0.885874544
LOC101904332	0.999996191	0.907069508	0.897767405	0.99999488	0.999985264	0.885874544
EPS15L1	0.999996191	0.896575712	0.899248502	0.99999488	0.999985264	0.885874544
MRPS25	0.999996191	0.948421431	0.902552409	0.99999488	0.999985264	0.885874544
MID2	0.999996191	0.932925766	0.907484111	0.99999488	0.999985264	0.885874544
C15H11orf94	0.999996191	0.972764622	0.910381378	0.99999488	0.999985264	0.885874544
UQCR11	0.999996191	0.893748757	0.923604668	0.99999488	0.999985264	0.885874544
C7H1orf35	0.999996191	0.925580392	0.923604668	0.99999488	0.999985264	0.885874544
VPS9D1	0.999996191	0.885718559	0.926552506	0.99999488	0.999985264	0.885874544
COX7A1	0.999996191	0.90024974	0.926552506	0.99999488	0.999985264	0.885874544
ENPP1	0.999996191	0.922102474	0.926552506	0.99999488	0.999985264	0.885874544
KCND3	0.999996191	0.920253543	0.930678591	0.99999488	0.999985264	0.885874544
LINS1	0.999996191	0.978510121	0.930678591	0.99999488	0.999985264	0.885874544
LOC107132288	0.999996191	0.936273761	0.945678616	0.99999488	0.999985264	0.885874544
RNF125	0.999996191	0.934871569	0.956565728	0.99999488	0.999985264	0.885874544
ZBTB46	0.999996191	0.956746116	0.956565728	0.99999488	0.999985264	0.885874544
RTL5	0.999996191	0.892008332	0.973406453	0.99999488	0.999985264	0.885874544
CPEB2	0.999996191	0.902408393	0.993594894	0.99999488	0.999985264	0.885874544
STK17A	0.999996191	0.88167071	0.998936876	0.99999488	0.999985264	0.885874544
MEGF9	0.999996191	0.946274461	0.999558179	0.99999488	0.999985264	0.885874544
TCF3	0.999996191	0.91407604	0.923902957	0.99999488	0.999985264	0.886185283
SLC35C2	0.999996191	0.904961534	0.920629231	0.99999488	0.999985264	0.886642513
MMRN1	0.999996191	0.95697765	0.884407415	0.99999488	0.999985264	0.88682376
MSRA	0.999996191	0.958098081	0.920629231	0.99999488	0.999985264	0.88682376
POLA1	0.999996191	0.999347049	0.939147816	0.99999488	0.999985264	0.88682376
RELL1	0.999996191	0.927997582	0.926552506	0.99999488	0.999985264	0.887077233
SMPD4	0.999996191	0.935588259	0.973406453	0.99999488	0.999985264	0.887158291
CHKB	0.999996191	0.970426668	0.939147816	0.99999488	0.999985264	0.887231766
LOC101903853	0.999996191	0.888041844	0.908181052	0.99999488	0.999985264	0.887277447
UBE2Z	0.999996191	0.919884594	0.926552506	0.99999488	0.999985264	0.887607259

TOR4A	0.999996191	0.999347049	0.871474302	0.99999488	0.999985264	0.887798625
MTMR6	0.999996191	0.985406717	0.889272129	0.99999488	0.999985264	0.887798625
LOC100848569	0.999996191	0.979263532	0.987198143	0.99999488	0.999985264	0.887798625
RIN3	0.999996191	0.935588259	0.880229444	0.99999488	0.999985264	0.888645453
N4BP2	0.999996191	0.919191546	0.884615251	0.99999488	0.999985264	0.888645453
LOC100335268	0.999996191	0.997975376	0.911922552	0.99999488	0.999985264	0.888645453
OSBPL6	0.999996191	0.883856603	0.933447488	0.99999488	0.999985264	0.888681581
KCTD12	0.999996191	0.924416082	0.874485384	0.99999488	0.999985264	0.888721578
NDUFV1	0.999996191	0.890789478	0.929207633	0.99999488	0.999985264	0.888828328
MRPL33	0.999996191	0.928525825	0.993594894	0.99999488	0.999985264	0.890260495
BOC	0.999996191	0.918704767	0.933992957	0.99999488	0.999985264	0.890433418
WTIP	0.999996191	0.905927857	0.948359914	0.99999488	0.999985264	0.890433418
MOB4	0.999996191	0.9582784	0.996715234	0.99999488	0.999985264	0.890433418
SEC62	0.999996191	0.955266582	0.911919459	0.99999488	0.999985264	0.89069589
BGN	0.999996191	0.899944331	0.922749791	0.99999488	0.999985264	0.89069589
NUTF2	0.999996191	0.926955605	0.932878547	0.99999488	0.999985264	0.89069589
PCDHA13	0.999996191	0.999992444	0.95230212	0.99999488	0.999985264	0.89069589
MOV10	0.999996191	0.943802122	0.988922732	0.99999488	0.999985264	0.891426382
POC5	0.999996191	0.938017364	0.946479014	0.99999488	0.999985264	0.892056797
SUPT6H	0.999996191	0.915313641	0.910036508	0.99999488	0.999985264	0.892187201
TSPAN4	0.999996191	0.90024974	0.994601277	0.99999488	0.999985264	0.892187201
FANCM	0.999996191	0.978089982	0.916975216	0.99999488	0.999985264	0.892231098
ARF5	0.999996191	0.999992444	0.882515776	0.99999488	0.999985264	0.892908035
CNIH3	0.999996191	0.935742738	0.898815524	0.99999488	0.999985264	0.892908035
NAGK	0.999996191	0.943802122	0.933447488	0.99999488	0.999985264	0.892908035
RNF38	0.999996191	0.913157854	0.998936876	0.99999488	0.999985264	0.892908035
MRPS2	0.999996191	0.938597964	0.907381775	0.99999488	0.999985264	0.89329705
LMNA	0.999996191	0.991488652	0.892943118	0.99999488	0.999985264	0.893392612
OCIAD1	0.999996191	0.999992444	0.940815039	0.99999488	0.999985264	0.893392612
NCAPG2	0.999996191	0.89076613	0.999558179	0.99999488	0.999985264	0.893392612
ATP5PF	0.999996191	0.91388073	0.964152013	0.99999488	0.999985264	0.893921142
NHSL2	0.999996191	0.999992444	0.890248427	0.99999488	0.999985264	0.894144329
LOC101902937	0.999996191	0.90116492	0.941118576	0.99999488	0.999985264	0.894144329
LOC101907523	0.999996191	0.874697957	0.965065403	0.99999488	0.999985264	0.894144329
LOC112442349	0.999996191	0.948421431	0.877742202	0.99999488	0.999985264	0.894174543
DCAF6	0.999996191	0.938597964	0.934941286	0.99999488	0.999985264	0.894174543
LAMTOR2	0.999996191	0.938597964	0.938203102	0.99999488	0.999985264	0.894174543
CCDC17	0.999996191	0.994177236	0.923089792	0.99999488	0.999985264	0.894236694
ITPA	0.999996191	0.933208464	0.886962419	0.99999488	0.999985264	0.894333557

FAM171A2	0.999996191	0.921549	0.923604668	0.99999488	0.999985264	0.894333557
NDUFA6	0.999996191	0.918254521	0.938559901	0.99999488	0.999985264	0.894420979
LOC101903424	0.999996191	0.921934673	0.944993804	0.99999488	0.999985264	0.894420979
GALNT11	0.999996191	0.916036681	0.884119589	0.99999488	0.999985264	0.894723749
MID1	0.999996191	0.961166521	0.884320747	0.99999488	0.999985264	0.894723749
PSMG4	0.999996191	0.998131745	0.887707591	0.99999488	0.999985264	0.894723749
TMEM145	0.999996191	0.911418256	0.898815524	0.99999488	0.999985264	0.894723749
SNX2	0.999996191	0.9409629	0.899576888	0.99999488	0.999985264	0.894723749
ZBTB2	0.999996191	0.967276215	0.904959653	0.99999488	0.999985264	0.894723749
WNT5B	0.999996191	0.999992444	0.914001994	0.99999488	0.999985264	0.894723749
KCTD21	0.999996191	0.937231686	0.915266146	0.99999488	0.999985264	0.894723749
ARL15	0.999996191	0.999992444	0.920629231	0.99999488	0.999985264	0.894723749
LOC781064	0.999996191	0.915367594	0.922569757	0.99999488	0.999985264	0.894723749
CENPH	0.999996191	0.928879317	0.923604668	0.99999488	0.999985264	0.894723749
PPP1R36	0.999996191	0.90116492	0.926552506	0.99999488	0.999985264	0.894723749
LOC101906134	0.999996191	0.919884594	0.926552506	0.99999488	0.999985264	0.894723749
FAT1	0.999996191	0.934445689	0.926552506	0.99999488	0.999985264	0.894723749
NDUFA8	0.999996191	0.935742738	0.927600835	0.99999488	0.999985264	0.894723749
ZNF609	0.999996191	0.909208194	0.930678591	0.99999488	0.999985264	0.894723749
LOC100296324	0.999996191	0.941495217	0.931507181	0.99999488	0.999985264	0.894723749
SLC39A13	0.999996191	0.937231686	0.935094475	0.99999488	0.999985264	0.894723749
MATN2	0.999996191	0.96566261	0.937822865	0.99999488	0.999985264	0.894723749
ABCB1	0.999996191	0.991488652	0.948420301	0.99999488	0.999985264	0.894723749
LOC101906837	0.999996191	0.904961534	0.948923067	0.99999488	0.999985264	0.894723749
MGC148714	0.999996191	0.925580392	0.95106368	0.99999488	0.999985264	0.894723749
LOC112442593	0.999996191	0.929230097	0.972362683	0.99999488	0.999985264	0.894723749
STX17	0.999996191	0.918892358	0.981388276	0.99999488	0.999985264	0.894723749
S100A2	0.999996191	0.9409629	0.998936876	0.99999488	0.999985264	0.894723749
SEPT7	0.999996191	0.90116492	0.999558179	0.99999488	0.999985264	0.894723749
IFT172	0.999996191	0.920253543	0.999558179	0.99999488	0.999985264	0.894723749
EAF1	0.999996191	0.994434609	0.924717963	0.99999488	0.999985264	0.895126936
NOS2	0.999996191	0.90116492	0.87398841	0.99999488	0.999985264	0.895349622
ABHD14A	0.999996191	0.985204209	0.877742202	0.99999488	0.999985264	0.895349622
LOC101902029	0.999996191	0.986637746	0.879620211	0.99999488	0.999985264	0.895349622
SLC5A2	0.999996191	0.918911961	0.912247957	0.99999488	0.999985264	0.895349622
MAP3K5	0.999996191	0.991488652	0.915215321	0.99999488	0.999985264	0.895349622
ANKS3	0.999996191	0.936562168	0.916975216	0.99999488	0.999985264	0.895349622
GJC2	0.999996191	0.9527938	0.923604668	0.99999488	0.999985264	0.895349622
KCTD2	0.999996191	0.973092298	0.938203102	0.99999488	0.999985264	0.895349622

TSPAN14	0.999996191	0.979065751	0.938203102	0.99999488	0.999985264	0.895349622
SDC2	0.999996191	0.9582784	0.978389389	0.99999488	0.999985264	0.895349622
LOC618939	0.999996191	0.885718559	0.999558179	0.99999488	0.999985264	0.895349622
SAO	0.999996191	0.873963285	0.876188482	0.99999488	0.999985264	0.895370805
SCAF1	0.999996191	0.957700659	0.922400936	0.99999488	0.999985264	0.895370805
PAPOLB	0.999996191	0.905019423	0.946479014	0.99999488	0.999985264	0.895370805
EGR3	0.999996191	0.912316394	0.999558179	0.99999488	0.999985264	0.895370805
RRM2B	0.999996191	0.999992444	0.93198623	0.99999488	0.999985264	0.895482135
PTPN23	0.999996191	0.896536444	0.91207077	0.99999488	0.999985264	0.89589047
AGTR2	0.999996191	0.946274461	0.920154807	0.99999488	0.999985264	0.89589047
SLC30A1	0.999996191	0.896996319	0.922400936	0.99999488	0.999985264	0.89589047
C1QTNF1	0.999996191	0.921661251	0.956565728	0.99999488	0.999985264	0.89589047
HNRNPA2B1	0.999996191	0.978154072	0.993594894	0.99999488	0.999985264	0.89589047
LOC100299503	0.999996191	0.978089982	0.944993804	0.99999488	0.999985264	0.896473596
NRARP	0.999996191	0.946274461	0.892933333	0.99999488	0.999985264	0.896480535
RWDD4	0.999996191	0.999992444	0.93198623	0.99999488	0.999985264	0.896480535
VPS41	0.999996191	0.90213106	0.92487482	0.99999488	0.999985264	0.896481747
LOC783202	0.999996191	0.907202013	0.905757315	0.99999488	0.999985264	0.896674499
DAGLA	0.999996191	0.918883775	0.892943118	0.99999488	0.999985264	0.89668604
COX8B	0.999996191	0.926955605	0.907484111	0.99999488	0.999985264	0.89668604
TNFAIP3	0.999996191	0.918332306	0.926552506	0.99999488	0.999985264	0.89668604
CLEC18C	0.999996191	0.9582784	0.926552506	0.99999488	0.999985264	0.89668604
DDRGK1	0.999996191	0.93377057	0.915117288	0.99999488	0.999985264	0.897014912
LOC107131289	0.999996191	0.986445172	0.975538142	0.99999488	0.999985264	0.897014912
TALDO1	0.999996191	0.938476452	0.887640584	0.99999488	0.999985264	0.897467714
CRBN	0.999996191	0.943802122	0.900148803	0.99999488	0.999985264	0.897467714
MFNG	0.999996191	0.999992444	0.910036508	0.99999488	0.999985264	0.897467714
LOC104972622	0.999996191	0.925580392	0.921065686	0.99999488	0.999985264	0.897467714
SIGIRR	0.999996191	0.919381083	0.923604668	0.99999488	0.999985264	0.897467714
GDE1	0.999996191	0.988862859	0.936816488	0.99999488	0.999985264	0.897467714
LOC783657	0.999996191	0.992977597	0.937004034	0.99999488	0.999985264	0.897467714
NAA30	0.999996191	0.999992444	0.938203102	0.99999488	0.999985264	0.897467714
PMPCA	0.999996191	0.92202441	0.947432352	0.99999488	0.999985264	0.897467714
NDUFA2	0.999996191	0.900368858	0.95106368	0.99999488	0.999985264	0.897467714
TXNRD2	0.999996191	0.921934673	0.964152013	0.99999488	0.999985264	0.897467714
WDR62	0.999996191	0.893642384	0.999558179	0.99999488	0.999985264	0.897467714
LOC101908111	0.999996191	0.991488652	0.998936876	0.99999488	0.999985264	0.897856757
ZNF146	0.999996191	0.999992444	0.881942256	0.99999488	0.999985264	0.897965262
LOC101907545	0.999996191	0.957073593	0.892982948	0.99999488	0.999985264	0.897965262

MGP	0.999996191	0.988170324	0.899576888	0.99999488	0.999985264	0.897965262
LOC787550	0.999996191	0.999992444	0.900148803	0.99999488	0.999985264	0.897965262
CILP	0.999996191	0.985406717	0.908181052	0.99999488	0.999985264	0.897965262
TLR3	0.999996191	0.992977597	0.908181052	0.99999488	0.999985264	0.897965262
LOC100299705	0.999996191	0.999992444	0.911063423	0.99999488	0.999985264	0.897965262
TCF24	0.999996191	0.974812121	0.915117288	0.99999488	0.999985264	0.897965262
NDUFA7	0.999996191	0.917849199	0.921646762	0.99999488	0.999985264	0.897965262
MRM2	0.999996191	0.999992444	0.925745065	0.99999488	0.999985264	0.897965262
LOC789157	0.999996191	0.984641223	0.935856933	0.99999488	0.999985264	0.897965262
DCTN4	0.999996191	0.998131745	0.938203102	0.99999488	0.999985264	0.897965262
RAB36	0.999996191	0.897406772	0.93955737	0.99999488	0.999985264	0.897965262
NUCB1	0.999996191	0.991365955	0.941118576	0.99999488	0.999985264	0.897965262
PIGC	0.999996191	0.904961534	0.95230212	0.99999488	0.999985264	0.897965262
EMC2	0.999996191	0.943802122	0.95230212	0.99999488	0.999985264	0.897965262
COPZ2	0.999996191	0.99398773	0.953903563	0.99999488	0.999985264	0.897965262
CTH	0.999996191	0.912518351	0.981541532	0.99999488	0.999985264	0.897965262
LOC112446457	0.999996191	0.916036681	0.999558179	0.99999488	0.999985264	0.897965262
MT3	0.999996191	0.999992444	0.877901599	0.99999488	0.999985264	0.898272644
GLIPR1	0.999996191	0.946274461	0.899576888	0.99999488	0.999985264	0.898272644
TAF5	0.999996191	0.999992444	0.921065686	0.99999488	0.999985264	0.899223071
LMBR1	0.999996191	0.888041844	0.934694748	0.99999488	0.999985264	0.899223071
TMEM131	0.999996191	0.906348981	0.95230212	0.99999488	0.999985264	0.899223071
AOC2	0.999996191	0.931488015	0.973303821	0.99999488	0.999985264	0.899223071
DEK	0.999996191	0.991488652	0.870264123	0.99999488	0.999985264	0.89935427
BAZ1B	0.999996191	0.9409629	0.88153193	0.99999488	0.999985264	0.89935427
CXHXorf36	0.999996191	0.918952092	0.887349218	0.99999488	0.999985264	0.89935427
LOC112447811	0.999996191	0.992977597	0.887626998	0.99999488	0.999985264	0.89935427
ACYP2	0.999996191	0.932593501	0.907381775	0.99999488	0.999985264	0.89935427
CTTNBP2NL	0.999996191	0.934781828	0.908181052	0.99999488	0.999985264	0.89935427
MPC2	0.999996191	0.916036681	0.912008683	0.99999488	0.999985264	0.89935427
LOC785804	0.999996191	0.915367594	0.914001994	0.99999488	0.999985264	0.89935427
IGDCC4	0.999996191	0.999992444	0.914001994	0.99999488	0.999985264	0.89935427
BCL2L12	0.999996191	0.999992444	0.922431991	0.99999488	0.999985264	0.89935427
ACTN4	0.999996191	0.9582784	0.926552506	0.99999488	0.999985264	0.89935427
BCL6	0.999996191	0.999992444	0.95106368	0.99999488	0.999985264	0.89935427
MORC3	0.999996191	0.9582784	0.964908146	0.99999488	0.999985264	0.89935427
LOC101905875	0.999996191	0.927853996	0.904140521	0.99999488	0.999985264	0.900681286
LOC101910153	0.999996191	0.999992444	0.926552506	0.99999488	0.999985264	0.900681286
LOC101908577	0.999996191	0.918883775	0.930678591	0.99999488	0.999985264	0.900681286

LOC112444276	0.999996191	0.999992444	0.933791093	0.99999488	0.999985264	0.900681286
FBXO6	0.999996191	0.946480858	0.951822586	0.99999488	0.999985264	0.900681286
LDHA	0.999996191	0.89880506	0.955141547	0.99999488	0.999985264	0.900681286
GDAP1	0.999996191	0.982981183	0.978389389	0.99999488	0.999985264	0.900681286
IRX5	0.999996191	0.931828419	0.930678591	0.99999488	0.999985264	0.900741642
LYPD6	0.999996191	0.999992444	0.898815524	0.99999488	0.999985264	0.900914171
LOC112446696	0.999996191	0.872223076	0.873452279	0.99999488	0.999985264	0.90109791
DVL3	0.999996191	0.893743321	0.877799478	0.99999488	0.999985264	0.90109791
LY96	0.999996191	0.925387304	0.886962419	0.99999488	0.999985264	0.90109791
PXMP2	0.999996191	0.88167071	0.892461717	0.99999488	0.999985264	0.90109791
SELENOW	0.999996191	0.928925667	0.895626254	0.99999488	0.999985264	0.90109791
MTPN	0.999996191	0.900867274	0.938203102	0.99999488	0.999985264	0.90109791
BCAR3	0.999996191	0.947709094	0.939147816	0.99999488	0.999985264	0.90109791
C15H11orf71	0.999996191	0.922700337	0.944085862	0.99999488	0.999985264	0.90109791
ZNF567	0.999996191	0.999992444	0.945766581	0.99999488	0.999985264	0.90109791
RELN	0.999996191	0.999992444	0.946479014	0.99999488	0.999985264	0.90109791
NUPL2	0.999996191	0.957700659	0.978389389	0.99999488	0.999985264	0.90109791
RAB2B	0.999996191	0.959754057	0.999558179	0.99999488	0.999985264	0.90109791
DGCR6L	0.999996191	0.935742738	0.888692542	0.99999488	0.999985264	0.901545041
ZFAND3	0.999996191	0.918911961	0.907015206	0.99999488	0.999985264	0.901545041
NUDT13	0.999996191	0.964044235	0.926552506	0.99999488	0.999985264	0.901545871
LOC112442997	0.999996191	0.887454823	0.870264123	0.99999488	0.999985264	0.901614166
GOLGA7	0.999996191	0.896536444	0.912008683	0.99999488	0.999985264	0.901614166
RNPS1	0.999996191	0.928525825	0.912008683	0.99999488	0.999985264	0.901614166
MS4A7	0.999996191	0.991365955	0.944871472	0.99999488	0.999985264	0.901614166
RYR3	0.999996191	0.934479017	0.879253779	0.99999488	0.999985264	0.901643249
SPRED1	0.999996191	0.915367594	0.904959653	0.99999488	0.999985264	0.901643249
DLC1	0.999996191	0.938597964	0.908181052	0.99999488	0.999985264	0.901643249
METTL22	0.999996191	0.902408393	0.970903823	0.99999488	0.999985264	0.901643249
LOC101902854	0.999996191	0.921431065	0.908181052	0.99999488	0.999985264	0.901754411
SCAMP5	0.999996191	0.986637746	0.913375522	0.99999488	0.999985264	0.901763402
CDH6	0.999996191	0.975677199	0.882515776	0.99999488	0.999985264	0.901809636
GTF2H2	0.999996191	0.999992444	0.898815524	0.99999488	0.999985264	0.901809636
LOC101906120	0.999996191	0.959773007	0.900890709	0.99999488	0.999985264	0.901809636
SYMPK	0.999996191	0.949283886	0.902527616	0.99999488	0.999985264	0.901809636
LMAN2L	0.999996191	0.999992444	0.909383349	0.99999488	0.999985264	0.901809636
DEPTOR	0.999996191	0.938587041	0.914449935	0.99999488	0.999985264	0.901809636
LAP3	0.999996191	0.938597964	0.914630535	0.99999488	0.999985264	0.901809636
LOC104970852	0.999996191	0.934886531	0.924604843	0.99999488	0.999985264	0.901809636

LOC521224	0.999996191	0.999992444	0.925520255	0.99999488	0.999985264	0.901809636
APEX2	0.999996191	0.957700659	0.92577351	0.99999488	0.999985264	0.901809636
COA3	0.999996191	0.915539284	0.926552506	0.99999488	0.999985264	0.901809636
MTCH1	0.999996191	0.991488652	0.932657945	0.99999488	0.999985264	0.901809636
GRB2	0.999996191	0.999992444	0.934941286	0.99999488	0.999985264	0.901809636
LOC112444847	0.999996191	0.981298804	0.938203102	0.99999488	0.999985264	0.901809636
XBP1	0.999996191	0.955266582	0.940815039	0.99999488	0.999985264	0.901809636
LOC104975091	0.999996191	0.961479426	0.951822586	0.99999488	0.999985264	0.901809636
CALU	0.999996191	0.978510121	0.962777771	0.99999488	0.999985264	0.901809636
NOV	0.999996191	0.946274461	0.963465247	0.99999488	0.999985264	0.901809636
LOC516742	0.999996191	0.915367594	0.982783495	0.99999488	0.999985264	0.901809636
LIN7C	0.999996191	0.992977597	0.994601277	0.99999488	0.999985264	0.901809636
SMAD2	0.999996191	0.913071503	0.999558179	0.99999488	0.999985264	0.901809636
CCDC57	0.999996191	0.941495217	0.999558179	0.99999488	0.999985264	0.901809636
CPXM2	0.999996191	0.89880506	0.912008683	0.99999488	0.999985264	0.902357112
MAGEH1	0.999996191	0.999992444	0.921010865	0.99999488	0.999985264	0.902508792
ZWINT	0.999996191	0.921408297	0.965059328	0.99999488	0.999985264	0.902508792
PRPF39	0.999996191	0.993316598	0.948420301	0.99999488	0.999985264	0.902590955
LOC107131293	0.999996191	0.88753482	0.907484111	0.99999488	0.999985264	0.902893979
LOC100336368	0.999996191	0.978089982	0.908181052	0.99999488	0.999985264	0.902893979
SLC27A3	0.999996191	0.999992444	0.933791093	0.99999488	0.999985264	0.902893979
SOD3	0.999996191	0.976393271	0.981388276	0.99999488	0.999985264	0.902893979
ZNF389	0.999996191	0.921934673	0.999558179	0.99999488	0.999985264	0.902893979
CMC4	0.999996191	0.957700659	0.999558179	0.99999488	0.999985264	0.902893979
DPEP3	0.999996191	0.999992444	0.907484111	0.99999488	0.999985264	0.902958012
TRAPPC5	0.999996191	0.941495217	0.924604843	0.99999488	0.999985264	0.902958012
PIN4	0.999996191	0.970426668	0.938203102	0.99999488	0.999985264	0.902958012
CEP250	0.999996191	0.90116492	0.999558179	0.99999488	0.999985264	0.902958012
NDUFA10	0.999996191	0.90213106	0.927600835	0.99999488	0.999985264	0.903243306
WIPI1	0.999996191	0.915367594	0.951822586	0.99999488	0.999985264	0.903445296
SAFB2	0.999996191	0.926675185	0.95230212	0.99999488	0.999985264	0.903445296
PHLDA1	0.999996191	0.988850552	0.938203102	0.99999488	0.999985264	0.903974039
SMYD3	0.999996191	0.999347049	0.88153193	0.99999488	0.999985264	0.90408677
LOC107131703	0.999996191	0.94984369	0.903957821	0.99999488	0.999985264	0.90408677
ORC4	0.999996191	0.987516216	0.914001994	0.99999488	0.999985264	0.90408677
CD55	0.999996191	0.951038017	0.945678616	0.99999488	0.999985264	0.904300404
GRSF1	0.999996191	0.985406717	0.949038056	0.99999488	0.999985264	0.904300404
LOC506408	0.999996191	0.924139925	0.999558179	0.99999488	0.999985264	0.904300404
AMY2B	0.999996191	0.948421431	0.945280564	0.99999488	0.999985264	0.904629573

PFKFB1	0.999996191	0.991488652	0.887673	0.99999488	0.999985264	0.904813143
C23H6orf132	0.999996191	0.956310879	0.908700926	0.99999488	0.999985264	0.904813143
ADGRD1	0.999996191	0.922700337	0.930678591	0.99999488	0.999985264	0.904813143
EXOC2	0.999996191	0.918892358	0.932762395	0.99999488	0.999985264	0.904813143
LOC789551	0.999996191	0.9582784	0.933447488	0.99999488	0.999985264	0.904813143
ZNF18	0.999996191	0.957700659	0.951099739	0.99999488	0.999985264	0.904813143
LOC101905219	0.999996191	0.991488652	0.957541703	0.99999488	0.999985264	0.904813143
THSD1	0.999996191	0.95697765	0.96210311	0.99999488	0.999985264	0.904813143
RDH5	0.999996191	0.978089982	0.998429524	0.99999488	0.999985264	0.904813143
LOC107131974	0.999996191	0.91407604	0.999558179	0.99999488	0.999985264	0.904813143
LOC112448518	0.999996191	0.958098081	0.890096618	0.99999488	0.999985264	0.904958457
LOC112447324	0.999996191	0.936273761	0.958627535	0.99999488	0.999985264	0.904958457
LOC112445177	0.999996191	0.921934673	0.980660791	0.99999488	0.999985264	0.904958457
NGEF	0.999996191	0.998131745	0.875823795	0.99999488	0.999985264	0.905099683
LOC100139363	0.999996191	0.90024974	0.881938573	0.99999488	0.999985264	0.905099683
APLNR	0.999996191	0.931336072	0.90339136	0.99999488	0.999985264	0.905099683
MCU	0.999996191	0.999992444	0.909383349	0.99999488	0.999985264	0.905099683
LOC107132410	0.999996191	0.89880506	0.916975216	0.99999488	0.999985264	0.905099683
NECAB2	0.999996191	0.948421431	0.934694748	0.99999488	0.999985264	0.905099683
SKA2	0.999996191	0.985406717	0.935094475	0.99999488	0.999985264	0.905099683
PI3	0.999996191	0.948421431	0.948946702	0.99999488	0.999985264	0.905099683
HIST1H2BD	0.999996191	0.9582784	0.95106368	0.99999488	0.999985264	0.905099683
TOMM7	0.999996191	0.941495217	0.964920281	0.99999488	0.999985264	0.905099683
LOC100174924	0.999996191	0.957700659	0.9808801	0.99999488	0.999985264	0.905099683
NKAP	0.999996191	0.938597964	0.981039752	0.99999488	0.999985264	0.905099683
GRIA1	0.999996191	0.90213106	0.992709008	0.99999488	0.999985264	0.905099683
TACC2	0.999996191	0.988170127	0.892943118	0.99999488	0.999985264	0.905391122
GCDH	0.999996191	0.949285285	0.927600835	0.99999488	0.999985264	0.905391122
CHMP7	0.999996191	0.9582784	0.944085862	0.99999488	0.999985264	0.905391122
BBS4	0.999996191	0.950729918	0.910426741	0.99999488	0.999985264	0.905557485
RBM4	0.999996191	0.945217876	0.935890635	0.99999488	0.999985264	0.905557485
RAD51D	0.999996191	0.88753482	0.875030741	0.99999488	0.999985264	0.906493565
C7H19orf38	0.999996191	0.921759937	0.900351064	0.99999488	0.999985264	0.906644037
UQCRFS1	0.999996191	0.890682633	0.909227985	0.99999488	0.999985264	0.906644037
FANCL	0.999996191	0.9582784	0.922076383	0.99999488	0.999985264	0.906644037
CHCHD2	0.999996191	0.893748757	0.922400936	0.99999488	0.999985264	0.906644037
NDUFC1	0.999996191	0.957700659	0.938905004	0.99999488	0.999985264	0.906644037
COX7A2	0.999996191	0.928611179	0.946479014	0.99999488	0.999985264	0.906644037
SLC39A2	0.999996191	0.935060764	0.953704305	0.99999488	0.999985264	0.906644037

WDR47	0.999996191	0.921549	0.960133832	0.99999488	0.999985264	0.906644037
PRRG1	0.999996191	0.935821833	0.985966356	0.99999488	0.999985264	0.906644037
ZC3H10	0.999996191	0.90116492	0.931726283	0.99999488	0.999985264	0.907296322
ADSS	0.999996191	0.92202441	0.944871472	0.99999488	0.999985264	0.907993548
MFSD14B	0.999996191	0.974812121	0.96131087	0.99999488	0.999985264	0.907993548
NME4	0.999996191	0.962968114	0.985966356	0.99999488	0.999985264	0.907993548
GLTP	0.999996191	0.999992444	0.895626254	0.99999488	0.999985264	0.908047942
EIF2B4	0.999996191	0.979032123	0.897767405	0.99999488	0.999985264	0.908047942
LOC101909384	0.999996191	0.916320187	0.911063423	0.99999488	0.999985264	0.908047942
JPT1	0.999996191	0.9582784	0.93198623	0.99999488	0.999985264	0.908047942
BRWD3	0.999996191	0.958967142	0.929849872	0.99999488	0.999985264	0.909412965
HUNK	0.999996191	0.893642384	0.917054062	0.99999488	0.999985264	0.909427764
JOSD2	0.999996191	0.971736716	0.944993804	0.99999488	0.999985264	0.909427764
RAPGEF5	0.999996191	0.946274461	0.963068909	0.99999488	0.999985264	0.909427764
CROCC	0.999996191	0.919884594	0.985363931	0.99999488	0.999985264	0.909427764
LOC112443328	0.999996191	0.918911961	0.993594894	0.99999488	0.999985264	0.909427764
LOC512684	0.999996191	0.915367594	0.999558179	0.99999488	0.999985264	0.909427764
GUSB	0.999996191	0.985293257	0.999558179	0.99999488	0.999985264	0.909427764
CMTM3	0.999996191	0.970426668	0.919618001	0.99999488	0.999985264	0.909912452
PRPF38B	0.999996191	0.927465399	0.884220809	0.99999488	0.999985264	0.910018813
COPE	0.999996191	0.981520287	0.884220809	0.99999488	0.999985264	0.910018813
LOC112447418	0.999996191	0.947399873	0.899576888	0.99999488	0.999985264	0.910018813
LOC101903615	0.999996191	0.922995823	0.902038586	0.99999488	0.999985264	0.910018813
LOC104973054	0.999996191	0.883624935	0.912008683	0.99999488	0.999985264	0.910018813
CDKN2AIP	0.999996191	0.971009675	0.923604668	0.99999488	0.999985264	0.910018813
COA5	0.999996191	0.991574265	0.936816488	0.99999488	0.999985264	0.910018813
EVI5	0.999996191	0.963737928	0.946479014	0.99999488	0.999985264	0.910018813
LOC101905708	0.999996191	0.999992444	0.95106368	0.99999488	0.999985264	0.910018813
INHBA	0.999996191	0.978510121	0.956565728	0.99999488	0.999985264	0.910018813
KHDRBS1	0.999996191	0.941329909	0.968232195	0.99999488	0.999985264	0.910018813
LMNB2	0.999996191	0.990248517	0.973284651	0.99999488	0.999985264	0.910018813
E2F1	0.999996191	0.942911665	0.976321912	0.99999488	0.999985264	0.910018813
TMEM199	0.999996191	0.935588259	0.999558179	0.99999488	0.999985264	0.910018813
SOX17	0.999996191	0.977665426	0.999558179	0.99999488	0.999985264	0.910018813
TAF2	0.999996191	0.946274461	0.954823586	0.99999488	0.999985264	0.91016113
NGLY1	0.999996191	0.892008332	0.889272129	0.99999488	0.999985264	0.911089313
TRAIP	0.999996191	0.929230097	0.923604668	0.99999488	0.999985264	0.911089313
EML1	0.999996191	0.949285285	0.926552506	0.99999488	0.999985264	0.911089313
EXO5	0.999996191	0.978089982	0.927365933	0.99999488	0.999985264	0.911089313

LOC101906914	0.999996191	0.892286715	0.927600835	0.99999488	0.999985264	0.911089313
TMEM121B	0.999996191	0.9582784	0.941118576	0.99999488	0.999985264	0.911089313
VASH1	0.999996191	0.922700337	0.944871472	0.99999488	0.999985264	0.911089313
LOC112442623	0.999996191	0.921759937	0.956565728	0.99999488	0.999985264	0.911089313
TRAF7	0.999996191	0.950729918	0.959552266	0.99999488	0.999985264	0.911089313
TSPAN13	0.999996191	0.946274461	0.962777771	0.99999488	0.999985264	0.911089313
LOC112447322	0.999996191	0.918883775	0.948069113	0.99999488	0.999985264	0.911779864
FGFR2	0.999996191	0.977354623	0.876758498	0.99999488	0.999985264	0.911997747
LOC100337390	0.999996191	0.9582784	0.887707591	0.99999488	0.999985264	0.911997747
LOC781565	0.999996191	0.981520287	0.916975216	0.99999488	0.999985264	0.911997747
ACOT7	0.999996191	0.9582784	0.919763144	0.99999488	0.999985264	0.911997747
RSF1	0.999996191	0.985406717	0.924866532	0.99999488	0.999985264	0.911997747
BMPR2	0.999996191	0.931488015	0.948359914	0.99999488	0.999985264	0.911997747
LOC101906923	0.999996191	0.915367594	0.95106368	0.99999488	0.999985264	0.911997747
SSNA1	0.999996191	0.938597964	0.952207545	0.99999488	0.999985264	0.911997747
PLEKHB1	0.999996191	0.991365955	0.954872702	0.99999488	0.999985264	0.911997747
PNMA8B	0.999996191	0.921550129	0.96210311	0.99999488	0.999985264	0.911997747
SHCBP1L	0.999996191	0.985406717	0.964920281	0.99999488	0.999985264	0.911997747
HIP1R	0.999996191	0.917849199	0.969292287	0.99999488	0.999985264	0.911997747
LOC101908154	0.999996191	0.94984369	0.981388276	0.99999488	0.999985264	0.911997747
BOLA2B	0.999996191	0.916036681	0.996391386	0.99999488	0.999985264	0.911997747
MZT1	0.999996191	0.985406717	0.998936876	0.99999488	0.999985264	0.911997747
LOC104970503	0.999996191	0.995476917	0.998936876	0.99999488	0.999985264	0.911997747
LOC104972584	0.999996191	0.990248517	0.999558179	0.99999488	0.999985264	0.911997747
USP20	0.999996191	0.911418256	0.956565728	0.99999488	0.999985264	0.912067992
NIT1	0.999996191	0.921759937	0.978423532	0.99999488	0.999985264	0.912067992
GSTA2	0.999996191	0.999992444	0.900890709	0.99999488	0.999985264	0.91208425
PAPD4	0.999996191	0.999992444	0.908181052	0.99999488	0.999985264	0.91208425
ALDH1A3	0.999996191	0.921549	0.998936876	0.99999488	0.999985264	0.91208425
RPS24	0.999996191	0.938597964	0.888926944	0.99999488	0.999985264	0.912262222
LOC101904757	0.999996191	0.957890487	0.915215321	0.99999488	0.999985264	0.912262222
KCNJ13	0.999996191	0.993316598	0.925520255	0.99999488	0.999985264	0.912262222
DROSHA	0.999996191	0.938597964	0.926552506	0.99999488	0.999985264	0.912262222
STON1	0.999996191	0.974812121	0.996715234	0.99999488	0.999985264	0.912262222
UHRF1BP1	0.999996191	0.917570301	0.923604668	0.99999488	0.999985264	0.912369911
LOC614785	0.999996191	0.998131745	0.875823795	0.99999488	0.999985264	0.912655166
PRELID2	0.999996191	0.934886531	0.946479014	0.99999488	0.999985264	0.913094861
SRSF10	0.999996191	0.999992444	0.948946702	0.99999488	0.999985264	0.913094861
EXOC3L1	0.999996191	0.956768356	0.969880768	0.99999488	0.999985264	0.913094861

RCN2	0.999996191	0.979263532	0.884220809	0.99999488	0.999985264	0.913527577
ADAMTSL1	0.999996191	0.999992444	0.904038351	0.99999488	0.999985264	0.913527577
CTNNB1	0.999996191	0.978089982	0.930678591	0.99999488	0.999985264	0.913527577
JAK2	0.999996191	0.991488652	0.909383349	0.99999488	0.999985264	0.913576575
PPP2R3A	0.999996191	0.9582784	0.93198623	0.99999488	0.999985264	0.913593134
NICN1	0.999996191	0.938017364	0.967163192	0.99999488	0.999985264	0.913593134
LOC100298453	0.999996191	0.970077079	0.973303821	0.99999488	0.999985264	0.913593134
MED10	0.999996191	0.921550129	0.985966356	0.99999488	0.999985264	0.913593134
RAPGEF1	0.999996191	0.978089982	0.998936876	0.99999488	0.999985264	0.913593134
LOC104975099	0.999996191	0.925580392	0.999558179	0.99999488	0.999985264	0.913593134
OAF	0.999996191	0.999992444	0.911018006	0.99999488	0.999985264	0.914756067
ISCU	0.999996191	0.946274461	0.923604668	0.99999488	0.999985264	0.914756067
TRIM65	0.999996191	0.983640273	0.938203102	0.99999488	0.999985264	0.914756067
LOC112445912	0.999996191	0.922102474	0.939147816	0.99999488	0.999985264	0.914756067
GBP4	0.999996191	0.936657629	0.947267164	0.99999488	0.999985264	0.914756067
TAF6L	0.999996191	0.928525825	0.904959653	0.99999488	0.999985264	0.914991968
IQCA1L	0.999996191	0.999992444	0.997859293	0.99999488	0.999985264	0.914991968
SELENOV	0.999996191	0.999992444	0.923604668	0.99999488	0.999985264	0.915039579
MCM3AP	0.999996191	0.925631263	0.998816213	0.99999488	0.999985264	0.915235751
NAA38	0.999996191	0.974812121	0.94634922	0.99999488	0.999985264	0.915351311
PLXND1	0.999996191	0.941495217	0.898427502	0.99999488	0.999985264	0.915476587
WDR20	0.999996191	0.950729918	0.901007502	0.99999488	0.999985264	0.915476587
PRELID3A	0.999996191	0.935588259	0.924866532	0.99999488	0.999985264	0.915476587
CDK5	0.999996191	0.946274461	0.926552506	0.99999488	0.999985264	0.915476587
LOC107132032	0.999996191	0.999992444	0.956643068	0.99999488	0.999985264	0.915476587
SMDT1	0.999996191	0.938597964	0.956565728	0.99999488	0.999985264	0.915574092
ZSWIM1	0.999996191	0.943802122	0.969880768	0.99999488	0.999985264	0.915574092
TIPRL	0.999996191	0.974812121	0.998936876	0.99999488	0.999985264	0.915574092
EIF2AK3	0.999996191	0.95697765	0.911131131	0.99999488	0.999985264	0.91564201
LOC112449075	0.999996191	0.999347049	0.930127869	0.99999488	0.999985264	0.916014896
LOC101907943	0.999996191	0.978510121	0.902527616	0.99999488	0.999985264	0.916021343
RPS27	0.999996191	0.957700659	0.920629231	0.99999488	0.999985264	0.916064976
COX17	0.999996191	0.938520118	0.95106368	0.99999488	0.999985264	0.916064976
BPHL	0.999996191	0.947212907	0.920629231	0.99999488	0.999985264	0.916081555
LOC112445193	0.999996191	0.942353856	0.94634922	0.99999488	0.999985264	0.916081555
FLVCR1	0.999996191	0.999992444	0.923604668	0.99999488	0.999985264	0.916338306
LOC101905167	0.999996191	0.9582784	0.999558179	0.99999488	0.999985264	0.916627874
GNPDA2	0.999996191	0.992977597	0.912014666	0.99999488	0.999985264	0.917028736
LOC782101	0.999996191	0.999992444	0.913211244	0.99999488	0.999985264	0.917028736

SORL1	0.999996191	0.940362847	0.925520255	0.99999488	0.999985264	0.917028736
IPO11	0.999996191	0.974812121	0.944932814	0.99999488	0.999985264	0.917028736
ARHGEF4	0.999996191	0.985161531	0.907484111	0.99999488	0.999985264	0.917056369
ACO2	0.999996191	0.916036681	0.911636823	0.99999488	0.999985264	0.918145254
FANCD2	0.999996191	0.957700659	0.926552506	0.99999488	0.999985264	0.918145254
SERTAD2	0.999996191	0.972660432	0.926552506	0.99999488	0.999985264	0.918145254
LOC104969028	0.999996191	0.999347049	0.893044539	0.99999488	0.999985264	0.918256134
MR1	0.999996191	0.922700337	0.895962167	0.99999488	0.999985264	0.918256134
DNMT3B	0.999996191	0.937511653	0.981039752	0.99999488	0.999985264	0.918256134
CDC42EP4	0.999996191	0.992977597	0.884320747	0.99999488	0.999985264	0.918343194
CYGB	0.999996191	0.917603394	0.921646762	0.99999488	0.999985264	0.918343194
RINT1	0.999996191	0.978089982	0.934501347	0.99999488	0.999985264	0.918343194
CDK17	0.999996191	0.978089982	0.944085862	0.99999488	0.999985264	0.918343194
BMI1	0.999996191	0.970426668	0.957541703	0.99999488	0.999985264	0.918356615
LTB4R	0.999996191	0.9582784	0.884320747	0.99999488	0.999985264	0.918400465
DHRS3	0.999996191	0.902408393	0.888545123	0.99999488	0.999985264	0.918400465
LOC101907606	0.999996191	0.915367594	0.888545123	0.99999488	0.999985264	0.918400465
S100A16	0.999996191	0.963737928	0.898248457	0.99999488	0.999985264	0.918400465
LACC1	0.999996191	0.999992444	0.907484111	0.99999488	0.999985264	0.918400465
FANCA	0.999996191	0.957700659	0.915215321	0.99999488	0.999985264	0.918400465
CEP104	0.999996191	0.938597964	0.916718376	0.99999488	0.999985264	0.918400465
COQ10A	0.999996191	0.957700659	0.923604668	0.99999488	0.999985264	0.918400465
TMEM147	0.999996191	0.943802122	0.924866532	0.99999488	0.999985264	0.918400465
CCDC9B	0.999996191	0.962968114	0.926552506	0.99999488	0.999985264	0.918400465
PPIC	0.999996191	0.913341599	0.931901017	0.99999488	0.999985264	0.918400465
IFITM3	0.999996191	0.979263532	0.939478907	0.99999488	0.999985264	0.918400465
MYO7A	0.999996191	0.980496345	0.946479014	0.99999488	0.999985264	0.918400465
LOC107133209	0.999996191	0.984068885	0.954823586	0.99999488	0.999985264	0.918400465
FABP5	0.999996191	0.985406717	0.95955996	0.99999488	0.999985264	0.918400465
HIST1H2AK	0.999996191	0.957700659	0.964920281	0.99999488	0.999985264	0.918400465
CDON	0.999996191	0.918648578	0.973303821	0.99999488	0.999985264	0.918400465
PRDX5	0.999996191	0.979263532	0.973406453	0.99999488	0.999985264	0.918400465
CNBP	0.999996191	0.921934673	0.998936876	0.99999488	0.999985264	0.918400465
THAP12	0.999996191	0.958579042	0.998936876	0.99999488	0.999985264	0.918400465
CD59	0.999996191	0.938597964	0.999558179	0.99999488	0.999985264	0.918400465
SLIT3	0.999996191	0.946274461	0.907484111	0.99999488	0.999985264	0.918417922
NR2F1	0.999996191	0.948421431	0.998936876	0.99999488	0.999985264	0.918417922
LOC107133343	0.999996191	0.938597964	0.999558179	0.99999488	0.999985264	0.918417922
C25H16orf91	0.999996191	0.999992444	0.944085862	0.99999488	0.999985264	0.918641886

QPRT	0.999996191	0.92976013	0.908181052	0.99999488	0.999985264	0.919441558
SCAF4	0.999996191	0.943454882	0.913194357	0.99999488	0.999985264	0.919441558
LOC107131498	0.999996191	0.972895283	0.900148803	0.99999488	0.999985264	0.919530287
IER5L	0.999996191	0.959733269	0.926552506	0.99999488	0.999985264	0.919699055
ARHGAP29	0.999996191	0.978426416	0.886192181	0.99999488	0.999985264	0.919755394
ACAT1	0.999996191	0.922102474	0.903957821	0.99999488	0.999985264	0.919755394
ACHE	0.999996191	0.941495217	0.908572237	0.99999488	0.999985264	0.919755394
TMA7	0.999996191	0.993849887	0.91207077	0.99999488	0.999985264	0.919755394
TRMT12	0.999996191	0.895685432	0.912247957	0.99999488	0.999985264	0.919755394
SATB2	0.999996191	0.991574265	0.916975216	0.99999488	0.999985264	0.919755394
PAM	0.999996191	0.992977597	0.925520255	0.99999488	0.999985264	0.919755394
LOC101907348	0.999996191	0.92475149	0.925745065	0.99999488	0.999985264	0.919755394
PBK	0.999996191	0.884739596	0.930678591	0.99999488	0.999985264	0.919755394
CBWD2	0.999996191	0.999992444	0.934694748	0.99999488	0.999985264	0.919755394
PITPNC1	0.999996191	0.999992444	0.939478907	0.99999488	0.999985264	0.919755394
CDK10	0.999996191	0.977354623	0.944871472	0.99999488	0.999985264	0.919755394
ARHGAP44	0.999996191	0.999992444	0.94634922	0.99999488	0.999985264	0.919755394
COQ7	0.999996191	0.987516216	0.946446014	0.99999488	0.999985264	0.919755394
LENG1	0.999996191	0.992977597	0.95230212	0.99999488	0.999985264	0.919755394
LOC533597	0.999996191	0.946480304	0.954872702	0.99999488	0.999985264	0.919755394
RAP2C	0.999996191	0.999992444	0.957541703	0.99999488	0.999985264	0.919755394
DOCK6	0.999996191	0.895304124	0.958627535	0.99999488	0.999985264	0.919755394
HMCES	0.999996191	0.991488652	0.962346588	0.99999488	0.999985264	0.919755394
CPLANE1	0.999996191	0.9582784	0.964152013	0.99999488	0.999985264	0.919755394
SLC38A5	0.999996191	0.943802122	0.964920281	0.99999488	0.999985264	0.919755394
DHX38	0.999996191	0.938597964	0.969880768	0.99999488	0.999985264	0.919755394
FKBP4	0.999996191	0.948037796	0.978389389	0.99999488	0.999985264	0.919755394
RHOV	0.999996191	0.981298804	0.980132104	0.99999488	0.999985264	0.919755394
LOC104969192	0.999996191	0.967374613	0.981715036	0.99999488	0.999985264	0.919755394
RN7SL1	0.999996191	0.921934673	0.996348351	0.99999488	0.999985264	0.919755394
WISP2	0.999996191	0.938777693	0.998816213	0.99999488	0.999985264	0.919755394
ADGB	0.999996191	0.978089982	0.998936876	0.99999488	0.999985264	0.919755394
NDUFAF2	0.999996191	0.93570298	0.999558179	0.99999488	0.999985264	0.919755394
GNAI1	0.999996191	0.993316598	0.882737471	0.99999488	0.999985264	0.919758204
HSPB7	0.999996191	0.999992444	0.884320747	0.99999488	0.999985264	0.919758204
RMND1	0.999996191	0.926818306	0.885948497	0.99999488	0.999985264	0.919758204
LOC112443139	0.999996191	0.883624935	0.891081265	0.99999488	0.999985264	0.919758204
NSUN6	0.999996191	0.985406717	0.899576888	0.99999488	0.999985264	0.919758204
KIAA1324	0.999996191	0.999992444	0.908181052	0.99999488	0.999985264	0.919758204

EGFLAM	0.999996191	0.9582784	0.911018006	0.99999488	0.999985264	0.919758204
USP48	0.999996191	0.969314233	0.913211244	0.99999488	0.999985264	0.919758204
THG1L	0.999996191	0.999992444	0.914092538	0.99999488	0.999985264	0.919758204
LOC101908046	0.999996191	0.999992444	0.915215321	0.99999488	0.999985264	0.919758204
LOC104976344	0.999996191	0.991365955	0.920154807	0.99999488	0.999985264	0.919758204
RTRAF	0.999996191	0.938597964	0.923604668	0.99999488	0.999985264	0.919758204
EHD3	0.999996191	0.925580392	0.925745065	0.99999488	0.999985264	0.919758204
LOC112444616	0.999996191	0.949285285	0.930678591	0.99999488	0.999985264	0.919758204
NDUFS7	0.999996191	0.95697765	0.931507181	0.99999488	0.999985264	0.919758204
HNRNPLL	0.999996191	0.943802122	0.93198623	0.99999488	0.999985264	0.919758204
LEMD3	0.999996191	0.976717114	0.935094475	0.99999488	0.999985264	0.919758204
CAMSAP3	0.999996191	0.999992444	0.935094475	0.99999488	0.999985264	0.919758204
SLC50A1	0.999996191	0.999992444	0.935136095	0.99999488	0.999985264	0.919758204
NUP160	0.999996191	0.980496345	0.938203102	0.99999488	0.999985264	0.919758204
ZSCAN23	0.999996191	0.985406717	0.938203102	0.99999488	0.999985264	0.919758204
COL23A1	0.999996191	0.946274461	0.939714392	0.99999488	0.999985264	0.919758204
PRORSD1	0.999996191	0.958098081	0.941118576	0.99999488	0.999985264	0.919758204
ATP5PO	0.999996191	0.92817225	0.941336964	0.99999488	0.999985264	0.919758204
RXFP3	0.999996191	0.936273761	0.941336964	0.99999488	0.999985264	0.919758204
LOC101907294	0.999996191	0.999992444	0.944085862	0.99999488	0.999985264	0.919758204
MITD1	0.999996191	0.943802122	0.951680496	0.99999488	0.999985264	0.919758204
LOC112449086	0.999996191	0.946274461	0.952386544	0.99999488	0.999985264	0.919758204
ANAPC15	0.999996191	0.994754253	0.95429596	0.99999488	0.999985264	0.919758204
RASL11B	0.999996191	0.928759674	0.977141934	0.99999488	0.999985264	0.919758204
AR	0.999996191	0.883624935	0.98286145	0.99999488	0.999985264	0.919758204
LOC112445939	0.999996191	0.921550129	0.985966356	0.99999488	0.999985264	0.919758204
E2F7	0.999996191	0.941495217	0.991285432	0.99999488	0.999985264	0.919758204
FUK	0.999996191	0.958098081	0.994879215	0.99999488	0.999985264	0.919758204
CFL2	0.999996191	0.919884594	0.995045982	0.99999488	0.999985264	0.919758204
LOC787858	0.999996191	0.927997582	0.995045982	0.99999488	0.999985264	0.919758204
SAYSD1	0.999996191	0.940762397	0.926552506	0.99999488	0.999985264	0.919792225
C10H14orf119	0.999996191	0.999992444	0.938203102	0.99999488	0.999985264	0.919792225
APOD	0.999996191	0.999992444	0.942686702	0.99999488	0.999985264	0.919792225
DNAJC7	0.999996191	0.993316598	0.95106368	0.99999488	0.999985264	0.919792225
IRF2BP1	0.999996191	0.978004343	0.923604668	0.99999488	0.999985264	0.919903716
CEP70	0.999996191	0.941876954	0.891893039	0.99999488	0.999985264	0.920531105
IL18	0.999996191	0.913160799	0.910426741	0.99999488	0.999985264	0.920531105
LOC614141	0.999996191	0.9582784	0.914827591	0.99999488	0.999985264	0.920531105
LOC112442657	0.999996191	0.990248517	0.917191523	0.99999488	0.999985264	0.920531105

CYP27B1	0.999996191	0.999992444	0.926552506	0.99999488	0.999985264	0.920531105
NID1	0.999996191	0.999992444	0.926552506	0.99999488	0.999985264	0.920531105
LOC101908759	0.999996191	0.991488652	0.927600835	0.99999488	0.999985264	0.920531105
NMRK1	0.999996191	0.999992444	0.93036537	0.99999488	0.999985264	0.920531105
TBC1D13	0.999996191	0.896507811	0.933447488	0.99999488	0.999985264	0.920531105
LOC112443140	0.999996191	0.974079639	0.935726137	0.99999488	0.999985264	0.920531105
PWWP2A	0.999996191	0.998011664	0.938203102	0.99999488	0.999985264	0.920531105
LOC112449358	0.999996191	0.999992444	0.950829165	0.99999488	0.999985264	0.920531105
LOC101902809	0.999996191	0.943802122	0.951822586	0.99999488	0.999985264	0.920531105
LOC112443015	0.999996191	0.945306277	0.956565728	0.99999488	0.999985264	0.920531105
NDUFB4	0.999996191	0.948700494	0.961024709	0.99999488	0.999985264	0.920531105
TGM2	0.999996191	0.998131745	0.973406453	0.99999488	0.999985264	0.920531105
PPP1R42	0.999996191	0.972764622	0.986180419	0.99999488	0.999985264	0.920531105
EHMT2	0.999996191	0.935060764	0.993594894	0.99999488	0.999985264	0.920531105
MIA	0.999996191	0.999992444	0.993594894	0.99999488	0.999985264	0.920531105
LOC100847122	0.999996191	0.921759937	0.999558179	0.99999488	0.999985264	0.920531105
SETD9	0.999996191	0.95830623	0.999558179	0.99999488	0.999985264	0.920531105
AP3S1	0.999996191	0.999992444	0.99035966	0.99999488	0.999985264	0.920650439
GPHN	0.999996191	0.999992444	0.945678616	0.99999488	0.999985264	0.920805856
ZSCAN12	0.999996191	0.976717114	0.884320747	0.99999488	0.999985264	0.921008981
MRPS9	0.999996191	0.914544898	0.900351064	0.99999488	0.999985264	0.921008981
LOC535280	0.999996191	0.999992444	0.930678591	0.99999488	0.999985264	0.921150136
MEIS2	0.999996191	0.926675185	0.946479014	0.99999488	0.999985264	0.921308173
LOC616948	0.999996191	0.893642384	0.90323157	0.99999488	0.999985264	0.921416283
CPXM1	0.999996191	0.991365955	0.907381775	0.99999488	0.999985264	0.921416283
CHMP3	0.999996191	0.938597964	0.988922732	0.99999488	0.999985264	0.921416283
TTC13	0.999996191	0.985406717	0.920629231	0.99999488	0.999985264	0.921760865
GADD45GIP1	0.999996191	0.921661251	0.892664791	0.99999488	0.999985264	0.921771959
LOC614129	0.999996191	0.966812935	0.913211244	0.99999488	0.999985264	0.921771959
ZBTB44	0.999996191	0.999992444	0.926552506	0.99999488	0.999985264	0.921771959
GZF1	0.999996191	0.941495217	0.930678591	0.99999488	0.999985264	0.921771959
ACADS	0.999996191	0.941495217	0.938905004	0.99999488	0.999985264	0.921771959
CHMP4A	0.999996191	0.925580392	0.946479014	0.99999488	0.999985264	0.921771959
TTK	0.999996191	0.913160799	0.951099739	0.99999488	0.999985264	0.921771959
FAM186B	0.999996191	0.999992444	0.952386544	0.99999488	0.999985264	0.921771959
NRADD	0.999996191	0.893642384	0.964908146	0.99999488	0.999985264	0.921991933
RPL10A	0.999996191	0.979263532	0.898125038	0.99999488	0.999985264	0.922063125
SMIM4	0.999996191	0.983883272	0.904959653	0.99999488	0.999985264	0.922063125
MDH1B	0.999996191	0.999992444	0.907724252	0.99999488	0.999985264	0.922063125

IL17RE	0.999996191	0.992977597	0.921096024	0.99999488	0.999985264	0.922063125
EMSY	0.999996191	0.991488652	0.925520255	0.99999488	0.999985264	0.922063125
ENTPD6	0.999996191	0.935742738	0.926552506	0.99999488	0.999985264	0.922063125
UBE2QL1	0.999996191	0.943802122	0.949038056	0.99999488	0.999985264	0.922403963
PARS2	0.999996191	0.886801652	0.907484111	0.99999488	0.999985264	0.923087984
NAPA	0.999996191	0.942353856	0.890592965	0.99999488	0.999985264	0.923146498
HSD3B7	0.999996191	0.982289317	0.892943118	0.99999488	0.999985264	0.923146498
PLSCR4	0.999996191	0.999992444	0.907484111	0.99999488	0.999985264	0.923146498
PYCR1	0.999996191	0.936273761	0.921065686	0.99999488	0.999985264	0.923146498
ZMIZ1	0.999996191	0.943036463	0.937874396	0.99999488	0.999985264	0.923146498
MIA3	0.999996191	0.936657629	0.946479014	0.99999488	0.999985264	0.923146498
RASL11A	0.999996191	0.896575712	0.999558179	0.99999488	0.999985264	0.923146498
LOC112441718	0.999996191	0.999992444	0.871958811	0.99999488	0.999985264	0.923360345
RAB8B	0.999996191	0.967520941	0.879481883	0.99999488	0.999985264	0.923360345
ITSN1	0.999996191	0.905690924	0.886962419	0.99999488	0.999985264	0.923360345
UNC119	0.999996191	0.958098081	0.892574927	0.99999488	0.999985264	0.923360345
COMMD6	0.999996191	0.94984369	0.900148803	0.99999488	0.999985264	0.923360345
ZNF569	0.999996191	0.999992444	0.906524923	0.99999488	0.999985264	0.923360345
ARL2	0.999996191	0.998131745	0.912247957	0.99999488	0.999985264	0.923360345
HDAC1	0.999996191	0.947015413	0.923604668	0.99999488	0.999985264	0.923360345
LOC783730	0.999996191	0.984068885	0.923604668	0.99999488	0.999985264	0.923360345
ESF1	0.999996191	0.985406717	0.924447826	0.99999488	0.999985264	0.923360345
STK25	0.999996191	0.92239337	0.925745065	0.99999488	0.999985264	0.923360345
NDUFAF7	0.999996191	0.940459751	0.926552506	0.99999488	0.999985264	0.923360345
AMMECR1L	0.999996191	0.941495217	0.926552506	0.99999488	0.999985264	0.923360345
MARK4	0.999996191	0.975951808	0.926552506	0.99999488	0.999985264	0.923360345
LOC104972290	0.999996191	0.938597964	0.927022752	0.99999488	0.999985264	0.923360345
DCAF8	0.999996191	0.921934673	0.927600835	0.99999488	0.999985264	0.923360345
LRP1	0.999996191	0.967276215	0.927600835	0.99999488	0.999985264	0.923360345
LOC101906008	0.999996191	0.934142668	0.930678591	0.99999488	0.999985264	0.923360345
LIMCH1	0.999996191	0.949285285	0.931907741	0.99999488	0.999985264	0.923360345
LOC107131573	0.999996191	0.999992444	0.933791093	0.99999488	0.999985264	0.923360345
LOC100295347	0.999996191	0.916036681	0.935094475	0.99999488	0.999985264	0.923360345
METTL9	0.999996191	0.919884594	0.946479014	0.99999488	0.999985264	0.923360345
STAT1	0.999996191	0.921550129	0.946479014	0.99999488	0.999985264	0.923360345
ZNF93	0.999996191	0.999992444	0.946479014	0.99999488	0.999985264	0.923360345
IGSF3	0.999996191	0.999992444	0.946832224	0.99999488	0.999985264	0.923360345
KIAA2013	0.999996191	0.934871569	0.95106368	0.99999488	0.999985264	0.923360345
HSP90B1	0.999996191	0.9527938	0.95106368	0.99999488	0.999985264	0.923360345

TAX1BP3	0.999996191	0.999992444	0.95106368	0.99999488	0.999985264	0.923360345
SYF2	0.999996191	0.914723992	0.956565728	0.99999488	0.999985264	0.923360345
CC2D1A	0.999996191	0.937231686	0.957541703	0.99999488	0.999985264	0.923360345
PDE1C	0.999996191	0.999992444	0.957541703	0.99999488	0.999985264	0.923360345
LOC112443502	0.999996191	0.991574265	0.960133832	0.99999488	0.999985264	0.923360345
LOC101907713	0.999996191	0.999992444	0.961850944	0.99999488	0.999985264	0.923360345
SLC25A36	0.999996191	0.999992444	0.962777771	0.99999488	0.999985264	0.923360345
GDF1	0.999996191	0.941495217	0.96870883	0.99999488	0.999985264	0.923360345
LOC104973105	0.999996191	0.919209432	0.971838819	0.99999488	0.999985264	0.923360345
ZDHHC4	0.999996191	0.941495217	0.973303821	0.99999488	0.999985264	0.923360345
MED28	0.999996191	0.951765021	0.973406453	0.99999488	0.999985264	0.923360345
SARAF	0.999996191	0.937231686	0.98480229	0.99999488	0.999985264	0.923360345
SAPCD2	0.999996191	0.921661251	0.986938092	0.99999488	0.999985264	0.923360345
NAPB	0.999996191	0.984714297	0.992080972	0.99999488	0.999985264	0.923360345
NRAP	0.999996191	0.95697765	0.992742113	0.99999488	0.999985264	0.923360345
LOC101902346	0.999996191	0.980496345	0.998816213	0.99999488	0.999985264	0.923360345
ETS2	0.999996191	0.903322573	0.999558179	0.99999488	0.999985264	0.923360345
POLD1	0.999996191	0.921549	0.999558179	0.99999488	0.999985264	0.923360345
CENPO	0.999996191	0.938587041	0.999558179	0.99999488	0.999985264	0.923360345
MAS1	0.999996191	0.955128586	0.999558179	0.99999488	0.999985264	0.923360345
CDH5	0.999996191	0.998131745	0.923604668	0.99999488	0.999985264	0.923465932
KIAA1468	0.999996191	0.986637746	0.912008683	0.99999488	0.999985264	0.923467533
PAXBP1	0.999996191	0.995532499	0.954620171	0.99999488	0.999985264	0.923544244
LOC101902812	0.999996191	0.9582784	0.981039752	0.99999488	0.999985264	0.923593784
DMAP1	0.999996191	0.946274461	0.887673	0.99999488	0.999985264	0.924201993
ST8SIA5	0.999996191	0.936273761	0.923604668	0.99999488	0.999985264	0.924201993
URGCP	0.999996191	0.999992444	0.935726137	0.99999488	0.999985264	0.924201993
CPNE3	0.999996191	0.935742738	0.938203102	0.99999488	0.999985264	0.924201993
TPRG1L	0.999996191	0.958098081	0.939501959	0.99999488	0.999985264	0.924201993
D2HGDH	0.999996191	0.9582784	0.965059328	0.99999488	0.999985264	0.924201993
LTC4S	0.999996191	0.935742738	0.968232195	0.99999488	0.999985264	0.924201993
ISCA1	0.999996191	0.948421431	0.985363931	0.99999488	0.999985264	0.924201993
C16H1orf116	0.999996191	0.999992444	0.912247957	0.99999488	0.999985264	0.924291134
DYNLRB1	0.999996191	0.943802122	0.944871472	0.99999488	0.999985264	0.924291134
NSRP1	0.999996191	0.999992444	0.902552409	0.99999488	0.999985264	0.924295622
GLRB	0.999996191	0.999992444	0.871557153	0.99999488	0.999985264	0.924461747
SHISA5	0.999996191	0.970426668	0.886962419	0.99999488	0.999985264	0.924461747
CD81	0.999996191	0.97993071	0.983419007	0.99999488	0.999985264	0.924461747
AZGP1	0.999996191	0.978089982	0.889744621	0.99999488	0.999985264	0.924984093

CSNK1D	0.999996191	0.978510121	0.908181052	0.99999488	0.999985264	0.925023825
IFITM2	0.999996191	0.89695121	0.926552506	0.99999488	0.999985264	0.925023825
MOSPD2	0.999996191	0.943802122	0.907381775	0.99999488	0.999985264	0.925146169
BOK	0.999996191	0.983640273	0.927600835	0.99999488	0.999985264	0.925146169
ANKRD33	0.999996191	0.991488652	0.926552506	0.99999488	0.999985264	0.925150883
RPL27A	0.999996191	0.925580392	0.878210077	0.99999488	0.999985264	0.925260365
HEATR5A	0.999996191	0.958967142	0.921262217	0.99999488	0.999985264	0.925260365
RSBN1	0.999996191	0.978089982	0.923089792	0.99999488	0.999985264	0.925260365
MDFIC	0.999996191	0.95697765	0.926552506	0.99999488	0.999985264	0.925260365
ATF7IP	0.999996191	0.999992444	0.926552506	0.99999488	0.999985264	0.925260365
LSM14A	0.999996191	0.999992444	0.93198623	0.99999488	0.999985264	0.925260365
SLC35A1	0.999996191	0.986445172	0.935094475	0.99999488	0.999985264	0.925260365
RBM43	0.999996191	0.956310879	0.938203102	0.99999488	0.999985264	0.925260365
BTG1	0.999996191	0.948421431	0.939147816	0.99999488	0.999985264	0.925260365
WDR5	0.999996191	0.934886531	0.946338395	0.99999488	0.999985264	0.925260365
LOC788724	0.999996191	0.934142668	0.95106368	0.99999488	0.999985264	0.925260365
GRID1	0.999996191	0.986637746	0.95230212	0.99999488	0.999985264	0.925260365
TRIM11	0.999996191	0.943802122	0.963068909	0.99999488	0.999985264	0.925260365
CEP78	0.999996191	0.938017364	0.973303821	0.99999488	0.999985264	0.925260365
NELFB	0.999996191	0.946274461	0.981039752	0.99999488	0.999985264	0.925260365
LOC112446375	0.999996191	0.970426668	0.991285432	0.99999488	0.999985264	0.925260365
POLQ	0.999996191	0.935274301	0.999558179	0.99999488	0.999985264	0.925260365
RPL23	0.999996191	0.937231686	0.898815524	0.99999488	0.999985264	0.925558146
HDAC9	0.999996191	0.999992444	0.908181052	0.99999488	0.999985264	0.925558146
GPRASP1	0.999996191	0.978510121	0.926552506	0.99999488	0.999985264	0.925558146
LOC112446699	0.999996191	0.921759937	0.958627535	0.99999488	0.999985264	0.925558146
LRRN1	0.999996191	0.982911005	0.973406453	0.99999488	0.999985264	0.925558146
LOC112444653	0.999996191	0.937231686	0.990780762	0.99999488	0.999985264	0.925558146
ARHGEF1	0.999996191	0.944868298	0.992742113	0.99999488	0.999985264	0.925558146
ETFBKMT	0.999996191	0.987860004	0.995045982	0.99999488	0.999985264	0.925558146
LOC100847269	0.999996191	0.930955919	0.926932508	0.99999488	0.999985264	0.925740594
SQLE	0.999996191	0.967276215	0.926552506	0.99999488	0.999985264	0.925789352
MANF	0.999996191	0.999992444	0.935844161	0.99999488	0.999985264	0.925789352
ATPAF1	0.999996191	0.916036681	0.926552506	0.99999488	0.999985264	0.926027375
NEURL3	0.999996191	0.937065208	0.996391386	0.99999488	0.999985264	0.926027375
TRERF1	0.999996191	0.885718559	0.886962419	0.99999488	0.999985264	0.926326154
PCM1	0.999996191	0.993849887	0.907484111	0.99999488	0.999985264	0.926326154
OSBP2	0.999996191	0.998131745	0.912008683	0.99999488	0.999985264	0.926326154
ERGIC3	0.999996191	0.999992444	0.917154286	0.99999488	0.999985264	0.926326154

SAMD8	0.999996191	0.994481342	0.929945671	0.99999488	0.999985264	0.926326154
ZNRF1	0.999996191	0.944181275	0.938203102	0.99999488	0.999985264	0.926326154
SH3BP5	0.999996191	0.95697765	0.938203102	0.99999488	0.999985264	0.926326154
LOC107133095	0.999996191	0.999992444	0.938203102	0.99999488	0.999985264	0.926326154
NDUFB2	0.999996191	0.974812121	0.938508265	0.99999488	0.999985264	0.926326154
LOC107132967	0.999996191	0.969314233	0.938905004	0.99999488	0.999985264	0.926326154
SLC44A2	0.999996191	0.991488652	0.938911935	0.99999488	0.999985264	0.926326154
DESI2	0.999996191	0.981520287	0.940815039	0.99999488	0.999985264	0.926326154
RAB2A	0.999996191	0.974812121	0.973406453	0.99999488	0.999985264	0.926326154
LOC101908149	0.999996191	0.910133637	0.995045982	0.99999488	0.999985264	0.926326154
TSNARE1	0.999996191	0.916036681	0.999558179	0.99999488	0.999985264	0.926326154
LOC112446036	0.999996191	0.999992444	0.908572237	0.99999488	0.999985264	0.926480606
TNFRSF11A	0.999996191	0.917537324	0.908700926	0.99999488	0.999985264	0.926480606
RCAN1	0.999996191	0.922995823	0.920629231	0.99999488	0.999985264	0.926480606
CITED1	0.999996191	0.946274461	0.922400936	0.99999488	0.999985264	0.926480606
SMIM37	0.999996191	0.999992444	0.924397528	0.99999488	0.999985264	0.926480606
GMEB2	0.999996191	0.998578163	0.926552506	0.99999488	0.999985264	0.926480606
GCH1	0.999996191	0.992117536	0.940815039	0.99999488	0.999985264	0.926480606
G0S2	0.999996191	0.991488652	0.944085862	0.99999488	0.999985264	0.926480606
COL13A1	0.999996191	0.905019423	0.969036336	0.99999488	0.999985264	0.926480606
CCT4	0.999996191	0.938597964	0.969880768	0.99999488	0.999985264	0.926480606
NF1	0.999996191	0.938597964	0.971111634	0.99999488	0.999985264	0.926480606
SMG8	0.999996191	0.9582784	0.981622418	0.99999488	0.999985264	0.926480606
LEPROTL1	0.999996191	0.9582784	0.998936876	0.99999488	0.999985264	0.926480606
LOC104971852	0.999996191	0.943802122	0.999558179	0.99999488	0.999985264	0.926480606
DST	0.999996191	0.938597964	0.926552506	0.99999488	0.999985264	0.92658818
NFU1	0.999996191	0.958967142	0.938203102	0.99999488	0.999985264	0.92658818
SEPT4	0.999996191	0.978510121	0.938905004	0.99999488	0.999985264	0.92658818
SEMA3B	0.999996191	0.927997582	0.999558179	0.99999488	0.999985264	0.92658818
TMEM161A	0.999996191	0.935742738	0.899576888	0.99999488	0.999985264	0.926596859
CRYZL1	0.999996191	0.978089982	0.923902957	0.99999488	0.999985264	0.926596859
VPS13A	0.999996191	0.981298804	0.933791093	0.99999488	0.999985264	0.926596859
DLG4	0.999996191	0.980496345	0.946479014	0.99999488	0.999985264	0.926596859
LOC112441566	0.999996191	0.917849199	0.900890709	0.99999488	0.999985264	0.926673043
CABP1	0.999996191	0.993540482	0.927600835	0.99999488	0.999985264	0.926673043
ABCC9	0.999996191	0.987262615	0.949038056	0.99999488	0.999985264	0.926673043
LOC112445029	0.999996191	0.950729918	0.988161083	0.99999488	0.999985264	0.926673043
UXS1	0.999996191	0.927997582	0.916975216	0.99999488	0.999985264	0.926849821
FBXL21	0.999996191	0.999992444	0.900890709	0.99999488	0.999985264	0.926885454

PNP	0.999996191	0.985406717	0.935094475	0.99999488	0.999985264	0.926885454
TBC1D19	0.999996191	0.999992444	0.94634922	0.99999488	0.999985264	0.926885454
GMPS	0.999996191	0.995021629	0.993594894	0.99999488	0.999985264	0.926944023
ZNF175	0.999996191	0.991488652	0.944031108	0.99999488	0.999985264	0.927472114
SLC17A9	0.999996191	0.893642384	0.971194521	0.99999488	0.999985264	0.927614625
LOC100847946	0.999996191	0.998962836	0.964582964	0.99999488	0.999985264	0.927656353
LRRC20	0.999996191	0.928525825	0.945678616	0.99999488	0.999985264	0.927915932
TSKS	0.999996191	0.957700659	0.926552506	0.99999488	0.999985264	0.927933967
HSBP1	0.999996191	0.974812121	0.926552506	0.99999488	0.999985264	0.927933967
GRIP1	0.999996191	0.93699217	0.869864092	0.99999488	0.999985264	0.927969856
MUM1	0.999996191	0.90116492	0.887626998	0.99999488	0.999985264	0.927969856
NAP1L5	0.999996191	0.9582784	0.890035877	0.99999488	0.999985264	0.927969856
RPA4	0.999996191	0.999992444	0.890248427	0.99999488	0.999985264	0.927969856
ZNF618	0.999996191	0.896996319	0.892943118	0.99999488	0.999985264	0.927969856
DLGAP1	0.999996191	0.946274461	0.907381775	0.99999488	0.999985264	0.927969856
TMEM192	0.999996191	0.999992444	0.908181052	0.99999488	0.999985264	0.927969856
RPL38	0.999996191	0.900867274	0.910426741	0.99999488	0.999985264	0.927969856
DDO	0.999996191	0.978089982	0.910426741	0.99999488	0.999985264	0.927969856
IGFBP3	0.999996191	0.941495217	0.911636823	0.99999488	0.999985264	0.927969856
KLRD1	0.999996191	0.928925667	0.916021503	0.99999488	0.999985264	0.927969856
LOC101907491	0.999996191	0.956531374	0.919618001	0.99999488	0.999985264	0.927969856
SRSF5	0.999996191	0.946274461	0.920154807	0.99999488	0.999985264	0.927969856
NODAL	0.999996191	0.938597964	0.920629231	0.99999488	0.999985264	0.927969856
C18H19orf33	0.999996191	0.999992444	0.920629231	0.99999488	0.999985264	0.927969856
JRKL	0.999996191	0.992977597	0.925604199	0.99999488	0.999985264	0.927969856
TPCN1	0.999996191	0.999992444	0.925745065	0.99999488	0.999985264	0.927969856
SLC37A3	0.999996191	0.925387304	0.926552506	0.99999488	0.999985264	0.927969856
DBNDD1	0.999996191	0.927997582	0.926552506	0.99999488	0.999985264	0.927969856
FAAP20	0.999996191	0.986637746	0.926552506	0.99999488	0.999985264	0.927969856
TPRKB	0.999996191	0.999992444	0.926552506	0.99999488	0.999985264	0.927969856
LOC104969981	0.999996191	0.9409629	0.927600835	0.99999488	0.999985264	0.927969856
ITM2C	0.999996191	0.986637746	0.927600835	0.99999488	0.999985264	0.927969856
TNFSF18	0.999996191	0.937231686	0.930678591	0.99999488	0.999985264	0.927969856
MACROD2	0.999996191	0.999347049	0.930678591	0.99999488	0.999985264	0.927969856
LOC101907615	0.999996191	0.992977597	0.935094475	0.99999488	0.999985264	0.927969856
RAMP3	0.999996191	0.999992444	0.935094475	0.99999488	0.999985264	0.927969856
LOC112443193	0.999996191	0.940618948	0.938203102	0.99999488	0.999985264	0.927969856
AP3S2	0.999996191	0.946480304	0.938911935	0.99999488	0.999985264	0.927969856
SLC29A3	0.999996191	0.964246761	0.938983799	0.99999488	0.999985264	0.927969856

LOC104975749	0.999996191	0.969534305	0.940815039	0.99999488	0.999985264	0.927969856
SUDS3	0.999996191	0.981298804	0.941782902	0.99999488	0.999985264	0.927969856
MED18	0.999996191	0.991365955	0.945280564	0.99999488	0.999985264	0.927969856
EHBP1	0.999996191	0.940884954	0.945678616	0.99999488	0.999985264	0.927969856
SDHA	0.999996191	0.948421431	0.946479014	0.99999488	0.999985264	0.927969856
CDC5L	0.999996191	0.999992444	0.948420301	0.99999488	0.999985264	0.927969856
LAMTOR4	0.999996191	0.978089982	0.949048582	0.99999488	0.999985264	0.927969856
NDP	0.999996191	0.974812121	0.95106368	0.99999488	0.999985264	0.927969856
LOC784841	0.999996191	0.980496345	0.95106368	0.99999488	0.999985264	0.927969856
DLGAP3	0.999996191	0.991488652	0.95106368	0.99999488	0.999985264	0.927969856
TAZ	0.999996191	0.938597964	0.95230212	0.99999488	0.999985264	0.927969856
PHLPP2	0.999996191	0.999992444	0.954823586	0.99999488	0.999985264	0.927969856
BANF1	0.999996191	0.978510121	0.95498522	0.99999488	0.999985264	0.927969856
LOC617692	0.999996191	0.991488652	0.956565728	0.99999488	0.999985264	0.927969856
LOC100847190	0.999996191	0.991488652	0.957286935	0.99999488	0.999985264	0.927969856
MAGOH	0.999996191	0.959773007	0.95758013	0.99999488	0.999985264	0.927969856
FAM98C	0.999996191	0.999992444	0.96000759	0.99999488	0.999985264	0.927969856
ZNF304	0.999996191	0.9582784	0.960133832	0.99999488	0.999985264	0.927969856
ZYX	0.999996191	0.999992444	0.963068909	0.99999488	0.999985264	0.927969856
ACTN3	0.999996191	0.985161531	0.964699599	0.99999488	0.999985264	0.927969856
LOC101904103	0.999996191	0.992977597	0.96684887	0.99999488	0.999985264	0.927969856
LRRC14	0.999996191	0.921549	0.973406453	0.99999488	0.999985264	0.927969856
SPIN2B	0.999996191	0.986637746	0.973406453	0.99999488	0.999985264	0.927969856
ETHE1	0.999996191	0.991574265	0.973406453	0.99999488	0.999985264	0.927969856
CHST12	0.999996191	0.999992444	0.973406453	0.99999488	0.999985264	0.927969856
COPS9	0.999996191	0.9582784	0.980355834	0.99999488	0.999985264	0.927969856
LGI4	0.999996191	0.9093283	0.980859619	0.99999488	0.999985264	0.927969856
ZDHHC16	0.999996191	0.999992444	0.981218677	0.99999488	0.999985264	0.927969856
ANGPTL2	0.999996191	0.951765021	0.981666445	0.99999488	0.999985264	0.927969856
SLC2A13	0.999996191	0.927447588	0.985966356	0.99999488	0.999985264	0.927969856
LOC112441455	0.999996191	0.985161531	0.988922732	0.99999488	0.999985264	0.927969856
MAGT1	0.999996191	0.961479426	0.993594894	0.99999488	0.999985264	0.927969856
SLC25A41	0.999996191	0.941495217	0.994601277	0.99999488	0.999985264	0.927969856
ATG2A	0.999996191	0.998131745	0.996348351	0.99999488	0.999985264	0.927969856
LOC512149	0.999996191	0.952346363	0.996391386	0.99999488	0.999985264	0.927969856
LOC101903913	0.999996191	0.999992444	0.997859293	0.99999488	0.999985264	0.927969856
CMKLR1	0.999996191	0.934515915	0.998235205	0.99999488	0.999985264	0.927969856
G3BP2	0.999996191	0.921759937	0.998936876	0.99999488	0.999985264	0.927969856
BAG4	0.999996191	0.9409629	0.999558179	0.99999488	0.999985264	0.927969856

ST6GAL2	0.999996191	0.943802122	0.927022752	0.99999488	0.999985264	0.928145377
THOC7	0.999996191	0.991365955	0.973303821	0.99999488	0.999985264	0.928145377
MAST2	0.999996191	0.9677397	0.926552506	0.99999488	0.999985264	0.928213338
UBR2	0.999996191	0.955266582	0.884298435	0.99999488	0.999985264	0.928634547
ACOT6	0.999996191	0.941495217	0.891552252	0.99999488	0.999985264	0.928634547
SMC3	0.999996191	0.991488652	0.894525066	0.99999488	0.999985264	0.928634547
RAB32	0.999996191	0.926955605	0.900148803	0.99999488	0.999985264	0.928634547
PPEF1	0.999996191	0.992977597	0.932284584	0.99999488	0.999985264	0.928634547
LOC107132883	0.999996191	0.992977597	0.945678616	0.99999488	0.999985264	0.928634547
PCBD1	0.999996191	0.99398773	0.94634922	0.99999488	0.999985264	0.928634547
FAM221A	0.999996191	0.999992444	0.94634922	0.99999488	0.999985264	0.928634547
GLIS2	0.999996191	0.898155217	0.947957719	0.99999488	0.999985264	0.928634547
IGSF9	0.999996191	0.992895985	0.95230212	0.99999488	0.999985264	0.928634547
DGCR2	0.999996191	0.94317872	0.956504686	0.99999488	0.999985264	0.928634547
RBP2	0.999996191	0.999992444	0.980859619	0.99999488	0.999985264	0.928634547
UNG	0.999996191	0.936392293	0.981388276	0.99999488	0.999985264	0.928634547
LOC505099	0.999996191	0.992977597	0.984444908	0.99999488	0.999985264	0.928634547
PLOD3	0.999996191	0.938597964	0.985363931	0.99999488	0.999985264	0.928634547
YPEL5	0.999996191	0.987516216	0.985501168	0.99999488	0.999985264	0.928634547
IFT80	0.999996191	0.90387438	0.988161083	0.99999488	0.999985264	0.928634547
PTGDS	0.999996191	0.916036681	0.899576888	0.99999488	0.999985264	0.928814001
CIB1	0.999996191	0.999992444	0.958304511	0.99999488	0.999985264	0.928814001
N6AMT1	0.999996191	0.972764622	0.944085862	0.99999488	0.999985264	0.928928703
U2AF1L4	0.999996191	0.961479426	0.995045982	0.99999488	0.999985264	0.928928703
FAM89B	0.999996191	0.999992444	0.946479014	0.99999488	0.999985264	0.928979564
SATB1	0.999996191	0.926655515	0.923604668	0.99999488	0.999985264	0.929111441
LOC101907487	0.999996191	0.999992444	0.95230212	0.99999488	0.999985264	0.929322121
PPP1R3E	0.999996191	0.938017364	0.999558179	0.99999488	0.999985264	0.929322121
TCEANC	0.999996191	0.980496345	0.909070782	0.99999488	0.999985264	0.929458454
MYH9	0.999996191	0.979530318	0.914001994	0.99999488	0.999985264	0.929458454
RPN2	0.999996191	0.992357549	0.918170048	0.99999488	0.999985264	0.929458454
ADRB2	0.999996191	0.993316598	0.927391044	0.99999488	0.999985264	0.929458454
EPHX2	0.999996191	0.94254418	0.927600835	0.99999488	0.999985264	0.929458454
MYL6B	0.999996191	0.938597964	0.932653752	0.99999488	0.999985264	0.929458454
LOC101909754	0.999996191	0.986637746	0.937542377	0.99999488	0.999985264	0.929458454
NLGN2	0.999996191	0.944868298	0.939147816	0.99999488	0.999985264	0.929458454
NHLRC3	0.999996191	0.993316598	0.939799459	0.99999488	0.999985264	0.929458454
SMIM11A	0.999996191	0.957700659	0.944085862	0.99999488	0.999985264	0.929458454
SLC7A1	0.999996191	0.999992444	0.959968577	0.99999488	0.999985264	0.929458454

TRIM9	0.999996191	0.943802122	0.980169282	0.99999488	0.999985264	0.929458454
AMER1	0.999996191	0.937231686	0.998936876	0.99999488	0.999985264	0.929458454
OSTM1	0.999996191	0.957700659	0.944993804	0.99999488	0.999985264	0.929515611
SLC19A3	0.999996191	0.991488652	0.954328478	0.99999488	0.999985264	0.929515611
YIPF4	0.999996191	0.999992444	0.921065686	0.99999488	0.999985264	0.929527103
SMCHD1	0.999996191	0.998131745	0.923604668	0.99999488	0.999985264	0.929527103
UBR1	0.999996191	0.999347049	0.923604668	0.99999488	0.999985264	0.929527103
PMVK	0.999996191	0.941495217	0.930678591	0.99999488	0.999985264	0.929527103
SLC26A7	0.999996191	0.981628252	0.935136095	0.99999488	0.999985264	0.929527103
PRRX2	0.999996191	0.938597964	0.938203102	0.99999488	0.999985264	0.929527103
LOC101905997	0.999996191	0.938597964	0.959575351	0.99999488	0.999985264	0.929527103
DSB	0.999996191	0.967919743	0.966991646	0.99999488	0.999985264	0.929527103
TANK	0.999996191	0.9582784	0.973406453	0.99999488	0.999985264	0.929527103
TRMT13	0.999996191	0.999992444	0.981039752	0.99999488	0.999985264	0.929527103
SPTAN1	0.999996191	0.940269477	0.938905004	0.99999488	0.999985264	0.929860062
LOC112442787	0.999996191	0.964246761	0.88687452	0.99999488	0.999985264	0.931163114
RFWD3	0.999996191	0.970426668	0.886962419	0.99999488	0.999985264	0.931163114
LOC101905779	0.999996191	0.882226341	0.888545123	0.99999488	0.999985264	0.931163114
GGT1	0.999996191	0.991488652	0.891081265	0.99999488	0.999985264	0.931163114
RPS14	0.999996191	0.998131745	0.892803866	0.99999488	0.999985264	0.931163114
BCS1L	0.999996191	0.913160799	0.898815524	0.99999488	0.999985264	0.931163114
LOC101904526	0.999996191	0.938597964	0.900148803	0.99999488	0.999985264	0.931163114
USF3	0.999996191	0.918883775	0.904522669	0.99999488	0.999985264	0.931163114
PAK1IP1	0.999996191	0.975677199	0.907484111	0.99999488	0.999985264	0.931163114
SLC39A10	0.999996191	0.941495217	0.909383349	0.99999488	0.999985264	0.931163114
BCLAF3	0.999996191	0.944481254	0.911018006	0.99999488	0.999985264	0.931163114
ATF7	0.999996191	0.999992444	0.911813157	0.99999488	0.999985264	0.931163114
DNAJC24	0.999996191	0.982981183	0.915445865	0.99999488	0.999985264	0.931163114
PKIA	0.999996191	0.998131745	0.919763144	0.99999488	0.999985264	0.931163114
IL15	0.999996191	0.935060764	0.920154807	0.99999488	0.999985264	0.931163114
FAM83H	0.999996191	0.938597964	0.920629231	0.99999488	0.999985264	0.931163114
MRPL40	0.999996191	0.991488652	0.921010865	0.99999488	0.999985264	0.931163114
YTHDC1	0.999996191	0.991488652	0.921065686	0.99999488	0.999985264	0.931163114
DKKL1	0.999996191	0.999347049	0.921065686	0.99999488	0.999985264	0.931163114
R3HCC1L	0.999996191	0.99873048	0.921646762	0.99999488	0.999985264	0.931163114
P2RX6	0.999996191	0.958098081	0.922400936	0.99999488	0.999985264	0.931163114
ICA1L	0.999996191	0.999992444	0.922400936	0.99999488	0.999985264	0.931163114
EXOC3L2	0.999996191	0.992977597	0.924447826	0.99999488	0.999985264	0.931163114
ZSCAN2	0.999996191	0.935060764	0.92487482	0.99999488	0.999985264	0.931163114

RAC3	0.999996191	0.90213106	0.925745065	0.99999488	0.999985264	0.931163114
WNT11	0.999996191	0.999992444	0.925745065	0.99999488	0.999985264	0.931163114
TMEM67	0.999996191	0.946274461	0.926552506	0.99999488	0.999985264	0.931163114
HYKK	0.999996191	0.949285285	0.926552506	0.99999488	0.999985264	0.931163114
LRIG1	0.999996191	0.952345011	0.926552506	0.99999488	0.999985264	0.931163114
CEP152	0.999996191	0.978510121	0.926552506	0.99999488	0.999985264	0.931163114
SESN2	0.999996191	0.999992444	0.926552506	0.99999488	0.999985264	0.931163114
BLVRB	0.999996191	0.921934673	0.927600835	0.99999488	0.999985264	0.931163114
PRPF18	0.999996191	0.901232581	0.929722337	0.99999488	0.999985264	0.931163114
PTDSS2	0.999996191	0.948225526	0.930678591	0.99999488	0.999985264	0.931163114
GHSR	0.999996191	0.94880978	0.930678591	0.99999488	0.999985264	0.931163114
TMEM214	0.999996191	0.967276215	0.930678591	0.99999488	0.999985264	0.931163114
LOC112448032	0.999996191	0.988850552	0.930678591	0.99999488	0.999985264	0.931163114
ERF	0.999996191	0.999347049	0.930678591	0.99999488	0.999985264	0.931163114
MPP2	0.999996191	0.972937948	0.933046493	0.99999488	0.999985264	0.931163114
LOC522610	0.999996191	0.97015318	0.934694748	0.99999488	0.999985264	0.931163114
CYBA	0.999996191	0.971819361	0.934694748	0.99999488	0.999985264	0.931163114
PYGO2	0.999996191	0.938476452	0.935726137	0.99999488	0.999985264	0.931163114
LOC100336897	0.999996191	0.893642384	0.938203102	0.99999488	0.999985264	0.931163114
ATP5F1C	0.999996191	0.941495217	0.938203102	0.99999488	0.999985264	0.931163114
DOCK7	0.999996191	0.999992444	0.938203102	0.99999488	0.999985264	0.931163114
RAB28	0.999996191	0.999992444	0.938203102	0.99999488	0.999985264	0.931163114
ZNF280D	0.999996191	0.999992444	0.938911935	0.99999488	0.999985264	0.931163114
CD44	0.999996191	0.999992444	0.939501959	0.99999488	0.999985264	0.931163114
DCTN5	0.999996191	0.915367594	0.940815039	0.99999488	0.999985264	0.931163114
RAD52	0.999996191	0.993316598	0.940815039	0.99999488	0.999985264	0.931163114
PIGX	0.999996191	0.935588259	0.941118576	0.99999488	0.999985264	0.931163114
HIBADH	0.999996191	0.950729918	0.945678616	0.99999488	0.999985264	0.931163114
LOC548613	0.999996191	0.999992444	0.945678616	0.99999488	0.999985264	0.931163114
KLHL4	0.999996191	0.991488652	0.945766581	0.99999488	0.999985264	0.931163114
FGF12	0.999996191	0.95697765	0.94634922	0.99999488	0.999985264	0.931163114
VKORC1	0.999996191	0.984366454	0.94634922	0.99999488	0.999985264	0.931163114
SLC7A2	0.999996191	0.992977597	0.94634922	0.99999488	0.999985264	0.931163114
TRMT10A	0.999996191	0.938597964	0.946479014	0.99999488	0.999985264	0.931163114
RNF144B	0.999996191	0.95697765	0.946479014	0.99999488	0.999985264	0.931163114
CSF2RA	0.999996191	0.974812121	0.946479014	0.99999488	0.999985264	0.931163114
COMMD4	0.999996191	0.999992444	0.946832224	0.99999488	0.999985264	0.931163114
KIF25	0.999996191	0.9582784	0.946900905	0.99999488	0.999985264	0.931163114
ATP6V1F	0.999996191	0.984068885	0.949038056	0.99999488	0.999985264	0.931163114

WDR54	0.999996191	0.90116492	0.95230212	0.99999488	0.999985264	0.931163114
SARS2	0.999996191	0.921934673	0.95230212	0.99999488	0.999985264	0.931163114
PRRG3	0.999996191	0.938017364	0.952547836	0.99999488	0.999985264	0.931163114
RYK	0.999996191	0.913160799	0.95401762	0.99999488	0.999985264	0.931163114
MEPCE	0.999996191	0.987559864	0.954620171	0.99999488	0.999985264	0.931163114
KLC1	0.999996191	0.961199335	0.954823586	0.99999488	0.999985264	0.931163114
RILPL1	0.999996191	0.961479426	0.954872702	0.99999488	0.999985264	0.931163114
COMTD1	0.999996191	0.978089982	0.956565728	0.99999488	0.999985264	0.931163114
MST1	0.999996191	0.992977597	0.957542358	0.99999488	0.999985264	0.931163114
SLC39A6	0.999996191	0.937231686	0.959396074	0.99999488	0.999985264	0.931163114
HS3ST3B1	0.999996191	0.992977597	0.962777771	0.99999488	0.999985264	0.931163114
EMC7	0.999996191	0.90116492	0.966991646	0.99999488	0.999985264	0.931163114
RBCK1	0.999996191	0.981298804	0.96752854	0.99999488	0.999985264	0.931163114
RIN1	0.999996191	0.93597782	0.968325332	0.99999488	0.999985264	0.931163114
ZNF572	0.999996191	0.999347049	0.968325332	0.99999488	0.999985264	0.931163114
KLC4	0.999996191	0.943802122	0.970903823	0.99999488	0.999985264	0.931163114
LDHC	0.999996191	0.980496345	0.973303821	0.99999488	0.999985264	0.931163114
ZFYVE27	0.999996191	0.978089982	0.973406453	0.99999488	0.999985264	0.931163114
LOC112442292	0.999996191	0.957700659	0.980660791	0.99999488	0.999985264	0.931163114
SFSWAP	0.999996191	0.946274461	0.9808801	0.99999488	0.999985264	0.931163114
LOC112446012	0.999996191	0.978089982	0.981622418	0.99999488	0.999985264	0.931163114
DCHS2	0.999996191	0.985406717	0.993585211	0.99999488	0.999985264	0.931163114
CEP170	0.999996191	0.917570301	0.997208738	0.99999488	0.999985264	0.931163114
CLTA	0.999996191	0.952346363	0.998208226	0.99999488	0.999985264	0.931163114
ABRAXAS2	0.999996191	0.94286486	0.998936876	0.99999488	0.999985264	0.931163114
LRP2BP	0.999996191	0.952346363	0.998936876	0.99999488	0.999985264	0.931163114
HSDL1	0.999996191	0.921264323	0.999558179	0.99999488	0.999985264	0.931163114
PURA	0.999996191	0.934142668	0.999558179	0.99999488	0.999985264	0.931163114
PRKAA2	0.999996191	0.943802122	0.999558179	0.99999488	0.999985264	0.931163114
PIGT	0.999996191	0.999992444	0.907484111	0.99999488	0.999985264	0.931270478
RASGEF1B	0.999996191	0.928759674	0.913211244	0.99999488	0.999985264	0.931270478
PANX2	0.999996191	0.999992444	0.925520255	0.99999488	0.999985264	0.931270478
H2AFZ	0.999996191	0.97587222	0.951822586	0.99999488	0.999985264	0.931471274
LOC101905510	0.999996191	0.893743321	0.99699426	0.99999488	0.999985264	0.931480862
MAP7	0.999996191	0.992977597	0.935094475	0.99999488	0.999985264	0.932008586
LOC104973485	0.999996191	0.985406717	0.97379223	0.99999488	0.999985264	0.932008586
USP37	0.999996191	0.969534305	0.956565728	0.99999488	0.999985264	0.93221452
LOC112444340	0.999996191	0.958967142	0.993594894	0.99999488	0.999985264	0.932425574
HIST3H2A	0.999996191	0.984068885	0.994601277	0.99999488	0.999985264	0.932425574

SMG5	0.999996191	0.943802122	0.886192181	0.99999488	0.999985264	0.932608891
PUDP	0.999996191	0.939104531	0.888215738	0.99999488	0.999985264	0.932608891
LOC112448155	0.999996191	0.938597964	0.892053976	0.99999488	0.999985264	0.932608891
SLITRK4	0.999996191	0.925580392	0.910426741	0.99999488	0.999985264	0.932608891
FAM133B	0.999996191	0.999992444	0.914001994	0.99999488	0.999985264	0.932608891
HIC1	0.999996191	0.991488652	0.921010865	0.99999488	0.999985264	0.932608891
LOC112442347	0.999996191	0.9582784	0.923953195	0.99999488	0.999985264	0.932608891
ADI1	0.999996191	0.95697765	0.926552506	0.99999488	0.999985264	0.932608891
LOC781439	0.999996191	0.978089982	0.929879425	0.99999488	0.999985264	0.932608891
LOC104974345	0.999996191	0.974812121	0.930678591	0.99999488	0.999985264	0.932608891
FKBP15	0.999996191	0.985161531	0.930678591	0.99999488	0.999985264	0.932608891
DCBLD1	0.999996191	0.97286093	0.93198623	0.99999488	0.999985264	0.932608891
DYSF	0.999996191	0.999992444	0.932901419	0.99999488	0.999985264	0.932608891
PHF20	0.999996191	0.90213106	0.937004034	0.99999488	0.999985264	0.932608891
CCDC51	0.999996191	0.907069508	0.938203102	0.99999488	0.999985264	0.932608891
NOP53	0.999996191	0.992977597	0.940667943	0.99999488	0.999985264	0.932608891
LOC100848339	0.999996191	0.974580033	0.945678616	0.99999488	0.999985264	0.932608891
LOC789694	0.999996191	0.97161271	0.946479014	0.99999488	0.999985264	0.932608891
APOPT1	0.999996191	0.992311836	0.946479014	0.99999488	0.999985264	0.932608891
SOCS5	0.999996191	0.983640273	0.951822586	0.99999488	0.999985264	0.932608891
RPAP3	0.999996191	0.999992444	0.954620171	0.99999488	0.999985264	0.932608891
TOMT	0.999996191	0.943802122	0.95477544	0.99999488	0.999985264	0.932608891
YWHAZ	0.999996191	0.957700659	0.955638134	0.99999488	0.999985264	0.932608891
PFDN5	0.999996191	0.986637746	0.957541703	0.99999488	0.999985264	0.932608891
NDUFA4	0.999996191	0.952639631	0.962142195	0.99999488	0.999985264	0.932608891
GRID2IP	0.999996191	0.999992444	0.962142195	0.99999488	0.999985264	0.932608891
SLC25A11	0.999996191	0.998131745	0.964152013	0.99999488	0.999985264	0.932608891
RUFY2	0.999996191	0.999992444	0.971111634	0.99999488	0.999985264	0.932608891
GCNT1	0.999996191	0.917849199	0.973406453	0.99999488	0.999985264	0.932608891
LOC112442754	0.999996191	0.946274461	0.978389389	0.99999488	0.999985264	0.932608891
ITPR3	0.999996191	0.883624935	0.993594894	0.99999488	0.999985264	0.932608891
PHAX	0.999996191	0.952603267	0.993594894	0.99999488	0.999985264	0.932608891
FAM57A	0.999996191	0.991488652	0.993594894	0.99999488	0.999985264	0.932608891
ZNF740	0.999996191	0.991488652	0.994601277	0.99999488	0.999985264	0.932608891
AOX2	0.999996191	0.935588259	0.998852661	0.99999488	0.999985264	0.932608891
TMEM167B	0.999996191	0.952603267	0.999558179	0.99999488	0.999985264	0.932608891
FBXO24	0.999996191	0.95830623	0.999558179	0.99999488	0.999985264	0.932608891
LOC101902301	0.999996191	0.967463091	0.999558179	0.99999488	0.999985264	0.932608891
HES1	0.999996191	0.993316598	0.999558179	0.99999488	0.999985264	0.932608891

PCDHGA8	0.999996191	0.954072335	0.923604668	0.99999488	0.999985264	0.932886425
LOC614741	0.999996191	0.915367594	0.939501959	0.99999488	0.999985264	0.932886425
DEAF1	0.999996191	0.922885449	0.946479014	0.99999488	0.999985264	0.932886425
LOC100139996	0.999996191	0.9582784	0.95230212	0.99999488	0.999985264	0.932886425
B9D1	0.999996191	0.984068885	0.95230212	0.99999488	0.999985264	0.932886425
UBE2D2	0.999996191	0.946274461	0.9808801	0.99999488	0.999985264	0.932886425
ABCA5	0.999996191	0.9582784	0.993594894	0.99999488	0.999985264	0.932886425
PYROXD2	0.999996191	0.956531374	0.998936876	0.99999488	0.999985264	0.932886425
NCBP1	0.999996191	0.999992444	0.910748743	0.99999488	0.999985264	0.933374538
FAM177A1	0.999996191	0.952639631	0.970903823	0.99999488	0.999985264	0.933417214
TOP2B	0.999996191	0.962665318	0.919763144	0.99999488	0.999985264	0.933698703
ZDHHC21	0.999996191	0.999347049	0.930678591	0.99999488	0.999985264	0.933698703
ZNF507	0.999996191	0.976813871	0.9426263	0.99999488	0.999985264	0.933698703
FBXL19	0.999996191	0.913581457	0.95106368	0.99999488	0.999985264	0.933698703
E2F8	0.999996191	0.938017364	0.954620171	0.99999488	0.999985264	0.933698703
MED23	0.999996191	0.999992444	0.95230212	0.99999488	0.999985264	0.933741383
LETM1	0.999996191	0.935060764	0.900890709	0.99999488	0.999985264	0.934109451
CPSF6	0.999996191	0.998131745	0.941336964	0.99999488	0.999985264	0.934109451
LOC100337044	0.999996191	0.935060764	0.994601277	0.99999488	0.999985264	0.934109451
PCED1A	0.999996191	0.941495217	0.999558179	0.99999488	0.999985264	0.934109451
AKT3	0.999996191	0.936273761	0.907381775	0.99999488	0.999985264	0.934204736
DISC1	0.999996191	0.937511653	0.925745065	0.99999488	0.999985264	0.934204736
MIA2	0.999996191	0.991488652	0.93198623	0.99999488	0.999985264	0.934204736
ZNF570	0.999996191	0.999992444	0.945797778	0.99999488	0.999985264	0.934204736
GLDC	0.999996191	0.978510121	0.981218677	0.99999488	0.999985264	0.934204736
GFOD1	0.999996191	0.95697765	0.94634922	0.99999488	0.999985264	0.934227564
IGF2R	0.999996191	0.927912616	0.884119589	0.99999488	0.999985264	0.934515869
SEC14L2	0.999996191	0.999992444	0.907484111	0.99999488	0.999985264	0.934515869
RFTN1	0.999996191	0.958967142	0.985363931	0.99999488	0.999985264	0.934621279
LOC789018	0.999996191	0.921549	0.893182079	0.99999488	0.999985264	0.934749052
CCDC194	0.999996191	0.919446414	0.894415423	0.99999488	0.999985264	0.934749052
C25H7orf50	0.999996191	0.943802122	0.900148803	0.99999488	0.999985264	0.934749052
AZI2	0.999996191	0.900867274	0.902552409	0.99999488	0.999985264	0.934749052
AGPAT2	0.999996191	0.938597964	0.902552409	0.99999488	0.999985264	0.934749052
NHSL1	0.999996191	0.999992444	0.908181052	0.99999488	0.999985264	0.934749052
ADORA1	0.999996191	0.97015318	0.920629231	0.99999488	0.999985264	0.934749052
LOC112446033	0.999996191	0.992977597	0.923604668	0.99999488	0.999985264	0.934749052
BIRC3	0.999996191	0.938587041	0.924866532	0.99999488	0.999985264	0.934749052
LOC101904492	0.999996191	0.999992444	0.925520255	0.99999488	0.999985264	0.934749052

LOC101907518	0.999996191	0.90116492	0.926552506	0.99999488	0.999985264	0.934749052
LOC783396	0.999996191	0.999992444	0.926552506	0.99999488	0.999985264	0.934749052
TPX2	0.999996191	0.958098081	0.930678591	0.99999488	0.999985264	0.934749052
RFC3	0.999996191	0.943802122	0.934668684	0.99999488	0.999985264	0.934749052
LOC100848886	0.999996191	0.958098081	0.938203102	0.99999488	0.999985264	0.934749052
YRDC	0.999996191	0.999992444	0.938203102	0.99999488	0.999985264	0.934749052
GHR	0.999996191	0.958098081	0.938559901	0.99999488	0.999985264	0.934749052
MCRIP1	0.999996191	0.9582784	0.938559901	0.99999488	0.999985264	0.934749052
ETV5	0.999996191	0.999992444	0.941118576	0.99999488	0.999985264	0.934749052
LOC101905053	0.999996191	0.998011664	0.944871472	0.99999488	0.999985264	0.934749052
NEDD8	0.999996191	0.9582784	0.944993804	0.99999488	0.999985264	0.934749052
PHYH	0.999996191	0.934479017	0.945797778	0.99999488	0.999985264	0.934749052
MIB1	0.999996191	0.940724055	0.946479014	0.99999488	0.999985264	0.934749052
MDM1	0.999996191	0.878186176	0.95230212	0.99999488	0.999985264	0.934749052
RAD51B	0.999996191	0.998131745	0.957541703	0.99999488	0.999985264	0.934749052
NRGN	0.999996191	0.985406717	0.962119257	0.99999488	0.999985264	0.934749052
CCL25	0.999996191	0.974580033	0.973303821	0.99999488	0.999985264	0.934749052
NR1I3	0.999996191	0.978510121	0.97379223	0.99999488	0.999985264	0.934749052
LOC101906177	0.999996191	0.999992444	0.981039752	0.99999488	0.999985264	0.934749052
TCF20	0.999996191	0.935588259	0.981388276	0.99999488	0.999985264	0.934749052
WDFY2	0.999996191	0.992110949	0.985363931	0.99999488	0.999985264	0.934749052
EHMT1	0.999996191	0.946480858	0.996715234	0.99999488	0.999985264	0.934749052
LOC515042	0.999996191	0.943802122	0.999558179	0.99999488	0.999985264	0.934749052
KIF5B	0.999996191	0.938597964	0.926552506	0.99999488	0.999985264	0.935106628
LOC100848939	0.999996191	0.991574265	0.938203102	0.99999488	0.999985264	0.935106628
IBA57	0.999996191	0.957700659	0.954571119	0.99999488	0.999985264	0.935106628
NFATC4	0.999996191	0.98811113	0.998936876	0.99999488	0.999985264	0.935106628
LOC104970589	0.999996191	0.95296201	0.999558179	0.99999488	0.999985264	0.935106628
PTTG1	0.999996191	0.999992444	0.95106368	0.99999488	0.999985264	0.935160451
LOC788201	0.999996191	0.999992444	0.884320747	0.99999488	0.999985264	0.935349027
KBTBD11	0.999996191	0.999992444	0.891893039	0.99999488	0.999985264	0.935349027
ANKRD42	0.999996191	0.991574265	0.910748743	0.99999488	0.999985264	0.935349027
VEZF1	0.999996191	0.999992444	0.920629231	0.99999488	0.999985264	0.935349027
INIP	0.999996191	0.970286235	0.922443171	0.99999488	0.999985264	0.935349027
CTSW	0.999996191	0.943802122	0.925745065	0.99999488	0.999985264	0.935349027
RHOBTB3	0.999996191	0.997051616	0.925745065	0.99999488	0.999985264	0.935349027
RABIF	0.999996191	0.999992444	0.926552506	0.99999488	0.999985264	0.935349027
LOC101904062	0.999996191	0.943802122	0.938203102	0.99999488	0.999985264	0.935349027
CENPU	0.999996191	0.999992444	0.938203102	0.99999488	0.999985264	0.935349027

BMP5	0.999996191	0.948700494	0.944932814	0.99999488	0.999985264	0.935349027
ISG20	0.999996191	0.970426668	0.945678616	0.99999488	0.999985264	0.935349027
MAOA	0.999996191	0.957700659	0.946479014	0.99999488	0.999985264	0.935349027
MRPL27	0.999996191	0.968240168	0.946832224	0.99999488	0.999985264	0.935349027
ZNF473	0.999996191	0.958967142	0.969880768	0.99999488	0.999985264	0.935349027
MTIF2	0.999996191	0.999992444	0.985202052	0.99999488	0.999985264	0.935349027
LOC101903645	0.999996191	0.978089982	0.999558179	0.99999488	0.999985264	0.935349027
SNAP91	0.999996191	0.935060764	0.923902957	0.99999488	0.999985264	0.935713496
ERN1	0.999996191	0.974812121	0.946479014	0.99999488	0.999985264	0.935713496
RBMS1	0.999996191	0.927997582	0.916975216	0.99999488	0.999985264	0.935834055
NDUFV2	0.999996191	0.943802122	0.960133832	0.99999488	0.999985264	0.936037155
FAM213B	0.999996191	0.974812121	0.94634922	0.99999488	0.999985264	0.936623492
LOC112443244	0.999996191	0.9582784	0.887673	0.99999488	0.999985264	0.936885223
LOC107131940	0.999996191	0.991488652	0.898248457	0.99999488	0.999985264	0.936885223
AMOT	0.999996191	0.929230097	0.923089792	0.99999488	0.999985264	0.936885223
PDGFD	0.999996191	0.96566261	0.933447488	0.99999488	0.999985264	0.936885223
JRK	0.999996191	0.970426668	0.940815039	0.99999488	0.999985264	0.936885223
CCSER2	0.999996191	0.994481342	0.944085862	0.99999488	0.999985264	0.936885223
KPNA3	0.999996191	0.99398773	0.998936876	0.99999488	0.999985264	0.936885223
RPS3	0.999996191	0.967276215	0.911882087	0.99999488	0.999985264	0.937108046
ANAPC13	0.999996191	0.958215923	0.925520255	0.99999488	0.999985264	0.937108046
GSC	0.999996191	0.995826426	0.927600835	0.99999488	0.999985264	0.937108046
ARHGAP35	0.999996191	0.946480858	0.931507181	0.99999488	0.999985264	0.937108046
FASTKD1	0.999996191	0.993316598	0.932876936	0.99999488	0.999985264	0.937108046
LDB2	0.999996191	0.999992444	0.944085862	0.99999488	0.999985264	0.937108046
VAMP2	0.999996191	0.999992444	0.946479014	0.99999488	0.999985264	0.937108046
UQCRH	0.999996191	0.943880584	0.956565728	0.99999488	0.999985264	0.937108046
CAAP1	0.999996191	0.9582784	0.9808801	0.99999488	0.999985264	0.937108046
PCBP3	0.999996191	0.991574265	0.988922732	0.99999488	0.999985264	0.937108046
ST3GAL1	0.999996191	0.999992444	0.923604668	0.99999488	0.999985264	0.937393304
GAB3	0.999996191	0.970286235	0.960133832	0.99999488	0.999985264	0.937393304
TNFAIP6	0.999996191	0.981298804	0.898815524	0.99999488	0.999985264	0.937880392
NFRKB	0.999996191	0.947032486	0.998936876	0.99999488	0.999985264	0.93801273
AIMP1	0.999996191	0.960538126	0.888700204	0.99999488	0.999985264	0.93845573
LOC789895	0.999996191	0.972260186	0.923604668	0.99999488	0.999985264	0.93845573
FAM160B1	0.999996191	0.993316598	0.958142113	0.99999488	0.999985264	0.93845573
UQCRB	0.999996191	0.9582784	0.964594039	0.99999488	0.999985264	0.93845573
KIAA1211	0.999996191	0.957700659	0.969880768	0.99999488	0.999985264	0.938625326
CPT2	0.999996191	0.937231686	0.963068909	0.99999488	0.999985264	0.938658403

AP1S1	0.999996191	0.980496345	0.930678591	0.99999488	0.999985264	0.938885777
FCER2	0.999996191	0.949285285	0.884119589	0.99999488	0.999985264	0.939035437
CD163	0.999996191	0.901839069	0.886192181	0.99999488	0.999985264	0.939035437
LRFN3	0.999996191	0.922700337	0.886962419	0.99999488	0.999985264	0.939035437
DOLPP1	0.999996191	0.978510121	0.890724014	0.99999488	0.999985264	0.939035437
LOC515150	0.999996191	0.920253543	0.891893039	0.99999488	0.999985264	0.939035437
CASC4	0.999996191	0.991335402	0.897767405	0.99999488	0.999985264	0.939035437
KIDINS220	0.999996191	0.893642384	0.900148803	0.99999488	0.999985264	0.939035437
S100PBP	0.999996191	0.965422267	0.900890709	0.99999488	0.999985264	0.939035437
CYB5B	0.999996191	0.946480858	0.907484111	0.99999488	0.999985264	0.939035437
LRRC8C	0.999996191	0.984068885	0.907484111	0.99999488	0.999985264	0.939035437
CXHXorf56	0.999996191	0.924817361	0.910426741	0.99999488	0.999985264	0.939035437
RASSF2	0.999996191	0.943802122	0.915215321	0.99999488	0.999985264	0.939035437
GFPT1	0.999996191	0.999992444	0.917154286	0.99999488	0.999985264	0.939035437
PSME4	0.999996191	0.981520287	0.918170048	0.99999488	0.999985264	0.939035437
RTL8C	0.999996191	0.991838765	0.919763144	0.99999488	0.999985264	0.939035437
NANS	0.999996191	0.991488652	0.920154807	0.99999488	0.999985264	0.939035437
COMMD3	0.999996191	0.924216699	0.920629231	0.99999488	0.999985264	0.939035437
LOC112444726	0.999996191	0.933454973	0.920629231	0.99999488	0.999985264	0.939035437
SLC38A3	0.999996191	0.9582784	0.920629231	0.99999488	0.999985264	0.939035437
MAP2K1	0.999996191	0.925580392	0.921065686	0.99999488	0.999985264	0.939035437
PREB	0.999996191	0.999347049	0.921065686	0.99999488	0.999985264	0.939035437
NECTIN2	0.999996191	0.999992444	0.921065686	0.99999488	0.999985264	0.939035437
PSMB5	0.999996191	0.935060764	0.921379317	0.99999488	0.999985264	0.939035437
ZNFX1	0.999996191	0.979263532	0.921629523	0.99999488	0.999985264	0.939035437
MCOLN1	0.999996191	0.999992444	0.921646762	0.99999488	0.999985264	0.939035437
LOC532875	0.999996191	0.938597964	0.922400936	0.99999488	0.999985264	0.939035437
LOC616720	0.999996191	0.941793392	0.923604668	0.99999488	0.999985264	0.939035437
RPS19	0.999996191	0.990248517	0.923604668	0.99999488	0.999985264	0.939035437
ZNF746	0.999996191	0.992977597	0.923604668	0.99999488	0.999985264	0.939035437
TGM3	0.999996191	0.926949474	0.924397528	0.99999488	0.999985264	0.939035437
ETNK1	0.999996191	0.993316598	0.924604843	0.99999488	0.999985264	0.939035437
WSCD2	0.999996191	0.901566382	0.925520255	0.99999488	0.999985264	0.939035437
ERGIC2	0.999996191	0.92462339	0.925520255	0.99999488	0.999985264	0.939035437
CD274	0.999996191	0.941876954	0.926552506	0.99999488	0.999985264	0.939035437
PIK3C2B	0.999996191	0.95697765	0.926552506	0.99999488	0.999985264	0.939035437
MECOM	0.999996191	0.973334182	0.926552506	0.99999488	0.999985264	0.939035437
LOC112445063	0.999996191	0.975951808	0.926552506	0.99999488	0.999985264	0.939035437
MEIS1	0.999996191	0.977354623	0.926552506	0.99999488	0.999985264	0.939035437

MNAT1	0.999996191	0.992977597	0.926552506	0.99999488	0.999985264	0.939035437
SPTBN4	0.999996191	0.952405695	0.927022752	0.99999488	0.999985264	0.939035437
SMARCB1	0.999996191	0.975677199	0.927600835	0.99999488	0.999985264	0.939035437
PPP4R3A	0.999996191	0.978089982	0.929188473	0.99999488	0.999985264	0.939035437
LARP1B	0.999996191	0.935274301	0.930678591	0.99999488	0.999985264	0.939035437
MAEA	0.999996191	0.941495217	0.930678591	0.99999488	0.999985264	0.939035437
CHD4	0.999996191	0.952346363	0.930678591	0.99999488	0.999985264	0.939035437
HINT3	0.999996191	0.9582784	0.930678591	0.99999488	0.999985264	0.939035437
UBAC1	0.999996191	0.9582784	0.930678591	0.99999488	0.999985264	0.939035437
RAB42	0.999996191	0.96566261	0.930678591	0.99999488	0.999985264	0.939035437
OXNAD1	0.999996191	0.991488652	0.930678591	0.99999488	0.999985264	0.939035437
G6PC3	0.999996191	0.979263532	0.931507181	0.99999488	0.999985264	0.939035437
EXOSC6	0.999996191	0.998131745	0.93198623	0.99999488	0.999985264	0.939035437
CUL5	0.999996191	0.999992444	0.93198623	0.99999488	0.999985264	0.939035437
FGFR1OP2	0.999996191	0.991488652	0.932653752	0.99999488	0.999985264	0.939035437
SNRNP27	0.999996191	0.996966166	0.932653752	0.99999488	0.999985264	0.939035437
CABCOCO1	0.999996191	0.967276215	0.932657945	0.99999488	0.999985264	0.939035437
LRRC3B	0.999996191	0.964591213	0.933447488	0.99999488	0.999985264	0.939035437
ABHD16A	0.999996191	0.999992444	0.933791093	0.99999488	0.999985264	0.939035437
NUP62	0.999996191	0.985406717	0.935094475	0.99999488	0.999985264	0.939035437
CALHM5	0.999996191	0.992895985	0.935726137	0.99999488	0.999985264	0.939035437
GALNT2	0.999996191	0.991488652	0.937822865	0.99999488	0.999985264	0.939035437
NFIX	0.999996191	0.957700659	0.938203102	0.99999488	0.999985264	0.939035437
MLF2	0.999996191	0.985296656	0.938203102	0.99999488	0.999985264	0.939035437
SRD5A1	0.999996191	0.991488652	0.938203102	0.99999488	0.999985264	0.939035437
LOC100848642	0.999996191	0.999992444	0.938203102	0.99999488	0.999985264	0.939035437
MAP3K13	0.999996191	0.999992444	0.938203102	0.99999488	0.999985264	0.939035437
ODR4	0.999996191	0.999992444	0.938203102	0.99999488	0.999985264	0.939035437
PKP2	0.999996191	0.999347049	0.938905004	0.99999488	0.999985264	0.939035437
DR1	0.999996191	0.999992444	0.938905004	0.99999488	0.999985264	0.939035437
FGF18	0.999996191	0.921934673	0.938911935	0.99999488	0.999985264	0.939035437
TMEM185B	0.999996191	0.940724055	0.939147816	0.99999488	0.999985264	0.939035437
LOC101906086	0.999996191	0.955128586	0.939147816	0.99999488	0.999985264	0.939035437
C6H4orf3	0.999996191	0.999992444	0.939147816	0.99999488	0.999985264	0.939035437
PEX11G	0.999996191	0.991488652	0.939478907	0.99999488	0.999985264	0.939035437
TMEM106B	0.999996191	0.999347049	0.939501959	0.99999488	0.999985264	0.939035437
CEBPD	0.999996191	0.9409629	0.940815039	0.99999488	0.999985264	0.939035437
C11H9orf78	0.999996191	0.952345011	0.941118576	0.99999488	0.999985264	0.939035437
COQ6	0.999996191	0.952346363	0.941336964	0.99999488	0.999985264	0.939035437

POLR2B	0.999996191	0.985406717	0.941336964	0.99999488	0.999985264	0.939035437
ABCB10	0.999996191	0.999992444	0.941336964	0.99999488	0.999985264	0.939035437
GTF2H5	0.999996191	0.922700337	0.941730265	0.99999488	0.999985264	0.939035437
ARHGEF40	0.999996191	0.946274461	0.942675769	0.99999488	0.999985264	0.939035437
LOC104974070	0.999996191	0.9527938	0.944085862	0.99999488	0.999985264	0.939035437
LOC104974667	0.999996191	0.95697765	0.944085862	0.99999488	0.999985264	0.939035437
XXYLT1	0.999996191	0.985406717	0.944085862	0.99999488	0.999985264	0.939035437
LOC112442079	0.999996191	0.934479017	0.945407489	0.99999488	0.999985264	0.939035437
SETDB1	0.999996191	0.946274461	0.945678616	0.99999488	0.999985264	0.939035437
KRAS	0.999996191	0.947121355	0.945678616	0.99999488	0.999985264	0.939035437
POLN	0.999996191	0.967463091	0.945678616	0.99999488	0.999985264	0.939035437
H2AFV	0.999996191	0.991365955	0.94634922	0.99999488	0.999985264	0.939035437
WDSUB1	0.999996191	0.999992444	0.94634922	0.99999488	0.999985264	0.939035437
BPNT1	0.999996191	0.926955605	0.946479014	0.99999488	0.999985264	0.939035437
AIFM1	0.999996191	0.938597964	0.946479014	0.99999488	0.999985264	0.939035437
FAM21A	0.999996191	0.941495217	0.946479014	0.99999488	0.999985264	0.939035437
DFFA	0.999996191	0.991488652	0.946479014	0.99999488	0.999985264	0.939035437
HERC4	0.999996191	0.999347049	0.946479014	0.99999488	0.999985264	0.939035437
VAMP8	0.999996191	0.999347049	0.946479014	0.99999488	0.999985264	0.939035437
TLL1	0.999996191	0.999992444	0.946479014	0.99999488	0.999985264	0.939035437
OMD	0.999996191	0.9582784	0.947187955	0.99999488	0.999985264	0.939035437
LOC531152	0.999996191	0.993316598	0.947226687	0.99999488	0.999985264	0.939035437
LOC112447350	0.999996191	0.999992444	0.948420301	0.99999488	0.999985264	0.939035437
MINDY4	0.999996191	0.999992444	0.948420301	0.99999488	0.999985264	0.939035437
EPM2A	0.999996191	0.935742738	0.950948716	0.99999488	0.999985264	0.939035437
LOC101905399	0.999996191	0.958579042	0.95106368	0.99999488	0.999985264	0.939035437
LOC112441887	0.999996191	0.938597964	0.951099739	0.99999488	0.999985264	0.939035437
PPP2R2B	0.999996191	0.882505302	0.951735967	0.99999488	0.999985264	0.939035437
TARBP2	0.999996191	0.926949474	0.95230212	0.99999488	0.999985264	0.939035437
NDUFAF3	0.999996191	0.941495217	0.95230212	0.99999488	0.999985264	0.939035437
RNASE12	0.999996191	0.942911665	0.95230212	0.99999488	0.999985264	0.939035437
VCAM1	0.999996191	0.955128586	0.95230212	0.99999488	0.999985264	0.939035437
BET1L	0.999996191	0.986637746	0.95230212	0.99999488	0.999985264	0.939035437
CAPN1	0.999996191	0.999992444	0.95230212	0.99999488	0.999985264	0.939035437
SETD4	0.999996191	0.999992444	0.954571119	0.99999488	0.999985264	0.939035437
FOXF1	0.999996191	0.935742738	0.954620171	0.99999488	0.999985264	0.939035437
LOC104968820	0.999996191	0.96895079	0.954620171	0.99999488	0.999985264	0.939035437
LOC101906021	0.999996191	0.896536444	0.95477544	0.99999488	0.999985264	0.939035437
RNF41	0.999996191	0.998131745	0.954864482	0.99999488	0.999985264	0.939035437

SSR1	0.999996191	0.893642384	0.956565728	0.99999488	0.999985264	0.939035437
GPSM1	0.999996191	0.960498589	0.956565728	0.99999488	0.999985264	0.939035437
FOXP4	0.999996191	0.991488652	0.956565728	0.99999488	0.999985264	0.939035437
FAM96B	0.999996191	0.999992444	0.956565728	0.99999488	0.999985264	0.939035437
RAPGEF4	0.999996191	0.921934673	0.956852976	0.99999488	0.999985264	0.939035437
GSKIP	0.999996191	0.998131745	0.956852976	0.99999488	0.999985264	0.939035437
LMO4	0.999996191	0.978089982	0.957117655	0.99999488	0.999985264	0.939035437
TRIM33	0.999996191	0.999992444	0.957541703	0.99999488	0.999985264	0.939035437
HSD17B4	0.999996191	0.918254521	0.960133832	0.99999488	0.999985264	0.939035437
RPA2	0.999996191	0.9582784	0.960133832	0.99999488	0.999985264	0.939035437
ZMYND19	0.999996191	0.993316598	0.960133832	0.99999488	0.999985264	0.939035437
AURKA	0.999996191	0.985058069	0.961734809	0.99999488	0.999985264	0.939035437
C23H6orf226	0.999996191	0.949285285	0.962119257	0.99999488	0.999985264	0.939035437
PFDN4	0.999996191	0.970426668	0.962346588	0.99999488	0.999985264	0.939035437
LOC100295750	0.999996191	0.998131745	0.962346588	0.99999488	0.999985264	0.939035437
PAPLN	0.999996191	0.91058693	0.964152013	0.99999488	0.999985264	0.939035437
LOC112448889	0.999996191	0.978089982	0.964582964	0.99999488	0.999985264	0.939035437
SF3B4	0.999996191	0.999992444	0.964908146	0.99999488	0.999985264	0.939035437
RBX1	0.999996191	0.947462841	0.966991646	0.99999488	0.999985264	0.939035437
HOXB4	0.999996191	0.935060764	0.968836394	0.99999488	0.999985264	0.939035437
LOC104975196	0.999996191	0.999992444	0.969342421	0.99999488	0.999985264	0.939035437
SMIM26	0.999996191	0.957700659	0.969880768	0.99999488	0.999985264	0.939035437
TMEFF2	0.999996191	0.985161531	0.969880768	0.99999488	0.999985264	0.939035437
LOC107133071	0.999996191	0.992977597	0.969880768	0.99999488	0.999985264	0.939035437
LOC112449618	0.999996191	0.993316598	0.969880768	0.99999488	0.999985264	0.939035437
ATXN3	0.999996191	0.999992444	0.969880768	0.99999488	0.999985264	0.939035437
ERI1	0.999996191	0.997975376	0.973406453	0.99999488	0.999985264	0.939035437
ZFYVE9	0.999996191	0.999347049	0.973406453	0.99999488	0.999985264	0.939035437
LOC112443011	0.999996191	0.999992444	0.973406453	0.99999488	0.999985264	0.939035437
MAN1B1	0.999996191	0.921549	0.977141934	0.99999488	0.999985264	0.939035437
ASF1A	0.999996191	0.895242264	0.979587501	0.99999488	0.999985264	0.939035437
LOC112441481	0.999996191	0.9582784	0.980660791	0.99999488	0.999985264	0.939035437
AMIGO1	0.999996191	0.991574265	0.980847826	0.99999488	0.999985264	0.939035437
BRCA1	0.999996191	0.94254418	0.980859619	0.99999488	0.999985264	0.939035437
ITGA2	0.999996191	0.982313546	0.980859619	0.99999488	0.999985264	0.939035437
LOC781913	0.999996191	0.999992444	0.9808801	0.99999488	0.999985264	0.939035437
ARHGEF39	0.999996191	0.9582784	0.981218677	0.99999488	0.999985264	0.939035437
LOC112441880	0.999996191	0.986018996	0.981622418	0.99999488	0.999985264	0.939035437
GINM1	0.999996191	0.986637746	0.981622418	0.99999488	0.999985264	0.939035437

LOC783421	0.999996191	0.9582784	0.981736235	0.99999488	0.999985264	0.939035437
ZNF165	0.999996191	0.978510121	0.991285432	0.99999488	0.999985264	0.939035437
PASK	0.999996191	0.976717114	0.993594894	0.99999488	0.999985264	0.939035437
SMIM14	0.999996191	0.999347049	0.994601277	0.99999488	0.999985264	0.939035437
EIF1AD	0.999996191	0.943802122	0.994626276	0.99999488	0.999985264	0.939035437
LOC100296121	0.999996191	0.967276215	0.996715234	0.99999488	0.999985264	0.939035437
LOC101904290	0.999996191	0.907069508	0.999558179	0.99999488	0.999985264	0.939035437
NPAT	0.999996191	0.943802122	0.999558179	0.99999488	0.999985264	0.939035437
ATAD1	0.999996191	0.946274461	0.999558179	0.99999488	0.999985264	0.939035437
MFAP1	0.999996191	0.950729918	0.999558179	0.99999488	0.999985264	0.939035437
LOC101910094	0.999996191	0.9582784	0.999558179	0.99999488	0.999985264	0.939035437
POLL	0.999996191	0.958967142	0.999558179	0.99999488	0.999985264	0.939035437
LOC112447599	0.999996191	0.963558676	0.999558179	0.99999488	0.999985264	0.939035437
RAB6A	0.999996191	0.96566261	0.999558179	0.99999488	0.999985264	0.939035437
ASAP1	0.999996191	0.989958965	0.999558179	0.99999488	0.999985264	0.939035437
RBPMS2	0.999996191	0.991488652	0.999558179	0.99999488	0.999985264	0.939035437
EID2	0.999996191	0.938597964	0.922400936	0.99999488	0.999985264	0.93938415
DDX39B	0.999996191	0.991488652	0.981218677	0.99999488	0.999985264	0.93938415
LANCL2	0.999996191	0.946274461	0.939147816	0.99999488	0.999985264	0.939456356
TDRD10	0.999996191	0.967919743	0.95230212	0.99999488	0.999985264	0.939657518
EFR3A	0.999996191	0.974812121	0.999558179	0.99999488	0.999985264	0.939657518
SGSM3	0.999996191	0.915971865	0.915033913	0.99999488	0.999985264	0.939708143
GPN1	0.999996191	0.97015318	0.936816488	0.99999488	0.999985264	0.939708143
LOC112446691	0.999996191	0.941495217	0.954620171	0.99999488	0.999985264	0.939708143
LOC781108	0.999996191	0.941495217	0.998936876	0.99999488	0.999985264	0.939708143
TMPO	0.999996191	0.991488652	0.934959203	0.99999488	0.999985264	0.939788879
PXDN	0.999996191	0.916036681	0.927600835	0.99999488	0.999985264	0.939855019
ERCC1	0.999996191	0.992977597	0.934501347	0.99999488	0.999985264	0.939855019
MAN1A1	0.999996191	0.944855558	0.938203102	0.99999488	0.999985264	0.939855019
NDUFB10	0.999996191	0.959455624	0.938905004	0.99999488	0.999985264	0.939855019
LOC537017	0.999996191	0.969534305	0.946479014	0.99999488	0.999985264	0.939855019
LOC101906235	0.999996191	0.999347049	0.999558179	0.99999488	0.999985264	0.939855019
KDM8	0.999996191	0.954790265	0.957541703	0.99999488	0.999985264	0.940137966
LOC112442280	0.999996191	0.999992444	0.95230212	0.99999488	0.999985264	0.940299347
LOC107133032	0.999996191	0.936392293	0.893044539	0.99999488	0.999985264	0.940358165
RELB	0.999996191	0.970426668	0.903957821	0.99999488	0.999985264	0.940358165
SRP72	0.999996191	0.957700659	0.910426741	0.99999488	0.999985264	0.940358165
LOC104968522	0.999996191	0.957700659	0.920629231	0.99999488	0.999985264	0.940358165
CHMP1B	0.999996191	0.916036681	0.925745065	0.99999488	0.999985264	0.940358165

CLSTN3	0.999996191	0.999347049	0.925745065	0.99999488	0.999985264	0.940358165
LOC781218	0.999996191	0.966782939	0.926552506	0.99999488	0.999985264	0.940358165
NPL	0.999996191	0.986637746	0.927600835	0.99999488	0.999985264	0.940358165
LOC112443463	0.999996191	0.992977597	0.930127869	0.99999488	0.999985264	0.940358165
TMEM154	0.999996191	0.958967142	0.933791093	0.99999488	0.999985264	0.940358165
FBXW5	0.999996191	0.999347049	0.938203102	0.99999488	0.999985264	0.940358165
TRUB1	0.999996191	0.999992444	0.938983799	0.99999488	0.999985264	0.940358165
PEMT	0.999996191	0.999992444	0.939147816	0.99999488	0.999985264	0.940358165
HUS1	0.999996191	0.999992444	0.93955737	0.99999488	0.999985264	0.940358165
LOC112445001	0.999996191	0.956655737	0.946479014	0.99999488	0.999985264	0.940358165
LOC616200	0.999996191	0.979756265	0.946479014	0.99999488	0.999985264	0.940358165
RBM33	0.999996191	0.985471417	0.946479014	0.99999488	0.999985264	0.940358165
COX20	0.999996191	0.997051616	0.946479014	0.99999488	0.999985264	0.940358165
ARSG	0.999996191	0.999992444	0.946479014	0.99999488	0.999985264	0.940358165
LOC112449560	0.999996191	0.999992444	0.947267164	0.99999488	0.999985264	0.940358165
SP3	0.999996191	0.993316598	0.948420301	0.99999488	0.999985264	0.940358165
TFPI2	0.999996191	0.927997582	0.949038056	0.99999488	0.999985264	0.940358165
SH3PXD2B	0.999996191	0.977354623	0.951680496	0.99999488	0.999985264	0.940358165
FAM89A	0.999996191	0.9582784	0.952207545	0.99999488	0.999985264	0.940358165
FAM208B	0.999996191	0.9582784	0.95230212	0.99999488	0.999985264	0.940358165
SUMO2	0.999996191	0.983781707	0.954823586	0.99999488	0.999985264	0.940358165
LOC112443213	0.999996191	0.966035002	0.956565728	0.99999488	0.999985264	0.940358165
LOC100849069	0.999996191	0.992977597	0.957541703	0.99999488	0.999985264	0.940358165
LOC100848212	0.999996191	0.997051616	0.964920281	0.99999488	0.999985264	0.940358165
OMP	0.999996191	0.9582784	0.973406453	0.99999488	0.999985264	0.940358165
GPC4	0.999996191	0.992977597	0.973661483	0.99999488	0.999985264	0.940358165
PRODH	0.999996191	0.957700659	0.981218677	0.99999488	0.999985264	0.940358165
EGR2	0.999996191	0.925580392	0.993594894	0.99999488	0.999985264	0.940358165
DLL4	0.999996191	0.999992444	0.993594894	0.99999488	0.999985264	0.940358165
FYTTD1	0.999996191	0.9582784	0.996776138	0.99999488	0.999985264	0.940358165
PACSIN1	0.999996191	0.958967142	0.999558179	0.99999488	0.999985264	0.940358165
LOC101904840	0.999996191	0.961479426	0.999558179	0.99999488	0.999985264	0.940358165
LAPTM4A	0.999996191	0.972764622	0.999558179	0.99999488	0.999985264	0.940358165
THBD	0.999996191	0.986637746	0.911018006	0.99999488	0.999985264	0.940465928
RNF31	0.999996191	0.9582784	0.994601277	0.99999488	0.999985264	0.940509936
LOC782812	0.999996191	0.95697765	0.941336964	0.99999488	0.999985264	0.940620954
PWP1	0.999996191	0.893642384	0.922400936	0.99999488	0.999985264	0.940731625
LOC100335467	0.999996191	0.982628449	0.946832224	0.99999488	0.999985264	0.940731625
PSCA	0.999996191	0.998131745	0.948420301	0.99999488	0.999985264	0.940731625

SLC25A17	0.999996191	0.9582784	0.946283845	0.99999488	0.999985264	0.94085445
CELA1	0.999996191	0.919884594	0.902552409	0.99999488	0.999985264	0.94099302
DHX15	0.999996191	0.938597964	0.873452279	0.99999488	0.999985264	0.941072433
LOC101904435	0.999996191	0.999992444	0.946479014	0.99999488	0.999985264	0.941072433
LOC101904701	0.999996191	0.9582784	0.957840205	0.99999488	0.999985264	0.941072433
DNAJA4	0.999996191	0.946274461	0.999558179	0.99999488	0.999985264	0.941072433
PALM2	0.999996191	0.9582784	0.999558179	0.99999488	0.999985264	0.941072433
FAM234B	0.999996191	0.995021629	0.960133832	0.99999488	0.999985264	0.941076534
SOSTDC1	0.999996191	0.962665318	0.945678616	0.99999488	0.999985264	0.941205126
TOMM6	0.999996191	0.943802122	0.946479014	0.99999488	0.999985264	0.941205126
SGK1	0.999996191	0.999992444	0.940815039	0.99999488	0.999985264	0.941265666
BCL2L11	0.999996191	0.956801903	0.941336964	0.99999488	0.999985264	0.941265666
B3GAT3	0.999996191	0.978510121	0.955919075	0.99999488	0.999985264	0.941265666
PTH1R	0.999996191	0.951765021	0.9808801	0.99999488	0.999985264	0.941265666
KARS	0.999996191	0.970077079	0.877901599	0.99999488	0.999985264	0.941583574
CWC25	0.999996191	0.999992444	0.891024373	0.99999488	0.999985264	0.941724934
RNPEP	0.999996191	0.926955605	0.900890709	0.99999488	0.999985264	0.941724934
SHISA7	0.999996191	0.941495217	0.907381775	0.99999488	0.999985264	0.941724934
ASB3	0.999996191	0.9582784	0.920154807	0.99999488	0.999985264	0.941724934
LOC107132958	0.999996191	0.921549	0.926552506	0.99999488	0.999985264	0.941724934
UBE2R2	0.999996191	0.9409629	0.926552506	0.99999488	0.999985264	0.941724934
NCKIPSD	0.999996191	0.943802122	0.926552506	0.99999488	0.999985264	0.941724934
LOC112443141	0.999996191	0.9582784	0.948329591	0.99999488	0.999985264	0.941724934
FAM122A	0.999996191	0.991488652	0.954872702	0.99999488	0.999985264	0.941724934
MTUS2	0.999996191	0.9582784	0.956565728	0.99999488	0.999985264	0.941724934
FBXO8	0.999996191	0.928759674	0.981039752	0.99999488	0.999985264	0.941724934
HSPD1	0.999996191	0.959809688	0.981458415	0.99999488	0.999985264	0.941724934
SGF29	0.999996191	0.919505104	0.892461717	0.99999488	0.999985264	0.94182698
HELZ	0.999996191	0.952335654	0.909070782	0.99999488	0.999985264	0.94182698
GRIN3A	0.999996191	0.999992444	0.91207077	0.99999488	0.999985264	0.94182698
AGTPBP1	0.999996191	0.999992444	0.929207633	0.99999488	0.999985264	0.94187247
LOC107132465	0.999996191	0.935742738	0.916975216	0.99999488	0.999985264	0.942095876
USF2	0.999996191	0.991574265	0.925745065	0.99999488	0.999985264	0.942112464
LOC101906477	0.999996191	0.985406717	0.939799459	0.99999488	0.999985264	0.942112464
MDGA2	0.999996191	0.999992444	0.95230212	0.99999488	0.999985264	0.94217554
DMXL1	0.999996191	0.95830623	0.939501959	0.99999488	0.999985264	0.942238579
PDZD11	0.999996191	0.962665318	0.95106368	0.99999488	0.999985264	0.942238579
LOC101903574	0.999996191	0.945955989	0.914449935	0.99999488	0.999985264	0.942340826
DDOST	0.999996191	0.999992444	0.947928805	0.99999488	0.999985264	0.942340826

FBXW7	0.999996191	0.936657629	0.957840205	0.99999488	0.999985264	0.942340826
WBP1	0.999996191	0.999992444	0.95106368	0.99999488	0.999985264	0.94303248
NT5M	0.999996191	0.949285285	0.88687452	0.99999488	0.999985264	0.943220018
LOC614923	0.999996191	0.970077079	0.907480055	0.99999488	0.999985264	0.943220018
PGS1	0.999996191	0.964246761	0.919763144	0.99999488	0.999985264	0.943220018
GTF3C3	0.999996191	0.999992444	0.923089792	0.99999488	0.999985264	0.943220018
LOC619159	0.999996191	0.999992444	0.926552506	0.99999488	0.999985264	0.943220018
PTPN3	0.999996191	0.948421431	0.927600835	0.99999488	0.999985264	0.943220018
PDE6C	0.999996191	0.999992444	0.930678591	0.99999488	0.999985264	0.943220018
POLR2J	0.999996191	0.9582784	0.933307828	0.99999488	0.999985264	0.943220018
LOC101903200	0.999996191	0.90024974	0.935726137	0.99999488	0.999985264	0.943220018
ACOT8	0.999996191	0.981520287	0.938203102	0.99999488	0.999985264	0.943220018
ABHD1	0.999996191	0.9582784	0.941118576	0.99999488	0.999985264	0.943220018
PHC1	0.999996191	0.999992444	0.941782902	0.99999488	0.999985264	0.943220018
LOC101907189	0.999996191	0.999992444	0.945280564	0.99999488	0.999985264	0.943220018
LOC101907017	0.999996191	0.999992444	0.95429596	0.99999488	0.999985264	0.943220018
RALGPS2	0.999996191	0.967374613	0.954328478	0.99999488	0.999985264	0.943220018
TRIP11	0.999996191	0.9582784	0.958627535	0.99999488	0.999985264	0.943220018
ABI3BP	0.999996191	0.967276215	0.971778256	0.99999488	0.999985264	0.943220018
ATG5	0.999996191	0.999992444	0.973406453	0.99999488	0.999985264	0.943220018
CCNB1	0.999996191	0.978510121	0.980660791	0.99999488	0.999985264	0.943220018
RNF11	0.999996191	0.999992444	0.981218677	0.99999488	0.999985264	0.943220018
MPZL2	0.999996191	0.991488652	0.981388276	0.99999488	0.999985264	0.943220018
LPIN3	0.999996191	0.929943407	0.999558179	0.99999488	0.999985264	0.943220018
C8G	0.999996191	0.979263532	0.999558179	0.99999488	0.999985264	0.943220018
ZFAT	0.999996191	0.941495217	0.96870883	0.99999488	0.999985264	0.94328177
SH3BP2	0.999996191	0.978510121	0.902336298	0.99999488	0.999985264	0.943492944
DECR1	0.999996191	0.935742738	0.921262217	0.99999488	0.999985264	0.943492944
SNX13	0.999996191	0.991671026	0.942911233	0.99999488	0.999985264	0.943633749
RPL31	0.999996191	0.991488652	0.945766581	0.99999488	0.999985264	0.943633749
TNFSF15	0.999996191	0.985406717	0.95230212	0.99999488	0.999985264	0.943633749
TMEM184B	0.999996191	0.999992444	0.93955737	0.99999488	0.999985264	0.943664078
LOC101902083	0.999996191	0.999992444	0.893711366	0.99999488	0.999985264	0.943860392
LANCL3	0.999996191	0.940459751	0.906591357	0.99999488	0.999985264	0.943860392
DDAH1	0.999996191	0.970426668	0.954823586	0.99999488	0.999985264	0.943860392
GOLGA1	0.999996191	0.992977597	0.982783495	0.99999488	0.999985264	0.943860392
ARSB	0.999996191	0.986637746	0.992337371	0.99999488	0.999985264	0.943860392
EAF2	0.999996191	0.9582784	0.998936876	0.99999488	0.999985264	0.943860392
DTX3	0.999996191	0.941495217	0.934501347	0.99999488	0.999985264	0.943865868

YIPF3	0.999996191	0.999992444	0.938203102	0.99999488	0.999985264	0.943905234
TAOK2	0.999996191	0.957700659	0.994601277	0.99999488	0.999985264	0.943905234
MAP2K2	0.999996191	0.999992444	0.93198623	0.99999488	0.999985264	0.943930915
RBMX2	0.999996191	0.941876954	0.97262931	0.99999488	0.999985264	0.944174932
PALM	0.999996191	0.978426416	0.960133832	0.99999488	0.999985264	0.94431248
ADAM17	0.999996191	0.935060764	0.913976361	0.99999488	0.999985264	0.944457836
CCDC85C	0.999996191	0.941495217	0.921010865	0.99999488	0.999985264	0.944457836
LGR6	0.999996191	0.999992444	0.930678591	0.99999488	0.999985264	0.944457836
NABP2	0.999996191	0.978154072	0.933992957	0.99999488	0.999985264	0.944457836
TRIM31	0.999996191	0.999992444	0.938203102	0.99999488	0.999985264	0.944457836
IFNGR1	0.999996191	0.944868298	0.939002524	0.99999488	0.999985264	0.944457836
LOC616400	0.999996191	0.991488652	0.939799459	0.99999488	0.999985264	0.944457836
EIF1B	0.999996191	0.953844371	0.941336964	0.99999488	0.999985264	0.944457836
LOC100847780	0.999996191	0.999992444	0.941336964	0.99999488	0.999985264	0.944457836
GPR62	0.999996191	0.9582784	0.944085862	0.99999488	0.999985264	0.944457836
MAMLD1	0.999996191	0.973092298	0.945678616	0.99999488	0.999985264	0.944457836
MED1	0.999996191	0.992977597	0.946479014	0.99999488	0.999985264	0.944457836
TMEM219	0.999996191	0.998131745	0.949038056	0.99999488	0.999985264	0.944457836
C5H12orf75	0.999996191	0.999992444	0.951735967	0.99999488	0.999985264	0.944457836
NSMCE1	0.999996191	0.992977597	0.95230212	0.99999488	0.999985264	0.944457836
RNF187	0.999996191	0.96781747	0.962346588	0.99999488	0.999985264	0.944457836
TBC1D8B	0.999996191	0.969314233	0.969880768	0.99999488	0.999985264	0.944457836
SHPRH	0.999996191	0.999992444	0.970903823	0.99999488	0.999985264	0.944457836
PLAGL1	0.999996191	0.927465399	0.973303821	0.99999488	0.999985264	0.944457836
SRRM5	0.999996191	0.999992444	0.900148803	0.99999488	0.999985264	0.94446315
TRPC6	0.999996191	0.986018996	0.922400936	0.99999488	0.999985264	0.94446315
MAPK8	0.999996191	0.984068885	0.938203102	0.99999488	0.999985264	0.94446315
CAMKK2	0.999996191	0.999992444	0.980660791	0.99999488	0.999985264	0.94446315
LOC107131992	0.999996191	0.974079639	0.938203102	0.99999488	0.999985264	0.944511198
HEATR6	0.999996191	0.985406717	0.941336964	0.99999488	0.999985264	0.944511198
PRELP	0.999996191	0.9582784	0.946479014	0.99999488	0.999985264	0.944511198
IFNAR1	0.999996191	0.970470739	0.951099739	0.99999488	0.999985264	0.944511198
CRTAM	0.999996191	0.938476452	0.988922732	0.99999488	0.999985264	0.944511198
KCNMB2	0.999996191	0.976717114	0.993594894	0.99999488	0.999985264	0.944511198
LOC104974330	0.999996191	0.998939143	0.999558179	0.99999488	0.999985264	0.944511198
LOC511847	0.999996191	0.999347049	0.940717701	0.99999488	0.999985264	0.944599417
LOC101902531	0.999996191	0.986637746	0.913976361	0.99999488	0.999985264	0.944906868
DENND2C	0.999996191	0.978154072	0.914001994	0.99999488	0.999985264	0.944906868
EPHX1	0.999996191	0.999992444	0.923604668	0.99999488	0.999985264	0.944906868

MPV17	0.999996191	0.937231686	0.929213642	0.99999488	0.999985264	0.944906868
SULT1A1	0.999996191	0.938777693	0.93198623	0.99999488	0.999985264	0.944906868
BRD4	0.999996191	0.998131745	0.938905004	0.99999488	0.999985264	0.944906868
CDK13	0.999996191	0.97015318	0.938911935	0.99999488	0.999985264	0.944906868
TGDS	0.999996191	0.999992444	0.939147816	0.99999488	0.999985264	0.944906868
RFX5	0.999996191	0.967919743	0.939432817	0.99999488	0.999985264	0.944906868
ERCC6L2	0.999996191	0.974079639	0.941782902	0.99999488	0.999985264	0.944906868
C11H9orf50	0.999996191	0.999992444	0.948069113	0.99999488	0.999985264	0.944906868
ARHGAP24	0.999996191	0.9582784	0.954872702	0.99999488	0.999985264	0.944906868
MRPS15	0.999996191	0.974812121	0.957541703	0.99999488	0.999985264	0.944906868
APOE	0.999996191	0.935742738	0.958627535	0.99999488	0.999985264	0.944906868
CBLN4	0.999996191	0.991488652	0.960133832	0.99999488	0.999985264	0.944906868
EML4	0.999996191	0.996105883	0.964152013	0.99999488	0.999985264	0.944906868
SUCO	0.999996191	0.991488652	0.964582964	0.99999488	0.999985264	0.944906868
MFSD14A	0.999996191	0.961479426	0.98023668	0.99999488	0.999985264	0.944906868
XKRX	0.999996191	0.998131745	0.981622418	0.99999488	0.999985264	0.944906868
LAMC1	0.999996191	0.999347049	0.998936876	0.99999488	0.999985264	0.944906868
FAM173A	0.999996191	0.970426668	0.945678616	0.99999488	0.999985264	0.944954215
ASCL4	0.999996191	0.9582784	0.95230212	0.99999488	0.999985264	0.944954215
CIZ1	0.999996191	0.972764622	0.981039752	0.99999488	0.999985264	0.945556076
INTS5	0.999996191	0.999992444	0.907484111	0.99999488	0.999985264	0.945570512
DECR2	0.999996191	0.999992444	0.993357864	0.99999488	0.999985264	0.945570512
NFAT5	0.999996191	0.991365955	0.879253779	0.99999488	0.999985264	0.945670698
PRRC1	0.999996191	0.949285285	0.902552409	0.99999488	0.999985264	0.945670698
SREBF2	0.999996191	0.999992444	0.902552409	0.99999488	0.999985264	0.945670698
PROCA1	0.999996191	0.991476708	0.903389425	0.99999488	0.999985264	0.945670698
LOC112442866	0.999996191	0.978510121	0.907381775	0.99999488	0.999985264	0.945670698
PODXL	0.999996191	0.992977597	0.908572237	0.99999488	0.999985264	0.945670698
FZD8	0.999996191	0.894928471	0.916975216	0.99999488	0.999985264	0.945670698
DDT	0.999996191	0.938587041	0.921646762	0.99999488	0.999985264	0.945670698
EIF3H	0.999996191	0.946274461	0.923177807	0.99999488	0.999985264	0.945670698
BDKRB2	0.999996191	0.999992444	0.923604668	0.99999488	0.999985264	0.945670698
DOCK10	0.999996191	0.943802122	0.926552506	0.99999488	0.999985264	0.945670698
INTS9	0.999996191	0.970426668	0.926552506	0.99999488	0.999985264	0.945670698
PCDH18	0.999996191	0.994481342	0.937822865	0.99999488	0.999985264	0.945670698
RHBDD3	0.999996191	0.999347049	0.938203102	0.99999488	0.999985264	0.945670698
LAPTM4B	0.999996191	0.94984369	0.938911935	0.99999488	0.999985264	0.945670698
PTPN21	0.999996191	0.970426668	0.939799459	0.99999488	0.999985264	0.945670698
WDR75	0.999996191	0.973092298	0.944993804	0.99999488	0.999985264	0.945670698

PLK3	0.999996191	0.998962836	0.944993804	0.99999488	0.999985264	0.945670698
PCDH17	0.999996191	0.946480858	0.946479014	0.99999488	0.999985264	0.945670698
ADHFE1	0.999996191	0.991488652	0.948923067	0.99999488	0.999985264	0.945670698
RAB11FIP4	0.999996191	0.985158008	0.949038056	0.99999488	0.999985264	0.945670698
HSD17B7	0.999996191	0.957700659	0.95106368	0.99999488	0.999985264	0.945670698
HDGFL3	0.999996191	0.974812121	0.951564604	0.99999488	0.999985264	0.945670698
AIP	0.999996191	0.999992444	0.95230212	0.99999488	0.999985264	0.945670698
GPM6B	0.999996191	0.999992444	0.95230212	0.99999488	0.999985264	0.945670698
JADE3	0.999996191	0.981298804	0.952547836	0.99999488	0.999985264	0.945670698
LOC515736	0.999996191	0.9582784	0.954571119	0.99999488	0.999985264	0.945670698
LOC101903478	0.999996191	0.964405281	0.954823586	0.99999488	0.999985264	0.945670698
TRAP1	0.999996191	0.946274461	0.956565728	0.99999488	0.999985264	0.945670698
SEC61B	0.999996191	0.975677199	0.956565728	0.99999488	0.999985264	0.945670698
ATP6V0D2	0.999996191	0.967276215	0.957541703	0.99999488	0.999985264	0.945670698
FAS	0.999996191	0.941495217	0.961597123	0.99999488	0.999985264	0.945670698
NAA20	0.999996191	0.9582784	0.964349087	0.99999488	0.999985264	0.945670698
CHIC1	0.999996191	0.935742738	0.966991646	0.99999488	0.999985264	0.945670698
KIF26A	0.999996191	0.94254418	0.968508342	0.99999488	0.999985264	0.945670698
ADCY4	0.999996191	0.9582784	0.96968839	0.99999488	0.999985264	0.945670698
LOC508131	0.999996191	0.999992444	0.970874203	0.99999488	0.999985264	0.945670698
PRNP	0.999996191	0.974812121	0.970945672	0.99999488	0.999985264	0.945670698
EXOSC3	0.999996191	0.97015318	0.973406453	0.99999488	0.999985264	0.945670698
CDCA2	0.999996191	0.991476708	0.973406453	0.99999488	0.999985264	0.945670698
LOC112442382	0.999996191	0.999992444	0.977141934	0.99999488	0.999985264	0.945670698
GNB1	0.999996191	0.998011664	0.977930966	0.99999488	0.999985264	0.945670698
TMEM265	0.999996191	0.893642384	0.977938265	0.99999488	0.999985264	0.945670698
LOC101902458	0.999996191	0.999992444	0.9808801	0.99999488	0.999985264	0.945670698
SNX30	0.999996191	0.974079639	0.981039752	0.99999488	0.999985264	0.945670698
CEP85	0.999996191	0.9582784	0.988799866	0.99999488	0.999985264	0.945670698
FUNDC1	0.999996191	0.992977597	0.988922732	0.99999488	0.999985264	0.945670698
OGG1	0.999996191	0.952345011	0.992709008	0.99999488	0.999985264	0.945670698
C24H18orf32	0.999996191	0.999347049	0.993594894	0.99999488	0.999985264	0.945670698
ASH2L	0.999996191	0.991488652	0.995045982	0.99999488	0.999985264	0.945670698
LOC101902030	0.999996191	0.978089982	0.998936876	0.99999488	0.999985264	0.945670698
MMS22L	0.999996191	0.9582784	0.999558179	0.99999488	0.999985264	0.945670698
FBP2	0.999996191	0.977354623	0.999558179	0.99999488	0.999985264	0.945670698
PDE4C	0.999996191	0.978510121	0.999558179	0.99999488	0.999985264	0.945670698
EDC3	0.999996191	0.985406717	0.999558179	0.99999488	0.999985264	0.945670698
PCSK7	0.999996191	0.999992444	0.919618001	0.99999488	0.999985264	0.945705257

DUSP23	0.999996191	0.999992444	0.929722337	0.99999488	0.999985264	0.945705257
NUDC	0.999996191	0.991574265	0.96210311	0.99999488	0.999985264	0.945705257
ALDH1L2	0.999996191	0.991488652	0.907381775	0.99999488	0.999985264	0.94572902
UBXN11	0.999996191	0.9582784	0.927600835	0.99999488	0.999985264	0.94572902
RPL9	0.999996191	0.987516216	0.946479014	0.99999488	0.999985264	0.94572902
NCKAP5L	0.999996191	0.9582784	0.956565728	0.99999488	0.999985264	0.94572902
C19H17orf80	0.999996191	0.976819696	0.998936876	0.99999488	0.999985264	0.94572902
MBOAT1	0.999996191	0.999992444	0.925745065	0.99999488	0.999985264	0.945742939
MYO19	0.999996191	0.924543195	0.922400936	0.99999488	0.999985264	0.945794274
LOC101905908	0.999996191	0.999992444	0.938905004	0.99999488	0.999985264	0.945794274
ZC3H13	0.999996191	0.992977597	0.94634922	0.99999488	0.999985264	0.945794274
LOC100294994	0.999996191	0.999992444	0.948420301	0.99999488	0.999985264	0.945794274
MIEN1	0.999996191	0.999992444	0.961024709	0.99999488	0.999985264	0.945794274
RAB3B	0.999996191	0.978510121	0.970903823	0.99999488	0.999985264	0.945794274
RIMKLB	0.999996191	0.977354623	0.973406453	0.99999488	0.999985264	0.945794274
FKBP1A	0.999996191	0.952345011	0.980859619	0.99999488	0.999985264	0.945794274
MRPL42	0.999996191	0.991488652	0.981039752	0.99999488	0.999985264	0.945794274
LOC101906067	0.999996191	0.989958965	0.999558179	0.99999488	0.999985264	0.945794274
SERF2	0.999996191	0.974812121	0.946479014	0.99999488	0.999985264	0.945838829
TMCO4	0.999996191	0.91507531	0.948359914	0.99999488	0.999985264	0.945838829
ZNF266	0.999996191	0.896996319	0.926552506	0.99999488	0.999985264	0.946072352
OGFRL1	0.999996191	0.926955605	0.973406453	0.99999488	0.999985264	0.946072352
PROX1	0.999996191	0.985293257	0.926552506	0.99999488	0.999985264	0.94639699
PPIP5K2	0.999996191	0.986637746	0.996715234	0.99999488	0.999985264	0.94639699
TMEM39B	0.999996191	0.999992444	0.932657945	0.99999488	0.999985264	0.946499396
LRAT	0.999996191	0.941793392	0.939147816	0.99999488	0.999985264	0.946629335
LOC101905593	0.999996191	0.999992444	0.901758346	0.99999488	0.999985264	0.946653006
ATP6AP2	0.999996191	0.991574265	0.973406453	0.99999488	0.999985264	0.946653006
ABI2	0.999996191	0.999992444	0.919763144	0.99999488	0.999985264	0.946666773
MYO5A	0.999996191	0.999992444	0.92487482	0.99999488	0.999985264	0.946666773
KLF9	0.999996191	0.949285285	0.926552506	0.99999488	0.999985264	0.946666773
ERO1B	0.999996191	0.999992444	0.94634922	0.99999488	0.999985264	0.946666773
TMX2	0.999996191	0.946274461	0.946479014	0.99999488	0.999985264	0.946666773
LOC112449106	0.999996191	0.999992444	0.96000759	0.99999488	0.999985264	0.946666773
HYLS1	0.999996191	0.998011664	0.964152013	0.99999488	0.999985264	0.946666773
ZNF639	0.999996191	0.999347049	0.973406453	0.99999488	0.999985264	0.946666773
ARCN1	0.999996191	0.943802122	0.981388276	0.99999488	0.999985264	0.946666773
C5H12orf45	0.999996191	0.996370892	0.992192736	0.99999488	0.999985264	0.946666773
HAUS2	0.999996191	0.97226888	0.999558179	0.99999488	0.999985264	0.946666773

LOC101903015	0.999996191	0.991488652	0.999558179	0.99999488	0.999985264	0.946666773
TTC36	0.999996191	0.945306277	0.892933333	0.99999488	0.999985264	0.9467439
GPAT2	0.999996191	0.999992444	0.902552409	0.99999488	0.999985264	0.9467439
LOC100848906	0.999996191	0.999992444	0.921065686	0.99999488	0.999985264	0.9467439
CXCL12	0.999996191	0.982313546	0.940815039	0.99999488	0.999985264	0.9467439
TPBGL	0.999996191	0.989547896	0.941336964	0.99999488	0.999985264	0.9467439
CFD	0.999996191	0.999992444	0.947957719	0.99999488	0.999985264	0.9467439
LIN7B	0.999996191	0.999347049	0.948420301	0.99999488	0.999985264	0.9467439
ADAM20	0.999996191	0.999347049	0.949038056	0.99999488	0.999985264	0.9467439
ANO3	0.999996191	0.981298804	0.950287431	0.99999488	0.999985264	0.9467439
KDM2A	0.999996191	0.978089982	0.951822586	0.99999488	0.999985264	0.9467439
PANK2	0.999996191	0.976819696	0.981039752	0.99999488	0.999985264	0.9467439
LVRN	0.999996191	0.981298804	0.981039752	0.99999488	0.999985264	0.9467439
G3BP1	0.999996191	0.946274461	0.996715234	0.99999488	0.999985264	0.9467439
LOC101904069	0.999996191	0.999992444	0.945678616	0.99999488	0.999985264	0.947012976
SELENOH	0.999996191	0.994481342	0.964152013	0.99999488	0.999985264	0.947012976
LOC112447495	0.999996191	0.95697765	0.979650244	0.99999488	0.999985264	0.947012976
HYAL2	0.999996191	0.992301181	0.938203102	0.99999488	0.999985264	0.947192047
NACAD	0.999996191	0.952346363	0.930678591	0.99999488	0.999985264	0.947400071
PBX4	0.999996191	0.958967142	0.938203102	0.99999488	0.999985264	0.947400071
ZNF668	0.999996191	0.998131745	0.930678591	0.99999488	0.999985264	0.947433142
LOC529399	0.999996191	0.978089982	0.954823586	0.99999488	0.999985264	0.947452706
IGFLR1	0.999996191	0.938597964	0.993594894	0.99999488	0.999985264	0.947452706
TCF7	0.999996191	0.9527938	0.938203102	0.99999488	0.999985264	0.947519413
LOC781254	0.999996191	0.970426668	0.993594894	0.99999488	0.999985264	0.947682139
WIPI2	0.999996191	0.994177236	0.961945138	0.99999488	0.999985264	0.9479986
RBM22	0.999996191	0.967276215	0.969880768	0.99999488	0.999985264	0.9479986
ATP2C1	0.999996191	0.957700659	0.994601277	0.99999488	0.999985264	0.9479986
LOC112443214	0.999996191	0.991488652	0.887626998	0.99999488	0.999985264	0.948213108
OOEP	0.999996191	0.938597964	0.900351064	0.99999488	0.999985264	0.948213108
COX4I2	0.999996191	0.998964359	0.911704194	0.99999488	0.999985264	0.948213108
PFDN6	0.999996191	0.985406717	0.916214824	0.99999488	0.999985264	0.948213108
LOC100848208	0.999996191	0.915367594	0.923953195	0.99999488	0.999985264	0.948213108
RNF152	0.999996191	0.922700337	0.929207633	0.99999488	0.999985264	0.948213108
KLHL11	0.999996191	0.992977597	0.930678591	0.99999488	0.999985264	0.948213108
LOC107132431	0.999996191	0.938597964	0.935937999	0.99999488	0.999985264	0.948213108
SPC24	0.999996191	0.946274461	0.938203102	0.99999488	0.999985264	0.948213108
SMG1	0.999996191	0.992977597	0.939147816	0.99999488	0.999985264	0.948213108
LOC107131769	0.999996191	0.9527938	0.939501959	0.99999488	0.999985264	0.948213108

MSRB1	0.999996191	0.988850552	0.941336964	0.99999488	0.999985264	0.948213108
CLCN4	0.999996191	0.993316598	0.944871472	0.99999488	0.999985264	0.948213108
CCDC58	0.999996191	0.90116492	0.945678616	0.99999488	0.999985264	0.948213108
WASHC1	0.999996191	0.999992444	0.945766581	0.99999488	0.999985264	0.948213108
THTPA	0.999996191	0.957700659	0.95106368	0.99999488	0.999985264	0.948213108
LOC526769	0.999996191	0.991488652	0.95230212	0.99999488	0.999985264	0.948213108
RPS27A	0.999996191	0.949285285	0.954620171	0.99999488	0.999985264	0.948213108
FAM104A	0.999996191	0.993540482	0.954620171	0.99999488	0.999985264	0.948213108
SCML1	0.999996191	0.999992444	0.955919075	0.99999488	0.999985264	0.948213108
REV1	0.999996191	0.985406717	0.956472932	0.99999488	0.999985264	0.948213108
MOB1A	0.999996191	0.991488652	0.956565728	0.99999488	0.999985264	0.948213108
FEM1B	0.999996191	0.999992444	0.956565728	0.99999488	0.999985264	0.948213108
FAR2	0.999996191	0.995532499	0.958627535	0.99999488	0.999985264	0.948213108
RTL8C	0.999996191	0.978510121	0.96290532	0.99999488	0.999985264	0.948213108
NUP54	0.999996191	0.992977597	0.963068909	0.99999488	0.999985264	0.948213108
CREG2	0.999996191	0.974812121	0.969880768	0.99999488	0.999985264	0.948213108
BDKRB1	0.999996191	0.941495217	0.973406453	0.99999488	0.999985264	0.948213108
SLC35E1	0.999996191	0.949285285	0.973406453	0.99999488	0.999985264	0.948213108
LOC101907570	0.999996191	0.999992444	0.973406453	0.99999488	0.999985264	0.948213108
CEP76	0.999996191	0.957700659	0.974727926	0.99999488	0.999985264	0.948213108
TARSL2	0.999996191	0.919884594	0.980660791	0.99999488	0.999985264	0.948213108
LOC112442264	0.999996191	0.978089982	0.983077441	0.99999488	0.999985264	0.948213108
CHMP4B	0.999996191	0.991488652	0.993594894	0.99999488	0.999985264	0.948213108
BAAT	0.999996191	0.986637746	0.996715234	0.99999488	0.999985264	0.948213108
LOC112441493	0.999996191	0.991488652	0.998523328	0.99999488	0.999985264	0.948213108
DUSP1	0.999996191	0.973092298	0.998844241	0.99999488	0.999985264	0.948213108
SRP14	0.999996191	0.965625946	0.998936876	0.99999488	0.999985264	0.948213108
ADD3	0.999996191	0.921549	0.999558179	0.99999488	0.999985264	0.948213108
RNF5	0.999996191	0.946274461	0.999558179	0.99999488	0.999985264	0.948213108
NKPD1	0.999996191	0.958967142	0.999558179	0.99999488	0.999985264	0.948213108
C16H1orf174	0.999996191	0.964246761	0.999558179	0.99999488	0.999985264	0.948213108
MAD2L1BP	0.999996191	0.991488652	0.999558179	0.99999488	0.999985264	0.948213108
RAB27B	0.999996191	0.991488652	0.999558179	0.99999488	0.999985264	0.948213108
LOC101903253	0.999996191	0.999992444	0.948946702	0.99999488	0.999985264	0.948334251
GSTA1	0.999996191	0.9582784	0.968235535	0.99999488	0.999985264	0.948347116
LOC112441607	0.999996191	0.991365955	0.944085862	0.99999488	0.999985264	0.948441365
ALOX12	0.999996191	0.999992444	0.979462526	0.99999488	0.999985264	0.948441365
HEPH	0.999996191	0.925387304	0.981218677	0.99999488	0.999985264	0.948441365
ELAC1	0.999996191	0.952639631	0.96684887	0.99999488	0.999985264	0.948455284

SERPINA5	0.999996191	0.976819696	0.953358777	0.99999488	0.999985264	0.94847204
TSTD1	0.999996191	0.938597964	0.998936876	0.99999488	0.999985264	0.94847204
GSN	0.999996191	0.999347049	0.926552506	0.99999488	0.999985264	0.94847852
WDR11	0.999996191	0.986637746	0.941118576	0.99999488	0.999985264	0.94847852
LOC100848148	0.999996191	0.986637746	0.973661483	0.99999488	0.999985264	0.94847852
NSD2	0.999996191	0.957073593	0.999558179	0.99999488	0.999985264	0.94847852
LOC101904227	0.999996191	0.958967142	0.945678616	0.99999488	0.999985264	0.948569712
LOC515578	0.999996191	0.999347049	0.946479014	0.99999488	0.999985264	0.948675165
TPMT	0.999996191	0.999992444	0.95230212	0.99999488	0.999985264	0.948675165
EVI5L	0.999996191	0.989958965	0.959396074	0.99999488	0.999985264	0.948675165
C10H15orf59	0.999996191	0.935742738	0.969880768	0.99999488	0.999985264	0.948675165
LOC101902926	0.999996191	0.991488652	0.998852661	0.99999488	0.999985264	0.949073334
AP3D1	0.999996191	0.974812121	0.930678591	0.99999488	0.999985264	0.94909885
FAM43A	0.999996191	0.999992444	0.911018006	0.99999488	0.999985264	0.94921096
GDPD4	0.999996191	0.991365955	0.94634922	0.99999488	0.999985264	0.94921096
LOC784322	0.999996191	0.946480858	0.999558179	0.99999488	0.999985264	0.94921096
VEGFA	0.999996191	0.991365955	0.938203102	0.99999488	0.999985264	0.949268951
CDKN3	0.999996191	0.958417033	0.998936876	0.99999488	0.999985264	0.949315689
FAM76B	0.999996191	0.999992444	0.916975216	0.99999488	0.999985264	0.949545831
LOC112445076	0.999996191	0.978510121	0.926552506	0.99999488	0.999985264	0.949545831
CEP97	0.999996191	0.999992444	0.939147816	0.99999488	0.999985264	0.949545831
KLHL26	0.999996191	0.993100184	0.952875254	0.99999488	0.999985264	0.949545831
UBR4	0.999996191	0.97505371	0.965690512	0.99999488	0.999985264	0.949545831
LOC112441491	0.999996191	0.998131745	0.997208738	0.99999488	0.999985264	0.949545831
FBXL8	0.999996191	0.971736716	0.999558179	0.99999488	0.999985264	0.949545831
IGHMBP2	0.999996191	0.994481342	0.999558179	0.99999488	0.999985264	0.949545831
PDPN	0.999996191	0.907956107	0.9808801	0.99999488	0.999985264	0.949643184
FAM161A	0.999996191	0.937626884	0.908122166	0.99999488	0.999985264	0.949718683
MRPL32	0.999996191	0.967276215	0.915266146	0.99999488	0.999985264	0.949718683
B4GAT1	0.999996191	0.946274461	0.919763144	0.99999488	0.999985264	0.949718683
ZNF518A	0.999996191	0.957700659	0.921010865	0.99999488	0.999985264	0.949718683
LOC100337323	0.999996191	0.942353856	0.925520255	0.99999488	0.999985264	0.949718683
LOC100848264	0.999996191	0.978426416	0.925520255	0.99999488	0.999985264	0.949718683
LOC104976574	0.999996191	0.913160799	0.927600835	0.99999488	0.999985264	0.949718683
SRSF2	0.999996191	0.95830623	0.930678591	0.99999488	0.999985264	0.949718683
GFI1	0.999996191	0.999992444	0.936914995	0.99999488	0.999985264	0.949718683
ULK1	0.999996191	0.915251309	0.938203102	0.99999488	0.999985264	0.949718683
MFSD3	0.999996191	0.978089982	0.944085862	0.99999488	0.999985264	0.949718683
LOC100848527	0.999996191	0.991488652	0.944085862	0.99999488	0.999985264	0.949718683

COPB1	0.999996191	0.992977597	0.945766581	0.99999488	0.999985264	0.949718683
MTSS1L	0.999996191	0.985406717	0.946479014	0.99999488	0.999985264	0.949718683
STAT6	0.999996191	0.991488652	0.946479014	0.99999488	0.999985264	0.949718683
RGS2	0.999996191	0.992977597	0.95230212	0.99999488	0.999985264	0.949718683
SEC24D	0.999996191	0.966804283	0.95405911	0.99999488	0.999985264	0.949718683
SEMA3A	0.999996191	0.958967142	0.954571119	0.99999488	0.999985264	0.949718683
BCL10	0.999996191	0.988850552	0.954864482	0.99999488	0.999985264	0.949718683
ADGRF5	0.999996191	0.999992444	0.956565728	0.99999488	0.999985264	0.949718683
FUNDC2	0.999996191	0.9582784	0.958627535	0.99999488	0.999985264	0.949718683
COA6	0.999996191	0.992977597	0.961024709	0.99999488	0.999985264	0.949718683
THOC2	0.999996191	0.991671026	0.962346588	0.99999488	0.999985264	0.949718683
PSMC3	0.999996191	0.999992444	0.968232195	0.99999488	0.999985264	0.949718683
SDHD	0.999996191	0.991488652	0.96968839	0.99999488	0.999985264	0.949718683
CAVIN4	0.999996191	0.991488652	0.973406453	0.99999488	0.999985264	0.949718683
HNRNPU	0.999996191	0.986445172	0.980660791	0.99999488	0.999985264	0.949718683
LOC101902968	0.999996191	0.992357549	0.980660791	0.99999488	0.999985264	0.949718683
LOC112443004	0.999996191	0.999992444	0.981593378	0.99999488	0.999985264	0.949718683
CTC1	0.999996191	0.961479426	0.982128584	0.99999488	0.999985264	0.949718683
ZCCHC10	0.999996191	0.999992444	0.98286145	0.99999488	0.999985264	0.949718683
PRIMPOL	0.999996191	0.948421431	0.983419007	0.99999488	0.999985264	0.949718683
FBXO45	0.999996191	0.973334182	0.984814673	0.99999488	0.999985264	0.949718683
SNX15	0.999996191	0.983506778	0.98545277	0.99999488	0.999985264	0.949718683
GPR137	0.999996191	0.991345032	0.988922732	0.99999488	0.999985264	0.949718683
AAAS	0.999996191	0.999992444	0.988922732	0.99999488	0.999985264	0.949718683
INHBB	0.999996191	0.974812121	0.989612502	0.99999488	0.999985264	0.949718683
SRRM1	0.999996191	0.957700659	0.992352317	0.99999488	0.999985264	0.949718683
CLDN1	0.999996191	0.991488652	0.994879215	0.99999488	0.999985264	0.949718683
ANKAR	0.999996191	0.999992444	0.995045982	0.99999488	0.999985264	0.949718683
ARID1B	0.999996191	0.998131745	0.998816213	0.99999488	0.999985264	0.949718683
TNIP2	0.999996191	0.946274461	0.998936876	0.99999488	0.999985264	0.949718683
ATMIN	0.999996191	0.993100184	0.998936876	0.99999488	0.999985264	0.949718683
SPTY2D1OS	0.999996191	0.966824751	0.999558179	0.99999488	0.999985264	0.949718683
LOC101907247	0.999996191	0.967374613	0.999558179	0.99999488	0.999985264	0.949718683
SUB1	0.999996191	0.999992444	0.999558179	0.99999488	0.999985264	0.949718683
TAF8	0.999996191	0.941760655	0.900351064	0.99999488	0.999985264	0.949764317
CHL1	0.999996191	0.928925667	0.919763144	0.99999488	0.999985264	0.949764317
LOC112449563	0.999996191	0.986637746	0.920154807	0.99999488	0.999985264	0.949764317
GCNT4	0.999996191	0.922102474	0.9222878	0.99999488	0.999985264	0.949764317
CAV3	0.999996191	0.993777777	0.922400936	0.99999488	0.999985264	0.949764317

MYO1C	0.999996191	0.995532499	0.924604843	0.99999488	0.999985264	0.949764317
CLASP2	0.999996191	0.938597964	0.926552506	0.99999488	0.999985264	0.949764317
LYRM7	0.999996191	0.999992444	0.930678591	0.99999488	0.999985264	0.949764317
LOC101905648	0.999996191	0.967463091	0.933447488	0.99999488	0.999985264	0.949764317
TCAM1	0.999996191	0.942353856	0.939147816	0.99999488	0.999985264	0.949764317
SMARCA2	0.999996191	0.93597782	0.945678616	0.99999488	0.999985264	0.949764317
LOC107132767	0.999996191	0.922700337	0.945766581	0.99999488	0.999985264	0.949764317
DPCD	0.999996191	0.991476708	0.946479014	0.99999488	0.999985264	0.949764317
UROD	0.999996191	0.9582784	0.946832224	0.99999488	0.999985264	0.949764317
MRPS6	0.999996191	0.957700659	0.95106368	0.99999488	0.999985264	0.949764317
ZDBF2	0.999996191	0.999992444	0.95106368	0.99999488	0.999985264	0.949764317
BEX2	0.999996191	0.973334182	0.951822586	0.99999488	0.999985264	0.949764317
AMZ2	0.999996191	0.944181275	0.952547836	0.99999488	0.999985264	0.949764317
LOC100335340	0.999996191	0.998131745	0.954823586	0.99999488	0.999985264	0.949764317
NOL7	0.999996191	0.978089982	0.958627535	0.99999488	0.999985264	0.949764317
CSNK1G3	0.999996191	0.999992444	0.958627535	0.99999488	0.999985264	0.949764317
PATL2	0.999996191	0.941306392	0.96210311	0.99999488	0.999985264	0.949764317
ZNF521	0.999996191	0.985406717	0.964349087	0.99999488	0.999985264	0.949764317
ZNF275	0.999996191	0.949285285	0.968232195	0.99999488	0.999985264	0.949764317
ACOX3	0.999996191	0.999992444	0.969880768	0.99999488	0.999985264	0.949764317
LOC101906135	0.999996191	0.991488652	0.970945672	0.99999488	0.999985264	0.949764317
UBA5	0.999996191	0.946274461	0.972362683	0.99999488	0.999985264	0.949764317
PLCD3	0.999996191	0.985406717	0.973303821	0.99999488	0.999985264	0.949764317
ASB8	0.999996191	0.971222251	0.973406453	0.99999488	0.999985264	0.949764317
LRRC74B	0.999996191	0.999992444	0.9808801	0.99999488	0.999985264	0.949764317
TARS2	0.999996191	0.981520287	0.981593378	0.99999488	0.999985264	0.949764317
ANKRD40	0.999996191	0.977354623	0.981666445	0.99999488	0.999985264	0.949764317
DYM	0.999996191	0.99398773	0.981764406	0.99999488	0.999985264	0.949764317
RIPK2	0.999996191	0.999992444	0.985363931	0.99999488	0.999985264	0.949764317
TMEM220	0.999996191	0.978510121	0.992709008	0.99999488	0.999985264	0.949764317
LSM6	0.999996191	0.926949474	0.999558179	0.99999488	0.999985264	0.949764317
NYAP1	0.999996191	0.941495217	0.999558179	0.99999488	0.999985264	0.949764317
MUTYH	0.999996191	0.991365955	0.999558179	0.99999488	0.999985264	0.949764317
TXNL4A	0.999996191	0.938520118	0.931507181	0.99999488	0.999985264	0.949894986
LARP1	0.999996191	0.904961534	0.888545123	0.99999488	0.999985264	0.950203425
APOBR	0.999996191	0.999347049	0.908181052	0.99999488	0.999985264	0.950203425
GOSR1	0.999996191	0.992977597	0.933791093	0.99999488	0.999985264	0.950203425
PHACTR2	0.999996191	0.961479426	0.946479014	0.99999488	0.999985264	0.950203425
ST3GAL3	0.999996191	0.999992444	0.951680496	0.99999488	0.999985264	0.950203425

NCAPD3	0.999996191	0.948421431	0.999558179	0.99999488	0.999985264	0.950203425
ABLIM3	0.999996191	0.991488652	0.956565728	0.99999488	0.999985264	0.950580945
MED24	0.999996191	0.994481342	0.96210311	0.99999488	0.999985264	0.950666747
SIGLEC5	0.999996191	0.938597964	0.902527616	0.99999488	0.999985264	0.950946633
MKKS	0.999996191	0.922700337	0.999558179	0.99999488	0.999985264	0.95096227
LOC112443143	0.999996191	0.949285285	0.891365486	0.99999488	0.999985264	0.95096765
ADGRG6	0.999996191	0.999992444	0.897767405	0.99999488	0.999985264	0.95096765
EFL1	0.999996191	0.918883775	0.904528319	0.99999488	0.999985264	0.95096765
MMAB	0.999996191	0.934871569	0.907381775	0.99999488	0.999985264	0.95096765
TSPAN11	0.999996191	0.959158523	0.920629231	0.99999488	0.999985264	0.95096765
IFT81	0.999996191	0.916036681	0.921010865	0.99999488	0.999985264	0.95096765
SYNPO2	0.999996191	0.999992444	0.921755524	0.99999488	0.999985264	0.95096765
SUCLG1	0.999996191	0.934142668	0.923604668	0.99999488	0.999985264	0.95096765
LSM4	0.999996191	0.959983117	0.923604668	0.99999488	0.999985264	0.95096765
NTN3	0.999996191	0.938597964	0.924568821	0.99999488	0.999985264	0.95096765
LOC101902807	0.999996191	0.948421431	0.926552506	0.99999488	0.999985264	0.95096765
LOC101906756	0.999996191	0.9582784	0.926552506	0.99999488	0.999985264	0.95096765
LOC104970908	0.999996191	0.9582784	0.926552506	0.99999488	0.999985264	0.95096765
LOC107132870	0.999996191	0.998181556	0.926552506	0.99999488	0.999985264	0.95096765
ARHGEF37	0.999996191	0.94984369	0.927600835	0.99999488	0.999985264	0.95096765
RAB11FIP5	0.999996191	0.967520941	0.927600835	0.99999488	0.999985264	0.95096765
LRP5	0.999996191	0.999992444	0.931507181	0.99999488	0.999985264	0.95096765
MDN1	0.999996191	0.958098081	0.93198623	0.99999488	0.999985264	0.95096765
MPV17L	0.999996191	0.974079639	0.932762395	0.99999488	0.999985264	0.95096765
LOC112446760	0.999996191	0.9582784	0.933791093	0.99999488	0.999985264	0.95096765
METTL18	0.999996191	0.999992444	0.933791093	0.99999488	0.999985264	0.95096765
LOC112441554	0.999996191	0.958967142	0.935726137	0.99999488	0.999985264	0.95096765
FCAR	0.999996191	0.969534305	0.936914995	0.99999488	0.999985264	0.95096765
VPS28	0.999996191	0.999347049	0.937874396	0.99999488	0.999985264	0.95096765
PHKA1	0.999996191	0.999992444	0.937874396	0.99999488	0.999985264	0.95096765
CYB5A	0.999996191	0.999992444	0.938203102	0.99999488	0.999985264	0.95096765
MRPL24	0.999996191	0.9582784	0.939147816	0.99999488	0.999985264	0.95096765
ELK4	0.999996191	0.985471417	0.939147816	0.99999488	0.999985264	0.95096765
SEC23IP	0.999996191	0.991488652	0.93955737	0.99999488	0.999985264	0.95096765
MLST8	0.999996191	0.985406717	0.940815039	0.99999488	0.999985264	0.95096765
GAPVD1	0.999996191	0.991488652	0.941336964	0.99999488	0.999985264	0.95096765
DCUN1D1	0.999996191	0.999992444	0.94212771	0.99999488	0.999985264	0.95096765
ITGA8	0.999996191	0.999992444	0.942675769	0.99999488	0.999985264	0.95096765
LOC112442265	0.999996191	0.999992444	0.944085862	0.99999488	0.999985264	0.95096765

KLHL36	0.999996191	0.995029734	0.944129251	0.99999488	0.999985264	0.95096765
S100A14	0.999996191	0.994481342	0.944168373	0.99999488	0.999985264	0.95096765
SNW1	0.999996191	0.992977597	0.944871472	0.99999488	0.999985264	0.95096765
EP300	0.999996191	0.979263532	0.944993804	0.99999488	0.999985264	0.95096765
ETFRF1	0.999996191	0.941760655	0.945678616	0.99999488	0.999985264	0.95096765
LOC112442649	0.999996191	0.985406717	0.945678616	0.99999488	0.999985264	0.95096765
KCTD20	0.999996191	0.999992444	0.945678616	0.99999488	0.999985264	0.95096765
LTBP4	0.999996191	0.999992444	0.94634922	0.99999488	0.999985264	0.95096765
SEMA4G	0.999996191	0.999992444	0.94634922	0.99999488	0.999985264	0.95096765
CSRP1	0.999996191	0.948421431	0.946479014	0.99999488	0.999985264	0.95096765
LOC112446024	0.999996191	0.95697765	0.946479014	0.99999488	0.999985264	0.95096765
OLFML2A	0.999996191	0.957700659	0.946479014	0.99999488	0.999985264	0.95096765
RNF227	0.999996191	0.998962836	0.946479014	0.99999488	0.999985264	0.95096765
CALY	0.999996191	0.999992444	0.946479014	0.99999488	0.999985264	0.95096765
ERG	0.999996191	0.999992444	0.946479014	0.99999488	0.999985264	0.95096765
SCYL3	0.999996191	0.999992444	0.946479014	0.99999488	0.999985264	0.95096765
LOC112441645	0.999996191	0.967276215	0.948420301	0.99999488	0.999985264	0.95096765
DUSP28	0.999996191	0.981298804	0.949038056	0.99999488	0.999985264	0.95096765
RHOJ	0.999996191	0.967276215	0.951099739	0.99999488	0.999985264	0.95096765
CDK6	0.999996191	0.9582784	0.951735967	0.99999488	0.999985264	0.95096765
GPATCH2L	0.999996191	0.949283886	0.95230212	0.99999488	0.999985264	0.95096765
KCNC3	0.999996191	0.957700659	0.95230212	0.99999488	0.999985264	0.95096765
BAG5	0.999996191	0.991488652	0.95230212	0.99999488	0.999985264	0.95096765
EGLN1	0.999996191	0.991488652	0.952386544	0.99999488	0.999985264	0.95096765
SUPT16H	0.999996191	0.993316598	0.952547836	0.99999488	0.999985264	0.95096765
ZDHHC7	0.999996191	0.952639631	0.954620171	0.99999488	0.999985264	0.95096765
GMDS	0.999996191	0.999347049	0.954620171	0.99999488	0.999985264	0.95096765
PRMT9	0.999996191	0.981520287	0.95477544	0.99999488	0.999985264	0.95096765
TRAPPC1	0.999996191	0.999992444	0.95477544	0.99999488	0.999985264	0.95096765
LRRC42	0.999996191	0.964405281	0.954823586	0.99999488	0.999985264	0.95096765
RAB4A	0.999996191	0.986445172	0.954864482	0.99999488	0.999985264	0.95096765
LOC786252	0.999996191	0.982628449	0.956472932	0.99999488	0.999985264	0.95096765
ATP6V1E1	0.999996191	0.991488652	0.957541703	0.99999488	0.999985264	0.95096765
DNASE1	0.999996191	0.96566261	0.958627535	0.99999488	0.999985264	0.95096765
CTNNAL1	0.999996191	0.999992444	0.958627535	0.99999488	0.999985264	0.95096765
PITX3	0.999996191	0.981520287	0.961024709	0.99999488	0.999985264	0.95096765
SPG11	0.999996191	0.999992444	0.962346588	0.99999488	0.999985264	0.95096765
COIL	0.999996191	0.992895985	0.964079616	0.99999488	0.999985264	0.95096765
GAN	0.999996191	0.999992444	0.966609561	0.99999488	0.999985264	0.95096765

RSPO1	0.999996191	0.998131745	0.969880768	0.99999488	0.999985264	0.95096765
OPLAH	0.999996191	0.978510121	0.970874203	0.99999488	0.999985264	0.95096765
LOC513969	0.999996191	0.999992444	0.970882851	0.99999488	0.999985264	0.95096765
CRTAP	0.999996191	0.999992444	0.971161101	0.99999488	0.999985264	0.95096765
PCK1	0.999996191	0.999992444	0.973303821	0.99999488	0.999985264	0.95096765
RTN2	0.999996191	0.935246749	0.978389389	0.99999488	0.999985264	0.95096765
C17H12orf49	0.999996191	0.992977597	0.9808801	0.99999488	0.999985264	0.95096765
CLMP	0.999996191	0.992977597	0.981218677	0.99999488	0.999985264	0.95096765
GALNS	0.999996191	0.998131745	0.983077441	0.99999488	0.999985264	0.95096765
EDRF1	0.999996191	0.999992444	0.985966356	0.99999488	0.999985264	0.95096765
AKNAD1	0.999996191	0.998011664	0.988161083	0.99999488	0.999985264	0.95096765
TAC3	0.999996191	0.90116492	0.988799866	0.99999488	0.999985264	0.95096765
LOC787269	0.999996191	0.978089982	0.992709008	0.99999488	0.999985264	0.95096765
DCUN1D4	0.999996191	0.997083202	0.992742113	0.99999488	0.999985264	0.95096765
MRFAP1L1	0.999996191	0.990248517	0.993594894	0.99999488	0.999985264	0.95096765
LOC782673	0.999996191	0.992977597	0.993594894	0.99999488	0.999985264	0.95096765
CTNNBIP1	0.999996191	0.999992444	0.993594894	0.99999488	0.999985264	0.95096765
CCNC	0.999996191	0.998131745	0.994601277	0.99999488	0.999985264	0.95096765
WDR31	0.999996191	0.9582784	0.996348351	0.99999488	0.999985264	0.95096765
TMOD2	0.999996191	0.931676157	0.998852661	0.99999488	0.999985264	0.95096765
AP4M1	0.999996191	0.926955605	0.998936876	0.99999488	0.999985264	0.95096765
LOC100336734	0.999996191	0.945217876	0.998936876	0.99999488	0.999985264	0.95096765
AKAP10	0.999996191	0.961479426	0.998936876	0.99999488	0.999985264	0.95096765
TCOF1	0.999996191	0.985406717	0.998936876	0.99999488	0.999985264	0.95096765
DDX52	0.999996191	0.991574265	0.998936876	0.99999488	0.999985264	0.95096765
CCDC91	0.999996191	0.999992444	0.998936876	0.99999488	0.999985264	0.95096765
C7H5orf24	0.999996191	0.9527938	0.999558179	0.99999488	0.999985264	0.95096765
PDLIM1	0.999996191	0.991488652	0.999558179	0.99999488	0.999985264	0.95096765
MPST	0.999996191	0.995532499	0.94634922	0.99999488	0.999985264	0.951196054
IGFBP6	0.999996191	0.992977597	0.95230212	0.99999488	0.999985264	0.951196054
PRAM1	0.999996191	0.981520287	0.984444908	0.99999488	0.999985264	0.951196054
MTA3	0.999996191	0.999992444	0.916975216	0.99999488	0.999985264	0.951197866
TBPL1	0.999996191	0.999992444	0.934299387	0.99999488	0.999985264	0.951197866
CLCC1	0.999996191	0.979263532	0.938203102	0.99999488	0.999985264	0.951197866
C13H20orf194	0.999996191	0.999347049	0.95230212	0.99999488	0.999985264	0.951197866
RPL22	0.999996191	0.991488652	0.980660791	0.99999488	0.999985264	0.951197866
DCTN1	0.999996191	0.896400219	0.988922732	0.99999488	0.999985264	0.951197866
GRAMD2A	0.999996191	0.89076613	0.992742113	0.99999488	0.999985264	0.951197866
SEC22B	0.999996191	0.933454973	0.907381775	0.99999488	0.999985264	0.951269302

LOC781612	0.999996191	0.991488652	0.926552506	0.99999488	0.999985264	0.951269302
LOC104974113	0.999996191	0.999992444	0.926552506	0.99999488	0.999985264	0.951269302
QSOX1	0.999996191	0.999992444	0.938203102	0.99999488	0.999985264	0.951269302
LOC112446427	0.999996191	0.978426416	0.95106368	0.99999488	0.999985264	0.951269302
ADGRF3	0.999996191	0.991365955	0.994601277	0.99999488	0.999985264	0.951269302
TACR2	0.999996191	0.964246761	0.998936876	0.99999488	0.999985264	0.951269302
APIP	0.999996191	0.991365955	0.998936876	0.99999488	0.999985264	0.951295996
RPN1	0.999996191	0.980496345	0.925520255	0.99999488	0.999985264	0.951301677
ATOH8	0.999996191	0.998962836	0.946479014	0.99999488	0.999985264	0.951301677
AP2S1	0.999996191	0.973759991	0.95230212	0.99999488	0.999985264	0.951301677
SLC38A1	0.999996191	0.974812121	0.9808801	0.99999488	0.999985264	0.951499563
PNKP	0.999996191	0.985293257	0.999558179	0.99999488	0.999985264	0.951499563
UBE2S	0.999996191	0.999992444	0.926552506	0.99999488	0.999985264	0.951863062
MTERF3	0.999996191	0.984366454	0.946479014	0.99999488	0.999985264	0.951863062
STK3	0.999996191	0.999992444	0.946479014	0.99999488	0.999985264	0.951863062
TMEM243	0.999996191	0.999992444	0.957117655	0.99999488	0.999985264	0.951863062
AKAP2	0.999996191	0.999347049	0.982128584	0.99999488	0.999985264	0.951863062
HOXB6	0.999996191	0.99949931	0.98253615	0.99999488	0.999985264	0.951863062
NMNAT1	0.999996191	0.95697765	0.991285432	0.99999488	0.999985264	0.951863062
SHPK	0.999996191	0.957700659	0.998936876	0.99999488	0.999985264	0.951863062
EBF2	0.999996191	0.93570298	0.948420301	0.99999488	0.999985264	0.951922741
LOC101903557	0.999996191	0.999992444	0.921065686	0.99999488	0.999985264	0.951993931
GFER	0.999996191	0.941495217	0.926552506	0.99999488	0.999985264	0.951993931
ANAPC1	0.999996191	0.959839652	0.938203102	0.99999488	0.999985264	0.951993931
LOC101902681	0.999996191	0.985406717	0.954864482	0.99999488	0.999985264	0.951993931
TMEM42	0.999996191	0.978510121	0.989872445	0.99999488	0.999985264	0.951993931
LOC785087	0.999996191	0.998131745	0.907484111	0.99999488	0.999985264	0.952124699
LOC104973073	0.999996191	0.999992444	0.94212771	0.99999488	0.999985264	0.952124699
ZNF420	0.999996191	0.999992444	0.95106368	0.99999488	0.999985264	0.952124699
C18H19orf81	0.999996191	0.91058693	0.95230212	0.99999488	0.999985264	0.952124699
METAP1D	0.999996191	0.985406717	0.973406453	0.99999488	0.999985264	0.952124699
SPCS1	0.999996191	0.986637746	0.980677819	0.99999488	0.999985264	0.952124699
LOC100848407	0.999996191	0.991488652	0.981039752	0.99999488	0.999985264	0.952124699
BLK	0.999996191	0.95697765	0.999558179	0.99999488	0.999985264	0.952124699
SPATA6	0.999996191	0.999992444	0.948254593	0.99999488	0.999985264	0.952422008
HPGD	0.999996191	0.943802122	0.980677819	0.99999488	0.999985264	0.952422008
ASB4	0.999996191	0.992977597	0.946283845	0.99999488	0.999985264	0.952637514
PCDHB14	0.999996191	0.9677397	0.963068909	0.99999488	0.999985264	0.952649963
LOC107131615	0.999996191	0.948257806	0.876758498	0.99999488	0.999985264	0.952669273

TPCN2	0.999996191	0.987516216	0.925520255	0.99999488	0.999985264	0.952669273
RAD23B	0.999996191	0.961479426	0.931008069	0.99999488	0.999985264	0.952669273
PPP1R18	0.999996191	0.989958965	0.935094475	0.99999488	0.999985264	0.952669273
ARID2	0.999996191	0.967063915	0.938559901	0.99999488	0.999985264	0.952669273
EMCN	0.999996191	0.978426416	0.938905004	0.99999488	0.999985264	0.952669273
GAS6	0.999996191	0.993316598	0.940815039	0.99999488	0.999985264	0.952669273
ZNF622	0.999996191	0.974812121	0.945678616	0.99999488	0.999985264	0.952669273
PYGO1	0.999996191	0.998131745	0.945678616	0.99999488	0.999985264	0.952669273
LOC112444681	0.999996191	0.999992444	0.94634922	0.99999488	0.999985264	0.952669273
VIPAS39	0.999996191	0.967276215	0.961490767	0.99999488	0.999985264	0.952669273
RELT	0.999996191	0.991488652	0.96210311	0.99999488	0.999985264	0.952669273
SLC9A7	0.999996191	0.978154072	0.965002113	0.99999488	0.999985264	0.952669273
CEP164	0.999996191	0.943802122	0.970903823	0.99999488	0.999985264	0.952669273
SPPL2A	0.999996191	0.983781707	0.97532818	0.99999488	0.999985264	0.952669273
TP53I11	0.999996191	0.949285285	0.980660791	0.99999488	0.999985264	0.952669273
DNAJB2	0.999996191	0.985406717	0.9808801	0.99999488	0.999985264	0.952669273
OAZ1	0.999996191	0.985406717	0.9808801	0.99999488	0.999985264	0.952669273
ALS2CL	0.999996191	0.984366454	0.988922732	0.99999488	0.999985264	0.952669273
KIAA0753	0.999996191	0.991476708	0.990780762	0.99999488	0.999985264	0.952669273
FBXO15	0.999996191	0.95697765	0.993594894	0.99999488	0.999985264	0.952669273
LOC100141253	0.999996191	0.999992444	0.993594894	0.99999488	0.999985264	0.952669273
LOC112442630	0.999996191	0.975951808	0.99397371	0.99999488	0.999985264	0.952669273
FASTKD5	0.999996191	0.986445172	0.998936876	0.99999488	0.999985264	0.952669273
CHCHD7	0.999996191	0.9582784	0.999558179	0.99999488	0.999985264	0.952669273
LOC784127	0.999996191	0.989872618	0.999558179	0.99999488	0.999985264	0.952669273
RTF1	0.999996191	0.937511653	0.926552506	0.99999488	0.999985264	0.952715074
PNRC1	0.999996191	0.938597964	0.882737471	0.99999488	0.999985264	0.952764661
KCNH4	0.999996191	0.999992444	0.938203102	0.99999488	0.999985264	0.952764661
ZDHHC8	0.999996191	0.991488652	0.944932814	0.99999488	0.999985264	0.952764661
ITPRIP	0.999996191	0.999992444	0.946479014	0.99999488	0.999985264	0.952764661
ZBTB7A	0.999996191	0.967276215	0.964152013	0.99999488	0.999985264	0.952764661
LOC112444920	0.999996191	0.998131745	0.981039752	0.99999488	0.999985264	0.952764661
ENPEP	0.999996191	0.999992444	0.991285432	0.99999488	0.999985264	0.952764661
CLP1	0.999996191	0.984366454	0.993594894	0.99999488	0.999985264	0.952764661
ORC3	0.999996191	0.985406717	0.998936876	0.99999488	0.999985264	0.952764661
AMHR2	0.999996191	0.991488652	0.999558179	0.99999488	0.999985264	0.952764661
LOC101906484	0.999996191	0.980867747	0.95230212	0.99999488	0.999985264	0.952851247
TENM4	0.999996191	0.935588259	0.914001994	0.99999488	0.999985264	0.952948574
KIAA1522	0.999996191	0.958967142	0.938905004	0.99999488	0.999985264	0.952948574

RNF135	0.999996191	0.96566261	0.939147816	0.99999488	0.999985264	0.952948574
TCIM	0.999996191	0.938597964	0.944085862	0.99999488	0.999985264	0.952948574
GANAB	0.999996191	0.883624935	0.945766581	0.99999488	0.999985264	0.952948574
C3H1orf123	0.999996191	0.97286093	0.946695467	0.99999488	0.999985264	0.952948574
LOX	0.999996191	0.948421431	0.95230212	0.99999488	0.999985264	0.952948574
ZBED8	0.999996191	0.992977597	0.973406453	0.99999488	0.999985264	0.952948574
SLC1A1	0.999996191	0.913160799	0.977265078	0.99999488	0.999985264	0.952948574
LOC104976804	0.999996191	0.970470739	0.981039752	0.99999488	0.999985264	0.952948574
CEP19	0.999996191	0.981298804	0.996391386	0.99999488	0.999985264	0.952948574
CETN4	0.999996191	0.994177236	0.962346588	0.99999488	0.999985264	0.953077417
POLM	0.999996191	0.999992444	0.981622418	0.99999488	0.999985264	0.953077417
LRRC27	0.999996191	0.935742738	0.926552506	0.99999488	0.999985264	0.953181772
NCOA1	0.999996191	0.991488652	0.941118576	0.99999488	0.999985264	0.953181772
MBIP	0.999996191	0.999347049	0.981039752	0.99999488	0.999985264	0.953181772
CCDC112	0.999996191	0.921934673	0.954872702	0.99999488	0.999985264	0.953252635
LOC112445968	0.999996191	0.957700659	0.999558179	0.99999488	0.999985264	0.953252635
TMEM8A	0.999996191	0.999992444	0.922400936	0.99999488	0.999985264	0.953254073
RPL14	0.999996191	0.999992444	0.938905004	0.99999488	0.999985264	0.953254073
TCN2	0.999996191	0.991671026	0.940815039	0.99999488	0.999985264	0.953254073
BASP1	0.999996191	0.992977597	0.941336964	0.99999488	0.999985264	0.953254073
CACNA2D1	0.999996191	0.999992444	0.949048582	0.99999488	0.999985264	0.953254073
LOC101902288	0.999996191	0.991488652	0.95106368	0.99999488	0.999985264	0.953254073
TPC3	0.999996191	0.999992444	0.965065403	0.99999488	0.999985264	0.953254073
SAR1A	0.999996191	0.941495217	0.998936876	0.99999488	0.999985264	0.953254073
SRCIN1	0.999996191	0.999992444	0.999558179	0.99999488	0.999985264	0.953254073
LOC112443499	0.999996191	0.991365955	0.980859619	0.99999488	0.999985264	0.953400133
CLDN5	0.999996191	0.987516216	0.998936876	0.99999488	0.999985264	0.953495274
PELP1	0.999996191	0.974812121	0.930678591	0.99999488	0.999985264	0.953565168
LLGL1	0.999996191	0.984068885	0.975344975	0.99999488	0.999985264	0.953565168
NXF1	0.999996191	0.983781707	0.96210311	0.99999488	0.999985264	0.953658739
LOC515227	0.999996191	0.99873048	0.913976361	0.99999488	0.999985264	0.953795193
PDK4	0.999996191	0.987705076	0.930678591	0.99999488	0.999985264	0.953795193
ARHGEF10	0.999996191	0.997051616	0.946479014	0.99999488	0.999985264	0.953795193
TGFB1	0.999996191	0.978510121	0.964152013	0.99999488	0.999985264	0.95389363
RWDD2B	0.999996191	0.992977597	0.964920281	0.99999488	0.999985264	0.95389363
ACAD9	0.999996191	0.946274461	0.900148803	0.99999488	0.999985264	0.953908163
SLC8A3	0.999996191	0.999992444	0.924866532	0.99999488	0.999985264	0.953908163
ABHD2	0.999996191	0.991365955	0.927022752	0.99999488	0.999985264	0.953908163
GPR182	0.999996191	0.986637746	0.94753587	0.99999488	0.999985264	0.953908163

RNF43	0.999996191	0.999992444	0.958627535	0.99999488	0.999985264	0.953908163
LOC101908166	0.999996191	0.997975376	0.907484111	0.99999488	0.999985264	0.95395615
COL4A1	0.999996191	0.994481342	0.946832224	0.99999488	0.999985264	0.95395615
GTPBP8	0.999996191	0.999992444	0.95230212	0.99999488	0.999985264	0.95395615
SNX7	0.999996191	0.999992444	0.970874203	0.99999488	0.999985264	0.95395615
RPL21	0.999996191	0.967276215	0.975538142	0.99999488	0.999985264	0.954042188
CENPQ	0.999996191	0.970426668	0.981388276	0.99999488	0.999985264	0.954042188
CISD3	0.999996191	0.974812121	0.987198143	0.99999488	0.999985264	0.954042188
LOC512323	0.999996191	0.978089982	0.992709008	0.99999488	0.999985264	0.954042188
LOC107132589	0.999996191	0.958098081	0.999558179	0.99999488	0.999985264	0.954042188
MPI	0.999996191	0.937231686	0.920629231	0.99999488	0.999985264	0.954101579
POLR3E	0.999996191	0.94984369	0.921646762	0.99999488	0.999985264	0.954519353
SZT2	0.999996191	0.89880506	0.925745065	0.99999488	0.999985264	0.954519353
LOC112444287	0.999996191	0.927853996	0.926552506	0.99999488	0.999985264	0.954519353
NEMP1	0.999996191	0.991488652	0.926552506	0.99999488	0.999985264	0.954519353
LOC112445889	0.999996191	0.9582784	0.932653752	0.99999488	0.999985264	0.954519353
LOC104974666	0.999996191	0.97993071	0.938203102	0.99999488	0.999985264	0.954519353
EIF4B	0.999996191	0.998131745	0.938203102	0.99999488	0.999985264	0.954519353
ANGEL2	0.999996191	0.999992444	0.946479014	0.99999488	0.999985264	0.954519353
PLEKHH2	0.999996191	0.961479426	0.948359914	0.99999488	0.999985264	0.954519353
GNPNAT1	0.999996191	0.974812121	0.948657401	0.99999488	0.999985264	0.954519353
ADIPOR1	0.999996191	0.991345032	0.948923067	0.99999488	0.999985264	0.954519353
ZMYM2	0.999996191	0.958967142	0.954620171	0.99999488	0.999985264	0.954519353
CFAP70	0.999996191	0.978089982	0.956565728	0.99999488	0.999985264	0.954519353
LOC107132849	0.999996191	0.985406717	0.958627535	0.99999488	0.999985264	0.954519353
SCN2A	0.999996191	0.991345032	0.960133832	0.99999488	0.999985264	0.954519353
UBE2L3	0.999996191	0.978089982	0.96138904	0.99999488	0.999985264	0.954519353
TUT1	0.999996191	0.895632001	0.964920281	0.99999488	0.999985264	0.954519353
NSD1	0.999996191	0.967276215	0.992709008	0.99999488	0.999985264	0.954519353
LOC104970162	0.999996191	0.994210852	0.999558179	0.99999488	0.999985264	0.954519353
FOXC2	0.999996191	0.998131745	0.999558179	0.99999488	0.999985264	0.954519353
MAP1S	0.999996191	0.992977597	0.93179776	0.99999488	0.999985264	0.954683883
PUS7L	0.999996191	0.999992444	0.911018006	0.99999488	0.999985264	0.954870155
GPR39	0.999996191	0.985293257	0.954620171	0.99999488	0.999985264	0.954870155
CCNH	0.999996191	0.999992444	0.960133832	0.99999488	0.999985264	0.954870155
APOO	0.999996191	0.948700494	0.973406453	0.99999488	0.999985264	0.954870155
COMMD8	0.999996191	0.999992444	0.973406453	0.99999488	0.999985264	0.954870155
LOC786553	0.999996191	0.978510121	0.983938648	0.99999488	0.999985264	0.954870155
XRCC1	0.999996191	0.918911961	0.98545277	0.99999488	0.999985264	0.954870155

AUP1	0.999996191	0.946274461	0.935094475	0.99999488	0.999985264	0.954912094
EMX2	0.999996191	0.991365955	0.945678616	0.99999488	0.999985264	0.954912094
C10H15orf61	0.999996191	0.968438183	0.965065403	0.99999488	0.999985264	0.954912094
PHF10	0.999996191	0.940459751	0.884320747	0.99999488	0.999985264	0.954944271
HERPUD2	0.999996191	0.982981183	0.956565728	0.99999488	0.999985264	0.954944271
LOC101907140	0.999996191	0.992977597	0.991285432	0.99999488	0.999985264	0.954944271
ZKSCAN7	0.999996191	0.9582784	0.935094475	0.99999488	0.999985264	0.955184323
DIABLO	0.999996191	0.928575646	0.939478907	0.99999488	0.999985264	0.955184323
FBXL15	0.999996191	0.977354623	0.946479014	0.99999488	0.999985264	0.955184323
YTHDC2	0.999996191	0.999992444	0.951822586	0.99999488	0.999985264	0.955184323
MRPS11	0.999996191	0.989078167	0.956472932	0.99999488	0.999985264	0.955184323
MTFR2	0.999996191	0.999347049	0.956565728	0.99999488	0.999985264	0.955184323
GLT8D1	0.999996191	0.973092298	0.96210311	0.99999488	0.999985264	0.955184323
SDF2	0.999996191	0.999992444	0.978389389	0.99999488	0.999985264	0.955184323
LOC509972	0.999996191	0.989549179	0.981039752	0.99999488	0.999985264	0.955184323
KLK10	0.999996191	0.916036681	0.998936876	0.99999488	0.999985264	0.955184323
LIN52	0.999996191	0.978089982	0.999558179	0.99999488	0.999985264	0.955184323
EIF2D	0.999996191	0.999992444	0.95935771	0.99999488	0.999985264	0.955236184
PTPRD	0.999996191	0.881001912	0.921010865	0.99999488	0.999985264	0.955280826
TMEM132B	0.999996191	0.962910915	0.941782902	0.99999488	0.999985264	0.955280826
MCM4	0.999996191	0.943802122	0.938203102	0.99999488	0.999985264	0.955326589
TADA3	0.999996191	0.992977597	0.999558179	0.99999488	0.999985264	0.955326589
LRMP	0.999996191	0.917537324	0.884119589	0.99999488	0.999985264	0.955374205
GAR1	0.999996191	0.90024974	0.892574927	0.99999488	0.999985264	0.955374205
LOC112444279	0.999996191	0.985406717	0.898815524	0.99999488	0.999985264	0.955374205
LOC782954	0.999996191	0.991488652	0.907484111	0.99999488	0.999985264	0.955374205
ZMAT3	0.999996191	0.976717114	0.921646762	0.99999488	0.999985264	0.955374205
WDR33	0.999996191	0.999992444	0.925520255	0.99999488	0.999985264	0.955374205
ABHD8	0.999996191	0.992977597	0.926552506	0.99999488	0.999985264	0.955374205
ZNF140	0.999996191	0.999347049	0.926552506	0.99999488	0.999985264	0.955374205
KLF16	0.999996191	0.999992444	0.926552506	0.99999488	0.999985264	0.955374205
TMEM256	0.999996191	0.957700659	0.938203102	0.99999488	0.999985264	0.955374205
TRMU	0.999996191	0.999992444	0.938911935	0.99999488	0.999985264	0.955374205
PPIP5K1	0.999996191	0.978089982	0.939147816	0.99999488	0.999985264	0.955374205
ENKUR	0.999996191	0.95697765	0.93955737	0.99999488	0.999985264	0.955374205
CARS2	0.999996191	0.941495217	0.940815039	0.99999488	0.999985264	0.955374205
HIC2	0.999996191	0.991488652	0.944871472	0.99999488	0.999985264	0.955374205
NCOA3	0.999996191	0.94984369	0.945678616	0.99999488	0.999985264	0.955374205
GVQW3	0.999996191	0.985406717	0.945678616	0.99999488	0.999985264	0.955374205

PXN	0.999996191	0.999992444	0.945678616	0.99999488	0.999985264	0.955374205
LOC112444300	0.999996191	0.97475736	0.945690614	0.99999488	0.999985264	0.955374205
MATR3	0.999996191	0.989917435	0.946479014	0.99999488	0.999985264	0.955374205
BRPF3	0.999996191	0.998131745	0.946479014	0.99999488	0.999985264	0.955374205
SLC7A6OS	0.999996191	0.999992444	0.946479014	0.99999488	0.999985264	0.955374205
NIPBL	0.999996191	0.998131745	0.948359914	0.99999488	0.999985264	0.955374205
DLG5	0.999996191	0.985406717	0.95106368	0.99999488	0.999985264	0.955374205
ICMT	0.999996191	0.944868298	0.95230212	0.99999488	0.999985264	0.955374205
PRKAR1B	0.999996191	0.970077079	0.95560271	0.99999488	0.999985264	0.955374205
FAM171B	0.999996191	0.958098081	0.956565728	0.99999488	0.999985264	0.955374205
ZFHX4	0.999996191	0.983640273	0.956565728	0.99999488	0.999985264	0.955374205
LOC112443767	0.999996191	0.999992444	0.956565728	0.99999488	0.999985264	0.955374205
SRPK2	0.999996191	0.991488652	0.957541703	0.99999488	0.999985264	0.955374205
HAUS6	0.999996191	0.999992444	0.957541703	0.99999488	0.999985264	0.955374205
FN3K	0.999996191	0.981298804	0.957840205	0.99999488	0.999985264	0.955374205
ARHGAP15	0.999996191	0.999992444	0.958627535	0.99999488	0.999985264	0.955374205
COL4A2	0.999996191	0.993316598	0.96210311	0.99999488	0.999985264	0.955374205
PLK1	0.999996191	0.918883775	0.962119257	0.99999488	0.999985264	0.955374205
SNHG4	0.999996191	0.936657629	0.962142195	0.99999488	0.999985264	0.955374205
GRIN2D	0.999996191	0.985161531	0.968398207	0.99999488	0.999985264	0.955374205
CYP2D14	0.999996191	0.991488652	0.979462526	0.99999488	0.999985264	0.955374205
BNIP3L	0.999996191	0.998131745	0.981039752	0.99999488	0.999985264	0.955374205
CCNYL1	0.999996191	0.974812121	0.98480229	0.99999488	0.999985264	0.955374205
COX4I1	0.999996191	0.975677199	0.993766105	0.99999488	0.999985264	0.955374205
EFCAB2	0.999996191	0.999992444	0.996129075	0.99999488	0.999985264	0.955374205
ECI1	0.999996191	0.976717114	0.998816213	0.99999488	0.999985264	0.955374205
TPD52	0.999996191	0.999992444	0.998936876	0.99999488	0.999985264	0.955374205
DTNB	0.999996191	0.960125322	0.999558179	0.99999488	0.999985264	0.955374205
KLHL29	0.999996191	0.967276215	0.999558179	0.99999488	0.999985264	0.955374205
PTGES	0.999996191	0.893642384	0.957541703	0.99999488	0.999985264	0.955561861
HMG20B	0.999996191	0.964162565	0.957840205	0.99999488	0.999985264	0.955561861
TRPV4	0.999996191	0.9582784	0.992709008	0.99999488	0.999985264	0.955753116
PUM1	0.999996191	0.992301181	0.945280564	0.99999488	0.999985264	0.955845777
ASB6	0.999996191	0.991488652	0.95106368	0.99999488	0.999985264	0.955845777
CHMP4C	0.999996191	0.967374613	0.991285432	0.99999488	0.999985264	0.955845777
RASSF3	0.999996191	0.986018996	0.998936876	0.99999488	0.999985264	0.955845777
MAPKAPK5	0.999996191	0.989547896	0.980660791	0.99999488	0.999985264	0.955880729
RBP1	0.999996191	0.999992444	0.988922732	0.99999488	0.999985264	0.956037421
RPS2	0.999996191	0.94984369	0.922400936	0.99999488	0.999985264	0.956283599

ZBTB26	0.999996191	0.999992444	0.930678591	0.99999488	0.999985264	0.956283599
ATP5F1B	0.999996191	0.943802122	0.95106368	0.99999488	0.999985264	0.956283599
LOC515089	0.999996191	0.999992444	0.95230212	0.99999488	0.999985264	0.956283599
RCCD1	0.999996191	0.993316598	0.981039752	0.99999488	0.999985264	0.956283599
PPP6C	0.999996191	0.985406717	0.999558179	0.99999488	0.999985264	0.956283599
CHRNE	0.999996191	0.999992444	0.944085862	0.99999488	0.999985264	0.956456894
LOC112445060	0.999996191	0.985058069	0.998816213	0.99999488	0.999985264	0.95646588
CCSER1	0.999996191	0.999992444	0.946479014	0.99999488	0.999985264	0.956548594
BICD2	0.999996191	0.963737928	0.948329591	0.99999488	0.999985264	0.956548594
TM6SF2	0.999996191	0.999992444	0.95230212	0.99999488	0.999985264	0.956548594
TRPM2	0.999996191	0.90024974	0.981218677	0.99999488	0.999985264	0.956548594
LOC112446879	0.999996191	0.999992444	0.999558179	0.99999488	0.999985264	0.956548594
MAP1LC3A	0.999996191	0.967535824	0.93088519	0.99999488	0.999985264	0.956550292
SYBU	0.999996191	0.999992444	0.933447488	0.99999488	0.999985264	0.956550292
MAGI2	0.999996191	0.981562714	0.938559901	0.99999488	0.999985264	0.956550292
PAPOLG	0.999996191	0.998131745	0.944085862	0.99999488	0.999985264	0.956550292
ATF2	0.999996191	0.999992444	0.946479014	0.99999488	0.999985264	0.956550292
LOC100847835	0.999996191	0.991488652	0.948420301	0.99999488	0.999985264	0.956550292
CHN1	0.999996191	0.978510121	0.961024709	0.99999488	0.999985264	0.956550292
LOC101909432	0.999996191	0.999992444	0.964079616	0.99999488	0.999985264	0.956550292
HEXB	0.999996191	0.972895283	0.964152013	0.99999488	0.999985264	0.956550292
LOC100336448	0.999996191	0.999992444	0.96684887	0.99999488	0.999985264	0.956550292
PTPN9	0.999996191	0.985204209	0.969880768	0.99999488	0.999985264	0.956550292
LOC614376	0.999996191	0.952345011	0.999558179	0.99999488	0.999985264	0.956550292
WWP2	0.999996191	0.995073696	0.999558179	0.99999488	0.999985264	0.956550292
CAMK2D	0.999996191	0.997051616	0.993303055	0.99999488	0.999985264	0.956895113
LOC107132270	0.999996191	0.958098081	0.939147816	0.99999488	0.999985264	0.956970213
TSSK1B	0.999996191	0.999992444	0.95106368	0.99999488	0.999985264	0.957057389
HTRA1	0.999996191	0.999992444	0.951822586	0.99999488	0.999985264	0.95715976
CCDC89	0.999996191	0.944855558	0.86638329	0.99999488	0.999985264	0.957171025
LOC104969353	0.999996191	0.999992444	0.897767405	0.99999488	0.999985264	0.957171025
FKBP3	0.999996191	0.946274461	0.921270971	0.99999488	0.999985264	0.957171025
RNF170	0.999996191	0.999992444	0.926552506	0.99999488	0.999985264	0.957171025
MEX3A	0.999996191	0.943802122	0.938203102	0.99999488	0.999985264	0.957171025
ADORA2B	0.999996191	0.985406717	0.939478907	0.99999488	0.999985264	0.957171025
CLNK	0.999996191	0.991345032	0.944993804	0.99999488	0.999985264	0.957171025
LMBRD2	0.999996191	0.999992444	0.945678616	0.99999488	0.999985264	0.957171025
LOC518623	0.999996191	0.992977597	0.94634922	0.99999488	0.999985264	0.957171025
LOC101903301	0.999996191	0.979263532	0.948420301	0.99999488	0.999985264	0.957171025

GPR135	0.999996191	0.999992444	0.951822586	0.99999488	0.999985264	0.957171025
RPL36	0.999996191	0.999992444	0.95230212	0.99999488	0.999985264	0.957171025
LOC104972409	0.999996191	0.999992444	0.956472932	0.99999488	0.999985264	0.957171025
LOC100335936	0.999996191	0.991365955	0.956565728	0.99999488	0.999985264	0.957171025
UQCC1	0.999996191	0.978154072	0.956852976	0.99999488	0.999985264	0.957171025
LOC112449590	0.999996191	0.992977597	0.956852976	0.99999488	0.999985264	0.957171025
DKK1	0.999996191	0.999992444	0.957541703	0.99999488	0.999985264	0.957171025
PAG1	0.999996191	0.920253543	0.960133832	0.99999488	0.999985264	0.957171025
SLC25A23	0.999996191	0.97161271	0.962142195	0.99999488	0.999985264	0.957171025
LOC112442307	0.999996191	0.936657629	0.96789269	0.99999488	0.999985264	0.957171025
MRPS36	0.999996191	0.993316598	0.973303821	0.99999488	0.999985264	0.957171025
SUGP1	0.999996191	0.962968114	0.973406453	0.99999488	0.999985264	0.957171025
METTL5	0.999996191	0.991488652	0.977318995	0.99999488	0.999985264	0.957171025
HEPACAM	0.999996191	0.880359107	0.980859619	0.99999488	0.999985264	0.957171025
DNMT1	0.999996191	0.989958965	0.98286145	0.99999488	0.999985264	0.957171025
STOM	0.999996191	0.989872618	0.98771613	0.99999488	0.999985264	0.957171025
NFIL3	0.999996191	0.980496345	0.998936876	0.99999488	0.999985264	0.957171025
SLK	0.999996191	0.998011664	0.998936876	0.99999488	0.999985264	0.957171025
THAP8	0.999996191	0.974812121	0.999558179	0.99999488	0.999985264	0.957171025
MAPK1IP1L	0.999996191	0.975677199	0.999558179	0.99999488	0.999985264	0.957171025
MMP17	0.999996191	0.984366454	0.999558179	0.99999488	0.999985264	0.957171025
FASN	0.999996191	0.937626884	0.925184579	0.99999488	0.999985264	0.957277881
FLII	0.999996191	0.992977597	0.930678591	0.99999488	0.999985264	0.957277881
PQLC1	0.999996191	0.999992444	0.933447488	0.99999488	0.999985264	0.957277881
CCNI2	0.999996191	0.999347049	0.950948716	0.99999488	0.999985264	0.957277881
MYLIP	0.999996191	0.999992444	0.964920281	0.99999488	0.999985264	0.957277881
PAIP2	0.999996191	0.999992444	0.971778256	0.99999488	0.999985264	0.957277881
MESP1	0.999996191	0.999992444	0.973303821	0.99999488	0.999985264	0.957277881
LOC112449367	0.999996191	0.963737928	0.993594894	0.99999488	0.999985264	0.957277881
IFT74	0.999996191	0.998131745	0.997208738	0.99999488	0.999985264	0.957277881
FOXD2	0.999996191	0.988850552	0.999558179	0.99999488	0.999985264	0.957277881
LOC787905	0.999996191	0.992977597	0.948420301	0.99999488	0.999985264	0.957310055
ISYNA1	0.999996191	0.927997582	0.998936876	0.99999488	0.999985264	0.957310055
TCEAL9	0.999996191	0.975113385	0.94634922	0.99999488	0.999985264	0.957620262
SIK1	0.999996191	0.953941295	0.934694748	0.99999488	0.999985264	0.957622829
C20H5orf51	0.999996191	0.999992444	0.964920281	0.99999488	0.999985264	0.957622829
PTPRU	0.999996191	0.999992444	0.946479014	0.99999488	0.999985264	0.957739231
FAM124A	0.999996191	0.943802122	0.981039752	0.99999488	0.999985264	0.957739231
SPAG9	0.999996191	0.991488652	0.992709008	0.99999488	0.999985264	0.957739231

LOC107132606	0.999996191	0.985406717	0.998852661	0.99999488	0.999985264	0.957739231
RNF20	0.999996191	0.994481342	0.956852976	0.99999488	0.999985264	0.957914799
CERCAM	0.999996191	0.9582784	0.954872702	0.99999488	0.999985264	0.957984797
MST1R	0.999996191	0.946480858	0.964594039	0.99999488	0.999985264	0.957984797
ATP5MG	0.999996191	0.979263532	0.984631623	0.99999488	0.999985264	0.957984797
RNF220	0.999996191	0.981298804	0.929207633	0.99999488	0.999985264	0.958083698
LOC112444588	0.999996191	0.998131745	0.944085862	0.99999488	0.999985264	0.958083698
CYB561	0.999996191	0.978510121	0.95230212	0.99999488	0.999985264	0.958083698
VAC14	0.999996191	0.999992444	0.953903563	0.99999488	0.999985264	0.958083698
FAM129C	0.999996191	0.999992444	0.962777771	0.99999488	0.999985264	0.958083698
PHLDB3	0.999996191	0.978510121	0.964920281	0.99999488	0.999985264	0.958083698
C18H19orf84	0.999996191	0.936273761	0.973303821	0.99999488	0.999985264	0.958083698
FMNL3	0.999996191	0.9582784	0.980558494	0.99999488	0.999985264	0.958083698
TMX4	0.999996191	0.970426668	0.98480229	0.99999488	0.999985264	0.958083698
LOC112442191	0.999996191	0.978510121	0.991285432	0.99999488	0.999985264	0.958083698
ZSCAN30	0.999996191	0.999992444	0.999558179	0.99999488	0.999985264	0.958083698
RAD9B	0.999996191	0.943737806	0.926552506	0.99999488	0.999985264	0.958106334
RPL26	0.999996191	0.9582784	0.901758346	0.99999488	0.999985264	0.958193903
MRPL37	0.999996191	0.926955605	0.920629231	0.99999488	0.999985264	0.958193903
LOC101905010	0.999996191	0.943802122	0.922431991	0.99999488	0.999985264	0.958193903
EXD2	0.999996191	0.991488652	0.938203102	0.99999488	0.999985264	0.958193903
SLC24A3	0.999996191	0.984068885	0.939147816	0.99999488	0.999985264	0.958193903
RPL18A	0.999996191	0.998131745	0.941118576	0.99999488	0.999985264	0.958193903
SMARCD2	0.999996191	0.999992444	0.944085862	0.99999488	0.999985264	0.958193903
HYOU1	0.999996191	0.993316598	0.945766581	0.99999488	0.999985264	0.958193903
CORO6	0.999996191	0.957700659	0.951680496	0.99999488	0.999985264	0.958193903
STRIP1	0.999996191	0.9582784	0.95230212	0.99999488	0.999985264	0.958193903
SEC31B	0.999996191	0.992977597	0.95230212	0.99999488	0.999985264	0.958193903
AHCYL1	0.999996191	0.993316598	0.956565728	0.99999488	0.999985264	0.958193903
AK4	0.999996191	0.999992444	0.960727367	0.99999488	0.999985264	0.958193903
CCDC160	0.999996191	0.964246761	0.967510773	0.99999488	0.999985264	0.958193903
AES	0.999996191	0.991488652	0.973406453	0.99999488	0.999985264	0.958193903
OTUD3	0.999996191	0.999992444	0.981039752	0.99999488	0.999985264	0.958193903
IGFALS	0.999996191	0.98964647	0.988922732	0.99999488	0.999985264	0.958193903
PPP2CA	0.999996191	0.9582784	0.998936876	0.99999488	0.999985264	0.958193903
TUFT1	0.999996191	0.982981183	0.999558179	0.99999488	0.999985264	0.958193903
TRIP13	0.999996191	0.998131745	0.999558179	0.99999488	0.999985264	0.958193903
KLHDC8B	0.999996191	0.9582784	0.946479014	0.99999488	0.999985264	0.958213857
DAB2IP	0.999996191	0.999992444	0.946479014	0.99999488	0.999985264	0.958213857

TOGARAM1	0.999996191	0.991365955	0.938203102	0.99999488	0.999985264	0.958704443
RBM20	0.999996191	0.999992444	0.920629231	0.99999488	0.999985264	0.958869789
TWIST1	0.999996191	0.961479426	0.923089792	0.99999488	0.999985264	0.958869789
LOC104971510	0.999996191	0.991488652	0.934941286	0.99999488	0.999985264	0.958869789
SYNGR2	0.999996191	0.999992444	0.939147816	0.99999488	0.999985264	0.958869789
LOC101904447	0.999996191	0.986445172	0.939799459	0.99999488	0.999985264	0.958869789
FUT11	0.999996191	0.986637746	0.946479014	0.99999488	0.999985264	0.958869789
MOGS	0.999996191	0.993640257	0.95477544	0.99999488	0.999985264	0.958869789
KLKB1	0.999996191	0.95122565	0.956472932	0.99999488	0.999985264	0.958869789
TYW5	0.999996191	0.999992444	0.961490767	0.99999488	0.999985264	0.958869789
SNAPIN	0.999996191	0.978089982	0.964349087	0.99999488	0.999985264	0.958869789
ZNF287	0.999996191	0.9582784	0.964594039	0.99999488	0.999985264	0.958869789
LOC104970103	0.999996191	0.999992444	0.973406453	0.99999488	0.999985264	0.958869789
ZKSCAN2	0.999996191	0.991488652	0.980859619	0.99999488	0.999985264	0.958869789
CPNE5	0.999996191	0.993316598	0.997859293	0.99999488	0.999985264	0.958869789
SH3KBP1	0.999996191	0.977354623	0.9808801	0.99999488	0.999985264	0.95893655
PEX16	0.999996191	0.9527938	0.93198623	0.99999488	0.999985264	0.958984414
GALNT7	0.999996191	0.9582784	0.944085862	0.99999488	0.999985264	0.958984414
LOC100847819	0.999996191	0.999992444	0.95106368	0.99999488	0.999985264	0.958984414
HERC1	0.999996191	0.992977597	0.95230212	0.99999488	0.999985264	0.958984414
SART1	0.999996191	0.991488652	0.956565728	0.99999488	0.999985264	0.958984414
CFDP2	0.999996191	0.921549	0.957541703	0.99999488	0.999985264	0.958984414
GEN1	0.999996191	0.986523286	0.958125965	0.99999488	0.999985264	0.958984414
ABCA7	0.999996191	0.999992444	0.977141934	0.99999488	0.999985264	0.958984414
LOC101906077	0.999996191	0.999992444	0.996391386	0.99999488	0.999985264	0.958984414
ATP6V1G1	0.999996191	0.998131745	0.999558179	0.99999488	0.999985264	0.958984414
WRAP73	0.999996191	0.982313546	0.999684753	0.99999488	0.999985264	0.958984414
LRP10	0.999996191	0.999347049	0.923604668	0.99999488	0.999985264	0.95905828
KIF26B	0.999996191	0.991488652	0.952207545	0.99999488	0.999985264	0.95905828
ARL6IP4	0.999996191	0.993316598	0.914063577	0.99999488	0.999985264	0.959062777
C28H10orf71	0.999996191	0.999992444	0.95106368	0.99999488	0.999985264	0.959062777
ANXA4	0.999996191	0.883624935	0.900351064	0.99999488	0.999985264	0.95956081
TMEM254	0.999996191	0.978510121	0.938203102	0.99999488	0.999985264	0.95956081
ZNF292	0.999996191	0.998131745	0.938203102	0.99999488	0.999985264	0.95956081
IL17REL	0.999996191	0.991488652	0.998429524	0.99999488	0.999985264	0.95956081
ZNF408	0.999996191	0.896996319	0.889744621	0.99999488	0.999985264	0.959651127
LMX1A	0.999996191	0.978510121	0.946479014	0.99999488	0.999985264	0.959804828
LOC112446390	0.999996191	0.985406717	0.93198623	0.99999488	0.999985264	0.959884589
VRK2	0.999996191	0.999992444	0.939147816	0.99999488	0.999985264	0.959884589

PLA1A	0.999996191	0.999992444	0.933791093	0.99999488	0.999985264	0.959920062
LOC101906009	0.999996191	0.999992444	0.938203102	0.99999488	0.999985264	0.959920062
ZNF782	0.999996191	0.999992444	0.951735967	0.99999488	0.999985264	0.959920062
RPL35A	0.999996191	0.991488652	0.952547836	0.99999488	0.999985264	0.959920062
CREB3L2	0.999996191	0.974812121	0.958627535	0.99999488	0.999985264	0.959920062
SIGLEC8	0.999996191	0.999992444	0.970945672	0.99999488	0.999985264	0.959920062
DCN	0.999996191	0.999992444	0.980859619	0.99999488	0.999985264	0.959920062
ATG4D	0.999996191	0.999992444	0.997859293	0.99999488	0.999985264	0.959920062
SAXO2	0.999996191	0.985406717	0.94634922	0.99999488	0.999985264	0.960005777
LOC101909140	0.999996191	0.935060764	0.913211244	0.99999488	0.999985264	0.960294693
MIC1	0.999996191	0.96566261	0.938203102	0.99999488	0.999985264	0.960294693
ZNF423	0.999996191	0.957700659	0.938911935	0.99999488	0.999985264	0.960294693
TPP2	0.999996191	0.952191526	0.944085862	0.99999488	0.999985264	0.960294693
STX16	0.999996191	0.999992444	0.95230212	0.99999488	0.999985264	0.960294693
LOC515333	0.999996191	0.999992444	0.955210387	0.99999488	0.999985264	0.960294693
LOC112445952	0.999996191	0.993316598	0.973406453	0.99999488	0.999985264	0.960294693
FAM212B	0.999996191	0.999992444	0.973406453	0.99999488	0.999985264	0.960294693
H3F3C	0.999996191	0.999992444	0.973406453	0.99999488	0.999985264	0.960294693
SSPN	0.999996191	0.999992444	0.981218677	0.99999488	0.999985264	0.960294693
CTSL	0.999996191	0.999347049	0.985363931	0.99999488	0.999985264	0.960294693
CEP290	0.999996191	0.999992444	0.995045982	0.99999488	0.999985264	0.960294693
LOC782560	0.999996191	0.916036681	0.998936876	0.99999488	0.999985264	0.960294693
OCLN	0.999996191	0.992977597	0.998936876	0.99999488	0.999985264	0.960294693
LOC101902221	0.999996191	0.995476917	0.999558179	0.99999488	0.999985264	0.960294693
TBC1D10C	0.999996191	0.991345032	0.877799478	0.99999488	0.999985264	0.960343814
DHCR24	0.999996191	0.957252213	0.886192181	0.99999488	0.999985264	0.960343814
EBD	0.999996191	0.999992444	0.890248427	0.99999488	0.999985264	0.960343814
LOC107131489	0.999996191	0.978089982	0.902552409	0.99999488	0.999985264	0.960343814
LOC112443176	0.999996191	0.893642384	0.910036508	0.99999488	0.999985264	0.960343814
EEA1	0.999996191	0.948421431	0.910748743	0.99999488	0.999985264	0.960343814
RGS6	0.999996191	0.992977597	0.920629231	0.99999488	0.999985264	0.960343814
GTPBP1	0.999996191	0.978089982	0.926552506	0.99999488	0.999985264	0.960343814
MTHFSD	0.999996191	0.999992444	0.926932508	0.99999488	0.999985264	0.960343814
TSPAN31	0.999996191	0.984641223	0.927600835	0.99999488	0.999985264	0.960343814
SLC12A4	0.999996191	0.989547896	0.930762164	0.99999488	0.999985264	0.960343814
RAB37	0.999996191	0.945217876	0.932376188	0.99999488	0.999985264	0.960343814
SYT3	0.999996191	0.999992444	0.932657945	0.99999488	0.999985264	0.960343814
PTGER1	0.999996191	0.9582784	0.933791093	0.99999488	0.999985264	0.960343814
MLC1	0.999996191	0.946274461	0.934668684	0.99999488	0.999985264	0.960343814

PYROXD1	0.999996191	0.999992444	0.934694748	0.99999488	0.999985264	0.960343814
DDR2	0.999996191	0.986445172	0.941118576	0.99999488	0.999985264	0.960343814
LOC107131531	0.999996191	0.999992444	0.941118576	0.99999488	0.999985264	0.960343814
SGCE	0.999996191	0.999992444	0.941118576	0.99999488	0.999985264	0.960343814
RNF14	0.999996191	0.981298804	0.941336964	0.99999488	0.999985264	0.960343814
PRR13	0.999996191	0.999992444	0.941336964	0.99999488	0.999985264	0.960343814
LOC112448762	0.999996191	0.999992444	0.941782902	0.99999488	0.999985264	0.960343814
KDM3A	0.999996191	0.935742738	0.944085862	0.99999488	0.999985264	0.960343814
THRSP	0.999996191	0.957700659	0.944871472	0.99999488	0.999985264	0.960343814
BAZ2A	0.999996191	0.963958704	0.945678616	0.99999488	0.999985264	0.960343814
RALA	0.999996191	0.991488652	0.945678616	0.99999488	0.999985264	0.960343814
PTGFRN	0.999996191	0.999992444	0.945678616	0.99999488	0.999985264	0.960343814
PSMC6	0.999996191	0.986637746	0.94634922	0.99999488	0.999985264	0.960343814
AP1AR	0.999996191	0.951029275	0.946479014	0.99999488	0.999985264	0.960343814
GAPDH	0.999996191	0.967520941	0.946479014	0.99999488	0.999985264	0.960343814
TBC1D17	0.999996191	0.972895283	0.946479014	0.99999488	0.999985264	0.960343814
LOC112447460	0.999996191	0.991488652	0.946479014	0.99999488	0.999985264	0.960343814
SIK2	0.999996191	0.999347049	0.946479014	0.99999488	0.999985264	0.960343814
TCFL5	0.999996191	0.95697765	0.948069113	0.99999488	0.999985264	0.960343814
TMEM9	0.999996191	0.999992444	0.948247859	0.99999488	0.999985264	0.960343814
CD7	0.999996191	0.991345032	0.948359914	0.99999488	0.999985264	0.960343814
BBS7	0.999996191	0.999992444	0.948420301	0.99999488	0.999985264	0.960343814
LRRC8A	0.999996191	0.999992444	0.949544705	0.99999488	0.999985264	0.960343814
BCL2L2	0.999996191	0.999992444	0.95106368	0.99999488	0.999985264	0.960343814
ZZZ3	0.999996191	0.999992444	0.95106368	0.99999488	0.999985264	0.960343814
RPUSD2	0.999996191	0.992977597	0.951564604	0.99999488	0.999985264	0.960343814
MAGEE2	0.999996191	0.986637746	0.951822586	0.99999488	0.999985264	0.960343814
MRS2	0.999996191	0.988170127	0.951822586	0.99999488	0.999985264	0.960343814
RABAC1	0.999996191	0.993316598	0.951822586	0.99999488	0.999985264	0.960343814
APOM	0.999996191	0.998181556	0.951822586	0.99999488	0.999985264	0.960343814
NONO	0.999996191	0.993316598	0.95230212	0.99999488	0.999985264	0.960343814
ADGRL2	0.999996191	0.998011664	0.95230212	0.99999488	0.999985264	0.960343814
ARF2	0.999996191	0.999992444	0.95230212	0.99999488	0.999985264	0.960343814
MBTPS1	0.999996191	0.999992444	0.95230212	0.99999488	0.999985264	0.960343814
PAFAH1B3	0.999996191	0.999992444	0.95230212	0.99999488	0.999985264	0.960343814
MCM10	0.999996191	0.999992444	0.952547836	0.99999488	0.999985264	0.960343814
B3GNT3	0.999996191	0.998131745	0.952875254	0.99999488	0.999985264	0.960343814
FAM69B	0.999996191	0.922885449	0.954620171	0.99999488	0.999985264	0.960343814
DDIT3	0.999996191	0.94254418	0.954620171	0.99999488	0.999985264	0.960343814

AKAIN1	0.999996191	0.995007119	0.954620171	0.99999488	0.999985264	0.960343814
PKP4	0.999996191	0.999992444	0.954620171	0.99999488	0.999985264	0.960343814
STXBP1	0.999996191	0.999347049	0.95477544	0.99999488	0.999985264	0.960343814
OTULINL	0.999996191	0.980496345	0.954823586	0.99999488	0.999985264	0.960343814
COG3	0.999996191	0.999992444	0.954872702	0.99999488	0.999985264	0.960343814
SUMF2	0.999996191	0.999992444	0.954872702	0.99999488	0.999985264	0.960343814
RNF128	0.999996191	0.985406717	0.956565728	0.99999488	0.999985264	0.960343814
ECHDC3	0.999996191	0.989547896	0.956565728	0.99999488	0.999985264	0.960343814
CLN8	0.999996191	0.999992444	0.956565728	0.99999488	0.999985264	0.960343814
LOC101905723	0.999996191	0.999992444	0.956565728	0.99999488	0.999985264	0.960343814
TSPAN6	0.999996191	0.999992444	0.956565728	0.99999488	0.999985264	0.960343814
LOC100847941	0.999996191	0.985406717	0.957117655	0.99999488	0.999985264	0.960343814
RETREG1	0.999996191	0.978510121	0.957541703	0.99999488	0.999985264	0.960343814
PID1	0.999996191	0.999992444	0.958627535	0.99999488	0.999985264	0.960343814
LOC104972390	0.999996191	0.943802122	0.960133832	0.99999488	0.999985264	0.960343814
CCZ1	0.999996191	0.999992444	0.960133832	0.99999488	0.999985264	0.960343814
TCTA	0.999996191	0.993316598	0.960727367	0.99999488	0.999985264	0.960343814
LOC527186	0.999996191	0.997051616	0.961024709	0.99999488	0.999985264	0.960343814
BRMS1L	0.999996191	0.999992444	0.961024709	0.99999488	0.999985264	0.960343814
CBY1	0.999996191	0.985406717	0.961490767	0.99999488	0.999985264	0.960343814
IL4R	0.999996191	0.991365955	0.961490767	0.99999488	0.999985264	0.960343814
PDGFRL	0.999996191	0.999992444	0.96210311	0.99999488	0.999985264	0.960343814
RBM42	0.999996191	0.997975376	0.962346588	0.99999488	0.999985264	0.960343814
FAM161B	0.999996191	0.9582784	0.963068909	0.99999488	0.999985264	0.960343814
TNFAIP1	0.999996191	0.96566261	0.963068909	0.99999488	0.999985264	0.960343814
BORCS7	0.999996191	0.999992444	0.96307344	0.99999488	0.999985264	0.960343814
CDH3	0.999996191	0.998011664	0.964920281	0.99999488	0.999985264	0.960343814
MSANTD1	0.999996191	0.992977597	0.96551632	0.99999488	0.999985264	0.960343814
FURIN	0.999996191	0.999992444	0.96684887	0.99999488	0.999985264	0.960343814
SLC41A2	0.999996191	0.999992444	0.96684887	0.99999488	0.999985264	0.960343814
SIRT5	0.999996191	0.970077079	0.966991646	0.99999488	0.999985264	0.960343814
C11H2orf40	0.999996191	0.993316598	0.968232195	0.99999488	0.999985264	0.960343814
TAOK1	0.999996191	0.999992444	0.968508342	0.99999488	0.999985264	0.960343814
GPSM2	0.999996191	0.992977597	0.96870883	0.99999488	0.999985264	0.960343814
PCNX1	0.999996191	0.992977597	0.969342421	0.99999488	0.999985264	0.960343814
ALKBH7	0.999996191	0.999992444	0.970903823	0.99999488	0.999985264	0.960343814
LOC785477	0.999996191	0.919884594	0.970945672	0.99999488	0.999985264	0.960343814
NDUFS4	0.999996191	0.992977597	0.971111634	0.99999488	0.999985264	0.960343814
SMG9	0.999996191	0.999992444	0.971111634	0.99999488	0.999985264	0.960343814

HDAC6	0.999996191	0.979263532	0.971778256	0.99999488	0.999985264	0.960343814
ACSS1	0.999996191	0.978089982	0.972362683	0.99999488	0.999985264	0.960343814
GPR107	0.999996191	0.972825253	0.973303821	0.99999488	0.999985264	0.960343814
SLC38A10	0.999996191	0.986637746	0.973303821	0.99999488	0.999985264	0.960343814
BOD1L1	0.999996191	0.986637746	0.973406453	0.99999488	0.999985264	0.960343814
SUPT7L	0.999996191	0.999992444	0.973406453	0.99999488	0.999985264	0.960343814
GATC	0.999996191	0.978089982	0.97379223	0.99999488	0.999985264	0.960343814
SSUH2	0.999996191	0.978089982	0.974075221	0.99999488	0.999985264	0.960343814
LOC104972407	0.999996191	0.974079639	0.977141934	0.99999488	0.999985264	0.960343814
DNAJC1	0.999996191	0.991488652	0.977141934	0.99999488	0.999985264	0.960343814
COL4A3BP	0.999996191	0.999992444	0.978047045	0.99999488	0.999985264	0.960343814
TAF10	0.999996191	0.992977597	0.979462526	0.99999488	0.999985264	0.960343814
DCHS1	0.999996191	0.999992444	0.979462526	0.99999488	0.999985264	0.960343814
LOC112449596	0.999996191	0.962968114	0.979745141	0.99999488	0.999985264	0.960343814
CCDC88C	0.999996191	0.991488652	0.980044975	0.99999488	0.999985264	0.960343814
SMIM30	0.999996191	0.999992444	0.980558494	0.99999488	0.999985264	0.960343814
UACA	0.999996191	0.991488652	0.980660791	0.99999488	0.999985264	0.960343814
TAMM41	0.999996191	0.999992444	0.980660791	0.99999488	0.999985264	0.960343814
NISCH	0.999996191	0.978510121	0.9808801	0.99999488	0.999985264	0.960343814
PTMA	0.999996191	0.999347049	0.981039752	0.99999488	0.999985264	0.960343814
CNST	0.999996191	0.988850552	0.981388276	0.99999488	0.999985264	0.960343814
SERPINH1	0.999996191	0.99398773	0.983419007	0.99999488	0.999985264	0.960343814
ARL4A	0.999996191	0.995532499	0.984309048	0.99999488	0.999985264	0.960343814
JMJD4	0.999996191	0.999992444	0.988922732	0.99999488	0.999985264	0.960343814
CCNB3	0.999996191	0.999992444	0.992742113	0.99999488	0.999985264	0.960343814
VPS26A	0.999996191	0.999992444	0.992742113	0.99999488	0.999985264	0.960343814
LOC112441654	0.999996191	0.993316598	0.993594894	0.99999488	0.999985264	0.960343814
ZNF350	0.999996191	0.938597964	0.994601277	0.99999488	0.999985264	0.960343814
BHLHE41	0.999996191	0.993316598	0.996366709	0.99999488	0.999985264	0.960343814
TDRKH	0.999996191	0.993777777	0.996715234	0.99999488	0.999985264	0.960343814
C21H15orf39	0.999996191	0.999992444	0.996776138	0.99999488	0.999985264	0.960343814
SUCLG2	0.999996191	0.958967142	0.998693674	0.99999488	0.999985264	0.960343814
LOC104970779	0.999996191	0.999992444	0.998816213	0.99999488	0.999985264	0.960343814
CCDC191	0.999996191	0.946480858	0.998936876	0.99999488	0.999985264	0.960343814
LOC101905046	0.999996191	0.981520287	0.998936876	0.99999488	0.999985264	0.960343814
RPRM	0.999996191	0.985058069	0.998936876	0.99999488	0.999985264	0.960343814
UVSSA	0.999996191	0.985406717	0.998936876	0.99999488	0.999985264	0.960343814
LOC100847802	0.999996191	0.998131745	0.998936876	0.99999488	0.999985264	0.960343814
CHURC1	0.999996191	0.999347049	0.998936876	0.99999488	0.999985264	0.960343814

ITPRIPL1	0.999996191	0.999992444	0.998936876	0.99999488	0.999985264	0.960343814
MYORG	0.999996191	0.999992444	0.998936876	0.99999488	0.999985264	0.960343814
FOS	0.999996191	0.978089982	0.999558179	0.99999488	0.999985264	0.960343814
CRACR2B	0.999996191	0.981298804	0.999558179	0.99999488	0.999985264	0.960343814
LRRC7	0.999996191	0.986637746	0.999558179	0.99999488	0.999985264	0.960343814
PRPF3	0.999996191	0.99398773	0.999558179	0.99999488	0.999985264	0.960343814
ANKRD34A	0.999996191	0.999992444	0.999558179	0.99999488	0.999985264	0.960343814
CNEP1R1	0.999996191	0.999992444	0.999558179	0.99999488	0.999985264	0.960343814
STRN4	0.999996191	0.999992444	0.999558179	0.99999488	0.999985264	0.960343814
TRIM32	0.999996191	0.999992444	0.999558179	0.99999488	0.999985264	0.960343814
GON7	0.999996191	0.998011664	0.941118576	0.99999488	0.999985264	0.960388164
HOMEZ	0.999996191	0.999992444	0.9808801	0.99999488	0.999985264	0.960388164
CASP2	0.999996191	0.999992444	0.898815524	0.99999488	0.999985264	0.960613782
KAT7	0.999996191	0.999992444	0.920629231	0.99999488	0.999985264	0.960613782
EMC1	0.999996191	0.943802122	0.923089792	0.99999488	0.999985264	0.960613782
MTREX	0.999996191	0.999992444	0.930678591	0.99999488	0.999985264	0.960613782
PRRX1	0.999996191	0.999992444	0.930678591	0.99999488	0.999985264	0.960613782
THRA	0.999996191	0.999992444	0.93198623	0.99999488	0.999985264	0.960613782
CCT6B	0.999996191	0.989547896	0.938203102	0.99999488	0.999985264	0.960613782
CTNNBL1	0.999996191	0.991365955	0.938559901	0.99999488	0.999985264	0.960613782
SUCLA2	0.999996191	0.937231686	0.941118576	0.99999488	0.999985264	0.960613782
ARPC1A	0.999996191	0.95697765	0.944168373	0.99999488	0.999985264	0.960613782
DDIT4L	0.999996191	0.999347049	0.946479014	0.99999488	0.999985264	0.960613782
LOC100337355	0.999996191	0.989547896	0.948420301	0.99999488	0.999985264	0.960613782
ANKRD37	0.999996191	0.998131745	0.948420301	0.99999488	0.999985264	0.960613782
LOC107133150	0.999996191	0.992977597	0.951822586	0.99999488	0.999985264	0.960613782
AP1S3	0.999996191	0.949285285	0.957541703	0.99999488	0.999985264	0.960613782
EOGT	0.999996191	0.999992444	0.960133832	0.99999488	0.999985264	0.960613782
PRR7	0.999996191	0.999992444	0.96542138	0.99999488	0.999985264	0.960613782
LOC100336941	0.999996191	0.883624935	0.973406453	0.99999488	0.999985264	0.960613782
SH3D19	0.999996191	0.975677199	0.979462526	0.99999488	0.999985264	0.960613782
FRS3	0.999996191	0.954281479	0.980660791	0.99999488	0.999985264	0.960613782
KTI12	0.999996191	0.999992444	0.985789565	0.99999488	0.999985264	0.960613782
TRMT44	0.999996191	0.974812121	0.994180047	0.99999488	0.999985264	0.960613782
RPAP1	0.999996191	0.967276215	0.998816213	0.99999488	0.999985264	0.960613782
DCTN3	0.999996191	0.981155176	0.96290532	0.99999488	0.999985264	0.960762541
STX8	0.999996191	0.96566261	0.973406453	0.99999488	0.999985264	0.960762541
ISL2	0.999996191	0.918892358	0.921065686	0.99999488	0.999985264	0.960811267
JARID2	0.999996191	0.946274461	0.941118576	0.99999488	0.999985264	0.960811267

GHITM	0.999996191	0.978510121	0.946283845	0.99999488	0.999985264	0.960811267
PRPF31	0.999996191	0.999992444	0.949038056	0.99999488	0.999985264	0.960811267
TMX1	0.999996191	0.999992444	0.95230212	0.99999488	0.999985264	0.960811267
RABGAP1	0.999996191	0.978089982	0.954620171	0.99999488	0.999985264	0.960811267
ACAA1	0.999996191	0.991335402	0.980169282	0.99999488	0.999985264	0.960811267
LOC784243	0.999996191	0.978510121	0.995045982	0.99999488	0.999985264	0.960811267
HAX1	0.999996191	0.999992444	0.998816213	0.99999488	0.999985264	0.960811267
PLEKHG7	0.999996191	0.9582784	0.998936876	0.99999488	0.999985264	0.960811267
GALK1	0.999996191	0.985204209	0.971849816	0.99999488	0.999985264	0.960921817
NUDT12	0.999996191	0.999992444	0.964152013	0.99999488	0.999985264	0.960933437
GCLM	0.999996191	0.962377515	0.942675769	0.99999488	0.999985264	0.96095955
AP3B1	0.999996191	0.978510121	0.996348351	0.99999488	0.999985264	0.96095955
TMEM170B	0.999996191	0.990367449	0.999558179	0.99999488	0.999985264	0.961394958
PTPRCAP	0.999996191	0.92239337	0.908181052	0.99999488	0.999985264	0.961808188
FOXP2	0.999996191	0.999992444	0.950948716	0.99999488	0.999985264	0.961808188
BCOR	0.999996191	0.999992444	0.95230212	0.99999488	0.999985264	0.961808188
FAM204A	0.999996191	0.992977597	0.969656316	0.99999488	0.999985264	0.961808188
LOC112447290	0.999996191	0.999992444	0.993585211	0.99999488	0.999985264	0.961808188
DYNLT1	0.999996191	0.978089982	0.998936876	0.99999488	0.999985264	0.961808188
LOC615959	0.999996191	0.999347049	0.998936876	0.99999488	0.999985264	0.961808188
SRBD1	0.999996191	0.95697765	0.938203102	0.99999488	0.999985264	0.9618678
WBP11	0.999996191	0.935588259	0.96210311	0.99999488	0.999985264	0.9618678
LOC100847554	0.999996191	0.981298804	0.96816546	0.99999488	0.999985264	0.9618678
GBF1	0.999996191	0.991574265	0.995045982	0.99999488	0.999985264	0.9618678
LOC104974272	0.999996191	0.940434564	0.904959653	0.99999488	0.999985264	0.961987885
TAF1C	0.999996191	0.985406717	0.984444908	0.99999488	0.999985264	0.961987885
CLDN20	0.999996191	0.991488652	0.999558179	0.99999488	0.999985264	0.961987885
JSRP1	0.999996191	0.990248517	0.930678591	0.99999488	0.999985264	0.962025387
C2H2orf76	0.999996191	0.991488652	0.941118576	0.99999488	0.999985264	0.962025387
PEX19	0.999996191	0.993316598	0.969880768	0.99999488	0.999985264	0.962025387
SLC25A42	0.999996191	0.957252213	0.973406453	0.99999488	0.999985264	0.962025387
LOC104973390	0.999996191	0.999992444	0.980660791	0.99999488	0.999985264	0.962025387
TXNL1	0.999996191	0.9582784	0.997859293	0.99999488	0.999985264	0.962025387
NKIRAS1	0.999996191	0.988850552	0.95230212	0.99999488	0.999985264	0.962052567
SH3RF3	0.999996191	0.992977597	0.966991646	0.99999488	0.999985264	0.962052567
RAB35	0.999996191	0.999992444	0.998936876	0.99999488	0.999985264	0.962052567
CENPN	0.999996191	0.989547896	0.965065403	0.99999488	0.999985264	0.962074154
INO80	0.999996191	0.988850552	0.963233239	0.99999488	0.999985264	0.962368568
PPIL4	0.999996191	0.999992444	0.95106368	0.99999488	0.999985264	0.962383033

PM20D2	0.999996191	0.909359235	0.922400936	0.99999488	0.999985264	0.962703352
CNOT10	0.999996191	0.999992444	0.945678616	0.99999488	0.999985264	0.962703352
GOT2	0.999996191	0.95697765	0.94634922	0.99999488	0.999985264	0.962703352
LTBP1	0.999996191	0.991345032	0.946900905	0.99999488	0.999985264	0.962703352
KRCC1	0.999996191	0.999992444	0.956565728	0.99999488	0.999985264	0.962703352
LOC786616	0.999996191	0.999347049	0.958125965	0.99999488	0.999985264	0.962703352
XKR6	0.999996191	0.999992444	0.972362683	0.99999488	0.999985264	0.962703352
PTTG1IP	0.999996191	0.999992444	0.973303821	0.99999488	0.999985264	0.962703352
RWDD3	0.999996191	0.999992444	0.999558179	0.99999488	0.999985264	0.962703352
SECISBP2L	0.999996191	0.994481342	0.956565728	0.99999488	0.999985264	0.962718528
LOC782418	0.999996191	0.918652095	0.958627535	0.99999488	0.999985264	0.962718528
LOC112448088	0.999996191	0.999992444	0.999558179	0.99999488	0.999985264	0.962718528
WWC2	0.999996191	0.96718649	0.946479014	0.99999488	0.999985264	0.963513949
NECTIN4	0.999996191	0.999992444	0.951822586	0.99999488	0.999985264	0.963513949
CNNM4	0.999996191	0.97015318	0.944871472	0.99999488	0.999985264	0.963558933
ARHGAP10	0.999996191	0.9582784	0.981622418	0.99999488	0.999985264	0.963607563
ZNF771	0.999996191	0.974812121	0.944085862	0.99999488	0.999985264	0.963727152
TBP	0.999996191	0.978510121	0.944085862	0.99999488	0.999985264	0.963727152
HMCN2	0.999996191	0.999992444	0.948420301	0.99999488	0.999985264	0.963903108
SLC1A3	0.999996191	0.999992444	0.957541703	0.99999488	0.999985264	0.963903108
EXOC6B	0.999996191	0.986637746	0.973406453	0.99999488	0.999985264	0.963903108
RPS10	0.999996191	0.994746565	0.996715234	0.99999488	0.999985264	0.963903108
L2HGDH	0.999996191	0.934142668	0.925745065	0.99999488	0.999985264	0.964201819
KLHL42	0.999996191	0.957700659	0.952207545	0.99999488	0.999985264	0.964201819
XRRA1	0.999996191	0.973912396	0.999558179	0.99999488	0.999985264	0.964201819
CPD	0.999996191	0.993540482	0.93198623	0.99999488	0.999985264	0.964333944
IPP	0.999996191	0.999992444	0.93198623	0.99999488	0.999985264	0.964333944
C15H11orf49	0.999996191	0.999992444	0.936517513	0.99999488	0.999985264	0.964333944
TOM1L1	0.999996191	0.983640273	0.939147816	0.99999488	0.999985264	0.964333944
ZNF112	0.999996191	0.999347049	0.946479014	0.99999488	0.999985264	0.964333944
RALY	0.999996191	0.999347049	0.95106368	0.99999488	0.999985264	0.964333944
LRG1	0.999996191	0.937231686	0.951099739	0.99999488	0.999985264	0.964333944
GSTM1	0.999996191	0.985406717	0.95230212	0.99999488	0.999985264	0.964333944
ZNF239	0.999996191	0.997051616	0.957117655	0.99999488	0.999985264	0.964333944
PDE6D	0.999996191	0.999992444	0.96551632	0.99999488	0.999985264	0.964333944
SDHAF2	0.999996191	0.986637746	0.968053992	0.99999488	0.999985264	0.964333944
TMEM41A	0.999996191	0.941793392	0.970945672	0.99999488	0.999985264	0.964333944
LOC614402	0.999996191	0.999992444	0.9808801	0.99999488	0.999985264	0.964333944
TMEM230	0.999996191	0.970426668	0.985966356	0.99999488	0.999985264	0.964333944

TXNDC9	0.999996191	0.986637746	0.991285432	0.99999488	0.999985264	0.964333944
TMCO6	0.999996191	0.972764622	0.992742113	0.99999488	0.999985264	0.964333944
BNIP1	0.999996191	0.999992444	0.999558179	0.99999488	0.999985264	0.964333944
FTCDNL1	0.999996191	0.985161531	0.962346588	0.99999488	0.999985264	0.964400909
L3MBTL3	0.999996191	0.969857319	0.88687452	0.99999488	0.999985264	0.964476713
TLDC2	0.999996191	0.905019423	0.892664791	0.99999488	0.999985264	0.964476713
PTPDC1	0.999996191	0.950729918	0.926552506	0.99999488	0.999985264	0.964476713
DDX39A	0.999996191	0.999992444	0.926552506	0.99999488	0.999985264	0.964476713
LOC101904962	0.999996191	0.999992444	0.931507181	0.99999488	0.999985264	0.964476713
LOC101905014	0.999996191	0.999992444	0.944031108	0.99999488	0.999985264	0.964476713
SUCNR1	0.999996191	0.999992444	0.94634922	0.99999488	0.999985264	0.964476713
RPS23	0.999996191	0.991488652	0.946479014	0.99999488	0.999985264	0.964476713
RAB38	0.999996191	0.999992444	0.95106368	0.99999488	0.999985264	0.964476713
TDP2	0.999996191	0.983506778	0.957541703	0.99999488	0.999985264	0.964476713
LOC112441525	0.999996191	0.986637746	0.957840205	0.99999488	0.999985264	0.964476713
RAD54L	0.999996191	0.988850552	0.959552266	0.99999488	0.999985264	0.964476713
PSEN2	0.999996191	0.998131745	0.959866698	0.99999488	0.999985264	0.964476713
SLC25A16	0.999996191	0.999992444	0.960133832	0.99999488	0.999985264	0.964476713
LOC100850437	0.999996191	0.991488652	0.961111949	0.99999488	0.999985264	0.964476713
KIAA0754	0.999996191	0.962968114	0.96307344	0.99999488	0.999985264	0.964476713
LOC112445989	0.999996191	0.999992444	0.965234684	0.99999488	0.999985264	0.964476713
HIGD1A	0.999996191	0.989872618	0.966991646	0.99999488	0.999985264	0.964476713
CRIPT	0.999996191	0.999992444	0.968232195	0.99999488	0.999985264	0.964476713
ARL2BP	0.999996191	0.991488652	0.973406453	0.99999488	0.999985264	0.964476713
IKBIP	0.999996191	0.999992444	0.973406453	0.99999488	0.999985264	0.964476713
TSC22D4	0.999996191	0.975951808	0.9808801	0.99999488	0.999985264	0.964476713
FBXW9	0.999996191	0.999992444	0.9808801	0.99999488	0.999985264	0.964476713
EVA1C	0.999996191	0.999992444	0.981715036	0.99999488	0.999985264	0.964476713
LOC101903851	0.999996191	0.986445172	0.983077441	0.99999488	0.999985264	0.964476713
CDCA5	0.999996191	0.989872618	0.993594894	0.99999488	0.999985264	0.964476713
LOC781298	0.999996191	0.991488652	0.993594894	0.99999488	0.999985264	0.964476713
LOC112444350	0.999996191	0.978426416	0.998936876	0.99999488	0.999985264	0.964476713
LEPROT	0.999996191	0.999347049	0.998936876	0.99999488	0.999985264	0.964476713
POLDIP2	0.999996191	0.985293257	0.999558179	0.99999488	0.999985264	0.964476713
EPHB2	0.999996191	0.978510121	0.907484111	0.99999488	0.999985264	0.964675807
LOC112446129	0.999996191	0.946274461	0.926552506	0.99999488	0.999985264	0.964675807
ELL	0.999996191	0.993316598	0.939147816	0.99999488	0.999985264	0.964675807
REEP5	0.999996191	0.999992444	0.954620171	0.99999488	0.999985264	0.964675807
AP5S1	0.999996191	0.991488652	0.958627535	0.99999488	0.999985264	0.964930578

IGFBP4	0.999996191	0.9582784	0.888545123	0.99999488	0.999985264	0.965048604
SRL	0.999996191	0.999992444	0.930678591	0.99999488	0.999985264	0.965048604
RPL37A	0.999996191	0.9582784	0.951565052	0.99999488	0.999985264	0.965048604
PTPRM	0.999996191	0.999992444	0.954620171	0.99999488	0.999985264	0.965048604
C8H8orf58	0.999996191	0.992977597	0.95955996	0.99999488	0.999985264	0.965048604
NKAIN2	0.999996191	0.931828419	0.96210311	0.99999488	0.999985264	0.965048604
SLC25A46	0.999996191	0.967919743	0.963068909	0.99999488	0.999985264	0.965048604
DGKA	0.999996191	0.991488652	0.973406453	0.99999488	0.999985264	0.965048604
RECQL5	0.999996191	0.999347049	0.993594894	0.99999488	0.999985264	0.965048604
RABGAP1L	0.999996191	0.992977597	0.998936876	0.99999488	0.999985264	0.965048604
PCNP	0.999996191	0.999992444	0.998936876	0.99999488	0.999985264	0.965048604
MAP6D1	0.999996191	0.904695096	0.999558179	0.99999488	0.999985264	0.965048604
NUP35	0.999996191	0.989958965	0.939147816	0.99999488	0.999985264	0.965138155
IL15RA	0.999996191	0.985406717	0.972381338	0.99999488	0.999985264	0.965257351
ALKAL2	0.999996191	0.989547896	0.948420301	0.99999488	0.999985264	0.965746168
ABRAXAS1	0.999996191	0.964246761	0.999558179	0.99999488	0.999985264	0.965746168
MFAP5	0.999996191	0.943802122	0.969880768	0.99999488	0.999985264	0.96577301
LOC785630	0.999996191	0.985406717	0.945678616	0.99999488	0.999985264	0.965920258
HOXA4	0.999996191	0.998131745	0.956472932	0.99999488	0.999985264	0.965920258
FUT7	0.999996191	0.949285285	0.961490767	0.99999488	0.999985264	0.965920258
ABI3	0.999996191	0.991488652	0.976143419	0.99999488	0.999985264	0.965920258
RAE1	0.999996191	0.961479426	0.946479014	0.99999488	0.999985264	0.966070399
FUZ	0.999996191	0.993316598	0.998936876	0.99999488	0.999985264	0.966070399
LOC112441778	0.999996191	0.985406717	0.969342421	0.99999488	0.999985264	0.966103507
SENP1	0.999996191	0.999992444	0.956504686	0.99999488	0.999985264	0.966522116
LOC789764	0.999996191	0.992977597	0.981388276	0.99999488	0.999985264	0.966522116
INTS10	0.999996191	0.957700659	0.877799478	0.99999488	0.999985264	0.966921967
SLC6A1	0.999996191	0.981520287	0.894185401	0.99999488	0.999985264	0.966921967
SPEG	0.999996191	0.985293257	0.898125038	0.99999488	0.999985264	0.966921967
GABRB1	0.999996191	0.999992444	0.907484111	0.99999488	0.999985264	0.966921967
SNRPA1	0.999996191	0.907956107	0.908181052	0.99999488	0.999985264	0.966921967
SEC24A	0.999996191	0.946274461	0.915117288	0.99999488	0.999985264	0.966921967
FIG4	0.999996191	0.985293257	0.920154807	0.99999488	0.999985264	0.966921967
BATF	0.999996191	0.921934673	0.922400936	0.99999488	0.999985264	0.966921967
RBFA	0.999996191	0.97015318	0.923604668	0.99999488	0.999985264	0.966921967
METTL6	0.999996191	0.985293257	0.923604668	0.99999488	0.999985264	0.966921967
ASL	0.999996191	0.987516216	0.923953195	0.99999488	0.999985264	0.966921967
LHFPL2	0.999996191	0.967276215	0.926552506	0.99999488	0.999985264	0.966921967
ZNF792	0.999996191	0.985293257	0.930678591	0.99999488	0.999985264	0.966921967

RPL23A	0.999996191	0.949644575	0.93198623	0.99999488	0.999985264	0.966921967
NSMF	0.999996191	0.956801903	0.93198623	0.99999488	0.999985264	0.966921967
SSB	0.999996191	0.957700659	0.932892118	0.99999488	0.999985264	0.966921967
GNL2	0.999996191	0.934905044	0.938203102	0.99999488	0.999985264	0.966921967
WDR48	0.999996191	0.962910915	0.938203102	0.99999488	0.999985264	0.966921967
HSF1	0.999996191	0.987516216	0.938203102	0.99999488	0.999985264	0.966921967
GNB2	0.999996191	0.998131745	0.938203102	0.99999488	0.999985264	0.966921967
SPSB2	0.999996191	0.999992444	0.938203102	0.99999488	0.999985264	0.966921967
RBSN	0.999996191	0.985293257	0.938905004	0.99999488	0.999985264	0.966921967
RETREG2	0.999996191	0.943802122	0.941118576	0.99999488	0.999985264	0.966921967
NEU1	0.999996191	0.981520287	0.941838477	0.99999488	0.999985264	0.966921967
USP39	0.999996191	0.991488652	0.944085862	0.99999488	0.999985264	0.966921967
SHLD2	0.999996191	0.999992444	0.944381639	0.99999488	0.999985264	0.966921967
LOC100297170	0.999996191	0.994210852	0.945280564	0.99999488	0.999985264	0.966921967
TUBE1	0.999996191	0.981520287	0.945766581	0.99999488	0.999985264	0.966921967
TSTA3	0.999996191	0.978154072	0.946479014	0.99999488	0.999985264	0.966921967
NADK	0.999996191	0.993100184	0.946479014	0.99999488	0.999985264	0.966921967
FRMD4B	0.999996191	0.957700659	0.948329591	0.99999488	0.999985264	0.966921967
ZKSCAN1	0.999996191	0.998131745	0.948946702	0.99999488	0.999985264	0.966921967
TMEM259	0.999996191	0.974812121	0.95106368	0.99999488	0.999985264	0.966921967
PCID2	0.999996191	0.992895985	0.951199173	0.99999488	0.999985264	0.966921967
RNASEH2B	0.999996191	0.969314233	0.951822586	0.99999488	0.999985264	0.966921967
OCRL	0.999996191	0.978089982	0.95230212	0.99999488	0.999985264	0.966921967
LOC539009	0.999996191	0.975677199	0.952336457	0.99999488	0.999985264	0.966921967
LOC112442386	0.999996191	0.974812121	0.952571879	0.99999488	0.999985264	0.966921967
CCNF	0.999996191	0.941495217	0.954620171	0.99999488	0.999985264	0.966921967
MDH1	0.999996191	0.966144902	0.954620171	0.99999488	0.999985264	0.966921967
OS9	0.999996191	0.991574265	0.954620171	0.99999488	0.999985264	0.966921967
ELP2	0.999996191	0.943802122	0.95477544	0.99999488	0.999985264	0.966921967
PPM1A	0.999996191	0.992977597	0.956504686	0.99999488	0.999985264	0.966921967
LOC101907084	0.999996191	0.985406717	0.956565728	0.99999488	0.999985264	0.966921967
SLC2A12	0.999996191	0.998131745	0.956565728	0.99999488	0.999985264	0.966921967
LOC112442683	0.999996191	0.999992444	0.956565728	0.99999488	0.999985264	0.966921967
ZBTB6	0.999996191	0.961081284	0.956852976	0.99999488	0.999985264	0.966921967
IFT27	0.999996191	0.993316598	0.956892468	0.99999488	0.999985264	0.966921967
C23H6orf89	0.999996191	0.991488652	0.957541703	0.99999488	0.999985264	0.966921967
SRRM2	0.999996191	0.978426416	0.957542358	0.99999488	0.999985264	0.966921967
TMTC1	0.999996191	0.998962836	0.959137142	0.99999488	0.999985264	0.966921967
LOC510362	0.999996191	0.999992444	0.960095471	0.99999488	0.999985264	0.966921967

MCEE	0.999996191	0.991488652	0.960133832	0.99999488	0.999985264	0.966921967
PTGER3	0.999996191	0.999347049	0.960133832	0.99999488	0.999985264	0.966921967
ANKRD2	0.999996191	0.991365955	0.960727367	0.99999488	0.999985264	0.966921967
CLSPN	0.999996191	0.91407604	0.961490767	0.99999488	0.999985264	0.966921967
COA1	0.999996191	0.993316598	0.96210311	0.99999488	0.999985264	0.966921967
DHDH	0.999996191	0.999992444	0.96210311	0.99999488	0.999985264	0.966921967
HMGN4	0.999996191	0.999992444	0.96210311	0.99999488	0.999985264	0.966921967
PPP2R5A	0.999996191	0.978089982	0.962142195	0.99999488	0.999985264	0.966921967
FAM110C	0.999996191	0.992977597	0.962346588	0.99999488	0.999985264	0.966921967
GAL3ST4	0.999996191	0.999347049	0.962346588	0.99999488	0.999985264	0.966921967
ZNF845	0.999996191	0.999992444	0.96290532	0.99999488	0.999985264	0.966921967
LOC782114	0.999996191	0.956655737	0.964152013	0.99999488	0.999985264	0.966921967
SNRNP40	0.999996191	0.999992444	0.964594039	0.99999488	0.999985264	0.966921967
TTC23	0.999996191	0.991365955	0.965714506	0.99999488	0.999985264	0.966921967
ARHGEF28	0.999996191	0.999992444	0.96684887	0.99999488	0.999985264	0.966921967
GXYLT1	0.999996191	0.970426668	0.967427257	0.99999488	0.999985264	0.966921967
LOC781100	0.999996191	0.938017364	0.968232195	0.99999488	0.999985264	0.966921967
LOC510185	0.999996191	0.991365955	0.968325332	0.99999488	0.999985264	0.966921967
LOC112443475	0.999996191	0.999992444	0.969036336	0.99999488	0.999985264	0.966921967
TAF1	0.999996191	0.999992444	0.969880768	0.99999488	0.999985264	0.966921967
NXPE3	0.999996191	0.961479426	0.970903823	0.99999488	0.999985264	0.966921967
KLB	0.999996191	0.999992444	0.970903823	0.99999488	0.999985264	0.966921967
ENTPD4	0.999996191	0.976717114	0.970945672	0.99999488	0.999985264	0.966921967
SMC1A	0.999996191	0.991488652	0.970945672	0.99999488	0.999985264	0.966921967
INF2	0.999996191	0.985406717	0.973406453	0.99999488	0.999985264	0.966921967
DACT1	0.999996191	0.993316598	0.973406453	0.99999488	0.999985264	0.966921967
FIS1	0.999996191	0.999992444	0.973406453	0.99999488	0.999985264	0.966921967
MYDGF	0.999996191	0.999992444	0.973406453	0.99999488	0.999985264	0.966921967
NRIP3	0.999996191	0.999992444	0.973406453	0.99999488	0.999985264	0.966921967
UBE2B	0.999996191	0.999992444	0.973406453	0.99999488	0.999985264	0.966921967
ADGRG1	0.999996191	0.999992444	0.973661483	0.99999488	0.999985264	0.966921967
POLH	0.999996191	0.961479426	0.973713465	0.99999488	0.999985264	0.966921967
DIAPH1	0.999996191	0.967276215	0.9808801	0.99999488	0.999985264	0.966921967
WDR7	0.999996191	0.987516216	0.981039752	0.99999488	0.999985264	0.966921967
LOC101905818	0.999996191	0.998131745	0.981039752	0.99999488	0.999985264	0.966921967
PRKACA	0.999996191	0.991345032	0.981388276	0.99999488	0.999985264	0.966921967
FRS2	0.999996191	0.991488652	0.981388276	0.99999488	0.999985264	0.966921967
CYB561D1	0.999996191	0.993540482	0.981388276	0.99999488	0.999985264	0.966921967
EXOC8	0.999996191	0.981298804	0.981622418	0.99999488	0.999985264	0.966921967

LOC534627	0.999996191	0.992977597	0.981622418	0.99999488	0.999985264	0.966921967
LOC112443816	0.999996191	0.976717114	0.983077441	0.99999488	0.999985264	0.966921967
SCAMP2	0.999996191	0.999992444	0.984444908	0.99999488	0.999985264	0.966921967
RGS3	0.999996191	0.882505302	0.98480229	0.99999488	0.999985264	0.966921967
NSF	0.999996191	0.985406717	0.985363931	0.99999488	0.999985264	0.966921967
DNASE1L1	0.999996191	0.999992444	0.985363931	0.99999488	0.999985264	0.966921967
PHF23	0.999996191	0.985293257	0.985796273	0.99999488	0.999985264	0.966921967
TSG101	0.999996191	0.999992444	0.988922732	0.99999488	0.999985264	0.966921967
MTFR1	0.999996191	0.992977597	0.990780762	0.99999488	0.999985264	0.966921967
LGALSL	0.999996191	0.991488652	0.992922546	0.99999488	0.999985264	0.966921967
PSMG1	0.999996191	0.993316598	0.993585211	0.99999488	0.999985264	0.966921967
NAPRT	0.999996191	0.998131745	0.994133066	0.99999488	0.999985264	0.966921967
OPHN1	0.999996191	0.946480858	0.995045982	0.99999488	0.999985264	0.966921967
LOC101906347	0.999996191	0.951765021	0.996715234	0.99999488	0.999985264	0.966921967
LCAT	0.999996191	0.998131745	0.997208738	0.99999488	0.999985264	0.966921967
MMP24	0.999996191	0.946274461	0.998936876	0.99999488	0.999985264	0.966921967
VPS52	0.999996191	0.952345011	0.998936876	0.99999488	0.999985264	0.966921967
ZFP36	0.999996191	0.978089982	0.998936876	0.99999488	0.999985264	0.966921967
FRY	0.999996191	0.999347049	0.998936876	0.99999488	0.999985264	0.966921967
RMDN2	0.999996191	0.999992444	0.998936876	0.99999488	0.999985264	0.966921967
RNASEK	0.999996191	0.999992444	0.998936876	0.99999488	0.999985264	0.966921967
SEM1	0.999996191	0.999992444	0.998936876	0.99999488	0.999985264	0.966921967
VAMP3	0.999996191	0.981451133	0.999072092	0.99999488	0.999985264	0.966921967
SRSF3	0.999996191	0.933850688	0.999558179	0.99999488	0.999985264	0.966921967
IL4I1	0.999996191	0.956655737	0.999558179	0.99999488	0.999985264	0.966921967
TBX2	0.999996191	0.978510121	0.999558179	0.99999488	0.999985264	0.966921967
TMEM101	0.999996191	0.991488652	0.999558179	0.99999488	0.999985264	0.966921967
MAD1L1	0.999996191	0.992977597	0.999558179	0.99999488	0.999985264	0.966921967
C14H8orf76	0.999996191	0.999992444	0.999558179	0.99999488	0.999985264	0.966921967
FAM168B	0.999996191	0.999992444	0.999558179	0.99999488	0.999985264	0.966921967
CCDC8	0.999996191	0.999992444	0.953457715	0.99999488	0.999985264	0.967045771
LOC101909173	0.999996191	0.992977597	0.960133832	0.99999488	0.999985264	0.967045771
FMN1	0.999996191	0.999992444	0.961024709	0.99999488	0.999985264	0.967045771
KCNJ16	0.999996191	0.9582784	0.98253615	0.99999488	0.999985264	0.967045771
AAR2	0.999996191	0.999992444	0.983938648	0.99999488	0.999985264	0.967045771
MAP7D1	0.999996191	0.992977597	0.954864482	0.99999488	0.999985264	0.967488698
SF3B6	0.999996191	0.985406717	0.999558179	0.99999488	0.999985264	0.967488698
FMNL2	0.999996191	0.958098081	0.941336964	0.99999488	0.999985264	0.967622257
RBMS2	0.999996191	0.961479426	0.893182079	0.99999488	0.999985264	0.967874696

LOC781421	0.999996191	0.941495217	0.915215321	0.99999488	0.999985264	0.967874696
SYNE1	0.999996191	0.938017364	0.926552506	0.99999488	0.999985264	0.967874696
SDF2L1	0.999996191	0.99490847	0.926552506	0.99999488	0.999985264	0.967874696
RBM41	0.999996191	0.999992444	0.926552506	0.99999488	0.999985264	0.967874696
MFSD2B	0.999996191	0.987559864	0.940815039	0.99999488	0.999985264	0.967874696
SAMD14	0.999996191	0.999347049	0.941336964	0.99999488	0.999985264	0.967874696
LYL1	0.999996191	0.941876954	0.944085862	0.99999488	0.999985264	0.967874696
RBM8A	0.999996191	0.951765021	0.948359914	0.99999488	0.999985264	0.967874696
UAP1	0.999996191	0.991488652	0.948359914	0.99999488	0.999985264	0.967874696
SLC22A17	0.999996191	0.979263532	0.95106368	0.99999488	0.999985264	0.967874696
FABP9	0.999996191	0.999992444	0.951114184	0.99999488	0.999985264	0.967874696
LOC100850875	0.999996191	0.999347049	0.954620171	0.99999488	0.999985264	0.967874696
JAK3	0.999996191	0.99949931	0.95477544	0.99999488	0.999985264	0.967874696
MOB1B	0.999996191	0.999347049	0.954823586	0.99999488	0.999985264	0.967874696
TDRD7	0.999996191	0.985406717	0.956565728	0.99999488	0.999985264	0.967874696
YIF1A	0.999996191	0.994135588	0.956565728	0.99999488	0.999985264	0.967874696
LSM11	0.999996191	0.999992444	0.956565728	0.99999488	0.999985264	0.967874696
NMI	0.999996191	0.999992444	0.956565728	0.99999488	0.999985264	0.967874696
AGAP3	0.999996191	0.978426416	0.959396074	0.99999488	0.999985264	0.967874696
NDUFS1	0.999996191	0.959773007	0.960804071	0.99999488	0.999985264	0.967874696
RTCA	0.999996191	0.978089982	0.960804071	0.99999488	0.999985264	0.967874696
STAU1	0.999996191	0.999992444	0.961024709	0.99999488	0.999985264	0.967874696
PLA2G4A	0.999996191	0.991488652	0.96210311	0.99999488	0.999985264	0.967874696
MYC	0.999996191	0.999992444	0.962142195	0.99999488	0.999985264	0.967874696
FAM229B	0.999996191	0.999992444	0.964079616	0.99999488	0.999985264	0.967874696
BACH2	0.999996191	0.999347049	0.971759453	0.99999488	0.999985264	0.967874696
ZNF518B	0.999996191	0.993316598	0.978389389	0.99999488	0.999985264	0.967874696
SFXN5	0.999996191	0.991574265	0.981039752	0.99999488	0.999985264	0.967874696
HINT2	0.999996191	0.993540482	0.981039752	0.99999488	0.999985264	0.967874696
DAB2	0.999996191	0.995532499	0.981622418	0.99999488	0.999985264	0.967874696
LOC107131939	0.999996191	0.982981183	0.985363931	0.99999488	0.999985264	0.967874696
ADAR	0.999996191	0.993316598	0.985363931	0.99999488	0.999985264	0.967874696
SLC6A3	0.999996191	0.999992444	0.993594894	0.99999488	0.999985264	0.967874696
TPM3	0.999996191	0.973759991	0.998936876	0.99999488	0.999985264	0.967874696
HOOK3	0.999996191	0.992357549	0.998936876	0.99999488	0.999985264	0.967874696
HGFAC	0.999996191	0.96566261	0.999558179	0.99999488	0.999985264	0.967874696
SLC6A14	0.999996191	0.998131745	0.999558179	0.99999488	0.999985264	0.967874696
LOC104976281	0.999996191	0.999992444	0.999558179	0.99999488	0.999985264	0.967874696
CTXN1	0.999996191	0.978089982	0.944871472	0.99999488	0.999985264	0.967991416

TSGA10IP	0.999996191	0.999992444	0.960133832	0.99999488	0.999985264	0.967991416
ARHGEF11	0.999996191	0.985406717	0.998936876	0.99999488	0.999985264	0.967991416
RAP2A	0.999996191	0.936273761	0.999558179	0.99999488	0.999985264	0.967991416
NDST2	0.999996191	0.999992444	0.999558179	0.99999488	0.999985264	0.967991416
TRIM68	0.999996191	0.960125322	0.929207633	0.99999488	0.999985264	0.968053232
LOC104976321	0.999996191	0.99490847	0.963068909	0.99999488	0.999985264	0.968053232
STAMBP	0.999996191	0.88167071	0.908181052	0.99999488	0.999985264	0.968059852
TIGD2	0.999996191	0.991488652	0.926552506	0.99999488	0.999985264	0.968059852
PLTP	0.999996191	0.999992444	0.944993804	0.99999488	0.999985264	0.968059852
TBC1D22A	0.999996191	0.970426668	0.945678616	0.99999488	0.999985264	0.968059852
ACRBP	0.999996191	0.993849887	0.946479014	0.99999488	0.999985264	0.968059852
B3GALT6	0.999996191	0.975677199	0.948359914	0.99999488	0.999985264	0.968059852
RB1CC1	0.999996191	0.991488652	0.956247715	0.99999488	0.999985264	0.968059852
PRDX2	0.999996191	0.992977597	0.956565728	0.99999488	0.999985264	0.968059852
LOC789587	0.999996191	0.999992444	0.961373544	0.99999488	0.999985264	0.968059852
BLVRA	0.999996191	0.999992444	0.96210311	0.99999488	0.999985264	0.968059852
SH3BGRL	0.999996191	0.999992444	0.969656316	0.99999488	0.999985264	0.968059852
LOC787257	0.999996191	0.978510121	0.969880768	0.99999488	0.999985264	0.968059852
TRMT112	0.999996191	0.999992444	0.971111634	0.99999488	0.999985264	0.968059852
AAED1	0.999996191	0.999992444	0.973661483	0.99999488	0.999985264	0.968059852
CAVIN2	0.999996191	0.999992444	0.9808801	0.99999488	0.999985264	0.968059852
GNG12	0.999996191	0.978089982	0.981666445	0.99999488	0.999985264	0.968059852
USP46	0.999996191	0.978089982	0.993594894	0.99999488	0.999985264	0.968059852
PRRT1B	0.999996191	0.991488652	0.993594894	0.99999488	0.999985264	0.968059852
EML3	0.999996191	0.978519912	0.999558179	0.99999488	0.999985264	0.968059852
LOC112441659	0.999996191	0.992977597	0.999558179	0.99999488	0.999985264	0.968059852
TMEM120B	0.999996191	0.998011664	0.999558179	0.99999488	0.999985264	0.968059852
ADAMTS1	0.999996191	0.999347049	0.999558179	0.99999488	0.999985264	0.968059852
RPS6KA5	0.999996191	0.913160799	0.903354205	0.99999488	0.999985264	0.968240454
ZC3H18	0.999996191	0.9582784	0.923604668	0.99999488	0.999985264	0.968240454
RBBP7	0.999996191	0.957252213	0.926552506	0.99999488	0.999985264	0.968240454
ZFPM1	0.999996191	0.943802122	0.939501959	0.99999488	0.999985264	0.968240454
ATXN7	0.999996191	0.999992444	0.941118576	0.99999488	0.999985264	0.968240454
ATP13A3	0.999996191	0.9582784	0.944168373	0.99999488	0.999985264	0.968240454
LOC107131607	0.999996191	0.943802122	0.944871472	0.99999488	0.999985264	0.968240454
TNK1	0.999996191	0.999992444	0.944871472	0.99999488	0.999985264	0.968240454
GGCX	0.999996191	0.943802122	0.95106368	0.99999488	0.999985264	0.968240454
PEAK1	0.999996191	0.961479426	0.95106368	0.99999488	0.999985264	0.968240454
RPS4X	0.999996191	0.994135588	0.951099739	0.99999488	0.999985264	0.968240454

RAB23	0.999996191	0.970426668	0.956565728	0.99999488	0.999985264	0.968240454
THRB	0.999996191	0.985406717	0.964920281	0.99999488	0.999985264	0.968240454
ACSF3	0.999996191	0.991488652	0.964920281	0.99999488	0.999985264	0.968240454
OTUB2	0.999996191	0.998131745	0.968235535	0.99999488	0.999985264	0.968240454
ALDH4A1	0.999996191	0.972764622	0.968841179	0.99999488	0.999985264	0.968240454
PPHLN1	0.999996191	0.999992444	0.969656316	0.99999488	0.999985264	0.968240454
CAPRIN2	0.999996191	0.992977597	0.970945672	0.99999488	0.999985264	0.968240454
RIF1	0.999996191	0.999992444	0.973406453	0.99999488	0.999985264	0.968240454
IL3RA	0.999996191	0.991365955	0.977141934	0.99999488	0.999985264	0.968240454
LOC100847782	0.999996191	0.999347049	0.977141934	0.99999488	0.999985264	0.968240454
SPG21	0.999996191	0.978089982	0.9808801	0.99999488	0.999985264	0.968240454
LOC101907404	0.999996191	0.999992444	0.981039752	0.99999488	0.999985264	0.968240454
PRMT6	0.999996191	0.984068885	0.98286145	0.99999488	0.999985264	0.968240454
LOC100295687	0.999996191	0.964044235	0.983938648	0.99999488	0.999985264	0.968240454
EXOSC7	0.999996191	0.978510121	0.984765193	0.99999488	0.999985264	0.968240454
LRCH1	0.999996191	0.992977597	0.985363931	0.99999488	0.999985264	0.968240454
ASXL3	0.999996191	0.998131745	0.988922732	0.99999488	0.999985264	0.968240454
MBNL3	0.999996191	0.999992444	0.992742113	0.99999488	0.999985264	0.968240454
PARVA	0.999996191	0.991488652	0.994601277	0.99999488	0.999985264	0.968240454
OTOP1	0.999996191	0.996730063	0.994601277	0.99999488	0.999985264	0.968240454
PIGU	0.999996191	0.978089982	0.994626276	0.99999488	0.999985264	0.968240454
LOC101904536	0.999996191	0.994464252	0.996139177	0.99999488	0.999985264	0.968240454
HYAL1	0.999996191	0.999992444	0.998936876	0.99999488	0.999985264	0.968240454
HACD3	0.999996191	0.961479426	0.999558179	0.99999488	0.999985264	0.968240454
PLBD1	0.999996191	0.985293257	0.999558179	0.99999488	0.999985264	0.968240454
LOC104974883	0.999996191	0.999992444	0.999558179	0.99999488	0.999985264	0.968240454
N4BP2L2	0.999996191	0.999992444	0.999558179	0.99999488	0.999985264	0.968254764
NDUFS2	0.999996191	0.978510121	0.992742113	0.99999488	0.999985264	0.968447167
HDGF	0.999996191	0.991574265	0.945766581	0.99999488	0.999985264	0.968495693
DENND2A	0.999996191	0.999992444	0.96307344	0.99999488	0.999985264	0.968495693
CDKL4	0.999996191	0.938597964	0.966991646	0.99999488	0.999985264	0.968495693
VPS51	0.999996191	0.973912396	0.96138904	0.99999488	0.999985264	0.968594994
NTSR2	0.999996191	0.999992444	0.961024709	0.99999488	0.999985264	0.96860672
EPC1	0.999996191	0.998131745	0.969880768	0.99999488	0.999985264	0.968615911
LAMTOR5	0.999996191	0.991488652	0.9808801	0.99999488	0.999985264	0.968644022
GPAT4	0.999996191	0.967463091	0.938905004	0.99999488	0.999985264	0.968647961
MFSD6	0.999996191	0.999992444	0.914764839	0.99999488	0.999985264	0.968685438
LOC112441886	0.999996191	0.994481342	0.909383349	0.99999488	0.999985264	0.968695729
FAM46A	0.999996191	0.936657629	0.911919459	0.99999488	0.999985264	0.968695729

TXNIP	0.999996191	0.948421431	0.93198623	0.99999488	0.999985264	0.968695729
NDRG1	0.999996191	0.9582784	0.93198623	0.99999488	0.999985264	0.968695729
BORCS5	0.999996191	0.986637746	0.933447488	0.99999488	0.999985264	0.968695729
MRPS14	0.999996191	0.94984369	0.938215065	0.99999488	0.999985264	0.968695729
XDH	0.999996191	0.972825253	0.945678616	0.99999488	0.999985264	0.968695729
OTUD5	0.999996191	0.97883539	0.946479014	0.99999488	0.999985264	0.968695729
LOC107131458	0.999996191	0.985293257	0.948420301	0.99999488	0.999985264	0.968695729
LOC512953	0.999996191	0.948155891	0.95106368	0.99999488	0.999985264	0.968695729
C19H17orf49	0.999996191	0.999992444	0.954620171	0.99999488	0.999985264	0.968695729
CCNQ	0.999996191	0.967919743	0.956472932	0.99999488	0.999985264	0.968695729
SPX	0.999996191	0.943802122	0.959182693	0.99999488	0.999985264	0.968695729
XRN1	0.999996191	0.999992444	0.959182693	0.99999488	0.999985264	0.968695729
YARS2	0.999996191	0.992977597	0.960133832	0.99999488	0.999985264	0.968695729
LOC101902839	0.999996191	0.954281479	0.964920281	0.99999488	0.999985264	0.968695729
FBXO46	0.999996191	0.966926067	0.964920281	0.99999488	0.999985264	0.968695729
PRRT1	0.999996191	0.993316598	0.964920281	0.99999488	0.999985264	0.968695729
CRKL	0.999996191	0.999992444	0.965065403	0.99999488	0.999985264	0.968695729
CISD1	0.999996191	0.987559864	0.968508342	0.99999488	0.999985264	0.968695729
LOC615454	0.999996191	0.999992444	0.969880768	0.99999488	0.999985264	0.968695729
LOC112448853	0.999996191	0.991365955	0.971833995	0.99999488	0.999985264	0.968695729
DDX55	0.999996191	0.999347049	0.973303821	0.99999488	0.999985264	0.968695729
ITGA5	0.999996191	0.998131745	0.973406453	0.99999488	0.999985264	0.968695729
IVD	0.999996191	0.991365955	0.979441303	0.99999488	0.999985264	0.968695729
HAGHL	0.999996191	0.997975376	0.9808801	0.99999488	0.999985264	0.968695729
DGKD	0.999996191	0.999992444	0.981039752	0.99999488	0.999985264	0.968695729
TCAIM	0.999996191	0.999347049	0.981622418	0.99999488	0.999985264	0.968695729
FRG1	0.999996191	0.999992444	0.985501168	0.99999488	0.999985264	0.968695729
NOSTRIN	0.999996191	0.999992444	0.99035966	0.99999488	0.999985264	0.968695729
MYEF2	0.999996191	0.991488652	0.996391386	0.99999488	0.999985264	0.968695729
SPRY1	0.999996191	0.957700659	0.998936876	0.99999488	0.999985264	0.968695729
ZBTB49	0.999996191	0.991488652	0.998936876	0.99999488	0.999985264	0.968695729
THAP1	0.999996191	0.99034118	0.999558179	0.99999488	0.999985264	0.968695729
LOC112447762	0.999996191	0.991488652	0.999558179	0.99999488	0.999985264	0.968695729
METRN	0.999996191	0.999992444	0.999558179	0.99999488	0.999985264	0.968695729
PLCG2	0.999996191	0.999992444	0.999558179	0.99999488	0.999985264	0.968695729
LOC101905293	0.999996191	0.883624935	0.874559186	0.99999488	0.999985264	0.968772302
LOC512464	0.999996191	0.943802122	0.874559186	0.99999488	0.999985264	0.968772302
FKBP11	0.999996191	0.935742738	0.908181052	0.99999488	0.999985264	0.968772302
STXBP6	0.999996191	0.991365955	0.915551437	0.99999488	0.999985264	0.968772302

PLD5	0.999996191	0.978426416	0.926552506	0.99999488	0.999985264	0.968772302
SLC25A34	0.999996191	0.999992444	0.926552506	0.99999488	0.999985264	0.968772302
MRPS18A	0.999996191	0.935060764	0.930678591	0.99999488	0.999985264	0.968772302
CDRT4	0.999996191	0.943802122	0.935094475	0.99999488	0.999985264	0.968772302
GLG1	0.999996191	0.974812121	0.945678616	0.99999488	0.999985264	0.968772302
LOC104974912	0.999996191	0.986637746	0.94634922	0.99999488	0.999985264	0.968772302
ANK1	0.999996191	0.999992444	0.946479014	0.99999488	0.999985264	0.968772302
LOC112447845	0.999996191	0.999992444	0.946479014	0.99999488	0.999985264	0.968772302
WDR91	0.999996191	0.986637746	0.948923067	0.99999488	0.999985264	0.968772302
IL10RB	0.999996191	0.978089982	0.95230212	0.99999488	0.999985264	0.968772302
LOC101905845	0.999996191	0.978510121	0.95230212	0.99999488	0.999985264	0.968772302
FSTL3	0.999996191	0.999992444	0.95230212	0.99999488	0.999985264	0.968772302
MTOR	0.999996191	0.991488652	0.954620171	0.99999488	0.999985264	0.968772302
SLC35F5	0.999996191	0.999992444	0.954620171	0.99999488	0.999985264	0.968772302
LOC100298923	0.999996191	0.993316598	0.954823586	0.99999488	0.999985264	0.968772302
THSD7A	0.999996191	0.999992444	0.954823586	0.99999488	0.999985264	0.968772302
LOC100295848	0.999996191	0.991574265	0.956472932	0.99999488	0.999985264	0.968772302
ZNF25	0.999996191	0.999992444	0.959314104	0.99999488	0.999985264	0.968772302
PBX2	0.999996191	0.991476708	0.960133832	0.99999488	0.999985264	0.968772302
LOC112447797	0.999996191	0.999992444	0.963465247	0.99999488	0.999985264	0.968772302
FBXO9	0.999996191	0.999992444	0.964594039	0.99999488	0.999985264	0.968772302
RNF166	0.999996191	0.974812121	0.964920281	0.99999488	0.999985264	0.968772302
CCDC47	0.999996191	0.941495217	0.965714506	0.99999488	0.999985264	0.968772302
FLYWCH1	0.999996191	0.991365955	0.966991646	0.99999488	0.999985264	0.968772302
SERINC5	0.999996191	0.991488652	0.970874203	0.99999488	0.999985264	0.968772302
TLE2	0.999996191	0.967919743	0.973661483	0.99999488	0.999985264	0.968772302
CD276	0.999996191	0.999992444	0.978389389	0.99999488	0.999985264	0.968772302
CDPF1	0.999996191	0.999992444	0.9808801	0.99999488	0.999985264	0.968772302
TP53I3	0.999996191	0.999992444	0.9808801	0.99999488	0.999985264	0.968772302
SLCO4C1	0.999996191	0.991488652	0.981388276	0.99999488	0.999985264	0.968772302
LOC101907658	0.999996191	0.999992444	0.981736235	0.99999488	0.999985264	0.968772302
SPIRE1	0.999996191	0.999992444	0.982216395	0.99999488	0.999985264	0.968772302
LOC101902385	0.999996191	0.978750639	0.985363931	0.99999488	0.999985264	0.968772302
NCALD	0.999996191	0.998131745	0.985966356	0.99999488	0.999985264	0.968772302
LZTS2	0.999996191	0.984366454	0.986938092	0.99999488	0.999985264	0.968772302
HOMER3	0.999996191	0.999347049	0.988799866	0.99999488	0.999985264	0.968772302
CATSPERE	0.999996191	0.928611179	0.988922732	0.99999488	0.999985264	0.968772302
DAZAP1	0.999996191	0.975462313	0.990216356	0.99999488	0.999985264	0.968772302
ZNF598	0.999996191	0.991488652	0.990862264	0.99999488	0.999985264	0.968772302

ZNF511	0.999996191	0.998131745	0.991992782	0.99999488	0.999985264	0.968772302
UBE2Q2	0.999996191	0.999992444	0.993594894	0.99999488	0.999985264	0.968772302
UBOX5	0.999996191	0.998131745	0.994048963	0.99999488	0.999985264	0.968772302
FAM216A	0.999996191	0.999992444	0.995045982	0.99999488	0.999985264	0.968772302
MEFV	0.999996191	0.941760655	0.998936876	0.99999488	0.999985264	0.968772302
SNX18	0.999996191	0.992977597	0.998936876	0.99999488	0.999985264	0.968772302
ITGA1	0.999996191	0.978510121	0.999558179	0.99999488	0.999985264	0.968772302
LOC100336564	0.999996191	0.99398773	0.999558179	0.99999488	0.999985264	0.968772302
LATS1	0.999996191	0.998131745	0.999558179	0.99999488	0.999985264	0.968772302
TMEM178A	0.999996191	0.998131745	0.999558179	0.99999488	0.999985264	0.968772302
CACNG4	0.999996191	0.999992444	0.999558179	0.99999488	0.999985264	0.968772302
LOC104975686	0.999996191	0.999992444	0.999558179	0.99999488	0.999985264	0.968772302
COL4A6	0.999996191	0.991574265	0.890592965	0.99999488	0.999985264	0.968787037
ISPD	0.999996191	0.919884594	0.945280564	0.99999488	0.999985264	0.968787037
CRIP2	0.999996191	0.915539284	0.908122166	0.99999488	0.999985264	0.968977894
ZNF543	0.999996191	0.9582784	0.938983799	0.99999488	0.999985264	0.968977894
SCAMP1	0.999996191	0.999992444	0.966991646	0.99999488	0.999985264	0.968977894
LNX1	0.999996191	0.999992444	0.981715036	0.99999488	0.999985264	0.968977894
USP11	0.999996191	0.986637746	0.952571879	0.99999488	0.999985264	0.969176867
AURKC	0.999996191	0.991365955	0.998936876	0.99999488	0.999985264	0.969224147
ST8SIA1	0.999996191	0.989872618	0.899576888	0.99999488	0.999985264	0.969444295
PTPN4	0.999996191	0.987559864	0.935094475	0.99999488	0.999985264	0.969444295
LOC783163	0.999996191	0.987516216	0.946479014	0.99999488	0.999985264	0.969444295
LOC514011	0.999996191	0.994434609	0.998936876	0.99999488	0.999985264	0.969551076
PDLIM2	0.999996191	0.999992444	0.96271079	0.99999488	0.999985264	0.969584602
PRPSAP1	0.999996191	0.943802122	0.926552506	0.99999488	0.999985264	0.969741463
LOC101906989	0.999996191	0.915367594	0.944982712	0.99999488	0.999985264	0.969741463
LOC613519	0.999996191	0.935742738	0.94634922	0.99999488	0.999985264	0.969741463
VTI1A	0.999996191	0.987559864	0.946479014	0.99999488	0.999985264	0.969741463
ARV1	0.999996191	0.999992444	0.957541703	0.99999488	0.999985264	0.969741463
CDK5RAP1	0.999996191	0.999992444	0.963779465	0.99999488	0.999985264	0.969741463
CELSR2	0.999996191	0.999347049	0.964152013	0.99999488	0.999985264	0.969741463
HNRNPUL1	0.999996191	0.999992444	0.968232195	0.99999488	0.999985264	0.969741463
SLC25A32	0.999996191	0.999992444	0.973406453	0.99999488	0.999985264	0.969741463
RPS17	0.999996191	0.961479426	0.977141934	0.99999488	0.999985264	0.969741463
PPP1R10	0.999996191	0.987559864	0.987678394	0.99999488	0.999985264	0.969741463
MPP6	0.999996191	0.978510121	0.998208226	0.99999488	0.999985264	0.969741463
LOC525426	0.999996191	0.974812121	0.895962167	0.99999488	0.999985264	0.969801844
RFX1	0.999996191	0.992977597	0.898815524	0.99999488	0.999985264	0.969801844

TOMM40L	0.999996191	0.941793392	0.903389425	0.99999488	0.999985264	0.969801844
CCDC115	0.999996191	0.926955605	0.907484111	0.99999488	0.999985264	0.969801844
TCF12	0.999996191	0.978510121	0.915197389	0.99999488	0.999985264	0.969801844
MFSD4B	0.999996191	0.973092298	0.927600835	0.99999488	0.999985264	0.969801844
RPL6	0.999996191	0.946274461	0.930678591	0.99999488	0.999985264	0.969801844
EIF2S3	0.999996191	0.992977597	0.933046493	0.99999488	0.999985264	0.969801844
PPM1L	0.999996191	0.978510121	0.94634922	0.99999488	0.999985264	0.969801844
OSBP	0.999996191	0.900384134	0.948359914	0.99999488	0.999985264	0.969801844
LOC104974260	0.999996191	0.940362847	0.948420301	0.99999488	0.999985264	0.969801844
ANGPT4	0.999996191	0.999992444	0.954872702	0.99999488	0.999985264	0.969801844
ADCY7	0.999996191	0.964246761	0.956565728	0.99999488	0.999985264	0.969801844
MPP1	0.999996191	0.991488652	0.971038643	0.99999488	0.999985264	0.969801844
LOC785760	0.999996191	0.999992444	0.973406453	0.99999488	0.999985264	0.969801844
SCN11A	0.999996191	0.999992444	0.977141934	0.99999488	0.999985264	0.969801844
ECHS1	0.999996191	0.989547896	0.978389389	0.99999488	0.999985264	0.969801844
ASMT	0.999996191	0.992977597	0.978389389	0.99999488	0.999985264	0.969801844
NRXN2	0.999996191	0.999992444	0.980677819	0.99999488	0.999985264	0.969801844
GLMP	0.999996191	0.999992444	0.981622418	0.99999488	0.999985264	0.969801844
LOC104969024	0.999996191	0.999347049	0.985363931	0.99999488	0.999985264	0.969801844
NME1	0.999996191	0.987559864	0.993585211	0.99999488	0.999985264	0.969801844
UBE3C	0.999996191	0.991365955	0.993594894	0.99999488	0.999985264	0.969801844
RPS27L	0.999996191	0.991488652	0.996715234	0.99999488	0.999985264	0.969801844
CEP135	0.999996191	0.991488652	0.997859293	0.99999488	0.999985264	0.969801844
PAQR6	0.999996191	0.999992444	0.998936876	0.99999488	0.999985264	0.969801844
TJAP1	0.999996191	0.999992444	0.998936876	0.99999488	0.999985264	0.969801844
RAD54B	0.999996191	0.967276215	0.999558179	0.99999488	0.999985264	0.969801844
SLC12A9	0.999996191	0.978510121	0.999558179	0.99999488	0.999985264	0.969801844
ACTL6A	0.999996191	0.991488652	0.999558179	0.99999488	0.999985264	0.969801844
DTX2	0.999996191	0.993316598	0.999558179	0.99999488	0.999985264	0.969801844
DHH	0.999996191	0.999992444	0.999558179	0.99999488	0.999985264	0.969801844
RIOX2	0.999996191	0.990367449	0.956565728	0.99999488	0.999985264	0.969837047
NPR3	0.999996191	0.992977597	0.998936876	0.99999488	0.999985264	0.969837047
MORN1	0.999996191	0.97161271	0.999558179	0.99999488	0.999985264	0.970275862
LOC531090	0.999996191	0.99873048	0.938203102	0.99999488	0.999985264	0.970594306
RSBN1L	0.999996191	0.999992444	0.945678616	0.99999488	0.999985264	0.970594306
LOC112442967	0.999996191	0.949285285	0.948420301	0.99999488	0.999985264	0.970594306
RAB24	0.999996191	0.999992444	0.95477544	0.99999488	0.999985264	0.970594306
LOC101903820	0.999996191	0.999992444	0.96271079	0.99999488	0.999985264	0.970594306
LOC100139325	0.999996191	0.9582784	0.973406453	0.99999488	0.999985264	0.970594306

OGN	0.999996191	0.999992444	0.978389389	0.99999488	0.999985264	0.970594306
LUZP6	0.999996191	0.991488652	0.998936876	0.99999488	0.999985264	0.970594306
CHD1L	0.999996191	0.999347049	0.997208738	0.99999488	0.999985264	0.970712898
RIBC1	0.999996191	0.970470739	0.927516734	0.99999488	0.999985264	0.970758558
LOC107131296	0.999996191	0.992977597	0.941118576	0.99999488	0.999985264	0.970758558
BOLA1	0.999996191	0.999992444	0.946479014	0.99999488	0.999985264	0.970758558
AVPR1A	0.999996191	0.985406717	0.956565728	0.99999488	0.999985264	0.970758558
CDK18	0.999996191	0.998798123	0.960133832	0.99999488	0.999985264	0.970758558
ANKRD16	0.999996191	0.992977597	0.967427257	0.99999488	0.999985264	0.970758558
CCDC6	0.999996191	0.982313546	0.998936876	0.99999488	0.999985264	0.970758558
MB21D2	0.999996191	0.980867747	0.944085862	0.99999488	0.999985264	0.970849664
PIAS3	0.999996191	0.992977597	0.945678616	0.99999488	0.999985264	0.970849664
RPS27P	0.999996191	0.999992444	0.959137142	0.99999488	0.999985264	0.970849664
MAP3K2	0.999996191	0.995476917	0.961024709	0.99999488	0.999985264	0.970849664
BTBD2	0.999996191	0.999992444	0.964152013	0.99999488	0.999985264	0.970849664
LOC112442949	0.999996191	0.999992444	0.969880768	0.99999488	0.999985264	0.970849664
ETFDH	0.999996191	0.982628449	0.980491974	0.99999488	0.999985264	0.970849664
LOC104969425	0.999996191	0.991488652	0.984309048	0.99999488	0.999985264	0.970849664
BABAM2	0.999996191	0.991488652	0.988922732	0.99999488	0.999985264	0.970849664
SUMO3	0.999996191	0.999992444	0.994601277	0.99999488	0.999985264	0.970849664
C7H19orf53	0.999996191	0.997975376	0.996139177	0.99999488	0.999985264	0.970849664
UVRAG	0.999996191	0.999992444	0.996391386	0.99999488	0.999985264	0.970849664
KIAA1549	0.999996191	0.991365955	0.998936876	0.99999488	0.999985264	0.970849664
FAM200A	0.999996191	0.978510121	0.999558179	0.99999488	0.999985264	0.970849664
SLC30A6	0.999996191	0.992977597	0.912008683	0.99999488	0.999985264	0.971123507
ADGRA1	0.999996191	0.999992444	0.954620171	0.99999488	0.999985264	0.971133624
DYNC1I1	0.999996191	0.994135588	0.973406453	0.99999488	0.999985264	0.971133624
LOC112442214	0.999996191	0.999992444	0.981039752	0.99999488	0.999985264	0.971133624
LOC112447066	0.999996191	0.996735402	0.958627535	0.99999488	0.999985264	0.971439528
TMEFF1	0.999996191	0.999992444	0.9808801	0.99999488	0.999985264	0.971620128
CCDC28A	0.999996191	0.941495217	0.962346588	0.99999488	0.999985264	0.971977425
NDN	0.999996191	0.999992444	0.956565728	0.99999488	0.999985264	0.972380059
KIAA0513	0.999996191	0.992117536	0.958125965	0.99999488	0.999985264	0.972380059
VTA1	0.999996191	0.999992444	0.964152013	0.99999488	0.999985264	0.972380059
LOC786372	0.999996191	0.974812121	0.986501204	0.99999488	0.999985264	0.972380059
DMTN	0.999996191	0.991488652	0.99534463	0.99999488	0.999985264	0.972380059
TIGAR	0.999996191	0.979263532	0.999558179	0.99999488	0.999985264	0.972380059
COPS5	0.999996191	0.938597964	0.924866532	0.99999488	0.999985264	0.972391591
EBI3	0.999996191	0.943802122	0.926552506	0.99999488	0.999985264	0.972391591

TMEM268	0.999996191	0.991365955	0.938203102	0.99999488	0.999985264	0.972391591
ARHGAP22	0.999996191	0.998131745	0.938905004	0.99999488	0.999985264	0.972391591
GLIPR2	0.999996191	0.991488652	0.944085862	0.99999488	0.999985264	0.972391591
LOC101902475	0.999996191	0.999992444	0.95106368	0.99999488	0.999985264	0.972391591
PTPN14	0.999996191	0.999992444	0.95106368	0.99999488	0.999985264	0.972391591
SERINC4	0.999996191	0.999992444	0.95106368	0.99999488	0.999985264	0.972391591
RAB10	0.999996191	0.915971865	0.95230212	0.99999488	0.999985264	0.972391591
P4HA3	0.999996191	0.96566261	0.95230212	0.99999488	0.999985264	0.972391591
CLEC16A	0.999996191	0.955266582	0.956565728	0.99999488	0.999985264	0.972391591
ITCH	0.999996191	0.999992444	0.956565728	0.99999488	0.999985264	0.972391591
GPS2	0.999996191	0.981298804	0.957542358	0.99999488	0.999985264	0.972391591
TEX14	0.999996191	0.97996284	0.960133832	0.99999488	0.999985264	0.972391591
ZW10	0.999996191	0.96566261	0.962346588	0.99999488	0.999985264	0.972391591
LOC101907622	0.999996191	0.999992444	0.964908146	0.99999488	0.999985264	0.972391591
SEC23A	0.999996191	0.999992444	0.96759319	0.99999488	0.999985264	0.972391591
LOC786978	0.999996191	0.980496345	0.969880768	0.99999488	0.999985264	0.972391591
KDELR1	0.999996191	0.992977597	0.969880768	0.99999488	0.999985264	0.972391591
DMTF1	0.999996191	0.999992444	0.969880768	0.99999488	0.999985264	0.972391591
GBP6	0.999996191	0.999992444	0.969880768	0.99999488	0.999985264	0.972391591
PLPP7	0.999996191	0.999992444	0.970945672	0.99999488	0.999985264	0.972391591
DCLK3	0.999996191	0.992977597	0.973406453	0.99999488	0.999985264	0.972391591
CAPN5	0.999996191	0.999992444	0.973406453	0.99999488	0.999985264	0.972391591
SYNDIG1	0.999996191	0.999992444	0.973661483	0.99999488	0.999985264	0.972391591
DHRS4	0.999996191	0.999992444	0.977141934	0.99999488	0.999985264	0.972391591
RFX7	0.999996191	0.991488652	0.980859619	0.99999488	0.999985264	0.972391591
KSR1	0.999996191	0.999992444	0.981458415	0.99999488	0.999985264	0.972391591
MIDN	0.999996191	0.999992444	0.981622418	0.99999488	0.999985264	0.972391591
KATNA1	0.999996191	0.97286093	0.983077441	0.99999488	0.999985264	0.972391591
PCP4L1	0.999996191	0.999992444	0.984444908	0.99999488	0.999985264	0.972391591
PIGY	0.999996191	0.9582784	0.98480229	0.99999488	0.999985264	0.972391591
MLLT10	0.999996191	0.998131745	0.987693608	0.99999488	0.999985264	0.972391591
CAPRIN1	0.999996191	0.998962836	0.991285432	0.99999488	0.999985264	0.972391591
SPRED3	0.999996191	0.998131745	0.993132892	0.99999488	0.999985264	0.972391591
NOL9	0.999996191	0.93825132	0.993594894	0.99999488	0.999985264	0.972391591
ADNP2	0.999996191	0.999992444	0.994916815	0.99999488	0.999985264	0.972391591
CHCHD5	0.999996191	0.999992444	0.996715234	0.99999488	0.999985264	0.972391591
HEG1	0.999996191	0.999992444	0.996715234	0.99999488	0.999985264	0.972391591
AGPAT5	0.999996191	0.992977597	0.998936876	0.99999488	0.999985264	0.972391591
LSM2	0.999996191	0.999992444	0.998936876	0.99999488	0.999985264	0.972391591

CENPS	0.999996191	0.993039239	0.999558179	0.99999488	0.999985264	0.972391591
GOLT1B	0.999996191	0.997426798	0.999558179	0.99999488	0.999985264	0.972391591
LYSMD2	0.999996191	0.986637746	0.96551632	0.99999488	0.999985264	0.972477182
NAIF1	0.999996191	0.999992444	0.967048079	0.99999488	0.999985264	0.972477182
ADIRF	0.999996191	0.999992444	0.973406453	0.99999488	0.999985264	0.972477182
TRAPPC10	0.999996191	0.991488652	0.987678394	0.99999488	0.999985264	0.972477182
LOC512175	0.999996191	0.992977597	0.998936876	0.99999488	0.999985264	0.972607184
RPS7	0.999996191	0.999992444	0.954823586	0.99999488	0.999985264	0.972662617
LOC104975006	0.999996191	0.990559471	0.956565728	0.99999488	0.999985264	0.972662617
PRR3	0.999996191	0.999992444	0.964152013	0.99999488	0.999985264	0.972662617
HPS1	0.999996191	0.991488652	0.96759319	0.99999488	0.999985264	0.972662617
SEPSECS	0.999996191	0.999992444	0.970945672	0.99999488	0.999985264	0.972662617
BCL2L13	0.999996191	0.999992444	0.980677819	0.99999488	0.999985264	0.972662617
KLRK1	0.999996191	0.999992444	0.981039752	0.99999488	0.999985264	0.972662617
LOC101904698	0.999996191	0.938597964	0.980558494	0.99999488	0.999985264	0.97276548
LOC101902551	0.999996191	0.999992444	0.986266701	0.99999488	0.999985264	0.97276548
COA7	0.999996191	0.962377515	0.925520255	0.99999488	0.999985264	0.972825932
SERAC1	0.999996191	0.985406717	0.94634922	0.99999488	0.999985264	0.972825932
CREBBP	0.999996191	0.993316598	0.94634922	0.99999488	0.999985264	0.972825932
CEACAM1	0.999996191	0.978089982	0.946479014	0.99999488	0.999985264	0.972825932
KIAA0141	0.999996191	0.991488652	0.968232195	0.99999488	0.999985264	0.972825932
LRRFIP2	0.999996191	0.999992444	0.996391386	0.99999488	0.999985264	0.972825932
TMEM200B	0.999996191	0.999992444	0.908181052	0.99999488	0.999985264	0.972919703
TECR	0.999996191	0.921661251	0.910426741	0.99999488	0.999985264	0.972919703
GNE	0.999996191	0.9582784	0.91207077	0.99999488	0.999985264	0.972919703
TBC1D12	0.999996191	0.999992444	0.937874396	0.99999488	0.999985264	0.972919703
EBF1	0.999996191	0.972764622	0.938203102	0.99999488	0.999985264	0.972919703
MARCH3	0.999996191	0.984068885	0.938203102	0.99999488	0.999985264	0.972919703
LOC112443728	0.999996191	0.996105883	0.938905004	0.99999488	0.999985264	0.972919703
WRN	0.999996191	0.999992444	0.938911935	0.99999488	0.999985264	0.972919703
CCT6A	0.999996191	0.936273761	0.95230212	0.99999488	0.999985264	0.972919703
CAMK2N1	0.999996191	0.998962836	0.95230212	0.99999488	0.999985264	0.972919703
GIPR	0.999996191	0.991488652	0.953058578	0.99999488	0.999985264	0.972919703
MYZAP	0.999996191	0.999992444	0.956565728	0.99999488	0.999985264	0.972919703
SNX9	0.999996191	0.991488652	0.957117655	0.99999488	0.999985264	0.972919703
LONRF1	0.999996191	0.999992444	0.961024709	0.99999488	0.999985264	0.972919703
CDH22	0.999996191	0.999992444	0.96210311	0.99999488	0.999985264	0.972919703
SHE	0.999996191	0.999992444	0.964186888	0.99999488	0.999985264	0.972919703
GTF3A	0.999996191	0.999992444	0.965065403	0.99999488	0.999985264	0.972919703

EPS8	0.999996191	0.985406717	0.96684887	0.99999488	0.999985264	0.972919703
PARK7	0.999996191	0.992977597	0.968235535	0.99999488	0.999985264	0.972919703
MGC137036	0.999996191	0.999347049	0.970945672	0.99999488	0.999985264	0.972919703
ABCA4	0.999996191	0.999992444	0.971778256	0.99999488	0.999985264	0.972919703
SLC8B1	0.999996191	0.999992444	0.973406453	0.99999488	0.999985264	0.972919703
PRPS2	0.999996191	0.97286093	0.980660791	0.99999488	0.999985264	0.972919703
LOC107132317	0.999996191	0.999992444	0.980660791	0.99999488	0.999985264	0.972919703
ORMDL1	0.999996191	0.999992444	0.980660791	0.99999488	0.999985264	0.972919703
SLC44A1	0.999996191	0.970470739	0.981039752	0.99999488	0.999985264	0.972919703
DEPDC1B	0.999996191	0.991335402	0.981218677	0.99999488	0.999985264	0.972919703
B4GALNT3	0.999996191	0.991365955	0.981218677	0.99999488	0.999985264	0.972919703
HIBCH	0.999996191	0.999347049	0.981218677	0.99999488	0.999985264	0.972919703
TMEM267	0.999996191	0.952345011	0.99035966	0.99999488	0.999985264	0.972919703
FPGT	0.999996191	0.999992444	0.992709008	0.99999488	0.999985264	0.972919703
LOC112442227	0.999996191	0.999992444	0.992939335	0.99999488	0.999985264	0.972919703
RUNDC1	0.999996191	0.999992444	0.994601277	0.99999488	0.999985264	0.972919703
LOC112442071	0.999996191	0.999347049	0.997208738	0.99999488	0.999985264	0.972919703
LOC104968964	0.999996191	0.999992444	0.998936876	0.99999488	0.999985264	0.972919703
MIER3	0.999996191	0.999992444	0.998936876	0.99999488	0.999985264	0.972919703
KDM4A	0.999996191	0.991365955	0.999558179	0.99999488	0.999985264	0.972919703
DENND4B	0.999996191	0.991488652	0.999558179	0.99999488	0.999985264	0.972919703
LOC112441644	0.999996191	0.998131745	0.999558179	0.99999488	0.999985264	0.972919703
NINJ1	0.999996191	0.999992444	0.999558179	0.99999488	0.999985264	0.972919703
MAPK6	0.999996191	0.992977597	0.999558179	0.99999488	0.999985264	0.973205276
FAM172A	0.999996191	0.993316598	0.999558179	0.99999488	0.999985264	0.973206401
ARNT2	0.999996191	0.991488652	0.926552506	0.99999488	0.999985264	0.973238011
CATSPER2	0.999996191	0.996003806	0.97843676	0.99999488	0.999985264	0.973238011
DAP3	0.999996191	0.9409629	0.938905004	0.99999488	0.999985264	0.973330026
C8H9orf3	0.999996191	0.952639631	0.941782902	0.99999488	0.999985264	0.973330026
LOC101903545	0.999996191	0.999992444	0.941782902	0.99999488	0.999985264	0.973330026
RAI14	0.999996191	0.946274461	0.95106368	0.99999488	0.999985264	0.973330026
CNPY2	0.999996191	0.992977597	0.956804823	0.99999488	0.999985264	0.973330026
ACADSB	0.999996191	0.999347049	0.957117655	0.99999488	0.999985264	0.973330026
LOC101902760	0.999996191	0.999347049	0.969880768	0.99999488	0.999985264	0.973330026
GLYCTK	0.999996191	0.994210852	0.970874203	0.99999488	0.999985264	0.973330026
CMC1	0.999996191	0.986637746	0.980660791	0.99999488	0.999985264	0.973330026
COQ5	0.999996191	0.991488652	0.981039752	0.99999488	0.999985264	0.973330026
NCSTN	0.999996191	0.999992444	0.982783495	0.99999488	0.999985264	0.973330026
EBAG9	0.999996191	0.999992444	0.983077441	0.99999488	0.999985264	0.973330026

GON4L	0.999996191	0.999992444	0.998936876	0.99999488	0.999985264	0.973330026
PKN3	0.999996191	0.986523286	0.999558179	0.99999488	0.999985264	0.973330026
CHRNA3	0.999996191	0.95697765	0.938911935	0.99999488	0.999985264	0.973404498
LOC104974937	0.999996191	0.977354623	0.923604668	0.99999488	0.999985264	0.973500697
PMPCB	0.999996191	0.990248517	0.956565728	0.99999488	0.999985264	0.973500697
HPS4	0.999996191	0.991488652	0.964349087	0.99999488	0.999985264	0.973500697
FOXO1	0.999996191	0.946274461	0.964920281	0.99999488	0.999985264	0.973500697
MICU1	0.999996191	0.999992444	0.994601277	0.99999488	0.999985264	0.973500697
APPBP2	0.999996191	0.999992444	0.996715234	0.99999488	0.999985264	0.973500697
LOC101907941	0.999996191	0.999992444	0.919763144	0.99999488	0.999985264	0.973588942
DHX32	0.999996191	0.999992444	0.95106368	0.99999488	0.999985264	0.973588942
LZTR1	0.999996191	0.994746565	0.96210311	0.99999488	0.999985264	0.974234571
LOC112442952	0.999996191	0.986637746	0.932653752	0.99999488	0.999985264	0.974376225
PRSS23	0.999996191	0.978510121	0.932657945	0.99999488	0.999985264	0.974376225
INTS11	0.999996191	0.9582784	0.933447488	0.99999488	0.999985264	0.974376225
CDC40	0.999996191	0.999992444	0.941118576	0.99999488	0.999985264	0.974376225
RPS13	0.999996191	0.978089982	0.948466212	0.99999488	0.999985264	0.974376225
SCAMP3	0.999996191	0.991365955	0.955112492	0.99999488	0.999985264	0.974376225
HVCN1	0.999996191	0.999992444	0.970945672	0.99999488	0.999985264	0.974376225
XIAP	0.999996191	0.991365955	0.962119257	0.99999488	0.999985264	0.974389007
ACTR1B	0.999996191	0.999992444	0.973303821	0.99999488	0.999985264	0.974389007
LMBRD1	0.999996191	0.999992444	0.998936876	0.99999488	0.999985264	0.974389007
ZDHHC24	0.999996191	0.999992444	0.981039752	0.99999488	0.999985264	0.974391767
AUNIP	0.999996191	0.945955989	0.944085862	0.99999488	0.999985264	0.974493911
XPO4	0.999996191	0.991574265	0.935094475	0.99999488	0.999985264	0.974732013
UQCRC2	0.999996191	0.9582784	0.963068909	0.99999488	0.999985264	0.974876921
LOC101904705	0.999996191	0.978510121	0.980660791	0.99999488	0.999985264	0.97513192
IMMT	0.999996191	0.931488015	0.93179776	0.99999488	0.999985264	0.97558997
PEX26	0.999996191	0.991365955	0.951099739	0.99999488	0.999985264	0.97558997
DHRS7B	0.999996191	0.985406717	0.946479014	0.99999488	0.999985264	0.975680198
LOC107131643	0.999996191	0.999992444	0.973406453	0.99999488	0.999985264	0.975680198
AKNA	0.999996191	0.995532499	0.977141934	0.99999488	0.999985264	0.975680198
ANGPTL6	0.999996191	0.951765021	0.951822586	0.99999488	0.999985264	0.975693116
CAMTA2	0.999996191	0.982313546	0.992742113	0.99999488	0.999985264	0.975693116
SRPK1	0.999996191	0.951765021	0.926552506	0.99999488	0.999985264	0.975709501
RCAN3	0.999996191	0.991488652	0.930678591	0.99999488	0.999985264	0.975709501
ARAF	0.999996191	0.999992444	0.954823586	0.99999488	0.999985264	0.975709501
DCDC2B	0.999996191	0.989958965	0.984444908	0.99999488	0.999985264	0.975709501
KDM5C	0.999996191	0.970426668	0.998936876	0.99999488	0.999985264	0.975829506

CYP7B1	0.999996191	0.999992444	0.983419007	0.99999488	0.999985264	0.975917298
STAT5B	0.999996191	0.999992444	0.981622418	0.99999488	0.999985264	0.975983925
KCNC2	0.999996191	0.998181556	0.986501204	0.99999488	0.999985264	0.975983925
CCRL2	0.999996191	0.915367594	0.903175962	0.99999488	0.999985264	0.976082276
ST3GAL4	0.999996191	0.9527938	0.946479014	0.99999488	0.999985264	0.976082276
NR5A2	0.999996191	0.986396187	0.954620171	0.99999488	0.999985264	0.976082276
TIE1	0.999996191	0.9582784	0.956472932	0.99999488	0.999985264	0.976082276
TTC30B	0.999996191	0.972764622	0.956565728	0.99999488	0.999985264	0.976082276
EEPD1	0.999996191	0.974812121	0.957541703	0.99999488	0.999985264	0.976082276
DDX5	0.999996191	0.978510121	0.957541703	0.99999488	0.999985264	0.976082276
HELQ	0.999996191	0.999992444	0.957541703	0.99999488	0.999985264	0.976082276
UNC119B	0.999996191	0.994481342	0.96290532	0.99999488	0.999985264	0.976082276
CALCRL	0.999996191	0.991488652	0.984444908	0.99999488	0.999985264	0.976082276
PDE5A	0.999996191	0.995476917	0.998936876	0.99999488	0.999985264	0.976082276
CDH24	0.999996191	0.985406717	0.999558179	0.99999488	0.999985264	0.976082276
ACADM	0.999996191	0.991488652	0.999558179	0.99999488	0.999985264	0.976082276
CNOT2	0.999996191	0.997083202	0.969880768	0.99999488	0.999985264	0.976119159
NKAIN3	0.999996191	0.999992444	0.982085804	0.99999488	0.999985264	0.976119159
LOC101906273	0.999996191	0.999992444	0.991285432	0.99999488	0.999985264	0.976119159
LOC112446127	0.999996191	0.999992444	0.995045982	0.99999488	0.999985264	0.97613839
B9D2	0.999996191	0.969789939	0.941782902	0.99999488	0.999985264	0.976404355
RANBP17	0.999996191	0.992977597	0.956504686	0.99999488	0.999985264	0.976404355
LOC100299757	0.999996191	0.991365955	0.992742113	0.99999488	0.999985264	0.976404355
ADRB1	0.999996191	0.992977597	0.994626276	0.99999488	0.999985264	0.976404355
CDK4	0.999996191	0.999992444	0.996715234	0.99999488	0.999985264	0.976404355
EFCAB14	0.999996191	0.991488652	0.999558179	0.99999488	0.999985264	0.976404355
DCK	0.999996191	0.999992444	0.93198623	0.99999488	0.999985264	0.976541077
RABGAP1L	0.999996191	0.978089982	0.922400936	0.99999488	0.999985264	0.976783794
ALMS1	0.999996191	0.952346363	0.927391044	0.99999488	0.999985264	0.976783794
LRRC40	0.999996191	0.999992444	0.938203102	0.99999488	0.999985264	0.976783794
C20H5orf22	0.999996191	0.999992444	0.939414234	0.99999488	0.999985264	0.976783794
TRPC4AP	0.999996191	0.978510121	0.940815039	0.99999488	0.999985264	0.976783794
TAF1B	0.999996191	0.9582784	0.944993804	0.99999488	0.999985264	0.976783794
LOC112448387	0.999996191	0.999992444	0.969880768	0.99999488	0.999985264	0.976783794
ROBO1	0.999996191	0.978089982	0.970945672	0.99999488	0.999985264	0.976783794
LAMP1	0.999996191	0.999992444	0.981666445	0.99999488	0.999985264	0.976783794
MASP1	0.999996191	0.999992444	0.994601277	0.99999488	0.999985264	0.976783794
RDX	0.999996191	0.998131745	0.998816213	0.99999488	0.999985264	0.976783794
SCARA5	0.999996191	0.958967142	0.999558179	0.99999488	0.999985264	0.976783794

LOC107132610	0.999996191	0.981520287	0.999558179	0.99999488	0.999985264	0.976783794
LOC507696	0.999996191	0.991488652	0.999558179	0.99999488	0.999985264	0.976783794
LOC100336013	0.999996191	0.993100184	0.999558179	0.99999488	0.999985264	0.976783794
HMG20A	0.999996191	0.99490847	0.999558179	0.99999488	0.999985264	0.976783794
PLCE1	0.999996191	0.970426668	0.903957821	0.99999488	0.999985264	0.976814573
FLRT3	0.999996191	0.944868298	0.913211244	0.99999488	0.999985264	0.976814573
MAP3K11	0.999996191	0.93094333	0.915215321	0.99999488	0.999985264	0.976814573
LYPD1	0.999996191	0.937182585	0.923089792	0.99999488	0.999985264	0.976814573
RBL1	0.999996191	0.919154078	0.926552506	0.99999488	0.999985264	0.976814573
VDAC2	0.999996191	0.921855042	0.926552506	0.99999488	0.999985264	0.976814573
ABCD2	0.999996191	0.999992444	0.926552506	0.99999488	0.999985264	0.976814573
TIFA	0.999996191	0.95697765	0.938203102	0.99999488	0.999985264	0.976814573
ZCCHC9	0.999996191	0.992977597	0.939799459	0.99999488	0.999985264	0.976814573
RIOK1	0.999996191	0.952639631	0.944085862	0.99999488	0.999985264	0.976814573
C5H12orf73	0.999996191	0.985406717	0.946479014	0.99999488	0.999985264	0.976814573
CDRT1	0.999996191	0.935742738	0.948420301	0.99999488	0.999985264	0.976814573
GADD45B	0.999996191	0.999992444	0.948420301	0.99999488	0.999985264	0.976814573
DEPDC7	0.999996191	0.999347049	0.949038056	0.99999488	0.999985264	0.976814573
EPHA1	0.999996191	0.991488652	0.95106368	0.99999488	0.999985264	0.976814573
NCL	0.999996191	0.949285285	0.951099739	0.99999488	0.999985264	0.976814573
SMCR8	0.999996191	0.991488652	0.954620171	0.99999488	0.999985264	0.976814573
NUDT22	0.999996191	0.999347049	0.954620171	0.99999488	0.999985264	0.976814573
RUFY1	0.999996191	0.985293257	0.957117655	0.99999488	0.999985264	0.976814573
MRPL47	0.999996191	0.999992444	0.960133832	0.99999488	0.999985264	0.976814573
ACTR6	0.999996191	0.999992444	0.961373544	0.99999488	0.999985264	0.976814573
SP1	0.999996191	0.992977597	0.962777771	0.99999488	0.999985264	0.976814573
MMP24OS	0.999996191	0.986637746	0.9639767	0.99999488	0.999985264	0.976814573
WRNIP1	0.999996191	0.992110949	0.964152013	0.99999488	0.999985264	0.976814573
CEBPZOS	0.999996191	0.999992444	0.96684887	0.99999488	0.999985264	0.976814573
RESP18	0.999996191	0.999992444	0.96684887	0.99999488	0.999985264	0.976814573
BOLA	0.999996191	0.993316598	0.966991646	0.99999488	0.999985264	0.976814573
LOC616254	0.999996191	0.999992444	0.968325332	0.99999488	0.999985264	0.976814573
NOL10	0.999996191	0.99805349	0.969342421	0.99999488	0.999985264	0.976814573
RPL32	0.999996191	0.999992444	0.96968839	0.99999488	0.999985264	0.976814573
HUWE1	0.999996191	0.991488652	0.969880768	0.99999488	0.999985264	0.976814573
GPATCH2	0.999996191	0.999992444	0.969880768	0.99999488	0.999985264	0.976814573
EGR1	0.999996191	0.924817361	0.970945672	0.99999488	0.999985264	0.976814573
UBE2Q1	0.999996191	0.973912396	0.971759453	0.99999488	0.999985264	0.976814573
PAQR4	0.999996191	0.999992444	0.972362683	0.99999488	0.999985264	0.976814573

LHFPL6	0.999996191	0.999992444	0.97532818	0.99999488	0.999985264	0.976814573
CAPZA1	0.999996191	0.934142668	0.975701948	0.99999488	0.999985264	0.976814573
DPYD	0.999996191	0.991574265	0.977141934	0.99999488	0.999985264	0.976814573
NOD1	0.999996191	0.999992444	0.977141934	0.99999488	0.999985264	0.976814573
HNF4A	0.999996191	0.999347049	0.980491974	0.99999488	0.999985264	0.976814573
LYST	0.999996191	0.999992444	0.9808801	0.99999488	0.999985264	0.976814573
SCOC	0.999996191	0.999992444	0.9808801	0.99999488	0.999985264	0.976814573
GLI1	0.999996191	0.999992444	0.981622418	0.99999488	0.999985264	0.976814573
HAUS5	0.999996191	0.999992444	0.981622418	0.99999488	0.999985264	0.976814573
S100G	0.999996191	0.991365955	0.983077441	0.99999488	0.999985264	0.976814573
THOC1	0.999996191	0.999992444	0.985363931	0.99999488	0.999985264	0.976814573
MRPL30	0.999996191	0.991574265	0.985966356	0.99999488	0.999985264	0.976814573
LOC104975111	0.999996191	0.999992444	0.985966356	0.99999488	0.999985264	0.976814573
LOC104969648	0.999996191	0.986637746	0.988922732	0.99999488	0.999985264	0.976814573
GYPC	0.999996191	0.999992444	0.988922732	0.99999488	0.999985264	0.976814573
SLAIN1	0.999996191	0.991574265	0.991992782	0.99999488	0.999985264	0.976814573
HLF	0.999996191	0.985406717	0.993594894	0.99999488	0.999985264	0.976814573
INTS12	0.999996191	0.959158523	0.995045982	0.99999488	0.999985264	0.976814573
LAMA4	0.999996191	0.992977597	0.996139177	0.99999488	0.999985264	0.976814573
LOC615768	0.999996191	0.977991484	0.996715234	0.99999488	0.999985264	0.976814573
AHSA1	0.999996191	0.989547896	0.996776138	0.99999488	0.999985264	0.976814573
CADM2	0.999996191	0.9582784	0.998936876	0.99999488	0.999985264	0.976814573
ME3	0.999996191	0.961479426	0.998936876	0.99999488	0.999985264	0.976814573
KIRREL3	0.999996191	0.991488652	0.998936876	0.99999488	0.999985264	0.976814573
CENPT	0.999996191	0.991488652	0.999558179	0.99999488	0.999985264	0.976814573
ALG14	0.999996191	0.992977597	0.999558179	0.99999488	0.999985264	0.976814573
LOC104974443	0.999996191	0.995029734	0.999558179	0.99999488	0.999985264	0.976814573
ZNF419	0.999996191	0.999347049	0.999558179	0.99999488	0.999985264	0.976814573
ZNF677	0.999996191	0.999992444	0.999558179	0.99999488	0.999985264	0.976814573
LOC101904810	0.999996191	0.999992444	0.999558179	0.99999488	0.999985264	0.977067252
LOC524181	0.999996191	0.991488652	0.988922732	0.99999488	0.999985264	0.977159914
MRPL13	0.999996191	0.991488652	0.990697261	0.99999488	0.999985264	0.977159914
LOC614882	0.999996191	0.991365955	0.930678591	0.99999488	0.999985264	0.977180137
VGLL4	0.999996191	0.982313546	0.938911935	0.99999488	0.999985264	0.977180137
LOC104974459	0.999996191	0.993777777	0.945678616	0.99999488	0.999985264	0.977180137
CASP3	0.999996191	0.978154072	0.958627535	0.99999488	0.999985264	0.977180137
RBM26	0.999996191	0.999992444	0.970874203	0.99999488	0.999985264	0.977180137
AFAP1L1	0.999996191	0.999992444	0.98023668	0.99999488	0.999985264	0.977180137
UST	0.999996191	0.999992444	0.980847826	0.99999488	0.999985264	0.977180137

LOC100337081	0.999996191	0.9582784	0.94634922	0.99999488	0.999985264	0.977185271
CCDC162P	0.999996191	0.999992444	0.946479014	0.99999488	0.999985264	0.977185271
TCF4	0.999996191	0.995073696	0.981388276	0.99999488	0.999985264	0.977185271
GOLPH3L	0.999996191	0.967520941	0.95106368	0.99999488	0.999985264	0.977209935
NUPR2	0.999996191	0.999347049	0.965234684	0.99999488	0.999985264	0.977209935
UBE2I	0.999996191	0.999992444	0.998936876	0.99999488	0.999985264	0.977209935
CCDC85B	0.999996191	0.999992444	0.958627535	0.99999488	0.999985264	0.977235025
PDE2A	0.999996191	0.999992444	0.933791093	0.99999488	0.999985264	0.977569033
LOC101907000	0.999996191	0.978089982	0.941118576	0.99999488	0.999985264	0.977569033
LOC101905706	0.999996191	0.981520287	0.946479014	0.99999488	0.999985264	0.977569033
TRIR	0.999996191	0.999347049	0.966991646	0.99999488	0.999985264	0.977569033
ARHGEF18	0.999996191	0.998011664	0.991285432	0.99999488	0.999985264	0.977569033
DAD1	0.999996191	0.992977597	0.993594894	0.99999488	0.999985264	0.977569033
NVL	0.999996191	0.999992444	0.996348351	0.99999488	0.999985264	0.977569033
TMC7	0.999996191	0.977354623	0.999558179	0.99999488	0.999985264	0.977569033
LOC101904529	0.999996191	0.935742738	0.886962419	0.99999488	0.999985264	0.977753483
LOC112449087	0.999996191	0.95697765	0.894525066	0.99999488	0.999985264	0.977753483
GPR55	0.999996191	0.91407604	0.900890709	0.99999488	0.999985264	0.977753483
ARIH2	0.999996191	0.985161531	0.904564	0.99999488	0.999985264	0.977753483
ZBTB34	0.999996191	0.956768356	0.907484111	0.99999488	0.999985264	0.977753483
SALL2	0.999996191	0.938597964	0.909070782	0.99999488	0.999985264	0.977753483
LOC112443159	0.999996191	0.972048346	0.914001994	0.99999488	0.999985264	0.977753483
KCTD13	0.999996191	0.992977597	0.914001994	0.99999488	0.999985264	0.977753483
DNAH12	0.999996191	0.954790265	0.914262201	0.99999488	0.999985264	0.977753483
SNAI1	0.999996191	0.999992444	0.916975216	0.99999488	0.999985264	0.977753483
GGNBP2	0.999996191	0.991488652	0.926552506	0.99999488	0.999985264	0.977753483
LOC107132924	0.999996191	0.999992444	0.926552506	0.99999488	0.999985264	0.977753483
CENPP	0.999996191	0.991488652	0.926932508	0.99999488	0.999985264	0.977753483
SEH1L	0.999996191	0.940135363	0.929188473	0.99999488	0.999985264	0.977753483
PRR15	0.999996191	0.979263532	0.930678591	0.99999488	0.999985264	0.977753483
SLC1A5	0.999996191	0.9582784	0.93198623	0.99999488	0.999985264	0.977753483
MRC1	0.999996191	0.962968114	0.932653752	0.99999488	0.999985264	0.977753483
SPTSSA	0.999996191	0.919884594	0.935094475	0.99999488	0.999985264	0.977753483
PRKAB2	0.999996191	0.991488652	0.935094475	0.99999488	0.999985264	0.977753483
LOC101903232	0.999996191	0.946480858	0.938905004	0.99999488	0.999985264	0.977753483
KPNA5	0.999996191	0.999992444	0.939799459	0.99999488	0.999985264	0.977753483
LOC112447846	0.999996191	0.937231686	0.941782902	0.99999488	0.999985264	0.977753483
FSD1L	0.999996191	0.938597964	0.944871472	0.99999488	0.999985264	0.977753483
WNT9A	0.999996191	0.942741936	0.945678616	0.99999488	0.999985264	0.977753483

SNN	0.999996191	0.970426668	0.94634922	0.99999488	0.999985264	0.977753483
LOC101902808	0.999996191	0.985406717	0.94634922	0.99999488	0.999985264	0.977753483
ASRGL1	0.999996191	0.925580392	0.946479014	0.99999488	0.999985264	0.977753483
MARK3	0.999996191	0.946274461	0.946479014	0.99999488	0.999985264	0.977753483
CCDC9	0.999996191	0.992977597	0.946479014	0.99999488	0.999985264	0.977753483
ARMC7	0.999996191	0.986637746	0.947267164	0.99999488	0.999985264	0.977753483
CHSY1	0.999996191	0.971114244	0.948946702	0.99999488	0.999985264	0.977753483
ID4	0.999996191	0.999992444	0.949038056	0.99999488	0.999985264	0.977753483
EHHADH	0.999996191	0.985406717	0.95083766	0.99999488	0.999985264	0.977753483
HOXB5	0.999996191	0.999992444	0.95106368	0.99999488	0.999985264	0.977753483
ZNF174	0.999996191	0.981298804	0.951822586	0.99999488	0.999985264	0.977753483
COQ4	0.999996191	0.993316598	0.952207545	0.99999488	0.999985264	0.977753483
LOC782293	0.999996191	0.992977597	0.95230212	0.99999488	0.999985264	0.977753483
INO80D	0.999996191	0.999992444	0.95230212	0.99999488	0.999985264	0.977753483
FAM162B	0.999996191	0.999992444	0.95477544	0.99999488	0.999985264	0.977753483
DUS1L	0.999996191	0.974079639	0.954823586	0.99999488	0.999985264	0.977753483
LOC107133459	0.999996191	0.999347049	0.954823586	0.99999488	0.999985264	0.977753483
KCNAB2	0.999996191	0.999992444	0.954872702	0.99999488	0.999985264	0.977753483
GDF5	0.999996191	0.978089982	0.956565728	0.99999488	0.999985264	0.977753483
CPLANE2	0.999996191	0.999992444	0.956565728	0.99999488	0.999985264	0.977753483
LOC107131623	0.999996191	0.978089982	0.957541703	0.99999488	0.999985264	0.977753483
HSPB1	0.999996191	0.999992444	0.957541703	0.99999488	0.999985264	0.977753483
C15H11orf74	0.999996191	0.999992444	0.957840205	0.99999488	0.999985264	0.977753483
PSMA6	0.999996191	0.932593501	0.958627535	0.99999488	0.999985264	0.977753483
LOC112442081	0.999996191	0.991365955	0.959314104	0.99999488	0.999985264	0.977753483
LOC104975979	0.999996191	0.991365955	0.96210311	0.99999488	0.999985264	0.977753483
LOC101906632	0.999996191	0.999992444	0.96210311	0.99999488	0.999985264	0.977753483
LOC101908535	0.999996191	0.985406717	0.962777771	0.99999488	0.999985264	0.977753483
LOC107132798	0.999996191	0.994135588	0.962777771	0.99999488	0.999985264	0.977753483
HIST1H1C	0.999996191	0.999992444	0.962777771	0.99999488	0.999985264	0.977753483
FAM160A1	0.999996191	0.999992444	0.964920281	0.99999488	0.999985264	0.977753483
VGLL3	0.999996191	0.938597964	0.965059328	0.99999488	0.999985264	0.977753483
RPP21	0.999996191	0.991464005	0.965234684	0.99999488	0.999985264	0.977753483
LOC112443001	0.999996191	0.999992444	0.96752854	0.99999488	0.999985264	0.977753483
PGM3	0.999996191	0.999992444	0.968232195	0.99999488	0.999985264	0.977753483
ERLEC1	0.999996191	0.991488652	0.968325332	0.99999488	0.999985264	0.977753483
AGO2	0.999996191	0.998131745	0.968508342	0.99999488	0.999985264	0.977753483
PITPNB	0.999996191	0.999992444	0.969572392	0.99999488	0.999985264	0.977753483
ADGRG5	0.999996191	0.999992444	0.96968839	0.99999488	0.999985264	0.977753483

ZC3HAV1	0.999996191	0.978089982	0.969880768	0.99999488	0.999985264	0.977753483
DNAJB11	0.999996191	0.999992444	0.970874203	0.99999488	0.999985264	0.977753483
SKIDA1	0.999996191	0.985406717	0.970903823	0.99999488	0.999985264	0.977753483
TXLNB	0.999996191	0.999992444	0.971759453	0.99999488	0.999985264	0.977753483
B4GALT4	0.999996191	0.999992444	0.97262931	0.99999488	0.999985264	0.977753483
TTF1	0.999996191	0.992895985	0.973303821	0.99999488	0.999985264	0.977753483
NBAS	0.999996191	0.991365955	0.973406453	0.99999488	0.999985264	0.977753483
EIF4EBP2	0.999996191	0.999992444	0.973406453	0.99999488	0.999985264	0.977753483
LOC104970105	0.999996191	0.999992444	0.973406453	0.99999488	0.999985264	0.977753483
LOC112448056	0.999996191	0.999992444	0.973406453	0.99999488	0.999985264	0.977753483
SPTA1	0.999996191	0.999992444	0.973406453	0.99999488	0.999985264	0.977753483
MIF4GD	0.999996191	0.999992444	0.977127779	0.99999488	0.999985264	0.977753483
CHP1	0.999996191	0.974812121	0.977141934	0.99999488	0.999985264	0.977753483
RBPJ	0.999996191	0.999992444	0.977832293	0.99999488	0.999985264	0.977753483
YIPF6	0.999996191	0.999992444	0.978389389	0.99999488	0.999985264	0.977753483
THYN1	0.999996191	0.998131745	0.979812868	0.99999488	0.999985264	0.977753483
TNKS2	0.999996191	0.997975376	0.980542624	0.99999488	0.999985264	0.977753483
MAPRE2	0.999996191	0.999992444	0.980660791	0.99999488	0.999985264	0.977753483
SVBP	0.999996191	0.999992444	0.980660791	0.99999488	0.999985264	0.977753483
DYNC1LI2	0.999996191	0.999347049	0.9808801	0.99999488	0.999985264	0.977753483
NUP107	0.999996191	0.999992444	0.9808801	0.99999488	0.999985264	0.977753483
ZNF260	0.999996191	0.999992444	0.9808801	0.99999488	0.999985264	0.977753483
ARPC5L	0.999996191	0.946274461	0.981039752	0.99999488	0.999985264	0.977753483
PPIH	0.999996191	0.972895283	0.981039752	0.99999488	0.999985264	0.977753483
CHCHD3	0.999996191	0.991488652	0.981039752	0.99999488	0.999985264	0.977753483
EEF1AKMT2	0.999996191	0.999992444	0.981039752	0.99999488	0.999985264	0.977753483
PRND	0.999996191	0.999992444	0.981218677	0.99999488	0.999985264	0.977753483
LATS2	0.999996191	0.992744518	0.981388276	0.99999488	0.999985264	0.977753483
LOC617698	0.999996191	0.993316598	0.981465954	0.99999488	0.999985264	0.977753483
NCAPH2	0.999996191	0.989958965	0.983888817	0.99999488	0.999985264	0.977753483
LOC101905711	0.999996191	0.999992444	0.984444908	0.99999488	0.999985264	0.977753483
TMEM14C	0.999996191	0.999992444	0.984704176	0.99999488	0.999985264	0.977753483
PPP1R9B	0.999996191	0.999347049	0.98480229	0.99999488	0.999985264	0.977753483
DBT	0.999996191	0.978510121	0.985363931	0.99999488	0.999985264	0.977753483
HAP1	0.999996191	0.989547896	0.985363931	0.99999488	0.999985264	0.977753483
AFG1L	0.999996191	0.991488652	0.985363931	0.99999488	0.999985264	0.977753483
UPK1B	0.999996191	0.993440468	0.985363931	0.99999488	0.999985264	0.977753483
APC	0.999996191	0.999347049	0.985363931	0.99999488	0.999985264	0.977753483
LOC100296211	0.999996191	0.999992444	0.98545277	0.99999488	0.999985264	0.977753483

BLOC1S2	0.999996191	0.94984369	0.985764826	0.99999488	0.999985264	0.977753483
AGER	0.999996191	0.998962836	0.985966356	0.99999488	0.999985264	0.977753483
PDGFRB	0.999996191	0.991488652	0.986266701	0.99999488	0.999985264	0.977753483
NQO1	0.999996191	0.999992444	0.988922732	0.99999488	0.999985264	0.977753483
HAT1	0.999996191	0.998131745	0.991285432	0.99999488	0.999985264	0.977753483
PROX2	0.999996191	0.973685368	0.991319768	0.99999488	0.999985264	0.977753483
CHERP	0.999996191	0.999992444	0.991615606	0.99999488	0.999985264	0.977753483
HNRNPH1	0.999996191	0.994210852	0.992742113	0.99999488	0.999985264	0.977753483
LOC790037	0.999996191	0.999992444	0.992939335	0.99999488	0.999985264	0.977753483
COX7A2L	0.999996191	0.978089982	0.993594894	0.99999488	0.999985264	0.977753483
PDE8B	0.999996191	0.985406717	0.993594894	0.99999488	0.999985264	0.977753483
DVL2	0.999996191	0.991488652	0.993594894	0.99999488	0.999985264	0.977753483
BAP1	0.999996191	0.992977597	0.993594894	0.99999488	0.999985264	0.977753483
RRAGA	0.999996191	0.936657629	0.994048963	0.99999488	0.999985264	0.977753483
UBE2N	0.999996191	0.9582784	0.994601277	0.99999488	0.999985264	0.977753483
EXOC1L	0.999996191	0.999992444	0.994601277	0.99999488	0.999985264	0.977753483
GAS7	0.999996191	0.999992444	0.994601277	0.99999488	0.999985264	0.977753483
GOLPH3	0.999996191	0.999347049	0.995045982	0.99999488	0.999985264	0.977753483
SLC9C2	0.999996191	0.999992444	0.995045982	0.99999488	0.999985264	0.977753483
EMP2	0.999996191	0.999992444	0.996348351	0.99999488	0.999985264	0.977753483
ETFB	0.999996191	0.989996767	0.996391386	0.99999488	0.999985264	0.977753483
NAGPA	0.999996191	0.999992444	0.996698354	0.99999488	0.999985264	0.977753483
VAMP7	0.999996191	0.991476708	0.998936876	0.99999488	0.999985264	0.977753483
HSPA4	0.999996191	0.991574265	0.998936876	0.99999488	0.999985264	0.977753483
DYRK1B	0.999996191	0.999347049	0.998936876	0.99999488	0.999985264	0.977753483
ZNF462	0.999996191	0.999347049	0.998936876	0.99999488	0.999985264	0.977753483
GINS4	0.999996191	0.999992444	0.998936876	0.99999488	0.999985264	0.977753483
TC2N	0.999996191	0.999992444	0.998936876	0.99999488	0.999985264	0.977753483
LOC112442048	0.999996191	0.978089982	0.999558179	0.99999488	0.999985264	0.977753483
LOC112444897	0.999996191	0.984714297	0.999558179	0.99999488	0.999985264	0.977753483
PIDD1	0.999996191	0.991488652	0.999558179	0.99999488	0.999985264	0.977753483
C3H1orf43	0.999996191	0.991574265	0.999558179	0.99999488	0.999985264	0.977753483
TRNP1	0.999996191	0.992409171	0.999558179	0.99999488	0.999985264	0.977753483
ECHDC2	0.999996191	0.992977597	0.999558179	0.99999488	0.999985264	0.977753483
TBC1D9	0.999996191	0.992977597	0.999558179	0.99999488	0.999985264	0.977753483
CREG1	0.999996191	0.993316598	0.999558179	0.99999488	0.999985264	0.977753483
METTL23	0.999996191	0.993316598	0.999558179	0.99999488	0.999985264	0.977753483
ATRN	0.999996191	0.995532499	0.999558179	0.99999488	0.999985264	0.977753483
L3MBTL1	0.999996191	0.998131745	0.999558179	0.99999488	0.999985264	0.977753483

NEK8	0.999996191	0.998962836	0.999558179	0.99999488	0.999985264	0.977753483
ENDOD1	0.999996191	0.999347049	0.999558179	0.99999488	0.999985264	0.977753483
MARCH8	0.999996191	0.999347049	0.999558179	0.99999488	0.999985264	0.977753483
CDC26	0.999996191	0.999992444	0.999558179	0.99999488	0.999985264	0.977753483
COG1	0.999996191	0.999992444	0.999558179	0.99999488	0.999985264	0.977753483
GAREM2	0.999996191	0.999992444	0.999558179	0.99999488	0.999985264	0.977753483
LOC101902895	0.999996191	0.999992444	0.999558179	0.99999488	0.999985264	0.977753483
NR3C1	0.999996191	0.991488652	0.991285432	0.99999488	0.999985264	0.977762079
PROM1	0.999996191	0.999992444	0.944085862	0.99999488	0.999985264	0.977821653
SPIN1	0.999996191	0.962115278	0.958627535	0.99999488	0.999985264	0.977821653
LOC615258	0.999996191	0.943802122	0.96684887	0.99999488	0.999985264	0.977821653
PPP1R14A	0.999996191	0.943405288	0.973303821	0.99999488	0.999985264	0.977821653
FNTA	0.999996191	0.992977597	0.979462526	0.99999488	0.999985264	0.977821653
PARL	0.999996191	0.938597964	0.980847826	0.99999488	0.999985264	0.977821653
ZNF789	0.999996191	0.960125322	0.980847826	0.99999488	0.999985264	0.977821653
LOC104975415	0.999996191	0.991365955	0.993594894	0.99999488	0.999985264	0.977821653
INTS7	0.999996191	0.991574265	0.999558179	0.99999488	0.999985264	0.977821653
CES2	0.999996191	0.999992444	0.877786216	0.99999488	0.999985264	0.977830614
KLHL33	0.999996191	0.938597964	0.932653752	0.99999488	0.999985264	0.978125202
CHST9	0.999996191	0.991488652	0.900148803	0.99999488	0.999985264	0.978249551
TRAPPC13	0.999996191	0.978089982	0.947565182	0.99999488	0.999985264	0.978249551
ASNSD1	0.999996191	0.992977597	0.972381338	0.99999488	0.999985264	0.978249551
WDR34	0.999996191	0.999992444	0.973406453	0.99999488	0.999985264	0.978249551
LOC100616098	0.999996191	0.998962836	0.981039752	0.99999488	0.999985264	0.978249551
LOC112442414	0.999996191	0.960125322	0.957541703	0.99999488	0.999985264	0.978523484
SETD5	0.999996191	0.972764622	0.890724268	0.99999488	0.999985264	0.978682689
LOC112443339	0.999996191	0.944868298	0.897043265	0.99999488	0.999985264	0.978682689
PSMC5	0.999996191	0.917849199	0.897767405	0.99999488	0.999985264	0.978682689
QRSL1	0.999996191	0.993316598	0.930678591	0.99999488	0.999985264	0.978682689
TSPEAR	0.999996191	0.999992444	0.939478907	0.99999488	0.999985264	0.978682689
FBXO32	0.999996191	0.978426416	0.944168373	0.99999488	0.999985264	0.978682689
PMAIP1	0.999996191	0.949285285	0.945678616	0.99999488	0.999985264	0.978682689
TXNL4B	0.999996191	0.991488652	0.951099739	0.99999488	0.999985264	0.978682689
LOC101903567	0.999996191	0.998131745	0.956565728	0.99999488	0.999985264	0.978682689
DSC2	0.999996191	0.992977597	0.969656316	0.99999488	0.999985264	0.978682689
TMEM159	0.999996191	0.999347049	0.969880768	0.99999488	0.999985264	0.978682689
DBI	0.999996191	0.967276215	0.971111634	0.99999488	0.999985264	0.978682689
LOC112449073	0.999996191	0.999992444	0.971778256	0.99999488	0.999985264	0.978682689
C6	0.999996191	0.973912396	0.977141934	0.99999488	0.999985264	0.978682689

DEF8	0.999996191	0.999992444	0.979745141	0.99999488	0.999985264	0.978682689
PCNA	0.999996191	0.999992444	0.98480229	0.99999488	0.999985264	0.978682689
KCMF1	0.999996191	0.985406717	0.985966356	0.99999488	0.999985264	0.978682689
SPESP1	0.999996191	0.997975376	0.991285432	0.99999488	0.999985264	0.978682689
FAM213A	0.999996191	0.985406717	0.992709008	0.99999488	0.999985264	0.978682689
SWAP70	0.999996191	0.999992444	0.993594894	0.99999488	0.999985264	0.978682689
LOC112442323	0.999996191	0.999992444	0.996401083	0.99999488	0.999985264	0.978682689
COMMD7	0.999996191	0.997051616	0.999558179	0.99999488	0.999985264	0.978682689
PRDM4	0.999996191	0.999992444	0.925520255	0.99999488	0.999985264	0.9787163
DPY19L1	0.999996191	0.992977597	0.946479014	0.99999488	0.999985264	0.9787163
TJP1	0.999996191	0.989547896	0.947432352	0.99999488	0.999985264	0.9787163
LOC112442865	0.999996191	0.999992444	0.95106368	0.99999488	0.999985264	0.9787163
SLC29A1	0.999996191	0.999992444	0.957541703	0.99999488	0.999985264	0.9787163
CDK14	0.999996191	0.999992444	0.962653182	0.99999488	0.999985264	0.9787163
IRS1	0.999996191	0.992977597	0.96290532	0.99999488	0.999985264	0.9787163
MAPK7	0.999996191	0.999992444	0.963068909	0.99999488	0.999985264	0.9787163
LOC107131792	0.999996191	0.948421431	0.964920281	0.99999488	0.999985264	0.9787163
STAG1	0.999996191	0.999992444	0.965690512	0.99999488	0.999985264	0.9787163
RN18S1	0.999996191	0.998011664	0.96968839	0.99999488	0.999985264	0.9787163
BAD	0.999996191	0.999992444	0.971833995	0.99999488	0.999985264	0.9787163
ARL6IP6	0.999996191	0.999992444	0.973406453	0.99999488	0.999985264	0.9787163
PNPLA2	0.999996191	0.999992444	0.973406453	0.99999488	0.999985264	0.9787163
LOC107132784	0.999996191	0.999992444	0.978389389	0.99999488	0.999985264	0.9787163
SLC40A1	0.999996191	0.999992444	0.981218677	0.99999488	0.999985264	0.9787163
NQO2	0.999996191	0.991365955	0.995045982	0.99999488	0.999985264	0.9787163
TMEM218	0.999996191	0.943802122	0.996139177	0.99999488	0.999985264	0.9787163
STRADA	0.999996191	0.999992444	0.999558179	0.99999488	0.999985264	0.9787163
RADIL	0.999996191	0.97286093	0.952875254	0.99999488	0.999985264	0.978896655
PHB	0.999996191	0.992977597	0.938203102	0.99999488	0.999985264	0.979046621
ATG9A	0.999996191	0.986637746	0.949038056	0.99999488	0.999985264	0.979046621
ZC3H12A	0.999996191	0.978510121	0.968232195	0.99999488	0.999985264	0.979046621
NPC1	0.999996191	0.978426416	0.968235535	0.99999488	0.999985264	0.979046621
TGFBR2	0.999996191	0.946274461	0.992742113	0.99999488	0.999985264	0.979046621
LOC534742	0.999996191	0.997083202	0.993594894	0.99999488	0.999985264	0.979046621
LOC107132515	0.999996191	0.9582784	0.995045982	0.99999488	0.999985264	0.979046621
AP1G1	0.999996191	0.979263532	0.998936876	0.99999488	0.999985264	0.979046621
H3F3B	0.999996191	0.999992444	0.999558179	0.99999488	0.999985264	0.979046621
ACSM1	0.999996191	0.996370892	0.985363931	0.99999488	0.999985264	0.979122913
IPO9	0.999996191	0.970426668	0.955919075	0.99999488	0.999985264	0.979193553

LOC112441611	0.999996191	0.999992444	0.971838819	0.99999488	0.999985264	0.979193553
RAPGEF2	0.999996191	0.999992444	0.999558179	0.99999488	0.999985264	0.979193553
LOC104969916	0.999996191	0.999992444	0.957541703	0.99999488	0.999985264	0.979232737
FZD7	0.999996191	0.992301181	0.963465247	0.99999488	0.999985264	0.979232737
SSR2	0.999996191	0.999992444	0.977141934	0.99999488	0.999985264	0.979232737
ARSK	0.999996191	0.999992444	0.977141934	0.99999488	0.999985264	0.979376051
PPM1K	0.999996191	0.9677397	0.869855015	0.99999488	0.999985264	0.979570541
LOC112442352	0.999996191	0.930409519	0.886962419	0.99999488	0.999985264	0.979961637
ECT2	0.999996191	0.964246761	0.954571119	0.99999488	0.999985264	0.979961637
LOC100847182	0.999996191	0.992977597	0.999558179	0.99999488	0.999985264	0.979961637
B4GALNT1	0.999996191	0.941329909	0.922400936	0.99999488	0.999985264	0.980032028
QSER1	0.999996191	0.993316598	0.951566587	0.99999488	0.999985264	0.980032028
LOC104976664	0.999996191	0.999992444	0.956565728	0.99999488	0.999985264	0.980032028
TRIM14	0.999996191	0.998131745	0.968232195	0.99999488	0.999985264	0.980032028
LOC112445178	0.999996191	0.99398773	0.98286145	0.99999488	0.999985264	0.980150333
LOC112441885	0.999996191	0.91507531	0.898815524	0.99999488	0.999985264	0.980223673
ZNF710	0.999996191	0.946274461	0.902862658	0.99999488	0.999985264	0.980745678
BTN3A3	0.999996191	0.978089982	0.992742113	0.99999488	0.999985264	0.98086554
KRT7	0.999996191	0.971009675	0.93615295	0.99999488	0.999985264	0.981027853
GMFB	0.999996191	0.999992444	0.938203102	0.99999488	0.999985264	0.981027853
CALN1	0.999996191	0.993316598	0.981388276	0.99999488	0.999985264	0.981027853
SHC1	0.999996191	0.999992444	0.973303821	0.99999488	0.999985264	0.981041874
HCFC1	0.999996191	0.999347049	0.994601277	0.99999488	0.999985264	0.981041874
DPH2	0.999996191	0.991488652	0.998936876	0.99999488	0.999985264	0.981041874
EID3	0.999996191	0.999347049	0.952414682	0.99999488	0.999985264	0.98114037
LHCGR	0.999996191	0.991488652	0.96210311	0.99999488	0.999985264	0.98114037
LAYN	0.999996191	0.999992444	0.977141934	0.99999488	0.999985264	0.98114037
ASB16	0.999996191	0.999992444	0.978389389	0.99999488	0.999985264	0.98114037
ZNHIT1	0.999996191	0.999992444	0.979745141	0.99999488	0.999985264	0.98114037
CCNG1	0.999996191	0.998131745	0.981388276	0.99999488	0.999985264	0.98114037
COL4A5	0.999996191	0.981520287	0.892943118	0.99999488	0.999985264	0.981216942
LOC782799	0.999996191	0.93094333	0.898815524	0.99999488	0.999985264	0.981216942
IGF1R	0.999996191	0.970426668	0.923604668	0.99999488	0.999985264	0.981216942
PPP1R37	0.999996191	0.998131745	0.923604668	0.99999488	0.999985264	0.981216942
TCEA3	0.999996191	0.993316598	0.924604843	0.99999488	0.999985264	0.981216942
P3H2	0.999996191	0.91388073	0.938203102	0.99999488	0.999985264	0.981216942
CUL4B	0.999996191	0.999992444	0.944031108	0.99999488	0.999985264	0.981216942
LOC112447140	0.999996191	0.999992444	0.948420301	0.99999488	0.999985264	0.981216942
NTN4	0.999996191	0.941495217	0.95106368	0.99999488	0.999985264	0.981216942

UGCG	0.999996191	0.978510121	0.951735967	0.99999488	0.999985264	0.981216942
LOC104968873	0.999996191	0.999992444	0.961024709	0.99999488	0.999985264	0.981216942
PPP2R5B	0.999996191	0.999347049	0.962346588	0.99999488	0.999985264	0.981216942
DIP2A	0.999996191	0.987516216	0.968232195	0.99999488	0.999985264	0.981216942
WNT10B	0.999996191	0.967919743	0.968325332	0.99999488	0.999985264	0.981216942
WASF3	0.999996191	0.991488652	0.972371941	0.99999488	0.999985264	0.981216942
SMAD4	0.999996191	0.993316598	0.973303821	0.99999488	0.999985264	0.981216942
ACAD10	0.999996191	0.979756265	0.973406453	0.99999488	0.999985264	0.981216942
LOC100299303	0.999996191	0.999992444	0.973406453	0.99999488	0.999985264	0.981216942
LOC100848478	0.999996191	0.999992444	0.973406453	0.99999488	0.999985264	0.981216942
PHTF2	0.999996191	0.999992444	0.973406453	0.99999488	0.999985264	0.981216942
OSTC	0.999996191	0.999992444	0.973661483	0.99999488	0.999985264	0.981216942
CST6	0.999996191	0.999992444	0.97558773	0.99999488	0.999985264	0.981216942
DPM3	0.999996191	0.999992444	0.976143419	0.99999488	0.999985264	0.981216942
KIAA0232	0.999996191	0.992977597	0.976925152	0.99999488	0.999985264	0.981216942
DHX34	0.999996191	0.999992444	0.979462526	0.99999488	0.999985264	0.981216942
KREMEN1	0.999996191	0.999992444	0.979462526	0.99999488	0.999985264	0.981216942
FBXO31	0.999996191	0.999992444	0.9808801	0.99999488	0.999985264	0.981216942
NADSYN1	0.999996191	0.999992444	0.981039752	0.99999488	0.999985264	0.981216942
KYAT3	0.999996191	0.992977597	0.981388276	0.99999488	0.999985264	0.981216942
LOC112442408	0.999996191	0.999992444	0.98286145	0.99999488	0.999985264	0.981216942
CIC	0.999996191	0.999992444	0.984444908	0.99999488	0.999985264	0.981216942
PRCC	0.999996191	0.999992444	0.995045982	0.99999488	0.999985264	0.981216942
CAV1	0.999996191	0.992977597	0.998936876	0.99999488	0.999985264	0.981216942
NDUFV3	0.999996191	0.994481342	0.998936876	0.99999488	0.999985264	0.981216942
FBXO17	0.999996191	0.999992444	0.998936876	0.99999488	0.999985264	0.981216942
PRUNE1	0.999996191	0.999992444	0.998936876	0.99999488	0.999985264	0.981216942
SYN3	0.999996191	0.992977597	0.999558179	0.99999488	0.999985264	0.981216942
ADGRE5	0.999996191	0.999992444	0.999558179	0.99999488	0.999985264	0.981216942
MIB2	0.999996191	0.999992444	0.999558179	0.99999488	0.999985264	0.981216942
USP9X	0.999996191	0.999992444	0.999558179	0.99999488	0.999985264	0.981216942
CCR7	0.999996191	0.991488652	0.932653752	0.99999488	0.999985264	0.98130784
FZD3	0.999996191	0.957700659	0.960143151	0.99999488	0.999985264	0.981635786
LOC615514	0.999996191	0.996599769	0.981039752	0.99999488	0.999985264	0.981635786
ZNF274	0.999996191	0.999992444	0.999558179	0.99999488	0.999985264	0.981635786
PAIP2B	0.999996191	0.993316598	0.996391386	0.99999488	0.999985264	0.981644492
ZNF500	0.999996191	0.999992444	0.999558179	0.99999488	0.999985264	0.981644492
LOC104969259	0.999996191	0.999992444	0.996348351	0.99999488	0.999985264	0.981646808
A2M	0.999996191	0.999992444	0.968232195	0.99999488	0.999985264	0.981702741

RRP1	0.999996191	0.938702715	0.892943118	0.99999488	0.999985264	0.981734058
NCOA6	0.999996191	0.952346363	0.901758346	0.99999488	0.999985264	0.981734058
SLC16A12	0.999996191	0.947110864	0.911636823	0.99999488	0.999985264	0.981734058
NPM1	0.999996191	0.928088831	0.923604668	0.99999488	0.999985264	0.981734058
FAM174B	0.999996191	0.985406717	0.926552506	0.99999488	0.999985264	0.981734058
MINK1	0.999996191	0.935060764	0.938203102	0.99999488	0.999985264	0.981734058
CA9	0.999996191	0.978089982	0.944085862	0.99999488	0.999985264	0.981734058
ODF2	0.999996191	0.946274461	0.945280564	0.99999488	0.999985264	0.981734058
LHFPL3	0.999996191	0.975677199	0.946479014	0.99999488	0.999985264	0.981734058
KCNC4	0.999996191	0.999992444	0.946479014	0.99999488	0.999985264	0.981734058
TMEM14A	0.999996191	0.991488652	0.95106368	0.99999488	0.999985264	0.981734058
NME3	0.999996191	0.985293257	0.957117655	0.99999488	0.999985264	0.981734058
TICAM1	0.999996191	0.999992444	0.95935771	0.99999488	0.999985264	0.981734058
LOC101906676	0.999996191	0.988819917	0.960133832	0.99999488	0.999985264	0.981734058
CCT2	0.999996191	0.96566261	0.966991646	0.99999488	0.999985264	0.981734058
LOC112444907	0.999996191	0.92462339	0.971833995	0.99999488	0.999985264	0.981734058
RARA	0.999996191	0.999992444	0.972381338	0.99999488	0.999985264	0.981734058
METTL8	0.999996191	0.999992444	0.973661483	0.99999488	0.999985264	0.981734058
C27H8orf48	0.999996191	0.999992444	0.9808801	0.99999488	0.999985264	0.981734058
LOC107133226	0.999996191	0.999992444	0.9808801	0.99999488	0.999985264	0.981734058
LOC112444333	0.999996191	0.998578163	0.980983885	0.99999488	0.999985264	0.981734058
DKK3	0.999996191	0.992977597	0.984444908	0.99999488	0.999985264	0.981734058
DARS2	0.999996191	0.999347049	0.988922732	0.99999488	0.999985264	0.981734058
LOC100847839	0.999996191	0.998131745	0.991285432	0.99999488	0.999985264	0.981734058
RDH8	0.999996191	0.993540482	0.992709008	0.99999488	0.999985264	0.981734058
ARNT	0.999996191	0.999992444	0.992709008	0.99999488	0.999985264	0.981734058
TMEM184C	0.999996191	0.999992444	0.992709008	0.99999488	0.999985264	0.981734058
POLR2H	0.999996191	0.992977597	0.992742113	0.99999488	0.999985264	0.981734058
DIAPH2	0.999996191	0.992301181	0.993594894	0.99999488	0.999985264	0.981734058
NARS	0.999996191	0.992977597	0.993594894	0.99999488	0.999985264	0.981734058
ERCC5	0.999996191	0.999992444	0.993594894	0.99999488	0.999985264	0.981734058
ARID3B	0.999996191	0.999992444	0.994601277	0.99999488	0.999985264	0.981734058
VAMP1	0.999996191	0.993316598	0.994626276	0.99999488	0.999985264	0.981734058
TMEM184A	0.999996191	0.935588259	0.998936876	0.99999488	0.999985264	0.981734058
CCND2	0.999996191	0.999347049	0.998936876	0.99999488	0.999985264	0.981734058
LOC101905813	0.999996191	0.936273761	0.999558179	0.99999488	0.999985264	0.981734058
LOC101906411	0.999996191	0.993316598	0.999558179	0.99999488	0.999985264	0.981734058
APPL1	0.999996191	0.993540482	0.999558179	0.99999488	0.999985264	0.981734058
C8H9orf85	0.999996191	0.996003806	0.999558179	0.99999488	0.999985264	0.981734058

CCNL2	0.999996191	0.997083202	0.999558179	0.99999488	0.999985264	0.981734058
TTLL4	0.999996191	0.999347049	0.999558179	0.99999488	0.999985264	0.981734058
C23H6orf52	0.999996191	0.999992444	0.999558179	0.99999488	0.999985264	0.981734058
PRKAB1	0.999996191	0.999992444	0.999558179	0.99999488	0.999985264	0.981734058
LOC101907797	0.999996191	0.9582784	0.961024709	0.99999488	0.999985264	0.981751532
DBNL	0.999996191	0.999992444	0.941118576	0.99999488	0.999985264	0.981855491
HOXA3	0.999996191	0.999992444	0.954620171	0.99999488	0.999985264	0.981855491
LOC104969067	0.999996191	0.992977597	0.973406453	0.99999488	0.999985264	0.981855491
LOC107131510	0.999996191	0.932580437	0.954823586	0.99999488	0.999985264	0.981905927
URB1	0.999996191	0.961874682	0.956565728	0.99999488	0.999985264	0.981905927
GLCCI1	0.999996191	0.943802122	0.916214824	0.99999488	0.999985264	0.982009663
MYF6	0.999996191	0.999992444	0.928642875	0.99999488	0.999985264	0.982009663
MAGI1	0.999996191	0.999992444	0.948359914	0.99999488	0.999985264	0.982009663
ZNF75A	0.999996191	0.997051616	0.987678394	0.99999488	0.999985264	0.982009663
BCL2L10	0.999996191	0.992977597	0.95477544	0.99999488	0.999985264	0.982151663
RPL15	0.999996191	0.999347049	0.962142195	0.99999488	0.999985264	0.982245784
SPTLC2	0.999996191	0.946480858	0.892053976	0.99999488	0.999985264	0.982250307
LOC107133190	0.999996191	0.928525825	0.908181052	0.99999488	0.999985264	0.982250307
PSMC4	0.999996191	0.938476452	0.920509464	0.99999488	0.999985264	0.982250307
EIF3F	0.999996191	0.953844371	0.921010865	0.99999488	0.999985264	0.982250307
C1QC	0.999996191	0.941495217	0.926552506	0.99999488	0.999985264	0.982250307
LOC101903629	0.999996191	0.941876954	0.926552506	0.99999488	0.999985264	0.982250307
BCAS1	0.999996191	0.970426668	0.926552506	0.99999488	0.999985264	0.982250307
PPP2R3C	0.999996191	0.999992444	0.928642875	0.99999488	0.999985264	0.982250307
KLHL30	0.999996191	0.989547896	0.930678591	0.99999488	0.999985264	0.982250307
TFPI	0.999996191	0.999347049	0.930678591	0.99999488	0.999985264	0.982250307
LOC104974269	0.999996191	0.971526813	0.932653752	0.99999488	0.999985264	0.982250307
VPS4A	0.999996191	0.948421431	0.934694748	0.99999488	0.999985264	0.982250307
ACOX1	0.999996191	0.999992444	0.938203102	0.99999488	0.999985264	0.982250307
A4GALT	0.999996191	0.999992444	0.941118576	0.99999488	0.999985264	0.982250307
DNAAF2	0.999996191	0.935742738	0.941336964	0.99999488	0.999985264	0.982250307
AGT	0.999996191	0.925580392	0.944031108	0.99999488	0.999985264	0.982250307
COBLL1	0.999996191	0.945217876	0.944993804	0.99999488	0.999985264	0.982250307
CYP4A11	0.999996191	0.985406717	0.94634922	0.99999488	0.999985264	0.982250307
LOC112443509	0.999996191	0.999992444	0.94634922	0.99999488	0.999985264	0.982250307
TCP11L2	0.999996191	0.994481342	0.946479014	0.99999488	0.999985264	0.982250307
LOC112448627	0.999996191	0.978510121	0.946900905	0.99999488	0.999985264	0.982250307
NFIB	0.999996191	0.978510121	0.948946702	0.99999488	0.999985264	0.982250307
LOC101906730	0.999996191	0.95697765	0.95106368	0.99999488	0.999985264	0.982250307

TSPAN32	0.999996191	0.957334182	0.951099739	0.99999488	0.999985264	0.982250307
ALDOC	0.999996191	0.976819696	0.951735967	0.99999488	0.999985264	0.982250307
MICAL1	0.999996191	0.991488652	0.951822586	0.99999488	0.999985264	0.982250307
ALDH7A1	0.999996191	0.967919743	0.95230212	0.99999488	0.999985264	0.982250307
WDR73	0.999996191	0.971736716	0.95230212	0.99999488	0.999985264	0.982250307
LOC100849046	0.999996191	0.999992444	0.95230212	0.99999488	0.999985264	0.982250307
GRAP2	0.999996191	0.999992444	0.954620171	0.99999488	0.999985264	0.982250307
TLE1	0.999996191	0.999992444	0.954823586	0.99999488	0.999985264	0.982250307
WASHC4	0.999996191	0.999992444	0.954872702	0.99999488	0.999985264	0.982250307
KCNMB3	0.999996191	0.992977597	0.956565728	0.99999488	0.999985264	0.982250307
TTC30A	0.999996191	0.999347049	0.956852976	0.99999488	0.999985264	0.982250307
NFX1	0.999996191	0.998131745	0.960133832	0.99999488	0.999985264	0.982250307
RAB3GAP1	0.999996191	0.9582784	0.961024709	0.99999488	0.999985264	0.982250307
LOC616942	0.999996191	0.94984369	0.961490767	0.99999488	0.999985264	0.982250307
REX1BD	0.999996191	0.999992444	0.964152013	0.99999488	0.999985264	0.982250307
LOC529125	0.999996191	0.982313546	0.964920281	0.99999488	0.999985264	0.982250307
C17H4orf33	0.999996191	0.999992444	0.965065403	0.99999488	0.999985264	0.982250307
LOC112444207	0.999996191	0.999992444	0.96551632	0.99999488	0.999985264	0.982250307
MEX3D	0.999996191	0.999992444	0.966164355	0.99999488	0.999985264	0.982250307
LOC614531	0.999996191	0.999347049	0.967427257	0.99999488	0.999985264	0.982250307
COG4	0.999996191	0.991488652	0.968232195	0.99999488	0.999985264	0.982250307
DPP7	0.999996191	0.999992444	0.968232195	0.99999488	0.999985264	0.982250307
LASP1	0.999996191	0.999992444	0.968232195	0.99999488	0.999985264	0.982250307
LOC100847180	0.999996191	0.999992444	0.968398207	0.99999488	0.999985264	0.982250307
PRKG1	0.999996191	0.999992444	0.969656316	0.99999488	0.999985264	0.982250307
GLI2	0.999996191	0.999992444	0.969834071	0.99999488	0.999985264	0.982250307
WDR83OS	0.999996191	0.991488652	0.969880768	0.99999488	0.999985264	0.982250307
LOC112448579	0.999996191	0.999992444	0.970874203	0.99999488	0.999985264	0.982250307
CSK	0.999996191	0.999992444	0.971838819	0.99999488	0.999985264	0.982250307
RNASEL	0.999996191	0.999992444	0.973303821	0.99999488	0.999985264	0.982250307
TCERG1	0.999996191	0.978089982	0.973406453	0.99999488	0.999985264	0.982250307
RPL11	0.999996191	0.993316598	0.973406453	0.99999488	0.999985264	0.982250307
C13H10orf113	0.999996191	0.994523667	0.973406453	0.99999488	0.999985264	0.982250307
LOC789258	0.999996191	0.999992444	0.973406453	0.99999488	0.999985264	0.982250307
ZNF41	0.999996191	0.999992444	0.973406453	0.99999488	0.999985264	0.982250307
LOC112445980	0.999996191	0.928879317	0.973406896	0.99999488	0.999985264	0.982250307
HNRNPM	0.999996191	0.999992444	0.974727926	0.99999488	0.999985264	0.982250307
LOC100336161	0.999996191	0.999992444	0.975701948	0.99999488	0.999985264	0.982250307
PEX14	0.999996191	0.994434609	0.977141934	0.99999488	0.999985264	0.982250307

TMEM110	0.999996191	0.999474142	0.977141934	0.99999488	0.999985264	0.982250307
TOPORS	0.999996191	0.999992444	0.978389389	0.99999488	0.999985264	0.982250307
LOC104976573	0.999996191	0.999992444	0.978894849	0.99999488	0.999985264	0.982250307
LOC100298868	0.999996191	0.993316598	0.979675621	0.99999488	0.999985264	0.982250307
ATP13A1	0.999996191	0.999992444	0.980677819	0.99999488	0.999985264	0.982250307
PANX1	0.999996191	0.978089982	0.9808801	0.99999488	0.999985264	0.982250307
ATP5PB	0.999996191	0.982911005	0.9808801	0.99999488	0.999985264	0.982250307
LOC101907648	0.999996191	0.996105883	0.9808801	0.99999488	0.999985264	0.982250307
MARCH2	0.999996191	0.998131745	0.9808801	0.99999488	0.999985264	0.982250307
COX19	0.999996191	0.999992444	0.9808801	0.99999488	0.999985264	0.982250307
ELOC	0.999996191	0.999992444	0.9808801	0.99999488	0.999985264	0.982250307
IGFBP5	0.999996191	0.999992444	0.9808801	0.99999488	0.999985264	0.982250307
NFKBIE	0.999996191	0.999992444	0.9808801	0.99999488	0.999985264	0.982250307
SUMF1	0.999996191	0.999992444	0.9808801	0.99999488	0.999985264	0.982250307
PSMD7	0.999996191	0.941495217	0.981218677	0.99999488	0.999985264	0.982250307
CLIP3	0.999996191	0.999992444	0.981218677	0.99999488	0.999985264	0.982250307
VWA8	0.999996191	0.993316598	0.981388276	0.99999488	0.999985264	0.982250307
LOC788405	0.999996191	0.991476708	0.981622418	0.99999488	0.999985264	0.982250307
NDUFB5	0.999996191	0.999347049	0.982216395	0.99999488	0.999985264	0.982250307
LOC512165	0.999996191	0.999992444	0.982216395	0.99999488	0.999985264	0.982250307
LOC112442296	0.999996191	0.999347049	0.98286145	0.99999488	0.999985264	0.982250307
DIDO1	0.999996191	0.991488652	0.983077441	0.99999488	0.999985264	0.982250307
BMP4	0.999996191	0.981298804	0.983361288	0.99999488	0.999985264	0.982250307
LOC112446369	0.999996191	0.976389135	0.984444908	0.99999488	0.999985264	0.982250307
FGD5	0.999996191	0.998131745	0.985363931	0.99999488	0.999985264	0.982250307
KCNK13	0.999996191	0.999992444	0.99035966	0.99999488	0.999985264	0.982250307
KBTBD3	0.999996191	0.999992444	0.990780762	0.99999488	0.999985264	0.982250307
JAG2	0.999996191	0.999347049	0.991285432	0.99999488	0.999985264	0.982250307
DLST	0.999996191	0.993316598	0.99176376	0.99999488	0.999985264	0.982250307
HECW1	0.999996191	0.999347049	0.991992782	0.99999488	0.999985264	0.982250307
PEX13	0.999996191	0.999992444	0.993496486	0.99999488	0.999985264	0.982250307
ESCO2	0.999996191	0.999347049	0.993594894	0.99999488	0.999985264	0.982250307
NOL8	0.999996191	0.999992444	0.993809467	0.99999488	0.999985264	0.982250307
JADE1	0.999996191	0.997051616	0.99397371	0.99999488	0.999985264	0.982250307
RAB5A	0.999996191	0.999347049	0.99397371	0.99999488	0.999985264	0.982250307
PCDHGC3	0.999996191	0.934142668	0.99436655	0.99999488	0.999985264	0.982250307
YES1	0.999996191	0.999992444	0.994601277	0.99999488	0.999985264	0.982250307
LOC107132487	0.999996191	0.957700659	0.995045982	0.99999488	0.999985264	0.982250307
DNAL4	0.999996191	0.999992444	0.995045982	0.99999488	0.999985264	0.982250307

GPR1	0.999996191	0.999992444	0.995045982	0.99999488	0.999985264	0.982250307
TNPO1	0.999996191	0.999992444	0.996348351	0.99999488	0.999985264	0.982250307
BIN1	0.999996191	0.999992444	0.996401083	0.99999488	0.999985264	0.982250307
LOC515823	0.999996191	0.999992444	0.996601949	0.99999488	0.999985264	0.982250307
ICAM1	0.999996191	0.969314233	0.998936876	0.99999488	0.999985264	0.982250307
INTU	0.999996191	0.974079639	0.998936876	0.99999488	0.999985264	0.982250307
SNX8	0.999996191	0.991365955	0.998936876	0.99999488	0.999985264	0.982250307
RDH11	0.999996191	0.991488652	0.998936876	0.99999488	0.999985264	0.982250307
FH	0.999996191	0.998131745	0.998936876	0.99999488	0.999985264	0.982250307
ALYREF	0.999996191	0.999992444	0.998936876	0.99999488	0.999985264	0.982250307
RASA1	0.999996191	0.999992444	0.998936876	0.99999488	0.999985264	0.982250307
TMEM41B	0.999996191	0.999992444	0.998936876	0.99999488	0.999985264	0.982250307
ARFGAP3	0.999996191	0.991365955	0.999558179	0.99999488	0.999985264	0.982250307
RALGAPA2	0.999996191	0.991488652	0.999558179	0.99999488	0.999985264	0.982250307
PELO	0.999996191	0.991574265	0.999558179	0.99999488	0.999985264	0.982250307
MTM1	0.999996191	0.992977597	0.999558179	0.99999488	0.999985264	0.982250307
NALCN	0.999996191	0.995029734	0.999558179	0.99999488	0.999985264	0.982250307
ARL3	0.999996191	0.996370892	0.999558179	0.99999488	0.999985264	0.982250307
C15H11orf96	0.999996191	0.999992444	0.999558179	0.99999488	0.999985264	0.982250307
ETV4	0.999996191	0.999992444	0.999558179	0.99999488	0.999985264	0.982250307
HSPA12B	0.999996191	0.999992444	0.999558179	0.99999488	0.999985264	0.982250307
LOC101904796	0.999996191	0.999992444	0.999558179	0.99999488	0.999985264	0.982250307
MKL1	0.999996191	0.999992444	0.999558179	0.99999488	0.999985264	0.982250307
TRAF1	0.999996191	0.998011664	0.994601277	0.99999488	0.999985264	0.982284749
PTEN	0.999996191	0.971009675	0.930678591	0.99999488	0.999985264	0.982482827
RPL4	0.999996191	0.991488652	0.94634922	0.99999488	0.999985264	0.982482827
MRPS31	0.999996191	0.931212837	0.95230212	0.99999488	0.999985264	0.982482827
PACS1	0.999996191	0.999992444	0.970874203	0.99999488	0.999985264	0.982482827
DGUOK	0.999996191	0.999992444	0.981218677	0.99999488	0.999985264	0.982482827
DCX	0.999996191	0.999992444	0.999558179	0.99999488	0.999985264	0.982482827
PLXNA4	0.999996191	0.999347049	0.995237198	0.99999488	0.999985264	0.982484944
LPCAT1	0.999996191	0.998962836	0.930678591	0.99999488	0.999985264	0.98261338
LOC101905265	0.999996191	0.999992444	0.961024709	0.99999488	0.999985264	0.982768193
LCOR	0.999996191	0.999992444	0.948420301	0.99999488	0.999985264	0.982781494
NFYA	0.999996191	0.984068885	0.969880768	0.99999488	0.999985264	0.982781494
LOC101906266	0.999996191	0.999347049	0.933046493	0.99999488	0.999985264	0.982826535
CHD3	0.999996191	0.999992444	0.964908146	0.99999488	0.999985264	0.982831822
PMEPA1	0.999996191	0.999992444	0.999558179	0.99999488	0.999985264	0.983101792
SNURF	0.999996191	0.999992444	0.94634922	0.99999488	0.999985264	0.983131484

DSTYK	0.999996191	0.998964359	0.962346588	0.99999488	0.999985264	0.983279119
RANBP9	0.999996191	0.937231686	0.973406453	0.99999488	0.999985264	0.983330992
LOC101905894	0.999996191	0.988170127	0.930678591	0.99999488	0.999985264	0.98341414
LOC107133302	0.999996191	0.946274461	0.935297807	0.99999488	0.999985264	0.98341414
TTBK2	0.999996191	0.992977597	0.938203102	0.99999488	0.999985264	0.98341414
GPR156	0.999996191	0.940269477	0.944168373	0.99999488	0.999985264	0.98341414
GAMT	0.999996191	0.94984369	0.945766581	0.99999488	0.999985264	0.98341414
CALHM6	0.999996191	0.999992444	0.949038056	0.99999488	0.999985264	0.98341414
CDKL5	0.999996191	0.974079639	0.95106368	0.99999488	0.999985264	0.98341414
LOC112445065	0.999996191	0.985293257	0.956565728	0.99999488	0.999985264	0.98341414
NUDT5	0.999996191	0.978089982	0.957541703	0.99999488	0.999985264	0.98341414
YY1	0.999996191	0.987559864	0.958627535	0.99999488	0.999985264	0.98341414
GRAP	0.999996191	0.992977597	0.964152013	0.99999488	0.999985264	0.98341414
LOC783942	0.999996191	0.999992444	0.964152013	0.99999488	0.999985264	0.98341414
NMUR1	0.999996191	0.991488652	0.964920281	0.99999488	0.999985264	0.98341414
RPL5	0.999996191	0.993316598	0.965674733	0.99999488	0.999985264	0.98341414
TRIM41	0.999996191	0.999347049	0.968232195	0.99999488	0.999985264	0.98341414
RPL34	0.999996191	0.999992444	0.973406453	0.99999488	0.999985264	0.98341414
MESP2	0.999996191	0.999992444	0.974410662	0.99999488	0.999985264	0.98341414
TRAPPC6A	0.999996191	0.999992444	0.975538142	0.99999488	0.999985264	0.98341414
ZCWPW1	0.999996191	0.999992444	0.977127779	0.99999488	0.999985264	0.98341414
RAB9B	0.999996191	0.991365955	0.9808801	0.99999488	0.999985264	0.98341414
HINFP	0.999996191	0.999992444	0.981039752	0.99999488	0.999985264	0.98341414
LMO1	0.999996191	0.9582784	0.981458415	0.99999488	0.999985264	0.98341414
SIX1	0.999996191	0.991476708	0.981666445	0.99999488	0.999985264	0.98341414
COG7	0.999996191	0.992977597	0.98286145	0.99999488	0.999985264	0.98341414
FGGY	0.999996191	0.999992444	0.98286145	0.99999488	0.999985264	0.98341414
LOC101905343	0.999996191	0.999992444	0.985363931	0.99999488	0.999985264	0.98341414
LOC107131368	0.999996191	0.999992444	0.992709008	0.99999488	0.999985264	0.98341414
YIPF1	0.999996191	0.999992444	0.992709008	0.99999488	0.999985264	0.98341414
ABHD17C	0.999996191	0.999992444	0.994601277	0.99999488	0.999985264	0.98341414
ELOVL7	0.999996191	0.999992444	0.994601277	0.99999488	0.999985264	0.98341414
GRASP	0.999996191	0.999992444	0.995045982	0.99999488	0.999985264	0.98341414
PTPRB	0.999996191	0.999992444	0.995045982	0.99999488	0.999985264	0.98341414
RABGGTB	0.999996191	0.999992444	0.996348351	0.99999488	0.999985264	0.98341414
CLK2	0.999996191	0.999992444	0.996391386	0.99999488	0.999985264	0.98341414
EIF1AX	0.999996191	0.992895985	0.996715234	0.99999488	0.999985264	0.98341414
NXT2	0.999996191	0.993316598	0.996715234	0.99999488	0.999985264	0.98341414
HESX1	0.999996191	0.999992444	0.997208738	0.99999488	0.999985264	0.98341414

CDC34	0.999996191	0.999992444	0.997230633	0.99999488	0.999985264	0.98341414
LOC107132070	0.999996191	0.992576047	0.998936876	0.99999488	0.999985264	0.98341414
LOC614922	0.999996191	0.998131745	0.998936876	0.99999488	0.999985264	0.98341414
IPO8	0.999996191	0.999992444	0.998936876	0.99999488	0.999985264	0.98341414
LOC100848699	0.999996191	0.999992444	0.998936876	0.99999488	0.999985264	0.98341414
LOC101905357	0.999996191	0.999992444	0.998936876	0.99999488	0.999985264	0.98341414
ZDHHC1	0.999996191	0.999992444	0.998936876	0.99999488	0.999985264	0.98341414
COL28A1	0.999996191	0.96895079	0.999558179	0.99999488	0.999985264	0.98341414
PTGER4	0.999996191	0.991488652	0.999558179	0.99999488	0.999985264	0.98341414
ZHX3	0.999996191	0.991488652	0.999558179	0.99999488	0.999985264	0.98341414
LOC112443142	0.999996191	0.991838765	0.999558179	0.99999488	0.999985264	0.98341414
CLNS1A	0.999996191	0.993316598	0.999558179	0.99999488	0.999985264	0.98341414
LOC112448253	0.999996191	0.998131745	0.999558179	0.99999488	0.999985264	0.98341414
SFT2D2	0.999996191	0.998131745	0.999558179	0.99999488	0.999985264	0.98341414
ACER1	0.999996191	0.999992444	0.999558179	0.99999488	0.999985264	0.98341414
DNAJC6	0.999996191	0.999992444	0.999558179	0.99999488	0.999985264	0.98341414
SNX12	0.999996191	0.999992444	0.999558179	0.99999488	0.999985264	0.98341414
TTC12	0.999996191	0.999992444	0.994601277	0.99999488	0.999985264	0.983438534
LOC618787	0.999996191	0.999417463	0.999558179	0.99999488	0.999985264	0.983444917
ATP6V1H	0.999996191	0.916036681	0.917909881	0.99999488	0.999985264	0.983479593
SLC4A3	0.999996191	0.957700659	0.936914995	0.99999488	0.999985264	0.983479593
EMC3	0.999996191	0.978510121	0.962346588	0.99999488	0.999985264	0.983479593
NDNF	0.999996191	0.999992444	0.970945672	0.99999488	0.999985264	0.983479593
SEMA3F	0.999996191	0.999347049	0.881851998	0.99999488	0.999985264	0.983542592
ALDH3B1	0.999996191	0.964246761	0.937874396	0.99999488	0.999985264	0.983542592
RUNX2	0.999996191	0.999347049	0.938203102	0.99999488	0.999985264	0.983542592
SNIP1	0.999996191	0.991488652	0.952408389	0.99999488	0.999985264	0.983542592
TRMT2B	0.999996191	0.999992444	0.964152013	0.99999488	0.999985264	0.983542592
CGRRF1	0.999996191	0.999992444	0.973406453	0.99999488	0.999985264	0.983542592
DLEC1	0.999996191	0.999992444	0.977141934	0.99999488	0.999985264	0.983542592
ZNF268	0.999996191	0.999992444	0.97868062	0.99999488	0.999985264	0.983542592
NXT1	0.999996191	0.999992444	0.9808801	0.99999488	0.999985264	0.983542592
LOC100336909	0.999996191	0.999992444	0.981039752	0.99999488	0.999985264	0.983542592
SRSF9	0.999996191	0.999992444	0.981218677	0.99999488	0.999985264	0.983542592
PLEKHA3	0.999996191	0.999992444	0.992709008	0.99999488	0.999985264	0.983542592
TPR	0.999996191	0.998131745	0.998936876	0.99999488	0.999985264	0.983542592
SMPD1	0.999996191	0.999992444	0.998936876	0.99999488	0.999985264	0.983542592
HES4	0.999996191	0.999347049	0.999558179	0.99999488	0.999985264	0.983542592
FAM228B	0.999996191	0.999992444	0.999558179	0.99999488	0.999985264	0.983542592

LOC107131649	0.999996191	0.999992444	0.927073042	0.99999488	0.999985264	0.983680505
ITPKB	0.999996191	0.999992444	0.97379223	0.99999488	0.999985264	0.983680505
SEMA7A	0.999996191	0.999992444	0.988922732	0.99999488	0.999985264	0.983680505
LOC112448075	0.999996191	0.992977597	0.985363931	0.99999488	0.999985264	0.983816776
EFTUD2	0.999996191	0.999992444	0.965065403	0.99999488	0.999985264	0.983858538
NEDD1	0.999996191	0.999992444	0.998936876	0.99999488	0.999985264	0.98388003
TRIM8	0.999996191	0.999992444	0.981039752	0.99999488	0.999985264	0.983940382
TRIM56	0.999996191	0.940269477	0.921755524	0.99999488	0.999985264	0.983972552
SDF4	0.999996191	0.991365955	0.971111634	0.99999488	0.999985264	0.983972552
LOC101906588	0.999996191	0.999992444	0.987678394	0.99999488	0.999985264	0.984027719
RMND5B	0.999996191	0.999992444	0.998936876	0.99999488	0.999985264	0.984027719
MAP1B	0.999996191	0.999992444	0.980301481	0.99999488	0.999985264	0.984273569
CCDC43	0.999996191	0.993316598	0.999558179	0.99999488	0.999985264	0.984273569
LETMD1	0.999996191	0.999992444	0.999558179	0.99999488	0.999985264	0.984273569
SIPA1	0.999996191	0.995476917	0.999558179	0.99999488	0.999985264	0.984303318
LOC101904039	0.999996191	0.999992444	0.938203102	0.99999488	0.999985264	0.984433375
LOC100848872	0.999996191	0.981520287	0.964152013	0.99999488	0.999985264	0.984657258
GOLGA5	0.999996191	0.991488652	0.930678591	0.99999488	0.999985264	0.984730283
PTAR1	0.999996191	0.999992444	0.958240365	0.99999488	0.999985264	0.984825523
PI4K2B	0.999996191	0.998131745	0.981388276	0.99999488	0.999985264	0.984825523
FMC1	0.999996191	0.994754253	0.962142195	0.99999488	0.999985264	0.984862316
LOC112441868	0.999996191	0.947399873	0.895962167	0.99999488	0.999985264	0.984869777
NEK7	0.999996191	0.919135712	0.956565728	0.99999488	0.999985264	0.984869777
LOC112446021	0.999996191	0.992977597	0.9808801	0.99999488	0.999985264	0.984869777
NBR1	0.999996191	0.992977597	0.991285432	0.99999488	0.999985264	0.984869777
RFK	0.999996191	0.992977597	0.992709008	0.99999488	0.999985264	0.984869777
LOC101904355	0.999996191	0.99398773	0.997230633	0.99999488	0.999985264	0.984869777
MOSPD3	0.999996191	0.999992444	0.999558179	0.99999488	0.999985264	0.985197857
NPHP4	0.999996191	0.91058693	0.930678591	0.99999488	0.999985264	0.985267066
CDADC1	0.999996191	0.964246761	0.981218677	0.99999488	0.999985264	0.985267066
LRRK1	0.999996191	0.999347049	0.985966356	0.99999488	0.999985264	0.985267066
E2F5	0.999996191	0.999992444	0.988922732	0.99999488	0.999985264	0.985267066
TPRG1	0.999996191	0.999992444	0.988022384	0.99999488	0.999985264	0.985274619
NCR3	0.999996191	0.999992444	0.963068909	0.99999488	0.999985264	0.985275814
FSTL1	0.999996191	0.989817451	0.961024709	0.99999488	0.999985264	0.985332098
EHD2	0.999996191	0.999992444	0.969880768	0.99999488	0.999985264	0.985332098
CLIP4	0.999996191	0.996105883	0.971778256	0.99999488	0.999985264	0.985332098
ARL1	0.999996191	0.991488652	0.980677819	0.99999488	0.999985264	0.985332098
PGRMC2	0.999996191	0.999992444	0.981039752	0.99999488	0.999985264	0.985332098

GRIA3	0.999996191	0.999992444	0.991285432	0.99999488	0.999985264	0.985332098
EVC	0.999996191	0.999992444	0.993594894	0.99999488	0.999985264	0.985332098
LOC107131710	0.999996191	0.999992444	0.916975216	0.99999488	0.999985264	0.98534845
LOC112448366	0.999996191	0.909577824	0.921379317	0.99999488	0.999985264	0.98534845
PTP4A2	0.999996191	0.957700659	0.922076383	0.99999488	0.999985264	0.98534845
IPO5	0.999996191	0.975677199	0.930678591	0.99999488	0.999985264	0.98534845
NAGLU	0.999996191	0.991365955	0.930678591	0.99999488	0.999985264	0.98534845
PLEC	0.999996191	0.938777693	0.938203102	0.99999488	0.999985264	0.98534845
USB1	0.999996191	0.999992444	0.94634922	0.99999488	0.999985264	0.98534845
MRPL53	0.999996191	0.941495217	0.946479014	0.99999488	0.999985264	0.98534845
C25H16orf89	0.999996191	0.977080378	0.946479014	0.99999488	0.999985264	0.98534845
UBA52	0.999996191	0.993316598	0.948420301	0.99999488	0.999985264	0.98534845
ARHGEF6	0.999996191	0.94095416	0.95106368	0.99999488	0.999985264	0.98534845
DDHD1	0.999996191	0.999347049	0.95230212	0.99999488	0.999985264	0.98534845
SLC35C1	0.999996191	0.991365955	0.956166665	0.99999488	0.999985264	0.98534845
GINS1	0.999996191	0.991365955	0.958627535	0.99999488	0.999985264	0.98534845
TUBGCP2	0.999996191	0.998131745	0.966609561	0.99999488	0.999985264	0.98534845
PWWP2B	0.999996191	0.999992444	0.970874203	0.99999488	0.999985264	0.98534845
ABHD12	0.999996191	0.999992444	0.970945672	0.99999488	0.999985264	0.98534845
LRPAP1	0.999996191	0.999992444	0.973406453	0.99999488	0.999985264	0.98534845
HMGXB4	0.999996191	0.974079639	0.976925152	0.99999488	0.999985264	0.98534845
LOC101906460	0.999996191	0.992977597	0.979462526	0.99999488	0.999985264	0.98534845
DCUN1D2	0.999996191	0.999992444	0.980132104	0.99999488	0.999985264	0.98534845
RBBP4	0.999996191	0.92239337	0.980169282	0.99999488	0.999985264	0.98534845
PAPPA	0.999996191	0.949285285	0.980169282	0.99999488	0.999985264	0.98534845
LOC100848122	0.999996191	0.999992444	0.981622418	0.99999488	0.999985264	0.98534845
CCDC106	0.999996191	0.999992444	0.993594894	0.99999488	0.999985264	0.98534845
SEC11A	0.999996191	0.999347049	0.995045982	0.99999488	0.999985264	0.98534845
TBCA	0.999996191	0.993316598	0.996348351	0.99999488	0.999985264	0.98534845
COPRS	0.999996191	0.999347049	0.996391386	0.99999488	0.999985264	0.98534845
LOC112446855	0.999996191	0.992977597	0.997208738	0.99999488	0.999985264	0.98534845
FUT10	0.999996191	0.999992444	0.998936876	0.99999488	0.999985264	0.98534845
LOC112443243	0.999996191	0.999992444	0.998936876	0.99999488	0.999985264	0.98534845
UBE2F	0.999996191	0.991488652	0.999558179	0.99999488	0.999985264	0.98534845
POU2F3	0.999996191	0.999347049	0.999558179	0.99999488	0.999985264	0.98534845
LOC112444869	0.999996191	0.999992444	0.999558179	0.99999488	0.999985264	0.98534845
CCL16	0.999996191	0.978089982	0.956565728	0.99999488	0.999985264	0.985469637
LOC107131225	0.999996191	0.999992444	0.999558179	0.99999488	0.999985264	0.985495054
CEND1	0.999996191	0.999992444	0.984765193	0.99999488	0.999985264	0.985600434

JCAD	0.999996191	0.925387304	0.907484111	0.99999488	0.999985264	0.98564799
AFAP1L2	0.999996191	0.999992444	0.941336964	0.99999488	0.999985264	0.98564799
LOC787287	0.999996191	0.981298804	0.946479014	0.99999488	0.999985264	0.98564799
BCAM	0.999996191	0.999992444	0.964920281	0.99999488	0.999985264	0.98564799
ONECUT2	0.999996191	0.999992444	0.968232195	0.99999488	0.999985264	0.98564799
ZNF10	0.999996191	0.991488652	0.969880768	0.99999488	0.999985264	0.98564799
CLEC9A	0.999996191	0.999992444	0.970945672	0.99999488	0.999985264	0.98564799
NUP214	0.999996191	0.999992444	0.981039752	0.99999488	0.999985264	0.98564799
LOC101906408	0.999996191	0.941760655	0.990042787	0.99999488	0.999985264	0.98564799
APP	0.999996191	0.999992444	0.991331566	0.99999488	0.999985264	0.98564799
LOC101907126	0.999996191	0.998131745	0.993606304	0.99999488	0.999985264	0.98564799
LOC107131848	0.999996191	0.970426668	0.996391386	0.99999488	0.999985264	0.98564799
RFC4	0.999996191	0.994210852	0.998936876	0.99999488	0.999985264	0.98564799
KIAA1614	0.999996191	0.991488652	0.999558179	0.99999488	0.999985264	0.98564799
NHLH1	0.999996191	0.921549	0.886962419	0.99999488	0.999985264	0.985664957
LOC101905770	0.999996191	0.946480858	0.947957719	0.99999488	0.999985264	0.985664957
SOCS1	0.999996191	0.981520287	0.95230212	0.99999488	0.999985264	0.985664957
AHSP	0.999996191	0.999992444	0.962653182	0.99999488	0.999985264	0.985664957
ADAP1	0.999996191	0.986637746	0.963068909	0.99999488	0.999985264	0.985664957
ORMDL2	0.999996191	0.999992444	0.964152013	0.99999488	0.999985264	0.985664957
LOC101903992	0.999996191	0.999992444	0.969292287	0.99999488	0.999985264	0.985664957
AVEN	0.999996191	0.998962836	0.984444908	0.99999488	0.999985264	0.985664957
ARSJ	0.999996191	0.999992444	0.993594894	0.99999488	0.999985264	0.985664957
DTD1	0.999996191	0.999992444	0.996391386	0.99999488	0.999985264	0.985664957
NDUFB6	0.999996191	0.999347049	0.997383664	0.99999488	0.999985264	0.985664957
LOC112448390	0.999996191	0.999992444	0.999558179	0.99999488	0.999985264	0.985664957
ADRA1A	0.999996191	0.957700659	0.923604668	0.99999488	0.999985264	0.985798686
PPCDC	0.999996191	0.991838765	0.973303821	0.99999488	0.999985264	0.985798686
LOC784208	0.999996191	0.999992444	0.980660791	0.99999488	0.999985264	0.985811281
SERPINB1	0.999996191	0.996966166	0.96684887	0.99999488	0.999985264	0.986136939
LOC101902705	0.999996191	0.991488652	0.893182079	0.99999488	0.999985264	0.986338245
TNFSF9	0.999996191	0.9582784	0.938905004	0.99999488	0.999985264	0.986338245
GLYAT	0.999996191	0.991574265	0.946479014	0.99999488	0.999985264	0.986338245
MEST	0.999996191	0.943802122	0.954864482	0.99999488	0.999985264	0.986338245
STC1	0.999996191	0.999992444	0.958627535	0.99999488	0.999985264	0.986338245
RPL28	0.999996191	0.992895985	0.968232195	0.99999488	0.999985264	0.986338245
NTAN1	0.999996191	0.999992444	0.969880768	0.99999488	0.999985264	0.986338245
ZFP62	0.999996191	0.999992444	0.972362683	0.99999488	0.999985264	0.986338245
CS	0.999996191	0.978510121	0.973406453	0.99999488	0.999985264	0.986338245

LOC107132237	0.999996191	0.999992444	0.973406453	0.99999488	0.999985264	0.986338245
RAB3IP	0.999996191	0.999992444	0.9808801	0.99999488	0.999985264	0.986338245
PAPD7	0.999996191	0.999347049	0.981039752	0.99999488	0.999985264	0.986338245
NATD1	0.999996191	0.999992444	0.981388276	0.99999488	0.999985264	0.986338245
HEATR5B	0.999996191	0.992977597	0.984765193	0.99999488	0.999985264	0.986338245
ASCC3	0.999996191	0.992977597	0.991285432	0.99999488	0.999985264	0.986338245
PDZD8	0.999996191	0.992977597	0.991755419	0.99999488	0.999985264	0.986338245
AGTRAP	0.999996191	0.999347049	0.992709008	0.99999488	0.999985264	0.986338245
LOC107132783	0.999996191	0.999992444	0.993594894	0.99999488	0.999985264	0.986338245
WWC3	0.999996191	0.999992444	0.993594894	0.99999488	0.999985264	0.986338245
VPS50	0.999996191	0.999992444	0.995045982	0.99999488	0.999985264	0.986338245
TMEM39A	0.999996191	0.998131745	0.998429524	0.99999488	0.999985264	0.986338245
FAM199X	0.999996191	0.999992444	0.998429524	0.99999488	0.999985264	0.986338245
UBE2K	0.999996191	0.992977597	0.998936876	0.99999488	0.999985264	0.986338245
PDCD5	0.999996191	0.999992444	0.998936876	0.99999488	0.999985264	0.986338245
LOC101909718	0.999996191	0.958967142	0.881942256	0.99999488	0.999985264	0.986408155
LOC511531	0.999996191	0.960498589	0.907484111	0.99999488	0.999985264	0.986408155
TM4SF5	0.999996191	0.999992444	0.907484111	0.99999488	0.999985264	0.986408155
LOC112446388	0.999996191	0.993316598	0.910426741	0.99999488	0.999985264	0.986408155
PSMD3	0.999996191	0.927997582	0.921379317	0.99999488	0.999985264	0.986408155
LOC104970966	0.999996191	0.972826018	0.926552506	0.99999488	0.999985264	0.986408155
LOC789569	0.999996191	0.991488652	0.930678591	0.99999488	0.999985264	0.986408155
LOC101904097	0.999996191	0.919209432	0.931367689	0.99999488	0.999985264	0.986408155
CBFA2T2	0.999996191	0.941495217	0.93198623	0.99999488	0.999985264	0.986408155
EML5	0.999996191	0.985406717	0.939147816	0.99999488	0.999985264	0.986408155
PTPA	0.999996191	0.993316598	0.939432817	0.99999488	0.999985264	0.986408155
HIST1H1E	0.999996191	0.974079639	0.93955737	0.99999488	0.999985264	0.986408155
LIPE	0.999996191	0.978510121	0.94634922	0.99999488	0.999985264	0.986408155
MTIF3	0.999996191	0.960498589	0.946479014	0.99999488	0.999985264	0.986408155
MFSD12	0.999996191	0.996105883	0.946479014	0.99999488	0.999985264	0.986408155
YIF1B	0.999996191	0.999347049	0.946832224	0.99999488	0.999985264	0.986408155
LOC107132300	0.999996191	0.964246761	0.95106368	0.99999488	0.999985264	0.986408155
EIF3G	0.999996191	0.999992444	0.95106368	0.99999488	0.999985264	0.986408155
EMC4	0.999996191	0.941495217	0.951735967	0.99999488	0.999985264	0.986408155
C1QB	0.999996191	0.9582784	0.951822586	0.99999488	0.999985264	0.986408155
GJA10	0.999996191	0.978510121	0.95230212	0.99999488	0.999985264	0.986408155
EPHA2	0.999996191	0.999992444	0.954174725	0.99999488	0.999985264	0.986408155
LOC100297152	0.999996191	0.991488652	0.954620171	0.99999488	0.999985264	0.986408155
CD19	0.999996191	0.89880506	0.958627535	0.99999488	0.999985264	0.986408155

AMH	0.999996191	0.999992444	0.958627535	0.99999488	0.999985264	0.986408155
SNX6	0.999996191	0.999992444	0.961024709	0.99999488	0.999985264	0.986408155
GALNT16	0.999996191	0.999992444	0.96140026	0.99999488	0.999985264	0.986408155
TCP1	0.999996191	0.935742738	0.962346588	0.99999488	0.999985264	0.986408155
ATP8B1	0.999996191	0.992977597	0.962346588	0.99999488	0.999985264	0.986408155
KRT10	0.999996191	0.991488652	0.964920281	0.99999488	0.999985264	0.986408155
TGIF2	0.999996191	0.998131745	0.972811368	0.99999488	0.999985264	0.986408155
RPL35	0.999996191	0.999992444	0.973303821	0.99999488	0.999985264	0.986408155
LOC515547	0.999996191	0.999992444	0.973406453	0.99999488	0.999985264	0.986408155
HDHD2	0.999996191	0.999104092	0.975790221	0.99999488	0.999985264	0.986408155
GPR137C	0.999996191	0.985058069	0.977141934	0.99999488	0.999985264	0.986408155
FRMD5	0.999996191	0.999992444	0.978389389	0.99999488	0.999985264	0.986408155
LOC100336976	0.999996191	0.998131745	0.980677819	0.99999488	0.999985264	0.986408155
PTS	0.999996191	0.999347049	0.980677819	0.99999488	0.999985264	0.986408155
RNF123	0.999996191	0.9582784	0.980859619	0.99999488	0.999985264	0.986408155
CCDC184	0.999996191	0.999992444	0.980859619	0.99999488	0.999985264	0.986408155
ZNF793	0.999996191	0.999992444	0.9808801	0.99999488	0.999985264	0.986408155
NPRL2	0.999996191	0.999347049	0.981039752	0.99999488	0.999985264	0.986408155
TBC1D25	0.999996191	0.999992444	0.981622418	0.99999488	0.999985264	0.986408155
CTU1	0.999996191	0.999347049	0.981736235	0.99999488	0.999985264	0.986408155
LOC101904642	0.999996191	0.972895283	0.981768327	0.99999488	0.999985264	0.986408155
SESN3	0.999996191	0.999992444	0.98286145	0.99999488	0.999985264	0.986408155
PPP1R8	0.999996191	0.964162565	0.984335914	0.99999488	0.999985264	0.986408155
NPHP3	0.999996191	0.999992444	0.984444908	0.99999488	0.999985264	0.986408155
MGMT	0.999996191	0.999992444	0.985966356	0.99999488	0.999985264	0.986408155
LOC112449505	0.999996191	0.999992444	0.987678394	0.99999488	0.999985264	0.986408155
LOC783988	0.999996191	0.95697765	0.988922732	0.99999488	0.999985264	0.986408155
LOC101904769	0.999996191	0.999992444	0.990154225	0.99999488	0.999985264	0.986408155
ATP1B3	0.999996191	0.999992444	0.990216356	0.99999488	0.999985264	0.986408155
HMGN3	0.999996191	0.999992444	0.991285432	0.99999488	0.999985264	0.986408155
CDC42SE2	0.999996191	0.999992444	0.992742113	0.99999488	0.999985264	0.986408155
FBXO38	0.999996191	0.999992444	0.993132892	0.99999488	0.999985264	0.986408155
AIDA	0.999996191	0.999347049	0.993594894	0.99999488	0.999985264	0.986408155
LOC541276	0.999996191	0.999992444	0.993594894	0.99999488	0.999985264	0.986408155
PAAF1	0.999996191	0.991488652	0.994601277	0.99999488	0.999985264	0.986408155
LOC616281	0.999996191	0.999347049	0.994601277	0.99999488	0.999985264	0.986408155
ENOX1	0.999996191	0.991488652	0.995045982	0.99999488	0.999985264	0.986408155
GPR153	0.999996191	0.999992444	0.996391386	0.99999488	0.999985264	0.986408155
ANKRD26	0.999996191	0.994481342	0.996776138	0.99999488	0.999985264	0.986408155

RHBDL1	0.999996191	0.999992444	0.997230633	0.99999488	0.999985264	0.986408155
ZNF641	0.999996191	0.961479426	0.998816213	0.99999488	0.999985264	0.986408155
RNF216	0.999996191	0.999992444	0.998852661	0.99999488	0.999985264	0.986408155
GNG5	0.999996191	0.992977597	0.998936876	0.99999488	0.999985264	0.986408155
HINT1	0.999996191	0.999347049	0.998936876	0.99999488	0.999985264	0.986408155
AP3M1	0.999996191	0.999992444	0.998936876	0.99999488	0.999985264	0.986408155
EGLN2	0.999996191	0.999992444	0.998936876	0.99999488	0.999985264	0.986408155
KCNJ12	0.999996191	0.999992444	0.998936876	0.99999488	0.999985264	0.986408155
LOC101906218	0.999996191	0.992977597	0.999558179	0.99999488	0.999985264	0.986408155
TNRC6B	0.999996191	0.994434609	0.999558179	0.99999488	0.999985264	0.986408155
LOC104973100	0.999996191	0.998131745	0.999558179	0.99999488	0.999985264	0.986408155
HDAC4	0.999996191	0.998659022	0.999558179	0.99999488	0.999985264	0.986408155
DLL1	0.999996191	0.999992444	0.999558179	0.99999488	0.999985264	0.986408155
LOC101905595	0.999996191	0.999992444	0.999558179	0.99999488	0.999985264	0.986408155
LOC784297	0.999996191	0.999992444	0.999558179	0.99999488	0.999985264	0.986408155
NUCKS1	0.999996191	0.999992444	0.999558179	0.99999488	0.999985264	0.986408155
ZNF22	0.999996191	0.999992444	0.999558179	0.99999488	0.999985264	0.986408155
LOC112441530	0.999996191	0.999992444	0.996715234	0.99999488	0.999985264	0.986506125
ZNF33B	0.999996191	0.991488652	0.937874396	0.99999488	0.999985264	0.986539714
KIAA0825	0.999996191	0.992808545	0.962346588	0.99999488	0.999985264	0.986539714
SLC37A4	0.999996191	0.989627874	0.964908146	0.99999488	0.999985264	0.986539714
CORIN	0.999996191	0.99873048	0.930678591	0.99999488	0.999985264	0.986584398
RAB39A	0.999996191	0.9582784	0.933046493	0.99999488	0.999985264	0.986584398
PTPN11	0.999996191	0.980496345	0.96210311	0.99999488	0.999985264	0.986584398
PEBP1	0.999996191	0.991488652	0.973661483	0.99999488	0.999985264	0.986584398
CCDC32	0.999996191	0.999992444	0.978389389	0.99999488	0.999985264	0.986584398
CTBP2	0.999996191	0.9968942	0.991285432	0.99999488	0.999985264	0.986584398
ATL3	0.999996191	0.999992444	0.999558179	0.99999488	0.999985264	0.986584398
DDB1	0.999996191	0.991574265	0.961024709	0.99999488	0.999985264	0.986633921
CCNT2	0.999996191	0.999992444	0.9808801	0.99999488	0.999985264	0.986633921
ANXA5	0.999996191	0.999992444	0.976321912	0.99999488	0.999985264	0.986757457
LOC112447303	0.999996191	0.999992444	0.9808801	0.99999488	0.999985264	0.986757457
ASB12	0.999996191	0.931828419	0.892943118	0.99999488	0.999985264	0.986787448
LCTL	0.999996191	0.926955605	0.89795961	0.99999488	0.999985264	0.986787448
DNPEP	0.999996191	0.957700659	0.933447488	0.99999488	0.999985264	0.986787448
RPS16	0.999996191	0.999992444	0.946479014	0.99999488	0.999985264	0.986787448
SEC11C	0.999996191	0.938597964	0.948420301	0.99999488	0.999985264	0.986787448
NUDT15	0.999996191	0.991365955	0.948420301	0.99999488	0.999985264	0.986787448
C1QTNF5	0.999996191	0.999992444	0.961024709	0.99999488	0.999985264	0.986787448

SLC25A14	0.999996191	0.999347049	0.969880768	0.99999488	0.999985264	0.986787448
RPA1	0.999996191	0.960786329	0.973406453	0.99999488	0.999985264	0.986787448
PCDH11X	0.999996191	0.999992444	0.973406453	0.99999488	0.999985264	0.986787448
LOC112448894	0.999996191	0.999992444	0.977930966	0.99999488	0.999985264	0.986787448
ATP11B	0.999996191	0.999992444	0.978842418	0.99999488	0.999985264	0.986787448
PNLDC1	0.999996191	0.999992444	0.980429215	0.99999488	0.999985264	0.986787448
LOC101906366	0.999996191	0.997426798	0.981039752	0.99999488	0.999985264	0.986787448
PAK1	0.999996191	0.992301181	0.985363931	0.99999488	0.999985264	0.986787448
NXPH2	0.999996191	0.999992444	0.985966356	0.99999488	0.999985264	0.986787448
STARD8	0.999996191	0.998131745	0.993594894	0.99999488	0.999985264	0.986787448
ATOX1	0.999996191	0.999992444	0.996139177	0.99999488	0.999985264	0.986787448
SLC4A5	0.999996191	0.991488652	0.998936876	0.99999488	0.999985264	0.986787448
KRBA1	0.999996191	0.999992444	0.998936876	0.99999488	0.999985264	0.986787448
NGRN	0.999996191	0.999992444	0.998936876	0.99999488	0.999985264	0.986787448
HECW2	0.999996191	0.984068885	0.999558179	0.99999488	0.999985264	0.986787448
CCNL1	0.999996191	0.989547896	0.999558179	0.99999488	0.999985264	0.986787448
PTDSS1	0.999996191	0.979756265	0.975701948	0.99999488	0.999985264	0.986825194
USP30	0.999996191	0.904961534	0.890248427	0.99999488	0.999985264	0.986873741
TESK1	0.999996191	0.958098081	0.907381775	0.99999488	0.999985264	0.986873741
LTBR	0.999996191	0.985406717	0.925745065	0.99999488	0.999985264	0.986873741
SLC46A3	0.999996191	0.943802122	0.926552506	0.99999488	0.999985264	0.986873741
LOC101902449	0.999996191	0.975677199	0.927600835	0.99999488	0.999985264	0.986873741
ADIPOR2	0.999996191	0.960180932	0.930678591	0.99999488	0.999985264	0.986873741
LOC100847999	0.999996191	0.9582784	0.940815039	0.99999488	0.999985264	0.986873741
MAP9	0.999996191	0.9582784	0.94634922	0.99999488	0.999985264	0.986873741
UFL1	0.999996191	0.983781707	0.946479014	0.99999488	0.999985264	0.986873741
LOC112441472	0.999996191	0.985293257	0.946479014	0.99999488	0.999985264	0.986873741
TMEM135	0.999996191	0.994481342	0.946479014	0.99999488	0.999985264	0.986873741
LOC789715	0.999996191	0.999347049	0.946479014	0.99999488	0.999985264	0.986873741
LOC100852077	0.999996191	0.999992444	0.946479014	0.99999488	0.999985264	0.986873741
TXN	0.999996191	0.980496345	0.948359914	0.99999488	0.999985264	0.986873741
GPR17	0.999996191	0.999992444	0.949038056	0.99999488	0.999985264	0.986873741
LOC112448524	0.999996191	0.991488652	0.95230212	0.99999488	0.999985264	0.986873741
CEP44	0.999996191	0.999992444	0.95230212	0.99999488	0.999985264	0.986873741
AARS	0.999996191	0.95967541	0.95405911	0.99999488	0.999985264	0.986873741
LOC107131566	0.999996191	0.992977597	0.956209414	0.99999488	0.999985264	0.986873741
USP8	0.999996191	0.985406717	0.957541703	0.99999488	0.999985264	0.986873741
CD72	0.999996191	0.999992444	0.96210311	0.99999488	0.999985264	0.986873741
NSG1	0.999996191	0.999992444	0.96210311	0.99999488	0.999985264	0.986873741

PSMA3	0.999996191	0.969096683	0.964920281	0.99999488	0.999985264	0.986873741
PON3	0.999996191	0.991488652	0.964920281	0.99999488	0.999985264	0.986873741
TMED8	0.999996191	0.918332306	0.965065403	0.99999488	0.999985264	0.986873741
NRTN	0.999996191	0.957252213	0.969880768	0.99999488	0.999985264	0.986873741
ALDH1A1	0.999996191	0.998131745	0.969880768	0.99999488	0.999985264	0.986873741
STK10	0.999996191	0.999992444	0.969880768	0.99999488	0.999985264	0.986873741
UBAP1	0.999996191	0.984068885	0.970945672	0.99999488	0.999985264	0.986873741
KLHL32	0.999996191	0.999992444	0.971759453	0.99999488	0.999985264	0.986873741
MAL2	0.999996191	0.999992444	0.973303821	0.99999488	0.999985264	0.986873741
RPS5	0.999996191	0.999992444	0.973406453	0.99999488	0.999985264	0.986873741
TMTC2	0.999996191	0.999992444	0.973661483	0.99999488	0.999985264	0.986873741
ELK3	0.999996191	0.992977597	0.976321912	0.99999488	0.999985264	0.986873741
LOC107131684	0.999996191	0.999992444	0.976475354	0.99999488	0.999985264	0.986873741
LOC112442263	0.999996191	0.991335402	0.979462526	0.99999488	0.999985264	0.986873741
PDPR	0.999996191	0.978154072	0.979745141	0.99999488	0.999985264	0.986873741
PLCH1	0.999996191	0.999992444	0.98023668	0.99999488	0.999985264	0.986873741
FAM129B	0.999996191	0.993777777	0.9808801	0.99999488	0.999985264	0.986873741
IL2RB	0.999996191	0.999992444	0.9808801	0.99999488	0.999985264	0.986873741
LOC100296627	0.999996191	0.999992444	0.9808801	0.99999488	0.999985264	0.986873741
RPS21	0.999996191	0.999992444	0.9808801	0.99999488	0.999985264	0.986873741
ZNF581	0.999996191	0.999992444	0.9808801	0.99999488	0.999985264	0.986873741
LOC104975925	0.999996191	0.999992444	0.981039752	0.99999488	0.999985264	0.986873741
PDCD4	0.999996191	0.999992444	0.981218677	0.99999488	0.999985264	0.986873741
ALOX5	0.999996191	0.999992444	0.981388276	0.99999488	0.999985264	0.986873741
N4BP2L1	0.999996191	0.999992444	0.981388276	0.99999488	0.999985264	0.986873741
LOC104973145	0.999996191	0.999992444	0.981715036	0.99999488	0.999985264	0.986873741
CCDC120	0.999996191	0.999992444	0.981736235	0.99999488	0.999985264	0.986873741
LOC112446402	0.999996191	0.99534056	0.98480229	0.99999488	0.999985264	0.986873741
ZCCHC24	0.999996191	0.999992444	0.985202052	0.99999488	0.999985264	0.986873741
POMGNT1	0.999996191	0.999992444	0.985363931	0.99999488	0.999985264	0.986873741
SLC20A2	0.999996191	0.999992444	0.985363931	0.99999488	0.999985264	0.986873741
PDE3B	0.999996191	0.999347049	0.985789565	0.99999488	0.999985264	0.986873741
ABCD3	0.999996191	0.999347049	0.985966356	0.99999488	0.999985264	0.986873741
SH3D21	0.999996191	0.999347049	0.988922732	0.99999488	0.999985264	0.986873741
GOPC	0.999996191	0.999992444	0.988922732	0.99999488	0.999985264	0.986873741
FBXL2	0.999996191	0.998131745	0.989872445	0.99999488	0.999985264	0.986873741
DCUN1D5	0.999996191	0.999992444	0.992709008	0.99999488	0.999985264	0.986873741
TRIM17	0.999996191	0.999992444	0.993296605	0.99999488	0.999985264	0.986873741
VPS29	0.999996191	0.999992444	0.993303055	0.99999488	0.999985264	0.986873741

ABTB2	0.999996191	0.991488652	0.993594894	0.99999488	0.999985264	0.986873741
ISOC1	0.999996191	0.999992444	0.993594894	0.99999488	0.999985264	0.986873741
LOC101905586	0.999996191	0.999992444	0.993594894	0.99999488	0.999985264	0.986873741
MAN1A2	0.999996191	0.999992444	0.993594894	0.99999488	0.999985264	0.986873741
TLE6	0.999996191	0.999992444	0.994601277	0.99999488	0.999985264	0.986873741
LOC101905588	0.999996191	0.992977597	0.995045982	0.99999488	0.999985264	0.986873741
LOC101902154	0.999996191	0.998131745	0.995045982	0.99999488	0.999985264	0.986873741
TST	0.999996191	0.999992444	0.995045982	0.99999488	0.999985264	0.986873741
EXOSC8	0.999996191	0.999992444	0.996348351	0.99999488	0.999985264	0.986873741
ACVR1B	0.999996191	0.993885692	0.996391386	0.99999488	0.999985264	0.986873741
S100A9	0.999996191	0.964044235	0.998936876	0.99999488	0.999985264	0.986873741
SGCB	0.999996191	0.991671026	0.998936876	0.99999488	0.999985264	0.986873741
MAP3K21	0.999996191	0.999992444	0.998936876	0.99999488	0.999985264	0.986873741
SUPT20H	0.999996191	0.999992444	0.998936876	0.99999488	0.999985264	0.986873741
POLA2	0.999996191	0.946274461	0.999558179	0.99999488	0.999985264	0.986873741
COMMD2	0.999996191	0.985406717	0.999558179	0.99999488	0.999985264	0.986873741
LOC112444278	0.999996191	0.987516216	0.999558179	0.99999488	0.999985264	0.986873741
C8H9orf152	0.999996191	0.991365955	0.999558179	0.99999488	0.999985264	0.986873741
LOC101906850	0.999996191	0.998181556	0.999558179	0.99999488	0.999985264	0.986873741
ERCC3	0.999996191	0.998495474	0.999558179	0.99999488	0.999985264	0.986873741
MTUS1	0.999996191	0.999347049	0.999558179	0.99999488	0.999985264	0.986873741
ZCRB1	0.999996191	0.999347049	0.999558179	0.99999488	0.999985264	0.986873741
LOC112448304	0.999996191	0.999992444	0.999558179	0.99999488	0.999985264	0.986873741
LOC785403	0.999996191	0.999992444	0.999558179	0.99999488	0.999985264	0.986873741
PPP2R5C	0.999996191	0.999992444	0.999558179	0.99999488	0.999985264	0.986873741
RPE	0.999996191	0.999992444	0.999558179	0.99999488	0.999985264	0.986873741
SARM1	0.999996191	0.999992444	0.999558179	0.99999488	0.999985264	0.986873741
ZNF583	0.999996191	0.999992444	0.999558179	0.99999488	0.999985264	0.986873741
EMD	0.999996191	0.919446414	0.923604668	0.99999488	0.999985264	0.987011114
ASAP2	0.999996191	0.95348666	0.928642875	0.99999488	0.999985264	0.987011114
MEMO1	0.999996191	0.948257806	0.939501959	0.99999488	0.999985264	0.987011114
TCTEX1D1	0.999996191	0.999992444	0.940815039	0.99999488	0.999985264	0.987011114
PPP4R4	0.999996191	0.949285285	0.962346588	0.99999488	0.999985264	0.987011114
PSMA4	0.999996191	0.946274461	0.964152013	0.99999488	0.999985264	0.987011114
MARF1	0.999996191	0.991488652	0.968232195	0.99999488	0.999985264	0.987011114
LOC100848568	0.999996191	0.998131745	0.968232195	0.99999488	0.999985264	0.987011114
IFT88	0.999996191	0.999992444	0.969880768	0.99999488	0.999985264	0.987011114
CDK19	0.999996191	0.992977597	0.990697261	0.99999488	0.999985264	0.987011114
KIRREL1	0.999996191	0.999347049	0.999558179	0.99999488	0.999985264	0.987011114

ZNF580	0.999996191	0.999347049	0.970874203	0.99999488	0.999985264	0.987282455
LOC104968479	0.999996191	0.998019747	0.999558179	0.99999488	0.999985264	0.987282455
TRIM38	0.999996191	0.971902659	0.945678616	0.99999488	0.999985264	0.987314146
DCAF4	0.999996191	0.978089982	0.956565728	0.99999488	0.999985264	0.987314146
PANK3	0.999996191	0.949285285	0.963233239	0.99999488	0.999985264	0.987314146
SEC61A2	0.999996191	0.991476708	0.981017751	0.99999488	0.999985264	0.987314146
LOC516355	0.999996191	0.999992444	0.983077441	0.99999488	0.999985264	0.987314146
USP40	0.999996191	0.999992444	0.984765193	0.99999488	0.999985264	0.987314146
TP53I13	0.999996191	0.999992444	0.986294333	0.99999488	0.999985264	0.987314146
M1AP	0.999996191	0.999992444	0.990216356	0.99999488	0.999985264	0.987314146
LOC112446360	0.999996191	0.999992444	0.996522781	0.99999488	0.999985264	0.987314146
TFE3	0.999996191	0.992576047	0.916975216	0.99999488	0.999985264	0.987365188
RPL37	0.999996191	0.989547896	0.954823586	0.99999488	0.999985264	0.987365188
H2AFX	0.999996191	0.999992444	0.960804071	0.99999488	0.999985264	0.987365188
TBCB	0.999996191	0.999992444	0.969880768	0.99999488	0.999985264	0.987365188
MCC	0.999996191	0.999992444	0.981039752	0.99999488	0.999985264	0.987365188
FAM96A	0.999996191	0.999992444	0.981218677	0.99999488	0.999985264	0.987365188
LOC112448373	0.999996191	0.999992444	0.981622418	0.99999488	0.999985264	0.987365188
LOC112447085	0.999996191	0.999992444	0.991331566	0.99999488	0.999985264	0.987365188
SS18L1	0.999996191	0.999347049	0.993594894	0.99999488	0.999985264	0.987365188
ARPC2	0.999996191	0.989872618	0.998852661	0.99999488	0.999985264	0.987365188
KL	0.999996191	0.999992444	0.999558179	0.99999488	0.999985264	0.987365188
SPICE1	0.999996191	0.985406717	0.959314104	0.99999488	0.999985264	0.987378272
TRAFD1	0.999996191	0.999992444	0.988626363	0.99999488	0.999985264	0.987378272
FAM84B	0.999996191	0.949285285	0.880594299	0.99999488	0.999985264	0.987454496
AKR1A1	0.999996191	0.965422267	0.923953195	0.99999488	0.999985264	0.987454496
LOC533308	0.999996191	0.991488652	0.925745065	0.99999488	0.999985264	0.987454496
LOC112449561	0.999996191	0.883624935	0.926552506	0.99999488	0.999985264	0.987454496
MARK1	0.999996191	0.970470739	0.926932508	0.99999488	0.999985264	0.987454496
MDC1	0.999996191	0.991365955	0.927600835	0.99999488	0.999985264	0.987454496
CCDC126	0.999996191	0.978089982	0.931367689	0.99999488	0.999985264	0.987454496
ALPL	0.999996191	0.973493284	0.931507181	0.99999488	0.999985264	0.987454496
LOC100848689	0.999996191	0.993316598	0.932010844	0.99999488	0.999985264	0.987454496
NEIL2	0.999996191	0.992977597	0.933447488	0.99999488	0.999985264	0.987454496
UBQLN4	0.999996191	0.978089982	0.933791093	0.99999488	0.999985264	0.987454496
LOC112449102	0.999996191	0.992977597	0.939501959	0.99999488	0.999985264	0.987454496
ACKR1	0.999996191	0.999992444	0.940815039	0.99999488	0.999985264	0.987454496
LOC112445150	0.999996191	0.9582784	0.94634922	0.99999488	0.999985264	0.987454496
SMTNL1	0.999996191	0.974812121	0.946479014	0.99999488	0.999985264	0.987454496

LOC614643	0.999996191	0.974812121	0.947957719	0.99999488	0.999985264	0.987454496
PCGF6	0.999996191	0.999992444	0.947957719	0.99999488	0.999985264	0.987454496
KDM2B	0.999996191	0.968240168	0.948420301	0.99999488	0.999985264	0.987454496
PCSK5	0.999996191	0.991335402	0.949047123	0.99999488	0.999985264	0.987454496
PSEN1	0.999996191	0.952346363	0.951822586	0.99999488	0.999985264	0.987454496
FAM173B	0.999996191	0.991488652	0.954620171	0.99999488	0.999985264	0.987454496
LOC107132748	0.999996191	0.999992444	0.955334115	0.99999488	0.999985264	0.987454496
LOC112442547	0.999996191	0.999992444	0.956565728	0.99999488	0.999985264	0.987454496
LOC112444288	0.999996191	0.941495217	0.957541703	0.99999488	0.999985264	0.987454496
RPS6KA2	0.999996191	0.991488652	0.958627535	0.99999488	0.999985264	0.987454496
N4BP3	0.999996191	0.991345032	0.960133832	0.99999488	0.999985264	0.987454496
FRAT1	0.999996191	0.991488652	0.960727367	0.99999488	0.999985264	0.987454496
LOC790271	0.999996191	0.991365955	0.961024709	0.99999488	0.999985264	0.987454496
COL27A1	0.999996191	0.968240168	0.961373544	0.99999488	0.999985264	0.987454496
MLLT11	0.999996191	0.935742738	0.96210311	0.99999488	0.999985264	0.987454496
NDUFB9	0.999996191	0.998131745	0.962777771	0.99999488	0.999985264	0.987454496
CIDEC	0.999996191	0.991488652	0.964152013	0.99999488	0.999985264	0.987454496
IMPACT	0.999996191	0.999992444	0.964152013	0.99999488	0.999985264	0.987454496
ATN1	0.999996191	0.991488652	0.964582964	0.99999488	0.999985264	0.987454496
EEF1AKMT3	0.999996191	0.937231686	0.96684887	0.99999488	0.999985264	0.987454496
TMEM143	0.999996191	0.991365955	0.96684887	0.99999488	0.999985264	0.987454496
LOC104969177	0.999996191	0.991488652	0.968232195	0.99999488	0.999985264	0.987454496
LOC107132175	0.999996191	0.991488652	0.970874203	0.99999488	0.999985264	0.987454496
ITIH5	0.999996191	0.992977597	0.970874203	0.99999488	0.999985264	0.987454496
TRMT61B	0.999996191	0.999992444	0.972420732	0.99999488	0.999985264	0.987454496
TOR1AIP1	0.999996191	0.997051616	0.97262931	0.99999488	0.999985264	0.987454496
RAB1B	0.999996191	0.999992444	0.97262931	0.99999488	0.999985264	0.987454496
EML2	0.999996191	0.99490847	0.972871571	0.99999488	0.999985264	0.987454496
NT5C	0.999996191	0.970286235	0.973303821	0.99999488	0.999985264	0.987454496
RBM4B	0.999996191	0.992977597	0.973406453	0.99999488	0.999985264	0.987454496
HOXC10	0.999996191	0.998962836	0.973406453	0.99999488	0.999985264	0.987454496
TMEM150B	0.999996191	0.998962836	0.973406453	0.99999488	0.999985264	0.987454496
CALR3	0.999996191	0.999992444	0.973406453	0.99999488	0.999985264	0.987454496
CPSF2	0.999996191	0.958224615	0.975701948	0.99999488	0.999985264	0.987454496
LOC101902959	0.999996191	0.999992444	0.975701948	0.99999488	0.999985264	0.987454496
MTO1	0.999996191	0.999992444	0.9808801	0.99999488	0.999985264	0.987454496
GPR146	0.999996191	0.999992444	0.981622418	0.99999488	0.999985264	0.987454496
OGFOD3	0.999996191	0.999992444	0.981622418	0.99999488	0.999985264	0.987454496
INTS3	0.999996191	0.991488652	0.98286145	0.99999488	0.999985264	0.987454496

PSTPIP2	0.999996191	0.998131745	0.982978515	0.99999488	0.999985264	0.987454496
RBM23	0.999996191	0.999992444	0.985363931	0.99999488	0.999985264	0.987454496
GGPS1	0.999996191	0.999992444	0.985966356	0.99999488	0.999985264	0.987454496
SPEF2	0.999996191	0.999992444	0.985966356	0.99999488	0.999985264	0.987454496
CARMIL1	0.999996191	0.999992444	0.986930681	0.99999488	0.999985264	0.987454496
RAB11A	0.999996191	0.991488652	0.988922732	0.99999488	0.999985264	0.987454496
FOXQ1	0.999996191	0.999992444	0.988922732	0.99999488	0.999985264	0.987454496
NKAPL	0.999996191	0.999992444	0.988922732	0.99999488	0.999985264	0.987454496
LGALS3BP	0.999996191	0.999992444	0.990697261	0.99999488	0.999985264	0.987454496
PPP6R3	0.999996191	0.999992444	0.991331566	0.99999488	0.999985264	0.987454496
PEX11A	0.999996191	0.999992444	0.992709008	0.99999488	0.999985264	0.987454496
UBE2E3	0.999996191	0.999992444	0.992709008	0.99999488	0.999985264	0.987454496
FAM78B	0.999996191	0.991365955	0.993594894	0.99999488	0.999985264	0.987454496
OSBPL5	0.999996191	0.991365955	0.993594894	0.99999488	0.999985264	0.987454496
FAM192A	0.999996191	0.998131745	0.993594894	0.99999488	0.999985264	0.987454496
PAM16	0.999996191	0.999347049	0.993594894	0.99999488	0.999985264	0.987454496
FAM76A	0.999996191	0.999992444	0.993594894	0.99999488	0.999985264	0.987454496
LOC786055	0.999996191	0.999992444	0.993594894	0.99999488	0.999985264	0.987454496
LRCH2	0.999996191	0.999992444	0.993594894	0.99999488	0.999985264	0.987454496
MYO6	0.999996191	0.999992444	0.993594894	0.99999488	0.999985264	0.987454496
HAUS1	0.999996191	0.999992444	0.994601277	0.99999488	0.999985264	0.987454496
KIAA1328	0.999996191	0.999992444	0.995045982	0.99999488	0.999985264	0.987454496
VPS35	0.999996191	0.996370892	0.996391386	0.99999488	0.999985264	0.987454496
ACP2	0.999996191	0.999992444	0.996715234	0.99999488	0.999985264	0.987454496
SPATA24	0.999996191	0.999347049	0.997208738	0.99999488	0.999985264	0.987454496
PCSK4	0.999996191	0.950786203	0.997230633	0.99999488	0.999985264	0.987454496
EPS8L2	0.999996191	0.954403227	0.998936876	0.99999488	0.999985264	0.987454496
FAM20B	0.999996191	0.991488652	0.998936876	0.99999488	0.999985264	0.987454496
LOC101906131	0.999996191	0.998131745	0.998936876	0.99999488	0.999985264	0.987454496
IRAK1BP1	0.999996191	0.999347049	0.998936876	0.99999488	0.999985264	0.987454496
LOC100848353	0.999996191	0.999992444	0.998936876	0.99999488	0.999985264	0.987454496
LOC104973517	0.999996191	0.999992444	0.998936876	0.99999488	0.999985264	0.987454496
OTULIN	0.999996191	0.999992444	0.998936876	0.99999488	0.999985264	0.987454496
PMP22	0.999996191	0.999992444	0.998936876	0.99999488	0.999985264	0.987454496
PCGF1	0.999996191	0.992977597	0.999558179	0.99999488	0.999985264	0.987454496
HASPIN	0.999996191	0.998131745	0.999558179	0.99999488	0.999985264	0.987454496
UNK	0.999996191	0.999347049	0.999558179	0.99999488	0.999985264	0.987454496
AMFR	0.999996191	0.999992444	0.999558179	0.99999488	0.999985264	0.987454496
HFE	0.999996191	0.999992444	0.999558179	0.99999488	0.999985264	0.987454496

HNRNPA3	0.999996191	0.999992444	0.999558179	0.99999488	0.999985264	0.987454496
LOC101905203	0.999996191	0.999992444	0.999558179	0.99999488	0.999985264	0.987454496
LOC104975612	0.999996191	0.999992444	0.999558179	0.99999488	0.999985264	0.987454496
LOC787812	0.999996191	0.999992444	0.999558179	0.99999488	0.999985264	0.987454496
MARCH7	0.999996191	0.999992444	0.999558179	0.99999488	0.999985264	0.987454496
LOC112447727	0.999996191	0.978426416	0.932653752	0.99999488	0.999985264	0.987457361
ACBD4	0.999996191	0.958098081	0.945407489	0.99999488	0.999985264	0.987457361
MB	0.999996191	0.925580392	0.946479014	0.99999488	0.999985264	0.987457361
LOC112446044	0.999996191	0.981628252	0.951822586	0.99999488	0.999985264	0.987457361
TTC9	0.999996191	0.978089982	0.95230212	0.99999488	0.999985264	0.987457361
PCGF3	0.999996191	0.992977597	0.95230212	0.99999488	0.999985264	0.987457361
LOC505600	0.999996191	0.999992444	0.95477544	0.99999488	0.999985264	0.987457361
DCAF12	0.999996191	0.986637746	0.956565728	0.99999488	0.999985264	0.987457361
KCNQ5	0.999996191	0.999992444	0.956852976	0.99999488	0.999985264	0.987457361
SULF2	0.999996191	0.978089982	0.960133832	0.99999488	0.999985264	0.987457361
ZNF696	0.999996191	0.926949474	0.961013192	0.99999488	0.999985264	0.987457361
LOC112449318	0.999996191	0.999992444	0.96210311	0.99999488	0.999985264	0.987457361
DHRS7	0.999996191	0.992977597	0.963068909	0.99999488	0.999985264	0.987457361
LOC783142	0.999996191	0.998131745	0.964594039	0.99999488	0.999985264	0.987457361
METTL17	0.999996191	0.999992444	0.96870883	0.99999488	0.999985264	0.987457361
LOC101903649	0.999996191	0.948037796	0.969880768	0.99999488	0.999985264	0.987457361
RPL10	0.999996191	0.999992444	0.971111634	0.99999488	0.999985264	0.987457361
TPM4	0.999996191	0.999992444	0.973406453	0.99999488	0.999985264	0.987457361
DLGAP4	0.999996191	0.999992444	0.975430932	0.99999488	0.999985264	0.987457361
UBE2V1	0.999996191	0.991488652	0.980491974	0.99999488	0.999985264	0.987457361
LOC101902469	0.999996191	0.999992444	0.980660791	0.99999488	0.999985264	0.987457361
LOC104975290	0.999996191	0.998131745	0.980859619	0.99999488	0.999985264	0.987457361
ZMYM3	0.999996191	0.999347049	0.9808801	0.99999488	0.999985264	0.987457361
PHYKPL	0.999996191	0.999992444	0.98286145	0.99999488	0.999985264	0.987457361
C14H8orf59	0.999996191	0.960125322	0.984444908	0.99999488	0.999985264	0.987457361
DAGLB	0.999996191	0.991335402	0.984444908	0.99999488	0.999985264	0.987457361
LOC112447087	0.999996191	0.999347049	0.984444908	0.99999488	0.999985264	0.987457361
HNRNPUL2	0.999996191	0.999992444	0.985363931	0.99999488	0.999985264	0.987457361
PJVK	0.999996191	0.999992444	0.985363931	0.99999488	0.999985264	0.987457361
ATXN7L3	0.999996191	0.999992444	0.986266701	0.99999488	0.999985264	0.987457361
LONP1	0.999996191	0.992977597	0.989872445	0.99999488	0.999985264	0.987457361
RFC2	0.999996191	0.999992444	0.99035966	0.99999488	0.999985264	0.987457361
RAB11FIP3	0.999996191	0.999347049	0.991285432	0.99999488	0.999985264	0.987457361
TRIP6	0.999996191	0.999992444	0.991955927	0.99999488	0.999985264	0.987457361

CCDC150	0.999996191	0.995532499	0.992742113	0.99999488	0.999985264	0.987457361
GATD3A	0.999996191	0.999992444	0.993594894	0.99999488	0.999985264	0.987457361
HGS	0.999996191	0.999992444	0.993594894	0.99999488	0.999985264	0.987457361
C7H19orf66	0.999996191	0.999992444	0.994601277	0.99999488	0.999985264	0.987457361
MRPL28	0.999996191	0.999992444	0.996715234	0.99999488	0.999985264	0.987457361
MARCH5	0.999996191	0.991365955	0.998936876	0.99999488	0.999985264	0.987457361
COQ8B	0.999996191	0.999992444	0.998936876	0.99999488	0.999985264	0.987457361
FBXO2	0.999996191	0.999992444	0.998936876	0.99999488	0.999985264	0.987457361
FXYD6	0.999996191	0.999992444	0.998936876	0.99999488	0.999985264	0.987457361
LOC104970173	0.999996191	0.999992444	0.998936876	0.99999488	0.999985264	0.987457361
LOC112443151	0.999996191	0.999992444	0.998936876	0.99999488	0.999985264	0.987457361
TSPAN15	0.999996191	0.999992444	0.998936876	0.99999488	0.999985264	0.987457361
ARL6IP5	0.999996191	0.995476917	0.999558179	0.99999488	0.999985264	0.987457361
ANTXR2	0.999996191	0.999347049	0.999558179	0.99999488	0.999985264	0.987457361
LOC101903385	0.999996191	0.999347049	0.999558179	0.99999488	0.999985264	0.987457361
RAD54L2	0.999996191	0.999347049	0.999558179	0.99999488	0.999985264	0.987457361
ZMYND11	0.999996191	0.999347049	0.999558179	0.99999488	0.999985264	0.987457361
LOC100848177	0.999996191	0.999992444	0.999558179	0.99999488	0.999985264	0.987457361
LOC101907132	0.999996191	0.999992444	0.999558179	0.99999488	0.999985264	0.987457361
LOC112444909	0.999996191	0.999992444	0.999558179	0.99999488	0.999985264	0.987457361
SSRP1	0.999996191	0.999992444	0.961490767	0.99999488	0.999985264	0.98780064
LOC100848324	0.999996191	0.999992444	0.970874203	0.99999488	0.999985264	0.98780064
KCNQ4	0.999996191	0.918883775	0.923902957	0.99999488	0.999985264	0.987872708
LOC101902542	0.999996191	0.978089982	0.951099739	0.99999488	0.999985264	0.987872708
FARP2	0.999996191	0.901232581	0.951735967	0.99999488	0.999985264	0.987872708
TSEN34	0.999996191	0.999992444	0.961490767	0.99999488	0.999985264	0.987872708
STAT3	0.999996191	0.999992444	0.973406453	0.99999488	0.999985264	0.987872708
ALDH9A1	0.999996191	0.974812121	0.985363931	0.99999488	0.999985264	0.987872708
KLHL7	0.999996191	0.999992444	0.991285432	0.99999488	0.999985264	0.987872708
UFC1	0.999996191	0.991488652	0.993594894	0.99999488	0.999985264	0.987872708
SIAH2	0.999996191	0.999347049	0.998936876	0.99999488	0.999985264	0.987872708
CGNL1	0.999996191	0.999992444	0.998936876	0.99999488	0.999985264	0.987872708
ASXL1	0.999996191	0.999992444	0.999558179	0.99999488	0.999985264	0.987872708
CXHXorf58	0.999996191	0.999992444	0.999558179	0.99999488	0.999985264	0.987872708
LOC101907893	0.999996191	0.999992444	0.999558179	0.99999488	0.999985264	0.987872708
SKP1	0.999996191	0.999992444	0.999558179	0.99999488	0.999985264	0.987872708
FAM131B	0.999996191	0.941876954	0.935094475	0.99999488	0.999985264	0.98795961
LOC101905686	0.999996191	0.991365955	0.944085862	0.99999488	0.999985264	0.98795961
KCTD7	0.999996191	0.992977597	0.902552409	0.99999488	0.999985264	0.988030484

FHOD1	0.999996191	0.931336072	0.936914995	0.99999488	0.999985264	0.988030484
NUDT6	0.999996191	0.999992444	0.964152013	0.99999488	0.999985264	0.988030484
RPL19	0.999996191	0.999992444	0.964908146	0.99999488	0.999985264	0.988030484
CNRIP1	0.999996191	0.998131745	0.968232195	0.99999488	0.999985264	0.988030484
RAB11FIP1	0.999996191	0.999992444	0.981039752	0.99999488	0.999985264	0.988030484
SLC9A3R2	0.999996191	0.999992444	0.981622418	0.99999488	0.999985264	0.988030484
EPN2	0.999996191	0.999992444	0.985363931	0.99999488	0.999985264	0.988030484
LOC104971814	0.999996191	0.999992444	0.992742113	0.99999488	0.999985264	0.988030484
GBA	0.999996191	0.999992444	0.994601277	0.99999488	0.999985264	0.988030484
ARID1A	0.999996191	0.992977597	0.995045982	0.99999488	0.999985264	0.988030484
RTN4RL2	0.999996191	0.992977597	0.999558179	0.99999488	0.999985264	0.988030484
MRPS33	0.999996191	0.999992444	0.999558179	0.99999488	0.999985264	0.988205529
CPSF7	0.999996191	0.992977597	0.957541703	0.99999488	0.999985264	0.988210632
PHF20L1	0.999996191	0.999992444	0.980496281	0.99999488	0.999985264	0.988507916
DDX20	0.999996191	0.999992444	0.997208738	0.99999488	0.999985264	0.988507916
VRK1	0.999996191	0.999992444	0.998816213	0.99999488	0.999985264	0.98854352
ZFP30	0.999996191	0.999992444	0.973406453	0.99999488	0.999985264	0.988655969
LOC407171	0.999996191	0.991488652	0.926552506	0.99999488	0.999985264	0.988767597
NEURL1B	0.999996191	0.991488652	0.992709008	0.99999488	0.999985264	0.988767597
NAB2	0.999996191	0.999992444	0.993594894	0.99999488	0.999985264	0.988767597
LOC112442246	0.999996191	0.999347049	0.999558179	0.99999488	0.999985264	0.988767597
LYRM2	0.999996191	0.999347049	0.999558179	0.99999488	0.999985264	0.988767597
SH3GLB2	0.999996191	0.978089982	0.948420301	0.99999488	0.999985264	0.988771632
ALKBH3	0.999996191	0.991488652	0.99176376	0.99999488	0.999985264	0.988771632
LOC782525	0.999996191	0.994754253	0.94166887	0.99999488	0.999985264	0.988817857
DDX23	0.999996191	0.993188604	0.946479014	0.99999488	0.999985264	0.988817857
CFAP53	0.999996191	0.999992444	0.949038056	0.99999488	0.999985264	0.988817857
PDK3	0.999996191	0.999992444	0.959137142	0.99999488	0.999985264	0.988817857
ABCB6	0.999996191	0.999347049	0.97868062	0.99999488	0.999985264	0.988817857
C27H4orf47	0.999996191	0.999347049	0.981218677	0.99999488	0.999985264	0.988817857
MFSD1	0.999996191	0.999992444	0.998936876	0.99999488	0.999985264	0.98885207
PCYT1B	0.999996191	0.996814721	0.999558179	0.99999488	0.999985264	0.988903869
LOC784980	0.999996191	0.984714297	0.958417011	0.99999488	0.999985264	0.988934034
SC5D	0.999996191	0.999992444	0.997208738	0.99999488	0.999985264	0.988934034
LOC107132971	0.999996191	0.999992444	0.997859293	0.99999488	0.999985264	0.988934034
LOC615663	0.999996191	0.999992444	0.999558179	0.99999488	0.999985264	0.98895368
ZNF699	0.999996191	0.999992444	0.95230212	0.99999488	0.999985264	0.989152768
SIRT6	0.999996191	0.999992444	0.978389389	0.99999488	0.999985264	0.989152768
IP6K3	0.999996191	0.996153506	0.978842418	0.99999488	0.999985264	0.989152768

TRIQK	0.999996191	0.999992444	0.985966356	0.99999488	0.999985264	0.989152768
SS18	0.999996191	0.979263532	0.925745065	0.99999488	0.999985264	0.989348186
YWHAG	0.999996191	0.999992444	0.998936876	0.99999488	0.999985264	0.989348186
LOC101908075	0.999996191	0.984366454	0.926134459	0.99999488	0.999985264	0.989354156
PKP1	0.999996191	0.925580392	0.926552506	0.99999488	0.999985264	0.989354156
TRAF3IP2	0.999996191	0.999347049	0.981218677	0.99999488	0.999985264	0.989354156
ARHGEF12	0.999996191	0.999992444	0.984814673	0.99999488	0.999985264	0.989354156
FAM151B	0.999996191	0.999992444	0.994601277	0.99999488	0.999985264	0.989354156
NIPAL4	0.999996191	0.999992444	0.999558179	0.99999488	0.999985264	0.989354156
LDLR	0.999996191	0.978089982	0.941118576	0.99999488	0.999985264	0.989539011
LOC781813	0.999996191	0.999992444	0.967165568	0.99999488	0.999985264	0.989539011
HEATR1	0.999996191	0.997975376	0.96870883	0.99999488	0.999985264	0.989539011
RNF217	0.999996191	0.999347049	0.970945672	0.99999488	0.999985264	0.989539011
CCDC166	0.999996191	0.999992444	0.9808801	0.99999488	0.999985264	0.989539011
ORAOV1	0.999996191	0.998131745	0.981218677	0.99999488	0.999985264	0.989539011
EFCC1	0.999996191	0.999992444	0.981622418	0.99999488	0.999985264	0.989539011
FAM167B	0.999996191	0.998011664	0.993594894	0.99999488	0.999985264	0.989539011
SLC25A6	0.999996191	0.999992444	0.997208738	0.99999488	0.999985264	0.989539011
PLCD1	0.999996191	0.999347049	0.998936876	0.99999488	0.999985264	0.989539011
GATAD1	0.999996191	0.999992444	0.998936876	0.99999488	0.999985264	0.989539011
METTL26	0.999996191	0.999992444	0.998936876	0.99999488	0.999985264	0.989539011
CCPG1	0.999996191	0.999992444	0.999558179	0.99999488	0.999985264	0.989539011
LOC112446668	0.999996191	0.999992444	0.999558179	0.99999488	0.999985264	0.989539011
C7H19orf71	0.999996191	0.940459751	0.877742202	0.99999488	0.999985264	0.989606676
CCDC134	0.999996191	0.992977597	0.938911935	0.99999488	0.999985264	0.989606676
EDN3	0.999996191	0.999347049	0.95230212	0.99999488	0.999985264	0.989606676
LOC104975007	0.999996191	0.999347049	0.970874203	0.99999488	0.999985264	0.989606676
RNF126	0.999996191	0.999347049	0.981388276	0.99999488	0.999985264	0.989606676
GALNT14	0.999996191	0.999992444	0.984309048	0.99999488	0.999985264	0.989606676
SNX29	0.999996191	0.993316598	0.992742113	0.99999488	0.999985264	0.989606676
POLR2M	0.999996191	0.999992444	0.998936876	0.99999488	0.999985264	0.989606676
AP4S1	0.999996191	0.999992444	0.999697966	0.99999488	0.999985264	0.989606676
JMJD1C	0.999996191	0.999992444	0.991285432	0.99999488	0.999985264	0.989635855
C3H1orf109	0.999996191	0.991488652	0.938203102	0.99999488	0.999985264	0.989647101
GHDC	0.999996191	0.999992444	0.988969839	0.99999488	0.999985264	0.989647101
LOC100848369	0.999996191	0.991488652	0.961024709	0.99999488	0.999985264	0.989675278
FLYWCH2	0.999996191	0.97883539	0.961490767	0.99999488	0.999985264	0.989675278
C1GALT1	0.999996191	0.991488652	0.96210311	0.99999488	0.999985264	0.989675278
SLC10A3	0.999996191	0.998131745	0.964152013	0.99999488	0.999985264	0.989675278

LOC505199	0.999996191	0.993100184	0.964920281	0.99999488	0.999985264	0.989675278
STK35	0.999996191	0.965712238	0.970874203	0.99999488	0.999985264	0.989675278
DEGS1	0.999996191	0.999347049	0.976993966	0.99999488	0.999985264	0.989675278
TMEM158	0.999996191	0.998131745	0.984444908	0.99999488	0.999985264	0.989675278
UBE2T	0.999996191	0.998131745	0.985779332	0.99999488	0.999985264	0.989675278
GGACT	0.999996191	0.999992444	0.995045982	0.99999488	0.999985264	0.989675278
SDHB	0.999996191	0.999992444	0.998936876	0.99999488	0.999985264	0.989675278
TXNDC17	0.999996191	0.999992444	0.998936876	0.99999488	0.999985264	0.989675278
GAB1	0.999996191	0.999992444	0.999558179	0.99999488	0.999985264	0.989675278
SLC35E4	0.999996191	0.999992444	0.999558179	0.99999488	0.999985264	0.989675278
RIDA	0.999996191	0.985406717	0.95106368	0.99999488	0.999985264	0.989785661
DNAJB6	0.999996191	0.999992444	0.999558179	0.99999488	0.999985264	0.989785661
TOMM22	0.999996191	0.982940915	0.9808801	0.99999488	0.999985264	0.98981123
FBXW2	0.999996191	0.993316598	0.985966356	0.99999488	0.999985264	0.98981123
LOC112444328	0.999996191	0.999992444	0.992709008	0.99999488	0.999985264	0.98981123
ZSCAN16	0.999996191	0.957719519	0.95230212	0.99999488	0.999985264	0.989813739
USP3	0.999996191	0.961222468	0.908181052	0.99999488	0.999985264	0.990559159
UNC80	0.999996191	0.934184493	0.912511938	0.99999488	0.999985264	0.990559159
LOC789352	0.999996191	0.985293257	0.94634922	0.99999488	0.999985264	0.990559159
C14H8orf33	0.999996191	0.9582784	0.95477544	0.99999488	0.999985264	0.990559159
RPP14	0.999996191	0.999992444	0.998936876	0.99999488	0.999985264	0.990559159
EDEM1	0.999996191	0.99490847	0.99035966	0.99999488	0.999985264	0.990681466
BCL9L	0.999996191	0.999992444	0.998936876	0.99999488	0.999985264	0.990796977
REL	0.999996191	0.999992444	0.985363931	0.99999488	0.999985264	0.99088878
RABEPK	0.999996191	0.993540482	0.999558179	0.99999488	0.999985264	0.990931698
ZMYND12	0.999996191	0.999992444	0.973406453	0.99999488	0.999985264	0.990960855
NDRG3	0.999996191	0.999992444	0.999558179	0.99999488	0.999985264	0.990960855
METRNL	0.999996191	0.999992444	0.954620171	0.99999488	0.999985264	0.991003085
LOC786173	0.999996191	0.999992444	0.991285432	0.99999488	0.999985264	0.991003085
CAMK2A	0.999996191	0.972298153	0.954174725	0.99999488	0.999985264	0.991007678
AK1	0.999996191	0.991488652	0.938203102	0.99999488	0.999985264	0.991014754
LOC101905365	0.999996191	0.991488652	0.945766581	0.99999488	0.999985264	0.991014754
LOC101907749	0.999996191	0.991365955	0.94634922	0.99999488	0.999985264	0.991014754
SMPX	0.999996191	0.999992444	0.958627535	0.99999488	0.999985264	0.991014754
MTF2	0.999996191	0.999992444	0.964920281	0.99999488	0.999985264	0.991014754
MYSM1	0.999996191	0.999992444	0.985363931	0.99999488	0.999985264	0.991014754
TMEM151A	0.999996191	0.985406717	0.993594894	0.99999488	0.999985264	0.991014754
LOC112442544	0.999996191	0.999992444	0.998235205	0.99999488	0.999985264	0.991014754
ZNF76	0.999996191	0.992977597	0.999558179	0.99999488	0.999985264	0.991014754

EFNA2	0.999996191	0.927997582	0.907484111	0.99999488	0.999985264	0.99105915
RBM38	0.999996191	0.96566261	0.948359914	0.99999488	0.999985264	0.99105915
LOC782566	0.999996191	0.991488652	0.95106368	0.99999488	0.999985264	0.99105915
ALDH1L1	0.999996191	0.999992444	0.954823586	0.99999488	0.999985264	0.99105915
LMTK2	0.999996191	0.992977597	0.965065403	0.99999488	0.999985264	0.99105915
LOC786914	0.999996191	0.928759674	0.973406453	0.99999488	0.999985264	0.99105915
CCDC71	0.999996191	0.999992444	0.978389389	0.99999488	0.999985264	0.99105915
LOC100336777	0.999996191	0.999992444	0.980660791	0.99999488	0.999985264	0.99105915
MORC4	0.999996191	0.998131745	0.981039752	0.99999488	0.999985264	0.99105915
LOC112447370	0.999996191	0.999992444	0.981039752	0.99999488	0.999985264	0.99105915
SH3RF1	0.999996191	0.999992444	0.981039752	0.99999488	0.999985264	0.99105915
FUT8	0.999996191	0.999992444	0.981622418	0.99999488	0.999985264	0.99105915
TAAR1	0.999996191	0.998011664	0.985966356	0.99999488	0.999985264	0.99105915
RNF24	0.999996191	0.998181556	0.986294333	0.99999488	0.999985264	0.99105915
RTN1	0.999996191	0.991365955	0.998936876	0.99999488	0.999985264	0.99105915
CSRNP3	0.999996191	0.999992444	0.998936876	0.99999488	0.999985264	0.99105915
LOC100300938	0.999996191	0.999992444	0.998936876	0.99999488	0.999985264	0.99105915
DNAAF4	0.999996191	0.999992444	0.999558179	0.99999488	0.999985264	0.99105915
LOC100847825	0.999996191	0.999992444	0.999558179	0.99999488	0.999985264	0.99105915
FLOT2	0.999996191	0.999992444	0.991285432	0.99999488	0.999985264	0.991193537
LOC100335642	0.999996191	0.941779521	0.938215065	0.99999488	0.999985264	0.991216524
SCML2	0.999996191	0.991488652	0.95106368	0.99999488	0.999985264	0.991216524
PTPN18	0.999996191	0.999992444	0.982085804	0.99999488	0.999985264	0.991216524
DLG3	0.999996191	0.999992444	0.982633104	0.99999488	0.999985264	0.991216524
AP5M1	0.999996191	0.999992444	0.983419007	0.99999488	0.999985264	0.991216524
GDPD3	0.999996191	0.998011664	0.990780762	0.99999488	0.999985264	0.991216524
LOC101903586	0.999996191	0.999992444	0.993594894	0.99999488	0.999985264	0.991216524
GTPBP10	0.999996191	0.999992444	0.994601277	0.99999488	0.999985264	0.991216524
EEF1D	0.999996191	0.991488652	0.998693674	0.99999488	0.999985264	0.991216524
HSPE1	0.999996191	0.998131745	0.998936876	0.99999488	0.999985264	0.991216524
LOC112441473	0.999996191	0.999992444	0.999558179	0.99999488	0.999985264	0.991216524
NUP210L	0.999996191	0.999992444	0.999558179	0.99999488	0.999985264	0.991216524
MFSD2A	0.999996191	0.962665318	0.982065621	0.99999488	0.999985264	0.991220625
TMEM136	0.999996191	0.987262615	0.985363931	0.99999488	0.999985264	0.991255817
LSM1	0.999996191	0.985406717	0.957117655	0.99999488	0.999985264	0.991416599
ZDHHC5	0.999996191	0.999992444	0.981593378	0.99999488	0.999985264	0.991416599
TEX2	0.999996191	0.999992444	0.999558179	0.99999488	0.999985264	0.991416599
UBQLN2	0.999996191	0.95697765	0.886962419	0.99999488	0.999985264	0.991488481
ARMH4	0.999996191	0.991365955	0.95230212	0.99999488	0.999985264	0.991488481

PCMTD1	0.999996191	0.972730896	0.969880768	0.99999488	0.999985264	0.991488481
FBXO48	0.999996191	0.999992444	0.986266701	0.99999488	0.999985264	0.991488481
LOC101906606	0.999996191	0.999347049	0.991285432	0.99999488	0.999985264	0.991488481
KTN1	0.999996191	0.999992444	0.994601277	0.99999488	0.999985264	0.991488481
RAB34	0.999996191	0.999992444	0.994626276	0.99999488	0.999985264	0.991488481
KIAA0556	0.999996191	0.999992444	0.996391386	0.99999488	0.999985264	0.991488481
CD9	0.999996191	0.999992444	0.998936876	0.99999488	0.999985264	0.991488481
FCRLA	0.999996191	0.925580392	0.907484111	0.99999488	0.999985264	0.991499904
FAM57B	0.999996191	0.980496345	0.946479014	0.99999488	0.999985264	0.991627052
PSMB7	0.999996191	0.9582784	0.938203102	0.99999488	0.999985264	0.991669822
LOC112443425	0.999996191	0.999992444	0.966991646	0.99999488	0.999985264	0.992257636
KIAA0355	0.999996191	0.999992444	0.999558179	0.99999488	0.999985264	0.992257636
LOC112446004	0.999996191	0.991365955	0.96307344	0.99999488	0.999985264	0.992644147
LOC112446701	0.999996191	0.999992444	0.979650244	0.99999488	0.999985264	0.992644147
LOC101902991	0.999996191	0.999992444	0.981218677	0.99999488	0.999985264	0.992644147
ENGASE	0.999996191	0.999992444	0.981940287	0.99999488	0.999985264	0.992644147
POLR3GL	0.999996191	0.998131745	0.985363931	0.99999488	0.999985264	0.992644147
LOC101906656	0.999996191	0.999992444	0.998936876	0.99999488	0.999985264	0.992644147
PAQR5	0.999996191	0.999992444	0.998936876	0.99999488	0.999985264	0.992644147
DERA	0.999996191	0.978089982	0.999558179	0.99999488	0.999985264	0.992644147
DNAJC18	0.999996191	0.998131745	0.999558179	0.99999488	0.999985264	0.992644147
RXRB	0.999996191	0.999992444	0.996139177	0.99999488	0.999985264	0.99265531
TMEM9B	0.999996191	0.974812121	0.999558179	0.99999488	0.999985264	0.99265531
CFAP97	0.999996191	0.992977597	0.9808801	0.99999488	0.999985264	0.992716012
PRIMA1	0.999996191	0.999992444	0.995045982	0.99999488	0.999985264	0.992716012
IL12RB2	0.999996191	0.999992444	0.996715234	0.99999488	0.999985264	0.992716012
MRPL17	0.999996191	0.92202441	0.907484111	0.99999488	0.999985264	0.992727599
LOC618220	0.999996191	0.926675185	0.913211244	0.99999488	0.999985264	0.992727599
NMB	0.999996191	0.958098081	0.946479014	0.99999488	0.999985264	0.992727599
SOX18	0.999996191	0.985406717	0.946479014	0.99999488	0.999985264	0.992727599
PIP4K2C	0.999996191	0.999992444	0.946479014	0.99999488	0.999985264	0.992727599
LOC101904173	0.999996191	0.961015497	0.947226687	0.99999488	0.999985264	0.992727599
UBA1	0.999996191	0.957700659	0.948420301	0.99999488	0.999985264	0.992727599
STAMBPL1	0.999996191	0.992977597	0.969880768	0.99999488	0.999985264	0.992727599
GLOD4	0.999996191	0.991488652	0.969913497	0.99999488	0.999985264	0.992727599
SAMD1	0.999996191	0.990367449	0.978842418	0.99999488	0.999985264	0.992727599
CRYZ	0.999996191	0.999992444	0.978993134	0.99999488	0.999985264	0.992727599
ENTR1	0.999996191	0.999992444	0.978993134	0.99999488	0.999985264	0.992727599
LOC104975299	0.999996191	0.995029734	0.980660791	0.99999488	0.999985264	0.992727599

PI4KA	0.999996191	0.999992444	0.980859619	0.99999488	0.999985264	0.992727599
FAM32A	0.999996191	0.999992444	0.981039752	0.99999488	0.999985264	0.992727599
LIPC	0.999996191	0.997830492	0.981622418	0.99999488	0.999985264	0.992727599
CYLD	0.999996191	0.999992444	0.984765193	0.99999488	0.999985264	0.992727599
PDS5B	0.999996191	0.999992444	0.989872445	0.99999488	0.999985264	0.992727599
PAF1	0.999996191	0.999992444	0.99035966	0.99999488	0.999985264	0.992727599
PRAF2	0.999996191	0.999992444	0.991992782	0.99999488	0.999985264	0.992727599
CENPL	0.999996191	0.999992444	0.993132892	0.99999488	0.999985264	0.992727599
FOSL2	0.999996191	0.999992444	0.993594894	0.99999488	0.999985264	0.992727599
PAXX	0.999996191	0.999992444	0.993594894	0.99999488	0.999985264	0.992727599
VN1R1	0.999996191	0.999992444	0.994601277	0.99999488	0.999985264	0.992727599
HPF1	0.999996191	0.999347049	0.998936876	0.99999488	0.999985264	0.992727599
KRT8	0.999996191	0.999992444	0.998936876	0.99999488	0.999985264	0.992727599
TRPC2	0.999996191	0.999347049	0.999558179	0.99999488	0.999985264	0.992727599
LOC112442278	0.999996191	0.999992444	0.999558179	0.99999488	0.999985264	0.992727599
LOC107132546	0.999996191	0.999992444	0.944085862	0.99999488	0.999985264	0.992791941
ATP2A1	0.999996191	0.987559864	0.973303821	0.99999488	0.999985264	0.992791941
PARPBP	0.999996191	0.998962836	0.977141934	0.99999488	0.999985264	0.992791941
C9H6orf120	0.999996191	0.999992444	0.981388276	0.99999488	0.999985264	0.992791941
KLHDC9	0.999996191	0.994481342	0.99035966	0.99999488	0.999985264	0.992791941
LOC783533	0.999996191	0.999992444	0.998936876	0.99999488	0.999985264	0.992791941
DIO3	0.999996191	0.999992444	0.999558179	0.99999488	0.999985264	0.992791941
LOC104974137	0.999996191	0.882022911	0.88153193	0.99999488	0.999985264	0.992853834
LOC788672	0.999996191	0.991476708	0.961850944	0.99999488	0.999985264	0.992853834
HBS1L	0.999996191	0.999992444	0.962777771	0.99999488	0.999985264	0.992853834
TMEM150A	0.999996191	0.999992444	0.963233239	0.99999488	0.999985264	0.992853834
LOC112445938	0.999996191	0.999992444	0.980169282	0.99999488	0.999985264	0.992853834
LOC509941	0.999996191	0.999992444	0.981388276	0.99999488	0.999985264	0.992853834
TTC21B	0.999996191	0.999992444	0.981388276	0.99999488	0.999985264	0.992853834
TDRD9	0.999996191	0.999992444	0.985363931	0.99999488	0.999985264	0.992853834
FAM92B	0.999996191	0.999992444	0.985966356	0.99999488	0.999985264	0.992853834
PTBP1	0.999996191	0.999992444	0.986938092	0.99999488	0.999985264	0.992853834
CTDNEP1	0.999996191	0.999992444	0.987348473	0.99999488	0.999985264	0.992853834
LRRC58	0.999996191	0.999992444	0.993132892	0.99999488	0.999985264	0.992853834
LOC112446667	0.999996191	0.999992444	0.995045982	0.99999488	0.999985264	0.992853834
R3HDM2	0.999996191	0.99241354	0.998936876	0.99999488	0.999985264	0.992853834
KLF6	0.999996191	0.999992444	0.998936876	0.99999488	0.999985264	0.992853834
LOC100140958	0.999996191	0.999992444	0.998936876	0.99999488	0.999985264	0.992853834
TCTEX1D2	0.999996191	0.991488652	0.999558179	0.99999488	0.999985264	0.992853834

LOC112442613	0.999996191	0.992977597	0.999558179	0.99999488	0.999985264	0.992853834
RPGR	0.999996191	0.999992444	0.999558179	0.99999488	0.999985264	0.992853834
TADA2B	0.999996191	0.999992444	0.999558179	0.99999488	0.999985264	0.992853834
LOC107131311	0.999996191	0.999347049	0.993594894	0.99999488	0.999985264	0.99290512
IMPAD1	0.999996191	0.999992444	0.998936876	0.99999488	0.999985264	0.99290512
ZNF283	0.999996191	0.999992444	0.970945672	0.99999488	0.999985264	0.993058999
CPNE1	0.999996191	0.957700659	0.877799478	0.99999488	0.999985264	0.993086089
CDYL2	0.999996191	0.986637746	0.926552506	0.99999488	0.999985264	0.993086089
ADPRM	0.999996191	0.991488652	0.941782902	0.99999488	0.999985264	0.993086089
PRDM11	0.999996191	0.962968114	0.944085862	0.99999488	0.999985264	0.993086089
FAM107A	0.999996191	0.961348688	0.94634922	0.99999488	0.999985264	0.993086089
LOC112449115	0.999996191	0.985161531	0.946479014	0.99999488	0.999985264	0.993086089
LOC112446010	0.999996191	0.977333066	0.949038056	0.99999488	0.999985264	0.993086089
PDAP1	0.999996191	0.999992444	0.952412901	0.99999488	0.999985264	0.993086089
PLA2G5	0.999996191	0.998131745	0.954823586	0.99999488	0.999985264	0.993086089
MRPL9	0.999996191	0.991488652	0.954872702	0.99999488	0.999985264	0.993086089
MCCD1	0.999996191	0.999992444	0.956166665	0.99999488	0.999985264	0.993086089
TXNDC16	0.999996191	0.988850552	0.956565728	0.99999488	0.999985264	0.993086089
ART4	0.999996191	0.999992444	0.957117655	0.99999488	0.999985264	0.993086089
MYO9A	0.999996191	0.999992444	0.957541703	0.99999488	0.999985264	0.993086089
MUT	0.999996191	0.985406717	0.95758013	0.99999488	0.999985264	0.993086089
DAAM1	0.999996191	0.999992444	0.962346588	0.99999488	0.999985264	0.993086089
ATP9A	0.999996191	0.985406717	0.963978721	0.99999488	0.999985264	0.993086089
SEMA5B	0.999996191	0.957700659	0.964152013	0.99999488	0.999985264	0.993086089
TMEM38A	0.999996191	0.998962836	0.964920281	0.99999488	0.999985264	0.993086089
GPR21	0.999996191	0.998011664	0.96684887	0.99999488	0.999985264	0.993086089
ITPRIPL2	0.999996191	0.978510121	0.968232195	0.99999488	0.999985264	0.993086089
NIPSNAP3A	0.999996191	0.998131745	0.969880768	0.99999488	0.999985264	0.993086089
TAF5L	0.999996191	0.994464252	0.971111634	0.99999488	0.999985264	0.993086089
GPR89A	0.999996191	0.999992444	0.973303821	0.99999488	0.999985264	0.993086089
APPL2	0.999996191	0.999347049	0.976143419	0.99999488	0.999985264	0.993086089
RAB20	0.999996191	0.9582784	0.979462526	0.99999488	0.999985264	0.993086089
GDPD1	0.999996191	0.991488652	0.979675621	0.99999488	0.999985264	0.993086089
FAM53C	0.999996191	0.999992444	0.980132104	0.99999488	0.999985264	0.993086089
PLEKHG1	0.999996191	0.999992444	0.980132104	0.99999488	0.999985264	0.993086089
SLC26A10	0.999996191	0.992977597	0.9808801	0.99999488	0.999985264	0.993086089
LOC101902048	0.999996191	0.994481342	0.9808801	0.99999488	0.999985264	0.993086089
AMDHD2	0.999996191	0.999992444	0.9808801	0.99999488	0.999985264	0.993086089
MALSU1	0.999996191	0.998962836	0.981039752	0.99999488	0.999985264	0.993086089

SFT2D3	0.999996191	0.999992444	0.981039752	0.99999488	0.999985264	0.993086089
CA5B	0.999996191	0.999992444	0.981388276	0.99999488	0.999985264	0.993086089
STK11	0.999996191	0.999992444	0.981388276	0.99999488	0.999985264	0.993086089
LOC616094	0.999996191	0.999992444	0.981622418	0.99999488	0.999985264	0.993086089
DUSP18	0.999996191	0.972544855	0.984444908	0.99999488	0.999985264	0.993086089
KIF13B	0.999996191	0.999992444	0.98480229	0.99999488	0.999985264	0.993086089
RPL7	0.999996191	0.992977597	0.985363931	0.99999488	0.999985264	0.993086089
FOXP1	0.999996191	0.998131745	0.988922732	0.99999488	0.999985264	0.993086089
GIGYF2	0.999996191	0.999992444	0.988922732	0.99999488	0.999985264	0.993086089
LOC112446799	0.999996191	0.999992444	0.989872445	0.99999488	0.999985264	0.993086089
PPIB	0.999996191	0.999992444	0.990780762	0.99999488	0.999985264	0.993086089
ARRDC2	0.999996191	0.999992444	0.991285432	0.99999488	0.999985264	0.993086089
LOC112441469	0.999996191	0.999992444	0.992742113	0.99999488	0.999985264	0.993086089
ERFE	0.999996191	0.985406717	0.993594894	0.99999488	0.999985264	0.993086089
PCBP4	0.999996191	0.992977597	0.993594894	0.99999488	0.999985264	0.993086089
LACTB2	0.999996191	0.999992444	0.993594894	0.99999488	0.999985264	0.993086089
SOD1	0.999996191	0.999992444	0.993594894	0.99999488	0.999985264	0.993086089
LOC783803	0.999996191	0.999992444	0.995045982	0.99999488	0.999985264	0.993086089
S1PR2	0.999996191	0.999992444	0.995045982	0.99999488	0.999985264	0.993086089
F8A1	0.999996191	0.999992444	0.996348351	0.99999488	0.999985264	0.993086089
S1PR1	0.999996191	0.999992444	0.996391386	0.99999488	0.999985264	0.993086089
LOC511161	0.999996191	0.991365955	0.996715234	0.99999488	0.999985264	0.993086089
NAGS	0.999996191	0.999992444	0.996715234	0.99999488	0.999985264	0.993086089
ROR1	0.999996191	0.985058069	0.998936876	0.99999488	0.999985264	0.993086089
SRA1	0.999996191	0.998131745	0.998936876	0.99999488	0.999985264	0.993086089
CHST8	0.999996191	0.999992444	0.998936876	0.99999488	0.999985264	0.993086089
LANCL1	0.999996191	0.999992444	0.998936876	0.99999488	0.999985264	0.993086089
LOC614208	0.999996191	0.999992444	0.998936876	0.99999488	0.999985264	0.993086089
NRBF2	0.999996191	0.999992444	0.998936876	0.99999488	0.999985264	0.993086089
POLD2	0.999996191	0.999992444	0.999072092	0.99999488	0.999985264	0.993086089
PPP3R1	0.999996191	0.948700494	0.999558179	0.99999488	0.999985264	0.993086089
ZSCAN25	0.999996191	0.990248517	0.999558179	0.99999488	0.999985264	0.993086089
LOC534630	0.999996191	0.992977597	0.999558179	0.99999488	0.999985264	0.993086089
PIGG	0.999996191	0.999347049	0.999558179	0.99999488	0.999985264	0.993086089
BRAP	0.999996191	0.999992444	0.999558179	0.99999488	0.999985264	0.993086089
DGKZ	0.999996191	0.999992444	0.999558179	0.99999488	0.999985264	0.993086089
ENOX2	0.999996191	0.999992444	0.999558179	0.99999488	0.999985264	0.993086089
KLHL22	0.999996191	0.999992444	0.999558179	0.99999488	0.999985264	0.993086089
LOC101903402	0.999996191	0.999992444	0.999558179	0.99999488	0.999985264	0.993086089

LOC101903682	0.999996191	0.999992444	0.999558179	0.99999488	0.999985264	0.993086089
MIPEP	0.999996191	0.999992444	0.999558179	0.99999488	0.999985264	0.993086089
PCMT1	0.999996191	0.999992444	0.999558179	0.99999488	0.999985264	0.993086089
RDM1	0.999996191	0.999992444	0.999558179	0.99999488	0.999985264	0.993086089
RNF181	0.999996191	0.999992444	0.999558179	0.99999488	0.999985264	0.993086089
SHISA6	0.999996191	0.999992444	0.999558179	0.99999488	0.999985264	0.993086089
SMIM15	0.999996191	0.999992444	0.999558179	0.99999488	0.999985264	0.993086089
PGK1	0.999996191	0.95697765	0.938203102	0.99999488	0.999985264	0.993125118
AQP11	0.999996191	0.999992444	0.944085862	0.99999488	0.999985264	0.993125118
LOC101907213	0.999996191	0.999992444	0.988922732	0.99999488	0.999985264	0.993125118
TBC1D2	0.999996191	0.999992444	0.993594894	0.99999488	0.999985264	0.993125118
LOC101904855	0.999996191	0.999992444	0.998936876	0.99999488	0.999985264	0.993125118
C25H7orf43	0.999996191	0.999992444	0.999558179	0.99999488	0.999985264	0.993125118
MRPL52	0.999996191	0.999992444	0.998936876	0.99999488	0.999985264	0.993316777
SLC22A15	0.999996191	0.991488652	0.95230212	0.99999488	0.999985264	0.993385369
LSM8	0.999996191	0.999992444	0.999558179	0.99999488	0.999985264	0.993385369
LOC101906358	0.999996191	0.999992444	0.999558179	0.99999488	0.999985264	0.993514542
LOC615112	0.999996191	0.999992444	0.886962419	0.99999488	0.999985264	0.993564907
STRAP	0.999996191	0.922102474	0.926552506	0.99999488	0.999985264	0.993564907
LOC101905525	0.999996191	0.994210852	0.927516734	0.99999488	0.999985264	0.993564907
RARS	0.999996191	0.95697765	0.935094475	0.99999488	0.999985264	0.993564907
EBNA1BP2	0.999996191	0.978089982	0.939501959	0.99999488	0.999985264	0.993564907
KMT2A	0.999996191	0.97015318	0.940815039	0.99999488	0.999985264	0.993564907
TCEAL8	0.999996191	0.9582784	0.946832224	0.99999488	0.999985264	0.993564907
LOC112446733	0.999996191	0.992977597	0.951822586	0.99999488	0.999985264	0.993564907
UBXN1	0.999996191	0.970426668	0.95230212	0.99999488	0.999985264	0.993564907
C24H18orf21	0.999996191	0.999992444	0.956565728	0.99999488	0.999985264	0.993564907
LOC107132302	0.999996191	0.993316598	0.961490767	0.99999488	0.999985264	0.993564907
CLPTM1	0.999996191	0.991365955	0.969880768	0.99999488	0.999985264	0.993564907
SLC45A4	0.999996191	0.946274461	0.973406453	0.99999488	0.999985264	0.993564907
FBXO22	0.999996191	0.999992444	0.973406453	0.99999488	0.999985264	0.993564907
UNC13C	0.999996191	0.991488652	0.977141934	0.99999488	0.999985264	0.993564907
ZNF697	0.999996191	0.999992444	0.977141934	0.99999488	0.999985264	0.993564907
POU2F1	0.999996191	0.999992444	0.979462526	0.99999488	0.999985264	0.993564907
TTI2	0.999996191	0.999992444	0.979675621	0.99999488	0.999985264	0.993564907
MAP2K7	0.999996191	0.999992444	0.980859619	0.99999488	0.999985264	0.993564907
CLIC3	0.999996191	0.999992444	0.9808801	0.99999488	0.999985264	0.993564907
LOC616051	0.999996191	0.999992444	0.981388276	0.99999488	0.999985264	0.993564907
GALT	0.999996191	0.999992444	0.981622418	0.99999488	0.999985264	0.993564907

COP1	0.999996191	0.999347049	0.981736235	0.99999488	0.999985264	0.993564907
EXOC5	0.999996191	0.999992444	0.984631623	0.99999488	0.999985264	0.993564907
YPEL4	0.999996191	0.980496345	0.98480229	0.99999488	0.999985264	0.993564907
APTX	0.999996191	0.999992444	0.98480229	0.99999488	0.999985264	0.993564907
CD99L2	0.999996191	0.999992444	0.98480229	0.99999488	0.999985264	0.993564907
STX4	0.999996191	0.999992444	0.985363931	0.99999488	0.999985264	0.993564907
FAM110B	0.999996191	0.999992444	0.99035966	0.99999488	0.999985264	0.993564907
EPB41L4A	0.999996191	0.994135588	0.990697261	0.99999488	0.999985264	0.993564907
HNRNPL	0.999996191	0.999992444	0.990780762	0.99999488	0.999985264	0.993564907
LOC112445944	0.999996191	0.999992444	0.991992782	0.99999488	0.999985264	0.993564907
SCAMP4	0.999996191	0.999992444	0.992709008	0.99999488	0.999985264	0.993564907
COX14	0.999996191	0.999992444	0.993585211	0.99999488	0.999985264	0.993564907
IGFBP7	0.999996191	0.999992444	0.993594894	0.99999488	0.999985264	0.993564907
PLB1	0.999996191	0.999992444	0.993594894	0.99999488	0.999985264	0.993564907
ARRDC1	0.999996191	0.999992444	0.994601277	0.99999488	0.999985264	0.993564907
LDLRAD3	0.999996191	0.999347049	0.995045982	0.99999488	0.999985264	0.993564907
BTBD8	0.999996191	0.999992444	0.995045982	0.99999488	0.999985264	0.993564907
SLC25A53	0.999996191	0.999992444	0.995045982	0.99999488	0.999985264	0.993564907
TGIF1	0.999996191	0.999992444	0.995045982	0.99999488	0.999985264	0.993564907
CA2	0.999996191	0.999992444	0.996715234	0.99999488	0.999985264	0.993564907
LOC104972417	0.999996191	0.999992444	0.998936678	0.99999488	0.999985264	0.993564907
PLAC9	0.999996191	0.999347049	0.998936876	0.99999488	0.999985264	0.993564907
BCAP29	0.999996191	0.999992444	0.998936876	0.99999488	0.999985264	0.993564907
FXYD7	0.999996191	0.999992444	0.998936876	0.99999488	0.999985264	0.993564907
NOL4L	0.999996191	0.999992444	0.998936876	0.99999488	0.999985264	0.993564907
TFEB	0.999996191	0.999992444	0.998936876	0.99999488	0.999985264	0.993564907
YOD1	0.999996191	0.999992444	0.998936876	0.99999488	0.999985264	0.993564907
KIAA0319L	0.999996191	0.991488652	0.999558179	0.99999488	0.999985264	0.993564907
ANKIB1	0.999996191	0.999347049	0.999558179	0.99999488	0.999985264	0.993564907
GNB5	0.999996191	0.999992444	0.999558179	0.99999488	0.999985264	0.993564907
LOC100848538	0.999996191	0.999992444	0.999558179	0.99999488	0.999985264	0.993564907
LOC104972567	0.999996191	0.999992444	0.999558179	0.99999488	0.999985264	0.993564907
MYLK2	0.999996191	0.999992444	0.999558179	0.99999488	0.999985264	0.993564907
MYMK	0.999996191	0.999992444	0.999558179	0.99999488	0.999985264	0.993564907
SCRN2	0.999996191	0.999992444	0.999558179	0.99999488	0.999985264	0.993564907
SDE2	0.999996191	0.999992444	0.999558179	0.99999488	0.999985264	0.993564907
SLC2A4RG	0.999996191	0.999992444	0.999558179	0.99999488	0.999985264	0.993564907
SNRPF	0.999996191	0.999992444	0.999558179	0.99999488	0.999985264	0.993564907
TAF6	0.999996191	0.999992444	0.999558179	0.99999488	0.999985264	0.993564907

TDRD6	0.999996191	0.999992444	0.999558179	0.99999488	0.999985264	0.993564907
TSPAN2	0.999996191	0.999992444	0.999558179	0.99999488	0.999985264	0.993564907
TTC31	0.999996191	0.999992444	0.999558179	0.99999488	0.999985264	0.993564907
PCGF2	0.999996191	0.999347049	0.964920281	0.99999488	0.999985264	0.993606543
POLK	0.999996191	0.999992444	0.973406453	0.99999488	0.999985264	0.993606543
SDHAF1	0.999996191	0.999992444	0.986930681	0.99999488	0.999985264	0.993606543
RBM24	0.999996191	0.991365955	0.990780762	0.99999488	0.999985264	0.993606543
PHF3	0.999996191	0.999992444	0.992709008	0.99999488	0.999985264	0.993606543
KCTD18	0.999996191	0.999992444	0.998936876	0.99999488	0.999985264	0.993606543
WDR59	0.999996191	0.999992444	0.998936876	0.99999488	0.999985264	0.993606543
KIF7	0.999996191	0.991574265	0.999558179	0.99999488	0.999985264	0.993606543
LOC112444842	0.999996191	0.999992444	0.999558179	0.99999488	0.999985264	0.993606543
STMN3	0.999996191	0.991488652	0.948359914	0.99999488	0.999985264	0.993701943
VPS26C	0.999996191	0.994481342	0.954823586	0.99999488	0.999985264	0.993718569
LOC112441843	0.999996191	0.958967142	0.964920281	0.99999488	0.999985264	0.993718569
FBN1	0.999996191	0.991866692	0.968841179	0.99999488	0.999985264	0.993718569
RCC1L	0.999996191	0.991574265	0.977141934	0.99999488	0.999985264	0.993718569
ERI2	0.999996191	0.999992444	0.981218677	0.99999488	0.999985264	0.993718569
STAT2	0.999996191	0.978510121	0.982388473	0.99999488	0.999985264	0.993718569
FBXL4	0.999996191	0.999992444	0.992742113	0.99999488	0.999985264	0.993718569
ORAI3	0.999996191	0.999992444	0.995045982	0.99999488	0.999985264	0.993718569
DRD1	0.999996191	0.999992444	0.999558179	0.99999488	0.999985264	0.993718569
SLC25A3	0.999996191	0.999347049	0.990862264	0.99999488	0.999985264	0.993740805
SHLD1	0.999996191	0.985406717	0.938203102	0.99999488	0.999985264	0.99382486
TRAPPC12	0.999996191	0.989289055	0.945678616	0.99999488	0.999985264	0.99382486
GPR174	0.999996191	0.944181275	0.948420301	0.99999488	0.999985264	0.99382486
INPP4A	0.999996191	0.999992444	0.95106368	0.99999488	0.999985264	0.99382486
ULK2	0.999996191	0.993316598	0.952571879	0.99999488	0.999985264	0.99382486
LOC101905801	0.999996191	0.991488652	0.960909242	0.99999488	0.999985264	0.99382486
SF3A3	0.999996191	0.987559864	0.961490767	0.99999488	0.999985264	0.99382486
TMEM187	0.999996191	0.999992444	0.969880768	0.99999488	0.999985264	0.99382486
MOCOS	0.999996191	0.999992444	0.971778256	0.99999488	0.999985264	0.99382486
INTS4	0.999996191	0.999992444	0.975768865	0.99999488	0.999985264	0.99382486
SYNJ2BP	0.999996191	0.999992444	0.981736235	0.99999488	0.999985264	0.99382486
SLC25A40	0.999996191	0.999992444	0.991319768	0.99999488	0.999985264	0.99382486
PRPF4	0.999996191	0.998011664	0.991403854	0.99999488	0.999985264	0.99382486
LOC112449059	0.999996191	0.999992444	0.993594894	0.99999488	0.999985264	0.99382486
TMEM53	0.999996191	0.999992444	0.993594894	0.99999488	0.999985264	0.99382486
MRGBP	0.999996191	0.999992444	0.998208226	0.99999488	0.999985264	0.99382486

ACCS	0.999996191	0.991488652	0.998936876	0.99999488	0.999985264	0.99382486
EIF4E3	0.999996191	0.998011664	0.998936876	0.99999488	0.999985264	0.99382486
TBC1D30	0.999996191	0.999992444	0.998936876	0.99999488	0.999985264	0.99382486
TBCD	0.999996191	0.999992444	0.998936876	0.99999488	0.999985264	0.99382486
LOC112443223	0.999996191	0.993316598	0.999558179	0.99999488	0.999985264	0.99382486
CRCP	0.999996191	0.998131745	0.999558179	0.99999488	0.999985264	0.99382486
PI4KB	0.999996191	0.999347049	0.999558179	0.99999488	0.999985264	0.99382486
LRRC55	0.999996191	0.999992444	0.999558179	0.99999488	0.999985264	0.99382486
ZNF784	0.999996191	0.999992444	0.999558179	0.99999488	0.999985264	0.99382486
LOC104975222	0.999996191	0.992977597	0.880594299	0.99999488	0.999985264	0.993879732
FAF2	0.999996191	0.999992444	0.9808801	0.99999488	0.999985264	0.993879732
LOC112447523	0.999996191	0.999992444	0.998936876	0.99999488	0.999985264	0.993879732
MGA	0.999996191	0.999992444	0.998936876	0.99999488	0.999985264	0.993879732
TBC1D1	0.999996191	0.999992444	0.998936876	0.99999488	0.999985264	0.993879732
FCHSD1	0.999996191	0.991488652	0.999558179	0.99999488	0.999985264	0.993879732
FGF9	0.999996191	0.999992444	0.999558179	0.99999488	0.999985264	0.993879732
CNN3	0.999996191	0.991488652	0.964152013	0.99999488	0.999985264	0.993883944
CAPN2	0.999996191	0.999992444	0.986180419	0.99999488	0.999985264	0.993883944
KCNAB1	0.999996191	0.999992444	0.999558179	0.99999488	0.999985264	0.993883944
LOC112444521	0.999996191	0.998011664	0.996348351	0.99999488	0.999985264	0.994042098
WIF1	0.999996191	0.945955989	0.945678616	0.99999488	0.999985264	0.994062777
LOC614423	0.999996191	0.941495217	0.94634922	0.99999488	0.999985264	0.994062777
SMIM20	0.999996191	0.999347049	0.990752015	0.99999488	0.999985264	0.994062777
RMI2	0.999996191	0.999992444	0.996715234	0.99999488	0.999985264	0.994062777
CDYL	0.999996191	0.999992444	0.999558179	0.99999488	0.999985264	0.994062777
SHBG	0.999996191	0.952639631	0.935094475	0.99999488	0.999985264	0.99417298
FXYD1	0.999996191	0.999992444	0.991285432	0.99999488	0.999985264	0.994243941
OCEL1	0.999996191	0.999992444	0.964152013	0.99999488	0.999985264	0.99424848
RRN3	0.999996191	0.999992444	0.998936876	0.99999488	0.999985264	0.994405348
DENND3	0.999996191	0.999992444	0.999558179	0.99999488	0.999985264	0.994405348
LOC112446795	0.999996191	0.999992444	0.981039752	0.99999488	0.999985264	0.994607516
GPR143	0.999996191	0.999992444	0.996715234	0.99999488	0.999985264	0.994607516
MAGED2	0.999996191	0.999992444	0.997208738	0.99999488	0.999985264	0.994607516
MAPK8IP1	0.999996191	0.999992444	0.997208738	0.99999488	0.999985264	0.994607516
SNX3	0.999996191	0.999992444	0.999558179	0.99999488	0.999985264	0.994607516
ALDH16A1	0.999996191	0.999992444	0.94634922	0.99999488	0.999985264	0.99475352
SCCPDH	0.999996191	0.991488652	0.970903823	0.99999488	0.999985264	0.99475352
ZNF672	0.999996191	0.999992444	0.999558179	0.99999488	0.999985264	0.99475352
ACAA2	0.999996191	0.999992444	0.999558179	0.99999488	0.999985264	0.994845153

IQCH	0.999996191	0.9582784	0.935726137	0.99999488	0.999985264	0.994845239
DLD	0.999996191	0.992977597	0.95230212	0.99999488	0.999985264	0.994845239
LOC112443469	0.999996191	0.991488652	0.954620171	0.99999488	0.999985264	0.994845239
SORD	0.999996191	0.976819696	0.955141547	0.99999488	0.999985264	0.994845239
TMEM8B	0.999996191	0.994434609	0.977141934	0.99999488	0.999985264	0.994845239
IDH1	0.999996191	0.999992444	0.9840454	0.99999488	0.999985264	0.994845239
C19H17orf75	0.999996191	0.999992444	0.986727257	0.99999488	0.999985264	0.994845239
STK16	0.999996191	0.999992444	0.988922732	0.99999488	0.999985264	0.994845239
SLC25A38	0.999996191	0.999347049	0.993594894	0.99999488	0.999985264	0.994845239
LOC101907174	0.999996191	0.999992444	0.993594894	0.99999488	0.999985264	0.994845239
SNAPC1	0.999996191	0.992977597	0.994601277	0.99999488	0.999985264	0.994845239
IBTK	0.999996191	0.999992444	0.994626276	0.99999488	0.999985264	0.994845239
MFAP3	0.999996191	0.999992444	0.992337371	0.99999488	0.999985264	0.994895174
PPFIBP2	0.999996191	0.938597964	0.92768724	0.99999488	0.999985264	0.995105522
MRPL38	0.999996191	0.999992444	0.999558179	0.99999488	0.999985264	0.995105522
PPCS	0.999996191	0.89880506	0.877799478	0.99999488	0.999985264	0.99512563
LOC788634	0.999996191	0.946274461	0.925520255	0.99999488	0.999985264	0.99512563
U2AF1	0.999996191	0.936273761	0.931241982	0.99999488	0.999985264	0.99512563
APMAP	0.999996191	0.999992444	0.933791093	0.99999488	0.999985264	0.99512563
ARHGEF12	0.999996191	0.938597964	0.939501959	0.99999488	0.999985264	0.99512563
RB1	0.999996191	0.998131745	0.944085862	0.99999488	0.999985264	0.99512563
S100A13	0.999996191	0.988170324	0.964920281	0.99999488	0.999985264	0.99512563
NR1D2	0.999996191	0.999992444	0.971111634	0.99999488	0.999985264	0.99512563
SEPT3	0.999996191	0.984366454	0.973406453	0.99999488	0.999985264	0.99512563
SYDE1	0.999996191	0.999992444	0.978389389	0.99999488	0.999985264	0.99512563
ASH1L	0.999996191	0.999992444	0.980847826	0.99999488	0.999985264	0.99512563
DOC2G	0.999996191	0.993316598	0.981218677	0.99999488	0.999985264	0.99512563
NSD3	0.999996191	0.999992444	0.981622418	0.99999488	0.999985264	0.99512563
PLCXD3	0.999996191	0.999992444	0.982783495	0.99999488	0.999985264	0.99512563
RBM14	0.999996191	0.999992444	0.987678394	0.99999488	0.999985264	0.99512563
AHCTF1	0.999996191	0.999992444	0.988626363	0.99999488	0.999985264	0.99512563
WDR45B	0.999996191	0.999992444	0.991285432	0.99999488	0.999985264	0.99512563
LOC112442867	0.999996191	0.999992444	0.995045982	0.99999488	0.999985264	0.99512563
QPCTL	0.999996191	0.999347049	0.998936876	0.99999488	0.999985264	0.99512563
LOC112442365	0.999996191	0.999992444	0.999558179	0.99999488	0.999985264	0.99512563
GRAMD2B	0.999996191	0.929943407	0.930127869	0.99999488	0.999985264	0.995136335
UBP1	0.999996191	0.999992444	0.988799866	0.99999488	0.999985264	0.99526359
NXN	0.999996191	0.999992444	0.991285432	0.99999488	0.999985264	0.995267431
SNRPD1	0.999996191	0.999347049	0.944085862	0.99999488	0.999985264	0.995270463

GKAP1	0.999996191	0.999992444	0.981622418	0.99999488	0.999985264	0.995270463
PNMA2	0.999996191	0.999347049	0.998936876	0.99999488	0.999985264	0.995270463
NAPG	0.999996191	0.999992444	0.998936876	0.99999488	0.999985264	0.995270463
ZBED4	0.999996191	0.999992444	0.998936876	0.99999488	0.999985264	0.995270463
BRD1	0.999996191	0.999347049	0.999558179	0.99999488	0.999985264	0.995270463
B4GALT6	0.999996191	0.999992444	0.999558179	0.99999488	0.999985264	0.995270463
COQ2	0.999996191	0.999992444	0.999558179	0.99999488	0.999985264	0.995270463
DNAJC4	0.999996191	0.999992444	0.999558179	0.99999488	0.999985264	0.995270463
ABR	0.999996191	0.938520118	0.924604843	0.99999488	0.999985264	0.995391635
E2F3	0.999996191	0.920768474	0.939147816	0.99999488	0.999985264	0.995391635
UBE4B	0.999996191	0.956801903	0.939147816	0.99999488	0.999985264	0.995391635
LOC785873	0.999996191	0.96728174	0.944085862	0.99999488	0.999985264	0.995391635
LPAR5	0.999996191	0.991488652	0.951735967	0.99999488	0.999985264	0.995391635
ITFG2	0.999996191	0.991488652	0.978389389	0.99999488	0.999985264	0.995391635
GPRC5B	0.999996191	0.999992444	0.9808801	0.99999488	0.999985264	0.995391635
ZNF576	0.999996191	0.995165487	0.981388276	0.99999488	0.999985264	0.995391635
SMIM17	0.999996191	0.999992444	0.981666445	0.99999488	0.999985264	0.995391635
TLNRD1	0.999996191	0.999992444	0.982216395	0.99999488	0.999985264	0.995391635
LOC100297420	0.999996191	0.998131745	0.98286145	0.99999488	0.999985264	0.995391635
CIB2	0.999996191	0.999992444	0.98480229	0.99999488	0.999985264	0.995391635
CCDC151	0.999996191	0.999992444	0.985363931	0.99999488	0.999985264	0.995391635
CMBL	0.999996191	0.999992444	0.987678394	0.99999488	0.999985264	0.995391635
CAMSAP1	0.999996191	0.999992444	0.993303055	0.99999488	0.999985264	0.995391635
EFEMP1	0.999996191	0.999992444	0.993594894	0.99999488	0.999985264	0.995391635
CYB5R1	0.999996191	0.999992444	0.994601277	0.99999488	0.999985264	0.995391635
VDAC1	0.999996191	0.999347049	0.994626276	0.99999488	0.999985264	0.995391635
NOP9	0.999996191	0.999992444	0.995045982	0.99999488	0.999985264	0.995391635
MINDY3	0.999996191	0.993316598	0.998936876	0.99999488	0.999985264	0.995391635
BBS1	0.999996191	0.999992444	0.998936876	0.99999488	0.999985264	0.995391635
KRTCAP2	0.999996191	0.999992444	0.998936876	0.99999488	0.999985264	0.995391635
PPWD1	0.999996191	0.999992444	0.998936876	0.99999488	0.999985264	0.995391635
RASGRP2	0.999996191	0.999992444	0.998936876	0.99999488	0.999985264	0.995391635
ALG1	0.999996191	0.999992444	0.999558179	0.99999488	0.999985264	0.995391635
ATG12	0.999996191	0.999992444	0.999558179	0.99999488	0.999985264	0.995391635
LOC107133075	0.999996191	0.999992444	0.999558179	0.99999488	0.999985264	0.995391635
RASSF7	0.999996191	0.999992444	0.999558179	0.99999488	0.999985264	0.995391635
ATG2B	0.999996191	0.999992444	0.985363931	0.99999488	0.999985264	0.995733391
IL27RA	0.999996191	0.999992444	0.988161083	0.99999488	0.999985264	0.995733391
REPIN1	0.999996191	0.87789341	0.875823795	0.99999488	0.999985264	0.996066257

IGF2BP2	0.999996191	0.89880506	0.890035877	0.99999488	0.999985264	0.996066257
FAM81A	0.999996191	0.992977597	0.898815524	0.99999488	0.999985264	0.996066257
SLC46A1	0.999996191	0.915367594	0.914630535	0.99999488	0.999985264	0.996066257
RAB6B	0.999996191	0.991488652	0.923604668	0.99999488	0.999985264	0.996066257
PSMD4	0.999996191	0.944868298	0.937874396	0.99999488	0.999985264	0.996066257
CPOX	0.999996191	0.958967142	0.938203102	0.99999488	0.999985264	0.996066257
GIGYF1	0.999996191	0.991488652	0.938203102	0.99999488	0.999985264	0.996066257
ZNF106	0.999996191	0.962968114	0.938905004	0.99999488	0.999985264	0.996066257
RMC1	0.999996191	0.985406717	0.939147816	0.99999488	0.999985264	0.996066257
ESRP2	0.999996191	0.999992444	0.939147816	0.99999488	0.999985264	0.996066257
ENO1	0.999996191	0.991488652	0.939501959	0.99999488	0.999985264	0.996066257
FAF1	0.999996191	0.921759937	0.941336964	0.99999488	0.999985264	0.996066257
HACD2	0.999996191	0.952346363	0.941336964	0.99999488	0.999985264	0.996066257
BRSK2	0.999996191	0.9527938	0.943440726	0.99999488	0.999985264	0.996066257
GPR176	0.999996191	0.978510121	0.944085862	0.99999488	0.999985264	0.996066257
LDLRAD4	0.999996191	0.991488652	0.944993804	0.99999488	0.999985264	0.996066257
LOC112449552	0.999996191	0.9582784	0.945766581	0.99999488	0.999985264	0.996066257
CNNM3	0.999996191	0.941495217	0.946479014	0.99999488	0.999985264	0.996066257
LOC104972045	0.999996191	0.992977597	0.948420301	0.99999488	0.999985264	0.996066257
SSU72	0.999996191	0.957700659	0.951565052	0.99999488	0.999985264	0.996066257
PLEKHO1	0.999996191	0.999992444	0.952547836	0.99999488	0.999985264	0.996066257
LOC104973551	0.999996191	0.999992444	0.954823586	0.99999488	0.999985264	0.996066257
MCCC1	0.999996191	0.977092593	0.956504686	0.99999488	0.999985264	0.996066257
TCEAL1	0.999996191	0.96566261	0.956565728	0.99999488	0.999985264	0.996066257
ABCD4	0.999996191	0.991488652	0.957117655	0.99999488	0.999985264	0.996066257
NPLOC4	0.999996191	0.963867256	0.960133832	0.99999488	0.999985264	0.996066257
FAM98B	0.999996191	0.999992444	0.965065403	0.99999488	0.999985264	0.996066257
RBM15	0.999996191	0.999992444	0.96551632	0.99999488	0.999985264	0.996066257
LOC112446740	0.999996191	0.991488652	0.96870883	0.99999488	0.999985264	0.996066257
FAM49A	0.999996191	0.985406717	0.969036336	0.99999488	0.999985264	0.996066257
CHAF1A	0.999996191	0.999992444	0.969656316	0.99999488	0.999985264	0.996066257
RPL17	0.999996191	0.999992444	0.969656316	0.99999488	0.999985264	0.996066257
CYTH1	0.999996191	0.999992444	0.96968839	0.99999488	0.999985264	0.996066257
NMNAT2	0.999996191	0.999992444	0.970945672	0.99999488	0.999985264	0.996066257
SLC25A35	0.999996191	0.991488652	0.971111634	0.99999488	0.999985264	0.996066257
LOC540707	0.999996191	0.999992444	0.973406453	0.99999488	0.999985264	0.996066257
ZNHIT6	0.999996191	0.991335402	0.976321912	0.99999488	0.999985264	0.996066257
VIM	0.999996191	0.937511653	0.978389389	0.99999488	0.999985264	0.996066257
SOGA1	0.999996191	0.998131745	0.978993134	0.99999488	0.999985264	0.996066257

XIRP1	0.999996191	0.999992444	0.980132104	0.99999488	0.999985264	0.996066257
HRH4	0.999996191	0.948421431	0.980677819	0.99999488	0.999985264	0.996066257
PKM	0.999996191	0.999992444	0.9808801	0.99999488	0.999985264	0.996066257
LOC783261	0.999996191	0.992977597	0.985363931	0.99999488	0.999985264	0.996066257
UNC50	0.999996191	0.999992444	0.985966356	0.99999488	0.999985264	0.996066257
ARF6	0.999996191	0.992977597	0.99035966	0.99999488	0.999985264	0.996066257
LOC100847363	0.999996191	0.999992444	0.99035966	0.99999488	0.999985264	0.996066257
CAMK2B	0.999996191	0.918883775	0.991285432	0.99999488	0.999985264	0.996066257
ALKBH2	0.999996191	0.999992444	0.992709008	0.99999488	0.999985264	0.996066257
APLN	0.999996191	0.999347049	0.993594894	0.99999488	0.999985264	0.996066257
SHB	0.999996191	0.999347049	0.993594894	0.99999488	0.999985264	0.996066257
ALDH2	0.999996191	0.999992444	0.993594894	0.99999488	0.999985264	0.996066257
CRAT	0.999996191	0.999992444	0.993594894	0.99999488	0.999985264	0.996066257
LOC112444626	0.999996191	0.999992444	0.993594894	0.99999488	0.999985264	0.996066257
LOC112447291	0.999996191	0.999992444	0.993594894	0.99999488	0.999985264	0.996066257
NCBP3	0.999996191	0.999992444	0.993594894	0.99999488	0.999985264	0.996066257
DLG2	0.999996191	0.999992444	0.995089012	0.99999488	0.999985264	0.996066257
LOC112446406	0.999996191	0.999992444	0.995089012	0.99999488	0.999985264	0.996066257
FNDC3B	0.999996191	0.999992444	0.996139177	0.99999488	0.999985264	0.996066257
GRIK5	0.999996191	0.992977597	0.996348351	0.99999488	0.999985264	0.996066257
ALG10	0.999996191	0.999992444	0.996348351	0.99999488	0.999985264	0.996066257
TRPC4	0.999996191	0.999992444	0.996348351	0.99999488	0.999985264	0.996066257
IKZF4	0.999996191	0.999992444	0.996391386	0.99999488	0.999985264	0.996066257
GPN2	0.999996191	0.999992444	0.996715234	0.99999488	0.999985264	0.996066257
PLXNA2	0.999996191	0.999992444	0.996715234	0.99999488	0.999985264	0.996066257
PPFIBP1	0.999996191	0.999992444	0.996715234	0.99999488	0.999985264	0.996066257
ODF2L	0.999996191	0.999992444	0.997859293	0.99999488	0.999985264	0.996066257
ZNF32	0.999996191	0.999992444	0.998344612	0.99999488	0.999985264	0.996066257
PPM1M	0.999996191	0.999992444	0.998816213	0.99999488	0.999985264	0.996066257
WASL	0.999996191	0.999992444	0.998816213	0.99999488	0.999985264	0.996066257
SGO1	0.999996191	0.991365955	0.998852661	0.99999488	0.999985264	0.996066257
GMEB1	0.999996191	0.999992444	0.998936876	0.99999488	0.999985264	0.996066257
GNRH2	0.999996191	0.999992444	0.998936876	0.99999488	0.999985264	0.996066257
LOC107132450	0.999996191	0.999992444	0.998936876	0.99999488	0.999985264	0.996066257
LOC112444635	0.999996191	0.999992444	0.998936876	0.99999488	0.999985264	0.996066257
LOC112447462	0.999996191	0.999992444	0.998936876	0.99999488	0.999985264	0.996066257
SCD5	0.999996191	0.999992444	0.998936876	0.99999488	0.999985264	0.996066257
TEAD4	0.999996191	0.999992444	0.998936876	0.99999488	0.999985264	0.996066257
TGFBRAP1	0.999996191	0.999992444	0.998936876	0.99999488	0.999985264	0.996066257

ZNF783	0.999996191	0.999992444	0.998936876	0.99999488	0.999985264	0.996066257
LOC112443431	0.999996191	0.978510121	0.999558179	0.99999488	0.999985264	0.996066257
PIGL	0.999996191	0.987516216	0.999558179	0.99999488	0.999985264	0.996066257
SMG7	0.999996191	0.996003806	0.999558179	0.99999488	0.999985264	0.996066257
ASB7	0.999996191	0.999992444	0.999558179	0.99999488	0.999985264	0.996066257
BUD13	0.999996191	0.999992444	0.999558179	0.99999488	0.999985264	0.996066257
DERL2	0.999996191	0.999992444	0.999558179	0.99999488	0.999985264	0.996066257
DRD2	0.999996191	0.999992444	0.999558179	0.99999488	0.999985264	0.996066257
DYNLL1	0.999996191	0.999992444	0.999558179	0.99999488	0.999985264	0.996066257
FKBP2	0.999996191	0.999992444	0.999558179	0.99999488	0.999985264	0.996066257
HARS2	0.999996191	0.999992444	0.999558179	0.99999488	0.999985264	0.996066257
LOC100847236	0.999996191	0.999992444	0.999558179	0.99999488	0.999985264	0.996066257
LOC104973382	0.999996191	0.999992444	0.999558179	0.99999488	0.999985264	0.996066257
LOC112444752	0.999996191	0.999992444	0.999558179	0.99999488	0.999985264	0.996066257
LOC112448773	0.999996191	0.999992444	0.999558179	0.99999488	0.999985264	0.996066257
METTL14	0.999996191	0.999992444	0.999558179	0.99999488	0.999985264	0.996066257
TGOLN2	0.999996191	0.999992444	0.999558179	0.99999488	0.999985264	0.996066257
VPS33A	0.999996191	0.999992444	0.999558179	0.99999488	0.999985264	0.996066257
LOC112449254	0.999996191	0.991488652	0.963068909	0.99999488	0.999985264	0.996113576
DPH5	0.999996191	0.991488652	0.922400936	0.99999488	0.999985264	0.99611491
ARRB1	0.999996191	0.969314233	0.938203102	0.99999488	0.999985264	0.99611491
AJUBA	0.999996191	0.978089982	0.95230212	0.99999488	0.999985264	0.99611491
CRLF3	0.999996191	0.999992444	0.983888817	0.99999488	0.999985264	0.99611491
DTWD2	0.999996191	0.984090956	0.985363931	0.99999488	0.999985264	0.99611491
DOCK1	0.999996191	0.999992444	0.985966356	0.99999488	0.999985264	0.99611491
BCLAF1	0.999996191	0.999992444	0.998936876	0.99999488	0.999985264	0.99611491
NTN5	0.999996191	0.999992444	0.998936876	0.99999488	0.999985264	0.99611491
ACTR10	0.999996191	0.999992444	0.999558179	0.99999488	0.999985264	0.99611491
LOC100297498	0.999996191	0.999992444	0.999558179	0.99999488	0.999985264	0.99611491
UBXN8	0.999996191	0.999992444	0.999558179	0.99999488	0.999985264	0.99611491
TBC1D10B	0.999996191	0.999992444	0.98480229	0.99999488	0.999985264	0.996135607
SDAD1	0.999996191	0.921264323	0.908181052	0.99999488	0.999985264	0.996156089
LOC112449602	0.999996191	0.993316598	0.914630535	0.99999488	0.999985264	0.996156089
LOC100300881	0.999996191	0.9582784	0.921379317	0.99999488	0.999985264	0.996156089
IP6K2	0.999996191	0.927447588	0.930678591	0.99999488	0.999985264	0.996156089
HDAC5	0.999996191	0.978154072	0.938203102	0.99999488	0.999985264	0.996156089
LOC782527	0.999996191	0.897451631	0.939147816	0.99999488	0.999985264	0.996156089
RNMT	0.999996191	0.968240168	0.94634922	0.99999488	0.999985264	0.996156089
MKS1	0.999996191	0.991365955	0.94634922	0.99999488	0.999985264	0.996156089

TMEM18	0.999996191	0.998011664	0.94634922	0.99999488	0.999985264	0.996156089
KLF13	0.999996191	0.999992444	0.951822586	0.99999488	0.999985264	0.996156089
LOC788414	0.999996191	0.981520287	0.95230212	0.99999488	0.999985264	0.996156089
CXCL9	0.999996191	0.999347049	0.954823586	0.99999488	0.999985264	0.996156089
PRIM1	0.999996191	0.994135588	0.956565728	0.99999488	0.999985264	0.996156089
PPIL1	0.999996191	0.960125322	0.957541703	0.99999488	0.999985264	0.996156089
LOC101903438	0.999996191	0.998131745	0.962142195	0.99999488	0.999985264	0.996156089
SLC1A7	0.999996191	0.999992444	0.962346588	0.99999488	0.999985264	0.996156089
DPF2	0.999996191	0.952639631	0.96290532	0.99999488	0.999985264	0.996156089
GORAB	0.999996191	0.991488652	0.963068909	0.99999488	0.999985264	0.996156089
NT5DC1	0.999996191	0.994753342	0.965065403	0.99999488	0.999985264	0.996156089
VAPB	0.999996191	0.967374613	0.969880768	0.99999488	0.999985264	0.996156089
LOC112444473	0.999996191	0.999992444	0.971967312	0.99999488	0.999985264	0.996156089
ALDOA	0.999996191	0.993316598	0.973364552	0.99999488	0.999985264	0.996156089
MYH14	0.999996191	0.997975376	0.973406453	0.99999488	0.999985264	0.996156089
SPNS1	0.999996191	0.999992444	0.980677819	0.99999488	0.999985264	0.996156089
TTLL7	0.999996191	0.999347049	0.980859619	0.99999488	0.999985264	0.996156089
PLXNB1	0.999996191	0.999992444	0.9808801	0.99999488	0.999985264	0.996156089
PXYLP1	0.999996191	0.999992444	0.9808801	0.99999488	0.999985264	0.996156089
RTN4R	0.999996191	0.993316598	0.981039752	0.99999488	0.999985264	0.996156089
EPN3	0.999996191	0.999347049	0.981039752	0.99999488	0.999985264	0.996156089
UHMK1	0.999996191	0.991488652	0.981218677	0.99999488	0.999985264	0.996156089
SNRPB2	0.999996191	0.938597964	0.982085804	0.99999488	0.999985264	0.996156089
LOC112444520	0.999996191	0.999992444	0.984444908	0.99999488	0.999985264	0.996156089
PBX1	0.999996191	0.999992444	0.985363931	0.99999488	0.999985264	0.996156089
VCP	0.999996191	0.999992444	0.985363931	0.99999488	0.999985264	0.996156089
LOC107132532	0.999996191	0.999992444	0.986180419	0.99999488	0.999985264	0.996156089
RACK1	0.999996191	0.999992444	0.987435853	0.99999488	0.999985264	0.996156089
LOC112441834	0.999996191	0.999992444	0.992709008	0.99999488	0.999985264	0.996156089
COLEC10	0.999996191	0.999992444	0.993594894	0.99999488	0.999985264	0.996156089
NIM1K	0.999996191	0.999992444	0.993594894	0.99999488	0.999985264	0.996156089
TMTC3	0.999996191	0.999992444	0.996348351	0.99999488	0.999985264	0.996156089
MAPRE3	0.999996191	0.999347049	0.996715234	0.99999488	0.999985264	0.996156089
TSPYL2	0.999996191	0.999992444	0.998344612	0.99999488	0.999985264	0.996156089
TMEM50B	0.999996191	0.997051616	0.998936876	0.99999488	0.999985264	0.996156089
MTMR10	0.999996191	0.999992444	0.998936876	0.99999488	0.999985264	0.996156089
PRPF38A	0.999996191	0.999992444	0.998936876	0.99999488	0.999985264	0.996156089
VPS33B	0.999996191	0.999992444	0.998936876	0.99999488	0.999985264	0.996156089
WDR17	0.999996191	0.992977597	0.999558179	0.99999488	0.999985264	0.996156089

LOC112446882	0.999996191	0.998131745	0.999558179	0.99999488	0.999985264	0.996156089
ERCC4	0.999996191	0.999347049	0.999558179	0.99999488	0.999985264	0.996156089
BUD31	0.999996191	0.999992444	0.999558179	0.99999488	0.999985264	0.996156089
C9H6orf203	0.999996191	0.999992444	0.999558179	0.99999488	0.999985264	0.996156089
LOC104976078	0.999996191	0.999992444	0.999558179	0.99999488	0.999985264	0.996156089
LOC112442805	0.999996191	0.999992444	0.999558179	0.99999488	0.999985264	0.996156089
PAFAH1B2	0.999996191	0.999992444	0.999558179	0.99999488	0.999985264	0.996156089
SAP30	0.999996191	0.999992444	0.999558179	0.99999488	0.999985264	0.996156089
PLSCR2	0.999996191	0.999992444	0.979462526	0.99999488	0.999985264	0.996313654
SDR42E1	0.999996191	0.999992444	0.991992782	0.99999488	0.999985264	0.996313654
NUP43	0.999996191	0.999992444	0.995045982	0.99999488	0.999985264	0.996313654
C24H18orf54	0.999996191	0.999992444	0.996715234	0.99999488	0.999985264	0.996313654
DNAH1	0.999996191	0.984068885	0.999558179	0.99999488	0.999985264	0.996313654
NTM	0.999996191	0.999992444	0.999558179	0.99999488	0.999985264	0.996313654
OTUD4	0.999996191	0.999992444	0.998936876	0.99999488	0.999985264	0.996448346
POMP	0.999996191	0.999992444	0.999558179	0.99999488	0.999985264	0.996448346
FBXO10	0.999996191	0.981155176	0.970945672	0.99999488	0.999985264	0.996517132
ATXN7L3B	0.999996191	0.998962836	0.999558179	0.99999488	0.999985264	0.996517132
MAP4	0.999996191	0.999992444	0.992047025	0.99999488	0.999985264	0.996659814
LOC107132192	0.999996191	0.999992444	0.998936876	0.99999488	0.999985264	0.996659814
LSM12	0.999996191	0.938777693	0.9840454	0.99999488	0.999985264	0.996758258
B3GNT9	0.999996191	0.999347049	0.970882851	0.99999488	0.999985264	0.996779387
FAM120C	0.999996191	0.999992444	0.995045982	0.99999488	0.999985264	0.996779387
LOC112443144	0.999996191	0.991488652	0.938203102	0.99999488	0.999985264	0.996792698
LOC528802	0.999996191	0.975462313	0.910748743	0.99999488	0.999985264	0.996887749
ENOSF1	0.999996191	0.958224615	0.935094475	0.99999488	0.999985264	0.996887749
PYGB	0.999996191	0.999992444	0.972381338	0.99999488	0.999985264	0.996887749
LOC100847831	0.999996191	0.990559471	0.973406453	0.99999488	0.999985264	0.996887749
LOC100295130	0.999996191	0.999992444	0.981388276	0.99999488	0.999985264	0.996887749
MORN2	0.999996191	0.995532499	0.998936876	0.99999488	0.999985264	0.996887749
ACBD5	0.999996191	0.999992444	0.998936876	0.99999488	0.999985264	0.996887749
LOC112442244	0.999996191	0.999992444	0.998936876	0.99999488	0.999985264	0.996887749
UBE2O	0.999996191	0.999992444	0.998936876	0.99999488	0.999985264	0.996887749
C25H7orf26	0.999996191	0.999992444	0.999558179	0.99999488	0.999985264	0.996887749
P2RY2	0.999996191	0.938597964	0.886192181	0.99999488	0.999985264	0.996929482
PSMB3	0.999996191	0.984068885	0.938203102	0.99999488	0.999985264	0.996929482
RNLS	0.999996191	0.952346363	0.941147648	0.99999488	0.999985264	0.996929482
LOC104974891	0.999996191	0.979263532	0.941336964	0.99999488	0.999985264	0.996929482
CIAPIN1	0.999996191	0.991488652	0.944085862	0.99999488	0.999985264	0.996929482

GALNT15	0.999996191	0.937626884	0.945678616	0.99999488	0.999985264	0.996929482
RNF25	0.999996191	0.993316598	0.95230212	0.99999488	0.999985264	0.996929482
SMG6	0.999996191	0.994365411	0.95230212	0.99999488	0.999985264	0.996929482
MEIOB	0.999996191	0.999347049	0.963233239	0.99999488	0.999985264	0.996929482
JPH4	0.999996191	0.999992444	0.964152013	0.99999488	0.999985264	0.996929482
C19H17orf100	0.999996191	0.992977597	0.965059328	0.99999488	0.999985264	0.996929482
TATDN2	0.999996191	0.999347049	0.968325332	0.99999488	0.999985264	0.996929482
ARL13B	0.999996191	0.999992444	0.970874203	0.99999488	0.999985264	0.996929482
PLEKHG5	0.999996191	0.998131745	0.973303821	0.99999488	0.999985264	0.996929482
TNFSF4	0.999996191	0.992977597	0.973406453	0.99999488	0.999985264	0.996929482
LOC101904520	0.999996191	0.999992444	0.973406453	0.99999488	0.999985264	0.996929482
LOC509155	0.999996191	0.999992444	0.979441303	0.99999488	0.999985264	0.996929482
TOR1B	0.999996191	0.974079639	0.9808801	0.99999488	0.999985264	0.996929482
DGKE	0.999996191	0.999347049	0.9808801	0.99999488	0.999985264	0.996929482
SLC33A1	0.999996191	0.977007804	0.981039752	0.99999488	0.999985264	0.996929482
TRIM23	0.999996191	0.999347049	0.981039752	0.99999488	0.999985264	0.996929482
PSMD13	0.999996191	0.999992444	0.981039752	0.99999488	0.999985264	0.996929482
CDKN2C	0.999996191	0.999992444	0.981622418	0.99999488	0.999985264	0.996929482
ERCC2	0.999996191	0.992977597	0.988922732	0.99999488	0.999985264	0.996929482
FAM50A	0.999996191	0.999992444	0.988922732	0.99999488	0.999985264	0.996929482
HBA	0.999996191	0.999992444	0.991285432	0.99999488	0.999985264	0.996929482
LOC112448364	0.999996191	0.999992444	0.994626276	0.99999488	0.999985264	0.996929482
FOXL1	0.999996191	0.999992444	0.995522557	0.99999488	0.999985264	0.996929482
METTL7A	0.999996191	0.999992444	0.996139177	0.99999488	0.999985264	0.996929482
PPFIA4	0.999996191	0.999992444	0.996348351	0.99999488	0.999985264	0.996929482
ACADVL	0.999996191	0.991488652	0.997230633	0.99999488	0.999985264	0.996929482
MTMR9	0.999996191	0.994434609	0.998936876	0.99999488	0.999985264	0.996929482
LOC101904187	0.999996191	0.999347049	0.998936876	0.99999488	0.999985264	0.996929482
MVB12A	0.999996191	0.999992444	0.998936876	0.99999488	0.999985264	0.996929482
PKDREJ	0.999996191	0.999992444	0.998936876	0.99999488	0.999985264	0.996929482
SFT2D1	0.999996191	0.999992444	0.998936876	0.99999488	0.999985264	0.996929482
HIRIP3	0.999996191	0.998131745	0.999558179	0.99999488	0.999985264	0.996929482
ACE	0.999996191	0.999992444	0.999558179	0.99999488	0.999985264	0.996929482
AXIN1	0.999996191	0.999992444	0.999558179	0.99999488	0.999985264	0.996929482
LOC104970145	0.999996191	0.999992444	0.999558179	0.99999488	0.999985264	0.996929482
RBM7	0.999996191	0.999992444	0.999558179	0.99999488	0.999985264	0.996929482
REXO1	0.999996191	0.999992444	0.999558179	0.99999488	0.999985264	0.996929482
SLC25A37	0.999996191	0.999992444	0.999558179	0.99999488	0.999985264	0.996929482
SSBP4	0.999996191	0.999992444	0.999558179	0.99999488	0.999985264	0.996929482

PYGL	0.999996191	0.991488652	0.927600835	0.99999488	0.999985264	0.996947451
POLR1C	0.999996191	0.955266582	0.933992957	0.99999488	0.999985264	0.996947451
CALCOCO2	0.999996191	0.938597964	0.908572237	0.99999488	0.999985264	0.996949674
PJA1	0.999996191	0.926521501	0.929207633	0.99999488	0.999985264	0.996949674
CSTF1	0.999996191	0.89880506	0.953457715	0.99999488	0.999985264	0.996949674
TANGO6	0.999996191	0.998495474	0.958627535	0.99999488	0.999985264	0.996949674
LOC100847609	0.999996191	0.993316598	0.973406453	0.99999488	0.999985264	0.996949674
MPLKIP	0.999996191	0.998131745	0.981388276	0.99999488	0.999985264	0.996949674
ABCB9	0.999996191	0.999992444	0.981622418	0.99999488	0.999985264	0.996949674
TRIM4	0.999996191	0.996680853	0.981666445	0.99999488	0.999985264	0.996949674
LOC104972830	0.999996191	0.999992444	0.991285432	0.99999488	0.999985264	0.996949674
LOC112445033	0.999996191	0.999992444	0.993594894	0.99999488	0.999985264	0.996949674
CDKN2AIPNL	0.999996191	0.999992444	0.996391386	0.99999488	0.999985264	0.996949674
C14H8orf89	0.999996191	0.999992444	0.997208738	0.99999488	0.999985264	0.996949674
FAM166A	0.999996191	0.999992444	0.997859293	0.99999488	0.999985264	0.996949674
TUBB3	0.999996191	0.999992444	0.998936876	0.99999488	0.999985264	0.996949674
ACVRL1	0.999996191	0.999992444	0.999558179	0.99999488	0.999985264	0.996949674
DSEL	0.999996191	0.999992444	0.999558179	0.99999488	0.999985264	0.996949674
LOC512978	0.999996191	0.999992444	0.999558179	0.99999488	0.999985264	0.996949674
RASGRF2	0.999996191	0.999992444	0.999558179	0.99999488	0.999985264	0.996949674
RNF44	0.999996191	0.999992444	0.999558179	0.99999488	0.999985264	0.996949674
LOC101906512	0.999996191	0.921549	0.983077441	0.99999488	0.999985264	0.996999374
SEPT11	0.999996191	0.999992444	0.990216356	0.99999488	0.999985264	0.996999374
ZC3H3	0.999996191	0.999992444	0.998936876	0.99999488	0.999985264	0.996999374
NUBPL	0.999996191	0.999992444	0.985966356	0.99999488	0.999985264	0.99715856
MAMSTR	0.999996191	0.92462339	0.876188482	0.99999488	0.999985264	0.997278082
LMF1	0.999996191	0.926955605	0.95106368	0.99999488	0.999985264	0.997419529
CST7	0.999996191	0.999992444	0.987678394	0.99999488	0.999985264	0.997419529
LOC101905033	0.999996191	0.999992444	0.992742113	0.99999488	0.999985264	0.997419529
LOC510613	0.999996191	0.999992444	0.996391386	0.99999488	0.999985264	0.997419529
UBB	0.999996191	0.999992444	0.996715234	0.99999488	0.999985264	0.997419529
PDHA1	0.999996191	0.999992444	0.998936876	0.99999488	0.999985264	0.997419529
SLC35D2	0.999996191	0.999992444	0.998936876	0.99999488	0.999985264	0.997419529
XYLT1	0.999996191	0.999992444	0.981736235	0.99999488	0.999985264	0.997580124
ST3GAL2	0.999996191	0.999992444	0.998936876	0.99999488	0.999985264	0.997580124
CSNK1E	0.999996191	0.992977597	0.880229444	0.99999488	0.999985264	0.997713313
ZC3H15	0.999996191	0.986445172	0.932657945	0.99999488	0.999985264	0.997713313
LRFN1	0.999996191	0.968438183	0.938203102	0.99999488	0.999985264	0.997713313
LOC101902141	0.999996191	0.957252213	0.946479014	0.99999488	0.999985264	0.997713313

RCBTB1	0.999996191	0.980496345	0.946479014	0.99999488	0.999985264	0.997713313
LOC101904891	0.999996191	0.993316598	0.946479014	0.99999488	0.999985264	0.997713313
C21H14orf28	0.999996191	0.999992444	0.948359914	0.99999488	0.999985264	0.997713313
CTIF	0.999996191	0.979756265	0.95106368	0.99999488	0.999985264	0.997713313
WDR27	0.999996191	0.967276215	0.95230212	0.99999488	0.999985264	0.997713313
CHCHD1	0.999996191	0.987262615	0.964186888	0.99999488	0.999985264	0.997713313
LOC101902174	0.999996191	0.999992444	0.964920281	0.99999488	0.999985264	0.997713313
FAM25A	0.999996191	0.992895985	0.96684887	0.99999488	0.999985264	0.997713313
F12	0.999996191	0.997051616	0.970945672	0.99999488	0.999985264	0.997713313
CITED4	0.999996191	0.992977597	0.973406453	0.99999488	0.999985264	0.997713313
SMYD2	0.999996191	0.999992444	0.976056868	0.99999488	0.999985264	0.997713313
LOC101908204	0.999996191	0.999992444	0.9808801	0.99999488	0.999985264	0.997713313
ZCCHC4	0.999996191	0.999992444	0.981039752	0.99999488	0.999985264	0.997713313
JPH1	0.999996191	0.999992444	0.981388276	0.99999488	0.999985264	0.997713313
GGT7	0.999996191	0.999992444	0.98286145	0.99999488	0.999985264	0.997713313
RTF2	0.999996191	0.993316598	0.985363931	0.99999488	0.999985264	0.997713313
RBM11	0.999996191	0.999992444	0.985789565	0.99999488	0.999985264	0.997713313
FAU	0.999996191	0.999992444	0.987678394	0.99999488	0.999985264	0.997713313
PGAM1	0.999996191	0.998181556	0.991285432	0.99999488	0.999985264	0.997713313
PRR5	0.999996191	0.999992444	0.992709008	0.99999488	0.999985264	0.997713313
PLPP5	0.999996191	0.999992444	0.993594894	0.99999488	0.999985264	0.997713313
LOC101907503	0.999996191	0.999992444	0.995045982	0.99999488	0.999985264	0.997713313
LPAR1	0.999996191	0.999992444	0.995522557	0.99999488	0.999985264	0.997713313
MRPL50	0.999996191	0.985406717	0.996391386	0.99999488	0.999985264	0.997713313
B3GNT2	0.999996191	0.999992444	0.997859293	0.99999488	0.999985264	0.997713313
AAMDC	0.999996191	0.999992444	0.998936876	0.99999488	0.999985264	0.997713313
AHDC1	0.999996191	0.999992444	0.998936876	0.99999488	0.999985264	0.997713313
RAD18	0.999996191	0.999992444	0.998936876	0.99999488	0.999985264	0.997713313
SLCO3A1	0.999996191	0.999992444	0.998936876	0.99999488	0.999985264	0.997713313
CCDC59	0.999996191	0.998011664	0.999558179	0.99999488	0.999985264	0.997713313
COMMD1	0.999996191	0.999992444	0.999558179	0.99999488	0.999985264	0.997713313
FYCO1	0.999996191	0.999992444	0.999558179	0.99999488	0.999985264	0.997713313
GPR180	0.999996191	0.999992444	0.999558179	0.99999488	0.999985264	0.997713313
PDCD6IP	0.999996191	0.999992444	0.999558179	0.99999488	0.999985264	0.997713313
CST3	0.999996191	0.999992444	0.999558179	0.99999488	0.999985264	0.997837162
MTERF4	0.999996191	0.956531374	0.922400936	0.99999488	0.999985264	0.997898038
SESTD1	0.999996191	0.992977597	0.96684887	0.99999488	0.999985264	0.997898038
GTF2I	0.999996191	0.998131745	0.9808801	0.99999488	0.999985264	0.997898038
PDCD2	0.999996191	0.999992444	0.994601277	0.99999488	0.999985264	0.997898038

STRN	0.999996191	0.999992444	0.999558179	0.99999488	0.999985264	0.997898038
ZFP3	0.999996191	0.999992444	0.999558179	0.99999488	0.999985264	0.997898038
RCBTB2	0.999996191	0.998011664	0.979462526	0.99999488	0.999985264	0.998016928
LOC112446007	0.999996191	0.999992444	0.996138614	0.99999488	0.999985264	0.998084571
CMTR1	0.999996191	0.999992444	0.998816213	0.99999488	0.999985264	0.99813111
C18H19orf47	0.999996191	0.943802122	0.945678616	0.99999488	0.999985264	0.998327828
CDC23	0.999996191	0.991574265	0.972381338	0.99999488	0.999985264	0.998327828
CDC14B	0.999996191	0.987516216	0.875823795	0.99999488	0.999985264	0.998331177
ARRDC3	0.999996191	0.935742738	0.901758346	0.99999488	0.999985264	0.998331177
SAMD10	0.999996191	0.991365955	0.923902957	0.99999488	0.999985264	0.998331177
TMPPE	0.999996191	0.943802122	0.927600835	0.99999488	0.999985264	0.998331177
OPRL1	0.999996191	0.999992444	0.940815039	0.99999488	0.999985264	0.998331177
MRPS17	0.999996191	0.978510121	0.948359914	0.99999488	0.999985264	0.998331177
SEC24C	0.999996191	0.958967142	0.948420301	0.99999488	0.999985264	0.998331177
YIPF5	0.999996191	0.991488652	0.958627535	0.99999488	0.999985264	0.998331177
CADM3	0.999996191	0.999992444	0.96210311	0.99999488	0.999985264	0.998331177
IGBP1	0.999996191	0.991365955	0.962777771	0.99999488	0.999985264	0.998331177
RAPH1	0.999996191	0.999992444	0.9639767	0.99999488	0.999985264	0.998331177
PHACTR3	0.999996191	0.993316598	0.964594039	0.99999488	0.999985264	0.998331177
LOC101903400	0.999996191	0.999992444	0.964920281	0.99999488	0.999985264	0.998331177
ZNF865	0.999996191	0.999992444	0.964920281	0.99999488	0.999985264	0.998331177
MFSD5	0.999996191	0.999992444	0.968235535	0.99999488	0.999985264	0.998331177
LOC613401	0.999996191	0.993100184	0.968841179	0.99999488	0.999985264	0.998331177
SIK3	0.999996191	0.991476708	0.969880768	0.99999488	0.999985264	0.998331177
ZNF358	0.999996191	0.999347049	0.969880768	0.99999488	0.999985264	0.998331177
ARFGEF3	0.999996191	0.991365955	0.970903823	0.99999488	0.999985264	0.998331177
C10H14orf93	0.999996191	0.992977597	0.978389389	0.99999488	0.999985264	0.998331177
ABCG2	0.999996191	0.991488652	0.979462526	0.99999488	0.999985264	0.998331177
TRAF2	0.999996191	0.998131745	0.9808801	0.99999488	0.999985264	0.998331177
FRMPD3	0.999996191	0.999992444	0.9808801	0.99999488	0.999985264	0.998331177
SSBP2	0.999996191	0.99398773	0.981039752	0.99999488	0.999985264	0.998331177
TRPC3	0.999996191	0.999992444	0.981039752	0.99999488	0.999985264	0.998331177
LOC101905786	0.999996191	0.999992444	0.98286145	0.99999488	0.999985264	0.998331177
PLPP2	0.999996191	0.999992444	0.983938648	0.99999488	0.999985264	0.998331177
PAIP1	0.999996191	0.987516216	0.985521458	0.99999488	0.999985264	0.998331177
FAAP100	0.999996191	0.999992444	0.986180419	0.99999488	0.999985264	0.998331177
CLEC3B	0.999996191	0.999992444	0.989872445	0.99999488	0.999985264	0.998331177
BIVM	0.999996191	0.999992444	0.992742113	0.99999488	0.999985264	0.998331177
TLCD1	0.999996191	0.999347049	0.993594894	0.99999488	0.999985264	0.998331177

RFX2	0.999996191	0.999992444	0.994626276	0.99999488	0.999985264	0.998331177
ACVR1C	0.999996191	0.999992444	0.995045982	0.99999488	0.999985264	0.998331177
COX16	0.999996191	0.999992444	0.995045982	0.99999488	0.999985264	0.998331177
VPS54	0.999996191	0.999992444	0.995045982	0.99999488	0.999985264	0.998331177
HSPA14	0.999996191	0.999992444	0.996139177	0.99999488	0.999985264	0.998331177
LOC112446716	0.999996191	0.999992444	0.996391386	0.99999488	0.999985264	0.998331177
RPL36AL	0.999996191	0.999992444	0.996715234	0.99999488	0.999985264	0.998331177
APOOL	0.999996191	0.999992444	0.998816213	0.99999488	0.999985264	0.998331177
SNAPC3	0.999996191	0.999992444	0.998816213	0.99999488	0.999985264	0.998331177
LOC112443007	0.999996191	0.999992444	0.998852661	0.99999488	0.999985264	0.998331177
PHKG2	0.999996191	0.999992444	0.998852661	0.99999488	0.999985264	0.998331177
LOC104975286	0.999996191	0.991574265	0.998936876	0.99999488	0.999985264	0.998331177
PRDM16	0.999996191	0.992977597	0.998936876	0.99999488	0.999985264	0.998331177
LOC112445041	0.999996191	0.998131745	0.998936876	0.99999488	0.999985264	0.998331177
BPGM	0.999996191	0.999992444	0.998936876	0.99999488	0.999985264	0.998331177
CETN3	0.999996191	0.999992444	0.998936876	0.99999488	0.999985264	0.998331177
CLCF1	0.999996191	0.999992444	0.998936876	0.99999488	0.999985264	0.998331177
DNPH1	0.999996191	0.999992444	0.998936876	0.99999488	0.999985264	0.998331177
DUSP22	0.999996191	0.999992444	0.998936876	0.99999488	0.999985264	0.998331177
GTF2A1	0.999996191	0.999992444	0.998936876	0.99999488	0.999985264	0.998331177
LOC100849023	0.999996191	0.999992444	0.998936876	0.99999488	0.999985264	0.998331177
RAB3IL1	0.999996191	0.999992444	0.998936876	0.99999488	0.999985264	0.998331177
ZNF180	0.999996191	0.999992444	0.998936876	0.99999488	0.999985264	0.998331177
BECN1	0.999996191	0.999992444	0.999215963	0.99999488	0.999985264	0.998331177
SLC26A1	0.999996191	0.985293257	0.999558179	0.99999488	0.999985264	0.998331177
TIMELESS	0.999996191	0.991365955	0.999558179	0.99999488	0.999985264	0.998331177
SMIM19	0.999996191	0.998131745	0.999558179	0.99999488	0.999985264	0.998331177
ERCC8	0.999996191	0.999992444	0.999558179	0.99999488	0.999985264	0.998331177
ERICH1	0.999996191	0.999992444	0.999558179	0.99999488	0.999985264	0.998331177
KLHL18	0.999996191	0.999992444	0.999558179	0.99999488	0.999985264	0.998331177
LINGO1	0.999996191	0.999992444	0.999558179	0.99999488	0.999985264	0.998331177
LOC100335190	0.999996191	0.999992444	0.999558179	0.99999488	0.999985264	0.998331177
LOC100847118	0.999996191	0.999992444	0.999558179	0.99999488	0.999985264	0.998331177
LOC112442704	0.999996191	0.999992444	0.999558179	0.99999488	0.999985264	0.998331177
LOC112448511	0.999996191	0.999992444	0.999558179	0.99999488	0.999985264	0.998331177
PLA2G15	0.999996191	0.999992444	0.999558179	0.99999488	0.999985264	0.998331177
TG	0.999996191	0.999992444	0.999558179	0.99999488	0.999985264	0.998331177
XRCC6	0.999996191	0.999992444	0.999558179	0.99999488	0.999985264	0.998331177
ZNF282	0.999996191	0.999992444	0.999558179	0.99999488	0.999985264	0.998331177

SIGLECL1	0.999996191	0.991488652	0.96210311	0.99999488	0.999985264	0.998401565
OTUD7B	0.999996191	0.999992444	0.999558179	0.99999488	0.999985264	0.998401565
ORC1	0.999996191	0.999347049	0.981218677	0.99999488	0.999985264	0.998411916
SLC52A2	0.999996191	0.999992444	0.999558179	0.99999488	0.999985264	0.998472552
HS2ST1	0.999996191	0.999992444	0.998936876	0.99999488	0.999985264	0.998486813
TPT1	0.999996191	0.993316598	0.973303821	0.99999488	0.999985264	0.998542595
LOC101903056	0.999996191	0.999992444	0.999558179	0.99999488	0.999985264	0.998555988
LRIG2	0.999996191	0.999992444	0.947565182	0.99999488	0.999985264	0.998556965
RPL24	0.999996191	0.985406717	0.957117655	0.99999488	0.999985264	0.998556965
AOC1	0.999996191	0.998131745	0.976475354	0.99999488	0.999985264	0.998556965
TNK2	0.999996191	0.999992444	0.982216395	0.99999488	0.999985264	0.998556965
NME6	0.999996191	0.999992444	0.985363931	0.99999488	0.999985264	0.998556965
OR51E1	0.999996191	0.999992444	0.985363931	0.99999488	0.999985264	0.998556965
AMACR	0.999996191	0.999992444	0.992337371	0.99999488	0.999985264	0.998556965
LOC112446775	0.999996191	0.999992444	0.993132892	0.99999488	0.999985264	0.998556965
LOC100138933	0.999996191	0.999992444	0.998936876	0.99999488	0.999985264	0.998556965
MYH6	0.999996191	0.999992444	0.998936876	0.99999488	0.999985264	0.998556965
QRICH1	0.999996191	0.999992444	0.998936876	0.99999488	0.999985264	0.998556965
ABCB7	0.999996191	0.999992444	0.999558179	0.99999488	0.999985264	0.998556965
GTF3C2	0.999996191	0.999992444	0.999558179	0.99999488	0.999985264	0.998556965
TMEM179B	0.999996191	0.999992444	0.999558179	0.99999488	0.999985264	0.998556965
TRIB1	0.999996191	0.936273761	0.872386998	0.99999488	0.999985264	0.998568387
LOC104971464	0.999996191	0.999992444	0.956565728	0.99999488	0.999985264	0.999010389
CHST14	0.999996191	0.999992444	0.998852661	0.99999488	0.999985264	0.999010389
GINS3	0.999996191	0.999992444	0.999558179	0.99999488	0.999985264	0.999027354
CTSD	0.999996191	0.999992444	0.998936876	0.99999488	0.999985264	0.999352226
LOC512440	0.999996191	0.999992444	0.999558179	0.99999488	0.999985264	0.999352226
BCL9	0.999996191	0.978089982	0.964152013	0.99999488	0.999985264	0.99939885
SRP54	0.999996191	0.99398773	0.9808801	0.99999488	0.999985264	0.99939885
BRCC3	0.999996191	0.999992444	0.998936876	0.99999488	0.999985264	0.99939885
COL11A2	0.999996191	0.999992444	0.998936876	0.99999488	0.999985264	0.999403993
ATP2C2	0.999996191	0.999992444	0.999558179	0.99999488	0.999985264	0.999403993
NXPE2	0.999996191	0.999347049	0.964920281	0.99999488	0.999985264	0.999412387
LOC104973803	0.999996191	0.999992444	0.992939335	0.99999488	0.999985264	0.999412387
PLAGL2	0.999996191	0.999992444	0.993594894	0.99999488	0.999985264	0.999412387
DDX28	0.999996191	0.999992444	0.999558179	0.99999488	0.999985264	0.999412387
RTN4IP1	0.999996191	0.999992444	0.999558179	0.99999488	0.999985264	0.999412387
TLR5	0.999996191	0.938520118	0.920509464	0.99999488	0.999985264	0.999422061
C11H2orf49	0.999996191	0.999992444	0.972381338	0.99999488	0.999985264	0.999422061

WDR89	0.999996191	0.941495217	0.923604668	0.99999488	0.999985264	0.999814909
LOC783255	0.999996191	0.998408496	0.988922732	0.99999488	0.999985264	0.999814909
GDPD5	0.999996191	0.993540482	0.996070666	0.99999488	0.999985264	0.999814909
OCIAD2	0.999996191	0.981298804	0.998936876	0.99999488	0.999985264	0.999874845
LOC112444867	0.999996191	0.999992444	0.999558179	0.99999488	0.999985264	0.999874845
EEF1A1	0.999996191	0.999992444	0.991331566	0.99999488	0.999985264	0.99990455
OVCA2	0.999996191	0.892008332	0.874136672	0.99999488	0.999985264	0.999960414
CHKA	0.999996191	0.932679143	0.877799478	0.99999488	0.999985264	0.999960414
AP2A2	0.999996191	0.946274461	0.877799478	0.99999488	0.999985264	0.999960414
RETSAT	0.999996191	0.925580392	0.880594299	0.99999488	0.999985264	0.999960414
TMCO1	0.999996191	0.921759937	0.884407415	0.99999488	0.999985264	0.999960414
SNRNP35	0.999996191	0.978089982	0.884407415	0.99999488	0.999985264	0.999960414
TEF	0.999996191	0.9582784	0.886192181	0.99999488	0.999985264	0.999960414
STON2	0.999996191	0.912485935	0.887626998	0.99999488	0.999985264	0.999960414
RAD1	0.999996191	0.9582784	0.887626998	0.99999488	0.999985264	0.999960414
B3GALT2	0.999996191	0.89880506	0.888545123	0.99999488	0.999985264	0.999960414
ASNS	0.999996191	0.938597964	0.89029356	0.99999488	0.999985264	0.999960414
ST6GALNAC3	0.999996191	0.915367594	0.894973732	0.99999488	0.999985264	0.999960414
DACT2	0.999996191	0.921550129	0.895644023	0.99999488	0.999985264	0.999960414
DNAI1	0.999996191	0.935742738	0.896177624	0.99999488	0.999985264	0.999960414
RAB19	0.999996191	0.921934673	0.898815524	0.99999488	0.999985264	0.999960414
ITGAV	0.999996191	0.916036681	0.899576888	0.99999488	0.999985264	0.999960414
LOC101902998	0.999996191	0.961479426	0.901414204	0.99999488	0.999985264	0.999960414
LOC107131424	0.999996191	0.946274461	0.902527616	0.99999488	0.999985264	0.999960414
LOC112447309	0.999996191	0.991488652	0.902527616	0.99999488	0.999985264	0.999960414
EIF2A	0.999996191	0.918952092	0.906584165	0.99999488	0.999985264	0.999960414
PIGB	0.999996191	0.938597964	0.907381775	0.99999488	0.999985264	0.999960414
STIM1	0.999996191	0.955128586	0.907381775	0.99999488	0.999985264	0.999960414
ENO2	0.999996191	0.991488652	0.907381775	0.99999488	0.999985264	0.999960414
LOC101902361	0.999996191	0.991488652	0.907381775	0.99999488	0.999985264	0.999960414
LPCAT3	0.999996191	0.921549	0.909383349	0.99999488	0.999985264	0.999960414
RARG	0.999996191	0.958967142	0.910426741	0.99999488	0.999985264	0.999960414
FAM214A	0.999996191	0.991671026	0.912008683	0.99999488	0.999985264	0.999960414
EIF3I	0.999996191	0.904695096	0.912511938	0.99999488	0.999985264	0.999960414
LOC101904691	0.999996191	0.893743321	0.913211244	0.99999488	0.999985264	0.999960414
TRMT10B	0.999996191	0.881001912	0.914001994	0.99999488	0.999985264	0.999960414
FAM13B	0.999996191	0.927072093	0.914001994	0.99999488	0.999985264	0.999960414
LOC112447858	0.999996191	0.9582784	0.914001994	0.99999488	0.999985264	0.999960414
CLMN	0.999996191	0.974407708	0.914001994	0.99999488	0.999985264	0.999960414

DMKN	0.999996191	0.936392293	0.915266146	0.99999488	0.999985264	0.999960414
BEND5	0.999996191	0.921934673	0.916021503	0.99999488	0.999985264	0.999960414
CGGBP1	0.999996191	0.978510121	0.916975216	0.99999488	0.999985264	0.999960414
TMEM229B	0.999996191	0.984068885	0.917191523	0.99999488	0.999985264	0.999960414
APOL3	0.999996191	0.999992444	0.919618001	0.99999488	0.999985264	0.999960414
MRGPRF	0.999996191	0.97015318	0.919763144	0.99999488	0.999985264	0.999960414
CHRDL1	0.999996191	0.90116492	0.920154807	0.99999488	0.999985264	0.999960414
ANKRD6	0.999996191	0.935742738	0.920629231	0.99999488	0.999985264	0.999960414
ZPBP	0.999996191	0.911418256	0.921646762	0.99999488	0.999985264	0.999960414
LOC112444593	0.999996191	0.921550129	0.922400936	0.99999488	0.999985264	0.999960414
LOC112447118	0.999996191	0.941495217	0.922400936	0.99999488	0.999985264	0.999960414
HIF3A	0.999996191	0.943802122	0.922400936	0.99999488	0.999985264	0.999960414
LOC614091	0.999996191	0.946480858	0.922400936	0.99999488	0.999985264	0.999960414
SND1	0.999996191	0.978510121	0.922431991	0.99999488	0.999985264	0.999960414
ENO4	0.999996191	0.921661251	0.923089792	0.99999488	0.999985264	0.999960414
SGPP1	0.999996191	0.946274461	0.923604668	0.99999488	0.999985264	0.999960414
TCEAL4	0.999996191	0.900532231	0.925520255	0.99999488	0.999985264	0.999960414
SSH1	0.999996191	0.893743321	0.925745065	0.99999488	0.999985264	0.999960414
AATF	0.999996191	0.91507531	0.925745065	0.99999488	0.999985264	0.999960414
LOC104975782	0.999996191	0.921759937	0.926552506	0.99999488	0.999985264	0.999960414
LOC112449258	0.999996191	0.935060764	0.926552506	0.99999488	0.999985264	0.999960414
WDFY3	0.999996191	0.941495217	0.926552506	0.99999488	0.999985264	0.999960414
PFKP	0.999996191	0.9582784	0.926552506	0.99999488	0.999985264	0.999960414
TWF1	0.999996191	0.967276215	0.926552506	0.99999488	0.999985264	0.999960414
PLPPR1	0.999996191	0.970470739	0.926552506	0.99999488	0.999985264	0.999960414
EIF2S2	0.999996191	0.921549	0.927600835	0.99999488	0.999985264	0.999960414
SERPING1	0.999996191	0.985406717	0.927600835	0.99999488	0.999985264	0.999960414
PUM3	0.999996191	0.991488652	0.929207633	0.99999488	0.999985264	0.999960414
PPP1R3G	0.999996191	0.9582784	0.929952093	0.99999488	0.999985264	0.999960414
HDDC2	0.999996191	0.925580392	0.930678591	0.99999488	0.999985264	0.999960414
TMEM51	0.999996191	0.928113285	0.930678591	0.99999488	0.999985264	0.999960414
LOC112444326	0.999996191	0.935060764	0.930678591	0.99999488	0.999985264	0.999960414
LOC112447103	0.999996191	0.935742738	0.930678591	0.99999488	0.999985264	0.999960414
TMEM249	0.999996191	0.941495217	0.930678591	0.99999488	0.999985264	0.999960414
CMTR2	0.999996191	0.96870516	0.930678591	0.99999488	0.999985264	0.999960414
ABCG1	0.999996191	0.984068885	0.930678591	0.99999488	0.999985264	0.999960414
RUSC2	0.999996191	0.999992444	0.930678591	0.99999488	0.999985264	0.999960414
NFKBIL1	0.999996191	0.973182877	0.93198623	0.99999488	0.999985264	0.999960414
FAM69C	0.999996191	0.988850552	0.93198623	0.99999488	0.999985264	0.999960414

YWHAE	0.999996191	0.938777693	0.932376188	0.99999488	0.999985264	0.999960414
PPM1J	0.999996191	0.938597964	0.932762395	0.99999488	0.999985264	0.999960414
EDNRA	0.999996191	0.967276215	0.933791093	0.99999488	0.999985264	0.999960414
MUM1L1	0.999996191	0.97286093	0.933791093	0.99999488	0.999985264	0.999960414
ZFYVE1	0.999996191	0.9582784	0.934941286	0.99999488	0.999985264	0.999960414
SEPT8	0.999996191	0.883624935	0.935136095	0.99999488	0.999985264	0.999960414
ZNF565	0.999996191	0.985406717	0.935856933	0.99999488	0.999985264	0.999960414
SMN2	0.999996191	0.941495217	0.936914995	0.99999488	0.999985264	0.999960414
LOC100848985	0.999996191	0.999992444	0.936914995	0.99999488	0.999985264	0.999960414
TRIM35	0.999996191	0.931127395	0.938203102	0.99999488	0.999985264	0.999960414
CPSF3	0.999996191	0.935742738	0.938203102	0.99999488	0.999985264	0.999960414
FITM1	0.999996191	0.941495217	0.938203102	0.99999488	0.999985264	0.999960414
LPIN2	0.999996191	0.943802122	0.938203102	0.99999488	0.999985264	0.999960414
ATP12A	0.999996191	0.94984369	0.938203102	0.99999488	0.999985264	0.999960414
WWTR1	0.999996191	0.9582784	0.938203102	0.99999488	0.999985264	0.999960414
LOC104968435	0.999996191	0.978510121	0.938203102	0.99999488	0.999985264	0.999960414
LOC101905533	0.999996191	0.922047448	0.938215065	0.99999488	0.999985264	0.999960414
LOC112449614	0.999996191	0.97671655	0.938905004	0.99999488	0.999985264	0.999960414
MRRF	0.999996191	0.932593501	0.939147816	0.99999488	0.999985264	0.999960414
LOC112449111	0.999996191	0.974812121	0.939147816	0.99999488	0.999985264	0.999960414
FAM210A	0.999996191	0.982981183	0.939147816	0.99999488	0.999985264	0.999960414
CD200	0.999996191	0.986445172	0.939147816	0.99999488	0.999985264	0.999960414
CDV3	0.999996191	0.991488652	0.939147816	0.99999488	0.999985264	0.999960414
HSF5	0.999996191	0.991488652	0.939147816	0.99999488	0.999985264	0.999960414
LOC112445927	0.999996191	0.999992444	0.939147816	0.99999488	0.999985264	0.999960414
LOC512863	0.999996191	0.959733269	0.939414234	0.99999488	0.999985264	0.999960414
ZNF286A	0.999996191	0.999992444	0.939478907	0.99999488	0.999985264	0.999960414
LOC112444904	0.999996191	0.952346363	0.939501959	0.99999488	0.999985264	0.999960414
NAA16	0.999996191	0.969314233	0.939501959	0.99999488	0.999985264	0.999960414
SCHIP1	0.999996191	0.985406717	0.939501959	0.99999488	0.999985264	0.999960414
C10H14orf1	0.999996191	0.992977597	0.93955737	0.99999488	0.999985264	0.999960414
NOX4	0.999996191	0.946480858	0.939799459	0.99999488	0.999985264	0.999960414
ERC2	0.999996191	0.999992444	0.939799459	0.99999488	0.999985264	0.999960414
EZR	0.999996191	0.970426668	0.941118576	0.99999488	0.999985264	0.999960414
CCDC25	0.999996191	0.9527938	0.941336964	0.99999488	0.999985264	0.999960414
MXI1	0.999996191	0.95697765	0.941336964	0.99999488	0.999985264	0.999960414
NUP93	0.999996191	0.964246761	0.941336964	0.99999488	0.999985264	0.999960414
ARL4D	0.999996191	0.992977597	0.941336964	0.99999488	0.999985264	0.999960414
LOC112448038	0.999996191	0.999992444	0.941336964	0.99999488	0.999985264	0.999960414

THNSL1	0.999996191	0.999992444	0.941336964	0.99999488	0.999985264	0.999960414
ZSCAN31	0.999996191	0.95697765	0.941782902	0.99999488	0.999985264	0.999960414
LOC112442843	0.999996191	0.976389135	0.942686702	0.99999488	0.999985264	0.999960414
RASIP1	0.999996191	0.999992444	0.944031108	0.99999488	0.999985264	0.999960414
PPARGC1A	0.999996191	0.949285285	0.944085862	0.99999488	0.999985264	0.999960414
LONRF2	0.999996191	0.9582784	0.944085862	0.99999488	0.999985264	0.999960414
LBH	0.999996191	0.958967142	0.944085862	0.99999488	0.999985264	0.999960414
LOC101905734	0.999996191	0.999347049	0.944085862	0.99999488	0.999985264	0.999960414
FBP1	0.999996191	0.983640273	0.944274964	0.99999488	0.999985264	0.999960414
FNDC3A	0.999996191	0.978089982	0.944932814	0.99999488	0.999985264	0.999960414
TMEM121	0.999996191	0.979263532	0.944993804	0.99999488	0.999985264	0.999960414
LOC104968807	0.999996191	0.987516216	0.945280564	0.99999488	0.999985264	0.999960414
TMEM169	0.999996191	0.935742738	0.945678616	0.99999488	0.999985264	0.999960414
CABYR	0.999996191	0.958224615	0.945678616	0.99999488	0.999985264	0.999960414
KLHL28	0.999996191	0.981520287	0.945797778	0.99999488	0.999985264	0.999960414
LOC101905666	0.999996191	0.985406717	0.946283845	0.99999488	0.999985264	0.999960414
LOC100140372	0.999996191	0.943802122	0.94634922	0.99999488	0.999985264	0.999960414
LOC112443503	0.999996191	0.949285285	0.94634922	0.99999488	0.999985264	0.999960414
ELMSAN1	0.999996191	0.978510121	0.94634922	0.99999488	0.999985264	0.999960414
ZNF227	0.999996191	0.991488652	0.94634922	0.99999488	0.999985264	0.999960414
LOC100848507	0.999996191	0.999992444	0.94634922	0.99999488	0.999985264	0.999960414
ANKLE1	0.999996191	0.9582784	0.946479014	0.99999488	0.999985264	0.999960414
KYAT1	0.999996191	0.964718326	0.946479014	0.99999488	0.999985264	0.999960414
DGKG	0.999996191	0.991365955	0.946479014	0.99999488	0.999985264	0.999960414
ABHD15	0.999996191	0.991488652	0.946479014	0.99999488	0.999985264	0.999960414
LOC104973826	0.999996191	0.992977597	0.946479014	0.99999488	0.999985264	0.999960414
FAM205C	0.999996191	0.993316598	0.946479014	0.99999488	0.999985264	0.999960414
DCPS	0.999996191	0.997051616	0.946479014	0.99999488	0.999985264	0.999960414
TMEM234	0.999996191	0.998131745	0.946479014	0.99999488	0.999985264	0.999960414
SHISAL1	0.999996191	0.999992444	0.946479014	0.99999488	0.999985264	0.999960414
CCDC149	0.999996191	0.971819361	0.946831016	0.99999488	0.999985264	0.999960414
CFAP300	0.999996191	0.958098081	0.946832224	0.99999488	0.999985264	0.999960414
EIF2B3	0.999996191	0.9582784	0.946832224	0.99999488	0.999985264	0.999960414
HSD17B1	0.999996191	0.999347049	0.947226687	0.99999488	0.999985264	0.999960414
UTP20	0.999996191	0.9582784	0.94753587	0.99999488	0.999985264	0.999960414
BRINP1	0.999996191	0.999992444	0.947674759	0.99999488	0.999985264	0.999960414
KIF17	0.999996191	0.930409519	0.948069113	0.99999488	0.999985264	0.999960414
CERK	0.999996191	0.985406717	0.948069113	0.99999488	0.999985264	0.999960414
TCP11L1	0.999996191	0.999992444	0.948359914	0.99999488	0.999985264	0.999960414

WNT2B	0.999996191	0.999992444	0.948359914	0.99999488	0.999985264	0.999960414
NRDE2	0.999996191	0.958967142	0.948420301	0.99999488	0.999985264	0.999960414
GPCPD1	0.999996191	0.985406717	0.948420301	0.99999488	0.999985264	0.999960414
NEURL2	0.999996191	0.985406717	0.948420301	0.99999488	0.999985264	0.999960414
LOC112446761	0.999996191	0.9582784	0.948923067	0.99999488	0.999985264	0.999960414
GTF2A2	0.999996191	0.991488652	0.948923067	0.99999488	0.999985264	0.999960414
NRK	0.999996191	0.978510121	0.948946702	0.99999488	0.999985264	0.999960414
EXOG	0.999996191	0.991488652	0.949010827	0.99999488	0.999985264	0.999960414
DNHD1	0.999996191	0.941495217	0.949038056	0.99999488	0.999985264	0.999960414
C3H1orf226	0.999996191	0.984366454	0.949038056	0.99999488	0.999985264	0.999960414
MSANTD4	0.999996191	0.999992444	0.949038056	0.99999488	0.999985264	0.999960414
DBNDD2	0.999996191	0.963958704	0.94983755	0.99999488	0.999985264	0.999960414
HPDL	0.999996191	0.974812121	0.95083766	0.99999488	0.999985264	0.999960414
ETNPPL	0.999996191	0.985064085	0.95106368	0.99999488	0.999985264	0.999960414
XPO5	0.999996191	0.988850552	0.95106368	0.99999488	0.999985264	0.999960414
BSDC1	0.999996191	0.991488652	0.95106368	0.99999488	0.999985264	0.999960414
PLA2G6	0.999996191	0.992977597	0.95106368	0.99999488	0.999985264	0.999960414
INTS8	0.999996191	0.997975376	0.95106368	0.99999488	0.999985264	0.999960414
PIGF	0.999996191	0.999992444	0.95106368	0.99999488	0.999985264	0.999960414
INCA1	0.999996191	0.974812121	0.951822586	0.99999488	0.999985264	0.999960414
TRAF3IP1	0.999996191	0.978089982	0.951822586	0.99999488	0.999985264	0.999960414
ICOS	0.999996191	0.991488652	0.951822586	0.99999488	0.999985264	0.999960414
LOC100335822	0.999996191	0.942353856	0.95230212	0.99999488	0.999985264	0.999960414
UNKL	0.999996191	0.943802122	0.95230212	0.99999488	0.999985264	0.999960414
CHRAC1	0.999996191	0.992977597	0.95230212	0.99999488	0.999985264	0.999960414
MAP3K7	0.999996191	0.999347049	0.95230212	0.99999488	0.999985264	0.999960414
HYI	0.999996191	0.999992444	0.95230212	0.99999488	0.999985264	0.999960414
LOC101907744	0.999996191	0.999992444	0.95230212	0.99999488	0.999985264	0.999960414
NCBP2	0.999996191	0.999992444	0.95230212	0.99999488	0.999985264	0.999960414
PGR	0.999996191	0.943802122	0.952336457	0.99999488	0.999985264	0.999960414
CD160	0.999996191	0.957700659	0.952386544	0.99999488	0.999985264	0.999960414
PPP1R1B	0.999996191	0.97161271	0.952547836	0.99999488	0.999985264	0.999960414
RFESD	0.999996191	0.991488652	0.952571879	0.99999488	0.999985264	0.999960414
ZFP1	0.999996191	0.991488652	0.952571879	0.99999488	0.999985264	0.999960414
WDCP	0.999996191	0.974812121	0.95401762	0.99999488	0.999985264	0.999960414
CBLN3	0.999996191	0.961479426	0.954620171	0.99999488	0.999985264	0.999960414
DSCC1	0.999996191	0.964162565	0.954620171	0.99999488	0.999985264	0.999960414
ATP9B	0.999996191	0.978089982	0.954620171	0.99999488	0.999985264	0.999960414
FLNB	0.999996191	0.992977597	0.954620171	0.99999488	0.999985264	0.999960414

CTDSPL	0.999996191	0.974079639	0.954714379	0.99999488	0.999985264	0.999960414
ABHD4	0.999996191	0.991488652	0.95477544	0.99999488	0.999985264	0.999960414
INTS6L	0.999996191	0.991488652	0.95477544	0.99999488	0.999985264	0.999960414
MYO1D	0.999996191	0.95830623	0.954823586	0.99999488	0.999985264	0.999960414
FLAD1	0.999996191	0.965625946	0.954823586	0.99999488	0.999985264	0.999960414
FBRS	0.999996191	0.982313546	0.954823586	0.99999488	0.999985264	0.999960414
CHCHD8	0.999996191	0.983781707	0.954823586	0.99999488	0.999985264	0.999960414
FAM117A	0.999996191	0.992977597	0.954823586	0.99999488	0.999985264	0.999960414
LOC107132278	0.999996191	0.993100184	0.954823586	0.99999488	0.999985264	0.999960414
LOC509034	0.999996191	0.986637746	0.954864482	0.99999488	0.999985264	0.999960414
IKZF2	0.999996191	0.985406717	0.954872702	0.99999488	0.999985264	0.999960414
RACGAP1	0.999996191	0.9582784	0.956504686	0.99999488	0.999985264	0.999960414
MSC	0.999996191	0.9582784	0.956565728	0.99999488	0.999985264	0.999960414
TADA1	0.999996191	0.967478685	0.956565728	0.99999488	0.999985264	0.999960414
SYNPO	0.999996191	0.974812121	0.956565728	0.99999488	0.999985264	0.999960414
ZC4H2	0.999996191	0.978089982	0.956565728	0.99999488	0.999985264	0.999960414
LOC781533	0.999996191	0.981520287	0.956565728	0.99999488	0.999985264	0.999960414
LOC107131323	0.999996191	0.985406717	0.956565728	0.99999488	0.999985264	0.999960414
FBXO21	0.999996191	0.991488652	0.956565728	0.99999488	0.999985264	0.999960414
VARS2	0.999996191	0.991488652	0.956565728	0.99999488	0.999985264	0.999960414
FAM181B	0.999996191	0.997975376	0.956565728	0.99999488	0.999985264	0.999960414
LOC100847765	0.999996191	0.999992444	0.956565728	0.99999488	0.999985264	0.999960414
LOC613444	0.999996191	0.999992444	0.956565728	0.99999488	0.999985264	0.999960414
PPP1R13L	0.999996191	0.999992444	0.956565728	0.99999488	0.999985264	0.999960414
RNFT1	0.999996191	0.999992444	0.956565728	0.99999488	0.999985264	0.999960414
LOC101908185	0.999996191	0.999992444	0.956852976	0.99999488	0.999985264	0.999960414
ISG20L2	0.999996191	0.998131745	0.957117655	0.99999488	0.999985264	0.999960414
TRNT1	0.999996191	0.974079639	0.957541703	0.99999488	0.999985264	0.999960414
KCTD6	0.999996191	0.991488652	0.957541703	0.99999488	0.999985264	0.999960414
PAPSS2	0.999996191	0.992977597	0.957541703	0.99999488	0.999985264	0.999960414
TNFRSF4	0.999996191	0.998131745	0.957541703	0.99999488	0.999985264	0.999960414
EEFSEC	0.999996191	0.999347049	0.957541703	0.99999488	0.999985264	0.999960414
FAM222B	0.999996191	0.999992444	0.957541703	0.99999488	0.999985264	0.999960414
LOC101902918	0.999996191	0.991488652	0.95758013	0.99999488	0.999985264	0.999960414
IZUMO1	0.999996191	0.991488652	0.957669868	0.99999488	0.999985264	0.999960414
LOC112442082	0.999996191	0.978510121	0.957840205	0.99999488	0.999985264	0.999960414
DDX1	0.999996191	0.9582784	0.958627535	0.99999488	0.999985264	0.999960414
AKAP13	0.999996191	0.97161271	0.958627535	0.99999488	0.999985264	0.999960414
DCTN2	0.999996191	0.986445172	0.958627535	0.99999488	0.999985264	0.999960414

SMOX	0.999996191	0.998131745	0.958627535	0.99999488	0.999985264	0.999960414
CABLES1	0.999996191	0.986637746	0.95935771	0.99999488	0.999985264	0.999960414
FADD	0.999996191	0.999992444	0.959396074	0.99999488	0.999985264	0.999960414
PUF60	0.999996191	0.999992444	0.959396074	0.99999488	0.999985264	0.999960414
SHMT2	0.999996191	0.985406717	0.959575351	0.99999488	0.999985264	0.999960414
KAT6A	0.999996191	0.938597964	0.960133832	0.99999488	0.999985264	0.999960414
PEX2	0.999996191	0.963717626	0.960133832	0.99999488	0.999985264	0.999960414
BICRA	0.999996191	0.974812121	0.960133832	0.99999488	0.999985264	0.999960414
RGMB	0.999996191	0.986637746	0.960133832	0.99999488	0.999985264	0.999960414
TBC1D16	0.999996191	0.99490847	0.960133832	0.99999488	0.999985264	0.999960414
DGAT1	0.999996191	0.999992444	0.960133832	0.99999488	0.999985264	0.999960414
LOC104975811	0.999996191	0.999992444	0.960133832	0.99999488	0.999985264	0.999960414
RPS6KB1	0.999996191	0.991488652	0.96050344	0.99999488	0.999985264	0.999960414
HPCAL1	0.999996191	0.999992444	0.960909242	0.99999488	0.999985264	0.999960414
EIF2B5	0.999996191	0.982940915	0.961024709	0.99999488	0.999985264	0.999960414
SARS	0.999996191	0.992977597	0.961024709	0.99999488	0.999985264	0.999960414
CAMK1D	0.999996191	0.999992444	0.961024709	0.99999488	0.999985264	0.999960414
SGK3	0.999996191	0.998962836	0.96131087	0.99999488	0.999985264	0.999960414
FDFT1	0.999996191	0.9677397	0.961490767	0.99999488	0.999985264	0.999960414
ASB9	0.999996191	0.991574265	0.961541561	0.99999488	0.999985264	0.999960414
LOC101906018	0.999996191	0.975677199	0.961613926	0.99999488	0.999985264	0.999960414
ASXL2	0.999996191	0.981298804	0.961734809	0.99999488	0.999985264	0.999960414
TMEM201	0.999996191	0.992977597	0.96210311	0.99999488	0.999985264	0.999960414
FAM168A	0.999996191	0.978089982	0.962142195	0.99999488	0.999985264	0.999960414
YBX2	0.999996191	0.985406717	0.962142195	0.99999488	0.999985264	0.999960414
MIPOL1	0.999996191	0.970426668	0.962346588	0.99999488	0.999985264	0.999960414
PLEKHM3	0.999996191	0.986018996	0.962346588	0.99999488	0.999985264	0.999960414
TLR9	0.999996191	0.993316598	0.962346588	0.99999488	0.999985264	0.999960414
LOC509006	0.999996191	0.999992444	0.962346588	0.99999488	0.999985264	0.999960414
MYOZ3	0.999996191	0.999992444	0.962346588	0.99999488	0.999985264	0.999960414
PLD1	0.999996191	0.990388693	0.962777771	0.99999488	0.999985264	0.999960414
UBXN4	0.999996191	0.991488652	0.96290532	0.99999488	0.999985264	0.999960414
ZNF689	0.999996191	0.991488652	0.96290532	0.99999488	0.999985264	0.999960414
KRT80	0.999996191	0.991574265	0.96290532	0.99999488	0.999985264	0.999960414
TSC1	0.999996191	0.992977597	0.96290532	0.99999488	0.999985264	0.999960414
EPHA7	0.999996191	0.999992444	0.96290532	0.99999488	0.999985264	0.999960414
NUP188	0.999996191	0.992977597	0.963068909	0.99999488	0.999985264	0.999960414
TARS	0.999996191	0.994135588	0.963068909	0.99999488	0.999985264	0.999960414
GCNT7	0.999996191	0.994481342	0.963068909	0.99999488	0.999985264	0.999960414

LOC104975960	0.999996191	0.999992444	0.963068909	0.99999488	0.999985264	0.999960414
TMEM205	0.999996191	0.999992444	0.963068909	0.99999488	0.999985264	0.999960414
LOC101904849	0.999996191	0.986637746	0.96307344	0.99999488	0.999985264	0.999960414
ADAMTS18	0.999996191	0.999992444	0.96307344	0.99999488	0.999985264	0.999960414
PLEKHD1	0.999996191	0.999347049	0.963233239	0.99999488	0.999985264	0.999960414
RGL1	0.999996191	0.99490847	0.964079616	0.99999488	0.999985264	0.999960414
LOC100847326	0.999996191	0.991488652	0.964152013	0.99999488	0.999985264	0.999960414
RBM45	0.999996191	0.991488652	0.964152013	0.99999488	0.999985264	0.999960414
FZD4	0.999996191	0.992977597	0.964152013	0.99999488	0.999985264	0.999960414
MAL	0.999996191	0.992977597	0.964152013	0.99999488	0.999985264	0.999960414
XRCC2	0.999996191	0.996814721	0.964152013	0.99999488	0.999985264	0.999960414
RPS9	0.999996191	0.999347049	0.964152013	0.99999488	0.999985264	0.999960414
LOC112447438	0.999996191	0.999992444	0.964152013	0.99999488	0.999985264	0.999960414
ZC3H6	0.999996191	0.999992444	0.964152013	0.99999488	0.999985264	0.999960414
ZNF584	0.999996191	0.999992444	0.964152013	0.99999488	0.999985264	0.999960414
PTGS1	0.999996191	0.970291664	0.964186888	0.99999488	0.999985264	0.999960414
PLAA	0.999996191	0.991476708	0.964396605	0.99999488	0.999985264	0.999960414
DHX33	0.999996191	0.998131745	0.964582964	0.99999488	0.999985264	0.999960414
C1RL	0.999996191	0.935588259	0.964594039	0.99999488	0.999985264	0.999960414
LOC112448021	0.999996191	0.958967142	0.964920281	0.99999488	0.999985264	0.999960414
IQCK	0.999996191	0.987559864	0.964920281	0.99999488	0.999985264	0.999960414
LOC107132189	0.999996191	0.991488652	0.964920281	0.99999488	0.999985264	0.999960414
PIM1	0.999996191	0.991488652	0.964920281	0.99999488	0.999985264	0.999960414
TSPOAP1	0.999996191	0.991488652	0.964920281	0.99999488	0.999985264	0.999960414
EXOC1	0.999996191	0.992977597	0.964920281	0.99999488	0.999985264	0.999960414
SLC12A6	0.999996191	0.992977597	0.964920281	0.99999488	0.999985264	0.999960414
P2RY14	0.999996191	0.998131745	0.964920281	0.99999488	0.999985264	0.999960414
S100A3	0.999996191	0.998131745	0.964920281	0.99999488	0.999985264	0.999960414
RIN2	0.999996191	0.999417463	0.964920281	0.99999488	0.999985264	0.999960414
KANK1	0.999996191	0.980496345	0.965059328	0.99999488	0.999985264	0.999960414
FBLN2	0.999996191	0.999992444	0.965059328	0.99999488	0.999985264	0.999960414
ADGRL3	0.999996191	0.961479426	0.965065403	0.99999488	0.999985264	0.999960414
AGPAT1	0.999996191	0.992977597	0.965065403	0.99999488	0.999985264	0.999960414
NFATC2	0.999996191	0.997975376	0.965065403	0.99999488	0.999985264	0.999960414
NDUFAF1	0.999996191	0.980377208	0.965161441	0.99999488	0.999985264	0.999960414
RPUSD4	0.999996191	0.992977597	0.965161441	0.99999488	0.999985264	0.999960414
DNAJC15	0.999996191	0.999992444	0.965214449	0.99999488	0.999985264	0.999960414
TUSC1	0.999996191	0.991574265	0.965642988	0.99999488	0.999985264	0.999960414
MTMR4	0.999996191	0.9582784	0.96684887	0.99999488	0.999985264	0.999960414

FAH	0.999996191	0.999992444	0.966991646	0.99999488	0.999985264	0.999960414
LOC104976448	0.999996191	0.999992444	0.966991646	0.99999488	0.999985264	0.999960414
KDM6B	0.999996191	0.999992444	0.967011468	0.99999488	0.999985264	0.999960414
DIMT1	0.999996191	0.978426416	0.968232195	0.99999488	0.999985264	0.999960414
CPEB1	0.999996191	0.992117536	0.968232195	0.99999488	0.999985264	0.999960414
C1QA	0.999996191	0.992977597	0.968232195	0.99999488	0.999985264	0.999960414
ERAS	0.999996191	0.992977597	0.968232195	0.99999488	0.999985264	0.999960414
TTC37	0.999996191	0.992977597	0.968232195	0.99999488	0.999985264	0.999960414
LOC787530	0.999996191	0.999347049	0.968232195	0.99999488	0.999985264	0.999960414
LOC104969611	0.999996191	0.999992444	0.968232195	0.99999488	0.999985264	0.999960414
MCAM	0.999996191	0.974812121	0.968325332	0.99999488	0.999985264	0.999960414
EPB41	0.999996191	0.985293257	0.968325332	0.99999488	0.999985264	0.999960414
ETV3	0.999996191	0.998011664	0.968335968	0.99999488	0.999985264	0.999960414
LOC104973058	0.999996191	0.978510121	0.968508342	0.99999488	0.999985264	0.999960414
DXO	0.999996191	0.969298476	0.96870883	0.99999488	0.999985264	0.999960414
UBE3D	0.999996191	0.999992444	0.968836394	0.99999488	0.999985264	0.999960414
AGPAT4	0.999996191	0.96566261	0.969036336	0.99999488	0.999985264	0.999960414
HNRNPH2	0.999996191	0.938597964	0.969656316	0.99999488	0.999985264	0.999960414
ADAMTSL5	0.999996191	0.991574265	0.969656316	0.99999488	0.999985264	0.999960414
PVRIG	0.999996191	0.999992444	0.969656316	0.99999488	0.999985264	0.999960414
SP2	0.999996191	0.999992444	0.969656316	0.99999488	0.999985264	0.999960414
COL5A3	0.999996191	0.991488652	0.96968839	0.99999488	0.999985264	0.999960414
STMN1	0.999996191	0.999992444	0.96968839	0.99999488	0.999985264	0.999960414
ZDHHC6	0.999996191	0.958098081	0.969880768	0.99999488	0.999985264	0.999960414
MGST1	0.999996191	0.986637746	0.969880768	0.99999488	0.999985264	0.999960414
WDR92	0.999996191	0.986637746	0.969880768	0.99999488	0.999985264	0.999960414
CAMTA1	0.999996191	0.992977597	0.969880768	0.99999488	0.999985264	0.999960414
LOC101905228	0.999996191	0.993316598	0.969880768	0.99999488	0.999985264	0.999960414
ANKEF1	0.999996191	0.993540482	0.969880768	0.99999488	0.999985264	0.999960414
NARS2	0.999996191	0.998131745	0.969880768	0.99999488	0.999985264	0.999960414
ZMIZ2	0.999996191	0.998131745	0.969880768	0.99999488	0.999985264	0.999960414
DAXX	0.999996191	0.998962836	0.969880768	0.99999488	0.999985264	0.999960414
CARHSP1	0.999996191	0.999992444	0.969880768	0.99999488	0.999985264	0.999960414
DNAH5	0.999996191	0.999992444	0.969880768	0.99999488	0.999985264	0.999960414
LOC101903397	0.999996191	0.999992444	0.969880768	0.99999488	0.999985264	0.999960414
LOC104969378	0.999996191	0.999992444	0.969880768	0.99999488	0.999985264	0.999960414
LOC513573	0.999996191	0.999992444	0.969880768	0.99999488	0.999985264	0.999960414
RUVBL2	0.999996191	0.999992444	0.969880768	0.99999488	0.999985264	0.999960414
LOC112442849	0.999996191	0.989958965	0.970874203	0.99999488	0.999985264	0.999960414

EIF3D	0.999996191	0.994754253	0.970874203	0.99999488	0.999985264	0.999960414
CYP39A1	0.999996191	0.998131745	0.970874203	0.99999488	0.999985264	0.999960414
LOC104972724	0.999996191	0.938597964	0.970903823	0.99999488	0.999985264	0.999960414
TECPR2	0.999996191	0.97993071	0.970945672	0.99999488	0.999985264	0.999960414
LTB	0.999996191	0.985406717	0.970945672	0.99999488	0.999985264	0.999960414
HARS	0.999996191	0.991365955	0.970945672	0.99999488	0.999985264	0.999960414
TMEM164	0.999996191	0.991365955	0.970945672	0.99999488	0.999985264	0.999960414
FNBP1	0.999996191	0.991488652	0.970945672	0.99999488	0.999985264	0.999960414
SLC25A19	0.999996191	0.994464252	0.970945672	0.99999488	0.999985264	0.999960414
LOC100141168	0.999996191	0.997083202	0.970945672	0.99999488	0.999985264	0.999960414
TTC27	0.999996191	0.999347049	0.970945672	0.99999488	0.999985264	0.999960414
LIX1L	0.999996191	0.999992444	0.970945672	0.99999488	0.999985264	0.999960414
MRPS35	0.999996191	0.999992444	0.971038643	0.99999488	0.999985264	0.999960414
LOC101904442	0.999996191	0.981298804	0.971111634	0.99999488	0.999985264	0.999960414
SULT1C4	0.999996191	0.982289317	0.971759453	0.99999488	0.999985264	0.999960414
LOC101904622	0.999996191	0.988850552	0.971759453	0.99999488	0.999985264	0.999960414
CANX	0.999996191	0.911418256	0.971778256	0.99999488	0.999985264	0.999960414
ZNF45	0.999996191	0.999347049	0.972362683	0.99999488	0.999985264	0.999960414
MYO18A	0.999996191	0.978089982	0.972381338	0.99999488	0.999985264	0.999960414
ZNF444	0.999996191	0.989872618	0.972381338	0.99999488	0.999985264	0.999960414
CLCN3	0.999996191	0.986637746	0.972521741	0.99999488	0.999985264	0.999960414
NOS1	0.999996191	0.991488652	0.972521741	0.99999488	0.999985264	0.999960414
ZNF263	0.999996191	0.999347049	0.97262931	0.99999488	0.999985264	0.999960414
LOC112441639	0.999996191	0.999992444	0.97262931	0.99999488	0.999985264	0.999960414
LOC781280	0.999996191	0.992977597	0.972871571	0.99999488	0.999985264	0.999960414
BMPR1A	0.999996191	0.972764622	0.973303821	0.99999488	0.999985264	0.999960414
LOC104975861	0.999996191	0.976061777	0.973303821	0.99999488	0.999985264	0.999960414
BEX3	0.999996191	0.985406717	0.973303821	0.99999488	0.999985264	0.999960414
FAM229A	0.999996191	0.992117536	0.973303821	0.99999488	0.999985264	0.999960414
ARHGAP18	0.999996191	0.992301181	0.973303821	0.99999488	0.999985264	0.999960414
RMDN1	0.999996191	0.998131745	0.973303821	0.99999488	0.999985264	0.999960414
APBA1	0.999996191	0.999992444	0.973303821	0.99999488	0.999985264	0.999960414
CPNE8	0.999996191	0.999992444	0.973303821	0.99999488	0.999985264	0.999960414
LOC101902856	0.999996191	0.999992444	0.973303821	0.99999488	0.999985264	0.999960414
PI15	0.999996191	0.999992444	0.973303821	0.99999488	0.999985264	0.999960414
LOC100139638	0.999996191	0.999347049	0.973364552	0.99999488	0.999985264	0.999960414
PPP3CC	0.999996191	0.987516216	0.973406453	0.99999488	0.999985264	0.999960414
QKI	0.999996191	0.991488652	0.973406453	0.99999488	0.999985264	0.999960414
MDP1	0.999996191	0.991574265	0.973406453	0.99999488	0.999985264	0.999960414

NCKAP5	0.999996191	0.992301181	0.973406453	0.99999488	0.999985264	0.999960414
LOC112445194	0.999996191	0.992977597	0.973406453	0.99999488	0.999985264	0.999960414
LOC613677	0.999996191	0.992977597	0.973406453	0.99999488	0.999985264	0.999960414
THUMPD2	0.999996191	0.992977597	0.973406453	0.99999488	0.999985264	0.999960414
TMEM98	0.999996191	0.992977597	0.973406453	0.99999488	0.999985264	0.999960414
SFRS18	0.999996191	0.993316598	0.973406453	0.99999488	0.999985264	0.999960414
ZBTB4	0.999996191	0.995532499	0.973406453	0.99999488	0.999985264	0.999960414
ERRFI1	0.999996191	0.998131745	0.973406453	0.99999488	0.999985264	0.999960414
UBE4A	0.999996191	0.998962836	0.973406453	0.99999488	0.999985264	0.999960414
SORBS3	0.999996191	0.999347049	0.973406453	0.99999488	0.999985264	0.999960414
ADAMTS4	0.999996191	0.999992444	0.973406453	0.99999488	0.999985264	0.999960414
ARHGEF38	0.999996191	0.999992444	0.973406453	0.99999488	0.999985264	0.999960414
ATAT1	0.999996191	0.999992444	0.973406453	0.99999488	0.999985264	0.999960414
CDK11B	0.999996191	0.999992444	0.973406453	0.99999488	0.999985264	0.999960414
LGR4	0.999996191	0.999992444	0.973406453	0.99999488	0.999985264	0.999960414
LOC101906367	0.999996191	0.999992444	0.973406453	0.99999488	0.999985264	0.999960414
LOC112449080	0.999996191	0.999992444	0.973406453	0.99999488	0.999985264	0.999960414
NSUN3	0.999996191	0.999992444	0.973406453	0.99999488	0.999985264	0.999960414
SOD2	0.999996191	0.999992444	0.973406453	0.99999488	0.999985264	0.999960414
TCF7L1	0.999996191	0.999992444	0.973406453	0.99999488	0.999985264	0.999960414
VPS45	0.999996191	0.999992444	0.973406453	0.99999488	0.999985264	0.999960414
ZNF548	0.999996191	0.999992444	0.973406453	0.99999488	0.999985264	0.999960414
HYAL3	0.999996191	0.991488652	0.973451777	0.99999488	0.999985264	0.999960414
IMPDH2	0.999996191	0.999992444	0.973451777	0.99999488	0.999985264	0.999960414
MLXIP	0.999996191	0.999992444	0.973661483	0.99999488	0.999985264	0.999960414
CEACAM19	0.999996191	0.993316598	0.973713465	0.99999488	0.999985264	0.999960414
PC	0.999996191	0.992977597	0.97379223	0.99999488	0.999985264	0.999960414
LOC112449548	0.999996191	0.999992444	0.974075221	0.99999488	0.999985264	0.999960414
ATXN2	0.999996191	0.999992444	0.974727926	0.99999488	0.999985264	0.999960414
TP53	0.999996191	0.999992444	0.975114359	0.99999488	0.999985264	0.999960414
ZSWIM5	0.999996191	0.978426416	0.975117552	0.99999488	0.999985264	0.999960414
PPARA	0.999996191	0.99995848	0.975211073	0.99999488	0.999985264	0.999960414
MFN2	0.999996191	0.998011664	0.975538142	0.99999488	0.999985264	0.999960414
C26H10orf143	0.999996191	0.991365955	0.97558773	0.99999488	0.999985264	0.999960414
SCRN3	0.999996191	0.998131745	0.975701948	0.99999488	0.999985264	0.999960414
ADAT1	0.999996191	0.999992444	0.976321912	0.99999488	0.999985264	0.999960414
LOC107133048	0.999996191	0.999992444	0.977127779	0.99999488	0.999985264	0.999960414
LRRC8E	0.999996191	0.993316598	0.977141934	0.99999488	0.999985264	0.999960414
PRKAG1	0.999996191	0.996622477	0.977141934	0.99999488	0.999985264	0.999960414

TMEM131L	0.999996191	0.997975376	0.977141934	0.99999488	0.999985264	0.999960414
FDXR	0.999996191	0.999347049	0.977141934	0.99999488	0.999985264	0.999960414
SYCE2	0.999996191	0.999347049	0.977141934	0.99999488	0.999985264	0.999960414
TAF4	0.999996191	0.999347049	0.977141934	0.99999488	0.999985264	0.999960414
LOC112443422	0.999996191	0.999992444	0.977141934	0.99999488	0.999985264	0.999960414
LOC112446708	0.999996191	0.999992444	0.977141934	0.99999488	0.999985264	0.999960414
RASSF1	0.999996191	0.999992444	0.977141934	0.99999488	0.999985264	0.999960414
VMAC	0.999996191	0.999992444	0.977141934	0.99999488	0.999985264	0.999960414
PDIK1L	0.999996191	0.993316598	0.977250076	0.99999488	0.999985264	0.999960414
TBC1D9B	0.999996191	0.999992444	0.977301149	0.99999488	0.999985264	0.999960414
SOX15	0.999996191	0.999992444	0.977930966	0.99999488	0.999985264	0.999960414
IPPK	0.999996191	0.970077079	0.978389389	0.99999488	0.999985264	0.999960414
ATP1B2	0.999996191	0.976717114	0.978389389	0.99999488	0.999985264	0.999960414
XPNPEP1	0.999996191	0.992977597	0.978389389	0.99999488	0.999985264	0.999960414
LHFPL1	0.999996191	0.994481342	0.978389389	0.99999488	0.999985264	0.999960414
CBFA2T3	0.999996191	0.985406717	0.978842418	0.99999488	0.999985264	0.999960414
RBBP9	0.999996191	0.999992444	0.978894849	0.99999488	0.999985264	0.999960414
LOC112441506	0.999996191	0.999992444	0.978993134	0.99999488	0.999985264	0.999960414
ZNF384	0.999996191	0.991476708	0.979441303	0.99999488	0.999985264	0.999960414
CACNA1C	0.999996191	0.998131745	0.979462526	0.99999488	0.999985264	0.999960414
GSTM2	0.999996191	0.999992444	0.979462526	0.99999488	0.999985264	0.999960414
MAU2	0.999996191	0.999992444	0.979462526	0.99999488	0.999985264	0.999960414
LOC112444502	0.999996191	0.992977597	0.979650244	0.99999488	0.999985264	0.999960414
LOC100337507	0.999996191	0.999992444	0.979650244	0.99999488	0.999985264	0.999960414
PRKCH	0.999996191	0.999992444	0.979650244	0.99999488	0.999985264	0.999960414
HECTD4	0.999996191	0.999992444	0.979745141	0.99999488	0.999985264	0.999960414
RHOBTB1	0.999996191	0.999992444	0.979745141	0.99999488	0.999985264	0.999960414
GDPGP1	0.999996191	0.999992444	0.979812868	0.99999488	0.999985264	0.999960414
PBDC1	0.999996191	0.999347049	0.979861819	0.99999488	0.999985264	0.999960414
TBCC	0.999996191	0.992977597	0.980169282	0.99999488	0.999985264	0.999960414
ZNF568	0.999996191	0.999992444	0.980429215	0.99999488	0.999985264	0.999960414
FTO	0.999996191	0.978510121	0.980660791	0.99999488	0.999985264	0.999960414
TCF7L2	0.999996191	0.979263532	0.980660791	0.99999488	0.999985264	0.999960414
UTP11	0.999996191	0.991488652	0.980660791	0.99999488	0.999985264	0.999960414
IL5RA	0.999996191	0.992977597	0.980660791	0.99999488	0.999985264	0.999960414
LOC112447824	0.999996191	0.992977597	0.980660791	0.99999488	0.999985264	0.999960414
CLK3	0.999996191	0.998011664	0.980660791	0.99999488	0.999985264	0.999960414
LOC107131660	0.999996191	0.998131745	0.980660791	0.99999488	0.999985264	0.999960414
CREBZF	0.999996191	0.999992444	0.980660791	0.99999488	0.999985264	0.999960414

GRM8	0.999996191	0.999992444	0.980660791	0.99999488	0.999985264	0.999960414
LOC101904339	0.999996191	0.999992444	0.980660791	0.99999488	0.999985264	0.999960414
SMPDL3B	0.999996191	0.999992444	0.980660791	0.99999488	0.999985264	0.999960414
ZBTB9	0.999996191	0.999992444	0.980660791	0.99999488	0.999985264	0.999960414
LOC107133166	0.999996191	0.99398773	0.980677819	0.99999488	0.999985264	0.999960414
GCK	0.999996191	0.999347049	0.980677819	0.99999488	0.999985264	0.999960414
DNM3	0.999996191	0.999992444	0.980677819	0.99999488	0.999985264	0.999960414
DQX1	0.999996191	0.999992444	0.980677819	0.99999488	0.999985264	0.999960414
SMAGP	0.999996191	0.999992444	0.980677819	0.99999488	0.999985264	0.999960414
NUP205	0.999996191	0.999347049	0.980847826	0.99999488	0.999985264	0.999960414
IL20RB	0.999996191	0.967535824	0.980859619	0.99999488	0.999985264	0.999960414
MCOLN2	0.999996191	0.991574265	0.980859619	0.99999488	0.999985264	0.999960414
TXNDC12	0.999996191	0.997759055	0.980859619	0.99999488	0.999985264	0.999960414
EXOSC5	0.999996191	0.999347049	0.980859619	0.99999488	0.999985264	0.999960414
AGBL5	0.999996191	0.999992444	0.980859619	0.99999488	0.999985264	0.999960414
LOC101905114	0.999996191	0.999992444	0.980859619	0.99999488	0.999985264	0.999960414
MMADHC	0.999996191	0.974812121	0.9808801	0.99999488	0.999985264	0.999960414
LACTB	0.999996191	0.992895985	0.9808801	0.99999488	0.999985264	0.999960414
KIAA0408	0.999996191	0.992977597	0.9808801	0.99999488	0.999985264	0.999960414
LOC104973767	0.999996191	0.992977597	0.9808801	0.99999488	0.999985264	0.999960414
PDE4DIP	0.999996191	0.992977597	0.9808801	0.99999488	0.999985264	0.999960414
LOC104970815	0.999996191	0.995476917	0.9808801	0.99999488	0.999985264	0.999960414
CBS	0.999996191	0.998011664	0.9808801	0.99999488	0.999985264	0.999960414
C1H3orf38	0.999996191	0.999347049	0.9808801	0.99999488	0.999985264	0.999960414
ESR1	0.999996191	0.999992444	0.9808801	0.99999488	0.999985264	0.999960414
LOC107132757	0.999996191	0.999992444	0.9808801	0.99999488	0.999985264	0.999960414
MPDZ	0.999996191	0.999992444	0.9808801	0.99999488	0.999985264	0.999960414
NOCT	0.999996191	0.999992444	0.9808801	0.99999488	0.999985264	0.999960414
SBK1	0.999996191	0.999992444	0.9808801	0.99999488	0.999985264	0.999960414
SIDT2	0.999996191	0.999992444	0.9808801	0.99999488	0.999985264	0.999960414
SLC4A1AP	0.999996191	0.999992444	0.9808801	0.99999488	0.999985264	0.999960414
TCHP	0.999996191	0.999992444	0.9808801	0.99999488	0.999985264	0.999960414
UAP1L1	0.999996191	0.999992444	0.9808801	0.99999488	0.999985264	0.999960414
SMARCD3	0.999996191	0.976485049	0.981039752	0.99999488	0.999985264	0.999960414
ABCD1	0.999996191	0.991574265	0.981039752	0.99999488	0.999985264	0.999960414
LOC782437	0.999996191	0.992977597	0.981039752	0.99999488	0.999985264	0.999960414
CLYBL	0.999996191	0.993316598	0.981039752	0.99999488	0.999985264	0.999960414
ACVR1	0.999996191	0.999347049	0.981039752	0.99999488	0.999985264	0.999960414
FAM126A	0.999996191	0.999347049	0.981039752	0.99999488	0.999985264	0.999960414

ITGA6	0.999996191	0.999347049	0.981039752	0.99999488	0.999985264	0.999960414
BBS2	0.999996191	0.999992444	0.981039752	0.99999488	0.999985264	0.999960414
DAPK1	0.999996191	0.999992444	0.981039752	0.99999488	0.999985264	0.999960414
DENND1B	0.999996191	0.999992444	0.981039752	0.99999488	0.999985264	0.999960414
DGKH	0.999996191	0.999992444	0.981039752	0.99999488	0.999985264	0.999960414
DIXDC1	0.999996191	0.999992444	0.981039752	0.99999488	0.999985264	0.999960414
LOC101905687	0.999996191	0.999992444	0.981039752	0.99999488	0.999985264	0.999960414
LOC101907998	0.999996191	0.999992444	0.981039752	0.99999488	0.999985264	0.999960414
MECP2	0.999996191	0.999992444	0.981039752	0.99999488	0.999985264	0.999960414
SETX	0.999996191	0.999992444	0.981039752	0.99999488	0.999985264	0.999960414
TMEM65	0.999996191	0.999992444	0.981039752	0.99999488	0.999985264	0.999960414
USE1	0.999996191	0.999992444	0.981039752	0.99999488	0.999985264	0.999960414
BMX	0.999996191	0.991365955	0.981218677	0.99999488	0.999985264	0.999960414
ETV1	0.999996191	0.993316598	0.981218677	0.99999488	0.999985264	0.999960414
DUS2	0.999996191	0.999347049	0.981218677	0.99999488	0.999985264	0.999960414
RPS6KC1	0.999996191	0.999347049	0.981218677	0.99999488	0.999985264	0.999960414
STK39	0.999996191	0.999347049	0.981218677	0.99999488	0.999985264	0.999960414
ARHGEF3	0.999996191	0.999992444	0.981218677	0.99999488	0.999985264	0.999960414
ATG4A	0.999996191	0.999992444	0.981218677	0.99999488	0.999985264	0.999960414
DCP2	0.999996191	0.999992444	0.981218677	0.99999488	0.999985264	0.999960414
OSMR	0.999996191	0.999992444	0.981218677	0.99999488	0.999985264	0.999960414
PREX2	0.999996191	0.999992444	0.981218677	0.99999488	0.999985264	0.999960414
SNX33	0.999996191	0.999992444	0.981218677	0.99999488	0.999985264	0.999960414
FAAP24	0.999996191	0.986445172	0.981388276	0.99999488	0.999985264	0.999960414
MTX3	0.999996191	0.998131745	0.981388276	0.99999488	0.999985264	0.999960414
AIF1L	0.999996191	0.999992444	0.981388276	0.99999488	0.999985264	0.999960414
FAM83G	0.999996191	0.999992444	0.981388276	0.99999488	0.999985264	0.999960414
LOC100847612	0.999996191	0.999992444	0.981388276	0.99999488	0.999985264	0.999960414
LOC510193	0.999996191	0.999992444	0.981388276	0.99999488	0.999985264	0.999960414
ZNF226	0.999996191	0.999992444	0.981388276	0.99999488	0.999985264	0.999960414
LOC112446407	0.999996191	0.999992444	0.981593378	0.99999488	0.999985264	0.999960414
LOC790218	0.999996191	0.96728174	0.981622418	0.99999488	0.999985264	0.999960414
CSNK2A1	0.999996191	0.978154072	0.981622418	0.99999488	0.999985264	0.999960414
DDX50	0.999996191	0.986637746	0.981622418	0.99999488	0.999985264	0.999960414
BLMH	0.999996191	0.992895985	0.981622418	0.99999488	0.999985264	0.999960414
TEX12	0.999996191	0.992977597	0.981622418	0.99999488	0.999985264	0.999960414
INSL3	0.999996191	0.997083202	0.981622418	0.99999488	0.999985264	0.999960414
CEP295NL	0.999996191	0.997975376	0.981622418	0.99999488	0.999985264	0.999960414
CEBPA	0.999996191	0.999347049	0.981622418	0.99999488	0.999985264	0.999960414

REEP6	0.999996191	0.999347049	0.981622418	0.99999488	0.999985264	0.999960414
BLOC1S4	0.999996191	0.999992444	0.981622418	0.99999488	0.999985264	0.999960414
LOC104973746	0.999996191	0.999992444	0.981622418	0.99999488	0.999985264	0.999960414
PSMG3	0.999996191	0.999992444	0.981622418	0.99999488	0.999985264	0.999960414
SUSD6	0.999996191	0.999992444	0.981622418	0.99999488	0.999985264	0.999960414
TOX2	0.999996191	0.999992444	0.981622418	0.99999488	0.999985264	0.999960414
USP16	0.999996191	0.999992444	0.981622418	0.99999488	0.999985264	0.999960414
ZFAND6	0.999996191	0.999992444	0.981622418	0.99999488	0.999985264	0.999960414
MEN1	0.999996191	0.999992444	0.981666445	0.99999488	0.999985264	0.999960414
MSRB2	0.999996191	0.999992444	0.981666445	0.99999488	0.999985264	0.999960414
LOC782688	0.999996191	0.999347049	0.981715036	0.99999488	0.999985264	0.999960414
GLUD1	0.999996191	0.999992444	0.981715036	0.99999488	0.999985264	0.999960414
PIK3R3	0.999996191	0.991365955	0.981736235	0.99999488	0.999985264	0.999960414
NACA	0.999996191	0.998131745	0.981736235	0.99999488	0.999985264	0.999960414
C1QTNF7	0.999996191	0.999992444	0.981736235	0.99999488	0.999985264	0.999960414
DUSP5	0.999996191	0.999992444	0.981736235	0.99999488	0.999985264	0.999960414
GSC2	0.999996191	0.999992444	0.981736235	0.99999488	0.999985264	0.999960414
TMEM102	0.999996191	0.999992444	0.981736235	0.99999488	0.999985264	0.999960414
LOC100140207	0.999996191	0.999992444	0.981764406	0.99999488	0.999985264	0.999960414
SERINC1	0.999996191	0.999992444	0.981940287	0.99999488	0.999985264	0.999960414
LOC112449558	0.999996191	0.999347049	0.982065621	0.99999488	0.999985264	0.999960414
ZNF330	0.999996191	0.999992444	0.982128584	0.99999488	0.999985264	0.999960414
TRIT1	0.999996191	0.998131745	0.982433474	0.99999488	0.999985264	0.999960414
TRRAP	0.999996191	0.999992444	0.982433474	0.99999488	0.999985264	0.999960414
EXOSC10	0.999996191	0.999992444	0.98253615	0.99999488	0.999985264	0.999960414
FAM208A	0.999996191	0.998131745	0.982783495	0.99999488	0.999985264	0.999960414
RBM12B	0.999996191	0.999992444	0.982783495	0.99999488	0.999985264	0.999960414
TMEM273	0.999996191	0.999992444	0.982783495	0.99999488	0.999985264	0.999960414
PRR18	0.999996191	0.999992444	0.982820713	0.99999488	0.999985264	0.999960414
ETV6	0.999996191	0.999992444	0.98286145	0.99999488	0.999985264	0.999960414
MOCS3	0.999996191	0.999992444	0.98286145	0.99999488	0.999985264	0.999960414
RHBDF2	0.999996191	0.999992444	0.98286145	0.99999488	0.999985264	0.999960414
RIMBP2	0.999996191	0.998131745	0.982978515	0.99999488	0.999985264	0.999960414
BBOF1	0.999996191	0.999992444	0.982978515	0.99999488	0.999985264	0.999960414
CCDC102B	0.999996191	0.966072944	0.983077441	0.99999488	0.999985264	0.999960414
NEK6	0.999996191	0.991476708	0.983077441	0.99999488	0.999985264	0.999960414
CEP83	0.999996191	0.999992444	0.983077441	0.99999488	0.999985264	0.999960414
FRAT2	0.999996191	0.999992444	0.983077441	0.99999488	0.999985264	0.999960414
MSANTD3	0.999996191	0.999347049	0.983212944	0.99999488	0.999985264	0.999960414

DNAJC10	0.999996191	0.999992444	0.983419007	0.99999488	0.999985264	0.999960414
SLC30A7	0.999996191	0.999992444	0.983419007	0.99999488	0.999985264	0.999960414
LOC100336104	0.999996191	0.987262615	0.983938648	0.99999488	0.999985264	0.999960414
LOC104968518	0.999996191	0.998131745	0.9840454	0.99999488	0.999985264	0.999960414
SIRT2	0.999996191	0.989547896	0.984309048	0.99999488	0.999985264	0.999960414
LOC100139345	0.999996191	0.958098081	0.984444908	0.99999488	0.999985264	0.999960414
FBXO44	0.999996191	0.969534305	0.984444908	0.99999488	0.999985264	0.999960414
ARHGAP21	0.999996191	0.976717114	0.984444908	0.99999488	0.999985264	0.999960414
FEZ1	0.999996191	0.991488652	0.984444908	0.99999488	0.999985264	0.999960414
CERS5	0.999996191	0.992977597	0.984444908	0.99999488	0.999985264	0.999960414
LOC507930	0.999996191	0.992977597	0.984444908	0.99999488	0.999985264	0.999960414
USP14	0.999996191	0.993316598	0.984444908	0.99999488	0.999985264	0.999960414
C21H15orf40	0.999996191	0.999992444	0.984444908	0.99999488	0.999985264	0.999960414
CKAP5	0.999996191	0.999992444	0.984444908	0.99999488	0.999985264	0.999960414
PIP5K1A	0.999996191	0.999992444	0.984444908	0.99999488	0.999985264	0.999960414
RFX3	0.999996191	0.999992444	0.984444908	0.99999488	0.999985264	0.999960414
SWT1	0.999996191	0.999992444	0.984444908	0.99999488	0.999985264	0.999960414
VAV3	0.999996191	0.999992444	0.984444908	0.99999488	0.999985264	0.999960414
YAP1	0.999996191	0.999992444	0.984444908	0.99999488	0.999985264	0.999960414
ZBTB41	0.999996191	0.999992444	0.984444908	0.99999488	0.999985264	0.999960414
LOC112444770	0.999996191	0.999992444	0.984619622	0.99999488	0.999985264	0.999960414
MAN1B1	0.999996191	0.999992444	0.984631623	0.99999488	0.999985264	0.999960414
MTHFD2L	0.999996191	0.999992444	0.984631623	0.99999488	0.999985264	0.999960414
CSGALNACT1	0.999996191	0.985406717	0.984765193	0.99999488	0.999985264	0.999960414
DPF1	0.999996191	0.985406717	0.984765193	0.99999488	0.999985264	0.999960414
CLN6	0.999996191	0.996966166	0.984765193	0.99999488	0.999985264	0.999960414
SERTAD4	0.999996191	0.997975376	0.98480229	0.99999488	0.999985264	0.999960414
LOC613664	0.999996191	0.999347049	0.98480229	0.99999488	0.999985264	0.999960414
LOC112442248	0.999996191	0.999992444	0.98480229	0.99999488	0.999985264	0.999960414
LOC788541	0.999996191	0.999992444	0.98480229	0.99999488	0.999985264	0.999960414
NANP	0.999996191	0.999992444	0.98480229	0.99999488	0.999985264	0.999960414
RILP	0.999996191	0.999992444	0.98480229	0.99999488	0.999985264	0.999960414
MYL4	0.999996191	0.991488652	0.984814673	0.99999488	0.999985264	0.999960414
LOC112448062	0.999996191	0.998131745	0.984814673	0.99999488	0.999985264	0.999960414
KCNK7	0.999996191	0.999992444	0.984814673	0.99999488	0.999985264	0.999960414
LIMD1	0.999996191	0.999992444	0.984814673	0.99999488	0.999985264	0.999960414
GDI2	0.999996191	0.954470324	0.985077073	0.99999488	0.999985264	0.999960414
CHTF8	0.999996191	0.999347049	0.985202052	0.99999488	0.999985264	0.999960414
SAMD12	0.999996191	0.999992444	0.985202052	0.99999488	0.999985264	0.999960414

OGA	0.999996191	0.991488652	0.985363931	0.99999488	0.999985264	0.999960414
LOC104971266	0.999996191	0.992977597	0.985363931	0.99999488	0.999985264	0.999960414
ANKRD50	0.999996191	0.993316598	0.985363931	0.99999488	0.999985264	0.999960414
RAB5B	0.999996191	0.998131745	0.985363931	0.99999488	0.999985264	0.999960414
LOC784866	0.999996191	0.999347049	0.985363931	0.99999488	0.999985264	0.999960414
AKT2	0.999996191	0.999992444	0.985363931	0.99999488	0.999985264	0.999960414
ALG6	0.999996191	0.999992444	0.985363931	0.99999488	0.999985264	0.999960414
B3GALNT1	0.999996191	0.999992444	0.985363931	0.99999488	0.999985264	0.999960414
C14H8orf88	0.999996191	0.999992444	0.985363931	0.99999488	0.999985264	0.999960414
C21H14orf132	0.999996191	0.999992444	0.985363931	0.99999488	0.999985264	0.999960414
C22H3orf49	0.999996191	0.999992444	0.985363931	0.99999488	0.999985264	0.999960414
CASP4	0.999996191	0.999992444	0.985363931	0.99999488	0.999985264	0.999960414
GDNF	0.999996191	0.999992444	0.985363931	0.99999488	0.999985264	0.999960414
HEY1	0.999996191	0.999992444	0.985363931	0.99999488	0.999985264	0.999960414
LOC112447041	0.999996191	0.999992444	0.985363931	0.99999488	0.999985264	0.999960414
NLGN1	0.999996191	0.999992444	0.985363931	0.99999488	0.999985264	0.999960414
SCFD1	0.999996191	0.999992444	0.985363931	0.99999488	0.999985264	0.999960414
SERTAD3	0.999996191	0.999992444	0.985363931	0.99999488	0.999985264	0.999960414
SLC35B2	0.999996191	0.999992444	0.985363931	0.99999488	0.999985264	0.999960414
LOC112443859	0.999996191	0.999992444	0.985393722	0.99999488	0.999985264	0.999960414
XKR4	0.999996191	0.991488652	0.985789565	0.99999488	0.999985264	0.999960414
PROCR	0.999996191	0.999992444	0.985789565	0.99999488	0.999985264	0.999960414
TIMP4	0.999996191	0.999992444	0.985789565	0.99999488	0.999985264	0.999960414
CDC123	0.999996191	0.999992444	0.985944206	0.99999488	0.999985264	0.999960414
TSTD2	0.999996191	0.996626851	0.985966356	0.99999488	0.999985264	0.999960414
ASAH1	0.999996191	0.999992444	0.985966356	0.99999488	0.999985264	0.999960414
CDK20	0.999996191	0.999992444	0.985966356	0.99999488	0.999985264	0.999960414
CDKN2D	0.999996191	0.999992444	0.985966356	0.99999488	0.999985264	0.999960414
CYSRT1	0.999996191	0.999992444	0.985966356	0.99999488	0.999985264	0.999960414
LNPK	0.999996191	0.999992444	0.985966356	0.99999488	0.999985264	0.999960414
LOC100336602	0.999996191	0.999992444	0.985966356	0.99999488	0.999985264	0.999960414
LOC100848869	0.999996191	0.999992444	0.985966356	0.99999488	0.999985264	0.999960414
MAN2B1	0.999996191	0.999992444	0.985966356	0.99999488	0.999985264	0.999960414
MARK2	0.999996191	0.999992444	0.985966356	0.99999488	0.999985264	0.999960414
NOP58	0.999996191	0.999992444	0.985966356	0.99999488	0.999985264	0.999960414
ZAP70	0.999996191	0.999992444	0.986050103	0.99999488	0.999985264	0.999960414
CSTF2	0.999996191	0.999992444	0.986180419	0.99999488	0.999985264	0.999960414
LPL	0.999996191	0.999992444	0.986180419	0.99999488	0.999985264	0.999960414
LRCH3	0.999996191	0.999992444	0.986180419	0.99999488	0.999985264	0.999960414

SPAAR	0.999996191	0.999347049	0.986266701	0.99999488	0.999985264	0.999960414
GIN1	0.999996191	0.999992444	0.986930681	0.99999488	0.999985264	0.999960414
LOC104972545	0.999996191	0.999992444	0.986938092	0.99999488	0.999985264	0.999960414
SV2A	0.999996191	0.999992444	0.986938092	0.99999488	0.999985264	0.999960414
GRINA	0.999996191	0.999992444	0.986948824	0.99999488	0.999985264	0.999960414
BLZF1	0.999996191	0.999992444	0.987198143	0.99999488	0.999985264	0.999960414
LOC531679	0.999996191	0.999992444	0.987661078	0.99999488	0.999985264	0.999960414
MPDU1	0.999996191	0.999992444	0.987678394	0.99999488	0.999985264	0.999960414
PACSIN3	0.999996191	0.999992444	0.987678394	0.99999488	0.999985264	0.999960414
TINF2	0.999996191	0.999992444	0.987678394	0.99999488	0.999985264	0.999960414
TRIM52	0.999996191	0.999992444	0.987678394	0.99999488	0.999985264	0.999960414
ZNF585A	0.999996191	0.999992444	0.987678394	0.99999488	0.999985264	0.999960414
LEO1	0.999996191	0.996966166	0.987691491	0.99999488	0.999985264	0.999960414
HIVEP1	0.999996191	0.999992444	0.987693608	0.99999488	0.999985264	0.999960414
CMPK1	0.999996191	0.999347049	0.987711058	0.99999488	0.999985264	0.999960414
LOC786015	0.999996191	0.998131745	0.987884324	0.99999488	0.999985264	0.999960414
LOC786258	0.999996191	0.979263532	0.988161083	0.99999488	0.999985264	0.999960414
RAP1GAP	0.999996191	0.991488652	0.988161083	0.99999488	0.999985264	0.999960414
DBR1	0.999996191	0.957700659	0.988249967	0.99999488	0.999985264	0.999960414
HRCT1	0.999996191	0.999992444	0.988256989	0.99999488	0.999985264	0.999960414
NUP133	0.999996191	0.999992444	0.988537521	0.99999488	0.999985264	0.999960414
TRAPPC2	0.999996191	0.999992444	0.988537521	0.99999488	0.999985264	0.999960414
DIS3L2	0.999996191	0.978510121	0.988922732	0.99999488	0.999985264	0.999960414
GINS2	0.999996191	0.991365955	0.988922732	0.99999488	0.999985264	0.999960414
CHMP5	0.999996191	0.991488652	0.988922732	0.99999488	0.999985264	0.999960414
GCLC	0.999996191	0.992977597	0.988922732	0.99999488	0.999985264	0.999960414
SMIM1	0.999996191	0.993885692	0.988922732	0.99999488	0.999985264	0.999960414
KMT2C	0.999996191	0.998131745	0.988922732	0.99999488	0.999985264	0.999960414
DGAT2	0.999996191	0.999347049	0.988922732	0.99999488	0.999985264	0.999960414
LOC104973431	0.999996191	0.999347049	0.988922732	0.99999488	0.999985264	0.999960414
ATP6AP1L	0.999996191	0.999992444	0.988922732	0.99999488	0.999985264	0.999960414
HILPDA	0.999996191	0.999992444	0.988922732	0.99999488	0.999985264	0.999960414
LOC100847143	0.999996191	0.999992444	0.988922732	0.99999488	0.999985264	0.999960414
LOC112442987	0.999996191	0.999992444	0.988922732	0.99999488	0.999985264	0.999960414
NUP88	0.999996191	0.999992444	0.988922732	0.99999488	0.999985264	0.999960414
NYX	0.999996191	0.999992444	0.988922732	0.99999488	0.999985264	0.999960414
PQLC3	0.999996191	0.999992444	0.988922732	0.99999488	0.999985264	0.999960414
RWDD2A	0.999996191	0.999992444	0.988922732	0.99999488	0.999985264	0.999960414
SLC9A8	0.999996191	0.999992444	0.988922732	0.99999488	0.999985264	0.999960414

LOC112443416	0.999996191	0.991365955	0.989367576	0.99999488	0.999985264	0.999960414
MIS12	0.999996191	0.999347049	0.989872445	0.99999488	0.999985264	0.999960414
RPRD1A	0.999996191	0.999992444	0.989872445	0.99999488	0.999985264	0.999960414
TNNT1	0.999996191	0.999992444	0.989872445	0.99999488	0.999985264	0.999960414
TTLL1	0.999996191	0.999992444	0.989872445	0.99999488	0.999985264	0.999960414
ZNF513	0.999996191	0.999992444	0.989872445	0.99999488	0.999985264	0.999960414
PKD2	0.999996191	0.999992444	0.990174738	0.99999488	0.999985264	0.999960414
LOC107132577	0.999996191	0.999992444	0.990201471	0.99999488	0.999985264	0.999960414
CAPN3	0.999996191	0.991488652	0.990216356	0.99999488	0.999985264	0.999960414
HOXA2	0.999996191	0.999992444	0.990216356	0.99999488	0.999985264	0.999960414
RHBDL2	0.999996191	0.999992444	0.990216356	0.99999488	0.999985264	0.999960414
XPNPEP2	0.999996191	0.999992444	0.990216356	0.99999488	0.999985264	0.999960414
LOC101906206	0.999996191	0.984366454	0.99035966	0.99999488	0.999985264	0.999960414
LOC112449516	0.999996191	0.992977597	0.99035966	0.99999488	0.999985264	0.999960414
DOK1	0.999996191	0.999347049	0.99035966	0.99999488	0.999985264	0.999960414
CHRM3	0.999996191	0.999992444	0.99035966	0.99999488	0.999985264	0.999960414
CTDSP1	0.999996191	0.999992444	0.99035966	0.99999488	0.999985264	0.999960414
KAT2A	0.999996191	0.999992444	0.99035966	0.99999488	0.999985264	0.999960414
SIPA1L1	0.999996191	0.999992444	0.99035966	0.99999488	0.999985264	0.999960414
LOC107132296	0.999996191	0.999992444	0.990697261	0.99999488	0.999985264	0.999960414
HSD11B1L	0.999996191	0.999992444	0.990752015	0.99999488	0.999985264	0.999960414
LOC112442611	0.999996191	0.999992444	0.990752015	0.99999488	0.999985264	0.999960414
ARHGAP31	0.999996191	0.999992444	0.990780762	0.99999488	0.999985264	0.999960414
LOC511937	0.999996191	0.999992444	0.990780762	0.99999488	0.999985264	0.999960414
LOC616427	0.999996191	0.999992444	0.990780762	0.99999488	0.999985264	0.999960414
HIST1H1E	0.999996191	0.988850552	0.990862264	0.99999488	0.999985264	0.999960414
RMDN3	0.999996191	0.999347049	0.991285432	0.99999488	0.999985264	0.999960414
ECE1	0.999996191	0.999992444	0.991285432	0.99999488	0.999985264	0.999960414
EXT1	0.999996191	0.999992444	0.991285432	0.99999488	0.999985264	0.999960414
KLHL38	0.999996191	0.999992444	0.991285432	0.99999488	0.999985264	0.999960414
LOC101903098	0.999996191	0.999992444	0.991285432	0.99999488	0.999985264	0.999960414
LOC101903114	0.999996191	0.999992444	0.991285432	0.99999488	0.999985264	0.999960414
LOC112446771	0.999996191	0.999992444	0.991285432	0.99999488	0.999985264	0.999960414
LOC112447769	0.999996191	0.999992444	0.991285432	0.99999488	0.999985264	0.999960414
LOC112448848	0.999996191	0.999992444	0.991285432	0.99999488	0.999985264	0.999960414
MXRA7	0.999996191	0.999992444	0.991285432	0.99999488	0.999985264	0.999960414
NFKBIB	0.999996191	0.999992444	0.991285432	0.99999488	0.999985264	0.999960414
PSMD10	0.999996191	0.999992444	0.991285432	0.99999488	0.999985264	0.999960414
SCAI	0.999996191	0.999992444	0.991615606	0.99999488	0.999985264	0.999960414

MAP3K7CL	0.999996191	0.999992444	0.991868009	0.99999488	0.999985264	0.999960414
CCDC22	0.999996191	0.999992444	0.991955927	0.99999488	0.999985264	0.999960414
ASB14	0.999996191	0.999992444	0.991972013	0.99999488	0.999985264	0.999960414
BAMBI	0.999996191	0.999992444	0.991992782	0.99999488	0.999985264	0.999960414
BCKDHB	0.999996191	0.999992444	0.991992782	0.99999488	0.999985264	0.999960414
BPI	0.999996191	0.999992444	0.991992782	0.99999488	0.999985264	0.999960414
C1QTNF9	0.999996191	0.999992444	0.991992782	0.99999488	0.999985264	0.999960414
DHX9	0.999996191	0.999992444	0.991992782	0.99999488	0.999985264	0.999960414
FGF16	0.999996191	0.999992444	0.991992782	0.99999488	0.999985264	0.999960414
HADHB	0.999996191	0.999992444	0.991992782	0.99999488	0.999985264	0.999960414
LOC101903928	0.999996191	0.999992444	0.991992782	0.99999488	0.999985264	0.999960414
ZNF202	0.999996191	0.999992444	0.991992782	0.99999488	0.999985264	0.999960414
C5H12orf4	0.999996191	0.999347049	0.992080972	0.99999488	0.999985264	0.999960414
LOC104975890	0.999996191	0.999992444	0.992300408	0.99999488	0.999985264	0.999960414
NCOA4	0.999996191	0.999992444	0.992337371	0.99999488	0.999985264	0.999960414
ANKRD61	0.999996191	0.964246761	0.992709008	0.99999488	0.999985264	0.999960414
TRIM62	0.999996191	0.992977597	0.992709008	0.99999488	0.999985264	0.999960414
KCNB2	0.999996191	0.99398773	0.992709008	0.99999488	0.999985264	0.999960414
CRTC3	0.999996191	0.99490847	0.992709008	0.99999488	0.999985264	0.999960414
LOC112442693	0.999996191	0.999347049	0.992709008	0.99999488	0.999985264	0.999960414
GPR161	0.999996191	0.999992444	0.992709008	0.99999488	0.999985264	0.999960414
JKAMP	0.999996191	0.999992444	0.992709008	0.99999488	0.999985264	0.999960414
LOC101905571	0.999996191	0.999992444	0.992709008	0.99999488	0.999985264	0.999960414
LOC618256	0.999996191	0.999992444	0.992709008	0.99999488	0.999985264	0.999960414
LONP2	0.999996191	0.999992444	0.992709008	0.99999488	0.999985264	0.999960414
NECTIN3	0.999996191	0.999992444	0.992709008	0.99999488	0.999985264	0.999960414
PLVAP	0.999996191	0.999992444	0.992709008	0.99999488	0.999985264	0.999960414
SPEF1	0.999996191	0.999992444	0.992709008	0.99999488	0.999985264	0.999960414
TFDP2	0.999996191	0.999992444	0.992709008	0.99999488	0.999985264	0.999960414
TTC8	0.999996191	0.999992444	0.992709008	0.99999488	0.999985264	0.999960414
TXLNG	0.999996191	0.999992444	0.992709008	0.99999488	0.999985264	0.999960414
MBD6	0.999996191	0.963024093	0.992742113	0.99999488	0.999985264	0.999960414
LOC781688	0.999996191	0.988850552	0.992742113	0.99999488	0.999985264	0.999960414
CTNNA3	0.999996191	0.998131745	0.992742113	0.99999488	0.999985264	0.999960414
ATP5S	0.999996191	0.999992444	0.992742113	0.99999488	0.999985264	0.999960414
C16H1orf53	0.999996191	0.999992444	0.992742113	0.99999488	0.999985264	0.999960414
CD109	0.999996191	0.999992444	0.992742113	0.99999488	0.999985264	0.999960414
CIRBP	0.999996191	0.999992444	0.992742113	0.99999488	0.999985264	0.999960414
LOC101904753	0.999996191	0.999992444	0.992742113	0.99999488	0.999985264	0.999960414

LOC101908123	0.999996191	0.999992444	0.992742113	0.99999488	0.999985264	0.999960414
LOC783926	0.999996191	0.999992444	0.992742113	0.99999488	0.999985264	0.999960414
NUBP1	0.999996191	0.999992444	0.992742113	0.99999488	0.999985264	0.999960414
UBTD1	0.999996191	0.999992444	0.992742113	0.99999488	0.999985264	0.999960414
ING3	0.999996191	0.999992444	0.992939335	0.99999488	0.999985264	0.999960414
LYZ1	0.999996191	0.999992444	0.992939335	0.99999488	0.999985264	0.999960414
LOC112443476	0.999996191	0.999992444	0.993132892	0.99999488	0.999985264	0.999960414
PRSS48	0.999996191	0.999992444	0.993132892	0.99999488	0.999985264	0.999960414
ST6GALNAC2	0.999996191	0.999992444	0.993132892	0.99999488	0.999985264	0.999960414
VPS13D	0.999996191	0.999992444	0.993132892	0.99999488	0.999985264	0.999960414
LOC514680	0.999996191	0.999992444	0.993228629	0.99999488	0.999985264	0.999960414
PLPP6	0.999996191	0.999992444	0.993228629	0.99999488	0.999985264	0.999960414
RANBP3	0.999996191	0.999992444	0.993303055	0.99999488	0.999985264	0.999960414
ING4	0.999996191	0.999992444	0.99340527	0.99999488	0.999985264	0.999960414
LOC783195	0.999996191	0.999992444	0.993484228	0.99999488	0.999985264	0.999960414
TTC17	0.999996191	0.999992444	0.993535718	0.99999488	0.999985264	0.999960414
LOC101906546	0.999996191	0.999992444	0.993585211	0.99999488	0.999985264	0.999960414
APCDD1L	0.999996191	0.980867747	0.993594894	0.99999488	0.999985264	0.999960414
USO1	0.999996191	0.991488652	0.993594894	0.99999488	0.999985264	0.999960414
MIOS	0.999996191	0.992977597	0.993594894	0.99999488	0.999985264	0.999960414
MXD3	0.999996191	0.992977597	0.993594894	0.99999488	0.999985264	0.999960414
C11H2orf42	0.999996191	0.997975376	0.993594894	0.99999488	0.999985264	0.999960414
AHNAK	0.999996191	0.998131745	0.993594894	0.99999488	0.999985264	0.999960414
LOC101905199	0.999996191	0.998501121	0.993594894	0.99999488	0.999985264	0.999960414
XPO7	0.999996191	0.999347049	0.993594894	0.99999488	0.999985264	0.999960414
ARL6	0.999996191	0.999992444	0.993594894	0.99999488	0.999985264	0.999960414
BCHE	0.999996191	0.999992444	0.993594894	0.99999488	0.999985264	0.999960414
BRD3	0.999996191	0.999992444	0.993594894	0.99999488	0.999985264	0.999960414
BRD7	0.999996191	0.999992444	0.993594894	0.99999488	0.999985264	0.999960414
CARD11	0.999996191	0.999992444	0.993594894	0.99999488	0.999985264	0.999960414
CD302	0.999996191	0.999992444	0.993594894	0.99999488	0.999985264	0.999960414
CRY2	0.999996191	0.999992444	0.993594894	0.99999488	0.999985264	0.999960414
CYBRD1	0.999996191	0.999992444	0.993594894	0.99999488	0.999985264	0.999960414
EMILIN2	0.999996191	0.999992444	0.993594894	0.99999488	0.999985264	0.999960414
FAM149A	0.999996191	0.999992444	0.993594894	0.99999488	0.999985264	0.999960414
GEMIN7	0.999996191	0.999992444	0.993594894	0.99999488	0.999985264	0.999960414
GPANK1	0.999996191	0.999992444	0.993594894	0.99999488	0.999985264	0.999960414
HCST	0.999996191	0.999992444	0.993594894	0.99999488	0.999985264	0.999960414
HNRNPDL	0.999996191	0.999992444	0.993594894	0.99999488	0.999985264	0.999960414

KDM1A	0.999996191	0.999992444	0.993594894	0.99999488	0.999985264	0.999960414
LEPR	0.999996191	0.999992444	0.993594894	0.99999488	0.999985264	0.999960414
LOC100337495	0.999996191	0.999992444	0.993594894	0.99999488	0.999985264	0.999960414
LOC100847759	0.999996191	0.999992444	0.993594894	0.99999488	0.999985264	0.999960414
LOC101905141	0.999996191	0.999992444	0.993594894	0.99999488	0.999985264	0.999960414
LOC101907577	0.999996191	0.999992444	0.993594894	0.99999488	0.999985264	0.999960414
LOC104971501	0.999996191	0.999992444	0.993594894	0.99999488	0.999985264	0.999960414
LOC112449615	0.999996191	0.999992444	0.993594894	0.99999488	0.999985264	0.999960414
LOC510798	0.999996191	0.999992444	0.993594894	0.99999488	0.999985264	0.999960414
LOC512617	0.999996191	0.999992444	0.993594894	0.99999488	0.999985264	0.999960414
LOC530348	0.999996191	0.999992444	0.993594894	0.99999488	0.999985264	0.999960414
LTB4R2	0.999996191	0.999992444	0.993594894	0.99999488	0.999985264	0.999960414
MAPKBP1	0.999996191	0.999992444	0.993594894	0.99999488	0.999985264	0.999960414
MICAL3	0.999996191	0.999992444	0.993594894	0.99999488	0.999985264	0.999960414
MMD	0.999996191	0.999992444	0.993594894	0.99999488	0.999985264	0.999960414
PANK4	0.999996191	0.999992444	0.993594894	0.99999488	0.999985264	0.999960414
PLIN1	0.999996191	0.999992444	0.993594894	0.99999488	0.999985264	0.999960414
RAB3D	0.999996191	0.999992444	0.993594894	0.99999488	0.999985264	0.999960414
RBFOX3	0.999996191	0.999992444	0.993594894	0.99999488	0.999985264	0.999960414
RHBDL3	0.999996191	0.999992444	0.993594894	0.99999488	0.999985264	0.999960414
RHOQ	0.999996191	0.999992444	0.993594894	0.99999488	0.999985264	0.999960414
ROBO4	0.999996191	0.999992444	0.993594894	0.99999488	0.999985264	0.999960414
SKI	0.999996191	0.999992444	0.993594894	0.99999488	0.999985264	0.999960414
SLC25A28	0.999996191	0.999992444	0.993594894	0.99999488	0.999985264	0.999960414
SOCS4	0.999996191	0.999992444	0.993594894	0.99999488	0.999985264	0.999960414
STIM2	0.999996191	0.999992444	0.993594894	0.99999488	0.999985264	0.999960414
THAP6	0.999996191	0.999992444	0.993594894	0.99999488	0.999985264	0.999960414
TMEM19	0.999996191	0.999992444	0.993594894	0.99999488	0.999985264	0.999960414
TTC7A	0.999996191	0.999992444	0.993594894	0.99999488	0.999985264	0.999960414
USP49	0.999996191	0.999992444	0.993594894	0.99999488	0.999985264	0.999960414
WNT7B	0.999996191	0.999992444	0.993594894	0.99999488	0.999985264	0.999960414
ZDHHC12	0.999996191	0.999992444	0.993594894	0.99999488	0.999985264	0.999960414
RFTN2	0.999996191	0.999992444	0.99397371	0.99999488	0.999985264	0.999960414
ESRRA	0.999996191	0.999992444	0.994152361	0.99999488	0.999985264	0.999960414
LOC785693	0.999996191	0.999992444	0.994152361	0.99999488	0.999985264	0.999960414
CCDC142	0.999996191	0.9582784	0.99436655	0.99999488	0.999985264	0.999960414
CCT3	0.999996191	0.994481342	0.99436655	0.99999488	0.999985264	0.999960414
CUX1	0.999996191	0.999992444	0.99436655	0.99999488	0.999985264	0.999960414
LOC782776	0.999996191	0.999992444	0.994539721	0.99999488	0.999985264	0.999960414

TNFRSF8	0.999996191	0.931336072	0.994601277	0.99999488	0.999985264	0.999960414
RXRG	0.999996191	0.998131745	0.994601277	0.99999488	0.999985264	0.999960414
FAM117B	0.999996191	0.999992444	0.994601277	0.99999488	0.999985264	0.999960414
FBXW8	0.999996191	0.999992444	0.994601277	0.99999488	0.999985264	0.999960414
FEZ2	0.999996191	0.999992444	0.994601277	0.99999488	0.999985264	0.999960414
KIFC3	0.999996191	0.999992444	0.994601277	0.99999488	0.999985264	0.999960414
LOC100847546	0.999996191	0.999992444	0.994601277	0.99999488	0.999985264	0.999960414
LOC100847613	0.999996191	0.999992444	0.994601277	0.99999488	0.999985264	0.999960414
LOC104975027	0.999996191	0.999992444	0.994601277	0.99999488	0.999985264	0.999960414
LOC112447802	0.999996191	0.999992444	0.994601277	0.99999488	0.999985264	0.999960414
MBLAC1	0.999996191	0.999992444	0.994601277	0.99999488	0.999985264	0.999960414
NDFIP2	0.999996191	0.999992444	0.994601277	0.99999488	0.999985264	0.999960414
PTGIS	0.999996191	0.999992444	0.994601277	0.99999488	0.999985264	0.999960414
RARB	0.999996191	0.999992444	0.994601277	0.99999488	0.999985264	0.999960414
SFXN4	0.999996191	0.999992444	0.994601277	0.99999488	0.999985264	0.999960414
SMIM33	0.999996191	0.999992444	0.994601277	0.99999488	0.999985264	0.999960414
SYTL4	0.999996191	0.999992444	0.994601277	0.99999488	0.999985264	0.999960414
UPF3A	0.999996191	0.999992444	0.994601277	0.99999488	0.999985264	0.999960414
VPS13B	0.999996191	0.999992444	0.994601277	0.99999488	0.999985264	0.999960414
ZNF814	0.999996191	0.999992444	0.994601277	0.99999488	0.999985264	0.999960414
CD80	0.999996191	0.992977597	0.994626276	0.99999488	0.999985264	0.999960414
PRX	0.999996191	0.994481342	0.994626276	0.99999488	0.999985264	0.999960414
COPS4	0.999996191	0.999992444	0.994626276	0.99999488	0.999985264	0.999960414
FAR1	0.999996191	0.999992444	0.994626276	0.99999488	0.999985264	0.999960414
HSPBAP1	0.999996191	0.999992444	0.994626276	0.99999488	0.999985264	0.999960414
LOC101905453	0.999996191	0.999992444	0.994626276	0.99999488	0.999985264	0.999960414
LOC112441543	0.999996191	0.999992444	0.994626276	0.99999488	0.999985264	0.999960414
RING1	0.999996191	0.999992444	0.994626276	0.99999488	0.999985264	0.999960414
SDHAF3	0.999996191	0.999992444	0.994626276	0.99999488	0.999985264	0.999960414
ZNF16	0.999996191	0.999992444	0.994626276	0.99999488	0.999985264	0.999960414
DUSP10	0.999996191	0.999992444	0.994879215	0.99999488	0.999985264	0.999960414
ADD1	0.999996191	0.999992444	0.994916815	0.99999488	0.999985264	0.999960414
SLCO2A1	0.999996191	0.999992444	0.994916815	0.99999488	0.999985264	0.999960414
SMC6	0.999996191	0.999992444	0.994916815	0.99999488	0.999985264	0.999960414
LOC781646	0.999996191	0.964246761	0.995045982	0.99999488	0.999985264	0.999960414
DCP1B	0.999996191	0.974812121	0.995045982	0.99999488	0.999985264	0.999960414
LOC784488	0.999996191	0.993316598	0.995045982	0.99999488	0.999985264	0.999960414
PRDM6	0.999996191	0.994481342	0.995045982	0.99999488	0.999985264	0.999960414
ECH1	0.999996191	0.999347049	0.995045982	0.99999488	0.999985264	0.999960414

AMBRA1	0.999996191	0.999992444	0.995045982	0.99999488	0.999985264	0.999960414
ATF5	0.999996191	0.999992444	0.995045982	0.99999488	0.999985264	0.999960414
ATXN1	0.999996191	0.999992444	0.995045982	0.99999488	0.999985264	0.999960414
CDR2L	0.999996191	0.999992444	0.995045982	0.99999488	0.999985264	0.999960414
CHEK2	0.999996191	0.999992444	0.995045982	0.99999488	0.999985264	0.999960414
DNTTIP1	0.999996191	0.999992444	0.995045982	0.99999488	0.999985264	0.999960414
EEF1B2	0.999996191	0.999992444	0.995045982	0.99999488	0.999985264	0.999960414
EPHB4	0.999996191	0.999992444	0.995045982	0.99999488	0.999985264	0.999960414
FEN1	0.999996191	0.999992444	0.995045982	0.99999488	0.999985264	0.999960414
HPCAL4	0.999996191	0.999992444	0.995045982	0.99999488	0.999985264	0.999960414
JUP	0.999996191	0.999992444	0.995045982	0.99999488	0.999985264	0.999960414
KIF21A	0.999996191	0.999992444	0.995045982	0.99999488	0.999985264	0.999960414
LOC100139990	0.999996191	0.999992444	0.995045982	0.99999488	0.999985264	0.999960414
LOC100296900	0.999996191	0.999992444	0.995045982	0.99999488	0.999985264	0.999960414
LOC100847357	0.999996191	0.999992444	0.995045982	0.99999488	0.999985264	0.999960414
LOC101901983	0.999996191	0.999992444	0.995045982	0.99999488	0.999985264	0.999960414
LOC101906966	0.999996191	0.999992444	0.995045982	0.99999488	0.999985264	0.999960414
LOC104972400	0.999996191	0.999992444	0.995045982	0.99999488	0.999985264	0.999960414
LRCH4	0.999996191	0.999992444	0.995045982	0.99999488	0.999985264	0.999960414
NOVA1	0.999996191	0.999992444	0.995045982	0.99999488	0.999985264	0.999960414
NRBP1	0.999996191	0.999992444	0.995045982	0.99999488	0.999985264	0.999960414
PHETA2	0.999996191	0.999992444	0.995045982	0.99999488	0.999985264	0.999960414
PHKB	0.999996191	0.999992444	0.995045982	0.99999488	0.999985264	0.999960414
RASA2	0.999996191	0.999992444	0.995045982	0.99999488	0.999985264	0.999960414
SLC22A5	0.999996191	0.999992444	0.995045982	0.99999488	0.999985264	0.999960414
SLC25A20	0.999996191	0.999992444	0.995045982	0.99999488	0.999985264	0.999960414
TMEM141	0.999996191	0.999992444	0.995045982	0.99999488	0.999985264	0.999960414
TPO	0.999996191	0.999992444	0.995045982	0.99999488	0.999985264	0.999960414
TRIM27	0.999996191	0.999992444	0.995045982	0.99999488	0.999985264	0.999960414
TTC3	0.999996191	0.999992444	0.995045982	0.99999488	0.999985264	0.999960414
TTC39A	0.999996191	0.999992444	0.995045982	0.99999488	0.999985264	0.999960414
ZBTB12	0.999996191	0.999992444	0.995045982	0.99999488	0.999985264	0.999960414
HCAR1	0.999996191	0.999992444	0.99534463	0.99999488	0.999985264	0.999960414
TRNAU1AP	0.999996191	0.999992444	0.99535777	0.99999488	0.999985264	0.999960414
RNPEPL1	0.999996191	0.999992444	0.995522557	0.99999488	0.999985264	0.999960414
SORBS1	0.999996191	0.999992444	0.995522557	0.99999488	0.999985264	0.999960414
CNIH4	0.999996191	0.999992444	0.995721518	0.99999488	0.999985264	0.999960414
CARS	0.999996191	0.999992444	0.996129075	0.99999488	0.999985264	0.999960414
NOS3	0.999996191	0.999992444	0.996139177	0.99999488	0.999985264	0.999960414

SMYD4	0.999996191	0.999992444	0.996139177	0.99999488	0.999985264	0.999960414
TBKBP1	0.999996191	0.998011664	0.996348351	0.99999488	0.999985264	0.999960414
TANC2	0.999996191	0.998659022	0.996348351	0.99999488	0.999985264	0.999960414
AFMID	0.999996191	0.999992444	0.996348351	0.99999488	0.999985264	0.999960414
ARHGAP6	0.999996191	0.999992444	0.996348351	0.99999488	0.999985264	0.999960414
ATR	0.999996191	0.999992444	0.996348351	0.99999488	0.999985264	0.999960414
CHRNA3	0.999996191	0.999992444	0.996348351	0.99999488	0.999985264	0.999960414
CLSTN2	0.999996191	0.999992444	0.996348351	0.99999488	0.999985264	0.999960414
DTWD1	0.999996191	0.999992444	0.996348351	0.99999488	0.999985264	0.999960414
EEF2	0.999996191	0.999992444	0.996348351	0.99999488	0.999985264	0.999960414
GNG3	0.999996191	0.999992444	0.996348351	0.99999488	0.999985264	0.999960414
HOXC6	0.999996191	0.999992444	0.996348351	0.99999488	0.999985264	0.999960414
ITGA11	0.999996191	0.999992444	0.996348351	0.99999488	0.999985264	0.999960414
LNPEP	0.999996191	0.999992444	0.996348351	0.99999488	0.999985264	0.999960414
LOC104968434	0.999996191	0.999992444	0.996348351	0.99999488	0.999985264	0.999960414
LOC104971613	0.999996191	0.999992444	0.996348351	0.99999488	0.999985264	0.999960414
LOC112446753	0.999996191	0.999992444	0.996348351	0.99999488	0.999985264	0.999960414
LOC510382	0.999996191	0.999992444	0.996348351	0.99999488	0.999985264	0.999960414
LOC785386	0.999996191	0.999992444	0.996348351	0.99999488	0.999985264	0.999960414
PTRHD1	0.999996191	0.999992444	0.996348351	0.99999488	0.999985264	0.999960414
SCG5	0.999996191	0.999992444	0.996348351	0.99999488	0.999985264	0.999960414
SCN3B	0.999996191	0.999992444	0.996348351	0.99999488	0.999985264	0.999960414
SPATA22	0.999996191	0.999992444	0.996348351	0.99999488	0.999985264	0.999960414
TAF13	0.999996191	0.999992444	0.996348351	0.99999488	0.999985264	0.999960414
TRDN	0.999996191	0.999992444	0.996348351	0.99999488	0.999985264	0.999960414
VPS72	0.999996191	0.999992444	0.996348351	0.99999488	0.999985264	0.999960414
CD58	0.999996191	0.998131745	0.996391386	0.99999488	0.999985264	0.999960414
SPPL3	0.999996191	0.999347049	0.996391386	0.99999488	0.999985264	0.999960414
CASP9	0.999996191	0.999992444	0.996391386	0.99999488	0.999985264	0.999960414
CHM	0.999996191	0.999992444	0.996391386	0.99999488	0.999985264	0.999960414
DNAJC12	0.999996191	0.999992444	0.996391386	0.99999488	0.999985264	0.999960414
ERCC6	0.999996191	0.999992444	0.996391386	0.99999488	0.999985264	0.999960414
FBXO47	0.999996191	0.999992444	0.996391386	0.99999488	0.999985264	0.999960414
GLB1L	0.999996191	0.999992444	0.996391386	0.99999488	0.999985264	0.999960414
GPAT3	0.999996191	0.999992444	0.996391386	0.99999488	0.999985264	0.999960414
GSTP1	0.999996191	0.999992444	0.996391386	0.99999488	0.999985264	0.999960414
KATNBL1	0.999996191	0.999992444	0.996391386	0.99999488	0.999985264	0.999960414
LOC100124497	0.999996191	0.999992444	0.996391386	0.99999488	0.999985264	0.999960414
LOC101906522	0.999996191	0.999992444	0.996391386	0.99999488	0.999985264	0.999960414

LOC112448474	0.999996191	0.999992444	0.996391386	0.99999488	0.999985264	0.999960414
LOC614617	0.999996191	0.999992444	0.996391386	0.99999488	0.999985264	0.999960414
LOC789192	0.999996191	0.999992444	0.996391386	0.99999488	0.999985264	0.999960414
LZTFL1	0.999996191	0.999992444	0.996391386	0.99999488	0.999985264	0.999960414
MAVS	0.999996191	0.999992444	0.996391386	0.99999488	0.999985264	0.999960414
PLCD4	0.999996191	0.999992444	0.996391386	0.99999488	0.999985264	0.999960414
PLEKHA8	0.999996191	0.999992444	0.996391386	0.99999488	0.999985264	0.999960414
RAB33B	0.999996191	0.999992444	0.996391386	0.99999488	0.999985264	0.999960414
SRSF4	0.999996191	0.999992444	0.996391386	0.99999488	0.999985264	0.999960414
STUM	0.999996191	0.999992444	0.996391386	0.99999488	0.999985264	0.999960414
TMEM81	0.999996191	0.999992444	0.996391386	0.99999488	0.999985264	0.999960414
WLS	0.999996191	0.999992444	0.996391386	0.99999488	0.999985264	0.999960414
ZBTB24	0.999996191	0.999992444	0.996391386	0.99999488	0.999985264	0.999960414
LOC100848766	0.999996191	0.999992444	0.996522781	0.99999488	0.999985264	0.999960414
ELMO1	0.999996191	0.999992444	0.996601949	0.99999488	0.999985264	0.999960414
NNAT	0.999996191	0.999992444	0.996676884	0.99999488	0.999985264	0.999960414
PEX10	0.999996191	0.985406717	0.996715234	0.99999488	0.999985264	0.999960414
MXD1	0.999996191	0.992977597	0.996715234	0.99999488	0.999985264	0.999960414
ADAMTS20	0.999996191	0.999992444	0.996715234	0.99999488	0.999985264	0.999960414
ADGRA3	0.999996191	0.999992444	0.996715234	0.99999488	0.999985264	0.999960414
BAIAP2	0.999996191	0.999992444	0.996715234	0.99999488	0.999985264	0.999960414
BARD1	0.999996191	0.999992444	0.996715234	0.99999488	0.999985264	0.999960414
CALD1	0.999996191	0.999992444	0.996715234	0.99999488	0.999985264	0.999960414
CLCN6	0.999996191	0.999992444	0.996715234	0.99999488	0.999985264	0.999960414
DPYSL3	0.999996191	0.999992444	0.996715234	0.99999488	0.999985264	0.999960414
ELFN2	0.999996191	0.999992444	0.996715234	0.99999488	0.999985264	0.999960414
GSPT2	0.999996191	0.999992444	0.996715234	0.99999488	0.999985264	0.999960414
LOC112442039	0.999996191	0.999992444	0.996715234	0.99999488	0.999985264	0.999960414
LOC112443853	0.999996191	0.999992444	0.996715234	0.99999488	0.999985264	0.999960414
LOC112444215	0.999996191	0.999992444	0.996715234	0.99999488	0.999985264	0.999960414
LOC615989	0.999996191	0.999992444	0.996715234	0.99999488	0.999985264	0.999960414
LOC787679	0.999996191	0.999992444	0.996715234	0.99999488	0.999985264	0.999960414
MBOAT7	0.999996191	0.999992444	0.996715234	0.99999488	0.999985264	0.999960414
NUP85	0.999996191	0.999992444	0.996715234	0.99999488	0.999985264	0.999960414
PDLIM4	0.999996191	0.999992444	0.996715234	0.99999488	0.999985264	0.999960414
PHC2	0.999996191	0.999992444	0.996715234	0.99999488	0.999985264	0.999960414
PLEKHA2	0.999996191	0.999992444	0.996715234	0.99999488	0.999985264	0.999960414
SBNO1	0.999996191	0.999992444	0.996715234	0.99999488	0.999985264	0.999960414
SFXN3	0.999996191	0.999992444	0.996715234	0.99999488	0.999985264	0.999960414

SLC35E3	0.999996191	0.999992444	0.996715234	0.99999488	0.999985264	0.999960414
SLC37A1	0.999996191	0.999992444	0.996715234	0.99999488	0.999985264	0.999960414
STX6	0.999996191	0.999992444	0.996715234	0.99999488	0.999985264	0.999960414
TADA2A	0.999996191	0.999992444	0.996715234	0.99999488	0.999985264	0.999960414
TRIM47	0.999996191	0.999992444	0.996715234	0.99999488	0.999985264	0.999960414
TULP4	0.999996191	0.999992444	0.996715234	0.99999488	0.999985264	0.999960414
VPS37D	0.999996191	0.999992444	0.996715234	0.99999488	0.999985264	0.999960414
WDR81	0.999996191	0.999992444	0.996715234	0.99999488	0.999985264	0.999960414
ZER1	0.999996191	0.999992444	0.996715234	0.99999488	0.999985264	0.999960414
PPM1D	0.999996191	0.999992444	0.996717067	0.99999488	0.999985264	0.999960414
ATP6V1D	0.999996191	0.999992444	0.996776138	0.99999488	0.999985264	0.999960414
LOC101907322	0.999996191	0.999992444	0.996776138	0.99999488	0.999985264	0.999960414
LOC112441500	0.999996191	0.999992444	0.996776138	0.99999488	0.999985264	0.999960414
PGBD2	0.999996191	0.999992444	0.996776138	0.99999488	0.999985264	0.999960414
PCOLCE2	0.999996191	0.999992444	0.996853874	0.99999488	0.999985264	0.999960414
RPS3A	0.999996191	0.999992444	0.996879305	0.99999488	0.999985264	0.999960414
HAND1	0.999996191	0.999992444	0.99699426	0.99999488	0.999985264	0.999960414
CAMLG	0.999996191	0.999992444	0.997153389	0.99999488	0.999985264	0.999960414
HS3ST4	0.999996191	0.999992444	0.997153389	0.99999488	0.999985264	0.999960414
UGP2	0.999996191	0.992977597	0.997208738	0.99999488	0.999985264	0.999960414
LOC107132092	0.999996191	0.994464252	0.997208738	0.99999488	0.999985264	0.999960414
GPC6	0.999996191	0.999992444	0.997208738	0.99999488	0.999985264	0.999960414
LOC112448078	0.999996191	0.999992444	0.997208738	0.99999488	0.999985264	0.999960414
MSH6	0.999996191	0.999992444	0.997208738	0.99999488	0.999985264	0.999960414
NBEAL1	0.999996191	0.999992444	0.997208738	0.99999488	0.999985264	0.999960414
PECR	0.999996191	0.999992444	0.997208738	0.99999488	0.999985264	0.999960414
SF1	0.999996191	0.999992444	0.997208738	0.99999488	0.999985264	0.999960414
SF3B2	0.999996191	0.999992444	0.997208738	0.99999488	0.999985264	0.999960414
SH3BGRL2	0.999996191	0.999992444	0.997208738	0.99999488	0.999985264	0.999960414
SIRT3	0.999996191	0.999992444	0.997208738	0.99999488	0.999985264	0.999960414
TOR3A	0.999996191	0.999992444	0.997208738	0.99999488	0.999985264	0.999960414
U2SURP	0.999996191	0.999992444	0.997208738	0.99999488	0.999985264	0.999960414
WDFY1	0.999996191	0.999992444	0.997208738	0.99999488	0.999985264	0.999960414
ATG101	0.999996191	0.999992444	0.997230633	0.99999488	0.999985264	0.999960414
ATP5F1A	0.999996191	0.999992444	0.997230633	0.99999488	0.999985264	0.999960414
PPP5C	0.999996191	0.998962836	0.997709094	0.99999488	0.999985264	0.999960414
DPF3	0.999996191	0.999992444	0.997832482	0.99999488	0.999985264	0.999960414
EPB41L2	0.999996191	0.999347049	0.997859293	0.99999488	0.999985264	0.999960414
UBE2J2	0.999996191	0.999347049	0.997859293	0.99999488	0.999985264	0.999960414

CHPT1	0.999996191	0.999992444	0.997859293	0.99999488	0.999985264	0.999960414
CWC27	0.999996191	0.999992444	0.997859293	0.99999488	0.999985264	0.999960414
LMLN	0.999996191	0.999992444	0.997859293	0.99999488	0.999985264	0.999960414
LOC100847284	0.999996191	0.999992444	0.997859293	0.99999488	0.999985264	0.999960414
LOC112444474	0.999996191	0.999992444	0.997859293	0.99999488	0.999985264	0.999960414
MRPS10	0.999996191	0.999992444	0.997859293	0.99999488	0.999985264	0.999960414
NIPAL3	0.999996191	0.999992444	0.997859293	0.99999488	0.999985264	0.999960414
PCIF1	0.999996191	0.999992444	0.997859293	0.99999488	0.999985264	0.999960414
UGGT1	0.999996191	0.999347049	0.99806407	0.99999488	0.999985264	0.999960414
LOC112447342	0.999996191	0.999992444	0.998208226	0.99999488	0.999985264	0.999960414
PPP1R13B	0.999996191	0.999992444	0.998208226	0.99999488	0.999985264	0.999960414
LOC511409	0.999996191	0.999992444	0.998235205	0.99999488	0.999985264	0.999960414
FAM118B	0.999996191	0.998131745	0.998284449	0.99999488	0.999985264	0.999960414
TGFBR3	0.999996191	0.999992444	0.998284449	0.99999488	0.999985264	0.999960414
LOC100300896	0.999996191	0.999992444	0.998344612	0.99999488	0.999985264	0.999960414
RPIA	0.999996191	0.985406717	0.998429524	0.99999488	0.999985264	0.999960414
LOC112446029	0.999996191	0.999992444	0.998429524	0.99999488	0.999985264	0.999960414
MMRN2	0.999996191	0.999992444	0.998523328	0.99999488	0.999985264	0.999960414
SLC39A1	0.999996191	0.999992444	0.998523328	0.99999488	0.999985264	0.999960414
SLC11A2	0.999996191	0.973092298	0.998693674	0.99999488	0.999985264	0.999960414
GALM	0.999996191	0.999992444	0.998693674	0.99999488	0.999985264	0.999960414
FAM185A	0.999996191	0.999992444	0.998737179	0.99999488	0.999985264	0.999960414
LOC112442040	0.999996191	0.999992444	0.998737179	0.99999488	0.999985264	0.999960414
AP2A1	0.999996191	0.999992444	0.998816213	0.99999488	0.999985264	0.999960414
BDH2	0.999996191	0.999992444	0.998816213	0.99999488	0.999985264	0.999960414
CENPV	0.999996191	0.999992444	0.998816213	0.99999488	0.999985264	0.999960414
COPB2	0.999996191	0.999992444	0.998816213	0.99999488	0.999985264	0.999960414
FGR	0.999996191	0.999992444	0.998816213	0.99999488	0.999985264	0.999960414
GPR19	0.999996191	0.999992444	0.998816213	0.99999488	0.999985264	0.999960414
LOC506181	0.999996191	0.999992444	0.998816213	0.99999488	0.999985264	0.999960414
RNF145	0.999996191	0.999992444	0.998816213	0.99999488	0.999985264	0.999960414
RORA	0.999996191	0.999992444	0.998816213	0.99999488	0.999985264	0.999960414
RPL18	0.999996191	0.999992444	0.998816213	0.99999488	0.999985264	0.999960414
SPATA6L	0.999996191	0.999992444	0.998816213	0.99999488	0.999985264	0.999960414
SRSF7	0.999996191	0.999992444	0.998816213	0.99999488	0.999985264	0.999960414
ANKS1A	0.999996191	0.999992444	0.998852661	0.99999488	0.999985264	0.999960414
CHD7	0.999996191	0.999992444	0.998852661	0.99999488	0.999985264	0.999960414
ELMOD3	0.999996191	0.999992444	0.998852661	0.99999488	0.999985264	0.999960414
GPR63	0.999996191	0.999992444	0.998852661	0.99999488	0.999985264	0.999960414

GRK6	0.999996191	0.999992444	0.998852661	0.99999488	0.999985264	0.999960414
LDB1	0.999996191	0.999992444	0.998852661	0.99999488	0.999985264	0.999960414
LOC107131846	0.999996191	0.999992444	0.998852661	0.99999488	0.999985264	0.999960414
LOC112441770	0.999996191	0.999992444	0.998852661	0.99999488	0.999985264	0.999960414
RPS8	0.999996191	0.999992444	0.998852661	0.99999488	0.999985264	0.999960414
SCARB1	0.999996191	0.999992444	0.998852661	0.99999488	0.999985264	0.999960414
SGSM2	0.999996191	0.999992444	0.998852661	0.99999488	0.999985264	0.999960414
THOC5	0.999996191	0.999992444	0.998852661	0.99999488	0.999985264	0.999960414
TMEM163	0.999996191	0.999992444	0.998852661	0.99999488	0.999985264	0.999960414
ZNF653	0.999996191	0.999992444	0.998852661	0.99999488	0.999985264	0.999960414
AXL	0.999996191	0.999992444	0.998936678	0.99999488	0.999985264	0.999960414
LOC101907613	0.999996191	0.95841099	0.998936876	0.99999488	0.999985264	0.999960414
NAPEPLD	0.999996191	0.979263532	0.998936876	0.99999488	0.999985264	0.999960414
LMOD3	0.999996191	0.980496345	0.998936876	0.99999488	0.999985264	0.999960414
LOC112448335	0.999996191	0.985204209	0.998936876	0.99999488	0.999985264	0.999960414
SET	0.999996191	0.985406717	0.998936876	0.99999488	0.999985264	0.999960414
CARD10	0.999996191	0.991488652	0.998936876	0.99999488	0.999985264	0.999960414
LOC531038	0.999996191	0.991488652	0.998936876	0.99999488	0.999985264	0.999960414
SOX13	0.999996191	0.991488652	0.998936876	0.99999488	0.999985264	0.999960414
FAM13A	0.999996191	0.991671026	0.998936876	0.99999488	0.999985264	0.999960414
LOC112442633	0.999996191	0.992977597	0.998936876	0.99999488	0.999985264	0.999960414
IGSF8	0.999996191	0.994477048	0.998936876	0.99999488	0.999985264	0.999960414
LOC101903793	0.999996191	0.996105883	0.998936876	0.99999488	0.999985264	0.999960414
S100A4	0.999996191	0.997554967	0.998936876	0.99999488	0.999985264	0.999960414
TRADD	0.999996191	0.997794477	0.998936876	0.99999488	0.999985264	0.999960414
LOC101907747	0.999996191	0.998011664	0.998936876	0.99999488	0.999985264	0.999960414
PSMC2	0.999996191	0.998011664	0.998936876	0.99999488	0.999985264	0.999960414
SWSAP1	0.999996191	0.998011664	0.998936876	0.99999488	0.999985264	0.999960414
CKS1B	0.999996191	0.998131745	0.998936876	0.99999488	0.999985264	0.999960414
MOB2	0.999996191	0.998131745	0.998936876	0.99999488	0.999985264	0.999960414
SRR	0.999996191	0.998131745	0.998936876	0.99999488	0.999985264	0.999960414
BTBD3	0.999996191	0.999347049	0.998936876	0.99999488	0.999985264	0.999960414
CERS2	0.999996191	0.999347049	0.998936876	0.99999488	0.999985264	0.999960414
GUCY1B1	0.999996191	0.999347049	0.998936876	0.99999488	0.999985264	0.999960414
OGDH	0.999996191	0.999347049	0.998936876	0.99999488	0.999985264	0.999960414
PDZD9	0.999996191	0.999347049	0.998936876	0.99999488	0.999985264	0.999960414
PSMD11	0.999996191	0.999347049	0.998936876	0.99999488	0.999985264	0.999960414
SPAG1	0.999996191	0.999347049	0.998936876	0.99999488	0.999985264	0.999960414
TTC28	0.999996191	0.999347049	0.998936876	0.99999488	0.999985264	0.999960414

ZNF133	0.999996191	0.999347049	0.998936876	0.99999488	0.999985264	0.999960414
ABHD11	0.999996191	0.999992444	0.998936876	0.99999488	0.999985264	0.999960414
ABHD14B	0.999996191	0.999992444	0.998936876	0.99999488	0.999985264	0.999960414
ACAP2	0.999996191	0.999992444	0.998936876	0.99999488	0.999985264	0.999960414
ADAL	0.999996191	0.999992444	0.998936876	0.99999488	0.999985264	0.999960414
ADAMTSL4	0.999996191	0.999992444	0.998936876	0.99999488	0.999985264	0.999960414
ADAP2	0.999996191	0.999992444	0.998936876	0.99999488	0.999985264	0.999960414
AJM1	0.999996191	0.999992444	0.998936876	0.99999488	0.999985264	0.999960414
AK7	0.999996191	0.999992444	0.998936876	0.99999488	0.999985264	0.999960414
ALG2	0.999996191	0.999992444	0.998936876	0.99999488	0.999985264	0.999960414
ALKBH6	0.999996191	0.999992444	0.998936876	0.99999488	0.999985264	0.999960414
AMN1	0.999996191	0.999992444	0.998936876	0.99999488	0.999985264	0.999960414
ANAPC5	0.999996191	0.999992444	0.998936876	0.99999488	0.999985264	0.999960414
ANO10	0.999996191	0.999992444	0.998936876	0.99999488	0.999985264	0.999960414
APEH	0.999996191	0.999992444	0.998936876	0.99999488	0.999985264	0.999960414
APOBEC3Z2	0.999996191	0.999992444	0.998936876	0.99999488	0.999985264	0.999960414
AQR	0.999996191	0.999992444	0.998936876	0.99999488	0.999985264	0.999960414
ARHGAP12	0.999996191	0.999992444	0.998936876	0.99999488	0.999985264	0.999960414
ATAD2	0.999996191	0.999992444	0.998936876	0.99999488	0.999985264	0.999960414
ATP11A	0.999996191	0.999992444	0.998936876	0.99999488	0.999985264	0.999960414
ATXN10	0.999996191	0.999992444	0.998936876	0.99999488	0.999985264	0.999960414
BBX	0.999996191	0.999992444	0.998936876	0.99999488	0.999985264	0.999960414
BCAP31	0.999996191	0.999992444	0.998936876	0.99999488	0.999985264	0.999960414
BCAS3	0.999996191	0.999992444	0.998936876	0.99999488	0.999985264	0.999960414
BCDIN3D	0.999996191	0.999992444	0.998936876	0.99999488	0.999985264	0.999960414
BCL7C	0.999996191	0.999992444	0.998936876	0.99999488	0.999985264	0.999960414
BORCS8	0.999996191	0.999992444	0.998936876	0.99999488	0.999985264	0.999960414
BRSK1	0.999996191	0.999992444	0.998936876	0.99999488	0.999985264	0.999960414
BRWD1	0.999996191	0.999992444	0.998936876	0.99999488	0.999985264	0.999960414
BTBD19	0.999996191	0.999992444	0.998936876	0.99999488	0.999985264	0.999960414
C1D	0.999996191	0.999992444	0.998936876	0.99999488	0.999985264	0.999960414
C5H12orf66	0.999996191	0.999992444	0.998936876	0.99999488	0.999985264	0.999960414
C8H9orf64	0.999996191	0.999992444	0.998936876	0.99999488	0.999985264	0.999960414
CALML6	0.999996191	0.999992444	0.998936876	0.99999488	0.999985264	0.999960414
CAMK1	0.999996191	0.999992444	0.998936876	0.99999488	0.999985264	0.999960414
CCNK	0.999996191	0.999992444	0.998936876	0.99999488	0.999985264	0.999960414
CD209	0.999996191	0.999992444	0.998936876	0.99999488	0.999985264	0.999960414
CD38	0.999996191	0.999992444	0.998936876	0.99999488	0.999985264	0.999960414
CDK5R1	0.999996191	0.999992444	0.998936876	0.99999488	0.999985264	0.999960414

CDO1	0.999996191	0.999992444	0.998936876	0.99999488	0.999985264	0.999960414
CERS1	0.999996191	0.999992444	0.998936876	0.99999488	0.999985264	0.999960414
CFAP54	0.999996191	0.999992444	0.998936876	0.99999488	0.999985264	0.999960414
CHRNA7	0.999996191	0.999992444	0.998936876	0.99999488	0.999985264	0.999960414
CLDN12	0.999996191	0.999992444	0.998936876	0.99999488	0.999985264	0.999960414
CLIC1	0.999996191	0.999992444	0.998936876	0.99999488	0.999985264	0.999960414
CLPB	0.999996191	0.999992444	0.998936876	0.99999488	0.999985264	0.999960414
CMTM4	0.999996191	0.999992444	0.998936876	0.99999488	0.999985264	0.999960414
CRACR2A	0.999996191	0.999992444	0.998936876	0.99999488	0.999985264	0.999960414
CRTC2	0.999996191	0.999992444	0.998936876	0.99999488	0.999985264	0.999960414
CTCF	0.999996191	0.999992444	0.998936876	0.99999488	0.999985264	0.999960414
CTF1	0.999996191	0.999992444	0.998936876	0.99999488	0.999985264	0.999960414
CYP26B1	0.999996191	0.999992444	0.998936876	0.99999488	0.999985264	0.999960414
CYYR1	0.999996191	0.999992444	0.998936876	0.99999488	0.999985264	0.999960414
DCLK1	0.999996191	0.999992444	0.998936876	0.99999488	0.999985264	0.999960414
DENND4A	0.999996191	0.999992444	0.998936876	0.99999488	0.999985264	0.999960414
DIAPH2	0.999996191	0.999992444	0.998936876	0.99999488	0.999985264	0.999960414
DICER1	0.999996191	0.999992444	0.998936876	0.99999488	0.999985264	0.999960414
DPP8	0.999996191	0.999992444	0.998936876	0.99999488	0.999985264	0.999960414
DPY19L3	0.999996191	0.999992444	0.998936876	0.99999488	0.999985264	0.999960414
DTNA	0.999996191	0.999992444	0.998936876	0.99999488	0.999985264	0.999960414
EIF5	0.999996191	0.999992444	0.998936876	0.99999488	0.999985264	0.999960414
ENTPD3	0.999996191	0.999992444	0.998936876	0.99999488	0.999985264	0.999960414
ENTPD7	0.999996191	0.999992444	0.998936876	0.99999488	0.999985264	0.999960414
ERLIN2	0.999996191	0.999992444	0.998936876	0.99999488	0.999985264	0.999960414
FAM20C	0.999996191	0.999992444	0.998936876	0.99999488	0.999985264	0.999960414
FBXO42	0.999996191	0.999992444	0.998936876	0.99999488	0.999985264	0.999960414
FKBP8	0.999996191	0.999992444	0.998936876	0.99999488	0.999985264	0.999960414
FOLH1B	0.999996191	0.999992444	0.998936876	0.99999488	0.999985264	0.999960414
FOXN3	0.999996191	0.999992444	0.998936876	0.99999488	0.999985264	0.999960414
FRMD8	0.999996191	0.999992444	0.998936876	0.99999488	0.999985264	0.999960414
FTSJ1	0.999996191	0.999992444	0.998936876	0.99999488	0.999985264	0.999960414
FUOM	0.999996191	0.999992444	0.998936876	0.99999488	0.999985264	0.999960414
FXR2	0.999996191	0.999992444	0.998936876	0.99999488	0.999985264	0.999960414
FZR1	0.999996191	0.999992444	0.998936876	0.99999488	0.999985264	0.999960414
GFRA3	0.999996191	0.999992444	0.998936876	0.99999488	0.999985264	0.999960414
GMNN	0.999996191	0.999992444	0.998936876	0.99999488	0.999985264	0.999960414
GNG2	0.999996191	0.999992444	0.998936876	0.99999488	0.999985264	0.999960414
GOLIM4	0.999996191	0.999992444	0.998936876	0.99999488	0.999985264	0.999960414

GPAA1	0.999996191	0.999992444	0.998936876	0.99999488	0.999985264	0.999960414
GRIPAP1	0.999996191	0.999992444	0.998936876	0.99999488	0.999985264	0.999960414
GSK3B	0.999996191	0.999992444	0.998936876	0.99999488	0.999985264	0.999960414
GTDC1	0.999996191	0.999992444	0.998936876	0.99999488	0.999985264	0.999960414
GYG2	0.999996191	0.999992444	0.998936876	0.99999488	0.999985264	0.999960414
HAUS4	0.999996191	0.999992444	0.998936876	0.99999488	0.999985264	0.999960414
HCFC2	0.999996191	0.999992444	0.998936876	0.99999488	0.999985264	0.999960414
HES7	0.999996191	0.999992444	0.998936876	0.99999488	0.999985264	0.999960414
HLCS	0.999996191	0.999992444	0.998936876	0.99999488	0.999985264	0.999960414
HPS5	0.999996191	0.999992444	0.998936876	0.99999488	0.999985264	0.999960414
HRASLS	0.999996191	0.999992444	0.998936876	0.99999488	0.999985264	0.999960414
HRH1	0.999996191	0.999992444	0.998936876	0.99999488	0.999985264	0.999960414
HS3ST1	0.999996191	0.999992444	0.998936876	0.99999488	0.999985264	0.999960414
HS3ST3A1	0.999996191	0.999992444	0.998936876	0.99999488	0.999985264	0.999960414
HS3ST6	0.999996191	0.999992444	0.998936876	0.99999488	0.999985264	0.999960414
IGF2	0.999996191	0.999992444	0.998936876	0.99999488	0.999985264	0.999960414
IMPDH1	0.999996191	0.999992444	0.998936876	0.99999488	0.999985264	0.999960414
INTS2	0.999996191	0.999992444	0.998936876	0.99999488	0.999985264	0.999960414
ISLR	0.999996191	0.999992444	0.998936876	0.99999488	0.999985264	0.999960414
KANTR	0.999996191	0.999992444	0.998936876	0.99999488	0.999985264	0.999960414
KCNIP2	0.999996191	0.999992444	0.998936876	0.99999488	0.999985264	0.999960414
KCNK6	0.999996191	0.999992444	0.998936876	0.99999488	0.999985264	0.999960414
KIAA1671	0.999996191	0.999992444	0.998936876	0.99999488	0.999985264	0.999960414
KIF24	0.999996191	0.999992444	0.998936876	0.99999488	0.999985264	0.999960414
KRT18	0.999996191	0.999992444	0.998936876	0.99999488	0.999985264	0.999960414
LDAH	0.999996191	0.999992444	0.998936876	0.99999488	0.999985264	0.999960414
LGALS12	0.999996191	0.999992444	0.998936876	0.99999488	0.999985264	0.999960414
LOC100138645	0.999996191	0.999992444	0.998936876	0.99999488	0.999985264	0.999960414
LOC100299025	0.999996191	0.999992444	0.998936876	0.99999488	0.999985264	0.999960414
LOC100299845	0.999996191	0.999992444	0.998936876	0.99999488	0.999985264	0.999960414
LOC100335205	0.999996191	0.999992444	0.998936876	0.99999488	0.999985264	0.999960414
LOC100337293	0.999996191	0.999992444	0.998936876	0.99999488	0.999985264	0.999960414
LOC100847890	0.999996191	0.999992444	0.998936876	0.99999488	0.999985264	0.999960414
LOC100847934	0.999996191	0.999992444	0.998936876	0.99999488	0.999985264	0.999960414
LOC100848077	0.999996191	0.999992444	0.998936876	0.99999488	0.999985264	0.999960414
LOC100848991	0.999996191	0.999992444	0.998936876	0.99999488	0.999985264	0.999960414
LOC101902644	0.999996191	0.999992444	0.998936876	0.99999488	0.999985264	0.999960414
LOC101902668	0.999996191	0.999992444	0.998936876	0.99999488	0.999985264	0.999960414
LOC101902838	0.999996191	0.999992444	0.998936876	0.99999488	0.999985264	0.999960414

LOC101902840	0.999996191	0.999992444	0.998936876	0.99999488	0.999985264	0.999960414
LOC101903205	0.999996191	0.999992444	0.998936876	0.99999488	0.999985264	0.999960414
LOC101903413	0.999996191	0.999992444	0.998936876	0.99999488	0.999985264	0.999960414
LOC101903831	0.999996191	0.999992444	0.998936876	0.99999488	0.999985264	0.999960414
LOC101903900	0.999996191	0.999992444	0.998936876	0.99999488	0.999985264	0.999960414
LOC101903988	0.999996191	0.999992444	0.998936876	0.99999488	0.999985264	0.999960414
LOC101904098	0.999996191	0.999992444	0.998936876	0.99999488	0.999985264	0.999960414
LOC101904270	0.999996191	0.999992444	0.998936876	0.99999488	0.999985264	0.999960414
LOC101904314	0.999996191	0.999992444	0.998936876	0.99999488	0.999985264	0.999960414
LOC101904498	0.999996191	0.999992444	0.998936876	0.99999488	0.999985264	0.999960414
LOC101905239	0.999996191	0.999992444	0.998936876	0.99999488	0.999985264	0.999960414
LOC101905254	0.999996191	0.999992444	0.998936876	0.99999488	0.999985264	0.999960414
LOC101906200	0.999996191	0.999992444	0.998936876	0.99999488	0.999985264	0.999960414
LOC101906513	0.999996191	0.999992444	0.998936876	0.99999488	0.999985264	0.999960414
LOC101906545	0.999996191	0.999992444	0.998936876	0.99999488	0.999985264	0.999960414
LOC101906855	0.999996191	0.999992444	0.998936876	0.99999488	0.999985264	0.999960414
LOC101907383	0.999996191	0.999992444	0.998936876	0.99999488	0.999985264	0.999960414
LOC101907549	0.999996191	0.999992444	0.998936876	0.99999488	0.999985264	0.999960414
LOC101907800	0.999996191	0.999992444	0.998936876	0.99999488	0.999985264	0.999960414
LOC101908205	0.999996191	0.999992444	0.998936876	0.99999488	0.999985264	0.999960414
LOC104968751	0.999996191	0.999992444	0.998936876	0.99999488	0.999985264	0.999960414
LOC104970628	0.999996191	0.999992444	0.998936876	0.99999488	0.999985264	0.999960414
LOC104970645	0.999996191	0.999992444	0.998936876	0.99999488	0.999985264	0.999960414
LOC104970698	0.999996191	0.999992444	0.998936876	0.99999488	0.999985264	0.999960414
LOC104971021	0.999996191	0.999992444	0.998936876	0.99999488	0.999985264	0.999960414
LOC104971057	0.999996191	0.999992444	0.998936876	0.99999488	0.999985264	0.999960414
LOC104972578	0.999996191	0.999992444	0.998936876	0.99999488	0.999985264	0.999960414
LOC104973252	0.999996191	0.999992444	0.998936876	0.99999488	0.999985264	0.999960414
LOC104973848	0.999996191	0.999992444	0.998936876	0.99999488	0.999985264	0.999960414
LOC104975004	0.999996191	0.999992444	0.998936876	0.99999488	0.999985264	0.999960414
LOC104975073	0.999996191	0.999992444	0.998936876	0.99999488	0.999985264	0.999960414
LOC107132196	0.999996191	0.999992444	0.998936876	0.99999488	0.999985264	0.999960414
LOC107132799	0.999996191	0.999992444	0.998936876	0.99999488	0.999985264	0.999960414
LOC107132942	0.999996191	0.999992444	0.998936876	0.99999488	0.999985264	0.999960414
LOC112441603	0.999996191	0.999992444	0.998936876	0.99999488	0.999985264	0.999960414
LOC112441638	0.999996191	0.999992444	0.998936876	0.99999488	0.999985264	0.999960414
LOC112441879	0.999996191	0.999992444	0.998936876	0.99999488	0.999985264	0.999960414
LOC112442083	0.999996191	0.999992444	0.998936876	0.99999488	0.999985264	0.999960414
LOC112442383	0.999996191	0.999992444	0.998936876	0.99999488	0.999985264	0.999960414

LOC112442384	0.999996191	0.999992444	0.998936876	0.99999488	0.999985264	0.999960414
LOC112442392	0.999996191	0.999992444	0.998936876	0.99999488	0.999985264	0.999960414
LOC112442625	0.999996191	0.999992444	0.998936876	0.99999488	0.999985264	0.999960414
LOC112442687	0.999996191	0.999992444	0.998936876	0.99999488	0.999985264	0.999960414
LOC112443512	0.999996191	0.999992444	0.998936876	0.99999488	0.999985264	0.999960414
LOC112444763	0.999996191	0.999992444	0.998936876	0.99999488	0.999985264	0.999960414
LOC112444846	0.999996191	0.999992444	0.998936876	0.99999488	0.999985264	0.999960414
LOC112444888	0.999996191	0.999992444	0.998936876	0.99999488	0.999985264	0.999960414
LOC112444924	0.999996191	0.999992444	0.998936876	0.99999488	0.999985264	0.999960414
LOC112445088	0.999996191	0.999992444	0.998936876	0.99999488	0.999985264	0.999960414
LOC112445982	0.999996191	0.999992444	0.998936876	0.99999488	0.999985264	0.999960414
LOC112445996	0.999996191	0.999992444	0.998936876	0.99999488	0.999985264	0.999960414
LOC112446001	0.999996191	0.999992444	0.998936876	0.99999488	0.999985264	0.999960414
LOC112446022	0.999996191	0.999992444	0.998936876	0.99999488	0.999985264	0.999960414
LOC112446381	0.999996191	0.999992444	0.998936876	0.99999488	0.999985264	0.999960414
LOC112446639	0.999996191	0.999992444	0.998936876	0.99999488	0.999985264	0.999960414
LOC112446777	0.999996191	0.999992444	0.998936876	0.99999488	0.999985264	0.999960414
LOC112447026	0.999996191	0.999992444	0.998936876	0.99999488	0.999985264	0.999960414
LOC112448090	0.999996191	0.999992444	0.998936876	0.99999488	0.999985264	0.999960414
LOC112448260	0.999996191	0.999992444	0.998936876	0.99999488	0.999985264	0.999960414
LOC112448453	0.999996191	0.999992444	0.998936876	0.99999488	0.999985264	0.999960414
LOC112448776	0.999996191	0.999992444	0.998936876	0.99999488	0.999985264	0.999960414
LOC112449282	0.999996191	0.999992444	0.998936876	0.99999488	0.999985264	0.999960414
LOC112449510	0.999996191	0.999992444	0.998936876	0.99999488	0.999985264	0.999960414
LOC504858	0.999996191	0.999992444	0.998936876	0.99999488	0.999985264	0.999960414
LOC505918	0.999996191	0.999992444	0.998936876	0.99999488	0.999985264	0.999960414
LOC508455	0.999996191	0.999992444	0.998936876	0.99999488	0.999985264	0.999960414
LOC509184	0.999996191	0.999992444	0.998936876	0.99999488	0.999985264	0.999960414
LOC510913	0.999996191	0.999992444	0.998936876	0.99999488	0.999985264	0.999960414
LOC520104	0.999996191	0.999992444	0.998936876	0.99999488	0.999985264	0.999960414
LOC613570	0.999996191	0.999992444	0.998936876	0.99999488	0.999985264	0.999960414
LOC614614	0.999996191	0.999992444	0.998936876	0.99999488	0.999985264	0.999960414
LOC618409	0.999996191	0.999992444	0.998936876	0.99999488	0.999985264	0.999960414
LOC781004	0.999996191	0.999992444	0.998936876	0.99999488	0.999985264	0.999960414
LOC781158	0.999996191	0.999992444	0.998936876	0.99999488	0.999985264	0.999960414
LOC781499	0.999996191	0.999992444	0.998936876	0.99999488	0.999985264	0.999960414
LOC781799	0.999996191	0.999992444	0.998936876	0.99999488	0.999985264	0.999960414
LOC783022	0.999996191	0.999992444	0.998936876	0.99999488	0.999985264	0.999960414
LOC783612	0.999996191	0.999992444	0.998936876	0.99999488	0.999985264	0.999960414

LOC784054	0.999996191	0.999992444	0.998936876	0.99999488	0.999985264	0.999960414
LOC784354	0.999996191	0.999992444	0.998936876	0.99999488	0.999985264	0.999960414
LOC784521	0.999996191	0.999992444	0.998936876	0.99999488	0.999985264	0.999960414
LOC785761	0.999996191	0.999992444	0.998936876	0.99999488	0.999985264	0.999960414
LOC786256	0.999996191	0.999992444	0.998936876	0.99999488	0.999985264	0.999960414
LOC787250	0.999996191	0.999992444	0.998936876	0.99999488	0.999985264	0.999960414
LOC790098	0.999996191	0.999992444	0.998936876	0.99999488	0.999985264	0.999960414
LPAR4	0.999996191	0.999992444	0.998936876	0.99999488	0.999985264	0.999960414
LPCAT4	0.999996191	0.999992444	0.998936876	0.99999488	0.999985264	0.999960414
LRRC69	0.999996191	0.999992444	0.998936876	0.99999488	0.999985264	0.999960414
LRSAM1	0.999996191	0.999992444	0.998936876	0.99999488	0.999985264	0.999960414
LSR	0.999996191	0.999992444	0.998936876	0.99999488	0.999985264	0.999960414
MAD2L2	0.999996191	0.999992444	0.998936876	0.99999488	0.999985264	0.999960414
MADD	0.999996191	0.999992444	0.998936876	0.99999488	0.999985264	0.999960414
MAP3K4	0.999996191	0.999992444	0.998936876	0.99999488	0.999985264	0.999960414
MCUB	0.999996191	0.999992444	0.998936876	0.99999488	0.999985264	0.999960414
MED16	0.999996191	0.999992444	0.998936876	0.99999488	0.999985264	0.999960414
MED22	0.999996191	0.999992444	0.998936876	0.99999488	0.999985264	0.999960414
MED7	0.999996191	0.999992444	0.998936876	0.99999488	0.999985264	0.999960414
MELK	0.999996191	0.999992444	0.998936876	0.99999488	0.999985264	0.999960414
METTL25	0.999996191	0.999992444	0.998936876	0.99999488	0.999985264	0.999960414
MEX3C	0.999996191	0.999992444	0.998936876	0.99999488	0.999985264	0.999960414
MGC127055	0.999996191	0.999992444	0.998936876	0.99999488	0.999985264	0.999960414
MGLL	0.999996191	0.999992444	0.998936876	0.99999488	0.999985264	0.999960414
MGST3	0.999996191	0.999992444	0.998936876	0.99999488	0.999985264	0.999960414
MINDY1	0.999996191	0.999992444	0.998936876	0.99999488	0.999985264	0.999960414
MKLN1	0.999996191	0.999992444	0.998936876	0.99999488	0.999985264	0.999960414
MNF1	0.999996191	0.999992444	0.998936876	0.99999488	0.999985264	0.999960414
MOCS1	0.999996191	0.999992444	0.998936876	0.99999488	0.999985264	0.999960414
MPHOSPH6	0.999996191	0.999992444	0.998936876	0.99999488	0.999985264	0.999960414
MRPL22	0.999996191	0.999992444	0.998936876	0.99999488	0.999985264	0.999960414
MRPL58	0.999996191	0.999992444	0.998936876	0.99999488	0.999985264	0.999960414
MXRA8	0.999996191	0.999992444	0.998936876	0.99999488	0.999985264	0.999960414
MYBPHL	0.999996191	0.999992444	0.998936876	0.99999488	0.999985264	0.999960414
MYCBP	0.999996191	0.999992444	0.998936876	0.99999488	0.999985264	0.999960414
NDC1	0.999996191	0.999992444	0.998936876	0.99999488	0.999985264	0.999960414
NDST3	0.999996191	0.999992444	0.998936876	0.99999488	0.999985264	0.999960414
NEDD4L	0.999996191	0.999992444	0.998936876	0.99999488	0.999985264	0.999960414
NOTCH1	0.999996191	0.999992444	0.998936876	0.99999488	0.999985264	0.999960414

NOTCH4	0.999996191	0.999992444	0.998936876	0.99999488	0.999985264	0.999960414
NPHP1	0.999996191	0.999992444	0.998936876	0.99999488	0.999985264	0.999960414
NSMCE3	0.999996191	0.999992444	0.998936876	0.99999488	0.999985264	0.999960414
NT5C2	0.999996191	0.999992444	0.998936876	0.99999488	0.999985264	0.999960414
NTRK2	0.999996191	0.999992444	0.998936876	0.99999488	0.999985264	0.999960414
NUDT7	0.999996191	0.999992444	0.998936876	0.99999488	0.999985264	0.999960414
NUP37	0.999996191	0.999992444	0.998936876	0.99999488	0.999985264	0.999960414
OGFR	0.999996191	0.999992444	0.998936876	0.99999488	0.999985264	0.999960414
OPA1	0.999996191	0.999992444	0.998936876	0.99999488	0.999985264	0.999960414
OSBPL10	0.999996191	0.999992444	0.998936876	0.99999488	0.999985264	0.999960414
OSBPL2	0.999996191	0.999992444	0.998936876	0.99999488	0.999985264	0.999960414
OTUB1	0.999996191	0.999992444	0.998936876	0.99999488	0.999985264	0.999960414
OTUD7A	0.999996191	0.999992444	0.998936876	0.99999488	0.999985264	0.999960414
PABPC5	0.999996191	0.999992444	0.998936876	0.99999488	0.999985264	0.999960414
PARN	0.999996191	0.999992444	0.998936876	0.99999488	0.999985264	0.999960414
PARP16	0.999996191	0.999992444	0.998936876	0.99999488	0.999985264	0.999960414
PCDHB11	0.999996191	0.999992444	0.998936876	0.99999488	0.999985264	0.999960414
PDCD10	0.999996191	0.999992444	0.998936876	0.99999488	0.999985264	0.999960414
PDGFC	0.999996191	0.999992444	0.998936876	0.99999488	0.999985264	0.999960414
PFDN1	0.999996191	0.999992444	0.998936876	0.99999488	0.999985264	0.999960414
PFKM	0.999996191	0.999992444	0.998936876	0.99999488	0.999985264	0.999960414
PGRMC1	0.999996191	0.999992444	0.998936876	0.99999488	0.999985264	0.999960414
PHF2	0.999996191	0.999992444	0.998936876	0.99999488	0.999985264	0.999960414
PHKA2	0.999996191	0.999992444	0.998936876	0.99999488	0.999985264	0.999960414
PIN1	0.999996191	0.999992444	0.998936876	0.99999488	0.999985264	0.999960414
PLBD2	0.999996191	0.999992444	0.998936876	0.99999488	0.999985264	0.999960414
PLCB3	0.999996191	0.999992444	0.998936876	0.99999488	0.999985264	0.999960414
PLEKHM1	0.999996191	0.999992444	0.998936876	0.99999488	0.999985264	0.999960414
POLR3C	0.999996191	0.999992444	0.998936876	0.99999488	0.999985264	0.999960414
POP7	0.999996191	0.999992444	0.998936876	0.99999488	0.999985264	0.999960414
PPOX	0.999996191	0.999992444	0.998936876	0.99999488	0.999985264	0.999960414
PPP1R12A	0.999996191	0.999992444	0.998936876	0.99999488	0.999985264	0.999960414
PPP1R12C	0.999996191	0.999992444	0.998936876	0.99999488	0.999985264	0.999960414
PPYR1	0.999996191	0.999992444	0.998936876	0.99999488	0.999985264	0.999960414
PRKAR2A	0.999996191	0.999992444	0.998936876	0.99999488	0.999985264	0.999960414
PRKCD	0.999996191	0.999992444	0.998936876	0.99999488	0.999985264	0.999960414
PRKD1	0.999996191	0.999992444	0.998936876	0.99999488	0.999985264	0.999960414
PRKRA	0.999996191	0.999992444	0.998936876	0.99999488	0.999985264	0.999960414
PRR14L	0.999996191	0.999992444	0.998936876	0.99999488	0.999985264	0.999960414

PRRC2B	0.999996191	0.999992444	0.998936876	0.99999488	0.999985264	0.999960414
PRTFDC1	0.999996191	0.999992444	0.998936876	0.99999488	0.999985264	0.999960414
PTGR2	0.999996191	0.999992444	0.998936876	0.99999488	0.999985264	0.999960414
PTPRA	0.999996191	0.999992444	0.998936876	0.99999488	0.999985264	0.999960414
QSOX2	0.999996191	0.999992444	0.998936876	0.99999488	0.999985264	0.999960414
R3HDM1	0.999996191	0.999992444	0.998936876	0.99999488	0.999985264	0.999960414
R3HDM4	0.999996191	0.999992444	0.998936876	0.99999488	0.999985264	0.999960414
RAB15	0.999996191	0.999992444	0.998936876	0.99999488	0.999985264	0.999960414
RASA3	0.999996191	0.999992444	0.998936876	0.99999488	0.999985264	0.999960414
RBM27	0.999996191	0.999992444	0.998936876	0.99999488	0.999985264	0.999960414
RCAN2	0.999996191	0.999992444	0.998936876	0.99999488	0.999985264	0.999960414
RGP1	0.999996191	0.999992444	0.998936876	0.99999488	0.999985264	0.999960414
RHPN2	0.999996191	0.999992444	0.998936876	0.99999488	0.999985264	0.999960414
RNF10	0.999996191	0.999992444	0.998936876	0.99999488	0.999985264	0.999960414
RNF169	0.999996191	0.999992444	0.998936876	0.99999488	0.999985264	0.999960414
RP9	0.999996191	0.999992444	0.998936876	0.99999488	0.999985264	0.999960414
RPL13	0.999996191	0.999992444	0.998936876	0.99999488	0.999985264	0.999960414
RPL26L1	0.999996191	0.999992444	0.998936876	0.99999488	0.999985264	0.999960414
RTTN	0.999996191	0.999992444	0.998936876	0.99999488	0.999985264	0.999960414
RUNDC3A	0.999996191	0.999992444	0.998936876	0.99999488	0.999985264	0.999960414
RYR1	0.999996191	0.999992444	0.998936876	0.99999488	0.999985264	0.999960414
SAMM50	0.999996191	0.999992444	0.998936876	0.99999488	0.999985264	0.999960414
SCLT1	0.999996191	0.999992444	0.998936876	0.99999488	0.999985264	0.999960414
SCNN1A	0.999996191	0.999992444	0.998936876	0.99999488	0.999985264	0.999960414
SDHC	0.999996191	0.999992444	0.998936876	0.99999488	0.999985264	0.999960414
SDR39U1	0.999996191	0.999992444	0.998936876	0.99999488	0.999985264	0.999960414
SETD1A	0.999996191	0.999992444	0.998936876	0.99999488	0.999985264	0.999960414
SF3A2	0.999996191	0.999992444	0.998936876	0.99999488	0.999985264	0.999960414
SH2D3C	0.999996191	0.999992444	0.998936876	0.99999488	0.999985264	0.999960414
SHF	0.999996191	0.999992444	0.998936876	0.99999488	0.999985264	0.999960414
SKAP2	0.999996191	0.999992444	0.998936876	0.99999488	0.999985264	0.999960414
SLC22A4	0.999996191	0.999992444	0.998936876	0.99999488	0.999985264	0.999960414
SLC25A21	0.999996191	0.999992444	0.998936876	0.99999488	0.999985264	0.999960414
SLC6A9	0.999996191	0.999992444	0.998936876	0.99999488	0.999985264	0.999960414
SLU7	0.999996191	0.999992444	0.998936876	0.99999488	0.999985264	0.999960414
SMAD3	0.999996191	0.999992444	0.998936876	0.99999488	0.999985264	0.999960414
SMKR1	0.999996191	0.999992444	0.998936876	0.99999488	0.999985264	0.999960414
SMO	0.999996191	0.999992444	0.998936876	0.99999488	0.999985264	0.999960414
SNRNP200	0.999996191	0.999992444	0.998936876	0.99999488	0.999985264	0.999960414

SNTB2	0.999996191	0.999992444	0.998936876	0.99999488	0.999985264	0.999960414
SNX14	0.999996191	0.999992444	0.998936876	0.99999488	0.999985264	0.999960414
SPATA2L	0.999996191	0.999992444	0.998936876	0.99999488	0.999985264	0.999960414
SPG7	0.999996191	0.999992444	0.998936876	0.99999488	0.999985264	0.999960414
SPTBN1	0.999996191	0.999992444	0.998936876	0.99999488	0.999985264	0.999960414
SRC	0.999996191	0.999992444	0.998936876	0.99999488	0.999985264	0.999960414
SRP9	0.999996191	0.999992444	0.998936876	0.99999488	0.999985264	0.999960414
SRSF1	0.999996191	0.999992444	0.998936876	0.99999488	0.999985264	0.999960414
SSBP3	0.999996191	0.999992444	0.998936876	0.99999488	0.999985264	0.999960414
SUGCT	0.999996191	0.999992444	0.998936876	0.99999488	0.999985264	0.999960414
SULF1	0.999996191	0.999992444	0.998936876	0.99999488	0.999985264	0.999960414
SYN1	0.999996191	0.999992444	0.998936876	0.99999488	0.999985264	0.999960414
SYNE3	0.999996191	0.999992444	0.998936876	0.99999488	0.999985264	0.999960414
SYT15	0.999996191	0.999992444	0.998936876	0.99999488	0.999985264	0.999960414
SYTL2	0.999996191	0.999992444	0.998936876	0.99999488	0.999985264	0.999960414
TAF3	0.999996191	0.999992444	0.998936876	0.99999488	0.999985264	0.999960414
TBC1D10A	0.999996191	0.999992444	0.998936876	0.99999488	0.999985264	0.999960414
TBCE	0.999996191	0.999992444	0.998936876	0.99999488	0.999985264	0.999960414
TBX6	0.999996191	0.999992444	0.998936876	0.99999488	0.999985264	0.999960414
TEDC1	0.999996191	0.999992444	0.998936876	0.99999488	0.999985264	0.999960414
TEK	0.999996191	0.999992444	0.998936876	0.99999488	0.999985264	0.999960414
TEX10	0.999996191	0.999992444	0.998936876	0.99999488	0.999985264	0.999960414
TEX35	0.999996191	0.999992444	0.998936876	0.99999488	0.999985264	0.999960414
TFAP4	0.999996191	0.999992444	0.998936876	0.99999488	0.999985264	0.999960414
THAP11	0.999996191	0.999992444	0.998936876	0.99999488	0.999985264	0.999960414
TICAM2	0.999996191	0.999992444	0.998936876	0.99999488	0.999985264	0.999960414
TMCO3	0.999996191	0.999992444	0.998936876	0.99999488	0.999985264	0.999960414
TMEM167A	0.999996191	0.999992444	0.998936876	0.99999488	0.999985264	0.999960414
TMEM175	0.999996191	0.999992444	0.998936876	0.99999488	0.999985264	0.999960414
TMEM183A	0.999996191	0.999992444	0.998936876	0.99999488	0.999985264	0.999960414
TMEM240	0.999996191	0.999992444	0.998936876	0.99999488	0.999985264	0.999960414
TMEM68	0.999996191	0.999992444	0.998936876	0.99999488	0.999985264	0.999960414
TMEM88B	0.999996191	0.999992444	0.998936876	0.99999488	0.999985264	0.999960414
TMOD1	0.999996191	0.999992444	0.998936876	0.99999488	0.999985264	0.999960414
TMTC4	0.999996191	0.999992444	0.998936876	0.99999488	0.999985264	0.999960414
TNPO3	0.999996191	0.999992444	0.998936876	0.99999488	0.999985264	0.999960414
TNRC6A	0.999996191	0.999992444	0.998936876	0.99999488	0.999985264	0.999960414
TNRC6C	0.999996191	0.999992444	0.998936876	0.99999488	0.999985264	0.999960414
TNS1	0.999996191	0.999992444	0.998936876	0.99999488	0.999985264	0.999960414

TP53RK	0.999996191	0.999992444	0.998936876	0.99999488	0.999985264	0.999960414
TPGS1	0.999996191	0.999992444	0.998936876	0.99999488	0.999985264	0.999960414
TRAPPC4	0.999996191	0.999992444	0.998936876	0.99999488	0.999985264	0.999960414
TRMT1L	0.999996191	0.999992444	0.998936876	0.99999488	0.999985264	0.999960414
TTC26	0.999996191	0.999992444	0.998936876	0.99999488	0.999985264	0.999960414
TUBGCP4	0.999996191	0.999992444	0.998936876	0.99999488	0.999985264	0.999960414
TXNDC15	0.999996191	0.999992444	0.998936876	0.99999488	0.999985264	0.999960414
UBALD2	0.999996191	0.999992444	0.998936876	0.99999488	0.999985264	0.999960414
UBAP1L	0.999996191	0.999992444	0.998936876	0.99999488	0.999985264	0.999960414
UFD1	0.999996191	0.999992444	0.998936876	0.99999488	0.999985264	0.999960414
URI1	0.999996191	0.999992444	0.998936876	0.99999488	0.999985264	0.999960414
USP36	0.999996191	0.999992444	0.998936876	0.99999488	0.999985264	0.999960414
VASH2	0.999996191	0.999992444	0.998936876	0.99999488	0.999985264	0.999960414
VPS37B	0.999996191	0.999992444	0.998936876	0.99999488	0.999985264	0.999960414
WDR61	0.999996191	0.999992444	0.998936876	0.99999488	0.999985264	0.999960414
XPO6	0.999996191	0.999992444	0.998936876	0.99999488	0.999985264	0.999960414
YJEFN3	0.999996191	0.999992444	0.998936876	0.99999488	0.999985264	0.999960414
YLPM1	0.999996191	0.999992444	0.998936876	0.99999488	0.999985264	0.999960414
ZBTB42	0.999996191	0.999992444	0.998936876	0.99999488	0.999985264	0.999960414
ZBTB8A	0.999996191	0.999992444	0.998936876	0.99999488	0.999985264	0.999960414
ZCCHC3	0.999996191	0.999992444	0.998936876	0.99999488	0.999985264	0.999960414
ZDHHC3	0.999996191	0.999992444	0.998936876	0.99999488	0.999985264	0.999960414
ZFHX3	0.999996191	0.999992444	0.998936876	0.99999488	0.999985264	0.999960414
ZFP69	0.999996191	0.999992444	0.998936876	0.99999488	0.999985264	0.999960414
ZHX1	0.999996191	0.999992444	0.998936876	0.99999488	0.999985264	0.999960414
ZNF322	0.999996191	0.999992444	0.998936876	0.99999488	0.999985264	0.999960414
ZNF366	0.999996191	0.999992444	0.998936876	0.99999488	0.999985264	0.999960414
ZNF367	0.999996191	0.999992444	0.998936876	0.99999488	0.999985264	0.999960414
ZNF445	0.999996191	0.999992444	0.998936876	0.99999488	0.999985264	0.999960414
ZNF575	0.999996191	0.999992444	0.998936876	0.99999488	0.999985264	0.999960414
ZNF613	0.999996191	0.999992444	0.998936876	0.99999488	0.999985264	0.999960414
ZNF629	0.999996191	0.999992444	0.998936876	0.99999488	0.999985264	0.999960414
ZNF683	0.999996191	0.999992444	0.998936876	0.99999488	0.999985264	0.999960414
ZNF830	0.999996191	0.999992444	0.998936876	0.99999488	0.999985264	0.999960414
ZSCAN4	0.999996191	0.999992444	0.998936876	0.99999488	0.999985264	0.999960414
ZSWIM8	0.999996191	0.999992444	0.998936876	0.99999488	0.999985264	0.999960414
LOC789733	0.999996191	0.999992444	0.99906766	0.99999488	0.999985264	0.999960414
KMT2B	0.999996191	0.999992444	0.999072092	0.99999488	0.999985264	0.999960414
CECR2	0.999996191	0.999992444	0.999268965	0.99999488	0.999985264	0.999960414

MCF2L	0.999996191	0.987516216	0.999558179	0.99999488	0.999985264	0.999960414
LZIC	0.999996191	0.991365955	0.999558179	0.99999488	0.999985264	0.999960414
CLUAP1	0.999996191	0.991488652	0.999558179	0.99999488	0.999985264	0.999960414
MAP3K14	0.999996191	0.991488652	0.999558179	0.99999488	0.999985264	0.999960414
CETN2	0.999996191	0.993316598	0.999558179	0.99999488	0.999985264	0.999960414
HOXB8	0.999996191	0.995476917	0.999558179	0.99999488	0.999985264	0.999960414
TICRR	0.999996191	0.996814721	0.999558179	0.99999488	0.999985264	0.999960414
KLF2	0.999996191	0.997051616	0.999558179	0.99999488	0.999985264	0.999960414
LOC112444147	0.999996191	0.998011664	0.999558179	0.99999488	0.999985264	0.999960414
ENPP6	0.999996191	0.998131745	0.999558179	0.99999488	0.999985264	0.999960414
GJB2	0.999996191	0.998131745	0.999558179	0.99999488	0.999985264	0.999960414
HOXC8	0.999996191	0.998131745	0.999558179	0.99999488	0.999985264	0.999960414
LOC101906398	0.999996191	0.998131745	0.999558179	0.99999488	0.999985264	0.999960414
RAP1B	0.999996191	0.998131745	0.999558179	0.99999488	0.999985264	0.999960414
CDH1	0.999996191	0.999347049	0.999558179	0.99999488	0.999985264	0.999960414
GATB	0.999996191	0.999347049	0.999558179	0.99999488	0.999985264	0.999960414
LOC101902232	0.999996191	0.999347049	0.999558179	0.99999488	0.999985264	0.999960414
LOC521656	0.999996191	0.999347049	0.999558179	0.99999488	0.999985264	0.999960414
LOC787057	0.999996191	0.999347049	0.999558179	0.99999488	0.999985264	0.999960414
RIMS3	0.999996191	0.999347049	0.999558179	0.99999488	0.999985264	0.999960414
VBP1	0.999996191	0.999347049	0.999558179	0.99999488	0.999985264	0.999960414
ZNF467	0.999996191	0.999347049	0.999558179	0.99999488	0.999985264	0.999960414
AADACL3	0.999996191	0.999992444	0.999558179	0.99999488	0.999985264	0.999960414
AAK1	0.999996191	0.999992444	0.999558179	0.99999488	0.999985264	0.999960414
ABCA1	0.999996191	0.999992444	0.999558179	0.99999488	0.999985264	0.999960414
ABCA10	0.999996191	0.999992444	0.999558179	0.99999488	0.999985264	0.999960414
ABCA13	0.999996191	0.999992444	0.999558179	0.99999488	0.999985264	0.999960414
ABCA9	0.999996191	0.999992444	0.999558179	0.99999488	0.999985264	0.999960414
ABCC10	0.999996191	0.999992444	0.999558179	0.99999488	0.999985264	0.999960414
ABCG4	0.999996191	0.999992444	0.999558179	0.99999488	0.999985264	0.999960414
ACACB	0.999996191	0.999992444	0.999558179	0.99999488	0.999985264	0.999960414
ACAD11	0.999996191	0.999992444	0.999558179	0.99999488	0.999985264	0.999960414
ACAD8	0.999996191	0.999992444	0.999558179	0.99999488	0.999985264	0.999960414
ACADL	0.999996191	0.999992444	0.999558179	0.99999488	0.999985264	0.999960414
ACBD6	0.999996191	0.999992444	0.999558179	0.99999488	0.999985264	0.999960414
ACE2	0.999996191	0.999992444	0.999558179	0.99999488	0.999985264	0.999960414
ACKR2	0.999996191	0.999992444	0.999558179	0.99999488	0.999985264	0.999960414
ACMSD	0.999996191	0.999992444	0.999558179	0.99999488	0.999985264	0.999960414
ACOT11	0.999996191	0.999992444	0.999558179	0.99999488	0.999985264	0.999960414

ACOT13	0.999996191	0.999992444	0.999558179	0.99999488	0.999985264	0.999960414
ACOX2	0.999996191	0.999992444	0.999558179	0.99999488	0.999985264	0.999960414
ACP1	0.999996191	0.999992444	0.999558179	0.99999488	0.999985264	0.999960414
ACP6	0.999996191	0.999992444	0.999558179	0.99999488	0.999985264	0.999960414
ACSBG1	0.999996191	0.999992444	0.999558179	0.99999488	0.999985264	0.999960414
ACSF2	0.999996191	0.999992444	0.999558179	0.99999488	0.999985264	0.999960414
ACSM2B	0.999996191	0.999992444	0.999558179	0.99999488	0.999985264	0.999960414
ACSS3	0.999996191	0.999992444	0.999558179	0.99999488	0.999985264	0.999960414
ACTR8	0.999996191	0.999992444	0.999558179	0.99999488	0.999985264	0.999960414
ACY1	0.999996191	0.999992444	0.999558179	0.99999488	0.999985264	0.999960414
ADAM15	0.999996191	0.999992444	0.999558179	0.99999488	0.999985264	0.999960414
ADAM9	0.999996191	0.999992444	0.999558179	0.99999488	0.999985264	0.999960414
ADAMTS14	0.999996191	0.999992444	0.999558179	0.99999488	0.999985264	0.999960414
ADAMTSL3	0.999996191	0.999992444	0.999558179	0.99999488	0.999985264	0.999960414
ADCY9	0.999996191	0.999992444	0.999558179	0.99999488	0.999985264	0.999960414
ADGRF1	0.999996191	0.999992444	0.999558179	0.99999488	0.999985264	0.999960414
ADH5	0.999996191	0.999992444	0.999558179	0.99999488	0.999985264	0.999960414
ADIPOQ	0.999996191	0.999992444	0.999558179	0.99999488	0.999985264	0.999960414
ADK	0.999996191	0.999992444	0.999558179	0.99999488	0.999985264	0.999960414
ADORA2A	0.999996191	0.999992444	0.999558179	0.99999488	0.999985264	0.999960414
ADPRHL2	0.999996191	0.999992444	0.999558179	0.99999488	0.999985264	0.999960414
ADSSL1	0.999996191	0.999992444	0.999558179	0.99999488	0.999985264	0.999960414
AFDN	0.999996191	0.999992444	0.999558179	0.99999488	0.999985264	0.999960414
AGA	0.999996191	0.999992444	0.999558179	0.99999488	0.999985264	0.999960414
AGK	0.999996191	0.999992444	0.999558179	0.99999488	0.999985264	0.999960414
AGL	0.999996191	0.999992444	0.999558179	0.99999488	0.999985264	0.999960414
AGPAT3	0.999996191	0.999992444	0.999558179	0.99999488	0.999985264	0.999960414
AIG1	0.999996191	0.999992444	0.999558179	0.99999488	0.999985264	0.999960414
AK3	0.999996191	0.999992444	0.999558179	0.99999488	0.999985264	0.999960414
AK9	0.999996191	0.999992444	0.999558179	0.99999488	0.999985264	0.999960414
AKAP1	0.999996191	0.999992444	0.999558179	0.99999488	0.999985264	0.999960414
AKAP17A	0.999996191	0.999992444	0.999558179	0.99999488	0.999985264	0.999960414
AKAP8	0.999996191	0.999992444	0.999558179	0.99999488	0.999985264	0.999960414
AKIRIN2	0.999996191	0.999992444	0.999558179	0.99999488	0.999985264	0.999960414
ALAD	0.999996191	0.999992444	0.999558179	0.99999488	0.999985264	0.999960414
ALDH6A1	0.999996191	0.999992444	0.999558179	0.99999488	0.999985264	0.999960414
ALPK2	0.999996191	0.999992444	0.999558179	0.99999488	0.999985264	0.999960414
AMPH	0.999996191	0.999992444	0.999558179	0.99999488	0.999985264	0.999960414
ANAPC16	0.999996191	0.999992444	0.999558179	0.99999488	0.999985264	0.999960414

ANAPC7	0.999996191	0.999992444	0.999558179	0.99999488	0.999985264	0.999960414
ANGEL1	0.999996191	0.999992444	0.999558179	0.99999488	0.999985264	0.999960414
ANGPT1	0.999996191	0.999992444	0.999558179	0.99999488	0.999985264	0.999960414
ANGPTL1	0.999996191	0.999992444	0.999558179	0.99999488	0.999985264	0.999960414
ANK2	0.999996191	0.999992444	0.999558179	0.99999488	0.999985264	0.999960414
ANKFY1	0.999996191	0.999992444	0.999558179	0.99999488	0.999985264	0.999960414
ANKH	0.999996191	0.999992444	0.999558179	0.99999488	0.999985264	0.999960414
ANKRD10	0.999996191	0.999992444	0.999558179	0.99999488	0.999985264	0.999960414
ANKRD13A	0.999996191	0.999992444	0.999558179	0.99999488	0.999985264	0.999960414
ANKRD13D	0.999996191	0.999992444	0.999558179	0.99999488	0.999985264	0.999960414
ANKRD28	0.999996191	0.999992444	0.999558179	0.99999488	0.999985264	0.999960414
ANKRD35	0.999996191	0.999992444	0.999558179	0.99999488	0.999985264	0.999960414
ANKRD46	0.999996191	0.999992444	0.999558179	0.99999488	0.999985264	0.999960414
ANKRD49	0.999996191	0.999992444	0.999558179	0.99999488	0.999985264	0.999960414
ANKRD9	0.999996191	0.999992444	0.999558179	0.99999488	0.999985264	0.999960414
ANTXR1	0.999996191	0.999992444	0.999558179	0.99999488	0.999985264	0.999960414
ANXA2	0.999996191	0.999992444	0.999558179	0.99999488	0.999985264	0.999960414
AOC3	0.999996191	0.999992444	0.999558179	0.99999488	0.999985264	0.999960414
APBB2	0.999996191	0.999992444	0.999558179	0.99999488	0.999985264	0.999960414
APOL3	0.999996191	0.999992444	0.999558179	0.99999488	0.999985264	0.999960414
AQP7	0.999996191	0.999992444	0.999558179	0.99999488	0.999985264	0.999960414
ARAP1	0.999996191	0.999992444	0.999558179	0.99999488	0.999985264	0.999960414
ARAP3	0.999996191	0.999992444	0.999558179	0.99999488	0.999985264	0.999960414
ARF4	0.999996191	0.999992444	0.999558179	0.99999488	0.999985264	0.999960414
ARFGEF2	0.999996191	0.999992444	0.999558179	0.99999488	0.999985264	0.999960414
ARHGAP17	0.999996191	0.999992444	0.999558179	0.99999488	0.999985264	0.999960414
ARHGAP32	0.999996191	0.999992444	0.999558179	0.99999488	0.999985264	0.999960414
ARHGAP39	0.999996191	0.999992444	0.999558179	0.99999488	0.999985264	0.999960414
ARHGEF7	0.999996191	0.999992444	0.999558179	0.99999488	0.999985264	0.999960414
ARID5B	0.999996191	0.999992444	0.999558179	0.99999488	0.999985264	0.999960414
ARL13A	0.999996191	0.999992444	0.999558179	0.99999488	0.999985264	0.999960414
ARL16	0.999996191	0.999992444	0.999558179	0.99999488	0.999985264	0.999960414
ARL5A	0.999996191	0.999992444	0.999558179	0.99999488	0.999985264	0.999960414
ARMC5	0.999996191	0.999992444	0.999558179	0.99999488	0.999985264	0.999960414
ARMCX5	0.999996191	0.999992444	0.999558179	0.99999488	0.999985264	0.999960414
ARMT1	0.999996191	0.999992444	0.999558179	0.99999488	0.999985264	0.999960414
ARPIN	0.999996191	0.999992444	0.999558179	0.99999488	0.999985264	0.999960414
AS3MT	0.999996191	0.999992444	0.999558179	0.99999488	0.999985264	0.999960414
ASB2	0.999996191	0.999992444	0.999558179	0.99999488	0.999985264	0.999960414

ASB5	0.999996191	0.999992444	0.999558179	0.99999488	0.999985264	0.999960414
ASPH	0.999996191	0.999992444	0.999558179	0.99999488	0.999985264	0.999960414
ASPN	0.999996191	0.999992444	0.999558179	0.99999488	0.999985264	0.999960414
ATAD5	0.999996191	0.999992444	0.999558179	0.99999488	0.999985264	0.999960414
ATF6B	0.999996191	0.999992444	0.999558179	0.99999488	0.999985264	0.999960414
ATF7IP2	0.999996191	0.999992444	0.999558179	0.99999488	0.999985264	0.999960414
ATG10	0.999996191	0.999992444	0.999558179	0.99999488	0.999985264	0.999960414
ATG16L2	0.999996191	0.999992444	0.999558179	0.99999488	0.999985264	0.999960414
ATG4C	0.999996191	0.999992444	0.999558179	0.99999488	0.999985264	0.999960414
ATG7	0.999996191	0.999992444	0.999558179	0.99999488	0.999985264	0.999960414
ATL1	0.999996191	0.999992444	0.999558179	0.99999488	0.999985264	0.999960414
ATM	0.999996191	0.999992444	0.999558179	0.99999488	0.999985264	0.999960414
ATP10A	0.999996191	0.999992444	0.999558179	0.99999488	0.999985264	0.999960414
ATP23	0.999996191	0.999992444	0.999558179	0.99999488	0.999985264	0.999960414
ATP2A3	0.999996191	0.999992444	0.999558179	0.99999488	0.999985264	0.999960414
ATP2B1	0.999996191	0.999992444	0.999558179	0.99999488	0.999985264	0.999960414
ATP2B4	0.999996191	0.999992444	0.999558179	0.99999488	0.999985264	0.999960414
ATP5MC2	0.999996191	0.999992444	0.999558179	0.99999488	0.999985264	0.999960414
ATP6V0A2	0.999996191	0.999992444	0.999558179	0.99999488	0.999985264	0.999960414
ATP8A1	0.999996191	0.999992444	0.999558179	0.99999488	0.999985264	0.999960414
ATRAID	0.999996191	0.999992444	0.999558179	0.99999488	0.999985264	0.999960414
ATRX	0.999996191	0.999992444	0.999558179	0.99999488	0.999985264	0.999960414
ATXN7L1	0.999996191	0.999992444	0.999558179	0.99999488	0.999985264	0.999960414
AUH	0.999996191	0.999992444	0.999558179	0.99999488	0.999985264	0.999960414
AVPR2	0.999996191	0.999992444	0.999558179	0.99999488	0.999985264	0.999960414
B4GALT1	0.999996191	0.999992444	0.999558179	0.99999488	0.999985264	0.999960414
BABAM1	0.999996191	0.999992444	0.999558179	0.99999488	0.999985264	0.999960414
BACE1	0.999996191	0.999992444	0.999558179	0.99999488	0.999985264	0.999960414
BACH1	0.999996191	0.999992444	0.999558179	0.99999488	0.999985264	0.999960414
BAHD1	0.999996191	0.999992444	0.999558179	0.99999488	0.999985264	0.999960414
BAIAP2L1	0.999996191	0.999992444	0.999558179	0.99999488	0.999985264	0.999960414
BANP	0.999996191	0.999992444	0.999558179	0.99999488	0.999985264	0.999960414
BAX	0.999996191	0.999992444	0.999558179	0.99999488	0.999985264	0.999960414
BBS10	0.999996191	0.999992444	0.999558179	0.99999488	0.999985264	0.999960414
BBS12	0.999996191	0.999992444	0.999558179	0.99999488	0.999985264	0.999960414
BCAR1	0.999996191	0.999992444	0.999558179	0.99999488	0.999985264	0.999960414
BCAS2	0.999996191	0.999992444	0.999558179	0.99999488	0.999985264	0.999960414
BCAT1	0.999996191	0.999992444	0.999558179	0.99999488	0.999985264	0.999960414
BCKDHA	0.999996191	0.999992444	0.999558179	0.99999488	0.999985264	0.999960414

BEX5	0.999996191	0.999992444	0.999558179	0.99999488	0.999985264	0.999960414
BFSP1	0.999996191	0.999992444	0.999558179	0.99999488	0.999985264	0.999960414
BICD1	0.999996191	0.999992444	0.999558179	0.99999488	0.999985264	0.999960414
BICRAL	0.999996191	0.999992444	0.999558179	0.99999488	0.999985264	0.999960414
BIN3	0.999996191	0.999992444	0.999558179	0.99999488	0.999985264	0.999960414
BIRC6	0.999996191	0.999992444	0.999558179	0.99999488	0.999985264	0.999960414
BLCAP	0.999996191	0.999992444	0.999558179	0.99999488	0.999985264	0.999960414
BLOC1S3	0.999996191	0.999992444	0.999558179	0.99999488	0.999985264	0.999960414
BLOC1S5	0.999996191	0.999992444	0.999558179	0.99999488	0.999985264	0.999960414
BMP3	0.999996191	0.999992444	0.999558179	0.99999488	0.999985264	0.999960414
BMP7	0.999996191	0.999992444	0.999558179	0.99999488	0.999985264	0.999960414
BNC2	0.999996191	0.999992444	0.999558179	0.99999488	0.999985264	0.999960414
BRMS1	0.999996191	0.999992444	0.999558179	0.99999488	0.999985264	0.999960414
BRPF1	0.999996191	0.999992444	0.999558179	0.99999488	0.999985264	0.999960414
BSCL2	0.999996191	0.999992444	0.999558179	0.99999488	0.999985264	0.999960414
BTBD1	0.999996191	0.999992444	0.999558179	0.99999488	0.999985264	0.999960414
BTC	0.999996191	0.999992444	0.999558179	0.99999488	0.999985264	0.999960414
BTD	0.999996191	0.999992444	0.999558179	0.99999488	0.999985264	0.999960414
C10H15orf41	0.999996191	0.999992444	0.999558179	0.99999488	0.999985264	0.999960414
C11H2orf68	0.999996191	0.999992444	0.999558179	0.99999488	0.999985264	0.999960414
C11H2orf92	0.999996191	0.999992444	0.999558179	0.99999488	0.999985264	0.999960414
C14H8orf82	0.999996191	0.999992444	0.999558179	0.99999488	0.999985264	0.999960414
C16H1orf112	0.999996191	0.999992444	0.999558179	0.99999488	0.999985264	0.999960414
C16H1orf159	0.999996191	0.999992444	0.999558179	0.99999488	0.999985264	0.999960414
C16H1orf21	0.999996191	0.999992444	0.999558179	0.99999488	0.999985264	0.999960414
C18H16orf70	0.999996191	0.999992444	0.999558179	0.99999488	0.999985264	0.999960414
C18H19orf12	0.999996191	0.999992444	0.999558179	0.99999488	0.999985264	0.999960414
C18H19orf54	0.999996191	0.999992444	0.999558179	0.99999488	0.999985264	0.999960414
C1H21orf58	0.999996191	0.999992444	0.999558179	0.99999488	0.999985264	0.999960414
C1H3orf33	0.999996191	0.999992444	0.999558179	0.99999488	0.999985264	0.999960414
C1H3orf58	0.999996191	0.999992444	0.999558179	0.99999488	0.999985264	0.999960414
C1H3orf70	0.999996191	0.999992444	0.999558179	0.99999488	0.999985264	0.999960414
C22H3orf14	0.999996191	0.999992444	0.999558179	0.99999488	0.999985264	0.999960414
C22H3orf18	0.999996191	0.999992444	0.999558179	0.99999488	0.999985264	0.999960414
C22H3orf20	0.999996191	0.999992444	0.999558179	0.99999488	0.999985264	0.999960414
C25H16orf58	0.999996191	0.999992444	0.999558179	0.99999488	0.999985264	0.999960414
C25H16orf71	0.999996191	0.999992444	0.999558179	0.99999488	0.999985264	0.999960414
C29H11orf54	0.999996191	0.999992444	0.999558179	0.99999488	0.999985264	0.999960414
C2CD2	0.999996191	0.999992444	0.999558179	0.99999488	0.999985264	0.999960414

C2CD3	0.999996191	0.999992444	0.999558179	0.99999488	0.999985264	0.999960414
C3H1orf216	0.999996191	0.999992444	0.999558179	0.99999488	0.999985264	0.999960414
C3H1orf50	0.999996191	0.999992444	0.999558179	0.99999488	0.999985264	0.999960414
C5AR2	0.999996191	0.999992444	0.999558179	0.99999488	0.999985264	0.999960414
C5H12orf10	0.999996191	0.999992444	0.999558179	0.99999488	0.999985264	0.999960414
C5H12orf56	0.999996191	0.999992444	0.999558179	0.99999488	0.999985264	0.999960414
C7H5orf15	0.999996191	0.999992444	0.999558179	0.99999488	0.999985264	0.999960414
C8H9orf40	0.999996191	0.999992444	0.999558179	0.99999488	0.999985264	0.999960414
C9H6orf163	0.999996191	0.999992444	0.999558179	0.99999488	0.999985264	0.999960414
CA10	0.999996191	0.999992444	0.999558179	0.99999488	0.999985264	0.999960414
CAB39L	0.999996191	0.999992444	0.999558179	0.99999488	0.999985264	0.999960414
CABLES2	0.999996191	0.999992444	0.999558179	0.99999488	0.999985264	0.999960414
CABP7	0.999996191	0.999992444	0.999558179	0.99999488	0.999985264	0.999960414
CACNG7	0.999996191	0.999992444	0.999558179	0.99999488	0.999985264	0.999960414
CACTIN	0.999996191	0.999992444	0.999558179	0.99999488	0.999985264	0.999960414
CALHM2	0.999996191	0.999992444	0.999558179	0.99999488	0.999985264	0.999960414
CAMKK1	0.999996191	0.999992444	0.999558179	0.99999488	0.999985264	0.999960414
CAMKMT	0.999996191	0.999992444	0.999558179	0.99999488	0.999985264	0.999960414
CASC1	0.999996191	0.999992444	0.999558179	0.99999488	0.999985264	0.999960414
CASC3	0.999996191	0.999992444	0.999558179	0.99999488	0.999985264	0.999960414
CASKIN1	0.999996191	0.999992444	0.999558179	0.99999488	0.999985264	0.999960414
CASKIN2	0.999996191	0.999992444	0.999558179	0.99999488	0.999985264	0.999960414
CASP6	0.999996191	0.999992444	0.999558179	0.99999488	0.999985264	0.999960414
CAST	0.999996191	0.999992444	0.999558179	0.99999488	0.999985264	0.999960414
CASTOR1	0.999996191	0.999992444	0.999558179	0.99999488	0.999985264	0.999960414
CAT	0.999996191	0.999992444	0.999558179	0.99999488	0.999985264	0.999960414
CBX1	0.999996191	0.999992444	0.999558179	0.99999488	0.999985264	0.999960414
CC2D2B	0.999996191	0.999992444	0.999558179	0.99999488	0.999985264	0.999960414
CCDC107	0.999996191	0.999992444	0.999558179	0.99999488	0.999985264	0.999960414
CCDC127	0.999996191	0.999992444	0.999558179	0.99999488	0.999985264	0.999960414
CCDC130	0.999996191	0.999992444	0.999558179	0.99999488	0.999985264	0.999960414
CCDC138	0.999996191	0.999992444	0.999558179	0.99999488	0.999985264	0.999960414
CCDC167	0.999996191	0.999992444	0.999558179	0.99999488	0.999985264	0.999960414
CCDC170	0.999996191	0.999992444	0.999558179	0.99999488	0.999985264	0.999960414
CCDC197	0.999996191	0.999992444	0.999558179	0.99999488	0.999985264	0.999960414
CCDC36	0.999996191	0.999992444	0.999558179	0.99999488	0.999985264	0.999960414
CCDC50	0.999996191	0.999992444	0.999558179	0.99999488	0.999985264	0.999960414
CCDC69	0.999996191	0.999992444	0.999558179	0.99999488	0.999985264	0.999960414
CCDC85A	0.999996191	0.999992444	0.999558179	0.99999488	0.999985264	0.999960414

CCDC88A	0.999996191	0.999992444	0.999558179	0.99999488	0.999985264	0.999960414
CCDC90B	0.999996191	0.999992444	0.999558179	0.99999488	0.999985264	0.999960414
CCDC92	0.999996191	0.999992444	0.999558179	0.99999488	0.999985264	0.999960414
CCDC96	0.999996191	0.999992444	0.999558179	0.99999488	0.999985264	0.999960414
CCDC97	0.999996191	0.999992444	0.999558179	0.99999488	0.999985264	0.999960414
CCL28	0.999996191	0.999992444	0.999558179	0.99999488	0.999985264	0.999960414
CCM2L	0.999996191	0.999992444	0.999558179	0.99999488	0.999985264	0.999960414
CCNE1	0.999996191	0.999992444	0.999558179	0.99999488	0.999985264	0.999960414
CCP110	0.999996191	0.999992444	0.999558179	0.99999488	0.999985264	0.999960414
CCR8	0.999996191	0.999992444	0.999558179	0.99999488	0.999985264	0.999960414
CCS	0.999996191	0.999992444	0.999558179	0.99999488	0.999985264	0.999960414
CD2AP	0.999996191	0.999992444	0.999558179	0.99999488	0.999985264	0.999960414
CD2BP2	0.999996191	0.999992444	0.999558179	0.99999488	0.999985264	0.999960414
CD34	0.999996191	0.999992444	0.999558179	0.99999488	0.999985264	0.999960414
CD46	0.999996191	0.999992444	0.999558179	0.99999488	0.999985264	0.999960414
CDC42BPA	0.999996191	0.999992444	0.999558179	0.99999488	0.999985264	0.999960414
CDCA7L	0.999996191	0.999992444	0.999558179	0.99999488	0.999985264	0.999960414
CDH13	0.999996191	0.999992444	0.999558179	0.99999488	0.999985264	0.999960414
CDH23	0.999996191	0.999992444	0.999558179	0.99999488	0.999985264	0.999960414
CDH7	0.999996191	0.999992444	0.999558179	0.99999488	0.999985264	0.999960414
CDK5RAP3	0.999996191	0.999992444	0.999558179	0.99999488	0.999985264	0.999960414
CDKAL1	0.999996191	0.999992444	0.999558179	0.99999488	0.999985264	0.999960414
CELF2	0.999996191	0.999992444	0.999558179	0.99999488	0.999985264	0.999960414
CELF6	0.999996191	0.999992444	0.999558179	0.99999488	0.999985264	0.999960414
CENPJ	0.999996191	0.999992444	0.999558179	0.99999488	0.999985264	0.999960414
CENPM	0.999996191	0.999992444	0.999558179	0.99999488	0.999985264	0.999960414
CEP41	0.999996191	0.999992444	0.999558179	0.99999488	0.999985264	0.999960414
CEP63	0.999996191	0.999992444	0.999558179	0.99999488	0.999985264	0.999960414
CFAP126	0.999996191	0.999992444	0.999558179	0.99999488	0.999985264	0.999960414
CGN	0.999996191	0.999992444	0.999558179	0.99999488	0.999985264	0.999960414
CHAMP1	0.999996191	0.999992444	0.999558179	0.99999488	0.999985264	0.999960414
CHD6	0.999996191	0.999992444	0.999558179	0.99999488	0.999985264	0.999960414
CHD8	0.999996191	0.999992444	0.999558179	0.99999488	0.999985264	0.999960414
CHD9	0.999996191	0.999992444	0.999558179	0.99999488	0.999985264	0.999960414
CHEK1	0.999996191	0.999992444	0.999558179	0.99999488	0.999985264	0.999960414
CHFR	0.999996191	0.999992444	0.999558179	0.99999488	0.999985264	0.999960414
CHIC2	0.999996191	0.999992444	0.999558179	0.99999488	0.999985264	0.999960414
CHML	0.999996191	0.999992444	0.999558179	0.99999488	0.999985264	0.999960414
CHRNA5	0.999996191	0.999992444	0.999558179	0.99999488	0.999985264	0.999960414

CHRNB4	0.999996191	0.999992444	0.999558179	0.99999488	0.999985264	0.999960414
CHST1	0.999996191	0.999992444	0.999558179	0.99999488	0.999985264	0.999960414
CHST15	0.999996191	0.999992444	0.999558179	0.99999488	0.999985264	0.999960414
CHST2	0.999996191	0.999992444	0.999558179	0.99999488	0.999985264	0.999960414
CHSY3	0.999996191	0.999992444	0.999558179	0.99999488	0.999985264	0.999960414
CHTOP	0.999996191	0.999992444	0.999558179	0.99999488	0.999985264	0.999960414
CIB4	0.999996191	0.999992444	0.999558179	0.99999488	0.999985264	0.999960414
CINP	0.999996191	0.999992444	0.999558179	0.99999488	0.999985264	0.999960414
CIPC	0.999996191	0.999992444	0.999558179	0.99999488	0.999985264	0.999960414
CIR1	0.999996191	0.999992444	0.999558179	0.99999488	0.999985264	0.999960414
CLCA3	0.999996191	0.999992444	0.999558179	0.99999488	0.999985264	0.999960414
CLCN2	0.999996191	0.999992444	0.999558179	0.99999488	0.999985264	0.999960414
CLDN12	0.999996191	0.999992444	0.999558179	0.99999488	0.999985264	0.999960414
CLEC1A	0.999996191	0.999992444	0.999558179	0.99999488	0.999985264	0.999960414
CLIP1	0.999996191	0.999992444	0.999558179	0.99999488	0.999985264	0.999960414
CLOCK	0.999996191	0.999992444	0.999558179	0.99999488	0.999985264	0.999960414
CLSTN1	0.999996191	0.999992444	0.999558179	0.99999488	0.999985264	0.999960414
CLTC	0.999996191	0.999992444	0.999558179	0.99999488	0.999985264	0.999960414
CLTRN	0.999996191	0.999992444	0.999558179	0.99999488	0.999985264	0.999960414
CMIP	0.999996191	0.999992444	0.999558179	0.99999488	0.999985264	0.999960414
CMTM7	0.999996191	0.999992444	0.999558179	0.99999488	0.999985264	0.999960414
CNMD	0.999996191	0.999992444	0.999558179	0.99999488	0.999985264	0.999960414
CNNM2	0.999996191	0.999992444	0.999558179	0.99999488	0.999985264	0.999960414
CNOT3	0.999996191	0.999992444	0.999558179	0.99999488	0.999985264	0.999960414
CNOT8	0.999996191	0.999992444	0.999558179	0.99999488	0.999985264	0.999960414
CNOT9	0.999996191	0.999992444	0.999558179	0.99999488	0.999985264	0.999960414
CNPY3	0.999996191	0.999992444	0.999558179	0.99999488	0.999985264	0.999960414
CNPY4	0.999996191	0.999992444	0.999558179	0.99999488	0.999985264	0.999960414
CNR1	0.999996191	0.999992444	0.999558179	0.99999488	0.999985264	0.999960414
CNTF	0.999996191	0.999992444	0.999558179	0.99999488	0.999985264	0.999960414
CNTN1	0.999996191	0.999992444	0.999558179	0.99999488	0.999985264	0.999960414
CNTNAP3	0.999996191	0.999992444	0.999558179	0.99999488	0.999985264	0.999960414
COCH	0.999996191	0.999992444	0.999558179	0.99999488	0.999985264	0.999960414
COG5	0.999996191	0.999992444	0.999558179	0.99999488	0.999985264	0.999960414
COL18A1	0.999996191	0.999992444	0.999558179	0.99999488	0.999985264	0.999960414
COMMD10	0.999996191	0.999992444	0.999558179	0.99999488	0.999985264	0.999960414
COMMD9	0.999996191	0.999992444	0.999558179	0.99999488	0.999985264	0.999960414
COQ3	0.999996191	0.999992444	0.999558179	0.99999488	0.999985264	0.999960414
CORO1B	0.999996191	0.999992444	0.999558179	0.99999488	0.999985264	0.999960414

COX15	0.999996191	0.999992444	0.999558179	0.99999488	0.999985264	0.999960414
CPED1	0.999996191	0.999992444	0.999558179	0.99999488	0.999985264	0.999960414
CPN2	0.999996191	0.999992444	0.999558179	0.99999488	0.999985264	0.999960414
CPSF4	0.999996191	0.999992444	0.999558179	0.99999488	0.999985264	0.999960414
CPTP	0.999996191	0.999992444	0.999558179	0.99999488	0.999985264	0.999960414
CREBL2	0.999996191	0.999992444	0.999558179	0.99999488	0.999985264	0.999960414
CRELD1	0.999996191	0.999992444	0.999558179	0.99999488	0.999985264	0.999960414
CRNKL1	0.999996191	0.999992444	0.999558179	0.99999488	0.999985264	0.999960414
CROT	0.999996191	0.999992444	0.999558179	0.99999488	0.999985264	0.999960414
CRY1	0.999996191	0.999992444	0.999558179	0.99999488	0.999985264	0.999960414
CRYBA4	0.999996191	0.999992444	0.999558179	0.99999488	0.999985264	0.999960414
CRYBG1	0.999996191	0.999992444	0.999558179	0.99999488	0.999985264	0.999960414
CSAD	0.999996191	0.999992444	0.999558179	0.99999488	0.999985264	0.999960414
CSDC2	0.999996191	0.999992444	0.999558179	0.99999488	0.999985264	0.999960414
CSDE1	0.999996191	0.999992444	0.999558179	0.99999488	0.999985264	0.999960414
CSF2RB	0.999996191	0.999992444	0.999558179	0.99999488	0.999985264	0.999960414
CSF3	0.999996191	0.999992444	0.999558179	0.99999488	0.999985264	0.999960414
CSNK1A1	0.999996191	0.999992444	0.999558179	0.99999488	0.999985264	0.999960414
CSNK1G1	0.999996191	0.999992444	0.999558179	0.99999488	0.999985264	0.999960414
CTDP1	0.999996191	0.999992444	0.999558179	0.99999488	0.999985264	0.999960414
CTDSP2	0.999996191	0.999992444	0.999558179	0.99999488	0.999985264	0.999960414
CTDSPL2	0.999996191	0.999992444	0.999558179	0.99999488	0.999985264	0.999960414
CTNNA1	0.999996191	0.999992444	0.999558179	0.99999488	0.999985264	0.999960414
CTNND1	0.999996191	0.999992444	0.999558179	0.99999488	0.999985264	0.999960414
CTR9	0.999996191	0.999992444	0.999558179	0.99999488	0.999985264	0.999960414
CTU2	0.999996191	0.999992444	0.999558179	0.99999488	0.999985264	0.999960414
CUL2	0.999996191	0.999992444	0.999558179	0.99999488	0.999985264	0.999960414
CUTC	0.999996191	0.999992444	0.999558179	0.99999488	0.999985264	0.999960414
CWC15	0.999996191	0.999992444	0.999558179	0.99999488	0.999985264	0.999960414
CYB5D1	0.999996191	0.999992444	0.999558179	0.99999488	0.999985264	0.999960414
CYFIP1	0.999996191	0.999992444	0.999558179	0.99999488	0.999985264	0.999960414
CYP2U1	0.999996191	0.999992444	0.999558179	0.99999488	0.999985264	0.999960414
CYP8B1	0.999996191	0.999992444	0.999558179	0.99999488	0.999985264	0.999960414
CYS1	0.999996191	0.999992444	0.999558179	0.99999488	0.999985264	0.999960414
CYTH3	0.999996191	0.999992444	0.999558179	0.99999488	0.999985264	0.999960414
DAAM2	0.999996191	0.999992444	0.999558179	0.99999488	0.999985264	0.999960414
DAB1	0.999996191	0.999992444	0.999558179	0.99999488	0.999985264	0.999960414
DCAF15	0.999996191	0.999992444	0.999558179	0.99999488	0.999985264	0.999960414
DCAF16	0.999996191	0.999992444	0.999558179	0.99999488	0.999985264	0.999960414

DCAF7	0.999996191	0.999992444	0.999558179	0.99999488	0.999985264	0.999960414
DCBLD2	0.999996191	0.999992444	0.999558179	0.99999488	0.999985264	0.999960414
DCLRE1A	0.999996191	0.999992444	0.999558179	0.99999488	0.999985264	0.999960414
DCLRE1C	0.999996191	0.999992444	0.999558179	0.99999488	0.999985264	0.999960414
DCUN1D3	0.999996191	0.999992444	0.999558179	0.99999488	0.999985264	0.999960414
DDAH2	0.999996191	0.999992444	0.999558179	0.99999488	0.999985264	0.999960414
DDB2	0.999996191	0.999992444	0.999558179	0.99999488	0.999985264	0.999960414
DDHD2	0.999996191	0.999992444	0.999558179	0.99999488	0.999985264	0.999960414
DDX19B	0.999996191	0.999992444	0.999558179	0.99999488	0.999985264	0.999960414
DDX42	0.999996191	0.999992444	0.999558179	0.99999488	0.999985264	0.999960414
DDX59	0.999996191	0.999992444	0.999558179	0.99999488	0.999985264	0.999960414
DENND4C	0.999996191	0.999992444	0.999558179	0.99999488	0.999985264	0.999960414
DEPDC5	0.999996191	0.999992444	0.999558179	0.99999488	0.999985264	0.999960414
DEPP1	0.999996191	0.999992444	0.999558179	0.99999488	0.999985264	0.999960414
DET1	0.999996191	0.999992444	0.999558179	0.99999488	0.999985264	0.999960414
DHFR	0.999996191	0.999992444	0.999558179	0.99999488	0.999985264	0.999960414
DHRS12	0.999996191	0.999992444	0.999558179	0.99999488	0.999985264	0.999960414
DHTKD1	0.999996191	0.999992444	0.999558179	0.99999488	0.999985264	0.999960414
DHX16	0.999996191	0.999992444	0.999558179	0.99999488	0.999985264	0.999960414
DHX8	0.999996191	0.999992444	0.999558179	0.99999488	0.999985264	0.999960414
DIO1	0.999996191	0.999992444	0.999558179	0.99999488	0.999985264	0.999960414
DIO2	0.999996191	0.999992444	0.999558179	0.99999488	0.999985264	0.999960414
DIS3L	0.999996191	0.999992444	0.999558179	0.99999488	0.999985264	0.999960414
DMXL2	0.999996191	0.999992444	0.999558179	0.99999488	0.999985264	0.999960414
DNAJB5	0.999996191	0.999992444	0.999558179	0.99999488	0.999985264	0.999960414
DNAJC13	0.999996191	0.999992444	0.999558179	0.99999488	0.999985264	0.999960414
DNAJC17	0.999996191	0.999992444	0.999558179	0.99999488	0.999985264	0.999960414
DNAJC9	0.999996191	0.999992444	0.999558179	0.99999488	0.999985264	0.999960414
DNAL1	0.999996191	0.999992444	0.999558179	0.99999488	0.999985264	0.999960414
DND1	0.999996191	0.999992444	0.999558179	0.99999488	0.999985264	0.999960414
DOC2B	0.999996191	0.999992444	0.999558179	0.99999488	0.999985264	0.999960414
DOCK11	0.999996191	0.999992444	0.999558179	0.99999488	0.999985264	0.999960414
DOCK3	0.999996191	0.999992444	0.999558179	0.99999488	0.999985264	0.999960414
DOCK9	0.999996191	0.999992444	0.999558179	0.99999488	0.999985264	0.999960414
DOPEY1	0.999996191	0.999992444	0.999558179	0.99999488	0.999985264	0.999960414
DOT1L	0.999996191	0.999992444	0.999558179	0.99999488	0.999985264	0.999960414
DPH6	0.999996191	0.999992444	0.999558179	0.99999488	0.999985264	0.999960414
DPY19L4	0.999996191	0.999992444	0.999558179	0.99999488	0.999985264	0.999960414
DPY30	0.999996191	0.999992444	0.999558179	0.99999488	0.999985264	0.999960414

DSN1	0.999996191	0.999992444	0.999558179	0.99999488	0.999985264	0.999960414
DUSP6	0.999996191	0.999992444	0.999558179	0.99999488	0.999985264	0.999960414
DUSP7	0.999996191	0.999992444	0.999558179	0.99999488	0.999985264	0.999960414
DUT	0.999996191	0.999992444	0.999558179	0.99999488	0.999985264	0.999960414
DYNC1H1	0.999996191	0.999992444	0.999558179	0.99999488	0.999985264	0.999960414
DZIP1	0.999996191	0.999992444	0.999558179	0.99999488	0.999985264	0.999960414
DZIP1L	0.999996191	0.999992444	0.999558179	0.99999488	0.999985264	0.999960414
DZIP3	0.999996191	0.999992444	0.999558179	0.99999488	0.999985264	0.999960414
E4F1	0.999996191	0.999992444	0.999558179	0.99999488	0.999985264	0.999960414
EBF3	0.999996191	0.999992444	0.999558179	0.99999488	0.999985264	0.999960414
ECHDC1	0.999996191	0.999992444	0.999558179	0.99999488	0.999985264	0.999960414
ECI2	0.999996191	0.999992444	0.999558179	0.99999488	0.999985264	0.999960414
ECM2	0.999996191	0.999992444	0.999558179	0.99999488	0.999985264	0.999960414
ECPAS	0.999996191	0.999992444	0.999558179	0.99999488	0.999985264	0.999960414
ECSCR	0.999996191	0.999992444	0.999558179	0.99999488	0.999985264	0.999960414
EDC4	0.999996191	0.999992444	0.999558179	0.99999488	0.999985264	0.999960414
EDEM2	0.999996191	0.999992444	0.999558179	0.99999488	0.999985264	0.999960414
EDEM3	0.999996191	0.999992444	0.999558179	0.99999488	0.999985264	0.999960414
EDN1	0.999996191	0.999992444	0.999558179	0.99999488	0.999985264	0.999960414
EEF1AKMT1	0.999996191	0.999992444	0.999558179	0.99999488	0.999985264	0.999960414
EFCAB5	0.999996191	0.999992444	0.999558179	0.99999488	0.999985264	0.999960414
EFCAB6	0.999996191	0.999992444	0.999558179	0.99999488	0.999985264	0.999960414
EFCAB7	0.999996191	0.999992444	0.999558179	0.99999488	0.999985264	0.999960414
EFCAB8	0.999996191	0.999992444	0.999558179	0.99999488	0.999985264	0.999960414
EFHD1	0.999996191	0.999992444	0.999558179	0.99999488	0.999985264	0.999960414
EFNA1	0.999996191	0.999992444	0.999558179	0.99999488	0.999985264	0.999960414
EFNA5	0.999996191	0.999992444	0.999558179	0.99999488	0.999985264	0.999960414
EGFL7	0.999996191	0.999992444	0.999558179	0.99999488	0.999985264	0.999960414
EGLN3	0.999996191	0.999992444	0.999558179	0.99999488	0.999985264	0.999960414
EHBP1L1	0.999996191	0.999992444	0.999558179	0.99999488	0.999985264	0.999960414
EHD4	0.999996191	0.999992444	0.999558179	0.99999488	0.999985264	0.999960414
EIF2AK1	0.999996191	0.999992444	0.999558179	0.99999488	0.999985264	0.999960414
EIF2AK4	0.999996191	0.999992444	0.999558179	0.99999488	0.999985264	0.999960414
EIF4ENIF1	0.999996191	0.999992444	0.999558179	0.99999488	0.999985264	0.999960414
EIF5A2	0.999996191	0.999992444	0.999558179	0.99999488	0.999985264	0.999960414
ELF4	0.999996191	0.999992444	0.999558179	0.99999488	0.999985264	0.999960414
ELOA	0.999996191	0.999992444	0.999558179	0.99999488	0.999985264	0.999960414
ELOVL4	0.999996191	0.999992444	0.999558179	0.99999488	0.999985264	0.999960414
ENDOU	0.999996191	0.999992444	0.999558179	0.99999488	0.999985264	0.999960414

ENO3	0.999996191	0.999992444	0.999558179	0.99999488	0.999985264	0.999960414
ENOPH1	0.999996191	0.999992444	0.999558179	0.99999488	0.999985264	0.999960414
ENTPD5	0.999996191	0.999992444	0.999558179	0.99999488	0.999985264	0.999960414
EP400	0.999996191	0.999992444	0.999558179	0.99999488	0.999985264	0.999960414
EPB41L1	0.999996191	0.999992444	0.999558179	0.99999488	0.999985264	0.999960414
EPG5	0.999996191	0.999992444	0.999558179	0.99999488	0.999985264	0.999960414
EPHX3	0.999996191	0.999992444	0.999558179	0.99999488	0.999985264	0.999960414
ERMAP	0.999996191	0.999992444	0.999558179	0.99999488	0.999985264	0.999960414
ERP44	0.999996191	0.999992444	0.999558179	0.99999488	0.999985264	0.999960414
ESYT1	0.999996191	0.999992444	0.999558179	0.99999488	0.999985264	0.999960414
ETV2	0.999996191	0.999992444	0.999558179	0.99999488	0.999985264	0.999960414
EWSR1	0.999996191	0.999992444	0.999558179	0.99999488	0.999985264	0.999960414
EXOC4	0.999996191	0.999992444	0.999558179	0.99999488	0.999985264	0.999960414
EXOSC1	0.999996191	0.999992444	0.999558179	0.99999488	0.999985264	0.999960414
EZH1	0.999996191	0.999992444	0.999558179	0.99999488	0.999985264	0.999960414
FA2H	0.999996191	0.999992444	0.999558179	0.99999488	0.999985264	0.999960414
FAM110A	0.999996191	0.999992444	0.999558179	0.99999488	0.999985264	0.999960414
FAM110D	0.999996191	0.999992444	0.999558179	0.99999488	0.999985264	0.999960414
FAM111B	0.999996191	0.999992444	0.999558179	0.99999488	0.999985264	0.999960414
FAM114A1	0.999996191	0.999992444	0.999558179	0.99999488	0.999985264	0.999960414
FAM122B	0.999996191	0.999992444	0.999558179	0.99999488	0.999985264	0.999960414
FAM133A	0.999996191	0.999992444	0.999558179	0.99999488	0.999985264	0.999960414
FAM151A	0.999996191	0.999992444	0.999558179	0.99999488	0.999985264	0.999960414
FAM155B	0.999996191	0.999992444	0.999558179	0.99999488	0.999985264	0.999960414
FAM162A	0.999996191	0.999992444	0.999558179	0.99999488	0.999985264	0.999960414
FAM184B	0.999996191	0.999992444	0.999558179	0.99999488	0.999985264	0.999960414
FAM193A	0.999996191	0.999992444	0.999558179	0.99999488	0.999985264	0.999960414
FAM196A	0.999996191	0.999992444	0.999558179	0.99999488	0.999985264	0.999960414
FAM241B	0.999996191	0.999992444	0.999558179	0.99999488	0.999985264	0.999960414
FAM45A	0.999996191	0.999992444	0.999558179	0.99999488	0.999985264	0.999960414
FAM71F2	0.999996191	0.999992444	0.999558179	0.99999488	0.999985264	0.999960414
FAM8A1	0.999996191	0.999992444	0.999558179	0.99999488	0.999985264	0.999960414
FAM92A	0.999996191	0.999992444	0.999558179	0.99999488	0.999985264	0.999960414
FAT4	0.999996191	0.999992444	0.999558179	0.99999488	0.999985264	0.999960414
FBF1	0.999996191	0.999992444	0.999558179	0.99999488	0.999985264	0.999960414
FBXL12	0.999996191	0.999992444	0.999558179	0.99999488	0.999985264	0.999960414
FBXL17	0.999996191	0.999992444	0.999558179	0.99999488	0.999985264	0.999960414
FBXL20	0.999996191	0.999992444	0.999558179	0.99999488	0.999985264	0.999960414
FBXO25	0.999996191	0.999992444	0.999558179	0.99999488	0.999985264	0.999960414

FBXO34	0.999996191	0.999992444	0.999558179	0.99999488	0.999985264	0.999960414
FBXO4	0.999996191	0.999992444	0.999558179	0.99999488	0.999985264	0.999960414
FBXO5	0.999996191	0.999992444	0.999558179	0.99999488	0.999985264	0.999960414
FBXW4	0.999996191	0.999992444	0.999558179	0.99999488	0.999985264	0.999960414
FCHSD2	0.999996191	0.999992444	0.999558179	0.99999488	0.999985264	0.999960414
FDX1	0.999996191	0.999992444	0.999558179	0.99999488	0.999985264	0.999960414
FECH	0.999996191	0.999992444	0.999558179	0.99999488	0.999985264	0.999960414
FER	0.999996191	0.999992444	0.999558179	0.99999488	0.999985264	0.999960414
FER1L5	0.999996191	0.999992444	0.999558179	0.99999488	0.999985264	0.999960414
FFAR4	0.999996191	0.999992444	0.999558179	0.99999488	0.999985264	0.999960414
FGD1	0.999996191	0.999992444	0.999558179	0.99999488	0.999985264	0.999960414
FGFRL1	0.999996191	0.999992444	0.999558179	0.99999488	0.999985264	0.999960414
FGL2	0.999996191	0.999992444	0.999558179	0.99999488	0.999985264	0.999960414
FIGNL1	0.999996191	0.999992444	0.999558179	0.99999488	0.999985264	0.999960414
FIGNL2	0.999996191	0.999992444	0.999558179	0.99999488	0.999985264	0.999960414
FIZ1	0.999996191	0.999992444	0.999558179	0.99999488	0.999985264	0.999960414
FLRT1	0.999996191	0.999992444	0.999558179	0.99999488	0.999985264	0.999960414
FLRT2	0.999996191	0.999992444	0.999558179	0.99999488	0.999985264	0.999960414
FLT1	0.999996191	0.999992444	0.999558179	0.99999488	0.999985264	0.999960414
FLT3LG	0.999996191	0.999992444	0.999558179	0.99999488	0.999985264	0.999960414
FMR1	0.999996191	0.999992444	0.999558179	0.99999488	0.999985264	0.999960414
FN3KRP	0.999996191	0.999992444	0.999558179	0.99999488	0.999985264	0.999960414
FOCAD	0.999996191	0.999992444	0.999558179	0.99999488	0.999985264	0.999960414
FOXF2	0.999996191	0.999992444	0.999558179	0.99999488	0.999985264	0.999960414
FOXK1	0.999996191	0.999992444	0.999558179	0.99999488	0.999985264	0.999960414
FRK	0.999996191	0.999992444	0.999558179	0.99999488	0.999985264	0.999960414
FRMD3	0.999996191	0.999992444	0.999558179	0.99999488	0.999985264	0.999960414
FRMD4A	0.999996191	0.999992444	0.999558179	0.99999488	0.999985264	0.999960414
FRMD6	0.999996191	0.999992444	0.999558179	0.99999488	0.999985264	0.999960414
FRMPD4	0.999996191	0.999992444	0.999558179	0.99999488	0.999985264	0.999960414
FSD2	0.999996191	0.999992444	0.999558179	0.99999488	0.999985264	0.999960414
FUCA2	0.999996191	0.999992444	0.999558179	0.99999488	0.999985264	0.999960414
FZD6	0.999996191	0.999992444	0.999558179	0.99999488	0.999985264	0.999960414
G6PC	0.999996191	0.999992444	0.999558179	0.99999488	0.999985264	0.999960414
GAA	0.999996191	0.999992444	0.999558179	0.99999488	0.999985264	0.999960414
GAB2	0.999996191	0.999992444	0.999558179	0.99999488	0.999985264	0.999960414
GABPA	0.999996191	0.999992444	0.999558179	0.99999488	0.999985264	0.999960414
GABRG3	0.999996191	0.999992444	0.999558179	0.99999488	0.999985264	0.999960414
GAREM1	0.999996191	0.999992444	0.999558179	0.99999488	0.999985264	0.999960414

GAT	0.999996191	0.999992444	0.999558179	0.99999488	0.999985264	0.999960414
GATA2	0.999996191	0.999992444	0.999558179	0.99999488	0.999985264	0.999960414
GATM	0.999996191	0.999992444	0.999558179	0.99999488	0.999985264	0.999960414
GBX1	0.999996191	0.999992444	0.999558179	0.99999488	0.999985264	0.999960414
GCC2	0.999996191	0.999992444	0.999558179	0.99999488	0.999985264	0.999960414
GCGR	0.999996191	0.999992444	0.999558179	0.99999488	0.999985264	0.999960414
GDAP2	0.999996191	0.999992444	0.999558179	0.99999488	0.999985264	0.999960414
GEMIN8	0.999996191	0.999992444	0.999558179	0.99999488	0.999985264	0.999960414
GGH	0.999996191	0.999992444	0.999558179	0.99999488	0.999985264	0.999960414
GGT5	0.999996191	0.999992444	0.999558179	0.99999488	0.999985264	0.999960414
GIT1	0.999996191	0.999992444	0.999558179	0.99999488	0.999985264	0.999960414
GIT2	0.999996191	0.999992444	0.999558179	0.99999488	0.999985264	0.999960414
GJA4	0.999996191	0.999992444	0.999558179	0.99999488	0.999985264	0.999960414
GJB6	0.999996191	0.999992444	0.999558179	0.99999488	0.999985264	0.999960414
GLB1	0.999996191	0.999992444	0.999558179	0.99999488	0.999985264	0.999960414
GLB1L3	0.999996191	0.999992444	0.999558179	0.99999488	0.999985264	0.999960414
GLE1	0.999996191	0.999992444	0.999558179	0.99999488	0.999985264	0.999960414
GLMN	0.999996191	0.999992444	0.999558179	0.99999488	0.999985264	0.999960414
GLRX2	0.999996191	0.999992444	0.999558179	0.99999488	0.999985264	0.999960414
GLS2	0.999996191	0.999992444	0.999558179	0.99999488	0.999985264	0.999960414
GMPR2	0.999996191	0.999992444	0.999558179	0.99999488	0.999985264	0.999960414
GNAO1	0.999996191	0.999992444	0.999558179	0.99999488	0.999985264	0.999960414
GNAQ	0.999996191	0.999992444	0.999558179	0.99999488	0.999985264	0.999960414
GNAS	0.999996191	0.999992444	0.999558179	0.99999488	0.999985264	0.999960414
GNAT2	0.999996191	0.999992444	0.999558179	0.99999488	0.999985264	0.999960414
GNB4	0.999996191	0.999992444	0.999558179	0.99999488	0.999985264	0.999960414
GNPAT	0.999996191	0.999992444	0.999558179	0.99999488	0.999985264	0.999960414
GPAM	0.999996191	0.999992444	0.999558179	0.99999488	0.999985264	0.999960414
GPATCH11	0.999996191	0.999992444	0.999558179	0.99999488	0.999985264	0.999960414
GPD1	0.999996191	0.999992444	0.999558179	0.99999488	0.999985264	0.999960414
GPKOW	0.999996191	0.999992444	0.999558179	0.99999488	0.999985264	0.999960414
GPR108	0.999996191	0.999992444	0.999558179	0.99999488	0.999985264	0.999960414
GPR157	0.999996191	0.999992444	0.999558179	0.99999488	0.999985264	0.999960414
GPR27	0.999996191	0.999992444	0.999558179	0.99999488	0.999985264	0.999960414
GPR31	0.999996191	0.999992444	0.999558179	0.99999488	0.999985264	0.999960414
GPR52	0.999996191	0.999992444	0.999558179	0.99999488	0.999985264	0.999960414
GRB14	0.999996191	0.999992444	0.999558179	0.99999488	0.999985264	0.999960414
GREB1	0.999996191	0.999992444	0.999558179	0.99999488	0.999985264	0.999960414
GRK2	0.999996191	0.999992444	0.999558179	0.99999488	0.999985264	0.999960414

GRK5	0.999996191	0.999992444	0.999558179	0.99999488	0.999985264	0.999960414
GSTCD	0.999996191	0.999992444	0.999558179	0.99999488	0.999985264	0.999960414
GSTZ1	0.999996191	0.999992444	0.999558179	0.99999488	0.999985264	0.999960414
GTF2E2	0.999996191	0.999992444	0.999558179	0.99999488	0.999985264	0.999960414
GTF2F2	0.999996191	0.999992444	0.999558179	0.99999488	0.999985264	0.999960414
GTF2IRD1	0.999996191	0.999992444	0.999558179	0.99999488	0.999985264	0.999960414
GTF3C4	0.999996191	0.999992444	0.999558179	0.99999488	0.999985264	0.999960414
GTPBP2	0.999996191	0.999992444	0.999558179	0.99999488	0.999985264	0.999960414
GULO	0.999996191	0.999992444	0.999558179	0.99999488	0.999985264	0.999960414
GULP1	0.999996191	0.999992444	0.999558179	0.99999488	0.999985264	0.999960414
H2B	0.999996191	0.999992444	0.999558179	0.99999488	0.999985264	0.999960414
HACD1	0.999996191	0.999992444	0.999558179	0.99999488	0.999985264	0.999960414
HACL1	0.999996191	0.999992444	0.999558179	0.99999488	0.999985264	0.999960414
HADH	0.999996191	0.999992444	0.999558179	0.99999488	0.999985264	0.999960414
HADHA	0.999996191	0.999992444	0.999558179	0.99999488	0.999985264	0.999960414
HAUS7	0.999996191	0.999992444	0.999558179	0.99999488	0.999985264	0.999960414
HBA1	0.999996191	0.999992444	0.999558179	0.99999488	0.999985264	0.999960414
HDAC8	0.999996191	0.999992444	0.999558179	0.99999488	0.999985264	0.999960414
HDDC3	0.999996191	0.999992444	0.999558179	0.99999488	0.999985264	0.999960414
HECTD2	0.999996191	0.999992444	0.999558179	0.99999488	0.999985264	0.999960414
HERC2	0.999996191	0.999992444	0.999558179	0.99999488	0.999985264	0.999960414
HERC3	0.999996191	0.999992444	0.999558179	0.99999488	0.999985264	0.999960414
HEXA	0.999996191	0.999992444	0.999558179	0.99999488	0.999985264	0.999960414
HGSNAT	0.999996191	0.999992444	0.999558179	0.99999488	0.999985264	0.999960414
HHAT	0.999996191	0.999992444	0.999558179	0.99999488	0.999985264	0.999960414
HIKESHI	0.999996191	0.999992444	0.999558179	0.99999488	0.999985264	0.999960414
HIP1	0.999996191	0.999992444	0.999558179	0.99999488	0.999985264	0.999960414
HIPK1	0.999996191	0.999992444	0.999558179	0.99999488	0.999985264	0.999960414
HIST1H1D	0.999996191	0.999992444	0.999558179	0.99999488	0.999985264	0.999960414
HIST1H2AC	0.999996191	0.999992444	0.999558179	0.99999488	0.999985264	0.999960414
HIST1H2BB	0.999996191	0.999992444	0.999558179	0.99999488	0.999985264	0.999960414
HIST1H2BL	0.999996191	0.999992444	0.999558179	0.99999488	0.999985264	0.999960414
HIST2H2AC	0.999996191	0.999992444	0.999558179	0.99999488	0.999985264	0.999960414
HIST2H2BE	0.999996191	0.999992444	0.999558179	0.99999488	0.999985264	0.999960414
HIVEP2	0.999996191	0.999992444	0.999558179	0.99999488	0.999985264	0.999960414
HLTF	0.999996191	0.999992444	0.999558179	0.99999488	0.999985264	0.999960414
HMBOX1	0.999996191	0.999992444	0.999558179	0.99999488	0.999985264	0.999960414
HNRNPA0	0.999996191	0.999992444	0.999558179	0.99999488	0.999985264	0.999960414
HNRNPA1	0.999996191	0.999992444	0.999558179	0.99999488	0.999985264	0.999960414

HNRNPC	0.999996191	0.999992444	0.999558179	0.99999488	0.999985264	0.999960414
HNRNPR	0.999996191	0.999992444	0.999558179	0.99999488	0.999985264	0.999960414
HOMER2	0.999996191	0.999992444	0.999558179	0.99999488	0.999985264	0.999960414
HOOK2	0.999996191	0.999992444	0.999558179	0.99999488	0.999985264	0.999960414
HOXA5	0.999996191	0.999992444	0.999558179	0.99999488	0.999985264	0.999960414
HOXB2	0.999996191	0.999992444	0.999558179	0.99999488	0.999985264	0.999960414
HOXB7	0.999996191	0.999992444	0.999558179	0.99999488	0.999985264	0.999960414
HOXC4	0.999996191	0.999992444	0.999558179	0.99999488	0.999985264	0.999960414
HP1BP3	0.999996191	0.999992444	0.999558179	0.99999488	0.999985264	0.999960414
HPS3	0.999996191	0.999992444	0.999558179	0.99999488	0.999985264	0.999960414
HRH2	0.999996191	0.999992444	0.999558179	0.99999488	0.999985264	0.999960414
HS1BP3	0.999996191	0.999992444	0.999558179	0.99999488	0.999985264	0.999960414
HS3ST2	0.999996191	0.999992444	0.999558179	0.99999488	0.999985264	0.999960414
HSCB	0.999996191	0.999992444	0.999558179	0.99999488	0.999985264	0.999960414
HSD17B8	0.999996191	0.999992444	0.999558179	0.99999488	0.999985264	0.999960414
HSDL2	0.999996191	0.999992444	0.999558179	0.99999488	0.999985264	0.999960414
HSPA12A	0.999996191	0.999992444	0.999558179	0.99999488	0.999985264	0.999960414
HTATIP2	0.999996191	0.999992444	0.999558179	0.99999488	0.999985264	0.999960414
HTR1E	0.999996191	0.999992444	0.999558179	0.99999488	0.999985264	0.999960414
HTRA4	0.999996191	0.999992444	0.999558179	0.99999488	0.999985264	0.999960414
IAH1	0.999996191	0.999992444	0.999558179	0.99999488	0.999985264	0.999960414
IARS	0.999996191	0.999992444	0.999558179	0.99999488	0.999985264	0.999960414
IARS2	0.999996191	0.999992444	0.999558179	0.99999488	0.999985264	0.999960414
ICE1	0.999996191	0.999992444	0.999558179	0.99999488	0.999985264	0.999960414
ICK	0.999996191	0.999992444	0.999558179	0.99999488	0.999985264	0.999960414
IDH3G	0.999996191	0.999992444	0.999558179	0.99999488	0.999985264	0.999960414
IDS	0.999996191	0.999992444	0.999558179	0.99999488	0.999985264	0.999960414
IFI47	0.999996191	0.999992444	0.999558179	0.99999488	0.999985264	0.999960414
IFT46	0.999996191	0.999992444	0.999558179	0.99999488	0.999985264	0.999960414
IFT52	0.999996191	0.999992444	0.999558179	0.99999488	0.999985264	0.999960414
IGSF1	0.999996191	0.999992444	0.999558179	0.99999488	0.999985264	0.999960414
IKBKB	0.999996191	0.999992444	0.999558179	0.99999488	0.999985264	0.999960414
IL13RA1	0.999996191	0.999992444	0.999558179	0.99999488	0.999985264	0.999960414
IL17RC	0.999996191	0.999992444	0.999558179	0.99999488	0.999985264	0.999960414
IL1R1	0.999996191	0.999992444	0.999558179	0.99999488	0.999985264	0.999960414
ILF2	0.999996191	0.999992444	0.999558179	0.99999488	0.999985264	0.999960414
ILF3	0.999996191	0.999992444	0.999558179	0.99999488	0.999985264	0.999960414
ILKAP	0.999996191	0.999992444	0.999558179	0.99999488	0.999985264	0.999960414
INAFM1	0.999996191	0.999992444	0.999558179	0.99999488	0.999985264	0.999960414

ING1	0.999996191	0.999992444	0.999558179	0.99999488	0.999985264	0.999960414
ING5	0.999996191	0.999992444	0.999558179	0.99999488	0.999985264	0.999960414
INO80C	0.999996191	0.999992444	0.999558179	0.99999488	0.999985264	0.999960414
INPP5E	0.999996191	0.999992444	0.999558179	0.99999488	0.999985264	0.999960414
INPP5K	0.999996191	0.999992444	0.999558179	0.99999488	0.999985264	0.999960414
INPPL1	0.999996191	0.999992444	0.999558179	0.99999488	0.999985264	0.999960414
INTS14	0.999996191	0.999992444	0.999558179	0.99999488	0.999985264	0.999960414
INVS	0.999996191	0.999992444	0.999558179	0.99999488	0.999985264	0.999960414
IPMK	0.999996191	0.999992444	0.999558179	0.99999488	0.999985264	0.999960414
IQCG	0.999996191	0.999992444	0.999558179	0.99999488	0.999985264	0.999960414
IREB2	0.999996191	0.999992444	0.999558179	0.99999488	0.999985264	0.999960414
IRF2BP2	0.999996191	0.999992444	0.999558179	0.99999488	0.999985264	0.999960414
IST1	0.999996191	0.999992444	0.999558179	0.99999488	0.999985264	0.999960414
ITFG1	0.999996191	0.999992444	0.999558179	0.99999488	0.999985264	0.999960414
ITGA7	0.999996191	0.999992444	0.999558179	0.99999488	0.999985264	0.999960414
ITGAE	0.999996191	0.999992444	0.999558179	0.99999488	0.999985264	0.999960414
ITGB4	0.999996191	0.999992444	0.999558179	0.99999488	0.999985264	0.999960414
ITGB5	0.999996191	0.999992444	0.999558179	0.99999488	0.999985264	0.999960414
ITM2B	0.999996191	0.999992444	0.999558179	0.99999488	0.999985264	0.999960414
ITPR1	0.999996191	0.999992444	0.999558179	0.99999488	0.999985264	0.999960414
ITPR2	0.999996191	0.999992444	0.999558179	0.99999488	0.999985264	0.999960414
IVNS1ABP	0.999996191	0.999992444	0.999558179	0.99999488	0.999985264	0.999960414
IWS1	0.999996191	0.999992444	0.999558179	0.99999488	0.999985264	0.999960414
JAG1	0.999996191	0.999992444	0.999558179	0.99999488	0.999985264	0.999960414
JAZF1	0.999996191	0.999992444	0.999558179	0.99999488	0.999985264	0.999960414
JMJD7	0.999996191	0.999992444	0.999558179	0.99999488	0.999985264	0.999960414
JMJD8	0.999996191	0.999992444	0.999558179	0.99999488	0.999985264	0.999960414
JTB	0.999996191	0.999992444	0.999558179	0.99999488	0.999985264	0.999960414
JUN	0.999996191	0.999992444	0.999558179	0.99999488	0.999985264	0.999960414
KANK3	0.999996191	0.999992444	0.999558179	0.99999488	0.999985264	0.999960414
KANSL2	0.999996191	0.999992444	0.999558179	0.99999488	0.999985264	0.999960414
KAT2B	0.999996191	0.999992444	0.999558179	0.99999488	0.999985264	0.999960414
KAT5	0.999996191	0.999992444	0.999558179	0.99999488	0.999985264	0.999960414
KAT6B	0.999996191	0.999992444	0.999558179	0.99999488	0.999985264	0.999960414
KAT8	0.999996191	0.999992444	0.999558179	0.99999488	0.999985264	0.999960414
KCNJ11	0.999996191	0.999992444	0.999558179	0.99999488	0.999985264	0.999960414
KCNMB4	0.999996191	0.999992444	0.999558179	0.99999488	0.999985264	0.999960414
KDM1B	0.999996191	0.999992444	0.999558179	0.99999488	0.999985264	0.999960414
KDM3B	0.999996191	0.999992444	0.999558179	0.99999488	0.999985264	0.999960414

KDM5A	0.999996191	0.999992444	0.999558179	0.99999488	0.999985264	0.999960414
KDSR	0.999996191	0.999992444	0.999558179	0.99999488	0.999985264	0.999960414
KHK	0.999996191	0.999992444	0.999558179	0.99999488	0.999985264	0.999960414
KHNYN	0.999996191	0.999992444	0.999558179	0.99999488	0.999985264	0.999960414
KHSRP	0.999996191	0.999992444	0.999558179	0.99999488	0.999985264	0.999960414
KIAA0895	0.999996191	0.999992444	0.999558179	0.99999488	0.999985264	0.999960414
KIF1C	0.999996191	0.999992444	0.999558179	0.99999488	0.999985264	0.999960414
KIF3B	0.999996191	0.999992444	0.999558179	0.99999488	0.999985264	0.999960414
KIN	0.999996191	0.999992444	0.999558179	0.99999488	0.999985264	0.999960414
KLF3	0.999996191	0.999992444	0.999558179	0.99999488	0.999985264	0.999960414
KLF8	0.999996191	0.999992444	0.999558179	0.99999488	0.999985264	0.999960414
KLHDC1	0.999996191	0.999992444	0.999558179	0.99999488	0.999985264	0.999960414
KLHDC10	0.999996191	0.999992444	0.999558179	0.99999488	0.999985264	0.999960414
KLHDC3	0.999996191	0.999992444	0.999558179	0.99999488	0.999985264	0.999960414
KLHL12	0.999996191	0.999992444	0.999558179	0.99999488	0.999985264	0.999960414
KLHL20	0.999996191	0.999992444	0.999558179	0.99999488	0.999985264	0.999960414
KLHL23	0.999996191	0.999992444	0.999558179	0.99999488	0.999985264	0.999960414
KLHL5	0.999996191	0.999992444	0.999558179	0.99999488	0.999985264	0.999960414
KLHL9	0.999996191	0.999992444	0.999558179	0.99999488	0.999985264	0.999960414
KLK4	0.999996191	0.999992444	0.999558179	0.99999488	0.999985264	0.999960414
KLRG1	0.999996191	0.999992444	0.999558179	0.99999488	0.999985264	0.999960414
KMT2D	0.999996191	0.999992444	0.999558179	0.99999488	0.999985264	0.999960414
KMT5A	0.999996191	0.999992444	0.999558179	0.99999488	0.999985264	0.999960414
KNSTRN	0.999996191	0.999992444	0.999558179	0.99999488	0.999985264	0.999960414
KXD1	0.999996191	0.999992444	0.999558179	0.99999488	0.999985264	0.999960414
L3HYPDH	0.999996191	0.999992444	0.999558179	0.99999488	0.999985264	0.999960414
LARP6	0.999996191	0.999992444	0.999558179	0.99999488	0.999985264	0.999960414
LCA5	0.999996191	0.999992444	0.999558179	0.99999488	0.999985264	0.999960414
LCLAT1	0.999996191	0.999992444	0.999558179	0.99999488	0.999985264	0.999960414
LCN6	0.999996191	0.999992444	0.999558179	0.99999488	0.999985264	0.999960414
LDHAL6B	0.999996191	0.999992444	0.999558179	0.99999488	0.999985264	0.999960414
LDHD	0.999996191	0.999992444	0.999558179	0.99999488	0.999985264	0.999960414
LEMD2	0.999996191	0.999992444	0.999558179	0.99999488	0.999985264	0.999960414
LETM2	0.999996191	0.999992444	0.999558179	0.99999488	0.999985264	0.999960414
LFNG	0.999996191	0.999992444	0.999558179	0.99999488	0.999985264	0.999960414
LGALS4	0.999996191	0.999992444	0.999558179	0.99999488	0.999985264	0.999960414
LHPP	0.999996191	0.999992444	0.999558179	0.99999488	0.999985264	0.999960414
LIAS	0.999996191	0.999992444	0.999558179	0.99999488	0.999985264	0.999960414
LIMA1	0.999996191	0.999992444	0.999558179	0.99999488	0.999985264	0.999960414

LIN37	0.999996191	0.999992444	0.999558179	0.99999488	0.999985264	0.999960414
LMO2	0.999996191	0.999992444	0.999558179	0.99999488	0.999985264	0.999960414
LNP1	0.999996191	0.999992444	0.999558179	0.99999488	0.999985264	0.999960414
LOC100138078	0.999996191	0.999992444	0.999558179	0.99999488	0.999985264	0.999960414
LOC100138131	0.999996191	0.999992444	0.999558179	0.99999488	0.999985264	0.999960414
LOC100138449	0.999996191	0.999992444	0.999558179	0.99999488	0.999985264	0.999960414
LOC100138633	0.999996191	0.999992444	0.999558179	0.99999488	0.999985264	0.999960414
LOC100139360	0.999996191	0.999992444	0.999558179	0.99999488	0.999985264	0.999960414
LOC100139732	0.999996191	0.999992444	0.999558179	0.99999488	0.999985264	0.999960414
LOC100140533	0.999996191	0.999992444	0.999558179	0.99999488	0.999985264	0.999960414
LOC100140873	0.999996191	0.999992444	0.999558179	0.99999488	0.999985264	0.999960414
LOC100140915	0.999996191	0.999992444	0.999558179	0.99999488	0.999985264	0.999960414
LOC100196898	0.999996191	0.999992444	0.999558179	0.99999488	0.999985264	0.999960414
LOC100294792	0.999996191	0.999992444	0.999558179	0.99999488	0.999985264	0.999960414
LOC100297097	0.999996191	0.999992444	0.999558179	0.99999488	0.999985264	0.999960414
LOC100297513	0.999996191	0.999992444	0.999558179	0.99999488	0.999985264	0.999960414
LOC100297616	0.999996191	0.999992444	0.999558179	0.99999488	0.999985264	0.999960414
LOC100298774	0.999996191	0.999992444	0.999558179	0.99999488	0.999985264	0.999960414
LOC100299281	0.999996191	0.999992444	0.999558179	0.99999488	0.999985264	0.999960414
LOC100300095	0.999996191	0.999992444	0.999558179	0.99999488	0.999985264	0.999960414
LOC100335177	0.999996191	0.999992444	0.999558179	0.99999488	0.999985264	0.999960414
LOC100335404	0.999996191	0.999992444	0.999558179	0.99999488	0.999985264	0.999960414
LOC100336532	0.999996191	0.999992444	0.999558179	0.99999488	0.999985264	0.999960414
LOC100336644	0.999996191	0.999992444	0.999558179	0.99999488	0.999985264	0.999960414
LOC100462699	0.999996191	0.999992444	0.999558179	0.99999488	0.999985264	0.999960414
LOC100847156	0.999996191	0.999992444	0.999558179	0.99999488	0.999985264	0.999960414
LOC100847320	0.999996191	0.999992444	0.999558179	0.99999488	0.999985264	0.999960414
LOC100847453	0.999996191	0.999992444	0.999558179	0.99999488	0.999985264	0.999960414
LOC100847490	0.999996191	0.999992444	0.999558179	0.99999488	0.999985264	0.999960414
LOC100847567	0.999996191	0.999992444	0.999558179	0.99999488	0.999985264	0.999960414
LOC100847573	0.999996191	0.999992444	0.999558179	0.99999488	0.999985264	0.999960414
LOC100847745	0.999996191	0.999992444	0.999558179	0.99999488	0.999985264	0.999960414
LOC100847773	0.999996191	0.999992444	0.999558179	0.99999488	0.999985264	0.999960414
LOC100847841	0.999996191	0.999992444	0.999558179	0.99999488	0.999985264	0.999960414
LOC100848007	0.999996191	0.999992444	0.999558179	0.99999488	0.999985264	0.999960414
LOC100848105	0.999996191	0.999992444	0.999558179	0.99999488	0.999985264	0.999960414
LOC100848171	0.999996191	0.999992444	0.999558179	0.99999488	0.999985264	0.999960414
LOC100848325	0.999996191	0.999992444	0.999558179	0.99999488	0.999985264	0.999960414
LOC100848357	0.999996191	0.999992444	0.999558179	0.99999488	0.999985264	0.999960414

LOC100848439	0.999996191	0.999992444	0.999558179	0.99999488	0.999985264	0.999960414
LOC100848469	0.999996191	0.999992444	0.999558179	0.99999488	0.999985264	0.999960414
LOC100848472	0.999996191	0.999992444	0.999558179	0.99999488	0.999985264	0.999960414
LOC100848492	0.999996191	0.999992444	0.999558179	0.99999488	0.999985264	0.999960414
LOC100848495	0.999996191	0.999992444	0.999558179	0.99999488	0.999985264	0.999960414
LOC100848570	0.999996191	0.999992444	0.999558179	0.99999488	0.999985264	0.999960414
LOC100848581	0.999996191	0.999992444	0.999558179	0.99999488	0.999985264	0.999960414
LOC100848721	0.999996191	0.999992444	0.999558179	0.99999488	0.999985264	0.999960414
LOC100848912	0.999996191	0.999992444	0.999558179	0.99999488	0.999985264	0.999960414
LOC100849067	0.999996191	0.999992444	0.999558179	0.99999488	0.999985264	0.999960414
LOC100849681	0.999996191	0.999992444	0.999558179	0.99999488	0.999985264	0.999960414
LOC100851323	0.999996191	0.999992444	0.999558179	0.99999488	0.999985264	0.999960414
LOC101901950	0.999996191	0.999992444	0.999558179	0.99999488	0.999985264	0.999960414
LOC101901960	0.999996191	0.999992444	0.999558179	0.99999488	0.999985264	0.999960414
LOC101902036	0.999996191	0.999992444	0.999558179	0.99999488	0.999985264	0.999960414
LOC101902067	0.999996191	0.999992444	0.999558179	0.99999488	0.999985264	0.999960414
LOC101902106	0.999996191	0.999992444	0.999558179	0.99999488	0.999985264	0.999960414
LOC101902124	0.999996191	0.999992444	0.999558179	0.99999488	0.999985264	0.999960414
LOC101902172	0.999996191	0.999992444	0.999558179	0.99999488	0.999985264	0.999960414
LOC101902290	0.999996191	0.999992444	0.999558179	0.99999488	0.999985264	0.999960414
LOC101902345	0.999996191	0.999992444	0.999558179	0.99999488	0.999985264	0.999960414
LOC101902360	0.999996191	0.999992444	0.999558179	0.99999488	0.999985264	0.999960414
LOC101902430	0.999996191	0.999992444	0.999558179	0.99999488	0.999985264	0.999960414
LOC101902440	0.999996191	0.999992444	0.999558179	0.99999488	0.999985264	0.999960414
LOC101902444	0.999996191	0.999992444	0.999558179	0.99999488	0.999985264	0.999960414
LOC101902663	0.999996191	0.999992444	0.999558179	0.99999488	0.999985264	0.999960414
LOC101902664	0.999996191	0.999992444	0.999558179	0.99999488	0.999985264	0.999960414
LOC101902768	0.999996191	0.999992444	0.999558179	0.99999488	0.999985264	0.999960414
LOC101902831	0.999996191	0.999992444	0.999558179	0.99999488	0.999985264	0.999960414
LOC101902861	0.999996191	0.999992444	0.999558179	0.99999488	0.999985264	0.999960414
LOC101902930	0.999996191	0.999992444	0.999558179	0.99999488	0.999985264	0.999960414
LOC101902983	0.999996191	0.999992444	0.999558179	0.99999488	0.999985264	0.999960414
LOC101902994	0.999996191	0.999992444	0.999558179	0.99999488	0.999985264	0.999960414
LOC101903097	0.999996191	0.999992444	0.999558179	0.99999488	0.999985264	0.999960414
LOC101903165	0.999996191	0.999992444	0.999558179	0.99999488	0.999985264	0.999960414
LOC101903281	0.999996191	0.999992444	0.999558179	0.99999488	0.999985264	0.999960414
LOC101903375	0.999996191	0.999992444	0.999558179	0.99999488	0.999985264	0.999960414
LOC101903572	0.999996191	0.999992444	0.999558179	0.99999488	0.999985264	0.999960414
LOC101903600	0.999996191	0.999992444	0.999558179	0.99999488	0.999985264	0.999960414

LOC101903678	0.999996191	0.999992444	0.999558179	0.99999488	0.999985264	0.999960414
LOC101903806	0.999996191	0.999992444	0.999558179	0.99999488	0.999985264	0.999960414
LOC101903976	0.999996191	0.999992444	0.999558179	0.99999488	0.999985264	0.999960414
LOC101903997	0.999996191	0.999992444	0.999558179	0.99999488	0.999985264	0.999960414
LOC101904084	0.999996191	0.999992444	0.999558179	0.99999488	0.999985264	0.999960414
LOC101904133	0.999996191	0.999992444	0.999558179	0.99999488	0.999985264	0.999960414
LOC101904156	0.999996191	0.999992444	0.999558179	0.99999488	0.999985264	0.999960414
LOC101904248	0.999996191	0.999992444	0.999558179	0.99999488	0.999985264	0.999960414
LOC101904377	0.999996191	0.999992444	0.999558179	0.99999488	0.999985264	0.999960414
LOC101904393	0.999996191	0.999992444	0.999558179	0.99999488	0.999985264	0.999960414
LOC101904396	0.999996191	0.999992444	0.999558179	0.99999488	0.999985264	0.999960414
LOC101904413	0.999996191	0.999992444	0.999558179	0.99999488	0.999985264	0.999960414
LOC101904614	0.999996191	0.999992444	0.999558179	0.99999488	0.999985264	0.999960414
LOC101904667	0.999996191	0.999992444	0.999558179	0.99999488	0.999985264	0.999960414
LOC101904822	0.999996191	0.999992444	0.999558179	0.99999488	0.999985264	0.999960414
LOC101904871	0.999996191	0.999992444	0.999558179	0.99999488	0.999985264	0.999960414
LOC101904902	0.999996191	0.999992444	0.999558179	0.99999488	0.999985264	0.999960414
LOC101904916	0.999996191	0.999992444	0.999558179	0.99999488	0.999985264	0.999960414
LOC101904947	0.999996191	0.999992444	0.999558179	0.99999488	0.999985264	0.999960414
LOC101904963	0.999996191	0.999992444	0.999558179	0.99999488	0.999985264	0.999960414
LOC101905049	0.999996191	0.999992444	0.999558179	0.99999488	0.999985264	0.999960414
LOC101905179	0.999996191	0.999992444	0.999558179	0.99999488	0.999985264	0.999960414
LOC101905188	0.999996191	0.999992444	0.999558179	0.99999488	0.999985264	0.999960414
LOC101905232	0.999996191	0.999992444	0.999558179	0.99999488	0.999985264	0.999960414
LOC101905390	0.999996191	0.999992444	0.999558179	0.99999488	0.999985264	0.999960414
LOC101905493	0.999996191	0.999992444	0.999558179	0.99999488	0.999985264	0.999960414
LOC101905498	0.999996191	0.999992444	0.999558179	0.99999488	0.999985264	0.999960414
LOC101905513	0.999996191	0.999992444	0.999558179	0.99999488	0.999985264	0.999960414
LOC101905668	0.999996191	0.999992444	0.999558179	0.99999488	0.999985264	0.999960414
LOC101905771	0.999996191	0.999992444	0.999558179	0.99999488	0.999985264	0.999960414
LOC101905821	0.999996191	0.999992444	0.999558179	0.99999488	0.999985264	0.999960414
LOC101905951	0.999996191	0.999992444	0.999558179	0.99999488	0.999985264	0.999960414
LOC101905977	0.999996191	0.999992444	0.999558179	0.99999488	0.999985264	0.999960414
LOC101906110	0.999996191	0.999992444	0.999558179	0.99999488	0.999985264	0.999960414
LOC101906167	0.999996191	0.999992444	0.999558179	0.99999488	0.999985264	0.999960414
LOC101906221	0.999996191	0.999992444	0.999558179	0.99999488	0.999985264	0.999960414
LOC101906276	0.999996191	0.999992444	0.999558179	0.99999488	0.999985264	0.999960414
LOC101906283	0.999996191	0.999992444	0.999558179	0.99999488	0.999985264	0.999960414
LOC101906315	0.999996191	0.999992444	0.999558179	0.99999488	0.999985264	0.999960414

LOC101906363	0.999996191	0.999992444	0.999558179	0.99999488	0.999985264	0.999960414
LOC101906364	0.999996191	0.999992444	0.999558179	0.99999488	0.999985264	0.999960414
LOC101906392	0.999996191	0.999992444	0.999558179	0.99999488	0.999985264	0.999960414
LOC101906451	0.999996191	0.999992444	0.999558179	0.99999488	0.999985264	0.999960414
LOC101906457	0.999996191	0.999992444	0.999558179	0.99999488	0.999985264	0.999960414
LOC101906472	0.999996191	0.999992444	0.999558179	0.99999488	0.999985264	0.999960414
LOC101906688	0.999996191	0.999992444	0.999558179	0.99999488	0.999985264	0.999960414
LOC101906836	0.999996191	0.999992444	0.999558179	0.99999488	0.999985264	0.999960414
LOC101907005	0.999996191	0.999992444	0.999558179	0.99999488	0.999985264	0.999960414
LOC101907006	0.999996191	0.999992444	0.999558179	0.99999488	0.999985264	0.999960414
LOC101907138	0.999996191	0.999992444	0.999558179	0.99999488	0.999985264	0.999960414
LOC101907195	0.999996191	0.999992444	0.999558179	0.99999488	0.999985264	0.999960414
LOC101907250	0.999996191	0.999992444	0.999558179	0.99999488	0.999985264	0.999960414
LOC101907327	0.999996191	0.999992444	0.999558179	0.99999488	0.999985264	0.999960414
LOC101907353	0.999996191	0.999992444	0.999558179	0.99999488	0.999985264	0.999960414
LOC101907514	0.999996191	0.999992444	0.999558179	0.99999488	0.999985264	0.999960414
LOC101907544	0.999996191	0.999992444	0.999558179	0.99999488	0.999985264	0.999960414
LOC101907641	0.999996191	0.999992444	0.999558179	0.99999488	0.999985264	0.999960414
LOC101907653	0.999996191	0.999992444	0.999558179	0.99999488	0.999985264	0.999960414
LOC101907688	0.999996191	0.999992444	0.999558179	0.99999488	0.999985264	0.999960414
LOC101907697	0.999996191	0.999992444	0.999558179	0.99999488	0.999985264	0.999960414
LOC101907835	0.999996191	0.999992444	0.999558179	0.99999488	0.999985264	0.999960414
LOC101907883	0.999996191	0.999992444	0.999558179	0.99999488	0.999985264	0.999960414
LOC101907920	0.999996191	0.999992444	0.999558179	0.99999488	0.999985264	0.999960414
LOC101907965	0.999996191	0.999992444	0.999558179	0.99999488	0.999985264	0.999960414
LOC101908034	0.999996191	0.999992444	0.999558179	0.99999488	0.999985264	0.999960414
LOC101909083	0.999996191	0.999992444	0.999558179	0.99999488	0.999985264	0.999960414
LOC104968634	0.999996191	0.999992444	0.999558179	0.99999488	0.999985264	0.999960414
LOC104968656	0.999996191	0.999992444	0.999558179	0.99999488	0.999985264	0.999960414
LOC104968671	0.999996191	0.999992444	0.999558179	0.99999488	0.999985264	0.999960414
LOC104969238	0.999996191	0.999992444	0.999558179	0.99999488	0.999985264	0.999960414
LOC104969299	0.999996191	0.999992444	0.999558179	0.99999488	0.999985264	0.999960414
LOC104969340	0.999996191	0.999992444	0.999558179	0.99999488	0.999985264	0.999960414
LOC104970809	0.999996191	0.999992444	0.999558179	0.99999488	0.999985264	0.999960414
LOC104970913	0.999996191	0.999992444	0.999558179	0.99999488	0.999985264	0.999960414
LOC104971030	0.999996191	0.999992444	0.999558179	0.99999488	0.999985264	0.999960414
LOC104971220	0.999996191	0.999992444	0.999558179	0.99999488	0.999985264	0.999960414
LOC104971296	0.999996191	0.999992444	0.999558179	0.99999488	0.999985264	0.999960414
LOC104971307	0.999996191	0.999992444	0.999558179	0.99999488	0.999985264	0.999960414

LOC104971503	0.999996191	0.999992444	0.999558179	0.99999488	0.999985264	0.999960414
LOC104971845	0.999996191	0.999992444	0.999558179	0.99999488	0.999985264	0.999960414
LOC104971926	0.999996191	0.999992444	0.999558179	0.99999488	0.999985264	0.999960414
LOC104972026	0.999996191	0.999992444	0.999558179	0.99999488	0.999985264	0.999960414
LOC104972346	0.999996191	0.999992444	0.999558179	0.99999488	0.999985264	0.999960414
LOC104972526	0.999996191	0.999992444	0.999558179	0.99999488	0.999985264	0.999960414
LOC104972542	0.999996191	0.999992444	0.999558179	0.99999488	0.999985264	0.999960414
LOC104972595	0.999996191	0.999992444	0.999558179	0.99999488	0.999985264	0.999960414
LOC104973050	0.999996191	0.999992444	0.999558179	0.99999488	0.999985264	0.999960414
LOC104973224	0.999996191	0.999992444	0.999558179	0.99999488	0.999985264	0.999960414
LOC104973285	0.999996191	0.999992444	0.999558179	0.99999488	0.999985264	0.999960414
LOC104973760	0.999996191	0.999992444	0.999558179	0.99999488	0.999985264	0.999960414
LOC104974034	0.999996191	0.999992444	0.999558179	0.99999488	0.999985264	0.999960414
LOC104974144	0.999996191	0.999992444	0.999558179	0.99999488	0.999985264	0.999960414
LOC104974473	0.999996191	0.999992444	0.999558179	0.99999488	0.999985264	0.999960414
LOC104974542	0.999996191	0.999992444	0.999558179	0.99999488	0.999985264	0.999960414
LOC104974758	0.999996191	0.999992444	0.999558179	0.99999488	0.999985264	0.999960414
LOC104974837	0.999996191	0.999992444	0.999558179	0.99999488	0.999985264	0.999960414
LOC104974890	0.999996191	0.999992444	0.999558179	0.99999488	0.999985264	0.999960414
LOC104974934	0.999996191	0.999992444	0.999558179	0.99999488	0.999985264	0.999960414
LOC104975022	0.999996191	0.999992444	0.999558179	0.99999488	0.999985264	0.999960414
LOC104975044	0.999996191	0.999992444	0.999558179	0.99999488	0.999985264	0.999960414
LOC104975162	0.999996191	0.999992444	0.999558179	0.99999488	0.999985264	0.999960414
LOC104975283	0.999996191	0.999992444	0.999558179	0.99999488	0.999985264	0.999960414
LOC104975324	0.999996191	0.999992444	0.999558179	0.99999488	0.999985264	0.999960414
LOC104975610	0.999996191	0.999992444	0.999558179	0.99999488	0.999985264	0.999960414
LOC104975666	0.999996191	0.999992444	0.999558179	0.99999488	0.999985264	0.999960414
LOC104975684	0.999996191	0.999992444	0.999558179	0.99999488	0.999985264	0.999960414
LOC104975788	0.999996191	0.999992444	0.999558179	0.99999488	0.999985264	0.999960414
LOC104975849	0.999996191	0.999992444	0.999558179	0.99999488	0.999985264	0.999960414
LOC104975911	0.999996191	0.999992444	0.999558179	0.99999488	0.999985264	0.999960414
LOC104975977	0.999996191	0.999992444	0.999558179	0.99999488	0.999985264	0.999960414
LOC104976062	0.999996191	0.999992444	0.999558179	0.99999488	0.999985264	0.999960414
LOC104976575	0.999996191	0.999992444	0.999558179	0.99999488	0.999985264	0.999960414
LOC104976614	0.999996191	0.999992444	0.999558179	0.99999488	0.999985264	0.999960414
LOC107131341	0.999996191	0.999992444	0.999558179	0.99999488	0.999985264	0.999960414
LOC107131356	0.999996191	0.999992444	0.999558179	0.99999488	0.999985264	0.999960414
LOC107131357	0.999996191	0.999992444	0.999558179	0.99999488	0.999985264	0.999960414
LOC107131494	0.999996191	0.999992444	0.999558179	0.99999488	0.999985264	0.999960414

LOC107131525	0.999996191	0.999992444	0.999558179	0.99999488	0.999985264	0.999960414
LOC107131619	0.999996191	0.999992444	0.999558179	0.99999488	0.999985264	0.999960414
LOC107131651	0.999996191	0.999992444	0.999558179	0.99999488	0.999985264	0.999960414
LOC107131652	0.999996191	0.999992444	0.999558179	0.99999488	0.999985264	0.999960414
LOC107131749	0.999996191	0.999992444	0.999558179	0.99999488	0.999985264	0.999960414
LOC107131834	0.999996191	0.999992444	0.999558179	0.99999488	0.999985264	0.999960414
LOC107131906	0.999996191	0.999992444	0.999558179	0.99999488	0.999985264	0.999960414
LOC107131919	0.999996191	0.999992444	0.999558179	0.99999488	0.999985264	0.999960414
LOC107131941	0.999996191	0.999992444	0.999558179	0.99999488	0.999985264	0.999960414
LOC107132093	0.999996191	0.999992444	0.999558179	0.99999488	0.999985264	0.999960414
LOC107132098	0.999996191	0.999992444	0.999558179	0.99999488	0.999985264	0.999960414
LOC107132121	0.999996191	0.999992444	0.999558179	0.99999488	0.999985264	0.999960414
LOC107132243	0.999996191	0.999992444	0.999558179	0.99999488	0.999985264	0.999960414
LOC107132251	0.999996191	0.999992444	0.999558179	0.99999488	0.999985264	0.999960414
LOC107132255	0.999996191	0.999992444	0.999558179	0.99999488	0.999985264	0.999960414
LOC107132262	0.999996191	0.999992444	0.999558179	0.99999488	0.999985264	0.999960414
LOC107132308	0.999996191	0.999992444	0.999558179	0.99999488	0.999985264	0.999960414
LOC107132327	0.999996191	0.999992444	0.999558179	0.99999488	0.999985264	0.999960414
LOC107132374	0.999996191	0.999992444	0.999558179	0.99999488	0.999985264	0.999960414
LOC107132386	0.999996191	0.999992444	0.999558179	0.99999488	0.999985264	0.999960414
LOC107132395	0.999996191	0.999992444	0.999558179	0.99999488	0.999985264	0.999960414
LOC107132486	0.999996191	0.999992444	0.999558179	0.99999488	0.999985264	0.999960414
LOC107132490	0.999996191	0.999992444	0.999558179	0.99999488	0.999985264	0.999960414
LOC107132556	0.999996191	0.999992444	0.999558179	0.99999488	0.999985264	0.999960414
LOC107132664	0.999996191	0.999992444	0.999558179	0.99999488	0.999985264	0.999960414
LOC107132672	0.999996191	0.999992444	0.999558179	0.99999488	0.999985264	0.999960414
LOC107132713	0.999996191	0.999992444	0.999558179	0.99999488	0.999985264	0.999960414
LOC107132735	0.999996191	0.999992444	0.999558179	0.99999488	0.999985264	0.999960414
LOC107132793	0.999996191	0.999992444	0.999558179	0.99999488	0.999985264	0.999960414
LOC107132877	0.999996191	0.999992444	0.999558179	0.99999488	0.999985264	0.999960414
LOC107132944	0.999996191	0.999992444	0.999558179	0.99999488	0.999985264	0.999960414
LOC107132987	0.999996191	0.999992444	0.999558179	0.99999488	0.999985264	0.999960414
LOC107133180	0.999996191	0.999992444	0.999558179	0.99999488	0.999985264	0.999960414
LOC107133289	0.999996191	0.999992444	0.999558179	0.99999488	0.999985264	0.999960414
LOC107133294	0.999996191	0.999992444	0.999558179	0.99999488	0.999985264	0.999960414
LOC112441456	0.999996191	0.999992444	0.999558179	0.99999488	0.999985264	0.999960414
LOC112441511	0.999996191	0.999992444	0.999558179	0.99999488	0.999985264	0.999960414
LOC112441568	0.999996191	0.999992444	0.999558179	0.99999488	0.999985264	0.999960414
LOC112441619	0.999996191	0.999992444	0.999558179	0.99999488	0.999985264	0.999960414

LOC112441629	0.999996191	0.999992444	0.999558179	0.99999488	0.999985264	0.999960414
LOC112441810	0.999996191	0.999992444	0.999558179	0.99999488	0.999985264	0.999960414
LOC112441827	0.999996191	0.999992444	0.999558179	0.99999488	0.999985264	0.999960414
LOC112441846	0.999996191	0.999992444	0.999558179	0.99999488	0.999985264	0.999960414
LOC112441859	0.999996191	0.999992444	0.999558179	0.99999488	0.999985264	0.999960414
LOC112441888	0.999996191	0.999992444	0.999558179	0.99999488	0.999985264	0.999960414
LOC112442038	0.999996191	0.999992444	0.999558179	0.99999488	0.999985264	0.999960414
LOC112442091	0.999996191	0.999992444	0.999558179	0.99999488	0.999985264	0.999960414
LOC112442208	0.999996191	0.999992444	0.999558179	0.99999488	0.999985264	0.999960414
LOC112442221	0.999996191	0.999992444	0.999558179	0.99999488	0.999985264	0.999960414
LOC112442223	0.999996191	0.999992444	0.999558179	0.99999488	0.999985264	0.999960414
LOC112442245	0.999996191	0.999992444	0.999558179	0.99999488	0.999985264	0.999960414
LOC112442254	0.999996191	0.999992444	0.999558179	0.99999488	0.999985264	0.999960414
LOC112442257	0.999996191	0.999992444	0.999558179	0.99999488	0.999985264	0.999960414
LOC112442312	0.999996191	0.999992444	0.999558179	0.99999488	0.999985264	0.999960414
LOC112442367	0.999996191	0.999992444	0.999558179	0.99999488	0.999985264	0.999960414
LOC112442538	0.999996191	0.999992444	0.999558179	0.99999488	0.999985264	0.999960414
LOC112442559	0.999996191	0.999992444	0.999558179	0.99999488	0.999985264	0.999960414
LOC112442585	0.999996191	0.999992444	0.999558179	0.99999488	0.999985264	0.999960414
LOC112442597	0.999996191	0.999992444	0.999558179	0.99999488	0.999985264	0.999960414
LOC112442602	0.999996191	0.999992444	0.999558179	0.99999488	0.999985264	0.999960414
LOC112442619	0.999996191	0.999992444	0.999558179	0.99999488	0.999985264	0.999960414
LOC112442656	0.999996191	0.999992444	0.999558179	0.99999488	0.999985264	0.999960414
LOC112442676	0.999996191	0.999992444	0.999558179	0.99999488	0.999985264	0.999960414
LOC112442702	0.999996191	0.999992444	0.999558179	0.99999488	0.999985264	0.999960414
LOC112442708	0.999996191	0.999992444	0.999558179	0.99999488	0.999985264	0.999960414
LOC112442715	0.999996191	0.999992444	0.999558179	0.99999488	0.999985264	0.999960414
LOC112442721	0.999996191	0.999992444	0.999558179	0.99999488	0.999985264	0.999960414
LOC112442802	0.999996191	0.999992444	0.999558179	0.99999488	0.999985264	0.999960414
LOC112443163	0.999996191	0.999992444	0.999558179	0.99999488	0.999985264	0.999960414
LOC112443170	0.999996191	0.999992444	0.999558179	0.99999488	0.999985264	0.999960414
LOC112443177	0.999996191	0.999992444	0.999558179	0.99999488	0.999985264	0.999960414
LOC112443178	0.999996191	0.999992444	0.999558179	0.99999488	0.999985264	0.999960414
LOC112443199	0.999996191	0.999992444	0.999558179	0.99999488	0.999985264	0.999960414
LOC112443215	0.999996191	0.999992444	0.999558179	0.99999488	0.999985264	0.999960414
LOC112443225	0.999996191	0.999992444	0.999558179	0.99999488	0.999985264	0.999960414
LOC112443240	0.999996191	0.999992444	0.999558179	0.99999488	0.999985264	0.999960414
LOC112443419	0.999996191	0.999992444	0.999558179	0.99999488	0.999985264	0.999960414
LOC112443437	0.999996191	0.999992444	0.999558179	0.99999488	0.999985264	0.999960414

LOC112443452	0.999996191	0.999992444	0.999558179	0.99999488	0.999985264	0.999960414
LOC112443783	0.999996191	0.999992444	0.999558179	0.99999488	0.999985264	0.999960414
LOC112443849	0.999996191	0.999992444	0.999558179	0.99999488	0.999985264	0.999960414
LOC112443864	0.999996191	0.999992444	0.999558179	0.99999488	0.999985264	0.999960414
LOC112443877	0.999996191	0.999992444	0.999558179	0.99999488	0.999985264	0.999960414
LOC112444171	0.999996191	0.999992444	0.999558179	0.99999488	0.999985264	0.999960414
LOC112444190	0.999996191	0.999992444	0.999558179	0.99999488	0.999985264	0.999960414
LOC112444194	0.999996191	0.999992444	0.999558179	0.99999488	0.999985264	0.999960414
LOC112444198	0.999996191	0.999992444	0.999558179	0.99999488	0.999985264	0.999960414
LOC112444281	0.999996191	0.999992444	0.999558179	0.99999488	0.999985264	0.999960414
LOC112444285	0.999996191	0.999992444	0.999558179	0.99999488	0.999985264	0.999960414
LOC112444289	0.999996191	0.999992444	0.999558179	0.99999488	0.999985264	0.999960414
LOC112444339	0.999996191	0.999992444	0.999558179	0.99999488	0.999985264	0.999960414
LOC112444463	0.999996191	0.999992444	0.999558179	0.99999488	0.999985264	0.999960414
LOC112444464	0.999996191	0.999992444	0.999558179	0.99999488	0.999985264	0.999960414
LOC112444484	0.999996191	0.999992444	0.999558179	0.99999488	0.999985264	0.999960414
LOC112444531	0.999996191	0.999992444	0.999558179	0.99999488	0.999985264	0.999960414
LOC112444613	0.999996191	0.999992444	0.999558179	0.99999488	0.999985264	0.999960414
LOC112444843	0.999996191	0.999992444	0.999558179	0.99999488	0.999985264	0.999960414
LOC112444871	0.999996191	0.999992444	0.999558179	0.99999488	0.999985264	0.999960414
LOC112444967	0.999996191	0.999992444	0.999558179	0.99999488	0.999985264	0.999960414
LOC112445007	0.999996191	0.999992444	0.999558179	0.99999488	0.999985264	0.999960414
LOC112445035	0.999996191	0.999992444	0.999558179	0.99999488	0.999985264	0.999960414
LOC112445052	0.999996191	0.999992444	0.999558179	0.99999488	0.999985264	0.999960414
LOC112445190	0.999996191	0.999992444	0.999558179	0.99999488	0.999985264	0.999960414
LOC112445915	0.999996191	0.999992444	0.999558179	0.99999488	0.999985264	0.999960414
LOC112445951	0.999996191	0.999992444	0.999558179	0.99999488	0.999985264	0.999960414
LOC112445971	0.999996191	0.999992444	0.999558179	0.99999488	0.999985264	0.999960414
LOC112445972	0.999996191	0.999992444	0.999558179	0.99999488	0.999985264	0.999960414
LOC112445985	0.999996191	0.999992444	0.999558179	0.99999488	0.999985264	0.999960414
LOC112445988	0.999996191	0.999992444	0.999558179	0.99999488	0.999985264	0.999960414
LOC112446034	0.999996191	0.999992444	0.999558179	0.99999488	0.999985264	0.999960414
LOC112446039	0.999996191	0.999992444	0.999558179	0.99999488	0.999985264	0.999960414
LOC112446053	0.999996191	0.999992444	0.999558179	0.99999488	0.999985264	0.999960414
LOC112446357	0.999996191	0.999992444	0.999558179	0.99999488	0.999985264	0.999960414
LOC112446452	0.999996191	0.999992444	0.999558179	0.99999488	0.999985264	0.999960414
LOC112446462	0.999996191	0.999992444	0.999558179	0.99999488	0.999985264	0.999960414
LOC112446642	0.999996191	0.999992444	0.999558179	0.99999488	0.999985264	0.999960414
LOC112446645	0.999996191	0.999992444	0.999558179	0.99999488	0.999985264	0.999960414

LOC112446676	0.999996191	0.999992444	0.999558179	0.99999488	0.999985264	0.999960414
LOC112446709	0.999996191	0.999992444	0.999558179	0.99999488	0.999985264	0.999960414
LOC112446725	0.999996191	0.999992444	0.999558179	0.99999488	0.999985264	0.999960414
LOC112446779	0.999996191	0.999992444	0.999558179	0.99999488	0.999985264	0.999960414
LOC112446793	0.999996191	0.999992444	0.999558179	0.99999488	0.999985264	0.999960414
LOC112446822	0.999996191	0.999992444	0.999558179	0.99999488	0.999985264	0.999960414
LOC112447027	0.999996191	0.999992444	0.999558179	0.99999488	0.999985264	0.999960414
LOC112447029	0.999996191	0.999992444	0.999558179	0.99999488	0.999985264	0.999960414
LOC112447030	0.999996191	0.999992444	0.999558179	0.99999488	0.999985264	0.999960414
LOC112447323	0.999996191	0.999992444	0.999558179	0.99999488	0.999985264	0.999960414
LOC112447328	0.999996191	0.999992444	0.999558179	0.99999488	0.999985264	0.999960414
LOC112447340	0.999996191	0.999992444	0.999558179	0.99999488	0.999985264	0.999960414
LOC112447346	0.999996191	0.999992444	0.999558179	0.99999488	0.999985264	0.999960414
LOC112447347	0.999996191	0.999992444	0.999558179	0.99999488	0.999985264	0.999960414
LOC112447411	0.999996191	0.999992444	0.999558179	0.99999488	0.999985264	0.999960414
LOC112447420	0.999996191	0.999992444	0.999558179	0.99999488	0.999985264	0.999960414
LOC112447433	0.999996191	0.999992444	0.999558179	0.99999488	0.999985264	0.999960414
LOC112447443	0.999996191	0.999992444	0.999558179	0.99999488	0.999985264	0.999960414
LOC112447461	0.999996191	0.999992444	0.999558179	0.99999488	0.999985264	0.999960414
LOC112447469	0.999996191	0.999992444	0.999558179	0.99999488	0.999985264	0.999960414
LOC112447473	0.999996191	0.999992444	0.999558179	0.99999488	0.999985264	0.999960414
LOC112447499	0.999996191	0.999992444	0.999558179	0.99999488	0.999985264	0.999960414
LOC112447506	0.999996191	0.999992444	0.999558179	0.99999488	0.999985264	0.999960414
LOC112447508	0.999996191	0.999992444	0.999558179	0.99999488	0.999985264	0.999960414
LOC112447510	0.999996191	0.999992444	0.999558179	0.99999488	0.999985264	0.999960414
LOC112447731	0.999996191	0.999992444	0.999558179	0.99999488	0.999985264	0.999960414
LOC112447735	0.999996191	0.999992444	0.999558179	0.99999488	0.999985264	0.999960414
LOC112447817	0.999996191	0.999992444	0.999558179	0.99999488	0.999985264	0.999960414
LOC112447832	0.999996191	0.999992444	0.999558179	0.99999488	0.999985264	0.999960414
LOC112447838	0.999996191	0.999992444	0.999558179	0.99999488	0.999985264	0.999960414
LOC112447842	0.999996191	0.999992444	0.999558179	0.99999488	0.999985264	0.999960414
LOC112448022	0.999996191	0.999992444	0.999558179	0.99999488	0.999985264	0.999960414
LOC112448030	0.999996191	0.999992444	0.999558179	0.99999488	0.999985264	0.999960414
LOC112448045	0.999996191	0.999992444	0.999558179	0.99999488	0.999985264	0.999960414
LOC112448057	0.999996191	0.999992444	0.999558179	0.99999488	0.999985264	0.999960414
LOC112448103	0.999996191	0.999992444	0.999558179	0.99999488	0.999985264	0.999960414
LOC112448169	0.999996191	0.999992444	0.999558179	0.99999488	0.999985264	0.999960414
LOC112448271	0.999996191	0.999992444	0.999558179	0.99999488	0.999985264	0.999960414
LOC112448368	0.999996191	0.999992444	0.999558179	0.99999488	0.999985264	0.999960414

LOC112448430	0.999996191	0.999992444	0.999558179	0.99999488	0.999985264	0.999960414
LOC112448488	0.999996191	0.999992444	0.999558179	0.99999488	0.999985264	0.999960414
LOC112448507	0.999996191	0.999992444	0.999558179	0.99999488	0.999985264	0.999960414
LOC112448531	0.999996191	0.999992444	0.999558179	0.99999488	0.999985264	0.999960414
LOC112448540	0.999996191	0.999992444	0.999558179	0.99999488	0.999985264	0.999960414
LOC112448760	0.999996191	0.999992444	0.999558179	0.99999488	0.999985264	0.999960414
LOC112448764	0.999996191	0.999992444	0.999558179	0.99999488	0.999985264	0.999960414
LOC112448770	0.999996191	0.999992444	0.999558179	0.99999488	0.999985264	0.999960414
LOC112448772	0.999996191	0.999992444	0.999558179	0.99999488	0.999985264	0.999960414
LOC112448832	0.999996191	0.999992444	0.999558179	0.99999488	0.999985264	0.999960414
LOC112448847	0.999996191	0.999992444	0.999558179	0.99999488	0.999985264	0.999960414
LOC112448863	0.999996191	0.999992444	0.999558179	0.99999488	0.999985264	0.999960414
LOC112449052	0.999996191	0.999992444	0.999558179	0.99999488	0.999985264	0.999960414
LOC112449053	0.999996191	0.999992444	0.999558179	0.99999488	0.999985264	0.999960414
LOC112449056	0.999996191	0.999992444	0.999558179	0.99999488	0.999985264	0.999960414
LOC112449092	0.999996191	0.999992444	0.999558179	0.99999488	0.999985264	0.999960414
LOC112449099	0.999996191	0.999992444	0.999558179	0.99999488	0.999985264	0.999960414
LOC112449245	0.999996191	0.999992444	0.999558179	0.99999488	0.999985264	0.999960414
LOC112449247	0.999996191	0.999992444	0.999558179	0.99999488	0.999985264	0.999960414
LOC112449266	0.999996191	0.999992444	0.999558179	0.99999488	0.999985264	0.999960414
LOC112449275	0.999996191	0.999992444	0.999558179	0.99999488	0.999985264	0.999960414
LOC112449338	0.999996191	0.999992444	0.999558179	0.99999488	0.999985264	0.999960414
LOC112449346	0.999996191	0.999992444	0.999558179	0.99999488	0.999985264	0.999960414
LOC112449360	0.999996191	0.999992444	0.999558179	0.99999488	0.999985264	0.999960414
LOC112449363	0.999996191	0.999992444	0.999558179	0.99999488	0.999985264	0.999960414
LOC507443	0.999996191	0.999992444	0.999558179	0.99999488	0.999985264	0.999960414
LOC509810	0.999996191	0.999992444	0.999558179	0.99999488	0.999985264	0.999960414
LOC512541	0.999996191	0.999992444	0.999558179	0.99999488	0.999985264	0.999960414
LOC516849	0.999996191	0.999992444	0.999558179	0.99999488	0.999985264	0.999960414
LOC518768	0.999996191	0.999992444	0.999558179	0.99999488	0.999985264	0.999960414
LOC518980	0.999996191	0.999992444	0.999558179	0.99999488	0.999985264	0.999960414
LOC519145	0.999996191	0.999992444	0.999558179	0.99999488	0.999985264	0.999960414
LOC519208	0.999996191	0.999992444	0.999558179	0.99999488	0.999985264	0.999960414
LOC520336	0.999996191	0.999992444	0.999558179	0.99999488	0.999985264	0.999960414
LOC521580	0.999996191	0.999992444	0.999558179	0.99999488	0.999985264	0.999960414
LOC523461	0.999996191	0.999992444	0.999558179	0.99999488	0.999985264	0.999960414
LOC523963	0.999996191	0.999992444	0.999558179	0.99999488	0.999985264	0.999960414
LOC527744	0.999996191	0.999992444	0.999558179	0.99999488	0.999985264	0.999960414
LOC528767	0.999996191	0.999992444	0.999558179	0.99999488	0.999985264	0.999960414

LOC529930	0.999996191	0.999992444	0.999558179	0.99999488	0.999985264	0.999960414
LOC533093	0.999996191	0.999992444	0.999558179	0.99999488	0.999985264	0.999960414
LOC534155	0.999996191	0.999992444	0.999558179	0.99999488	0.999985264	0.999960414
LOC534391	0.999996191	0.999992444	0.999558179	0.99999488	0.999985264	0.999960414
LOC534520	0.999996191	0.999992444	0.999558179	0.99999488	0.999985264	0.999960414
LOC539069	0.999996191	0.999992444	0.999558179	0.99999488	0.999985264	0.999960414
LOC540014	0.999996191	0.999992444	0.999558179	0.99999488	0.999985264	0.999960414
LOC614695	0.999996191	0.999992444	0.999558179	0.99999488	0.999985264	0.999960414
LOC614914	0.999996191	0.999992444	0.999558179	0.99999488	0.999985264	0.999960414
LOC615223	0.999996191	0.999992444	0.999558179	0.99999488	0.999985264	0.999960414
LOC615521	0.999996191	0.999992444	0.999558179	0.99999488	0.999985264	0.999960414
LOC615792	0.999996191	0.999992444	0.999558179	0.99999488	0.999985264	0.999960414
LOC616295	0.999996191	0.999992444	0.999558179	0.99999488	0.999985264	0.999960414
LOC616304	0.999996191	0.999992444	0.999558179	0.99999488	0.999985264	0.999960414
LOC616538	0.999996191	0.999992444	0.999558179	0.99999488	0.999985264	0.999960414
LOC616957	0.999996191	0.999992444	0.999558179	0.99999488	0.999985264	0.999960414
LOC617224	0.999996191	0.999992444	0.999558179	0.99999488	0.999985264	0.999960414
LOC617648	0.999996191	0.999992444	0.999558179	0.99999488	0.999985264	0.999960414
LOC617654	0.999996191	0.999992444	0.999558179	0.99999488	0.999985264	0.999960414
LOC618633	0.999996191	0.999992444	0.999558179	0.99999488	0.999985264	0.999960414
LOC619026	0.999996191	0.999992444	0.999558179	0.99999488	0.999985264	0.999960414
LOC619156	0.999996191	0.999992444	0.999558179	0.99999488	0.999985264	0.999960414
LOC751811	0.999996191	0.999992444	0.999558179	0.99999488	0.999985264	0.999960414
LOC780963	0.999996191	0.999992444	0.999558179	0.99999488	0.999985264	0.999960414
LOC781059	0.999996191	0.999992444	0.999558179	0.99999488	0.999985264	0.999960414
LOC781379	0.999996191	0.999992444	0.999558179	0.99999488	0.999985264	0.999960414
LOC781576	0.999996191	0.999992444	0.999558179	0.99999488	0.999985264	0.999960414
LOC781692	0.999996191	0.999992444	0.999558179	0.99999488	0.999985264	0.999960414
LOC781728	0.999996191	0.999992444	0.999558179	0.99999488	0.999985264	0.999960414
LOC781770	0.999996191	0.999992444	0.999558179	0.99999488	0.999985264	0.999960414
LOC781982	0.999996191	0.999992444	0.999558179	0.99999488	0.999985264	0.999960414
LOC782024	0.999996191	0.999992444	0.999558179	0.99999488	0.999985264	0.999960414
LOC782032	0.999996191	0.999992444	0.999558179	0.99999488	0.999985264	0.999960414
LOC782120	0.999996191	0.999992444	0.999558179	0.99999488	0.999985264	0.999960414
LOC782159	0.999996191	0.999992444	0.999558179	0.99999488	0.999985264	0.999960414
LOC782202	0.999996191	0.999992444	0.999558179	0.99999488	0.999985264	0.999960414
LOC782258	0.999996191	0.999992444	0.999558179	0.99999488	0.999985264	0.999960414
LOC782305	0.999996191	0.999992444	0.999558179	0.99999488	0.999985264	0.999960414
LOC782385	0.999996191	0.999992444	0.999558179	0.99999488	0.999985264	0.999960414

LOC782470	0.999996191	0.999992444	0.999558179	0.99999488	0.999985264	0.999960414
LOC782755	0.999996191	0.999992444	0.999558179	0.99999488	0.999985264	0.999960414
LOC782987	0.999996191	0.999992444	0.999558179	0.99999488	0.999985264	0.999960414
LOC783060	0.999996191	0.999992444	0.999558179	0.99999488	0.999985264	0.999960414
LOC783185	0.999996191	0.999992444	0.999558179	0.99999488	0.999985264	0.999960414
LOC783294	0.999996191	0.999992444	0.999558179	0.99999488	0.999985264	0.999960414
LOC783301	0.999996191	0.999992444	0.999558179	0.99999488	0.999985264	0.999960414
LOC783376	0.999996191	0.999992444	0.999558179	0.99999488	0.999985264	0.999960414
LOC783461	0.999996191	0.999992444	0.999558179	0.99999488	0.999985264	0.999960414
LOC783539	0.999996191	0.999992444	0.999558179	0.99999488	0.999985264	0.999960414
LOC784058	0.999996191	0.999992444	0.999558179	0.99999488	0.999985264	0.999960414
LOC784088	0.999996191	0.999992444	0.999558179	0.99999488	0.999985264	0.999960414
LOC784357	0.999996191	0.999992444	0.999558179	0.99999488	0.999985264	0.999960414
LOC784464	0.999996191	0.999992444	0.999558179	0.99999488	0.999985264	0.999960414
LOC784473	0.999996191	0.999992444	0.999558179	0.99999488	0.999985264	0.999960414
LOC784522	0.999996191	0.999992444	0.999558179	0.99999488	0.999985264	0.999960414
LOC784659	0.999996191	0.999992444	0.999558179	0.99999488	0.999985264	0.999960414
LOC784735	0.999996191	0.999992444	0.999558179	0.99999488	0.999985264	0.999960414
LOC784808	0.999996191	0.999992444	0.999558179	0.99999488	0.999985264	0.999960414
LOC784966	0.999996191	0.999992444	0.999558179	0.99999488	0.999985264	0.999960414
LOC785216	0.999996191	0.999992444	0.999558179	0.99999488	0.999985264	0.999960414
LOC785445	0.999996191	0.999992444	0.999558179	0.99999488	0.999985264	0.999960414
LOC785605	0.999996191	0.999992444	0.999558179	0.99999488	0.999985264	0.999960414
LOC785843	0.999996191	0.999992444	0.999558179	0.99999488	0.999985264	0.999960414
LOC786303	0.999996191	0.999992444	0.999558179	0.99999488	0.999985264	0.999960414
LOC786332	0.999996191	0.999992444	0.999558179	0.99999488	0.999985264	0.999960414
LOC786363	0.999996191	0.999992444	0.999558179	0.99999488	0.999985264	0.999960414
LOC786417	0.999996191	0.999992444	0.999558179	0.99999488	0.999985264	0.999960414
LOC786435	0.999996191	0.999992444	0.999558179	0.99999488	0.999985264	0.999960414
LOC786489	0.999996191	0.999992444	0.999558179	0.99999488	0.999985264	0.999960414
LOC786783	0.999996191	0.999992444	0.999558179	0.99999488	0.999985264	0.999960414
LOC786930	0.999996191	0.999992444	0.999558179	0.99999488	0.999985264	0.999960414
LOC787074	0.999996191	0.999992444	0.999558179	0.99999488	0.999985264	0.999960414
LOC787237	0.999996191	0.999992444	0.999558179	0.99999488	0.999985264	0.999960414
LOC787497	0.999996191	0.999992444	0.999558179	0.99999488	0.999985264	0.999960414
LOC787714	0.999996191	0.999992444	0.999558179	0.99999488	0.999985264	0.999960414
LOC787875	0.999996191	0.999992444	0.999558179	0.99999488	0.999985264	0.999960414
LOC788142	0.999996191	0.999992444	0.999558179	0.99999488	0.999985264	0.999960414
LOC788183	0.999996191	0.999992444	0.999558179	0.99999488	0.999985264	0.999960414

LOC788205	0.999996191	0.999992444	0.999558179	0.99999488	0.999985264	0.999960414
LOC789384	0.999996191	0.999992444	0.999558179	0.99999488	0.999985264	0.999960414
LOC789494	0.999996191	0.999992444	0.999558179	0.99999488	0.999985264	0.999960414
LOC789626	0.999996191	0.999992444	0.999558179	0.99999488	0.999985264	0.999960414
LOC789960	0.999996191	0.999992444	0.999558179	0.99999488	0.999985264	0.999960414
LOC789996	0.999996191	0.999992444	0.999558179	0.99999488	0.999985264	0.999960414
LOC789997	0.999996191	0.999992444	0.999558179	0.99999488	0.999985264	0.999960414
LOC790266	0.999996191	0.999992444	0.999558179	0.99999488	0.999985264	0.999960414
LOC790871	0.999996191	0.999992444	0.999558179	0.99999488	0.999985264	0.999960414
LOC790886	0.999996191	0.999992444	0.999558179	0.99999488	0.999985264	0.999960414
LRP12	0.999996191	0.999992444	0.999558179	0.99999488	0.999985264	0.999960414
LRP8	0.999996191	0.999992444	0.999558179	0.99999488	0.999985264	0.999960414
LRRC28	0.999996191	0.999992444	0.999558179	0.99999488	0.999985264	0.999960414
LRRC32	0.999996191	0.999992444	0.999558179	0.99999488	0.999985264	0.999960414
LRRC75A	0.999996191	0.999992444	0.999558179	0.99999488	0.999985264	0.999960414
LRRFIP1	0.999996191	0.999992444	0.999558179	0.99999488	0.999985264	0.999960414
LRRN4CL	0.999996191	0.999992444	0.999558179	0.99999488	0.999985264	0.999960414
LSM3	0.999996191	0.999992444	0.999558179	0.99999488	0.999985264	0.999960414
LSM7	0.999996191	0.999992444	0.999558179	0.99999488	0.999985264	0.999960414
LYPLAL1	0.999996191	0.999992444	0.999558179	0.99999488	0.999985264	0.999960414
LYSMD4	0.999996191	0.999992444	0.999558179	0.99999488	0.999985264	0.999960414
LYVE1	0.999996191	0.999992444	0.999558179	0.99999488	0.999985264	0.999960414
LZTS3	0.999996191	0.999992444	0.999558179	0.99999488	0.999985264	0.999960414
MACO1	0.999996191	0.999992444	0.999558179	0.99999488	0.999985264	0.999960414
MACROD1	0.999996191	0.999992444	0.999558179	0.99999488	0.999985264	0.999960414
MAFA	0.999996191	0.999992444	0.999558179	0.99999488	0.999985264	0.999960414
MAGOHB	0.999996191	0.999992444	0.999558179	0.99999488	0.999985264	0.999960414
MALL	0.999996191	0.999992444	0.999558179	0.99999488	0.999985264	0.999960414
MAML1	0.999996191	0.999992444	0.999558179	0.99999488	0.999985264	0.999960414
MAN2A1	0.999996191	0.999992444	0.999558179	0.99999488	0.999985264	0.999960414
MAN2C1	0.999996191	0.999992444	0.999558179	0.99999488	0.999985264	0.999960414
MANEA	0.999996191	0.999992444	0.999558179	0.99999488	0.999985264	0.999960414
MANSC4	0.999996191	0.999992444	0.999558179	0.99999488	0.999985264	0.999960414
MAP10	0.999996191	0.999992444	0.999558179	0.99999488	0.999985264	0.999960414
MAP1LC3B	0.999996191	0.999992444	0.999558179	0.99999488	0.999985264	0.999960414
MAP2K5	0.999996191	0.999992444	0.999558179	0.99999488	0.999985264	0.999960414
MAP3K20	0.999996191	0.999992444	0.999558179	0.99999488	0.999985264	0.999960414
MAP3K3	0.999996191	0.999992444	0.999558179	0.99999488	0.999985264	0.999960414
MAP3K8	0.999996191	0.999992444	0.999558179	0.99999488	0.999985264	0.999960414

MAPK12	0.999996191	0.999992444	0.999558179	0.99999488	0.999985264	0.999960414
MAPK8IP3	0.999996191	0.999992444	0.999558179	0.99999488	0.999985264	0.999960414
MAPKAP1	0.999996191	0.999992444	0.999558179	0.99999488	0.999985264	0.999960414
MARC2	0.999996191	0.999992444	0.999558179	0.99999488	0.999985264	0.999960414
MARCH9	0.999996191	0.999992444	0.999558179	0.99999488	0.999985264	0.999960414
MARS2	0.999996191	0.999992444	0.999558179	0.99999488	0.999985264	0.999960414
MAST3	0.999996191	0.999992444	0.999558179	0.99999488	0.999985264	0.999960414
MBD1	0.999996191	0.999992444	0.999558179	0.99999488	0.999985264	0.999960414
MBD2	0.999996191	0.999992444	0.999558179	0.99999488	0.999985264	0.999960414
MBD4	0.999996191	0.999992444	0.999558179	0.99999488	0.999985264	0.999960414
MBD5	0.999996191	0.999992444	0.999558179	0.99999488	0.999985264	0.999960414
MCCC2	0.999996191	0.999992444	0.999558179	0.99999488	0.999985264	0.999960414
MCF2	0.999996191	0.999992444	0.999558179	0.99999488	0.999985264	0.999960414
MCM7	0.999996191	0.999992444	0.999558179	0.99999488	0.999985264	0.999960414
MCTP1	0.999996191	0.999992444	0.999558179	0.99999488	0.999985264	0.999960414
MDFI	0.999996191	0.999992444	0.999558179	0.99999488	0.999985264	0.999960414
MED13L	0.999996191	0.999992444	0.999558179	0.99999488	0.999985264	0.999960414
MED14	0.999996191	0.999992444	0.999558179	0.99999488	0.999985264	0.999960414
MED15	0.999996191	0.999992444	0.999558179	0.99999488	0.999985264	0.999960414
MED20	0.999996191	0.999992444	0.999558179	0.99999488	0.999985264	0.999960414
MED25	0.999996191	0.999992444	0.999558179	0.99999488	0.999985264	0.999960414
MED26	0.999996191	0.999992444	0.999558179	0.99999488	0.999985264	0.999960414
MED4	0.999996191	0.999992444	0.999558179	0.99999488	0.999985264	0.999960414
MELTF	0.999996191	0.999992444	0.999558179	0.99999488	0.999985264	0.999960414
METTL27	0.999996191	0.999992444	0.999558179	0.99999488	0.999985264	0.999960414
MFAP3L	0.999996191	0.999992444	0.999558179	0.99999488	0.999985264	0.999960414
MFF	0.999996191	0.999992444	0.999558179	0.99999488	0.999985264	0.999960414
MFSD10	0.999996191	0.999992444	0.999558179	0.99999488	0.999985264	0.999960414
MGARP	0.999996191	0.999992444	0.999558179	0.99999488	0.999985264	0.999960414
MGAT5	0.999996191	0.999992444	0.999558179	0.99999488	0.999985264	0.999960414
MGME1	0.999996191	0.999992444	0.999558179	0.99999488	0.999985264	0.999960414
MICU2	0.999996191	0.999992444	0.999558179	0.99999488	0.999985264	0.999960414
MIGA1	0.999996191	0.999992444	0.999558179	0.99999488	0.999985264	0.999960414
MIS18A	0.999996191	0.999992444	0.999558179	0.99999488	0.999985264	0.999960414
MISP3	0.999996191	0.999992444	0.999558179	0.99999488	0.999985264	0.999960414
MKRN2	0.999996191	0.999992444	0.999558179	0.99999488	0.999985264	0.999960414
MKRN3	0.999996191	0.999992444	0.999558179	0.99999488	0.999985264	0.999960414
MLH1	0.999996191	0.999992444	0.999558179	0.99999488	0.999985264	0.999960414
MLH3	0.999996191	0.999992444	0.999558179	0.99999488	0.999985264	0.999960414

MLKL	0.999996191	0.999992444	0.999558179	0.99999488	0.999985264	0.999960414
MLLT6	0.999996191	0.999992444	0.999558179	0.99999488	0.999985264	0.999960414
MLPH	0.999996191	0.999992444	0.999558179	0.99999488	0.999985264	0.999960414
MME	0.999996191	0.999992444	0.999558179	0.99999488	0.999985264	0.999960414
MMS19	0.999996191	0.999992444	0.999558179	0.99999488	0.999985264	0.999960414
MNT	0.999996191	0.999992444	0.999558179	0.99999488	0.999985264	0.999960414
MOB3B	0.999996191	0.999992444	0.999558179	0.99999488	0.999985264	0.999960414
MOCS2	0.999996191	0.999992444	0.999558179	0.99999488	0.999985264	0.999960414
MOGAT1	0.999996191	0.999992444	0.999558179	0.99999488	0.999985264	0.999960414
MORC2	0.999996191	0.999992444	0.999558179	0.99999488	0.999985264	0.999960414
MORF4L2	0.999996191	0.999992444	0.999558179	0.99999488	0.999985264	0.999960414
MORN4	0.999996191	0.999992444	0.999558179	0.99999488	0.999985264	0.999960414
MOSMO	0.999996191	0.999992444	0.999558179	0.99999488	0.999985264	0.999960414
MRAS	0.999996191	0.999992444	0.999558179	0.99999488	0.999985264	0.999960414
MRE11	0.999996191	0.999992444	0.999558179	0.99999488	0.999985264	0.999960414
MRI1	0.999996191	0.999992444	0.999558179	0.99999488	0.999985264	0.999960414
MRNIP	0.999996191	0.999992444	0.999558179	0.99999488	0.999985264	0.999960414
MRPL10	0.999996191	0.999992444	0.999558179	0.99999488	0.999985264	0.999960414
MRPL3	0.999996191	0.999992444	0.999558179	0.99999488	0.999985264	0.999960414
MRPL39	0.999996191	0.999992444	0.999558179	0.99999488	0.999985264	0.999960414
MRPL45	0.999996191	0.999992444	0.999558179	0.99999488	0.999985264	0.999960414
MRPS28	0.999996191	0.999992444	0.999558179	0.99999488	0.999985264	0.999960414
MS4A13	0.999996191	0.999992444	0.999558179	0.99999488	0.999985264	0.999960414
MSH2	0.999996191	0.999992444	0.999558179	0.99999488	0.999985264	0.999960414
MSH3	0.999996191	0.999992444	0.999558179	0.99999488	0.999985264	0.999960414
MSL3	0.999996191	0.999992444	0.999558179	0.99999488	0.999985264	0.999960414
MSX2	0.999996191	0.999992444	0.999558179	0.99999488	0.999985264	0.999960414
MT1E	0.999996191	0.999992444	0.999558179	0.99999488	0.999985264	0.999960414
MTA2	0.999996191	0.999992444	0.999558179	0.99999488	0.999985264	0.999960414
MTF1	0.999996191	0.999992444	0.999558179	0.99999488	0.999985264	0.999960414
MTHFR	0.999996191	0.999992444	0.999558179	0.99999488	0.999985264	0.999960414
MTHFS	0.999996191	0.999992444	0.999558179	0.99999488	0.999985264	0.999960414
MTMR11	0.999996191	0.999992444	0.999558179	0.99999488	0.999985264	0.999960414
MTMR3	0.999996191	0.999992444	0.999558179	0.99999488	0.999985264	0.999960414
MTMR9	0.999996191	0.999992444	0.999558179	0.99999488	0.999985264	0.999960414
MTX2	0.999996191	0.999992444	0.999558179	0.99999488	0.999985264	0.999960414
MUC20	0.999996191	0.999992444	0.999558179	0.99999488	0.999985264	0.999960414
MUS81	0.999996191	0.999992444	0.999558179	0.99999488	0.999985264	0.999960414
MYCBPAP	0.999996191	0.999992444	0.999558179	0.99999488	0.999985264	0.999960414

MYO10	0.999996191	0.999992444	0.999558179	0.99999488	0.999985264	0.999960414
MYO1B	0.999996191	0.999992444	0.999558179	0.99999488	0.999985264	0.999960414
MYO1H	0.999996191	0.999992444	0.999558179	0.99999488	0.999985264	0.999960414
MYO9B	0.999996191	0.999992444	0.999558179	0.99999488	0.999985264	0.999960414
MYOF	0.999996191	0.999992444	0.999558179	0.99999488	0.999985264	0.999960414
NAA35	0.999996191	0.999992444	0.999558179	0.99999488	0.999985264	0.999960414
NAALAD2	0.999996191	0.999992444	0.999558179	0.99999488	0.999985264	0.999960414
NAALADL2	0.999996191	0.999992444	0.999558179	0.99999488	0.999985264	0.999960414
NADK2	0.999996191	0.999992444	0.999558179	0.99999488	0.999985264	0.999960414
NAP1L3	0.999996191	0.999992444	0.999558179	0.99999488	0.999985264	0.999960414
NASP	0.999996191	0.999992444	0.999558179	0.99999488	0.999985264	0.999960414
NBEAL2	0.999996191	0.999992444	0.999558179	0.99999488	0.999985264	0.999960414
NCOR1	0.999996191	0.999992444	0.999558179	0.99999488	0.999985264	0.999960414
NCR3LG1	0.999996191	0.999992444	0.999558179	0.99999488	0.999985264	0.999960414
NDFIP1	0.999996191	0.999992444	0.999558179	0.99999488	0.999985264	0.999960414
NDRG2	0.999996191	0.999992444	0.999558179	0.99999488	0.999985264	0.999960414
NDUFA12	0.999996191	0.999992444	0.999558179	0.99999488	0.999985264	0.999960414
NEBL	0.999996191	0.999992444	0.999558179	0.99999488	0.999985264	0.999960414
NECAP1	0.999996191	0.999992444	0.999558179	0.99999488	0.999985264	0.999960414
NECAP2	0.999996191	0.999992444	0.999558179	0.99999488	0.999985264	0.999960414
NECTIN1	0.999996191	0.999992444	0.999558179	0.99999488	0.999985264	0.999960414
NEK1	0.999996191	0.999992444	0.999558179	0.99999488	0.999985264	0.999960414
NEK3	0.999996191	0.999992444	0.999558179	0.99999488	0.999985264	0.999960414
NELFCD	0.999996191	0.999992444	0.999558179	0.99999488	0.999985264	0.999960414
NFATC2IP	0.999996191	0.999992444	0.999558179	0.99999488	0.999985264	0.999960414
NFS1	0.999996191	0.999992444	0.999558179	0.99999488	0.999985264	0.999960414
NGDN	0.999996191	0.999992444	0.999558179	0.99999488	0.999985264	0.999960414
NHLRC2	0.999996191	0.999992444	0.999558179	0.99999488	0.999985264	0.999960414
NHS	0.999996191	0.999992444	0.999558179	0.99999488	0.999985264	0.999960414
NIPAL1	0.999996191	0.999992444	0.999558179	0.99999488	0.999985264	0.999960414
NIT2	0.999996191	0.999992444	0.999558179	0.99999488	0.999985264	0.999960414
NME7	0.999996191	0.999992444	0.999558179	0.99999488	0.999985264	0.999960414
NNT	0.999996191	0.999992444	0.999558179	0.99999488	0.999985264	0.999960414
NOBOX	0.999996191	0.999992444	0.999558179	0.99999488	0.999985264	0.999960414
NOL3	0.999996191	0.999992444	0.999558179	0.99999488	0.999985264	0.999960414
NOS1AP	0.999996191	0.999992444	0.999558179	0.99999488	0.999985264	0.999960414
NOSIP	0.999996191	0.999992444	0.999558179	0.99999488	0.999985264	0.999960414
NOTCH2	0.999996191	0.999992444	0.999558179	0.99999488	0.999985264	0.999960414
NOVA2	0.999996191	0.999992444	0.999558179	0.99999488	0.999985264	0.999960414

NPAS2	0.999996191	0.999992444	0.999558179	0.99999488	0.999985264	0.999960414
NPRL3	0.999996191	0.999992444	0.999558179	0.99999488	0.999985264	0.999960414
NPTN	0.999996191	0.999992444	0.999558179	0.99999488	0.999985264	0.999960414
NR2C1	0.999996191	0.999992444	0.999558179	0.99999488	0.999985264	0.999960414
NR6A1	0.999996191	0.999992444	0.999558179	0.99999488	0.999985264	0.999960414
NRDC	0.999996191	0.999992444	0.999558179	0.99999488	0.999985264	0.999960414
NRP1	0.999996191	0.999992444	0.999558179	0.99999488	0.999985264	0.999960414
NSMAF	0.999996191	0.999992444	0.999558179	0.99999488	0.999985264	0.999960414
NSUN4	0.999996191	0.999992444	0.999558179	0.99999488	0.999985264	0.999960414
NTNG2	0.999996191	0.999992444	0.999558179	0.99999488	0.999985264	0.999960414
NTPCR	0.999996191	0.999992444	0.999558179	0.99999488	0.999985264	0.999960414
NUDCD2	0.999996191	0.999992444	0.999558179	0.99999488	0.999985264	0.999960414
NUDT1	0.999996191	0.999992444	0.999558179	0.99999488	0.999985264	0.999960414
NUDT2	0.999996191	0.999992444	0.999558179	0.99999488	0.999985264	0.999960414
NUFIP1	0.999996191	0.999992444	0.999558179	0.99999488	0.999985264	0.999960414
NUP58	0.999996191	0.999992444	0.999558179	0.99999488	0.999985264	0.999960414
NXPE4	0.999996191	0.999992444	0.999558179	0.99999488	0.999985264	0.999960414
OAT	0.999996191	0.999992444	0.999558179	0.99999488	0.999985264	0.999960414
OLA1	0.999996191	0.999992444	0.999558179	0.99999488	0.999985264	0.999960414
OMA1	0.999996191	0.999992444	0.999558179	0.99999488	0.999985264	0.999960414
OPTN	0.999996191	0.999992444	0.999558179	0.99999488	0.999985264	0.999960414
ORAI1	0.999996191	0.999992444	0.999558179	0.99999488	0.999985264	0.999960414
ORC2	0.999996191	0.999992444	0.999558179	0.99999488	0.999985264	0.999960414
ORC5	0.999996191	0.999992444	0.999558179	0.99999488	0.999985264	0.999960414
ORMDL3	0.999996191	0.999992444	0.999558179	0.99999488	0.999985264	0.999960414
OSBPL3	0.999996191	0.999992444	0.999558179	0.99999488	0.999985264	0.999960414
OSGEP	0.999996191	0.999992444	0.999558179	0.99999488	0.999985264	0.999960414
OSGEPL1	0.999996191	0.999992444	0.999558179	0.99999488	0.999985264	0.999960414
OTOGL	0.999996191	0.999992444	0.999558179	0.99999488	0.999985264	0.999960414
OTOR	0.999996191	0.999992444	0.999558179	0.99999488	0.999985264	0.999960414
OVGP1	0.999996191	0.999992444	0.999558179	0.99999488	0.999985264	0.999960414
OXA1L	0.999996191	0.999992444	0.999558179	0.99999488	0.999985264	0.999960414
OXCT1	0.999996191	0.999992444	0.999558179	0.99999488	0.999985264	0.999960414
OXSR1	0.999996191	0.999992444	0.999558179	0.99999488	0.999985264	0.999960414
P2RX4	0.999996191	0.999992444	0.999558179	0.99999488	0.999985264	0.999960414
PABPC4L	0.999996191	0.999992444	0.999558179	0.99999488	0.999985264	0.999960414
PACSIN2	0.999996191	0.999992444	0.999558179	0.99999488	0.999985264	0.999960414
PAFAH1B1	0.999996191	0.999992444	0.999558179	0.99999488	0.999985264	0.999960414
PAICSP	0.999996191	0.999992444	0.999558179	0.99999488	0.999985264	0.999960414

PALMD	0.999996191	0.999992444	0.999558179	0.99999488	0.999985264	0.999960414
PAPSS1	0.999996191	0.999992444	0.999558179	0.99999488	0.999985264	0.999960414
PARG	0.999996191	0.999992444	0.999558179	0.99999488	0.999985264	0.999960414
PARP1	0.999996191	0.999992444	0.999558179	0.99999488	0.999985264	0.999960414
PARP2	0.999996191	0.999992444	0.999558179	0.99999488	0.999985264	0.999960414
PARP4	0.999996191	0.999992444	0.999558179	0.99999488	0.999985264	0.999960414
PARP8	0.999996191	0.999992444	0.999558179	0.99999488	0.999985264	0.999960414
PARVB	0.999996191	0.999992444	0.999558179	0.99999488	0.999985264	0.999960414
PATJ	0.999996191	0.999992444	0.999558179	0.99999488	0.999985264	0.999960414
PATZ1	0.999996191	0.999992444	0.999558179	0.99999488	0.999985264	0.999960414
PAXIP1	0.999996191	0.999992444	0.999558179	0.99999488	0.999985264	0.999960414
PBX3	0.999996191	0.999992444	0.999558179	0.99999488	0.999985264	0.999960414
PCCA	0.999996191	0.999992444	0.999558179	0.99999488	0.999985264	0.999960414
PCCB	0.999996191	0.999992444	0.999558179	0.99999488	0.999985264	0.999960414
PCDH9	0.999996191	0.999992444	0.999558179	0.99999488	0.999985264	0.999960414
PCDHGB4	0.999996191	0.999992444	0.999558179	0.99999488	0.999985264	0.999960414
PCK2	0.999996191	0.999992444	0.999558179	0.99999488	0.999985264	0.999960414
PCNT	0.999996191	0.999992444	0.999558179	0.99999488	0.999985264	0.999960414
PCSK6	0.999996191	0.999992444	0.999558179	0.99999488	0.999985264	0.999960414
PCYOX1	0.999996191	0.999992444	0.999558179	0.99999488	0.999985264	0.999960414
PCYOX1L	0.999996191	0.999992444	0.999558179	0.99999488	0.999985264	0.999960414
PDCD2L	0.999996191	0.999992444	0.999558179	0.99999488	0.999985264	0.999960414
PDE10A	0.999996191	0.999992444	0.999558179	0.99999488	0.999985264	0.999960414
PDE6A	0.999996191	0.999992444	0.999558179	0.99999488	0.999985264	0.999960414
PDE9A	0.999996191	0.999992444	0.999558179	0.99999488	0.999985264	0.999960414
PDHB	0.999996191	0.999992444	0.999558179	0.99999488	0.999985264	0.999960414
PDSS2	0.999996191	0.999992444	0.999558179	0.99999488	0.999985264	0.999960414
PDZD2	0.999996191	0.999992444	0.999558179	0.99999488	0.999985264	0.999960414
PEAR1	0.999996191	0.999992444	0.999558179	0.99999488	0.999985264	0.999960414
PECAM1	0.999996191	0.999992444	0.999558179	0.99999488	0.999985264	0.999960414
PEG10	0.999996191	0.999992444	0.999558179	0.99999488	0.999985264	0.999960414
PEX1	0.999996191	0.999992444	0.999558179	0.99999488	0.999985264	0.999960414
PEX12	0.999996191	0.999992444	0.999558179	0.99999488	0.999985264	0.999960414
PEX3	0.999996191	0.999992444	0.999558179	0.99999488	0.999985264	0.999960414
PEX7	0.999996191	0.999992444	0.999558179	0.99999488	0.999985264	0.999960414
PFKFB2	0.999996191	0.999992444	0.999558179	0.99999488	0.999985264	0.999960414
PGAM2	0.999996191	0.999992444	0.999558179	0.99999488	0.999985264	0.999960414
PGAP1	0.999996191	0.999992444	0.999558179	0.99999488	0.999985264	0.999960414
PGAP2	0.999996191	0.999992444	0.999558179	0.99999488	0.999985264	0.999960414

PGBD1	0.999996191	0.999992444	0.999558179	0.99999488	0.999985264	0.999960414
PGM1	0.999996191	0.999992444	0.999558179	0.99999488	0.999985264	0.999960414
PGPEP1	0.999996191	0.999992444	0.999558179	0.99999488	0.999985264	0.999960414
PHACTR1	0.999996191	0.999992444	0.999558179	0.99999488	0.999985264	0.999960414
PHF1	0.999996191	0.999992444	0.999558179	0.99999488	0.999985264	0.999960414
PHF12	0.999996191	0.999992444	0.999558179	0.99999488	0.999985264	0.999960414
PHF19	0.999996191	0.999992444	0.999558179	0.99999488	0.999985264	0.999960414
PHF5A	0.999996191	0.999992444	0.999558179	0.99999488	0.999985264	0.999960414
PHF6	0.999996191	0.999992444	0.999558179	0.99999488	0.999985264	0.999960414
PHOSPHO2	0.999996191	0.999992444	0.999558179	0.99999488	0.999985264	0.999960414
PHRF1	0.999996191	0.999992444	0.999558179	0.99999488	0.999985264	0.999960414
PIAS4	0.999996191	0.999992444	0.999558179	0.99999488	0.999985264	0.999960414
PICK1	0.999996191	0.999992444	0.999558179	0.99999488	0.999985264	0.999960414
PIEZO1	0.999996191	0.999992444	0.999558179	0.99999488	0.999985264	0.999960414
PIGH	0.999996191	0.999992444	0.999558179	0.99999488	0.999985264	0.999960414
PIGK	0.999996191	0.999992444	0.999558179	0.99999488	0.999985264	0.999960414
PIGP	0.999996191	0.999992444	0.999558179	0.99999488	0.999985264	0.999960414
PIGV	0.999996191	0.999992444	0.999558179	0.99999488	0.999985264	0.999960414
PIH1D1	0.999996191	0.999992444	0.999558179	0.99999488	0.999985264	0.999960414
PIK3CB	0.999996191	0.999992444	0.999558179	0.99999488	0.999985264	0.999960414
PIK3IP1	0.999996191	0.999992444	0.999558179	0.99999488	0.999985264	0.999960414
PINK1	0.999996191	0.999992444	0.999558179	0.99999488	0.999985264	0.999960414
PIP4K2B	0.999996191	0.999992444	0.999558179	0.99999488	0.999985264	0.999960414
PIP4P1	0.999996191	0.999992444	0.999558179	0.99999488	0.999985264	0.999960414
PIP4P2	0.999996191	0.999992444	0.999558179	0.99999488	0.999985264	0.999960414
PIP5K1B	0.999996191	0.999992444	0.999558179	0.99999488	0.999985264	0.999960414
PITRM1	0.999996191	0.999992444	0.999558179	0.99999488	0.999985264	0.999960414
PJA2	0.999996191	0.999992444	0.999558179	0.99999488	0.999985264	0.999960414
PKN1	0.999996191	0.999992444	0.999558179	0.99999488	0.999985264	0.999960414
PLA2G12A	0.999996191	0.999992444	0.999558179	0.99999488	0.999985264	0.999960414
PLA2G16	0.999996191	0.999992444	0.999558179	0.99999488	0.999985264	0.999960414
PLCG1	0.999996191	0.999992444	0.999558179	0.99999488	0.999985264	0.999960414
PLEKHA5	0.999996191	0.999992444	0.999558179	0.99999488	0.999985264	0.999960414
PLEKHA7	0.999996191	0.999992444	0.999558179	0.99999488	0.999985264	0.999960414
PLEKHG3	0.999996191	0.999992444	0.999558179	0.99999488	0.999985264	0.999960414
PLGRKT	0.999996191	0.999992444	0.999558179	0.99999488	0.999985264	0.999960414
PLK2	0.999996191	0.999992444	0.999558179	0.99999488	0.999985264	0.999960414
PLPP3	0.999996191	0.999992444	0.999558179	0.99999488	0.999985264	0.999960414
PLPP4	0.999996191	0.999992444	0.999558179	0.99999488	0.999985264	0.999960414

PLPPR3	0.999996191	0.999992444	0.999558179	0.99999488	0.999985264	0.999960414
PLPPR5	0.999996191	0.999992444	0.999558179	0.99999488	0.999985264	0.999960414
PMEL	0.999996191	0.999992444	0.999558179	0.99999488	0.999985264	0.999960414
PNPLA6	0.999996191	0.999992444	0.999558179	0.99999488	0.999985264	0.999960414
PNPLA8	0.999996191	0.999992444	0.999558179	0.99999488	0.999985264	0.999960414
POFUT1	0.999996191	0.999992444	0.999558179	0.99999488	0.999985264	0.999960414
POGK	0.999996191	0.999992444	0.999558179	0.99999488	0.999985264	0.999960414
POGZ	0.999996191	0.999992444	0.999558179	0.99999488	0.999985264	0.999960414
POLB	0.999996191	0.999992444	0.999558179	0.99999488	0.999985264	0.999960414
POLD3	0.999996191	0.999992444	0.999558179	0.99999488	0.999985264	0.999960414
POLE2	0.999996191	0.999992444	0.999558179	0.99999488	0.999985264	0.999960414
POLE4	0.999996191	0.999992444	0.999558179	0.99999488	0.999985264	0.999960414
POLR2G	0.999996191	0.999992444	0.999558179	0.99999488	0.999985264	0.999960414
POMC	0.999996191	0.999992444	0.999558179	0.99999488	0.999985264	0.999960414
PON2	0.999996191	0.999992444	0.999558179	0.99999488	0.999985264	0.999960414
POPDC3	0.999996191	0.999992444	0.999558179	0.99999488	0.999985264	0.999960414
PPA2	0.999996191	0.999992444	0.999558179	0.99999488	0.999985264	0.999960414
PPARG	0.999996191	0.999992444	0.999558179	0.99999488	0.999985264	0.999960414
PPFIA1	0.999996191	0.999992444	0.999558179	0.99999488	0.999985264	0.999960414
PPFIA3	0.999996191	0.999992444	0.999558179	0.99999488	0.999985264	0.999960414
PPM1H	0.999996191	0.999992444	0.999558179	0.99999488	0.999985264	0.999960414
PPM1N	0.999996191	0.999992444	0.999558179	0.99999488	0.999985264	0.999960414
PPP1R16B	0.999996191	0.999992444	0.999558179	0.99999488	0.999985264	0.999960414
PPP1R21	0.999996191	0.999992444	0.999558179	0.99999488	0.999985264	0.999960414
PPP2R3B	0.999996191	0.999992444	0.999558179	0.99999488	0.999985264	0.999960414
PPP3CB	0.999996191	0.999992444	0.999558179	0.99999488	0.999985264	0.999960414
PPP4C	0.999996191	0.999992444	0.999558179	0.99999488	0.999985264	0.999960414
PPP4R1	0.999996191	0.999992444	0.999558179	0.99999488	0.999985264	0.999960414
PPP6R1	0.999996191	0.999992444	0.999558179	0.99999488	0.999985264	0.999960414
PPT1	0.999996191	0.999992444	0.999558179	0.99999488	0.999985264	0.999960414
PRDM10	0.999996191	0.999992444	0.999558179	0.99999488	0.999985264	0.999960414
PRDM8	0.999996191	0.999992444	0.999558179	0.99999488	0.999985264	0.999960414
PRDX1	0.999996191	0.999992444	0.999558179	0.99999488	0.999985264	0.999960414
PRDX6	0.999996191	0.999992444	0.999558179	0.99999488	0.999985264	0.999960414
PRELID3B	0.999996191	0.999992444	0.999558179	0.99999488	0.999985264	0.999960414
PRICKLE3	0.999996191	0.999992444	0.999558179	0.99999488	0.999985264	0.999960414
PRIM2	0.999996191	0.999992444	0.999558179	0.99999488	0.999985264	0.999960414
PRKAA1	0.999996191	0.999992444	0.999558179	0.99999488	0.999985264	0.999960414
PRKAR2B	0.999996191	0.999992444	0.999558179	0.99999488	0.999985264	0.999960414

PRKCE	0.999996191	0.999992444	0.999558179	0.99999488	0.999985264	0.999960414
PRKCG	0.999996191	0.999992444	0.999558179	0.99999488	0.999985264	0.999960414
PRKD3	0.999996191	0.999992444	0.999558179	0.99999488	0.999985264	0.999960414
PRKDC	0.999996191	0.999992444	0.999558179	0.99999488	0.999985264	0.999960414
PRKN	0.999996191	0.999992444	0.999558179	0.99999488	0.999985264	0.999960414
PRL	0.999996191	0.999992444	0.999558179	0.99999488	0.999985264	0.999960414
PROB1	0.999996191	0.999992444	0.999558179	0.99999488	0.999985264	0.999960414
PROKR2	0.999996191	0.999992444	0.999558179	0.99999488	0.999985264	0.999960414
PROSER1	0.999996191	0.999992444	0.999558179	0.99999488	0.999985264	0.999960414
PROSER3	0.999996191	0.999992444	0.999558179	0.99999488	0.999985264	0.999960414
PRPF6	0.999996191	0.999992444	0.999558179	0.99999488	0.999985264	0.999960414
PRR14	0.999996191	0.999992444	0.999558179	0.99999488	0.999985264	0.999960414
PRR29	0.999996191	0.999992444	0.999558179	0.99999488	0.999985264	0.999960414
PRSS2	0.999996191	0.999992444	0.999558179	0.99999488	0.999985264	0.999960414
PRSS33	0.999996191	0.999992444	0.999558179	0.99999488	0.999985264	0.999960414
PRSS42	0.999996191	0.999992444	0.999558179	0.99999488	0.999985264	0.999960414
PSD3	0.999996191	0.999992444	0.999558179	0.99999488	0.999985264	0.999960414
PSMA2	0.999996191	0.999992444	0.999558179	0.99999488	0.999985264	0.999960414
PSMB1	0.999996191	0.999992444	0.999558179	0.99999488	0.999985264	0.999960414
PSMD5	0.999996191	0.999992444	0.999558179	0.99999488	0.999985264	0.999960414
PSPC1	0.999996191	0.999992444	0.999558179	0.99999488	0.999985264	0.999960414
PSPN	0.999996191	0.999992444	0.999558179	0.99999488	0.999985264	0.999960414
PSTK	0.999996191	0.999992444	0.999558179	0.99999488	0.999985264	0.999960414
PTBP2	0.999996191	0.999992444	0.999558179	0.99999488	0.999985264	0.999960414
PTCH1	0.999996191	0.999992444	0.999558179	0.99999488	0.999985264	0.999960414
PTER	0.999996191	0.999992444	0.999558179	0.99999488	0.999985264	0.999960414
PTGDR2	0.999996191	0.999992444	0.999558179	0.99999488	0.999985264	0.999960414
PTK2	0.999996191	0.999992444	0.999558179	0.99999488	0.999985264	0.999960414
PTMA	0.999996191	0.999992444	0.999558179	0.99999488	0.999985264	0.999960414
PTPN1	0.999996191	0.999992444	0.999558179	0.99999488	0.999985264	0.999960414
PTPRH	0.999996191	0.999992444	0.999558179	0.99999488	0.999985264	0.999960414
PWWP2B	0.999996191	0.999992444	0.999558179	0.99999488	0.999985264	0.999960414
PXK	0.999996191	0.999992444	0.999558179	0.99999488	0.999985264	0.999960414
PYCR3	0.999996191	0.999992444	0.999558179	0.99999488	0.999985264	0.999960414
PYURF	0.999996191	0.999992444	0.999558179	0.99999488	0.999985264	0.999960414
QARS	0.999996191	0.999992444	0.999558179	0.99999488	0.999985264	0.999960414
RAB11FIP2	0.999996191	0.999992444	0.999558179	0.99999488	0.999985264	0.999960414
RAB27A	0.999996191	0.999992444	0.999558179	0.99999488	0.999985264	0.999960414
RAB33A	0.999996191	0.999992444	0.999558179	0.99999488	0.999985264	0.999960414

RAB3C	0.999996191	0.999992444	0.999558179	0.99999488	0.999985264	0.999960414
RAB4B	0.999996191	0.999992444	0.999558179	0.99999488	0.999985264	0.999960414
RABL2B	0.999996191	0.999992444	0.999558179	0.99999488	0.999985264	0.999960414
RAD9A	0.999996191	0.999992444	0.999558179	0.99999488	0.999985264	0.999960414
RAI2	0.999996191	0.999992444	0.999558179	0.99999488	0.999985264	0.999960414
RALGPS1	0.999996191	0.999992444	0.999558179	0.99999488	0.999985264	0.999960414
RAMMET	0.999996191	0.999992444	0.999558179	0.99999488	0.999985264	0.999960414
RAMP2	0.999996191	0.999992444	0.999558179	0.99999488	0.999985264	0.999960414
RAN	0.999996191	0.999992444	0.999558179	0.99999488	0.999985264	0.999960414
RANBP10	0.999996191	0.999992444	0.999558179	0.99999488	0.999985264	0.999960414
RAP1GDS1	0.999996191	0.999992444	0.999558179	0.99999488	0.999985264	0.999960414
RAPGEF3	0.999996191	0.999992444	0.999558179	0.99999488	0.999985264	0.999960414
RARS2	0.999996191	0.999992444	0.999558179	0.99999488	0.999985264	0.999960414
RASAL1	0.999996191	0.999992444	0.999558179	0.99999488	0.999985264	0.999960414
RASEF	0.999996191	0.999992444	0.999558179	0.99999488	0.999985264	0.999960414
RAVER1	0.999996191	0.999992444	0.999558179	0.99999488	0.999985264	0.999960414
RBAK	0.999996191	0.999992444	0.999558179	0.99999488	0.999985264	0.999960414
RBBP8	0.999996191	0.999992444	0.999558179	0.99999488	0.999985264	0.999960414
RBM15B	0.999996191	0.999992444	0.999558179	0.99999488	0.999985264	0.999960414
RBM6	0.999996191	0.999992444	0.999558179	0.99999488	0.999985264	0.999960414
RBMS3	0.999996191	0.999992444	0.999558179	0.99999488	0.999985264	0.999960414
RBP4	0.999996191	0.999992444	0.999558179	0.99999488	0.999985264	0.999960414
RC3H2	0.999996191	0.999992444	0.999558179	0.99999488	0.999985264	0.999960414
RCC2	0.999996191	0.999992444	0.999558179	0.99999488	0.999985264	0.999960414
RCE1	0.999996191	0.999992444	0.999558179	0.99999488	0.999985264	0.999960414
RCOR3	0.999996191	0.999992444	0.999558179	0.99999488	0.999985264	0.999960414
RDH14	0.999996191	0.999992444	0.999558179	0.99999488	0.999985264	0.999960414
RECK	0.999996191	0.999992444	0.999558179	0.99999488	0.999985264	0.999960414
RELA	0.999996191	0.999992444	0.999558179	0.99999488	0.999985264	0.999960414
REM1	0.999996191	0.999992444	0.999558179	0.99999488	0.999985264	0.999960414
RERG	0.999996191	0.999992444	0.999558179	0.99999488	0.999985264	0.999960414
REST	0.999996191	0.999992444	0.999558179	0.99999488	0.999985264	0.999960414
REV3L	0.999996191	0.999992444	0.999558179	0.99999488	0.999985264	0.999960414
REXO5	0.999996191	0.999992444	0.999558179	0.99999488	0.999985264	0.999960414
RGS11	0.999996191	0.999992444	0.999558179	0.99999488	0.999985264	0.999960414
RGS13	0.999996191	0.999992444	0.999558179	0.99999488	0.999985264	0.999960414
RGS19	0.999996191	0.999992444	0.999558179	0.99999488	0.999985264	0.999960414
RHBDD2	0.999996191	0.999992444	0.999558179	0.99999488	0.999985264	0.999960414
RHNO1	0.999996191	0.999992444	0.999558179	0.99999488	0.999985264	0.999960414

RHOB	0.999996191	0.999992444	0.999558179	0.99999488	0.999985264	0.999960414
RIC3	0.999996191	0.999992444	0.999558179	0.99999488	0.999985264	0.999960414
RIPOR1	0.999996191	0.999992444	0.999558179	0.99999488	0.999985264	0.999960414
RIPPLY3	0.999996191	0.999992444	0.999558179	0.99999488	0.999985264	0.999960414
RNASE13	0.999996191	0.999992444	0.999558179	0.99999488	0.999985264	0.999960414
RNASE4	0.999996191	0.999992444	0.999558179	0.99999488	0.999985264	0.999960414
RNASEH2A	0.999996191	0.999992444	0.999558179	0.99999488	0.999985264	0.999960414
RND3	0.999996191	0.999992444	0.999558179	0.99999488	0.999985264	0.999960414
RNF111	0.999996191	0.999992444	0.999558179	0.99999488	0.999985264	0.999960414
RNF113A	0.999996191	0.999992444	0.999558179	0.99999488	0.999985264	0.999960414
RNF130	0.999996191	0.999992444	0.999558179	0.99999488	0.999985264	0.999960414
RNF141	0.999996191	0.999992444	0.999558179	0.99999488	0.999985264	0.999960414
RNF144A	0.999996191	0.999992444	0.999558179	0.99999488	0.999985264	0.999960414
RNF149	0.999996191	0.999992444	0.999558179	0.99999488	0.999985264	0.999960414
RNF157	0.999996191	0.999992444	0.999558179	0.99999488	0.999985264	0.999960414
RNF180	0.999996191	0.999992444	0.999558179	0.99999488	0.999985264	0.999960414
RNF215	0.999996191	0.999992444	0.999558179	0.99999488	0.999985264	0.999960414
RNF219	0.999996191	0.999992444	0.999558179	0.99999488	0.999985264	0.999960414
RNF26	0.999996191	0.999992444	0.999558179	0.99999488	0.999985264	0.999960414
RNF6	0.999996191	0.999992444	0.999558179	0.99999488	0.999985264	0.999960414
RNF8	0.999996191	0.999992444	0.999558179	0.99999488	0.999985264	0.999960414
RNFT2	0.999996191	0.999992444	0.999558179	0.99999488	0.999985264	0.999960414
RNGTT	0.999996191	0.999992444	0.999558179	0.99999488	0.999985264	0.999960414
RORB	0.999996191	0.999992444	0.999558179	0.99999488	0.999985264	0.999960414
RPAIN	0.999996191	0.999992444	0.999558179	0.99999488	0.999985264	0.999960414
RPGRIP1	0.999996191	0.999992444	0.999558179	0.99999488	0.999985264	0.999960414
RPL27	0.999996191	0.999992444	0.999558179	0.99999488	0.999985264	0.999960414
RPP25	0.999996191	0.999992444	0.999558179	0.99999488	0.999985264	0.999960414
RPP25L	0.999996191	0.999992444	0.999558179	0.99999488	0.999985264	0.999960414
RPRML	0.999996191	0.999992444	0.999558179	0.99999488	0.999985264	0.999960414
RRAS2	0.999996191	0.999992444	0.999558179	0.99999488	0.999985264	0.999960414
RREB1	0.999996191	0.999992444	0.999558179	0.99999488	0.999985264	0.999960414
RRH	0.999996191	0.999992444	0.999558179	0.99999488	0.999985264	0.999960414
RRM1	0.999996191	0.999992444	0.999558179	0.99999488	0.999985264	0.999960414
RRNAD1	0.999996191	0.999992444	0.999558179	0.99999488	0.999985264	0.999960414
RRP1B	0.999996191	0.999992444	0.999558179	0.99999488	0.999985264	0.999960414
RSPH3	0.999996191	0.999992444	0.999558179	0.99999488	0.999985264	0.999960414
RSRC1	0.999996191	0.999992444	0.999558179	0.99999488	0.999985264	0.999960414
RTKN	0.999996191	0.999992444	0.999558179	0.99999488	0.999985264	0.999960414

RTL9	0.999996191	0.999992444	0.999558179	0.99999488	0.999985264	0.999960414
RTN3	0.999996191	0.999992444	0.999558179	0.99999488	0.999985264	0.999960414
RTN4	0.999996191	0.999992444	0.999558179	0.99999488	0.999985264	0.999960414
SAAL1	0.999996191	0.999992444	0.999558179	0.99999488	0.999985264	0.999960414
SAMD13	0.999996191	0.999992444	0.999558179	0.99999488	0.999985264	0.999960414
SAMD4A	0.999996191	0.999992444	0.999558179	0.99999488	0.999985264	0.999960414
SARNP	0.999996191	0.999992444	0.999558179	0.99999488	0.999985264	0.999960414
SART3	0.999996191	0.999992444	0.999558179	0.99999488	0.999985264	0.999960414
SASH1	0.999996191	0.999992444	0.999558179	0.99999488	0.999985264	0.999960414
SBDS	0.999996191	0.999992444	0.999558179	0.99999488	0.999985264	0.999960414
SCAP	0.999996191	0.999992444	0.999558179	0.99999488	0.999985264	0.999960414
SCARA3	0.999996191	0.999992444	0.999558179	0.99999488	0.999985264	0.999960414
SCARF1	0.999996191	0.999992444	0.999558179	0.99999488	0.999985264	0.999960414
SCFD2	0.999996191	0.999992444	0.999558179	0.99999488	0.999985264	0.999960414
SCNM1	0.999996191	0.999992444	0.999558179	0.99999488	0.999985264	0.999960414
SCP2D1	0.999996191	0.999992444	0.999558179	0.99999488	0.999985264	0.999960414
SCUBE3	0.999996191	0.999992444	0.999558179	0.99999488	0.999985264	0.999960414
SDCCAG8	0.999996191	0.999992444	0.999558179	0.99999488	0.999985264	0.999960414
SDSL	0.999996191	0.999992444	0.999558179	0.99999488	0.999985264	0.999960414
SEC22C	0.999996191	0.999992444	0.999558179	0.99999488	0.999985264	0.999960414
SEC61G	0.999996191	0.999992444	0.999558179	0.99999488	0.999985264	0.999960414
SEL1L	0.999996191	0.999992444	0.999558179	0.99999488	0.999985264	0.999960414
SEMA3C	0.999996191	0.999992444	0.999558179	0.99999488	0.999985264	0.999960414
SEMA5A	0.999996191	0.999992444	0.999558179	0.99999488	0.999985264	0.999960414
SEMA6D	0.999996191	0.999992444	0.999558179	0.99999488	0.999985264	0.999960414
SENP2	0.999996191	0.999992444	0.999558179	0.99999488	0.999985264	0.999960414
SENP3	0.999996191	0.999992444	0.999558179	0.99999488	0.999985264	0.999960414
SENP5	0.999996191	0.999992444	0.999558179	0.99999488	0.999985264	0.999960414
SEPT2	0.999996191	0.999992444	0.999558179	0.99999488	0.999985264	0.999960414
SERF1A	0.999996191	0.999992444	0.999558179	0.99999488	0.999985264	0.999960414
SERP2	0.999996191	0.999992444	0.999558179	0.99999488	0.999985264	0.999960414
SETBP1	0.999996191	0.999992444	0.999558179	0.99999488	0.999985264	0.999960414
SETD1B	0.999996191	0.999992444	0.999558179	0.99999488	0.999985264	0.999960414
SETD6	0.999996191	0.999992444	0.999558179	0.99999488	0.999985264	0.999960414
SF3A1	0.999996191	0.999992444	0.999558179	0.99999488	0.999985264	0.999960414
SFMBT1	0.999996191	0.999992444	0.999558179	0.99999488	0.999985264	0.999960414
SFPQ	0.999996191	0.999992444	0.999558179	0.99999488	0.999985264	0.999960414
SGMS2	0.999996191	0.999992444	0.999558179	0.99999488	0.999985264	0.999960414
SGO2	0.999996191	0.999992444	0.999558179	0.99999488	0.999985264	0.999960414

SGSM1	0.999996191	0.999992444	0.999558179	0.99999488	0.999985264	0.999960414
SGTA	0.999996191	0.999992444	0.999558179	0.99999488	0.999985264	0.999960414
SH2D7	0.999996191	0.999992444	0.999558179	0.99999488	0.999985264	0.999960414
SH3BP4	0.999996191	0.999992444	0.999558179	0.99999488	0.999985264	0.999960414
SHANK3	0.999996191	0.999992444	0.999558179	0.99999488	0.999985264	0.999960414
SHISAL2A	0.999996191	0.999992444	0.999558179	0.99999488	0.999985264	0.999960414
SHQ1	0.999996191	0.999992444	0.999558179	0.99999488	0.999985264	0.999960414
SHROOM1	0.999996191	0.999992444	0.999558179	0.99999488	0.999985264	0.999960414
SHROOM4	0.999996191	0.999992444	0.999558179	0.99999488	0.999985264	0.999960414
SIKE1	0.999996191	0.999992444	0.999558179	0.99999488	0.999985264	0.999960414
SIL1	0.999996191	0.999992444	0.999558179	0.99999488	0.999985264	0.999960414
SIN3A	0.999996191	0.999992444	0.999558179	0.99999488	0.999985264	0.999960414
SIN3B	0.999996191	0.999992444	0.999558179	0.99999488	0.999985264	0.999960414
SKA1	0.999996191	0.999992444	0.999558179	0.99999488	0.999985264	0.999960414
SLC10A6	0.999996191	0.999992444	0.999558179	0.99999488	0.999985264	0.999960414
SLC10A7	0.999996191	0.999992444	0.999558179	0.99999488	0.999985264	0.999960414
SLC12A7	0.999996191	0.999992444	0.999558179	0.99999488	0.999985264	0.999960414
SLC16A10	0.999996191	0.999992444	0.999558179	0.99999488	0.999985264	0.999960414
SLC16A2	0.999996191	0.999992444	0.999558179	0.99999488	0.999985264	0.999960414
SLC22A3	0.999996191	0.999992444	0.999558179	0.99999488	0.999985264	0.999960414
SLC23A3	0.999996191	0.999992444	0.999558179	0.99999488	0.999985264	0.999960414
SLC25A29	0.999996191	0.999992444	0.999558179	0.99999488	0.999985264	0.999960414
SLC25A37	0.999996191	0.999992444	0.999558179	0.99999488	0.999985264	0.999960414
SLC25A44	0.999996191	0.999992444	0.999558179	0.99999488	0.999985264	0.999960414
SLC26A6	0.999996191	0.999992444	0.999558179	0.99999488	0.999985264	0.999960414
SLC27A1	0.999996191	0.999992444	0.999558179	0.99999488	0.999985264	0.999960414
SLC27A4	0.999996191	0.999992444	0.999558179	0.99999488	0.999985264	0.999960414
SLC27A5	0.999996191	0.999992444	0.999558179	0.99999488	0.999985264	0.999960414
SLC27A6	0.999996191	0.999992444	0.999558179	0.99999488	0.999985264	0.999960414
SLC30A9	0.999996191	0.999992444	0.999558179	0.99999488	0.999985264	0.999960414
SLC35A2	0.999996191	0.999992444	0.999558179	0.99999488	0.999985264	0.999960414
SLC35B3	0.999996191	0.999992444	0.999558179	0.99999488	0.999985264	0.999960414
SLC35B4	0.999996191	0.999992444	0.999558179	0.99999488	0.999985264	0.999960414
SLC35D1	0.999996191	0.999992444	0.999558179	0.99999488	0.999985264	0.999960414
SLC35E2	0.999996191	0.999992444	0.999558179	0.99999488	0.999985264	0.999960414
SLC35F6	0.999996191	0.999992444	0.999558179	0.99999488	0.999985264	0.999960414
SLC36A1	0.999996191	0.999992444	0.999558179	0.99999488	0.999985264	0.999960414
SLC38A7	0.999996191	0.999992444	0.999558179	0.99999488	0.999985264	0.999960414
SLC39A11	0.999996191	0.999992444	0.999558179	0.99999488	0.999985264	0.999960414

SLC39A9	0.999996191	0.999992444	0.999558179	0.99999488	0.999985264	0.999960414
SLC43A3	0.999996191	0.999992444	0.999558179	0.99999488	0.999985264	0.999960414
SLC4A10	0.999996191	0.999992444	0.999558179	0.99999488	0.999985264	0.999960414
SLC4A4	0.999996191	0.999992444	0.999558179	0.99999488	0.999985264	0.999960414
SLC4A7	0.999996191	0.999992444	0.999558179	0.99999488	0.999985264	0.999960414
SLCO5A1	0.999996191	0.999992444	0.999558179	0.99999488	0.999985264	0.999960414
SLFNL1	0.999996191	0.999992444	0.999558179	0.99999488	0.999985264	0.999960414
SLIRP	0.999996191	0.999992444	0.999558179	0.99999488	0.999985264	0.999960414
SLX4IP	0.999996191	0.999992444	0.999558179	0.99999488	0.999985264	0.999960414
SMAP1	0.999996191	0.999992444	0.999558179	0.99999488	0.999985264	0.999960414
SMARCD1	0.999996191	0.999992444	0.999558179	0.99999488	0.999985264	0.999960414
SMARCE1	0.999996191	0.999992444	0.999558179	0.99999488	0.999985264	0.999960414
SMCO3	0.999996191	0.999992444	0.999558179	0.99999488	0.999985264	0.999960414
SMIM7	0.999996191	0.999992444	0.999558179	0.99999488	0.999985264	0.999960414
SMIM8	0.999996191	0.999992444	0.999558179	0.99999488	0.999985264	0.999960414
SMOC1	0.999996191	0.999992444	0.999558179	0.99999488	0.999985264	0.999960414
SMPD2	0.999996191	0.999992444	0.999558179	0.99999488	0.999985264	0.999960414
SMPD5	0.999996191	0.999992444	0.999558179	0.99999488	0.999985264	0.999960414
SMS	0.999996191	0.999992444	0.999558179	0.99999488	0.999985264	0.999960414
SMURF1	0.999996191	0.999992444	0.999558179	0.99999488	0.999985264	0.999960414
SNRPC	0.999996191	0.999992444	0.999558179	0.99999488	0.999985264	0.999960414
SNRPE	0.999996191	0.999992444	0.999558179	0.99999488	0.999985264	0.999960414
SNRPN	0.999996191	0.999992444	0.999558179	0.99999488	0.999985264	0.999960414
SNUPN	0.999996191	0.999992444	0.999558179	0.99999488	0.999985264	0.999960414
SNX1	0.999996191	0.999992444	0.999558179	0.99999488	0.999985264	0.999960414
SNX25	0.999996191	0.999992444	0.999558179	0.99999488	0.999985264	0.999960414
SNX27	0.999996191	0.999992444	0.999558179	0.99999488	0.999985264	0.999960414
SNX31	0.999996191	0.999992444	0.999558179	0.99999488	0.999985264	0.999960414
SPA17	0.999996191	0.999992444	0.999558179	0.99999488	0.999985264	0.999960414
SPAG16	0.999996191	0.999992444	0.999558179	0.99999488	0.999985264	0.999960414
SPAM1	0.999996191	0.999992444	0.999558179	0.99999488	0.999985264	0.999960414
SPATA21	0.999996191	0.999992444	0.999558179	0.99999488	0.999985264	0.999960414
SPATS2	0.999996191	0.999992444	0.999558179	0.99999488	0.999985264	0.999960414
SPATS2L	0.999996191	0.999992444	0.999558179	0.99999488	0.999985264	0.999960414
SPC25	0.999996191	0.999992444	0.999558179	0.99999488	0.999985264	0.999960414
SPCS2	0.999996191	0.999992444	0.999558179	0.99999488	0.999985264	0.999960414
SPIN4	0.999996191	0.999992444	0.999558179	0.99999488	0.999985264	0.999960414
SPINDOC	0.999996191	0.999992444	0.999558179	0.99999488	0.999985264	0.999960414
SPR	0.999996191	0.999992444	0.999558179	0.99999488	0.999985264	0.999960414

SPRTN	0.999996191	0.999992444	0.999558179	0.99999488	0.999985264	0.999960414
SPRY4	0.999996191	0.999992444	0.999558179	0.99999488	0.999985264	0.999960414
SPSB3	0.999996191	0.999992444	0.999558179	0.99999488	0.999985264	0.999960414
SRD5A3	0.999996191	0.999992444	0.999558179	0.99999488	0.999985264	0.999960414
SRI	0.999996191	0.999992444	0.999558179	0.99999488	0.999985264	0.999960414
SRMS	0.999996191	0.999992444	0.999558179	0.99999488	0.999985264	0.999960414
SRRT	0.999996191	0.999992444	0.999558179	0.99999488	0.999985264	0.999960414
SSH3	0.999996191	0.999992444	0.999558179	0.99999488	0.999985264	0.999960414
SSX2IP	0.999996191	0.999992444	0.999558179	0.99999488	0.999985264	0.999960414
ST6GALNAC5	0.999996191	0.999992444	0.999558179	0.99999488	0.999985264	0.999960414
ST6GALNAC6	0.999996191	0.999992444	0.999558179	0.99999488	0.999985264	0.999960414
ST7	0.999996191	0.999992444	0.999558179	0.99999488	0.999985264	0.999960414
STAG3	0.999996191	0.999992444	0.999558179	0.99999488	0.999985264	0.999960414
STAM2	0.999996191	0.999992444	0.999558179	0.99999488	0.999985264	0.999960414
STARD3NL	0.999996191	0.999992444	0.999558179	0.99999488	0.999985264	0.999960414
STAU2	0.999996191	0.999992444	0.999558179	0.99999488	0.999985264	0.999960414
STK11IP	0.999996191	0.999992444	0.999558179	0.99999488	0.999985264	0.999960414
STMP1	0.999996191	0.999992444	0.999558179	0.99999488	0.999985264	0.999960414
STN1	0.999996191	0.999992444	0.999558179	0.99999488	0.999985264	0.999960414
STOML1	0.999996191	0.999992444	0.999558179	0.99999488	0.999985264	0.999960414
STRADB	0.999996191	0.999992444	0.999558179	0.99999488	0.999985264	0.999960414
STX10	0.999996191	0.999992444	0.999558179	0.99999488	0.999985264	0.999960414
STX11	0.999996191	0.999992444	0.999558179	0.99999488	0.999985264	0.999960414
STX3	0.999996191	0.999992444	0.999558179	0.99999488	0.999985264	0.999960414
STXBP4	0.999996191	0.999992444	0.999558179	0.99999488	0.999985264	0.999960414
STYXL1	0.999996191	0.999992444	0.999558179	0.99999488	0.999985264	0.999960414
SULT1B1	0.999996191	0.999992444	0.999558179	0.99999488	0.999985264	0.999960414
SUN1	0.999996191	0.999992444	0.999558179	0.99999488	0.999985264	0.999960414
SUPT5H	0.999996191	0.999992444	0.999558179	0.99999488	0.999985264	0.999960414
SUV39H1	0.999996191	0.999992444	0.999558179	0.99999488	0.999985264	0.999960414
SYCE1L	0.999996191	0.999992444	0.999558179	0.99999488	0.999985264	0.999960414
SYCP2	0.999996191	0.999992444	0.999558179	0.99999488	0.999985264	0.999960414
SYN2	0.999996191	0.999992444	0.999558179	0.99999488	0.999985264	0.999960414
SYNGR3	0.999996191	0.999992444	0.999558179	0.99999488	0.999985264	0.999960414
SYNJ1	0.999996191	0.999992444	0.999558179	0.99999488	0.999985264	0.999960414
SYNRG	0.999996191	0.999992444	0.999558179	0.99999488	0.999985264	0.999960414
SYT12	0.999996191	0.999992444	0.999558179	0.99999488	0.999985264	0.999960414
SYT7	0.999996191	0.999992444	0.999558179	0.99999488	0.999985264	0.999960414
TAF12	0.999996191	0.999992444	0.999558179	0.99999488	0.999985264	0.999960414

TAF7	0.999996191	0.999992444	0.999558179	0.99999488	0.999985264	0.999960414
TAF9B	0.999996191	0.999992444	0.999558179	0.99999488	0.999985264	0.999960414
TAL1	0.999996191	0.999992444	0.999558179	0.99999488	0.999985264	0.999960414
TANGO2	0.999996191	0.999992444	0.999558179	0.99999488	0.999985264	0.999960414
TAPBPL	0.999996191	0.999992444	0.999558179	0.99999488	0.999985264	0.999960414
TASP1	0.999996191	0.999992444	0.999558179	0.99999488	0.999985264	0.999960414
TBC1D20	0.999996191	0.999992444	0.999558179	0.99999488	0.999985264	0.999960414
TBC1D2B	0.999996191	0.999992444	0.999558179	0.99999488	0.999985264	0.999960414
TBC1D31	0.999996191	0.999992444	0.999558179	0.99999488	0.999985264	0.999960414
TBC1D5	0.999996191	0.999992444	0.999558179	0.99999488	0.999985264	0.999960414
TBC1D7	0.999996191	0.999992444	0.999558179	0.99999488	0.999985264	0.999960414
TBC1D8	0.999996191	0.999992444	0.999558179	0.99999488	0.999985264	0.999960414
TBCCD1	0.999996191	0.999992444	0.999558179	0.99999488	0.999985264	0.999960414
TBCEL	0.999996191	0.999992444	0.999558179	0.99999488	0.999985264	0.999960414
TBCK	0.999996191	0.999992444	0.999558179	0.99999488	0.999985264	0.999960414
TBX18	0.999996191	0.999992444	0.999558179	0.99999488	0.999985264	0.999960414
TCEANC2	0.999996191	0.999992444	0.999558179	0.99999488	0.999985264	0.999960414
TCF25	0.999996191	0.999992444	0.999558179	0.99999488	0.999985264	0.999960414
TCTN1	0.999996191	0.999992444	0.999558179	0.99999488	0.999985264	0.999960414
TCTN2	0.999996191	0.999992444	0.999558179	0.99999488	0.999985264	0.999960414
TCTN3	0.999996191	0.999992444	0.999558179	0.99999488	0.999985264	0.999960414
TDG	0.999996191	0.999992444	0.999558179	0.99999488	0.999985264	0.999960414
TDRP	0.999996191	0.999992444	0.999558179	0.99999488	0.999985264	0.999960414
TEN1	0.999996191	0.999992444	0.999558179	0.99999488	0.999985264	0.999960414
TENM1	0.999996191	0.999992444	0.999558179	0.99999488	0.999985264	0.999960414
TEX261	0.999996191	0.999992444	0.999558179	0.99999488	0.999985264	0.999960414
TEX30	0.999996191	0.999992444	0.999558179	0.99999488	0.999985264	0.999960414
TFPT	0.999996191	0.999992444	0.999558179	0.99999488	0.999985264	0.999960414
TGS1	0.999996191	0.999992444	0.999558179	0.99999488	0.999985264	0.999960414
THADA	0.999996191	0.999992444	0.999558179	0.99999488	0.999985264	0.999960414
THAP5	0.999996191	0.999992444	0.999558179	0.99999488	0.999985264	0.999960414
THEM4	0.999996191	0.999992444	0.999558179	0.99999488	0.999985264	0.999960414
THOC6	0.999996191	0.999992444	0.999558179	0.99999488	0.999985264	0.999960414
THRAP3	0.999996191	0.999992444	0.999558179	0.99999488	0.999985264	0.999960414
TIAM2	0.999996191	0.999992444	0.999558179	0.99999488	0.999985264	0.999960414
TIGD7	0.999996191	0.999992444	0.999558179	0.99999488	0.999985264	0.999960414
TIPIN	0.999996191	0.999992444	0.999558179	0.99999488	0.999985264	0.999960414
TKFC	0.999996191	0.999992444	0.999558179	0.99999488	0.999985264	0.999960414
TLDC1	0.999996191	0.999992444	0.999558179	0.99999488	0.999985264	0.999960414

TLK2	0.999996191	0.999992444	0.999558179	0.99999488	0.999985264	0.999960414
TLN2	0.999996191	0.999992444	0.999558179	0.99999488	0.999985264	0.999960414
TM2D2	0.999996191	0.999992444	0.999558179	0.99999488	0.999985264	0.999960414
TM2D3	0.999996191	0.999992444	0.999558179	0.99999488	0.999985264	0.999960414
TM4SF1	0.999996191	0.999992444	0.999558179	0.99999488	0.999985264	0.999960414
TM6SF1	0.999996191	0.999992444	0.999558179	0.99999488	0.999985264	0.999960414
TM7SF3	0.999996191	0.999992444	0.999558179	0.99999488	0.999985264	0.999960414
TM9SF1	0.999996191	0.999992444	0.999558179	0.99999488	0.999985264	0.999960414
TMBIM6	0.999996191	0.999992444	0.999558179	0.99999488	0.999985264	0.999960414
TMEM11	0.999996191	0.999992444	0.999558179	0.99999488	0.999985264	0.999960414
TMEM116	0.999996191	0.999992444	0.999558179	0.99999488	0.999985264	0.999960414
TMEM117	0.999996191	0.999992444	0.999558179	0.99999488	0.999985264	0.999960414
TMEM123	0.999996191	0.999992444	0.999558179	0.99999488	0.999985264	0.999960414
TMEM134	0.999996191	0.999992444	0.999558179	0.99999488	0.999985264	0.999960414
TMEM233	0.999996191	0.999992444	0.999558179	0.99999488	0.999985264	0.999960414
TMEM241	0.999996191	0.999992444	0.999558179	0.99999488	0.999985264	0.999960414
TMEM246	0.999996191	0.999992444	0.999558179	0.99999488	0.999985264	0.999960414
TMEM255A	0.999996191	0.999992444	0.999558179	0.99999488	0.999985264	0.999960414
TMEM260	0.999996191	0.999992444	0.999558179	0.99999488	0.999985264	0.999960414
TMEM44	0.999996191	0.999992444	0.999558179	0.99999488	0.999985264	0.999960414
TMEM47	0.999996191	0.999992444	0.999558179	0.99999488	0.999985264	0.999960414
TMEM63B	0.999996191	0.999992444	0.999558179	0.99999488	0.999985264	0.999960414
TMEM74	0.999996191	0.999992444	0.999558179	0.99999488	0.999985264	0.999960414
TMEM74B	0.999996191	0.999992444	0.999558179	0.99999488	0.999985264	0.999960414
TMEM86B	0.999996191	0.999992444	0.999558179	0.99999488	0.999985264	0.999960414
TMEM87B	0.999996191	0.999992444	0.999558179	0.99999488	0.999985264	0.999960414
TMLHE	0.999996191	0.999992444	0.999558179	0.99999488	0.999985264	0.999960414
TNFRSF1A	0.999996191	0.999992444	0.999558179	0.99999488	0.999985264	0.999960414
TNIP1	0.999996191	0.999992444	0.999558179	0.99999488	0.999985264	0.999960414
TNKS1BP1	0.999996191	0.999992444	0.999558179	0.99999488	0.999985264	0.999960414
TNNI3	0.999996191	0.999992444	0.999558179	0.99999488	0.999985264	0.999960414
TNS2	0.999996191	0.999992444	0.999558179	0.99999488	0.999985264	0.999960414
TNS3	0.999996191	0.999992444	0.999558179	0.99999488	0.999985264	0.999960414
TOP3A	0.999996191	0.999992444	0.999558179	0.99999488	0.999985264	0.999960414
TP53INP2	0.999996191	0.999992444	0.999558179	0.99999488	0.999985264	0.999960414
TPRA1	0.999996191	0.999992444	0.999558179	0.99999488	0.999985264	0.999960414
TPST2	0.999996191	0.999992444	0.999558179	0.99999488	0.999985264	0.999960414
TRAM1L1	0.999996191	0.999992444	0.999558179	0.99999488	0.999985264	0.999960414
TRAPPC11	0.999996191	0.999992444	0.999558179	0.99999488	0.999985264	0.999960414

TRAPPC2L	0.999996191	0.999992444	0.999558179	0.99999488	0.999985264	0.999960414
TRIAP1	0.999996191	0.999992444	0.999558179	0.99999488	0.999985264	0.999960414
TRIM16	0.999996191	0.999992444	0.999558179	0.99999488	0.999985264	0.999960414
TRIM3	0.999996191	0.999992444	0.999558179	0.99999488	0.999985264	0.999960414
TRIM45	0.999996191	0.999992444	0.999558179	0.99999488	0.999985264	0.999960414
TRIO	0.999996191	0.999992444	0.999558179	0.99999488	0.999985264	0.999960414
TRMT11	0.999996191	0.999992444	0.999558179	0.99999488	0.999985264	0.999960414
TRPM6	0.999996191	0.999992444	0.999558179	0.99999488	0.999985264	0.999960414
TRPS1	0.999996191	0.999992444	0.999558179	0.99999488	0.999985264	0.999960414
TSEN15	0.999996191	0.999992444	0.999558179	0.99999488	0.999985264	0.999960414
TSEN2	0.999996191	0.999992444	0.999558179	0.99999488	0.999985264	0.999960414
TSGA10	0.999996191	0.999992444	0.999558179	0.99999488	0.999985264	0.999960414
TSNAX	0.999996191	0.999992444	0.999558179	0.99999488	0.999985264	0.999960414
TSPAN12	0.999996191	0.999992444	0.999558179	0.99999488	0.999985264	0.999960414
TSPAN17	0.999996191	0.999992444	0.999558179	0.99999488	0.999985264	0.999960414
TSPAN3	0.999996191	0.999992444	0.999558179	0.99999488	0.999985264	0.999960414
TSPAN33	0.999996191	0.999992444	0.999558179	0.99999488	0.999985264	0.999960414
TSPAN5	0.999996191	0.999992444	0.999558179	0.99999488	0.999985264	0.999960414
TSPYL5	0.999996191	0.999992444	0.999558179	0.99999488	0.999985264	0.999960414
TSTD3	0.999996191	0.999992444	0.999558179	0.99999488	0.999985264	0.999960414
TTC5	0.999996191	0.999992444	0.999558179	0.99999488	0.999985264	0.999960414
TTC7B	0.999996191	0.999992444	0.999558179	0.99999488	0.999985264	0.999960414
TTF2	0.999996191	0.999992444	0.999558179	0.99999488	0.999985264	0.999960414
TTI1	0.999996191	0.999992444	0.999558179	0.99999488	0.999985264	0.999960414
TTLL9	0.999996191	0.999992444	0.999558179	0.99999488	0.999985264	0.999960414
TUBB1	0.999996191	0.999992444	0.999558179	0.99999488	0.999985264	0.999960414
TULP3	0.999996191	0.999992444	0.999558179	0.99999488	0.999985264	0.999960414
TWSG1	0.999996191	0.999992444	0.999558179	0.99999488	0.999985264	0.999960414
TYRO3	0.999996191	0.999992444	0.999558179	0.99999488	0.999985264	0.999960414
TYW1	0.999996191	0.999992444	0.999558179	0.99999488	0.999985264	0.999960414
UBAC2	0.999996191	0.999992444	0.999558179	0.99999488	0.999985264	0.999960414
UBALD1	0.999996191	0.999992444	0.999558179	0.99999488	0.999985264	0.999960414
UBAP2L	0.999996191	0.999992444	0.999558179	0.99999488	0.999985264	0.999960414
UBC	0.999996191	0.999992444	0.999558179	0.99999488	0.999985264	0.999960414
UBE2A	0.999996191	0.999992444	0.999558179	0.99999488	0.999985264	0.999960414
UBE2D1	0.999996191	0.999992444	0.999558179	0.99999488	0.999985264	0.999960414
UBE2D4	0.999996191	0.999992444	0.999558179	0.99999488	0.999985264	0.999960414
UBE2E1	0.999996191	0.999992444	0.999558179	0.99999488	0.999985264	0.999960414
UBE2E2	0.999996191	0.999992444	0.999558179	0.99999488	0.999985264	0.999960414

UBE2J1	0.999996191	0.999992444	0.999558179	0.99999488	0.999985264	0.999960414
UBE2L6	0.999996191	0.999992444	0.999558179	0.99999488	0.999985264	0.999960414
UBTD2	0.999996191	0.999992444	0.999558179	0.99999488	0.999985264	0.999960414
UBTF	0.999996191	0.999992444	0.999558179	0.99999488	0.999985264	0.999960414
UBXN2A	0.999996191	0.999992444	0.999558179	0.99999488	0.999985264	0.999960414
UBXN2B	0.999996191	0.999992444	0.999558179	0.99999488	0.999985264	0.999960414
UBXN6	0.999996191	0.999992444	0.999558179	0.99999488	0.999985264	0.999960414
UCHL5	0.999996191	0.999992444	0.999558179	0.99999488	0.999985264	0.999960414
UGDH	0.999996191	0.999992444	0.999558179	0.99999488	0.999985264	0.999960414
ULBP21	0.999996191	0.999992444	0.999558179	0.99999488	0.999985264	0.999960414
UNC45B	0.999996191	0.999992444	0.999558179	0.99999488	0.999985264	0.999960414
UPP1	0.999996191	0.999992444	0.999558179	0.99999488	0.999985264	0.999960414
UPRT	0.999996191	0.999992444	0.999558179	0.99999488	0.999985264	0.999960414
UROC1	0.999996191	0.999992444	0.999558179	0.99999488	0.999985264	0.999960414
UROS	0.999996191	0.999992444	0.999558179	0.99999488	0.999985264	0.999960414
USF1	0.999996191	0.999992444	0.999558179	0.99999488	0.999985264	0.999960414
USP27X	0.999996191	0.999992444	0.999558179	0.99999488	0.999985264	0.999960414
USP31	0.999996191	0.999992444	0.999558179	0.99999488	0.999985264	0.999960414
USP33	0.999996191	0.999992444	0.999558179	0.99999488	0.999985264	0.999960414
USP45	0.999996191	0.999992444	0.999558179	0.99999488	0.999985264	0.999960414
UTP23	0.999996191	0.999992444	0.999558179	0.99999488	0.999985264	0.999960414
UTP3	0.999996191	0.999992444	0.999558179	0.99999488	0.999985264	0.999960414
VAMP4	0.999996191	0.999992444	0.999558179	0.99999488	0.999985264	0.999960414
VDAC3	0.999996191	0.999992444	0.999558179	0.99999488	0.999985264	0.999960414
VEGFB	0.999996191	0.999992444	0.999558179	0.99999488	0.999985264	0.999960414
VKORC1L1	0.999996191	0.999992444	0.999558179	0.99999488	0.999985264	0.999960414
VPS11	0.999996191	0.999992444	0.999558179	0.99999488	0.999985264	0.999960414
VPS25	0.999996191	0.999992444	0.999558179	0.99999488	0.999985264	0.999960414
VPS35L	0.999996191	0.999992444	0.999558179	0.99999488	0.999985264	0.999960414
VPS36	0.999996191	0.999992444	0.999558179	0.99999488	0.999985264	0.999960414
VPS39	0.999996191	0.999992444	0.999558179	0.99999488	0.999985264	0.999960414
VPS4B	0.999996191	0.999992444	0.999558179	0.99999488	0.999985264	0.999960414
VPS53	0.999996191	0.999992444	0.999558179	0.99999488	0.999985264	0.999960414
VRK3	0.999996191	0.999992444	0.999558179	0.99999488	0.999985264	0.999960414
VWA1	0.999996191	0.999992444	0.999558179	0.99999488	0.999985264	0.999960414
VWA2	0.999996191	0.999992444	0.999558179	0.99999488	0.999985264	0.999960414
VWA5B2	0.999996191	0.999992444	0.999558179	0.99999488	0.999985264	0.999960414
WASHC3	0.999996191	0.999992444	0.999558179	0.99999488	0.999985264	0.999960414
WASHC5	0.999996191	0.999992444	0.999558179	0.99999488	0.999985264	0.999960414

WDPCP	0.999996191	0.999992444	0.999558179	0.99999488	0.999985264	0.999960414
WDR25	0.999996191	0.999992444	0.999558179	0.99999488	0.999985264	0.999960414
WDR26	0.999996191	0.999992444	0.999558179	0.99999488	0.999985264	0.999960414
WDR37	0.999996191	0.999992444	0.999558179	0.99999488	0.999985264	0.999960414
WDR41	0.999996191	0.999992444	0.999558179	0.99999488	0.999985264	0.999960414
WDR45	0.999996191	0.999992444	0.999558179	0.99999488	0.999985264	0.999960414
WDR60	0.999996191	0.999992444	0.999558179	0.99999488	0.999985264	0.999960414
WDR70	0.999996191	0.999992444	0.999558179	0.99999488	0.999985264	0.999960414
WDR78	0.999996191	0.999992444	0.999558179	0.99999488	0.999985264	0.999960414
WDR82	0.999996191	0.999992444	0.999558179	0.99999488	0.999985264	0.999960414
WDR93	0.999996191	0.999992444	0.999558179	0.99999488	0.999985264	0.999960414
WDYHV1	0.999996191	0.999992444	0.999558179	0.99999488	0.999985264	0.999960414
WHAMM	0.999996191	0.999992444	0.999558179	0.99999488	0.999985264	0.999960414
WIPF2	0.999996191	0.999992444	0.999558179	0.99999488	0.999985264	0.999960414
WIPF3	0.999996191	0.999992444	0.999558179	0.99999488	0.999985264	0.999960414
WNT8B	0.999996191	0.999992444	0.999558179	0.99999488	0.999985264	0.999960414
XAB2	0.999996191	0.999992444	0.999558179	0.99999488	0.999985264	0.999960414
XKR8	0.999996191	0.999992444	0.999558179	0.99999488	0.999985264	0.999960414
XPC	0.999996191	0.999992444	0.999558179	0.99999488	0.999985264	0.999960414
XPNPEP3	0.999996191	0.999992444	0.999558179	0.99999488	0.999985264	0.999960414
XRN2	0.999996191	0.999992444	0.999558179	0.99999488	0.999985264	0.999960414
YAE1D1	0.999996191	0.999992444	0.999558179	0.99999488	0.999985264	0.999960414
YEATS4	0.999996191	0.999992444	0.999558179	0.99999488	0.999985264	0.999960414
YPEL2	0.999996191	0.999992444	0.999558179	0.99999488	0.999985264	0.999960414
YWHAB	0.999996191	0.999992444	0.999558179	0.99999488	0.999985264	0.999960414
ZBED1	0.999996191	0.999992444	0.999558179	0.99999488	0.999985264	0.999960414
ZBED3	0.999996191	0.999992444	0.999558179	0.99999488	0.999985264	0.999960414
ZBTB3	0.999996191	0.999992444	0.999558179	0.99999488	0.999985264	0.999960414
ZBTB39	0.999996191	0.999992444	0.999558179	0.99999488	0.999985264	0.999960414
ZBTB48	0.999996191	0.999992444	0.999558179	0.99999488	0.999985264	0.999960414
ZC2HC1C	0.999996191	0.999992444	0.999558179	0.99999488	0.999985264	0.999960414
ZC3H4	0.999996191	0.999992444	0.999558179	0.99999488	0.999985264	0.999960414
ZC3H7A	0.999996191	0.999992444	0.999558179	0.99999488	0.999985264	0.999960414
ZC3HAV1L	0.999996191	0.999992444	0.999558179	0.99999488	0.999985264	0.999960414
ZCCHC7	0.999996191	0.999992444	0.999558179	0.99999488	0.999985264	0.999960414
ZCCHC8	0.999996191	0.999992444	0.999558179	0.99999488	0.999985264	0.999960414
ZCWPW2	0.999996191	0.999992444	0.999558179	0.99999488	0.999985264	0.999960414
ZDHHC15	0.999996191	0.999992444	0.999558179	0.99999488	0.999985264	0.999960414
ZDHHC19	0.999996191	0.999992444	0.999558179	0.99999488	0.999985264	0.999960414

ZEB1	0.999996191	0.999992444	0.999558179	0.99999488	0.999985264	0.999960414
ZEB2	0.999996191	0.999992444	0.999558179	0.99999488	0.999985264	0.999960414
ZFAND1	0.999996191	0.999992444	0.999558179	0.99999488	0.999985264	0.999960414
ZFAND2B	0.999996191	0.999992444	0.999558179	0.99999488	0.999985264	0.999960414
ZFAND4	0.999996191	0.999992444	0.999558179	0.99999488	0.999985264	0.999960414
ZFHX2	0.999996191	0.999992444	0.999558179	0.99999488	0.999985264	0.999960414
ZFP28	0.999996191	0.999992444	0.999558179	0.99999488	0.999985264	0.999960414
ZFP36L2	0.999996191	0.999992444	0.999558179	0.99999488	0.999985264	0.999960414
ZFP91	0.999996191	0.999992444	0.999558179	0.99999488	0.999985264	0.999960414
ZFYVE26	0.999996191	0.999992444	0.999558179	0.99999488	0.999985264	0.999960414
ZHX2	0.999996191	0.999992444	0.999558179	0.99999488	0.999985264	0.999960414
ZKSCAN4	0.999996191	0.999992444	0.999558179	0.99999488	0.999985264	0.999960414
ZKSCAN8	0.999996191	0.999992444	0.999558179	0.99999488	0.999985264	0.999960414
ZNF132	0.999996191	0.999992444	0.999558179	0.99999488	0.999985264	0.999960414
ZNF142	0.999996191	0.999992444	0.999558179	0.99999488	0.999985264	0.999960414
ZNF154	0.999996191	0.999992444	0.999558179	0.99999488	0.999985264	0.999960414
ZNF169	0.999996191	0.999992444	0.999558179	0.99999488	0.999985264	0.999960414
ZNF177	0.999996191	0.999992444	0.999558179	0.99999488	0.999985264	0.999960414
ZNF189	0.999996191	0.999992444	0.999558179	0.99999488	0.999985264	0.999960414
ZNF197	0.999996191	0.999992444	0.999558179	0.99999488	0.999985264	0.999960414
ZNF2	0.999996191	0.999992444	0.999558179	0.99999488	0.999985264	0.999960414
ZNF200	0.999996191	0.999992444	0.999558179	0.99999488	0.999985264	0.999960414
ZNF205	0.999996191	0.999992444	0.999558179	0.99999488	0.999985264	0.999960414
ZNF212	0.999996191	0.999992444	0.999558179	0.99999488	0.999985264	0.999960414
ZNF214	0.999996191	0.999992444	0.999558179	0.99999488	0.999985264	0.999960414
ZNF23	0.999996191	0.999992444	0.999558179	0.99999488	0.999985264	0.999960414
ZNF235	0.999996191	0.999992444	0.999558179	0.99999488	0.999985264	0.999960414
ZNF236	0.999996191	0.999992444	0.999558179	0.99999488	0.999985264	0.999960414
ZNF24	0.999996191	0.999992444	0.999558179	0.99999488	0.999985264	0.999960414
ZNF280C	0.999996191	0.999992444	0.999558179	0.99999488	0.999985264	0.999960414
ZNF281	0.999996191	0.999992444	0.999558179	0.99999488	0.999985264	0.999960414
ZNF3	0.999996191	0.999992444	0.999558179	0.99999488	0.999985264	0.999960414
ZNF300	0.999996191	0.999992444	0.999558179	0.99999488	0.999985264	0.999960414
ZNF316	0.999996191	0.999992444	0.999558179	0.99999488	0.999985264	0.999960414
ZNF317	0.999996191	0.999992444	0.999558179	0.99999488	0.999985264	0.999960414
ZNF319	0.999996191	0.999992444	0.999558179	0.99999488	0.999985264	0.999960414
ZNF329	0.999996191	0.999992444	0.999558179	0.99999488	0.999985264	0.999960414
ZNF331	0.999996191	0.999992444	0.999558179	0.99999488	0.999985264	0.999960414
ZNF333	0.999996191	0.999992444	0.999558179	0.99999488	0.999985264	0.999960414

ZNF346	0.999996191	0.999992444	0.999558179	0.99999488	0.999985264	0.999960414
ZNF35	0.999996191	0.999992444	0.999558179	0.99999488	0.999985264	0.999960414
ZNF382	0.999996191	0.999992444	0.999558179	0.99999488	0.999985264	0.999960414
ZNF397	0.999996191	0.999992444	0.999558179	0.99999488	0.999985264	0.999960414
ZNF398	0.999996191	0.999992444	0.999558179	0.99999488	0.999985264	0.999960414
ZNF449	0.999996191	0.999992444	0.999558179	0.99999488	0.999985264	0.999960414
ZNF48	0.999996191	0.999992444	0.999558179	0.99999488	0.999985264	0.999960414
ZNF496	0.999996191	0.999992444	0.999558179	0.99999488	0.999985264	0.999960414
ZNF512	0.999996191	0.999992444	0.999558179	0.99999488	0.999985264	0.999960414
ZNF514	0.999996191	0.999992444	0.999558179	0.99999488	0.999985264	0.999960414
ZNF524	0.999996191	0.999992444	0.999558179	0.99999488	0.999985264	0.999960414
ZNF526	0.999996191	0.999992444	0.999558179	0.99999488	0.999985264	0.999960414
ZNF554	0.999996191	0.999992444	0.999558179	0.99999488	0.999985264	0.999960414
ZNF574	0.999996191	0.999992444	0.999558179	0.99999488	0.999985264	0.999960414
ZNF579	0.999996191	0.999992444	0.999558179	0.99999488	0.999985264	0.999960414
ZNF592	0.999996191	0.999992444	0.999558179	0.99999488	0.999985264	0.999960414
ZNF596	0.999996191	0.999992444	0.999558179	0.99999488	0.999985264	0.999960414
ZNF597	0.999996191	0.999992444	0.999558179	0.99999488	0.999985264	0.999960414
ZNF606	0.999996191	0.999992444	0.999558179	0.99999488	0.999985264	0.999960414
ZNF614	0.999996191	0.999992444	0.999558179	0.99999488	0.999985264	0.999960414
ZNF623	0.999996191	0.999992444	0.999558179	0.99999488	0.999985264	0.999960414
ZNF646	0.999996191	0.999992444	0.999558179	0.99999488	0.999985264	0.999960414
ZNF655	0.999996191	0.999992444	0.999558179	0.99999488	0.999985264	0.999960414
ZNF664	0.999996191	0.999992444	0.999558179	0.99999488	0.999985264	0.999960414
ZNF687	0.999996191	0.999992444	0.999558179	0.99999488	0.999985264	0.999960414
ZNF70	0.999996191	0.999992444	0.999558179	0.99999488	0.999985264	0.999960414
ZNF703	0.999996191	0.999992444	0.999558179	0.99999488	0.999985264	0.999960414
ZNF713	0.999996191	0.999992444	0.999558179	0.99999488	0.999985264	0.999960414
ZNF774	0.999996191	0.999992444	0.999558179	0.99999488	0.999985264	0.999960414
ZNF787	0.999996191	0.999992444	0.999558179	0.99999488	0.999985264	0.999960414
ZNF8	0.999996191	0.999992444	0.999558179	0.99999488	0.999985264	0.999960414
ZNF81	0.999996191	0.999992444	0.999558179	0.99999488	0.999985264	0.999960414
ZNF821	0.999996191	0.999992444	0.999558179	0.99999488	0.999985264	0.999960414
ZNF853	0.999996191	0.999992444	0.999558179	0.99999488	0.999985264	0.999960414
ZNF891	0.999996191	0.999992444	0.999558179	0.99999488	0.999985264	0.999960414
ZNRF3	0.999996191	0.999992444	0.999558179	0.99999488	0.999985264	0.999960414
ZRANB3	0.999996191	0.999992444	0.999558179	0.99999488	0.999985264	0.999960414
ZSCAN20	0.999996191	0.999992444	0.999558179	0.99999488	0.999985264	0.999960414
ZSWIM3	0.999996191	0.999992444	0.999558179	0.99999488	0.999985264	0.999960414

ZSWIM9	0.999996191	0.999992444	0.999558179	0.99999488	0.999985264	0.999960414
ZXDB	0.999996191	0.999992444	0.999558179	0.99999488	0.999985264	0.999960414
ZXDC	0.999996191	0.999992444	0.999558179	0.99999488	0.999985264	0.999960414
ZZEF1	0.999996191	0.999992444	0.999558179	0.99999488	0.999985264	0.999960414
GNG10	0.999996191	0.999992444	0.999597443	0.99999488	0.999985264	0.999960414
INO80E	0.999996191	0.999992444	0.999597443	0.99999488	0.999985264	0.999960414
PFKL	0.999996191	0.999992444	0.999597443	0.99999488	0.999985264	0.999960414
LOC101903356	0.999996191	0.999992444	0.999672138	0.99999488	0.999985264	0.999960414
METTL2A	0.999996191	0.999992444	0.999684753	0.99999488	0.999985264	0.999960414
CRAMP1	0.999996191	0.999992444	0.999697966	0.99999488	0.999985264	0.999960414
FHIT	0.999996191	0.999992444	0.999697966	0.99999488	0.999985264	0.999960414
LOC100848815	0.999996191	0.999992444	0.999697966	0.99999488	0.999985264	0.999960414
LOC107131452	0.999996191	0.999992444	0.999697966	0.99999488	0.999985264	0.999960414
LOC112447399	0.999996191	0.999992444	0.999697966	0.99999488	0.999985264	0.999960414
LOC615271	0.999996191	0.999992444	0.999697966	0.99999488	0.999985264	0.999960414
MTRF1L	0.999996191	0.999992444	0.999697966	0.99999488	0.999985264	0.999960414
PIGS	0.999996191	0.999992444	0.999697966	0.99999488	0.999985264	0.999960414
PIM2	0.999996191	0.999992444	0.999697966	0.99999488	0.999985264	0.999960414
TRIM2	0.999996191	0.999992444	0.999697966	0.99999488	0.999985264	0.999960414
ZC3HC1	0.999996191	0.999992444	0.999697966	0.99999488	0.999985264	0.999960414
LOC107131455	0.999996191	0.999992444	0.999705939	0.99999488	0.999985264	0.999960414
SYT4	0.999996191	0.999992444	0.999705939	0.99999488	0.999985264	0.999960414
TRMO	0.999996191	0.999992444	0.999705939	0.99999488	0.999985264	0.999960414
ARHGEF15	0.999996191	0.999992444	0.999936313	0.99999488	0.999985264	0.999960414
SPIN2	0.999996191	0.999992444	0.999936313	0.99999488	0.999985264	0.999960414
CRADD	0.999996191	0.999992444	0.999949305	0.99999488	0.999985264	0.999960414
FMO5	0.999996191	0.999992444	0.999949305	0.99999488	0.999985264	0.999960414
KLRF2	0.999996191	0.999992444	0.999949305	0.99999488	0.999985264	0.999960414
LOC100848138	0.999996191	0.999992444	0.999949305	0.99999488	0.999985264	0.999960414
LOC112446417	0.999996191	0.999992444	0.999949305	0.99999488	0.999985264	0.999960414
LOC788293	0.999996191	0.999992444	0.999949305	0.99999488	0.999985264	0.999960414
SNRPG	0.999996191	0.999992444	0.999949305	0.99999488	0.999985264	0.999960414
ZNF691	0.999996191	0.999992444	0.999949305	0.99999488	0.999985264	0.999960414
RHOD	NaN	NaN	NaN	NaN	NaN	0.211012174
LOC508459	NaN	NaN	NaN	NaN	NaN	0.25556725
AMZ1	NaN	NaN	NaN	NaN	NaN	0.390688306
SVOP	NaN	NaN	NaN	NaN	NaN	0.500888858
REC8	NaN	NaN	NaN	NaN	NaN	0.52928764
GOLGA7B	NaN	NaN	NaN	NaN	NaN	0.541323364

ST8SIA2	NaN	NaN	NaN	NaN	NaN	0.588139353
LOC112444652	NaN	NaN	NaN	NaN	NaN	0.601946079
CXCL11	NaN	NaN	NaN	NaN	NaN	0.6155859
SPCS3	NaN	NaN	NaN	NaN	NaN	0.657047929
LOC100300115	NaN	NaN	NaN	NaN	NaN	0.743798578
LOC784266	NaN	NaN	NaN	NaN	NaN	0.772479356
CD5L	NaN	NaN	NaN	NaN	NaN	0.827190118
SOCS3	NaN	NaN	NaN	NaN	NaN	0.838478213
TMEM216	NaN	NaN	NaN	NaN	NaN	0.850842307
MT1A	NaN	NaN	NaN	NaN	NaN	0.877707746
ACP5	NaN	NaN	NaN	NaN	NaN	0.903974039
LOC112443862	NaN	NaN	NaN	NaN	NaN	0.90444661
LGR5	NaN	NaN	NaN	NaN	NaN	0.925492402
FOSL1	NaN	NaN	NaN	NaN	NaN	0.931163114
LCN2	NaN	NaN	NaN	NaN	NaN	0.939035437
SLC9A2	NaN	NaN	NaN	NaN	NaN	0.939035437
CR2	NaN	NaN	NaN	NaN	NaN	0.961808188
TIMD4	NaN	NaN	NaN	NaN	NaN	0.966921967
CCR9	NaN	NaN	NaN	NaN	NaN	0.98534845
LOC101901948	NaN	NaN	NaN	NaN	NaN	0.999960414
SECTM1A	NaN	NaN	NaN	NaN	NaN	0.999960414
SPTB	NaN	NaN	NaN	NaN	NaN	0.999960414
ADAM8	NaN	NaN	NaN	NaN	0.119428945	NaN
ART3	NaN	NaN	NaN	NaN	0.164984228	NaN
DPP10	NaN	NaN	NaN	NaN	0.240033377	NaN
SAMD9	NaN	NaN	NaN	NaN	0.258000341	NaN
ACBD7	NaN	NaN	NaN	NaN	0.306037672	NaN
CXCL8	NaN	NaN	NaN	NaN	0.323980448	NaN
TRIB3	NaN	NaN	NaN	NaN	0.357979213	NaN
IL1B	NaN	NaN	NaN	NaN	0.425292769	NaN
CDH19	NaN	NaN	NaN	NaN	0.456336784	NaN
PLP1	NaN	NaN	NaN	NaN	0.535395397	NaN
OSM	NaN	NaN	NaN	NaN	0.545924011	NaN
SOX10	NaN	NaN	NaN	NaN	0.546848192	NaN
COL2A1	NaN	NaN	NaN	NaN	0.554002562	NaN
LOC112441507	NaN	NaN	NaN	NaN	0.566923246	NaN
ADAMTS19	NaN	NaN	NaN	NaN	0.588838485	NaN
LUZP2	NaN	NaN	NaN	NaN	0.635384007	NaN
MMP12	NaN	NaN	NaN	NaN	0.655410555	NaN

TTYH1	NaN	NaN	NaN	NaN	0.664686023	NaN
LOC100298356	NaN	NaN	NaN	NaN	0.694282601	NaN
PCDH10	NaN	NaN	NaN	NaN	0.708864153	NaN
MX1	NaN	NaN	NaN	NaN	0.729522487	NaN
SCUBE1	NaN	NaN	NaN	NaN	0.740690296	NaN
MPZ	NaN	NaN	NaN	NaN	0.741080831	NaN
LOC100848536	NaN	NaN	NaN	NaN	0.755784104	NaN
LOC101902787	NaN	NaN	NaN	NaN	0.77926164	NaN
OAS1Y	NaN	NaN	NaN	NaN	0.847202014	NaN
CXHXorf57	NaN	NaN	NaN	NaN	0.901104314	NaN
FOXD1	NaN	NaN	NaN	NaN	0.901763837	NaN
TMPRSS5	NaN	NaN	NaN	NaN	0.908980492	NaN
LOC507055	NaN	NaN	NaN	NaN	0.934591321	NaN
LOC100847981	NaN	NaN	NaN	NaN	0.944638139	NaN
SLC35F1	NaN	NaN	NaN	NaN	0.965672975	NaN
BOLA	NaN	NaN	NaN	NaN	0.999985264	NaN
C19H17orf58	NaN	NaN	NaN	NaN	0.999985264	NaN
CADM4	NaN	NaN	NaN	NaN	0.999985264	NaN
CCL17	NaN	NaN	NaN	NaN	0.999985264	NaN
DDX58	NaN	NaN	NaN	NaN	0.999985264	NaN
DEFB7	NaN	NaN	NaN	NaN	0.999985264	NaN
DNAH9	NaN	NaN	NaN	NaN	0.999985264	NaN
FOXD3	NaN	NaN	NaN	NaN	0.999985264	NaN
FREM2	NaN	NaN	NaN	NaN	0.999985264	NaN
GABRB3	NaN	NaN	NaN	NaN	0.999985264	NaN
GRIK4	NaN	NaN	NaN	NaN	0.999985264	NaN
GYS2	NaN	NaN	NaN	NaN	0.999985264	NaN
IFIH1	NaN	NaN	NaN	NaN	0.999985264	NaN
IGSF11	NaN	NaN	NaN	NaN	0.999985264	NaN
IGSF9B	NaN	NaN	NaN	NaN	0.999985264	NaN
ISG15	NaN	NaN	NaN	NaN	0.999985264	NaN
JAKMIP2	NaN	NaN	NaN	NaN	0.999985264	NaN
LGI1	NaN	NaN	NaN	NaN	0.999985264	NaN
LOC101905509	NaN	NaN	NaN	NaN	0.999985264	NaN
LOC112441557	NaN	NaN	NaN	NaN	0.999985264	NaN
LOC505033	NaN	NaN	NaN	NaN	0.999985264	NaN
LOC509283	NaN	NaN	NaN	NaN	0.999985264	NaN
PDZRN4	NaN	NaN	NaN	NaN	0.999985264	NaN
RSAD2	NaN	NaN	NaN	NaN	0.999985264	NaN

SGCG	NaN	NaN	NaN	NaN			0.999985264	NaN
SNAP25	NaN	NaN	NaN	NaN			0.999985264	NaN
SNCA	NaN	NaN	NaN	NaN			0.999985264	NaN
SPOCK1	NaN	NaN	NaN	NaN			0.999985264	NaN
TNFRSF11B	NaN	NaN	NaN	NaN			0.999985264	NaN
WNT6	NaN	NaN	NaN	NaN			0.999985264	NaN
PPM1E	NaN	NaN	NaN		0.011172292	NaN		NaN
LOC616830	NaN	NaN	NaN		0.322360579	NaN		NaN
LAMC3	NaN	NaN	NaN		0.489113434	NaN		NaN
LOC104968484	NaN	NaN	NaN		0.543066914	NaN		NaN
TGM5	NaN	NaN	NaN		0.548239725	NaN		NaN
CERS3	NaN	NaN	NaN		0.695630809	NaN		NaN
FCGR2A	NaN	NaN	NaN		0.803237525	NaN		NaN
LOC101905242	NaN	NaN	NaN		0.831276941	NaN		NaN
DPP4	NaN	NaN	NaN		0.869065101	NaN		NaN
ATP10B	NaN	NaN	NaN		0.870282231	NaN		NaN
LOC783106	NaN	NaN	NaN		0.880829722	NaN		NaN
G6PD	NaN	NaN	NaN		0.917553107	NaN		NaN
SERHL2	NaN	NaN	NaN		0.921764051	NaN		NaN
MID1IP1	NaN	NaN	NaN		0.931835237	NaN		NaN
ARHGAP11A	NaN	NaN	NaN		0.949602784	NaN		NaN
SLC25A4	NaN	NaN	NaN		0.949602784	NaN		NaN
SPRY3	NaN	NaN	NaN		0.949602784	NaN		NaN
KIF2C	NaN	NaN	NaN		0.959611215	NaN		NaN
MARCO	NaN	NaN	NaN		0.964626155	NaN		NaN
SNRNP25	NaN	NaN	NaN		0.967551965	NaN		NaN
SLC16A1	NaN	NaN	NaN		0.969770561	NaN		NaN
HMOX1	NaN	NaN	NaN		0.98846246	NaN		NaN
MPC1	NaN	NaN	NaN		0.991596938	NaN		NaN
YDJC	NaN	NaN	NaN		0.99579057	NaN		NaN
ABAT	NaN	NaN	NaN		0.99999488	NaN		NaN
ACOT2	NaN	NaN	NaN		0.99999488	NaN		NaN
ALB	NaN	NaN	NaN		0.99999488	NaN		NaN
BIRC5	NaN	NaN	NaN		0.99999488	NaN		NaN
BOLA3	NaN	NaN	NaN		0.99999488	NaN		NaN
BSG	NaN	NaN	NaN		0.99999488	NaN		NaN
BUB1B	NaN	NaN	NaN		0.99999488	NaN		NaN
CATHL5	NaN	NaN	NaN		0.99999488	NaN		NaN
CCDC73	NaN	NaN	NaN		0.99999488	NaN		NaN

CDC6	NaN	NaN	NaN	0.99999488	NaN	NaN
CDCA8	NaN	NaN	NaN	0.99999488	NaN	NaN
CDKN2A	NaN	NaN	NaN	0.99999488	NaN	NaN
CKB	NaN	NaN	NaN	0.99999488	NaN	NaN
COBL	NaN	NaN	NaN	0.99999488	NaN	NaN
CYP21	NaN	NaN	NaN	0.99999488	NaN	NaN
CYP46A1	NaN	NaN	NaN	0.99999488	NaN	NaN
ELOVL6	NaN	NaN	NaN	0.99999488	NaN	NaN
FAM49B	NaN	NaN	NaN	0.99999488	NaN	NaN
FGB	NaN	NaN	NaN	0.99999488	NaN	NaN
GPT2	NaN	NaN	NaN	0.99999488	NaN	NaN
GSS	NaN	NaN	NaN	0.99999488	NaN	NaN
GSTM1	NaN	NaN	NaN	0.99999488	NaN	NaN
IRX1	NaN	NaN	NaN	0.99999488	NaN	NaN
KCNA4	NaN	NaN	NaN	0.99999488	NaN	NaN
KCNK2	NaN	NaN	NaN	0.99999488	NaN	NaN
KCNN2	NaN	NaN	NaN	0.99999488	NaN	NaN
KIF11	NaN	NaN	NaN	0.99999488	NaN	NaN
KIF20A	NaN	NaN	NaN	0.99999488	NaN	NaN
KLHL31	NaN	NaN	NaN	0.99999488	NaN	NaN
LDHB	NaN	NaN	NaN	0.99999488	NaN	NaN
LIN7A	NaN	NaN	NaN	0.99999488	NaN	NaN
LOC100138641	NaN	NaN	NaN	0.99999488	NaN	NaN
LOC100301224	NaN	NaN	NaN	0.99999488	NaN	NaN
LOC100851369	NaN	NaN	NaN	0.99999488	NaN	NaN
LOC101907335	NaN	NaN	NaN	0.99999488	NaN	NaN
LOC104973965	NaN	NaN	NaN	0.99999488	NaN	NaN
LOC104974444	NaN	NaN	NaN	0.99999488	NaN	NaN
LOC112443013	NaN	NaN	NaN	0.99999488	NaN	NaN
LOC112445090	NaN	NaN	NaN	0.99999488	NaN	NaN
LOC112448034	NaN	NaN	NaN	0.99999488	NaN	NaN
LOC513210	NaN	NaN	NaN	0.99999488	NaN	NaN
LOC618297	NaN	NaN	NaN	0.99999488	NaN	NaN
MLXIPL	NaN	NaN	NaN	0.99999488	NaN	NaN
PARD6B	NaN	NaN	NaN	0.99999488	NaN	NaN
PBLD	NaN	NaN	NaN	0.99999488	NaN	NaN
PCLAF	NaN	NaN	NaN	0.99999488	NaN	NaN
PIR	NaN	NaN	NaN	0.99999488	NaN	NaN
PTCH2	NaN	NaN	NaN	0.99999488	NaN	NaN

PTPRQ	NaN	NaN	NaN			0.99999488	NaN	NaN
RRM2	NaN	NaN	NaN			0.99999488	NaN	NaN
SCP2	NaN	NaN	NaN			0.99999488	NaN	NaN
SH3RF2	NaN	NaN	NaN			0.99999488	NaN	NaN
SLC13A3	NaN	NaN	NaN			0.99999488	NaN	NaN
SLC16A7	NaN	NaN	NaN			0.99999488	NaN	NaN
SLC5A9	NaN	NaN	NaN			0.99999488	NaN	NaN
SRXN1	NaN	NaN	NaN			0.99999488	NaN	NaN
TDH	NaN	NaN	NaN			0.99999488	NaN	NaN
TECTB	NaN	NaN	NaN			0.99999488	NaN	NaN
TKT	NaN	NaN	NaN			0.99999488	NaN	NaN
TMEM179	NaN	NaN	NaN			0.99999488	NaN	NaN
TRIM44	NaN	NaN	NaN			0.99999488	NaN	NaN
UBE2C	NaN	NaN	NaN			0.99999488	NaN	NaN
UCHL3	NaN	NaN	NaN			0.99999488	NaN	NaN
UCMA	NaN	NaN	NaN			0.99999488	NaN	NaN
UHRF1	NaN	NaN	NaN			0.99999488	NaN	NaN
LOC100847119	NaN	NaN		0.006093607	NaN		NaN	NaN
LOC100297192	NaN	NaN		0.042065882	NaN		NaN	NaN
PRSS35	NaN	NaN		0.139653829	NaN		NaN	NaN
LOC112442062	NaN	NaN		0.179774584	NaN		NaN	NaN
FAM83D	NaN	NaN		0.224307217	NaN		NaN	NaN
EEF1A2	NaN	NaN		0.311949976	NaN		NaN	NaN
MUSK	NaN	NaN		0.387533191	NaN		NaN	NaN
P2RX1	NaN	NaN		0.405095497	NaN		NaN	NaN
PTGIR	NaN	NaN		0.525355249	NaN		NaN	NaN
MAPK4	NaN	NaN		0.575199502	NaN		NaN	NaN
SIX2	NaN	NaN		0.580363758	NaN		NaN	NaN
LOC100847724	NaN	NaN		0.598765064	NaN		NaN	NaN
PPDPFL	NaN	NaN		0.683588966	NaN		NaN	NaN
COL24A1	NaN	NaN		0.718226122	NaN		NaN	NaN
HAND2	NaN	NaN		0.730374662	NaN		NaN	NaN
HP	NaN	NaN		0.75028318	NaN		NaN	NaN
LOC509911	NaN	NaN		0.787006358	NaN		NaN	NaN
OPCML	NaN	NaN		0.829269849	NaN		NaN	NaN
HPSE2	NaN	NaN		0.857021454	NaN		NaN	NaN
NPFFR2	NaN	NaN		0.877799478	NaN		NaN	NaN
LOC511683	NaN	NaN		0.886962419	NaN		NaN	NaN
LOC112441777	NaN	NaN		0.893442581	NaN		NaN	NaN

LOC104974455	NaN	NaN	0.895962167	NaN	NaN	NaN
LOC112447816	NaN	NaN	0.933177263	NaN	NaN	NaN
PHF21B	NaN	NaN	0.95230212	NaN	NaN	NaN
LOC112446726	NaN	NaN	0.954864482	NaN	NaN	NaN
LOC104976942	NaN	NaN	0.961490767	NaN	NaN	NaN
LOC100847415	NaN	NaN	0.969880768	NaN	NaN	NaN
RASL12	NaN	NaN	0.972362683	NaN	NaN	NaN
DMRT3	NaN	NaN	0.973303821	NaN	NaN	NaN
ADRA2C	NaN	NaN	0.981039752	NaN	NaN	NaN
ACTA1	NaN	NaN	0.982065621	NaN	NaN	NaN
A2ML1	NaN	NaN	0.98286145	NaN	NaN	NaN
LOC516421	NaN	NaN	0.998936876	NaN	NaN	NaN
C10H15orf62	NaN	NaN	0.999558179	NaN	NaN	NaN
LOC785161	NaN	NaN	0.999558179	NaN	NaN	NaN
LOC514978	NaN	0.002395692	NaN	NaN	NaN	NaN
TMPRSS2	NaN	0.10000994	NaN	NaN	NaN	NaN
PLA2G2D1	NaN	0.205058781	NaN	NaN	NaN	NaN
BCL2L15	NaN	0.205375425	NaN	NaN	NaN	NaN
LOC789829	NaN	0.225280182	NaN	NaN	NaN	NaN
CKMT1A	NaN	0.235570183	NaN	NaN	NaN	NaN
UCP1	NaN	0.270621658	NaN	NaN	NaN	NaN
LOC101906743	NaN	0.287853656	NaN	NaN	NaN	NaN
CHAD	NaN	0.337920983	NaN	NaN	NaN	NaN
OAS1Z	NaN	0.38625193	NaN	NaN	NaN	NaN
CDH17	NaN	0.398462449	NaN	NaN	NaN	NaN
CA12	NaN	0.411165859	NaN	NaN	NaN	NaN
LOC781796	NaN	0.447339842	NaN	NaN	NaN	NaN
VIL1	NaN	0.447339842	NaN	NaN	NaN	NaN
ALDOB	NaN	0.614974807	NaN	NaN	NaN	NaN
DNAJB13	NaN	0.736960298	NaN	NaN	NaN	NaN
LOC530653	NaN	0.775729202	NaN	NaN	NaN	NaN
APOA4	NaN	0.78130286	NaN	NaN	NaN	NaN
RIPK4	NaN	0.797034906	NaN	NaN	NaN	NaN
LOC616782	NaN	0.816100073	NaN	NaN	NaN	NaN
MYO1A	NaN	0.827123416	NaN	NaN	NaN	NaN
TUBA1D	NaN	0.835638623	NaN	NaN	NaN	NaN
FZD5	NaN	0.835868428	NaN	NaN	NaN	NaN
LOC104974214	NaN	0.838314316	NaN	NaN	NaN	NaN
SLC6A8	NaN	0.847051507	NaN	NaN	NaN	NaN

LOC100139885	NaN	0.850692013	NaN	NaN	NaN	NaN
LTF	NaN	0.859484941	NaN	NaN	NaN	NaN
MISP	NaN	0.862811597	NaN	NaN	NaN	NaN
HNF4G	NaN	0.893748757	NaN	NaN	NaN	NaN
CHGA	NaN	0.921759937	NaN	NaN	NaN	NaN
KIAA1211L	NaN	0.925580392	NaN	NaN	NaN	NaN
SLC9A3	NaN	0.933208464	NaN	NaN	NaN	NaN
CRB2	NaN	0.934142668	NaN	NaN	NaN	NaN
FAM3B	NaN	0.938476452	NaN	NaN	NaN	NaN
ST14	NaN	0.952639631	NaN	NaN	NaN	NaN
CLDN3	NaN	0.967276215	NaN	NaN	NaN	NaN
PDZK1	NaN	0.978089982	NaN	NaN	NaN	NaN
PLEK2	NaN	0.985406717	NaN	NaN	NaN	NaN
CCL5	NaN	0.991365955	NaN	NaN	NaN	NaN
LOC101903734	NaN	0.991365955	NaN	NaN	NaN	NaN
GRIA2	NaN	0.992977597	NaN	NaN	NaN	NaN
LOC781736	NaN	0.99873048	NaN	NaN	NaN	NaN
SLC51B	NaN	0.999422917	NaN	NaN	NaN	NaN
BAIAP2L2	NaN	0.999992444	NaN	NaN	NaN	NaN
DSG2	NaN	0.999992444	NaN	NaN	NaN	NaN
GUCY2C	NaN	0.999992444	NaN	NaN	NaN	NaN
LOC100140226	NaN	0.999992444	NaN	NaN	NaN	NaN
LOC112447079	NaN	0.999992444	NaN	NaN	NaN	NaN
SLC15A1	NaN	0.999992444	NaN	NaN	NaN	NaN
TMEM45B	NaN	0.999992444	NaN	NaN	NaN	NaN
ACTN2	NaN	NaN	NaN	NaN	NaN	NaN
C4BPA	NaN	NaN	NaN	NaN	NaN	NaN
CA13	NaN	NaN	NaN	NaN	NaN	NaN
CFHR5	NaN	NaN	NaN	NaN	NaN	NaN
FCGBP	NaN	NaN	NaN	NaN	NaN	NaN
GSTA3	NaN	NaN	NaN	NaN	NaN	NaN
IGLL1	NaN	NaN	NaN	NaN	NaN	NaN
IGSF5	NaN	NaN	NaN	NaN	NaN	NaN
LOC100139670	NaN	NaN	NaN	NaN	NaN	NaN
LOC100297779	NaN	NaN	NaN	NaN	NaN	NaN
LOC101903284	NaN	NaN	NaN	NaN	NaN	NaN
LOC107131864	NaN	NaN	NaN	NaN	NaN	NaN
LOC107131942	NaN	NaN	NaN	NaN	NaN	NaN
LOC112446680	NaN	NaN	NaN	NaN	NaN	NaN

LOC515676	NaN	NaN	NaN	NaN	NaN	NaN
LOC519274	NaN	NaN	NaN	NaN	NaN	NaN
LOC615051	NaN	NaN	NaN	NaN	NaN	NaN
MEF2B	NaN	NaN	NaN	NaN	NaN	NaN
MEGF11	NaN	NaN	NaN	NaN	NaN	NaN
MOXD1	NaN	NaN	NaN	NaN	NaN	NaN
MX2	NaN	NaN	NaN	NaN	NaN	NaN
NAALADL1	NaN	NaN	NaN	NaN	NaN	NaN
PEBP4	NaN	NaN	NaN	NaN	NaN	NaN
PKD2L1	NaN	NaN	NaN	NaN	NaN	NaN
SPHAR	NaN	NaN	NaN	NaN	NaN	NaN
ZBTB37	NaN	NaN	NaN	NaN	NaN	NaN
ZDHHC23	NaN	NaN	NaN	NaN	NaN	NaN
