# Supplementary material for: Genes associated with body weight gain and feed intake identified by meta-analysis of the mesenteric fat from crossbred beef steers
Source: PLoS One. 2020 Jan 7;15(1):e0227154. doi: 10.1371/journal.pone.0227154 (PMC6946124; doi:10.1371/journal.pone.0227154)
Supplement: S6 Table — Jackknife 1 P-value gives the adjusted P-value for the meta-analysis with Cohort 1 removed, Jackknife 2 P-value gives the adjusted P-value for the meta-analysis with Cohort 2 removed, and so on. Yellow cells indicate jackknife analyses where the P-value was insignificant, i.e. the gene failed to pass the jackknife analysis. (PDF) [file pone.0227154.s006.pdf]

Supplemental Table 6. Jackknife sensitivity analysis results for the DEGs associated with the gain by intake interaction. Yellow cells indicate jackknife analyses where the P-value was insignificant, i.e. the gene failed to pass the jackknife analysis.

Gene	Original P-value	Jackknife 1 P-value	Jackknife 2 P-value	Jackknife 3 P-value	Jackknife 4 P-value	Jackknife 5 P-value
LOC100300716	4.27E-05	0.025140695	0.006391975	0.046426379	2.07E-05	2.72E-05
PTAFR	0.000466231	0.002103816	0.000404612	0.216038971	0.005444437	0.021972873
FST	0.002606736	0.093934033	0.012662975	0.012965047	0.005191068	0.051555143
AACS	0.016756576	0.021680662	0.006391975	0.046426379	0.557535797	0.05600716
H19	0.02277232	0.143024392	0.105089027	0.078055201	0.090081289	0.015993852
PDLIM5	0.026224822	0.025140695	0.812258905	0.024526301	0.090081289	0.017971283
MRPL14	0.026224822	0.261833331	0.035013691	0.024526301	0.348579075	0.021972873
RCOR2	0.026224822	0.033794357	0.02066225	0.024526301	0.557535797	0.084765086
HPS6	0.036476044	0.025140695	0.035013691	0.024526301	0.886279726	0.065043003
IL18BP	0.042765245	0.025140695	0.041123306	0.225840846	0.029455319	0.610416296
GLCE	0.048500326	0.031095541	0.035013691	0.925901365	0.129183245	0.021972873
JCHAIN	0.05109403	0.246642896	0.123436199	0.285302249	0.090081289	0.024379244
PDE4B	0.062160292	0.040498382	0.035013691	0.95175364	0.090081289	0.051555143
FCER1A	0.062160292	0.084165947	0.099028551	0.167313685	0.191201528	0.16831485
RASSF9	0.062160292	0.040498382	0.035013691	0.720889501	0.387398824	0.051555143
CREB3L1	0.062160292	0.047913864	0.099028551	0.279362388	0.428386725	0.051555143
OLFML3	0.062160292	0.047913864	0.055822562	0.232472259	0.575621913	0.051555143
CD5	0.06233403	0.226249144	0.035013691	0.046426379	0.348579075	0.175271238
TRIP12	0.075500256	0.047913864	0.053379604	0.720889501	0.412271251	0.051555143
LOC101906455	0.07610185	0.047913864	0.849077738	0.091112279	0.090081289	0.089880277
SPP1	0.07610185	0.044250974	0.053379604	0.311184619	0.090081289	0.728276429
KCNK12	0.07610185	0.051343645	0.468416519	0.438283629	0.105653569	0.068652486
FAM210B	0.07610185	0.043265835	0.053379604	0.915749182	0.105653569	0.078244821
CHI3L1	0.07610185	0.060448731	0.702276506	0.216038971	0.145233548	0.051555143
KPNA1	0.07610185	0.05334035	0.549314874	0.348670606	0.16600146	0.051555143
LOC782367	0.07610185	0.297475421	0.114346425	0.089438851	0.204360066	0.16831485
PTK7	0.07610185	0.047913864	0.242438335	0.563012044	0.286254203	0.051555143
SRPX	0.07610185	0.084573771	0.115105377	0.216038971	0.674717419	0.051555143
PDE4D	0.07610185	0.104751445	0.053379604	0.089438851	0.882754591	0.051555143
BTAF1	0.07610185	0.073999168	0.041123306	0.059449008	0.99989309	0.051555143
LOC112445144	0.080573338	0.047913864	0.047063836	0.438283629	0.819670067	0.051555143
CAB39	0.081045291	0.047913864	0.602931071	0.321633652	0.204360066	0.073148059
TNNT3	0.081045291	0.084573771	0.198823488	0.472816297	0.316291551	0.0808165
LOC789607	0.081045291	0.093934033	0.144935647	0.089438851	0.87465791	0.051555143
LOC100138922	0.093279052	0.05334035	0.051143992	0.830830186	0.467659476	0.051555143

LOC107131807	0.094153623	0.05334035	0.056459917	0.999939566	0.090081289	0.084765086
SYS1	0.094153623	0.05334035	0.917092799	0.224725341	0.105653569	0.051555143
MXRA5	0.094153623	0.05334035	0.29012228	0.825933581	0.129183245	0.051555143
KCNA3	0.094153623	0.105526596	0.053379604	0.216038971	0.145233548	0.77208617
EPM2AIP1	0.094153623	0.060448731	0.053379604	0.781241796	0.348579075	0.109906112
ELOVL3	0.094153623	0.093934033	0.067211301	0.6044852	0.348579075	0.169732915
FAM124B	0.094153623	0.051343645	0.055822562	0.806922554	0.422902715	0.073148059
INSIG1	0.094153623	0.155296686	0.06054081	0.089438851	0.476180257	0.307062312
RNF165	0.094153623	0.05334035	0.076227561	0.438283629	0.725609902	0.089880277
CCDC117	0.094153623	0.103894006	0.068071904	0.089438851	0.99989309	0.067778583
LOC107132475	0.102491005	0.05334035	0.114346425	0.147903015	0.666979426	0.349092138
CBARP	0.103606391	0.093934033	0.053379604	0.721039637	0.519859295	0.089880277
TIMM13	0.132842195	0.16406369	0.114346425	0.348670606	0.472422117	0.175271238
SH2B2	0.149143959	0.129009789	0.066147321	0.835232007	0.380986498	0.089880277
EMB	0.156636123	0.226249144	0.236666325	0.750804902	0.145233548	0.089880277
WAPL	0.156636123	0.093934033	0.318905523	0.216038971	0.809298212	0.088999547
ACSL6	0.162432078	0.096267072	0.144935647	0.225840846	0.412271251	0.578719809
CDCA4	0.170925284	0.093934033	0.335119813	0.472816297	0.148898015	0.477051336
ADAMTS17	0.170925284	0.133031513	0.442490839	0.731974857	0.1494725	0.137045199
COL5A1	0.170925284	0.142585466	0.442490839	0.727339718	0.204360066	0.093246673
ADAM12	0.170925284	0.104751445	0.529161538	0.638365897	0.286254203	0.11000926
NID2	0.170925284	0.153057998	0.114346425	0.279362388	0.309965206	0.661937165
LOC101906101	0.170925284	0.16406369	0.159737651	0.731974857	0.348579075	0.137045199
UNC5A	0.170925284	0.104751445	0.209869752	0.806922554	0.412271251	0.084765086
WDR13	0.170925284	0.105526596	0.744499269	0.216038971	0.412271251	0.093246673
SVEP1	0.170925284	0.155296686	0.29012228	0.328938275	0.639769128	0.093246673
DDX3X	0.170925284	0.146009135	0.252685075	0.422062779	0.675325659	0.089880277
INTS6	0.170925284	0.093934033	0.132558004	0.167313685	0.99989309	0.088999547
HSPH1	0.170925284	0.164916074	0.105978862	0.17319008	0.99989309	0.089880277
MZF1	0.174083393	0.093934033	0.682591947	0.721039637	0.1494725	0.088999547
HSD11B2	0.184784934	0.287735709	0.099028551	0.587474951	0.575621913	0.089880277
MAPRE1	0.202091222	0.093934033	0.827125538	0.472816297	0.244493745	0.089880277
CREM	0.203923321	0.208370547	0.114346425	0.225840846	0.99989309	0.089880277
ZNF684	0.207432027	0.093934033	0.198823488	0.486299109	0.779159678	0.157631956
MAT2A	0.209377655	0.153888814	0.245005989	0.820228145	0.348579075	0.089880277
TTC38	0.212125102	0.146009135	0.318905523	0.841828571	0.16600146	0.169732915
KCTD10	0.212125102	0.120203792	0.976719499	0.279362388	0.191201528	0.089880277
LOC101902413	0.212125102	0.146009135	0.226649284	0.721039637	0.222805789	0.366284508

LOC101906565	0.212125102	0.105526596	0.90002174	0.299077338	0.245989122	0.152705879
PUS10	0.212125102	0.093934033	0.947268948	0.225840846	0.286254203	0.111581674
LOC101905979	0.212125102	0.104751445	0.726668529	0.376311552	0.328429317	0.185234614
GPT	0.212125102	0.120203792	0.132558004	0.948172741	0.343392796	0.158542897
GALNT5	0.212125102	0.105526596	0.726668529	0.503800558	0.348579075	0.089880277
PAK5	0.212125102	0.404916922	0.142738185	0.472816297	0.429633082	0.17340875
KBTBD8	0.213411088	0.129009789	0.144935647	0.820228145	0.191201528	0.474949941
NTNG1	0.213411088	0.865599955	0.137312219	0.376311552	0.239369296	0.109906112
KCP	0.213411088	0.410916047	0.159737651	0.664346372	0.348579075	0.157631956
PTGS2	0.213411088	0.166689794	0.373939346	0.5065118	0.348579075	0.26883667
LYZ	0.213411088	0.146009135	0.258881153	0.742618602	0.487161254	0.138494038
CCDC188	0.213411088	0.105526596	0.684005375	0.225840846	0.570932145	0.157631956
ADCK5	0.213603781	0.129009789	0.751496241	0.575695951	0.316291551	0.09411184
MAP2K6	0.213603781	0.153057998	0.115105377	0.969717497	0.3195083	0.159467692
COL21A1	0.213603781	0.142585466	0.318905523	0.519421263	0.55440643	0.176823639
SLC30A4	0.213603781	0.120203792	0.193448607	0.445520524	0.906183446	0.168494828
CXCL13	0.216044443	0.258471892	0.134220431	0.763060676	0.286254203	0.316567316
GZMB	0.222059928	0.238519097	0.176891968	0.855177907	0.353874732	0.106946256
NEIL1	0.222700534	0.184134038	0.142738185	0.412632272	0.996250445	0.111581674
LOC112441594	0.223249614	0.133031513	0.80326876	0.40149038	0.286254203	0.176823639
COL6A5	0.226093936	0.26690502	0.170943533	0.577746499	0.422902715	0.297377614
CLN5	0.226388048	0.146009135	0.226649284	0.999939566	0.222805789	0.111581674
ANXA9	0.226388048	0.234754876	0.147165838	0.851394268	0.286254203	0.23037647
GIMAP7	0.226388048	0.171042729	0.209869752	0.472816297	0.348579075	0.550542614
SMU1	0.226388048	0.16406369	0.276229568	0.820228145	0.422902715	0.11856704
MTFP1	0.226388048	0.261833331	0.147165838	0.441752472	0.538639002	0.368219725
LOC112447770	0.229493737	0.404916922	0.198823488	0.331327902	0.348579075	0.419654233
HOGA1	0.229493737	0.29140192	0.132558004	0.376311552	0.886279726	0.163208215
LOC506828	0.230223033	0.16406369	0.411196743	0.820228145	0.204360066	0.184970544
GCAT	0.230223033	0.146009135	0.170943533	0.225840846	0.99989309	0.175271238
CACNA1G	0.233160518	0.129009789	0.29012228	0.969717497	0.286254203	0.140713373
MVD	0.238494849	0.142585466	0.170943533	0.225840846	0.784855148	0.578699613
TPBG	0.24243725	0.400929909	0.413677672	0.638365897	0.3195083	0.137045199
CACNA1A	0.24872476	0.155185635	0.631413719	0.303656584	0.581593891	0.176823639
KHDC4	0.24872476	0.158098084	0.198823488	0.225840846	0.99989309	0.222265603
TRAF3	0.250374562	0.160225733	0.17468289	0.513544	0.642463841	0.361339031
SDS	0.250589278	0.129009789	0.300083198	0.709187504	0.570932145	0.243463286
SLC14A1	0.254586668	0.261833331	0.443645982	0.321633652	0.472422117	0.31118166

RYBP	0.254586668	0.155296686	0.381755045	0.438283629	0.77278187	0.169732915
CYP4V2	0.25627978	0.538091051	0.373939346	0.472816297	0.348579075	0.142106596
HSPB3	0.26548619	0.142585466	0.198823488	0.850828215	0.642463841	0.136745313
KLK7	0.276226636	0.173576127	0.442819817	0.781241796	0.348579075	0.182332401
JAML	0.276226636	0.404916922	0.258881153	0.472816297	0.457751601	0.29669025
WT1	0.276226636	0.155296686	0.720067907	0.462440418	0.513734713	0.16831485
SH3TC1	0.276226636	0.170847421	0.29012228	0.781241796	0.550221271	0.169732915
LOC112445943	0.276226636	0.390662449	0.198823488	0.303656584	0.60923233	0.356054774
SNX16	0.276333119	0.144480598	0.226649284	0.90419464	0.47208906	0.200537512
NIP7	0.28370758	0.16406369	0.703042171	0.328938275	0.422902715	0.307062312
NIF3L1	0.28370758	0.158098084	0.17468289	0.306696545	0.753418479	0.610416296
ANKRD17	0.28370758	0.199705433	0.193448607	0.306696545	0.99989309	0.142106596
SLC6A16	0.284142595	0.841629926	0.170943533	0.596023251	0.286254203	0.169732915
ADAM19	0.284142595	0.155296686	0.258881153	0.927618239	0.286254203	0.335320707
WDR90	0.284142595	0.146009135	0.84311048	0.472816297	0.296442957	0.263186262
MYLK	0.284142595	0.804860126	0.318905523	0.34433942	0.298761465	0.185012125
ECM1	0.284142595	0.146009135	0.455548287	0.721039637	0.3195083	0.493149999
IMPA2	0.284142595	0.142585466	0.209869752	0.781241796	0.328429317	0.611777732
SCYL2	0.284142595	0.153057998	0.746957417	0.638365897	0.348579075	0.182332401
TP53INP1	0.284142595	0.209294085	0.198823488	0.883885658	0.348579075	0.284081535
ITGAD	0.284142595	0.143024392	0.413677672	0.5065118	0.422902715	0.555358122
TMEM236	0.284142595	0.146009135	0.198823488	0.825933581	0.428386725	0.376125338
ICAM3	0.284142595	0.16406369	0.206790374	0.731974857	0.487161254	0.407408224
IPO7	0.284142595	0.184134038	0.601873896	0.5065118	0.570932145	0.176823639
CD93	0.284142595	0.193758262	0.17468289	0.788938193	0.713082855	0.174119693
C4H7orf25	0.284142595	0.146009135	0.318905523	0.438283629	0.780497593	0.344621529
SLC32A1	0.284142595	0.146009135	0.595695785	0.303656584	0.784855148	0.243463286
ALKBH1	0.284142595	0.277352418	0.198823488	0.279362388	0.99989309	0.16831485
TOP1	0.284142595	0.187297679	0.236666325	0.303656584	0.99989309	0.210807578
PRMT7	0.284142595	0.153057998	0.170943533	0.306696545	0.99989309	0.273167681
TFRC	0.288456178	0.419875993	0.620860761	0.328938275	0.348579075	0.262589794
SOX4	0.28915433	0.16099824	0.198823488	0.999939566	0.286254203	0.16831485
POSTN	0.28915433	0.315941541	0.378250914	0.875409325	0.3195083	0.169732915
TAS1R3	0.28915433	0.341210765	0.607812429	0.721039637	0.3195083	0.175271238
XK	0.28915433	0.210298923	0.541411887	0.843411019	0.3195083	0.175271238
GSE1	0.28915433	0.155296686	0.29012228	0.976007283	0.3195083	0.27364105
HAS3	0.28915433	0.16406369	0.198823488	0.999939566	0.320563941	0.170471271
H1F0	0.28915433	0.16517468	0.193448607	0.999939566	0.348579075	0.187244829

TUBB	0.28915433	0.261087908	0.226649284	0.820228145	0.348579075	0.378863449
CLINT1	0.28915433	0.6343277	0.242438335	0.422062779	0.348579075	0.383793067
LOC615278	0.28915433	0.261833331	0.193448607	0.709187504	0.348579075	0.610416296
SMTNL2	0.28915433	0.171042729	0.198823488	0.958879502	0.467659476	0.187772376
WDR86	0.28915433	0.305298878	0.482588959	0.650028997	0.530828834	0.182332401
LOC101907985	0.28915433	0.146009135	0.198823488	0.9846464	0.534940753	0.16831485
IL7R	0.28915433	0.261833331	0.258881153	0.472161229	0.550221271	0.520677492
PTN	0.28915433	0.246223229	0.442819817	0.720889501	0.55440643	0.16831485
PLEKHA6	0.28915433	0.158098084	0.684005375	0.457214186	0.55440643	0.243463286
L3MBTL2	0.28915433	0.153057998	0.325582863	0.438283629	0.570932145	0.637306705
PUM2	0.28915433	0.261833331	0.29012228	0.742618602	0.608328153	0.17340875
LOC784052	0.28915433	0.208396065	0.30853667	0.438283629	0.981777076	0.17340875
PKNOX1	0.28915433	0.277352418	0.198823488	0.303656584	0.99989309	0.16831485
PYCR2	0.28915433	0.155296686	0.247055391	0.376311552	0.99989309	0.16831485
TPST1	0.28915433	0.153888814	0.240130639	0.376311552	0.99989309	0.175271238
GSTT2	0.28915433	0.218676107	0.271251081	0.321633652	0.99989309	0.214735852
TYSND1	0.28915433	0.155185635	0.198823488	0.299077338	0.99989309	0.238340156
NCR1	0.29170627	0.226249144	0.198823488	0.832191555	0.487161254	0.321747892
PIEZO2	0.294388099	0.204093734	0.392704601	0.898441424	0.428386725	0.16831485
DCAF1	0.294388099	0.225358651	0.198823488	0.34433942	0.99989309	0.16831485
MZB1	0.294902391	0.261833331	0.545279742	0.781241796	0.348579075	0.210807578
SMPD3	0.294902391	0.158098084	0.295722872	0.925901365	0.348579075	0.289685975
IGFBP2	0.294902391	0.158098084	0.768676249	0.462440418	0.559664539	0.184970544
CCR2	0.294902391	0.257077268	0.230311454	0.472816297	0.581593891	0.598502354
JAK1	0.294902391	0.155185635	0.266052579	0.90419464	0.659374254	0.16831485
IFRD1	0.294902391	0.24639569	0.295722872	0.5065118	0.730791761	0.326183134
SMARCA5	0.294902391	0.190726663	0.269208198	0.664203576	0.934859184	0.174119693
C7H19orf25	0.294902391	0.226249144	0.198823488	0.638365897	0.954501767	0.182332401
PLEKHJ1	0.305803826	0.182202816	0.684005375	0.5065118	0.348579075	0.419654233
ARL11	0.305803826	0.16406369	0.638691336	0.807614341	0.374904764	0.175271238
RXFP1	0.305803826	0.310288662	0.242438335	0.472816297	0.472422117	0.662706224
PLIN5	0.305803826	0.333820614	0.198823488	0.685074116	0.581593891	0.355244522
LOC100139916	0.305803826	0.462191552	0.242438335	0.560545614	0.662444459	0.215517697
KCNMA1	0.305977787	0.857705533	0.276229568	0.472816297	0.348579075	0.169732915
ESRP1	0.305977787	0.177317951	0.350169568	0.5065118	0.977155757	0.169732915
GNAI3	0.309169727	0.258471892	0.726668529	0.664346372	0.412271251	0.17340875
ELP3	0.317224879	0.26690502	0.468416519	0.331327902	0.412271251	0.686549574
LOC112441505	0.323051977	0.16406369	0.212043424	0.995118128	0.348579075	0.329101162

LOC107131896	0.326821624	0.16406369	0.620720409	0.638365897	0.589093408	0.27364105
NUDT9	0.328211021	0.225358651	0.434565865	0.820228145	0.514157633	0.222265603
KYNU	0.329734104	0.258011751	0.318905523	0.691510355	0.320551553	0.703742328
SLC23A2	0.329734104	0.171042729	0.500943297	0.841828571	0.516169075	0.195330621
ACTR3	0.329734104	0.166689794	0.823527402	0.5065118	0.534028907	0.175271238
LOC100850659	0.329734104	0.261833331	0.404798158	0.5065118	0.725609902	0.289685975
LOC112441499	0.330371651	0.353585531	0.620720409	0.742618602	0.348579075	0.200537512
COL5A2	0.330371651	0.261833331	0.545279742	0.781241796	0.348579075	0.307062312
HK2	0.330371651	0.353410732	0.337996237	0.438283629	0.348579075	0.764546301
KCNG1	0.330371651	0.393843824	0.243275165	0.846715063	0.422902715	0.262589794
TMED10	0.330371651	0.16406369	0.870054336	0.486299109	0.468751669	0.204271649
CYP2B6	0.330371651	0.310288662	0.368565765	0.732079395	0.66198161	0.175271238
LRRC46	0.330371651	0.204093734	0.295722872	0.5065118	0.96532164	0.27364105
CABIN1	0.330638071	0.173576127	0.335119813	0.820228145	0.72279174	0.175271238
NR3C2	0.330999554	0.655438106	0.242052174	0.493705305	0.353874732	0.447705793
MARC1	0.330999554	0.21695436	0.384546147	0.969717497	0.374904764	0.176823639
ELOF1	0.330999554	0.454401393	0.245005989	0.472816297	0.856913734	0.205163612
PRLR	0.332872069	0.846959498	0.328200044	0.472816297	0.334078335	0.26033195
C2CD2L	0.332872069	0.568497289	0.420304634	0.457214186	0.412271251	0.352446668
DIAPH3	0.332872069	0.225391163	0.678204563	0.781241796	0.422902715	0.182332401
LOC100847365	0.332872069	0.271813256	0.318905523	0.5065118	0.550221271	0.606366962
BCL11B	0.332872069	0.313970142	0.245005989	0.438283629	0.609212557	0.651173954
PPP1CB	0.332872069	0.208370547	0.620860761	0.720889501	0.611314139	0.175271238
GAL	0.332872069	0.246223229	0.22938661	0.721039637	0.750160769	0.350053671
C1QTNF6	0.335539476	0.246223229	0.46756118	0.841828571	0.348579075	0.341504205
SLC31A2	0.335539476	0.277352418	0.295722872	0.788938193	0.369127403	0.536475491
CCDC84	0.335539476	0.261833331	0.820767684	0.472816297	0.422902715	0.232026619
PLEKHG4	0.335539476	0.226249144	0.581540603	0.797488058	0.530828834	0.185234614
TRPC1	0.335539476	0.211465569	0.245005989	0.438283629	0.67894608	0.752857325
CEBPZ	0.335539476	0.305298878	0.250847491	0.742618602	0.713082855	0.262589794
NUDT18	0.335539476	0.166689794	0.455548287	0.381793624	0.955470665	0.348115023
DLX3	0.338588731	0.26690502	0.370075267	0.980418209	0.348579075	0.187244829
CSPG5	0.338588731	0.246642896	0.271251081	0.493705305	0.941107925	0.352446668
NUP98	0.338588731	0.258518679	0.321108831	0.421232674	0.99989309	0.1808177
SFRP1	0.340843069	0.534103098	0.29012228	0.472816297	0.348579075	0.613207282
CALM3	0.342504635	0.476356516	0.29012228	0.438283629	0.880626834	0.230042241
AKR1B1	0.343592771	0.426522272	0.515161241	0.781241796	0.352734074	0.20637797
APLP1	0.343592771	0.588244908	0.269208198	0.498059975	0.72279174	0.183533815

TMEM203	0.343592771	0.209294085	0.564018208	0.650028997	0.750160769	0.210807578
LOC101907041	0.343592771	0.171224422	0.330250811	0.720889501	0.839893529	0.316575757
CKS2	0.348040759	0.188356195	0.750089824	0.721039637	0.348579075	0.317385564
CPM	0.348868896	0.246223229	0.396975946	0.758703434	0.348579075	0.637306705
LOC100297044	0.349738334	0.226249144	0.617376463	0.853352358	0.422902715	0.185012125
FAM136A	0.349738334	0.261833331	0.318905523	0.472816297	0.472422117	0.783134114
HAS1	0.353324284	0.184134038	0.668846123	0.90419464	0.348579075	0.210807578
TMEM245	0.353324284	0.557135186	0.492002127	0.638365897	0.422902715	0.243463286
COL3A1	0.353324284	0.260281276	0.592801724	0.788938193	0.422902715	0.295536455
MAGEL2	0.353324284	0.310589896	0.381755045	0.846115604	0.422902715	0.316575757
LCMT2	0.353324284	0.190688945	0.612353108	0.6044852	0.422902715	0.610416296
SLC1A2	0.353324284	0.24467056	0.29012228	0.946344632	0.467659476	0.296580029
LOC783540	0.353324284	0.370119057	0.318905523	0.438283629	0.490281392	0.725528824
TRAPPC8	0.353324284	0.246223229	0.381755045	0.908496281	0.570932145	0.187244829
ATP7A	0.353324284	0.336061895	0.475476772	0.685074116	0.596576866	0.286011737
THBS4	0.353324284	0.209294085	0.247055391	0.638365897	0.725609902	0.610416296
TCAF1	0.359485848	0.24639569	0.335119813	0.894144195	0.412271251	0.430901994
TROAP	0.359485848	0.331135793	0.678204563	0.664346372	0.530828834	0.212158021
LOC513659	0.360804263	0.197152392	0.295722872	0.44042983	0.99989309	0.243463286
SLC16A3	0.364475038	0.341307707	0.245005989	0.721039637	0.719753534	0.341562774
C18H16orf86	0.369559939	0.197152392	0.765074242	0.724487925	0.348579075	0.341562774
TMEM100	0.37633555	0.204093734	0.807843373	0.437482642	0.659374254	0.296580029
TPPP	0.378800931	0.258471892	0.396975946	0.424471094	0.99989309	0.33509675
ZMYND8	0.380977595	0.291484811	0.440363537	0.850828215	0.348579075	0.438979833
LOC101908206	0.380977595	0.208396065	0.295722872	0.999939566	0.391174668	0.266095269
LOC514181	0.380977595	0.560714408	0.300083198	0.709187504	0.422902715	0.419654233
PTI	0.380977595	0.332079848	0.384015984	0.732079395	0.422902715	0.567131921
P3H1	0.380977595	0.246223229	0.648198992	0.841828571	0.452246871	0.205163612
LOC100300806	0.380977595	0.261833331	0.442357041	0.92064557	0.472422117	0.243463286
RFXANK	0.380977595	0.24639569	0.703042171	0.747176491	0.519859295	0.233834838
LAMB3	0.380977595	0.442555387	0.269208198	0.806922554	0.575855826	0.26883667
ME2	0.380977595	0.208396065	0.341615276	0.860663136	0.576919855	0.361120421
ADM5	0.380977595	0.230659206	0.411555219	0.846715063	0.614338501	0.27364105
BBS9	0.380977595	0.332213949	0.27522625	0.493705305	0.643180575	0.660829605
NUAK1	0.380977595	0.204093734	0.398476708	0.650028997	0.939361296	0.317385564
SMUG1	0.38217746	0.26690502	0.638691336	0.438283629	0.348579075	0.752857325
LOC617565	0.38217746	0.24639569	0.373939346	0.982140778	0.374904764	0.316541336
SIRPA	0.38217746	0.601979156	0.29012228	0.412632272	0.465175996	0.642568004

CLASRP	0.386010235	0.204093734	0.827350832	0.729345432	0.374904764	0.284081535
APC2	0.386010235	0.57068927	0.532777091	0.710581246	0.422902715	0.222265603
LOC781001	0.386010235	0.247499799	0.844009594	0.638365897	0.476180257	0.243463286
LOC112441884	0.386010235	0.588244908	0.381755045	0.573702655	0.537139644	0.338215064
FNDC1	0.386010235	0.208396065	0.483333483	0.884946934	0.551236163	0.286011737
SLIT1	0.386010235	0.277352418	0.411555219	0.438283629	0.904396685	0.425703712
ZNF428	0.389777135	0.310589896	0.335119813	0.990049248	0.422902715	0.231041655
CENPB	0.389777135	0.246223229	0.747084388	0.493705305	0.618467008	0.332863994
RRS1	0.392159065	0.261833331	0.598843922	0.710581246	0.550221271	0.417648084
HDC	0.392368099	0.464010019	0.507971264	0.781241796	0.364322083	0.326183134
SAP30L	0.392368099	0.823074953	0.335119813	0.46847415	0.453219141	0.335320707
ANTXRL	0.392368099	0.246223229	0.78825731	0.438283629	0.467321269	0.550542614
CD248	0.392368099	0.261833331	0.306476792	0.998542029	0.487161254	0.243463286
TMCC1	0.392368099	0.226650133	0.263713067	0.948547383	0.575855826	0.33509675
INAFM2	0.392368099	0.26928316	0.729587161	0.472816297	0.725609902	0.255350095
BEND6	0.392368099	0.272804303	0.266052579	0.781241796	0.784855148	0.33512303
SIX4	0.392368099	0.253018751	0.529161538	0.638365897	0.91011204	0.233834838
CIITA	0.392368099	0.209294085	0.335119813	0.709187504	0.952172109	0.335320707
CCL2	0.392952328	0.226249144	0.318905523	0.999939566	0.374904764	0.324293992
FAM118A	0.393001841	0.301558487	0.281557813	0.638365897	0.559664539	0.746913739
ACAN	0.393218332	0.261833331	0.479530839	0.798715973	0.575855826	0.341562774
TARBP1	0.395189872	0.246223229	0.620720409	0.878330291	0.422902715	0.289685975
CKAP4	0.395189872	0.258471892	0.335119813	0.915749182	0.472422117	0.419654233
GCC1	0.395189872	0.404916922	0.289698626	0.775761952	0.575855826	0.4568517
EHF	0.395189872	0.333820614	0.295722872	0.437482642	0.99989309	0.344621529
SCIN	0.395569854	0.310589896	0.484315892	0.835232007	0.353874732	0.46663273
BMP1	0.395569854	0.319738829	0.610765273	0.915749182	0.369127403	0.262589794
BMPER	0.395569854	0.425397899	0.328393245	0.781241796	0.391174668	0.610416296
SYNM	0.395569854	0.823588167	0.404798158	0.5065118	0.409513357	0.297377614
WNT4	0.395569854	0.277352418	0.63818281	0.891273781	0.422902715	0.233735366
IFI27	0.395569854	0.310288662	0.829843435	0.5065118	0.422902715	0.379405841
ZNF182	0.395569854	0.246642896	0.500525108	0.913413181	0.422902715	0.383793067
LOC512867	0.395569854	0.277352418	0.348383378	0.781241796	0.422902715	0.651173954
RRAGD	0.395569854	0.246642896	0.556529139	0.893831952	0.429633082	0.338215064
TSR1	0.395569854	0.404916922	0.373939346	0.691510355	0.467659476	0.660829605
CA3	0.395569854	0.458781098	0.468416519	0.806922554	0.472422117	0.284081535
CD4	0.395569854	0.27349965	0.38056815	0.6044852	0.534940753	0.784080446
LOC104976020	0.395569854	0.37198719	0.295722872	0.595982966	0.537139644	0.77208617

CTHRC1	0.395569854	0.26690502	0.63818281	0.788938193	0.552293096	0.256295184
SIAE	0.395569854	0.405306771	0.29012228	0.755141399	0.559664539	0.539135678
CLASP1	0.395569854	0.251435522	0.849077738	0.493705305	0.566460478	0.292549586
SRGAP3	0.395569854	0.820796062	0.304589413	0.441752472	0.575621913	0.338215064
CNOT7	0.395569854	0.358502077	0.632743297	0.721039637	0.575855826	0.253506135
PTPRT	0.395569854	0.392589008	0.318905523	0.459769756	0.579030436	0.738110854
LRRC8B	0.395569854	0.291051348	0.373939346	0.820228145	0.580642237	0.366284508
PPP1R3B	0.395569854	0.563639555	0.442357041	0.5065118	0.581593891	0.33509675
LAT	0.395569854	0.330337809	0.491092787	0.674893583	0.603246896	0.493149999
DNAJB4	0.395569854	0.507234108	0.341051552	0.486299109	0.659905012	0.486576625
HOXD3	0.395569854	0.310288662	0.378250914	0.806922554	0.682242009	0.316575757
SBF1	0.395569854	0.24639569	0.820767684	0.493705305	0.68375459	0.269208894
RABGEF1	0.395569854	0.246642896	0.570590624	0.754030944	0.709792998	0.27364105
LOC100139881	0.395569854	0.341210765	0.411555219	0.754030944	0.742316849	0.27364105
PIK3R6	0.395569854	0.246642896	0.335119813	0.861272209	0.807113699	0.250997799
MYO1E	0.395569854	0.246223229	0.318905523	0.720889501	0.808924032	0.523061787
ZFX	0.395569854	0.419875993	0.477104301	0.5065118	0.818457184	0.27364105
AP2B1	0.395569854	0.246642896	0.551458879	0.700857388	0.83417924	0.286011737
KCNH1	0.395569854	0.405306771	0.318905523	0.505314011	0.861762366	0.449106162
ELF3	0.395569854	0.435636214	0.474941169	0.486299109	0.918209674	0.240691816
ECD	0.395569854	0.240061186	0.378250914	0.438283629	0.925981327	0.610416296
CCAR1	0.395569854	0.258518679	0.573880698	0.486299109	0.975291792	0.27364105
AFF4	0.395569854	0.261833331	0.511586767	0.486299109	0.99989309	0.233834838
ARMCX3	0.395569854	0.332213949	0.381755045	0.5065118	0.99989309	0.269208894
KIF14	0.397516223	0.72843033	0.318905523	0.591336191	0.55440643	0.326183134
TENM2	0.399274067	0.538091051	0.49888896	0.638365897	0.575855826	0.316567316
PIPOX	0.399274067	0.351597799	0.300054559	0.721039637	0.943768595	0.27364105
KCNMB1	0.400856805	0.538770068	0.573880698	0.462440418	0.55440643	0.438979833
RASGRP3	0.400856805	0.246223229	0.332413548	0.825933581	0.652021057	0.509959298
TSHZ3	0.400856805	0.280877535	0.392704601	0.820228145	0.685145778	0.329103701
TMEM120A	0.400856805	0.331135793	0.315950029	0.721039637	0.797393203	0.400813158
CCSAP	0.401114753	0.411098122	0.318905523	0.472816297	0.412271251	0.908939618
NDST1	0.401114753	0.344954476	0.295722872	0.818984341	0.519859295	0.588661483
BAHCC1	0.401114753	0.244923348	0.481390179	0.826315685	0.679015559	0.321747892
STX12	0.402303612	0.332213949	0.442490839	0.721039637	0.702383614	0.383793067
RAB7B	0.402303612	0.246642896	0.318905523	0.5065118	0.99989309	0.417648084
ALPK3	0.405622048	0.293243938	0.300996052	0.486299109	0.422902715	0.974017283
ZNF131	0.405622048	0.310288662	0.305181318	0.438283629	0.99989309	0.281391471

CHST13	0.406198422	0.313970142	0.331052055	0.846715063	0.68375459	0.329103701
TBL1XR1	0.4070887	0.306785657	0.618763457	0.915749182	0.422902715	0.258178188
ZNF469	0.4070887	0.246223229	0.638691336	0.794713619	0.550221271	0.352700661
CNOT1	0.4070887	0.374774278	0.335119813	0.472816297	0.99989309	0.243463286
TCEA1	0.408539878	0.258471892	0.423354776	0.638365897	0.534028907	0.809650729
LOC107131975	0.420935737	0.246223229	0.949977753	0.5065118	0.422902715	0.365183219
CENPA	0.420935737	0.344954476	0.63818281	0.808533625	0.513734713	0.281391471
COL1A1	0.420935737	0.261833331	0.660379966	0.820228145	0.557652444	0.286011737
LOC104969384	0.420935737	0.310288662	0.524936203	0.788938193	0.557652444	0.419654233
DGKI	0.420935737	0.311661697	0.455548287	0.915749182	0.575855826	0.267335219
LSAMP	0.420935737	0.817040135	0.411196743	0.472816297	0.61025542	0.27364105
PPIF	0.420935737	0.258518679	0.442836461	0.5065118	0.731693646	0.66854638
PAQR7	0.420935737	0.258011751	0.425452649	0.769006129	0.825598281	0.365183219
AQP9	0.420935737	0.246642896	0.318905523	0.664203576	0.860908683	0.611777732
PTGES3	0.420935737	0.419875993	0.440343439	0.640285187	0.87483668	0.296580029
TMEM64	0.420935737	0.310288662	0.549314874	0.564064573	0.954741113	0.27364105
TYRP1	0.421013884	0.329026062	0.315950029	0.640139053	0.659374254	0.730588392
CFI	0.424359175	0.673034156	0.538419273	0.741347517	0.412271251	0.266095269
MFSD9	0.424359175	0.426923729	0.335119813	0.5065118	0.696261246	0.635645816
PRAG1	0.424359175	0.45311986	0.318905523	0.747317032	0.713082855	0.378863449
RAP1A	0.424949106	0.310288662	0.558576546	0.925901365	0.465175996	0.284081535
KIF4A	0.424949106	0.300798943	0.623085363	0.750804902	0.579030436	0.367140897
CCNJ	0.42503764	0.567752026	0.318905523	0.820228145	0.566460478	0.27364105
ATP13A2	0.42503764	0.373363257	0.611257543	0.685435089	0.696261246	0.33509675
FAM160A2	0.42503764	0.246223229	0.373939346	0.794713619	0.876274511	0.352446668
GPBP1	0.426472581	0.365354616	0.335119813	0.472816297	0.99989309	0.352446668
LOC788801	0.429233744	0.333820614	0.384546147	0.841828571	0.582277536	0.438979833
LRRC24	0.429233744	0.425397899	0.459957269	0.795834665	0.685145778	0.266095269
LOC516494	0.429233744	0.249704163	0.545279742	0.781241796	0.804250204	0.307062312
FLNC	0.436189841	0.761788537	0.442490839	0.638365897	0.422902715	0.38743116
LOC112442610	0.436189841	0.258518679	0.632743297	0.754030944	0.519859295	0.55361803
CENPF	0.436285732	0.268415585	0.753103921	0.818984341	0.530828834	0.269208894
ACAP1	0.436307929	0.277352418	0.747367965	0.781241796	0.550221271	0.286011737
HGH1	0.436307929	0.319488714	0.349112061	0.472816297	0.686348774	0.799940894
TIMP1	0.436307929	0.261833331	0.545279742	0.778545211	0.747513637	0.355733415
CPZ	0.437482113	0.333820614	0.621963024	0.781241796	0.540480584	0.378863449
STOX2	0.437924932	0.310589896	0.318905523	0.90419464	0.741475707	0.316575757
ARMCX4	0.440653954	0.265280172	0.335119813	0.939292364	0.536775392	0.503077635

CFB	0.440653954	0.466972398	0.694542317	0.700857388	0.550221271	0.289685975
USPL1	0.440653954	0.340337237	0.374692393	0.472816297	0.99989309	0.27364105
IGF1	0.441677425	0.286759172	0.620860761	0.781241796	0.487161254	0.542853655
DIRAS2	0.443080868	0.258471892	0.318905523	0.820228145	0.422902715	0.831041995
AOX1	0.443290302	0.353585531	0.318905523	0.612578766	0.748253784	0.667744852
KLRG2	0.444009844	0.39484082	0.941593855	0.493705305	0.422902715	0.307221656
PAN2	0.444009844	0.261833331	0.968954353	0.5065118	0.422902715	0.365817513
GDF6	0.444009844	0.261833331	0.713802232	0.742618602	0.519859295	0.474949941
ZAR1L	0.444009844	0.330337809	0.662688842	0.664346372	0.534940753	0.578699613
VASN	0.444009844	0.258518679	0.373939346	0.999939566	0.581593891	0.27364105
RHOH	0.444009844	0.277352418	0.331145954	0.765249395	0.623887134	0.68555259
PSRC1	0.444009844	0.258471892	0.384587737	0.883885658	0.836838903	0.284081535
PREX1	0.447299164	0.473863398	0.384546147	0.472816297	0.422902715	0.891908825
PALB2	0.447299164	0.341210765	0.883726795	0.638365897	0.487161254	0.306655736
SH3BGR	0.447299164	0.562255493	0.545279742	0.472816297	0.516169075	0.635645816
ZBTB40	0.447299164	0.297475421	0.620720409	0.721039637	0.519859295	0.637306705
TTLL11	0.447299164	0.277352418	0.764057751	0.751870003	0.530828834	0.391156448
LOC404103	0.447299164	0.466972398	0.482377258	0.721039637	0.534940753	0.567131921
ADAMTS2	0.447299164	0.426522272	0.716657925	0.731974857	0.537139644	0.307062312
PICALM	0.447299164	0.277352418	0.481247285	0.983569039	0.549542833	0.286011737
RRAGC	0.447299164	0.310288662	0.598843922	0.915749182	0.550221271	0.27385068
SEC16B	0.447299164	0.277352418	0.631413719	0.814297309	0.551236163	0.421262316
CYP20A1	0.447299164	0.644204975	0.341615276	0.78508807	0.566460478	0.300399428
FLT3	0.447299164	0.266787121	0.500525108	0.721039637	0.580642237	0.656643244
PCDH19	0.447299164	0.277352418	0.371334403	0.638365897	0.581593891	0.866253786
GLIS3	0.447299164	0.464010019	0.442357041	0.721039637	0.765330204	0.317385564
ARFGEF1	0.447299164	0.393843824	0.318905523	0.820228145	0.855938808	0.296580029
PCF11	0.447299164	0.404916922	0.381755045	0.472816297	0.910770324	0.567131921
ZBTB8OS	0.447299164	0.534103098	0.411555219	0.5065118	0.934859184	0.306655736
LOC783577	0.447299164	0.336061895	0.46756118	0.721039637	0.960835287	0.27364105
EMC9	0.447299164	0.34163007	0.442819817	0.486299109	0.99989309	0.27364105
ZNF451	0.447299164	0.358502077	0.368565765	0.539075052	0.99989309	0.289685975
GTF2H1	0.447299164	0.359690277	0.337996237	0.664346372	0.99989309	0.321747892
EN1	0.448524169	0.336061895	0.944612298	0.591987222	0.426742624	0.329103701
CAPN8	0.448524169	0.426522272	0.757787642	0.770428353	0.472422117	0.295536455
FARSB	0.448524169	0.300798943	0.343433547	0.638365897	0.472422117	0.95401375
GLULP	0.448524169	0.268415585	0.434565865	0.999939566	0.519859295	0.355733415
FHL5	0.448524169	0.688099473	0.455548287	0.475138817	0.519859295	0.610416296

POLR1B	0.448524169	0.26690502	0.620720409	0.638365897	0.530828834	0.782430721
CHMP6	0.448524169	0.432788109	0.415167574	0.721039637	0.550221271	0.653436888
DAZAP2	0.448524169	0.321108986	0.894449269	0.638365897	0.551236163	0.284081535
ACTR2	0.448524169	0.330337809	0.743936447	0.721039637	0.551236163	0.419654233
GPR162	0.448524169	0.261833331	0.545279742	0.956154344	0.557535797	0.316575757
CKAP2L	0.448524169	0.271813256	0.806311697	0.737540682	0.557535797	0.318078419
LOC514457	0.448524169	0.287222999	0.726668529	0.670054175	0.570932145	0.516100157
SNTA1	0.448524169	0.268415585	0.440343439	0.720889501	0.579030436	0.788131005
TRAF3IP3	0.448524169	0.334993741	0.411196743	0.721039637	0.582641456	0.673339985
RALGAPB	0.448524169	0.305298878	0.631413719	0.851394268	0.601402099	0.284081535
EZH2	0.448524169	0.330337809	0.702886073	0.731974857	0.6333098	0.329101162
VASP	0.448524169	0.330337809	0.620720409	0.636067868	0.6333098	0.615356919
ARFGAP1	0.448524169	0.63203115	0.350879394	0.5065118	0.696261246	0.48476321
FAM91A1	0.448524169	0.258518679	0.777178978	0.650028997	0.702383614	0.344621529
SIGLEC11	0.448524169	0.647086084	0.335119813	0.587474951	0.72279174	0.427381522
TM9SF2	0.448524169	0.336061895	0.545279742	0.811000597	0.745796372	0.284081535
CDK8	0.448524169	0.404637771	0.335615902	0.820228145	0.790028797	0.31938132
LOC101903647	0.448524169	0.323476519	0.696685689	0.664346372	0.790063341	0.292549586
CBX2	0.448524169	0.280877535	0.335119813	0.806922554	0.939361296	0.344621529
SGK2	0.448524169	0.390662449	0.335119813	0.747998327	0.957605284	0.322282065
CENPC	0.448524169	0.358502077	0.479530839	0.486299109	0.996250445	0.416023813
LOC112444603	0.448524169	0.343679785	0.395460724	0.493705305	0.99989309	0.326183134
SLC16A14	0.448524169	0.344954476	0.341615276	0.664346372	0.99989309	0.378863449
CCR4	0.449157916	0.538091051	0.335119813	0.502870122	0.925981327	0.447180581
LOC112442226	0.44936511	0.404916922	0.491326142	0.714257651	0.725609902	0.474949941
ATP6V0A1	0.451597191	0.261833331	0.620720409	0.925931623	0.422902715	0.449106162
TRAM2	0.451597191	0.308825456	0.42911507	0.969717497	0.557652444	0.355718162
LOC107131704	0.451716774	0.404916922	0.825888497	0.587976629	0.422902715	0.507772366
RGS7	0.451716774	0.688099473	0.400656656	0.664346372	0.429633082	0.600168258
MYB	0.451716774	0.46456927	0.386752537	0.820228145	0.730791761	0.307062312
PLD4	0.451906632	0.502718799	0.520659464	0.721039637	0.581593891	0.447180581
CAPZB	0.454714738	0.341210765	0.828407935	0.731974857	0.487161254	0.33509675
MRPL16	0.454714738	0.359701392	0.335119813	0.5065118	0.68375459	0.833139817
PTCD2	0.454714738	0.365314964	0.440343439	0.638365897	0.696143233	0.684905734
SLAIN2	0.454714738	0.305298878	0.712526829	0.691923003	0.804250204	0.316541336
PRKAR1A	0.454714738	0.310288662	0.663761588	0.721039637	0.809298212	0.316575757
TRA2A	0.454714738	0.310288662	0.479530839	0.638365897	0.934379881	0.523061787
LOC100847509	0.454714738	0.404916922	0.442357041	0.493705305	0.99989309	0.348996078

CHST6	0.45513569	0.277352418	0.632743297	0.781241796	0.678920037	0.401092887
EIF2S1	0.455952571	0.277352418	0.765074242	0.781241796	0.519859295	0.447513267
GUCD1	0.457902267	0.330337809	0.827125538	0.721039637	0.487161254	0.379405841
RIPOR3	0.461464748	0.595087191	0.491326142	0.489078985	0.614338501	0.580759255
MEG3	0.464885191	0.426522272	0.674142655	0.842538664	0.514157633	0.307062312
AARSD1	0.465001625	0.344954476	0.442490839	0.650028997	0.538639002	0.856870625
IL18R1	0.465001625	0.310288662	0.547390233	0.513544	0.77975903	0.640138785
LOC782951	0.467249149	0.405306771	0.392711371	0.5065118	0.99989309	0.323106305
LOC782348	0.467703654	0.291051348	0.719216596	0.5065118	0.538639002	0.752900996
CAMK2G	0.468688936	0.42732587	0.484315892	0.781241796	0.659374254	0.474949941
LOC112444310	0.468688936	0.357803681	0.713802232	0.721039637	0.702383614	0.321747892
KIFC2	0.468763014	0.305298878	0.619290318	0.960986457	0.487161254	0.328386532
DHRS1	0.468763014	0.509590069	0.632855053	0.493705305	0.550221271	0.610416296
M6PR	0.468763014	0.287735709	0.699166169	0.873939476	0.566460478	0.31118166
PCDH1	0.468763014	0.287222999	0.335119813	0.925931623	0.779159678	0.347308394
PCYT2	0.468763014	0.333228543	0.381755045	0.5065118	0.99989309	0.564529177
KCNH8	0.471945547	0.404916922	0.479530839	0.781241796	0.570932145	0.583304321
EFNB2	0.473109108	0.310288662	0.423354776	0.999939566	0.550221271	0.307062312
ZNF391	0.473109108	0.277352418	0.678204563	0.820228145	0.550221271	0.498137139
CCL22	0.473109108	0.459680594	0.444483187	0.923873451	0.573056154	0.29669025
STPG4	0.473448064	0.57320535	0.628637065	0.720889501	0.534940753	0.378863449
CDKN1A	0.473448064	0.277352418	0.551625304	0.88501388	0.657566173	0.419654233
MRPS24	0.473448064	0.343913484	0.392711371	0.493705305	0.99989309	0.446807465
DUSP16	0.473448064	0.329301793	0.348383378	0.577109306	0.99989309	0.555681086
TMEM35A	0.474069858	0.617732204	0.400656656	0.710581246	0.534028907	0.610416296
SELL	0.476502244	0.388260068	0.597303102	0.721039637	0.578685166	0.610882384
COL1A2	0.47687011	0.277352418	0.685222626	0.811000597	0.678920037	0.344621529
FSCN1	0.477194245	0.300798943	0.440343439	0.99879985	0.472422117	0.474949941
PBXIP1	0.477194245	0.404916922	0.51477511	0.539075052	0.513021438	0.870895425
FAM167A	0.477194245	0.310288662	0.400656656	0.754030944	0.516169075	0.862912171
DCAF13	0.477194245	0.277352418	0.573295201	0.917869295	0.519859295	0.516100157
ABT1	0.477194245	0.32817301	0.731855498	0.731974857	0.549542833	0.545791366
ATG4B	0.477194245	0.30449549	0.34137673	0.919247117	0.566460478	0.610882384
GIMAP7	0.477194245	0.46456927	0.345188109	0.776207435	0.638572006	0.614421218
CBX6	0.477194245	0.340824889	0.468416519	0.830830186	0.797429056	0.349622284
IL2RA	0.477194245	0.370119057	0.384015984	0.619137099	0.954741113	0.578699613
DNAJC27	0.477194245	0.426522272	0.494015611	0.638365897	0.964172455	0.365817513
CBL	0.477194245	0.305298878	0.545279742	0.721039637	0.968795608	0.341677582

DDX21	0.477194245	0.297475421	0.545279742	0.654787691	0.99989309	0.419654233
FABP1	0.477572768	0.671602771	0.495716287	0.513544	0.750160769	0.35129924
MAP3K12	0.477694159	0.340824889	0.632855053	0.908496281	0.472422117	0.432516585
STEAP2	0.477694159	0.487116492	0.440343439	0.850828215	0.576003049	0.419654233
DLGAP5	0.477694159	0.336061895	0.744499269	0.78508807	0.581593891	0.344621529
PAK2	0.477694159	0.330337809	0.707441133	0.750804902	0.725609902	0.329103701
LOC112441663	0.477694159	0.316853613	0.350879394	0.932656599	0.814605912	0.31118166
TARDBP	0.477694159	0.310288662	0.350879394	0.6044852	0.846122929	0.77208617
MTAP	0.477694159	0.277352418	0.500684374	0.776207435	0.889941524	0.457231394
NFYB	0.477694159	0.432788109	0.373939346	0.5065118	0.99989309	0.343697535
HOXA9	0.477769975	0.300798943	0.875669731	0.721039637	0.581593891	0.322282065
ODC1	0.479056366	0.310288662	0.623085363	0.788938193	0.725609902	0.387718302
GPR35	0.479056366	0.419875993	0.684005375	0.605239919	0.746187115	0.438291938
GMPR	0.479865957	0.449728959	0.722604787	0.788938193	0.534940753	0.341499166
CAPZA2	0.480197593	0.39484082	0.620720409	0.92064557	0.487161254	0.355733415
GPR171	0.480197593	0.494665274	0.420304634	0.806922554	0.685145778	0.421401315
GPX3	0.480197593	0.310288662	0.477104301	0.781241796	0.700903924	0.633384404
ACLY	0.480197593	0.310288662	0.40419952	0.664203576	0.964521207	0.588661483
MUSTN1	0.481526798	0.538415775	0.620720409	0.5065118	0.538639002	0.647432409
LOC112442023	0.481526798	0.358502077	0.825888497	0.692399817	0.6333098	0.341562774
TTR	0.483620623	0.494451722	0.546505003	0.5065118	0.534940753	0.788131005
CDK3	0.483620623	0.310288662	0.847659392	0.692399817	0.557652444	0.449106162
MTCL1	0.485560133	0.336061895	0.597307213	0.944544762	0.513021438	0.419654233
SPATA46	0.485560133	0.321108986	0.974832809	0.553735811	0.530828834	0.356054774
MTR	0.485560133	0.315941541	0.50205214	0.95964071	0.534028907	0.468599414
LOC782706	0.485560133	0.353585531	0.381755045	0.943539568	0.534028907	0.567131921
SELENOK	0.485560133	0.341210765	0.684005375	0.868090726	0.55440643	0.361120421
STK32B	0.485560133	0.411098122	0.651174146	0.741347517	0.601402099	0.474949941
LOC101907219	0.485560133	0.340337237	0.545631821	0.664346372	0.604778634	0.77208617
P2RY6	0.485560133	0.332079848	0.545279742	0.700857388	0.675656796	0.704974208
HOXA6	0.485560133	0.426522272	0.491092787	0.781241796	0.676199383	0.522495274
LUZP1	0.485560133	0.530214567	0.475476772	0.5065118	0.866920896	0.507387413
LOC100848703	0.485560133	0.289038121	0.434565865	0.5065118	0.99989309	0.419654233
FAIM2	0.487536897	0.296238139	0.373939346	0.856178306	0.66198161	0.651139949
FBN2	0.488699389	0.358502077	0.545279742	0.820228145	0.472422117	0.684905734
LHX6	0.488699389	0.406246837	0.5957071	0.868417977	0.519859295	0.498031245
DEPDC1	0.488699389	0.376484768	0.783662574	0.765249395	0.550221271	0.387718302
NDOR1	0.488699389	0.464176632	0.625367433	0.685074116	0.818457184	0.341677582

ABL1	0.488699389	0.310288662	0.384500746	0.638365897	0.99989309	0.394269201
SNPH	0.488853455	0.340824889	0.539146924	0.86288219	0.534940753	0.610416296
KIF21B	0.488853455	0.350505932	0.547390233	0.721039637	0.622490325	0.684905734
PSTPIP1	0.488886791	0.353585531	0.515161241	0.664346372	0.84234617	0.610416296
FGG	0.489822932	0.462191552	0.652359766	0.741905872	0.487161254	0.579379269
UTP15	0.493679232	0.310288662	0.707441133	0.835232007	0.55440643	0.45454353
LOC107131817	0.494278403	0.347262685	0.384015984	0.769006129	0.513021438	0.886992214
JPH2	0.494278403	0.766762596	0.479530839	0.721039637	0.519859295	0.437463305
MAP1LC3C	0.494278403	0.603785862	0.400656656	0.908054165	0.534940753	0.355733415
RGS16	0.494278403	0.49642959	0.373939346	0.93471064	0.577671494	0.355733415
PMS2	0.494544578	0.300798943	0.394733855	0.685074116	0.99989309	0.328386532
PGF	0.496352109	0.560898132	0.534985399	0.806922554	0.611314139	0.347308394
ASGR1	0.497083705	0.48029058	0.384500746	0.721039637	0.780497593	0.579379269
PLCB4	0.498010064	0.568497289	0.460374163	0.539075052	0.537139644	0.787080595
TMEM176B	0.499158885	0.411376371	0.495716287	0.928623745	0.484768737	0.516100157
UNC5C	0.499158885	0.701581467	0.618763457	0.721039637	0.537139644	0.346829441
CLIC4	0.499158885	0.314682036	0.546505003	0.99879985	0.55440643	0.326183134
VAT1	0.499158885	0.333228543	0.529161538	0.899336497	0.60923233	0.516100157
USP2	0.499158885	0.333196297	0.479530839	0.792591369	0.819670067	0.50847245
SNRPA	0.499904283	0.459453253	0.392711371	0.904990622	0.654494581	0.449106162
C5H12orf29	0.499904283	0.310288662	0.479530839	0.731974857	0.68375459	0.75292536
INO80B	0.499904283	0.565609316	0.443645982	0.64154788	0.952172109	0.341562774
UTP4	0.500122946	0.300999909	0.398476708	0.700857388	0.99989309	0.487013094
FKBP10	0.502933971	0.310589896	0.665743961	0.940779565	0.538639002	0.361339031
BID	0.502933971	0.400929909	0.395541169	0.989391869	0.550221271	0.474949941
RAB21	0.502933971	0.310288662	0.957379098	0.691923003	0.566460478	0.329103701
NKTR	0.502933971	0.310288662	0.620860761	0.820228145	0.581593891	0.585177964
KBTBD7	0.502933971	0.310288662	0.573880698	0.781241796	0.582845642	0.683895124
TMEM126B	0.502933971	0.336061895	0.468416519	0.519966378	0.60923233	0.928123265
C2	0.502933971	0.426522272	0.689299245	0.731974857	0.741060815	0.33509675
TGFBI	0.502933971	0.310288662	0.573880698	0.820228145	0.84234617	0.378863449
CHD1	0.502933971	0.382781759	0.383538833	0.5065118	0.99989309	0.35601804
CD79A	0.503317583	0.490341542	0.420304634	0.89088601	0.519859295	0.579379269
LOC781339	0.503317583	0.659488126	0.546505003	0.650028997	0.519859295	0.632484935
KCTD17	0.503317583	0.310589896	0.479530839	0.995800463	0.549542833	0.474949941
CDC42EP3	0.503317583	0.62522808	0.651248606	0.5065118	0.553667127	0.567131921
ACER2	0.503317583	0.358502077	0.434565865	0.96095927	0.576003049	0.474949941
MMP23	0.503317583	0.408156624	0.651248606	0.825933581	0.66198161	0.348115023

NOA1	0.503317583	0.343913484	0.442357041	0.591336191	0.685145778	0.877623013
LOC530077	0.503317583	0.329026062	0.442490839	0.894144195	0.702383614	0.550542614
MCMBP	0.503317583	0.400929909	0.440343439	0.807296971	0.917088283	0.372140749
BHLHE40	0.503317583	0.404916922	0.482377258	0.670054175	0.99989309	0.3807892
LSMEM1	0.505622778	0.462191552	0.549314874	0.825933581	0.487161254	0.610416296
ITK	0.505622778	0.388260068	0.459957269	0.612578766	0.742316849	0.789577614
POLR2K	0.505875917	0.385897798	0.892122482	0.714257651	0.534028907	0.39950546
P2RY13	0.507614692	0.579665846	0.435794403	0.781241796	0.557535797	0.578699613
HJURP	0.507771216	0.450661146	0.719335065	0.784305663	0.530828834	0.474949941
EBPL	0.507771216	0.310288662	0.545279742	0.91329897	0.675325659	0.493149999
LIF	0.507771216	0.340337237	0.46756118	0.931750296	0.766792351	0.33509675
ZNF19	0.509203815	0.405306771	0.440343439	0.638365897	0.964150118	0.591154525
SMAD1	0.509339286	0.404916922	0.500525108	0.951929772	0.550221271	0.446807465
ZNF831	0.511015458	0.537186174	0.400656656	0.519556778	0.678920037	0.785275816
NPC2	0.514580973	0.353585531	0.79610462	0.87452213	0.519534276	0.33509675
TSR2	0.516866083	0.336061895	0.885830884	0.788938193	0.513021438	0.361120421
LOC782177	0.516866083	0.458781098	0.520659464	0.882049207	0.540480627	0.55361803
DMRT2	0.516866083	0.574094539	0.538132307	0.721039637	0.557535797	0.610416296
RRBP1	0.516866083	0.454401393	0.581540603	0.908496281	0.566460478	0.398331041
PKDCC	0.516866083	0.32817301	0.566214106	0.934591536	0.575855826	0.48476321
TSC22D3	0.516866083	0.390662449	0.468416519	0.959786126	0.576003049	0.4568517
GJA1	0.516866083	0.458781098	0.461725549	0.931268564	0.576164442	0.419654233
CCNDBP1	0.516866083	0.315941541	0.738217747	0.781241796	0.60923233	0.543098314
ZNF75D	0.516866083	0.310288662	0.880204512	0.750893959	0.611314139	0.344621529
RGS12	0.516866083	0.358502077	0.548448623	0.956154344	0.642463841	0.33509675
LOC509118	0.516866083	0.390662449	0.491092787	0.971220023	0.652448205	0.33509675
ERO1A	0.516866083	0.371567288	0.814444866	0.691923003	0.741060815	0.352446668
CASQ1	0.516866083	0.568497289	0.460374163	0.664203576	0.745796372	0.578699613
RBM39	0.516866083	0.353585531	0.479530839	0.664346372	0.954501767	0.610882384
GBP5	0.516866083	0.310288662	0.573380215	0.781241796	0.964150118	0.361120421
GMPPB	0.517958653	0.578451435	0.404798158	0.720889501	0.581593891	0.683474857
KNTC1	0.517958653	0.310288662	0.738217747	0.731974857	0.857710467	0.344621529
H2AFY2	0.519454621	0.382781759	0.621963024	0.638365897	0.99989309	0.341499166
TUBA1A	0.520257462	0.551037136	0.55673062	0.820228145	0.550221271	0.48476321
APOC3	0.520257462	0.426798084	0.917876971	0.638365897	0.559664539	0.378863449
ASAH2	0.520257462	0.310288662	0.413677672	0.999939566	0.582845642	0.425703712
LOC112448166	0.520257462	0.394411867	0.425452649	0.721039637	0.934859184	0.580034896
CENPX	0.520257462	0.376731512	0.404798158	0.655549413	0.99989309	0.365290024

MOB3A	0.522270737	0.388260068	0.573880698	0.691510355	0.599125199	0.806054104
DAPK3	0.522270737	0.494451722	0.447448455	0.721039637	0.732120393	0.610651912
NAV1	0.522872531	0.502718799	0.777178978	0.758703434	0.550221271	0.361120421
ZDHHC2	0.522872531	0.321108986	0.632743297	0.946344632	0.550221271	0.438979833
ZNF711	0.522872531	0.358502077	0.703042171	0.788938193	0.68375459	0.446807465
DPYSL5	0.522872531	0.514566709	0.600123126	0.709187504	0.765330204	0.474949941
LGALS3	0.528533644	0.505355809	0.618763457	0.789823574	0.519534276	0.610416296
DTX4	0.528533644	0.382781759	0.937363055	0.731974857	0.519859295	0.361339031
ATP6V1C2	0.528533644	0.688099473	0.400672166	0.915526452	0.519859295	0.378863449
POU2AF1	0.528533644	0.454613627	0.577831019	0.820228145	0.534940753	0.635645816
SEMA4F	0.528533644	0.572780725	0.620720409	0.842538664	0.550221271	0.361120421
ARL4C	0.528533644	0.419875993	0.532777091	0.915749182	0.55440643	0.535254324
PLXNA3	0.528533644	0.432162553	0.668846123	0.842538664	0.570932145	0.449106162
LOC533307	0.528533644	0.420056896	0.493359376	0.915749182	0.571258463	0.543098314
LOC112447626	0.528533644	0.875022483	0.607119516	0.6044852	0.576003049	0.361339031
VPS18	0.528533644	0.383748398	0.479530839	0.721039637	0.576003049	0.859973633
CD2	0.528533644	0.505355809	0.440343439	0.709187504	0.6333098	0.77208617
RIMS1	0.528533644	0.651481516	0.445754452	0.75922259	0.652021057	0.507387413
SEPT1	0.528533644	0.411098122	0.620720409	0.691510355	0.68375459	0.671528522
CUL3	0.528533644	0.494451722	0.705547551	0.709187504	0.740575942	0.384542553
RASAL3	0.528533644	0.542133539	0.446781144	0.670054175	0.769784115	0.610882384
ATAD3A	0.528533644	0.419875993	0.404798158	0.577746499	0.779159678	0.839419627
KIAA1217	0.528533644	0.442555387	0.618763457	0.778545211	0.819670067	0.393276679
TSSC4	0.528533644	0.490341542	0.400656656	0.577746499	0.860908683	0.706354992
CYP1B1	0.528533644	0.492812851	0.411555219	0.650028997	0.904995537	0.614421218
MFSD8	0.528533644	0.332213949	0.442357041	0.806922554	0.939994656	0.543098314
LOC101906178	0.528533644	0.39139161	0.545279742	0.664346372	0.99989309	0.352700661
NDUFA1	0.528533644	0.315941541	0.435794403	0.721039637	0.99989309	0.392527953
TIMM10	0.528533644	0.313970142	0.435794403	0.760589144	0.99989309	0.457231394
PIM3	0.528533644	0.330337809	0.482588959	0.638365897	0.99989309	0.474949941
ILVBL	0.528533644	0.333228543	0.458618441	0.573702655	0.99989309	0.639250492
LOC101906024	0.5296719	0.340337237	0.545279742	0.999939566	0.519859295	0.457231394
SLC18B1	0.5296719	0.341210765	0.765785685	0.820228145	0.519859295	0.55361803
CD28	0.5296719	0.358502077	0.598843922	0.914862928	0.550221271	0.588661483
LOC100140586	0.5296719	0.818774879	0.620860761	0.692399817	0.559664539	0.347308394
ZNF410	0.5296719	0.520074008	0.442490839	0.806922554	0.676199383	0.572789272
CDC20	0.5296719	0.358502077	0.685222626	0.846715063	0.702383614	0.371151838
SUPT4H1	0.5296719	0.340824889	0.794216389	0.710581246	0.702383614	0.503077635

BOP1	0.5296719	0.458781098	0.400656656	0.638365897	0.702383614	0.847189049
MED11	0.532789424	0.494665274	0.497403314	0.781241796	0.846122929	0.442202033
NEXN	0.532855626	0.490341542	0.555589198	0.729345432	0.570932145	0.704011905
LSM14B	0.532855626	0.48077029	0.610765273	0.721039637	0.846122929	0.432516585
ACAP3	0.533114487	0.405306771	0.673737267	0.943323364	0.519859295	0.383793067
IFFO1	0.533114487	0.777334441	0.513743573	0.706360738	0.696261246	0.365817513
SAC3D1	0.533114487	0.50719125	0.611553159	0.721039637	0.713082855	0.544692674
PFN2	0.533114487	0.479254481	0.684005375	0.692399817	0.819670067	0.407011685
MYH3	0.534137589	0.332079848	0.619290318	0.999939566	0.549542833	0.365817513
PHACTR4	0.534137589	0.426522272	0.513743573	0.714257651	0.55440643	0.866612872
B4GALT7	0.534137589	0.432788109	0.411555219	0.664346372	0.763613029	0.806054104
LOC101904923	0.534137589	0.388260068	0.510933406	0.806997685	0.904151325	0.4568517
ZNF335	0.5341399	0.384747951	0.855712722	0.669727908	0.713691227	0.38845723
PSMD12	0.536144169	0.330337809	0.678204563	0.709187504	0.925981327	0.50419947
UBN1	0.536635304	0.340895455	0.715979316	0.638365897	0.99989309	0.352387644
MED12	0.536669175	0.464176632	0.510329362	0.720889501	0.781256945	0.635645816
MPV17L2	0.540328736	0.426522272	0.51477511	0.781241796	0.725609902	0.627553513
RASAL2	0.541041939	0.336061895	0.440363537	0.709187504	0.892149112	0.759107825
ELL2	0.541533002	0.388260068	0.460374163	0.999939566	0.614338501	0.355733415
IMPA1	0.541533002	0.390662449	0.478072091	0.681139802	0.627119169	0.908939618
FZD2	0.541533002	0.376484768	0.684005375	0.914862928	0.6333098	0.365290024
COL16A1	0.541533002	0.450661146	0.726668529	0.794713619	0.652021057	0.410169963
TCF19	0.541533002	0.411098122	0.765074242	0.806698028	0.675656796	0.352446668
AKAP7	0.541533002	0.464965392	0.51477511	0.663620175	0.99989309	0.378969231
GMPPA	0.542628559	0.462191552	0.699166169	0.908496281	0.538639002	0.378863449
MKX	0.542628559	0.692025761	0.515161241	0.772430178	0.552331099	0.555681086
FBLN7	0.542628559	0.404916922	0.620720409	0.925901365	0.570932145	0.467459971
WDR19	0.542628559	0.401248462	0.765074242	0.788938193	0.622490325	0.446807465
ARHGDIA	0.542628559	0.600050072	0.570590624	0.721039637	0.675656796	0.550542614
LMOD1	0.542628559	0.579771417	0.611257543	0.664346372	0.702383614	0.572789272
LOC112445197	0.542721982	0.388260068	0.744490351	0.721039637	0.852745499	0.376125338
POLR3B	0.543384952	0.487116492	0.460374163	0.842538664	0.704551568	0.550542614
DHX35	0.543384952	0.438385292	0.769285605	0.664346372	0.77975903	0.419654233
SERPINE2	0.543384952	0.382781759	0.632743297	0.830877858	0.819670067	0.365183219
AFTPH	0.543384952	0.406246837	0.468416519	0.851394268	0.899048045	0.416023813
TRIM36	0.543384952	0.336061895	0.520659464	0.691877804	0.943768595	0.667185675
SCN8A	0.543384952	0.411376371	0.477908341	0.700857388	0.996250445	0.567131921
AMT	0.545272914	0.330337809	0.765074242	0.781241796	0.55440643	0.63363599

C2CD4B	0.546272319	0.340824889	0.541411887	0.954423369	0.742316849	0.378863449
LOC101903289	0.546851594	0.353642889	0.500525108	0.982140778	0.538639002	0.610416296
NLRC3	0.546851594	0.529335263	0.598843922	0.846715063	0.581593891	0.478678586
LOC107131398	0.546851594	0.46726452	0.827125538	0.713056741	0.627119169	0.446807465
NKD1	0.546851594	0.400929909	0.435794403	0.962840284	0.692179478	0.474949941
LOC112449261	0.546851594	0.473863398	0.442819817	0.914862928	0.713082855	0.469450527
ACSM5	0.546851594	0.404916922	0.632743297	0.825933581	0.713082855	0.474949941
HBB	0.546851594	0.471726524	0.620860761	0.638365897	0.762655357	0.642154165
GP9	0.546851594	0.393843824	0.547390233	0.825933581	0.839134872	0.474949941
UFSP1	0.546851594	0.494451722	0.443645982	0.692399817	0.892412659	0.616913081
SLMAP	0.548615111	0.762756164	0.599201355	0.663620175	0.538639002	0.610416296
SCARB2	0.548615111	0.350505932	0.617037621	0.999939566	0.559664539	0.383793067
OIT3	0.548615111	0.490341542	0.442357041	0.984506451	0.559664539	0.473401438
HSPB11	0.548615111	0.435300125	0.477104301	0.685074116	0.618467008	0.888121679
CXXC5	0.548615111	0.4835064	0.547390233	0.742618602	0.627119169	0.704011905
MED9	0.548615111	0.450661146	0.675040193	0.843411019	0.6333098	0.4568517
UCK2	0.548615111	0.426522272	0.455548287	0.654787691	0.634936594	0.920229508
RAPGEFL1	0.548615111	0.488637812	0.725374309	0.806922554	0.649744166	0.387718302
FBXO28	0.548615111	0.435300125	0.754897816	0.806445529	0.675325659	0.378616142
NDRG4	0.548615111	0.490341542	0.601873896	0.6044852	0.675656796	0.77208617
STAM	0.548615111	0.353642889	0.678204563	0.83476264	0.758224343	0.427381522
HIPK3	0.548615111	0.365354616	0.569321962	0.788938193	0.961835052	0.417648084
DCXR	0.548615111	0.464965392	0.528333149	0.664346372	0.99989309	0.442687436
SFRP2	0.548999961	0.506366149	0.621963024	0.915749182	0.550221271	0.418451026
MEG8	0.548999961	0.392037341	0.82177309	0.825933581	0.550221271	0.434515473
PTPN2	0.548999961	0.382781759	0.607812429	0.788938193	0.934379881	0.429644157
ATXN1L	0.54932821	0.437950914	0.495716287	0.966133452	0.549542833	0.564168606
DERL1	0.54932821	0.432788109	0.845425011	0.731974857	0.557652444	0.474949941
TMEM242	0.54932821	0.804875341	0.71043466	0.64154788	0.566460478	0.361339031
P4HB	0.54932821	0.534344991	0.495716287	0.781241796	0.566460478	0.710381708
LOC112447082	0.54932821	0.722790338	0.496119495	0.692399817	0.580642237	0.637306705
ISCA2	0.54932821	0.341210765	0.88950849	0.781241796	0.608328153	0.393072341
CSNK1B	0.54932821	0.340337237	0.722352424	0.721039637	0.611314139	0.719551722
LOC101909196	0.54932821	0.464010019	0.848444291	0.692399817	0.614931961	0.469450527
GDF11	0.54932821	0.370119057	0.481247285	0.931989854	0.642463841	0.602586916
DENR	0.54932821	0.490341542	0.600123126	0.721039637	0.68375459	0.660829605
STXBP3	0.54932821	0.473793567	0.538132307	0.825933581	0.702383614	0.564529177
MED6	0.54932821	0.492812851	0.431888752	0.638365897	0.939361296	0.661937165

TNFSF10	0.54932821	0.436522146	0.435794403	0.844510397	0.96532164	0.36894905
PADI1	0.54932821	0.453259111	0.460374163	0.781241796	0.982014614	0.446807465
ZSWIM6	0.54932821	0.374774278	0.431888752	0.664346372	0.99989309	0.401598248
NUDT8	0.54932821	0.382781759	0.545279742	0.638365897	0.99989309	0.419654233
BCL7A	0.549716787	0.412213237	0.632743297	0.691923003	0.99989309	0.37226337
USP4	0.550187372	0.360591115	0.703042171	0.984289715	0.540480627	0.378863449
LENG8	0.550187372	0.344954476	0.826667106	0.781241796	0.549542833	0.610416296
CARD9	0.550187372	0.487116492	0.842478086	0.721039637	0.550221271	0.493149999
TMSB4X	0.550187372	0.341210765	0.950638965	0.704390487	0.55440643	0.503077635
SLC4A11	0.550187372	0.556505404	0.602931071	0.721039637	0.576003049	0.671528522
CXCL16	0.550187372	0.438385292	0.51477511	0.990571725	0.60461463	0.421262316
MYOM1	0.550187372	0.494451722	0.545279742	0.638365897	0.614338501	0.86143104
KCNS3	0.550187372	0.464010019	0.493081734	0.650028997	0.643180575	0.887248119
PRPF19	0.550187372	0.408910233	0.620720409	0.721039637	0.652448205	0.770391484
LAIR1	0.550187372	0.440318475	0.573880698	0.841828571	0.675325659	0.600077084
P2RY10	0.550187372	0.376323391	0.592245048	0.846715063	0.675467149	0.63363599
TNNT2	0.550187372	0.464010019	0.684005375	0.811000597	0.702383614	0.439845685
CLDN10	0.550187372	0.557966843	0.597303102	0.720889501	0.868380278	0.447513267
CAND1	0.550187372	0.420056896	0.63818281	0.700857388	0.904995537	0.539135678
FFAR2	0.550187372	0.432788109	0.532777091	0.638365897	0.977155757	0.61405517
LOC107133024	0.550187372	0.350505932	0.468416519	0.754030944	0.99989309	0.419654233
GTF2H4	0.550226773	0.388260068	0.620720409	0.664346372	0.972917711	0.578699613
LOC534967	0.550226773	0.458781098	0.468416519	0.664346372	0.99989309	0.491624392
CDS1	0.550579691	0.536568815	0.573880698	0.908054165	0.566460478	0.488111929
WFDC3	0.553466743	0.419875993	0.869529186	0.732079395	0.647468461	0.393072341
PLEKHA6	0.553466743	0.340824889	0.769285605	0.730465064	0.80664102	0.474949941
GALNT10	0.554921566	0.711124077	0.749405258	0.669727908	0.538639002	0.474949941
MYH11	0.554921566	0.876057665	0.545279742	0.709187504	0.55440643	0.495124073
HSD17B11	0.554921566	0.404916922	0.545279742	0.943539568	0.678920037	0.474949941
LOC101905514	0.554921566	0.401951083	0.620860761	0.751870003	0.742316849	0.642154165
C13H20orf202	0.557606549	0.426522272	0.510933406	0.998542029	0.550221271	0.508971078
PLPPR2	0.557606549	0.404916922	0.473962011	0.999939566	0.581593891	0.484287317
RNF185	0.557606549	0.365314964	0.722951238	0.915749182	0.6333098	0.419654233
FGF2	0.557606549	0.404916922	0.479530839	0.913413181	0.702383614	0.59580789
SHANK2	0.557606549	0.665563964	0.479530839	0.674893583	0.846122929	0.539135678
DUSP26	0.557606549	0.437950914	0.481247285	0.825933581	0.921504737	0.469816868
LOC783686	0.557606549	0.358502077	0.443180215	0.776207435	0.928131565	0.66915423
TMEM200A	0.557606549	0.425397899	0.494015611	0.638365897	0.99989309	0.38845723

HOXD4	0.55814318	0.550965714	0.581737542	0.781241796	0.741060815	0.520677492
PIGZ	0.559223374	0.358308875	0.620720409	0.638365897	0.550221271	0.970702553
TMEM119	0.559223374	0.358502077	0.600123126	0.995118128	0.552199893	0.523061787
TMEM138	0.559223374	0.353410732	0.674989274	0.700857388	0.6333098	0.839419627
ANKRD13B	0.559223374	0.442555387	0.764057751	0.806997685	0.635486252	0.4568517
FCHO2	0.559223374	0.553493197	0.479530839	0.893831952	0.675325659	0.499155216
CHAC1	0.559223374	0.868963165	0.547390233	0.691510355	0.685145778	0.378863449
PHYHD1	0.559223374	0.411376371	0.479530839	0.99879985	0.685145778	0.379405841
CENPW	0.559223374	0.473526978	0.772990161	0.781241796	0.696261246	0.378863449
IL17B	0.559223374	0.63144743	0.61759981	0.664346372	0.702383614	0.600077084
GPR4	0.559223374	0.588244908	0.524936203	0.721039637	0.702383614	0.642154165
SMARCC1	0.559223374	0.359690277	0.632855053	0.781241796	0.804250204	0.580920535
GLI3	0.559223374	0.458781098	0.726668529	0.760188917	0.819670067	0.378863449
ZNF862	0.559223374	0.536970867	0.66128476	0.714257651	0.83627631	0.455659884
SPOUT1	0.559223374	0.411376371	0.442357041	0.683202337	0.899048045	0.78510176
NLRP3	0.559223374	0.432788109	0.482588959	0.700857388	0.947146038	0.657118951
CCDC61	0.559223374	0.398815025	0.607812429	0.709187504	0.99989309	0.474949941
LY75	0.560896062	0.435300125	0.481351254	0.720889501	0.753062237	0.800658496
DDX18	0.560896062	0.412430776	0.684005375	0.796330272	0.813531278	0.447180581
ANP32E	0.562396249	0.393843824	0.976241809	0.709187504	0.550221271	0.454197422
GPX7	0.562439494	0.382781759	0.692119389	0.931268564	0.550221271	0.539135678
LOC100140431	0.562439494	0.388260068	0.747084388	0.90419464	0.550221271	0.544692674
CCNJL	0.562439494	0.701581467	0.632743297	0.796330272	0.571258463	0.408678342
TP53BP2	0.562439494	0.514208066	0.479530839	0.88747242	0.587069217	0.617575248
CADM1	0.562439494	0.426522272	0.618763457	0.721039637	0.601402099	0.834254546
SAV1	0.562439494	0.473526978	0.481247285	0.987561095	0.63496351	0.455659884
RPL22L1	0.562439494	0.356524425	0.651248606	0.781241796	0.676199383	0.707492534
RSPH10B	0.562439494	0.405306771	0.852758896	0.692399817	0.725609902	0.474949941
RBBP6	0.562439494	0.435300125	0.581540603	0.738189414	0.838993207	0.627994005
C25H16orf72	0.562439494	0.473863398	0.549314874	0.770892936	0.850852167	0.576908474
OSBPL11	0.562439494	0.358502077	0.442490839	0.943323364	0.925981327	0.391156448
SNX17	0.562439494	0.400929909	0.512077812	0.721039637	0.99989309	0.588661483
GPR137B	0.562977102	0.458781098	0.632855053	0.913413181	0.55440643	0.55361803
LSMEM2	0.562977102	0.369400037	0.659491881	0.863958691	0.780497593	0.447180581
LOC789374	0.563476436	0.374893127	0.834643686	0.65732455	0.551236163	0.763686154
KLF15	0.563476436	0.390061613	0.620720409	0.999939566	0.55440643	0.435648157
TFDP1	0.563476436	0.584982353	0.685222626	0.781241796	0.581593891	0.503077635
ZNF608	0.563476436	0.353976731	0.74784538	0.796330272	0.741060815	0.51887862

PNO1	0.563476436	0.404916922	0.694542317	0.841828571	0.742316849	0.4568517
RND2	0.563476436	0.400929909	0.545279742	0.720889501	0.755089404	0.802270182
TPD52L2	0.563476436	0.538091051	0.468416519	0.750804902	0.83627631	0.610416296
ACAT2	0.563476436	0.435300125	0.459957269	0.683202337	0.847784631	0.812509877
MSTO1	0.563476436	0.419875993	0.479530839	0.685074116	0.870287145	0.794529985
ICOSLG	0.563476436	0.582472344	0.545279742	0.721039637	0.889271428	0.478543685
SLIT2	0.563476436	0.388260068	0.682822841	0.794713619	0.934859184	0.383793067
AEBP2	0.563476436	0.487116492	0.473168228	0.638365897	0.99989309	0.588661483
CASTOR2	0.563845487	0.360591115	0.768676249	0.811000597	0.730791761	0.449106162
MOG	0.565156448	0.411376371	0.696685689	0.638365897	0.573056154	0.870895425
RRP7A	0.565327835	0.588244908	0.468416519	0.781241796	0.575855826	0.761409946
LOC104970180	0.565327835	0.55750845	0.468416519	0.670054175	0.611314139	0.872175131
TMEM206	0.565327835	0.473863398	0.473078104	0.716604372	0.941064037	0.650973086
TM7SF2	0.565327835	0.463039039	0.51477511	0.683202337	0.982014614	0.61405517
RERE	0.565603572	0.353585531	0.524936203	0.638365897	0.566460478	0.99797558
ABCC5	0.567407781	0.373363257	0.63818281	0.784305663	0.550221271	0.829807823
ZC3H12D	0.567407781	0.390662449	0.747093618	0.811000597	0.55440643	0.64896659
ACKR4	0.567407781	0.716335802	0.477908341	0.90419464	0.557652444	0.451630294
FBXW12	0.567407781	0.425397899	0.836294915	0.721039637	0.614338501	0.580920535
ITGB7	0.567407781	0.505355809	0.617037621	0.721039637	0.685145778	0.694702496
STX18	0.567407781	0.405306771	0.709521163	0.820228145	0.7817968	0.435648157
GPR183	0.567407781	0.358502077	0.511586767	0.724487925	0.888595211	0.757509276
SEMA6B	0.56848222	0.438160875	0.617376463	0.995800463	0.566460478	0.419654233
CLECL1	0.56848222	0.462191552	0.504377317	0.904990622	0.659374254	0.619060217
SDK1	0.568924228	0.404916922	0.720067907	0.846715063	0.601402099	0.578699613
CDC73	0.568924228	0.426923729	0.551625304	0.825933581	0.895183012	0.493149999
TMEM176A	0.569764505	0.514208066	0.620720409	0.883885658	0.550221271	0.607830667
DUOXA2	0.570919148	0.358502077	0.747093618	0.914862928	0.675325659	0.419654233
CBX7	0.570919148	0.488637812	0.546505003	0.69337983	0.675325659	0.845803016
SMAP2	0.570919148	0.358308875	0.512985904	0.720889501	0.882754591	0.795637468
IDI1	0.570919148	0.396956826	0.460374163	0.788938193	0.906183446	0.6608989
BDP1	0.570919148	0.411098122	0.499401055	0.664346372	0.99989309	0.468599414
BDH1	0.570919148	0.381899719	0.469204177	0.742618602	0.99989309	0.567131921
SLC16A6	0.5710197	0.595931157	0.556244714	0.721039637	0.550221271	0.791259029
KMT5C	0.5710197	0.382781759	0.765074242	0.908054165	0.55440643	0.522495274
ADGRF2	0.5710197	0.740948281	0.515161241	0.78508807	0.713082855	0.419654233
NIN	0.5710197	0.411804906	0.529161538	0.883885658	0.808944674	0.559070967
SEPHS2	0.5710197	0.404916922	0.556529139	0.740509526	0.954501767	0.610416296

NF2	0.5710197	0.422259435	0.482329815	0.721039637	0.99989309	0.487526214
RIOX1	0.5710197	0.432162553	0.515161241	0.721039637	0.99989309	0.555681086
ACSM3	0.575678311	0.36694325	0.924266781	0.692399817	0.685145778	0.515290598
OXTR	0.577807657	0.473526978	0.684005375	0.862604827	0.725609902	0.404171086
SPRN	0.580365932	0.420056896	0.789039067	0.841828571	0.566460478	0.550542614
FARSA	0.580365932	0.537186174	0.541411887	0.714257651	0.569491169	0.880404833
ARHGEF26	0.580365932	0.826827062	0.632743297	0.664346372	0.570932145	0.567131921
SAE1	0.580365932	0.466972398	0.694190265	0.691510355	0.579030436	0.812509877
PROKR1	0.580365932	0.396956826	0.58489603	0.820228145	0.6333098	0.77208617
KIF22	0.580365932	0.52442418	0.751496241	0.820228145	0.657566173	0.421262316
CNTNAP1	0.580365932	0.587833611	0.673737267	0.778545211	0.660341896	0.545791366
LOC112442634	0.580365932	0.562255493	0.477908341	0.917869295	0.675325659	0.50638682
ADAMTS7	0.580365932	0.505355809	0.632855053	0.882551906	0.676199383	0.474949941
AFAP1	0.580365932	0.437293399	0.469204177	0.847813982	0.741060815	0.670243498
HECTD3	0.580365932	0.479254481	0.864879592	0.670054175	0.762655357	0.418451026
DOLK	0.580365932	0.468564925	0.620720409	0.670054175	0.766792351	0.757327233
GZMK	0.580365932	0.505355809	0.46756118	0.672528632	0.91011204	0.731709303
LOC112442284	0.580365932	0.442555387	0.678204563	0.721039637	0.912507538	0.550542614
TUBG2	0.580365932	0.475771869	0.500525108	0.820228145	0.954501767	0.4568517
YTHDF2	0.580365932	0.418766973	0.602496975	0.778545211	0.96532164	0.51887862
MMP28	0.580365932	0.471868414	0.480608818	0.721039637	0.99989309	0.419654233
DNAJA1	0.580365932	0.488637812	0.509263975	0.664346372	0.99989309	0.462198866
RSRC2	0.586328793	0.406246837	0.632855053	0.729345432	0.976970807	0.523061787
SEC24B	0.586328793	0.537295148	0.529161538	0.664346372	0.99989309	0.438979833
VSIG10	0.590986672	0.427197858	0.520659464	0.781241796	0.61025542	0.880272231
TLL2	0.590986672	0.405306771	0.620720409	0.958879502	0.67894608	0.474949941
FILIP1L	0.590986672	0.444747926	0.468416519	0.781241796	0.685145778	0.840150874
GNGT2	0.590986672	0.692547228	0.479530839	0.788938193	0.750160769	0.498961382
LTN1	0.590986672	0.488637812	0.684005375	0.781241796	0.858282183	0.447513267
ZNF318	0.591457684	0.373363257	0.652359766	0.806922554	0.55440643	0.832108368
ARL14EP	0.591457684	0.479254481	0.735516022	0.915749182	0.575855826	0.412133814
KIF1BP	0.591817674	0.688099473	0.620720409	0.721039637	0.589511049	0.636570258
TAOK3	0.591817674	0.393843824	0.491092787	0.721039637	0.969323945	0.701364325
INPP5J	0.592691607	0.549065127	0.746686237	0.847813982	0.581593891	0.419654233
LOC107132469	0.594646879	0.568497289	0.611257543	0.692399817	0.611314139	0.796002422
RGS18	0.59484223	0.373363257	0.605653985	0.942280741	0.576003049	0.653387719
OPTC	0.595962414	0.37198719	0.620860761	0.720889501	0.55440643	0.961336964
SMIM3	0.595962414	0.506366149	0.481390179	0.806922554	0.580642237	0.830456671

NPTX1	0.595962414	0.406246837	0.620720409	0.95964071	0.581593891	0.572789272
RBM12	0.595962414	0.507234108	0.798686086	0.740026105	0.614338501	0.55361803
CCDC158	0.595962414	0.595931157	0.49119595	0.915749182	0.675325659	0.487526214
PKLR	0.595962414	0.578451435	0.632190849	0.750804902	0.675325659	0.635211097
ELFN1	0.595962414	0.411098122	0.620860761	0.925210703	0.675656796	0.55361803
IL33	0.595962414	0.575232052	0.545279742	0.89088601	0.68375459	0.516100157
FAM198A	0.595962414	0.383324576	0.722604787	0.825933581	0.702383614	0.600077084
MAGED1	0.595962414	0.404916922	0.605995935	0.991978984	0.713143057	0.419654233
WDR6	0.595962414	0.390662449	0.495716287	0.861272209	0.943768595	0.563734201
LOC782779	0.595962414	0.479254481	0.620720409	0.709187504	0.99989309	0.493149999
DBF4B	0.595962414	0.383324576	0.500943297	0.664346372	0.99989309	0.507387413
LNX2	0.595962414	0.473863398	0.479530839	0.721039637	0.99989309	0.610416296
CX3CR1	0.596630518	0.426522272	0.620860761	0.995118128	0.559664539	0.520677492
LOC104970537	0.596630518	0.394411867	0.833567938	0.776207435	0.566460478	0.671528522
ITIH4	0.596630518	0.701581467	0.623512949	0.806922554	0.570932145	0.550052252
TBC1D22B	0.596630518	0.393843824	0.511586767	0.999939566	0.570932145	0.610416296
TESPA1	0.596630518	0.415275887	0.591811466	0.854955257	0.575855826	0.808302424
LOC101903026	0.596630518	0.398815025	0.849077738	0.883885658	0.576003049	0.447180581
LOC112441484	0.596630518	0.473863398	0.924266781	0.741347517	0.576003049	0.474949941
BHLHA15	0.596630518	0.464010019	0.726668529	0.851394268	0.576003049	0.607436462
RGS4	0.596630518	0.62584388	0.545279742	0.928836449	0.580642237	0.4568517
DCTN6	0.596630518	0.411376371	0.765074242	0.841828571	0.580642237	0.607328652
C6H4orf48	0.596630518	0.559785028	0.607119516	0.923832787	0.581593891	0.507772366
PPP1R2	0.596630518	0.419595013	0.843427302	0.781241796	0.587069217	0.578699613
TUBB2A	0.596630518	0.473863398	0.879215964	0.664346372	0.622490325	0.610416296
LOC112449613	0.596630518	0.462191552	0.507386236	0.721039637	0.623122621	0.943685681
FN1	0.596630518	0.453259111	0.516623782	0.999939566	0.657434382	0.478543685
IQGAP3	0.596630518	0.542880911	0.720067907	0.820228145	0.659374254	0.474949941
LMNB1	0.596630518	0.473863398	0.684005375	0.846715063	0.675325659	0.564529177
NCEH1	0.596630518	0.404916922	0.688553093	0.87452213	0.675325659	0.595702749
DNAH10	0.596630518	0.443474229	0.813256265	0.721039637	0.675325659	0.635661027
TET3	0.596630518	0.464965392	0.612353108	0.871411597	0.675467149	0.640138785
SEPT5	0.596630518	0.559754831	0.746957417	0.781241796	0.675656796	0.474949941
FCRL5	0.596630518	0.538091051	0.524936203	0.781241796	0.675889904	0.752731247
MYRF	0.596630518	0.432788109	0.825888497	0.820228145	0.685145778	0.457231394
INSR	0.596630518	0.411098122	0.570590624	0.915749182	0.702383614	0.610416296
PAPPA2	0.596630518	0.400548351	0.776990008	0.741347517	0.702383614	0.637776476
ZNF341	0.596630518	0.382781759	0.777178978	0.87630412	0.713082855	0.435648157

LOC100300510	0.596630518	0.701581467	0.484315892	0.720889501	0.713082855	0.671528522
GIMAP4	0.596630518	0.456669239	0.524936203	0.775761952	0.713082855	0.830456671
CDC42EP2	0.596630518	0.458781098	0.62144899	0.924489924	0.725609902	0.467459971
NINJ2	0.596630518	0.550965714	0.775306135	0.692399817	0.730791761	0.523061787
EXTL1	0.596630518	0.494451722	0.520659464	0.87452213	0.730791761	0.609066537
CHUK	0.596630518	0.583641966	0.625367433	0.785145388	0.741060815	0.507387413
COL7A1	0.596630518	0.419875993	0.637126628	0.781241796	0.742316849	0.678644983
COL6A1	0.596630518	0.54984186	0.632743297	0.842538664	0.750220157	0.451842351
NAPSA	0.596630518	0.437076272	0.592801724	0.760188917	0.763613029	0.764546301
PDLIM7	0.596630518	0.425397899	0.486992317	0.985181062	0.789627785	0.474949941
PDZD3	0.596630518	0.730944867	0.612611059	0.664346372	0.814605912	0.516100157
FTSJ3	0.596630518	0.569044188	0.620720409	0.721039637	0.825598281	0.567131921
VEGFC	0.596630518	0.453259111	0.482588959	0.948172741	0.836838903	0.454706429
BCL11A	0.596630518	0.400929909	0.545279742	0.820228145	0.846122929	0.671528522
LOC784769	0.596630518	0.443474229	0.633986838	0.846115604	0.849382765	0.457231394
PDCD1	0.596630518	0.393843824	0.699760739	0.781241796	0.881823169	0.550052252
SHISA4	0.596630518	0.425397899	0.678070701	0.820228145	0.883643824	0.469450527
PDGFA	0.596630518	0.436312524	0.561129392	0.781241796	0.888595211	0.660829605
ASPSCR1	0.596630518	0.505355809	0.738217747	0.692399817	0.912512577	0.481755863
SOBP	0.596630518	0.587833611	0.617037621	0.721039637	0.925981327	0.478678586
RANBP6	0.596630518	0.419875993	0.618763457	0.727579206	0.932197133	0.647432409
USP53	0.596630518	0.426522272	0.55673062	0.850465555	0.952172109	0.474949941
EBP	0.596630518	0.473526978	0.545279742	0.742618602	0.956624006	0.610882384
LOC112445030	0.596630518	0.456669239	0.632855053	0.721039637	0.968795608	0.550542614
HDAC10	0.596630518	0.454401393	0.709561629	0.721039637	0.972917711	0.446548697
LOC112443504	0.596630518	0.454401393	0.617037621	0.685074116	0.972917711	0.640138785
ARIH1	0.596630518	0.473863398	0.557464877	0.721039637	0.99989309	0.473495286
PSPH	0.596630518	0.473793567	0.602496975	0.700857388	0.99989309	0.578699613
DES	0.5972934	0.871030989	0.620720409	0.710581246	0.559664539	0.572789272
CCDC114	0.5972934	0.437950914	0.712526829	0.968293092	0.566460478	0.474949941
PPDPF	0.5972934	0.406246837	0.63818281	0.996601241	0.575855826	0.508971078
HSPA13	0.5972934	0.419875993	0.810425784	0.806922554	0.702383614	0.51887862
CHRNA1	0.5972934	0.765341142	0.58489603	0.714257651	0.718425007	0.572628211
SLC9A1	0.5972934	0.432788109	0.678204563	0.806922554	0.915496086	0.474949941
C17H22orf39	0.5972934	0.401951083	0.499056056	0.713901319	0.99989309	0.474949941
CSTB	0.597434784	0.507048734	0.573880698	0.781241796	0.6333098	0.788131005
OGFOD2	0.597434784	0.550141073	0.538132307	0.691510355	0.99989309	0.55361803
PLEKHA1	0.597937176	0.494451722	0.726668529	0.908054165	0.614338501	0.474949941

LOC100298890	0.597937176	0.464965392	0.744369186	0.758703434	0.675656796	0.653387719
RGS14	0.597937176	0.573324334	0.620860761	0.691923003	0.789365673	0.651139949
AKAP8L	0.597937176	0.400929909	0.724199536	0.761349001	0.804250204	0.636570258
EIF1	0.601870002	0.390662449	0.726668529	0.915749182	0.676199383	0.523061787
MON1B	0.602679027	0.400929909	0.806311697	0.806922554	0.566460478	0.665449472
CCBE1	0.604281217	0.404916922	0.812770091	0.721039637	0.90399271	0.474949941
POLD4	0.604293466	0.436312524	0.715979316	0.897446122	0.675325659	0.546389196
GPR173	0.604342459	0.426522272	0.484315892	0.98636843	0.818457184	0.474949941
RABEP1	0.604342459	0.404916922	0.573295201	0.78508807	0.99989309	0.501985625
ZNF436	0.604356254	0.473863398	0.601873896	0.999939566	0.566460478	0.4568517
LOC112448523	0.604356254	0.406246837	0.726668529	0.95175364	0.570932145	0.54521041
PLIN2	0.604356254	0.404916922	0.788848487	0.862060767	0.571258463	0.610882384
DFFB	0.604356254	0.432788109	0.919108971	0.778545211	0.575855826	0.55361803
CPSF1	0.604356254	0.405306771	0.883982433	0.850828215	0.580642237	0.4568517
LOC101902869	0.604356254	0.68147929	0.632855053	0.734194666	0.581593891	0.66854638
LIG1	0.604356254	0.404916922	0.90002174	0.826315685	0.60923233	0.474949941
PIGO	0.604356254	0.464010019	0.860197593	0.820228145	0.627119169	0.446591403
TMEM109	0.604356254	0.404916922	0.897665623	0.806922554	0.6333098	0.478543685
TMEM35B	0.604356254	0.628485621	0.689299245	0.825933581	0.6333098	0.478543685
RCN3	0.604356254	0.46456927	0.66128476	0.934591536	0.6333098	0.536475491
XYLB	0.604356254	0.479254481	0.551458879	0.846115604	0.650629171	0.77208617
MFSD6L	0.604356254	0.490341542	0.594753564	0.992137653	0.659374254	0.4568517
SDC1	0.604356254	0.454401393	0.549314874	0.999939566	0.659374254	0.486525843
PLN	0.604356254	0.897918739	0.66128476	0.692399817	0.664794646	0.438979833
MKI67	0.604356254	0.454401393	0.771402466	0.846043369	0.675325659	0.523061787
LOC101906739	0.604356254	0.506366149	0.633986838	0.928836449	0.682242009	0.446807465
THAP3	0.604356254	0.494451722	0.885274776	0.685074116	0.685145778	0.567131921
TPGS2	0.604356254	0.471726524	0.726668529	0.709187504	0.696261246	0.752900996
MAP4K3	0.604356254	0.494210805	0.66128476	0.90419464	0.701078953	0.54521041
DTL	0.604356254	0.693153864	0.545279742	0.796330272	0.702383614	0.567131921
AKAP4	0.604356254	0.494248266	0.545279742	0.88747242	0.703447227	0.642154165
ZNF296	0.604356254	0.567752026	0.721333834	0.788938193	0.706443708	0.520937232
HENMT1	0.604356254	0.426356475	0.812770091	0.709731242	0.713082855	0.671720185
STAP2	0.604356254	0.404916922	0.620720409	0.945929971	0.725609902	0.550542614
EOMES	0.604356254	0.494451722	0.561939653	0.806922554	0.730791761	0.701784819
SCG3	0.604356254	0.437076272	0.611257543	0.984968973	0.742316849	0.447513267
MPPE1	0.604356254	0.619184305	0.644103972	0.738189414	0.742316849	0.579379269
SLC25A51	0.604356254	0.629715977	0.750089824	0.721039637	0.745432249	0.479527244

LOC112441542	0.604356254	0.490341542	0.545279742	0.781241796	0.750220157	0.764546301
COL6A2	0.604356254	0.516673361	0.660379966	0.868090726	0.766792351	0.447180581
STBD1	0.604356254	0.642122145	0.620720409	0.721039637	0.766792351	0.610416296
PPL	0.604356254	0.434767437	0.852758896	0.740690231	0.77278187	0.474949941
SRGN	0.604356254	0.612773311	0.546505003	0.720889501	0.7817968	0.671720185
CNTFR	0.604356254	0.81808696	0.517310352	0.715279959	0.819670067	0.515839823
BNIP2	0.604356254	0.419875993	0.765074242	0.820228145	0.83417924	0.447180581
TRIM66	0.604356254	0.425397899	0.620720409	0.721039637	0.84102894	0.765130735
PPM1F	0.604356254	0.400929909	0.622707492	0.731974857	0.849776159	0.757327233
COQ10B	0.604356254	0.538091051	0.625367433	0.781241796	0.861762366	0.55361803
PANK1	0.604356254	0.419875993	0.549314874	0.700857388	0.889271428	0.839340243
CNKSR3	0.604356254	0.655710881	0.610765273	0.709187504	0.902843706	0.55361803
MYH10	0.604356254	0.494451722	0.545279742	0.878289777	0.904151325	0.457231394
LOC100848246	0.604356254	0.420056896	0.620720409	0.69621924	0.907192767	0.770391484
PI4K2A	0.604356254	0.435300125	0.750089824	0.721039637	0.934859184	0.535096243
ZNF484	0.604356254	0.426522272	0.513743573	0.710581246	0.941107925	0.812208548
GCHFR	0.604356254	0.411098122	0.600123126	0.842538664	0.954741113	0.540636511
C18H19orf48	0.604356254	0.619374114	0.524936203	0.721039637	0.970733065	0.550845146
RERGL	0.604356254	0.453259111	0.600123126	0.731974857	0.981777076	0.625532034
CNOT4	0.604356254	0.475373997	0.605016625	0.721039637	0.99989309	0.454706429
C15H11orf87	0.604356254	0.405438493	0.515689199	0.775761952	0.99989309	0.457231394
PMM1	0.604356254	0.471868414	0.500943297	0.721039637	0.99989309	0.501985625
KIAA1024	0.604356254	0.464176632	0.512985904	0.737540682	0.99989309	0.503077635
NRM	0.604356254	0.404916922	0.520659464	0.75922259	0.99989309	0.508203007
STK38	0.604356254	0.499960638	0.491092787	0.716992579	0.99989309	0.579379269
PTPN12	0.604356254	0.506366149	0.514686689	0.709187504	0.99989309	0.612490982
CD69	0.604356254	0.400929909	0.547390233	0.781241796	0.99989309	0.616913081
TMEM82	0.604356254	0.406246837	0.545279742	0.720889501	0.99989309	0.671720185
C25H16orf45	0.604572826	0.502718799	0.825888497	0.820228145	0.571258463	0.550542614
LDB3	0.604731088	0.934248552	0.598843922	0.731974857	0.575855826	0.516100157
AHR	0.604731088	0.435300125	0.570590624	0.971220023	0.575855826	0.661861937
SPIB	0.604731088	0.502885369	0.526382013	0.943323364	0.581593891	0.654251319
MROH6	0.604731088	0.505355809	0.873750661	0.788165915	0.614338501	0.462198866
MYBL2	0.604731088	0.419156188	0.979231344	0.721039637	0.625198	0.474949941
LOC782675	0.604731088	0.882736993	0.500943297	0.729345432	0.6333098	0.610416296
PPP2CB	0.604731088	0.476356516	0.620860761	0.917869295	0.68375459	0.578699613
PABPN1	0.604731088	0.420056896	0.820767684	0.820228145	0.696261246	0.550542614
CP	0.604731088	0.436312524	0.765074242	0.721039637	0.696261246	0.703742328

BMP2	0.604731088	0.541005316	0.747084388	0.820228145	0.717414242	0.474949941
PGLYRP2	0.604731088	0.426522272	0.64822617	0.794713619	0.7326067	0.731709303
PADI2	0.604731088	0.404916922	0.66679673	0.825933581	0.750160769	0.66854638
SMIM10L1	0.604731088	0.494248266	0.633986838	0.91329897	0.763613029	0.462075603
UNC13B	0.604731088	0.543699592	0.678204563	0.721039637	0.766792351	0.647432409
ABTB1	0.604731088	0.42732587	0.79610462	0.825933581	0.766932246	0.451462446
TAPT1	0.604731088	0.494451722	0.598843922	0.847774989	0.846122929	0.560772099
LOC112448395	0.604731088	0.419875993	0.694542317	0.781241796	0.887132523	0.593724238
HPSE	0.604731088	0.406246837	0.66128476	0.721039637	0.941064037	0.671528522
ATG9B	0.604731088	0.747164473	0.51477511	0.709187504	0.954501767	0.474949941
LPGAT1	0.604731088	0.425892926	0.625367433	0.806922554	0.996250445	0.51887862
MZT2B	0.604731088	0.534344991	0.516623782	0.685074116	0.99989309	0.508203007
HCN4	0.604731088	0.595625745	0.524936203	0.714257651	0.99989309	0.51887862
ZBTB11	0.604731088	0.490341542	0.545279742	0.721039637	0.99989309	0.523061787
WDR43	0.604731088	0.419875993	0.620720409	0.781241796	0.99989309	0.55361803
GPX4	0.604731088	0.453259111	0.520659464	0.692399817	0.99989309	0.578699613
DEDD2	0.604731088	0.52442418	0.591451674	0.691510355	0.99989309	0.579379269
LOC785629	0.604731088	0.456669239	0.515161241	0.788165915	0.99989309	0.594117809
PCOLCE	0.606301196	0.503247136	0.684005375	0.846715063	0.819670067	0.45454353
BUB1	0.607294552	0.494451722	0.747093618	0.841828571	0.750160769	0.4568517
ITGB8	0.609480904	0.458781098	0.547390233	0.925004412	0.66198161	0.683895124
ESM1	0.609480904	0.464010019	0.827125538	0.806922554	0.725609902	0.474949941
HHEX	0.609480904	0.502718799	0.518916624	0.983939542	0.7326067	0.493149999
SLC7A8	0.609480904	0.404916922	0.694422859	0.910808102	0.765898761	0.543098314
CARNS1	0.609480904	0.600960324	0.598843922	0.781241796	0.766648996	0.636570258
ASPHD2	0.609480904	0.427663241	0.751496241	0.78508807	0.766792351	0.607436462
DHX58	0.609480904	0.428968716	0.787740344	0.731974857	0.846122929	0.572789272
RIOK2	0.609480904	0.55176988	0.611257543	0.841828571	0.862637717	0.487526214
ANPEP	0.609480904	0.540649036	0.557974073	0.714257651	0.939361296	0.671528522
RIC1	0.609480904	0.436312524	0.546505003	0.709187504	0.99989309	0.516168837
ELP1	0.609520737	0.494451722	0.848646852	0.806922554	0.640280368	0.497799452
PET100	0.609520737	0.712838989	0.615875797	0.768226168	0.784855148	0.503077635
ENKD1	0.611259489	0.406246837	0.832431595	0.850828215	0.575855826	0.607436462
RTEL1	0.611259489	0.4835064	0.919108971	0.806922554	0.576003049	0.467459971
DENND1A	0.611259489	0.464010019	0.539146924	0.709187504	0.609004508	0.984609406
CENPE	0.611259489	0.473526978	0.775786014	0.913319839	0.6333098	0.474949941
TMEM87A	0.611259489	0.519805717	0.678204563	0.946344632	0.6333098	0.474949941
GTF3C1	0.611259489	0.432788109	0.735054113	0.91329897	0.6333098	0.588661483

SLC49A3	0.611259489	0.473526978	0.845336233	0.820228145	0.675325659	0.474949941
TXK	0.611259489	0.479254481	0.545279742	0.820228145	0.682242009	0.823195389
ERI3	0.611259489	0.405306771	0.773281026	0.720889501	0.975291792	0.542853655
RCOR1	0.611259489	0.420056896	0.547390233	0.820228145	0.99989309	0.508971078
C5AR1	0.611259489	0.562255493	0.538132307	0.691877804	0.99989309	0.610416296
LOC615733	0.612643118	0.530282658	0.632855053	0.776207435	0.701136511	0.752857325
TCAF2	0.612799716	0.438385292	0.703042171	0.925931623	0.576164442	0.611777732
P3H4	0.612799716	0.426522272	0.632743297	0.999939566	0.581593891	0.474949941
RBM47	0.612799716	0.458781098	0.678204563	0.846715063	0.581593891	0.770391484
ATP6AP1	0.612799716	0.534344991	0.733231491	0.855137785	0.608328153	0.578699613
FGF11	0.612799716	0.955798423	0.611257543	0.721039637	0.659374254	0.474949941
CEP95	0.612799716	0.409964496	0.870054336	0.848115681	0.659374254	0.503077635
C29H11orf24	0.612799716	0.568025614	0.611257543	0.806922554	0.659374254	0.740271903
FREM1	0.612799716	0.595792756	0.726668529	0.692399817	0.675656796	0.732453932
HEATR4	0.612799716	0.484750909	0.722352424	0.78508807	0.676199383	0.705712189
LOC100848263	0.612799716	0.406246837	0.744490351	0.89088601	0.678920037	0.611777732
CEPT1	0.612799716	0.545732987	0.684005375	0.850828215	0.702383614	0.55361803
LOC107131209	0.612799716	0.464965392	0.685222626	0.924396934	0.72279174	0.498031245
ATF6	0.612799716	0.479254481	0.812043145	0.754030944	0.725609902	0.591016795
TRMT1	0.612799716	0.567752026	0.577571931	0.788938193	0.727443327	0.704011905
GPBP1L1	0.612799716	0.473526978	0.726668529	0.781241796	0.745432249	0.651173954
C7H5orf63	0.612799716	0.462191552	0.556529139	0.700857388	0.745432249	0.927412916
FASLG	0.612799716	0.688099473	0.51477511	0.770892936	0.747513637	0.661937165
NSMCE4A	0.612799716	0.458781098	0.591811466	0.992137653	0.750160769	0.474949941
CDCA3	0.612799716	0.567752026	0.707441133	0.820228145	0.758167492	0.474949941
SDCBP	0.612799716	0.458781098	0.678204563	0.925901365	0.763613029	0.457231394
PKN2	0.612799716	0.447739746	0.638223753	0.925931623	0.775432213	0.491256306
TRAPPC9	0.612799716	0.473526978	0.545279742	0.776207435	0.777396446	0.837501229
LOC781663	0.612799716	0.506366149	0.694542317	0.851394268	0.780497593	0.474949941
TP53BP1	0.612799716	0.494451722	0.616878782	0.88747242	0.808517124	0.578699613
C11H9orf116	0.612799716	0.404916922	0.573880698	0.924489924	0.814605912	0.610416296
PPP1R12B	0.612799716	0.506366149	0.632855053	0.781241796	0.819670067	0.637306705
LOC788425	0.612799716	0.442584324	0.765074242	0.720889501	0.83627631	0.651139949
MERTK	0.612799716	0.426522272	0.684005375	0.754030944	0.87465791	0.677152076
ITPKC	0.612799716	0.435300125	0.583731628	0.825933581	0.892149112	0.647432409
SLC7A6	0.612799716	0.490341542	0.589475107	0.820228145	0.902569851	0.614421218
CXCR4	0.612799716	0.502718799	0.532777091	0.806922554	0.925981327	0.642154165
PRR11	0.612799716	0.582472344	0.651174146	0.721039637	0.927828188	0.567131921

NOL11	0.612799716	0.494451722	0.632190849	0.691923003	0.960835287	0.660829605
SCML4	0.612799716	0.537295148	0.520659464	0.731974857	0.983089554	0.636570258
LOC100847995	0.612799716	0.432788109	0.757235816	0.721039637	0.998276725	0.481755863
MED13	0.612799716	0.473863398	0.598843922	0.692399817	0.99989309	0.474949941
LOC617475	0.612799716	0.454807376	0.632190849	0.707990872	0.99989309	0.474949941
RANBP2	0.612799716	0.4835064	0.632743297	0.781241796	0.99989309	0.474949941
TMF1	0.612799716	0.505355809	0.504377317	0.709187504	0.99989309	0.497799452
DNAJC25	0.612799716	0.454401393	0.699920157	0.721039637	0.99989309	0.540636511
MRPS21	0.612799716	0.411376371	0.652359766	0.721039637	0.99989309	0.55361803
NDEL1	0.612799716	0.479254481	0.598843922	0.754030944	0.99989309	0.610416296
ENDOG	0.612799716	0.426522272	0.55673062	0.721039637	0.99989309	0.63363599
MRPL57	0.612799716	0.505355809	0.565627771	0.69621924	0.99989309	0.660829605
FANCF	0.612799716	0.412213237	0.545279742	0.729652612	0.99989309	0.691363988
AASDHPPT	0.615019795	0.430578239	0.620720409	0.820228145	0.750160769	0.764546301
POP4	0.615424913	0.464010019	0.746957417	0.820228145	0.675325659	0.66854638
DARS	0.616776139	0.415275887	0.64445174	0.781241796	0.638822105	0.882322567
FKBP7	0.616776139	0.426522272	0.682591947	0.956154344	0.725609902	0.503077635
FNDC10	0.616776139	0.514566709	0.600123126	0.825933581	0.943768595	0.539135678
TMED3	0.617871977	0.616586256	0.744369186	0.836785013	0.581593891	0.567131921
SMTN	0.617871977	0.857034402	0.620860761	0.731974857	0.581593891	0.610416296
FAM166B	0.617871977	0.534344991	0.515689199	0.883885658	0.581593891	0.828376685
ARPC5	0.617871977	0.464010019	0.929605682	0.806922554	0.594664259	0.508971078
LOC112446791	0.617871977	0.868963165	0.548852488	0.721039637	0.606036044	0.660829605
CTSB	0.617871977	0.534344991	0.661590836	0.943323364	0.60923233	0.550542614
ZNRD1	0.617871977	0.411376371	0.929043558	0.726002049	0.6333098	0.642154165
LOC527388	0.617871977	0.490384759	0.70435078	0.820228145	0.6333098	0.729817953
CCDC168	0.617871977	0.413361832	0.830033146	0.795476498	0.63496351	0.66854638
NEK2	0.617871977	0.450665155	0.871859013	0.850828215	0.642463841	0.474949941
MIS18BP1	0.617871977	0.425397899	0.844065668	0.871894706	0.657566173	0.536475491
NPPC	0.617871977	0.625663019	0.655246594	0.721039637	0.659374254	0.753067712
RAD50	0.617871977	0.538091051	0.750089824	0.820228145	0.676199383	0.579379269
PCNX3	0.617871977	0.411098122	0.899530079	0.820228145	0.68375459	0.493149999
LOC781022	0.617871977	0.533749385	0.81284583	0.809634958	0.68375459	0.51887862
KPNA2	0.617871977	0.494451722	0.616881105	0.781241796	0.68375459	0.84763005
AAGAB	0.617871977	0.609819443	0.620860761	0.781241796	0.68653192	0.703742328
SLX1A	0.617871977	0.487116492	0.79610462	0.806922554	0.702383614	0.579379269
JPT2	0.617871977	0.450665155	0.722951238	0.820228145	0.702383614	0.683895124
RAD23A	0.617871977	0.534103098	0.707441133	0.820228145	0.703786371	0.610416296

SRRM3	0.617871977	0.502718799	0.545279742	0.910808102	0.713082855	0.66915423
ANLN	0.617871977	0.411376371	0.726668529	0.865118174	0.713130507	0.637306705
DRAM2	0.617871977	0.443012007	0.662688842	0.777706486	0.727364064	0.799314198
BYSL	0.617871977	0.48029058	0.683668554	0.781241796	0.730791761	0.728959145
ADM2	0.617871977	0.435888764	0.754897816	0.781241796	0.766792351	0.637306705
TUBB4B	0.617871977	0.493191436	0.621963024	0.704390487	0.766792351	0.855778243
EMILIN1	0.617871977	0.567752026	0.529161538	0.858949322	0.771516579	0.633384404
GPRC5A	0.617871977	0.537295148	0.632855053	0.811000597	0.846122929	0.583720387
JMY	0.617871977	0.655947947	0.548852488	0.781241796	0.87483668	0.588661483
PURG	0.617871977	0.551867132	0.573880698	0.781241796	0.884679766	0.635211097
NSDHL	0.617871977	0.514208066	0.524936203	0.742618602	0.910770324	0.757327233
PRKD2	0.617871977	0.701581467	0.545279742	0.720889501	0.911283882	0.609824407
CBX4	0.617871977	0.426522272	0.726668529	0.781241796	0.954501767	0.545791366
MACF1	0.617871977	0.563639555	0.542182681	0.731974857	0.957605284	0.646642632
SIRT1	0.617871977	0.551867132	0.607119516	0.781241796	0.975291792	0.543098314
MGC152281	0.617871977	0.494451722	0.703042171	0.731974857	0.996250445	0.501829475
POLR2F	0.617871977	0.568497289	0.591811466	0.700857388	0.99989309	0.550542614
VHL	0.617871977	0.420056896	0.665743961	0.700857388	0.99989309	0.578699613
PYGM	0.620360611	0.795993324	0.620860761	0.731974857	0.675325659	0.611777732
NR2C2AP	0.620571756	0.601692739	0.620720409	0.781241796	0.581593891	0.808631013
LOC104971345	0.620571756	0.490341542	0.841835423	0.781241796	0.582845642	0.645838677
ANKRD13C	0.620571756	0.442555387	0.872397372	0.721039637	0.612294326	0.752857325
AGPS	0.620571756	0.462191552	0.685222626	0.915749182	0.622490325	0.651488215
PRG4	0.620571756	0.543274277	0.817256644	0.781241796	0.642463841	0.604227527
LOC101903383	0.620571756	0.504573248	0.600123126	0.904990622	0.652021057	0.728276429
CCL11	0.620571756	0.516029379	0.675040193	0.915749182	0.660550162	0.609824407
LOC783797	0.620571756	0.589137013	0.545279742	0.781241796	0.66198161	0.828602505
PDGFRA	0.620571756	0.669648359	0.610765273	0.781241796	0.713691227	0.651488215
HMGCS1	0.620571756	0.496432649	0.615488018	0.803538703	0.725609902	0.788318946
KBTBD2	0.620571756	0.619184305	0.620860761	0.731974857	0.752741558	0.698487167
HNRNPH3	0.620571756	0.448380044	0.589825708	0.925004412	0.753418479	0.642154165
KCNH2	0.620571756	0.692987203	0.632190849	0.781241796	0.766792351	0.567131921
UBLCP1	0.620571756	0.421889095	0.611257543	0.781241796	0.773626517	0.862403446
WFIKKN2	0.620571756	0.485870931	0.757337101	0.721039637	0.848896892	0.633384404
TACO1	0.620571756	0.525690188	0.632743297	0.731974857	0.885690198	0.671528522
ZNF12	0.620571756	0.425397899	0.551625304	0.781241796	0.895183012	0.808138656
PTGFR	0.620571756	0.46726452	0.55673062	0.883885658	0.910770324	0.59947988
BAG3	0.620571756	0.710155842	0.583731628	0.721039637	0.927804504	0.552730018

GID8	0.620571756	0.426522272	0.63818281	0.732079395	0.947146038	0.706152771
MLF1	0.620571756	0.491746055	0.620720409	0.721039637	0.975291792	0.651488215
RLF	0.620571756	0.538091051	0.547390233	0.721039637	0.99989309	0.567131921
VWA3B	0.620571756	0.432788109	0.620860761	0.731974857	0.99989309	0.648987851
SLC16A9	0.621255732	0.507322509	0.618763457	0.806922554	0.954501767	0.567131921
MOK	0.62126847	0.425397899	0.827125538	0.781241796	0.611314139	0.745987575
ANAPC10	0.621738391	0.464965392	0.827568561	0.721039637	0.925981327	0.523061787
PRRC2C	0.622012575	0.465780053	0.570590624	0.721039637	0.99989309	0.611777732
LIPA	0.623819155	0.505355809	0.684005375	0.845816079	0.614338501	0.730588392
TAGLN	0.623819155	0.536568815	0.683668554	0.720889501	0.685145778	0.840150874
SLFN11	0.623819155	0.506366149	0.633986838	0.860663136	0.780497593	0.599490586
ZNF638	0.623819155	0.490341542	0.661898431	0.721039637	0.99989309	0.550052252
RALBP1	0.624166466	0.473526978	0.804700844	0.820228145	0.719753534	0.578699613
PIK3C3	0.624166466	0.743422331	0.617037621	0.784305663	0.742316849	0.564506858
NUP210	0.624166466	0.420056896	0.733231491	0.806922554	0.766792351	0.671720185
UGGT2	0.62435533	0.420056896	0.701564796	0.89088601	0.915496086	0.481755863
THOP1	0.624581868	0.487116492	0.520659464	0.721039637	0.822907584	0.886992214
EXD1	0.62537954	0.443474229	0.660379966	0.893831952	0.675325659	0.732184848
FLCN	0.62537954	0.54331542	0.598843922	0.721039637	0.790063341	0.809650729
GPNMB	0.625490156	0.46039787	0.633986838	0.857959288	0.611314139	0.809650729
CATSPERD	0.625490156	0.432788109	0.817256644	0.841828571	0.6333098	0.642154165
SPAG5	0.625490156	0.432788109	0.827125538	0.915749182	0.675325659	0.474949941
OTUD6B	0.625490156	0.426923729	0.678070701	0.992137653	0.675325659	0.557327019
MYL9	0.625490156	0.63512774	0.678070701	0.720889501	0.713082855	0.732375703
FCN1	0.625490156	0.520020743	0.545279742	0.724487925	0.906183446	0.78893698
CD8B	0.625929275	0.735625249	0.581350014	0.742618602	0.861762366	0.567131921
PRKCQ	0.626210164	0.502718799	0.555591179	0.820228145	0.702383614	0.839340243
LOC101904976	0.626210164	0.538091051	0.766352455	0.721039637	0.947146038	0.498137139
LRRC66	0.627597656	0.66071365	0.618763457	0.89088601	0.622490325	0.610882384
RAB12	0.627597656	0.435888764	0.941312064	0.767938166	0.695415649	0.540400894
CELF1	0.628229988	0.481533098	0.918576062	0.721039637	0.63496351	0.640100696
SNCG	0.628399042	0.462191552	0.678204563	0.781241796	0.892966823	0.669225252
CRABP2	0.628835775	0.434767437	0.662688842	0.939292364	0.72279174	0.602586916
UGT8	0.628835775	0.636332133	0.532777091	0.750804902	0.725609902	0.80261571
LSP1	0.628835775	0.504999582	0.838488596	0.796330272	0.76279367	0.474949941
BAK1	0.628835775	0.543839253	0.600123126	0.731974857	0.868810704	0.755204495
PTP4A3	0.628835775	0.438825702	0.55673062	0.959009349	0.899048045	0.51887862
LOC100337213	0.628835775	0.703437628	0.545279742	0.714257651	0.970733065	0.580920535

EIF3A	0.628835775	0.435300125	0.662483244	0.781241796	0.993370551	0.610416296
JMJD6	0.628835775	0.487116492	0.621963024	0.709187504	0.99989309	0.478543685
LOC518526	0.629083513	0.476356516	0.598843922	0.714257651	0.614338501	0.989128955
NEK4	0.629083513	0.538091051	0.621963024	0.760188917	0.705101251	0.823139458
SMARCC2	0.629083513	0.505355809	0.620720409	0.779232964	0.86558233	0.728276429
CFAP100	0.629383427	0.502718799	0.776574729	0.825933581	0.750220157	0.540636511
NFE2	0.630303845	0.693153864	0.712526829	0.715279959	0.713691227	0.640642131
IKZF1	0.630303845	0.597404414	0.538657314	0.760188917	0.737449785	0.826868779
GSDME	0.630303845	0.573020585	0.578013891	0.939292364	0.750160769	0.516100157
METTL13	0.630303845	0.588244908	0.620720409	0.820228145	0.780831493	0.614421218
ANOS1	0.63047349	0.462191552	0.688145488	0.796330272	0.886279726	0.636570258
HSF2	0.63047349	0.502885369	0.592801724	0.731974857	0.99989309	0.477051336
LOC112448070	0.630511054	0.522228461	0.765074242	0.721039637	0.611314139	0.821467423
CIT	0.630511054	0.525690188	0.73065099	0.925931623	0.659374254	0.516168837
DBF4	0.630511054	0.499960638	0.806311697	0.893831952	0.659374254	0.520677492
CLTB	0.630511054	0.488637812	0.703042171	0.788938193	0.819670067	0.647432409
ZFC3H1	0.630511054	0.490341542	0.735302276	0.760188917	0.915042549	0.588661483
TAB2	0.630511054	0.507048734	0.588314456	0.714257651	0.99989309	0.507772366
WEE1	0.630511054	0.479254481	0.549314874	0.781052987	0.99989309	0.635645816
LOC112443175	0.630738085	0.444747926	0.545279742	0.946344632	0.609677074	0.802399829
FARS2	0.630738085	0.458781098	0.544411443	0.738189414	0.86558233	0.877623013
LPP	0.630778165	0.694933558	0.62764537	0.87630412	0.611314139	0.588661483
LOC782598	0.630778165	0.445425026	0.705023135	0.915749182	0.642463841	0.66915423
ARL8B	0.630778165	0.426356475	0.712526829	0.958879502	0.676199383	0.579379269
MEG9	0.630778165	0.425397899	0.817256644	0.915749182	0.685145778	0.51887862
CARMIL2	0.630778165	0.432788109	0.808333956	0.721039637	0.858282183	0.645838677
HBQ1	0.630778165	0.504573248	0.611257543	0.720889501	0.99989309	0.567131921
ADRA1D	0.63100647	0.496432649	0.583057712	0.915749182	0.727364064	0.671720185
CCDC78	0.631271609	0.479254481	0.812585086	0.781241796	0.685145778	0.678496087
OVOS2	0.63143858	0.538091051	0.55673062	0.999939566	0.6333098	0.487174962
S1PR5	0.63143858	0.563639555	0.800682903	0.820228145	0.666979426	0.56318581
TOP2A	0.63143858	0.494451722	0.773846486	0.891584014	0.713082855	0.516100157
ZNF276	0.63143858	0.426522272	0.929958748	0.781241796	0.725609902	0.550542614
NOG	0.63143858	0.427663241	0.884325235	0.721039637	0.804250204	0.588661483
LRRC3	0.63143858	0.432775862	0.707441133	0.846715063	0.87465791	0.588971976
COLEC12	0.63143858	0.509590069	0.611257543	0.820228145	0.99989309	0.543098314
CD22	0.631461993	0.711564038	0.620860761	0.846115604	0.618467008	0.616913081
LOC104974460	0.631461993	0.637634861	0.63818281	0.721039637	0.661802294	0.829389486

ABCF2	0.631461993	0.490341542	0.545279742	0.806922554	0.676199383	0.914440253
PSMF1	0.631461993	0.459792626	0.863172962	0.851394268	0.686348774	0.503077635
SMAD7	0.631461993	0.462191552	0.979851274	0.721039637	0.701136511	0.498031245
NCAPG	0.631461993	0.448739356	0.844065668	0.883885658	0.7120086	0.487526214
MAP4K1	0.631461993	0.487116492	0.735302276	0.75922259	0.753418479	0.735805669
RPRD2	0.631461993	0.534103098	0.670969728	0.820686724	0.943768595	0.498031245
VSTM2L	0.631506134	0.443012007	0.747084388	0.781241796	0.960835287	0.567131921
LOC101907540	0.632441964	0.432775862	0.819529308	0.742618602	0.975291792	0.487526214
CD79B	0.632582515	0.553720138	0.651174146	0.781241796	0.6333098	0.829463053
SLC2A3	0.632582515	0.557135186	0.566214106	0.999939566	0.657566173	0.540969064
LOC112444164	0.632582515	0.502885369	0.764481234	0.731974857	0.949074145	0.550052252
NFKBID	0.633631594	0.453259111	0.817256644	0.87452213	0.725609902	0.539135678
FABP7	0.634013938	0.538091051	0.546505003	0.721039637	0.60923233	0.981090047
VCPIP1	0.634013938	0.464965392	0.748723724	0.963153458	0.623122621	0.550542614
ITGA2B	0.634013938	0.445425026	0.918681165	0.820228145	0.6333098	0.578699613
E2F2	0.634013938	0.554772918	0.806311697	0.806922554	0.675325659	0.593987552
RUVBL1	0.634013938	0.557135186	0.600123126	0.737540682	0.702383614	0.879160297
ZSCAN29	0.634013938	0.454401393	0.618763457	0.88747242	0.704991203	0.783856727
CD164L2	0.634013938	0.830448181	0.556529139	0.754030944	0.713082855	0.636570258
FTL	0.634013938	0.511649327	0.717649445	0.915749182	0.725609902	0.550052252
ACSL3	0.634013938	0.454613627	0.678204563	0.932404322	0.766792351	0.564529177
MRPL19	0.634013938	0.48029058	0.611257543	0.770425979	0.771516579	0.866482265
GIMAP7	0.634013938	0.636332133	0.584928427	0.781241796	0.780497593	0.714231652
C2CD5	0.634013938	0.505355809	0.583731628	0.754030944	0.819670067	0.845048764
SUPV3L1	0.634013938	0.582627279	0.621963024	0.721039637	0.838889011	0.748131898
SF3B1	0.634013938	0.4835064	0.706787562	0.781241796	0.857733715	0.671012712
ERH	0.634013938	0.455667522	0.744369186	0.781241796	0.87465791	0.647432409
ATRNL1	0.634013938	0.516673361	0.764481234	0.730465064	0.899048045	0.588661483
LOC104974923	0.634013938	0.506711792	0.620860761	0.885300782	0.932081893	0.535178421
MEGF8	0.634013938	0.485870931	0.707441133	0.805070058	0.934859184	0.578699613
ABCD4	0.634013938	0.506366149	0.632743297	0.820228145	0.943901678	0.576908474
EPDR1	0.634013938	0.605987327	0.620860761	0.727579206	0.99989309	0.55361803
TPPP3	0.634013938	0.473526978	0.606619944	0.784305663	0.99989309	0.555681086
SECISBP2	0.634013938	0.442555387	0.620720409	0.781241796	0.99989309	0.635645816
HOXA7	0.635216713	0.609096933	0.638691336	0.721039637	0.99989309	0.516100157
DNAJC2	0.635257691	0.471726524	0.592801724	0.788938193	0.622490325	0.95342487
AVPI1	0.636333126	0.462191552	0.607119516	0.781241796	0.766792351	0.872493553
CNDP2	0.637156567	0.636332133	0.685222626	0.908804713	0.642463841	0.550542614

FAHD2A	0.637156567	0.567752026	0.620720409	0.741905872	0.889271428	0.703742328
EPC2	0.637207387	0.763751563	0.556529139	0.721039637	0.899048045	0.610416296
THPO	0.63793472	0.494451722	0.764481234	0.784305663	0.846122929	0.588661483
SHOC2	0.638799684	0.479254481	0.651248606	0.806922554	0.99989309	0.51887862
TIMM8B	0.638799684	0.479254481	0.620860761	0.746346194	0.99989309	0.55361803
CIDEA	0.638799684	0.534103098	0.620860761	0.754030944	0.99989309	0.55361803
LOC784007	0.639363277	0.63203115	0.699166169	0.915749182	0.614338501	0.550542614
EIF4A3	0.641121069	0.462191552	0.880277219	0.84302258	0.621908963	0.59580789
PIF1	0.641121069	0.522228461	0.705858477	0.841828571	0.6333098	0.752900996
CNN1	0.641121069	0.790637585	0.675040193	0.720889501	0.63496351	0.705712189
RBM3	0.641121069	0.543274277	0.573880698	0.999939566	0.642463841	0.547791473
KIF18A	0.641121069	0.537186174	0.765785685	0.78508807	0.642463841	0.728276429
RRP15	0.641121069	0.529887686	0.747084388	0.820228145	0.682242009	0.671398444
KIF20B	0.641121069	0.491746055	0.744490351	0.916026707	0.702383614	0.554838062
PAQR8	0.641121069	0.54984186	0.610765273	0.721039637	0.702383614	0.916525501
EIF5A	0.641121069	0.564571365	0.620860761	0.721039637	0.742316849	0.864571107
LOC104968422	0.641121069	0.462191552	0.694542317	0.855137785	0.760712035	0.66915423
MAGEF1	0.641121069	0.499960638	0.570555042	0.995118128	0.779159678	0.524575704
C3H1orf162	0.641121069	0.479254481	0.764481234	0.820228145	0.779159678	0.625125637
YTHDF3	0.641121069	0.527109198	0.63818281	0.925931623	0.782662718	0.535298505
SGPL1	0.641121069	0.499960638	0.620860761	0.956154344	0.790063341	0.523061787
LOC101907276	0.641121069	0.487116492	0.759671935	0.846715063	0.804250204	0.56099024
TMEM209	0.641121069	0.435636214	0.620720409	0.721039637	0.819670067	0.910619304
CAMK1G	0.641121069	0.557966843	0.551625304	0.721039637	0.934859184	0.784671496
CYP4B1	0.641121069	0.443474229	0.558576546	0.983569039	0.939994656	0.493149999
AMPD3	0.641121069	0.473793567	0.632743297	0.781241796	0.99989309	0.583720387
MED19	0.641121069	0.505355809	0.601873896	0.720889501	0.99989309	0.646642632
PLD2	0.641450308	0.462191552	0.712526829	0.99879985	0.614338501	0.55361803
FANCG	0.641450308	0.450398	0.893763648	0.806922554	0.631282463	0.642154165
PLEKHG2	0.641450308	0.487116492	0.709561629	0.99483611	0.642463841	0.543439888
TOMM70	0.641450308	0.567752026	0.713284223	0.796330272	0.652448205	0.752857325
FBXO33	0.641450308	0.494451722	0.573880698	0.999939566	0.670071417	0.610882384
PPP1R14B	0.641450308	0.648099013	0.611257543	0.731974857	0.675325659	0.856870625
GTF2E1	0.641450308	0.599491268	0.817256644	0.781241796	0.67894608	0.587775177
LOC112441502	0.641450308	0.494451722	0.858865175	0.721039637	0.684777964	0.694702496
ATIC	0.641450308	0.549065127	0.675040193	0.749462451	0.702383614	0.833139817
GSDMD	0.641450308	0.595931157	0.759608199	0.825933581	0.708132496	0.55361803
C29H11orf95	0.641450308	0.479254481	0.620720409	0.917869295	0.766932246	0.647432409

MNS1	0.641450308	0.435300125	0.75548993	0.846715063	0.834745492	0.602586916
CLEC10A	0.641450308	0.516029379	0.62764537	0.850828215	0.856442535	0.627380177
SELENOP	0.641450308	0.457610754	0.545279742	0.992692773	0.866920896	0.55361803
TRAPPC3	0.641450308	0.547184103	0.771550992	0.775761952	0.882754591	0.550542614
TMEM97	0.641450308	0.534344991	0.612353108	0.760188917	0.889271428	0.770391484
PKIB	0.641450308	0.536568815	0.605653985	0.730465064	0.899048045	0.795243197
ADNP	0.641450308	0.568753762	0.545279742	0.820228145	0.932197133	0.642154165
ALG9	0.641450308	0.502718799	0.726668529	0.781241796	0.945774918	0.590036846
RGS1	0.641450308	0.494248266	0.627822502	0.751870003	0.99989309	0.497810188
LOC100336414	0.641450308	0.490384759	0.598843922	0.721039637	0.99989309	0.550542614
ANKRD23	0.64169626	0.506366149	0.945706364	0.727339718	0.675325659	0.578699613
CLK4	0.64169626	0.568497289	0.61759981	0.721039637	0.961835052	0.692674475
SCEL	0.642462389	0.573298366	0.849077738	0.721039637	0.819670067	0.543098314
FICD	0.643615661	0.703437628	0.810723656	0.731974857	0.6333098	0.607436462
API5	0.643615661	0.517228833	0.814444866	0.820228145	0.675325659	0.640642131
PLEKHF1	0.643615661	0.538091051	0.633986838	0.891563211	0.675325659	0.728700419
LOC100298530	0.643615661	0.488405939	0.699166169	0.820228145	0.675656796	0.812509877
ADAM33	0.643615661	0.533749385	0.765074242	0.825933581	0.67894608	0.647432409
KIF1B	0.643615661	0.435300125	0.632743297	0.999939566	0.685145778	0.568609729
RAET1L	0.643615661	0.488637812	0.547390233	0.999939566	0.7120086	0.550542614
ZNF566	0.643615661	0.553493197	0.735302276	0.806922554	0.730791761	0.653387719
ANKRD63	0.643615661	0.504238181	0.632855053	0.88747242	0.745432249	0.681177433
SOX5	0.643615661	0.462191552	0.651174146	0.781241796	0.766792351	0.844335246
GIMAP8	0.643615661	0.494451722	0.615875797	0.915749182	0.819670067	0.637306705
WBP2	0.643615661	0.491016411	0.675040193	0.915749182	0.85647293	0.539135678
TMEM30A	0.643615661	0.549065127	0.806221631	0.779232964	0.87483668	0.516100157
PLXDC1	0.643615661	0.737937244	0.617037621	0.784305663	0.897363817	0.523061787
SLC25A13	0.643615661	0.517228833	0.583057712	0.781241796	0.904428762	0.769244117
USP24	0.643615661	0.520020743	0.631413719	0.820228145	0.967609842	0.578699613
PLPBP	0.643615661	0.538091051	0.621963024	0.781241796	0.98152792	0.610416296
MRPS18C	0.643615661	0.464010019	0.583057712	0.820228145	0.99989309	0.550052252
PEX11B	0.643615661	0.435636214	0.620860761	0.781241796	0.99989309	0.578699613
KDM7A	0.643615661	0.542976882	0.557464877	0.806922554	0.99989309	0.588661483
SLAMF1	0.643615661	0.502718799	0.620720409	0.731974857	0.99989309	0.610416296
BRIX1	0.643615661	0.473863398	0.646622914	0.721039637	0.99989309	0.651173954
PLSCR3	0.64372732	0.516586591	0.651174146	0.99879985	0.6333098	0.56099024
LONRF3	0.64372732	0.48029058	0.733993364	0.90419464	0.84234617	0.523061787
LOC104970450	0.64372732	0.502718799	0.73016804	0.820228145	0.913229811	0.55361803

CACNB3	0.64372732	0.538091051	0.591811466	0.732079395	0.978732102	0.727921992
ZNF207	0.64372732	0.464010019	0.685222626	0.731974857	0.99989309	0.523061787
METTL1	0.64675873	0.534103098	0.632855053	0.825933581	0.641204758	0.845803016
TATDN1	0.64675873	0.583641966	0.598843922	0.992746023	0.659374254	0.580920535
CACNB2	0.64675873	0.921280256	0.632743297	0.721039637	0.702383614	0.55361803
ADAMTS10	0.64675873	0.448739356	0.872444023	0.811000597	0.72279174	0.610416296
TMED4	0.64675873	0.458781098	0.850046649	0.843512648	0.761764417	0.540969064
DMD	0.64675873	0.542150706	0.673737267	0.781241796	0.762655357	0.770391484
CMTM6	0.64675873	0.452704583	0.884197487	0.807614341	0.804257626	0.508971078
NT5E	0.64675873	0.54984186	0.733231491	0.721039637	0.99989309	0.503077635
STXBP5	0.64675873	0.692547228	0.620720409	0.721039637	0.99989309	0.51621774
ANGPTL8	0.64675873	0.490341542	0.591811466	0.778545211	0.99989309	0.607328652
ABI1	0.646768009	0.473863398	0.699166169	0.781241796	0.99989309	0.551729166
CRYL1	0.646836729	0.479254481	0.684005375	0.998542029	0.6333098	0.588661483
ENTPD2	0.646836729	0.48029058	0.549314874	0.999939566	0.675656796	0.503284966
MTHFD1L	0.646836729	0.616586256	0.620720409	0.781241796	0.678920037	0.832858909
CD53	0.646836729	0.537186174	0.63818281	0.943323364	0.702383614	0.610882384
TRANK1	0.646836729	0.443474229	0.827125538	0.91329897	0.713082855	0.55361803
TACC3	0.646836729	0.555700238	0.773846486	0.830830186	0.713082855	0.602586916
GABARAPL1	0.646836729	0.459453253	0.556529139	0.99483611	0.713082855	0.671528522
FEM1A	0.646836729	0.479254481	0.659491881	0.909743798	0.755968957	0.669225252
LOC107132921	0.646836729	0.450661146	0.793430641	0.849183739	0.77278187	0.599490586
PARP6	0.646836729	0.473526978	0.844065668	0.795834665	0.779159678	0.591016795
PROS1	0.646836729	0.522805425	0.744490351	0.904990622	0.790028797	0.516382876
LOC101903248	0.646836729	0.560898132	0.726668529	0.883885658	0.794387408	0.516100157
FAM207A	0.646836729	0.637203338	0.623085363	0.775761952	0.837323996	0.675179275
UBR3	0.646836729	0.525690188	0.65719823	0.915749182	0.875054206	0.523061787
CUBN	0.646836729	0.50714456	0.66128476	0.843411019	0.875054206	0.63363599
LYAR	0.646836729	0.506366149	0.55673062	0.750804902	0.99989309	0.542672123
ADAMTS9	0.646836729	0.567752026	0.557464877	0.721039637	0.99989309	0.55361803
CDK1	0.646836729	0.453214948	0.588471859	0.883885658	0.99989309	0.581523252
TERC	0.646836729	0.454401393	0.64445174	0.721039637	0.99989309	0.708819884
USP42	0.647654911	0.514208066	0.583057712	0.731974857	0.99989309	0.516100157
STYK1	0.648563835	0.489814027	0.725755479	0.92064557	0.659374254	0.659349202
POMT1	0.648563835	0.553668207	0.842605286	0.729652612	0.725609902	0.642154165
HNRNPK	0.648563835	0.462191552	0.733231491	0.903607716	0.759114886	0.636570258
LOC101903038	0.648563835	0.505355809	0.610765273	0.893559455	0.954501767	0.579379269
LOC516108	0.648563835	0.635240156	0.610765273	0.763060676	0.964150118	0.610882384

EFCAB11	0.648565299	0.72843033	0.668846123	0.825933581	0.713691227	0.567131921
FGF10	0.648565299	0.553512189	0.615875797	0.721039637	0.77278187	0.891000792
FASTKD2	0.648565299	0.502718799	0.555589198	0.731974857	0.943768595	0.84491737
MCRIP2	0.648565299	0.536568815	0.598843922	0.721039637	0.99989309	0.610416296
FOXK2	0.648565299	0.484750909	0.726668529	0.721039637	0.99989309	0.610882384
IMP3	0.648604814	0.55176988	0.754101752	0.721039637	0.725609902	0.784080446
MDM4	0.649572411	0.893839642	0.589475107	0.82300162	0.675325659	0.567131921
MYL12A	0.649572411	0.507048734	0.765074242	0.90419464	0.68375459	0.610882384
FLNA	0.649572411	0.693153864	0.678204563	0.781241796	0.713691227	0.671720185
CD63	0.649572411	0.471726524	0.80193314	0.903607716	0.741060815	0.568609729
SLC39A8	0.649572411	0.769091087	0.55673062	0.788938193	0.819670067	0.614421218
FAAH	0.649572411	0.494451722	0.843427302	0.721039637	0.871845329	0.61405517
RAB9A	0.649572411	0.534344991	0.675040193	0.825933581	0.939361296	0.583304321
ATPAF2	0.649572411	0.583641966	0.600123126	0.742618602	0.961879452	0.684595958
ECSIT	0.649572411	0.514208066	0.555589198	0.721039637	0.99989309	0.627092396
LSS	0.649572411	0.464176632	0.555591179	0.727579206	0.99989309	0.794529985
PRPF40B	0.650098551	0.449728959	0.726668529	0.91329897	0.685145778	0.705712189
SMARCAL1	0.650098551	0.651481516	0.748016329	0.781241796	0.769784115	0.588661483
CCDC77	0.650686215	0.445425026	0.747084388	0.891273781	0.675325659	0.747674417
LOC618456	0.650782322	0.456669239	0.720067907	0.996084773	0.713082855	0.516382876
MEIS3	0.651056764	0.556152946	0.707441133	0.910808102	0.713082855	0.600077084
TAF4B	0.651056764	0.453259111	0.600123126	0.943323364	0.925981327	0.59580789
NUCB2	0.651260515	0.454401393	0.632855053	0.935763381	0.814605912	0.635211097
LOC104976082	0.652056023	0.537186174	0.662483244	0.915749182	0.659374254	0.722309684
FCMR	0.652056023	0.690829991	0.620720409	0.729652612	0.674717419	0.86143104
CRELD2	0.652056023	0.744066065	0.570590624	0.826315685	0.675325659	0.728141571
GTSE1	0.652056023	0.641318552	0.640276611	0.850828215	0.685145778	0.66854638
NDUFAF6	0.652056023	0.464176632	0.662890316	0.866251502	0.702383614	0.813759229
IRF8	0.652056023	0.484838844	0.556529139	0.825933581	0.713143057	0.914122611
GEMIN4	0.652056023	0.537186174	0.579518125	0.934174776	0.725609902	0.691363988
VMA21	0.652056023	0.473526978	0.707750661	0.785145388	0.745432249	0.832189969
LOC101904942	0.652056023	0.48029058	0.758814705	0.778545211	0.766792351	0.770391484
NMD3	0.652056023	0.464010019	0.632855053	0.784021174	0.766792351	0.875441396
LOC540403	0.652056023	0.511608561	0.844065668	0.75922259	0.769784115	0.632484935
GDI1	0.652056023	0.604295899	0.755534187	0.811430538	0.773626517	0.567131921
IDH3A	0.652056023	0.538091051	0.611257543	0.778545211	0.790028797	0.862912171
PGGHG	0.652056023	0.471868414	0.827125538	0.846715063	0.808924032	0.55361803
MICALL1	0.652056023	0.6608873	0.611257543	0.825933581	0.808924032	0.642154165

RDH13	0.652056023	0.568497289	0.618763457	0.721039637	0.87483668	0.822691858
TROVE2	0.652056023	0.542133539	0.747093618	0.825933581	0.91011204	0.515839823
KCNE4	0.652056023	0.557135186	0.713797253	0.806922554	0.934379881	0.567131921
C18H16orf87	0.652056023	0.50714456	0.632743297	0.915749182	0.939361296	0.516382876
GPR141	0.652056023	0.487116492	0.620720409	0.776207435	0.954501767	0.77208617
LOC112442253	0.652056023	0.487116492	0.703042171	0.842538664	0.96532164	0.550542614
DNMBP	0.652056023	0.54984186	0.620720409	0.732079395	0.988693274	0.685672629
ABCA6	0.652056023	0.655710881	0.620720409	0.731974857	0.99989309	0.550542614
ARHGAP40	0.652056023	0.458781098	0.697476919	0.721039637	0.99989309	0.684902922
C22H3orf67	0.652602583	0.57320535	0.765074242	0.86950142	0.725609902	0.550542614
CD1D	0.652821499	0.568497289	0.620720409	0.915749182	0.818344379	0.588661483
LOC101904794	0.653128282	0.464010019	0.774385549	0.97504565	0.642463841	0.574812527
ACTB	0.653128282	0.450692562	0.99304917	0.781241796	0.659374254	0.543098314
KCNJ8	0.653128282	0.55176988	0.55673062	0.95964071	0.702383614	0.671528522
IL9R	0.653128282	0.57320535	0.703042171	0.915749182	0.725609902	0.567131921
TMED6	0.653128282	0.458781098	0.620860761	0.850828215	0.819670067	0.789797484
DNM1	0.653128282	0.509590069	0.758406163	0.820228145	0.821928612	0.610882384
SPDYA	0.653128282	0.583843515	0.684005375	0.721039637	0.893052667	0.725528824
PEX6	0.653128282	0.450665155	0.836294915	0.785145388	0.932197133	0.55361803
NUFIP2	0.653128282	0.537295148	0.611257543	0.734194666	0.99989309	0.567131921
SAFB	0.653128282	0.473863398	0.64445174	0.781241796	0.99989309	0.637306705
CDC45	0.653395783	0.534344991	0.832483532	0.904990622	0.659374254	0.55361803
SLC38A8	0.655429075	0.479254481	0.925442336	0.781241796	0.670071417	0.647432409
TSPYL1	0.656178292	0.534103098	0.849077738	0.820228145	0.700903924	0.607436462
PTX3	0.657548822	0.50714456	0.620943849	0.727339718	0.941107925	0.809650729
SMIM12	0.657763462	0.562255493	0.694422859	0.781241796	0.99989309	0.550542614
DTX1	0.657796623	0.494451722	0.675040193	0.977470078	0.675325659	0.640642131
BLOC1S6	0.657821662	0.490341542	0.886176337	0.843411019	0.713082855	0.550052252
TEAD2	0.657821662	0.567752026	0.607812429	0.820228145	0.762253517	0.795243197
RXYLT1	0.657821662	0.538091051	0.696685689	0.846715063	0.876274511	0.594117809
LOC107131239	0.657821662	0.4835064	0.632743297	0.893559455	0.896933443	0.645838677
MRPL4	0.657821662	0.632464232	0.620720409	0.778545211	0.961382403	0.625532034
GRAMD4	0.658226805	0.464010019	0.850020542	0.910808102	0.651594606	0.614421218
LOC104973519	0.658226805	0.536568815	0.798686086	0.918910148	0.652021057	0.567131921
SH2D2A	0.658226805	0.596971844	0.66128476	0.781241796	0.652021057	0.868108836
KCND1	0.658226805	0.496432649	0.646427644	0.999939566	0.659374254	0.567131921
MAFG	0.658226805	0.505355809	0.681766822	0.915749182	0.662444459	0.747414217
PTPRG	0.658226805	0.466972398	0.788848487	0.820228145	0.666155381	0.794556896

LMAN1	0.658226805	0.502885369	0.573880698	0.820228145	0.669967615	0.957896427
LOC101907574	0.658226805	0.488637812	0.599201355	0.918161195	0.674717419	0.852634896
SIT1	0.658226805	0.55750845	0.573880698	0.749462451	0.674717419	0.98016619
FKBP1B	0.658226805	0.514208066	0.726668529	0.990571725	0.675325659	0.545791366
EMP1	0.658226805	0.4835064	0.745118667	0.99879985	0.675325659	0.550542614
CA11	0.658226805	0.557135186	0.694422859	0.974992862	0.675325659	0.579379269
SLC31A1	0.658226805	0.631868333	0.684005375	0.788938193	0.675325659	0.809650729
ARHGEF5	0.658226805	0.538091051	0.926613927	0.820228145	0.675656796	0.550542614
GPC5	0.658226805	0.488637812	0.628260967	0.846715063	0.675656796	0.893400499
KLHL17	0.658226805	0.514208066	0.870054336	0.86288354	0.675889904	0.564529177
PNCK	0.658226805	0.86764843	0.581822604	0.858031724	0.676199383	0.578699613
LOXL1	0.658226805	0.537186174	0.620720409	0.999939566	0.67894608	0.544692674
CALCOCO1	0.658226805	0.537186174	0.621552502	0.851394268	0.67894608	0.844622232
GPR68	0.658226805	0.490341542	0.817670412	0.93471064	0.68375459	0.539135678
NFE2L1	0.658226805	0.4835064	0.620720409	0.998542029	0.68375459	0.653387719
DUOX2	0.658226805	0.506366149	0.812043145	0.925931623	0.694012377	0.550542614
GPR155	0.658226805	0.538091051	0.682591947	0.953508052	0.694012377	0.610882384
KLK8	0.658226805	0.488405939	0.774057931	0.934591536	0.696261246	0.606037484
EIF4EBP1	0.658226805	0.469378134	0.583057712	0.999939566	0.702383614	0.55361803
SLC2A9	0.658226805	0.538091051	0.875669731	0.797488058	0.702383614	0.610416296
LOC101905925	0.658226805	0.629715977	0.788938503	0.781241796	0.702383614	0.636570258
AMD1	0.658226805	0.80659559	0.65719823	0.775761952	0.702383614	0.651173954
LOC104973739	0.658226805	0.488637812	0.741982235	0.89088601	0.702383614	0.733357065
FGD6	0.658226805	0.663106292	0.620720409	0.820228145	0.702383614	0.787474205
EIF6	0.658226805	0.479254481	0.788848487	0.914862928	0.704551568	0.635211097
NUSAP1	0.658226805	0.466972398	0.830125816	0.928836449	0.713082855	0.542853655
WDR74	0.658226805	0.520020743	0.699166169	0.883885658	0.713082855	0.701784819
UBASH3A	0.658226805	0.557135186	0.579518125	0.731974857	0.718371887	0.955585296
GLUL	0.658226805	0.458781098	0.600123126	0.999939566	0.721611529	0.575609565
LRFN4	0.658226805	0.52442418	0.751496241	0.846715063	0.725609902	0.667185675
PDSS1	0.658226805	0.507234108	0.63818281	0.820228145	0.725609902	0.84491737
DNAJC16	0.658226805	0.600050072	0.632743297	0.731974857	0.737866415	0.865926817
ARHGAP28	0.658226805	0.476356516	0.641216042	0.999939566	0.738661337	0.567131921
EXD3	0.658226805	0.473793567	0.829843435	0.908496281	0.742316849	0.557327019
TSC22D1	0.658226805	0.54331542	0.684005375	0.850828215	0.745432249	0.717273603
DHODH	0.658226805	0.534344991	0.616881105	0.778545211	0.745432249	0.928442671
ASPM	0.658226805	0.581298031	0.757337101	0.893559455	0.745796372	0.55361803
LOC101904265	0.658226805	0.595931157	0.750089824	0.820228145	0.746187115	0.611777732

MSX1	0.658226805	0.543699592	0.632855053	0.931268564	0.748847452	0.636570258
COMMD5	0.658226805	0.586639262	0.620720409	0.964134647	0.750160769	0.567131921
INCENP	0.658226805	0.476356516	0.896272282	0.749462451	0.750160769	0.637306705
ZNF470	0.658226805	0.557966843	0.707441133	0.868417977	0.750160769	0.637306705
NOD2	0.658226805	0.487116492	0.588633621	0.788938193	0.750160769	0.941925704
LOC789148	0.658226805	0.603785862	0.776574729	0.825933581	0.750220157	0.567131921
GADD45A	0.658226805	0.462191552	0.746957417	0.96776512	0.757810174	0.549984078
PHF21A	0.658226805	0.464010019	0.744369186	0.892229846	0.760712035	0.66854638
THY1	0.658226805	0.473863398	0.788848487	0.915749182	0.769784115	0.550542614
MTHFD2	0.658226805	0.624850517	0.607812429	0.820228145	0.779159678	0.742259651
LOC530973	0.658226805	0.464965392	0.809322753	0.778545211	0.779159678	0.757327233
PIMREG	0.658226805	0.469378134	0.828407935	0.891273781	0.780497593	0.55361803
ABHD16B	0.658226805	0.553493197	0.605653985	0.959727203	0.784855148	0.610416296
ZCCHC13	0.658226805	0.567752026	0.573880698	0.963153458	0.790725614	0.60535862
C3	0.658226805	0.553512189	0.827186499	0.820228145	0.810368126	0.51887862
CTNS	0.658226805	0.637203338	0.620860761	0.910808102	0.819670067	0.567131921
LOC784289	0.658226805	0.534103098	0.581822604	0.973377993	0.821169338	0.588661483
MEGF6	0.658226805	0.615918697	0.811402052	0.781241796	0.821928612	0.550542614
ABCC2	0.658226805	0.641720043	0.620860761	0.788938193	0.832446464	0.692562513
GOLGB1	0.658226805	0.538091051	0.684005375	0.846627306	0.84234617	0.639128578
PPA1	0.658226805	0.490341542	0.63818281	0.742618602	0.84234617	0.886520861
PIK3R5	0.658226805	0.462191552	0.662890316	0.837962878	0.846122929	0.788411132
PXMP4	0.658226805	0.533146197	0.632190849	0.741347517	0.846122929	0.860827207
KPNA4	0.658226805	0.506366149	0.735302276	0.87630412	0.850852167	0.578699613
TFCP2	0.658226805	0.46456927	0.620860761	0.925901365	0.87465791	0.661937165
SH2D1A	0.658226805	0.583843515	0.632274215	0.763060676	0.87465791	0.764546301
LOC101903752	0.658226805	0.487116492	0.678070701	0.934591536	0.87483668	0.550542614
KIF18B	0.658226805	0.489247524	0.783662574	0.868090726	0.881257517	0.538873029
TMEM156	0.658226805	0.568497289	0.694542317	0.742618602	0.883643824	0.729817953
ARMCX2	0.658226805	0.507234108	0.583731628	0.924489924	0.899048045	0.636570258
HMBS	0.658226805	0.645207132	0.632855053	0.730465064	0.925981327	0.671528522
ZNF148	0.658226805	0.488637812	0.87146798	0.721039637	0.934859184	0.567131921
CNTN4	0.658226805	0.847749382	0.573880698	0.749462451	0.952172109	0.543098314
XYLT2	0.658226805	0.538091051	0.623085363	0.915749182	0.952172109	0.550542614
LOC107131416	0.658226805	0.462191552	0.620860761	0.75922259	0.952172109	0.853233759
GOT1L1	0.658226805	0.669648359	0.684005375	0.741905872	0.961382403	0.554838062
MYCBP2	0.658226805	0.538091051	0.744490351	0.729345432	0.972917711	0.636570258
RIOK3	0.658226805	0.506711792	0.754897816	0.806922554	0.976090711	0.550542614

SLTM	0.658226805	0.462191552	0.685222626	0.811000597	0.98152792	0.640642131
ACOT4	0.658226805	0.468564925	0.617037621	0.850828215	0.991174265	0.642154165
LOC100849865	0.658226805	0.63203115	0.632855053	0.721039637	0.99989309	0.523061787
PPP1CC	0.658226805	0.640189848	0.581737542	0.778545211	0.99989309	0.523061787
PPTC7	0.658226805	0.487116492	0.618763457	0.890511543	0.99989309	0.539135678
RIPK1	0.658226805	0.553512189	0.610765273	0.765249395	0.99989309	0.547791473
FUBP1	0.658226805	0.462191552	0.610765273	0.729652612	0.99989309	0.550542614
COL22A1	0.658226805	0.479254481	0.598843922	0.721039637	0.99989309	0.567131921
HABP4	0.658226805	0.578451435	0.596627782	0.825933581	0.99989309	0.575609565
LOC112446470	0.658226805	0.560898132	0.63818281	0.789271151	0.99989309	0.581523252
HRAS	0.658226805	0.538091051	0.620720409	0.830830186	0.99989309	0.590403901
RALGAPA1	0.658226805	0.578451435	0.612611059	0.737540682	0.99989309	0.610416296
HMGB3	0.658226805	0.454401393	0.682591947	0.741905872	0.99989309	0.610416296
FBXO16	0.658226805	0.486209063	0.649291546	0.825933581	0.99989309	0.610416296
PPP1R1A	0.658226805	0.48029058	0.596627782	0.830877858	0.99989309	0.637306705
LOC786352	0.658226805	0.490341542	0.620720409	0.721039637	0.99989309	0.647432409
AASDH	0.658324081	0.675748768	0.685222626	0.820228145	0.813964572	0.610416296
HIST1H3G	0.658325317	0.479254481	0.678204563	0.842538664	0.678920037	0.87900534
AKAP6	0.658325317	0.72843033	0.652359766	0.732079395	0.720084803	0.771518355
LARS	0.658325317	0.534344991	0.749405258	0.781241796	0.889271428	0.671528522
PTPRS	0.659323304	0.534344991	0.744490351	0.826315685	0.957605284	0.539362901
MRPL23	0.660137662	0.567752026	0.620860761	0.828524115	0.846122929	0.729817953
USP12	0.660980075	0.479541039	0.765074242	0.934591536	0.678920037	0.637306705
ACTA2	0.660980075	0.665563964	0.670235176	0.741347517	0.725609902	0.812682271
LAMP3	0.660980075	0.505355809	0.619290318	0.769895471	0.737866415	0.95460851
LOC107131330	0.660980075	0.488637812	0.74784538	0.87452213	0.886279726	0.588661483
CPAMD8	0.660980075	0.490341542	0.753656036	0.758703434	0.905719399	0.728276429
GFPT2	0.661427959	0.505355809	0.788848487	0.806922554	0.952172109	0.553758001
CCNA2	0.661497164	0.490341542	0.751496241	0.915749182	0.745432249	0.633384404
MIIP	0.661497164	0.488637812	0.921135532	0.721039637	0.77278187	0.642154165
TMEM250	0.661497164	0.4835064	0.632743297	0.729345432	0.99989309	0.727921992
SELENBP1	0.662168356	0.68147929	0.668846123	0.75922259	0.99989309	0.550542614
CAPN10	0.662641564	0.629715977	0.836294915	0.820228145	0.725609902	0.55361803
CHTF18	0.66288407	0.487116492	0.892360225	0.89088601	0.676199383	0.567131921
CEP57	0.66288407	0.651481516	0.611257543	0.737540682	0.819670067	0.832097423
TBXA2R	0.66288407	0.502953903	0.605653985	0.721039637	0.99989309	0.567131921
SLC6A17	0.663477159	0.798909583	0.685222626	0.806922554	0.675467149	0.637306705
EVPL	0.663477159	0.473863398	0.948628899	0.846115604	0.675656796	0.553225333

RPP38	0.665234058	0.685777004	0.632190849	0.820228145	0.83627631	0.642154165
SERPINF1	0.665234058	0.490341542	0.746957417	0.913413181	0.89629383	0.549734587
GLI4	0.665234058	0.467718175	0.618763457	0.825933581	0.99989309	0.581523252
EVL	0.665860751	0.568025614	0.684005375	0.943240775	0.741475707	0.583091156
SHMT1	0.665860751	0.52442418	0.632743297	0.925265292	0.84234617	0.635645816
METAP2	0.665860751	0.536568815	0.632743297	0.820228145	0.986955873	0.640138785
RNASE1	0.665969198	0.507234108	0.813541285	0.788938193	0.702383614	0.764546301
GDF9	0.665969198	0.747499345	0.620860761	0.781241796	0.723334634	0.75292536
HECTD1	0.665969198	0.537493495	0.703042171	0.939446567	0.825710291	0.540636511
ETF1	0.666110165	0.53701889	0.747700432	0.90419464	0.713082855	0.651173954
SUSD4	0.666110165	0.466972398	0.888610896	0.820228145	0.725609902	0.636570258
ABCA2	0.667127415	0.530282658	0.788848487	0.922713162	0.713691227	0.56099024
EPB41L5	0.667218427	0.534344991	0.61759981	0.806922554	0.809298212	0.863757526
BAZ1A	0.667218427	0.515489146	0.726668529	0.754030944	0.99989309	0.583720387
SLF1	0.668562525	0.488637812	0.837473402	0.781241796	0.685145778	0.800008791
CKAP2	0.668562525	0.473863398	0.81284583	0.915749182	0.762655357	0.569645194
DNAJC30	0.668562525	0.537186174	0.611257543	0.811000597	0.99989309	0.612910364
GRHL2	0.668652441	0.488637812	0.720067907	0.925931623	0.704551568	0.681177433
NAT14	0.668652441	0.476356516	0.696685689	0.781241796	0.707777957	0.921734133
CEMIP	0.668883934	0.550965714	0.678204563	0.908496281	0.821928612	0.635211097
PRDM5	0.668883934	0.473793567	0.822800372	0.850828215	0.892149112	0.550542614
CFAP46	0.669748817	0.582627279	0.632743297	0.825933581	0.987694035	0.588661483
FYN	0.67007254	0.613737651	0.632855053	0.846715063	0.952172109	0.567131921
STAB1	0.670640586	0.560447714	0.685222626	0.955687359	0.675889904	0.637306705
RBM5	0.670640586	0.488637812	0.684005375	0.771850653	0.702383614	0.946314386
ADAMTS12	0.670640586	0.560374186	0.620860761	0.999939566	0.713082855	0.55361803
DHX40	0.670640586	0.609819443	0.62764537	0.883885658	0.804250204	0.660829605
ACSL1	0.67068216	0.524212852	0.632743297	0.727339718	0.887132523	0.875441396
STARD9	0.671337619	0.490341542	0.755534187	0.806922554	0.728674856	0.812509877
GFRA1	0.671337619	0.573020585	0.788938503	0.825933581	0.780831493	0.588661483
SYNE1	0.671337619	0.563639555	0.684005375	0.781241796	0.887539821	0.742259651
C17H12orf65	0.671337619	0.651387022	0.675040193	0.754030944	0.954501767	0.636570258
MLYCD	0.671337619	0.553512189	0.619290318	0.724487925	0.99989309	0.635645816
CLK1	0.671485166	0.494451722	0.73016804	0.781241796	0.849382765	0.77821816
PENK	0.671485166	0.494451722	0.62764537	0.886966548	0.99989309	0.543098314
SENP6	0.672810087	0.494451722	0.615875797	0.788938193	0.99989309	0.611777732
TRIP10	0.673136335	0.522385364	0.751496241	0.754030944	0.957605284	0.660829605
DACT3	0.674024934	0.554589145	0.66128476	0.913319839	0.934859184	0.55361803

LOC788175	0.674189627	0.911196851	0.709561629	0.727339718	0.676552979	0.633384404
LOC506495	0.674189627	0.551867132	0.842605286	0.915749182	0.682242009	0.550542614
DEFB4A	0.674189627	0.567752026	0.773281026	0.864540217	0.685145778	0.661861937
LOC617905	0.674189627	0.562255493	0.817256644	0.781241796	0.685145778	0.759107825
RAB3GAP2	0.674189627	0.536970867	0.620860761	0.999939566	0.745432249	0.55361803
PODXL2	0.674189627	0.494665274	0.617376463	0.925901365	0.83417924	0.712710718
WSB2	0.674189627	0.499960638	0.765074242	0.915749182	0.836838903	0.55361803
TMED5	0.674189627	0.479254481	0.765074242	0.904990622	0.836971947	0.610416296
DUSP11	0.674189627	0.560374186	0.684005375	0.806922554	0.844253767	0.752791853
NAA25	0.674189627	0.567752026	0.733993364	0.781241796	0.972048096	0.588309899
TENT5C	0.674189627	0.490341542	0.620720409	0.742618602	0.99989309	0.55361803
PPP4R2	0.674189627	0.48029058	0.620720409	0.806922554	0.99989309	0.662706224
RECQL4	0.674225816	0.652542331	0.789686719	0.90419464	0.675325659	0.550845146
SAMD3	0.674225816	0.499960638	0.684005375	0.731974857	0.766648996	0.934653208
PCYT1A	0.674225816	0.514208066	0.709521163	0.903607716	0.928131565	0.578719809
GATAD2A	0.674601376	0.538091051	0.62764537	0.99879985	0.675325659	0.651173954
IL11RA	0.674601376	0.978453686	0.620720409	0.792591369	0.67894608	0.55361803
MS4A1	0.674601376	0.655710881	0.612611059	0.806922554	0.67894608	0.882322567
GCNA	0.674601376	0.4835064	0.759671935	0.806922554	0.683256838	0.867424829
TRPM1	0.674601376	0.567752026	0.682591947	0.992137653	0.68375459	0.602586916
CNOT11	0.674601376	0.50714456	0.879515214	0.841828571	0.68375459	0.640642131
MTBP	0.674601376	0.767950704	0.617837276	0.742618602	0.696143233	0.84491737
RHOF	0.674601376	0.579665846	0.788322476	0.806997685	0.696261246	0.725528824
PRCP	0.674601376	0.496691318	0.611257543	0.999939566	0.700903924	0.639684299
PTHLH	0.674601376	0.691880741	0.620720409	0.963153458	0.702383614	0.578699613
GDF10	0.674601376	0.545732987	0.703042171	0.820228145	0.713082855	0.823139458
DNER	0.674601376	0.506366149	0.618763457	0.943323364	0.713691227	0.77208617
FABP4	0.674601376	0.688511424	0.65719823	0.919247117	0.719753534	0.588661483
ATP6V1C1	0.674601376	0.473863398	0.678204563	0.99879985	0.725609902	0.633384404
CCDC141	0.674601376	0.547317493	0.64109875	0.883885658	0.725609902	0.792329436
PDPK1	0.674601376	0.473863398	0.751496241	0.958879502	0.762655357	0.581523252
LOC786039	0.674601376	0.527128233	0.632190849	0.86288354	0.766792351	0.812622197
ESPNL	0.674601376	0.473863398	0.849077738	0.88649616	0.780831493	0.572789272
RBPMS	0.674601376	0.558856137	0.725755479	0.788938193	0.804250204	0.766542864
EPHA4	0.674601376	0.473526978	0.598843922	0.992137653	0.819670067	0.671720185
FBXL5	0.674601376	0.5497363	0.63818281	0.90419464	0.83417924	0.671720185
CCDC186	0.674601376	0.825215718	0.632855053	0.781241796	0.834198722	0.610882384
SLC43A2	0.674601376	0.610559852	0.712526829	0.731974857	0.861762366	0.75292536

ZFYVE21	0.674601376	0.538052744	0.607119516	0.969717497	0.875054206	0.602586916
CCL1	0.674601376	0.742728851	0.598843922	0.78508807	0.896242324	0.657118951
RET	0.674601376	0.478717027	0.684005375	0.940921143	0.899048045	0.589238861
SPRYD7	0.674601376	0.473863398	0.779697898	0.841828571	0.902843706	0.633384404
ZCCHC17	0.674601376	0.582931029	0.684005375	0.820228145	0.907192767	0.651139949
SPNS2	0.674601376	0.494248266	0.678204563	0.935763381	0.934379881	0.55361803
FDX2	0.674601376	0.652876871	0.640276611	0.78508807	0.934859184	0.645838677
LRP6	0.674601376	0.544802725	0.620860761	0.781241796	0.99989309	0.550052252
C7H19orf70	0.674601376	0.543699592	0.632743297	0.741905872	0.99989309	0.564168606
UHRF1BP1L	0.674601376	0.527128233	0.678070701	0.794713619	0.99989309	0.567131921
TSR3	0.674601376	0.600960324	0.620720409	0.781241796	0.99989309	0.637306705
BPTF	0.674601376	0.562255493	0.632190849	0.781241796	0.99989309	0.660829605
ANKRD12	0.674601376	0.557966843	0.632855053	0.729652612	0.99989309	0.683895124
TOMM5	0.674601376	0.556152946	0.63818281	0.778545211	0.99989309	0.683895124
HS6ST1	0.674908571	0.494451722	0.620720409	0.741905872	0.77278187	0.972293581
LOC100336282	0.674981982	0.693051877	0.611257543	0.883885658	0.675467149	0.77208617
LOC104970249	0.674981982	0.844390603	0.780363715	0.781241796	0.678920037	0.55361803
CXCR6	0.674981982	0.600050072	0.598843922	0.781241796	0.67894608	0.945759045
ATP6V0E1	0.674981982	0.507234108	0.879215964	0.888642721	0.68653192	0.588661483
VCL	0.674981982	0.529335263	0.751496241	0.925901365	0.686757807	0.651173954
TAX1BP1	0.674981982	0.55176988	0.861838588	0.850828215	0.696143233	0.610416296
FAT3	0.674981982	0.76090129	0.678204563	0.826315685	0.702383614	0.656486178
FBLIM1	0.674981982	0.494451722	0.620720409	0.999939566	0.702383614	0.66915423
TMEM202	0.674981982	0.617732204	0.615875797	0.956154344	0.713691227	0.651173954
TMEM107	0.674981982	0.6608873	0.618763457	0.919247117	0.754922627	0.637306705
RUNX1	0.674981982	0.505355809	0.744490351	0.841828571	0.819670067	0.71963823
LOC101904177	0.674981982	0.529887686	0.72395094	0.731974857	0.83627631	0.849772722
USP15	0.674981982	0.63203115	0.712526829	0.87452213	0.849825976	0.564529177
ARHGAP20	0.674981982	0.613737651	0.870911433	0.731974857	0.853884483	0.561617312
CNTLN	0.674981982	0.553493197	0.775786014	0.806922554	0.925981327	0.595201312
MAF1	0.674981982	0.473863398	0.735302276	0.820228145	0.954501767	0.66915423
KIF23	0.674981982	0.505355809	0.620720409	0.901909542	0.988693274	0.635211097
SOX7	0.674981982	0.63780285	0.632190849	0.788938193	0.99989309	0.564813705
USP34	0.674981982	0.553512189	0.612353108	0.781241796	0.99989309	0.610416296
CUL7	0.675258953	0.530282658	0.939683463	0.820228145	0.675889904	0.588661483
LMBR1L	0.675258953	0.479254481	0.927460291	0.850828215	0.742316849	0.550542614
CCDC18	0.675653448	0.553493197	0.726668529	0.841828571	0.676199383	0.84491737
TONSL	0.675653448	0.550965714	0.921319817	0.841828571	0.67894608	0.581523252

SPTLC1	0.675653448	0.499960638	0.80326876	0.934591536	0.67894608	0.645838677
PAWR	0.675653448	0.755940768	0.685222626	0.868417977	0.67894608	0.648987851
LRRC45	0.675653448	0.799579962	0.76749973	0.820228145	0.68375459	0.557327019
ZNF512B	0.675653448	0.502718799	0.726668529	0.999939566	0.68375459	0.579379269
DNMT3A	0.675653448	0.523087485	0.78825731	0.946344632	0.68375459	0.610882384
VWCE	0.675653448	0.574094539	0.614381915	0.999939566	0.68375459	0.611777732
CD1B	0.675653448	0.595931157	0.945188764	0.781241796	0.685145778	0.579379269
LOC513508	0.675653448	0.478717027	0.97162329	0.778545211	0.685145778	0.651173954
LOC101902537	0.675653448	0.507234108	0.885434576	0.731974857	0.685145778	0.786737722
CALM	0.675653448	0.490341542	0.957575736	0.781241796	0.686757807	0.661861937
MADCAM1	0.675653448	0.553493197	0.602496975	0.999939566	0.691978508	0.612910364
GNAT1	0.675653448	0.496432649	0.750421005	0.820228145	0.694012377	0.867440033
RABEP2	0.675653448	0.488637812	0.749405258	0.99879985	0.696261246	0.607436462
LOC101903604	0.675653448	0.543274277	0.93050447	0.781241796	0.696261246	0.633384404
CYSLTR2	0.675653448	0.655438106	0.620860761	0.971220023	0.700991562	0.619864252
LOC614625	0.675653448	0.562255493	0.632855053	0.999939566	0.702383614	0.564168606
HELB	0.675653448	0.553512189	0.623085363	0.999939566	0.702383614	0.588661483
TNFAIP8L3	0.675653448	0.593314312	0.620860761	0.99879985	0.702383614	0.590403901
GNS	0.675653448	0.504238181	0.755534187	0.937531834	0.702383614	0.646642632
ZNF432	0.675653448	0.918371039	0.607119516	0.75922259	0.702383614	0.741983203
LOC101905099	0.675653448	0.488637812	0.89007492	0.781241796	0.702383614	0.77208617
CDC27	0.675653448	0.490341542	0.820549834	0.806922554	0.702383614	0.821220576
C16H1orf105	0.675653448	0.600050072	0.761854021	0.731974857	0.702383614	0.853233759
LOC101902059	0.675653448	0.538091051	0.648198992	0.847813982	0.702383614	0.878152258
MMP19	0.675653448	0.550965714	0.70435078	0.806922554	0.702383614	0.886992214
MAFK	0.675653448	0.600050072	0.747093618	0.90419464	0.711025129	0.650757255
ACTG2	0.675653448	0.839461242	0.698225674	0.777706486	0.711949405	0.674719861
LOC112446717	0.675653448	0.4835064	0.757235816	0.984100122	0.713082855	0.611777732
ZNF652	0.675653448	0.479254481	0.899530079	0.843411019	0.713082855	0.642154165
SLC35B1	0.675653448	0.639238577	0.63818281	0.741347517	0.713082855	0.902660792
GALNT1	0.675653448	0.91994024	0.63818281	0.781241796	0.713255123	0.610416296
ADAMTS6	0.675653448	0.505355809	0.828407935	0.925901365	0.713691227	0.578719809
LRRC51	0.675653448	0.504999582	0.632743297	0.995118128	0.713691227	0.677152076
OAS2	0.675653448	0.505355809	0.763592007	0.850828215	0.713691227	0.783134114
SNX5	0.675653448	0.612773311	0.638691336	0.915749182	0.725609902	0.671528522
CARD6	0.675653448	0.4835064	0.767599201	0.820228145	0.732120393	0.841665278
E2F6	0.675653448	0.749070017	0.744369186	0.843411019	0.741060815	0.571839367
LOC100295712	0.675653448	0.479254481	0.952331743	0.806922554	0.741060815	0.607328652

LOC101908760	0.675653448	0.534344991	0.838877266	0.820228145	0.741060815	0.692674475
ALDH1A2	0.675653448	0.52442418	0.921135532	0.820228145	0.742316849	0.581523252
WNK2	0.675653448	0.74796249	0.632855053	0.784305663	0.742316849	0.770391484
LOC536097	0.675653448	0.733883576	0.620720409	0.781241796	0.742316849	0.812408228
CD3EAP	0.675653448	0.502718799	0.685222626	0.86288354	0.742316849	0.84491737
NDUFAF4	0.675653448	0.542150706	0.757337101	0.731974857	0.742316849	0.86307729
TBRG4	0.675653448	0.652542331	0.620860761	0.738908397	0.742316849	0.908939618
MSMO1	0.675653448	0.505355809	0.706787562	0.915749182	0.748252736	0.729696557
FGD2	0.675653448	0.619374114	0.74784538	0.820228145	0.750160769	0.671720185
TEAD1	0.675653448	0.693153864	0.63818281	0.919247117	0.753062237	0.58254695
DCAF11	0.675653448	0.490341542	0.62144899	0.999939566	0.759114886	0.550542614
RHBDF1	0.675653448	0.505355809	0.914685423	0.820228145	0.766792351	0.567131921
LOC515551	0.675653448	0.537186174	0.632743297	0.999939566	0.766792351	0.567131921
SH3GL3	0.675653448	0.747179932	0.751496241	0.820228145	0.766792351	0.579379269
LOC104974348	0.675653448	0.479254481	0.825960826	0.841828571	0.766792351	0.704011905
CD3E	0.675653448	0.682470071	0.631413719	0.75922259	0.766792351	0.83849759
LOC112449100	0.675653448	0.677340516	0.849077738	0.778545211	0.769784115	0.588661483
GALNT6	0.675653448	0.568497289	0.684005375	0.820228145	0.769784115	0.777933888
HMGCLL1	0.675653448	0.593994283	0.788938503	0.788938193	0.77278187	0.671720185
PRUNE2	0.675653448	0.559785028	0.735921892	0.883885658	0.779159678	0.645838677
PDE12	0.675653448	0.490341542	0.623085363	0.781241796	0.77975903	0.956410517
DALRD3	0.675653448	0.63203115	0.743146522	0.781241796	0.781918601	0.748594275
CYB5R2	0.675653448	0.7116845	0.607812429	0.820228145	0.819670067	0.722689871
STT3B	0.675653448	0.48029058	0.712526829	0.990049248	0.834198722	0.567131921
PACRGL	0.675653448	0.48029058	0.694190265	0.781241796	0.834198722	0.893400499
ERCC6L	0.675653448	0.563639555	0.806825406	0.851394268	0.84234617	0.55361803
CHD2	0.675653448	0.522228461	0.699361465	0.841828571	0.856178094	0.747414217
TPSB2	0.675653448	0.502718799	0.675040193	0.925901365	0.85647293	0.646784897
LHFPL4	0.675653448	0.675573824	0.620720409	0.750804902	0.85647293	0.826868779
THUMPD1	0.675653448	0.556152946	0.715669669	0.846715063	0.856651628	0.678496087
KCTD1	0.675653448	0.593994283	0.632175215	0.946344632	0.864593182	0.567131921
OSER1	0.675653448	0.499960638	0.808440332	0.86288354	0.86558233	0.581443154
CRYBG2	0.675653448	0.583843515	0.744490351	0.790649254	0.86785481	0.683895124
CENPBD1	0.675653448	0.675957273	0.684005375	0.794956735	0.87465791	0.660829605
LOC100196897	0.675653448	0.583641966	0.620720409	0.959727203	0.882754591	0.567131921
ZBTB14	0.675653448	0.551037136	0.755534187	0.905146083	0.886279726	0.55361803
MEOX2	0.675653448	0.518056987	0.678204563	0.935763381	0.887132523	0.610416296
PSMA7	0.675653448	0.587833611	0.632743297	0.781241796	0.91011204	0.792329436

SNX11	0.675653448	0.487770351	0.738217747	0.781241796	0.925981327	0.792329436
RNF19B	0.675653448	0.487116492	0.621963024	0.945929971	0.932081893	0.636570258
NOXO1	0.675653448	0.547317493	0.632743297	0.750804902	0.939361296	0.858042054
LRRTM2	0.675653448	0.63203115	0.750089824	0.781241796	0.943768595	0.588661483
ZFPM2	0.675653448	0.560898132	0.699166169	0.871894706	0.954501767	0.578699613
RAB43	0.675653448	0.534103098	0.600123126	0.925004412	0.954501767	0.616913081
MSL2	0.675653448	0.553512189	0.682591947	0.785145388	0.954501767	0.703742328
MAIP1	0.675653448	0.520719436	0.670235176	0.788938193	0.954501767	0.77208617
CNTN3	0.675653448	0.534103098	0.832483532	0.781241796	0.961382403	0.579379269
SNX19	0.675653448	0.494451722	0.620860761	0.806922554	0.961835052	0.80787758
OLFML2B	0.675653448	0.538091051	0.684005375	0.851394268	0.964521207	0.627553513
MMP15	0.675653448	0.701581467	0.611257543	0.781241796	0.981777076	0.637306705
DCAF5	0.675653448	0.506366149	0.651174146	0.75922259	0.988693274	0.799314198
PNPLA3	0.675653448	0.505355809	0.620720409	0.760589144	0.998120751	0.821774001
SUGT1	0.675653448	0.588244908	0.701483892	0.820228145	0.998276725	0.55361803
H2AFY	0.675653448	0.691175262	0.611257543	0.781241796	0.99989309	0.55361803
C1R	0.675653448	0.520020743	0.642916091	0.781241796	0.99989309	0.55361803
MYL12B	0.675653448	0.563639555	0.755534187	0.781241796	0.99989309	0.56501485
COX7C	0.675653448	0.543699592	0.611257543	0.781241796	0.99989309	0.567131921
NDUFB3	0.675653448	0.514566709	0.620720409	0.781241796	0.99989309	0.567131921
HHIPL1	0.675653448	0.629715977	0.620720409	0.826315685	0.99989309	0.569645194
PIK3CA	0.675653448	0.567471709	0.709561629	0.820228145	0.99989309	0.574399459
NUP153	0.675653448	0.591604402	0.633986838	0.738189414	0.99989309	0.579379269
NDUFA3	0.675653448	0.494451722	0.610765273	0.781241796	0.99989309	0.580920535
UQCRQ	0.675653448	0.557135186	0.620720409	0.732079395	0.99989309	0.591016795
ZNF800	0.675653448	0.57320535	0.632274215	0.737540682	0.99989309	0.610416296
SLC25A15	0.675653448	0.489814027	0.63616512	0.731974857	0.99989309	0.610882384
ANKRD11	0.675653448	0.50714456	0.753656036	0.781241796	0.99989309	0.61405517
GPR65	0.675653448	0.562255493	0.63818281	0.781241796	0.99989309	0.640138785
MAST4	0.675653448	0.611256318	0.616881105	0.737540682	0.99989309	0.661937165
DNAJC3	0.675653448	0.490341542	0.632855053	0.746668767	0.99989309	0.705712189
EPHB1	0.67710828	0.751946504	0.633986838	0.78508807	0.907192767	0.635211097
TWIST2	0.677136473	0.908709906	0.615856183	0.835232007	0.761764417	0.579379269
PRPS1	0.677303215	0.499960638	0.8549878	0.915749182	0.67894608	0.627380177
ARF1	0.677303215	0.507322509	0.90002174	0.90419464	0.685145778	0.588661483
EBF4	0.677303215	0.850723096	0.632855053	0.843411019	0.686853362	0.642154165
PLAU	0.677303215	0.490341542	0.617376463	0.999939566	0.72279174	0.557810503
B3GALT1	0.677303215	0.769065415	0.820767684	0.778545211	0.725609902	0.593724238

CD99	0.677303215	0.49642959	0.762423061	0.781241796	0.745432249	0.875441396
CNGA3	0.677303215	0.81668556	0.703042171	0.788938193	0.745497678	0.640138785
NFIC	0.677303215	0.506366149	0.720067907	0.91329897	0.780831493	0.710580013
MINDY2	0.677303215	0.543699592	0.817256644	0.846715063	0.834605872	0.610416296
NOX5	0.677303215	0.636332133	0.620720409	0.917869295	0.860806019	0.610882384
HAPLN1	0.677303215	0.553512189	0.746957417	0.868090726	0.875418728	0.627380177
TIMP3	0.677303215	0.563639555	0.688413073	0.842538664	0.90448127	0.671720185
PRDM2	0.677303215	0.583843515	0.675040193	0.825933581	0.92188432	0.691363988
SPON1	0.677303215	0.534344991	0.772990161	0.843411019	0.939361296	0.590316781
USP54	0.677303215	0.653341013	0.620860761	0.820228145	0.939361296	0.66915423
PPP1R3D	0.677303215	0.538091051	0.752868635	0.781241796	0.943768595	0.691363988
DNAJC11	0.677303215	0.549040188	0.632743297	0.781241796	0.972176983	0.788142857
HERPUD1	0.677303215	0.502885369	0.620860761	0.781241796	0.99989309	0.568609729
TMEM94	0.677303215	0.538091051	0.726668529	0.772430178	0.99989309	0.624508998
SEC31A	0.677303215	0.50714456	0.62144899	0.850828215	0.99989309	0.671398444
XPOT	0.677985198	0.505355809	0.618763457	0.75922259	0.713082855	0.997922692
LIMS2	0.677985198	0.600050072	0.709521163	0.806922554	0.784855148	0.77208617
LOC526488	0.677985198	0.568239497	0.668846123	0.775761952	0.902843706	0.828602505
STK36	0.677985198	0.595931157	0.765074242	0.784305663	0.904995537	0.640138785
MPP5	0.679588796	0.536970867	0.63818281	0.995800463	0.745796372	0.639684299
ASS1	0.679588796	0.593994283	0.747084388	0.756865703	0.998276725	0.628138194
WWP1	0.680558178	0.49642959	0.941593855	0.760188917	0.742316849	0.671720185
ATP5MD	0.680558178	0.557135186	0.651599878	0.781241796	0.99989309	0.57551997
ANXA7	0.680694097	0.50714456	0.886282865	0.893831952	0.68375459	0.627553513
ANKRD22	0.680694097	0.505355809	0.758988656	0.915749182	0.68375459	0.76332064
LOC781494	0.680694097	0.701581467	0.620720409	0.908054165	0.68375459	0.765573509
GLDN	0.680694097	0.559785028	0.774248983	0.847196289	0.702383614	0.757327233
POMGNT2	0.680694097	0.565007103	0.764057751	0.850305862	0.702383614	0.763746661
KIAA1257	0.680694097	0.552267835	0.844065668	0.90419464	0.708132496	0.610416296
LOC618541	0.680694097	0.582931029	0.631413719	0.738189414	0.72279174	0.96558836
WRB	0.680694097	0.538091051	0.916702	0.846715063	0.741060815	0.567131921
MAP2K3	0.680694097	0.583843515	0.765074242	0.835254768	0.742316849	0.723028401
GNB1L	0.680694097	0.507234108	0.904691312	0.781241796	0.747513637	0.692802214
LOC506989	0.680694097	0.502718799	0.648198992	0.75922259	0.765898761	0.971025918
ANGPTL5	0.680694097	0.710155842	0.618763457	0.841828571	0.769018758	0.757327233
TUBB6	0.680694097	0.688099473	0.726668529	0.806922554	0.87483668	0.635413074
SLC45A3	0.680694097	0.514208066	0.720067907	0.913413181	0.892149112	0.636570258
RANGRF	0.680694097	0.6663481	0.620860761	0.781241796	0.904995537	0.77208617

HECA	0.680694097	0.549628602	0.633986838	0.850828215	0.975291792	0.660829605
SYTL3	0.680694097	0.632709306	0.624662047	0.806922554	0.975291792	0.671528522
SPOPL	0.680694097	0.549040188	0.685222626	0.892229846	0.983089554	0.578699613
ATF1	0.680694097	0.534103098	0.64109875	0.781241796	0.99989309	0.567131921
HSP90AA1	0.680694097	0.568239497	0.683668554	0.806997685	0.99989309	0.600155479
GBGT1	0.681509991	0.553493197	0.624662047	0.90419464	0.892268822	0.725528824
CYP2R1	0.681864285	0.487116492	0.765074242	0.914862928	0.925981327	0.588661483
ACIN1	0.682579715	0.506019626	0.844065668	0.846715063	0.804250204	0.637306705
MSL1	0.682579715	0.490341542	0.707441133	0.820228145	0.934859184	0.770391484
SLX4	0.683431255	0.490341542	0.855743966	0.820228145	0.849382765	0.640138785
ARIH2OS	0.683431255	0.595087191	0.705858477	0.838641474	0.954501767	0.611777732
TAB3	0.684191005	0.494451722	0.73016804	0.934591536	0.689368096	0.770391484
SIX5	0.684191005	0.504999582	0.682591947	0.995118128	0.87465791	0.567131921
TEC	0.684191005	0.487116492	0.684005375	0.802640473	0.99989309	0.567131921
CDK16	0.684536632	0.516586591	0.729587161	0.943323364	0.887061423	0.567131921
LOC529792	0.684678013	0.568497289	0.780363715	0.849060614	0.721123232	0.712710718
USP10	0.685760639	0.517228833	0.747093618	0.837964092	0.74589961	0.823195389
NDUFS3	0.685760639	0.559785028	0.618763457	0.788938193	0.99989309	0.610416296
TMEM70	0.686343481	0.490341542	0.657330797	0.820228145	0.905719399	0.856870625
NLRX1	0.686343481	0.567471709	0.620720409	0.806922554	0.952672789	0.794556896
SYNE2	0.686380606	0.680835681	0.685222626	0.794713619	0.728674856	0.812509877
ALDH18A1	0.687040344	0.524212852	0.618763457	0.846715063	0.983634477	0.753138357
SLC25A33	0.687310604	0.494451722	0.620860761	0.999939566	0.696261246	0.611777732
CHP2	0.687310604	0.541005316	0.726668529	0.940611812	0.745796372	0.661937165
LOC104973322	0.687310604	0.553493197	0.620720409	0.781241796	0.766331537	0.956410517
VAV2	0.687310604	0.490341542	0.788848487	0.908054165	0.83417924	0.642154165
UBE2H	0.687310604	0.505355809	0.682591947	0.841828571	0.860908683	0.831206878
NPTXR	0.687310604	0.595666095	0.617376463	0.975849638	0.904151325	0.569645194
LOC112445176	0.687310604	0.560898132	0.636736341	0.916305829	0.961835052	0.588661483
LOC100336208	0.687310604	0.534344991	0.66128476	0.846115604	0.99989309	0.579379269
RSPRY1	0.687310604	0.49642959	0.678204563	0.843411019	0.99989309	0.610416296
ROBO2	0.687310604	0.557135186	0.620860761	0.749462451	0.99989309	0.642154165
HARBI1	0.687310604	0.567845198	0.684005375	0.820228145	0.99989309	0.646642632
ABCB8	0.687310604	0.543699592	0.620860761	0.742618602	0.99989309	0.764546301
NUS1	0.687755267	0.499960638	0.840850593	0.788938193	0.952672789	0.616913081
GCA	0.688301397	0.496432649	0.747093618	0.901287343	0.74589961	0.774684541
WDR47	0.688491515	0.558701855	0.753656036	0.820228145	0.946536678	0.637306705
KCNJ2	0.689575365	0.921046401	0.620720409	0.88747242	0.702383614	0.567131921

LOC101906754	0.689575365	0.488637812	0.733231491	0.883885658	0.713691227	0.846367069
DNAJA3	0.689575365	0.538091051	0.628260967	0.825933581	0.90399271	0.834008567
KLHL15	0.689575365	0.588244908	0.639921149	0.758703434	0.99989309	0.627553513
MED29	0.689724496	0.506366149	0.678204563	0.781241796	0.99989309	0.567131921
PPARD	0.690283268	0.538091051	0.618763457	0.820228145	0.964521207	0.807277693
EPHB3	0.690692119	0.516586591	0.69131755	0.999939566	0.689368096	0.579379269
TMEM92	0.690692119	0.671879214	0.724199536	0.915749182	0.702383614	0.640642131
CCR5	0.690692119	0.688099473	0.618763457	0.781241796	0.719753534	0.912101047
CHRNB1	0.690692119	0.600538394	0.684005375	0.915749182	0.72279174	0.752857325
MTDH	0.690692119	0.553493197	0.878050811	0.806922554	0.75144456	0.661937165
MAPK8IP2	0.690692119	0.748058967	0.810723656	0.742618602	0.819670067	0.610416296
ZSWIM2	0.690692119	0.544639002	0.648198992	0.820228145	0.908091868	0.812509877
SCAPER	0.690692119	0.653112351	0.63616512	0.781241796	0.954501767	0.742259651
BEND7	0.690692119	0.582985895	0.620720409	0.89088601	0.96730957	0.648987851
MRPL55	0.690692119	0.668175004	0.620720409	0.781241796	0.998276725	0.694702496
CXCR1	0.690692119	0.534344991	0.812043145	0.754030944	0.99989309	0.615356919
LRRN3	0.690915592	0.544802725	0.828407935	0.806922554	0.730791761	0.77208617
LOC539166	0.690965496	0.665563964	0.839601684	0.846715063	0.694012377	0.611777732
CDAN1	0.690965496	0.505355809	0.845237542	0.78508807	0.694246867	0.845969723
LOC104970821	0.690965496	0.537295148	0.765074242	0.931268564	0.702383614	0.671720185
NIPAL2	0.690965496	0.655710881	0.654865457	0.994309225	0.72279174	0.582317254
TM4SF19	0.690965496	0.634031195	0.678204563	0.90419464	0.725609902	0.759107825
LOC112448454	0.690965496	0.72477102	0.747084388	0.763060676	0.741060815	0.769994723
STK40	0.690965496	0.567752026	0.726668529	0.788938193	0.769784115	0.849769587
TGM1	0.690965496	0.944755173	0.685222626	0.754030944	0.780497593	0.579379269
SYNGAP1	0.690965496	0.637203338	0.726668529	0.925004412	0.812342522	0.567131921
LOC107132820	0.690965496	0.692547228	0.621963024	0.776207435	0.84234617	0.840150874
C4A	0.690965496	0.589137013	0.726668529	0.915749182	0.883643824	0.578719809
SYT9	0.690965496	0.536568815	0.713797253	0.914862928	0.887132523	0.645838677
NUAK2	0.690965496	0.550965714	0.706787562	0.943323364	0.892506554	0.576908474
SH3GLB1	0.690965496	0.505355809	0.838488596	0.846715063	0.902843706	0.601095203
ATG3	0.690965496	0.601801243	0.65285332	0.841828571	0.927804504	0.704011905
PELI2	0.690965496	0.553512189	0.665743961	0.781241796	0.954501767	0.798051736
DEFB13	0.690965496	0.536970867	0.64196075	0.781241796	0.996250445	0.803688478
ASB10	0.690965496	0.567752026	0.620720409	0.781241796	0.99989309	0.77208617
LOC100335514	0.691515499	0.529887686	0.788938503	0.760188917	0.769784115	0.86143104
KCNK1	0.691515499	0.63203115	0.618763457	0.825933581	0.934859184	0.752900996
MARCH6	0.691515499	0.563639555	0.777178978	0.820228145	0.949531814	0.610882384

TOMM40	0.691515499	0.609199122	0.63616512	0.775761952	0.972176983	0.77208617
QPCT	0.691572345	0.631868333	0.870054336	0.850828215	0.696143233	0.598156294
TTC4	0.691572345	0.536568815	0.750089824	0.855385038	0.696143233	0.84491737
ARRDC5	0.691572345	0.540649036	0.781294783	0.806922554	0.700991562	0.862351065
LOC783033	0.691572345	0.501940404	0.74784538	0.992137653	0.702383614	0.657118951
CMAS	0.691572345	0.699626135	0.76974432	0.820228145	0.704551568	0.694702496
BMP6	0.691572345	0.543699592	0.780363715	0.929762437	0.725609902	0.637306705
OIP5	0.691572345	0.642501337	0.882898206	0.825933581	0.741060815	0.567131921
HOXB3	0.691572345	0.499949267	0.746957417	0.958879502	0.741060815	0.671720185
AVIL	0.691572345	0.537186174	0.63818281	0.999939566	0.747513637	0.642154165
AP5Z1	0.691572345	0.615179529	0.836294915	0.825933581	0.850852167	0.567131921
MEAF6	0.691572345	0.553512189	0.764481234	0.89088601	0.85489951	0.635645816
AZGP1	0.691572345	0.665803806	0.63818281	0.904074458	0.86558233	0.642154165
ZNF775	0.691572345	0.612450407	0.620720409	0.846715063	0.925981327	0.752358594
SPEN	0.691572345	0.629715977	0.66128476	0.88747242	0.934859184	0.623807852
RNF121	0.691572345	0.578451435	0.694542317	0.898441424	0.941064037	0.61405517
SBF2	0.691572345	0.556152946	0.665743961	0.90419464	0.943768595	0.651173954
CXCR2	0.691572345	0.579263781	0.726668529	0.776207435	0.943901678	0.763746661
LOC100140403	0.691572345	0.537186174	0.620720409	0.863958691	0.952672789	0.77208617
TRAM1	0.691572345	0.490341542	0.845237542	0.781241796	0.954501767	0.64641911
LLGL2	0.691572345	0.669648359	0.665792807	0.788938193	0.957663949	0.66854638
ATE1	0.691572345	0.692987203	0.625367433	0.811000597	0.975291792	0.642154165
ZNF768	0.691572345	0.537295148	0.670235176	0.908496281	0.988693274	0.614421218
ACOT9	0.691572345	0.600538394	0.633986838	0.87452213	0.99989309	0.588661483
VNN2	0.691572345	0.550965714	0.632743297	0.846715063	0.99989309	0.671528522
DNASE1L3	0.691572345	0.544802725	0.621963024	0.758703434	0.99989309	0.76332064
LRRC59	0.692065367	0.595625745	0.638691336	0.793761252	0.702383614	0.940917403
PPIL3	0.692065367	0.553493197	0.692220903	0.788938193	0.99989309	0.583304321
FGF1	0.6926671	0.767950704	0.783875251	0.868090726	0.692179478	0.578699613
USP35	0.6926671	0.534344991	0.787740344	0.964413123	0.692179478	0.636570258
LRRC8D	0.6926671	0.519805717	0.620720409	0.769025565	0.696261246	0.998928226
BCL7B	0.6926671	0.514208066	0.939091808	0.847813982	0.710892232	0.612490982
TRIB2	0.6926671	0.595931157	0.620720409	0.999334425	0.713082855	0.657118951
WDR18	0.6926671	0.506484882	0.621300072	0.775761952	0.741060815	0.996564988
NT5DC2	0.6926671	0.83978684	0.765074242	0.781241796	0.745796372	0.610882384
PRC1	0.6926671	0.553512189	0.838927319	0.87452213	0.762655357	0.636570258
IFI6	0.6926671	0.567845198	0.757235816	0.925931623	0.778366344	0.610416296
NME2	0.6926671	0.648099013	0.63818281	0.781241796	0.846122929	0.845024318

NEGR1	0.6926671	0.636332133	0.699166169	0.760589144	0.876253597	0.805239446
UBL3	0.6926671	0.49642959	0.64109875	0.931750296	0.99989309	0.567131921
LAMTOR1	0.6926671	0.600050072	0.699166169	0.830830186	0.99989309	0.588661483
MRPL54	0.6926671	0.583641966	0.64445174	0.811000597	0.99989309	0.645838677
SLC7A11	0.693087189	0.595931157	0.665743961	0.925931623	0.702383614	0.770391484
LOC104971817	0.693087189	0.557135186	0.832483532	0.894194221	0.725609902	0.645838677
PER3	0.693269946	0.552267835	0.836294915	0.858274325	0.702383614	0.723641471
LOC519309	0.693269946	0.553493197	0.817256644	0.788938193	0.702383614	0.840150874
TIRAP	0.693269946	0.600050072	0.694542317	0.861272209	0.975819525	0.595201312
LRRC17	0.693702098	0.506366149	0.727543729	0.999939566	0.702383614	0.583720387
LOC112445242	0.693702098	0.825301941	0.678204563	0.915749182	0.702383614	0.599490586
VANGL2	0.693702098	0.55750845	0.733231491	0.99879985	0.702383614	0.610882384
HR	0.693702098	0.618982432	0.872470014	0.846715063	0.702383614	0.636570258
C17H4orf46	0.693702098	0.529981214	0.828569127	0.846715063	0.702383614	0.794529985
RSAD1	0.693702098	0.499960638	0.934958503	0.90419464	0.704551568	0.610416296
DDX6	0.693702098	0.534103098	0.984936232	0.805665137	0.7120086	0.590316781
GYG1	0.693702098	0.600050072	0.829843435	0.825933581	0.713082855	0.731709303
LOC618367	0.693702098	0.497480167	0.726668529	0.919203654	0.713082855	0.823139458
DPH3	0.693702098	0.502718799	0.716657925	0.999939566	0.719912953	0.576908474
C11H2orf50	0.693702098	0.542133539	0.844065668	0.913413181	0.72279174	0.652355711
LOC107132301	0.693702098	0.968123234	0.651599878	0.820228145	0.725609902	0.595702749
LOC524810	0.693702098	0.574094539	0.893763648	0.851394268	0.725609902	0.60535862
SLC2A10	0.693702098	0.600050072	0.620720409	0.992339205	0.725609902	0.703914683
KIAA0040	0.693702098	0.567752026	0.739676418	0.915749182	0.725609902	0.76360318
SMYD5	0.693702098	0.567752026	0.684005375	0.792764425	0.725609902	0.934775196
SP100	0.693702098	0.499960638	0.62144899	0.776207435	0.725609902	0.998928226
CALM2	0.693702098	0.557135186	0.96880775	0.776207435	0.726686506	0.645838677
MCHR1	0.693702098	0.57320535	0.720067907	0.944558402	0.727364064	0.671720185
GRK3	0.693702098	0.55750845	0.63818281	0.914402649	0.728674856	0.840150874
ROBO3	0.693702098	0.55750845	0.685222626	0.910808102	0.738661337	0.815459921
SH3PXD2A	0.693702098	0.578451435	0.655038495	0.998542029	0.741060815	0.651173954
SAMD15	0.693702098	0.544639002	0.788938503	0.913413181	0.741060815	0.708819884
BAALC	0.693702098	0.559785028	0.853686558	0.781052987	0.741060815	0.806054104
TRMT6	0.693702098	0.523000612	0.684005375	0.820228145	0.741060815	0.940683751
SLC9A5	0.693702098	0.505355809	0.79610462	0.958879502	0.742316849	0.636570258
C2H2orf72	0.693702098	0.534103098	0.788938503	0.946344632	0.742316849	0.645838677
EIF4E	0.693702098	0.525690188	0.926825953	0.806922554	0.742316849	0.693975957
SYT2	0.693702098	0.507048734	0.713802232	0.999939566	0.745432249	0.635211097

LLPH	0.693702098	0.560898132	0.827125538	0.806922554	0.745796372	0.784671496
PHOSPHO1	0.693702098	0.682847958	0.765074242	0.858021281	0.74589961	0.66048464
ACTG1	0.693702098	0.615920476	0.914685423	0.820228145	0.747513637	0.588661483
BCL2L1	0.693702098	0.534103098	0.748310092	0.962142137	0.748847452	0.653655829
AMIGO2	0.693702098	0.693153864	0.709210234	0.915749182	0.750977672	0.615544321
SOCS6	0.693702098	0.530325237	0.820767684	0.937531834	0.752504864	0.595201312
MYBPC2	0.693702098	0.629715977	0.769285605	0.915749182	0.753418479	0.614421218
REEP2	0.693702098	0.608096264	0.707441133	0.846043369	0.758039654	0.807277693
NCAPD2	0.693702098	0.528592712	0.795258121	0.904074458	0.76054683	0.712710718
EI24	0.693702098	0.567752026	0.765074242	0.90419464	0.762655357	0.671720185
HAPLN3	0.693702098	0.538091051	0.620720409	0.999939566	0.766792351	0.607436462
RBFOX2	0.693702098	0.637634861	0.665743961	0.846715063	0.766792351	0.80844231
TECPR1	0.693702098	0.534103098	0.869455175	0.90419464	0.769784115	0.610882384
EGR1	0.693702098	0.557135186	0.777178978	0.841338807	0.769784115	0.77208617
GSDMB	0.693702098	0.642677274	0.620720409	0.820228145	0.769784115	0.880642012
FLVCR2	0.693702098	0.77396808	0.694422859	0.90419464	0.77278187	0.600077084
GATA5	0.693702098	0.591370069	0.790109639	0.913413181	0.77278187	0.610416296
UBE2W	0.693702098	0.625663019	0.825888497	0.88501388	0.775461217	0.609374985
LOC112446002	0.693702098	0.520705766	0.632743297	0.928836449	0.779159678	0.828602505
PRR12	0.693702098	0.567752026	0.757161143	0.959786126	0.780497593	0.595201312
C1QL3	0.693702098	0.553512189	0.633986838	0.788938193	0.780497593	0.95470473
LOC112442053	0.693702098	0.533749385	0.788848487	0.942384819	0.780831493	0.610416296
LIPJ	0.693702098	0.511608561	0.620860761	0.850828215	0.782284336	0.926872824
SIRPB1	0.693702098	0.737937244	0.620720409	0.781241796	0.784855148	0.859973633
SIGIRR	0.693702098	0.557135186	0.703042171	0.984306983	0.788042205	0.613207282
DNAJC5	0.693702098	0.567752026	0.747084388	0.913413181	0.788042205	0.671528522
STARD3	0.693702098	0.568239497	0.932521008	0.806922554	0.790063341	0.580920535
SLC38A4	0.693702098	0.659132414	0.620720409	0.914862928	0.804250204	0.728567358
MANBAP1	0.693702098	0.502718799	0.806311697	0.91329897	0.805400552	0.673808763
LOC112441478	0.693702098	0.559754831	0.850952986	0.883313728	0.808944674	0.609417643
SEMA3E	0.693702098	0.692025761	0.620720409	0.904990622	0.813582383	0.704974208
EPB42	0.693702098	0.790637585	0.632743297	0.841828571	0.814605912	0.680544964
EVC2	0.693702098	0.551037136	0.770051055	0.892377118	0.819670067	0.671720185
SLAMF8	0.693702098	0.521384007	0.706787562	0.925931623	0.819670067	0.740271903
KLK12	0.693702098	0.524481002	0.696685689	0.820228145	0.834198722	0.867440033
GXYLT2	0.693702098	0.544802725	0.733231491	0.971220023	0.836838903	0.577658632
C7H19orf57	0.693702098	0.582472344	0.620720409	0.99879985	0.836838903	0.602586916
FMOD	0.693702098	0.629715977	0.754897816	0.892229846	0.84234617	0.610882384

LOC112441476	0.693702098	0.502718799	0.806311697	0.772771256	0.849382765	0.833805452
PRR33	0.693702098	0.507048734	0.850243032	0.857959288	0.854242533	0.640138785
STX1A	0.693702098	0.758051794	0.620720409	0.90419464	0.85647293	0.611777732
DDX56	0.693702098	0.560898132	0.708651879	0.850828215	0.867149605	0.759107825
KRT23	0.693702098	0.581298031	0.632190849	0.995118128	0.87465791	0.598680695
CPNE9	0.693702098	0.567752026	0.805449488	0.781241796	0.87465791	0.764546301
PQBP1	0.693702098	0.644538289	0.727732528	0.781241796	0.881257517	0.76935309
LOC107132853	0.693702098	0.538091051	0.628260967	0.78508807	0.886279726	0.912396352
PI16	0.693702098	0.514566709	0.827125538	0.904990622	0.887539821	0.574096987
PPP1R15B	0.693702098	0.595850779	0.703042171	0.89088601	0.887539821	0.661937165
MAGI3	0.693702098	0.538091051	0.715669669	0.806922554	0.893989986	0.826868779
TSHZ2	0.693702098	0.499960638	0.792827466	0.910808102	0.90399271	0.607436462
SLC39A3	0.693702098	0.568025614	0.709521163	0.841828571	0.915496086	0.742259651
MYCT1	0.693702098	0.556152946	0.620943849	0.995118128	0.932081893	0.578699613
DGKQ	0.693702098	0.537295148	0.892122482	0.788938193	0.932197133	0.581523252
EPAS1	0.693702098	0.525336112	0.806221631	0.825933581	0.934859184	0.648987851
SLC39A14	0.693702098	0.534344991	0.620943849	0.934591536	0.939994656	0.704011905
IL1A	0.693702098	0.619374114	0.672328922	0.916305829	0.943768595	0.583091156
LURAP1	0.693702098	0.557966843	0.620860761	0.916305829	0.943768595	0.666847686
LIPG	0.693702098	0.661476635	0.620720409	0.788938193	0.943768595	0.792329436
LOC787234	0.693702098	0.514208066	0.694190265	0.781241796	0.945983467	0.859973633
RNF146	0.693702098	0.514208066	0.684005375	0.940921143	0.951073785	0.633384404
LOC112443510	0.693702098	0.616586256	0.703042171	0.820228145	0.952672789	0.683895124
SNRK	0.693702098	0.700682675	0.620860761	0.781241796	0.952821886	0.750665174
MBLAC2	0.693702098	0.55176988	0.670696706	0.806922554	0.954501767	0.814576296
LOC112446756	0.693702098	0.537295148	0.631435199	0.850828215	0.955813674	0.792329436
HSPA2	0.693702098	0.550965714	0.65719823	0.934591536	0.962610771	0.610882384
CRYAB	0.693702098	0.557135186	0.623085363	0.971220023	0.96532164	0.578699613
CDK5RAP2	0.693702098	0.600050072	0.703042171	0.779764419	0.982800653	0.734655423
CCDC93	0.693702098	0.609819443	0.628247134	0.781241796	0.987816158	0.797202346
BICC1	0.693702098	0.507322509	0.765074242	0.850828215	0.988693274	0.607328652
GNA12	0.693702098	0.534344991	0.684005375	0.850828215	0.988693274	0.693975957
CCDC15	0.693702098	0.560898132	0.729587161	0.864540217	0.99989309	0.578699613
LOC101905312	0.693702098	0.541005316	0.827125538	0.758703434	0.99989309	0.579379269
DMPK	0.693702098	0.507048734	0.726668529	0.781241796	0.99989309	0.579379269
NAT9	0.693702098	0.502718799	0.63818281	0.883885658	0.99989309	0.579379269
LOC112446426	0.693702098	0.568025614	0.620720409	0.92064557	0.99989309	0.580034896
STRN3	0.693702098	0.534344991	0.712526829	0.825933581	0.99989309	0.580920535

SDHAF4	0.693702098	0.499960638	0.631413719	0.886726226	0.99989309	0.580920535
CYREN	0.693702098	0.549999039	0.63818281	0.776207435	0.99989309	0.58203053
TK2	0.693702098	0.731268229	0.625367433	0.781241796	0.99989309	0.583091156
ST8SIA4	0.693702098	0.538091051	0.726668529	0.835232007	0.99989309	0.583720387
SPRYD3	0.693702098	0.697755907	0.699166169	0.781241796	0.99989309	0.588661483
BOLA	0.693702098	0.506366149	0.80193314	0.820228145	0.99989309	0.60396541
C16H1orf115	0.693702098	0.538091051	0.633986838	0.910808102	0.99989309	0.604410267
WAC	0.693702098	0.557135186	0.651248606	0.781241796	0.99989309	0.605287862
NEDD4	0.693702098	0.624948373	0.652359766	0.806922554	0.99989309	0.60717846
VANGL1	0.693702098	0.560168665	0.620720409	0.826315685	0.99989309	0.609824407
POLR2A	0.693702098	0.567752026	0.62144899	0.806922554	0.99989309	0.610416296
CCNI	0.693702098	0.600050072	0.727543729	0.806997685	0.99989309	0.614421218
SIRT4	0.693702098	0.537493495	0.726668529	0.842538664	0.99989309	0.617575248
UBR5	0.693702098	0.551037136	0.621963024	0.781241796	0.99989309	0.624218577
RPS6KB2	0.693702098	0.553456417	0.746957417	0.775761952	0.99989309	0.633384404
SLC25A45	0.693702098	0.511608561	0.703042171	0.883885658	0.99989309	0.63363599
CBX8	0.693702098	0.544739082	0.647411184	0.781241796	0.99989309	0.635211097
KIAA1147	0.693702098	0.573020585	0.632743297	0.844510397	0.99989309	0.636079141
UBAP2	0.693702098	0.502718799	0.62144899	0.936835069	0.99989309	0.636570258
PTGES2	0.693702098	0.629956157	0.622707492	0.781241796	0.99989309	0.642154165
LOC100847410	0.693702098	0.567752026	0.679460199	0.850828215	0.99989309	0.647432409
NDUFS8	0.693702098	0.583843515	0.620720409	0.75922259	0.99989309	0.651173954
DOK7	0.693702098	0.595087191	0.620720409	0.781241796	0.99989309	0.651173954
LOC104975244	0.693702098	0.553512189	0.757787642	0.788938193	0.99989309	0.652355711
RRP8	0.693702098	0.50714456	0.678204563	0.806922554	0.99989309	0.710580013
MTX1	0.693702098	0.559136042	0.631413719	0.781241796	0.99989309	0.712710718
MVK	0.693702098	0.595271828	0.620860761	0.781241796	0.99989309	0.742259651
DEXI	0.693702098	0.551037136	0.620720409	0.820228145	0.99989309	0.789743203
LOC101902293	0.693771403	0.529887686	0.802857717	0.843512648	0.780497593	0.77208617
UBFD1	0.693771403	0.748058967	0.69467582	0.781241796	0.804250204	0.770391484
KLHL25	0.693887307	0.72477102	0.620860761	0.849183739	0.836838903	0.745987575
LOC782021	0.693887307	0.603785862	0.639631378	0.850828215	0.857710467	0.799147792
VCAN	0.693887307	0.502718799	0.765074242	0.943323364	0.875054206	0.610882384
KRIT1	0.693887307	0.782196458	0.726668529	0.776207435	0.939361296	0.61405517
STX7	0.694024636	0.51925133	0.86719452	0.931268564	0.750160769	0.585177964
PHF14	0.694516714	0.581298031	0.684005375	0.999939566	0.727830623	0.630788956
LOC782264	0.694516714	0.540649036	0.620860761	0.999939566	0.748847452	0.610416296
SLC25A5	0.694516714	0.640136201	0.670235176	0.781241796	0.748847452	0.918220726

CAPS2	0.694516714	0.632550803	0.632743297	0.99879985	0.814605912	0.579379269
CXCL5	0.694516714	0.514208066	0.63818281	0.915749182	0.988693274	0.66915423
LOC107131516	0.694516714	0.536568815	0.788938503	0.781241796	0.99989309	0.660829605
SEC61B	0.69481038	0.688099473	0.780363715	0.806922554	0.750160769	0.728276429
MRPL43	0.69481038	0.6188407	0.712526829	0.794956735	0.925981327	0.764546301
CERS4	0.69481038	0.612773311	0.648198992	0.891273781	0.96532164	0.640138785
LOC104976061	0.695246138	0.635240156	0.703042171	0.982809512	0.750160769	0.609824407
FERMT1	0.695246138	0.566780371	0.750089824	0.806922554	0.839043209	0.815459921
PATL1	0.695748245	0.553512189	0.81284583	0.781052987	0.972917711	0.671720185
MED27	0.69582707	0.538091051	0.699166169	0.873519404	0.908708309	0.764546301
CCL8	0.69582707	0.63512774	0.753656036	0.776207435	0.99989309	0.610882384
GIMAP6	0.69618048	0.549040188	0.66128476	0.846715063	0.868380278	0.849772722
RAB7A	0.69618048	0.755866871	0.726668529	0.781052987	0.95956556	0.612910364
F5	0.696256628	0.50714456	0.64445174	0.807614341	0.99989309	0.738110854
MMP2	0.696968505	0.516586591	0.726668529	0.913413181	0.899048045	0.705712189
ADIG	0.696968505	0.608532438	0.685222626	0.858274325	0.94217472	0.671720185
YBX3	0.69719848	0.506484882	0.709561629	0.982140778	0.881823169	0.633384404
APRT	0.69719848	0.578451435	0.632855053	0.820228145	0.939361296	0.826868779
ATP6V0B	0.697242531	0.617063658	0.870054336	0.910808102	0.710892232	0.588661483
SFI1	0.697242531	0.567845198	0.90495551	0.905146083	0.713082855	0.588661483
FRRS1L	0.697242531	0.537186174	0.774385549	0.931989854	0.733904817	0.704367673
MPHOSPH9	0.697242531	0.543699592	0.99115315	0.786808276	0.741017354	0.579379269
EPYC	0.697242531	0.534344991	0.695426006	0.999939566	0.741060815	0.590316781
SNAP23	0.697242531	0.511649327	0.852758896	0.820228145	0.941064037	0.639684299
SREK1	0.697242531	0.511608561	0.739676418	0.90419464	0.943901678	0.667474347
ASB13	0.697242531	0.506366149	0.620860761	0.806922554	0.947146038	0.908939618
NUP50	0.697242531	0.574891833	0.757787642	0.845236547	0.996250445	0.589238861
DSE	0.697399974	0.599807217	0.758406163	0.866960502	0.952821886	0.588661483
LOXL2	0.697958695	0.510190474	0.750603333	0.990571725	0.769784115	0.640138785
NAP1L1	0.697958695	0.843640278	0.631413719	0.820228145	0.892150832	0.633384404
FAM222A	0.698050606	0.567752026	0.631435199	0.992137653	0.750160769	0.740271903
SERP1	0.700280567	0.559441889	0.878603452	0.781241796	0.762655357	0.763656348
PTPRO	0.700870335	0.636332133	0.781294783	0.820228145	0.954501767	0.588661483
UBR7	0.701067658	0.505355809	0.90166788	0.883869828	0.819670067	0.610416296
HELLS	0.701086678	0.586282126	0.684005375	0.999939566	0.723334634	0.610416296
VEGFD	0.701086678	0.835832963	0.729587161	0.781241796	0.865955438	0.611777732
PLEKHH3	0.701212349	0.637634861	0.726668529	0.915749182	0.827743342	0.640138785
CORO1C	0.701212349	0.505355809	0.878603452	0.90419464	0.856442535	0.588661483

CCDC189	0.701212349	0.629715977	0.869529186	0.781241796	0.904151325	0.599490586
PRPF8	0.701212349	0.547184103	0.688413073	0.820228145	0.99989309	0.607436462
LOC786726	0.701410215	0.538091051	0.777178978	0.914402649	0.769784115	0.725620954
SQSTM1	0.701879764	0.588244908	0.754897816	0.943323364	0.742316849	0.651139949
F3	0.701932336	0.534103098	0.682591947	0.965590385	0.745796372	0.777537507
THNSL2	0.701932336	0.553493197	0.699166169	0.943323364	0.932197133	0.616913081
DNLZ	0.701932336	0.669039484	0.684005375	0.781241796	0.957605284	0.743377609
SRRD	0.701932336	0.538091051	0.750089824	0.850828215	0.99989309	0.610416296
LOC112449072	0.702617699	0.790637585	0.788848487	0.858021281	0.704551568	0.607436462
BTBD11	0.702617699	0.905638138	0.633986838	0.781241796	0.707777957	0.77208617
CNTROB	0.702617699	0.509590069	0.939091808	0.908496281	0.710892232	0.610882384
SELP	0.702617699	0.553512189	0.709521163	0.949947918	0.741060815	0.753613607
ZNRF2	0.702617699	0.583763975	0.810425784	0.915749182	0.74589961	0.642154165
ELAVL1	0.702617699	0.589137013	0.892122482	0.825933581	0.74589961	0.651173954
MKL2	0.702617699	0.872969587	0.632855053	0.869637097	0.747513637	0.642154165
LOC104973229	0.702617699	0.63203115	0.765074242	0.820228145	0.80664102	0.764546301
KIAA0586	0.702617699	0.711594168	0.765074242	0.820228145	0.80841171	0.671528522
PSAT1	0.702617699	0.661476635	0.685222626	0.806922554	0.813531278	0.840150874
DMAC1	0.702617699	0.567845198	0.74784538	0.863958691	0.846122929	0.750665174
CHKB	0.702617699	0.6861152	0.715979316	0.855343521	0.917145033	0.627553513
LDLRAP1	0.702617699	0.687403165	0.794216389	0.781241796	0.932081893	0.642154165
ANKMY2	0.702617699	0.656553906	0.747084388	0.850828215	0.952821886	0.583720387
SKP2	0.703885589	0.549065127	0.684005375	0.915749182	0.824698899	0.79628721
GPATCH3	0.70430214	0.762150099	0.631350327	0.781241796	0.987215089	0.693975957
GCSH	0.70430415	0.586243836	0.632743297	0.806922554	0.99989309	0.671528522
B3GNT6	0.704625304	0.636332133	0.812043145	0.925901365	0.704551568	0.61405517
GSPT1	0.704625304	0.538091051	0.751496241	0.917869295	0.712561815	0.799935743
TTC33	0.704625304	0.530886586	0.628625476	0.999939566	0.713082855	0.77208617
BBS5	0.704625304	0.537186174	0.63818281	0.943591298	0.727364064	0.858777168
NAV2	0.704625304	0.567471709	0.632743297	0.99879985	0.7817968	0.671720185
RBBP5	0.704625304	0.625663019	0.880277219	0.820228145	0.818457184	0.610416296
LUC7L	0.704625304	0.534344991	0.834569848	0.875093568	0.819670067	0.681177433
MVP	0.704625304	0.581298031	0.685222626	0.875889425	0.83417924	0.808798209
F2RL2	0.704625304	0.691880741	0.684005375	0.844446125	0.846122929	0.745987575
ZNF705A	0.704625304	0.514208066	0.699166169	0.956154344	0.859775628	0.694702496
ASCL2	0.704625304	0.588244908	0.802857717	0.820228145	0.934859184	0.640138785
DPM1	0.704625304	0.567752026	0.621963024	0.851394268	0.934859184	0.826929951
GPC3	0.704625304	0.510190474	0.856105201	0.841828571	0.964150118	0.588661483

ASNA1	0.704625304	0.61754698	0.706787562	0.820228145	0.972917711	0.692802214
NBEA	0.704625304	0.673034156	0.621963024	0.82652165	0.988693274	0.694702496
AKIP1	0.704625304	0.50714456	0.765591612	0.820228145	0.99989309	0.588661483
RABL6	0.704625304	0.705118484	0.620943849	0.841828571	0.99989309	0.635645816
UTP18	0.704770739	0.578451435	0.735921892	0.928018321	0.727364064	0.748417897
LPAR6	0.704770739	0.507048734	0.621963024	0.999939566	0.939361296	0.616913081
STK19	0.705153147	0.523000612	0.870054336	0.88053803	0.765961167	0.684905734
SYDE2	0.705153147	0.834869027	0.625367433	0.825933581	0.837323996	0.694702496
PLEKHB2	0.706101732	0.583843515	0.787740344	0.91329897	0.741060815	0.710580013
NKAIN1	0.706101732	0.553493197	0.633986838	0.999939566	0.839893529	0.588661483
FAP	0.706101732	0.534344991	0.715979316	0.883885658	0.856651628	0.809650729
FAHD1	0.706101732	0.583641966	0.699166169	0.87630412	0.952821886	0.677152076
ASTE1	0.706101732	0.583843515	0.726668529	0.784305663	0.99989309	0.636570258
CBR4	0.706101732	0.567752026	0.678204563	0.87630412	0.99989309	0.645838677
MRVI1	0.706437616	0.81105622	0.712526829	0.825933581	0.718950706	0.728567358
ELAVL3	0.706437616	0.63203115	0.849077738	0.820228145	0.745796372	0.714683388
SPC24	0.706437616	0.615879066	0.743505042	0.915749182	0.750160769	0.732184848
FERMT2	0.706437616	0.644204975	0.845237542	0.842538664	0.83627631	0.607436462
FBXO3	0.706437616	0.625663019	0.754101752	0.851175396	0.875054206	0.66915423
SIVA1	0.706437616	0.688099473	0.64109875	0.914402649	0.961382403	0.588661483
RPRD1B	0.706437616	0.583843515	0.781294783	0.841828571	0.964150118	0.610882384
TAGLN2	0.706437616	0.629715977	0.678070701	0.883885658	0.982014614	0.636570258
PAQR3	0.706437616	0.511608561	0.62764537	0.937539418	0.988693274	0.681177433
LOC515697	0.706437616	0.692987203	0.684005375	0.781241796	0.99989309	0.610416296
NFKB1	0.706437616	0.595087191	0.675040193	0.784305663	0.99989309	0.610416296
ZNF385D	0.706437616	0.536746779	0.670235176	0.925265292	0.99989309	0.610416296
HSPA4L	0.706437616	0.599807217	0.70435078	0.788938193	0.99989309	0.694702496
TMEM223	0.706886108	0.553493197	0.668857004	0.835232007	0.99989309	0.653313672
MAP3K1	0.707004579	0.615914135	0.827125538	0.910808102	0.713691227	0.661861937
TMBIM4	0.707004579	0.568025614	0.849077738	0.915749182	0.762655357	0.610416296
LAMP2	0.707004579	0.538091051	0.685222626	0.999939566	0.766792351	0.636570258
AMPD2	0.707004579	0.653112351	0.631350327	0.820228145	0.784855148	0.893961809
FAM234A	0.707004579	0.669648359	0.677728208	0.807296971	0.99989309	0.588661483
CHAF1B	0.707004579	0.578451435	0.69160052	0.781241796	0.99989309	0.630578915
DIRAS1	0.707004579	0.536568815	0.712526829	0.873939476	0.99989309	0.636570258
MYL6	0.707004579	0.568025614	0.765074242	0.784305663	0.99989309	0.677152076
PCDHGA2	0.707145235	0.816119178	0.632190849	0.886726226	0.87483668	0.614421218
LOC112445051	0.707327817	0.579545624	0.715979316	0.820228145	0.753418479	0.896934364

IFNLR1	0.707327817	0.730944867	0.678204563	0.91329897	0.860908683	0.627553513
MCM8	0.707349635	0.950844365	0.685222626	0.806997685	0.762655357	0.610416296
WDR87	0.707349635	0.563639555	0.757235816	0.781241796	0.784855148	0.895218005
DCTN4	0.707349635	0.750335462	0.744490351	0.873519404	0.795700715	0.633384404
VPS16	0.707349635	0.563639555	0.879515214	0.87630412	0.808944674	0.610882384
SERPINB5	0.707756556	0.779501962	0.849077738	0.781241796	0.766792351	0.610416296
TNRC18	0.707756556	0.511608561	0.65719823	0.944558402	0.818344379	0.8125889
PIP5K1C	0.707756556	0.668175004	0.696685689	0.820228145	0.819670067	0.811943134
TTPA	0.707756556	0.553493197	0.675040193	0.999939566	0.823462814	0.610882384
CFDP1	0.707756556	0.616586256	0.882898206	0.825933581	0.84234617	0.604410267
LOC514507	0.707756556	0.776573165	0.787740344	0.778545211	0.84234617	0.660829605
LOC104971162	0.707756556	0.585680732	0.817256644	0.846715063	0.861762366	0.661861937
GSTO1	0.707756556	0.553493197	0.685222626	0.796330272	0.866920896	0.911263177
ZBTB7B	0.707756556	0.625663019	0.685222626	0.956154344	0.87483668	0.610416296
EFEMP2	0.707756556	0.52442418	0.765074242	0.925901365	0.943768595	0.609824407
ASZ1	0.707756556	0.632709306	0.678070701	0.851394268	0.99989309	0.588661483
ARHGEF19	0.707756556	0.567752026	0.685222626	0.820228145	0.99989309	0.591016795
LOC100126544	0.707756556	0.703437628	0.684005375	0.835007451	0.99989309	0.610416296
NDUFS6	0.707756556	0.6861152	0.63818281	0.78508807	0.99989309	0.635211097
LOC107131843	0.708032997	0.541897864	0.632855053	0.788938193	0.99989309	0.752900996
BNIP3	0.70806699	0.655438106	0.622639736	0.973377993	0.742316849	0.74008849
RPF1	0.70806699	0.686014664	0.745242261	0.846715063	0.750160769	0.770391484
MAT2B	0.708901945	0.522228461	0.894447907	0.842538664	0.745432249	0.742259651
CFH	0.708901945	0.528592712	0.773281026	0.892741454	0.970733065	0.635211097
BCL2	0.709148354	0.557966843	0.699166169	0.999939566	0.713082855	0.607436462
LGALS8	0.709148354	0.553751933	0.726668529	0.999939566	0.713691227	0.636570258
LOC615559	0.709148354	0.63203115	0.744933814	0.992137653	0.720269226	0.611777732
C1H21orf91	0.709148354	0.6608873	0.894547778	0.781241796	0.72279174	0.710648363
WNT2	0.709148354	0.937978608	0.684005375	0.806922554	0.725609902	0.66854638
GABBR1	0.709148354	0.536568815	0.811009079	0.806922554	0.725609902	0.896934364
HDLBP	0.709148354	0.657500304	0.642916091	0.841828571	0.746187115	0.8905829
EPHB2	0.709148354	0.648099013	0.685222626	0.915749182	0.750160769	0.770391484
TUBA1B	0.709148354	0.692928782	0.712526829	0.846715063	0.750160769	0.791259029
ARFGAP2	0.709148354	0.538091051	0.764481234	0.915749182	0.763230439	0.77208617
ZSWIM4	0.709148354	0.567845198	0.632855053	0.999939566	0.769784115	0.637306705
NAA15	0.709148354	0.582985895	0.765074242	0.892229846	0.804250204	0.738775793
ATP7B	0.709148354	0.800441059	0.744933814	0.84302258	0.819670067	0.614421218
LOC112445999	0.709148354	0.538091051	0.715979316	0.924876334	0.861524746	0.742259651

SLC46A2	0.709148354	0.632709306	0.64445174	0.916026707	0.910770324	0.671528522
PSMB6	0.709148354	0.612450407	0.690468174	0.835254768	0.916423755	0.784080446
GIPC3	0.709148354	0.624948373	0.79610462	0.861272209	0.928701823	0.595702749
LOC101903501	0.709148354	0.655438106	0.632855053	0.87630412	0.96532164	0.671720185
LITAF	0.709148354	0.568239497	0.739676418	0.879874842	0.970439271	0.642154165
NKRF	0.709148354	0.550965714	0.76749973	0.807809786	0.99989309	0.671720185
SLC25A1	0.709148354	0.550673085	0.623085363	0.781241796	0.99989309	0.707492534
SPRYD4	0.709148354	0.553493197	0.682591947	0.806922554	0.99989309	0.752358594
RGS7BP	0.709148354	0.538091051	0.685222626	0.781241796	0.99989309	0.770391484
YEATS2	0.709148354	0.55176988	0.684005375	0.806922554	0.99989309	0.792329436
LOC112449523	0.709507115	0.6136689	0.765074242	0.934591536	0.747513637	0.6608989
CYSLTR1	0.709507115	0.707802804	0.632743297	0.99419346	0.747527514	0.63363599
EIF4EBP3	0.709507115	0.612450407	0.652359766	0.826315685	0.766792351	0.911696156
LOC101907152	0.709507115	0.707874469	0.678204563	0.781241796	0.818457184	0.868025807
SLC26A2	0.709507115	0.561653393	0.62764537	0.999939566	0.833004048	0.610416296
ASMTL	0.709507115	0.612450407	0.726668529	0.811000597	0.952172109	0.753067712
PRKCI	0.709507115	0.57847388	0.632743297	0.806922554	0.99989309	0.591154525
CCL21	0.709790194	0.571420811	0.66128476	0.999939566	0.727364064	0.705712189
CAPN11	0.709790194	0.903820329	0.678204563	0.891273781	0.742316849	0.610416296
LOC101906280	0.709790194	0.60290293	0.678204563	0.992137653	0.766792351	0.671528522
YARS	0.709790194	0.567752026	0.724199536	0.785145388	0.769976034	0.937591162
IDUA	0.709790194	0.842797231	0.673737267	0.903607716	0.771827245	0.635645816
TMEM165	0.709790194	0.522228461	0.632743297	0.785145388	0.780497593	0.9956013
NLRP12	0.709790194	0.557966843	0.685222626	0.99879985	0.784855148	0.660829605
LOC783224	0.709790194	0.878594098	0.694422859	0.820228145	0.809298212	0.637306705
SAPCD1	0.709790194	0.567752026	0.922126495	0.781241796	0.881823169	0.637306705
ST3GAL5	0.709790194	0.543699592	0.670235176	0.999939566	0.886279726	0.610882384
IFT20	0.709790194	0.81250936	0.733231491	0.794713619	0.91011204	0.616913081
SAP18	0.709790194	0.629715977	0.765074242	0.826315685	0.910770324	0.671720185
RPS19BP1	0.709790194	0.704817952	0.684005375	0.820228145	0.919854965	0.740271903
PGP	0.709790194	0.62464979	0.661590836	0.784305663	0.954501767	0.839419627
POLR3H	0.709790194	0.619184305	0.719216596	0.788938193	0.988120779	0.742451714
TK1	0.709790194	0.72843033	0.632743297	0.788938193	0.99989309	0.610416296
AQP1	0.709790194	0.624067943	0.73016804	0.820228145	0.99989309	0.610882384
MIEF2	0.709790194	0.633706991	0.726668529	0.781241796	0.99989309	0.651173954
LOC101903832	0.709790194	0.567752026	0.632855053	0.836785013	0.99989309	0.691363988
MRPS7	0.709790194	0.550965714	0.632855053	0.843411019	0.99989309	0.799137321
UMAD1	0.71143298	0.615959252	0.694542317	0.995118128	0.750160769	0.646642632

CC2D2A	0.711704739	0.625615428	0.735921892	0.925004412	0.784855148	0.681247744
ADO	0.71212465	0.595931157	0.738217747	0.843411019	0.713691227	0.886992214
LOC112447408	0.71212465	0.593689466	0.777638604	0.925901365	0.745432249	0.681655965
LOC112447819	0.71212465	0.93453191	0.682591947	0.806922554	0.849382765	0.599490586
ZNF438	0.714127103	0.567845198	0.694190265	0.935163227	0.725609902	0.823435077
FOXO4	0.714354343	0.71282706	0.638691336	0.904990622	0.766792351	0.77208617
OSR1	0.714354343	0.701505998	0.64822617	0.90419464	0.961835052	0.613207282
ATXN2L	0.714354343	0.527088167	0.813000643	0.788165915	0.99989309	0.689916216
MITF	0.714599482	0.538091051	0.631350327	0.983971658	0.750160769	0.840150874
MYOM3	0.714599482	0.769091087	0.693120773	0.850828215	0.784855148	0.727921992
SSC5D	0.714599482	0.646557232	0.772990161	0.93471064	0.790063341	0.607436462
LOC107131675	0.714599482	0.748529544	0.812770091	0.781241796	0.834198722	0.657118951
POLR2I	0.714599482	0.690829991	0.845237542	0.828524115	0.838889011	0.607436462
LOC112442664	0.714599482	0.629715977	0.633986838	0.806997685	0.86558233	0.897818829
DCP1A	0.714599482	0.600050072	0.790504275	0.811000597	0.988828259	0.636570258
TAB1	0.714599482	0.521384007	0.638691336	0.788165915	0.99989309	0.610416296
HOXD9	0.714599482	0.589137013	0.660379966	0.87630412	0.99989309	0.671528522
LOC101906511	0.714599482	0.557135186	0.726668529	0.781241796	0.99989309	0.76332064
CRYM	0.714931146	0.702457626	0.633986838	0.90419464	0.779159678	0.792329436
ADRM1	0.714931146	0.569044188	0.726668529	0.841828571	0.933983913	0.784080446
DHX36	0.714931146	0.603474998	0.685222626	0.784305663	0.99989309	0.770391484
ACTR5	0.715125893	0.6608873	0.788848487	0.781241796	0.99989309	0.610416296
LOC104970711	0.715457387	0.538091051	0.787740344	0.982367414	0.741060815	0.66915423
AMOTL2	0.715457387	0.769817928	0.879215964	0.788938193	0.745796372	0.615751088
LRRC4C	0.715457387	0.583641966	0.638691336	0.91329897	0.750160769	0.886992214
GUCA1A	0.715457387	0.703437628	0.693454224	0.915749182	0.819670067	0.66915423
OSBPL7	0.715457387	0.553493197	0.883982433	0.830830186	0.838889011	0.671528522
AK6	0.715457387	0.731268229	0.651248606	0.842538664	0.84234617	0.775116427
DBN1	0.715457387	0.625663019	0.665743961	0.989087699	0.847612185	0.639128578
SUZ12	0.715457387	0.642677274	0.750089824	0.915749182	0.849825976	0.635211097
CCNE2	0.715457387	0.669648359	0.703042171	0.820228145	0.856651628	0.815402066
RASSF5	0.715457387	0.560898132	0.632743297	0.915749182	0.882754591	0.813759229
LOC104976232	0.715457387	0.583843515	0.870054336	0.781241796	0.89629383	0.703742328
LOC101902527	0.715457387	0.634420495	0.817256644	0.781241796	0.927804504	0.704974208
GMEB2	0.715457387	0.760200908	0.73016804	0.781241796	0.949645557	0.671528522
SOX9	0.715457387	0.553512189	0.684005375	0.956154344	0.972917711	0.61405517
DCAF10	0.715457387	0.72477102	0.707441133	0.781241796	0.99989309	0.610416296
SETD2	0.715457387	0.614559394	0.632743297	0.806922554	0.99989309	0.637306705

NOC4L	0.716063816	0.567752026	0.713797253	0.841828571	0.753062237	0.911696156
SELENOO	0.716238004	0.601801243	0.694542317	0.787056428	0.99989309	0.759308648
LPIN1	0.716364169	0.534344991	0.684005375	0.989418929	0.838889011	0.743377609
EXTL2	0.716364169	0.693544464	0.70435078	0.903607716	0.84038133	0.694702496
LOC613460	0.716899825	0.736325439	0.764057751	0.919247117	0.725609902	0.633384404
LOC100848799	0.716899825	0.578451435	0.694190265	0.87452213	0.725609902	0.910353954
PNMA1	0.716899825	0.666543433	0.790109639	0.781241796	0.741060815	0.849769587
ANXA8L1	0.716899825	0.619374114	0.828569127	0.90419464	0.766792351	0.653387719
APH1B	0.716899825	0.551037136	0.684005375	0.811000597	0.784855148	0.957877422
ENY2	0.716899825	0.567752026	0.7079185	0.861272209	0.99989309	0.635211097
SHKBP1	0.716899825	0.567752026	0.63818281	0.788938193	0.99989309	0.671528522
TSLP	0.716899825	0.545732987	0.712526829	0.788938193	0.99989309	0.749880183
TM9SF3	0.716899825	0.537186174	0.682591947	0.841828571	0.99989309	0.770391484
POU3F1	0.718519787	0.688099473	0.706787562	0.820686724	0.808924032	0.830923241
FDPS	0.718519787	0.665524216	0.632743297	0.788938193	0.943768595	0.858538874
SLC25A26	0.718519787	0.553512189	0.659491881	0.818984341	0.99989309	0.609458315
TXNDC5	0.71859068	0.693544464	0.729587161	0.990049248	0.725609902	0.610882384
LOC788648	0.71859068	0.544739082	0.80326876	0.990571725	0.725609902	0.654251319
RTCB	0.71859068	0.543699592	0.753656036	0.946344632	0.725609902	0.797202346
BATF3	0.71859068	0.890427603	0.682591947	0.796330272	0.727364064	0.787474205
RPP40	0.71859068	0.533844215	0.716657925	0.826315685	0.730791761	0.972216653
CBLL1	0.71859068	0.990179479	0.688413073	0.788938193	0.732864869	0.610416296
TRIM25	0.71859068	0.6861152	0.937363055	0.820228145	0.741060815	0.610416296
TMEM248	0.71859068	0.524212852	0.747084388	0.999939566	0.741060815	0.671720185
CYR61	0.71859068	0.550965714	0.812770091	0.968306132	0.742316849	0.650757255
LOC508933	0.71859068	0.684832051	0.817256644	0.925901365	0.745432249	0.610416296
RGL3	0.71859068	0.537295148	0.882898206	0.841338807	0.745432249	0.785919503
RPF2	0.71859068	0.568025614	0.828449612	0.864540217	0.746187115	0.77208617
LOC107133276	0.71859068	0.629715977	0.827350832	0.940863121	0.747527514	0.607436462
ATP13A4	0.71859068	0.694672395	0.632855053	0.990049248	0.753418479	0.671528522
ZFAND5	0.71859068	0.629715977	0.694190265	0.999939566	0.762253517	0.610882384
IDE	0.71859068	0.529762119	0.684005375	0.838641474	0.765330204	0.965134939
DYNLRB2	0.71859068	0.600050072	0.751496241	0.964841815	0.766792351	0.659862452
DSTN	0.71859068	0.761788537	0.765074242	0.805631094	0.773626517	0.764546301
PAMR1	0.71859068	0.689368188	0.911757786	0.820228145	0.780497593	0.606284793
ADGRL1	0.71859068	0.710028977	0.638223753	0.958849311	0.7817968	0.66854638
LOC101902288	0.71859068	0.693153864	0.788848487	0.806997685	0.7817968	0.770391484
KIF15	0.71859068	0.562255493	0.827278747	0.943323364	0.782284336	0.636570258

LOC112444309	0.71859068	0.609819443	0.702276506	0.811000597	0.784855148	0.915899294
SPATA5L1	0.71859068	0.600960324	0.921146002	0.843411019	0.790063341	0.611777732
LOC112448084	0.71859068	0.537186174	0.685222626	0.883885658	0.804250204	0.911696156
CYTH2	0.71859068	0.549065127	0.684005375	0.781241796	0.804250204	0.986389023
F13A1	0.71859068	0.61754698	0.675040193	0.90419464	0.808924032	0.850612178
LOC101904344	0.71859068	0.680267573	0.814444866	0.820228145	0.818457184	0.708819884
SLC29A2	0.71859068	0.581298031	0.632743297	0.999939566	0.825710291	0.654251319
MANBAL	0.71859068	0.636332133	0.817993104	0.915749182	0.829012825	0.610882384
LOC618169	0.71859068	0.553512189	0.707441133	0.995800463	0.835860597	0.661937165
USP38	0.71859068	0.583641966	0.820549834	0.925901365	0.839134872	0.611777732
SERINC2	0.71859068	0.585193727	0.939683463	0.806922554	0.84234617	0.636570258
LOC107133049	0.71859068	0.808513267	0.673737267	0.915749182	0.846122929	0.610416296
LOC515358	0.71859068	0.600050072	0.694190265	0.959210368	0.854368278	0.66854638
LOC112445044	0.71859068	0.561653393	0.726668529	0.990571725	0.85647293	0.636079141
SNU13	0.71859068	0.534103098	0.840938565	0.917869295	0.875054206	0.635211097
CALM1	0.71859068	0.538091051	0.843427302	0.893235972	0.875530861	0.66915423
CHDH	0.71859068	0.544739082	0.733776767	0.943539568	0.882754591	0.677152076
TOP3B	0.71859068	0.562255493	0.880204512	0.842538664	0.899048045	0.636570258
PTPRR	0.71859068	0.708581322	0.755534187	0.825058801	0.899048045	0.66854638
IL17RB	0.71859068	0.587833611	0.684005375	0.915749182	0.899048045	0.752900996
NUBP2	0.71859068	0.769817928	0.668846123	0.811000597	0.905719399	0.763746661
RASGRP1	0.71859068	0.693544464	0.63818281	0.781241796	0.910393751	0.873118284
SLC48A1	0.71859068	0.559785028	0.744369186	0.943323364	0.9215167	0.637306705
RHPN1	0.71859068	0.536568815	0.750089824	0.971220023	0.92188432	0.612910364
LOC112449284	0.71859068	0.567845198	0.832483532	0.781241796	0.928701823	0.763460317
ZNF414	0.71859068	0.537186174	0.757787642	0.956154344	0.939361296	0.610416296
MPZL3	0.71859068	0.567845198	0.720067907	0.915749182	0.946536678	0.671720185
SEC63	0.71859068	0.535160666	0.844065668	0.850828215	0.946944212	0.642154165
FBXL6	0.71859068	0.54984186	0.855743966	0.841429084	0.947146038	0.642154165
RENBP	0.71859068	0.560898132	0.726668529	0.925931623	0.954501767	0.642154165
LOC100335608	0.71859068	0.538091051	0.774555223	0.846115604	0.957663949	0.738110854
TMEM263	0.71859068	0.542976882	0.730446027	0.90419464	0.961588929	0.722309684
TMEM38B	0.71859068	0.534344991	0.813000643	0.903607716	0.964150118	0.619060217
TIMM23	0.71859068	0.595931157	0.694190265	0.820228145	0.96532164	0.794529985
HMGXB3	0.71859068	0.567752026	0.878603452	0.806922554	0.968795608	0.610882384
RFFL	0.71859068	0.578451435	0.726668529	0.794713619	0.982014614	0.79339547
SOST	0.71859068	0.53478796	0.755534187	0.788938193	0.982800653	0.812509877
CCDC174	0.71859068	0.72843033	0.633986838	0.820228145	0.988693274	0.719551722

SDR16C5	0.71859068	0.560898132	0.632743297	0.863958691	0.988693274	0.80252768
PPCS	0.71859068	0.562255493	0.632743297	0.825933581	0.988693274	0.852772378
LOC112443415	0.71859068	0.562255493	0.812043145	0.837490975	0.98871637	0.637306705
LOC100297099	0.71859068	0.632709306	0.685222626	0.881507912	0.996250445	0.648987851
RDH10	0.71859068	0.66621239	0.699166169	0.806922554	0.996250445	0.719551722
PLXDC2	0.71859068	0.574094539	0.754101752	0.836672323	0.99989309	0.607436462
TRIM34	0.71859068	0.543699592	0.649291546	0.88747242	0.99989309	0.607436462
SCRG1	0.71859068	0.583843515	0.722352424	0.806922554	0.99989309	0.610416296
LAMA2	0.71859068	0.722790338	0.746957417	0.806922554	0.99989309	0.610416296
HSP90AB1	0.71859068	0.570533541	0.678204563	0.806997685	0.99989309	0.610416296
LOC618076	0.71859068	0.537186174	0.633986838	0.806922554	0.99989309	0.612490982
LRRC34	0.71859068	0.617454372	0.747093618	0.806997685	0.99989309	0.613207282
SRGAP1	0.71859068	0.538091051	0.77992581	0.843411019	0.99989309	0.616913081
CHORDC1	0.71859068	0.526218028	0.673737267	0.781241796	0.99989309	0.627790264
LOC530102	0.71859068	0.619184305	0.800718134	0.803538703	0.99989309	0.636570258
CRIP1	0.71859068	0.703437628	0.675040193	0.825933581	0.99989309	0.636570258
NDUFA5	0.71859068	0.629715977	0.688413073	0.841828571	0.99989309	0.639128578
EPB41L3	0.71859068	0.555093165	0.63818281	0.946344632	0.99989309	0.645838677
EIPR1	0.71859068	0.649200791	0.73016804	0.788938193	0.99989309	0.689075996
FOXP3	0.71859068	0.6663481	0.63818281	0.820228145	0.99989309	0.692802214
TBL2	0.71859068	0.537186174	0.726668529	0.861131837	0.99989309	0.705712189
DNAJC28	0.71859068	0.53072311	0.685222626	0.850828215	0.99989309	0.759308648
HSPA1A	0.71859068	0.600960324	0.63818281	0.820228145	0.99989309	0.783479395
ANO9	0.71859068	0.542133539	0.632855053	0.825933581	0.99989309	0.80787758
LOC785745	0.718852434	0.562255493	0.905017554	0.917869295	0.725609902	0.636570258
AP4E1	0.718852434	0.603844039	0.754897816	0.995118128	0.747513637	0.635645816
LOC100848883	0.718852434	0.655710881	0.659491881	0.788938193	0.748847452	0.961506391
RSU1	0.718852434	0.612773311	0.937930242	0.846715063	0.750160769	0.616913081
LOC112446013	0.718852434	0.624948373	0.808333956	0.806922554	0.750160769	0.851366694
CHCHD6	0.718852434	0.665563964	0.845237542	0.846998569	0.750220157	0.694269806
PHF11	0.718852434	0.536568815	0.852758896	0.858274325	0.762655357	0.796002422
MFAP2	0.718852434	0.636332133	0.808333956	0.924489924	0.778366344	0.637306705
ROR2	0.718852434	0.549065127	0.751496241	0.998335031	0.779159678	0.651173954
FABP3	0.718852434	0.693153864	0.747093618	0.888642721	0.780497593	0.742259651
NEU3	0.718852434	0.534103098	0.744490351	0.820228145	0.784855148	0.941925704
HSF4	0.718852434	0.567752026	0.921420528	0.868090726	0.797393203	0.633384404
GIMAP5	0.718852434	0.589137013	0.678204563	0.915749182	0.811505026	0.847189049
SEMA4A	0.718852434	0.596400907	0.712526829	0.87630412	0.853884483	0.823139458

MAN2A2	0.718852434	0.608346598	0.764057751	0.905388347	0.87483668	0.687121026
TRHDE	0.718852434	0.704519382	0.750089824	0.811000597	0.87483668	0.752900996
C8H9orf131	0.718852434	0.560898132	0.773281026	0.820228145	0.881823169	0.831206878
SCN1B	0.718852434	0.6861152	0.755534187	0.90419464	0.896242324	0.633384404
TH	0.718852434	0.534103098	0.765074242	0.855177907	0.896242324	0.806469522
CDK2	0.718852434	0.549065127	0.706787562	0.843411019	0.906183446	0.868108836
GID4	0.718852434	0.608717568	0.707441133	0.90419464	0.925981327	0.742259651
LOC100848598	0.718852434	0.543699592	0.63818281	0.891273781	0.934859184	0.86252043
GJC1	0.718852434	0.563639555	0.675040193	0.908804713	0.957605284	0.767960884
METAP1	0.718852434	0.538091051	0.755534187	0.931268564	0.960296839	0.633384404
GET4	0.718852434	0.689503192	0.665743961	0.843411019	0.961382403	0.735313642
MBNL2	0.718852434	0.567752026	0.651248606	0.990571725	0.961835052	0.621269165
FBXW11	0.718852434	0.583411145	0.760400875	0.876107068	0.98152792	0.645838677
LOC100848684	0.718852434	0.67497759	0.707441133	0.829151867	0.997488969	0.66854638
LOC101906134	0.718852434	0.608096264	0.744490351	0.850828215	0.99989309	0.614421218
UPF2	0.718852434	0.559785028	0.748016329	0.794713619	0.99989309	0.636570258
SELENOW	0.718852434	0.600050072	0.675040193	0.90419464	0.99989309	0.642154165
DPM2	0.718852434	0.591604402	0.751496241	0.825933581	0.99989309	0.661861937
CSGALNACT2	0.718852434	0.538091051	0.69131755	0.781241796	0.99989309	0.66854638
SLC16A13	0.718852434	0.693560633	0.706787562	0.781241796	0.99989309	0.66854638
CAV2	0.718852434	0.55176988	0.684005375	0.910675605	0.99989309	0.683474857
HIF1A	0.718852434	0.550233457	0.684005375	0.90419464	0.99989309	0.731709303
LOC101906006	0.718881088	0.542133539	0.694190265	0.806922554	0.99989309	0.636570258
GCSAML	0.719037563	0.551867132	0.751496241	0.943323364	0.882754591	0.674719861
LOC104974850	0.719823254	0.589334554	0.684005375	0.796330272	0.742316849	0.983453112
SETD7	0.719823254	0.537186174	0.755324843	0.995800463	0.745432249	0.709387613
LTV1	0.719823254	0.642501337	0.731086678	0.806922554	0.846122929	0.859973633
PA2G4	0.719823254	0.553512189	0.689481932	0.90419464	0.986976797	0.747230622
CXCL10	0.720228029	0.738328793	0.765074242	0.841828571	0.727364064	0.770391484
LOC509513	0.720228029	0.72843033	0.684005375	0.806922554	0.77278187	0.879725389
ZMYM4	0.720228029	0.803903736	0.63818281	0.811000597	0.99989309	0.610882384
CEP128	0.720228029	0.629715977	0.665743961	0.781241796	0.99989309	0.806469522
ZNF184	0.72075596	0.676166653	0.687699922	0.925004412	0.730791761	0.799993234
TMEM132E	0.72075596	0.534344991	0.699166169	0.966070428	0.742316849	0.846893818
LIG3	0.72075596	0.570927544	0.633986838	0.99879985	0.747527514	0.773402056
LOC112442295	0.72075596	0.581298031	0.893863239	0.855137785	0.750160769	0.697499202
SREK1IP1	0.72075596	0.665563964	0.747093618	0.943323364	0.766792351	0.66854638
LOC104975460	0.72075596	0.547807041	0.717649445	0.936102593	0.780497593	0.826868779

PLXNA1	0.72075596	0.625663019	0.709561629	0.820228145	0.821928612	0.893961809
KRTCAP3	0.72075596	0.555093165	0.878050811	0.825933581	0.83417924	0.75292536
FOXN2	0.72075596	0.646924712	0.747093618	0.806922554	0.902843706	0.80787758
NFE2L2	0.72075596	0.538091051	0.638071191	0.99879985	0.988693274	0.610882384
CHID1	0.72075596	0.653341013	0.642916091	0.820228145	0.996250445	0.788131005
NDUFB7	0.72075596	0.612450407	0.684005375	0.788938193	0.99989309	0.610416296
HNRNPF	0.72075596	0.586056515	0.633986838	0.825933581	0.99989309	0.610416296
ANKHD1	0.72075596	0.703437628	0.63818281	0.811000597	0.99989309	0.614724725
C1S	0.72075596	0.583641966	0.694422859	0.841828571	0.99989309	0.640642131
ZNF609	0.72075596	0.559785028	0.752494129	0.820228145	0.99989309	0.647432409
TSPYL6	0.72075596	0.595087191	0.63818281	0.820228145	0.99989309	0.661861937
PRPF40A	0.72075596	0.595931157	0.765074242	0.781241796	0.99989309	0.712413606
FADS2	0.721482914	0.688099473	0.744490351	0.903607716	0.750160769	0.77208617
SNF8	0.721482914	0.593994283	0.820549834	0.915749182	0.86122464	0.636570258
IKBKE	0.722480767	0.765341142	0.764057751	0.868090726	0.796614241	0.659862452
ATP6V0A4	0.722480767	0.568497289	0.638431555	0.999939566	0.804250204	0.734980295
TUBG1	0.722480767	0.693487404	0.744490351	0.841828571	0.87465791	0.752900996
NR4A2	0.722480767	0.63203115	0.788938503	0.806997685	0.957605284	0.690873643
LOC112443526	0.722641813	0.60480946	0.879968243	0.91329897	0.794314038	0.610416296
TEX22	0.723540403	0.537295148	0.98846962	0.820228145	0.784855148	0.61405517
CUX2	0.723652314	0.704817952	0.684005375	0.99879985	0.745796372	0.61405517
RGCC	0.723652314	0.553493197	0.772990161	0.999939566	0.750160769	0.610882384
CAND2	0.723652314	0.568239497	0.63818281	0.914402649	0.823462814	0.886992214
MGC139164	0.724347143	0.595931157	0.965416289	0.847813982	0.742316849	0.610416296
YKT6	0.725192432	0.593994283	0.707441133	0.841828571	0.819670067	0.902660792
HSD11B1	0.725301494	0.650344958	0.733231491	0.939292364	0.823462814	0.66915423
SIRPB2	0.725777771	0.567752026	0.701483892	0.811000597	0.99989309	0.728276429
MRPL12	0.725939399	0.639676128	0.678070701	0.784305663	0.99989309	0.735313642
LOC101907369	0.72603936	0.553493197	0.685222626	0.956154344	0.76054683	0.84491737
LOC104969670	0.72603936	0.849834516	0.765074242	0.851394268	0.780497593	0.610416296
MCUR1	0.72603936	0.567752026	0.953045982	0.806922554	0.838889011	0.647971989
LOC101906664	0.72603936	0.600050072	0.712526829	0.90419464	0.910770324	0.766359829
ITGA3	0.72603936	0.562255493	0.684005375	0.825933581	0.939361296	0.884168604
ANXA11	0.726240619	0.610068686	0.774555223	0.873519404	0.87483668	0.745987575
HSPA1L	0.726240619	0.556152946	0.63818281	0.811000597	0.99989309	0.712066047
CDS2	0.726659186	0.619585357	0.832483532	0.919247117	0.732120393	0.683474857
TRAF4	0.726659186	0.601801243	0.747093618	0.956154344	0.799665755	0.692802214
LOC783854	0.726659186	0.542133539	0.746686237	0.84302258	0.84234617	0.895287865

AKAP11	0.726659186	0.658193849	0.810425784	0.788938193	0.993370551	0.646020737
VTI1B	0.727409899	0.693153864	0.766184475	0.905185614	0.745432249	0.740271903
ZMPSTE24	0.727409899	0.600050072	0.829843435	0.820228145	0.748847452	0.849769587
RPS6KA3	0.727409899	0.567752026	0.747093618	0.996601241	0.779159678	0.663217328
SOX8	0.727409899	0.552342771	0.66128476	0.999939566	0.846122929	0.645838677
LOC518134	0.727409899	0.604125493	0.693454224	0.915749182	0.996250445	0.652355711
FOPNL	0.727409899	0.538091051	0.757235816	0.863958691	0.99989309	0.636570258
AHI1	0.727409899	0.562255493	0.63818281	0.781241796	0.99989309	0.637306705
B3GNTL1	0.727409899	0.553512189	0.812770091	0.811000597	0.99989309	0.650973086
TDRD12	0.727547589	0.595931157	0.79610462	0.925004412	0.745796372	0.749141396
CX3CL1	0.727547589	0.781154903	0.703042171	0.956154344	0.762655357	0.611777732
ZKSCAN5	0.727547589	0.538091051	0.746730313	0.781517824	0.766792351	0.982559673
ADGRE3	0.727547589	0.780290359	0.684005375	0.956154344	0.807226912	0.610416296
LOC112446759	0.727547589	0.553493197	0.773281026	0.925265292	0.838130422	0.752358594
LYPLA2	0.727547589	0.669648359	0.703042171	0.781241796	0.84234617	0.908939618
IQCA1	0.727547589	0.6861152	0.679460199	0.947615714	0.87483668	0.660829605
RPS6KA4	0.727547589	0.689128491	0.693120773	0.915749182	0.89629383	0.692674475
BRD3OS	0.728801464	0.642501337	0.777178978	0.842538664	0.7817968	0.812509877
LOC100847495	0.729390744	0.543699592	0.806825406	0.910808102	0.941064037	0.66854638
ARHGEF10L	0.729390744	0.692987203	0.787740344	0.835232007	0.961382403	0.614421218
SLC4A8	0.729390744	0.647660219	0.63818281	0.915749182	0.99989309	0.61405517
PDE3A	0.729466082	0.948273015	0.744933814	0.788938193	0.745796372	0.66854638
ANKRD33B	0.729466082	0.541586725	0.850243032	0.983971658	0.750160769	0.636570258
INPP1	0.729466082	0.72843033	0.750089824	0.892229846	0.765330204	0.742259651
LOC104975814	0.729466082	0.653033143	0.735921892	0.995118128	0.765898761	0.636570258
TIGIT	0.729466082	0.688099473	0.715979316	0.781241796	0.780497593	0.918220726
ARMCX1	0.729466082	0.549065127	0.72395094	0.915749182	0.809298212	0.85958325
PXDC1	0.729466082	0.567752026	0.691758173	0.95175364	0.819670067	0.798051736
SLAMF6	0.729466082	0.588244908	0.684005375	0.949947918	0.837323996	0.77208617
NHP2	0.729466082	0.675748768	0.685222626	0.788938193	0.864593182	0.898279031
CYTIP	0.729466082	0.540483502	0.651248606	0.918161195	0.87483668	0.869171706
FUBP3	0.729466082	0.72477102	0.726668529	0.861272209	0.934859184	0.657673143
RAD51	0.729466082	0.547317493	0.827125538	0.848643625	0.952821886	0.700756592
CCNT1	0.729466082	0.730317314	0.759608199	0.846115604	0.954501767	0.610882384
LOC782343	0.729466082	0.595087191	0.789868841	0.861272209	0.957605284	0.661861937
IFI44	0.729466082	0.540483502	0.835301782	0.861272209	0.975291792	0.646642632
REC114	0.729466082	0.679258786	0.668846123	0.830830186	0.986955873	0.76507496
LOC508666	0.729466082	0.583843515	0.661590836	0.842538664	0.996250445	0.823139458

KIAA1109	0.729466082	0.637634861	0.684005375	0.825933581	0.99989309	0.690181038
RAC1	0.729778769	0.541005316	0.894449269	0.943323364	0.77278187	0.611777732
CAPS	0.729778769	0.893512526	0.752868635	0.806922554	0.778366344	0.671528522
LOC112449547	0.729778769	0.539320181	0.678070701	0.999939566	0.805400552	0.671720185
LOC112446383	0.729778769	0.551251552	0.843427302	0.928623745	0.807026238	0.671720185
CYB5RL	0.729778769	0.632709306	0.678070701	0.998542029	0.807226912	0.678496087
LOC104969028	0.729778769	0.780290359	0.685222626	0.925901365	0.819670067	0.647432409
ELP5	0.729778769	0.657613355	0.709561629	0.959786126	0.819670067	0.671528522
FAIM	0.729778769	0.538091051	0.959646613	0.806922554	0.837323996	0.694702496
LOC112447005	0.729778769	0.828797861	0.726668529	0.83476264	0.84234617	0.692802214
CD3D	0.729778769	0.741061871	0.694190265	0.784305663	0.84234617	0.867440033
SORBS2	0.729778769	0.695114938	0.725660838	0.826315685	0.846122929	0.815459921
LOC101909003	0.729778769	0.600050072	0.817993104	0.825933581	0.849825976	0.790039686
DUSP4	0.729778769	0.567752026	0.921225977	0.784305663	0.87465791	0.738110854
GALNT17	0.729778769	0.578451435	0.767160534	0.958879502	0.87483668	0.637306705
PLCXD2	0.729778769	0.730317314	0.651248606	0.891273781	0.87483668	0.766589049
CNN2	0.729778769	0.604218287	0.685222626	0.995118128	0.899048045	0.636570258
CD6	0.729778769	0.840444202	0.640276611	0.806922554	0.904151325	0.764732114
CLEC5A	0.729778769	0.688099473	0.694422859	0.925901365	0.934859184	0.636570258
TBC1D15	0.729778769	0.603474998	0.709561629	0.963829131	0.935641613	0.636570258
GPR132	0.729778769	0.776972642	0.703042171	0.820228145	0.956624006	0.66854638
MRPS18A	0.729778769	0.595931157	0.730664367	0.836672323	0.961879452	0.778016848
DAPK2	0.729778769	0.550965714	0.695426006	0.893559455	0.99989309	0.612910364
DYNC1LI1	0.729778769	0.538091051	0.755362619	0.843512648	0.99989309	0.618734857
C1QTNF3	0.729778769	0.557135186	0.858865175	0.806922554	0.99989309	0.636570258
CCL19	0.729778769	0.567752026	0.726668529	0.919247117	0.99989309	0.640642131
NPEPL1	0.729778769	0.562255493	0.751496241	0.781517824	0.99989309	0.66854638
KBTBD6	0.729778769	0.589137013	0.73016804	0.825933581	0.99989309	0.66854638
FAM120B	0.729778769	0.538091051	0.707441133	0.843411019	0.99989309	0.701784819
TMEM177	0.729778769	0.545732987	0.651599878	0.784305663	0.99989309	0.705712189
ARVCF	0.729778769	0.742728851	0.675040193	0.788938193	0.99989309	0.740271903
NEMP2	0.730120637	0.60290293	0.945229187	0.785145388	0.883643824	0.636570258
LOC112443428	0.730120637	0.557966843	0.820767684	0.89088601	0.957605284	0.64896659
WASF1	0.730366536	0.883267666	0.694542317	0.846715063	0.741060815	0.745987575
LTBP2	0.730366536	0.831242188	0.684005375	0.846715063	0.819670067	0.749691032
CSTF2T	0.730679549	0.576666287	0.726668529	0.806997685	0.857710467	0.922263023
PDLIM3	0.731265941	0.820350377	0.730682825	0.842538664	0.741060815	0.77208617
SAP130	0.731265941	0.538091051	0.662483244	0.999939566	0.750160769	0.659225631

IRF4	0.731265941	0.688099473	0.678204563	0.934591536	0.760712035	0.792329436
NECAB3	0.731265941	0.568025614	0.73016804	0.934591536	0.789627785	0.809650729
MASTL	0.731265941	0.568025614	0.827125538	0.915749182	0.819670067	0.727250186
APBB3	0.731265941	0.592843957	0.843427302	0.851394268	0.834198722	0.752900996
KCNG2	0.731265941	0.586530238	0.726668529	0.996601241	0.857710467	0.640642131
PLCB1	0.731265941	0.634420495	0.81284583	0.846715063	0.870258866	0.735235065
SLC9B2	0.731265941	0.73244249	0.645597693	0.90419464	0.889271428	0.752900996
EPOR	0.731265941	0.556152946	0.763480043	0.982143397	0.928131565	0.612490982
SPTSSA	0.731265941	0.583843515	0.750089824	0.825933581	0.934859184	0.823139458
RBM18	0.731265941	0.632709306	0.838838955	0.820228145	0.952172109	0.653387719
RABL3	0.731265941	0.600050072	0.765074242	0.883885658	0.952172109	0.692674475
ADCY5	0.731265941	0.665524216	0.788938503	0.843411019	0.954501767	0.645838677
HDAC2	0.731265941	0.545334109	0.726668529	0.820228145	0.98152792	0.859481777
MIER1	0.731265941	0.6663481	0.685222626	0.820228145	0.988693274	0.778016848
SSBP1	0.731265941	0.601662433	0.726668529	0.806922554	0.99989309	0.708662616
LOC101903540	0.731265941	0.622507225	0.699166169	0.788938193	0.99989309	0.797413393
TEP1	0.731809802	0.567471709	0.894414265	0.951929772	0.741060815	0.615356919
ZMYND15	0.731809802	0.603419875	0.756911784	0.995800463	0.750160769	0.661937165
ZNF605	0.731809802	0.576666287	0.777178978	0.850828215	0.750160769	0.888617204
LOC786586	0.731809802	0.557135186	0.828407935	0.992368879	0.760472812	0.637306705
ZNF827	0.731809802	0.852313021	0.757235816	0.863818844	0.766792351	0.642318097
THBS3	0.731809802	0.595087191	0.812043145	0.990571725	0.769784115	0.628138194
LOC509415	0.731809802	0.685980044	0.717447819	0.946344632	0.804250204	0.696941321
DYNLL2	0.731809802	0.656553906	0.846650632	0.907767578	0.819670067	0.635211097
ZMAT2	0.731809802	0.557966843	0.926727295	0.820228145	0.872085861	0.678496087
LOC781989	0.731809802	0.553512189	0.755534187	0.990571725	0.877393875	0.645838677
RMND5A	0.731809802	0.687002479	0.715979316	0.871894706	0.886279726	0.770391484
TRIP4	0.731809802	0.72809533	0.726668529	0.915749182	0.904151325	0.636570258
POR	0.731809802	0.578451435	0.66128476	0.788938193	0.91011204	0.957877422
CRLF1	0.731809802	0.64691986	0.787740344	0.88501388	0.933637471	0.651683366
LOC513329	0.731809802	0.560898132	0.730682825	0.863958691	0.939361296	0.825219059
MARCKS	0.731809802	0.637203338	0.685010486	0.976519231	0.943768595	0.614421218
MDM2	0.731809802	0.538091051	0.684005375	0.995118128	0.943768595	0.673471687
UNC80	0.731809802	0.591502729	0.731086678	0.811000597	0.947146038	0.854193997
KY	0.732385151	0.568497289	0.744932729	0.985181062	0.84234617	0.684902922
ITPKA	0.732585002	0.982051974	0.684005375	0.788938193	0.747513637	0.701784819
MND1	0.732585002	0.559785028	0.85972234	0.843411019	0.753418479	0.839419627
CLIP2	0.732585002	0.560714408	0.763592007	0.999939566	0.762655357	0.637306705

GK	0.732585002	0.676274012	0.739978345	0.959786126	0.762655357	0.692674475
LOC112447316	0.732585002	0.747555258	0.794216389	0.86288354	0.771511186	0.694702496
ZFP2	0.732585002	0.623400308	0.777178978	0.820228145	0.790063341	0.869290956
GRP	0.732585002	0.92813228	0.764057751	0.806997685	0.818457184	0.61405517
PDIA6	0.732585002	0.688099473	0.712526829	0.963153458	0.819670067	0.656248868
PDZD7	0.732585002	0.720945114	0.744490351	0.844510397	0.838889011	0.77208617
TMEM59	0.732585002	0.692025761	0.715979316	0.946344632	0.84102894	0.667744852
IL6	0.732585002	0.839399097	0.684005375	0.784305663	0.846122929	0.813001848
CSPP1	0.732585002	0.573298366	0.849077738	0.806922554	0.870258866	0.799935743
DKK2	0.732585002	0.731268229	0.648366719	0.847813982	0.87465791	0.832858909
NIPA1	0.732585002	0.617063658	0.762423061	0.972367384	0.87483668	0.611777732
TGFBR1	0.732585002	0.568025614	0.922126495	0.788938193	0.887061423	0.719551722
TMEM50A	0.732585002	0.552267835	0.958420472	0.807296971	0.889271428	0.645838677
CCNB2	0.732585002	0.632709306	0.793430641	0.917869295	0.89629383	0.635645816
BCAT2	0.732585002	0.658608874	0.754101752	0.807296971	0.899048045	0.828475368
BASP1	0.732585002	0.747164473	0.750089824	0.820228145	0.91011204	0.714683388
TMEM59L	0.732585002	0.675837248	0.724199536	0.934591536	0.941107925	0.614575887
RAB29	0.732585002	0.600050072	0.684005375	0.992137653	0.956624006	0.615356919
ASCC1	0.732585002	0.635240156	0.678204563	0.820228145	0.99989309	0.654251319
DOK5	0.732585002	0.625615428	0.699166169	0.843512648	0.99989309	0.660829605
LOC112444864	0.732585002	0.557966843	0.678204563	0.87452213	0.99989309	0.661937165
SPOP	0.732585002	0.547800063	0.733231491	0.820228145	0.99989309	0.678496087
SUSD5	0.732585002	0.644224672	0.687699922	0.820228145	0.99989309	0.688024753
LOC104969027	0.732585002	0.642501337	0.755534187	0.820228145	0.99989309	0.701784819
KIAA1143	0.732585002	0.567752026	0.712526829	0.89088601	0.99989309	0.723028401
AHSA2	0.732585002	0.567752026	0.665743961	0.788938193	0.99989309	0.753613607
ADSL	0.732585002	0.567752026	0.648198992	0.78508807	0.99989309	0.871780952
HDAC7	0.732669216	0.550560637	0.821908722	0.934591536	0.831259131	0.703742328
ADH6	0.732830507	0.567471709	0.648198992	0.998542029	0.83417924	0.77208617
TMEM71	0.732859098	0.557966843	0.651174146	0.924489924	0.810368126	0.908939618
GNB3	0.732904351	0.567752026	0.819529308	0.983569039	0.779159678	0.650757255
FUT1	0.732904351	0.567752026	0.661590836	0.999939566	0.784855148	0.671720185
TNFRSF25	0.732904351	0.568497289	0.766352455	0.89088601	0.857710467	0.812509877
CUL1	0.732904351	0.675748768	0.78825731	0.90419464	0.933637471	0.633384404
NCOA6	0.732904351	0.64691986	0.684005375	0.820228145	0.99989309	0.80844231
SCNN1D	0.733113711	0.550965714	0.994425789	0.78508807	0.819670067	0.636570258
LOC100336941	0.733113711	0.55750845	0.820549834	0.90419464	0.819670067	0.788131005
TMEM132A	0.733113711	0.541005316	0.795199152	0.820228145	0.819670067	0.912101047

FLT4	0.733113711	0.567752026	0.724199536	0.999939566	0.836838903	0.650499364
KIAA1551	0.733113711	0.632913728	0.768676249	0.864719155	0.981938411	0.64896659
CAVIN3	0.733113711	0.595792756	0.827125538	0.841338807	0.99989309	0.642318097
DIP2B	0.733308661	0.562255493	0.654810863	0.999939566	0.819670067	0.637306705
UBE2QL1	0.733308661	0.619005993	0.765074242	0.956154344	0.830518034	0.661861937
LOC531747	0.733308661	0.559754831	0.777178978	0.788938193	0.83627631	0.933730876
SH3GL2	0.733308661	0.55176988	0.709561629	0.913413181	0.886279726	0.852755805
CMSS1	0.733308661	0.568239497	0.760913563	0.89088601	0.899048045	0.794556896
CDK2AP2	0.733308661	0.693153864	0.759671935	0.842538664	0.991591622	0.637306705
LOC100849050	0.733308661	0.655710881	0.733231491	0.850828215	0.99989309	0.637306705
MLX	0.733308661	0.555430518	0.699166169	0.846715063	0.99989309	0.753138357
SAT2	0.734205678	0.70263626	0.774057931	0.925901365	0.745796372	0.712710718
ABCA3	0.734205678	0.655710881	0.684005375	0.908804713	0.74589961	0.90525134
SNRNP70	0.734205678	0.595958344	0.827350832	0.992137653	0.748847452	0.636570258
RPLP1	0.734205678	0.633093764	0.726668529	0.999939566	0.750160769	0.672067469
HK3	0.734205678	0.603785862	0.765074242	0.925901365	0.750160769	0.79339547
LOC100848895	0.734205678	0.594528548	0.843427302	0.869637988	0.750160769	0.813759229
CCDC102A	0.734205678	0.550965714	0.768676249	0.999939566	0.761764417	0.627380177
LOC112445031	0.734205678	0.669648359	0.777638604	0.983038839	0.762655357	0.633384404
RGL2	0.734205678	0.567752026	0.926613927	0.922713162	0.762655357	0.636570258
LOC101907302	0.734205678	0.568025614	0.694542317	0.999939566	0.765961167	0.653655829
BTLA	0.734205678	0.873566716	0.744490351	0.803538703	0.766792351	0.771541268
TDP1	0.734205678	0.606614518	0.933102499	0.87630412	0.767200321	0.642154165
KATNAL1	0.734205678	0.619005993	0.877947964	0.794956735	0.769784115	0.828663852
BTBD9	0.734205678	0.543699592	0.764057751	0.843512648	0.769784115	0.940917403
MAMDC4	0.734205678	0.564994938	0.914406673	0.90419464	0.779159678	0.693146856
TGFB1I1	0.734205678	0.623400308	0.744490351	0.841828571	0.77975903	0.908939618
CROCC2	0.734205678	0.563639555	0.933963997	0.894144195	0.782043033	0.650757255
RETREG3	0.734205678	0.7164915	0.694190265	0.788938193	0.804250204	0.923788376
CEP295	0.734205678	0.619184305	0.90166788	0.891273781	0.804257626	0.646784897
LOC539893	0.734205678	0.55750845	0.872397372	0.846115604	0.807226912	0.80787758
NUMA1	0.734205678	0.567752026	0.769174658	0.806922554	0.808924032	0.940683751
DDX11	0.734205678	0.597619549	0.812770091	0.90419464	0.819670067	0.766542864
LXN	0.734205678	0.652876871	0.682591947	0.990571725	0.829351305	0.712413606
USP28	0.734205678	0.609819443	0.806221631	0.849315934	0.83417924	0.80787758
C3H1orf109	0.734205678	0.691175262	0.733231491	0.846715063	0.836838903	0.831041995
AMMECR1	0.734205678	0.599807217	0.747806937	0.946344632	0.838889011	0.733909633
MAFF	0.734205678	0.642439727	0.827125538	0.925440563	0.84234617	0.636570258

UBE2V2	0.734205678	0.603474998	0.773281026	0.915749182	0.84234617	0.757327233
LARP4	0.734205678	0.567752026	0.726668529	0.980840984	0.860908683	0.749531513
CHCHD4	0.734205678	0.679258786	0.764057751	0.842538664	0.864262913	0.792329436
LOC101905499	0.734205678	0.619184305	0.744490351	0.90419464	0.86975774	0.78965579
UBQLN1	0.734205678	0.579714663	0.777178978	0.956154344	0.87465791	0.661861937
LOC112443226	0.734205678	0.553512189	0.695426006	0.967063428	0.876859226	0.774602575
SLA2	0.734205678	0.703437628	0.694542317	0.904990622	0.883643824	0.75292536
LOC101905956	0.734205678	0.693544464	0.788139177	0.886659234	0.887805568	0.661861937
TBC1D32	0.734205678	0.81808696	0.828407935	0.788938193	0.899048045	0.615544321
COMMD4	0.734205678	0.740647787	0.744277834	0.91329897	0.904151325	0.640138785
RCAN1	0.734205678	0.556505404	0.726668529	0.971220023	0.904151325	0.71509671
PSMA1	0.734205678	0.562255493	0.701483892	0.870741954	0.904151325	0.886992214
PCDH12	0.734205678	0.560898132	0.673737267	0.999939566	0.915496086	0.651173954
MOSPD2	0.734205678	0.619184305	0.694422859	0.969677561	0.927804504	0.671528522
GPR83	0.734205678	0.619184305	0.685222626	0.806922554	0.927804504	0.911475796
STK24	0.734205678	0.619374114	0.726668529	0.951606954	0.941064037	0.646642632
CEP192	0.734205678	0.559785028	0.836294915	0.917869295	0.941107925	0.633384404
NAIP	0.734205678	0.601979156	0.759671935	0.820228145	0.943768595	0.834008567
RUNX3	0.734205678	0.560168665	0.685222626	0.87452213	0.952172109	0.865926817
SNRNP48	0.734205678	0.547317493	0.751496241	0.806922554	0.952172109	0.886992214
TMUB1	0.734205678	0.581298031	0.733231491	0.954980798	0.954501767	0.649837366
FYB1	0.734205678	0.616586256	0.678070701	0.841828571	0.954501767	0.866482265
LOC783466	0.734205678	0.543699592	0.73016804	0.958879502	0.957605284	0.671528522
ZNF143	0.734205678	0.619184305	0.812770091	0.806922554	0.95983326	0.759729981
SYNGR1	0.734205678	0.81808696	0.726668529	0.806922554	0.960835287	0.66854638
PROCA1	0.734205678	0.69873818	0.684005375	0.86288354	0.961382403	0.749141396
FOXS1	0.734205678	0.578451435	0.726668529	0.825933581	0.975291792	0.833139817
RPGRIP1L	0.734205678	0.763751563	0.694542317	0.820228145	0.976970807	0.704948464
ACYP1	0.734205678	0.595931157	0.81284583	0.843512648	0.996250445	0.66854638
XRCC3	0.734205678	0.635562415	0.827125538	0.788938193	0.99989309	0.627380177
IL6R	0.734205678	0.562255493	0.670235176	0.925931623	0.99989309	0.627380177
NUDT14	0.734205678	0.782865867	0.707441133	0.820228145	0.99989309	0.636570258
PIK3C2A	0.734205678	0.632464232	0.678204563	0.925931623	0.99989309	0.636570258
GOLGA7	0.734205678	0.578451435	0.73016804	0.908054165	0.99989309	0.639128578
KCNK17	0.734205678	0.55176988	0.684005375	0.794713619	0.99989309	0.642154165
KANSL1	0.734205678	0.693544464	0.694542317	0.806922554	0.99989309	0.642154165
COG2	0.734205678	0.595087191	0.707441133	0.806922554	0.99989309	0.646642632
NKD2	0.734205678	0.579771417	0.726668529	0.820228145	0.99989309	0.648894737

TREX1	0.734205678	0.610965998	0.726668529	0.915749182	0.99989309	0.64896659
IDH2	0.734205678	0.653723309	0.7079185	0.883885658	0.99989309	0.660829605
ELF1	0.734205678	0.550965714	0.657805748	0.808819492	0.99989309	0.661861937
KIAA1191	0.734205678	0.548073502	0.781294783	0.90419464	0.99989309	0.66854638
TMBIM7	0.734205678	0.674009696	0.678070701	0.825933581	0.99989309	0.66915423
C13H20orf27	0.734205678	0.735625249	0.726668529	0.806922554	0.99989309	0.673471687
INPP5A	0.734205678	0.671439983	0.747084388	0.811000597	0.99989309	0.712413606
ATP5MC3	0.734205678	0.655710881	0.684005375	0.796330272	0.99989309	0.734664544
RSRP1	0.734205678	0.579665846	0.775711284	0.820228145	0.99989309	0.742259651
TIMM17A	0.734205678	0.568497289	0.701483892	0.86288354	0.99989309	0.788131005
AKR7A2	0.734205678	0.567752026	0.684005375	0.810346558	0.99989309	0.809650729
LOC522763	0.73470691	0.562255493	0.712526829	0.849060614	0.764607568	0.968039618
MYOZ1	0.73470691	0.560898132	0.673737267	0.995800463	0.810511507	0.812509877
DLX4	0.73470691	0.67537609	0.917876971	0.841828571	0.819670067	0.640642131
ACD	0.73470691	0.553512189	0.926613927	0.908496281	0.836838903	0.640642131
MRC2	0.73470691	0.600538394	0.844316758	0.925265292	0.850852167	0.640642131
ANO1	0.73470691	0.747179932	0.820549834	0.820228145	0.881823169	0.66854638
GCH1	0.73470691	0.703437628	0.733231491	0.928836449	0.882754591	0.651173954
EIF4G2	0.73470691	0.568025614	0.927460291	0.860663136	0.886279726	0.636570258
TIMM10B	0.73470691	0.581298031	0.744933814	0.925004412	0.896933443	0.770391484
ANO6	0.73470691	0.573324334	0.812043145	0.935763381	0.902633397	0.647432409
LOC100848315	0.73470691	0.615179529	0.875405854	0.891273781	0.904151325	0.627380177
LOC112448105	0.73470691	0.650107532	0.702650233	0.915749182	0.917288642	0.759107825
TMEM72	0.73470691	0.911196851	0.707441133	0.806698028	0.954501767	0.633238667
PVR	0.73470691	0.632464232	0.707441133	0.825933581	0.95956556	0.837249706
KIF5C	0.73470691	0.557966843	0.685222626	0.99879985	0.96532164	0.636570258
SLBP	0.73470691	0.573608051	0.694190265	0.928836449	0.972917711	0.740271903
POLR3A	0.73470691	0.568025614	0.678204563	0.820228145	0.972917711	0.91299942
ADA	0.73470691	0.629715977	0.688145488	0.86937758	0.99989309	0.622773569
TCF15	0.73470691	0.551867132	0.684148439	0.87630412	0.99989309	0.635661027
STARD5	0.73470691	0.757368351	0.66128476	0.825933581	0.99989309	0.637306705
TNFAIP3	0.73470691	0.589137013	0.747084388	0.820228145	0.99989309	0.642154165
ROCK2	0.73470691	0.661476635	0.755534187	0.850828215	0.99989309	0.659225631
BAZ1B	0.73470691	0.671855274	0.689106738	0.846715063	0.99989309	0.682748733
RBM28	0.73470691	0.553512189	0.694422859	0.89088601	0.99989309	0.759308648
LOC100848307	0.735013532	0.629715977	0.696685689	0.915749182	0.87465791	0.809548115
OVCA2	0.735051413	0.557135186	0.677544154	0.850828215	0.95956556	0.899551847
CCDC80	0.735160081	0.678391216	0.882898206	0.892229846	0.757810174	0.661861937

SAP25	0.735160081	0.612773311	0.845237542	0.820228145	0.780497593	0.846367069
PNISR	0.735160081	0.550965714	0.789118983	0.92064557	0.814933542	0.807579326
KCTD12	0.735160081	0.619374114	0.665743961	0.999939566	0.881823169	0.652355711
LANCL3	0.735160081	0.587990449	0.684005375	0.928836449	0.962262333	0.752900996
MTFR1L	0.735160081	0.600050072	0.787740344	0.820228145	0.99989309	0.75292536
BORCS6	0.735160081	0.627697325	0.684005375	0.806997685	0.99989309	0.839419627
RAB5C	0.735394176	0.936703425	0.738217747	0.820228145	0.766792351	0.684595958
MYCN	0.735394176	0.563639555	0.776574729	0.995118128	0.766792351	0.719551722
MAGED4B	0.735394176	0.600050072	0.765074242	0.999939566	0.769784115	0.637306705
CDH2	0.735394176	0.567752026	0.733776767	0.995800463	0.769784115	0.769994723
ZBTB5	0.735394176	0.848132141	0.685222626	0.90419464	0.780497593	0.728276429
CRK	0.735394176	0.553493197	0.787740344	0.999939566	0.798575605	0.637306705
UBE2G2	0.735394176	0.560898132	0.930710426	0.915749182	0.814302842	0.622773569
EFS	0.735394176	0.571961522	0.755498962	0.999939566	0.819670067	0.642154165
ARC	0.735394176	0.841617281	0.842478086	0.803538703	0.819670067	0.646642632
ZNF706	0.735394176	0.604997439	0.801296181	0.976950878	0.84234617	0.636570258
LOC112448743	0.735394176	0.617732204	0.885396428	0.820228145	0.84234617	0.76360318
ANO8	0.735394176	0.692461545	0.827125538	0.91329897	0.850852167	0.636570258
LOC101902122	0.735394176	0.563639555	0.678070701	0.868090726	0.87022617	0.942421464
TBL3	0.735394176	0.634632759	0.834793268	0.795834665	0.934859184	0.770391484
COLGALT1	0.735394176	0.603785862	0.685222626	0.958849311	0.939361296	0.721206498
ZMAT5	0.735394176	0.851167599	0.684005375	0.796330272	0.99989309	0.633384404
ESPN	0.735394176	0.724518891	0.696685689	0.842538664	0.99989309	0.642154165
MIF	0.735394176	0.638242621	0.678204563	0.806922554	0.99989309	0.671720185
UBA2	0.735394176	0.655710881	0.683668554	0.825933581	0.99989309	0.708819884
LOC101903193	0.735394176	0.578638921	0.684005375	0.841828571	0.99989309	0.769704643
LOC101907405	0.735394176	0.549999039	0.685519162	0.850828215	0.99989309	0.77208617
IFRD2	0.735394176	0.619374114	0.678204563	0.820228145	0.99989309	0.839340243
PAX5	0.735505242	0.644435679	0.678204563	0.925931623	0.766792351	0.868108836
BGLAP	0.735505242	0.588893909	0.661590836	0.999939566	0.778366344	0.636570258
DNAJB9	0.735562107	0.753193121	0.765074242	0.796330272	0.96532164	0.687737779
MID2	0.735562107	0.606521634	0.726668529	0.842538664	0.99989309	0.645838677
RRAS	0.735988512	0.600050072	0.788848487	0.915749182	0.795200669	0.796593386
CCDC86	0.736244515	0.747899938	0.675040193	0.908054165	0.819670067	0.809650729
LSG1	0.736244515	0.702217402	0.698225674	0.864435381	0.99989309	0.649372601
THBS2	0.7363336	0.559785028	0.834364497	0.936573639	0.766932246	0.759729981
LOC101902083	0.7363336	0.905760551	0.720067907	0.806922554	0.78387531	0.77208617
SNX4	0.736566956	0.600960324	0.726668529	0.99879985	0.896242324	0.633384404

APOLD1	0.737341662	0.557135186	0.726668529	0.999939566	0.819670067	0.671528522
FNBP4	0.737341662	0.59911295	0.87214481	0.868417977	0.883643824	0.682943723
FGF7	0.737365203	0.593994283	0.80193314	0.994309225	0.750160769	0.682748733
ABHD17B	0.737365203	0.61754698	0.726668529	0.878289777	0.750160769	0.926872824
LOC100297240	0.737365203	0.560898132	0.976719499	0.893831952	0.761764417	0.637306705
LEP	0.737365203	0.777762359	0.726668529	0.90419464	0.766792351	0.77208617
ADRA2A	0.737365203	0.639901253	0.817256644	0.886726226	0.769784115	0.808138656
LOC104974666	0.737365203	0.688099473	0.731855498	0.949947918	0.778366344	0.752900996
LOC112447305	0.737365203	0.576666287	0.694422859	0.86288354	0.77975903	0.967376295
UMPS	0.737365203	0.567845198	0.726668529	0.846115604	0.784855148	0.957877422
SNX22	0.737365203	0.583843515	0.982659943	0.826315685	0.790063341	0.637306705
C22H3orf22	0.737365203	0.559754831	0.933755755	0.820228145	0.795200669	0.788131005
PVALB	0.737365203	0.661476635	0.684005375	0.972329985	0.804250204	0.77208617
LOC107132382	0.737365203	0.600050072	0.705547551	0.996084773	0.814605912	0.75292536
LOC112448354	0.737365203	0.72477102	0.789039067	0.904990622	0.819670067	0.683474857
OLFML2A	0.737365203	0.638484396	0.765074242	0.92064557	0.819670067	0.764546301
INPP5F	0.737365203	0.601979156	0.765074242	0.806922554	0.819670067	0.935655141
TREML2	0.737365203	0.595271828	0.757337101	0.825933581	0.821928612	0.923871657
SGCA	0.737365203	0.816119178	0.843427302	0.820228145	0.823462814	0.66048464
ABHD13	0.737365203	0.637278759	0.843427302	0.91329897	0.85995837	0.66854638
FAM84A	0.737365203	0.770808357	0.788938503	0.886659234	0.86122464	0.641488392
AP1M1	0.737365203	0.595931157	0.750089824	0.820228145	0.862637717	0.926872824
LOC100336476	0.737365203	0.642412048	0.92300328	0.806997685	0.86785481	0.684905734
ARFIP1	0.737365203	0.688099473	0.726668529	0.883885658	0.86975774	0.811943134
LOC112447381	0.737365203	0.600050072	0.859931483	0.803538703	0.871556014	0.823435077
COL6A3	0.737365203	0.578451435	0.787015778	0.963153458	0.87483668	0.66854638
LGI2	0.737365203	0.632709306	0.726668529	0.995118128	0.875054206	0.648678306
LOC783604	0.737365203	0.655138935	0.890801024	0.858021281	0.883643824	0.633384404
HNRNPAB	0.737365203	0.603555122	0.883874752	0.825933581	0.883643824	0.730179678
CLEC4A	0.737365203	0.724249725	0.755534187	0.88747242	0.889093274	0.704011905
ACSL5	0.737365203	0.64691986	0.665743961	0.825933581	0.892966823	0.930707319
WAS	0.737365203	0.710155842	0.726668529	0.820228145	0.896242324	0.840150874
LOC100847708	0.737365203	0.624067943	0.759201829	0.820228145	0.899048045	0.867505247
CENPI	0.737365203	0.701581467	0.715979316	0.797039431	0.899048045	0.881550981
TARBP2	0.737365203	0.595931157	0.787740344	0.91329897	0.905719399	0.749691032
EIF2B2	0.737365203	0.688099473	0.73016804	0.825933581	0.91011204	0.836564988
GIMAP7	0.737365203	0.79236511	0.684005375	0.811000597	0.910770324	0.809650729
CDC25B	0.737365203	0.615879066	0.750089824	0.915749182	0.925981327	0.75292536

PPP1R7	0.737365203	0.626607857	0.726668529	0.924489924	0.930229436	0.74008849
RAC2	0.737365203	0.695114938	0.72395094	0.807296971	0.934859184	0.846367069
SIMC1	0.737365203	0.642412048	0.751496241	0.874047001	0.951791592	0.75292536
PARP10	0.737365203	0.573324334	0.920449324	0.820228145	0.952821886	0.661937165
RNF182	0.737365203	0.765341142	0.703042171	0.898958429	0.954501767	0.650499364
MTMR14	0.737365203	0.557966843	0.694190265	0.955560327	0.961382403	0.752358594
SORT1	0.737365203	0.646924712	0.750089824	0.925004412	0.96413701	0.640138785
FAM126B	0.737365203	0.83971475	0.722951238	0.820228145	0.975291792	0.646642632
CLPP	0.737365203	0.642501337	0.703042171	0.88501388	0.978272166	0.770391484
RNF208	0.737365203	0.655710881	0.726668529	0.820228145	0.99989309	0.632127688
FASTK	0.737365203	0.68147929	0.777178978	0.841828571	0.99989309	0.633384404
SAG	0.737365203	0.636332133	0.670235176	0.847813982	0.99989309	0.635038367
DYNC1I2	0.737365203	0.567752026	0.673062359	0.89088601	0.99989309	0.636570258
NPAS3	0.737365203	0.655202329	0.668846123	0.934591536	0.99989309	0.636570258
MORF4L1	0.737365203	0.553512189	0.820767684	0.89088601	0.99989309	0.645838677
RAD21	0.737365203	0.62522808	0.764057751	0.862651556	0.99989309	0.66915423
GLO1	0.737365203	0.632709306	0.699166169	0.90419464	0.99989309	0.671720185
PPIA	0.737365203	0.567752026	0.726668529	0.825933581	0.99989309	0.712413606
RBM25	0.737365203	0.55176988	0.744490351	0.915749182	0.99989309	0.727921992
IMPG2	0.737365203	0.563639555	0.684005375	0.825933581	0.99989309	0.742259651
TIMM44	0.737365203	0.626773146	0.678204563	0.820228145	0.99989309	0.758090697
SLA	0.737365203	0.615879066	0.717649445	0.793761252	0.99989309	0.77208617
ALAS1	0.737365203	0.625009304	0.709521163	0.822789354	0.99989309	0.77208617
MRPS34	0.737365203	0.652542331	0.694190265	0.811000597	0.99989309	0.773957499
CCHCR1	0.737369607	0.737937244	0.788139177	0.846115604	0.808944674	0.77208617
RTN4RL1	0.737369607	0.908688859	0.726668529	0.850828215	0.860806019	0.637306705
LOC101902851	0.737369607	0.595931157	0.726668529	0.814297309	0.881823169	0.939426596
RIC8B	0.737369607	0.629715977	0.873750661	0.87630412	0.933637471	0.633384404
PHF10	0.737369607	0.665803806	0.684005375	0.868417977	0.975291792	0.788131005
LOC112444461	0.737369607	0.582472344	0.703042171	0.806922554	0.982014614	0.901965781
VPS37A	0.737547477	0.556152946	0.960137022	0.915749182	0.766792351	0.635661027
LOC787851	0.737547477	0.693153864	0.849077738	0.83855098	0.846122929	0.725528824
ISPD	0.737547477	0.557135186	0.765074242	0.847813982	0.987414096	0.798482976
DYRK1A	0.737547477	0.665563964	0.788848487	0.820228145	0.99989309	0.637306705
LOC112446690	0.737547477	0.574424407	0.673737267	0.90419464	0.99989309	0.74008849
GABRA1	0.737582099	0.698870039	0.699166169	0.958879502	0.765961167	0.77288364
LOC539973	0.737582099	0.584982353	0.819114812	0.915749182	0.766792351	0.809108223
UTP6	0.737582099	0.653341013	0.810824842	0.943323364	0.775358813	0.694702496

CTPS1	0.737582099	0.646924712	0.749513157	0.825933581	0.790063341	0.91601693
ALG11	0.737582099	0.595931157	0.843427302	0.915749182	0.79576416	0.759107825
POU6F1	0.737582099	0.665803806	0.726668529	0.836785013	0.797393203	0.922263023
SLC43A1	0.737582099	0.666075265	0.896272282	0.884946934	0.83417924	0.636570258
FOSB	0.737582099	0.583843515	0.943227209	0.87630412	0.83627631	0.640642131
SCARF2	0.737582099	0.637203338	0.698225674	0.999939566	0.84234617	0.636570258
STK38L	0.737582099	0.600050072	0.685222626	0.999939566	0.87483668	0.635211097
UBE3A	0.737582099	0.619374114	0.777178978	0.893831952	0.87483668	0.793566607
RRP12	0.737582099	0.600538394	0.688413073	0.842538664	0.87483668	0.94076264
NLRC5	0.737582099	0.568025614	0.976719499	0.795299953	0.881823169	0.66854638
P4HA2	0.737582099	0.614103064	0.751496241	0.994309225	0.899048045	0.635211097
IRF3	0.737582099	0.693153864	0.726668529	0.937531834	0.943768595	0.642154165
EFR3B	0.737582099	0.648099013	0.750089824	0.927618239	0.952172109	0.64896659
LOC107131542	0.737582099	0.573020585	0.70435078	0.99879985	0.954501767	0.640138785
MANEAL	0.737582099	0.659488126	0.774057931	0.841828571	0.959394756	0.75292536
PLSCR4	0.737582099	0.838405969	0.694422859	0.820228145	0.97668994	0.685938484
MAX	0.737582099	0.593450768	0.73016804	0.952995342	0.99989309	0.635645816
OST4	0.737582099	0.630939532	0.820767684	0.814297309	0.99989309	0.645838677
SSTR1	0.737582099	0.632709306	0.788848487	0.87630412	0.99989309	0.645838677
MAML2	0.737582099	0.728008849	0.678204563	0.868090726	0.99989309	0.652016115
SLC2A4	0.737582099	0.583843515	0.715979316	0.82652165	0.99989309	0.671528522
FES	0.737582099	0.631868333	0.743973038	0.825933581	0.99989309	0.694702496
KDM4C	0.737582099	0.693052904	0.71043466	0.820228145	0.99989309	0.772244344
CDIPT	0.737582099	0.669648359	0.706787562	0.820228145	0.99989309	0.799147792
PCNX4	0.737582099	0.608096264	0.711013049	0.806922554	0.99989309	0.865159042
LOC783776	0.737811472	0.894203989	0.788848487	0.825933581	0.766792351	0.671528522
EXT2	0.737811472	0.563639555	0.819094028	0.952020618	0.886279726	0.66854638
TIA1	0.737811472	0.567752026	0.807843373	0.915749182	0.954501767	0.694702496
HSF2BP	0.737811472	0.567752026	0.712526829	0.90419464	0.99989309	0.646642632
FAM71E1	0.737827825	0.611461281	0.842748697	0.915749182	0.766792351	0.765573509
TPK1	0.737827825	0.847082229	0.712526829	0.892741454	0.875054206	0.661861937
GNG7	0.737827825	0.557966843	0.709521163	0.982140778	0.952172109	0.725528824
GPR88	0.737827825	0.584982353	0.699166169	0.883885658	0.99989309	0.637306705
LOC101902786	0.737827825	0.703437628	0.69131755	0.865118174	0.99989309	0.660829605
AGPAT2	0.737827825	0.644204975	0.694542317	0.850828215	0.99989309	0.742259651
TBC1D14	0.737835279	0.681991477	0.678070701	0.850828215	0.83627631	0.918220726
NFKBIA	0.737835279	0.554589145	0.705547551	0.971220023	0.999592263	0.677152076
CD226	0.739279807	0.657500304	0.765074242	0.820228145	0.946944212	0.813345852

LOC112446659	0.739279807	0.600960324	0.764057751	0.846715063	0.99989309	0.752857325
MREG	0.73944838	0.705118484	0.757235816	0.904990622	0.881823169	0.727656584
FAM20A	0.73944838	0.72843033	0.709521163	0.904074458	0.913650683	0.75292536
SETD5	0.73944838	0.727892714	0.724199536	0.820686724	0.943768595	0.800008791
CNTN2	0.740238183	0.619585357	0.758406163	0.999939566	0.77278187	0.666847686
EIF3B	0.740238183	0.611256318	0.744369186	0.922782374	0.809298212	0.845024318
DERL3	0.740238183	0.584982353	0.878603452	0.841828571	0.814605912	0.816194685
MUC16	0.740238183	0.698870039	0.857036033	0.864540217	0.818457184	0.692674475
EPHA5	0.740238183	0.800382588	0.703042171	0.925901365	0.885921829	0.646642632
MFSD14B	0.740238183	0.688099473	0.816249708	0.886614433	0.941107925	0.645838677
LOC112442377	0.740238183	0.626635021	0.684005375	0.805665137	0.99989309	0.881550981
MRPL36	0.740397848	0.710486439	0.730446027	0.825933581	0.98871637	0.752900996
STK17A	0.740945322	0.567752026	0.912681633	0.951606954	0.760712035	0.645838677
CD68	0.740945322	0.655710881	0.773281026	0.915749182	0.769784115	0.812509877
TPM1	0.740945322	0.792090907	0.765074242	0.796330272	0.7817968	0.847256027
ATP6V0C	0.740945322	0.730317314	0.79610462	0.936573639	0.819670067	0.635413074
EID2	0.740945322	0.636332133	0.733487658	0.943323364	0.876253597	0.752358594
NAGK	0.740945322	0.67537609	0.776574729	0.927618239	0.886279726	0.661861937
TBC1D10C	0.740945322	0.709162177	0.682591947	0.825933581	0.976970807	0.817471068
CBR1	0.740945322	0.77295675	0.684005375	0.850828215	0.99989309	0.642154165
PGD	0.740945322	0.616586256	0.696685689	0.820228145	0.99989309	0.800008791
LOC781726	0.741156706	0.603555122	0.982223562	0.825933581	0.769784115	0.657118951
LOC112442080	0.741578249	0.600960324	0.684005375	0.971220023	0.808502843	0.847256027
LOC107132531	0.741578249	0.562255493	0.858972712	0.841828571	0.954501767	0.752900996
LOC101904449	0.741578249	0.568025614	0.90806894	0.854497387	0.956624006	0.637306705
NIFK	0.741578249	0.600538394	0.750089824	0.842538664	0.999592263	0.796934582
ATP6V1E1	0.741960853	0.72477102	0.777638604	0.90419464	0.860709045	0.704011905
LOC527796	0.742488225	0.582985895	0.744490351	0.89088601	0.933781163	0.845803016
RAB10	0.742624753	0.567752026	0.788376748	0.955560327	0.799088145	0.784671496
ERICH3	0.742624753	0.583843515	0.838927319	0.806922554	0.846122929	0.888396511
PDK1	0.742624753	0.583411145	0.720067907	0.971220023	0.934859184	0.735604419
C23H6orf201	0.743890857	0.944420015	0.712526829	0.893235972	0.769784115	0.645838677
ATF3	0.743890857	0.790637585	0.817256644	0.89088601	0.804250204	0.660829605
RBM34	0.743890857	0.646924712	0.812770091	0.910808102	0.868380278	0.734807976
ICA1	0.744042401	0.72784212	0.882890706	0.868417977	0.790063341	0.651173954
RRAD	0.744042401	0.929573947	0.682591947	0.87452213	0.808944674	0.704011905
LOC100140121	0.744042401	0.562255493	0.979231344	0.806922554	0.875054206	0.671528522
LOC112445995	0.744042401	0.72477102	0.781294783	0.846115604	0.964150118	0.661861937

NDUFA11	0.744042401	0.642412048	0.694190265	0.806922554	0.99989309	0.660829605
COX6B1	0.744042401	0.603497985	0.696685689	0.811000597	0.99989309	0.660829605
EFHB	0.744042401	0.567752026	0.678204563	0.90419464	0.99989309	0.671528522
MRPL11	0.744042401	0.644204975	0.787740344	0.820228145	0.99989309	0.694702496
MPHOSPH10	0.744042401	0.595931157	0.738217747	0.891273781	0.99989309	0.740271903
LOC782418	0.744092166	0.582627279	0.765074242	0.915749182	0.907192767	0.797272671
CRB3	0.744433069	0.775921167	0.820549834	0.850828215	0.904428762	0.636570258
PLOD1	0.744433069	0.615879066	0.765074242	0.965097245	0.934859184	0.640138785
AVL9	0.744513165	0.578451435	0.827125538	0.999334425	0.7817968	0.640642131
TRAT1	0.744522283	0.557135186	0.678204563	0.850828215	0.99989309	0.858042054
LOC112447526	0.744636649	0.683518638	0.882890706	0.91329897	0.821228548	0.636570258
TIPARP	0.744636649	0.568239497	0.694190265	0.909940206	0.99989309	0.636570258
CLEC4D	0.745185815	0.795993324	0.684005375	0.915749182	0.957605284	0.637306705
LOC104975034	0.745185815	0.761767541	0.726668529	0.858274325	0.960835287	0.703812878
FCHO1	0.745185815	0.560168665	0.827125538	0.808710858	0.99989309	0.727921992
RSL1D1	0.745185815	0.573080237	0.774248983	0.820228145	0.99989309	0.773957499
LOC107131642	0.745234861	0.692025761	0.747084388	0.913413181	0.779159678	0.83849759
ZNF688	0.745234861	0.589137013	0.932275241	0.908054165	0.807226912	0.663483489
MOB4	0.745234861	0.665563964	0.882898206	0.913413181	0.83417924	0.639128578
TMEM251	0.745234861	0.595625745	0.795263308	0.992368879	0.835925794	0.661937165
MAZ	0.745234861	0.57867355	0.788848487	0.990571725	0.84102894	0.691363988
CASZ1	0.745234861	0.57867355	0.726668529	0.999939566	0.846122929	0.708785032
PDIA5	0.745234861	0.567752026	0.793430641	0.999939566	0.853884483	0.637306705
CLPX	0.745234861	0.578451435	0.744490351	0.999939566	0.8594734	0.651173954
TRAIP	0.745234861	0.617454372	0.750089824	0.939292364	0.952672789	0.682748733
RAB5IF	0.745234861	0.669648359	0.726668529	0.841828571	0.975375626	0.796002422
TOX4	0.745234861	0.613737651	0.882898206	0.820228145	0.99989309	0.637306705
ZCCHC11	0.745234861	0.682847958	0.735054113	0.842538664	0.99989309	0.757327233
HSD17B12	0.745234861	0.583843515	0.703042171	0.820228145	0.99989309	0.857727168
DNTTIP2	0.746402766	0.588244908	0.796782264	0.847813982	0.857710467	0.86921618
TAF6L	0.746402766	0.600765597	0.684005375	0.915749182	0.99989309	0.667185675
HINT3	0.746667842	0.665733407	0.750089824	0.846715063	0.99989309	0.742259651
MRPL34	0.746667842	0.6663481	0.720067907	0.820228145	0.99989309	0.752900996
RAB14	0.746996699	0.568497289	0.883334551	0.914402649	0.904151325	0.660829605
LOC112449531	0.747212605	0.671602771	0.747084388	0.841828571	0.99989309	0.752857325
ACOD1	0.747602448	0.708270551	0.765074242	0.939292364	0.762655357	0.752857325
TSN	0.747602448	0.641416336	0.765074242	0.835254768	0.766792351	0.930707319
GLYR1	0.747602448	0.710155842	0.958420472	0.806997685	0.804250204	0.645838677

EMC8	0.747602448	0.67221723	0.726668529	0.841828571	0.875054206	0.887248119
LOC782479	0.747602448	0.738328793	0.737352631	0.919247117	0.947146038	0.637306705
HOXB4	0.747602448	0.589137013	0.79220963	0.915749182	0.961382403	0.678644983
CPA3	0.74765791	0.611256318	0.70435078	0.820228145	0.99989309	0.867117589
ARRDC3	0.74765791	0.609819443	0.69363613	0.811000597	0.99989309	0.86921618
GNA13	0.747712517	0.559785028	0.846650632	0.995118128	0.769784115	0.671528522
SLC26A11	0.747712517	0.686399081	0.747093618	0.91329897	0.875054206	0.773957499
CA4	0.747712517	0.692461545	0.707441133	0.806922554	0.875054206	0.933678522
CNTRL	0.747712517	0.588244908	0.863512509	0.915749182	0.934859184	0.640642131
TLE3	0.747712517	0.563665535	0.707441133	0.908496281	0.99989309	0.697857798
ABCB4	0.747712517	0.656553906	0.685222626	0.806922554	0.99989309	0.851366694
BAZ2B	0.747943783	0.655710881	0.838488596	0.915749182	0.814933542	0.725528824
TPM2	0.747943783	0.82890642	0.763080797	0.825933581	0.819670067	0.773957499
LOC112444505	0.747943783	0.583843515	0.707441133	0.840835021	0.99989309	0.696027936
MGAT4A	0.748194726	0.695850654	0.769285605	0.990571725	0.766792351	0.663483489
CYB5R4	0.748194726	0.656553906	0.900128576	0.904990622	0.766792351	0.691363988
ENPP4	0.748194726	0.76090129	0.888610896	0.83855098	0.766792351	0.701784819
SURF4	0.748194726	0.761788537	0.735302276	0.925004412	0.766792351	0.770391484
GUCY1A1	0.748194726	0.625663019	0.830013482	0.883885658	0.766792351	0.852095955
LOC112444622	0.748194726	0.629715977	0.774248983	0.915749182	0.769784115	0.859447707
LOC107131567	0.748194726	0.885981636	0.827125538	0.806922554	0.7817968	0.703941754
SPTBN5	0.748194726	0.568497289	0.844065668	0.915749182	0.804250204	0.807579326
SAR1B	0.748194726	0.619184305	0.749397932	0.97378567	0.809298212	0.77208617
MYNN	0.748194726	0.962934461	0.678204563	0.891273781	0.819670067	0.647432409
LOC526966	0.748194726	0.600050072	0.726668529	0.851394268	0.83417924	0.943138585
ARMC1	0.748194726	0.624948373	0.830013482	0.883885658	0.834198722	0.809650729
MAB21L2	0.748194726	0.963478319	0.699760739	0.850828215	0.838889011	0.648987851
MTMR12	0.748194726	0.693773777	0.733231491	0.868090726	0.847784631	0.859973633
FBXO8	0.748194726	0.567752026	0.832483532	0.943539568	0.849825976	0.728276429
SLAMF9	0.748194726	0.62464979	0.682822841	0.841828571	0.853884483	0.964188691
LOC112442386	0.748194726	0.642501337	0.773281026	0.915749182	0.862637717	0.77288364
CHMP1B	0.748194726	0.617732204	0.747806937	0.958879502	0.87483668	0.755246621
LBR	0.748194726	0.703437628	0.845237542	0.841828571	0.887132523	0.723641471
KNL1	0.748194726	0.567752026	0.826873746	0.95175364	0.904995537	0.671528522
MGAT4C	0.748194726	0.648099013	0.726668529	0.87630412	0.906183446	0.858042054
GMPS	0.748194726	0.732522135	0.843427302	0.850828215	0.932081893	0.637306705
COA5	0.748194726	0.711124077	0.755534187	0.912712485	0.943768595	0.671720185
TRMT61A	0.748194726	0.679258786	0.694422859	0.806922554	0.943768595	0.908939618

SMYD3	0.748194726	0.803903736	0.684005375	0.910808102	0.96532164	0.654251319
LOC112443250	0.748194726	0.578451435	0.750089824	0.846715063	0.96730957	0.859447707
LOC511386	0.748194726	0.567752026	0.89007492	0.820228145	0.972917711	0.707492534
ATP2A2	0.748194726	0.567752026	0.870367989	0.874189736	0.975291792	0.671528522
TNIP2	0.748194726	0.608096264	0.868936984	0.820228145	0.975291792	0.717273603
FAM160B1	0.748194726	0.7116845	0.765074242	0.87452213	0.976657193	0.661937165
USP32	0.748194726	0.6861152	0.726668529	0.86288354	0.982014614	0.763686154
GPR45	0.748194726	0.871412404	0.720067907	0.806922554	0.987855669	0.66854638
MB	0.748194726	0.562255493	0.765074242	0.841695395	0.996250445	0.844335246
SCAF8	0.748194726	0.687403165	0.684005375	0.850305862	0.99989309	0.64804033
LOC100336868	0.748194726	0.655710881	0.834793268	0.820228145	0.99989309	0.651139949
ME1	0.748194726	0.601915561	0.719216596	0.892229846	0.99989309	0.651173954
DNAJC14	0.748194726	0.645930592	0.765074242	0.825933581	0.99989309	0.660829605
WBP4	0.748194726	0.63203115	0.757337101	0.850828215	0.99989309	0.747829293
SGIP1	0.748194726	0.619374114	0.788848487	0.820228145	0.99989309	0.764546301
ZNF555	0.748194726	0.619374114	0.726668529	0.841828571	0.99989309	0.764546301
SOGA3	0.748194726	0.583641966	0.694542317	0.915749182	0.99989309	0.77208617
TMEM126A	0.748194726	0.600050072	0.733231491	0.825933581	0.99989309	0.788142857
PABPC4	0.748194726	0.568497289	0.684005375	0.820228145	0.99989309	0.789905519
DHX57	0.748560516	0.583843515	0.790109639	0.87452213	0.775461217	0.912380696
AP1G2	0.748560516	0.629715977	0.899100566	0.915749182	0.779960806	0.671720185
LOC101906120	0.748560516	0.679999905	0.712526829	0.999939566	0.78999884	0.671528522
DGCR8	0.748560516	0.629715977	0.957575736	0.891273781	0.799530855	0.642154165
REEP1	0.748560516	0.639676128	0.957802423	0.883885658	0.804250204	0.637306705
LOC781977	0.748560516	0.871113425	0.787740344	0.878289777	0.814605912	0.646589553
CREBRF	0.748560516	0.768980848	0.709521163	0.995118128	0.814605912	0.647432409
S100A1	0.748560516	0.57320535	0.775151646	0.984769774	0.814605912	0.77208617
P4HA3	0.748560516	0.63144743	0.770871239	0.925931623	0.825710291	0.796002422
RANBP1	0.748560516	0.731268229	0.726668529	0.842538664	0.83417924	0.878896178
GRPEL1	0.748560516	0.6861152	0.765074242	0.915749182	0.837323996	0.792329436
NDC80	0.748560516	0.595931157	0.89007492	0.938519355	0.84234617	0.642154165
LOC788736	0.748560516	0.603785862	0.707441133	0.995118128	0.84234617	0.775254902
TOR2A	0.748560516	0.600050072	0.747084388	0.999939566	0.846122929	0.651173954
SERTM1	0.748560516	0.851167599	0.684005375	0.820228145	0.846122929	0.86143104
FBXO41	0.748560516	0.642501337	0.828737895	0.806997685	0.857710467	0.865827457
ST6GALNAC4	0.748560516	0.568025614	0.937544913	0.90419464	0.861524746	0.671528522
LOC784148	0.748560516	0.600050072	0.726668529	0.946344632	0.87465791	0.822691858
C26H10orf82	0.748560516	0.886652502	0.703042171	0.819543775	0.87483668	0.790039686

CSE1L	0.748560516	0.747899938	0.80326876	0.846043369	0.875644404	0.75292536
LOC101903877	0.748560516	0.573020585	0.747093618	0.88747242	0.881823169	0.905220694
PITPNA	0.748560516	0.605987327	0.787015778	0.943323364	0.90399271	0.725620954
FBXL7	0.748560516	0.81717488	0.694422859	0.854955257	0.907192767	0.770391484
GRB10	0.748560516	0.67497759	0.735502764	0.86288354	0.925981327	0.841346557
KMT5B	0.748560516	0.855151723	0.684005375	0.820228145	0.932081893	0.778022404
NCLN	0.748560516	0.734994631	0.684005375	0.844446125	0.932197133	0.86143104
ALMS1	0.748560516	0.675748768	0.720067907	0.873519404	0.934380317	0.840150874
MMP16	0.748560516	0.636332133	0.757337101	0.907949786	0.934859184	0.775254902
ZBTB16	0.748560516	0.625663019	0.685222626	0.956154344	0.939361296	0.774602575
LOC112446351	0.748560516	0.631379878	0.726668529	0.996601241	0.941064037	0.642154165
LEF1	0.748560516	0.61365658	0.764057751	0.846715063	0.941064037	0.849769587
CLEC12A	0.748560516	0.640473376	0.754897816	0.820228145	0.943901678	0.873520548
DENND1C	0.748560516	0.691175262	0.787740344	0.865082156	0.946944212	0.729572018
KLF11	0.748560516	0.692547228	0.685222626	0.820228145	0.949645557	0.891000792
VPS8	0.748560516	0.637175486	0.759671935	0.904990622	0.952172109	0.764546301
RWDD1	0.748560516	0.601801243	0.751496241	0.915749182	0.954501767	0.7658475
ALKBH8	0.748560516	0.921280256	0.685222626	0.820228145	0.957605284	0.671528522
ADA2	0.748560516	0.613737651	0.720067907	0.90419464	0.961382403	0.823139458
LOC101905875	0.748560516	0.651279405	0.684005375	0.982140778	0.972917711	0.671133446
RBKS	0.748560516	0.688099473	0.694190265	0.949947918	0.975291792	0.661861937
RFWD3	0.748560516	0.661476635	0.684005375	0.934591536	0.98152792	0.74008849
KIT	0.748560516	0.689826702	0.726668529	0.87452213	0.988693274	0.764546301
DEDD	0.748560516	0.683461871	0.739676418	0.91329897	0.993321574	0.677383279
ELF2	0.748560516	0.80403784	0.747084388	0.820228145	0.996737199	0.671720185
PGLS	0.748560516	0.692987203	0.685559247	0.820686724	0.998276725	0.840150874
IL1RAP	0.748560516	0.617063658	0.696685689	0.811000597	0.99989309	0.637306705
PGM2	0.748560516	0.665733407	0.747084388	0.90419464	0.99989309	0.651683366
LOC100299503	0.748560516	0.696309568	0.775786014	0.868090726	0.99989309	0.652355711
EEF2KMT	0.748560516	0.692987203	0.733231491	0.908496281	0.99989309	0.661861937
TRPT1	0.748560516	0.632709306	0.697476919	0.866960502	0.99989309	0.66854638
PTPN13	0.748560516	0.693153864	0.774385549	0.841828571	0.99989309	0.671528522
LOC789694	0.748560516	0.670188815	0.757235816	0.820228145	0.99989309	0.681177433
TMEM88	0.748560516	0.573020585	0.685222626	0.913413181	0.99989309	0.694702496
TIMM21	0.748560516	0.589382558	0.689481932	0.850828215	0.99989309	0.742259651
LSM4	0.748560516	0.680267573	0.703042171	0.825933581	0.99989309	0.765492321
KPNB1	0.748560516	0.578638921	0.759671935	0.820228145	0.99989309	0.770391484
DPH7	0.748560516	0.688099473	0.709521163	0.825933581	0.99989309	0.770391484

LIX1	0.748560516	0.6861152	0.684005375	0.850828215	0.99989309	0.77208617
MTCH2	0.748560516	0.604125493	0.733993364	0.86288354	0.99989309	0.797730509
TYK2	0.748560516	0.595625745	0.733231491	0.855137785	0.99989309	0.809432201
ERBIN	0.748560516	0.584982353	0.684005375	0.826315685	0.99989309	0.814576296
TMEM168	0.748560516	0.567752026	0.768676249	0.820228145	0.99989309	0.840150874
ZFP41	0.748560516	0.567752026	0.751496241	0.83476264	0.99989309	0.845803016
LCK	0.74898516	0.790637585	0.684005375	0.852776101	0.77975903	0.890898561
CENPN	0.74898516	0.685777004	0.765074242	0.908496281	0.925981327	0.75292536
RECQL	0.74898516	0.578451435	0.891455327	0.914862928	0.941064037	0.640138785
DUSP14	0.74898516	0.587833611	0.894547778	0.884946934	0.954501767	0.645838677
TUB	0.74898516	0.567471709	0.702276506	0.928623745	0.99989309	0.694702496
GAS8	0.749565631	0.747499345	0.754897816	0.838641474	0.988693274	0.707492534
NR2C2	0.749565631	0.575412942	0.724199536	0.825933581	0.99989309	0.703742328
LOC616840	0.749986114	0.828600257	0.699920157	0.948416955	0.795200669	0.694702496
PHPT1	0.749986114	0.658193849	0.73016804	0.820228145	0.99989309	0.642154165
LOC101902656	0.750020181	0.673034156	0.709731835	0.99879985	0.804250204	0.742259651
OGT	0.750020181	0.567752026	0.892122482	0.910808102	0.813531278	0.77208617
CACUL1	0.750020181	0.57068927	0.92153058	0.945301766	0.819670067	0.640138785
CC2D1B	0.750020181	0.779501962	0.79610462	0.925931623	0.819670067	0.640642131
C29H11orf80	0.750020181	0.711020048	0.777178978	0.891563211	0.819670067	0.805038934
HSDL1	0.750020181	0.568025614	0.919108971	0.915749182	0.820037676	0.724507872
BZW1	0.750020181	0.575412942	0.812770091	0.867865861	0.838889011	0.889622239
NT5DC3	0.750020181	0.603785862	0.820120352	0.956154344	0.84234617	0.722309684
HDAC9	0.750020181	0.936550733	0.74784538	0.808629099	0.84234617	0.725528824
LURAP1L	0.750020181	0.647660219	0.751496241	0.97666622	0.849825976	0.738110854
SMC4	0.750020181	0.608096264	0.858865175	0.946344632	0.87483668	0.642154165
ANKRD55	0.750020181	0.600050072	0.78151514	0.995118128	0.882754591	0.653387719
OSCAR	0.750020181	0.787707638	0.763592007	0.92064557	0.886279726	0.640138785
WFS1	0.750020181	0.703437628	0.82710681	0.87487639	0.904151325	0.710580013
LOC104973054	0.750020181	0.595087191	0.739676418	0.983569039	0.934368424	0.728276429
SYAP1	0.750020181	0.567752026	0.879215964	0.924489924	0.934859184	0.646642632
LOC112444279	0.750020181	0.747164473	0.684005375	0.917869295	0.939361296	0.752900996
PURB	0.750020181	0.600538394	0.790109639	0.956154344	0.943768595	0.653655829
MYOCD	0.750020181	0.790637585	0.73016804	0.806997685	0.952172109	0.795637468
TTC19	0.750020181	0.582472344	0.747084388	0.846715063	0.952172109	0.888617204
LAX1	0.750020181	0.595931157	0.684005375	0.999939566	0.963031162	0.671720185
CPEB4	0.750020181	0.72843033	0.846650632	0.820228145	0.964150118	0.654411795
ATP5ME	0.750020181	0.688099473	0.707441133	0.807614341	0.99989309	0.641763222

COX5B	0.750020181	0.609819443	0.712113371	0.841828571	0.99989309	0.651139949
TRUB2	0.750020181	0.658193849	0.733231491	0.846715063	0.99989309	0.651173954
ZCCHC6	0.750020181	0.635815471	0.803883983	0.850828215	0.99989309	0.651173954
LOC112447360	0.750020181	0.651387022	0.69131755	0.95175364	0.99989309	0.651173954
CCDC62	0.750020181	0.583843515	0.79610462	0.820228145	0.99989309	0.661861937
DNAJB14	0.750020181	0.568239497	0.814444866	0.820228145	0.99989309	0.666104994
NIPSNAP1	0.750020181	0.608096264	0.70435078	0.886659234	0.99989309	0.692674475
USF3	0.750020181	0.647086084	0.747084388	0.826909448	0.99989309	0.765527601
PDHX	0.750020181	0.574094539	0.684005375	0.915749182	0.99989309	0.767960884
LARS2	0.750020181	0.619374114	0.685222626	0.811000597	0.99989309	0.798051736
GPS1	0.750020181	0.636332133	0.715979316	0.864683403	0.99989309	0.808302424
TUFM	0.750020181	0.617063658	0.685222626	0.825933581	0.99989309	0.809650729
RBM48	0.750048694	0.737374533	0.694190265	0.996084773	0.781855055	0.723141819
CAP2	0.750048694	0.934248552	0.765074242	0.846715063	0.782662718	0.66854638
CD300LB	0.750048694	0.747499345	0.729587161	0.925901365	0.797462197	0.786737722
LOC512869	0.750048694	0.608346598	0.685222626	0.915749182	0.99989309	0.659225631
TRA2B	0.750048694	0.595931157	0.684005375	0.846715063	0.99989309	0.686965009
TECR	0.750048694	0.629715977	0.684005375	0.846115604	0.99989309	0.828663852
MRPL33	0.750102821	0.626635021	0.879215964	0.850305862	0.994688893	0.640138785
TRAK1	0.750581954	0.571758501	0.926791789	0.915749182	0.821821919	0.696593441
LOC617785	0.750581954	0.747499345	0.701483892	0.992137653	0.87483668	0.654251319
RINL	0.750581954	0.661476635	0.844065668	0.861272209	0.927804504	0.732184848
PLCL2	0.750581954	0.567752026	0.843870009	0.90419464	0.981777076	0.696365784
ACAD9	0.750581954	0.684042078	0.715979316	0.87630412	0.981938411	0.792329436
ZBED5	0.750581954	0.567752026	0.787740344	0.914862928	0.99989309	0.659862452
SUOX	0.750581954	0.593994283	0.696685689	0.846715063	0.99989309	0.745987575
LOC789748	0.750851131	0.687113907	0.777178978	0.825933581	0.779159678	0.918255496
GLT8D2	0.750851131	0.780290359	0.748016329	0.9565815	0.819670067	0.660829605
PNN	0.750851131	0.567845198	0.832431595	0.915749182	0.954501767	0.715756042
SON	0.750851131	0.665563964	0.703042171	0.850828215	0.99989309	0.678496087
NR2F6	0.75106112	0.84043321	0.70435078	0.844510397	0.996250445	0.671164434
LOC101904357	0.752133701	0.608096264	0.724199536	0.820228145	0.99989309	0.651173954
LOC104969050	0.752456624	0.615959252	0.79610462	0.807614341	0.835802242	0.941925704
LOC101907843	0.752456624	0.588244908	0.788848487	0.99879985	0.86785481	0.659456873
METTL21A	0.752567287	0.913690438	0.735302276	0.869637988	0.889271428	0.642154165
ROCK1	0.752567287	0.734994631	0.74784538	0.915749182	0.899048045	0.738110854
LOC100337457	0.752567287	0.588893909	0.712526829	0.915749182	0.915496086	0.877623013
THBS1	0.752567287	0.666543433	0.715979316	0.841828571	0.99989309	0.74008849

LOC107131471	0.752954828	0.600050072	0.817993104	0.868090726	0.821928612	0.878391388
LOC112448737	0.753126215	0.567752026	0.773281026	0.846715063	0.849825976	0.941925704
XKR5	0.753316627	0.605658706	0.703042171	0.999939566	0.819670067	0.66854638
FHOD3	0.753316627	0.739873533	0.726668529	0.989391869	0.885921829	0.640642131
TTC25	0.753316627	0.738981601	0.693120773	0.917869295	0.99989309	0.661861937
NPHP4	0.753326567	0.588244908	0.777178978	0.948120268	0.788042205	0.840150874
TNC	0.753326567	0.890554452	0.713802232	0.883885658	0.808924032	0.764546301
AURKB	0.753326567	0.886044896	0.702276506	0.878289777	0.814605912	0.77208617
IZUMO4	0.753326567	0.567752026	0.764481234	0.959287984	0.814605912	0.847189049
PGM5	0.753326567	0.83744789	0.812770091	0.846715063	0.819670067	0.710580013
KCNT1	0.753326567	0.615179529	0.845237542	0.925901365	0.819670067	0.76360318
MRPL15	0.753326567	0.691175262	0.827125538	0.90419464	0.823462814	0.770391484
PTPRCAP	0.753326567	0.611623727	0.735502764	0.968796024	0.83417924	0.821467423
TTLL12	0.753326567	0.629715977	0.685222626	0.999939566	0.846122929	0.752358594
REPS1	0.753326567	0.578451435	0.806939645	0.995118128	0.868287608	0.681247744
NEO1	0.753326567	0.655710881	0.751930447	0.992368879	0.87465791	0.681177433
WISP1	0.753326567	0.600538394	0.904682623	0.92064557	0.875054206	0.650757255
LOC112447844	0.753326567	0.63512774	0.735302276	0.851175396	0.896933443	0.908939618
SHCBP1	0.753326567	0.567845198	0.825888497	0.925901365	0.897363817	0.77208617
TMEM45A	0.753326567	0.567752026	0.751496241	0.999939566	0.904151325	0.660428801
BCL6B	0.753326567	0.907649596	0.696685689	0.90419464	0.912365272	0.645838677
SLC1A5	0.753326567	0.6861152	0.751033189	0.849315934	0.943768595	0.829389486
PLAC8	0.753326567	0.568025614	0.819114812	0.850828215	0.943768595	0.84491737
SEC11C	0.753326567	0.638499519	0.788848487	0.811000597	0.947146038	0.863578778
LCP1	0.753326567	0.704817952	0.777178978	0.90419464	0.949645557	0.697990115
LOC787858	0.753326567	0.584982353	0.870054336	0.915749182	0.952172109	0.661861937
KIAA1211	0.753326567	0.699417603	0.810425784	0.850828215	0.955780925	0.723028401
DENND2D	0.753326567	0.582065511	0.790504275	0.850828215	0.961835052	0.837312175
DIRC2	0.753326567	0.575232052	0.693454224	0.99879985	0.961879452	0.703742328
ZNF385B	0.753326567	0.567752026	0.786602178	0.835254768	0.96532164	0.868745583
ANAPC15	0.753326567	0.738328793	0.771402466	0.90419464	0.970733065	0.646642632
TMEM69	0.753326567	0.632709306	0.731086678	0.949947918	0.975291792	0.692802214
EMC6	0.753326567	0.742728851	0.753656036	0.875093568	0.981938411	0.681247744
ABCF3	0.753326567	0.665803806	0.735302276	0.830830186	0.988693274	0.840150874
SNAI2	0.753326567	0.748082375	0.699920157	0.842538664	0.99989309	0.645838677
NUP160	0.753326567	0.711594168	0.764057751	0.825933581	0.99989309	0.660829605
KCTD15	0.753326567	0.568497289	0.764057751	0.915749182	0.99989309	0.671720185
FXR1	0.753326567	0.710486439	0.719335065	0.90419464	0.99989309	0.683895124

ZNHIT2	0.753326567	0.609819443	0.806825406	0.841828571	0.99989309	0.697990115
MFSD13A	0.753326567	0.72477102	0.73911052	0.838800638	0.99989309	0.705712189
CSNK2A2	0.753326567	0.619184305	0.699760739	0.913319839	0.99989309	0.728276429
ZBTB47	0.753326567	0.642412048	0.744933814	0.846715063	0.99989309	0.752900996
PER1	0.753326567	0.63203115	0.707441133	0.846715063	0.99989309	0.839419627
PNPO	0.753326567	0.595931157	0.69363613	0.86288354	0.99989309	0.873405973
PTGES	0.754087891	0.582066282	0.838080059	0.926405541	0.887132523	0.750665174
LOC104970976	0.754200825	0.619184305	0.74784538	0.936880771	0.99989309	0.663483489
PITHD1	0.754234909	0.568497289	0.892122482	0.990571725	0.804250204	0.642154165
LOC112444585	0.754234909	0.916898138	0.74784538	0.820228145	0.837323996	0.759729981
LOXL3	0.754234909	0.589334554	0.777178978	0.949947918	0.988693274	0.660829605
LOC112444206	0.754313804	0.588244908	0.751496241	0.915749182	0.862637717	0.88667499
SCRIB	0.754449572	0.587990449	0.954826554	0.915749182	0.808949108	0.651139949
CEBPG	0.754449572	0.595087191	0.873750661	0.913319839	0.86785481	0.754897098
HEBP1	0.754449572	0.568025614	0.795701822	0.994309225	0.943768595	0.645838677
DYNC2H1	0.754449572	0.72843033	0.707441133	0.883885658	0.99989309	0.705712189
SIGLEC1	0.754449572	0.63203115	0.703042171	0.809634958	0.99989309	0.77208617
TSSK2	0.754702779	0.595931157	0.685222626	0.858021281	0.99989309	0.725528824
LY6G5B	0.754845309	0.587833611	0.846782643	0.925265292	0.819670067	0.784080446
CD27	0.75531405	0.568025614	0.79610462	0.92064557	0.886279726	0.824778321
LOC101903868	0.756056574	0.691175262	0.694190265	0.99879985	0.875054206	0.697857798
RBMX2	0.756165541	0.610068686	0.862585131	0.90419464	0.83627631	0.794529985
CATSPERG	0.756165541	0.568025614	0.83314199	0.971220023	0.91011204	0.671528522
FMO3	0.7563917	0.609819443	0.757787642	0.934591536	0.952172109	0.753138357
CKMT2	0.756619817	0.691175262	0.703042171	0.820228145	0.799665755	0.978467321
ASF1A	0.7567154	0.596593582	0.859931483	0.820228145	0.997854738	0.764546301
HAGH	0.7567154	0.613737651	0.694422859	0.846715063	0.99989309	0.692674475
METTL16	0.7567154	0.579263781	0.777178978	0.915749182	0.99989309	0.6995315
MRPS23	0.757040929	0.601801243	0.75246943	0.851394268	0.99989309	0.764546301
FBXO11	0.757159855	0.655710881	0.696560245	0.931268564	0.99989309	0.728276429
PALLD	0.757415594	0.694526901	0.746957417	0.992368879	0.823462814	0.707492534
DGKB	0.757415594	0.7116845	0.699166169	0.820228145	0.943768595	0.90211634
MINOS1	0.757415594	0.665563964	0.735054113	0.820228145	0.99989309	0.651173954
ARID4B	0.757929626	0.618528036	0.764057751	0.925931623	0.99989309	0.671133446
NAMPT	0.758016905	0.686576166	0.858972712	0.934591536	0.77975903	0.692802214
NR2F2	0.758016905	0.687113907	0.694542317	0.999939566	0.809298212	0.660829605
CBR3	0.758016905	0.72477102	0.726668529	0.87452213	0.905719399	0.839419627
LOC101904087	0.758016905	0.653408806	0.776574729	0.980625409	0.910770324	0.660829605

NUPR1	0.758016905	0.568497289	0.744499269	0.992137653	0.961835052	0.703914683
PAICS	0.758016905	0.635815471	0.870054336	0.825933581	0.972917711	0.729817953
DMBT1	0.758215884	0.629715977	0.712526829	0.925059533	0.813531278	0.908939618
FANCM	0.758215884	0.655710881	0.765074242	0.945301766	0.932197133	0.703914683
KCNE3	0.758215884	0.711172851	0.792827466	0.904990622	0.941064037	0.694840199
PSD	0.758215884	0.595931157	0.788938503	0.825933581	0.99989309	0.752358594
ANKLE2	0.758219404	0.642501337	0.886176337	0.904769083	0.908708309	0.661937165
GLRX	0.758219404	0.765341142	0.788938503	0.82300162	0.943768595	0.759729981
KAZN	0.758219404	0.582985895	0.694190265	0.949947918	0.952172109	0.839340243
USP7	0.758219404	0.584982353	0.735302276	0.990049248	0.988693274	0.678128476
LOC100847454	0.758219404	0.629715977	0.746957417	0.909940206	0.99989309	0.763746661
CXXC1	0.758402943	0.595931157	0.909002826	0.962142137	0.779159678	0.663483489
DLX5	0.758402943	0.93086109	0.788938503	0.825933581	0.790028797	0.703742328
ATXN7L2	0.758402943	0.588893909	0.789182336	0.995118128	0.795200669	0.770391484
HNRNPD	0.758402943	0.605333364	0.765074242	0.999939566	0.797462197	0.747414217
FTH1	0.758402943	0.706231276	0.897316161	0.90419464	0.804250204	0.671720185
LOC112446481	0.758402943	0.59054411	0.803883983	0.820228145	0.804250204	0.968245211
WDR53	0.758402943	0.579665846	0.794072677	0.999939566	0.822146466	0.683895124
CABP1	0.758402943	0.806439163	0.7079185	0.934591536	0.823462814	0.755246621
RBM10	0.758402943	0.615179529	0.939683463	0.891273781	0.846122929	0.692674475
PDXK	0.758402943	0.6799453	0.688145488	0.976950878	0.846122929	0.815459921
ARHGEF12	0.758402943	0.63512774	0.803398613	0.903607716	0.850879779	0.845803016
PLA2G2C	0.758402943	0.611461281	0.772990161	0.99879985	0.866920896	0.694840199
DDX54	0.758402943	0.72843033	0.685222626	0.916446425	0.87483668	0.839494796
LOC768255	0.758402943	0.625663019	0.702276506	0.908496281	0.881823169	0.917524589
LOC786065	0.758402943	0.600050072	0.778658634	0.971220023	0.886279726	0.752358594
LOC112442745	0.758402943	0.804875341	0.707441133	0.925004412	0.892149112	0.727921992
SCMH1	0.758402943	0.832239565	0.699920157	0.841699279	0.892506554	0.837249706
CCT5	0.758402943	0.618528036	0.757235816	0.89088601	0.961382403	0.830456671
BRI3BP	0.758402943	0.685495173	0.751496241	0.850828215	0.96532164	0.809650729
STARD7	0.758402943	0.595931157	0.793034745	0.94373839	0.96730957	0.671720185
TMEM170A	0.758402943	0.600050072	0.788848487	0.95175364	0.972917711	0.663483489
MTG1	0.758402943	0.583181278	0.833533313	0.904990622	0.99989309	0.651173954
LOC100297399	0.758402943	0.678391216	0.724199536	0.925931623	0.99989309	0.661861937
NGF	0.758402943	0.586530238	0.782758498	0.925931623	0.99989309	0.678496087
CLU	0.758402943	0.693051877	0.73016804	0.825933581	0.99989309	0.701784819
MMAB	0.758402943	0.632208593	0.707441133	0.868090726	0.99989309	0.759119384
PMM2	0.758402943	0.71492723	0.733231491	0.836785013	0.99989309	0.76935309

ITGB1BP2	0.758402943	0.588244908	0.744499269	0.901638646	0.99989309	0.820483064
NPR2	0.758402943	0.598504547	0.81285201	0.820228145	0.99989309	0.822574444
NUMB	0.758402943	0.583641966	0.712526829	0.820228145	0.99989309	0.908939618
TNFAIP2	0.75858032	0.6861152	0.765074242	0.883885658	0.820727319	0.884387948
MCFD2	0.75858032	0.589137013	0.899539089	0.915749182	0.821928612	0.759308648
SQOR	0.75858032	0.631379878	0.722352424	0.943323364	0.84234617	0.864688571
LOC101903261	0.75858032	0.593314312	0.706787562	0.943323364	0.861524746	0.895218005
S100A2	0.75858032	0.688099473	0.860010936	0.915749182	0.87483668	0.671720185
LAP3	0.75858032	0.603555122	0.733487658	0.999939566	0.91011204	0.691363988
FAM189A2	0.75858032	0.687403165	0.757235816	0.982140778	0.932081893	0.650973086
SINHCAF	0.75858032	0.689053394	0.726668529	0.934591536	0.947146038	0.743377609
REEP4	0.75858032	0.845979391	0.712526829	0.900737606	0.954501767	0.66915423
FBXL22	0.75858032	0.81105622	0.758406163	0.858006538	0.954501767	0.683895124
TMEM189	0.75858032	0.625663019	0.765074242	0.983569039	0.961382403	0.650973086
LOC529196	0.75858032	0.675748768	0.770051055	0.883885658	0.99989309	0.684905734
MT1E	0.759152291	0.63203115	0.733231491	0.837066215	0.947146038	0.912101047
EFNA4	0.759469742	0.632709306	0.810425784	0.891273781	0.784855148	0.886992214
RUSC1	0.759469742	0.735625249	0.766184475	0.883885658	0.99989309	0.655092706
UEVLD	0.759573248	0.632304442	0.84405284	0.922782374	0.809298212	0.784671496
TMED7	0.759573248	0.648099013	0.806311697	0.89088601	0.823462814	0.864219261
USF2	0.759573248	0.723119554	0.751496241	0.943240775	0.843348001	0.761409946
ZPBP2	0.759573248	0.688016924	0.712526829	0.820228145	0.882754591	0.94588513
SUSD3	0.759573248	0.778320571	0.838488596	0.8968247	0.887061423	0.648987851
NT5C3B	0.759573248	0.635240156	0.807113974	0.925901365	0.941064037	0.712066047
ATP5PD	0.759573248	0.63512774	0.750421005	0.850828215	0.99989309	0.650757255
TRIM11	0.759573248	0.601801243	0.806825406	0.86288354	0.99989309	0.66915423
LOC786474	0.759573248	0.715519139	0.698225674	0.820228145	0.99989309	0.812509877
DNAJC21	0.75965071	0.574891833	0.9343664	0.850828215	0.784855148	0.831157143
ORAI2	0.75965071	0.69635562	0.708560005	0.820228145	0.808944674	0.981759054
LOC785842	0.75965071	0.638242621	0.703042171	0.999939566	0.809298212	0.671528522
AGBL2	0.75965071	0.642677274	0.891014698	0.931989854	0.814933542	0.671720185
AK2	0.75965071	0.575625328	0.685222626	0.913413181	0.846122929	0.968059826
TIE1	0.75965071	0.648099013	0.751496241	0.946344632	0.868303349	0.799935743
DHX29	0.75965071	0.59605585	0.733231491	0.842538664	0.868380278	0.971025918
POLG	0.75965071	0.604361932	0.945417678	0.90419464	0.87465791	0.661937165
SLC35G2	0.75965071	0.626607857	0.844065668	0.956154344	0.87483668	0.671528522
PRPS2	0.75965071	0.669738807	0.820767684	0.903607716	0.902569851	0.77208617
BAG1	0.75965071	0.588244908	0.870175297	0.910808102	0.904151325	0.761409946

SCPEP1	0.75965071	0.612773311	0.832483532	0.943161565	0.925981327	0.692674475
TERF2IP	0.75965071	0.703437628	0.843427302	0.843512648	0.932081893	0.752900996
POP1	0.75965071	0.619005993	0.750603333	0.825933581	0.939361296	0.923064313
PCED1B	0.75965071	0.655149112	0.764057751	0.87452213	0.981736785	0.792329436
LOC100847700	0.75965071	0.747164473	0.73016804	0.843512648	0.996250445	0.77208617
BZW2	0.75965071	0.5692164	0.746041436	0.87630412	0.999592263	0.864219261
LOC787122	0.75965071	0.655589933	0.702276506	0.846715063	0.99989309	0.657118951
BLOC1S1	0.75965071	0.693153864	0.721682634	0.846715063	0.99989309	0.703742328
LYN	0.75965071	0.608346598	0.752494129	0.851175396	0.99989309	0.707492534
ATP1B1	0.75965071	0.655710881	0.699166169	0.869637988	0.99989309	0.740271903
LOC100335990	0.75965071	0.665563964	0.707441133	0.915749182	0.99989309	0.742259651
ADM	0.75965071	0.604997439	0.709521163	0.917185893	0.99989309	0.764546301
NACC1	0.75965071	0.665563964	0.694190265	0.820228145	0.99989309	0.766742189
TUBB4A	0.75965071	0.581298031	0.713802232	0.863958691	0.99989309	0.800008791
LOC100141145	0.759654078	0.765341142	0.878603452	0.850828215	0.7817968	0.752857325
KDELC1	0.759654078	0.671879214	0.751496241	0.958879502	0.947146038	0.682748733
PRAP1	0.759654078	0.589137013	0.733487658	0.915749182	0.99989309	0.691363988
SEC23B	0.759952144	0.698870039	0.894449269	0.820228145	0.87465791	0.771597542
PPP1R11	0.759953306	0.575473391	0.904691312	0.915749182	0.943901678	0.64804033
CREB1	0.760064658	0.665803806	0.762423061	0.858274325	0.99989309	0.649372601
ADD2	0.761036221	0.912112179	0.735054113	0.87630412	0.805400552	0.757327233
ADGRD1	0.761036221	0.588244908	0.751496241	0.995118128	0.939361296	0.728276429
CLBA1	0.761036221	0.626850404	0.788848487	0.915749182	0.950183356	0.772065505
ZNF326	0.761036221	0.583641966	0.707441133	0.904990622	0.99989309	0.725528824
RGN	0.761118113	0.771098738	0.702276506	0.917869295	0.972917711	0.692674475
UBA6	0.761118113	0.835276066	0.726668529	0.843411019	0.977155757	0.728276429
SPSB1	0.761163536	0.81808696	0.779087149	0.87630412	0.874640903	0.742259651
ARHGAP27	0.761163536	0.600960324	0.817256644	0.914862928	0.926899298	0.795637468
TDRD3	0.761163536	0.751946504	0.747084388	0.915749182	0.934859184	0.742259651
LOC107131728	0.761163536	0.72477102	0.733487658	0.843411019	0.975291792	0.813577244
DENND5A	0.761163536	0.600538394	0.765074242	0.932656599	0.99989309	0.671720185
LOC516442	0.76157528	0.578451435	0.832483532	0.820228145	0.814605912	0.956610132
CFL2	0.76157528	0.579545624	0.880204512	0.969717497	0.838889011	0.684905734
TNFRSF21	0.76157528	0.963737744	0.703042171	0.863958691	0.841022011	0.703742328
CCDC136	0.76157528	0.583843515	0.747084388	0.948547383	0.861524746	0.866253786
SEMA6C	0.76157528	0.629715977	0.764057751	0.999939566	0.868380278	0.6868458
IQCC	0.76157528	0.693544464	0.712526829	0.999939566	0.87465791	0.648987851
ESAM	0.76157528	0.702026685	0.776574729	0.87630412	0.887539821	0.832858909

USP19	0.76157528	0.653112351	0.853222116	0.915749182	0.925981327	0.687121026
MICAL2	0.76157528	0.730838712	0.765074242	0.908496281	0.954501767	0.705712189
CYSTM1	0.76157528	0.587833611	0.765074242	0.990571725	0.96532164	0.671133446
SLC12A2	0.76157528	0.648099013	0.726668529	0.820228145	0.99989309	0.652209333
SHARPIN	0.76157528	0.599687292	0.885434576	0.850828215	0.99989309	0.667744852
OXSM	0.76157528	0.588244908	0.69131755	0.846715063	0.99989309	0.679674648
UQCR10	0.76157528	0.625663019	0.720067907	0.856178306	0.99989309	0.688426764
NEDD9	0.76157528	0.683315591	0.747093618	0.846115604	0.99989309	0.742259651
SAMD4B	0.76157528	0.588244908	0.747093618	0.825933581	0.99989309	0.752358594
DNAAF3	0.76157528	0.641493941	0.747084388	0.846715063	0.99989309	0.798051736
SHLD1	0.76157528	0.696257932	0.733231491	0.820228145	0.99989309	0.84491737
LMX1B	0.762171579	0.679258786	0.712526829	0.90419464	0.819670067	0.934775196
LOC510520	0.762171579	0.693544464	0.768676249	0.940921143	0.852745499	0.770391484
LOC107132228	0.762171579	0.582985895	0.886282865	0.908804713	0.972917711	0.661937165
LOC104970812	0.762171579	0.671602771	0.726668529	0.820228145	0.99989309	0.784340268
STK26	0.762171579	0.600960324	0.765785685	0.865118174	0.99989309	0.788411132
SYNJ2	0.762230341	0.739918356	0.777178978	0.820228145	0.99989309	0.712710718
ADRB2	0.762471011	0.779536052	0.726668529	0.955687359	0.862637717	0.708819884
BTG3	0.762471011	0.63349599	0.887130994	0.86288354	0.868380278	0.777494305
TEPSIN	0.763292903	0.647086084	0.739676418	0.820228145	0.99989309	0.742259651
RHEB	0.76331918	0.608346598	0.966157152	0.825933581	0.823462814	0.770391484
SYCP3	0.76331918	0.599807217	0.880277219	0.898441424	0.84234617	0.812622197
LOC504773	0.76331918	0.747106203	0.693120773	0.951929772	0.86558233	0.788142857
LOC112444773	0.76331918	0.768563436	0.747093618	0.873939476	0.882754591	0.828602505
PPP6R2	0.76331918	0.651481516	0.835301782	0.956154344	0.902843706	0.657673143
CHRNA3	0.76331918	0.62464979	0.779087149	0.908496281	0.925981327	0.839698265
ZNF521	0.76331918	0.688099473	0.788848487	0.925004412	0.927804504	0.728276429
PPP2R5E	0.76331918	0.747899938	0.873750661	0.820228145	0.957910867	0.6608989
LRRC10B	0.76331918	0.619184305	0.801087628	0.850828215	0.99989309	0.685342585
DENND6A	0.763677287	0.643302108	0.784107501	0.982140778	0.892149112	0.704974208
RXRA	0.763677287	0.653161725	0.733231491	0.845686584	0.99989309	0.6950206
GARS	0.763782949	0.703437628	0.747084388	0.820228145	0.846122929	0.943138585
LOC515042	0.763782949	0.588244908	0.933740801	0.883885658	0.904995537	0.714737045
TET1	0.763782949	0.583843515	0.734661757	0.999939566	0.928701823	0.717517382
LOC100337355	0.763782949	0.703437628	0.763592007	0.860663136	0.982577194	0.77208617
RNASE6	0.763782949	0.655710881	0.733231491	0.87452213	0.998276725	0.832189969
SPARCL1	0.764278729	0.742963341	0.773281026	0.883885658	0.83417924	0.840150874
TLR10	0.764510112	0.688099473	0.744499269	0.837066215	0.99989309	0.684229586

GPR50	0.764678782	0.743422331	0.787740344	0.945301766	0.819670067	0.725506681
CAGE1	0.764693092	0.68147929	0.779087149	0.915749182	0.979654999	0.706354992
CDKL1	0.765243395	0.847082229	0.818479704	0.87630412	0.846122929	0.678496087
LOC785568	0.765243395	0.735625249	0.850751395	0.895106644	0.910770324	0.663483489
ZNF624	0.765243395	0.76090129	0.750089824	0.88747242	0.930836804	0.783479395
LOC101906989	0.765243395	0.595931157	0.817256644	0.873939476	0.940638859	0.847196533
PNPT1	0.765243395	0.615179529	0.735302276	0.854497387	0.99989309	0.776420145
NAA40	0.765378919	0.695850654	0.765074242	0.846715063	0.970439271	0.809432201
WDR12	0.765405322	0.612773311	0.770051055	0.925931623	0.846122929	0.86307729
PET117	0.765405322	0.579263781	0.699760739	0.999939566	0.910770324	0.691363988
LOC112449324	0.765405322	0.578451435	0.757787642	0.825933581	0.99989309	0.671528522
SYT1	0.765405322	0.594493124	0.707441133	0.91329897	0.99989309	0.74008849
PLRG1	0.765566274	0.698870039	0.772990161	0.982463694	0.789627785	0.757343511
C26H10orf62	0.765588835	0.598654687	0.787740344	0.999939566	0.849382765	0.724507872
C15H11orf58	0.765697437	0.589137013	0.894829955	0.921320709	0.84234617	0.753138357
FOXM1	0.765697437	0.589137013	0.919796896	0.952024081	0.846122929	0.653387719
OTUD1	0.765697437	0.62464979	0.851048846	0.934591536	0.904151325	0.696593441
LOC104972216	0.765697437	0.621440227	0.885434576	0.832608616	0.957605284	0.764546301
PTPDC1	0.765697437	0.644204975	0.770571624	0.915526452	0.966214002	0.770391484
CDR2	0.765697437	0.692987203	0.777178978	0.820228145	0.99989309	0.743464215
SRP72	0.765697437	0.64351615	0.7261904	0.898441424	0.99989309	0.761409946
BVES	0.765789883	0.778839781	0.750139062	0.825933581	0.864995027	0.875441396
TUBA1C	0.765872624	0.728894485	0.854240468	0.908054165	0.881823169	0.671720185
SLAMF7	0.765879663	0.690592057	0.795258121	0.915749182	0.881823169	0.78917391
NDUFAF5	0.766354217	0.747164473	0.715979316	0.820228145	0.99989309	0.763746661
METTL3	0.766614075	0.702217402	0.757337101	0.825933581	0.99989309	0.655092706
LOC100138864	0.766835168	0.655438106	0.849077738	0.946344632	0.904151325	0.651173954
LOC112441605	0.7676442	0.687113907	0.73016804	0.999939566	0.84234617	0.682748733
TTLL3	0.7676442	0.691175262	0.825960826	0.925004412	0.87465791	0.742259651
PFKFB4	0.7676442	0.853448902	0.715979316	0.917869295	0.881823169	0.714123227
VSIG2	0.7676442	0.684832051	0.777178978	0.841828571	0.904151325	0.88494083
POLR3K	0.7676442	0.665524216	0.757235816	0.841828571	0.99989309	0.703742328
THEM6	0.7676442	0.675748768	0.702872593	0.825933581	0.99989309	0.723028401
LOC112441885	0.7676442	0.617454372	0.715979316	0.846715063	0.99989309	0.845866317
CXHXorf36	0.767849013	0.642677274	0.724199536	0.999939566	0.910770324	0.699553866
SEPT9	0.767849013	0.705889653	0.733231491	0.830830186	0.999592263	0.840150874
DMAC2	0.768049958	0.613737651	0.744933814	0.915749182	0.99989309	0.704226189
IL1RN	0.768384851	0.922434012	0.754101752	0.844446125	0.87483668	0.71509671

NLK	0.768458581	0.588244908	0.806311697	0.940921143	0.939994656	0.770391484
IMMP2L	0.768687039	0.583641966	0.827278747	0.919247117	0.95827048	0.759308648
RASSF10	0.768848811	0.699340689	0.707441133	0.992137653	0.843348001	0.786737722
POLA1	0.768848811	0.836653211	0.762423061	0.908496281	0.931194129	0.66854638
TMEM37	0.769083092	0.720945114	0.847037539	0.91329897	0.90448127	0.671528522
MCEMP1	0.769727306	0.698870039	0.787740344	0.846115604	0.880531631	0.872175131
ZRANB1	0.769727306	0.611461281	0.820767684	0.915749182	0.949645557	0.770391484
TMEM25	0.769727306	0.693544464	0.85972234	0.891584014	0.952172109	0.66854638
FBXW7	0.769727306	0.624067943	0.836294915	0.86288354	0.99989309	0.717517382
CAPN7	0.769727306	0.62464979	0.788848487	0.871380923	0.99989309	0.753138357
AZIN1	0.770308696	0.614559394	0.921146002	0.88747242	0.87483668	0.757327233
CYP51A1	0.770449132	0.684832051	0.754897816	0.999939566	0.804250204	0.671528522
LOC101904768	0.770449132	0.595931157	0.872397372	0.841828571	0.853884483	0.886992214
FCRLA	0.770449132	0.616586256	0.699166169	0.982140778	0.87465791	0.864294309
CDC37L1	0.770449132	0.619184305	0.802847755	0.971220023	0.954501767	0.66854638
TMEM160	0.770449132	0.600050072	0.765074242	0.858274325	0.99989309	0.752857325
SPAG8	0.771305335	0.63512774	0.827952023	0.908496281	0.997854738	0.701784819
LOC533921	0.771305335	0.589137013	0.806311697	0.846715063	0.99989309	0.6608989
RGS20	0.771670291	0.595087191	0.820549834	0.931268564	0.957605284	0.742259651
WDR97	0.771700661	0.679258786	0.789804786	0.892229846	0.902569851	0.834936698
FBXL14	0.771700661	0.88311684	0.726668529	0.89088601	0.902843706	0.739362521
SURF6	0.771700661	0.68571684	0.713802232	0.850465555	0.940638859	0.912376736
CIART	0.771700661	0.655710881	0.74784538	0.953653186	0.954501767	0.748594275
WDR3	0.771700661	0.584982353	0.751496241	0.881591838	0.997854738	0.868177389
QDPR	0.771766759	0.730317314	0.741982235	0.841828571	0.99989309	0.77208617
CPT1A	0.771766759	0.608346598	0.722951238	0.90419464	0.99989309	0.859447707
NFKBIZ	0.772233278	0.703437628	0.81284583	0.925931623	0.804564502	0.788142857
LOC101905267	0.772233278	0.705684763	0.847037539	0.841828571	0.809298212	0.86143104
DPP3	0.772233278	0.737410753	0.706787562	0.843512648	0.928701823	0.905220694
CHST10	0.772233278	0.865490181	0.765074242	0.851394268	0.934859184	0.697990115
GPN3	0.772233278	0.6861152	0.821106115	0.915749182	0.970439271	0.661937165
COL12A1	0.772233278	0.6861152	0.747093618	0.943161565	0.988693274	0.694840199
IL1RL1	0.772233278	0.632709306	0.774248983	0.897446122	0.99989309	0.660829605
USP6NL	0.772233278	0.705458146	0.705023135	0.846115604	0.99989309	0.671720185
ATP1A1	0.772233278	0.595087191	0.726668529	0.924489924	0.99989309	0.689117201
TMEM266	0.772233278	0.619184305	0.720067907	0.820228145	0.99989309	0.770391484
TMEM208	0.772233278	0.655710881	0.726668529	0.88501388	0.99989309	0.840150874
SHOX2	0.772779122	0.886909612	0.74784538	0.88747242	0.889271428	0.708819884

LOC107132949	0.772779122	0.725594888	0.820767684	0.908496281	0.943768595	0.693146856
LPCAT3	0.772917043	0.595271828	0.720067907	0.86288354	0.896242324	0.973195541
EME2	0.772917043	0.665563964	0.930262306	0.861272209	0.939994656	0.660829605
SLC5A6	0.772917043	0.595087191	0.751496241	0.825933581	0.946944212	0.952631575
LOC101907566	0.772917043	0.767950704	0.765074242	0.846115604	0.99989309	0.66854638
CILP2	0.772924961	0.619184305	0.793430641	0.841828571	0.86558233	0.940683751
SH3TC2	0.773140985	0.629715977	0.803106522	0.87630412	0.882754591	0.886992214
PHLDB2	0.773472188	0.692987203	0.703042171	0.846715063	0.814933542	0.980172286
GLP1R	0.773472188	0.829503998	0.747084388	0.904990622	0.823462814	0.806453168
C24H18orf25	0.773595357	0.713953236	0.832431595	0.846715063	0.941107925	0.770391484
LOC100847870	0.773595357	0.687403165	0.817993104	0.891273781	0.99989309	0.66854638
TMBIM1	0.773635663	0.603844039	0.814444866	0.990571725	0.934379881	0.674653824
F11R	0.773635663	0.759619476	0.712526829	0.853352358	0.957605284	0.840150874
LOC101905262	0.773851315	0.669179367	0.834793268	0.82652165	0.99989309	0.722309684
MCRS1	0.773851315	0.691964288	0.773281026	0.825933581	0.99989309	0.80787758
LOC101902407	0.773926933	0.632709306	0.795258121	0.999939566	0.804250204	0.734406433
LOC616944	0.773926933	0.626695103	0.70435078	0.999939566	0.804257626	0.682748733
POU2F2	0.773926933	0.676166653	0.777638604	0.915749182	0.805400552	0.880404833
LOC104976276	0.773926933	0.911106863	0.720067907	0.915749182	0.809298212	0.761409946
SLIT3	0.773926933	0.703437628	0.735921892	0.999939566	0.814605912	0.73862615
SMARCAD1	0.773926933	0.600538394	0.781294783	0.925901365	0.814605912	0.903961701
TMEM182	0.773926933	0.583843515	0.858394457	0.820228145	0.814605912	0.948306629
MTPN	0.773926933	0.589334554	0.817993104	0.999939566	0.819670067	0.691363988
NDE1	0.773926933	0.780641867	0.744490351	0.984968973	0.837323996	0.704367673
SLC8A1	0.773926933	0.942608215	0.787740344	0.86950142	0.838889011	0.660829605
CACNA1D	0.773926933	0.921280256	0.724199536	0.934591536	0.838993207	0.661861937
NSUN5	0.773926933	0.703437628	0.879215964	0.891273781	0.84102894	0.764546301
SLC23A1	0.773926933	0.769817928	0.755324843	0.875889425	0.84234617	0.86143104
LOC112447371	0.773926933	0.669648359	0.913909017	0.921209664	0.8594734	0.661861937
ASAP3	0.773926933	0.625663019	0.953426945	0.858021281	0.862637717	0.74540456
C5H12orf71	0.773926933	0.669648359	0.705547551	0.999939566	0.868303349	0.694840199
MFAP5	0.773926933	0.661476635	0.827125538	0.943161565	0.87465791	0.757327233
IFITM3	0.773926933	0.676166653	0.92153058	0.91329897	0.876859226	0.660829605
STEAP4	0.773926933	0.6188407	0.748723724	0.999939566	0.880437125	0.671398444
LOC112446699	0.773926933	0.615179529	0.827125538	0.992137653	0.8815575	0.703721528
CD59	0.773926933	0.607259348	0.929303679	0.923578108	0.899048045	0.661861937
LOC112442047	0.773926933	0.647086084	0.806221631	0.846115604	0.899048045	0.896934364
NRAS	0.773926933	0.624948373	0.908165509	0.919247117	0.928131565	0.660829605

IQANK1	0.773926933	0.619184305	0.969618195	0.841338807	0.933983913	0.671528522
CYFIP2	0.773926933	0.762756164	0.747084388	0.837066215	0.941064037	0.86143104
HAS2	0.773926933	0.661743864	0.702872593	0.999939566	0.94294964	0.663483489
ANGPTL4	0.773926933	0.853252069	0.713802232	0.888642721	0.94294964	0.759107825
ID2	0.773926933	0.692547228	0.709521163	0.999939566	0.943768595	0.660829605
ABHD5	0.773926933	0.595931157	0.753656036	0.971220023	0.943901678	0.788131005
FAM193B	0.773926933	0.600960324	0.836294915	0.914862928	0.947826027	0.775254902
POLE	0.773926933	0.603497985	0.817256644	0.915749182	0.954501767	0.79628721
SASH3	0.773926933	0.695866639	0.709561629	0.891273781	0.957910867	0.863755488
PSMC5	0.773926933	0.669648359	0.726668529	0.915749182	0.960835287	0.834735448
LOC101904275	0.773926933	0.615918697	0.733487658	0.843411019	0.961835052	0.936439483
PHETA1	0.773926933	0.921280256	0.74784538	0.832608616	0.964150118	0.661937165
TXNIP	0.773926933	0.611256318	0.712526829	0.976519231	0.975291792	0.780611431
IQGAP1	0.773926933	0.589137013	0.850243032	0.892229846	0.997004421	0.752900996
PIAS1	0.773926933	0.897375356	0.735302276	0.825933581	0.99989309	0.661937165
PIGC	0.773926933	0.616586256	0.817993104	0.931989854	0.99989309	0.661937165
IRAK3	0.773926933	0.688099473	0.827186499	0.89088601	0.99989309	0.664012011
ZYG11B	0.773926933	0.6608873	0.882898206	0.850828215	0.99989309	0.66854638
ZBTB22	0.773926933	0.688099473	0.757787642	0.883885658	0.99989309	0.66854638
LOC524576	0.773926933	0.588244908	0.712526829	0.984090361	0.99989309	0.66915423
DUSP15	0.773926933	0.635240156	0.707441133	0.934267153	0.99989309	0.675179275
FNIP1	0.773926933	0.693153864	0.707441133	0.919247117	0.99989309	0.683474857
MARK4	0.773926933	0.72843033	0.761854021	0.891273781	0.99989309	0.702979594
STRIP2	0.773926933	0.636332133	0.722352424	0.919764668	0.99989309	0.703372675
ACYP2	0.773926933	0.633388589	0.726668529	0.942235206	0.99989309	0.712710718
LOC781064	0.773926933	0.669648359	0.712526829	0.915749182	0.99989309	0.727921992
LOC100848226	0.773926933	0.703437628	0.756911784	0.830830186	0.99989309	0.77208617
LOC101908014	0.773926933	0.625663019	0.828407935	0.850828215	0.99989309	0.77208617
LIPT1	0.773926933	0.600538394	0.744933814	0.925440563	0.99989309	0.773957499
MFSD4B	0.773926933	0.687403165	0.751496241	0.841828571	0.99989309	0.812509877
KLF7	0.773926933	0.653723309	0.7079185	0.846715063	0.99989309	0.872224159
LOC616063	0.773926933	0.625663019	0.712526829	0.846715063	0.99989309	0.886992214
MYO7A	0.773960792	0.74401284	0.793430641	0.868090726	0.99989309	0.684905734
COX5A	0.77398107	0.669648359	0.726668529	0.843411019	0.99989309	0.704011905
ZDHHC13	0.774341876	0.997277632	0.747084388	0.825933581	0.814933542	0.66854638
GGA1	0.774341876	0.619184305	0.79610462	0.999939566	0.824099979	0.661747342
ST6GALNAC3	0.774341876	0.632709306	0.751930447	0.908496281	0.832446464	0.937591162
IRGQ	0.774341876	0.63203115	0.786602178	0.999939566	0.84102894	0.683895124

AKTIP	0.774341876	0.609829017	0.834793268	0.99879985	0.846122929	0.697990115
AGAP2	0.774341876	0.688099473	0.761854021	0.999939566	0.863589901	0.660829605
POLDIP3	0.774341876	0.6663481	0.879215964	0.925931623	0.87483668	0.694702496
PDRG1	0.774341876	0.651481516	0.83314199	0.919247117	0.880626834	0.788131005
S100A5	0.774341876	0.612773311	0.726668529	0.87452213	0.882917206	0.968245211
LOC101905779	0.774341876	0.586639262	0.726668529	0.999939566	0.895183012	0.76195131
LOC112444348	0.774341876	0.693069654	0.744369186	0.925004412	0.897363817	0.840150874
LOC112442677	0.774341876	0.609819443	0.991693262	0.820228145	0.904151325	0.66854638
ADAM17	0.774341876	0.692461545	0.764057751	0.925931623	0.904151325	0.798051736
CCDC157	0.774341876	0.668925462	0.752494129	0.999939566	0.91011204	0.673808763
NYNRIN	0.774341876	0.597768231	0.819114812	0.99879985	0.910770324	0.66915423
OSBPL1A	0.774341876	0.595931157	0.712526829	0.999939566	0.925981327	0.743377609
PSMB2	0.774341876	0.701581467	0.765074242	0.87630412	0.925981327	0.857727168
LOC788745	0.774341876	0.636332133	0.827125538	0.846115604	0.925981327	0.875441396
MRPL20	0.774341876	0.693153864	0.814444866	0.87630412	0.928131565	0.809650729
LOC100139549	0.774341876	0.688099473	0.814444866	0.915749182	0.934859184	0.752900996
FAM46A	0.774341876	0.615879066	0.764057751	0.925901365	0.939361296	0.840150874
MTRF1	0.774341876	0.588244908	0.709561629	0.883885658	0.943901678	0.95470473
NOP10	0.774341876	0.693454767	0.793430641	0.913319839	0.952172109	0.763746661
ADAMTS15	0.774341876	0.7116845	0.749513157	0.908054165	0.964150118	0.788131005
FBXO6	0.774341876	0.693051877	0.836257071	0.876107068	0.975819525	0.728276429
LOC112442218	0.774341876	0.615179529	0.788848487	0.915749182	0.982014614	0.78392265
N4BP1	0.774341876	0.668175004	0.765074242	0.948172741	0.98871637	0.683895124
DDX46	0.774341876	0.655149112	0.765074242	0.915526452	0.99989309	0.661861937
XBP1	0.774341876	0.653408806	0.793871071	0.890130444	0.99989309	0.671528522
ATP5IF1	0.774341876	0.629715977	0.715979316	0.883885658	0.99989309	0.684595958
SLC4A2	0.774341876	0.63512774	0.787740344	0.915749182	0.99989309	0.684905734
FOXRED1	0.774341876	0.682470071	0.726668529	0.828524115	0.99989309	0.694840199
LOC783963	0.774341876	0.731268229	0.825888497	0.836672323	0.99989309	0.705712189
ZNF215	0.774341876	0.600538394	0.724199536	0.915749182	0.99989309	0.740271903
UIMC1	0.774341876	0.600050072	0.830022042	0.846715063	0.99989309	0.747674417
ZFP90	0.774341876	0.619184305	0.722951238	0.892229846	0.99989309	0.764546301
DHRS11	0.774341876	0.762933238	0.707441133	0.88501388	0.99989309	0.77208617
CLCN7	0.775009461	0.7116845	0.73016804	0.998542029	0.83627631	0.76360318
CEP350	0.775319596	0.672239777	0.703042171	0.847774989	0.99989309	0.683895124
SHTN1	0.776218825	0.71492723	0.765074242	0.915749182	0.880683246	0.821774001
TIMM17B	0.776218825	0.5994967	0.766049441	0.850828215	0.99989309	0.677152076
PRRX2	0.776452405	0.668175004	0.777178978	0.998542029	0.819670067	0.759107825

LOC104973139	0.776452405	0.675837248	0.941312064	0.86288354	0.846122929	0.740271903
ZNF644	0.776452405	0.730838712	0.703042171	0.820228145	0.99989309	0.784080446
NDUFAF8	0.776452405	0.700679806	0.735302276	0.843411019	0.99989309	0.823435077
DRG1	0.77649915	0.600538394	0.789868841	0.891273781	0.808944674	0.954110387
PPM1G	0.77649915	0.665563964	0.941319062	0.86288354	0.809298212	0.77208617
LOXL4	0.77649915	0.862527737	0.717447819	0.934591536	0.814933542	0.770391484
LOC101906916	0.77649915	0.619374114	0.747084388	0.999939566	0.819670067	0.671398444
RNF34	0.77649915	0.600538394	0.849077738	0.846115604	0.836838903	0.927529392
EMC2	0.77649915	0.686122011	0.830125816	0.992137653	0.838889011	0.677152076
TRAF6	0.77649915	0.77414098	0.765074242	0.925931623	0.84234617	0.77208617
LOC101906512	0.77649915	0.61754698	0.825888497	0.89088601	0.860908683	0.895218005
NUF2	0.77649915	0.595850779	0.886282865	0.981022805	0.86558233	0.673471687
SLC25A12	0.77649915	0.612773311	0.730682825	0.999939566	0.866920896	0.671133446
RAB2B	0.77649915	0.72477102	0.942023913	0.846715063	0.87483668	0.66915423
OLFM1	0.77649915	0.601801243	0.802847755	0.99879985	0.87483668	0.747674417
C7H19orf71	0.77649915	0.6390296	0.726668529	0.92064557	0.884679766	0.908645305
LOC104976247	0.77649915	0.723008622	0.827125538	0.837066215	0.892149112	0.847196533
TMEM225B	0.77649915	0.776522777	0.713802232	0.924489924	0.89629383	0.807277693
MACROD2	0.77649915	0.761903756	0.757235816	0.926405541	0.904151325	0.75292536
LOC107131699	0.77649915	0.629715977	0.880277219	0.825933581	0.928701823	0.84491737
ESD	0.77649915	0.701581467	0.93399639	0.846715063	0.934379881	0.663757654
METTL6	0.77649915	0.759143476	0.787740344	0.842538664	0.943768595	0.812509877
LOC112444290	0.77649915	0.589137013	0.773625269	0.850828215	0.96532164	0.908939618
ZBTB18	0.77649915	0.595931157	0.839622619	0.943510519	0.98152792	0.678496087
CDKN2B	0.77649915	0.692987203	0.765074242	0.908496281	0.99989309	0.671528522
YTHDF1	0.77649915	0.629715977	0.773281026	0.924489924	0.99989309	0.671528522
LOC614129	0.77649915	0.726229532	0.747084388	0.904990622	0.99989309	0.742259651
CYCS	0.77649915	0.734072007	0.747084388	0.84302258	0.99989309	0.790039686
NT5M	0.77649915	0.7116845	0.726668529	0.846115604	0.99989309	0.80787758
IGF2BP2	0.77649915	0.595931157	0.729982594	0.847813982	0.99989309	0.895218005
ATP1A2	0.776570521	0.918371039	0.738217747	0.858006538	0.87483668	0.769244117
CHST7	0.776570521	0.655138935	0.707441133	0.997308645	0.99989309	0.661937165
PSENEN	0.776831004	0.608532438	0.825960826	0.91329897	0.99989309	0.728276429
RAB22A	0.776859081	0.589374064	0.850243032	0.99879985	0.850852167	0.677383279
AZI2	0.777167739	0.625663019	0.754101752	0.999939566	0.814933542	0.77208617
A1CF	0.777167739	0.904539095	0.746957417	0.854497387	0.819670067	0.812682271
HSPB7	0.777167739	0.911230889	0.733231491	0.90419464	0.83417924	0.770391484
FAM171A2	0.777167739	0.619184305	0.773846486	0.999939566	0.85647293	0.677152076

NCS1	0.777167739	0.652542331	0.828569127	0.949411254	0.894300232	0.747674417
ITGA10	0.777167739	0.60290293	0.892122482	0.871894706	0.934859184	0.792329436
ZMAT1	0.777167739	0.693051877	0.703042171	0.843411019	0.952172109	0.935937948
GAK	0.777167739	0.62584388	0.939683463	0.86288354	0.954501767	0.673471687
LIMS1	0.777167739	0.747499345	0.715979316	0.925528817	0.96532164	0.763746661
CSRP1	0.777167739	0.726229532	0.775711284	0.825933581	0.972048096	0.840150874
ANKZF1	0.777167739	0.619184305	0.922161207	0.846115604	0.975291792	0.722309684
LOC112446757	0.777167739	0.610559852	0.757787642	0.846115604	0.99989309	0.671720185
TCF3	0.777167739	0.63203115	0.786602178	0.846715063	0.99989309	0.682748733
DHX30	0.777167739	0.655438106	0.788848487	0.87630412	0.99989309	0.720353522
MCOLN3	0.777196827	0.700796351	0.765074242	0.946344632	0.809298212	0.840150874
TOMM7	0.777196827	0.651481516	0.850339392	0.915749182	0.986955873	0.66854638
NINL	0.777559047	0.59054411	0.947268948	0.87630412	0.960835287	0.671720185
NAV3	0.777635446	0.695850654	0.868936984	0.958879502	0.838889011	0.66854638
FGFR1OP	0.777635446	0.913075885	0.719216596	0.86288354	0.981938411	0.671528522
FAM160B2	0.777636841	0.610266204	0.726668529	0.91329897	0.99989309	0.701784819
LOC112444314	0.777692089	0.600050072	0.850020542	0.820228145	0.809298212	0.961336964
ADAM1A	0.777692089	0.625663019	0.879968243	0.959727203	0.839134872	0.722309684
CD40	0.777733316	0.653112351	0.724199536	0.958879502	0.87465791	0.868108836
SPART	0.777733316	0.7116845	0.768676249	0.934591536	0.886279726	0.780615615
EPHA3	0.777733316	0.629715977	0.709561629	0.89088601	0.915155902	0.95579175
VLDLR	0.777733316	0.600960324	0.878603452	0.835254768	0.99989309	0.720716439
LOC104974272	0.777733316	0.661476635	0.758406163	0.846115604	0.99989309	0.845024318
MAP2K4	0.777756208	0.62522808	0.994425789	0.844510397	0.838889011	0.671528522
MRPL41	0.777756208	0.72477102	0.744499269	0.850828215	0.99989309	0.701868607
MOB3C	0.778555104	0.688955241	0.747093618	0.90419464	0.99989309	0.737313924
ADCK2	0.778628853	0.625663019	0.757235816	0.92064557	0.964521207	0.832189969
SAMSN1	0.778628853	0.684832051	0.726668529	0.915749182	0.99989309	0.753138357
TMEM128	0.778860907	0.648099013	0.87146798	0.943323364	0.880265141	0.710580013
UFM1	0.778861113	0.629956157	0.830022042	0.846715063	0.819670067	0.941925704
FCF1	0.778861113	0.639901253	0.962754771	0.891781866	0.881823169	0.66854638
OSGIN2	0.778886545	0.615754699	0.84207783	0.999939566	0.8165034	0.681177433
LOC514257	0.778886545	0.820796062	0.755534187	0.87452213	0.939361296	0.77208617
RNF115	0.778886545	0.646924712	0.806825406	0.990059245	0.941064037	0.671720185
TRMT10B	0.778886545	0.609199122	0.779087149	0.843512648	0.952672789	0.922709797
HSPG2	0.778989069	0.593314312	0.765074242	0.999939566	0.912507538	0.66915423
TRIM24	0.778989069	0.786188116	0.713802232	0.956154344	0.940638859	0.717273603
SULT1A1	0.778989069	0.651481516	0.707441133	0.995800463	0.941064037	0.77208617

MYADM	0.778989069	0.617454372	0.941593855	0.825933581	0.946536678	0.765600997
NDUFB2	0.778989069	0.703437628	0.747084388	0.891413337	0.99989309	0.699838787
SLC6A1	0.779012442	0.693153864	0.751496241	0.956154344	0.818457184	0.849769587
EPRS	0.779012442	0.619005993	0.757235816	0.825933581	0.89629383	0.979792753
GABRE	0.779012442	0.647660219	0.965750046	0.825933581	0.926794253	0.712413606
LOC112446663	0.779012442	0.663180847	0.747093618	0.915749182	0.961835052	0.833139817
SCAF11	0.779012442	0.760547392	0.744490351	0.82652165	0.99989309	0.671398444
NDUFS5	0.779012442	0.701370638	0.733993364	0.875889425	0.99989309	0.671528522
COQ7	0.779012442	0.737374533	0.765074242	0.90419464	0.99989309	0.671528522
LOC101905887	0.779012442	0.693153864	0.712526829	0.841828571	0.99989309	0.701784819
KDM5B	0.779012442	0.613737651	0.735054113	0.948120268	0.99989309	0.715023149
PKNOX2	0.779012442	0.704817952	0.709561629	0.90419464	0.99989309	0.777494305
IRAK1	0.779179328	0.704817952	0.863172962	0.86288354	0.87483668	0.808138656
COX8A	0.779179328	0.63203115	0.738217747	0.868090726	0.99989309	0.66915423
LOC100847345	0.77928902	0.831242188	0.747084388	0.848268459	0.875644404	0.856870625
WDR27	0.77928902	0.695850654	0.814794516	0.841828571	0.881823169	0.893961809
KCNG3	0.77928902	0.625663019	0.726668529	0.999939566	0.934859184	0.671133446
SOS1	0.77928902	0.707140461	0.788938503	0.825933581	0.99989309	0.677383279
GGA2	0.779444219	0.747899938	0.861838588	0.825933581	0.84234617	0.840150874
LMCD1	0.779595625	0.704817952	0.724042354	0.87630412	0.955392067	0.882322567
LOC112446689	0.779754548	0.661476635	0.726668529	0.940779565	0.99989309	0.66854638
TUBGCP5	0.779808017	0.600538394	0.879515214	0.996601241	0.814605912	0.699838787
PALM3	0.779808017	0.710028977	0.849077738	0.936573639	0.83417924	0.742259651
LOC112443751	0.779808017	0.604683229	0.882898206	0.92064557	0.845171429	0.808138656
CDC42	0.779808017	0.595931157	0.930262306	0.915749182	0.861762366	0.770391484
MINPP1	0.779808017	0.77414098	0.726668529	0.825933581	0.934859184	0.899551847
DYM	0.779808017	0.737937244	0.827125538	0.915749182	0.941107925	0.699680053
TAGAP	0.779808017	0.684832051	0.733231491	0.86288354	0.952172109	0.911284706
LOC112448014	0.779808017	0.62584388	0.827125538	0.928836449	0.978272166	0.728276429
ROM1	0.779808017	0.619374114	0.842478086	0.90419464	0.99989309	0.66915423
SNAPC5	0.779808017	0.651481516	0.744490351	0.89088601	0.99989309	0.703721528
PIAS2	0.779808017	0.666610276	0.733993364	0.914402649	0.99989309	0.707534696
ZNF446	0.779808017	0.688099473	0.733487658	0.865118174	0.99989309	0.728276429
TPI1	0.779808017	0.730838712	0.709561629	0.825933581	0.99989309	0.849772722
CRIP2	0.779808017	0.600050072	0.738217747	0.861272209	0.99989309	0.853233759
LOC100295797	0.779953915	0.707140461	0.803398613	0.915749182	0.819670067	0.858042054
GNG11	0.779953915	0.742298467	0.741982235	0.925931623	0.849382765	0.846367069
GMCL1	0.779953915	0.688099473	0.721561325	0.996601241	0.934859184	0.749691032

ACSS2	0.779953915	0.640962756	0.726668529	0.825933581	0.99989309	0.823435077
AGMO	0.780117664	0.665563964	0.747084388	0.999939566	0.819670067	0.681177433
GNAI1	0.780117664	0.802441232	0.709210234	0.971220023	0.857710467	0.770391484
LOC101905167	0.780117664	0.670188815	0.918378682	0.915749182	0.881823169	0.68555259
EIF2B1	0.780117664	0.665524216	0.751496241	0.886614433	0.933637471	0.908939618
LOC112444355	0.780117664	0.629704189	0.739676418	0.846715063	0.99989309	0.694702496
DYNLRB1	0.780117664	0.63512774	0.806825406	0.90419464	0.99989309	0.700756592
ADAT2	0.780117664	0.842797231	0.764481234	0.828096959	0.99989309	0.705712189
WDR77	0.780161183	0.684164826	0.748016329	0.925931623	0.819670067	0.908939618
NFKB2	0.780522655	0.710155842	0.713802232	0.915749182	0.99989309	0.671528522
CCDC47	0.780776808	0.655710881	0.814705151	0.925931623	0.87483668	0.827241584
HK1	0.780776808	0.687403165	0.788848487	0.972489653	0.943901678	0.684902922
AMMECR1L	0.780776808	0.655710881	0.751496241	0.867865861	0.99989309	0.673339985
EDF1	0.780776808	0.688099473	0.747084388	0.90419464	0.99989309	0.742259651
CACYBP	0.780776808	0.642501337	0.758987377	0.891273781	0.99989309	0.752900996
ALDH5A1	0.780776808	0.703437628	0.726668529	0.923832787	0.99989309	0.752900996
NUDCD1	0.780776808	0.63203115	0.788848487	0.908804713	0.99989309	0.753138357
TMEM108	0.781097287	0.881079118	0.778658634	0.90419464	0.819670067	0.764546301
NANOS1	0.781097287	0.599807217	0.757787642	0.999939566	0.87465791	0.803682823
POLR3G	0.781097287	0.615179529	0.753656036	0.936573639	0.889402797	0.888617204
IRAK4	0.781097287	0.600960324	0.909511376	0.915749182	0.930303931	0.734664544
STAMBP	0.781097287	0.603555122	0.770051055	0.956154344	0.954501767	0.797730509
IMP4	0.781097287	0.72843033	0.735785958	0.825933581	0.960835287	0.908785004
LOC101902570	0.781097287	0.767950704	0.883274745	0.828524115	0.96532164	0.671528522
STAC2	0.781097287	0.760918029	0.747084388	0.903607716	0.99989309	0.683895124
PEMT	0.781097287	0.785383224	0.726668529	0.90419464	0.99989309	0.684177013
RHOT1	0.781097287	0.750745995	0.781294783	0.850465555	0.99989309	0.694702496
ROGDI	0.781097287	0.665563964	0.801865674	0.841338807	0.99989309	0.701784819
PSMC3IP	0.781235782	0.707140461	0.836294915	0.92064557	0.821928612	0.799314198
LOC112446456	0.781235782	0.688099473	0.937270184	0.88747242	0.84234617	0.752647234
CDK15	0.781235782	0.686014664	0.982659943	0.843512648	0.846122929	0.683895124
CDH11	0.781235782	0.665803806	0.836294915	0.990571725	0.86558233	0.696365784
RNF112	0.781235782	0.665563964	0.855743966	0.924489924	0.932377891	0.728959145
GDE1	0.781235782	0.788961546	0.775597966	0.915749182	0.943768595	0.706354992
MTUS2	0.781235782	0.63203115	0.787740344	0.922782374	0.99989309	0.701784819
RNF2	0.781235782	0.747499345	0.726668529	0.915749182	0.99989309	0.731709303
LOC101902366	0.781262921	0.629715977	0.874092011	0.990571725	0.840559272	0.699838787
RRP9	0.781262921	0.752588921	0.788848487	0.850828215	0.846122929	0.886992214

NOC2L	0.781262921	0.769313383	0.712526829	0.86288354	0.899234751	0.908939618
NOL6	0.781477095	0.665803806	0.812043145	0.838800638	0.837323996	0.950199992
LOC100335822	0.781477095	0.6188407	0.793078777	0.925931623	0.846122929	0.887248119
GNA15	0.781477095	0.930225567	0.726668529	0.888081401	0.866920896	0.768633933
C14H8orf37	0.781477095	0.921280256	0.747806937	0.87630412	0.899048045	0.740271903
PSD4	0.781477095	0.707383119	0.733993364	0.983569039	0.91011204	0.770391484
CFAP20	0.781477095	0.616586256	0.939091808	0.893831952	0.954501767	0.686482558
AQP3	0.781477095	0.625663019	0.825888497	0.919247117	0.982577194	0.761020046
MSRA	0.781477095	0.730838712	0.733993364	0.92064557	0.99989309	0.66854638
FGD3	0.781477095	0.642501337	0.765074242	0.825933581	0.99989309	0.837798115
PACS2	0.781477095	0.609819443	0.712526829	0.903607716	0.99989309	0.838324968
WNT5A	0.781952322	0.670188815	0.817256644	0.999939566	0.819670067	0.673339985
LOC104975673	0.782575154	0.687403165	0.882898206	0.829382062	0.990560223	0.752358594
PPP1CA	0.782575154	0.659488126	0.808228186	0.843411019	0.99989309	0.773402056
FITM1	0.782575154	0.636332133	0.763592007	0.825933581	0.99989309	0.90175726
NDUFB8	0.782822831	0.629715977	0.726668529	0.915749182	0.99989309	0.704974208
NR1H2	0.782861261	0.6663481	0.747084388	0.992426863	0.819670067	0.846367069
LOC112449102	0.782861261	0.809783026	0.817256644	0.835232007	0.83417924	0.86252043
YPEL3	0.782861261	0.617694439	0.712526829	0.841828571	0.85995837	0.998636142
SZT2	0.782861261	0.600050072	0.772319988	0.946319248	0.954501767	0.828376685
MEF2D	0.782861261	0.632304442	0.757235816	0.850828215	0.975291792	0.908939618
PLPPR4	0.782861261	0.615879066	0.748723724	0.999939566	0.982014614	0.682748733
ZPR1	0.782861261	0.598654687	0.787015778	0.992137653	0.988693274	0.694702496
SERF2	0.782861261	0.6861152	0.763592007	0.90419464	0.99989309	0.722689871
ZNF618	0.782861261	0.63512774	0.746686237	0.908054165	0.99989309	0.723641471
CCNY	0.783454088	0.637634861	0.871859013	0.948120268	0.819670067	0.783479395
MCM6	0.783454088	0.625663019	0.712526829	0.999939566	0.838993207	0.834550008
AKIRIN1	0.783454088	0.724056735	0.850243032	0.919247117	0.843348001	0.765527601
CHRM1	0.783454088	0.641720043	0.880277219	0.990571725	0.846122929	0.671720185
DCP1B	0.783454088	0.670978167	0.866834495	0.866960502	0.846122929	0.869171706
NFATC1	0.783454088	0.637634861	0.777178978	0.999939566	0.8594734	0.717273603
CD96	0.783454088	0.655149112	0.744490351	0.952024081	0.86122464	0.884387948
NPNT	0.783454088	0.838815395	0.744369186	0.916305829	0.87465791	0.780615615
PPP4R3B	0.783454088	0.651279405	0.805580394	0.939292364	0.87483668	0.839419627
LOC101907803	0.783454088	0.665803806	0.726668529	0.999939566	0.882754591	0.728276429
SELENON	0.783454088	0.63203115	0.81285201	0.995118128	0.899048045	0.74008849
MMP25	0.783454088	0.771765977	0.788848487	0.908496281	0.926794253	0.770391484
PRDX4	0.783454088	0.600050072	0.831320191	0.996084773	0.927804504	0.697857798

PRSS53	0.783454088	0.633706991	0.845425011	0.89088601	0.934379881	0.843733465
KANK2	0.783454088	0.671906999	0.721561325	0.999939566	0.934859184	0.66915423
REEP3	0.783454088	0.6608873	0.921225977	0.913413181	0.941107925	0.66854638
LOC101906818	0.783454088	0.628127963	0.832483532	0.951929772	0.943768595	0.747674417
OGFRL1	0.783454088	0.603474998	0.868861706	0.925901365	0.952172109	0.755204495
STK17B	0.783454088	0.675810425	0.720067907	0.873519404	0.952172109	0.934775196
RYR2	0.783454088	0.731268229	0.751496241	0.86288354	0.954501767	0.86307729
LOC101905876	0.783454088	0.635815471	0.777178978	0.90419464	0.956624006	0.866253786
ADD3	0.783454088	0.612773311	0.93050447	0.847774989	0.957605284	0.764546301
TOR1AIP2	0.783454088	0.639901253	0.777178978	0.959786126	0.961382403	0.754339777
FAM13C	0.783454088	0.652876871	0.712526829	0.983971658	0.975038198	0.787474205
SRRM1	0.783454088	0.619184305	0.851867246	0.915749182	0.98152792	0.747829293
DTYMK	0.783454088	0.688099473	0.712526829	0.956154344	0.99989309	0.66854638
SMIM11A	0.783454088	0.703437628	0.802847755	0.90419464	0.99989309	0.682369514
FOXJ3	0.783454088	0.692547228	0.777178978	0.838371152	0.99989309	0.692674475
PPFIA2	0.783454088	0.636332133	0.823456027	0.90419464	0.99989309	0.692802214
ARMH3	0.783454088	0.603474998	0.775711284	0.92064557	0.99989309	0.696365784
TOPBP1	0.783454088	0.63146711	0.747093618	0.850465555	0.99989309	0.701907624
CYC1	0.783454088	0.668175004	0.712526829	0.826315685	0.99989309	0.705712189
MFGE8	0.783454088	0.629715977	0.764057751	0.846115604	0.99989309	0.71963823
ZDHHC18	0.783454088	0.63203115	0.73016804	0.915749182	0.99989309	0.742259651
MRPL27	0.783454088	0.693051877	0.775786014	0.883885658	0.99989309	0.745987575
DHX15	0.783454088	0.693153864	0.73016804	0.841828571	0.99989309	0.763656348
SLC16A5	0.783454088	0.617063658	0.776954726	0.848158235	0.99989309	0.764546301
PAPOLA	0.783454088	0.63203115	0.752037298	0.909743798	0.99989309	0.77208617
BUD23	0.783454088	0.689053394	0.722352424	0.88501388	0.99989309	0.846367069
REPIN1	0.783454088	0.601662433	0.726668529	0.891273781	0.99989309	0.908939618
LOC101906526	0.783967886	0.914162656	0.74784538	0.846715063	0.910770324	0.770484731
TMEM173	0.784823266	0.625009304	0.847037539	0.995118128	0.819670067	0.761409946
ALPK1	0.784823266	0.655710881	0.849077738	0.931989854	0.934859184	0.742259651
ZFR	0.784823266	0.629715977	0.788848487	0.942235206	0.972917711	0.77208617
LOC104974345	0.784823266	0.685446639	0.750421005	0.850828215	0.99989309	0.742451714
SPTLC2	0.785327499	0.7164915	0.763858703	0.87630412	0.999592263	0.812509877
JADE3	0.785327499	0.739753419	0.807113974	0.853475385	0.99989309	0.712710718
LOC789337	0.785494535	0.6608873	0.751257888	0.865046898	0.99989309	0.750665174
LRRN2	0.785695107	0.637634861	0.899195818	0.915749182	0.832446464	0.803879331
TMEM198	0.785695107	0.81808696	0.757235816	0.925901365	0.857710467	0.778672431
PTH1R	0.785695107	0.665524216	0.825888497	0.990049248	0.87483668	0.744571654

C18H16orf46	0.785695107	0.648099013	0.839833549	0.849183739	0.910770324	0.887248119
SHROOM2	0.785695107	0.619374114	0.810425784	0.958879502	0.975291792	0.732184848
CAMK2N1	0.787010935	0.747899938	0.76145258	0.888642721	0.99989309	0.770426407
DLAT	0.787010935	0.650344958	0.751496241	0.850828215	0.99989309	0.839340243
NOL12	0.787060793	0.724333213	0.761854021	0.943323364	0.836838903	0.846367069
PTPRE	0.787060793	0.619184305	0.736386431	0.939027148	0.846122929	0.940683751
GALNT12	0.787060793	0.88311684	0.825888497	0.892229846	0.880531631	0.684595958
MGC157082	0.787060793	0.600960324	0.868936984	0.841828571	0.926480902	0.905220694
DCTD	0.787060793	0.601692739	0.751496241	0.828394676	0.975291792	0.959625205
REV1	0.787060793	0.722790338	0.769528869	0.836785013	0.99989309	0.75292536
LOC112444633	0.787204173	0.978215444	0.806221631	0.841828571	0.84234617	0.671528522
HSD17B14	0.787204173	0.747555258	0.929303679	0.868417977	0.846122929	0.728700419
LOC112448808	0.787204173	0.616586256	0.890264995	0.924489924	0.87465791	0.78965579
ZDHHC7	0.787204173	0.642501337	0.793430641	0.994652359	0.896111613	0.76360318
TMCO1	0.787204173	0.685777004	0.755534187	0.846715063	0.904151325	0.946717155
MAPK9	0.787204173	0.868963165	0.726668529	0.87630412	0.939361296	0.799314198
ASF1B	0.787204173	0.665524216	0.726668529	0.992829869	0.99989309	0.692674475
KDM2A	0.787204173	0.703437628	0.780363715	0.915749182	0.99989309	0.723028401
BTBD10	0.787204173	0.755397146	0.787740344	0.842538664	0.99989309	0.728567358
MIEF1	0.787204173	0.605333364	0.788938503	0.90419464	0.99989309	0.849772722
TESK2	0.787246686	0.636332133	0.726668529	0.883398728	0.883643824	0.980346257
HPGDS	0.787246686	0.668833349	0.776954726	0.912830144	0.910393751	0.882699301
SAFB2	0.787246686	0.601801243	0.834793268	0.846715063	0.99989309	0.691363988
ATP6V1B2	0.787285636	0.608346598	0.803771265	0.999939566	0.846122929	0.770391484
LOC112444896	0.78741467	0.680267573	0.965518535	0.89088601	0.846122929	0.704011905
NRBP2	0.78741467	0.694355537	0.891014698	0.846715063	0.96532164	0.738152854
LOC107131703	0.78741467	0.7116845	0.759671935	0.90419464	0.99989309	0.671528522
AEN	0.78741467	0.651481516	0.788938503	0.915749182	0.99989309	0.76360318
CAVIN1	0.787671173	0.642501337	0.859931483	0.922713162	0.833004048	0.844335246
ILK	0.787671173	0.636332133	0.827125538	0.913413181	0.899048045	0.866253786
RHOT2	0.787671173	0.780290359	0.897144948	0.846115604	0.902843706	0.729817953
LOC112446798	0.787671173	0.603785862	0.858674427	0.91329897	0.904995537	0.849772722
SOX6	0.787671173	0.786188116	0.829843435	0.91329897	0.927804504	0.710319037
MDFIC2	0.787671173	0.686122011	0.713802232	0.825933581	0.968477804	0.95543
OARD1	0.787671173	0.854362191	0.74784538	0.84302258	0.99989309	0.671528522
VPS9D1	0.787671173	0.610559852	0.808228186	0.986710389	0.99989309	0.672755858
PARD3	0.787671173	0.674771172	0.726668529	0.837398381	0.99989309	0.682748733
G3BP1	0.787671173	0.632709306	0.882890706	0.858021281	0.99989309	0.752857325

SLC15A2	0.787671173	0.7116845	0.726668529	0.90419464	0.99989309	0.775116427
BRF2	0.787671173	0.619184305	0.850092739	0.846715063	0.99989309	0.788142857
ATAD2B	0.788471704	0.651481516	0.750421005	0.913413181	0.99989309	0.696709089
PARD3B	0.788947979	0.62584388	0.788848487	0.86288354	0.99989309	0.840150874
PRMT5	0.789170324	0.629715977	0.733776767	0.850828215	0.99989309	0.77208617
LRRC71	0.789434268	0.775921167	0.744490351	0.919247117	0.8594734	0.859447707
GTPBP3	0.789461858	0.665803806	0.93710335	0.868090726	0.952172109	0.705712189
LOC101906235	0.789655213	0.76090129	0.891455327	0.915749182	0.846342589	0.691363988
CEP250	0.789655213	0.637203338	0.933963997	0.922713162	0.849382765	0.742259651
GNA14	0.789655213	0.743422331	0.777638604	0.925775859	0.859775628	0.829389486
VSIG4	0.789655213	0.716304797	0.812043145	0.915749182	0.943768595	0.770391484
ENKUR	0.789655213	0.692987203	0.783875251	0.91329897	0.982014614	0.792329436
TTC32	0.789655213	0.601801243	0.812770091	0.936573639	0.99989309	0.691363988
PODN	0.789655213	0.670324428	0.777178978	0.841828571	0.99989309	0.752900996
LOC100847304	0.789655213	0.699626135	0.759671935	0.86950142	0.99989309	0.812208548
ARPC4	0.789919551	0.706231276	0.869529186	0.888642721	0.84234617	0.840150874
SUV39H2	0.789919551	0.632709306	0.764481234	0.91888654	0.954501767	0.865926817
KCNQ1	0.789919551	0.608346598	0.811009079	0.846115604	0.99989309	0.788131005
DUSP3	0.790075842	0.831242188	0.891051885	0.834221775	0.846122929	0.763746661
LOC782456	0.790075842	0.820000472	0.751496241	0.928836449	0.846122929	0.800008791
DNAJA2	0.790075842	0.617063658	0.879215964	0.943323364	0.853884483	0.792329436
NYAP1	0.790075842	0.647660219	0.916702	0.908054165	0.881823169	0.777494305
GPALPP1	0.790075842	0.827609894	0.829843435	0.915749182	0.899048045	0.671720185
ESS2	0.790075842	0.718469663	0.758406163	0.841338807	0.906139319	0.940673472
PDE1A	0.790075842	0.688099473	0.806825406	0.946344632	0.91011204	0.77208617
LOC112444479	0.790075842	0.690829991	0.751491252	0.869637097	0.932197133	0.926872824
BANK1	0.790075842	0.607716533	0.726668529	0.995118128	0.952172109	0.833155047
RNF19A	0.790075842	0.692987203	0.78256495	0.936763538	0.972917711	0.752900996
THUMPD3	0.790075842	0.646924712	0.735054113	0.915749182	0.988693274	0.868430302
DDR1	0.790075842	0.608346598	0.747093618	0.917869295	0.996250445	0.868430302
PPID	0.790075842	0.616586256	0.719216596	0.91329897	0.99989309	0.694702496
SLC5A3	0.790075842	0.613737651	0.757235816	0.915749182	0.99989309	0.752857325
LIME1	0.790075842	0.623482618	0.768676249	0.846715063	0.99989309	0.770391484
MMAA	0.790075842	0.625663019	0.751496241	0.846115604	0.99989309	0.844335246
CFAP44	0.790304609	0.61754698	0.851617203	0.87630412	0.857710467	0.914440253
CEP55	0.790304609	0.758051794	0.788848487	0.988915501	0.882754591	0.692674475
LOC101906408	0.790304609	0.659488126	0.879968243	0.873519404	0.910770324	0.832189969
HRH4	0.790304609	0.63203115	0.843427302	0.915749182	0.912512577	0.844335246

SLC46A1	0.790304609	0.655710881	0.773281026	0.88747242	0.934859184	0.912101047
POLG2	0.790304609	0.63203115	0.846444468	0.965097245	0.952172109	0.701784819
MTMR1	0.790304609	0.606614518	0.726668529	0.999939566	0.952172109	0.75292536
GNL2	0.790304609	0.681764999	0.817256644	0.925931623	0.952172109	0.770391484
ASL	0.790304609	0.747164473	0.738217747	0.915749182	0.954501767	0.813741671
MGC126945	0.790304609	0.737937244	0.750089824	0.915749182	0.99989309	0.684905734
YWHAH	0.790304609	0.655710881	0.743146522	0.946344632	0.99989309	0.742259651
LOC112444926	0.79052169	0.71282706	0.816249708	0.846715063	0.853884483	0.912376736
NFRKB	0.79052169	0.693454767	0.894874079	0.875093568	0.933637471	0.764546301
PNMA8B	0.79052169	0.692547228	0.812770091	0.934591536	0.964150118	0.735313642
STAC	0.79052169	0.655710881	0.755534187	0.969717497	0.99989309	0.740271903
PRPSAP1	0.791404333	0.665803806	0.754101752	0.99879985	0.83417924	0.835752988
SCHIP1	0.791404333	0.769313383	0.777638604	0.854497387	0.849382765	0.90843033
FAM186B	0.791404333	0.776972642	0.858972712	0.87630412	0.857710467	0.792329436
GFI1	0.791404333	0.851167599	0.777178978	0.88747242	0.890942773	0.790039686
PRPF18	0.791404333	0.619374114	0.769285605	0.99879985	0.904151325	0.785275816
LRRN4	0.791404333	0.653341013	0.939683463	0.910808102	0.905719399	0.709387613
ADPGK	0.791404333	0.632709306	0.810425784	0.917869295	0.926899298	0.86143104
DST	0.791404333	0.701581467	0.757780111	0.982367414	0.932081893	0.770391484
NSA2	0.791404333	0.629715977	0.84311048	0.959727203	0.934859184	0.756815771
PDE1C	0.791404333	0.821588096	0.812770091	0.908496281	0.939994656	0.707492534
NMT1	0.791404333	0.653341013	0.764057751	0.851394268	0.947146038	0.939507593
BDKRB2	0.791404333	0.89272885	0.720067907	0.90419464	0.947826027	0.764546301
RHOBTB2	0.791404333	0.693281931	0.777178978	0.925265292	0.954501767	0.798051736
TXN	0.791404333	0.755940768	0.757787642	0.850828215	0.955813674	0.86307729
STAR	0.791404333	0.755210284	0.777638604	0.915749182	0.957605284	0.763746661
ENO4	0.791404333	0.623400308	0.765074242	0.87452213	0.96532164	0.924845138
LOC112447857	0.791404333	0.646924712	0.747093618	0.992368879	0.966405324	0.766795378
C18H19orf18	0.791404333	0.609819443	0.764057751	0.915749182	0.997981612	0.868108836
PSMB10	0.791404333	0.625663019	0.820549834	0.919247117	0.998276725	0.773957499
PKD1L2	0.791404333	0.750546265	0.747084388	0.915749182	0.99989309	0.678496087
PTPN13	0.791404333	0.619005993	0.870054336	0.88747242	0.99989309	0.692802214
ABCC1	0.791404333	0.693153864	0.757235816	0.835254768	0.99989309	0.694840199
BTBD7	0.791404333	0.74214695	0.777178978	0.883885658	0.99989309	0.715285936
ZMYM5	0.791404333	0.677470151	0.757787642	0.868090726	0.99989309	0.770391484
IPCEF1	0.791404333	0.716302962	0.717447819	0.843411019	0.99989309	0.783479395
RAPGEF4	0.791404333	0.688099473	0.825960826	0.841828571	0.99989309	0.784608137
RAB3A	0.791404333	0.617063658	0.787740344	0.925004412	0.99989309	0.785275816

DCAKD	0.791404333	0.7116845	0.726668529	0.87452213	0.99989309	0.833139817
PSMD9	0.792145214	0.704923066	0.817256644	0.915749182	0.99989309	0.705712189
AOAH	0.792571725	0.604295899	0.765074242	0.830877858	0.996250445	0.945759045
ZNF770	0.792571725	0.608346598	0.753656036	0.888642721	0.99989309	0.874335327
LOC101902346	0.79283216	0.742728851	0.882898206	0.861272209	0.968795608	0.694702496
LOC112442307	0.792882615	0.60970017	0.81747245	0.956154344	0.926899298	0.816194685
LOC112444726	0.792882615	0.626773146	0.729587161	0.86950142	0.99989309	0.764546301
TMPRSS6	0.792978648	0.912292519	0.827125538	0.843411019	0.934379881	0.690181038
NDUFB1	0.792978648	0.703437628	0.755362619	0.837066215	0.99989309	0.692802214
UQCRC1	0.793288004	0.617454372	0.744490351	0.865118174	0.99989309	0.742259651
SERPINE1	0.793521237	0.655438106	0.817256644	0.928623745	0.962258842	0.77208617
CEP164	0.793558522	0.637634861	0.846650632	0.933991524	0.899048045	0.808631013
LOC101903758	0.793558522	0.710588617	0.773281026	0.917869295	0.99989309	0.682748733
RNH1	0.793558522	0.72477102	0.751496241	0.846115604	0.99989309	0.74008849
MECR	0.793558522	0.653408806	0.73016804	0.904990622	0.99989309	0.85703477
SELENOI	0.793920632	0.637203338	0.979096894	0.915749182	0.833084402	0.678496087
SFSWAP	0.793920632	0.60970017	0.886481882	0.915749182	0.943768595	0.788142857
NUDT17	0.793920632	0.669648359	0.787740344	0.908496281	0.975782017	0.840150874
LOC112444931	0.793920632	0.651481516	0.817256644	0.846715063	0.99989309	0.753138357
LOC781197	0.793920632	0.7861604	0.73016804	0.864540217	0.99989309	0.815459921
TMEM104	0.793920632	0.722790338	0.759671935	0.843411019	0.99989309	0.824410712
ZUP1	0.793920632	0.6608873	0.797593435	0.87630412	0.99989309	0.829389486
HTR6	0.793922017	0.629715977	0.812043145	0.858274325	0.99989309	0.728276429
SORL1	0.793982744	0.668705903	0.751496241	0.999939566	0.933637471	0.688426764
VARS	0.794310028	0.642439727	0.750089824	0.843411019	0.860908683	0.994307746
BREH1	0.794310028	0.686014664	0.743369461	0.999939566	0.883643824	0.701784819
CRBN	0.794310028	0.670729787	0.751496241	0.999939566	0.954501767	0.683474857
MRPS16	0.794310028	0.764525656	0.750089824	0.843411019	0.99989309	0.738110854
IL10RA	0.794310028	0.7116845	0.735054113	0.843411019	0.99989309	0.838324968
ADARB1	0.79461984	0.612450407	0.765074242	0.999939566	0.889271428	0.764546301
PUSL1	0.794705446	0.693544464	0.726668529	0.883885658	0.86558233	0.971219102
CERCAM	0.794705446	0.63512774	0.812043145	0.994309225	0.880572558	0.784671496
LOC112448582	0.794705446	0.693153864	0.73016804	0.87630412	0.952172109	0.930707319
KCNK3	0.794705446	0.838815395	0.788848487	0.860663136	0.975291792	0.742259651
LOC112448744	0.794705446	0.63203115	0.756911784	0.83476264	0.996250445	0.943138585
SELENOT	0.794760941	0.632709306	0.765074242	0.850828215	0.875418728	0.983427394
CORO2B	0.794760941	0.822988334	0.765074242	0.915749182	0.952172109	0.737883632
LOC100848148	0.795861823	0.71492723	0.817256644	0.915749182	0.996250445	0.738110854

EFNB1	0.795957589	0.694355537	0.836294915	0.982140778	0.84234617	0.76360318
PGBD5	0.795957589	0.973158835	0.773779465	0.840664471	0.84234617	0.764546301
CYP1A1	0.795957589	0.746081288	0.735174555	0.999939566	0.872085861	0.735313642
EIF4E2	0.795957589	0.688099473	0.902548377	0.86950142	0.881823169	0.821467423
GDPD3	0.795957589	0.686576166	0.827125538	0.913413181	0.949535684	0.812509877
ALG5	0.795957589	0.7116845	0.76749973	0.850828215	0.98152792	0.866253786
RPA3	0.795957589	0.66339516	0.757235816	0.995800463	0.99989309	0.681177433
PDE1B	0.795957589	0.692987203	0.747084388	0.850828215	0.99989309	0.740906006
MRPL21	0.795957589	0.693153864	0.754897816	0.842538664	0.99989309	0.763746661
S1PR4	0.796181715	0.637634861	0.755362619	0.841828571	0.99989309	0.908785004
LOC112441481	0.796732724	0.635815471	0.827186499	0.915749182	0.99989309	0.704011905
C25H7orf50	0.796761627	0.72843033	0.751496241	0.958879502	0.941064037	0.77208617
CXHXorf56	0.796761627	0.629715977	0.747700432	0.935763381	0.99989309	0.738152854
ETFBKMT	0.796807581	0.755866871	0.919108971	0.847813982	0.886279726	0.76360318
SFN	0.796807581	0.68147929	0.832483532	0.86950142	0.962262333	0.840150874
MAGEH1	0.796807581	0.883267666	0.751496241	0.850828215	0.99989309	0.682369514
SLC22A16	0.797001734	0.825215718	0.85122003	0.846115604	0.904151325	0.77208617
COMMD3	0.797001734	0.639901253	0.733231491	0.90419464	0.99989309	0.792329436
S100A12	0.797048707	0.665803806	0.74941488	0.982908486	0.99989309	0.747674417
SLC25A39	0.797048707	0.655619314	0.732087134	0.90419464	0.99989309	0.786737722
INSL6	0.797048707	0.680835681	0.752494129	0.850828215	0.99989309	0.844352265
LOC112442189	0.797438844	0.866675066	0.921420669	0.841828571	0.84102894	0.701784819
IL12RB1	0.797438844	0.611535729	0.979347606	0.868090726	0.857566272	0.77208617
LOC100141070	0.797438844	0.641416336	0.817993104	0.915749182	0.905719399	0.871129794
NUDCD3	0.797438844	0.687403165	0.843836079	0.936880771	0.943768595	0.746894056
NUMBL	0.797438844	0.686399081	0.812770091	0.868090726	0.99989309	0.694702496
FHDC1	0.797970487	0.637634861	0.773281026	0.999939566	0.874640903	0.701364325
PLK1	0.797970487	0.693544464	0.812770091	0.925901365	0.881823169	0.840150874
LOC101907944	0.797970487	0.751946504	0.744369186	0.99879985	0.926899298	0.697857798
SYNC	0.797970487	0.931105147	0.753279014	0.886726226	0.933637471	0.729037507
SREBF1	0.797970487	0.753368216	0.765074242	0.850828215	0.949645557	0.875853001
SELENOM	0.797970487	0.629715977	0.755362619	0.999939566	0.952172109	0.681177433
KIZ	0.797970487	0.767675287	0.747093618	0.846115604	0.99989309	0.714260956
TRAF7	0.797970487	0.72843033	0.845237542	0.843411019	0.99989309	0.757327233
LOC101902468	0.797970487	0.675748768	0.827125538	0.850828215	0.99989309	0.77208617
CCDC171	0.798388698	0.857034402	0.828827918	0.841828571	0.837323996	0.840150874
SYNPO2	0.798388698	0.911279338	0.807113974	0.843411019	0.838889011	0.826868779
ZNF550	0.798388698	0.6390296	0.993665588	0.87452213	0.84102894	0.725620954

NEK9	0.798388698	0.629715977	0.958420472	0.936573639	0.84234617	0.705712189
IGDCC4	0.798388698	0.90249621	0.757787642	0.95964071	0.846122929	0.683895124
LOC782938	0.798388698	0.693544464	0.911119875	0.947581168	0.846122929	0.704011905
LOC522610	0.798388698	0.803903736	0.788848487	0.939292364	0.846122929	0.787474205
LOC100847791	0.798388698	0.742728851	0.733484336	0.998542029	0.846122929	0.799314198
CHCHD7	0.798388698	0.691175262	0.926613927	0.908496281	0.849382765	0.78355885
LOC100848639	0.798388698	0.619005993	0.909170161	0.956154344	0.850081404	0.770391484
MNAT1	0.798388698	0.818806929	0.787693453	0.951929772	0.857710467	0.757327233
APEX1	0.798388698	0.7116845	0.750089824	0.883885658	0.857710467	0.961506391
PPP2R1B	0.798388698	0.749070017	0.751496241	0.992368879	0.868149867	0.77208617
APOBEC3H	0.798388698	0.689368188	0.748576908	0.994309225	0.874640903	0.839494796
SLC6A6	0.798388698	0.86764843	0.744499269	0.976950878	0.87465791	0.703914683
CNBP	0.798388698	0.612450407	0.92582321	0.958879502	0.87465791	0.732184848
SCLY	0.798388698	0.719478857	0.787740344	0.990571725	0.87465791	0.770391484
ARMC4	0.798388698	0.9136091	0.758406163	0.925931623	0.87483668	0.707492534
IQCN	0.798388698	0.632709306	0.812770091	0.883885658	0.87483668	0.952238962
FLOT1	0.798388698	0.703437628	0.777178978	0.915749182	0.883643824	0.891000792
HSPB8	0.798388698	0.851167599	0.827125538	0.873939476	0.885921829	0.777494305
LOC107131418	0.798388698	0.668175004	0.788848487	0.915749182	0.886068777	0.902660792
PFKFB3	0.798388698	0.742728851	0.91546474	0.913413181	0.899048045	0.690287753
PSMC1	0.798388698	0.704817952	0.77586539	0.940921143	0.899048045	0.840150874
TRPV4	0.798388698	0.68147929	0.858865175	0.930538015	0.902569851	0.778022404
LOC101905510	0.798388698	0.629704189	0.933629769	0.897446122	0.902843706	0.799993234
CACNA1B	0.798388698	0.958564557	0.735302276	0.910808102	0.904151325	0.706152771
ASNS	0.798388698	0.709451322	0.733231491	0.915749182	0.904151325	0.908939618
LAMA3	0.798388698	0.887725794	0.726668529	0.914862928	0.91011204	0.788829963
TNFRSF18	0.798388698	0.913175252	0.726668529	0.850828215	0.912365272	0.833139817
ANKS6	0.798388698	0.72477102	0.842605286	0.926078322	0.915496086	0.753138357
SLC25A43	0.798388698	0.632709306	0.79610462	0.995800463	0.925981327	0.770391484
XRCC4	0.798388698	0.884220302	0.753279014	0.850828215	0.934859184	0.813741671
CHMP3	0.798388698	0.655619314	0.882898206	0.956154344	0.939361296	0.697857798
LOC104972821	0.798388698	0.671602771	0.757337101	0.921761002	0.943768595	0.880404833
TAF5	0.798388698	0.939681897	0.765074242	0.87630412	0.946944212	0.684905734
SCO1	0.798388698	0.655710881	0.776954726	0.999939566	0.947652461	0.700618632
CPSF2	0.798388698	0.642501337	0.844065668	0.908054165	0.949645557	0.844335246
OFD1	0.798388698	0.806076007	0.878603452	0.873939476	0.952172109	0.683895124
OTUD5	0.798388698	0.690829991	0.808333956	0.915749182	0.954501767	0.826868779
PIK3C2B	0.798388698	0.688130143	0.74784538	0.99879985	0.954585906	0.723641471

MID1	0.798388698	0.769817928	0.740319216	0.990049248	0.957910867	0.707492534
INPP5D	0.798388698	0.651481516	0.752494129	0.915749182	0.962262333	0.88667499
LOC788599	0.798388698	0.751946504	0.761854021	0.842538664	0.964150118	0.893961809
ERBB2	0.798388698	0.739312113	0.747084388	0.868090726	0.964521207	0.888617204
ELN	0.798388698	0.631868333	0.751930447	0.999939566	0.96532164	0.705712189
LOC112442298	0.798388698	0.842797231	0.747084388	0.874189736	0.96532164	0.799147792
UCP2	0.798388698	0.685517925	0.820767684	0.925931623	0.972663468	0.770391484
PHTF1	0.798388698	0.670978167	0.765074242	0.998542029	0.980286089	0.701784819
ADCK1	0.798388698	0.655149112	0.850092739	0.88747242	0.981777076	0.811688797
LOC100848504	0.798388698	0.62522808	0.729587161	0.99879985	0.982577194	0.788142857
DIDO1	0.798388698	0.704817952	0.834793268	0.898433137	0.983935609	0.77208617
FFAR3	0.798388698	0.693153864	0.808228186	0.916305829	0.988693274	0.77208617
FAM131B	0.798388698	0.63203115	0.765074242	0.913413181	0.988693274	0.878040551
IER2	0.798388698	0.688099473	0.844065668	0.915749182	0.996250445	0.735394214
SEMA6A	0.798388698	0.738981601	0.726668529	0.928836449	0.998276725	0.792591216
SMDT1	0.798388698	0.691175262	0.79610462	0.915749182	0.99989309	0.682748733
CEMIP2	0.798388698	0.629715977	0.764057751	0.956154344	0.99989309	0.685789462
ATP5MPL	0.798388698	0.688099473	0.738217747	0.908054165	0.99989309	0.691103775
GOLGA4	0.798388698	0.626607857	0.776574729	0.908479268	0.99989309	0.694702496
EIF2AK3	0.798388698	0.686576166	0.726668529	0.924489924	0.99989309	0.694702496
HEXIM1	0.798388698	0.623400308	0.828407935	0.934591536	0.99989309	0.701784819
NDUFA13	0.798388698	0.665563964	0.726668529	0.893831952	0.99989309	0.704011905
ATP5MF	0.798388698	0.679258786	0.726668529	0.842538664	0.99989309	0.707492534
UBL4A	0.798388698	0.73244249	0.788848487	0.846715063	0.99989309	0.714123227
LEMD3	0.798388698	0.727981349	0.765074242	0.864540217	0.99989309	0.719551722
BEND3	0.798388698	0.656556642	0.806221631	0.8968247	0.99989309	0.722309684
TMEM80	0.798388698	0.615179529	0.850243032	0.893559455	0.99989309	0.725528824
LOC112447459	0.798388698	0.7116845	0.765074242	0.925004412	0.99989309	0.728276429
UBL5	0.798388698	0.669648359	0.845237542	0.915749182	0.99989309	0.738110854
RSF1	0.798388698	0.785163144	0.733231491	0.891273781	0.99989309	0.740271903
KAT14	0.798388698	0.625663019	0.774248983	0.942235206	0.99989309	0.752857325
MRPL51	0.798388698	0.623400308	0.788848487	0.870157143	0.99989309	0.752900996
NDUFC2	0.798388698	0.636332133	0.765074242	0.89088601	0.99989309	0.75292536
KIAA1958	0.798388698	0.659488126	0.726668529	0.855137785	0.99989309	0.7566392
PIGQ	0.798388698	0.621440227	0.747806937	0.846115604	0.99989309	0.764546301
BOD1L1	0.798388698	0.685980044	0.819664302	0.915749182	0.99989309	0.766359829
ANAPC11	0.798388698	0.80403784	0.750089824	0.850828215	0.99989309	0.770391484
LOC100848208	0.798388698	0.692987203	0.765074242	0.925004412	0.99989309	0.770391484

GYS1	0.798388698	0.72477102	0.738217747	0.846715063	0.99989309	0.77208617
PLEKHM2	0.798388698	0.677340516	0.726668529	0.915749182	0.99989309	0.77208617
PEAK1	0.798388698	0.624067943	0.765074242	0.915749182	0.99989309	0.777494305
POLR2E	0.798388698	0.702477628	0.757787642	0.87452213	0.99989309	0.778022404
DOCK10	0.798388698	0.636332133	0.750089824	0.915749182	0.99989309	0.783479395
UTRN	0.798388698	0.689368188	0.733231491	0.850828215	0.99989309	0.792329436
BRD2	0.798388698	0.68147929	0.782651196	0.90419464	0.99989309	0.808138656
PHLDA2	0.798388698	0.639676128	0.774385549	0.883885658	0.99989309	0.809650729
LOC101904121	0.798388698	0.722790338	0.735302276	0.91329897	0.99989309	0.809650729
FBXO32	0.798388698	0.685980044	0.757201498	0.863958691	0.99989309	0.823435077
FIBP	0.798388698	0.703437628	0.726668529	0.892229846	0.99989309	0.840150874
NOTCH3	0.798388698	0.62464979	0.726668529	0.946344632	0.99989309	0.840150874
KIF16B	0.798388698	0.63203115	0.735916174	0.922782374	0.99989309	0.856870625
CD84	0.798582069	0.731268229	0.843427302	0.925931623	0.908091868	0.76360318
TALDO1	0.798582069	0.71282706	0.726668529	0.915749182	0.99989309	0.728276429
RTRAF	0.798582069	0.629715977	0.775786014	0.91329897	0.99989309	0.752900996
TP63	0.798582069	0.705458146	0.750089824	0.92359825	0.99989309	0.798051736
TMEM186	0.798582069	0.642501337	0.744490351	0.846115604	0.99989309	0.891000792
RPP30	0.798803829	0.629715977	0.846650632	0.90419464	0.99989309	0.76935309
OXLD1	0.798935683	0.629715977	0.726668529	0.932656599	0.99989309	0.784080446
EFL1	0.799420767	0.644538289	0.743265842	0.957928699	0.99989309	0.77208617
ERMP1	0.801362722	0.72843033	0.726668529	0.943323364	0.954501767	0.844171661
SRRM2	0.801362722	0.655138935	0.808333956	0.919247117	0.996250445	0.80787758
LRP2BP	0.801362722	0.66621239	0.917876971	0.846115604	0.99989309	0.706152771
GDF1	0.801828329	0.685517925	0.828407935	0.999334425	0.857710467	0.740271903
TLCD2	0.801828329	0.753193121	0.868936984	0.943323364	0.87483668	0.712413606
LOC533597	0.801828329	0.629715977	0.845092405	0.999939566	0.87483668	0.740271903
ZFYVE9	0.801828329	0.794355325	0.827125538	0.928623745	0.886279726	0.752358594
CWF19L1	0.801828329	0.703437628	0.851617203	0.904990622	0.975291792	0.763746661
FAM169A	0.801828329	0.727892714	0.827125538	0.864540217	0.987694035	0.809650729
ARFRP1	0.801828329	0.736566631	0.726668529	0.90419464	0.99989309	0.833139817
MATN4	0.801828329	0.624067943	0.753656036	0.91329897	0.99989309	0.847256027
EXOSC4	0.801828329	0.769313383	0.731086678	0.842538664	0.99989309	0.863099382
IL3RA	0.801868931	0.704817952	0.845237542	0.925931623	0.846122929	0.830909703
ZNF354A	0.801868931	0.687113907	0.87214481	0.994309225	0.849825976	0.712710718
CD164	0.801868931	0.648099013	0.817256644	0.982367414	0.850879779	0.845803016
NUB1	0.801868931	0.674009696	0.828407935	0.990571725	0.852745499	0.795637468
ISY1	0.801868931	0.758702769	0.942004125	0.850828215	0.853884483	0.76507496

GPR17	0.801868931	0.803903736	0.787740344	0.913413181	0.856651628	0.85244017
HOPX	0.801868931	0.665563964	0.773281026	0.999939566	0.866920896	0.785679752
LOC511229	0.801868931	0.629715977	0.779087149	0.999939566	0.87483668	0.771597542
SETDB1	0.801868931	0.619184305	0.838877266	0.995800463	0.876859226	0.788131005
HSH2D	0.801868931	0.750546265	0.828569127	0.913413181	0.881823169	0.837048016
KCNJ15	0.801868931	0.805157515	0.849077738	0.846115604	0.892149112	0.832858909
LOC101907348	0.801868931	0.717508618	0.820549834	0.955687359	0.892506554	0.777933888
TMEM265	0.801868931	0.624067943	0.85972234	0.928836449	0.899048045	0.844335246
PHGDH	0.801868931	0.693153864	0.801209802	0.977470078	0.932081893	0.77208617
LOC101904595	0.801868931	0.623796638	0.953045982	0.904990622	0.932197133	0.756105719
COPZ1	0.801868931	0.678989647	0.759671935	0.964413123	0.932197133	0.845803016
SEPHS1	0.801868931	0.665803806	0.777178978	0.99879985	0.934859184	0.764546301
LOC104976574	0.801868931	0.650007851	0.773281026	0.99879985	0.935641613	0.770391484
HID1	0.801868931	0.763751563	0.788848487	0.943323364	0.941107925	0.757327233
PSMD14	0.801868931	0.673034156	0.764481234	0.873939476	0.943901678	0.941925704
MCM5	0.801868931	0.640189848	0.738217747	0.999939566	0.946944212	0.789577614
LRRC3B	0.801868931	0.780641867	0.823351722	0.858274325	0.947146038	0.823139458
GLT8D1	0.801868931	0.680267573	0.81285201	0.957928699	0.949645557	0.77208617
LIFR	0.801868931	0.673034156	0.788848487	0.99879985	0.954501767	0.702779403
ELP4	0.801868931	0.631843853	0.73016804	0.925004412	0.954501767	0.930707319
FBXO32	0.801868931	0.851167599	0.815870524	0.846715063	0.957605284	0.770391484
RBMS2	0.801868931	0.688099473	0.748016329	0.948120268	0.95956556	0.839340243
LOC782609	0.801868931	0.866803532	0.765074242	0.919247117	0.960835287	0.689496537
TMEM144	0.801868931	0.83971475	0.726668529	0.93471064	0.961382403	0.752857325
RHOBTB3	0.801868931	0.790637585	0.726668529	0.978220863	0.964150118	0.723641471
ENTPD1	0.801868931	0.655710881	0.748016329	0.846715063	0.998120751	0.943138585
FDFT1	0.801868931	0.629715977	0.781404227	0.87630412	0.998276725	0.908939618
PRRG1	0.801868931	0.681991477	0.843427302	0.925004412	0.99989309	0.692674475
LOC112442652	0.801868931	0.747499345	0.73016804	0.928921135	0.99989309	0.714260956
ENPP1	0.801868931	0.692025761	0.754101752	0.848051361	0.99989309	0.717940464
LOC104974070	0.801868931	0.655438106	0.776574729	0.948120268	0.99989309	0.725620954
ZC3H13	0.801868931	0.730792193	0.765074242	0.891273781	0.99989309	0.752857325
EP300	0.801868931	0.703437628	0.751496241	0.892229846	0.99989309	0.753138357
STUB1	0.801868931	0.747555258	0.726668529	0.908496281	0.99989309	0.761409946
HIGD1A	0.801868931	0.72477102	0.802693537	0.915749182	0.99989309	0.761409946
FAM3C	0.801868931	0.786856496	0.73016804	0.87487639	0.99989309	0.770391484
C23H6orf132	0.801868931	0.636332133	0.788848487	0.908804713	0.99989309	0.77208617
TM4SF18	0.802003733	0.626635021	0.757787642	0.999939566	0.957605284	0.77208617

SUFU	0.802563923	0.72809533	0.733231491	0.915749182	0.933983913	0.902660792
SCRN1	0.802965871	0.796591413	0.878603452	0.915749182	0.85647293	0.752358594
LOC104974678	0.802965871	0.908857456	0.788938503	0.891273781	0.882754591	0.770391484
ISCA1	0.802965871	0.665733407	0.858276354	0.982140778	0.925981327	0.728276429
EDA	0.802965871	0.868963165	0.727803501	0.892229846	0.949645557	0.826868779
CCDC57	0.802965871	0.619585357	0.939683463	0.915749182	0.964150118	0.701784819
TRMU	0.802965871	0.827609894	0.751496241	0.915749182	0.964872582	0.770391484
COPZ2	0.802965871	0.824119076	0.803883983	0.90419464	0.968477804	0.747674417
COX11	0.802965871	0.803995245	0.726668529	0.918313511	0.999592263	0.77208617
GBA2	0.802965871	0.651481516	0.727732528	0.95964071	0.99989309	0.712413606
IGIP	0.803269166	0.682470071	0.788938503	0.925931623	0.99989309	0.773957499
DNAJC8	0.803411228	0.6861152	0.98846962	0.869637988	0.86558233	0.707492534
POLR2J	0.803418662	0.691175262	0.765074242	0.846715063	0.99989309	0.749531513
ARHGEF17	0.803508808	0.809341818	0.773846486	0.915749182	0.977379814	0.752900996
MFNG	0.803812977	0.921961076	0.750089824	0.846715063	0.99989309	0.696941321
TRABD	0.803942707	0.691357843	0.965875422	0.915749182	0.846122929	0.71157802
RAP1GAP2	0.803942707	0.742728851	0.777178978	0.999939566	0.846122929	0.730588392
MLLT1	0.803942707	0.653341013	0.744933814	0.990571725	0.846122929	0.911526437
ICE2	0.803942707	0.852463941	0.787740344	0.913413181	0.847784631	0.825124845
TOMM20	0.803942707	0.657500304	0.926613927	0.936880771	0.857710467	0.769640373
IL17RE	0.803942707	0.838405969	0.764057751	0.992137653	0.86122464	0.717273603
HNRNPH2	0.803942707	0.665803806	0.85404379	0.904990622	0.86558233	0.896228048
ZNF503	0.803942707	0.89141259	0.777178978	0.89088601	0.871556014	0.82036646
LOC101904477	0.803942707	0.655710881	0.744490351	0.999939566	0.87483668	0.728276429
SCN3A	0.803942707	0.673034156	0.735302276	0.999939566	0.876274511	0.764546301
PPIL2	0.803942707	0.703437628	0.926613927	0.914343375	0.882754591	0.759107825
HBP1	0.803942707	0.740948281	0.75246943	0.91329897	0.883643824	0.922229044
PRMT1	0.803942707	0.684832051	0.827125538	0.935763381	0.887061423	0.846987044
ESPL1	0.803942707	0.688130143	0.882898206	0.982140778	0.889271428	0.694702496
RCC1	0.803942707	0.700682675	0.753656036	0.864540217	0.892966823	0.968751621
ALDH1A3	0.803942707	0.641493941	0.894449269	0.982140778	0.893052667	0.728276429
RBBP4	0.803942707	0.625663019	0.878603452	0.908496281	0.902843706	0.873520548
MS4A2	0.803942707	0.642677274	0.802847755	0.931268564	0.90399271	0.893961809
SELENOF	0.803942707	0.6608873	0.806311697	0.842538664	0.908708309	0.968039618
CTSZ	0.803942707	0.843588854	0.789182336	0.897891011	0.910770324	0.800276945
EHMT2	0.803942707	0.688099473	0.892122482	0.934591536	0.911961812	0.742259651
KCTD3	0.803942707	0.648555447	0.733231491	0.864540217	0.929095808	0.989313458
LOC101906870	0.803942707	0.747899938	0.849077738	0.846715063	0.932081893	0.859973633

YPEL1	0.803942707	0.703437628	0.757235816	0.999939566	0.937283243	0.728567358
PTPN7	0.803942707	0.790416788	0.74784538	0.908054165	0.939361296	0.868108836
MET	0.803942707	0.94610099	0.777178978	0.86288354	0.943768595	0.708662616
LOC104971374	0.803942707	0.624164321	0.918681165	0.910808102	0.943768595	0.794558849
LOC104975626	0.803942707	0.629704189	0.751496241	0.999939566	0.943901678	0.798018153
ZNF383	0.803942707	0.790637585	0.747084388	0.925265292	0.949645557	0.812509877
VAPA	0.803942707	0.619374114	0.86796189	0.988949923	0.952172109	0.722309684
GNPTG	0.803942707	0.718302402	0.81284583	0.843645855	0.952672789	0.899771153
RIPOR2	0.803942707	0.868963165	0.747093618	0.842538664	0.957605284	0.856870625
KRT19	0.803942707	0.75328994	0.930710426	0.850828215	0.957663949	0.694702496
SARAF	0.803942707	0.686576166	0.827125538	0.962142137	0.957910867	0.742259651
RAB44	0.803942707	0.629715977	0.790396664	0.91329897	0.959936656	0.908939618
LOC112443235	0.803942707	0.66403328	0.747945974	0.888642721	0.962262333	0.941275898
SRP14	0.803942707	0.674717949	0.883982433	0.919247117	0.975819525	0.722309684
BRAT1	0.803942707	0.6390296	0.933102499	0.843411019	0.976970807	0.79339547
IFI27L2	0.803942707	0.852281996	0.751496241	0.915153525	0.981509235	0.753138357
TSPAN4	0.803942707	0.670324428	0.92153058	0.883885658	0.998276725	0.727250186
ST13	0.803942707	0.629715977	0.788848487	0.944728796	0.99989309	0.699680053
UBL7	0.803942707	0.710155842	0.739676418	0.862060767	0.99989309	0.704011905
NDUFAB1	0.803942707	0.665563964	0.754101752	0.851394268	0.99989309	0.71509671
HDHD5	0.803942707	0.780290359	0.735302276	0.86297965	0.99989309	0.73862615
TMEM63A	0.803942707	0.63512774	0.891014698	0.850923823	0.99989309	0.741704592
MRPL18	0.803942707	0.76837825	0.765074242	0.89419838	0.99989309	0.742451714
S100A10	0.803942707	0.710155842	0.758406163	0.846715063	0.99989309	0.752900996
ATF4	0.803942707	0.710486439	0.799265002	0.913413181	0.99989309	0.752900996
TMEM219	0.803942707	0.769817928	0.757235816	0.923832787	0.99989309	0.752900996
CCDC153	0.803942707	0.77295675	0.759375097	0.843411019	0.99989309	0.7566392
FAM219B	0.803942707	0.804875341	0.733231491	0.846115604	0.99989309	0.759107825
MPC2	0.803942707	0.690829991	0.747084388	0.915749182	0.99989309	0.759308648
THRSP	0.803942707	0.688099473	0.777178978	0.915749182	0.99989309	0.770391484
ORM1	0.803942707	0.7116845	0.747093618	0.925931623	0.99989309	0.77208617
CCR10	0.803942707	0.655710881	0.817256644	0.850828215	0.99989309	0.788131005
LOC101907084	0.803942707	0.742728851	0.815548119	0.888642721	0.99989309	0.788131005
LOC101906086	0.803942707	0.619585357	0.806825406	0.905816196	0.99989309	0.788131005
NOC3L	0.803942707	0.66071365	0.747093618	0.944558402	0.99989309	0.794558849
KDM6A	0.803942707	0.731268229	0.726668529	0.851175396	0.99989309	0.809650729
DUSP2	0.803942707	0.629715977	0.815548119	0.883885658	0.99989309	0.855778243
PER2	0.803942707	0.629715977	0.733231491	0.915749182	0.99989309	0.878417122

TMEM222	0.803942707	0.740108813	0.729587161	0.846715063	0.99989309	0.883302043
LPIN3	0.804358341	0.62464979	0.985552223	0.87268432	0.860908683	0.788131005
DFFA	0.804358341	0.824676261	0.812043145	0.951929772	0.864593182	0.745987575
BOLA2B	0.804358341	0.655710881	0.911867523	0.943323364	0.925981327	0.728276429
LOC107132465	0.804358341	0.665803806	0.726668529	0.999939566	0.943768595	0.789577614
ZFAT	0.804358341	0.685980044	0.821908722	0.846715063	0.99989309	0.77208617
TCTEX1D1	0.804601856	0.852463941	0.764481234	0.893831952	0.850879779	0.872224159
PPP1R3C	0.804601856	0.840313358	0.843427302	0.915749182	0.866920896	0.752900996
GABARAP	0.804601856	0.661476635	0.921612776	0.972367384	0.87483668	0.701784819
LOC506408	0.804601856	0.645930592	0.939683463	0.925901365	0.87483668	0.763746661
NHLRC1	0.804601856	0.661476635	0.777638604	0.999939566	0.881823169	0.788142857
LOC112447402	0.804601856	0.696881713	0.740319216	0.999939566	0.910770324	0.789577614
EARS2	0.804601856	0.736066622	0.764057751	0.915749182	0.934379881	0.872224159
LOC104975006	0.804601856	0.715484373	0.751496241	0.982367414	0.953263639	0.788131005
RELT	0.804601856	0.728017748	0.776574729	0.958849311	0.972048096	0.757327233
PDS5A	0.804601856	0.651481516	0.820767684	0.888642721	0.99989309	0.770391484
SEC24A	0.804601856	0.693544464	0.734250356	0.918395075	0.99989309	0.840961242
USP5	0.804601856	0.758051794	0.727732528	0.850828215	0.99989309	0.86143104
TTC21A	0.804650519	0.63512774	0.950638965	0.90419464	0.90478776	0.787719474
PCBD2	0.804650519	0.740413436	0.777178978	0.908804713	0.99989309	0.708662616
CHMP1A	0.804650519	0.646924712	0.811009079	0.915749182	0.99989309	0.710580013
PEG3	0.805025931	0.7861604	0.758406163	0.90419464	0.99989309	0.701784819
STRA6	0.805353359	0.671602771	0.906925281	0.948172741	0.899048045	0.738152854
COPS8	0.805353359	0.644204975	0.879726737	0.951929772	0.975819525	0.694702496
C11H9orf50	0.805353359	0.812575653	0.765074242	0.934591536	0.996250445	0.702779403
TERF2	0.805353359	0.671879214	0.788848487	0.860663136	0.99989309	0.821456459
TTC22	0.80543337	0.72477102	0.779087149	0.993811478	0.899048045	0.77208617
POT1	0.80543337	0.884220302	0.744369186	0.874047001	0.99989309	0.700618632
LOC100335467	0.80543337	0.693454767	0.765074242	0.992137653	0.99989309	0.701868607
ACOT8	0.80543337	0.7116845	0.756911784	0.908804713	0.99989309	0.770391484
PMVK	0.805583624	0.679258786	0.757787642	0.999939566	0.954501767	0.753138357
ZBTB17	0.8055922	0.655710881	0.845237542	0.999939566	0.849382765	0.742259651
FAM43B	0.8055922	0.636075633	0.73016804	0.956154344	0.852745499	0.96024204
PIK3R2	0.8055922	0.692987203	0.819114812	0.999939566	0.853281511	0.728276429
CROCC	0.8055922	0.637203338	0.89248682	0.990571725	0.853640563	0.765573509
PSAP	0.8055922	0.685495173	0.788938503	0.999939566	0.857733715	0.728276429
ULK3	0.8055922	0.63203115	0.945417678	0.878289777	0.869068239	0.857727168
SH3YL1	0.8055922	0.650107532	0.92153058	0.956154344	0.874640903	0.759729981

RACGAP1	0.8055922	0.703437628	0.829843435	0.90419464	0.87465791	0.911696156
LOC512005	0.8055922	0.685495173	0.880277219	0.908804713	0.87483668	0.86143104
LOC100141266	0.8055922	0.808022384	0.808333956	0.945929971	0.875054206	0.770426407
PNPLA7	0.8055922	0.677521241	0.944612298	0.915749182	0.875748951	0.764441047
ALDH1B1	0.8055922	0.675748768	0.961742979	0.916305829	0.882917206	0.707492534
MSRB3	0.8055922	0.750546265	0.81284583	0.917869295	0.886279726	0.849504243
GPN1	0.8055922	0.761788537	0.806311697	0.992368879	0.887805568	0.728000197
COX7B2	0.8055922	0.838843184	0.751496241	0.925931623	0.892150832	0.823435077
PCSK4	0.8055922	0.647660219	0.894447907	0.90419464	0.907192767	0.859973633
POLQ	0.8055922	0.647086084	0.957802423	0.925901365	0.908091868	0.707492534
VASH1	0.8055922	0.69363319	0.827125538	0.999939566	0.91011204	0.697857798
LOC104974667	0.8055922	0.6608873	0.777178978	0.999939566	0.91011204	0.748417897
MFSD12	0.8055922	0.744066065	0.761593203	0.959786126	0.912507538	0.831832432
FNBP1L	0.8055922	0.843333021	0.773281026	0.934591536	0.921504737	0.764546301
LAMA5	0.8055922	0.64691986	0.747084388	0.992368879	0.925981327	0.871129794
BCCIP	0.8055922	0.671602771	0.802857717	0.915749182	0.926480902	0.902660792
BATF	0.8055922	0.626861326	0.766352455	0.994365581	0.934228164	0.84763005
CORO1A	0.8055922	0.762756164	0.735502764	0.883885658	0.934859184	0.933763401
TFE3	0.8055922	0.782865867	0.765074242	0.89088601	0.938025821	0.878851638
CCDC71L	0.8055922	0.839289192	0.87214481	0.846115604	0.939361296	0.766359829
TTC39B	0.8055922	0.629715977	0.784016889	0.91329897	0.941064037	0.940683751
DDX52	0.8055922	0.785163144	0.895065905	0.845686584	0.941107925	0.77208617
TIFA	0.8055922	0.743422331	0.802857717	0.907553418	0.94294964	0.855692379
CPT1C	0.8055922	0.73599132	0.84311048	0.943323364	0.943768595	0.743377609
PHLPP2	0.8055922	0.786258422	0.844065668	0.917869295	0.954501767	0.704367673
PRAM1	0.8055922	0.769817928	0.883274745	0.908054165	0.954501767	0.706152771
ADGB	0.8055922	0.718469663	0.931173336	0.87630412	0.954501767	0.723028401
EIF4A1	0.8055922	0.718231253	0.844065668	0.849315934	0.954501767	0.865926817
PRRX1	0.8055922	0.836026119	0.733231491	0.951929772	0.957605284	0.754897098
ELL3	0.8055922	0.644224672	0.831430643	0.915749182	0.957605284	0.849772722
PFDN2	0.8055922	0.736964914	0.827125538	0.941207767	0.959936656	0.745987575
SBNO2	0.8055922	0.72477102	0.850106729	0.87630412	0.960296839	0.823139458
GALNT9	0.8055922	0.795534928	0.849077738	0.846715063	0.962262333	0.792329436
CFAP69	0.8055922	0.736066622	0.857417456	0.915749182	0.964150118	0.729817953
PKMYT1	0.8055922	0.782196458	0.849077738	0.884121831	0.972917711	0.76360318
LOC515578	0.8055922	0.739918356	0.777638604	0.934591536	0.975782017	0.77208617
LOC101905743	0.8055922	0.632709306	0.76749973	0.999939566	0.976657193	0.703742328
AEBP1	0.8055922	0.754663123	0.777178978	0.946344632	0.982014614	0.752857325

CYP2J2	0.8055922	0.823588167	0.827350832	0.903607716	0.982577194	0.714817255
UBE2D3	0.8055922	0.679006126	0.850106729	0.93026568	0.988693274	0.752900996
SPRED2	0.8055922	0.687113907	0.76749973	0.918607898	0.988693274	0.859973633
KIF3A	0.8055922	0.703437628	0.744933814	0.96095927	0.996250445	0.798051736
LOC101907729	0.8055922	0.703437628	0.812585086	0.868090726	0.99989309	0.697990115
CTSV	0.8055922	0.842797231	0.748723724	0.916305829	0.99989309	0.699838787
PAK3	0.8055922	0.801968936	0.74784538	0.91329897	0.99989309	0.703914683
IRAK2	0.8055922	0.68568006	0.746957417	0.931268564	0.99989309	0.704011905
TSEN54	0.8055922	0.723119554	0.765074242	0.934591536	0.99989309	0.704011905
THAP9	0.8055922	0.670188815	0.861838588	0.875889425	0.99989309	0.707492534
TBC1D13	0.8055922	0.655710881	0.802847755	0.958879502	0.99989309	0.707492534
UBE2Z	0.8055922	0.680195281	0.787740344	0.990571725	0.99989309	0.709387613
RAD51D	0.8055922	0.652542331	0.740319216	0.925901365	0.99989309	0.710580013
ARF5	0.8055922	0.849834516	0.747084388	0.883885658	0.99989309	0.712413606
LOC104971683	0.8055922	0.652542331	0.832431595	0.956303465	0.99989309	0.722309684
HSPA8	0.8055922	0.704923066	0.772990161	0.875889425	0.99989309	0.725528824
DHPS	0.8055922	0.636614658	0.788848487	0.932404322	0.99989309	0.727921992
CUEDC2	0.8055922	0.730838712	0.738217747	0.850828215	0.99989309	0.728276429
LOC104968411	0.8055922	0.6608873	0.886373777	0.891273781	0.99989309	0.729006658
CD101	0.8055922	0.775921167	0.765074242	0.904074458	0.99989309	0.734406433
RTP4	0.8055922	0.631764076	0.885434576	0.913413181	0.99989309	0.739614903
DUSP12	0.8055922	0.693153864	0.750089824	0.892741454	0.99989309	0.745987575
FAM46B	0.8055922	0.675810425	0.788938503	0.915749182	0.99989309	0.753138357
SLC25A17	0.8055922	0.691175262	0.788938503	0.908054165	0.99989309	0.759107825
LOC107133284	0.8055922	0.731268229	0.788938503	0.897446122	0.99989309	0.764546301
RBMS1	0.8055922	0.647660219	0.751496241	0.990300191	0.99989309	0.764546301
TAF1	0.8055922	0.736066622	0.788848487	0.908496281	0.99989309	0.766359829
AP3S2	0.8055922	0.700682675	0.809322753	0.925004412	0.99989309	0.770391484
TSTD1	0.8055922	0.688099473	0.885354035	0.858274325	0.99989309	0.771597542
ZNHIT3	0.8055922	0.655710881	0.777178978	0.87630412	0.99989309	0.77208617
GRAMD1C	0.8055922	0.661476635	0.747084388	0.990571725	0.99989309	0.774602575
HSPA6	0.8055922	0.774326932	0.798686086	0.891273781	0.99989309	0.776333209
SMARCA1	0.8055922	0.659488126	0.76145258	0.846715063	0.99989309	0.787474205
LOC112444616	0.8055922	0.713885977	0.765074242	0.915749182	0.99989309	0.792329436
LOC101902839	0.8055922	0.655710881	0.827350832	0.850828215	0.99989309	0.796934582
JSRP1	0.8055922	0.769817928	0.747084388	0.873939476	0.99989309	0.812682271
TRIM13	0.8055922	0.637278759	0.820767684	0.888642721	0.99989309	0.827803438
GFER	0.8055922	0.72843033	0.765074242	0.915749182	0.99989309	0.837048016

KMT2A	0.8055922	0.692547228	0.757235816	0.90419464	0.99989309	0.839788983
PRDM15	0.8055922	0.653341013	0.877133001	0.850828215	0.99989309	0.840150874
ELP6	0.8055922	0.668175004	0.741982235	0.915749182	0.99989309	0.880404833
PDF	0.8055922	0.693153864	0.751496241	0.846715063	0.99989309	0.936791233
C3H1orf52	0.806193943	0.629715977	0.747093618	0.999939566	0.925981327	0.753138357
WRAP53	0.806440431	0.677340516	0.809332251	0.996601241	0.926858478	0.770426407
PANK3	0.806451729	0.635240156	0.843427302	0.925901365	0.952172109	0.847196533
MEF2A	0.806518575	0.703437628	0.836294915	0.925931623	0.986955873	0.758090697
TMEM167B	0.806614235	0.673782715	0.956880455	0.915749182	0.857710467	0.770391484
PRKAB2	0.806614235	0.730944867	0.825960826	0.915749182	0.875054206	0.866836199
FCGR2B	0.806614235	0.89490916	0.750089824	0.848392493	0.889302351	0.886992214
PFDN6	0.806614235	0.828797861	0.754101752	0.89088601	0.960835287	0.84491737
ALG13	0.80676408	0.691175262	0.817256644	0.915749182	0.861748588	0.924885155
SDK2	0.80676408	0.642501337	0.951742137	0.925901365	0.87483668	0.76507496
LOC107132225	0.80676408	0.731268229	0.829843435	0.984100122	0.883643824	0.764546301
CCAR2	0.80676408	0.665563964	0.968455468	0.915749182	0.904151325	0.705712189
LOC511617	0.80676408	0.701581467	0.733231491	0.908054165	0.925981327	0.958540316
LOC107132852	0.80676408	0.744494437	0.774555223	0.946344632	0.934859184	0.821467423
CCDC191	0.80676408	0.632709306	0.886282865	0.945929971	0.940638859	0.77208617
SPATA9	0.80676408	0.761788537	0.870054336	0.92064557	0.941107925	0.731995209
YWHAQ	0.80676408	0.658897564	0.828407935	0.858021281	0.99989309	0.702851213
DLGAP1	0.80676408	0.693153864	0.733231491	0.990300191	0.99989309	0.76360318
LOC112442049	0.80676408	0.629715977	0.831320191	0.916305829	0.99989309	0.789577614
POLR1A	0.80676408	0.693544464	0.735174555	0.847813982	0.99989309	0.933678522
LOC783497	0.806825111	0.686112625	0.891021219	0.925004412	0.947146038	0.76360318
INTS13	0.806825111	0.836038392	0.761720705	0.917869295	0.99989309	0.703372675
ARL2	0.80682817	0.751946504	0.765074242	0.89088601	0.99989309	0.791787698
LOC101907133	0.807187121	0.74401284	0.788938503	0.888727169	0.954501767	0.872224159
LOC112441525	0.807501549	0.672471408	0.79610462	0.942235206	0.99989309	0.790463232
MCL1	0.807640593	0.688099473	0.836110462	0.995118128	0.904995537	0.753067712
KCNJ10	0.807640593	0.692987203	0.841835423	0.943323364	0.941064037	0.794529985
DLG4	0.807640593	0.704817952	0.827125538	0.958879502	0.988693274	0.709387613
LOC112444841	0.807640593	0.688099473	0.914685423	0.904990622	0.99989309	0.704347031
RBM4	0.807640593	0.731331179	0.775711284	0.87630412	0.99989309	0.77208617
SSU72	0.807660542	0.668175004	0.782862233	0.850828215	0.99989309	0.875441396
TBX1	0.807690507	0.7164915	0.810425784	0.999939566	0.858282183	0.740205409
GABPB1	0.807690507	0.900467607	0.788938503	0.847813982	0.993399243	0.752857325
SUPT6H	0.807690507	0.68374798	0.805580394	0.925901365	0.99989309	0.708819884

NADK	0.807690507	0.738981601	0.744369186	0.937115321	0.99989309	0.770391484
SYBU	0.807690507	0.811755592	0.747093618	0.854497387	0.99989309	0.792329436
BTF3	0.807784037	0.669039484	0.783791102	0.999939566	0.947146038	0.707492534
MEX3A	0.807784037	0.692928782	0.757235816	0.963829131	0.968477804	0.828602505
HGF	0.807942711	0.786258422	0.828407935	0.915749182	0.862637717	0.84491737
VPS41	0.807942711	0.636332133	0.813256265	0.999939566	0.866920896	0.737883632
LY86	0.807942711	0.982703363	0.749513157	0.883885658	0.87483668	0.77020109
LOC112448833	0.807942711	0.820959665	0.86719452	0.925901365	0.886279726	0.724507872
ASCC2	0.807942711	0.926619092	0.747093618	0.916305829	0.887061423	0.778407119
TSSK1B	0.807942711	0.785163144	0.802847755	0.943323364	0.899048045	0.799935743
ZNF106	0.807942711	0.703437628	0.768138655	0.93471064	0.899048045	0.895218005
NMT2	0.807942711	0.873566716	0.843733648	0.908496281	0.901846473	0.745392593
AP3B2	0.807942711	0.693153864	0.747093618	0.940921143	0.907192767	0.92035958
MAGT1	0.807942711	0.704093198	0.92153058	0.908496281	0.91011204	0.773957499
LOC789231	0.807942711	0.6861152	0.79610462	0.999939566	0.927804504	0.701784819
RHBG	0.807942711	0.914162656	0.788938503	0.915749182	0.939361296	0.709387613
LOC100848025	0.807942711	0.680195281	0.739676418	0.999939566	0.943768595	0.701784819
NPAT	0.807942711	0.661476635	0.894547778	0.925931623	0.947146038	0.770391484
C19H17orf80	0.807942711	0.673034156	0.896272282	0.90419464	0.961382403	0.799147792
TOLLIP	0.807942711	0.693544464	0.750089824	0.99879985	0.972917711	0.764546301
ZNF385A	0.807942711	0.944780259	0.754101752	0.883885658	0.979654999	0.714231652
ZC3H11A	0.807942711	0.694355537	0.764057751	0.956154344	0.988693274	0.809108223
NDUFB11	0.807942711	0.720945114	0.777178978	0.86937758	0.99989309	0.705712189
YJU2	0.807942711	0.677340516	0.820767684	0.850465555	0.99989309	0.728276429
ZNF507	0.807942711	0.703437628	0.787740344	0.851394268	0.99989309	0.740271903
RBCK1	0.807942711	0.703437628	0.846650632	0.904546259	0.99989309	0.745987575
CMC2	0.807942711	0.728588754	0.760400875	0.850828215	0.99989309	0.77208617
LOC107133095	0.807942711	0.882542824	0.765074242	0.851175396	0.99989309	0.777933888
GATD1	0.807942711	0.669648359	0.751496241	0.915749182	0.99989309	0.783479395
MRPL46	0.807942711	0.7116845	0.763592007	0.913319839	0.99989309	0.788411132
SORCS2	0.807942711	0.693153864	0.844009594	0.908054165	0.99989309	0.791259029
ARMC6	0.807942711	0.693153864	0.746957417	0.916305829	0.99989309	0.873520548
KIAA0930	0.808232164	0.747555258	0.879515214	0.943323364	0.875054206	0.752900996
LOC101905525	0.808232164	0.786258422	0.746957417	0.946344632	0.882917206	0.865159042
RNF7	0.808232164	0.767670077	0.880277219	0.915749182	0.939361296	0.747674417
COX17	0.808232164	0.703437628	0.802847755	0.913413181	0.99989309	0.706354992
SH2B3	0.808232164	0.688599369	0.784347647	0.850828215	0.99989309	0.77208617
LOC112442032	0.808595131	0.7116845	0.789039067	0.999939566	0.899048045	0.740271903

KAT7	0.808595131	0.843377488	0.765074242	0.910808102	0.925981327	0.840150874
POLR2C	0.808939503	0.644204975	0.933963997	0.985342672	0.859775628	0.735313642
TAC3	0.808939503	0.686122011	0.855743966	0.943161565	0.861762366	0.856870625
LOC112447010	0.808939503	0.688304784	0.762423061	0.850828215	0.862637717	0.995411183
HMGA1	0.808939503	0.786258422	0.842478086	0.98259522	0.866920896	0.732184848
UBE2D2	0.808939503	0.63203115	0.899539089	0.981022805	0.87465791	0.787474205
PRPSAP2	0.808939503	0.926435857	0.811673049	0.921235651	0.87483668	0.705712189
EIF4H	0.808939503	0.637634861	0.910982029	0.95964071	0.87483668	0.784080446
JAM3	0.808939503	0.655438106	0.855743966	0.919247117	0.87483668	0.895287865
LOC100336368	0.808939503	0.796591413	0.747806937	0.999939566	0.886279726	0.752900996
AP4M1	0.808939503	0.665524216	0.95712241	0.866960502	0.886279726	0.839340243
CRTAM	0.808939503	0.698870039	0.894547778	0.915749182	0.887061423	0.821456459
NUDT3	0.808939503	0.675550965	0.878050811	0.956634362	0.889271428	0.793940734
THAP12	0.808939503	0.693153864	0.893763648	0.953508052	0.902843706	0.750665174
HLX	0.808939503	0.691175262	0.802847755	0.999939566	0.902843706	0.770426407
PINX1	0.808939503	0.851167599	0.754101752	0.85428936	0.906183446	0.908939618
PUS7	0.808939503	0.715305297	0.846650632	0.89088601	0.915914249	0.877813306
LMBR1	0.808939503	0.632709306	0.825178565	0.999939566	0.92188432	0.742259651
FAM98A	0.808939503	0.685446639	0.810425784	0.846715063	0.93842945	0.955585296
POLR3F	0.808939503	0.82783173	0.747806937	0.956154344	0.941064037	0.773957499
SMARCA2	0.808939503	0.688099473	0.79610462	0.995118128	0.941064037	0.774602575
TNFSF9	0.808939503	0.693544464	0.772990161	0.951929772	0.941107925	0.859973633
FDXACB1	0.808939503	0.704923066	0.777178978	0.925901365	0.941107925	0.87428828
LOC104973099	0.808939503	0.955798423	0.739676418	0.89088601	0.941922158	0.77208617
AATF	0.808939503	0.6663481	0.80193314	0.90419464	0.943768595	0.932267465
RPP21	0.808939503	0.72843033	0.81285201	0.935763381	0.947146038	0.80787758
PRELID1	0.808939503	0.771098738	0.765074242	0.90419464	0.961835052	0.869579703
LOC112446734	0.808939503	0.72843033	0.847037539	0.915749182	0.975291792	0.77208617
NELFB	0.808939503	0.688099473	0.875669731	0.90419464	0.99989309	0.706617665
GRAMD1A	0.808939503	0.741353552	0.826600651	0.846715063	0.99989309	0.707492534
ATP5PF	0.808939503	0.700679806	0.827125538	0.88226398	0.99989309	0.714260956
TSC22D2	0.808939503	0.723243905	0.788892061	0.851175396	0.99989309	0.723641471
LOC101904332	0.808939503	0.636332133	0.753656036	0.87630412	0.99989309	0.732184848
LOC104975663	0.808939503	0.642412048	0.807563733	0.905816196	0.99989309	0.759107825
SEC24D	0.808939503	0.677389817	0.76749973	0.925931623	0.99989309	0.761409946
ARHGAP33	0.808939503	0.661749528	0.825888497	0.931268564	0.99989309	0.76935309
TRIM7	0.808939503	0.804860126	0.80326876	0.86288354	0.99989309	0.770391484
GZMA	0.808939503	0.632709306	0.923072506	0.873519404	0.99989309	0.77208617

MRPL2	0.808939503	0.747555258	0.755534187	0.896408418	0.99989309	0.77208617
MEDAG	0.808939503	0.769817928	0.812043145	0.846715063	0.99989309	0.775546211
GTF2H5	0.808939503	0.648099013	0.788938503	0.883885658	0.99989309	0.787474205
PCM1	0.808939503	0.786829125	0.765074242	0.891635304	0.99989309	0.788411132
PRPF4B	0.808939503	0.681991477	0.795199152	0.942235206	0.99989309	0.788411132
CLASP2	0.808939503	0.71963672	0.765074242	0.87630412	0.99989309	0.792329436
ATP13A3	0.808939503	0.673034156	0.752494129	0.956154344	0.99989309	0.795637468
LOC112444889	0.808939503	0.754137665	0.79610462	0.851394268	0.99989309	0.809650729
LOC112443850	0.808939503	0.636332133	0.750089824	0.87452213	0.99989309	0.836944254
MOSPD1	0.809061805	0.689503192	0.877998684	0.935763381	0.864593182	0.840150874
LOC616199	0.809061805	0.779536052	0.763592007	0.999939566	0.87465791	0.75292536
COL25A1	0.809061805	0.769817928	0.787015778	0.90419464	0.887539821	0.912101047
LOC101902361	0.809061805	0.7116845	0.747700432	0.910808102	0.939361296	0.942421464
ARHGEF39	0.809061805	0.661476635	0.859774281	0.992368879	0.952172109	0.71509671
HOXB9	0.809061805	0.747899938	0.739676418	0.990049248	0.954501767	0.796593386
LOC101904103	0.809061805	0.80931829	0.832483532	0.893831952	0.972048096	0.771597542
SPP2	0.809061805	0.707802804	0.827350832	0.915749182	0.975291792	0.813759229
PFDN4	0.809061805	0.647086084	0.827125538	0.907949786	0.99989309	0.742259651
MSN	0.809061805	0.692987203	0.857864225	0.908496281	0.99989309	0.764546301
LOC112444588	0.809093178	0.780641867	0.763858703	0.999939566	0.899048045	0.752358594
CDC42EP5	0.809093178	0.675748768	0.809322753	0.995800463	0.899048045	0.812622197
LOC112443339	0.809093178	0.763478948	0.776954726	0.898441424	0.928701823	0.908939618
CLEC4G	0.809093178	0.704817952	0.823486909	0.909000216	0.956624006	0.86307729
LOC100848940	0.809093178	0.747164473	0.788938503	0.925931623	0.99989309	0.752900996
CCND1	0.809093178	0.727186688	0.80671639	0.925901365	0.99989309	0.770391484
LARP1B	0.809093178	0.676166653	0.740300128	0.925931623	0.99989309	0.788131005
DNMT1	0.809525904	0.730949525	0.849077738	0.990571725	0.910770324	0.707492534
CD160	0.809525904	0.687103484	0.827350832	0.850828215	0.913229811	0.95460851
CUL9	0.809525904	0.665563964	0.927460291	0.949947918	0.925981327	0.728276429
PPP1R14C	0.809525904	0.652236067	0.925587088	0.942508938	0.925981327	0.763746661
WDHD1	0.809525904	0.688099473	0.827350832	0.948120268	0.988693274	0.770391484
PRELID3A	0.809525904	0.681991477	0.765074242	0.90419464	0.99989309	0.704011905
PRICKLE1	0.809525904	0.655710881	0.777939926	0.927451836	0.99989309	0.742259651
LOC101905866	0.809525904	0.710969641	0.753656036	0.982367414	0.99989309	0.747230622
NDUFA7	0.809525904	0.700729482	0.747806937	0.858021281	0.99989309	0.763746661
GREB1L	0.809525904	0.655710881	0.871638215	0.850828215	0.99989309	0.764546301
IL34	0.809525904	0.710155842	0.899530079	0.865118174	0.99989309	0.765492321
PLAG1	0.809525904	0.785406526	0.764057751	0.850997716	0.99989309	0.849504243

BFAR	0.809525904	0.632709306	0.807843373	0.87487639	0.99989309	0.904593245
MRPL17	0.810106112	0.705458146	0.789686719	0.956154344	0.874640903	0.877623013
SNAPC4	0.810106112	0.634632759	0.969390163	0.935163227	0.87465791	0.742259651
CCDC34	0.810106112	0.72843033	0.856769954	0.850465555	0.904151325	0.908118871
LOC112447756	0.810106112	0.684832051	0.849077738	0.951929772	0.949645557	0.786737722
PISD	0.810106112	0.792090907	0.802857717	0.915749182	0.998684149	0.764546301
NFX1	0.810106112	0.740948281	0.765074242	0.904990622	0.99989309	0.792329436
STYX	0.810106112	0.639901253	0.750089824	0.925901365	0.99989309	0.798051736
CDC16	0.810106112	0.652236067	0.744490351	0.858021281	0.99989309	0.893622929
IGSF10	0.810487451	0.703437628	0.751496241	0.995118128	0.934859184	0.836652666
CYP27A1	0.810487451	0.899387334	0.747084388	0.914862928	0.939361296	0.813001848
TTK	0.810487451	0.639164228	0.848444291	0.982554614	0.95956556	0.770391484
EXOC3L2	0.810487451	0.782865867	0.744369186	0.995118128	0.986955873	0.715665393
ATP6V1F	0.810487451	0.731268229	0.787015778	0.918607898	0.99989309	0.728276429
RABGGTA	0.810487451	0.703437628	0.765074242	0.889138988	0.99989309	0.740271903
MPG	0.810487451	0.687403165	0.750139062	0.915749182	0.99989309	0.753067712
ACADS	0.810487451	0.693454767	0.765074242	0.915749182	0.99989309	0.771541268
SHC2	0.810487451	0.669039484	0.744490351	0.995112305	0.99989309	0.77821816
PHLPP1	0.810487451	0.648099013	0.750089824	0.975529024	0.99989309	0.840150874
MAP3K6	0.810495678	0.632709306	0.976719499	0.90419464	0.925981327	0.770391484
ENSA	0.810673803	0.674551689	0.829843435	0.931268564	0.886279726	0.893961809
LOC101902084	0.810673803	0.900467607	0.79610462	0.932656599	0.907192767	0.719551722
LOC112448816	0.810673803	0.636332133	0.900128576	0.925440563	0.951073785	0.799314198
EHD3	0.810673803	0.688099473	0.775306135	0.945929971	0.99989309	0.74008849
LOC101906756	0.810673803	0.703437628	0.74941488	0.850828215	0.99989309	0.77208617
BORCS5	0.810673803	0.730944867	0.765074242	0.90419464	0.99989309	0.845591203
VDAC2	0.810673803	0.686014664	0.76749973	0.883885658	0.99989309	0.856495922
LOC100847376	0.810673803	0.731268229	0.765074242	0.850828215	0.99989309	0.902122572
AP1AR	0.810779413	0.693153864	0.788938503	0.999939566	0.875054206	0.788142857
FAM177A1	0.810779413	0.670188815	0.880204512	0.953508052	0.905719399	0.796593386
PATL2	0.810779413	0.687403165	0.878603452	0.953144955	0.934859184	0.770391484
SPIRE1	0.810779413	0.792663811	0.843427302	0.928623745	0.939361296	0.753138357
PMS1	0.810779413	0.705023968	0.793430641	0.87106187	0.941107925	0.940917403
EMC10	0.810779413	0.699793718	0.747084388	0.908054165	0.99989309	0.752857325
IER5L	0.810779413	0.7116845	0.791822794	0.943323364	0.99989309	0.764546301
QPRT	0.810779413	0.692987203	0.762423061	0.915749182	0.99989309	0.765492321
LOC100139548	0.810779413	0.764525656	0.79610462	0.84783497	0.99989309	0.799147792
WDR36	0.810779413	0.636332133	0.802951601	0.868417977	0.99989309	0.840150874

RPS6KA5	0.810779413	0.665142095	0.751496241	0.915749182	0.99989309	0.845803016
NOX4	0.810779413	0.635815471	0.765074242	0.868417977	0.99989309	0.942421464
APOBEC2	0.81112644	0.694486794	0.923516626	0.93471064	0.910770324	0.742259651
AP2A2	0.81112644	0.763751563	0.751496241	0.86288354	0.949645557	0.941925704
SRPK3	0.811130023	0.692547228	0.777178978	0.924489924	0.934859184	0.912101047
GOSR2	0.811130023	0.686014664	0.744490351	0.868872346	0.99989309	0.882699301
LOC101905403	0.811349762	0.647086084	0.866690722	0.915749182	0.943768595	0.873118284
NUDT4	0.811349762	0.72843033	0.757787642	0.999939566	0.949760006	0.742259651
TGFB3	0.811349762	0.679258786	0.788848487	0.925210703	0.99989309	0.730588392
LOC781261	0.811522083	0.894203989	0.780363715	0.934591536	0.881823169	0.770391484
NACAD	0.811522083	0.6663481	0.777178978	0.999939566	0.910770324	0.800008791
TRMT5	0.811522083	0.692987203	0.750089824	0.91329897	0.962791364	0.936439483
ZNRF1	0.811522083	0.72843033	0.754897816	0.995118128	0.993370551	0.749691032
LOC112446033	0.811522083	0.824676261	0.777178978	0.90419464	0.99989309	0.712066047
RNF13	0.811522083	0.800441059	0.753656036	0.925901365	0.99989309	0.716278422
HSD17B10	0.811522083	0.677470151	0.747093618	0.917869295	0.99989309	0.758090697
ARID1B	0.811707405	0.738056281	0.878603452	0.90419464	0.99989309	0.707492534
LMO3	0.812553724	0.841044548	0.817256644	0.908496281	0.941107925	0.792329436
TOP2B	0.812553724	0.707016816	0.746686237	0.883885658	0.99989309	0.770265362
IER3	0.812644243	0.681991477	0.764481234	0.999939566	0.933637471	0.815459921
CLN3	0.813123334	0.841044548	0.939683463	0.850828215	0.87483668	0.75292536
TMEM150C	0.813123334	0.72477102	0.749513157	0.99879985	0.962262333	0.770391484
FLRT3	0.813123334	0.688099473	0.74618805	0.908496281	0.99989309	0.893400499
GHR	0.813564568	0.693153864	0.760400875	0.999939566	0.954501767	0.728276429
COPG1	0.813564568	0.762756164	0.744369186	0.925901365	0.98152792	0.859973633
NDUFB4	0.813564568	0.748534108	0.777178978	0.88747242	0.99989309	0.77208617
USP21	0.813626742	0.648099013	0.849132683	0.99879985	0.871556014	0.798051736
CDYL2	0.813626742	0.718302288	0.748016329	0.987503851	0.87483668	0.892466834
TAOK2	0.813626742	0.636673491	0.92153058	0.946344632	0.880683246	0.809650729
CDK9	0.813626742	0.640189848	0.944612298	0.934591536	0.882754591	0.786737722
FBXO36	0.813626742	0.926435857	0.758406163	0.951606954	0.904151325	0.717517382
LOC101906240	0.813626742	0.781154903	0.82199268	0.963706639	0.904151325	0.770391484
ARGLU1	0.813626742	0.680267573	0.878050811	0.992746023	0.908708309	0.750102841
RPA2	0.813626742	0.769817928	0.788848487	0.956154344	0.9187105	0.812623355
ZFP36L1	0.813626742	0.736964914	0.753656036	0.999939566	0.922811199	0.774602575
NCAPD3	0.813626742	0.661476635	0.968992166	0.891273781	0.934859184	0.770426407
LCORL	0.813626742	0.738328793	0.787740344	0.982554614	0.934859184	0.799314198
RIBC1	0.813626742	0.732522135	0.751496241	0.9846464	0.934859184	0.845803016

IQSEC2	0.813626742	0.663180847	0.769285605	0.999939566	0.941064037	0.784080446
SPEG	0.813626742	0.785406526	0.747093618	0.954423369	0.941064037	0.840150874
USP47	0.813626742	0.692547228	0.90002174	0.95175364	0.952172109	0.714260956
OGG1	0.813626742	0.739753419	0.883726795	0.90419464	0.952172109	0.793572843
PKHD1L1	0.813626742	0.785406526	0.744490351	0.982140778	0.952172109	0.80261571
EIF4A2	0.813626742	0.853340226	0.787740344	0.892229846	0.983634477	0.787719474
GTF3C5	0.813626742	0.701581467	0.76749973	0.956154344	0.988693274	0.820483064
NCAPH	0.813626742	0.805132292	0.807843373	0.929346072	0.996250445	0.723028401
COX6A1	0.813626742	0.653723309	0.747084388	0.915749182	0.99989309	0.708785032
MED1	0.813626742	0.732654391	0.776954726	0.903607716	0.99989309	0.734406433
COMMD6	0.813626742	0.727741548	0.775786014	0.906725112	0.99989309	0.750665174
B3GALT4	0.813626742	0.693153864	0.812043145	0.883398728	0.99989309	0.765573509
HSPA5	0.813626742	0.747899938	0.749513157	0.927618239	0.99989309	0.77208617
SLC10A1	0.813626742	0.701581467	0.754897816	0.951929772	0.99989309	0.788411132
TOR1A	0.813626742	0.7116845	0.774385549	0.850828215	0.99989309	0.867440033
NHSL2	0.813772295	0.967758952	0.747084388	0.948547383	0.869637547	0.714123227
HACD2	0.813772295	0.693181652	0.812770091	0.924489924	0.905719399	0.906895944
SRSF6	0.813772295	0.688099473	0.85972234	0.892741454	0.99989309	0.770391484
WDR48	0.814019731	0.686576166	0.765074242	0.999939566	0.899048045	0.845969723
SEC61A1	0.814019731	0.785163144	0.777178978	0.886726226	0.913166496	0.919645668
LOC785477	0.814019731	0.636332133	0.858476637	0.949947918	0.925981327	0.851594246
DRG2	0.814108258	0.700682675	0.757235816	0.956154344	0.943901678	0.876189885
LIN9	0.814108258	0.707802804	0.750089824	0.934591536	0.956624006	0.894901464
PIGW	0.814108258	0.638499519	0.8895568	0.904990622	0.96532164	0.847196533
PDK4	0.814108258	0.747179932	0.751496241	0.95692783	0.99989309	0.740271903
LOC783641	0.814108258	0.641837566	0.836294915	0.915749182	0.99989309	0.742655778
LOC107132767	0.814108258	0.693051877	0.844065668	0.934591536	0.99989309	0.764546301
LOC100139764	0.814108258	0.711594168	0.830033146	0.891273781	0.99989309	0.788131005
NAA10	0.814108258	0.7116845	0.747093618	0.864540217	0.99989309	0.859487774
TMEM17	0.814262367	0.899387334	0.844065668	0.919247117	0.882754591	0.723641471
VEGFA	0.814262367	0.761788537	0.765074242	0.99879985	0.933637471	0.768077698
BACE2	0.814386802	0.763751563	0.806311697	0.99879985	0.87483668	0.763746661
RAB19	0.814386802	0.679006126	0.777638604	0.915749182	0.87483668	0.972487907
DPEP3	0.814386802	0.885007606	0.76145258	0.990059245	0.902843706	0.719551722
SNRPD1	0.814386802	0.823692881	0.788848487	0.915749182	0.907192767	0.855785545
LOC100141253	0.814386802	0.844758245	0.899195818	0.864540217	0.910770324	0.764546301
LOC112444326	0.814386802	0.66121103	0.836294915	0.868417977	0.925981327	0.956610132
IL17D	0.814386802	0.681181887	0.958420472	0.858006538	0.928881366	0.823139458

LMNB2	0.814386802	0.779536052	0.836294915	0.926078322	0.932197133	0.792329436
ACBD3	0.814386802	0.655710881	0.817670412	0.858006538	0.99989309	0.742259651
DNAJC19	0.814386802	0.747555258	0.757787642	0.908496281	0.99989309	0.763460317
MTERF4	0.814386802	0.647660219	0.765074242	0.913413181	0.99989309	0.911696156
DISP1	0.814462006	0.693153864	0.858865175	0.99879985	0.904151325	0.725528824
S100A8	0.814582942	0.703437628	0.772990161	0.915749182	0.99989309	0.784080446
N4BP2	0.815266121	0.695866639	0.747084388	0.90419464	0.99989309	0.727515552
POPDC2	0.815487738	0.808839416	0.787015778	0.853492367	0.892268822	0.937591162
CIDEB	0.815487738	0.733883576	0.87146798	0.959009349	0.90399271	0.764546301
ARHGEF1	0.815487738	0.640575107	0.907270422	0.995570827	0.91011204	0.728276429
RGS2	0.815487738	0.694355537	0.845237542	0.981022805	0.941107925	0.77208617
LOC101902531	0.815487738	0.777046516	0.764481234	0.995800463	0.943768595	0.764546301
LOC101906828	0.815487738	0.693153864	0.787740344	0.915749182	0.952821886	0.910920424
DISC1	0.815487738	0.712213007	0.806221631	0.995800463	0.976657193	0.715665393
ANXA6	0.815487738	0.739753419	0.765074242	0.850828215	0.977981414	0.936523789
SFXN2	0.815487738	0.650945381	0.752494129	0.919276287	0.99989309	0.735313642
COX7ALP1	0.815487738	0.686014664	0.777178978	0.928836449	0.99989309	0.742259651
BLVRB	0.815487738	0.688099473	0.747084388	0.943323364	0.99989309	0.761409946
MIC1	0.815487738	0.72477102	0.80193314	0.925931623	0.99989309	0.770391484
CHCHD2	0.815487738	0.65252936	0.787740344	0.915749182	0.99989309	0.77208617
EIF3CL	0.815487738	0.655710881	0.757235816	0.977216943	0.99989309	0.842016958
GTPBP6	0.815487738	0.661476635	0.751930447	0.87452213	0.99989309	0.86143104
BCAS1	0.815487738	0.707383119	0.757787642	0.910808102	0.99989309	0.870895425
GLP2R	0.815501688	0.638242621	0.979231344	0.91329897	0.934859184	0.751320135
ZDHHC4	0.815501688	0.690592057	0.875669731	0.87452213	0.99989309	0.732184848
TTC1	0.815501688	0.655589933	0.832731001	0.934591536	0.99989309	0.77208617
DCAF8	0.815644036	0.665733407	0.802857717	0.999939566	0.887132523	0.753088421
HIPK2	0.815644036	0.665563964	0.764057751	0.999939566	0.947826027	0.733424412
LOC112447474	0.81593866	0.692547228	0.803398613	0.915749182	0.910770324	0.928057673
ANKRA2	0.81593866	0.700959888	0.787740344	0.982140778	0.941064037	0.840150874
LRRN1	0.81593866	0.747499345	0.878603452	0.916026707	0.96532164	0.752377875
BEND5	0.816322841	0.641493941	0.776954726	0.962142137	0.882754591	0.939479667
IL17RA	0.816322841	0.685777004	0.815870524	0.990571725	0.887805568	0.851651757
SLC38A5	0.816322841	0.704817952	0.883726795	0.982140778	0.925981327	0.728276429
UQCR11	0.816609591	0.688099473	0.769822168	0.850828215	0.99989309	0.759729981
ACOT6	0.817914963	0.751946504	0.764057751	0.91329897	0.99989309	0.814576296
CDON	0.818033367	0.719622555	0.885434576	0.915749182	0.992440234	0.74008849
PDK2	0.818033367	0.659488126	0.789686719	0.91329897	0.99989309	0.714737045

ALOX15	0.818101836	0.680267573	0.846650632	0.999939566	0.941064037	0.728276429
G0S2	0.818101836	0.840874016	0.844065668	0.908496281	0.960296839	0.752900996
POLR2B	0.818101836	0.780082505	0.827125538	0.925004412	0.98871637	0.759308648
AIMP2	0.818464499	0.747499345	0.838488596	0.857792038	0.883619726	0.937591162
WDR35	0.818611351	0.747555258	0.765074242	0.99879985	0.881257517	0.822691858
EIF3E	0.819250974	0.939870925	0.750089824	0.915749182	0.943768595	0.764441047
OAZ1	0.819250974	0.688099473	0.84311048	0.972367384	0.968795608	0.770391484
ZNF792	0.819250974	0.719840239	0.754419313	0.958879502	0.996250445	0.830923241
CDC5L	0.819250974	0.823692881	0.819114812	0.861272209	0.99989309	0.717273603
ACOT2	0.819250974	0.644538289	0.777178978	0.90419464	0.99989309	0.723641471
CCDC30	0.819250974	0.677470151	0.914685423	0.858274325	0.99989309	0.734406433
TLR7	0.819250974	0.80403784	0.759671935	0.850828215	0.99989309	0.770391484
ZNF384	0.819396416	0.67497759	0.81285201	0.915749182	0.964150118	0.895218005
PIN4	0.819396416	0.762941584	0.789039067	0.883885658	0.99989309	0.752358594
NDUFA9	0.819396416	0.692987203	0.751496241	0.892229846	0.99989309	0.770391484
TAF8	0.819539966	0.710155842	0.777178978	0.995118128	0.877297732	0.859973633
NHLH1	0.819540694	0.678209697	0.757787642	0.925931623	0.957910867	0.926872824
CLSTN3	0.819540694	0.837382091	0.747093618	0.89088601	0.99989309	0.788142857
QTRT1	0.819898011	0.724540534	0.878603452	0.915749182	0.991830391	0.766589049
IKZF5	0.820167994	0.763478948	0.747093618	0.917869295	0.889271428	0.942800958
TIMM22	0.820167994	0.7116845	0.771402466	0.86288354	0.99989309	0.759308648
SETD3	0.820167994	0.675810425	0.827125538	0.951177494	0.99989309	0.792329436
CNP	0.820622893	0.693153864	0.789868841	0.940863121	0.943768595	0.88494083
RMI1	0.820622893	0.717132341	0.779087149	0.925919277	0.961879452	0.870895425
LOC112443141	0.820622893	0.659488126	0.787740344	0.982367414	0.99989309	0.723909814
MSS51	0.820667343	0.645207132	0.803771265	0.939292364	0.943768595	0.911696156
GART	0.820667343	0.652542331	0.788848487	0.90419464	0.957605284	0.956610132
UNC79	0.820667343	0.852463941	0.750089824	0.908496281	0.99989309	0.732184848
ZGPAT	0.820667343	0.702457626	0.76886194	0.943323364	0.99989309	0.78965579
CAPG	0.821051893	0.737937244	0.805449488	0.908054165	0.882917206	0.940683751
NOX1	0.821051893	0.680267573	0.880277219	0.999939566	0.892149112	0.731709303
PNPLA4	0.821051893	0.886967296	0.748576908	0.915749182	0.99989309	0.75292536
RASSF8	0.821051893	0.703437628	0.815548119	0.90419464	0.99989309	0.770391484
ENO2	0.821538779	0.804875341	0.747084388	0.919764668	0.87483668	0.928799366
ANAPC13	0.821538779	0.76090129	0.777178978	0.91329897	0.99989309	0.790039686
TNFRSF6B	0.821774158	0.718231253	0.783875251	0.992137653	0.87483668	0.86307729
MTA3	0.822492944	0.930469765	0.750089824	0.915749182	0.897363817	0.826868779
CDK2AP1	0.822492944	0.693544464	0.74784538	0.984915557	0.99989309	0.772409274

CHN2	0.82252914	0.796591413	0.787740344	0.992368879	0.883643824	0.79180744
UBIAD1	0.82252914	0.692928782	0.845237542	0.963829131	0.892966823	0.844622232
LOC112443479	0.82252914	0.887629766	0.817256644	0.948725226	0.902569851	0.725528824
OGFOD1	0.82252914	0.751946504	0.802847755	0.87630412	0.904151325	0.943685681
B4GALT3	0.82252914	0.693544464	0.757235816	0.905147767	0.941064037	0.968245211
NCKAP1	0.82252914	0.825768191	0.827350832	0.934591536	0.957605284	0.737883632
LOC101907189	0.82252914	0.852281996	0.790109639	0.934591536	0.973197369	0.728276429
AUP1	0.82252914	0.747899938	0.790109639	0.916305829	0.996250445	0.837501229
PHLDA3	0.82252914	0.790637585	0.777178978	0.915749182	0.998276725	0.812509877
IFT80	0.82252914	0.659488126	0.92582321	0.915749182	0.999592263	0.752857325
TXNRD3	0.82252914	0.708270551	0.856729427	0.861272209	0.99989309	0.725528824
PCBD1	0.82252914	0.780290359	0.801865674	0.925440563	0.99989309	0.742259651
NRF1	0.82252914	0.747499345	0.789686719	0.863958691	0.99989309	0.770391484
NELL1	0.82252914	0.652876871	0.765074242	0.968694679	0.99989309	0.770391484
MTERF2	0.82252914	0.677340516	0.765074242	0.87630412	0.99989309	0.792329436
RIDA	0.822657369	0.692547228	0.755534187	0.972378561	0.988693274	0.849769587
TEFM	0.82285846	0.682470071	0.868936984	0.891273781	0.882754591	0.941214045
TRPA1	0.82285846	0.924433008	0.765785685	0.915749182	0.904151325	0.809650729
SEC22B	0.82285846	0.707874469	0.798686086	0.943240775	0.962610771	0.849769587
LSM1	0.82285846	0.731268229	0.817993104	0.908054165	0.962791364	0.875441396
ZNF365	0.82285846	0.669648359	0.755362619	0.999939566	0.975291792	0.735313642
ETFRF1	0.82285846	0.677340516	0.814444866	0.956154344	0.975291792	0.838324968
LRRC8C	0.82285846	0.820796062	0.748723724	0.931989854	0.979654999	0.817471068
LOC786948	0.82285846	0.64691986	0.783791102	0.955560327	0.994688893	0.868745583
KLHL42	0.82285846	0.701581467	0.817256644	0.968306132	0.996250445	0.770891305
SDC2	0.82285846	0.657500304	0.8868125	0.901456922	0.99989309	0.748169845
PPP3CA	0.82285846	0.768680549	0.846650632	0.871894706	0.99989309	0.759107825
RND1	0.82285846	0.705889653	0.929958748	0.864540217	0.99989309	0.764546301
LOC100336669	0.82285846	0.688251757	0.787740344	0.90419464	0.99989309	0.770391484
ERF	0.82285846	0.812575653	0.765074242	0.87452213	0.99989309	0.783479395
TNFSF8	0.82285846	0.764097231	0.755534187	0.908496281	0.99989309	0.797813391
ZNF667	0.82285846	0.648099013	0.763592007	0.955560327	0.99989309	0.837249706
ACO2	0.822965407	0.719516802	0.747093618	0.87630412	0.99989309	0.800276945
MRO	0.822979254	0.719478857	0.777178978	0.949947918	0.926899298	0.88667499
SAA3	0.822991492	0.911106863	0.788938503	0.915749182	0.882754591	0.831041995
TMEM129	0.822991492	0.747555258	0.838488596	0.908054165	0.883643824	0.911696156
STON2	0.822991492	0.673034156	0.768676249	0.98189469	0.883643824	0.926872824
CXHXorf38	0.822991492	0.703437628	0.832483532	0.915749182	0.892150832	0.925494614

FBXL19	0.822991492	0.669648359	0.878050811	0.995118128	0.896242324	0.78355885
SYF2	0.822991492	0.665563964	0.855743966	0.995118128	0.899048045	0.809432201
PLXNB3	0.822991492	0.928284948	0.844065668	0.883885658	0.902843706	0.770391484
CEP170	0.822991492	0.702217402	0.92153058	0.925901365	0.904995537	0.795243197
LOC100849237	0.822991492	0.648099013	0.747093618	0.991837175	0.907192767	0.937591162
HCRTR1	0.822991492	0.747499345	0.780363715	0.999939566	0.910770324	0.759729981
LOC790218	0.822991492	0.646924712	0.87146798	0.925901365	0.932197133	0.880642012
PRRT1	0.822991492	0.769817928	0.846650632	0.917869295	0.933637471	0.834285507
CPEB2	0.822991492	0.655710881	0.880277219	0.99879985	0.934859184	0.728276429
NKAPD1	0.822991492	0.710155842	0.939683463	0.917869295	0.934859184	0.75292536
AARS2	0.822991492	0.700679806	0.976719499	0.886966548	0.934859184	0.758090697
PBK	0.822991492	0.671805407	0.836294915	0.999939566	0.939361296	0.769244117
LOC104969833	0.822991492	0.823965712	0.871859013	0.92064557	0.94294964	0.740622409
MICU3	0.822991492	0.72843033	0.76749973	0.999939566	0.943768595	0.780615615
CSNK2B	0.822991492	0.785163144	0.806311697	0.951929772	0.943768595	0.792329436
FAM96A	0.822991492	0.81250936	0.798507072	0.917869295	0.943901678	0.840150874
SPG11	0.822991492	0.786188116	0.810425784	0.979043693	0.952172109	0.752857325
LOC782054	0.822991492	0.707874469	0.829784147	0.956154344	0.960835287	0.808302424
LOC112446761	0.822991492	0.673034156	0.795356854	0.90419464	0.961382403	0.942421464
PLCL1	0.822991492	0.847082229	0.845237542	0.910808102	0.961835052	0.752900996
DTX2	0.822991492	0.761788537	0.883334551	0.892229846	0.961835052	0.799940894
ITGB1	0.822991492	0.721725677	0.88950849	0.905816196	0.964150118	0.805809435
SLC44A3	0.822991492	0.688099473	0.762423061	0.974590296	0.971927196	0.866482265
DDA1	0.822991492	0.76090129	0.879215964	0.915749182	0.977981285	0.75292536
FBN1	0.822991492	0.688099473	0.777178978	0.983569039	0.979654999	0.836652666
TMEM230	0.822991492	0.6861152	0.885481718	0.8968247	0.982014614	0.845748713
IGSF9	0.822991492	0.80403784	0.858865175	0.915749182	0.988693274	0.723028401
SSR1	0.822991492	0.6608873	0.873750661	0.91329897	0.990044405	0.849772722
STX17	0.822991492	0.653341013	0.926613927	0.936573639	0.994688893	0.728276429
FAM122A	0.822991492	0.821982753	0.823456027	0.915749182	0.996250445	0.768077698
F2RL1	0.822991492	0.665563964	0.827125538	0.92064557	0.99989309	0.725528824
PRR19	0.822991492	0.646945543	0.765074242	0.943323364	0.99989309	0.725528824
FBLN1	0.822991492	0.668175004	0.939683463	0.90419464	0.99989309	0.732184848
PCBP2	0.822991492	0.676166569	0.849077738	0.945336628	0.99989309	0.738110854
NRARP	0.822991492	0.689368188	0.762319981	0.891273781	0.99989309	0.740271903
NDUFA8	0.822991492	0.740647787	0.765074242	0.90419464	0.99989309	0.751677609
CACHD1	0.822991492	0.809942574	0.807152675	0.908496281	0.99989309	0.752900996
LOC101907545	0.822991492	0.703437628	0.747093618	0.948608757	0.99989309	0.75292536

LOC785804	0.822991492	0.699793718	0.751496241	0.956154344	0.99989309	0.770391484
MTG2	0.822991492	0.659488126	0.759748444	0.891273781	0.99989309	0.77208617
CFDP2	0.822991492	0.654850332	0.863172962	0.904990622	0.99989309	0.783479395
LOC101903478	0.822991492	0.655138935	0.827350832	0.915749182	0.99989309	0.789797484
AKAP9	0.822991492	0.688099473	0.852758896	0.893559455	0.99989309	0.790608202
POLR2L	0.822991492	0.747164473	0.757787642	0.904990622	0.99989309	0.796002422
LOC101903200	0.822991492	0.646945543	0.833955994	0.946344632	0.99989309	0.798431187
LENG9	0.822991492	0.669648359	0.755534187	0.991837175	0.99989309	0.805809435
CRYBG3	0.822991492	0.703437628	0.748576908	0.883885658	0.99989309	0.8125889
LOC112442288	0.822991492	0.655710881	0.777178978	0.904990622	0.99989309	0.838612096
GEM	0.822991492	0.781154903	0.751930447	0.925901365	0.99989309	0.839340243
LOC101903616	0.822991492	0.693487404	0.777178978	0.915749182	0.99989309	0.839419627
ADAMTS16	0.822991492	0.730582684	0.76749973	0.919247117	0.99989309	0.840150874
PDCD6	0.822991492	0.693454767	0.838488596	0.87452213	0.99989309	0.848704126
MMACHC	0.822991492	0.702989234	0.788848487	0.854497387	0.99989309	0.86307729
PSMA4	0.822991492	0.669747548	0.843427302	0.87630412	0.99989309	0.86307729
GPR176	0.822991492	0.674551689	0.777178978	0.925901365	0.99989309	0.879725389
TFB1M	0.822991492	0.652542331	0.747093618	0.858021281	0.99989309	0.912101047
C10H14orf1	0.822991492	0.745665426	0.751496241	0.883885658	0.99989309	0.921381434
FYTTD1	0.823056616	0.6861152	0.917876971	0.946344632	0.880265141	0.812682271
DCLK2	0.823056616	0.650624388	0.755324843	0.925931623	0.904995537	0.981411983
MINK1	0.823056616	0.674510077	0.802857717	0.99879985	0.905719399	0.842461327
GALNT15	0.823056616	0.665803806	0.788938503	0.96754757	0.91011204	0.909990551
MS4A8	0.823056616	0.751946504	0.832431595	0.990300191	0.943768595	0.752900996
VAT1L	0.823056616	0.705776259	0.796933111	0.99879985	0.988693274	0.729817953
PDZD11	0.823056616	0.703437628	0.819404084	0.925901365	0.99989309	0.77208617
DPF2	0.823056616	0.688099473	0.838488596	0.90419464	0.99989309	0.875441396
PDZD4	0.823186788	0.780290359	0.817256644	0.959727203	0.910770324	0.807277693
KRI1	0.8233769	0.695866639	0.877998684	0.908496281	0.938265656	0.875441396
PTPN22	0.8233769	0.72843033	0.755534187	0.868090726	0.954501767	0.969551744
CD86	0.8233769	0.737374533	0.750089824	0.912712485	0.972917711	0.934458012
OSBPL8	0.8233769	0.647660219	0.834793268	0.995118128	0.997981612	0.764546301
GPATCH2L	0.8233769	0.702457626	0.832483532	0.915749182	0.99989309	0.815459921
DUSP8	0.823391562	0.736066622	0.764057751	0.883885658	0.910770324	0.98016619
PYM1	0.823391562	0.759619476	0.777178978	0.958879502	0.925981327	0.86143104
NLGN3	0.823391562	0.751994319	0.747806937	0.915749182	0.943768595	0.936690389
LPAR2	0.823391562	0.775921167	0.828407935	0.915749182	0.964150118	0.815459921
FARP2	0.823391562	0.681991477	0.827125538	0.87630412	0.982014614	0.928799366

CTSC	0.823391562	0.756776837	0.850751395	0.855137785	0.99989309	0.734664544
LOC100196901	0.823391562	0.912112179	0.74784538	0.90419464	0.99989309	0.743464215
ZBTB34	0.823391562	0.706231276	0.765074242	0.924489924	0.99989309	0.84491737
CEP126	0.823391562	0.763751563	0.788848487	0.890546185	0.99989309	0.859973633
LOC786733	0.823557201	0.769817928	0.774248983	0.928623745	0.904151325	0.905325866
IL7	0.823653185	0.655710881	0.765074242	0.999939566	0.886279726	0.821914747
LOC101905801	0.823653185	0.756805777	0.79610462	0.888642721	0.99989309	0.875441396
MLLT10	0.823731895	0.740948281	0.852758896	0.946344632	0.910770324	0.8125889
GADD45G	0.823756645	0.675748768	0.828407935	0.992368879	0.940918626	0.827631181
HMGN1	0.823756645	0.740948281	0.87146798	0.880286519	0.941064037	0.875960923
ASIC2	0.823756645	0.655710881	0.787740344	0.86288354	0.941064037	0.991281092
UBE2N	0.82385159	0.653341013	0.879376765	0.934591536	0.952172109	0.84491737
CDKL4	0.823885749	0.719516802	0.812258905	0.956154344	0.883643824	0.887248119
PFKP	0.823885749	0.655138935	0.765074242	0.951606954	0.934859184	0.942421464
RHOA	0.823885749	0.703437628	0.870920678	0.984335993	0.946536678	0.753138357
GJD3	0.823885749	0.685517925	0.807843373	0.915749182	0.99989309	0.759308648
RINT1	0.823885749	0.661476635	0.812770091	0.925210703	0.99989309	0.765573509
NIPSNAP2	0.823885749	0.688099473	0.806311697	0.915749182	0.99989309	0.777933888
KIAA2026	0.823885749	0.77396808	0.788848487	0.883885658	0.99989309	0.845803016
KMT2E	0.823885749	0.769817928	0.751496241	0.915749182	0.99989309	0.859481777
BRK1	0.823911041	0.747899938	0.820767684	0.915749182	0.99989309	0.740271903
STAT5A	0.824023023	0.652542331	0.777178978	0.86288354	0.988693274	0.978656049
BLOC1S2	0.824239363	0.684832051	0.84207783	0.937531834	0.943768595	0.870895425
ZGRF1	0.824243478	0.67546099	0.920607829	0.947615714	0.907640102	0.797730509
ANKRD40	0.824243478	0.692025761	0.843427302	0.999939566	0.941064037	0.728567358
TOMM40L	0.824243478	0.757368351	0.8045653	0.888642721	0.996250445	0.879725389
APLNR	0.824243478	0.668175004	0.750089824	0.99879985	0.99989309	0.728276429
NDUFB10	0.824243478	0.703437628	0.775786014	0.915749182	0.99989309	0.788411132
RGS6	0.824243478	0.810519292	0.774555223	0.87630412	0.99989309	0.812509877
SPATA20	0.824271618	0.909085943	0.880277219	0.865046898	0.91011204	0.764546301
LRWD1	0.824575734	0.669648359	0.963643451	0.945301766	0.882917206	0.757327233
LOC107132870	0.824575734	0.857034402	0.750089824	0.956154344	0.911283882	0.832703434
COX10	0.824575734	0.693544464	0.774385549	0.868090726	0.961835052	0.97084193
ABRAXAS2	0.824575734	0.655138935	0.92153058	0.943539568	0.999592263	0.728276429
PPM1L	0.824575734	0.699537133	0.765074242	0.95964071	0.99989309	0.812509877
TRMT10C	0.824607188	0.665142095	0.788848487	0.915749182	0.902843706	0.978656049
LOC104975593	0.824823227	0.707140461	0.817256644	0.999939566	0.886279726	0.747674417
LOC104975590	0.824823227	0.687403165	0.846650632	0.90419464	0.886279726	0.949527038

TCEAL4	0.824823227	0.703437628	0.811009079	0.90419464	0.901432895	0.963430383
TCF20	0.824823227	0.689053394	0.899195818	0.98860254	0.904151325	0.770391484
ZNF407	0.824823227	0.691357843	0.827125538	0.915749182	0.926899298	0.93691717
PGR	0.824823227	0.704923066	0.820767684	0.868090726	0.927804504	0.96163007
CAAP1	0.824823227	0.665524216	0.846782643	0.998542029	0.965219542	0.754897098
RASGEF1B	0.824823227	0.720945114	0.796677998	0.99879985	0.990424444	0.728276429
LOC112447302	0.824823227	0.747499345	0.927460291	0.87630412	0.996250445	0.759107825
SMIM37	0.824823227	0.901922021	0.763370546	0.86288354	0.99989309	0.77208617
KPTN	0.824823227	0.699417603	0.759671935	0.921477746	0.99989309	0.794529985
MRPL53	0.824823227	0.6663481	0.810425784	0.921284421	0.99989309	0.881439164
EVA1A	0.825203536	0.677470151	0.815118489	0.999939566	0.943768595	0.743464215
CLTA	0.82535347	0.692025761	0.902141181	0.985342672	0.887132523	0.77208617
MGAT2	0.82535347	0.693153864	0.788848487	0.908496281	0.904151325	0.9759685
ZDHHC17	0.82535347	0.821124254	0.757235816	0.915749182	0.99989309	0.794529985
PPP6C	0.825602816	0.735625249	0.926613927	0.925931623	0.932081893	0.759093273
NXF1	0.825602816	0.658193849	0.838838955	0.924489924	0.99989309	0.80844231
SIGMAR1	0.825602816	0.737937244	0.765074242	0.87452213	0.99989309	0.921718583
PRMT6	0.825772063	0.712908612	0.886481882	0.891273781	0.954501767	0.858042054
MESP1	0.82587231	0.876169307	0.812770091	0.915749182	0.954501767	0.788131005
POC5	0.82587231	0.675728311	0.86360816	0.998542029	0.961382403	0.730588392
IL4I1	0.826036743	0.693153864	0.966157152	0.868090726	0.899048045	0.840150874
SNED1	0.826036743	0.651481516	0.897590515	0.943323364	0.954501767	0.809650729
GIPC2	0.826036743	0.695114938	0.870054336	0.983151855	0.960835287	0.75292536
LOC104968435	0.826036743	0.766403118	0.81285201	0.883885658	0.982073092	0.884387948
RNF31	0.826036743	0.717871715	0.914685423	0.912712485	0.988693274	0.770131172
GHSR	0.826036743	0.669648359	0.844316758	0.940863121	0.999356955	0.828663852
NOV	0.826036743	0.750546265	0.843427302	0.90419464	0.99989309	0.745987575
LOC101902922	0.826387577	0.912112179	0.802951601	0.916305829	0.928830848	0.77208617
LOC112444904	0.826387577	0.689053394	0.817256644	0.883885658	0.961382403	0.944524573
CEP131	0.826849421	0.688099473	0.807563733	0.999939566	0.957605284	0.77208617
FAM174A	0.827254022	0.747899938	0.870054336	0.915749182	0.982014614	0.773402056
GDPD4	0.827254022	0.769313383	0.779087149	0.959727203	0.999592263	0.773957499
TMA7	0.827254022	0.836487169	0.765074242	0.891273781	0.99989309	0.729817953
METTL9	0.827254022	0.703437628	0.828407935	0.956154344	0.99989309	0.735235065
LOC100848941	0.827254022	0.712838989	0.81285201	0.904990622	0.99989309	0.764546301
TTC13	0.827254022	0.810158911	0.765074242	0.883885658	0.99989309	0.806054104
ACO1	0.827384261	0.707874469	0.832483532	0.999939566	0.884679766	0.753138357
LOC104968476	0.827384261	0.703437628	0.914668913	0.883885658	0.90448127	0.889201226

ARL8A	0.827384261	0.761949365	0.917876971	0.934591536	0.906183446	0.759107825
BNC1	0.827384261	0.703437628	0.962209895	0.908496281	0.941064037	0.764546301
TRARG1	0.827384261	0.815963293	0.777178978	0.990049248	0.952172109	0.770391484
C3H1orf54	0.827384261	0.955798423	0.787740344	0.868090726	0.964172455	0.770391484
GLRX5	0.827384261	0.732654391	0.787740344	0.865264265	0.99989309	0.759729981
NDUFA2	0.827384261	0.703437628	0.788848487	0.89088601	0.99989309	0.770391484
ARMCX6	0.827384261	0.713953236	0.751496241	0.99879985	0.99989309	0.770391484
KALRN	0.827384261	0.820000472	0.764481234	0.883885658	0.99989309	0.788131005
C25H16orf54	0.827426711	0.722790338	0.777178978	0.915749182	0.99989309	0.845628259
LOC101902128	0.827758338	0.740540908	0.863172962	0.915749182	0.882754591	0.893961809
SEMA5B	0.827758338	0.693300643	0.877133001	0.908054165	0.883643824	0.926872824
LOC101908113	0.827758338	0.907878616	0.760913563	0.903607716	0.899048045	0.887252664
CHCHD10	0.827758338	0.735625249	0.768676249	0.91329897	0.99989309	0.763746661
ARFIP2	0.827758338	0.757368351	0.753656036	0.915749182	0.99989309	0.862788295
DNM2	0.827758338	0.693153864	0.762319981	0.915749182	0.99989309	0.912396352
SCD	0.827758338	0.657396352	0.765440147	0.865046898	0.99989309	0.921254858
REPS2	0.828338461	0.703437628	0.812770091	0.935763381	0.941064037	0.895218005
BRD9	0.828701019	0.883267666	0.775786014	0.913413181	0.961835052	0.817471068
KRT24	0.829006953	0.795421065	0.760400875	0.948416955	0.99989309	0.747674417
HM13	0.829336355	0.785383224	0.757787642	0.936154294	0.894638153	0.917546198
NT5C	0.829336355	0.699537133	0.817256644	0.988949923	0.902843706	0.865997479
LMX1A	0.829336355	0.704817952	0.787740344	0.999939566	0.927806098	0.785975261
SCN11A	0.829336355	0.897918739	0.812043145	0.919247117	0.932033253	0.785275816
CHPF2	0.829336355	0.688099473	0.757787642	0.956154344	0.941064037	0.934458012
LOC112444288	0.829336355	0.655619314	0.824817082	0.958879502	0.952821886	0.880404833
AGFG2	0.829336355	0.6861152	0.755534187	0.934591536	0.99989309	0.783134114
MEIOB	0.829336355	0.778839781	0.795199152	0.868090726	0.99989309	0.864219261
TLE4	0.829336355	0.665563964	0.753656036	0.91329897	0.99989309	0.957877422
SPON2	0.829386515	0.866803532	0.802857717	0.951929772	0.904151325	0.787719474
SLC39A7	0.829386515	0.866803532	0.755534187	0.913413181	0.939361296	0.882170826
ZNF829	0.829760236	0.672069372	0.819404084	0.897446122	0.99989309	0.76360318
KDELC2	0.830178837	0.886967296	0.838927319	0.865118174	0.883643824	0.866253786
ARSI	0.83020475	0.730406881	0.87146798	0.953648387	0.904151325	0.819814885
TSPAN9	0.830220249	0.840313358	0.773281026	0.99419346	0.943768595	0.749141396
FKBP9	0.830220249	0.680267573	0.754897816	0.903607716	0.99989309	0.820483064
PPP1R16A	0.830221601	0.685980044	0.894414265	0.908054165	0.99989309	0.770391484
TEX9	0.830317149	0.926435857	0.803398613	0.940921143	0.907192767	0.745045717
NCAPG2	0.830317149	0.655710881	0.97381056	0.921761002	0.932197133	0.766359829

SLC15A4	0.830317149	0.685980044	0.750089824	0.916305829	0.99989309	0.731995209
LOC101904601	0.830317149	0.659488126	0.787740344	0.914402649	0.99989309	0.752900996
NCK1	0.830396371	0.6608873	0.751496241	0.999939566	0.988693274	0.810707901
DGKA	0.830626548	0.693153864	0.820767684	0.946344632	0.99989309	0.770391484
PRPF38B	0.83076861	0.700659415	0.777178978	0.92064557	0.99989309	0.774602575
CENPO	0.831350568	0.693544464	0.939683463	0.931750296	0.94217472	0.763746661
UBA7	0.831415381	0.679549691	0.883726795	0.915749182	0.99989309	0.757327233
LOC112448269	0.831415381	0.662366902	0.777178978	0.873519404	0.99989309	0.809650729
LOC112445029	0.831744751	0.680835681	0.912223566	0.990571725	0.905719399	0.770391484
CCR3	0.831744751	0.994376473	0.765074242	0.86288354	0.934859184	0.76360318
RAD9B	0.831744751	0.689368188	0.765074242	0.99879985	0.952821886	0.845803016
PANX1	0.831744751	0.6861152	0.825888497	0.990049248	0.961835052	0.826370041
RRAGA	0.831744751	0.655619314	0.894829955	0.908054165	0.965219542	0.872224159
HTRA2	0.831744751	0.747179932	0.850106729	0.915749182	0.99989309	0.745987575
RPRM	0.831744751	0.693544464	0.883077722	0.925901365	0.99989309	0.764546301
BCORL1	0.831744751	0.665563964	0.777178978	0.981022805	0.99989309	0.836652666
ZBTB2	0.831755962	0.81250936	0.801087628	0.90419464	0.99989309	0.738110854
METTL22	0.831755962	0.673034156	0.836294915	0.908496281	0.99989309	0.764546301
LRFN3	0.831755962	0.685980044	0.756911784	0.908496281	0.99989309	0.839419627
ABHD8	0.832021742	0.798799934	0.788848487	0.951929772	0.99989309	0.74008849
DECR1	0.832021742	0.677470151	0.751496241	0.925901365	0.99989309	0.8125889
BBS7	0.832021742	0.834741258	0.788938503	0.91329897	0.99989309	0.817471068
SSR4	0.832066921	0.692547228	0.858865175	0.990571725	0.952172109	0.783134114
FUNDC2	0.832462856	0.693544464	0.817256644	0.939292364	0.99989309	0.747674417
RETREG2	0.832657429	0.661476635	0.801865674	0.999939566	0.899048045	0.832659179
TXNRD2	0.832657429	0.675748768	0.882890706	0.869637988	0.99989309	0.752358594
LRRC41	0.832992128	0.907594268	0.767160534	0.943323364	0.943901678	0.770391484
VIPR2	0.832992128	0.744527629	0.877816623	0.934591536	0.988693274	0.75292536
RPS4Y1	0.833177354	0.690592057	0.856812671	0.935163227	0.908708309	0.887248119
RMRP	0.833177354	0.663180847	0.765074242	0.969717497	0.954501767	0.924645458
APEX2	0.833177354	0.687403165	0.827125538	0.92064557	0.99989309	0.770391484
NID1	0.833177354	0.903820329	0.787740344	0.868090726	0.99989309	0.777933888
SEC14L1	0.833177354	0.775921167	0.777178978	0.928623745	0.99989309	0.783479395
EIF3J	0.833347167	0.703437628	0.852758896	0.992692773	0.904151325	0.811943134
ZDHHC20	0.833347167	0.666533003	0.856105201	0.995118128	0.939361296	0.806469522
GTF2B	0.833347167	0.6608873	0.929958748	0.915749182	0.99989309	0.742451714
ATP5F1D	0.833347167	0.688099473	0.752969568	0.898441424	0.99989309	0.77208617
CLP1	0.833458852	0.720945114	0.828737895	0.935163227	0.99989309	0.764546301

ZSCAN2	0.833461859	0.72843033	0.833567938	0.999939566	0.933775851	0.742259651
COL8A1	0.833758773	0.899142992	0.765074242	0.990049248	0.888598655	0.77208617
VSIG10L	0.833758773	0.718231253	0.976869041	0.91329897	0.889271428	0.770391484
NCKAP5L	0.833758773	0.670188815	0.838871111	0.999939566	0.892149112	0.792329436
ZC4H2	0.833758773	0.68147929	0.814444866	0.931268564	0.90448127	0.945862772
HDAC3	0.833758773	0.838843184	0.808333956	0.995800463	0.904995537	0.745392593
LOC104973105	0.833758773	0.736964914	0.846650632	0.980955077	0.91011204	0.815459921
LOC100139345	0.833758773	0.684832051	0.866753941	0.897891011	0.934859184	0.939426596
SLC7A4	0.833758773	0.659488126	0.820549834	0.943323364	0.943768595	0.921582976
REP15	0.833758773	0.668175004	0.773281026	0.990571725	0.943901678	0.903581348
VIPR1	0.833758773	0.848136412	0.856997596	0.910808102	0.952672789	0.783479395
GIPC1	0.833758773	0.803903736	0.765074242	0.987143706	0.960835287	0.792329436
LRRC47	0.833758773	0.671879214	0.844065668	0.946344632	0.961382403	0.86307729
BTG1	0.833758773	0.688251757	0.817256644	0.999939566	0.970377899	0.757327233
LOC112446010	0.833758773	0.730317314	0.770051055	0.942235206	0.975291792	0.884387948
CYB5R3	0.833758773	0.705046225	0.787015778	0.995800463	0.975375626	0.80787758
RER1	0.833758773	0.699507369	0.827125538	0.915749182	0.976970807	0.891000792
GEMIN5	0.833758773	0.72477102	0.939683463	0.89088601	0.98152792	0.77208617
PP2D1	0.833758773	0.768680549	0.79610462	0.934174776	0.988693274	0.837249706
MLLT3	0.833758773	0.840078234	0.764481234	0.915749182	0.996250445	0.839494796
ISCU	0.833758773	0.733388284	0.786290681	0.958879502	0.99989309	0.740271903
RBX1	0.833758773	0.688099473	0.836294915	0.903607716	0.99989309	0.741458429
PARD6A	0.833758773	0.810519292	0.838488596	0.863958691	0.99989309	0.752857325
SLC39A6	0.833758773	0.707874469	0.843472659	0.904990622	0.99989309	0.752900996
FKBP1A	0.833758773	0.688099473	0.883726795	0.91329897	0.99989309	0.75292536
LAGE3	0.833758773	0.699537133	0.836257071	0.943323364	0.99989309	0.75292536
SLC8A2	0.833758773	0.835130235	0.795199152	0.896291232	0.99989309	0.755204495
LOC101902854	0.833758773	0.692987203	0.777178978	0.873421154	0.99989309	0.76360318
PTPRF	0.833758773	0.693544464	0.882898206	0.919247117	0.99989309	0.76360318
CDK5	0.833758773	0.707802804	0.770051055	0.91329897	0.99989309	0.76935309
CEBPD	0.833758773	0.665524216	0.818793839	0.990571725	0.99989309	0.770265362
GGN	0.833758773	0.688099473	0.843427302	0.89088601	0.99989309	0.77208617
PARS2	0.833758773	0.669039484	0.812770091	0.88501388	0.99989309	0.808302424
DNASE1	0.833758773	0.6861152	0.85122003	0.915749182	0.99989309	0.821467423
PRRC1	0.833758773	0.710028977	0.757787642	0.955560327	0.99989309	0.827241584
LOC101904574	0.833758773	0.692987203	0.780363715	0.925265292	0.99989309	0.829662533
PAPOLB	0.833804056	0.659488126	0.845237542	0.908054165	0.99989309	0.752358594
LOC101906779	0.83384748	0.675810425	0.933102499	0.905185614	0.941107925	0.86252043

OSR2	0.83384748	0.693153864	0.764057751	0.999939566	0.961382403	0.747230622
LOC100141258	0.83384748	0.86741371	0.770051055	0.90419464	0.99989309	0.749691032
PDXP	0.83384748	0.692928782	0.827125538	0.90419464	0.99989309	0.752358594
LOC107131792	0.83384748	0.70088693	0.856769954	0.921652467	0.99989309	0.799147792
NAT10	0.833885631	0.835809337	0.772990161	0.898441424	0.887132523	0.942868422
RTF1	0.833885631	0.693544464	0.757787642	0.875889425	0.99989309	0.844622232
LIG4	0.833965712	0.758051794	0.959159138	0.903607716	0.92188432	0.772530815
CDK12	0.833965712	0.747499345	0.839378262	0.91329897	0.99989309	0.77208617
NMRAL1	0.833965712	0.718302288	0.777178978	0.915749182	0.99989309	0.833139817
DOPEY2	0.834061336	0.71963672	0.833531187	0.925004412	0.960835287	0.869171706
MYCL	0.834061336	0.796591413	0.812770091	0.908054165	0.99989309	0.777537507
KLHL21	0.834064358	0.813511912	0.765074242	0.959009349	0.899048045	0.871630129
LOC112445912	0.834064358	0.703437628	0.845237542	0.934591536	0.99989309	0.742259651
LOC104970589	0.834072288	0.710486439	0.954463909	0.896408418	0.954501767	0.792329436
LOC112443614	0.834664888	0.790637585	0.80329874	0.910808102	0.954501767	0.891000792
CRIM1	0.834664888	0.80403784	0.849077738	0.915749182	0.977010303	0.796934582
ARHGEF9	0.834664888	0.688099473	0.817993104	0.999939566	0.988693274	0.764546301
VTA1	0.834664888	0.78070503	0.827125538	0.913413181	0.999592263	0.838324968
COX20	0.83473563	0.81808696	0.808333956	0.914862928	0.99989309	0.747674417
DEPDC7	0.83473563	0.76167712	0.79610462	0.915749182	0.99989309	0.799314198
MANBA	0.834847173	0.705458146	0.788848487	0.949947918	0.99989309	0.737883632
NFYC	0.834944948	0.747179932	0.853222116	0.91329897	0.934859184	0.887248119
NAP1L5	0.834944948	0.751946504	0.787740344	0.908054165	0.99989309	0.78510176
RABGAP1	0.834944948	0.797209625	0.836294915	0.868090726	0.99989309	0.803732909
ANKRD27	0.835015733	0.737937244	0.755534187	0.908496281	0.99989309	0.893961809
LTB	0.835148307	0.7116845	0.845237542	0.915749182	0.904995537	0.918220726
FOXJ2	0.835148307	0.835645515	0.834793268	0.90419464	0.957605284	0.839419627
LOC101903526	0.835148307	0.718302402	0.843427302	0.908054165	0.962262333	0.902122572
NUDT16L1	0.835148307	0.787965627	0.787740344	0.908496281	0.972917711	0.893961809
ZFYVE1	0.835148307	0.703437628	0.777178978	0.916305829	0.996290884	0.915447631
HIGD2A	0.835148307	0.693153864	0.770871239	0.925901365	0.99989309	0.755204495
STX8	0.835148307	0.688251757	0.843733648	0.908496281	0.99989309	0.770426407
BOLA1	0.835148307	0.897327046	0.765074242	0.904990622	0.99989309	0.792329436
CCDC28A	0.835148307	0.683518638	0.813541285	0.925901365	0.99989309	0.840150874
CDH15	0.835148307	0.669648359	0.81284583	0.87452213	0.99989309	0.875441396
ADGRB2	0.835275014	0.688099473	0.796847019	0.999939566	0.922811199	0.752358594
CAD	0.835275014	0.66071365	0.911743083	0.956154344	0.972917711	0.77208617
NRIP3	0.835275014	0.808517017	0.822417045	0.922713162	0.99989309	0.763746661

BCKDK	0.835275014	0.734188031	0.761854021	0.88501388	0.99989309	0.770391484
LOC112442851	0.835741152	0.708437597	0.918576062	0.915749182	0.906791802	0.86143104
LOC101903564	0.835741152	0.703437628	0.939683463	0.925004412	0.925981327	0.796593386
PEX5	0.835741152	0.803995245	0.765074242	0.936573639	0.925981327	0.896538019
LOC101908104	0.835741152	0.677470151	0.909170161	0.925901365	0.961382403	0.827925468
SPTLC3	0.835741152	0.707874469	0.823456027	0.883885658	0.966423767	0.941925704
LOC107133071	0.835741152	0.733386687	0.834569848	0.988949923	0.996250445	0.75292536
ATP5F1E	0.835741152	0.688099473	0.765074242	0.883885658	0.99989309	0.763656348
DCTN3	0.835741152	0.712645214	0.823527402	0.915749182	0.99989309	0.770391484
KLF12	0.836054017	0.6608873	0.765074242	0.919247117	0.99989309	0.839419627
KLK13	0.83629246	0.711124077	0.849077738	0.869637988	0.892149112	0.968245211
REXO2	0.83629246	0.855979085	0.80326876	0.905816196	0.961835052	0.845628259
PARP12	0.83629246	0.693153864	0.974158329	0.897446122	0.986976797	0.743377609
VAV1	0.836418891	0.750112192	0.753656036	0.908054165	0.99989309	0.867440033
SLCO1C1	0.836820752	0.679258786	0.790109639	0.923873451	0.99989309	0.76360318
SENP8	0.837213602	0.669648359	0.786602178	0.999939566	0.99989309	0.77208617
MED10	0.837255071	0.692987203	0.875669731	0.99879985	0.941269773	0.763746661
SLC2A6	0.837255347	0.669648359	0.757235816	0.999939566	0.904995537	0.850612178
PSMG2	0.837255347	0.711594168	0.787740344	0.919247117	0.915496086	0.958475633
FBXL3	0.837416289	0.868789501	0.870054336	0.904769083	0.899048045	0.828376685
LOC107131424	0.83755349	0.739918356	0.773281026	0.915749182	0.925981327	0.955585296
PGS1	0.83755349	0.836487169	0.75548993	0.928836449	0.961835052	0.86143104
UBAC1	0.83755349	0.742728851	0.764481234	0.922782374	0.99989309	0.770391484
DCTPP1	0.83755349	0.76013532	0.755534187	0.915749182	0.99989309	0.86179903
GNL3L	0.837719755	0.72843033	0.798686086	0.91329897	0.99989309	0.86143104
KIF13A	0.837845196	0.747164473	0.812770091	0.995118128	0.91822454	0.828376685
TTC39C	0.838080776	0.707802804	0.827125538	0.938867707	0.949645557	0.887457549
FBXW5	0.838080776	0.835276066	0.770051055	0.87487639	0.99989309	0.752358594
COX7A1	0.838080776	0.697949203	0.765074242	0.964413123	0.99989309	0.75292536
IQCH	0.838080776	0.700682675	0.757235816	0.943323364	0.99989309	0.895287865
FCAR	0.838317703	0.731268229	0.787740344	0.999939566	0.906223387	0.794529985
PFN1	0.838317703	0.893430006	0.839833549	0.90419464	0.952172109	0.799935743
ITFG2	0.838317703	0.693153864	0.793871071	0.990049248	0.975375626	0.847189049
LOC614785	0.838317703	0.905210378	0.754101752	0.915749182	0.99989309	0.806054104
GPAT2	0.838317703	0.812502616	0.765074242	0.91329897	0.99989309	0.86307729
CACNA1H	0.83837719	0.932024312	0.777178978	0.919247117	0.934859184	0.799314198
LGALS1	0.83837719	0.792663811	0.839622619	0.939292364	0.99989309	0.740271903
ADGRV1	0.838470663	0.818435706	0.766184475	0.999939566	0.915017227	0.752900996

COLQ	0.838470663	0.77575347	0.784107501	0.999939566	0.934859184	0.770391484
LAMC2	0.838470663	0.747499345	0.878050811	0.89088601	0.954501767	0.879725389
ZNF219	0.838470663	0.71963672	0.832483532	0.948547383	0.964836226	0.844622232
SLCO4A1	0.838470663	0.701581467	0.765074242	0.943323364	0.99989309	0.749691032
MRPL24	0.838470663	0.76145014	0.757235816	0.931268564	0.99989309	0.807886012
RTL5	0.838611102	0.699626135	0.80326876	0.990049248	0.99989309	0.77208617
RAF1	0.838731164	0.699100407	0.787740344	0.990049248	0.915496086	0.90843033
AP1G1	0.838731164	0.7116845	0.886282865	0.91329897	0.987694035	0.839419627
LOC100850276	0.83901468	0.736964914	0.832431595	0.943323364	0.925981327	0.881550981
SDF2L1	0.839126218	0.843640278	0.765074242	0.915749182	0.968477804	0.86143104
TM2D1	0.839258006	0.825215718	0.760400875	0.915749182	0.99989309	0.770391484
LOC112447103	0.839350509	0.693544464	0.765074242	0.984100122	0.897238235	0.941925704
SLC35A5	0.839350509	0.766403118	0.844162118	0.99879985	0.897978818	0.77208617
CD52	0.839350509	0.823692881	0.765074242	0.915749182	0.941064037	0.914440253
TXNL1	0.839350509	0.673034156	0.885354035	0.990049248	0.943768595	0.799935743
MIB1	0.839350509	0.66403328	0.828407935	0.999939566	0.952172109	0.770426407
LIN7C	0.839350509	0.730317314	0.939683463	0.925901365	0.954501767	0.752358594
UPF3B	0.839350509	0.700682675	0.916702	0.86950142	0.956885889	0.886992214
OSBPL6	0.839350509	0.687403165	0.777178978	0.999939566	0.960296839	0.770391484
LOC112447118	0.839350509	0.774697122	0.788848487	0.90419464	0.961835052	0.925420938
SEC13	0.839350509	0.934248552	0.776574729	0.925901365	0.966423767	0.752900996
COL26A1	0.839350509	0.889794524	0.754897816	0.927644708	0.975291792	0.809650729
FSTL1	0.839350509	0.703437628	0.781294783	0.996601241	0.988693274	0.817471068
NPEPPS	0.839350509	0.72843033	0.802847755	0.936835069	0.99989309	0.752900996
RAC3	0.839350509	0.6663481	0.81285201	0.903607716	0.99989309	0.75292536
C7H5orf30	0.839350509	0.6861152	0.850952986	0.943323364	0.99989309	0.770391484
MAN1A1	0.839350509	0.693454767	0.806221631	0.90419464	0.99989309	0.77208617
BCLAF3	0.839350509	0.715519139	0.765074242	0.989391869	0.99989309	0.796511119
TPRN	0.839350509	0.700682675	0.808942589	0.925901365	0.99989309	0.805216326
LYRM9	0.839350509	0.685777004	0.793443916	0.919247117	0.99989309	0.806054104
WHRN	0.839350509	0.693959692	0.834569848	0.904990622	0.99989309	0.808138656
CCDC51	0.839350509	0.665563964	0.806311697	0.915749182	0.99989309	0.812622197
DOLPP1	0.839350509	0.852463941	0.755534187	0.886966548	0.99989309	0.863755488
C1QBP	0.839350509	0.701581467	0.769024417	0.892229846	0.99989309	0.914440253
LOC101908166	0.839517409	0.883267666	0.759671935	0.924489924	0.941107925	0.86307729
ERGIC2	0.839517409	0.688251757	0.787015778	0.944558402	0.99989309	0.86143104
NCOA3	0.839518675	0.72477102	0.842605286	0.977216943	0.902843706	0.86307729
LOC785087	0.839518675	0.86515799	0.761854021	0.992137653	0.934859184	0.785975261

SP140L	0.839518675	0.693544464	0.958420472	0.92064557	0.960835287	0.759901657
FADS1	0.839518675	0.748534108	0.763592007	0.913413181	0.996250445	0.930707319
LOC112448378	0.839518675	0.674551689	0.770051055	0.982554614	0.997488969	0.882322567
BARX1	0.839518675	0.707140461	0.873750661	0.904990622	0.99989309	0.746004817
COA3	0.839518675	0.688099473	0.808731835	0.915749182	0.99989309	0.763239138
SLC6A2	0.839518675	0.674510077	0.879215964	0.904074458	0.99989309	0.786737722
COL4A6	0.839518675	0.885981636	0.769285605	0.915749182	0.99989309	0.788829963
MRPL49	0.839518675	0.693153864	0.840938565	0.929812579	0.99989309	0.798482976
LOC112441452	0.839703607	0.692547228	0.765074242	0.999939566	0.894300232	0.77208617
DIS3L2	0.839703607	0.700796351	0.87214481	0.908496281	0.894638153	0.942421464
TSPAN13	0.839703607	0.747899938	0.86719452	0.999939566	0.899048045	0.752358594
TSPYL4	0.839703607	0.962934461	0.787740344	0.943323364	0.899048045	0.753067712
SBSPON	0.839703607	0.692547228	0.810425784	0.992137653	0.912365272	0.891140839
LOC112447309	0.839703607	0.780290359	0.760400875	0.883885658	0.927804504	0.982045172
TMEM229B	0.839703607	0.703437628	0.793430641	0.934267153	0.933637471	0.943138585
C5H12orf75	0.839703607	0.90249621	0.833955994	0.915749182	0.941064037	0.788411132
LOC112449602	0.839703607	0.866813771	0.755534187	0.904990622	0.943768595	0.913166854
ADCY4	0.839703607	0.692461545	0.827125538	0.999939566	0.952172109	0.795243197
LOC531462	0.839703607	0.761788537	0.757787642	0.99879985	0.952172109	0.840150874
ATP6V1H	0.839703607	0.709162177	0.787740344	0.992829869	0.952172109	0.862788295
RBM4B	0.839703607	0.73984736	0.845237542	0.925901365	0.952172109	0.868108836
RCCD1	0.839703607	0.771765977	0.879215964	0.915749182	0.952672789	0.826868779
RBM33	0.839703607	0.77295675	0.879215964	0.892229846	0.95956556	0.858538874
CARD19	0.839703607	0.780290359	0.850174739	0.952695586	0.961835052	0.77208617
LOC101903232	0.839703607	0.665563964	0.764057751	0.999939566	0.970733065	0.844622232
ADTRP	0.839703607	0.758750448	0.846782643	0.971220023	0.988693274	0.752857325
IPO5	0.839703607	0.7635426	0.765074242	0.990571725	0.999592263	0.80792472
LAMTOR5	0.839703607	0.703437628	0.827125538	0.990571725	0.99989309	0.758281397
SMPD4	0.839703607	0.747899938	0.914489705	0.90419464	0.99989309	0.763460317
VAMP8	0.839703607	0.836487169	0.787693453	0.89088601	0.99989309	0.763746661
MAOB	0.839703607	0.669179367	0.918576062	0.892741454	0.99989309	0.764546301
CALU	0.839703607	0.80055527	0.817601111	0.953508052	0.99989309	0.766542864
DHRS9	0.839703607	0.782865867	0.805580394	0.925931623	0.99989309	0.770391484
ARID4A	0.839703607	0.731331179	0.81284583	0.915749182	0.99989309	0.77208617
OTULINL	0.839703607	0.665563964	0.79610462	0.990049248	0.99989309	0.788142857
HDGFL3	0.839703607	0.76090129	0.834793268	0.908496281	0.99989309	0.796593386
LOC112448853	0.839703607	0.702457626	0.834793268	0.915749182	0.99989309	0.799940894
PIGY	0.839703607	0.665563964	0.87214481	0.938519355	0.99989309	0.815459921

RUM1	0.839703607	0.674551689	0.844065668	0.883885658	0.99989309	0.822691858
MSI2	0.839703607	0.693153864	0.764057751	0.915749182	0.99989309	0.85433331
RRAGB	0.839703607	0.704175579	0.805580394	0.90419464	0.99989309	0.854831489
CCR6	0.839703607	0.7164915	0.827125538	0.925004412	0.99989309	0.854890867
COQ8A	0.839703607	0.766108651	0.76749973	0.90419464	0.99989309	0.875441396
URM1	0.839703607	0.79139205	0.765785685	0.915749182	0.99989309	0.878152258
KLF14	0.839703607	0.710155842	0.827125538	0.915749182	0.99989309	0.88494083
FBRS	0.839703607	0.703437628	0.788892061	0.90419464	0.99989309	0.909990551
DANCR	0.839852237	0.693544464	0.861835782	0.946344632	0.939361296	0.870895425
HERPUD2	0.839852237	0.703437628	0.788938503	0.999939566	0.957605284	0.747674417
CLMP	0.839852237	0.774697122	0.846650632	0.946668073	0.96532164	0.788311337
TSPAN18	0.839852237	0.682470071	0.79610462	0.999939566	0.99989309	0.747414217
LRP11	0.839852237	0.747899938	0.884189563	0.91329897	0.99989309	0.792329436
MRPL44	0.840362858	0.770427846	0.765074242	0.915749182	0.99989309	0.822691858
SIAH1	0.840443133	0.851167599	0.765074242	0.888642721	0.899048045	0.957877422
KCNMB3	0.840443133	0.871412404	0.880277219	0.87452213	0.904151325	0.846367069
KIF3C	0.840443133	0.747899938	0.776954726	0.995800463	0.904396685	0.871166844
HHIP	0.840443133	0.668175004	0.975854775	0.90419464	0.91011204	0.850612178
TP53I3	0.840443133	0.828417266	0.827125538	0.988140196	0.917145033	0.783479395
MTMR7	0.840443133	0.98958941	0.777178978	0.91329897	0.934859184	0.752358594
POLD1	0.840443133	0.673034156	0.9728362	0.943240775	0.934859184	0.761409946
LOC619159	0.840443133	0.907691022	0.765785685	0.956154344	0.941107925	0.789577614
VOPP1	0.840443133	0.854944719	0.911743083	0.91329897	0.949645557	0.747414217
SGSH	0.840443133	0.767670077	0.788848487	0.999939566	0.952672789	0.764546301
LOC104970387	0.840443133	0.7635426	0.79610462	0.999939566	0.954501767	0.749880183
CDH6	0.840443133	0.841958822	0.79610462	0.946344632	0.954501767	0.813345852
SEMA3F	0.840443133	0.914162656	0.765074242	0.90419464	0.954501767	0.865159042
LOC112445980	0.840443133	0.688099473	0.869529186	0.934267153	0.954501767	0.875441396
PPP1R14A	0.840443133	0.740948281	0.879215964	0.87452213	0.965638808	0.888396511
MMS22L	0.840443133	0.685368354	0.977782604	0.904990622	0.996250445	0.752857325
COPS9	0.840443133	0.74401284	0.817256644	0.936573639	0.99989309	0.743464215
SELENOV	0.840443133	0.898231471	0.788938503	0.893831952	0.99989309	0.745987575
LOC615610	0.840443133	0.666543433	0.812258905	0.981022805	0.99989309	0.747414217
ITSN2	0.840443133	0.747555258	0.836671539	0.934591536	0.99989309	0.763656348
KDR	0.840443133	0.734767216	0.830022042	0.959009349	0.99989309	0.763847251
FAM114A2	0.840443133	0.760200908	0.801865674	0.96095927	0.99989309	0.769553374
OLFML1	0.840443133	0.755210284	0.777638604	0.996775429	0.99989309	0.770391484
LOC100335744	0.840443133	0.669648359	0.76749973	0.915749182	0.99989309	0.77208617

COA6	0.840443133	0.771098738	0.789039067	0.937781149	0.99989309	0.77208617
MYC	0.840443133	0.796591413	0.777178978	0.977043589	0.99989309	0.773402056
C8H9orf72	0.840443133	0.704923066	0.793430641	0.915749182	0.99989309	0.797730509
HUWE1	0.840443133	0.72843033	0.814444866	0.90419464	0.99989309	0.799314198
FZD8	0.840443133	0.69592751	0.765074242	0.984968973	0.99989309	0.810707901
BPNT1	0.840443133	0.727437191	0.806221631	0.922713162	0.99989309	0.815459921
ABHD10	0.840443133	0.759546301	0.79610462	0.915749182	0.99989309	0.829389486
COQ10A	0.840443133	0.711594168	0.844065668	0.931268564	0.99989309	0.829807823
PAK6	0.840443133	0.731268229	0.770051055	0.87630412	0.99989309	0.852755805
PPIH	0.840443133	0.734188031	0.87214481	0.891584014	0.99989309	0.856870625
MED30	0.840443133	0.669648359	0.817256644	0.949947918	0.99989309	0.859973633
TSSK3	0.840452639	0.680132416	0.98039137	0.922713162	0.905068358	0.788142857
MED28	0.840452639	0.738981601	0.879515214	0.995118128	0.907192767	0.77208617
APOL3	0.840452639	0.914616699	0.786436049	0.883313728	0.908708309	0.905858488
ARID1A	0.840452639	0.744527629	0.87146798	0.946344632	0.910770324	0.846367069
EEF1E1	0.840452639	0.7116845	0.814444866	0.940863121	0.94217472	0.914440253
NFYA	0.840452639	0.699417603	0.827125538	0.995800463	0.943901678	0.839419627
MMP9	0.840452639	0.68865036	0.880277219	0.958879502	0.943901678	0.840150874
MYEF2	0.840452639	0.703437628	0.880277219	0.969717497	0.952672789	0.803732909
COLGALT2	0.840452639	0.769313383	0.770051055	0.925901365	0.960296839	0.912101047
B4GALNT1	0.840452639	0.743422331	0.766352455	0.943323364	0.961382403	0.90952934
BTG2	0.840452639	0.771165031	0.885354035	0.904990622	0.988693274	0.812509877
RELL1	0.840452639	0.719840239	0.79610462	0.937781149	0.99989309	0.76360318
GRHL1	0.840452639	0.826048291	0.765074242	0.956154344	0.99989309	0.764546301
SRD5A1	0.840452639	0.780082505	0.765074242	0.942125631	0.99989309	0.770391484
LOC613401	0.840452639	0.71282706	0.787740344	0.931268564	0.99989309	0.837249706
NLRC4	0.840576373	0.679549691	0.792827466	0.90419464	0.99989309	0.898273855
LOC515169	0.840965416	0.706231276	0.79610462	0.995118128	0.90448127	0.893961809
LOC100849652	0.840965416	0.852463941	0.765074242	0.90419464	0.934859184	0.933730876
SELENOS	0.840965416	0.693544464	0.991406769	0.893559455	0.954501767	0.752358594
LOC107132534	0.840965416	0.719840239	0.788938503	0.894976239	0.954501767	0.972267965
BICDL1	0.840965416	0.838405969	0.850243032	0.91329897	0.965219542	0.812509877
DBNDD1	0.840965416	0.693544464	0.812770091	0.925265292	0.99989309	0.753138357
TMEM67	0.840965416	0.724518891	0.765074242	0.999939566	0.99989309	0.773957499
LOC515828	0.840965416	0.686576166	0.881292098	0.90419464	0.99989309	0.877730428
HSPBP1	0.841172283	0.717044998	0.819114812	0.999939566	0.904995537	0.796775592
SEC16A	0.841172283	0.695114938	0.798686086	0.969956798	0.904995537	0.934578283
ATP2C1	0.841172283	0.694933558	0.904682623	0.998102387	0.91011204	0.770391484

GEMIN6	0.841172283	0.6861152	0.84311048	0.915749182	0.925981327	0.956610132
DZANK1	0.841172283	0.765341142	0.787740344	0.94524673	0.945845518	0.897902197
C17H12orf49	0.841172283	0.763751563	0.857486511	0.975710367	0.961382403	0.76935309
TWISTNB	0.841172283	0.68147929	0.774057931	0.974992862	0.973705865	0.910619304
LRG1	0.841172283	0.678391216	0.846782643	0.951929772	0.982073092	0.86143104
FLNB	0.841172283	0.723119554	0.777178978	0.951929772	0.988693274	0.886992214
MKNK1	0.841172283	0.740540908	0.884189563	0.90419464	0.996250445	0.84491737
ADPRH	0.841172283	0.719478857	0.892122482	0.915749182	0.99989309	0.77208617
LOC100847818	0.841172283	0.670188815	0.87146798	0.958879502	0.99989309	0.799314198
EPN1	0.841172283	0.854656328	0.788848487	0.904074458	0.99989309	0.829034472
ARHGAP45	0.841172283	0.877749555	0.780363715	0.87630412	0.99989309	0.86143104
PSMB4	0.841172283	0.751946504	0.79610462	0.925931623	0.99989309	0.86143104
PSMA3	0.841172283	0.695866639	0.817256644	0.925004412	0.99989309	0.865159042
NOL9	0.84122325	0.747499345	0.926613927	0.888642721	0.927804504	0.871166844
PLK4	0.84122325	0.700682675	0.933102499	0.946344632	0.932081893	0.800276945
ABLIM1	0.84122325	0.76167712	0.787015778	0.942235206	0.934379881	0.922670883
RAB18	0.84122325	0.7116845	0.886282865	0.99879985	0.934859184	0.753067712
CSF1	0.84122325	0.681991477	0.774555223	0.999939566	0.954827567	0.77208617
CEP85	0.84122325	0.737374533	0.863172962	0.934267153	0.995037199	0.811943134
LOC101902937	0.84122325	0.711594168	0.772990161	0.908496281	0.99989309	0.770391484
MAF	0.84122325	0.823692881	0.833533313	0.883885658	0.99989309	0.770891305
MCEE	0.84122325	0.727892714	0.765074242	0.956154344	0.99989309	0.774602575
BCS1L	0.84122325	0.739918356	0.773846486	0.898441424	0.99989309	0.847196533
TLR6	0.841361337	0.936134921	0.817993104	0.893831952	0.904563451	0.847189049
PPARGC1A	0.841361337	0.692547228	0.757787642	0.982140778	0.975375626	0.910619304
CENPK	0.841443173	0.676166569	0.918050385	0.992137653	0.899048045	0.812509877
SRGAP2	0.841443173	0.747164473	0.842605286	0.999939566	0.943768595	0.76507496
TESC	0.841443173	0.693454767	0.775306135	0.999939566	0.986955873	0.764546301
COX7B	0.841443173	0.707383119	0.765440147	0.883885658	0.99989309	0.77208617
PXMP2	0.841443173	0.67537609	0.764057751	0.956154344	0.99989309	0.790110917
LOC101907916	0.841719673	0.822936459	0.777178978	0.956154344	0.902569851	0.8851281
RNF125	0.841719673	0.680267573	0.878603452	0.999939566	0.910393751	0.773402056
DCAF17	0.841719673	0.703437628	0.814444866	0.8780831	0.918449569	0.991281092
LAPTM4A	0.841719673	0.763751563	0.958420472	0.91329897	0.925981327	0.788142857
SLC7A5	0.841719673	0.89571555	0.892122482	0.883885658	0.94294964	0.783134114
STAG2	0.841719673	0.805157515	0.791475833	0.914862928	0.99989309	0.771518355
PPIL1	0.841719673	0.693544464	0.817256644	0.87630412	0.99989309	0.897633315
LOC407171	0.841719673	0.761788537	0.784655813	0.908496281	0.99989309	0.905858488

MCM2	0.841767588	0.755999274	0.843304408	0.995118128	0.943901678	0.784340268
COPS2	0.841936785	0.740948281	0.908823466	0.971220023	0.899048045	0.794529985
GPER1	0.842328884	0.740601865	0.768676249	0.915749182	0.99989309	0.764546301
ATP6V1G1	0.842426761	0.780290359	0.944976702	0.931268564	0.899048045	0.764546301
NKPD1	0.842426761	0.722790338	0.984700021	0.915749182	0.899048045	0.771597542
TMEM8A	0.842426761	0.880103565	0.795199152	0.944558402	0.904151325	0.844622232
ABHD6	0.842426761	0.823692881	0.771550992	0.99879985	0.925981327	0.812509877
SH2D5	0.842426761	0.803995245	0.773281026	0.924489924	0.925981327	0.935937948
SFR1	0.842426761	0.769817928	0.812770091	0.999939566	0.926899298	0.788131005
LOC404051	0.842426761	0.761788537	0.817256644	0.999939566	0.934379881	0.796002422
MESD	0.842426761	0.824676261	0.916702	0.915749182	0.940638859	0.777349536
SETDB2	0.842426761	0.691457949	0.787740344	0.999939566	0.949644914	0.763746661
LOC112441839	0.842426761	0.716335802	0.830033146	0.995536195	0.954501767	0.8125889
LOC531090	0.842426761	0.81987904	0.795258121	0.935763381	0.960835287	0.857269299
KCNA5	0.842426761	0.733386687	0.808333956	0.965590385	0.98152792	0.849772722
AP1B1	0.842426761	0.82674215	0.765074242	0.883885658	0.988693274	0.926872824
CSNK1G2	0.842426761	0.911106863	0.820767684	0.90419464	0.998276725	0.772530815
ZCCHC14	0.842426761	0.73599132	0.802857717	0.998542029	0.99989309	0.752900996
SNAPIN	0.842426761	0.688099473	0.828569127	0.946344632	0.99989309	0.771597542
VEZF1	0.842426761	0.857949626	0.776954726	0.89088601	0.99989309	0.775116427
LOC781710	0.842426761	0.800441059	0.787740344	0.876107068	0.99989309	0.783134114
IGFLR1	0.842426761	0.681219724	0.937544913	0.908496281	0.99989309	0.799314198
PPIL6	0.842426761	0.80403784	0.788938503	0.915749182	0.99989309	0.847189049
PTGER1	0.84257676	0.699626135	0.812043145	0.999939566	0.922811199	0.807160598
SUN2	0.84257676	0.690011276	0.838992284	0.999939566	0.94217472	0.753067712
NEXMIF	0.84257676	0.916401332	0.787015778	0.934591536	0.961835052	0.77208617
BAAT	0.84257676	0.688099473	0.918681165	0.925901365	0.989970625	0.808302424
CCDC9B	0.84257676	0.737937244	0.787740344	0.91329897	0.99989309	0.76507496
ZNF582	0.84257676	0.687403165	0.787740344	0.913413181	0.99989309	0.77208617
AP3M2	0.84257676	0.680267573	0.918576062	0.886966548	0.99989309	0.792329436
PM20D1	0.84257676	0.692547228	0.767599201	0.996118855	0.99989309	0.812682271
LIMD2	0.84257676	0.717132341	0.767047792	0.915749182	0.99989309	0.891000792
FASTKD5	0.84272183	0.780223998	0.87214481	0.951929772	0.934859184	0.810707901
CEP89	0.84272183	0.702457626	0.92582321	0.943323364	0.954501767	0.792591216
MRPS14	0.84272183	0.747499345	0.788938503	0.916305829	0.99989309	0.822882339
MRPS30	0.84272183	0.681991477	0.776954726	0.915749182	0.99989309	0.961465235
ITIH1	0.842773853	0.721805248	0.834569848	0.943323364	0.99989309	0.817015539
ABHD18	0.843361701	0.757476878	0.875669731	0.966882988	0.959936656	0.77208617

RPAP1	0.84347097	0.744690834	0.925587088	0.925931623	0.904151325	0.837048016
CYP2S1	0.84347097	0.811206862	0.879515214	0.892377118	0.952172109	0.859973633
ATP10D	0.844090906	0.688099473	0.845237542	0.999939566	0.960835287	0.788142857
PTPMT1	0.844090906	0.731268229	0.887385205	0.908804713	0.99989309	0.789905519
CAVIN4	0.844275481	0.695753905	0.849077738	0.995800463	0.99989309	0.759107825
SFMBT2	0.844412738	0.747499345	0.787399925	0.999939566	0.908708309	0.753138357
FAM69B	0.844571762	0.693153864	0.853222116	0.995800463	0.964150118	0.792329436
XPO4	0.844571762	0.871109705	0.761720705	0.90419464	0.997488969	0.884097654
NACC2	0.844571762	0.72843033	0.788848487	0.971220023	0.99989309	0.806469522
WWOX	0.84463075	0.712197118	0.802458996	0.999939566	0.907910153	0.784080446
NSD2	0.84463075	0.685980044	0.959320266	0.932656599	0.941107925	0.795637468
SRSF3	0.84463075	0.703437628	0.939683463	0.883885658	0.961382403	0.864680268
LOC101906195	0.84463075	0.699417603	0.920607829	0.958849311	0.962262333	0.77208617
WSCD2	0.84463075	0.685980044	0.802847755	0.992137653	0.999356955	0.859447707
THYN1	0.84463075	0.753193121	0.806825406	0.951929772	0.99989309	0.794529985
EMSY	0.84463075	0.841992867	0.764481234	0.90419464	0.99989309	0.809650729
IL17RD	0.84463075	0.685910529	0.765074242	0.919764668	0.99989309	0.943138585
GPM6A	0.844636208	0.852463941	0.899624092	0.915749182	0.952672789	0.753067712
AMZ2	0.844636208	0.72843033	0.838877266	0.995118128	0.988693274	0.770391484
MPV17	0.844636208	0.71282706	0.779087149	0.925004412	0.99989309	0.770391484
LOC107131367	0.8449611	0.69592751	0.949190017	0.893596954	0.904151325	0.897011069
SLC25A22	0.8449611	0.831974458	0.765074242	0.975529024	0.905068358	0.876183804
RGS9	0.8449611	0.785406526	0.849077738	0.95175364	0.907192767	0.854936659
LOC100299242	0.8449611	0.690592057	0.817256644	0.999939566	0.913166496	0.865997479
LOC112443444	0.8449611	0.801779928	0.788848487	0.925901365	0.926899298	0.923064313
G3BP2	0.8449611	0.683518638	0.914685423	0.989686311	0.934859184	0.808138656
SVIL	0.8449611	0.874935142	0.836294915	0.963829131	0.939361296	0.763656348
FAM129A	0.8449611	0.855861376	0.904445148	0.883885658	0.941107925	0.819521456
NCF4	0.8449611	0.762715669	0.907270422	0.90419464	0.943768595	0.865159042
PPP1R37	0.8449611	0.875022483	0.787740344	0.8968247	0.943901678	0.908939618
LOC112447599	0.8449611	0.711594168	0.952331743	0.940921143	0.949645557	0.764546301
PRR5L	0.8449611	0.693544464	0.827125538	0.999939566	0.949645557	0.794529985
DSC1	0.8449611	0.717132341	0.808333956	0.999939566	0.952172109	0.799940894
CHD1L	0.8449611	0.743422331	0.891455327	0.943323364	0.952172109	0.813577244
FBXO46	0.8449611	0.703437628	0.857065866	0.956154344	0.954501767	0.861807035
TES	0.8449611	0.785163144	0.817670412	0.908646404	0.954501767	0.923064313
GTF3C6	0.8449611	0.685980044	0.788848487	0.956154344	0.954501767	0.938535773
ATG13	0.8449611	0.692928782	0.806221631	0.925901365	0.961835052	0.943138585

VAMP3	0.8449611	0.676587388	0.90166788	0.991862855	0.972917711	0.770391484
ABCD3	0.8449611	0.695850654	0.857036033	0.951929772	0.975291792	0.847450944
GRIK3	0.8449611	0.769313383	0.776574729	0.915526452	0.980286089	0.935385789
LUC7L3	0.8449611	0.688099473	0.850243032	0.990571725	0.99989309	0.757327233
TRAPPC1	0.8449611	0.820796062	0.798347523	0.915749182	0.99989309	0.763656348
SMAD6	0.8449611	0.692547228	0.853222116	0.915749182	0.99989309	0.771541268
HDHD3	0.8449611	0.693544464	0.883726795	0.936573639	0.99989309	0.77208617
TMCC3	0.8449611	0.731550432	0.798686086	0.972489653	0.99989309	0.77208617
LOC104969159	0.8449611	0.692025761	0.939683463	0.915749182	0.99989309	0.777387039
SNN	0.8449611	0.693544464	0.775306135	0.999939566	0.99989309	0.788131005
PPRC1	0.8449611	0.704817952	0.795388144	0.889138988	0.99989309	0.795243197
BCNT2	0.8449611	0.757368351	0.81285201	0.923832787	0.99989309	0.795637468
RETN	0.8449611	0.780641867	0.798686086	0.922782374	0.99989309	0.798051736
TPX2	0.8449611	0.707140461	0.779087149	0.965097245	0.99989309	0.799940894
RMND1	0.8449611	0.703437628	0.765074242	0.891273781	0.99989309	0.809650729
LRIG3	0.8449611	0.736964914	0.765074242	0.995800463	0.99989309	0.819930249
SF3B3	0.8449611	0.693153864	0.79018754	0.90419464	0.99989309	0.828602505
LOC514189	0.8449611	0.693153864	0.795423149	0.943323364	0.99989309	0.862403446
C16H1orf74	0.8449611	0.743422331	0.832483532	0.89088601	0.99989309	0.866612872
SERPINA5	0.8449611	0.780290359	0.788938503	0.915749182	0.99989309	0.868108836
ARHGAP30	0.8449611	0.76090129	0.788938503	0.908054165	0.99989309	0.908939618
LOC101906317	0.8449611	0.703437628	0.827350832	0.91329897	0.99989309	0.916351432
PLAT	0.845183391	0.680835681	0.773281026	0.89088601	0.99989309	0.864688571
MED17	0.845254293	0.675837248	0.839666249	0.90419464	0.99989309	0.943138585
ZNF654	0.845285898	0.743422331	0.786290681	0.906248002	0.99989309	0.77208617
AXIN2	0.84531466	0.916740848	0.849077738	0.908496281	0.982014614	0.75292536
EME1	0.84605889	0.7116845	0.82199268	0.999939566	0.904995537	0.832097423
PLEKHH2	0.84605889	0.693153864	0.820767684	0.999939566	0.906386254	0.821467423
IL18	0.84605889	0.685446639	0.789760976	0.999939566	0.932081893	0.753138357
WDR76	0.84605889	0.677340516	0.788848487	0.999939566	0.932197133	0.876904341
TRIM28	0.84605889	0.680267573	0.79610462	0.999939566	0.941064037	0.77208617
FTO	0.84605889	0.703437628	0.878050811	0.915749182	0.941107925	0.912101047
TREM2	0.84605889	0.795806916	0.849077738	0.921320709	0.943768595	0.86729863
CCZ1	0.84605889	0.814136857	0.802857717	0.994309225	0.943901678	0.808302424
LRRC40	0.84605889	0.926435857	0.788848487	0.90419464	0.943901678	0.86143104
CD72	0.84605889	0.851167599	0.788938503	0.96095927	0.949645557	0.823139458
LOC100296952	0.84605889	0.827459094	0.820549834	0.995800463	0.954501767	0.753138357
LOC512464	0.84605889	0.769313383	0.776574729	0.99879985	0.957605284	0.825601599

NOB1	0.84605889	0.790637585	0.77070006	0.915749182	0.960835287	0.936563391
FARP1	0.84605889	0.693544464	0.939683463	0.95964071	0.961835052	0.756168541
USP43	0.84605889	0.973158835	0.788938503	0.910808102	0.96532164	0.764546301
KDM2B	0.84605889	0.692987203	0.78825731	0.99879985	0.972917711	0.86179903
MSLN	0.84605889	0.733883576	0.939091808	0.925901365	0.975291792	0.764546301
RHOU	0.84605889	0.847082229	0.862946056	0.92064557	0.979654999	0.771597542
PPP2CA	0.84605889	0.701581467	0.939683463	0.928623745	0.979654999	0.77208617
UBE2R2	0.84605889	0.694933558	0.825888497	0.995118128	0.98152792	0.839043244
DBR1	0.84605889	0.690829991	0.878603452	0.883398728	0.996250445	0.924845138
TRIM35	0.84605889	0.681764999	0.827125538	0.925901365	0.999592263	0.917968123
TANC1	0.84605889	0.747899938	0.839833549	0.903607716	0.99989309	0.75292536
ATP8B4	0.84605889	0.711594168	0.918681165	0.91329897	0.99989309	0.753088421
MAPK11	0.84605889	0.688099473	0.878603452	0.925004412	0.99989309	0.753613607
LOC104969545	0.84605889	0.751946504	0.810425784	0.90419464	0.99989309	0.757327233
CYP3A4	0.84605889	0.763059076	0.784107501	0.943323364	0.99989309	0.763656348
IL32	0.84605889	0.834316926	0.768676249	0.915749182	0.99989309	0.765600997
DTD2	0.84605889	0.759619476	0.787015778	0.888642721	0.99989309	0.770391484
MIGA2	0.84605889	0.790637585	0.771550992	0.89088601	0.99989309	0.77208617
ISOC2	0.84605889	0.720945114	0.84405284	0.908496281	0.99989309	0.77208617
TCF21	0.84605889	0.851167599	0.773281026	0.915749182	0.99989309	0.77208617
EXOC6B	0.84605889	0.761788537	0.84311048	0.915749182	0.99989309	0.77208617
LOC104975749	0.84605889	0.710155842	0.85972234	0.919247117	0.99989309	0.77208617
ZCCHC10	0.84605889	0.836026119	0.844065668	0.925440563	0.99989309	0.77208617
NDUFC1	0.84605889	0.755210284	0.80326876	0.89088601	0.99989309	0.774633112
TOGARAM1	0.84605889	0.80403784	0.787740344	0.915749182	0.99989309	0.793566607
BORCS7	0.84605889	0.808839416	0.834793268	0.934591536	0.99989309	0.796002422
SYVN1	0.84605889	0.859941746	0.777178978	0.944558402	0.99989309	0.796002422
RB1CC1	0.84605889	0.747164473	0.812770091	0.973377993	0.99989309	0.796002422
SP7	0.84605889	0.685495173	0.774248983	0.990571725	0.99989309	0.807160598
TSTA3	0.84605889	0.77295675	0.803398613	0.91329897	0.99989309	0.809370236
LOC101904435	0.84605889	0.821124254	0.788376748	0.915749182	0.99989309	0.829463053
MATR3	0.84605889	0.798909583	0.825178565	0.897446122	0.99989309	0.831041995
SH3BP1	0.84605889	0.736066622	0.787740344	0.915749182	0.99989309	0.840150874
MUL1	0.84605889	0.72477102	0.832431595	0.953929637	0.99989309	0.840150874
CADM3	0.84605889	0.871858734	0.788848487	0.90419464	0.99989309	0.84491737
AGER	0.84605889	0.694486794	0.878050811	0.917869295	0.99989309	0.84491737
PARP11	0.84605889	0.691175262	0.852758896	0.919247117	0.99989309	0.858042054
RNF14	0.84605889	0.692987203	0.828407935	0.925901365	0.99989309	0.859447134

ZNF641	0.84605889	0.731268229	0.874092011	0.907949786	0.99989309	0.86307729
COPS6	0.84605889	0.742963341	0.817256644	0.910808102	0.99989309	0.86307729
BCO2	0.84605889	0.693544464	0.823456027	0.915749182	0.99989309	0.897818829
IRF1	0.84605889	0.703437628	0.803771265	0.915749182	0.99989309	0.901689556
PAN3	0.84605889	0.766408271	0.765074242	0.883885658	0.99989309	0.914122611
MT2A	0.84605889	0.684832051	0.765074242	0.925210703	0.99989309	0.934451585
LOC112445002	0.846171961	0.725594888	0.893863239	0.926078322	0.941107925	0.86307729
LOC112441554	0.846277467	0.743422331	0.776574729	0.90419464	0.99989309	0.802399829
SCYL1	0.846607594	0.936134921	0.861835782	0.915749182	0.934859184	0.763746661
PALD1	0.846607594	0.801942311	0.870054336	0.908054165	0.943768595	0.880404833
ABCC9	0.846607594	0.778839781	0.814794516	0.990571725	0.99989309	0.759901657
ADCY3	0.84686416	0.7164915	0.844316758	0.990049248	0.99989309	0.788411132
CLHC1	0.847326916	0.747164473	0.777178978	0.940921143	0.979654999	0.912380696
LARP4B	0.847344217	0.69635562	0.780363715	0.999939566	0.934859184	0.789577614
KPNA6	0.847344217	0.680267573	0.85972234	0.992137653	0.975291792	0.832858909
LOC101907487	0.847344217	0.876057665	0.845237542	0.88747242	0.99989309	0.763656348
CPXM1	0.847344217	0.805132292	0.765074242	0.990049248	0.99989309	0.770391484
RPS6KA6	0.847367254	0.739918356	0.85972234	0.999939566	0.908091868	0.777494305
FAM120A	0.847367254	0.790637585	0.808228186	0.915749182	0.908091868	0.952887889
MDK	0.847367254	0.852463941	0.852794393	0.990049248	0.910770324	0.757327233
ACTL6A	0.847367254	0.754663123	0.962209895	0.915749182	0.917088283	0.808450403
H1FX	0.847367254	0.778839781	0.834643686	0.999939566	0.925981327	0.770391484
ABRAXAS1	0.847367254	0.7116845	0.941319062	0.925901365	0.925981327	0.843747482
ZFP1	0.847367254	0.724540534	0.820767684	0.915749182	0.932081893	0.961506391
RAB6A	0.847367254	0.693544464	0.97420715	0.95175364	0.932197133	0.76360318
LOC101906837	0.847367254	0.695866639	0.855743966	0.999939566	0.932197133	0.77208617
SIGLEC8	0.847367254	0.94751312	0.849077738	0.884946934	0.934379881	0.812185902
EML6	0.847367254	0.938064973	0.81285201	0.925865589	0.934859184	0.795637468
LOC788672	0.847367254	0.730317314	0.79610462	0.999939566	0.943901678	0.840150874
B3GAT1	0.847367254	0.945105738	0.788848487	0.946344632	0.949645557	0.764546301
MFAP1	0.847367254	0.710155842	0.951850716	0.925265292	0.95091153	0.803688478
PTMS	0.847367254	0.909085943	0.829843435	0.91329897	0.952172109	0.835370067
HEMK1	0.847367254	0.76167712	0.870911433	0.90419464	0.952172109	0.910589559
LOC101907483	0.847367254	0.736921399	0.878050811	0.904990622	0.952172109	0.912376736
SNHG12	0.847367254	0.703437628	0.827125538	0.999939566	0.954501767	0.764546301
KIAA1324	0.847367254	0.959845131	0.817256644	0.915749182	0.954501767	0.770391484
LOC101902561	0.847367254	0.747499345	0.787693453	0.999939566	0.954501767	0.832189969
DYRK2	0.847367254	0.693153864	0.817256644	0.992692773	0.954501767	0.88363494

BUB3	0.847367254	0.693153864	0.820549834	0.925901365	0.954501767	0.948277018
RNF214	0.847367254	0.682847958	0.787015778	0.886966548	0.954501767	0.998074562
ATRIP	0.847367254	0.698870039	0.869529186	0.90419464	0.956624006	0.942421464
MIER2	0.847367254	0.771098738	0.933629769	0.925931623	0.961382403	0.764546301
LOC101906923	0.847367254	0.703437628	0.806221631	0.999939566	0.961382403	0.77208617
LOC784322	0.847367254	0.704817952	0.965357634	0.904990622	0.972917711	0.808138656
CDC42EP4	0.847367254	0.80070147	0.776574729	0.992368879	0.972917711	0.823139458
ACOT7	0.847367254	0.761788537	0.777178978	0.999334425	0.975291792	0.812509877
MEIS2	0.847367254	0.705458146	0.856997596	0.995118128	0.982014614	0.777933888
FANCC	0.847367254	0.703437628	0.806825406	0.915749182	0.987731629	0.948277018
DYRK3	0.847367254	0.868963165	0.849077738	0.915749182	0.988693274	0.777933888
LOC613664	0.847367254	0.761392036	0.849077738	0.925931623	0.996250445	0.845061099
PWWP2A	0.847367254	0.795534928	0.8045653	0.992368879	0.99989309	0.753067712
OPA3	0.847367254	0.733883576	0.765074242	0.972367384	0.99989309	0.753138357
SLC38A1	0.847367254	0.701581467	0.849077738	0.915749182	0.99989309	0.770391484
TMEM131	0.847367254	0.692547228	0.788848487	0.946344632	0.99989309	0.770391484
PUDP	0.847367254	0.722790338	0.777178978	0.971220023	0.99989309	0.770391484
DAGLA	0.847367254	0.708437597	0.777178978	0.995118128	0.99989309	0.770391484
LIMK2	0.847367254	0.7116845	0.780363715	0.956154344	0.99989309	0.784080446
LOC101906001	0.847367254	0.779536052	0.849077738	0.921209664	0.99989309	0.791259029
NES	0.847367254	0.771331117	0.805449488	0.90419464	0.99989309	0.798018153
CYHR1	0.847367254	0.809942574	0.765074242	0.895106644	0.99989309	0.806054104
FAM212A	0.847367254	0.683461871	0.777638604	0.992746023	0.99989309	0.815459921
N6AMT1	0.847367254	0.72477102	0.805449488	0.956154344	0.99989309	0.824778321
UBXN11	0.847367254	0.771165031	0.765074242	0.990049248	0.99989309	0.845803016
MARF1	0.847367254	0.747164473	0.827125538	0.943323364	0.99989309	0.846943306
MDH1	0.847367254	0.737374533	0.811673049	0.893831952	0.99989309	0.84764189
NOP2	0.847367254	0.681991477	0.784132314	0.903607716	0.99989309	0.86143104
LOC790271	0.847367254	0.68147929	0.845237542	0.936573639	0.99989309	0.878152258
GAPDH	0.847367254	0.792318612	0.765074242	0.904990622	0.99989309	0.881039089
KIAA0408	0.847367254	0.735625249	0.788848487	0.943323364	0.99989309	0.884097654
NAE1	0.847367254	0.701581467	0.765074242	0.915749182	0.99989309	0.891000792
HCLS1	0.847367254	0.72843033	0.765074242	0.90419464	0.99989309	0.928799803
GFM1	0.847367254	0.680267573	0.765074242	0.91329897	0.99989309	0.935655141
TIMM9	0.847367254	0.716302962	0.777638604	0.908054165	0.99989309	0.95342487
BIRC2	0.84756528	0.728017748	0.906597758	0.943323364	0.941064037	0.84491737
ATP6V0D1	0.84756528	0.83744789	0.836294915	0.936880771	0.957605284	0.825114751
RGS22	0.84756528	0.766086544	0.766184475	0.990049248	0.962262333	0.875441396

ZNF839	0.84756528	0.792318612	0.843427302	0.914862928	0.99989309	0.770391484
LOC101903424	0.84756528	0.731268229	0.838877266	0.929346072	0.99989309	0.772244344
LOC614923	0.84756528	0.779536052	0.774385549	0.892229846	0.99989309	0.835783772
FAM198B	0.84756528	0.692987203	0.87214481	0.898441424	0.99989309	0.875441396
INTS10	0.84756528	0.769817928	0.793430641	0.917869295	0.99989309	0.905229516
LOC112441457	0.847637724	0.765341142	0.870054336	0.915749182	0.990044405	0.85958325
ZNF502	0.847782396	0.744882039	0.770051055	0.916305829	0.99989309	0.763847251
RBM43	0.847782396	0.780290359	0.814444866	0.990049248	0.99989309	0.770391484
SIRT5	0.847782396	0.687403165	0.852758896	0.931268564	0.99989309	0.847450944
LOC101905041	0.847803886	0.836487169	0.86719452	0.958879502	0.943768595	0.770391484
EDNRB	0.847803886	0.755210284	0.777178978	0.928836449	0.99989309	0.759729981
MPPED2	0.847803886	0.802701235	0.844065668	0.925931623	0.99989309	0.795637468
FAM47E	0.847872845	0.925277949	0.800718134	0.908054165	0.952172109	0.85958325
LOC112443006	0.847872845	0.693153864	0.812258905	0.924489924	0.961835052	0.956610132
BMS1	0.848154481	0.68147929	0.806311697	0.904769083	0.99989309	0.836944254
SPAG7	0.848154481	0.736566631	0.828407935	0.90419464	0.99989309	0.886992214
CAMTA1	0.848154481	0.73599132	0.839397412	0.908054165	0.99989309	0.912101047
CABCOCO1	0.848249695	0.72477102	0.827186499	0.90419464	0.99989309	0.809650729
PCED1A	0.84829262	0.719969167	0.993278977	0.915749182	0.91011204	0.764546301
TMED2	0.848611821	0.686122011	0.968419607	0.904990622	0.939361296	0.86307729
LOC112442683	0.849450944	0.841958822	0.765074242	0.940921143	0.99989309	0.784080446
C2H2orf69	0.849525284	0.771098738	0.880277219	0.99879985	0.930303931	0.759729981
LRFN1	0.849866453	0.747555258	0.773281026	0.915749182	0.99989309	0.872175131
PTPRC	0.850109337	0.747499345	0.802458996	0.892741454	0.99989309	0.908939618
LOC104976804	0.850297488	0.703437628	0.930892662	0.915749182	0.908708309	0.905220694
YAF2	0.850297488	0.76145014	0.838080059	0.99879985	0.925981327	0.823435077
TMEM170B	0.850297488	0.72843033	0.933102499	0.982140778	0.934859184	0.770391484
TIPRL	0.850297488	0.684832051	0.959822136	0.967063428	0.939361296	0.772530815
PSMD1	0.850297488	0.685517925	0.817993104	0.946344632	0.939994656	0.957140928
OGA	0.850297488	0.710217093	0.878603452	0.925931623	0.941107925	0.906178214
NDRG1	0.850297488	0.692547228	0.79610462	0.999939566	0.972917711	0.847189049
HOMER3	0.850297488	0.767950704	0.849077738	0.990571725	0.975038198	0.77208617
PRR15L	0.850297488	0.92505272	0.773846486	0.916305829	0.99672832	0.809650729
BRD4	0.850297488	0.791182736	0.76749973	0.914862928	0.99989309	0.773957499
GATAD2B	0.850297488	0.768980848	0.832483532	0.915749182	0.99989309	0.794529985
ARRDC4	0.850297488	0.715519139	0.777178978	0.934591536	0.99989309	0.802771083
BRPF3	0.850297488	0.81250936	0.787740344	0.915749182	0.99989309	0.821467423
PDIA4	0.850297488	0.795466803	0.765074242	0.979043693	0.99989309	0.844622232

MYL5	0.850300372	0.844031777	0.880277219	0.935742691	0.934859184	0.793566607
EVA1C	0.850406272	0.782865867	0.845425011	0.934591536	0.99989309	0.770391484
ZRSR2	0.850406272	0.875803224	0.836294915	0.905388347	0.99989309	0.806453168
POLRMT	0.850543009	0.739918356	0.94821101	0.915749182	0.91011204	0.859973633
LOC101907491	0.850543009	0.770427846	0.787740344	0.907949786	0.99989309	0.764546301
SCAMP1	0.850543009	0.928119925	0.812770091	0.915749182	0.99989309	0.768077698
ANKRD42	0.850543009	0.693454767	0.829843435	0.931268564	0.99989309	0.847189049
PQLC2	0.850543009	0.790637585	0.780363715	0.88747242	0.99989309	0.89533127
LSM12	0.850931729	0.727201976	0.883724665	0.915749182	0.910393751	0.934775196
SLC25A46	0.850931729	0.691175262	0.852758896	0.99879985	0.910770324	0.865159042
SCN4B	0.850931729	0.835276066	0.787693453	0.994365581	0.927804504	0.845024318
MYBBP1A	0.850931729	0.72843033	0.827125538	0.915749182	0.939361296	0.964143145
SLC4A3	0.850931729	0.703437628	0.788938503	0.999939566	0.941064037	0.886520861
EXOC8	0.850931729	0.703437628	0.90166788	0.934591536	0.943901678	0.871787716
EFEMP1	0.850931729	0.785406526	0.827125538	0.989391869	0.954501767	0.829389486
POLA2	0.850931729	0.691175262	0.920017619	0.913413181	0.954501767	0.906178214
TECPR2	0.850931729	0.684832051	0.830125816	0.915749182	0.957605284	0.968245211
MCM3	0.850931729	0.695753905	0.882898206	0.992368879	0.964150118	0.797054978
MFSD11	0.850931729	0.842368986	0.788139177	0.908054165	0.964521207	0.918156909
PROCR	0.850931729	0.759619476	0.817256644	0.919671703	0.966214002	0.921449509
SERBP1	0.850931729	0.700682675	0.837084949	0.940921143	0.977379814	0.908939618
SERTAD2	0.850931729	0.79449527	0.788848487	0.969717497	0.99989309	0.770391484
LOC100847190	0.850931729	0.828153513	0.798686086	0.971220023	0.99989309	0.770391484
ESF1	0.850931729	0.72843033	0.827475817	0.914862928	0.99989309	0.77208617
ZSWIM1	0.850931729	0.750745995	0.883982433	0.88747242	0.99989309	0.77378997
PLAUR	0.850931729	0.711124077	0.814444866	0.995800463	0.99989309	0.773957499
XDH	0.850931729	0.719478857	0.795701822	0.992368879	0.99989309	0.788318946
OBSCN	0.850931729	0.763751563	0.90495551	0.915749182	0.99989309	0.792329436
EMC3	0.850931729	0.751700705	0.82199268	0.95964071	0.99989309	0.812509877
INTS1	0.850931729	0.708270551	0.845237542	0.907553418	0.99989309	0.820127764
TRIM65	0.850931729	0.693544464	0.878603452	0.915749182	0.99989309	0.83849759
GLG1	0.850931729	0.703546093	0.845237542	0.968306132	0.99989309	0.841189457
CYB5B	0.850931729	0.703437628	0.840306601	0.934591536	0.99989309	0.84491737
JDP2	0.850931729	0.703437628	0.819664302	0.91329897	0.99989309	0.880212606
RBM8A	0.851187732	0.703437628	0.832431595	0.922782374	0.99989309	0.859973633
PSMC4	0.851187732	0.710155842	0.806825406	0.915749182	0.99989309	0.895218005
DUSP23	0.851215232	0.81808696	0.787740344	0.925901365	0.99989309	0.805809435
TMSB10	0.85122941	0.885181785	0.858865175	0.963829131	0.917145033	0.764546301

TMEM258	0.85122941	0.899387334	0.846650632	0.92064557	0.952172109	0.799314198
RAB11A	0.85122941	0.693959692	0.849077738	0.990571725	0.998276725	0.822869041
DUS4L	0.851370176	0.693544464	0.793078777	0.999939566	0.913229811	0.88674203
SGCB	0.851370176	0.747499345	0.909170161	0.925865589	0.934859184	0.863755488
SRF	0.851370176	0.904077835	0.87214481	0.90419464	0.968076673	0.791259029
COPS7B	0.851370176	0.711594168	0.936308309	0.915749182	0.99989309	0.788142857
JAK2	0.851370176	0.812502616	0.771402466	0.90419464	0.99989309	0.798051736
DENND2A	0.851370176	0.753384944	0.819529308	0.945929971	0.99989309	0.821467423
SACS	0.851370176	0.78164144	0.773281026	0.88747242	0.99989309	0.908939618
LOC505099	0.851419	0.886628459	0.855743966	0.898441424	0.996250445	0.80787758
CDC40	0.851952321	0.888994602	0.854279129	0.934591536	0.910770324	0.813001848
FAM107A	0.851952321	0.689503192	0.844065668	0.997612654	0.910770324	0.891908825
MBNL1	0.851952321	0.692987203	0.780363715	0.999939566	0.925981327	0.838303925
PITPNM2	0.851952321	0.728017748	0.777178978	0.999939566	0.927416857	0.898018497
CANX	0.851952321	0.711594168	0.875669731	0.915749182	0.928701823	0.945759045
CSRNP1	0.851952321	0.790637585	0.829374927	0.999939566	0.934859184	0.764546301
C19H17orf53	0.851952321	0.847082229	0.899195818	0.943323364	0.934859184	0.769994723
NCAM1	0.851952321	0.797362398	0.825601262	0.999939566	0.938077562	0.805015002
CCNB1IP1	0.851952321	0.709339485	0.933102499	0.975529024	0.941064037	0.794558849
UBA5	0.851952321	0.739860832	0.875669731	0.944392799	0.941107925	0.876262605
DIRAS3	0.851952321	0.921280256	0.819094028	0.931268564	0.943768595	0.813345852
LOC615768	0.851952321	0.781751024	0.846650632	0.946344632	0.943768595	0.868108836
MFSD6	0.851952321	0.851923106	0.814796982	0.915749182	0.947146038	0.90259329
CRYBB1	0.851952321	0.761903756	0.845336233	0.995800463	0.949645557	0.810707901
LOC616903	0.851952321	0.743422331	0.892122482	0.934591536	0.949645557	0.859973633
MED31	0.851952321	0.736511232	0.842478086	0.99879985	0.952172109	0.826929951
NCOR2	0.851952321	0.693544464	0.878603452	0.984968973	0.952672789	0.854493916
LTC4S	0.851952321	0.704817952	0.870585788	0.99879985	0.954501767	0.799314198
LOC527186	0.851952321	0.826048291	0.894547778	0.90419464	0.954501767	0.846367069
FRMD4B	0.851952321	0.710155842	0.838488596	0.999939566	0.960835287	0.788131005
LOC101906067	0.851952321	0.77396808	0.958420472	0.90419464	0.961382403	0.799935743
CD19	0.851952321	0.693153864	0.913413399	0.903607716	0.961382403	0.920942379
RP2	0.851952321	0.785406526	0.819114812	0.995946943	0.961835052	0.80787758
SLC35G1	0.851952321	0.742728851	0.765785685	0.995687815	0.963031162	0.887248119
GSAP	0.851952321	0.796591413	0.829843435	0.943323364	0.964150118	0.859973633
NPC1	0.851952321	0.688099473	0.802857717	0.999939566	0.972917711	0.837416527
LOC112446129	0.851952321	0.80403784	0.814444866	0.915749182	0.972917711	0.908939618
GALNS	0.851952321	0.809783026	0.880277219	0.936573639	0.979654999	0.788131005

ATP2B2	0.851952321	0.816088312	0.832431595	0.951929772	0.979654999	0.821774001
IFI35	0.851952321	0.710486439	0.845425011	0.915749182	0.981938411	0.935655141
RPA1	0.851952321	0.688099473	0.919108971	0.895106644	0.986071704	0.908939618
KCTD2	0.851952321	0.780290359	0.843427302	0.992368879	0.988693274	0.77208617
ALDH3B1	0.851952321	0.809942574	0.820767684	0.934591536	0.988693274	0.857435073
TOMM22	0.851952321	0.722790338	0.875201284	0.943323364	0.996250445	0.843747482
FAM213B	0.851952321	0.738328793	0.831320191	0.90419464	0.99989309	0.764546301
LRRK2	0.851952321	0.903878399	0.788938503	0.915749182	0.99989309	0.765492321
LOC112444340	0.851952321	0.750287778	0.929958748	0.912712485	0.99989309	0.770391484
NDUFS7	0.851952321	0.779208532	0.772990161	0.915749182	0.99989309	0.770391484
ATP5PO	0.851952321	0.709830012	0.777178978	0.915749182	0.99989309	0.770391484
LAMTOR4	0.851952321	0.764097231	0.806221631	0.915749182	0.99989309	0.770391484
LIMCH1	0.851952321	0.717508618	0.832431595	0.95964071	0.99989309	0.770391484
HAUS3	0.851952321	0.785406526	0.79610462	0.933991524	0.99989309	0.77208617
CCR1	0.851952321	0.842797231	0.836294915	0.943323364	0.99989309	0.77208617
PRKAG3	0.851952321	0.810519292	0.788848487	0.995118128	0.99989309	0.77208617
FAM204A	0.851952321	0.747555258	0.801296181	0.954818475	0.99989309	0.773130327
SNAP91	0.851952321	0.693544464	0.817256644	0.946344632	0.99989309	0.776802855
ARHGEF40	0.851952321	0.693544464	0.788938503	0.999939566	0.99989309	0.777494305
DNAJA4	0.851952321	0.704817952	0.958420472	0.91329897	0.99989309	0.788142857
LTB4R	0.851952321	0.776126383	0.765074242	0.943323364	0.99989309	0.788142857
DDX19A	0.851952321	0.724540534	0.914685423	0.925440563	0.99989309	0.795243197
RAB31	0.851952321	0.763751563	0.883982433	0.929346072	0.99989309	0.795637468
OGN	0.851952321	0.771331117	0.84405284	0.91888654	0.99989309	0.80787758
SMG1	0.851952321	0.764097231	0.810124821	0.903607716	0.99989309	0.809650729
LDHC	0.851952321	0.830448181	0.827125538	0.90419464	0.99989309	0.809650729
R3HCC1L	0.851952321	0.798799934	0.765074242	0.913319839	0.99989309	0.810707901
ARHGEF2	0.851952321	0.693544464	0.844009594	0.943323364	0.99989309	0.823139458
LRIG1	0.851952321	0.739918356	0.788848487	0.992137653	0.99989309	0.826868779
SARS2	0.851952321	0.704817952	0.869455175	0.908496281	0.99989309	0.831041995
CLCN4	0.851952321	0.754663123	0.777178978	0.990571725	0.99989309	0.83675435
WWC2	0.851952321	0.701581467	0.790109639	0.994365581	0.99989309	0.839340243
STK16	0.851952321	0.790637585	0.828569127	0.925931623	0.99989309	0.840150874
N4BP3	0.851952321	0.717508618	0.789039067	0.943323364	0.99989309	0.840150874
HEY2	0.851952321	0.72477102	0.820549834	0.925931623	0.99989309	0.844335246
PGAM5	0.851952321	0.707383119	0.79018754	0.95175364	0.99989309	0.873271935
PHACTR3	0.851952321	0.762941584	0.788848487	0.915749182	0.99989309	0.888617204
L2HGDH	0.851952321	0.718866883	0.787740344	0.91329897	0.99989309	0.893961809

GRHPR	0.851952321	0.702990859	0.788848487	0.89088601	0.99989309	0.895287865
SRP19	0.851952321	0.747499345	0.843427302	0.915749182	0.99989309	0.897011069
NTN4	0.851952321	0.776313457	0.843427302	0.90419464	0.99989309	0.901173172
PIH1D2	0.852224036	0.737374533	0.883726795	0.943510519	0.954501767	0.85958325
SLC25A30	0.852224036	0.769817928	0.852758896	0.946344632	0.954501767	0.863755488
ROMO1	0.852224036	0.737937244	0.819582814	0.916305829	0.99989309	0.765492321
ASB8	0.852224036	0.754914873	0.852758896	0.919247117	0.99989309	0.809650729
ATP6V1A	0.852521916	0.72477102	0.903930649	0.982140778	0.943768595	0.815459921
SUGP1	0.852582385	0.692547228	0.849077738	0.90419464	0.99989309	0.794529985
ABHD3	0.852688984	0.694933558	0.880277219	0.999939566	0.927804504	0.799993234
LOC101907642	0.852688984	0.968312904	0.790109639	0.908054165	0.943768595	0.839419627
PGM2L1	0.852688984	0.702457626	0.974158329	0.925901365	0.954501767	0.782430721
ABCA5	0.852688984	0.747499345	0.878050811	0.992368879	0.982014614	0.766359829
SP4	0.852688984	0.85749413	0.843427302	0.925931623	0.99989309	0.770391484
LOC107132606	0.852688984	0.688251757	0.904655347	0.943323364	0.99989309	0.770391484
ZNF33B	0.853106249	0.899387334	0.85546132	0.908496281	0.926794253	0.86307729
P4HA1	0.853129468	0.782865867	0.76749973	0.934591536	0.99989309	0.794529985
TRPC6	0.853129468	0.719969167	0.771402466	0.915749182	0.99989309	0.854721711
AURKAIP1	0.853129468	0.755940768	0.773281026	0.892377118	0.99989309	0.85958325
AFF2	0.853409454	0.785152571	0.92153058	0.915749182	0.975375626	0.808302424
RAB3GAP1	0.853657599	0.730228653	0.856769954	0.98636843	0.932033253	0.872224159
CAPZA1	0.853657599	0.695866639	0.908165509	0.915749182	0.935830921	0.928123265
PRF1	0.853657599	0.7164915	0.843427302	0.915749182	0.949645557	0.960712754
MZT1	0.853657599	0.688099473	0.965469111	0.956154344	0.952172109	0.777349536
ARID3A	0.853657599	0.692547228	0.883982433	0.994309225	0.952172109	0.840150874
CNIH3	0.853657599	0.779536052	0.806221631	0.999939566	0.952672789	0.788411132
ACVR2A	0.853657599	0.7116845	0.810425784	0.99879985	0.952821886	0.875441396
WDR7	0.853657599	0.726229532	0.843427302	0.996084773	0.997981612	0.797202346
LOC522540	0.853657599	0.781154903	0.79610462	0.893559455	0.99989309	0.76935309
FUNDC1	0.853657599	0.810519292	0.892122482	0.915749182	0.99989309	0.77208617
LOC101902029	0.853657599	0.836487169	0.775285424	0.973377993	0.99989309	0.77208617
CSRNP3	0.853657599	0.785383224	0.845425011	0.919247117	0.99989309	0.817471068
SGSM3	0.853657599	0.687403165	0.817256644	0.91329897	0.99989309	0.859447707
YWHAE	0.853657599	0.697755907	0.827125538	0.956154344	0.99989309	0.893961809
LRRC57	0.85375852	0.861568371	0.901591079	0.943323364	0.934859184	0.770131172
INHA	0.85375852	0.717508618	0.96472755	0.919671703	0.934859184	0.834936698
GREM1	0.85375852	0.763751563	0.880277219	0.946668073	0.939361296	0.86143104
P2RY1	0.85375852	0.703437628	0.846782643	0.96095927	0.943768595	0.908939618

RALGDS	0.85375852	0.810488979	0.880375205	0.982554614	0.954501767	0.764546301
GPATCH8	0.85375852	0.838405969	0.924266781	0.90419464	0.968076673	0.800008791
CCDC137	0.85375852	0.691175262	0.820549834	0.946344632	0.996250445	0.926576964
RNF103	0.85375852	0.851167599	0.814444866	0.915749182	0.999592263	0.86143104
AUTS2	0.85375852	0.689503192	0.79018754	0.908054165	0.99989309	0.770391484
NAPB	0.85375852	0.692987203	0.939683463	0.934591536	0.99989309	0.77208617
KIAA0895L	0.85375852	0.711172851	0.767697587	0.999939566	0.99989309	0.773402056
TTC9B	0.85375852	0.956093076	0.777178978	0.912712485	0.99989309	0.794958283
ATPAF1	0.85375852	0.691175262	0.827125538	0.948120268	0.99989309	0.795637468
INTS11	0.85375852	0.765341142	0.817993104	0.915749182	0.99989309	0.833155047
SLC38A2	0.85375852	0.857034402	0.774057931	0.910808102	0.99989309	0.845803016
ATP8B1	0.85375852	0.803683367	0.812770091	0.891273781	0.99989309	0.866099047
DNPEP	0.85375852	0.688099473	0.777178978	0.960986457	0.99989309	0.883906472
CD48	0.85375852	0.823588167	0.773281026	0.893559455	0.99989309	0.886238304
UBASH3B	0.85375852	0.813459746	0.778658634	0.904990622	0.99989309	0.908939618
TIMM50	0.85375852	0.747555258	0.787740344	0.925931623	0.99989309	0.908939618
WRAP73	0.853829209	0.708270551	0.989332994	0.918607898	0.941064037	0.77208617
STAMBPL1	0.853829209	0.730838712	0.808333956	0.999939566	0.981777076	0.826868779
LOC112449302	0.85388236	0.693281931	0.812043145	0.91329897	0.95827048	0.986862062
CCRL2	0.85395348	0.719478857	0.807884028	0.981022805	0.99989309	0.859973633
LYRM1	0.854159103	0.703437628	0.843427302	0.953648387	0.923218785	0.942421464
GPR174	0.854159103	0.733883576	0.844316758	0.922713162	0.941269773	0.943138585
GALNT3	0.854159103	0.851167599	0.773846486	0.982140778	0.943768595	0.863755488
SEPT10	0.854159103	0.801968936	0.828407935	0.925901365	0.95091153	0.908939618
NPM3	0.854159103	0.716304797	0.777178978	0.913413181	0.966423767	0.986389023
ABCB1	0.854159103	0.883267666	0.886176337	0.91329897	0.986955873	0.77208617
SH3BP5L	0.854159103	0.758051794	0.900342462	0.904990622	0.99989309	0.77208617
SMURF2	0.854159103	0.740948281	0.801865674	0.915749182	0.99989309	0.866482265
LOC101903713	0.854159103	0.691175262	0.921135532	0.913413181	0.99989309	0.873118284
LOC112443484	0.854214693	0.778839781	0.967873529	0.925901365	0.932197133	0.770391484
LOC104969496	0.854214693	0.967758952	0.787740344	0.915749182	0.943768595	0.839419627
PGK1	0.854405785	0.738981601	0.788848487	0.915749182	0.99989309	0.886992214
YME1L1	0.854405785	0.72843033	0.802711728	0.893559455	0.99989309	0.964143145
UBR2	0.854632063	0.758051794	0.779087149	0.943323364	0.99989309	0.77208617
RNF38	0.854846456	0.691880741	0.960200856	0.992137653	0.934859184	0.769244117
TBRG1	0.854846456	0.703546093	0.970904484	0.915749182	0.94294964	0.83849759
SLC12A9	0.854846456	0.7116845	0.961899356	0.921477746	0.947146038	0.831041995
RCSD1	0.854846456	0.695045653	0.860520146	0.973207466	0.95983326	0.878152258

LAPTM5	0.854846456	0.820796062	0.849077738	0.976950878	0.977155757	0.77208617
MED23	0.854846456	0.936134921	0.838838955	0.903607716	0.99989309	0.763746661
ZNF628	0.854846456	0.820796062	0.788848487	0.934591536	0.99989309	0.766306355
TMEM132C	0.854846456	0.708969489	0.832483532	0.908496281	0.99989309	0.77208617
GAS1	0.854846456	0.703437628	0.892122482	0.958879502	0.99989309	0.77208617
HEBP2	0.854846456	0.730317314	0.788848487	0.977995577	0.99989309	0.77208617
GSTP1	0.854846456	0.740182637	0.87146798	0.913319839	0.99989309	0.783479395
LETM1	0.854846456	0.753270374	0.780363715	0.913413181	0.99989309	0.795637468
MRPL30	0.854846456	0.73599132	0.844162118	0.925004412	0.99989309	0.799314198
GUK1	0.854846456	0.732815436	0.777178978	0.898441424	0.99989309	0.819521456
DHRS13	0.854846456	0.780290359	0.787740344	0.891273781	0.99989309	0.891908825
ICOS	0.855047481	0.725666405	0.827125538	0.945929971	0.952172109	0.937591162
SMIM4	0.855047481	0.795582607	0.781677833	0.940779565	0.99989309	0.821467423
ULK4	0.855300474	0.861841373	0.788848487	0.990571725	0.943901678	0.834936698
LOC107132410	0.855300474	0.693487404	0.843427302	0.999939566	0.949074145	0.783479395
MPP2	0.855300474	0.79591781	0.787740344	0.999939566	0.957605284	0.77208617
LRBA	0.855300474	0.71282706	0.776954726	0.919247117	0.99989309	0.80105809
TMED1	0.855300474	0.831141011	0.779087149	0.934591536	0.99989309	0.829389486
SLC22A17	0.855300474	0.781287182	0.820767684	0.915749182	0.99989309	0.856870625
MEST	0.855300474	0.7116845	0.827125538	0.968293092	0.99989309	0.86143104
GPR18	0.855445719	0.703437628	0.771402466	0.999939566	0.925981327	0.86307729
BLK	0.855445719	0.693153864	0.950801642	0.969717497	0.932197133	0.817471068
ADGRG6	0.855445719	0.944314627	0.769822168	0.943323364	0.935361969	0.844171661
LOC101908048	0.855445719	0.747555258	0.860197593	0.999939566	0.941064037	0.791259029
IQCK	0.855445719	0.688251757	0.817256644	0.99879985	0.954501767	0.898273855
TMEM106C	0.855445719	0.831242188	0.789868841	0.892229846	0.954501767	0.961506391
GPR37L1	0.855445719	0.703437628	0.838488596	0.990571725	0.962610771	0.878152258
FAM131A	0.855445719	0.785406526	0.819529308	0.90419464	0.96532164	0.948277018
PRKAA2	0.855445719	0.717871715	0.976869041	0.919484872	0.972917711	0.769244117
LOC789764	0.855445719	0.798185691	0.802847755	0.995800463	0.976657193	0.817471068
ZNF572	0.855445719	0.782865867	0.87146798	0.977216943	0.976970807	0.78510176
SF3B5	0.855445719	0.795993324	0.909170161	0.908496281	0.977981414	0.84491737
ACAD10	0.855445719	0.703437628	0.820767684	0.999939566	0.988693274	0.813345852
TBC1D8B	0.855445719	0.688099473	0.873750661	0.99879985	0.99989309	0.764546301
IK	0.855445719	0.707802804	0.899530079	0.945929971	0.99989309	0.775254902
NDUFV1	0.855445719	0.713953236	0.802857717	0.908496281	0.99989309	0.776139261
PTPRD	0.855445719	0.692987203	0.850952986	0.999939566	0.99989309	0.777494305
DOK6	0.855445719	0.703437628	0.78403766	0.919247117	0.99989309	0.778672431

SOCS5	0.855445719	0.748534108	0.87214481	0.934591536	0.99989309	0.784340268
UNG	0.855445719	0.726229532	0.923888516	0.91329897	0.99989309	0.799935743
SENP1	0.855445719	0.869907246	0.819529308	0.924489924	0.99989309	0.809650729
LRRC61	0.855445719	0.913171043	0.812770091	0.910808102	0.99989309	0.817764803
LOC101906366	0.855445719	0.710155842	0.820549834	0.959210368	0.99989309	0.818405353
EEA1	0.855445719	0.72477102	0.777178978	0.99879985	0.99989309	0.821467423
NLN	0.855445719	0.881642454	0.788938503	0.904074458	0.99989309	0.864571107
NFAT5	0.855445719	0.785406526	0.772319988	0.925901365	0.99989309	0.868059993
LOC104972065	0.855445719	0.769817928	0.812258905	0.943323364	0.99989309	0.872224159
LOC101904062	0.855445719	0.701581467	0.880277219	0.904990622	0.99989309	0.873520548
KCNB2	0.855445719	0.710155842	0.836414702	0.934591536	0.99989309	0.9071195
CEP112	0.855445719	0.732522135	0.788848487	0.915749182	0.99989309	0.908939618
GRB7	0.856124106	0.691357843	0.813027794	0.929346072	0.99989309	0.86252043
HMMR	0.856136123	0.694806652	0.989332994	0.915749182	0.968477804	0.777494305
COL14A1	0.856136123	0.688251757	0.850339392	0.94524673	0.99989309	0.773130327
SOGA1	0.856241478	0.74724898	0.814444866	0.999939566	0.932081893	0.856529344
LOC104975974	0.856321547	0.692461545	0.936308309	0.943323364	0.949645557	0.86143104
LOC786512	0.856440058	0.712908612	0.793430641	0.999939566	0.95091153	0.77208617
ADIRF	0.856677945	0.867949895	0.844009594	0.925901365	0.99989309	0.764546301
LAMTOR3	0.856707965	0.7116845	0.899195818	0.998542029	0.932197133	0.817471068
CBLN4	0.856707965	0.768680549	0.835091365	0.956154344	0.99989309	0.773957499
DNASE2	0.857051912	0.852463941	0.845237542	0.951929772	0.941107925	0.837416527
LAG3	0.857051912	0.798909583	0.909170161	0.90419464	0.954501767	0.875853001
IL16	0.857051912	0.763059076	0.787015778	0.919247117	0.954501767	0.969981358
DHDDS	0.857051912	0.769313383	0.845237542	0.917869295	0.966132692	0.91107007
LOC529399	0.857051912	0.736996221	0.84710326	0.999939566	0.972917711	0.77208617
NRP2	0.857051912	0.730317314	0.959646613	0.91329897	0.981777076	0.815459921
ABCE1	0.857062062	0.701180826	0.868936984	0.925067841	0.996250445	0.908939618
LOC101906410	0.857062062	0.761178476	0.773281026	0.99879985	0.99989309	0.794529985
JUNB	0.857062062	0.706231276	0.92153058	0.90419464	0.99989309	0.877623013
ZKSCAN2	0.857101792	0.820796062	0.899195818	0.925901365	0.941107925	0.840150874
MECOM	0.857101792	0.806009257	0.773625269	0.999939566	0.952821886	0.81350208
LOC112442787	0.857101792	0.790637585	0.819114812	0.994365581	0.954501767	0.845803016
SATB1	0.857101792	0.742728851	0.808333956	0.931268564	0.99989309	0.837501229
MRPS18B	0.857101792	0.743556979	0.827125538	0.925901365	0.99989309	0.847091529
TOMM6	0.857101792	0.779536052	0.829843435	0.915153525	0.99989309	0.865926817
ACACA	0.857101792	0.69592751	0.777178978	0.90419464	0.99989309	0.897902772
TXLNA	0.857150989	0.783416338	0.801865674	0.923578108	0.99989309	0.867440033

IFT43	0.857241119	0.703437628	0.88230201	0.922782374	0.975291792	0.908939618
PBRM1	0.857241119	0.955798423	0.812770091	0.910808102	0.99989309	0.773957499
TMEM214	0.857241119	0.71282706	0.788848487	0.915749182	0.99989309	0.792329436
HDDC2	0.857428905	0.751946504	0.849077738	0.908496281	0.939361296	0.963676648
CD82	0.857428905	0.747899938	0.839622619	0.999939566	0.941064037	0.822642815
LOC101905127	0.857428905	0.690592057	0.887130994	0.943323364	0.975291792	0.884387948
LOC101902449	0.857428905	0.694355537	0.788848487	0.998542029	0.975291792	0.90525134
COBLL1	0.857428905	0.743422331	0.827125538	0.904990622	0.99989309	0.856870625
RAD23B	0.857655017	0.702457626	0.863484223	0.989527018	0.934859184	0.896934364
FSTL4	0.857655017	0.693454767	0.842478086	0.934591536	0.972176983	0.942421464
PRR16	0.857655017	0.761178476	0.882898206	0.958849311	0.99989309	0.770426407
CBFB	0.857669049	0.72843033	0.920449324	0.924489924	0.99989309	0.765573509
IL17REL	0.857871054	0.820796062	0.929958748	0.90419464	0.99989309	0.770391484
KIDINS220	0.857871054	0.701581467	0.786436049	0.908054165	0.99989309	0.868745583
RIMKLB	0.857899533	0.803903736	0.827125538	0.99879985	0.932197133	0.83849759
C13H20orf96	0.857899533	0.731268229	0.915636025	0.999939566	0.934379881	0.770391484
ALOX5AP	0.857899533	0.87421443	0.832431595	0.943591298	0.934859184	0.858042054
SH2B1	0.857899533	0.693544464	0.987006595	0.91329897	0.983342337	0.792329436
ALS2CL	0.857899533	0.762941584	0.852758896	0.943323364	0.997854738	0.858538874
LOC112445078	0.857899533	0.731268229	0.886176337	0.992137653	0.99989309	0.765130735
PCGF6	0.857899533	0.802763306	0.812043145	0.934591536	0.99989309	0.87900534
TADA3	0.857903167	0.730135629	0.951850716	0.92064557	0.957663949	0.834254546
EPB41	0.857903167	0.703437628	0.817256644	0.990300191	0.962262333	0.910243726
AP4B1	0.857903167	0.71963672	0.812043145	0.99879985	0.996250445	0.851366694
SUMO1	0.857903167	0.703437628	0.951607422	0.925210703	0.99989309	0.77208617
TXN2	0.857903167	0.710155842	0.794216389	0.919247117	0.99989309	0.788131005
STT3A	0.857903167	0.747555258	0.828407935	0.91329897	0.99989309	0.882322567
PAGR1	0.858140352	0.780641867	0.903407548	0.936835069	0.934859184	0.859973633
ZMYND19	0.858140352	0.868275677	0.913354785	0.915749182	0.952172109	0.799935743
SEPT7	0.858140352	0.730944867	0.960137022	0.925440563	0.961879452	0.799314198
LOC107131289	0.858140352	0.868238903	0.868461814	0.925265292	0.99989309	0.770391484
NUTF2	0.858140352	0.722790338	0.85122003	0.995118128	0.99989309	0.788142857
MDGA2	0.858277196	0.845979391	0.827125538	0.915749182	0.99989309	0.812208548
YPEL5	0.85830478	0.72477102	0.938306386	0.948120268	0.960835287	0.812509877
MYBPH	0.858632248	0.899387334	0.820549834	0.908496281	0.99989309	0.832097423
ZC2HC1A	0.859238414	0.692928782	0.871859013	0.999939566	0.954501767	0.823435077
IDH3B	0.859238414	0.724738403	0.787740344	0.915749182	0.99989309	0.77208617
LOC781254	0.859238414	0.691175262	0.909522865	0.994309225	0.99989309	0.77208617

LOC783202	0.859238414	0.703437628	0.806825406	0.915749182	0.99989309	0.798051736
LCN1	0.859238414	0.7116845	0.777178978	0.999939566	0.99989309	0.809650729
TSACC	0.859238414	0.936134921	0.787740344	0.915749182	0.99989309	0.817471068
FASN	0.859238414	0.709791644	0.777178978	0.915749182	0.99989309	0.865926817
TARS	0.859331834	0.739753419	0.817256644	0.982140778	0.952672789	0.911696156
PAK1IP1	0.859331834	0.838405969	0.784107501	0.999939566	0.981777076	0.770391484
PEX11G	0.859331834	0.813371202	0.777178978	0.925931623	0.99989309	0.77208617
MN1	0.859331834	0.700679806	0.905832059	0.934591536	0.99989309	0.848704126
PDIA3	0.85945975	0.703437628	0.803398613	0.999939566	0.996250445	0.811943134
PJA1	0.859491272	0.71963672	0.838488596	0.908496281	0.99989309	0.930058266
MKL1	0.859970098	0.81668556	0.906079535	0.956154344	0.954501767	0.776200563
MFN1	0.859970098	0.780061391	0.808228186	0.91329897	0.99989309	0.826868779
NCKAP5	0.860577238	0.731268229	0.832483532	0.943539568	0.952672789	0.937632097
SDC4	0.860783436	0.7116845	0.939683463	0.908054165	0.99989309	0.829568933
TRRAP	0.860783436	0.785406526	0.828407935	0.908496281	0.99989309	0.869171706
HMGB1	0.861613051	0.747499345	0.974193101	0.91329897	0.972917711	0.792329436
DTNB	0.861613051	0.692987203	0.963643451	0.913413181	0.99989309	0.793572843
SSSCA1	0.861804963	0.710155842	0.831320191	0.928836449	0.99989309	0.91332511
CAP1	0.862280943	0.824119076	0.906597758	0.936573639	0.932081893	0.837249706
LOC100847122	0.862280943	0.72843033	0.979036577	0.914728628	0.943768595	0.835767164
LOC101903629	0.862280943	0.747555258	0.826667106	0.979729115	0.943901678	0.910619304
PDE7B	0.862280943	0.995965505	0.774555223	0.913413181	0.95983326	0.776200563
LOC614741	0.862280943	0.747106203	0.864741549	0.998542029	0.970377899	0.7986242
TCF24	0.862280943	0.762150099	0.827125538	0.999939566	0.972917711	0.799314198
LOC104968964	0.862280943	0.858423555	0.886282865	0.914862928	0.99989309	0.77208617
LOC100848939	0.862280943	0.774326932	0.844702985	0.952020618	0.99989309	0.799993234
SLC22A18	0.862280943	0.835809337	0.810425784	0.908054165	0.99989309	0.803879331
MFSD14A	0.862431771	0.704817952	0.894547778	0.960986457	0.99989309	0.797675006
TMEM9B	0.862499277	0.693153864	0.92332596	0.978328182	0.939361296	0.859973633
CD3G	0.862499277	0.919351156	0.777178978	0.915749182	0.954501767	0.902660792
ZNF181	0.862499277	0.740182637	0.783791102	0.999939566	0.961382403	0.817848737
BEGAIN	0.862563017	0.761788537	0.844316758	0.995118128	0.957605284	0.840150874
PARP9	0.862563017	0.747499345	0.939683463	0.904990622	0.99989309	0.833139817
MRPS25	0.862819585	0.834944503	0.788848487	0.910929308	0.99989309	0.792329436
FUT7	0.862830976	0.693153864	0.851048846	0.999939566	0.951073785	0.806054104
RAD52	0.862830976	0.876057665	0.834793268	0.971220023	0.960835287	0.793448996
RASGRP4	0.862830976	0.779536052	0.843427302	0.943323364	0.98152792	0.875441396
HPD	0.862830976	0.72809533	0.780363715	0.943539568	0.99989309	0.788411132

HSP90B1	0.862830976	0.751946504	0.787015778	0.99879985	0.99989309	0.78859079
EMP3	0.862930545	0.864821455	0.788848487	0.999939566	0.934859184	0.793627474
FBXL2	0.862930545	0.742728851	0.832041802	0.994309225	0.988693274	0.845866317
SMARCA4	0.863073706	0.72477102	0.870054336	0.998675745	0.936067236	0.856495922
MYH15	0.863073706	0.838405969	0.812043145	0.995800463	0.943768595	0.837249706
UHMK1	0.863073706	0.693153864	0.834643686	0.999939566	0.961382403	0.862912171
HTR2A	0.863073706	0.733727362	0.843427302	0.955560327	0.99989309	0.77208617
FANCD2	0.863073706	0.751946504	0.843427302	0.949930337	0.99989309	0.813713324
ATP8B2	0.863073706	0.7116845	0.904682623	0.933991524	0.99989309	0.849772722
WNK4	0.863073706	0.707140461	0.836294915	0.925901365	0.99989309	0.869171706
SIK3	0.863073706	0.737937244	0.819114812	0.975529024	0.99989309	0.875441396
C8H9orf3	0.863175131	0.77021507	0.788848487	0.999939566	0.941064037	0.873520548
LOC104970930	0.863175131	0.693153864	0.817982471	0.999939566	0.99989309	0.840150874
SOX12	0.863731879	0.730838712	0.879467505	0.999939566	0.934379881	0.821467423
LOC112441654	0.863731879	0.800441059	0.853686558	0.998542029	0.947146038	0.795637468
IFIT3	0.863731879	0.7116845	0.828407935	0.934591536	0.99989309	0.776802855
TSFM	0.863731879	0.766403118	0.788938503	0.915749182	0.99989309	0.911526437
IL6ST	0.864029522	0.77414098	0.787740344	0.95964071	0.943901678	0.941925704
THAP8	0.864029522	0.711594168	0.942004125	0.971220023	0.972917711	0.77208617
MPST	0.864029522	0.833841519	0.777178978	0.915749182	0.99989309	0.80787758
FMNL1	0.864036205	0.809568433	0.817256644	0.915749182	0.99989309	0.812509877
CD55	0.864036205	0.77414098	0.877133001	0.914402649	0.99989309	0.840150874
LEPROTL1	0.864193026	0.712838989	0.962209895	0.940921143	0.962337158	0.796002422
KIF1A	0.864204564	0.692987203	0.939683463	0.992137653	0.932081893	0.829807823
VSTM1	0.86464359	0.867722813	0.870054336	0.965525444	0.939994656	0.794529985
SND1	0.86464359	0.810976764	0.814444866	0.912566341	0.954501767	0.956986009
ARRB1	0.86464359	0.783416338	0.850046649	0.945929971	0.981777076	0.862481601
CDKN2AIP	0.86464359	0.844390603	0.827125538	0.968293092	0.99989309	0.77208617
SEC22A	0.86464359	0.841044548	0.847037539	0.915749182	0.99989309	0.799940894
PAXBP1	0.86464359	0.697997787	0.882898206	0.990049248	0.99989309	0.799940894
JOSD2	0.86464359	0.851167599	0.798686086	0.90419464	0.99989309	0.818819752
AADAT	0.86464359	0.717508618	0.788848487	0.915749182	0.99989309	0.859487774
GLS	0.86464359	0.703437628	0.784655813	0.925901365	0.99989309	0.862788295
IGSF6	0.86464359	0.747106203	0.813027794	0.93471064	0.99989309	0.86307729
MROH1	0.864753854	0.703437628	0.991406769	0.925901365	0.934859184	0.792329436
SLC22A23	0.864753854	0.709791644	0.853063495	0.999939566	0.964172455	0.785454907
SIGLEC15	0.864753854	0.769817928	0.805449488	0.969717497	0.99989309	0.770391484
PKD1	0.864753854	0.701581467	0.886711634	0.99045019	0.99989309	0.770391484

LOC101909718	0.864753854	0.835130235	0.777178978	0.924489924	0.99989309	0.833139817
IFI30	0.864753854	0.761788537	0.788848487	0.943323364	0.99989309	0.859973633
FMNL2	0.864753854	0.701581467	0.825960826	0.915749182	0.99989309	0.862912171
ATP5PB	0.864753854	0.750323987	0.808333956	0.925775859	0.99989309	0.865997479
LOC786258	0.864753854	0.717508618	0.874092011	0.931989854	0.99989309	0.866253786
TTLL5	0.864753854	0.72477102	0.845237542	0.922713162	0.99989309	0.871787716
LOC112443144	0.864753854	0.731268229	0.845425011	0.908496281	0.99989309	0.944524573
TMEM185A	0.864938887	0.717871715	0.860134949	0.916990541	0.99989309	0.918220726
MSC	0.865009058	0.703437628	0.844065668	0.99879985	0.939361296	0.893961809
WASF2	0.865009058	0.737937244	0.821908722	0.999939566	0.961382403	0.840150874
RAB37	0.865009058	0.700145515	0.787740344	0.999939566	0.996787268	0.86307729
SLC30A5	0.865009058	0.780290359	0.863172962	0.90419464	0.99989309	0.829807823
PRDX2	0.865009058	0.812502616	0.787740344	0.915749182	0.99989309	0.843672601
CRTAC1	0.865073709	0.841992867	0.787740344	0.999939566	0.980286089	0.770391484
GMFB	0.865073709	0.836026119	0.810425784	0.922713162	0.982014614	0.906529452
NPM1	0.865209582	0.719516802	0.80671639	0.925901365	0.99989309	0.859707535
LPCAT2	0.865359706	0.828746588	0.828407935	0.999939566	0.932197133	0.77208617
QRICH2	0.865359706	0.764525656	0.892122482	0.925901365	0.932197133	0.908939618
LOC618737	0.865359706	0.812371469	0.880277219	0.925210703	0.941107925	0.884387948
PLEKHF2	0.865359706	0.732983608	0.836414702	0.999939566	0.955813674	0.792329436
RAB2A	0.865359706	0.790637585	0.924266781	0.925004412	0.96532164	0.826868779
VAMP7	0.865359706	0.703437628	0.886282865	0.99879985	0.996250445	0.787719474
LOC507581	0.865359706	0.72843033	0.855743966	0.99879985	0.99989309	0.788142857
LOC100174924	0.865359706	0.703437628	0.929958748	0.928836449	0.99989309	0.82533924
ANAPC1	0.865359706	0.76090129	0.788938503	0.925931623	0.99989309	0.840150874
LOC112447858	0.865686545	0.779226683	0.827125538	0.90419464	0.99989309	0.94425343
MPND	0.865731694	0.872137621	0.894414265	0.925931623	0.943768595	0.812509877
CHMP4A	0.865731694	0.704817952	0.880277219	0.99879985	0.99989309	0.77208617
LYNX1	0.865731694	0.705458146	0.79610462	0.915749182	0.99989309	0.821774001
NF1	0.865886534	0.72477102	0.834793268	0.91329897	0.99989309	0.784608137
KANSL1L	0.865922583	0.795582607	0.777178978	0.915749182	0.99989309	0.905220694
DMXL1	0.866248643	0.782360068	0.832764285	0.999939566	0.941064037	0.794529985
SSR3	0.866248643	0.747164473	0.870780616	0.999939566	0.947070238	0.788131005
CDKN2AIPNL	0.866248643	0.811402363	0.845237542	0.943323364	0.99989309	0.840150874
GNL3	0.866352778	0.798799934	0.814444866	0.914862928	0.999445023	0.936563391
NCL	0.866352778	0.700682675	0.868936984	0.925265292	0.99989309	0.832858909
PRNP	0.866352778	0.747555258	0.881837962	0.954423369	0.99989309	0.834936698
SKIDA1	0.866352778	0.736066622	0.807843373	0.942280741	0.99989309	0.840150874

HEATR1	0.866352778	0.799579962	0.846949487	0.90419464	0.99989309	0.86307729
MCCC1	0.866352778	0.703437628	0.805580394	0.96095927	0.99989309	0.906529452
TMX4	0.866440271	0.693544464	0.894449269	0.999939566	0.961382403	0.770391484
CCT4	0.866941876	0.710155842	0.832431595	0.915749182	0.99989309	0.794529985
PSME3	0.867090343	0.805212705	0.828569127	0.925901365	0.934859184	0.942421464
ARMC10	0.867090343	0.693544464	0.981977161	0.945301766	0.95827048	0.77208617
POLR1E	0.867090343	0.747899938	0.820767684	0.958879502	0.99989309	0.86252043
KIF17	0.86716246	0.751946504	0.870054336	0.904990622	0.934859184	0.968594601
SPECC1	0.867807999	0.712213007	0.855743966	0.999939566	0.961382403	0.809650729
ICMT	0.867807999	0.726229532	0.860197593	0.942384819	0.99989309	0.870895425
TAF1A	0.8686962	0.737937244	0.975292061	0.915749182	0.939361296	0.840150874
LOC101904701	0.868789912	0.693544464	0.880277219	0.996775429	0.943768595	0.867440033
TMEM106A	0.868820355	0.747164473	0.892122482	0.990571725	0.943768595	0.840150874
CD1E	0.868820355	0.739873533	0.965750046	0.939292364	0.952172109	0.798051736
HSF5	0.868820355	0.7164915	0.817256644	0.956154344	0.964150118	0.94425343
CIZ1	0.868820355	0.742728851	0.908927423	0.971220023	0.996250445	0.795637468
HIBADH	0.868820355	0.693544464	0.828407935	0.957209279	0.99989309	0.787474205
EMC7	0.868820355	0.705889653	0.896272282	0.968293092	0.99989309	0.788311337
LAMTOR2	0.868820355	0.800441059	0.823456027	0.918385514	0.99989309	0.792329436
CALR	0.868820355	0.765341142	0.851617203	0.981022805	0.99989309	0.80261571
PSME4	0.868820355	0.736066622	0.788848487	0.956154344	0.99989309	0.848704126
FKBP14	0.868820355	0.780223998	0.836294915	0.917869295	0.99989309	0.879725389
ADGRL3	0.86894845	0.76863346	0.87214481	0.931268564	0.972663468	0.887248119
CHRD	0.86894845	0.740948281	0.939683463	0.915749182	0.99989309	0.808586684
BATF2	0.86894845	0.703437628	0.92153058	0.905146083	0.99989309	0.832858909
LOC112443328	0.86894845	0.780641867	0.825960826	0.948547383	0.99989309	0.845969723
PCDH17	0.869217192	0.72843033	0.82707168	0.999939566	0.954501767	0.795637468
QKI	0.869533314	0.72477102	0.829843435	0.99879985	0.941064037	0.897011069
HMGCR	0.869533314	0.693544464	0.892122482	0.999939566	0.976657193	0.78965579
MANF	0.869533314	0.882993091	0.808333956	0.931268564	0.99989309	0.77208617
LOC112441880	0.869533314	0.785978032	0.894874079	0.956154344	0.99989309	0.77208617
TRIM21	0.869533314	0.780641867	0.944612298	0.908496281	0.99989309	0.773402056
DDX17	0.869533314	0.775217746	0.840938565	0.91329897	0.99989309	0.775254902
PGAP3	0.869533314	0.700517321	0.817256644	0.922713162	0.99989309	0.787474205
LOC104972290	0.869533314	0.747499345	0.828569127	0.942280741	0.99989309	0.799940894
DHRS3	0.869533314	0.76013532	0.777638604	0.915749182	0.99989309	0.802067495
LOC101902435	0.869533314	0.744066065	0.964900522	0.904546259	0.99989309	0.806469522
UBE2V1	0.869533314	0.780082505	0.831430643	0.924297153	0.99989309	0.849769587

METRNL	0.869533314	0.80055527	0.788848487	0.976519231	0.99989309	0.86143104
EPHA2	0.869533314	0.87285861	0.787015778	0.922713162	0.99989309	0.874394916
UHRF2	0.869533314	0.72843033	0.863172962	0.915749182	0.99989309	0.897818829
PDCD1LG2	0.869533314	0.780290359	0.844065668	0.90419464	0.99989309	0.901689556
HACE1	0.869533314	0.761788537	0.779087149	0.917869295	0.99989309	0.94425343
ANO3	0.869912035	0.710155842	0.825888497	0.998542029	0.99989309	0.771541268
LOC784697	0.869912035	0.787707638	0.844162118	0.931268564	0.99989309	0.773957499
TMEM178B	0.869912035	0.80403784	0.845425011	0.917869295	0.99989309	0.862403446
C17H12orf43	0.869912035	0.805157515	0.788848487	0.90419464	0.99989309	0.961506391
DLK2	0.870040396	0.72843033	0.816129315	0.999939566	0.934859184	0.895287865
LOC112447362	0.870040396	0.717508618	0.860120877	0.999939566	0.935641613	0.858587492
LOC112441616	0.870040396	0.922434012	0.811009079	0.943323364	0.941107925	0.859973633
SURF2	0.870040396	0.790637585	0.882898206	0.977216943	0.943768595	0.840961242
KIFC1	0.870040396	0.704817952	0.942004125	0.992137653	0.954501767	0.788190443
LOC787905	0.870040396	0.763751563	0.878603452	0.946344632	0.957605284	0.880404833
LOC107131992	0.870040396	0.747164473	0.844162118	0.999939566	0.957910867	0.806453168
ASB11	0.870040396	0.72809533	0.939683463	0.945929971	0.964150118	0.834008567
LOC100847999	0.870040396	0.710155842	0.787740344	0.998542029	0.96532164	0.926872824
THSD4	0.870040396	0.951440129	0.79610462	0.946344632	0.982014614	0.780612804
CUL4A	0.870040396	0.755210284	0.820767684	0.972489653	0.988693274	0.893961809
RNF128	0.870040396	0.742298467	0.806221631	0.999939566	0.998276725	0.796002422
FAM104A	0.870040396	0.791704113	0.820549834	0.943161565	0.99989309	0.77208617
LOC100295347	0.870040396	0.711594168	0.788938503	0.915749182	0.99989309	0.792329436
FUK	0.870040396	0.740540908	0.939683463	0.922713162	0.99989309	0.795243197
STRIP1	0.870040396	0.7116845	0.846782643	0.956154344	0.99989309	0.821914747
SSB	0.870040396	0.737374533	0.80326876	0.959009349	0.99989309	0.822691858
NAPA	0.870040396	0.747164473	0.806311697	0.925901365	0.99989309	0.834285507
MARCH5	0.870040396	0.69592751	0.882898206	0.978403606	0.99989309	0.835866152
PASK	0.870040396	0.71282706	0.942314296	0.91329897	0.99989309	0.845866317
LOC101908214	0.870040396	0.829503998	0.788848487	0.919247117	0.99989309	0.849772722
SLITRK5	0.870040396	0.767675287	0.850020542	0.914402649	0.99989309	0.908939618
EDNRA	0.870387331	0.823588167	0.846782643	0.95964071	0.988693274	0.829807823
COPA	0.870387331	0.72477102	0.832431595	0.915749182	0.99989309	0.787474205
HOXB6	0.87043827	0.767797556	0.893863239	0.995800463	0.943901678	0.806054104
LOC104971852	0.87043827	0.747499345	0.957379098	0.953648387	0.954501767	0.795637468
ATAD1	0.87043827	0.719478857	0.965750046	0.947581168	0.954501767	0.799314198
NSUN2	0.87043827	0.762756164	0.87146798	0.91329897	0.957605284	0.943138585
LUZP6	0.87043827	0.747164473	0.922147089	0.990571725	0.96532164	0.77821816

LOC782114	0.87043827	0.72477102	0.878603452	0.990049248	0.996250445	0.83849759
FNTB	0.87043827	0.871682971	0.787740344	0.917869295	0.99989309	0.812509877
CD244	0.87043827	0.779501962	0.832431595	0.917869295	0.99989309	0.834254546
KATNA1	0.87043827	0.708507038	0.865007702	0.925901365	0.99989309	0.847256027
LOC100848872	0.87043827	0.72477102	0.845237542	0.910808102	0.99989309	0.877601804
CIP2A	0.87050614	0.785406526	0.818793839	0.999939566	0.939361296	0.815459921
CRLF3	0.87050614	0.848269044	0.843427302	0.971220023	0.941064037	0.859487774
PTP4A2	0.87050614	0.795993324	0.819161886	0.951929772	0.941064037	0.935147648
FHL2	0.87050614	0.834741258	0.806221631	0.999939566	0.943768595	0.828602505
TDRD10	0.87050614	0.806439163	0.81285201	0.999939566	0.946944212	0.845803016
CXCL3	0.87050614	0.742298467	0.843427302	0.908054165	0.952172109	0.983453112
SQLE	0.87050614	0.750913074	0.86052939	0.99879985	0.952672789	0.839419627
RCHY1	0.87050614	0.731268229	0.87146798	0.990571725	0.954501767	0.871787716
LOC101904891	0.87050614	0.747555258	0.817256644	0.98221491	0.954501767	0.926872824
LOC104975007	0.87050614	0.868963165	0.850020542	0.90419464	0.956007499	0.908939618
MTURN	0.87050614	0.76145014	0.87146798	0.99879985	0.956624006	0.812509877
GNPDA1	0.87050614	0.831094721	0.933963997	0.925931623	0.957605284	0.795243197
PGPEP1	0.87050614	0.761788537	0.918576062	0.925901365	0.957605284	0.871787716
ZBTB1	0.87050614	0.742728851	0.805580394	0.999939566	0.961382403	0.845803016
RASSF3	0.87050614	0.701581467	0.924266781	0.994309225	0.961835052	0.813001848
MATK	0.87050614	0.804860126	0.878603452	0.933991524	0.961835052	0.868108836
VGLL4	0.87050614	0.832852787	0.839378262	0.971220023	0.96532164	0.844622232
UCKL1	0.87050614	0.851167599	0.856997596	0.990059245	0.968076673	0.77208617
PLEKHA4	0.87050614	0.79449527	0.91546474	0.93471064	0.977981414	0.822882339
RSL24D1	0.87050614	0.737937244	0.827350832	0.999939566	0.979670554	0.812509877
EFCAB14	0.87050614	0.742298467	0.952255572	0.943323364	0.988693274	0.785975261
DENND4B	0.87050614	0.72843033	0.930262306	0.946344632	0.998276725	0.815459921
RAB1A	0.87050614	0.774697122	0.788848487	0.965097245	0.998276725	0.908939618
S100A14	0.87050614	0.779536052	0.802847755	0.983569039	0.99989309	0.77208617
LOC511847	0.87050614	0.821588096	0.849077738	0.916446425	0.99989309	0.783479395
AMOT	0.87050614	0.703437628	0.829843435	0.9846464	0.99989309	0.784671496
EPC1	0.87050614	0.817040135	0.828407935	0.908054165	0.99989309	0.809650729
TRIM5	0.87050614	0.771331117	0.933102499	0.90419464	0.99989309	0.837386109
UGCG	0.87050614	0.713621189	0.79610462	0.968293092	0.99989309	0.845866317
LOC112442079	0.87050614	0.774326932	0.817993104	0.934591536	0.99989309	0.86307729
FSBP	0.87050614	0.710155842	0.787740344	0.977470078	0.99989309	0.866836199
C10H15orf48	0.87050614	0.747499345	0.843427302	0.919247117	0.99989309	0.906529452
AIF1	0.87050614	0.713936417	0.788848487	0.928836449	0.99989309	0.908939618

LOC101905666	0.87050614	0.7116845	0.828407935	0.915749182	0.99989309	0.928374551
LOC525426	0.87050614	0.701581467	0.820549834	0.93471064	0.99989309	0.934653208
GJC2	0.870626258	0.751946504	0.858394457	0.956154344	0.99989309	0.773164323
UBE2M	0.870626258	0.86515799	0.836414702	0.925004412	0.99989309	0.845803016
GIGYF1	0.870771047	0.769817928	0.879215964	0.91329897	0.934859184	0.956610132
NSD1	0.870771047	0.69873818	0.899624092	0.999939566	0.943768595	0.77208617
MGRN1	0.870771047	0.72477102	0.943891967	0.919247117	0.947146038	0.887457549
KRBA2	0.870771047	0.807261713	0.957802423	0.939292364	0.952672789	0.77208617
LOC100335751	0.870771047	0.741607738	0.971104162	0.943323364	0.956624006	0.784080446
LOC112446760	0.870771047	0.747179932	0.802951601	0.999939566	0.962791364	0.864516178
TDP2	0.870771047	0.780641867	0.849077738	0.988949923	0.964150118	0.858777168
VAMP5	0.870771047	0.80403784	0.788938503	0.999939566	0.970439271	0.77208617
RNASE12	0.870771047	0.791922306	0.878050811	0.990571725	0.98152792	0.792329436
BMPR2	0.870771047	0.72477102	0.879515214	0.999939566	0.982014614	0.77208617
LOC101909140	0.870771047	0.805157515	0.817993104	0.998542029	0.986955873	0.829389486
WBP1	0.870771047	0.934248552	0.806311697	0.915749182	0.99989309	0.772065505
ZNF529	0.870771047	0.852281996	0.917876971	0.91483028	0.99989309	0.77208617
SLC19A2	0.870771047	0.7164915	0.882898206	0.999939566	0.99989309	0.77208617
RNPEP	0.870771047	0.735625249	0.80671639	0.919247117	0.99989309	0.788131005
LOC787257	0.870771047	0.701581467	0.831320191	0.985342672	0.99989309	0.792329436
PDE6D	0.870771047	0.846959498	0.858394457	0.910808102	0.99989309	0.812622197
SLC37A3	0.870771047	0.720945114	0.809322753	0.971220023	0.99989309	0.812682271
RNF20	0.870771047	0.808197752	0.802857717	0.908496281	0.99989309	0.839340243
ACP4	0.870771047	0.735625249	0.79610462	0.915749182	0.99989309	0.855653734
ESYT2	0.870771047	0.717132341	0.909511376	0.915749182	0.99989309	0.86143104
GLYAT	0.870771047	0.750745995	0.787740344	0.915749182	0.99989309	0.872175131
STX2	0.870771047	0.736066622	0.852758896	0.951929772	0.99989309	0.872175131
LYPLA1	0.870771047	0.7116845	0.827350832	0.969458182	0.99989309	0.901173172
LOC107131510	0.870997248	0.80389399	0.886282865	0.91329897	0.95983326	0.908939618
RPS27L	0.870997248	0.710155842	0.880277219	0.956154344	0.99989309	0.775254902
ZFYVE19	0.870997248	0.831242188	0.787015778	0.992829869	0.99989309	0.794529985
CREBBP	0.870997248	0.80055527	0.788848487	0.915749182	0.99989309	0.843747482
PRR15	0.870997248	0.703437628	0.836294915	0.943323364	0.99989309	0.912101047
FAM43A	0.871178865	0.922264623	0.787740344	0.965590385	0.941064037	0.860827207
RNF114	0.871178865	0.758051794	0.854082742	0.999939566	0.975291792	0.788142857
YTHDC1	0.871178865	0.825215718	0.788848487	0.905146083	0.99989309	0.832426534
GCLM	0.871259181	0.71742407	0.844065668	0.999939566	0.968477804	0.840150874
LOC531557	0.871259181	0.705118484	0.849233704	0.990571725	0.99989309	0.816121377

LOC112442979	0.871267202	0.769313383	0.787015778	0.934591536	0.99989309	0.941925704
SCAF4	0.871383726	0.703437628	0.817256644	0.908496281	0.99989309	0.806054104
RRP36	0.87171284	0.769091087	0.875669731	0.908496281	0.939361296	0.966567675
C23H6orf62	0.87171284	0.72477102	0.81285201	0.915749182	0.941064037	0.99797558
PIK3CG	0.87171284	0.707383119	0.962790365	0.990049248	0.943768595	0.791259029
TFAM	0.87171284	0.704817952	0.966919864	0.958879502	0.943768595	0.822691858
AGPAT5	0.87171284	0.743422331	0.907071712	0.995118128	0.943901678	0.836944254
ZFP57	0.87171284	0.936570494	0.885396428	0.915749182	0.952172109	0.799935743
RANGAP1	0.87171284	0.77021507	0.828407935	0.995118128	0.952172109	0.88667499
CLGN	0.87171284	0.704923066	0.871859013	0.92064557	0.952172109	0.968245211
CUL5	0.87171284	0.766086544	0.878603452	0.961983611	0.952821886	0.880404833
STPG1	0.87171284	0.767675287	0.914489705	0.987342814	0.954501767	0.815459921
CITED2	0.87171284	0.748909634	0.81505267	0.999939566	0.960835287	0.799314198
SYNJ2BP	0.87171284	0.907436461	0.852758896	0.91329897	0.961382403	0.870731971
CDC42EP1	0.87171284	0.850514868	0.831320191	0.968306132	0.977981285	0.840150874
SHC4	0.87171284	0.885604065	0.793430641	0.99879985	0.98152792	0.774684541
LOC101903992	0.87171284	0.790637585	0.823456027	0.99879985	0.983359692	0.837501229
AGFG1	0.87171284	0.731268229	0.807884028	0.956154344	0.988693274	0.943138585
ZNF696	0.87171284	0.7116845	0.854977705	0.943323364	0.997854738	0.916777309
MAATS1	0.87171284	0.703437628	0.951607422	0.908496281	0.998276725	0.886992214
LOC107132032	0.87171284	0.81808696	0.893763648	0.924489924	0.99989309	0.77208617
ZNF423	0.87171284	0.776126383	0.838838955	0.99879985	0.99989309	0.773957499
LOC101906426	0.87171284	0.753368216	0.820767684	0.983688085	0.99989309	0.775254902
SCUBE2	0.87171284	0.903657308	0.818793839	0.935163227	0.99989309	0.776333209
SCN9A	0.87171284	0.737937244	0.939091808	0.925901365	0.99989309	0.778407119
SH3PXD2B	0.87171284	0.711124077	0.812770091	0.992137653	0.99989309	0.792329436
GOSR1	0.87171284	0.779536052	0.807843373	0.999939566	0.99989309	0.792329436
ST3GAL1	0.87171284	0.913075885	0.856997596	0.92064557	0.99989309	0.793566607
RBM22	0.87171284	0.72370825	0.832483532	0.984968973	0.99989309	0.793627474
CSKMT	0.87171284	0.754812081	0.836294915	0.999939566	0.99989309	0.797730509
NDUFS4	0.87171284	0.792090907	0.817256644	0.914862928	0.99989309	0.803879331
PRPF39	0.87171284	0.753368216	0.883982433	0.956154344	0.99989309	0.805038934
SDHA	0.87171284	0.74401284	0.8045653	0.926078322	0.99989309	0.809650729
TOM1L2	0.87171284	0.792663811	0.787740344	0.913413181	0.99989309	0.822691858
PTRH2	0.87171284	0.791704113	0.853232343	0.958879502	0.99989309	0.832291764
CFL1	0.87171284	0.835197362	0.892635738	0.915749182	0.99989309	0.83394957
LOC112447087	0.87171284	0.730792193	0.83722246	0.99879985	0.99989309	0.837312175
DDX41	0.87171284	0.74401284	0.788938503	0.919276287	0.99989309	0.839419627

C15H11orf74	0.87171284	0.834316926	0.814444866	0.945301766	0.99989309	0.839728308
EXOSC2	0.87171284	0.699507369	0.926613927	0.90419464	0.99989309	0.84491737
CARS2	0.87171284	0.730944867	0.828407935	0.915749182	0.99989309	0.855778243
TPP2	0.87171284	0.742728851	0.821958182	0.990571725	0.99989309	0.857727168
NUDT22	0.87171284	0.852463941	0.788848487	0.934591536	0.99989309	0.863757526
ARL6IP4	0.87171284	0.894203989	0.788848487	0.925901365	0.99989309	0.870895425
LOC104972390	0.87171284	0.721805248	0.860520146	0.915749182	0.99989309	0.872224159
SUSD2	0.87171284	0.704817952	0.827125538	0.995800463	0.99989309	0.872224159
THBD	0.87171284	0.761767541	0.788938503	0.990059245	0.99989309	0.875441396
RFNG	0.87171284	0.775921167	0.836353375	0.951929772	0.99989309	0.877959203
PRRT4	0.87171284	0.72477102	0.829843435	0.943003813	0.99989309	0.886992214
CAMSAP2	0.87171284	0.710155842	0.905017554	0.915749182	0.99989309	0.887248119
LOC101905228	0.87171284	0.768980848	0.827125538	0.951929772	0.99989309	0.893961809
GTF2A2	0.87171284	0.710338031	0.817256644	0.980295672	0.99989309	0.912101047
NRK	0.87171284	0.763059076	0.827125538	0.915749182	0.99989309	0.925449698
PPAN	0.87171284	0.724518891	0.788848487	0.915749182	0.99989309	0.945618717
LRPPRC	0.87171284	0.72843033	0.79018754	0.915749182	0.99989309	0.979606084
DNMT3B	0.871844663	0.76090129	0.941593855	0.962142137	0.949645557	0.812509877
COPG2	0.871844663	0.9136091	0.788848487	0.959287984	0.99989309	0.773402056
HERC4	0.871844663	0.812575653	0.787740344	0.999939566	0.99989309	0.788131005
LOC112446369	0.871873379	0.72843033	0.875669731	0.981022805	0.99989309	0.858042054
PNKD	0.872024887	0.747555258	0.857417456	0.995800463	0.954501767	0.864219261
SLC7A2	0.872024887	0.907670026	0.858865175	0.946344632	0.96413701	0.795637468
PEX19	0.872024887	0.840874016	0.877230238	0.93471064	0.975291792	0.844171661
PITPNB	0.872024887	0.905977412	0.787740344	0.934591536	0.976970807	0.877623013
ADAM11	0.872024887	0.80403784	0.827125538	0.931268564	0.99989309	0.802270182
PHYH	0.872024887	0.709830012	0.840938565	0.999939566	0.99989309	0.845803016
CCNL2	0.872658637	0.711594168	0.951850716	0.934591536	0.954741113	0.864680268
TOMT	0.873010249	0.703437628	0.86859301	0.921761002	0.99989309	0.788131005
PEX16	0.873010249	0.74214695	0.787740344	0.914862928	0.99989309	0.866482265
PWP2	0.873154388	0.852463941	0.845237542	0.91329897	0.975291792	0.908939618
LOC112444920	0.873154388	0.766838126	0.902141181	0.924489924	0.998276725	0.868108836
SKIL	0.873265248	0.843422478	0.817256644	0.937531834	0.996250445	0.884838629
SRP68	0.873472879	0.747106203	0.885995229	0.939292364	0.975483283	0.89366754
CD36	0.873825451	0.885181785	0.869455175	0.990571725	0.943768595	0.773402056
WWC1	0.873825451	0.761788537	0.993278977	0.915749182	0.943768595	0.796593386
TSC1	0.873825451	0.769186282	0.874092011	0.915749182	0.954501767	0.943685681
LOC112448736	0.873825451	0.726229532	0.922147089	0.908496281	0.964521207	0.934653208

LOC510536	0.873825451	0.866977641	0.867451783	0.908054165	0.96532164	0.897011069
CALCRL	0.873825451	0.786829125	0.845237542	0.995800463	0.982014614	0.829389486
WDR24	0.873825451	0.810096755	0.886282865	0.908804713	0.986071704	0.896538019
TSKU	0.873825451	0.857034402	0.825888497	0.990571725	0.99989309	0.775254902
SLC35C2	0.873825451	0.763068721	0.787740344	0.971220023	0.99989309	0.788131005
LOC100616098	0.873825451	0.766403118	0.830013482	0.925901365	0.99989309	0.792329436
TMEM256	0.873825451	0.728017748	0.801865674	0.998542029	0.99989309	0.792329436
CAV1	0.873825451	0.703437628	0.881432424	0.980418209	0.99989309	0.799314198
SH3BP5	0.873825451	0.786829125	0.812585086	0.924489924	0.99989309	0.799940894
NEDD8	0.873825451	0.770808357	0.844611045	0.913413181	0.99989309	0.8141247
HSPB6	0.873825451	0.703437628	0.806311697	0.999939566	0.99989309	0.816440237
NOS2	0.873825451	0.720945114	0.788938503	0.981022805	0.99989309	0.826868779
CIAO1	0.873825451	0.703437628	0.85972234	0.953653186	0.99989309	0.839419627
CEP85L	0.873825451	0.897375356	0.788938503	0.913413181	0.99989309	0.84491737
GATC	0.873825451	0.758702769	0.877998684	0.925901365	0.99989309	0.84491737
SRSF5	0.873825451	0.710155842	0.810425784	0.908054165	0.99989309	0.855653734
PSMA5	0.873825451	0.745665426	0.828569127	0.915749182	0.99989309	0.87114736
LOC100139891	0.873825451	0.747555258	0.879215964	0.915749182	0.99989309	0.872224159
CCDC89	0.873825451	0.769186282	0.788938503	0.908496281	0.99989309	0.911696156
MYH7	0.873825451	0.710155842	0.814131145	0.956154344	0.99989309	0.931126954
PRKX	0.873883447	0.827668363	0.850106729	0.955560327	0.943768595	0.887248119
TELO2	0.873883447	0.707874469	0.998442742	0.91329897	0.947826027	0.809650729
PLEK	0.873883447	0.780290359	0.883982433	0.995946943	0.975291792	0.788131005
LYPD3	0.873883447	0.72843033	0.929303679	0.946344632	0.99989309	0.805847707
SCCPDH	0.873902617	0.736066622	0.81284583	0.958879502	0.99989309	0.867440033
CISD2	0.874104611	0.764525656	0.879215964	0.908054165	0.970439271	0.94425343
CCDC12	0.874104611	0.747082543	0.886176337	0.990571725	0.99989309	0.809650729
LRAT	0.874354293	0.828600257	0.857943088	0.942384819	0.943768595	0.902122572
STIL	0.874354293	0.864929219	0.83314199	0.975529024	0.954501767	0.853233759
MGAT4B	0.874354293	0.779208532	0.857417456	0.999939566	0.977155757	0.77208617
TUBD1	0.874354293	0.7164915	0.79220963	0.999939566	0.99989309	0.795637468
GORASP1	0.874354293	0.818833112	0.832483532	0.93290597	0.99989309	0.808138656
ERI1	0.874354293	0.836487169	0.894414265	0.915749182	0.99989309	0.809432201
ATOH8	0.874354293	0.803903736	0.844065668	0.959727203	0.99989309	0.847848978
METTL8	0.874482396	0.825215718	0.803398613	0.990571725	0.996250445	0.86143104
SMAD2	0.874574762	0.737937244	0.98567143	0.951929772	0.941064037	0.778022404
SECISBP2L	0.874574762	0.765341142	0.850106729	0.999939566	0.941064037	0.854831489
RANBP9	0.874574762	0.71963672	0.916526594	0.983038839	0.941064037	0.875441396

MRM3	0.874574762	0.795534928	0.855743966	0.919247117	0.947826027	0.943138585
LOC112445925	0.874574762	0.732035954	0.827125538	0.999939566	0.949645557	0.80787758
LOC112444498	0.874574762	0.914162656	0.836294915	0.908054165	0.954501767	0.908939618
TULP2	0.874574762	0.711594168	0.980734833	0.91329897	0.960835287	0.86307729
FAM149B1	0.874574762	0.820993717	0.845237542	0.995800463	0.964150118	0.823435077
ENDOV	0.874574762	0.763751563	0.827186499	0.999939566	0.972917711	0.853233759
ANGPT2	0.874574762	0.972418749	0.843427302	0.915749182	0.975291792	0.788142857
KIF26A	0.874574762	0.707802804	0.846650632	0.999939566	0.99989309	0.776550567
ACHE	0.874574762	0.733386687	0.814444866	0.940387993	0.99989309	0.794556896
POC1A	0.874574762	0.722790338	0.898889441	0.915749182	0.99989309	0.796002422
THTPA	0.874574762	0.72843033	0.819404084	0.945301766	0.99989309	0.800008791
NAXE	0.874574762	0.779536052	0.788938503	0.910808102	0.99989309	0.812622197
LOC101902760	0.874574762	0.826739573	0.849077738	0.925004412	0.99989309	0.825331577
ZBTB21	0.874574762	0.761788537	0.829843435	0.943323364	0.99989309	0.831206878
FIG4	0.874574762	0.81808696	0.825960826	0.914862928	0.99989309	0.837249706
KLKB1	0.874574762	0.72843033	0.878603452	0.925901365	0.99989309	0.86143104
ARCN1	0.874574762	0.711594168	0.929303679	0.915749182	0.99989309	0.872224159
LOC112447313	0.874574762	0.738981601	0.819529308	0.956154344	0.99989309	0.882322567
IP6K2	0.874574762	0.703437628	0.845237542	0.91329897	0.99989309	0.887248119
CD300A	0.874574762	0.79236511	0.832483532	0.908804713	0.99989309	0.893961809
SLC33A1	0.874574762	0.747499345	0.883982433	0.915749182	0.99989309	0.912101047
FAM217B	0.874574762	0.710155842	0.802951601	0.956154344	0.99989309	0.94734477
LOC101907813	0.874711288	0.869907246	0.806221631	0.995118128	0.962791364	0.839340243
NCALD	0.874711288	0.771165031	0.883982433	0.937781149	0.99989309	0.823139458
ITGB3	0.874853227	0.851167599	0.849077738	0.925004412	0.941107925	0.915253587
EXO5	0.874853227	0.776217482	0.854082742	0.999939566	0.972917711	0.798982351
ABCD2	0.874853227	0.90039743	0.788848487	0.925931623	0.982014614	0.893961809
LOC104975091	0.874853227	0.761788537	0.832483532	0.999939566	0.99989309	0.788142857
FRS3	0.874853227	0.748534108	0.872587089	0.956154344	0.99989309	0.789905519
LOC614226	0.874853227	0.823588167	0.795258121	0.982140778	0.99989309	0.795637468
HIC1	0.874853227	0.708033106	0.849077738	0.9846464	0.99989309	0.845803016
PELI1	0.874853227	0.786258422	0.849077738	0.95964071	0.99989309	0.86143104
LOC104974050	0.874853227	0.7164915	0.837473402	0.931750296	0.99989309	0.908939618
RTL8C	0.87495229	0.862527737	0.828407935	0.914728628	0.99989309	0.814576296
HEATR6	0.875018414	0.763478948	0.825888497	0.995118128	0.99989309	0.811943134
FXN	0.875018414	0.722790338	0.89265635	0.944002443	0.99989309	0.837501229
EGR2	0.875027148	0.7164915	0.927460291	0.91329897	0.99989309	0.845981344
CARD10	0.875133408	0.771098738	0.903214794	0.925004412	0.961879452	0.898551094

NDP	0.875288364	0.804875341	0.832293589	0.926078322	0.99989309	0.78965579
TMEM232	0.875359474	0.780290359	0.93798101	0.915749182	0.943768595	0.901689556
PRKCB	0.875359474	0.755210284	0.878603452	0.995946943	0.957605284	0.845591203
ADAM22	0.875359474	0.80403784	0.951092876	0.91329897	0.964872582	0.849772722
BCL2L13	0.875359474	0.828417266	0.788848487	0.990571725	0.99989309	0.794529985
GSTA1	0.875359474	0.731268229	0.879215964	0.925901365	0.99989309	0.802067495
NAA20	0.875359474	0.763751563	0.846782643	0.925901365	0.99989309	0.837312175
COPE	0.875359474	0.76013532	0.81284583	0.917869295	0.99989309	0.839419627
MPRIP	0.875359474	0.7116845	0.814705151	0.995118128	0.99989309	0.840150874
AKNA	0.875359474	0.728008849	0.861838588	0.948120268	0.99989309	0.853233759
SCAMP3	0.875359474	0.779208532	0.844065668	0.915749182	0.99989309	0.862912171
LOC112441545	0.875504349	0.995965505	0.80326876	0.924489924	0.941107925	0.80787758
CSTF3	0.875504349	0.771098738	0.845336233	0.915749182	0.943768595	0.98016619
GABRB1	0.875504349	0.944755173	0.806221631	0.956154344	0.946536678	0.845803016
LOC112442719	0.875504349	0.736511232	0.978215635	0.915749182	0.947826027	0.86307729
ZNF704	0.875504349	0.841958822	0.803398613	0.915749182	0.949531814	0.974379635
MAPT	0.875504349	0.944780259	0.790109639	0.934591536	0.949645557	0.875441396
PHC3	0.875504349	0.934248552	0.844162118	0.908496281	0.949645557	0.891908825
CDH4	0.875504349	0.747899938	0.827186499	0.999939566	0.952172109	0.86307729
ARFGEF3	0.875504349	0.794823268	0.828407935	0.990049248	0.952172109	0.908939618
LOC783541	0.875504349	0.840541876	0.802951601	0.999939566	0.953320369	0.809650729
TEAD3	0.875504349	0.911279338	0.845237542	0.990571725	0.954501767	0.783479395
CISH	0.875504349	0.731268229	0.982659943	0.925901365	0.954501767	0.828602505
RDH16	0.875504349	0.742728851	0.814444866	0.956154344	0.954501767	0.966567675
LIPT2	0.875504349	0.897918739	0.85972234	0.922713162	0.957605284	0.869171706
PAFAH2	0.875504349	0.851167599	0.85972234	0.990571725	0.960835287	0.812509877
ZNF74	0.875504349	0.712908612	0.958420472	0.956154344	0.960835287	0.840961242
ELP2	0.875504349	0.761788537	0.886176337	0.956154344	0.960835287	0.887248119
SNAP29	0.875504349	0.846695169	0.820767684	0.940863121	0.960835287	0.917150226
LOC112446796	0.875504349	0.780290359	0.84311048	0.999939566	0.961879452	0.784080446
TRMT12	0.875504349	0.740343022	0.852758896	0.999939566	0.964172455	0.845580904
RARRES2	0.875504349	0.825273463	0.879215964	0.925931623	0.96532164	0.886992214
GJA10	0.875504349	0.730838712	0.878603452	0.940921143	0.96532164	0.928057673
MAPK6	0.875504349	0.785890605	0.939683463	0.940921143	0.975291792	0.815459921
LOC101906607	0.875504349	0.731268229	0.849077738	0.925931623	0.975291792	0.957122667
HASPIN	0.875504349	0.706917161	0.921146002	0.990571725	0.98152792	0.844622232
ZNF304	0.875504349	0.821588096	0.924266781	0.915749182	0.982014614	0.861807035
AFF3	0.875504349	0.770808357	0.820549834	0.916305829	0.983935609	0.968245211

E2F1	0.875504349	0.790637585	0.894829955	0.994309225	0.988693274	0.775546211
RBL2	0.875504349	0.782865867	0.803771265	0.999939566	0.988693274	0.80252768
BCL9	0.875504349	0.733883576	0.844162118	0.999939566	0.988693274	0.852634896
LOC107132300	0.875504349	0.747555258	0.827125538	0.958879502	0.993321574	0.928442671
CDK10	0.875504349	0.81250936	0.87214481	0.988949923	0.996250445	0.812198201
TMEM267	0.875504349	0.719478857	0.951278495	0.943323364	0.996250445	0.832189969
LOC516599	0.875504349	0.7116845	0.821908722	0.995118128	0.996250445	0.911696156
MTCP1	0.875504349	0.711594168	0.978892556	0.91329897	0.997186167	0.846367069
TMEM218	0.875504349	0.742069534	0.95721973	0.915749182	0.997488969	0.856495922
GSC	0.875504349	0.920347742	0.839666249	0.925210703	0.997854738	0.834254546
LOC101907648	0.875504349	0.796591413	0.869455175	0.995118128	0.997981612	0.799314198
EIF3M	0.875504349	0.842511984	0.793430641	0.99879985	0.99989309	0.773130327
WBP11	0.875504349	0.724861785	0.918681165	0.99483611	0.99989309	0.776420145
ACTRT3	0.875504349	0.968237269	0.814444866	0.915749182	0.99989309	0.780092074
CDKN3	0.875504349	0.731268229	0.932690637	0.969717497	0.99989309	0.784080446
WDSUB1	0.875504349	0.831242188	0.858276354	0.987143706	0.99989309	0.784080446
RBP2	0.875504349	0.885981636	0.916930247	0.915749182	0.99989309	0.788131005
COX7A2	0.875504349	0.747164473	0.799265002	0.919247117	0.99989309	0.788131005
FAM96B	0.875504349	0.838405969	0.852758896	0.928836449	0.99989309	0.788131005
LOC112441887	0.875504349	0.719478857	0.844162118	0.973377993	0.99989309	0.788131005
ZNF518B	0.875504349	0.771098738	0.828407935	0.99879985	0.99989309	0.788142857
GOLM1	0.875504349	0.717373027	0.87146798	0.9846464	0.99989309	0.789905519
MAP3K5	0.875504349	0.890427603	0.794779482	0.956154344	0.99989309	0.790039686
SNX2	0.875504349	0.737937244	0.844065668	0.990571725	0.99989309	0.792329436
MLC1	0.875504349	0.747899938	0.814444866	0.956154344	0.99989309	0.793572843
LOC101907606	0.875504349	0.750287778	0.79610462	0.995687815	0.99989309	0.794529985
ASGR2	0.875504349	0.747164473	0.806221631	0.999939566	0.99989309	0.794727196
EYA3	0.875504349	0.72843033	0.819529308	0.915749182	0.99989309	0.795637468
RNF5	0.875504349	0.7116845	0.965491674	0.919247117	0.99989309	0.796002422
LOC112442593	0.875504349	0.731268229	0.882898206	0.918161195	0.99989309	0.799314198
SNX10	0.875504349	0.769817928	0.886282865	0.963153458	0.99989309	0.800276945
ZNF135	0.875504349	0.88418172	0.850020542	0.915749182	0.99989309	0.80252768
PAM	0.875504349	0.803903736	0.832483532	0.952024081	0.99989309	0.812509877
SOD1	0.875504349	0.764525656	0.836294915	0.984968973	0.99989309	0.8125889
SS18L1	0.875504349	0.72843033	0.848237641	0.971220023	0.99989309	0.812682271
ASCL4	0.875504349	0.736649498	0.875669731	0.908496281	0.99989309	0.814637245
EVA1B	0.875504349	0.790637585	0.845237542	0.934267153	0.99989309	0.815056305
MYD88	0.875504349	0.717044998	0.814444866	0.99879985	0.99989309	0.815459921

GALNT11	0.875504349	0.785406526	0.788938503	0.946344632	0.99989309	0.827631181
ZNF420	0.875504349	0.852463941	0.855743966	0.943323364	0.99989309	0.828376685
FAM173B	0.875504349	0.759546301	0.813203969	0.98470348	0.99989309	0.831041995
SERINC5	0.875504349	0.786792359	0.806311697	0.989154268	0.99989309	0.834550008
ZNHIT6	0.875504349	0.830448181	0.893763648	0.915749182	0.99989309	0.83849759
LRIF1	0.875504349	0.823692881	0.788848487	0.915749182	0.99989309	0.839419627
SETMAR	0.875504349	0.862267429	0.806221631	0.91329897	0.99989309	0.840150874
LOC616948	0.875504349	0.712908612	0.839601684	0.925004412	0.99989309	0.840150874
SUCLG1	0.875504349	0.741061871	0.811009079	0.934591536	0.99989309	0.844622232
XG	0.875504349	0.703437628	0.868936984	0.915749182	0.99989309	0.84491737
MYO5A	0.875504349	0.957353974	0.788848487	0.917869295	0.99989309	0.84491737
TCEAL9	0.875504349	0.7116845	0.828407935	0.999939566	0.99989309	0.845866317
SAYSD1	0.875504349	0.754663123	0.869529186	0.915749182	0.99989309	0.849105061
DAPP1	0.875504349	0.709451322	0.893544538	0.948120268	0.99989309	0.852634896
PEBP1	0.875504349	0.72477102	0.817256644	0.943323364	0.99989309	0.853233759
FAM49A	0.875504349	0.737937244	0.844162118	0.998542029	0.99989309	0.855453999
TNFSF4	0.875504349	0.726229532	0.802847755	0.995118128	0.99989309	0.862259367
FCHSD1	0.875504349	0.711594168	0.930134801	0.925901365	0.99989309	0.864294309
PHLDB1	0.875504349	0.866813771	0.878603452	0.908054165	0.99989309	0.864571107
SPX	0.875504349	0.718866883	0.810425784	0.949930337	0.99989309	0.865159042
LOC537848	0.875504349	0.835276066	0.819094028	0.91329897	0.99989309	0.868108836
PGA5	0.875504349	0.763751563	0.850020542	0.934591536	0.99989309	0.868108836
LOC112447353	0.875504349	0.821588096	0.806311697	0.956154344	0.99989309	0.872493553
SRPX2	0.875504349	0.727727075	0.806939645	0.99879985	0.99989309	0.882322567
GCFC2	0.875504349	0.736964914	0.880277219	0.948891906	0.99989309	0.884097654
NFXL1	0.875504349	0.780290359	0.882898206	0.909370705	0.99989309	0.88676448
LEO1	0.875504349	0.780061391	0.849077738	0.917869295	0.99989309	0.901173172
AARS	0.875504349	0.74796249	0.856812671	0.915749182	0.99989309	0.901320431
KDM3A	0.875504349	0.703437628	0.79610462	0.93471064	0.99989309	0.901965781
C23H6orf136	0.875504349	0.704067623	0.89007492	0.910808102	0.99989309	0.902660792
PSMD8	0.875504349	0.774326932	0.842605286	0.915749182	0.99989309	0.911263177
TIMM8A	0.875504349	0.779501962	0.820742891	0.943323364	0.99989309	0.917968123
CPOX	0.875504349	0.705889653	0.802847755	0.992137653	0.99989309	0.926872824
EDIL3	0.875504349	0.742728851	0.803398613	0.908496281	0.99989309	0.928799803
RUBCNL	0.875504349	0.794355325	0.812770091	0.913413181	0.99989309	0.940917403
PLS3	0.875715531	0.782196458	0.957578315	0.909743798	0.99989309	0.839419627
GPR75	0.875729229	0.72843033	0.856769954	0.925004412	0.99989309	0.799940894
SYNCRIP	0.875729229	0.731268229	0.812043145	0.943880976	0.99989309	0.902660792

CD163	0.875729229	0.780892456	0.803771265	0.915749182	0.99989309	0.905220694
CORO2A	0.876188606	0.953940554	0.808303251	0.9846464	0.964150118	0.78218588
FANCL	0.876222069	0.742728851	0.792827466	0.999939566	0.99989309	0.809953778
IGF2BP3	0.876249731	0.852463941	0.850046649	0.910808102	0.943901678	0.944892687
GAS2	0.876249731	0.757851881	0.988836461	0.936573639	0.952172109	0.777933888
LOC509034	0.876249731	0.747555258	0.805580394	0.946344632	0.968477804	0.970801789
DLGAP3	0.876249731	0.798909583	0.863138583	0.99879985	0.972917711	0.810707901
FUCA1	0.876249731	0.862527737	0.845237542	0.962391675	0.977379814	0.842187877
REX1BD	0.876249731	0.831242188	0.80326876	0.93026568	0.99989309	0.846367069
LOC104972797	0.876249731	0.711594168	0.820767684	0.925931623	0.99989309	0.889622239
SRBD1	0.876314163	0.72477102	0.810425784	0.999939566	0.999592263	0.840150874
CTPS2	0.876323246	0.821124254	0.853222116	0.999939566	0.967609842	0.792329436
MRPS2	0.876323246	0.780082505	0.808440332	0.956154344	0.99989309	0.834735448
SLC9A9	0.876446362	0.820796062	0.827125538	0.917869295	0.99989309	0.799147792
BNIP1	0.876741674	0.829255735	0.953826265	0.915749182	0.954501767	0.834254546
FKRP	0.876741674	0.708437597	0.97311093	0.915749182	0.99989309	0.797675006
PALM2	0.876741674	0.724861785	0.929958748	0.917869295	0.99989309	0.809650729
CREB3	0.876741674	0.71282706	0.789868841	0.995118128	0.99989309	0.815459921
FABP2	0.876741674	0.844086067	0.870054336	0.915749182	0.99989309	0.862403446
DGKG	0.877094404	0.847919882	0.836294915	0.917869295	0.943768595	0.948277018
CLEC11A	0.877100659	0.803903736	0.90166788	0.956154344	0.98152792	0.838324968
LARP7	0.877170538	0.810519292	0.806221631	0.92064557	0.99989309	0.840150874
PVRIG	0.877186458	0.885991833	0.795258121	0.915749182	0.964521207	0.940917403
MEX3B	0.877186458	0.717508618	0.886282865	0.999939566	0.996250445	0.786737722
CDCA2	0.877230313	0.80403784	0.885061883	0.999939566	0.961382403	0.777933888
WDR75	0.877453194	0.717044998	0.832483532	0.917869295	0.99989309	0.854831489
TWF1	0.877748089	0.769091087	0.845425011	0.92064557	0.961835052	0.963996128
C26H10orf88	0.877748089	0.849926024	0.933963997	0.917869295	0.964521207	0.830456671
SUCLA2	0.877748089	0.769783999	0.828407935	0.990571725	0.996250445	0.89063727
ZMYM2	0.877748089	0.737374533	0.846650632	0.91329897	0.99989309	0.791259029
CHD4	0.877748089	0.739918356	0.792827466	0.999939566	0.99989309	0.829807823
UBE2L3	0.877748089	0.770808357	0.828407935	0.931989854	0.99989309	0.837798115
TTF1	0.877748089	0.780641867	0.883726795	0.943323364	0.99989309	0.839419627
UFL1	0.877748089	0.747164473	0.802951601	0.953508052	0.99989309	0.84491737
AASS	0.877748089	0.820993717	0.892122482	0.915749182	0.99989309	0.859973633
SLC38A9	0.877748089	0.755210284	0.788938503	0.949947918	0.99989309	0.864571107
CEP135	0.877748089	0.780061391	0.929958748	0.91030227	0.99989309	0.867431709
TLDC2	0.877748089	0.771098738	0.834364497	0.949947918	0.99989309	0.872175131

CLYBL	0.877748089	0.72843033	0.810425784	0.999939566	0.99989309	0.872493553
PARM1	0.877749104	0.722481078	0.827125538	0.935763381	0.99989309	0.859973633
MSRB1	0.877929943	0.844031777	0.832483532	0.915749182	0.99989309	0.858042054
LOC101902428	0.877929943	0.786258422	0.815870524	0.915749182	0.99989309	0.888617204
EML2	0.878289867	0.757476878	0.844065668	0.943323364	0.99989309	0.863755488
TNFRSF19	0.878462787	0.776313457	0.817993104	0.918313511	0.947652461	0.989313458
ATG5	0.878462787	0.866514089	0.892122482	0.963829131	0.954501767	0.809650729
ZMIZ1	0.878462787	0.710155842	0.850243032	0.999939566	0.954501767	0.813345852
LOC104973073	0.878462787	0.997390201	0.80326876	0.915749182	0.954501767	0.813759229
SLC46A3	0.878462787	0.710155842	0.826667106	0.999939566	0.954501767	0.934660101
MEOX1	0.878462787	0.824676261	0.817256644	0.999939566	0.957605284	0.789577614
LOC783045	0.878462787	0.744066065	0.966157152	0.96095927	0.957663949	0.805198358
RPAP2	0.878462787	0.730317314	0.926613927	0.995118128	0.961382403	0.829463053
LOC107131134	0.878462787	0.767950704	0.808333956	0.999939566	0.975291792	0.848289476
BAG2	0.878462787	0.904764822	0.921135532	0.915749182	0.983342337	0.788142857
PPIP5K2	0.878462787	0.737004023	0.944592818	0.9565815	0.986955873	0.830456671
KDM5C	0.878462787	0.732798765	0.9343664	0.958879502	0.988693274	0.84491737
PDAP1	0.878462787	0.90874814	0.838080059	0.934591536	0.996081327	0.845949552
SLC25A10	0.878462787	0.855151723	0.817256644	0.99879985	0.997488969	0.809650729
LOC781612	0.878462787	0.868963165	0.814444866	0.995118128	0.99989309	0.77821816
LOC100296324	0.878462787	0.790637585	0.790396664	0.925901365	0.99989309	0.78859079
CMKLR1	0.878462787	0.708437597	0.93798101	0.925931623	0.99989309	0.796002422
LOC784841	0.878462787	0.838405969	0.832483532	0.995118128	0.99989309	0.809650729
VIRMA	0.878462787	0.840505408	0.79018754	0.915749182	0.99989309	0.8125889
PPP2R2A	0.878462787	0.823692881	0.798686086	0.915749182	0.99989309	0.821456459
ATP5MC1	0.878462787	0.730944867	0.839378262	0.915749182	0.99989309	0.823139458
NUDT16	0.878462787	0.927298992	0.827125538	0.915749182	0.99989309	0.826868779
GPI	0.878462787	0.7116845	0.806825406	0.915749182	0.99989309	0.834550008
CLEC9A	0.878462787	0.79738652	0.819404084	0.99879985	0.99989309	0.834735448
SLC19A3	0.878462787	0.732522135	0.902584766	0.971220023	0.99989309	0.845866317
IRX5	0.878462787	0.747164473	0.812770091	0.999939566	0.99989309	0.84763005
LOC107132247	0.878462787	0.805157515	0.92153058	0.915749182	0.99989309	0.861807035
S100A16	0.878462787	0.707802804	0.828407935	0.995118128	0.99989309	0.865159042
SNRPD3	0.878462787	0.810519292	0.885434576	0.915749182	0.99989309	0.868177389
DDX55	0.878462787	0.792190608	0.879968243	0.915749182	0.99989309	0.871780952
MRPL37	0.878462787	0.818711217	0.788938503	0.91329897	0.99989309	0.880404833
EAF2	0.878743405	0.769817928	0.977782604	0.934591536	0.952672789	0.8125889
LOC112449516	0.878743405	0.824676261	0.89007492	0.917869295	0.956624006	0.908939618

CPEB1	0.878743405	0.754202934	0.849077738	0.990571725	0.957605284	0.910353954
DDX47	0.878743405	0.715519139	0.885354035	0.939915888	0.961835052	0.943138585
RELB	0.878743405	0.823692881	0.809322753	0.999939566	0.962791364	0.828663852
TMEM252	0.878743405	0.800441059	0.960137022	0.925901365	0.972917711	0.812509877
SCN2A	0.878743405	0.75177936	0.855911319	0.999939566	0.975291792	0.810707901
SERPINA5	0.878743405	0.827609894	0.875669731	0.925931623	0.975291792	0.893961809
TCIM	0.878743405	0.836487169	0.810425784	0.99879985	0.981938411	0.852476672
COL15A1	0.878743405	0.780290359	0.806311697	0.999939566	0.997981612	0.789577614
CTTNBP2	0.878743405	0.735625249	0.894447907	0.985181062	0.99989309	0.783134114
FAM173A	0.878743405	0.766403118	0.827125538	0.925931623	0.99989309	0.783479395
UQCRB	0.878743405	0.747555258	0.849459857	0.944558402	0.99989309	0.788142857
NDUFA4	0.878743405	0.74214695	0.812043145	0.924489924	0.99989309	0.798051736
RAPGEF6	0.878743405	0.776182952	0.814444866	0.91329897	0.99989309	0.809548115
SPAG9	0.878743405	0.771098738	0.87214481	0.985342672	0.99989309	0.812198201
ITPRIP	0.878743405	0.852463941	0.812585086	0.956154344	0.99989309	0.812509877
PLOD2	0.878743405	0.7164915	0.86637689	0.963829131	0.99989309	0.8125889
RBM20	0.878743405	0.887165309	0.815870524	0.977470078	0.99989309	0.821467423
TAF1D	0.878743405	0.720945114	0.951850716	0.943240775	0.99989309	0.826868779
MUC1	0.878743405	0.798956241	0.847037539	0.95964071	0.99989309	0.829807823
MBTPS2	0.878743405	0.80925601	0.849077738	0.943323364	0.99989309	0.835866152
RGMA	0.878743405	0.76090129	0.848890018	0.955560327	0.99989309	0.839494796
FOXF1	0.878743405	0.72477102	0.849077738	0.99879985	0.99989309	0.840961242
ZNF622	0.878743405	0.735465232	0.848018557	0.925931623	0.99989309	0.842461327
AP2S1	0.878743405	0.80320642	0.79610462	0.913413181	0.99989309	0.859973633
LOC107131531	0.878743405	0.80403784	0.834793268	0.95964071	0.99989309	0.86307729
COPS5	0.878743405	0.743422331	0.827125538	0.95175364	0.99989309	0.873118284
COIL	0.878743405	0.718302288	0.896272282	0.943323364	0.99989309	0.886992214
SLC35A4	0.878743405	0.828797861	0.838838955	0.910808102	0.99989309	0.906876302
ZNF280B	0.878743405	0.808197752	0.828407935	0.915749182	0.99989309	0.935655141
ANKLE1	0.878743405	0.717132341	0.822800372	0.915749182	0.99989309	0.955585296
LBP	0.878743405	0.718469663	0.832483532	0.914402649	0.99989309	0.979810204
AGGF1	0.879064974	0.737937244	0.820767684	0.999939566	0.981938411	0.815459921
ERO1B	0.879064974	0.916172682	0.836294915	0.925440563	0.99989309	0.847450944
GPR34	0.879271085	0.841629926	0.806221631	0.936573639	0.99989309	0.897011069
FAM76A	0.879304241	0.796591413	0.859796267	0.999939566	0.954501767	0.839419627
PARL	0.879304241	0.728358862	0.870054336	0.998251353	0.960296839	0.89321306
LOC521224	0.879304241	0.921043975	0.843427302	0.990049248	0.961835052	0.790039686
ZNF266	0.879304241	0.775921167	0.882471228	0.99879985	0.975291792	0.825813467

GORASP2	0.879304241	0.77069491	0.946659074	0.910808102	0.988693274	0.883302043
KIF26B	0.879304241	0.771098738	0.844316758	0.99879985	0.991830391	0.858042054
FAM214B	0.879304241	0.769313383	0.846650632	0.995800463	0.996250445	0.86143104
LYSMD3	0.879304241	0.813459746	0.83314199	0.925931623	0.99989309	0.788142857
WDR20	0.879304241	0.711594168	0.827125538	0.915749182	0.99989309	0.792329436
TTC9C	0.879304241	0.740182637	0.850106729	0.919247117	0.99989309	0.792329436
LOC504548	0.879304241	0.963478319	0.804449716	0.916305829	0.99989309	0.793566607
EIF1B	0.879304241	0.765434632	0.830022042	0.956154344	0.99989309	0.798051736
TMEM121B	0.879304241	0.833111922	0.855991442	0.936573639	0.99989309	0.806453168
ZC3H14	0.879304241	0.755354791	0.845336233	0.990571725	0.99989309	0.812198201
LOC101904769	0.879304241	0.962934461	0.84311048	0.915749182	0.99989309	0.812509877
CDC7	0.879304241	0.737937244	0.849077738	0.960986457	0.99989309	0.812509877
ZBTB44	0.879304241	0.808303695	0.838016689	0.971220023	0.99989309	0.812622197
RHBDD3	0.879304241	0.842667989	0.814444866	0.918908983	0.99989309	0.822642815
XIRP1	0.879304241	0.854362191	0.819529308	0.925931623	0.99989309	0.84491737
TANGO6	0.879304241	0.842797231	0.856997596	0.925901365	0.99989309	0.855069409
TXLNG	0.879304241	0.783416338	0.834904664	0.998542029	0.99989309	0.858744772
TMX2	0.879304241	0.780944257	0.844065668	0.946344632	0.99989309	0.859487774
LOC101904698	0.879304241	0.780290359	0.901025618	0.915749182	0.99989309	0.85958325
PSMD7	0.879304241	0.714281434	0.917092799	0.973785871	0.99989309	0.86143104
ZNF10	0.879304241	0.743422331	0.870911433	0.916305829	0.99989309	0.86307729
ACTR1A	0.879304241	0.780061391	0.911743083	0.925004412	0.99989309	0.868108836
DCAF4	0.879304241	0.7164915	0.819114812	0.984915557	0.99989309	0.871787716
ISM1	0.879304241	0.74638208	0.86719452	0.928623745	0.99989309	0.882170826
MFSD2B	0.879304241	0.771098738	0.859931483	0.960986457	0.99989309	0.886992214
GALE	0.879304241	0.859450771	0.821254591	0.915749182	0.99989309	0.893961809
LOC100297152	0.879304241	0.742298467	0.846650632	0.955560327	0.99989309	0.903583219
DOC2A	0.879722392	0.72809533	0.974158329	0.913413181	0.98571085	0.869171706
EGFLAM	0.879722392	0.747499345	0.843427302	0.938519355	0.99989309	0.788131005
DESI1	0.880019685	0.8232491	0.820549834	0.956303465	0.99989309	0.870079716
PSMD2	0.880187741	0.742728851	0.817256644	0.96095927	0.961382403	0.968462588
LOC783730	0.88022356	0.847082229	0.839601684	0.915749182	0.99989309	0.799935743
RAVER2	0.880427087	0.72843033	0.806221631	0.971220023	0.99989309	0.796002422
COQ9	0.880427087	0.717132341	0.79610462	0.928623745	0.99989309	0.806453168
KMT2C	0.880427087	0.76167712	0.855712722	0.925931623	0.99989309	0.908118871
PDCL	0.880710509	0.785406526	0.905374144	0.925004412	0.99989309	0.818819752
LOC100336976	0.880710509	0.747899938	0.817256644	0.955560327	0.99989309	0.842461327
SYCE2	0.880710509	0.771765977	0.837473402	0.913413181	0.99989309	0.889622239

LOC107131715	0.881429447	0.719840239	0.862585131	0.990491321	0.99989309	0.821467423
NKAP	0.881430638	0.810049386	0.953198168	0.949947918	0.952172109	0.815459921
NEMF	0.881430638	0.762756164	0.904682623	0.999939566	0.954501767	0.828550207
NMB	0.881467327	0.751946504	0.817256644	0.999939566	0.947146038	0.893961809
HAUS8	0.881467327	0.718231253	0.877816623	0.956154344	0.99989309	0.822574444
CWC22	0.881489038	0.893639679	0.80326876	0.915749182	0.99989309	0.840150874
JAKMIP3	0.881544502	0.918976923	0.877133001	0.915749182	0.964150118	0.863891806
GTSF1	0.881544502	0.736066622	0.878603452	0.99879985	0.99989309	0.802875906
LOC101905706	0.881623699	0.774326932	0.81285201	0.999939566	0.95091153	0.846367069
PRMT3	0.881623699	0.751946504	0.843427302	0.923832787	0.95091153	0.986716086
FAM161A	0.881623699	0.747899938	0.835091365	0.999939566	0.952821886	0.828550207
TET2	0.881623699	0.72843033	0.832483532	0.999939566	0.952821886	0.86143104
WDR83	0.881623699	0.968237269	0.80326876	0.925004412	0.957910867	0.870895425
FBH1	0.881623699	0.744066065	0.962209895	0.925440563	0.964521207	0.866482265
ARG2	0.881623699	0.810173561	0.855743966	0.99879985	0.976970807	0.821774001
NODAL	0.881623699	0.785978032	0.817256644	0.992368879	0.99989309	0.799940894
MRPL13	0.881623699	0.750546265	0.849077738	0.928836449	0.99989309	0.83849759
OSTC	0.881623699	0.876057665	0.827125538	0.959009349	0.99989309	0.859973633
DCUN1D4	0.881883762	0.780290359	0.922126495	0.990049248	0.964150118	0.828602505
SNX12	0.881883762	0.786188116	0.920607829	0.977470078	0.98152792	0.825331577
NTN1	0.881883762	0.747179932	0.894449269	0.919247117	0.99989309	0.788131005
LOC101904378	0.881883762	0.957254162	0.820767684	0.949947918	0.99989309	0.792329436
KRT7	0.881883762	0.835276066	0.863742167	0.944558402	0.99989309	0.812682271
LOC100336734	0.881883762	0.725594888	0.951607422	0.943539568	0.99989309	0.839419627
DPCD	0.881883762	0.771165031	0.858865175	0.959410646	0.99989309	0.839419627
LOC104969409	0.881883762	0.972786652	0.802951601	0.913319839	0.99989309	0.840150874
PMPCB	0.881883762	0.738981601	0.832483532	0.995118128	0.99989309	0.840150874
LOC615454	0.881883762	0.877206497	0.842605286	0.956154344	0.99989309	0.845969723
MINDY3	0.881883762	0.723119554	0.890801024	0.990571725	0.99989309	0.86143104
KCNK13	0.881883762	0.769313383	0.886282865	0.915749182	0.99989309	0.866253786
CDK20	0.881883762	0.754663123	0.838080059	0.915749182	0.99989309	0.868108836
LOC112443469	0.881883762	0.736066622	0.794216389	0.956154344	0.99989309	0.889622239
STS	0.881883762	0.726229532	0.801316658	0.924489924	0.99989309	0.926872824
ZWILCH	0.881883762	0.71963672	0.849077738	0.915749182	0.99989309	0.971554741
PID1	0.881921633	0.926435857	0.820767684	0.934591536	0.99989309	0.826868779
LOC614208	0.881921633	0.875022483	0.867451783	0.925931623	0.99989309	0.832291916
RASIP1	0.881921633	0.886967296	0.802951601	0.919671703	0.99989309	0.906065482
MTFR2	0.881949054	0.88418172	0.858263809	0.969717497	0.993321574	0.809650729

PSEN2	0.881949054	0.813230451	0.806825406	0.965097245	0.99989309	0.791259029
PHAX	0.882022357	0.742728851	0.929958748	0.99879985	0.962791364	0.80844231
CDKN1C	0.882657466	0.72843033	0.827125538	0.992368879	0.99989309	0.792329436
YBX2	0.882818681	0.789863026	0.847441894	0.951929772	0.979654999	0.921582976
SERAC1	0.8829371	0.776182952	0.817601111	0.999939566	0.954501767	0.847256027
LOC101903615	0.882939388	0.749195479	0.827125538	0.999939566	0.99989309	0.811943134
DNAJC1	0.882939388	0.810205594	0.850952986	0.915749182	0.99989309	0.835370067
ADORA1	0.882939388	0.829255735	0.827125538	0.948120268	0.99989309	0.837501229
INPP4B	0.882939388	0.765761724	0.827350832	0.915749182	0.99989309	0.956986009
PLCE1	0.88294185	0.882706523	0.817993104	0.990300191	0.951073785	0.880404833
PHLDB3	0.88294185	0.731268229	0.857065866	0.999939566	0.954741113	0.812509877
SPG21	0.88294185	0.722790338	0.878603452	0.999939566	0.955780925	0.823435077
DYNLT3	0.88294185	0.779501962	0.879515214	0.981022805	0.972917711	0.8770378
APLF	0.88294185	0.932024312	0.850106729	0.91329897	0.972917711	0.883302043
PARP3	0.88294185	0.73599132	0.988788601	0.925901365	0.975291792	0.812509877
LOC101906632	0.88294185	0.803903736	0.844316758	0.999939566	0.982014614	0.819007
MAPK1	0.88294185	0.737937244	0.874092011	0.994309225	0.988693274	0.875441396
PPIC	0.88294185	0.71282706	0.870585788	0.999939566	0.997488969	0.794529985
LOC100848484	0.88294185	0.717132341	0.836294915	0.915749182	0.99989309	0.810707901
GPR20	0.88294185	0.795302167	0.828407935	0.95175364	0.99989309	0.837148498
PHF8	0.88294185	0.776217482	0.829696084	0.943323364	0.99989309	0.837312175
PSIP1	0.88294185	0.786660393	0.828407935	0.999939566	0.99989309	0.840150874
B3GAT3	0.88294185	0.825301941	0.802847755	0.915749182	0.99989309	0.84491737
CCL25	0.88294185	0.837455678	0.810425784	0.942125631	0.99989309	0.84491737
TGM3	0.88294185	0.720920448	0.79610462	0.981022805	0.99989309	0.859973633
LOC786987	0.88294185	0.780641867	0.816129315	0.969717497	0.99989309	0.869171706
ADD1	0.88294185	0.740948281	0.848018557	0.990571725	0.99989309	0.885060677
CASP8AP2	0.88294185	0.71282706	0.843427302	0.943807256	0.99989309	0.908939618
CNNM3	0.88294185	0.764097231	0.836294915	0.943323364	0.99989309	0.910353954
GFM2	0.88294185	0.760730252	0.827125538	0.91329897	0.99989309	0.923064313
CNPPD1	0.883385601	0.828417266	0.795701822	0.940247932	0.99989309	0.876330039
SOCS7	0.883393443	0.835276066	0.817256644	0.915749182	0.99989309	0.808138656
LOC512672	0.883393443	0.780290359	0.832483532	0.956154344	0.99989309	0.878896178
PAAF1	0.883803243	0.737374533	0.87312855	0.946344632	0.99989309	0.820932605
C28H10orf71	0.884166692	0.818806929	0.81284583	0.999939566	0.99989309	0.793566607
TCEAL8	0.884578392	0.765341142	0.846650632	0.994719152	0.954501767	0.912396352
SLC19A1	0.884578392	0.839399097	0.843427302	0.948547383	0.964239946	0.911696156
LOC112449258	0.884578392	0.737374533	0.878603452	0.923578108	0.981938411	0.957877422

MMP14	0.884578392	0.753193121	0.858972712	0.943323364	0.99989309	0.796002422
MTMR9	0.884578392	0.748889217	0.887441226	0.958879502	0.99989309	0.839494796
RADIL	0.884578392	0.737937244	0.869529186	0.973377993	0.99989309	0.864809768
DUSP19	0.884578392	0.77414098	0.837967179	0.989391869	0.99989309	0.887248119
MAPK8	0.884578392	0.921280256	0.806118979	0.917869295	0.99989309	0.891000792
INTS6L	0.884626845	0.74401284	0.831320191	0.996775429	0.978165544	0.913230542
DUSP28	0.884626845	0.77396808	0.817993104	0.992137653	0.99989309	0.795637468
BIRC3	0.884626845	0.749070017	0.829843435	0.995118128	0.99989309	0.879160297
DDX31	0.884628654	0.73599132	0.93050447	0.958879502	0.952172109	0.90259329
LOC101909754	0.884628654	0.832607032	0.858865175	0.99879985	0.952672789	0.844335246
LOC101905770	0.884628654	0.72812452	0.869529186	0.995536195	0.957605284	0.914122611
CDH24	0.884628654	0.750487364	0.942661406	0.992137653	0.960296839	0.823435077
ABLIM2	0.884628654	0.736566631	0.944612298	0.925004412	0.961835052	0.910353954
ZNF34	0.884628654	0.765434632	0.847037539	0.915749182	0.962791364	0.983453112
FKBP11	0.884628654	0.730944867	0.836257071	0.99879985	0.972176983	0.918124352
TNFSF15	0.884628654	0.825289499	0.885434576	0.995118128	0.975291792	0.812509877
CTSA	0.884628654	0.873482516	0.810124821	0.946344632	0.979654999	0.912101047
WDR92	0.884628654	0.737937244	0.843427302	0.945929971	0.98152792	0.964143145
PRKCZ	0.884628654	0.780641867	0.847037539	0.999939566	0.981938411	0.845803016
LAPTM4B	0.884628654	0.774697122	0.84311048	0.999939566	0.987694035	0.812509877
ZNF354C	0.884628654	0.747106203	0.85972234	0.999939566	0.988693274	0.808138656
RDH11	0.884628654	0.81250936	0.909002826	0.947581168	0.988693274	0.852755805
ERAS	0.884628654	0.723005264	0.869455175	0.915749182	0.988693274	0.978924127
LOC101903820	0.884628654	0.88311684	0.838992284	0.977470078	0.996250445	0.840150874
LYZ2	0.884628654	0.883927618	0.838080059	0.915749182	0.996250445	0.912396352
JAGN1	0.884628654	0.73984736	0.906597758	0.999939566	0.99989309	0.788131005
HPGD	0.884628654	0.727186688	0.870774839	0.995118128	0.99989309	0.792329436
KLF5	0.884628654	0.844176756	0.832483532	0.956154344	0.99989309	0.794529985
TMEFF2	0.884628654	0.747179932	0.8895568	0.925931623	0.99989309	0.799935743
EAF1	0.884628654	0.739753419	0.87146798	0.958879502	0.99989309	0.799940894
ARHGAP10	0.884628654	0.743422331	0.915874142	0.996775429	0.99989309	0.80787758
LOC112443499	0.884628654	0.73517853	0.870054336	0.995118128	0.99989309	0.808138656
GINM1	0.884628654	0.804875341	0.899195818	0.958879502	0.99989309	0.809650729
SNX15	0.884628654	0.769817928	0.896272282	0.915749182	0.99989309	0.812509877
FZD10	0.884628654	0.72477102	0.878050811	0.992368879	0.99989309	0.813703101
LOC104976344	0.884628654	0.840078234	0.827125538	0.943323364	0.99989309	0.823139458
DDIAS	0.884628654	0.72843033	0.933963997	0.956154344	0.99989309	0.823139458
NDUFA10	0.884628654	0.731268229	0.810425784	0.927618239	0.99989309	0.830456671

IGFALS	0.884628654	0.747164473	0.899195818	0.914402649	0.99989309	0.830923241
ZBED8	0.884628654	0.780290359	0.894449269	0.989391869	0.99989309	0.834008567
CCDC6	0.884628654	0.724540534	0.885481718	0.995118128	0.99989309	0.84491737
MAML3	0.884628654	0.764097231	0.885812254	0.932656599	0.99989309	0.845803016
AP3B1	0.884628654	0.780641867	0.893763648	0.917869295	0.99989309	0.859487774
FAM50A	0.884628654	0.902832493	0.864640429	0.915749182	0.99989309	0.859973633
FITM2	0.884628654	0.715305297	0.845237542	0.995118128	0.99989309	0.859973633
DCTN5	0.884628654	0.77414098	0.857943088	0.935763381	0.99989309	0.86143104
UBD	0.884628654	0.848132141	0.846650632	0.925931623	0.99989309	0.86307729
EHD1	0.884628654	0.766086544	0.843427302	0.978124976	0.99989309	0.869857598
GTF3C3	0.884628654	0.91557476	0.817256644	0.916305829	0.99989309	0.870895425
SLC25A16	0.884628654	0.794355325	0.87242852	0.962775522	0.99989309	0.8711678
CCDC88B	0.884628654	0.769313383	0.860134949	0.925265292	0.99989309	0.877623013
DUS1L	0.884628654	0.751946504	0.832483532	0.937070243	0.99989309	0.884387948
LOC112442392	0.884628654	0.774326932	0.870054336	0.956154344	0.99989309	0.887248119
C7H19orf44	0.884628654	0.736964914	0.88330023	0.925004412	0.99989309	0.888695935
TMEM204	0.884628654	0.726229532	0.887130994	0.956154344	0.99989309	0.890898561
LOC505600	0.884628654	0.808513267	0.841835423	0.952024081	0.99989309	0.892802259
STAT2	0.884628654	0.720945114	0.880277219	0.92064557	0.99989309	0.894901464
LOC789867	0.884628654	0.755397146	0.815548119	0.956154344	0.99989309	0.895218005
RARS	0.884628654	0.777469821	0.830013482	0.913413181	0.99989309	0.898273855
TRPM2	0.884628654	0.719478857	0.863172962	0.913413181	0.99989309	0.908939618
COA7	0.884628654	0.72843033	0.836294915	0.990571725	0.99989309	0.911696156
LOC101905593	0.884628654	0.771098738	0.850243032	0.915749182	0.99989309	0.912380696
LOC112442952	0.884628654	0.852463941	0.830013482	0.913413181	0.99989309	0.930925458
TADA1	0.884628654	0.753055981	0.837473402	0.915749182	0.99989309	0.968245211
EFNA2	0.884866253	0.786188116	0.858972712	0.995800463	0.954501767	0.88667499
SYP	0.884968139	0.897327046	0.834643686	0.915749182	0.99989309	0.865159042
KCNA2	0.885007921	0.782196458	0.802857717	0.963829131	0.99989309	0.829389486
EPOP	0.885025067	0.84185344	0.842478086	0.919247117	0.99989309	0.925494614
GPC1	0.885108809	0.85749413	0.814444866	0.925004412	0.99989309	0.802771083
RNMT	0.885294162	0.774326932	0.827125538	0.983569039	0.952821886	0.955585296
CSNK2A1	0.885294162	0.762756164	0.911743083	0.922782374	0.961382403	0.940917403
HSBP1	0.885294162	0.855151723	0.845237542	0.939292364	0.99989309	0.795637468
H3F3C	0.885294162	0.824676261	0.862585131	0.917869295	0.99989309	0.798018153
DNASE1L1	0.885294162	0.851167599	0.878603452	0.960357834	0.99989309	0.806054104
LOC104976321	0.885294162	0.795993324	0.83314199	0.924489924	0.99989309	0.812509877
GDPD5	0.885294162	0.747106203	0.868936984	0.951177494	0.99989309	0.873118284

TCTA	0.885552926	0.810519292	0.803771265	0.943323364	0.99989309	0.799314198
SAMD5	0.885574099	0.851167599	0.844065668	0.995800463	0.99989309	0.789577614
ZNF362	0.885574099	0.751529702	0.879215964	0.956154344	0.99989309	0.794529985
BHLHE41	0.885574099	0.81808696	0.904920398	0.915749182	0.99989309	0.805038934
ZDBF2	0.885574099	0.911106863	0.820767684	0.92064557	0.99989309	0.840150874
ALDH4A1	0.885708684	0.740948281	0.827125538	0.931268564	0.99989309	0.854750035
TBCA	0.885782064	0.806076007	0.884197487	0.995118128	0.99989309	0.809650729
MRTO4	0.885989412	0.866803532	0.81285201	0.943323364	0.952821886	0.94425343
LOC112445150	0.885989412	0.726229532	0.802857717	0.999939566	0.960296839	0.908939618
MICALL2	0.885989412	0.747499345	0.982659255	0.943323364	0.961382403	0.822574444
SPDL1	0.885989412	0.768450917	0.802847755	0.999939566	0.975291792	0.868108836
KRCC1	0.885989412	0.859882995	0.843427302	0.992154129	0.981938411	0.84491737
PSMA6	0.885989412	0.719622555	0.852758896	0.99879985	0.983634477	0.896718993
LAMB2	0.885989412	0.74401284	0.87214481	0.995118128	0.988693274	0.880781298
TSPO	0.885989412	0.739860832	0.844065668	0.933991524	0.99989309	0.792329436
CIB1	0.885989412	0.839641196	0.849132683	0.990571725	0.99989309	0.792329436
AKAP2	0.885989412	0.790637585	0.884325235	0.949947918	0.99989309	0.797130253
IP6K1	0.885989412	0.831242188	0.810425784	0.921477746	0.99989309	0.812509877
RILPL1	0.885989412	0.824016465	0.886282865	0.928836449	0.99989309	0.812509877
LOC112449406	0.885989412	0.751946504	0.890264995	0.99879985	0.99989309	0.815459921
ATP5F1C	0.885989412	0.780290359	0.817256644	0.931989854	0.99989309	0.823139458
USP50	0.885989412	0.823187795	0.939091808	0.925901365	0.99989309	0.837501229
TRIM59	0.885989412	0.782196458	0.828569127	0.988949923	0.99989309	0.840150874
ARHGAP22	0.885989412	0.868963165	0.812770091	0.958879502	0.99989309	0.844622232
YIF1B	0.885989412	0.853649912	0.814444866	0.915749182	0.99989309	0.845803016
SGCE	0.885989412	0.869907246	0.843427302	0.925931623	0.99989309	0.855692379
SCAND1	0.885989412	0.866803532	0.823351722	0.943510519	0.99989309	0.868108836
NT5DC1	0.885989412	0.742963341	0.827125538	0.99879985	0.99989309	0.869290956
TRIM68	0.885989412	0.803683367	0.817256644	0.915749182	0.99989309	0.877623013
SRPK1	0.885989412	0.747179932	0.808333956	0.946344632	0.99989309	0.912101047
LOC100296832	0.885989412	0.766408271	0.841124495	0.973377993	0.99989309	0.922670883
STK32C	0.886202788	0.80403784	0.827125538	0.999939566	0.952172109	0.805038934
RHBDD1	0.886202788	0.80403784	0.878603452	0.999939566	0.954501767	0.803605488
NFATC4	0.886202788	0.840874016	0.945417678	0.956154344	0.957605284	0.797407409
AOX2	0.886202788	0.76167712	0.985160065	0.956154344	0.957910867	0.792329436
LOC112442265	0.886202788	0.973158835	0.843427302	0.92064557	0.961835052	0.84491737
LSM10	0.886202788	0.761657408	0.93798101	0.977216943	0.965517213	0.850181558
HEATR5B	0.886202788	0.824119076	0.863172962	0.992368879	0.98152792	0.85227092

HPDL	0.886202788	0.780290359	0.806221631	0.951929772	0.98152792	0.96826636
LOC534181	0.886202788	0.719869488	0.802951601	0.999939566	0.988120779	0.912101047
ZBTB49	0.886202788	0.808513267	0.924266781	0.990571725	0.988693274	0.794558849
EIF3I	0.886202788	0.780290359	0.831430643	0.990049248	0.988693274	0.91727446
NDUFAF3	0.886202788	0.72477102	0.823304404	0.925901365	0.99989309	0.799935743
UQCC3	0.886202788	0.80403784	0.803771265	0.999939566	0.99989309	0.800658496
KRT79	0.886202788	0.820796062	0.855743966	0.915749182	0.99989309	0.82036646
ARHGAP5	0.886202788	0.807417478	0.852758896	0.915749182	0.99989309	0.823139458
LOC104973485	0.886202788	0.747555258	0.92332596	0.943323364	0.99989309	0.828663852
PPP1R3F	0.886202788	0.764525656	0.844065668	0.915749182	0.99989309	0.829389486
HCN2	0.886202788	0.874669928	0.878603452	0.925901365	0.99989309	0.858042054
TMEM269	0.886202788	0.913075885	0.856997596	0.915749182	0.99989309	0.858777168
LOC112446036	0.886202788	0.852523001	0.853222116	0.914402649	0.99989309	0.859487774
LOC107131623	0.886202788	0.743422331	0.844065668	0.998963067	0.99989309	0.862481601
ZNF576	0.886202788	0.750100112	0.849077738	0.915749182	0.99989309	0.872224159
OAZ2	0.886202788	0.747179932	0.87630259	0.980295672	0.99989309	0.87900534
TNFSF12	0.886202788	0.786856496	0.843427302	0.940921143	0.99989309	0.897818829
LOC112449115	0.886202788	0.72843033	0.834643686	0.94783387	0.99989309	0.934653208
TCHP	0.886202788	0.804528018	0.806825406	0.927086602	0.99989309	0.946314386
LOC112449596	0.88626245	0.780061391	0.929958748	0.931268564	0.99989309	0.802875906
LOC789587	0.88626422	0.933264454	0.868936984	0.960986457	0.952172109	0.825331577
DLL4	0.88626422	0.914252497	0.887130994	0.962775522	0.954501767	0.805257609
ZFAND3	0.88626422	0.747106203	0.845425011	0.999939566	0.954501767	0.817719556
TNFRSF11A	0.88626422	0.72843033	0.832483532	0.999939566	0.954501767	0.849769587
C22H3orf62	0.88626422	0.798799934	0.844162118	0.999939566	0.954501767	0.877623013
LOC112441683	0.88626422	0.725594888	0.987842204	0.915749182	0.954501767	0.888617204
SRSF11	0.88626422	0.791182736	0.899195818	0.980418209	0.954501767	0.891000792
FAM161B	0.88626422	0.80403784	0.941593855	0.917869295	0.954501767	0.902122572
IFT57	0.88626422	0.83304503	0.861038179	0.958879502	0.954501767	0.911696156
BRIP1	0.88626422	0.771331117	0.939683463	0.925004412	0.954501767	0.926576964
REEP5	0.88626422	0.871412404	0.833064774	0.999939566	0.954585906	0.84491737
TINAGL1	0.88626422	0.824016465	0.885354035	0.99879985	0.955470665	0.834936698
C1QTNF2	0.88626422	0.747555258	0.854240468	0.999939566	0.956624006	0.808138656
KLF9	0.88626422	0.759619476	0.848946904	0.999939566	0.957605284	0.826868779
GNPNAT1	0.88626422	0.730228653	0.886176337	0.999939566	0.957605284	0.891000792
TRPV2	0.88626422	0.823012828	0.8895568	0.956154344	0.957605284	0.893400499
LOC107131530	0.88626422	0.785406526	0.843465477	0.915749182	0.957605284	0.99016699
ZNF740	0.88626422	0.746081288	0.950399873	0.99879985	0.957910867	0.815459921

MAP2	0.88626422	0.795421065	0.86637689	0.95952938	0.960835287	0.927412916
DACT2	0.88626422	0.739873533	0.817256644	0.987342814	0.960835287	0.98016619
GON7	0.88626422	0.851167599	0.845237542	0.999939566	0.961382403	0.808138656
ARRB2	0.88626422	0.880674338	0.845237542	0.99879985	0.961382403	0.829096716
CGGBP1	0.88626422	0.837455678	0.812770091	0.931268564	0.961382403	0.978515834
TIMP2	0.88626422	0.763751563	0.85665551	0.999939566	0.961835052	0.821774001
NEURL1	0.88626422	0.935324849	0.806221631	0.924489924	0.961835052	0.933183326
EMILIN3	0.88626422	0.955798423	0.850106729	0.941467481	0.962262333	0.837249706
LOC100299712	0.88626422	0.835408071	0.838488596	0.999939566	0.962262333	0.856474735
LOC104973058	0.88626422	0.735826208	0.858972712	0.915749182	0.964150118	0.993612847
HTRA3	0.88626422	0.733727362	0.870054336	0.999939566	0.96532164	0.840150874
SIX1	0.88626422	0.739918356	0.849077738	0.999939566	0.96532164	0.865926817
IPO4	0.88626422	0.851167599	0.833656166	0.945929971	0.96532164	0.930774946
GPATCH4	0.88626422	0.847451823	0.828569127	0.956154344	0.966405324	0.922872513
SPARC	0.88626422	0.852463941	0.899195818	0.990571725	0.972917711	0.812622197
ATP6AP2	0.88626422	0.780082505	0.879515214	0.999939566	0.975291792	0.809941826
DNHD1	0.88626422	0.73280582	0.882898206	0.934591536	0.975291792	0.963996128
TMEM187	0.88626422	0.820796062	0.886282865	0.915749182	0.976970807	0.93636925
GNG2	0.88626422	0.820796062	0.87146798	0.982140778	0.98152792	0.878152258
FCER2	0.88626422	0.81808696	0.817256644	0.999939566	0.981938411	0.840150874
BTRC	0.88626422	0.736964914	0.820873572	0.999939566	0.982577194	0.902660792
SPHK1	0.88626422	0.83744789	0.856729427	0.999939566	0.983935609	0.799935743
BHLHE22	0.88626422	0.751946504	0.838488596	0.999939566	0.987855669	0.877623013
TTYH3	0.88626422	0.775921167	0.957578315	0.955547884	0.988120779	0.823139458
NEURL4	0.88626422	0.72477102	0.979635589	0.947615714	0.988693274	0.82835984
LOC101904442	0.88626422	0.751946504	0.870054336	0.99879985	0.988693274	0.884387948
YIPF5	0.88626422	0.731268229	0.90002174	0.921326109	0.988693274	0.957877422
CRYZL1	0.88626422	0.77414098	0.849077738	0.999939566	0.993321574	0.794529985
SLC16A11	0.88626422	0.79449527	0.806825406	0.999939566	0.994688893	0.824842482
TPRG1L	0.88626422	0.738648674	0.847037539	0.999939566	0.996250445	0.813345852
TYROBP	0.88626422	0.855979085	0.880457248	0.977216943	0.996250445	0.822691858
LOC618289	0.88626422	0.785163144	0.875405854	0.925004412	0.996250445	0.940683751
LOC112444778	0.88626422	0.763751563	0.849077738	0.933991524	0.996250445	0.961506391
TMEM229A	0.88626422	0.72809533	0.817256644	0.951929772	0.996290884	0.984491523
ARF3	0.88626422	0.811559721	0.817256644	0.94783387	0.997186167	0.943138585
ADGRG5	0.88626422	0.939870925	0.843427302	0.956154344	0.999592263	0.813345852
TMEM200C	0.88626422	0.786829125	0.886282865	0.956154344	0.999592263	0.885826563
YIPF2	0.88626422	0.813371202	0.878603452	0.916305829	0.99989309	0.793764114

RNF41	0.88626422	0.825215718	0.878603452	0.915749182	0.99989309	0.794529985
UBXN7	0.88626422	0.812502616	0.845237542	0.982140778	0.99989309	0.795637468
LOC100295848	0.88626422	0.800441059	0.810425784	0.999939566	0.99989309	0.796002422
S1PR3	0.88626422	0.792190608	0.844316758	0.956154344	0.99989309	0.796934582
MICU1	0.88626422	0.81668556	0.872397372	0.992137653	0.99989309	0.797130253
HOOK3	0.88626422	0.763751563	0.882898206	0.925931623	0.99989309	0.797202346
HMCN1	0.88626422	0.835809337	0.817993104	0.999939566	0.99989309	0.798051736
IL2RG	0.88626422	0.780290359	0.827125538	0.983688085	0.99989309	0.799147792
LMAN2L	0.88626422	0.875022483	0.844162118	0.967333752	0.99989309	0.799935743
PRELID2	0.88626422	0.761949365	0.836294915	0.999939566	0.99989309	0.799993234
ERCC3	0.88626422	0.769817928	0.939091808	0.934591536	0.99989309	0.800658496
HMGCL	0.88626422	0.726229532	0.830022042	0.999939566	0.99989309	0.80261571
PLPP1	0.88626422	0.758051794	0.88330023	0.99879985	0.99989309	0.806054104
LOC782293	0.88626422	0.835276066	0.849077738	0.990049248	0.99989309	0.806469522
CHMP2A	0.88626422	0.813511912	0.828449612	0.943591298	0.99989309	0.80787758
TMEM147	0.88626422	0.766086544	0.817993104	0.943323364	0.99989309	0.808138656
SETD9	0.88626422	0.835408071	0.883726795	0.95964071	0.99989309	0.808341957
SPSB4	0.88626422	0.920967949	0.845237542	0.915749182	0.99989309	0.809650729
TRERF1	0.88626422	0.72843033	0.836294915	0.999939566	0.99989309	0.809650729
GZF1	0.88626422	0.796591413	0.828569127	0.922782374	0.99989309	0.812509877
SMNDC1	0.88626422	0.780641867	0.833736568	0.924489924	0.99989309	0.812509877
SYPL2	0.88626422	0.905977412	0.858865175	0.927084627	0.99989309	0.812509877
SLITRK4	0.88626422	0.750913074	0.828569127	0.999939566	0.99989309	0.812509877
HINT2	0.88626422	0.811755592	0.808333956	0.99879985	0.99989309	0.813342306
ZBP1	0.88626422	0.72843033	0.945417678	0.916305829	0.99989309	0.813345852
NDUFA6	0.88626422	0.786258422	0.817993104	0.928836449	0.99989309	0.813577244
ARRDC1	0.88626422	0.852463941	0.863172962	0.956154344	0.99989309	0.814739833
ATMIN	0.88626422	0.852281996	0.926613927	0.934591536	0.99989309	0.815459921
CDRT4	0.88626422	0.810976764	0.828407935	0.995118128	0.99989309	0.815459921
ENC1	0.88626422	0.790637585	0.853222116	0.925931623	0.99989309	0.820994656
SMCO4	0.88626422	0.844579208	0.883874752	0.97136787	0.99989309	0.821467423
LOC787287	0.88626422	0.820559187	0.827125538	0.995118128	0.99989309	0.822691858
STK3	0.88626422	0.820742284	0.832431595	0.928623745	0.99989309	0.822882339
LOC112443193	0.88626422	0.747499345	0.843427302	0.915749182	0.99989309	0.823139458
NCBP1	0.88626422	0.920540438	0.827125538	0.915749182	0.99989309	0.825601599
TNKS	0.88626422	0.920347742	0.812770091	0.925059533	0.99989309	0.826868779
GPD1L	0.88626422	0.72843033	0.914477011	0.934591536	0.99989309	0.826868779
EXOC6	0.88626422	0.905573962	0.899530079	0.915749182	0.99989309	0.828737285

SLC7A1	0.88626422	0.781287182	0.894547778	0.919247117	0.99989309	0.829389486
LOC100335642	0.88626422	0.746644403	0.843465477	0.999939566	0.99989309	0.829389486
MBTD1	0.88626422	0.944420015	0.829843435	0.92064557	0.99989309	0.832189969
NCOA1	0.88626422	0.817040135	0.805580394	0.940668335	0.99989309	0.832189969
RGS3	0.88626422	0.736092667	0.939683463	0.948725226	0.99989309	0.832189969
SIK1	0.88626422	0.774117125	0.849077738	0.995800463	0.99989309	0.832189969
GPSM1	0.88626422	0.780290359	0.875669731	0.951929772	0.99989309	0.832858909
SLCO3A1	0.88626422	0.779256669	0.878603452	0.975529024	0.99989309	0.832858909
SSPN	0.88626422	0.886366574	0.87101857	0.955687359	0.99989309	0.835264004
ANKRD45	0.88626422	0.763751563	0.823456027	0.99879985	0.99989309	0.837048016
ESCO1	0.88626422	0.781154903	0.87146798	0.928836449	0.99989309	0.83849759
C29H11orf68	0.88626422	0.751946504	0.879215964	0.922782374	0.99989309	0.839340243
PHACTR2	0.88626422	0.736594462	0.832431595	0.941467481	0.99989309	0.839340243
LOC112441566	0.88626422	0.72477102	0.810425784	0.946668073	0.99989309	0.839340243
MDH2	0.88626422	0.735625249	0.809322753	0.915749182	0.99989309	0.839419627
CLEC18C	0.88626422	0.767950704	0.880277219	0.969717497	0.99989309	0.839447517
LOC112446018	0.88626422	0.835276066	0.808228186	0.915749182	0.99989309	0.840150874
STRBP	0.88626422	0.845907464	0.824495067	0.934591536	0.99989309	0.840150874
CLK2	0.88626422	0.742963341	0.887130994	0.958879502	0.99989309	0.840150874
CNTNAP2	0.88626422	0.732654391	0.925587088	0.992368879	0.99989309	0.842016958
NUP155	0.88626422	0.888994602	0.80326876	0.923578108	0.99989309	0.844622232
PPP1R9B	0.88626422	0.747499345	0.878603452	0.995800463	0.99989309	0.844622232
PSMC3	0.88626422	0.890427603	0.845237542	0.937531834	0.99989309	0.84491737
HDHD2	0.88626422	0.806820745	0.844162118	0.95964071	0.99989309	0.84491737
SMAD5	0.88626422	0.738981601	0.829843435	0.990571725	0.99989309	0.84491737
CBLN2	0.88626422	0.94883251	0.838877266	0.915749182	0.99989309	0.845628259
LOC112449280	0.88626422	0.789041282	0.840938565	0.999939566	0.99989309	0.845803016
EPN3	0.88626422	0.780061391	0.849077738	0.99879985	0.99989309	0.845949552
CDK18	0.88626422	0.812503168	0.834569848	0.915749182	0.99989309	0.846367069
C24H18orf32	0.88626422	0.743422331	0.939683463	0.948891906	0.99989309	0.846367069
TAF1C	0.88626422	0.771098738	0.891014698	0.982140778	0.99989309	0.847450944
IRS2	0.88626422	0.790637585	0.886282865	0.959786126	0.99989309	0.84763005
GLOD4	0.88626422	0.77069491	0.846650632	0.998542029	0.99989309	0.847810523
ZSCAN16	0.88626422	0.824676261	0.915636025	0.915749182	0.99989309	0.856870625
SAR1A	0.88626422	0.769186282	0.957417441	0.915749182	0.99989309	0.856870625
ERCC4	0.88626422	0.743422331	0.942004125	0.915749182	0.99989309	0.858744772
XRCC5	0.88626422	0.804860126	0.894414265	0.944558402	0.99989309	0.859973633
SNAPC1	0.88626422	0.775921167	0.892122482	0.956154344	0.99989309	0.859973633

AKAP5	0.88626422	0.823692881	0.845237542	0.975529024	0.99989309	0.860755302
EIF1AX	0.88626422	0.786258422	0.859774281	0.968694679	0.99989309	0.860827207
ATXN7L3	0.88626422	0.806660599	0.836294915	0.992368879	0.99989309	0.860827207
LOC104969611	0.88626422	0.838843184	0.824856882	0.992339205	0.99989309	0.862403446
RPUSD2	0.88626422	0.799436659	0.848155676	0.925901365	0.99989309	0.86307729
MRS2	0.88626422	0.785978032	0.850952986	0.983569039	0.99989309	0.864571107
SMIM20	0.88626422	0.731268229	0.845237542	0.948547383	0.99989309	0.865159042
VDAC1	0.88626422	0.781579221	0.842478086	0.973377993	0.99989309	0.866482265
RNF123	0.88626422	0.743422331	0.884073083	0.915749182	0.99989309	0.866612872
ADCY7	0.88626422	0.775921167	0.860197593	0.92064557	0.99989309	0.868108836
CDC37	0.88626422	0.83744789	0.820873572	0.992426863	0.99989309	0.868108836
PSMB5	0.88626422	0.775921167	0.831710876	0.934591536	0.99989309	0.868177389
ANK1	0.88626422	0.921393399	0.806311697	0.940779565	0.99989309	0.869171706
COX7A2L	0.88626422	0.776182952	0.846650632	0.949947918	0.99989309	0.870895425
ATN1	0.88626422	0.747899938	0.831320191	0.999334425	0.99989309	0.873520548
PDP2	0.88626422	0.767106625	0.879726737	0.915749182	0.99989309	0.880404833
POLH	0.88626422	0.810423202	0.89007492	0.925901365	0.99989309	0.886992214
LOC107132487	0.88626422	0.742728851	0.849077738	0.990571725	0.99989309	0.886992214
MON1A	0.88626422	0.828850649	0.833567938	0.943323364	0.99989309	0.889201226
NSMF	0.88626422	0.7635426	0.828569127	0.915749182	0.99989309	0.892214703
CPSF7	0.88626422	0.747164473	0.838765859	0.915749182	0.99989309	0.89260699
SRP54	0.88626422	0.761788537	0.871847658	0.925901365	0.99989309	0.893961809
LOC101906230	0.88626422	0.780290359	0.827125538	0.925901365	0.99989309	0.894901464
LOC100298923	0.88626422	0.72843033	0.878603452	0.943323364	0.99989309	0.895218005
OCEL1	0.88626422	0.820796062	0.836294915	0.945929971	0.99989309	0.895218005
CXCL2	0.88626422	0.736566631	0.836294915	0.993970682	0.99989309	0.895218005
LOC104971464	0.88626422	0.839278952	0.808303251	0.93471064	0.99989309	0.901811882
NSFL1C	0.88626422	0.835408071	0.817256644	0.950665515	0.99989309	0.906529452
COPS3	0.88626422	0.747106203	0.869529186	0.959009349	0.99989309	0.908939618
LOC112444775	0.88626422	0.808197752	0.820549834	0.978369589	0.99989309	0.910619304
STK35	0.88626422	0.730944867	0.832483532	0.915749182	0.99989309	0.911696156
SLC25A19	0.88626422	0.767154522	0.820767684	0.943323364	0.99989309	0.91727446
MMADHC	0.88626422	0.735158583	0.855978798	0.934591536	0.99989309	0.92035958
HSPA9	0.88626422	0.730582684	0.817670412	0.96095927	0.99989309	0.924845138
FAM107B	0.88626422	0.745665426	0.820767684	0.919247117	0.99989309	0.926872824
SHMT2	0.88626422	0.835276066	0.806825406	0.921320709	0.99989309	0.927529392
FADD	0.88626422	0.820796062	0.830013482	0.915749182	0.99989309	0.935937948
PRKCA	0.88626422	0.782865867	0.806221631	0.990571725	0.99989309	0.937591162

DEF6	0.88626422	0.770427846	0.828569127	0.915749182	0.99989309	0.940917403
NCOA2	0.88626422	0.804860126	0.817256644	0.915749182	0.99989309	0.944524573
ODF2	0.886533144	0.740948281	0.909506646	0.992154129	0.988693274	0.870895425
DDX10	0.886533144	0.868963165	0.807843373	0.931268564	0.99989309	0.927573435
DCBLD1	0.886536117	0.813371202	0.810425784	0.922713162	0.99989309	0.839419627
TNK1	0.887222017	0.900584769	0.882898206	0.944728796	0.954501767	0.868108836
MYL4	0.887222017	0.747179932	0.878050811	0.943323364	0.954501767	0.97345607
VPS35	0.887222017	0.761788537	0.899539089	0.99879985	0.956717959	0.867440033
SOX11	0.887222017	0.790637585	0.85972234	0.981022805	0.961382403	0.92920113
MORN2	0.887222017	0.730228653	0.883982433	0.999939566	0.972917711	0.868108836
SHCBP1L	0.887222017	0.926435857	0.868936984	0.946319248	0.974406708	0.845803016
GSTA2	0.887222017	0.937904802	0.817256644	0.980840984	0.996250445	0.838303925
TLL1	0.887222017	0.956958713	0.836294915	0.931268564	0.996250445	0.847450944
MAOA	0.887222017	0.741897509	0.843427302	0.925901365	0.99989309	0.797730509
RPS6KL1	0.887222017	0.751946504	0.843427302	0.915749182	0.99989309	0.808586684
LOC787904	0.887222017	0.810488979	0.899623367	0.925931623	0.99989309	0.812509877
LOC101905014	0.887222017	0.900467607	0.8450479	0.971220023	0.99989309	0.813713324
PDGFD	0.887222017	0.808839416	0.843427302	0.99483611	0.99989309	0.817719556
EMC4	0.887222017	0.72843033	0.870054336	0.99879985	0.99989309	0.829389486
ASTN2	0.887222017	0.785406526	0.941041568	0.954423369	0.99989309	0.830456671
FGF14	0.887222017	0.887725794	0.919939634	0.915749182	0.99989309	0.832097423
GHITM	0.887222017	0.779501962	0.828407935	0.998542029	0.99989309	0.832858909
HEATR5A	0.887222017	0.796591413	0.832483532	0.97136787	0.99989309	0.834550008
RGS10	0.887222017	0.834316926	0.810425784	0.994309225	0.99989309	0.858042054
TBPL1	0.887222017	0.908178836	0.810425784	0.943323364	0.99989309	0.858538874
RNF168	0.887222017	0.876348847	0.842478086	0.943323364	0.99989309	0.862259367
DBI	0.887222017	0.747499345	0.834793268	0.917869295	0.99989309	0.881039089
XPO7	0.887222017	0.728008849	0.859931483	0.998542029	0.99989309	0.891000792
P4HTM	0.887222017	0.739918356	0.878603452	0.931268564	0.99989309	0.909768235
SLC35C1	0.887222017	0.835276066	0.869455175	0.915749182	0.99989309	0.911663291
FBL	0.887222017	0.823012828	0.817256644	0.943323364	0.99989309	0.940683751
MIPOL1	0.887222017	0.72843033	0.827350832	0.934591536	0.99989309	0.98704566
ALKBH5	0.887428978	0.72809533	0.939683463	0.999939566	0.960835287	0.797202346
LOC101905708	0.887455	0.845123271	0.891014698	0.995800463	0.975291792	0.809650729
NOP16	0.887455	0.728023504	0.857604355	0.99879985	0.99989309	0.859447707
INHBB	0.887457067	0.773083896	0.882312543	0.999939566	0.972048096	0.845803016
BRCA1	0.887457067	0.781444941	0.960137022	0.95175364	0.972917711	0.843672601
APBA3	0.887482519	0.751946504	0.917092799	0.998542029	0.99989309	0.834008567

LOC101904705	0.887482519	0.747899938	0.883860423	0.915749182	0.99989309	0.839340243
GPRIN3	0.88760785	0.747499345	0.855743966	0.999939566	0.996250445	0.853764016
JRKL	0.88760785	0.810519292	0.843304408	0.951929772	0.99989309	0.809548115
HEPH	0.88760785	0.743422331	0.88330023	0.995118128	0.99989309	0.834735448
PIGA	0.88767585	0.734188031	0.939683463	0.925901365	0.99989309	0.809941826
ARHGAP9	0.887741618	0.924799163	0.856589093	0.936102593	0.965219542	0.872175131
LOC101906008	0.887741618	0.803995245	0.858972712	0.999939566	0.979654999	0.802399829
INPP5B	0.887741618	0.72812452	0.828407935	0.999939566	0.996250445	0.847189049
YARS2	0.887741618	0.813476598	0.817256644	0.928836449	0.99989309	0.845803016
LOC101907603	0.887741618	0.771098738	0.850952986	0.99879985	0.99989309	0.856495922
LOC100336897	0.887741618	0.751946504	0.822417045	0.916305829	0.99989309	0.887248119
RNF139	0.887857039	0.855979085	0.971104162	0.925901365	0.955392067	0.813345852
PDE6C	0.887857039	0.990179479	0.839666249	0.925901365	0.955392067	0.832858909
CCDC32	0.887857039	0.855979085	0.844316758	0.99879985	0.982014614	0.839494796
GGT1	0.887857039	0.913171043	0.839833549	0.995118128	0.983935609	0.812622197
C7H19orf53	0.887857039	0.778839781	0.88330023	0.98221491	0.99989309	0.798737807
GNB1	0.887857039	0.781154903	0.882898206	0.999939566	0.99989309	0.799314198
ARSA	0.887857039	0.746902965	0.870774839	0.993948479	0.99989309	0.812509877
MEIS1	0.887857039	0.828417266	0.812770091	0.999939566	0.99989309	0.837501229
NREP	0.887857039	0.751946504	0.810425784	0.999939566	0.99989309	0.840150874
LOC100298868	0.887857039	0.788961546	0.846650632	0.995118128	0.99989309	0.85958325
ANGPTL2	0.887857039	0.742963341	0.957802423	0.945301766	0.99989309	0.86307729
LOC107133032	0.887857039	0.785383224	0.827125538	0.949947918	0.99989309	0.902324068
PTCD3	0.887857039	0.77295675	0.810425784	0.937531834	0.99989309	0.982514294
RNASET2	0.887876693	0.806152572	0.878603452	0.999939566	0.988693274	0.820483064
RHOV	0.888020655	0.751946504	0.957379098	0.981022805	0.954501767	0.862709032
OSTF1	0.888020655	0.810519292	0.906804311	0.999939566	0.957605284	0.826868779
MIR3064	0.888020655	0.742728851	0.912031396	0.956154344	0.957605284	0.940683751
KIAA1614	0.888020655	0.736066622	0.939091808	0.992741182	0.960835287	0.86885756
CCNF	0.888020655	0.747555258	0.87146798	0.99879985	0.961382403	0.908815767
LOC101905757	0.888020655	0.808839416	0.838838955	0.916305829	0.961382403	0.988323336
RNF150	0.888020655	0.770427846	0.868936984	0.999939566	0.961835052	0.84491737
FXYD3	0.888020655	0.80403784	0.953045982	0.983569039	0.961879452	0.813345852
ARAP3	0.888020655	0.825215718	0.918681165	0.943539568	0.961879452	0.889622239
SLC35A3	0.888020655	0.79591781	0.922147089	0.995118128	0.964521207	0.83849759
KCTD11	0.888020655	0.780281604	0.817601111	0.991029787	0.96532164	0.956959747
ALG3	0.888020655	0.866803532	0.812585086	0.915749182	0.96730957	0.98016619
GDI2	0.888020655	0.750546265	0.889578981	0.958849311	0.970439271	0.939800211

CCNQ	0.888020655	0.739753419	0.834569848	0.999939566	0.972917711	0.859973633
EIF4G3	0.888020655	0.836038392	0.872651958	0.949930337	0.98152792	0.908182968
NOXA1	0.888020655	0.814136857	0.972401758	0.919247117	0.981777076	0.840150874
TCP11	0.888020655	0.93164553	0.886282865	0.934591536	0.982771074	0.829662533
OSTM1	0.888020655	0.758051794	0.879968243	0.999939566	0.983342337	0.806469522
DDX39B	0.888020655	0.751946504	0.924266781	0.999939566	0.988693274	0.812682271
CHMP5	0.888020655	0.746081288	0.883334551	0.995118128	0.988693274	0.895218005
LHFPL2	0.888020655	0.855861376	0.845237542	0.925901365	0.988693274	0.939507593
ETS1	0.888020655	0.791704113	0.929958748	0.986394228	0.990560223	0.839340243
TBL1X	0.888020655	0.878904218	0.827125538	0.915749182	0.99989309	0.799940894
DTX3	0.888020655	0.733883576	0.833656166	0.999939566	0.99989309	0.807579326
ADRB3	0.888020655	0.932024312	0.849077738	0.959009349	0.99989309	0.80787758
NSRP1	0.888020655	0.883378562	0.830033146	0.980418209	0.99989309	0.810707901
NIPBL	0.888020655	0.851167599	0.817256644	0.915749182	0.99989309	0.813001848
COA1	0.888020655	0.848986275	0.850243032	0.925931623	0.99989309	0.823139458
ABCC3	0.888020655	0.747555258	0.939091808	0.99419346	0.99989309	0.82533924
PPT2	0.888020655	0.790637585	0.930262306	0.946344632	0.99989309	0.833139817
RPAP3	0.888020655	0.838405969	0.894829955	0.925004412	0.99989309	0.834008567
STOM	0.888020655	0.751946504	0.939967226	0.949947918	0.99989309	0.839419627
TMEM53	0.888020655	0.820796062	0.845237542	0.991029787	0.99989309	0.839419627
AOC2	0.888020655	0.80403784	0.892258313	0.943323364	0.99989309	0.840150874
KIAA1549	0.888020655	0.739918356	0.92736775	0.992368879	0.99989309	0.840150874
FAM129B	0.888020655	0.737937244	0.828449612	0.999939566	0.99989309	0.840150874
TAMM41	0.888020655	0.911347642	0.827350832	0.943323364	0.99989309	0.842461327
NICN1	0.888020655	0.735826208	0.957379098	0.915749182	0.99989309	0.849769587
MORC4	0.888020655	0.760132527	0.820767684	0.954980798	0.99989309	0.853233759
RNF144B	0.888020655	0.737937244	0.860197593	0.999939566	0.99989309	0.856870625
HSPB2	0.888020655	0.8232491	0.920449324	0.919247117	0.99989309	0.862709032
ZBTB6	0.888020655	0.747499345	0.884189563	0.925901365	0.99989309	0.86307729
POMK	0.888020655	0.821124254	0.833656166	0.98470348	0.99989309	0.864219261
LOC101905786	0.888020655	0.85692481	0.849132683	0.953648387	0.99989309	0.866156441
NFASC	0.888020655	0.845854159	0.825178565	0.993970682	0.99989309	0.868108836
COQ4	0.888020655	0.771098738	0.822189888	0.916305829	0.99989309	0.875441396
XPR1	0.888020655	0.730231821	0.844316758	0.925004412	0.99989309	0.875441396
LOC101904039	0.888020655	0.913075885	0.855743966	0.925901365	0.99989309	0.875441396
ARAP2	0.888020655	0.750913074	0.827125538	0.995118128	0.99989309	0.879725389
ADGRL4	0.888020655	0.945105738	0.817256644	0.924489924	0.99989309	0.884387948
TRIM38	0.888020655	0.740343022	0.820767684	0.999939566	0.99989309	0.90211634

PLVAP	0.888020655	0.89141259	0.841111553	0.919247117	0.99989309	0.911475796
LOC112448627	0.888020655	0.839278952	0.835301782	0.943323364	0.99989309	0.926872824
PHB2	0.888020655	0.795993324	0.83851491	0.915749182	0.99989309	0.929688889
LOC101904590	0.888020655	0.769817928	0.820767684	0.958879502	0.99989309	0.933730876
LOC789569	0.888020655	0.851167599	0.820767684	0.925931623	0.99989309	0.934653208
MDC1	0.888020655	0.762756164	0.829843435	0.985048108	0.99989309	0.940683751
MRM1	0.888020655	0.736511232	0.828569127	0.946344632	0.99989309	0.954935833
KLHDC2	0.888020655	0.747899938	0.878603452	0.915749182	0.99989309	0.956959747
CEP57L1	0.888038369	0.858731648	0.87146798	0.943323364	0.971348001	0.908939618
EIF4B	0.888038369	0.90249621	0.838080059	0.946344632	0.99989309	0.812509877
POP5	0.888038369	0.780892456	0.853659206	0.943323364	0.99989309	0.911058479
PSMD6	0.888038369	0.790289887	0.851617203	0.925901365	0.99989309	0.956610132
EOGT	0.888107726	0.883378562	0.864741549	0.990571725	0.99989309	0.799935743
ERP29	0.888139591	0.762756164	0.814705151	0.956154344	0.964150118	0.989128955
TCN1	0.888139591	0.792090907	0.939683463	0.992137653	0.975291792	0.823195389
CDH8	0.888139591	0.769313383	0.937191211	0.919247117	0.990044405	0.921381434
FKBP3	0.888139591	0.835601822	0.853222116	0.954423369	0.99989309	0.842945521
CDT1	0.888407602	0.796591413	0.855712722	0.999939566	0.99989309	0.849772722
TMEM220	0.888475489	0.795993324	0.867451783	0.999939566	0.972917711	0.812509877
LOC112441650	0.888475489	0.753871999	0.96993432	0.946344632	0.99989309	0.809108223
FAM234B	0.888475489	0.909853448	0.829843435	0.985815315	0.99989309	0.837501229
LOC112442997	0.888475489	0.769874274	0.820767684	0.915749182	0.99989309	0.853233759
ZNF532	0.888475489	0.751946504	0.850020542	0.999939566	0.99989309	0.881439164
GRIP1	0.888475489	0.847082229	0.817256644	0.992137653	0.99989309	0.89547073
LOC107131224	0.88850622	0.736066622	0.81747245	0.915749182	0.99989309	0.998074562
ANG	0.888560917	0.81808696	0.828407935	0.999939566	0.99989309	0.814576296
TRIP11	0.888560917	0.771098738	0.855743966	0.915749182	0.99989309	0.823139458
C16H1orf53	0.888560917	0.774326932	0.828407935	0.995800463	0.99989309	0.86307729
TTC30B	0.888560917	0.747499345	0.849077738	0.915749182	0.99989309	0.879160297
ANKRD61	0.888560917	0.747499345	0.875669731	0.924489924	0.99989309	0.963996128
MAPK1IP1L	0.888598455	0.771098738	0.968466017	0.95986631	0.99989309	0.799314198
ITGB2	0.888636707	0.838843184	0.883334551	0.995800463	0.988693274	0.826868779
MFHAS1	0.888912571	0.779765764	0.810425784	0.99879985	0.964150118	0.948277018
SPTAN1	0.888912571	0.733937303	0.885481718	0.999939566	0.968795608	0.867440033
KCND3	0.888912571	0.761788537	0.87146798	0.999939566	0.99989309	0.799993234
HIF1AN	0.888912571	0.753384944	0.885481718	0.990571725	0.99989309	0.860827207
BRB	0.888912571	0.88311684	0.886282865	0.931989854	0.99989309	0.862912171
LOC616868	0.888934991	0.893512526	0.886282865	0.962142137	0.961382403	0.854493916

USP14	0.888934991	0.751946504	0.870054336	0.99879985	0.96532164	0.911116909
LOC100140372	0.888934991	0.780061391	0.921319817	0.919671703	0.975819525	0.941889677
CDC42SE2	0.888934991	0.857005855	0.849077738	0.999939566	0.99989309	0.808138656
PTPA	0.888934991	0.829503998	0.831320191	0.93192216	0.99989309	0.893356178
GNG12	0.888946878	0.747106203	0.933102499	0.995800463	0.961835052	0.866253786
NUP210L	0.888946878	0.847082229	0.940785296	0.947289671	0.962791364	0.844622232
HUNK	0.888946878	0.766086544	0.828407935	0.999939566	0.965219542	0.812623355
ADGRB1	0.888946878	0.766152858	0.936383207	0.943323364	0.968477804	0.912376736
DECR2	0.888946878	0.914162656	0.87214481	0.976950878	0.996250445	0.812509877
DYRK1B	0.888946878	0.800441059	0.926613927	0.95964071	0.996250445	0.86143104
RAMP1	0.888946878	0.776522777	0.838080059	0.917869295	0.99989309	0.812408228
POLL	0.888946878	0.782865867	0.987419404	0.915749182	0.99989309	0.812509877
WIPF1	0.888946878	0.804875341	0.873350964	0.915749182	0.99989309	0.812682271
SYPL1	0.888946878	0.737937244	0.862585131	0.999939566	0.99989309	0.815459921
MRPS6	0.888946878	0.769817928	0.864165322	0.999939566	0.99989309	0.828602505
COX8B	0.888946878	0.781579221	0.854156608	0.923832787	0.99989309	0.839419627
APPL1	0.888946878	0.785383224	0.904682623	0.922782374	0.99989309	0.840150874
ATRN	0.888946878	0.800441059	0.90002174	0.925901365	0.99989309	0.840150874
CFAP36	0.888946878	0.800441059	0.939683463	0.943240775	0.99989309	0.840150874
PPIL4	0.888946878	0.852463941	0.820549834	0.931268564	0.99989309	0.84491737
PRODH	0.888946878	0.792663811	0.817256644	0.999939566	0.99989309	0.84491737
MMP24	0.888946878	0.739860832	0.966157152	0.946344632	0.99989309	0.845803016
LMO4	0.888946878	0.76090129	0.874372526	0.999939566	0.99989309	0.849504243
RNF25	0.888946878	0.88311684	0.883334551	0.915749182	0.99989309	0.854831489
DIAPH2	0.888946878	0.770427846	0.844065668	0.998542029	0.99989309	0.859973633
NSF	0.888946878	0.785163144	0.915636025	0.915749182	0.99989309	0.86307729
PARP14	0.888946878	0.739873533	0.957578315	0.925004412	0.99989309	0.86307729
AHSP	0.888946878	0.886701868	0.827186499	0.927267062	0.99989309	0.870079716
HES6	0.888946878	0.736050235	0.827125538	0.994365581	0.99989309	0.870764093
EBI3	0.888946878	0.751946504	0.827125538	0.99879985	0.99989309	0.895218005
PABPC1L2A	0.888946878	0.776972642	0.836294915	0.99879985	0.99989309	0.901689556
PDCD11	0.888946878	0.763901726	0.828407935	0.943323364	0.99989309	0.908118871
CCDC149	0.888946878	0.747499345	0.847659392	0.946344632	0.99989309	0.923064313
PIM1	0.888946878	0.821588096	0.827125538	0.915749182	0.99989309	0.941889677
MMP11	0.888981078	0.820000472	0.860710878	0.999939566	0.957605284	0.809941826
PRDM1	0.888981078	0.747499345	0.828449612	0.915749182	0.962262333	0.998928226
PTGR1	0.888981078	0.769313383	0.979231344	0.977216943	0.965219542	0.808138656
MAGI1	0.888981078	0.94751312	0.838838955	0.917503169	0.965517213	0.910353954

C27H8orf48	0.888981078	0.881396756	0.87146798	0.99879985	0.968795608	0.815459921
CXCL9	0.888981078	0.827609894	0.880375205	0.966882988	0.975291792	0.897861362
MED21	0.888981078	0.832026744	0.873750661	0.940921143	0.975291792	0.933763401
ABO	0.888981078	0.83971475	0.880277219	0.969717497	0.976503658	0.884097654
TSG101	0.888981078	0.868963165	0.91094712	0.990571725	0.98152792	0.800276945
DYNC2LI1	0.888981078	0.877420806	0.844065668	0.94524673	0.982014614	0.918220726
COPS7A	0.888981078	0.80403784	0.870054336	0.983569039	0.99989309	0.799935743
LOC100848527	0.888981078	0.824988091	0.814705151	0.999939566	0.99989309	0.806054104
SLC41A3	0.888981078	0.734529459	0.969618195	0.924489924	0.99989309	0.813345852
LOC100299201	0.888981078	0.790406748	0.880277219	0.925901365	0.99989309	0.815459921
KCNC2	0.888981078	0.821588096	0.907621715	0.971220023	0.99989309	0.828602505
USP39	0.888981078	0.81808696	0.817256644	0.990571725	0.99989309	0.829034472
RABGAP1L	0.888981078	0.841992867	0.827125538	0.925901365	0.99989309	0.829389486
COL8A2	0.888981078	0.800441059	0.878603452	0.990571725	0.99989309	0.833579083
TMEM161B	0.888981078	0.780290359	0.863172962	0.95964071	0.99989309	0.837196519
LOC104974057	0.888981078	0.838843184	0.833111583	0.921326109	0.99989309	0.839419627
MTHFSD	0.888981078	0.93181855	0.836294915	0.931989854	0.99989309	0.840150874
DGCR6L	0.888981078	0.80403784	0.819404084	0.953508052	0.99989309	0.840150874
LOC107133459	0.888981078	0.790637585	0.827186499	0.999939566	0.99989309	0.840150874
DNAJB1	0.888981078	0.843103375	0.827125538	0.946344632	0.99989309	0.84491737
TNFAIP8L1	0.888981078	0.813371202	0.915208497	0.969717497	0.99989309	0.856012024
AP1S1	0.888981078	0.796591413	0.850106729	0.943323364	0.99989309	0.873520548
SLC15A3	0.888981078	0.809254208	0.836294915	0.934591536	0.99989309	0.892802259
LOC101903851	0.888981078	0.864821455	0.852758896	0.939292364	0.99989309	0.893961809
TTLL7	0.888981078	0.820816566	0.849077738	0.949947918	0.99989309	0.908815767
FAM174B	0.888981078	0.733388284	0.842478086	0.995946943	0.99989309	0.908939618
SPIRE2	0.888981078	0.763751563	0.850106729	0.98470348	0.99989309	0.916525501
HPN	0.888981078	0.785406526	0.838838955	0.989686311	0.99989309	0.922301228
LOC112441886	0.888981078	0.910596218	0.817256644	0.925931623	0.99989309	0.923064313
LPL	0.888981078	0.840874016	0.846421774	0.919671703	0.99989309	0.942943347
PSME2	0.888981078	0.80931829	0.836671539	0.939292364	0.99989309	0.95188818
WDR73	0.888982534	0.780892456	0.849077738	0.941020528	0.99989309	0.853233759
UQCRFS1	0.888982534	0.764097231	0.827125538	0.925931623	0.99989309	0.856870625
CACNB1	0.88908405	0.80055527	0.859796267	0.995118128	0.960835287	0.911696156
PRKG2	0.88908405	0.836038392	0.878603452	0.969717497	0.99989309	0.833155047
DDT	0.88908405	0.742963341	0.827125538	0.925004412	0.99989309	0.840150874
HEATR3	0.88908405	0.823588167	0.817256644	0.998542029	0.99989309	0.887248119
WDR46	0.88908405	0.765341142	0.817256644	0.959727203	0.99989309	0.956610132

CHMP4B	0.889413418	0.820350377	0.96880775	0.956154344	0.960493616	0.827997078
GVQW3	0.889413418	0.790637585	0.886481882	0.982367414	0.960835287	0.91332511
HSBP1L1	0.889413418	0.785406526	0.850106729	0.999939566	0.961382403	0.84491737
LOC100847819	0.889413418	0.910086997	0.817993104	0.999939566	0.961835052	0.849772722
BCL3	0.889413418	0.810488979	0.828569127	0.999939566	0.961835052	0.908227076
CCNH	0.889413418	0.898000467	0.910123657	0.949947918	0.96413701	0.849817459
LOC786914	0.889413418	0.770864836	0.914477011	0.934591536	0.964150118	0.946344585
LLGL1	0.889413418	0.823187795	0.92153058	0.99879985	0.96532164	0.80787758
CCNB3	0.889413418	0.936134921	0.894414265	0.946344632	0.975291792	0.815459921
LOC615663	0.889413418	0.842136201	0.9343664	0.982140778	0.975291792	0.821456459
CCDC112	0.889413418	0.818063528	0.827350832	0.990571725	0.982014614	0.935607719
DHX34	0.889413418	0.927203323	0.838838955	0.998069032	0.988693274	0.806469522
TNRC6B	0.889413418	0.792190608	0.947660114	0.967063428	0.988693274	0.840150874
SNW1	0.889413418	0.835130235	0.817256644	0.915749182	0.99989309	0.80261571
SETD4	0.889413418	0.851167599	0.880277219	0.953508052	0.99989309	0.803732909
CUEDC1	0.889413418	0.811755592	0.878050811	0.995118128	0.99989309	0.80787758
MPHOSPH8	0.889413418	0.764525656	0.879215964	0.966882988	0.99989309	0.809650729
ARID2	0.889413418	0.810096755	0.817256644	0.945929971	0.99989309	0.812509877
GDF5	0.889413418	0.826937434	0.905017554	0.934591536	0.99989309	0.813188922
NEURL1B	0.889413418	0.776972642	0.89495244	0.99879985	0.99989309	0.832291916
LOC112449254	0.889413418	0.81808696	0.855743966	0.964134647	0.99989309	0.837048016
UROD	0.889413418	0.771098738	0.817993104	0.928836449	0.99989309	0.840150874
SUMO3	0.889413418	0.761788537	0.908165509	0.94850175	0.99989309	0.843813191
POLR3GL	0.889413418	0.790637585	0.870054336	0.995800463	0.99989309	0.844622232
LYSMD1	0.889413418	0.750068475	0.976719499	0.931268564	0.99989309	0.84491737
HOXD8	0.889413418	0.820796062	0.842605286	0.982140778	0.99989309	0.84491737
LOC104970145	0.889413418	0.775921167	0.886176337	0.953648387	0.99989309	0.859973633
TLE2	0.889413418	0.738981601	0.845237542	0.999939566	0.99989309	0.864846278
GRWD1	0.889413418	0.743422331	0.879968243	0.999939566	0.99989309	0.865926817
LOC104969425	0.889413418	0.823012828	0.929958748	0.934591536	0.99989309	0.866099047
TCAM1	0.889413418	0.739918356	0.843427302	0.924489924	0.99989309	0.867505247
LOC616400	0.889413418	0.76167712	0.849077738	0.999939566	0.99989309	0.868025807
GCNT7	0.889413418	0.786258422	0.849077738	0.995800463	0.99989309	0.868108836
SMIM13	0.889413418	0.841044548	0.904682623	0.915749182	0.99989309	0.872224159
KDELR2	0.889413418	0.775463154	0.922126495	0.934591536	0.99989309	0.873271935
ALDH7A1	0.889413418	0.751946504	0.81285201	0.981022805	0.99989309	0.893400499
LOC107132092	0.889413418	0.810096755	0.87214481	0.940921143	0.99989309	0.924754279
LONRF2	0.889413418	0.750745995	0.878603452	0.923832787	0.99989309	0.933678522

DCPS	0.889413418	0.751946504	0.812770091	0.990571725	0.99989309	0.960805387
PRICKLE2	0.889413418	0.769817928	0.819529308	0.918607898	0.99989309	0.980980823
C3H1orf226	0.889413418	0.76167712	0.817256644	0.917869295	0.99989309	0.998074562
LOC784251	0.889448537	0.885253121	0.878603452	0.975529024	0.975291792	0.859973633
LOC112442191	0.889448537	0.771098738	0.883726795	0.999939566	0.988693274	0.859323448
SPCS1	0.889448537	0.813371202	0.884197487	0.925004412	0.99989309	0.823139458
FAM98C	0.889448537	0.886909612	0.90166788	0.946344632	0.99989309	0.826868779
VPS13C	0.889448537	0.819900146	0.849132683	0.918607898	0.99989309	0.849769587
DSEL	0.889448537	0.763751563	0.944976702	0.938519355	0.99989309	0.872175131
CPNE1	0.889448537	0.84219422	0.81747245	0.917869295	0.99989309	0.942421464
LOC782560	0.889634728	0.740601865	0.979231344	0.915749182	0.961835052	0.914440253
LOC107131948	0.889634728	0.763751563	0.965750046	0.958879502	0.972917711	0.859447707
LOC101904097	0.889634728	0.763751563	0.828407935	0.999939566	0.979654999	0.926576964
METTL5	0.889634728	0.743422331	0.878603452	0.999939566	0.99989309	0.815459921
LMBRD1	0.889634728	0.781154903	0.882898206	0.990300191	0.99989309	0.844335246
PERP	0.889634728	0.786258422	0.885354035	0.934591536	0.99989309	0.859973633
LOC101903557	0.889634728	0.953226245	0.830013482	0.925901365	0.99989309	0.86143104
MYH9	0.889634728	0.840313358	0.844162803	0.917869295	0.99989309	0.864272734
CCDC82	0.889634728	0.757256262	0.818501962	0.992368879	0.99989309	0.865997479
CREB5	0.889634728	0.795993324	0.851617203	0.916305829	0.99989309	0.909768235
EXOC3L1	0.889982366	0.761178476	0.958420472	0.998542029	0.975291792	0.812509877
HLF	0.889982366	0.766403118	0.921225977	0.999939566	0.980286089	0.812208548
UNC45A	0.889982366	0.866803532	0.880277219	0.925004412	0.99989309	0.810805433
LOC783838	0.889982366	0.955798423	0.828449612	0.92064557	0.99989309	0.838324968
ACER3	0.889982366	0.767200099	0.849077738	0.948120268	0.99989309	0.860015978
BDKRB1	0.889982366	0.769874274	0.814444866	0.999939566	0.99989309	0.872224159
GNAL	0.889982366	0.748534108	0.843427302	0.999939566	0.99989309	0.873520548
TRIAP1	0.889982366	0.809942574	0.875669731	0.956154344	0.99989309	0.874885645
DPP6	0.889982366	0.842667989	0.812770091	0.924489924	0.99989309	0.943138585
SLC6A20	0.889982366	0.775411968	0.838080059	0.945929971	0.99989309	0.956610132
LOC101905533	0.890113764	0.80055527	0.902141181	0.943323364	0.972917711	0.930707319
CCT8	0.890643333	0.775411968	0.844316758	0.947581168	0.972917711	0.980638112
PLCD3	0.890643333	0.810976764	0.847037539	0.99879985	0.99989309	0.825331577
OPHN1	0.890643333	0.739753419	0.886176337	0.99879985	0.99989309	0.847256027
TCP1	0.890808285	0.779536052	0.878603452	0.948891906	0.99989309	0.933316009
ZNF76	0.890981583	0.771098738	0.976869041	0.925931623	0.964150118	0.878417122
LOC101902895	0.890981583	0.818806929	0.919796896	0.995118128	0.99989309	0.806054104
TNXB	0.890981583	0.76414114	0.914668913	0.931989854	0.99989309	0.812509877

IQSEC1	0.890981583	0.838405969	0.828407935	0.990049248	0.99989309	0.812509877
MOB1A	0.890981583	0.781154903	0.8895568	0.928836449	0.99989309	0.839419627
HTR7	0.890981583	0.951985518	0.834793268	0.955560327	0.99989309	0.845803016
B3GNT8	0.890981583	0.762941584	0.857943088	0.999939566	0.99989309	0.845969723
EPHA1	0.890981583	0.769313383	0.836294915	0.999939566	0.99989309	0.86143104
PGM3	0.890981583	0.87401231	0.863760763	0.958879502	0.99989309	0.866253786
RAE1	0.890981583	0.764525656	0.858865175	0.995800463	0.99989309	0.873520548
ACSS1	0.890981583	0.842667989	0.817993104	0.919247117	0.99989309	0.888645056
MFSD4A	0.890981583	0.751946504	0.817256644	0.99879985	0.99989309	0.908939618
HTR2B	0.89098278	0.975341721	0.827125538	0.919247117	0.961835052	0.905705129
APOPT1	0.89098278	0.876555705	0.834115242	0.999939566	0.99989309	0.812509877
CHN1	0.891113023	0.77295675	0.851617203	0.999939566	0.972917711	0.809650729
TUBB2B	0.891113023	0.906538642	0.930262306	0.915749182	0.99989309	0.84491737
JAM2	0.891275308	0.875022483	0.857289591	0.973377993	0.987816158	0.882322567
LOC104972045	0.891327043	0.827609894	0.817256644	0.99879985	0.975291792	0.912396352
SLC25A25	0.891332261	0.757368351	0.965712499	0.951929772	0.996250445	0.860313207
LMTK2	0.891444389	0.761178476	0.820549834	0.958849311	0.99989309	0.876009151
ASAP2	0.891618462	0.758051794	0.857943088	0.99879985	0.972917711	0.928123265
LRP4	0.891618462	0.90339131	0.844065668	0.997612654	0.998276725	0.828602505
CACFD1	0.891618462	0.796591413	0.828569127	0.999939566	0.99989309	0.840150874
LOC112444341	0.891618462	0.785163144	0.832041802	0.995946943	0.99989309	0.84491737
TBC1D9B	0.891618462	0.825856868	0.832483532	0.99879985	0.99989309	0.884387948
LCP2	0.891618462	0.897918739	0.83314199	0.925931623	0.99989309	0.911696156
CAMK2B	0.891777563	0.804860126	0.882898206	0.915749182	0.979654999	0.968462588
CDC42SE1	0.891777563	0.78070503	0.880277219	0.956154344	0.99989309	0.846367069
PIP5K1A	0.891777563	0.893512526	0.817256644	0.92064557	0.99989309	0.896877362
LOC100337044	0.892540655	0.771165031	0.956508857	0.931268564	0.99989309	0.864571107
LOC614423	0.892608135	0.769817928	0.880277219	0.985048108	0.99989309	0.826868779
NLGN4X	0.892632057	0.767106625	0.87146798	0.971220023	0.977981285	0.950199992
EPHX2	0.892987445	0.750546265	0.814444866	0.99879985	0.99989309	0.813001848
NAA50	0.892987445	0.742728851	0.943748342	0.958879502	0.99989309	0.846879092
TPR	0.893101986	0.77021507	0.899195818	0.967063428	0.99989309	0.814637245
TMEM18	0.893101986	0.795993324	0.838765859	0.925901365	0.99989309	0.94091418
CPSF3	0.893101986	0.747499345	0.856812671	0.925931623	0.99989309	0.982045172
ALDH3A2	0.893157967	0.739918356	0.875669731	0.946344632	0.99989309	0.812682271
LHCGR	0.893157967	0.785978032	0.84311048	0.999939566	0.99989309	0.845024318
TCAP	0.893169731	0.852463941	0.920449324	0.926731827	0.980286089	0.895218005
FZD9	0.893169731	0.792190608	0.942004125	0.943323364	0.98152792	0.887457549

DTNBP1	0.893169731	0.835809337	0.832431595	0.999939566	0.988545919	0.859973633
CARD14	0.893169731	0.948631073	0.839622619	0.956154344	0.99989309	0.812682271
LOC513779	0.893169731	0.737937244	0.872397372	0.999939566	0.99989309	0.821467423
MUTYH	0.893347076	0.835130235	0.962790365	0.956154344	0.961382403	0.837048016
RASD1	0.893347076	0.785978032	0.827125538	0.999939566	0.961382403	0.864219261
RAB20	0.893347076	0.764097231	0.884325235	0.999939566	0.975291792	0.886992214
ITGA8	0.893347076	0.97572611	0.873750661	0.934591536	0.978272166	0.815459921
DNAAF5	0.893347076	0.762756164	0.877948633	0.999939566	0.99989309	0.815459921
LOC101904069	0.893347076	0.907229289	0.845425011	0.990571725	0.99989309	0.821467423
LOC112443143	0.893347076	0.744066065	0.838877266	0.999939566	0.99989309	0.833139817
DOCK7	0.893347076	0.876169307	0.827125538	0.943323364	0.99989309	0.834254546
NDUFV2	0.893347076	0.750745995	0.822923265	0.943323364	0.99989309	0.837048016
GLI1	0.893347076	0.83744789	0.833955994	0.993811478	0.99989309	0.837312175
CMC4	0.893347076	0.753193121	0.992643455	0.916305829	0.99989309	0.844622232
RFC3	0.893347076	0.764525656	0.844162118	0.969717497	0.99989309	0.84491737
FAM208B	0.893347076	0.769817928	0.880277219	0.942384819	0.99989309	0.845803016
ANKRD39	0.893347076	0.820737014	0.825888497	0.931268564	0.99989309	0.846987044
PRDM4	0.893347076	0.927630122	0.834569848	0.925901365	0.99989309	0.856870625
NSD3	0.893347076	0.847919882	0.831292358	0.925901365	0.99989309	0.86143104
ATF7	0.893347076	0.890554452	0.838838955	0.925960563	0.99989309	0.863755488
ENTPD6	0.893347076	0.820796062	0.817993104	0.915749182	0.99989309	0.865159042
TRIM4	0.893347076	0.747179932	0.850106729	0.985405486	0.99989309	0.886520861
TRMT2A	0.893347076	0.79878182	0.863560863	0.934591536	0.99989309	0.891000792
ZMAT3	0.893347076	0.790637585	0.844065668	0.943539568	0.99989309	0.897095338
MRPS35	0.893347076	0.780290359	0.836294915	0.966203886	0.99989309	0.909054478
LOC112449087	0.893347076	0.743556979	0.817993104	0.965590385	0.99989309	0.96322378
DNM1L	0.893347076	0.758702769	0.843427302	0.915749182	0.99989309	0.989128955
DDHD1	0.893493982	0.790637585	0.842478086	0.971679871	0.99989309	0.929046416
MIF4GD	0.893788802	0.872125506	0.844162118	0.99483611	0.99989309	0.830456671
ZSCAN21	0.893810369	0.763751563	0.919609671	0.917869295	0.99989309	0.920754358
KRBA1	0.894260164	0.851167599	0.914406673	0.971220023	0.999592263	0.84491737
LOC509972	0.894260164	0.836106877	0.836294915	0.99879985	0.99989309	0.809941826
LOC104974912	0.894260164	0.808513267	0.828407935	0.990571725	0.99989309	0.831041995
KCNK7	0.894260164	0.751946504	0.870054336	0.998542029	0.99989309	0.90259329
ARMC12	0.894260164	0.779536052	0.850751395	0.943323364	0.99989309	0.957877422
GSTT4	0.894300821	0.818435706	0.904305179	0.995118128	0.99989309	0.837148498
AMDHD1	0.894389975	0.921046401	0.942004125	0.925059533	0.993370551	0.808866655
TMEM14C	0.894389975	0.81808696	0.856812671	0.990571725	0.99989309	0.821467423

LY6E	0.894389975	0.747106203	0.87214481	0.925901365	0.99989309	0.867440033
FPGS	0.894389975	0.779765764	0.857417456	0.917869295	0.99989309	0.873118284
LCTL	0.894389975	0.80403784	0.817256644	0.990571725	0.99989309	0.891000792
LOC101902475	0.894717325	0.832239565	0.91546474	0.931268564	0.962262333	0.930528039
TMOD4	0.894717325	0.741353552	0.849077738	0.992368879	0.964521207	0.968245211
FKTN	0.894717325	0.956843573	0.842605286	0.915749182	0.966779894	0.92035958
MDGA1	0.894717325	0.842230087	0.875669731	0.999939566	0.975291792	0.813577244
TMEM199	0.894717325	0.744066065	0.994425789	0.934591536	0.982577194	0.840150874
CDYL	0.894717325	0.780641867	0.942004125	0.971220023	0.990424444	0.86307729
TBX3	0.894717325	0.860449455	0.883726795	0.924489924	0.99989309	0.832097423
GAPDHS	0.894717325	0.798883115	0.962754771	0.925004412	0.99989309	0.86307729
CLN6	0.894717325	0.758588486	0.846782643	0.934591536	0.99989309	0.941925704
ZNF274	0.894767302	0.866803532	0.939683463	0.956154344	0.961382403	0.84491737
LOC785403	0.894767302	0.889410709	0.930520213	0.946344632	0.968477804	0.847189049
SIPA1	0.894767302	0.804875341	0.941312064	0.990571725	0.975291792	0.840150874
RAI1	0.894767302	0.759439869	0.882898206	0.999939566	0.975819525	0.880404833
ELOVL1	0.894767302	0.805483225	0.875669731	0.999939566	0.976970807	0.864219261
FAM241A	0.894767302	0.786856496	0.836294915	0.999939566	0.979654999	0.832189969
LOC101902301	0.894767302	0.855151723	0.939683463	0.922782374	0.981187819	0.882170826
LOC100847269	0.894767302	0.81250936	0.827125538	0.999939566	0.98152792	0.84491737
ZNF782	0.894767302	0.92725556	0.880277219	0.925004412	0.988693274	0.878152258
DOCK6	0.894767302	0.761788537	0.929958748	0.990491321	0.998276725	0.872175131
LOC101903574	0.894767302	0.769817928	0.836414702	0.999939566	0.99989309	0.808755724
LOC101905908	0.894767302	0.965239369	0.852758896	0.928623745	0.99989309	0.809650729
SLC30A2	0.894767302	0.779208532	0.817993104	0.999939566	0.99989309	0.832189969
MMP24OS	0.894767302	0.751946504	0.842478086	0.962142137	0.99989309	0.833139817
SNHG4	0.894767302	0.753193121	0.843427302	0.980955077	0.99989309	0.833139817
C8H9orf152	0.894767302	0.776313457	0.942396659	0.934591536	0.99989309	0.837312175
IPO11	0.894767302	0.877180988	0.897712517	0.925004412	0.99989309	0.839419627
LOC112446127	0.894767302	0.809254208	0.892122482	0.959009349	0.99989309	0.839419627
LIN54	0.894767302	0.79441406	0.844065668	0.925004412	0.99989309	0.843214053
XRN1	0.894767302	0.823692881	0.842415578	0.928623745	0.99989309	0.846943306
MATN2	0.894767302	0.740182637	0.877998684	0.999939566	0.99989309	0.862145862
L3MBTL1	0.894767302	0.741353552	0.939683463	0.940921143	0.99989309	0.86307729
ARIH2	0.894767302	0.911196851	0.846650632	0.95964071	0.99989309	0.864680268
TBCD	0.894767302	0.829255735	0.883334551	0.946668073	0.99989309	0.868108836
LOC100139363	0.894767302	0.80403784	0.828449612	0.990571725	0.99989309	0.875441396
PPIE	0.894767302	0.747555258	0.941319062	0.922782374	0.99989309	0.879410856

GNG5	0.894767302	0.742728851	0.939091808	0.915749182	0.99989309	0.886992214
CDK6	0.894767302	0.852463941	0.912095646	0.93290597	0.99989309	0.887399945
LEPR	0.894767302	0.747164473	0.832483532	0.988140196	0.99989309	0.908939618
FANCE	0.894767302	0.76090129	0.836294915	0.917869295	0.99989309	0.929173783
RB1	0.894767302	0.804875341	0.820549834	0.943323364	0.99989309	0.937591162
STIM1	0.894767302	0.747499345	0.844009594	0.925265292	0.99989309	0.99064193
SRSF12	0.895254816	0.79738652	0.853686558	0.99879985	0.99989309	0.885060677
EHHADH	0.895254816	0.775921167	0.827125538	0.946344632	0.99989309	0.89063727
PSMB7	0.895309904	0.823187795	0.842478086	0.956154344	0.99989309	0.872141555
LOC101905156	0.895334841	0.973734713	0.92153058	0.921761002	0.961835052	0.809650729
NUP93	0.895334841	0.808513267	0.879148792	0.917869295	0.962262333	0.983453112
EDC3	0.895334841	0.771331117	0.953045982	0.994365581	0.964150118	0.856870625
LGALSL	0.895334841	0.780290359	0.92153058	0.999939566	0.964150118	0.856870625
P2RX5	0.895334841	0.808513267	0.842605286	0.999939566	0.975819525	0.858042054
ALG8	0.895334841	0.799412839	0.882898206	0.996601241	0.979654999	0.888617204
LTBP1	0.895334841	0.815071683	0.817256644	0.999939566	0.981777076	0.834008567
APOBR	0.895334841	0.962934461	0.85972234	0.917869295	0.982014614	0.88494083
ZC2HC1C	0.895334841	0.831141011	0.893544538	0.978328182	0.986955873	0.889622239
VPS26A	0.895334841	0.851167599	0.916702	0.996118855	0.988693274	0.809650729
RAD54L	0.895334841	0.795534928	0.842478086	0.999939566	0.988693274	0.844335246
PRKAR1B	0.895334841	0.769817928	0.879215964	0.999939566	0.988693274	0.869171706
PGGT1B	0.895334841	0.837455678	0.911202784	0.990049248	0.994688893	0.854831489
LOC101905046	0.895334841	0.776182952	0.920449324	0.999939566	0.995037199	0.840150874
LOC101905997	0.895334841	0.74401284	0.836294915	0.946344632	0.99989309	0.809650729
SEC62	0.895334841	0.76145014	0.870780616	0.999939566	0.99989309	0.809650729
U2AF2	0.895334841	0.804875341	0.882890706	0.999939566	0.99989309	0.809650729
PPP1R36	0.895334841	0.744494437	0.834643686	0.999939566	0.99989309	0.812622197
PAIP2B	0.895334841	0.784957951	0.903453422	0.999939566	0.99989309	0.821456459
SOSTDC1	0.895334841	0.77396808	0.846650632	0.999939566	0.99989309	0.827585683
PDLIM1	0.895334841	0.794823268	0.926613927	0.928623745	0.99989309	0.834254546
LOC112443509	0.895334841	0.914910569	0.820549834	0.987514339	0.99989309	0.839419627
EMX2	0.895334841	0.834011972	0.817993104	0.999939566	0.99989309	0.846367069
CIDEC	0.895334841	0.78164144	0.844065668	0.999939566	0.99989309	0.846367069
HSD17B4	0.895334841	0.823074953	0.899539089	0.95964071	0.99989309	0.849769587
LOC101907247	0.895334841	0.835276066	0.953692935	0.92064557	0.99989309	0.852562859
AP1S3	0.895334841	0.761788537	0.887130994	0.928836449	0.99989309	0.858042054
DNAJC7	0.895334841	0.924799163	0.845237542	0.925901365	0.99989309	0.859973633
DNAH17	0.895334841	0.74401284	0.843427302	0.999939566	0.99989309	0.859973633

CYTH1	0.895334841	0.834007213	0.836294915	0.990049248	0.99989309	0.860313207
NMUR1	0.895334841	0.76090129	0.860197593	0.9565815	0.99989309	0.860827207
STK25	0.895334841	0.748140931	0.856812671	0.982809512	0.99989309	0.862860542
ALKBH3	0.895334841	0.747179932	0.86796189	0.999939566	0.99989309	0.864219261
LOC101905114	0.895334841	0.824119076	0.843427302	0.976007283	0.99989309	0.865159042
LOC101902721	0.895334841	0.808517017	0.880277219	0.942235206	0.99989309	0.865997479
VEPH1	0.895334841	0.762756164	0.87146798	0.999939566	0.99989309	0.866253786
CPT2	0.895334841	0.747555258	0.849077738	0.962775522	0.99989309	0.866482265
ATP5F1B	0.895334841	0.763059076	0.827125538	0.934591536	0.99989309	0.875441396
RTTN	0.895334841	0.841992867	0.877346012	0.946344632	0.99989309	0.877623013
RIT1	0.895334841	0.906538642	0.844065668	0.934591536	0.99989309	0.878152258
SUPT3H	0.895334841	0.832892045	0.817256644	0.934591536	0.99989309	0.878570508
PHKG1	0.895334841	0.820556554	0.820767684	0.918313511	0.99989309	0.882322567
UNC119B	0.895334841	0.794823268	0.85792584	0.955560327	0.99989309	0.882322567
TSPAN31	0.895334841	0.78897006	0.84405284	0.999939566	0.99989309	0.888724443
C23H6orf47	0.895334841	0.767797556	0.878603452	0.949947918	0.99989309	0.906529452
LOC112442414	0.895334841	0.798799934	0.888610896	0.916305829	0.99989309	0.908939618
YBEY	0.895334841	0.866813771	0.834347048	0.924297153	0.99989309	0.926872824
DBNL	0.895334841	0.813371202	0.87214481	0.934591536	0.99989309	0.938553439
NDUFAF1	0.895334841	0.762756164	0.85292501	0.934591536	0.99989309	0.943138585
UBXN4	0.895334841	0.758582896	0.843427302	0.942235206	0.99989309	0.95401375
NOP56	0.895334841	0.776313457	0.860010936	0.925901365	0.99989309	0.958814003
CDIP1	0.895390233	0.854656328	0.91546474	0.925265292	0.99989309	0.893961809
TWNK	0.895523814	0.79441406	0.886282865	0.946344632	0.99989309	0.912376736
LOC112448777	0.895526716	0.761788537	0.90166788	0.915749182	0.964150118	0.986862062
MANSC1	0.895526716	0.767661556	0.827125538	0.999939566	0.975291792	0.844171661
ZMYND11	0.895526716	0.80931829	0.929958748	0.996084773	0.976657193	0.842945521
ZNF729	0.895526716	0.770371538	0.965712499	0.972489653	0.980653438	0.858538874
FLYWCH1	0.895526716	0.889816737	0.884197487	0.925004412	0.983089554	0.918803168
LOC101907682	0.895526716	0.82737092	0.905832059	0.990571725	0.983935609	0.868108836
TSNARE1	0.895526716	0.751946504	0.985552223	0.915749182	0.99989309	0.811547112
BOC	0.895526716	0.774326932	0.909170161	0.965590385	0.99989309	0.815459921
HMG20B	0.895526716	0.780290359	0.867451783	0.935742691	0.99989309	0.822642815
PIK3R4	0.895526716	0.748507328	0.820767684	0.995118128	0.99989309	0.825601599
NECAB2	0.895526716	0.790637585	0.893763648	0.943323364	0.99989309	0.826868779
TSPAN7	0.895526716	0.859941746	0.93050447	0.924489924	0.99989309	0.833155047
FAM206A	0.895526716	0.843103375	0.939091808	0.925901365	0.99989309	0.835362105
HNRNPA3	0.895526716	0.836487169	0.941593855	0.925931623	0.99989309	0.838324968

GNL1	0.895526716	0.855690015	0.817256644	0.995118128	0.99989309	0.83849759
LRRC7	0.895526716	0.79738652	0.927722996	0.946344632	0.99989309	0.840150874
TMC4	0.895526716	0.785978032	0.838712347	0.999334425	0.99989309	0.840150874
NLRP1	0.895526716	0.796591413	0.915636025	0.986438861	0.99989309	0.84491737
ARL6IP5	0.895526716	0.749070017	0.905832059	0.997614724	0.99989309	0.84491737
CADPS2	0.895526716	0.938378242	0.829784147	0.928836449	0.99989309	0.849769587
LOC101904042	0.895526716	0.817040135	0.897622604	0.917869295	0.99989309	0.850612178
PRPF3	0.895526716	0.765341142	0.962209895	0.93290597	0.99989309	0.853233759
LOC112443139	0.895526716	0.762756164	0.844065668	0.958879502	0.99989309	0.853233759
SLC38A3	0.895526716	0.856092464	0.849077738	0.995118128	0.99989309	0.858538874
B3GNT7	0.895526716	0.805132292	0.820767684	0.995118128	0.99989309	0.859973633
PML	0.895526716	0.750329158	0.882898206	0.987342814	0.99989309	0.86143104
PLA2G4B	0.895526716	0.763478948	0.911743083	0.995302856	0.99989309	0.866099047
TRAPPC6B	0.895526716	0.787965627	0.880277219	0.990049248	0.99989309	0.875441396
LYL1	0.895526716	0.780290359	0.879215964	0.943323364	0.99989309	0.885826563
KIAA0825	0.895526716	0.83671984	0.845237542	0.98636843	0.99989309	0.89635579
LOC112446740	0.895526716	0.77921146	0.845425011	0.934591536	0.99989309	0.902244322
LOC112444907	0.895526716	0.751946504	0.92153058	0.952024081	0.99989309	0.930707319
GAMT	0.895526716	0.755999274	0.844009594	0.943323364	0.99989309	0.930925458
PSMD3	0.895526716	0.785406526	0.853222116	0.959786126	0.99989309	0.933763401
MGST1	0.895526716	0.780105406	0.846782643	0.957351094	0.99989309	0.934653208
FNBP1	0.895526716	0.751946504	0.905017554	0.931989854	0.99989309	0.954859132
ZSCAN31	0.895526716	0.762941584	0.829843435	0.943323364	0.99989309	0.968039618
SLC39A10	0.895532718	0.820796062	0.838151539	0.925265292	0.99989309	0.874335327
LOC112442071	0.895664718	0.855979085	0.914685423	0.956154344	0.999356955	0.865926817
NAA38	0.895689385	0.780290359	0.830033146	0.995118128	0.99989309	0.815459921
LDLR	0.895689385	0.769817928	0.861838588	0.967063428	0.99989309	0.913230542
CDCA7	0.895827227	0.743422331	0.848018557	0.999939566	0.970439271	0.894974898
LOC101902390	0.895827227	0.878594098	0.847441894	0.925931623	0.99989309	0.895287865
PIK3R1	0.895869574	0.751946504	0.843427302	0.999939566	0.975375626	0.862860542
SIPA1L1	0.895869574	0.880036391	0.839666249	0.943323364	0.995037199	0.942421464
SCRN2	0.895869574	0.857034402	0.933963997	0.939292364	0.999592263	0.863578778
UTP25	0.895869574	0.798491587	0.909472681	0.995118128	0.99989309	0.832189969
MIER3	0.895869574	0.836487169	0.922147089	0.953508052	0.99989309	0.835370067
ERCC1	0.895869574	0.858230868	0.819094028	0.994309225	0.99989309	0.840150874
LOC112449563	0.895869574	0.77414098	0.836294915	0.999939566	0.99989309	0.840150874
DENND6B	0.895869574	0.767950704	0.979231344	0.921501634	0.99989309	0.846367069
ERAL1	0.895869574	0.786660393	0.820767684	0.921209664	0.99989309	0.858538874

FILIP1	0.895869574	0.920423305	0.89248682	0.925931623	0.99989309	0.86307729
TMEM30B	0.895869574	0.796591413	0.939683463	0.918161195	0.99989309	0.908939618
SESTD1	0.895869574	0.765341142	0.839666249	0.923832787	0.99989309	0.912376736
MKS1	0.895869574	0.747499345	0.848900897	0.958849311	0.99989309	0.926872824
C5H12orf66	0.895869574	0.84357015	0.879515214	0.925901365	0.99989309	0.934458012
RETREG1	0.895925605	0.755866871	0.863172962	0.999939566	0.982014614	0.864440643
ARPP19	0.895925605	0.792090907	0.848890018	0.999939566	0.99989309	0.829389486
LOC101904520	0.895925605	0.904077835	0.829843435	0.925004412	0.99989309	0.86307729
DDO	0.895944738	0.771098738	0.834569848	0.999939566	0.987816158	0.86307729
TACC1	0.896068576	0.756540592	0.930710426	0.999939566	0.970439271	0.839340243
S100PBP	0.896068576	0.88311684	0.86637689	0.999939566	0.972176983	0.845949552
LOC101905586	0.896068576	0.936809216	0.868936984	0.992368879	0.976970807	0.821467423
LOC112447011	0.896068576	0.860271574	0.833736568	0.999939566	0.988693274	0.815459921
GBF1	0.896068576	0.838405969	0.8895568	0.931989854	0.99989309	0.828376685
FOXO6	0.896068576	0.745665426	0.886282865	0.958879502	0.99989309	0.840157695
ERC1	0.896068576	0.811804876	0.820549834	0.919247117	0.99989309	0.84491737
FGFR3	0.896068576	0.9347557	0.875669731	0.933849042	0.99989309	0.845024318
SLC8A3	0.896068576	0.944420015	0.845237542	0.924489924	0.99989309	0.845969723
TIGD5	0.896068576	0.758702769	0.830013482	0.94783387	0.99989309	0.860827207
RNASEH1	0.896068576	0.778839781	0.863172962	0.995800463	0.99989309	0.868108836
SLCO4C1	0.896068576	0.867722813	0.893763648	0.925901365	0.99989309	0.908939618
SLC27A5	0.896068576	0.80931829	0.886282865	0.948172741	0.99989309	0.912385767
MYZAP	0.896147381	0.988649234	0.843427302	0.917869295	0.988120779	0.865159042
MED18	0.896147381	0.824676261	0.865793123	0.922713162	0.99989309	0.852562859
PTRH1	0.896162378	0.808839416	0.843427302	0.925931623	0.99989309	0.937591162
TMED8	0.896162378	0.763618945	0.909472681	0.922782374	0.99989309	0.961506391
KLHL29	0.896192079	0.747555258	0.984700021	0.928836449	0.96532164	0.901689556
LOC101905343	0.896192079	0.838405969	0.915066299	0.963829131	0.972048096	0.895218005
ITGAV	0.896192079	0.787152924	0.841048308	0.92064557	0.982800653	0.998074562
LOC505479	0.896192079	0.780641867	0.832483532	0.956154344	0.988693274	0.983453112
SMPX	0.896192079	0.965239369	0.820767684	0.934591536	0.993399243	0.902122572
BEX3	0.896192079	0.763751563	0.853222116	0.995800463	0.994688893	0.941925704
RARRES1	0.896192079	0.813459746	0.90002174	0.944558402	0.996250445	0.928442671
RBP1	0.896192079	0.882516719	0.920017619	0.946344632	0.99989309	0.8125889
SEC11A	0.896192079	0.835276066	0.882728714	0.999939566	0.99989309	0.813345852
LOC101906469	0.896192079	0.831242188	0.919034552	0.998542029	0.99989309	0.815459921
LOC112441644	0.896192079	0.747164473	0.93145152	0.999939566	0.99989309	0.821774001
NSUN6	0.896192079	0.769817928	0.828569127	0.999939566	0.99989309	0.822642815

FZD7	0.896192079	0.749070017	0.852758896	0.999939566	0.99989309	0.822691858
LOC101905630	0.896192079	0.751946504	0.872651958	0.980032491	0.99989309	0.825601599
ELAC1	0.896192079	0.770808357	0.885834459	0.925004412	0.99989309	0.829807823
LOC616200	0.896192079	0.832239565	0.827219802	0.928623745	0.99989309	0.829807823
SNAPC2	0.896192079	0.881353204	0.882898206	0.925901365	0.99989309	0.839340243
LVRN	0.896192079	0.851559446	0.939683463	0.937070243	0.99989309	0.839419627
LOC100847946	0.896192079	0.767950704	0.915874142	0.99419346	0.99989309	0.839494796
IFNAR2	0.896192079	0.790637585	0.845237542	0.999939566	0.99989309	0.840150874
PNRC2	0.896192079	0.830448181	0.881647103	0.992368879	0.99989309	0.842461327
C3H1orf210	0.896192079	0.888994602	0.870774839	0.925265292	0.99989309	0.849769587
GLIPR1	0.896192079	0.873045394	0.850046649	0.934591536	0.99989309	0.858042054
JOSD1	0.896192079	0.820988267	0.874057431	0.972367384	0.99989309	0.858042054
NPW	0.896192079	0.771098738	0.820767684	0.999939566	0.99989309	0.858777168
SMIM30	0.896192079	0.780082505	0.894449269	0.992368879	0.99989309	0.859481777
LOC100847782	0.896192079	0.859941746	0.883726795	0.919247117	0.99989309	0.868108836
CEP120	0.896192079	0.80403784	0.880277219	0.925931623	0.99989309	0.868177389
PPP1R12A	0.896192079	0.854944719	0.886282865	0.92064557	0.99989309	0.880404833
ITPRIPL2	0.896192079	0.751946504	0.827125538	0.999939566	0.99989309	0.884646285
ENPP5	0.896192079	0.786258422	0.849077738	0.934591536	0.99989309	0.893961809
SAMHD1	0.896192079	0.837455678	0.870054336	0.949947918	0.99989309	0.896228048
LOC112443528	0.896192079	0.78070503	0.882898206	0.931268564	0.99989309	0.908939618
LOC112444600	0.896192079	0.747106203	0.929958748	0.953648387	0.99989309	0.92689297
NUDT21	0.896192079	0.771098738	0.877998684	0.943323364	0.99989309	0.94425343
IKZF3	0.896267375	0.810519292	0.842478086	0.925931623	0.991830391	0.988323336
CARNMT1	0.896267375	0.761788537	0.929958748	0.99879985	0.996250445	0.862403446
CD8A	0.896271396	0.823012828	0.848018557	0.96415791	0.987816158	0.95470473
TMEM39B	0.896271396	0.988153273	0.859931483	0.925440563	0.99989309	0.8125889
CBLB	0.896271396	0.77414098	0.844162118	0.925004412	0.99989309	0.877623013
OSBP2	0.896329751	0.920423305	0.838838955	0.924489924	0.99989309	0.823139458
LOC104974443	0.896495189	0.747899938	0.967302577	0.995118128	0.982014614	0.840150874
CD300E	0.896495189	0.880674338	0.882898206	0.956154344	0.988693274	0.893961809
LPIN2	0.896495189	0.790637585	0.836294915	0.99879985	0.988693274	0.941925704
LOC101906312	0.896495189	0.823588167	0.865007702	0.998542029	0.996250445	0.893961809
GYPC	0.896495189	0.808110762	0.880277219	0.990571725	0.99989309	0.815459921
TTC14	0.896495189	0.771098738	0.926613927	0.935073052	0.99989309	0.822691858
NFATC3	0.896495189	0.782865867	0.875669731	0.955687359	0.99989309	0.833139817
CTH	0.896495189	0.80403784	0.965750046	0.956154344	0.99989309	0.833139817
NQO2	0.896495189	0.76167712	0.906597758	0.983569039	0.99989309	0.849772722

RBM19	0.896495189	0.982017699	0.832483532	0.917869295	0.99989309	0.868108836
LOC100336161	0.896495189	0.890554452	0.825502814	0.940921143	0.99989309	0.872224159
BMP2K	0.896495189	0.763751563	0.908821939	0.925210703	0.99989309	0.908939618
ARHGEF6	0.896495189	0.805132292	0.896272282	0.928623745	0.99989309	0.908939618
CHRAC1	0.896495189	0.859941746	0.844611045	0.925265292	0.99989309	0.935154223
GPATCH2	0.896619313	0.79738652	0.843427302	0.969717497	0.99989309	0.84491737
VPS28	0.896756258	0.817237509	0.827125538	0.971220023	0.99989309	0.84491737
SYT11	0.896769882	0.877420806	0.891455327	0.948120268	0.99989309	0.865997479
CTC1	0.89705536	0.780290359	0.962209895	0.922782374	0.998276725	0.911284706
ARHGEF12	0.89705536	0.813476598	0.832483532	0.99419346	0.99989309	0.832189969
CRISPLD2	0.89705536	0.767670077	0.827125538	0.981704194	0.99989309	0.840150874
PCBP4	0.89705536	0.792090907	0.908528428	0.997308645	0.99989309	0.847189049
MSI1	0.89705536	0.777206577	0.844316758	0.999939566	0.99989309	0.877623013
B9D2	0.89705536	0.747499345	0.875669731	0.990049248	0.99989309	0.928086248
TMEM136	0.89705536	0.780290359	0.899539089	0.958879502	0.99989309	0.934775196
TGFBR3L	0.89726198	0.780641867	0.880277219	0.99879985	0.982014614	0.908939618
C7H5orf24	0.89726198	0.834869027	0.983372282	0.924489924	0.988693274	0.84491737
NPLOC4	0.89726198	0.79738652	0.879726737	0.990571725	0.988693274	0.924845138
TWIST1	0.89726198	0.806152572	0.827125538	0.996512319	0.99989309	0.815459921
IRF7	0.89726198	0.758702769	0.980734833	0.923832787	0.99989309	0.840150874
ELK4	0.89726198	0.841044548	0.832483532	0.925901365	0.99989309	0.864571107
NIM1K	0.89726198	0.776269089	0.878603452	0.95060093	0.99989309	0.866004383
COPB1	0.89726198	0.883267666	0.899816306	0.925865589	0.99989309	0.875441396
VCAM1	0.89726198	0.771165031	0.921146002	0.969717497	0.99989309	0.88198647
MPDU1	0.89726198	0.81250936	0.836294915	0.971220023	0.99989309	0.883080515
SEH1L	0.89726198	0.823692881	0.878603452	0.971220023	0.99989309	0.908785004
FAAP24	0.89726198	0.805157515	0.838488596	0.943323364	0.99989309	0.943138585
CCDC17	0.897434397	0.785406526	0.878603452	0.999939566	0.999592263	0.859973633
SMAD4	0.897454907	0.758051794	0.916263052	0.933387241	0.99989309	0.918220726
SYTL1	0.897457197	0.810220398	0.951850716	0.99879985	0.966066172	0.823139458
BTBD6	0.897457197	0.879851444	0.880277219	0.95964071	0.977038808	0.906323179
ST3GAL6	0.897457197	0.843422478	0.886282865	0.946344632	0.979654999	0.935607719
PRRG4	0.897457197	0.839399097	0.906597758	0.969717497	0.985955764	0.893961809
ACOX1	0.897457197	0.922796598	0.825888497	0.99879985	0.994943664	0.86143104
CTCFL	0.897457197	0.817434986	0.884189563	0.938519355	0.998276725	0.948277018
GON4L	0.897457197	0.886967296	0.894874079	0.982140778	0.99989309	0.813345852
SUPT16H	0.897457197	0.823187795	0.865535471	0.925901365	0.99989309	0.815459921
PCBP1	0.897457197	0.76090129	0.927460291	0.95060093	0.99989309	0.815459921

ADHFE1	0.897457197	0.848132141	0.838488596	0.946344632	0.99989309	0.816251097
ELMO3	0.897457197	0.835809337	0.980734833	0.920877669	0.99989309	0.826868779
PNKP	0.897457197	0.785406526	0.974559286	0.936573639	0.99989309	0.826868779
ZDHHC9	0.897457197	0.859528714	0.875669731	0.946344632	0.99989309	0.826868779
SLC25A27	0.897457197	0.785978032	0.914685423	0.922782374	0.99989309	0.830923241
LOC100139115	0.897457197	0.840313358	0.843427302	0.992137653	0.99989309	0.832189969
NUPR2	0.897457197	0.835809337	0.842605286	0.999939566	0.99989309	0.839494796
RBPMS2	0.897457197	0.750068475	0.981670362	0.925901365	0.99989309	0.840150874
LOC104971510	0.897457197	0.897918739	0.836650368	0.99879985	0.99989309	0.840150874
LOC112448893	0.897457197	0.782865867	0.988111832	0.925210703	0.99989309	0.842187877
CDCA5	0.897457197	0.769186282	0.92489263	0.99879985	0.99989309	0.84491737
C5H22orf23	0.897457197	0.76090129	0.887214835	0.943323364	0.99989309	0.849772722
PRDX1	0.897457197	0.813868782	0.886282865	0.993545419	0.99989309	0.849772722
TRIM41	0.897457197	0.790637585	0.844316758	0.99879985	0.99989309	0.86143104
APOO	0.897457197	0.771098738	0.880277219	0.934591536	0.99989309	0.868177389
DEGS1	0.897457197	0.780946412	0.846650632	0.982140778	0.99989309	0.868745583
FUT4	0.897457197	0.781287182	0.939683463	0.956154344	0.99989309	0.872175131
WDR83OS	0.897457197	0.782865867	0.849077738	0.943323364	0.99989309	0.872872291
LOC107132431	0.897457197	0.775921167	0.850849709	0.925931623	0.99989309	0.873520548
HOXC10	0.897457197	0.808513267	0.87146798	0.990571725	0.99989309	0.885758502
GNE	0.897457197	0.81808696	0.860430781	0.926405541	0.99989309	0.891000792
FAM221A	0.897457197	0.955798423	0.825888497	0.943323364	0.99989309	0.891908825
COMMD2	0.897457197	0.779208532	0.950801642	0.934174776	0.99989309	0.89260699
TAF4	0.897457197	0.805157515	0.838080059	0.925775859	0.99989309	0.908939618
SARS	0.897457197	0.853448902	0.832483532	0.990049248	0.99989309	0.917968123
TFB2M	0.897457197	0.747555258	0.867451783	0.999939566	0.99989309	0.925355626
TATDN3	0.897457197	0.769313383	0.880277219	0.977470078	0.99989309	0.934458012
LOC112447727	0.897457197	0.785890605	0.852758896	0.936573639	0.99989309	0.943138585
TRIL	0.897457197	0.761949365	0.863172962	0.925440563	0.99989309	0.948816455
TNFRSF8	0.897457197	0.747499345	0.882898206	0.934591536	0.99989309	0.964143145
TMEM135	0.897564275	0.747555258	0.852758896	0.999939566	0.988693274	0.924885155
RAB40B	0.897576143	0.965016829	0.826667106	0.919207975	0.968477804	0.941925704
NUDT15	0.897576143	0.798956241	0.879215964	0.99879985	0.973705865	0.908939618
TMTC1	0.897576143	0.798956241	0.87214481	0.999939566	0.97668994	0.872175131
DEPTOR	0.897576143	0.823187795	0.852758896	0.999939566	0.99989309	0.814576296
SYNDIG1	0.897576143	0.856914648	0.867541376	0.972367384	0.99989309	0.833139817
ID1	0.897576143	0.883378562	0.829843435	0.990571725	0.99989309	0.834008567
TRAPPC5	0.897576143	0.830709707	0.828407935	0.943323364	0.99989309	0.85177107

MAPK3	0.897576143	0.81668556	0.883726795	0.976950878	0.99989309	0.856870625
LOC101904498	0.897576143	0.808513267	0.846650632	0.995800463	0.99989309	0.862403446
TUBE1	0.897576143	0.801968936	0.906597758	0.952227936	0.99989309	0.919645668
MCRIP1	0.89791131	0.838405969	0.850020542	0.923832787	0.99989309	0.859973633
ACAT1	0.897990288	0.795993324	0.828407935	0.977216943	0.99989309	0.845803016
FAM69A	0.898046615	0.893512526	0.845237542	0.925901365	0.99989309	0.84491737
PRRT2	0.898067666	0.778574724	0.840938565	0.999939566	0.99989309	0.839419627
DHCR24	0.898067666	0.771765977	0.82765733	0.995118128	0.99989309	0.943138585
PTP4A1	0.898073363	0.765341142	0.966157152	0.992741182	0.972917711	0.856870625
NARS	0.898073363	0.814633312	0.893763648	0.995118128	0.997854738	0.881039089
IFT172	0.898073363	0.769313383	0.994425789	0.936573639	0.99989309	0.813732245
MRPS33	0.898073363	0.779536052	0.921612776	0.995800463	0.99989309	0.822691858
ITPA	0.898073363	0.841958822	0.83722246	0.936573639	0.99989309	0.822882339
G6PC3	0.898073363	0.855151723	0.838488596	0.925004412	0.99989309	0.834008567
RAB28	0.898073363	0.851167599	0.878050811	0.987143706	0.99989309	0.840150874
CDC14A	0.898073363	0.862267429	0.844065668	0.999939566	0.99989309	0.844622232
SCO2	0.898073363	0.852463941	0.825888497	0.943323364	0.99989309	0.859973633
CCDC124	0.898073363	0.775463154	0.880277219	0.992402807	0.99989309	0.86143104
LOC101905813	0.898073363	0.751946504	0.974698466	0.939292364	0.99989309	0.880404833
XIAP	0.898073363	0.834869027	0.827125538	0.95175364	0.99989309	0.897633315
WNT9B	0.898073363	0.911152896	0.843427302	0.925901365	0.99989309	0.931495481
KANK1	0.898073363	0.771098738	0.862946056	0.956154344	0.99989309	0.946717739
ABCF1	0.898073363	0.77849524	0.849077738	0.925931623	0.99989309	0.977961448
HAP1	0.89814859	0.790637585	0.850046649	0.992154129	0.99989309	0.864680268
EML3	0.898197674	0.777708835	0.957886297	0.958879502	0.99989309	0.840150874
PTPRK	0.898223985	0.899387334	0.846650632	0.946344632	0.99989309	0.908939618
SPOCK2	0.89842977	0.786024086	0.828449612	0.990571725	0.99989309	0.877623013
CCDC58	0.898601102	0.775921167	0.864135894	0.959009349	0.99989309	0.893961809
PCK1	0.89878927	0.974986418	0.903130395	0.93471064	0.975291792	0.823139458
SMIM14	0.89878927	0.804875341	0.939683463	0.99879985	0.986955873	0.840150874
ZNF311	0.89878927	0.861568371	0.939683463	0.946344632	0.990424444	0.866253786
LYPD6	0.89878927	0.944755173	0.832483532	0.990571725	0.99989309	0.814637245
FAT1	0.89878927	0.770427846	0.827125538	0.931268564	0.99989309	0.815459921
PSKH1	0.89878927	0.836487169	0.861038179	0.99879985	0.99989309	0.825601599
STARD8	0.89878927	0.821124254	0.899195818	0.98470348	0.99989309	0.832858909
ZNF516	0.89878927	0.768563436	0.860430781	0.925901365	0.99989309	0.835370067
LOC101904536	0.89878927	0.835408071	0.909170161	0.98470348	0.99989309	0.839340243
BANF1	0.89878927	0.780290359	0.892122482	0.944002443	0.99989309	0.840150874

PTGER3	0.89878927	0.781287182	0.828407935	0.999939566	0.99989309	0.840150874
TAF11	0.89878927	0.758051794	0.962176387	0.985776562	0.99989309	0.844335246
BMI1	0.89878927	0.780061391	0.917066863	0.999939566	0.99989309	0.847450944
SELPLG	0.89878927	0.914162656	0.846650632	0.924489924	0.99989309	0.858042054
LOC524181	0.89878927	0.80403784	0.917876971	0.925901365	0.99989309	0.85958325
LRCH1	0.89878927	0.835276066	0.860240602	0.992829869	0.99989309	0.86307729
ECHDC3	0.89878927	0.832239565	0.845237542	0.974382211	0.99989309	0.863578778
PREB	0.89878927	0.926435857	0.892122482	0.919276287	0.99989309	0.866253786
PDE2A	0.89878927	0.947959005	0.83722246	0.930483452	0.99989309	0.885198927
ADAMTS5	0.89878927	0.776126383	0.863172962	0.999939566	0.99989309	0.908939618
LOC510860	0.89878927	0.747555258	0.825888497	0.981022805	0.99989309	0.918666628
LOC101905734	0.89878927	0.883267666	0.827125538	0.92064557	0.99989309	0.942421464
LOC101904810	0.898856199	0.810519292	0.942004125	0.934591536	0.99989309	0.829389486
JPH4	0.898856199	0.839289192	0.827125538	0.972489653	0.99989309	0.908939618
PPARA	0.898856199	0.798956241	0.861838588	0.953648387	0.99989309	0.937218904
LOC112442082	0.89890323	0.747899938	0.870321375	0.99879985	0.975291792	0.943866443
TMEM14A	0.89890323	0.868312459	0.870054336	0.999939566	0.99989309	0.825114751
TCOF1	0.89890323	0.795171452	0.939683463	0.992137653	0.99989309	0.826868779
FN3K	0.89890323	0.778839781	0.828569127	0.995118128	0.99989309	0.837249706
NXPE3	0.89890323	0.792090907	0.907270422	0.990049248	0.99989309	0.859447707
LOC101905818	0.89890323	0.840313358	0.933102499	0.92064557	0.99989309	0.893961809
PLEKHG5	0.89890323	0.851167599	0.878603452	0.943510519	0.99989309	0.924845138
CHTF8	0.89890323	0.852463941	0.843427302	0.943323364	0.99989309	0.935655141
CLCN5	0.89890323	0.80403784	0.871344378	0.932203219	0.99989309	0.950199992
TCF23	0.898961074	0.966794964	0.868936984	0.922713162	0.99989309	0.840150874
LOC100847609	0.899220801	0.820000472	0.87214481	0.993811478	0.996250445	0.912396352
CDPF1	0.899378839	0.952713646	0.878603452	0.940921143	0.99989309	0.829034472
MLIP	0.899378839	0.923642562	0.863049062	0.923832787	0.99989309	0.86143104
MTMR6	0.89947558	0.758588486	0.858394457	0.999939566	0.979654999	0.85958325
LOC112448038	0.89947558	0.934248552	0.827125538	0.956154344	0.987816158	0.923064313
OSBPL5	0.89947558	0.758750448	0.882890706	0.999939566	0.997854738	0.875441396
ALS2	0.89947558	0.810519292	0.838488596	0.999939566	0.99989309	0.820356896
LIN52	0.89947558	0.785406526	0.994425789	0.922713162	0.99989309	0.821774001
LOC112444333	0.89947558	0.790637585	0.844065668	0.995800463	0.99989309	0.840150874
KLHL8	0.89947558	0.763751563	0.844065668	0.999334425	0.99989309	0.847256027
RIMKLA	0.89947558	0.878594098	0.924266781	0.921761002	0.99989309	0.855653734
ELK3	0.89947558	0.765493178	0.826600651	0.999939566	0.99989309	0.859973633
LOC101908359	0.89947558	0.748889217	0.849077738	0.999939566	0.99989309	0.864219261

ACBD4	0.89947558	0.771098738	0.832431595	0.999939566	0.99989309	0.887457549
GUCY1B1	0.89947558	0.771864943	0.89248682	0.992137653	0.99989309	0.895218005
ANXA1	0.899831718	0.808839416	0.8895568	0.958849311	0.99989309	0.868108836
SGF29	0.899875313	0.824988091	0.827186499	0.999334425	0.997488969	0.928923695
CCDC88C	0.900097118	0.776217482	0.850020542	0.999939566	0.99989309	0.839419627
CLCC1	0.900097118	0.849718733	0.850020542	0.996601241	0.99989309	0.872175131
TNFAIP1	0.90017924	0.798799934	0.849233704	0.969717497	0.99989309	0.911696156
RICTOR	0.900284434	0.910596218	0.827125538	0.934591536	0.99989309	0.926872824
ARHGAP19	0.90046392	0.799579962	0.836294915	0.99879985	0.99989309	0.886992214
TNFRSF17	0.900683673	0.92229715	0.910978655	0.951929772	0.972917711	0.866156441
FFAR4	0.900683673	0.858230868	0.91094712	0.959410646	0.975819525	0.902660792
ST6GAL1	0.900683673	0.782196458	0.830013482	0.999939566	0.986955873	0.837249706
LOC112445177	0.900683673	0.77396808	0.876296093	0.990491321	0.99989309	0.828768167
RIPK2	0.900683673	0.810205594	0.930262306	0.995118128	0.99989309	0.829389486
LOC112447324	0.900683673	0.817040135	0.885354035	0.983038839	0.99989309	0.830923241
AGTRAP	0.900683673	0.779765764	0.87214481	0.999939566	0.99989309	0.83394957
CCSER1	0.900683673	0.926435857	0.832431595	0.966070428	0.99989309	0.839419627
AMHR2	0.900683673	0.768980848	0.982930137	0.967063428	0.99989309	0.840150874
HOXC9	0.900683673	0.83744789	0.907270422	0.954980798	0.99989309	0.85958325
BOD1	0.900683673	0.780290359	0.829843435	0.999939566	0.99989309	0.859973633
GINS1	0.900683673	0.785978032	0.828407935	0.958879502	0.99989309	0.868108836
COQ6	0.900683673	0.769817928	0.85972234	0.925931623	0.99989309	0.871780952
LOC104975925	0.900683673	0.79441406	0.869529186	0.971220023	0.99989309	0.872224159
MYOC	0.900683673	0.913171043	0.845237542	0.951929772	0.99989309	0.881742037
WRN	0.900683673	0.934248552	0.848890018	0.925440563	0.99989309	0.912101047
RAB5B	0.900683673	0.750335462	0.874092011	0.946344632	0.99989309	0.912376736
TAF5L	0.900683673	0.798799934	0.844065668	0.951929772	0.99989309	0.913230542
HTT	0.900683673	0.769313383	0.849077738	0.925901365	0.99989309	0.956610132
LOC112445063	0.901144727	0.799579962	0.87214481	0.999939566	0.996250445	0.893961809
IBA57	0.901144727	0.790637585	0.845237542	0.925931623	0.99989309	0.82036646
DLK1	0.901144727	0.90249621	0.904682623	0.956154344	0.99989309	0.846367069
ZBED4	0.90123925	0.851167599	0.882890706	0.992235612	0.99989309	0.870895425
LOC112448153	0.90123925	0.780290359	0.863742167	0.976950878	0.99989309	0.954110387
NAP1L4	0.901473769	0.877420806	0.827125538	0.934591536	0.99989309	0.859707535
NEPRO	0.901766628	0.780290359	0.877998684	0.927618239	0.986955873	0.989947227
C15H11orf49	0.901766628	0.935230802	0.827125538	0.963153458	0.99989309	0.840150874
TSPYL2	0.901794724	0.79738652	0.87146798	0.995118128	0.99989309	0.875441396
BRF1	0.901929827	0.781154903	0.92153058	0.966070428	0.99989309	0.846367069

FUS	0.901961247	0.828133113	0.868936984	0.924489924	0.99989309	0.954334316
MOCS2	0.902100664	0.918306418	0.887640011	0.943323364	0.99989309	0.868237691
ITGA9	0.90210852	0.944420015	0.834793268	0.999939566	0.975291792	0.830509603
CTSH	0.90210852	0.831242188	0.847784725	0.999939566	0.975291792	0.86307729
CADM2	0.90210852	0.835276066	0.920449324	0.971517408	0.975291792	0.908939618
NIPA2	0.90210852	0.762756164	0.87214481	0.999939566	0.983634477	0.864219261
EDN3	0.90210852	0.843422478	0.849077738	0.999939566	0.987855669	0.886992214
LOC101903015	0.90210852	0.898629793	0.93050447	0.943323364	0.988693274	0.871129794
SNRPB	0.90210852	0.781444941	0.952255572	0.934591536	0.988693274	0.932014575
CXADR	0.90210852	0.769817928	0.869529186	0.99879985	0.988693274	0.9369759
CCER2	0.90210852	0.951616411	0.874092011	0.963829131	0.996250445	0.847450944
EBD	0.90210852	0.944420015	0.828569127	0.925265292	0.996250445	0.942512132
LOC107132589	0.90210852	0.76090129	0.972141593	0.975133428	0.996558365	0.865159042
CCDC146	0.90210852	0.86415368	0.844065668	0.999939566	0.99989309	0.822642815
MIEN1	0.90210852	0.88311684	0.879215964	0.966133452	0.99989309	0.825601599
DNAH2	0.90210852	0.811402363	0.844009594	0.990049248	0.99989309	0.832189969
FBXO38	0.90210852	0.841044548	0.865192015	0.99879985	0.99989309	0.832858909
SLITRK6	0.90210852	0.766408271	0.827186499	0.994309225	0.99989309	0.833139817
FKBP5	0.90210852	0.765434632	0.835693067	0.999939566	0.99989309	0.834550008
SRFBP1	0.90210852	0.790637585	0.884197487	0.936573639	0.99989309	0.835767164
TMEM158	0.90210852	0.808513267	0.875669731	0.999939566	0.99989309	0.837249706
EVI5	0.90210852	0.761178476	0.892348336	0.999939566	0.99989309	0.837312175
ARRDC2	0.90210852	0.780641867	0.845237542	0.999939566	0.99989309	0.83849759
LOC786616	0.90210852	0.821588096	0.838838955	0.992402807	0.99989309	0.839340243
ZCCHC24	0.90210852	0.897657501	0.849077738	0.990571725	0.99989309	0.839419627
CYP2D14	0.90210852	0.821124254	0.857289591	0.999939566	0.99989309	0.839419627
MRPL40	0.90210852	0.915717447	0.836671539	0.944558402	0.99989309	0.839494796
LOC112445193	0.90210852	0.774326932	0.827125538	0.946344632	0.99989309	0.840150874
USHBP1	0.90210852	0.810096755	0.844065668	0.999939566	0.99989309	0.840150874
PHIP	0.90210852	0.833331159	0.845237542	0.943323364	0.99989309	0.844335246
GRSF1	0.90210852	0.83744789	0.94991125	0.971220023	0.99989309	0.845591203
LOC101905319	0.90210852	0.828417266	0.827125538	0.948120268	0.99989309	0.846367069
SNRNP27	0.90210852	0.763751563	0.843427302	0.965590385	0.99989309	0.846367069
PPEF1	0.90210852	0.843861385	0.880277219	0.925901365	0.99989309	0.853233759
NDUFAF7	0.90210852	0.751946504	0.850952986	0.943323364	0.99989309	0.853233759
TMEM42	0.90210852	0.817040135	0.93737555	0.969717497	0.99989309	0.853233759
TRAF2	0.90210852	0.821297608	0.87146798	0.925901365	0.99989309	0.86307729
LOC112441645	0.90210852	0.769313383	0.87214481	0.958879502	0.99989309	0.86307729

ACVR2B	0.90210852	0.831242188	0.883982433	0.972367384	0.99989309	0.86307729
CD151	0.90210852	0.851167599	0.843427302	0.995946943	0.99989309	0.865159042
F2R	0.90210852	0.837455678	0.844009594	0.999939566	0.99989309	0.866099047
C27H4orf47	0.90210852	0.751946504	0.862901296	0.999939566	0.99989309	0.872493553
LOC104972407	0.90210852	0.785163144	0.880277219	0.996601241	0.99989309	0.875963854
ZBTB10	0.90210852	0.839289192	0.827125538	0.992137653	0.99989309	0.886992214
MEPCE	0.90210852	0.820796062	0.853222116	0.998542029	0.99989309	0.886992214
TRAF3IP2	0.90210852	0.792190608	0.834917445	0.99879985	0.99989309	0.887248119
CIAPIN1	0.90210852	0.877749555	0.845237542	0.990571725	0.99989309	0.892214703
TMEM192	0.90210852	0.763735296	0.883726795	0.995118128	0.99989309	0.892802259
LOC112442401	0.90210852	0.813230451	0.880277219	0.971220023	0.99989309	0.893961809
NT5C3A	0.90210852	0.820000472	0.828410622	0.99879985	0.99989309	0.908645305
MCM9	0.90210852	0.785163144	0.827125538	0.999939566	0.99989309	0.908939618
NRIP1	0.90210852	0.820000472	0.878603452	0.92064557	0.99989309	0.918803168
GTF2H3	0.90210852	0.825215718	0.888554785	0.931268564	0.99989309	0.920942379
ITGB1BP1	0.90210852	0.769313383	0.868936984	0.971220023	0.99989309	0.928190067
CYBC1	0.90210852	0.92813228	0.827186499	0.924489924	0.99989309	0.930925458
AHCY	0.90210852	0.823012828	0.842605286	0.943323364	0.99989309	0.953940638
RUSC2	0.90210852	0.91761409	0.830013482	0.925004412	0.99989309	0.957877422
EEF1AKMT3	0.90210852	0.785406526	0.839601684	0.931989854	0.99989309	0.968245211
LOC112444936	0.90210852	0.751946504	0.856729427	0.956154344	0.99989309	0.978656049
LOC101902665	0.90210852	0.784957951	0.870054336	0.928623745	0.99989309	0.979606084
SGPP1	0.90210852	0.80403784	0.827125538	0.960878389	0.99989309	0.981164039
GPD2	0.902209683	0.882275117	0.874623951	0.932404322	0.99989309	0.867440033
SPAG1	0.902209683	0.831242188	0.886176337	0.943323364	0.99989309	0.932014575
SSH1	0.902209683	0.780290359	0.8895568	0.925004412	0.99989309	0.956610132
ZC3H6	0.902282994	0.972563829	0.842478086	0.924489924	0.979654999	0.920980794
NXPH2	0.902282994	0.851167599	0.868936984	0.990049248	0.99989309	0.901320431
MUT	0.902420841	0.762150099	0.827125538	0.958879502	0.99989309	0.908118871
ANXA5	0.902431755	0.823012828	0.868936984	0.999939566	0.99989309	0.832097423
CSTF1	0.902431755	0.771165031	0.939683463	0.931268564	0.99989309	0.922872513
ASPA	0.902445803	0.9789789	0.84593343	0.949947918	0.99989309	0.839697035
GEMIN8	0.902450905	0.808513267	0.902584766	0.992368879	0.99989309	0.878851638
ENDOD1	0.902555386	0.820350377	0.979231344	0.963153458	0.975375626	0.841340974
RAB3B	0.902555386	0.828797861	0.879215964	0.999939566	0.979499404	0.84491737
ARNTL	0.902555386	0.785978032	0.878050811	0.999939566	0.979654999	0.908939618
GLRX3	0.902555386	0.769817928	0.863890803	0.99879985	0.979654999	0.948277018
ORC4	0.902555386	0.885981636	0.851617203	0.999939566	0.98152792	0.847189049

HEPACAM	0.902555386	0.77414098	0.980734833	0.981022805	0.981777076	0.856870625
HMGXB4	0.902555386	0.866123629	0.849077738	0.980418209	0.988693274	0.940673472
SOX13	0.902555386	0.76090129	0.90002174	0.953144955	0.988693274	0.973195541
LPCAT1	0.902555386	0.967758952	0.882898206	0.934591536	0.999592263	0.858777168
MGAT5B	0.902555386	0.936134921	0.849077738	0.925004412	0.999592263	0.935154223
CGREF1	0.902555386	0.950861808	0.838488596	0.99879985	0.99989309	0.826868779
ARL5B	0.902555386	0.87314795	0.944202372	0.946344632	0.99989309	0.830456671
FBXO45	0.902555386	0.785383224	0.939683463	0.995118128	0.99989309	0.832097423
HEXDC	0.902555386	0.769817928	0.953198168	0.928836449	0.99989309	0.833139817
TMEM62	0.902555386	0.823588167	0.831320191	0.925265292	0.99989309	0.838324968
GALNT2	0.902555386	0.801782332	0.831430643	0.951929772	0.99989309	0.839340243
GPC4	0.902555386	0.882706523	0.863172962	0.994309225	0.99989309	0.840150874
NRN1	0.902555386	0.944959989	0.84405284	0.93471064	0.99989309	0.84491737
FBXO30	0.902555386	0.901256223	0.827186499	0.956154344	0.99989309	0.84491737
LOC787397	0.902555386	0.820796062	0.871109615	0.999939566	0.99989309	0.846367069
MARCH3	0.902555386	0.834819546	0.909522865	0.946344632	0.99989309	0.847450944
GLYCTK	0.902555386	0.785406526	0.829843435	0.951929772	0.99989309	0.849769587
LOC112448253	0.902555386	0.8232491	0.942004125	0.943240775	0.99989309	0.856870625
COMMD8	0.902555386	0.79449527	0.899195818	0.999939566	0.99989309	0.859323448
LOC534627	0.902555386	0.852880948	0.855033582	0.994309225	0.99989309	0.861596496
AIFM1	0.902555386	0.792663811	0.829843435	0.925931623	0.99989309	0.862788295
LOC104969097	0.902555386	0.769091087	0.929303679	0.995118128	0.99989309	0.866253786
MAMLD1	0.902555386	0.79878182	0.879726737	0.931989854	0.99989309	0.866612872
PLEKHG6	0.902555386	0.8232491	0.828407935	0.999939566	0.99989309	0.876183804
GNPDA2	0.902555386	0.803903736	0.867451783	0.999939566	0.99989309	0.876183804
MRPL35	0.902555386	0.823588167	0.842478086	0.943161565	0.99989309	0.884387948
HYOU1	0.902555386	0.838405969	0.838838955	0.97136787	0.99989309	0.88494083
FRG1	0.902555386	0.857034402	0.89248682	0.956154344	0.99989309	0.886992214
PDP1	0.902555386	0.779648098	0.883726795	0.943323364	0.99989309	0.887399945
FGFR1	0.902555386	0.804173299	0.831320191	0.999939566	0.99989309	0.895218005
APP	0.902555386	0.755318304	0.926613927	0.946344632	0.99989309	0.902660792
LOC112445982	0.902555386	0.780290359	0.870780616	0.976519231	0.99989309	0.908939618
PHF13	0.902555386	0.884768652	0.844065668	0.946344632	0.99989309	0.910353954
LGR4	0.902555386	0.886967296	0.845237542	0.931268564	0.99989309	0.936563391
LOC512863	0.902555386	0.778839781	0.828407935	0.925901365	0.99989309	0.942455807
CENPQ	0.90270055	0.78689911	0.92153058	0.937360017	0.99989309	0.872872291
GAPT	0.90270055	0.770408403	0.957379098	0.946344632	0.99989309	0.918666628
MTX3	0.90270055	0.766403118	0.878050811	0.959727203	0.99989309	0.926872824

CHTOP	0.90270055	0.794823268	0.884189563	0.934591536	0.99989309	0.940683751
LOC614732	0.902794713	0.794059256	0.939091808	0.99879985	0.998276725	0.855778243
PCSK1N	0.902794713	0.840313358	0.845237542	0.95964071	0.99989309	0.83849759
LOC101902030	0.902794713	0.794823268	0.962176387	0.923832787	0.99989309	0.840150874
ITGAX	0.902805834	0.83793617	0.924266781	0.990571725	0.98152792	0.880272231
LOC533308	0.902805834	0.886628459	0.832483532	0.99879985	0.996250445	0.893961809
POLE3	0.902805834	0.755866871	0.944612298	0.992137653	0.996250445	0.896877362
CDC25A	0.902805834	0.842797231	0.849077738	0.992368879	0.99989309	0.826868779
FAM89B	0.902805834	0.911196851	0.886282865	0.969717497	0.99989309	0.84491737
E2F8	0.902805834	0.769817928	0.891014698	0.999939566	0.99989309	0.84491737
SERPIND1	0.902805834	0.834819546	0.914668913	0.951929772	0.99989309	0.86307729
ZNF22	0.902805834	0.817066389	0.939683463	0.953508052	0.99989309	0.868108836
RBBP9	0.902805834	0.825301941	0.843427302	0.95964071	0.99989309	0.909369594
DNAAF2	0.902805834	0.82890642	0.844065668	0.944728796	0.99989309	0.943138585
RMDN3	0.903082655	0.832852787	0.893863239	0.994719152	0.99989309	0.839788983
RNF181	0.903082655	0.845452558	0.885867136	0.976456131	0.99989309	0.86143104
MRPS11	0.903082655	0.863339074	0.850046649	0.925004412	0.99989309	0.866482265
EAPP	0.903082655	0.905977412	0.850952986	0.985181062	0.99989309	0.873520548
TBC1D23	0.903082655	0.829476146	0.864627365	0.999334425	0.99989309	0.876009151
SIRT2	0.903082655	0.788961546	0.844065668	0.996601241	0.99989309	0.908939618
ADPRM	0.903166573	0.882706523	0.87146798	0.956154344	0.99989309	0.912101047
TRIM63	0.9032591	0.817066389	0.883860423	0.999939566	0.996250445	0.890898561
PRR13	0.9032591	0.825215718	0.862585131	0.995800463	0.99989309	0.836085591
CD14	0.9032591	0.880674338	0.87146798	0.948172741	0.99989309	0.844255149
KATNB1	0.9032591	0.882736993	0.832431595	0.940921143	0.99989309	0.859973633
LOC112443837	0.9032591	0.860329184	0.829843435	0.935763381	0.99989309	0.86143104
DDIT4L	0.9032591	0.867138744	0.870920678	0.941467481	0.99989309	0.86307729
SLC17A9	0.9032591	0.81259481	0.926613927	0.956154344	0.99989309	0.883329104
FAM78B	0.9032591	0.786258422	0.892122482	0.992137653	0.99989309	0.913230542
BRD8	0.9032591	0.771098738	0.94454396	0.925931623	0.99989309	0.945593145
PKN3	0.903267193	0.781154903	0.939683463	0.99879985	0.981777076	0.872721005
WDR62	0.903267193	0.77414098	0.992165187	0.953648387	0.981938411	0.855692379
SPRED3	0.903267193	0.794405568	0.911743083	0.999939566	0.982014614	0.84491737
SCAMP2	0.903267193	0.780290359	0.91546474	0.999939566	0.988693274	0.869171706
METTL18	0.903267193	0.959119521	0.855743966	0.951177494	0.988693274	0.891000792
PDPN	0.903267193	0.776126383	0.958420472	0.956154344	0.988693274	0.917524589
ABCD4	0.903267193	0.851167599	0.875669731	0.959786126	0.988693274	0.943685681
WDR44	0.903267193	0.769817928	0.843427302	0.999939566	0.996250445	0.957877422

RNF223	0.903267193	0.806189585	0.830125816	0.926967136	0.996250445	0.998636142
LOC100126043	0.903267193	0.827459094	0.879215964	0.999939566	0.997488969	0.875960923
CNIH1	0.903267193	0.789863026	0.947475618	0.946344632	0.999592263	0.923871657
RNASE10	0.903267193	0.778653225	0.853686558	0.984618049	0.999592263	0.975972322
ETFA	0.903267193	0.773993079	0.856997596	0.925265292	0.99989309	0.830909703
LOC512486	0.903267193	0.794823268	0.89007492	0.995800463	0.99989309	0.832189969
CCDC68	0.903267193	0.767675287	0.899539089	0.999939566	0.99989309	0.832189969
SLC2A13	0.903267193	0.76090129	0.958977478	0.925901365	0.99989309	0.832659179
TUSC2	0.903267193	0.809942574	0.98082053	0.95964071	0.99989309	0.832858909
ALKBH7	0.903267193	0.889064624	0.834793268	0.971517408	0.99989309	0.833139817
SPPL2A	0.903267193	0.765341142	0.917876971	0.999939566	0.99989309	0.833139817
LOC112448373	0.903267193	0.882275117	0.828407935	0.999939566	0.99989309	0.836564988
FURIN	0.903267193	0.912112179	0.87146798	0.944558402	0.99989309	0.837501229
GCDH	0.903267193	0.794823268	0.848018557	0.933913424	0.99989309	0.839419627
ICAM1	0.903267193	0.784957951	0.965750046	0.943323364	0.99989309	0.839419627
KLC1	0.903267193	0.835408071	0.86719452	0.943323364	0.99989309	0.839494796
TYMS	0.903267193	0.85749413	0.942004125	0.954423369	0.99989309	0.839494796
LOC112442280	0.903267193	0.976925958	0.886282865	0.925004412	0.99989309	0.840150874
UBE2K	0.903267193	0.813371202	0.89265635	0.990049248	0.99989309	0.840150874
SPHK2	0.903267193	0.840874016	0.828744606	0.946344632	0.99989309	0.84491737
SLF2	0.903267193	0.821987045	0.834643686	0.996601241	0.99989309	0.84491737
OSGIN1	0.903267193	0.868963165	0.862946056	0.999939566	0.99989309	0.851651757
HMGB2	0.903267193	0.855979085	0.839833549	0.999939566	0.99989309	0.856495922
GSTM1	0.903267193	0.784936368	0.84311048	0.999939566	0.99989309	0.858538874
LOC104969719	0.903267193	0.784957951	0.991406769	0.931989854	0.99989309	0.859323448
PTMA	0.903267193	0.876057665	0.904249961	0.981022805	0.99989309	0.859323448
HBA	0.903267193	0.851167599	0.832483532	0.982140778	0.99989309	0.859973633
LOC112442623	0.903267193	0.779536052	0.835301782	0.999939566	0.99989309	0.860313207
NKIRAS1	0.903267193	0.765434632	0.850106729	0.944558402	0.99989309	0.86143104
DIP2A	0.903267193	0.774697122	0.870054336	0.999939566	0.99989309	0.862145862
PPIG	0.903267193	0.862941799	0.846650632	0.956154344	0.99989309	0.862481601
LOC107131939	0.903267193	0.7635426	0.849233704	0.99879985	0.99989309	0.862481601
CDK7	0.903267193	0.796591413	0.829843435	0.925059533	0.99989309	0.86307729
TRIO	0.903267193	0.810519292	0.917876971	0.99879985	0.99989309	0.86307729
FRRS1	0.903267193	0.849785039	0.832431595	0.971220023	0.99989309	0.866612872
LOC112443130	0.903267193	0.820559187	0.966260688	0.928596748	0.99989309	0.868108836
LOC101907965	0.903267193	0.823588167	0.879215964	0.934591536	0.99989309	0.868108836
ZBTB7A	0.903267193	0.780946412	0.878603452	0.947615714	0.99989309	0.869857598

SEC61G	0.903267193	0.790637585	0.967489175	0.959410646	0.99989309	0.870895425
COL4A5	0.903267193	0.935324849	0.836294915	0.972367384	0.99989309	0.872224159
EIF4E3	0.903267193	0.789486407	0.914406673	0.989087699	0.99989309	0.875441396
GSKIP	0.903267193	0.761788537	0.937930242	0.99879985	0.99989309	0.875441396
ACADSB	0.903267193	0.868963165	0.836257071	0.972367384	0.99989309	0.877495052
ERCC5	0.903267193	0.871113425	0.849077738	0.982809512	0.99989309	0.87900534
PORCN	0.903267193	0.851167599	0.870054336	0.990300191	0.99989309	0.884387948
NBR1	0.903267193	0.825768191	0.83722246	0.999939566	0.99989309	0.884646285
SFRP5	0.903267193	0.779536052	0.844316758	0.999939566	0.99989309	0.887248119
ACVR1B	0.903267193	0.781444941	0.877346012	0.928596748	0.99989309	0.893400499
ACSL4	0.903267193	0.790637585	0.880277219	0.998542029	0.99989309	0.906178214
GLB1L	0.903267193	0.769817928	0.87214481	0.9846464	0.99989309	0.908939618
GALC	0.903267193	0.786188116	0.833955994	0.990571725	0.99989309	0.923064313
TLR4	0.903267193	0.821124254	0.831320191	0.925901365	0.99989309	0.92689297
LOC107133473	0.903267193	0.800441059	0.878603452	0.925901365	0.99989309	0.92920113
CBS	0.903267193	0.81808696	0.838080059	0.943539568	0.99989309	0.934775196
ATL2	0.903267193	0.778839781	0.939612562	0.925440563	0.99989309	0.940683751
MAU2	0.903267193	0.80931829	0.887130994	0.958879502	0.99989309	0.942868422
CYP4A11	0.903267193	0.786258422	0.844065668	0.938899383	0.99989309	0.944606747
SLC35E3	0.903267193	0.823371359	0.87146798	0.961377855	0.99989309	0.954110387
AGPAT1	0.903267193	0.792149275	0.828407935	0.971220023	0.99989309	0.954334316
FBXO44	0.903267193	0.775921167	0.863560863	0.956634362	0.99989309	0.974139155
E2F3	0.903267193	0.811804876	0.855743966	0.946344632	0.99989309	0.981164039
LOC112442843	0.903267193	0.810488979	0.838877266	0.940863121	0.99989309	0.991281092
UFC1	0.903335304	0.81724461	0.875669731	0.959410646	0.99989309	0.868108836
LOC101907138	0.903352033	0.846959498	0.925852918	0.925931623	0.99989309	0.901689556
SMOC2	0.903450211	0.824676261	0.843427302	0.999939566	0.99989309	0.916089952
MRPS12	0.903450211	0.835130235	0.836294915	0.943323364	0.99989309	0.961506391
TMEM145	0.903472445	0.810519292	0.850046649	0.999939566	0.99989309	0.839494796
ZNF213	0.903472445	0.769817928	0.859614039	0.926522831	0.99989309	0.840150874
LOC104975196	0.903472445	0.878594098	0.918233663	0.954875446	0.99989309	0.853233759
IDNK	0.903472445	0.820000472	0.924266781	0.990049248	0.99989309	0.856870625
MTM1	0.903472445	0.780290359	0.950801642	0.994365581	0.99989309	0.865926817
ASH1L	0.903472445	0.866803532	0.840938565	0.928623745	0.99989309	0.867440033
LRRC1	0.903472445	0.763751563	0.879726737	0.996084773	0.99989309	0.877294997
MEFV	0.903562943	0.780290359	0.989332994	0.963153458	0.988693274	0.847189049
PSCA	0.903562943	0.765572369	0.941888866	0.981022805	0.988693274	0.924294935
LOC101903400	0.903562943	0.880674338	0.870054336	0.963829131	0.988693274	0.935385789

METTL4	0.903562943	0.893839642	0.850751395	0.97136787	0.99989309	0.829389486
LOC112442613	0.903562943	0.781444941	0.962790365	0.934591536	0.99989309	0.845628259
PAPLN	0.903562943	0.77295675	0.879515214	0.981022805	0.99989309	0.845803016
RIPK3	0.903562943	0.866803532	0.959705869	0.925901365	0.99989309	0.855653734
MAP2K1	0.903562943	0.771331117	0.846782643	0.995800463	0.99989309	0.86143104
PGAM1	0.903562943	0.805157515	0.850855725	0.977216943	0.99989309	0.875441396
PDPR	0.903562943	0.820796062	0.931265798	0.971220023	0.99989309	0.902660792
ELOC	0.903562943	0.886967296	0.84405284	0.943323364	0.99989309	0.904312348
CD37	0.903562943	0.852463941	0.849077738	0.943323364	0.99989309	0.908939618
KRT10	0.903562943	0.831242188	0.844316758	0.937531834	0.99989309	0.931901179
CABLES1	0.903562943	0.765434632	0.870774839	0.939292364	0.99989309	0.986716086
LOC112449505	0.903676327	0.853448902	0.8895568	0.94925691	0.99989309	0.839494796
FOSL2	0.903751423	0.829548444	0.868936984	0.99879985	0.99989309	0.859487774
SLC1A7	0.903751423	0.901922021	0.830125816	0.992137653	0.99989309	0.911696156
LOC107133343	0.903837397	0.762756164	0.994425789	0.943323364	0.99989309	0.828376685
C23H6orf52	0.903837397	0.854037042	0.878603452	0.977470078	0.99989309	0.872175131
LOC112444351	0.90383981	0.825856868	0.899195818	0.934591536	0.99989309	0.849772722
CEBPZOS	0.90383981	0.824676261	0.844065668	0.973377993	0.99989309	0.866253786
ZNF710	0.90383981	0.846959498	0.844065668	0.991978984	0.99989309	0.912396352
MEMO1	0.90405664	0.813371202	0.846650632	0.925901365	0.99989309	0.95676005
CREB3L4	0.904396687	0.774326932	0.979635589	0.936835069	0.99989309	0.839494796
FAN1	0.904396687	0.843333021	0.852758896	0.94783387	0.99989309	0.887248119
CDRT1	0.904646859	0.760918029	0.852758896	0.947615714	0.99989309	0.891140839
PEX10	0.904790665	0.76145014	0.958420472	0.927267062	0.996250445	0.953604648
LOC101907622	0.904790665	0.964105444	0.845237542	0.943323364	0.99989309	0.829807823
LOC781439	0.904790665	0.855979085	0.850106729	0.977470078	0.99989309	0.840287208
RSBN1	0.904790665	0.897918739	0.844065668	0.925004412	0.99989309	0.859487774
LOC534578	0.904790665	0.767200099	0.906925281	0.999939566	0.99989309	0.865159042
MTPAP	0.904790665	0.785383224	0.836294915	0.999939566	0.99989309	0.872224159
MAP1S	0.904790665	0.780290359	0.874057431	0.98636843	0.99989309	0.90259329
ZNF286A	0.904790665	0.920347742	0.839601684	0.931268564	0.99989309	0.914440253
AGRN	0.904790665	0.855151723	0.851048846	0.943323364	0.99989309	0.934775196
LOC104975054	0.904790665	0.80931829	0.851617203	0.951929772	0.99989309	0.969551744
HSPA4	0.904919875	0.77414098	0.899539089	0.947581168	0.99989309	0.830456671
MAK16	0.905007804	0.768548562	0.925442336	0.943323364	0.99989309	0.961506391
RCL1	0.905045872	0.781154903	0.844065668	0.999939566	0.998276725	0.867440033
IARS	0.905045872	0.80320642	0.918576062	0.925901365	0.99989309	0.961506391
LOC112443244	0.905091648	0.852281996	0.838080059	0.999939566	0.99989309	0.86143104

CST7	0.905091648	0.775921167	0.843427302	0.999939566	0.99989309	0.868108836
NXPE2	0.905091648	0.838405969	0.854976005	0.95986631	0.99989309	0.912385767
TUBGCP6	0.905495342	0.76414114	0.979231344	0.992368879	0.988693274	0.858042054
TPMT	0.905569685	0.907594268	0.849077738	0.989391869	0.99989309	0.84491737
DPH1	0.905569685	0.80403784	0.836294915	0.995118128	0.99989309	0.849769587
FAM53B	0.905569685	0.840313358	0.855379431	0.951929772	0.99989309	0.867440033
CGRRF1	0.905569685	0.855163746	0.870054336	0.996084773	0.99989309	0.880404833
LOC112442271	0.905569685	0.828600257	0.847441894	0.995800463	0.99989309	0.891908825
BLOC1S5	0.905569685	0.778839781	0.943882376	0.971220023	0.99989309	0.908939618
POLR3D	0.905569685	0.883267666	0.850243032	0.925931623	0.99989309	0.962255684
KCNT2	0.90562955	0.761788537	0.930892662	0.995118128	0.99989309	0.832097423
SNX24	0.90562955	0.964616377	0.929303679	0.927618239	0.99989309	0.832858909
LOC785630	0.90562955	0.786188116	0.880204512	0.99879985	0.99989309	0.899551847
EGR3	0.905678883	0.823588167	0.959506858	0.995118128	0.996250445	0.839494796
LOC112444300	0.905678883	0.803903736	0.8549878	0.963153458	0.99989309	0.86252043
SELENOH	0.905678883	0.911347642	0.835301782	0.931989854	0.99989309	0.870895425
CCT2	0.905678883	0.769313383	0.879148792	0.998542029	0.99989309	0.878152258
ARHGAP24	0.905678883	0.832674547	0.845237542	0.969717497	0.99989309	0.88363494
EMC1	0.905678883	0.817040135	0.846782643	0.995118128	0.99989309	0.908939618
MRC1	0.905678883	0.867425848	0.843427302	0.934591536	0.99989309	0.930707319
SMIM26	0.906073265	0.763751563	0.869455175	0.999939566	0.99989309	0.839419627
WDFY2	0.906073265	0.892520818	0.85792584	0.99879985	0.99989309	0.852634896
NRXN1	0.906073265	0.868963165	0.832483532	0.99879985	0.99989309	0.908939618
LOC112447041	0.906073265	0.771098738	0.89265635	0.990571725	0.99989309	0.935655141
MAP7	0.906402613	0.796591413	0.858247297	0.999939566	0.99989309	0.829389486
DCSTAMP	0.906402613	0.823692881	0.875669731	0.999939566	0.99989309	0.864219261
ST3GAL3	0.906402613	0.842797231	0.878603452	0.999939566	0.99989309	0.865926817
ELL	0.906402613	0.912112179	0.880277219	0.936573639	0.99989309	0.888396511
CD38	0.906402613	0.875449288	0.892122482	0.969717497	0.99989309	0.905220694
STK4	0.906402613	0.838405969	0.849077738	0.925901365	0.99989309	0.942421464
LOC104973100	0.906534359	0.809942574	0.878603452	0.995800463	0.99989309	0.85958325
ETNK2	0.906534359	0.771098738	0.885354035	0.999939566	0.99989309	0.864219261
CBFA2T2	0.906534359	0.771496954	0.844162118	0.999939566	0.99989309	0.933730876
RFLNB	0.906911337	0.830709707	0.882890706	0.999939566	0.982014614	0.832189969
ADORA2B	0.906911337	0.843422478	0.853686558	0.951929772	0.99989309	0.829807823
TMEM238	0.906911337	0.911106863	0.926613927	0.940921143	0.99989309	0.830923241
SMC5	0.906911337	0.834007213	0.90002174	0.956154344	0.99989309	0.831832432
LOC786614	0.906911337	0.841044548	0.869455175	0.943323364	0.99989309	0.833139817

PEX11A	0.906911337	0.81250936	0.87214481	0.999939566	0.99989309	0.833139817
NAXD	0.906911337	0.862527737	0.850155832	0.925901365	0.99989309	0.837048016
LOC101903645	0.906911337	0.851167599	0.967302577	0.953436496	0.99989309	0.839419627
MRPS36	0.906911337	0.841657074	0.87146798	0.943323364	0.99989309	0.847848978
DACT1	0.906911337	0.785999739	0.845237542	0.999939566	0.99989309	0.859481777
THAP7	0.906911337	0.868963165	0.844316758	0.931989854	0.99989309	0.884387948
LOC104975299	0.906911337	0.790637585	0.850243032	0.994365581	0.99989309	0.886992214
ADAT1	0.906911337	0.866803532	0.875669731	0.948120268	0.99989309	0.917150226
SNX13	0.906911337	0.77414098	0.894447907	0.995800463	0.99989309	0.934653208
CXHXorf21	0.906911337	0.810976764	0.85972234	0.956154344	0.99989309	0.953604648
LOC100848205	0.906911337	0.808022384	0.879515214	0.951964262	0.99989309	0.973195541
CCND3	0.907038133	0.763751563	0.850855725	0.999939566	0.99989309	0.88667499
LOC112447359	0.907178872	0.800441059	0.994425789	0.925931623	0.99989309	0.83849759
BAG5	0.907389006	0.843333021	0.879215964	0.999939566	0.982014614	0.870351337
ZSCAN26	0.907389006	0.780641867	0.939683463	0.99483611	0.996250445	0.905220694
SPIDR	0.907389006	0.824994464	0.918576062	0.990491321	0.996250445	0.911526437
EPS8L2	0.907389006	0.786258422	0.957379098	0.995118128	0.99672832	0.868745583
LOC104974020	0.907389006	0.975899801	0.899195818	0.934591536	0.997488969	0.846279751
GAL3ST4	0.907389006	0.871682971	0.836294915	0.999939566	0.999592263	0.859973633
ZNF835	0.907389006	0.91994024	0.831852803	0.950461476	0.999592263	0.95470473
LOC112449614	0.907389006	0.834741258	0.838838955	0.988949923	0.999592263	0.969981358
BPHL	0.907389006	0.852463941	0.834793268	0.994309225	0.99989309	0.830509603
DIP2C	0.907389006	0.995316112	0.844316758	0.928836449	0.99989309	0.833016105
SYNPR	0.907389006	0.956958713	0.883982433	0.925931623	0.99989309	0.834936698
TLN1	0.907389006	0.831242188	0.863172962	0.999939566	0.99989309	0.839340243
SMC1A	0.907389006	0.80931829	0.852548589	0.956154344	0.99989309	0.840150874
TEX264	0.907389006	0.964270094	0.836294915	0.990049248	0.99989309	0.840150874
LOC101904013	0.907389006	0.975287816	0.844065668	0.990300191	0.99989309	0.840150874
PCP4L1	0.907389006	0.830738666	0.844065668	0.990571725	0.99989309	0.840150874
PIP4K2A	0.907389006	0.804875341	0.842211815	0.939292364	0.99989309	0.844622232
ATG2B	0.907389006	0.856765056	0.885481718	0.982140778	0.99989309	0.84491737
ATP11C	0.907389006	0.864606958	0.844162118	0.937070243	0.99989309	0.845803016
SUMF2	0.907389006	0.942048064	0.836414702	0.963153458	0.99989309	0.847189049
HECW1	0.907389006	0.79713597	0.882898206	0.980295672	0.99989309	0.849504243
MRGBP	0.907389006	0.851167599	0.901550297	0.992368879	0.99989309	0.849504243
PWWP2B	0.907389006	0.836487169	0.852758896	0.998542029	0.99989309	0.859487774
EIF3K	0.907389006	0.836487169	0.850046649	0.999939566	0.99989309	0.86143104
TNFSF18	0.907389006	0.785978032	0.877133001	0.999939566	0.99989309	0.86307729

UBE3B	0.907389006	0.803903736	0.874092011	0.944002443	0.99989309	0.863755488
CCL24	0.907389006	0.805157515	0.872397372	0.973377993	0.99989309	0.864680268
LTA4H	0.907389006	0.896591631	0.856997596	0.99879985	0.99989309	0.865926817
LYRM4	0.907389006	0.809942574	0.8895568	0.983700077	0.99989309	0.866482265
ALG12	0.907389006	0.85439477	0.845237542	0.925931623	0.99989309	0.868108836
RBM14	0.907389006	0.823187795	0.849077738	0.976950878	0.99989309	0.870895425
GPR55	0.907389006	0.795421065	0.857486511	0.975529024	0.99989309	0.872175131
GRIA3	0.907389006	0.920818159	0.855743966	0.992368879	0.99989309	0.872175131
NSMCE1	0.907389006	0.873075317	0.87214481	0.999939566	0.99989309	0.872224159
FGF18	0.907389006	0.805132292	0.900128576	0.99879985	0.99989309	0.877623013
RAB24	0.907389006	0.879770617	0.878050811	0.96095927	0.99989309	0.878152258
COASY	0.907389006	0.795993324	0.845237542	0.943323364	0.99989309	0.883302043
NANS	0.907389006	0.859941746	0.849077738	0.999939566	0.99989309	0.883302043
ARL13B	0.907389006	0.934508	0.849077738	0.947581168	0.99989309	0.886520861
GRO1	0.907389006	0.826048291	0.87214481	0.990571725	0.99989309	0.888396511
LOC112442292	0.907389006	0.90393667	0.849077738	0.934591536	0.99989309	0.889159914
IRF2BPL	0.907389006	0.790621653	0.89007492	0.943323364	0.99989309	0.891312894
C18H19orf47	0.907389006	0.841044548	0.922147089	0.943240775	0.99989309	0.908939618
LOC101905588	0.907389006	0.823074953	0.937270184	0.958879502	0.99989309	0.908939618
LOC531038	0.907389006	0.781154903	0.939091808	0.971220023	0.99989309	0.917524589
LOC112446733	0.907389006	0.785163144	0.838488596	0.99879985	0.99989309	0.922263023
LOC101906018	0.907389006	0.775411968	0.87214481	0.99879985	0.99989309	0.925739558
CKAP5	0.907389006	0.817040135	0.84410203	0.962775522	0.99989309	0.926872824
TBKBP1	0.907389006	0.808513267	0.892122482	0.940921143	0.99989309	0.933730876
RPUSD1	0.907389006	0.763751563	0.850020542	0.995800463	0.99989309	0.934775196
GSK3B	0.907389006	0.779501962	0.878050811	0.971220023	0.99989309	0.940683751
RPL7L1	0.907389006	0.771506919	0.893732737	0.965097245	0.99989309	0.95470473
LOC112442215	0.907389006	0.84664709	0.870054336	0.956154344	0.99989309	0.957877422
FAM222B	0.907389006	0.87401231	0.84207783	0.963153458	0.99989309	0.958540316
IFITM1	0.907389006	0.847082229	0.844065668	0.943323364	0.99989309	0.960712754
LOC112445888	0.907509586	0.800441059	0.874092011	0.999939566	0.99989309	0.859481777
GRN	0.90763221	0.876057665	0.868936984	0.998542029	0.99989309	0.877916603
E2F7	0.907786118	0.775463154	0.95126311	0.995118128	0.99989309	0.842461327
WDR1	0.908038128	0.792816405	0.931173336	0.999939566	0.99989309	0.862860542
ZCCHC9	0.908338117	0.765434632	0.846650632	0.99879985	0.99989309	0.886520861
PABPC1	0.908383997	0.835276066	0.845237542	0.99879985	0.99989309	0.840150874
CHST11	0.908514139	0.988649234	0.844065668	0.96095927	0.99989309	0.844335246
SOWAHA	0.90877971	0.91994024	0.836294915	0.995118128	0.987816158	0.912376736

ID4	0.908822911	0.967758952	0.909170161	0.943323364	0.985452335	0.86252043
KCTD7	0.908822911	0.866803532	0.839601684	0.956154344	0.987731629	0.981164039
RAP1B	0.908822911	0.806152572	0.941593855	0.995118128	0.987855669	0.88667499
ALDH1L2	0.908822911	0.852463941	0.880277219	0.999939566	0.988693274	0.849772722
TMEM50B	0.908822911	0.847423683	0.926613927	0.949947918	0.988693274	0.933763401
DPY19L1	0.908822911	0.776126383	0.931397502	0.951177494	0.988693274	0.964722523
VAPB	0.908822911	0.786258422	0.913828295	0.999939566	0.990044405	0.87428828
LOC518775	0.908822911	0.781444941	0.951850716	0.990571725	0.996250445	0.902660792
XRCC1	0.908822911	0.809942574	0.965750046	0.943161565	0.998276725	0.911696156
FEM1B	0.908822911	0.936134921	0.844065668	0.971220023	0.99989309	0.832858909
LOC112448856	0.908822911	0.773461585	0.978855687	0.992154129	0.99989309	0.832858909
SF3A3	0.908822911	0.820796062	0.885434576	0.999939566	0.99989309	0.833139817
ITGBL1	0.908822911	0.851167599	0.939683463	0.930202113	0.99989309	0.837249706
AAMP	0.908822911	0.842797231	0.880277219	0.999939566	0.99989309	0.839340243
USP37	0.908822911	0.841657074	0.89145542	0.942235206	0.99989309	0.840150874
NRG2	0.908822911	0.966852427	0.845425011	0.977470078	0.99989309	0.843747482
SFXN1	0.908822911	0.866803532	0.848018557	0.995946943	0.99989309	0.84491737
CDK5RAP1	0.908822911	0.907594268	0.840938565	0.946344632	0.99989309	0.846367069
CNPY2	0.908822911	0.786660393	0.879215964	0.998526871	0.99989309	0.847848978
SLC25A37	0.908822911	0.831242188	0.888554785	0.999939566	0.99989309	0.849772722
DMWD	0.908822911	0.771165031	0.914685423	0.928836449	0.99989309	0.852095955
MGAT1	0.908822911	0.795534928	0.860197593	0.995118128	0.99989309	0.853233759
LOC107131403	0.908822911	0.821588096	0.843733648	0.995800463	0.99989309	0.857241194
GLIS2	0.908822911	0.769817928	0.939683463	0.99879985	0.99989309	0.857659295
MYBL1	0.908822911	0.922434012	0.892122482	0.980418209	0.99989309	0.85958325
KLHL11	0.908822911	0.899387334	0.878603452	0.934591536	0.99989309	0.86143104
PTDSS2	0.908822911	0.810096755	0.868936984	0.990571725	0.99989309	0.86143104
TDRKH	0.908822911	0.880103565	0.870054336	0.999939566	0.99989309	0.86307729
FOXP2	0.908822911	0.851167599	0.839666249	0.982554614	0.99989309	0.866253786
SEC23A	0.908822911	0.904764822	0.872397372	0.996270281	0.99989309	0.868025807
FAM216A	0.908822911	0.919351156	0.937270184	0.928623745	0.99989309	0.868108836
LOC104970103	0.908822911	0.873030987	0.878050811	0.999939566	0.99989309	0.868108836
NGRN	0.908822911	0.88418172	0.92332596	0.971220023	0.99989309	0.869171706
FAM117B	0.908822911	0.899387334	0.839039559	0.972489653	0.99989309	0.869171706
ZMYND12	0.908822911	0.879686372	0.845425011	0.987143706	0.99989309	0.873520548
SPTY2D1OS	0.908822911	0.79236511	0.950801642	0.992137653	0.99989309	0.877623013
PRUNE1	0.908822911	0.81668556	0.939683463	0.954248582	0.99989309	0.87771261
HELZ	0.908822911	0.775411968	0.840938565	0.946344632	0.99989309	0.877740826

TTPAL	0.908822911	0.876057665	0.962209895	0.925931623	0.99989309	0.880404833
NAT1	0.908822911	0.805954385	0.89265635	0.956154344	0.99989309	0.885826563
GPR89A	0.908822911	0.857034402	0.849077738	0.999939566	0.99989309	0.88609792
GAS2L1	0.908822911	0.841044548	0.834569848	0.964413123	0.99989309	0.887457549
PHF20	0.908822911	0.771331117	0.912031396	0.986030331	0.99989309	0.888375847
WFDC1	0.908822911	0.904539095	0.90002174	0.937531834	0.99989309	0.889622239
MYO9A	0.908822911	0.921280256	0.836294915	0.934591536	0.99989309	0.8905829
BCKDHB	0.908822911	0.808513267	0.838080059	0.971220023	0.99989309	0.891000792
ITGA6	0.908822911	0.820796062	0.901454778	0.980002938	0.99989309	0.893961809
FXYD1	0.908822911	0.938538223	0.87531282	0.926405541	0.99989309	0.895218005
KLHL13	0.908822911	0.775921167	0.853686558	0.992201432	0.99989309	0.899291343
CWC25	0.908822911	0.834869027	0.846009738	0.946344632	0.99989309	0.906178214
DOCK4	0.908822911	0.974986418	0.834793268	0.943323364	0.99989309	0.908939618
LOC112442248	0.908822911	0.775921167	0.87146798	0.999939566	0.99989309	0.912101047
TLNRD1	0.908822911	0.780290359	0.891014698	0.990571725	0.99989309	0.914440253
PLD3	0.908822911	0.888994602	0.848890018	0.990049248	0.99989309	0.921254858
ATF5	0.908822911	0.848136412	0.843427302	0.93471064	0.99989309	0.93636925
PBDC1	0.908822911	0.80403784	0.856128945	0.968293092	0.99989309	0.940683751
FGF16	0.908822911	0.77295675	0.844065668	0.992426863	0.99989309	0.940917403
LOC112441770	0.908822911	0.882930807	0.847441894	0.958879502	0.99989309	0.943738005
C19H17orf113	0.908822911	0.782954474	0.880277219	0.975529024	0.99989309	0.954935833
LOC781688	0.908822911	0.770808357	0.88950849	0.946344632	0.99989309	0.980668392
USP13	0.908822911	0.769817928	0.845336233	0.925931623	0.99989309	0.9956013
TOB2	0.908822911	0.781287182	0.853222116	0.928623745	0.99989309	0.998636142
LOC101905053	0.908909081	0.930890453	0.914406673	0.958879502	0.987731629	0.878417122
WDTC1	0.908909081	0.834011972	0.855743966	0.940921143	0.988120779	0.991281092
PAPSS2	0.908909081	0.845255059	0.870054336	0.999939566	0.988693274	0.897793801
TMTC2	0.908909081	0.935793939	0.919108971	0.969717497	0.993399243	0.858042054
GPR21	0.908909081	0.852281996	0.878603452	0.999939566	0.996250445	0.849769587
LOC100335268	0.908909081	0.973649843	0.845237542	0.990049248	0.996250445	0.86143104
LOC112445007	0.908909081	0.81808696	0.944612298	0.99879985	0.996250445	0.86307729
SFRS18	0.908909081	0.831242188	0.886282865	0.953648387	0.997004421	0.96435585
SESN1	0.908909081	0.784957951	0.869529186	0.999939566	0.997455339	0.839340243
PDCL3	0.908909081	0.854944719	0.950801642	0.956154344	0.998276725	0.888121679
PPAT	0.908909081	0.876919789	0.877134281	0.956634362	0.998276725	0.946344585
FTCDNL1	0.908909081	0.80403784	0.87481753	0.999939566	0.999445023	0.859447707
LEPROT	0.908909081	0.868963165	0.945200638	0.934591536	0.99989309	0.839419627
RBFOX1	0.908909081	0.844031777	0.836294915	0.999939566	0.99989309	0.839419627

KCNS2	0.908909081	0.972786652	0.87531282	0.951929772	0.99989309	0.840150874
EFCAB2	0.908909081	0.868963165	0.962569922	0.965590385	0.99989309	0.840150874
EFHC1	0.908909081	0.820796062	0.86360816	0.999939566	0.99989309	0.840150874
IL23A	0.908909081	0.989993945	0.835301782	0.925931623	0.99989309	0.842016958
LOC107131293	0.908909081	0.771098738	0.856812671	0.995800463	0.99989309	0.844622232
PLA2R1	0.908909081	0.82890642	0.842478086	0.999939566	0.99989309	0.845024318
LOC112448773	0.908909081	0.854944719	0.939683463	0.980032491	0.99989309	0.845580904
ATP5MG	0.908909081	0.792090907	0.848657934	0.945929971	0.99989309	0.846367069
RDH8	0.908909081	0.797209625	0.90002174	0.999939566	0.99989309	0.847189049
PCTP	0.908909081	0.781579221	0.880277219	0.959009349	0.99989309	0.847848978
SNRPF	0.908909081	0.805157515	0.976161819	0.97136787	0.99989309	0.84959042
BRCA2	0.908909081	0.771496954	0.97420715	0.990571725	0.99989309	0.854831489
IL20RA	0.908909081	0.835276066	0.952208316	0.958879502	0.99989309	0.858042054
HPF1	0.908909081	0.824676261	0.930520213	0.938519355	0.99989309	0.859487774
SOS2	0.908909081	0.825215718	0.943891967	0.959009349	0.99989309	0.859973633
RSPO2	0.908909081	0.964270094	0.838488596	0.995118128	0.99989309	0.859973633
RALA	0.908909081	0.841958822	0.926613927	0.951929772	0.99989309	0.86143104
CSPG4	0.908909081	0.855151723	0.909170161	0.971220023	0.99989309	0.862788295
FBP2	0.908909081	0.776126383	0.965416289	0.995118128	0.99989309	0.862860542
LRRC2	0.908909081	0.897375356	0.834904664	0.975529024	0.99989309	0.862912171
TMEM106B	0.908909081	0.8232491	0.849077738	0.999939566	0.99989309	0.864571107
PPP2R3A	0.908909081	0.780946412	0.869529186	0.999939566	0.99989309	0.866482265
EHBP1	0.908909081	0.80403784	0.858865175	0.998542029	0.99989309	0.868108836
LOC512627	0.908909081	0.823012828	0.944485232	0.969717497	0.99989309	0.868430302
DNAJC18	0.908909081	0.775921167	0.940785296	0.979043693	0.99989309	0.871780952
XCR1	0.908909081	0.816866927	0.957802423	0.937531834	0.99989309	0.87428828
LOC512953	0.908909081	0.780223998	0.836257071	0.934591536	0.99989309	0.882322567
CCDC9	0.908909081	0.907878616	0.847441894	0.934591536	0.99989309	0.886520861
LOC511531	0.908909081	0.885981636	0.843465477	0.955078298	0.99989309	0.886992214
PCGF5	0.908909081	0.920347742	0.878050811	0.969717497	0.99989309	0.887248119
KIF7	0.908909081	0.804875341	0.937930242	0.995800463	0.99989309	0.887457549
PSMC6	0.908909081	0.775921167	0.886282865	0.99879985	0.99989309	0.898791601
TNFAIP8L2	0.908909081	0.860449455	0.849077738	0.934591536	0.99989309	0.908939618
ZNF214	0.908909081	0.834869027	0.92332596	0.936835069	0.99989309	0.908939618
GOT2	0.908909081	0.778839781	0.843427302	0.963153458	0.99989309	0.908939618
CRYBA4	0.908909081	0.794355325	0.939683463	0.988949923	0.99989309	0.908939618
LOC112448524	0.908909081	0.80403784	0.864627365	0.999939566	0.99989309	0.911632967
DDIT3	0.908909081	0.785152571	0.879968243	0.928623745	0.99989309	0.911696156

ZNF653	0.908909081	0.785406526	0.88330023	0.984677276	0.99989309	0.911696156
ZNF879	0.908909081	0.855151723	0.834569848	0.995118128	0.99989309	0.911696156
LOC101909173	0.908909081	0.775921167	0.939683463	0.976133321	0.99989309	0.918255496
GATA3	0.908909081	0.832607032	0.850243032	0.925931623	0.99989309	0.922872513
ZNF599	0.908909081	0.769313383	0.85972234	0.999939566	0.99989309	0.923724782
LOC101908185	0.908909081	0.914162656	0.838080059	0.98470348	0.99989309	0.936439483
MRPS31	0.908909081	0.825215718	0.859796267	0.949947918	0.99989309	0.968245211
ATOX1	0.909034543	0.824676261	0.87214481	0.995118128	0.99989309	0.840150874
SUB1	0.909580927	0.779208532	0.982028743	0.990571725	0.988693274	0.864571107
ZHX3	0.909580927	0.796591413	0.965365809	0.940921143	0.99989309	0.865926817
PTOV1	0.909580927	0.888368527	0.844162118	0.940921143	0.99989309	0.868108836
PPP3R1	0.909580927	0.798286118	0.980734833	0.928264268	0.99989309	0.872224159
PRKACB	0.909580927	0.820796062	0.904682623	0.940921143	0.99989309	0.968059826
LOC107131940	0.909597131	0.852281996	0.843472659	0.994944652	0.99989309	0.915110778
TTC27	0.909626645	0.811559721	0.852758896	0.977470078	0.99989309	0.906529452
UBA3	0.909811576	0.786856496	0.836414702	0.961749608	0.99989309	0.962255684
PIGU	0.909846771	0.781444941	0.921225977	0.999939566	0.998276725	0.875441396
RPS23	0.909952445	0.893847586	0.838838955	0.999939566	0.99989309	0.839419627
LOC100850875	0.909952445	0.88418172	0.842605286	0.999939566	0.99989309	0.845969723
RBSN	0.909952445	0.83744789	0.862797408	0.996601241	0.99989309	0.878152258
LOC104968873	0.909952445	0.908709906	0.843427302	0.999939566	0.99989309	0.886992214
LOC112442263	0.909952445	0.795421065	0.855743966	0.999939566	0.99989309	0.890650763
EIF2B3	0.909979605	0.781154903	0.875669731	0.944558402	0.99989309	0.956610132
CNOT2	0.910151403	0.771098738	0.852758896	0.956154344	0.99989309	0.859323448
EBF2	0.910151403	0.806152572	0.922147089	0.992368879	0.99989309	0.885565926
NAA35	0.910151403	0.800441059	0.951607422	0.946344632	0.99989309	0.920521546
TMA16	0.910292757	0.820796062	0.916263052	0.973377993	0.99989309	0.84491737
CCSER2	0.910353884	0.821124254	0.862797408	0.999939566	0.99989309	0.837249706
ZBTB32	0.910353884	0.817434986	0.96880775	0.990049248	0.99989309	0.86143104
CHCHD3	0.910381783	0.83971475	0.870774839	0.96095927	0.99989309	0.868108836
TRMT44	0.910487848	0.771098738	0.970218925	0.926405541	0.99989309	0.926872824
TFG	0.910710673	0.90249621	0.872651958	0.943161565	0.99989309	0.95154358
NFU1	0.910887181	0.785163144	0.868936984	0.999939566	0.99989309	0.858042054
ANKRD26	0.910887181	0.779536052	0.939683463	0.99879985	0.99989309	0.866099047
NMNAT2	0.910887181	0.922331228	0.894449269	0.931268564	0.99989309	0.870209136
LRRC49	0.910887181	0.839399097	0.849077738	0.946344632	0.99989309	0.906529452
CDK5R1	0.910887181	0.82783173	0.843427302	0.988949923	0.99989309	0.908939618
CCDC102B	0.910887181	0.780641867	0.927456842	0.958879502	0.99989309	0.923064313

LOC790009	0.910984402	0.908192144	0.880204512	0.946344632	0.99989309	0.890957889
SP3	0.910992145	0.928119925	0.904682623	0.943323364	0.99989309	0.840150874
SMG9	0.910992145	0.963291559	0.930710426	0.93290597	0.99989309	0.845061099
LOC100848443	0.910992145	0.974986418	0.843427302	0.966882988	0.99989309	0.862788295
RCN1	0.910992145	0.779536052	0.892635738	0.999939566	0.99989309	0.875853001
OOEP	0.910992145	0.855634182	0.845237542	0.943323364	0.99989309	0.881039089
LHFPL1	0.910992145	0.80320642	0.853686558	0.999939566	0.99989309	0.894901464
WDR17	0.91111936	0.81808696	0.957802423	0.971220023	0.996250445	0.906529452
LOC107131769	0.91111936	0.786258422	0.850106729	0.943539568	0.99989309	0.859973633
SMAD9	0.91111936	0.901922021	0.843427302	0.999939566	0.99989309	0.873520548
COQ5	0.911490349	0.780290359	0.863172962	0.999939566	0.99989309	0.847256027
DEK	0.911516727	0.899429403	0.852758896	0.958879502	0.99989309	0.859447707
SPPL2B	0.911516727	0.831242188	0.906597758	0.967063428	0.99989309	0.912101047
STK39	0.911521179	0.831242188	0.878603452	0.943323364	0.996250445	0.983453112
LOC104974260	0.91167486	0.813371202	0.84710326	0.99879985	0.99989309	0.858538874
LMTK3	0.91167486	0.771098738	0.87146798	0.984968973	0.99989309	0.935655141
IGFBP4	0.91167486	0.792001722	0.868936984	0.99879985	0.99989309	0.937591162
OTUD4	0.911790314	0.843422478	0.892122482	0.977043589	0.99989309	0.845803016
FASTKD1	0.911815089	0.819130992	0.918576062	0.953508052	0.99989309	0.849772722
ZNF567	0.911815089	0.944605763	0.909170161	0.948120268	0.99989309	0.853233759
LAMC1	0.911815089	0.89539921	0.939683463	0.962775522	0.99989309	0.859973633
LOC100847171	0.911815089	0.883592664	0.944764946	0.956154344	0.99989309	0.869171706
LOC112448863	0.911815089	0.820816566	0.93145152	0.982140778	0.99989309	0.876189885
THOC7	0.911815089	0.775921167	0.939683463	0.949947918	0.99989309	0.893961809
LOC112444921	0.911827325	0.780641867	0.970904484	0.99879985	0.988693274	0.859487774
RTL8C	0.911906768	0.835130235	0.856812671	0.958879502	0.99989309	0.83849759
HTR1B	0.911906768	0.913625328	0.890801024	0.934591536	0.99989309	0.861949792
INCA1	0.911910464	0.822260127	0.853222116	0.998542029	0.99989309	0.940917403
ACTN4	0.912095712	0.838405969	0.917079579	0.999939566	0.988693274	0.886520861
AURKA	0.912095712	0.871113425	0.917876971	0.999939566	0.996250445	0.839419627
PPP4R4	0.912095712	0.808513267	0.929958748	0.999939566	0.996250445	0.865159042
SERTAD1	0.912095712	0.904077835	0.921319817	0.995800463	0.997981612	0.845866317
HINT1	0.912095712	0.789863026	0.882898206	0.987029316	0.99989309	0.842461327
ATG12	0.912095712	0.794309826	0.966157152	0.99879985	0.99989309	0.845803016
SERINC3	0.912095712	0.821588096	0.892122482	0.995800463	0.99989309	0.864219261
ACAA1	0.912095712	0.835276066	0.841180422	0.999939566	0.99989309	0.866253786
LOC104976274	0.912095712	0.771098738	0.987842204	0.943323364	0.99989309	0.868108836
INSIG2	0.912095712	0.832852787	0.942004125	0.983569039	0.99989309	0.893961809

LOC101904227	0.912095712	0.797209625	0.929303679	0.994309225	0.99989309	0.905220694
C10H14orf93	0.912095712	0.806439163	0.875669731	0.995118128	0.99989309	0.914233769
ZC3H15	0.912095712	0.804875341	0.894829955	0.953929637	0.99989309	0.947577032
AGT	0.912095712	0.81250936	0.875669731	0.931268564	0.99989309	0.980144374
PLCH2	0.912359651	0.820796062	0.87214481	0.958879502	0.99989309	0.908939618
ABHD11	0.912548322	0.851167599	0.843427302	0.99879985	0.99989309	0.890898561
NCAPH2	0.912548322	0.821588096	0.842478086	0.996601241	0.99989309	0.891000792
SLC25A35	0.912548322	0.795421065	0.846650632	0.990571725	0.99989309	0.907965478
TOMM34	0.912548322	0.79441406	0.870054336	0.931268564	0.99989309	0.998074562
FKBP2	0.912673807	0.87401231	0.909170161	0.966252636	0.99989309	0.853411611
LOC112442091	0.912940792	0.848986275	0.941593855	0.946344632	0.996250445	0.926872824
LOC101907797	0.912940792	0.804860126	0.850243032	0.934065538	0.99989309	0.90175726
ERGIC1	0.912940792	0.825856868	0.848646852	0.999939566	0.99989309	0.908939618
TMX3	0.912988956	0.859941746	0.894829955	0.96336515	0.99989309	0.867440033
LOC100337323	0.912988956	0.808517017	0.882898206	0.931268564	0.99989309	0.868108836
CHSY1	0.912988956	0.780944257	0.844065668	0.951929772	0.99989309	0.893961809
SOX15	0.912988956	0.843966059	0.850952986	0.943323364	0.99989309	0.989128955
LTBP3	0.913008195	0.800441059	0.921531707	0.99879985	0.99989309	0.839419627
HMGN5	0.913008195	0.785383224	0.939683463	0.971220023	0.99989309	0.847189049
S100B	0.913008195	0.841657074	0.883982433	0.963153458	0.99989309	0.847256027
SKA3	0.913141517	0.781287182	0.937270184	0.960986457	0.99989309	0.860827207
SLC17A5	0.913173596	0.890427603	0.885434576	0.995118128	0.998684149	0.896685501
GMDS	0.913173596	0.866803532	0.852758896	0.966882988	0.99989309	0.840150874
RBM17	0.913173596	0.820796062	0.957911933	0.992368879	0.99989309	0.840961242
BNIP3L	0.913173596	0.80403784	0.92153058	0.999939566	0.99989309	0.84491737
GLIPR2	0.913173596	0.835130235	0.850243032	0.962775522	0.99989309	0.845024318
INTS4	0.913173596	0.936365238	0.855743966	0.938899383	0.99989309	0.845580904
HSPD1	0.913173596	0.77414098	0.858674427	0.999939566	0.99989309	0.845803016
MLST8	0.913173596	0.885991833	0.843427302	0.947320716	0.99989309	0.846367069
LOC101905399	0.913173596	0.831242188	0.899195818	0.999939566	0.99989309	0.847256027
SPTBN4	0.913173596	0.855151723	0.849132683	0.934591536	0.99989309	0.856012024
TMEM33	0.913173596	0.849834516	0.922147089	0.951929772	0.99989309	0.859447707
LOC112441472	0.913173596	0.796591413	0.85404379	0.999939566	0.99989309	0.86143104
ARHGAP44	0.913173596	0.818806929	0.893863239	0.994309225	0.99989309	0.868430302
FLII	0.913173596	0.813230451	0.844065668	0.999939566	0.99989309	0.875441396
DHRSX	0.913173596	0.8232491	0.939091808	0.943323364	0.99989309	0.877601804
LOC112445033	0.913173596	0.838815395	0.883726795	0.944558402	0.99989309	0.886520861
PPP6R1	0.913173596	0.821588096	0.95712241	0.972367384	0.99989309	0.887457549

RPL23A	0.913173596	0.856222211	0.886282865	0.992137653	0.99989309	0.90525134
P2RY14	0.913173596	0.844031777	0.845237542	0.999939566	0.99989309	0.920834985
MYO19	0.913173596	0.780290359	0.883726795	0.992829869	0.99989309	0.922263023
QSER1	0.913173596	0.911106863	0.872397372	0.934591536	0.99989309	0.928123265
LOC101907998	0.913173596	0.886019911	0.891973662	0.940611812	0.99989309	0.936563391
LOC107132278	0.913173596	0.88418172	0.83722246	0.949947918	0.99989309	0.961506391
LOC785873	0.913173596	0.877420806	0.844065668	0.934591536	0.99989309	0.98016619
LOC508131	0.913655925	0.936099262	0.927460291	0.990049248	0.990044405	0.840150874
KERA	0.913687473	0.856914648	0.947475618	0.93290597	0.99989309	0.859973633
STAP1	0.913687473	0.936134921	0.855959143	0.956154344	0.99989309	0.912101047
NET1	0.913687473	0.778320571	0.878050811	0.939292364	0.99989309	0.981164039
DESI2	0.913765973	0.866803532	0.891455327	0.931989854	0.99989309	0.845024318
LOC107131607	0.913765973	0.795993324	0.896272282	0.999939566	0.99989309	0.85958325
ZBED6CL	0.913797864	0.852281996	0.962469014	0.943323364	0.99989309	0.86307729
TDRD7	0.913797864	0.785964175	0.858820545	0.99705757	0.99989309	0.886992214
LOC100847320	0.913830852	0.944420015	0.899195818	0.971220023	0.99989309	0.844622232
RNF4	0.913839264	0.866803532	0.8895568	0.956154344	0.99989309	0.840150874
LOC101906266	0.913839264	0.9347557	0.858865175	0.977470078	0.99989309	0.908939618
FAM69C	0.913839264	0.809875903	0.8895568	0.943323364	0.99989309	0.945618717
SLC7A10	0.914166118	0.810519292	0.939683463	0.999939566	0.99989309	0.859447707
CYB5A	0.914166118	0.787965627	0.845237542	0.995118128	0.99989309	0.873146162
PKP1	0.914326739	0.827609894	0.860010936	0.999939566	0.99989309	0.933678522
LOC101907213	0.914439863	0.810519292	0.891311171	0.933849042	0.99989309	0.882322567
DENND5B	0.914528411	0.790637585	0.865690138	0.999939566	0.99989309	0.847256027
HSPA14	0.914528411	0.804875341	0.860134949	0.992137653	0.99989309	0.86143104
LOC112448805	0.914580227	0.848798576	0.849077738	0.999939566	0.996250445	0.845803016
CCDC14	0.914580227	0.797748803	0.911743083	0.999939566	0.996250445	0.891000792
SLC11A2	0.914580227	0.792190608	0.953911996	0.946344632	0.996250445	0.952631575
DHRS4	0.914580227	0.90874814	0.844316758	0.990571725	0.99989309	0.839419627
EPSTI1	0.914580227	0.805132292	0.982223562	0.940779565	0.99989309	0.840150874
MCAT	0.914580227	0.824119076	0.843427302	0.99879985	0.99989309	0.840150874
ELOVL7	0.914580227	0.834316926	0.855743966	0.999939566	0.99989309	0.840150874
VPS33A	0.914580227	0.806152572	0.929345124	0.999939566	0.99989309	0.842187877
ADIPOR1	0.914580227	0.790637585	0.870054336	0.999939566	0.99989309	0.844622232
ENPEP	0.914580227	0.956751311	0.894414265	0.958849311	0.99989309	0.84491737
LANCL2	0.914580227	0.831242188	0.875669731	0.999939566	0.99989309	0.84491737
LSM6	0.914580227	0.780641867	0.988788601	0.933384737	0.99989309	0.845866317
CUTA	0.914580227	0.875022483	0.899195818	0.936573639	0.99989309	0.845969723

POLR2M	0.914580227	0.798799934	0.908927423	0.999939566	0.99989309	0.846367069
TTC12	0.914580227	0.864479552	0.889170207	0.999939566	0.99989309	0.847189049
GDAP1	0.914580227	0.843333021	0.911119875	0.999939566	0.99989309	0.849504243
ACTN1	0.914580227	0.834316926	0.934837042	0.982554614	0.99989309	0.849772722
GAPVD1	0.914580227	0.906538642	0.858972712	0.936573639	0.99989309	0.864440643
LOC100847604	0.914580227	0.806798154	0.993747807	0.934065538	0.99989309	0.865159042
TMEM132B	0.914580227	0.827459094	0.851048846	0.999939566	0.99989309	0.866482265
PYCR1	0.914580227	0.83744789	0.879968243	0.995800463	0.99989309	0.868108836
SACM1L	0.914580227	0.829690146	0.870054336	0.999939566	0.99989309	0.868108836
NR5A2	0.914580227	0.779536052	0.843427302	0.999939566	0.99989309	0.87114736
PFKFB1	0.914580227	0.847926598	0.844702985	0.949947918	0.99989309	0.872224159
DNAJC12	0.914580227	0.936134921	0.850106729	0.954423369	0.99989309	0.889367841
GSTM3	0.914580227	0.775411968	0.938195001	0.99879985	0.99989309	0.892192495
GCNT2	0.914580227	0.820796062	0.914685423	0.988140196	0.99989309	0.896877362
CTBP1	0.914580227	0.821124254	0.851617203	0.928623745	0.99989309	0.899551847
FAM53C	0.914580227	0.868963165	0.86360816	0.93471064	0.99989309	0.900168812
SWSAP1	0.914580227	0.774966356	0.911119875	0.996084773	0.99989309	0.902122572
LOC112443485	0.914580227	0.820988267	0.90002174	0.999939566	0.99989309	0.905858488
TEAD4	0.914580227	0.904539095	0.919108971	0.934174776	0.99989309	0.906178214
ETV2	0.914580227	0.836487169	0.894547778	0.956154344	0.99989309	0.909768235
LPCAT4	0.914580227	0.780290359	0.899195818	0.999939566	0.99989309	0.911470632
CDKL5	0.914580227	0.83744789	0.845237542	0.97006045	0.99989309	0.918220726
RMDN1	0.914580227	0.786258422	0.844065668	0.980002938	0.99989309	0.922229044
GOLGA5	0.914580227	0.785978032	0.84593343	0.99879985	0.99989309	0.926872824
ZDHHC6	0.914580227	0.786188116	0.914685423	0.98829462	0.99989309	0.956610132
RBM45	0.914580227	0.787707638	0.848444291	0.943323364	0.99989309	0.968059826
P2RY8	0.914580227	0.805157515	0.846782643	0.928623745	0.99989309	0.968245211
RAP2C	0.91458982	0.81105622	0.926613927	0.999939566	0.99989309	0.866253786
SYK	0.914599381	0.836487169	0.853686558	0.986229255	0.99989309	0.868108836
LOC112448271	0.914599381	0.820796062	0.961742979	0.992137653	0.99989309	0.875441396
SMG7	0.914599664	0.785383224	0.985222638	0.949947918	0.997488969	0.908939618
CTXN1	0.914599664	0.852463941	0.858865175	0.935163227	0.99989309	0.911696156
HCFC1R1	0.914648497	0.811209935	0.846650632	0.928836449	0.99989309	0.843747482
APELA	0.914648497	0.876739423	0.958420472	0.956154344	0.99989309	0.868108836
PLXNA4	0.914648497	0.88418172	0.939683463	0.931989854	0.99989309	0.920980794
FAM102B	0.914648497	0.813829586	0.860134949	0.999939566	0.99989309	0.933678522
SPATA5	0.914648497	0.851167599	0.844316758	0.952024081	0.99989309	0.986882901
GPATCH1	0.914734147	0.925360181	0.889578981	0.996601241	0.996250445	0.864219261

EIF3G	0.914734147	0.840313358	0.846650632	0.999939566	0.996369173	0.908939618
ARNT2	0.914745029	0.904764822	0.84311048	0.995118128	0.996250445	0.941925704
LOC101903997	0.914745029	0.924433008	0.880277219	0.9846464	0.99989309	0.872224159
VPS4A	0.914745029	0.842511984	0.865202013	0.995118128	0.99989309	0.908939618
SLC12A6	0.914745029	0.855151723	0.856589093	0.954423369	0.99989309	0.95676005
DHX37	0.914897529	0.823074953	0.879467505	0.981017664	0.99989309	0.926534325
FOXC1	0.915206766	0.911279338	0.9343664	0.936573639	0.99989309	0.870895425
ARHGAP23	0.915343212	0.799412839	0.868936984	0.946344632	0.99989309	0.942421464
LOC112448894	0.915407911	0.955353499	0.908823466	0.969717497	0.99989309	0.840150874
STIP1	0.915407911	0.77414098	0.853222116	0.942235206	0.99989309	0.858042054
LOC788201	0.915407911	0.977990642	0.853063495	0.959727203	0.99989309	0.86143104
PTBP3	0.915407911	0.874785912	0.89248682	0.943323364	0.99989309	0.868108836
LOC112443015	0.915407911	0.779256669	0.849406005	0.999939566	0.99989309	0.868430302
EVI2A	0.915407911	0.867722813	0.843427302	0.940921143	0.99989309	0.935655141
DQX1	0.915407911	0.812502616	0.875669731	0.980418209	0.99989309	0.948277018
NEURL2	0.915407911	0.861552326	0.842478086	0.958879502	0.99989309	0.961468136
SGK1	0.915577515	0.8554287	0.869529186	0.999939566	0.99989309	0.84763005
LOC104973252	0.915585468	0.823588167	0.905832059	0.981022805	0.99989309	0.929537775
FBXO24	0.915588416	0.795993324	0.998904268	0.946344632	0.99989309	0.842461327
CCDC183	0.915588416	0.852463941	0.960398536	0.973377993	0.99989309	0.856495922
TMEM150A	0.915588416	0.976925958	0.843427302	0.943323364	0.99989309	0.864219261
ACKR1	0.915588416	0.949008068	0.871859013	0.984090361	0.99989309	0.868108836
HRC	0.915588416	0.80403784	0.845237542	0.999939566	0.99989309	0.872175131
LOC615899	0.915588416	0.774697122	0.879515214	0.999939566	0.99989309	0.876189885
PRKAG2	0.915588416	0.876220907	0.869529186	0.974898047	0.99989309	0.949206487
CCNB1	0.915638789	0.796591413	0.942004125	0.999939566	0.99989309	0.84503021
PREPL	0.915638789	0.786258422	0.857943088	0.988140196	0.99989309	0.847256027
LOC112446691	0.915638789	0.859528714	0.929303679	0.969717497	0.99989309	0.86307729
CD2BP2	0.915638789	0.859941746	0.912023718	0.956154344	0.99989309	0.871231167
SDHAF2	0.915638789	0.866675066	0.87146798	0.956154344	0.99989309	0.883302043
LOC112442630	0.915638789	0.838405969	0.852758896	0.995118128	0.99989309	0.902660792
NLE1	0.915638789	0.843103375	0.868936984	0.999939566	0.99989309	0.905111733
DUS3L	0.915638789	0.816887624	0.960137022	0.93471064	0.99989309	0.908939618
WASF3	0.915638789	0.893639679	0.868936984	0.956154344	0.99989309	0.929667741
PTGER2	0.915638789	0.82919708	0.904682623	0.933991524	0.99989309	0.956610132
ZNF75D	0.915653209	0.852463941	0.941319062	0.956154344	0.99989309	0.864440643
CIPC	0.915653209	0.885181785	0.879215964	0.959727203	0.99989309	0.908939618
ICA1L	0.915653209	0.93453191	0.899195818	0.937781149	0.99989309	0.912380696

ITM2C	0.915809113	0.873098735	0.844065668	0.999939566	0.99989309	0.859973633
LOC508153	0.915809113	0.780892456	0.844065668	0.959786126	0.99989309	0.868430302
FBXL21	0.915809113	0.855634182	0.870054336	0.995118128	0.99989309	0.881439164
RPS6KC1	0.915809113	0.823371359	0.880277219	0.956874599	0.99989309	0.896115638
WIF1	0.915809113	0.852463941	0.885354035	0.934591536	0.99989309	0.908939618
SRCIN1	0.915813314	0.924066031	0.937979342	0.990059245	0.996250445	0.846943306
BRI3	0.915813314	0.835130235	0.882898206	0.999939566	0.998276725	0.86307729
KIAA1755	0.915813314	0.835536917	0.8895568	0.958879502	0.998599842	0.973494928
CD81	0.915813314	0.880711088	0.87531282	0.990571725	0.99989309	0.84045465
SHROOM3	0.915813314	0.795466803	0.987842204	0.990571725	0.99989309	0.841035892
MAPK15	0.915813314	0.94610099	0.939091808	0.944728796	0.99989309	0.84491737
TMSB15B	0.915813314	0.978453686	0.843304408	0.995800463	0.99989309	0.84491737
HDGFL2	0.915813314	0.834869027	0.849077738	0.998542029	0.99989309	0.84491737
RTL6	0.915813314	0.810519292	0.909170161	0.999939566	0.99989309	0.845591203
PDE6G	0.915813314	0.780061391	0.97381056	0.939292364	0.99989309	0.845866317
SLC25A23	0.915813314	0.797408811	0.891014698	0.99879985	0.99989309	0.845969723
NEK8	0.915813314	0.834007213	0.962209895	0.992137653	0.99989309	0.849769587
SERPINI1	0.915813314	0.949965815	0.870054336	0.943323364	0.99989309	0.85958325
LOC107132335	0.915813314	0.934531829	0.852758896	0.987143706	0.99989309	0.85958325
PTPRJ	0.915813314	0.854656328	0.905017554	0.951929772	0.99989309	0.86143104
CHURC1	0.915813314	0.782865867	0.962209895	0.992829869	0.99989309	0.862259367
UPK1B	0.915813314	0.916725199	0.947268948	0.956154344	0.99989309	0.86307729
LAMP1	0.915813314	0.837455678	0.899530079	0.999939566	0.99989309	0.86307729
TRPC4	0.915813314	0.951616411	0.878050811	0.946344632	0.99989309	0.867978129
POU2F1	0.915813314	0.951426413	0.871859013	0.94524673	0.99989309	0.868108836
ATP13A1	0.915813314	0.931248531	0.852758896	0.956154344	0.99989309	0.868108836
S100A11	0.915813314	0.813230451	0.897144948	0.959410646	0.99989309	0.868108836
MYO5C	0.915813314	0.780105406	0.9233932	0.999939566	0.99989309	0.868108836
PIDD1	0.915813314	0.798757458	0.98662331	0.965097245	0.99989309	0.872493553
NDUFB6	0.915813314	0.836487169	0.868936984	0.946344632	0.99989309	0.873271935
C23H6orf89	0.915813314	0.833277156	0.914685423	0.935742691	0.99989309	0.875441396
PSMD13	0.915813314	0.834741258	0.845336233	0.990300191	0.99989309	0.880404833
AFF1	0.915813314	0.782196458	0.846782643	0.999939566	0.99989309	0.880404833
GXYLT1	0.915813314	0.820959665	0.90936727	0.995800463	0.99989309	0.886087239
RUNDC3A	0.915813314	0.912112179	0.894874079	0.940921143	0.99989309	0.88667499
ADI1	0.915813314	0.841657074	0.842478086	0.998542029	0.99989309	0.886992214
TIGAR	0.915813314	0.827609894	0.97311093	0.933772215	0.99989309	0.887248119
GTF2IRD2	0.915813314	0.820796062	0.886282865	0.999939566	0.99989309	0.894951353

TPP1	0.915813314	0.796591413	0.880204512	0.999939566	0.99989309	0.896718993
LOC112448579	0.915813314	0.905977412	0.87146798	0.943323364	0.99989309	0.901689556
SLC10A3	0.915813314	0.870855329	0.843427302	0.992137653	0.99989309	0.902660792
LOC101906363	0.915813314	0.800441059	0.93050447	0.990571725	0.99989309	0.906065482
LOC101902808	0.915813314	0.808513267	0.86637689	0.958879502	0.99989309	0.908939618
KRAS	0.915813314	0.88311684	0.852758896	0.990059245	0.99989309	0.908939618
LOC514011	0.915813314	0.819448169	0.921612776	0.934591536	0.99989309	0.909765334
MOGS	0.915813314	0.835276066	0.849077738	0.995570827	0.99989309	0.910353954
LOC112444352	0.915813314	0.879100594	0.948304067	0.934591536	0.99989309	0.918220726
STMN1	0.915813314	0.886909612	0.863172962	0.991978984	0.99989309	0.921619793
LGALS12	0.915813314	0.828417266	0.922126495	0.986394228	0.99989309	0.926872824
TTLL1	0.915813314	0.889592783	0.849077738	0.95964071	0.99989309	0.930707319
LOC100848689	0.915813314	0.814136857	0.887391534	0.99879985	0.99989309	0.941889677
THNSL1	0.915813314	0.918306418	0.843427302	0.949947918	0.99989309	0.948277018
LOC100847326	0.915813314	0.823371359	0.879215964	0.980759595	0.99989309	0.972487907
STX6	0.915813314	0.813371202	0.87214481	0.968293092	0.99989309	0.98016619
UBQLN2	0.915813314	0.786258422	0.850751395	0.967063428	0.99989309	0.989313458
NUCKS1	0.916136101	0.885376959	0.988585687	0.942235206	0.996250445	0.847256027
BORA	0.916136101	0.80403784	0.976719499	0.99879985	0.99989309	0.842187877
STX5	0.916136101	0.847228759	0.891115063	0.999939566	0.99989309	0.845024318
SLC26A7	0.916136101	0.820796062	0.91546474	0.999939566	0.99989309	0.845628259
CDK13	0.916136101	0.818806929	0.849077738	0.956154344	0.99989309	0.86143104
H2AFZ	0.916136101	0.908183362	0.93145152	0.992137653	0.99989309	0.86143104
CEP170B	0.916136101	0.852377308	0.851383008	0.999939566	0.99989309	0.862788295
KMT2B	0.916136101	0.81808696	0.882898206	0.990571725	0.99989309	0.872224159
OAF	0.916136101	0.963291559	0.857289591	0.984968973	0.99989309	0.873520548
LOC104972888	0.916136101	0.813476598	0.843427302	0.998542029	0.99989309	0.873520548
RNF187	0.916136101	0.779765764	0.909170161	0.935144099	0.99989309	0.875441396
XPO5	0.916136101	0.857917583	0.85122003	0.999939566	0.99989309	0.875441396
ERLEC1	0.916136101	0.805003206	0.87146798	0.999939566	0.99989309	0.875441396
CIC	0.916136101	0.81808696	0.904691312	0.999939566	0.99989309	0.884097654
VPS13B	0.916136101	0.868963165	0.849077738	0.943323364	0.99989309	0.88667499
UBE4B	0.916136101	0.800441059	0.905832059	0.992137653	0.99989309	0.903579191
NEMP1	0.916136101	0.911106863	0.886373777	0.958879502	0.99989309	0.911284706
AOX4	0.916136101	0.841657074	0.908927423	0.994365581	0.99989309	0.913387533
FLYWCH2	0.916136101	0.780290359	0.856812671	0.982140778	0.99989309	0.919948949
GALNT14	0.916136101	0.965239369	0.862585131	0.935742691	0.99989309	0.934458012
LOC782950	0.916136101	0.940039358	0.843427302	0.943323364	0.99989309	0.945759045

ZSWIM8	0.916136101	0.852463941	0.884197487	0.951929772	0.99989309	0.948277018
SDAD1	0.916136101	0.81808696	0.845237542	0.998542029	0.99989309	0.964143145
RFX1	0.916136101	0.796591413	0.870054336	0.995118128	0.99989309	0.975019116
LOC786978	0.916214087	0.785978032	0.891455327	0.999939566	0.997981612	0.875441396
GK5	0.916214087	0.810519292	0.872397372	0.999939566	0.998276725	0.908182968
CSRP2	0.916214087	0.932838587	0.911547015	0.93471064	0.998276725	0.933577604
IRF9	0.916214087	0.839399097	0.985016728	0.934174776	0.99989309	0.840150874
PUM1	0.916214087	0.855151723	0.844316758	0.943323364	0.99989309	0.843747482
PDCD5	0.916214087	0.851167599	0.902141181	0.957928699	0.99989309	0.844352265
TOPORS	0.916214087	0.89272885	0.852758896	0.934591536	0.99989309	0.84491737
ERN1	0.916214087	0.790637585	0.878603452	0.956154344	0.99989309	0.84491737
KRR1	0.916214087	0.835130235	0.850106729	0.971220023	0.99989309	0.84491737
ANKRD2	0.916214087	0.852463941	0.843465477	0.99419346	0.99989309	0.84491737
IQCB1	0.916214087	0.913075885	0.865007702	0.942235206	0.99989309	0.845024318
KDM4B	0.916214087	0.798799934	0.886282865	0.995118128	0.99989309	0.845024318
EIF1AD	0.916214087	0.817066389	0.984192956	0.946344632	0.99989309	0.845803016
CCDC28B	0.916214087	0.86741371	0.985757941	0.949947918	0.99989309	0.845969723
PPP6R3	0.916214087	0.852463941	0.869529186	0.96095927	0.99989309	0.846367069
MPP7	0.916214087	0.972563829	0.908821787	0.968694679	0.99989309	0.846367069
PAK4	0.916214087	0.786188116	0.870920678	0.999939566	0.99989309	0.846879092
MGC148714	0.916214087	0.786856496	0.849077738	0.940921143	0.99989309	0.846943306
LOC101902809	0.916214087	0.780290359	0.929303679	0.999939566	0.99989309	0.847189049
JRK	0.916214087	0.851167599	0.86859301	0.951929772	0.99989309	0.847196533
APOE	0.916214087	0.81668556	0.844316758	0.999939566	0.99989309	0.847196533
INO80	0.916214087	0.80403784	0.879968243	0.936573639	0.99989309	0.847256027
BAG4	0.916214087	0.80403784	0.963111703	0.999939566	0.99989309	0.847256027
CEP104	0.916214087	0.810519292	0.8549878	0.999939566	0.99989309	0.849769587
OBSL1	0.916214087	0.822988334	0.880277219	0.999939566	0.99989309	0.849769587
CNNM4	0.916214087	0.787707638	0.904682623	0.965590385	0.99989309	0.849772722
MLF2	0.916214087	0.864821455	0.878050811	0.981022805	0.99989309	0.850612178
RNF152	0.916214087	0.80403784	0.907270422	0.999939566	0.99989309	0.853939716
LOC112441473	0.916214087	0.884711627	0.878603452	0.977642102	0.99989309	0.854493916
LOC783657	0.916214087	0.917634342	0.894829955	0.936573639	0.99989309	0.854831489
PANX2	0.916214087	0.922331228	0.845237542	0.999939566	0.99989309	0.854831489
NELFE	0.916214087	0.810096755	0.909522865	0.999939566	0.99989309	0.856474735
B2M	0.916214087	0.885181785	0.933740801	0.982140778	0.99989309	0.859323448
WDFY4	0.916214087	0.841629926	0.880277219	0.99879985	0.99989309	0.859447707
OXNAD1	0.916214087	0.912112179	0.847441894	0.934591536	0.99989309	0.85958325

SEC31B	0.916214087	0.844510795	0.856997596	0.958879502	0.99989309	0.859973633
CFLAR	0.916214087	0.779208532	0.847037539	0.999939566	0.99989309	0.859973633
MTFR1	0.916214087	0.788182825	0.917013548	0.999939566	0.99989309	0.859973633
LOC104971021	0.916214087	0.956958713	0.892122482	0.951177494	0.99989309	0.86143104
RAD54B	0.916214087	0.789863026	0.995304601	0.943323364	0.99989309	0.861807035
LOC100335828	0.916214087	0.907594268	0.893544538	0.998542029	0.99989309	0.862912171
ZNF146	0.916214087	0.889929882	0.845237542	0.943323364	0.99989309	0.86307729
TRIP13	0.916214087	0.809942574	0.968934792	0.990571725	0.99989309	0.86307729
SALL2	0.916214087	0.804860126	0.844065668	0.999939566	0.99989309	0.86307729
NR1H3	0.916214087	0.823588167	0.879968243	0.999939566	0.99989309	0.86307729
BECN1	0.916214087	0.804173299	0.8895568	0.999939566	0.99989309	0.86307729
SPATA6	0.916214087	0.972786652	0.849077738	0.992368879	0.99989309	0.863755488
LRRC42	0.916214087	0.785163144	0.877133001	0.999939566	0.99989309	0.864219261
LOC107132192	0.916214087	0.971712051	0.891455327	0.962142137	0.99989309	0.864371447
AP5S1	0.916214087	0.836487169	0.850106729	0.959727203	0.99989309	0.865159042
SH3BGRL	0.916214087	0.858046677	0.878050811	0.999939566	0.99989309	0.865159042
SDHB	0.916214087	0.820000472	0.853222116	0.999939566	0.99989309	0.865926817
PPP2R5A	0.916214087	0.808513267	0.896272282	0.999939566	0.99989309	0.865926817
PFDN1	0.916214087	0.820796062	0.889913771	0.977216943	0.99989309	0.868108836
AAAS	0.916214087	0.88311684	0.939683463	0.992137653	0.99989309	0.868108836
RHOJ	0.916214087	0.80931829	0.855743966	0.999939566	0.99989309	0.868108836
NCOA4	0.916214087	0.789834039	0.870054336	0.999939566	0.99989309	0.868108836
SLC2A12	0.916214087	0.883927618	0.849077738	0.963153458	0.99989309	0.868430302
LOC781100	0.916214087	0.828417266	0.892122482	0.958879502	0.99989309	0.869171706
NOL7	0.916214087	0.835276066	0.894829955	0.994309225	0.99989309	0.869171706
CAPNS1	0.916214087	0.804528018	0.899195818	0.99879985	0.99989309	0.869171706
ERBB4	0.916214087	0.898231471	0.87531282	0.997043216	0.99989309	0.870895425
DAG1	0.916214087	0.820796062	0.883726795	0.934591536	0.99989309	0.872224159
ZNF639	0.916214087	0.835832963	0.944485232	0.939292364	0.99989309	0.872224159
LOC786055	0.916214087	0.908709906	0.866690722	0.966203886	0.99989309	0.873520548
OPLAH	0.916214087	0.788848861	0.93050447	0.981022805	0.99989309	0.873520548
LOC101903913	0.916214087	0.852377308	0.987419404	0.940921143	0.99989309	0.875441396
NARF	0.916214087	0.823692881	0.886282865	0.975849638	0.99989309	0.875441396
LOC112443011	0.916214087	0.913171043	0.929958748	0.977470078	0.99989309	0.876534787
TMEM140	0.916214087	0.926435857	0.855074038	0.951606954	0.99989309	0.877730428
LONRF1	0.916214087	0.956288424	0.850020542	0.937531834	0.99989309	0.878152258
NDUFB5	0.916214087	0.823721951	0.86796189	0.972367384	0.99989309	0.880404833
PLEKHG1	0.916214087	0.965239369	0.844065668	0.969717497	0.99989309	0.884387948

CLSPN	0.916214087	0.810488979	0.933963997	0.999939566	0.99989309	0.88609792
PLXND1	0.916214087	0.786188116	0.858972712	0.999939566	0.99989309	0.886210131
IL21R	0.916214087	0.973649843	0.880277219	0.934591536	0.99989309	0.88676448
RASL10B	0.916214087	0.842650422	0.849077738	0.96095927	0.99989309	0.886992214
DNAJB11	0.916214087	0.846959498	0.845237542	0.972367384	0.99989309	0.886992214
SLC35E1	0.916214087	0.78070503	0.939683463	0.995800463	0.99989309	0.886992214
FGL1	0.916214087	0.81808696	0.850106729	0.999939566	0.99989309	0.887248119
LZTS2	0.916214087	0.791922306	0.864236102	0.999939566	0.99989309	0.887248119
NFIA	0.916214087	0.796591413	0.962209895	0.948172741	0.99989309	0.888396511
CMBL	0.916214087	0.818435706	0.858972712	0.95964071	0.99989309	0.888617204
ATXN7	0.916214087	0.978453686	0.846650632	0.934591536	0.99989309	0.888695935
URI1	0.916214087	0.817211917	0.870054336	0.999939566	0.99989309	0.892466834
SETX	0.916214087	0.897327046	0.899816306	0.981022805	0.99989309	0.893400499
AGO3	0.916214087	0.792190608	0.844065668	0.93471064	0.99989309	0.893961809
RAD54L2	0.916214087	0.862527737	0.89145542	0.974400952	0.99989309	0.893961809
ZNF275	0.916214087	0.828417266	0.856997596	0.990049248	0.99989309	0.893961809
LOC107131331	0.916214087	0.890503475	0.89145542	0.969717497	0.99989309	0.894438452
CHCHD5	0.916214087	0.938791776	0.875669731	0.934591536	0.99989309	0.895218005
CCNL1	0.916214087	0.834869027	0.921135532	0.940921143	0.99989309	0.895218005
LOC100296627	0.916214087	0.899387334	0.901550297	0.953648387	0.99989309	0.897633315
PSMB3	0.916214087	0.874441163	0.868936984	0.958879502	0.99989309	0.897818829
RNF167	0.916214087	0.841044548	0.879515214	0.998542029	0.99989309	0.898018497
RABEPK	0.916214087	0.824016465	0.939689648	0.989391869	0.99989309	0.90259329
ELOVL5	0.916214087	0.838843184	0.845425011	0.949947918	0.99989309	0.905705129
LOC101905897	0.916214087	0.784936368	0.844065668	0.999939566	0.99989309	0.906963595
LOC112448474	0.916214087	0.862267429	0.850020542	0.999939566	0.99989309	0.907965478
PPCDC	0.916214087	0.80403784	0.88330023	0.937781149	0.99989309	0.908182968
AFMID	0.916214087	0.90249621	0.883726795	0.934591536	0.99989309	0.908939618
WDR55	0.916214087	0.855151723	0.846650632	0.948120268	0.99989309	0.908939618
TCEANC	0.916214087	0.842667989	0.879215964	0.959727203	0.99989309	0.908939618
NUP85	0.916214087	0.854362191	0.906804686	0.995118128	0.99989309	0.908939618
POFUT2	0.916214087	0.782342112	0.868936984	0.999939566	0.99989309	0.910920424
MGME1	0.916214087	0.780290359	0.976719499	0.943591298	0.99989309	0.911696156
MPZL1	0.916214087	0.832852787	0.877998684	0.992368879	0.99989309	0.912101047
EPHX3	0.916214087	0.864821455	0.89007492	0.958879502	0.99989309	0.912380696
MKRN1	0.916214087	0.792090907	0.877133001	0.999939566	0.99989309	0.91299942
MSRB2	0.916214087	0.816119178	0.853686558	0.99879985	0.99989309	0.914440253
SNCAIP	0.916214087	0.843103375	0.849132683	0.999939566	0.99989309	0.917524589

MAP1A	0.916214087	0.808513267	0.856769954	0.999939566	0.99989309	0.917968123
MBD3	0.916214087	0.882275117	0.892122482	0.971220023	0.99989309	0.918668679
TIMM29	0.916214087	0.784020152	0.942004125	0.995800463	0.99989309	0.920849243
LATS2	0.916214087	0.838843184	0.907457888	0.995800463	0.99989309	0.920942379
KCNJ16	0.916214087	0.783052602	0.855743966	0.959111196	0.99989309	0.920980794
LOC616720	0.916214087	0.877180988	0.870920678	0.956154344	0.99989309	0.924845138
ZBTB26	0.916214087	0.778839781	0.880277219	0.982140778	0.99989309	0.926576964
BCDIN3D	0.916214087	0.840444202	0.933102499	0.949947918	0.99989309	0.932492907
GEMIN7	0.916214087	0.780290359	0.886373777	0.992741182	0.99989309	0.934451585
CHD5	0.916214087	0.8554287	0.844065668	0.946668073	0.99989309	0.934775196
RASD2	0.916214087	0.785406526	0.843427302	0.999939566	0.99989309	0.935651847
LOC100847831	0.916214087	0.796591413	0.846650632	0.958879502	0.99989309	0.935655141
PUS7L	0.916214087	0.823588167	0.845237542	0.969717497	0.99989309	0.936563391
CYTH4	0.916214087	0.886943153	0.863172962	0.937531834	0.99989309	0.937408144
URB2	0.916214087	0.780290359	0.87242852	0.97136787	0.99989309	0.941785987
NDUFA4L2	0.916214087	0.795534928	0.844065668	0.998542029	0.99989309	0.942455807
GPR37	0.916214087	0.780290359	0.856997596	0.999939566	0.99989309	0.942800958
NANP	0.916214087	0.877180988	0.902548377	0.93471064	0.99989309	0.945759045
SCNN1A	0.916214087	0.78070503	0.930262306	0.963829131	0.99989309	0.949206487
PDE7A	0.916214087	0.885981636	0.879726737	0.943323364	0.99989309	0.95470473
LOC781646	0.916214087	0.80403784	0.8895568	0.99879985	0.99989309	0.95470473
ELAC2	0.916214087	0.804875341	0.877133001	0.975529024	0.99989309	0.954935833
EXOC1	0.916214087	0.786829125	0.873750661	0.967063428	0.99989309	0.956610132
SET	0.916214087	0.795466803	0.930710426	0.956154344	0.99989309	0.957877422
CTTN	0.916214087	0.80320642	0.87146798	0.939292364	0.99989309	0.961506391
HOMER1	0.916214087	0.790964441	0.920449324	0.943323364	0.99989309	0.961506391
MALT1	0.916214087	0.817040135	0.922147089	0.948547383	0.99989309	0.968245211
LOC112442598	0.916214087	0.781287182	0.85665551	0.990059245	0.99989309	0.97356849
TEX12	0.916214087	0.81808696	0.88330023	0.951606954	0.99989309	0.977961448
KCNH3	0.916214087	0.827609894	0.87146798	0.971220023	0.99989309	0.983427394
LOC100296205	0.916214087	0.820796062	0.856997596	0.969717497	0.99989309	0.989313458
LOC101904691	0.916214087	0.795302167	0.849077738	0.9846464	0.99989309	0.995145616
SNX30	0.916415387	0.820796062	0.921420528	0.949947918	0.99989309	0.844622232
FBLN5	0.916415387	0.945028184	0.870054336	0.957928699	0.99989309	0.855653734
LOC101908339	0.916415387	0.916894642	0.851617203	0.996601241	0.99989309	0.856870625
RAB34	0.916415387	0.809783026	0.883334551	0.999939566	0.99989309	0.857718781
CLIC2	0.916415387	0.841044548	0.908165509	0.999939566	0.99989309	0.858042054
TRNP1	0.916415387	0.81808696	0.953198168	0.995800463	0.99989309	0.860808821

DNAH1	0.916415387	0.782096871	0.972485306	0.943323364	0.99989309	0.864440643
C8G	0.916415387	0.785406526	0.982659943	0.944558402	0.99989309	0.869171706
ABCC11	0.916415387	0.785406526	0.850952986	0.999939566	0.99989309	0.869562039
LOC112447523	0.916415387	0.871412404	0.926613927	0.99879985	0.99989309	0.872175131
PRADC1	0.916415387	0.866803532	0.852758896	0.956154344	0.99989309	0.872224159
IGSF8	0.916415387	0.826739573	0.939683463	0.999939566	0.99989309	0.877813306
LOC107132952	0.916415387	0.80403784	0.929958748	0.992137653	0.99989309	0.883329104
OXR1	0.916415387	0.803903736	0.862585131	0.999939566	0.99989309	0.88494083
ANGPT4	0.916415387	0.913075885	0.87214481	0.998542029	0.99989309	0.893961809
SOAT1	0.916415387	0.832852787	0.916526594	0.999939566	0.99989309	0.894974898
BAMBI	0.916415387	0.835408071	0.870920678	0.999939566	0.99989309	0.895218005
MEGF10	0.916415387	0.883378562	0.844065668	0.999939566	0.99989309	0.899551847
IFT122	0.916415387	0.821588096	0.898889441	0.956154344	0.99989309	0.902660792
PLAA	0.916415387	0.858919611	0.878050811	0.999939566	0.99989309	0.908939618
GJA5	0.916415387	0.785163144	0.958420472	0.977470078	0.99989309	0.910137184
UNC13C	0.916415387	0.792398364	0.89145542	0.939391201	0.99989309	0.911475796
LOC104975027	0.916415387	0.820796062	0.880204512	0.943539568	0.99989309	0.917968123
FAM219A	0.916415387	0.80403784	0.940831329	0.990049248	0.99989309	0.920942379
DDR2	0.916415387	0.871858734	0.870054336	0.992368879	0.99989309	0.928799366
LOC789352	0.916415387	0.820000472	0.85972234	0.99879985	0.99989309	0.930707319
LOC100300881	0.916415387	0.86778334	0.926613927	0.951929772	0.99989309	0.940683751
CCDC25	0.916415387	0.80403784	0.883726795	0.994309225	0.99989309	0.941445869
C29H11orf98	0.916415387	0.840874016	0.903453422	0.943323364	0.99989309	0.974086897
MYO18A	0.916457134	0.804528018	0.925442336	0.995800463	0.99989309	0.927573435
RAD51AP1	0.916569669	0.803903736	0.921225977	0.999939566	0.99989309	0.86143104
LDLRAD3	0.916607095	0.809254208	0.875669731	0.992137653	0.99989309	0.893961809
UXS1	0.916698416	0.790637585	0.845336233	0.999939566	0.99989309	0.876009151
USP22	0.916760471	0.783416338	0.953198168	0.99879985	0.99989309	0.854493916
PAIP2	0.916760471	0.836487169	0.926727295	0.999939566	0.99989309	0.886992214
WDR89	0.9167934	0.803903736	0.851617203	0.955560327	0.99989309	0.98016619
CLDN1	0.91693347	0.881642454	0.939683463	0.964413123	0.99989309	0.84503021
PROX1	0.91693347	0.810423202	0.87146798	0.999939566	0.99989309	0.852640131
PLEC	0.91693347	0.824676261	0.921225977	0.99879985	0.99989309	0.86143104
MGAT3	0.91693347	0.928119925	0.872397372	0.995946943	0.99989309	0.862145862
MGP	0.91693347	0.899387334	0.870054336	0.956142205	0.99989309	0.86307729
NPY5R	0.91693347	0.965239369	0.914489705	0.962142137	0.99989309	0.86307729
HMGN2	0.91693347	0.85749413	0.941319062	0.995118128	0.99989309	0.869171706
MTIF3	0.91693347	0.827310227	0.880391595	0.956154344	0.99989309	0.928374551

SKA2	0.916938244	0.791862736	0.846650632	0.999939566	0.99989309	0.858042054
GABBR2	0.916938244	0.798491587	0.844065668	0.973207466	0.99989309	0.974379635
C3H1orf123	0.917065616	0.808513267	0.884189563	0.966882988	0.99989309	0.853233759
UPF3A	0.917147158	0.855979085	0.868533597	0.999939566	0.99989309	0.875441396
TNK2	0.917147158	0.879100594	0.872470014	0.992692773	0.99989309	0.901422892
LOC104974269	0.917147158	0.785383224	0.856812671	0.999939566	0.99989309	0.979792753
UQCRH	0.917189227	0.79441406	0.847037539	0.992137653	0.99989309	0.84491737
UBXN10	0.917189227	0.808513267	0.885354035	0.999939566	0.99989309	0.84491737
SSBP4	0.917189227	0.80931829	0.957578315	0.999939566	0.99989309	0.855653734
PRIMPOL	0.917189227	0.803995245	0.935247964	0.990491321	0.99989309	0.86143104
LOC101906012	0.917189227	0.822173925	0.872397372	0.960878389	0.99989309	0.862403446
MRPL9	0.917189227	0.851167599	0.856812671	0.995118128	0.99989309	0.865159042
UBE3C	0.917189227	0.788961546	0.886282865	0.959624281	0.99989309	0.866482265
POLR1D	0.917189227	0.790637585	0.850092739	0.999939566	0.99989309	0.896877362
SPATA13	0.917189227	0.794823268	0.879726737	0.982140778	0.99989309	0.943685681
TSKS	0.917225501	0.835130235	0.879215964	0.958879502	0.99989309	0.845803016
SLC10A5	0.917288489	0.80403784	0.873750661	0.946344632	0.99989309	0.936192693
MTRR	0.917516765	0.975287816	0.846650632	0.990049248	0.99989309	0.895218005
FNIP2	0.917666952	0.802811033	0.968455468	0.99879985	0.99989309	0.847352863
ENHO	0.917781233	0.887725794	0.908821787	0.935247713	0.99989309	0.875441396
SUGCT	0.917781233	0.845256475	0.892122482	0.999939566	0.99989309	0.887248119
CYB561A3	0.917781233	0.926435857	0.870911433	0.951929772	0.99989309	0.95470473
MGST2	0.917827635	0.835276066	0.86637689	0.999939566	0.99989309	0.86307729
TBC1D1	0.917827635	0.82750276	0.929958748	0.999939566	0.99989309	0.868108836
C7	0.917827635	0.790637585	0.882898206	0.999939566	0.99989309	0.895218005
SLC25A28	0.917827635	0.908709906	0.89265635	0.965097245	0.99989309	0.928374551
PROZ	0.917965615	0.820796062	0.985552223	0.946344632	0.99989309	0.847196533
ISOC1	0.917965615	0.835276066	0.870054336	0.990571725	0.99989309	0.858042054
FUT8	0.917965615	0.823074953	0.857065866	0.999939566	0.99989309	0.859447707
PRKG1	0.917965615	0.903820329	0.922147089	0.995118128	0.99989309	0.859973633
RBP7	0.917965615	0.920423305	0.879515214	0.994365581	0.99989309	0.866007808
LRRC8A	0.917965615	0.95320487	0.845237542	0.988892611	0.99989309	0.869171706
ZNF236	0.917965615	0.821124254	0.932952899	0.95175364	0.99989309	0.880404833
ANKRD44	0.917965615	0.886943153	0.845425011	0.958879502	0.99989309	0.884387948
RAB11FIP5	0.917965615	0.842565421	0.846650632	0.999939566	0.99989309	0.886992214
LOC112445011	0.917965615	0.953748191	0.911743083	0.939292364	0.99989309	0.895194567
LOC100849681	0.917965615	0.798799934	0.933102499	0.959786126	0.99989309	0.908939618
STEAP3	0.917965615	0.856658152	0.845237542	0.999939566	0.99989309	0.908939618

TTYH2	0.917965615	0.963478319	0.880277219	0.951929772	0.99989309	0.911696156
DOK3	0.917965615	0.794823268	0.87242852	0.981022805	0.99989309	0.969545443
USP48	0.918081971	0.785163144	0.855743966	0.944558402	0.99989309	0.853233759
KCNH4	0.918081971	0.91994024	0.886176337	0.999939566	0.99989309	0.859973633
ZNF93	0.918081971	0.798799934	0.92582321	0.999939566	0.99989309	0.862796381
HORMAD2	0.918081971	0.81808696	0.863172962	0.985181062	0.99989309	0.871780952
MFSD10	0.918081971	0.886732397	0.951092876	0.956154344	0.99989309	0.888396511
DIABLO	0.918081971	0.810519292	0.921122957	0.96095927	0.99989309	0.889159914
CTU1	0.918081971	0.80931829	0.883726795	0.956154344	0.99989309	0.911632967
OMD	0.918081971	0.783416338	0.88950849	0.999939566	0.99989309	0.942421464
UBE4A	0.918081971	0.827609894	0.845336233	0.999939566	0.99989309	0.944606747
RPS6KB1	0.918081971	0.835408071	0.850046649	0.999939566	0.99989309	0.949206487
HECTD4	0.918081971	0.871359854	0.858972712	0.969717497	0.99989309	0.961506391
FAM210A	0.918081971	0.812575653	0.850339392	0.990049248	0.99989309	0.986389023
LOC107132724	0.918172905	0.851167599	0.915066299	0.999939566	0.99989309	0.858042054
SKP1	0.918172905	0.790637585	0.93145152	0.999939566	0.99989309	0.868108836
ZNF140	0.918172905	0.818806929	0.878050811	0.999939566	0.99989309	0.895218005
AAMDC	0.918216482	0.803928056	0.892258313	0.99879985	0.99989309	0.875441396
EIF4EBP2	0.918395682	0.798799934	0.878603452	0.999939566	0.99989309	0.857727168
PDE3B	0.918395682	0.821588096	0.874412734	0.999939566	0.99989309	0.86143104
GSTT1	0.918395682	0.86415368	0.869529186	0.943323364	0.99989309	0.874394916
LOC101905367	0.918395682	0.899387334	0.846650632	0.973377993	0.99989309	0.880404833
ZNF692	0.918444555	0.798984731	0.994425789	0.963153458	0.99989309	0.847091529
LOC785408	0.918444555	0.818806929	0.983706867	0.95175364	0.99989309	0.901422892
LOC112444328	0.918458277	0.86696578	0.917092799	0.990571725	0.99989309	0.906529452
CORO6	0.918619045	0.86287074	0.939683463	0.976430606	0.99989309	0.858777168
ATXN7L3B	0.918733028	0.785978032	0.942642657	0.949947918	0.99989309	0.912376736
HOXC5	0.918848141	0.87795323	0.941593855	0.955687359	0.99989309	0.845969723
RILPL2	0.918848141	0.79449527	0.850952986	0.999939566	0.99989309	0.849504243
LOC537017	0.918848141	0.786380465	0.850106729	0.992137653	0.99989309	0.856870625
TINF2	0.918848141	0.823588167	0.878258655	0.999939566	0.99989309	0.868108836
TSPAN6	0.918848141	0.980639049	0.856812671	0.967063428	0.99989309	0.889159914
RAB8A	0.918848141	0.837540819	0.926618528	0.946344632	0.99989309	0.961506391
GGPS1	0.918954363	0.823012828	0.877133001	0.999939566	0.99989309	0.869857598
TFPI	0.918954363	0.876057665	0.872444023	0.999939566	0.99989309	0.891000792
FAM110B	0.918954363	0.950464104	0.855379431	0.940921143	0.99989309	0.893961809
PDZD8	0.918954363	0.808513267	0.92921168	0.990571725	0.99989309	0.896685501
LOC618071	0.918954363	0.871412404	0.855743966	0.971220023	0.99989309	0.926872824

PRMT2	0.918954363	0.789863026	0.853222116	0.999939566	0.99989309	0.926872824
PTS	0.91902794	0.80055527	0.874092011	0.999939566	0.99989309	0.845024318
PTPN11	0.91902794	0.835276066	0.90002174	0.999939566	0.99989309	0.845803016
SNTB1	0.91902794	0.785406526	0.966157152	0.951929772	0.99989309	0.845866317
LOC112446716	0.91902794	0.955798423	0.865007702	0.999939566	0.99989309	0.84815789
CARM1	0.91902794	0.840874016	0.882890706	0.999939566	0.99989309	0.848231635
ZDHHC16	0.91902794	0.886672311	0.932435007	0.994365581	0.99989309	0.85958325
LOC513767	0.91902794	0.851167599	0.868936984	0.999939566	0.99989309	0.86143104
MPZL2	0.91902794	0.796591413	0.961742979	0.990571725	0.99989309	0.86252043
SAMD11	0.91902794	0.796591413	0.882898206	0.999939566	0.99989309	0.875072542
LOC784127	0.91902794	0.861552326	0.926613927	0.999939566	0.99989309	0.875441396
DYNLT1	0.91902794	0.798286118	0.975939166	0.980418209	0.99989309	0.876009151
LOC107133075	0.91902794	0.883378562	0.870054336	0.999939566	0.99989309	0.887457549
ZNF668	0.91902794	0.823187795	0.862585131	0.998542029	0.99989309	0.893961809
LOC112444909	0.91902794	0.805157515	0.958420472	0.947615714	0.99989309	0.895287865
TMEM88B	0.91902794	0.850825188	0.918576062	0.990571725	0.99989309	0.9071195
UQCRC2	0.91902794	0.786258422	0.849077738	0.95964071	0.99989309	0.908939618
NECTIN4	0.91902794	0.970179915	0.863172962	0.940921143	0.99989309	0.910353954
SH2D3C	0.91902794	0.884444922	0.860430781	0.973377993	0.99989309	0.914131819
DAGLB	0.91902794	0.880674338	0.917876971	0.951929772	0.99989309	0.916385428
MAN1B1	0.91902794	0.840078234	0.858169115	0.99879985	0.99989309	0.922263023
DSTYK	0.91902794	0.899163209	0.852758896	0.984100122	0.99989309	0.927584908
KCNIP3	0.91902794	0.842797231	0.903165947	0.995687815	0.99989309	0.935163166
ESRRG	0.91902794	0.885181785	0.85792584	0.946344632	0.99989309	0.948277018
LOC510454	0.91902794	0.829476146	0.846009738	0.992368879	0.99989309	0.98609047
PPP1R8	0.919068268	0.800441059	0.942314296	0.956154344	0.99989309	0.941806801
CCL3	0.919205216	0.798757458	0.951742137	0.994309225	0.99989309	0.865926817
LOC112445927	0.919205216	0.918976923	0.87214481	0.952020618	0.99989309	0.957877422
ZNF133	0.919495359	0.809942574	0.92153058	0.971220023	0.99989309	0.937591162
DPH5	0.919495359	0.909237134	0.852758896	0.990049248	0.99989309	0.948721648
MTMR10	0.919629823	0.78897006	0.891014698	0.999939566	0.99989309	0.86143104
LYPD1	0.919629823	0.842011139	0.845336233	0.956154344	0.99989309	0.887457549
WDPCP	0.919629823	0.806820745	0.929483934	0.995118128	0.99989309	0.912376736
PRR5	0.919652221	0.788182825	0.906804686	0.999939566	0.99989309	0.902660792
COX18	0.919652221	0.795421065	0.886282865	0.99879985	0.99989309	0.946344585
LOC100336602	0.920161006	0.881079118	0.87146798	0.999939566	0.99989309	0.880404833
FAM19A5	0.920161006	0.835408071	0.894414265	0.958879502	0.99989309	0.948277018
PLEKHM3	0.920161006	0.825856868	0.883726795	0.99879985	0.99989309	0.95470473

DDX39A	0.920202607	0.812502616	0.870054336	0.988915501	0.99989309	0.932440538
PEX13	0.92045235	0.804875341	0.945124666	0.982140778	0.99989309	0.912101047
RIN1	0.920506192	0.786380465	0.953045982	0.95964071	0.99989309	0.888617204
MPI	0.920506192	0.812960487	0.845425011	0.992137653	0.99989309	0.897818829
COG1	0.920555987	0.910232617	0.93710335	0.990571725	0.99989309	0.86143104
PDGFRB	0.920617649	0.821588096	0.877133001	0.999939566	0.99989309	0.873520548
HYI	0.920617649	0.965239369	0.880277219	0.956154344	0.99989309	0.880383788
TUBGCP2	0.920617649	0.851167599	0.855743966	0.978207036	0.99989309	0.897633315
GPR161	0.920617649	0.823692881	0.87214481	0.999939566	0.99989309	0.905220694
CASP1	0.920617649	0.97799108	0.857486511	0.95175364	0.99989309	0.911696156
FAM124A	0.920617649	0.798799934	0.87146798	0.995118128	0.99989309	0.937408144
SLCO2B1	0.920665837	0.921280256	0.849077738	0.998542029	0.99989309	0.921381434
ENOX1	0.920679354	0.824676261	0.903930649	0.978220863	0.99989309	0.908939618
KLB	0.921001274	0.855861376	0.858972712	0.999939566	0.99989309	0.854831489
LOC782688	0.92108208	0.841958822	0.897144948	0.988949923	0.99989309	0.928442671
LOC100337108	0.921379809	0.988725451	0.858865175	0.995118128	0.99989309	0.845969723
LOC112448082	0.921379809	0.800441059	0.863172962	0.999939566	0.99989309	0.848704126
LOC789157	0.921379809	0.789863026	0.931259164	0.999939566	0.99989309	0.857659295
UBE2G1	0.921379809	0.793795775	0.980442556	0.995118128	0.99989309	0.859973633
PEF1	0.921379809	0.804860126	0.885354035	0.995946943	0.99989309	0.86143104
KLHL3	0.921379809	0.936134921	0.884325235	0.999939566	0.99989309	0.86307729
LRRFIP2	0.921379809	0.862527737	0.950841028	0.973690015	0.99989309	0.865926817
LSM8	0.921379809	0.810519292	0.932690637	0.971220023	0.99989309	0.867440033
UCHL1	0.921379809	0.811206862	0.87146798	0.949947918	0.99989309	0.868108836
ITSN1	0.921379809	0.831242188	0.869455175	0.949947918	0.99989309	0.868430302
AHCTF1	0.921379809	0.892078189	0.868936984	0.948891906	0.99989309	0.86921618
LOC783680	0.921379809	0.921280256	0.918497922	0.988949923	0.99989309	0.873271935
LOC789258	0.921379809	0.904764822	0.871859013	0.943323364	0.99989309	0.875441396
PAXX	0.921379809	0.946246178	0.853646409	0.990049248	0.99989309	0.87900534
IQCA1L	0.921379809	0.850723096	0.993278977	0.939292364	0.99989309	0.882322567
LOC101904749	0.921379809	0.935324849	0.908821939	0.984618049	0.99989309	0.88667499
CLDN4	0.921379809	0.892859755	0.939683463	0.943323364	0.99989309	0.889159914
CA5B	0.921379809	0.823692881	0.870054336	0.997612654	0.99989309	0.892466834
SPIN1	0.921379809	0.825215718	0.917876971	0.96095927	0.99989309	0.893961809
ERLIN1	0.921379809	0.837455678	0.939612562	0.99879985	0.99989309	0.895218005
SORD	0.921379809	0.820796062	0.850243032	0.99879985	0.99989309	0.895287865
SIPA1L3	0.921379809	0.803903736	0.987433396	0.95964071	0.99989309	0.897818829
MCOLN1	0.921379809	0.924799142	0.87146798	0.995118128	0.99989309	0.9071195

MAP4K5	0.921379809	0.91796081	0.850092739	0.996601241	0.99989309	0.908939618
E4F1	0.921379809	0.816597835	0.929303679	0.971220023	0.99989309	0.90952934
MLEC	0.921379809	0.821124254	0.873750661	0.948120268	0.99989309	0.910619304
PXK	0.921379809	0.805110306	0.976719499	0.947581168	0.99989309	0.912101047
NDUFS1	0.921379809	0.805757597	0.848890018	0.946344632	0.99989309	0.916385428
EGFL7	0.921379809	0.897327046	0.879515214	0.958879502	0.99989309	0.917968123
PSTPIP2	0.921379809	0.848986275	0.878603452	0.978403606	0.99989309	0.917968123
DCUN1D1	0.921379809	0.808513267	0.917876971	0.99419346	0.99989309	0.918220726
MARK1	0.921379809	0.881079118	0.877998684	0.992368879	0.99989309	0.921381434
PHC2	0.921379809	0.806076007	0.88330023	0.958879502	0.99989309	0.924342462
WDR54	0.921379809	0.80931829	0.906925281	0.999939566	0.99989309	0.926335372
MYO1F	0.921379809	0.885981636	0.845425011	0.943323364	0.99989309	0.926534325
LOC112441506	0.921379809	0.792190608	0.868936984	0.990571725	0.99989309	0.939966658
RNF219	0.921379809	0.817040135	0.939683463	0.982140778	0.99989309	0.942455807
BFSP2	0.921379809	0.841958822	0.87146798	0.99879985	0.99989309	0.951047219
PLA2G6	0.921379809	0.901922021	0.864165322	0.962142137	0.99989309	0.96074434
SPATA16	0.921379809	0.842797231	0.853686558	0.990571725	0.99989309	0.963996128
TMEM234	0.921379809	0.796591413	0.861835782	0.956154344	0.99989309	0.969981358
LOC101902918	0.921379809	0.840313358	0.852758896	0.956154344	0.99989309	0.988323336
SNRNP35	0.921379809	0.808513267	0.852758896	0.971220023	0.99989309	0.998636142
LOC784451	0.921492802	0.795171452	0.848646852	0.999939566	0.99989309	0.866612872
USP11	0.921536136	0.842797231	0.850020542	0.95964071	0.99989309	0.914233769
ULK1	0.92156705	0.810760953	0.905017554	0.995800463	0.99989309	0.929042184
SH3GLB2	0.921747497	0.87225939	0.849077738	0.943323364	0.99989309	0.926872824
PI15	0.921747497	0.855151723	0.899539089	0.946344632	0.99989309	0.968245211
FNTA	0.921880014	0.820816566	0.878050811	0.999939566	0.99989309	0.859487774
C2H2orf76	0.921880014	0.901922021	0.847037539	0.992373112	0.99989309	0.908939618
TMEM43	0.921880014	0.86415368	0.887130994	0.979667111	0.99989309	0.917524589
LOC112446044	0.921880014	0.81808696	0.849077738	0.958879502	0.99989309	0.935655141
CCDC85C	0.921991708	0.854410916	0.878050811	0.970617557	0.99989309	0.847256027
LOC112448518	0.921991708	0.798799934	0.880277219	0.943323364	0.99989309	0.859447707
VPS13A	0.921991708	0.86415368	0.893544538	0.96095927	0.99989309	0.865926817
MIA3	0.921991708	0.801002064	0.87214481	0.943733182	0.99989309	0.865997479
PRKCSH	0.921991708	0.790637585	0.978892556	0.992746023	0.99989309	0.870895425
IVNS1ABP	0.921991708	0.934508	0.917876971	0.958849311	0.99989309	0.886992214
NIPAL3	0.921991708	0.828417266	0.856997596	0.972367384	0.99989309	0.891000792
FAM76B	0.921991708	0.905977412	0.880204512	0.96095927	0.99989309	0.906529452
LOC101904396	0.921991708	0.852463941	0.879968243	0.971220023	0.99989309	0.908939618

LOC789035	0.921991708	0.799436659	0.849077738	0.943323364	0.99989309	0.913230542
CAPN6	0.921991708	0.972786652	0.87214481	0.956154344	0.99989309	0.91596005
PIP5K1B	0.921991708	0.880711088	0.925442336	0.964413123	0.99989309	0.941925704
WDR4	0.921991708	0.909853448	0.846650632	0.96095927	0.99989309	0.974348283
ADAMTSL5	0.921991708	0.81808696	0.880277219	0.992137653	0.99989309	0.978921738
C11H2orf42	0.921991708	0.80320642	0.86652249	0.954423369	0.99989309	0.993612847
HINFP	0.922168352	0.806686588	0.870054336	0.999939566	0.99989309	0.863099382
GTDC1	0.922168352	0.88311684	0.899629961	0.998542029	0.99989309	0.875441396
ADNP2	0.922168352	0.934248552	0.904691312	0.983971658	0.99989309	0.906529452
LOC112445065	0.922168352	0.820796062	0.89741279	0.995118128	0.99989309	0.908939618
VRK2	0.922180039	0.905977412	0.847037539	0.99270018	0.99989309	0.881039089
TMEM41B	0.922288689	0.80055527	0.951742137	0.999939566	0.99989309	0.868108836
LOC101905357	0.922507101	0.9136091	0.915843145	0.999939566	0.99989309	0.847256027
DEPDC1B	0.922507101	0.840078234	0.914685423	0.999939566	0.99989309	0.858042054
ABCB10	0.922507101	0.921393399	0.886282865	0.963829131	0.99989309	0.864809768
SHBG	0.922507101	0.803995245	0.864741549	0.999939566	0.99989309	0.875441396
UBN2	0.922507101	0.851167599	0.921225977	0.951177494	0.99989309	0.934653208
TCP11L1	0.922507101	0.825215718	0.846650632	0.999939566	0.99989309	0.934775196
USO1	0.922507101	0.798799934	0.940785296	0.943323364	0.99989309	0.971025918
KLHL41	0.922547615	0.974348229	0.878603452	0.955755829	0.99989309	0.859973633
OPRL1	0.922547615	0.920347742	0.857065866	0.941467481	0.99989309	0.96767215
LOC510613	0.922978828	0.802701235	0.875669731	0.999939566	0.99989309	0.868192873
ZADH2	0.923028971	0.842654254	0.875669731	0.959410646	0.99989309	0.943685681
LOC100847182	0.923194112	0.803903736	0.989527187	0.994309225	0.99989309	0.86143104
PTPN9	0.923684192	0.795865937	0.850020542	0.99879985	0.99989309	0.851651757
ATP6V1E2	0.923684192	0.994365533	0.880277219	0.946344632	0.99989309	0.852755805
ZSCAN25	0.923684192	0.831242188	0.987006595	0.992368879	0.99989309	0.856870625
SMG8	0.923684192	0.83744789	0.886282865	0.990491321	0.99989309	0.863755488
GUF1	0.923684192	0.855979085	0.887130994	0.971220023	0.99989309	0.888471585
LOC615989	0.923684192	0.981087742	0.861835782	0.943323364	0.99989309	0.891000792
SIRT3	0.923684192	0.83744789	0.878603452	0.999939566	0.99989309	0.895218005
ZBTB38	0.923684192	0.954298318	0.883726795	0.958879502	0.99989309	0.90175726
LZTS1	0.923684192	0.823588167	0.852758896	0.999939566	0.99989309	0.935154223
DAP3	0.923684192	0.87401231	0.863138583	0.988140196	0.99989309	0.940006665
HIGD1B	0.923684192	0.931756108	0.878050811	0.949947918	0.99989309	0.955585296
CPT1B	0.923698487	0.900502778	0.852758896	0.999939566	0.99989309	0.86143104
BLZF1	0.923698487	0.907594268	0.904682623	0.995118128	0.99989309	0.869171706
MARCKSL1	0.923698487	0.790637585	0.849077738	0.999939566	0.99989309	0.869773801

LOC107132317	0.923962888	0.835130235	0.892122482	0.943323364	0.99989309	0.859973633
LOC101904057	0.923962888	0.79441406	0.976782463	0.984968973	0.99989309	0.873118284
TCF7L2	0.923964206	0.79738652	0.870054336	0.959786126	0.99989309	0.941925704
LOC515227	0.924088979	0.87401231	0.886282865	0.965590385	0.99989309	0.948277018
LOC786553	0.924177228	0.821987045	0.926613927	0.999939566	0.99989309	0.854217772
FKBP4	0.924254343	0.791922306	0.896272282	0.992154129	0.99989309	0.868108836
PPP4R3A	0.924254343	0.861882481	0.855743966	0.942235206	0.99989309	0.887248119
APOD	0.924254343	0.968447746	0.882898206	0.971220023	0.99989309	0.895218005
TMEM185B	0.924254343	0.822900782	0.937363055	0.99879985	0.99989309	0.918291722
LOC101907000	0.924254343	0.798799934	0.849077738	0.951929772	0.99989309	0.921582976
GINS2	0.924254343	0.798799934	0.92153058	0.999939566	0.99989309	0.932613047
LOC107132283	0.924254343	0.882930807	0.939683463	0.945929971	0.99989309	0.940683751
HYAL3	0.924254343	0.821124254	0.879515214	0.992137653	0.99989309	0.964143145
C21H14orf28	0.924254343	0.835276066	0.897665623	0.975529024	0.99989309	0.964188691
MGA	0.92432369	0.860329184	0.894432335	0.942384819	0.99989309	0.85958325
TCERG1	0.92432369	0.792090907	0.939683463	0.999334425	0.99989309	0.88667499
CEP152	0.92432369	0.893161917	0.850243032	0.980418209	0.99989309	0.911696156
LOC104969140	0.924512171	0.820796062	0.970218925	0.943323364	0.99989309	0.857269299
SLC25A11	0.924512171	0.934571318	0.862797408	0.958879502	0.99989309	0.859323448
FOLR2	0.924512171	0.883267666	0.941312064	0.999939566	0.99989309	0.859487774
TTN	0.924512171	0.82783173	0.994425789	0.943323364	0.99989309	0.859973633
TGOLN2	0.924512171	0.835130235	0.941593855	0.997612654	0.99989309	0.859973633
SAMD8	0.924512171	0.875345836	0.916702	0.951929772	0.99989309	0.86143104
NDST2	0.924512171	0.842759163	0.96993432	0.990049248	0.99989309	0.862403446
EML4	0.924512171	0.937978608	0.914685423	0.99879985	0.99989309	0.862860542
SLC22A31	0.924512171	0.979916117	0.870054336	0.985476949	0.99989309	0.862912171
SDC3	0.924512171	0.82750276	0.877816623	0.999939566	0.99989309	0.865159042
EGLN1	0.924512171	0.892520818	0.879215964	0.998542029	0.99989309	0.867440033
LOC787679	0.924512171	0.905210378	0.880277219	0.999939566	0.99989309	0.869171706
FAM81A	0.924512171	0.965829305	0.849077738	0.968293092	0.99989309	0.874970609
SUCO	0.924512171	0.911196851	0.919939634	0.990300191	0.99989309	0.87585551
CRB1	0.924512171	0.867425848	0.983332897	0.946344632	0.99989309	0.876517132
RRM2B	0.924512171	0.79738652	0.929303679	0.999939566	0.99989309	0.880404833
ATXN10	0.924512171	0.898903115	0.869529186	0.994309225	0.99989309	0.895218005
NCF1	0.924512171	0.913075885	0.885434576	0.943161565	0.99989309	0.904937451
SATB2	0.924512171	0.934248552	0.855978798	0.943323364	0.99989309	0.906178214
FAM20B	0.924512171	0.831242188	0.959646613	0.987342814	0.99989309	0.906529452
RRP1	0.924512171	0.899387334	0.855033582	0.971220023	0.99989309	0.913558791

EID1	0.924512171	0.834316926	0.892258313	0.995118128	0.99989309	0.930669597
EIF2S2	0.924512171	0.80925601	0.909170161	0.999939566	0.99989309	0.943685681
KIFAP3	0.924512171	0.841629926	0.917876971	0.951929772	0.99989309	0.96546833
BSDC1	0.924512171	0.820796062	0.849077738	0.99879985	0.99989309	0.979606084
DUSP10	0.924646216	0.852214392	0.871859013	0.943323364	0.99989309	0.908939618
CLPTM1L	0.924727486	0.849718733	0.869455175	0.980295672	0.99989309	0.912101047
LOC112449080	0.924727486	0.820796062	0.852758896	0.992137653	0.99989309	0.979517983
PMPCA	0.924749034	0.823692881	0.879515214	0.971220023	0.99989309	0.857727168
NBEAL1	0.924749034	0.867202036	0.849077738	0.963153458	0.99989309	0.91596005
C20H5orf22	0.924749034	0.90249621	0.880277219	0.985433712	0.99989309	0.919948949
RFX5	0.924798371	0.842565421	0.863138583	0.999939566	0.99989309	0.862145862
LOC112446882	0.925064031	0.81668556	0.985552223	0.944558402	0.99989309	0.901320431
MEF2C	0.925241345	0.95320487	0.944976702	0.945021186	0.99989309	0.849772722
KCTD21	0.925309588	0.895762772	0.915208497	0.962852957	0.99989309	0.862788295
MYPOP	0.925309588	0.855151723	0.858394457	0.962142137	0.99989309	0.867440033
CAMK1	0.925309588	0.881304554	0.917876971	0.998542029	0.99989309	0.911696156
LOC112443416	0.925309588	0.834316926	0.926727295	0.956154344	0.99989309	0.942868422
PTCD1	0.925333651	0.86415368	0.987100092	0.946344632	0.99989309	0.868108836
CS	0.925333651	0.798956241	0.858865175	0.947581168	0.99989309	0.926872824
EEPD1	0.925372895	0.81808696	0.909472681	0.94257281	0.99989309	0.874885645
SLC35F2	0.925372895	0.907878616	0.914685423	0.990571725	0.99989309	0.887248119
CDKN1B	0.925372895	0.957228764	0.880277219	0.943323364	0.99989309	0.888617204
LOC107131944	0.925372895	0.874669928	0.892258313	0.999939566	0.99989309	0.902660792
LOC112443159	0.925372895	0.828417266	0.878603452	0.963153458	0.99989309	0.912380696
MUM1L1	0.925372895	0.796591413	0.890801024	0.943323364	0.99989309	0.996455238
C11H2orf40	0.92551859	0.866123629	0.866310262	0.990049248	0.99989309	0.855653734
EFR3A	0.92551859	0.820559187	0.994425789	0.971220023	0.99989309	0.865926817
LOC100336381	0.92551859	0.851167599	0.875669731	0.999718667	0.99989309	0.92689297
MTREX	0.92551859	0.912112179	0.86719452	0.943323364	0.99989309	0.931886764
NBN	0.92551859	0.876057665	0.909170161	0.9846464	0.99989309	0.943138585
CRTC3	0.92551859	0.835276066	0.904364967	0.946344632	0.99989309	0.988323336
WNT5B	0.925545254	0.972418749	0.894414265	0.995118128	0.99989309	0.856870625
PRR14L	0.925545254	0.839289192	0.913203127	0.999939566	0.99989309	0.856870625
PTDSS1	0.925545254	0.831207892	0.894449269	0.999939566	0.99989309	0.86179903
ACTN3	0.925545254	0.800441059	0.926613927	0.999939566	0.99989309	0.873520548
LOC787812	0.925720406	0.934248552	0.962209895	0.945929971	0.99989309	0.861807035
ISLR2	0.925721999	0.842565421	0.92332596	0.999939566	0.99989309	0.852634896
SARM1	0.925721999	0.876057665	0.92153058	0.999939566	0.99989309	0.858042054

ANAPC16	0.925721999	0.825768191	0.939091808	0.999939566	0.99989309	0.865159042
COL11A2	0.925721999	0.930890453	0.897889644	0.968694679	0.99989309	0.866482265
HUS1	0.925721999	0.928685678	0.878258655	0.946344632	0.99989309	0.868025807
FAM168B	0.925721999	0.88311684	0.926727295	0.946344632	0.99989309	0.86921618
HYAL2	0.925721999	0.840505408	0.87214481	0.999939566	0.99989309	0.875441396
LOC100299757	0.925721999	0.825215718	0.922147089	0.99879985	0.99989309	0.887248119
AR	0.925721999	0.795421065	0.981442587	0.945929971	0.99989309	0.888695935
LOC112447392	0.925721999	0.855151723	0.941593855	0.971220023	0.99989309	0.911696156
CAMK2A	0.925721999	0.818648608	0.859614039	0.999939566	0.99989309	0.919975503
TP53	0.925721999	0.81808696	0.850243032	0.999939566	0.99989309	0.923064313
C1QTNF9	0.925721999	0.795534928	0.883726795	0.98636843	0.99989309	0.968245211
LOC615514	0.925958361	0.821124254	0.880277219	0.999939566	0.99989309	0.849772722
RNF43	0.925958361	0.959119521	0.859614039	0.956154344	0.99989309	0.86143104
EFCC1	0.925958361	0.852281996	0.86637689	0.999939566	0.99989309	0.866253786
L3MBTL3	0.925958361	0.829548444	0.877998684	0.999939566	0.99989309	0.868108836
PTPN21	0.925958361	0.839641196	0.880277219	0.999939566	0.99989309	0.873520548
IFNGR2	0.925958361	0.855151723	0.877998684	0.956154344	0.99989309	0.877623013
GPX8	0.925958361	0.848106858	0.899195818	0.999939566	0.99989309	0.893961809
IGSF3	0.925958361	0.804875341	0.942004125	0.956154344	0.99989309	0.912101047
LOC100295130	0.925958361	0.884185138	0.849077738	0.999939566	0.99989309	0.924984776
ZNF527	0.925958361	0.821124254	0.886282865	0.945163905	0.99989309	0.948277018
NME4	0.925973525	0.912112179	0.862179776	0.999939566	0.99989309	0.865159042
VIM	0.926066339	0.808839416	0.861160919	0.999939566	0.99989309	0.958219037
MGC137036	0.926082144	0.81808696	0.8895568	0.999939566	0.99989309	0.855692379
CHST8	0.926082144	0.977732731	0.92153058	0.943323364	0.99989309	0.857727168
AJUBA	0.926082144	0.827609894	0.89265635	0.956154344	0.99989309	0.859973633
KCMF1	0.926082144	0.818435706	0.886176337	0.999939566	0.99989309	0.859973633
DPT	0.926082144	0.960112278	0.870054336	0.999939566	0.99989309	0.860827207
MED24	0.926082144	0.900584769	0.91546474	0.980955077	0.99989309	0.86143104
BDH2	0.926082144	0.92757729	0.873750661	0.995118128	0.99989309	0.86143104
RBM38	0.926082144	0.887957103	0.933963997	0.959009349	0.99989309	0.861807035
LHFPL6	0.926082144	0.851167599	0.870774839	0.99879985	0.99989309	0.862788295
DDX20	0.926082144	0.949250535	0.92153058	0.95175364	0.99989309	0.86307729
FMNL3	0.926082144	0.820796062	0.887214835	0.999939566	0.99989309	0.867505247
YPEL4	0.926082144	0.823692881	0.922147089	0.943510519	0.99989309	0.868108836
FOXO3	0.926082144	0.800441059	0.882898206	0.999939566	0.99989309	0.872224159
SLC6A4	0.926082144	0.852281996	0.849459857	0.995118128	0.99989309	0.873118284
CDC23	0.926082144	0.798799934	0.87146798	0.999939566	0.99989309	0.874985656

LOC101906411	0.926082144	0.800441059	0.962209895	0.985054722	0.99989309	0.878152258
SMC3	0.926082144	0.851167599	0.879215964	0.994309225	0.99989309	0.887248119
LOC112446402	0.926082144	0.899387334	0.894449269	0.999939566	0.99989309	0.890898561
TAOK1	0.926082144	0.945105738	0.861838588	0.948725226	0.99989309	0.893400499
C9H6orf120	0.926082144	0.809942574	0.878603452	0.999939566	0.99989309	0.896934364
ENGASE	0.926082144	0.920289742	0.880277219	0.995680081	0.99989309	0.897011069
PIN1	0.926082144	0.83793617	0.901550297	0.995118128	0.99989309	0.899551847
BAHD1	0.926082144	0.871113425	0.905047103	0.995118128	0.99989309	0.905220694
USP20	0.926082144	0.818806929	0.894449269	0.956154344	0.99989309	0.908939618
ZNF793	0.926082144	0.83094123	0.92153058	0.97144093	0.99989309	0.908939618
LOC507696	0.926082144	0.834869027	0.899771699	0.999939566	0.99989309	0.909990551
LOC101906392	0.926082144	0.809942574	0.930262306	0.999939566	0.99989309	0.914440253
TXNDC15	0.926082144	0.841044548	0.93737555	0.956154344	0.99989309	0.919731102
LOC112446708	0.926082144	0.836487169	0.853686558	0.986383435	0.99989309	0.930318133
LOC101906347	0.926082144	0.811755592	0.94197861	0.982140778	0.99989309	0.934653208
RAPH1	0.926082144	0.966852427	0.852758896	0.953648387	0.99989309	0.940683751
TSPEAR	0.926082144	0.954352142	0.87214481	0.946344632	0.99989309	0.941925704
UBQLN4	0.926082144	0.864821455	0.869529186	0.955042328	0.99989309	0.959625205
ABCD1	0.926082144	0.796591413	0.872397372	0.952020618	0.99989309	0.961506391
PDIK1L	0.926082144	0.809942574	0.939683463	0.967822229	0.99989309	0.968039618
LOC513580	0.926165869	0.820796062	0.858865175	0.990049248	0.99989309	0.981411983
GRID1	0.926598759	0.919351156	0.936383207	0.995800463	0.99989309	0.856870625
LOC112448791	0.926598759	0.801968936	0.900109169	0.958879502	0.99989309	0.86307729
RPTOR	0.926598759	0.799579962	0.852758896	0.983569039	0.99989309	0.940683751
CTF1	0.926598759	0.817040135	0.879215964	0.99879985	0.99989309	0.942455807
EVI2B	0.926598759	0.851167599	0.861838588	0.953648387	0.99989309	0.943138585
COL4A3BP	0.926696401	0.841629926	0.921225977	0.999939566	0.99989309	0.865159042
RAB26	0.926706214	0.890427603	0.899624092	0.963153458	0.99989309	0.86307729
HSPA12B	0.926706214	0.827609894	0.95126311	0.952024081	0.99989309	0.877623013
LOC100141185	0.926706214	0.835536917	0.926613927	0.99879985	0.99989309	0.923498231
C10H15orf65	0.926706214	0.839461242	0.870920678	0.943323364	0.99989309	0.991051281
FAF1	0.927203967	0.806009257	0.930262306	0.956154344	0.99989309	0.958475633
MS4A7	0.927279837	0.957950474	0.939091808	0.971220023	0.99989309	0.862259367
LPAR1	0.927279837	0.974348229	0.869529186	0.995800463	0.99989309	0.862351065
LOC101902551	0.927279837	0.883267666	0.904849074	0.945929971	0.99989309	0.86921618
SLC20A1	0.927279837	0.817040135	0.938653997	0.980840984	0.99989309	0.911526437
GABARAPL2	0.927279837	0.842797231	0.91546474	0.99879985	0.99989309	0.924788226
ZNF287	0.927279837	0.877499021	0.917876971	0.992137653	0.99989309	0.931515831

TBCC	0.927279837	0.808517017	0.886881381	0.992339205	0.99989309	0.933763401
ARMT1	0.927279837	0.809501344	0.947475618	0.956154344	0.99989309	0.942421464
C23H6orf141	0.927279837	0.827609894	0.855743966	0.999939566	0.99989309	0.942455807
DOCK2	0.927279837	0.858230868	0.856812671	0.943323364	0.99989309	0.948277018
MARS	0.927279837	0.882993091	0.863890803	0.963153458	0.99989309	0.986389023
RAB35	0.927408499	0.832239565	0.957379098	0.999939566	0.99989309	0.858815268
MAGOH	0.927408499	0.796591413	0.953045982	0.995118128	0.99989309	0.862860542
CENPT	0.927408499	0.823187795	0.985552223	0.943323364	0.99989309	0.868108836
ACSM1	0.927408499	0.871412404	0.892323611	0.995118128	0.99989309	0.868177389
LAS1L	0.927408499	0.870087004	0.914406673	0.981022805	0.99989309	0.869171706
RAB5A	0.927408499	0.848986275	0.925587088	0.999939566	0.99989309	0.871630129
LOC100847835	0.927408499	0.848398356	0.86523939	0.983569039	0.99989309	0.874885645
SLC2A8	0.927408499	0.836026119	0.970904484	0.992368879	0.99989309	0.886992214
LOC100337495	0.927408499	0.886732397	0.909170161	0.99879985	0.99989309	0.886992214
C18H16orf74	0.927408499	0.860329184	0.917876971	0.99879985	0.99989309	0.89471811
IDH1	0.927408499	0.80403784	0.885434576	0.999939566	0.99989309	0.908182968
AKT2	0.927408499	0.852463941	0.882898206	0.990571725	0.99989309	0.908939618
NFKBIL1	0.927408499	0.926435857	0.912054602	0.983569039	0.99989309	0.910619304
MRPS10	0.927408499	0.841629926	0.894447907	0.999939566	0.99989309	0.911526437
LOC112449172	0.927408499	0.838843184	0.937270184	0.977216943	0.99989309	0.912396352
LOC104975666	0.927408499	0.907594268	0.85972234	0.990059245	0.99989309	0.912396352
AGBL5	0.927408499	0.89763446	0.880277219	0.967063428	0.99989309	0.925449698
ACOX3	0.927408499	0.838843184	0.965169397	0.95964071	0.99989309	0.930379536
SYT15	0.927408499	0.808942497	0.882890706	0.95964071	0.99989309	0.957877422
LOC107131458	0.927408499	0.941972432	0.875669731	0.943323364	0.99989309	0.961506391
FBXL8	0.927573797	0.80320642	0.941593855	0.998608191	0.99989309	0.856870625
WDR33	0.927573797	0.910232617	0.850106729	0.95175364	0.99989309	0.926147535
MRPL42	0.927573797	0.842797231	0.910123657	0.969717497	0.99989309	0.929046416
B4GALT5	0.927581474	0.80403784	0.878603452	0.959410646	0.99989309	0.873271935
PKIG	0.927581474	0.942608215	0.90002174	0.962775522	0.99989309	0.908939618
BRD1	0.927587398	0.823692881	0.91546474	0.968293092	0.99989309	0.897818829
SYN2	0.927607971	0.948215967	0.908927423	0.946344632	0.99989309	0.881044982
LOC100336589	0.927607971	0.971310681	0.860430781	0.952024081	0.99989309	0.887248119
LOC101908149	0.927607971	0.835276066	0.894547778	0.995687815	0.99989309	0.897818829
RBM24	0.927607971	0.907594268	0.932340777	0.946344632	0.99989309	0.943138585
ATP2C2	0.927652411	0.834869027	0.929958748	0.999939566	0.99989309	0.862403446
HGFAC	0.927652411	0.79713597	0.999541752	0.943539568	0.99989309	0.864571107
TRAPPC6A	0.927652411	0.934248552	0.872397372	0.956553353	0.99989309	0.870895425

CDK17	0.927652411	0.926618064	0.909170161	0.952024081	0.99989309	0.873520548
RTKN2	0.927652411	0.808513267	0.87146798	0.999939566	0.99989309	0.877623013
MSANTD1	0.927652411	0.843640278	0.90166788	0.999939566	0.99989309	0.879494654
POLD2	0.927652411	0.821124254	0.897665623	0.990571725	0.99989309	0.887248119
LOC100848212	0.927652411	0.866803532	0.878603452	0.999939566	0.99989309	0.893400499
LOC614617	0.927652411	0.905432526	0.869529186	0.982809512	0.99989309	0.901422892
NTAN1	0.927652411	0.809783026	0.855743966	0.995687815	0.99989309	0.90259329
CPXM2	0.927652411	0.852011434	0.917876971	0.999939566	0.99989309	0.908939618
LOC101904614	0.927652411	0.809254208	0.889420065	0.984968973	0.99989309	0.909768235
TAP1	0.927652411	0.842136201	0.896272282	0.955547884	0.99989309	0.910619304
RNF44	0.927652411	0.810519292	0.959159138	0.983934517	0.99989309	0.918666628
UBE2Q1	0.927652411	0.809304604	0.921612776	0.992746023	0.99989309	0.922709797
SUPT20H	0.927652411	0.831141011	0.962209895	0.956154344	0.99989309	0.92689297
ARHGAP1	0.927652411	0.876057665	0.894449269	0.99879985	0.99989309	0.932267465
FBXL18	0.927652411	0.838815395	0.939683463	0.990571725	0.99989309	0.937591162
LOC511713	0.927652411	0.871412404	0.875669731	0.983971658	0.99989309	0.941785987
TRIM8	0.927724174	0.821124254	0.91546474	0.944558402	0.99989309	0.908939618
CSRNP2	0.927853654	0.90249621	0.886282865	0.949947918	0.99989309	0.910619304
DOHH	0.9279073	0.804875341	0.937270184	0.992137653	0.99989309	0.923064313
LOC101906367	0.928207123	0.854656328	0.884197487	0.943323364	0.99989309	0.99064193
SDHD	0.928268418	0.801968936	0.909170161	0.990049248	0.99989309	0.858861717
KLHDC9	0.928268418	0.800441059	0.876075512	0.98636843	0.99989309	0.908939618
POLR2D	0.928268418	0.812575653	0.896272282	0.995800463	0.99989309	0.908939618
PITPNC1	0.928268418	0.881795385	0.858972712	0.987342814	0.99989309	0.920754358
LOC100337507	0.928268418	0.862267429	0.879515214	0.999939566	0.99989309	0.920942379
QTRT2	0.928268418	0.841935322	0.873350964	0.956154344	0.99989309	0.957877422
IQGAP2	0.928474371	0.814432322	0.877998684	0.946344632	0.99989309	0.862912171
DRAP1	0.928474371	0.862267429	0.939683463	0.968293092	0.99989309	0.886992214
PIGH	0.928474371	0.881079118	0.92153058	0.958879502	0.99989309	0.895218005
GGTA1	0.928512485	0.831242188	0.92153058	0.999939566	0.99989309	0.86143104
ITPKB	0.928512485	0.91994024	0.864236102	0.999939566	0.99989309	0.867440033
N4BP2L2	0.928512485	0.936134921	0.926613927	0.982140778	0.99989309	0.868108836
PPIP5K1	0.928512485	0.867425848	0.869455175	0.992137653	0.99989309	0.875441396
PRRC2A	0.928512485	0.80403784	0.898304171	0.99879985	0.99989309	0.882322567
LOC104971814	0.928512485	0.831242188	0.910978655	0.999939566	0.99989309	0.893961809
ZDHHC3	0.928512485	0.885981636	0.87214481	0.995680081	0.99989309	0.895218005
LOC112446001	0.928512485	0.920606262	0.920449324	0.952024081	0.99989309	0.898273855
ASCC3	0.928512485	0.825856868	0.853232343	0.969717497	0.99989309	0.91008914

UAP1	0.928512485	0.840874016	0.892258313	0.977216943	0.99989309	0.911696156
HAUS4	0.928512485	0.875345836	0.902179915	0.953508052	0.99989309	0.922229044
SSH2	0.928512485	0.839289192	0.879726737	0.958879502	0.99989309	0.933763401
DCTN1	0.928512485	0.834869027	0.937979342	0.971220023	0.99989309	0.953940638
LOC104973382	0.928740997	0.957071555	0.909170161	0.998675745	0.99989309	0.86143104
UST	0.928789262	0.883267666	0.891455327	0.951929772	0.99989309	0.858538874
GSTK1	0.928789262	0.825768191	0.878603452	0.999939566	0.99989309	0.858815268
LOC788405	0.928789262	0.804875341	0.914685423	0.994365581	0.99989309	0.86307729
MBOAT2	0.928789262	0.884185138	0.858972712	0.999939566	0.99989309	0.865997479
MARCH2	0.928789262	0.829476146	0.874092011	0.998542029	0.99989309	0.866099047
LOC101904840	0.928789262	0.80055527	0.969732581	0.999939566	0.99989309	0.866482265
RBL1	0.928789262	0.844031777	0.918965426	0.999939566	0.99989309	0.870351337
LOC107132360	0.928789262	0.907878616	0.859931483	0.991837175	0.99989309	0.875441396
PLCG2	0.928789262	0.912112179	0.979231344	0.945929971	0.99989309	0.877623013
GORAB	0.928789262	0.83744789	0.875201284	0.999939566	0.99989309	0.878152258
ABLIM3	0.928789262	0.900584769	0.883274745	0.992137653	0.99989309	0.888617204
SRPK2	0.928789262	0.820796062	0.911743083	0.99879985	0.99989309	0.891511718
ZMYM3	0.928789262	0.840571636	0.909002826	0.999939566	0.99989309	0.891908825
SIRT7	0.928789262	0.897327046	0.968934792	0.956154344	0.99989309	0.895218005
U2AF1	0.928789262	0.813459746	0.904751055	0.999939566	0.99989309	0.897742414
MPV17L	0.928789262	0.810135334	0.879215964	0.992154129	0.99989309	0.908939618
DAZAP1	0.928789262	0.836487169	0.869455175	0.999939566	0.99989309	0.911526437
SHISA7	0.928789262	0.824119076	0.879376765	0.983569039	0.99989309	0.917968123
KIF21A	0.928789262	0.835276066	0.880277219	0.999939566	0.99989309	0.941925704
KAT6A	0.928789262	0.820816566	0.902141181	0.989391869	0.99989309	0.981578785
TAZ	0.928837421	0.810096755	0.9343664	0.981022805	0.99989309	0.868108836
MSR1	0.928837421	0.873297522	0.910703333	0.999939566	0.99989309	0.884387948
C25H7orf43	0.928852999	0.830193776	0.939683463	0.999939566	0.99989309	0.858042054
ABR	0.92888107	0.806152572	0.861838588	0.999939566	0.99989309	0.893961809
GNPTAB	0.92888107	0.821588096	0.931775341	0.991029787	0.99989309	0.902244322
RAB3IL1	0.929012855	0.876169307	0.894874079	0.999939566	0.99989309	0.875441396
GOLT1B	0.929055687	0.817875214	0.994524327	0.965097245	0.99989309	0.860827207
LOC787550	0.929134277	0.820796062	0.89145542	0.999939566	0.99989309	0.887457549
PIGM	0.929252137	0.852377308	0.86360816	0.995800463	0.99989309	0.937591162
KCTD20	0.929305965	0.952226848	0.855911319	0.995118128	0.99989309	0.867440033
TUBB1	0.929305965	0.867262498	0.943891967	0.963829131	0.99989309	0.89260699
AKAP10	0.929305965	0.852463941	0.962857767	0.946344632	0.99989309	0.902660792
MAP3K7	0.929305965	0.81808696	0.856729427	0.999939566	0.99989309	0.983453112

SUCNR1	0.929316203	0.841629926	0.864832658	0.995118128	0.99989309	0.906178214
CORO7	0.929465314	0.993511686	0.89248682	0.958879502	0.99989309	0.86307729
ALDOA	0.929482506	0.852222691	0.870175297	0.992137653	0.99989309	0.901173172
STOML2	0.929511479	0.841044548	0.870054336	0.977616954	0.99989309	0.910353954
ABI3BP	0.929579651	0.820124064	0.939683463	0.996601241	0.99989309	0.86143104
LOC780968	0.929579651	0.803995245	0.91546474	0.995118128	0.99989309	0.86252043
MAN1A2	0.929579651	0.835613722	0.899539089	0.953648387	0.99989309	0.863755488
ELOB	0.929579651	0.80403784	0.883726795	0.947581168	0.99989309	0.865926817
CSMD2	0.929579651	0.992032812	0.87214481	0.944558402	0.99989309	0.866253786
TEX2	0.929579651	0.825215718	0.939683463	0.999939566	0.99989309	0.867440033
KITLG	0.929579651	0.800441059	0.94991125	0.99879985	0.99989309	0.867978129
SPATA24	0.929579651	0.828417266	0.868936984	0.999939566	0.99989309	0.870895425
METTL26	0.929579651	0.937626904	0.892122482	0.956154344	0.99989309	0.871780952
LOC112446423	0.929579651	0.831141011	0.914668913	0.982477333	0.99989309	0.873118284
LOC100297420	0.929579651	0.803995245	0.878603452	0.999939566	0.99989309	0.875441396
SP110	0.929579651	0.822900782	0.970904484	0.990059245	0.99989309	0.877623013
LOC100140533	0.929579651	0.809942574	0.915891679	0.996084773	0.99989309	0.880272231
TIMMDC1	0.929579651	0.820796062	0.941312064	0.995800463	0.99989309	0.880404833
RPN2	0.929579651	0.881823401	0.885434576	0.999939566	0.99989309	0.884387948
LOC101906077	0.929579651	0.835276066	0.925343447	0.994309225	0.99989309	0.886210131
CHST3	0.929579651	0.835276066	0.963643451	0.9846464	0.99989309	0.887457549
LOC101907615	0.929579651	0.813459746	0.909170161	0.999939566	0.99989309	0.893961809
MFSD2A	0.929579651	0.831242188	0.939683463	0.999939566	0.99989309	0.893961809
LOC783255	0.929579651	0.820796062	0.921135532	0.99419346	0.99989309	0.897818829
ZNF280D	0.929579651	0.912112179	0.886481882	0.990571725	0.99989309	0.901320431
MRPL47	0.929579651	0.912112179	0.87146798	0.999939566	0.99989309	0.902295033
CCNG1	0.929579651	0.840365131	0.904854349	0.999939566	0.99989309	0.902660792
TOX	0.929579651	0.924510975	0.882898206	0.99879985	0.99989309	0.908939618
WDR11	0.929579651	0.813511912	0.909511376	0.999939566	0.99989309	0.908939618
UBE2B	0.929579651	0.838405969	0.930710426	0.999939566	0.99989309	0.908939618
UGP2	0.929579651	0.825215718	0.943480836	0.949947918	0.99989309	0.914440253
MAD1L1	0.929579651	0.844263736	0.979231344	0.956154344	0.99989309	0.914440253
NHSL1	0.929579651	0.908192144	0.852758896	0.999939566	0.99989309	0.919645668
NUBPL	0.929579651	0.877420806	0.909522865	0.99879985	0.99989309	0.928123265
SERINC4	0.929579651	0.880711088	0.917876971	0.958849311	0.99989309	0.935655141
MXD4	0.929579651	0.825215718	0.917876971	0.999939566	0.99989309	0.935937948
ATP2A1	0.929579651	0.832607032	0.858865175	0.999939566	0.99989309	0.943866443
BRINP1	0.929579651	0.950928726	0.877777356	0.944558402	0.99989309	0.955130073

CCDC134	0.929579651	0.918306418	0.878603452	0.959786126	0.99989309	0.959625205
WDFY3	0.929579651	0.831974458	0.864236102	0.971685879	0.99989309	0.968245211
IFFO2	0.929579651	0.852313021	0.858865175	0.990571725	0.99989309	0.98016619
RPL37A	0.929788777	0.838405969	0.913413399	0.999939566	0.99989309	0.862481601
SERPINH1	0.929788777	0.838815395	0.893863239	0.999939566	0.99989309	0.884387948
GHDC	0.929788777	0.805157515	0.915891679	0.999939566	0.99989309	0.908939618
MB21D2	0.929788777	0.866813771	0.860197593	0.999939566	0.99989309	0.912380696
GCN1	0.929788777	0.82783173	0.929958748	0.977470078	0.99989309	0.958814003
LOC100300938	0.929789182	0.823692881	0.878050811	0.995118128	0.99989309	0.880404833
B3GNT5	0.929789182	0.83978684	0.896272282	0.999939566	0.99989309	0.901173172
OAT	0.929789182	0.922434012	0.906597758	0.992137653	0.99989309	0.902660792
KPNA3	0.929910915	0.848398356	0.989408632	0.968306132	0.99989309	0.865159042
MYL6B	0.929910915	0.803995245	0.87146798	0.999939566	0.99989309	0.875441396
KIAA2012	0.929910915	0.80925601	0.860197593	0.999939566	0.99989309	0.889159914
ARHGAP29	0.929910915	0.963478319	0.856236544	0.946344632	0.99989309	0.919731102
ZKSCAN1	0.929914182	0.926435857	0.879515214	0.956154344	0.99989309	0.861807035
LOC100141168	0.929914182	0.866803532	0.882898206	0.992154129	0.99989309	0.866482265
ADGRG2	0.929914182	0.839289192	0.887391534	0.971220023	0.99989309	0.873271935
TAAR1	0.929914182	0.82890642	0.899100566	0.956154344	0.99989309	0.875441396
FRMD5	0.929914182	0.955798423	0.878603452	0.972489653	0.99989309	0.897902197
LOC781499	0.929914182	0.82890642	0.899195818	0.956154344	0.99989309	0.908939618
CABP7	0.929914182	0.866813771	0.926613927	0.995800463	0.99989309	0.908939618
LOC112442611	0.929914182	0.938064973	0.875669731	0.995118128	0.99989309	0.914440253
OCRL	0.929914182	0.804528018	0.870321375	0.996601241	0.99989309	0.925494614
TRAPPC2	0.929914182	0.838263859	0.903453422	0.966203886	0.99989309	0.945759045
CD300LG	0.930158529	0.971426434	0.929303679	0.973945516	0.99989309	0.862145862
KLHDC7A	0.930158529	0.864541566	0.939683463	0.990571725	0.99989309	0.874985656
JAG2	0.930206641	0.844031777	0.869455175	0.999939566	0.99989309	0.865997479
ANKRD37	0.930206641	0.817040135	0.880277219	0.999939566	0.99989309	0.895287865
LOC101902440	0.930206641	0.876169307	0.870774839	0.981022805	0.99989309	0.912396352
ARHGAP31	0.930206641	0.926435857	0.856589093	0.995118128	0.99989309	0.920942379
TRAPPC13	0.930206641	0.820796062	0.883726795	0.984677276	0.99989309	0.935655141
CHST9	0.930206641	0.821527913	0.87146798	0.958879502	0.99989309	0.973494928
ZNF282	0.930211922	0.851167599	0.939683463	0.99879985	0.99989309	0.870895425
PMEPA1	0.930277011	0.934531829	0.951742137	0.96999145	0.99989309	0.859973633
SUSD1	0.930277011	0.926435857	0.877230238	0.991029787	0.99989309	0.879160297
CRTAP	0.930277011	0.855151723	0.914489705	0.999939566	0.99989309	0.891000792
EHMT1	0.930313971	0.821588096	0.984700021	0.99419346	0.99989309	0.86143104

BCL2L14	0.930313971	0.876057665	0.92153058	0.999939566	0.99989309	0.86307729
LOC101906656	0.930313971	0.982051974	0.922147089	0.953510787	0.99989309	0.884387948
LOC107133289	0.930313971	0.918306418	0.963643451	0.956154344	0.99989309	0.894951353
RHOQ	0.930313971	0.823074953	0.90936727	0.999939566	0.99989309	0.901422892
NPR3	0.930313971	0.891986447	0.957379098	0.963528138	0.99989309	0.906178214
MAGI2	0.930313971	0.825215718	0.883726795	0.999939566	0.99989309	0.906665513
RECQL5	0.930313971	0.885999002	0.969618195	0.95175364	0.99989309	0.909768235
COG4	0.930313971	0.828797861	0.87214481	0.956154344	0.99989309	0.914440253
PINK1	0.930387182	0.899474896	0.886282865	0.99879985	0.99989309	0.888294113
LOC112441718	0.930387182	0.967758952	0.870054336	0.992137653	0.99989309	0.912396352
ZNF395	0.930804814	0.914738515	0.987419404	0.95164126	0.99989309	0.862912171
TLCD1	0.930804814	0.905834401	0.949770432	0.985181062	0.99989309	0.86307729
SNX7	0.930804814	0.89272885	0.917013548	0.999939566	0.99989309	0.864219261
LOC512165	0.930804814	0.852463941	0.944592818	0.995118128	0.99989309	0.867104748
JMJD1C	0.930804814	0.915978689	0.870054336	0.99879985	0.99989309	0.868108836
ZNF462	0.930804814	0.876919789	0.885434576	0.976950878	0.99989309	0.870895425
AKAIN1	0.930804814	0.90039743	0.87146798	0.995800463	0.99989309	0.870895425
LOC104975044	0.930804814	0.838843184	0.943891967	0.989527018	0.99989309	0.872224159
LOC101907943	0.930804814	0.823588167	0.878050811	0.987143706	0.99989309	0.875441396
MYH14	0.930804814	0.848986275	0.879215964	0.999939566	0.99989309	0.875441396
GANAB	0.930804814	0.820350377	0.921319817	0.999939566	0.99989309	0.88494083
CSF2RA	0.930804814	0.890503475	0.931397502	0.992137653	0.99989309	0.886992214
BMF	0.930804814	0.803903736	0.89248682	0.999939566	0.99989309	0.886992214
LDHA	0.930804814	0.820816566	0.938195001	0.999939566	0.99989309	0.886992214
NDUFS2	0.930804814	0.809942574	0.880277219	0.97144093	0.99989309	0.887248119
LOC112441494	0.930804814	0.901922021	0.879726737	0.995118128	0.99989309	0.893961809
PAK1	0.930804814	0.806686588	0.917092799	0.999939566	0.99989309	0.896934364
LOC107132515	0.930804814	0.826048291	0.905832059	0.992368879	0.99989309	0.897818829
ADK	0.930804814	0.838843184	0.926613927	0.992368879	0.99989309	0.900729479
SUV39H1	0.930804814	0.885981636	0.914685423	0.990571725	0.99989309	0.902669631
LOC784054	0.930804814	0.921280256	0.89248682	0.971220023	0.99989309	0.908939618
ASB12	0.930804814	0.813371202	0.856769954	0.99879985	0.99989309	0.908939618
B3GNT3	0.930804814	0.861730561	0.884189563	0.999939566	0.99989309	0.908939618
KIF27	0.930804814	0.88689462	0.922161207	0.995118128	0.99989309	0.914742013
PCMTD2	0.930804814	0.897327046	0.92582321	0.980418209	0.99989309	0.924984776
MIA2	0.930804814	0.892564295	0.919108971	0.971220023	0.99989309	0.930707319
HSPB1	0.930804814	0.836487169	0.884325235	0.999939566	0.99989309	0.931886764
CEP295NL	0.930804814	0.878594098	0.873750661	0.999939566	0.99989309	0.939507593

TNFRSF12A	0.930804814	0.918976923	0.855084629	0.94850175	0.99989309	0.940917403
LOC100848324	0.930804814	0.81808696	0.939612562	0.990049248	0.99989309	0.955585296
TMEM273	0.930804814	0.835276066	0.919708765	0.97821933	0.99989309	0.966375116
LRMP	0.930804814	0.843333021	0.870054336	0.995536195	0.99989309	0.968039618
LOC101904849	0.930804814	0.832239565	0.867451783	0.992137653	0.99989309	0.981411983
DMKN	0.930804814	0.891031143	0.873750661	0.958879502	0.99989309	0.983453112
CHL1	0.930895161	0.820796062	0.877133001	0.999939566	0.99989309	0.88609792
CEACAM1	0.931019073	0.886909612	0.883544865	0.971220023	0.99989309	0.94091418
VCPKMT	0.931131647	0.822260127	0.999323478	0.956154344	0.99989309	0.868430302
TRIM33	0.931131647	0.907878616	0.914489705	0.958879502	0.99989309	0.871183939
FKBPL	0.931131647	0.969202872	0.867166602	0.998542029	0.99989309	0.877740826
PI4KB	0.931131647	0.813459746	0.974698466	0.949947918	0.99989309	0.883302043
UNKL	0.931131647	0.810205594	0.90936727	0.99879985	0.99989309	0.908939618
NEK6	0.931131647	0.813511912	0.910978655	0.956142205	0.99989309	0.978656049
LOC100295750	0.931236539	0.820796062	0.939683463	0.946344632	0.99989309	0.859973633
BNC2	0.931236539	0.880103565	0.958420472	0.995118128	0.99989309	0.870895425
MBOAT1	0.931236539	0.852214392	0.883982433	0.992741182	0.99989309	0.871166844
PITX3	0.931236539	0.855979085	0.891014698	0.999939566	0.99989309	0.872224159
LOC617692	0.931236539	0.851559446	0.890801024	0.969717497	0.99989309	0.875441396
FGD5	0.931236539	0.835276066	0.899530079	0.999939566	0.99989309	0.875441396
CISD1	0.931236539	0.820796062	0.870911433	0.969717497	0.99989309	0.876534787
DNAJC13	0.931236539	0.903657308	0.94454396	0.961749608	0.99989309	0.923064313
GTPBP4	0.931236539	0.843422478	0.875669731	0.99879985	0.99989309	0.926872824
ZNF227	0.931236539	0.841657074	0.87146798	0.95060093	0.99989309	0.998636142
FBXO2	0.931256772	0.834819546	0.893732737	0.999939566	0.99989309	0.870895425
VDR	0.931342001	0.808513267	0.944060947	0.967063428	0.99989309	0.895287865
RASL11A	0.931342001	0.847082229	0.972401758	0.95964071	0.99989309	0.907965478
LBH	0.931342001	0.903820329	0.87146798	0.985181062	0.99989309	0.956610132
CTDSPL	0.931342001	0.845123271	0.890548045	0.995946943	0.99989309	0.957959714
LOC512323	0.931809502	0.885253121	0.974193101	0.992957926	0.99989309	0.86143104
LOC505199	0.931809502	0.913625328	0.922147089	0.99879985	0.99989309	0.86307729
BABAM2	0.931809502	0.820559187	0.902685881	0.999939566	0.99989309	0.86307729
SCRN3	0.931809502	0.88311684	0.914668913	0.999939566	0.99989309	0.867440033
LOC786252	0.931809502	0.86515799	0.87214481	0.999939566	0.99989309	0.868108836
LOC786173	0.931809502	0.899387334	0.885274776	0.948120268	0.99989309	0.872224159
LOC101906358	0.931809502	0.811402363	0.959159138	0.992137653	0.99989309	0.878040551
DYSF	0.931809502	0.855151723	0.857943088	0.982140778	0.99989309	0.879550676
LOC100336564	0.931809502	0.881079118	0.987419404	0.955547884	0.99989309	0.886520861

FGFR2	0.931809502	0.955798423	0.85792584	0.990571725	0.99989309	0.886992214
RNASEH2B	0.931809502	0.835130235	0.909170161	0.956154344	0.99989309	0.887248119
LOC101906850	0.931809502	0.820796062	0.967302577	0.956154344	0.99989309	0.890898561
SHLD2	0.931809502	0.835276066	0.864135894	0.999939566	0.99989309	0.895287865
RYR1	0.931809502	0.963426589	0.878050811	0.995118128	0.99989309	0.9071195
NDFIP2	0.931809502	0.837799788	0.883982433	0.956154344	0.99989309	0.908939618
PARPBP	0.931809502	0.825856868	0.878258655	0.999939566	0.99989309	0.918220726
SLC11A1	0.931809502	0.820816566	0.902179915	0.999939566	0.99989309	0.92689297
TIMP4	0.931809502	0.835809337	0.904682623	0.956154344	0.99989309	0.940917403
NLGN1	0.931809502	0.831242188	0.885481718	0.992137653	0.99989309	0.948277018
RNF135	0.931987312	0.80931829	0.880277219	0.998542029	0.99989309	0.859973633
SIK2	0.931987312	0.893639679	0.861838588	0.999939566	0.99989309	0.865997479
NRXN2	0.932159687	0.857034402	0.93050447	0.99879985	0.99989309	0.897818829
TMCC2	0.932279115	0.925520582	0.879215964	0.999939566	0.99989309	0.865926817
MAP3K9	0.932279115	0.920347742	0.914685423	0.951929772	0.99989309	0.960985422
CYYR1	0.932337077	0.941502351	0.887364739	0.995946943	0.99989309	0.898791601
LOC101903678	0.932337077	0.875022483	0.941319062	0.969717497	0.99989309	0.908939618
XCL2	0.932337077	0.867722813	0.87146798	0.952024081	0.99989309	0.991281092
ADIPOR2	0.93240381	0.81808696	0.873750661	0.999939566	0.99989309	0.908939618
LOC788414	0.93240381	0.842797231	0.870780616	0.981022805	0.99989309	0.911263177
ZNF358	0.93240381	0.821982753	0.861838588	0.999939566	0.99989309	0.915447631
SLC16A12	0.93240381	0.882437001	0.861160919	0.992368879	0.99989309	0.977961448
TOM1	0.93246758	0.826048291	0.957802423	0.951929772	0.99989309	0.865666823
BICD2	0.93246758	0.835276066	0.887595661	0.999939566	0.99989309	0.870895425
MEA1	0.93246758	0.843166272	0.965416289	0.998542029	0.99989309	0.873118284
MTSS1L	0.93246758	0.829690146	0.877133001	0.999939566	0.99989309	0.88200747
TGIF2	0.93246758	0.867722813	0.86637689	0.949142271	0.99989309	0.905267129
LOC101908154	0.93246758	0.922434012	0.916702	0.95175364	0.99989309	0.908939618
ROR1	0.933094101	0.806439163	0.893544538	0.999939566	0.99989309	0.942421464
LOC112447032	0.933094101	0.878594098	0.939683463	0.946344632	0.99989309	0.961465235
SLC27A3	0.933268401	0.983065474	0.902179915	0.946344632	0.99989309	0.86143104
C14H8orf59	0.933268401	0.820796062	0.959705869	0.988115459	0.99989309	0.86143104
CLNS1A	0.933268401	0.840444202	0.966334813	0.995800463	0.99989309	0.864219261
COX4I1	0.933268401	0.834819546	0.875669731	0.998542029	0.99989309	0.868108836
TMEM110	0.933268401	0.86415368	0.87214481	0.999939566	0.99989309	0.868108836
LOC107133190	0.933268401	0.858230868	0.878603452	0.999939566	0.99989309	0.868108836
PODNL1	0.933268401	0.88418172	0.899195818	0.999939566	0.99989309	0.868108836
THOC1	0.933268401	0.852463941	0.893763648	0.999939566	0.99989309	0.872175131

BROX	0.933268401	0.869509858	0.910978655	0.999939566	0.99989309	0.875441396
TGIF1	0.933268401	0.877749555	0.871883815	0.977656613	0.99989309	0.876262605
SPRY2	0.933268401	0.852281996	0.94897867	0.990049248	0.99989309	0.886992214
CMYA5	0.933268401	0.80925601	0.918965426	0.995118128	0.99989309	0.887248119
GTPBP2	0.933268401	0.900467607	0.893544538	0.999939566	0.99989309	0.893961809
PLA2G12A	0.933268401	0.885981636	0.92153058	0.995118128	0.99989309	0.895218005
VAMP2	0.933268401	0.820959665	0.926613927	0.99879985	0.99989309	0.895218005
PALM	0.933268401	0.820796062	0.939683463	0.999939566	0.99989309	0.908939618
SLC9A8	0.933268401	0.945105738	0.892122482	0.959786126	0.99989309	0.936565114
RMC1	0.933268401	0.855979085	0.858972712	0.999939566	0.99989309	0.937591162
CLK3	0.933268401	0.842511984	0.875503395	0.992137653	0.99989309	0.950959274
RPN1	0.933268401	0.820796062	0.883874752	0.99879985	0.99989309	0.960712754
TRDMT1	0.933268401	0.834399235	0.863172962	0.999939566	0.99989309	0.973195541
GRIN1	0.933268401	0.823588167	0.872397372	0.992137653	0.99989309	0.97356849
FBXW2	0.933283578	0.817040135	0.914685423	0.999939566	0.99989309	0.868108836
LOC112446645	0.933283578	0.951985518	0.882898206	0.998542029	0.99989309	0.873520548
PRKCH	0.933283578	0.831242188	0.878603452	0.999939566	0.99989309	0.945759045
TMEM181	0.933283578	0.834869027	0.884189563	0.949947918	0.99989309	0.968245211
PUS1	0.933286163	0.911347642	0.979231344	0.963829131	0.99989309	0.868108836
FAM101A	0.933286163	0.891549709	0.865007702	0.95175364	0.99989309	0.871780952
SMC2	0.93332433	0.810701997	0.970034593	0.999939566	0.99989309	0.86307729
CEP68	0.93332433	0.8104985	0.858865175	0.999939566	0.99989309	0.870895425
SDF2	0.93332433	0.834011972	0.939091808	0.969956798	0.99989309	0.872224159
PRKRIP1	0.93332433	0.936099262	0.887130994	0.969717497	0.99989309	0.882322567
VDAC3	0.93332433	0.843037817	0.897665623	0.990049248	0.99989309	0.905220694
MAPKBP1	0.93332433	0.866916992	0.894414265	0.992368879	0.99989309	0.910619304
UBTD1	0.93332433	0.844510795	0.893763648	0.999939566	0.99989309	0.912101047
LOC100337053	0.93332433	0.855151723	0.951850716	0.956154344	0.99989309	0.926872824
LOC101902232	0.93332433	0.810519292	0.976719499	0.98639059	0.99989309	0.928231607
ARMC9	0.93332433	0.893623223	0.89007492	0.990571725	0.99989309	0.941925704
PUS3	0.93332433	0.861568371	0.883647437	0.995800463	0.99989309	0.942421464
VPS45	0.93332433	0.883267666	0.868936984	0.956154344	0.99989309	0.943138585
FRMD6	0.93373051	0.860449455	0.89007492	0.983569039	0.99989309	0.908939618
NSG1	0.933740119	0.965239369	0.88230201	0.992137653	0.99989309	0.868108836
LOC104974669	0.933740119	0.823692881	0.861838588	0.999939566	0.99989309	0.962255684
ZNF593	0.933740119	0.893512526	0.917876971	0.96095927	0.99989309	0.966338859
EBAG9	0.933761995	0.870087004	0.965518535	0.994365581	0.99989309	0.893961809
LOC112442296	0.933793144	0.908183362	0.877998684	0.990059245	0.99989309	0.875441396

LOC107131311	0.933793144	0.852281996	0.906752332	0.999939566	0.99989309	0.891511718
ZNF613	0.933793144	0.852463941	0.909522865	0.963829131	0.99989309	0.912101047
MXD3	0.933793144	0.820076805	0.942004125	0.999939566	0.99989309	0.923064313
LOC100848264	0.933793144	0.887725794	0.872651958	0.990571725	0.99989309	0.925494614
PIK3CD	0.933793144	0.867138744	0.857486511	0.956154344	0.99989309	0.939966658
NRROS	0.9338401	0.850723096	0.882898206	0.999939566	0.99989309	0.866482265
LOC100140958	0.933945226	0.88418172	0.947660114	0.999939566	0.99989309	0.866253786
EMP2	0.933945226	0.884397857	0.939091808	0.998542029	0.99989309	0.867376048
LMO1	0.933945226	0.808513267	0.939683463	0.996084773	0.99989309	0.872175131
CSDE1	0.933945226	0.82750276	0.937191211	0.999939566	0.99989309	0.880404833
MRPS9	0.933945226	0.835408071	0.870466659	0.981022805	0.99989309	0.893961809
PHKB	0.933945226	0.856897628	0.858972712	0.990491321	0.99989309	0.956610132
PLAGL1	0.934021103	0.810519292	0.941319062	0.972367384	0.99989309	0.86143104
LOC112446007	0.934027657	0.8554287	0.887130994	0.999939566	0.99989309	0.8851281
HAX1	0.93403125	0.962934461	0.86360816	0.999939566	0.99989309	0.86143104
MGST3	0.93403125	0.877420806	0.909506646	0.995118128	0.99989309	0.864571107
GBE1	0.93403125	0.962934461	0.864443977	0.999939566	0.99989309	0.867505247
SLC50A1	0.93403125	0.982212778	0.894547778	0.982140778	0.99989309	0.88479494
LOC618787	0.93403125	0.820796062	0.962754771	0.999939566	0.99989309	0.887248119
BLVRA	0.93403125	0.91994024	0.872397372	0.999939566	0.99989309	0.89124006
MILR1	0.93403125	0.835896803	0.926613927	0.960357834	0.99989309	0.908939618
ZSCAN30	0.93403125	0.820959665	0.987842204	0.981022805	0.99989309	0.912101047
IFIT2	0.93403125	0.838405969	0.90002174	0.965590385	0.99989309	0.926872824
SERTAD4	0.93403125	0.831242188	0.899530079	0.999939566	0.99989309	0.942421464
EPB41L2	0.93403125	0.820000472	0.921225977	0.956154344	0.99989309	0.963768949
RIMBP2	0.93403125	0.88311684	0.933102499	0.95175364	0.99989309	0.96544761
LOC104974891	0.93403125	0.852829558	0.870054336	0.995118128	0.99989309	0.97183638
HIVEP3	0.93403125	0.80931829	0.87146798	0.948172741	0.99989309	0.991281092
KIAA0513	0.934044388	0.834316926	0.870054336	0.999939566	0.99989309	0.86307729
ANKRD34A	0.934044388	0.823187795	0.979096894	0.99879985	0.99989309	0.863755488
GOLPH3	0.934044388	0.837455678	0.939417893	0.956154344	0.99989309	0.864219261
LOC112444869	0.934044388	0.838405969	0.960137022	0.995800463	0.99989309	0.864219261
LOC101902807	0.934044388	0.848136412	0.861838588	0.999939566	0.99989309	0.866836199
HYPK	0.934044388	0.967515137	0.917092799	0.992402807	0.99989309	0.868108836
WDR34	0.934044388	0.877749555	0.879152963	0.999939566	0.99989309	0.868108836
NUP54	0.934044388	0.855979085	0.87146798	0.999939566	0.99989309	0.869162232
CDC26	0.934044388	0.88311684	0.899195818	0.999939566	0.99989309	0.870531681
SFXN3	0.934044388	0.908709906	0.914668913	0.999939566	0.99989309	0.871630129

CDNF	0.934044388	0.921393399	0.937930242	0.963528138	0.99989309	0.872224159
DHRS7	0.934044388	0.854656328	0.869529186	0.999939566	0.99989309	0.875441396
KYAT3	0.934044388	0.815354163	0.893544538	0.956154344	0.99989309	0.883302043
ATP1B3	0.934044388	0.96412538	0.892122482	0.990571725	0.99989309	0.886992214
FBXO17	0.934044388	0.942048064	0.870054336	0.999939566	0.99989309	0.887248119
RPL22	0.934044388	0.902136721	0.950443857	0.995118128	0.99989309	0.890898561
KIAA0141	0.934044388	0.825301941	0.906804311	0.999939566	0.99989309	0.891000792
LOC101902174	0.934044388	0.926435857	0.87214481	0.999939566	0.99989309	0.891908825
SCAI	0.934044388	0.881079118	0.877133001	0.999939566	0.99989309	0.902660792
CYB561	0.934044388	0.862527737	0.871859013	0.999939566	0.99989309	0.905220694
KCNN4	0.934044388	0.857034402	0.930892662	0.999939566	0.99989309	0.905220694
NAB1	0.934044388	0.989661212	0.87214481	0.95964071	0.99989309	0.90525134
PAPD5	0.934044388	0.875803224	0.87146798	0.981022805	0.99989309	0.906445281
BTN3A3	0.934044388	0.818711217	0.958420472	0.969717497	0.99989309	0.912101047
LOC101906226	0.934044388	0.893639679	0.863172962	0.995118128	0.99989309	0.922709797
LRRC20	0.934044388	0.810519292	0.929303679	0.999939566	0.99989309	0.928653932
TAL1	0.934044388	0.868963165	0.929958748	0.996601241	0.99989309	0.930318133
PHB	0.934044388	0.879686372	0.8868125	0.986030331	0.99989309	0.930707319
NR1D2	0.934044388	0.907878616	0.868936984	0.959727203	0.99989309	0.931466775
GALM	0.934044388	0.852463941	0.87146798	0.999939566	0.99989309	0.935655141
CETN2	0.934044388	0.811804876	0.965750046	0.959009349	0.99989309	0.939426596
TMEM11	0.934044388	0.813371202	0.965416289	0.990571725	0.99989309	0.940683751
VMP1	0.934044388	0.89460994	0.880277219	0.999939566	0.99989309	0.940683751
TBC1D24	0.934044388	0.836653211	0.963986266	0.972367384	0.99989309	0.941925704
ABCB9	0.934044388	0.857034402	0.878050811	0.999939566	0.99989309	0.941925704
LOC786015	0.934044388	0.838843184	0.886282865	0.992368879	0.99989309	0.943777356
LOC101908075	0.934044388	0.831242188	0.878603452	0.999939566	0.99989309	0.943891002
PQLC1	0.934044388	0.913075885	0.877998684	0.960986457	0.99989309	0.946682284
NXPH3	0.934044388	0.842654254	0.887130994	0.994365581	0.99989309	0.956610132
SGK3	0.934044388	0.844510795	0.861838588	0.992137653	0.99989309	0.958540316
LOC104975635	0.934044388	0.866977641	0.878603452	0.963829131	0.99989309	0.961606931
RHOC	0.934044388	0.837455678	0.894874079	0.973785871	0.99989309	0.976323853
NTRK3	0.934044388	0.835276066	0.882898206	0.959786126	0.99989309	0.978656049
LMF1	0.934044388	0.821124254	0.929958748	0.956154344	0.99989309	0.98016619
FIS1	0.934160237	0.880711088	0.914685423	0.995118128	0.99989309	0.866600727
POMP	0.934160237	0.818587254	0.962209895	0.999939566	0.99989309	0.870895425
ERCC8	0.934160237	0.956843573	0.87146798	0.990571725	0.99989309	0.908939618
SIDT1	0.934160237	0.866803532	0.899195818	0.982140778	0.99989309	0.956610132

EFTUD2	0.934181522	0.846959498	0.933629769	0.994365581	0.99989309	0.95470473
SPAST	0.93419712	0.874669928	0.870585788	0.951715131	0.99989309	0.868430302
HOXB5	0.93419712	0.908192144	0.87146798	0.999939566	0.99989309	0.922301228
AIMP1	0.934239804	0.852463941	0.879515214	0.999939566	0.99989309	0.887248119
SMIM17	0.934239804	0.958403824	0.89248682	0.956154344	0.99989309	0.917968123
TOR1AIP1	0.934324912	0.842797231	0.870054336	0.999939566	0.99989309	0.911696156
ADAMTSL4	0.934379159	0.877749555	0.899539089	0.999939566	0.99989309	0.889622239
LOC101906606	0.934379159	0.828417266	0.93145152	0.956154344	0.99989309	0.912101047
PPP1R15A	0.934379159	0.969537549	0.878603452	0.956154344	0.99989309	0.935607719
USP46	0.934392963	0.821588096	0.908823466	0.980418209	0.99989309	0.87114736
INIP	0.934392963	0.903178998	0.924266781	0.948608757	0.99989309	0.930058266
SNRPD2	0.934403723	0.907594268	0.93710335	0.992426863	0.99989309	0.891000792
ASXL3	0.934403723	0.919351156	0.880277219	0.999939566	0.99989309	0.901320431
EIF2S3	0.934861228	0.936581054	0.878050811	0.995880311	0.99989309	0.864219261
CAV3	0.934861228	0.902136721	0.875669731	0.969717497	0.99989309	0.872224159
RWDD3	0.934861228	0.86415368	0.962209895	0.974382211	0.99989309	0.876189885
GRAP	0.934861228	0.813371202	0.886282865	0.984100122	0.99989309	0.90175726
FBLL1	0.934861228	0.885796876	0.918576062	0.992137653	0.99989309	0.914344594
PSMA2	0.934861228	0.831198698	0.915891679	0.995118128	0.99989309	0.91596005
VKORC1L1	0.934861228	0.823187795	0.958420472	0.99879985	0.99989309	0.918291722
ENO1	0.934861228	0.852463941	0.878050811	0.9846464	0.99989309	0.944524573
COX14	0.934992759	0.857929799	0.885434576	0.97378567	0.99989309	0.884097654
LOC530929	0.934992759	0.827609894	0.878603452	0.999939566	0.99989309	0.923064313
SAMD1	0.934992759	0.831242188	0.860197593	0.999939566	0.99989309	0.92920113
ZNF180	0.935257695	0.964616377	0.920449324	0.99483611	0.99989309	0.86307729
PPP1R10	0.935257695	0.827609894	0.869455175	0.976950878	0.99989309	0.875441396
NADSYN1	0.935257695	0.841044548	0.909170161	0.998542029	0.99989309	0.875441396
POU2F3	0.935257695	0.832239565	0.965712499	0.992137653	0.99989309	0.884673942
AK4	0.935257695	0.886967296	0.91094712	0.999939566	0.99989309	0.885361631
LGALS3BP	0.935257695	0.926630033	0.870054336	0.969717497	0.99989309	0.886992214
EXOSC3	0.935257695	0.842565421	0.917876971	0.999939566	0.99989309	0.894901464
YWHAB	0.935257695	0.841044548	0.957379098	0.967063428	0.99989309	0.917968123
TMC6	0.935257695	0.817747418	0.952085491	0.962142137	0.99989309	0.935655141
TENM4	0.935257695	0.835276066	0.876075512	0.959786126	0.99989309	0.945593145
GTF3A	0.93527259	0.897657501	0.870054336	0.999939566	0.99989309	0.869857598
TRPM7	0.935331099	0.852463941	0.896272282	0.955560327	0.99989309	0.86307729
RPGR	0.935331099	0.936134921	0.941593855	0.96520291	0.99989309	0.86307729
GATA6	0.935331099	0.837455678	0.892348336	0.992368879	0.99989309	0.86307729

ABI3	0.935331099	0.835130235	0.880277219	0.999939566	0.99989309	0.864219261
SLC20A2	0.935331099	0.845470117	0.878603452	0.99879985	0.99989309	0.865159042
PTPRN2	0.935331099	0.867722813	0.947268948	0.999939566	0.99989309	0.865159042
MCM3AP	0.935331099	0.820796062	0.994425789	0.98470348	0.99989309	0.866253786
D2HGDH	0.935331099	0.828133113	0.952208316	0.956154344	0.99989309	0.868108836
LOC112443243	0.935331099	0.934248552	0.93145152	0.99879985	0.99989309	0.868108836
HAGHL	0.935331099	0.878594098	0.87214481	0.995118128	0.99989309	0.869171706
SZRD1	0.935331099	0.8104985	0.907733757	0.976950878	0.99989309	0.871166844
LOC100139325	0.935331099	0.813371202	0.950443857	0.99879985	0.99989309	0.871231167
LOC112442704	0.935331099	0.824119076	0.965416289	0.990571725	0.99989309	0.872224159
PHKA1	0.935331099	0.955798423	0.894414265	0.999939566	0.99989309	0.872493553
ROBO1	0.935331099	0.820796062	0.896272282	0.999939566	0.99989309	0.887248119
LOC512617	0.935331099	0.904539095	0.885354035	0.983569039	0.99989309	0.889622239
DHX9	0.935331099	0.922752283	0.87214481	0.956154344	0.99989309	0.893961809
IFT140	0.935331099	0.887383289	0.978892556	0.949947918	0.99989309	0.897818829
ALDH1A1	0.935331099	0.852377308	0.867166602	0.999939566	0.99989309	0.901422892
PNMA8A	0.935331099	0.835276066	0.939683463	0.973207466	0.99989309	0.90823073
LOC104970779	0.935331099	0.916740848	0.914668913	0.998542029	0.99989309	0.908939618
MTHFS	0.935331099	0.907229289	0.900109169	0.99879985	0.99989309	0.908939618
NCKAP1L	0.935331099	0.918306418	0.886282865	0.956154344	0.99989309	0.91008914
MOSPD3	0.935331099	0.934248552	0.941319062	0.962142137	0.99989309	0.912101047
STMN3	0.935331099	0.90339131	0.878050811	0.991837175	0.99989309	0.918291722
DGAT2	0.935331099	0.838169368	0.879968243	0.953510787	0.99989309	0.926872824
C8H9orf40	0.935331099	0.914252497	0.875669731	0.999939566	0.99989309	0.934653208
PLD5	0.935331099	0.909085943	0.880277219	0.999939566	0.99989309	0.937591162
MTHFD1	0.935331099	0.922434012	0.879215964	0.95175364	0.99989309	0.941925704
TTI1	0.935331099	0.823692881	0.894449269	0.999939566	0.99989309	0.943685681
SYT17	0.935331099	0.872282604	0.894449269	0.960123975	0.99989309	0.95470473
HTATIP2	0.935331099	0.8232491	0.904682623	0.99879985	0.99989309	0.956610132
SLC18A2	0.935331099	0.833369167	0.939683463	0.953508052	0.99989309	0.958540316
PROKR2	0.935331099	0.840313358	0.917092799	0.995800463	0.99989309	0.959256161
LOC786974	0.935331099	0.828417266	0.900128576	0.998542029	0.99989309	0.973195541
AGO2	0.935337969	0.823187795	0.861838588	0.98189469	0.99989309	0.876189885
PTTG1	0.935551653	0.890284639	0.917876971	0.999939566	0.99989309	0.863465757
VPS33B	0.935551653	0.939870925	0.912223566	0.971220023	0.99989309	0.866600727
PTEN	0.935551653	0.839641196	0.880277219	0.99879985	0.99989309	0.867716339
NTRK2	0.935551653	0.976302681	0.879215964	0.992154129	0.99989309	0.871712083
CLEC2B	0.935551653	0.838405969	0.869529186	0.999939566	0.99989309	0.871780952

MAP3K13	0.935551653	0.900467607	0.877998684	0.998542029	0.99989309	0.873118284
YIPF4	0.935551653	0.862791034	0.911743083	0.999939566	0.99989309	0.908939618
LOC101907174	0.935685775	0.885796876	0.882898206	0.996230812	0.99989309	0.86307729
TMEM39A	0.93576533	0.838405969	0.939683463	0.994309225	0.99989309	0.863755488
IGHMBP2	0.93576533	0.831242188	0.994425789	0.985048108	0.99989309	0.864219261
LOC101906484	0.93576533	0.838815395	0.867451783	0.999939566	0.99989309	0.864219261
GIGYF2	0.93576533	0.823588167	0.880277219	0.992235612	0.99989309	0.865159042
EIF3H	0.93576533	0.880711088	0.886881381	0.999939566	0.99989309	0.868108836
THSD1	0.93576533	0.836487169	0.921135532	0.999939566	0.99989309	0.868108836
EPM2A	0.93576533	0.835130235	0.939683463	0.974665397	0.99989309	0.869171706
GGACT	0.93576533	0.831242188	0.891455327	0.99879985	0.99989309	0.872175131
FABP5	0.93576533	0.904764822	0.957802423	0.967172536	0.99989309	0.873118284
MAP4K2	0.93576533	0.83793617	0.892122482	0.999939566	0.99989309	0.873118284
LOC515823	0.93576533	0.899387334	0.962176387	0.965590385	0.99989309	0.873520548
DIO3	0.93576533	0.939870925	0.920449324	0.959410646	0.99989309	0.877623013
LNX1	0.93576533	0.908192144	0.882898206	0.999939566	0.99989309	0.880272231
TXNDC9	0.93576533	0.845899886	0.949727203	0.998069032	0.99989309	0.881039089
RARA	0.93576533	0.820796062	0.869529186	0.999939566	0.99989309	0.882322567
CNOT6L	0.93576533	0.893639679	0.875669731	0.965097245	0.99989309	0.884608788
HDAC4	0.93576533	0.820556554	0.976479691	0.976950878	0.99989309	0.88494083
PLXNC1	0.93576533	0.938378242	0.880277219	0.986030331	0.99989309	0.886520861
DGKD	0.93576533	0.834011972	0.932521008	0.990049248	0.99989309	0.887248119
TMEM175	0.93576533	0.866803532	0.899195818	0.990571725	0.99989309	0.887248119
PPM1A	0.93576533	0.819900146	0.871109615	0.999939566	0.99989309	0.887248119
BGN	0.93576533	0.852463941	0.878603452	0.999939566	0.99989309	0.887457549
BCL2L12	0.93576533	0.855151723	0.926613927	0.990049248	0.99989309	0.891511718
FAM166A	0.93576533	0.922434012	0.921146002	0.951604306	0.99989309	0.894788061
TAX1BP3	0.93576533	0.885181785	0.95783333	0.959727203	0.99989309	0.895218005
RIC8A	0.93576533	0.913075885	0.862585131	0.993948479	0.99989309	0.902660792
LOC541276	0.93576533	0.934571318	0.921135532	0.963829131	0.99989309	0.90525134
ETFB	0.93576533	0.82919708	0.882898206	0.998251353	0.99989309	0.90628585
GRIA1	0.93576533	0.820796062	0.875669731	0.999939566	0.99989309	0.906895944
STON1	0.93576533	0.881823401	0.941319062	0.99879985	0.99989309	0.908939618
SYTL4	0.93576533	0.867425848	0.870920678	0.999939566	0.99989309	0.908939618
NXT2	0.93576533	0.820796062	0.909069696	0.999939566	0.99989309	0.908939618
ADORA3	0.93576533	0.939027995	0.886282865	0.99879985	0.99989309	0.911632967
BACH1	0.93576533	0.90249621	0.93145152	0.954423369	0.99989309	0.911696156
R3HDM2	0.93576533	0.810976764	0.933102499	0.990300191	0.99989309	0.912376736

LOC107132175	0.93576533	0.823602091	0.877998684	0.990049248	0.99989309	0.926872824
CDKN2C	0.93576533	0.820796062	0.899195818	0.965097245	0.99989309	0.928653932
MRPS5	0.93576533	0.834869027	0.868936984	0.956154344	0.99989309	0.930707319
LOC112443816	0.93576533	0.862941799	0.930262306	0.992137653	0.99989309	0.934653208
CDKL2	0.93576533	0.835809337	0.886282865	0.983569039	0.99989309	0.942455807
XRN2	0.93576533	0.828600257	0.939683463	0.995118128	0.99989309	0.961506391
LOC104975811	0.93576533	0.854362191	0.882898206	0.995118128	0.99989309	0.983525373
NELFA	0.935833031	0.842797231	0.957379098	0.994309225	0.99989309	0.863891806
MAPKAPK2	0.935833031	0.820796062	0.968455468	0.985815315	0.99989309	0.886992214
UBR1	0.935833031	0.929536223	0.878603452	0.956154344	0.99989309	0.893961809
ZNF830	0.935833031	0.921280256	0.885354035	0.999939566	0.99989309	0.902660792
COMMD10	0.935833031	0.821982753	0.888610896	0.995118128	0.99989309	0.943685681
PRDX5	0.935999613	0.90005089	0.915581825	0.9846464	0.99989309	0.887248119
LOC512978	0.935999613	0.871682971	0.948628899	0.977470078	0.99989309	0.896685501
MFSD3	0.935999613	0.91158897	0.917876971	0.969717497	0.99989309	0.911526437
MRPL32	0.935999613	0.924421023	0.878603452	0.98636843	0.99989309	0.917968123
ATP6V1D	0.936008325	0.834011972	0.906925281	0.999939566	0.99989309	0.896149571
PEX2	0.936008325	0.830709707	0.923202492	0.981022805	0.99989309	0.979233909
TBX6	0.936031638	0.889410709	0.87146798	0.982908486	0.99989309	0.940683751
NPRL3	0.93603997	0.88311684	0.882890706	0.999939566	0.99989309	0.886992214
CTNNAL1	0.936306943	0.875022483	0.868936984	0.999939566	0.99989309	0.866253786
FAM3A	0.936306943	0.934248552	0.886282865	0.990049248	0.99989309	0.910353954
EPPK1	0.936325046	0.821124254	0.987842204	0.977043589	0.99989309	0.866612872
LOC781913	0.936325046	0.944605763	0.91546474	0.971220023	0.99989309	0.87114736
INPP4A	0.936448543	0.944314627	0.87146798	0.999939566	0.99989309	0.895218005
RUFY3	0.93657281	0.837455678	0.868936984	0.978630043	0.99989309	0.895218005
LOC104973803	0.937045637	0.945105738	0.899530079	0.998542029	0.99989309	0.870895425
ARL1	0.937045637	0.855979085	0.939683463	0.99879985	0.99989309	0.914440253
WDR61	0.937045637	0.81808696	0.920449324	0.996601241	0.99989309	0.942195382
HAUS2	0.937050965	0.882930807	0.994425789	0.956154344	0.99989309	0.869162232
DERA	0.937103584	0.824676261	0.967489175	0.971220023	0.99989309	0.957877422
CRCP	0.937135786	0.834869027	0.959320266	0.992137653	0.99989309	0.912376736
TMEM60	0.937139078	0.838815395	0.921225977	0.956154344	0.99989309	0.867440033
RAB4A	0.937139078	0.823371359	0.920449324	0.969982152	0.99989309	0.870895425
ATL3	0.937139078	0.861552326	0.972874677	0.99879985	0.99989309	0.872175131
FAM160A1	0.937139078	0.988649234	0.867451783	0.992137653	0.99989309	0.873520548
ATG4D	0.937139078	0.857034402	0.957802423	0.999939566	0.99989309	0.875441396
NHLRC2	0.937139078	0.921393399	0.933102499	0.981022805	0.99989309	0.876009151

GRAMD2A	0.937139078	0.820796062	0.988752364	0.990059245	0.99989309	0.878011255
KRT8	0.937139078	0.921280256	0.958420472	0.965097245	0.99989309	0.878851638
RFC5	0.937139078	0.838405969	0.957802423	0.999939566	0.99989309	0.882322567
NRGN	0.937139078	0.886909612	0.922147089	0.99879985	0.99989309	0.888396511
KLHL33	0.937139078	0.875345836	0.904682623	0.99879985	0.99989309	0.895218005
PHETA2	0.937139078	0.851167599	0.886176337	0.999939566	0.99989309	0.902660792
DNAJC17	0.937139078	0.852463941	0.963643451	0.998542029	0.99989309	0.906529452
LOC101902757	0.937139078	0.837455678	0.939683463	0.999939566	0.99989309	0.908939618
SLC12A4	0.937139078	0.821588096	0.872470014	0.995118128	0.99989309	0.914440253
ST6GAL2	0.937139078	0.823965712	0.927079582	0.999939566	0.99989309	0.914440253
DLD	0.937139078	0.866813771	0.878603452	0.992829869	0.99989309	0.923871657
LOC112446777	0.937139078	0.842797231	0.922126495	0.995118128	0.99989309	0.926872824
THRA	0.937139078	0.913690438	0.873750661	0.975529024	0.99989309	0.927901387
LOC531679	0.937139078	0.849834516	0.870774839	0.999939566	0.99989309	0.939507593
SNX20	0.937139078	0.858423555	0.909170161	0.977470078	0.99989309	0.941925704
AGO1	0.937139078	0.838815395	0.870492132	0.999939566	0.99989309	0.943138585
CRTC2	0.937139078	0.835276066	0.908821787	0.956154344	0.99989309	0.944722345
FAM25A	0.937139078	0.820000472	0.894414265	0.981022805	0.99989309	0.95470473
COL28A1	0.937139078	0.851167599	0.882890706	0.999939566	0.99989309	0.95470473
CLUH	0.937139078	0.845979391	0.878603452	0.993811478	0.99989309	0.955585296
DPP9	0.937139078	0.926613567	0.875669731	0.959786126	0.99989309	0.981164039
SPATA7	0.937139078	0.818650481	0.90166788	0.968293092	0.99989309	0.991281092
RNLS	0.937193791	0.840313358	0.869455175	0.958879502	0.99989309	0.943138585
LOC100848575	0.93732181	0.847749382	0.939683463	0.998542029	0.99989309	0.893961809
QPCTL	0.93732181	0.834869027	0.917876971	0.963829131	0.99989309	0.923064313
LOC104970105	0.937377316	0.82890642	0.903453422	0.999939566	0.99989309	0.888617204
MYH6	0.937377316	0.932974678	0.892871521	0.999939566	0.99989309	0.891000792
EIF5B	0.937377316	0.848556568	0.878603452	0.992137653	0.99989309	0.96544761
MAPK8IP1	0.937497742	0.835809337	0.915891679	0.999939566	0.99989309	0.872175131
LOC112443502	0.937497742	0.820796062	0.92153058	0.992368879	0.99989309	0.88667499
USP1	0.937497742	0.924799142	0.877133001	0.955687359	0.99989309	0.888396511
LOC104969981	0.937497742	0.81808696	0.904682623	0.999939566	0.99989309	0.890898561
LOC101903649	0.937497742	0.831141011	0.96636908	0.971220023	0.99989309	0.891908825
ANKIB1	0.937497742	0.853258514	0.887130994	0.998542029	0.99989309	0.920942379
SMAD3	0.937497742	0.836487169	0.891570161	0.999939566	0.99989309	0.936521866
TMEM41A	0.937555558	0.834011972	0.96201942	0.9846464	0.99989309	0.940683751
GRAMD2B	0.937578734	0.86415368	0.927460291	0.995118128	0.99989309	0.865926817
NAGPA	0.937578734	0.855979085	0.922147089	0.994309225	0.99989309	0.866253786

PIGX	0.937578734	0.860449455	0.8895568	0.999939566	0.99989309	0.868108836
NGLY1	0.937578734	0.818806929	0.86796189	0.983569039	0.99989309	0.86921618
VAC14	0.937578734	0.879686372	0.887130994	0.999939566	0.99989309	0.870895425
LPAR3	0.937578734	0.835408071	0.909002826	0.995536195	0.99989309	0.872175131
STAU1	0.937578734	0.86415368	0.882898206	0.999939566	0.99989309	0.876262605
FH	0.937578734	0.84670433	0.888610896	0.995946943	0.99989309	0.878040551
EXOC1L	0.937578734	0.855151723	0.914685423	0.982367414	0.99989309	0.911696156
CYBRD1	0.937578734	0.834316926	0.880255347	0.961525281	0.99989309	0.912101047
TFEC	0.937578734	0.914162656	0.92153058	0.982140778	0.99989309	0.914440253
PKP4	0.937578734	0.911279338	0.886282865	0.971220023	0.99989309	0.928799366
PPFIBP2	0.937578734	0.829099159	0.900128576	0.968306132	0.99989309	0.942421464
SLC22A15	0.937578734	0.910503919	0.894547778	0.961525281	0.99989309	0.967796552
CHM	0.937578734	0.866605676	0.874057431	0.984968973	0.99989309	0.982559673
CD200R1L	0.937884572	0.969870198	0.922147089	0.98860254	0.99989309	0.865159042
LOC107131772	0.937884572	0.883378562	0.988740473	0.992137653	0.99989309	0.865159042
AP3M1	0.937884572	0.868963165	0.957417441	0.999939566	0.99989309	0.87114736
BCL6	0.937884572	0.934248552	0.926183278	0.99879985	0.99989309	0.876517132
FOXP1	0.937884572	0.841629926	0.90002174	0.999939566	0.99989309	0.877623013
FOXO1	0.937884572	0.820796062	0.87265573	0.999939566	0.99989309	0.887248119
NEURL3	0.937884572	0.818435706	0.880277219	0.999939566	0.99989309	0.891000792
SH3D19	0.937884572	0.851923106	0.945455812	0.999939566	0.99989309	0.892214703
ZNF543	0.937884572	0.835276066	0.878050811	0.956303465	0.99989309	0.908785004
GPRASP1	0.937884572	0.905621601	0.879726737	0.99879985	0.99989309	0.908785004
PIK3CB	0.937884572	0.839461242	0.941593855	0.999939566	0.99989309	0.908939618
LOC112442081	0.937884572	0.842667989	0.868936984	0.990571725	0.99989309	0.910619304
MTA2	0.937884572	0.884084518	0.878603452	0.995118128	0.99989309	0.924845138
PSMB9	0.937884572	0.886967296	0.878603452	0.999334425	0.99989309	0.935163166
CD58	0.937884572	0.820559187	0.933116459	0.999939566	0.99989309	0.935655141
TXNDC17	0.937884572	0.94883251	0.885481718	0.956154344	0.99989309	0.937923672
LOC614207	0.937884572	0.888994602	0.880277219	0.960986457	0.99989309	0.940917403
DCP2	0.937884572	0.907878616	0.891455327	0.99419346	0.99989309	0.942421464
EXOSC5	0.937884572	0.871412404	0.870916127	0.999939566	0.99989309	0.943866443
APBA1	0.937884572	0.857005855	0.878603452	0.992137653	0.99989309	0.965332394
DTWD2	0.937884572	0.90249621	0.921225977	0.956154344	0.99989309	0.972698857
AMER1	0.93802457	0.909270621	0.98662331	0.958849311	0.99989309	0.865926817
TMEM159	0.93802457	0.880843163	0.870911433	0.969717497	0.99989309	0.867376048
PDGFB	0.93802457	0.911368806	0.882898206	0.994652359	0.99989309	0.869171706
GPR160	0.93802457	0.877723422	0.877230238	0.999939566	0.99989309	0.872224159

CTSD	0.93802457	0.9136091	0.917876971	0.999939566	0.99989309	0.87428828
NKAIN3	0.93802457	0.967515137	0.899195818	0.959786126	0.99989309	0.875441396
BLOC1S4	0.93802457	0.892580117	0.909170161	0.992137653	0.99989309	0.875441396
LOC107131566	0.93802457	0.878720916	0.882898206	0.999939566	0.99989309	0.875441396
RNF11	0.93802457	0.824676261	0.962209895	0.999939566	0.99989309	0.875441396
DIAPH1	0.93802457	0.893512526	0.977612552	0.990491321	0.99989309	0.883080515
ZNF500	0.93802457	0.828417266	0.994425789	0.96095927	0.99989309	0.891000792
USP3	0.93802457	0.868503809	0.872470014	0.99879985	0.99989309	0.894901464
PSMD11	0.93802457	0.838405969	0.93050447	0.999939566	0.99989309	0.898273855
VGF	0.93802457	0.851167599	0.915636025	0.999939566	0.99989309	0.906529452
ERRFI1	0.93802457	0.838843184	0.882898206	0.995118128	0.99989309	0.908939618
RBM23	0.93802457	0.872969587	0.89248682	0.999939566	0.99989309	0.908939618
CERS2	0.93802457	0.890427603	0.937835937	0.999939566	0.99989309	0.908939618
ZFP30	0.93802457	0.874341511	0.87214481	0.999939566	0.99989309	0.912101047
FAM171B	0.93802457	0.81808696	0.899195818	0.999939566	0.99989309	0.912376736
LOC101904447	0.93802457	0.851167599	0.8895568	0.990049248	0.99989309	0.914046542
LOC787891	0.93802457	0.899474896	0.937930242	0.990491321	0.99989309	0.922229044
FOS	0.93802457	0.8554287	0.899195818	0.983038839	0.99989309	0.922301228
YTHDC2	0.93802457	0.95559169	0.877580131	0.981022805	0.99989309	0.923064313
MCTS1	0.93802457	0.825856868	0.87214481	0.995800463	0.99989309	0.926872824
MBD6	0.93802457	0.827536765	0.970904484	0.995800463	0.99989309	0.927529392
WDR45B	0.93802457	0.835130235	0.931397502	0.998542029	0.99989309	0.929537775
FIGN	0.93802457	0.851629339	0.939091808	0.956154344	0.99989309	0.933763401
AQP11	0.93802457	0.958564557	0.868936984	0.998542029	0.99989309	0.934775196
LOC101902991	0.93802457	0.823692881	0.926613927	0.999939566	0.99989309	0.937591162
PARP16	0.93802457	0.83793617	0.8895568	0.99879985	0.99989309	0.943138585
BPI	0.93802457	0.914616699	0.92153058	0.990049248	0.99989309	0.945759045
IPO13	0.93802457	0.866803532	0.911330721	0.990049248	0.99989309	0.96435585
HDAC5	0.93802457	0.837799788	0.880277219	0.983448487	0.99989309	0.978565009
KYAT1	0.93802457	0.823692881	0.870054336	0.992368879	0.99989309	0.989947227
RNFT1	0.93802457	0.829033708	0.890398089	0.955560327	0.99989309	0.99497777
LOC104974137	0.93802457	0.828133113	0.890264995	0.982367414	0.99989309	0.997366598
MTA1	0.938375846	0.820796062	0.993278977	0.995800463	0.99989309	0.873051618
ETHE1	0.938375846	0.93453191	0.87214481	0.956154344	0.99989309	0.910353954
CERS6	0.93858033	0.88469581	0.89007492	0.999939566	0.99989309	0.908939618
SSNA1	0.938637917	0.882930807	0.904682623	0.992692773	0.99989309	0.871780952
LOC614141	0.938805212	0.954891113	0.917876971	0.999939566	0.99989309	0.866612872
LOC518961	0.938805212	0.823692881	0.887130994	0.999939566	0.99989309	0.893961809

LOC107132883	0.938924585	0.835130235	0.894447907	0.996084773	0.99989309	0.868108836
LMNA	0.938924585	0.83744789	0.878603452	0.999939566	0.99989309	0.869171706
LOC101903402	0.938924585	0.847619966	0.957379098	0.99879985	0.99989309	0.870895425
CDCP1	0.938924585	0.838405969	0.967489175	0.999939566	0.99989309	0.871129794
ALKBH4	0.938924585	0.883378562	0.905832059	0.992741182	0.99989309	0.871780952
SGMS2	0.938924585	0.924799142	0.933963997	0.981022805	0.99989309	0.875441396
LOC613822	0.938924585	0.915717447	0.962209895	0.995118128	0.99989309	0.880404833
DNAJB2	0.938924585	0.8232491	0.931265798	0.999939566	0.99989309	0.882322567
ACTR3B	0.938924585	0.838843184	0.941593855	0.956154344	0.99989309	0.884387948
METTL14	0.938924585	0.964616377	0.89265635	0.992692773	0.99989309	0.887248119
INPP5K	0.938924585	0.955483597	0.924266781	0.995118128	0.99989309	0.887248119
RNF227	0.938924585	0.904764822	0.879215964	0.99879985	0.99989309	0.887248119
C3H1orf43	0.938924585	0.820988267	0.942004125	0.999939566	0.99989309	0.888617204
ASB4	0.938924585	0.883476578	0.882898206	0.990049248	0.99989309	0.890898561
LOC112449086	0.938924585	0.835809337	0.870054336	0.990571725	0.99989309	0.891511718
AHSA1	0.938924585	0.820269509	0.886282865	0.985048108	0.99989309	0.893622929
RALGAPA2	0.938924585	0.831242188	0.985552223	0.971220023	0.99989309	0.893961809
AXIN1	0.938924585	0.824676261	0.93050447	0.972367384	0.99989309	0.896934364
MPP6	0.938924585	0.846678309	0.904682623	0.981022805	0.99989309	0.902122572
SLC25A34	0.938924585	0.908018498	0.877816623	0.999939566	0.99989309	0.904984293
LOC101910153	0.938924585	0.921280256	0.939683463	0.990049248	0.99989309	0.905220694
TNFAIP6	0.938924585	0.848986275	0.869455175	0.999939566	0.99989309	0.905267129
ESR1	0.938924585	0.864459672	0.882890706	0.973377993	0.99989309	0.906529452
DPF3	0.938924585	0.883927618	0.892122482	0.984100122	0.99989309	0.906529452
AKAP12	0.938924585	0.881727487	0.88330023	0.995946943	0.99989309	0.906963595
SLC35F6	0.938924585	0.904764822	0.939091808	0.999939566	0.99989309	0.908182968
ZBTB33	0.938924585	0.914616699	0.912223566	0.956154344	0.99989309	0.908939618
SP140	0.938924585	0.828600257	0.974832809	0.989418929	0.99989309	0.908939618
SPINT2	0.938924585	0.885245308	0.966157152	0.990049248	0.99989309	0.908939618
TOM1L1	0.938924585	0.871914169	0.882898206	0.999939566	0.99989309	0.909990551
TBC1D10B	0.938924585	0.835276066	0.880277219	0.99879985	0.99989309	0.910619304
LOC101906513	0.938924585	0.840313358	0.89145542	0.999939566	0.99989309	0.910619304
MYRIP	0.938924585	0.972786652	0.891307181	0.992137653	0.99989309	0.912101047
SBNO1	0.938924585	0.863826371	0.892122482	0.983038839	0.99989309	0.914440253
NCOA7	0.938924585	0.841044548	0.939683463	0.985181062	0.99989309	0.918666628
LOC107133226	0.938924585	0.867722813	0.919997767	0.999939566	0.99989309	0.92035958
NUDT19	0.938924585	0.825215718	0.915216087	0.995800463	0.99989309	0.922229044
LOC104975684	0.938924585	0.897918739	0.942314296	0.958879502	0.99989309	0.924845138

LOC100335553	0.938924585	0.851167599	0.93710335	0.966252636	0.99989309	0.944123524
TNFSF13B	0.938924585	0.853448902	0.908208688	0.99879985	0.99989309	0.95342487
SEL1L3	0.938924585	0.882706523	0.91546474	0.986394228	0.99989309	0.955817715
SAP30BP	0.938924585	0.845256475	0.915636025	0.999939566	0.99989309	0.956610132
PCDH20	0.938924585	0.861568371	0.886282865	0.995118128	0.99989309	0.962555912
DNAH12	0.938924585	0.880674338	0.875669731	0.999334425	0.99989309	0.968245211
SPPL3	0.938924585	0.846157703	0.893763648	0.956154344	0.99989309	0.974139155
C1RL	0.938924585	0.818806929	0.922257272	0.956154344	0.99989309	0.992671204
LOC786363	0.93899044	0.893927942	0.932641597	0.999939566	0.99989309	0.885060677
XKR6	0.939239618	0.988153273	0.880277219	0.990341344	0.99989309	0.881439164
LOC107132757	0.939340935	0.954059509	0.870054336	0.99879985	0.99989309	0.936273494
KNOP1	0.939340935	0.883378562	0.886373777	0.999939566	0.99989309	0.949206487
SBDS	0.939433229	0.862267429	0.915636025	0.999939566	0.99989309	0.933678522
HMOX2	0.93947879	0.840313358	0.878050811	0.999939566	0.99989309	0.873118284
ARHGEF28	0.93947879	0.969202872	0.891051885	0.999939566	0.99989309	0.884387948
PRDX3	0.93947879	0.820796062	0.88950849	0.97136787	0.99989309	0.886992214
PKDREJ	0.93947879	0.891430331	0.929958748	0.979043693	0.99989309	0.906178214
PLEKHH1	0.93947879	0.864541566	0.869529186	0.999939566	0.99989309	0.908939618
DKK3	0.93947879	0.866803532	0.924266781	0.999939566	0.99989309	0.908939618
LOC112446042	0.939484402	0.93526537	0.880277219	0.992137653	0.99989309	0.936563391
LOC101902204	0.9395534	0.920347742	0.870054336	0.987143706	0.99989309	0.891000792
CARF	0.939800432	0.94610099	0.899624092	0.990049248	0.99989309	0.867440033
SMARCB1	0.939800432	0.934531829	0.92153058	0.99879985	0.99989309	0.887248119
TIAL1	0.939800432	0.929573947	0.959198396	0.984769774	0.99989309	0.897011069
TPC3	0.939800432	0.992968229	0.878603452	0.96095927	0.99989309	0.908939618
PPME1	0.939800432	0.914616699	0.879515214	0.967333752	0.99989309	0.908939618
ALG10	0.939800432	0.931636882	0.923967596	0.972367384	0.99989309	0.912598075
LOC529125	0.939800432	0.821124254	0.878603452	0.973377993	0.99989309	0.931126954
CASP2	0.939800432	0.922264623	0.878603452	0.995800463	0.99989309	0.940683751
RNF122	0.9398649	0.911106603	0.896272282	0.971220023	0.99989309	0.868108836
RRN3	0.9398649	0.852463941	0.939775098	0.977470078	0.99989309	0.872224159
PSPN	0.9398649	0.851309641	0.939683463	0.966070428	0.99989309	0.957707888
UTP14A	0.93989625	0.978453686	0.882898206	0.995118128	0.99989309	0.886992214
LOC107132911	0.940130041	0.855151723	0.924457409	0.971220023	0.99989309	0.86934024
FBXO31	0.940130041	0.879391812	0.896009596	0.99879985	0.99989309	0.871787716
ABHD14B	0.940130041	0.926435857	0.87146798	0.99879985	0.99989309	0.878152258
C16H1orf174	0.940130041	0.834316926	0.979231344	0.992368879	0.99989309	0.912376736
GSN	0.940130041	0.855151723	0.879726737	0.99879985	0.99989309	0.923064313

NUP35	0.940130041	0.899142992	0.899539089	0.96095927	0.99989309	0.957140928
LOC100294723	0.940130041	0.821588096	0.874057431	0.992829869	0.99989309	0.984449688
C8H9orf85	0.940574335	0.828600257	0.961742979	0.999939566	0.99989309	0.868108836
C25H16orf89	0.94090711	0.882552594	0.879215964	0.999939566	0.99989309	0.868108836
ABHD1	0.94090711	0.839399097	0.883726795	0.999939566	0.99989309	0.868108836
CARMIL1	0.94090711	0.884768652	0.886282865	0.971220023	0.99989309	0.869171706
MYMK	0.94090711	0.932516219	0.955434173	0.998542029	0.99989309	0.870895425
MESP2	0.94090711	0.967113913	0.886162626	0.992137653	0.99989309	0.87114736
ADAMTS3	0.94090711	0.84999555	0.874724243	0.95964071	0.99989309	0.872224159
IPO8	0.94090711	0.978453686	0.942004125	0.963153458	0.99989309	0.877623013
LOC101905390	0.94090711	0.928119925	0.933102499	0.99879985	0.99989309	0.878152258
TTLL4	0.94090711	0.82890642	0.969618195	0.982367414	0.99989309	0.880884375
FAM172A	0.94090711	0.834011972	0.985552223	0.994365581	0.99989309	0.88609792
NPRL2	0.94090711	0.854944719	0.878603452	0.988140196	0.99989309	0.892192495
MRFAP1L1	0.94090711	0.88311684	0.948009116	0.992137653	0.99989309	0.893961809
LOC783378	0.94090711	0.959119521	0.879726737	0.99879985	0.99989309	0.897011069
LOC112445972	0.94090711	0.920347742	0.899195818	0.999939566	0.99989309	0.898871685
GPBAR1	0.94090711	0.96931064	0.893763648	0.995118128	0.99989309	0.905220694
C8H8orf58	0.94090711	0.827609894	0.917876971	0.990571725	0.99989309	0.908939618
MAP3K11	0.94090711	0.825215718	0.883647437	0.999939566	0.99989309	0.912101047
EXTL3	0.94090711	0.835276066	0.916702	0.995800463	0.99989309	0.912396352
VPS26C	0.94090711	0.882504547	0.878603452	0.984618049	0.99989309	0.923064313
C19H17orf49	0.94090711	0.913175252	0.872651958	0.982140778	0.99989309	0.925494614
PAXIP1	0.94090711	0.875449288	0.91234361	0.990571725	0.99989309	0.941925704
LOC112445889	0.94090711	0.827609894	0.870054336	0.999939566	0.99989309	0.95470473
PTBP2	0.94090711	0.90249621	0.9014981	0.984968973	0.99989309	0.970702553
ZNF419	0.940995995	0.836653211	0.953911996	0.999939566	0.99989309	0.870895425
PAPD7	0.940995995	0.835197362	0.882898206	0.999939566	0.99989309	0.87428828
SCN2B	0.940995995	0.974986418	0.899539089	0.992368879	0.99989309	0.876517132
KIF2A	0.940995995	0.836026119	0.892122482	0.999334425	0.99989309	0.884387948
NOP9	0.940995995	0.896278848	0.917066863	0.959727203	0.99989309	0.886992214
PSEN1	0.940995995	0.834316926	0.927460291	0.999939566	0.99989309	0.886992214
CATSPERE	0.940995995	0.823187795	0.980442556	0.999939566	0.99989309	0.889622239
ATG2A	0.940995995	0.835276066	0.976719499	0.999939566	0.99989309	0.893961809
SNTB2	0.940995995	0.851167599	0.91546474	0.994365581	0.99989309	0.899551847
IVD	0.940995995	0.852377308	0.872397372	0.963829131	0.99989309	0.906065482
LTB4R2	0.940995995	0.921280256	0.8895568	0.990049248	0.99989309	0.909768235
ANKRD31	0.940995995	0.899048532	0.879148792	0.989391869	0.99989309	0.914440253

PRR3	0.940995995	0.936134921	0.873750661	0.995118128	0.99989309	0.930707319
SLITRK2	0.940995995	0.824676261	0.920449324	0.990049248	0.99989309	0.933763401
ZNF789	0.940995995	0.84043321	0.909442165	0.992368879	0.99989309	0.934775196
DCAF12	0.940995995	0.843966059	0.880277219	0.99879985	0.99989309	0.940917403
FXYD5	0.940995995	0.93519674	0.878050811	0.969717497	0.99989309	0.943685681
CHP1	0.940995995	0.85749413	0.87214481	0.999939566	0.99989309	0.943685681
CCT7	0.940995995	0.867204257	0.908165509	0.996084773	0.99989309	0.945759045
ALAD	0.940995995	0.851167599	0.899195818	0.990049248	0.99989309	0.948763249
LOC112442967	0.940995995	0.820796062	0.939683463	0.996084773	0.99989309	0.950601157
LOC783612	0.940995995	0.868963165	0.882898206	0.976950878	0.99989309	0.961506391
SMG6	0.940995995	0.875022483	0.883647437	0.99879985	0.99989309	0.96544761
STC1	0.941198191	0.974875445	0.921612776	0.994365581	0.99989309	0.868108836
CLEC7A	0.941198191	0.884220302	0.878603452	0.999939566	0.99989309	0.868108836
ATG16L2	0.941198191	0.885181785	0.931173336	0.969717497	0.99989309	0.900168812
ING2	0.941198191	0.907594268	0.894547778	0.982140778	0.99989309	0.955585296
LZIC	0.941212402	0.843422478	0.978892556	0.998542029	0.99989309	0.888617204
ARHGEF18	0.941212402	0.883920212	0.951607422	0.963829131	0.99989309	0.89321306
ZCWPW2	0.941212402	0.911196851	0.883726795	0.983569039	0.99989309	0.910353954
SH2D3A	0.941212402	0.952464938	0.880277219	0.992137653	0.99989309	0.942421464
GLI2	0.941259056	0.89763446	0.942004125	0.985181062	0.99989309	0.940683751
PSMD4	0.941259056	0.864466447	0.88950849	0.990571725	0.99989309	0.955585296
ACAA2	0.941317606	0.852463941	0.880277219	0.999939566	0.99989309	0.876189885
TCP11L2	0.941317606	0.824676261	0.891014698	0.999939566	0.99989309	0.886520861
MOV10	0.941650441	0.876057665	0.994425789	0.975529024	0.99989309	0.872175131
LOC100848569	0.941650441	0.89400848	0.939683463	0.969744477	0.99989309	0.873520548
SAXO2	0.941650441	0.873566716	0.880277219	0.998542029	0.99989309	0.875441396
SLC2A1	0.941650441	0.849718733	0.87242852	0.999939566	0.99989309	0.877916603
IFNGR1	0.941650441	0.843588854	0.893544538	0.999939566	0.99989309	0.881923424
LOC101902754	0.941650441	0.828417266	0.875669731	0.999939566	0.99989309	0.882322567
TPRA1	0.941650441	0.904764822	0.894547778	0.999939566	0.99989309	0.886992214
SASS6	0.941650441	0.835276066	0.979635589	0.999939566	0.99989309	0.889622239
PCDHGA8	0.941650441	0.84869594	0.878603452	0.995118128	0.99989309	0.893622929
BOK	0.941650441	0.91458438	0.886282865	0.992368879	0.99989309	0.895287865
ZNF48	0.941650441	0.829503998	0.941593855	0.999939566	0.99989309	0.897633315
LOC112447303	0.941650441	0.991043872	0.88950849	0.974992862	0.99989309	0.897818829
PRKCE	0.941650441	0.982963758	0.883982433	0.977216943	0.99989309	0.897818829
ASXL1	0.941650441	0.821588096	0.971981006	0.995118128	0.99989309	0.905220694
ITGA5	0.941650441	0.851167599	0.917013548	0.999939566	0.99989309	0.90525134

LOC783803	0.941650441	0.897918739	0.909170161	0.99879985	0.99989309	0.906529452
LOC101907383	0.941650441	0.903820329	0.902588138	0.999939566	0.99989309	0.908939618
FBXO21	0.941650441	0.866803532	0.870911433	0.977216943	0.99989309	0.911696156
LOC112445041	0.941650441	0.824676261	0.947475618	0.990049248	0.99989309	0.914440253
LOC100298774	0.941650441	0.897657501	0.942004125	0.971220023	0.99989309	0.926872824
LOC101902469	0.941650441	0.938538223	0.883860423	0.985181062	0.99989309	0.926872824
FCGR3A	0.941650441	0.90249621	0.885047308	0.999939566	0.99989309	0.926872824
TCTEX1D2	0.941650441	0.852463941	0.908165509	0.956154344	0.99989309	0.940917403
LOC112443476	0.941650441	0.866803532	0.87214481	0.999939566	0.99989309	0.942421464
IMMT	0.941650441	0.843422478	0.884199387	0.990300191	0.99989309	0.942455807
DGKE	0.941650441	0.839461242	0.929958748	0.99879985	0.99989309	0.95470473
CTSO	0.941650441	0.822173925	0.892122482	0.992137653	0.99989309	0.968059826
CNOT10	0.941650441	0.835130235	0.904682623	0.995800463	0.99989309	0.968245211
KCNQ4	0.941650441	0.826937434	0.878603452	0.995118128	0.99989309	0.984609406
NCSTN	0.941673877	0.878594098	0.909170161	0.990049248	0.99989309	0.912101047
PSMB1	0.941673877	0.874669928	0.939683463	0.993948479	0.99989309	0.942421464
TUBGCP3	0.941731581	0.855979085	0.958420472	0.995118128	0.99989309	0.893961809
MYF6	0.941731581	0.972418749	0.877998684	0.959009349	0.99989309	0.897633315
TMEM52	0.941731581	0.881079118	0.967873529	0.996512319	0.99989309	0.905220694
PLCH1	0.941731581	0.841263423	0.87312855	0.999939566	0.99989309	0.905220694
RECK	0.941731581	0.898419542	0.90002174	0.990049248	0.99989309	0.9071195
CACNB4	0.941731581	0.858142249	0.885434576	0.999939566	0.99989309	0.909768235
DMAP1	0.941731581	0.899142992	0.883647437	0.959009349	0.99989309	0.92035958
SLC44A1	0.941731581	0.851167599	0.958420472	0.995118128	0.99989309	0.940683751
LOC112446639	0.941731581	0.914252497	0.894874079	0.982140778	0.99989309	0.941794462
MSANTD4	0.941731581	0.824676261	0.879215964	0.995118128	0.99989309	0.981164039
CHCHD8	0.941731581	0.827609894	0.872397372	0.990059245	0.99989309	0.996574245
RASGRP2	0.94188748	0.950443845	0.919997767	0.990571725	0.99989309	0.868108836
ERAP1	0.94188748	0.825215718	0.980744205	0.977555889	0.99989309	0.875441396
PPP1R3E	0.94188748	0.825301941	0.968230268	0.999939566	0.99989309	0.876262605
PRKACA	0.94188748	0.842654254	0.899539089	0.999939566	0.99989309	0.879550676
PNPLA2	0.94188748	0.899387334	0.880277219	0.999939566	0.99989309	0.882322567
HIST2H2BE	0.94188748	0.838405969	0.896272282	0.999939566	0.99989309	0.888617204
LOC100847951	0.94188748	0.965027449	0.894547778	0.999939566	0.99989309	0.895218005
ABCC10	0.94188748	0.825215718	0.976719499	0.999939566	0.99989309	0.905220694
SCML2	0.94188748	0.847919882	0.87146798	0.956154344	0.99989309	0.906065482
MORN1	0.94188748	0.834869027	0.957379098	0.99879985	0.99989309	0.908113351
LOC107132524	0.94188748	0.870855329	0.912071401	0.999939566	0.99989309	0.908939618

NECAP1	0.94188748	0.851167599	0.937544913	0.999939566	0.99989309	0.909768235
DDX27	0.94188748	0.835408071	0.909522865	0.981022805	0.99989309	0.914046542
RAB11B	0.94188748	0.823692881	0.907270422	0.999939566	0.99989309	0.917524589
ACP2	0.94188748	0.934248552	0.941593855	0.982140778	0.99989309	0.926872824
ROBO4	0.94188748	0.911152896	0.886282865	0.959009349	0.99989309	0.940683751
NGDN	0.94188748	0.835809337	0.961899356	0.956154344	0.99989309	0.945759045
TIGD4	0.94188748	0.897375356	0.879515214	0.999939566	0.99989309	0.95470473
LOC112446024	0.94188748	0.829033708	0.961742979	0.959410646	0.99989309	0.964820716
NOP14	0.94188748	0.871412404	0.87146798	0.976646737	0.99989309	0.996455238
ZNHIT1	0.942126225	0.93077794	0.902141181	0.995118128	0.99989309	0.870351337
HNRNPLL	0.942126225	0.845979391	0.880277219	0.990571725	0.99989309	0.880272231
FADS6	0.942126225	0.914735022	0.89145542	0.999939566	0.99989309	0.886992214
LACC1	0.942126225	0.988630403	0.894414265	0.990571725	0.99989309	0.895287865
LOC112444276	0.942126225	0.834316926	0.914489705	0.999939566	0.99989309	0.905220694
RTN2	0.942126225	0.857034402	0.899539089	0.999939566	0.99989309	0.926872824
PREX2	0.942126225	0.912112179	0.885354035	0.973377993	0.99989309	0.938643464
LOC107133048	0.942126225	0.949008068	0.873750661	0.99879985	0.99989309	0.941925704
UGGT1	0.942126225	0.851167599	0.912071401	0.995118128	0.99989309	0.983453112
LOC104969378	0.942461781	0.885981636	0.884189563	0.999939566	0.99989309	0.962255684
PAPPA	0.942590231	0.877749555	0.92921168	0.99879985	0.99989309	0.918220726
LOC784808	0.942704416	0.940764123	0.962754771	0.956154344	0.99989309	0.887938432
ADAMTSL2	0.942870281	0.831242188	0.990350221	0.967063428	0.99989309	0.889622239
TJAP1	0.94297429	0.895061601	0.967873529	0.990571725	0.99989309	0.875441396
GMIP	0.94297429	0.950091107	0.880277219	0.958849311	0.99989309	0.930318133
TSGA10IP	0.94297429	0.837455678	0.958420472	0.99879985	0.99989309	0.943138585
LOC101908034	0.94316589	0.842797231	0.93145152	0.992137653	0.99989309	0.935636428
DDX24	0.94316589	0.831539903	0.947660114	0.995800463	0.99989309	0.941925704
HIP1R	0.943269589	0.838405969	0.899195818	0.999939566	0.99989309	0.87114736
LOC112446457	0.943269589	0.836487169	0.992915726	0.976519231	0.99989309	0.906178214
SRSF2	0.943358665	0.858933073	0.883982433	0.995118128	0.99989309	0.935655141
ADCY6	0.943698991	0.860449455	0.880277219	0.992746023	0.99989309	0.908939618
CWC15	0.943723682	0.836487169	0.962209895	0.999939566	0.99989309	0.896934364
TSC22D4	0.943971052	0.828600257	0.919785713	0.990571725	0.99989309	0.908939618
PCDH7	0.944077404	0.899387334	0.899539089	0.999939566	0.99989309	0.879550676
ATP5S	0.944077404	0.851167599	0.878603452	0.999939566	0.99989309	0.908939618
NEK7	0.944077404	0.851167599	0.965416289	0.99879985	0.99989309	0.923788376
ZC3H8	0.944077404	0.838843184	0.894449269	0.987143706	0.99989309	0.940683751
PSME1	0.944077404	0.9136091	0.886282865	0.99879985	0.99989309	0.950601157

NHEJ1	0.944077404	0.886019911	0.897316161	0.959009349	0.99989309	0.969102473
NR2F1	0.944088199	0.889410709	0.874092011	0.999939566	0.99989309	0.891000792
PLA2G16	0.944132705	0.835276066	0.965491674	0.99879985	0.99989309	0.877623013
FGGY	0.94440559	0.920347742	0.88428155	0.999939566	0.99989309	0.875441396
LOC100848991	0.94440559	0.937978608	0.924476109	0.992137653	0.99989309	0.887248119
SNX21	0.94440559	0.871412404	0.882898206	0.989313742	0.99989309	0.887457549
SNX25	0.94440559	0.855979085	0.914406673	0.999939566	0.99989309	0.891520514
PHF23	0.94440559	0.823692881	0.944976702	0.958879502	0.99989309	0.895218005
SLC35F5	0.94440559	0.868963165	0.877998684	0.999939566	0.99989309	0.906895944
GKAP1	0.94440559	0.843640278	0.914685423	0.999939566	0.99989309	0.908939618
GGNBP2	0.94440559	0.89539921	0.931173336	0.999939566	0.99989309	0.912376736
GPLD1	0.94440559	0.934571318	0.884189563	0.99879985	0.99989309	0.920754358
LOC112444763	0.94440559	0.842565421	0.91234361	0.989391869	0.99989309	0.991281092
RMND5B	0.944438795	0.956843573	0.940785296	0.973377993	0.99989309	0.887248119
NOP53	0.944438795	0.899142992	0.924266781	0.999939566	0.99989309	0.895287865
LOC107131619	0.944438795	0.914616699	0.951850716	0.98470348	0.99989309	0.935154223
VPS37C	0.944469361	0.93453191	0.951850716	0.992137653	0.99989309	0.887248119
NRADD	0.944469361	0.834011972	0.975287067	0.99419346	0.99989309	0.90843033
VIT	0.944472546	0.9136091	0.899539089	0.971220023	0.99989309	0.872224159
THSD7A	0.944472546	0.959119521	0.879968243	0.99879985	0.99989309	0.875441396
IFITM3	0.944472546	0.91994024	0.939091808	0.999939566	0.99989309	0.875441396
MYLK2	0.944472546	0.901667799	0.939091808	0.995118128	0.99989309	0.878152258
HMG20A	0.944472546	0.86415368	0.982659943	0.996850715	0.99989309	0.879725389
TMEM184A	0.944472546	0.838815395	0.962176387	0.998542029	0.99989309	0.880404833
PLCXD3	0.944472546	0.936134921	0.894874079	0.99879985	0.99989309	0.883906472
ANKAR	0.944472546	0.938064973	0.96993432	0.982809512	0.99989309	0.884387948
RTF2	0.944472546	0.847082229	0.939683463	0.982367414	0.99989309	0.886520861
MAP3K10	0.944472546	0.922434012	0.914406673	0.990571725	0.99989309	0.886992214
NHLRC3	0.944472546	0.972786652	0.892122482	0.973785871	0.99989309	0.887248119
COG6	0.944472546	0.93453191	0.930710426	0.960357834	0.99989309	0.892192495
DVL1	0.944472546	0.9136091	0.886282865	0.990571725	0.99989309	0.893961809
LOC100851323	0.944472546	0.90249621	0.962777992	0.997612654	0.99989309	0.903527718
THOC6	0.944472546	0.839641196	0.95896293	0.990300191	0.99989309	0.904593245
RPS6KA2	0.944472546	0.879497786	0.878603452	0.995118128	0.99989309	0.9071195
LOC112442687	0.944472546	0.887725794	0.907423125	0.999939566	0.99989309	0.908939618
PCYT1B	0.944472546	0.82890642	0.967302577	0.999939566	0.99989309	0.908939618
FAM151A	0.944472546	0.884185138	0.880277219	0.999939566	0.99989309	0.91008914
APAF1	0.944472546	0.85692481	0.875669731	0.994652359	0.99989309	0.911263177

DLG1	0.944472546	0.838405969	0.922147089	0.958879502	0.99989309	0.911696156
MLLT11	0.944472546	0.841044548	0.924266781	0.967063428	0.99989309	0.934653208
LOC101906021	0.944472546	0.830738666	0.97381056	0.990571725	0.99989309	0.938553439
LOC784087	0.944472546	0.829099159	0.968954353	0.965097245	0.99989309	0.940917403
ARPC1A	0.944472546	0.82890642	0.930224138	0.998542029	0.99989309	0.941925704
LOC104974934	0.944472546	0.868963165	0.947660114	0.990049248	0.99989309	0.942455807
IKZF2	0.944472546	0.866977641	0.942908478	0.995118128	0.99989309	0.945258337
SRSF4	0.944472546	0.82890642	0.929303679	0.994365581	0.99989309	0.95470473
CDKN2D	0.944472546	0.855979085	0.880277219	0.973377993	0.99989309	0.98016619
C26H10orf143	0.944472546	0.885374928	0.921135532	0.983939542	0.99989309	0.981090047
LOC101901950	0.944472546	0.8554287	0.92332596	0.968306132	0.99989309	0.991281092
TFIP11	0.944472546	0.845452558	0.878603452	0.980418209	0.99989309	0.991281092
LOC107132971	0.944476598	0.825273463	0.883982433	0.999939566	0.99989309	0.870895425
ETS2	0.944476598	0.846914612	0.965270177	0.999939566	0.99989309	0.87114736
EPHX1	0.944476598	0.933412624	0.920859924	0.999939566	0.99989309	0.880404833
TPRG1	0.944476598	0.992273495	0.87516419	0.99879985	0.99989309	0.882322567
LOC112448368	0.944476598	0.890503475	0.989332994	0.967063428	0.99989309	0.886992214
ITPR3	0.944476598	0.839289192	0.976719499	0.999939566	0.99989309	0.886992214
MAP1B	0.944476598	0.88469581	0.883010895	0.999939566	0.99989309	0.887457549
TOR4A	0.944476598	0.843544538	0.878603452	0.969717497	0.99989309	0.893961809
GPR63	0.944476598	0.893839642	0.883982433	0.99879985	0.99989309	0.902660792
LOC616281	0.944476598	0.862791034	0.957379098	0.973207466	0.99989309	0.906178214
CRTC1	0.944476598	0.877206497	0.911157179	0.999939566	0.99989309	0.908939618
BBS12	0.944476598	0.824676261	0.930262306	0.999939566	0.99989309	0.909940901
LOC107132783	0.944476598	0.926435857	0.943891967	0.981022805	0.99989309	0.912376736
DNAJC24	0.944476598	0.848328614	0.886282865	0.990571725	0.99989309	0.923064313
EIF5	0.944476598	0.852463941	0.938306386	0.999939566	0.99989309	0.92920113
LOC520336	0.944476598	0.838405969	0.962209895	0.99879985	0.99989309	0.934451585
IBTK	0.944476598	0.834316926	0.939683463	0.999939566	0.99989309	0.934775196
MTHFD2L	0.944476598	0.853023763	0.892122482	0.99879985	0.99989309	0.939966658
CCDC115	0.944476598	0.881079118	0.879215964	0.992137653	0.99989309	0.940683751
SLC40A1	0.944476598	0.913075885	0.939091808	0.992137653	0.99989309	0.940683751
RCBTB1	0.944476598	0.833277156	0.920449324	0.969717497	0.99989309	0.943138585
TMEM19	0.944476598	0.840874016	0.917876971	0.99879985	0.99989309	0.958475633
LOC784521	0.944476598	0.883378562	0.899539089	0.999939566	0.99989309	0.961506391
PHKA2	0.944476598	0.867722813	0.878603452	0.97378567	0.99989309	0.96544761
LOC101902705	0.944476598	0.864821455	0.87974371	0.994309225	0.99989309	0.968245211
LOC104975222	0.944476598	0.832852787	0.90166788	0.99879985	0.99989309	0.981411983

IMPACT	0.944600876	0.889410709	0.882898206	0.999939566	0.99989309	0.886992214
MINDY1	0.944600876	0.94883251	0.919108971	0.999939566	0.99989309	0.895218005
FRS2	0.944600876	0.883226808	0.939683463	0.982140778	0.99989309	0.910353954
LOC101904642	0.944600876	0.842230087	0.892122482	0.999939566	0.99989309	0.912380696
ZMYM1	0.944600876	0.918306418	0.882898206	0.999939566	0.99989309	0.914122611
WSB1	0.944600876	0.838843184	0.927079582	0.99879985	0.99989309	0.943138585
SYT7	0.944600876	0.840505408	0.957575736	0.990571725	0.99989309	0.950601157
PWP1	0.944600876	0.855634182	0.950569634	0.994309225	0.99989309	0.954012991
CD200	0.944600876	0.842797231	0.93050447	0.999939566	0.99989309	0.954853707
LPXN	0.944600876	0.926435857	0.878603452	0.983151855	0.99989309	0.961506391
NENF	0.944600876	0.857034402	0.956706081	0.982140778	0.99989309	0.968245211
TIMELESS	0.944600876	0.838263859	0.939683463	0.992137653	0.99989309	0.968462588
LOC101905199	0.944600876	0.831198698	0.891455327	0.99879985	0.99989309	0.984491523
MAP6	0.944655116	0.847082229	0.873750661	0.995800463	0.99989309	0.934458012
TMEM249	0.944655116	0.84869594	0.883982433	0.990059245	0.99989309	0.954581551
LOC104975977	0.944657837	0.832607032	0.958420472	0.999939566	0.99989309	0.904593245
SEPSECS	0.944683812	0.857738813	0.939091808	0.996084773	0.99989309	0.92689297
APCDD1	0.944884038	0.839289192	0.931397502	0.99879985	0.99989309	0.872224159
PCNP	0.944884038	0.82890642	0.965712499	0.999939566	0.99989309	0.875441396
TLR5	0.944884038	0.885796876	0.931173336	0.999939566	0.99989309	0.87900534
INHBA	0.944884038	0.959119521	0.879515214	0.999334425	0.99989309	0.886992214
DNAJC4	0.944884038	0.828352909	0.957802423	0.997612654	0.99989309	0.895287865
PAM16	0.944884038	0.888994602	0.906804686	0.95964071	0.99989309	0.926872824
NUBP1	0.944884038	0.920347742	0.891311171	0.969717497	0.99989309	0.934653208
ZAP70	0.944884038	0.859941746	0.878050811	0.992746023	0.99989309	0.987775991
MAP1LC3A	0.944900159	0.834011972	0.891014698	0.995800463	0.99989309	0.899551847
PAOX	0.944900159	0.849475321	0.926613927	0.999939566	0.99989309	0.912101047
B4GALT2	0.944975834	0.932024312	0.88300827	0.999939566	0.99989309	0.872224159
RDM1	0.944975834	0.871113425	0.991817553	0.985181062	0.99989309	0.881039089
ZNF112	0.945008546	0.904764822	0.875669731	0.995118128	0.99989309	0.941925704
NAPRT	0.945035234	0.852463941	0.934152444	0.981022805	0.99989309	0.888617204
GPR156	0.945035234	0.86778334	0.910703333	0.999939566	0.99989309	0.891000792
MST1R	0.945035234	0.859752721	0.890801024	0.97136787	0.99989309	0.906529452
ARHGEF25	0.945035234	0.838405969	0.882898206	0.999939566	0.99989309	0.937591162
KCNAB1	0.945233029	0.840313358	0.960095031	0.990571725	0.99989309	0.873520548
KLHDC4	0.945233029	0.965027449	0.946564234	0.995118128	0.99989309	0.875441396
POC1B	0.945233029	0.852463941	0.903461267	0.971622187	0.99989309	0.970786053
NUP205	0.945233029	0.871412404	0.878050811	0.973377993	0.99989309	0.991281092

RPL15	0.94527195	0.908192144	0.916702	0.998307633	0.99989309	0.918803168
HRASLS	0.94527195	0.838405969	0.923976352	0.969717497	0.99989309	0.965769212
DAP	0.945354369	0.853940139	0.960137022	0.988949923	0.99989309	0.873118284
CNEP1R1	0.945354369	0.844031777	0.989408632	0.981022805	0.99989309	0.908785004
ARHGAP21	0.945354369	0.834869027	0.899539089	0.977470078	0.99989309	0.943777356
LOC104973848	0.945368188	0.861730561	0.886282865	0.999939566	0.99989309	0.879550676
PFAS	0.945542141	0.852463941	0.965518535	0.990571725	0.99989309	0.936439483
ZNF45	0.945542141	0.905808353	0.878603452	0.982140778	0.99989309	0.988297209
WARS2	0.945583722	0.832852787	0.956706081	0.996140327	0.99989309	0.872175131
C2CD3	0.945583722	0.945805877	0.939683463	0.969717497	0.99989309	0.872224159
YLPM1	0.945583722	0.871113425	0.894547778	0.999939566	0.99989309	0.872224159
HPS4	0.945583722	0.912112179	0.886282865	0.999939566	0.99989309	0.872493553
GAN	0.945583722	0.967113913	0.883982433	0.995946943	0.99989309	0.873520548
SLC6A14	0.945583722	0.920347742	0.976719499	0.992137653	0.99989309	0.875441396
LOC104970628	0.945583722	0.896278848	0.89007492	0.999939566	0.99989309	0.875441396
ALDH1L1	0.945583722	0.977584325	0.910978655	0.999939566	0.99989309	0.878152258
CCM2L	0.945583722	0.934248552	0.947614537	0.995118128	0.99989309	0.879494654
DPYD	0.945583722	0.852463941	0.90166788	0.999939566	0.99989309	0.881550981
LOC112442805	0.945583722	0.851167599	0.987842204	0.990571725	0.99989309	0.884387948
KLF6	0.945583722	0.835130235	0.90002174	0.999939566	0.99989309	0.884387948
HNRNPL	0.945583722	0.99134261	0.880277219	0.99879985	0.99989309	0.884673942
PDLIM2	0.945583722	0.885796876	0.902179915	0.990571725	0.99989309	0.88609889
SNX3	0.945583722	0.883927618	0.950569634	0.992368879	0.99989309	0.886992214
IDO1	0.945583722	0.829548444	0.923072506	0.999939566	0.99989309	0.886992214
AMFR	0.945583722	0.866564777	0.96644151	0.999939566	0.99989309	0.886992214
CHMP2B	0.945583722	0.88418172	0.922126495	0.999939566	0.99989309	0.887248119
SNHG3	0.945583722	0.834741258	0.92332596	0.999939566	0.99989309	0.887457549
CCDC66	0.945583722	0.828417266	0.991406769	0.95964071	0.99989309	0.889622239
FBXL15	0.945583722	0.859941746	0.877998684	0.990059245	0.99989309	0.891908825
CDADC1	0.945583722	0.861552326	0.944638172	0.999939566	0.99989309	0.891908825
PABPC5	0.945583722	0.980930691	0.886282865	0.99879985	0.99989309	0.893961809
EDRF1	0.945583722	0.963478319	0.877998684	0.965097245	0.99989309	0.895218005
GOLPH3L	0.945583722	0.831242188	0.879376765	0.999939566	0.99989309	0.895218005
TRAPPC2L	0.945583722	0.886943153	0.900109169	0.999939566	0.99989309	0.895218005
NIT1	0.945583722	0.845979391	0.878603452	0.999939566	0.99989309	0.897011069
CPNE2	0.945583722	0.851167599	0.925587088	0.999939566	0.99989309	0.899551847
PRKAB1	0.945583722	0.836391898	0.977612552	0.999939566	0.99989309	0.90175726
PBX1	0.945583722	0.859945547	0.886176337	0.999939566	0.99989309	0.90525134

KIAA0319L	0.945583722	0.838405969	0.991938331	0.984100122	0.99989309	0.906802901
C5H12orf73	0.945583722	0.871653212	0.923976352	0.95964071	0.99989309	0.908939618
TBCB	0.945583722	0.928119925	0.879515214	0.980418209	0.99989309	0.908939618
LOC101903385	0.945583722	0.844031777	0.951850716	0.984968973	0.99989309	0.908939618
LOC112446021	0.945583722	0.831242188	0.882898206	0.999939566	0.99989309	0.908939618
FOXRED2	0.945583722	0.851167599	0.904682623	0.994365581	0.99989309	0.911470632
LRP8	0.945583722	0.920347742	0.906941752	0.999939566	0.99989309	0.911696156
MAP9	0.945583722	0.835381515	0.921225977	0.983151855	0.99989309	0.913230542
AQR	0.945583722	0.882275117	0.880277219	0.969717497	0.99989309	0.917968123
XRRA1	0.945583722	0.875641453	0.944160795	0.999939566	0.99989309	0.918291722
SYNJ1	0.945583722	0.866803532	0.921146002	0.999939566	0.99989309	0.926872824
CTSK	0.945583722	0.936134921	0.918576062	0.982140778	0.99989309	0.928653932
MLXIP	0.945583722	0.857005855	0.887235549	0.99879985	0.99989309	0.934775196
GNAI2	0.945583722	0.851167599	0.952255572	0.992137653	0.99989309	0.93691717
HLCS	0.945583722	0.847082229	0.899539089	0.99879985	0.99989309	0.939800211
ABHD17A	0.945583722	0.873098735	0.918965426	0.981022805	0.99989309	0.940917403
NAGA	0.945583722	0.90039743	0.882898206	0.992368879	0.99989309	0.940917403
HMGN4	0.945583722	0.888994602	0.944976702	0.967063428	0.99989309	0.942421464
POLR2G	0.945583722	0.93077794	0.92153058	0.967333752	0.99989309	0.942455807
ERFE	0.945583722	0.867722813	0.939683463	0.95964071	0.99989309	0.943138585
PIK3AP1	0.945583722	0.867749813	0.921319817	0.990049248	0.99989309	0.946314386
HS3ST1	0.945583722	0.852463941	0.961899356	0.992368879	0.99989309	0.95470473
GCNT3	0.945583722	0.841629926	0.916100231	0.999939566	0.99989309	0.95470473
RNPC3	0.945583722	0.852463941	0.939683463	0.980418209	0.99989309	0.957877422
CUL4B	0.945583722	0.899387334	0.878603452	0.995118128	0.99989309	0.957877422
LOC112447085	0.945583722	0.892341046	0.904682623	0.99879985	0.99989309	0.95929043
MARCH1	0.945583722	0.842797231	0.875669731	0.99879985	0.99989309	0.965332394
CMPK1	0.945583722	0.866813771	0.887391534	0.990571725	0.99989309	0.98016619
HIVEP1	0.945583722	0.897375356	0.882898206	0.990571725	0.99989309	0.983427394
LOC104971266	0.945583722	0.829548444	0.924266781	0.961525281	0.99989309	0.984449688
TTC37	0.945583722	0.833277156	0.899195818	0.977216943	0.99989309	0.988297209
TMEM164	0.945583722	0.835276066	0.886282865	0.999939566	0.99989309	0.988297209
LOC112442849	0.945583722	0.871030989	0.882898206	0.976950878	0.99989309	0.995411183
HPS5	0.945627747	0.855979085	0.899195818	0.996775429	0.99989309	0.906529452
KLHL23	0.945627747	0.936550733	0.909170161	0.99879985	0.99989309	0.940683751
ST3GAL4	0.945627747	0.864541566	0.942004125	0.983569039	0.99989309	0.974348283
HAUS7	0.945704534	0.897327046	0.894414265	0.99879985	0.99989309	0.90259329
IER5	0.945704534	0.855634182	0.914668913	0.999939566	0.99989309	0.921381434

LOC782566	0.945714787	0.909573683	0.905017554	0.999939566	0.99989309	0.87900534
ABHD4	0.94575414	0.847749382	0.879376765	0.999939566	0.99989309	0.976175609
DSB	0.94580165	0.91358069	0.929958748	0.987143706	0.99989309	0.893961809
LOC107131848	0.94580165	0.835276066	0.980442556	0.99879985	0.99989309	0.926872824
SPRED1	0.94580165	0.866803532	0.929303679	0.990491321	0.99989309	0.933730876
BANP	0.94580165	0.83971475	0.905047103	0.990571725	0.99989309	0.944738447
TESK1	0.94580165	0.834399235	0.880277219	0.999939566	0.99989309	0.983453112
LOC104970852	0.945808303	0.883998738	0.879215964	0.999939566	0.99989309	0.877223954
DARS2	0.945808303	0.836391898	0.897665623	0.999939566	0.99989309	0.877623013
GAREM2	0.945808303	0.908192144	0.987949796	0.982140778	0.99989309	0.88667499
NAA60	0.945808303	0.859882995	0.886282865	0.969717497	0.99989309	0.88676448
CEP162	0.945808303	0.843434807	0.894449269	0.999939566	0.99989309	0.893400499
USP9X	0.945808303	0.894203989	0.958420472	0.99879985	0.99989309	0.9044785
NTMT1	0.945808303	0.851167599	0.882898206	0.999939566	0.99989309	0.908024508
SLC25A37	0.945808303	0.830001751	0.941319062	0.999939566	0.99989309	0.908939618
CORIN	0.945808303	0.897323728	0.880277219	0.990049248	0.99989309	0.911696156
MNF1	0.945808303	0.926435857	0.878050811	0.995118128	0.99989309	0.911696156
ARHGEF37	0.945808303	0.889592783	0.922147089	0.976950878	0.99989309	0.912376736
ANKRD46	0.945808303	0.866813771	0.939683463	0.995687815	0.99989309	0.932854449
ASMT	0.945808303	0.886909612	0.918576062	0.989391869	0.99989309	0.933763401
CARHSP1	0.945808303	0.899387334	0.882898206	0.962142137	0.99989309	0.943138585
PYURF	0.945808303	0.938064973	0.894874079	0.963153458	0.99989309	0.943685681
MOB2	0.945808303	0.836487169	0.933102499	0.969717497	0.99989309	0.959240045
FEN1	0.945808303	0.90039743	0.885434576	0.985342672	0.99989309	0.967690297
ATAT1	0.945808303	0.852222691	0.879942201	0.999939566	0.99989309	0.978467321
MCTP2	0.945808303	0.853448902	0.899195818	0.999939566	0.99989309	0.979606084
ABCG2	0.945808303	0.876364777	0.899195818	0.994144632	0.99989309	0.983452055
GPCPD1	0.945808303	0.868963165	0.909002826	0.990571725	0.99989309	0.983453112
EPS15	0.946261533	0.851167599	0.887130994	0.988949923	0.99989309	0.873271935
COQ2	0.946261533	0.866803532	0.92153058	0.99879985	0.99989309	0.875441396
TSC2	0.946261533	0.868963165	0.999323478	0.959727203	0.99989309	0.881550981
MARK3	0.946261533	0.868963165	0.894449269	0.975064606	0.99989309	0.90525134
LOC615183	0.946261533	0.982017699	0.905017554	0.964254095	0.99989309	0.906529452
CYP3A5	0.946261533	0.861630666	0.886282865	0.999939566	0.99989309	0.908939618
SEMA7A	0.946453505	0.843333021	0.93000135	0.99879985	0.99989309	0.895218005
RAB1B	0.946746222	0.874626842	0.899195818	0.999939566	0.99989309	0.908939618
YIF1A	0.946757877	0.872884948	0.92153058	0.970252567	0.99989309	0.906529452
IMMP1L	0.946757877	0.852463941	0.904305179	0.965097245	0.99989309	0.912101047

PI3	0.946757877	0.835276066	0.976719499	0.990571725	0.99989309	0.937591162
LOC104975004	0.946757877	0.913075885	0.916700687	0.999939566	0.99989309	0.940683751
MFSD5	0.946757877	0.835276066	0.901550297	0.995118128	0.99989309	0.969545443
ZFP3	0.946822037	0.867656476	0.987842204	0.99879985	0.99989309	0.875441396
HIST3H2A	0.947091546	0.871113425	0.993927956	0.980418209	0.99989309	0.897818829
SLC35A1	0.947091546	0.932156311	0.91546474	0.977555889	0.99989309	0.905220694
LOC518080	0.947091546	0.889064624	0.914668913	0.999939566	0.99989309	0.920687336
TBC1D16	0.947091546	0.905977412	0.880277219	0.99879985	0.99989309	0.940522025
EED	0.947139038	0.95126822	0.953045982	0.990049248	0.99989309	0.875441396
LOC515418	0.947139038	0.922752283	0.96880775	0.99879985	0.99989309	0.875441396
DCX	0.947139038	0.899387334	0.954463909	0.999939566	0.99989309	0.875441396
LOC112446012	0.947139038	0.840444202	0.958420472	0.99879985	0.99989309	0.876060028
ARSE	0.947139038	0.958403824	0.886282865	0.99879985	0.99989309	0.88363494
XAF1	0.947139038	0.837455678	0.98662331	0.990571725	0.99989309	0.884387948
HTR4	0.947139038	0.867107408	0.894414265	0.999939566	0.99989309	0.886520861
FAM167B	0.947139038	0.843333021	0.894547778	0.999939566	0.99989309	0.886992214
ZBTB12	0.947139038	0.832070244	0.911119875	0.999939566	0.99989309	0.886992214
SLC30A1	0.947139038	0.866813771	0.899629961	0.999939566	0.99989309	0.891908825
WFIKKN1	0.947139038	0.846901876	0.905832059	0.999939566	0.99989309	0.893088787
LOC112445939	0.947139038	0.836487169	0.891014698	0.971220023	0.99989309	0.893961809
NDUFB9	0.947139038	0.902036896	0.880277219	0.965590385	0.99989309	0.895218005
ZNF32	0.947139038	0.851167599	0.91094712	0.999939566	0.99989309	0.895218005
SS18L2	0.947139038	0.877420806	0.961899356	0.999939566	0.99989309	0.898273855
YOD1	0.947139038	0.912785459	0.879726737	0.992137653	0.99989309	0.90259329
LOC101904239	0.947139038	0.901247881	0.939683463	0.995118128	0.99989309	0.90525134
EPS15L1	0.947139038	0.876243021	0.894547778	0.969717497	0.99989309	0.905858488
CCDC142	0.947139038	0.867722813	0.979231344	0.969717497	0.99989309	0.908939618
LOC112448848	0.947139038	0.978453686	0.89007492	0.992137653	0.99989309	0.908939618
CLCN2	0.947139038	0.880711088	0.962209895	0.996775429	0.99989309	0.908939618
LOC785693	0.947139038	0.883378562	0.883726795	0.999939566	0.99989309	0.908939618
SMTNL1	0.947139038	0.901922021	0.924266781	0.982140778	0.99989309	0.909940901
C1H21orf62	0.947139038	0.985326169	0.87974371	0.980933282	0.99989309	0.911696156
PRPF4	0.947139038	0.847919882	0.882898206	0.999939566	0.99989309	0.912101047
BTBD8	0.947139038	0.905210378	0.942004125	0.999939566	0.99989309	0.914233769
TMEM86A	0.947139038	0.881079118	0.914668913	0.999939566	0.99989309	0.924090522
DNAJC10	0.947139038	0.885253121	0.884325235	0.99879985	0.99989309	0.926872824
SLC26A6	0.947139038	0.913171043	0.962209895	0.975849638	0.99989309	0.933763401
ACP6	0.947139038	0.913075885	0.939091808	0.981922957	0.99989309	0.940683751

GRINA	0.947139038	0.862267429	0.879215964	0.999939566	0.99989309	0.943138585
KCNN3	0.947139038	0.833277156	0.90936727	0.999939566	0.99989309	0.95470473
MARK2	0.947139038	0.894203989	0.899530079	0.963829131	0.99989309	0.964143145
TAF15	0.947139038	0.866803532	0.880277219	0.976950878	0.99989309	0.991051281
BUD13	0.947342634	0.836026119	0.983143823	0.981022805	0.99989309	0.895218005
CPNE8	0.947445566	0.905834401	0.902141181	0.990049248	0.99989309	0.908939618
FAAP20	0.947542763	0.950091107	0.885355884	0.969717497	0.99989309	0.889130845
TMEM151A	0.947699633	0.914509221	0.972211632	0.988949923	0.99989309	0.881550981
UBE2J2	0.947948225	0.852463941	0.929303679	0.99879985	0.99989309	0.964143145
MMRN1	0.948202052	0.929536223	0.878603452	0.999939566	0.99989309	0.882613477
HSD3B7	0.948202052	0.871113425	0.882898206	0.999939566	0.99989309	0.911696156
ARL10	0.948202052	0.844031777	0.908821787	0.999939566	0.99989309	0.95470473
TRADD	0.948202052	0.893839642	0.897816959	0.977216943	0.99989309	0.968245211
LOC100848886	0.948216225	0.866803532	0.879968243	0.995946943	0.99989309	0.889622239
B4GALT6	0.948216225	0.886909612	0.957578315	0.999939566	0.99989309	0.891000792
THAP4	0.948216225	0.939651596	0.882898206	0.983569039	0.99989309	0.912101047
NIPSNAP3A	0.948216225	0.888994602	0.886282865	0.990571725	0.99989309	0.940683751
APTX	0.948260158	0.899387334	0.904691312	0.971220023	0.99989309	0.900168812
SNX18	0.94842413	0.835276066	0.939091808	0.999939566	0.99989309	0.885803926
PELI3	0.948481201	0.913171043	0.921135532	0.99879985	0.99989309	0.876907918
DVL2	0.948481201	0.839641196	0.945417678	0.99879985	0.99989309	0.901422892
HDAC11	0.948481201	0.868963165	0.926613927	0.992339205	0.99989309	0.902122572
CPE	0.948481201	0.856092464	0.878603452	0.999939566	0.99989309	0.908939618
KIF25	0.948481201	0.920262533	0.916702	0.982140778	0.99989309	0.917524589
GRID2	0.948481201	0.968312904	0.879968243	0.985824667	0.99989309	0.935607719
MAPK14	0.948481201	0.867204257	0.899539089	0.977216943	0.99989309	0.941925704
BRAF	0.948481201	0.851167599	0.909442165	0.96196115	0.99989309	0.946294331
ADAP1	0.948667577	0.908709906	0.902141181	0.999939566	0.99989309	0.886992214
LOC112441834	0.948667577	0.897657501	0.929958748	0.992137653	0.99989309	0.922263023
CYB561D2	0.948667577	0.837799788	0.884189563	0.99879985	0.99989309	0.923064313
COX19	0.948667577	0.926435857	0.929958748	0.999334425	0.99989309	0.923064313
GGCX	0.948667577	0.838405969	0.93050447	0.999939566	0.99989309	0.943138585
AGAP3	0.948669795	0.860449455	0.929303679	0.999939566	0.99989309	0.875441396
EIF2A	0.948812871	0.861568371	0.892122482	0.972367384	0.99989309	0.998671064
ZNF565	0.949038352	0.843101091	0.89007492	0.988115459	0.99989309	0.983453112
ALKBH6	0.949281437	0.927298992	0.89248682	0.992368879	0.99989309	0.877623013
NUP214	0.949281437	0.913171043	0.892122482	0.998542029	0.99989309	0.87900534
RDX	0.949281437	0.837455678	0.96880775	0.999939566	0.99989309	0.880404833

TVP23B	0.949281437	0.854944719	0.958420472	0.990571725	0.99989309	0.883080515
GATD3A	0.949281437	0.881795385	0.925587088	0.999939566	0.99989309	0.884097654
CENPJ	0.949281437	0.852463941	0.966157152	0.995118128	0.99989309	0.884387948
CNOT3	0.949281437	0.83744789	0.922147089	0.999939566	0.99989309	0.884387948
PROSER3	0.949281437	0.874967248	0.977612552	0.999939566	0.99989309	0.886992214
APIP	0.949281437	0.852463941	0.976719499	0.999939566	0.99989309	0.888617204
GM2A	0.949281437	0.974591667	0.929958748	0.982908486	0.99989309	0.889622239
LACTB2	0.949281437	0.834227824	0.878603452	0.999939566	0.99989309	0.893961809
RPL26	0.949281437	0.9347557	0.886176337	0.999939566	0.99989309	0.895218005
CCDC8	0.949281437	0.892859755	0.886282865	0.999939566	0.99989309	0.905220694
PTTG1IP	0.949281437	0.897918739	0.950569634	0.999939566	0.99989309	0.908939618
MED12L	0.949281437	0.955139334	0.879376765	0.999939566	0.99989309	0.914122611
CYB5D2	0.949281437	0.835276066	0.919939634	0.995118128	0.99989309	0.917968123
TMEM154	0.949281437	0.914910569	0.922161207	0.982140778	0.99989309	0.922263023
GCLC	0.949281437	0.853070404	0.886282865	0.999939566	0.99989309	0.926872824
WIPF3	0.949281437	0.897657501	0.909170161	0.999939566	0.99989309	0.934775196
MYO1C	0.949281437	0.881079118	0.892122482	0.997612654	0.99989309	0.941925704
COG8	0.949281437	0.838405969	0.977782604	0.994309225	0.99989309	0.942421464
LOC112441639	0.949281437	0.959119521	0.914668913	0.981022805	0.99989309	0.948277018
SLC25A14	0.949281437	0.929536223	0.914685423	0.9846464	0.99989309	0.957877422
GSTM2	0.949281437	0.90249621	0.882898206	0.999939566	0.99989309	0.96826636
TMEM8B	0.949281437	0.899387334	0.886176337	0.995118128	0.99989309	0.979792753
ELF4	0.949281437	0.834399235	0.916702	0.98657182	0.99989309	0.986389023
LY6D	0.949311964	0.904764822	0.929958748	0.969717497	0.99989309	0.886520861
LRRC58	0.949311964	0.868963165	0.893763648	0.99879985	0.99989309	0.890650763
SYMPK	0.949373232	0.897918739	0.887391534	0.990571725	0.99989309	0.880404833
DYNC1LI2	0.949373232	0.907670026	0.93050447	0.999939566	0.99989309	0.88667499
HS3ST3B1	0.949540881	0.847082229	0.906804311	0.999939566	0.99989309	0.879550676
FAM126A	0.949591597	0.833277156	0.880277219	0.999939566	0.99989309	0.991281092
NAGLU	0.949690708	0.882736993	0.908927423	0.992137653	0.99989309	0.913230542
IL4R	0.949889107	0.849834516	0.892122482	0.992339205	0.99989309	0.887248119
LOC112448260	0.949889107	0.884084518	0.918576062	0.999939566	0.99989309	0.920489812
FAM92B	0.950016086	0.89400848	0.878603452	0.976950878	0.99989309	0.879494654
UVRAG	0.950016086	0.857034402	0.924266781	0.999939566	0.99989309	0.887248119
XXYLT1	0.950016086	0.897375356	0.893763648	0.999939566	0.99989309	0.895218005
LOC786372	0.950016086	0.857917583	0.926613927	0.999939566	0.99989309	0.906178214
RPL12	0.950016086	0.866803532	0.929958748	0.999939566	0.99989309	0.908939618
SORBS1	0.950016086	0.913657073	0.893863239	0.999939566	0.99989309	0.917968123

C7H19orf24	0.950016086	0.852313021	0.878603452	0.976950878	0.99989309	0.993714265
LOC782101	0.950059302	0.843640278	0.944612298	0.999939566	0.99989309	0.945759045
DUSP6	0.950076057	0.888994602	0.929958748	0.999939566	0.99989309	0.887248119
STRN4	0.950076057	0.924433008	0.962754771	0.995800463	0.99989309	0.893622929
SYT3	0.950076057	0.988725451	0.886282865	0.963829131	0.99989309	0.930707319
CREG1	0.950130117	0.855151723	0.919997767	0.999939566	0.99989309	0.888471585
AK3	0.950130117	0.862267429	0.894414265	0.999939566	0.99989309	0.909990551
ETNPPL	0.950150857	0.841657074	0.909170161	0.999939566	0.99989309	0.936439483
ASB1	0.950242708	0.851167599	0.904682623	0.990571725	0.99989309	0.891511718
SUCLG2	0.950291193	0.866813771	0.884199387	0.999939566	0.99989309	0.936439483
CLNK	0.950291193	0.908178836	0.886176337	0.964413123	0.99989309	0.936563391
MAN2C1	0.950354172	0.858230868	0.929958748	0.995800463	0.99989309	0.949206487
LOC101905595	0.950376013	0.906831279	0.989332994	0.981022805	0.99989309	0.891000792
PSMC2	0.950376013	0.877420806	0.922161207	0.992137653	0.99989309	0.956959747
VASH2	0.95051324	0.851167599	0.892348336	0.999939566	0.99989309	0.926872824
ETFDH	0.950613649	0.851167599	0.886282865	0.996447295	0.99989309	0.887248119
RABAC1	0.950613649	0.93453191	0.896272282	0.966070428	0.99989309	0.887457549
LOC101901983	0.950613649	0.871412404	0.943891967	0.99879985	0.99989309	0.888396511
LOC112447460	0.950613649	0.897918739	0.950638965	0.965097245	0.99989309	0.924845138
REV3L	0.950647653	0.868963165	0.933963997	0.971220023	0.99989309	0.926484322
LOC107132748	0.950711049	0.974986418	0.882898206	0.999939566	0.99989309	0.886520861
EPS8	0.950712881	0.841657074	0.919108971	0.9846464	0.99989309	0.908442273
HIKESHI	0.950911256	0.851167599	0.92153058	0.999939566	0.99989309	0.933763401
DKK1	0.950974438	0.963478319	0.883334551	0.999939566	0.99989309	0.880404833
KLHL38	0.950974438	0.866803532	0.894414265	0.990049248	0.99989309	0.88667499
CCNE1	0.950974438	0.90249621	0.959575405	0.999939566	0.99989309	0.886992214
F12	0.950974438	0.883267666	0.891152365	0.999939566	0.99989309	0.887248119
ZDHHC1	0.950974438	0.921280256	0.959646613	0.990049248	0.99989309	0.9071195
LOC107132121	0.950974438	0.955798423	0.943891967	0.992137653	0.99989309	0.908939618
LOC112444847	0.950974438	0.972418749	0.914406673	0.998542029	0.99989309	0.909940901
ZNF277	0.950974438	0.938064973	0.90002174	0.997612654	0.99989309	0.912101047
PSTK	0.950974438	0.928119925	0.939683463	0.994309225	0.99989309	0.928799366
LDB1	0.950974438	0.887165309	0.912223566	0.999939566	0.99989309	0.935385789
LOC101903326	0.950974438	0.841277186	0.939091808	0.999939566	0.99989309	0.940683751
HEYL	0.950974438	0.836487169	0.95126311	0.99879985	0.99989309	0.95470473
NEFH	0.951094414	0.840874016	0.905333362	0.99879985	0.99989309	0.881439164
LOC107132532	0.951094414	0.937851991	0.922126495	0.973282056	0.99989309	0.949206487
LOC104975099	0.951160871	0.855151723	0.937765241	0.999939566	0.99989309	0.887457549

TRPC2	0.951160871	0.90485456	0.942004125	0.999939566	0.99989309	0.908939618
LOC104970645	0.951160871	0.892564295	0.880277219	0.999939566	0.99989309	0.940683751
GCNT4	0.951160871	0.857034402	0.879726737	0.999939566	0.99989309	0.961506391
LOC104975073	0.951160871	0.879686372	0.8895568	0.999939566	0.99989309	0.98016619
ALG14	0.95131678	0.899387334	0.92153058	0.982554614	0.99989309	0.918220726
MITD1	0.95131678	0.851167599	0.985552223	0.968106154	0.99989309	0.940683751
PES1	0.95131678	0.918306418	0.880277219	0.99879985	0.99989309	0.971283342
CHAC2	0.951486783	0.913075885	0.917245456	0.999939566	0.99989309	0.893317233
ERAP2	0.951486783	0.857034402	0.884325235	0.999939566	0.99989309	0.905220694
SNX9	0.951486783	0.854362191	0.919939634	0.999939566	0.99989309	0.934458012
LOC112449059	0.951486783	0.861074758	0.938306386	0.992829869	0.99989309	0.961606931
PRPF31	0.951792344	0.982588455	0.883726795	0.990571725	0.99989309	0.887248119
LOC104974113	0.951792344	0.876169307	0.884325235	0.992368879	0.99989309	0.940683751
PLCB3	0.951868815	0.939027995	0.926613927	0.999939566	0.99989309	0.884387948
TOB1	0.951868815	0.912112179	0.933629769	0.999939566	0.99989309	0.893961809
YWHAZ	0.951868815	0.867722813	0.937191211	0.999939566	0.99989309	0.905220694
EVC	0.951868815	0.90249621	0.937363055	0.998542029	0.99989309	0.906529452
SP2	0.951868815	0.883378562	0.929958748	0.969717497	0.99989309	0.988297209
KHDRBS3	0.95189423	0.838843184	0.880255347	0.99879985	0.99989309	0.895218005
LOC112447385	0.95189423	0.871359854	0.884197487	0.992154129	0.99989309	0.899551847
SMIM7	0.95189423	0.838843184	0.990107362	0.995946943	0.99989309	0.90259329
LRRC55	0.95189423	0.841629926	0.970108169	0.99879985	0.99989309	0.911696156
HAUS1	0.95189423	0.922434012	0.883726795	0.999939566	0.99989309	0.914440253
HRH2	0.95189423	0.837620957	0.933102499	0.999939566	0.99989309	0.924788226
SNAP47	0.95189423	0.889724071	0.935247964	0.998542029	0.99989309	0.936563391
HIF3A	0.95189423	0.873566716	0.879515214	0.999939566	0.99989309	0.955585296
NTF3	0.95189423	0.852463941	0.89007492	0.999939566	0.99989309	0.957877422
PPP2R5D	0.951897504	0.840418048	0.926613927	0.999939566	0.99989309	0.900701704
ZC3H7B	0.951908522	0.842565421	0.93050447	0.99879985	0.99989309	0.902268895
CTNND2	0.951908522	0.882542824	0.893763648	0.995118128	0.99989309	0.943685681
ZCRB1	0.951922726	0.836487169	0.989702604	0.998542029	0.99989309	0.884387948
LOC100297056	0.951922726	0.853070404	0.937930242	0.999939566	0.99989309	0.884387948
SLC7A7	0.951922726	0.920347742	0.879515214	0.999939566	0.99989309	0.885515447
KXD1	0.951922726	0.91994024	0.926613927	0.999939566	0.99989309	0.88609792
THAP5	0.951922726	0.903820329	0.880277219	0.99879985	0.99989309	0.886992214
SAMD14	0.951922726	0.845472161	0.923072506	0.999939566	0.99989309	0.887248119
CD320	0.951922726	0.89141259	0.933102499	0.999939566	0.99989309	0.887457549
LOC509941	0.951922726	0.934248552	0.879376765	0.999939566	0.99989309	0.889159914

LOC107133150	0.951922726	0.881079118	0.883982433	0.999939566	0.99989309	0.889622239
ARSB	0.951922726	0.873984972	0.894414265	0.999939566	0.99989309	0.893961809
LOC786139	0.951922726	0.839641196	0.880457248	0.993488944	0.99989309	0.897011069
LONP1	0.951922726	0.857207609	0.920449324	0.999939566	0.99989309	0.901320431
LOC101907893	0.951922726	0.881079118	0.979231344	0.980955077	0.99989309	0.906529452
TIPIN	0.951922726	0.866803532	0.939417893	0.999939566	0.99989309	0.907965478
LOC107132098	0.951922726	0.926618064	0.904305179	0.999939566	0.99989309	0.908939618
TNKS2	0.951922726	0.843546903	0.937363055	0.999939566	0.99989309	0.908939618
CASK	0.951922726	0.852463941	0.939683463	0.999939566	0.99989309	0.909990551
ZNF784	0.951922726	0.842797231	0.982659255	0.98470348	0.99989309	0.912101047
LOC112443463	0.951922726	0.836487169	0.880277219	0.999939566	0.99989309	0.920942379
CPTP	0.951922726	0.857207609	0.894414265	0.992368879	0.99989309	0.930058266
PPP1R42	0.951922726	0.866803532	0.933963997	0.999939566	0.99989309	0.940673472
LOC100296900	0.951922726	0.911152896	0.894547778	0.99879985	0.99989309	0.940683751
SLC24A3	0.951922726	0.898920732	0.918292936	0.999939566	0.99989309	0.941116399
LOC784866	0.951922726	0.86301242	0.886282865	0.999939566	0.99989309	0.952989112
PHF1	0.951922726	0.939177233	0.886176337	0.995800463	0.99989309	0.954334316
LOC515570	0.951922726	0.866803532	0.880277219	0.999939566	0.99989309	0.955585296
SARDH	0.951922726	0.849410515	0.92153058	0.968306132	0.99989309	0.983453112
SYTL2	0.951922726	0.869882004	0.880277219	0.99879985	0.99989309	0.995411183
APLN	0.951937945	0.852463941	0.939091808	0.999939566	0.99989309	0.886992214
LOC104975022	0.951937945	0.899387334	0.939683463	0.999939566	0.99989309	0.908785004
RBM6	0.952044962	0.854944719	0.967302577	0.999939566	0.99989309	0.902295033
HNRNPA2B1	0.952044962	0.954606567	0.891455327	0.998542029	0.99989309	0.902959588
SULF2	0.952044962	0.876919789	0.880277219	0.999939566	0.99989309	0.908939618
POLK	0.952111923	0.973158835	0.880277219	0.999939566	0.99989309	0.909990551
TXNL4A	0.952188938	0.892711879	0.921420528	0.981022805	0.99989309	0.882170826
LOC112446375	0.952188938	0.917191385	0.991113951	0.990300191	0.99989309	0.883185575
CALCOCO2	0.952188938	0.897375356	0.882890706	0.999939566	0.99989309	0.88363494
PFDN5	0.952188938	0.883267666	0.962569922	0.999939566	0.99989309	0.886520861
LOC101904579	0.952188938	0.848986275	0.952208316	0.969432809	0.99989309	0.887248119
LOC104972584	0.952188938	0.907691022	0.957802423	0.969717497	0.99989309	0.889622239
POGLUT1	0.952188938	0.877420806	0.883874752	0.9846464	0.99989309	0.895218005
CISD3	0.952188938	0.861552326	0.918965426	0.999939566	0.99989309	0.897011069
ARAF	0.952188938	0.897375356	0.884325235	0.999939566	0.99989309	0.900264185
MTSS1	0.952188938	0.967758952	0.882898206	0.983569039	0.99989309	0.903313922
THG1L	0.952188938	0.862527737	0.896272282	0.999939566	0.99989309	0.906065482
MAGED2	0.952188938	0.925360181	0.916100231	0.999939566	0.99989309	0.909940901

LOC112449510	0.952188938	0.83744789	0.925587088	0.999939566	0.99989309	0.912101047
TBC1D9	0.952188938	0.899387334	0.92153058	0.999939566	0.99989309	0.914283173
MEX3C	0.952188938	0.844390603	0.927460291	0.998542029	0.99989309	0.914440253
ZDHHC21	0.952188938	0.851167599	0.921531707	0.999939566	0.99989309	0.928595999
LOC100847947	0.952188938	0.855151723	0.918681165	0.995118128	0.99989309	0.933678522
PGRMC1	0.952188938	0.849717639	0.906597758	0.992137653	0.99989309	0.936439483
GALK2	0.952188938	0.898231471	0.951742137	0.98860254	0.99989309	0.937591162
AKT3	0.952188938	0.842797231	0.923516626	0.981022805	0.99989309	0.938054315
LOC112444897	0.952188938	0.881079118	0.959198396	0.990300191	0.99989309	0.940683751
KANSL2	0.952188938	0.883378562	0.947268948	0.983038839	0.99989309	0.942455807
MPHOSPH6	0.952188938	0.841629926	0.929958748	0.992235612	0.99989309	0.943138585
LOC100848507	0.952188938	0.924433008	0.909170161	0.99879985	0.99989309	0.953604648
SPINDOC	0.952188938	0.838843184	0.939683463	0.99879985	0.99989309	0.957877422
NSUN3	0.952188938	0.843640278	0.904682623	0.982367414	0.99989309	0.963996128
RPSA	0.952188938	0.850240161	0.909366752	0.999939566	0.99989309	0.968059826
LOC101906546	0.952188938	0.884768652	0.90002174	0.995800463	0.99989309	0.968245211
CLTC	0.952202385	0.855979085	0.980734833	0.991978984	0.99989309	0.92920113
DNAL4	0.952257661	0.883576091	0.892122482	0.982367414	0.99989309	0.880404833
EWSR1	0.952257661	0.875022483	0.958977478	0.999939566	0.99989309	0.884387948
FHL3	0.952257661	0.865765403	0.926613927	0.999939566	0.99989309	0.885683692
UPK3B	0.952257661	0.838405969	0.999917831	0.976519231	0.99989309	0.886520861
ALOX12	0.952257661	0.963478319	0.92730429	0.998542029	0.99989309	0.88667499
COG3	0.952257661	0.83744789	0.882898206	0.999939566	0.99989309	0.886992214
SLC28A1	0.952257661	0.847082229	0.900109169	0.990049248	0.99989309	0.887457549
TIGD7	0.952257661	0.880103565	0.933102499	0.999939566	0.99989309	0.891908825
LOC104976664	0.952257661	0.989661212	0.89145542	0.981022805	0.99989309	0.893961809
SPAAR	0.952257661	0.83971475	0.939683463	0.999939566	0.99989309	0.893961809
C20H5orf34	0.952257661	0.878843255	0.957575736	0.99879985	0.99989309	0.895218005
TNPO1	0.952257661	0.843433874	0.947118233	0.999939566	0.99989309	0.896934364
ADGRF3	0.952257661	0.836487169	0.994425789	0.992829869	0.99989309	0.906065482
ANAPC5	0.952257661	0.848398356	0.918965426	0.999939566	0.99989309	0.906287255
LOC112448090	0.952257661	0.881823401	0.919626586	0.998675745	0.99989309	0.908939618
SKAP1	0.952257661	0.848986275	0.965416289	0.99879985	0.99989309	0.908939618
NUP133	0.952257661	0.926618064	0.891492421	0.981022805	0.99989309	0.910353954
CASP3	0.952257661	0.854262144	0.939683463	0.999939566	0.99989309	0.911263177
DDOST	0.952257661	0.926435857	0.894449269	0.999939566	0.99989309	0.911696156
LOC112447301	0.952257661	0.89272885	0.92153058	0.990049248	0.99989309	0.912385767
CACNG4	0.952257661	0.924510975	0.970109291	0.982809512	0.99989309	0.916385428

LOC101905219	0.952257661	0.847082229	0.966157152	0.992339205	0.99989309	0.918803168
AKT1S1	0.952257661	0.898819658	0.882898206	0.990571725	0.99989309	0.920383473
FRY	0.952257661	0.957353974	0.929958748	0.992368879	0.99989309	0.921582976
VSIR	0.952257661	0.842797231	0.899771699	0.984506451	0.99989309	0.92546728
LOC112444350	0.952257661	0.882706523	0.969811559	0.992137653	0.99989309	0.926872824
PAICSP	0.952257661	0.920818159	0.919939634	0.995118128	0.99989309	0.926872824
FER1L5	0.952257661	0.841044548	0.937270184	0.999939566	0.99989309	0.926872824
ZNF189	0.952257661	0.892564295	0.939683463	0.990571725	0.99989309	0.940683751
ORC1	0.952257661	0.875345836	0.897144948	0.999939566	0.99989309	0.940917403
TAF1B	0.952257661	0.855979085	0.931775341	0.999939566	0.99989309	0.941275898
GALNT18	0.952257661	0.839278952	0.926613927	0.999939566	0.99989309	0.941806801
HTR1E	0.952257661	0.885991833	0.955710723	0.990571725	0.99989309	0.943138585
KLHL6	0.952257661	0.944314627	0.884073083	0.990049248	0.99989309	0.944123524
LOC112449615	0.952257661	0.905808353	0.882898206	0.999939566	0.99989309	0.94425343
LOC781280	0.952257661	0.90339131	0.943891967	0.977043589	0.99989309	0.955585296
LOC101907835	0.952257661	0.901922021	0.939683463	0.969717497	0.99989309	0.955817715
WDCP	0.952257661	0.851167599	0.933102499	0.999334425	0.99989309	0.97815722
NUDT7	0.952257661	0.864821455	0.917876971	0.985181062	0.99989309	0.98016619
LOC618268	0.952257661	0.836487169	0.880277219	0.989087699	0.99989309	0.998928226
NOCT	0.952303334	0.963426589	0.908927423	0.981922957	0.99989309	0.911696156
ARSH	0.952375528	0.930469765	0.939091808	0.984968973	0.99989309	0.886520861
DONSON	0.952375528	0.859528714	0.965712499	0.995118128	0.99989309	0.887248119
KRTCAP2	0.952375528	0.840444202	0.939612562	0.995800463	0.99989309	0.895218005
LOC107131974	0.952375528	0.838843184	0.990107362	0.977470078	0.99989309	0.908939618
KIAA1328	0.952375528	0.943562922	0.942761121	0.998766683	0.99989309	0.912302996
CYLD	0.952375528	0.924999357	0.916283427	0.999939566	0.99989309	0.918666628
CTSF	0.952375528	0.838405969	0.886282865	0.999939566	0.99989309	0.943866443
TNIK	0.952375528	0.851167599	0.892122482	0.990049248	0.99989309	0.972216653
LOC101902968	0.952382028	0.937851991	0.979231344	0.990491321	0.99989309	0.896877362
CLEC4E	0.952382028	0.885376959	0.88330023	0.995118128	0.99989309	0.901422892
ADRA1A	0.952382028	0.942048064	0.939683463	0.983038839	0.99989309	0.908939618
UBE2E2	0.952382028	0.901922021	0.899732746	0.999939566	0.99989309	0.91332511
KATNBL1	0.952382028	0.85749413	0.894449269	0.990049248	0.99989309	0.940917403
MAD2L2	0.952382028	0.845510608	0.942004125	0.999939566	0.99989309	0.941925704
DUS2	0.952382028	0.838405969	0.886176337	0.987342814	0.99989309	0.961506391
LOC100847612	0.952382028	0.889592783	0.899539089	0.969717497	0.99989309	0.984651205
DNM3	0.952382028	0.934248552	0.880277219	0.984506451	0.99989309	0.985788947
CASP4	0.952382028	0.862267429	0.886176337	0.989087699	0.99989309	0.99797558

LYST	0.952445294	0.944780259	0.884325235	0.999939566	0.99989309	0.908785004
CACNG5	0.952475553	0.929536223	0.951278495	0.999939566	0.99989309	0.884387948
ACVR1C	0.952475553	0.901781061	0.880277219	0.999939566	0.99989309	0.884752599
CELSR1	0.952475553	0.90249621	0.917876971	0.999939566	0.99989309	0.886520861
PAPD4	0.952475553	0.994172236	0.911119875	0.992137653	0.99989309	0.88667499
DCAF6	0.952475553	0.905977412	0.882898206	0.999939566	0.99989309	0.886900013
LOC112446668	0.952475553	0.924799142	0.939091808	0.999939566	0.99989309	0.886992214
GPR137	0.952475553	0.91994024	0.939091808	0.982559568	0.99989309	0.887248119
LCMT1	0.952475553	0.918371039	0.968419607	0.998542029	0.99989309	0.887248119
NISCH	0.952475553	0.85439477	0.950801642	0.987614745	0.99989309	0.888695935
LRMDA	0.952475553	0.997825512	0.884197487	0.998542029	0.99989309	0.889159914
SLC25A32	0.952475553	0.891817085	0.90002174	0.999939566	0.99989309	0.890708894
ITCH	0.952475553	0.878843255	0.884189563	0.999939566	0.99989309	0.893961809
ITPK1	0.952475553	0.933412624	0.894449269	0.999939566	0.99989309	0.893961809
LOC101904468	0.952475553	0.908363698	0.947268948	0.992829869	0.99989309	0.895218005
SH3RF1	0.952475553	0.957071555	0.909170161	0.994719152	0.99989309	0.895218005
ADGRL2	0.952475553	0.911196851	0.886282865	0.999939566	0.99989309	0.895218005
PHF3	0.952475553	0.911347642	0.903407548	0.969717497	0.99989309	0.896877362
SHPK	0.952475553	0.878720916	0.898639163	0.999939566	0.99989309	0.897011069
CNMD	0.952475553	0.868963165	0.985552223	0.998542029	0.99989309	0.899551847
S100G	0.952475553	0.872513862	0.911743083	0.999939566	0.99989309	0.900887042
SOWAHC	0.952475553	0.864456026	0.906804311	0.999939566	0.99989309	0.901896586
DNAAF4	0.952475553	0.911279338	0.966157152	0.994309225	0.99989309	0.902268895
STRADA	0.952475553	0.854944719	0.994425789	0.992137653	0.99989309	0.905212874
LOC107133209	0.952475553	0.902136721	0.970904484	0.995800463	0.99989309	0.905220694
HDAC1	0.952475553	0.851167599	0.914406673	0.999939566	0.99989309	0.905220694
IQCD	0.952475553	0.852463941	0.987419404	0.995946943	0.99989309	0.90525134
BAD	0.952475553	0.950915448	0.926825953	0.999939566	0.99989309	0.908815767
MAP2K7	0.952475553	0.982588455	0.914685423	0.988949923	0.99989309	0.908939618
TRIM23	0.952475553	0.924799142	0.918576062	0.990571725	0.99989309	0.908939618
DCHS2	0.952475553	0.955798423	0.925022236	0.99879985	0.99989309	0.908939618
SGO2	0.952475553	0.944420015	0.941319062	0.99879985	0.99989309	0.908939618
DKKL1	0.952475553	0.985646138	0.886176337	0.999939566	0.99989309	0.908939618
NAT8L	0.952475553	0.855979085	0.917092799	0.999939566	0.99989309	0.908939618
GRB2	0.952475553	0.846959498	0.957379098	0.999939566	0.99989309	0.908939618
UNK	0.952475553	0.855151723	0.979231344	0.999939566	0.99989309	0.908939618
CHCHD1	0.952475553	0.854262144	0.92153058	0.999939566	0.99989309	0.910353954
LOC511937	0.952475553	0.974986418	0.886282865	0.99879985	0.99989309	0.911696156

NOL4L	0.952475553	0.862527737	0.882898206	0.999939566	0.99989309	0.911696156
UAP1L1	0.952475553	0.885376959	0.899195818	0.999939566	0.99989309	0.912101047
SMO	0.952475553	0.855861376	0.942761121	0.999939566	0.99989309	0.912101047
SH3D21	0.952475553	0.90874814	0.944670105	0.990571725	0.99989309	0.912396352
PTPRM	0.952475553	0.911799689	0.905374144	0.992137653	0.99989309	0.912396352
LOC100139144	0.952475553	0.921280256	0.914685423	0.996512319	0.99989309	0.913230542
LOC788724	0.952475553	0.862527737	0.957802423	0.99879985	0.99989309	0.914046542
CALR3	0.952475553	0.873566716	0.883982433	0.999939566	0.99989309	0.914344594
SLC23A3	0.952475553	0.859903298	0.929958748	0.99879985	0.99989309	0.920942379
FAM133B	0.952475553	0.863775454	0.909522865	0.999939566	0.99989309	0.923788376
CHRNA7	0.952475553	0.913690438	0.921531707	0.999939566	0.99989309	0.923788376
DXO	0.952475553	0.901712734	0.95896293	0.992368879	0.99989309	0.926534325
RNF166	0.952475553	0.852463941	0.959198396	0.999939566	0.99989309	0.926872824
SRPRA	0.952475553	0.877420806	0.959822136	0.999939566	0.99989309	0.92767015
SCP2D1	0.952475553	0.853258514	0.942642657	0.999939566	0.99989309	0.928799366
CDC34	0.952475553	0.925360181	0.882898206	0.990571725	0.99989309	0.933763401
PIGN	0.952475553	0.942608215	0.922147089	0.97964593	0.99989309	0.935020624
TICRR	0.952475553	0.861730561	0.987006595	0.9846464	0.99989309	0.935655141
LOC112449073	0.952475553	0.971235331	0.927460291	0.985181062	0.99989309	0.939465834
OAS1X	0.952475553	0.902136721	0.880457248	0.995118128	0.99989309	0.940429581
PARN	0.952475553	0.840313358	0.939091808	0.999939566	0.99989309	0.940683751
ARMC7	0.952475553	0.893915663	0.886373777	0.999939566	0.99989309	0.940917403
SEMA4C	0.952475553	0.843422478	0.886176337	0.999939566	0.99989309	0.941925704
JCAD	0.952475553	0.883267666	0.915581825	0.99879985	0.99989309	0.942868422
CSF1R	0.952475553	0.964616377	0.882898206	0.980840984	0.99989309	0.943685681
LOC781412	0.952475553	0.905977412	0.968954353	0.97136787	0.99989309	0.949334126
SMPDL3B	0.952475553	0.881079118	0.915843145	0.998542029	0.99989309	0.95342487
EBNA1BP2	0.952475553	0.901922021	0.92332596	0.99879985	0.99989309	0.95543
RNF113A	0.952475553	0.838843184	0.931173336	0.999939566	0.99989309	0.955585296
SCARA5	0.952475553	0.851167599	0.957802423	0.972367384	0.99989309	0.957877422
RBM15	0.952475553	0.866803532	0.909170161	0.999939566	0.99989309	0.961068561
MSANTD3	0.952475553	0.884397857	0.894449269	0.999939566	0.99989309	0.96544761
H4	0.952475553	0.848132141	0.915636025	0.999939566	0.99989309	0.966334828
CCT6A	0.952475553	0.839641196	0.939091808	0.999939566	0.99989309	0.966375116
BCHE	0.952475553	0.89539921	0.892258313	0.995118128	0.99989309	0.974379635
SOD2	0.952475553	0.862527737	0.890801024	0.999939566	0.99989309	0.98016619
SLC30A7	0.952475553	0.851167599	0.912031396	0.982140778	0.99989309	0.985399216
IPPK	0.952475553	0.859941746	0.950801642	0.973785871	0.99989309	0.988323336

LOC100847546	0.952475553	0.886909612	0.886176337	0.995118128	0.99989309	0.990291862
C2H2orf88	0.952475553	0.907691022	0.917092799	0.97157433	0.99989309	0.993259399
RNF24	0.952606555	0.852463941	0.90002174	0.999939566	0.99989309	0.908939618
YEATS4	0.952606555	0.855151723	0.926613927	0.99879985	0.99989309	0.981164039
LOC112442559	0.953003914	0.911347642	0.961668028	0.99879985	0.99989309	0.911696156
LOC112447762	0.953003914	0.911279338	0.947268948	0.99419346	0.99989309	0.955585296
RETSAT	0.953163949	0.885999002	0.888610896	0.999334425	0.99989309	0.949206487
UPRT	0.953163949	0.881079118	0.95721973	0.983038839	0.99989309	0.972487907
ARPC5L	0.953163949	0.840313358	0.890801024	0.999939566	0.99989309	0.97815722
ITGA1	0.953216701	0.851167599	0.993278977	0.99419346	0.99989309	0.89063727
ABHD12	0.953216701	0.927162932	0.89007492	0.971220023	0.99989309	0.924845138
IMPDH2	0.953216701	0.882993091	0.904682623	0.996084773	0.99989309	0.96163007
ZNF280C	0.953216701	0.911230889	0.939683463	0.98470348	0.99989309	0.966332158
SERINC1	0.95327979	0.871682971	0.939091808	0.999939566	0.99989309	0.908939618
EPHB4	0.953339869	0.920347742	0.945417678	0.987143706	0.99989309	0.954732204
ABCA7	0.953358659	0.932838587	0.943891967	0.999939566	0.99989309	0.888695935
LOC104974144	0.953358659	0.972786652	0.931173336	0.987143706	0.99989309	0.908939618
RFC1	0.953358659	0.889410709	0.933102499	0.992137653	0.99989309	0.926872824
PARK7	0.953358659	0.905210378	0.902141181	0.998542029	0.99989309	0.935655141
NGFR	0.953358659	0.839641196	0.939683463	0.999939566	0.99989309	0.956959747
AP2A1	0.953486084	0.907878616	0.917876971	0.989391869	0.99989309	0.926872824
NR4A3	0.953496501	0.94883251	0.958420472	0.990571725	0.99989309	0.887248119
LOC101903600	0.953496501	0.873566716	0.987842204	0.995800463	0.99989309	0.893961809
ZBTB9	0.953592092	0.971426434	0.884189563	0.971220023	0.99989309	0.98016619
LOC101902221	0.953598138	0.852463941	0.998442742	0.973207466	0.99989309	0.901689556
CASD1	0.953598138	0.907229289	0.883874752	0.999939566	0.99989309	0.902660792
DPYSL3	0.953598138	0.918976923	0.910618298	0.999939566	0.99989309	0.908939618
PRPF38A	0.953598138	0.92813228	0.92582321	0.990049248	0.99989309	0.910619304
LOC112443151	0.953598138	0.852463941	0.94821101	0.999939566	0.99989309	0.912101047
LOC751811	0.953598138	0.871113425	0.91546474	0.999939566	0.99989309	0.919731102
GOLIM4	0.953598138	0.888164274	0.897316161	0.999939566	0.99989309	0.942421464
LOC101904355	0.953598138	0.85749413	0.929738756	0.976950878	0.99989309	0.950199992
C3AR1	0.953598138	0.944420015	0.882898206	0.99879985	0.99989309	0.96163007
ANGPTL6	0.953598138	0.874669928	0.929958748	0.977555889	0.99989309	0.983453112
TSSK4	0.953598138	0.899474896	0.911743083	0.992137653	0.99989309	0.988323336
AHNAK2	0.953702581	0.907691022	0.92153058	0.999939566	0.99989309	0.895218005
NUDCD2	0.953702581	0.881079118	0.926613927	0.995118128	0.99989309	0.911468373
SERPING1	0.953702581	0.905210378	0.909170161	0.999939566	0.99989309	0.912396352

LOC100124497	0.953702581	0.9136091	0.91546474	0.990571725	0.99989309	0.943685681
CFAP53	0.953702581	0.859941746	0.887130994	0.987143706	0.99989309	0.96826636
COP1	0.953716791	0.861552326	0.894414265	0.999939566	0.99989309	0.892802259
TICAM2	0.953716791	0.895909584	0.914489705	0.999939566	0.99989309	0.893961809
STRN	0.953716791	0.91994024	0.926018972	0.991662302	0.99989309	0.896877362
ASXL2	0.953716791	0.86652066	0.927697701	0.995946943	0.99989309	0.97491665
PLA2G4A	0.953736782	0.916329514	0.908821939	0.999939566	0.99989309	0.908118871
NAPG	0.953813527	0.882542824	0.93798101	0.999939566	0.99989309	0.88667499
KIAA0753	0.953813527	0.907594268	0.962209895	0.982140778	0.99989309	0.886992214
DYNC1I1	0.953813527	0.911230889	0.929958748	0.999939566	0.99989309	0.886992214
KLF4	0.953813527	0.922434012	0.905374144	0.999939566	0.99989309	0.887248119
SUDS3	0.953813527	0.859941746	0.929303679	0.999939566	0.99989309	0.889159914
LAMB1	0.953813527	0.852313021	0.930520213	0.992368879	0.99989309	0.889622239
ZFPM1	0.953813527	0.866813771	0.941873695	0.999939566	0.99989309	0.890394899
BRAP	0.953813527	0.87401231	0.959159138	0.99879985	0.99989309	0.895218005
PLA2G15	0.953813527	0.857034402	0.961552493	0.999939566	0.99989309	0.895218005
SLC41A2	0.953813527	0.873299679	0.899530079	0.992137653	0.99989309	0.895287865
ERBB3	0.953813527	0.885981636	0.92153058	0.999939566	0.99989309	0.900168812
DUSP1	0.953813527	0.876220907	0.899195818	0.999939566	0.99989309	0.90211634
BLNK	0.953813527	0.962123724	0.908821787	0.999939566	0.99989309	0.902660792
CEP78	0.953813527	0.843422478	0.951742137	0.999939566	0.99989309	0.902660792
FAM118B	0.953813527	0.847082229	0.951742137	0.999939566	0.99989309	0.902660792
HNRNPH1	0.953813527	0.847423683	0.959377939	0.992137653	0.99989309	0.905229516
NOSTRIN	0.953813527	0.930469765	0.897590515	0.999939566	0.99989309	0.906665513
NQO1	0.953813527	0.84501381	0.935247964	0.99879985	0.99989309	0.908939618
PYGO2	0.953813527	0.883267666	0.912681633	0.999939566	0.99989309	0.908939618
CDH7	0.953813527	0.885796876	0.922147089	0.999939566	0.99989309	0.908939618
TEX35	0.953813527	0.877749555	0.89248682	0.999939566	0.99989309	0.914233769
MACROD1	0.953813527	0.926529317	0.889631743	0.999939566	0.99989309	0.920942379
LOC101902458	0.953813527	0.86415368	0.982223562	0.992137653	0.99989309	0.921381434
LOC112444463	0.953813527	0.96471209	0.939683463	0.990571725	0.99989309	0.922263023
SDHAF1	0.953813527	0.905934691	0.936383207	0.995800463	0.99989309	0.922876577
SF1	0.953813527	0.855151723	0.93145152	0.995118128	0.99989309	0.926872824
SIL1	0.953813527	0.916740848	0.883726795	0.999939566	0.99989309	0.926872824
SPATS2	0.953813527	0.852463941	0.965750046	0.999939566	0.99989309	0.926872824
SERPINB6	0.953813527	0.90249621	0.903622442	0.990491321	0.99989309	0.92920113
CHPT1	0.953813527	0.881079118	0.906925281	0.999939566	0.99989309	0.934653208
WDR81	0.953813527	0.852313021	0.939091808	0.993948479	0.99989309	0.935323128

TMEM81	0.953813527	0.882736993	0.904920398	0.999939566	0.99989309	0.935323128
ERCC6L2	0.953813527	0.907691022	0.929958748	0.999939566	0.99989309	0.935385789
PDE8B	0.953813527	0.841044548	0.921135532	0.999939566	0.99989309	0.935655141
STRAP	0.953813527	0.855861376	0.926613927	0.99879985	0.99989309	0.94091418
CANT1	0.953813527	0.913171043	0.945417678	0.973377993	0.99989309	0.942455807
PYROXD2	0.953813527	0.926435857	0.953045982	0.990571725	0.99989309	0.944606747
DMTN	0.953813527	0.897375356	0.892775521	0.982554614	0.99989309	0.949206487
SMG5	0.953813527	0.900335526	0.886176337	0.990049248	0.99989309	0.952631575
SPA17	0.953813527	0.87784497	0.953045982	0.995118128	0.99989309	0.954215255
LOC101903545	0.953813527	0.887725794	0.884325235	0.990571725	0.99989309	0.954334316
TSPAN11	0.953813527	0.907878616	0.885434576	0.995800463	0.99989309	0.95470473
PAPSS1	0.953813527	0.866803532	0.894449269	0.999939566	0.99989309	0.955585296
LOC112441868	0.953813527	0.913171043	0.899195818	0.994568382	0.99989309	0.956610132
S100A13	0.953813527	0.911279338	0.894547778	0.995118128	0.99989309	0.956610132
LOC784357	0.953813527	0.859528714	0.949770432	0.999939566	0.99989309	0.956959747
LOC107132958	0.953813527	0.87314795	0.939683463	0.972489653	0.99989309	0.957122667
ZW10	0.953813527	0.842667989	0.948746792	0.981022805	0.99989309	0.957877422
ATP12A	0.953813527	0.868963165	0.893544538	0.999939566	0.99989309	0.961506391
TRIM62	0.953813527	0.936134921	0.918681165	0.981022805	0.99989309	0.962255684
ULK2	0.953813527	0.921280256	0.88330023	0.999939566	0.99989309	0.962255684
RBFA	0.953813527	0.890427603	0.884197487	0.995800463	0.99989309	0.965053654
ECHDC1	0.953813527	0.846678309	0.951742137	0.985776562	0.99989309	0.966832455
LOC112444502	0.953813527	0.893326814	0.939091808	0.990049248	0.99989309	0.973195541
ZFP64	0.953813527	0.855979085	0.920449324	0.995118128	0.99989309	0.979233909
THEMIS2	0.953813527	0.848798576	0.899195818	0.999939566	0.99989309	0.979606084
RGMB	0.953813527	0.844031777	0.884325235	0.999939566	0.99989309	0.99064193
DSCC1	0.953813527	0.869904696	0.909170161	0.992746023	0.99989309	0.992358083
RDH5	0.953832839	0.852463941	0.9840633	0.99879985	0.99989309	0.891000792
LOC112449318	0.953832839	0.983048937	0.884325235	0.999939566	0.99989309	0.908645305
PCNX1	0.953832839	0.924999357	0.899195818	0.973207466	0.99989309	0.908939618
ST8SIA1	0.953832839	0.978453686	0.911119875	0.9846464	0.99989309	0.912380696
TACC2	0.953832839	0.848398356	0.923072506	0.99419346	0.99989309	0.92689297
ZBTB45	0.953832839	0.891661444	0.914685423	0.99879985	0.99989309	0.940683751
ANKS1A	0.953832839	0.924433008	0.894414265	0.999939566	0.99989309	0.943138585
HSD17B7	0.953832839	0.891430331	0.887391534	0.995680081	0.99989309	0.960377169
HS3ST6	0.953925259	0.880711088	0.886282865	0.999939566	0.99989309	0.940463549
LOC617141	0.954023093	0.926435857	0.892122482	0.999939566	0.99989309	0.912679756
SGO1	0.954111346	0.89618217	0.962790365	0.999939566	0.99989309	0.893961809

CWF19L2	0.954111346	0.984082087	0.886373777	0.990571725	0.99989309	0.906802901
PLBD2	0.954111346	0.953748191	0.886282865	0.999939566	0.99989309	0.931016589
YJEFN3	0.954111346	0.88311684	0.930520213	0.977470078	0.99989309	0.940683751
ZC3H18	0.954123775	0.866813771	0.883874752	0.99879985	0.99989309	0.906043653
PIKFYVE	0.954162565	0.900467607	0.978832388	0.999939566	0.99989309	0.895218005
ADAM10	0.954162565	0.900467607	0.930710426	0.990571725	0.99989309	0.937591162
LOC112446454	0.954162565	0.951362434	0.937270184	0.99483611	0.99989309	0.941925704
LOC104972827	0.954162565	0.923841618	0.939091808	0.990049248	0.99989309	0.963768949
KIAA0391	0.954162565	0.86515799	0.892122482	0.982140778	0.99989309	0.998928226
BRCC3	0.954165187	0.862941799	0.90166788	0.99879985	0.99989309	0.908939618
RAD51B	0.954165187	0.851167599	0.941593855	0.999939566	0.99989309	0.91601693
PARVA	0.954181789	0.871682971	0.979851274	0.995118128	0.99989309	0.911696156
B4GAT1	0.954649226	0.852463941	0.939091808	0.993948479	0.99989309	0.910353954
ACCS	0.954649226	0.874441163	0.961742979	0.995118128	0.99989309	0.942421464
IL1R1	0.954975787	0.886732397	0.963643451	0.998542029	0.99989309	0.905915871
CDC42BPA	0.954975787	0.88311684	0.958420472	0.999939566	0.99989309	0.917968123
ETAA1	0.954975787	0.92505272	0.960137022	0.995118128	0.99989309	0.929046416
GADD45B	0.954975787	0.860463255	0.896272282	0.999939566	0.99989309	0.940917403
C6	0.954975787	0.897657501	0.886176337	0.999939566	0.99989309	0.944606747
PQLC3	0.954975787	0.908402307	0.931775341	0.990571725	0.99989309	0.955130073
TMOD2	0.954975787	0.851167599	0.933963997	0.981022805	0.99989309	0.960985422
ATG4A	0.954975787	0.877420806	0.887130994	0.999939566	0.99989309	0.980920516
HEY1	0.955027195	0.843101091	0.89007492	0.999939566	0.99989309	0.943685681
LOC107131429	0.955027195	0.861416597	0.939091808	0.982809512	0.99989309	0.981164039
METTL17	0.955202269	0.913075885	0.89248682	0.984968973	0.99989309	0.941827389
ANKRD13D	0.955212285	0.866803532	0.972485306	0.995118128	0.99989309	0.943848108
GIN1	0.955212285	0.868963165	0.899195818	0.99879985	0.99989309	0.948336587
ACTR10	0.955296177	0.866803532	0.972608996	0.999939566	0.99989309	0.895218005
NCOA5	0.955296177	0.886909612	0.90002174	0.977555889	0.99989309	0.921734133
FAM117A	0.955296177	0.938064973	0.886282865	0.999939566	0.99989309	0.9369759
TCEAL1	0.955296177	0.883378562	0.926010642	0.995118128	0.99989309	0.986389023
TDRP	0.955300367	0.855151723	0.943891967	0.99879985	0.99989309	0.925494614
KIF13B	0.95531051	0.921046401	0.933102499	0.983688085	0.99989309	0.911468373
ACP1	0.95531051	0.862527737	0.894547778	0.994539387	0.99989309	0.92035958
SMARCD3	0.95531051	0.851167599	0.939091808	0.98829462	0.99989309	0.978467321
AGMAT	0.955350899	0.849884547	0.892122482	0.999939566	0.99989309	0.893961809
FADS3	0.955350899	0.867972894	0.917876971	0.999939566	0.99989309	0.909768235
SMN2	0.955350899	0.866675066	0.92153058	0.999939566	0.99989309	0.940683751

ARHGEF3	0.955548549	0.959119521	0.899195818	0.990571725	0.99989309	0.912376736
ZNF175	0.955575855	0.851167599	0.975361542	0.999939566	0.99989309	0.90198911
LHPP	0.955575855	0.894715081	0.917876971	0.99879985	0.99989309	0.914440253
BBC3	0.955575855	0.875345836	0.888610896	0.999939566	0.99989309	0.924297091
LOC616427	0.955575855	0.885495456	0.90106167	0.999939566	0.99989309	0.942195382
NFATC2	0.955575855	0.842797231	0.929409032	0.999939566	0.99989309	0.944606747
B4GALT1	0.955575855	0.928119925	0.92582321	0.99483611	0.99989309	0.950090995
BCLAF1	0.955755359	0.915257946	0.897144948	0.999939566	0.99989309	0.893961809
UBE2Q2	0.955834353	0.859941746	0.959159138	0.999939566	0.99989309	0.893961809
SPICE1	0.955881535	0.903657308	0.926613927	0.999939566	0.99989309	0.949334126
CBX5	0.95588212	0.880674338	0.96644151	0.990049248	0.99989309	0.895287865
PWWP2B	0.95588212	0.896440437	0.967489175	0.992154129	0.99989309	0.917968123
LARP1	0.955954735	0.852281996	0.892258313	0.999939566	0.99989309	0.956610132
CTSS	0.956023259	0.936134921	0.957578315	0.995687815	0.99989309	0.895287865
ASIP	0.956401591	0.868963165	0.999541752	0.982140778	0.99989309	0.893961809
MCC	0.956401591	0.982614603	0.893763648	0.996084773	0.99989309	0.895218005
SIAH2	0.956401591	0.89272885	0.971981006	0.990300191	0.99989309	0.897011069
NUDT5	0.956401591	0.96471209	0.911119875	0.990571725	0.99989309	0.914440253
CFAP161	0.956401591	0.866803532	0.903358864	0.992137653	0.99989309	0.928190067
ZNF25	0.956401591	0.851167599	0.91546474	0.999939566	0.99989309	0.933678522
ATRAID	0.956401591	0.87793677	0.906925281	0.999939566	0.99989309	0.936439483
TPM3	0.956401591	0.883215952	0.968954353	0.992154129	0.99989309	0.942421464
TCTN2	0.956401591	0.843422478	0.933102499	0.999939566	0.99989309	0.955585296
HSPBAP1	0.956401591	0.848556568	0.933102499	0.99879985	0.99989309	0.984628302
LOC101905711	0.956479378	0.885181785	0.910978655	0.999939566	0.99989309	0.904937451
ZZZ3	0.956518456	0.936859329	0.892122482	0.999939566	0.99989309	0.905229516
LOC789551	0.956615587	0.885991833	0.914685423	0.999939566	0.99989309	0.908939618
CPD	0.956615587	0.984102025	0.911743083	0.995118128	0.99989309	0.910342303
RAPGEF1	0.956803411	0.849834516	0.976719499	0.999752367	0.99989309	0.906178214
LOC112443012	0.956803411	0.864821455	0.939683463	0.992137653	0.99989309	0.935154223
ZNF367	0.956803411	0.87314795	0.929303679	0.999939566	0.99989309	0.935655141
ATP9A	0.956803411	0.862267429	0.904691312	0.992137653	0.99989309	0.966567675
SGMS1	0.956964649	0.852689372	0.884189563	0.999939566	0.99989309	0.902660792
SCOC	0.956964649	0.9242917	0.939612562	0.999939566	0.99989309	0.919853984
TPM4	0.956976179	0.929536223	0.918681165	0.986058146	0.99989309	0.887248119
KIAA0100	0.956976179	0.844031777	0.905832059	0.982554614	0.99989309	0.998636142
FBP1	0.956976179	0.843640278	0.900109169	0.991978984	0.99989309	0.998636142
FJX1	0.957029529	0.854362191	0.929958748	0.999939566	0.99989309	0.911146927

TTC7A	0.957039214	0.916725199	0.885064018	0.999939566	0.99989309	0.917524589
GPR158	0.957210948	0.926435857	0.943501903	0.999939566	0.99989309	0.909768235
MRPS27	0.957210948	0.867380289	0.89007492	0.987143706	0.99989309	0.989313458
SEC23IP	0.957230123	0.877749555	0.917092799	0.998542029	0.99989309	0.927427936
SLC4A10	0.957324867	0.869907246	0.96880775	0.98636843	0.99989309	0.95342487
RPS6KA1	0.957353077	0.976925958	0.896272282	0.994365581	0.99989309	0.941925704
LOC112447492	0.957353523	0.939870925	0.959646613	0.996118855	0.99989309	0.911475796
RAB13	0.957355481	0.893512526	0.88997646	0.999939566	0.99989309	0.888396511
NRBF2	0.957426294	0.964962641	0.942004125	0.995800463	0.99989309	0.897818829
RUNDC1	0.957559314	0.967237998	0.95826379	0.990049248	0.99989309	0.892802259
CLN8	0.957564304	0.897918739	0.909002826	0.981022805	0.99989309	0.893961809
FOXC2	0.957564304	0.945105738	0.894547778	0.998542029	0.99989309	0.895218005
KIF5B	0.957564304	0.877420806	0.915636025	0.999939566	0.99989309	0.926872824
MRPS15	0.957679073	0.851167599	0.892122482	0.99879985	0.99989309	0.908939618
ZNF197	0.957781673	0.918306418	0.922126495	0.99879985	0.99989309	0.889411149
TRPM4	0.957781673	0.881795385	0.933963997	0.999939566	0.99989309	0.90525134
NBEAL2	0.957862872	0.885181785	0.939683463	0.994309225	0.99989309	0.979606084
KDM4A	0.958011152	0.864459672	0.971981006	0.995800463	0.99989309	0.943138585
IMPAD1	0.958169549	0.934571318	0.959646613	0.983569039	0.99989309	0.924342462
NCOR1	0.958502339	0.921280256	0.939683463	0.998542029	0.99989309	0.908939618
LATS1	0.958502339	0.941769611	0.948411239	0.992137653	0.99989309	0.918666628
SEMA4D	0.958502339	0.941288692	0.918681165	0.990571725	0.99989309	0.968245211
DDIT4	0.958572482	0.855151723	0.939683463	0.999939566	0.99989309	0.891000792
LOC101903253	0.958572482	0.88157531	0.926613927	0.999939566	0.99989309	0.893961809
MBP	0.958572482	0.936134921	0.914489705	0.999939566	0.99989309	0.896041888
PRR7	0.958572482	0.906831279	0.967983981	0.982140778	0.99989309	0.901320431
LOC101907017	0.958572482	0.88418172	0.89248682	0.999939566	0.99989309	0.906529452
AFG3L2	0.958572482	0.858423555	0.91546474	0.982140778	0.99989309	0.9071195
NUDT13	0.958572482	0.908709906	0.945417678	0.998542029	0.99989309	0.9071195
TIGD2	0.958572482	0.874669928	0.886282865	0.990571725	0.99989309	0.939966658
HPRT1	0.958995079	0.885981636	0.942004125	0.988140196	0.99989309	0.982045172
HCFC2	0.959095802	0.95126822	0.917876971	0.992137653	0.99989309	0.908939618
PKM	0.959362657	0.883592664	0.90495551	0.999939566	0.99989309	0.893961809
ST8SIA5	0.959362657	0.874669928	0.902141181	0.999939566	0.99989309	0.895218005
MGMT	0.959362657	0.872750098	0.919609671	0.999939566	0.99989309	0.895218005
LUM	0.959362657	0.864541566	0.902141181	0.999939566	0.99989309	0.898663406
ERLIN2	0.959362657	0.881079118	0.921319817	0.999939566	0.99989309	0.90525134
MAMSTR	0.959362657	0.928694297	0.915636025	0.999939566	0.99989309	0.906529452

ART4	0.959362657	0.91796081	0.919785713	0.999939566	0.99989309	0.918249775
UBAP1	0.959362657	0.852463941	0.951850716	0.995800463	0.99989309	0.943138585
CLDND1	0.959362657	0.872606795	0.942314296	0.992137653	0.99989309	0.952921369
DUSP5	0.959362657	0.911237043	0.931397502	0.99879985	0.99989309	0.964143145
LOC104974330	0.959439617	0.866123629	0.979231344	0.991029787	0.99989309	0.891000792
CCNYL1	0.959439617	0.850712398	0.919108971	0.998542029	0.99989309	0.893961809
FCRL1	0.959439617	0.924799142	0.962176387	0.99879985	0.99989309	0.893961809
ZDHHC12	0.959439617	0.972418749	0.926613927	0.999939566	0.99989309	0.893961809
SLC25A36	0.959439617	0.871858734	0.953045982	0.999939566	0.99989309	0.893961809
ABI2	0.959439617	0.977732731	0.901701313	0.999939566	0.99989309	0.89635579
ZNF81	0.959439617	0.90421287	0.939683463	0.999939566	0.99989309	0.896934364
LOC112441469	0.959439617	0.994365533	0.88950849	0.99419346	0.99989309	0.898279031
ARSK	0.959439617	0.996467699	0.909522865	0.992137653	0.99989309	0.898791601
LOC783261	0.959439617	0.871682971	0.902179915	0.999939566	0.99989309	0.901320431
KLC4	0.959439617	0.866803532	0.888610896	0.999939566	0.99989309	0.902295033
DCTN2	0.959439617	0.855979085	0.906914073	0.999939566	0.99989309	0.908939618
CA9	0.959439617	0.885181785	0.978879234	0.99879985	0.99989309	0.910353954
LOC101906508	0.959439617	0.921961076	0.933102499	0.999939566	0.99989309	0.911470632
CFAP54	0.959439617	0.927298992	0.929303679	0.984527252	0.99989309	0.912376736
POLR3C	0.959439617	0.883476578	0.939683463	0.999939566	0.99989309	0.912376736
MYCBP	0.959439617	0.878594098	0.929958748	0.999939566	0.99989309	0.918668679
SLC3A2	0.959439617	0.880711088	0.943891967	0.992829869	0.99989309	0.919645668
QSOX2	0.959439617	0.962934461	0.89007492	0.99879985	0.99989309	0.923064313
DROSHA	0.959439617	0.882542824	0.915636025	0.999939566	0.99989309	0.923064313
C13H20orf194	0.959439617	0.925191675	0.924266781	0.995680081	0.99989309	0.923871657
UCHL5	0.959439617	0.879044895	0.930262306	0.999939566	0.99989309	0.924537498
MPLKIP	0.959439617	0.882542824	0.894414265	0.999939566	0.99989309	0.924788226
TMEM143	0.959439617	0.848398356	0.893763648	0.990059245	0.99989309	0.927412916
DOT1L	0.959439617	0.883378562	0.98730572	0.992368879	0.99989309	0.934458012
C1QC	0.959439617	0.855151723	0.894547778	0.999939566	0.99989309	0.937591162
BABAM1	0.959439617	0.9136091	0.914489705	0.994365581	0.99989309	0.940683751
TRIB1	0.959439617	0.920347742	0.893863239	0.999939566	0.99989309	0.940917403
GLRB	0.959439617	0.96412538	0.886176337	0.999939566	0.99989309	0.941925704
ADAM20	0.959439617	0.881079118	0.922147089	0.995118128	0.99989309	0.942512132
CAPN15	0.959439617	0.864541566	0.989332994	0.990571725	0.99989309	0.943138585
C10H14orf119	0.959439617	0.962934461	0.886282865	0.992368879	0.99989309	0.943685681
KMT2D	0.959439617	0.887725794	0.939683463	0.997612654	0.99989309	0.945759045
DKC1	0.959439617	0.934248552	0.939683463	0.990571725	0.99989309	0.948277018

C14H8orf33	0.959439617	0.918371039	0.899195818	0.990049248	0.99989309	0.949527038
CTNNA3	0.959439617	0.854944719	0.906597758	0.992368879	0.99989309	0.971554741
IL5RA	0.959439617	0.868963165	0.911743083	0.99879985	0.99989309	0.97519644
KDM6B	0.959439617	0.88469581	0.909170161	0.99879985	0.99989309	0.98016619
FAM205C	0.959439617	0.883378562	0.90166788	0.998542029	0.99989309	0.989128955
IGF1R	0.959439617	0.885991833	0.926613927	0.984100122	0.99989309	0.9956013
RSPO3	0.959493482	0.919224637	0.936775151	0.999939566	0.99989309	0.893961809
NOS1	0.959493482	0.916413195	0.886282865	0.992137653	0.99989309	0.95342487
STAT5B	0.959577842	0.865366022	0.972401758	0.995118128	0.99989309	0.934775196
ADGRE5	0.959662539	0.936134921	0.982680848	0.995118128	0.99989309	0.891908825
MCOLN2	0.959662539	0.852463941	0.929958748	0.99879985	0.99989309	0.896685501
WIPI2	0.959690579	0.936134921	0.886881381	0.999939566	0.99989309	0.902660792
IRF2	0.959690579	0.884185138	0.994425789	0.995118128	0.99989309	0.908939618
ISYNA1	0.959690579	0.877749555	0.941593855	0.999939566	0.99989309	0.953300242
ARHGEF16	0.95978808	0.971068039	0.939080693	0.996775429	0.99989309	0.889367841
LOC100296211	0.95978808	0.936750204	0.941593855	0.990049248	0.99989309	0.893961809
LOC781224	0.95978808	0.871858734	0.96880775	0.995118128	0.99989309	0.906529452
CLIP3	0.95978808	0.852377308	0.924266781	0.999939566	0.99989309	0.924845138
OCSTAMP	0.95978808	0.885253121	0.97381056	0.99879985	0.99989309	0.933678522
ZSCAN23	0.95978808	0.949169888	0.925442336	0.990571725	0.99989309	0.943685681
LONP2	0.95978808	0.868963165	0.897665623	0.999939566	0.99989309	0.948277018
KCTD6	0.95978808	0.897327046	0.921531707	0.992154129	0.99989309	0.992464397
CARS	0.959812395	0.934248552	0.88950849	0.990571725	0.99989309	0.943138585
MAML1	0.959867285	0.908192144	0.939683463	0.998542029	0.99989309	0.891140839
NUP107	0.959867285	0.934531829	0.93798101	0.99419346	0.99989309	0.891511718
COQ8B	0.959867285	0.91843896	0.962790365	0.988915501	0.99989309	0.891908825
NATD1	0.959867285	0.936134921	0.908927423	0.999939566	0.99989309	0.893961809
TRIR	0.959867285	0.913625328	0.925587088	0.999939566	0.99989309	0.893961809
LOC112443004	0.959867285	0.953619177	0.919997767	0.981731033	0.99989309	0.895194567
FAM162A	0.959867285	0.905808353	0.890801024	0.995800463	0.99989309	0.895218005
VTI1A	0.959867285	0.982061045	0.886881381	0.998542029	0.99989309	0.89635579
LOC516355	0.959867285	0.890427603	0.914685423	0.999939566	0.99989309	0.896877362
STXBP1	0.959867285	0.88418172	0.908821939	0.999939566	0.99989309	0.901422892
IGFBP7	0.959867285	0.875420427	0.939091808	0.999939566	0.99989309	0.904984293
COPRS	0.959867285	0.868789501	0.931397502	0.996601241	0.99989309	0.907018349
GLDC	0.959867285	0.91994024	0.941593855	0.999939566	0.99989309	0.908785004
PLK3	0.959867285	0.965239369	0.926613927	0.992154129	0.99989309	0.908939618
EID3	0.959867285	0.897327046	0.929958748	0.99879985	0.99989309	0.912376736

PLXNB1	0.959867285	0.970586744	0.886282865	0.999939566	0.99989309	0.912396352
RYR3	0.959867285	0.889929882	0.918681165	0.999939566	0.99989309	0.914440253
TRIOBP	0.959867285	0.880103565	0.899530079	0.999939566	0.99989309	0.918946855
LOC781298	0.959867285	0.918306418	0.924266781	0.99879985	0.99989309	0.920687336
TRIM9	0.959867285	0.907878616	0.896272282	0.999939566	0.99989309	0.925181465
NPHP3	0.959867285	0.880227868	0.929958748	0.998542029	0.99989309	0.935655141
LOC783942	0.959867285	0.900339302	0.938306386	0.999939566	0.99989309	0.937591162
SFT2D1	0.959867285	0.904764822	0.952085491	0.996601241	0.99989309	0.940683751
LOC112447351	0.959867285	0.854362191	0.989408632	0.990571725	0.99989309	0.941889677
WASHC4	0.959867285	0.909573683	0.918681165	0.999939566	0.99989309	0.943138585
SLC30A9	0.959867285	0.866813771	0.939683463	0.999939566	0.99989309	0.944524573
C22H3orf49	0.959867285	0.875449288	0.939091808	0.990571725	0.99989309	0.949206487
TMEM134	0.959867285	0.875022483	0.911119875	0.99879985	0.99989309	0.955585296
TNS1	0.959867285	0.862267429	0.947475618	0.999939566	0.99989309	0.957122667
CFAP97	0.959867285	0.913075885	0.927460291	0.99879985	0.99989309	0.961506391
VCP	0.959867285	0.883267666	0.940785296	0.999939566	0.99989309	0.961506391
TCF7L1	0.959867285	0.900467607	0.905832059	0.995118128	0.99989309	0.96163007
ZFAND6	0.959867285	0.88311684	0.901550297	0.999939566	0.99989309	0.963768949
CCR7	0.959867285	0.899387334	0.911157179	0.989391869	0.99989309	0.974139155
FIGNL2	0.959867285	0.887165309	0.917876971	0.984100122	0.99989309	0.979352545
TNFRSF4	0.959867285	0.888074023	0.960137022	0.988705838	0.99989309	0.98016619
TREML1	0.959867285	0.867380289	0.930892662	0.99879985	0.99989309	0.981396128
HPCAL4	0.959867285	0.86415368	0.890801024	0.99879985	0.99989309	0.981411983
LOC112444888	0.959867285	0.851167599	0.962754771	0.990049248	0.99989309	0.98361583
SH2D1B	0.960201206	0.857005855	0.939683463	0.992368879	0.99989309	0.961506391
ATG9A	0.960201206	0.876057665	0.921612776	0.99879985	0.99989309	0.990085862
EIF3L	0.960201206	0.907594268	0.90002174	0.990049248	0.99989309	0.998074562
HSD11B1L	0.960272455	0.855151723	0.886282865	0.999939566	0.99989309	0.941925704
LOC615959	0.960440686	0.873566716	0.925587088	0.999939566	0.99989309	0.895218005
MRPL22	0.960440686	0.918306418	0.918576062	0.999939566	0.99989309	0.96435585
LGMN	0.960505403	0.955798423	0.94897867	0.999939566	0.99989309	0.891000792
RFT1	0.960515498	0.852463941	0.941319062	0.992137653	0.99989309	0.906529452
MAP3K14	0.960515498	0.876169307	0.965712499	0.999939566	0.99989309	0.908939618
REXO1	0.960515498	0.849718733	0.978892556	0.99879985	0.99989309	0.920942379
SAT1	0.960515498	0.926435857	0.914685423	0.999939566	0.99989309	0.941925704
REEP6	0.960515498	0.892711879	0.892122482	0.990571725	0.99989309	0.944809797
LOC782922	0.960831744	0.893512526	0.976719499	0.990049248	0.99989309	0.920942379
SLAIN1	0.960831744	0.884768652	0.939091808	0.994309225	0.99989309	0.941925704

C24H18orf21	0.960902178	0.965239369	0.939683463	0.990049248	0.99989309	0.895218005
ECT2	0.961176291	0.852749415	0.93145152	0.999939566	0.99989309	0.893961809
VSTM4	0.961176291	0.852463941	0.91234361	0.999939566	0.99989309	0.901173172
ARL2BP	0.961176291	0.903820329	0.939091808	0.999939566	0.99989309	0.903944392
C18H19orf54	0.961176291	0.901166705	0.899539089	0.998963067	0.99989309	0.908939618
LOC101906569	0.961176291	0.886019911	0.957802423	0.999939566	0.99989309	0.914440253
NDST3	0.961176291	0.886967296	0.91546474	0.992368879	0.99989309	0.917968123
LOC101906717	0.961176291	0.852463941	0.949190017	0.999939566	0.99989309	0.923064313
UBE2E1	0.961176291	0.906409999	0.967873529	0.998542029	0.99989309	0.92689297
POLDIP2	0.961176291	0.852377308	0.918965426	0.999939566	0.99989309	0.929537775
NME9	0.961176291	0.901169294	0.891014698	0.99879985	0.99989309	0.930925458
NPR1	0.961176291	0.924799142	0.925587088	0.982367414	0.99989309	0.934660101
WWP2	0.961176291	0.862527737	0.958420472	0.990059245	0.99989309	0.93691717
LOC112447824	0.961176291	0.921280256	0.93050447	0.994309225	0.99989309	0.937771281
LOC112443425	0.961176291	0.936134921	0.914489705	0.99879985	0.99989309	0.946302385
NOLC1	0.961176291	0.852281996	0.932432094	0.99879985	0.99989309	0.946376229
FZD4	0.961176291	0.876057665	0.899539089	0.999939566	0.99989309	0.947398404
TBK1	0.961176291	0.87401231	0.909170161	0.999939566	0.99989309	0.950959274
C22H3orf14	0.961176291	0.904764822	0.907270422	0.995800463	0.99989309	0.983453112
MFSD1	0.961312308	0.893678247	0.942004125	0.999939566	0.99989309	0.893961809
RFK	0.961427252	0.866803532	0.931173336	0.992368879	0.99989309	0.906178214
TMEM259	0.961427252	0.913075885	0.913650484	0.999939566	0.99989309	0.944892687
MASP1	0.961537564	0.974986418	0.924808476	0.996775429	0.99989309	0.919645668
NR1I3	0.961537564	0.959845131	0.93050447	0.982140778	0.99989309	0.93691717
RNF220	0.961537564	0.924799142	0.909170161	0.992137653	0.99989309	0.940683751
NYX	0.961771646	0.871113425	0.894414265	0.999939566	0.99989309	0.908939618
SGCD	0.961772121	0.908857456	0.917092799	0.999939566	0.99989309	0.901173172
CMTR2	0.961808227	0.897657501	0.914668913	0.990571725	0.99989309	0.98016619
CAPRIN1	0.961967952	0.851167599	0.938306386	0.99879985	0.99989309	0.954653099
ORAOV1	0.962012151	0.899387334	0.921135532	0.999939566	0.99989309	0.893400499
TTC23	0.962012151	0.874441163	0.917876971	0.999939566	0.99989309	0.911696156
AHNAK	0.962012151	0.852829558	0.937270184	0.999939566	0.99989309	0.971554741
TLE6	0.962194944	0.890554452	0.946882059	0.999939566	0.99989309	0.893961809
CEP72	0.962194944	0.980682689	0.892348336	0.999939566	0.99989309	0.895218005
SLC9C2	0.962194944	0.938064973	0.899539089	0.999939566	0.99989309	0.895218005
RAPGEF5	0.962194944	0.907594268	0.917876971	0.999939566	0.99989309	0.905220694
RNPEPL1	0.962194944	0.920818159	0.896272282	0.999939566	0.99989309	0.906065482
APOOL	0.962194944	0.862527737	0.917876971	0.990571725	0.99989309	0.909054478

LOC112447802	0.962194944	0.985326169	0.914685423	0.992368879	0.99989309	0.910353954
PTRHD1	0.962194944	0.96471209	0.909472681	0.992235612	0.99989309	0.910619304
PELO	0.962194944	0.8554287	0.991406769	0.999939566	0.99989309	0.912376736
CWC27	0.962194944	0.897375356	0.938306386	0.990049248	0.99989309	0.912380696
HNRNPU	0.962194944	0.871412404	0.961742979	0.982140778	0.99989309	0.914440253
LOC617654	0.962194944	0.852463941	0.939091808	0.99879985	0.99989309	0.914440253
PACSIN2	0.962194944	0.875022483	0.939683463	0.999939566	0.99989309	0.917968123
TMEM127	0.962194944	0.89460994	0.972485306	0.998542029	0.99989309	0.918666628
LOC101904855	0.962194944	0.907594268	0.965416289	0.991837175	0.99989309	0.926853716
LOC112442214	0.962194944	0.948666776	0.944612298	0.99669458	0.99989309	0.92689297
TMOD3	0.962194944	0.872513862	0.918965426	0.99879985	0.99989309	0.92920113
LOC101906364	0.962194944	0.928119925	0.926613927	0.999939566	0.99989309	0.933678522
KANK3	0.962194944	0.940161597	0.923976352	0.999939566	0.99989309	0.934653208
JUND	0.962194944	0.911196851	0.941593855	0.990571725	0.99989309	0.935607719
KREMEN1	0.962194944	0.921280256	0.93050447	0.99879985	0.99989309	0.938643464
ZNF217	0.962194944	0.885796876	0.918576062	0.989686311	0.99989309	0.940683751
FANCA	0.962194944	0.868963165	0.957578315	0.999939566	0.99989309	0.943138585
LOC100847490	0.962194944	0.94883251	0.892323611	0.999939566	0.99989309	0.954334316
MRPS17	0.962194944	0.854944719	0.914489705	0.999939566	0.99989309	0.956554661
LOC104975861	0.962194944	0.855861376	0.939091808	0.998542029	0.99989309	0.991281092
LOC104973826	0.962194944	0.902136721	0.894449269	0.990571725	0.99989309	0.998074562
LCAT	0.962310591	0.952464938	0.892258313	0.988915501	0.99989309	0.908939618
BCOR	0.962310591	0.941266539	0.911743083	0.984769774	0.99989309	0.910619304
WDR59	0.962310591	0.980639049	0.936383207	0.992137653	0.99989309	0.920826497
C21H15orf39	0.962310591	0.927695076	0.969618195	0.9846464	0.99989309	0.922872513
PRSS2	0.962310591	0.884768652	0.922161207	0.999939566	0.99989309	0.926872824
RAN	0.962310591	0.897375356	0.965169397	0.995118128	0.99989309	0.956610132
EML5	0.962310591	0.880103565	0.894449269	0.992137653	0.99989309	0.981411983
CHIC1	0.962312583	0.852463941	0.899195818	0.992368879	0.99989309	0.969981358
ARID5A	0.962384831	0.911196851	0.911119875	0.995118128	0.99989309	0.912101047
LOC101907294	0.962424119	0.908192144	0.917092799	0.999939566	0.99989309	0.915323299
HP1BP3	0.962731786	0.875022483	0.957802423	0.990571725	0.99989309	0.94425343
PROSER2	0.962857989	0.86084613	0.989332994	0.999939566	0.99989309	0.908939618
PDE8A	0.962857989	0.866675066	0.891051885	0.999939566	0.99989309	0.911696156
NIPAL4	0.962857989	0.868963165	0.917066863	0.999939566	0.99989309	0.943738005
NOTCH4	0.962857989	0.920967949	0.899530079	0.998542029	0.99989309	0.955585296
KCNC4	0.962857989	0.863248726	0.912223566	0.999939566	0.99989309	0.964143145
DERL2	0.963128329	0.96761188	0.941312064	0.999939566	0.99989309	0.900168812

LOC107132270	0.963128329	0.87401231	0.892122482	0.999939566	0.99989309	0.908939618
KANTR	0.963128329	0.885604065	0.896272282	0.999939566	0.99989309	0.908939618
LOC100139996	0.963128329	0.852214392	0.907270422	0.999939566	0.99989309	0.908939618
PHF19	0.963128329	0.921280256	0.944863023	0.999939566	0.99989309	0.910353954
RPE	0.963128329	0.878843255	0.979635589	0.99879985	0.99989309	0.911876023
PRORSD1	0.963128329	0.882706523	0.904682623	0.999939566	0.99989309	0.914211806
DNAJC9	0.963128329	0.957071555	0.957379098	0.992137653	0.99989309	0.928123265
LOC104968434	0.963128329	0.908453308	0.93050447	0.999939566	0.99989309	0.930707319
CMPK2	0.963128329	0.881079118	0.909522865	0.990571725	0.99989309	0.932613047
LDLRAD4	0.963128329	0.963291559	0.92153058	0.989391869	0.99989309	0.93636925
LOC112444626	0.963128329	0.931640193	0.89007492	0.990571725	0.99989309	0.942421464
LOC101902840	0.963128329	0.861552326	0.959646613	0.999939566	0.99989309	0.953604648
LOC101904339	0.963128329	0.902686006	0.899195818	0.992235612	0.99989309	0.95470473
NUDT6	0.963128329	0.912112179	0.929958748	0.995118128	0.99989309	0.963996128
LOC112445035	0.963128329	0.855151723	0.918576062	0.99879985	0.99989309	0.982559673
INTS8	0.963128329	0.921961076	0.898889441	0.995118128	0.99989309	0.986819555
SV2B	0.963132565	0.90760125	0.904920398	0.999939566	0.99989309	0.901689556
ITGA2	0.963243869	0.924510975	0.945417678	0.996084773	0.99989309	0.898852666
GNPAT	0.963243869	0.866977641	0.962754771	0.999939566	0.99989309	0.912376736
CELA1	0.963243869	0.877393801	0.905401375	0.999939566	0.99989309	0.956610132
LOC112448453	0.963243869	0.972418749	0.899732746	0.992368879	0.99989309	0.960805387
LOC101907744	0.963243869	0.926435857	0.921319817	0.996084773	0.99989309	0.980144374
TMEM101	0.963342613	0.911196851	0.939683463	0.999939566	0.99989309	0.89471811
TPCN1	0.963342613	0.999377692	0.911743083	0.992137653	0.99989309	0.895218005
COMMD1	0.963342613	0.922004954	0.949977753	0.998542029	0.99989309	0.898791601
HVCN1	0.963342613	0.912112179	0.939683463	0.990049248	0.99989309	0.902660792
ZWINT	0.963342613	0.854944719	0.9785457	0.999939566	0.99989309	0.909768235
CRAT	0.963342613	0.905432526	0.91036839	0.990571725	0.99989309	0.912380696
MPP1	0.963342613	0.85749413	0.899195818	0.999939566	0.99989309	0.922263023
UBE2J1	0.963342613	0.89141259	0.988585687	0.990571725	0.99989309	0.924845138
LOC112444613	0.963342613	0.912785459	0.940785296	0.999939566	0.99989309	0.92546728
TP53INP2	0.963342613	0.885181785	0.961742979	0.990049248	0.99989309	0.926853716
ZFP62	0.963342613	0.9347557	0.933629769	0.994365581	0.99989309	0.928653932
RNF10	0.963342613	0.903820329	0.922126495	0.998542029	0.99989309	0.935385789
NXT1	0.963342613	0.861730561	0.939091808	0.999939566	0.99989309	0.941925704
LOC100140207	0.963342613	0.934248552	0.892122482	0.999939566	0.99989309	0.943685681
ECH1	0.963342613	0.852463941	0.914477011	0.999939566	0.99989309	0.946314386
SS18	0.963342613	0.895221573	0.913703629	0.99879985	0.99989309	0.948277018

REXO4	0.963342613	0.974986418	0.909170161	0.990571725	0.99989309	0.95470473
LOC104968522	0.963342613	0.90249621	0.926267643	0.999939566	0.99989309	0.961506391
IREB2	0.963342613	0.855151723	0.947990122	0.999939566	0.99989309	0.964820716
LOC781158	0.963342613	0.89454843	0.900109169	0.999939566	0.99989309	0.969981358
NABP1	0.963342613	0.904764822	0.926613927	0.995800463	0.99989309	0.986862062
MYCBPAP	0.96337463	0.90393667	0.944612298	0.999939566	0.99989309	0.902660792
MED20	0.96337463	0.96412538	0.92153058	0.992137653	0.99989309	0.905220694
ANGEL2	0.96337463	0.954015268	0.918576062	0.999939566	0.99989309	0.90525134
ACAP2	0.96337463	0.926435857	0.936383207	0.997612654	0.99989309	0.905267129
LOC107132395	0.96337463	0.894967094	0.962790365	0.999939566	0.99989309	0.906665513
PCDHB8	0.96337463	0.934248552	0.972874677	0.990049248	0.99989309	0.908939618
HES4	0.96337463	0.882542824	0.939091808	0.99879985	0.99989309	0.908939618
VPS52	0.96337463	0.852463941	0.93050447	0.999939566	0.99989309	0.908939618
EGFR	0.96337463	0.875449288	0.891455327	0.999939566	0.99989309	0.914440253
BCAP31	0.96337463	0.882582114	0.917876971	0.999939566	0.99989309	0.917968123
CRACR2B	0.96337463	0.877180988	0.939683463	0.999939566	0.99989309	0.917968123
LOC104973517	0.96337463	0.85749413	0.962209895	0.999939566	0.99989309	0.917968123
CCL16	0.96337463	0.857917583	0.929538682	0.999939566	0.99989309	0.926872824
AIP	0.96337463	0.866803532	0.951850716	0.999939566	0.99989309	0.931016589
FAM21A	0.96337463	0.893839642	0.925442336	0.999939566	0.99989309	0.931126954
PTGDR	0.96337463	0.925360181	0.921039185	0.984100122	0.99989309	0.972487907
C18H19orf84	0.96337463	0.873098735	0.909170161	0.990049248	0.99989309	0.97680108
GNB4	0.96337463	0.88311684	0.945417678	0.995118128	0.99989309	0.983453112
DDX1	0.96337463	0.856914648	0.921135532	0.995118128	0.99989309	0.988323336
LOC613677	0.963573166	0.877016814	0.960623345	0.999939566	0.99989309	0.931495481
LOC781379	0.963608879	0.863248726	0.99304917	0.995946943	0.99989309	0.895218005
FAM129C	0.963608879	0.979617176	0.939091808	0.999939566	0.99989309	0.895218005
TSPAN3	0.963608879	0.968237269	0.965712499	0.995118128	0.99989309	0.901422892
ECE2	0.963608879	0.891806038	0.968230268	0.999939566	0.99989309	0.905220694
SLC25A3	0.963608879	0.883592664	0.920017619	0.994365581	0.99989309	0.911284706
VPS25	0.963608879	0.855979085	0.951850716	0.998542029	0.99989309	0.912376736
LINS1	0.963608879	0.945105738	0.935024946	0.99879985	0.99989309	0.918249775
HERC6	0.963608879	0.873566716	0.987842204	0.990049248	0.99989309	0.920834985
ST5	0.963608879	0.920347742	0.918681165	0.999939566	0.99989309	0.936563391
FMN1	0.963619783	0.965241881	0.894449269	0.998542029	0.99989309	0.90259329
UBE2F	0.963756587	0.916725199	0.966742389	0.999939566	0.99989309	0.901173172
C15H11orf96	0.963849801	0.942048064	0.967302577	0.99879985	0.99989309	0.908939618
YWHAG	0.963849801	0.88311684	0.957379098	0.999939566	0.99989309	0.910353954

LOC101905571	0.963849801	0.868108717	0.899195818	0.999939566	0.99989309	0.923064313
BIVM	0.963849801	0.952464938	0.909170161	0.995800463	0.99989309	0.926872824
GPX1	0.963849801	0.892520818	0.953045982	0.995118128	0.99989309	0.946344585
LOC101902856	0.963849801	0.913075885	0.921936757	0.999939566	0.99989309	0.96163007
NOL10	0.963890334	0.874669928	0.979231344	0.998542029	0.99989309	0.895218005
PPM1K	0.963890334	0.903657308	0.899624092	0.999939566	0.99989309	0.918220726
PCSK7	0.963890334	0.879391812	0.92153058	0.992402807	0.99989309	0.968245211
DNAH5	0.963890334	0.956688135	0.899195818	0.984968973	0.99989309	0.99064193
F8A1	0.963963798	0.859941746	0.922051039	0.999939566	0.99989309	0.897011069
LOC112443142	0.963963798	0.883681659	0.994326396	0.983688085	0.99989309	0.923064313
KCNMB2	0.964012028	0.852463941	0.941593855	0.995800463	0.99989309	0.902660792
PREP	0.964012028	0.855151723	0.939683463	0.999939566	0.99989309	0.98016619
ETV5	0.964067773	0.974256477	0.892122482	0.99879985	0.99989309	0.895218005
LOC112447473	0.964067773	0.955798423	0.944863023	0.994309225	0.99989309	0.922636514
LOC509810	0.964067773	0.890581051	0.926613927	0.990059245	0.99989309	0.960712754
RBPJ	0.964114517	0.893512526	0.927460291	0.995800463	0.99989309	0.914233769
CAVIN2	0.964128162	0.888164274	0.941593855	0.999939566	0.99989309	0.895218005
RGS5	0.964215149	0.873045394	0.958340249	0.998542029	0.99989309	0.906178214
NPY1R	0.964215149	0.942048064	0.899539089	0.995118128	0.99989309	0.98016619
GTPBP8	0.964224313	0.938064973	0.90002174	0.999939566	0.99989309	0.90175726
LOC112442365	0.964311873	0.893639679	0.933102499	0.999939566	0.99989309	0.922709797
ZNF583	0.964328974	0.926618064	0.987639174	0.995118128	0.99989309	0.897574464
LOC515736	0.964328974	0.909926193	0.942004125	0.999939566	0.99989309	0.92035958
AFAP1L1	0.964328974	0.934248552	0.942004125	0.999939566	0.99989309	0.936439483
SIDT2	0.964328974	0.885181785	0.914685423	0.999939566	0.99989309	0.942421464
LOC101904529	0.964328974	0.921280256	0.911743083	0.990049248	0.99989309	0.943138585
ZBED3	0.964328974	0.868963165	0.916702	0.999939566	0.99989309	0.966375116
MCU	0.964330401	0.925360181	0.92582321	0.999939566	0.99989309	0.897011069
NASP	0.964330401	0.872513862	0.918576062	0.999939566	0.99989309	0.9071195
RFXAP	0.964330401	0.969942982	0.909170161	0.990300191	0.99989309	0.910353954
RALY	0.964330401	0.854262144	0.921531707	0.999939566	0.99989309	0.927412916
ABL2	0.964330401	0.858046677	0.92153058	0.99483611	0.99989309	0.933763401
SMYD4	0.964338935	0.872277331	0.899530079	0.999939566	0.99989309	0.908939618
SP1	0.964351014	0.914162656	0.909170161	0.999939566	0.99989309	0.912101047
TNNT1	0.964525705	0.975117302	0.893544538	0.988949923	0.99989309	0.940683751
ZNF16	0.964525705	0.855979085	0.922126495	0.999939566	0.99989309	0.958814003
FEM1C	0.964604538	0.901922021	0.965712499	0.998542029	0.99989309	0.950577985
NTPCR	0.964678258	0.899387334	0.939683463	0.999939566	0.99989309	0.914440253

SHANK1	0.964678258	0.87795323	0.962209895	0.998102387	0.99989309	0.974139155
HAT1	0.964724769	0.963478319	0.957389865	0.991978984	0.99989309	0.899551847
MRPL52	0.964764765	0.963478319	0.939683463	0.995118128	0.99989309	0.922263023
ZSCAN12	0.964764765	0.883628587	0.894547778	0.999939566	0.99989309	0.933678522
SNAI1	0.964764765	0.986681109	0.892258313	0.99879985	0.99989309	0.940917403
OTOR	0.964764765	0.906025962	0.930520213	0.995118128	0.99989309	0.943792032
CALN1	0.964783133	0.972786652	0.941593855	0.984968973	0.99989309	0.908939618
CCDC184	0.964783133	0.962934461	0.918317407	0.998526871	0.99989309	0.908939618
KLHDC8B	0.964783133	0.855979085	0.930710426	0.999939566	0.99989309	0.908939618
HCFC1	0.964783133	0.884939495	0.942004125	0.999939566	0.99989309	0.908939618
LOC614424	0.964783133	0.854944719	0.963226207	0.999939566	0.99989309	0.908939618
GPS2	0.964783133	0.857034402	0.915216087	0.995118128	0.99989309	0.909768235
PRR14	0.964783133	0.893266617	0.949727203	0.999939566	0.99989309	0.912101047
COTL1	0.964783133	0.938885124	0.921225977	0.992137653	0.99989309	0.912376736
FUT11	0.964783133	0.902136721	0.90002174	0.999939566	0.99989309	0.917470253
CEP76	0.964783133	0.87401231	0.940785296	0.990300191	0.99989309	0.919731102
DPY30	0.964783133	0.855979085	0.989332994	0.999939566	0.99989309	0.926872824
AGPAT3	0.964783133	0.882930807	0.916100231	0.999939566	0.99989309	0.935655141
AMY2B	0.964783133	0.883628587	0.984700021	0.992137653	0.99989309	0.937591162
C14H8orf88	0.964783133	0.873566716	0.92153058	0.99879985	0.99989309	0.942421464
LOC107131615	0.964783133	0.970979488	0.917876971	0.995118128	0.99989309	0.95470473
VPS53	0.964783133	0.881079118	0.941873695	0.995880311	0.99989309	0.957877422
FZD1	0.964783133	0.859941746	0.922366987	0.992368879	0.99989309	0.963996128
ITGA4	0.964783133	0.854656328	0.957578315	0.990491321	0.99989309	0.965964276
PRKAG1	0.964783133	0.868963165	0.946659074	0.995118128	0.99989309	0.968245211
MKKS	0.964783133	0.89272885	0.909522865	0.999939566	0.99989309	0.968245211
ACVR1	0.964783133	0.90249621	0.899624092	0.995800463	0.99989309	0.972487907
EIF2AK2	0.964783133	0.855979085	0.90002174	0.990571725	0.99989309	0.983453112
LOC100139638	0.964783133	0.91458438	0.896272282	0.987143706	0.99989309	0.998636142
RIF1	0.964823875	0.921280256	0.929958748	0.998408583	0.99989309	0.905936524
RWDD2A	0.964823875	0.967515137	0.89265635	0.999939566	0.99989309	0.908939618
POMGNT1	0.964823875	0.899387334	0.930710426	0.992137653	0.99989309	0.926872824
CCDC22	0.964823875	0.866803532	0.931173336	0.99879985	0.99989309	0.983453112
UBE2T	0.964935355	0.924046689	0.922147089	0.98636843	0.99989309	0.96163007
TUSC1	0.964935355	0.862527737	0.916100231	0.990300191	0.99989309	0.9956013
CSTB	0.964968741	0.94883251	0.947475618	0.999939566	0.99989309	0.916175407
C1QTNF7	0.964968741	0.908192144	0.899629961	0.996601241	0.99989309	0.921381434
SHPRH	0.965015974	0.965829305	0.962209895	0.990571725	0.99989309	0.896149571

RFX7	0.965015974	0.875449288	0.970108169	0.999939566	0.99989309	0.908939618
CENPU	0.965015974	0.924799142	0.926613927	0.990571725	0.99989309	0.910353954
ARPC3	0.965015974	0.874441163	0.975361542	0.999939566	0.99989309	0.926872824
SPATA2	0.965015974	0.931095524	0.929958748	0.990049248	0.99989309	0.945258337
LOC107131846	0.965015974	0.957353974	0.90002174	0.995118128	0.99989309	0.972487907
MAPKAPK5	0.965081186	0.897327046	0.957802423	0.999939566	0.99989309	0.930229476
RBBP7	0.965081186	0.855409006	0.921225977	0.999939566	0.99989309	0.952631575
PRKD1	0.965081186	0.936134921	0.929605682	0.995800463	0.99989309	0.957877422
PGAM2	0.965081186	0.907670026	0.904691312	0.99879985	0.99989309	0.973915852
WIPF2	0.96508442	0.859941746	0.97311093	0.999334425	0.99989309	0.922229044
LOC101907570	0.96520886	0.944314627	0.979635589	0.989391869	0.99989309	0.899771153
KLC2	0.96520886	0.95781929	0.92921168	0.999939566	0.99989309	0.901965781
CCDC160	0.96520886	0.90039743	0.966955806	0.999939566	0.99989309	0.903961701
CD276	0.96520886	0.957071555	0.932432094	0.999939566	0.99989309	0.905229516
LOC112442374	0.96520886	0.855151723	0.999917831	0.990386269	0.99989309	0.905494754
GALNT4	0.96520886	0.960365676	0.958357122	0.995118128	0.99989309	0.90926165
DAB2	0.96520886	0.877749555	0.911743083	0.992339205	0.99989309	0.909940901
LRCH4	0.96520886	0.920540438	0.894829955	0.987342814	0.99989309	0.912101047
NACA	0.96520886	0.90249621	0.939683463	0.992368879	0.99989309	0.913166854
TCEANC2	0.96520886	0.926618064	0.939683463	0.999939566	0.99989309	0.914657697
DAD1	0.96520886	0.91271182	0.944612298	0.990049248	0.99989309	0.926872824
MOSMO	0.96520886	0.945105738	0.941888866	0.995118128	0.99989309	0.926872824
ZFP28	0.96520886	0.885604065	0.939091808	0.99879985	0.99989309	0.926872824
LOC531152	0.96520886	0.874441163	0.914489705	0.999939566	0.99989309	0.928799366
ATF2	0.96520886	0.920347742	0.919609671	0.999939566	0.99989309	0.928799366
RPIA	0.96520886	0.857917583	0.96993432	0.998542029	0.99989309	0.932613047
ANTXR2	0.96520886	0.90249621	0.987006595	0.992137653	0.99989309	0.933763401
SCN7A	0.96520886	0.875803224	0.905017554	0.999939566	0.99989309	0.934458012
INTU	0.96520886	0.86764843	0.990493758	0.988949923	0.99989309	0.937343676
PTPRB	0.96520886	0.934135475	0.968934792	0.987143706	0.99989309	0.940917403
RNASEH2A	0.96520886	0.91557476	0.924266781	0.999939566	0.99989309	0.942421464
BTNL9	0.96520886	0.93453191	0.946659074	0.990049248	0.99989309	0.94425343
TXNDC16	0.96520886	0.893266617	0.893863239	0.999939566	0.99989309	0.94588513
PRELID3B	0.96520886	0.93086109	0.925587088	0.999939566	0.99989309	0.946717155
EXOC3	0.96520886	0.944314627	0.923072506	0.994365581	0.99989309	0.953940638
DNAJC15	0.96520886	0.957071555	0.905017554	0.999939566	0.99989309	0.95470473
SNX6	0.96520886	0.938064973	0.917758314	0.995118128	0.99989309	0.956959747
WDFY1	0.96520886	0.85749413	0.914685423	0.990571725	0.99989309	0.961336964

ANXA4	0.96520886	0.857034402	0.941593855	0.999939566	0.99989309	0.968039618
ETV3	0.96520886	0.924799142	0.909170161	0.990300191	0.99989309	0.97792865
DPF1	0.96520886	0.90393667	0.91355109	0.995118128	0.99989309	0.982559673
GAR1	0.96520886	0.888994602	0.939689648	0.990571725	0.99989309	0.988323336
INTS12	0.96520886	0.906955073	0.906925281	0.995118128	0.99989309	0.991281092
SMS	0.965359888	0.899387334	0.951850716	0.998542029	0.99989309	0.912101047
APPL2	0.965359888	0.876057665	0.918050385	0.995118128	0.99989309	0.957877422
LRRC14	0.965359888	0.8554287	0.914489705	0.98636843	0.99989309	0.961506391
ANKS3	0.965540735	0.919422509	0.958367037	0.990571725	0.99989309	0.928374551
LOC112444474	0.965540735	0.874030037	0.927115509	0.999939566	0.99989309	0.933183326
NUP62	0.965540735	0.877749555	0.913909611	0.992154129	0.99989309	0.943138585
DCAF15	0.965540735	0.926435857	0.931397502	0.999939566	0.99989309	0.944892687
LRP10	0.965540735	0.893639679	0.919108971	0.999939566	0.99989309	0.973153155
PRX	0.965540735	0.90249621	0.947268948	0.992137653	0.99989309	0.981164039
LOC104975960	0.965540735	0.871682971	0.905017554	0.99879985	0.99989309	0.991934523
SLC39A2	0.965550625	0.860449455	0.929303679	0.999939566	0.99989309	0.898273855
FLRT1	0.965550625	0.884220302	0.939091808	0.99879985	0.99989309	0.928123265
LOC101908759	0.965550625	0.98743321	0.909522865	0.992137653	0.99989309	0.936439483
CEP70	0.965550625	0.878720916	0.915891679	0.999939566	0.99989309	0.940683751
PXYLP1	0.965660636	0.936809216	0.904682623	0.990059245	0.99989309	0.943685681
LOC101907195	0.965740631	0.881396756	0.970904484	0.999939566	0.99989309	0.902660792
C11H9orf16	0.965740631	0.931441145	0.956568695	0.992829869	0.99989309	0.908939618
LOC112443163	0.965740631	0.924799163	0.904682623	0.999939566	0.99989309	0.946314386
A4GALT	0.965740631	0.990179479	0.90002174	0.992339205	0.99989309	0.953893582
DDRGK1	0.965795164	0.908709906	0.944592818	0.999939566	0.99989309	0.897818829
DCN	0.965795164	0.871359854	0.962209895	0.998526871	0.99989309	0.901422892
TACR2	0.965795164	0.885181785	0.993665588	0.992154129	0.99989309	0.902083541
SFXN5	0.965795164	0.901922021	0.914685423	0.999939566	0.99989309	0.902268895
C15H11orf71	0.965795164	0.883267666	0.910123657	0.999939566	0.99989309	0.902660792
ETV4	0.965795164	0.897327046	0.985596108	0.999939566	0.99989309	0.904877584
LRRK1	0.965795164	0.911347642	0.957802423	0.994309225	0.99989309	0.906178214
LOC100848665	0.965795164	0.903820329	0.899539089	0.999939566	0.99989309	0.9071195
TGFB2	0.965795164	0.890427603	0.915581825	0.999939566	0.99989309	0.908939618
MBIP	0.965795164	0.874669928	0.962209895	0.999939566	0.99989309	0.91008914
SDR39U1	0.965795164	0.904764822	0.922126495	0.999939566	0.99989309	0.911427827
GNAZ	0.965795164	0.860271574	0.989408632	0.999939566	0.99989309	0.912101047
EFHD2	0.965795164	0.927162932	0.946779183	0.99879985	0.99989309	0.914440253
LOC789895	0.965795164	0.899917012	0.925442336	0.999939566	0.99989309	0.914440253

GANC	0.965795164	0.857207609	0.933963997	0.999939566	0.99989309	0.917968123
ADCYAP1R1	0.965795164	0.998683615	0.90002174	0.990571725	0.99989309	0.918255496
ARFGAP3	0.965795164	0.86415368	0.974681091	0.995880311	0.99989309	0.928799366
LOC112442757	0.965795164	0.911279338	0.97420715	0.995118128	0.99989309	0.932282574
LOC101910045	0.965795164	0.920347742	0.961899356	0.994365581	0.99989309	0.936439483
CCM2	0.965795164	0.991043872	0.908647463	0.995118128	0.99989309	0.937591162
QSOX1	0.965795164	0.920347742	0.921319817	0.999939566	0.99989309	0.937591162
LY96	0.965795164	0.929730109	0.899530079	0.998542029	0.99989309	0.938054315
ZFP36	0.965795164	0.903820329	0.960137022	0.999939566	0.99989309	0.938054315
CBX3	0.965795164	0.881642454	0.930520213	0.999939566	0.99989309	0.938643464
TCHH	0.965795164	0.938064973	0.919997767	0.999939566	0.99989309	0.939426596
SIGLEC10	0.965795164	0.906025962	0.926613927	0.989391869	0.99989309	0.941925704
ZHX2	0.965795164	0.918976923	0.929958748	0.999939566	0.99989309	0.941925704
KCTD14	0.965795164	0.936134921	0.899539089	0.999939566	0.99989309	0.942421464
TTC9	0.965795164	0.885796876	0.930710426	0.999939566	0.99989309	0.943866443
TEX10	0.965795164	0.886173201	0.907456407	0.990300191	0.99989309	0.944606747
LOC112449552	0.965795164	0.89272885	0.933102499	0.999939566	0.99989309	0.948277018
FAM162B	0.965795164	0.866813771	0.919997767	0.999939566	0.99989309	0.949744931
FLAD1	0.965795164	0.881642454	0.924266781	0.999939566	0.99989309	0.95470473
GPAT3	0.965795164	0.90039743	0.919997767	0.999939566	0.99989309	0.955585296
GREB1	0.965795164	0.92161844	0.894547778	0.999939566	0.99989309	0.957707888
MYLK3	0.965795164	0.885253121	0.939091808	0.995118128	0.99989309	0.957877422
LOC101906167	0.965795164	0.871682971	0.918576062	0.999939566	0.99989309	0.957877422
PDXDC1	0.965795164	0.918306418	0.919108971	0.999939566	0.99989309	0.95929043
RANBP3	0.965795164	0.879686372	0.917092799	0.998542029	0.99989309	0.971554741
HARS	0.965795164	0.920347742	0.930710426	0.99879985	0.99989309	0.976757738
NRIP2	0.965795164	0.926435857	0.921146002	0.990571725	0.99989309	0.98016619
LOC112448515	0.965795164	0.908709906	0.954826554	0.988949923	0.99989309	0.986389023
LOC100848766	0.965795164	0.876169307	0.91546474	0.991837175	0.99989309	0.998074562
NPL	0.965871699	0.977732731	0.926010642	0.999939566	0.99989309	0.901689556
SLC5A2	0.965871699	0.897375356	0.931397502	0.999939566	0.99989309	0.901689556
SENP3	0.965871699	0.90596729	0.952255572	0.999939566	0.99989309	0.901689556
POLN	0.965871699	0.883378562	0.904682623	0.995118128	0.99989309	0.902660792
SESN2	0.965871699	0.95781929	0.899539089	0.995800463	0.99989309	0.902660792
LOC104976293	0.965871699	0.872085303	0.903407548	0.999939566	0.99989309	0.904877584
COX4I2	0.965871699	0.990668708	0.913463626	0.99879985	0.99989309	0.905220694
B4GALT4	0.965871699	0.883378562	0.929958748	0.999939566	0.99989309	0.90525134
GPR39	0.965871699	0.921280256	0.939683463	0.999939566	0.99989309	0.90525134

LOC112449106	0.965871699	0.973666167	0.957417441	0.99879985	0.99989309	0.905267129
GTF2E2	0.965871699	0.868312459	0.970486383	0.999939566	0.99989309	0.905267129
WIPI1	0.965871699	0.874441163	0.9785457	0.999939566	0.99989309	0.906065482
CTCF	0.965871699	0.964234693	0.915636025	0.999939566	0.99989309	0.906178214
FAM228B	0.965871699	0.90485456	0.97381056	0.999939566	0.99989309	0.906895944
ESCO2	0.965871699	0.89571555	0.957802423	0.999939566	0.99989309	0.907018349
ATF7IP	0.965871699	0.970179915	0.939683463	0.999939566	0.99989309	0.9071195
HTRA1	0.965871699	0.872513862	0.940168084	0.999939566	0.99989309	0.9071195
ATF6B	0.965871699	0.866803532	0.968419607	0.999939566	0.99989309	0.9071195
VPS26B	0.965871699	0.920347742	0.899530079	0.999939566	0.99989309	0.908785004
LOC112445178	0.965871699	0.926618064	0.922126495	0.990049248	0.99989309	0.908939618
TMPPE	0.965871699	0.877420806	0.93050447	0.990049248	0.99989309	0.908939618
ACER1	0.965871699	0.965027449	0.945417678	0.990049248	0.99989309	0.908939618
ELK1	0.965871699	0.988649234	0.909170161	0.990571725	0.99989309	0.908939618
KCNJ12	0.965871699	0.946142324	0.968455468	0.990571725	0.99989309	0.908939618
LOC100847567	0.965871699	0.90339131	0.980442556	0.992137653	0.99989309	0.908939618
LYSMD2	0.965871699	0.871030989	0.92153058	0.992368879	0.99989309	0.908939618
DLG5	0.965871699	0.930890453	0.951607422	0.995118128	0.99989309	0.908939618
TSPAN14	0.965871699	0.934248552	0.968230268	0.995118128	0.99989309	0.908939618
AP3S1	0.965871699	0.964270094	0.960726438	0.998542029	0.99989309	0.908939618
PRKAA1	0.965871699	0.961481835	0.906887887	0.99879985	0.99989309	0.908939618
TRIM27	0.965871699	0.929573947	0.910978655	0.99879985	0.99989309	0.908939618
TUG1	0.965871699	0.950091107	0.93737555	0.99879985	0.99989309	0.908939618
DUT	0.965871699	0.924160227	0.944060947	0.99879985	0.99989309	0.908939618
RTN1	0.965871699	0.866813771	0.977920946	0.99879985	0.99989309	0.908939618
XPA	0.965871699	0.927298992	0.899195818	0.999939566	0.99989309	0.908939618
LOC532875	0.965871699	0.893639679	0.906278164	0.999939566	0.99989309	0.908939618
IFT88	0.965871699	0.876057665	0.914489705	0.999939566	0.99989309	0.908939618
TMEM184C	0.965871699	0.955353499	0.919263031	0.999939566	0.99989309	0.908939618
ZFHX3	0.965871699	0.911196851	0.9260538	0.999939566	0.99989309	0.908939618
FAM78A	0.965871699	0.963480908	0.939091808	0.999939566	0.99989309	0.908939618
RORA	0.965871699	0.88469581	0.943891967	0.999939566	0.99989309	0.908939618
WDR31	0.965871699	0.962934461	0.948213748	0.999939566	0.99989309	0.908939618
ZFAND4	0.965871699	0.892242414	0.962209895	0.999939566	0.99989309	0.908939618
COL27A1	0.965871699	0.897375356	0.966157152	0.999939566	0.99989309	0.908939618
CAMLG	0.965871699	0.912112179	0.966157152	0.999939566	0.99989309	0.908939618
UBXN8	0.965871699	0.907670026	0.96993432	0.999939566	0.99989309	0.908939618
WLS	0.965871699	0.944314627	0.907265706	0.999939566	0.99989309	0.909768235

SRSF7	0.965871699	0.899387334	0.92153058	0.999939566	0.99989309	0.909768235
LOC100847839	0.965871699	0.881079118	0.909442165	0.99879985	0.99989309	0.909990551
LOC112446879	0.965871699	0.873297522	0.995918981	0.992368879	0.99989309	0.910353954
GPR1	0.965871699	0.871030989	0.958420472	0.99879985	0.99989309	0.910353954
THRB	0.965871699	0.890427603	0.939091808	0.999939566	0.99989309	0.910619304
LOC101906588	0.965871699	0.988649234	0.92332596	0.992368879	0.99989309	0.911526437
TENM3	0.965871699	0.881079118	0.942004125	0.994309225	0.99989309	0.911526437
DDAH1	0.965871699	0.903657308	0.897743967	0.999939566	0.99989309	0.911526437
ETNK1	0.965871699	0.899387334	0.917876971	0.999939566	0.99989309	0.911526437
LOC101904308	0.965871699	0.893839642	0.958420472	0.999939566	0.99989309	0.911526437
GNA11	0.965871699	0.878843255	0.987842204	0.999939566	0.99989309	0.911632967
CTNNBIP1	0.965871699	0.912138473	0.927460291	0.999939566	0.99989309	0.911696156
LOC107132784	0.965871699	0.882930807	0.942004125	0.999939566	0.99989309	0.911696156
STX4	0.965871699	0.859937298	0.908821787	0.999939566	0.99989309	0.912101047
TBP	0.965871699	0.921280256	0.91546474	0.999939566	0.99989309	0.912101047
CLDN11	0.965871699	0.922488799	0.919939634	0.999939566	0.99989309	0.912101047
DVL3	0.965871699	0.886019911	0.919997767	0.999939566	0.99989309	0.912101047
LOC101902211	0.965871699	0.905210378	0.976161819	0.999939566	0.99989309	0.912302996
PLAC8	0.965871699	0.93453191	0.921225977	0.99879985	0.99989309	0.912376736
PRRG3	0.965871699	0.873098735	0.92153058	0.99879985	0.99989309	0.912380696
TAF2	0.965871699	0.899142992	0.90002174	0.999939566	0.99989309	0.912396352
LOC101906273	0.965871699	0.976754814	0.917876971	0.999939566	0.99989309	0.913230542
FLRT2	0.965871699	0.868963165	0.966157152	0.999939566	0.99989309	0.91332511
KCNK4	0.965871699	0.959119521	0.90002174	0.995112305	0.99989309	0.914440253
MRAS	0.965871699	0.946142324	0.93050447	0.999939566	0.99989309	0.914440253
PPP2R5C	0.965871699	0.871412404	0.990493758	0.990049248	0.99989309	0.917524589
PRRT1B	0.965871699	0.866803532	0.928536078	0.99879985	0.99989309	0.917524589
LOC518495	0.965871699	0.90339131	0.906804311	0.999939566	0.99989309	0.917524589
EIF2B5	0.965871699	0.911368806	0.91546474	0.999939566	0.99989309	0.918220726
LOC789018	0.965871699	0.936134921	0.939091808	0.999334425	0.99989309	0.92035958
LOC107131919	0.965871699	0.908709906	0.928784316	0.998542029	0.99989309	0.920942379
ARSJ	0.965871699	0.957556126	0.941784822	0.999939566	0.99989309	0.921582976
SLC2A4RG	0.965871699	0.928844077	0.922147089	0.990571725	0.99989309	0.922263023
LOC100847825	0.965871699	0.867202036	0.987842204	0.999939566	0.99989309	0.922263023
PTGFRN	0.965871699	0.875345836	0.929605682	0.999939566	0.99989309	0.922872513
RSBN1L	0.965871699	0.963478319	0.929303679	0.999939566	0.99989309	0.923064313
LOC101902861	0.965871699	0.885253121	0.930262306	0.999939566	0.99989309	0.924294935
PANK2	0.965871699	0.901667799	0.927166634	0.995800463	0.99989309	0.924845138

BRMS1L	0.965871699	0.901922021	0.939683463	0.998542029	0.99989309	0.924885155
KLHL32	0.965871699	0.974986418	0.908927423	0.999939566	0.99989309	0.924885155
FAM193A	0.965871699	0.911196851	0.926613927	0.999939566	0.99989309	0.924885155
TCAIM	0.965871699	0.862527737	0.939612562	0.999939566	0.99989309	0.925181465
HERC3	0.965871699	0.919085792	0.947247352	0.999939566	0.99989309	0.926164895
DTNA	0.965871699	0.920347742	0.970904484	0.990049248	0.99989309	0.926872824
SLFNL1	0.965871699	0.956958713	0.95826379	0.992368879	0.99989309	0.926872824
ANP32B	0.965871699	0.920347742	0.926613927	0.995800463	0.99989309	0.926872824
KLK4	0.965871699	0.963478319	0.92582321	0.999939566	0.99989309	0.926872824
TBX18	0.965871699	0.885181785	0.989702604	0.998251353	0.99989309	0.92689297
LOC101904187	0.965871699	0.883378562	0.968954353	0.999939566	0.99989309	0.928374551
PLPP6	0.965871699	0.907878616	0.927460291	0.992137653	0.99989309	0.928799366
BTF3L4	0.965871699	0.901256223	0.979231344	0.999939566	0.99989309	0.928799366
PCDH18	0.965871699	0.971712051	0.909069696	0.999939566	0.99989309	0.929046416
GRPEL2	0.965871699	0.879606268	0.908821787	0.99879985	0.99989309	0.92920113
TPD52	0.965871699	0.866803532	0.98846962	0.999939566	0.99989309	0.92920113
ERP44	0.965871699	0.866883797	0.937270184	0.990571725	0.99989309	0.93072624
LOC513969	0.965871699	0.89618217	0.904691312	0.99879985	0.99989309	0.932613047
ALDH9A1	0.965871699	0.877180988	0.927460291	0.999939566	0.99989309	0.932613047
ABCB6	0.965871699	0.899387334	0.899530079	0.999939566	0.99989309	0.933316009
PARD6G	0.965871699	0.868312459	0.92153058	0.999939566	0.99989309	0.933409694
MAP3K2	0.965871699	0.920347742	0.931173336	0.992368879	0.99989309	0.933678522
LOC112444871	0.965871699	0.920540438	0.958420472	0.992137653	0.99989309	0.934458012
RXFP4	0.965871699	0.98833023	0.939683463	0.990571725	0.99989309	0.934653208
HBEGF	0.965871699	0.882706523	0.927460291	0.99879985	0.99989309	0.934653208
OSBPL10	0.965871699	0.907594268	0.939683463	0.990049248	0.99989309	0.934775196
NABP2	0.965871699	0.942048064	0.913413399	0.999939566	0.99989309	0.934775196
NFIX	0.965871699	0.866675066	0.953045982	0.999939566	0.99989309	0.934775196
EIF2D	0.965871699	0.91761409	0.960137022	0.999939566	0.99989309	0.934775196
CRYZ	0.965871699	0.894203989	0.899195818	0.999939566	0.99989309	0.935154223
LOC789960	0.965871699	0.927298992	0.967810154	0.999939566	0.99989309	0.935607719
MAS1	0.965871699	0.955798423	0.971440944	0.992137653	0.99989309	0.935655141
MYORG	0.965871699	0.916172682	0.976719499	0.99879985	0.99989309	0.935655141
GLCCI1	0.965871699	0.911786925	0.937270184	0.999939566	0.99989309	0.935655141
IPO9	0.965871699	0.859945547	0.939683463	0.999939566	0.99989309	0.935655141
LOC782987	0.965871699	0.889064624	0.93710335	0.999939566	0.99989309	0.935937948
LOC112442585	0.965871699	0.957645111	0.939683463	0.995118128	0.99989309	0.936439483
ADAM15	0.965871699	0.874669928	0.939683463	0.999939566	0.99989309	0.936563391

SPI1	0.965871699	0.959119521	0.939683463	0.990571725	0.99989309	0.937591162
SPTA1	0.965871699	0.866803532	0.963422261	0.990571725	0.99989309	0.937591162
PPFIBP1	0.965871699	0.89539921	0.942761121	0.999939566	0.99989309	0.937591162
ARF2	0.965871699	0.916622103	0.942146285	0.999939566	0.99989309	0.938643464
ZNF383	0.965871699	0.992194873	0.921420528	0.992154129	0.99989309	0.939800211
TIAM2	0.965871699	0.881079118	0.937930242	0.99924173	0.99989309	0.939800211
LOC781770	0.965871699	0.914616699	0.904682623	0.999939566	0.99989309	0.939966658
LOC104972567	0.965871699	0.962934461	0.959320266	0.992137653	0.99989309	0.940683751
LOC112442257	0.965871699	0.866675066	0.935674734	0.999939566	0.99989309	0.940683751
TPT1	0.965871699	0.899387334	0.919997767	0.999939566	0.99989309	0.941445869
PSD3	0.965871699	0.950091107	0.939683463	0.992137653	0.99989309	0.941925704
LOC112446795	0.965871699	0.957353974	0.899771699	0.995118128	0.99989309	0.941925704
BOLA	0.965871699	0.908192144	0.958557986	0.995118128	0.99989309	0.941925704
FAH	0.965871699	0.911196851	0.906278164	0.999939566	0.99989309	0.941925704
COLEC10	0.965871699	0.86415368	0.911119875	0.999939566	0.99989309	0.941925704
VAMP4	0.965871699	0.884768652	0.96544733	0.999939566	0.99989309	0.941925704
LY9	0.965871699	0.913075885	0.909170161	0.990049248	0.99989309	0.942421464
MRPS22	0.965871699	0.877420806	0.902141181	0.999939566	0.99989309	0.943138585
JADE2	0.965871699	0.970179915	0.906887887	0.999939566	0.99989309	0.943138585
RNPS1	0.965871699	0.893639679	0.910406632	0.999939566	0.99989309	0.943391881
SOWAHD	0.965871699	0.901712734	0.908927423	0.995118128	0.99989309	0.943685681
THAP6	0.965871699	0.868963165	0.926727295	0.999939566	0.99989309	0.943685681
LSR	0.965871699	0.894935852	0.931397502	0.999939566	0.99989309	0.943685681
C25H16orf71	0.965871699	0.947392706	0.933102499	0.999939566	0.99989309	0.943685681
ATP11A	0.965871699	0.955798423	0.93710335	0.999939566	0.99989309	0.943685681
GLUD1	0.965871699	0.908192144	0.915581825	0.999939566	0.99989309	0.946294331
NFIB	0.965871699	0.949008068	0.908821787	0.999939566	0.99989309	0.946717155
ARL6IP1	0.965871699	0.877749555	0.978795062	0.992339205	0.99989309	0.948277018
GRK4	0.965871699	0.888994602	0.98730572	0.994719152	0.99989309	0.948277018
STARD13	0.965871699	0.882542824	0.897316161	0.99879985	0.99989309	0.948277018
IL13RA1	0.965871699	0.878843255	0.942314296	0.99879985	0.99989309	0.948277018
ADRA2B	0.965871699	0.911347642	0.957417441	0.999939566	0.99989309	0.948277018
VNN1	0.965871699	0.883267666	0.90495551	0.994309225	0.99989309	0.950959274
LOC101907749	0.965871699	0.920347742	0.927079582	0.999939566	0.99989309	0.952238962
RCBTB2	0.965871699	0.882706523	0.909170161	0.994309225	0.99989309	0.95342487
GRAP2	0.965871699	0.957902362	0.921122957	0.995800463	0.99989309	0.95342487
LOC107132302	0.965871699	0.876057665	0.899539089	0.999939566	0.99989309	0.95342487
LOC781218	0.965871699	0.912112179	0.936675073	0.999939566	0.99989309	0.95342487

MPDZ	0.965871699	0.942048064	0.914406673	0.999939566	0.99989309	0.953604648
AMH	0.965871699	0.876223815	0.932340777	0.995800463	0.99989309	0.95401375
LOC112443417	0.965871699	0.912112179	0.929958748	0.990571725	0.99989309	0.954110387
ZNF852	0.965871699	0.9136091	0.90166788	0.999939566	0.99989309	0.954581551
ZNF592	0.965871699	0.89618217	0.939683463	0.995118128	0.99989309	0.95470473
LOC101903900	0.965871699	0.9136091	0.958420472	0.995118128	0.99989309	0.95470473
DZIP1L	0.965871699	0.912839817	0.937270184	0.999939566	0.99989309	0.95470473
RFX3	0.965871699	0.929536223	0.905832059	0.999939566	0.99989309	0.955585296
EXOC7	0.965871699	0.924299073	0.929605682	0.999939566	0.99989309	0.955585296
PGM1	0.965871699	0.914162656	0.918024782	0.999939566	0.99989309	0.955817715
ACRBP	0.965871699	0.965239369	0.942004125	0.990491321	0.99989309	0.956410517
GFPT1	0.965871699	0.91458438	0.899530079	0.99879985	0.99989309	0.956610132
SLCO5A1	0.965871699	0.901922021	0.945417678	0.999939566	0.99989309	0.956610132
ING3	0.965871699	0.955798423	0.904682623	0.994309225	0.99989309	0.956670063
SRSF9	0.965871699	0.970237188	0.939683463	0.990049248	0.99989309	0.956959747
NOP58	0.965871699	0.868963165	0.906752332	0.995118128	0.99989309	0.957140928
SDCCAG8	0.965871699	0.885181785	0.987006595	0.990571725	0.99989309	0.957877422
ZNF771	0.965871699	0.890427603	0.91546474	0.998542029	0.99989309	0.957877422
LOC614376	0.965871699	0.877420806	0.97381056	0.990571725	0.99989309	0.957910156
LOC100848331	0.965871699	0.890554452	0.899624092	0.999939566	0.99989309	0.961506391
LOC101906545	0.965871699	0.893623223	0.942004125	0.993488944	0.99989309	0.961606931
RUNX1T1	0.965871699	0.860289966	0.930710426	0.992368879	0.99989309	0.968039618
SPRTN	0.965871699	0.904642208	0.93798101	0.990571725	0.99989309	0.968059826
MMRN2	0.965871699	0.933338749	0.951850716	0.992368879	0.99989309	0.968245211
EPHA7	0.965871699	0.876555705	0.907551309	0.999939566	0.99989309	0.969981358
ZKSCAN8	0.965871699	0.883267666	0.954313013	0.999939566	0.99989309	0.970405322
DDX49	0.965871699	0.94883251	0.921420528	0.997612654	0.99989309	0.970702553
C11H2orf92	0.965871699	0.897657501	0.904682623	0.999939566	0.99989309	0.973195384
KCNIP2	0.965871699	0.93015407	0.941319062	0.990049248	0.99989309	0.976454461
S100A9	0.965871699	0.911196851	0.913413399	0.992368879	0.99989309	0.978924127
B3GALNT1	0.965871699	0.892078189	0.903830224	0.999939566	0.99989309	0.980144374
GTPBP1	0.965871699	0.865366022	0.941593855	0.994365581	0.99989309	0.98016619
ABHD15	0.965871699	0.866803532	0.931892015	0.99578044	0.99989309	0.98016619
PCDHGC3	0.965871699	0.868963165	0.944612298	0.99879985	0.99989309	0.98016619
LOC100847765	0.965871699	0.968447746	0.917876971	0.990571725	0.99989309	0.982066213
RRNAD1	0.965871699	0.883267666	0.930262306	0.992137653	0.99989309	0.983453112
LOC112442633	0.965871699	0.875311947	0.90002174	0.995118128	0.99989309	0.983453112
ERCC6	0.965871699	0.883378562	0.939091808	0.99879985	0.99989309	0.983453112

BoLA	0.965871699	0.907878616	0.931173336	0.994309225	0.99989309	0.983525373
UBA1	0.965871699	0.905977412	0.903622442	0.999939566	0.99989309	0.98410581
EZH1	0.965871699	0.921280256	0.916702	0.99879985	0.99989309	0.988323336
GRM8	0.965871699	0.860271574	0.939683463	0.99879985	0.99989309	0.989313458
CCL4	0.965871699	0.944273956	0.907270422	0.990049248	0.99989309	0.995063891
CYP39A1	0.965871699	0.862267429	0.929958748	0.995118128	0.99989309	0.996455238
BMX	0.965871699	0.90249621	0.927460291	0.992368879	0.99989309	0.998074562
ZNF689	0.965871699	0.862669791	0.920449324	0.99879985	0.99989309	0.998074562
PRSS48	0.965871699	0.883267666	0.911119875	0.995800463	0.99989309	0.998636142
LOC101903114	0.965871699	0.883927618	0.90166788	0.99879985	0.99989309	0.998691798
LOC783142	0.965941339	0.986245673	0.921225977	0.999939566	0.99989309	0.909768235
TMEM150B	0.966026486	0.920818159	0.910978655	0.999939566	0.99989309	0.912101047
BCL2L2	0.966030633	0.974986418	0.939091808	0.99879985	0.99989309	0.912101047
TMTC3	0.966030633	0.886173201	0.958420472	0.999939566	0.99989309	0.948174718
LOC112447027	0.966077441	0.866675066	0.941688757	0.999939566	0.99989309	0.907276081
LOC112443140	0.966077441	0.911279338	0.920449324	0.99879985	0.99989309	0.908939618
ZNF672	0.966077441	0.934248552	0.96596172	0.99879985	0.99989309	0.908939618
FLVCR1	0.966077441	0.944755173	0.904682623	0.999939566	0.99989309	0.908939618
AGTR2	0.966077441	0.955798423	0.905047103	0.999939566	0.99989309	0.908939618
CCDC69	0.966077441	0.922331228	0.921135532	0.999939566	0.99989309	0.908939618
PLPP7	0.966077441	0.965239369	0.93050447	0.999939566	0.99989309	0.908939618
STMP1	0.966077441	0.866803532	0.941319062	0.999939566	0.99989309	0.908939618
RHBDL1	0.966077441	0.936750204	0.965518535	0.999939566	0.99989309	0.908939618
METAP1D	0.966077441	0.9136091	0.960137022	0.999939566	0.99989309	0.912180128
ITGB3BP	0.966077441	0.96471209	0.962176387	0.99879985	0.99989309	0.912396352
LOC618733	0.966077441	0.900458021	0.939683463	0.999939566	0.99989309	0.915253587
LOC101903301	0.966077441	0.897918739	0.947268948	0.990571725	0.99989309	0.922229044
NAA30	0.966077441	0.866813771	0.967489175	0.999939566	0.99989309	0.922670883
CTDNEP1	0.966077441	0.941626019	0.90936727	0.999939566	0.99989309	0.923396585
LOC100138645	0.966077441	0.922663208	0.906597758	0.995800463	0.99989309	0.92546728
PCDH11X	0.966077441	0.899387334	0.939683463	0.999939566	0.99989309	0.92546728
SEMA3D	0.966077441	0.944755173	0.907621715	0.999939566	0.99989309	0.930528039
JPT1	0.966077441	0.937142804	0.961736888	0.999939566	0.99989309	0.934653208
ZNF585A	0.966077441	0.926619092	0.947268948	0.99879985	0.99989309	0.940917403
SPESP1	0.966077441	0.876057665	0.939683463	0.999939566	0.99989309	0.941925704
GADD45GIP1	0.966077441	0.888994602	0.905832059	0.999939566	0.99989309	0.942039558
CHEK2	0.966077441	0.96104457	0.939683463	0.991662302	0.99989309	0.942421464
AMDHD2	0.966077441	0.903820329	0.904682623	0.999939566	0.99989309	0.94425343

FCGR1A	0.966077441	0.957019895	0.923202492	0.99879985	0.99989309	0.955585296
ZNF329	0.966077441	0.886574129	0.934958503	0.995118128	0.99989309	0.956410517
ETV1	0.966077441	0.899917012	0.939683463	0.995118128	0.99989309	0.956610132
LOC107132924	0.966077441	0.965239369	0.921122957	0.991029787	0.99989309	0.968059826
C13H10orf113	0.966114479	0.868963165	0.926613927	0.999939566	0.99989309	0.911696156
LOC107132944	0.966148658	0.906264336	0.962209895	0.990571725	0.99989309	0.964746062
LNPK	0.966170739	0.924393439	0.910978655	0.999939566	0.99989309	0.946717155
LOC104975607	0.966484098	0.963478319	0.918050385	0.995118128	0.99989309	0.948763249
RUFY2	0.966536319	0.866977641	0.977612552	0.999939566	0.99989309	0.924788226
OSBPL3	0.966536319	0.877420806	0.926825953	0.999939566	0.99989309	0.942455807
CSGALNACT1	0.966536319	0.944314627	0.915874142	0.999939566	0.99989309	0.954732204
LOC112447328	0.966836906	0.890427603	0.958420472	0.996084773	0.99989309	0.908785004
KIAA0232	0.966836906	0.872513862	0.92153058	0.999939566	0.99989309	0.908939618
DUSP22	0.966836906	0.91994024	0.939683463	0.999939566	0.99989309	0.908939618
GRAMD1B	0.966836906	0.877180988	0.958420472	0.999939566	0.99989309	0.908939618
LOC782799	0.966836906	0.921280256	0.939091808	0.992829869	0.99989309	0.910619304
TRMT13	0.966836906	0.919351156	0.990107362	0.99879985	0.99989309	0.911632967
BET1	0.966836906	0.911347642	0.939683463	0.99879985	0.99989309	0.912376736
SLC44A2	0.966836906	0.939870925	0.965568718	0.994719152	0.99989309	0.915382752
NUP58	0.966836906	0.889724071	0.962209895	0.999939566	0.99989309	0.917968123
RPS4X	0.966836906	0.942048064	0.92153058	0.999939566	0.99989309	0.921582976
PCMT1	0.966836906	0.871858734	0.962209895	0.999939566	0.99989309	0.923871657
OCLN	0.966836906	0.945105738	0.940785296	0.99879985	0.99989309	0.924845138
TMEFF1	0.966836906	0.959138652	0.908165509	0.999939566	0.99989309	0.926872824
CREBL2	0.966836906	0.89272885	0.982223562	0.999939566	0.99989309	0.930669597
CHEK1	0.966836906	0.868963165	0.976719499	0.999939566	0.99989309	0.930707319
MDFI	0.966836906	0.972786652	0.908165509	0.995118128	0.99989309	0.934653208
GOLGA1	0.966836906	0.913171043	0.990107362	0.990571725	0.99989309	0.937591162
LOC112449075	0.966836906	0.962934461	0.933963997	0.999939566	0.99989309	0.938643464
ZYX	0.966836906	0.875022483	0.987842204	0.991862855	0.99989309	0.943848108
LOC100852077	0.966836906	0.972786652	0.915636025	0.992692773	0.99989309	0.95470473
KDM3B	0.966836906	0.949625762	0.909522865	0.995800463	0.99989309	0.956610132
MARCH8	0.966836906	0.876057665	0.969311025	0.992368879	0.99989309	0.957877422
R3HDM1	0.966836906	0.901922021	0.91546474	0.999939566	0.99989309	0.961506391
LOC104971503	0.966836906	0.935793939	0.951850716	0.994365581	0.99989309	0.968245211
BTBD3	0.966836906	0.910596218	0.977612552	0.990571725	0.99989309	0.968751621
TTC8	0.966836906	0.933334539	0.939683463	0.990571725	0.99989309	0.970959759
EBF1	0.966836906	0.877420806	0.909170161	0.99879985	0.99989309	0.977961448

CTBP2	0.966937006	0.901922021	0.942004125	0.999939566	0.99989309	0.937591162
LPAR4	0.966937006	0.920967949	0.921225977	0.994309225	0.99989309	0.956453747
STAU2	0.966965636	0.878085977	0.93798101	0.999939566	0.99989309	0.941275898
DIMT1	0.966965636	0.873566716	0.95721973	0.99879985	0.99989309	0.987721456
NONO	0.967084069	0.920818159	0.93050447	0.999939566	0.99989309	0.917968123
MTMR9	0.967261665	0.957902362	0.939080693	0.999939566	0.99989309	0.906529452
KLHL4	0.967261665	0.911435389	0.943891967	0.999939566	0.99989309	0.908939618
CSNK1D	0.967261665	0.884768652	0.915636025	0.995118128	0.99989309	0.911696156
SPR	0.967261665	0.928601644	0.939683463	0.999939566	0.99989309	0.912101047
ENOSF1	0.967261665	0.877723422	0.929958748	0.999939566	0.99989309	0.912385767
LOC112449099	0.967261665	0.905977412	0.92582321	0.995118128	0.99989309	0.914440253
CCDC59	0.967261665	0.907594268	0.985552223	0.999939566	0.99989309	0.916117913
LOC101907713	0.967261665	0.91501681	0.937930242	0.999939566	0.99989309	0.92035958
PRND	0.967261665	0.899474896	0.907270422	0.999939566	0.99989309	0.922709797
BCAR3	0.967261665	0.963478319	0.929958748	0.999939566	0.99989309	0.923871657
ZC3H3	0.967261665	0.928844077	0.947118233	0.999939566	0.99989309	0.926872824
ADCY2	0.967261665	0.91557476	0.966955806	0.999939566	0.99989309	0.92689297
C9H6orf203	0.967261665	0.907594268	0.97420715	0.995118128	0.99989309	0.930707319
CFAP300	0.967261665	0.919224637	0.913463626	0.999939566	0.99989309	0.935655141
TTBK2	0.967261665	0.947806191	0.917876971	0.995800463	0.99989309	0.936563391
MARVELD1	0.967261665	0.877420806	0.987842204	0.994652359	0.99989309	0.93691717
TTC3	0.967261665	0.882706523	0.92332596	0.999939566	0.99989309	0.94091418
BTK	0.967261665	0.920347742	0.904682623	0.999939566	0.99989309	0.940917403
MTMR4	0.967261665	0.926435857	0.951850716	0.995118128	0.99989309	0.941925704
TSEN34	0.967261665	0.936809216	0.929303679	0.999939566	0.99989309	0.941925704
RFTN1	0.967261665	0.883267666	0.962209895	0.995800463	0.99989309	0.942421464
GATAD1	0.967261665	0.944314627	0.929958748	0.995118128	0.99989309	0.946376229
C1H21orf2	0.967261665	0.868963165	0.938195001	0.999939566	0.99989309	0.946586915
ANKRD35	0.967261665	0.920347742	0.909170161	0.994365581	0.99989309	0.948277018
COL13A1	0.967261665	0.888313577	0.921531707	0.999939566	0.99989309	0.949206487
LOC101904962	0.967261665	0.873566716	0.914489705	0.992368879	0.99989309	0.950338641
FAM171A1	0.967261665	0.868963165	0.958420472	0.999939566	0.99989309	0.957122667
MBOAT7	0.967261665	0.905210378	0.92921168	0.999939566	0.99989309	0.957877422
AQP4	0.967261665	0.913075885	0.919997767	0.999939566	0.99989309	0.95929043
TNPO3	0.967261665	0.878720916	0.931397502	0.999939566	0.99989309	0.961506391
TBC1D31	0.967261665	0.900467607	0.983517715	0.992692773	0.99989309	0.967238897
FAM241B	0.967261665	0.914162656	0.930710426	0.999939566	0.99989309	0.968039618
DBP	0.967261665	0.920347742	0.947268948	0.998542029	0.99989309	0.97815722

STARD10	0.967261665	0.895762772	0.962469014	0.992368879	0.99989309	0.986389023
SDE2	0.967332944	0.991541192	0.921319817	0.995800463	0.99989309	0.908939618
CREB3L2	0.967332944	0.871412404	0.91546474	0.999939566	0.99989309	0.936563391
GRIK2	0.967352229	0.952464938	0.933963997	0.999939566	0.99989309	0.930707319
C22H3orf20	0.967352229	0.885181785	0.939417893	0.999939566	0.99989309	0.973195541
PRR22	0.967502994	0.907670026	0.957802423	0.998542029	0.99989309	0.914122611
LOC510382	0.967502994	0.982017699	0.914489705	0.994365581	0.99989309	0.918608642
BCAP29	0.967585266	0.961634772	0.943891967	0.999939566	0.99989309	0.908939618
LOC534742	0.967585266	0.90249621	0.958420472	0.999939566	0.99989309	0.917968123
PRDM10	0.967585266	0.886909612	0.963643451	0.999939566	0.99989309	0.926872824
TRIM56	0.967585266	0.903657308	0.916702	0.999939566	0.99989309	0.983453112
TBC1D10A	0.967585266	0.882706523	0.929958748	0.992137653	0.99989309	0.99289587
WNT16	0.96763119	0.907878616	0.958420472	0.999939566	0.99989309	0.923064313
TLK1	0.96763119	0.925360181	0.918681165	0.995118128	0.99989309	0.926872824
TCFL5	0.96763119	0.886223703	0.939683463	0.999939566	0.99989309	0.928690415
XPNPEP2	0.96763119	0.914162656	0.926613927	0.999939566	0.99989309	0.938643464
ARL4D	0.96763119	0.964014612	0.908647463	0.998542029	0.99989309	0.943848108
SRL	0.96763119	0.96412538	0.917066863	0.992137653	0.99989309	0.98016619
EFNA1	0.967705101	0.985784205	0.926613927	0.999939566	0.99989309	0.908939618
TRAF5	0.967705101	0.871359854	0.958420472	0.998542029	0.99989309	0.949627777
AFAP1L2	0.967721524	0.962063344	0.951742137	0.999939566	0.99989309	0.931391375
KIRREL1	0.967721524	0.921280256	0.933963997	0.999939566	0.99989309	0.942421464
GDPD1	0.967949735	0.883267666	0.942642657	0.999939566	0.99989309	0.908939618
EXOC2	0.967949735	0.875022483	0.944160795	0.999939566	0.99989309	0.908939618
SPTSSB	0.967949735	0.885604065	0.904682623	0.990571725	0.99989309	0.910920424
ZBTB7C	0.967949735	0.869509858	0.906925281	0.999939566	0.99989309	0.911696156
MIA	0.967949735	0.911196851	0.993278977	0.99879985	0.99989309	0.920942379
LOC104974837	0.967949735	0.870855329	0.978879234	0.992137653	0.99989309	0.921381434
TBC1D19	0.967949735	0.924799142	0.978892556	0.999939566	0.99989309	0.930528039
ST7	0.967949735	0.91761409	0.959320266	0.99879985	0.99989309	0.943685681
ING4	0.967949735	0.90249621	0.909170161	0.999939566	0.99989309	0.991281092
VPS50	0.96801054	0.994810749	0.941593855	0.998542029	0.99989309	0.907505511
LOC112449590	0.96801054	0.877749555	0.933104095	0.995118128	0.99989309	0.908939618
C1QTNF1	0.96801054	0.904764822	0.989845175	0.996775429	0.99989309	0.908939618
LOC104971845	0.96801054	0.878843255	0.985552223	0.99879985	0.99989309	0.908939618
PAFAH1B2	0.96801054	0.924510975	0.987842204	0.99879985	0.99989309	0.908939618
PMP2	0.96801054	0.923981714	0.907265706	0.999939566	0.99989309	0.908939618
MUM1	0.96801054	0.879044895	0.917092799	0.999939566	0.99989309	0.908939618

TMEM120B	0.96801054	0.913171043	0.958420472	0.999939566	0.99989309	0.908939618
LOC112443240	0.96801054	0.883378562	0.977612552	0.999939566	0.99989309	0.908939618
NFIL3	0.96801054	0.890427603	0.979096894	0.999939566	0.99989309	0.908939618
RASSF2	0.96801054	0.924433008	0.916526594	0.99879985	0.99989309	0.910353954
SRRT	0.96801054	0.906461079	0.947660114	0.99879985	0.99989309	0.910920424
PDS5B	0.96801054	0.950091107	0.922147089	0.999939566	0.99989309	0.911468373
LOC518623	0.96801054	0.944314627	0.908821939	0.999939566	0.99989309	0.911696156
KIAA0355	0.96801054	0.899387334	0.921225977	0.999939566	0.99989309	0.912101047
LOC101905151	0.96801054	0.996722893	0.941319062	0.995118128	0.99989309	0.912376736
CCDC166	0.96801054	0.913171043	0.917092799	0.995118128	0.99989309	0.912385767
ENG	0.96801054	0.900467607	0.93078193	0.999939566	0.99989309	0.912679756
ARMC2	0.96801054	0.942048064	0.982680848	0.998542029	0.99989309	0.914283173
RTN4R	0.96801054	0.886366574	0.941593855	0.995800463	0.99989309	0.914657697
LOC112448366	0.96801054	0.87225939	0.918576062	0.999939566	0.99989309	0.918220726
MIS18A	0.96801054	0.962934461	0.922147089	0.999939566	0.99989309	0.918608642
ECHDC2	0.96801054	0.88311684	0.944856216	0.999939566	0.99989309	0.918608642
SSBP3	0.96801054	0.90249621	0.939683463	0.992137653	0.99989309	0.919967118
ARNT	0.96801054	0.939870925	0.93198207	0.994309225	0.99989309	0.92035958
H3F3B	0.96801054	0.914162656	0.985552223	0.99879985	0.99989309	0.920754358
ZNF511	0.96801054	0.945105738	0.923976352	0.999939566	0.99989309	0.921582976
MAN2B1	0.96801054	0.9136091	0.909522865	0.99879985	0.99989309	0.921794978
GDPGP1	0.96801054	0.994365533	0.939683463	0.995800463	0.99989309	0.923064313
LOC784243	0.96801054	0.885991833	0.941593855	0.999939566	0.99989309	0.924045829
MAPK13	0.96801054	0.883267666	0.968230268	0.999939566	0.99989309	0.924316735
LOC100847118	0.96801054	0.934571318	0.98730572	0.996775429	0.99989309	0.924788226
CCT6B	0.96801054	0.934248552	0.931397502	0.999939566	0.99989309	0.924788226
ALDH6A1	0.96801054	0.907878616	0.93870579	0.999939566	0.99989309	0.924885155
CDH5	0.96801054	0.928119925	0.914685423	0.999939566	0.99989309	0.925181465
N4BP2L1	0.96801054	0.929573947	0.93145152	0.999939566	0.99989309	0.926853716
SEM1	0.96801054	0.952464938	0.982659943	0.995118128	0.99989309	0.926872824
NAAA	0.96801054	0.913075885	0.906914073	0.999939566	0.99989309	0.926872824
SSUH2	0.96801054	0.899387334	0.941593855	0.999939566	0.99989309	0.926872824
SLK	0.96801054	0.945110652	0.965518535	0.999939566	0.99989309	0.926872824
EXOSC9	0.96801054	0.883105062	0.994425789	0.99483611	0.99989309	0.92689297
DCBLD2	0.96801054	0.894935852	0.953045982	0.999939566	0.99989309	0.927584908
PTCH1	0.96801054	0.89413223	0.916702	0.999939566	0.99989309	0.928057673
LOC527744	0.96801054	0.886943153	0.98168496	0.99879985	0.99989309	0.92920113
SVBP	0.96801054	0.96104457	0.926613927	0.999939566	0.99989309	0.931095589

DGUOK	0.96801054	0.918311236	0.914685423	0.999939566	0.99989309	0.933678522
CHD3	0.96801054	0.937043243	0.917876971	0.999939566	0.99989309	0.935937948
FRYL	0.96801054	0.936365238	0.917876971	0.999939566	0.99989309	0.936563391
ADAM9	0.96801054	0.914162656	0.926613927	0.999939566	0.99989309	0.936563391
VPS51	0.96801054	0.876243021	0.927460291	0.992368879	0.99989309	0.940683751
C24H18orf54	0.96801054	0.956843573	0.959320266	0.995118128	0.99989309	0.941925704
FAM114A1	0.96801054	0.942236132	0.909522865	0.999939566	0.99989309	0.941925704
LOC112444681	0.96801054	0.934248552	0.933963997	0.999939566	0.99989309	0.941925704
ESRRA	0.96801054	0.920347742	0.930262306	0.992154129	0.99989309	0.943138585
LOC112444924	0.96801054	0.952464938	0.909170161	0.999939566	0.99989309	0.94425343
CALHM5	0.96801054	0.963478319	0.915636025	0.99419346	0.99989309	0.949206487
ACE	0.96801054	0.965027449	0.959646613	0.995118128	0.99989309	0.950199992
PCGF1	0.96801054	0.901922021	0.929958748	0.999939566	0.99989309	0.950959274
NBAS	0.96801054	0.926435857	0.953045982	0.999939566	0.99989309	0.951978897
PTPN4	0.96801054	0.920347742	0.939683463	0.999939566	0.99989309	0.95470473
LOC781256	0.96801054	0.882275117	0.948628899	0.995800463	0.99989309	0.956410517
ANKRD50	0.96801054	0.893678247	0.926613927	0.999939566	0.99989309	0.956431159
HNRNPR	0.96801054	0.89113211	0.979096894	0.995118128	0.99989309	0.956610132
LOC614614	0.96801054	0.918306418	0.937979342	0.99879985	0.99989309	0.956610132
LOC112448520	0.96801054	0.982963758	0.922161207	0.998542029	0.99989309	0.956670063
ASNSD1	0.96801054	0.88418172	0.958420472	0.999939566	0.99989309	0.956959747
MFN2	0.96801054	0.907284287	0.919108971	0.999939566	0.99989309	0.957877422
TMEM200B	0.96801054	0.886366574	0.92921168	0.999939566	0.99989309	0.957877422
GGT5	0.96801054	0.880674338	0.93050447	0.999939566	0.99989309	0.958678078
LOC112445968	0.96801054	0.90249621	0.919997767	0.992829869	0.99989309	0.959625205
LOC101903905	0.96801054	0.886740912	0.959705869	0.998542029	0.99989309	0.960712754
SENP2	0.96801054	0.890427603	0.929958748	0.99879985	0.99989309	0.960985422
LOC101905845	0.96801054	0.955798423	0.939683463	0.992137653	0.99989309	0.961506391
HACD3	0.96801054	0.922434012	0.966157152	0.996601241	0.99989309	0.963756876
ALPL	0.96801054	0.871113425	0.930892662	0.999939566	0.99989309	0.964003802
SCARF1	0.96801054	0.901399893	0.93198207	0.999939566	0.99989309	0.965332394
LOC107132798	0.96801054	0.907878616	0.919785713	0.999939566	0.99989309	0.968245211
BCR	0.96801054	0.91843896	0.92153058	0.999939566	0.99989309	0.972204129
LOC104972031	0.96801054	0.899163209	0.965750046	0.999939566	0.99989309	0.972204129
CCDC150	0.96801054	0.882993091	0.970904484	0.995800463	0.99989309	0.974348283
BST1	0.96801054	0.890427603	0.933102499	0.99879985	0.99989309	0.97815722
LOC513894	0.96801054	0.957353974	0.93710335	0.990571725	0.99989309	0.979233909
CALHM6	0.96801054	0.881079118	0.915636025	0.999939566	0.99989309	0.98016619

SEC22C	0.96801054	0.909926193	0.924266781	0.999939566	0.99989309	0.98016619
CAPN3	0.96801054	0.889410709	0.944485232	0.992339205	0.99989309	0.981090047
TTC5	0.96801054	0.914735022	0.937765241	0.999939566	0.99989309	0.982045172
PSMD5	0.96801054	0.89539921	0.958420472	0.99879985	0.99989309	0.982559673
GSTP1	0.96801054	0.88469581	0.960398536	0.998542029	0.99989309	0.983453112
SRR	0.96801054	0.875022483	0.95206243	0.999939566	0.99989309	0.983453112
ZNF226	0.96801054	0.935793939	0.906925281	0.99879985	0.99989309	0.988323336
LOC104973154	0.96801054	0.92989323	0.904682623	0.992137653	0.99989309	0.991281092
LOC112443176	0.96801054	0.903820329	0.943196053	0.995118128	0.99989309	0.993612847
LOC782527	0.96801054	0.876057665	0.93145152	0.995800463	0.99989309	0.997673689
SORBS3	0.96801054	0.878474251	0.909170161	0.995118128	0.99989309	0.998928226
TSPAN2	0.96834646	0.886909612	0.944612298	0.999939566	0.99989309	0.940683751
ZNF330	0.96834646	0.916172682	0.91291082	0.999939566	0.99989309	0.968245211
ADCY9	0.968562323	0.939473539	0.925587088	0.999939566	0.99989309	0.938643464
DHDH	0.96866057	0.971839996	0.92153058	0.999939566	0.99989309	0.910619304
EEF1AKMT2	0.96866057	0.926435857	0.918050385	0.998542029	0.99989309	0.934653208
RNASE4	0.968705611	0.885991833	0.916014573	0.999939566	0.99989309	0.972787584
FAM192A	0.968835486	0.877749555	0.966157152	0.992402807	0.99989309	0.908939618
PDCD4	0.968835486	0.911894421	0.933963997	0.99879985	0.99989309	0.908939618
LOC112442538	0.968835486	0.911347642	0.968537205	0.999939566	0.99989309	0.908939618
UBTD2	0.968835486	0.922264623	0.957379098	0.999939566	0.99989309	0.909768235
HNF4A	0.968835486	0.911894421	0.919997767	0.999939566	0.99989309	0.910619304
LOC112445076	0.968835486	0.949625762	0.922147089	0.99879985	0.99989309	0.912101047
LOC107132713	0.968835486	0.899387334	0.947268948	0.999939566	0.99989309	0.912434521
LOC112446799	0.968835486	0.959721325	0.908165509	0.999939566	0.99989309	0.914440253
RBM12B	0.968835486	0.962934461	0.933629769	0.998542029	0.99989309	0.926872824
CHMP4C	0.968835486	0.89618217	0.940168084	0.995118128	0.99989309	0.92920113
RBM42	0.968835486	0.897657501	0.970904484	0.998542029	0.99989309	0.935154223
LOC511161	0.968835486	0.871412404	0.929958748	0.999939566	0.99989309	0.941925704
ACY1	0.968835486	0.913075885	0.920661055	0.999939566	0.99989309	0.943138585
AKAP13	0.968835486	0.874669928	0.925442336	0.995800463	0.99989309	0.948277018
CXCL14	0.968835486	0.964014612	0.914406673	0.999939566	0.99989309	0.948277018
LOC112441456	0.968835486	0.897327046	0.980442556	0.99879985	0.99989309	0.954110387
SPG7	0.968835486	0.883007259	0.92153058	0.99879985	0.99989309	0.956610132
ABHD2	0.968835486	0.963478319	0.907551309	0.992368879	0.99989309	0.957877422
GMFG	0.968835486	0.948631073	0.917876971	0.995118128	0.99989309	0.959625205
LOC781799	0.968835486	0.890427603	0.962209895	0.999939566	0.99989309	0.973195541
LOC100848568	0.968835486	0.873195229	0.939683463	0.99879985	0.99989309	0.981164039

SERTAD3	0.968835486	0.947080604	0.926613927	0.994309225	0.99989309	0.989947227
SRC	0.968862112	0.913075885	0.921319817	0.999939566	0.99989309	0.911358878
RPS26	0.968914023	0.875022483	0.926613927	0.999939566	0.99989309	0.908939618
LOC107132196	0.968914023	0.912112179	0.975183307	0.995118128	0.99989309	0.96767215
CRNKL1	0.968948267	0.883267666	0.948213748	0.998542029	0.99989309	0.908939618
RAB36	0.968948267	0.921280256	0.919939634	0.992137653	0.99989309	0.940683751
CRABP1	0.968948267	0.885981636	0.949163196	0.999939566	0.99989309	0.956610132
LOC104974890	0.969387422	0.977732731	0.920449324	0.999939566	0.99989309	0.914440253
LOC112446771	0.969387422	0.95320487	0.961742979	0.999939566	0.99989309	0.917968123
LOC101902644	0.969387422	0.887763891	0.926613927	0.999939566	0.99989309	0.922936098
LOC101902998	0.969387422	0.972786652	0.918576062	0.99879985	0.99989309	0.926872824
KCNA6	0.969387422	0.885999002	0.925587088	0.999939566	0.99989309	0.937591162
DCDC2B	0.969387422	0.9347557	0.965712499	0.995118128	0.99989309	0.962255684
VGLL3	0.969538579	0.930890453	0.93710335	0.999939566	0.99989309	0.923064313
LOC112448762	0.969538579	0.918976923	0.945012822	0.999939566	0.99989309	0.942421464
PITPNM3	0.969830995	0.876739423	0.92921168	0.999939566	0.99989309	0.923064313
ARL3	0.969936127	0.883378562	0.994425789	0.992154129	0.99989309	0.908939618
FUT10	0.969936127	0.885604065	0.968455468	0.999939566	0.99989309	0.908939618
DOPEY1	0.969936127	0.953693331	0.921135532	0.999939566	0.99989309	0.910619304
LAMA4	0.969936127	0.922256822	0.939091808	0.999939566	0.99989309	0.911696156
TMEM26	0.969936127	0.921046401	0.917092799	0.996084773	0.99989309	0.912101047
GFOD1	0.969936127	0.903878399	0.939683463	0.999939566	0.99989309	0.912101047
KLF13	0.969936127	0.950091107	0.908821787	0.999939566	0.99989309	0.912380696
RPL30	0.969936127	0.939027995	0.953045982	0.999939566	0.99989309	0.917968123
ING1	0.969936127	0.913075885	0.965416289	0.995687815	0.99989309	0.919079129
LOC104972622	0.969936127	0.936134921	0.947660114	0.999939566	0.99989309	0.92035958
LOC100335936	0.969936127	0.934248552	0.939683463	0.999939566	0.99989309	0.920942379
UBXN2A	0.969936127	0.885181785	0.980442556	0.999939566	0.99989309	0.922229044
NOSIP	0.969936127	0.899387334	0.941688757	0.999939566	0.99989309	0.924845138
NARFL	0.969936127	0.924510975	0.941593855	0.996084773	0.99989309	0.926872824
R3HCC1	0.969936127	0.89113211	0.968419607	0.999939566	0.99989309	0.926872824
TEDC2	0.969936127	0.879686372	0.995624898	0.999939566	0.99989309	0.926872824
ECHS1	0.969936127	0.876057665	0.92582321	0.999939566	0.99989309	0.928123265
KHSRP	0.969936127	0.911237043	0.930710426	0.99879985	0.99989309	0.928374551
SMIM33	0.969936127	0.881079118	0.909442165	0.999939566	0.99989309	0.928442671
LRRC8E	0.969936127	0.877180988	0.922816581	0.999939566	0.99989309	0.933409694
CPNE3	0.969936127	0.914252497	0.952331743	0.999939566	0.99989309	0.935655141
FBXO15	0.969936127	0.907878616	0.92921168	0.999939566	0.99989309	0.936479857

OLFM2	0.969936127	0.99817889	0.914685423	0.992137653	0.99989309	0.938054315
EFCAB8	0.969936127	0.938064973	0.950801642	0.999939566	0.99989309	0.939800211
LOC101909384	0.969936127	0.931381024	0.909170161	0.999939566	0.99989309	0.940683751
MTIF2	0.969936127	0.968123234	0.942004125	0.996118855	0.99989309	0.941806801
WTAP	0.969936127	0.901256223	0.914477011	0.99879985	0.99989309	0.941889677
ANKEF1	0.969936127	0.887725794	0.914477011	0.999939566	0.99989309	0.942421464
HIST1H2BD	0.969936127	0.912112179	0.937270184	0.998542029	0.99989309	0.943685681
FAT2	0.969936127	0.88469581	0.929303679	0.999939566	0.99989309	0.943685681
LOC784738	0.969936127	0.884444922	0.953045982	0.999939566	0.99989309	0.953940638
LOC112444171	0.969936127	0.911196851	0.968934792	0.994719312	0.99989309	0.958722356
ADGRA1	0.969936127	0.974986418	0.939683463	0.995687815	0.99989309	0.958814003
OSBPL2	0.969936127	0.889929882	0.962209895	0.999939566	0.99989309	0.961506391
KCNAB2	0.969936127	0.984479682	0.919997767	0.995118128	0.99989309	0.963595366
CEP63	0.969936127	0.91761409	0.968954353	0.992137653	0.99989309	0.979893615
ATF7IP2	0.969936127	0.881079118	0.969618195	0.999334425	0.99989309	0.98016619
FOCAD	0.969936127	0.882504547	0.917876971	0.995800463	0.99989309	0.998636142
CXHXorf58	0.970036834	0.897375356	0.978879234	0.999939566	0.99989309	0.912376736
LYRM7	0.970036834	0.892242414	0.939683463	0.999939566	0.99989309	0.923788376
LOC782120	0.970167268	0.957254162	0.925442336	0.995118128	0.99989309	0.912101047
U2AF1L4	0.970167268	0.876919789	0.966157152	0.99879985	0.99989309	0.912376736
TMBIM6	0.970167268	0.881944078	0.965750046	0.999939566	0.99989309	0.936563391
LRRC4	0.970167268	0.897918739	0.959198396	0.995118128	0.99989309	0.991281092
SAPCD2	0.970183324	0.899429403	0.933629769	0.99879985	0.99989309	0.910353954
DPP7	0.970183324	0.993959968	0.92582321	0.999939566	0.99989309	0.910619304
LOC100299705	0.970183324	0.971068039	0.93923481	0.999939566	0.99989309	0.910619304
ITGAM	0.970183324	0.956958713	0.942642657	0.999939566	0.99989309	0.912101047
GINS4	0.970183324	0.899387334	0.909170161	0.999939566	0.99989309	0.916525501
ERMARD	0.970183324	0.970179915	0.944976702	0.999939566	0.99989309	0.917968123
XRCC6	0.970183324	0.901256223	0.959646613	0.999939566	0.99989309	0.92035958
SERPINB1	0.970183324	0.921280256	0.921225977	0.999939566	0.99989309	0.920942379
NDNF	0.970183324	0.986681109	0.921319817	0.992368879	0.99989309	0.928374551
ENTR1	0.970183324	0.939027995	0.924266781	0.999939566	0.99989309	0.928799366
MRPL58	0.970183324	0.934248552	0.926613927	0.999939566	0.99989309	0.92920113
GAB3	0.970183324	0.881079118	0.965712499	0.999939566	0.99989309	0.932267465
TM2D3	0.970183324	0.899917012	0.939683463	0.99879985	0.99989309	0.933763401
CAMSAP3	0.970183324	0.977732731	0.95126311	0.996775429	0.99989309	0.934653208
GBA	0.970183324	0.974986418	0.933102499	0.999939566	0.99989309	0.940683751
EPN2	0.970183324	0.875803224	0.930710426	0.999939566	0.99989309	0.942421464

C17H4orf33	0.970183324	0.883267666	0.941312064	0.999939566	0.99989309	0.942455807
AMBRA1	0.970183324	0.924799163	0.922147089	0.999939566	0.99989309	0.94588513
PACSIN3	0.970183324	0.965239369	0.91546474	0.999939566	0.99989309	0.95470473
DHCR7	0.970183324	0.90591801	0.917876971	0.998542029	0.99989309	0.955585296
RWDD2B	0.970183324	0.893512526	0.924266781	0.999939566	0.99989309	0.956610132
ENAH	0.970183324	0.944755173	0.918135077	0.999939566	0.99989309	0.96435585
TRPV1	0.970183324	0.885181785	0.933102499	0.999939566	0.99989309	0.974139155
FASTKD3	0.970183324	0.874669928	0.985552223	0.992368879	0.99989309	0.980714682
CSF2RB	0.970183324	0.905977412	0.939683463	0.999939566	0.99989309	0.981164039
MXI1	0.970183324	0.899474896	0.916702	0.999939566	0.99989309	0.989128955
RAB39A	0.970183324	0.932156311	0.93050447	0.995118128	0.99989309	0.994620117
PPP1R13L	0.970183324	0.936134921	0.922147089	0.992368879	0.99989309	0.995411183
LOC100336104	0.970183324	0.907878616	0.93145152	0.995118128	0.99989309	0.998074562
SLC45A4	0.970225234	0.918306418	0.950638965	0.999939566	0.99989309	0.908939618
IER3IP1	0.970225234	0.922264623	0.976719499	0.999939566	0.99989309	0.908939618
KDM1A	0.970225234	0.899429403	0.91546474	0.999939566	0.99989309	0.922229044
UQCC1	0.970225234	0.899387334	0.92153058	0.998963067	0.99989309	0.924984776
SETD1A	0.970251618	0.945105738	0.940831329	0.99879985	0.99989309	0.928799366
GGA3	0.970281421	0.913171043	0.98567143	0.992692773	0.99989309	0.909990551
NSMCE2	0.970281421	0.939870925	0.937930242	0.999939566	0.99989309	0.91963933
LOC781692	0.970281421	0.958271889	0.960623345	0.994652359	0.99989309	0.92035958
LOC107131368	0.970281421	0.988649234	0.939683463	0.999939566	0.99989309	0.921770545
SF3B6	0.970281421	0.899917012	0.997081057	0.995118128	0.99989309	0.923064313
ZCCHC18	0.970281421	0.936365238	0.968956634	0.999939566	0.99989309	0.928057673
TBC1D4	0.970281421	0.944422127	0.937546616	0.999939566	0.99989309	0.928374551
ZBTB46	0.970281421	0.957071555	0.939091808	0.999939566	0.99989309	0.928653932
LOC107132308	0.970281421	0.875803224	0.939091808	0.999939566	0.99989309	0.928923695
IP6K3	0.970281421	0.916894642	0.918576062	0.999939566	0.99989309	0.934775196
VMAC	0.970281421	0.926435857	0.909522865	0.999939566	0.99989309	0.940683751
LOC784058	0.970281421	0.888201529	0.991406769	0.99879985	0.99989309	0.948277018
BSN	0.970281421	0.931095524	0.939683463	0.999939566	0.99989309	0.949744931
CCT3	0.970281421	0.909853448	0.937854949	0.99879985	0.99989309	0.95470473
BIN2	0.970281421	0.921280256	0.933102499	0.994744628	0.99989309	0.968245211
LOC112442597	0.970281421	0.876057665	0.975212837	0.99879985	0.99989309	0.972487907
CD80	0.970281421	0.890427603	0.933647928	0.998069032	0.99989309	0.983427394
C18H19orf81	0.970281421	0.884220302	0.976719499	0.995118128	0.99989309	0.983453112
ATP1B2	0.970281421	0.9347557	0.910978655	0.999939566	0.99989309	0.984449688
SDHC	0.970307575	0.88418172	0.926010642	0.999939566	0.99989309	0.943138585

ZNF177	0.97031946	0.926429754	0.939683463	0.999939566	0.99989309	0.933763401
EMD	0.97043897	0.885181785	0.950365732	0.999939566	0.99989309	0.924845138
NECTIN1	0.97043897	0.920347742	0.910978655	0.999939566	0.99989309	0.954334316
KANK4	0.97050833	0.899474896	0.935247964	0.999939566	0.99989309	0.911058479
IKBKB	0.97050833	0.913075885	0.918576062	0.999939566	0.99989309	0.922936098
IDH3G	0.97052428	0.888074023	0.951742137	0.996775429	0.99989309	0.926872824
LOC101905977	0.97052428	0.918306418	0.922126495	0.999939566	0.99989309	0.932613047
WTIP	0.97052428	0.887165309	0.929958748	0.999939566	0.99989309	0.932613047
TPBGL	0.97052428	0.942887777	0.937765241	0.999939566	0.99989309	0.943138585
TMCO6	0.97052428	0.907594268	0.989702604	0.998069032	0.99989309	0.95342487
AXL	0.97052428	0.897375356	0.921225977	0.99879985	0.99989309	0.99797558
PLIN4	0.970742699	0.887725794	0.933102499	0.999939566	0.99989309	0.934458012
RGS2	0.970742699	0.881823401	0.978215635	0.999939566	0.99989309	0.935655141
LOC101907549	0.970742699	0.898903115	0.926613927	0.999939566	0.99989309	0.938553439
LOC101902664	0.970742699	0.888331955	0.941792355	0.999939566	0.99989309	0.969808185
DPH6	0.970742699	0.924510975	0.962176387	0.993822494	0.99989309	0.972144361
SLC38A6	0.970778111	0.885991833	0.939683463	0.999939566	0.99989309	0.961506391
PBX2	0.970977881	0.944420015	0.947268948	0.999939566	0.99989309	0.948832722
DHFR	0.97107065	0.913075885	0.937772325	0.999939566	0.99989309	0.929046416
TRPC4AP	0.971114025	0.930016722	0.946573665	0.992368879	0.99989309	0.983453112
PXN	0.971336203	0.90249621	0.917092799	0.999939566	0.99989309	0.926872824
SLC25A53	0.971480858	0.921280256	0.914685423	0.999939566	0.99989309	0.943138585
PDE11A	0.971480858	0.877420806	0.929958748	0.999939566	0.99989309	0.944606747
LOC101902172	0.971547447	0.884768652	0.978892556	0.998542029	0.99989309	0.936137631
PAIP1	0.971547447	0.89272885	0.957578315	0.999939566	0.99989309	0.940673472
SYT5	0.971547447	0.892242414	0.939683463	0.999939566	0.99989309	0.943138585
ARPIN	0.971547447	0.945105738	0.921225977	0.999939566	0.99989309	0.952578814
ILF2	0.971547447	0.897918739	0.946885095	0.999939566	0.99989309	0.978467321
ZNF18	0.97156475	0.957902362	0.929958748	0.998542029	0.99989309	0.918291722
BAG6	0.971655754	0.878843255	0.939091808	0.999939566	0.99989309	0.978656049
STXBP2	0.971691053	0.974986418	0.92332596	0.999939566	0.99989309	0.917524589
LOC104969648	0.971691053	0.885181785	0.92332596	0.995118128	0.99989309	0.93636925
PRTFDC1	0.971691053	0.893639679	0.92153058	0.999939566	0.99989309	0.940683751
VPS13D	0.971691053	0.957950474	0.915638324	0.998542029	0.99989309	0.942421464
KANSL3	0.971691053	0.978473197	0.914406673	0.999939566	0.99989309	0.963893419
TFAP4	0.971691053	0.909176324	0.91542603	0.999939566	0.99989309	0.970702553
LHFPL3	0.971691053	0.889498035	0.922126495	0.999939566	0.99989309	0.976454461
LOC112444147	0.971691053	0.907594268	0.91234361	0.999939566	0.99989309	0.98016619

LOC112443475	0.971747047	0.921280256	0.926613927	0.999939566	0.99989309	0.914283173
M1AP	0.971747047	0.945105738	0.929958748	0.998542029	0.99989309	0.941925704
DNTTIP1	0.971747047	0.914162656	0.922126495	0.999939566	0.99989309	0.97815722
CLIC5	0.97176306	0.89272885	0.967489175	0.999939566	0.99989309	0.911696156
FDX1	0.971807107	0.899474896	0.979096894	0.999939566	0.99989309	0.910353954
C8H9orf64	0.971807107	0.96471209	0.92153058	0.999939566	0.99989309	0.911526437
ALDH16A1	0.971973532	0.907670026	0.921612776	0.999939566	0.99989309	0.943685681
ADAMTSL3	0.971973532	0.936099262	0.966157152	0.99879985	0.99989309	0.95342487
RPGRIP1	0.972072262	0.929536223	0.96993432	0.995800463	0.99989309	0.929537775
LOC781304	0.972095436	0.957950474	0.917876971	0.999939566	0.99989309	0.911696156
CASP8	0.972095436	0.926435857	0.953198168	0.999939566	0.99989309	0.912101047
CCDC91	0.972095436	0.877420806	0.985552223	0.999939566	0.99989309	0.912396352
TGFB1	0.972095436	0.894935852	0.968406955	0.99879985	0.99989309	0.927412916
LOC112449561	0.972095436	0.877749555	0.927079582	0.999939566	0.99989309	0.93270341
FRMPD3	0.972095436	0.946034281	0.937270184	0.999939566	0.99989309	0.942421464
MDM1	0.972095436	0.882542824	0.976719499	0.999939566	0.99989309	0.964143145
PSMD10	0.972095436	0.934248552	0.916100231	0.99879985	0.99989309	0.967640694
LOC539069	0.972095436	0.909086769	0.922349023	0.995118128	0.99989309	0.972336599
ZSCAN20	0.972114373	0.941309212	0.927460291	0.999939566	0.99989309	0.920521546
EEF1B2	0.97211572	0.922752283	0.927460291	0.999939566	0.99989309	0.926872824
PRKAR2A	0.97211572	0.89618217	0.937191211	0.999939566	0.99989309	0.933763401
NRDC	0.97211572	0.899387334	0.947475618	0.999939566	0.99989309	0.967640694
TP53I13	0.972420433	0.88099298	0.939683463	0.999939566	0.99989309	0.911263177
LOC107133180	0.972420433	0.974591667	0.939612562	0.999939566	0.99989309	0.911696156
FAM180A	0.972420433	0.959119521	0.921225977	0.999939566	0.99989309	0.912101047
PLEKHB1	0.972420433	0.962428993	0.967043892	0.998542029	0.99989309	0.912302996
TRAPPC10	0.972420433	0.893927942	0.927456842	0.999939566	0.99989309	0.912396352
LOC614531	0.972420433	0.944755173	0.922349023	0.999939566	0.99989309	0.914046542
NAB2	0.972420433	0.945105738	0.926613927	0.999939566	0.99989309	0.917524589
LOC112443147	0.972420433	0.953364061	0.976719499	0.99879985	0.99989309	0.920383473
CXCL12	0.972420433	0.939027995	0.939683463	0.999939566	0.99989309	0.920942379
GPIHBP1	0.972420433	0.991043872	0.951850716	0.995800463	0.99989309	0.921381434
RPL38	0.972420433	0.89272885	0.939683463	0.999939566	0.99989309	0.921381434
HADH	0.972420433	0.899387334	0.926613927	0.999939566	0.99989309	0.923064313
KLHL7	0.972420433	0.933914617	0.941593855	0.999939566	0.99989309	0.926872824
FNDC3A	0.972420433	0.905210378	0.948799259	0.999939566	0.99989309	0.926872824
METTL27	0.972420433	0.89272885	0.975183307	0.999939566	0.99989309	0.930528039
MYO1B	0.972420433	0.955483597	0.939683463	0.999939566	0.99989309	0.930774946

PDE5A	0.972420433	0.93181855	0.987419404	0.999939566	0.99989309	0.931515831
FBXL12	0.972420433	0.889064624	0.985135994	0.999939566	0.99989309	0.932809657
DOCK8	0.972420433	0.948215967	0.942004125	0.999939566	0.99989309	0.933183326
ZNF569	0.972420433	0.989736861	0.915636025	0.999939566	0.99989309	0.933730876
LRP1	0.972420433	0.887725794	0.953198168	0.999939566	0.99989309	0.934122809
SC5D	0.972420433	0.885181785	0.950801642	0.992829869	0.99989309	0.934458012
HSPE1	0.972420433	0.89141259	0.929303679	0.999939566	0.99989309	0.935607719
NMI	0.972420433	0.988649234	0.926613927	0.999939566	0.99989309	0.936563391
SLC9A3R1	0.972420433	0.972786652	0.929958748	0.999939566	0.99989309	0.937591162
PLIN1	0.972420433	0.920818159	0.933102499	0.999939566	0.99989309	0.937829933
LRRC74B	0.972420433	0.995289146	0.93050447	0.998542029	0.99989309	0.940917403
PEAR1	0.972420433	0.920347742	0.917092799	0.999939566	0.99989309	0.940917403
NMNAT1	0.972420433	0.899474896	0.949727203	0.999939566	0.99989309	0.940917403
SH2D4A	0.972420433	0.976925958	0.939683463	0.999939566	0.99989309	0.941925704
SEMA3B	0.972420433	0.894203989	0.987208945	0.999939566	0.99989309	0.941925704
TMEM68	0.972420433	0.928119925	0.939683463	0.999939566	0.99989309	0.942455807
SAAL1	0.972420433	0.939027995	0.914668913	0.999939566	0.99989309	0.943138585
LOC107131684	0.972420433	0.883007259	0.941593855	0.999939566	0.99989309	0.943777356
MAPKAPK3	0.972420433	0.955798423	0.933629769	0.999939566	0.99989309	0.945759045
TAF13	0.972420433	0.911347642	0.941319062	0.998542029	0.99989309	0.95470473
B4GALNT3	0.972420433	0.886732397	0.939091808	0.99879985	0.99989309	0.956610132
MXRA7	0.972420433	0.920347742	0.948411239	0.999939566	0.99989309	0.956670063
GSR	0.972420433	0.947806191	0.941319062	0.999939566	0.99989309	0.957140928
KCTD8	0.972420433	0.90249621	0.926613927	0.999939566	0.99989309	0.963996128
PYCARD	0.972420433	0.970179915	0.929958748	0.994652359	0.99989309	0.964143145
LOC613660	0.972420433	0.96471209	0.933102499	0.995800463	0.99989309	0.964594479
ARPC2	0.972420433	0.935176956	0.966919864	0.998542029	0.99989309	0.968059826
SYNGR3	0.972420433	0.941972432	0.944612298	0.99879985	0.99989309	0.970702553
SCML1	0.972420433	0.897918739	0.982712567	0.995118128	0.99989309	0.97356849
ZSWIM5	0.972420433	0.913171043	0.930520213	0.995800463	0.99989309	0.978656049
LGR6	0.972420433	0.881823401	0.945553946	0.999939566	0.99989309	0.979606084
AGPAT4	0.972420433	0.88418172	0.916022652	0.998542029	0.99989309	0.981411983
MT1E	0.972420433	0.93453191	0.926613927	0.995800463	0.99989309	0.99064193
SH3GL1	0.972420433	0.883378562	0.937191211	0.995118128	0.99989309	0.995411183
FBXO40	0.972420433	0.887725794	0.925587088	0.998542029	0.99989309	0.998636142
LCLAT1	0.972488054	0.939870925	0.987208945	0.995118128	0.99989309	0.917968123
NCBP3	0.972488054	0.926435857	0.926613927	0.99879985	0.99989309	0.934775196
HRCT1	0.972488054	0.955798423	0.933963997	0.999939566	0.99989309	0.963756876

ARHGAP25	0.972488054	0.974875445	0.914685423	0.995118128	0.99989309	0.97815722
RASL11B	0.972714358	0.905977412	0.969618195	0.999939566	0.99989309	0.935607719
CENPH	0.973086273	0.936330626	0.965296906	0.995118128	0.99989309	0.918608642
HADHB	0.97314149	0.891817085	0.937270184	0.999939566	0.99989309	0.914283173
PLXNB2	0.97314149	0.897918739	0.962209895	0.999939566	0.99989309	0.91601693
FAM8A1	0.97314149	0.900458021	0.916702	0.999939566	0.99989309	0.926872824
CAMTA2	0.97314149	0.90249621	0.985222638	0.999939566	0.99989309	0.952238962
LOC784980	0.97314149	0.91271182	0.937311143	0.999939566	0.99989309	0.958275359
NEIL2	0.97314149	0.908167107	0.921135532	0.998542029	0.99989309	0.991281092
DSC2	0.973275624	0.886909612	0.914406673	0.999939566	0.99989309	0.923064313
HGSNAT	0.973275624	0.944314627	0.965169397	0.999939566	0.99989309	0.927529392
IGFBP6	0.973427213	0.923357593	0.962754771	0.999939566	0.99989309	0.912101047
OR51E1	0.973427213	0.923729244	0.939683463	0.995800463	0.99989309	0.95470473
HOXA2	0.973700405	0.926435857	0.930262306	0.99879985	0.99989309	0.912396352
LOC534155	0.973700405	0.929573947	0.937627891	0.999939566	0.99989309	0.912396352
TKFC	0.973700405	0.966331409	0.961742979	0.993488944	0.99989309	0.914283173
SMCHD1	0.973700405	0.994810749	0.933963997	0.999939566	0.99989309	0.916117913
GPR19	0.973700405	0.914162656	0.917876971	0.999939566	0.99989309	0.926872824
LYRM2	0.973700405	0.897375356	0.925000236	0.999939566	0.99989309	0.928442671
GLS2	0.973700405	0.922434012	0.982223562	0.99879985	0.99989309	0.936183569
RTN4IP1	0.973700405	0.925360181	0.930710426	0.994309225	0.99989309	0.937591162
NOD1	0.973700405	0.95320487	0.951742137	0.999939566	0.99989309	0.942455807
DAXX	0.973700405	0.88311684	0.951742137	0.995800463	0.99989309	0.98016619
CTXND1	0.973700405	0.928292661	0.939091808	0.995946943	0.99989309	0.991281092
RTN4	0.973704882	0.906352322	0.937544913	0.999939566	0.99989309	0.979792753
DNAJB6	0.973738857	0.913075885	0.9840633	0.999939566	0.99989309	0.917524589
LOC104972545	0.973738857	0.936134921	0.933629769	0.999939566	0.99989309	0.927412916
SMIM19	0.973738857	0.893512526	0.980734833	0.999939566	0.99989309	0.933763401
TNS3	0.973738857	0.899474896	0.931259908	0.999939566	0.99989309	0.937591162
GTF2A1	0.973738857	0.91994024	0.958420472	0.998542029	0.99989309	0.940683751
FLI1	0.973738857	0.963478319	0.917876971	0.999939566	0.99989309	0.940917403
SLC10A7	0.973738857	0.924635955	0.969644737	0.99879985	0.99989309	0.941925704
BRMS1	0.973738857	0.926618064	0.957379098	0.995118128	0.99989309	0.942421464
SEMA3G	0.973738857	0.890427603	0.953198168	0.999939566	0.99989309	0.949527038
NDN	0.973738857	0.901922021	0.979500641	0.997612654	0.99989309	0.97792865
AMOTL1	0.973738857	0.924799142	0.958420472	0.99879985	0.99989309	0.981411983
LOC524650	0.973823019	0.952990784	0.922147089	0.995118128	0.99989309	0.922709797
PRMT9	0.973823019	0.899387334	0.93710335	0.999939566	0.99989309	0.923871657

LOC100848570	0.97398024	0.914735022	0.952085491	0.999939566	0.99989309	0.912598075
PIGB	0.97398024	0.926435857	0.921225977	0.999939566	0.99989309	0.943138585
DDX51	0.974046174	0.91557476	0.939683463	0.999939566	0.99989309	0.968245211
HDAC6	0.974126131	0.905432526	0.960726438	0.999939566	0.99989309	0.941889677
PMEL	0.974126131	0.980621528	0.930892662	0.99879985	0.99989309	0.95470473
PSMG3	0.974126131	0.944755173	0.917092799	0.99879985	0.99989309	0.956610132
TARSL2	0.974126131	0.907691022	0.919796896	0.999939566	0.99989309	0.981164039
OPTN	0.974232827	0.903820329	0.929303679	0.999939566	0.99989309	0.912101047
MSH6	0.974232827	0.987156012	0.925442336	0.994365581	0.99989309	0.915447631
LOC104970908	0.974232827	0.962321963	0.950801642	0.999939566	0.99989309	0.935154223
COL4A1	0.974232827	0.972786652	0.921936757	0.999939566	0.99989309	0.943138585
APMAP	0.974232827	0.908709906	0.917876971	0.995118128	0.99989309	0.993259231
EPB41L4A	0.974468189	0.911196851	0.965712499	0.998069032	0.99989309	0.963756876
USP16	0.974468189	0.899142992	0.918797372	0.999939566	0.99989309	0.981164039
LOC107131710	0.974468189	0.924433008	0.921135532	0.995118128	0.99989309	0.998074562
LOC101907006	0.974476309	0.936550733	0.984700021	0.995118128	0.99989309	0.921381434
GCK	0.974476309	0.889064624	0.916100231	0.999939566	0.99989309	0.937591162
PPP2R3C	0.974476309	0.897918739	0.933102499	0.999939566	0.99989309	0.977961448
LOC112442693	0.974476309	0.912965518	0.956880455	0.999939566	0.99989309	0.979606084
ZNF568	0.974476309	0.959119521	0.931397502	0.994309225	0.99989309	0.989128955
EGLN2	0.974557145	0.920347742	0.966626132	0.999939566	0.99989309	0.926872824
LOC515547	0.974557145	0.882930807	0.922147089	0.999939566	0.99989309	0.935493683
AHCYL2	0.974557145	0.893512526	0.939683463	0.999939566	0.99989309	0.943138585
FBLN2	0.974557145	0.924999357	0.941319062	0.999939566	0.99989309	0.956610132
SMKR1	0.974557145	0.924421023	0.931397502	0.995118128	0.99989309	0.991281092
LOC104975111	0.974980865	0.974591667	0.922126495	0.999939566	0.99989309	0.940917403
PCOLCE2	0.975005022	0.971839996	0.920449324	0.999939566	0.99989309	0.940917403
AMIGO1	0.97502316	0.908183362	0.96636908	0.999939566	0.99989309	0.914440253
SKAP2	0.97502316	0.921280256	0.947475618	0.999939566	0.99989309	0.915323299
MST1	0.97502316	0.98743321	0.928956273	0.99879985	0.99989309	0.916067231
COMT	0.97502316	0.883267666	0.988041385	0.999939566	0.99989309	0.918220726
USB1	0.97502316	0.991739536	0.919939634	0.998251353	0.99989309	0.919645668
ZNF292	0.97502316	0.978453686	0.916922165	0.999939566	0.99989309	0.925394029
GGH	0.97502316	0.934874752	0.939091808	0.999939566	0.99989309	0.926853716
MAPRE2	0.97502316	0.98618789	0.958420472	0.995118128	0.99989309	0.934775196
C18H16orf70	0.97502316	0.900070302	0.93050447	0.999939566	0.99989309	0.938643464
KCNC3	0.97502316	0.908709906	0.939683463	0.994337604	0.99989309	0.939966658
UPP1	0.97502316	0.924799142	0.93798101	0.999939566	0.99989309	0.943138585

TBCE	0.97502316	0.897375356	0.929303679	0.996601241	0.99989309	0.94425343
LOC104968518	0.97502316	0.899387334	0.944060947	0.999939566	0.99989309	0.956959747
NUP188	0.97502316	0.9347557	0.957379098	0.995118128	0.99989309	0.983453112
CHST2	0.975164391	0.963478319	0.957802423	0.999939566	0.99989309	0.950601157
LOX	0.975168018	0.911347642	0.966157152	0.99879985	0.99989309	0.955817715
MRPL1	0.975168018	0.886121828	0.941319062	0.99879985	0.99989309	0.974379635
LOC787309	0.975306294	0.936859329	0.93050447	0.999939566	0.99989309	0.937591162
LOC107133294	0.975306294	0.934248552	0.944976702	0.99879985	0.99989309	0.968163235
GOLGA2	0.975345262	0.949169888	0.941593855	0.999939566	0.99989309	0.912889185
TRAP1	0.975345262	0.889141991	0.949727203	0.99879985	0.99989309	0.917524589
FAM19A3	0.975345262	0.986245673	0.917876971	0.999939566	0.99989309	0.918220726
TTC31	0.975345262	0.905808353	0.976719499	0.999939566	0.99989309	0.922709797
SLC8B1	0.975345262	0.911347642	0.947268948	0.999939566	0.99989309	0.925720758
PLEKHA8	0.975345262	0.94610099	0.93050447	0.999939566	0.99989309	0.926335372
MFAP3L	0.975345262	0.885253121	0.939683463	0.999939566	0.99989309	0.926872824
LOC112441778	0.975345262	0.88418172	0.946047129	0.99483611	0.99989309	0.92689297
LOC107131273	0.975345262	0.993062628	0.941593855	0.99879985	0.99989309	0.92920113
MCM4	0.975345262	0.922434012	0.958420472	0.999939566	0.99989309	0.932613047
NKIRAS2	0.975345262	0.897375356	0.985552223	0.999939566	0.99989309	0.935655141
CYP7B1	0.975345262	0.905210378	0.939683463	0.999939566	0.99989309	0.936439483
LMF2	0.975345262	0.937978608	0.992056611	0.995118128	0.99989309	0.937591162
CHRNB4	0.975345262	0.942230793	0.9785457	0.999939566	0.99989309	0.940673472
TGFBRAP1	0.975345262	0.905934691	0.945417678	0.995118128	0.99989309	0.940683751
ABHD14A	0.975345262	0.942048064	0.922126495	0.999939566	0.99989309	0.940683751
LOC112442352	0.975345262	0.929536223	0.926613927	0.999939566	0.99989309	0.940917403
LYZ1	0.975345262	0.971812321	0.936383207	0.999334425	0.99989309	0.941925704
ENDOU	0.975345262	0.911106863	0.964668297	0.999939566	0.99989309	0.942868422
KTI12	0.975345262	0.903820329	0.979635589	0.999939566	0.99989309	0.943138585
YBX1	0.975345262	0.908709906	0.944485232	0.999939566	0.99989309	0.943685681
SLC4A5	0.975345262	0.944314627	0.92153058	0.999939566	0.99989309	0.952887889
CLEC6A	0.975345262	0.939027995	0.936450438	0.995118128	0.99989309	0.95470473
LOC521580	0.975345262	0.974348229	0.943891967	0.996775429	0.99989309	0.95470473
BICRA	0.975345262	0.884397857	0.939683463	0.99879985	0.99989309	0.95470473
ARHGEF7	0.975345262	0.93453191	0.929958748	0.999939566	0.99989309	0.955974956
CENPL	0.975345262	0.962321963	0.93710335	0.999939566	0.99989309	0.958219037
PECR	0.975345262	0.929536223	0.944160795	0.999939566	0.99989309	0.960985422
PGBD2	0.975345262	0.962428993	0.929303679	0.99879985	0.99989309	0.961506391
RASSF1	0.975345262	0.933351936	0.939091808	0.999939566	0.99989309	0.961506391

RIOX2	0.975345262	0.90249621	0.93050447	0.995800463	0.99989309	0.96163007
ZNF703	0.975345262	0.939309165	0.919108971	0.999939566	0.99989309	0.968245211
LOC101906397	0.975345262	0.884768652	0.932641597	0.999939566	0.99989309	0.968245211
NBDY	0.975345262	0.893623223	0.975527089	0.995118128	0.99989309	0.969981358
CIR1	0.975345262	0.913075885	0.917876971	0.999939566	0.99989309	0.972828434
PYROXD1	0.975345262	0.911230889	0.942004125	0.99879985	0.99989309	0.982066213
SOCS1	0.975345262	0.926435857	0.926018972	0.995118128	0.99989309	0.983453112
SYCP2	0.975345262	0.901712734	0.951850716	0.99879985	0.99989309	0.986389023
URB1	0.975345262	0.90249621	0.951742137	0.999939566	0.99989309	0.986862062
POLR1C	0.975345262	0.90249621	0.918292936	0.995800463	0.99989309	0.99064193
SLC35D1	0.975345262	0.929102966	0.939683463	0.998542029	0.99989309	0.993714265
MSANTD2	0.975345262	0.909237134	0.919108971	0.999939566	0.99989309	0.998636142
VARS2	0.97548302	0.958621801	0.948628899	0.999939566	0.99989309	0.933678522
LOC112443216	0.97548302	0.934531829	0.939683463	0.999939566	0.99989309	0.939966658
TUBA3E	0.97548302	0.921280256	0.935236722	0.999939566	0.99989309	0.943138585
FAM151B	0.97548302	0.908709906	0.917876971	0.999939566	0.99989309	0.943891002
HIST1H1E	0.97548302	0.934248552	0.957802423	0.998542029	0.99989309	0.956959747
MAL	0.97548302	0.927298992	0.921225977	0.999939566	0.99989309	0.957877422
SWI5	0.97548302	0.911347642	0.92153058	0.999939566	0.99989309	0.961336964
LOC101906009	0.97548302	0.963478319	0.93050447	0.998542029	0.99989309	0.961506391
C1GALT1C1	0.97548302	0.939297398	0.942004125	0.995118128	0.99989309	0.968059826
TNS2	0.97548302	0.884768652	0.971981006	0.99879985	0.99989309	0.985625041
PCDHA13	0.97564491	0.999968084	0.926372704	0.995118128	0.99989309	0.914046542
IL15	0.97564491	0.893512526	0.92153058	0.999939566	0.99989309	0.914046542
AP2M1	0.97564491	0.95751653	0.985222638	0.995118128	0.99989309	0.914440253
LOC112442323	0.97564491	0.967113913	0.966157152	0.999939566	0.99989309	0.914440253
LOC101902812	0.97564491	0.914735022	0.939683463	0.999939566	0.99989309	0.915323299
WBP1L	0.97564491	0.89141259	0.989702604	0.999939566	0.99989309	0.91596005
ID3	0.97564491	0.979916117	0.953692935	0.99879985	0.99989309	0.917524589
LSM11	0.97564491	0.944420015	0.92153058	0.999939566	0.99989309	0.917524589
NDRG3	0.97564491	0.98618789	0.957379098	0.999939566	0.99989309	0.917524589
WNT9A	0.97564491	0.905977412	0.987419404	0.999939566	0.99989309	0.917524589
MBNL3	0.97564491	0.962934461	0.926613927	0.999939566	0.99989309	0.917968123
ACADVL	0.97564491	0.913075885	0.939683463	0.999939566	0.99989309	0.917968123
DAAM1	0.97564491	0.988649234	0.957379098	0.999939566	0.99989309	0.917968123
RNASEK	0.97564491	0.949787238	0.976782463	0.999939566	0.99989309	0.917968123
LOC104974459	0.97564491	0.94858039	0.929958748	0.999939566	0.99989309	0.918220726
LOC104972595	0.97564491	0.96471209	0.944976702	0.999939566	0.99989309	0.918220726

LOC100297498	0.97564491	0.893512526	0.926613927	0.999939566	0.99989309	0.918666628
SCAMP5	0.97564491	0.992194873	0.944976702	0.99879985	0.99989309	0.918803168
GLMP	0.97564491	0.983065474	0.929958748	0.999939566	0.99989309	0.92035958
LOC100297170	0.97564491	0.913075885	0.939683463	0.999939566	0.99989309	0.92035958
CD1A	0.97564491	0.894203989	0.920161008	0.999939566	0.99989309	0.920383473
PDCD6IP	0.97564491	0.924541936	0.98082053	0.999939566	0.99989309	0.920834985
SERPINB8	0.97564491	0.920347742	0.921146002	0.999939566	0.99989309	0.920849243
ZNF518A	0.97564491	0.903657308	0.92153058	0.996601241	0.99989309	0.921794978
SHISAL2A	0.97564491	0.964234693	0.984700021	0.99879985	0.99989309	0.922229044
TBC1D30	0.97564491	0.925807756	0.939683463	0.999939566	0.99989309	0.922229044
FBXW8	0.97564491	0.955798423	0.939683463	0.999939566	0.99989309	0.922229044
LOC112442347	0.97564491	0.923365173	0.929303679	0.999939566	0.99989309	0.922263023
MIB2	0.97564491	0.911196851	0.987208945	0.999939566	0.99989309	0.922263023
VRK3	0.97564491	0.933412624	0.933963997	0.999939566	0.99989309	0.922690867
CYBB	0.97564491	0.978833989	0.958420472	0.999939566	0.99989309	0.922709797
RAB3IP	0.97564491	0.892363836	0.921420528	0.999939566	0.99989309	0.922872513
PDHA1	0.97564491	0.893512526	0.917876971	0.999939566	0.99989309	0.923064313
TMEM231	0.97564491	0.890284639	0.93710335	0.999939566	0.99989309	0.923064313
LOC112448381	0.97564491	0.924510975	0.96993432	0.999939566	0.99989309	0.923871657
PCBP3	0.97564491	0.976925958	0.969644737	0.998542029	0.99989309	0.924788226
MAGEE2	0.97564491	0.896388152	0.926613927	0.999939566	0.99989309	0.924788226
LOC104970966	0.97564491	0.955798423	0.939683463	0.99879985	0.99989309	0.924845138
LOC112447070	0.97564491	0.912112179	0.942004125	0.99879985	0.99989309	0.924845138
CTNNB1	0.97564491	0.912112179	0.962275739	0.999939566	0.99989309	0.924845138
KDELR3	0.97564491	0.893512526	0.921135532	0.999939566	0.99989309	0.92546728
THOC5	0.97564491	0.992032812	0.92153058	0.999939566	0.99989309	0.92546728
PDCD2	0.97564491	0.982614603	0.93710335	0.999939566	0.99989309	0.926484322
RAMP3	0.97564491	0.991043872	0.939683463	0.998542029	0.99989309	0.926872824
NFE2L3	0.97564491	0.992657849	0.92153058	0.999939566	0.99989309	0.926872824
BTBD2	0.97564491	0.926435857	0.929958748	0.999939566	0.99989309	0.926872824
CALML4	0.97564491	0.934248552	0.929958748	0.999939566	0.99989309	0.926872824
PJVK	0.97564491	0.974875445	0.93050447	0.999939566	0.99989309	0.926872824
ZNF346	0.97564491	0.90249621	0.937004587	0.999939566	0.99989309	0.926872824
ABCA1	0.97564491	0.913075885	0.939683463	0.999939566	0.99989309	0.926872824
LOC112444846	0.97564491	0.968440623	0.942004125	0.999939566	0.99989309	0.926872824
LOC614091	0.97564491	0.930469765	0.944976702	0.999939566	0.99989309	0.926872824
ORMDL3	0.97564491	0.885991833	0.968419607	0.999939566	0.99989309	0.926872824
ITPRIPL1	0.97564491	0.921280256	0.982223562	0.999939566	0.99989309	0.926872824

CHMP7	0.97564491	0.893512526	0.989408632	0.999939566	0.99989309	0.926872824
PXDN	0.97564491	0.936134921	0.939091808	0.999939566	0.99989309	0.92689297
JAG1	0.97564491	0.91994024	0.93050447	0.999939566	0.99989309	0.927412916
ATG4C	0.97564491	0.90249621	0.962569922	0.999939566	0.99989309	0.927412916
CAMKMT	0.97564491	0.89539921	0.969311025	0.999939566	0.99989309	0.927412916
LIPE	0.97564491	0.904077835	0.929958748	0.999939566	0.99989309	0.928123265
LMAN2	0.97564491	0.972786652	0.930979234	0.999939566	0.99989309	0.928123265
RPL7A	0.97564491	0.900312059	0.939683463	0.999939566	0.99989309	0.928799366
MYO5B	0.97564491	0.885981636	0.98567143	0.999939566	0.99989309	0.928799366
CYFIP1	0.97564491	0.956843573	0.93050447	0.999939566	0.99989309	0.92920113
LOC101906131	0.97564491	0.891817085	0.978892556	0.999939566	0.99989309	0.92920113
ENTPD4	0.97564491	0.922767609	0.974832809	0.999939566	0.99989309	0.930058266
FIP1L1	0.97564491	0.898000467	0.929303679	0.999939566	0.99989309	0.930318133
LOC107132617	0.97564491	0.921716092	0.9343664	0.999939566	0.99989309	0.930707319
BRWD3	0.97564491	0.913171043	0.939612562	0.999939566	0.99989309	0.930707319
LOC112449618	0.97564491	0.896356718	0.958420472	0.999939566	0.99989309	0.930707319
ZNF260	0.97564491	0.911347642	0.957575736	0.99879985	0.99989309	0.930774946
RASSF4	0.97564491	0.974591667	0.969311025	0.99879985	0.99989309	0.931886764
LIX1L	0.97564491	0.99950308	0.922147089	0.995687815	0.99989309	0.932014575
RHBDL2	0.97564491	0.919633395	0.927460291	0.999939566	0.99989309	0.932613047
LOC107133268	0.97564491	0.964616377	0.942004125	0.999939566	0.99989309	0.933316009
PHF7	0.97564491	0.94858039	0.93000135	0.999939566	0.99989309	0.933437144
GRIPAP1	0.97564491	0.959119521	0.971104162	0.995118128	0.99989309	0.933763401
MAP4K4	0.97564491	0.912292519	0.923202492	0.999939566	0.99989309	0.933763401
LOC112445060	0.97564491	0.914414818	0.926613927	0.999939566	0.99989309	0.933763401
CDV3	0.97564491	0.944420015	0.939683463	0.999939566	0.99989309	0.933763401
SENP7	0.97564491	0.938538223	0.939091808	0.997612654	0.99989309	0.934059568
ACE2	0.97564491	0.897657501	0.93050447	0.999939566	0.99989309	0.934122809
LOC528767	0.97564491	0.91557476	0.9343664	0.999939566	0.99989309	0.934458012
NAA16	0.97564491	0.962428993	0.965875422	0.999939566	0.99989309	0.934458012
MED16	0.97564491	0.931623635	0.959320266	0.999939566	0.99989309	0.934487064
RARS2	0.97564491	0.885981636	0.925587088	0.999939566	0.99989309	0.934578283
ENPP2	0.97564491	0.974590874	0.960137022	0.999939566	0.99989309	0.934653208
LOC100299303	0.97564491	0.996120899	0.939091808	0.998542029	0.99989309	0.934775196
TRIM17	0.97564491	0.935990876	0.959320266	0.999939566	0.99989309	0.934775196
LOC787269	0.97564491	0.901922021	0.923072506	0.999939566	0.99989309	0.935385789
GOT1	0.97564491	0.893161917	0.976719499	0.995118128	0.99989309	0.935655141
LOC107132450	0.97564491	0.903489502	0.92153058	0.999939566	0.99989309	0.935655141

DCLK1	0.97564491	0.978453686	0.928195567	0.999939566	0.99989309	0.935655141
SLC25A20	0.97564491	0.921280256	0.929303679	0.999939566	0.99989309	0.935655141
CCNI2	0.97564491	0.9347557	0.929958748	0.999939566	0.99989309	0.935655141
UBE2O	0.97564491	0.920347742	0.958420472	0.999939566	0.99989309	0.935655141
ADAMTS14	0.97564491	0.923729244	0.959320266	0.999939566	0.99989309	0.935655141
WWC3	0.97564491	0.911196851	0.962209895	0.999939566	0.99989309	0.935655141
RAP2B	0.97564491	0.897657501	0.974681091	0.999939566	0.99989309	0.935655141
DNAI1	0.97564491	0.963478319	0.922147089	0.999939566	0.99989309	0.935937948
PELP1	0.97564491	0.896591631	0.944485232	0.999939566	0.99989309	0.935937948
MRPL28	0.97564491	0.897077431	0.927079582	0.999939566	0.99989309	0.935964382
BCAM	0.97564491	0.918306418	0.93798101	0.999939566	0.99989309	0.935964382
LANCL1	0.97564491	0.905653284	0.97381056	0.995800463	0.99989309	0.936077435
NDUFA12	0.97564491	0.956134433	0.929958748	0.999939566	0.99989309	0.936137631
LOC101902444	0.97564491	0.936365238	0.974698466	0.999060254	0.99989309	0.936439483
RPL9	0.97564491	0.957645111	0.966157152	0.999939566	0.99989309	0.936439483
BIN1	0.97564491	0.944755173	0.939683463	0.99879985	0.99989309	0.936563391
LOC104968751	0.97564491	0.965392055	0.923072506	0.999939566	0.99989309	0.936563391
GNRH2	0.97564491	0.899387334	0.994425789	0.995118128	0.99989309	0.936565114
RIN3	0.97564491	0.897375356	0.933102499	0.999939566	0.99989309	0.93691717
MYO1G	0.97564491	0.920818159	0.922126495	0.999939566	0.99989309	0.937218904
SMARCD2	0.97564491	0.970524066	0.939683463	0.995118128	0.99989309	0.937591162
LOC101902385	0.97564491	0.887725794	0.941319062	0.99879985	0.99989309	0.937591162
LOC112441607	0.97564491	0.957353974	0.930710426	0.999939566	0.99989309	0.937591162
CDH13	0.97564491	0.913075885	0.939091808	0.999939566	0.99989309	0.937591162
PLPP2	0.97564491	0.908839246	0.966157152	0.999939566	0.99989309	0.937591162
RAB27B	0.97564491	0.934531829	0.966157152	0.999939566	0.99989309	0.937591162
TMOD1	0.97564491	0.926435857	0.967043892	0.999939566	0.99989309	0.937591162
TMTC4	0.97564491	0.886909612	0.923072506	0.999939566	0.99989309	0.938054315
HES7	0.97564491	0.90249621	0.924266781	0.999939566	0.99989309	0.938054315
SH3KBP1	0.97564491	0.903657308	0.951092876	0.995800463	0.99989309	0.939191109
ZNF623	0.97564491	0.894203989	0.989408632	0.999939566	0.99989309	0.939800211
TSPOAP1	0.97564491	0.921280256	0.945417678	0.999939566	0.99989309	0.939966658
MAP2K5	0.97564491	0.921280256	0.976719499	0.999939566	0.99989309	0.939966658
LOC787237	0.97564491	0.920423305	0.978892556	0.995118128	0.99989309	0.940683751
CPLANE1	0.97564491	0.884768652	0.994425789	0.995800463	0.99989309	0.940683751
LOC618939	0.97564491	0.896591631	0.994835068	0.999518323	0.99989309	0.940917403
KARS	0.97564491	0.936134921	0.921225977	0.999939566	0.99989309	0.941275898
OTUB1	0.97564491	0.959119521	0.931397502	0.99879985	0.99989309	0.941925704

EXOC4	0.97564491	0.916725199	0.951850716	0.99879985	0.99989309	0.941925704
PCDHB14	0.97564491	0.893639679	0.919997767	0.999939566	0.99989309	0.941925704
MTO1	0.97564491	0.984479682	0.930710426	0.999939566	0.99989309	0.941925704
CLPTM1	0.97564491	0.922434012	0.941593855	0.999939566	0.99989309	0.941925704
KDSR	0.97564491	0.904334308	0.942004125	0.999939566	0.99989309	0.941925704
CLCF1	0.97564491	0.90249621	0.947268948	0.999939566	0.99989309	0.941925704
SPECC1L	0.97564491	0.887561218	0.994425789	0.999939566	0.99989309	0.941925704
ATXN2	0.97564491	0.922434012	0.922161207	0.99879985	0.99989309	0.942421464
PLEKHA5	0.97564491	0.907691022	0.939612562	0.999939566	0.99989309	0.942421464
NME1	0.97564491	0.893512526	0.951607422	0.999939566	0.99989309	0.942421464
CYB561D1	0.97564491	0.957353974	0.922126495	0.999939566	0.99989309	0.942455807
LOC781533	0.97564491	0.905116327	0.927460291	0.999939566	0.99989309	0.942800958
NAIF1	0.97564491	0.985457646	0.939683463	0.999939566	0.99989309	0.942868422
TRMT10A	0.97564491	0.885181785	0.976719499	0.995118128	0.99989309	0.943138585
IL12RB2	0.97564491	0.955798423	0.977612552	0.995118128	0.99989309	0.943138585
SUPT7L	0.97564491	0.982703363	0.929958748	0.995302856	0.99989309	0.943138585
MBD5	0.97564491	0.942048064	0.917876971	0.995800463	0.99989309	0.943138585
POLM	0.97564491	0.94883251	0.925442336	0.99879985	0.99989309	0.943138585
TTC17	0.97564491	0.955798423	0.929303679	0.99879985	0.99989309	0.943138585
ANGPTL7	0.97564491	0.93453191	0.923072506	0.999939566	0.99989309	0.943138585
LOC101902490	0.97564491	0.93519674	0.959705869	0.999939566	0.99989309	0.943138585
SMYD2	0.97564491	0.90249621	0.933963997	0.999939566	0.99989309	0.943685681
GNB2	0.97564491	0.940311886	0.978879234	0.999939566	0.99989309	0.943685681
ZNF165	0.97564491	0.923841618	0.960726438	0.99879985	0.99989309	0.94425343
BCL2L10	0.97564491	0.899387334	0.968303964	0.999939566	0.99989309	0.94425343
PKIA	0.97564491	0.988649234	0.922366987	0.999939566	0.99989309	0.944606747
PAQR6	0.97564491	0.984247605	0.952208316	0.99879985	0.99989309	0.945759045
GPAA1	0.97564491	0.913171043	0.92153058	0.999939566	0.99989309	0.945759045
CHRNE	0.97564491	0.971812321	0.92921168	0.999939566	0.99989309	0.945759045
POFUT1	0.97564491	0.918306418	0.930262306	0.999939566	0.99989309	0.945759045
IGFBP5	0.97564491	0.945105738	0.95783333	0.999939566	0.99989309	0.945759045
DOK1	0.97564491	0.899387334	0.959351558	0.999939566	0.99989309	0.946294331
SH3BGRL3	0.97564491	0.982833172	0.939683463	0.995800463	0.99989309	0.946314386
LOC101902983	0.97564491	0.913175252	0.939683463	0.999939566	0.99989309	0.946314386
SLC36A4	0.97564491	0.932156311	0.966157152	0.999939566	0.99989309	0.948277018
LOC101905894	0.97564491	0.982963758	0.947268948	0.997612654	0.99989309	0.949012719
DOCK9	0.97564491	0.968246192	0.937270184	0.999939566	0.99989309	0.950199992
JAK3	0.97564491	0.89548617	0.939683463	0.999939566	0.99989309	0.950577985

LOC101903438	0.97564491	0.979466937	0.926613927	0.995800463	0.99989309	0.950601157
YIPF6	0.97564491	0.888993371	0.960137022	0.999939566	0.99989309	0.951216293
PROM1	0.97564491	0.982963758	0.926613927	0.999939566	0.99989309	0.951819529
ZNF536	0.97564491	0.918306418	0.926613927	0.999939566	0.99989309	0.95342487
LOC100300483	0.97564491	0.988153273	0.930710426	0.995118128	0.99989309	0.954334316
LOC516849	0.97564491	0.944314627	0.939683463	0.999939566	0.99989309	0.954334316
LOC101904753	0.97564491	0.907670026	0.919796896	0.999939566	0.99989309	0.95470473
ZNF382	0.97564491	0.893512526	0.979635589	0.99879985	0.99989309	0.95549381
CCL14	0.97564491	0.905140702	0.930710426	0.999939566	0.99989309	0.955585296
VPS29	0.97564491	0.907878616	0.958420472	0.999939566	0.99989309	0.955585296
GEMIN2	0.97564491	0.900467607	0.962209895	0.99879985	0.99989309	0.95579175
LOC510362	0.97564491	0.929573947	0.939683463	0.999939566	0.99989309	0.95640184
VIPAS39	0.97564491	0.928119925	0.967452429	0.999939566	0.99989309	0.956410517
KHDRBS1	0.97564491	0.934248552	0.978892556	0.995118128	0.99989309	0.956610132
GCC2	0.97564491	0.907594268	0.964720224	0.995800463	0.99989309	0.956610132
TLK2	0.97564491	0.911230889	0.942642657	0.99879985	0.99989309	0.956610132
HNRNPM	0.97564491	0.893915663	0.951850716	0.99879985	0.99989309	0.956610132
LOC100138933	0.97564491	0.899474896	0.950365732	0.999939566	0.99989309	0.956610132
XPO6	0.97564491	0.920347742	0.916702	0.998542029	0.99989309	0.956959747
LOC107133166	0.97564491	0.885181785	0.922147089	0.999939566	0.99989309	0.957877422
KIRREL3	0.97564491	0.929262063	0.957802423	0.999939566	0.99989309	0.957877422
MTCH1	0.97564491	0.924799142	0.975212837	0.999939566	0.99989309	0.957877422
PJA2	0.97564491	0.911106863	0.979096894	0.999939566	0.99989309	0.960985422
LOC101906200	0.97564491	0.91994024	0.968455468	0.999939566	0.99989309	0.961506391
ODR4	0.97564491	0.974986418	0.953426945	0.998542029	0.99989309	0.961606931
ZNF697	0.97564491	0.942048064	0.926613927	0.999939566	0.99989309	0.962255684
TSNAX	0.97564491	0.965027449	0.929958748	0.995118128	0.99989309	0.963996128
SEMA4G	0.97564491	0.89539921	0.971981006	0.999939566	0.99989309	0.9640547
OS9	0.97564491	0.885376959	0.950801642	0.999939566	0.99989309	0.964143145
VWF	0.97564491	0.988649234	0.919108971	0.995800463	0.99989309	0.964169813
PON3	0.97564491	0.899387334	0.931173336	0.999939566	0.99989309	0.964188691
PPP1R13B	0.97564491	0.986245673	0.919108971	0.998542029	0.99989309	0.965969659
NEK1	0.97564491	0.885604065	0.921225977	0.999939566	0.99989309	0.965969659
LOC101906283	0.97564491	0.93453191	0.95826379	0.99879985	0.99989309	0.967017962
CPPED1	0.97564491	0.929705559	0.942004125	0.999939566	0.99989309	0.967634187
FBXO7	0.97564491	0.934248552	0.93145152	0.999939566	0.99989309	0.967796552
RAB9B	0.97564491	0.920818159	0.924266781	0.998542029	0.99989309	0.968059826
LOC112442866	0.97564491	0.965239369	0.93078193	0.99879985	0.99989309	0.968059826

ZNF345	0.97564491	0.945028184	0.917092799	0.999939566	0.99989309	0.968163235
WARS	0.97564491	0.955798423	0.930262306	0.995800463	0.99989309	0.968245211
ZBTB48	0.97564491	0.909181154	0.974870423	0.998542029	0.99989309	0.968245211
SMARCE1	0.97564491	0.899387334	0.968954353	0.999939566	0.99989309	0.968245211
ZCCHC3	0.97564491	0.926435857	0.93050447	0.995800463	0.99989309	0.968857843
LOC100849046	0.97564491	0.929618781	0.929605682	0.999939566	0.99989309	0.969981358
GAREM1	0.97564491	0.973797778	0.937363055	0.995800463	0.99989309	0.972182019
TFDP2	0.97564491	0.951616411	0.922126495	0.999939566	0.99989309	0.972487907
POLE4	0.97564491	0.900467607	0.966157152	0.996601241	0.99989309	0.972787584
ZNF398	0.97564491	0.930469765	0.93145152	0.99879985	0.99989309	0.97345607
CD83	0.97564491	0.97099048	0.939683463	0.99879985	0.99989309	0.975972322
LOC784354	0.97564491	0.902602466	0.937765241	0.999939566	0.99989309	0.976757328
PLA2G5	0.97564491	0.913075885	0.930520213	0.999939566	0.99989309	0.977961448
RNF40	0.97564491	0.890427603	0.975212837	0.99879985	0.99989309	0.97815722
SRSF1	0.97564491	0.91994024	0.96596172	0.999939566	0.99989309	0.978346338
APOA1	0.97564491	0.907878616	0.951778266	0.995118128	0.99989309	0.978565009
FDXR	0.97564491	0.932156311	0.922816581	0.999939566	0.99989309	0.98016619
LOC101908123	0.97564491	0.921393399	0.933102499	0.999939566	0.99989309	0.98016619
TMUB2	0.97564491	0.886967296	0.92153058	0.999939566	0.99989309	0.980638112
ITIH3	0.97564491	0.912112179	0.929958748	0.999939566	0.99989309	0.981090047
MAN1C1	0.97564491	0.936134921	0.930710426	0.999939566	0.99989309	0.981164039
TMEM254	0.97564491	0.900792674	0.959822136	0.99879985	0.99989309	0.981411983
ERICH1	0.97564491	0.970237188	0.924266781	0.996775429	0.99989309	0.982074417
SMAGP	0.97564491	0.969023271	0.924476109	0.995800463	0.99989309	0.982559673
ZCCHC8	0.97564491	0.923680159	0.96993432	0.995118128	0.99989309	0.983453112
LOC788467	0.97564491	0.970524066	0.917876971	0.995946943	0.99989309	0.983453112
CYP8B1	0.97564491	0.955798423	0.92489263	0.99879985	0.99989309	0.983453112
RNF180	0.97564491	0.90249621	0.945417678	0.99879985	0.99989309	0.983453112
MAFB	0.97564491	0.938076005	0.929958748	0.999939566	0.99989309	0.983453112
LOC112443223	0.97564491	0.911196851	0.960137022	0.999939566	0.99989309	0.984449688
UTP20	0.97564491	0.920818159	0.927460291	0.99879985	0.99989309	0.984491523
GALR3	0.97564491	0.959119521	0.925442336	0.99879985	0.99989309	0.985517088
TRAPPC12	0.97564491	0.944755173	0.926613927	0.995800463	0.99989309	0.986389023
LOC618256	0.97564491	0.924799142	0.921420528	0.999939566	0.99989309	0.986389023
S100A3	0.97564491	0.934248552	0.947268948	0.99879985	0.99989309	0.986862062
BCAS2	0.97564491	0.921280256	0.947268948	0.99879985	0.99989309	0.98704566
PUM3	0.97564491	0.913171043	0.942004125	0.99879985	0.99989309	0.988323336
ATG14	0.97564491	0.940039358	0.950801642	0.998542029	0.99989309	0.989128955

TLR9	0.97564491	0.891817085	0.941638728	0.999939566	0.99989309	0.989780996
GDNF	0.97564491	0.927298992	0.920449324	0.999939566	0.99989309	0.990291862
THADA	0.97564491	0.91557476	0.941593855	0.999939566	0.99989309	0.99064193
XPNPEP1	0.97564491	0.901256223	0.947320947	0.99879985	0.99989309	0.991612651
LOC112441500	0.97564491	0.893639679	0.92153058	0.99857016	0.99989309	0.998074562
LOC100848077	0.97564491	0.924799142	0.921319817	0.99879985	0.99989309	0.998074562
EIF4G1	0.97564491	0.940039358	0.919997767	0.998542029	0.99989309	0.998636142
LOC781565	0.975728694	0.942236132	0.953826265	0.999939566	0.99989309	0.926872824
SENP5	0.975728694	0.921280256	0.92153058	0.999939566	0.99989309	0.937218904
VBP1	0.975728694	0.903820329	0.978067202	0.999939566	0.99989309	0.957140928
AOC1	0.975728694	0.92813228	0.924266781	0.998542029	0.99989309	0.96826636
EEF1D	0.975914236	0.914162656	0.929958748	0.998542029	0.99989309	0.973153155
TNNI2	0.975925449	0.907878616	0.929303679	0.999939566	0.99989309	0.931125957
LOC101906206	0.975925449	0.908709906	0.939683463	0.999939566	0.99989309	0.968059826
LOC100847719	0.976173684	0.934248552	0.960137022	0.99879985	0.99989309	0.944605169
LOC112442408	0.976409895	0.970524066	0.951006905	0.999939566	0.99989309	0.921254858
LOC100848011	0.976409895	0.970393237	0.957379098	0.999939566	0.99989309	0.929046416
THAP2	0.976409895	0.936279501	0.989332994	0.99879985	0.99989309	0.932267465
ASB7	0.976409895	0.913075885	0.993665588	0.99879985	0.99989309	0.932613047
KLF16	0.976409895	0.936859329	0.942761121	0.999939566	0.99989309	0.935655141
TMEM233	0.976409895	0.924799142	0.972211632	0.999939566	0.99989309	0.936137631
KIAA1468	0.976409895	0.974256477	0.933102499	0.999939566	0.99989309	0.937488554
SOX18	0.976409895	0.925360181	0.937835937	0.999939566	0.99989309	0.938553439
ISG20	0.976409895	0.922264623	0.977612552	0.99879985	0.99989309	0.940683751
SFT2D2	0.976409895	0.939027995	0.951850716	0.999939566	0.99989309	0.940966947
PTGDR2	0.976409895	0.920347742	0.959377939	0.99879985	0.99989309	0.941925704
FAM84B	0.976409895	0.979812776	0.929958748	0.999939566	0.99989309	0.941925704
HNRNPUL2	0.976409895	0.899474896	0.943690845	0.99879985	0.99989309	0.942421464
LOC507930	0.976409895	0.907878616	0.958420472	0.999939566	0.99989309	0.942421464
LOC112443001	0.976409895	0.936134921	0.921313359	0.999939566	0.99989309	0.943138585
ATP5F1A	0.976409895	0.908586813	0.92153058	0.998542029	0.99989309	0.943777356
TRAPPC4	0.976409895	0.920347742	0.962569922	0.999939566	0.99989309	0.945593145
NUDT12	0.976409895	0.911279338	0.92153058	0.999939566	0.99989309	0.946225397
LOC100336013	0.976409895	0.920347742	0.933963997	0.999939566	0.99989309	0.946682284
PTGR2	0.976409895	0.909926193	0.933102499	0.999939566	0.99989309	0.95470473
LOC112449358	0.976409895	0.949008068	0.939683463	0.999939566	0.99989309	0.95470473
CD34	0.976409895	0.92813228	0.931397502	0.999939566	0.99989309	0.955585296
LSM5	0.976409895	0.902136721	0.987208945	0.99879985	0.99989309	0.956610132

TSPYL5	0.976409895	0.901781061	0.928099904	0.999939566	0.99989309	0.956610132
RPS2	0.976409895	0.907594268	0.939091808	0.999939566	0.99989309	0.957877422
ASB2	0.976409895	0.959119521	0.966919864	0.995800463	0.99989309	0.961506391
OXSR1	0.976409895	0.912112179	0.932641597	0.999939566	0.99989309	0.967796552
TTC28	0.976409895	0.913075885	0.959320266	0.99879985	0.99989309	0.984408406
SNX27	0.976409895	0.921280256	0.929043558	0.999939566	0.99989309	0.984967003
ARL14EPL	0.976409895	0.897424386	0.966334813	0.99879985	0.99989309	0.988323336
LOC112446667	0.976424994	0.983065474	0.939683463	0.999939566	0.99989309	0.922872513
LOC112447443	0.976424994	0.924799163	0.976850508	0.999939566	0.99989309	0.946717155
C28H1orf198	0.976459555	0.909085943	0.978440841	0.999939566	0.99989309	0.941794462
STAM2	0.976588913	0.96412538	0.927460291	0.999939566	0.99989309	0.941925704
LOC112443853	0.976684111	0.952440271	0.976719499	0.999939566	0.99989309	0.936439483
LOC101903682	0.976692234	0.972786652	0.929958748	0.999939566	0.99989309	0.92920113
FBXW9	0.976692234	0.90249621	0.987639174	0.999939566	0.99989309	0.943685681
ACOX2	0.976692234	0.937978608	0.92153058	0.999939566	0.99989309	0.946314386
SRP9	0.976692234	0.927298992	0.963186571	0.999939566	0.99989309	0.94787004
MACO1	0.976692234	0.89548617	0.953045982	0.999939566	0.99989309	0.957877422
MAGOHB	0.976692234	0.957071555	0.939683463	0.999939566	0.99989309	0.968245211
USP25	0.976699614	0.996395607	0.942642657	0.995687815	0.99989309	0.941925704
SMIM5	0.976712449	0.922752283	0.921531707	0.999939566	0.99989309	0.967851966
ZFHX4	0.976813603	0.934248552	0.951742137	0.999939566	0.99989309	0.926335372
LOC104972830	0.976813603	0.967515137	0.926613927	0.999939566	0.99989309	0.937218904
GPAT4	0.976813603	0.936099262	0.922147089	0.999939566	0.99989309	0.941925704
LOC112449111	0.976813603	0.93453191	0.929303679	0.999939566	0.99989309	0.941925704
LOC104973145	0.976813603	0.929705559	0.944485232	0.999939566	0.99989309	0.946302385
LOC788634	0.976813603	0.9136091	0.932923087	0.99879985	0.99989309	0.986389023
DHX32	0.976846153	0.94883251	0.927455484	0.999939566	0.99989309	0.925449698
EPG5	0.976846153	0.994365533	0.929958748	0.999939566	0.99989309	0.930707319
LOC112443214	0.976846153	0.899142992	0.927460291	0.999939566	0.99989309	0.935607719
LOC112449560	0.976846153	0.982614603	0.958420472	0.999939566	0.99989309	0.937488554
RFESD	0.976846153	0.941117166	0.926613927	0.999939566	0.99989309	0.94588513
CCDC190	0.977038963	0.944314627	0.945553946	0.999939566	0.99989309	0.95470473
LOC112447322	0.977038963	0.907649596	0.982223562	0.99879985	0.99989309	0.968245211
VAV3	0.977038963	0.903657308	0.939683463	0.999939566	0.99989309	0.983525373
HBA1	0.97707002	0.897327046	0.957379098	0.999939566	0.99989309	0.968245211
RARB	0.977242614	0.955798423	0.933102499	0.999939566	0.99989309	0.956610132
NEDD1	0.977432355	0.999368101	0.937363055	0.999939566	0.99989309	0.921582976
MAP1LC3B	0.977432355	0.909573683	0.962790365	0.999939566	0.99989309	0.931976782

RNF216	0.977432355	0.920818159	0.971104162	0.999939566	0.99989309	0.944606747
LGALS9	0.977432355	0.986851582	0.929303679	0.999939566	0.99989309	0.955817715
LOC104971030	0.977432355	0.902136721	0.98730572	0.999939566	0.99989309	0.956610132
TMEM237	0.977432355	0.916894642	0.959159138	0.995880311	0.99989309	0.981164039
FAM229A	0.977432355	0.901922021	0.93050447	0.999939566	0.99989309	0.981164039
PAPOLG	0.977435337	0.982963758	0.941593855	0.998542029	0.99989309	0.920942379
SMCR8	0.977435337	0.899387334	0.929958748	0.999939566	0.99989309	0.926872824
ANGEL1	0.977435337	0.96471209	0.970904484	0.999939566	0.99989309	0.926872824
STAG1	0.977435337	0.994377463	0.922147089	0.998542029	0.99989309	0.928442671
LOC101903056	0.977435337	0.907878616	0.990057983	0.999939566	0.99989309	0.930707319
BBS4	0.977435337	0.944605763	0.93145152	0.999939566	0.99989309	0.932613047
KIN	0.977435337	0.947158785	0.965712499	0.995800463	0.99989309	0.934775196
SMPD1	0.977435337	0.959119521	0.976974632	0.99879985	0.99989309	0.935607719
ANKRD49	0.977435337	0.944420015	0.931265798	0.999939566	0.99989309	0.936563391
ACADM	0.977435337	0.906745647	0.940785296	0.999939566	0.99989309	0.941445869
LOC101908111	0.977435337	0.923186077	0.994425789	0.998542029	0.99989309	0.944606747
TUBA8	0.977435337	0.926435857	0.985552223	0.995800463	0.99989309	0.94588513
BMP4	0.977435337	0.914162656	0.979231344	0.999939566	0.99989309	0.950981777
BTBD1	0.977435337	0.952464938	0.957802423	0.999939566	0.99989309	0.951454721
ZKSCAN7	0.977435337	0.950614847	0.947268948	0.999939566	0.99989309	0.95342487
TFPI2	0.977435337	0.923729244	0.959822136	0.999939566	0.99989309	0.953940638
ZNF404	0.977435337	0.962934461	0.937270184	0.999939566	0.99989309	0.95470473
LOC112441807	0.977435337	0.914616699	0.93145152	0.999939566	0.99989309	0.956610132
RIPOR1	0.977435337	0.920610511	0.965750046	0.999939566	0.99989309	0.956610132
MDN1	0.977435337	0.908457499	0.939683463	0.99879985	0.99989309	0.957877422
TTC39A	0.977435337	0.965239369	0.939683463	0.999939566	0.99989309	0.958545695
PRIMA1	0.977435337	0.927298992	0.939683463	0.999939566	0.99989309	0.959256161
CHD6	0.977435337	0.921280256	0.946659074	0.999939566	0.99989309	0.960712754
EXOC5	0.977435337	0.941288692	0.945417678	0.999939566	0.99989309	0.961506391
ANKRD33	0.977435337	0.972786652	0.953198168	0.99879985	0.99989309	0.964143145
MYSM1	0.977435337	0.911200661	0.978892556	0.999939566	0.99989309	0.981578785
LOC615223	0.977435337	0.911279338	0.979635589	0.998542029	0.99989309	0.983453112
SPAG16	0.977454617	0.921280256	0.939683463	0.999939566	0.99989309	0.932583856
LOC515333	0.977489551	0.920347742	0.939091808	0.999939566	0.99989309	0.926872824
SAMM50	0.977489551	0.898616436	0.926613927	0.999939566	0.99989309	0.95470473
ARL16	0.977533842	0.993887397	0.927079582	0.999939566	0.99989309	0.925449698
BIRC6	0.977533842	0.974073092	0.925442336	0.99879985	0.99989309	0.926534325
BCL10	0.977533842	0.905977412	0.958420472	0.999939566	0.99989309	0.926872824

LOC101903586	0.977533842	0.932065148	0.927460291	0.999939566	0.99989309	0.927529392
NDUFV3	0.977533842	0.915717447	0.929303679	0.999939566	0.99989309	0.927901387
LOC101907132	0.977533842	0.953372138	0.987419404	0.999939566	0.99989309	0.928123265
AP5M1	0.977533842	0.966921779	0.929843151	0.999334425	0.99989309	0.928799366
SHISA6	0.977533842	0.91994024	0.990107362	0.999939566	0.99989309	0.929537775
RAVER1	0.977533842	0.921280256	0.942004125	0.999939566	0.99989309	0.929582058
HES1	0.977533842	0.974591667	0.959575405	0.99879985	0.99989309	0.930459544
C23H6orf106	0.977533842	0.948562569	0.925587088	0.999939566	0.99989309	0.930707319
NVL	0.977533842	0.928601644	0.927460291	0.999939566	0.99989309	0.932014575
PDLIM4	0.977533842	0.90249621	0.933963997	0.999939566	0.99989309	0.934653208
FAM110A	0.977533842	0.965881793	0.947268948	0.99879985	0.99989309	0.934775196
LOC112442636	0.977533842	0.905432526	0.932340777	0.999939566	0.99989309	0.934775196
HEXB	0.977533842	0.920172204	0.963643451	0.999939566	0.99989309	0.935655141
BET1L	0.977533842	0.970179915	0.967676552	0.999939566	0.99989309	0.935655141
ZFYVE16	0.977533842	0.934248552	0.941593855	0.999334425	0.99989309	0.93691717
LOC101905188	0.977533842	0.915913147	0.978879234	0.999939566	0.99989309	0.937591162
AJM1	0.977533842	0.935793939	0.933963997	0.999939566	0.99989309	0.939507593
C20H5orf51	0.977533842	0.903820329	0.962209895	0.999939566	0.99989309	0.939507593
FAM102A	0.977533842	0.922767609	0.941888866	0.999939566	0.99989309	0.939966658
LOC512175	0.977533842	0.921046401	0.944592818	0.999939566	0.99989309	0.939966658
LOC112441493	0.977533842	0.912292519	0.933963997	0.999939566	0.99989309	0.940683751
PAFAH1B1	0.977533842	0.903820329	0.973488065	0.999939566	0.99989309	0.940683751
AKAP8	0.977533842	0.963478319	0.944764946	0.998542029	0.99989309	0.940917403
PYGO1	0.977533842	0.908857456	0.945124666	0.999939566	0.99989309	0.940917403
BAZ2A	0.977533842	0.912112179	0.939683463	0.99879985	0.99989309	0.941925704
SVIP	0.977533842	0.990914937	0.939683463	0.999939566	0.99989309	0.941925704
CLCN3	0.977533842	0.918371039	0.967489175	0.999939566	0.99989309	0.942041594
PLAC9	0.977533842	0.911347642	0.931173336	0.999939566	0.99989309	0.942421464
LMO7	0.977533842	0.965027449	0.937270184	0.999939566	0.99989309	0.942421464
TUBGCP4	0.977533842	0.906025962	0.957578315	0.99879985	0.99989309	0.94313171
LOC112441655	0.977533842	0.947959005	0.961899356	0.999939566	0.99989309	0.943138585
TICAM1	0.977533842	0.955798423	0.939683463	0.999939566	0.99989309	0.95342487
ZNF445	0.977533842	0.907878616	0.958420472	0.999939566	0.99989309	0.953940638
EXOSC10	0.977533842	0.920818159	0.929605682	0.999939566	0.99989309	0.954596759
LOC112446406	0.977533842	0.912112179	0.926613927	0.995800463	0.99989309	0.95460851
FCER1G	0.977533842	0.974986418	0.926613927	0.99879985	0.99989309	0.95470473
DOCK1	0.977533842	0.942236132	0.926613927	0.999939566	0.99989309	0.95470473
SPEF2	0.977533842	0.939027995	0.93050447	0.999939566	0.99989309	0.95470473

KCNJ11	0.977533842	0.919224637	0.966157152	0.999939566	0.99989309	0.955649834
RASA2	0.977533842	0.956843573	0.925587088	0.99879985	0.99989309	0.957122667
LOC782673	0.977533842	0.903216497	0.994425789	0.998542029	0.99989309	0.957877422
SARNP	0.977533842	0.901922021	0.991406769	0.999939566	0.99989309	0.957877422
PNPLA6	0.977533842	0.901781061	0.970904484	0.999939566	0.99989309	0.958540316
NECTIN3	0.977533842	0.942236132	0.931173336	0.99879985	0.99989309	0.961465235
KAT6B	0.977533842	0.942048064	0.927460291	0.99879985	0.99989309	0.961506391
C1QB	0.977533842	0.899387334	0.929605682	0.999939566	0.99989309	0.961506391
SHQ1	0.977533842	0.952464938	0.953045982	0.999939566	0.99989309	0.961506391
LOC100336644	0.977533842	0.899917012	0.957379098	0.999939566	0.99989309	0.963996128
CHAF1A	0.977533842	0.897918739	0.929303679	0.999939566	0.99989309	0.964143145
OTOP1	0.977533842	0.913075885	0.953045982	0.995946943	0.99989309	0.965332394
RAB3D	0.977533842	0.911347642	0.924476109	0.999939566	0.99989309	0.968245211
CMIP	0.977533842	0.926435857	0.933629769	0.999939566	0.99989309	0.968245211
CHD7	0.977533842	0.96104457	0.925587088	0.999939566	0.99989309	0.975556309
FRZB	0.977533842	0.900467607	0.929958748	0.999939566	0.99989309	0.9759685
PANK4	0.977533842	0.970586744	0.926613927	0.99879985	0.99989309	0.977961448
CASC1	0.977533842	0.911279338	0.958420472	0.999939566	0.99989309	0.978924127
GSK3A	0.977533842	0.91557476	0.929958748	0.999939566	0.99989309	0.979810204
DENND1B	0.977533842	0.924799142	0.952255572	0.997612654	0.99989309	0.980144374
MYBPHL	0.977533842	0.90249621	0.939683463	0.999939566	0.99989309	0.98016619
USP8	0.977533842	0.937978608	0.95206243	0.999939566	0.99989309	0.983453112
MTMR11	0.977533842	0.913171043	0.966157152	0.996084773	0.99989309	0.986396639
ZNF283	0.977533842	0.89539921	0.976161819	0.99879985	0.99989309	0.991281092
GMNN	0.977533842	0.93453191	0.930892662	0.99879985	0.99989309	0.994392175
LOC615112	0.977533842	0.941635802	0.931397502	0.99879985	0.99989309	0.995063891
LOC107132942	0.977533842	0.904077835	0.944638172	0.999939566	0.99989309	0.99797558
LETMD1	0.977555322	0.942036047	0.944612298	0.999939566	0.99989309	0.928799366
ZBED1	0.977555322	0.913171043	0.943891967	0.999939566	0.99989309	0.93898607
SOD3	0.977634507	0.924799142	0.979231344	0.99879985	0.99989309	0.926872824
ZNF746	0.977751265	0.933036383	0.929958748	0.999939566	0.99989309	0.933409694
RAPGEF2	0.977809832	0.956958713	0.948562003	0.999939566	0.99989309	0.935655141
SLC36A1	0.977809832	0.963478319	0.939683463	0.999939566	0.99989309	0.940683751
LOC104976078	0.977809832	0.923729244	0.978832388	0.999939566	0.99989309	0.941889677
CNOT9	0.977809832	0.960454429	0.942761121	0.999939566	0.99989309	0.951216293
LOC509184	0.977809832	0.9136091	0.931397502	0.999939566	0.99989309	0.972487907
FBXO22	0.977809832	0.934248552	0.958420472	0.999939566	0.99989309	0.983453112
CRACR2A	0.977832848	0.942882453	0.939683463	0.999939566	0.99989309	0.944635363

TRIM32	0.977865023	0.963478319	0.951006905	0.99879985	0.99989309	0.924845138
WNT10B	0.977865023	0.913075885	0.944460278	0.999939566	0.99989309	0.924984776
LOC100847120	0.977865023	0.994511934	0.926613927	0.999939566	0.99989309	0.926872824
ISG20L2	0.977865023	0.926435857	0.933102499	0.999939566	0.99989309	0.926872824
TMEM243	0.977865023	0.921280256	0.941873695	0.999939566	0.99989309	0.926872824
CCDC151	0.977865023	0.938109437	0.943420215	0.999939566	0.99989309	0.926872824
ASH2L	0.977865023	0.967758952	0.94991125	0.999939566	0.99989309	0.926872824
RNF111	0.977865023	0.936134921	0.958420472	0.999939566	0.99989309	0.926872824
METTL24	0.977865023	0.89618217	0.962268274	0.999939566	0.99989309	0.926872824
SLC38A10	0.977865023	0.922434012	0.939683463	0.999939566	0.99989309	0.92689297
ADAR	0.977865023	0.919351156	0.982680848	0.999939566	0.99989309	0.927412916
PSMG4	0.977865023	0.906409999	0.939683463	0.999939566	0.99989309	0.927901387
CNOT6	0.977865023	0.91458438	0.957802423	0.999939566	0.99989309	0.928374551
CGNL1	0.977865023	0.939423963	0.958420472	0.999939566	0.99989309	0.928442671
LOC783920	0.977865023	0.944605763	0.939091808	0.999939566	0.99989309	0.928799366
BRD7	0.977865023	0.899474896	0.941312064	0.999939566	0.99989309	0.929183819
PPP1R1B	0.977865023	0.911196851	0.953045982	0.999939566	0.99989309	0.929582058
HARS2	0.977865023	0.908178836	0.963762816	0.999939566	0.99989309	0.929636267
STK32A	0.977865023	0.952027829	0.950801642	0.999939566	0.99989309	0.930058266
RPS25	0.977865023	0.921046401	0.97420715	0.999939566	0.99989309	0.930707319
ZZEF1	0.977865023	0.94883251	0.93050447	0.999939566	0.99989309	0.931125957
RNF215	0.977865023	0.93453191	0.959198396	0.999939566	0.99989309	0.931495481
PODXL	0.977865023	0.957071555	0.951850716	0.999939566	0.99989309	0.931886764
SNRPB2	0.977865023	0.907878616	0.997081057	0.999939566	0.99989309	0.932250711
UBE3D	0.977865023	0.99950308	0.939683463	0.99879985	0.99989309	0.932613047
GPKOW	0.977865023	0.94610099	0.943501903	0.999939566	0.99989309	0.932613047
LOC104969238	0.977865023	0.939870925	0.965169397	0.999939566	0.99989309	0.933316009
PPM1D	0.977865023	0.942236132	0.939091808	0.999939566	0.99989309	0.933577604
ARL6IP6	0.977865023	0.968123234	0.937363055	0.996601241	0.99989309	0.933678522
CAMK2N2	0.977865023	0.984479682	0.959198396	0.999939566	0.99989309	0.933678522
LOC100848407	0.977865023	0.922434012	0.937270184	0.999939566	0.99989309	0.934458012
LOC781108	0.977865023	0.922434012	0.967873529	0.999939566	0.99989309	0.934578283
ACBD5	0.977865023	0.958621801	0.93710335	0.999939566	0.99989309	0.934653208
TRPM3	0.977865023	0.914616699	0.947268948	0.999939566	0.99989309	0.934653208
LOC101904573	0.977865023	0.948631073	0.979231344	0.999939566	0.99989309	0.934653208
MAPRE3	0.977865023	0.907670026	0.979231344	0.999939566	0.99989309	0.934775196
TXLNB	0.977865023	0.999560732	0.939091808	0.996601241	0.99989309	0.935154223
THOC2	0.977865023	0.926435857	0.959198396	0.99879985	0.99989309	0.9352241

SRMS	0.977865023	0.929536223	0.990965255	0.999939566	0.99989309	0.935655141
FUCA2	0.977865023	0.918851896	0.939683463	0.999939566	0.99989309	0.935937948
TSPAN15	0.977865023	0.935324849	0.958420472	0.999939566	0.99989309	0.935937948
SLC9A7	0.977865023	0.926435857	0.932104138	0.999939566	0.99989309	0.936439483
LOC104968634	0.977865023	0.964014612	0.960137022	0.999939566	0.99989309	0.936690389
GBX1	0.977865023	0.920347742	0.970904484	0.999939566	0.99989309	0.93691717
LDAH	0.977865023	0.957353974	0.926613927	0.999939566	0.99989309	0.937218904
LOC101903126	0.977865023	0.978453686	0.976719499	0.998542029	0.99989309	0.937591162
FRMPD4	0.977865023	0.978473197	0.957417441	0.999939566	0.99989309	0.937591162
SRD5A3	0.977865023	0.931073006	0.962209895	0.999939566	0.99989309	0.937591162
PHF12	0.977865023	0.9347557	0.93050447	0.999939566	0.99989309	0.937727112
LOC112441603	0.977865023	0.939027995	0.939683463	0.999939566	0.99989309	0.938054315
SPN	0.977865023	0.944755173	0.958420472	0.999939566	0.99989309	0.938054315
FGD1	0.977865023	0.929705559	0.985016728	0.999939566	0.99989309	0.939966658
CCDC96	0.977865023	0.930469765	0.9861363	0.99879985	0.99989309	0.940683751
LOC112445001	0.977865023	0.913171043	0.957379098	0.999939566	0.99989309	0.940683751
C5H12orf56	0.977865023	0.926435857	0.994425789	0.999939566	0.99989309	0.940683751
PHTF2	0.977865023	0.912112179	0.951092876	0.999939566	0.99989309	0.94091418
SGTB	0.977865023	0.964516367	0.942761121	0.999939566	0.99989309	0.940917403
LOC614402	0.977865023	0.962123724	0.974832809	0.999939566	0.99989309	0.940917403
RAB30	0.977865023	0.90339131	0.929605682	0.999939566	0.99989309	0.941889677
LOC101904757	0.977865023	0.967515137	0.958420472	0.999939566	0.99989309	0.941889677
CLIC3	0.977865023	0.959119521	0.963422261	0.998542029	0.99989309	0.941925704
TGS1	0.977865023	0.911196851	0.978215635	0.999939566	0.99989309	0.941925704
ONECUT2	0.977865023	0.982963758	0.927460291	0.999939566	0.99989309	0.942421464
KLHL26	0.977865023	0.907670026	0.93050447	0.999939566	0.99989309	0.942421464
PHF24	0.977865023	0.934248552	0.939683463	0.999939566	0.99989309	0.942421464
LOC100848995	0.977865023	0.94544531	0.962209895	0.999939566	0.99989309	0.942421464
LRP12	0.977865023	0.911106863	0.967413848	0.999939566	0.99989309	0.942685052
LOC790098	0.977865023	0.921280256	0.939091808	0.999939566	0.99989309	0.942800958
BBIP1	0.977865023	0.980431776	0.959159138	0.99879985	0.99989309	0.943138585
LOC112441602	0.977865023	0.978309093	0.929958748	0.999939566	0.99989309	0.943138585
LOC101902430	0.977865023	0.908453308	0.933963997	0.999939566	0.99989309	0.943138585
LOC112442245	0.977865023	0.941544643	0.939683463	0.999939566	0.99989309	0.943138585
PIP4P2	0.977865023	0.928315012	0.970218925	0.999939566	0.99989309	0.943138585
CAPN5	0.977865023	0.972284293	0.933629769	0.999939566	0.99989309	0.943489123
C29H11orf54	0.977865023	0.915717447	0.97368009	0.999939566	0.99989309	0.943777356
LRRC27	0.977865023	0.944420015	0.965169397	0.999939566	0.99989309	0.94425343

GATB	0.977865023	0.962934461	0.965416289	0.999939566	0.99989309	0.944596144
TXNRD1	0.977865023	0.95751653	0.985222638	0.99879985	0.99989309	0.944606747
FAM83H	0.977865023	0.926435857	0.939683463	0.999939566	0.99989309	0.945759045
FBXO5	0.977865023	0.941288692	0.965539389	0.999939566	0.99989309	0.94588513
GYG2	0.977865023	0.962934461	0.937270184	0.999939566	0.99989309	0.946344585
ZDHHC15	0.977865023	0.918306418	0.927460291	0.999939566	0.99989309	0.946717155
LOC574091	0.977865023	0.988960148	0.926018972	0.999334425	0.99989309	0.948816455
ZNF783	0.977865023	0.912112179	0.931173336	0.999939566	0.99989309	0.949206487
LOC104973760	0.977865023	0.931571093	0.976719499	0.999939566	0.99989309	0.949206487
TRIP6	0.977865023	0.955798423	0.937627891	0.999939566	0.99989309	0.952801738
IST1	0.977865023	0.945105738	0.930892662	0.999939566	0.99989309	0.95342487
HTRA4	0.977865023	0.898000467	0.933963997	0.999939566	0.99989309	0.954110387
RAB27A	0.977865023	0.978453686	0.930892662	0.999939566	0.99989309	0.95460851
PLIN3	0.977865023	0.95320487	0.933102499	0.999939566	0.99989309	0.95470473
ZNF629	0.977865023	0.94610099	0.951850716	0.999939566	0.99989309	0.95470473
TUBB3	0.977865023	0.90249621	0.962209895	0.999939566	0.99989309	0.95470473
PRKDC	0.977865023	0.901735113	0.976719499	0.999939566	0.99989309	0.95470473
NTSR2	0.977865023	0.981278008	0.961742979	0.99879985	0.99989309	0.954732204
NCK2	0.977865023	0.936134921	0.942004125	0.999939566	0.99989309	0.955585296
CTR9	0.977865023	0.956134433	0.941319062	0.998542029	0.99989309	0.956610132
JUP	0.977865023	0.922434012	0.94454396	0.99879985	0.99989309	0.956610132
RNF157	0.977865023	0.900502778	0.946659074	0.999939566	0.99989309	0.956610132
FAM32A	0.977865023	0.962226361	0.926613927	0.999939566	0.99989309	0.956959747
CDK5RAP3	0.977865023	0.913075885	0.933963997	0.999939566	0.99989309	0.956959747
RN7SL1	0.977865023	0.955798423	0.953198168	0.999939566	0.99989309	0.958219037
XKRX	0.977865023	0.956958713	0.939683463	0.999939566	0.99989309	0.959625205
PCSK5	0.977865023	0.956843573	0.939683463	0.999939566	0.99989309	0.961506391
GPRC5B	0.977865023	0.944194974	0.942004125	0.999939566	0.99989309	0.961506391
DBNDD2	0.977865023	0.913075885	0.958420472	0.999939566	0.99989309	0.961506391
SETD6	0.977865023	0.956751311	0.939683463	0.999939566	0.99989309	0.964143145
LOC101907658	0.977865023	0.900257356	0.962209895	0.999939566	0.99989309	0.964143145
ARHGAP4	0.977865023	0.974591667	0.958420472	0.996775429	0.99989309	0.965053654
LIMA1	0.977865023	0.907691022	0.950801642	0.999939566	0.99989309	0.966375116
TMEM167A	0.977865023	0.926435857	0.966157152	0.999939566	0.99989309	0.967376295
LOC101906477	0.977865023	0.926618064	0.944863023	0.99879985	0.99989309	0.968039618
SCAP	0.977865023	0.921280256	0.929605682	0.99879985	0.99989309	0.968245211
MTUS1	0.977865023	0.926618064	0.942004125	0.999939566	0.99989309	0.968245211
CLDN5	0.977865023	0.957645111	0.957379098	0.999939566	0.99989309	0.968245211

QRSL1	0.977865023	0.936099262	0.931909128	0.998542029	0.99989309	0.970137981
C1QA	0.977865023	0.911279338	0.93145152	0.999939566	0.99989309	0.972487907
LOC112444770	0.977865023	0.967113913	0.929303679	0.999939566	0.99989309	0.97345607
LOC107132486	0.977865023	0.926619092	0.944160795	0.999939566	0.99989309	0.97792865
GSPT2	0.977865023	0.912112179	0.938306386	0.999939566	0.99989309	0.979233909
ELMOD3	0.977865023	0.924799142	0.971472903	0.999939566	0.99989309	0.979792753
LOC783988	0.977865023	0.922434012	0.951742137	0.999939566	0.99989309	0.980144374
LOC101906855	0.977865023	0.911106863	0.963643451	0.998542029	0.99989309	0.98016619
LOC101904270	0.977865023	0.930890453	0.965037144	0.999060254	0.99989309	0.98016619
HDGF	0.977865023	0.949625762	0.927722996	0.999939566	0.99989309	0.980179583
LOC112446390	0.977865023	0.959119521	0.939091808	0.999939566	0.99989309	0.981164039
LOC112445194	0.977865023	0.926435857	0.948789172	0.999939566	0.99989309	0.981578785
ASB3	0.977865023	0.934531829	0.950801642	0.999939566	0.99989309	0.982045172
RAMMET	0.977865023	0.936134921	0.941593855	0.999939566	0.99989309	0.982066213
CABYR	0.977865023	0.909731475	0.953822515	0.999939566	0.99989309	0.983427394
MIOS	0.977865023	0.9136091	0.97368009	0.999939566	0.99989309	0.983427394
GPAM	0.977865023	0.936951369	0.939683463	0.998542029	0.99989309	0.983453112
LOC617224	0.977865023	0.899387334	0.926613927	0.99879985	0.99989309	0.983453112
LOC101906730	0.977865023	0.949250535	0.951742137	0.999939566	0.99989309	0.983453112
DNAJB5	0.977865023	0.944780259	0.966157152	0.998542029	0.99989309	0.984491523
NARS2	0.977865023	0.960378334	0.937544913	0.99879985	0.99989309	0.989128955
B3GALT6	0.977865023	0.907691022	0.972104746	0.996775429	0.99989309	0.991281092
BMP7	0.977865023	0.897327046	0.925852918	0.999939566	0.99989309	0.991281092
LOC100847613	0.977865023	0.966331409	0.926372704	0.999939566	0.99989309	0.991281092
RUNX2	0.977865023	0.950928726	0.941319062	0.996118855	0.99989309	0.993612847
ARF6	0.977865023	0.924510975	0.945188764	0.998542029	0.99989309	0.993612847
SPATA6L	0.977865023	0.930469765	0.929958748	0.998542029	0.99989309	0.995145616
HIST1H1E	0.977865023	0.936134921	0.927079582	0.999939566	0.99989309	0.998074562
ZCCHC2	0.977865023	0.899387334	0.939091808	0.999939566	0.99989309	0.998074562
P3H2	0.977865023	0.897657501	0.930710426	0.999939566	0.99989309	0.998928226
CSNK1E	0.977865023	0.908192144	0.925442336	0.998542029	0.99989309	0.999973052
TTC36	0.978025526	0.955798423	0.929303679	0.999939566	0.99989309	0.931515831
NKAIN2	0.978025526	0.916239838	0.990493758	0.99879985	0.99989309	0.95342487
UPF1	0.978025526	0.916505879	0.941593855	0.999939566	0.99989309	0.957877422
STXBP6	0.978025526	0.921280256	0.963643451	0.999939566	0.99989309	0.970077808
KIF24	0.978045392	0.910596218	0.983706867	0.99879985	0.99989309	0.961506391
MCCD1	0.978071707	0.966852427	0.957379098	0.99879985	0.99989309	0.937591162
PDSS2	0.978121664	0.942184053	0.965518535	0.999939566	0.99989309	0.939966658

TXNDC11	0.978121664	0.955798423	0.929958748	0.999939566	0.99989309	0.943138585
DLL1	0.97825529	0.970167557	0.965819637	0.999939566	0.99989309	0.926872824
ZEB1	0.97825529	0.926435857	0.950801642	0.999939566	0.99989309	0.957213184
TST	0.978268	0.954015268	0.939683463	0.998542029	0.99989309	0.926872824
FKBP8	0.978268	0.91994024	0.944612298	0.999939566	0.99989309	0.929374767
NALCN	0.978268	0.90249621	0.989705268	0.999939566	0.99989309	0.930528039
LOC101908204	0.978268	0.992027017	0.929958748	0.999939566	0.99989309	0.934653208
TOR3A	0.978268	0.950091107	0.941312064	0.999939566	0.99989309	0.935595867
MCTP1	0.978268	0.994365533	0.95478223	0.999939566	0.99989309	0.935607719
S100A4	0.978268	0.90249621	0.979231344	0.999939566	0.99989309	0.936563391
FOXL1	0.978268	0.90249621	0.929605682	0.999939566	0.99989309	0.93691717
LOC104976281	0.978268	0.994299253	0.926613927	0.99879985	0.99989309	0.937662242
UBE2I	0.978268	0.94610099	0.990949901	0.998542029	0.99989309	0.938643464
LOC782954	0.978268	0.993223749	0.933629769	0.999939566	0.99989309	0.939080935
B3GALNT2	0.978268	0.944755173	0.926613927	0.999939566	0.99989309	0.940683751
DDX28	0.978268	0.957902362	0.958420472	0.999939566	0.99989309	0.941889677
GRK6	0.978268	0.964013919	0.93000135	0.999939566	0.99989309	0.941925704
C15H11orf94	0.978268	0.933412624	0.929958748	0.999939566	0.99989309	0.943138585
GLA	0.978268	0.921280256	0.963643451	0.999939566	0.99989309	0.943138585
LMOD3	0.978268	0.908586813	0.939683463	0.999939566	0.99989309	0.943489123
LOC112443452	0.978268	0.963478319	0.939683463	0.999939566	0.99989309	0.956959747
LOC112447845	0.978268	0.967758952	0.939683463	0.999939566	0.99989309	0.957140928
CDC42BPG	0.978268	0.902136721	0.967983981	0.999939566	0.99989309	0.961465235
LOC112442721	0.978268	0.906538642	0.977612552	0.99879985	0.99989309	0.961506391
FAM83F	0.978268	0.989736861	0.928099904	0.99879985	0.99989309	0.964143145
LOC112446793	0.978268	0.91557476	0.959377939	0.99879985	0.99989309	0.968039618
SSFA2	0.978268	0.956751311	0.929303679	0.999939566	0.99989309	0.96826636
LOC112442246	0.978268	0.934248552	0.931397502	0.999939566	0.99989309	0.96826636
SPCS2	0.978268	0.900335526	0.991406769	0.999939566	0.99989309	0.972487907
PCDHB11	0.978268	0.935324849	0.954313013	0.999939566	0.99989309	0.974017283
OGDH	0.978268	0.90760125	0.933116459	0.999939566	0.99989309	0.98016619
KLHL30	0.978268	0.907594268	0.97368009	0.99879985	0.99989309	0.985517088
MPEG1	0.978268	0.920818159	0.929958748	0.998542029	0.99989309	0.989947227
LOC112443422	0.978268	0.907691022	0.935247964	0.999939566	0.99989309	0.991612651
SDHAF3	0.978268	0.922234794	0.929958748	0.99879985	0.99989309	0.998928226
AIG1	0.978492448	0.928119925	0.939683463	0.999939566	0.99989309	0.948277018
L1CAM	0.978492448	0.942048064	0.962209895	0.999939566	0.99989309	0.965358544
LOC112442040	0.978492448	0.920818159	0.957802423	0.999939566	0.99989309	0.983453112

RNF39	0.978650854	0.911106863	0.993747807	0.999939566	0.99989309	0.934458012
AGL	0.978650854	0.9347557	0.96993432	0.99879985	0.99989309	0.956610132
NUDC	0.978769031	0.926435857	0.976575956	0.999939566	0.99989309	0.927584908
LOC101907514	0.978769031	0.974348229	0.974158329	0.99879985	0.99989309	0.928123265
LOC112442278	0.978769031	0.977728722	0.939683463	0.999939566	0.99989309	0.930318133
LOC101907140	0.978769031	0.988649234	0.961742979	0.999939566	0.99989309	0.930318133
HNRNPUL1	0.978769031	0.938064973	0.957501184	0.999939566	0.99989309	0.930707319
OTUB2	0.978769031	0.913075885	0.962209895	0.999939566	0.99989309	0.930774946
ABTB2	0.978769031	0.923186077	0.939683463	0.999939566	0.99989309	0.931901179
B3GALT2	0.978769031	0.926435857	0.952333346	0.999939566	0.99989309	0.933678522
MAEA	0.978769031	0.922434012	0.928099904	0.999939566	0.99989309	0.934775196
KLHL36	0.978769031	0.981087742	0.929958748	0.999939566	0.99989309	0.935636428
SNX8	0.978769031	0.903657308	0.942314296	0.999939566	0.99989309	0.935655141
LOC100847876	0.978769031	0.955188246	0.933102499	0.999939566	0.99989309	0.937591162
SFPQ	0.978769031	0.933783911	0.959320266	0.99879985	0.99989309	0.940683751
GALT	0.978769031	0.982051974	0.951850716	0.999939566	0.99989309	0.940683751
ZCWPW1	0.978769031	0.906917416	0.93710335	0.999939566	0.99989309	0.941925704
INO80C	0.978769031	0.959138652	0.976782463	0.99879985	0.99989309	0.943138585
LINGO1	0.978769031	0.924799142	0.947268948	0.999939566	0.99989309	0.943138585
PAX8	0.978769031	0.99950308	0.93145152	0.99879985	0.99989309	0.946314386
CTSL	0.978769031	0.995289146	0.929958748	0.99879985	0.99989309	0.953940638
CRHR2	0.978769031	0.96471209	0.939683463	0.999939566	0.99989309	0.95470473
B3GNT9	0.978769031	0.936134921	0.95461277	0.999939566	0.99989309	0.95470473
C16H1orf116	0.978769031	0.94610099	0.939683463	0.999939566	0.99989309	0.957877422
HCST	0.978769031	0.972418749	0.939683463	0.999939566	0.99989309	0.957877422
B9D1	0.978769031	0.962934461	0.953692935	0.999939566	0.99989309	0.957877422
LOC112447140	0.978769031	0.939681897	0.941593855	0.999939566	0.99989309	0.968039618
NME6	0.978769031	0.935324849	0.940785296	0.99879985	0.99989309	0.968059826
TNKS1BP1	0.978769031	0.918306418	0.957379098	0.999939566	0.99989309	0.968245211
LZTFL1	0.978769031	0.974591667	0.939683463	0.99879985	0.99989309	0.970702553
ADCY8	0.978769031	0.9347557	0.941593855	0.999939566	0.99989309	0.97792865
FUOM	0.978769031	0.914616699	0.958420472	0.999939566	0.99989309	0.979233909
TRMT61B	0.978769031	0.921280256	0.972485306	0.99879985	0.99989309	0.979606084
LOC101903988	0.978769031	0.903820329	0.939683463	0.999939566	0.99989309	0.98016619
RASGEF1A	0.978769031	0.949008068	0.944976702	0.999939566	0.99989309	0.98016619
TXNL4B	0.978769031	0.937904802	0.929958748	0.999939566	0.99989309	0.982066213
LRIG2	0.978769031	0.963089817	0.939683463	0.999939566	0.99989309	0.989313458
EXOG	0.978769031	0.955798423	0.933102499	0.99879985	0.99989309	0.991281092

SCFD2	0.978769031	0.920967949	0.929958748	0.999939566	0.99989309	0.993612847
CBFA2T3	0.978769031	0.914735022	0.959705869	0.99879985	0.99989309	0.996493376
HOXC6	0.978769031	0.925807756	0.93145152	0.999939566	0.99989309	0.998636142
LOC101903928	0.978811516	0.949625762	0.93050447	0.999939566	0.99989309	0.967045036
LOC100336448	0.979120707	0.922434012	0.951742137	0.998542029	0.99989309	0.940917403
COL23A1	0.979120707	0.938064973	0.953045982	0.999939566	0.99989309	0.954334316
FRMD8	0.979132511	0.913075885	0.939683463	0.99879985	0.99989309	0.986716086
SFRP4	0.97918697	0.959138652	0.976719499	0.999939566	0.99989309	0.938643464
POP7	0.979234726	0.913171043	0.965258393	0.999939566	0.99989309	0.931515831
LTBR	0.979234726	0.934531829	0.929958748	0.999939566	0.99989309	0.993612847
HMGN3	0.979284307	0.968440623	0.939683463	0.999939566	0.99989309	0.930774946
ZNF132	0.979284307	0.983435595	0.979096894	0.99879985	0.99989309	0.934653208
KLHL5	0.979284307	0.942048064	0.939683463	0.999939566	0.99989309	0.953940638
VPS72	0.979284307	0.9136091	0.957578315	0.999939566	0.99989309	0.954334316
LOC614882	0.979439378	0.913075885	0.950801642	0.999939566	0.99989309	0.94425343
STK10	0.979439378	0.907670026	0.939683463	0.999939566	0.99989309	0.958540316
RCN2	0.979439378	0.90339131	0.944060947	0.999939566	0.99989309	0.973338476
BAIAP2	0.979439378	0.933783911	0.939683463	0.999939566	0.99989309	0.974379635
ZNF333	0.979439378	0.913171043	0.958420472	0.999939566	0.99989309	0.983453112
RPH3AL	0.97946062	0.963480908	0.945417678	0.999939566	0.99989309	0.95470473
TLDC1	0.979650742	0.91458438	0.994425789	0.99879985	0.99989309	0.937591162
BEX2	0.979650742	0.915863788	0.972909109	0.999939566	0.99989309	0.955585296
RAB11FIP1	0.979650742	0.947806191	0.939683463	0.999939566	0.99989309	0.957877422
PTPN23	0.979650742	0.920347742	0.974698466	0.999939566	0.99989309	0.958814003
OSBP	0.979650742	0.90249621	0.950957834	0.999939566	0.99989309	0.962118346
SLC22A5	0.979650742	0.920347742	0.939091808	0.999939566	0.99989309	0.971025918
CRY2	0.979650742	0.921046401	0.939683463	0.99879985	0.99989309	0.976757738
CENPV	0.979650742	0.972418749	0.929958748	0.999939566	0.99989309	0.981578785
C5H12orf57	0.979650742	0.929573947	0.953045982	0.999939566	0.99989309	0.991281092
LOC101906676	0.979736753	0.910540517	0.93710335	0.999939566	0.99989309	0.941925704
LOC100847554	0.980029483	0.959138652	0.933963997	0.999939566	0.99989309	0.933763401
CALY	0.980029483	0.938910419	0.976719499	0.999939566	0.99989309	0.962255684
WDR82	0.980029483	0.916172682	0.965750046	0.99879985	0.99989309	0.968245211
LRCH2	0.980081283	0.972786652	0.965568718	0.999939566	0.99989309	0.943138585
LIMK1	0.980081283	0.959873186	0.937979342	0.999939566	0.99989309	0.97519644
ITGAE	0.980305107	0.936550733	0.958420472	0.999939566	0.99989309	0.930707319
MOCS1	0.980305107	0.912325342	0.939683463	0.999939566	0.99989309	0.933678522
INTS7	0.980305107	0.916725199	0.994425789	0.99879985	0.99989309	0.933763401

LOC112447080	0.980305107	0.926435857	0.951742137	0.999939566	0.99989309	0.934458012
DDX50	0.980305107	0.909085943	0.953788559	0.999939566	0.99989309	0.936439483
YRDC	0.980305107	0.922434012	0.974681091	0.999939566	0.99989309	0.937218904
LOC100847236	0.980305107	0.906765346	0.995184296	0.99879985	0.99989309	0.939966658
DRD2	0.980305107	0.918306418	0.97311093	0.99879985	0.99989309	0.940683751
S1PR2	0.980305107	0.907670026	0.960213582	0.999939566	0.99989309	0.940683751
DOK2	0.980305107	0.967237998	0.973306845	0.999939566	0.99989309	0.940683751
LOC112447340	0.980305107	0.926435857	0.967489175	0.999939566	0.99989309	0.941806801
GAS7	0.980305107	0.945105738	0.942004125	0.999939566	0.99989309	0.941925704
RGP1	0.980305107	0.945105738	0.943891967	0.999939566	0.99989309	0.941925704
HOXA3	0.980305107	0.986245673	0.93145152	0.999939566	0.99989309	0.942421464
PLA2G2D4	0.980305107	0.922434012	0.978832388	0.999939566	0.99989309	0.942421464
SUGP2	0.980305107	0.920347742	0.999323478	0.999939566	0.99989309	0.942421464
AS3MT	0.980305107	0.931381024	0.930710426	0.999939566	0.99989309	0.94425343
LOC112442243	0.980305107	0.996308856	0.939683463	0.99879985	0.99989309	0.95342487
PLP2	0.980305107	0.911152896	0.967489175	0.999939566	0.99989309	0.956410517
EPHB6	0.980305107	0.971839996	0.960137022	0.99879985	0.99989309	0.956610132
COG7	0.980305107	0.905977412	0.968230268	0.999939566	0.99989309	0.957707888
LOC512684	0.980305107	0.913075885	0.995148246	0.99879985	0.99989309	0.961506391
TTF2	0.980305107	0.962934461	0.969390163	0.999939566	0.99989309	0.961506391
ARHGAP42	0.980305107	0.919134026	0.941593855	0.999939566	0.99989309	0.964143145
UNC5B	0.980305107	0.936134921	0.962517983	0.99879985	0.99989309	0.968245211
LOC104969353	0.980305107	0.904764822	0.939683463	0.99879985	0.99989309	0.969981358
PLEKHO1	0.980305107	0.953748191	0.947284323	0.999939566	0.99989309	0.970702553
CD300LF	0.980305107	0.912112179	0.979231344	0.99879985	0.99989309	0.98016619
CHKA	0.980305107	0.94883251	0.939683463	0.99879985	0.99989309	0.984105954
CLEC14A	0.980369693	0.965239369	0.952356493	0.999939566	0.99989309	0.958540316
PICK1	0.980447697	0.969202872	0.958420472	0.99879985	0.99989309	0.942421464
RHOB	0.980447697	0.907691022	0.953198168	0.999939566	0.99989309	0.962668316
LOC784966	0.980465336	0.959119521	0.958977478	0.999939566	0.99989309	0.930669597
COL5A3	0.980465336	0.93453191	0.962209895	0.999939566	0.99989309	0.939800211
AMACR	0.980465336	0.934248552	0.939683463	0.999939566	0.99989309	0.940917403
ETV6	0.980465336	0.979264749	0.929958748	0.999939566	0.99989309	0.942421464
TRMT112	0.980465336	0.938064973	0.967676552	0.999939566	0.99989309	0.942868422
LOC112443849	0.980465336	0.913171043	0.97381056	0.999939566	0.99989309	0.943138585
HSCB	0.980465336	0.922434012	0.939683463	0.999939566	0.99989309	0.95470473
WDR5	0.980465336	0.940502427	0.959705869	0.999939566	0.99989309	0.95908735
ATG16L1	0.980575805	0.934248552	0.994425789	0.999939566	0.99989309	0.945258337

MED13L	0.980575805	0.908709906	0.941593855	0.999939566	0.99989309	0.95470473
DUSP18	0.980575805	0.913075885	0.939683463	0.999939566	0.99989309	0.961506391
ATP23	0.980607372	0.926435857	0.979231344	0.999939566	0.99989309	0.933730876
SNX1	0.980607372	0.942048064	0.968406955	0.999939566	0.99989309	0.935323128
NUPL2	0.980607372	0.904334308	0.933963997	0.999939566	0.99989309	0.936137631
SLIRP	0.980607372	0.905977412	0.962369613	0.999939566	0.99989309	0.937591162
PRDX6	0.980607372	0.911150428	0.941688757	0.999939566	0.99989309	0.937891903
LOC112444752	0.980607372	0.936134921	0.994425789	0.99879985	0.99989309	0.95470473
PPP2R5B	0.980607372	0.970537329	0.942004125	0.999939566	0.99989309	0.961506391
LOC616094	0.980607372	0.939027995	0.965416289	0.999939566	0.99989309	0.961506391
UCK1	0.980607372	0.968237269	0.940785296	0.999939566	0.99989309	0.96826636
PHF2	0.980642805	0.941909203	0.930710426	0.999939566	0.99989309	0.955585296
PLD1	0.980642805	0.94256935	0.967302577	0.999939566	0.99989309	0.957122667
LOC616304	0.980642805	0.982521985	0.968419607	0.99879985	0.99989309	0.958219037
COQ3	0.980695603	0.926618064	0.947391711	0.999939566	0.99989309	0.981411983
RIC3	0.980735499	0.914252497	0.987842204	0.999939566	0.99989309	0.934578283
KL	0.980735499	0.907670026	0.979231344	0.999939566	0.99989309	0.943138585
CERS5	0.980735499	0.913075885	0.966157152	0.999939566	0.99989309	0.958678078
RORB	0.980780209	0.957071555	0.985552223	0.999939566	0.99989309	0.930379536
FYB2	0.980780209	0.949787238	0.9343664	0.999939566	0.99989309	0.930707319
LOC100847861	0.980780209	0.942882453	0.985179619	0.999939566	0.99989309	0.930707319
FSD1L	0.980780209	0.907594268	0.965712499	0.999939566	0.99989309	0.934122809
IFNAR1	0.980780209	0.918976923	0.933102499	0.999939566	0.99989309	0.938553439
FGF9	0.980780209	0.912785459	0.989408632	0.999939566	0.99989309	0.941794462
PCNA	0.980780209	0.974986418	0.939683463	0.999939566	0.99989309	0.941925704
VKORC1	0.980780209	0.904764822	0.96880775	0.999939566	0.99989309	0.941925704
PLEKHA3	0.980780209	0.970971524	0.981567842	0.999939566	0.99989309	0.943138585
LOC101904916	0.980780209	0.911417623	0.985552223	0.999939566	0.99989309	0.943138585
SLC10A6	0.980780209	0.927298992	0.939683463	0.999939566	0.99989309	0.945759045
MYO10	0.980780209	0.962360138	0.965712499	0.999939566	0.99989309	0.949206487
CCDC167	0.980780209	0.956958713	0.95826379	0.999939566	0.99989309	0.950338641
RPLP0	0.980780209	0.913075885	0.984180716	0.999939566	0.99989309	0.95470473
DHRS12	0.980780209	0.944755173	0.941312064	0.999939566	0.99989309	0.955585296
LOC100847802	0.980780209	0.963505854	0.975320606	0.999939566	0.99989309	0.956410517
FANCI	0.980780209	0.924799142	0.948817336	0.999939566	0.99989309	0.957122667
MLH3	0.980780209	0.969145732	0.950484603	0.999939566	0.99989309	0.958522585
ZNF646	0.980780209	0.921280256	0.965712499	0.999939566	0.99989309	0.961454986
SLC43A3	0.980780209	0.914162656	0.951850716	0.999939566	0.99989309	0.961506391

LOC107132490	0.980780209	0.95320487	0.982659943	0.999939566	0.99989309	0.961506391
PLA1A	0.980780209	0.968237269	0.939091808	0.999939566	0.99989309	0.968245211
GPM6B	0.980780209	0.965239369	0.933963997	0.999939566	0.99989309	0.969981358
TMEM87B	0.980780209	0.911106863	0.990107362	0.999939566	0.99989309	0.976188983
FIZ1	0.980780209	0.913171043	0.980442556	0.999939566	0.99989309	0.979233909
TNPO2	0.980780209	0.922770857	0.962209895	0.99879985	0.99989309	0.984408406
LOC101905254	0.980780209	0.920540438	0.939683463	0.999939566	0.99989309	0.991281092
UGDH	0.980878603	0.907878616	0.958571632	0.999939566	0.99989309	0.934458012
LOC104975686	0.980878603	0.934248552	0.962209895	0.999939566	0.99989309	0.936137631
ARHGAP12	0.980878603	0.913625328	0.97420715	0.999939566	0.99989309	0.957249831
CELSR2	0.981022662	0.974986418	0.947660114	0.999939566	0.99989309	0.943012787
SIGLECL1	0.981022662	0.946722291	0.942004125	0.999939566	0.99989309	0.95342487
ZNF777	0.981058032	0.911106863	0.96880775	0.999939566	0.99989309	0.941925704
NCDN	0.981058032	0.959138652	0.939683463	0.999939566	0.99989309	0.978467321
TGM2	0.981126135	0.965239369	0.930892662	0.999939566	0.99989309	0.936439483
LOC100849023	0.981126135	0.955798423	0.957802423	0.999939566	0.99989309	0.937343676
FAXC	0.981126135	0.908192144	0.979096894	0.999939566	0.99989309	0.940475772
RPL31	0.981126135	0.930225567	0.966157152	0.999939566	0.99989309	0.940683751
ADSS	0.981126135	0.921280256	0.947660114	0.99879985	0.99989309	0.940917403
SLC25A41	0.981126135	0.942048064	0.951850716	0.999939566	0.99989309	0.942421464
DOCK11	0.981126135	0.939309165	0.966157152	0.999939566	0.99989309	0.942421464
LOC101907322	0.981126135	0.989736861	0.939091808	0.999939566	0.99989309	0.942455807
LOC107131573	0.981126135	0.953748191	0.981442587	0.999939566	0.99989309	0.945618717
AAR2	0.981126135	0.907594268	0.979231344	0.999939566	0.99989309	0.946314386
GEN1	0.981126135	0.938064973	0.965712499	0.999939566	0.99989309	0.95342487
CTNNBL1	0.981126135	0.921280256	0.951850716	0.999939566	0.99989309	0.95470473
FRAT1	0.981126135	0.918976923	0.965037144	0.999939566	0.99989309	0.954732204
ITGB4	0.981126135	0.962321963	0.962209895	0.999939566	0.99989309	0.955585296
KDM1B	0.981126135	0.941288692	0.943882376	0.999939566	0.99989309	0.957877422
SAP30	0.981126135	0.996120899	0.93050447	0.999939566	0.99989309	0.961506391
EZR	0.981126135	0.927162932	0.939683463	0.999939566	0.99989309	0.968059826
LOC100847357	0.981126135	0.955798423	0.933963997	0.999939566	0.99989309	0.983453112
LOC107132697	0.981126135	0.923981714	0.960398536	0.999939566	0.99989309	0.991281092
ATP2B4	0.981151793	0.957645111	0.962209895	0.999939566	0.99989309	0.931125957
SPATA22	0.981230282	0.947806191	0.957578315	0.999939566	0.99989309	0.937591162
CEND1	0.981230282	0.929730109	0.942004125	0.999939566	0.99989309	0.941925704
PTBP1	0.981230282	0.911279338	0.937712442	0.99879985	0.99989309	0.942421464
RAB33B	0.981282686	0.942048064	0.970804335	0.999939566	0.99989309	0.963756876

DHX33	0.981282692	0.920818159	0.939683463	0.999939566	0.99989309	0.998074562
AIDA	0.98129074	0.91761409	0.958420472	0.999939566	0.99989309	0.940917403
MGC127055	0.981361644	0.967515137	0.939091808	0.999939566	0.99989309	0.937591162
PAFAH1B3	0.981361644	0.916401332	0.954293634	0.999939566	0.99989309	0.942421464
CACNA1C	0.981361644	0.959666592	0.93710335	0.999939566	0.99989309	0.943138585
RASA1	0.981361644	0.957902362	0.956880455	0.999939566	0.99989309	0.943138585
LOC112446388	0.981361644	0.913075885	0.965712499	0.999939566	0.99989309	0.998027038
KCNN1	0.981402076	0.944959989	0.994425789	0.999939566	0.99989309	0.931886764
GTF3C2	0.981402076	0.921280256	0.971104162	0.999939566	0.99989309	0.933763401
FUZ	0.981402076	0.924799142	0.994872102	0.99879985	0.99989309	0.934458012
CNKSR1	0.981402076	0.913075885	0.967452429	0.999939566	0.99989309	0.934653208
SNRPA1	0.981402076	0.936134921	0.961899356	0.999939566	0.99989309	0.934775196
LOC112442649	0.981402076	0.971426434	0.957417441	0.999939566	0.99989309	0.935582793
LOC100336909	0.981402076	0.977500512	0.968230268	0.999939566	0.99989309	0.935636428
LOC104976062	0.981402076	0.96471209	0.957379098	0.999939566	0.99989309	0.935655141
CHRNA3	0.981402076	0.963478319	0.962209895	0.999939566	0.99989309	0.936137631
LOC534630	0.981402076	0.934531829	0.993747807	0.999939566	0.99989309	0.936439483
LOC784914	0.981402076	0.936134921	0.976161819	0.999939566	0.99989309	0.936563391
VRK1	0.981402076	0.924799142	0.979231344	0.999939566	0.99989309	0.936563391
FAM57A	0.981402076	0.947392706	0.994425789	0.999939566	0.99989309	0.936563391
PPP1R35	0.981402076	0.971068039	0.930892662	0.999939566	0.99989309	0.937591162
LOC107132189	0.981402076	0.936134921	0.962209895	0.999939566	0.99989309	0.937591162
ARAP1	0.981402076	0.948093381	0.979635589	0.999939566	0.99989309	0.937591162
TEDC1	0.981402076	0.907691022	0.935247964	0.999939566	0.99989309	0.938054315
STAT6	0.981402076	0.936550733	0.952717357	0.99879985	0.99989309	0.939078982
TCF4	0.981402076	0.946142324	0.962209895	0.999939566	0.99989309	0.939507593
ANKRD16	0.981402076	0.966403817	0.960137022	0.999939566	0.99989309	0.939800211
ABCA4	0.981402076	0.991528092	0.939683463	0.999939566	0.99989309	0.940683751
HIP1	0.981402076	0.994567583	0.953692935	0.999939566	0.99989309	0.940683751
TMEM121	0.981402076	0.963478319	0.939683463	0.999939566	0.99989309	0.940917403
TP53I11	0.981402076	0.9136091	0.944060947	0.999939566	0.99989309	0.940917403
KIAA1324L	0.981402076	0.963478319	0.951742137	0.999939566	0.99989309	0.940917403
SMIM15	0.981402076	0.94610099	0.989845175	0.999939566	0.99989309	0.940917403
STRADB	0.981402076	0.921865542	0.968466017	0.999939566	0.99989309	0.941275898
LOC100848339	0.981402076	0.942608215	0.93710335	0.999939566	0.99989309	0.941925704
ZNF322	0.981402076	0.977732731	0.939683463	0.999939566	0.99989309	0.942421464
GAS2L2	0.981402076	0.952464938	0.941593855	0.999939566	0.99989309	0.942421464
PC	0.981402076	0.932024312	0.950569634	0.999939566	0.99989309	0.942421464

DIAPH2	0.981402076	0.922434012	0.962209895	0.999939566	0.99989309	0.942421464
AKIRIN2	0.981402076	0.924510975	0.991113951	0.999939566	0.99989309	0.942421464
UBA52	0.981402076	0.911969123	0.947268948	0.999939566	0.99989309	0.942800958
RAPGEF3	0.981402076	0.953619177	0.950638965	0.999939566	0.99989309	0.943138585
AGTPBP1	0.981402076	0.968312904	0.953045982	0.999939566	0.99989309	0.943138585
PLGRKT	0.981402076	0.965239369	0.959646613	0.999939566	0.99989309	0.943138585
RTN3	0.981402076	0.907878616	0.965712499	0.999939566	0.99989309	0.943685681
LOC512149	0.981402076	0.944605763	0.981394466	0.99879985	0.99989309	0.943866443
LOC112442382	0.981402076	0.926529317	0.958420472	0.999939566	0.99989309	0.945759045
SLC25A21	0.981402076	0.911106863	0.962209895	0.999939566	0.99989309	0.946682284
LOC619026	0.981402076	0.911196851	0.933629769	0.999939566	0.99989309	0.948174718
COL4A2	0.981402076	0.957902362	0.939091808	0.999939566	0.99989309	0.948306629
PSMG1	0.981402076	0.922434012	0.975881144	0.999939566	0.99989309	0.949206487
MTERF3	0.981402076	0.937626904	0.939689648	0.999939566	0.99989309	0.950199992
LOC782024	0.981402076	0.922434012	0.976719499	0.999939566	0.99989309	0.950981777
CDH3	0.981402076	0.923186077	0.938306386	0.999939566	0.99989309	0.95188818
VWA5A	0.981402076	0.933412624	0.966157152	0.999939566	0.99989309	0.952631575
ST7L	0.981402076	0.967515137	0.958420472	0.999939566	0.99989309	0.95342487
RAB40C	0.981402076	0.911075771	0.982659255	0.99879985	0.99989309	0.95470473
ZNF467	0.981402076	0.911279338	0.978892556	0.999939566	0.99989309	0.955585296
KLHDC10	0.981402076	0.921961076	0.991406769	0.999939566	0.99989309	0.955585296
LOC112445944	0.981402076	0.921280256	0.945417678	0.99879985	0.99989309	0.956431159
LOC112447342	0.981402076	0.974986418	0.933647928	0.999939566	0.99989309	0.956610132
VPS35L	0.981402076	0.968440623	0.939683463	0.999939566	0.99989309	0.956610132
COX16	0.981402076	0.965239369	0.943891967	0.999939566	0.99989309	0.956610132
RORC	0.981402076	0.91196167	0.992165187	0.999939566	0.99989309	0.957877422
ALYREF	0.981402076	0.934248552	0.992850925	0.999939566	0.99989309	0.957877422
PGAP2	0.981402076	0.914162656	0.967302577	0.999939566	0.99989309	0.960712754
SLC25A6	0.981402076	0.962934461	0.958420472	0.99879985	0.99989309	0.961506391
RYK	0.981402076	0.920347742	0.994425789	0.99879985	0.99989309	0.961506391
HMBOX1	0.981402076	0.936134921	0.957379098	0.999939566	0.99989309	0.961506391
DCUN1D3	0.981402076	0.914735022	0.979231344	0.999939566	0.99989309	0.961506391
LOC101903281	0.981402076	0.913075885	0.990693603	0.999939566	0.99989309	0.961506391
LOC782305	0.981402076	0.926435857	0.937270184	0.999939566	0.99989309	0.963768949
SH3BP2	0.981402076	0.988153273	0.939683463	0.999939566	0.99989309	0.964143145
TCTN3	0.981402076	0.934248552	0.939683463	0.999939566	0.99989309	0.965134939
LOC790101	0.981402076	0.908192144	0.952208316	0.999939566	0.99989309	0.966375116
LOC100299025	0.981402076	0.966291707	0.940168084	0.99879985	0.99989309	0.966567675

PIGT	0.981402076	0.982051974	0.957578315	0.999939566	0.99989309	0.966567675
ERI2	0.981402076	0.991526443	0.941593855	0.999939566	0.99989309	0.967376295
SAMD12	0.981402076	0.936134921	0.943891967	0.999939566	0.99989309	0.967578505
LOC112442312	0.981402076	0.955798423	0.962209895	0.999939566	0.99989309	0.968039618
DCUN1D5	0.981402076	0.926435857	0.990107362	0.999939566	0.99989309	0.968059826
E2F4	0.981402076	0.913075885	0.985552223	0.999939566	0.99989309	0.968245211
CLIP4	0.981402076	0.909853448	0.941319062	0.999939566	0.99989309	0.970702553
LOC112444278	0.981402076	0.907670026	0.989408632	0.999939566	0.99989309	0.970959759
LOC785216	0.981402076	0.937978608	0.939683463	0.999939566	0.99989309	0.971283342
AADACL3	0.981402076	0.938064973	0.978215635	0.99879985	0.99989309	0.971402244
CDC123	0.981402076	0.984082087	0.931397502	0.999939566	0.99989309	0.972487907
LOC104969024	0.981402076	0.907594268	0.976719499	0.999939566	0.99989309	0.973195071
LOC522174	0.981402076	0.976925958	0.962268274	0.999939566	0.99989309	0.973494928
DGKZ	0.981402076	0.907878616	0.978879234	0.999939566	0.99989309	0.974258846
REM1	0.981402076	0.913075885	0.937191211	0.999939566	0.99989309	0.975019116
ITGA11	0.981402076	0.921961076	0.957379098	0.999939566	0.99989309	0.975454487
LOC112445989	0.981402076	0.974590874	0.93710335	0.999939566	0.99989309	0.977961448
CNRIP1	0.981402076	0.920540438	0.939091808	0.999939566	0.99989309	0.97815722
GPD1	0.981402076	0.936650943	0.939683463	0.99879985	0.99989309	0.978845762
C5H12orf45	0.981402076	0.962144114	0.939091808	0.999939566	0.99989309	0.979606084
LOC104969299	0.981402076	0.919134026	0.970034593	0.999939566	0.99989309	0.979606084
PROSER1	0.981402076	0.96471209	0.939683463	0.99879985	0.99989309	0.981090047
LOC101907577	0.981402076	0.939870925	0.933102499	0.999939566	0.99989309	0.983453112
TJP1	0.981402076	0.964901162	0.933825064	0.999939566	0.99989309	0.983453112
CD247	0.981402076	0.978250532	0.935848895	0.999939566	0.99989309	0.983453112
HBS1L	0.981402076	0.915913147	0.939091808	0.999939566	0.99989309	0.983453112
PFKFB2	0.981402076	0.957645111	0.953426945	0.999939566	0.99989309	0.984491523
FAAP100	0.981402076	0.934248552	0.939683463	0.999939566	0.99989309	0.984967003
SGSM1	0.981402076	0.97099048	0.951742137	0.999939566	0.99989309	0.986389023
MAP6D1	0.981402076	0.907878616	0.944485232	0.999939566	0.99989309	0.986862062
INTS5	0.981402076	0.944605763	0.951850716	0.999939566	0.99989309	0.987292804
EMG1	0.981402076	0.922434012	0.960726438	0.999939566	0.99989309	0.989947227
LOC518980	0.981402076	0.938064973	0.945417678	0.999939566	0.99989309	0.990291862
TUBA4A	0.981402076	0.972563829	0.939683463	0.999939566	0.99989309	0.99064193
LOC107131649	0.981402076	0.914162656	0.939683463	0.999939566	0.99989309	0.995063891
LOC523963	0.981454401	0.934248552	0.978440841	0.999939566	0.99989309	0.942421464
HIPK1	0.981489945	0.952464938	0.947391711	0.99879985	0.99989309	0.935607719
SYNE3	0.981489945	0.952990784	0.980744205	0.999939566	0.99989309	0.935655141

HESX1	0.981489945	0.937904802	0.95896293	0.999939566	0.99989309	0.936183569
PGRMC2	0.981489945	0.922256822	0.962209895	0.999939566	0.99989309	0.937591162
KLF3	0.981489945	0.974591667	0.933102499	0.999939566	0.99989309	0.940917403
CD74	0.981489945	0.992561812	0.939683463	0.999939566	0.99989309	0.941925704
TRUB1	0.981489945	0.922004954	0.963762816	0.999939566	0.99989309	0.941925704
PARP4	0.981489945	0.956288424	0.939683463	0.999939566	0.99989309	0.943138585
ADGRB3	0.981489945	0.994365533	0.940785296	0.999939566	0.99989309	0.943138585
ALG1	0.981489945	0.939027995	0.963643451	0.999939566	0.99989309	0.943685681
SLC25A38	0.981489945	0.942048064	0.989408632	0.999939566	0.99989309	0.946302385
PPP2R1A	0.981489945	0.944314627	0.958420472	0.999939566	0.99989309	0.949206487
TEKT3	0.981489945	0.963478319	0.945417678	0.999939566	0.99989309	0.953274474
ZDHHC8	0.981489945	0.921736842	0.939683463	0.999939566	0.99989309	0.955585296
SPIN2B	0.981489945	0.975673897	0.944128941	0.999939566	0.99989309	0.961506391
LOC540707	0.981489945	0.951616411	0.937311143	0.999939566	0.99989309	0.970702553
LOC101903098	0.981489945	0.926435857	0.939091808	0.999939566	0.99989309	0.974379635
LAT2	0.981489945	0.924799142	0.942004125	0.999939566	0.99989309	0.98016619
ILKAP	0.981489945	0.934248552	0.943501903	0.999939566	0.99989309	0.981578785
NPAS2	0.981489945	0.909085943	0.967294804	0.999939566	0.99989309	0.983212331
TRAF3IP1	0.981489945	0.920347742	0.970034593	0.999939566	0.99989309	0.984491523
ZFAND2B	0.981489945	0.921280256	0.964720224	0.999939566	0.99989309	0.989128955
LOC112447411	0.981489945	0.916274085	0.957501184	0.999939566	0.99989309	0.991051281
LOC101906135	0.981600132	0.910111393	0.979231344	0.999939566	0.99989309	0.940683751
MAP3K3	0.981600132	0.924510975	0.939683463	0.999939566	0.99989309	0.960712754
FNDC4	0.981690486	0.990334877	0.967619307	0.999939566	0.99989309	0.944606747
S1PR1	0.981724631	0.950844365	0.962209895	0.999939566	0.99989309	0.935385789
ADH5	0.981724631	0.963517553	0.9728362	0.99879985	0.99989309	0.936563391
IGBP1	0.981724631	0.918371039	0.939683463	0.999939566	0.99989309	0.942455807
GFRA3	0.981724631	0.93453191	0.975212837	0.999939566	0.99989309	0.986389023
ZNF845	0.981759008	0.913690438	0.939091808	0.999939566	0.99989309	0.942421464
LOC101903567	0.981759008	0.972418749	0.939683463	0.999939566	0.99989309	0.943138585
GFRA4	0.981759008	0.924799163	0.961899356	0.999939566	0.99989309	0.94501615
NR3C1	0.981759008	0.921280256	0.939683463	0.999939566	0.99989309	0.947399958
MCPH1	0.981759008	0.988649234	0.958420472	0.999939566	0.99989309	0.948277018
CUX1	0.981759008	0.917191385	0.942004125	0.999939566	0.99989309	0.954110387
SLC41A1	0.981759008	0.91761409	0.964645626	0.999939566	0.99989309	0.958606081
ARL4A	0.981759008	0.993887397	0.933102499	0.999939566	0.99989309	0.962510872
EMCN	0.981759008	0.959119521	0.939683463	0.999939566	0.99989309	0.973757028
LOC101908205	0.981759008	0.936134921	0.96636908	0.999939566	0.99989309	0.978565009

MGAT5	0.981759008	0.955353499	0.965712499	0.999939566	0.99989309	0.983427394
C7H1orf35	0.981985096	0.962934461	0.947475618	0.99879985	0.99989309	0.935655141
HOMEZ	0.981985096	0.955798423	0.970236012	0.999939566	0.99989309	0.935655141
TSPAN33	0.981985096	0.939870925	0.976719499	0.999939566	0.99989309	0.935655141
ZFP69	0.981985096	0.959119521	0.944485232	0.999939566	0.99989309	0.936183569
RMDN2	0.981985096	0.991043872	0.959646613	0.999939566	0.99989309	0.936439483
BEX5	0.981985096	0.934248552	0.969058758	0.999939566	0.99989309	0.936439483
CPSF6	0.981985096	0.912112179	0.944485232	0.999939566	0.99989309	0.936563391
CHD8	0.981985096	0.92813228	0.939683463	0.999939566	0.99989309	0.93691717
MRPL39	0.981985096	0.923186077	0.940785296	0.999939566	0.99989309	0.937591162
LYSMD4	0.981985096	0.916401332	0.977447568	0.999939566	0.99989309	0.938054315
AURKC	0.981985096	0.91796081	0.99245091	0.99879985	0.99989309	0.940683751
RERG	0.981985096	0.921280256	0.964744483	0.999939566	0.99989309	0.940683751
ZSCAN4	0.981985096	0.922434012	0.975183307	0.999939566	0.99989309	0.941889677
DOK4	0.981985096	0.914162656	0.967302577	0.999939566	0.99989309	0.941925704
ICE1	0.981985096	0.959348362	0.969985235	0.99879985	0.99989309	0.942421464
UBAP2L	0.981985096	0.91557476	0.958420472	0.999939566	0.99989309	0.942421464
LOC100300896	0.981985096	0.940026055	0.965518535	0.999939566	0.99989309	0.942421464
FMO4	0.981985096	0.94883251	0.989408632	0.99879985	0.99989309	0.943138585
SNX31	0.981985096	0.953748191	0.939683463	0.999939566	0.99989309	0.943138585
AP1S2	0.981985096	0.935324849	0.958420472	0.999939566	0.99989309	0.943138585
RPS13	0.981985096	0.938064973	0.959720192	0.999939566	0.99989309	0.943138585
LOC100848439	0.981985096	0.977728722	0.972909109	0.999939566	0.99989309	0.943138585
GLRX2	0.981985096	0.944314627	0.976869041	0.999939566	0.99989309	0.943138585
CRAMP1	0.981985096	0.920818159	0.994425789	0.999939566	0.99989309	0.943138585
LOC112446407	0.981985096	0.976925958	0.941593855	0.999939566	0.99989309	0.943685681
LOC101905453	0.981985096	0.962934461	0.939683463	0.999939566	0.99989309	0.944123524
CD47	0.981985096	0.976925958	0.962209895	0.999939566	0.99989309	0.944606747
CD46	0.981985096	0.921565868	0.993665588	0.999939566	0.99989309	0.944606747
LOC104970913	0.981985096	0.962934461	0.979635589	0.999939566	0.99989309	0.945258337
LOC112448056	0.981985096	0.956134433	0.958420472	0.999939566	0.99989309	0.945438446
EML1	0.981985096	0.928601644	0.979231344	0.999939566	0.99989309	0.946034824
LOC101907320	0.981985096	0.967137663	0.933647928	0.999939566	0.99989309	0.946314386
DYNLL1	0.981985096	0.9347557	0.996559603	0.999939566	0.99989309	0.946314386
LOC112442254	0.981985096	0.959119521	0.96712176	0.999939566	0.99989309	0.946717155
PCYOX1L	0.981985096	0.944691113	0.958420472	0.999939566	0.99989309	0.947991649
ARHGAP6	0.981985096	0.976925958	0.961742979	0.999939566	0.99989309	0.949206487
PDCD7	0.981985096	0.913075885	0.988752364	0.999939566	0.99989309	0.949206487

WDR26	0.981985096	0.94812255	0.963986266	0.999939566	0.99989309	0.95188818
HACL1	0.981985096	0.954015268	0.939683463	0.999939566	0.99989309	0.952887889
PIGG	0.981985096	0.916172682	0.999323478	0.999939566	0.99989309	0.953298732
TMEM116	0.981985096	0.938064973	0.935644121	0.999939566	0.99989309	0.95342487
UBE2E3	0.981985096	0.929730109	0.939683463	0.999939566	0.99989309	0.954110387
CHSY3	0.981985096	0.978453686	0.943891967	0.999939566	0.99989309	0.954581551
LOC518768	0.981985096	0.925360181	0.974559286	0.99879985	0.99989309	0.95470473
LOC112441511	0.981985096	0.922663208	0.939683463	0.999939566	0.99989309	0.95470473
CSF3	0.981985096	0.988649234	0.950801642	0.999939566	0.99989309	0.95470473
ADGRG1	0.981985096	0.957353974	0.95206243	0.999939566	0.99989309	0.95470473
FZD6	0.981985096	0.962934461	0.979231344	0.999939566	0.99989309	0.95470473
LOC101910094	0.981985096	0.914162656	0.980734833	0.999939566	0.99989309	0.95470473
ABRACL	0.981985096	0.947959005	0.958420472	0.999939566	0.99989309	0.954935833
ZNF350	0.981985096	0.924510975	0.939683463	0.999939566	0.99989309	0.955817715
C16H1orf159	0.981985096	0.921393399	0.953426945	0.999939566	0.99989309	0.955817715
ZNF7	0.981985096	0.944314627	0.994425789	0.999939566	0.99989309	0.956011611
ERG	0.981985096	0.986245673	0.962790365	0.99879985	0.99989309	0.956610132
SASH1	0.981985096	0.936134921	0.939683463	0.999939566	0.99989309	0.956610132
TAF10	0.981985096	0.934248552	0.947959246	0.999939566	0.99989309	0.956610132
MCF2	0.981985096	0.964270411	0.967043892	0.999939566	0.99989309	0.957122667
OGFOD3	0.981985096	0.937978608	0.941873695	0.999939566	0.99989309	0.957877422
TM4SF1	0.981985096	0.950091107	0.951850716	0.999939566	0.99989309	0.957877422
E2F5	0.981985096	0.984479682	0.953045982	0.999939566	0.99989309	0.957877422
C18H19orf12	0.981985096	0.91796081	0.98846962	0.999939566	0.99989309	0.957877422
IRS1	0.981985096	0.913075885	0.957444566	0.999939566	0.99989309	0.958475633
EMID1	0.981985096	0.914616699	0.979231344	0.999939566	0.99989309	0.958540316
DDB2	0.981985096	0.9347557	0.958420472	0.999939566	0.99989309	0.958814003
LOC104974812	0.981985096	0.9136091	0.942004125	0.999939566	0.99989309	0.961506391
LOC107131643	0.981985096	0.946142324	0.970904484	0.999939566	0.99989309	0.961506391
DLGAP4	0.981985096	0.944780259	0.93798101	0.999939566	0.99989309	0.961606931
LOC112447350	0.981985096	0.989661212	0.951742137	0.999939566	0.99989309	0.962118346
TMEM141	0.981985096	0.920172204	0.947268948	0.999939566	0.99989309	0.963996128
LOC100849587	0.981985096	0.918306418	0.979231344	0.999939566	0.99989309	0.965332394
LOC112448075	0.981985096	0.936134921	0.974681091	0.999939566	0.99989309	0.966375116
ZNF853	0.981985096	0.989426681	0.939091808	0.999939566	0.99989309	0.968245211
LOC112448103	0.981985096	0.972786652	0.962369613	0.999939566	0.99989309	0.968245211
DBT	0.981985096	0.914162656	0.939683463	0.999939566	0.99989309	0.96826636
SRM	0.981985096	0.946722291	0.941593855	0.999939566	0.99989309	0.969033954

GRASP	0.981985096	0.976925958	0.941319062	0.999939566	0.99989309	0.969545443
LOC112442083	0.981985096	0.926618064	0.951278495	0.999939566	0.99989309	0.970786053
VPS39	0.981985096	0.962428993	0.958420472	0.999939566	0.99989309	0.971025918
LOC112446753	0.981985096	0.982963758	0.933963997	0.999939566	0.99989309	0.972698857
VPS11	0.981985096	0.922434012	0.947268948	0.999939566	0.99989309	0.973195541
DZIP3	0.981985096	0.926435857	0.959646613	0.999939566	0.99989309	0.974017283
LOC509006	0.981985096	0.969202872	0.958420472	0.99879985	0.99989309	0.97510237
NMRK1	0.981985096	0.913625328	0.979851274	0.999939566	0.99989309	0.975972322
C19H17orf100	0.981985096	0.942608215	0.962777992	0.999939566	0.99989309	0.976175609
BRPF1	0.981985096	0.920347742	0.956918934	0.999939566	0.99989309	0.97792865
LDB2	0.981985096	0.911279338	0.962209895	0.999939566	0.99989309	0.978467321
POPDC3	0.981985096	0.924799142	0.950801642	0.999939566	0.99989309	0.978656049
RASGRF2	0.981985096	0.936134921	0.963651986	0.999939566	0.99989309	0.979233909
SLC22A3	0.981985096	0.929734246	0.961742979	0.999939566	0.99989309	0.979810204
LOC112443767	0.981985096	0.929102966	0.944060947	0.999939566	0.99989309	0.98016619
ARMH4	0.981985096	0.98743321	0.944485232	0.999939566	0.99989309	0.98016619
XPO1	0.981985096	0.959119521	0.948628899	0.999939566	0.99989309	0.98016619
DCK	0.981985096	0.933412624	0.953692935	0.999939566	0.99989309	0.98016619
ANKRD10	0.981985096	0.921280256	0.985135994	0.999939566	0.99989309	0.98016619
LOC112444531	0.981985096	0.950861808	0.968455468	0.999939566	0.99989309	0.981164039
UBB	0.981985096	0.955353499	0.97311093	0.99879985	0.99989309	0.982045172
LOC112442384	0.981985096	0.957071555	0.944976702	0.999939566	0.99989309	0.982045172
KIAA0754	0.981985096	0.924799142	0.939683463	0.999939566	0.99989309	0.982559673
CCDC126	0.981985096	0.916725199	0.939683463	0.999939566	0.99989309	0.983453112
GRK5	0.981985096	0.957645111	0.939683463	0.999939566	0.99989309	0.983453112
ASB14	0.981985096	0.939870925	0.958420472	0.999939566	0.99989309	0.983453112
LOC101904592	0.981985096	0.922434012	0.961281956	0.999939566	0.99989309	0.985788947
DPAGT1	0.981985096	0.944420015	0.939683463	0.999939566	0.99989309	0.98609047
LOC511936	0.981985096	0.914252497	0.945417678	0.999939566	0.99989309	0.98609047
TNFRSF1B	0.981985096	0.942608215	0.959705869	0.999939566	0.99989309	0.986389023
LOC112445190	0.981985096	0.920347742	0.95721973	0.99879985	0.99989309	0.991281092
RNGTT	0.981985096	0.940502427	0.958420472	0.99879985	0.99989309	0.993612847
LOC784488	0.981985096	0.912112179	0.979096894	0.999939566	0.99989309	0.995411183
FAM214A	0.981985096	0.92538929	0.939683463	0.999939566	0.99989309	0.998074562
TPO	0.981985096	0.94858039	0.941319062	0.999939566	0.99989309	0.998074562
TUT1	0.981985096	0.944420015	0.953045982	0.999939566	0.99989309	0.998074562
HOMER2	0.981985096	0.934248552	0.945417678	0.999939566	0.99989309	0.998636142
PLPPR1	0.981985096	0.934248552	0.945417678	0.999939566	0.99989309	0.998636142

NFKBIB	0.981985096	0.93077794	0.947268948	0.999939566	0.99989309	0.998636142
UROC1	0.981998279	0.965239369	0.939683463	0.999939566	0.99989309	0.941073767
PDE4A	0.981998279	0.938064973	0.968466017	0.999939566	0.99989309	0.984449688
DEPDC5	0.982003684	0.920347742	0.990107362	0.999939566	0.99989309	0.954565653
SNRPN	0.982003684	0.918306418	0.979099287	0.999939566	0.99989309	0.968039618
LOC104972346	0.982003684	0.934248552	0.968230268	0.999939566	0.99989309	0.980920516
POLI	0.982003684	0.965239369	0.942004125	0.999939566	0.99989309	0.982559673
UBE2A	0.982097999	0.962934461	0.969058758	0.999939566	0.99989309	0.943138585
LOC112448889	0.982097999	0.918306418	0.937270184	0.999939566	0.99989309	0.969455971
LOC101902106	0.982097999	0.944780259	0.976719499	0.999939566	0.99989309	0.973494928
LOC101903413	0.982157945	0.976765472	0.942004125	0.999939566	0.99989309	0.940683751
CREG2	0.982157945	0.952464938	0.979231344	0.999939566	0.99989309	0.945759045
LOC615792	0.982157945	0.959284624	0.962209895	0.999334425	0.99989309	0.950199992
FMC1	0.982157945	0.936993468	0.957379098	0.999939566	0.99989309	0.95470473
SH3BGRL2	0.982157945	0.962063344	0.939683463	0.999939566	0.99989309	0.961506391
DNAJB12	0.982157945	0.924799142	0.957379098	0.999939566	0.99989309	0.996564988
PRDM8	0.982159971	0.944273956	0.982223562	0.999939566	0.99989309	0.969942217
NTN5	0.982252708	0.956843573	0.976782463	0.999939566	0.99989309	0.942455807
SCAMP4	0.982252708	0.934248552	0.965169397	0.999939566	0.99989309	0.956431159
HIBCH	0.982340676	0.94610099	0.939612562	0.999939566	0.99989309	0.939108967
RBP4	0.982340676	0.988649234	0.955524646	0.999939566	0.99989309	0.942421464
GTF2H2	0.982340676	0.978453686	0.967676552	0.999939566	0.99989309	0.942421464
USP40	0.982340676	0.941117166	0.965518535	0.999939566	0.99989309	0.98016619
SULT1C4	0.982455763	0.936134921	0.958420472	0.999939566	0.99989309	0.96435585
BRD3	0.982593854	0.918306418	0.949343925	0.999939566	0.99989309	0.961506391
TMEM268	0.982593854	0.913625328	0.939683463	0.999939566	0.99989309	0.98016619
LOC101909083	0.982593939	0.952283714	0.992056611	0.999939566	0.99989309	0.948277018
LCA5	0.982643035	0.965239369	0.944638172	0.999939566	0.99989309	0.968245211
RFX2	0.982678509	0.921280256	0.942004125	0.999939566	0.99989309	0.94365313
ARHGEF11	0.982678509	0.929536223	0.987842204	0.999939566	0.99989309	0.949744931
HYLS1	0.982765326	0.952464938	0.944060947	0.999939566	0.99989309	0.93691717
CERK	0.982815353	0.955353499	0.962872914	0.999939566	0.99989309	0.941925704
LOC100848912	0.982815353	0.937043243	0.972874677	0.999939566	0.99989309	0.948336587
LOC112448078	0.982964678	0.971680593	0.976972382	0.999939566	0.99989309	0.941925704
LOC783185	0.98300809	0.941288692	0.966157152	0.999939566	0.99989309	0.941925704
LOC107131489	0.98300809	0.966403817	0.944592818	0.999939566	0.99989309	0.95470473
TEX261	0.983060722	0.94883251	0.950801642	0.999939566	0.99989309	0.937591162
ZNF174	0.983060722	0.982051974	0.939683463	0.999939566	0.99989309	0.938054315

LOC112447495	0.983060722	0.925397192	0.965518535	0.999939566	0.99989309	0.939426596
EP400	0.983060722	0.934248552	0.941593855	0.999939566	0.99989309	0.940673472
SLC25A42	0.983060722	0.920347742	0.939091808	0.999939566	0.99989309	0.940683751
TANGO2	0.983060722	0.96044854	0.957379098	0.999939566	0.99989309	0.940683751
PRELP	0.983060722	0.921736842	0.958420472	0.999939566	0.99989309	0.940683751
H3F3A	0.983060722	0.936134921	0.994425789	0.999939566	0.99989309	0.940683751
NINJ1	0.983060722	0.965239369	0.970904484	0.999939566	0.99989309	0.94076264
RC3H1	0.983060722	0.963480908	0.979231344	0.999939566	0.99989309	0.940917403
LOC101906177	0.983060722	0.953748191	0.994425789	0.999939566	0.99989309	0.940917403
NR6A1	0.983060722	0.980930691	0.945417678	0.999939566	0.99989309	0.941785987
USP31	0.983060722	0.915978689	0.939683463	0.999939566	0.99989309	0.941925704
RBM26	0.983060722	0.965239369	0.939683463	0.999939566	0.99989309	0.941925704
LOC100335340	0.983060722	0.98618789	0.939683463	0.999939566	0.99989309	0.941925704
CHERP	0.983060722	0.948215967	0.945012822	0.999939566	0.99989309	0.941925704
GUSB	0.983060722	0.99134261	0.963762816	0.999939566	0.99989309	0.941925704
SCUBE3	0.983060722	0.938064973	0.964720224	0.999939566	0.99989309	0.941925704
HMCN2	0.983060722	0.98893515	0.965169397	0.999939566	0.99989309	0.941925704
CCPG1	0.983060722	0.941266539	0.966157152	0.999939566	0.99989309	0.941925704
FAM92A	0.983060722	0.923680159	0.978669366	0.999939566	0.99989309	0.941925704
HACD1	0.983060722	0.971839996	0.979231344	0.999939566	0.99989309	0.941925704
SPRY4	0.983060722	0.955798423	0.988788601	0.999939566	0.99989309	0.941925704
NOL3	0.983060722	0.916725199	0.962209895	0.999939566	0.99989309	0.942195382
PF4	0.983060722	0.98743321	0.939091808	0.999939566	0.99989309	0.942421464
P2RX6	0.983060722	0.962428993	0.944686498	0.999939566	0.99989309	0.942421464
GPR62	0.983060722	0.921736842	0.951850716	0.999939566	0.99989309	0.942421464
LOC112448021	0.983060722	0.929536223	0.988111832	0.999939566	0.99989309	0.942421464
KIAA1522	0.983060722	0.916725199	0.962209895	0.999939566	0.99989309	0.942455807
PIBF1	0.983060722	0.944420015	0.939091808	0.999939566	0.99989309	0.943138585
PLEKHM1	0.983060722	0.931095524	0.939683463	0.999939566	0.99989309	0.943138585
WNT2B	0.983060722	0.992656764	0.939683463	0.999939566	0.99989309	0.943138585
LOC509155	0.983060722	0.997237491	0.939683463	0.999939566	0.99989309	0.943138585
LOC112447811	0.983060722	0.999368101	0.950332796	0.999939566	0.99989309	0.943138585
CD44	0.983060722	0.98618789	0.953911392	0.999939566	0.99989309	0.943138585
LOC112447290	0.983060722	0.928601644	0.978215635	0.999939566	0.99989309	0.943138585
INF2	0.983060722	0.949008068	0.981114194	0.999939566	0.99989309	0.943685681
HERC1	0.983060722	0.976925958	0.939683463	0.999939566	0.99989309	0.94425343
LOC112445996	0.983060722	0.939027995	0.958420472	0.999939566	0.99989309	0.94425343
RING1	0.983060722	0.936134921	0.944255223	0.999939566	0.99989309	0.944663458

FAM110D	0.983060722	0.972227933	0.956872818	0.999939566	0.99989309	0.944892687
G6PC	0.983060722	0.966852427	0.941319062	0.999939566	0.99989309	0.945235365
LOC101902681	0.983060722	0.956843573	0.958420472	0.999939566	0.99989309	0.945258337
NR4A1	0.983060722	0.949335109	0.989332994	0.999939566	0.99989309	0.945258337
SERPINB9	0.983060722	0.972786652	0.976869041	0.999939566	0.99989309	0.945438446
YY1	0.983060722	0.929262063	0.941593855	0.999939566	0.99989309	0.945759045
TOE1	0.983060722	0.920347742	0.97381056	0.999939566	0.99989309	0.94588513
TBC1D17	0.983060722	0.916725199	0.942314296	0.999939566	0.99989309	0.946034824
CDK4	0.983060722	0.965239369	0.989845175	0.999939566	0.99989309	0.946302385
CCNT2	0.983060722	0.942048064	0.939683463	0.999939566	0.99989309	0.946314386
RALGPS1	0.983060722	0.992273495	0.962369613	0.999939566	0.99989309	0.946717155
PIGP	0.983060722	0.91796081	0.959705869	0.999939566	0.99989309	0.947398404
UBXN1	0.983060722	0.922264623	0.966777694	0.999939566	0.99989309	0.948277018
ZNF655	0.983060722	0.955798423	0.965712499	0.999939566	0.99989309	0.949120514
MARVELD2	0.983060722	0.967758952	0.942004125	0.999939566	0.99989309	0.950601157
MED7	0.983060722	0.923841618	0.941593855	0.999939566	0.99989309	0.952631575
MAPKAP1	0.983060722	0.925461739	0.957417441	0.999939566	0.99989309	0.95342487
JKAMP	0.983060722	0.913171043	0.990107362	0.999939566	0.99989309	0.95342487
LRR1	0.983060722	0.959356497	0.99115315	0.999939566	0.99989309	0.954110387
IFITM5	0.983060722	0.964516367	0.962588495	0.999939566	0.99989309	0.95470473
MDP1	0.983060722	0.955798423	0.967489175	0.999939566	0.99989309	0.95470473
RNF26	0.983060722	0.918976923	0.969811559	0.999939566	0.99989309	0.95470473
MGLL	0.983060722	0.923729244	0.939091808	0.999939566	0.99989309	0.955585296
FAR2	0.983060722	0.974590874	0.942004125	0.999939566	0.99989309	0.956554727
GARNL3	0.983060722	0.934248552	0.944612298	0.999939566	0.99989309	0.956610132
ARHGAP39	0.983060722	0.965239369	0.960137022	0.999939566	0.99989309	0.956610132
TIAM1	0.983060722	0.97528575	0.979231344	0.999939566	0.99989309	0.956610132
FAM135A	0.983060722	0.981332269	0.979231344	0.999939566	0.99989309	0.956670063
KHK	0.983060722	0.921736842	0.952255572	0.999939566	0.99989309	0.957122667
CCDC90B	0.983060722	0.934045324	0.994425789	0.999939566	0.99989309	0.957122667
SDF4	0.983060722	0.955798423	0.960137022	0.999939566	0.99989309	0.957877422
PPP1R18	0.983060722	0.978453686	0.941319062	0.999939566	0.99989309	0.958475633
MAPK8IP3	0.983060722	0.927162932	0.958420472	0.999939566	0.99989309	0.958540316
TGFBR3	0.983060722	0.934248552	0.941319062	0.999939566	0.99989309	0.958814003
C5AR2	0.983060722	0.91458438	0.994304212	0.999939566	0.99989309	0.95908735
WSCD1	0.983060722	0.942048064	0.939683463	0.999939566	0.99989309	0.959501399
DLST	0.983060722	0.918306418	0.939091808	0.999939566	0.99989309	0.959625205
ACSS3	0.983060722	0.962934461	0.950801642	0.999939566	0.99989309	0.960712754

TCTN1	0.983060722	0.971068039	0.952717357	0.999939566	0.99989309	0.961336964
LOC104972400	0.983060722	0.928601644	0.942004125	0.999939566	0.99989309	0.961506391
PMAIP1	0.983060722	0.962123724	0.950638965	0.999939566	0.99989309	0.961506391
IFT22	0.983060722	0.957645111	0.975881144	0.999939566	0.99989309	0.961506391
CHODL	0.983060722	0.946142324	0.985552223	0.999939566	0.99989309	0.961506391
NKAPL	0.983060722	0.944755173	0.989332994	0.999939566	0.99989309	0.961506391
COMMD7	0.983060722	0.962934461	0.974158329	0.999939566	0.99989309	0.96163007
LOC112444967	0.983060722	0.918371039	0.993278977	0.999939566	0.99989309	0.962411861
SUMO2	0.983060722	0.932064285	0.979231344	0.999939566	0.99989309	0.963135727
ANKRD6	0.983060722	0.929573947	0.946551014	0.999939566	0.99989309	0.963996128
TM9SF1	0.983060722	0.922331228	0.949977753	0.999939566	0.99989309	0.963996128
SLC22A4	0.983060722	0.925277949	0.962209895	0.999939566	0.99989309	0.96435585
RPL26L1	0.983060722	0.956958713	0.970108169	0.999939566	0.99989309	0.966375116
BMP3	0.983060722	0.971207468	0.97381056	0.999939566	0.99989309	0.966832455
IFT81	0.983060722	0.925461739	0.939091808	0.999939566	0.99989309	0.968039618
ADSSL1	0.983060722	0.970167557	0.951607422	0.999939566	0.99989309	0.968039618
FAXDC2	0.983060722	0.974403012	0.979096894	0.999939566	0.99989309	0.968039618
MME	0.983060722	0.926147555	0.979231344	0.999939566	0.99989309	0.968245211
LOC101907523	0.983060722	0.922964639	0.987842204	0.999939566	0.99989309	0.968245211
COL4A4	0.983060722	0.920347742	0.994425789	0.999939566	0.99989309	0.968245211
LOC528802	0.983060722	0.918851896	0.964720224	0.999939566	0.99989309	0.969808185
CCDC71	0.983060722	0.925807756	0.951673671	0.999939566	0.99989309	0.97084193
POLB	0.983060722	0.936968495	0.947475618	0.999939566	0.99989309	0.971554741
RCC1L	0.983060722	0.941643123	0.946573665	0.999939566	0.99989309	0.972487907
LOC112442865	0.983060722	0.979791607	0.947660114	0.999939566	0.99989309	0.972487907
ZNF268	0.983060722	0.920479003	0.971104162	0.999939566	0.99989309	0.973463195
VPS37B	0.983060722	0.944605763	0.939683463	0.999939566	0.99989309	0.975923557
LOC782202	0.983060722	0.954241168	0.947268948	0.999939566	0.99989309	0.976950162
COX15	0.983060722	0.935793939	0.949977753	0.999939566	0.99989309	0.978287053
PSMB8	0.983060722	0.949169888	0.941593855	0.999939566	0.99989309	0.978467321
LOC787554	0.983060722	0.913171043	0.968466017	0.999939566	0.99989309	0.979606084
TPST2	0.983060722	0.950153076	0.945455812	0.999939566	0.99989309	0.979717242
OSMR	0.983060722	0.962934461	0.941593855	0.999939566	0.99989309	0.980144374
LOC104969916	0.983060722	0.934248552	0.939091808	0.999939566	0.99989309	0.98016619
DENND2C	0.983060722	0.989445679	0.939683463	0.999939566	0.99989309	0.98016619
HNRNPC	0.983060722	0.936134921	0.979231344	0.999939566	0.99989309	0.98016619
IGF2R	0.983060722	0.918371039	0.939091808	0.999939566	0.99989309	0.980841407
LOC107132799	0.983060722	0.913625328	0.959720192	0.999939566	0.99989309	0.981164039

GAB2	0.983060722	0.971839996	0.948628899	0.999939566	0.99989309	0.981578785
LOC510185	0.983060722	0.924799142	0.974360603	0.999939566	0.99989309	0.981578785
LOC107132537	0.983060722	0.98547302	0.957802423	0.999939566	0.99989309	0.982045172
RAP1GDS1	0.983060722	0.956134433	0.958420472	0.999939566	0.99989309	0.983453112
LOC112443503	0.983060722	0.944959989	0.964645626	0.999939566	0.99989309	0.983453112
PLSCR2	0.983060722	0.96471209	0.939683463	0.999939566	0.99989309	0.984449688
ATP9B	0.983060722	0.922434012	0.939683463	0.999939566	0.99989309	0.986389023
DACH1	0.983060722	0.979916117	0.939091808	0.999939566	0.99989309	0.986529689
C5H12orf4	0.983060722	0.93587722	0.962209895	0.999939566	0.99989309	0.986862062
LOC782525	0.983060722	0.924799142	0.979099287	0.999939566	0.99989309	0.988200507
LGI4	0.983060722	0.939870925	0.939683463	0.999939566	0.99989309	0.988571083
LOC104974034	0.983060722	0.920423305	0.951850716	0.999939566	0.99989309	0.989313458
RALB	0.983060722	0.950464104	0.959198396	0.999939566	0.99989309	0.989780996
RND3	0.983060722	0.963478319	0.95721973	0.999939566	0.99989309	0.990275559
LOC112441682	0.983060722	0.922434012	0.9840633	0.999939566	0.99989309	0.990291862
LOC101905293	0.983060722	0.914616699	0.944976702	0.999939566	0.99989309	0.990396832
MCF2L	0.983060722	0.930688185	0.94821101	0.999939566	0.99989309	0.991281092
KMT5A	0.983060722	0.936134921	0.959320266	0.999939566	0.99989309	0.991281092
LARP6	0.983060722	0.965458817	0.939683463	0.999939566	0.99989309	0.994550936
AK5	0.983060722	0.943855442	0.952414818	0.999939566	0.99989309	0.994888756
LOC511409	0.983060722	0.942048064	0.957802423	0.999939566	0.99989309	0.996455238
LOC100848405	0.983060722	0.926435857	0.941319062	0.999939566	0.99989309	0.998636142
PIGF	0.983060722	0.918306418	0.950476553	0.999939566	0.99989309	0.998636142
GTPBP10	0.983105849	0.988153273	0.939683463	0.999939566	0.99989309	0.940683751
LOC112447462	0.983105849	0.920347742	0.966157152	0.999939566	0.99989309	0.940917403
HES2	0.983105849	0.966852427	0.939683463	0.999939566	0.99989309	0.941275898
EFCAB7	0.983105849	0.918306418	0.982930137	0.999939566	0.99989309	0.943685681
ALOX5	0.983105849	0.97572611	0.951607422	0.999939566	0.99989309	0.946314386
LOC783504	0.983105849	0.962428993	0.961899356	0.999939566	0.99989309	0.948277018
RAB23	0.983105849	0.942048064	0.965750046	0.999939566	0.99989309	0.948277018
LOC112442227	0.983105849	0.931073006	0.98945074	0.999939566	0.99989309	0.948911861
BRWD1	0.983105849	0.965239369	0.957379098	0.999939566	0.99989309	0.949206487
LOC101907518	0.983105849	0.946801118	0.979231344	0.999939566	0.99989309	0.949206487
POLD3	0.983105849	0.983757161	0.948411239	0.999939566	0.99989309	0.951047219
MVB12B	0.983105849	0.920172204	0.942004125	0.999939566	0.99989309	0.954334316
FAM212B	0.983105849	0.94883251	0.979231344	0.999939566	0.99989309	0.955585296
SCARB1	0.983105849	0.948273015	0.966626132	0.999939566	0.99989309	0.968059826
KBTBD3	0.983105849	0.945105738	0.946551014	0.999939566	0.99989309	0.969981358

ABHD16A	0.983105849	0.924999357	0.957802423	0.999939566	0.99989309	0.97815722
LOC112444520	0.983105849	0.921280256	0.939683463	0.999939566	0.99989309	0.979233909
ZNF154	0.983105849	0.981226249	0.942004125	0.999939566	0.99989309	0.982045172
OTOGL	0.983105849	0.93453191	0.939091808	0.999939566	0.99989309	0.983453112
ZFAND2A	0.983105849	0.936134921	0.959646613	0.999939566	0.99989309	0.983453112
GDAP2	0.983107366	0.949625762	0.98846962	0.999939566	0.99989309	0.942421464
PBX3	0.983218275	0.954015268	0.959822136	0.999939566	0.99989309	0.98016619
ITGA7	0.983508564	0.922434012	0.962209895	0.999939566	0.99989309	0.949206487
C25H16orf58	0.983508564	0.924799142	0.995404976	0.999939566	0.99989309	0.957877422
LOC785843	0.983508564	0.926435857	0.962209895	0.999939566	0.99989309	0.958606081
TF	0.983508564	0.920423305	0.968406955	0.999939566	0.99989309	0.961506391
ZNF317	0.983508564	0.967731788	0.974109812	0.999939566	0.99989309	0.968240941
LOC512541	0.983508564	0.920423305	0.970867579	0.999939566	0.99989309	0.968245211
TTC30A	0.983508564	0.956843573	0.965518535	0.999939566	0.99989309	0.973494928
RHBDL3	0.983508564	0.918311236	0.957379098	0.999939566	0.99989309	0.983453112
ELMSAN1	0.983508564	0.92161844	0.958357122	0.999939566	0.99989309	0.983453112
LOC101905179	0.983508564	0.920522986	0.980734833	0.999939566	0.99989309	0.983453112
ATP6V0A2	0.983508564	0.926435857	0.944592818	0.999939566	0.99989309	0.998928226
LOC101905723	0.983557795	0.969537549	0.939683463	0.999939566	0.99989309	0.941925704
LOC507443	0.983557795	0.974348229	0.963643451	0.999939566	0.99989309	0.942421464
LOC101904268	0.983557795	0.970237188	0.965169397	0.999939566	0.99989309	0.943685681
ATG10	0.983557795	0.948273015	0.968406955	0.999939566	0.99989309	0.945759045
GTF2IRD1	0.983557795	0.941288692	0.979231344	0.999939566	0.99989309	0.946717155
MADD	0.983557795	0.963480908	0.947660114	0.999939566	0.99989309	0.961506391
CACNA1F	0.983557795	0.945105738	0.990107362	0.999939566	0.99989309	0.961506391
FER	0.983557795	0.924999357	0.950801642	0.999939566	0.99989309	0.963996128
DHX16	0.983557795	0.925130457	0.939683463	0.999939566	0.99989309	0.964143145
LOC107131498	0.983557795	0.93181855	0.939683463	0.999939566	0.99989309	0.965332394
RHOBTB1	0.983557795	0.921280256	0.940785296	0.999939566	0.99989309	0.968059826
CNTN1	0.983557795	0.989736861	0.939683463	0.999939566	0.99989309	0.968245211
ACOT11	0.983557795	0.981226249	0.956508857	0.999939566	0.99989309	0.971399402
LOC100848177	0.983557795	0.968312904	0.942501338	0.999939566	0.99989309	0.973195071
PALMD	0.983557795	0.925752434	0.965169397	0.999939566	0.99989309	0.98016619
FAM122B	0.983557795	0.942608215	0.95237206	0.999939566	0.99989309	0.996564988
CLMN	0.983557795	0.932024312	0.945417678	0.999939566	0.99989309	0.997755698
FGFR1OP2	0.983557795	0.921393399	0.941593855	0.999939566	0.999961511	0.940683751
ATP6V0D2	0.983589637	0.944780259	0.977371811	0.999939566	0.99989309	0.940683751
NKG7	0.983589637	0.997390201	0.942004125	0.999939566	0.99989309	0.940917403

ANO10	0.983589637	0.933412624	0.962209895	0.999939566	0.99989309	0.940917403
LOC112442223	0.983589637	0.96471209	0.939683463	0.999939566	0.99989309	0.941445869
LOC101903976	0.983589637	0.964234693	0.982930137	0.999939566	0.99989309	0.941912576
BTC	0.983589637	0.986032534	0.941593855	0.999939566	0.99989309	0.941925704
LOC101905686	0.983589637	0.939027995	0.955325896	0.999939566	0.99989309	0.941925704
PDCD10	0.983589637	0.928999919	0.966157152	0.999939566	0.99989309	0.941925704
FAM199X	0.983589637	0.962934461	0.968934792	0.999939566	0.99989309	0.941925704
TWF2	0.983589637	0.957353974	0.942004125	0.999939566	0.99989309	0.942421464
LOC100297725	0.983589637	0.920347742	0.995412965	0.999939566	0.99989309	0.942421464
LOC101907503	0.983589637	0.926613567	0.954463909	0.999939566	0.99989309	0.942800958
RAD9A	0.983589637	0.971313644	0.958420472	0.999939566	0.99989309	0.943026933
FOXP4	0.983589637	0.920423305	0.941593855	0.999939566	0.99989309	0.943138585
LOC112442544	0.983589637	0.930469765	0.944754534	0.999939566	0.99989309	0.943138585
ANXA3	0.983589637	0.936550733	0.960137022	0.999939566	0.99989309	0.943138585
PPP1R26	0.983589637	0.949965815	0.968406955	0.999939566	0.99989309	0.943138585
YIPF1	0.983589637	0.99134261	0.975361542	0.999939566	0.99989309	0.943138585
RUBCN	0.983589637	0.982963758	0.98846962	0.999939566	0.99989309	0.943138585
LOC783421	0.983589637	0.936134921	0.990107362	0.999939566	0.99989309	0.943138585
LOC784208	0.983589637	0.967668715	0.941319062	0.999939566	0.99989309	0.943685681
PIK3IP1	0.983589637	0.959119521	0.957379098	0.999939566	0.99989309	0.943685681
LOC104975559	0.983589637	0.978309093	0.987842204	0.999939566	0.99989309	0.946314386
C6H4orf19	0.983589637	0.937978608	0.968406955	0.999939566	0.99989309	0.94734477
RNF169	0.983589637	0.965239369	0.954313013	0.999939566	0.99989309	0.947577032
BEAN1	0.983589637	0.936134921	0.962790365	0.999939566	0.99989309	0.948277018
LOC104968479	0.983589637	0.936134921	0.989586949	0.999939566	0.99989309	0.948277018
SEMA6D	0.983589637	0.921280256	0.968419607	0.999939566	0.99989309	0.948817495
KDELR1	0.983589637	0.92813228	0.945706364	0.999939566	0.99989309	0.953604648
LOC101907581	0.983589637	0.962934461	0.972401758	0.999939566	0.99989309	0.95401375
LOC100139360	0.983589637	0.98395047	0.939683463	0.999939566	0.99989309	0.95470473
WDR45	0.983589637	0.936134921	0.963643451	0.999939566	0.99989309	0.95470473
LOC616254	0.983589637	0.934248552	0.942004125	0.999939566	0.99989309	0.955585296
FAM180B	0.983589637	0.980930691	0.965568718	0.999939566	0.99989309	0.955817715
C11H9orf78	0.983589637	0.936134921	0.939683463	0.999939566	0.99989309	0.956610132
LOC104975676	0.983589637	0.988649234	0.942146285	0.999939566	0.99989309	0.956610132
PILRA	0.983589637	0.929573947	0.941593855	0.999939566	0.99989309	0.957877422
HPCAL1	0.983589637	0.945870442	0.959646613	0.999939566	0.99989309	0.958814003
EEF1G	0.983589637	0.9347557	0.962777992	0.999939566	0.99989309	0.959625205
NUP88	0.983589637	0.929536223	0.945417678	0.999939566	0.99989309	0.960805387

LOC112444207	0.983589637	0.930688185	0.945317503	0.999939566	0.99989309	0.960985422
DTWD1	0.983589637	0.98618789	0.943891967	0.999939566	0.99989309	0.961454986
SETBP1	0.983589637	0.945105738	0.957578315	0.999939566	0.99989309	0.965053654
KRT80	0.983589637	0.941266539	0.945455812	0.999939566	0.99989309	0.968059826
LOC101903375	0.983589637	0.962934461	0.943420215	0.999939566	0.99989309	0.968245211
LTBP4	0.983589637	0.985855411	0.958420472	0.999939566	0.99989309	0.968245211
PHACTR1	0.983589637	0.934531829	0.960137022	0.999939566	0.99989309	0.968245211
CYB5R1	0.983589637	0.942048064	0.969430048	0.999939566	0.99989309	0.968245211
PEPD	0.983589637	0.979916117	0.976719499	0.999939566	0.99989309	0.969981358
LOC101906472	0.983589637	0.920347742	0.987949796	0.999939566	0.99989309	0.972787584
DOC2B	0.983589637	0.955798423	0.957578315	0.999939566	0.99989309	0.973915852
FBXO10	0.983589637	0.936750204	0.981442587	0.999939566	0.99989309	0.978656049
LOC112442383	0.983589637	0.938886519	0.965365809	0.999939566	0.99989309	0.981411983
ANGPT1	0.983589637	0.956958713	0.951092876	0.999939566	0.99989309	0.982559673
MFAP3	0.983589637	0.939437235	0.965416289	0.999939566	0.99989309	0.983453112
FAM98B	0.983589637	0.960095183	0.966626132	0.999939566	0.99989309	0.983453112
PDK3	0.983589637	0.949008068	0.950638965	0.999939566	0.99989309	0.984609406
INAFM1	0.983589637	0.96412538	0.965416289	0.999939566	0.99989309	0.984609406
TLR2	0.983589637	0.94883251	0.944592818	0.999939566	0.99989309	0.986353216
OPA1	0.983589637	0.947806191	0.951006905	0.999939566	0.99989309	0.986389023
LOC112441619	0.983589637	0.930469765	0.963643451	0.999939566	0.99989309	0.986819555
GLE1	0.983589637	0.963517553	0.958420472	0.999939566	0.99989309	0.989128955
LOC112448335	0.983589637	0.933412624	0.939683463	0.999939566	0.99989309	0.990275559
ORC3	0.983589637	0.933171026	0.940785296	0.999939566	0.99989309	0.991281092
SPATS2L	0.983589637	0.944314627	0.944976702	0.999939566	0.99989309	0.991281092
PRRC2B	0.983589637	0.934531829	0.959320266	0.999939566	0.99989309	0.991281092
COPB2	0.983589637	0.930030848	0.96880775	0.999939566	0.99989309	0.991281092
ADGRA2	0.983589637	0.926435857	0.950638965	0.999939566	0.99989309	0.993259399
ATP6AP1L	0.983589637	0.959666592	0.962790365	0.999939566	0.99989309	0.993259399
SLC30A6	0.983589637	0.91994024	0.970114658	0.999939566	0.99989309	0.998074562
ACOT13	0.983636347	0.967515137	0.958420472	0.999939566	0.99989309	0.953690321
GATA2	0.983636347	0.966921779	0.961142538	0.999939566	0.99989309	0.957122667
LOC107132386	0.983636347	0.94883251	0.939683463	0.999939566	0.99989309	0.967851966
ATG7	0.983636347	0.932678969	0.941319062	0.999939566	0.99989309	0.973494928
NTNG2	0.983636347	0.91994024	0.94821101	0.999939566	0.99989309	0.97815722
LOC112444635	0.98364777	0.977500512	0.939683463	0.999939566	0.99989309	0.942685052
LMLN	0.98364777	0.920347742	0.944612298	0.999939566	0.99989309	0.957877422
EDEM2	0.98364777	0.934248552	0.947475618	0.999939566	0.99989309	0.958814003

CINP	0.983807424	0.920347742	0.959320266	0.999939566	0.99989309	0.988323336
CERS1	0.983860733	0.934248552	0.979231344	0.999939566	0.99989309	0.941925704
KCNB1	0.983860733	0.924986626	0.942004125	0.999939566	0.99989309	0.970702553
LOC101905203	0.983884921	0.965239369	0.983736538	0.999939566	0.99989309	0.943685681
LOC100297097	0.983884921	0.936134921	0.959705869	0.999939566	0.99989309	0.975556309
LOC104975890	0.983888725	0.935793939	0.963643451	0.999939566	0.99989309	0.98016619
ZNF185	0.983914886	0.999946057	0.939683463	0.999939566	0.99989309	0.943866443
PPP2R2C	0.983932968	0.922659577	0.962569922	0.999939566	0.99989309	0.956610132
UXT	0.984067307	0.936750204	0.945888703	0.999939566	0.99989309	0.981090047
LOC101903806	0.984395529	0.933412624	0.96993432	0.999939566	0.99989309	0.96544761
CPQ	0.98448751	0.934531829	0.939683463	0.999939566	0.99989309	0.95470473
LOC112443215	0.98448751	0.94610099	0.939683463	0.999939566	0.99989309	0.998074562
LOC617875	0.984665518	0.954059509	0.944976702	0.999939566	0.99989309	0.98016619
LOC100848478	0.984681879	0.921280256	0.941319062	0.999939566	0.99989309	0.941925704
IKBIP	0.984681879	0.936650943	0.96880775	0.999939566	0.99989309	0.941925704
CUL2	0.984681879	0.989661212	0.96880775	0.999939566	0.99989309	0.941925704
GRIN2D	0.984681879	0.93015407	0.980170426	0.999939566	0.99989309	0.941925704
LOC523461	0.984681879	0.934248552	0.982659943	0.999939566	0.99989309	0.941925704
FIGNL1	0.984681879	0.925277949	0.983143823	0.999939566	0.99989309	0.941925704
BLM	0.984681879	0.962934461	0.990949901	0.999939566	0.99989309	0.941925704
PPARGC1B	0.984681879	0.953940554	0.959377939	0.999939566	0.99989309	0.942421464
SLC37A2	0.984681879	0.971426434	0.959705869	0.999939566	0.99989309	0.942421464
RMI2	0.984681879	0.922264623	0.970108169	0.999939566	0.99989309	0.942421464
COMTD1	0.984681879	0.934531829	0.975183307	0.999939566	0.99989309	0.942421464
SPTY2D1	0.984681879	0.930594744	0.98846962	0.999939566	0.99989309	0.942421464
VPS37D	0.984681879	0.928119925	0.958420472	0.999939566	0.99989309	0.942455807
LOC112441543	0.984681879	0.929573947	0.961742979	0.999939566	0.99989309	0.942455807
LOC101902124	0.984681879	0.952464938	0.96939872	0.999939566	0.99989309	0.942455807
C25H7orf26	0.984681879	0.952464938	0.979635589	0.999939566	0.99989309	0.942455807
PITPNM1	0.984681879	0.964279453	0.941593855	0.999939566	0.99989309	0.943138585
FXYD7	0.984681879	0.994365533	0.941593855	0.999939566	0.99989309	0.943138585
CASC4	0.984681879	0.938064973	0.942004125	0.999939566	0.99989309	0.943138585
AAK1	0.984681879	0.944755173	0.942314296	0.999939566	0.99989309	0.943138585
LOC100847941	0.984681879	0.936134921	0.952717357	0.999939566	0.99989309	0.943138585
RAD51C	0.984681879	0.999377692	0.958420472	0.999939566	0.99989309	0.943138585
TMCO4	0.984681879	0.924510975	0.958977478	0.999939566	0.99989309	0.943138585
GMEB1	0.984681879	0.95881154	0.959646613	0.999939566	0.99989309	0.943138585
LOC104972409	0.984681879	0.942882453	0.966260688	0.999939566	0.99989309	0.943138585

LETM2	0.984681879	0.98618789	0.978215635	0.999939566	0.99989309	0.943138585
RXFP3	0.984681879	0.954059509	0.987949796	0.999939566	0.99989309	0.943138585
TOP3A	0.984681879	0.938064973	0.99245091	0.999939566	0.99989309	0.943138585
EXOSC7	0.984681879	0.936134921	0.995799138	0.999939566	0.99989309	0.943138585
NFATC2IP	0.984681879	0.971426434	0.987118563	0.999939566	0.99989309	0.943685681
LSM3	0.984681879	0.958735381	0.965712499	0.999939566	0.99989309	0.943792032
GALK1	0.984681879	0.935176956	0.962754771	0.999939566	0.99989309	0.943848108
CGN	0.984681879	0.974348229	0.994425789	0.999939566	0.99989309	0.943848108
LOC540014	0.984681879	0.99950308	0.942004125	0.999939566	0.99989309	0.943866443
DIS3L	0.984681879	0.99677427	0.979160316	0.999939566	0.99989309	0.94425343
LOC101904084	0.984681879	0.972418749	0.984083818	0.999939566	0.99989309	0.94425343
LOC783533	0.984681879	0.925277949	0.98417194	0.999939566	0.99989309	0.944422403
PMF1	0.984681879	0.971839996	0.979231344	0.999939566	0.99989309	0.944524573
TTLL9	0.984681879	0.983288516	0.959646613	0.999939566	0.99989309	0.944606747
FAM110C	0.984681879	0.974348229	0.994425789	0.999939566	0.99989309	0.944606747
RSPO1	0.984681879	0.999368101	0.959705869	0.999939566	0.99989309	0.944738447
LOC101906522	0.984681879	0.926144389	0.965491674	0.999939566	0.99989309	0.945438446
SF3B4	0.984681879	0.940026055	0.994425789	0.999939566	0.99989309	0.945593145
LOC112441455	0.984681879	0.955798423	0.994425789	0.999939566	0.99989309	0.945593145
LOC101909432	0.984681879	0.973915123	0.939683463	0.999939566	0.99989309	0.945759045
PPP2R3B	0.984681879	0.926435857	0.980772074	0.999939566	0.99989309	0.94588513
IGSF1	0.984681879	0.944194974	0.96880775	0.999939566	0.99989309	0.945888785
SHE	0.984681879	0.992194873	0.96201942	0.999939566	0.99989309	0.946376229
IARS2	0.984681879	0.935793939	0.971070575	0.999939566	0.99989309	0.946376229
ESYT1	0.984681879	0.955798423	0.942004125	0.999939566	0.99989309	0.946717155
LOC539009	0.984681879	0.926435857	0.953198168	0.999939566	0.99989309	0.946717155
MIDN	0.984681879	0.988725451	0.953692935	0.999939566	0.99989309	0.946717155
APBB2	0.984681879	0.956288424	0.965712499	0.999939566	0.99989309	0.946717155
CLOCK	0.984681879	0.938064973	0.982223562	0.999939566	0.99989309	0.946717155
MYOF	0.984681879	0.944194974	0.957379098	0.999939566	0.99989309	0.94734477
HIVEP2	0.984681879	0.936550733	0.945188764	0.999939566	0.99989309	0.947398404
HPS1	0.984681879	0.922234794	0.942004125	0.999939566	0.99989309	0.948277018
ZNF389	0.984681879	0.948994748	0.954313013	0.999939566	0.99989309	0.948277018
LRP3	0.984681879	0.957228764	0.957802423	0.999939566	0.99989309	0.948277018
CDH20	0.984681879	0.983048937	0.958357122	0.999939566	0.99989309	0.948277018
ELFN2	0.984681879	0.988649234	0.961736888	0.999939566	0.99989309	0.948277018
IRAK1BP1	0.984681879	0.935962337	0.979231344	0.999939566	0.99989309	0.948277018
SEPT8	0.984681879	0.924352846	0.989642163	0.999939566	0.99989309	0.948277018

SMIM10	0.984681879	0.957645111	0.959705869	0.999939566	0.99989309	0.949206487
KIF9	0.984681879	0.984479682	0.942004125	0.999939566	0.99989309	0.950409777
EFHD1	0.984681879	0.936968495	0.965169397	0.999939566	0.99989309	0.950601157
GTF3C4	0.984681879	0.93164553	0.991113951	0.999939566	0.99989309	0.950601157
MOB1B	0.984681879	0.942236132	0.980734833	0.999939566	0.99989309	0.950981777
CCNC	0.984681879	0.924510975	0.970486383	0.999939566	0.99989309	0.951047219
CDO1	0.984681879	0.933983042	0.970108169	0.999939566	0.99989309	0.952631575
LOC112443859	0.984681879	0.962934461	0.958571632	0.999939566	0.99989309	0.952784536
LOC112448531	0.984681879	0.968237269	0.957379098	0.999939566	0.99989309	0.952887889
LOC112445985	0.984681879	0.942048064	0.989332994	0.999939566	0.99989309	0.952921369
CEP83	0.984681879	0.936134921	0.959705869	0.999939566	0.99989309	0.95342487
UFSP2	0.984681879	0.928119925	0.985552223	0.999939566	0.99989309	0.95342487
AQP7	0.984681879	0.955798423	0.95577595	0.999939566	0.99989309	0.953940638
AKR1C4	0.984681879	0.92813228	0.979231344	0.999939566	0.99989309	0.953940638
CNST	0.984681879	0.941502351	0.966157152	0.999939566	0.99989309	0.95401375
SHC1	0.984681879	0.94883251	0.951850716	0.999939566	0.99989309	0.954110387
EBF3	0.984681879	0.924799163	0.962275739	0.999939566	0.99989309	0.954334316
ASB16	0.984681879	0.977485755	0.965712499	0.999939566	0.99989309	0.95470473
OMP	0.984681879	0.923186077	0.967302577	0.999939566	0.99989309	0.95470473
B3GNT2	0.984681879	0.929730109	0.979231344	0.999939566	0.99989309	0.95470473
KCNMB4	0.984681879	0.945105738	0.985016728	0.999939566	0.99989309	0.95470473
GPR182	0.984681879	0.937978608	0.957802423	0.999939566	0.99989309	0.954732204
MRM2	0.984681879	0.936134921	0.947475618	0.999939566	0.99989309	0.955020719
NOM1	0.984681879	0.982963758	0.957802423	0.999939566	0.99989309	0.955585296
USP36	0.984681879	0.926435857	0.959705869	0.999939566	0.99989309	0.955585296
LOC101907641	0.984681879	0.959666592	0.962209895	0.999939566	0.99989309	0.955585296
RPL17	0.984681879	0.965239369	0.963643451	0.999939566	0.99989309	0.955585296
KTN1	0.984681879	0.942048064	0.968230268	0.999939566	0.99989309	0.955585296
PKN1	0.984681879	0.942450381	0.968419607	0.999939566	0.99989309	0.955585296
RXRB	0.984681879	0.96412538	0.972211632	0.999939566	0.99989309	0.955585296
CEP290	0.984681879	0.939027995	0.995206789	0.999939566	0.99989309	0.955585296
STX3	0.984681879	0.924799142	0.958420472	0.999939566	0.99989309	0.95579175
FGF12	0.984681879	0.968246192	0.97420715	0.999939566	0.99989309	0.956410517
NFS1	0.984681879	0.923599439	0.951278495	0.999939566	0.99989309	0.956610132
PTK2	0.984681879	0.968312904	0.965712499	0.999939566	0.99989309	0.956610132
KRT18	0.984681879	0.970179915	0.979851274	0.999939566	0.99989309	0.956610132
UBTF	0.984681879	0.933412624	0.982659943	0.999939566	0.99989309	0.956610132
KAT8	0.984681879	0.962428993	0.976782463	0.999939566	0.99989309	0.956670063

ADAMTS4	0.984681879	0.959617383	0.944612298	0.999939566	0.99989309	0.956959747
ZNF300	0.984681879	0.956843573	0.953045982	0.999939566	0.99989309	0.956959747
LOC781421	0.984681879	0.925360181	0.958420472	0.999939566	0.99989309	0.956959747
TMEM86B	0.984681879	0.964270094	0.977447568	0.999939566	0.99989309	0.957140928
LOC101907920	0.984681879	0.922434012	0.989332994	0.999939566	0.99989309	0.957140928
ZC3H4	0.984681879	0.974986418	0.947268948	0.999939566	0.99989309	0.957707888
KHNYN	0.984681879	0.936570494	0.958420472	0.999939566	0.99989309	0.957877422
MAL2	0.984681879	0.922434012	0.961046149	0.999939566	0.99989309	0.957877422
SERPINC1	0.984681879	0.985603736	0.966157152	0.999939566	0.99989309	0.957877422
SLC29A3	0.984681879	0.952464938	0.985552223	0.999939566	0.99989309	0.957896427
CST6	0.984681879	0.935793939	0.942004125	0.999939566	0.99989309	0.957910156
DDX42	0.984681879	0.986851582	0.979347606	0.999939566	0.99989309	0.958183082
MMS19	0.984681879	0.922434012	0.978215635	0.999939566	0.99989309	0.95861387
PPA2	0.984681879	0.923981714	0.958420472	0.999939566	0.99989309	0.959256161
TRNT1	0.984681879	0.932838587	0.961777102	0.999939566	0.99989309	0.959423981
TRAK2	0.984681879	0.926435857	0.98082053	0.999939566	0.99989309	0.960985422
BBS1	0.984681879	0.972563829	0.985552223	0.999939566	0.99989309	0.960985422
LOC104968820	0.984681879	0.956843573	0.95721973	0.999939566	0.99989309	0.961336964
PLPP5	0.984681879	0.962934461	0.941319062	0.999939566	0.99989309	0.961506391
KIAA1841	0.984681879	0.987713536	0.944976702	0.999939566	0.99989309	0.961506391
WISP2	0.984681879	0.942608215	0.947268948	0.999939566	0.99989309	0.961506391
TJP2	0.984681879	0.978651934	0.957379098	0.999939566	0.99989309	0.961506391
COL18A1	0.984681879	0.922234794	0.958420472	0.999939566	0.99989309	0.961506391
LNP1	0.984681879	0.935793939	0.958420472	0.999939566	0.99989309	0.961506391
LOC100850437	0.984681879	0.951198224	0.962176387	0.999939566	0.99989309	0.961506391
USF1	0.984681879	0.943312617	0.976869041	0.999939566	0.99989309	0.961506391
LOC112448753	0.984681879	0.963480908	0.977782604	0.999939566	0.99989309	0.961506391
COPS4	0.984681879	0.971207468	0.978288029	0.999939566	0.99989309	0.961506391
KIAA0556	0.984681879	0.944755173	0.979851274	0.999939566	0.99989309	0.961606931
TNRC6C	0.984681879	0.959119521	0.972141593	0.999939566	0.99989309	0.96163007
LOC788142	0.984681879	0.988649234	0.97363516	0.999939566	0.99989309	0.962411861
LOC783294	0.984681879	0.932156311	0.965712499	0.999939566	0.99989309	0.963135727
C10H15orf61	0.984681879	0.936134921	0.947268948	0.999939566	0.99989309	0.963996128
ADGRA3	0.984681879	0.978473197	0.961742979	0.999939566	0.99989309	0.963996128
SNX14	0.984681879	0.983288516	0.965712499	0.999939566	0.99989309	0.964143145
MXD1	0.984681879	0.926435857	0.966157152	0.999939566	0.99989309	0.965332394
HS3ST4	0.984681879	0.951440129	0.953198168	0.999939566	0.99989309	0.966085124
CAMSAP1	0.984681879	0.939027995	0.967489175	0.999939566	0.99989309	0.967824119

AGO4	0.984681879	0.924433008	0.941319062	0.999939566	0.99989309	0.967945141
LOC100336369	0.984681879	0.938076005	0.976719499	0.999939566	0.99989309	0.968059826
LOC613570	0.984681879	0.938064973	0.947614537	0.999939566	0.99989309	0.968245211
LOC107132897	0.984681879	0.956843573	0.958420472	0.999939566	0.99989309	0.968245211
CREBZF	0.984681879	0.972418749	0.978892556	0.999939566	0.99989309	0.968245211
AGTR1	0.984681879	0.951616411	0.991235675	0.999939566	0.99989309	0.968245211
MTMR2	0.984681879	0.972418749	0.965518535	0.999939566	0.99989309	0.96826636
P2RX3	0.984681879	0.955798423	0.975526636	0.999939566	0.99989309	0.968462588
CD99L2	0.984681879	0.945182201	0.960137022	0.999939566	0.99989309	0.969551744
PDHB	0.984681879	0.941972432	0.941593855	0.999939566	0.99989309	0.970602984
HDDC3	0.984681879	0.955798423	0.95123967	0.999939566	0.99989309	0.970702553
RCAN3	0.984681879	0.974591667	0.960137022	0.999939566	0.99989309	0.970702553
CLIC1	0.984681879	0.969412264	0.942004125	0.999939566	0.99989309	0.970959759
PTGER4	0.984681879	0.94610099	0.982223562	0.999939566	0.99989309	0.970959759
IGF2	0.984681879	0.949008068	0.947475618	0.999939566	0.99989309	0.971025918
LOC112447506	0.984681879	0.922434012	0.957379098	0.999939566	0.99989309	0.971025918
DICER1	0.984681879	0.928844077	0.954463909	0.999939566	0.99989309	0.971560107
MRPS28	0.984681879	0.952069509	0.958977478	0.999939566	0.99989309	0.972231714
LOC782385	0.984681879	0.962225222	0.944976702	0.999939566	0.99989309	0.972487907
TRIM45	0.984681879	0.962321963	0.976972382	0.999939566	0.99989309	0.972487907
SAO	0.984681879	0.93077794	0.945417678	0.999939566	0.99989309	0.973463195
IDS	0.984681879	0.926618064	0.994425789	0.999939566	0.99989309	0.97519644
PACS1	0.984681879	0.978215444	0.951278495	0.999939566	0.99989309	0.975972322
LOC104975788	0.984681879	0.96412538	0.982659943	0.999939566	0.99989309	0.976175609
PPP1R12C	0.984681879	0.94883251	0.969311025	0.999939566	0.99989309	0.97815722
ZBTB42	0.984681879	0.963478319	0.958420472	0.999939566	0.99989309	0.978378356
ANXA2	0.984681879	0.968440623	0.951850716	0.999939566	0.99989309	0.978515834
FBXO25	0.984681879	0.985457646	0.941593855	0.999939566	0.99989309	0.978656049
SESN3	0.984681879	0.964105444	0.95896293	0.999939566	0.99989309	0.978656049
RTKN	0.984681879	0.935793939	0.96993432	0.999939566	0.99989309	0.979233909
DTX3L	0.984681879	0.948273015	0.959506858	0.999939566	0.99989309	0.979606084
HECTD2	0.984681879	0.93181855	0.965169397	0.999939566	0.99989309	0.979606084
C10H15orf41	0.984681879	0.962934461	0.966157152	0.999939566	0.99989309	0.98016619
PDZD9	0.984681879	0.927630122	0.942004125	0.999939566	0.99989309	0.980920516
LOC112447370	0.984681879	0.958622299	0.971981006	0.999939566	0.99989309	0.981164039
LOC112447842	0.984681879	0.947158785	0.97381056	0.999939566	0.99989309	0.981164039
LOC112441611	0.984681879	0.923186077	0.983474414	0.999939566	0.99989309	0.981164039
LOC104974473	0.984681879	0.937904802	0.987854175	0.999939566	0.99989309	0.981411983

ZBTB25	0.984681879	0.926435857	0.980744205	0.999939566	0.99989309	0.981578785
MMD	0.984681879	0.926380879	0.939683463	0.999939566	0.99989309	0.981759054
RGL1	0.984681879	0.949169888	0.942761121	0.999939566	0.99989309	0.982045172
BLMH	0.984681879	0.924799142	0.972070815	0.999939566	0.99989309	0.982045172
CDC14B	0.984681879	0.958621801	0.953198168	0.999939566	0.99989309	0.982066213
INTS9	0.984681879	0.98618789	0.941312064	0.999939566	0.99989309	0.983453112
PNRC1	0.984681879	0.93526537	0.941319062	0.999939566	0.99989309	0.983453112
OXCT1	0.984681879	0.922264623	0.951850716	0.999939566	0.99989309	0.983453112
BRSK1	0.984681879	0.953748191	0.953426945	0.999939566	0.99989309	0.983453112
ELMO1	0.984681879	0.978309093	0.953911392	0.999939566	0.99989309	0.983453112
PPP3CB	0.984681879	0.945105738	0.987842204	0.999939566	0.99989309	0.983453112
LOC107132398	0.984681879	0.958488235	0.98082053	0.999939566	0.99989309	0.983525373
NCBP2	0.984681879	0.971068039	0.941636822	0.999939566	0.99989309	0.98361583
TOX2	0.984681879	0.956134433	0.954819004	0.999939566	0.99989309	0.983836142
MON2	0.984681879	0.947392706	0.965416289	0.999939566	0.99989309	0.984449688
LOC112446029	0.984681879	0.934248552	0.958420472	0.999939566	0.99989309	0.984538433
TLE1	0.984681879	0.924799142	0.978215635	0.999939566	0.99989309	0.985625041
MCCC2	0.984681879	0.926435857	0.944754534	0.999939566	0.99989309	0.986389023
METTL15	0.984681879	0.972786652	0.941593855	0.999939566	0.99989309	0.987210851
GRIP2	0.984681879	0.926618064	0.944976702	0.999939566	0.99989309	0.988323336
RILP	0.984681879	0.922434012	0.965712499	0.999939566	0.99989309	0.98894622
MAMDC2	0.984681879	0.929573947	0.947268948	0.999939566	0.99989309	0.989128955
LOC112443170	0.984681879	0.951616411	0.970904484	0.999939566	0.99989309	0.989313458
PCGF2	0.984681879	0.938108408	0.95896293	0.999939566	0.99989309	0.99064193
TRIM3	0.984681879	0.939437235	0.961742979	0.999939566	0.99989309	0.991051281
CLUAP1	0.984681879	0.921280256	0.948628899	0.999939566	0.99989309	0.991281092
ISL2	0.984681879	0.930469765	0.95126311	0.999939566	0.99989309	0.991281092
TEX14	0.984681879	0.963478319	0.951742137	0.999939566	0.99989309	0.991281092
EEFSEC	0.984681879	0.924510975	0.951850716	0.999939566	0.99989309	0.991281092
ACACB	0.984681879	0.928601644	0.958420472	0.999939566	0.99989309	0.991281092
SCFD1	0.984681879	0.924433008	0.970218925	0.999939566	0.99989309	0.991281092
EHBP1L1	0.984681879	0.955353499	0.962209895	0.999939566	0.99989309	0.993259399
LOC789715	0.984681879	0.947806191	0.952255572	0.999939566	0.99989309	0.993612847
LOC112442048	0.984681879	0.93453191	0.952717357	0.999939566	0.99989309	0.993612847
SIGLEC5	0.984681879	0.970237188	0.941312064	0.999939566	0.99989309	0.995145616
ASRGL1	0.984681879	0.922434012	0.982223562	0.999939566	0.99989309	0.996564988
PUF60	0.984681879	0.940502427	0.961742979	0.999939566	0.99989309	0.998074562
CLDN12	0.984681879	0.929573947	0.961899356	0.999939566	0.99989309	0.998074562

TNRC6A	0.984681879	0.941756431	0.962209895	0.999939566	0.99989309	0.998074562
SPTBN1	0.984681879	0.93448389	0.941319062	0.999939566	0.99989309	0.998636142
HSD17B1	0.984681879	0.931664255	0.944638172	0.999939566	0.99989309	0.998636142
ALG6	0.984681879	0.926435857	0.950881054	0.999939566	0.99989309	0.998636142
SEC24C	0.984681879	0.926435857	0.972104746	0.999939566	0.99989309	0.998636142
LRSAM1	0.984851618	0.940039358	0.957802423	0.999939566	0.99989309	0.957877422
LDHAL6B	0.984950365	0.959119521	0.987208945	0.999939566	0.99989309	0.948277018
STAT4	0.985066755	0.958962779	0.950801642	0.999939566	0.99989309	0.943138585
CAT	0.985066755	0.962934461	0.951850716	0.999939566	0.99989309	0.943138585
LOC101904492	0.985066755	0.925807756	0.95721973	0.999939566	0.99989309	0.943138585
RNF141	0.985066755	0.928601644	0.985016728	0.999939566	0.99989309	0.943138585
LOC112448304	0.985066755	0.929262063	0.991406769	0.999939566	0.99989309	0.943138585
ZNF598	0.985066755	0.94751312	0.99245091	0.999939566	0.99989309	0.943138585
SMOX	0.985066755	0.957353974	0.942761121	0.999939566	0.99989309	0.943685681
FLT3LG	0.985066755	0.959119521	0.991235675	0.999939566	0.99989309	0.943792032
TBC1D7	0.985066755	0.939309165	0.949977753	0.999939566	0.99989309	0.943848108
HIST2H2AA4	0.985066755	0.927298992	0.990086898	0.999939566	0.99989309	0.944738447
TM6SF1	0.985066755	0.94610099	0.990107362	0.999939566	0.99989309	0.945759045
LOC112447438	0.985066755	0.926435857	0.963643451	0.999939566	0.99989309	0.945812207
LOC112443419	0.985066755	0.976925958	0.958420472	0.999939566	0.99989309	0.946302385
RXRG	0.985066755	0.952226848	0.96880775	0.999939566	0.99989309	0.946302385
UBE2S	0.985066755	0.960112278	0.984700021	0.999939566	0.99989309	0.946314386
LOC617648	0.985066755	0.985784205	0.9583959	0.999939566	0.99989309	0.946717739
C18H19orf33	0.985066755	0.99950308	0.957379098	0.999939566	0.99989309	0.948277018
DSP	0.985066755	0.95320487	0.977612552	0.999939566	0.99989309	0.948277018
PCK2	0.985066755	0.963517553	0.945417678	0.999939566	0.99989309	0.949246206
TRAFD1	0.985066755	0.932847328	0.94821101	0.999939566	0.99989309	0.949527038
TM7SF3	0.985066755	0.965239369	0.969618195	0.999939566	0.99989309	0.95342487
LOC787714	0.985066755	0.967758952	0.991235675	0.999939566	0.99989309	0.95342487
SLC39A13	0.985066755	0.93453191	0.992165187	0.999939566	0.99989309	0.954334316
NXN	0.985066755	0.994365533	0.944592818	0.999939566	0.99989309	0.95470473
CSAD	0.985066755	0.96412538	0.958420472	0.999939566	0.99989309	0.95470473
RAD17	0.985066755	0.962063344	0.962569922	0.999939566	0.99989309	0.95470473
LOC100335190	0.985066755	0.951616411	0.985222638	0.999939566	0.99989309	0.95470473
GNAQ	0.985066755	0.953382411	0.969311025	0.999939566	0.99989309	0.954935833
LOC112448364	0.985066755	0.926435857	0.965416289	0.999939566	0.99989309	0.955130073
LOC100848469	0.985066755	0.956843573	0.967873529	0.999939566	0.99989309	0.955585296
LOC616957	0.985066755	0.942048064	0.951850716	0.999939566	0.99989309	0.95579175

LOC107132577	0.985066755	0.992593205	0.944612298	0.999939566	0.99989309	0.955817715
LOC786435	0.985066755	0.991043872	0.942910339	0.999939566	0.99989309	0.956410517
PRR18	0.985066755	0.974986418	0.958420472	0.999939566	0.99989309	0.956410517
LOC783376	0.985066755	0.929536223	0.993278977	0.999939566	0.99989309	0.956410517
MAP4	0.985066755	0.959119521	0.957417441	0.999939566	0.99989309	0.956610132
TAF6	0.985066755	0.934248552	0.994425789	0.999939566	0.99989309	0.957122667
EFCAB5	0.985066755	0.944780259	0.995380703	0.999939566	0.99989309	0.957122667
PEX26	0.985066755	0.957514396	0.943501903	0.999939566	0.99989309	0.957877422
YPEL2	0.985066755	0.987713536	0.962209895	0.999939566	0.99989309	0.957877422
FLT1	0.985066755	0.92813228	0.991406769	0.999939566	0.99989309	0.957877422
PLEKHD1	0.985066755	0.945631802	0.950332796	0.999939566	0.99989309	0.958814003
SV2A	0.985066755	0.963478319	0.959575405	0.999939566	0.99989309	0.96074434
TSEN2	0.985066755	0.959119521	0.943891967	0.999939566	0.99989309	0.961506391
ERC2	0.985066755	0.999560732	0.944485232	0.999939566	0.99989309	0.961506391
PRDM11	0.985066755	0.924510975	0.947061359	0.999939566	0.99989309	0.961506391
LOC112444190	0.985066755	0.967515137	0.962209895	0.999939566	0.99989309	0.961506391
FERMT3	0.985066755	0.988649234	0.96644151	0.999939566	0.99989309	0.961506391
BIN3	0.985066755	0.976006372	0.976161819	0.999939566	0.99989309	0.961506391
TMEM179B	0.985066755	0.934248552	0.988788601	0.999939566	0.99989309	0.961506391
TRMT11	0.985066755	0.929573947	0.98082053	0.999939566	0.99989309	0.961829864
ZNF496	0.985066755	0.967758952	0.96987949	0.999939566	0.99989309	0.963996128
FAM89A	0.985066755	0.936750204	0.977782604	0.999939566	0.99989309	0.963996128
LOC112448057	0.985066755	0.970167557	0.960137022	0.999939566	0.99989309	0.96544761
YES1	0.985066755	0.95697941	0.966626132	0.999939566	0.99989309	0.96544761
HEXIM2	0.985066755	0.956843573	0.944592818	0.999939566	0.99989309	0.968039618
NFAM1	0.985066755	0.942882453	0.9746097	0.999939566	0.99989309	0.968245211
LOC616051	0.985066755	0.928601644	0.97368009	0.999939566	0.99989309	0.969981358
LOC100848472	0.985066755	0.962934461	0.974681091	0.999939566	0.99989309	0.969981358
LOC619156	0.985066755	0.948549713	0.980734833	0.999939566	0.99989309	0.969981358
LOC101906451	0.985066755	0.980930691	0.950801642	0.999939566	0.99989309	0.970702553
SHISAL1	0.985066755	0.998683615	0.944638172	0.999939566	0.99989309	0.970959759
LOC534520	0.985066755	0.936134921	0.98674157	0.999939566	0.99989309	0.971171364
IKBKG	0.985066755	0.948556367	0.944612298	0.999939566	0.99989309	0.971554741
LOC618409	0.985066755	0.960454429	0.948213748	0.999939566	0.99989309	0.974219208
TPCN2	0.985066755	0.939309165	0.983706867	0.999939566	0.99989309	0.974348283
LOC104968671	0.985066755	0.946409197	0.969385259	0.999939566	0.99989309	0.97491746
FRK	0.985066755	0.939870925	0.957575736	0.999939566	0.99989309	0.975877807
NAGS	0.985066755	0.995182748	0.944863023	0.999939566	0.99989309	0.976454461

LMBRD2	0.985066755	0.959666592	0.958420472	0.999939566	0.99989309	0.976918757
PPM1N	0.985066755	0.97365566	0.967489175	0.999939566	0.99989309	0.97815722
ZEB2	0.985066755	0.946142324	0.957575736	0.999939566	0.99989309	0.978565009
SCG5	0.985066755	0.965239369	0.96636908	0.999939566	0.99989309	0.978924127
EXOSC6	0.985066755	0.968814321	0.959198396	0.999939566	0.99989309	0.979606084
DLEC1	0.985066755	0.92813228	0.965750046	0.999939566	0.99989309	0.979810204
TUSC3	0.985066755	0.934248552	0.943891967	0.999939566	0.99989309	0.98016619
LOC112441638	0.985066755	0.955798423	0.950601737	0.999939566	0.99989309	0.98016619
CA10	0.985066755	0.992657849	0.951742137	0.999939566	0.99989309	0.98016619
LOC789733	0.985066755	0.990914937	0.953045982	0.999939566	0.99989309	0.98016619
CA8	0.985066755	0.978309093	0.953198168	0.999939566	0.99989309	0.98016619
ENOX2	0.985066755	0.992657849	0.958420472	0.999939566	0.99989309	0.98016619
ICK	0.985066755	0.936134921	0.954465618	0.999939566	0.99989309	0.981164039
RAMP2	0.985066755	0.936570494	0.959646613	0.999939566	0.99989309	0.981164039
LOC112445965	0.985066755	0.934571318	0.94821101	0.999939566	0.99989309	0.981411983
LOC100337390	0.985066755	0.976925958	0.952208316	0.999939566	0.99989309	0.981411983
DEAF1	0.985066755	0.9347557	0.993927956	0.999939566	0.99989309	0.982066213
LOC786930	0.985066755	0.941288692	0.958420472	0.999939566	0.99989309	0.983427394
RNF217	0.985066755	0.958403824	0.958420472	0.999939566	0.99989309	0.983453112
CDC42BPB	0.985066755	0.928119925	0.976869041	0.999939566	0.99989309	0.984491523
LOC112445938	0.985066755	0.982614603	0.965169397	0.999939566	0.99989309	0.985415682
PLCG1	0.985066755	0.959119521	0.958420472	0.999939566	0.99989309	0.985788947
GATM	0.985066755	0.986162538	0.950638965	0.999939566	0.99989309	0.986716086
PTPN1	0.985066755	0.955353499	0.951092876	0.999939566	0.99989309	0.989313458
RHEX	0.985066755	0.937241331	0.978879234	0.999939566	0.99989309	0.99064193
ZNF557	0.985066755	0.959119521	0.952203776	0.999939566	0.99989309	0.991281092
ANAPC4	0.985066755	0.926435857	0.959705869	0.999939566	0.99989309	0.991612651
ANAPC7	0.985066755	0.92813228	0.962209895	0.999939566	0.99989309	0.993612847
ZBTB4	0.985066755	0.962934461	0.951887368	0.999939566	0.99989309	0.995411183
LOC104972724	0.985066755	0.926636777	0.974832809	0.999939566	0.99989309	0.995411183
CCDC88A	0.985066755	0.944755173	0.950638965	0.999939566	0.99989309	0.998636142
THUMPD2	0.985082458	0.974591667	0.993665588	0.999939566	0.99989309	0.945759045
RAB42	0.985082458	0.972212701	0.946573665	0.999939566	0.99989309	0.95470473
NOL8	0.985082458	0.966403817	0.98730572	0.999939566	0.99989309	0.972787584
ELMO2	0.985082458	0.946142324	0.962754771	0.999939566	0.99989309	0.991281092
ZNF408	0.985082458	0.93453191	0.97381056	0.999939566	0.99989309	0.998074562
NIT2	0.985148885	0.945105738	0.967489175	0.999939566	0.99989309	0.945997682
RC3H2	0.985148885	0.967515137	0.983706867	0.999939566	0.99989309	0.968245211

KCNK6	0.985148885	0.956843573	0.961899356	0.999939566	0.99989309	0.9956013
LOC614522	0.985152527	0.998683615	0.958420472	0.999939566	0.99989309	0.944524573
KAZALD1	0.985152527	0.932678969	0.998685166	0.999939566	0.99989309	0.944606747
ANKRD52	0.985152527	0.937104809	0.968406955	0.999939566	0.99989309	0.944722345
FRMPD1	0.985152527	0.944605763	0.947268948	0.999939566	0.99989309	0.945759045
TARS2	0.985152527	0.939027995	0.965750046	0.999939566	0.99989309	0.945759045
MINDY4	0.985152527	0.959119521	0.970904484	0.999939566	0.99989309	0.945759045
G2E3	0.985152527	0.967731788	0.953045982	0.999939566	0.99989309	0.94588513
KLRK1	0.985152527	0.998683615	0.958420472	0.999939566	0.99989309	0.945920273
JADE1	0.985152527	0.944780259	0.987949796	0.999939566	0.99989309	0.946015694
ALDH2	0.985152527	0.989445679	0.950638965	0.999939566	0.99989309	0.946344585
PRKN	0.985152527	0.965239369	0.975881144	0.999939566	0.99989309	0.946344585
RAB38	0.985152527	0.996722893	0.965518535	0.999939566	0.99989309	0.949206487
MED14	0.985152527	0.959119521	0.977612552	0.999939566	0.99989309	0.949206487
CA2	0.985152527	0.929536223	0.978879234	0.999939566	0.99989309	0.949206487
EGLN3	0.985152527	0.940161597	0.987419404	0.999939566	0.99989309	0.950959274
OSCP1	0.985152527	0.934248552	0.972401758	0.999939566	0.99989309	0.952631575
TRPC3	0.985152527	0.976925958	0.951850716	0.999939566	0.99989309	0.95342487
PHLDA1	0.985152527	0.93453191	0.980744205	0.999939566	0.99989309	0.954110387
WDR5B	0.985152527	0.959119521	0.959320266	0.999939566	0.99989309	0.954334316
NCF2	0.985152527	0.98362464	0.962209895	0.999939566	0.99989309	0.954565653
LOC112442715	0.985152527	0.929705559	0.944612298	0.999939566	0.99989309	0.95470473
KLHL20	0.985152527	0.976925958	0.963643451	0.999939566	0.99989309	0.95470473
CCND2	0.985152527	0.936134921	0.968478091	0.999939566	0.99989309	0.9547529
DNPH1	0.985152527	0.976925958	0.962209895	0.999939566	0.99989309	0.955544309
KDM5A	0.985152527	0.988649234	0.944612298	0.999939566	0.99989309	0.955585296
PRKRA	0.985152527	0.936134921	0.953692935	0.999939566	0.99989309	0.955585296
ZNF597	0.985152527	0.944314627	0.966157152	0.999939566	0.99989309	0.955585296
CCS	0.985152527	0.990586436	0.970904484	0.999939566	0.99989309	0.955585296
STIM2	0.985152527	0.982017699	0.969542472	0.999939566	0.99989309	0.956610132
C14H8orf76	0.985152527	0.980930691	0.983907159	0.999939566	0.99989309	0.956610132
KCTD18	0.985152527	0.965239369	0.965712499	0.999939566	0.99989309	0.957249831
ZNF473	0.985152527	0.949787238	0.991113951	0.999939566	0.99989309	0.957877422
LOC785605	0.985152527	0.965239369	0.963643451	0.999939566	0.99989309	0.958814003
LOC107131357	0.985152527	0.988598186	0.958420472	0.999939566	0.99989309	0.95908735
FAM184B	0.985152527	0.965239369	0.991235675	0.999939566	0.99989309	0.95983084
DCLRE1B	0.985152527	0.943855442	0.958420472	0.999939566	0.99989309	0.961454986
NDRG2	0.985152527	0.955798423	0.965416289	0.999939566	0.99989309	0.961454986

LOC104973431	0.985152527	0.93453191	0.962754771	0.999939566	0.99989309	0.961506391
ANKH	0.985152527	0.930225567	0.987949796	0.999939566	0.99989309	0.961506391
TMX1	0.985152527	0.933412624	0.944060947	0.999939566	0.99989309	0.96544761
EEF2K	0.985152527	0.962934461	0.989332994	0.999939566	0.99989309	0.966567675
APCDD1L	0.985152527	0.941972432	0.958420472	0.999939566	0.99989309	0.967796552
JMJD8	0.985152527	0.97572611	0.965712499	0.999939566	0.99989309	0.968059826
POGK	0.985152527	0.955798423	0.959351558	0.999939566	0.99989309	0.968245211
HOXA4	0.985152527	0.962934461	0.962209895	0.999939566	0.99989309	0.968245211
CAMK2D	0.985152527	0.952283714	0.958977478	0.999939566	0.99989309	0.96826636
ERGIC3	0.985152527	0.971530984	0.959377939	0.999939566	0.99989309	0.96826636
LOC112444194	0.985152527	0.944420015	0.953692935	0.999939566	0.99989309	0.969981358
APH1A	0.985152527	0.950861808	0.958420472	0.999939566	0.99989309	0.972487907
DIO2	0.985152527	0.945105738	0.947660114	0.999939566	0.99989309	0.973195071
LOC101905687	0.985152527	0.958621801	0.947475618	0.999939566	0.99989309	0.974379635
ZBTB8A	0.985152527	0.988725451	0.954463909	0.999939566	0.99989309	0.976454461
LNPEP	0.985152527	0.931095524	0.953045982	0.999939566	0.99989309	0.977961448
ZNF319	0.985152527	0.982051974	0.957578315	0.999939566	0.99989309	0.978515834
LOC112448155	0.985152527	0.936134921	0.944612298	0.999939566	0.99989309	0.979606084
UACA	0.985152527	0.944420015	0.95783333	0.999939566	0.99989309	0.980144374
IPMK	0.985152527	0.936099262	0.975212837	0.999939566	0.99989309	0.980144374
TMEM44	0.985152527	0.944755173	0.978892556	0.999939566	0.99989309	0.980144374
PURA	0.985152527	0.938538223	0.957417441	0.999939566	0.99989309	0.98016619
ITM2B	0.985152527	0.934525482	0.952208316	0.999939566	0.99989309	0.981411983
FAT4	0.985152527	0.963478319	0.945417678	0.999939566	0.99989309	0.983453112
MAP3K21	0.985152527	0.965881793	0.960213582	0.999939566	0.99989309	0.983453112
MCAM	0.985152527	0.939744361	0.96596172	0.999939566	0.99989309	0.983453112
MTMR3	0.985152527	0.957353974	0.964668297	0.999939566	0.99989309	0.984449688
SMPDL3A	0.985152527	0.967237998	0.962209895	0.999939566	0.99989309	0.986389023
RN18S1	0.985152527	0.98743321	0.952976177	0.999939566	0.99989309	0.988323336
RTL9	0.985152527	0.940039358	0.944863023	0.999939566	0.99989309	0.989243676
DGKH	0.985152527	0.979916117	0.957802423	0.999939566	0.99989309	0.991281092
LOC107132556	0.985152527	0.939437235	0.965169397	0.999939566	0.99989309	0.991281092
CLIP1	0.985152527	0.965882863	0.954313013	0.999939566	0.99989309	0.991612651
C21H14orf132	0.985152527	0.969537549	0.960623345	0.999939566	0.99989309	0.99244992
FGR	0.985152527	0.939027995	0.959822136	0.999939566	0.99989309	0.995736528
SPEF1	0.985152527	0.939309165	0.967413848	0.999939566	0.99989309	0.996455238
TBX21	0.985152527	0.964270094	0.950638965	0.999939566	0.99989309	0.998074562
TAF9	0.985152527	0.934248552	0.958420472	0.999939566	0.99989309	0.998074562

OCIAD2	0.985152527	0.930890453	0.965712499	0.999939566	0.99989309	0.998074562
PCNT	0.985152527	0.938064973	0.966157152	0.999939566	0.99989309	0.998074562
TRNAU1AP	0.985152527	0.959119521	0.957379098	0.999939566	0.99989309	0.998372556
PLEKHG7	0.985317629	0.941069307	0.985135994	0.999939566	0.99989309	0.946682284
LOC112449282	0.985317629	0.944194974	0.96644151	0.999939566	0.99989309	0.948277018
ZMIZ2	0.985317629	0.934248552	0.979231344	0.999939566	0.99989309	0.949206487
TANK	0.985317629	0.971426434	0.952356493	0.999939566	0.99989309	0.949627777
NRAP	0.985317629	0.958403824	0.951742137	0.999939566	0.99989309	0.952921369
GFOD2	0.985317629	0.93087237	0.963075987	0.999939566	0.99989309	0.95342487
ZFHX2	0.985317629	0.975346785	0.97420715	0.999939566	0.99989309	0.953604648
ZNF239	0.985317629	0.967113913	0.96596172	0.999939566	0.99989309	0.954334316
LOC112448032	0.985317629	0.966388183	0.962209895	0.999939566	0.99989309	0.95470473
CFAP70	0.985317629	0.936134921	0.969618195	0.999939566	0.99989309	0.95470473
DET1	0.985317629	0.941972432	0.958420472	0.999939566	0.99989309	0.955585296
IL20RB	0.985317629	0.933412624	0.982049749	0.999939566	0.99989309	0.955585296
APC	0.985317629	0.963478319	0.962209895	0.999939566	0.99989309	0.956410517
DHX8	0.985317629	0.950091107	0.957802423	0.999939566	0.99989309	0.956610132
CCDC121	0.985317629	0.947959005	0.956880455	0.999939566	0.99989309	0.956959747
NSUN4	0.985317629	0.97980576	0.976719499	0.999939566	0.99989309	0.957122667
NLGN2	0.985317629	0.949008068	0.973488065	0.999939566	0.99989309	0.957877422
ADAL	0.985317629	0.948631073	0.957379098	0.999939566	0.99989309	0.958540316
ST3GAL2	0.985317629	0.936160765	0.97420715	0.999939566	0.99989309	0.959256161
LOC107132288	0.985317629	0.985118714	0.96450055	0.999939566	0.99989309	0.961506391
TCF25	0.985317629	0.929573947	0.958420472	0.999939566	0.99989309	0.961606931
AUNIP	0.985317629	0.941635802	0.957379098	0.999939566	0.99989309	0.962255684
FAM187A	0.985317629	0.972786652	0.965118448	0.999939566	0.99989309	0.965186727
LOC101907005	0.985317629	0.942887777	0.97102147	0.999939566	0.99989309	0.96676428
ANKRD28	0.985317629	0.937957883	0.979096894	0.999939566	0.99989309	0.967238897
EIF4ENIF1	0.985317629	0.936134921	0.959646613	0.999939566	0.99989309	0.968245211
TNFSF14	0.985317629	0.941502351	0.972141593	0.999939566	0.99989309	0.968245211
SLC9A3R2	0.985317629	0.948273015	0.965416289	0.999939566	0.99989309	0.969981358
LOC112443512	0.985317629	0.969537549	0.959705869	0.999939566	0.99989309	0.973494928
C23H6orf226	0.985317629	0.942887777	0.972211632	0.999939566	0.99989309	0.978924127
PEX7	0.985317629	0.939870925	0.970108169	0.999939566	0.99989309	0.979893615
SRSF10	0.985317629	0.954241168	0.982659943	0.999939566	0.99989309	0.980005987
IWS1	0.985317629	0.942882453	0.962569922	0.999939566	0.99989309	0.981411983
TP53RK	0.985317629	0.944314627	0.969311025	0.999939566	0.99989309	0.981411983
LOC104973224	0.985317629	0.957353974	0.95126311	0.999939566	0.99989309	0.981578785

ZCCHC4	0.985317629	0.982703363	0.965365809	0.999939566	0.99989309	0.984651205
PEA15	0.985317629	0.934880166	0.980442556	0.999939566	0.99989309	0.991281092
RAB15	0.985317629	0.963478319	0.95126311	0.999939566	0.99989309	0.997366598
ALG2	0.985317629	0.930469765	0.98567143	0.999939566	0.99989309	0.99797558
TMEM255A	0.985317629	0.936330626	0.958420472	0.999939566	0.99989309	0.998928226
BCAR1	0.985318921	0.982963758	0.950801642	0.999939566	0.99989309	0.95342487
LOC614922	0.985318921	0.944605763	0.985552223	0.999939566	0.99989309	0.954110387
TM6SF2	0.985318921	0.955798423	0.953045982	0.999939566	0.99989309	0.954732204
TMEM184B	0.985318921	0.936134921	0.950638965	0.999939566	0.99989309	0.956610132
CCDC127	0.985318921	0.944314627	0.985214581	0.999939566	0.99989309	0.956610132
THAP1	0.985318921	0.962934461	0.976719499	0.999939566	0.99989309	0.957122667
KCTD9	0.985318921	0.929705559	0.994425789	0.999939566	0.99989309	0.963662162
LOC104970173	0.985318921	0.933363497	0.990347993	0.999939566	0.99989309	0.963996128
RTCA	0.985318921	0.941309212	0.963643451	0.999939566	0.99989309	0.967606536
CYSRT1	0.985318921	0.957645111	0.960137022	0.999939566	0.99989309	0.96826636
IGFBP3	0.985318921	0.972418749	0.947118233	0.999939566	0.99989309	0.970959759
EDEM3	0.985318921	0.977672793	0.96880775	0.999939566	0.99989309	0.978467321
LOC101905648	0.985318921	0.944314627	0.975854775	0.999939566	0.99989309	0.983453112
LOC104969177	0.985318921	0.955798423	0.947268948	0.999939566	0.99989309	0.988323336
ZBTB39	0.985318921	0.944058852	0.945888459	0.999939566	0.99989309	0.997911209
TMEM98	0.985427122	0.936134921	0.962209895	0.999939566	0.99989309	0.959256161
SFXN4	0.985427122	0.956134433	0.972211632	0.999939566	0.99989309	0.968245211
TMEM183A	0.985427122	0.970537329	0.947558764	0.999939566	0.99989309	0.991281092
FAM229B	0.985454301	0.936134921	0.953045982	0.999939566	0.99989309	0.952784536
DLG3	0.985454301	0.957071555	0.962569922	0.999939566	0.99989309	0.966832455
MTF2	0.985454301	0.963465197	0.95721973	0.999939566	0.99989309	0.970702553
LOC107132610	0.985454301	0.933412624	0.958420472	0.999939566	0.99989309	0.983453112
RNF138	0.985675299	0.964270094	0.958420472	0.999939566	0.99989309	0.95470473
VPS4B	0.985675299	0.935793939	0.965416289	0.999939566	0.99989309	0.961506391
LOC112447846	0.985675299	0.952464938	0.976575956	0.999939566	0.99989309	0.968039618
SHF	0.98569058	0.98362464	0.960137022	0.999939566	0.99989309	0.965593291
LOC782755	0.985705798	0.942048064	0.967873529	0.999939566	0.99989309	0.957877422
LOC112446360	0.985705798	0.960112278	0.962754771	0.999939566	0.99989309	0.968245211
C7H5orf15	0.985827525	0.947959005	0.988721867	0.999939566	0.99989309	0.954762004
TRIM31	0.985827525	0.999377692	0.959705869	0.999939566	0.99989309	0.956610132
APOM	0.985827525	0.936134921	0.956176987	0.999939566	0.99989309	0.960805387
LOC112442625	0.985827525	0.976925958	0.97363516	0.999939566	0.99989309	0.961506391
HOXB2	0.985827525	0.955798423	0.96201942	0.999939566	0.99989309	0.96826636

SGSM2	0.985827525	0.949169888	0.979231344	0.999939566	0.99989309	0.969981358
LOC112446696	0.985827525	0.932472944	0.958420472	0.999939566	0.99989309	0.97491665
AIFM3	0.985827525	0.949008068	0.963643451	0.999939566	0.99989309	0.98016619
BBS10	0.985827525	0.947392706	0.957802423	0.999939566	0.99989309	0.989128955
TC2N	0.985840799	0.986245673	0.961742979	0.999939566	0.99989309	0.960805387
LOC100196898	0.985842138	0.982703363	0.959320266	0.999939566	0.99989309	0.985775405
GSC2	0.98612941	0.936365238	0.957802423	0.999939566	0.99989309	0.999899229
LOC104972578	0.986338052	0.999934642	0.957578315	0.999939566	0.99989309	0.948277018
TEN1	0.986338052	0.939177233	0.960137022	0.999939566	0.99989309	0.948277018
RPL23	0.986338052	0.959138652	0.965712499	0.999939566	0.99989309	0.951533962
AHCYL1	0.986338052	0.9347557	0.960137022	0.999939566	0.99989309	0.951654149
NUP62CL	0.986338052	0.962934461	0.984717389	0.999939566	0.99989309	0.95342487
LOC101903165	0.986338052	0.936134921	0.994425789	0.999939566	0.99989309	0.953940638
NAF1	0.986338052	0.986032534	0.965712499	0.999939566	0.99989309	0.95470473
CACTIN	0.986338052	0.9347557	0.989332994	0.999939566	0.99989309	0.954935833
PLCD1	0.986338052	0.959119521	0.984700021	0.999939566	0.99989309	0.956959747
CKS1B	0.986338052	0.936099262	0.98730572	0.999939566	0.99989309	0.963189777
LOC101905239	0.986338052	0.972786652	0.959646613	0.999939566	0.99989309	0.968245211
EEF1AKMT1	0.986338052	0.970586744	0.994425789	0.999939566	0.99989309	0.968245211
POLR3E	0.986338052	0.936134921	0.957379098	0.999939566	0.99989309	0.983453112
LOC104973551	0.986338052	0.968312904	0.950638965	0.999939566	0.99989309	0.991281092
ZHX1	0.986338052	0.952027829	0.964645626	0.999939566	0.99989309	0.993685604
ORC6	0.986338052	0.938998528	0.952414818	0.999939566	0.99989309	0.998928226
LOC112447735	0.986341401	0.96471209	0.996948653	0.999939566	0.99989309	0.953940638
TTC7B	0.986341401	0.935793939	0.958420472	0.999939566	0.99989309	0.979792753
LOC104972542	0.986341401	0.934248552	0.957379098	0.999939566	0.99989309	0.98016619
MTFMT	0.986378216	0.954059509	0.966157152	0.999939566	0.99989309	0.950199992
LSM7	0.986378216	0.980639049	0.95721973	0.999939566	0.99989309	0.950959274
SPRY1	0.986378216	0.979812776	0.975212837	0.999939566	0.99989309	0.951533962
SLC37A1	0.986378216	0.972786652	0.965169397	0.999939566	0.99989309	0.95470473
LOC617698	0.986378216	0.967758952	0.960137022	0.999939566	0.99989309	0.961506391
LOC112441827	0.986378216	0.970179915	0.952976177	0.999939566	0.99989309	0.961606931
LOC101902360	0.986378216	0.965239369	0.976719499	0.999939566	0.99989309	0.963662162
PAQR4	0.986378216	0.985784205	0.967983981	0.999939566	0.99989309	0.966773782
BCL2L11	0.986378216	0.934248552	0.958420472	0.999939566	0.99989309	0.96826636
GPC6	0.986378216	0.978215444	0.957379098	0.999939566	0.99989309	0.970589606
ST6GALNAC6	0.986378216	0.936134921	0.96636908	0.999939566	0.99989309	0.970959759
CDK11B	0.986378216	0.996082675	0.957379098	0.999939566	0.99989309	0.972216653

SDSL	0.986378216	0.965829305	0.953045982	0.999939566	0.99989309	0.981164039
CD24	0.986378216	0.939437235	0.953045982	0.999939566	0.99989309	0.993612847
SPC25	0.986378216	0.962321963	0.962209895	0.999939566	0.99989309	0.9956013
MIS12	0.986453393	0.938064973	0.979231344	0.999939566	0.99989309	0.961506391
CHST1	0.986453393	0.971426434	0.980532396	0.999939566	0.99989309	0.963996128
KIAA0895	0.986453393	0.944314627	0.949770432	0.999939566	0.99989309	0.998636142
XPC	0.986527195	0.955798423	0.98846962	0.999939566	0.99989309	0.986389023
LOC104972843	0.986733585	0.942048064	0.988788601	0.999939566	0.99989309	0.968594601
C17H5orf52	0.986733585	0.951979252	0.953045982	0.999939566	0.99989309	0.991281092
CNN3	0.986747331	0.938064973	0.957333624	0.999939566	0.99989309	0.983453112
EXD2	0.986775468	0.994810749	0.976719499	0.999939566	0.99989309	0.952631575
PPM1H	0.986775468	0.932838587	0.989332994	0.999939566	0.99989309	0.95470473
URGCP	0.986775468	0.997390201	0.96993432	0.999939566	0.99989309	0.956610132
VAMP1	0.986775468	0.95320487	0.991113951	0.999939566	0.99989309	0.965053654
GGT7	0.986775468	0.98743321	0.966626132	0.999939566	0.99989309	0.97815722
GBP6	0.986775468	0.965239369	0.950801642	0.999939566	0.99989309	0.983453112
EMILIN2	0.986793352	0.999669688	0.951850716	0.999939566	0.99989309	0.950959274
ZNF248	0.986793352	0.94883251	0.980385041	0.999939566	0.99989309	0.977961448
UBE2D4	0.986793352	0.978287344	0.95126311	0.999939566	0.99989309	0.988323336
LOC100462699	0.986793352	0.955483597	0.958420472	0.999939566	0.99989309	0.989549426
RBBP8	0.986793352	0.95126822	0.967489175	0.999939566	0.99989309	0.991051281
ZNF41	0.986885907	0.973323731	0.96644151	0.999939566	0.99989309	0.96546833
PPHLN1	0.98704687	0.946142324	0.951742137	0.999939566	0.99989309	0.953604648
GBP4	0.98704687	0.949365661	0.953198168	0.999939566	0.99989309	0.956959747
SLU7	0.98704687	0.94883251	0.960137022	0.999939566	0.99989309	0.95929043
DDX19B	0.98704687	0.972786652	0.957578315	0.999939566	0.99989309	0.961506391
INO80D	0.98704687	0.994365533	0.951850716	0.999939566	0.99989309	0.979893615
PEX1	0.98704687	0.9347557	0.951850716	0.999939566	0.99989309	0.998880954
GRTP1	0.987060052	0.986245673	0.981977161	0.999939566	0.99989309	0.95470473
ZNF774	0.987060052	0.982963758	0.987419404	0.999939566	0.99989309	0.95470473
ZNF200	0.987060052	0.965027449	0.972485306	0.999939566	0.99989309	0.957122667
LOC510913	0.987060052	0.988649234	0.978879234	0.999939566	0.99989309	0.957877422
TBC1D2	0.987060052	0.974986418	0.988585687	0.999939566	0.99989309	0.957877422
ZFP36L2	0.987060052	0.951616411	0.953911392	0.999939566	0.99989309	0.96163007
ZCCHC7	0.987060052	0.972563829	0.959705869	0.999939566	0.99989309	0.965358544
RPL28	0.987060052	0.962063344	0.953692935	0.999939566	0.99989309	0.968245211
LOC101906218	0.987060052	0.942608215	0.980734833	0.999939566	0.99989309	0.968245211
SNAPC3	0.987060052	0.940502318	0.977612552	0.999939566	0.99989309	0.96826636

HIC2	0.987060052	0.970167557	0.960726438	0.999939566	0.99989309	0.971025918
RABGAP1L	0.987060052	0.974986418	0.957379098	0.999939566	0.99989309	0.977151753
OSBPL9	0.987060052	0.987713536	0.965518535	0.999939566	0.99989309	0.978565009
GFAP	0.987060052	0.962123724	0.95896293	0.999939566	0.99989309	0.983453112
AFG1L	0.987060052	0.960365676	0.976719499	0.999939566	0.99989309	0.983453112
PHKG2	0.987064182	0.967758952	0.950801642	0.999939566	0.99989309	0.956610132
ZNF713	0.987064182	0.961481835	0.958357122	0.999939566	0.99989309	0.969981358
LOC100848122	0.987064182	0.962934461	0.982659943	0.999939566	0.99989309	0.97815722
TAP2	0.987064182	0.939027995	0.976719499	0.999939566	0.99989309	0.984449688
SRA1	0.987064182	0.938064973	0.98567143	0.999939566	0.99989309	0.98609047
LOC784464	0.987064182	0.950861808	0.956880455	0.999939566	0.99989309	0.988323336
HCK	0.987064182	0.9347557	0.957379098	0.999939566	0.99989309	0.995411183
LOC112446004	0.987065525	0.954015268	0.958420472	0.999939566	0.99989309	0.951533962
ZBTB43	0.987065525	0.938064973	0.951607422	0.999939566	0.99989309	0.957877422
LOC101906914	0.987065525	0.936859329	0.988111832	0.999939566	0.99989309	0.968245211
LOC104976448	0.987065525	0.974986418	0.958420472	0.999939566	0.99989309	0.983056851
BBS2	0.987065525	0.947202901	0.95721973	0.999939566	0.99989309	0.983427394
PAG1	0.987065525	0.937904802	0.962209895	0.999939566	0.99989309	0.983453112
SNRNP200	0.987065525	0.94610099	0.967873529	0.999939566	0.99989309	0.983453112
ITPR1	0.987065525	0.980930691	0.962754771	0.999939566	0.99989309	0.988323336
GABPA	0.987065525	0.936134921	0.965416289	0.999939566	0.99989309	0.991281092
MISP3	0.98715536	0.962123724	0.989527187	0.999939566	0.99989309	0.950601157
LOC104975849	0.98715536	0.935324849	0.958420472	0.999939566	0.99989309	0.950981777
METTL7A	0.98715536	0.998683615	0.960623345	0.999939566	0.99989309	0.951978897
GNG10	0.98715536	0.939309165	0.994425789	0.999939566	0.99989309	0.952578814
LOC101904248	0.98715536	0.957071555	0.964645626	0.999939566	0.99989309	0.952631575
MCUB	0.98715536	0.948215967	0.971981006	0.999939566	0.99989309	0.952784536
MAST2	0.98715536	0.991459863	0.976972382	0.999939566	0.99989309	0.953604648
TMEM61	0.98715536	0.984479682	0.951278495	0.999939566	0.99989309	0.954110387
ATG101	0.98715536	0.938064973	0.965416289	0.999939566	0.99989309	0.95470473
RAB3C	0.98715536	0.983065474	0.976719499	0.999939566	0.99989309	0.95470473
JAZF1	0.98715536	0.995289146	0.951742137	0.999939566	0.99989309	0.955585296
LIAS	0.98715536	0.936650943	0.959705869	0.999939566	0.99989309	0.955585296
CTGF	0.98715536	0.997390201	0.953692935	0.999939566	0.99989309	0.956610132
CCL26	0.98715536	0.988649234	0.959159138	0.999939566	0.99989309	0.956610132
ITFG1	0.98715536	0.939309165	0.99115315	0.999939566	0.99989309	0.956610132
ALKBH2	0.98715536	0.986245673	0.956941766	0.999939566	0.99989309	0.957877422
FAM45A	0.98715536	0.963478319	0.958420472	0.999939566	0.99989309	0.957877422

ADORA2A	0.98715536	0.944314627	0.971472903	0.999939566	0.99989309	0.957877422
HS1BP3	0.98715536	0.971839996	0.962209895	0.999939566	0.99989309	0.958540316
LOC112446855	0.98715536	0.950675035	0.982223562	0.999939566	0.99989309	0.958606081
WDR37	0.98715536	0.959119521	0.962209895	0.999939566	0.99989309	0.960985422
CENPS	0.98715536	0.963478319	0.965416289	0.999939566	0.99989309	0.960985422
SNUPN	0.98715536	0.974986418	0.969058758	0.999939566	0.99989309	0.961068561
INPP5E	0.98715536	0.944755173	0.995148246	0.999939566	0.99989309	0.961506391
HNRNPA1	0.98715536	0.956843573	0.960137022	0.999939566	0.99989309	0.963996128
TXNDC12	0.98715536	0.970588681	0.971472903	0.999939566	0.99989309	0.963996128
SEC14L2	0.98715536	0.941969309	0.970904484	0.999939566	0.99989309	0.965118001
BCKDHA	0.98715536	0.944043161	0.95721973	0.999939566	0.99989309	0.96544761
GPR153	0.98715536	0.941620046	0.989845175	0.999939566	0.99989309	0.967640694
NFKBIE	0.98715536	0.986681109	0.967955062	0.999939566	0.99989309	0.968039618
GSTCD	0.98715536	0.960365676	0.989408632	0.999939566	0.99989309	0.968059826
LOC112447030	0.98715536	0.973158835	0.986895815	0.999939566	0.99989309	0.968245211
HAUS5	0.98715536	0.936365238	0.987639174	0.999939566	0.99989309	0.968245211
CTDSPL2	0.98715536	0.952941054	0.993665588	0.999939566	0.99989309	0.968245211
PTGDS	0.98715536	0.946142324	0.952255572	0.999939566	0.99989309	0.968462588
PDZRN3	0.98715536	0.937978608	0.962209895	0.999939566	0.99989309	0.97356849
ZFPL1	0.98715536	0.934531829	0.952356493	0.999939566	0.99989309	0.976757738
AP3D1	0.98715536	0.994365533	0.953045982	0.999939566	0.99989309	0.978565009
DAB1	0.98715536	0.955798423	0.985552223	0.999939566	0.99989309	0.978565009
DOCK5	0.98715536	0.939027995	0.959822136	0.999939566	0.99989309	0.978924127
PHOSPHO2	0.98715536	0.98718343	0.957379098	0.999939566	0.99989309	0.981164039
POMC	0.98715536	0.960219676	0.958420472	0.999939566	0.99989309	0.981578785
EXOSC1	0.98715536	0.959119521	0.962209895	0.999939566	0.99989309	0.982045172
FAM208A	0.98715536	0.94883251	0.9728362	0.999939566	0.99989309	0.983453112
DPY19L4	0.98715536	0.935324849	0.95726941	0.999939566	0.99989309	0.985977667
TNFRSF1A	0.98715536	0.9347557	0.965712499	0.999939566	0.99989309	0.986389023
PTAR1	0.98715536	0.965239369	0.952840808	0.999939566	0.99989309	0.987311417
ORMDL2	0.98715536	0.94544531	0.965712499	0.999939566	0.99989309	0.988323336
IRF2BP1	0.98715536	0.939027995	0.98567143	0.999939566	0.99989309	0.988323336
PYCR3	0.98715536	0.950928726	0.96712176	0.999939566	0.99989309	0.989947227
LOC107132070	0.98715536	0.94883251	0.959005679	0.999939566	0.99989309	0.990085862
SRRM5	0.98715536	0.936134921	0.980532396	0.999939566	0.99989309	0.991281092
BCAS4	0.98715536	0.942048064	0.953198168	0.999939566	0.99989309	0.998074562
NPTN	0.987240981	0.978453686	0.990493758	0.999939566	0.99989309	0.95470473
IZUMO1	0.987240981	0.940502427	0.951742137	0.999939566	0.99989309	0.955585296

GPR146	0.987240981	0.965239369	0.979851274	0.999939566	0.99989309	0.969981358
MDH1B	0.987240981	0.960825265	0.972909109	0.999939566	0.99989309	0.97491665
LOC101903205	0.987292265	0.994365533	0.958357122	0.999939566	0.99989309	0.978656049
RPRD1A	0.987292265	0.948318821	0.970904484	0.999939566	0.99989309	0.98016619
TRAF1	0.987321206	0.950091107	0.957379098	0.999939566	0.99989309	0.952592006
BSCL2	0.987321206	0.986361805	0.958420472	0.999939566	0.99989309	0.981090047
FAM13A	0.987321988	0.950928726	0.977782604	0.999939566	0.99989309	0.972267965
UBC	0.987321988	0.952464938	0.961899356	0.999939566	0.99989309	0.993612847
PON2	0.987635736	0.944780259	0.979099287	0.999939566	0.99989309	0.961506391
CIB4	0.987692623	0.989661212	0.970904484	0.999939566	0.99989309	0.952631575
PPM1J	0.987692623	0.936134921	0.976719499	0.999939566	0.99989309	0.956610132
ZNF677	0.987692623	0.974875445	0.995918981	0.999939566	0.99989309	0.957877422
LOC107131341	0.987692623	0.949008068	0.958357122	0.999939566	0.99989309	0.982045172
MBD2	0.987692623	0.939027995	0.959320266	0.999939566	0.99989309	0.988323336
BTD	0.987692623	0.964234693	0.97911312	0.999939566	0.99989309	0.988571083
TRIM52	0.987692623	0.967255704	0.965712499	0.999939566	0.99989309	0.991584567
ASAH1	0.98779429	0.956843573	0.959646613	0.999939566	0.99989309	0.953940638
SPSB3	0.98779429	0.947806191	0.976719499	0.999939566	0.99989309	0.953940638
GNG3	0.98779429	0.963240186	0.978215635	0.999939566	0.99989309	0.954110387
ACAD11	0.98779429	0.991043872	0.960137022	0.999939566	0.99989309	0.95470473
LOC107132546	0.98779429	0.966794964	0.957802423	0.999939566	0.99989309	0.954952212
TBC1D8	0.98779429	0.970843742	0.958420472	0.999939566	0.99989309	0.956410517
ECI1	0.98779429	0.955798423	0.954463909	0.999939566	0.99989309	0.956610132
LOC100848721	0.98779429	0.947392706	0.960213582	0.999939566	0.99989309	0.956610132
AOC3	0.98779429	0.972786652	0.992915726	0.999939566	0.99989309	0.956610132
CCDC197	0.98779429	0.963478319	0.954306201	0.999939566	0.99989309	0.956959747
SMC6	0.98779429	0.989335307	0.957379098	0.999939566	0.99989309	0.957122667
C1QTNF5	0.98779429	0.981087742	0.965169397	0.999939566	0.99989309	0.957122667
BUD31	0.98779429	0.974591667	0.990107362	0.999939566	0.99989309	0.957877422
ZNF142	0.98779429	0.942882453	0.991235675	0.999939566	0.99989309	0.957877422
SOCS2	0.98779429	0.994377463	0.958420472	0.999939566	0.99989309	0.958475633
HELQ	0.98779429	0.986032534	0.965819637	0.999939566	0.99989309	0.958540316
HSD17B8	0.98779429	0.943095297	0.962176387	0.999939566	0.99989309	0.961230194
ZNF574	0.98779429	0.970179915	0.965365809	0.999939566	0.99989309	0.961336964
CYP2U1	0.98779429	0.971207468	0.960726438	0.999939566	0.99989309	0.961506391
CILP	0.98779429	0.962063344	0.965750046	0.999939566	0.99989309	0.961506391
FIBIN	0.98779429	0.964013919	0.979096894	0.999939566	0.99989309	0.961506391
PRKAR2B	0.98779429	0.944780259	0.987118563	0.999939566	0.99989309	0.961506391

LOC785503	0.98779429	0.945631802	0.994425789	0.999939566	0.99989309	0.961506391
THOC3	0.98779429	0.959119521	0.994425789	0.999939566	0.99989309	0.961506391
TBC1D5	0.98779429	0.988725451	0.970904484	0.999939566	0.99989309	0.964224424
JMJD4	0.98779429	0.953748191	0.996075433	0.999939566	0.99989309	0.965053654
LRRFIP1	0.98779429	0.974986418	0.97420715	0.999939566	0.99989309	0.96544761
LOC104971296	0.98779429	0.971839996	0.953045982	0.999939566	0.99989309	0.965530607
ATP5MC2	0.98779429	0.939870925	0.970034593	0.999939566	0.99989309	0.968059826
RPA4	0.98779429	0.982614603	0.959646613	0.999939566	0.99989309	0.968245211
TRIM16	0.98779429	0.943878234	0.985552223	0.999939566	0.99989309	0.968245211
MRPS26	0.98779429	0.944420015	0.993665588	0.999939566	0.99989309	0.968245211
ZNF35	0.98779429	0.955798423	0.994425789	0.999939566	0.99989309	0.972487907
PARVG	0.98779429	0.978453686	0.958420472	0.999939566	0.99989309	0.973494928
CDKAL1	0.98779429	0.993959968	0.962209895	0.999939566	0.99989309	0.974258846
CYP27B1	0.98779429	0.978453686	0.974193101	0.999939566	0.99989309	0.974258846
LOC112447418	0.98779429	0.936808499	0.971104162	0.999939566	0.99989309	0.97519644
LOC107132967	0.98779429	0.970537329	0.96880775	0.999939566	0.99989309	0.975454487
RITA1	0.98779429	0.972786652	0.983474414	0.999939566	0.99989309	0.976757738
LOC107131941	0.98779429	0.982588455	0.96472755	0.999939566	0.99989309	0.978656049
LOC112449558	0.98779429	0.951198224	0.96880775	0.999939566	0.99989309	0.978924127
LOC112448430	0.98779429	0.943878234	0.989845175	0.999939566	0.99989309	0.980144374
LOC112443213	0.98779429	0.946142324	0.959320266	0.999939566	0.99989309	0.98016619
USP18	0.98779429	0.944755173	0.961281956	0.999939566	0.99989309	0.98016619
LPAR5	0.98779429	0.970179915	0.970034593	0.999939566	0.99989309	0.98016619
AREL1	0.98779429	0.939027995	0.982659943	0.999939566	0.99989309	0.98016619
NTN3	0.98779429	0.936134921	0.959320266	0.999939566	0.99989309	0.980841407
RNASEH2C	0.98779429	0.982614603	0.960137022	0.999939566	0.99989309	0.981411983
MORC3	0.98779429	0.988649234	0.959705869	0.999939566	0.99989309	0.983453112
RABIF	0.98779429	0.984082087	0.965118448	0.999939566	0.99989309	0.984491523
ZNF683	0.98779429	0.957645111	0.957379098	0.999939566	0.99989309	0.985788947
SEPT3	0.98779429	0.948666776	0.98730572	0.999939566	0.99989309	0.986389023
CFP	0.98779429	0.978981583	0.958420472	0.999939566	0.99989309	0.988323336
KBTBD11	0.98779429	0.990586436	0.957379098	0.999939566	0.99989309	0.99064193
SPATA21	0.98779429	0.94883251	0.959646613	0.999939566	0.99989309	0.99064193
MYH7B	0.98779429	0.980614912	0.958420472	0.999939566	0.99989309	0.991281092
LOC101907747	0.98779429	0.936134921	0.979231344	0.999939566	0.99989309	0.994307746
LOC112448540	0.98779429	0.938910419	0.965416289	0.999939566	0.99989309	0.995145616
APOL3	0.98779429	0.938064973	0.957379098	0.999939566	0.99989309	0.996564988
LOC101905010	0.98779429	0.949008068	0.957379098	0.999939566	0.99989309	0.998074562

TSPAN32	0.98779429	0.941643123	0.95896293	0.999939566	0.99989309	0.998074562
PSPC1	0.98779429	0.969870198	0.954306201	0.999939566	0.99989309	0.998636142
SAMD10	0.98779429	0.936859329	0.969618195	0.999939566	0.99989309	0.998636142
MIPEP	0.987907853	0.962123724	0.994425789	0.999939566	0.99989309	0.95470473
TNIP1	0.987961777	0.953320039	0.962209895	0.999939566	0.99989309	0.95470473
KLHL12	0.987961777	0.966291707	0.967162643	0.999939566	0.99989309	0.95470473
LOC784735	0.987961777	0.955798423	0.968406955	0.999939566	0.99989309	0.95470473
SLC1A1	0.987961777	0.967515137	0.989527187	0.999939566	0.99989309	0.95470473
LRRCC1	0.987961777	0.945463721	0.994425789	0.999939566	0.99989309	0.95470473
TWSG1	0.987961777	0.964270094	0.966157152	0.999939566	0.99989309	0.955585296
AAED1	0.987961777	0.962063344	0.988788601	0.999939566	0.99989309	0.955585296
C25H16orf91	0.987961777	0.994810749	0.988041385	0.999939566	0.99989309	0.955974956
LOC615271	0.987961777	0.939027995	0.998524485	0.999939566	0.99989309	0.95640184
RIMS3	0.987961777	0.951547595	0.989527187	0.999939566	0.99989309	0.956610132
NTM	0.987961777	0.974590874	0.965416289	0.999939566	0.99989309	0.957588442
MANEA	0.987961777	0.965239369	0.957379098	0.999939566	0.99989309	0.957877422
LOC781982	0.987961777	0.972786652	0.983706867	0.999939566	0.99989309	0.958814003
FEZ2	0.987961777	0.958557467	0.996948653	0.999939566	0.99989309	0.959256161
LOC514680	0.987961777	0.969537549	0.957578315	0.999939566	0.99989309	0.960985422
LOC104975162	0.987961777	0.974256477	0.976161819	0.999939566	0.99989309	0.960985422
LOC101905029	0.987961777	0.959119521	0.965416289	0.999939566	0.99989309	0.961506391
F8	0.987961777	0.967758952	0.954043686	0.999939566	0.99989309	0.961606931
LOC784297	0.987961777	0.986245673	0.985757941	0.999939566	0.99989309	0.961606931
ARHGAP17	0.987961777	0.956843573	0.977612552	0.999939566	0.99989309	0.962118346
ZNF575	0.987961777	0.971231193	0.991235675	0.999939566	0.99989309	0.962118346
SLC26A1	0.987961777	0.95320487	0.996948653	0.999939566	0.99989309	0.962118346
LOC100848419	0.987961777	0.947959005	0.958257805	0.999939566	0.99989309	0.963996128
EPB41L4B	0.987961777	0.983288516	0.957575736	0.999939566	0.99989309	0.964188691
SPNS1	0.987961777	0.977584325	0.979231344	0.999939566	0.99989309	0.96435585
TMEM38A	0.987961777	0.952464938	0.957578315	0.999939566	0.99989309	0.964820716
TMEM74	0.987961777	0.990685275	0.976972382	0.999939566	0.99989309	0.965332394
LOC112448488	0.987961777	0.962934461	0.984700021	0.999939566	0.99989309	0.96544761
FOLH1B	0.987961777	0.969418414	0.958420472	0.999939566	0.99989309	0.966338859
TAF3	0.987961777	0.962934461	0.955325896	0.999939566	0.99989309	0.96676428
TTL	0.987961777	0.962934461	0.962209895	0.999939566	0.99989309	0.968059826
LOC535280	0.987961777	0.963517553	0.984192956	0.999939566	0.99989309	0.968245211
LOC104968656	0.987961777	0.971812321	0.987537609	0.999939566	0.99989309	0.968245211
OTULIN	0.987961777	0.944314627	0.994425789	0.999939566	0.99989309	0.968245211

TRIM14	0.987961777	0.981226249	0.967413848	0.999939566	0.99989309	0.969942217
FBRSL1	0.987961777	0.957071555	0.965712499	0.999939566	0.99989309	0.970077808
YAE1D1	0.987961777	0.970167557	0.983706867	0.999939566	0.99989309	0.970351684
LOC788334	0.987961777	0.965027449	0.954900313	0.999939566	0.99989309	0.971025918
VWA8	0.987961777	0.944422127	0.969311025	0.999939566	0.99989309	0.972487907
TADA2B	0.987961777	0.957645111	0.982659943	0.999939566	0.99989309	0.973184786
ZNF524	0.987961777	0.951252603	0.958420472	0.999939566	0.99989309	0.973494928
ATP11B	0.987961777	0.949008068	0.963643451	0.999939566	0.99989309	0.979606084
HMCES	0.987961777	0.977618814	0.967873529	0.999939566	0.99989309	0.98016619
ACKR2	0.987961777	0.980682689	0.969058758	0.999939566	0.99989309	0.981090047
PPT1	0.987961777	0.944755173	0.971981006	0.999939566	0.99989309	0.981164039
HERC5	0.987961777	0.950091107	0.975183307	0.999939566	0.99989309	0.982066213
ABCA10	0.987961777	0.954105281	0.967983981	0.999939566	0.99989309	0.983453112
CENPM	0.987961777	0.937043243	0.987419404	0.999939566	0.99989309	0.983453112
RUNDC3B	0.987961777	0.963291559	0.959575405	0.999939566	0.99989309	0.983836142
POGZ	0.987961777	0.985603736	0.961899356	0.999939566	0.99989309	0.985517088
GCNT1	0.987961777	0.957902362	0.970218925	0.999939566	0.99989309	0.986819555
KAT2A	0.987961777	0.974348229	0.95826379	0.999939566	0.99989309	0.995411183
CAMK4	0.987961777	0.967237998	0.961899356	0.999939566	0.99989309	0.995411183
RBM41	0.987961777	0.960160968	0.976719499	0.999939566	0.99989309	0.996455238
LCN6	0.987961777	0.955798423	0.969311025	0.999939566	0.99989309	0.998074562
LOC112443877	0.987961777	0.948215967	0.958420472	0.999939566	0.99989309	0.998636142
TAPBPL	0.988120696	0.965239369	0.985552223	0.999939566	0.99989309	0.955585296
CSNK1G3	0.988172096	0.974986418	0.972909109	0.999939566	0.99989309	0.956986009
ARHGEF4	0.988172096	0.971426434	0.959198396	0.999939566	0.99989309	0.970702553
CCDC162P	0.988279445	0.962063344	0.969008372	0.999939566	0.99989309	0.963996128
LOC112448390	0.988360986	0.991878011	0.96712176	0.999939566	0.99989309	0.956610132
WDR70	0.988360986	0.959119521	0.963643451	0.999939566	0.99989309	0.99797558
SFT2D3	0.988382133	0.963478319	0.959646613	0.999939566	0.99989309	0.972487907
ADAMTS20	0.988382133	0.972418749	0.962209895	0.999939566	0.99989309	0.978924127
ZNF512	0.988382133	0.960521833	0.974832809	0.999939566	0.99989309	0.982567823
RNF6	0.988526013	0.996120899	0.967302577	0.999939566	0.99989309	0.957877422
RBM27	0.988526013	0.955798423	0.962209895	0.999939566	0.99989309	0.968245211
SMIM15	0.988533651	0.974986418	0.965169397	0.999939566	0.99989309	0.961506391
RPP14	0.98860466	0.942236132	0.993665588	0.999939566	0.99989309	0.956670063
TMEM178A	0.98860466	0.953940554	0.962209895	0.999939566	0.99989309	0.957122667
FBXW4	0.98860466	0.976766304	0.959159138	0.999939566	0.99989309	0.961336964
HS3ST3A1	0.98860466	0.99950308	0.962569922	0.999939566	0.99989309	0.961506391

CNR1	0.98860466	0.974590874	0.966919864	0.999939566	0.99989309	0.961506391
GALNT16	0.98860466	0.976925958	0.962176387	0.999939566	0.99989309	0.963996128
ARHGAP35	0.98860466	0.943380191	0.957547697	0.999939566	0.99989309	0.964188691
DGCR2	0.98860466	0.944780259	0.990693603	0.999939566	0.99989309	0.966811132
PFKM	0.98860466	0.939744361	0.965118448	0.999939566	0.99989309	0.968245211
GLB1L3	0.98860466	0.978833989	0.968406955	0.999939566	0.99989309	0.968245211
PRSS33	0.98860466	0.944780259	0.972378657	0.999939566	0.99989309	0.973494928
LRRC32	0.98860466	0.98743321	0.965518535	0.999939566	0.99989309	0.974017283
LOC112442264	0.98860466	0.990611325	0.962588495	0.999939566	0.99989309	0.978656049
MAP2K2	0.98860466	0.993959968	0.957379098	0.999939566	0.99989309	0.979233909
LOC512248	0.98860466	0.950746819	0.957417441	0.999939566	0.99989309	0.979606084
ZNF169	0.98860466	0.97908188	0.958420472	0.999939566	0.99989309	0.979792753
CTDSP2	0.98860466	0.956843573	0.988442544	0.999939566	0.99989309	0.98016619
ORAI3	0.98860466	0.962934461	0.985552223	0.999939566	0.99989309	0.980346257
PARP8	0.98860466	0.948732806	0.98662331	0.999939566	0.99989309	0.981090047
DPY19L3	0.98860466	0.949169888	0.958420472	0.999939566	0.99989309	0.983453112
OTUD7A	0.98860466	0.942048064	0.959159138	0.999939566	0.99989309	0.983453112
RGS19	0.98860466	0.95320487	0.979851274	0.999939566	0.99989309	0.983453112
KIAA2013	0.98860466	0.944605763	0.992542989	0.999939566	0.99989309	0.983453112
VEGFB	0.98860466	0.95320487	0.984035647	0.999939566	0.99989309	0.983913629
NRDE2	0.98860466	0.966291707	0.966420021	0.999939566	0.99989309	0.989313458
LOC786256	0.98860466	0.976760806	0.969502154	0.999939566	0.99989309	0.991612651
CLPB	0.98860466	0.955798423	0.958420472	0.999939566	0.99989309	0.998074562
RESP18	0.988757914	0.996237308	0.96880775	0.999939566	0.99989309	0.955585296
ZNF263	0.988757914	0.981332269	0.967489175	0.999939566	0.99989309	0.958183842
C1D	0.988947718	0.953940554	0.989332994	0.999939566	0.99989309	0.957122667
NTS	0.988947718	0.988725451	0.962209895	0.999939566	0.99989309	0.96544761
LOC104973746	0.988947718	0.97653184	0.958420472	0.999939566	0.99989309	0.966375116
PEX14	0.988947718	0.956267412	0.959198396	0.999939566	0.99989309	0.968245211
ZNF444	0.988947718	0.974986418	0.989332994	0.999939566	0.99989309	0.968462588
HECW2	0.988947718	0.964234693	0.965416289	0.999939566	0.99989309	0.988454337
KPNA5	0.988969796	0.962934461	0.958420472	0.999939566	0.99989309	0.957877422
FBXO34	0.988969796	0.962934461	0.987842204	0.999939566	0.99989309	0.969545443
SNRPC	0.988969796	0.962934461	0.987854175	0.999939566	0.99989309	0.986006158
MALSU1	0.989012309	0.957228764	0.958420472	0.999939566	0.99989309	0.955585296
CD9	0.989012309	0.939437235	0.989845175	0.999939566	0.99989309	0.96826636
USP45	0.98902957	0.975673897	0.962209895	0.999939566	0.99989309	0.996493376
PPP3CC	0.989043069	0.948273015	0.969311025	0.999939566	0.99989309	0.962118346

GPR107	0.989045248	0.945105738	0.965518535	0.999939566	0.99989309	0.981164039
CGAS	0.989144397	0.948215967	0.985396677	0.999939566	0.99989309	0.97458336
JTB	0.989204415	0.967707663	0.966157152	0.999939566	0.99989309	0.958963004
NUP43	0.989437693	0.951616411	0.962209895	0.999939566	0.99989309	0.956610132
CAPRIN2	0.989437693	0.944314627	0.958420472	0.999939566	0.99989309	0.957877422
LOC100849067	0.989437693	0.957645111	0.966157152	0.999939566	0.99989309	0.958540316
PI4KA	0.989437693	0.969023271	0.961899356	0.999939566	0.99989309	0.968039618
LOC101907857	0.989437693	0.997390201	0.960137022	0.999939566	0.99989309	0.97084193
PAF1	0.989437693	0.967758952	0.968406955	0.999939566	0.99989309	0.972487907
MAP3K4	0.989437693	0.94883251	0.958420472	0.999939566	0.99989309	0.972698857
SMPD2	0.989437693	0.951616411	0.990057983	0.999939566	0.99989309	0.974348283
C1H3orf33	0.989437693	0.944755173	0.968419607	0.999939566	0.99989309	0.978656049
RP9	0.989437693	0.950091107	0.964720224	0.999939566	0.99989309	0.996455238
SLC29A1	0.989445259	0.945105738	0.988585687	0.999939566	0.99989309	0.96544761
NRP1	0.989445259	0.970524066	0.980734833	0.999939566	0.99989309	0.968245211
CRYBG1	0.989558254	0.962123724	0.976719499	0.999939566	0.99989309	0.957896427
KEAP1	0.989558254	0.944422127	0.963643451	0.999939566	0.99989309	0.961336964
PCMTD1	0.989558254	0.945110652	0.982659255	0.999939566	0.99989309	0.981164039
LOC101902742	0.989558254	0.94883251	0.97363516	0.999939566	0.99989309	0.993612847
LOC101904667	0.989558254	0.954105281	0.980734833	0.999939566	0.99989309	0.993612847
SHROOM4	0.989558254	0.963478319	0.957802423	0.999939566	0.99989309	0.998636142
LOC783539	0.989648657	0.980930691	0.9671104	0.999939566	0.99989309	0.962422762
BMPR1A	0.989653527	0.942236132	0.994425789	0.999939566	0.99989309	0.962118346
PDCD2L	0.989653527	0.955798423	0.959159138	0.999939566	0.99989309	0.981090047
IL27RA	0.989653527	0.972786652	0.97381056	0.999939566	0.99989309	0.981411983
LOC529930	0.989657497	0.980248308	0.994425789	0.999939566	0.99989309	0.968039618
C1GALT1	0.989686308	0.983065474	0.959646613	0.999939566	0.99989309	0.956610132
TEX30	0.989686308	0.971419443	0.977774487	0.999939566	0.99989309	0.956610132
ARHGAP15	0.989686308	0.988649234	0.977782604	0.999939566	0.99989309	0.956610132
PLEKHA7	0.989686308	0.972052877	0.990107362	0.999939566	0.99989309	0.956610132
RWDD4	0.989686308	0.98833023	0.990603131	0.999939566	0.99989309	0.956610132
GCGR	0.989686308	0.98743321	0.988111832	0.999939566	0.99989309	0.956670063
FCHSD2	0.989686308	0.97908188	0.97420715	0.999939566	0.99989309	0.956959747
PDE4C	0.989686308	0.955798423	0.9840633	0.999939566	0.99989309	0.956959747
LOC112442367	0.989686308	0.950652683	0.976719499	0.999939566	0.99989309	0.957122667
PRR36	0.989686308	0.998683615	0.96201942	0.999939566	0.99989309	0.957140928
DNAJC6	0.989686308	0.985326169	0.988585687	0.999939566	0.99989309	0.957877422
UHRF1BP1	0.989686308	0.956843573	0.987118563	0.999939566	0.99989309	0.958540316

RPLP2	0.989686308	0.950861808	0.962209895	0.999939566	0.99989309	0.960364326
RIPPLY3	0.989686308	0.968123234	0.958420472	0.999939566	0.99989309	0.961506391
ACADL	0.989686308	0.971210729	0.962718273	0.999939566	0.99989309	0.961506391
LOC783461	0.989686308	0.972563829	0.989702604	0.999939566	0.99989309	0.961506391
PLPPR5	0.989686308	0.94883251	0.990493758	0.999939566	0.99989309	0.961506391
PITRM1	0.989686308	0.971068039	0.981977161	0.999939566	0.99989309	0.961606931
HADHA	0.989686308	0.954015268	0.961263525	0.999939566	0.99989309	0.961829864
LOC112448760	0.989686308	0.96104457	0.997140639	0.999939566	0.99989309	0.962118346
ZSWIM7	0.989686308	0.981205835	0.966157152	0.999939566	0.99989309	0.964143145
TSTD3	0.989686308	0.974591667	0.989408632	0.999939566	0.99989309	0.96435585
LSM2	0.989686308	0.978453686	0.985552223	0.999939566	0.99989309	0.96596646
GPR157	0.989686308	0.976925958	0.961742979	0.999939566	0.99989309	0.967011366
HILPDA	0.989686308	0.964013919	0.969618195	0.999939566	0.99989309	0.967011366
RPL36A	0.989686308	0.956843573	0.958420472	0.999939566	0.99989309	0.967376295
PPP5C	0.989686308	0.94883251	0.957575736	0.999939566	0.99989309	0.968059826
C6H4orf3	0.989686308	0.963517553	0.975183307	0.999939566	0.99989309	0.968059826
FN3KRP	0.989686308	0.988153273	0.958420472	0.999939566	0.99989309	0.968245211
DDB1	0.989686308	0.967515137	0.965712499	0.999939566	0.99989309	0.968245211
GIT2	0.989686308	0.942608215	0.967489175	0.999939566	0.99989309	0.968245211
MOCS3	0.989686308	0.97833257	0.969058758	0.999939566	0.99989309	0.968245211
PRCC	0.989686308	0.943855442	0.974193101	0.999939566	0.99989309	0.968245211
DIS3	0.989686308	0.959138652	0.958420472	0.999939566	0.99989309	0.96826636
TTC26	0.989686308	0.948562569	0.963643451	0.999939566	0.99989309	0.96826636
LOC101907688	0.989686308	0.955798423	0.994425789	0.999939566	0.99989309	0.96826636
MLPH	0.989686308	0.99002957	0.970108169	0.999939566	0.99989309	0.969942217
ZRANB3	0.989686308	0.990179479	0.965365809	0.999939566	0.99989309	0.969981358
RNASEL	0.989686308	0.988649234	0.957578315	0.999939566	0.99989309	0.970702553
ST6GALNAC2	0.989686308	0.946142324	0.979231344	0.999939566	0.99989309	0.97084193
LOC112442987	0.989686308	0.963478319	0.96880775	0.999939566	0.99989309	0.971025918
CCDC107	0.989686308	0.968312904	0.987842204	0.999939566	0.99989309	0.973757028
GRIK5	0.989686308	0.944314627	0.967302577	0.999939566	0.99989309	0.97510237
PTPN6	0.989686308	0.979916117	0.962569922	0.999939566	0.99989309	0.978467321
LOC104973285	0.989686308	0.94883251	0.958420472	0.999939566	0.99989309	0.978656049
FLOT2	0.989686308	0.959119521	0.962209895	0.999939566	0.99989309	0.978845762
LOC100847374	0.989686308	0.994810749	0.958420472	0.999939566	0.99989309	0.979792753
WASHC3	0.989686308	0.972418749	0.959159138	0.999939566	0.99989309	0.980144374
HNRNPA0	0.989686308	0.952464938	0.968934792	0.999939566	0.99989309	0.98016619
IFT27	0.989686308	0.958964646	0.976719499	0.999939566	0.99989309	0.98016619

SSRP1	0.989686308	0.980614912	0.966546415	0.999939566	0.99989309	0.980346257
SLC37A4	0.989686308	0.986245673	0.979231344	0.999939566	0.99989309	0.981164039
LOC781004	0.989686308	0.944780259	0.995624898	0.999939566	0.99989309	0.981411983
AGAP1	0.989686308	0.95781929	0.963643451	0.999939566	0.99989309	0.983453112
LOC100139990	0.989686308	0.946142324	0.968934792	0.999939566	0.99989309	0.983453112
GTF2F1	0.989686308	0.952464938	0.987419404	0.999939566	0.99989309	0.983453112
IRF5	0.989686308	0.967375256	0.958420472	0.999939566	0.99989309	0.984980785
KCNQ5	0.989686308	0.995965505	0.959720192	0.999939566	0.99989309	0.986389023
LOC781813	0.989686308	0.960409459	0.959377939	0.999939566	0.99989309	0.988323336
LOC107131660	0.989686308	0.96471209	0.966157152	0.999939566	0.99989309	0.988390891
SFMBT1	0.989686308	0.972786652	0.96993432	0.999939566	0.99989309	0.989128955
HOOK2	0.989686308	0.982703363	0.966157152	0.999939566	0.99989309	0.989313458
C1H3orf58	0.989686308	0.965239369	0.968406955	0.999939566	0.99989309	0.989313458
NGEF	0.989686308	0.984247605	0.966919864	0.999939566	0.99989309	0.989947227
MOCOS	0.989686308	0.957514396	0.96880775	0.999939566	0.99989309	0.989967429
STAT3	0.989686308	0.950928726	0.969896907	0.999939566	0.99989309	0.990501884
SMIM8	0.989686308	0.968123234	0.958420472	0.999939566	0.99989309	0.991281092
LOC101902542	0.989686308	0.958486398	0.969311025	0.999939566	0.99989309	0.991281092
VWA5B2	0.989686308	0.978453686	0.976719499	0.999939566	0.99989309	0.991569266
ITIH5	0.989686308	0.959119521	0.96993432	0.999939566	0.99989309	0.995870224
LOC104968807	0.989686308	0.945105738	0.958420472	0.999939566	0.99989309	0.998636142
LOC112449548	0.989710238	0.965049112	0.959159138	0.999939566	0.99989309	0.996455238
CD302	0.989762586	0.945105738	0.958420472	0.999939566	0.99989309	0.981164039
ORC5	0.989814108	0.970327631	0.974722101	0.999939566	0.99989309	0.983453112
CSTF2	0.989902676	0.962934461	0.962209895	0.999939566	0.99989309	0.961506391
FECH	0.989902676	0.948562569	0.968406955	0.999939566	0.99989309	0.964188691
ZNF664	0.989989326	0.963291559	0.979635589	0.999939566	0.99989309	0.96676428
PLB1	0.989989326	0.997390201	0.960142531	0.999939566	0.99989309	0.970649246
LOC781728	0.989989326	0.963251672	0.979096894	0.999939566	0.99989309	0.970959759
SLC4A1AP	0.989989326	0.990685275	0.958420472	0.999939566	0.99989309	0.991281092
BTBD19	0.989989326	0.94883251	0.963956982	0.999939566	0.99989309	0.998636142
CELF6	0.990006492	0.959119521	0.987842204	0.999939566	0.99989309	0.971554741
ZNF23	0.990006492	0.948666776	0.987842204	0.999939566	0.99989309	0.981090047
LOC107131356	0.990006492	0.963478319	0.978879234	0.999939566	0.99989309	0.983056851
SDR42E1	0.990006492	0.944314627	0.961899356	0.999939566	0.99989309	0.984491523
LOC787074	0.990006492	0.957353974	0.965750046	0.999939566	0.99989309	0.998636142
LOC104974937	0.990015797	0.982590111	0.972485306	0.999939566	0.99989309	0.956670063
WNK1	0.990015797	0.968312904	0.972211632	0.999939566	0.99989309	0.960805387

CLSTN2	0.990015797	0.962934461	0.988442544	0.999939566	0.99989309	0.983453112
LOC101907255	0.990015797	0.986985275	0.962754771	0.999939566	0.99989309	0.990291862
LOC504858	0.990015797	0.965881793	0.961899356	0.999939566	0.99989309	0.991051281
LOC510193	0.990015797	0.952464938	0.969311025	0.999939566	0.99989309	0.99845476
ME3	0.990110248	0.958621801	0.959646613	0.999939566	0.99989309	0.957122667
PDE10A	0.990110248	0.965241881	0.985552223	0.999939566	0.99989309	0.957122667
CAB39L	0.990110248	0.944201598	0.958420472	0.999939566	0.99989309	0.964143145
THRAP3	0.990110248	0.948631073	0.967302577	0.999939566	0.99989309	0.964143145
LOC107132994	0.990110248	0.974986418	0.962790365	0.999939566	0.99989309	0.96544761
OLR1	0.990110248	0.973158835	0.965270177	0.999939566	0.99989309	0.970702553
PLBD1	0.990110248	0.949008068	0.999323478	0.999939566	0.99989309	0.978565009
CFD	0.990110248	0.970537329	0.96880775	0.999939566	0.99989309	0.981164039
MED4	0.990110248	0.962177291	0.962790365	0.999939566	0.99989309	0.983453112
PCCA	0.990110248	0.951616411	0.961899356	0.999939566	0.99989309	0.991281092
LOC112444346	0.990110248	0.952464938	0.972211632	0.999939566	0.99989309	0.998074562
ACSF2	0.990182028	0.957228764	0.977896563	0.999939566	0.99989309	0.96163007
GTF2F2	0.990182028	0.965458817	0.967302577	0.999939566	0.99989309	0.995145616
RNF145	0.99026951	0.998683615	0.962275739	0.999939566	0.99989309	0.957877422
ZNF687	0.99026951	0.978165562	0.964645626	0.999939566	0.99989309	0.973195541
TBC1D25	0.99026951	0.984903461	0.985989185	0.999939566	0.99989309	0.981578785
CBLN3	0.99026951	0.964234693	0.972211632	0.999939566	0.99989309	0.984449688
SMAP1	0.99027832	0.955798423	0.995380703	0.999939566	0.99989309	0.978924127
LOC107132735	0.990296241	0.953147332	0.971981006	0.999939566	0.99989309	0.98016619
SHISA3	0.990561626	0.945105738	0.994425789	0.999939566	0.99989309	0.978656049
PHC1	0.990672715	0.982614603	0.961263525	0.999939566	0.99989309	0.957707888
AFDN	0.990672715	0.959138652	0.978215635	0.999939566	0.99989309	0.959240045
RAB8B	0.990672715	0.944314627	0.965518535	0.999939566	0.99989309	0.959875701
NAALAD2	0.990672715	0.973158835	0.95896293	0.999939566	0.99989309	0.96163007
PPWD1	0.990672715	0.951616411	0.985135994	0.999939566	0.99989309	0.96544761
LOC112448764	0.990672715	0.982521985	0.962176387	0.999939566	0.99989309	0.968857843
PPM1M	0.990672715	0.955798423	0.987208945	0.999939566	0.99989309	0.977401745
MRPL50	0.990672715	0.974986418	0.978892556	0.999939566	0.99989309	0.982066213
SLC25A44	0.990672715	0.972213699	0.972742538	0.999939566	0.99989309	0.982978165
PTPRZ1	0.990672715	0.96412538	0.960137022	0.999939566	0.99989309	0.986389023
LRCH3	0.990672715	0.982963758	0.972485306	0.999939566	0.99989309	0.989128955
OSGEP	0.990672715	0.963478319	0.979231344	0.999939566	0.99989309	0.989745849
KCNK5	0.990672715	0.957645111	0.962209895	0.999939566	0.99989309	0.993612847
LOC100847180	0.990672715	0.981226249	0.958420472	0.999939566	0.99989309	0.995411183

LOC101907800	0.990672715	0.944420015	0.972088763	0.999939566	0.99989309	0.996564988
LOC782966	0.990773879	0.94610099	0.995726315	0.999939566	0.99989309	0.983453112
SIN3A	0.990903194	0.979466937	0.969644737	0.999939566	0.99989309	0.961606931
RHBDD2	0.990903194	0.988153273	0.976719499	0.999939566	0.99989309	0.979606084
GIPR	0.990903194	0.949008068	0.962209895	0.999939566	0.99989309	0.981411983
LOC112448847	0.990903194	0.945631802	0.98846962	0.999939566	0.99989309	0.983453112
LOC112442547	0.991014779	0.94610099	0.965416289	0.999939566	0.99989309	0.972204129
LSM14A	0.991101017	0.986245673	0.959320266	0.999939566	0.99989309	0.957877422
ECSCR	0.991101017	0.993336983	0.959159138	0.999939566	0.99989309	0.958540316
LOC107132045	0.991101017	0.967758952	0.966157152	0.999939566	0.99989309	0.961336964
RUFY1	0.991101017	0.953382411	0.959646613	0.999939566	0.99989309	0.962118346
ARID3B	0.991101017	0.984082087	0.960726438	0.999939566	0.99989309	0.964143145
WASHC5	0.991101017	0.965239369	0.976719499	0.999939566	0.99989309	0.968245211
CEP19	0.991101017	0.974986418	0.978215635	0.999939566	0.99989309	0.971025918
LOC112448511	0.991101017	0.952464938	0.98730572	0.999939566	0.99989309	0.971025918
STYXL1	0.991101017	0.991895983	0.967302577	0.999939566	0.99989309	0.973153155
CYB5D1	0.991101017	0.967758952	0.962302575	0.999939566	0.99989309	0.973195541
LZTS3	0.991101017	0.983291297	0.96880775	0.999939566	0.99989309	0.973195541
TPRKB	0.991101017	0.990334877	0.974832809	0.999939566	0.99989309	0.973195541
UBAC2	0.991101017	0.986245673	0.965750046	0.999939566	0.99989309	0.978467321
INSL3	0.991101017	0.973158835	0.97381056	0.999939566	0.99989309	0.978656049
REXO5	0.991101017	0.964234693	0.961742979	0.999939566	0.99989309	0.979606084
LOC112442713	0.991101017	0.948318821	0.989408632	0.999939566	0.99989309	0.98016619
CAMKK2	0.991101017	0.985457646	0.990107362	0.999939566	0.99989309	0.980920516
PCYOX1	0.991101017	0.957353974	0.973488065	0.999939566	0.99989309	0.981164039
CRELD1	0.991101017	0.991528092	0.965750046	0.999939566	0.99989309	0.982045172
ENTPD3	0.991101017	0.967758952	0.965712499	0.999939566	0.99989309	0.983453112
PRSS23	0.991101017	0.98743321	0.969644737	0.999939566	0.99989309	0.983453112
CTDSP1	0.991101017	0.982833172	0.966157152	0.999939566	0.99989309	0.984449688
PHYKPL	0.991101017	0.955798423	0.976719499	0.999939566	0.99989309	0.984449688
NNAT	0.991101017	0.989445679	0.980325894	0.999939566	0.99989309	0.984491523
CCDC138	0.991101017	0.982963758	0.962777992	0.999939566	0.99989309	0.986819555
LOC100848581	0.991101017	0.962123724	0.962790365	0.999939566	0.99989309	0.988323336
APLP2	0.991101017	0.949250535	0.96636908	0.999939566	0.99989309	0.99064193
YAP1	0.991101017	0.965027449	0.985179619	0.999939566	0.99989309	0.991612651
IQCG	0.991101017	0.962427097	0.982223562	0.999939566	0.99989309	0.993612847
RLIM	0.991101017	0.964013919	0.959320266	0.999939566	0.99989309	0.995411183
TBC1D22A	0.991101017	0.971207468	0.962369613	0.999939566	0.99989309	0.996455238

LOC100847284	0.991101017	0.977341253	0.96636908	0.999939566	0.99989309	0.997366598
IL15RA	0.991101017	0.95018477	0.965169397	0.999939566	0.99989309	0.998074562
PPFIA3	0.991101017	0.969870198	0.961742979	0.999939566	0.99989309	0.998636142
SLC12A7	0.991162344	0.963478319	0.965712499	0.999939566	0.99989309	0.983056851
LOC107132296	0.99132998	0.969020522	0.9840633	0.999939566	0.99989309	0.983453112
LOC101905365	0.991331269	0.95320487	0.985321206	0.999939566	0.99989309	0.961336964
PYGL	0.991331269	0.947959005	0.968954353	0.999939566	0.99989309	0.96544761
H2AFJ	0.991331269	0.955798423	0.968455468	0.999939566	0.99989309	0.972487907
LOC112445952	0.991331269	0.950091107	0.993665588	0.999939566	0.99989309	0.97792865
FAM213A	0.991331269	0.965881793	0.974832809	0.999939566	0.99989309	0.979606084
LOC101903097	0.991331269	0.96471209	0.979231344	0.999939566	0.99989309	0.98016619
ABHD17C	0.991359022	0.973158835	0.991113951	0.999939566	0.99989309	0.962392673
EHD4	0.991359022	0.976150941	0.965169397	0.999939566	0.99989309	0.979088347
LOC505918	0.991359022	0.998890671	0.961742979	0.999939566	0.99989309	0.982066213
LOC789384	0.991359022	0.989445679	0.967302577	0.999939566	0.99989309	0.991281092
LOC112449245	0.991374782	0.981093825	0.965416289	0.999939566	0.99989309	0.983453112
LOC101904133	0.99143208	0.9789789	0.977447568	0.999939566	0.99989309	0.983690417
MAN2A1	0.99151117	0.972418749	0.962209895	0.999939566	0.99989309	0.965530607
SLC39A9	0.991560958	0.967515137	0.979231344	0.999939566	0.99989309	0.988323336
LOC101905232	0.991576976	0.988708263	0.976719499	0.999939566	0.99989309	0.960712754
GPR137C	0.991576976	0.947080604	0.966919864	0.999939566	0.99989309	0.961506391
LOC101907613	0.991576976	0.95320487	0.979231344	0.999939566	0.99989309	0.984609406
AKR1A1	0.991621414	0.976925958	0.959822136	0.999939566	0.99989309	0.968059826
GPRC5C	0.991621414	0.998683615	0.967873529	0.999939566	0.99989309	0.968245211
FMR1	0.991621414	0.965239369	0.978855687	0.999939566	0.99989309	0.968245211
RPL3	0.991621414	0.972418749	0.965712499	0.999939566	0.99989309	0.979792753
LOC112442740	0.991621414	0.966291707	0.972909109	0.999939566	0.99989309	0.98016619
NCR3LG1	0.991621414	0.972786652	0.993665588	0.999939566	0.99989309	0.982045172
TM2D2	0.991807762	0.982703363	0.993590563	0.999939566	0.99989309	0.961506391
BMP5	0.991807762	0.94883251	0.960137022	0.999939566	0.99989309	0.981411983
LOC101902067	0.991844012	0.971839996	0.980734833	0.999939566	0.99989309	0.960601779
RFC2	0.991893273	0.955048292	0.98039137	0.999939566	0.99989309	0.96163007
LOC112446034	0.991893273	0.964105444	0.962517983	0.999939566	0.99989309	0.968245211
GPR31	0.991893273	0.946142324	0.97420715	0.999939566	0.99989309	0.998880954
PIAS3	0.991963749	0.94883251	0.962790365	0.999939566	0.99989309	0.983453112
ZNF366	0.992034344	0.971680593	0.972485306	0.999939566	0.99989309	0.962118346
NDC1	0.992034344	0.964270094	0.970034593	0.999939566	0.99989309	0.97815722
PLTP	0.992058012	0.982051974	0.979231344	0.999939566	0.99989309	0.983453112

ACAD8	0.992126504	0.970524066	0.990107362	0.999939566	0.99989309	0.961506391
ACSF3	0.992126504	0.955798423	0.967955062	0.999939566	0.99989309	0.96163007
CSK	0.992126504	0.980598499	0.965712499	0.999939566	0.99989309	0.962255684
C9H6orf163	0.992126504	0.966921779	0.979635589	0.999939566	0.99989309	0.962255684
HYAL1	0.992126504	0.956134433	0.98082053	0.999939566	0.99989309	0.962392673
ASPH	0.992126504	0.964901162	0.978892556	0.999939566	0.99989309	0.964143145
FBXO4	0.992126504	0.952464938	0.974832809	0.999939566	0.99989309	0.965358544
LOC780963	0.992126504	0.982614603	0.965712499	0.999939566	0.99989309	0.968059826
TSHZ1	0.992126504	0.970237188	0.993301613	0.999939566	0.99989309	0.968059826
ZNF205	0.992126504	0.967113913	0.994425789	0.999939566	0.99989309	0.968059826
LOC100296121	0.992126504	0.94883251	0.999917831	0.999939566	0.99989309	0.968240941
FXYD6	0.992126504	0.956843573	0.962209895	0.999939566	0.99989309	0.968245211
ATM	0.992126504	0.978453686	0.962569922	0.999939566	0.99989309	0.968245211
LRP5	0.992126504	0.98743321	0.966157152	0.999939566	0.99989309	0.968245211
RELA	0.992126504	0.96216882	0.985757941	0.999939566	0.99989309	0.968245211
CHST12	0.992126504	0.94883251	0.989527187	0.999939566	0.99989309	0.968245211
KLK10	0.992126504	0.965027449	0.999323478	0.999939566	0.99989309	0.968245211
SLC52A2	0.992126504	0.990586436	0.963643451	0.999939566	0.99989309	0.968949613
CPED1	0.992126504	0.949008068	0.982659943	0.999939566	0.99989309	0.970589606
TRABD2B	0.992126504	0.967758952	0.978879234	0.999939566	0.99989309	0.970959759
PAQR5	0.992126504	0.986245673	0.974193101	0.999939566	0.99989309	0.972487907
PNP	0.992126504	0.958403824	0.987419404	0.999939566	0.99989309	0.973195071
TAF7	0.992126504	0.991043872	0.988788601	0.999939566	0.99989309	0.973195541
SLC16A10	0.992126504	0.962934461	0.965712499	0.999939566	0.99989309	0.97345607
DOC2G	0.992126504	0.959119521	0.985552223	0.999939566	0.99989309	0.973494928
LOC101902926	0.992126504	0.94883251	0.999695103	0.999939566	0.99989309	0.974379635
BRSK2	0.992126504	0.957071555	0.979635589	0.999939566	0.99989309	0.979233909
GAT	0.992126504	0.963480908	0.960137022	0.999939566	0.99989309	0.979606084
LOC104970698	0.992126504	0.999377692	0.965750046	0.999939566	0.99989309	0.979792753
TYW5	0.992126504	0.952873433	0.968954353	0.999939566	0.99989309	0.979792753
CDK14	0.992126504	0.992032812	0.962209895	0.999939566	0.99989309	0.98016619
DCLRE1A	0.992126504	0.990217839	0.978879234	0.999939566	0.99989309	0.98016619
NSMCE3	0.992126504	0.949883838	0.962754771	0.999939566	0.99989309	0.981090047
CEP97	0.992126504	0.968447746	0.979231344	0.999939566	0.99989309	0.981411983
ADGRF5	0.992126504	0.971839996	0.979231344	0.999939566	0.99989309	0.982045172
FSTL3	0.992126504	0.98743321	0.961263525	0.999939566	0.99989309	0.982066213
LOC101907353	0.992126504	0.978981583	0.987433396	0.999939566	0.99989309	0.982066213
LOC112442208	0.992126504	0.961300305	0.98846962	0.999939566	0.99989309	0.982066213

MAN1B1	0.992126504	0.964234693	0.965169397	0.999939566	0.99989309	0.982978165
ZNF70	0.992126504	0.982568993	0.972485306	0.999939566	0.99989309	0.983453112
MAFA	0.992126504	0.994280343	0.96993432	0.999939566	0.99989309	0.984294754
FBXO47	0.992126504	0.949008068	0.966157152	0.999939566	0.99989309	0.984609406
LOC100847934	0.992126504	0.968447746	0.991693262	0.999939566	0.99989309	0.984609406
LOC112448030	0.992126504	0.971068039	0.985552223	0.999939566	0.99989309	0.984651205
ZC3H12A	0.992126504	0.974986418	0.962209895	0.999939566	0.99989309	0.989313458
CHRM3	0.992126504	0.978453686	0.961742979	0.999939566	0.99989309	0.990825993
CDK19	0.992126504	0.974348229	0.965416289	0.999939566	0.99989309	0.991281092
TMEM117	0.992126504	0.964105444	0.979635589	0.999939566	0.99989309	0.991281092
SNIP1	0.992126504	0.957353974	0.990693603	0.999939566	0.99989309	0.995411183
TRIM37	0.992126504	0.968406819	0.980734833	0.999939566	0.99989309	0.997366598
GLMN	0.992126504	0.957071555	0.976028919	0.999939566	0.99989309	0.998074562
KBTBD4	0.992126504	0.962934461	0.962754771	0.999939566	0.99989309	0.998636142
LOC107132237	0.992363196	0.989773581	0.962777992	0.999939566	0.99989309	0.963662162
LOC112442657	0.992363196	0.979073808	0.965416289	0.999939566	0.99989309	0.970137981
LOC785760	0.992653535	0.967758952	0.97382176	0.999939566	0.99989309	0.989128955
ELOA	0.992905079	0.955798423	0.975526636	0.999939566	0.99989309	0.989128955
LIN7B	0.993000424	0.972786652	0.979231344	0.999939566	0.99989309	0.961915069
GULP1	0.993000424	0.952283714	0.98730572	0.999939566	0.99989309	0.963662162
RHOG	0.993000424	0.970237188	0.97420715	0.999939566	0.99989309	0.966536623
IPP	0.993000424	0.988649234	0.985552223	0.999939566	0.99989309	0.977961448
ECE1	0.993000424	0.971068039	0.962569922	0.999939566	0.99989309	0.98016619
DOCK3	0.993000424	0.980930691	0.970109291	0.999939566	0.99989309	0.983453112
FAM181B	0.993000424	0.972786652	0.962569922	0.999939566	0.99989309	0.989128955
CCDC194	0.993000424	0.956843573	0.963643451	0.999939566	0.99989309	0.998074562
NUP37	0.993016621	0.965239369	0.977447568	0.999939566	0.99989309	0.988323336
ANK3	0.993287744	0.988813991	0.962209895	0.999939566	0.99989309	0.970801789
PTPN14	0.993321595	0.98618789	0.962209895	0.999939566	0.99989309	0.968245211
EDC4	0.99333138	0.989773581	0.984700021	0.999939566	0.99989309	0.972487907
ARHGAP26	0.99339939	0.951616411	0.993278977	0.999939566	0.99989309	0.961606931
LOC101904258	0.99339939	0.964270094	0.989332994	0.999939566	0.99989309	0.974139155
KLF2	0.99339939	0.9789789	0.974681091	0.999939566	0.99989309	0.99064193
CDH23	0.99339939	0.984102025	0.962275739	0.999939566	0.99989309	0.995411183
CH25H	0.99339939	0.962934461	0.985596108	0.999939566	0.99989309	0.998074562
SCN3B	0.993418047	0.99935653	0.962754771	0.999939566	0.99989309	0.96826636
LOC112442262	0.993418047	0.983434022	0.986895815	0.999939566	0.99989309	0.969256573
ZNF316	0.993418047	0.965027449	0.981442587	0.999939566	0.99989309	0.973195541

SCX	0.993418047	0.956751311	0.993665588	0.999939566	0.99989309	0.974139155
KIF3B	0.993418047	0.956843573	0.985135994	0.999939566	0.99989309	0.982045172
POMT2	0.993418047	0.972786652	0.989845175	0.999939566	0.99989309	0.982978165
TRDN	0.993418047	0.993887397	0.962209895	0.999939566	0.99989309	0.983453112
PLEKHA2	0.993418047	0.955798423	0.967489175	0.999939566	0.99989309	0.983836142
LOC783195	0.993418047	0.95781929	0.965712499	0.999939566	0.99989309	0.988323336
ADGRF1	0.993418047	0.988960148	0.976149562	0.999939566	0.99989309	0.988323336
LOC100847695	0.993418047	0.978165562	0.976972382	0.999939566	0.99989309	0.988323336
LOC101905141	0.993418047	0.978453686	0.987842204	0.999939566	0.99989309	0.989780996
SYN1	0.993418047	0.981226249	0.965416289	0.999939566	0.99989309	0.991281092
SNX29	0.993418047	0.954015268	0.977612552	0.999939566	0.99989309	0.993612847
ALPK2	0.993418047	0.973168324	0.967413848	0.999939566	0.99989309	0.997620729
CFAP298	0.993427527	0.995706781	0.965712499	0.999939566	0.99989309	0.96435585
EIF3D	0.993676337	0.970586744	0.969311025	0.999939566	0.99989309	0.96435585
ATRX	0.993676337	0.993959968	0.962790365	0.999939566	0.99989309	0.966085124
FAM196A	0.993676337	0.98743321	0.994589668	0.999939566	0.99989309	0.968039618
AUH	0.993676337	0.952464938	0.966955806	0.999939566	0.99989309	0.968245211
KSR1	0.993676337	0.963478319	0.985321206	0.999939566	0.99989309	0.968245211
INTS14	0.993676337	0.985784205	0.981205716	0.999939566	0.99989309	0.969014529
LOC784473	0.993676337	0.963480908	0.994425789	0.999939566	0.99989309	0.969981358
BCL9L	0.993676337	0.955798423	0.976719499	0.999939566	0.99989309	0.973494928
PLPP3	0.993676337	0.965239369	0.98846962	0.999939566	0.99989309	0.974017283
COMMD9	0.993676337	0.949625762	0.965518535	0.999939566	0.99989309	0.977961448
DGAT1	0.993676337	0.996120899	0.971104162	0.999939566	0.99989309	0.978845762
SEPT4	0.993676337	0.992740593	0.966955806	0.999939566	0.99989309	0.979606084
DCUN1D2	0.993676337	0.968447746	0.981773988	0.999939566	0.99989309	0.979606084
UBALD2	0.993676337	0.978450937	0.962209895	0.999939566	0.99989309	0.98016619
DHRS7B	0.993676337	0.98743321	0.962517983	0.999939566	0.99989309	0.98016619
CHPF	0.993676337	0.989661212	0.979231344	0.999939566	0.99989309	0.98016619
RAP2A	0.993676337	0.955353499	0.98567143	0.999939566	0.99989309	0.98016619
SPATA2L	0.993676337	0.989445679	0.976719499	0.999939566	0.99989309	0.981411983
LOC112448772	0.993676337	0.982963758	0.985321206	0.999939566	0.99989309	0.982045172
CIB2	0.993676337	0.976925958	0.966157152	0.999939566	0.99989309	0.983453112
GPR108	0.993676337	0.969442016	0.978215635	0.999939566	0.99989309	0.986389023
HEXA	0.993676337	0.955353499	0.981977161	0.999939566	0.99989309	0.987210851
TMC8	0.993676337	0.974986418	0.968455468	0.999939566	0.99989309	0.989128955
RARG	0.99376699	0.969412264	0.965169397	0.999939566	0.99989309	0.988323336
UVSSA	0.993833963	0.957071555	0.999541752	0.999939566	0.99989309	0.969942217

LOC101906276	0.993892672	0.96593642	0.998442742	0.999939566	0.99989309	0.96544761
MLH1	0.993892672	0.967731788	0.965518535	0.999939566	0.99989309	0.968059826
CCDC36	0.993892672	0.974986418	0.989408632	0.999939566	0.99989309	0.969981358
HDAC8	0.993892672	0.982311628	0.976719499	0.999939566	0.99989309	0.971554741
TRPM6	0.993892672	0.974590874	0.963643451	0.999939566	0.99989309	0.985006822
MRNIP	0.993892672	0.96412538	0.97381056	0.999939566	0.99989309	0.998074562
LOC107132243	0.993975057	0.958488235	0.976782463	0.999939566	0.99989309	0.96826636
RAB33A	0.994037193	0.952764957	0.974681091	0.999939566	0.99989309	0.982045172
LOC101902141	0.994083222	0.98618789	0.993278977	0.999939566	0.99989309	0.96558836
LOC513573	0.994083222	0.996848511	0.962209895	0.999939566	0.99989309	0.968245211
WASL	0.994083222	0.994365533	0.962209895	0.999939566	0.99989309	0.96826636
RSRC1	0.994083222	0.96471209	0.974722101	0.999939566	0.99989309	0.973195541
UBAP1L	0.994083222	0.963478319	0.967302577	0.999939566	0.99989309	0.998636142
PRICKLE3	0.994224673	0.98618789	0.987433396	0.999939566	0.99989309	0.962255684
TM4SF5	0.994224673	0.987156012	0.97420715	0.999939566	0.99989309	0.967376295
SPIN4	0.99429481	0.977722338	0.974681091	0.999939566	0.99989309	0.96435585
IFI44L	0.99429481	0.974591667	0.980734833	0.999939566	0.99989309	0.965053654
RRP1B	0.99429481	0.953940554	0.985552223	0.999939566	0.99989309	0.968039618
LOC101902043	0.99429481	0.988153273	0.987433396	0.999939566	0.99989309	0.968039618
RAB4B	0.99429481	0.981226249	0.974193101	0.999939566	0.99989309	0.968059826
LOC107132262	0.99429481	0.963478319	0.965750046	0.999939566	0.99989309	0.968245211
UTP23	0.99429481	0.955798423	0.991583737	0.999939566	0.99989309	0.968245211
SEL1L	0.99429481	0.955798423	0.994425789	0.999939566	0.99989309	0.968245211
LOC112441879	0.99429481	0.978460366	0.970904484	0.999939566	0.99989309	0.970959759
EXOSC8	0.99429481	0.967758952	0.995607212	0.999939566	0.99989309	0.971005601
AMN1	0.99429481	0.953748191	0.965518535	0.999939566	0.99989309	0.972487907
TMEM123	0.99429481	0.982703363	0.962790365	0.999939566	0.99989309	0.978656049
ZNF526	0.99429481	0.991895983	0.97381056	0.999939566	0.99989309	0.982045172
C3H1orf50	0.99429481	0.95781929	0.982659943	0.999939566	0.99989309	0.983453112
RRAS2	0.99429481	0.985855411	0.962209895	0.999939566	0.99989309	0.984449688
SULF1	0.99429481	0.982703363	0.970922031	0.999939566	0.99989309	0.986006158
TEX11	0.99429481	0.974591667	0.965712499	0.999939566	0.99989309	0.989313458
PPP1R3G	0.99429481	0.989426681	0.965750046	0.999939566	0.99989309	0.995145616
ATP8A1	0.99429481	0.974986418	0.966626132	0.999939566	0.99989309	0.996564988
TMEM115	0.99429481	0.95881154	0.969811559	0.999939566	0.99989309	0.999676639
RPRML	0.994518383	0.982614603	0.989642163	0.999939566	0.99989309	0.981164039
GSTA4	0.994560569	0.962934461	0.979231344	0.999939566	0.99989309	0.962255684
DCHS1	0.994560569	0.956843573	0.994425789	0.999939566	0.99989309	0.964722523

ARHGEF10	0.994560569	0.978309093	0.968419607	0.999939566	0.99989309	0.964820716
ATP2B1	0.994560569	0.980639049	0.991406769	0.999939566	0.99989309	0.965118001
TRAM1L1	0.994560569	0.993887397	0.968969547	0.999939566	0.99989309	0.96544761
C16H1orf112	0.994560569	0.966291707	0.994425789	0.999939566	0.99989309	0.965530607
CHAMP1	0.994560569	0.982703363	0.979231344	0.999939566	0.99989309	0.965959871
PATJ	0.994560569	0.99134261	0.980772074	0.999939566	0.99989309	0.968039618
TCIRG1	0.994560569	0.985457646	0.973509969	0.999939566	0.99989309	0.968245211
RBFOX3	0.994560569	0.996148299	0.976782463	0.999939566	0.99989309	0.968245211
GPN2	0.994560569	0.989661212	0.978855687	0.999939566	0.99989309	0.968245211
ARL15	0.994560569	0.978676027	0.984700021	0.999939566	0.99989309	0.968245211
LOC101902907	0.994560569	0.987156012	0.98730572	0.999939566	0.99989309	0.970536819
CCDC97	0.994560569	0.957353974	0.985179619	0.999939566	0.99989309	0.970702553
BLCAP	0.994560569	0.972786652	0.986838559	0.999939566	0.99989309	0.970702553
LOC786303	0.994560569	0.963478319	0.994425789	0.999939566	0.99989309	0.970702553
LOC104973390	0.994560569	0.992155457	0.963643451	0.999939566	0.99989309	0.971554741
TJP3	0.994560569	0.970237188	0.994425789	0.999939566	0.99989309	0.971554741
LOC112448088	0.994560569	0.974986418	0.994425789	0.999939566	0.99989309	0.975972322
LOC100299845	0.994560569	0.979515768	0.987419404	0.999939566	0.99989309	0.976757738
SART3	0.994560569	0.962428993	0.975881144	0.999939566	0.99989309	0.977961448
SLC26A9	0.994560569	0.967113913	0.994425789	0.999939566	0.99989309	0.977961448
LOC112442221	0.994560569	0.958621801	0.993278977	0.999939566	0.99989309	0.97815722
IFT46	0.994560569	0.954015268	0.976719499	0.999939566	0.99989309	0.979352545
LRRC25	0.994560569	0.973158835	0.994425789	0.999939566	0.99989309	0.979810204
LOC104975324	0.994560569	0.978453686	0.97420715	0.999939566	0.99989309	0.98016619
ERMAP	0.994560569	0.962934461	0.967302577	0.999939566	0.99989309	0.981164039
RFC4	0.994560569	0.970167557	0.992850925	0.999939566	0.99989309	0.981411983
GNB5	0.994560569	0.971153682	0.993665588	0.999939566	0.99989309	0.982045172
STAG3	0.994560569	0.976006372	0.989332994	0.999939566	0.99989309	0.982066213
FOXF2	0.994560569	0.959119521	0.989845175	0.999939566	0.99989309	0.982514294
PLOD3	0.994560569	0.955798423	0.989642163	0.999939566	0.99989309	0.983427394
LOC112449367	0.994560569	0.955798423	0.97420715	0.999939566	0.99989309	0.983453112
ACSM2B	0.994560569	0.995289146	0.978892556	0.999939566	0.99989309	0.983453112
LOC104970162	0.994560569	0.964105444	0.981005633	0.999939566	0.99989309	0.983453112
ADRB1	0.994560569	0.979100028	0.988740473	0.999939566	0.99989309	0.983453112
LOC101902036	0.994560569	0.955798423	0.96993432	0.999939566	0.99989309	0.984449688
TMEM51	0.994560569	0.96412538	0.989527187	0.999939566	0.99989309	0.984491523
LOC100336777	0.994560569	0.966291707	0.965270177	0.999939566	0.99989309	0.985919775
TMEM169	0.994560569	0.96593642	0.965712499	0.999939566	0.99989309	0.988323336

PCDH9	0.994560569	0.970586744	0.967302577	0.999939566	0.99989309	0.988323336
LOC112441843	0.994560569	0.955798423	0.987273256	0.999939566	0.99989309	0.988323336
LOC112448770	0.994560569	0.972418749	0.994425789	0.999939566	0.99989309	0.988323336
LOC112443225	0.994560569	0.953748191	0.967676552	0.999939566	0.99989309	0.989128955
FAS	0.994560569	0.989661212	0.962790365	0.999939566	0.99989309	0.991281092
LOC100848105	0.994560569	0.967758952	0.96993432	0.999939566	0.99989309	0.991281092
ZNF579	0.994560569	0.96412538	0.978215635	0.999939566	0.99989309	0.991281092
DRAM1	0.994560569	0.956958713	0.979231344	0.999939566	0.99989309	0.991281092
SHROOM1	0.994560569	0.978228439	0.962569922	0.999939566	0.99989309	0.995518609
ZBTB3	0.994560569	0.953748191	0.97381056	0.999939566	0.99989309	0.997620729
ATR	0.99462265	0.993504895	0.965169397	0.999939566	0.99989309	0.966567675
UBR4	0.99462265	0.972133808	0.994425789	0.999939566	0.99989309	0.968245211
LOC112444867	0.99462265	0.966388183	0.990107362	0.999939566	0.99989309	0.983453112
RAB11FIP2	0.99462265	0.989445679	0.97420715	0.999939566	0.99989309	0.983525373
HGS	0.99462265	0.971421486	0.979231344	0.999939566	0.99989309	0.988323336
SLC16A2	0.99462265	0.957353974	0.96880775	0.999939566	0.99989309	0.989549426
H2B	0.99462265	0.970179915	0.98846962	0.999939566	0.99989309	0.991281092
PPOX	0.994642012	0.961455063	0.985552223	0.999939566	0.99989309	0.988323336
HHLA2	0.994788199	0.99950308	0.967294804	0.999939566	0.99989309	0.969981358
LOC100295687	0.994788199	0.965239369	0.98567143	0.999939566	0.99989309	0.972216653
LOC100138633	0.994788199	0.974986418	0.990107362	0.999939566	0.99989309	0.98016619
LOC101903831	0.994788199	0.995678646	0.979635589	0.999939566	0.99989309	0.983453112
LOC112444281	0.994788199	0.967758952	0.979635589	0.999939566	0.99989309	0.984651205
ALCAM	0.994788199	0.974591667	0.966157152	0.999939566	0.99989309	0.988200507
TRMT2B	0.994788199	0.991210574	0.967413848	0.999939566	0.99989309	0.990275559
BAIAP2L1	0.99479473	0.985603736	0.989642163	0.999939566	0.99989309	0.968245211
LOC112442867	0.99479473	0.988882365	0.98039137	0.999939566	0.99989309	0.96826636
LOC112444198	0.994871419	0.992194873	0.966577195	0.999939566	0.99989309	0.966832455
LEKR1	0.994871419	0.997246787	0.966157152	0.999939566	0.99989309	0.969942217
LOC101906457	0.994871419	0.986681109	0.976719499	0.999939566	0.99989309	0.981578785
LOC100336869	0.994876813	0.965239369	0.981567842	0.999939566	0.99989309	0.968039618
LOC112448169	0.994886058	0.974986418	0.990107362	0.999939566	0.99989309	0.965964276
LOC101906966	0.994886058	0.956134433	0.973306845	0.999939566	0.99989309	0.967824119
LOC112448045	0.994886058	0.956267412	0.98567143	0.999939566	0.99989309	0.968039618
CASKIN1	0.994886058	0.963480908	0.994425789	0.999939566	0.99989309	0.968039618
LOC101902768	0.994886058	0.982051974	0.994425789	0.999939566	0.99989309	0.968163235
GOPC	0.994886058	0.956958713	0.965712499	0.999939566	0.99989309	0.968245211
MARS2	0.994886058	0.960454429	0.979096894	0.999939566	0.99989309	0.968245211

OVGP1	0.994886058	0.958557467	0.989702604	0.999939566	0.99989309	0.968245211
LOC104974749	0.994886058	0.968237269	0.990350221	0.999939566	0.99989309	0.968245211
AJAP1	0.994886058	0.982212778	0.994425789	0.999939566	0.99989309	0.968245211
LOC112449619	0.994886058	0.957071555	0.985552223	0.999939566	0.99989309	0.96826636
LOC790266	0.994886058	0.967237998	0.987842204	0.999939566	0.99989309	0.969808185
FOXD2	0.994886058	0.982051974	0.973493452	0.999939566	0.99989309	0.970702553
ZC3H7A	0.994886058	0.997390201	0.976719499	0.999939566	0.99989309	0.971025918
DZIP1	0.994886058	0.997845497	0.979231344	0.999939566	0.99989309	0.972787584
RAB6B	0.994886058	0.991720467	0.969311025	0.999939566	0.99989309	0.973195541
IFI47	0.994886058	0.967758952	0.978892556	0.999939566	0.99989309	0.973195541
SLC13A5	0.994886058	0.98718343	0.976575956	0.999939566	0.99989309	0.97510237
CCDC43	0.994886058	0.96471209	0.996948653	0.999939566	0.99989309	0.977961448
ZNF581	0.994886058	0.995965505	0.976719499	0.999939566	0.99989309	0.978467321
ALDOC	0.994886058	0.982614603	0.967873529	0.999939566	0.99989309	0.978565009
ACKR3	0.994886058	0.974986418	0.982930137	0.999939566	0.99989309	0.978656049
CEACAM19	0.994886058	0.966852427	0.976719499	0.999939566	0.99989309	0.98016619
LOC112446779	0.994886058	0.968312904	0.978215635	0.999939566	0.99989309	0.98016619
LRRC28	0.994886058	0.961455063	0.987842204	0.999939566	0.99989309	0.98016619
RPL34	0.994886058	0.973202823	0.985552223	0.999939566	0.99989309	0.981090047
AGK	0.994886058	0.967707663	0.976869041	0.999939566	0.99989309	0.981164039
LOC107132877	0.994886058	0.989445679	0.979231344	0.999939566	0.99989309	0.981164039
CAPN1	0.994886058	0.980639049	0.966157152	0.999939566	0.99989309	0.981411983
HAVCR2	0.994886058	0.998683615	0.970218925	0.999939566	0.99989309	0.981411983
TMEM102	0.994886058	0.982614603	0.965518535	0.999939566	0.99989309	0.981578785
ECM2	0.994886058	0.960825265	0.989408632	0.999939566	0.99989309	0.982066213
LOC507787	0.994886058	0.973158835	0.965712499	0.999939566	0.99989309	0.983453112
SMIM1	0.994886058	0.967515137	0.973730711	0.999939566	0.99989309	0.983453112
ZSWIM3	0.994886058	0.988153273	0.979231344	0.999939566	0.99989309	0.983453112
LOC104969340	0.994886058	0.962934461	0.984936232	0.999939566	0.99989309	0.983453112
NDUFAF2	0.994886058	0.978165562	0.989043089	0.999939566	0.99989309	0.983690417
PRKCG	0.994886058	0.982703363	0.965518535	0.999939566	0.99989309	0.984651205
CD7	0.994886058	0.982051974	0.976719499	0.999939566	0.99989309	0.986389023
GPANK1	0.994886058	0.977722338	0.976719499	0.999939566	0.99989309	0.988323336
HOXB8	0.994886058	0.959119521	0.979231344	0.999939566	0.99989309	0.988323336
LOC104971926	0.994886058	0.95931759	0.980744205	0.999939566	0.99989309	0.988323336
SOCS4	0.994886058	0.971812321	0.96880775	0.999939566	0.99989309	0.989128955
LOC112441568	0.994886058	0.960454429	0.97368009	0.999939566	0.99989309	0.989128955
LOC112447469	0.994886058	0.965239369	0.993278977	0.999939566	0.99989309	0.989719747

OXA1L	0.994886058	0.957902362	0.980734833	0.999939566	0.99989309	0.990291862
FAM189B	0.994886058	0.976925958	0.978879234	0.999939566	0.99989309	0.991051281
CLDN20	0.994886058	0.970237188	0.966157152	0.999939566	0.99989309	0.99112148
BCL2A1	0.994886058	0.966921779	0.983706867	0.999939566	0.99989309	0.991281092
MSH3	0.994886058	0.963478319	0.974845535	0.999939566	0.99989309	0.991612651
WDR91	0.994886058	0.966218643	0.965416289	0.999939566	0.99989309	0.992671204
LOC107131906	0.994886058	0.958735381	0.966157152	0.999939566	0.99989309	0.993612847
GTF2I	0.994886058	0.957071555	0.966260688	0.999939566	0.99989309	0.994262941
LOC614695	0.994886058	0.962063344	0.965491674	0.999939566	0.99989309	0.995145616
SLC39A11	0.994886058	0.974073092	0.974559286	0.999939566	0.99989309	0.995145616
USP30	0.994886058	0.96412538	0.968406955	0.999939566	0.99989309	0.995518609
ARPC1B	0.994886058	0.978309093	0.96712176	0.999939566	0.99989309	0.996383947
LOC101906398	0.994886058	0.963478319	0.970108169	0.999939566	0.99989309	0.996564988
SUSD6	0.994886058	0.962934461	0.976161819	0.999939566	0.99989309	0.997620729
LOC788183	0.994886058	0.962428993	0.967452429	0.999939566	0.99989309	0.998074562
LOC101903793	0.994886058	0.963478319	0.989408632	0.999939566	0.99989309	0.998074562
LOC100297676	0.994886058	0.95781929	0.967489175	0.999939566	0.99989309	0.998636142
ITGB5	0.994886058	0.963517553	0.971981006	0.999939566	0.99989309	0.998636142
BBOF1	0.994886058	0.963478319	0.97420715	0.999939566	0.99989309	0.998928226
LOC112446381	0.995022675	0.972786652	0.994425789	0.999939566	0.99989309	0.97491746
C10H15orf59	0.995030296	0.963478319	0.999461814	0.999939566	0.99989309	0.968245211
TRPS1	0.995030296	0.967137663	0.99115315	0.999939566	0.99989309	0.969981358
HHAT	0.995030296	0.965239369	0.976719499	0.999939566	0.99989309	0.977961448
LOC104975415	0.995030296	0.959348617	0.98567143	0.999939566	0.99989309	0.978515834
PHF5A	0.995030296	0.961431799	0.991235675	0.999939566	0.99989309	0.981090047
CDR2L	0.995030296	0.957412778	0.991876172	0.999939566	0.99989309	0.981578785
LOC101902959	0.995030296	0.972563829	0.965712499	0.999939566	0.99989309	0.983453112
FAM185A	0.995030296	0.99677427	0.980734833	0.999939566	0.99989309	0.983453112
ZNF513	0.995030296	0.997152467	0.967043892	0.999939566	0.99989309	0.989313458
LOC101906688	0.995030296	0.967758952	0.967413848	0.999939566	0.99989309	0.998074562
LOC782258	0.995030296	0.962934461	0.978215635	0.999939566	0.99989309	0.998074562
LOC100848495	0.995059176	0.98743321	0.994425789	0.999939566	0.99989309	0.967011366
PTPN18	0.995059176	0.991043872	0.966157152	0.999939566	0.99989309	0.968039618
MBLAC1	0.995059176	0.987801409	0.96880775	0.999939566	0.99989309	0.968039618
DLC1	0.995059176	0.988649234	0.979231344	0.999939566	0.99989309	0.968059826
ADPRHL2	0.995059176	0.971313644	0.966157152	0.999939566	0.99989309	0.968245211
TMEM205	0.995059176	0.982051974	0.966919864	0.999939566	0.99989309	0.968245211
MAST3	0.995059176	0.968312904	0.987842204	0.999939566	0.99989309	0.968245211

ENTPD7	0.995059176	0.988960148	0.990350221	0.999939566	0.99989309	0.968245211
LOC112447433	0.995059176	0.974986418	0.995799138	0.999939566	0.99989309	0.968245211
LOC112444842	0.995059176	0.961481835	0.999541752	0.999939566	0.99989309	0.968245211
MRPL38	0.995059176	0.965829305	0.973488065	0.999939566	0.99989309	0.969545443
LOC100847363	0.995059176	0.972418749	0.97381056	0.999939566	0.99989309	0.971025918
TSPAN5	0.995059176	0.985784205	0.982659124	0.999939566	0.99989309	0.972204129
FAM120C	0.995059176	0.997762456	0.977371811	0.999939566	0.99989309	0.973195071
SHB	0.995059176	0.962934461	0.974698466	0.999939566	0.99989309	0.973195541
IFITM2	0.995059176	0.974986418	0.981715764	0.999939566	0.99989309	0.973463195
ARMC8	0.995059176	0.98743321	0.965819637	0.999939566	0.99989309	0.973847791
MARCH7	0.995059176	0.976925958	0.980734833	0.999939566	0.99989309	0.974017283
EIF2B4	0.995059176	0.965239369	0.987891679	0.999939566	0.99989309	0.976757738
FHOD1	0.995059176	0.965881793	0.968230268	0.999939566	0.99989309	0.977961448
GSTZ1	0.995059176	0.994365533	0.980734833	0.999939566	0.99989309	0.977961448
LOC789388	0.995059176	0.979812776	0.990107362	0.999939566	0.99989309	0.977961448
CEBPA	0.995059176	0.971068039	0.978855687	0.999939566	0.99989309	0.97815722
RPS6	0.995059176	0.977500512	0.971981006	0.999939566	0.99989309	0.978368368
SYNRG	0.995059176	0.974986418	0.980385041	0.999939566	0.99989309	0.979606084
UNC13D	0.995059176	0.972418749	0.967489175	0.999939566	0.99989309	0.98016619
SPAM1	0.995059176	0.959119521	0.970904484	0.999939566	0.99989309	0.98016619
TATDN2	0.995059176	0.970179915	0.989332994	0.999939566	0.99989309	0.98016619
LOC101904377	0.995059176	0.972418749	0.990107362	0.999939566	0.99989309	0.98016619
RAB11FIP4	0.995059176	0.964234693	0.967489175	0.999939566	0.99989309	0.981164039
TCEA3	0.995059176	0.978453686	0.970218925	0.999939566	0.99989309	0.981164039
TMEM65	0.995059176	0.974986418	0.976719499	0.999939566	0.99989309	0.981164039
AK7	0.995059176	0.96283175	0.980772074	0.999939566	0.99989309	0.981164039
LOC100297513	0.995059176	0.979916117	0.972378657	0.999939566	0.99989309	0.981578785
LRPAP1	0.995059176	0.983065474	0.979096894	0.999939566	0.99989309	0.981759054
ZNF281	0.995059176	0.969870198	0.966157152	0.999939566	0.99989309	0.982045172
ARF4	0.995059176	0.97099048	0.987842204	0.999939566	0.99989309	0.982066213
LOC101907697	0.995059176	0.990612677	0.982930137	0.999939566	0.99989309	0.982978165
SLC25A40	0.995059176	0.988605499	0.984700021	0.999939566	0.99989309	0.983427394
HRH1	0.995059176	0.96412538	0.970904484	0.999939566	0.99989309	0.983453112
APEH	0.995059176	0.967758952	0.970904484	0.999939566	0.99989309	0.983453112
KLHL9	0.995059176	0.972418749	0.978215635	0.999939566	0.99989309	0.983453112
LOC786332	0.995059176	0.966130596	0.987419404	0.999939566	0.99989309	0.983453112
LOC787057	0.995059176	0.967758952	0.991113951	0.999939566	0.99989309	0.983453112
LOC104972526	0.995059176	0.978262597	0.991693262	0.999939566	0.99989309	0.983453112

RAD1	0.995059176	0.989445679	0.987842204	0.999939566	0.99989309	0.983525373
TCF7	0.995059176	0.959119521	0.972401758	0.999939566	0.99989309	0.983726883
CASTOR1	0.995059176	0.974986418	0.96636908	0.999939566	0.99989309	0.984449688
TBCCD1	0.995059176	0.968237269	0.975212837	0.999939566	0.99989309	0.984449688
CCDC85B	0.995059176	0.987331445	0.975527089	0.999939566	0.99989309	0.984491523
LOC112449300	0.995059176	0.969870198	0.976719499	0.999939566	0.99989309	0.984491523
ZNF580	0.995059176	0.974591667	0.97311093	0.999939566	0.99989309	0.984609406
CPEB3	0.995059176	0.980930691	0.972401758	0.999939566	0.99989309	0.984651205
LOC112446701	0.995059176	0.973158835	0.981346992	0.999939566	0.99989309	0.986707375
SLC45A2	0.995059176	0.974591667	0.969311025	0.999939566	0.99989309	0.986862062
LOC107132664	0.995059176	0.974986418	0.988585687	0.999939566	0.99989309	0.987210851
ZNFX1	0.995059176	0.982588455	0.966157152	0.999939566	0.99989309	0.988323336
ATL1	0.995059176	0.991555439	0.967413848	0.999939566	0.99989309	0.988323336
LOC112445052	0.995059176	0.960602651	0.980772074	0.999939566	0.99989309	0.988571083
SEPT6	0.995059176	0.967113913	0.988432243	0.999939566	0.99989309	0.988571083
NAPEPLD	0.995059176	0.965807767	0.993665588	0.999939566	0.99989309	0.989272534
UNC93B1	0.995059176	0.993247261	0.970218925	0.999939566	0.99989309	0.989313458
STEAP1	0.995059176	0.99134261	0.978215635	0.999939566	0.99989309	0.99064193
LOC783163	0.995059176	0.959119521	0.96880775	0.999939566	0.99989309	0.991051281
LOC112449052	0.995059176	0.976925958	0.975183307	0.999939566	0.99989309	0.991281092
WIZ	0.995059176	0.978215444	0.981715764	0.999939566	0.99989309	0.991281092
DRD1	0.995059176	0.99002957	0.967294804	0.999939566	0.99989309	0.993685604
INTS3	0.995059176	0.976925958	0.980744205	0.999939566	0.99989309	0.994687715
LCOR	0.995059176	0.980639049	0.967413848	0.999939566	0.99989309	0.996455238
MECP2	0.995059176	0.983065474	0.972378657	0.999939566	0.99989309	0.99797558
LOC101904622	0.995059176	0.968447746	0.979096894	0.999939566	0.99989309	0.99797558
MKLN1	0.995059176	0.964234693	0.978879234	0.999939566	0.99989309	0.998027038
NT5C2	0.995059176	0.9764701	0.976869041	0.999939566	0.99989309	0.998074562
LOC112447838	0.995059176	0.967515137	0.979231344	0.999939566	0.99989309	0.998636142
APBB1	0.995059176	0.969642797	0.979347606	0.999939566	0.99989309	0.998636142
UTP3	0.995059176	0.95781929	0.979851274	0.999939566	0.99989309	0.998928226
LACTB	0.995059176	0.96412538	0.981945999	0.999939566	0.99989309	0.998928226
STUM	0.995161368	0.977500512	0.976719499	0.999939566	0.99989309	0.983453112
FAF2	0.995162703	0.967758952	0.979231344	0.999939566	0.99989309	0.99797558
TNNI3	0.995268046	0.989661212	0.977612552	0.999939566	0.99989309	0.968245211
MAP3K8	0.995268046	0.999377692	0.985552223	0.999939566	0.99989309	0.968245211
LOC100848353	0.995268046	0.972133808	0.993301613	0.999939566	0.99989309	0.968245211
RTL1	0.995268046	0.964962641	0.969732581	0.999939566	0.99989309	0.96826636

METTL23	0.995268046	0.972786652	0.971070575	0.999939566	0.99989309	0.968462588
LOC100847890	0.995268046	0.99950308	0.976719499	0.999939566	0.99989309	0.968751621
UFD1	0.995268046	0.963251672	0.974832809	0.999939566	0.99989309	0.969808185
RBM15B	0.995268046	0.976760806	0.982223562	0.999939566	0.99989309	0.970702553
AIFM2	0.995268046	0.988725451	0.987419404	0.999939566	0.99989309	0.974379635
SCIMP	0.995268046	0.97918681	0.993278977	0.999939566	0.99989309	0.974379635
GLB1	0.995268046	0.997152467	0.988788601	0.999939566	0.99989309	0.978245923
LOC101903795	0.995268046	0.98111573	0.987006595	0.999939566	0.99989309	0.978467321
LOC112442949	0.995268046	0.981463822	0.968455468	0.999939566	0.99989309	0.979233909
ZDHHC19	0.995268046	0.99950308	0.96993432	0.999939566	0.99989309	0.979352545
PHF6	0.995268046	0.998683615	0.96636908	0.999939566	0.99989309	0.979606084
LOC107132327	0.995268046	0.96412538	0.99054854	0.999939566	0.99989309	0.980144374
TDRD9	0.995268046	0.964234693	0.967302577	0.999939566	0.99989309	0.98016619
FEZ1	0.995268046	0.973158835	0.976719499	0.999939566	0.99989309	0.98016619
NECAP2	0.995268046	0.994810749	0.980442556	0.999939566	0.99989309	0.98016619
LOC782159	0.995268046	0.988153273	0.994425789	0.999939566	0.99989309	0.98016619
KNSTRN	0.995268046	0.965553878	0.999541752	0.999939566	0.99989309	0.98016619
ZFAND1	0.995268046	0.959119521	0.994425789	0.999939566	0.99989309	0.982074417
SLC1A4	0.995268046	0.978453686	0.993665588	0.999939566	0.99989309	0.982559673
HOXC8	0.995268046	0.959119521	0.994425789	0.999939566	0.99989309	0.983690417
TMPO	0.995268046	0.981087742	0.978855687	0.999939566	0.99989309	0.985842624
ZKSCAN4	0.995268046	0.978250325	0.968934792	0.999939566	0.99989309	0.989313458
LOC112444521	0.995268046	0.977732731	0.979231344	0.999939566	0.99989309	0.989549426
LOC101905498	0.995268046	0.98743321	0.985552223	0.999939566	0.99989309	0.989947227
TLN2	0.995268046	0.967515137	0.985989185	0.999939566	0.99989309	0.989947227
RIOK1	0.995268046	0.959119521	0.991406769	0.999939566	0.99989309	0.991135253
LOC101904963	0.995268046	0.976302681	0.979461445	0.999939566	0.99989309	0.991281092
LOC510798	0.995268046	0.965553878	0.966546415	0.999939566	0.99989309	0.991612651
ZNF891	0.995268046	0.964270094	0.985552223	0.999939566	0.99989309	0.993985914
ILF3	0.995268046	0.959138652	0.985552223	0.999939566	0.99989309	0.996383947
LOC101902048	0.995268046	0.967078509	0.9785457	0.999939566	0.99989309	0.996455238
LOC100337293	0.995280579	0.997390201	0.973488065	0.999939566	0.99989309	0.97084193
TUFT1	0.995280579	0.963478319	0.998904268	0.999939566	0.99989309	0.982045172
ORAI1	0.995380341	0.964198256	0.980734833	0.999939566	0.99989309	0.98016619
RBMS3	0.995380341	0.974986418	0.969529627	0.999939566	0.99989309	0.981090047
LOC101904098	0.995416523	0.96509773	0.979231344	0.999939566	0.99989309	0.991281092
STX10	0.995486527	0.987856147	0.970108169	0.999939566	0.99989309	0.969981358
RPL21	0.995486527	0.972786652	0.991235675	0.999939566	0.99989309	0.978656049

R3HDM4	0.995486527	0.962934461	0.983372282	0.999939566	0.99989309	0.98016619
BPGM	0.995486527	0.987790525	0.969311025	0.999939566	0.99989309	0.989128955
TTI2	0.995486527	0.980614912	0.974193101	0.999939566	0.99989309	0.989947227
PPP1R21	0.995509387	0.973158835	0.98846962	0.999939566	0.99989309	0.98016619
MTHFR	0.995509387	0.972563829	0.966867772	0.999939566	0.99989309	0.998074562
LOC112444593	0.995559216	0.981322782	0.97265144	0.999939566	0.99989309	0.998074562
RBM7	0.995573815	0.965239369	0.99115315	0.999939566	0.99989309	0.970786053
PPP1R9A	0.995573815	0.992032812	0.978215635	0.999939566	0.99989309	0.982514294
FNDC3B	0.995656008	0.968440623	0.969618195	0.999939566	0.99989309	0.978467321
LARGE1	0.995656008	0.967731788	0.990107362	0.999939566	0.99989309	0.980144374
AKAP1	0.995759723	0.99677427	0.968406955	0.999939566	0.99989309	0.973494928
CCDC125	0.995759723	0.962934461	0.980442556	0.999939566	0.99989309	0.979088347
GPATCH11	0.995759723	0.96412538	0.972485306	0.999939566	0.99989309	0.98016619
ATAD5	0.995759723	0.970524066	0.968406955	0.999939566	0.99989309	0.981090047
PRSS42	0.995759723	0.960597088	0.979635589	0.999939566	0.99989309	0.981411983
MSX2	0.995759723	0.963480908	0.987842204	0.999939566	0.99989309	0.981578785
KLHDC1	0.995759723	0.978453686	0.994425789	0.999939566	0.99989309	0.989313458
LOC100848642	0.99580676	0.999968084	0.971472903	0.999939566	0.99989309	0.969981358
RHPN2	0.99580676	0.977732731	0.972104746	0.999939566	0.99989309	0.969981358
SMOC1	0.99580676	0.982614603	0.992056611	0.999939566	0.99989309	0.970702553
P2RX7	0.99580676	0.967758952	0.996842235	0.999939566	0.99989309	0.970702553
LOC526769	0.99580676	0.970524066	0.976719499	0.999939566	0.99989309	0.970801789
LOC615258	0.99580676	0.967758952	0.970108169	0.999939566	0.99989309	0.97084193
TPGS1	0.99580676	0.970237188	0.976719499	0.999939566	0.99989309	0.970959759
DCAF16	0.99580676	0.992194873	0.993278977	0.999939566	0.99989309	0.971025918
RPL5	0.99580676	0.988708263	0.990107362	0.999939566	0.99989309	0.971907111
VWA2	0.99580676	0.975673897	0.994182378	0.999939566	0.99989309	0.972182019
FAM200A	0.99580676	0.994567583	0.982223562	0.999939566	0.99989309	0.972267965
APBB1IP	0.99580676	0.988649234	0.96880775	0.999939566	0.99989309	0.972648667
QARS	0.99580676	0.969418414	0.999323478	0.999939566	0.99989309	0.97280723
REST	0.99580676	0.991043872	0.969644737	0.999939566	0.99989309	0.973195071
GPR143	0.99580676	0.963478319	0.979231344	0.999939566	0.99989309	0.973195071
ARID5B	0.99580676	0.974986418	0.98730572	0.999939566	0.99989309	0.973195541
BAP1	0.99580676	0.968447746	0.993278977	0.999939566	0.99989309	0.973195541
ZNF674	0.99580676	0.975341721	0.994425789	0.999939566	0.99989309	0.973195541
LOC101902207	0.99580676	0.974256477	0.995726315	0.999939566	0.99989309	0.973195541
FBF1	0.99580676	0.965553878	0.999323478	0.999939566	0.99989309	0.973195541
AMPH	0.99580676	0.963779898	0.999917831	0.999939566	0.99989309	0.973195541

WDR93	0.99580676	0.973158835	0.970218925	0.999939566	0.99989309	0.973494928
RPS28	0.99580676	0.974875445	0.979231344	0.999939566	0.99989309	0.973861448
NDFIP1	0.99580676	0.967758952	0.999887981	0.999939566	0.99989309	0.974139155
ANTXR1	0.99580676	0.98743321	0.979231344	0.999939566	0.99989309	0.974379635
CST3	0.99580676	0.985306507	0.995723789	0.999939566	0.99989309	0.97491746
CACNA2D1	0.99580676	0.978215444	0.975212837	0.999939566	0.99989309	0.97519644
CRISPLD1	0.99580676	0.987628894	0.994425789	0.999939566	0.99989309	0.977151753
EHD2	0.99580676	0.965239369	0.968954353	0.999939566	0.99989309	0.977961448
CCP110	0.99580676	0.978309093	0.978892556	0.999939566	0.99989309	0.977961448
PTER	0.99580676	0.966516118	0.979720857	0.999939566	0.99989309	0.977961448
LOC616295	0.99580676	0.990586436	0.98846962	0.999939566	0.99989309	0.977961448
LOC104971501	0.99580676	0.972786652	0.994425789	0.999939566	0.99989309	0.977961448
MAP3K20	0.99580676	0.988725451	0.995726315	0.999939566	0.99989309	0.977961448
HFE	0.99580676	0.966921779	0.999323478	0.999939566	0.99989309	0.977961448
LOC101907886	0.99580676	0.983288516	0.979231344	0.999939566	0.99989309	0.97815722
LOC100847759	0.99580676	0.984280548	0.972909109	0.999939566	0.99989309	0.978467321
CCDC106	0.99580676	0.967515137	0.983094355	0.999939566	0.99989309	0.978467321
SCYL3	0.99580676	0.972786652	0.983474414	0.999939566	0.99989309	0.978467321
MTF1	0.99580676	0.968246192	0.976161819	0.999939566	0.99989309	0.978656049
RAI2	0.99580676	0.989445679	0.980385041	0.999939566	0.99989309	0.978656049
HAND1	0.99580676	0.964112964	0.976719499	0.999939566	0.99989309	0.978921738
PLEKHG3	0.99580676	0.988649234	0.994425789	0.999939566	0.99989309	0.979088347
LOC101902994	0.99580676	0.98743321	0.978215635	0.999939566	0.99989309	0.979352545
CABLES2	0.99580676	0.979916117	0.968406955	0.999939566	0.99989309	0.979606084
GJC3	0.99580676	0.976150941	0.987433396	0.999939566	0.99989309	0.979606084
PRIM1	0.99580676	0.979916117	0.989332994	0.999939566	0.99989309	0.979606084
GRID2IP	0.99580676	0.992194873	0.976719499	0.999939566	0.99989309	0.979810204
DDX59	0.99580676	0.962934461	0.988788601	0.999939566	0.99989309	0.980144374
LOC101905951	0.99580676	0.963478319	0.970904484	0.999939566	0.99989309	0.98016619
C7H19orf38	0.99580676	0.971839996	0.971981006	0.999939566	0.99989309	0.98016619
IQCE	0.99580676	0.989773581	0.97381056	0.999939566	0.99989309	0.98016619
GAA	0.99580676	0.994365533	0.97381056	0.999939566	0.99989309	0.98016619
BCAS3	0.99580676	0.974986418	0.977612552	0.999939566	0.99989309	0.98016619
LOC112448387	0.99580676	0.993223749	0.978879234	0.999939566	0.99989309	0.98016619
LOC101905668	0.99580676	0.965239369	0.978892556	0.999939566	0.99989309	0.98016619
RPS11	0.99580676	0.977732731	0.982223562	0.999939566	0.99989309	0.98016619
LOC101908535	0.99580676	0.978309093	0.98567143	0.999939566	0.99989309	0.98016619
LOC101903853	0.99580676	0.970237188	0.987842204	0.999939566	0.99989309	0.98016619

SEMA4B	0.99580676	0.990586436	0.994425789	0.999939566	0.99989309	0.98016619
KLHL18	0.99580676	0.962934461	0.999541752	0.999939566	0.99989309	0.98016619
BAX	0.99580676	0.985784205	0.99054854	0.999939566	0.99989309	0.980841407
ADAMTS1	0.99580676	0.974986418	0.995918981	0.999939566	0.99989309	0.980841407
MAD2L1BP	0.99580676	0.993511686	0.984700021	0.999939566	0.99989309	0.981090047
DIO1	0.99580676	0.982017699	0.989862418	0.999939566	0.99989309	0.981090047
DCLK3	0.99580676	0.986245673	0.972211632	0.999939566	0.99989309	0.981164039
SERP2	0.99580676	0.963478319	0.987419404	0.999939566	0.99989309	0.981164039
CROT	0.99580676	0.997450916	0.975212837	0.999939566	0.99989309	0.981411983
MEGF9	0.99580676	0.972418749	0.980734833	0.999939566	0.99989309	0.981411983
RPL27A	0.99580676	0.980639049	0.985552223	0.999939566	0.99989309	0.981411983
PHF20L1	0.99580676	0.974348229	0.990693603	0.999939566	0.99989309	0.981411983
GPR52	0.99580676	0.993887397	0.991235675	0.999939566	0.99989309	0.981411983
NME7	0.99580676	0.968814321	0.976719499	0.999939566	0.99989309	0.982045172
LOC107132093	0.99580676	0.969418414	0.992165187	0.999939566	0.99989309	0.982045172
UBALD1	0.99580676	0.972786652	0.997511268	0.999939566	0.99989309	0.982045172
LOC101902668	0.99580676	0.997450916	0.971981006	0.999939566	0.99989309	0.982066213
FAM57B	0.99580676	0.994365533	0.975212837	0.999939566	0.99989309	0.982066213
ITPR2	0.99580676	0.984479682	0.987208945	0.999939566	0.99989309	0.982066213
LOC104975911	0.99580676	0.992273495	0.990107362	0.999939566	0.99989309	0.982066213
SLX4IP	0.99580676	0.980930691	0.976972382	0.999939566	0.99989309	0.982559673
CTNNA1	0.99580676	0.972418749	0.990107362	0.999939566	0.99989309	0.983056851
LOC101904822	0.99580676	0.981332269	0.969732581	0.999939566	0.99989309	0.983160549
QRICH1	0.99580676	0.965349377	0.968954353	0.999939566	0.99989309	0.983453112
P2RY12	0.99580676	0.978453686	0.970108169	0.999939566	0.99989309	0.983453112
LOC101905033	0.99580676	0.99950308	0.97311093	0.999939566	0.99989309	0.983453112
LOC101903397	0.99580676	0.994810749	0.976719499	0.999939566	0.99989309	0.983453112
PPFIA1	0.99580676	0.993959968	0.978215635	0.999939566	0.99989309	0.983453112
JMJD7	0.99580676	0.96412538	0.979231344	0.999939566	0.99989309	0.983453112
CARD11	0.99580676	0.988605499	0.979635589	0.999939566	0.99989309	0.983453112
SIPA1L2	0.99580676	0.965027449	0.987842204	0.999939566	0.99989309	0.983453112
ANKRD29	0.99580676	0.965239369	0.989332994	0.999939566	0.99989309	0.983453112
AES	0.99580676	0.961455063	0.993665588	0.999939566	0.99989309	0.983453112
CEP44	0.99580676	0.988649234	0.994425789	0.999939566	0.99989309	0.983453112
RPL36AL	0.99580676	0.972786652	0.995918981	0.999939566	0.99989309	0.983453112
APOBEC3Z2	0.99580676	0.976925958	0.995918981	0.999939566	0.99989309	0.983453112
RRH	0.99580676	0.979812776	0.976869041	0.999939566	0.99989309	0.983512424
TCF12	0.99580676	0.964616377	0.968954353	0.999939566	0.99989309	0.983525373

LOC112444289	0.99580676	0.986681109	0.97381056	0.999939566	0.99989309	0.983525373
ANK2	0.99580676	0.976760806	0.980734833	0.999939566	0.99989309	0.983836142
TGDS	0.99580676	0.99950308	0.974193101	0.999939566	0.99989309	0.984449688
ENPP6	0.99580676	0.980930691	0.980772074	0.999939566	0.99989309	0.984491523
RBM11	0.99580676	0.965239369	0.97363516	0.999939566	0.99989309	0.984609406
LOC112449363	0.99580676	0.988500804	0.97381056	0.999939566	0.99989309	0.984609406
LOC508455	0.99580676	0.994365533	0.976869041	0.999939566	0.99989309	0.984609406
ERCC2	0.99580676	0.965239369	0.97381056	0.999939566	0.99989309	0.985210182
XKR4	0.99580676	0.972786652	0.989332994	0.999939566	0.99989309	0.985577866
LOC613519	0.99580676	0.972786652	0.97311093	0.999939566	0.99989309	0.98609047
TTC21B	0.99580676	0.99677427	0.97629841	0.999939566	0.99989309	0.986265953
TSTD2	0.99580676	0.965239369	0.98730572	0.999939566	0.99989309	0.986389023
GPR27	0.99580676	0.964270094	0.991235675	0.999939566	0.99989309	0.986389023
PNMA2	0.99580676	0.974986418	0.983143823	0.999939566	0.99989309	0.986819555
LOC614643	0.99580676	0.964234693	0.983555077	0.999939566	0.99989309	0.986819555
IRX3	0.99580676	0.974875445	0.995379298	0.999939566	0.99989309	0.986819555
LOC112446039	0.99580676	0.965239369	0.987208945	0.999939566	0.99989309	0.986948049
TMEM201	0.99580676	0.986032534	0.979096894	0.999939566	0.99989309	0.98704566
CPNE5	0.99580676	0.976925958	0.979096894	0.999939566	0.99989309	0.987311417
KLHL28	0.99580676	0.983065474	0.983555077	0.999939566	0.99989309	0.987721456
ATAD2	0.99580676	0.978250532	0.97381056	0.999939566	0.99989309	0.988323336
RBAK	0.99580676	0.974986418	0.976719499	0.999939566	0.99989309	0.988323336
WDYHV1	0.99580676	0.972563829	0.988442544	0.999939566	0.99989309	0.988323336
LOC112443728	0.99580676	0.971285029	0.989527187	0.999939566	0.99989309	0.988323336
PMP22	0.99580676	0.997762456	0.96993432	0.999939566	0.99989309	0.988571083
LOC112448062	0.99580676	0.969024576	0.987419404	0.999939566	0.99989309	0.988571083
RPUSD3	0.99580676	0.982963758	0.981977161	0.999939566	0.99989309	0.989128955
ANP32A	0.99580676	0.982614603	0.983094355	0.999939566	0.99989309	0.989128955
ZC3HC1	0.99580676	0.971426434	0.994425789	0.999939566	0.99989309	0.989128955
L3HYPDH	0.99580676	0.989773581	0.979231344	0.999939566	0.99989309	0.989313458
CCNG2	0.99580676	0.966852427	0.984700021	0.999939566	0.99989309	0.989313458
RNF8	0.99580676	0.981087742	0.970108169	0.999939566	0.99989309	0.99064193
RNF130	0.99580676	0.974986418	0.970108169	0.999939566	0.99989309	0.990822928
ZNF212	0.99580676	0.977732731	0.984700021	0.999939566	0.99989309	0.991051281
LOC107131494	0.99580676	0.986100049	0.984700021	0.999939566	0.99989309	0.991051281
FRA10AC1	0.99580676	0.971839996	0.975212837	0.999939566	0.99989309	0.991281092
LOC112442619	0.99580676	0.980930691	0.978879234	0.999939566	0.99989309	0.991281092
SSBP2	0.99580676	0.972786652	0.978892556	0.999939566	0.99989309	0.991281092

LOC104971613	0.99580676	0.977728722	0.985552223	0.999939566	0.99989309	0.991281092
UTP11	0.99580676	0.990914937	0.98730572	0.999939566	0.99989309	0.991281092
LOC782437	0.99580676	0.972563829	0.987208945	0.999939566	0.99989309	0.991569266
NOVA2	0.99580676	0.990194936	0.985824904	0.999939566	0.99989309	0.991612651
USP49	0.99580676	0.974986418	0.978215635	0.999939566	0.99989309	0.992186241
ACTR8	0.99580676	0.963480908	0.993278977	0.999939566	0.99989309	0.993612847
SLC27A1	0.99580676	0.965239369	0.994425789	0.999939566	0.99989309	0.993612847
CIRBP	0.99580676	0.980930691	0.98082053	0.999939566	0.99989309	0.993685604
SGTA	0.99580676	0.972786652	0.987419404	0.999939566	0.99989309	0.995145616
LOC112447031	0.99580676	0.979812776	0.973509969	0.999939566	0.99989309	0.995411183
SIRT6	0.99580676	0.966218643	0.976719499	0.999939566	0.99989309	0.996383947
DENND4A	0.99580676	0.986245673	0.984700021	0.999939566	0.99989309	0.997620729
HIST2H2AC	0.99580676	0.980639049	0.968478091	0.999939566	0.99989309	0.997673689
LOC100848906	0.99580676	0.962934461	0.969058758	0.999939566	0.99989309	0.998636142
PCGF3	0.99580676	0.974986418	0.974681091	0.999939566	0.99989309	0.998636142
LOC112446709	0.99580676	0.977207079	0.983701698	0.999939566	0.99989309	0.998636142
RGS11	0.99580676	0.972133808	0.979096894	0.999939566	0.99989309	0.998880954
METTL25	0.99580676	0.963374115	0.971104162	0.999939566	0.99989309	0.999921012
LOC787530	0.995948642	0.965239369	0.976719499	0.999939566	0.99989309	0.983453112
CYGB	0.995975746	0.975341721	0.980744205	0.999939566	0.99989309	0.981411983
VN1R1	0.995975746	0.98743321	0.993278977	0.999939566	0.99989309	0.983453112
MRPL45	0.995975746	0.968246192	0.98567143	0.999939566	0.99989309	0.984491523
C28H1orf131	0.995975746	0.970179915	0.996942087	0.999939566	0.99989309	0.986389023
EGFL8	0.995975746	0.992273495	0.970089535	0.999939566	0.99989309	0.991612651
KLF10	0.995975746	0.988649234	0.97311093	0.999939566	0.99989309	0.998074562
CMTM8	0.995975746	0.973158835	0.97102147	0.999939566	0.99989309	0.998636142
LOC104971220	0.996049318	0.991555439	0.970108169	0.999939566	0.99989309	0.983453112
TOR1B	0.996049318	0.971712051	0.974193101	0.999939566	0.99989309	0.998074562
LOC512440	0.99606619	0.996430877	0.985552223	0.999939566	0.99989309	0.97491746
PGBD1	0.99606619	0.98618789	0.972401758	0.999939566	0.99989309	0.975877807
GPR180	0.99606619	0.978281781	0.994425789	0.999939566	0.99989309	0.976454461
ADIPOQ	0.99606619	0.96471209	0.989408632	0.999939566	0.99989309	0.976891454
LOC101905821	0.99606619	0.975287816	0.985222638	0.999939566	0.99989309	0.97815722
LOC786489	0.99606619	0.971426434	0.993665588	0.999939566	0.99989309	0.97815722
LOC101904902	0.99606619	0.971812321	0.98846962	0.999939566	0.99989309	0.978467321
MAP7D1	0.99606619	0.965016829	0.985552223	0.999939566	0.99989309	0.978656049
ADAP2	0.99606619	0.978309093	0.987842204	0.999939566	0.99989309	0.979233909
HNRNPDL	0.99606619	0.967237998	0.978661024	0.999939566	0.99989309	0.979606084

ECPAS	0.99606619	0.965027449	0.983517715	0.999939566	0.99989309	0.979606084
RELN	0.99606619	0.974129875	0.970904484	0.999939566	0.99989309	0.98016619
ZXDB	0.99606619	0.980639049	0.972401758	0.999939566	0.99989309	0.98016619
PLK2	0.99606619	0.994810749	0.983372282	0.999939566	0.99989309	0.98016619
CBWD2	0.99606619	0.980682689	0.98846962	0.999939566	0.99989309	0.98016619
ZNF699	0.99606619	0.976925958	0.989408632	0.999939566	0.99989309	0.98016619
FAM155B	0.99606619	0.967137663	0.989642163	0.999939566	0.99989309	0.98016619
LOC112442754	0.99606619	0.966852427	0.99115315	0.999939566	0.99989309	0.98016619
MKRN2	0.99606619	0.977584325	0.991406769	0.999939566	0.99989309	0.98016619
ENOPH1	0.99606619	0.996722893	0.984700021	0.999939566	0.99989309	0.981090047
FZR1	0.99606619	0.980930691	0.983474414	0.999939566	0.99989309	0.981411983
LOC104970809	0.99606619	0.986162538	0.993278977	0.999939566	0.99989309	0.981411983
CMC1	0.99606619	0.988068991	0.994425789	0.999939566	0.99989309	0.981411983
CHST15	0.99606619	0.983065474	0.980734833	0.999939566	0.99989309	0.982066213
HEG1	0.99606619	0.988649234	0.985552223	0.999939566	0.99989309	0.983427394
P2RY2	0.99606619	0.968591287	0.979635589	0.999939566	0.99989309	0.983453112
LOC781381	0.99606619	0.976925958	0.98846962	0.999939566	0.99989309	0.983453112
LOC101901960	0.99606619	0.96577513	0.989332994	0.999939566	0.99989309	0.983453112
CAMKK1	0.99606619	0.992032812	0.989332994	0.999939566	0.99989309	0.983453112
TBCEL	0.99606619	0.967515137	0.998751041	0.999939566	0.99989309	0.983453112
CLDN15	0.99606619	0.969442016	0.991235675	0.999939566	0.99989309	0.984449688
WNT11	0.99606619	0.973666167	0.970904484	0.999939566	0.99989309	0.986389023
SF3A2	0.99606619	0.989405018	0.989702604	0.999939566	0.99989309	0.986389023
UBP1	0.99606619	0.967731788	0.991406769	0.999939566	0.99989309	0.986389023
PLPPR3	0.99606619	0.981093825	0.988836461	0.999939566	0.99989309	0.986862062
LOC613444	0.99606619	0.98618789	0.991235675	0.999939566	0.99989309	0.986862062
ZNF514	0.99606619	0.997450916	0.9785457	0.999939566	0.99989309	0.98704566
NOTCH2	0.99606619	0.972133808	0.972909109	0.999939566	0.99989309	0.987593346
LOC100848538	0.99606619	0.978309093	0.976719499	0.999939566	0.99989309	0.989128955
GDA	0.99606619	0.965881793	0.996569581	0.999939566	0.99989309	0.989128955
LOC101907661	0.99606619	0.989773581	0.991406769	0.999939566	0.99989309	0.989313458
NIPAL1	0.99606619	0.967515137	0.990107362	0.999939566	0.99989309	0.989967429
GMPR2	0.99606619	0.982614603	0.976719499	0.999939566	0.99989309	0.990291862
PRAF2	0.99606619	0.966852427	0.975183307	0.999939566	0.99989309	0.99064193
PTPRU	0.99606619	0.984479682	0.976479691	0.999939566	0.99989309	0.99064193
FGD4	0.99606619	0.991043872	0.979231344	0.999939566	0.99989309	0.99064193
RNF170	0.99606619	0.982703363	0.971067776	0.999939566	0.99989309	0.991281092
HIRIP3	0.99606619	0.967137663	0.975320606	0.999939566	0.99989309	0.991281092

LOC101908046	0.99606619	0.989845579	0.979851274	0.999939566	0.99989309	0.991281092
LOC112444287	0.99606619	0.992740593	0.980734833	0.999939566	0.99989309	0.991612651
PECAM1	0.99606619	0.994894651	0.979231344	0.999939566	0.99989309	0.991651986
CELF2	0.99606619	0.971210729	0.971981006	0.999939566	0.99989309	0.993612847
RCE1	0.99606619	0.986245673	0.973488065	0.999939566	0.99989309	0.993612847
LOC101905493	0.99606619	0.971812321	0.987208945	0.999939566	0.99989309	0.995145616
CD180	0.99606619	0.982017699	0.989527187	0.999939566	0.99989309	0.995282085
LOC112446462	0.99606619	0.992032812	0.970904484	0.999939566	0.99989309	0.996455238
LOC107131323	0.99606619	0.974256477	0.974360603	0.999939566	0.99989309	0.996455238
TMC7	0.99606619	0.972786652	0.970904484	0.999939566	0.99989309	0.998636142
USP33	0.99606619	0.981093825	0.978215635	0.999939566	0.99989309	0.998636142
ZRANB2	0.99606619	0.965829305	0.990870021	0.999939566	0.99989309	0.998636142
NNT	0.99606619	0.969145732	0.97311093	0.999939566	0.99989309	0.998928226
TBC1D2B	0.996082706	0.98618789	0.991406769	0.999939566	0.99989309	0.974741192
CATSPER2	0.996082706	0.982017699	0.987639174	0.999939566	0.99989309	0.975019116
FTSJ1	0.996082706	0.972418749	0.980589955	0.999939566	0.99989309	0.97815722
LOC789494	0.996082706	0.988649234	0.976719499	0.999939566	0.99989309	0.978565009
LOC506181	0.996082706	0.97653184	0.97311093	0.999939566	0.99989309	0.98016619
SAMD13	0.996082706	0.992032812	0.977447568	0.999939566	0.99989309	0.98016619
CHD9	0.996082706	0.981336605	0.979096894	0.999939566	0.99989309	0.98016619
GNAS	0.996082706	0.973740425	0.994425789	0.999939566	0.99989309	0.98016619
LOC112441530	0.996082706	0.973947352	0.988788601	0.999939566	0.99989309	0.981164039
HS3ST2	0.996082706	0.988153273	0.977371811	0.999939566	0.99989309	0.982045172
ZNF621	0.996082706	0.984479682	0.994425789	0.999939566	0.99989309	0.983427394
TMEM161A	0.996082706	0.99134261	0.97381056	0.999939566	0.99989309	0.983453112
RABL2B	0.996082706	0.989736861	0.99304917	0.999939566	0.99989309	0.983453112
LOC104974758	0.996082706	0.970586744	0.993665588	0.999939566	0.99989309	0.983453112
TBC1D20	0.996082706	0.978215444	0.994425789	0.999939566	0.99989309	0.983453112
DHTKD1	0.996082706	0.982963758	0.994425789	0.999939566	0.99989309	0.983453112
LOC112447347	0.996082706	0.974256477	0.996569581	0.999939566	0.99989309	0.983453112
PCSK6	0.996082706	0.996696059	0.979096894	0.999939566	0.99989309	0.98361583
LOC101902154	0.996082706	0.974591667	0.999323478	0.999939566	0.99989309	0.984491523
LGALS4	0.996082706	0.985457646	0.972874677	0.999939566	0.99989309	0.986389023
CD209	0.996082706	0.974073092	0.987842204	0.999939566	0.99989309	0.986716086
CCDC120	0.996082706	0.968246192	0.977371811	0.999939566	0.99989309	0.988323336
LOC101907404	0.996082706	0.986245673	0.987842204	0.999939566	0.99989309	0.988323336
TSGA10	0.996082706	0.992261995	0.989408632	0.999939566	0.99989309	0.988323336
LOC107133302	0.996082706	0.967515137	0.990107362	0.999939566	0.99989309	0.988323336

RALGPS2	0.996082706	0.970237188	0.99304917	0.999939566	0.99989309	0.989313458
ZNF24	0.996082706	0.97021399	0.974572591	0.999939566	0.99989309	0.991281092
PYGB	0.996082706	0.974986418	0.978832388	0.999939566	0.99989309	0.993612847
FRAT2	0.996082706	0.992657849	0.97420715	0.999939566	0.99989309	0.994437241
RPAIN	0.996082706	0.979466937	0.983555077	0.999939566	0.99989309	0.995145616
RRM1	0.996082706	0.974348229	0.990693603	0.999939566	0.99989309	0.995145616
DMTF1	0.996082706	0.966388183	0.995412965	0.999939566	0.99989309	0.996564988
HIST1H2AC	0.996082706	0.967758952	0.976161819	0.999939566	0.99989309	0.998636142
KIF1C	0.996210322	0.985784205	0.980734833	0.999939566	0.99989309	0.976454461
TSPAN17	0.996210322	0.981205835	0.974681091	0.999939566	0.99989309	0.98016619
LOC104971307	0.996210322	0.97572611	0.97381056	0.999939566	0.99989309	0.981411983
RPS10	0.996210322	0.977500512	0.985016728	0.999939566	0.99989309	0.983453112
RNASE13	0.996210322	0.972418749	0.987208945	0.999939566	0.99989309	0.983512424
CETN4	0.996210322	0.978287344	0.982387959	0.999939566	0.99989309	0.984609406
TNFSF13	0.996210322	0.993887397	0.97381056	0.999939566	0.99989309	0.989947227
ISLR	0.996210322	0.985603736	0.992056611	0.999939566	0.99989309	0.991281092
OLA1	0.996210322	0.972133808	0.983706867	0.999939566	0.99989309	0.995145616
LOC515150	0.996210322	0.971712051	0.976719499	0.999939566	0.99989309	0.998928226
LOC781059	0.996242236	0.972786652	0.994425789	0.999939566	0.99989309	0.978924127
SRI	0.996242236	0.975961032	0.982049749	0.999939566	0.99989309	0.980920516
ELMOD2	0.996242236	0.978453686	0.979231344	0.999939566	0.99989309	0.985842624
UBOX5	0.996242236	0.989661212	0.979635589	0.999939566	0.99989309	0.986353216
RTN4RL2	0.996242236	0.974986418	0.97381056	0.999939566	0.99989309	0.986389023
GIT1	0.996242236	0.986681109	0.985552223	0.999939566	0.99989309	0.989947227
CACNG7	0.996242236	0.982017699	0.974832809	0.999939566	0.99989309	0.991281092
MICAL1	0.996242236	0.97111768	0.988789822	0.999939566	0.99989309	0.998074562
SIN3B	0.996242236	0.966291707	0.975526636	0.999939566	0.99989309	0.998928226
LOC112449266	0.996265017	0.998833374	0.979231344	0.999939566	0.99989309	0.986389023
LOC107132796	0.99655492	0.996120899	0.979096894	0.999939566	0.99989309	0.981396128
LOC104970503	0.99655492	0.972418749	0.98952691	0.999939566	0.99989309	0.996564988
TRMT1L	0.996645867	0.99817889	0.979096894	0.999939566	0.99989309	0.98016619
NTHL1	0.996645867	0.980930691	0.999323478	0.999939566	0.99989309	0.98016619
KAT2B	0.996645867	0.974986418	0.976869041	0.999939566	0.99989309	0.981164039
LOC789192	0.996645867	0.982963758	0.974158329	0.999939566	0.99989309	0.988323336
C1H3orf70	0.996679955	0.972786652	0.991406769	0.999939566	0.99989309	0.978656049
PLEKHO2	0.996679955	0.978453686	0.979231344	0.999939566	0.99989309	0.982045172
ZNF821	0.996709291	0.986245673	0.991235675	0.999939566	0.99989309	0.981759054
RAI14	0.996709291	0.966868197	0.995624898	0.999939566	0.99989309	0.985788947

LOC112446357	0.996709291	0.981611503	0.983555077	0.999939566	0.99989309	0.995411183
AHDC1	0.996717263	0.972418749	0.989332994	0.999939566	0.99989309	0.983453112
LOC616942	0.996717263	0.982017699	0.983706867	0.999939566	0.99989309	0.99797558
CTDP1	0.99675082	0.976925958	0.990493758	0.999939566	0.99989309	0.984105954
SMCO3	0.99675082	0.970179915	0.979635589	0.999939566	0.99989309	0.991281092
LOC100848325	0.99675082	0.995344604	0.985552223	0.999939566	0.99989309	0.991281092
PPFIA4	0.996762294	0.992657849	0.976161819	0.999939566	0.99989309	0.98016619
ARL5A	0.996762294	0.967515137	0.994425789	0.999939566	0.99989309	0.983453112
NAP1L3	0.996762294	0.983053672	0.985135994	0.999939566	0.99989309	0.993612847
SSH3	0.996762294	0.991895983	0.976575956	0.999939566	0.99989309	0.99797558
KLHL24	0.996828813	0.992032812	0.985552223	0.999939566	0.99989309	0.983453112
GABRG3	0.996828813	0.971839996	0.990107362	0.999939566	0.99989309	0.996455238
JPH1	0.996828813	0.968312904	0.981346992	0.999939566	0.99989309	0.998636142
PNPLA8	0.996865657	0.997845497	0.993278977	0.999939566	0.99989309	0.979233909
JARID2	0.996865657	0.970237188	0.994425789	0.999939566	0.99989309	0.981411983
ARMC5	0.996865657	0.982017699	0.979231344	0.999939566	0.99989309	0.982045172
LOC112442228	0.996865657	0.989002009	0.97381056	0.999939566	0.99989309	0.993259399
MYO9B	0.996865657	0.968312904	0.977359505	0.999939566	0.99989309	0.995145616
FAM133A	0.996982683	0.982614603	0.994425789	0.999939566	0.99989309	0.978656049
CRLS1	0.996982683	0.970843742	0.975939166	0.999939566	0.99989309	0.979233909
ARL6	0.996982683	0.992273495	0.985757941	0.999939566	0.99989309	0.979517983
CMTM3	0.996982683	0.996749824	0.978879234	0.999939566	0.99989309	0.98016619
LOC100848699	0.996982683	0.990586436	0.988111832	0.999939566	0.99989309	0.98016619
RPL39	0.996982683	0.986360806	0.993301613	0.999939566	0.99989309	0.98016619
GGCT	0.996982683	0.972418749	0.999323478	0.999939566	0.99989309	0.98016619
TMEM47	0.996982683	0.999377692	0.988302976	0.999939566	0.99989309	0.981411983
LOC112449360	0.996982683	0.998984661	0.98846962	0.999939566	0.99989309	0.981411983
LOC786417	0.996982683	0.993175055	0.979231344	0.999939566	0.99989309	0.981578785
CLCN6	0.996982683	0.991878011	0.983332897	0.999939566	0.99989309	0.981578785
USE1	0.996982683	0.971426434	0.981174295	0.999939566	0.99989309	0.98199344
MYO1D	0.996982683	0.98547302	0.988322814	0.999939566	0.99989309	0.982559673
SYT4	0.996982683	0.972418749	0.998442742	0.999939566	0.99989309	0.982559673
AP4S1	0.996982683	0.974591667	0.999157119	0.999939566	0.99989309	0.983427394
UROS	0.996982683	0.973168324	0.980734833	0.999939566	0.99989309	0.983453112
RNF144A	0.996982683	0.971068039	0.985552223	0.999939566	0.99989309	0.983453112
LOC100294994	0.996982683	0.997264953	0.988788601	0.999939566	0.99989309	0.983453112
PCDHGB4	0.996982683	0.971068039	0.989845175	0.999939566	0.99989309	0.983453112
LOC107131225	0.996982683	0.989661212	0.991235675	0.999939566	0.99989309	0.984253502

CALML6	0.996982683	0.982017699	0.993278977	0.999939566	0.99989309	0.984253502
DHH	0.996982683	0.973666167	0.976869041	0.999939566	0.99989309	0.985842624
PPIB	0.996982683	0.982963758	0.982659943	0.999939566	0.99989309	0.986006158
KIAA1671	0.996982683	0.973158835	0.989702604	0.999939566	0.99989309	0.986389023
CETN3	0.996982683	0.992032812	0.975212837	0.999939566	0.99989309	0.989313458
ABCG4	0.996982683	0.991720467	0.991693262	0.999939566	0.99989309	0.99064193
ZNF2	0.996982683	0.977500512	0.97420715	0.999939566	0.99989309	0.991281092
LOC112442802	0.996982683	0.968237269	0.976869041	0.999939566	0.99989309	0.991281092
LOC520104	0.996982683	0.986032534	0.976869041	0.999939566	0.99989309	0.991281092
MLKL	0.996982683	0.986245673	0.985552223	0.999939566	0.99989309	0.991281092
LOC101907126	0.996982683	0.990179479	0.988149496	0.999939566	0.99989309	0.991281092
NEBL	0.996982683	0.988649234	0.99115315	0.999939566	0.99989309	0.99244992
LOC533093	0.996982683	0.967758952	0.975183307	0.999939566	0.99989309	0.993612847
ZPBP	0.996982683	0.970179915	0.984936232	0.999939566	0.99989309	0.997366598
LRRC69	0.996982683	0.974875445	0.983706867	0.999939566	0.99989309	0.997620729
LRRC72	0.996982683	0.996120899	0.974832809	0.999939566	0.99989309	0.998074562
LOC519208	0.996982683	0.973158835	0.989845175	0.999939566	0.99989309	0.998074562
LGALS7	0.996982683	0.979073808	0.989845175	0.999939566	0.99989309	0.998119692
CLEC16A	0.996982683	0.972418749	0.979461445	0.999939566	0.99989309	0.998636142
TBX2	0.996999903	0.976925958	0.978215635	0.999939566	0.99989309	0.98016619
NECTIN2	0.996999903	0.997390201	0.978215635	0.999939566	0.99989309	0.98016619
LOC112442039	0.996999903	0.970237188	0.979096894	0.999939566	0.99989309	0.98016619
NUDT1	0.996999903	0.997390201	0.979635589	0.999939566	0.99989309	0.98016619
ZNF584	0.996999903	0.995023283	0.980385041	0.999939566	0.99989309	0.98016619
RPS29	0.996999903	0.982963758	0.980734833	0.999939566	0.99989309	0.98016619
CMTM7	0.996999903	0.971712051	0.981134567	0.999939566	0.99989309	0.98016619
PLAGL2	0.996999903	0.969442016	0.983517715	0.999939566	0.99989309	0.98016619
LOC112446427	0.996999903	0.974986418	0.985016728	0.999939566	0.99989309	0.98016619
LOC112448832	0.996999903	0.991555439	0.987208945	0.999939566	0.99989309	0.98016619
RPL35A	0.996999903	0.98395047	0.989408632	0.999939566	0.99989309	0.98016619
LOC107131834	0.996999903	0.973158835	0.99304917	0.999939566	0.99989309	0.98016619
TYW3	0.996999903	0.972786652	0.994425789	0.999939566	0.99989309	0.98016619
MUS81	0.996999903	0.971426434	0.998751041	0.999939566	0.99989309	0.98016619
TSPAN12	0.996999903	0.994365533	0.976719499	0.999939566	0.99989309	0.981164039
LOC107132255	0.996999903	0.995965505	0.979231344	0.999939566	0.99989309	0.981164039
LOC112442708	0.996999903	0.98111573	0.98168496	0.999939566	0.99989309	0.981164039
CCDC85A	0.996999903	0.988153273	0.983706867	0.999939566	0.99989309	0.981164039
PRR29	0.996999903	0.979466937	0.985222638	0.999939566	0.99989309	0.981164039

LOC787250	0.996999903	0.978453686	0.976972382	0.999939566	0.99989309	0.981411983
NHS	0.996999903	0.990668708	0.982659255	0.999939566	0.99989309	0.981411983
ZDHHC14	0.996999903	0.994365533	0.985552223	0.999939566	0.99989309	0.981411983
LOC107131749	0.996999903	0.972418749	0.988788601	0.999939566	0.99989309	0.981411983
LOC100138078	0.996999903	0.985002724	0.983706867	0.999939566	0.99989309	0.981421439
LOC112447510	0.996999903	0.991526013	0.976719499	0.999939566	0.99989309	0.981578785
RPS17	0.996999903	0.971068039	0.990107362	0.999939566	0.99989309	0.981871771
PCCB	0.996999903	0.981226249	0.980734833	0.999939566	0.99989309	0.982045172
PIP4K2C	0.996999903	0.999704064	0.976719499	0.999939566	0.99989309	0.982066213
HIST1H2AK	0.996999903	0.972418749	0.995918981	0.999939566	0.99989309	0.982066213
HS2ST1	0.996999903	0.977584325	0.980734833	0.999939566	0.99989309	0.983056851
LOC101904796	0.996999903	0.983048937	0.977782604	0.999939566	0.99989309	0.983427394
PLA2G7	0.996999903	0.988960148	0.997794783	0.999939566	0.99989309	0.983427394
EVI5L	0.996999903	0.98743321	0.976719499	0.999939566	0.99989309	0.983453112
TEF	0.996999903	0.985784205	0.977612552	0.999939566	0.99989309	0.983453112
EIF2AK1	0.996999903	0.98618789	0.979096894	0.999939566	0.99989309	0.983453112
EEF2	0.996999903	0.998683615	0.979231344	0.999939566	0.99989309	0.983453112
SLC1A3	0.996999903	0.989736861	0.981715764	0.999939566	0.99989309	0.983453112
PRDM6	0.996999903	0.992032812	0.988788601	0.999939566	0.99989309	0.983453112
LOC101907250	0.996999903	0.972786652	0.989702604	0.999939566	0.99989309	0.983453112
CAPN2	0.996999903	0.974986418	0.989771742	0.999939566	0.99989309	0.983453112
AP5B1	0.996999903	0.999377692	0.989771742	0.999939566	0.99989309	0.983453112
TMED9	0.996999903	0.972786652	0.991406769	0.999939566	0.99989309	0.983453112
PM20D2	0.996999903	0.974256477	0.994425789	0.999939566	0.99989309	0.983453112
SLC4A7	0.996999903	0.978453686	0.994425789	0.999939566	0.99989309	0.983453112
ASB6	0.996999903	0.99134261	0.994425789	0.999939566	0.99989309	0.983453112
ZC3H10	0.996999903	0.986245673	0.982028743	0.999939566	0.99989309	0.983525373
GULO	0.996999903	0.978833989	0.987419404	0.999939566	0.99989309	0.983850983
CCDC92	0.996999903	0.972418749	0.980170426	0.999939566	0.99989309	0.98398551
LOC548613	0.996999903	0.988649234	0.978282952	0.999939566	0.99989309	0.984065808
DUSP7	0.996999903	0.988725451	0.978832388	0.999939566	0.99989309	0.984449688
PIEZO1	0.996999903	0.988153273	0.991406769	0.999939566	0.99989309	0.984449688
KCTD5	0.996999903	0.975341721	0.998524485	0.999939566	0.99989309	0.984491523
DLG2	0.996999903	0.972786652	0.985552223	0.999939566	0.99989309	0.984609406
LMO2	0.996999903	0.999368101	0.987419404	0.999939566	0.99989309	0.984609406
TASP1	0.996999903	0.991720467	0.985552223	0.999939566	0.99989309	0.985266349
LOC788541	0.996999903	0.986032534	0.987006595	0.999939566	0.99989309	0.985399216
CPN2	0.996999903	0.984102025	0.977612552	0.999939566	0.99989309	0.985565158

SHANK3	0.996999903	0.9734097	0.987842204	0.999939566	0.99989309	0.985788947
LOC112449247	0.996999903	0.996722893	0.993665588	0.999939566	0.99989309	0.985788947
LOC101902831	0.996999903	0.980930691	0.979231344	0.999939566	0.99989309	0.986006158
TMEM9	0.996999903	0.970911808	0.976869041	0.999939566	0.99989309	0.986389023
SLC35E4	0.996999903	0.989445679	0.977447568	0.999939566	0.99989309	0.986389023
LOC100848007	0.996999903	0.990914937	0.9785457	0.999939566	0.99989309	0.986389023
ZFP91	0.996999903	0.982833172	0.994425789	0.999939566	0.99989309	0.986389023
FHIT	0.996999903	0.992194873	0.994425789	0.999939566	0.99989309	0.986389023
NR1D1	0.996999903	0.974986418	0.994835068	0.999939566	0.99989309	0.98704566
SLCO2A1	0.996999903	0.971615208	0.989408632	0.999939566	0.99989309	0.987210851
KLRG1	0.996999903	0.992032812	0.976785552	0.999939566	0.99989309	0.988323336
CASKIN2	0.996999903	0.994365533	0.987639174	0.999939566	0.99989309	0.988323336
LOC112443783	0.996999903	0.997390201	0.987854175	0.999939566	0.99989309	0.988323336
MED22	0.996999903	0.996148299	0.988789822	0.999939566	0.99989309	0.988323336
TEK	0.996999903	0.994365533	0.991986182	0.999939566	0.99989309	0.988323336
LOC101904871	0.996999903	0.971615208	0.978282952	0.999939566	0.99989309	0.98894622
BACH2	0.996999903	0.971839996	0.994425789	0.999939566	0.99989309	0.989243676
HYKK	0.996999903	0.971839996	0.98567143	0.999939566	0.99989309	0.989313458
LOC112447420	0.996999903	0.988649234	0.990038008	0.999939566	0.99989309	0.989719747
NOBOX	0.996999903	0.983065474	0.994425789	0.999939566	0.99989309	0.990275559
C1H3orf38	0.996999903	0.992032812	0.978215635	0.999939566	0.99989309	0.99064193
ABCA13	0.996999903	0.982590111	0.987842204	0.999939566	0.99989309	0.99064193
SNURF	0.996999903	0.973158835	0.976719499	0.999939566	0.99989309	0.991051281
CPLANE2	0.996999903	0.972786652	0.994425789	0.999939566	0.99989309	0.991051281
LOC787497	0.996999903	0.979237036	0.976719499	0.999939566	0.99989309	0.991281092
ACTR6	0.996999903	0.993887397	0.979851274	0.999939566	0.99989309	0.991281092
CALD1	0.996999903	0.981463822	0.981276558	0.999939566	0.99989309	0.991281092
ZMYM6	0.996999903	0.988153273	0.987419404	0.999939566	0.99989309	0.991281092
LOC789997	0.996999903	0.988153273	0.987433396	0.999939566	0.99989309	0.991281092
SYNGR2	0.996999903	0.972786652	0.990693603	0.999939566	0.99989309	0.991281092
SYNPO	0.996999903	0.982703363	0.991406769	0.999939566	0.99989309	0.991281092
ING5	0.996999903	0.982614603	0.987842204	0.999939566	0.99989309	0.991305896
FBXO42	0.996999903	0.971077172	0.995624898	0.999939566	0.99989309	0.992643051
PPP1R16B	0.996999903	0.973649843	0.976885897	0.999939566	0.99989309	0.993612847
LOC112441629	0.996999903	0.972418749	0.987639174	0.999939566	0.99989309	0.993612847
TM9SF4	0.996999903	0.98833023	0.98082053	0.999939566	0.99989309	0.995145616
LOC112441659	0.996999903	0.995965505	0.977612552	0.999939566	0.99989309	0.995411183
CMTM4	0.996999903	0.977732731	0.990693603	0.999939566	0.99989309	0.995411183

TCN2	0.996999903	0.98743321	0.980734833	0.999939566	0.99989309	0.9956013
CYTH3	0.996999903	0.974986418	0.987842204	0.999939566	0.99989309	0.995757227
LOC112448776	0.996999903	0.988649234	0.990693603	0.999939566	0.99989309	0.996383947
LOC101904314	0.996999903	0.987539286	0.983474414	0.999939566	0.99989309	0.996455238
MTX2	0.996999903	0.970524066	0.98730572	0.999939566	0.99989309	0.997366598
CLCA3	0.996999903	0.991043872	0.979851274	0.999939566	0.99989309	0.997922692
XYLT1	0.996999903	0.974591667	0.979231344	0.999939566	0.99989309	0.998074562
LOC101907327	0.996999903	0.976925958	0.979231344	0.999939566	0.99989309	0.998074562
PCIF1	0.996999903	0.98743321	0.987208945	0.999939566	0.99989309	0.998074562
LOC101904290	0.996999903	0.974986418	0.991693262	0.999939566	0.99989309	0.99845476
H2AFX	0.996999903	0.992194873	0.979231344	0.999939566	0.99989309	0.998636142
FOXN3	0.996999903	0.974073092	0.985160065	0.999939566	0.99989309	0.998636142
ZNF394	0.996999903	0.977732731	0.989527187	0.999939566	0.99989309	0.998636142
OGFR	0.996999903	0.974591667	0.990870021	0.999939566	0.99989309	0.998636142
ARL13A	0.996999903	0.974403012	0.979231344	0.999939566	0.99989309	0.998928226
ZNRF3	0.996999903	0.974986418	0.985552223	0.999939566	0.99989309	0.998928226
PDGFC	0.997003397	0.988649234	0.978879234	0.999939566	0.99989309	0.983453112
FBXL20	0.997082295	0.98377426	0.994425789	0.999939566	0.99989309	0.980920516
SF3A1	0.997082295	0.982267858	0.983474414	0.999939566	0.99989309	0.981164039
PIGK	0.997082295	0.986032534	0.987208945	0.999939566	0.99989309	0.983453112
LOC512286	0.997082295	0.974875445	0.993278977	0.999939566	0.99989309	0.983453112
MKNK2	0.997082295	0.991720467	0.989408632	0.999939566	0.99989309	0.989128955
SCARA3	0.997082295	0.988737715	0.993665588	0.999939566	0.99989309	0.989313458
DDX5	0.997082295	0.975673897	0.994546187	0.999939566	0.99989309	0.989313458
CDH22	0.997082295	0.971426434	0.982930137	0.999939566	0.99989309	0.991281092
CNIH4	0.997082295	0.986245673	0.978215635	0.999939566	0.99989309	0.998213311
LOC112444484	0.997082345	0.982703363	0.980734833	0.999939566	0.99989309	0.982514294
CENPP	0.997175672	0.972133808	0.979851274	0.999939566	0.99989309	0.983453112
HSF1	0.997175672	0.986245673	0.984936232	0.999939566	0.99989309	0.988323336
PIH1D1	0.997175672	0.981624931	0.979720857	0.999939566	0.99989309	0.998074562
GINS3	0.997205222	0.972563829	0.995380703	0.999939566	0.99989309	0.981090047
HIST1H2BL	0.997205222	0.992032812	0.979231344	0.999939566	0.99989309	0.981164039
CASS4	0.997205222	0.995289146	0.989845175	0.999939566	0.99989309	0.981411983
HNMT	0.997205222	0.978676027	0.985552223	0.999939566	0.99989309	0.983453112
PTPRA	0.997205222	0.988649234	0.983966304	0.999939566	0.99989309	0.985788947
RPL36	0.997205222	0.984479682	0.979500641	0.999939566	0.99989309	0.986389023
RNFT2	0.997205222	0.992032812	0.985552223	0.999939566	0.99989309	0.988297209
ARHGDIB	0.997205222	0.99950308	0.979231344	0.999939566	0.99989309	0.988323336

C1H21orf58	0.997205222	0.988725451	0.985552223	0.999939566	0.99989309	0.988323336
CNPY4	0.997205222	0.978453686	0.985552223	0.999939566	0.99989309	0.989128955
C22H3orf18	0.997205222	0.971839996	0.991113951	0.999939566	0.99989309	0.990085862
AKT1	0.997205222	0.97572611	0.98567143	0.999939566	0.99989309	0.991934523
LOC112445951	0.997212942	0.992027017	0.979635589	0.999939566	0.99989309	0.98016619
ZNF814	0.997212942	0.99950308	0.978892556	0.999939566	0.99989309	0.980179583
CHFR	0.997212942	0.998250479	0.978892556	0.999939566	0.99989309	0.983453112
LOC100847143	0.997212942	0.991895983	0.994425789	0.999939566	0.99989309	0.989947227
SLC35E2	0.997212942	0.977584325	0.987842204	0.999939566	0.99989309	0.998074562
CITED4	0.997308747	0.976204049	0.979231344	0.999939566	0.99989309	0.988323336
TMEM131L	0.997342341	0.976150941	0.988322814	0.999939566	0.99989309	0.983453112
LOC101906221	0.997342341	0.98618789	0.978892556	0.999939566	0.99989309	0.998928226
UBE2D1	0.997481166	0.980930691	0.993266164	0.999939566	0.99989309	0.990501884
SLC35B4	0.997691374	0.998683615	0.985552223	0.999939566	0.99989309	0.985517088
PHRF1	0.997729164	0.978453686	0.991406769	0.999939566	0.99989309	0.995094611
PPM1B	0.997740751	0.992657849	0.985552223	0.999939566	0.99989309	0.981164039
RPS3A	0.997740751	0.982051974	0.995184296	0.999939566	0.99989309	0.981164039
FAM168A	0.997740751	0.991109612	0.989332994	0.999939566	0.99989309	0.981411983
CBX1	0.997740751	0.990194936	0.995380703	0.999939566	0.99989309	0.981411983
RPS27	0.997740751	0.982017699	0.990107362	0.999939566	0.99989309	0.981578785
LYPLAL1	0.997740751	0.981343316	0.985552223	0.999939566	0.99989309	0.982066213
ARHGAP32	0.997740751	0.98362464	0.985396677	0.999939566	0.99989309	0.982074417
REL	0.997740751	0.982568993	0.982728455	0.999939566	0.99989309	0.983453112
HOXB7	0.997740751	0.982963758	0.985552223	0.999939566	0.99989309	0.983453112
PKP2	0.997740751	0.984082087	0.98567143	0.999939566	0.99989309	0.983453112
MORC2	0.997740751	0.990179479	0.98730572	0.999939566	0.99989309	0.983453112
ABCA9	0.997740751	0.988725451	0.987517415	0.999939566	0.99989309	0.983453112
ADAMTSL1	0.997740751	0.981087742	0.989527187	0.999939566	0.99989309	0.983453112
OSGEPL1	0.997740751	0.981093825	0.989702604	0.999939566	0.99989309	0.983453112
DNAL1	0.997740751	0.976150941	0.994425789	0.999939566	0.99989309	0.983453112
RASEF	0.997740751	0.996120899	0.994425789	0.999939566	0.99989309	0.983453112
ORC2	0.997740751	0.973158835	0.99576701	0.999939566	0.99989309	0.983453112
LOC784522	0.997740751	0.972786652	0.999480442	0.999939566	0.99989309	0.983453112
DENND3	0.997740751	0.974073092	0.999906387	0.999939566	0.99989309	0.983453112
LOC786783	0.997740751	0.995368676	0.987854175	0.999939566	0.99989309	0.983512424
BFSP1	0.997740751	0.983065474	0.985222638	0.999939566	0.99989309	0.984491523
NEK3	0.997740751	0.98743321	0.987433396	0.999939566	0.99989309	0.984491523
HLTF	0.997740751	0.995965505	0.979231344	0.999939566	0.99989309	0.984609406

ARFGEF2	0.997740751	0.973158835	0.984936232	0.999939566	0.99989309	0.985517088
LOC104975612	0.997740751	0.98743321	0.982223562	0.999939566	0.99989309	0.986389023
LIPC	0.997740751	0.974986418	0.990107362	0.999939566	0.99989309	0.986389023
HCAR1	0.997740751	0.99464069	0.979231344	0.999939566	0.99989309	0.986839567
LOC112446676	0.997740751	0.980930691	0.987419404	0.999939566	0.99989309	0.986862062
RPL27	0.997740751	0.976302681	0.985222638	0.999939566	0.99989309	0.988323336
GFRA2	0.997740751	0.985457646	0.995380703	0.999939566	0.99989309	0.988323336
LOC521656	0.997740751	0.985002724	0.996569581	0.999939566	0.99989309	0.988323336
SOX17	0.997740751	0.998683615	0.980442556	0.999939566	0.99989309	0.989128955
LOC790886	0.997740751	0.989661212	0.979635589	0.999939566	0.99989309	0.989313458
SLC7A6OS	0.997740751	0.982963758	0.980744205	0.999939566	0.99989309	0.990291862
IRF2BP2	0.997740751	0.985138775	0.978892556	0.999939566	0.99989309	0.991281092
LOC112446053	0.997740751	0.982703363	0.982049749	0.999939566	0.99989309	0.991281092
CC2D2B	0.997740751	0.991895983	0.994425789	0.999939566	0.99989309	0.991281092
RANBP17	0.997740751	0.987628894	0.985552223	0.999939566	0.99989309	0.99244992
LOC112446775	0.997740751	0.977341253	0.979851274	0.999939566	0.99989309	0.993612847
DR1	0.997740751	0.976150941	0.980744205	0.999939566	0.99989309	0.993612847
SMPD5	0.997740751	0.978287344	0.991113951	0.999939566	0.99989309	0.993612847
FAM111B	0.997740751	0.981226249	0.994425789	0.999939566	0.99989309	0.994772186
CUTC	0.997740751	0.979515768	0.979099287	0.999939566	0.99989309	0.996564988
LOC100847773	0.997740751	0.976925958	0.994425789	0.999939566	0.99989309	0.99797558
DDAH2	0.997740751	0.978188325	0.982049749	0.999939566	0.99989309	0.998074562
LOC781741	0.997740751	0.989661212	0.989332994	0.999939566	0.99989309	0.998074562
CNNM2	0.997740751	0.974986418	0.994425789	0.999939566	0.99989309	0.998074562
TDG	0.997740751	0.978215444	0.981715764	0.999939566	0.99989309	0.998636142
CLEC3B	0.997740751	0.984903461	0.987842204	0.999939566	0.99989309	0.998636142
CYP26B1	0.997740751	0.980614912	0.979231344	0.999939566	0.99989309	0.998928226
INTS2	0.997740751	0.982703363	0.98567143	0.999939566	0.99989309	0.998928226
ZC3H12C	0.997740751	0.983065474	0.979231344	0.999939566	0.99989309	0.999899229
GRIN3A	0.997770075	0.97833257	0.979231344	0.999939566	0.99989309	0.981578785
CCDC3	0.997770075	0.98743321	0.994425789	0.999939566	0.99989309	0.982066213
HIST1H2BB	0.997770075	0.995182748	0.979231344	0.999939566	0.99989309	0.983427394
RASAL1	0.997770075	0.975287816	0.981977161	0.999939566	0.99989309	0.983453112
HERC2	0.997770075	0.982051974	0.985222638	0.999939566	0.99989309	0.983453112
MYDGF	0.997770075	0.994810749	0.987842204	0.999939566	0.99989309	0.983453112
ZNF614	0.997770075	0.983065474	0.98730572	0.999939566	0.99989309	0.985958645
SMARCD1	0.997770075	0.995965505	0.990107362	0.999939566	0.99989309	0.986716086
LOC100847573	0.997770075	0.984102025	0.979231344	0.999939566	0.99989309	0.988297209

LOC112448022	0.997770075	0.978215444	0.979231344	0.999939566	0.99989309	0.989128955
RPL19	0.997770075	0.989661212	0.98730572	0.999939566	0.99989309	0.989128955
CORO1B	0.997770075	0.982963758	0.988111832	0.999939566	0.99989309	0.989128955
SEPT11	0.997770075	0.979073808	0.979231344	0.999939566	0.99989309	0.989967429
LOC112441888	0.997770075	0.991555439	0.982659943	0.999939566	0.99989309	0.991281092
TFEB	0.997770075	0.978453686	0.98567143	0.999939566	0.99989309	0.991281092
POLR2H	0.997770075	0.977732731	0.988585687	0.999939566	0.99989309	0.991281092
KLHL22	0.997770075	0.979916117	0.989332994	0.999939566	0.99989309	0.991281092
WASHC1	0.997770075	0.976938431	0.991235675	0.999939566	0.99989309	0.991281092
POLE2	0.997770075	0.998816456	0.979231344	0.999939566	0.99989309	0.994307746
STAT1	0.997770075	0.985326169	0.988789822	0.999939566	0.99989309	0.995411183
PDE9A	0.997770075	0.982614603	0.987842204	0.999939566	0.99989309	0.997366598
CLTRN	0.997770075	0.986681109	0.989408632	0.999939566	0.99989309	0.998074562
NPDC1	0.997770075	0.979466937	0.991235675	0.999939566	0.99989309	0.998074562
SLC4A4	0.997770075	0.974986418	0.982659943	0.999939566	0.99989309	0.998928226
DDX23	0.997927431	0.977033454	0.983750815	0.999939566	0.99989309	0.999676639
UNC45B	0.998078877	0.994365533	0.990107362	0.999939566	0.99989309	0.984449688
LIMD1	0.998101595	0.986032534	0.987842204	0.999939566	0.99989309	0.991281092
MGARP	0.998101595	0.976925958	0.98567143	0.999939566	0.99989309	0.998636142
LOC784088	0.998248978	0.988605499	0.988740473	0.999939566	0.99989309	0.983056851
LOC783301	0.998248978	0.989661212	0.98730572	0.999939566	0.99989309	0.986389023
ALKAL2	0.998248978	0.978453686	0.993301613	0.999939566	0.99989309	0.986389023
SLC6A3	0.998248978	0.998683615	0.987842204	0.999939566	0.99989309	0.987452279
LOC112441810	0.998248978	0.982963758	0.987433396	0.999939566	0.99989309	0.991281092
C21H15orf40	0.998438289	0.981709588	0.985552223	0.999939566	0.99989309	0.983056851
JUN	0.998438289	0.99950308	0.982930137	0.999939566	0.99989309	0.983453112
PRRG2	0.998438289	0.999239602	0.984890088	0.999939566	0.99989309	0.983453112
SETD1B	0.998438289	0.981226249	0.991113951	0.999939566	0.99989309	0.983453112
KCNJ13	0.998438289	0.991878011	0.992542989	0.999939566	0.99989309	0.983453112
SSR2	0.998438289	0.988500804	0.994425789	0.999939566	0.99989309	0.983453112
LOC101902930	0.998438289	0.995678646	0.994425789	0.999939566	0.99989309	0.983453112
RANBP10	0.998438289	0.99950308	0.987842204	0.999939566	0.99989309	0.983525373
LOC112444339	0.998438289	0.984479682	0.990107362	0.999939566	0.99989309	0.984449688
LOC107131296	0.998438289	0.99950308	0.993747807	0.999939566	0.99989309	0.984449688
NUCB1	0.998438289	0.988960148	0.985552223	0.999939566	0.99989309	0.984609406
MELK	0.998438289	0.98743321	0.989702604	0.999939566	0.99989309	0.984967003
MFF	0.998438289	0.984082087	0.983706867	0.999939566	0.99989309	0.984980785
SEMA5A	0.998438289	0.982963758	0.988788601	0.999939566	0.99989309	0.985788947

MVB12A	0.998438289	0.981332269	0.994425789	0.999939566	0.99989309	0.985788947
PPARG	0.998438289	0.990217839	0.989332994	0.999939566	0.99989309	0.986389023
PTK2B	0.998438289	0.987713536	0.99245091	0.999939566	0.99989309	0.986389023
OMA1	0.998438289	0.978121478	0.991406769	0.999939566	0.99989309	0.986862062
SWAP70	0.998438289	0.992657849	0.985824904	0.999939566	0.99989309	0.988323336
FKBP15	0.998438289	0.99950308	0.988585687	0.999939566	0.99989309	0.988323336
H2AFV	0.998438289	0.978556875	0.994425789	0.999939566	0.99989309	0.988323336
ANGPTL1	0.998438289	0.98743321	0.995624898	0.999939566	0.99989309	0.988569171
TRMT9B	0.998438289	0.980639049	0.997590067	0.999939566	0.99989309	0.988835991
AKAP17A	0.998438289	0.977500512	0.984995192	0.999939566	0.99989309	0.99064193
KLHL2	0.998438289	0.992657849	0.985135994	0.999939566	0.99989309	0.99064193
MDFIC	0.998438289	0.992591147	0.993665588	0.999939566	0.99989309	0.99064193
LOC789996	0.998438289	0.980682689	0.999323478	0.999939566	0.99989309	0.99064193
MBD1	0.998438289	0.974986418	0.99115315	0.999939566	0.99989309	0.991051281
BMT2	0.998438289	0.980930691	0.987842204	0.999939566	0.99989309	0.991281092
RAB11FIP3	0.998438289	0.978453686	0.989642163	0.999939566	0.99989309	0.991281092
VPS36	0.998438289	0.991043872	0.989771742	0.999939566	0.99989309	0.991281092
SNX33	0.998438289	0.990586436	0.989845175	0.999939566	0.99989309	0.991281092
IAH1	0.998438289	0.992657849	0.991235675	0.999939566	0.99989309	0.991281092
LOC112444285	0.998438289	0.988649234	0.991972449	0.999939566	0.99989309	0.991281092
LOC104973050	0.998438289	0.976925958	0.994425789	0.999939566	0.99989309	0.991281092
LOC112449056	0.998438289	0.992273495	0.991693262	0.999939566	0.99989309	0.991305896
CRIPT	0.998438289	0.976754814	0.99576701	0.999939566	0.99989309	0.991612651
WDR78	0.998438289	0.988153273	0.984700021	0.999939566	0.99989309	0.991934523
FOXQ1	0.998438289	0.998683615	0.985118862	0.999939566	0.99989309	0.991986031
SUN1	0.998438289	0.990507797	0.994425789	0.999939566	0.99989309	0.992186241
LOC112444152	0.998438289	0.987713536	0.989845175	0.999939566	0.99989309	0.993259399
NR2C1	0.998438289	0.999239602	0.985552223	0.999939566	0.99989309	0.995145616
TRAPPC11	0.998438289	0.982963758	0.991817553	0.999939566	0.99989309	0.995411183
STARD3NL	0.998438289	0.988649234	0.983706867	0.999939566	0.99989309	0.995518609
NME3	0.998438289	0.987628894	0.986780789	0.999939566	0.99989309	0.995518609
PIAS4	0.998438289	0.994810749	0.983706867	0.999939566	0.99989309	0.996455238
LOC112446022	0.998438289	0.982963758	0.987273256	0.999939566	0.99989309	0.996455238
EPB41L1	0.998438289	0.988725451	0.989332994	0.999939566	0.99989309	0.996574245
MYOZ3	0.998438289	0.976925958	0.982223562	0.999939566	0.99989309	0.99797558
RREB1	0.998438289	0.977732731	0.985552223	0.999939566	0.99989309	0.99797558
SWT1	0.998438289	0.998250479	0.982659124	0.999939566	0.99989309	0.998074562
MELTF	0.998438289	0.981332269	0.987639174	0.999939566	0.99989309	0.998074562

RCOR3	0.998438289	0.991399746	0.987949796	0.999939566	0.99989309	0.998074562
LOC112444215	0.998438289	0.987713536	0.989702604	0.999939566	0.99989309	0.998074562
ZFYVE26	0.998438289	0.98743321	0.99115315	0.999939566	0.99989309	0.998074562
LOC101907653	0.998438289	0.981226249	0.980734833	0.999939566	0.99989309	0.998928226
PRDM16	0.998438289	0.975899801	0.982223562	0.999939566	0.99989309	0.998928226
CEP41	0.998438289	0.989445679	0.983555077	0.999939566	0.99989309	0.998928226
RCAN2	0.998438289	0.9789789	0.984083149	0.999939566	0.99989309	0.998928226
MTERF1	0.998438289	0.982653545	0.987842204	0.999939566	0.99989309	0.998928226
PARG	0.998438289	0.974986418	0.987842204	0.999939566	0.99989309	0.999019116
AATK	0.998482577	0.983805026	0.994425789	0.999939566	0.99989309	0.98361583
PRKD3	0.998511951	0.979351487	0.982049749	0.999939566	0.99989309	0.983453112
PTPN3	0.998511951	0.989773581	0.985552223	0.999939566	0.99989309	0.984609406
SNRNP40	0.998598299	0.989002009	0.982659124	0.999939566	0.99989309	0.983453112
ACVRL1	0.998598299	0.9789789	0.988788601	0.999939566	0.99989309	0.983453112
LOC112447066	0.998598299	0.982017699	0.989332994	0.999939566	0.99989309	0.983453112
MRPL10	0.998598299	0.984479682	0.989332994	0.999939566	0.99989309	0.983453112
TDRD6	0.998598299	0.987790525	0.989845175	0.999939566	0.99989309	0.983453112
C16H1orf21	0.998598299	0.98743321	0.99466875	0.999939566	0.99989309	0.983453112
CFAP126	0.998598299	0.976502785	0.991406769	0.999939566	0.99989309	0.983525373
C14H8orf89	0.998598299	0.999368101	0.986164239	0.999939566	0.99989309	0.983836142
PIP4P1	0.998598299	0.981133654	0.993278977	0.999939566	0.99989309	0.984449688
SYN3	0.998598299	0.985663256	0.987419404	0.999939566	0.99989309	0.984491523
MYO6	0.998598299	0.992593205	0.987433396	0.999939566	0.99989309	0.984491523
LOC100848171	0.998598299	0.982051974	0.994425789	0.999939566	0.99989309	0.985266349
FRMD4A	0.998598299	0.988725451	0.98846962	0.999939566	0.99989309	0.985788947
DCAF7	0.998598299	0.97908188	0.982222583	0.999939566	0.99989309	0.985919775
DPH2	0.998598299	0.981226249	0.983474414	0.999939566	0.99989309	0.986389023
LOC785761	0.998598299	0.987478313	0.990107362	0.999939566	0.99989309	0.986389023
LOC112444843	0.998598299	0.986245673	0.991235675	0.999939566	0.99989309	0.986389023
CSDC2	0.998598299	0.988649234	0.994340822	0.999939566	0.99989309	0.987210851
IL1RAPL2	0.998598299	0.98743321	0.994425789	0.999939566	0.99989309	0.988323336
FRMD3	0.998598299	0.978799707	0.985552223	0.999939566	0.99989309	0.989313458
CD109	0.998598299	0.992194873	0.987949796	0.999939566	0.99989309	0.991051281
NOS3	0.998598299	0.994810749	0.982223562	0.999939566	0.99989309	0.991281092
MAPK7	0.998598299	0.979466937	0.989951768	0.999939566	0.99989309	0.991281092
LOC101904526	0.998598299	0.994365533	0.993278977	0.999939566	0.99989309	0.991281092
MS4A13	0.998598299	0.98743321	0.99576701	0.999939566	0.99989309	0.991281092
PLXNA2	0.998598299	0.979916117	0.994425789	0.999939566	0.99989309	0.995038485

LOC785386	0.998598299	0.982963758	0.987419404	0.999939566	0.99989309	0.995411183
ZNF235	0.998598299	0.982963758	0.992850925	0.999939566	0.99989309	0.996383947
EIF2AK4	0.998598299	0.978890304	0.985552223	0.999939566	0.99989309	0.998074562
TYW1	0.998598299	0.983288516	0.98567143	0.999939566	0.99989309	0.998074562
PIK3R3	0.998598299	0.99134261	0.988041385	0.999939566	0.99989309	0.998074562
PTPRH	0.998598299	0.977844751	0.9840633	0.999939566	0.99989309	0.998523935
LOC112447832	0.998598299	0.98618789	0.982222583	0.999939566	0.99989309	0.998636142
TRIT1	0.998598299	0.979569387	0.98662331	0.999939566	0.99989309	0.998928226
LOC100337081	0.998598299	0.980614912	0.989845175	0.999939566	0.99989309	0.998928226
ZFYVE27	0.998703995	0.989445679	0.996265044	0.999939566	0.99989309	0.991281092
GPSM2	0.998842876	0.99677427	0.988788601	0.999939566	0.99989309	0.98361583
LOC104974883	0.998842876	0.995289146	0.990107362	0.999939566	0.99989309	0.98609047
FAM149A	0.998842876	0.998683615	0.989332994	0.999939566	0.99989309	0.986389023
LOC100337328	0.998842876	0.980621528	0.985552223	0.999939566	0.99989309	0.988323336
AIF1L	0.998842876	0.98833023	0.993860602	0.999939566	0.99989309	0.989313458
GAB1	0.998842876	0.992593205	0.987208945	0.999939566	0.99989309	0.991281092
LOC112441859	0.998842876	0.982703363	0.994425789	0.999939566	0.99989309	0.993612847
LOC107132849	0.998842876	0.983048937	0.997638343	0.999939566	0.99989309	0.99417788
DND1	0.998842876	0.990914937	0.983706867	0.999939566	0.99989309	0.996746451
MTOR	0.998842876	0.994365533	0.985222638	0.999939566	0.99989309	0.998074562
SLC39A1	0.998842876	0.987713536	0.987419404	0.999939566	0.99989309	0.998074562
FAM13B	0.998842876	0.98618789	0.995380703	0.999939566	0.99989309	0.998074562
NCAM2	0.998861762	0.988598186	0.990965255	0.999939566	0.99989309	0.995736528
LOC112447461	0.998987617	0.998683615	0.990693603	0.999939566	0.99989309	0.986389023
INO80E	0.998987617	0.982963758	0.994425789	0.999939566	0.99989309	0.991051281
HSDL2	0.999218952	0.98618789	0.989332994	0.999939566	0.99989309	0.983525373
PARP2	0.999218952	0.984593167	0.994580782	0.999939566	0.99989309	0.983525373
TENM1	0.999218952	0.982614603	0.99576701	0.999939566	0.99989309	0.983836142
GNAO1	0.999218952	0.989782307	0.985552223	0.999939566	0.99989309	0.984491523
GPR151	0.999218952	0.996722893	0.985135994	0.999939566	0.99989309	0.986353216
RPL35	0.999218952	0.986245673	0.990107362	0.999939566	0.99989309	0.986389023
GAS2L3	0.999218952	0.989736861	0.995723789	0.999939566	0.99989309	0.986389023
MYLIP	0.999218952	0.996722893	0.993079801	0.999939566	0.99989309	0.987210851
LOC104969192	0.999218952	0.99950308	0.988041385	0.999939566	0.99989309	0.988571083
LOC112449565	0.999218952	0.990217839	0.996942087	0.999939566	0.99989309	0.989780996
SKA1	0.999218952	0.992194873	0.989332994	0.999939566	0.99989309	0.990291862
ZC3HAV1	0.999218952	0.984102025	0.984700021	0.999939566	0.99989309	0.991281092
LOC112448507	0.999218952	0.982703363	0.988585687	0.999939566	0.99989309	0.991788784

STX11	0.999218952	0.982963758	0.994425789	0.999939566	0.99989309	0.993259399
LOC789626	0.999218952	0.989661212	0.985757941	0.999939566	0.99989309	0.993612847
MXRA8	0.999218952	0.981093825	0.993665588	0.999939566	0.99989309	0.993612847
ZNF570	0.999218952	0.980930691	0.990319523	0.999939566	0.99989309	0.995145616
CRKL	0.999218952	0.986851582	0.990493758	0.999939566	0.99989309	0.9956013
LOC781576	0.999218952	0.982087212	0.998021785	0.999939566	0.99989309	0.996455238
SHISA5	0.999218952	0.982833172	0.985321206	0.999939566	0.99989309	0.998074562
IL10RB	0.999218952	0.981239373	0.985552223	0.999939566	0.99989309	0.998074562
KLF8	0.999218952	0.990217839	0.992643455	0.999939566	0.99989309	0.998074562
LOC100335177	0.999218952	0.982614603	0.987208945	0.999939566	0.99989309	0.998928226
GPHN	0.999304672	0.982017699	0.985055964	0.999939566	0.99989309	0.989128955
SLC35B3	0.999304672	0.978453686	0.994425789	0.999939566	0.99989309	0.995145616
RPP25	0.999304672	0.991739536	0.9861363	0.999939566	0.99989309	0.998880954
FBXO9	0.999362691	0.996848511	0.989332994	0.999939566	0.99989309	0.984491523
PPP4C	0.999362691	0.99950308	0.988836461	0.999939566	0.99989309	0.986389023
RNF126	0.999362691	0.994810749	0.993278977	0.999939566	0.99989309	0.986389023
SEPT2	0.999362691	0.979916117	0.994425789	0.999939566	0.99989309	0.989128955
UNC50	0.999362691	0.98743321	0.994425789	0.999939566	0.99989309	0.991051281
SORCS1	0.999362691	0.98743321	0.985135994	0.999939566	0.99989309	0.991281092
MEX3D	0.999362691	0.978651934	0.991406769	0.999939566	0.99989309	0.9956013
IKZF4	0.999362691	0.980930691	0.999541752	0.999939566	0.99989309	0.9956013
CHIC2	0.999362691	0.988153273	0.987842204	0.999939566	0.99989309	0.998636142
ANKRD24	0.999390171	0.989445679	0.987842204	0.999939566	0.99989309	0.991281092
ATP2A3	0.99939915	0.982703363	0.989642163	0.999939566	0.99989309	0.985788947
FCGRT	0.99939915	0.999377692	0.988788601	0.999939566	0.99989309	0.993612847
RUVBL2	0.99939915	0.994810749	0.987208945	0.999939566	0.99989309	0.998636142
FYCO1	0.999459585	0.98833023	0.98567143	0.999939566	0.99989309	0.984449688
AVPR1A	0.999459585	0.980639049	0.989408632	0.999939566	0.99989309	0.984449688
FPGT	0.999459585	0.991988698	0.997140639	0.999939566	0.99989309	0.989128955
MTRF1L	0.999459585	0.982703363	0.990107362	0.999939566	0.99989309	0.991281092
ANKRD54	0.999459585	0.981709588	0.993278977	0.999939566	0.99989309	0.991281092
RACK1	0.999459585	0.994377463	0.994425789	0.999939566	0.99989309	0.991281092
MRRF	0.999459585	0.982963758	0.988322814	0.999939566	0.99989309	0.991934523
MARC2	0.999459585	0.988153273	0.993665588	0.999939566	0.99989309	0.99244992
LOC619131	0.999459585	0.991043872	0.99576701	0.999939566	0.99989309	0.993612847
LOC104973767	0.999459585	0.995182748	0.988585687	0.999939566	0.99989309	0.993985914
A2M	0.999459585	0.988153273	0.987419404	0.999939566	0.99989309	0.9956013
LOC783060	0.999459585	0.98833023	0.993665588	0.999939566	0.99989309	0.998074562

RPS3	0.999461065	0.994810749	0.987419404	0.999939566	0.99989309	0.989313458
SLC27A4	0.999461065	0.992593205	0.994425789	0.999939566	0.99989309	0.990026807
LOC784659	0.999461065	0.98743321	0.98567143	0.999939566	0.99989309	0.996564988
RAD18	0.999592796	0.988153273	0.998303405	0.999939566	0.99989309	0.985188807
PLPP4	0.999592796	0.98743321	0.987949796	0.999939566	0.99989309	0.985775405
CEBPB	0.999592796	0.988153273	0.994425789	0.999939566	0.99989309	0.985788947
PCID2	0.999592796	0.998683615	0.994425789	0.999939566	0.99989309	0.985919775
LOC618220	0.999592796	0.987156012	0.988041385	0.999939566	0.99989309	0.986006158
CTU2	0.999592796	0.991043872	0.996948653	0.999939566	0.99989309	0.986862062
HSPA12A	0.999592796	0.991043872	0.987273256	0.999939566	0.99989309	0.98704566
ZNF548	0.999592796	0.996722893	0.991406769	0.999939566	0.99989309	0.988297209
ENTPD5	0.999592796	0.992602165	0.990965255	0.999939566	0.99989309	0.988571083
LOC782776	0.999592796	0.99950308	0.989332994	0.999939566	0.99989309	0.989313458
SLC35B2	0.999592796	0.990668708	0.996948653	0.999939566	0.99989309	0.989313458
ECI2	0.999592796	0.996722893	0.994425789	0.999939566	0.99989309	0.989947227
LRRN4CL	0.999592796	0.983065474	0.985552223	0.999939566	0.99989309	0.991051281
KLRD1	0.999592796	0.982963758	0.990107362	0.999939566	0.99989309	0.991281092
LOC104975290	0.999592796	0.991895983	0.99304917	0.999939566	0.99989309	0.991281092
NCKIPSD	0.999592796	0.982963758	0.994425789	0.999939566	0.99989309	0.991281092
LOC100849069	0.999592796	0.981226249	0.999323478	0.999939566	0.99989309	0.991281092
LENG1	0.999592796	0.984247605	0.991234064	0.999939566	0.99989309	0.991612651
MR1	0.999592796	0.981332269	0.991406769	0.999939566	0.99989309	0.99244992
SCAF1	0.999592796	0.982051974	0.988788601	0.999939566	0.99989309	0.993036675
NRCAM	0.999592796	0.995706781	0.989408632	0.999939566	0.99989309	0.993612847
LOC100294792	0.999592796	0.994495614	0.995607212	0.999939566	0.99989309	0.993612847
LOC100138131	0.999592796	0.99714596	0.992165187	0.999939566	0.99989309	0.995145616
LOC104972417	0.999592796	0.988649234	0.987639174	0.999939566	0.99989309	0.995411183
LOC112445988	0.999592796	0.993223749	0.990107362	0.999939566	0.99989309	0.995411183
MCM10	0.999592796	0.992032812	0.993278977	0.999939566	0.99989309	0.995411183
LOC783926	0.999592796	0.982017699	0.994425789	0.999939566	0.99989309	0.996383947
RPUSD4	0.999592796	0.988153273	0.987419404	0.999939566	0.99989309	0.996455238
FZD3	0.999592796	0.987713536	0.988740473	0.999939566	0.99989309	0.996455238
TMCO3	0.999592796	0.981093825	0.998434485	0.999939566	0.99989309	0.996455238
LOC104974542	0.999592796	0.992194873	0.984717389	0.999939566	0.99989309	0.996746451
MARCH9	0.999592796	0.989770494	0.985016728	0.999939566	0.99989309	0.997620729
RABGGTB	0.999592796	0.982963758	0.985989185	0.999939566	0.99989309	0.998074562
IMPDH1	0.999592796	0.986681109	0.98730572	0.999939566	0.99989309	0.998074562
MANSC4	0.999592796	0.994365533	0.993747807	0.999939566	0.99989309	0.998074562

LOC101906315	0.999592796	0.988960148	0.995184296	0.999939566	0.99989309	0.998074562
RPL13	0.999592796	0.986245673	0.987208945	0.999939566	0.99989309	0.998636142
LOC104971057	0.999592796	0.991541192	0.987419404	0.999939566	0.99989309	0.998636142
TERF1	0.999592796	0.995706781	0.987433396	0.999939566	0.99989309	0.998636142
CD2AP	0.999592796	0.983408703	0.995412965	0.999939566	0.99989309	0.998636142
GJB2	0.999634776	0.992656129	0.990107362	0.999939566	0.99989309	0.991281092
LOC112447817	0.999634776	0.985999612	0.987208945	0.999939566	0.99989309	0.993612847
RPL8	0.999634776	0.985120788	0.992383565	0.999939566	0.99989309	0.993612847
LOC112444653	0.999634776	0.984479682	0.994425789	0.999939566	0.99989309	0.998074562
MOB3B	0.999641406	0.986032534	0.988788601	0.999939566	0.99989309	0.98704566
CNTNAP3	0.999678105	0.992593205	0.98567143	0.999939566	0.99989309	0.998074562
LOC112443431	0.999682441	0.982963758	0.994425789	0.999939566	0.99989309	0.990275559
LOC112445915	0.999863206	0.98618789	0.993278977	0.999939566	0.99989309	0.98704566
LZTR1	0.999863206	0.984082087	0.989332994	0.999939566	0.99989309	0.988323336
LOC100847453	0.999863206	0.998683615	0.991235675	0.999939566	0.99989309	0.988323336
BARD1	0.999863206	0.982833172	0.991583737	0.999939566	0.99989309	0.988323336
CTTNBP2NL	0.999863206	0.99950308	0.992915726	0.999939566	0.99989309	0.988323336
RPL6	0.999863206	0.995965505	0.993665588	0.999939566	0.99989309	0.988323336
MORF4L2	0.999863206	0.991895983	0.994425789	0.999939566	0.99989309	0.988323336
LOC107131452	0.999863206	0.986032534	0.999323478	0.999939566	0.99989309	0.988323336
PIM2	0.999863206	0.984102025	0.999917831	0.999939566	0.99989309	0.988323336
LOC100847156	0.999863206	0.987790525	0.991111137	0.999939566	0.99989309	0.988569171
SEMA3C	0.999863206	0.994810749	0.989332994	0.999939566	0.99989309	0.989128955
VWA1	0.999863206	0.999934642	0.990107362	0.999939566	0.99989309	0.989128955
RPS14	0.999863206	0.99965623	0.992554953	0.999939566	0.99989309	0.989128955
LOC790871	0.999863206	0.984097339	0.993665588	0.999939566	0.99989309	0.989128955
LOC100847780	0.999863206	0.998683615	0.994425789	0.999939566	0.99989309	0.989128955
CNKSR2	0.999863206	0.99134261	0.995918981	0.999939566	0.99989309	0.989128955
CNTNAP4	0.999863206	0.997390201	0.998442742	0.999939566	0.99989309	0.989128955
EDN1	0.999863206	0.988153273	0.989332994	0.999939566	0.99989309	0.989313458
LOC112443177	0.999863206	0.987478313	0.99576701	0.999939566	0.99989309	0.989313458
LOC112447499	0.999863206	0.996430877	0.995799138	0.999939566	0.99989309	0.989313458
DPYSL2	0.999863206	0.991720467	0.994425789	0.999939566	0.99989309	0.989947227
LOC112442602	0.999863206	0.998517125	0.989702604	0.999939566	0.99989309	0.990085862
LOC112447797	0.999863206	0.994365533	0.993665588	0.999939566	0.99989309	0.990085862
UBXN6	0.999863206	0.986681109	0.987854175	0.999939566	0.99989309	0.990291862
PROB1	0.999863206	0.988649234	0.994425789	0.999939566	0.99989309	0.990501884
ZXDC	0.999863206	0.992032812	0.998683403	0.999939566	0.99989309	0.99064193

LOC112446417	0.999863206	0.989661212	0.999336747	0.999939566	0.99989309	0.99064193
CDCA7L	0.999863206	0.995706781	0.990107362	0.999939566	0.99989309	0.991051281
CHRNA5	0.999863206	0.991895983	0.994425789	0.999939566	0.99989309	0.991051281
LOC788205	0.999863206	0.989335307	0.995723789	0.999939566	0.99989309	0.991051281
PRIM2	0.999863206	0.99002957	0.994425789	0.999939566	0.99989309	0.99112148
PDGFRL	0.999863206	0.987951604	0.995723789	0.999939566	0.99989309	0.99112148
GOLGA3	0.999863206	0.99817889	0.999323478	0.999939566	0.99989309	0.99112148
TAF9B	0.999863206	0.995965505	0.987639174	0.999939566	0.99989309	0.991281092
EDEM1	0.999863206	0.998938672	0.987639174	0.999939566	0.99989309	0.991281092
BCL7C	0.999863206	0.996430877	0.988041385	0.999939566	0.99989309	0.991281092
LOC100336532	0.999863206	0.992032812	0.988149496	0.999939566	0.99989309	0.991281092
CLSTN1	0.999863206	0.98618789	0.988752364	0.999939566	0.99989309	0.991281092
LOC100299281	0.999863206	0.992657849	0.988752364	0.999939566	0.99989309	0.991281092
MICU2	0.999863206	0.988649234	0.989332994	0.999939566	0.99989309	0.991281092
NRTN	0.999863206	0.994365533	0.989332994	0.999939566	0.99989309	0.991281092
LOC101905265	0.999863206	0.988153273	0.989408632	0.999939566	0.99989309	0.991281092
LOC112449275	0.999863206	0.992656764	0.989408632	0.999939566	0.99989309	0.991281092
AK1	0.999863206	0.999560732	0.989408632	0.999939566	0.99989309	0.991281092
ZNF606	0.999863206	0.999239602	0.989702604	0.999939566	0.99989309	0.991281092
LOC101904393	0.999863206	0.997024303	0.990107362	0.999939566	0.99989309	0.991281092
IFIT5	0.999863206	0.998683615	0.990237299	0.999939566	0.99989309	0.991281092
MEN1	0.999863206	0.987713536	0.990965255	0.999939566	0.99989309	0.991281092
NRBP1	0.999863206	0.993644379	0.991406769	0.999939566	0.99989309	0.991281092
LOC782032	0.999863206	0.999205683	0.991406769	0.999939566	0.99989309	0.991281092
LOC107131652	0.999863206	0.999377692	0.991406769	0.999939566	0.99989309	0.991281092
LOC112445971	0.999863206	0.991089429	0.99304917	0.999939566	0.99989309	0.991281092
PLCB2	0.999863206	0.99950308	0.99304917	0.999939566	0.99989309	0.991281092
RPL4	0.999863206	0.992968229	0.993278977	0.999939566	0.99989309	0.991281092
DMXL2	0.999863206	0.998683615	0.993278977	0.999939566	0.99989309	0.991281092
PIGL	0.999863206	0.982703363	0.993665588	0.999939566	0.99989309	0.991281092
LOC534391	0.999863206	0.994567583	0.993747807	0.999939566	0.99989309	0.991281092
CBY1	0.999863206	0.998517125	0.993747807	0.999939566	0.99989309	0.991281092
ATXN3	0.999863206	0.988649234	0.994182484	0.999939566	0.99989309	0.991281092
RPL18	0.999863206	0.986032534	0.994425789	0.999939566	0.99989309	0.991281092
LOC101904173	0.999863206	0.988153273	0.994425789	0.999939566	0.99989309	0.991281092
ABCB7	0.999863206	0.990217839	0.994425789	0.999939566	0.99989309	0.991281092
RGS13	0.999863206	0.992194873	0.994425789	0.999939566	0.99989309	0.991281092
LOC107132793	0.999863206	0.992656764	0.994425789	0.999939566	0.99989309	0.991281092

SPIN2	0.999863206	0.992657849	0.994425789	0.999939566	0.99989309	0.991281092
THEM4	0.999863206	0.994622291	0.994425789	0.999939566	0.99989309	0.991281092
FGFRL1	0.999863206	0.995289146	0.994425789	0.999939566	0.99989309	0.991281092
LRRC75A	0.999863206	0.995965505	0.994425789	0.999939566	0.99989309	0.991281092
LOC104975286	0.999863206	0.998683615	0.994425789	0.999939566	0.99989309	0.991281092
MRGPRF	0.999863206	0.998683615	0.994425789	0.999939566	0.99989309	0.991281092
LOC790037	0.999863206	0.99950308	0.994425789	0.999939566	0.99989309	0.991281092
LOC783022	0.999863206	0.994280343	0.994546187	0.999939566	0.99989309	0.991281092
MCM7	0.999863206	0.987713536	0.995799138	0.999939566	0.99989309	0.991281092
RAP1GAP	0.999863206	0.99114351	0.995918981	0.999939566	0.99989309	0.991281092
LOC534913	0.999863206	0.98618789	0.996265044	0.999939566	0.99989309	0.991281092
CCL28	0.999863206	0.994365533	0.996948006	0.999939566	0.99989309	0.991281092
MIGA1	0.999863206	0.988725451	0.996948653	0.999939566	0.99989309	0.991281092
C2CD2	0.999863206	0.989661212	0.997589693	0.999939566	0.99989309	0.991281092
TYRO3	0.999863206	0.992194873	0.998021785	0.999939566	0.99989309	0.991281092
ESRP2	0.999863206	0.994365533	0.998303405	0.999939566	0.99989309	0.991281092
TSEN15	0.999863206	0.988649234	0.999323478	0.999939566	0.99989309	0.991281092
CCNK	0.999863206	0.988714924	0.999323478	0.999939566	0.99989309	0.991281092
LOC100335404	0.999863206	0.992544107	0.999323478	0.999939566	0.99989309	0.991281092
RPL7	0.999863206	0.995182748	0.994425789	0.999939566	0.99989309	0.991305896
IFI16	0.999863206	0.99950308	0.998442742	0.999939566	0.99989309	0.991431199
ARHGAP18	0.999863206	0.992273495	0.994425789	0.999939566	0.99989309	0.991569266
RPP25L	0.999863206	0.99950308	0.988585687	0.999939566	0.99989309	0.991612651
KLHDC3	0.999863206	0.988649234	0.991406769	0.999939566	0.99989309	0.992354835
PRKCD	0.999863206	0.992740593	0.993278977	0.999939566	0.99989309	0.992358083
CCR8	0.999863206	0.982963758	0.987842204	0.999939566	0.99989309	0.99244992
TANC2	0.999863206	0.994810749	0.995624898	0.999939566	0.99989309	0.992464397
LIN37	0.999863206	0.990586436	0.991406769	0.999939566	0.99989309	0.99246916
PEX12	0.999863206	0.991043872	0.995412965	0.999939566	0.99989309	0.99246916
TGFBR2	0.999863206	0.984082087	0.987842204	0.999939566	0.99989309	0.992643051
LOC104976614	0.999863206	0.998683615	0.994425789	0.999939566	0.99989309	0.992671204
SLC38A7	0.999863206	0.998683615	0.993665588	0.999939566	0.99989309	0.99289587
RASSF7	0.999863206	0.990194936	0.991111137	0.999939566	0.99989309	0.993259399
LOC100139732	0.999863206	0.997845497	0.994425789	0.999939566	0.99989309	0.993259399
SBK1	0.999863206	0.997252752	0.999541752	0.999939566	0.99989309	0.993259399
CLEC1A	0.999863206	0.99950308	0.990107362	0.999939566	0.99989309	0.9933204
LOC112447026	0.999863206	0.994377463	0.987639174	0.999939566	0.99989309	0.993612847
GPR135	0.999863206	0.988153273	0.989332994	0.999939566	0.99989309	0.993612847

LEMD2	0.999863206	0.992273495	0.990493758	0.999939566	0.99989309	0.993612847
TBXAS1	0.999863206	0.993959968	0.990693603	0.999939566	0.99989309	0.993612847
MRE11	0.999863206	0.99950308	0.990693603	0.999939566	0.99989309	0.993612847
LOC100850436	0.999863206	0.995965505	0.991406769	0.999939566	0.99989309	0.993612847
EIF3F	0.999863206	0.989773581	0.991693262	0.999939566	0.99989309	0.993612847
TULP3	0.999863206	0.988649234	0.99245091	0.999939566	0.99989309	0.993612847
LOC101904156	0.999863206	0.99950308	0.993278977	0.999939566	0.99989309	0.993612847
SH3BP4	0.999863206	0.988725451	0.994425789	0.999939566	0.99989309	0.993612847
NCR3	0.999863206	0.998646269	0.994425789	0.999939566	0.99989309	0.993612847
COCH	0.999863206	0.999247641	0.994425789	0.999939566	0.99989309	0.993612847
PIP4K2B	0.999863206	0.991442953	0.995380703	0.999939566	0.99989309	0.993612847
LOC112442244	0.999863206	0.992593205	0.996760789	0.999939566	0.99989309	0.993612847
PPYR1	0.999863206	0.994810749	0.998314144	0.999939566	0.99989309	0.993612847
MMP17	0.999863206	0.989002009	0.999323478	0.999939566	0.99989309	0.993612847
UBXN2B	0.999863206	0.99002957	0.999323478	0.999939566	0.99989309	0.993612847
DIXDC1	0.999863206	0.994810749	0.999480442	0.999939566	0.99989309	0.993612847
XKR8	0.999863206	0.99677427	0.999541752	0.999939566	0.99989309	0.993612847
DENND4C	0.999863206	0.994376473	0.990107362	0.999939566	0.99989309	0.993714265
LOC783396	0.999863206	0.989661212	0.994425789	0.999939566	0.99989309	0.993714265
SUMF1	0.999863206	0.991043872	0.994425789	0.999939566	0.99989309	0.993714265
U2SURP	0.999863206	0.994810749	0.994425789	0.999939566	0.99989309	0.993714265
CC2D1A	0.999863206	0.988649234	0.998303405	0.999939566	0.99989309	0.99417067
LOC100848492	0.999863206	0.988153273	0.989770806	0.999939566	0.99989309	0.994307746
ANKRD13A	0.999863206	0.990611325	0.994425789	0.999939566	0.99989309	0.994388872
LOC112442676	0.999863206	0.988153273	0.999323478	0.999939566	0.99989309	0.994388872
ZNF691	0.999863206	0.997390201	0.999555497	0.999939566	0.99989309	0.994388872
LOC112447029	0.999863206	0.999968084	0.99115315	0.999939566	0.99989309	0.994437241
RPS15	0.999863206	0.991043872	0.994326396	0.999939566	0.99989309	0.994437241
LOC112442656	0.999863206	0.99950308	0.994835068	0.999939566	0.99989309	0.994437241
SCD5	0.999863206	0.991988698	0.993665588	0.999939566	0.99989309	0.994620117
LOC107132851	0.999863206	0.989445679	0.988788601	0.999939566	0.99989309	0.994702849
LOC101905771	0.999863206	0.99950308	0.993665588	0.999939566	0.99989309	0.994822605
ATP6V0E2	0.999863206	0.987632059	0.987949796	0.999939566	0.99989309	0.995063891
FAM20C	0.999863206	0.999669688	0.990107362	0.999939566	0.99989309	0.995063891
RSPH3	0.999863206	0.995678646	0.995624898	0.999939566	0.99989309	0.995063891
ASB5	0.999863206	0.988153273	0.989845175	0.999939566	0.99989309	0.995094611
LOC615521	0.999863206	0.994365533	0.994425789	0.999939566	0.99989309	0.995094611
SF3B2	0.999863206	0.98743321	0.989408632	0.999939566	0.99989309	0.995145616

LOC100298453	0.999863206	0.991555439	0.989642163	0.999939566	0.99989309	0.995145616
ANKRD9	0.999863206	0.994365533	0.991406769	0.999939566	0.99989309	0.995145616
LOC107132374	0.999863206	0.988649234	0.994425789	0.999939566	0.99989309	0.995145616
LOC104970815	0.999863206	0.994365533	0.994425789	0.999939566	0.99989309	0.995145616
METRN	0.999863206	0.995965505	0.994425789	0.999939566	0.99989309	0.995145616
PKD2	0.999863206	0.991043872	0.994524327	0.999939566	0.99989309	0.995145616
MED25	0.999863206	0.994810749	0.998442742	0.999939566	0.99989309	0.995145616
LOC101906460	0.999863206	0.991043872	0.999555497	0.999939566	0.99989309	0.995145616
FAM71F2	0.999863206	0.990914937	0.991235675	0.999939566	0.99989309	0.995227365
RFTN2	0.999863206	0.992656739	0.987949796	0.999939566	0.99989309	0.995282085
LOC112444473	0.999863206	0.99950308	0.990107362	0.999939566	0.99989309	0.995411183
LOC112447508	0.999863206	0.995688571	0.991693262	0.999939566	0.99989309	0.995411183
SLC27A6	0.999863206	0.991720467	0.993747807	0.999939566	0.99989309	0.995411183
ILDR2	0.999863206	0.991043872	0.994182484	0.999939566	0.99989309	0.995411183
PTGS1	0.999863206	0.991043872	0.994425789	0.999939566	0.99989309	0.995411183
RPS16	0.999863206	0.994365533	0.994425789	0.999939566	0.99989309	0.995411183
SART1	0.999863206	0.99950308	0.995184296	0.999939566	0.99989309	0.995411183
ZDHHC24	0.999863206	0.994810749	0.995380703	0.999939566	0.99989309	0.995411183
HOXC4	0.999863206	0.991541192	0.995412965	0.999939566	0.99989309	0.995411183
SEMA3A	0.999863206	0.988649234	0.995918981	0.999939566	0.99989309	0.995411183
KDM8	0.999863206	0.988960148	0.995918981	0.999939566	0.99989309	0.995411183
RIN2	0.999863206	0.989445679	0.995918981	0.999939566	0.99989309	0.995411183
ZFP37	0.999863206	0.996082675	0.996948653	0.999939566	0.99989309	0.995411183
RPS15A	0.999863206	0.999377692	0.998904268	0.999939566	0.99989309	0.995411183
FGL2	0.999863206	0.992273495	0.990493758	0.999939566	0.99989309	0.995518609
EEF1A1	0.999863206	0.996848511	0.994425789	0.999939566	0.99989309	0.995518609
MBTPS1	0.999863206	0.99950308	0.994425789	0.999939566	0.99989309	0.995518609
HOXA5	0.999863206	0.99134261	0.995918981	0.999939566	0.99989309	0.995518609
ZNF331	0.999863206	0.994365533	0.997454076	0.999939566	0.99989309	0.995518609
LOC100848815	0.999863206	0.995289146	0.992850925	0.999939566	0.99989309	0.9956013
HIST1H1C	0.999863206	0.998510693	0.994425789	0.999939566	0.99989309	0.9956013
ZNF787	0.999863206	0.995965505	0.996888611	0.999939566	0.99989309	0.9956013
ATP10A	0.999863206	0.995965505	0.988752364	0.999939566	0.99989309	0.995736528
LOC785445	0.999863206	0.998510693	0.993278977	0.999939566	0.99989309	0.995736528
RPS7	0.999863206	0.993210584	0.994425789	0.999939566	0.99989309	0.995736528
ZDHHC5	0.999863206	0.986874771	0.994425789	0.999939566	0.99989309	0.995994863
OTUD3	0.999863206	0.991526443	0.995380703	0.999939566	0.99989309	0.996052162
OTUD7B	0.999863206	0.99950308	0.994425789	0.999939566	0.99989309	0.996108962

LOC107131651	0.999863206	0.994365533	0.994425789	0.999939566	0.99989309	0.996383947
LOC100140873	0.999863206	0.988649234	0.998303405	0.999939566	0.99989309	0.996383947
MKRN3	0.999863206	0.987790525	0.999323478	0.999939566	0.99989309	0.996383947
ATXN7L1	0.999863206	0.992656739	0.989227873	0.999939566	0.99989309	0.996455238
HACD4	0.999863206	0.984479682	0.989332994	0.999939566	0.99989309	0.996455238
LOC101902838	0.999863206	0.996430877	0.991406769	0.999939566	0.99989309	0.996455238
LOC101904413	0.999863206	0.99134261	0.994425789	0.999939566	0.99989309	0.996455238
CHML	0.999863206	0.994810749	0.994425789	0.999939566	0.99989309	0.996455238
DYNC1H1	0.999863206	0.994810749	0.994425789	0.999939566	0.99989309	0.996455238
EFCAB6	0.999863206	0.987900945	0.996569581	0.999939566	0.99989309	0.996455238
LOC104975283	0.999863206	0.998683615	0.998751041	0.999939566	0.99989309	0.996455238
LOC112445088	0.999863206	0.992032812	0.999480442	0.999939566	0.99989309	0.996455238
ARMCX5	0.999863206	0.997623168	0.994425789	0.999939566	0.99989309	0.996493376
LOC782470	0.999863206	0.998890671	0.994425789	0.999939566	0.99989309	0.996493376
RPL36A	0.999863206	0.994377463	0.989332994	0.999939566	0.99989309	0.996564988
DTD1	0.999863206	0.988649234	0.990237299	0.999939566	0.99989309	0.996564988
LOC112442038	0.999863206	0.998683615	0.993278977	0.999939566	0.99989309	0.996564988
DSN1	0.999863206	0.990730535	0.993665588	0.999939566	0.99989309	0.996564988
ZNF3	0.999863206	0.995289146	0.994425789	0.999939566	0.99989309	0.996564988
SYT12	0.999863206	0.998683615	0.994425789	0.999939566	0.99989309	0.996564988
LOC112449346	0.999863206	0.999239602	0.994425789	0.999939566	0.99989309	0.996564988
SPSB2	0.999863206	0.99950308	0.995380703	0.999939566	0.99989309	0.996564988
LOC101902841	0.999863206	0.998683615	0.995918981	0.999939566	0.99989309	0.996564988
LOC787102	0.999863206	0.987628894	0.988788601	0.999939566	0.99989309	0.996574245
BLOC1S3	0.999863206	0.99470505	0.989702604	0.999939566	0.99989309	0.996574245
RPL14	0.999863206	0.999239602	0.991406769	0.999939566	0.99989309	0.996574245
ZNF554	0.999863206	0.999377692	0.994425789	0.999939566	0.99989309	0.996574245
EXT1	0.999863206	0.991878011	0.995412965	0.999939566	0.99989309	0.996574245
PARVB	0.999863206	0.992273495	0.994425789	0.999939566	0.99989309	0.997366598
C7H19orf66	0.999863206	0.998683615	0.999096118	0.999939566	0.99989309	0.997366598
OCIAD1	0.999863206	0.996848511	0.999323478	0.999939566	0.99989309	0.997366598
USP27X	0.999863206	0.994172236	0.999541752	0.999939566	0.99989309	0.997366598
WDR41	0.999863206	0.992273495	0.991406769	0.999939566	0.99989309	0.997620729
BICRAL	0.999863206	0.999704064	0.991406769	0.999939566	0.99989309	0.997620729
STK11IP	0.999863206	0.994897366	0.994425789	0.999939566	0.99989309	0.997620729
PABPC4L	0.999863206	0.999937207	0.994425789	0.999939566	0.99989309	0.997620729
CHRDL1	0.999863206	0.988708263	0.995258343	0.999939566	0.99989309	0.997620729
CECR2	0.999863206	0.992657849	0.995380703	0.999939566	0.99989309	0.997620729

WDR25	0.999863206	0.998510693	0.996760789	0.999939566	0.99989309	0.997620729
CYBA	0.999863206	0.997390201	0.99814724	0.999939566	0.99989309	0.997620729
PI4K2B	0.999863206	0.994365533	0.998303405	0.999939566	0.99989309	0.997620729
DPP8	0.999863206	0.99950308	0.999323478	0.999939566	0.99989309	0.997620729
ZNF596	0.999863206	0.992194873	0.994425789	0.999939566	0.99989309	0.997673689
LOC100140915	0.999863206	0.992273495	0.990107362	0.999939566	0.99989309	0.997783035
LOC101907883	0.999863206	0.998683615	0.994425789	0.999939566	0.99989309	0.99781381
XRCC2	0.999863206	0.992194873	0.989332994	0.999939566	0.99989309	0.997888186
TAPBP	0.999863206	0.99950308	0.995206789	0.999939566	0.99989309	0.997888186
CITED1	0.999863206	0.990668708	0.987842204	0.999939566	0.99989309	0.99797558
C19H17orf75	0.999863206	0.998683615	0.988788601	0.999939566	0.99989309	0.99797558
RPS5	0.999863206	0.994810749	0.990107362	0.999939566	0.99989309	0.99797558
LOC101905049	0.999863206	0.990586436	0.991406769	0.999939566	0.99989309	0.99797558
PLCD4	0.999863206	0.997390201	0.993665588	0.999939566	0.99989309	0.99797558
ZBTB24	0.999863206	0.994365533	0.994425789	0.999939566	0.99989309	0.99797558
MSL3	0.999863206	0.999617109	0.994425789	0.999939566	0.99989309	0.99797558
BORCS8	0.999863206	0.999617109	0.994546187	0.999939566	0.99989309	0.99797558
CRY1	0.999863206	0.998683615	0.999323478	0.999939566	0.99989309	0.99797558
CYS1	0.999863206	0.994897366	0.998524485	0.999939566	0.99989309	0.998026001
INVS	0.999863206	0.99134261	0.994425789	0.999939566	0.99989309	0.998061675
ZNF75A	0.999863206	0.997390201	0.988788601	0.999939566	0.99989309	0.998074562
MICAL3	0.999863206	0.986162538	0.989332994	0.999939566	0.99989309	0.998074562
MBD4	0.999863206	0.988605499	0.989332994	0.999939566	0.99989309	0.998074562
RPS20	0.999863206	0.99134261	0.989332994	0.999939566	0.99989309	0.998074562
LOC112442349	0.999863206	0.989661212	0.989408632	0.999939566	0.99989309	0.998074562
LOC515089	0.999863206	0.995289146	0.989408632	0.999939566	0.99989309	0.998074562
SERGEF	0.999863206	0.98833023	0.98945074	0.999939566	0.99989309	0.998074562
PBX4	0.999863206	0.998683615	0.989702604	0.999939566	0.99989309	0.998074562
NEU1	0.999863206	0.994365533	0.989771742	0.999939566	0.99989309	0.998074562
FA2H	0.999863206	0.995965505	0.989771742	0.999939566	0.99989309	0.998074562
GJA4	0.999863206	0.989661212	0.990107362	0.999939566	0.99989309	0.998074562
RASA3	0.999863206	0.988708263	0.990237299	0.999939566	0.99989309	0.998074562
LOC112449092	0.999863206	0.988649234	0.990693603	0.999939566	0.99989309	0.998074562
IFT74	0.999863206	0.996749824	0.990693603	0.999939566	0.99989309	0.998074562
ABCG1	0.999863206	0.994810749	0.990965255	0.999939566	0.99989309	0.998074562
PEX3	0.999863206	0.995965505	0.99115315	0.999939566	0.99989309	0.998074562
RPS27P	0.999863206	0.986245673	0.991406769	0.999939566	0.99989309	0.998074562
LOC112447346	0.999863206	0.988605499	0.991406769	0.999939566	0.99989309	0.998074562

LOC530348	0.999863206	0.994365533	0.991406769	0.999939566	0.99989309	0.998074562
LOC101905513	0.999863206	0.995965505	0.991406769	0.999939566	0.99989309	0.998074562
LOC112449338	0.999863206	0.996430877	0.991583737	0.999939566	0.99989309	0.998074562
TCEA2	0.999863206	0.986032534	0.992850925	0.999939566	0.99989309	0.998074562
RCC2	0.999863206	0.99950308	0.99304917	0.999939566	0.99989309	0.998074562
CALHM2	0.999863206	0.996722893	0.993278977	0.999939566	0.99989309	0.998074562
LOC112446642	0.999863206	0.987542027	0.993665588	0.999939566	0.99989309	0.998074562
CDH1	0.999863206	0.988649234	0.993665588	0.999939566	0.99989309	0.998074562
NXPE4	0.999863206	0.991895983	0.993665588	0.999939566	0.99989309	0.998074562
LAYN	0.999863206	0.992194873	0.993665588	0.999939566	0.99989309	0.998074562
ASPN	0.999863206	0.999377692	0.993665588	0.999939566	0.99989309	0.998074562
LOC107131525	0.999863206	0.990179479	0.993747807	0.999939566	0.99989309	0.998074562
CD274	0.999863206	0.991491771	0.993747807	0.999939566	0.99989309	0.998074562
CTIF	0.999863206	0.985907982	0.994425789	0.999939566	0.99989309	0.998074562
LOC100300095	0.999863206	0.988649234	0.994425789	0.999939566	0.99989309	0.998074562
ULBP21	0.999863206	0.988649234	0.994425789	0.999939566	0.99989309	0.998074562
PFKL	0.999863206	0.991043872	0.994425789	0.999939566	0.99989309	0.998074562
TLR3	0.999863206	0.991895983	0.994425789	0.999939566	0.99989309	0.998074562
ACTR1B	0.999863206	0.992032812	0.994425789	0.999939566	0.99989309	0.998074562
ZBTB41	0.999863206	0.992194873	0.994425789	0.999939566	0.99989309	0.998074562
CHST14	0.999863206	0.992656129	0.994425789	0.999939566	0.99989309	0.998074562
LOC100847841	0.999863206	0.994365533	0.994425789	0.999939566	0.99989309	0.998074562
LOC100848985	0.999863206	0.994365533	0.994425789	0.999939566	0.99989309	0.998074562
LOC101903572	0.999863206	0.994365533	0.994425789	0.999939566	0.99989309	0.998074562
LOC104972026	0.999863206	0.99470505	0.994425789	0.999939566	0.99989309	0.998074562
CAST	0.999863206	0.994810749	0.994425789	0.999939566	0.99989309	0.998074562
MLLT6	0.999863206	0.996148299	0.994425789	0.999939566	0.99989309	0.998074562
SLC25A29	0.999863206	0.996430877	0.994425789	0.999939566	0.99989309	0.998074562
TMEM260	0.999863206	0.996430877	0.994425789	0.999939566	0.99989309	0.998074562
DEPP1	0.999863206	0.99677427	0.994425789	0.999939566	0.99989309	0.998074562
LOC101904947	0.999863206	0.998683615	0.994425789	0.999939566	0.99989309	0.998074562
ARSG	0.999863206	0.99950308	0.994425789	0.999939566	0.99989309	0.998074562
CNTF	0.999863206	0.99950308	0.994425789	0.999939566	0.99989309	0.998074562
C3H1orf216	0.999863206	0.999504573	0.994425789	0.999939566	0.99989309	0.998074562
RPS19	0.999863206	0.999946057	0.994425789	0.999939566	0.99989309	0.998074562
LOC104974516	0.999863206	0.997943794	0.994546187	0.999939566	0.99989309	0.998074562
TLR8	0.999863206	0.994810749	0.994872102	0.999939566	0.99989309	0.998074562
STOML1	0.999863206	0.996308856	0.995206789	0.999939566	0.99989309	0.998074562

UBE2L6	0.999863206	0.99950308	0.995304601	0.999939566	0.99989309	0.998074562
LOC101902345	0.999863206	0.994365533	0.995380703	0.999939566	0.99989309	0.998074562
NUFIP1	0.999863206	0.995316112	0.995380703	0.999939566	0.99989309	0.998074562
LOC101907941	0.999863206	0.996205976	0.995380703	0.999939566	0.99989309	0.998074562
ANG2	0.999863206	0.996722893	0.995380703	0.999939566	0.99989309	0.998074562
LOC104975979	0.999863206	0.997762456	0.995380703	0.999939566	0.99989309	0.998074562
ASB9	0.999863206	0.999560732	0.995380703	0.999939566	0.99989309	0.998074562
ENO3	0.999863206	0.99950308	0.995509735	0.999939566	0.99989309	0.998074562
ST6GALNAC5	0.999863206	0.992194873	0.995607212	0.999939566	0.99989309	0.998074562
INPPL1	0.999863206	0.996120899	0.99576701	0.999939566	0.99989309	0.998074562
PATZ1	0.999863206	0.996722893	0.99576701	0.999939566	0.99989309	0.998074562
LOC112447399	0.999863206	0.99950308	0.99576701	0.999939566	0.99989309	0.998074562
NSMAF	0.999863206	0.995965505	0.995799138	0.999939566	0.99989309	0.998074562
TMEM74B	0.999863206	0.998816456	0.995799138	0.999939566	0.99989309	0.998074562
SYCE1L	0.999863206	0.999377692	0.995918981	0.999939566	0.99989309	0.998074562
BCAT1	0.999863206	0.997390201	0.996265044	0.999939566	0.99989309	0.998074562
LOC112441846	0.999863206	0.996722893	0.99650745	0.999939566	0.99989309	0.998074562
ACMSD	0.999863206	0.99950308	0.99650745	0.999939566	0.99989309	0.998074562
IL17RC	0.999863206	0.99464069	0.996571983	0.999939566	0.99989309	0.998074562
LFNG	0.999863206	0.996722893	0.996948653	0.999939566	0.99989309	0.998074562
PRPF6	0.999863206	0.99950308	0.997081057	0.999939566	0.99989309	0.998074562
BICD1	0.999863206	0.996120899	0.997454076	0.999939566	0.99989309	0.998074562
NELFCD	0.999863206	0.997845497	0.997511268	0.999939566	0.99989309	0.998074562
MALL	0.999863206	0.996696059	0.997586307	0.999939566	0.99989309	0.998074562
SAMD4A	0.999863206	0.996722893	0.997586307	0.999939566	0.99989309	0.998074562
ACBD6	0.999863206	0.994365533	0.997771235	0.999939566	0.99989309	0.998074562
MED15	0.999863206	0.997450916	0.997794783	0.999939566	0.99989309	0.998074562
RPS12	0.999863206	0.998683615	0.998021785	0.999939566	0.99989309	0.998074562
KCTD13	0.999863206	0.995368676	0.998157453	0.999939566	0.99989309	0.998074562
SIKE1	0.999863206	0.991043872	0.998303405	0.999939566	0.99989309	0.998074562
TFPT	0.999863206	0.996430877	0.998303405	0.999939566	0.99989309	0.998074562
LOC104969067	0.999863206	0.995965505	0.998314144	0.999939566	0.99989309	0.998074562
P2RX4	0.999863206	0.998683615	0.998442742	0.999939566	0.99989309	0.998074562
PPP4R1	0.999863206	0.99950308	0.998442742	0.999939566	0.99989309	0.998074562
NUDT2	0.999863206	0.999560732	0.998442742	0.999939566	0.99989309	0.998074562
WRNIP1	0.999863206	0.992027017	0.998559634	0.999939566	0.99989309	0.998074562
DPM3	0.999863206	0.999669688	0.998570532	0.999939566	0.99989309	0.998074562
NEDD4L	0.999863206	0.99950308	0.998904268	0.999939566	0.99989309	0.998074562

MAPK12	0.999863206	0.988649234	0.999205675	0.999939566	0.99989309	0.998074562
CMTR1	0.999863206	0.986361805	0.999323478	0.999939566	0.99989309	0.998074562
PPP2R2B	0.999863206	0.994474771	0.999323478	0.999939566	0.99989309	0.998074562
LOC101906110	0.999863206	0.996722893	0.999323478	0.999939566	0.99989309	0.998074562
WNT8B	0.999863206	0.996722893	0.999323478	0.999939566	0.99989309	0.998074562
LOC101908577	0.999863206	0.998280358	0.999541752	0.999939566	0.99989309	0.998074562
LOC104975782	0.999863206	0.994365533	0.999695103	0.999939566	0.99989309	0.998074562
ARHGEF15	0.999863206	0.994810749	0.999906387	0.999939566	0.99989309	0.998074562
TMEM241	0.999863206	0.997256297	0.999917831	0.999939566	0.99989309	0.998074562
THAP11	0.999863206	0.994810749	0.994425789	0.999939566	0.99989309	0.99809973
LOC101903356	0.999863206	0.997390201	0.995380703	0.999939566	0.99989309	0.998119692
RPS24	0.999863206	0.997193023	0.994425789	0.999939566	0.99989309	0.998135288
C14H8orf82	0.999863206	0.998683615	0.995380703	0.999939566	0.99989309	0.998135288
MRPL3	0.999863206	0.992273495	0.994425789	0.999939566	0.99989309	0.998523935
AK8	0.999863206	0.985457646	0.985552223	0.999939566	0.99989309	0.998636142
LOC107132251	0.999863206	0.984097339	0.987759344	0.999939566	0.99989309	0.998636142
LOC112441491	0.999863206	0.995678646	0.987842204	0.999939566	0.99989309	0.998636142
TMEM163	0.999863206	0.988153273	0.988462404	0.999939566	0.99989309	0.998636142
PARP1	0.999863206	0.98833023	0.988788601	0.999939566	0.99989309	0.998636142
ASAP1	0.999863206	0.99950308	0.988788601	0.999939566	0.99989309	0.998636142
CASC3	0.999863206	0.98618789	0.989408632	0.999939566	0.99989309	0.998636142
CAMK1D	0.999863206	0.990208174	0.989451484	0.999939566	0.99989309	0.998636142
TADA2A	0.999863206	0.989661212	0.989702604	0.999939566	0.99989309	0.998636142
UNC119	0.999863206	0.997845497	0.990107362	0.999939566	0.99989309	0.998636142
LOC614914	0.999863206	0.995965505	0.990965255	0.999939566	0.99989309	0.998636142
SNRPE	0.999863206	0.988960148	0.991235675	0.999939566	0.99989309	0.998636142
TBC1D12	0.999863206	0.990586436	0.991406769	0.999939566	0.99989309	0.998636142
GJB6	0.999863206	0.991043872	0.991406769	0.999939566	0.99989309	0.998636142
PROX2	0.999863206	0.99817889	0.991406769	0.999939566	0.99989309	0.998636142
RHNO1	0.999863206	0.991895983	0.99245091	0.999939566	0.99989309	0.998636142
SLC26A10	0.999863206	0.998683615	0.99245091	0.999939566	0.99989309	0.998636142
LOC112443437	0.999863206	0.988342173	0.993665588	0.999939566	0.99989309	0.998636142
AK9	0.999863206	0.99326478	0.993665588	0.999939566	0.99989309	0.998636142
CASP6	0.999863206	0.994810749	0.993665588	0.999939566	0.99989309	0.998636142
DAB2IP	0.999863206	0.994810749	0.993665588	0.999939566	0.99989309	0.998636142
TRIM2	0.999863206	0.992032812	0.993747807	0.999939566	0.99989309	0.998636142
LOC508628	0.999863206	0.996430877	0.993747807	0.999939566	0.99989309	0.998636142
TNFAIP8	0.999863206	0.98743321	0.994425789	0.999939566	0.99989309	0.998636142

CCDC50	0.999863206	0.988153273	0.994425789	0.999939566	0.99989309	0.998636142
LOC100848369	0.999863206	0.988649234	0.994425789	0.999939566	0.99989309	0.998636142
ORMDL1	0.999863206	0.990194936	0.994425789	0.999939566	0.99989309	0.998636142
CASP9	0.999863206	0.990586436	0.994425789	0.999939566	0.99989309	0.998636142
XPNPEP3	0.999863206	0.990914937	0.994425789	0.999939566	0.99989309	0.998636142
APPBP2	0.999863206	0.991895983	0.994425789	0.999939566	0.99989309	0.998636142
GALNT7	0.999863206	0.992032812	0.994425789	0.999939566	0.99989309	0.998636142
MUC20	0.999863206	0.994365533	0.994425789	0.999939566	0.99989309	0.998636142
CCDC130	0.999863206	0.995258648	0.994425789	0.999939566	0.99989309	0.998636142
LOC104976573	0.999863206	0.995289146	0.994425789	0.999939566	0.99989309	0.998636142
RNF149	0.999863206	0.995289146	0.994425789	0.999939566	0.99989309	0.998636142
TMEM63B	0.999863206	0.995678646	0.994425789	0.999939566	0.99989309	0.998636142
LOC101906836	0.999863206	0.995706781	0.994425789	0.999939566	0.99989309	0.998636142
ANKFY1	0.999863206	0.995965505	0.994425789	0.999939566	0.99989309	0.998636142
GLTP	0.999863206	0.995965505	0.994425789	0.999939566	0.99989309	0.998636142
LOC112443199	0.999863206	0.995965505	0.994425789	0.999939566	0.99989309	0.998636142
MED26	0.999863206	0.995965505	0.994425789	0.999939566	0.99989309	0.998636142
DDHD2	0.999863206	0.996941493	0.994425789	0.999939566	0.99989309	0.998636142
LOC112442702	0.999863206	0.997024303	0.994425789	0.999939566	0.99989309	0.998636142
RPL29	0.999863206	0.998280358	0.994425789	0.999939566	0.99989309	0.998636142
FAR1	0.999863206	0.998683615	0.994425789	0.999939566	0.99989309	0.998636142
LOC790312	0.999863206	0.998683615	0.994425789	0.999939566	0.99989309	0.998636142
SEC61A2	0.999863206	0.998683615	0.994425789	0.999939566	0.99989309	0.998636142
CPSF4	0.999863206	0.999239602	0.994425789	0.999939566	0.99989309	0.998636142
ARV1	0.999863206	0.999368101	0.994425789	0.999939566	0.99989309	0.998636142
LOC112444464	0.999863206	0.99950308	0.994425789	0.999939566	0.99989309	0.998636142
LOC618633	0.999863206	0.99950308	0.994425789	0.999939566	0.99989309	0.998636142
PNLDC1	0.999863206	0.99950308	0.994425789	0.999939566	0.99989309	0.998636142
PDZD2	0.999863206	0.994365533	0.995034854	0.999939566	0.99989309	0.998636142
C5H12orf10	0.999863206	0.993959968	0.995206789	0.999939566	0.99989309	0.998636142
FXR2	0.999863206	0.997390201	0.995206789	0.999939566	0.99989309	0.998636142
CTNND1	0.999863206	0.998683615	0.995206789	0.999939566	0.99989309	0.998636142
LOC519145	0.999863206	0.994810749	0.995380703	0.999939566	0.99989309	0.998636142
GABPB2	0.999863206	0.995965505	0.995380703	0.999939566	0.99989309	0.998636142
FAM83G	0.999863206	0.994365533	0.995624898	0.999939566	0.99989309	0.998636142
LOC100847745	0.999863206	0.994365533	0.995624898	0.999939566	0.99989309	0.998636142
DAPK1	0.999863206	0.999669688	0.995624898	0.999939566	0.99989309	0.998636142
XAB2	0.999863206	0.998993812	0.995723789	0.999939566	0.99989309	0.998636142

SH2D7	0.999863206	0.990179479	0.995726315	0.999939566	0.99989309	0.998636142
WWTR1	0.999863206	0.996749824	0.995726315	0.999939566	0.99989309	0.998636142
STK11	0.999863206	0.998683615	0.99576701	0.999939566	0.99989309	0.998636142
LOC112449053	0.999863206	0.99950308	0.99576701	0.999939566	0.99989309	0.998636142
WDR60	0.999863206	0.992396479	0.995799138	0.999939566	0.99989309	0.998636142
TMEM246	0.999863206	0.996848511	0.995799138	0.999939566	0.99989309	0.998636142
SREBF2	0.999863206	0.999669688	0.995893428	0.999939566	0.99989309	0.998636142
FSD2	0.999863206	0.994228143	0.995918981	0.999939566	0.99989309	0.998636142
STXBP4	0.999863206	0.994810749	0.995918981	0.999939566	0.99989309	0.998636142
TAF12	0.999863206	0.998683615	0.995918981	0.999939566	0.99989309	0.998636142
WHAMM	0.999863206	0.998683615	0.995918981	0.999939566	0.99989309	0.998636142
FABP9	0.999863206	0.99950308	0.995918981	0.999939566	0.99989309	0.998636142
GAS6	0.999863206	0.99965623	0.996194628	0.999939566	0.99989309	0.998636142
COMP	0.999863206	0.994172236	0.996222454	0.999939566	0.99989309	0.998636142
LOC788293	0.999863206	0.999368101	0.996265044	0.999939566	0.99989309	0.998636142
RPL10A	0.999863206	0.99950308	0.996265044	0.999939566	0.99989309	0.998636142
IL2RB	0.999863206	0.999377692	0.996336651	0.999939566	0.99989309	0.998636142
LYVE1	0.999863206	0.996696059	0.996569581	0.999939566	0.99989309	0.998636142
BACE1	0.999863206	0.994365533	0.996760789	0.999939566	0.99989309	0.998636142
SUPT5H	0.999863206	0.999937207	0.996930554	0.999939566	0.99989309	0.998636142
LOC112443178	0.999863206	0.992194873	0.997138402	0.999939566	0.99989309	0.998636142
LOC107132987	0.999863206	0.99950308	0.997138402	0.999939566	0.99989309	0.998636142
CNPY3	0.999863206	0.998683615	0.997327968	0.999939566	0.99989309	0.998636142
LOC100848357	0.999863206	0.989445679	0.997511268	0.999939566	0.99989309	0.998636142
TMEM240	0.999863206	0.992032812	0.998303405	0.999939566	0.99989309	0.998636142
STX16	0.999863206	0.99950308	0.998314144	0.999939566	0.99989309	0.998636142
RPS9	0.999863206	0.996848511	0.998442742	0.999939566	0.99989309	0.998636142
LOC516742	0.999863206	0.999239602	0.998442742	0.999939566	0.99989309	0.998636142
ZNF461	0.999863206	0.99950308	0.998442742	0.999939566	0.99989309	0.998636142
PTMA	0.999863206	0.994365533	0.998524485	0.999939566	0.99989309	0.998636142
RPL18A	0.999863206	0.999368101	0.998524485	0.999939566	0.99989309	0.998636142
LOC616538	0.999863206	0.999968084	0.998524485	0.999939566	0.99989309	0.998636142
C11H2orf68	0.999863206	0.99950308	0.998685166	0.999939566	0.99989309	0.998636142
STN1	0.999863206	0.995706781	0.998751041	0.999939566	0.99989309	0.998636142
RPL37	0.999863206	0.99950308	0.998751041	0.999939566	0.99989309	0.998636142
FOXK1	0.999863206	0.999395226	0.998904268	0.999939566	0.99989309	0.998636142
RPL24	0.999863206	0.999968084	0.998904268	0.999939566	0.99989309	0.998636142
EPCAM	0.999863206	0.99950308	0.998951793	0.999939566	0.99989309	0.998636142

LOC112447291	0.999863206	0.999560732	0.999096118	0.999939566	0.99989309	0.998636142
CCDC170	0.999863206	0.999968084	0.999096118	0.999939566	0.99989309	0.998636142
GRK2	0.999863206	0.99817889	0.999157119	0.999939566	0.99989309	0.998636142
LOC782812	0.999863206	0.991590911	0.999323478	0.999939566	0.99989309	0.998636142
LOC112443007	0.999863206	0.994810749	0.999323478	0.999939566	0.99989309	0.998636142
DCLRE1C	0.999863206	0.996120899	0.999323478	0.999939566	0.99989309	0.998636142
KLRF2	0.999863206	0.99677427	0.999323478	0.999939566	0.99989309	0.998636142
SLC35D2	0.999863206	0.998510693	0.999323478	0.999939566	0.99989309	0.998636142
NSL1	0.999863206	0.999368101	0.999323478	0.999939566	0.99989309	0.998636142
LASP1	0.999863206	0.99950308	0.999323478	0.999939566	0.99989309	0.998636142
LOC112446452	0.999863206	0.99950308	0.999323478	0.999939566	0.99989309	0.998636142
LOC100335205	0.999863206	0.999239602	0.999509969	0.999939566	0.99989309	0.998636142
PIGV	0.999863206	0.997390201	0.999541752	0.999939566	0.99989309	0.998636142
VPS54	0.999863206	0.999247641	0.999541752	0.999939566	0.99989309	0.998636142
FBXL4	0.999863206	0.99950308	0.999541752	0.999939566	0.99989309	0.998636142
LOC100848138	0.999863206	0.99950308	0.999541752	0.999939566	0.99989309	0.998636142
METTL2A	0.999863206	0.999617109	0.999555497	0.999939566	0.99989309	0.998636142
MAP3K7CL	0.999863206	0.996120899	0.999695103	0.999939566	0.99989309	0.998636142
DEF8	0.999863206	0.997390201	0.999695103	0.999939566	0.99989309	0.998636142
NAALADL2	0.999863206	0.999164935	0.999695103	0.999939566	0.99989309	0.998636142
LOC100848869	0.999863206	0.999239602	0.999695103	0.999939566	0.99989309	0.998636142
ZNF397	0.999863206	0.999395226	0.999695103	0.999939566	0.99989309	0.998636142
AVEN	0.999863206	0.99950308	0.999695103	0.999939566	0.99989309	0.998636142
SERF1A	0.999863206	0.996722893	0.999860751	0.999939566	0.99989309	0.998636142
CASP7	0.999863206	0.99950308	0.999906387	0.999939566	0.99989309	0.998636142
LOC104975610	0.999863206	0.99950308	0.999906387	0.999939566	0.99989309	0.998636142
CNOT8	0.999863206	0.997845497	0.999917831	0.999939566	0.99989309	0.998636142
KIFC3	0.999863206	0.99134261	0.989408632	0.999939566	0.99989309	0.998671064
AVPR2	0.999863206	0.999368101	0.999695103	0.999939566	0.99989309	0.998671064
DHX38	0.999863206	0.997264953	0.995918981	0.999939566	0.99989309	0.998708896
RPL13A	0.999863206	0.99950308	0.995184296	0.999939566	0.99989309	0.998880954
ADAM23	0.999863206	0.999164935	0.999323478	0.999939566	0.99989309	0.998880954
SLC35A2	0.999863206	0.999560732	0.999323478	0.999939566	0.99989309	0.998880954
ZNF8	0.999863206	0.994365533	0.986687875	0.999939566	0.99989309	0.998928226
DAAM2	0.999863206	0.990914937	0.98846962	0.999939566	0.99989309	0.998928226
ACSBG1	0.999863206	0.995965505	0.988788601	0.999939566	0.99989309	0.998928226
SLC6A9	0.999863206	0.988649234	0.989702604	0.999939566	0.99989309	0.998928226
ADRA1B	0.999863206	0.993511686	0.989702604	0.999939566	0.99989309	0.998928226

ADAMTS18	0.999863206	0.988649234	0.989845175	0.999939566	0.99989309	0.998928226
SCNM1	0.999863206	0.993258296	0.989866814	0.999939566	0.99989309	0.998928226
LOC782057	0.999863206	0.989661212	0.990038008	0.999939566	0.99989309	0.998928226
NOVA1	0.999863206	0.992032812	0.991406769	0.999939566	0.99989309	0.998928226
RDH14	0.999863206	0.994365533	0.991406769	0.999939566	0.99989309	0.998928226
WNT7B	0.999863206	0.992194873	0.993278977	0.999939566	0.99989309	0.998928226
MNT	0.999863206	0.992656129	0.993747807	0.999939566	0.99989309	0.998928226
GRB14	0.999863206	0.988153273	0.994425789	0.999939566	0.99989309	0.998928226
LOC101902663	0.999863206	0.988153273	0.994425789	0.999939566	0.99989309	0.998928226
TRIM47	0.999863206	0.991895983	0.994425789	0.999939566	0.99989309	0.998928226
RHBDF2	0.999863206	0.992032812	0.994425789	0.999939566	0.99989309	0.998928226
SSX2IP	0.999863206	0.993887397	0.994425789	0.999939566	0.99989309	0.998928226
ARHGEF38	0.999863206	0.994810749	0.994425789	0.999939566	0.99989309	0.998928226
RAB32	0.999863206	0.994810749	0.994425789	0.999939566	0.99989309	0.998928226
SKI	0.999863206	0.996848511	0.994425789	0.999939566	0.99989309	0.998928226
TBCK	0.999863206	0.997390201	0.994425789	0.999939566	0.99989309	0.998928226
LOC112447323	0.999863206	0.99817889	0.994425789	0.999939566	0.99989309	0.998928226
KAT5	0.999863206	0.998683615	0.994425789	0.999939566	0.99989309	0.998928226
ATXN1	0.999863206	0.999377692	0.994425789	0.999939566	0.99989309	0.998928226
LOC112447769	0.999863206	0.990194936	0.994546187	0.999939566	0.99989309	0.998928226
PACSIN1	0.999863206	0.999368101	0.994580782	0.999939566	0.99989309	0.998928226
ZC3HAV1L	0.999863206	0.998683615	0.994835068	0.999939566	0.99989309	0.998928226
LST1	0.999863206	0.99950308	0.995120045	0.999939566	0.99989309	0.998928226
LOC101902290	0.999863206	0.995289146	0.995213797	0.999939566	0.99989309	0.998928226
LOC112446725	0.999863206	0.998683615	0.995723789	0.999939566	0.99989309	0.998928226
LOC112446822	0.999863206	0.99950308	0.995726315	0.999939566	0.99989309	0.998928226
CSNK1G1	0.999863206	0.998683615	0.99576701	0.999939566	0.99989309	0.998928226
PDE6A	0.999863206	0.99950308	0.99576701	0.999939566	0.99989309	0.998928226
SH3RF3	0.999863206	0.997450916	0.995918981	0.999939566	0.99989309	0.998928226
LOC100335635	0.999863206	0.998510693	0.995918981	0.999939566	0.99989309	0.998928226
LOC787875	0.999863206	0.994377463	0.996569581	0.999939566	0.99989309	0.998928226
TULP4	0.999863206	0.99935653	0.996760789	0.999939566	0.99989309	0.998928226
TG	0.999863206	0.998683615	0.996948653	0.999939566	0.99989309	0.998928226
LOC107131455	0.999863206	0.999377692	0.996948653	0.999939566	0.99989309	0.998928226
CTSW	0.999863206	0.994365533	0.997032737	0.999939566	0.99989309	0.998928226
LOC100297616	0.999863206	0.99950308	0.997138402	0.999939566	0.99989309	0.998928226
PRL	0.999863206	0.997264953	0.997653182	0.999939566	0.99989309	0.998928226
SULT1B1	0.999863206	0.999164935	0.998021785	0.999939566	0.99989309	0.998928226

LOC100138449	0.999863206	0.999560732	0.998021785	0.999939566	0.99989309	0.998928226
HPS3	0.999863206	0.997390201	0.998128369	0.999939566	0.99989309	0.998928226
EIF5A2	0.999863206	0.99950308	0.998303405	0.999939566	0.99989309	0.998928226
LOC104976575	0.999863206	0.998683615	0.998442742	0.999939566	0.99989309	0.998928226
CES2	0.999863206	0.99950308	0.998442742	0.999939566	0.99989309	0.998928226
MT3	0.999863206	0.999560732	0.998442742	0.999939566	0.99989309	0.998928226
HIST1H2BI	0.999863206	0.99950308	0.998524485	0.999939566	0.99989309	0.998928226
MSH2	0.999863206	0.99950308	0.998524485	0.999939566	0.99989309	0.998928226
ODF2L	0.999863206	0.99950308	0.998904268	0.999939566	0.99989309	0.998928226
RPL32	0.999863206	0.99950308	0.998904268	0.999939566	0.99989309	0.998928226
FAU	0.999863206	0.999704064	0.999048305	0.999939566	0.99989309	0.998928226
PEG10	0.999863206	0.998683615	0.999157119	0.999939566	0.99989309	0.998928226
AKNAD1	0.999863206	0.999368101	0.999205675	0.999939566	0.99989309	0.998928226
IFT52	0.999863206	0.995289146	0.999323478	0.999939566	0.99989309	0.998928226
ZSWIM9	0.999863206	0.998683615	0.999323478	0.999939566	0.99989309	0.998928226
AGA	0.999863206	0.99950308	0.999323478	0.999939566	0.99989309	0.998928226
MFAP4	0.999863206	0.99950308	0.999323478	0.999939566	0.99989309	0.998928226
NADK2	0.999863206	0.99950308	0.999323478	0.999939566	0.99989309	0.998928226
RPL10	0.999863206	0.99950308	0.999323478	0.999939566	0.99989309	0.998928226
PGAP1	0.999863206	0.999946057	0.999323478	0.999939566	0.99989309	0.998928226
LDHD	0.999863206	0.999946057	0.999336747	0.999939566	0.99989309	0.998928226
FBXL17	0.999863206	0.99950308	0.999419938	0.999939566	0.99989309	0.998928226
RPL11	0.999863206	0.99950308	0.999480442	0.999939566	0.99989309	0.998928226
TRMO	0.999863206	0.995965505	0.999541752	0.999939566	0.99989309	0.998928226
YIPF3	0.999863206	0.999368101	0.999541752	0.999939566	0.99989309	0.998928226
C11H2orf49	0.999863206	0.99950308	0.999541752	0.999939566	0.99989309	0.998928226
LOC112447435	0.999863206	0.99950308	0.999541752	0.999939566	0.99989309	0.998928226
TRIQK	0.999863206	0.99950308	0.999541752	0.999939566	0.99989309	0.998928226
FMO5	0.999863206	0.999560732	0.999541752	0.999939566	0.99989309	0.998928226
RPS27A	0.999863206	0.999560948	0.999541752	0.999939566	0.99989309	0.998928226
MORN4	0.999863206	0.999669688	0.999541752	0.999939566	0.99989309	0.998928226
RPS18	0.999863206	0.99950308	0.999555497	0.999939566	0.99989309	0.998928226
MYO1H	0.999863206	0.999669688	0.999555497	0.999939566	0.99989309	0.998928226
LOC107132672	0.999863206	0.99950308	0.999567544	0.999939566	0.99989309	0.998928226
RPS21	0.999863206	0.999560732	0.999695103	0.999939566	0.99989309	0.998928226
GNAT2	0.999863206	0.99965623	0.999695103	0.999939566	0.99989309	0.998928226
MAVS	0.999863206	0.99965623	0.999695103	0.999939566	0.99989309	0.998928226
HAUS6	0.999863206	0.99950308	0.999906387	0.999939566	0.99989309	0.998928226

MAP10	0.999863206	0.99950308	0.999906387	0.999939566	0.99989309	0.998928226
SNRPG	0.999863206	0.99950308	0.999906387	0.999939566	0.99989309	0.998928226
ZNF449	0.999863206	0.998890671	0.999917831	0.999939566	0.99989309	0.998928226
PTGIS	0.999863206	0.999560732	0.999917831	0.999939566	0.99989309	0.998928226
ZER1	0.999863206	0.992657849	0.994425789	0.999939566	0.99989309	0.999315048
PDE4DIP	0.999863206	0.99950308	0.999653084	0.999939566	0.99989309	0.999315048
NOTCH1	0.999863206	0.99950308	0.999323478	0.999939566	0.99989309	0.999584634
ZNF202	0.999863206	0.986245673	0.993421266	0.999939566	0.99989309	0.999662468
CLDN12	0.999863206	0.990334877	0.987639174	0.999939566	0.99989309	0.999676639
ITGAL	0.999863206	0.991245446	0.991406769	0.999939566	0.99989309	0.999676639
ZNF865	0.999863206	0.994810749	0.994425789	0.999939566	0.99989309	0.999676639
SYDE1	0.999863206	0.99950308	0.999157119	0.999939566	0.99989309	0.999676639
LOC112443864	0.999863206	0.99950308	0.999323478	0.999939566	0.99989309	0.999676639
LOC104969259	0.999863206	0.999560948	0.999323478	0.999939566	0.99989309	0.999676639
CRADD	0.999863206	0.99950308	0.999906387	0.999939566	0.99989309	0.999676639
EFNA5	0.999863206	0.99950308	0.999323478	0.999939566	0.99989309	0.999797893
COG5	0.999863206	0.991720467	0.993278977	0.999939566	0.99989309	0.999899229
LOC101907544	0.999863206	0.991491771	0.994425789	0.999939566	0.99989309	0.999899229
NOS1AP	0.999863206	0.995706781	0.994425789	0.999939566	0.99989309	0.999899229
BBX	0.999863206	0.99950308	0.999323478	0.999939566	0.99989309	0.999899229
SCLT1	0.999863206	0.99950308	0.999323478	0.999939566	0.99989309	0.999899229
NPHP1	0.999863206	0.998683615	0.996569581	0.999939566	0.99989309	0.999973052
ELOVL4	0.999863206	0.989736861	0.989332994	0.999939566	0.999961511	0.990822928
MOGAT1	0.999863206	0.99950308	0.999323478	0.999939566	0.999961511	0.998928226
HIST1H1D	0.99990517	0.999560732	0.999541752	0.999939566	0.99989309	0.998928226
CSNK1A1	0.99990517	0.99950308	0.999917831	0.999939566	0.99989309	0.999676639
PIGS	0.99990517	0.99950308	0.999695103	0.999939566	0.99989309	0.999899229
SMURF1	0.99990517	0.99950308	0.999555497	0.999939566	0.99989309	0.999973052
RPS8	0.999956775	0.999669688	0.999906387	0.999939566	0.99989309	0.999676639
MRI1	0.999956775	0.999669688	0.999917831	0.999939566	0.99989309	0.999676639
LOC112447731	0.999956775	0.999704064	0.999917831	0.999939566	0.99989309	0.999899229
TMLHE	0.999974781	0.999987074	0.999917831	0.999939566	0.99989309	0.999899229
FBXO48	0.999974781	0.999937207	0.999917831	0.999939566	0.999961511	0.999899229
MRPL48	0.999974781	0.999968084	0.999917831	0.999939566	0.999961511	0.999899229
IL1B	NaN	NaN	NaN	NaN	0.348579075	NaN
CXCL8	NaN	NaN	NaN	NaN	0.472422117	NaN
ADAMTS19	NaN	NaN	NaN	NaN	0.476180257	NaN
OSM	NaN	NaN	NaN	NaN	0.538639002	NaN

SAMD9	NaN	NaN	NaN	NaN	0.550221271	NaN
ACBD7	NaN	NaN	NaN	NaN	0.581593891	NaN
ADAM8	NaN	NaN	NaN	NaN	0.676199383	NaN
FOXD1	NaN	NaN	NaN	NaN	0.766792351	NaN
TRIB3	NaN	NaN	NaN	NaN	0.850852167	NaN
IGSF9B	NaN	NaN	NaN	NaN	0.86558233	NaN
COL2A1	NaN	NaN	NaN	NaN	0.870258866	NaN
DPP10	NaN	NaN	NaN	NaN	0.886279726	NaN
MMP12	NaN	NaN	NaN	NaN	0.941269773	NaN
LOC101905509	NaN	NaN	NaN	NaN	0.943768595	NaN
PDZRN4	NaN	NaN	NaN	NaN	0.943901678	NaN
MX1	NaN	NaN	NaN	NaN	0.957390375	NaN
LOC100298356	NaN	NaN	NaN	NaN	0.975291792	NaN
LOC100848536	NaN	NaN	NaN	NaN	0.975291792	NaN
DEFB7	NaN	NaN	NaN	NaN	0.976970807	NaN
LOC112441507	NaN	NaN	NaN	NaN	0.982014614	NaN
C19H17orf58	NaN	NaN	NaN	NaN	0.996250445	NaN
TTYH1	NaN	NaN	NaN	NaN	0.996250445	NaN
ART3	NaN	NaN	NaN	NaN	0.99989309	NaN
BOLA	NaN	NaN	NaN	NaN	0.99989309	NaN
CADM4	NaN	NaN	NaN	NaN	0.99989309	NaN
CCL17	NaN	NaN	NaN	NaN	0.99989309	NaN
CDH19	NaN	NaN	NaN	NaN	0.99989309	NaN
CXHXorf57	NaN	NaN	NaN	NaN	0.99989309	NaN
DDX58	NaN	NaN	NaN	NaN	0.99989309	NaN
DNAH9	NaN	NaN	NaN	NaN	0.99989309	NaN
FOXD3	NaN	NaN	NaN	NaN	0.99989309	NaN
FREM2	NaN	NaN	NaN	NaN	0.99989309	NaN
GABRB3	NaN	NaN	NaN	NaN	0.99989309	NaN
GRIK4	NaN	NaN	NaN	NaN	0.99989309	NaN
GYS2	NaN	NaN	NaN	NaN	0.99989309	NaN
IFIH1	NaN	NaN	NaN	NaN	0.99989309	NaN
IGSF11	NaN	NaN	NaN	NaN	0.99989309	NaN
ISG15	NaN	NaN	NaN	NaN	0.99989309	NaN
JAKMIP2	NaN	NaN	NaN	NaN	0.99989309	NaN
LGI1	NaN	NaN	NaN	NaN	0.99989309	NaN
LOC100847981	NaN	NaN	NaN	NaN	0.99989309	NaN
LOC101902787	NaN	NaN	NaN	NaN	0.99989309	NaN

LOC112441557	NaN	NaN	NaN	NaN	0.99989309	NaN
LOC505033	NaN	NaN	NaN	NaN	0.99989309	NaN
LOC507055	NaN	NaN	NaN	NaN	0.99989309	NaN
LOC509283	NaN	NaN	NaN	NaN	0.99989309	NaN
LUZP2	NaN	NaN	NaN	NaN	0.99989309	NaN
MPZ	NaN	NaN	NaN	NaN	0.99989309	NaN
OAS1Y	NaN	NaN	NaN	NaN	0.99989309	NaN
PCDH10	NaN	NaN	NaN	NaN	0.99989309	NaN
PLP1	NaN	NaN	NaN	NaN	0.99989309	NaN
RSAD2	NaN	NaN	NaN	NaN	0.99989309	NaN
SCUBE1	NaN	NaN	NaN	NaN	0.99989309	NaN
SGCG	NaN	NaN	NaN	NaN	0.99989309	NaN
SLC35F1	NaN	NaN	NaN	NaN	0.99989309	NaN
SNAP25	NaN	NaN	NaN	NaN	0.99989309	NaN
SNCA	NaN	NaN	NaN	NaN	0.99989309	NaN
SOX10	NaN	NaN	NaN	NaN	0.99989309	NaN
SPOCK1	NaN	NaN	NaN	NaN	0.99989309	NaN
TMPRSS5	NaN	NaN	NaN	NaN	0.99989309	NaN
TNFRSF11B	NaN	NaN	NaN	NaN	0.99989309	NaN
WNT6	NaN	NaN	NaN	NaN	0.99989309	NaN
LOC508459	NaN	NaN	NaN	NaN	NaN	0.210229938
CXCL11	NaN	NaN	NaN	NaN	NaN	0.417648084
REC8	NaN	NaN	NaN	NaN	NaN	0.419654233
SVOP	NaN	NaN	NaN	NaN	NaN	0.447180581
AMZ1	NaN	NaN	NaN	NaN	NaN	0.557327019
RHOD	NaN	NaN	NaN	NaN	NaN	0.559833535
TMEM216	NaN	NaN	NaN	NaN	NaN	0.588661483
ST8SIA2	NaN	NaN	NaN	NaN	NaN	0.699838787
GOLGA7B	NaN	NaN	NaN	NaN	NaN	0.752857325
LOC100300115	NaN	NaN	NaN	NaN	NaN	0.75292536
CD5L	NaN	NaN	NaN	NaN	NaN	0.789577614
SPCS3	NaN	NaN	NaN	NaN	NaN	0.798051736
SOCS3	NaN	NaN	NaN	NaN	NaN	0.805038934
LOC112444652	NaN	NaN	NaN	NaN	NaN	0.846367069
TIMD4	NaN	NaN	NaN	NaN	NaN	0.851651757
LOC112443862	NaN	NaN	NaN	NaN	NaN	0.858604851
LCN2	NaN	NaN	NaN	NaN	NaN	0.86143104
MT1A	NaN	NaN	NaN	NaN	NaN	0.865926817

SLC9A2	NaN	NaN	NaN	NaN	NaN	0.868430302
LOC101901948	NaN	NaN	NaN	NaN	NaN	0.875853001
SPTB	NaN	NaN	NaN	NaN	NaN	0.909406094
LOC784266	NaN	NaN	NaN	NaN	NaN	0.923064313
CR2	NaN	NaN	NaN	NaN	NaN	0.925739558
FOSL1	NaN	NaN	NaN	NaN	NaN	0.928653932
SECTM1A	NaN	NaN	NaN	NaN	NaN	0.971219102
ACP5	NaN	NaN	NaN	NaN	NaN	0.979233909
LGR5	NaN	NaN	NaN	NaN	NaN	0.986389023
CCR9	NaN	NaN	NaN	NaN	NaN	0.998636142
LOC616830	NaN	NaN	NaN	0.438283629	NaN	NaN
TGM5	NaN	NaN	NaN	0.5065118	NaN	NaN
LOC104968484	NaN	NaN	NaN	0.669727908	NaN	NaN
SPRY3	NaN	NaN	NaN	0.691877804	NaN	NaN
MID1IP1	NaN	NaN	NaN	0.709187504	NaN	NaN
SLC16A1	NaN	NaN	NaN	0.721039637	NaN	NaN
PPM1E	NaN	NaN	NaN	0.742618602	NaN	NaN
KIF2C	NaN	NaN	NaN	0.781241796	NaN	NaN
LAMC3	NaN	NaN	NaN	0.781241796	NaN	NaN
LOC101905242	NaN	NaN	NaN	0.781241796	NaN	NaN
SNRNP25	NaN	NaN	NaN	0.788938193	NaN	NaN
ARHGAP11A	NaN	NaN	NaN	0.806922554	NaN	NaN
MARCO	NaN	NaN	NaN	0.806922554	NaN	NaN
SRXN1	NaN	NaN	NaN	0.806997685	NaN	NaN
BIRC5	NaN	NaN	NaN	0.820228145	NaN	NaN
YDJC	NaN	NaN	NaN	0.836785013	NaN	NaN
PCLAF	NaN	NaN	NaN	0.846715063	NaN	NaN
UBE2C	NaN	NaN	NaN	0.855385038	NaN	NaN
MLXIPL	NaN	NaN	NaN	0.861272209	NaN	NaN
FCGR2A	NaN	NaN	NaN	0.87630412	NaN	NaN
GSS	NaN	NaN	NaN	0.878289777	NaN	NaN
KIF20A	NaN	NaN	NaN	0.883143297	NaN	NaN
ABAT	NaN	NaN	NaN	0.891273781	NaN	NaN
BOLA3	NaN	NaN	NaN	0.90419464	NaN	NaN
G6PD	NaN	NaN	NaN	0.90419464	NaN	NaN
LOC100138641	NaN	NaN	NaN	0.908054165	NaN	NaN
TRIM44	NaN	NaN	NaN	0.908054165	NaN	NaN
ELOVL6	NaN	NaN	NaN	0.91329897	NaN	NaN

LOC783106	NaN	NaN	NaN	0.91329897	NaN	NaN
KLHL31	NaN	NaN	NaN	0.913413181	NaN	NaN
CDC6	NaN	NaN	NaN	0.915749182	NaN	NaN
HMOX1	NaN	NaN	NaN	0.915749182	NaN	NaN
SERHL2	NaN	NaN	NaN	0.915749182	NaN	NaN
CDCA8	NaN	NaN	NaN	0.917869295	NaN	NaN
TECTB	NaN	NaN	NaN	0.924396934	NaN	NaN
UCHL3	NaN	NaN	NaN	0.924489924	NaN	NaN
LOC104973965	NaN	NaN	NaN	0.925004412	NaN	NaN
LOC618297	NaN	NaN	NaN	0.925931623	NaN	NaN
BUB1B	NaN	NaN	NaN	0.937743794	NaN	NaN
KCNK2	NaN	NaN	NaN	0.939603188	NaN	NaN
ACOT2	NaN	NaN	NaN	0.943323364	NaN	NaN
PBLD	NaN	NaN	NaN	0.943323364	NaN	NaN
BSG	NaN	NaN	NaN	0.944010005	NaN	NaN
KIF11	NaN	NaN	NaN	0.950850938	NaN	NaN
CERS3	NaN	NaN	NaN	0.956154344	NaN	NaN
SLC25A4	NaN	NaN	NaN	0.956154344	NaN	NaN
DPP4	NaN	NaN	NaN	0.958879502	NaN	NaN
TKT	NaN	NaN	NaN	0.965097245	NaN	NaN
FAM49B	NaN	NaN	NaN	0.969223465	NaN	NaN
RRM2	NaN	NaN	NaN	0.972367384	NaN	NaN
UHRF1	NaN	NaN	NaN	0.981022805	NaN	NaN
PARD6B	NaN	NaN	NaN	0.984968973	NaN	NaN
GPT2	NaN	NaN	NaN	0.990571725	NaN	NaN
MPC1	NaN	NaN	NaN	0.990571725	NaN	NaN
TDH	NaN	NaN	NaN	0.992137653	NaN	NaN
SLC16A7	NaN	NaN	NaN	0.995118128	NaN	NaN
ALB	NaN	NaN	NaN	0.995800463	NaN	NaN
GSTM1	NaN	NaN	NaN	0.998706052	NaN	NaN
SH3RF2	NaN	NaN	NaN	0.99879985	NaN	NaN
ATP10B	NaN	NaN	NaN	0.999939566	NaN	NaN
CATHL5	NaN	NaN	NaN	0.999939566	NaN	NaN
CCDC73	NaN	NaN	NaN	0.999939566	NaN	NaN
CDKN2A	NaN	NaN	NaN	0.999939566	NaN	NaN
CKB	NaN	NaN	NaN	0.999939566	NaN	NaN
COBL	NaN	NaN	NaN	0.999939566	NaN	NaN
CYP21	NaN	NaN	NaN	0.999939566	NaN	NaN

CYP46A1	NaN	NaN	NaN	0.999939566	NaN	NaN
FGB	NaN	NaN	NaN	0.999939566	NaN	NaN
IRX1	NaN	NaN	NaN	0.999939566	NaN	NaN
KCNA4	NaN	NaN	NaN	0.999939566	NaN	NaN
KCNN2	NaN	NaN	NaN	0.999939566	NaN	NaN
LDHB	NaN	NaN	NaN	0.999939566	NaN	NaN
LIN7A	NaN	NaN	NaN	0.999939566	NaN	NaN
LOC100301224	NaN	NaN	NaN	0.999939566	NaN	NaN
LOC100851369	NaN	NaN	NaN	0.999939566	NaN	NaN
LOC101907335	NaN	NaN	NaN	0.999939566	NaN	NaN
LOC104974444	NaN	NaN	NaN	0.999939566	NaN	NaN
LOC112443013	NaN	NaN	NaN	0.999939566	NaN	NaN
LOC112445090	NaN	NaN	NaN	0.999939566	NaN	NaN
LOC112448034	NaN	NaN	NaN	0.999939566	NaN	NaN
LOC513210	NaN	NaN	NaN	0.999939566	NaN	NaN
PIR	NaN	NaN	NaN	0.999939566	NaN	NaN
PTCH2	NaN	NaN	NaN	0.999939566	NaN	NaN
PTPRQ	NaN	NaN	NaN	0.999939566	NaN	NaN
SCP2	NaN	NaN	NaN	0.999939566	NaN	NaN
SLC13A3	NaN	NaN	NaN	0.999939566	NaN	NaN
SLC5A9	NaN	NaN	NaN	0.999939566	NaN	NaN
TMEM179	NaN	NaN	NaN	0.999939566	NaN	NaN
UCMA	NaN	NaN	NaN	0.999939566	NaN	NaN
LOC100847119	NaN	NaN	0.041123306	NaN	NaN	NaN
FAM83D	NaN	NaN	0.198823488	NaN	NaN	NaN
LOC100297192	NaN	NaN	0.236666325	NaN	NaN	NaN
LOC112442062	NaN	NaN	0.318905523	NaN	NaN	NaN
LOC100847724	NaN	NaN	0.468416519	NaN	NaN	NaN
EEF1A2	NaN	NaN	0.514686689	NaN	NaN	NaN
PTGIR	NaN	NaN	0.516623782	NaN	NaN	NaN
HP	NaN	NaN	0.620720409	NaN	NaN	NaN
MUSK	NaN	NaN	0.620720409	NaN	NaN	NaN
PRSS35	NaN	NaN	0.620860761	NaN	NaN	NaN
MAPK4	NaN	NaN	0.622639736	NaN	NaN	NaN
COL24A1	NaN	NaN	0.702650233	NaN	NaN	NaN
LOC104974455	NaN	NaN	0.726668529	NaN	NaN	NaN
SIX2	NaN	NaN	0.744490351	NaN	NaN	NaN
P2RX1	NaN	NaN	0.748576908	NaN	NaN	NaN

LOC509911	NaN	NaN	0.810425784	NaN	NaN	NaN
LOC112441777	NaN	NaN	0.81285201	NaN	NaN	NaN
HPSE2	NaN	NaN	0.819320854	NaN	NaN	NaN
RASL12	NaN	NaN	0.832431595	NaN	NaN	NaN
LOC104976942	NaN	NaN	0.843427302	NaN	NaN	NaN
OPCML	NaN	NaN	0.859931483	NaN	NaN	NaN
LOC112447816	NaN	NaN	0.863172962	NaN	NaN	NaN
ACTA1	NaN	NaN	0.871859013	NaN	NaN	NaN
DMRT3	NaN	NaN	0.883982433	NaN	NaN	NaN
A2ML1	NaN	NaN	0.90166788	NaN	NaN	NaN
NPFFR2	NaN	NaN	0.904249961	NaN	NaN	NaN
ADRA2C	NaN	NaN	0.909170161	NaN	NaN	NaN
PHF21B	NaN	NaN	0.914685423	NaN	NaN	NaN
LOC100847415	NaN	NaN	0.933825064	NaN	NaN	NaN
LOC112446726	NaN	NaN	0.964720224	NaN	NaN	NaN
LOC516421	NaN	NaN	0.966577195	NaN	NaN	NaN
C10H15orf62	NaN	NaN	0.967983981	NaN	NaN	NaN
PPDPFL	NaN	NaN	0.970108169	NaN	NaN	NaN
LOC511683	NaN	NaN	0.979096894	NaN	NaN	NaN
HAND2	NaN	NaN	0.987419404	NaN	NaN	NaN
LOC785161	NaN	NaN	0.990693603	NaN	NaN	NaN
LOC514978	NaN	0.040498382	NaN	NaN	NaN	NaN
PLA2G2D1	NaN	0.313970142	NaN	NaN	NaN	NaN
TMPRSS2	NaN	0.390662449	NaN	NaN	NaN	NaN
LOC101906743	NaN	0.453040666	NaN	NaN	NaN	NaN
LOC781796	NaN	0.453214948	NaN	NaN	NaN	NaN
VIL1	NaN	0.517132694	NaN	NaN	NaN	NaN
CA12	NaN	0.553493197	NaN	NaN	NaN	NaN
LOC789829	NaN	0.557135186	NaN	NaN	NaN	NaN
ST14	NaN	0.595087191	NaN	NaN	NaN	NaN
MYO1A	NaN	0.600538394	NaN	NaN	NaN	NaN
CKMT1A	NaN	0.612450407	NaN	NaN	NaN	NaN
CDH17	NaN	0.629715977	NaN	NaN	NaN	NaN
FZD5	NaN	0.636332133	NaN	NaN	NaN	NaN
RIPK4	NaN	0.636332133	NaN	NaN	NaN	NaN
CHAD	NaN	0.648099013	NaN	NaN	NaN	NaN
APOA4	NaN	0.659488126	NaN	NaN	NaN	NaN
HNF4G	NaN	0.671879214	NaN	NaN	NaN	NaN

CHGA	NaN	0.675957273	NaN	NaN	NaN	NaN
BCL2L15	NaN	0.685980044	NaN	NaN	NaN	NaN
UCP1	NaN	0.703437628	NaN	NaN	NaN	NaN
PLEK2	NaN	0.711124077	NaN	NaN	NaN	NaN
LOC104974214	NaN	0.747555258	NaN	NaN	NaN	NaN
LOC101903734	NaN	0.77026764	NaN	NaN	NaN	NaN
PDZK1	NaN	0.776573165	NaN	NaN	NaN	NaN
CRB2	NaN	0.783114106	NaN	NaN	NaN	NaN
GRIA2	NaN	0.820350377	NaN	NaN	NaN	NaN
ALDOB	NaN	0.840505408	NaN	NaN	NaN	NaN
LOC616782	NaN	0.841629926	NaN	NaN	NaN	NaN
LOC100139885	NaN	0.843588854	NaN	NaN	NaN	NaN
KIAA1211L	NaN	0.866803532	NaN	NaN	NaN	NaN
SLC51B	NaN	0.871113425	NaN	NaN	NaN	NaN
FAM3B	NaN	0.871412404	NaN	NaN	NaN	NaN
BAIAP2L2	NaN	0.874669928	NaN	NaN	NaN	NaN
MISP	NaN	0.878225441	NaN	NaN	NaN	NaN
OAS1Z	NaN	0.885181785	NaN	NaN	NaN	NaN
LOC112447079	NaN	0.885796876	NaN	NaN	NaN	NaN
LTF	NaN	0.899387334	NaN	NaN	NaN	NaN
DNAJB13	NaN	0.90249621	NaN	NaN	NaN	NaN
SLC6A8	NaN	0.908192144	NaN	NaN	NaN	NaN
LOC100140226	NaN	0.913075885	NaN	NaN	NaN	NaN
LOC530653	NaN	0.926618064	NaN	NaN	NaN	NaN
SLC9A3	NaN	0.928601644	NaN	NaN	NaN	NaN
SLC15A1	NaN	0.934248552	NaN	NaN	NaN	NaN
DSG2	NaN	0.937978608	NaN	NaN	NaN	NaN
TUBA1D	NaN	0.944238043	NaN	NaN	NaN	NaN
GUCY2C	NaN	0.972133808	NaN	NaN	NaN	NaN
CCL5	NaN	0.978228439	NaN	NaN	NaN	NaN
TMEM45B	NaN	0.985457646	NaN	NaN	NaN	NaN
LOC781736	NaN	0.986851582	NaN	NaN	NaN	NaN
CLDN3	NaN	0.998683615	NaN	NaN	NaN	NaN
ACTN2	NaN	NaN	NaN	NaN	NaN	NaN
C4BPA	NaN	NaN	NaN	NaN	NaN	NaN
CA13	NaN	NaN	NaN	NaN	NaN	NaN
CFHR5	NaN	NaN	NaN	NaN	NaN	NaN
FCGBP	NaN	NaN	NaN	NaN	NaN	NaN

GSTA3	NaN	NaN	NaN	NaN	NaN	NaN
IGLL1	NaN	NaN	NaN	NaN	NaN	NaN
IGSF5	NaN	NaN	NaN	NaN	NaN	NaN
LOC100139670	NaN	NaN	NaN	NaN	NaN	NaN
LOC100297779	NaN	NaN	NaN	NaN	NaN	NaN
LOC101903284	NaN	NaN	NaN	NaN	NaN	NaN
LOC107131864	NaN	NaN	NaN	NaN	NaN	NaN
LOC107131942	NaN	NaN	NaN	NaN	NaN	NaN
LOC112446680	NaN	NaN	NaN	NaN	NaN	NaN
LOC515676	NaN	NaN	NaN	NaN	NaN	NaN
LOC519274	NaN	NaN	NaN	NaN	NaN	NaN
LOC615051	NaN	NaN	NaN	NaN	NaN	NaN
MEF2B	NaN	NaN	NaN	NaN	NaN	NaN
MEGF11	NaN	NaN	NaN	NaN	NaN	NaN
MOXD1	NaN	NaN	NaN	NaN	NaN	NaN
MX2	NaN	NaN	NaN	NaN	NaN	NaN
NAALADL1	NaN	NaN	NaN	NaN	NaN	NaN
PEBP4	NaN	NaN	NaN	NaN	NaN	NaN
PKD2L1	NaN	NaN	NaN	NaN	NaN	NaN
SPHAR	NaN	NaN	NaN	NaN	NaN	NaN
ZBTB37	NaN	NaN	NaN	NaN	NaN	NaN
ZDHHC23	NaN	NaN	NaN	NaN	NaN	NaN
